# Supplementary material for: Mendelian Randomization Mediation Analysis Reveals the Impact of Dietary Preferences on Preeclampsia and Fetal Growth Restriction via Immune Modulation
Source: Food Sci Nutr. 2025 Nov 26;13(12):e71280. doi: 10.1002/fsn3.71280 (PMC12648301; doi:10.1002/fsn3.71280)
Supplement: Supplementary file 1 — Data S1: fsn371280‐sup‐0001‐Supinfo1.zip. [file FSN3-13-e71280-s001.zip › fsn371280-sup-0007-Supinfo7@Supplementary Material 2 _Immune Cells_to_PE and FGR_MR_Analysis .docx]

| id.exposure | id.outcome | outcome | exposure | method | nsnp | b | se | pval | lo_ci | up_ci | or | or_lci95 | or_uci95 | p_fdr |
| --- | --- | --- | --- | --- | --- | --- | --- | --- | --- | --- | --- | --- | --- | --- |
| ebi-a-GCST90001391 | finngen_R11_O15_PREECLAMPS | Pre-eclampsia \|\| id:finngen_R11_O15_PREECLAMPS | IgD+ B cell %B cell \|\| id:ebi-a-GCST90001391 | Inverse variance weighted | 17 | -0.003185815 | 0.0247209321920827 | 0.897459609350111 | -0.051638842 | 0.045267212264178 | 0.996819254491031 | 0.949671786545768 | 1.04630740872933 | 0.986667691552896 |
| ebi-a-GCST90001391 | finngen_R11_O15_PREECLAMPS | Pre-eclampsia \|\| id:finngen_R11_O15_PREECLAMPS | IgD+ B cell %B cell \|\| id:ebi-a-GCST90001391 | MR Egger | 17 | 0.00965412540634559 | 0.0392647800864402 | 0.809113576976724 | -0.067304844 | 0.0866130943757684 | 1.00970087680186 | 0.93491015654867 | 1.09047468729832 | 0.99380795269851 |
| ebi-a-GCST90001391 | finngen_R11_O15_PREECLAMPS | Pre-eclampsia \|\| id:finngen_R11_O15_PREECLAMPS | IgD+ B cell %B cell \|\| id:ebi-a-GCST90001391 | Weighted median | 17 | 0.00640231685597513 | 0.0349128434228963 | 0.854499875792806 | -0.062026856 | 0.0748314899648519 | 1.00642285549477 | 0.939857645397529 | 1.07770253189122 | 0.964912412213677 |
| ebi-a-GCST90001391 | finngen_R11_O15_PREECLAMPS | Pre-eclampsia \|\| id:finngen_R11_O15_PREECLAMPS | IgD+ B cell %B cell \|\| id:ebi-a-GCST90001391 | Weighted mode | 17 | 0.0152731163790829 | 0.0380511429697542 | 0.693447448781131 | -0.059307124 | 0.0898533565998011 | 1.01539034648367 | 0.942417285888503 | 1.09401384203198 | 0.959313411571287 |
| ebi-a-GCST90001392 | finngen_R11_O15_PREECLAMPS | Pre-eclampsia \|\| id:finngen_R11_O15_PREECLAMPS | IgD+ CD38+ B cell Absolute Count \|\| id:ebi-a-GCST90001392 | Inverse variance weighted | 29 | 0.00388835990132823 | 0.00924572423673676 | 0.674078136302201 | -0.01423326 | 0.0220099794053323 | 1.00389592938046 | 0.985867554366206 | 1.02225398590641 | 0.950857618427329 |
| ebi-a-GCST90001392 | finngen_R11_O15_PREECLAMPS | Pre-eclampsia \|\| id:finngen_R11_O15_PREECLAMPS | IgD+ CD38+ B cell Absolute Count \|\| id:ebi-a-GCST90001392 | MR Egger | 29 | -0.000933247 | 0.0100867987134208 | 0.926966720437164 | -0.020703372 | 0.0188368786079588 | 0.999067188469078 | 0.979509471075078 | 1.0190154118469 | 0.99380795269851 |
| ebi-a-GCST90001392 | finngen_R11_O15_PREECLAMPS | Pre-eclampsia \|\| id:finngen_R11_O15_PREECLAMPS | IgD+ CD38+ B cell Absolute Count \|\| id:ebi-a-GCST90001392 | Weighted median | 29 | -0.003503386 | 0.0108855873715403 | 0.747576128478156 | -0.024839137 | 0.01783236569051 | 0.996502744137156 | 0.9754668161157 | 1.01799231164733 | 0.957984984509652 |
| ebi-a-GCST90001392 | finngen_R11_O15_PREECLAMPS | Pre-eclampsia \|\| id:finngen_R11_O15_PREECLAMPS | IgD+ CD38+ B cell Absolute Count \|\| id:ebi-a-GCST90001392 | Weighted mode | 29 | 0.00185090488313633 | 0.00927950960133295 | 0.843343218251408 | -0.016336934 | 0.0200387437017489 | 1.00185261886489 | 0.983795790019325 | 1.02024086716891 | 0.977585971930092 |
| ebi-a-GCST90001393 | finngen_R11_O15_PREECLAMPS | Pre-eclampsia \|\| id:finngen_R11_O15_PREECLAMPS | IgD+ CD38dim B cell %B cell \|\| id:ebi-a-GCST90001393 | Inverse variance weighted | 15 | -0.001967879 | 0.0261710449649601 | 0.940061153492054 | -0.053263128 | 0.049327368737815 | 0.998034055611653 | 0.948130500425201 | 1.05056421633303 | 0.986667691552896 |
| ebi-a-GCST90001393 | finngen_R11_O15_PREECLAMPS | Pre-eclampsia \|\| id:finngen_R11_O15_PREECLAMPS | IgD+ CD38dim B cell %B cell \|\| id:ebi-a-GCST90001393 | MR Egger | 15 | 0.0440472962680912 | 0.0384572241838113 | 0.272709124300612 | -0.031328863 | 0.119423455668361 | 1.04503177982285 | 0.969156800721604 | 1.12684698701652 | 0.99380795269851 |
| ebi-a-GCST90001393 | finngen_R11_O15_PREECLAMPS | Pre-eclampsia \|\| id:finngen_R11_O15_PREECLAMPS | IgD+ CD38dim B cell %B cell \|\| id:ebi-a-GCST90001393 | Weighted median | 15 | 0.0163482100345473 | 0.035789245383262 | 0.647821177960149 | -0.053798711 | 0.0864951309857408 | 1.01648257322081 | 0.947622833437037 | 1.09034605879435 | 0.940845681141708 |
| ebi-a-GCST90001393 | finngen_R11_O15_PREECLAMPS | Pre-eclampsia \|\| id:finngen_R11_O15_PREECLAMPS | IgD+ CD38dim B cell %B cell \|\| id:ebi-a-GCST90001393 | Weighted mode | 15 | 0.0162977053354901 | 0.0358835029720141 | 0.65665827492389 | -0.05403396 | 0.0866293711606377 | 1.01643123737072 | 0.947399931789882 | 1.09049243686467 | 0.959313411571287 |
| ebi-a-GCST90001394 | finngen_R11_O15_PREECLAMPS | Pre-eclampsia \|\| id:finngen_R11_O15_PREECLAMPS | IgD+ CD38dim B cell Absolute Count \|\| id:ebi-a-GCST90001394 | Inverse variance weighted | 25 | 0.00313669945736915 | 0.0101056521791117 | 0.756263940456562 | -0.016670379 | 0.0229437777284281 | 1.00314162404675 | 0.983467803037764 | 1.02320901079515 | 0.970051434846584 |
| ebi-a-GCST90001394 | finngen_R11_O15_PREECLAMPS | Pre-eclampsia \|\| id:finngen_R11_O15_PREECLAMPS | IgD+ CD38dim B cell Absolute Count \|\| id:ebi-a-GCST90001394 | MR Egger | 25 | 0.00197601456572858 | 0.0120021434604538 | 0.87066693529116 | -0.021548187 | 0.0255002157482179 | 1.00197796816908 | 0.978682316943167 | 1.02582812759101 | 0.99380795269851 |
| ebi-a-GCST90001394 | finngen_R11_O15_PREECLAMPS | Pre-eclampsia \|\| id:finngen_R11_O15_PREECLAMPS | IgD+ CD38dim B cell Absolute Count \|\| id:ebi-a-GCST90001394 | Weighted median | 25 | 0.00327465753950294 | 0.0124540280705531 | 0.792597109966642 | -0.021135237 | 0.0276845525577871 | 1.00328002508787 | 0.979086546419667 | 1.02807133079414 | 0.957984984509652 |
| ebi-a-GCST90001394 | finngen_R11_O15_PREECLAMPS | Pre-eclampsia \|\| id:finngen_R11_O15_PREECLAMPS | IgD+ CD38dim B cell Absolute Count \|\| id:ebi-a-GCST90001394 | Weighted mode | 25 | -0.00056526 | 0.0107807340124648 | 0.95861814254781 | -0.021695498 | 0.0205649788915532 | 0.99943489995633 | 0.978538156087879 | 1.02077789510439 | 0.990180645930572 |
| ebi-a-GCST90001395 | finngen_R11_O15_PREECLAMPS | Pre-eclampsia \|\| id:finngen_R11_O15_PREECLAMPS | IgD+ CD38- B cell %B cell \|\| id:ebi-a-GCST90001395 | Inverse variance weighted | 22 | 0.019333052742129 | 0.0126015482962866 | 0.124985193476463 | -0.005365982 | 0.0440320874028507 | 1.0195211463924 | 0.994648389245756 | 1.0450158861962 | 0.820779257935285 |
| ebi-a-GCST90001395 | finngen_R11_O15_PREECLAMPS | Pre-eclampsia \|\| id:finngen_R11_O15_PREECLAMPS | IgD+ CD38- B cell %B cell \|\| id:ebi-a-GCST90001395 | MR Egger | 22 | 0.021893680494223 | 0.0141592457267095 | 0.137722830371311 | -0.005858441 | 0.0496458021185737 | 1.02213510579392 | 0.994158686073549 | 1.0508988043173 | 0.97261503632431 |
| ebi-a-GCST90001395 | finngen_R11_O15_PREECLAMPS | Pre-eclampsia \|\| id:finngen_R11_O15_PREECLAMPS | IgD+ CD38- B cell %B cell \|\| id:ebi-a-GCST90001395 | Weighted median | 22 | 0.0203887216689284 | 0.0175675171357123 | 0.245807818151042 | -0.014043612 | 0.0548210552549246 | 1.02059799148281 | 0.986054539596306 | 1.05635156919939 | 0.940845681141708 |
| ebi-a-GCST90001395 | finngen_R11_O15_PREECLAMPS | Pre-eclampsia \|\| id:finngen_R11_O15_PREECLAMPS | IgD+ CD38- B cell %B cell \|\| id:ebi-a-GCST90001395 | Weighted mode | 22 | 0.015845091715914 | 0.0147366103449991 | 0.294475408759495 | -0.013038665 | 0.0447288479921123 | 1.0159712908462 | 0.987045970584054 | 1.04574426580449 | 0.872755742358858 |
| ebi-a-GCST90001396 | finngen_R11_O15_PREECLAMPS | Pre-eclampsia \|\| id:finngen_R11_O15_PREECLAMPS | IgD+ CD38- B cell Absolute Count \|\| id:ebi-a-GCST90001396 | Inverse variance weighted | 18 | -0.030829766 | 0.0357926517369856 | 0.389048920117257 | -0.100983363 | 0.0393238318197739 | 0.969640625231719 | 0.903948071754586 | 1.04010724894274 | 0.885079385681701 |
| ebi-a-GCST90001396 | finngen_R11_O15_PREECLAMPS | Pre-eclampsia \|\| id:finngen_R11_O15_PREECLAMPS | IgD+ CD38- B cell Absolute Count \|\| id:ebi-a-GCST90001396 | MR Egger | 18 | 0.0935225879333897 | 0.0771769312130721 | 0.243183802072299 | -0.057744197 | 0.244789373111011 | 1.09803540541178 | 0.943891366570357 | 1.27735224014039 | 0.99380795269851 |
| ebi-a-GCST90001396 | finngen_R11_O15_PREECLAMPS | Pre-eclampsia \|\| id:finngen_R11_O15_PREECLAMPS | IgD+ CD38- B cell Absolute Count \|\| id:ebi-a-GCST90001396 | Weighted median | 18 | -0.015264705 | 0.0432021234913208 | 0.723839344487509 | -0.099940867 | 0.0694114570323683 | 0.98485121004636 | 0.904890925320499 | 1.07187714981918 | 0.949957918887556 |
| ebi-a-GCST90001396 | finngen_R11_O15_PREECLAMPS | Pre-eclampsia \|\| id:finngen_R11_O15_PREECLAMPS | IgD+ CD38- B cell Absolute Count \|\| id:ebi-a-GCST90001396 | Weighted mode | 18 | -0.010226353 | 0.0584758883483879 | 0.863238646528466 | -0.124839094 | 0.104386387904568 | 0.98982575810453 | 0.882638912684375 | 1.11002927395017 | 0.977585971930092 |
| ebi-a-GCST90001397 | finngen_R11_O15_PREECLAMPS | Pre-eclampsia \|\| id:finngen_R11_O15_PREECLAMPS | Unswitched memory B cell %B cell \|\| id:ebi-a-GCST90001397 | Inverse variance weighted | 31 | -0.004857307 | 0.0147099553230648 | 0.741244743574968 | -0.03368882 | 0.0239742052983993 | 0.995154470504573 | 0.966872329578855 | 1.02426389696828 | 0.96638925304053 |
| ebi-a-GCST90001397 | finngen_R11_O15_PREECLAMPS | Pre-eclampsia \|\| id:finngen_R11_O15_PREECLAMPS | Unswitched memory B cell %B cell \|\| id:ebi-a-GCST90001397 | MR Egger | 31 | 0.021937305557552 | 0.0195949332745613 | 0.272097112437153 | -0.016468764 | 0.0603433747756921 | 1.0221796974753 | 0.983666105039117 | 1.06220121703709 | 0.99380795269851 |
| ebi-a-GCST90001397 | finngen_R11_O15_PREECLAMPS | Pre-eclampsia \|\| id:finngen_R11_O15_PREECLAMPS | Unswitched memory B cell %B cell \|\| id:ebi-a-GCST90001397 | Weighted median | 31 | -0.004739142 | 0.0232705064716658 | 0.838623544182861 | -0.050349335 | 0.0408710506196043 | 0.995272069950125 | 0.950897185042927 | 1.04171776802356 | 0.964912412213677 |
| ebi-a-GCST90001397 | finngen_R11_O15_PREECLAMPS | Pre-eclampsia \|\| id:finngen_R11_O15_PREECLAMPS | Unswitched memory B cell %B cell \|\| id:ebi-a-GCST90001397 | Weighted mode | 31 | 0.00146063508665617 | 0.0232055431287943 | 0.950229114746895 | -0.044022229 | 0.046943499619093 | 1.00146170233364 | 0.956932685153371 | 1.04806279145983 | 0.990180645930572 |
| ebi-a-GCST90001398 | finngen_R11_O15_PREECLAMPS | Pre-eclampsia \|\| id:finngen_R11_O15_PREECLAMPS | Unswitched memory B cell Absolute Count \|\| id:ebi-a-GCST90001398 | Inverse variance weighted | 20 | -0.019154513 | 0.02733117688354 | 0.48340901795222 | -0.07272362 | 0.0344145938113214 | 0.981027769105188 | 0.929857789008501 | 1.035013628 | 0.898694924660397 |
| ebi-a-GCST90001398 | finngen_R11_O15_PREECLAMPS | Pre-eclampsia \|\| id:finngen_R11_O15_PREECLAMPS | Unswitched memory B cell Absolute Count \|\| id:ebi-a-GCST90001398 | MR Egger | 20 | -0.024751361 | 0.0489929324479778 | 0.619546215583849 | -0.120777508 | 0.0712747870886705 | 0.975552442738176 | 0.886231116897589 | 1.07387627266353 | 0.99380795269851 |
| ebi-a-GCST90001398 | finngen_R11_O15_PREECLAMPS | Pre-eclampsia \|\| id:finngen_R11_O15_PREECLAMPS | Unswitched memory B cell Absolute Count \|\| id:ebi-a-GCST90001398 | Weighted median | 20 | -0.02330641 | 0.0398885431682286 | 0.559026345605477 | -0.101487955 | 0.0548751345311379 | 0.976963086570324 | 0.903492062120167 | 1.05640869747239 | 0.940845681141708 |
| ebi-a-GCST90001398 | finngen_R11_O15_PREECLAMPS | Pre-eclampsia \|\| id:finngen_R11_O15_PREECLAMPS | Unswitched memory B cell Absolute Count \|\| id:ebi-a-GCST90001398 | Weighted mode | 20 | -0.023990755 | 0.0442611336567413 | 0.594100054414228 | -0.110742577 | 0.0627610672549344 | 0.976294735841919 | 0.895169156688325 | 1.0647723998432 | 0.955353022288269 |
| ebi-a-GCST90001399 | finngen_R11_O15_PREECLAMPS | Pre-eclampsia \|\| id:finngen_R11_O15_PREECLAMPS | IgD- CD27- B cell %B cell \|\| id:ebi-a-GCST90001399 | Inverse variance weighted | 15 | 0.0219845387834096 | 0.0301449686668307 | 0.465821963327481 | -0.0370996 | 0.0810686773703978 | 1.02222797946006 | 0.963580158174992 | 1.08444537086579 | 0.898694924660397 |
| ebi-a-GCST90001399 | finngen_R11_O15_PREECLAMPS | Pre-eclampsia \|\| id:finngen_R11_O15_PREECLAMPS | IgD- CD27- B cell %B cell \|\| id:ebi-a-GCST90001399 | MR Egger | 15 | 0.0226502869039089 | 0.0586950966412235 | 0.705813898773288 | -0.092392103 | 0.137692676320707 | 1.02290875240262 | 0.911747580930292 | 1.14762280441068 | 0.99380795269851 |
| ebi-a-GCST90001399 | finngen_R11_O15_PREECLAMPS | Pre-eclampsia \|\| id:finngen_R11_O15_PREECLAMPS | IgD- CD27- B cell %B cell \|\| id:ebi-a-GCST90001399 | Weighted median | 15 | 0.0366323572202927 | 0.0348022661605871 | 0.292531051829991 | -0.031580084 | 0.104844798895043 | 1.03731159061082 | 0.968913358448856 | 1.11053824021806 | 0.940845681141708 |
| ebi-a-GCST90001399 | finngen_R11_O15_PREECLAMPS | Pre-eclampsia \|\| id:finngen_R11_O15_PREECLAMPS | IgD- CD27- B cell %B cell \|\| id:ebi-a-GCST90001399 | Weighted mode | 15 | 0.0341080625850728 | 0.0362316672312153 | 0.36246720860403 | -0.036906005 | 0.105122130358255 | 1.03469641265611 | 0.963766720163304 | 1.11084627012438 | 0.907825101194024 |
| ebi-a-GCST90001400 | finngen_R11_O15_PREECLAMPS | Pre-eclampsia \|\| id:finngen_R11_O15_PREECLAMPS | IgD+ B cell Absolute Count \|\| id:ebi-a-GCST90001400 | Inverse variance weighted | 14 | 0.00639779705373454 | 0.0136419552230197 | 0.639084304179354 | -0.020340435 | 0.0331360292908531 | 1.00641830667277 | 0.979865035985819 | 1.03369114195105 | 0.942541573457101 |
| ebi-a-GCST90001400 | finngen_R11_O15_PREECLAMPS | Pre-eclampsia \|\| id:finngen_R11_O15_PREECLAMPS | IgD+ B cell Absolute Count \|\| id:ebi-a-GCST90001400 | MR Egger | 14 | -0.00372963 | 0.016557905342245 | 0.825576875800313 | -0.036183124 | 0.0287238645377643 | 0.996277316498127 | 0.964463660477863 | 1.02914037308251 | 0.99380795269851 |
| ebi-a-GCST90001400 | finngen_R11_O15_PREECLAMPS | Pre-eclampsia \|\| id:finngen_R11_O15_PREECLAMPS | IgD+ B cell Absolute Count \|\| id:ebi-a-GCST90001400 | Weighted median | 14 | 0.000177479540749058 | 0.0134865740923681 | 0.989500365525313 | -0.026256206 | 0.0266111647617905 | 1.00017749529117 | 0.974085491399888 | 1.02696840361444 | 0.993577976921709 |
| ebi-a-GCST90001400 | finngen_R11_O15_PREECLAMPS | Pre-eclampsia \|\| id:finngen_R11_O15_PREECLAMPS | IgD+ B cell Absolute Count \|\| id:ebi-a-GCST90001400 | Weighted mode | 14 | -0.000543817 | 0.0121553556668898 | 0.964995375925544 | -0.024368314 | 0.0232806802591084 | 0.999456330993586 | 0.975926196317814 | 1.02355379057564 | 0.992140112238499 |
| ebi-a-GCST90001401 | finngen_R11_O15_PREECLAMPS | Pre-eclampsia \|\| id:finngen_R11_O15_PREECLAMPS | IgD- CD27- B cell Absolute Count \|\| id:ebi-a-GCST90001401 | Inverse variance weighted | 26 | 0.0176006264939341 | 0.0264769650940311 | 0.506208862673507 | -0.034294225 | 0.0694954780782351 | 1.01775643025945 | 0.966287156882382 | 1.07196721384194 | 0.898694924660397 |
| ebi-a-GCST90001401 | finngen_R11_O15_PREECLAMPS | Pre-eclampsia \|\| id:finngen_R11_O15_PREECLAMPS | IgD- CD27- B cell Absolute Count \|\| id:ebi-a-GCST90001401 | MR Egger | 26 | 0.073610997967191 | 0.0592046748706867 | 0.225755047104132 | -0.042430165 | 0.189652160713737 | 1.07638800692197 | 0.95845739726764 | 1.20882904628667 | 0.99380795269851 |
| ebi-a-GCST90001401 | finngen_R11_O15_PREECLAMPS | Pre-eclampsia \|\| id:finngen_R11_O15_PREECLAMPS | IgD- CD27- B cell Absolute Count \|\| id:ebi-a-GCST90001401 | Weighted median | 26 | 0.0289470644523187 | 0.0346776124424119 | 0.403859661119039 | -0.039021056 | 0.0969151848394461 | 1.02937010276271 | 0.961730458799329 | 1.10176692311958 | 0.940845681141708 |
| ebi-a-GCST90001401 | finngen_R11_O15_PREECLAMPS | Pre-eclampsia \|\| id:finngen_R11_O15_PREECLAMPS | IgD- CD27- B cell Absolute Count \|\| id:ebi-a-GCST90001401 | Weighted mode | 26 | 0.0339576762317817 | 0.0442551820012396 | 0.450080598059239 | -0.05278248 | 0.120697832954211 | 1.03454082013563 | 0.948586326075095 | 1.12828393063109 | 0.933945646747087 |
| ebi-a-GCST90001402 | finngen_R11_O15_PREECLAMPS | Pre-eclampsia \|\| id:finngen_R11_O15_PREECLAMPS | Switched memory B cell %B cell \|\| id:ebi-a-GCST90001402 | Inverse variance weighted | 13 | -0.001746494 | 0.0284321864426444 | 0.951019435595999 | -0.057473579 | 0.0539805917049021 | 0.998255030509997 | 0.944146835218551 | 1.05546411719724 | 0.990306563277315 |
| ebi-a-GCST90001402 | finngen_R11_O15_PREECLAMPS | Pre-eclampsia \|\| id:finngen_R11_O15_PREECLAMPS | Switched memory B cell %B cell \|\| id:ebi-a-GCST90001402 | MR Egger | 13 | -0.002700299 | 0.044011061531674 | 0.95217707772617 | -0.08896198 | 0.0835613813497685 | 0.997303343276337 | 0.914880356797594 | 1.08715194409864 | 0.99380795269851 |
| ebi-a-GCST90001402 | finngen_R11_O15_PREECLAMPS | Pre-eclampsia \|\| id:finngen_R11_O15_PREECLAMPS | Switched memory B cell %B cell \|\| id:ebi-a-GCST90001402 | Weighted median | 13 | 0.0028388059116682 | 0.0396948728254229 | 0.942987343889835 | -0.074963145 | 0.0806407566494971 | 1.00284283913678 | 0.927777679106106 | 1.08398141349648 | 0.981835244750373 |
| ebi-a-GCST90001402 | finngen_R11_O15_PREECLAMPS | Pre-eclampsia \|\| id:finngen_R11_O15_PREECLAMPS | Switched memory B cell %B cell \|\| id:ebi-a-GCST90001402 | Weighted mode | 13 | 0.0149997475986136 | 0.0479019215116365 | 0.759554003791158 | -0.078888019 | 0.108887513761421 | 1.01511280839981 | 0.924143405556442 | 1.11503691697815 | 0.959313411571287 |
| ebi-a-GCST90001403 | finngen_R11_O15_PREECLAMPS | Pre-eclampsia \|\| id:finngen_R11_O15_PREECLAMPS | Switched memory B cell Absolute Count \|\| id:ebi-a-GCST90001403 | Inverse variance weighted | 22 | 0.00928298712542338 | 0.0212031844456352 | 0.661523305210707 | -0.032275254 | 0.0508412286388684 | 1.00932620768552 | 0.968240033078726 | 1.05215582780804 | 0.948183404135347 |
| ebi-a-GCST90001403 | finngen_R11_O15_PREECLAMPS | Pre-eclampsia \|\| id:finngen_R11_O15_PREECLAMPS | Switched memory B cell Absolute Count \|\| id:ebi-a-GCST90001403 | MR Egger | 22 | -0.012125648 | 0.0298196491974766 | 0.688596223302587 | -0.070572161 | 0.0463208643108882 | 0.987947571311812 | 0.931860493540157 | 1.04741043367222 | 0.99380795269851 |
| ebi-a-GCST90001403 | finngen_R11_O15_PREECLAMPS | Pre-eclampsia \|\| id:finngen_R11_O15_PREECLAMPS | Switched memory B cell Absolute Count \|\| id:ebi-a-GCST90001403 | Weighted median | 22 | 0.00489561075952844 | 0.0310292582317101 | 0.874634872506907 | -0.055921735 | 0.0657129568936802 | 1.00490761384136 | 0.945613141052601 | 1.06792013405423 | 0.964912412213677 |
| ebi-a-GCST90001403 | finngen_R11_O15_PREECLAMPS | Pre-eclampsia \|\| id:finngen_R11_O15_PREECLAMPS | Switched memory B cell Absolute Count \|\| id:ebi-a-GCST90001403 | Weighted mode | 22 | 0.00844774261546211 | 0.0271196994553742 | 0.758493113278672 | -0.044706868 | 0.0616023535479954 | 1.00848352548362 | 0.956277756060446 | 1.06353934798374 | 0.959313411571287 |
| ebi-a-GCST90001404 | finngen_R11_O15_PREECLAMPS | Pre-eclampsia \|\| id:finngen_R11_O15_PREECLAMPS | Plasma Blast-Plasma Cell %B cell \|\| id:ebi-a-GCST90001404 | Inverse variance weighted | 26 | -0.025117929 | 0.0155268424699794 | 0.105725104578699 | -0.05555054 | 0.00531468252635762 | 0.975194901765513 | 0.945964213472592 | 1.00532883050444 | 0.820779257935285 |
| ebi-a-GCST90001404 | finngen_R11_O15_PREECLAMPS | Pre-eclampsia \|\| id:finngen_R11_O15_PREECLAMPS | Plasma Blast-Plasma Cell %B cell \|\| id:ebi-a-GCST90001404 | MR Egger | 26 | -0.028602458 | 0.0202344686969405 | 0.170333935527247 | -0.068262017 | 0.0110571006027021 | 0.971802720039969 | 0.934015713807951 | 1.01111845626964 | 0.99380795269851 |
| ebi-a-GCST90001404 | finngen_R11_O15_PREECLAMPS | Pre-eclampsia \|\| id:finngen_R11_O15_PREECLAMPS | Plasma Blast-Plasma Cell %B cell \|\| id:ebi-a-GCST90001404 | Weighted median | 26 | -0.014970402 | 0.0203756486680974 | 0.462509868340963 | -0.054906674 | 0.0249658691477149 | 0.985141097139591 | 0.946573484097422 | 1.0252801262427 | 0.940845681141708 |
| ebi-a-GCST90001404 | finngen_R11_O15_PREECLAMPS | Pre-eclampsia \|\| id:finngen_R11_O15_PREECLAMPS | Plasma Blast-Plasma Cell %B cell \|\| id:ebi-a-GCST90001404 | Weighted mode | 26 | -0.01744591 | 0.0184842863262113 | 0.354292397117898 | -0.053675111 | 0.0187832915928932 | 0.982705389148726 | 0.947739966962947 | 1.01896080731573 | 0.899262994073554 |
| ebi-a-GCST90001405 | finngen_R11_O15_PREECLAMPS | Pre-eclampsia \|\| id:finngen_R11_O15_PREECLAMPS | Plasma Blast-Plasma Cell Absolute Count \|\| id:ebi-a-GCST90001405 | Inverse variance weighted | 24 | -0.003666129 | 0.0127020617020687 | 0.772868574293806 | -0.02856217 | 0.0212299118991346 | 0.996340583009223 | 0.971841872884989 | 1.02145686973156 | 0.977824840984872 |
| ebi-a-GCST90001405 | finngen_R11_O15_PREECLAMPS | Pre-eclampsia \|\| id:finngen_R11_O15_PREECLAMPS | Plasma Blast-Plasma Cell Absolute Count \|\| id:ebi-a-GCST90001405 | MR Egger | 24 | -0.003202861 | 0.0149112340181755 | 0.831905604717139 | -0.03242888 | 0.0260231572811483 | 0.996802262294466 | 0.968091297968288 | 1.02636471601453 | 0.99380795269851 |
| ebi-a-GCST90001405 | finngen_R11_O15_PREECLAMPS | Pre-eclampsia \|\| id:finngen_R11_O15_PREECLAMPS | Plasma Blast-Plasma Cell Absolute Count \|\| id:ebi-a-GCST90001405 | Weighted median | 24 | -0.009489815 | 0.0163944159471206 | 0.562694197435763 | -0.04162287 | 0.022643240081851 | 0.990555071022066 | 0.959231466916712 | 1.02290154417206 | 0.940845681141708 |
| ebi-a-GCST90001405 | finngen_R11_O15_PREECLAMPS | Pre-eclampsia \|\| id:finngen_R11_O15_PREECLAMPS | Plasma Blast-Plasma Cell Absolute Count \|\| id:ebi-a-GCST90001405 | Weighted mode | 24 | -0.006627339 | 0.0138362164762245 | 0.636471844062644 | -0.033746323 | 0.0204916454725391 | 0.993394573555426 | 0.966816732589664 | 1.02070304071599 | 0.959313411571287 |
| ebi-a-GCST90001406 | finngen_R11_O15_PREECLAMPS | Pre-eclampsia \|\| id:finngen_R11_O15_PREECLAMPS | Memory B cell %B cell \|\| id:ebi-a-GCST90001406 | Inverse variance weighted | 28 | 0.00562474452669154 | 0.0172699058666428 | 0.744654140195823 | -0.028224271 | 0.0394737600253115 | 1.00564059310298 | 0.972170312769641 | 1.04026320204673 | 0.96638925304053 |
| ebi-a-GCST90001406 | finngen_R11_O15_PREECLAMPS | Pre-eclampsia \|\| id:finngen_R11_O15_PREECLAMPS | Memory B cell %B cell \|\| id:ebi-a-GCST90001406 | MR Egger | 28 | 0.0178089225855408 | 0.0254762460413469 | 0.490733486181272 | -0.03212452 | 0.0677423648265807 | 1.01796844702644 | 0.968385991481354 | 1.07008958024707 | 0.99380795269851 |
| ebi-a-GCST90001406 | finngen_R11_O15_PREECLAMPS | Pre-eclampsia \|\| id:finngen_R11_O15_PREECLAMPS | Memory B cell %B cell \|\| id:ebi-a-GCST90001406 | Weighted median | 28 | 0.0196462691384656 | 0.0245559499421335 | 0.423675189721004 | -0.028483393 | 0.0677759310250473 | 1.0198405271469 | 0.971918434906368 | 1.07012549968914 | 0.940845681141708 |
| ebi-a-GCST90001406 | finngen_R11_O15_PREECLAMPS | Pre-eclampsia \|\| id:finngen_R11_O15_PREECLAMPS | Memory B cell %B cell \|\| id:ebi-a-GCST90001406 | Weighted mode | 28 | 0.0243496815402459 | 0.0229655776274005 | 0.298415422784365 | -0.020662851 | 0.0693622136899508 | 1.02464855593761 | 0.979549163306594 | 1.07182436830525 | 0.875408265683677 |
| ebi-a-GCST90001407 | finngen_R11_O15_PREECLAMPS | Pre-eclampsia \|\| id:finngen_R11_O15_PREECLAMPS | Memory B cell Absolute Count \|\| id:ebi-a-GCST90001407 | Inverse variance weighted | 17 | -0.006840841 | 0.0125217310481439 | 0.584847719169427 | -0.031383433 | 0.0177017522394484 | 0.993182504671119 | 0.969103914951299 | 1.01785935684138 | 0.915399120505076 |
| ebi-a-GCST90001407 | finngen_R11_O15_PREECLAMPS | Pre-eclampsia \|\| id:finngen_R11_O15_PREECLAMPS | Memory B cell Absolute Count \|\| id:ebi-a-GCST90001407 | MR Egger | 17 | -0.000527936 | 0.0144343046071535 | 0.971306134732658 | -0.028819173 | 0.0277633013208485 | 0.999472203624363 | 0.971592138927864 | 1.02815229332759 | 0.99380795269851 |
| ebi-a-GCST90001407 | finngen_R11_O15_PREECLAMPS | Pre-eclampsia \|\| id:finngen_R11_O15_PREECLAMPS | Memory B cell Absolute Count \|\| id:ebi-a-GCST90001407 | Weighted median | 17 | -0.006170378 | 0.0138058393505633 | 0.654918606546557 | -0.033229823 | 0.0208890675819621 | 0.993848620140002 | 0.967316222841758 | 1.02110877128933 | 0.940845681141708 |
| ebi-a-GCST90001407 | finngen_R11_O15_PREECLAMPS | Pre-eclampsia \|\| id:finngen_R11_O15_PREECLAMPS | Memory B cell Absolute Count \|\| id:ebi-a-GCST90001407 | Weighted mode | 17 | -0.006353424 | 0.0131617931080529 | 0.635834673760822 | -0.032150539 | 0.0194436904257862 | 0.993666716256773 | 0.968360795468658 | 1.01963395009053 | 0.959313411571287 |
| ebi-a-GCST90001408 | finngen_R11_O15_PREECLAMPS | Pre-eclampsia \|\| id:finngen_R11_O15_PREECLAMPS | Naive-mature B cell %B cell \|\| id:ebi-a-GCST90001408 | Inverse variance weighted | 28 | -0.007620028 | 0.0167670478333724 | 0.649494546276859 | -0.040483442 | 0.0252433859475433 | 0.992408931003686 | 0.96032506586544 | 1.02556469818718 | 0.942541573457101 |
| ebi-a-GCST90001408 | finngen_R11_O15_PREECLAMPS | Pre-eclampsia \|\| id:finngen_R11_O15_PREECLAMPS | Naive-mature B cell %B cell \|\| id:ebi-a-GCST90001408 | MR Egger | 28 | -0.008604485 | 0.0232192693237307 | 0.713956035816025 | -0.054114253 | 0.0369052828460033 | 0.991432427605521 | 0.947323865816739 | 1.0375947381631 | 0.99380795269851 |
| ebi-a-GCST90001408 | finngen_R11_O15_PREECLAMPS | Pre-eclampsia \|\| id:finngen_R11_O15_PREECLAMPS | Naive-mature B cell %B cell \|\| id:ebi-a-GCST90001408 | Weighted median | 28 | -0.004351684 | 0.0209589684295023 | 0.835518860772269 | -0.045431262 | 0.0367278938798792 | 0.995657770616101 | 0.955585284988208 | 1.0374106966292 | 0.964912412213677 |
| ebi-a-GCST90001408 | finngen_R11_O15_PREECLAMPS | Pre-eclampsia \|\| id:finngen_R11_O15_PREECLAMPS | Naive-mature B cell %B cell \|\| id:ebi-a-GCST90001408 | Weighted mode | 28 | -0.015917049 | 0.0190362817961134 | 0.410413722910034 | -0.053228162 | 0.0213940628836957 | 0.984208957356739 | 0.948163653115687 | 1.02162455664502 | 0.930105377419646 |
| ebi-a-GCST90001409 | finngen_R11_O15_PREECLAMPS | Pre-eclampsia \|\| id:finngen_R11_O15_PREECLAMPS | Naive-mature B cell Absolute Count \|\| id:ebi-a-GCST90001409 | Inverse variance weighted | 22 | -0.013355426 | 0.0149282430144115 | 0.370978732149736 | -0.042614783 | 0.0159039298105393 | 0.986733361504029 | 0.958280465087315 | 1.01603107041979 | 0.885079385681701 |
| ebi-a-GCST90001409 | finngen_R11_O15_PREECLAMPS | Pre-eclampsia \|\| id:finngen_R11_O15_PREECLAMPS | Naive-mature B cell Absolute Count \|\| id:ebi-a-GCST90001409 | MR Egger | 22 | -0.007388142 | 0.0181806085011499 | 0.688782684473144 | -0.043022134 | 0.0282458508627743 | 0.992639083430931 | 0.957890187448679 | 1.02864854746974 | 0.99380795269851 |
| ebi-a-GCST90001409 | finngen_R11_O15_PREECLAMPS | Pre-eclampsia \|\| id:finngen_R11_O15_PREECLAMPS | Naive-mature B cell Absolute Count \|\| id:ebi-a-GCST90001409 | Weighted median | 22 | -0.02078336 | 0.0200983335605748 | 0.301097026118646 | -0.060176093 | 0.0186093742345396 | 0.979431125992829 | 0.941598709738792 | 1.01878360775334 | 0.940845681141708 |
| ebi-a-GCST90001409 | finngen_R11_O15_PREECLAMPS | Pre-eclampsia \|\| id:finngen_R11_O15_PREECLAMPS | Naive-mature B cell Absolute Count \|\| id:ebi-a-GCST90001409 | Weighted mode | 22 | -0.019461223 | 0.0196354477920316 | 0.332907064913846 | -0.0579467 | 0.0190242550751813 | 0.98072692449431 | 0.943700245065019 | 1.01920636924472 | 0.888599269773114 |
| ebi-a-GCST90001410 | finngen_R11_O15_PREECLAMPS | Pre-eclampsia \|\| id:finngen_R11_O15_PREECLAMPS | IgD- CD38+ B cell %B cell \|\| id:ebi-a-GCST90001410 | Inverse variance weighted | 19 | 0.00948328607785061 | 0.0354957320463502 | 0.789340630074707 | -0.060088349 | 0.0790549208886969 | 1.00952839491585 | 0.941681333556289 | 1.08226375932559 | 0.977824840984872 |
| ebi-a-GCST90001410 | finngen_R11_O15_PREECLAMPS | Pre-eclampsia \|\| id:finngen_R11_O15_PREECLAMPS | IgD- CD38+ B cell %B cell \|\| id:ebi-a-GCST90001410 | MR Egger | 19 | -0.028669106 | 0.0779551171043657 | 0.71758781392067 | -0.181461136 | 0.124122923340997 | 0.971737953354295 | 0.834050659461874 | 1.13215503072491 | 0.99380795269851 |
| ebi-a-GCST90001410 | finngen_R11_O15_PREECLAMPS | Pre-eclampsia \|\| id:finngen_R11_O15_PREECLAMPS | IgD- CD38+ B cell %B cell \|\| id:ebi-a-GCST90001410 | Weighted median | 19 | 0.0238284969273309 | 0.0464832551745895 | 0.608213387403576 | -0.067278683 | 0.114935677069526 | 1.02411466401682 | 0.934934614443825 | 1.12180127770561 | 0.940845681141708 |
| ebi-a-GCST90001410 | finngen_R11_O15_PREECLAMPS | Pre-eclampsia \|\| id:finngen_R11_O15_PREECLAMPS | IgD- CD38+ B cell %B cell \|\| id:ebi-a-GCST90001410 | Weighted mode | 19 | 0.0231844508190151 | 0.0641644308308734 | 0.722060084013984 | -0.102577834 | 0.148946735247527 | 1.02345529930642 | 0.902507901571548 | 1.16061116789608 | 0.959313411571287 |
| ebi-a-GCST90001411 | finngen_R11_O15_PREECLAMPS | Pre-eclampsia \|\| id:finngen_R11_O15_PREECLAMPS | IgD+ CD24+ B cell %B cell \|\| id:ebi-a-GCST90001411 | Inverse variance weighted | 20 | -0.035662341 | 0.0318501752208693 | 0.262845648924656 | -0.098088684 | 0.0267640028854321 | 0.964966068393901 | 0.906568502085556 | 1.02712537553363 | 0.885079385681701 |
| ebi-a-GCST90001411 | finngen_R11_O15_PREECLAMPS | Pre-eclampsia \|\| id:finngen_R11_O15_PREECLAMPS | IgD+ CD24+ B cell %B cell \|\| id:ebi-a-GCST90001411 | MR Egger | 20 | -0.002547277 | 0.0658283264392883 | 0.969558904720108 | -0.131570797 | 0.12647624271434 | 0.997455964450698 | 0.876717204056119 | 1.13482249055374 | 0.99380795269851 |
| ebi-a-GCST90001411 | finngen_R11_O15_PREECLAMPS | Pre-eclampsia \|\| id:finngen_R11_O15_PREECLAMPS | IgD+ CD24+ B cell %B cell \|\| id:ebi-a-GCST90001411 | Weighted median | 20 | -0.021419196 | 0.0428308951127518 | 0.617013450920532 | -0.105367751 | 0.0625293581608085 | 0.978808565666462 | 0.899993488502538 | 1.06452571097611 | 0.940845681141708 |
| ebi-a-GCST90001411 | finngen_R11_O15_PREECLAMPS | Pre-eclampsia \|\| id:finngen_R11_O15_PREECLAMPS | IgD+ CD24+ B cell %B cell \|\| id:ebi-a-GCST90001411 | Weighted mode | 20 | 0.00633155691908144 | 0.0569660044335279 | 0.912666109398676 | -0.105321812 | 0.117984925608796 | 1.00635164359653 | 0.900034834172578 | 1.12522714912526 | 0.985463701581141 |
| ebi-a-GCST90001412 | finngen_R11_O15_PREECLAMPS | Pre-eclampsia \|\| id:finngen_R11_O15_PREECLAMPS | IgD+ CD24+ B cell Absolute Count \|\| id:ebi-a-GCST90001412 | Inverse variance weighted (multiplicative random effects) | 23 | 0.0184337938560763 | 0.0411136702607669 | 0.653892199928536 | -0.062149 | 0.0990165875671795 | 1.01860474504477 | 0.939742854809846 | 1.10408461348469 | 0.94279131784568 |
| ebi-a-GCST90001412 | finngen_R11_O15_PREECLAMPS | Pre-eclampsia \|\| id:finngen_R11_O15_PREECLAMPS | IgD+ CD24+ B cell Absolute Count \|\| id:ebi-a-GCST90001412 | MR Egger | 23 | 0.0537702733219622 | 0.0802546427531015 | 0.510161430997007 | -0.103528826 | 0.211069373118041 | 1.05524215703283 | 0.901650030975951 | 1.23499802775363 | 0.99380795269851 |
| ebi-a-GCST90001412 | finngen_R11_O15_PREECLAMPS | Pre-eclampsia \|\| id:finngen_R11_O15_PREECLAMPS | IgD+ CD24+ B cell Absolute Count \|\| id:ebi-a-GCST90001412 | Weighted median | 23 | -0.028664586 | 0.0450855926677126 | 0.524918847970395 | -0.117032347 | 0.0597031759532363 | 0.971742346113492 | 0.889556417945962 | 1.06152141469628 | 0.940845681141708 |
| ebi-a-GCST90001412 | finngen_R11_O15_PREECLAMPS | Pre-eclampsia \|\| id:finngen_R11_O15_PREECLAMPS | IgD+ CD24+ B cell Absolute Count \|\| id:ebi-a-GCST90001412 | Weighted mode | 23 | -0.038015438 | 0.0566918947886696 | 0.50947841400583 | -0.149131552 | 0.0731006759557969 | 0.962698078807203 | 0.861455781544537 | 1.07583884256648 | 0.934398527469092 |
| ebi-a-GCST90001413 | finngen_R11_O15_PREECLAMPS | Pre-eclampsia \|\| id:finngen_R11_O15_PREECLAMPS | IgD- CD24- B cell %B cell \|\| id:ebi-a-GCST90001413 | Inverse variance weighted | 17 | -0.01888048 | 0.0189458055763328 | 0.318982022809109 | -0.056014259 | 0.0182532988075984 | 0.981296639688817 | 0.945525653495166 | 1.01842090852323 | 0.885079385681701 |
| ebi-a-GCST90001413 | finngen_R11_O15_PREECLAMPS | Pre-eclampsia \|\| id:finngen_R11_O15_PREECLAMPS | IgD- CD24- B cell %B cell \|\| id:ebi-a-GCST90001413 | MR Egger | 17 | -0.01369684 | 0.025043518646273 | 0.592476654886807 | -0.062782137 | 0.0353884562117956 | 0.98639653458275 | 0.939148057127101 | 1.03602207985527 | 0.99380795269851 |
| ebi-a-GCST90001413 | finngen_R11_O15_PREECLAMPS | Pre-eclampsia \|\| id:finngen_R11_O15_PREECLAMPS | IgD- CD24- B cell %B cell \|\| id:ebi-a-GCST90001413 | Weighted median | 17 | -0.005523171 | 0.0298020507825301 | 0.85297133102777 | -0.063935191 | 0.0528888480976304 | 0.994492053232846 | 0.938065792694941 | 1.05431244977175 | 0.964912412213677 |
| ebi-a-GCST90001413 | finngen_R11_O15_PREECLAMPS | Pre-eclampsia \|\| id:finngen_R11_O15_PREECLAMPS | IgD- CD24- B cell %B cell \|\| id:ebi-a-GCST90001413 | Weighted mode | 17 | -0.02561343 | 0.0302542637442154 | 0.409699434001717 | -0.084911787 | 0.0336849269564101 | 0.974711811151567 | 0.918593312772071 | 1.03425868835397 | 0.930105377419646 |
| ebi-a-GCST90001414 | finngen_R11_O15_PREECLAMPS | Pre-eclampsia \|\| id:finngen_R11_O15_PREECLAMPS | IgD- CD24- B cell Absolute Count \|\| id:ebi-a-GCST90001414 | Inverse variance weighted | 18 | 0.0515282201235502 | 0.0257657499186504 | 0.0455140106304596 | 0.00102735028299539 | 0.102029089964105 | 1.05287889824549 | 1.00102787818806 | 1.10741568594193 | 0.781760498894217 |
| ebi-a-GCST90001414 | finngen_R11_O15_PREECLAMPS | Pre-eclampsia \|\| id:finngen_R11_O15_PREECLAMPS | IgD- CD24- B cell Absolute Count \|\| id:ebi-a-GCST90001414 | MR Egger | 18 | 0.0721065641261108 | 0.0422580093761281 | 0.107275853094927 | -0.010719134 | 0.154932262503322 | 1.07476986987349 | 0.989338110946218 | 1.16757886955663 | 0.97261503632431 |
| ebi-a-GCST90001414 | finngen_R11_O15_PREECLAMPS | Pre-eclampsia \|\| id:finngen_R11_O15_PREECLAMPS | IgD- CD24- B cell Absolute Count \|\| id:ebi-a-GCST90001414 | Weighted median | 18 | 0.0749282105921599 | 0.0377599195353054 | 0.0472188589790545 | 0.000918768302961365 | 0.148937652881358 | 1.07780677299721 | 1.00091919049985 | 1.16060062684834 | 0.750369258993236 |
| ebi-a-GCST90001414 | finngen_R11_O15_PREECLAMPS | Pre-eclampsia \|\| id:finngen_R11_O15_PREECLAMPS | IgD- CD24- B cell Absolute Count \|\| id:ebi-a-GCST90001414 | Weighted mode | 18 | 0.0942844796811945 | 0.0421912472769052 | 0.0391517394594097 | 0.0115896350184603 | 0.176979324343929 | 1.09887230830018 | 1.01165705504448 | 1.19360641427631 | 0.838871423749761 |
| ebi-a-GCST90001415 | finngen_R11_O15_PREECLAMPS | Pre-eclampsia \|\| id:finngen_R11_O15_PREECLAMPS | IgD+ CD24- B cell %B cell \|\| id:ebi-a-GCST90001415 | Inverse variance weighted | 19 | 0.00391539022958024 | 0.0166000670199567 | 0.813536707087688 | -0.028620741 | 0.0364515215886954 | 1.00392306538371 | 0.971784952649459 | 1.03712402467399 | 0.977824840984872 |
| ebi-a-GCST90001415 | finngen_R11_O15_PREECLAMPS | Pre-eclampsia \|\| id:finngen_R11_O15_PREECLAMPS | IgD+ CD24- B cell %B cell \|\| id:ebi-a-GCST90001415 | MR Egger | 19 | 0.011525076568881 | 0.0227754623235503 | 0.619334168773809 | -0.03311483 | 0.0561649827230396 | 1.01159174614164 | 0.967427463916151 | 1.05777218347668 | 0.99380795269851 |
| ebi-a-GCST90001415 | finngen_R11_O15_PREECLAMPS | Pre-eclampsia \|\| id:finngen_R11_O15_PREECLAMPS | IgD+ CD24- B cell %B cell \|\| id:ebi-a-GCST90001415 | Weighted median | 19 | 0.00364699361576055 | 0.023464481124037 | 0.876485381604224 | -0.042343389 | 0.0496373766188732 | 1.00365365198886 | 0.958540571392687 | 1.05088995000704 | 0.964912412213677 |
| ebi-a-GCST90001415 | finngen_R11_O15_PREECLAMPS | Pre-eclampsia \|\| id:finngen_R11_O15_PREECLAMPS | IgD+ CD24- B cell %B cell \|\| id:ebi-a-GCST90001415 | Weighted mode | 19 | 0.00133245881378058 | 0.0210637473603462 | 0.950257935130957 | -0.039952486 | 0.0426174036400591 | 1.00133334693144 | 0.960835091174273 | 1.0435385644081 | 0.990180645930572 |
| ebi-a-GCST90001416 | finngen_R11_O15_PREECLAMPS | Pre-eclampsia \|\| id:finngen_R11_O15_PREECLAMPS | IgD+ CD24- B cell Absolute Count \|\| id:ebi-a-GCST90001416 | Inverse variance weighted | 22 | 0.000883320180310454 | 0.00956021535426218 | 0.926383872577034 | -0.017854702 | 0.0196213422746643 | 1.00088371042248 | 0.982303748844368 | 1.01981510603781 | 0.986667691552896 |
| ebi-a-GCST90001416 | finngen_R11_O15_PREECLAMPS | Pre-eclampsia \|\| id:finngen_R11_O15_PREECLAMPS | IgD+ CD24- B cell Absolute Count \|\| id:ebi-a-GCST90001416 | MR Egger | 22 | -0.002206781 | 0.011477999304842 | 0.84947589509264 | -0.024703659 | 0.0202900979603601 | 0.997795652473209 | 0.975598978865137 | 1.02049734128726 | 0.99380795269851 |
| ebi-a-GCST90001416 | finngen_R11_O15_PREECLAMPS | Pre-eclampsia \|\| id:finngen_R11_O15_PREECLAMPS | IgD+ CD24- B cell Absolute Count \|\| id:ebi-a-GCST90001416 | Weighted median | 22 | -0.00182 | 0.0126015309264773 | 0.885163367883502 | -0.026519001 | 0.022879000433637 | 0.998181655013769 | 0.973829540121215 | 1.02314273223013 | 0.964912412213677 |
| ebi-a-GCST90001416 | finngen_R11_O15_PREECLAMPS | Pre-eclampsia \|\| id:finngen_R11_O15_PREECLAMPS | IgD+ CD24- B cell Absolute Count \|\| id:ebi-a-GCST90001416 | Weighted mode | 22 | -0.003975018 | 0.0106472686346617 | 0.712639933616773 | -0.024843664 | 0.0168936286360165 | 0.996032872038023 | 0.975462399596357 | 1.01703713294416 | 0.959313411571287 |
| ebi-a-GCST90001417 | finngen_R11_O15_PREECLAMPS | Pre-eclampsia \|\| id:finngen_R11_O15_PREECLAMPS | CD24+ CD27+ B cell %B cell \|\| id:ebi-a-GCST90001417 | Inverse variance weighted | 26 | -0.01325304 | 0.0171263320495878 | 0.439025426017257 | -0.046820651 | 0.0203145703485865 | 0.986834394386785 | 0.954258527283852 | 1.02052231559997 | 0.898694924660397 |
| ebi-a-GCST90001417 | finngen_R11_O15_PREECLAMPS | Pre-eclampsia \|\| id:finngen_R11_O15_PREECLAMPS | CD24+ CD27+ B cell %B cell \|\| id:ebi-a-GCST90001417 | MR Egger | 26 | -0.044088484 | 0.0257519351821699 | 0.0997822236434794 | -0.094562277 | 0.00638530884749064 | 0.956869286000323 | 0.909771074987909 | 1.00640573839187 | 0.97261503632431 |
| ebi-a-GCST90001417 | finngen_R11_O15_PREECLAMPS | Pre-eclampsia \|\| id:finngen_R11_O15_PREECLAMPS | CD24+ CD27+ B cell %B cell \|\| id:ebi-a-GCST90001417 | Weighted median | 26 | -0.012294649 | 0.0244612658831073 | 0.615233534627843 | -0.060238731 | 0.0356494316625898 | 0.987780620944238 | 0.941539732407377 | 1.03629249146846 | 0.940845681141708 |
| ebi-a-GCST90001417 | finngen_R11_O15_PREECLAMPS | Pre-eclampsia \|\| id:finngen_R11_O15_PREECLAMPS | CD24+ CD27+ B cell %B cell \|\| id:ebi-a-GCST90001417 | Weighted mode | 26 | -0.015499278 | 0.022793430445963 | 0.502760272367709 | -0.060174402 | 0.0291758452096371 | 0.984620217189966 | 0.941600302157165 | 1.02960562977538 | 0.934398527469092 |
| ebi-a-GCST90001418 | finngen_R11_O15_PREECLAMPS | Pre-eclampsia \|\| id:finngen_R11_O15_PREECLAMPS | CD24+ CD27+ B cell Absolute Count \|\| id:ebi-a-GCST90001418 | Inverse variance weighted | 26 | -0.010129725 | 0.0116885885428058 | 0.386142746951487 | -0.033039358 | 0.0127799088983709 | 0.989921408215588 | 0.967500479772506 | 1.01286192093047 | 0.885079385681701 |
| ebi-a-GCST90001418 | finngen_R11_O15_PREECLAMPS | Pre-eclampsia \|\| id:finngen_R11_O15_PREECLAMPS | CD24+ CD27+ B cell Absolute Count \|\| id:ebi-a-GCST90001418 | MR Egger | 26 | -0.004756752 | 0.0134833474212933 | 0.727327585728062 | -0.031184113 | 0.0216706093763495 | 0.995254543856419 | 0.96929709692027 | 1.02190712240246 | 0.99380795269851 |
| ebi-a-GCST90001418 | finngen_R11_O15_PREECLAMPS | Pre-eclampsia \|\| id:finngen_R11_O15_PREECLAMPS | CD24+ CD27+ B cell Absolute Count \|\| id:ebi-a-GCST90001418 | Weighted median | 26 | -0.005866466 | 0.0142305439016957 | 0.680160083425238 | -0.033758332 | 0.0220253998740336 | 0.994150707939196 | 0.966805122054415 | 1.02226974966355 | 0.943447857654363 |
| ebi-a-GCST90001418 | finngen_R11_O15_PREECLAMPS | Pre-eclampsia \|\| id:finngen_R11_O15_PREECLAMPS | CD24+ CD27+ B cell Absolute Count \|\| id:ebi-a-GCST90001418 | Weighted mode | 26 | -0.005201428 | 0.0133804152156413 | 0.700763693700199 | -0.031427042 | 0.02102418562537 | 0.994812075806844 | 0.969061654655583 | 1.02124675083017 | 0.959313411571287 |
| ebi-a-GCST90001419 | finngen_R11_O15_PREECLAMPS | Pre-eclampsia \|\| id:finngen_R11_O15_PREECLAMPS | CD20- B cell %B cell \|\| id:ebi-a-GCST90001419 | Inverse variance weighted | 19 | 0.039141753776667 | 0.0206643059670321 | 0.058202157589511 | -0.001360286 | 0.07964379347205 | 1.03991788549022 | 0.99864063885081 | 1.08290126246706 | 0.789997151112252 |
| ebi-a-GCST90001419 | finngen_R11_O15_PREECLAMPS | Pre-eclampsia \|\| id:finngen_R11_O15_PREECLAMPS | CD20- B cell %B cell \|\| id:ebi-a-GCST90001419 | MR Egger | 19 | 0.0469852017101182 | 0.0295480627445286 | 0.130229229931937 | -0.010929001 | 0.104899404689394 | 1.0481064987811 | 0.989130503292784 | 1.11059888369655 | 0.97261503632431 |
| ebi-a-GCST90001419 | finngen_R11_O15_PREECLAMPS | Pre-eclampsia \|\| id:finngen_R11_O15_PREECLAMPS | CD20- B cell %B cell \|\| id:ebi-a-GCST90001419 | Weighted median | 19 | 0.0661799015904731 | 0.0329757221403847 | 0.0447573250946796 | 0.00154748619531908 | 0.130812316985627 | 1.06841891013879 | 1.00154868416995 | 1.13975384879888 | 0.743581923732063 |
| ebi-a-GCST90001419 | finngen_R11_O15_PREECLAMPS | Pre-eclampsia \|\| id:finngen_R11_O15_PREECLAMPS | CD20- B cell %B cell \|\| id:ebi-a-GCST90001419 | Weighted mode | 19 | 0.0479172728160317 | 0.0301421832040879 | 0.129310355503104 | -0.011161406 | 0.106995951896044 | 1.0490838639807 | 0.988900651133717 | 1.11292974921489 | 0.872755742358858 |
| ebi-a-GCST90001420 | finngen_R11_O15_PREECLAMPS | Pre-eclampsia \|\| id:finngen_R11_O15_PREECLAMPS | IgD- CD38+ B cell Absolute Count \|\| id:ebi-a-GCST90001420 | Inverse variance weighted | 21 | -0.01127488 | 0.0162107502607502 | 0.486730274914762 | -0.043047951 | 0.020498190011785 | 0.988788442755038 | 0.957865458349291 | 1.02070972076896 | 0.898694924660397 |
| ebi-a-GCST90001420 | finngen_R11_O15_PREECLAMPS | Pre-eclampsia \|\| id:finngen_R11_O15_PREECLAMPS | IgD- CD38+ B cell Absolute Count \|\| id:ebi-a-GCST90001420 | MR Egger | 21 | -0.012724792 | 0.0204534199203725 | 0.541247641768433 | -0.052813495 | 0.0273639110737477 | 0.98735582588439 | 0.948556906578445 | 1.02774174131979 | 0.99380795269851 |
| ebi-a-GCST90001420 | finngen_R11_O15_PREECLAMPS | Pre-eclampsia \|\| id:finngen_R11_O15_PREECLAMPS | IgD- CD38+ B cell Absolute Count \|\| id:ebi-a-GCST90001420 | Weighted median | 21 | 0.00774391600195618 | 0.0221821760971056 | 0.72701023202624 | -0.035733149 | 0.0512209811522832 | 1.00777397766772 | 0.964897742915727 | 1.0525554625045 | 0.950705688034314 |
| ebi-a-GCST90001420 | finngen_R11_O15_PREECLAMPS | Pre-eclampsia \|\| id:finngen_R11_O15_PREECLAMPS | IgD- CD38+ B cell Absolute Count \|\| id:ebi-a-GCST90001420 | Weighted mode | 21 | -0.002595863 | 0.0208908246943895 | 0.902350970257298 | -0.04354188 | 0.0383501530881901 | 0.997407503026873 | 0.957392457929216 | 1.03909501151288 | 0.98361388732834 |
| ebi-a-GCST90001421 | finngen_R11_O15_PREECLAMPS | Pre-eclampsia \|\| id:finngen_R11_O15_PREECLAMPS | CD20- B cell Absolute Count \|\| id:ebi-a-GCST90001421 | Inverse variance weighted | 22 | 0.0137918869428205 | 0.013832021605991 | 0.318716737983948 | -0.013318875 | 0.0409026492905629 | 1.01388743376723 | 0.986769428345817 | 1.04175068544062 | 0.885079385681701 |
| ebi-a-GCST90001421 | finngen_R11_O15_PREECLAMPS | Pre-eclampsia \|\| id:finngen_R11_O15_PREECLAMPS | CD20- B cell Absolute Count \|\| id:ebi-a-GCST90001421 | MR Egger | 22 | 0.00824949335242813 | 0.016831281463252 | 0.629376423868747 | -0.024739818 | 0.0412388050204021 | 1.0082836141847 | 0.975563702818535 | 1.04210093476845 | 0.99380795269851 |
| ebi-a-GCST90001421 | finngen_R11_O15_PREECLAMPS | Pre-eclampsia \|\| id:finngen_R11_O15_PREECLAMPS | CD20- B cell Absolute Count \|\| id:ebi-a-GCST90001421 | Weighted median | 22 | 0.0135276704792053 | 0.020271210166047 | 0.504558727839114 | -0.026203901 | 0.0532592424046575 | 1.01361958340177 | 0.974136441527853 | 1.05470303343149 | 0.940845681141708 |
| ebi-a-GCST90001421 | finngen_R11_O15_PREECLAMPS | Pre-eclampsia \|\| id:finngen_R11_O15_PREECLAMPS | CD20- B cell Absolute Count \|\| id:ebi-a-GCST90001421 | Weighted mode | 22 | 0.0117503287037238 | 0.014667171974952 | 0.432027207094058 | -0.016997328 | 0.0404979857746297 | 1.01181963500731 | 0.983146311237351 | 1.04132921222869 | 0.933945646747087 |
| ebi-a-GCST90001422 | finngen_R11_O15_PREECLAMPS | Pre-eclampsia \|\| id:finngen_R11_O15_PREECLAMPS | CD20- CD38- B cell %B cell \|\| id:ebi-a-GCST90001422 | Inverse variance weighted | 23 | -0.047358223 | 0.0165678945060477 | 0.00425738454363065 | -0.079831296 | -0.01488515 | 0.953745682563191 | 0.923272092549716 | 0.985225086242867 | 0.447221286871335 |
| ebi-a-GCST90001422 | finngen_R11_O15_PREECLAMPS | Pre-eclampsia \|\| id:finngen_R11_O15_PREECLAMPS | CD20- CD38- B cell %B cell \|\| id:ebi-a-GCST90001422 | MR Egger | 23 | -0.064941207 | 0.0220499555882855 | 0.00772991739214776 | -0.108159119 | -0.021723294 | 0.937122558470791 | 0.89748477777914 | 0.978510957888307 | 0.565056961366001 |
| ebi-a-GCST90001422 | finngen_R11_O15_PREECLAMPS | Pre-eclampsia \|\| id:finngen_R11_O15_PREECLAMPS | CD20- CD38- B cell %B cell \|\| id:ebi-a-GCST90001422 | Weighted median | 23 | -0.056477722 | 0.0232869592767432 | 0.0152959945243934 | -0.102120162 | -0.010835282 | 0.945087538664337 | 0.902921047941267 | 0.989223208136704 | 0.598716529366782 |
| ebi-a-GCST90001422 | finngen_R11_O15_PREECLAMPS | Pre-eclampsia \|\| id:finngen_R11_O15_PREECLAMPS | CD20- CD38- B cell %B cell \|\| id:ebi-a-GCST90001422 | Weighted mode | 23 | -0.055301878 | 0.0223229391131574 | 0.0213978372480378 | -0.099054839 | -0.011548917 | 0.946199467879538 | 0.905693039521083 | 0.98851751525985 | 0.744848525157888 |
| ebi-a-GCST90001423 | finngen_R11_O15_PREECLAMPS | Pre-eclampsia \|\| id:finngen_R11_O15_PREECLAMPS | CD20- CD38- B cell Absolute Count \|\| id:ebi-a-GCST90001423 | Inverse variance weighted | 14 | -0.035268572 | 0.035535735765805 | 0.320962544585782 | -0.104918614 | 0.0343814704666981 | 0.965346116854644 | 0.900397799618047 | 1.03497934548669 | 0.885079385681701 |
| ebi-a-GCST90001423 | finngen_R11_O15_PREECLAMPS | Pre-eclampsia \|\| id:finngen_R11_O15_PREECLAMPS | CD20- CD38- B cell Absolute Count \|\| id:ebi-a-GCST90001423 | MR Egger | 14 | -0.052112613 | 0.0673730635509045 | 0.454198714523262 | -0.184163818 | 0.0799385910600459 | 0.949221965616176 | 0.831799528880114 | 1.08322054620699 | 0.99380795269851 |
| ebi-a-GCST90001423 | finngen_R11_O15_PREECLAMPS | Pre-eclampsia \|\| id:finngen_R11_O15_PREECLAMPS | CD20- CD38- B cell Absolute Count \|\| id:ebi-a-GCST90001423 | Weighted median | 14 | -0.032852974 | 0.0494038401121162 | 0.506058003339397 | -0.1296845 | 0.063978553044183 | 0.967680823781993 | 0.878372513564976 | 1.06606953457012 | 0.940845681141708 |
| ebi-a-GCST90001423 | finngen_R11_O15_PREECLAMPS | Pre-eclampsia \|\| id:finngen_R11_O15_PREECLAMPS | CD20- CD38- B cell Absolute Count \|\| id:ebi-a-GCST90001423 | Weighted mode | 14 | -0.023296138 | 0.0588633839548914 | 0.698696105202773 | -0.13866837 | 0.0920760950197054 | 0.97697312252081 | 0.870516669965126 | 1.09644825315786 | 0.959313411571287 |
| ebi-a-GCST90001424 | finngen_R11_O15_PREECLAMPS | Pre-eclampsia \|\| id:finngen_R11_O15_PREECLAMPS | IgD+ B cell %Lymphocyte \|\| id:ebi-a-GCST90001424 | Inverse variance weighted | 18 | -0.002640281 | 0.0128980439487608 | 0.837803400694392 | -0.027920447 | 0.0226398850444102 | 0.99736320138139 | 0.972465726061771 | 1.02289811230483 | 0.977824840984872 |
| ebi-a-GCST90001424 | finngen_R11_O15_PREECLAMPS | Pre-eclampsia \|\| id:finngen_R11_O15_PREECLAMPS | IgD+ B cell %Lymphocyte \|\| id:ebi-a-GCST90001424 | MR Egger | 18 | 0.000621132084477263 | 0.0154542136305223 | 0.968437496423748 | -0.029669127 | 0.0309113908003011 | 1.00062132502696 | 0.97076668125797 | 1.0313941088308 | 0.99380795269851 |
| ebi-a-GCST90001424 | finngen_R11_O15_PREECLAMPS | Pre-eclampsia \|\| id:finngen_R11_O15_PREECLAMPS | IgD+ B cell %Lymphocyte \|\| id:ebi-a-GCST90001424 | Weighted median | 18 | 0.00417614051418201 | 0.0166238692196268 | 0.801649040984966 | -0.028406643 | 0.0367589241846506 | 1.00418487274042 | 0.971993032112155 | 1.03744288829874 | 0.959373517227545 |
| ebi-a-GCST90001424 | finngen_R11_O15_PREECLAMPS | Pre-eclampsia \|\| id:finngen_R11_O15_PREECLAMPS | IgD+ B cell %Lymphocyte \|\| id:ebi-a-GCST90001424 | Weighted mode | 18 | 0.00177014480569337 | 0.0142431181367345 | 0.902550724444678 | -0.026146367 | 0.029686656353693 | 1.00177171243685 | 0.974192489791958 | 1.03013169815441 | 0.98361388732834 |
| ebi-a-GCST90001425 | finngen_R11_O15_PREECLAMPS | Pre-eclampsia \|\| id:finngen_R11_O15_PREECLAMPS | IgD- CD38+ B cell %lymphocyte \|\| id:ebi-a-GCST90001425 | Inverse variance weighted | 30 | 0.00172655280470454 | 0.0147827486864192 | 0.9070224164323 | -0.027247635 | 0.0307007402300861 | 1.00172804415517 | 0.973120233424716 | 1.03117686795542 | 0.986667691552896 |
| ebi-a-GCST90001425 | finngen_R11_O15_PREECLAMPS | Pre-eclampsia \|\| id:finngen_R11_O15_PREECLAMPS | IgD- CD38+ B cell %lymphocyte \|\| id:ebi-a-GCST90001425 | MR Egger | 30 | 6.09372874024696e-05 | 0.0187934497934447 | 0.99743587568583 | -0.036774224 | 0.036896098882554 | 1.00006093914412 | 0.963893734558999 | 1.03758520897471 | 0.997699814990973 |
| ebi-a-GCST90001425 | finngen_R11_O15_PREECLAMPS | Pre-eclampsia \|\| id:finngen_R11_O15_PREECLAMPS | IgD- CD38+ B cell %lymphocyte \|\| id:ebi-a-GCST90001425 | Weighted median | 30 | 0.0101332801770024 | 0.0234683494988259 | 0.665897716935759 | -0.035864685 | 0.0561312451947012 | 1.01018479572068 | 0.96477083276988 | 1.05773649745965 | 0.940845681141708 |
| ebi-a-GCST90001425 | finngen_R11_O15_PREECLAMPS | Pre-eclampsia \|\| id:finngen_R11_O15_PREECLAMPS | IgD- CD38+ B cell %lymphocyte \|\| id:ebi-a-GCST90001425 | Weighted mode | 30 | 0.0114379762508022 | 0.0197415793698748 | 0.566803343492849 | -0.027255519 | 0.0501314718157567 | 1.01150364001588 | 0.97311256070021 | 1.05140931798184 | 0.939633861977944 |
| ebi-a-GCST90001426 | finngen_R11_O15_PREECLAMPS | Pre-eclampsia \|\| id:finngen_R11_O15_PREECLAMPS | IgD- CD38dim B cell %lymphocyte \|\| id:ebi-a-GCST90001426 | Inverse variance weighted | 23 | 0.0226398012768868 | 0.0180457667261426 | 0.209632409622054 | -0.012729902 | 0.0580095040601263 | 1.0228980266192 | 0.987350780966973 | 1.05972506735318 | 0.855687495048373 |
| ebi-a-GCST90001426 | finngen_R11_O15_PREECLAMPS | Pre-eclampsia \|\| id:finngen_R11_O15_PREECLAMPS | IgD- CD38dim B cell %lymphocyte \|\| id:ebi-a-GCST90001426 | MR Egger | 23 | 0.0117866578711141 | 0.022926157814284 | 0.612540223653538 | -0.033148611 | 0.0567219271871107 | 1.01185639423991 | 0.967394782969402 | 1.05836146792264 | 0.99380795269851 |
| ebi-a-GCST90001426 | finngen_R11_O15_PREECLAMPS | Pre-eclampsia \|\| id:finngen_R11_O15_PREECLAMPS | IgD- CD38dim B cell %lymphocyte \|\| id:ebi-a-GCST90001426 | Weighted median | 23 | -0.014769896 | 0.0241975127661284 | 0.541604133426454 | -0.062197021 | 0.032657228682191 | 0.985338643548149 | 0.939697728026237 | 1.03319632847106 | 0.940845681141708 |
| ebi-a-GCST90001426 | finngen_R11_O15_PREECLAMPS | Pre-eclampsia \|\| id:finngen_R11_O15_PREECLAMPS | IgD- CD38dim B cell %lymphocyte \|\| id:ebi-a-GCST90001426 | Weighted mode | 23 | 0.0123567872291084 | 0.0217753154966315 | 0.576142102948798 | -0.030322831 | 0.055036405602506 | 1.01243344775829 | 0.970132294070892 | 1.0565790793733 | 0.948158355498791 |
| ebi-a-GCST90001427 | finngen_R11_O15_PREECLAMPS | Pre-eclampsia \|\| id:finngen_R11_O15_PREECLAMPS | IgD- CD38- B cell %lymphocyte \|\| id:ebi-a-GCST90001427 | Inverse variance weighted | 28 | 0.018131691250044 | 0.0279622810470939 | 0.516704343405528 | -0.03667438 | 0.072937762102348 | 1.01829706837408 | 0.963989979049721 | 1.07566358779105 | 0.898694924660397 |
| ebi-a-GCST90001427 | finngen_R11_O15_PREECLAMPS | Pre-eclampsia \|\| id:finngen_R11_O15_PREECLAMPS | IgD- CD38- B cell %lymphocyte \|\| id:ebi-a-GCST90001427 | MR Egger | 28 | -0.041615407 | 0.0471525496057979 | 0.385556286133737 | -0.134034404 | 0.0508035903236347 | 0.959238626193509 | 0.874559975609695 | 1.05211622718058 | 0.99380795269851 |
| ebi-a-GCST90001427 | finngen_R11_O15_PREECLAMPS | Pre-eclampsia \|\| id:finngen_R11_O15_PREECLAMPS | IgD- CD38- B cell %lymphocyte \|\| id:ebi-a-GCST90001427 | Weighted median | 28 | 0.00134644113912431 | 0.0380493852611504 | 0.971771411776258 | -0.073230354 | 0.0759232362509792 | 1.00134734799796 | 0.929386717443331 | 1.07887975212394 | 0.990670780705477 |
| ebi-a-GCST90001427 | finngen_R11_O15_PREECLAMPS | Pre-eclampsia \|\| id:finngen_R11_O15_PREECLAMPS | IgD- CD38- B cell %lymphocyte \|\| id:ebi-a-GCST90001427 | Weighted mode | 28 | -0.011685593 | 0.0413215587435801 | 0.779489617120957 | -0.092675848 | 0.0693046621347019 | 0.988382418363706 | 0.911488913240702 | 1.0717626849209 | 0.961305639715923 |
| ebi-a-GCST90001428 | finngen_R11_O15_PREECLAMPS | Pre-eclampsia \|\| id:finngen_R11_O15_PREECLAMPS | IgD- CD38dim B cell %B cell \|\| id:ebi-a-GCST90001428 | Inverse variance weighted | 25 | 0.000556038815196393 | 0.0122079750215322 | 0.963671171034544 | -0.023371592 | 0.0244836698573994 | 1.00055619343344 | 0.97689940809279 | 1.02478585607139 | 0.992671503887443 |
| ebi-a-GCST90001428 | finngen_R11_O15_PREECLAMPS | Pre-eclampsia \|\| id:finngen_R11_O15_PREECLAMPS | IgD- CD38dim B cell %B cell \|\| id:ebi-a-GCST90001428 | MR Egger | 25 | -0.001377397 | 0.0145728700895313 | 0.925516396831828 | -0.029940222 | 0.0271854285666013 | 0.998623551366717 | 0.970503546396623 | 1.027558324 | 0.99380795269851 |
| ebi-a-GCST90001428 | finngen_R11_O15_PREECLAMPS | Pre-eclampsia \|\| id:finngen_R11_O15_PREECLAMPS | IgD- CD38dim B cell %B cell \|\| id:ebi-a-GCST90001428 | Weighted median | 25 | -0.005514529 | 0.017188276798974 | 0.748338615687368 | -0.039203551 | 0.0281744938953333 | 0.99450064847135 | 0.961554963599908 | 1.02857514884761 | 0.957984984509652 |
| ebi-a-GCST90001428 | finngen_R11_O15_PREECLAMPS | Pre-eclampsia \|\| id:finngen_R11_O15_PREECLAMPS | IgD- CD38dim B cell %B cell \|\| id:ebi-a-GCST90001428 | Weighted mode | 25 | -0.001478011 | 0.0154715459047164 | 0.924686330153409 | -0.031802241 | 0.028846219075473 | 0.998523080822411 | 0.968698132037038 | 1.02926630078085 | 0.986782054514076 |
| ebi-a-GCST90001429 | finngen_R11_O15_PREECLAMPS | Pre-eclampsia \|\| id:finngen_R11_O15_PREECLAMPS | IgD+ CD38+ B cell %lymphocyte \|\| id:ebi-a-GCST90001429 | Inverse variance weighted | 25 | 0.0371737343737274 | 0.0207992085275283 | 0.0738943811084399 | -0.003592714 | 0.0779401830876829 | 1.03787331944683 | 0.99641373173599 | 1.08105799118493 | 0.789997151112252 |
| ebi-a-GCST90001429 | finngen_R11_O15_PREECLAMPS | Pre-eclampsia \|\| id:finngen_R11_O15_PREECLAMPS | IgD+ CD38+ B cell %lymphocyte \|\| id:ebi-a-GCST90001429 | MR Egger | 25 | 0.0269526949070258 | 0.0310921722884098 | 0.394970242846988 | -0.033987963 | 0.0878933525923089 | 1.02731920418355 | 0.966583139543064 | 1.09187167053549 | 0.99380795269851 |
| ebi-a-GCST90001429 | finngen_R11_O15_PREECLAMPS | Pre-eclampsia \|\| id:finngen_R11_O15_PREECLAMPS | IgD+ CD38+ B cell %lymphocyte \|\| id:ebi-a-GCST90001429 | Weighted median | 25 | 0.0289796212561475 | 0.030298256607162 | 0.338830704481996 | -0.030404962 | 0.088364204206185 | 1.02940361630876 | 0.970052619844284 | 1.09238590112736 | 0.940845681141708 |
| ebi-a-GCST90001429 | finngen_R11_O15_PREECLAMPS | Pre-eclampsia \|\| id:finngen_R11_O15_PREECLAMPS | IgD+ CD38+ B cell %lymphocyte \|\| id:ebi-a-GCST90001429 | Weighted mode | 25 | 0.0332468632043039 | 0.0270443003706329 | 0.23085802924432 | -0.019759966 | 0.0862536919307444 | 1.03380571633485 | 0.980433983023598 | 1.09008283844941 | 0.872755742358858 |
| ebi-a-GCST90001430 | finngen_R11_O15_PREECLAMPS | Pre-eclampsia \|\| id:finngen_R11_O15_PREECLAMPS | IgD+ CD38dim B cell %lymphocyte \|\| id:ebi-a-GCST90001430 | Inverse variance weighted | 34 | -0.000987547 | 0.0103768288889743 | 0.924181012748319 | -0.021326132 | 0.0193510376826675 | 0.999012940524279 | 0.978899662428978 | 1.01953948258448 | 0.986667691552896 |
| ebi-a-GCST90001430 | finngen_R11_O15_PREECLAMPS | Pre-eclampsia \|\| id:finngen_R11_O15_PREECLAMPS | IgD+ CD38dim B cell %lymphocyte \|\| id:ebi-a-GCST90001430 | MR Egger | 34 | 0.00487672362778445 | 0.011964531188557 | 0.686282669685253 | -0.018573758 | 0.0283272047573562 | 1.0048886341981 | 0.98159767172967 | 1.02873223543936 | 0.99380795269851 |
| ebi-a-GCST90001430 | finngen_R11_O15_PREECLAMPS | Pre-eclampsia \|\| id:finngen_R11_O15_PREECLAMPS | IgD+ CD38dim B cell %lymphocyte \|\| id:ebi-a-GCST90001430 | Weighted median | 34 | 0.00572829924232801 | 0.0127087078024788 | 0.652178294483071 | -0.019180768 | 0.0306373665351865 | 1.00574473732086 | 0.981002012392349 | 1.03111152053788 | 0.940845681141708 |
| ebi-a-GCST90001430 | finngen_R11_O15_PREECLAMPS | Pre-eclampsia \|\| id:finngen_R11_O15_PREECLAMPS | IgD+ CD38dim B cell %lymphocyte \|\| id:ebi-a-GCST90001430 | Weighted mode | 34 | 0.0030651784740271 | 0.0106198285014443 | 0.774671473848282 | -0.017749685 | 0.0238800423368579 | 1.00306988093697 | 0.982406912387646 | 1.0241674537871 | 0.961305639715923 |
| ebi-a-GCST90001431 | finngen_R11_O15_PREECLAMPS | Pre-eclampsia \|\| id:finngen_R11_O15_PREECLAMPS | IgD+ CD38- B cell %lymphocyte \|\| id:ebi-a-GCST90001431 | Inverse variance weighted | 20 | -0.004858846 | 0.0184605498550114 | 0.792395217792376 | -0.041041523 | 0.0313238319395936 | 0.995152939319862 | 0.959789275318009 | 1.03181958592821 | 0.977824840984872 |
| ebi-a-GCST90001431 | finngen_R11_O15_PREECLAMPS | Pre-eclampsia \|\| id:finngen_R11_O15_PREECLAMPS | IgD+ CD38- B cell %lymphocyte \|\| id:ebi-a-GCST90001431 | MR Egger | 20 | -0.007838781 | 0.0224793583330888 | 0.731352493679652 | -0.051898324 | 0.0362207611042384 | 0.992191861896433 | 0.949425396128358 | 1.03688472504312 | 0.99380795269851 |
| ebi-a-GCST90001431 | finngen_R11_O15_PREECLAMPS | Pre-eclampsia \|\| id:finngen_R11_O15_PREECLAMPS | IgD+ CD38- B cell %lymphocyte \|\| id:ebi-a-GCST90001431 | Weighted median | 20 | -0.014362705 | 0.0263651028638758 | 0.585917235491833 | -0.066038307 | 0.0373128961561532 | 0.985739946157693 | 0.936095004547692 | 1.03801776179809 | 0.940845681141708 |
| ebi-a-GCST90001431 | finngen_R11_O15_PREECLAMPS | Pre-eclampsia \|\| id:finngen_R11_O15_PREECLAMPS | IgD+ CD38- B cell %lymphocyte \|\| id:ebi-a-GCST90001431 | Weighted mode | 20 | -0.013860898 | 0.020848250457229 | 0.514137822741704 | -0.054723469 | 0.0270016729623724 | 0.98623472200938 | 0.946746916790821 | 1.0273695215126 | 0.934398527469092 |
| ebi-a-GCST90001432 | finngen_R11_O15_PREECLAMPS | Pre-eclampsia \|\| id:finngen_R11_O15_PREECLAMPS | Unswitched memory B cell %lymphocyte \|\| id:ebi-a-GCST90001432 | Inverse variance weighted | 22 | -0.000363517 | 0.0250235169977785 | 0.988409514659057 | -0.049409611 | 0.0486825759317374 | 0.99963654868053 | 0.951791185987877 | 1.0498870384272 | 0.995223645914365 |
| ebi-a-GCST90001432 | finngen_R11_O15_PREECLAMPS | Pre-eclampsia \|\| id:finngen_R11_O15_PREECLAMPS | Unswitched memory B cell %lymphocyte \|\| id:ebi-a-GCST90001432 | MR Egger | 22 | 0.0017505806068657 | 0.0448407086509176 | 0.969245585687533 | -0.086137208 | 0.0896383695626641 | 1.00175211376761 | 0.917468338267675 | 1.09377866851803 | 0.99380795269851 |
| ebi-a-GCST90001432 | finngen_R11_O15_PREECLAMPS | Pre-eclampsia \|\| id:finngen_R11_O15_PREECLAMPS | Unswitched memory B cell %lymphocyte \|\| id:ebi-a-GCST90001432 | Weighted median | 22 | -0.008760899 | 0.0367287943053046 | 0.811470478769777 | -0.080749336 | 0.0632275378354782 | 0.99127736584639 | 0.922424881324716 | 1.06526920070519 | 0.961297867845628 |
| ebi-a-GCST90001432 | finngen_R11_O15_PREECLAMPS | Pre-eclampsia \|\| id:finngen_R11_O15_PREECLAMPS | Unswitched memory B cell %lymphocyte \|\| id:ebi-a-GCST90001432 | Weighted mode | 22 | -0.033173592 | 0.0463340242553491 | 0.481898513719171 | -0.123988279 | 0.0576410956767407 | 0.967370617344354 | 0.883390194681456 | 1.05933472766091 | 0.933945646747087 |
| ebi-a-GCST90001433 | finngen_R11_O15_PREECLAMPS | Pre-eclampsia \|\| id:finngen_R11_O15_PREECLAMPS | IgD- CD27- B cell %lymphocyte \|\| id:ebi-a-GCST90001433 | Inverse variance weighted | 18 | -0.005863185 | 0.0287254705941186 | 0.838266746454506 | -0.062165107 | 0.0504387373921523 | 0.994153969952815 | 0.939727718040856 | 1.05173242950994 | 0.977824840984872 |
| ebi-a-GCST90001433 | finngen_R11_O15_PREECLAMPS | Pre-eclampsia \|\| id:finngen_R11_O15_PREECLAMPS | IgD- CD27- B cell %lymphocyte \|\| id:ebi-a-GCST90001433 | MR Egger | 18 | 0.13415613259339 | 0.0704709672900713 | 0.0750940119534308 | -0.003966963 | 0.27227922848193 | 1.14357135446803 | 0.996040894709503 | 1.3129535641619 | 0.914895378965965 |
| ebi-a-GCST90001433 | finngen_R11_O15_PREECLAMPS | Pre-eclampsia \|\| id:finngen_R11_O15_PREECLAMPS | IgD- CD27- B cell %lymphocyte \|\| id:ebi-a-GCST90001433 | Weighted median | 18 | 0.0135716009335997 | 0.0400095841741941 | 0.734452480006707 | -0.064847184 | 0.0919903859150201 | 1.01366411314875 | 0.937210673176168 | 1.09635428158692 | 0.956980500401294 |
| ebi-a-GCST90001433 | finngen_R11_O15_PREECLAMPS | Pre-eclampsia \|\| id:finngen_R11_O15_PREECLAMPS | IgD- CD27- B cell %lymphocyte \|\| id:ebi-a-GCST90001433 | Weighted mode | 18 | 0.0164520387995011 | 0.0454079843578013 | 0.721580792519205 | -0.072547611 | 0.105451688140792 | 1.01658811883023 | 0.930021466780283 | 1.11121241848811 | 0.959313411571287 |
| ebi-a-GCST90001434 | finngen_R11_O15_PREECLAMPS | Pre-eclampsia \|\| id:finngen_R11_O15_PREECLAMPS | Switched memory B cell %lymphocyte \|\| id:ebi-a-GCST90001434 | Inverse variance weighted | 18 | 0.0167263619753538 | 0.0277679586909981 | 0.546933226601813 | -0.037698837 | 0.07115156100971 | 1.01686703076577 | 0.963002918014805 | 1.07374395125406 | 0.905435115099076 |
| ebi-a-GCST90001434 | finngen_R11_O15_PREECLAMPS | Pre-eclampsia \|\| id:finngen_R11_O15_PREECLAMPS | Switched memory B cell %lymphocyte \|\| id:ebi-a-GCST90001434 | MR Egger | 18 | 0.00674022164644355 | 0.0445902436096441 | 0.881739613744862 | -0.080656656 | 0.0941370991213459 | 1.00676298806185 | 0.922510375636322 | 1.09871036781791 | 0.99380795269851 |
| ebi-a-GCST90001434 | finngen_R11_O15_PREECLAMPS | Pre-eclampsia \|\| id:finngen_R11_O15_PREECLAMPS | Switched memory B cell %lymphocyte \|\| id:ebi-a-GCST90001434 | Weighted median | 18 | 0.0325080843072576 | 0.0327013239012415 | 0.320178677770035 | -0.031586511 | 0.096602679153691 | 1.03304224454113 | 0.968907132149536 | 1.10142266848529 | 0.940845681141708 |
| ebi-a-GCST90001434 | finngen_R11_O15_PREECLAMPS | Pre-eclampsia \|\| id:finngen_R11_O15_PREECLAMPS | Switched memory B cell %lymphocyte \|\| id:ebi-a-GCST90001434 | Weighted mode | 18 | 0.0376177763479859 | 0.0333721507325862 | 0.275308862209387 | -0.027791639 | 0.103027191783855 | 1.03833428110022 | 0.972590995637517 | 1.10852155134462 | 0.872755742358858 |
| ebi-a-GCST90001435 | finngen_R11_O15_PREECLAMPS | Pre-eclampsia \|\| id:finngen_R11_O15_PREECLAMPS | Plasma Blast-Plasma Cell %lymphocyte \|\| id:ebi-a-GCST90001435 | Inverse variance weighted | 18 | 0.00479217021735873 | 0.0107638316650211 | 0.656167683724406 | -0.01630494 | 0.0258892802808001 | 1.004803671 | 0.983827266173299 | 1.02622731858247 | 0.944209796855396 |
| ebi-a-GCST90001435 | finngen_R11_O15_PREECLAMPS | Pre-eclampsia \|\| id:finngen_R11_O15_PREECLAMPS | Plasma Blast-Plasma Cell %lymphocyte \|\| id:ebi-a-GCST90001435 | MR Egger | 18 | 0.0128316191605231 | 0.0124173694791254 | 0.316802776044199 | -0.011506425 | 0.0371696633396088 | 1.01291429764012 | 0.988559520714238 | 1.03786909423774 | 0.99380795269851 |
| ebi-a-GCST90001435 | finngen_R11_O15_PREECLAMPS | Pre-eclampsia \|\| id:finngen_R11_O15_PREECLAMPS | Plasma Blast-Plasma Cell %lymphocyte \|\| id:ebi-a-GCST90001435 | Weighted median | 18 | 0.00888811467902717 | 0.0148442299523503 | 0.549333678766958 | -0.020206576 | 0.0379828053856338 | 1.00892773125554 | 0.979996208671475 | 1.03871337244907 | 0.940845681141708 |
| ebi-a-GCST90001435 | finngen_R11_O15_PREECLAMPS | Pre-eclampsia \|\| id:finngen_R11_O15_PREECLAMPS | Plasma Blast-Plasma Cell %lymphocyte \|\| id:ebi-a-GCST90001435 | Weighted mode | 18 | 0.00889087190742316 | 0.0119454770458266 | 0.466866873232165 | -0.014522263 | 0.0323040069172433 | 1.00893051310356 | 0.985582676360086 | 1.03283144548648 | 0.933945646747087 |
| ebi-a-GCST90001436 | finngen_R11_O15_PREECLAMPS | Pre-eclampsia \|\| id:finngen_R11_O15_PREECLAMPS | Memory B cell %lymphocyte \|\| id:ebi-a-GCST90001436 | Inverse variance weighted | 28 | -0.004577978 | 0.0128994785561466 | 0.722667220276841 | -0.029860956 | 0.0207049997222242 | 0.99543248472209 | 0.970580477347174 | 1.02092083528026 | 0.959893708083249 |
| ebi-a-GCST90001436 | finngen_R11_O15_PREECLAMPS | Pre-eclampsia \|\| id:finngen_R11_O15_PREECLAMPS | Memory B cell %lymphocyte \|\| id:ebi-a-GCST90001436 | MR Egger | 28 | -0.008878809 | 0.0151083027674445 | 0.561818635272176 | -0.038491083 | 0.0207334639802388 | 0.991160490785527 | 0.962240285125061 | 1.02094989544792 | 0.99380795269851 |
| ebi-a-GCST90001436 | finngen_R11_O15_PREECLAMPS | Pre-eclampsia \|\| id:finngen_R11_O15_PREECLAMPS | Memory B cell %lymphocyte \|\| id:ebi-a-GCST90001436 | Weighted median | 28 | -0.006728481 | 0.0135539694482099 | 0.619597083410435 | -0.033294261 | 0.0198372987398258 | 0.993294104168298 | 0.967253892129321 | 1.0200353654856 | 0.940845681141708 |
| ebi-a-GCST90001436 | finngen_R11_O15_PREECLAMPS | Pre-eclampsia \|\| id:finngen_R11_O15_PREECLAMPS | Memory B cell %lymphocyte \|\| id:ebi-a-GCST90001436 | Weighted mode | 28 | -0.009679471 | 0.0113304902206274 | 0.400463443609866 | -0.031887232 | 0.0125282894813963 | 0.990367223948398 | 0.968615804610038 | 1.01260709726509 | 0.923466174381111 |
| ebi-a-GCST90001437 | finngen_R11_O15_PREECLAMPS | Pre-eclampsia \|\| id:finngen_R11_O15_PREECLAMPS | Naive-mature B cell %lymphocyte \|\| id:ebi-a-GCST90001437 | Inverse variance weighted | 22 | 0.0138068201018028 | 0.0330515076232444 | 0.676139805038148 | -0.050974135 | 0.0785877750433618 | 1.01390257442252 | 0.95030324996039 | 1.08175830237712 | 0.950857618427329 |
| ebi-a-GCST90001437 | finngen_R11_O15_PREECLAMPS | Pre-eclampsia \|\| id:finngen_R11_O15_PREECLAMPS | Naive-mature B cell %lymphocyte \|\| id:ebi-a-GCST90001437 | MR Egger | 22 | -0.057902637 | 0.0794103006018446 | 0.474358357932235 | -0.213546826 | 0.0977415520108098 | 0.943741828340157 | 0.807714336952042 | 1.10267776342777 | 0.99380795269851 |
| ebi-a-GCST90001437 | finngen_R11_O15_PREECLAMPS | Pre-eclampsia \|\| id:finngen_R11_O15_PREECLAMPS | Naive-mature B cell %lymphocyte \|\| id:ebi-a-GCST90001437 | Weighted median | 22 | 0.000876760088242098 | 0.0412358289300819 | 0.983036581268344 | -0.079945465 | 0.0816989847912026 | 1.00087714455472 | 0.923166690264995 | 1.08512912029404 | 0.992447515164719 |
| ebi-a-GCST90001437 | finngen_R11_O15_PREECLAMPS | Pre-eclampsia \|\| id:finngen_R11_O15_PREECLAMPS | Naive-mature B cell %lymphocyte \|\| id:ebi-a-GCST90001437 | Weighted mode | 22 | -0.041347854 | 0.0624270451948404 | 0.514957963093791 | -0.163704863 | 0.0810091542435547 | 0.959495307285085 | 0.848992554090885 | 1.08438082320749 | 0.934398527469092 |
| ebi-a-GCST90001438 | finngen_R11_O15_PREECLAMPS | Pre-eclampsia \|\| id:finngen_R11_O15_PREECLAMPS | IgD- CD38dim B cell Absolute Count \|\| id:ebi-a-GCST90001438 | Inverse variance weighted | 17 | 0.023143835899851 | 0.0340372775316146 | 0.496532603924233 | -0.043569228 | 0.0898568998618156 | 1.02341373259629 | 0.957366275184849 | 1.09401771841654 | 0.898694924660397 |
| ebi-a-GCST90001438 | finngen_R11_O15_PREECLAMPS | Pre-eclampsia \|\| id:finngen_R11_O15_PREECLAMPS | IgD- CD38dim B cell Absolute Count \|\| id:ebi-a-GCST90001438 | MR Egger | 17 | -0.005892475 | 0.0629566601599147 | 0.926669036522728 | -0.129287529 | 0.117502579131606 | 0.994124851798849 | 0.878721271638218 | 1.12468453064941 | 0.99380795269851 |
| ebi-a-GCST90001438 | finngen_R11_O15_PREECLAMPS | Pre-eclampsia \|\| id:finngen_R11_O15_PREECLAMPS | IgD- CD38dim B cell Absolute Count \|\| id:ebi-a-GCST90001438 | Weighted median | 17 | 0.0391032904051048 | 0.0501414241979671 | 0.435473292944107 | -0.059173901 | 0.13738048183312 | 1.03987788751143 | 0.942542845739277 | 1.14726457881826 | 0.940845681141708 |
| ebi-a-GCST90001438 | finngen_R11_O15_PREECLAMPS | Pre-eclampsia \|\| id:finngen_R11_O15_PREECLAMPS | IgD- CD38dim B cell Absolute Count \|\| id:ebi-a-GCST90001438 | Weighted mode | 17 | 0.051994116974604 | 0.0576732430492785 | 0.380677811530703 | -0.061045439 | 0.16503367335119 | 1.05336954549535 | 0.940780490301886 | 1.17943283349873 | 0.920254054953283 |
| ebi-a-GCST90001439 | finngen_R11_O15_PREECLAMPS | Pre-eclampsia \|\| id:finngen_R11_O15_PREECLAMPS | IgD+ CD24+ B cell %lymphocyte \|\| id:ebi-a-GCST90001439 | Inverse variance weighted | 15 | -0.020240404 | 0.0426007200719836 | 0.634702289813575 | -0.103737815 | 0.0632570077791639 | 0.979963058376768 | 0.9014616162415 | 1.06530059459113 | 0.942541573457101 |
| ebi-a-GCST90001439 | finngen_R11_O15_PREECLAMPS | Pre-eclampsia \|\| id:finngen_R11_O15_PREECLAMPS | IgD+ CD24+ B cell %lymphocyte \|\| id:ebi-a-GCST90001439 | MR Egger | 15 | 0.0423384936770208 | 0.0990588614180895 | 0.67607700755773 | -0.151816875 | 0.236493862056476 | 1.04324755169073 | 0.859145597629551 | 1.26679978005077 | 0.99380795269851 |
| ebi-a-GCST90001439 | finngen_R11_O15_PREECLAMPS | Pre-eclampsia \|\| id:finngen_R11_O15_PREECLAMPS | IgD+ CD24+ B cell %lymphocyte \|\| id:ebi-a-GCST90001439 | Weighted median | 15 | -0.004720574 | 0.0532005250636007 | 0.929295119340069 | -0.108993603 | 0.0995524547831948 | 0.995290550058192 | 0.896736153483078 | 1.10467641478206 | 0.977431269406605 |
| ebi-a-GCST90001439 | finngen_R11_O15_PREECLAMPS | Pre-eclampsia \|\| id:finngen_R11_O15_PREECLAMPS | IgD+ CD24+ B cell %lymphocyte \|\| id:ebi-a-GCST90001439 | Weighted mode | 15 | -0.040485356 | 0.0711422877538179 | 0.578320881788945 | -0.17992424 | 0.098953528477904 | 0.960323227843145 | 0.835333494283012 | 1.1040149931096 | 0.948158355498791 |
| ebi-a-GCST90001440 | finngen_R11_O15_PREECLAMPS | Pre-eclampsia \|\| id:finngen_R11_O15_PREECLAMPS | IgD- CD24- B cell %lymphocyte \|\| id:ebi-a-GCST90001440 | Inverse variance weighted | 24 | 0.0380312645572852 | 0.0223396185838643 | 0.088677912171537 | -0.005754388 | 0.0818169169816593 | 1.0387637088583 | 0.994262136910919 | 1.08525709949441 | 0.802698559103897 |
| ebi-a-GCST90001440 | finngen_R11_O15_PREECLAMPS | Pre-eclampsia \|\| id:finngen_R11_O15_PREECLAMPS | IgD- CD24- B cell %lymphocyte \|\| id:ebi-a-GCST90001440 | MR Egger | 24 | 0.0403414250987264 | 0.0332300868132156 | 0.237618490557187 | -0.024789545 | 0.105472395252629 | 1.04116619378485 | 0.975515192422434 | 1.11123542872618 | 0.99380795269851 |
| ebi-a-GCST90001440 | finngen_R11_O15_PREECLAMPS | Pre-eclampsia \|\| id:finngen_R11_O15_PREECLAMPS | IgD- CD24- B cell %lymphocyte \|\| id:ebi-a-GCST90001440 | Weighted median | 24 | 0.0376423530260622 | 0.0313945647487638 | 0.23052460286392 | -0.023890994 | 0.0991756999336393 | 1.03835980022117 | 0.97639213667415 | 1.10426030097697 | 0.940845681141708 |
| ebi-a-GCST90001440 | finngen_R11_O15_PREECLAMPS | Pre-eclampsia \|\| id:finngen_R11_O15_PREECLAMPS | IgD- CD24- B cell %lymphocyte \|\| id:ebi-a-GCST90001440 | Weighted mode | 24 | 0.0114739960475792 | 0.0336256828964401 | 0.736030248237859 | -0.054432342 | 0.0773803345246018 | 1.01154007482761 | 0.947022579937375 | 1.08045293180857 | 0.959313411571287 |
| ebi-a-GCST90001441 | finngen_R11_O15_PREECLAMPS | Pre-eclampsia \|\| id:finngen_R11_O15_PREECLAMPS | IgD+ CD24- B cell %lymphocyte \|\| id:ebi-a-GCST90001441 | Inverse variance weighted | 23 | -0.004241306 | 0.0303361083758879 | 0.888809729943993 | -0.063700078 | 0.0552174664342717 | 0.995767675653308 | 0.938286369684123 | 1.05677040178022 | 0.986383922906939 |
| ebi-a-GCST90001441 | finngen_R11_O15_PREECLAMPS | Pre-eclampsia \|\| id:finngen_R11_O15_PREECLAMPS | IgD+ CD24- B cell %lymphocyte \|\| id:ebi-a-GCST90001441 | MR Egger | 23 | -0.027535667 | 0.0683528080259068 | 0.691133103934541 | -0.16150717 | 0.106435837171886 | 0.972839984080086 | 0.850860430527746 | 1.1123065554217 | 0.99380795269851 |
| ebi-a-GCST90001441 | finngen_R11_O15_PREECLAMPS | Pre-eclampsia \|\| id:finngen_R11_O15_PREECLAMPS | IgD+ CD24- B cell %lymphocyte \|\| id:ebi-a-GCST90001441 | Weighted median | 23 | -0.013382068 | 0.0480438557530844 | 0.78059893580935 | -0.107548026 | 0.0807838887947458 | 0.986707073320188 | 0.898033392681336 | 1.08413657718579 | 0.957984984509652 |
| ebi-a-GCST90001441 | finngen_R11_O15_PREECLAMPS | Pre-eclampsia \|\| id:finngen_R11_O15_PREECLAMPS | IgD+ CD24- B cell %lymphocyte \|\| id:ebi-a-GCST90001441 | Weighted mode | 23 | -0.02150964 | 0.0566663071795768 | 0.707893430509944 | -0.132575602 | 0.089556321879047 | 0.978720042373643 | 0.875836716363695 | 1.09368893019335 | 0.959313411571287 |
| ebi-a-GCST90001442 | finngen_R11_O15_PREECLAMPS | Pre-eclampsia \|\| id:finngen_R11_O15_PREECLAMPS | CD24+ CD27+ B cell %lymphocyte \|\| id:ebi-a-GCST90001442 | Inverse variance weighted | 24 | -0.004969148 | 0.0106458604311186 | 0.640665790480671 | -0.025835035 | 0.0158967382393756 | 0.995043177586647 | 0.974495834395526 | 1.01602376358632 | 0.942541573457101 |
| ebi-a-GCST90001442 | finngen_R11_O15_PREECLAMPS | Pre-eclampsia \|\| id:finngen_R11_O15_PREECLAMPS | CD24+ CD27+ B cell %lymphocyte \|\| id:ebi-a-GCST90001442 | MR Egger | 24 | -0.004079052 | 0.0123727440530735 | 0.744759816750993 | -0.028329631 | 0.0201715258611854 | 0.995929255551599 | 0.972067890442212 | 1.02037634594878 | 0.99380795269851 |
| ebi-a-GCST90001442 | finngen_R11_O15_PREECLAMPS | Pre-eclampsia \|\| id:finngen_R11_O15_PREECLAMPS | CD24+ CD27+ B cell %lymphocyte \|\| id:ebi-a-GCST90001442 | Weighted median | 24 | -0.00796855 | 0.0134803821382984 | 0.554438715834305 | -0.034390099 | 0.0184529992583564 | 0.992063114996568 | 0.966194519862532 | 1.01862430794652 | 0.940845681141708 |
| ebi-a-GCST90001442 | finngen_R11_O15_PREECLAMPS | Pre-eclampsia \|\| id:finngen_R11_O15_PREECLAMPS | CD24+ CD27+ B cell %lymphocyte \|\| id:ebi-a-GCST90001442 | Weighted mode | 24 | -0.008067899 | 0.010968842402405 | 0.469452351458386 | -0.02956683 | 0.0134310317671065 | 0.991964558809931 | 0.970865992061601 | 1.01352163324364 | 0.933945646747087 |
| ebi-a-GCST90001443 | finngen_R11_O15_PREECLAMPS | Pre-eclampsia \|\| id:finngen_R11_O15_PREECLAMPS | CD20- B cell %lymphocyte \|\| id:ebi-a-GCST90001443 | Inverse variance weighted | 18 | 0.022875012657503 | 0.0166143707627169 | 0.168567632099679 | -0.009689154 | 0.0554391793524281 | 1.02313865217409 | 0.990357634579584 | 1.05700472740537 | 0.85505992597664 |
| ebi-a-GCST90001443 | finngen_R11_O15_PREECLAMPS | Pre-eclampsia \|\| id:finngen_R11_O15_PREECLAMPS | CD20- B cell %lymphocyte \|\| id:ebi-a-GCST90001443 | MR Egger | 18 | 0.025266342360199 | 0.0213565177895425 | 0.254061479067389 | -0.016592433 | 0.0671251172277023 | 1.02558824174384 | 0.983544463708129 | 1.06942927383032 | 0.99380795269851 |
| ebi-a-GCST90001443 | finngen_R11_O15_PREECLAMPS | Pre-eclampsia \|\| id:finngen_R11_O15_PREECLAMPS | CD20- B cell %lymphocyte \|\| id:ebi-a-GCST90001443 | Weighted median | 18 | 0.0282814457516448 | 0.0189300876514048 | 0.135177071798495 | -0.008821526 | 0.0653844175483981 | 1.02868516275213 | 0.99121726945346 | 1.0675693379009 | 0.88227178111339 |
| ebi-a-GCST90001443 | finngen_R11_O15_PREECLAMPS | Pre-eclampsia \|\| id:finngen_R11_O15_PREECLAMPS | CD20- B cell %lymphocyte \|\| id:ebi-a-GCST90001443 | Weighted mode | 18 | 0.0264783487886038 | 0.0181462404507711 | 0.162750683804547 | -0.009088282 | 0.0620449800721152 | 1.02683201486398 | 0.990952891117563 | 1.06401020290735 | 0.872755742358858 |
| ebi-a-GCST90001444 | finngen_R11_O15_PREECLAMPS | Pre-eclampsia \|\| id:finngen_R11_O15_PREECLAMPS | CD20- CD38- B cell %lymphocyte \|\| id:ebi-a-GCST90001444 | Inverse variance weighted | 23 | -0.047361605 | 0.0209064086010001 | 0.0234874789135563 | -0.088338166 | -0.006385044 | 0.953742457229561 | 0.915451250057336 | 0.993635296980925 | 0.708667785226302 |
| ebi-a-GCST90001444 | finngen_R11_O15_PREECLAMPS | Pre-eclampsia \|\| id:finngen_R11_O15_PREECLAMPS | CD20- CD38- B cell %lymphocyte \|\| id:ebi-a-GCST90001444 | MR Egger | 23 | -0.055179301 | 0.0308187229217565 | 0.0878109421050031 | -0.115583998 | 0.00522539627292183 | 0.946315457760955 | 0.890845740207613 | 1.00523907246683 | 0.936133620820556 |
| ebi-a-GCST90001444 | finngen_R11_O15_PREECLAMPS | Pre-eclampsia \|\| id:finngen_R11_O15_PREECLAMPS | CD20- CD38- B cell %lymphocyte \|\| id:ebi-a-GCST90001444 | Weighted median | 23 | -0.055871791 | 0.0335931476902296 | 0.0962743840706844 | -0.121714361 | 0.00997077802636935 | 0.945660369866755 | 0.885401237581789 | 1.01002065185607 | 0.88227178111339 |
| ebi-a-GCST90001444 | finngen_R11_O15_PREECLAMPS | Pre-eclampsia \|\| id:finngen_R11_O15_PREECLAMPS | CD20- CD38- B cell %lymphocyte \|\| id:ebi-a-GCST90001444 | Weighted mode | 23 | -0.050052489 | 0.0268453219422761 | 0.0756525693354468 | -0.10266932 | 0.00256434227270888 | 0.951179496982661 | 0.90242533839172 | 1.00256763301061 | 0.872755742358858 |
| ebi-a-GCST90001445 | finngen_R11_O15_PREECLAMPS | Pre-eclampsia \|\| id:finngen_R11_O15_PREECLAMPS | IgD- CD38- B cell %B cell \|\| id:ebi-a-GCST90001445 | Inverse variance weighted (multiplicative random effects) | 12 | 0.0416876645071411 | 0.0494377600589071 | 0.399096858865122 | -0.055210345 | 0.138585674222599 | 1.04256879665343 | 0.946286080275069 | 1.1486480868866 | 0.885079385681701 |
| ebi-a-GCST90001445 | finngen_R11_O15_PREECLAMPS | Pre-eclampsia \|\| id:finngen_R11_O15_PREECLAMPS | IgD- CD38- B cell %B cell \|\| id:ebi-a-GCST90001445 | MR Egger | 12 | -0.017680055 | 0.0904517041532988 | 0.848944197711707 | -0.194965395 | 0.159605284847286 | 0.982475319856479 | 0.82286313238521 | 1.17304775987366 | 0.99380795269851 |
| ebi-a-GCST90001445 | finngen_R11_O15_PREECLAMPS | Pre-eclampsia \|\| id:finngen_R11_O15_PREECLAMPS | IgD- CD38- B cell %B cell \|\| id:ebi-a-GCST90001445 | Weighted median | 12 | -0.015930719 | 0.0561042126513785 | 0.776449684507344 | -0.125894976 | 0.0940335378135979 | 0.984195503758665 | 0.881707442557526 | 1.09859658982699 | 0.957984984509652 |
| ebi-a-GCST90001445 | finngen_R11_O15_PREECLAMPS | Pre-eclampsia \|\| id:finngen_R11_O15_PREECLAMPS | IgD- CD38- B cell %B cell \|\| id:ebi-a-GCST90001445 | Weighted mode | 12 | -0.045395896 | 0.0617186248265021 | 0.477407345904384 | -0.166364401 | 0.075572608497667 | 0.955619081007555 | 0.846737626077142 | 1.07850153325125 | 0.933945646747087 |
| ebi-a-GCST90001446 | finngen_R11_O15_PREECLAMPS | Pre-eclampsia \|\| id:finngen_R11_O15_PREECLAMPS | IgD- CD38- B cell Absolute Count \|\| id:ebi-a-GCST90001446 | Inverse variance weighted | 14 | -0.004085257 | 0.0240077217523582 | 0.864880941513965 | -0.051140391 | 0.0429698779206815 | 0.995923076595504 | 0.950145268992791 | 1.04390644974414 | 0.981050218604997 |
| ebi-a-GCST90001446 | finngen_R11_O15_PREECLAMPS | Pre-eclampsia \|\| id:finngen_R11_O15_PREECLAMPS | IgD- CD38- B cell Absolute Count \|\| id:ebi-a-GCST90001446 | MR Egger | 14 | -0.041787278 | 0.0290675850025254 | 0.1761120017098 | -0.098759744 | 0.0151851889707046 | 0.959073775317012 | 0.905960344070068 | 1.01530106976701 | 0.99380795269851 |
| ebi-a-GCST90001446 | finngen_R11_O15_PREECLAMPS | Pre-eclampsia \|\| id:finngen_R11_O15_PREECLAMPS | IgD- CD38- B cell Absolute Count \|\| id:ebi-a-GCST90001446 | Weighted median | 14 | -0.005769928 | 0.0299137283076598 | 0.847048536119472 | -0.064400836 | 0.0528609790311714 | 0.994246685615975 | 0.937629088764149 | 1.05428306747745 | 0.964912412213677 |
| ebi-a-GCST90001446 | finngen_R11_O15_PREECLAMPS | Pre-eclampsia \|\| id:finngen_R11_O15_PREECLAMPS | IgD- CD38- B cell Absolute Count \|\| id:ebi-a-GCST90001446 | Weighted mode | 14 | -0.00791929 | 0.0275171273496429 | 0.778040533714775 | -0.06185286 | 0.0460142791999799 | 0.992111984561971 | 0.940021191323884 | 1.04708936244859 | 0.961305639715923 |
| ebi-a-GCST90001447 | finngen_R11_O15_PREECLAMPS | Pre-eclampsia \|\| id:finngen_R11_O15_PREECLAMPS | IgD+ CD38+ B cell %B cell \|\| id:ebi-a-GCST90001447 | Inverse variance weighted | 25 | -0.002270896 | 0.019776529602089 | 0.908581582441695 | -0.041032894 | 0.0364911018572328 | 0.997731680371114 | 0.959797557672067 | 1.03716507513378 | 0.986667691552896 |
| ebi-a-GCST90001447 | finngen_R11_O15_PREECLAMPS | Pre-eclampsia \|\| id:finngen_R11_O15_PREECLAMPS | IgD+ CD38+ B cell %B cell \|\| id:ebi-a-GCST90001447 | MR Egger | 25 | 0.00921331590430483 | 0.0297479070700052 | 0.759567809953266 | -0.049092582 | 0.0675192137615149 | 1.00925588914574 | 0.952092978990643 | 1.06985081525885 | 0.99380795269851 |
| ebi-a-GCST90001447 | finngen_R11_O15_PREECLAMPS | Pre-eclampsia \|\| id:finngen_R11_O15_PREECLAMPS | IgD+ CD38+ B cell %B cell \|\| id:ebi-a-GCST90001447 | Weighted median | 25 | 0.0109596734697917 | 0.0236022165762554 | 0.642397486083868 | -0.035300671 | 0.0572200179592523 | 1.01101995069605 | 0.965315130334932 | 1.05888875931199 | 0.940845681141708 |
| ebi-a-GCST90001447 | finngen_R11_O15_PREECLAMPS | Pre-eclampsia \|\| id:finngen_R11_O15_PREECLAMPS | IgD+ CD38+ B cell %B cell \|\| id:ebi-a-GCST90001447 | Weighted mode | 25 | 0.00372057440019424 | 0.0211734044383807 | 0.861988729732449 | -0.037779298 | 0.0452204470994203 | 1.0037275043289 | 0.962925436723032 | 1.04625847913508 | 0.977585971930092 |
| ebi-a-GCST90001448 | finngen_R11_O15_PREECLAMPS | Pre-eclampsia \|\| id:finngen_R11_O15_PREECLAMPS | CD11c+ monocyte Absolute Count \|\| id:ebi-a-GCST90001448 | Inverse variance weighted | 15 | 0.0210424569799666 | 0.0230142477657012 | 0.360547127554871 | -0.024065469 | 0.0661503826007408 | 1.02126541056215 | 0.976221795751656 | 1.06838737195744 | 0.885079385681701 |
| ebi-a-GCST90001448 | finngen_R11_O15_PREECLAMPS | Pre-eclampsia \|\| id:finngen_R11_O15_PREECLAMPS | CD11c+ monocyte Absolute Count \|\| id:ebi-a-GCST90001448 | MR Egger | 15 | 0.0233570253515655 | 0.0364280566435557 | 0.532545863698183 | -0.048041966 | 0.0947560163729347 | 1.02363193686742 | 0.953093789018996 | 1.09939058909769 | 0.99380795269851 |
| ebi-a-GCST90001448 | finngen_R11_O15_PREECLAMPS | Pre-eclampsia \|\| id:finngen_R11_O15_PREECLAMPS | CD11c+ monocyte Absolute Count \|\| id:ebi-a-GCST90001448 | Weighted median | 15 | 0.0142554497716047 | 0.0309750701557607 | 0.645355930562752 | -0.046455688 | 0.0749665872768957 | 1.01435754324832 | 0.954606860426186 | 1.07784813644163 | 0.940845681141708 |
| ebi-a-GCST90001448 | finngen_R11_O15_PREECLAMPS | Pre-eclampsia \|\| id:finngen_R11_O15_PREECLAMPS | CD11c+ monocyte Absolute Count \|\| id:ebi-a-GCST90001448 | Weighted mode | 15 | 0.0132002107110679 | 0.0291858243199611 | 0.657994883997483 | -0.044004005 | 0.0704044263781916 | 1.01328771810724 | 0.956950124922174 | 1.07294201957546 | 0.959313411571287 |
| ebi-a-GCST90001449 | finngen_R11_O15_PREECLAMPS | Pre-eclampsia \|\| id:finngen_R11_O15_PREECLAMPS | CD11c+ monocyte %monocyte \|\| id:ebi-a-GCST90001449 | Inverse variance weighted | 20 | 0.016416320288118 | 0.0224171333570377 | 0.463978577878288 | -0.027521261 | 0.060353901667912 | 1.01655180846441 | 0.972853998395524 | 1.06221239877367 | 0.898694924660397 |
| ebi-a-GCST90001449 | finngen_R11_O15_PREECLAMPS | Pre-eclampsia \|\| id:finngen_R11_O15_PREECLAMPS | CD11c+ monocyte %monocyte \|\| id:ebi-a-GCST90001449 | MR Egger | 20 | -0.020126931 | 0.0398231846248499 | 0.61940532490862 | -0.098180372 | 0.0579265112931024 | 0.980074264024705 | 0.906485384029943 | 1.05963712148705 | 0.99380795269851 |
| ebi-a-GCST90001449 | finngen_R11_O15_PREECLAMPS | Pre-eclampsia \|\| id:finngen_R11_O15_PREECLAMPS | CD11c+ monocyte %monocyte \|\| id:ebi-a-GCST90001449 | Weighted median | 20 | 0.0153609180246462 | 0.0338181126117504 | 0.64966932076566 | -0.050922583 | 0.081644418743677 | 1.01547950334099 | 0.950352241394476 | 1.08506991070232 | 0.940845681141708 |
| ebi-a-GCST90001449 | finngen_R11_O15_PREECLAMPS | Pre-eclampsia \|\| id:finngen_R11_O15_PREECLAMPS | CD11c+ monocyte %monocyte \|\| id:ebi-a-GCST90001449 | Weighted mode | 20 | 0.0174067915240045 | 0.0342436412981797 | 0.617078841546559 | -0.049710745 | 0.0845243284684368 | 1.01755917259073 | 0.951504611765644 | 1.08819931813273 | 0.959313411571287 |
| ebi-a-GCST90001450 | finngen_R11_O15_PREECLAMPS | Pre-eclampsia \|\| id:finngen_R11_O15_PREECLAMPS | CD62L- monocyte Absolute Count \|\| id:ebi-a-GCST90001450 | Inverse variance weighted | 19 | -0.006230631 | 0.0194468934116178 | 0.748671120926663 | -0.044346542 | 0.0318852802594863 | 0.993788739302713 | 0.956622390271245 | 1.03239906195886 | 0.96638925304053 |
| ebi-a-GCST90001450 | finngen_R11_O15_PREECLAMPS | Pre-eclampsia \|\| id:finngen_R11_O15_PREECLAMPS | CD62L- monocyte Absolute Count \|\| id:ebi-a-GCST90001450 | MR Egger | 19 | -0.008607542 | 0.0256945108019994 | 0.741726652482337 | -0.058968783 | 0.041753699461289 | 0.991429397116377 | 0.942736198204377 | 1.04263764490928 | 0.99380795269851 |
| ebi-a-GCST90001450 | finngen_R11_O15_PREECLAMPS | Pre-eclampsia \|\| id:finngen_R11_O15_PREECLAMPS | CD62L- monocyte Absolute Count \|\| id:ebi-a-GCST90001450 | Weighted median | 19 | -0.008070104 | 0.0215978086033034 | 0.708661883563175 | -0.050401809 | 0.0342616010779874 | 0.991962372083132 | 0.950847289070506 | 1.03485529058056 | 0.948776258030552 |
| ebi-a-GCST90001450 | finngen_R11_O15_PREECLAMPS | Pre-eclampsia \|\| id:finngen_R11_O15_PREECLAMPS | CD62L- monocyte Absolute Count \|\| id:ebi-a-GCST90001450 | Weighted mode | 19 | -0.00793671 | 0.0168107687840059 | 0.64251383120167 | -0.040885817 | 0.025012396967488 | 0.99209470267349 | 0.959938732695388 | 1.02532783140143 | 0.959313411571287 |
| ebi-a-GCST90001451 | finngen_R11_O15_PREECLAMPS | Pre-eclampsia \|\| id:finngen_R11_O15_PREECLAMPS | CD62L- monocyte %monocyte \|\| id:ebi-a-GCST90001451 | Inverse variance weighted (multiplicative random effects) | 23 | -0.045913832 | 0.0245087248439749 | 0.0610177457011794 | -0.093950933 | 0.00212326826823759 | 0.955124259385333 | 0.910327428071494 | 1.00212552399853 | 0.789997151112252 |
| ebi-a-GCST90001451 | finngen_R11_O15_PREECLAMPS | Pre-eclampsia \|\| id:finngen_R11_O15_PREECLAMPS | CD62L- monocyte %monocyte \|\| id:ebi-a-GCST90001451 | MR Egger | 23 | 0.0192259478029652 | 0.0561928793795691 | 0.735644466391561 | -0.090912096 | 0.129363991386921 | 1.01941195648953 | 0.913097972535701 | 1.13810430894719 | 0.99380795269851 |
| ebi-a-GCST90001451 | finngen_R11_O15_PREECLAMPS | Pre-eclampsia \|\| id:finngen_R11_O15_PREECLAMPS | CD62L- monocyte %monocyte \|\| id:ebi-a-GCST90001451 | Weighted median | 23 | -0.042760852 | 0.0317102041264547 | 0.177501252480736 | -0.104912852 | 0.0193911481759702 | 0.958140500139078 | 0.900402987486981 | 1.01958037763623 | 0.940845681141708 |
| ebi-a-GCST90001451 | finngen_R11_O15_PREECLAMPS | Pre-eclampsia \|\| id:finngen_R11_O15_PREECLAMPS | CD62L- monocyte %monocyte \|\| id:ebi-a-GCST90001451 | Weighted mode | 23 | -0.026975726 | 0.0404383055727511 | 0.511655550721993 | -0.106234805 | 0.0522833533238254 | 0.973384869572435 | 0.899213483894057 | 1.05367426232254 | 0.934398527469092 |
| ebi-a-GCST90001452 | finngen_R11_O15_PREECLAMPS | Pre-eclampsia \|\| id:finngen_R11_O15_PREECLAMPS | CD11c+ CD62L- monocyte Absolute Count \|\| id:ebi-a-GCST90001452 | Inverse variance weighted | 22 | 0.0281964462037659 | 0.0163411487688182 | 0.084439673652781 | -0.003832205 | 0.0602250977906495 | 1.02859772869436 | 0.996175128145079 | 1.06207559050914 | 0.791351300515166 |
| ebi-a-GCST90001452 | finngen_R11_O15_PREECLAMPS | Pre-eclampsia \|\| id:finngen_R11_O15_PREECLAMPS | CD11c+ CD62L- monocyte Absolute Count \|\| id:ebi-a-GCST90001452 | MR Egger | 22 | 0.0217670634270677 | 0.0227591400666001 | 0.350284146463366 | -0.022840851 | 0.0663749779576038 | 1.02200569423762 | 0.977418026396081 | 1.0686273537489 | 0.99380795269851 |
| ebi-a-GCST90001452 | finngen_R11_O15_PREECLAMPS | Pre-eclampsia \|\| id:finngen_R11_O15_PREECLAMPS | CD11c+ CD62L- monocyte Absolute Count \|\| id:ebi-a-GCST90001452 | Weighted median | 22 | 0.0332916315496205 | 0.0220698388048497 | 0.131434953538245 | -0.009965253 | 0.0765485156071259 | 1.03385199914215 | 0.990084236095654 | 1.07955456431179 | 0.88227178111339 |
| ebi-a-GCST90001452 | finngen_R11_O15_PREECLAMPS | Pre-eclampsia \|\| id:finngen_R11_O15_PREECLAMPS | CD11c+ CD62L- monocyte Absolute Count \|\| id:ebi-a-GCST90001452 | Weighted mode | 22 | 0.0211341869523722 | 0.0200575009403228 | 0.304002687285605 | -0.018178515 | 0.060446888795405 | 1.02135909550688 | 0.981985717637608 | 1.06231117544583 | 0.875408265683677 |
| ebi-a-GCST90001453 | finngen_R11_O15_PREECLAMPS | Pre-eclampsia \|\| id:finngen_R11_O15_PREECLAMPS | CD11c+ CD62L- monocyte %monocyte \|\| id:ebi-a-GCST90001453 | Inverse variance weighted | 18 | 0.0453053694951783 | 0.0322442107055915 | 0.160000497505234 | -0.017893283 | 0.108504022478138 | 1.04634733368453 | 0.982265850750938 | 1.11460939202124 | 0.85505992597664 |
| ebi-a-GCST90001453 | finngen_R11_O15_PREECLAMPS | Pre-eclampsia \|\| id:finngen_R11_O15_PREECLAMPS | CD11c+ CD62L- monocyte %monocyte \|\| id:ebi-a-GCST90001453 | MR Egger | 18 | 0.0661873616282548 | 0.0664164706611187 | 0.33381633761161 | -0.063988921 | 0.196363644124047 | 1.06842688061396 | 0.938015391869938 | 1.2169693686399 | 0.99380795269851 |
| ebi-a-GCST90001453 | finngen_R11_O15_PREECLAMPS | Pre-eclampsia \|\| id:finngen_R11_O15_PREECLAMPS | CD11c+ CD62L- monocyte %monocyte \|\| id:ebi-a-GCST90001453 | Weighted median | 18 | 0.0467848965627846 | 0.0427968427252211 | 0.27431227338007 | -0.037096915 | 0.130666708304218 | 1.04789657867918 | 0.963582745029779 | 1.13958790282571 | 0.940845681141708 |
| ebi-a-GCST90001453 | finngen_R11_O15_PREECLAMPS | Pre-eclampsia \|\| id:finngen_R11_O15_PREECLAMPS | CD11c+ CD62L- monocyte %monocyte \|\| id:ebi-a-GCST90001453 | Weighted mode | 18 | 0.0584655852899163 | 0.0523366186565573 | 0.279488825278001 | -0.044114187 | 0.161045357856769 | 1.06020849829865 | 0.956844691744987 | 1.17473825121481 | 0.872755742358858 |
| ebi-a-GCST90001454 | finngen_R11_O15_PREECLAMPS | Pre-eclampsia \|\| id:finngen_R11_O15_PREECLAMPS | CD62L- HLA DR++ monocyte Absolute Count \|\| id:ebi-a-GCST90001454 | Inverse variance weighted | 18 | 0.0160734175712769 | 0.0314661608997638 | 0.609479923921697 | -0.045600258 | 0.077747092934814 | 1.01620328984489 | 0.955423809088241 | 1.08084926968383 | 0.92242199665996 |
| ebi-a-GCST90001454 | finngen_R11_O15_PREECLAMPS | Pre-eclampsia \|\| id:finngen_R11_O15_PREECLAMPS | CD62L- HLA DR++ monocyte Absolute Count \|\| id:ebi-a-GCST90001454 | MR Egger | 18 | -0.01925577 | 0.0756063702764181 | 0.80221129169557 | -0.167444256 | 0.128932715481006 | 0.980928437832329 | 0.845823765572544 | 1.13761357780818 | 0.99380795269851 |
| ebi-a-GCST90001454 | finngen_R11_O15_PREECLAMPS | Pre-eclampsia \|\| id:finngen_R11_O15_PREECLAMPS | CD62L- HLA DR++ monocyte Absolute Count \|\| id:ebi-a-GCST90001454 | Weighted median | 18 | -0.003528778 | 0.0448484062501983 | 0.937285279391453 | -0.091431655 | 0.0843740979586554 | 0.996477440529285 | 0.912623687704387 | 1.08803584967371 | 0.978793627478789 |
| ebi-a-GCST90001454 | finngen_R11_O15_PREECLAMPS | Pre-eclampsia \|\| id:finngen_R11_O15_PREECLAMPS | CD62L- HLA DR++ monocyte Absolute Count \|\| id:ebi-a-GCST90001454 | Weighted mode | 18 | -0.02436799 | 0.0623897129994264 | 0.700960954670486 | -0.146651827 | 0.097915847962043 | 0.975926512945669 | 0.863594605406262 | 1.10286997244759 | 0.959313411571287 |
| ebi-a-GCST90001455 | finngen_R11_O15_PREECLAMPS | Pre-eclampsia \|\| id:finngen_R11_O15_PREECLAMPS | CD62L- HLA DR++ monocyte %monocyte \|\| id:ebi-a-GCST90001455 | Inverse variance weighted | 11 | 0.0141140734605556 | 0.0379400007225063 | 0.709885133271778 | -0.060248328 | 0.0884764748766679 | 1.01421414725741 | 0.941530696158395 | 1.09250855090976 | 0.957428104099021 |
| ebi-a-GCST90001455 | finngen_R11_O15_PREECLAMPS | Pre-eclampsia \|\| id:finngen_R11_O15_PREECLAMPS | CD62L- HLA DR++ monocyte %monocyte \|\| id:ebi-a-GCST90001455 | MR Egger | 11 | -0.006523078 | 0.0971296145808011 | 0.947923819298064 | -0.196897122 | 0.183850966769154 | 0.993498151278093 | 0.821275119790159 | 1.20183669614292 | 0.99380795269851 |
| ebi-a-GCST90001455 | finngen_R11_O15_PREECLAMPS | Pre-eclampsia \|\| id:finngen_R11_O15_PREECLAMPS | CD62L- HLA DR++ monocyte %monocyte \|\| id:ebi-a-GCST90001455 | Weighted median | 11 | -0.005377514 | 0.0493380439691141 | 0.913207843744838 | -0.10208008 | 0.0913250523928303 | 0.99463691915792 | 0.902957239988392 | 1.09562508293823 | 0.971695682354406 |
| ebi-a-GCST90001455 | finngen_R11_O15_PREECLAMPS | Pre-eclampsia \|\| id:finngen_R11_O15_PREECLAMPS | CD62L- HLA DR++ monocyte %monocyte \|\| id:ebi-a-GCST90001455 | Weighted mode | 11 | -0.007927809 | 0.0636755934644694 | 0.903384116337574 | -0.132731972 | 0.116876354248101 | 0.992103533255379 | 0.875699772599426 | 1.123980446 | 0.98361388732834 |
| ebi-a-GCST90001456 | finngen_R11_O15_PREECLAMPS | Pre-eclampsia \|\| id:finngen_R11_O15_PREECLAMPS | CD11c+ HLA DR++ monocyte Absolute Count \|\| id:ebi-a-GCST90001456 | Inverse variance weighted | 17 | 0.0383863005819476 | 0.0309292316953578 | 0.21456843786083 | -0.022234994 | 0.0990075947048488 | 1.03913257287219 | 0.97801038192253 | 1.1040746846484 | 0.855687495048373 |
| ebi-a-GCST90001456 | finngen_R11_O15_PREECLAMPS | Pre-eclampsia \|\| id:finngen_R11_O15_PREECLAMPS | CD11c+ HLA DR++ monocyte Absolute Count \|\| id:ebi-a-GCST90001456 | MR Egger | 17 | 0.0334046201644052 | 0.049985147597552 | 0.514100857980423 | -0.064566269 | 0.131375509455607 | 1.03396881924697 | 0.937473986621012 | 1.14039593037494 | 0.99380795269851 |
| ebi-a-GCST90001456 | finngen_R11_O15_PREECLAMPS | Pre-eclampsia \|\| id:finngen_R11_O15_PREECLAMPS | CD11c+ HLA DR++ monocyte Absolute Count \|\| id:ebi-a-GCST90001456 | Weighted median | 17 | 0.00923860810323638 | 0.0390588702362635 | 0.813021162274659 | -0.067316778 | 0.0857939937663129 | 1.00928141576927 | 0.934898999400463 | 1.08958184453131 | 0.961297867845628 |
| ebi-a-GCST90001456 | finngen_R11_O15_PREECLAMPS | Pre-eclampsia \|\| id:finngen_R11_O15_PREECLAMPS | CD11c+ HLA DR++ monocyte Absolute Count \|\| id:ebi-a-GCST90001456 | Weighted mode | 17 | -0.005617148 | 0.0466070747278791 | 0.905570651398048 | -0.096967015 | 0.0857327183658991 | 0.994398598578044 | 0.907585942754489 | 1.08951508201297 | 0.98361388732834 |
| ebi-a-GCST90001457 | finngen_R11_O15_PREECLAMPS | Pre-eclampsia \|\| id:finngen_R11_O15_PREECLAMPS | CD11c+ HLA DR++ monocyte %monocyte \|\| id:ebi-a-GCST90001457 | Inverse variance weighted | 17 | 0.000433670322379749 | 0.017359452635344 | 0.980069482939338 | -0.033590857 | 0.0344581974876541 | 1.00043376437095 | 0.966967051666644 | 1.03505875941518 | 0.994537505425743 |
| ebi-a-GCST90001457 | finngen_R11_O15_PREECLAMPS | Pre-eclampsia \|\| id:finngen_R11_O15_PREECLAMPS | CD11c+ HLA DR++ monocyte %monocyte \|\| id:ebi-a-GCST90001457 | MR Egger | 17 | 0.00576452300408264 | 0.0206122949106165 | 0.783554601127216 | -0.034635575 | 0.0461646210288909 | 1.00578116983846 | 0.96595737111805 | 1.04724679561247 | 0.99380795269851 |
| ebi-a-GCST90001457 | finngen_R11_O15_PREECLAMPS | Pre-eclampsia \|\| id:finngen_R11_O15_PREECLAMPS | CD11c+ HLA DR++ monocyte %monocyte \|\| id:ebi-a-GCST90001457 | Weighted median | 17 | -0.000557976 | 0.0203994432213501 | 0.97817856499018 | -0.040540885 | 0.0394249324680043 | 0.999442179393954 | 0.96026990311254 | 1.04021240977566 | 0.99075236588769 |
| ebi-a-GCST90001457 | finngen_R11_O15_PREECLAMPS | Pre-eclampsia \|\| id:finngen_R11_O15_PREECLAMPS | CD11c+ HLA DR++ monocyte %monocyte \|\| id:ebi-a-GCST90001457 | Weighted mode | 17 | 0.00178758300287374 | 0.019647282672721 | 0.928634852296416 | -0.036721091 | 0.040296257041407 | 1.00178918168182 | 0.963944950747693 | 1.04111916739259 | 0.989551132694869 |
| ebi-a-GCST90001458 | finngen_R11_O15_PREECLAMPS | Pre-eclampsia \|\| id:finngen_R11_O15_PREECLAMPS | Myeloid Dendritic Cell Absolute Count \|\| id:ebi-a-GCST90001458 | Inverse variance weighted | 23 | -0.01790861 | 0.0162030793085441 | 0.269047083249781 | -0.049666646 | 0.0138494251817743 | 0.982250795898438 | 0.951546573770914 | 1.01394577274301 | 0.885079385681701 |
| ebi-a-GCST90001458 | finngen_R11_O15_PREECLAMPS | Pre-eclampsia \|\| id:finngen_R11_O15_PREECLAMPS | Myeloid Dendritic Cell Absolute Count \|\| id:ebi-a-GCST90001458 | MR Egger | 23 | -0.026278424 | 0.0213190991208195 | 0.231338213400898 | -0.068063858 | 0.015507010496187 | 0.974063849311137 | 0.934200815423089 | 1.01562786808865 | 0.99380795269851 |
| ebi-a-GCST90001458 | finngen_R11_O15_PREECLAMPS | Pre-eclampsia \|\| id:finngen_R11_O15_PREECLAMPS | Myeloid Dendritic Cell Absolute Count \|\| id:ebi-a-GCST90001458 | Weighted median | 23 | -0.015611084 | 0.0211811945793085 | 0.461106746403527 | -0.057126225 | 0.0259040575084708 | 0.984510137484118 | 0.944474845275834 | 1.02624248348925 | 0.940845681141708 |
| ebi-a-GCST90001458 | finngen_R11_O15_PREECLAMPS | Pre-eclampsia \|\| id:finngen_R11_O15_PREECLAMPS | Myeloid Dendritic Cell Absolute Count \|\| id:ebi-a-GCST90001458 | Weighted mode | 23 | -0.019214799 | 0.019360020900616 | 0.331745728827366 | -0.05716044 | 0.0187308423325466 | 0.98096862889083 | 0.944442531230569 | 1.01890736497658 | 0.888599269773114 |
| ebi-a-GCST90001459 | finngen_R11_O15_PREECLAMPS | Pre-eclampsia \|\| id:finngen_R11_O15_PREECLAMPS | Myeloid Dendritic Cell %Dendritic Cell \|\| id:ebi-a-GCST90001459 | Inverse variance weighted | 26 | -0.044938318 | 0.0216457632586182 | 0.0378866296627101 | -0.087364014 | -0.002512622 | 0.956056451349825 | 0.916343472941345 | 0.997490531834811 | 0.781760498894217 |
| ebi-a-GCST90001459 | finngen_R11_O15_PREECLAMPS | Pre-eclampsia \|\| id:finngen_R11_O15_PREECLAMPS | Myeloid Dendritic Cell %Dendritic Cell \|\| id:ebi-a-GCST90001459 | MR Egger | 26 | -0.071827557 | 0.0365912487076628 | 0.0613403150163304 | -0.143546404 | -0.000108709 | 0.930691373253197 | 0.866280600026906 | 0.999891296447155 | 0.862303274556491 |
| ebi-a-GCST90001459 | finngen_R11_O15_PREECLAMPS | Pre-eclampsia \|\| id:finngen_R11_O15_PREECLAMPS | Myeloid Dendritic Cell %Dendritic Cell \|\| id:ebi-a-GCST90001459 | Weighted median | 26 | -0.075670362 | 0.0319726778516786 | 0.0179465264493511 | -0.13833681 | -0.013003913 | 0.927121771089355 | 0.870805346195126 | 0.98708027251277 | 0.598716529366782 |
| ebi-a-GCST90001459 | finngen_R11_O15_PREECLAMPS | Pre-eclampsia \|\| id:finngen_R11_O15_PREECLAMPS | Myeloid Dendritic Cell %Dendritic Cell \|\| id:ebi-a-GCST90001459 | Weighted mode | 26 | -0.070738655 | 0.0332086382467787 | 0.043183844166026 | -0.135827586 | -0.005649724 | 0.931705357074486 | 0.872993135995821 | 0.994366205882116 | 0.838871423749761 |
| ebi-a-GCST90001460 | finngen_R11_O15_PREECLAMPS | Pre-eclampsia \|\| id:finngen_R11_O15_PREECLAMPS | Plasmacytoid Dendritic Cell Absolute Count \|\| id:ebi-a-GCST90001460 | Inverse variance weighted | 31 | 0.00857988389373298 | 0.0136777503764059 | 0.530470697240884 | -0.018228507 | 0.0353882746314885 | 1.00861679659091 | 0.981936627480476 | 1.03602189173408 | 0.904087927705763 |
| ebi-a-GCST90001460 | finngen_R11_O15_PREECLAMPS | Pre-eclampsia \|\| id:finngen_R11_O15_PREECLAMPS | Plasmacytoid Dendritic Cell Absolute Count \|\| id:ebi-a-GCST90001460 | MR Egger | 31 | 0.00279766175930117 | 0.0181130694967625 | 0.87832059452633 | -0.032703954 | 0.0382992779729557 | 1.00280157886702 | 0.967825037472998 | 1.03904214877914 | 0.99380795269851 |
| ebi-a-GCST90001460 | finngen_R11_O15_PREECLAMPS | Pre-eclampsia \|\| id:finngen_R11_O15_PREECLAMPS | Plasmacytoid Dendritic Cell Absolute Count \|\| id:ebi-a-GCST90001460 | Weighted median | 31 | 0.0197249994842355 | 0.0189395851001965 | 0.297657719570532 | -0.017396587 | 0.0568465862806206 | 1.01992082270504 | 0.982753859628499 | 1.05849341052759 | 0.940845681141708 |
| ebi-a-GCST90001460 | finngen_R11_O15_PREECLAMPS | Pre-eclampsia \|\| id:finngen_R11_O15_PREECLAMPS | Plasmacytoid Dendritic Cell Absolute Count \|\| id:ebi-a-GCST90001460 | Weighted mode | 31 | 0.023495530184996 | 0.0162442608830192 | 0.158435924339058 | -0.008343221 | 0.0553342815157137 | 1.02377372465725 | 0.991691486931028 | 1.05689385571127 | 0.872755742358858 |
| ebi-a-GCST90001461 | finngen_R11_O15_PREECLAMPS | Pre-eclampsia \|\| id:finngen_R11_O15_PREECLAMPS | Dendritic Cell Absolute Count \|\| id:ebi-a-GCST90001461 | Inverse variance weighted | 21 | -0.00691487 | 0.0269389794711689 | 0.79742085993732 | -0.05971527 | 0.0458855297138564 | 0.993108982653154 | 0.942032720554539 | 1.04695455890938 | 0.977824840984872 |
| ebi-a-GCST90001461 | finngen_R11_O15_PREECLAMPS | Pre-eclampsia \|\| id:finngen_R11_O15_PREECLAMPS | Dendritic Cell Absolute Count \|\| id:ebi-a-GCST90001461 | MR Egger | 21 | -0.074513347 | 0.0443187839376169 | 0.109068702867153 | -0.161378163 | 0.0123514698202281 | 0.928195085637281 | 0.850970204623309 | 1.012428064 | 0.97261503632431 |
| ebi-a-GCST90001461 | finngen_R11_O15_PREECLAMPS | Pre-eclampsia \|\| id:finngen_R11_O15_PREECLAMPS | Dendritic Cell Absolute Count \|\| id:ebi-a-GCST90001461 | Weighted median | 21 | -0.063041843 | 0.0374693134594415 | 0.0924733589486774 | -0.136481697 | 0.0103980118120413 | 0.938904186704491 | 0.872422288103151 | 1.01045225899478 | 0.88227178111339 |
| ebi-a-GCST90001461 | finngen_R11_O15_PREECLAMPS | Pre-eclampsia \|\| id:finngen_R11_O15_PREECLAMPS | Dendritic Cell Absolute Count \|\| id:ebi-a-GCST90001461 | Weighted mode | 21 | -0.054724452 | 0.0384585452238759 | 0.170163592995214 | -0.1301032 | 0.020654297111347 | 0.946745986425465 | 0.878004816001928 | 1.02086907324066 | 0.872755742358858 |
| ebi-a-GCST90001462 | finngen_R11_O15_PREECLAMPS | Pre-eclampsia \|\| id:finngen_R11_O15_PREECLAMPS | CD62L- Dendritic Cell Absolute Count \|\| id:ebi-a-GCST90001462 | Inverse variance weighted | 17 | 0.0163091930586311 | 0.0173842087500594 | 0.348161488386308 | -0.017763856 | 0.0503822422087475 | 1.01644291391843 | 0.982392991090015 | 1.05167301337182 | 0.885079385681701 |
| ebi-a-GCST90001462 | finngen_R11_O15_PREECLAMPS | Pre-eclampsia \|\| id:finngen_R11_O15_PREECLAMPS | CD62L- Dendritic Cell Absolute Count \|\| id:ebi-a-GCST90001462 | MR Egger | 17 | 0.0138245786920485 | 0.0219646402757005 | 0.538555033065915 | -0.029226116 | 0.0568752736324214 | 1.01392058006276 | 0.971196836251789 | 1.05852377633599 | 0.99380795269851 |
| ebi-a-GCST90001462 | finngen_R11_O15_PREECLAMPS | Pre-eclampsia \|\| id:finngen_R11_O15_PREECLAMPS | CD62L- Dendritic Cell Absolute Count \|\| id:ebi-a-GCST90001462 | Weighted median | 17 | 0.0361539277589385 | 0.0230586404329257 | 0.116901582602784 | -0.009041007 | 0.0813488630074729 | 1.03681542888393 | 0.990999739528124 | 1.08474925945352 | 0.88227178111339 |
| ebi-a-GCST90001462 | finngen_R11_O15_PREECLAMPS | Pre-eclampsia \|\| id:finngen_R11_O15_PREECLAMPS | CD62L- Dendritic Cell Absolute Count \|\| id:ebi-a-GCST90001462 | Weighted mode | 17 | 0.029423626417753 | 0.0187586087438361 | 0.136317884275621 | -0.007343247 | 0.0661904995556717 | 1.02986077831141 | 0.992683649041693 | 1.06843023326522 | 0.872755742358858 |
| ebi-a-GCST90001463 | finngen_R11_O15_PREECLAMPS | Pre-eclampsia \|\| id:finngen_R11_O15_PREECLAMPS | CD62L- Dendritic Cell %Dendritic Cell \|\| id:ebi-a-GCST90001463 | Inverse variance weighted | 23 | -0.005779743 | 0.0121621186859585 | 0.634626627420676 | -0.029617495 | 0.0180580100729158 | 0.994236928027737 | 0.970816804648446 | 1.01822204181147 | 0.942541573457101 |
| ebi-a-GCST90001463 | finngen_R11_O15_PREECLAMPS | Pre-eclampsia \|\| id:finngen_R11_O15_PREECLAMPS | CD62L- Dendritic Cell %Dendritic Cell \|\| id:ebi-a-GCST90001463 | MR Egger | 23 | -0.006651958 | 0.0151086442884532 | 0.664236400700769 | -0.0362649 | 0.0229609852224708 | 0.993370117711849 | 0.964384793737328 | 1.0232266178096 | 0.99380795269851 |
| ebi-a-GCST90001463 | finngen_R11_O15_PREECLAMPS | Pre-eclampsia \|\| id:finngen_R11_O15_PREECLAMPS | CD62L- Dendritic Cell %Dendritic Cell \|\| id:ebi-a-GCST90001463 | Weighted median | 23 | -0.008849187 | 0.0181175572563602 | 0.625244167191431 | -0.0443596 | 0.0266612248356109 | 0.991189851433037 | 0.956609899069143 | 1.02701981501535 | 0.940845681141708 |
| ebi-a-GCST90001463 | finngen_R11_O15_PREECLAMPS | Pre-eclampsia \|\| id:finngen_R11_O15_PREECLAMPS | CD62L- Dendritic Cell %Dendritic Cell \|\| id:ebi-a-GCST90001463 | Weighted mode | 23 | -0.005220238 | 0.0142750056926593 | 0.718088509838703 | -0.033199249 | 0.0227587728658685 | 0.994793363473723 | 0.967345797268334 | 1.02301972966225 | 0.959313411571287 |
| ebi-a-GCST90001464 | finngen_R11_O15_PREECLAMPS | Pre-eclampsia \|\| id:finngen_R11_O15_PREECLAMPS | CD86+ myeloid Dendritic Cell Absolute Count \|\| id:ebi-a-GCST90001464 | Inverse variance weighted | 21 | -0.004850883 | 0.0155779485448188 | 0.755500960465126 | -0.035383662 | 0.0256818962790505 | 0.995160863662151 | 0.965235021194089 | 1.02601451752097 | 0.970051434846584 |
| ebi-a-GCST90001464 | finngen_R11_O15_PREECLAMPS | Pre-eclampsia \|\| id:finngen_R11_O15_PREECLAMPS | CD86+ myeloid Dendritic Cell Absolute Count \|\| id:ebi-a-GCST90001464 | MR Egger | 21 | -0.01056613 | 0.020014025007393 | 0.603655352498864 | -0.049793619 | 0.0286613588709234 | 0.989489495321852 | 0.951425760289499 | 1.02907604798758 | 0.99380795269851 |
| ebi-a-GCST90001464 | finngen_R11_O15_PREECLAMPS | Pre-eclampsia \|\| id:finngen_R11_O15_PREECLAMPS | CD86+ myeloid Dendritic Cell Absolute Count \|\| id:ebi-a-GCST90001464 | Weighted median | 21 | -0.015703647 | 0.0196615087438953 | 0.424464391511263 | -0.054240204 | 0.0228329102643212 | 0.984419012483094 | 0.947204556839223 | 1.02309557649508 | 0.940845681141708 |
| ebi-a-GCST90001464 | finngen_R11_O15_PREECLAMPS | Pre-eclampsia \|\| id:finngen_R11_O15_PREECLAMPS | CD86+ myeloid Dendritic Cell Absolute Count \|\| id:ebi-a-GCST90001464 | Weighted mode | 21 | -0.015382286 | 0.0183137230584044 | 0.410875083582729 | -0.051277184 | 0.0205126108153674 | 0.984735416701033 | 0.950015305396565 | 1.02072444032949 | 0.930105377419646 |
| ebi-a-GCST90001465 | finngen_R11_O15_PREECLAMPS | Pre-eclampsia \|\| id:finngen_R11_O15_PREECLAMPS | CD86+ myeloid Dendritic Cell %Dendritic Cell \|\| id:ebi-a-GCST90001465 | Inverse variance weighted | 23 | -0.012103205 | 0.00922992853439879 | 0.189756426446445 | -0.030193865 | 0.00598745458694495 | 0.987969743846353 | 0.970257416100475 | 1.00600541522143 | 0.855687495048373 |
| ebi-a-GCST90001465 | finngen_R11_O15_PREECLAMPS | Pre-eclampsia \|\| id:finngen_R11_O15_PREECLAMPS | CD86+ myeloid Dendritic Cell %Dendritic Cell \|\| id:ebi-a-GCST90001465 | MR Egger | 23 | -0.008869807 | 0.0115722395421768 | 0.451925765158501 | -0.031551397 | 0.0138117820841603 | 0.991169413277646 | 0.968941154581776 | 1.01390760540169 | 0.99380795269851 |
| ebi-a-GCST90001465 | finngen_R11_O15_PREECLAMPS | Pre-eclampsia \|\| id:finngen_R11_O15_PREECLAMPS | CD86+ myeloid Dendritic Cell %Dendritic Cell \|\| id:ebi-a-GCST90001465 | Weighted median | 23 | -0.007187294 | 0.0122026985829841 | 0.555866516791185 | -0.031104583 | 0.0167299951340663 | 0.992838472741358 | 0.969374187412152 | 1.0168707252118 | 0.940845681141708 |
| ebi-a-GCST90001465 | finngen_R11_O15_PREECLAMPS | Pre-eclampsia \|\| id:finngen_R11_O15_PREECLAMPS | CD86+ myeloid Dendritic Cell %Dendritic Cell \|\| id:ebi-a-GCST90001465 | Weighted mode | 23 | -0.008074714 | 0.00998213009360217 | 0.427224904457403 | -0.027639689 | 0.0114902604931253 | 0.991957798446966 | 0.972738791692462 | 1.01155652709987 | 0.933945646747087 |
| ebi-a-GCST90001466 | finngen_R11_O15_PREECLAMPS | Pre-eclampsia \|\| id:finngen_R11_O15_PREECLAMPS | CD86+ plasmacytoid Dendritic Cell Absolute Count \|\| id:ebi-a-GCST90001466 | Inverse variance weighted | 20 | 0.0291726344087515 | 0.0266893845136622 | 0.274375093409005 | -0.023138559 | 0.0814838280555294 | 1.02960232392202 | 0.977127084408807 | 1.08489567256956 | 0.885079385681701 |
| ebi-a-GCST90001466 | finngen_R11_O15_PREECLAMPS | Pre-eclampsia \|\| id:finngen_R11_O15_PREECLAMPS | CD86+ plasmacytoid Dendritic Cell Absolute Count \|\| id:ebi-a-GCST90001466 | MR Egger | 20 | -0.057387548 | 0.0433138354285853 | 0.201770912359272 | -0.142282665 | 0.0275075698666759 | 0.944228065153741 | 0.867376044969833 | 1.02788939606349 | 0.99380795269851 |
| ebi-a-GCST90001466 | finngen_R11_O15_PREECLAMPS | Pre-eclampsia \|\| id:finngen_R11_O15_PREECLAMPS | CD86+ plasmacytoid Dendritic Cell Absolute Count \|\| id:ebi-a-GCST90001466 | Weighted median | 20 | -0.004320006 | 0.0351631647852277 | 0.902221121038012 | -0.073239809 | 0.0645997968745705 | 0.99568931169941 | 0.929377930030493 | 1.06673202945536 | 0.967043459646315 |
| ebi-a-GCST90001466 | finngen_R11_O15_PREECLAMPS | Pre-eclampsia \|\| id:finngen_R11_O15_PREECLAMPS | CD86+ plasmacytoid Dendritic Cell Absolute Count \|\| id:ebi-a-GCST90001466 | Weighted mode | 20 | -0.01579583 | 0.0353137198440028 | 0.659715936798175 | -0.085010721 | 0.0534190604093742 | 0.984328269366209 | 0.918502436735823 | 1.05487160743608 | 0.959313411571287 |
| ebi-a-GCST90001467 | finngen_R11_O15_PREECLAMPS | Pre-eclampsia \|\| id:finngen_R11_O15_PREECLAMPS | CD86+ plasmacytoid Dendritic Cell %Dendritic Cell \|\| id:ebi-a-GCST90001467 | Inverse variance weighted | 26 | 0.012181603398845 | 0.0266372404847496 | 0.647444817879864 | -0.040027388 | 0.0643905947489541 | 1.01225610132359 | 0.960763125458329 | 1.06650889018875 | 0.942541573457101 |
| ebi-a-GCST90001467 | finngen_R11_O15_PREECLAMPS | Pre-eclampsia \|\| id:finngen_R11_O15_PREECLAMPS | CD86+ plasmacytoid Dendritic Cell %Dendritic Cell \|\| id:ebi-a-GCST90001467 | MR Egger | 26 | 0.0409361982761469 | 0.0473875132959748 | 0.396216257801795 | -0.051943328 | 0.133815724336258 | 1.04178563570561 | 0.949382668938048 | 1.14318213958609 | 0.99380795269851 |
| ebi-a-GCST90001467 | finngen_R11_O15_PREECLAMPS | Pre-eclampsia \|\| id:finngen_R11_O15_PREECLAMPS | CD86+ plasmacytoid Dendritic Cell %Dendritic Cell \|\| id:ebi-a-GCST90001467 | Weighted median | 26 | -0.005460506 | 0.0356141116848626 | 0.878142609919321 | -0.075264164 | 0.0643431533788314 | 0.994554375937699 | 0.927498441870608 | 1.06645829474592 | 0.964912412213677 |
| ebi-a-GCST90001467 | finngen_R11_O15_PREECLAMPS | Pre-eclampsia \|\| id:finngen_R11_O15_PREECLAMPS | CD86+ plasmacytoid Dendritic Cell %Dendritic Cell \|\| id:ebi-a-GCST90001467 | Weighted mode | 26 | -0.015440001 | 0.0499027374693249 | 0.759579927423534 | -0.113249367 | 0.0823693640393775 | 0.984678584315447 | 0.892927965725375 | 1.08585681222541 | 0.959313411571287 |
| ebi-a-GCST90001468 | finngen_R11_O15_PREECLAMPS | Pre-eclampsia \|\| id:finngen_R11_O15_PREECLAMPS | CD62L- myeloid Dendritic Cell Absolute Count \|\| id:ebi-a-GCST90001468 | Inverse variance weighted | 15 | -0.014366405 | 0.0263816483100983 | 0.586055798079857 | -0.066074435 | 0.0373416259535445 | 0.985736299639125 | 0.936061185588964 | 1.03804758426647 | 0.915399120505076 |
| ebi-a-GCST90001468 | finngen_R11_O15_PREECLAMPS | Pre-eclampsia \|\| id:finngen_R11_O15_PREECLAMPS | CD62L- myeloid Dendritic Cell Absolute Count \|\| id:ebi-a-GCST90001468 | MR Egger | 15 | -0.080269198 | 0.038439289593517 | 0.0570191836683915 | -0.155610206 | -0.004928191 | 0.922867878570403 | 0.855892747381172 | 0.995083933007949 | 0.862303274556491 |
| ebi-a-GCST90001468 | finngen_R11_O15_PREECLAMPS | Pre-eclampsia \|\| id:finngen_R11_O15_PREECLAMPS | CD62L- myeloid Dendritic Cell Absolute Count \|\| id:ebi-a-GCST90001468 | Weighted median | 15 | -0.05517691 | 0.029384096901092 | 0.0604110772233888 | -0.11276974 | 0.00241591962292692 | 0.946317719789293 | 0.893356340461212 | 1.00241884030831 | 0.771214851704188 |
| ebi-a-GCST90001468 | finngen_R11_O15_PREECLAMPS | Pre-eclampsia \|\| id:finngen_R11_O15_PREECLAMPS | CD62L- myeloid Dendritic Cell Absolute Count \|\| id:ebi-a-GCST90001468 | Weighted mode | 15 | -0.05099981 | 0.0302294213017323 | 0.113730054413924 | -0.110249476 | 0.00824985555975864 | 0.950278850903283 | 0.89561067410608 | 1.00828397939249 | 0.872755742358858 |
| ebi-a-GCST90001469 | finngen_R11_O15_PREECLAMPS | Pre-eclampsia \|\| id:finngen_R11_O15_PREECLAMPS | CD62L- myeloid Dendritic Cell %Dendritic Cell \|\| id:ebi-a-GCST90001469 | Inverse variance weighted | 24 | -0.015123362 | 0.0140874569597322 | 0.28303164613609 | -0.042734778 | 0.0124880533166918 | 0.984990421399953 | 0.958165482968844 | 1.01256635465881 | 0.885079385681701 |
| ebi-a-GCST90001469 | finngen_R11_O15_PREECLAMPS | Pre-eclampsia \|\| id:finngen_R11_O15_PREECLAMPS | CD62L- myeloid Dendritic Cell %Dendritic Cell \|\| id:ebi-a-GCST90001469 | MR Egger | 24 | -0.034899545 | 0.0197392810537146 | 0.0909240589305876 | -0.073588536 | 0.00378944564780153 | 0.965702420811862 | 0.929053887358294 | 1.0037966346749 | 0.936133620820556 |
| ebi-a-GCST90001469 | finngen_R11_O15_PREECLAMPS | Pre-eclampsia \|\| id:finngen_R11_O15_PREECLAMPS | CD62L- myeloid Dendritic Cell %Dendritic Cell \|\| id:ebi-a-GCST90001469 | Weighted median | 24 | -0.020349605 | 0.0201930411739765 | 0.313572875780475 | -0.059927966 | 0.0192287553723439 | 0.979856050522297 | 0.94183237506616 | 1.01941481856335 | 0.940845681141708 |
| ebi-a-GCST90001469 | finngen_R11_O15_PREECLAMPS | Pre-eclampsia \|\| id:finngen_R11_O15_PREECLAMPS | CD62L- myeloid Dendritic Cell %Dendritic Cell \|\| id:ebi-a-GCST90001469 | Weighted mode | 24 | -0.028195261 | 0.0168569135209959 | 0.107951470542241 | -0.061234811 | 0.00484428987294688 | 0.972198516173666 | 0.940602349943263 | 1.00485604241504 | 0.872755742358858 |
| ebi-a-GCST90001470 | finngen_R11_O15_PREECLAMPS | Pre-eclampsia \|\| id:finngen_R11_O15_PREECLAMPS | CD62L- plasmacytoid Dendritic Cell Absolute Count \|\| id:ebi-a-GCST90001470 | Inverse variance weighted | 25 | 0.000673822969408942 | 0.0210463992094292 | 0.974459236253206 | -0.040577119 | 0.0419247654198901 | 1.0006740500391 | 0.960235108822582 | 1.04281602 | 0.994537505425743 |
| ebi-a-GCST90001470 | finngen_R11_O15_PREECLAMPS | Pre-eclampsia \|\| id:finngen_R11_O15_PREECLAMPS | CD62L- plasmacytoid Dendritic Cell Absolute Count \|\| id:ebi-a-GCST90001470 | MR Egger | 25 | 0.0278544632689866 | 0.0345944089053144 | 0.428965856990999 | -0.039950578 | 0.0956595047234029 | 1.02824602596599 | 0.960836924283217 | 1.10038432453415 | 0.99380795269851 |
| ebi-a-GCST90001470 | finngen_R11_O15_PREECLAMPS | Pre-eclampsia \|\| id:finngen_R11_O15_PREECLAMPS | CD62L- plasmacytoid Dendritic Cell Absolute Count \|\| id:ebi-a-GCST90001470 | Weighted median | 25 | 0.023070117079599 | 0.0319955444182052 | 0.470883948431813 | -0.03964115 | 0.0857813841392813 | 1.02333829052408 | 0.96113428033113 | 1.08956810539725 | 0.940845681141708 |
| ebi-a-GCST90001470 | finngen_R11_O15_PREECLAMPS | Pre-eclampsia \|\| id:finngen_R11_O15_PREECLAMPS | CD62L- plasmacytoid Dendritic Cell Absolute Count \|\| id:ebi-a-GCST90001470 | Weighted mode | 25 | 0.0202390887791593 | 0.0344454285389474 | 0.562309198036679 | -0.047273951 | 0.0877521287154962 | 1.02044528788107 | 0.953826060042249 | 1.09171748307293 | 0.939633861977944 |
| ebi-a-GCST90001471 | finngen_R11_O15_PREECLAMPS | Pre-eclampsia \|\| id:finngen_R11_O15_PREECLAMPS | CD62L- plasmacytoid Dendritic Cell %Dendritic Cell \|\| id:ebi-a-GCST90001471 | Inverse variance weighted | 18 | -0.01250068 | 0.0175620652761345 | 0.476588550676825 | -0.046922328 | 0.0219209675513225 | 0.987577128556148 | 0.95416150602882 | 1.02216299723346 | 0.898694924660397 |
| ebi-a-GCST90001471 | finngen_R11_O15_PREECLAMPS | Pre-eclampsia \|\| id:finngen_R11_O15_PREECLAMPS | CD62L- plasmacytoid Dendritic Cell %Dendritic Cell \|\| id:ebi-a-GCST90001471 | MR Egger | 18 | -0.023617336 | 0.024250474549311 | 0.344605197456878 | -0.071148266 | 0.0239135945890636 | 0.97665937110286 | 0.931323798567593 | 1.0242018174883 | 0.99380795269851 |
| ebi-a-GCST90001471 | finngen_R11_O15_PREECLAMPS | Pre-eclampsia \|\| id:finngen_R11_O15_PREECLAMPS | CD62L- plasmacytoid Dendritic Cell %Dendritic Cell \|\| id:ebi-a-GCST90001471 | Weighted median | 18 | -0.025456787 | 0.0249534971018302 | 0.307648259812157 | -0.074365642 | 0.0234520671224067 | 0.974864504683166 | 0.928332194985792 | 1.02372922928273 | 0.940845681141708 |
| ebi-a-GCST90001471 | finngen_R11_O15_PREECLAMPS | Pre-eclampsia \|\| id:finngen_R11_O15_PREECLAMPS | CD62L- plasmacytoid Dendritic Cell %Dendritic Cell \|\| id:ebi-a-GCST90001471 | Weighted mode | 18 | -0.02365387 | 0.022170837470892 | 0.300938760046228 | -0.067108712 | 0.0198009709740613 | 0.976623689561881 | 0.935093540004499 | 1.01999831055284 | 0.875408265683677 |
| ebi-a-GCST90001472 | finngen_R11_O15_PREECLAMPS | Pre-eclampsia \|\| id:finngen_R11_O15_PREECLAMPS | CD62L- CD86+ myeloid Dendritic Cell Absolute Count \|\| id:ebi-a-GCST90001472 | Inverse variance weighted | 19 | -0.025812714 | 0.0185558101694512 | 0.16419957176294 | -0.062182102 | 0.0105566737767072 | 0.974517585867863 | 0.939711747738222 | 1.01061259205443 | 0.85505992597664 |
| ebi-a-GCST90001472 | finngen_R11_O15_PREECLAMPS | Pre-eclampsia \|\| id:finngen_R11_O15_PREECLAMPS | CD62L- CD86+ myeloid Dendritic Cell Absolute Count \|\| id:ebi-a-GCST90001472 | MR Egger | 19 | -0.056467705 | 0.023647396741043 | 0.0288226209312563 | -0.102816602 | -0.010118807 | 0.945097006255039 | 0.902292436713276 | 0.989932215863264 | 0.839403552053506 |
| ebi-a-GCST90001472 | finngen_R11_O15_PREECLAMPS | Pre-eclampsia \|\| id:finngen_R11_O15_PREECLAMPS | CD62L- CD86+ myeloid Dendritic Cell Absolute Count \|\| id:ebi-a-GCST90001472 | Weighted median | 19 | -0.047846687 | 0.0222532364401612 | 0.0315472926321623 | -0.091463031 | -0.004230344 | 0.953279925769431 | 0.91259505348441 | 0.995778591397434 | 0.721097500353414 |
| ebi-a-GCST90001472 | finngen_R11_O15_PREECLAMPS | Pre-eclampsia \|\| id:finngen_R11_O15_PREECLAMPS | CD62L- CD86+ myeloid Dendritic Cell Absolute Count \|\| id:ebi-a-GCST90001472 | Weighted mode | 19 | -0.042810609 | 0.0218368045600524 | 0.0656039116963595 | -0.085610746 | -1.05E-05 | 0.958092826936765 | 0.91795147792499 | 0.999989527880788 | 0.871935626364341 |
| ebi-a-GCST90001473 | finngen_R11_O15_PREECLAMPS | Pre-eclampsia \|\| id:finngen_R11_O15_PREECLAMPS | CD62L- CD86+ myeloid Dendritic Cell %Dendritic Cell \|\| id:ebi-a-GCST90001473 | Inverse variance weighted | 18 | -0.010507912 | 0.0195730247563857 | 0.591366510515089 | -0.048871041 | 0.02785521643703 | 0.989547103155653 | 0.952303930316039 | 1.02824680040833 | 0.91586635420875 |
| ebi-a-GCST90001473 | finngen_R11_O15_PREECLAMPS | Pre-eclampsia \|\| id:finngen_R11_O15_PREECLAMPS | CD62L- CD86+ myeloid Dendritic Cell %Dendritic Cell \|\| id:ebi-a-GCST90001473 | MR Egger | 18 | -0.043687957 | 0.0233761677767959 | 0.080054077384969 | -0.089505246 | 0.00212933173338762 | 0.95725261474688 | 0.914383468300256 | 1.00213160037014 | 0.928881437593846 |
| ebi-a-GCST90001473 | finngen_R11_O15_PREECLAMPS | Pre-eclampsia \|\| id:finngen_R11_O15_PREECLAMPS | CD62L- CD86+ myeloid Dendritic Cell %Dendritic Cell \|\| id:ebi-a-GCST90001473 | Weighted median | 18 | -0.042409922 | 0.0237866114818478 | 0.0745972935566716 | -0.089031681 | 0.00421183607727267 | 0.958476798896217 | 0.91481659087354 | 1.00422071832466 | 0.86014060819805 |
| ebi-a-GCST90001473 | finngen_R11_O15_PREECLAMPS | Pre-eclampsia \|\| id:finngen_R11_O15_PREECLAMPS | CD62L- CD86+ myeloid Dendritic Cell %Dendritic Cell \|\| id:ebi-a-GCST90001473 | Weighted mode | 18 | -0.033899964 | 0.0190588282314052 | 0.093175866645324 | -0.071255267 | 0.00345533920732633 | 0.966668201299023 | 0.931224150561639 | 1.00346131577355 | 0.872755742358858 |
| ebi-a-GCST90001474 | finngen_R11_O15_PREECLAMPS | Pre-eclampsia \|\| id:finngen_R11_O15_PREECLAMPS | Plasmacytoid Dendritic Cell %Dendritic Cell \|\| id:ebi-a-GCST90001474 | Inverse variance weighted | 24 | -0.02851457 | 0.0188447656204113 | 0.130246776909019 | -0.065450311 | 0.00842117052646291 | 0.971888133545844 | 0.936645586861707 | 1.00845672832566 | 0.820779257935285 |
| ebi-a-GCST90001474 | finngen_R11_O15_PREECLAMPS | Pre-eclampsia \|\| id:finngen_R11_O15_PREECLAMPS | Plasmacytoid Dendritic Cell %Dendritic Cell \|\| id:ebi-a-GCST90001474 | MR Egger | 24 | -0.068209726 | 0.0259045863919226 | 0.0151870090939671 | -0.118982715 | -0.017436736 | 0.934064555717656 | 0.887823146653087 | 0.982714403805626 | 0.693856477980621 |
| ebi-a-GCST90001474 | finngen_R11_O15_PREECLAMPS | Pre-eclampsia \|\| id:finngen_R11_O15_PREECLAMPS | Plasmacytoid Dendritic Cell %Dendritic Cell \|\| id:ebi-a-GCST90001474 | Weighted median | 24 | -0.074503946 | 0.0275991618203109 | 0.00694436920914081 | -0.128598303 | -0.020409589 | 0.928203811586009 | 0.879327117742954 | 0.979797277325238 | 0.507633389188193 |
| ebi-a-GCST90001474 | finngen_R11_O15_PREECLAMPS | Pre-eclampsia \|\| id:finngen_R11_O15_PREECLAMPS | Plasmacytoid Dendritic Cell %Dendritic Cell \|\| id:ebi-a-GCST90001474 | Weighted mode | 24 | -0.084670273 | 0.0367629563610449 | 0.0306535967123585 | -0.156725667 | -0.012614878 | 0.918815192917188 | 0.854938564371798 | 0.98746435582278 | 0.838871423749761 |
| ebi-a-GCST90001475 | finngen_R11_O15_PREECLAMPS | Pre-eclampsia \|\| id:finngen_R11_O15_PREECLAMPS | HLA DR++ monocyte %monocyte \|\| id:ebi-a-GCST90001475 | Inverse variance weighted | 21 | 0.0362728072253498 | 0.0227338198105714 | 0.110590664140321 | -0.00828548 | 0.0808310940540698 | 1.03693869227548 | 0.991748750380244 | 1.08418775534199 | 0.820779257935285 |
| ebi-a-GCST90001475 | finngen_R11_O15_PREECLAMPS | Pre-eclampsia \|\| id:finngen_R11_O15_PREECLAMPS | HLA DR++ monocyte %monocyte \|\| id:ebi-a-GCST90001475 | MR Egger | 21 | -0.001741911 | 0.0408090599069977 | 0.966398489945162 | -0.081727668 | 0.0782438466955444 | 0.998259605523795 | 0.921522884568535 | 1.08138631900292 | 0.99380795269851 |
| ebi-a-GCST90001475 | finngen_R11_O15_PREECLAMPS | Pre-eclampsia \|\| id:finngen_R11_O15_PREECLAMPS | HLA DR++ monocyte %monocyte \|\| id:ebi-a-GCST90001475 | Weighted median | 21 | 0.0160612035468297 | 0.0309371402524199 | 0.603651924286659 | -0.044575591 | 0.0766979984415726 | 1.01619087798886 | 0.95640330154637 | 1.07971595124999 | 0.940845681141708 |
| ebi-a-GCST90001475 | finngen_R11_O15_PREECLAMPS | Pre-eclampsia \|\| id:finngen_R11_O15_PREECLAMPS | HLA DR++ monocyte %monocyte \|\| id:ebi-a-GCST90001475 | Weighted mode | 21 | 0.00575828399366052 | 0.0330475436932082 | 0.863426627359207 | -0.059014902 | 0.0705314696323486 | 1.00577489477884 | 0.942692721380128 | 1.07307833828015 | 0.977585971930092 |
| ebi-a-GCST90001476 | finngen_R11_O15_PREECLAMPS | Pre-eclampsia \|\| id:finngen_R11_O15_PREECLAMPS | HLA DR++ monocyte %leukocyte \|\| id:ebi-a-GCST90001476 | Inverse variance weighted | 8 | 0.0722073972609298 | 0.0341403163043888 | 0.0344283788786573 | 0.00529237730432768 | 0.139122417217532 | 1.07487824775262 | 1.00530640667174 | 1.14926478118925 | 0.781760498894217 |
| ebi-a-GCST90001476 | finngen_R11_O15_PREECLAMPS | Pre-eclampsia \|\| id:finngen_R11_O15_PREECLAMPS | HLA DR++ monocyte %leukocyte \|\| id:ebi-a-GCST90001476 | MR Egger | 8 | -0.000702064 | 0.062615960951825 | 0.991417635777393 | -0.123429348 | 0.122025219051297 | 0.999298181975277 | 0.883884087322809 | 1.12978259346623 | 0.995857943572658 |
| ebi-a-GCST90001476 | finngen_R11_O15_PREECLAMPS | Pre-eclampsia \|\| id:finngen_R11_O15_PREECLAMPS | HLA DR++ monocyte %leukocyte \|\| id:ebi-a-GCST90001476 | Weighted median | 8 | 0.0504432586466168 | 0.0461768667578953 | 0.274660658806194 | -0.0400634 | 0.140949917492092 | 1.05173718467063 | 0.960728526841767 | 1.15136698319472 | 0.940845681141708 |
| ebi-a-GCST90001476 | finngen_R11_O15_PREECLAMPS | Pre-eclampsia \|\| id:finngen_R11_O15_PREECLAMPS | HLA DR++ monocyte %leukocyte \|\| id:ebi-a-GCST90001476 | Weighted mode | 8 | 0.0483088401740448 | 0.0505790082231661 | 0.371331054300215 | -0.050826016 | 0.14744369629145 | 1.04949473141355 | 0.95044401825398 | 1.15886803442479 | 0.919136986043459 |
| ebi-a-GCST90001477 | finngen_R11_O15_PREECLAMPS | Pre-eclampsia \|\| id:finngen_R11_O15_PREECLAMPS | HLA DR++ monocyte Absolute Count \|\| id:ebi-a-GCST90001477 | Inverse variance weighted | 23 | -0.007650058 | 0.0166903900297888 | 0.646700256660963 | -0.040363222 | 0.0250631067425135 | 0.992379129500269 | 0.960440522494199 | 1.02537982686342 | 0.942541573457101 |
| ebi-a-GCST90001477 | finngen_R11_O15_PREECLAMPS | Pre-eclampsia \|\| id:finngen_R11_O15_PREECLAMPS | HLA DR++ monocyte Absolute Count \|\| id:ebi-a-GCST90001477 | MR Egger | 23 | -0.020283215 | 0.0225240001099404 | 0.378057442659188 | -0.064430255 | 0.0238638254898879 | 0.979921105915907 | 0.937601505053896 | 1.0241508451549 | 0.99380795269851 |
| ebi-a-GCST90001477 | finngen_R11_O15_PREECLAMPS | Pre-eclampsia \|\| id:finngen_R11_O15_PREECLAMPS | HLA DR++ monocyte Absolute Count \|\| id:ebi-a-GCST90001477 | Weighted median | 23 | -0.016910048 | 0.0245461016642351 | 0.490880074854935 | -0.065020408 | 0.0312003109480316 | 0.983232124044629 | 0.937048340297271 | 1.03169214242098 | 0.940845681141708 |
| ebi-a-GCST90001477 | finngen_R11_O15_PREECLAMPS | Pre-eclampsia \|\| id:finngen_R11_O15_PREECLAMPS | HLA DR++ monocyte Absolute Count \|\| id:ebi-a-GCST90001477 | Weighted mode | 23 | -0.027318843 | 0.023738143091146 | 0.262152727329121 | -0.073845603 | 0.0192079177831268 | 0.973050941892853 | 0.928815088909795 | 1.01939357663743 | 0.872755742358858 |
| ebi-a-GCST90001478 | finngen_R11_O15_PREECLAMPS | Pre-eclampsia \|\| id:finngen_R11_O15_PREECLAMPS | CD4 regulatory T cell %CD4+ T cell \|\| id:ebi-a-GCST90001478 | Inverse variance weighted | 15 | -0.040663735 | 0.0198284131823894 | 0.0402882684909956 | -0.079527425 | -0.001800045 | 0.960151941080571 | 0.923552691260311 | 0.998201573862282 | 0.781760498894217 |
| ebi-a-GCST90001478 | finngen_R11_O15_PREECLAMPS | Pre-eclampsia \|\| id:finngen_R11_O15_PREECLAMPS | CD4 regulatory T cell %CD4+ T cell \|\| id:ebi-a-GCST90001478 | MR Egger | 15 | -0.053367476 | 0.0291060942137234 | 0.0897101347371977 | -0.110415421 | 0.00368046846932255 | 0.948031569435224 | 0.895462064408305 | 1.00368724971023 | 0.936133620820556 |
| ebi-a-GCST90001478 | finngen_R11_O15_PREECLAMPS | Pre-eclampsia \|\| id:finngen_R11_O15_PREECLAMPS | CD4 regulatory T cell %CD4+ T cell \|\| id:ebi-a-GCST90001478 | Weighted median | 15 | -0.046105486 | 0.0274336866421567 | 0.0928375758602271 | -0.099875511 | 0.0076645401501092 | 0.954941224264168 | 0.904950066912107 | 1.00769398792444 | 0.88227178111339 |
| ebi-a-GCST90001478 | finngen_R11_O15_PREECLAMPS | Pre-eclampsia \|\| id:finngen_R11_O15_PREECLAMPS | CD4 regulatory T cell %CD4+ T cell \|\| id:ebi-a-GCST90001478 | Weighted mode | 15 | -0.048057683 | 0.0242414052609986 | 0.0674059280775812 | -0.095570838 | -0.000544529 | 0.953078808698552 | 0.908853978268683 | 0.999455619175068 | 0.872755742358858 |
| ebi-a-GCST90001479 | finngen_R11_O15_PREECLAMPS | Pre-eclampsia \|\| id:finngen_R11_O15_PREECLAMPS | CD4 regulatory T cell %T cell \|\| id:ebi-a-GCST90001479 | Inverse variance weighted (multiplicative random effects) | 16 | -0.004318696 | 0.0315573310704719 | 0.891147476544488 | -0.066171065 | 0.0575336729031591 | 0.995690616162301 | 0.93597073886171 | 1.05922093709827 | 0.986383922906939 |
| ebi-a-GCST90001479 | finngen_R11_O15_PREECLAMPS | Pre-eclampsia \|\| id:finngen_R11_O15_PREECLAMPS | CD4 regulatory T cell %T cell \|\| id:ebi-a-GCST90001479 | MR Egger | 16 | -0.059951237 | 0.0445747288064804 | 0.200022105125184 | -0.147317705 | 0.0274152319145622 | 0.941810458395347 | 0.863019748161629 | 1.02779448724357 | 0.99380795269851 |
| ebi-a-GCST90001479 | finngen_R11_O15_PREECLAMPS | Pre-eclampsia \|\| id:finngen_R11_O15_PREECLAMPS | CD4 regulatory T cell %T cell \|\| id:ebi-a-GCST90001479 | Weighted median | 16 | -0.022974475 | 0.0364370310111645 | 0.528350865167873 | -0.094391056 | 0.0484421058565114 | 0.977287428789468 | 0.909926860564605 | 1.04963460236496 | 0.940845681141708 |
| ebi-a-GCST90001479 | finngen_R11_O15_PREECLAMPS | Pre-eclampsia \|\| id:finngen_R11_O15_PREECLAMPS | CD4 regulatory T cell %T cell \|\| id:ebi-a-GCST90001479 | Weighted mode | 16 | -0.019776284 | 0.0346049818442089 | 0.576133659034678 | -0.087602049 | 0.048049480011363 | 0.9804179835685 | 0.916125377368627 | 1.04922256958477 | 0.948158355498791 |
| ebi-a-GCST90001480 | finngen_R11_O15_PREECLAMPS | Pre-eclampsia \|\| id:finngen_R11_O15_PREECLAMPS | Resting CD4 regulatory T cell Absolute Count \|\| id:ebi-a-GCST90001480 | Inverse variance weighted | 25 | 0.0187612128872858 | 0.0214305701286335 | 0.381333665921935 | -0.023242705 | 0.0607651303394074 | 1.0189383102284 | 0.977025326488165 | 1.06264930079443 | 0.885079385681701 |
| ebi-a-GCST90001480 | finngen_R11_O15_PREECLAMPS | Pre-eclampsia \|\| id:finngen_R11_O15_PREECLAMPS | Resting CD4 regulatory T cell Absolute Count \|\| id:ebi-a-GCST90001480 | MR Egger | 25 | 0.0285251738604723 | 0.035884980170582 | 0.434794984497624 | -0.041809387 | 0.0988597349948131 | 1.0289359127976 | 0.959052570775881 | 1.10391144855399 | 0.99380795269851 |
| ebi-a-GCST90001480 | finngen_R11_O15_PREECLAMPS | Pre-eclampsia \|\| id:finngen_R11_O15_PREECLAMPS | Resting CD4 regulatory T cell Absolute Count \|\| id:ebi-a-GCST90001480 | Weighted median | 25 | 0.0206581003641254 | 0.0294022487889138 | 0.48230335865585 | -0.036970307 | 0.0782865079903965 | 1.02087295587119 | 0.963704749956738 | 1.08143245332759 | 0.940845681141708 |
| ebi-a-GCST90001480 | finngen_R11_O15_PREECLAMPS | Pre-eclampsia \|\| id:finngen_R11_O15_PREECLAMPS | Resting CD4 regulatory T cell Absolute Count \|\| id:ebi-a-GCST90001480 | Weighted mode | 25 | 0.0129661721011519 | 0.0286347693809057 | 0.654750967970068 | -0.043157976 | 0.069090320087727 | 1.01305059740704 | 0.957760075119399 | 1.07153298573112 | 0.959313411571287 |
| ebi-a-GCST90001481 | finngen_R11_O15_PREECLAMPS | Pre-eclampsia \|\| id:finngen_R11_O15_PREECLAMPS | Resting CD4 regulatory T cell %CD4 regulatory T cell \|\| id:ebi-a-GCST90001481 | Inverse variance weighted | 29 | 0.0179913964184679 | 0.00961418817142224 | 0.0612982357591991 | -0.000852412 | 0.0368352052344555 | 1.0181542165793 | 0.999147950802722 | 1.03752202854978 | 0.789997151112252 |
| ebi-a-GCST90001481 | finngen_R11_O15_PREECLAMPS | Pre-eclampsia \|\| id:finngen_R11_O15_PREECLAMPS | Resting CD4 regulatory T cell %CD4 regulatory T cell \|\| id:ebi-a-GCST90001481 | MR Egger | 29 | 0.018784998610906 | 0.0123877905510924 | 0.141036916516793 | -0.005495071 | 0.0430650680910472 | 1.01896254670167 | 0.994519999415963 | 1.04400582410659 | 0.97261503632431 |
| ebi-a-GCST90001481 | finngen_R11_O15_PREECLAMPS | Pre-eclampsia \|\| id:finngen_R11_O15_PREECLAMPS | Resting CD4 regulatory T cell %CD4 regulatory T cell \|\| id:ebi-a-GCST90001481 | Weighted median | 29 | 0.0175782129412204 | 0.0115161697694445 | 0.126911760159781 | -0.00499348 | 0.0401499056893315 | 1.01773361897769 | 0.995018966887343 | 1.04096680934394 | 0.88227178111339 |
| ebi-a-GCST90001481 | finngen_R11_O15_PREECLAMPS | Pre-eclampsia \|\| id:finngen_R11_O15_PREECLAMPS | Resting CD4 regulatory T cell %CD4 regulatory T cell \|\| id:ebi-a-GCST90001481 | Weighted mode | 29 | 0.0176443858842344 | 0.00994996650876626 | 0.0870561647758929 | -0.001857548 | 0.0371463202414163 | 1.01780096763477 | 0.998144175702472 | 1.03784486744033 | 0.872755742358858 |
| ebi-a-GCST90001482 | finngen_R11_O15_PREECLAMPS | Pre-eclampsia \|\| id:finngen_R11_O15_PREECLAMPS | Resting CD4 regulatory T cell %CD4+ T cell \|\| id:ebi-a-GCST90001482 | Inverse variance weighted | 32 | 0.0120273703362079 | 0.00900315668362495 | 0.181580024866744 | -0.005618817 | 0.0296735574361128 | 1.01209999000398 | 0.994396939263326 | 1.03011820463257 | 0.855687495048373 |
| ebi-a-GCST90001482 | finngen_R11_O15_PREECLAMPS | Pre-eclampsia \|\| id:finngen_R11_O15_PREECLAMPS | Resting CD4 regulatory T cell %CD4+ T cell \|\| id:ebi-a-GCST90001482 | MR Egger | 32 | 0.0225805581983193 | 0.0109682291831925 | 0.0482953855852326 | 0.00108282899926198 | 0.0440782873973765 | 1.02283742878606 | 1.00108341547025 | 1.0450641670397 | 0.862303274556491 |
| ebi-a-GCST90001482 | finngen_R11_O15_PREECLAMPS | Pre-eclampsia \|\| id:finngen_R11_O15_PREECLAMPS | Resting CD4 regulatory T cell %CD4+ T cell \|\| id:ebi-a-GCST90001482 | Weighted median | 32 | 0.0165971220562794 | 0.0121017749422652 | 0.170231043549125 | -0.007122357 | 0.0403166009431193 | 1.01673561944499 | 0.992902947042806 | 1.04114034803405 | 0.940845681141708 |
| ebi-a-GCST90001482 | finngen_R11_O15_PREECLAMPS | Pre-eclampsia \|\| id:finngen_R11_O15_PREECLAMPS | Resting CD4 regulatory T cell %CD4+ T cell \|\| id:ebi-a-GCST90001482 | Weighted mode | 32 | 0.0135069343470616 | 0.0101842206900039 | 0.194442230525311 | -0.006454138 | 0.0334680068994692 | 1.01359856507007 | 0.993566645008026 | 1.0340343612318 | 0.872755742358858 |
| ebi-a-GCST90001483 | finngen_R11_O15_PREECLAMPS | Pre-eclampsia \|\| id:finngen_R11_O15_PREECLAMPS | CD39+ resting CD4 regulatory T cell Absolute Count \|\| id:ebi-a-GCST90001483 | Inverse variance weighted | 29 | 0.0161943583902065 | 0.0120552623211237 | 0.179160769748761 | -0.007433956 | 0.0398226725396088 | 1.0163261977351 | 0.992593607745668 | 1.04062622622425 | 0.855687495048373 |
| ebi-a-GCST90001483 | finngen_R11_O15_PREECLAMPS | Pre-eclampsia \|\| id:finngen_R11_O15_PREECLAMPS | CD39+ resting CD4 regulatory T cell Absolute Count \|\| id:ebi-a-GCST90001483 | MR Egger | 29 | 0.00691138884166392 | 0.0160791437617383 | 0.670726349626461 | -0.024603733 | 0.0384265106146709 | 1.00693532760779 | 0.975696471813602 | 1.03917435726702 | 0.99380795269851 |
| ebi-a-GCST90001483 | finngen_R11_O15_PREECLAMPS | Pre-eclampsia \|\| id:finngen_R11_O15_PREECLAMPS | CD39+ resting CD4 regulatory T cell Absolute Count \|\| id:ebi-a-GCST90001483 | Weighted median | 29 | 0.0151681190273324 | 0.0169561576746612 | 0.371028063015569 | -0.01806595 | 0.0484021880696685 | 1.01528373878316 | 0.982096260959342 | 1.04959270411089 | 0.940845681141708 |
| ebi-a-GCST90001483 | finngen_R11_O15_PREECLAMPS | Pre-eclampsia \|\| id:finngen_R11_O15_PREECLAMPS | CD39+ resting CD4 regulatory T cell Absolute Count \|\| id:ebi-a-GCST90001483 | Weighted mode | 29 | 0.0161194363424531 | 0.0137825044386891 | 0.252038875850834 | -0.010894272 | 0.0431331450422836 | 1.01625005534759 | 0.989164855315273 | 1.04407689925943 | 0.872755742358858 |
| ebi-a-GCST90001484 | finngen_R11_O15_PREECLAMPS | Pre-eclampsia \|\| id:finngen_R11_O15_PREECLAMPS | CD39+ resting CD4 regulatory T cell %resting CD4 regulatory T cell \|\| id:ebi-a-GCST90001484 | Inverse variance weighted | 29 | 0.0059142125760607 | 0.0115255874261575 | 0.607855053030958 | -0.016675939 | 0.0285043639313295 | 1.0059317360601 | 0.983462335005892 | 1.02891450093695 | 0.92242199665996 |
| ebi-a-GCST90001484 | finngen_R11_O15_PREECLAMPS | Pre-eclampsia \|\| id:finngen_R11_O15_PREECLAMPS | CD39+ resting CD4 regulatory T cell %resting CD4 regulatory T cell \|\| id:ebi-a-GCST90001484 | MR Egger | 29 | -0.001448127 | 0.0164391088386144 | 0.930455001741607 | -0.03366878 | 0.0307725266316296 | 0.998552921337449 | 0.966891705461589 | 1.03125089508917 | 0.99380795269851 |
| ebi-a-GCST90001484 | finngen_R11_O15_PREECLAMPS | Pre-eclampsia \|\| id:finngen_R11_O15_PREECLAMPS | CD39+ resting CD4 regulatory T cell %resting CD4 regulatory T cell \|\| id:ebi-a-GCST90001484 | Weighted median | 29 | 0.0223372728281102 | 0.0154113089565549 | 0.147223706010507 | -0.007868893 | 0.0525434383829578 | 1.02258861767081 | 0.992161985962854 | 1.05394834289594 | 0.917572019373545 |
| ebi-a-GCST90001484 | finngen_R11_O15_PREECLAMPS | Pre-eclampsia \|\| id:finngen_R11_O15_PREECLAMPS | CD39+ resting CD4 regulatory T cell %resting CD4 regulatory T cell \|\| id:ebi-a-GCST90001484 | Weighted mode | 29 | 0.016848355247857 | 0.0137322074922245 | 0.230068667656126 | -0.010066771 | 0.0437634819326171 | 1.01699108926956 | 0.989983728906145 | 1.04473522690768 | 0.872755742358858 |
| ebi-a-GCST90001485 | finngen_R11_O15_PREECLAMPS | Pre-eclampsia \|\| id:finngen_R11_O15_PREECLAMPS | CD39+ resting CD4 regulatory T cell %CD4 regulatory T cell \|\| id:ebi-a-GCST90001485 | Inverse variance weighted | 34 | 0.019385350253623 | 0.00806974286777282 | 0.0162956145649674 | 0.00356865423278824 | 0.0352020462744577 | 1.01957446620551 | 1.00357502946071 | 1.03582897304336 | 0.588591724205005 |
| ebi-a-GCST90001485 | finngen_R11_O15_PREECLAMPS | Pre-eclampsia \|\| id:finngen_R11_O15_PREECLAMPS | CD39+ resting CD4 regulatory T cell %CD4 regulatory T cell \|\| id:ebi-a-GCST90001485 | MR Egger | 34 | 0.0187662911564091 | 0.010260728199554 | 0.0767413151774189 | -0.001344736 | 0.038877318427535 | 1.01894348468449 | 0.998656167637744 | 1.03964293079657 | 0.919637727781856 |
| ebi-a-GCST90001485 | finngen_R11_O15_PREECLAMPS | Pre-eclampsia \|\| id:finngen_R11_O15_PREECLAMPS | CD39+ resting CD4 regulatory T cell %CD4 regulatory T cell \|\| id:ebi-a-GCST90001485 | Weighted median | 34 | 0.0208329654578054 | 0.0113097523611162 | 0.0654699558622883 | -0.001334149 | 0.0430000800855931 | 1.02105148652518 | 0.998666740411366 | 1.043937978 | 0.811161656531063 |
| ebi-a-GCST90001485 | finngen_R11_O15_PREECLAMPS | Pre-eclampsia \|\| id:finngen_R11_O15_PREECLAMPS | CD39+ resting CD4 regulatory T cell %CD4 regulatory T cell \|\| id:ebi-a-GCST90001485 | Weighted mode | 34 | 0.0193611721825437 | 0.0093114127271025 | 0.0454373475529241 | 0.00111080323742278 | 0.0376115411276646 | 1.0195498151596 | 1.00111142040784 | 1.03832780687739 | 0.838871423749761 |
| ebi-a-GCST90001486 | finngen_R11_O15_PREECLAMPS | Pre-eclampsia \|\| id:finngen_R11_O15_PREECLAMPS | Activated CD4 regulatory T cell Absolute Count \|\| id:ebi-a-GCST90001486 | Inverse variance weighted | 15 | -0.002247961 | 0.0158388834269735 | 0.887137855438004 | -0.033292173 | 0.0287962500817474 | 0.997754563337968 | 0.967255912284873 | 1.02921487066449 | 0.986383922906939 |
| ebi-a-GCST90001486 | finngen_R11_O15_PREECLAMPS | Pre-eclampsia \|\| id:finngen_R11_O15_PREECLAMPS | Activated CD4 regulatory T cell Absolute Count \|\| id:ebi-a-GCST90001486 | MR Egger | 15 | -0.017431173 | 0.0197129296114308 | 0.392621428138839 | -0.056068515 | 0.0212061685828092 | 0.982719870550317 | 0.945474354028711 | 1.02143261724591 | 0.99380795269851 |
| ebi-a-GCST90001486 | finngen_R11_O15_PREECLAMPS | Pre-eclampsia \|\| id:finngen_R11_O15_PREECLAMPS | Activated CD4 regulatory T cell Absolute Count \|\| id:ebi-a-GCST90001486 | Weighted median | 15 | -0.005828767 | 0.0217575358973831 | 0.788779008899786 | -0.048473537 | 0.036816003238064 | 0.994188187185373 | 0.952682549353596 | 1.03750210624681 | 0.957984984509652 |
| ebi-a-GCST90001486 | finngen_R11_O15_PREECLAMPS | Pre-eclampsia \|\| id:finngen_R11_O15_PREECLAMPS | Activated CD4 regulatory T cell Absolute Count \|\| id:ebi-a-GCST90001486 | Weighted mode | 15 | -0.013119962 | 0.018163751613158 | 0.481992544437744 | -0.048720915 | 0.022480991653327 | 0.986965730020634 | 0.952446906568554 | 1.02273559346696 | 0.933945646747087 |
| ebi-a-GCST90001487 | finngen_R11_O15_PREECLAMPS | Pre-eclampsia \|\| id:finngen_R11_O15_PREECLAMPS | Activated CD4 regulatory T cell %CD4 regulatory T cell \|\| id:ebi-a-GCST90001487 | Inverse variance weighted (multiplicative random effects) | 28 | -0.011138792 | 0.0198834926813623 | 0.575340984748329 | -0.050110438 | 0.0278328535147886 | 0.988923014507835 | 0.951124378620062 | 1.0282238060622 | 0.915399120505076 |
| ebi-a-GCST90001487 | finngen_R11_O15_PREECLAMPS | Pre-eclampsia \|\| id:finngen_R11_O15_PREECLAMPS | Activated CD4 regulatory T cell %CD4 regulatory T cell \|\| id:ebi-a-GCST90001487 | MR Egger | 28 | 0.0245869197166136 | 0.0267671567570381 | 0.366775320328324 | -0.027876708 | 0.0770505469604083 | 1.02489167052933 | 0.972508262358488 | 1.08009667061645 | 0.99380795269851 |
| ebi-a-GCST90001487 | finngen_R11_O15_PREECLAMPS | Pre-eclampsia \|\| id:finngen_R11_O15_PREECLAMPS | Activated CD4 regulatory T cell %CD4 regulatory T cell \|\| id:ebi-a-GCST90001487 | Weighted median | 28 | 0.00390367064811298 | 0.0223604677489313 | 0.861410361150182 | -0.039922846 | 0.0477301874360184 | 1.0039112998945 | 0.960863570626128 | 1.04888761408566 | 0.964912412213677 |
| ebi-a-GCST90001487 | finngen_R11_O15_PREECLAMPS | Pre-eclampsia \|\| id:finngen_R11_O15_PREECLAMPS | Activated CD4 regulatory T cell %CD4 regulatory T cell \|\| id:ebi-a-GCST90001487 | Weighted mode | 28 | -9.96E-05 | 0.0198922986127767 | 0.996042942349711 | -0.039088478 | 0.0388893327620239 | 0.99990043243816 | 0.96166561932375 | 1.03965542148953 | 0.996042942349711 |
| ebi-a-GCST90001488 | finngen_R11_O15_PREECLAMPS | Pre-eclampsia \|\| id:finngen_R11_O15_PREECLAMPS | Activated CD4 regulatory T cell %CD4+ T cell \|\| id:ebi-a-GCST90001488 | Inverse variance weighted | 18 | -0.010481241 | 0.0243441971933404 | 0.666800174161967 | -0.058195868 | 0.0372333853939607 | 0.989573495699101 | 0.943465135082609 | 1.03793523149575 | 0.950857618427329 |
| ebi-a-GCST90001488 | finngen_R11_O15_PREECLAMPS | Pre-eclampsia \|\| id:finngen_R11_O15_PREECLAMPS | Activated CD4 regulatory T cell %CD4+ T cell \|\| id:ebi-a-GCST90001488 | MR Egger | 18 | -0.034744693 | 0.0354438890146364 | 0.341542277814428 | -0.104214715 | 0.0347253294829423 | 0.965851973565906 | 0.901031811194908 | 1.03533529366075 | 0.99380795269851 |
| ebi-a-GCST90001488 | finngen_R11_O15_PREECLAMPS | Pre-eclampsia \|\| id:finngen_R11_O15_PREECLAMPS | Activated CD4 regulatory T cell %CD4+ T cell \|\| id:ebi-a-GCST90001488 | Weighted median | 18 | -0.024914326 | 0.0282518630392113 | 0.377849875003457 | -0.080287978 | 0.0304593254325888 | 0.975393474188034 | 0.922850547755673 | 1.03092795664731 | 0.940845681141708 |
| ebi-a-GCST90001488 | finngen_R11_O15_PREECLAMPS | Pre-eclampsia \|\| id:finngen_R11_O15_PREECLAMPS | Activated CD4 regulatory T cell %CD4+ T cell \|\| id:ebi-a-GCST90001488 | Weighted mode | 18 | -0.02008261 | 0.0267368767160732 | 0.462852029524416 | -0.072486889 | 0.0323216680430989 | 0.980117702124867 | 0.930077941126212 | 1.03284968661371 | 0.933945646747087 |
| ebi-a-GCST90001489 | finngen_R11_O15_PREECLAMPS | Pre-eclampsia \|\| id:finngen_R11_O15_PREECLAMPS | CD39+ activated CD4 regulatory T cell Absolute Count \|\| id:ebi-a-GCST90001489 | Inverse variance weighted | 30 | 0.00605475004867913 | 0.00839284695677956 | 0.470652435095268 | -0.01039523 | 0.0225047300839671 | 1.00607311709834 | 0.989658613682677 | 1.02275987189308 | 0.898694924660397 |
| ebi-a-GCST90001489 | finngen_R11_O15_PREECLAMPS | Pre-eclampsia \|\| id:finngen_R11_O15_PREECLAMPS | CD39+ activated CD4 regulatory T cell Absolute Count \|\| id:ebi-a-GCST90001489 | MR Egger | 30 | 0.00458900320630722 | 0.0104857214532701 | 0.665002453880277 | -0.015963011 | 0.0251410172547166 | 1.00459954880661 | 0.984163722769367 | 1.02545971784306 | 0.99380795269851 |
| ebi-a-GCST90001489 | finngen_R11_O15_PREECLAMPS | Pre-eclampsia \|\| id:finngen_R11_O15_PREECLAMPS | CD39+ activated CD4 regulatory T cell Absolute Count \|\| id:ebi-a-GCST90001489 | Weighted median | 30 | 0.0057689090647166 | 0.0106726563496266 | 0.588830406619918 | -0.015149497 | 0.0266873155099848 | 1.00578558126533 | 0.984964678956357 | 1.02704661100448 | 0.940845681141708 |
| ebi-a-GCST90001489 | finngen_R11_O15_PREECLAMPS | Pre-eclampsia \|\| id:finngen_R11_O15_PREECLAMPS | CD39+ activated CD4 regulatory T cell Absolute Count \|\| id:ebi-a-GCST90001489 | Weighted mode | 30 | 0.00758120831534825 | 0.00864828202874664 | 0.38790019846069 | -0.009369424 | 0.0245318410916917 | 1.00761001843426 | 0.990674331832641 | 1.02483522245997 | 0.920286500917063 |
| ebi-a-GCST90001490 | finngen_R11_O15_PREECLAMPS | Pre-eclampsia \|\| id:finngen_R11_O15_PREECLAMPS | CD39+ activated CD4 regulatory T cell %activated CD4 regulatory T cell \|\| id:ebi-a-GCST90001490 | Inverse variance weighted | 24 | -0.010463525 | 0.0183287244315883 | 0.568080163434277 | -0.046387824 | 0.0254607753602021 | 0.98959102771169 | 0.954671645417236 | 1.02578766932947 | 0.910672367259773 |
| ebi-a-GCST90001490 | finngen_R11_O15_PREECLAMPS | Pre-eclampsia \|\| id:finngen_R11_O15_PREECLAMPS | CD39+ activated CD4 regulatory T cell %activated CD4 regulatory T cell \|\| id:ebi-a-GCST90001490 | MR Egger | 24 | 0.00783099850086655 | 0.0358211133709124 | 0.828965995749489 | -0.062378384 | 0.0780403807078549 | 1.0078617409653 | 0.939527317696083 | 1.08116631604977 | 0.99380795269851 |
| ebi-a-GCST90001490 | finngen_R11_O15_PREECLAMPS | Pre-eclampsia \|\| id:finngen_R11_O15_PREECLAMPS | CD39+ activated CD4 regulatory T cell %activated CD4 regulatory T cell \|\| id:ebi-a-GCST90001490 | Weighted median | 24 | -0.016304075 | 0.0262741400952214 | 0.534904326379507 | -0.06780139 | 0.0351932394784866 | 0.983828116926426 | 0.934446045762689 | 1.0358198507491 | 0.940845681141708 |
| ebi-a-GCST90001490 | finngen_R11_O15_PREECLAMPS | Pre-eclampsia \|\| id:finngen_R11_O15_PREECLAMPS | CD39+ activated CD4 regulatory T cell %activated CD4 regulatory T cell \|\| id:ebi-a-GCST90001490 | Weighted mode | 24 | -0.009754991 | 0.0260556948504184 | 0.711543798094501 | -0.060824153 | 0.0413141708321551 | 0.990292434513426 | 0.940988695284677 | 1.04217947651097 | 0.959313411571287 |
| ebi-a-GCST90001491 | finngen_R11_O15_PREECLAMPS | Pre-eclampsia \|\| id:finngen_R11_O15_PREECLAMPS | CD39+ activated CD4 regulatory T cell %CD4 regulatory T cell \|\| id:ebi-a-GCST90001491 | Inverse variance weighted | 21 | 0.00710316461397149 | 0.0114410683893664 | 0.534699627016124 | -0.015321329 | 0.0295276586571297 | 1.00712845192559 | 0.984795444998064 | 1.02996792260756 | 0.904087927705763 |
| ebi-a-GCST90001491 | finngen_R11_O15_PREECLAMPS | Pre-eclampsia \|\| id:finngen_R11_O15_PREECLAMPS | CD39+ activated CD4 regulatory T cell %CD4 regulatory T cell \|\| id:ebi-a-GCST90001491 | MR Egger | 21 | 0.0208102019025042 | 0.0175911405712607 | 0.251403715103334 | -0.013668434 | 0.0552888374221752 | 1.02102824402775 | 0.986424555268666 | 1.05684582721934 | 0.99380795269851 |
| ebi-a-GCST90001491 | finngen_R11_O15_PREECLAMPS | Pre-eclampsia \|\| id:finngen_R11_O15_PREECLAMPS | CD39+ activated CD4 regulatory T cell %CD4 regulatory T cell \|\| id:ebi-a-GCST90001491 | Weighted median | 21 | 0.016780969574974 | 0.0152622954149179 | 0.271547869839893 | -0.013133129 | 0.0466950685882131 | 1.01692256094963 | 0.986952733810751 | 1.0478024524796 | 0.940845681141708 |
| ebi-a-GCST90001491 | finngen_R11_O15_PREECLAMPS | Pre-eclampsia \|\| id:finngen_R11_O15_PREECLAMPS | CD39+ activated CD4 regulatory T cell %CD4 regulatory T cell \|\| id:ebi-a-GCST90001491 | Weighted mode | 21 | 0.0135995063053088 | 0.0128370756960003 | 0.302048333061749 | -0.011561162 | 0.0387601746694694 | 1.0136924002173 | 0.98850541137283 | 1.03952115024967 | 0.875408265683677 |
| ebi-a-GCST90001492 | finngen_R11_O15_PREECLAMPS | Pre-eclampsia \|\| id:finngen_R11_O15_PREECLAMPS | Secreting CD4 regulatory T cell Absolute Count \|\| id:ebi-a-GCST90001492 | Inverse variance weighted | 22 | -0.005369085 | 0.00771168914537049 | 0.486286806312127 | -0.020483995 | 0.00974582606550021 | 0.994645303114372 | 0.979724376461128 | 1.00979347128323 | 0.898694924660397 |
| ebi-a-GCST90001492 | finngen_R11_O15_PREECLAMPS | Pre-eclampsia \|\| id:finngen_R11_O15_PREECLAMPS | Secreting CD4 regulatory T cell Absolute Count \|\| id:ebi-a-GCST90001492 | MR Egger | 22 | -0.009349789 | 0.00964466705574725 | 0.34391122067172 | -0.028253337 | 0.00955375832865526 | 0.990693784271149 | 0.972142056507727 | 1.00959954116098 | 0.99380795269851 |
| ebi-a-GCST90001492 | finngen_R11_O15_PREECLAMPS | Pre-eclampsia \|\| id:finngen_R11_O15_PREECLAMPS | Secreting CD4 regulatory T cell Absolute Count \|\| id:ebi-a-GCST90001492 | Weighted median | 22 | 0.00440579183575747 | 0.0101708189271422 | 0.664884257901561 | -0.015529013 | 0.0243405969329561 | 1.00441551160579 | 0.984590940142971 | 1.02463924745015 | 0.940845681141708 |
| ebi-a-GCST90001492 | finngen_R11_O15_PREECLAMPS | Pre-eclampsia \|\| id:finngen_R11_O15_PREECLAMPS | Secreting CD4 regulatory T cell Absolute Count \|\| id:ebi-a-GCST90001492 | Weighted mode | 22 | -0.000761314 | 0.00885352219729907 | 0.932288976462734 | -0.018114218 | 0.0165915892283321 | 0.999238975447812 | 0.982048858506839 | 1.0167299940373 | 0.990180645930572 |
| ebi-a-GCST90001493 | finngen_R11_O15_PREECLAMPS | Pre-eclampsia \|\| id:finngen_R11_O15_PREECLAMPS | Secreting CD4 regulatory T cell %CD4 regulatory T cell \|\| id:ebi-a-GCST90001493 | Inverse variance weighted | 31 | -0.019927114 | 0.0141178882147001 | 0.158103178755691 | -0.047598175 | 0.00774394643925857 | 0.980270118218462 | 0.953516856674361 | 1.00777400834164 | 0.85505992597664 |
| ebi-a-GCST90001493 | finngen_R11_O15_PREECLAMPS | Pre-eclampsia \|\| id:finngen_R11_O15_PREECLAMPS | Secreting CD4 regulatory T cell %CD4 regulatory T cell \|\| id:ebi-a-GCST90001493 | MR Egger | 31 | -0.028216361 | 0.0180962454601759 | 0.129787606037542 | -0.063685003 | 0.00725227960272137 | 0.972178002154604 | 0.938300515206564 | 1.00727864107082 | 0.97261503632431 |
| ebi-a-GCST90001493 | finngen_R11_O15_PREECLAMPS | Pre-eclampsia \|\| id:finngen_R11_O15_PREECLAMPS | Secreting CD4 regulatory T cell %CD4 regulatory T cell \|\| id:ebi-a-GCST90001493 | Weighted median | 31 | -0.0129184 | 0.0159868116000986 | 0.419052541242633 | -0.044252551 | 0.0184157505064127 | 0.987164684145243 | 0.95671230834234 | 1.018586366 | 0.940845681141708 |
| ebi-a-GCST90001493 | finngen_R11_O15_PREECLAMPS | Pre-eclampsia \|\| id:finngen_R11_O15_PREECLAMPS | Secreting CD4 regulatory T cell %CD4 regulatory T cell \|\| id:ebi-a-GCST90001493 | Weighted mode | 31 | -0.011223996 | 0.0142718341810272 | 0.43777600945188 | -0.039196791 | 0.0167487989562648 | 0.988838758001851 | 0.961561463851977 | 1.01688984644789 | 0.933945646747087 |
| ebi-a-GCST90001494 | finngen_R11_O15_PREECLAMPS | Pre-eclampsia \|\| id:finngen_R11_O15_PREECLAMPS | Secreting CD4 regulatory T cell %CD4+ T cell \|\| id:ebi-a-GCST90001494 | Inverse variance weighted | 32 | -0.003416013 | 0.00541146943672663 | 0.527874319341743 | -0.014022493 | 0.0071904672126317 | 0.996589815050669 | 0.986075364240623 | 1.00721638069478 | 0.904087927705763 |
| ebi-a-GCST90001494 | finngen_R11_O15_PREECLAMPS | Pre-eclampsia \|\| id:finngen_R11_O15_PREECLAMPS | Secreting CD4 regulatory T cell %CD4+ T cell \|\| id:ebi-a-GCST90001494 | MR Egger | 32 | -0.004909027 | 0.00593099549115606 | 0.414381859645343 | -0.016533778 | 0.00671572412298709 | 0.995103002540997 | 0.983602154516935 | 1.00673832516403 | 0.99380795269851 |
| ebi-a-GCST90001494 | finngen_R11_O15_PREECLAMPS | Pre-eclampsia \|\| id:finngen_R11_O15_PREECLAMPS | Secreting CD4 regulatory T cell %CD4+ T cell \|\| id:ebi-a-GCST90001494 | Weighted median | 32 | -0.000897421 | 0.00654823007890256 | 0.890992993494161 | -0.013731952 | 0.0119371102702156 | 0.999102981877078 | 0.986361901521989 | 1.0120086419147 | 0.964912412213677 |
| ebi-a-GCST90001494 | finngen_R11_O15_PREECLAMPS | Pre-eclampsia \|\| id:finngen_R11_O15_PREECLAMPS | Secreting CD4 regulatory T cell %CD4+ T cell \|\| id:ebi-a-GCST90001494 | Weighted mode | 32 | -0.001280986 | 0.00524772226504747 | 0.808759190732274 | -0.011566522 | 0.00900454928215745 | 0.998719833755466 | 0.988500113059335 | 1.00904521219481 | 0.961305639715923 |
| ebi-a-GCST90001495 | finngen_R11_O15_PREECLAMPS | Pre-eclampsia \|\| id:finngen_R11_O15_PREECLAMPS | CD39+ secreting CD4 regulatory T cell Absolute Count \|\| id:ebi-a-GCST90001495 | Inverse variance weighted | 26 | 0.0201010009333139 | 0.0125444493734893 | 0.109071144106167 | -0.00448612 | 0.044688121705353 | 1.02030438651808 | 0.995523927766343 | 1.04570167739088 | 0.820779257935285 |
| ebi-a-GCST90001495 | finngen_R11_O15_PREECLAMPS | Pre-eclampsia \|\| id:finngen_R11_O15_PREECLAMPS | CD39+ secreting CD4 regulatory T cell Absolute Count \|\| id:ebi-a-GCST90001495 | MR Egger | 26 | 0.0311240055308436 | 0.020296170745492 | 0.138232675579214 | -0.008656489 | 0.070904500192008 | 1.03161342172509 | 0.991380870393222 | 1.07347870396291 | 0.97261503632431 |
| ebi-a-GCST90001495 | finngen_R11_O15_PREECLAMPS | Pre-eclampsia \|\| id:finngen_R11_O15_PREECLAMPS | CD39+ secreting CD4 regulatory T cell Absolute Count \|\| id:ebi-a-GCST90001495 | Weighted median | 26 | 0.0246293568683507 | 0.0161065585641083 | 0.126227064932201 | -0.006939498 | 0.0561982116540029 | 1.02493516493554 | 0.993084524797724 | 1.05780733269952 | 0.88227178111339 |
| ebi-a-GCST90001495 | finngen_R11_O15_PREECLAMPS | Pre-eclampsia \|\| id:finngen_R11_O15_PREECLAMPS | CD39+ secreting CD4 regulatory T cell Absolute Count \|\| id:ebi-a-GCST90001495 | Weighted mode | 26 | 0.0208511930062754 | 0.0143786704418085 | 0.15944850964693 | -0.007331001 | 0.0490333870722202 | 1.02107009796026 | 0.992695805183069 | 1.05025541510807 | 0.872755742358858 |
| ebi-a-GCST90001496 | finngen_R11_O15_PREECLAMPS | Pre-eclampsia \|\| id:finngen_R11_O15_PREECLAMPS | CD39+ secreting CD4 regulatory T cell %secreting CD4 regulatory T cell \|\| id:ebi-a-GCST90001496 | Inverse variance weighted | 28 | 0.00629835745024403 | 0.0114708717513152 | 0.582954666415978 | -0.016184551 | 0.0287812660828218 | 1.00631823381109 | 0.983945714953036 | 1.02919944902551 | 0.915399120505076 |
| ebi-a-GCST90001496 | finngen_R11_O15_PREECLAMPS | Pre-eclampsia \|\| id:finngen_R11_O15_PREECLAMPS | CD39+ secreting CD4 regulatory T cell %secreting CD4 regulatory T cell \|\| id:ebi-a-GCST90001496 | MR Egger | 28 | -0.001783907 | 0.01537041379185 | 0.90849590536903 | -0.031909918 | 0.0283421036163111 | 0.998217682801378 | 0.968593830585166 | 1.02874756249001 | 0.99380795269851 |
| ebi-a-GCST90001496 | finngen_R11_O15_PREECLAMPS | Pre-eclampsia \|\| id:finngen_R11_O15_PREECLAMPS | CD39+ secreting CD4 regulatory T cell %secreting CD4 regulatory T cell \|\| id:ebi-a-GCST90001496 | Weighted median | 28 | -0.014161732 | 0.0160906366185706 | 0.378792927998104 | -0.04569938 | 0.0173759157482013 | 0.985938073607076 | 0.955329110258741 | 1.01752775514687 | 0.940845681141708 |
| ebi-a-GCST90001496 | finngen_R11_O15_PREECLAMPS | Pre-eclampsia \|\| id:finngen_R11_O15_PREECLAMPS | CD39+ secreting CD4 regulatory T cell %secreting CD4 regulatory T cell \|\| id:ebi-a-GCST90001496 | Weighted mode | 28 | -0.001810568 | 0.0144286816185148 | 0.901070131650338 | -0.030090784 | 0.0264696480667966 | 0.998191070183805 | 0.970357436738743 | 1.02682308072314 | 0.98361388732834 |
| ebi-a-GCST90001497 | finngen_R11_O15_PREECLAMPS | Pre-eclampsia \|\| id:finngen_R11_O15_PREECLAMPS | CD39+ secreting CD4 regulatory T cell %CD4 regulatory T cell \|\| id:ebi-a-GCST90001497 | Inverse variance weighted | 22 | 0.000249056004537368 | 0.0121697490942862 | 0.983672295226042 | -0.023603652 | 0.0241017642293384 | 1.00024908702156 | 0.976672735124616 | 1.02439455930937 | 0.994556635975431 |
| ebi-a-GCST90001497 | finngen_R11_O15_PREECLAMPS | Pre-eclampsia \|\| id:finngen_R11_O15_PREECLAMPS | CD39+ secreting CD4 regulatory T cell %CD4 regulatory T cell \|\| id:ebi-a-GCST90001497 | MR Egger | 22 | 0.000806014412006676 | 0.0161228506743578 | 0.960624624520317 | -0.030794773 | 0.032406801733748 | 1.00080633932891 | 0.969674556144631 | 1.03293762066244 | 0.99380795269851 |
| ebi-a-GCST90001497 | finngen_R11_O15_PREECLAMPS | Pre-eclampsia \|\| id:finngen_R11_O15_PREECLAMPS | CD39+ secreting CD4 regulatory T cell %CD4 regulatory T cell \|\| id:ebi-a-GCST90001497 | Weighted median | 22 | -0.014128856 | 0.0174215779015076 | 0.417367117896274 | -0.048275148 | 0.0200174369105424 | 0.985970488084314 | 0.952871569856594 | 1.02021912934135 | 0.940845681141708 |
| ebi-a-GCST90001497 | finngen_R11_O15_PREECLAMPS | Pre-eclampsia \|\| id:finngen_R11_O15_PREECLAMPS | CD39+ secreting CD4 regulatory T cell %CD4 regulatory T cell \|\| id:ebi-a-GCST90001497 | Weighted mode | 22 | -0.002505221 | 0.0145436315230005 | 0.864885926121247 | -0.031010739 | 0.0260002969824444 | 0.997497914644123 | 0.969465162333443 | 1.02634125327873 | 0.977585971930092 |
| ebi-a-GCST90001498 | finngen_R11_O15_PREECLAMPS | Pre-eclampsia \|\| id:finngen_R11_O15_PREECLAMPS | Activated & resting CD4 regulatory T cell Absolute Count \|\| id:ebi-a-GCST90001498 | Inverse variance weighted | 19 | -0.018306777 | 0.0314176890306699 | 0.560101915085406 | -0.079885448 | 0.0432718930238901 | 0.9818597736876 | 0.923222097289066 | 1.04422177287208 | 0.90609555750305 |
| ebi-a-GCST90001498 | finngen_R11_O15_PREECLAMPS | Pre-eclampsia \|\| id:finngen_R11_O15_PREECLAMPS | Activated & resting CD4 regulatory T cell Absolute Count \|\| id:ebi-a-GCST90001498 | MR Egger | 19 | 0.0225367343941683 | 0.0692891630545051 | 0.748955952850617 | -0.113270025 | 0.158343493980998 | 1.02279260514108 | 0.892909519495428 | 1.17156855234606 | 0.99380795269851 |
| ebi-a-GCST90001498 | finngen_R11_O15_PREECLAMPS | Pre-eclampsia \|\| id:finngen_R11_O15_PREECLAMPS | Activated & resting CD4 regulatory T cell Absolute Count \|\| id:ebi-a-GCST90001498 | Weighted median | 19 | 0.000118174721797582 | 0.0424062831258353 | 0.997776516661934 | -0.08299814 | 0.0832344896484348 | 1.00011818170471 | 0.920352858886326 | 1.08679662122923 | 0.998487310447797 |
| ebi-a-GCST90001498 | finngen_R11_O15_PREECLAMPS | Pre-eclampsia \|\| id:finngen_R11_O15_PREECLAMPS | Activated & resting CD4 regulatory T cell Absolute Count \|\| id:ebi-a-GCST90001498 | Weighted mode | 19 | 0.0141429865629032 | 0.0594369539258733 | 0.814607938020015 | -0.102353443 | 0.130639416257615 | 1.01424347175878 | 0.902710438473525 | 1.13955680156397 | 0.965118967086923 |
| ebi-a-GCST90001499 | finngen_R11_O15_PREECLAMPS | Pre-eclampsia \|\| id:finngen_R11_O15_PREECLAMPS | Activated & resting CD4 regulatory T cell %CD4 regulatory T cell \|\| id:ebi-a-GCST90001499 | Inverse variance weighted | 29 | 0.0213009938485872 | 0.0140657103720236 | 0.129926544631671 | -0.006267798 | 0.0488697861777534 | 1.02152947945773 | 0.993751803193821 | 1.05008360643715 | 0.820779257935285 |
| ebi-a-GCST90001499 | finngen_R11_O15_PREECLAMPS | Pre-eclampsia \|\| id:finngen_R11_O15_PREECLAMPS | Activated & resting CD4 regulatory T cell %CD4 regulatory T cell \|\| id:ebi-a-GCST90001499 | MR Egger | 29 | 0.0282626114718861 | 0.0179831974860869 | 0.127687024291316 | -0.006984456 | 0.0635096785446165 | 1.02866578839044 | 0.99303987902152 | 1.0655697989164 | 0.97261503632431 |
| ebi-a-GCST90001499 | finngen_R11_O15_PREECLAMPS | Pre-eclampsia \|\| id:finngen_R11_O15_PREECLAMPS | Activated & resting CD4 regulatory T cell %CD4 regulatory T cell \|\| id:ebi-a-GCST90001499 | Weighted median | 29 | 0.0126583723860772 | 0.0160049629221618 | 0.429000626943839 | -0.018711355 | 0.0440280997135143 | 1.01273882870542 | 0.981462615695245 | 1.0450117190058 | 0.940845681141708 |
| ebi-a-GCST90001499 | finngen_R11_O15_PREECLAMPS | Pre-eclampsia \|\| id:finngen_R11_O15_PREECLAMPS | Activated & resting CD4 regulatory T cell %CD4 regulatory T cell \|\| id:ebi-a-GCST90001499 | Weighted mode | 29 | 0.0108931770471081 | 0.0148647565692327 | 0.469759682801943 | -0.018241746 | 0.0400280999228041 | 1.01095272372127 | 0.981923627722673 | 1.04084002130572 | 0.933945646747087 |
| ebi-a-GCST90001500 | finngen_R11_O15_PREECLAMPS | Pre-eclampsia \|\| id:finngen_R11_O15_PREECLAMPS | Activated & resting CD4 regulatory T cell %CD4+ T cell \|\| id:ebi-a-GCST90001500 | Inverse variance weighted | 26 | 0.025168799148507 | 0.0163305412778593 | 0.123265600670237 | -0.006839062 | 0.0571766600531112 | 1.02548820745178 | 0.993184271404145 | 1.05884284910784 | 0.820779257935285 |
| ebi-a-GCST90001500 | finngen_R11_O15_PREECLAMPS | Pre-eclampsia \|\| id:finngen_R11_O15_PREECLAMPS | Activated & resting CD4 regulatory T cell %CD4+ T cell \|\| id:ebi-a-GCST90001500 | MR Egger | 26 | 0.0281670963578189 | 0.0227165130600217 | 0.226984881202754 | -0.016357269 | 0.0726914619554615 | 1.02856753995251 | 0.98377578443593 | 1.0753986843156 | 0.99380795269851 |
| ebi-a-GCST90001500 | finngen_R11_O15_PREECLAMPS | Pre-eclampsia \|\| id:finngen_R11_O15_PREECLAMPS | Activated & resting CD4 regulatory T cell %CD4+ T cell \|\| id:ebi-a-GCST90001500 | Weighted median | 26 | 0.0239632642158071 | 0.0237586391525553 | 0.313160433724188 | -0.022603669 | 0.0705301969548155 | 1.02425269047369 | 0.977649880420363 | 1.07307697259833 | 0.940845681141708 |
| ebi-a-GCST90001500 | finngen_R11_O15_PREECLAMPS | Pre-eclampsia \|\| id:finngen_R11_O15_PREECLAMPS | Activated & resting CD4 regulatory T cell %CD4+ T cell \|\| id:ebi-a-GCST90001500 | Weighted mode | 26 | 0.0247469095848104 | 0.0217623050136311 | 0.26625964004132 | -0.017907208 | 0.0674010274115273 | 1.02505565593026 | 0.982252173035711 | 1.06972438096751 | 0.872755742358858 |
| ebi-a-GCST90001501 | finngen_R11_O15_PREECLAMPS | Pre-eclampsia \|\| id:finngen_R11_O15_PREECLAMPS | Activated & secreting CD4 regulatory T cell Absolute Count \|\| id:ebi-a-GCST90001501 | Inverse variance weighted | 22 | -0.001516229 | 0.00695053923828303 | 0.827315777380204 | -0.015139285 | 0.012106828319877 | 0.998484920306671 | 0.984974737355083 | 1.01218041262353 | 0.977824840984872 |
| ebi-a-GCST90001501 | finngen_R11_O15_PREECLAMPS | Pre-eclampsia \|\| id:finngen_R11_O15_PREECLAMPS | Activated & secreting CD4 regulatory T cell Absolute Count \|\| id:ebi-a-GCST90001501 | MR Egger | 22 | -0.004511287 | 0.00861337852653072 | 0.606205790027855 | -0.021393509 | 0.0123709344350656 | 0.995498873095585 | 0.978833708518986 | 1.01244777096411 | 0.99380795269851 |
| ebi-a-GCST90001501 | finngen_R11_O15_PREECLAMPS | Pre-eclampsia \|\| id:finngen_R11_O15_PREECLAMPS | Activated & secreting CD4 regulatory T cell Absolute Count \|\| id:ebi-a-GCST90001501 | Weighted median | 22 | 0.00469647034644674 | 0.0110969726724864 | 0.672134126768775 | -0.017053596 | 0.0264465367845201 | 1.00470751604847 | 0.983090993387941 | 1.0267993497993 | 0.941872178433165 |
| ebi-a-GCST90001501 | finngen_R11_O15_PREECLAMPS | Pre-eclampsia \|\| id:finngen_R11_O15_PREECLAMPS | Activated & secreting CD4 regulatory T cell Absolute Count \|\| id:ebi-a-GCST90001501 | Weighted mode | 22 | 0.000610401228947066 | 0.00922485533588937 | 0.947869032222773 | -0.017470315 | 0.0186911176872902 | 1.00061058756169 | 0.982681405904226 | 1.01886689004689 | 0.990180645930572 |
| ebi-a-GCST90001502 | finngen_R11_O15_PREECLAMPS | Pre-eclampsia \|\| id:finngen_R11_O15_PREECLAMPS | Activated & secreting CD4 regulatory T cell %CD4 regulatory T cell \|\| id:ebi-a-GCST90001502 | Inverse variance weighted (multiplicative random effects) | 36 | -0.003568478 | 0.00793957669952052 | 0.653103869359247 | -0.019130048 | 0.0119930923574131 | 0.996437881477108 | 0.981051769826901 | 1.01206529785658 | 0.94279131784568 |
| ebi-a-GCST90001502 | finngen_R11_O15_PREECLAMPS | Pre-eclampsia \|\| id:finngen_R11_O15_PREECLAMPS | Activated & secreting CD4 regulatory T cell %CD4 regulatory T cell \|\| id:ebi-a-GCST90001502 | MR Egger | 36 | -0.010328111 | 0.0093314310579366 | 0.276146137677097 | -0.028617716 | 0.00796149372264789 | 0.989725040645794 | 0.971787892405494 | 1.00799327068852 | 0.99380795269851 |
| ebi-a-GCST90001502 | finngen_R11_O15_PREECLAMPS | Pre-eclampsia \|\| id:finngen_R11_O15_PREECLAMPS | Activated & secreting CD4 regulatory T cell %CD4 regulatory T cell \|\| id:ebi-a-GCST90001502 | Weighted median | 36 | 0.00222531583168353 | 0.0094506837859764 | 0.813846972856748 | -0.016298024 | 0.0207486560521973 | 1.00222779368462 | 0.983834069812227 | 1.02096540591001 | 0.961297867845628 |
| ebi-a-GCST90001502 | finngen_R11_O15_PREECLAMPS | Pre-eclampsia \|\| id:finngen_R11_O15_PREECLAMPS | Activated & secreting CD4 regulatory T cell %CD4 regulatory T cell \|\| id:ebi-a-GCST90001502 | Weighted mode | 36 | -0.003217917 | 0.00679110970103598 | 0.63855473560151 | -0.016528492 | 0.0100926575824327 | 0.99678725451558 | 0.98360735361237 | 1.01014376022678 | 0.959313411571287 |
| ebi-a-GCST90001503 | finngen_R11_O15_PREECLAMPS | Pre-eclampsia \|\| id:finngen_R11_O15_PREECLAMPS | Activated & secreting CD4 regulatory T cell %CD4+ T cell \|\| id:ebi-a-GCST90001503 | Inverse variance weighted | 32 | -0.003300532 | 0.00461704380206416 | 0.474696634363612 | -0.012349938 | 0.00574887397822628 | 0.99670490889405 | 0.987726009784542 | 1.00576543046608 | 0.898694924660397 |
| ebi-a-GCST90001503 | finngen_R11_O15_PREECLAMPS | Pre-eclampsia \|\| id:finngen_R11_O15_PREECLAMPS | Activated & secreting CD4 regulatory T cell %CD4+ T cell \|\| id:ebi-a-GCST90001503 | MR Egger | 32 | -0.002970763 | 0.00506376797026648 | 0.561814209524185 | -0.012895748 | 0.00695422261486722 | 0.997033645741912 | 0.987187046048996 | 1.00697845937098 | 0.99380795269851 |
| ebi-a-GCST90001503 | finngen_R11_O15_PREECLAMPS | Pre-eclampsia \|\| id:finngen_R11_O15_PREECLAMPS | Activated & secreting CD4 regulatory T cell %CD4+ T cell \|\| id:ebi-a-GCST90001503 | Weighted median | 32 | -0.001369496 | 0.00615165260566805 | 0.823829334698001 | -0.013426735 | 0.0106877429141713 | 0.998631441139034 | 0.986663001238474 | 1.01074506085628 | 0.964912412213677 |
| ebi-a-GCST90001503 | finngen_R11_O15_PREECLAMPS | Pre-eclampsia \|\| id:finngen_R11_O15_PREECLAMPS | Activated & secreting CD4 regulatory T cell %CD4+ T cell \|\| id:ebi-a-GCST90001503 | Weighted mode | 32 | -0.000618057 | 0.00531921837529354 | 0.908248916251319 | -0.011043725 | 0.00980761118696968 | 0.999382134129173 | 0.989017033214253 | 1.00985586342295 | 0.984862613465304 |
| ebi-a-GCST90001504 | finngen_R11_O15_PREECLAMPS | Pre-eclampsia \|\| id:finngen_R11_O15_PREECLAMPS | CD25++ CD4+ T cell Absolute Count \|\| id:ebi-a-GCST90001504 | Inverse variance weighted | 23 | 0.00130407147002573 | 0.0153493128429734 | 0.932293509551209 | -0.028780582 | 0.0313887246422537 | 1.00130492214096 | 0.971629634399527 | 1.03188654566237 | 0.986667691552896 |
| ebi-a-GCST90001504 | finngen_R11_O15_PREECLAMPS | Pre-eclampsia \|\| id:finngen_R11_O15_PREECLAMPS | CD25++ CD4+ T cell Absolute Count \|\| id:ebi-a-GCST90001504 | MR Egger | 23 | -0.001645462 | 0.021271089899179 | 0.939072054982843 | -0.043336799 | 0.0400458737583504 | 0.998355890587064 | 0.957588821130895 | 1.04085852118949 | 0.99380795269851 |
| ebi-a-GCST90001504 | finngen_R11_O15_PREECLAMPS | Pre-eclampsia \|\| id:finngen_R11_O15_PREECLAMPS | CD25++ CD4+ T cell Absolute Count \|\| id:ebi-a-GCST90001504 | Weighted median | 23 | 0.00471864470357396 | 0.0233841006839663 | 0.840082001989144 | -0.041114193 | 0.0505514820441478 | 1.00472979503875 | 0.959719530786209 | 1.05185101340142 | 0.964912412213677 |
| ebi-a-GCST90001504 | finngen_R11_O15_PREECLAMPS | Pre-eclampsia \|\| id:finngen_R11_O15_PREECLAMPS | CD25++ CD4+ T cell Absolute Count \|\| id:ebi-a-GCST90001504 | Weighted mode | 23 | -0.00185999 | 0.0190145267514964 | 0.922961443733313 | -0.039128462 | 0.0354084826016108 | 0.998141738877804 | 0.961627168407952 | 1.03604282784505 | 0.986782054514076 |
| ebi-a-GCST90001505 | finngen_R11_O15_PREECLAMPS | Pre-eclampsia \|\| id:finngen_R11_O15_PREECLAMPS | CD25++ CD4+ T cell %CD4+ T cell \|\| id:ebi-a-GCST90001505 | Inverse variance weighted | 26 | -0.020101688 | 0.0192193210130155 | 0.295602401036806 | -0.057771557 | 0.0175681814061586 | 0.980099004147997 | 0.943865542319536 | 1.01772340959842 | 0.885079385681701 |
| ebi-a-GCST90001505 | finngen_R11_O15_PREECLAMPS | Pre-eclampsia \|\| id:finngen_R11_O15_PREECLAMPS | CD25++ CD4+ T cell %CD4+ T cell \|\| id:ebi-a-GCST90001505 | MR Egger | 26 | -0.02825549 | 0.0281266611504582 | 0.325120630423431 | -0.083383746 | 0.0268727653977918 | 0.972139962586733 | 0.919998033618985 | 1.02723709434528 | 0.99380795269851 |
| ebi-a-GCST90001505 | finngen_R11_O15_PREECLAMPS | Pre-eclampsia \|\| id:finngen_R11_O15_PREECLAMPS | CD25++ CD4+ T cell %CD4+ T cell \|\| id:ebi-a-GCST90001505 | Weighted median | 26 | -0.030772032 | 0.0278482057757558 | 0.269163244233767 | -0.085354516 | 0.0238104508459417 | 0.96969660721676 | 0.918186715001873 | 1.024096183 | 0.940845681141708 |
| ebi-a-GCST90001505 | finngen_R11_O15_PREECLAMPS | Pre-eclampsia \|\| id:finngen_R11_O15_PREECLAMPS | CD25++ CD4+ T cell %CD4+ T cell \|\| id:ebi-a-GCST90001505 | Weighted mode | 26 | -0.026541829 | 0.0264145402988349 | 0.324611229617806 | -0.078314328 | 0.0252306697457244 | 0.973807309363946 | 0.924673729805989 | 1.02555165698242 | 0.888599269773114 |
| ebi-a-GCST90001506 | finngen_R11_O15_PREECLAMPS | Pre-eclampsia \|\| id:finngen_R11_O15_PREECLAMPS | CD25++ CD4+ T cell %T cell \|\| id:ebi-a-GCST90001506 | Inverse variance weighted | 19 | -0.027312042 | 0.0207282076323167 | 0.187628600541389 | -0.067939329 | 0.0133152445076012 | 0.973057558879506 | 0.934317157429854 | 1.013404287 | 0.855687495048373 |
| ebi-a-GCST90001506 | finngen_R11_O15_PREECLAMPS | Pre-eclampsia \|\| id:finngen_R11_O15_PREECLAMPS | CD25++ CD4+ T cell %T cell \|\| id:ebi-a-GCST90001506 | MR Egger | 19 | -0.038646738 | 0.0285563860989489 | 0.1936674776576 | -0.094617255 | 0.0173237787539349 | 0.962090519142822 | 0.909721059253094 | 1.01747470569106 | 0.99380795269851 |
| ebi-a-GCST90001506 | finngen_R11_O15_PREECLAMPS | Pre-eclampsia \|\| id:finngen_R11_O15_PREECLAMPS | CD25++ CD4+ T cell %T cell \|\| id:ebi-a-GCST90001506 | Weighted median | 19 | -0.030533528 | 0.0308831294728257 | 0.322819804918527 | -0.091064462 | 0.029997405976871 | 0.969927911982831 | 0.9129588582533 | 1.03045186093409 | 0.940845681141708 |
| ebi-a-GCST90001506 | finngen_R11_O15_PREECLAMPS | Pre-eclampsia \|\| id:finngen_R11_O15_PREECLAMPS | CD25++ CD4+ T cell %T cell \|\| id:ebi-a-GCST90001506 | Weighted mode | 19 | -0.030981282 | 0.0307837516854494 | 0.327553600011809 | -0.091317436 | 0.0293548709672972 | 0.96949371956371 | 0.912727932533605 | 1.02978997220387 | 0.888599269773114 |
| ebi-a-GCST90001507 | finngen_R11_O15_PREECLAMPS | Pre-eclampsia \|\| id:finngen_R11_O15_PREECLAMPS | CD25++ CD45RA+ CD4 not regulatory T cell Absolute Count \|\| id:ebi-a-GCST90001507 | Inverse variance weighted | 26 | 0.0124504058645424 | 0.0126806034104502 | 0.326175484142405 | -0.012403577 | 0.0373043885490248 | 1.01252823483298 | 0.987673030477077 | 1.03800893078835 | 0.885079385681701 |
| ebi-a-GCST90001507 | finngen_R11_O15_PREECLAMPS | Pre-eclampsia \|\| id:finngen_R11_O15_PREECLAMPS | CD25++ CD45RA+ CD4 not regulatory T cell Absolute Count \|\| id:ebi-a-GCST90001507 | MR Egger | 26 | 0.00805436386764956 | 0.0171585780403818 | 0.643015105162084 | -0.025576449 | 0.0416851768267978 | 1.00808688751677 | 0.974747857529081 | 1.04256620307875 | 0.99380795269851 |
| ebi-a-GCST90001507 | finngen_R11_O15_PREECLAMPS | Pre-eclampsia \|\| id:finngen_R11_O15_PREECLAMPS | CD25++ CD45RA+ CD4 not regulatory T cell Absolute Count \|\| id:ebi-a-GCST90001507 | Weighted median | 26 | 0.0101545982621754 | 0.0172874282747895 | 0.556936535061894 | -0.023728761 | 0.0440379576807627 | 1.01020633115575 | 0.976550552281002 | 1.04502202074788 | 0.940845681141708 |
| ebi-a-GCST90001507 | finngen_R11_O15_PREECLAMPS | Pre-eclampsia \|\| id:finngen_R11_O15_PREECLAMPS | CD25++ CD45RA+ CD4 not regulatory T cell Absolute Count \|\| id:ebi-a-GCST90001507 | Weighted mode | 26 | 0.00716521865066055 | 0.0149108014410037 | 0.635022783921348 | -0.022059952 | 0.0363903894750278 | 1.00719095025062 | 0.978181589180703 | 1.03706062502812 | 0.959313411571287 |
| ebi-a-GCST90001508 | finngen_R11_O15_PREECLAMPS | Pre-eclampsia \|\| id:finngen_R11_O15_PREECLAMPS | CD25++ CD45RA+ CD4 not regulatory T cell %CD4+ T cell \|\| id:ebi-a-GCST90001508 | Inverse variance weighted | 32 | -0.007580293 | 0.00888351404765129 | 0.393493554207892 | -0.024991981 | 0.00983139451642483 | 0.992448364946501 | 0.975317733508409 | 1.00987988144326 | 0.885079385681701 |
| ebi-a-GCST90001508 | finngen_R11_O15_PREECLAMPS | Pre-eclampsia \|\| id:finngen_R11_O15_PREECLAMPS | CD25++ CD45RA+ CD4 not regulatory T cell %CD4+ T cell \|\| id:ebi-a-GCST90001508 | MR Egger | 32 | 0.000533778956681827 | 0.0109722367138893 | 0.961522140028941 | -0.020971805 | 0.0220393629159049 | 1.00053392144202 | 0.9792465740349 | 1.02228402375852 | 0.99380795269851 |
| ebi-a-GCST90001508 | finngen_R11_O15_PREECLAMPS | Pre-eclampsia \|\| id:finngen_R11_O15_PREECLAMPS | CD25++ CD45RA+ CD4 not regulatory T cell %CD4+ T cell \|\| id:ebi-a-GCST90001508 | Weighted median | 32 | -0.003369459 | 0.0126861588091807 | 0.790546389202149 | -0.02823433 | 0.0214954127221575 | 0.996636211711252 | 0.972160533915234 | 1.02172810337594 | 0.957984984509652 |
| ebi-a-GCST90001508 | finngen_R11_O15_PREECLAMPS | Pre-eclampsia \|\| id:finngen_R11_O15_PREECLAMPS | CD25++ CD45RA+ CD4 not regulatory T cell %CD4+ T cell \|\| id:ebi-a-GCST90001508 | Weighted mode | 32 | -0.001295381 | 0.010781001587905 | 0.905136501502781 | -0.022426144 | 0.0198353824019365 | 0.998705457933075 | 0.977823452828866 | 1.02003341075506 | 0.98361388732834 |
| ebi-a-GCST90001509 | finngen_R11_O15_PREECLAMPS | Pre-eclampsia \|\| id:finngen_R11_O15_PREECLAMPS | CD25++ CD45RA+ CD4 not regulatory T cell %T cell \|\| id:ebi-a-GCST90001509 | Inverse variance weighted | 27 | -0.001733565 | 0.00956578809517704 | 0.856190577508149 | -0.020482509 | 0.0170153799071943 | 0.998267936996113 | 0.979725832291927 | 1.0171609660462 | 0.977824840984872 |
| ebi-a-GCST90001509 | finngen_R11_O15_PREECLAMPS | Pre-eclampsia \|\| id:finngen_R11_O15_PREECLAMPS | CD25++ CD45RA+ CD4 not regulatory T cell %T cell \|\| id:ebi-a-GCST90001509 | MR Egger | 27 | -0.006311563 | 0.0121281078346779 | 0.607357841051541 | -0.030082655 | 0.0174595279722083 | 0.993708312694054 | 0.970365324940368 | 1.01761283646233 | 0.99380795269851 |
| ebi-a-GCST90001509 | finngen_R11_O15_PREECLAMPS | Pre-eclampsia \|\| id:finngen_R11_O15_PREECLAMPS | CD25++ CD45RA+ CD4 not regulatory T cell %T cell \|\| id:ebi-a-GCST90001509 | Weighted median | 27 | -0.003303375 | 0.0128285808464536 | 0.796791888620887 | -0.028447393 | 0.0218406434894078 | 0.996702075170517 | 0.971953423938349 | 1.02208089624696 | 0.957984984509652 |
| ebi-a-GCST90001509 | finngen_R11_O15_PREECLAMPS | Pre-eclampsia \|\| id:finngen_R11_O15_PREECLAMPS | CD25++ CD45RA+ CD4 not regulatory T cell %T cell \|\| id:ebi-a-GCST90001509 | Weighted mode | 27 | -0.00235642 | 0.0110815391688206 | 0.833263270035107 | -0.024076236 | 0.0193633971614242 | 0.997646354567754 | 0.976211284106215 | 1.01955208363893 | 0.977585971930092 |
| ebi-a-GCST90001510 | finngen_R11_O15_PREECLAMPS | Pre-eclampsia \|\| id:finngen_R11_O15_PREECLAMPS | CD25++ CD45RA- CD4 not regulatory T cell Absolute Count \|\| id:ebi-a-GCST90001510 | Inverse variance weighted | 20 | 0.00571620770820004 | 0.00984175612469183 | 0.561367327505736 | -0.013573634 | 0.025006049712596 | 1.00573257639756 | 0.98651807207929 | 1.02532132340499 | 0.90609555750305 |
| ebi-a-GCST90001510 | finngen_R11_O15_PREECLAMPS | Pre-eclampsia \|\| id:finngen_R11_O15_PREECLAMPS | CD25++ CD45RA- CD4 not regulatory T cell Absolute Count \|\| id:ebi-a-GCST90001510 | MR Egger | 20 | 0.00499812065963411 | 0.0136616382948891 | 0.718740714711545 | -0.02177869 | 0.0317749317176167 | 1.00501063210058 | 0.978456752965812 | 1.03228514451317 | 0.99380795269851 |
| ebi-a-GCST90001510 | finngen_R11_O15_PREECLAMPS | Pre-eclampsia \|\| id:finngen_R11_O15_PREECLAMPS | CD25++ CD45RA- CD4 not regulatory T cell Absolute Count \|\| id:ebi-a-GCST90001510 | Weighted median | 20 | 0.0109902742692622 | 0.0134351536692628 | 0.413343647960979 | -0.015342627 | 0.0373231754610174 | 1.01105088918819 | 0.984774471547007 | 1.03802843195396 | 0.940845681141708 |
| ebi-a-GCST90001510 | finngen_R11_O15_PREECLAMPS | Pre-eclampsia \|\| id:finngen_R11_O15_PREECLAMPS | CD25++ CD45RA- CD4 not regulatory T cell Absolute Count \|\| id:ebi-a-GCST90001510 | Weighted mode | 20 | 0.0061220313627377 | 0.0108970333456089 | 0.580814922649799 | -0.015236154 | 0.0274802167201311 | 1.00614080929688 | 0.984879328950391 | 1.02786128043873 | 0.948158355498791 |
| ebi-a-GCST90001511 | finngen_R11_O15_PREECLAMPS | Pre-eclampsia \|\| id:finngen_R11_O15_PREECLAMPS | CD25++ CD45RA- CD4 not regulatory T cell %CD4+ T cell \|\| id:ebi-a-GCST90001511 | Inverse variance weighted | 24 | 0.00195147996181037 | 0.0149948851292937 | 0.896453257261046 | -0.027438495 | 0.0313414548152259 | 1.00195338533806 | 0.97293452115643 | 1.03183776971667 | 0.986667691552896 |
| ebi-a-GCST90001511 | finngen_R11_O15_PREECLAMPS | Pre-eclampsia \|\| id:finngen_R11_O15_PREECLAMPS | CD25++ CD45RA- CD4 not regulatory T cell %CD4+ T cell \|\| id:ebi-a-GCST90001511 | MR Egger | 24 | 0.00444531905675488 | 0.0186442976100607 | 0.813756128911055 | -0.032097504 | 0.0409881423724738 | 1.00445521414435 | 0.968412153166296 | 1.04183975172452 | 0.99380795269851 |
| ebi-a-GCST90001511 | finngen_R11_O15_PREECLAMPS | Pre-eclampsia \|\| id:finngen_R11_O15_PREECLAMPS | CD25++ CD45RA- CD4 not regulatory T cell %CD4+ T cell \|\| id:ebi-a-GCST90001511 | Weighted median | 24 | 0.0102350034926764 | 0.01588646787281 | 0.519407351192166 | -0.020902474 | 0.041372480523384 | 1.01028756029424 | 0.979314468987595 | 1.0422402474462 | 0.940845681141708 |
| ebi-a-GCST90001511 | finngen_R11_O15_PREECLAMPS | Pre-eclampsia \|\| id:finngen_R11_O15_PREECLAMPS | CD25++ CD45RA- CD4 not regulatory T cell %CD4+ T cell \|\| id:ebi-a-GCST90001511 | Weighted mode | 24 | 0.00134567346187731 | 0.0137129936037548 | 0.922678260438277 | -0.025531794 | 0.0282231409252367 | 1.00134657928668 | 0.974791385954278 | 1.02862518719073 | 0.986782054514076 |
| ebi-a-GCST90001512 | finngen_R11_O15_PREECLAMPS | Pre-eclampsia \|\| id:finngen_R11_O15_PREECLAMPS | CD25++ CD45RA- CD4 not regulatory T cell %T cell \|\| id:ebi-a-GCST90001512 | Inverse variance weighted | 28 | 0.000540743273809283 | 0.0113969911482618 | 0.962157653469616 | -0.021797359 | 0.0228788459244023 | 1.00054088950181 | 0.978438486348301 | 1.02314257414514 | 0.992671503887443 |
| ebi-a-GCST90001512 | finngen_R11_O15_PREECLAMPS | Pre-eclampsia \|\| id:finngen_R11_O15_PREECLAMPS | CD25++ CD45RA- CD4 not regulatory T cell %T cell \|\| id:ebi-a-GCST90001512 | MR Egger | 28 | 0.00233838967138695 | 0.0145384917875118 | 0.873461634935059 | -0.026157054 | 0.0308338335749102 | 1.00234112583684 | 0.974182078175269 | 1.03131411986734 | 0.99380795269851 |
| ebi-a-GCST90001512 | finngen_R11_O15_PREECLAMPS | Pre-eclampsia \|\| id:finngen_R11_O15_PREECLAMPS | CD25++ CD45RA- CD4 not regulatory T cell %T cell \|\| id:ebi-a-GCST90001512 | Weighted median | 28 | 0.00176038532986662 | 0.0155171776136211 | 0.909675766722107 | -0.028653283 | 0.0321740534525641 | 1.00176193571775 | 0.971753329665269 | 1.03269723418251 | 0.971329194634319 |
| ebi-a-GCST90001512 | finngen_R11_O15_PREECLAMPS | Pre-eclampsia \|\| id:finngen_R11_O15_PREECLAMPS | CD25++ CD45RA- CD4 not regulatory T cell %T cell \|\| id:ebi-a-GCST90001512 | Weighted mode | 28 | 0.00158115406794479 | 0.0122161408660211 | 0.897975592400408 | -0.022362482 | 0.0255247901653461 | 1.00158240475113 | 0.977885704804816 | 1.02585333702907 | 0.98361388732834 |
| ebi-a-GCST90001513 | finngen_R11_O15_PREECLAMPS | Pre-eclampsia \|\| id:finngen_R11_O15_PREECLAMPS | CD4 regulatory T cell Absolute Count \|\| id:ebi-a-GCST90001513 | Inverse variance weighted | 20 | 0.0110844700051339 | 0.0239957405801592 | 0.644128006206948 | -0.035947182 | 0.058116121542246 | 1.01114613035629 | 0.964691245651213 | 1.05983805859492 | 0.942541573457101 |
| ebi-a-GCST90001513 | finngen_R11_O15_PREECLAMPS | Pre-eclampsia \|\| id:finngen_R11_O15_PREECLAMPS | CD4 regulatory T cell Absolute Count \|\| id:ebi-a-GCST90001513 | MR Egger | 20 | 0.00107499545306567 | 0.0396024387929965 | 0.978643027530471 | -0.076545785 | 0.0786957754873389 | 1.00107557346778 | 0.9263105025795 | 1.08187513906314 | 0.99380795269851 |
| ebi-a-GCST90001513 | finngen_R11_O15_PREECLAMPS | Pre-eclampsia \|\| id:finngen_R11_O15_PREECLAMPS | CD4 regulatory T cell Absolute Count \|\| id:ebi-a-GCST90001513 | Weighted median | 20 | -0.018376761 | 0.0310523181330837 | 0.553984549745102 | -0.079239304 | 0.0424857829001802 | 0.981791062437943 | 0.923818824283438 | 1.0434012221289 | 0.940845681141708 |
| ebi-a-GCST90001513 | finngen_R11_O15_PREECLAMPS | Pre-eclampsia \|\| id:finngen_R11_O15_PREECLAMPS | CD4 regulatory T cell Absolute Count \|\| id:ebi-a-GCST90001513 | Weighted mode | 20 | -0.018175352 | 0.0311841886393541 | 0.566855705750128 | -0.079296361 | 0.0429456581649765 | 0.981988823980039 | 0.923766115345848 | 1.04388116689112 | 0.939633861977944 |
| ebi-a-GCST90001514 | finngen_R11_O15_PREECLAMPS | Pre-eclampsia \|\| id:finngen_R11_O15_PREECLAMPS | Hematopoietic Stem Cell Absolute Count \|\| id:ebi-a-GCST90001514 | Inverse variance weighted | 19 | 0.0109426648417002 | 0.0172582747645198 | 0.526046084694699 | -0.022883554 | 0.0447688833801589 | 1.01100275477996 | 0.97737628900278 | 1.04578613342006 | 0.904087927705763 |
| ebi-a-GCST90001514 | finngen_R11_O15_PREECLAMPS | Pre-eclampsia \|\| id:finngen_R11_O15_PREECLAMPS | Hematopoietic Stem Cell Absolute Count \|\| id:ebi-a-GCST90001514 | MR Egger | 19 | 0.0289838058231252 | 0.0251764566744201 | 0.265574126367534 | -0.020362049 | 0.0783296609049886 | 1.02940792392615 | 0.979843857337974 | 1.08147912129681 | 0.99380795269851 |
| ebi-a-GCST90001514 | finngen_R11_O15_PREECLAMPS | Pre-eclampsia \|\| id:finngen_R11_O15_PREECLAMPS | Hematopoietic Stem Cell Absolute Count \|\| id:ebi-a-GCST90001514 | Weighted median | 19 | 0.00474447326360784 | 0.025496423294136 | 0.852378986380131 | -0.045228516 | 0.0547174629201144 | 1.00475574609772 | 0.955779045696001 | 1.05624214494181 | 0.964912412213677 |
| ebi-a-GCST90001514 | finngen_R11_O15_PREECLAMPS | Pre-eclampsia \|\| id:finngen_R11_O15_PREECLAMPS | Hematopoietic Stem Cell Absolute Count \|\| id:ebi-a-GCST90001514 | Weighted mode | 19 | -0.01124086 | 0.0269498208737878 | 0.681535219343976 | -0.064062509 | 0.0415807892204934 | 0.988822082708191 | 0.937946367979463 | 1.04245737776851 | 0.959313411571287 |
| ebi-a-GCST90001515 | finngen_R11_O15_PREECLAMPS | Pre-eclampsia \|\| id:finngen_R11_O15_PREECLAMPS | Immature Myeloid-Derived Suppressor Cells Absolute Count \|\| id:ebi-a-GCST90001515 | Inverse variance weighted | 22 | -0.048040833 | 0.0172945726676167 | 0.00547285913484939 | -0.081938196 | -0.014143471 | 0.953094868261729 | 0.921328898986054 | 0.985956078124272 | 0.500082503446863 |
| ebi-a-GCST90001515 | finngen_R11_O15_PREECLAMPS | Pre-eclampsia \|\| id:finngen_R11_O15_PREECLAMPS | Immature Myeloid-Derived Suppressor Cells Absolute Count \|\| id:ebi-a-GCST90001515 | MR Egger | 22 | -0.01234837 | 0.0330113926066632 | 0.712293084154948 | -0.077050699 | 0.0523539595559807 | 0.987727558315714 | 0.925842912678867 | 1.05374866091859 | 0.99380795269851 |
| ebi-a-GCST90001515 | finngen_R11_O15_PREECLAMPS | Pre-eclampsia \|\| id:finngen_R11_O15_PREECLAMPS | Immature Myeloid-Derived Suppressor Cells Absolute Count \|\| id:ebi-a-GCST90001515 | Weighted median | 22 | -0.018626357 | 0.0251166754966782 | 0.458333809928037 | -0.067855041 | 0.0306023267747667 | 0.981546041347508 | 0.934395912696623 | 1.03107539127022 | 0.940845681141708 |
| ebi-a-GCST90001515 | finngen_R11_O15_PREECLAMPS | Pre-eclampsia \|\| id:finngen_R11_O15_PREECLAMPS | Immature Myeloid-Derived Suppressor Cells Absolute Count \|\| id:ebi-a-GCST90001515 | Weighted mode | 22 | -0.010179791 | 0.0306672905115451 | 0.743222558987692 | -0.070287681 | 0.049928098299056 | 0.989871847597609 | 0.93212562695865 | 1.05119551091344 | 0.959313411571287 |
| ebi-a-GCST90001516 | finngen_R11_O15_PREECLAMPS | Pre-eclampsia \|\| id:finngen_R11_O15_PREECLAMPS | Immature Myeloid-Derived Suppressor Cells %CD33dim HLA DR- CD66b- \|\| id:ebi-a-GCST90001516 | Inverse variance weighted | 19 | -0.027103119 | 0.0154564572231823 | 0.0795137615278896 | -0.057397775 | 0.00319153688662322 | 0.973260874397854 | 0.944218407775486 | 1.00319663526292 | 0.789997151112252 |
| ebi-a-GCST90001516 | finngen_R11_O15_PREECLAMPS | Pre-eclampsia \|\| id:finngen_R11_O15_PREECLAMPS | Immature Myeloid-Derived Suppressor Cells %CD33dim HLA DR- CD66b- \|\| id:ebi-a-GCST90001516 | MR Egger | 19 | -0.014675704 | 0.0230108125618035 | 0.532117818560853 | -0.059776897 | 0.0304254887091282 | 0.985431459358015 | 0.941974667956638 | 1.03089307401329 | 0.99380795269851 |
| ebi-a-GCST90001516 | finngen_R11_O15_PREECLAMPS | Pre-eclampsia \|\| id:finngen_R11_O15_PREECLAMPS | Immature Myeloid-Derived Suppressor Cells %CD33dim HLA DR- CD66b- \|\| id:ebi-a-GCST90001516 | Weighted median | 19 | -0.027383366 | 0.0235380789436006 | 0.244681632328893 | -0.073518001 | 0.0187512684694415 | 0.97298815918369 | 0.929119420572076 | 1.01892817753046 | 0.940845681141708 |
| ebi-a-GCST90001516 | finngen_R11_O15_PREECLAMPS | Pre-eclampsia \|\| id:finngen_R11_O15_PREECLAMPS | Immature Myeloid-Derived Suppressor Cells %CD33dim HLA DR- CD66b- \|\| id:ebi-a-GCST90001516 | Weighted mode | 19 | -0.024040441 | 0.0209213605270786 | 0.265561107633153 | -0.065046308 | 0.0169654256828181 | 0.976246228634418 | 0.937024071052462 | 1.01711015582818 | 0.872755742358858 |
| ebi-a-GCST90001517 | finngen_R11_O15_PREECLAMPS | Pre-eclampsia \|\| id:finngen_R11_O15_PREECLAMPS | CD33+ HLA DR+ Absolute Count \|\| id:ebi-a-GCST90001517 | Inverse variance weighted | 30 | -0.024976537 | 0.00858193696492744 | 0.00361011233450393 | -0.041797133 | -0.00815594 | 0.975332796299967 | 0.959064323209552 | 0.991877229209019 | 0.447221286871335 |
| ebi-a-GCST90001517 | finngen_R11_O15_PREECLAMPS | Pre-eclampsia \|\| id:finngen_R11_O15_PREECLAMPS | CD33+ HLA DR+ Absolute Count \|\| id:ebi-a-GCST90001517 | MR Egger | 30 | -0.029782789 | 0.0103496207482556 | 0.00758503288830292 | -0.050068045 | -0.009497532 | 0.970656348167824 | 0.951164699936742 | 0.99054742706616 | 0.565056961366001 |
| ebi-a-GCST90001517 | finngen_R11_O15_PREECLAMPS | Pre-eclampsia \|\| id:finngen_R11_O15_PREECLAMPS | CD33+ HLA DR+ Absolute Count \|\| id:ebi-a-GCST90001517 | Weighted median | 30 | -0.02853471 | 0.0117126008268312 | 0.0148408234956498 | -0.051491407 | -0.005578012 | 0.971868560307492 | 0.949811811368203 | 0.994437516157613 | 0.598716529366782 |
| ebi-a-GCST90001517 | finngen_R11_O15_PREECLAMPS | Pre-eclampsia \|\| id:finngen_R11_O15_PREECLAMPS | CD33+ HLA DR+ Absolute Count \|\| id:ebi-a-GCST90001517 | Weighted mode | 30 | -0.029176117 | 0.0108101738835311 | 0.0114788252769974 | -0.050364058 | -0.007988177 | 0.97124539615763 | 0.95088318461628 | 0.992043644076069 | 0.720210568263475 |
| ebi-a-GCST90001518 | finngen_R11_O15_PREECLAMPS | Pre-eclampsia \|\| id:finngen_R11_O15_PREECLAMPS | CD33+ HLA DR+ CD14- Absolute Count \|\| id:ebi-a-GCST90001518 | Inverse variance weighted | 29 | -0.024400205 | 0.00700418561488987 | 0.000494606427772848 | -0.038128409 | -0.010672001 | 0.975895073623264 | 0.962589328123138 | 0.989384742690941 | 0.137722935518292 |
| ebi-a-GCST90001518 | finngen_R11_O15_PREECLAMPS | Pre-eclampsia \|\| id:finngen_R11_O15_PREECLAMPS | CD33+ HLA DR+ CD14- Absolute Count \|\| id:ebi-a-GCST90001518 | MR Egger | 29 | -0.02169816 | 0.00824763821280227 | 0.0139008253722873 | -0.037863531 | -0.005532789 | 0.978535551960393 | 0.962844330732432 | 0.994482488900401 | 0.677433556476132 |
| ebi-a-GCST90001518 | finngen_R11_O15_PREECLAMPS | Pre-eclampsia \|\| id:finngen_R11_O15_PREECLAMPS | CD33+ HLA DR+ CD14- Absolute Count \|\| id:ebi-a-GCST90001518 | Weighted median | 29 | -0.023461717 | 0.00954652833547574 | 0.0139861985168416 | -0.042172912 | -0.004750521 | 0.976811369533578 | 0.958703994604354 | 0.995260744734703 | 0.598716529366782 |
| ebi-a-GCST90001518 | finngen_R11_O15_PREECLAMPS | Pre-eclampsia \|\| id:finngen_R11_O15_PREECLAMPS | CD33+ HLA DR+ CD14- Absolute Count \|\| id:ebi-a-GCST90001518 | Weighted mode | 29 | -0.024332036 | 0.00896452619674595 | 0.0112411783029618 | -0.041902507 | -0.006761564 | 0.975961601855024 | 0.958963268172932 | 0.99326124358254 | 0.720210568263475 |
| ebi-a-GCST90001519 | finngen_R11_O15_PREECLAMPS | Pre-eclampsia \|\| id:finngen_R11_O15_PREECLAMPS | CD33+ HLA DR+ CD14- %CD33+ HLA DR+ \|\| id:ebi-a-GCST90001519 | Inverse variance weighted | 19 | 0.00917854530788282 | 0.0152745991484608 | 0.547904906490471 | -0.020759669 | 0.039116759638866 | 1.00922079732662 | 0.979454329501422 | 1.03989189396411 | 0.905435115099076 |
| ebi-a-GCST90001519 | finngen_R11_O15_PREECLAMPS | Pre-eclampsia \|\| id:finngen_R11_O15_PREECLAMPS | CD33+ HLA DR+ CD14- %CD33+ HLA DR+ \|\| id:ebi-a-GCST90001519 | MR Egger | 19 | 0.00642946980269457 | 0.0231005919241106 | 0.784119936974697 | -0.03884769 | 0.0517066299739514 | 1.00645018321195 | 0.961897204198432 | 1.05306675896982 | 0.99380795269851 |
| ebi-a-GCST90001519 | finngen_R11_O15_PREECLAMPS | Pre-eclampsia \|\| id:finngen_R11_O15_PREECLAMPS | CD33+ HLA DR+ CD14- %CD33+ HLA DR+ \|\| id:ebi-a-GCST90001519 | Weighted median | 19 | 0.0128088518966639 | 0.0228999007611321 | 0.575929063511805 | -0.032074954 | 0.0576926573884828 | 1.01289123661556 | 0.968433991749427 | 1.05938935018098 | 0.940845681141708 |
| ebi-a-GCST90001519 | finngen_R11_O15_PREECLAMPS | Pre-eclampsia \|\| id:finngen_R11_O15_PREECLAMPS | CD33+ HLA DR+ CD14- %CD33+ HLA DR+ \|\| id:ebi-a-GCST90001519 | Weighted mode | 19 | 0.0163465707739728 | 0.0216343387985451 | 0.459672469735012 | -0.026056733 | 0.0587498748191211 | 1.01648090694237 | 0.974279813959894 | 1.06050994732086 | 0.933945646747087 |
| ebi-a-GCST90001520 | finngen_R11_O15_PREECLAMPS | Pre-eclampsia \|\| id:finngen_R11_O15_PREECLAMPS | CD33+ HLA DR+ CD14dim Absolute Count \|\| id:ebi-a-GCST90001520 | Inverse variance weighted | 28 | -0.025768854 | 0.0105306025325613 | 0.0144033023701168 | -0.046408835 | -0.005128873 | 0.974560329470152 | 0.954651587618154 | 0.994884257351556 | 0.588591724205005 |
| ebi-a-GCST90001520 | finngen_R11_O15_PREECLAMPS | Pre-eclampsia \|\| id:finngen_R11_O15_PREECLAMPS | CD33+ HLA DR+ CD14dim Absolute Count \|\| id:ebi-a-GCST90001520 | MR Egger | 28 | -0.032789425 | 0.0132525018094549 | 0.0202003961723592 | -0.058764328 | -0.006814521 | 0.967742320920612 | 0.942928964848313 | 0.993208645204223 | 0.716086784616111 |
| ebi-a-GCST90001520 | finngen_R11_O15_PREECLAMPS | Pre-eclampsia \|\| id:finngen_R11_O15_PREECLAMPS | CD33+ HLA DR+ CD14dim Absolute Count \|\| id:ebi-a-GCST90001520 | Weighted median | 28 | -0.026413725 | 0.0150986325110403 | 0.0802198671274886 | -0.056007045 | 0.00317959423736968 | 0.973932065728334 | 0.945532474395955 | 1.00318465450891 | 0.875234669704391 |
| ebi-a-GCST90001520 | finngen_R11_O15_PREECLAMPS | Pre-eclampsia \|\| id:finngen_R11_O15_PREECLAMPS | CD33+ HLA DR+ CD14dim Absolute Count \|\| id:ebi-a-GCST90001520 | Weighted mode | 28 | -0.027172987 | 0.012495431655011 | 0.0385901727532429 | -0.051664033 | -0.002681941 | 0.973192877623576 | 0.94964786392423 | 0.997321652621359 | 0.838871423749761 |
| ebi-a-GCST90001521 | finngen_R11_O15_PREECLAMPS | Pre-eclampsia \|\| id:finngen_R11_O15_PREECLAMPS | CD33+ HLA DR+ CD14dim %CD33+ HLA DR+ \|\| id:ebi-a-GCST90001521 | Inverse variance weighted (multiplicative random effects) | 25 | -0.035432443 | 0.0212564203260562 | 0.0955332074446498 | -0.077095027 | 0.00623014099413138 | 0.965187937378655 | 0.925801873544243 | 1.00624958868885 | 0.802698559103897 |
| ebi-a-GCST90001521 | finngen_R11_O15_PREECLAMPS | Pre-eclampsia \|\| id:finngen_R11_O15_PREECLAMPS | CD33+ HLA DR+ CD14dim %CD33+ HLA DR+ \|\| id:ebi-a-GCST90001521 | MR Egger | 25 | -0.010916951 | 0.031809799085212 | 0.734569226963415 | -0.073264158 | 0.0514302548333896 | 0.989142422283828 | 0.929355301350129 | 1.05277575771089 | 0.99380795269851 |
| ebi-a-GCST90001521 | finngen_R11_O15_PREECLAMPS | Pre-eclampsia \|\| id:finngen_R11_O15_PREECLAMPS | CD33+ HLA DR+ CD14dim %CD33+ HLA DR+ \|\| id:ebi-a-GCST90001521 | Weighted median | 25 | -0.027986175 | 0.0236523554528658 | 0.236718029376286 | -0.074344792 | 0.0183724415138768 | 0.9724018099944 | 0.928351550593943 | 1.01854225317492 | 0.940845681141708 |
| ebi-a-GCST90001521 | finngen_R11_O15_PREECLAMPS | Pre-eclampsia \|\| id:finngen_R11_O15_PREECLAMPS | CD33+ HLA DR+ CD14dim %CD33+ HLA DR+ \|\| id:ebi-a-GCST90001521 | Weighted mode | 25 | -0.009388873 | 0.0250851937926743 | 0.711482946368507 | -0.058555853 | 0.0397781069242703 | 0.990655064941397 | 0.943125562778899 | 1.04057985110951 | 0.959313411571287 |
| ebi-a-GCST90001522 | finngen_R11_O15_PREECLAMPS | Pre-eclampsia \|\| id:finngen_R11_O15_PREECLAMPS | CD33- HLA DR- Absolute Count \|\| id:ebi-a-GCST90001522 | Inverse variance weighted | 21 | 0.00747630412793844 | 0.019206836694232 | 0.697089565682884 | -0.030169096 | 0.0451217040486332 | 1.00750432146817 | 0.970281449165066 | 1.04615517348138 | 0.950911139356934 |
| ebi-a-GCST90001522 | finngen_R11_O15_PREECLAMPS | Pre-eclampsia \|\| id:finngen_R11_O15_PREECLAMPS | CD33- HLA DR- Absolute Count \|\| id:ebi-a-GCST90001522 | MR Egger | 21 | 0.0156554566059669 | 0.0413250127918474 | 0.709008893718536 | -0.065341568 | 0.0966524816779878 | 1.01577864528552 | 0.936747445338495 | 1.10147752348045 | 0.99380795269851 |
| ebi-a-GCST90001522 | finngen_R11_O15_PREECLAMPS | Pre-eclampsia \|\| id:finngen_R11_O15_PREECLAMPS | CD33- HLA DR- Absolute Count \|\| id:ebi-a-GCST90001522 | Weighted median | 21 | 0.00489668014089585 | 0.0281245116243827 | 0.861781210075152 | -0.050227363 | 0.0600207229246859 | 1.00490868847141 | 0.951013175049216 | 1.06185855113214 | 0.964912412213677 |
| ebi-a-GCST90001522 | finngen_R11_O15_PREECLAMPS | Pre-eclampsia \|\| id:finngen_R11_O15_PREECLAMPS | CD33- HLA DR- Absolute Count \|\| id:ebi-a-GCST90001522 | Weighted mode | 21 | 0.0055471541584422 | 0.0489416167470842 | 0.91088921430781 | -0.090378415 | 0.101472722982727 | 1.00556256810607 | 0.913585405735301 | 1.10679972778489 | 0.985000023164215 |
| ebi-a-GCST90001523 | finngen_R11_O15_PREECLAMPS | Pre-eclampsia \|\| id:finngen_R11_O15_PREECLAMPS | CD33- HLA DR+ Absolute Count \|\| id:ebi-a-GCST90001523 | Inverse variance weighted | 36 | -0.004847118 | 0.0101856851347715 | 0.63416310303976 | -0.024811061 | 0.0151168252221053 | 0.995164610675508 | 0.975494203998878 | 1.01523166235242 | 0.942541573457101 |
| ebi-a-GCST90001523 | finngen_R11_O15_PREECLAMPS | Pre-eclampsia \|\| id:finngen_R11_O15_PREECLAMPS | CD33- HLA DR+ Absolute Count \|\| id:ebi-a-GCST90001523 | MR Egger | 36 | 0.00126924147274091 | 0.0133506101161173 | 0.924817400600007 | -0.024897954 | 0.0274364373003309 | 1.00127004730059 | 0.975409443235804 | 1.02781628225324 | 0.99380795269851 |
| ebi-a-GCST90001523 | finngen_R11_O15_PREECLAMPS | Pre-eclampsia \|\| id:finngen_R11_O15_PREECLAMPS | CD33- HLA DR+ Absolute Count \|\| id:ebi-a-GCST90001523 | Weighted median | 36 | 0.00805568218885877 | 0.0142271532645337 | 0.571245025579628 | -0.019829538 | 0.0359409025873448 | 1.00808821649997 | 0.980365773969263 | 1.03659458462281 | 0.940845681141708 |
| ebi-a-GCST90001523 | finngen_R11_O15_PREECLAMPS | Pre-eclampsia \|\| id:finngen_R11_O15_PREECLAMPS | CD33- HLA DR+ Absolute Count \|\| id:ebi-a-GCST90001523 | Weighted mode | 36 | 0.00380014685870672 | 0.0132906272935716 | 0.77661970103532 | -0.022249483 | 0.029849776354107 | 1.00380737657187 | 0.97799621153913 | 1.03029974694313 | 0.961305639715923 |
| ebi-a-GCST90001524 | finngen_R11_O15_PREECLAMPS | Pre-eclampsia \|\| id:finngen_R11_O15_PREECLAMPS | Granulocytic Myeloid-Derived Suppressor Cells Absolute Count \|\| id:ebi-a-GCST90001524 | Inverse variance weighted (multiplicative random effects) | 21 | 0.0260151344795412 | 0.0290039784370489 | 0.369745303215774 | -0.030832663 | 0.082862932216157 | 1.02635648172707 | 0.969637815534954 | 1.08639288887677 | 0.885079385681701 |
| ebi-a-GCST90001524 | finngen_R11_O15_PREECLAMPS | Pre-eclampsia \|\| id:finngen_R11_O15_PREECLAMPS | Granulocytic Myeloid-Derived Suppressor Cells Absolute Count \|\| id:ebi-a-GCST90001524 | MR Egger | 21 | 0.115273315911928 | 0.0540006828915851 | 0.0460323164213204 | 0.00943197744442112 | 0.221114654379435 | 1.12218010534028 | 1.0094765987223 | 1.24746644985669 | 0.862303274556491 |
| ebi-a-GCST90001524 | finngen_R11_O15_PREECLAMPS | Pre-eclampsia \|\| id:finngen_R11_O15_PREECLAMPS | Granulocytic Myeloid-Derived Suppressor Cells Absolute Count \|\| id:ebi-a-GCST90001524 | Weighted median | 21 | 0.0633809625161177 | 0.0300204954500701 | 0.0347502812292497 | 0.00454079143398022 | 0.122221133598255 | 1.0654326518305 | 1.0045511164494 | 1.13000395599455 | 0.721097500353414 |
| ebi-a-GCST90001524 | finngen_R11_O15_PREECLAMPS | Pre-eclampsia \|\| id:finngen_R11_O15_PREECLAMPS | Granulocytic Myeloid-Derived Suppressor Cells Absolute Count \|\| id:ebi-a-GCST90001524 | Weighted mode | 21 | 0.0835117972409146 | 0.0389397941908851 | 0.0444478488928702 | 0.00718980062677975 | 0.159833793855049 | 1.0870980399747 | 1.00721570929881 | 1.1733158424818 | 0.838871423749761 |
| ebi-a-GCST90001525 | finngen_R11_O15_PREECLAMPS | Pre-eclampsia \|\| id:finngen_R11_O15_PREECLAMPS | CD33dim HLA DR+ CD11b+ Absolute Count \|\| id:ebi-a-GCST90001525 | Inverse variance weighted | 27 | -0.015629339 | 0.0173754848371667 | 0.368383632685446 | -0.049685289 | 0.0184266114935364 | 0.984492165494027 | 0.951528833910531 | 1.01859742908248 | 0.885079385681701 |
| ebi-a-GCST90001525 | finngen_R11_O15_PREECLAMPS | Pre-eclampsia \|\| id:finngen_R11_O15_PREECLAMPS | CD33dim HLA DR+ CD11b+ Absolute Count \|\| id:ebi-a-GCST90001525 | MR Egger | 27 | -0.011634167 | 0.0292906739535096 | 0.694593037085944 | -0.069043888 | 0.0457755538487108 | 0.988433248128604 | 0.933285719089946 | 1.04683942550706 | 0.99380795269851 |
| ebi-a-GCST90001525 | finngen_R11_O15_PREECLAMPS | Pre-eclampsia \|\| id:finngen_R11_O15_PREECLAMPS | CD33dim HLA DR+ CD11b+ Absolute Count \|\| id:ebi-a-GCST90001525 | Weighted median | 27 | -0.013604624 | 0.0233528553913825 | 0.560184226597558 | -0.05937622 | 0.0321669728758709 | 0.98648750095473 | 0.942352170480646 | 1.03268992211643 | 0.940845681141708 |
| ebi-a-GCST90001525 | finngen_R11_O15_PREECLAMPS | Pre-eclampsia \|\| id:finngen_R11_O15_PREECLAMPS | CD33dim HLA DR+ CD11b+ Absolute Count \|\| id:ebi-a-GCST90001525 | Weighted mode | 27 | -0.014817695 | 0.0237766446110889 | 0.538579517833187 | -0.061419918 | 0.0317845289238525 | 0.985291547285328 | 0.940428254144913 | 1.03229505161414 | 0.935508728341156 |
| ebi-a-GCST90001526 | finngen_R11_O15_PREECLAMPS | Pre-eclampsia \|\| id:finngen_R11_O15_PREECLAMPS | CD33dim HLA DR+ CD11b+ %CD33dim HLA DR+ \|\| id:ebi-a-GCST90001526 | Inverse variance weighted | 24 | -0.000237601 | 0.0108832667533069 | 0.982582153756927 | -0.021568804 | 0.0210936018916661 | 0.999762427280053 | 0.978662139496811 | 1.02131764442714 | 0.994556635975431 |
| ebi-a-GCST90001526 | finngen_R11_O15_PREECLAMPS | Pre-eclampsia \|\| id:finngen_R11_O15_PREECLAMPS | CD33dim HLA DR+ CD11b+ %CD33dim HLA DR+ \|\| id:ebi-a-GCST90001526 | MR Egger | 24 | -0.021722914 | 0.0155176624147707 | 0.175492670272972 | -0.052137532 | 0.00869170451489968 | 0.978511329459492 | 0.949198312579735 | 1.00872958705366 | 0.99380795269851 |
| ebi-a-GCST90001526 | finngen_R11_O15_PREECLAMPS | Pre-eclampsia \|\| id:finngen_R11_O15_PREECLAMPS | CD33dim HLA DR+ CD11b+ %CD33dim HLA DR+ \|\| id:ebi-a-GCST90001526 | Weighted median | 24 | -0.022033353 | 0.0173307335437656 | 0.203605693463107 | -0.056001591 | 0.0119348842970543 | 0.978207607909805 | 0.945537631354914 | 1.01200638921313 | 0.940845681141708 |
| ebi-a-GCST90001526 | finngen_R11_O15_PREECLAMPS | Pre-eclampsia \|\| id:finngen_R11_O15_PREECLAMPS | CD33dim HLA DR+ CD11b+ %CD33dim HLA DR+ \|\| id:ebi-a-GCST90001526 | Weighted mode | 24 | -0.016180403 | 0.0150030045636689 | 0.292001907015907 | -0.045586292 | 0.0132254855101924 | 0.98394979612011 | 0.955437152069371 | 1.01331332907443 | 0.872755742358858 |
| ebi-a-GCST90001527 | finngen_R11_O15_PREECLAMPS | Pre-eclampsia \|\| id:finngen_R11_O15_PREECLAMPS | CD33dim HLA DR+ CD11b- Absolute Count \|\| id:ebi-a-GCST90001527 | Inverse variance weighted | 28 | -0.004239464 | 0.0106738201643619 | 0.691232227762882 | -0.025160152 | 0.0166812234150862 | 0.995769509734988 | 0.975153727063906 | 1.01682113188793 | 0.950911139356934 |
| ebi-a-GCST90001527 | finngen_R11_O15_PREECLAMPS | Pre-eclampsia \|\| id:finngen_R11_O15_PREECLAMPS | CD33dim HLA DR+ CD11b- Absolute Count \|\| id:ebi-a-GCST90001527 | MR Egger | 28 | -0.00583158 | 0.0133224279838424 | 0.66519943619205 | -0.031943539 | 0.0202803790832666 | 0.994185390891609 | 0.968561266847532 | 1.02048742324722 | 0.99380795269851 |
| ebi-a-GCST90001527 | finngen_R11_O15_PREECLAMPS | Pre-eclampsia \|\| id:finngen_R11_O15_PREECLAMPS | CD33dim HLA DR+ CD11b- Absolute Count \|\| id:ebi-a-GCST90001527 | Weighted median | 28 | -0.009326773 | 0.016189681326491 | 0.564551854985406 | -0.041058549 | 0.0224050021135848 | 0.990716586157688 | 0.959772934858297 | 1.02265787921271 | 0.940845681141708 |
| ebi-a-GCST90001527 | finngen_R11_O15_PREECLAMPS | Pre-eclampsia \|\| id:finngen_R11_O15_PREECLAMPS | CD33dim HLA DR+ CD11b- Absolute Count \|\| id:ebi-a-GCST90001527 | Weighted mode | 28 | -0.003564793 | 0.0127803493532656 | 0.782425650538534 | -0.028614278 | 0.0214846913824628 | 0.996441552982515 | 0.971791233361769 | 1.02171714914059 | 0.961305639715923 |
| ebi-a-GCST90001528 | finngen_R11_O15_PREECLAMPS | Pre-eclampsia \|\| id:finngen_R11_O15_PREECLAMPS | CD33dim HLA DR+ CD11b- %CD33dim HLA DR+ \|\| id:ebi-a-GCST90001528 | Inverse variance weighted | 24 | -0.007580219 | 0.00898499247697371 | 0.39886315589928 | -0.025190804 | 0.010030366462623 | 0.992448438610712 | 0.975123836702454 | 1.01008083920051 | 0.885079385681701 |
| ebi-a-GCST90001528 | finngen_R11_O15_PREECLAMPS | Pre-eclampsia \|\| id:finngen_R11_O15_PREECLAMPS | CD33dim HLA DR+ CD11b- %CD33dim HLA DR+ \|\| id:ebi-a-GCST90001528 | MR Egger | 24 | 0.00510973811168355 | 0.0125332857023429 | 0.687438958852439 | -0.019455502 | 0.0296749780882756 | 1.00512281508729 | 0.980732534986545 | 1.03011966807327 | 0.99380795269851 |
| ebi-a-GCST90001528 | finngen_R11_O15_PREECLAMPS | Pre-eclampsia \|\| id:finngen_R11_O15_PREECLAMPS | CD33dim HLA DR+ CD11b- %CD33dim HLA DR+ \|\| id:ebi-a-GCST90001528 | Weighted median | 24 | -0.002889121 | 0.014581449266851 | 0.842938091089087 | -0.031468761 | 0.0256905199428014 | 0.997115048872397 | 0.969021227057463 | 1.02602336556333 | 0.964912412213677 |
| ebi-a-GCST90001528 | finngen_R11_O15_PREECLAMPS | Pre-eclampsia \|\| id:finngen_R11_O15_PREECLAMPS | CD33dim HLA DR+ CD11b- %CD33dim HLA DR+ \|\| id:ebi-a-GCST90001528 | Weighted mode | 24 | 0.00358027302625163 | 0.0126996121038658 | 0.780526106302734 | -0.021310967 | 0.0284715127498285 | 1.00358668985944 | 0.978914507422559 | 1.02888070043513 | 0.961305639715923 |
| ebi-a-GCST90001529 | finngen_R11_O15_PREECLAMPS | Pre-eclampsia \|\| id:finngen_R11_O15_PREECLAMPS | CD66b++ myeloid cell Absolute Count \|\| id:ebi-a-GCST90001529 | Inverse variance weighted | 16 | -0.030489189 | 0.0255252111693088 | 0.23229278971034 | -0.080518603 | 0.0195402248486067 | 0.969970918324185 | 0.922637739654131 | 1.01973238461648 | 0.884406402490929 |
| ebi-a-GCST90001529 | finngen_R11_O15_PREECLAMPS | Pre-eclampsia \|\| id:finngen_R11_O15_PREECLAMPS | CD66b++ myeloid cell Absolute Count \|\| id:ebi-a-GCST90001529 | MR Egger | 16 | -0.042987234 | 0.0454306403778353 | 0.360084362290103 | -0.132031289 | 0.0460568215871763 | 0.957923619269719 | 0.87631357594315 | 1.04713390907723 | 0.99380795269851 |
| ebi-a-GCST90001529 | finngen_R11_O15_PREECLAMPS | Pre-eclampsia \|\| id:finngen_R11_O15_PREECLAMPS | CD66b++ myeloid cell Absolute Count \|\| id:ebi-a-GCST90001529 | Weighted median | 16 | -0.049359672 | 0.031618120548599 | 0.11849550529543 | -0.111331188 | 0.0126118446777547 | 0.951838718771911 | 0.894642405143971 | 1.01269170938477 | 0.88227178111339 |
| ebi-a-GCST90001529 | finngen_R11_O15_PREECLAMPS | Pre-eclampsia \|\| id:finngen_R11_O15_PREECLAMPS | CD66b++ myeloid cell Absolute Count \|\| id:ebi-a-GCST90001529 | Weighted mode | 16 | -0.072077103 | 0.0433903737242757 | 0.117439543251761 | -0.157122235 | 0.0129680297011933 | 0.930459152041011 | 0.854599590197388 | 1.01305247925161 | 0.872755742358858 |
| ebi-a-GCST90001530 | finngen_R11_O15_PREECLAMPS | Pre-eclampsia \|\| id:finngen_R11_O15_PREECLAMPS | Monocytic Myeloid-Derived Suppressor Cells Absolute Count \|\| id:ebi-a-GCST90001530 | Inverse variance weighted (multiplicative random effects) | 19 | -0.007804487 | 0.0215527993464509 | 0.717269737145711 | -0.050047974 | 0.0344389998373596 | 0.992225889051833 | 0.951183791694696 | 1.03503888890981 | 0.959745573017418 |
| ebi-a-GCST90001530 | finngen_R11_O15_PREECLAMPS | Pre-eclampsia \|\| id:finngen_R11_O15_PREECLAMPS | Monocytic Myeloid-Derived Suppressor Cells Absolute Count \|\| id:ebi-a-GCST90001530 | MR Egger | 19 | -0.042552914 | 0.0416518209017696 | 0.32127361719769 | -0.124190482 | 0.0390846554441524 | 0.958339755046351 | 0.883211588515425 | 1.03985850960815 | 0.99380795269851 |
| ebi-a-GCST90001530 | finngen_R11_O15_PREECLAMPS | Pre-eclampsia \|\| id:finngen_R11_O15_PREECLAMPS | Monocytic Myeloid-Derived Suppressor Cells Absolute Count \|\| id:ebi-a-GCST90001530 | Weighted median | 19 | -0.011915271 | 0.0233521126650951 | 0.609880670745882 | -0.057685412 | 0.0338548701410217 | 0.98815543505055 | 0.943946855551973 | 1.03443446850508 | 0.940845681141708 |
| ebi-a-GCST90001530 | finngen_R11_O15_PREECLAMPS | Pre-eclampsia \|\| id:finngen_R11_O15_PREECLAMPS | Monocytic Myeloid-Derived Suppressor Cells Absolute Count \|\| id:ebi-a-GCST90001530 | Weighted mode | 19 | -0.019843351 | 0.0228005216758972 | 0.395593570867543 | -0.064532374 | 0.0248456712049746 | 0.980352232201574 | 0.937505763179929 | 1.02515689708689 | 0.920286500917063 |
| ebi-a-GCST90001531 | finngen_R11_O15_PREECLAMPS | Pre-eclampsia \|\| id:finngen_R11_O15_PREECLAMPS | CD33dim HLA DR- Absolute Count \|\| id:ebi-a-GCST90001531 | Inverse variance weighted | 24 | -0.021438339 | 0.00796366946570264 | 0.00710211727268354 | -0.037047131 | -0.005829547 | 0.978789828495329 | 0.963630716983657 | 0.994187411713822 | 0.548313994322415 |
| ebi-a-GCST90001531 | finngen_R11_O15_PREECLAMPS | Pre-eclampsia \|\| id:finngen_R11_O15_PREECLAMPS | CD33dim HLA DR- Absolute Count \|\| id:ebi-a-GCST90001531 | MR Egger | 24 | -0.027815857 | 0.00994888840151053 | 0.010533729217937 | -0.047315678 | -0.008316035 | 0.972567442248193 | 0.953786260907949 | 0.991718447297366 | 0.592319696793224 |
| ebi-a-GCST90001531 | finngen_R11_O15_PREECLAMPS | Pre-eclampsia \|\| id:finngen_R11_O15_PREECLAMPS | CD33dim HLA DR- Absolute Count \|\| id:ebi-a-GCST90001531 | Weighted median | 24 | -0.018302509 | 0.0114469474369756 | 0.109843120206234 | -0.040738526 | 0.00413350797391146 | 0.981863964739128 | 0.960080133143825 | 1.00414206270095 | 0.88227178111339 |
| ebi-a-GCST90001531 | finngen_R11_O15_PREECLAMPS | Pre-eclampsia \|\| id:finngen_R11_O15_PREECLAMPS | CD33dim HLA DR- Absolute Count \|\| id:ebi-a-GCST90001531 | Weighted mode | 24 | -0.023993712 | 0.0100366945580317 | 0.0253975573592891 | -0.043665633 | -0.00432179 | 0.976291849093492 | 0.957273984901098 | 0.99568753527222 | 0.790577658379112 |
| ebi-a-GCST90001532 | finngen_R11_O15_PREECLAMPS | Pre-eclampsia \|\| id:finngen_R11_O15_PREECLAMPS | Basophil Absolute Count \|\| id:ebi-a-GCST90001532 | Inverse variance weighted | 23 | -0.019297029 | 0.00813022328045089 | 0.0176207234455336 | -0.035232266 | -0.003361791 | 0.980887967131993 | 0.965381164716131 | 0.996643853464088 | 0.588591724205005 |
| ebi-a-GCST90001532 | finngen_R11_O15_PREECLAMPS | Pre-eclampsia \|\| id:finngen_R11_O15_PREECLAMPS | Basophil Absolute Count \|\| id:ebi-a-GCST90001532 | MR Egger | 23 | -0.031087936 | 0.00982330496758769 | 0.00467024394832819 | -0.050341614 | -0.011834259 | 0.969390324636601 | 0.950904526584304 | 0.988235490764323 | 0.565056961366001 |
| ebi-a-GCST90001532 | finngen_R11_O15_PREECLAMPS | Pre-eclampsia \|\| id:finngen_R11_O15_PREECLAMPS | Basophil Absolute Count \|\| id:ebi-a-GCST90001532 | Weighted median | 23 | -0.01831409 | 0.0112700078244362 | 0.104156372564372 | -0.040403305 | 0.00377512541053731 | 0.981852593914198 | 0.960402025844721 | 1.00378226017185 | 0.88227178111339 |
| ebi-a-GCST90001532 | finngen_R11_O15_PREECLAMPS | Pre-eclampsia \|\| id:finngen_R11_O15_PREECLAMPS | Basophil Absolute Count \|\| id:ebi-a-GCST90001532 | Weighted mode | 23 | -0.025511474 | 0.00902563722761399 | 0.0098239924694255 | -0.043201723 | -0.007821225 | 0.974811193966099 | 0.957718176875893 | 0.992209281211911 | 0.720210568263475 |
| ebi-a-GCST90001533 | finngen_R11_O15_PREECLAMPS | Pre-eclampsia \|\| id:finngen_R11_O15_PREECLAMPS | Basophil %CD33dim HLA DR- CD66b- \|\| id:ebi-a-GCST90001533 | Inverse variance weighted | 19 | 0.00859763234110626 | 0.0219977548858973 | 0.695914504101828 | -0.034517967 | 0.051713231917465 | 1.00863469813191 | 0.966070981905954 | 1.05307371128002 | 0.950911139356934 |
| ebi-a-GCST90001533 | finngen_R11_O15_PREECLAMPS | Pre-eclampsia \|\| id:finngen_R11_O15_PREECLAMPS | Basophil %CD33dim HLA DR- CD66b- \|\| id:ebi-a-GCST90001533 | MR Egger | 19 | -0.00682494 | 0.0448307968724128 | 0.880791620100521 | -0.094693301 | 0.0810434222920186 | 0.993198297428437 | 0.909651880604608 | 1.08441798345879 | 0.99380795269851 |
| ebi-a-GCST90001533 | finngen_R11_O15_PREECLAMPS | Pre-eclampsia \|\| id:finngen_R11_O15_PREECLAMPS | Basophil %CD33dim HLA DR- CD66b- \|\| id:ebi-a-GCST90001533 | Weighted median | 19 | -0.032153181 | 0.0247063845759819 | 0.193117529910044 | -0.080577695 | 0.0162713328529758 | 0.968358236715982 | 0.92258322098649 | 1.01640443190915 | 0.940845681141708 |
| ebi-a-GCST90001533 | finngen_R11_O15_PREECLAMPS | Pre-eclampsia \|\| id:finngen_R11_O15_PREECLAMPS | Basophil %CD33dim HLA DR- CD66b- \|\| id:ebi-a-GCST90001533 | Weighted mode | 19 | -0.035517757 | 0.0252147136272835 | 0.175989522078002 | -0.084938596 | 0.0139030813656941 | 0.965105596365979 | 0.918568686412487 | 1.01400017866347 | 0.872755742358858 |
| ebi-a-GCST90001534 | finngen_R11_O15_PREECLAMPS | Pre-eclampsia \|\| id:finngen_R11_O15_PREECLAMPS | CD45RA- CD4+ T cell Absolute Count \|\| id:ebi-a-GCST90001534 | Inverse variance weighted | 14 | -0.01980129 | 0.0116995893338038 | 0.0905549949428304 | -0.042732485 | 0.00312990512884102 | 0.980393467972215 | 0.958167679954545 | 1.00313480839615 | 0.802698559103897 |
| ebi-a-GCST90001534 | finngen_R11_O15_PREECLAMPS | Pre-eclampsia \|\| id:finngen_R11_O15_PREECLAMPS | CD45RA- CD4+ T cell Absolute Count \|\| id:ebi-a-GCST90001534 | MR Egger | 14 | -0.013565756 | 0.013556365695868 | 0.336727446799688 | -0.040136233 | 0.0130047208461005 | 0.986525844272571 | 0.960658557145882 | 1.01308964998884 | 0.99380795269851 |
| ebi-a-GCST90001534 | finngen_R11_O15_PREECLAMPS | Pre-eclampsia \|\| id:finngen_R11_O15_PREECLAMPS | CD45RA- CD4+ T cell Absolute Count \|\| id:ebi-a-GCST90001534 | Weighted median | 14 | -0.017642805 | 0.0163519579329177 | 0.28061382821734 | -0.049692643 | 0.0144070323040158 | 0.9825119177916 | 0.951521836655014 | 1.01451131378766 | 0.940845681141708 |
| ebi-a-GCST90001534 | finngen_R11_O15_PREECLAMPS | Pre-eclampsia \|\| id:finngen_R11_O15_PREECLAMPS | CD45RA- CD4+ T cell Absolute Count \|\| id:ebi-a-GCST90001534 | Weighted mode | 14 | -0.016378153 | 0.0133009465194182 | 0.240002659177002 | -0.042448008 | 0.00969170219788174 | 0.983755239732407 | 0.958440295301737 | 1.00973881883408 | 0.872755742358858 |
| ebi-a-GCST90001535 | finngen_R11_O15_PREECLAMPS | Pre-eclampsia \|\| id:finngen_R11_O15_PREECLAMPS | CD45RA- CD4+ T cell %CD4+ T cell \|\| id:ebi-a-GCST90001535 | Inverse variance weighted (multiplicative random effects) | 30 | -0.01350785 | 0.0219646705919764 | 0.538567431129087 | -0.056558604 | 0.0295429046947925 | 0.986582971941038 | 0.945011101417401 | 1.029983626 | 0.905040901506581 |
| ebi-a-GCST90001535 | finngen_R11_O15_PREECLAMPS | Pre-eclampsia \|\| id:finngen_R11_O15_PREECLAMPS | CD45RA- CD4+ T cell %CD4+ T cell \|\| id:ebi-a-GCST90001535 | MR Egger | 30 | -0.025328137 | 0.0336185249606374 | 0.457499329274437 | -0.091220446 | 0.0405641719173511 | 0.974989929256332 | 0.912816462045449 | 1.04139813607342 | 0.99380795269851 |
| ebi-a-GCST90001535 | finngen_R11_O15_PREECLAMPS | Pre-eclampsia \|\| id:finngen_R11_O15_PREECLAMPS | CD45RA- CD4+ T cell %CD4+ T cell \|\| id:ebi-a-GCST90001535 | Weighted median | 30 | -0.020967923 | 0.0267063813229358 | 0.432378621268888 | -0.073312431 | 0.0313765840468882 | 0.979250375141082 | 0.929310439517297 | 1.0318740180214 | 0.940845681141708 |
| ebi-a-GCST90001535 | finngen_R11_O15_PREECLAMPS | Pre-eclampsia \|\| id:finngen_R11_O15_PREECLAMPS | CD45RA- CD4+ T cell %CD4+ T cell \|\| id:ebi-a-GCST90001535 | Weighted mode | 30 | -0.016944555 | 0.0260943519073754 | 0.521217841930468 | -0.068089485 | 0.0342003749101383 | 0.983198196716495 | 0.93417687542391 | 1.03479193229645 | 0.934398527469092 |
| ebi-a-GCST90001536 | finngen_R11_O15_PREECLAMPS | Pre-eclampsia \|\| id:finngen_R11_O15_PREECLAMPS | CD45RA- CD4+ T cell %T cell \|\| id:ebi-a-GCST90001536 | Inverse variance weighted (multiplicative random effects) | 26 | -0.014727831 | 0.00917010450275783 | 0.108258504859021 | -0.032701236 | 0.00324557401452221 | 0.985380093210773 | 0.967827668816771 | 1.0032508465925 | 0.820779257935285 |
| ebi-a-GCST90001536 | finngen_R11_O15_PREECLAMPS | Pre-eclampsia \|\| id:finngen_R11_O15_PREECLAMPS | CD45RA- CD4+ T cell %T cell \|\| id:ebi-a-GCST90001536 | MR Egger | 26 | -0.00994713 | 0.0105068341334099 | 0.353212057272472 | -0.030540524 | 0.0106462653941031 | 0.990102179555358 | 0.969921125790517 | 1.01070313852716 | 0.99380795269851 |
| ebi-a-GCST90001536 | finngen_R11_O15_PREECLAMPS | Pre-eclampsia \|\| id:finngen_R11_O15_PREECLAMPS | CD45RA- CD4+ T cell %T cell \|\| id:ebi-a-GCST90001536 | Weighted median | 26 | -0.004808362 | 0.0102307109914601 | 0.638360040947722 | -0.024860556 | 0.0152438313104325 | 0.995203179434603 | 0.975445922844827 | 1.01536061114307 | 0.940845681141708 |
| ebi-a-GCST90001536 | finngen_R11_O15_PREECLAMPS | Pre-eclampsia \|\| id:finngen_R11_O15_PREECLAMPS | CD45RA- CD4+ T cell %T cell \|\| id:ebi-a-GCST90001536 | Weighted mode | 26 | -0.009296805 | 0.00679451230870549 | 0.183396539208803 | -0.022614049 | 0.00402043951815658 | 0.990746277070363 | 0.977639732263172 | 1.00402853232703 | 0.872755742358858 |
| ebi-a-GCST90001537 | finngen_R11_O15_PREECLAMPS | Pre-eclampsia \|\| id:finngen_R11_O15_PREECLAMPS | Central Memory CD4+ T cell Absolute Count \|\| id:ebi-a-GCST90001537 | Inverse variance weighted (multiplicative random effects) | 27 | 0.0246160627017938 | 0.0224260984543615 | 0.272356265108007 | -0.01933909 | 0.0685712156723422 | 1.02492153936732 | 0.980846710271877 | 1.07097689257469 | 0.885079385681701 |
| ebi-a-GCST90001537 | finngen_R11_O15_PREECLAMPS | Pre-eclampsia \|\| id:finngen_R11_O15_PREECLAMPS | Central Memory CD4+ T cell Absolute Count \|\| id:ebi-a-GCST90001537 | MR Egger | 27 | 0.013792394076061 | 0.0318143564430144 | 0.668349632532018 | -0.048563745 | 0.0761485327043692 | 1.01388794794338 | 0.952596614525753 | 1.07912284728894 | 0.99380795269851 |
| ebi-a-GCST90001537 | finngen_R11_O15_PREECLAMPS | Pre-eclampsia \|\| id:finngen_R11_O15_PREECLAMPS | Central Memory CD4+ T cell Absolute Count \|\| id:ebi-a-GCST90001537 | Weighted median | 27 | 0.0422475151035301 | 0.0297982004876522 | 0.156252825186505 | -0.016156958 | 0.100651988059328 | 1.04315264283408 | 0.983972865666502 | 1.10589171126649 | 0.937437665462528 |
| ebi-a-GCST90001537 | finngen_R11_O15_PREECLAMPS | Pre-eclampsia \|\| id:finngen_R11_O15_PREECLAMPS | Central Memory CD4+ T cell Absolute Count \|\| id:ebi-a-GCST90001537 | Weighted mode | 27 | 0.0379304346453985 | 0.0275269046235395 | 0.179966486446699 | -0.016022298 | 0.0918831677075359 | 1.03865897568527 | 0.98410537581884 | 1.09623673874756 | 0.872755742358858 |
| ebi-a-GCST90001538 | finngen_R11_O15_PREECLAMPS | Pre-eclampsia \|\| id:finngen_R11_O15_PREECLAMPS | Central Memory CD4+ T cell %CD4+ T cell \|\| id:ebi-a-GCST90001538 | Inverse variance weighted | 32 | -0.007578701 | 0.00656673975398355 | 0.248457483088536 | -0.020449511 | 0.00529210849050743 | 0.992449944518325 | 0.979758161897499 | 1.00530613643152 | 0.885079385681701 |
| ebi-a-GCST90001538 | finngen_R11_O15_PREECLAMPS | Pre-eclampsia \|\| id:finngen_R11_O15_PREECLAMPS | Central Memory CD4+ T cell %CD4+ T cell \|\| id:ebi-a-GCST90001538 | MR Egger | 32 | -0.008605939 | 0.00733995682945449 | 0.250231728442103 | -0.022992255 | 0.00578037617223719 | 0.99143098588042 | 0.977270053092191 | 1.00579711478286 | 0.99380795269851 |
| ebi-a-GCST90001538 | finngen_R11_O15_PREECLAMPS | Pre-eclampsia \|\| id:finngen_R11_O15_PREECLAMPS | Central Memory CD4+ T cell %CD4+ T cell \|\| id:ebi-a-GCST90001538 | Weighted median | 32 | -0.007948451 | 0.00821426321755181 | 0.33322412744064 | -0.024048407 | 0.00815150516805813 | 0.992083054667909 | 0.976238452186187 | 1.00818481914447 | 0.940845681141708 |
| ebi-a-GCST90001538 | finngen_R11_O15_PREECLAMPS | Pre-eclampsia \|\| id:finngen_R11_O15_PREECLAMPS | Central Memory CD4+ T cell %CD4+ T cell \|\| id:ebi-a-GCST90001538 | Weighted mode | 32 | -0.007404558 | 0.00696717653262708 | 0.296092235437752 | -0.021060224 | 0.00625110828934983 | 0.992622788485742 | 0.979159994137867 | 1.00627068724223 | 0.872755742358858 |
| ebi-a-GCST90001539 | finngen_R11_O15_PREECLAMPS | Pre-eclampsia \|\| id:finngen_R11_O15_PREECLAMPS | Central Memory CD4+ T cell %T cell \|\| id:ebi-a-GCST90001539 | Inverse variance weighted | 25 | 0.0323252259697433 | 0.0206567157464698 | 0.117611677621479 | -0.008161937 | 0.0728123888328241 | 1.03285336142366 | 0.991871281277281 | 1.07552873678368 | 0.820779257935285 |
| ebi-a-GCST90001539 | finngen_R11_O15_PREECLAMPS | Pre-eclampsia \|\| id:finngen_R11_O15_PREECLAMPS | Central Memory CD4+ T cell %T cell \|\| id:ebi-a-GCST90001539 | MR Egger | 25 | 0.0206525769377711 | 0.0330758333211316 | 0.538509453321462 | -0.044176056 | 0.0854812102471891 | 1.02086731717017 | 0.956785494461389 | 1.08924109458086 | 0.99380795269851 |
| ebi-a-GCST90001539 | finngen_R11_O15_PREECLAMPS | Pre-eclampsia \|\| id:finngen_R11_O15_PREECLAMPS | Central Memory CD4+ T cell %T cell \|\| id:ebi-a-GCST90001539 | Weighted median | 25 | 0.0281085658182779 | 0.0335178513305341 | 0.401685509017309 | -0.037586423 | 0.0938035544261247 | 1.02850733910125 | 0.963111179369242 | 1.09834395991325 | 0.940845681141708 |
| ebi-a-GCST90001539 | finngen_R11_O15_PREECLAMPS | Pre-eclampsia \|\| id:finngen_R11_O15_PREECLAMPS | Central Memory CD4+ T cell %T cell \|\| id:ebi-a-GCST90001539 | Weighted mode | 25 | 0.0292418248596473 | 0.0292006670097451 | 0.326619211644717 | -0.027991482 | 0.0864751321987478 | 1.02967356503563 | 0.972396649174413 | 1.09032425341381 | 0.888599269773114 |
| ebi-a-GCST90001540 | finngen_R11_O15_PREECLAMPS | Pre-eclampsia \|\| id:finngen_R11_O15_PREECLAMPS | Naive CD4+ T cell Absolute Count \|\| id:ebi-a-GCST90001540 | Inverse variance weighted (multiplicative random effects) | 24 | 0.0284477981771273 | 0.0355765942029886 | 0.423930313305536 | -0.041282326 | 0.098177922814985 | 1.02885630125827 | 0.959558183036176 | 1.10315904481108 | 0.898694924660397 |
| ebi-a-GCST90001540 | finngen_R11_O15_PREECLAMPS | Pre-eclampsia \|\| id:finngen_R11_O15_PREECLAMPS | Naive CD4+ T cell Absolute Count \|\| id:ebi-a-GCST90001540 | MR Egger | 24 | 0.0609787813840187 | 0.0727231887612216 | 0.410765299371775 | -0.081558669 | 0.203516231356013 | 1.06287636118258 | 0.921678634683582 | 1.22570505233482 | 0.99380795269851 |
| ebi-a-GCST90001540 | finngen_R11_O15_PREECLAMPS | Pre-eclampsia \|\| id:finngen_R11_O15_PREECLAMPS | Naive CD4+ T cell Absolute Count \|\| id:ebi-a-GCST90001540 | Weighted median | 24 | 0.0392007774478044 | 0.0399747200290756 | 0.326770696495456 | -0.039149674 | 0.117551228704792 | 1.03997926707297 | 0.961606771026396 | 1.12473924740276 | 0.940845681141708 |
| ebi-a-GCST90001540 | finngen_R11_O15_PREECLAMPS | Pre-eclampsia \|\| id:finngen_R11_O15_PREECLAMPS | Naive CD4+ T cell Absolute Count \|\| id:ebi-a-GCST90001540 | Weighted mode | 24 | 0.059678644243476 | 0.0830248924130002 | 0.479501201976665 | -0.103050145 | 0.222407433372956 | 1.06149537408044 | 0.902081737561454 | 1.24908019115886 | 0.933945646747087 |
| ebi-a-GCST90001541 | finngen_R11_O15_PREECLAMPS | Pre-eclampsia \|\| id:finngen_R11_O15_PREECLAMPS | Naive CD4+ T cell %CD4+ T cell \|\| id:ebi-a-GCST90001541 | Inverse variance weighted (multiplicative random effects) | 35 | 0.00190758462966043 | 0.0231043325594868 | 0.934198278408843 | -0.043376907 | 0.0471920764462546 | 1.00190940522668 | 0.957550414411102 | 1.04832334796601 | 0.986667691552896 |
| ebi-a-GCST90001541 | finngen_R11_O15_PREECLAMPS | Pre-eclampsia \|\| id:finngen_R11_O15_PREECLAMPS | Naive CD4+ T cell %CD4+ T cell \|\| id:ebi-a-GCST90001541 | MR Egger | 35 | 0.0185407320736589 | 0.0385955465300519 | 0.63412278193291 | -0.057106539 | 0.0941880032725605 | 1.01871367864511 | 0.944493438501321 | 1.09876629816015 | 0.99380795269851 |
| ebi-a-GCST90001541 | finngen_R11_O15_PREECLAMPS | Pre-eclampsia \|\| id:finngen_R11_O15_PREECLAMPS | Naive CD4+ T cell %CD4+ T cell \|\| id:ebi-a-GCST90001541 | Weighted median | 35 | 0.0268570531921007 | 0.0305537273364201 | 0.379395475366212 | -0.033028252 | 0.086742358771484 | 1.02722095431155 | 0.967511224701075 | 1.09061565596074 | 0.940845681141708 |
| ebi-a-GCST90001541 | finngen_R11_O15_PREECLAMPS | Pre-eclampsia \|\| id:finngen_R11_O15_PREECLAMPS | Naive CD4+ T cell %CD4+ T cell \|\| id:ebi-a-GCST90001541 | Weighted mode | 35 | 0.032678426756227 | 0.0288522103964071 | 0.265300661415395 | -0.023871906 | 0.0892287591331849 | 1.03321823047547 | 0.976410774479763 | 1.09333073711281 | 0.872755742358858 |
| ebi-a-GCST90001542 | finngen_R11_O15_PREECLAMPS | Pre-eclampsia \|\| id:finngen_R11_O15_PREECLAMPS | Effector Memory CD4+ T cell Absolute Count \|\| id:ebi-a-GCST90001542 | Inverse variance weighted | 22 | -0.018904799 | 0.0110825462631936 | 0.0880421059563011 | -0.04062659 | 0.00281699182297035 | 0.981272776090095 | 0.960187607120933 | 1.00282096327274 | 0.802698559103897 |
| ebi-a-GCST90001542 | finngen_R11_O15_PREECLAMPS | Pre-eclampsia \|\| id:finngen_R11_O15_PREECLAMPS | Effector Memory CD4+ T cell Absolute Count \|\| id:ebi-a-GCST90001542 | MR Egger | 22 | -0.019485586 | 0.0142297941051271 | 0.186071154486517 | -0.047375983 | 0.00840480998961157 | 0.980703030492648 | 0.953728744469345 | 1.00844022956716 | 0.99380795269851 |
| ebi-a-GCST90001542 | finngen_R11_O15_PREECLAMPS | Pre-eclampsia \|\| id:finngen_R11_O15_PREECLAMPS | Effector Memory CD4+ T cell Absolute Count \|\| id:ebi-a-GCST90001542 | Weighted median | 22 | -0.019921649 | 0.0153985478234272 | 0.195756774575028 | -0.050102803 | 0.0102595046934021 | 0.980275475822031 | 0.951131640503073 | 1.01031231385579 | 0.940845681141708 |
| ebi-a-GCST90001542 | finngen_R11_O15_PREECLAMPS | Pre-eclampsia \|\| id:finngen_R11_O15_PREECLAMPS | Effector Memory CD4+ T cell Absolute Count \|\| id:ebi-a-GCST90001542 | Weighted mode | 22 | -0.019480361 | 0.0126578918926146 | 0.138739934248573 | -0.044289829 | 0.00532910679124548 | 0.980708154814862 | 0.956676644243826 | 1.00534333173837 | 0.872755742358858 |
| ebi-a-GCST90001543 | finngen_R11_O15_PREECLAMPS | Pre-eclampsia \|\| id:finngen_R11_O15_PREECLAMPS | Effector Memory CD4+ T cell %CD4+ T cell \|\| id:ebi-a-GCST90001543 | Inverse variance weighted | 28 | -0.023667853 | 0.0183916753652929 | 0.19813661174726 | -0.059715537 | 0.0123798306093715 | 0.976610033882117 | 0.942032469022933 | 1.01245677791602 | 0.855687495048373 |
| ebi-a-GCST90001543 | finngen_R11_O15_PREECLAMPS | Pre-eclampsia \|\| id:finngen_R11_O15_PREECLAMPS | Effector Memory CD4+ T cell %CD4+ T cell \|\| id:ebi-a-GCST90001543 | MR Egger | 28 | -0.037759916 | 0.0244168777824856 | 0.134078287096519 | -0.085616996 | 0.0100971646138279 | 0.962944100766887 | 0.917945740522143 | 1.01014831298668 | 0.97261503632431 |
| ebi-a-GCST90001543 | finngen_R11_O15_PREECLAMPS | Pre-eclampsia \|\| id:finngen_R11_O15_PREECLAMPS | Effector Memory CD4+ T cell %CD4+ T cell \|\| id:ebi-a-GCST90001543 | Weighted median | 28 | -0.024897859 | 0.0284625085360593 | 0.381704597382725 | -0.080684376 | 0.0308886575525667 | 0.975409536072099 | 0.922484803929034 | 1.03137066215953 | 0.940845681141708 |
| ebi-a-GCST90001543 | finngen_R11_O15_PREECLAMPS | Pre-eclampsia \|\| id:finngen_R11_O15_PREECLAMPS | Effector Memory CD4+ T cell %CD4+ T cell \|\| id:ebi-a-GCST90001543 | Weighted mode | 28 | -0.028830345 | 0.0239388322473364 | 0.238912198248904 | -0.075750456 | 0.0180897659879456 | 0.971581283897055 | 0.927047516451823 | 1.01825437689753 | 0.872755742358858 |
| ebi-a-GCST90001544 | finngen_R11_O15_PREECLAMPS | Pre-eclampsia \|\| id:finngen_R11_O15_PREECLAMPS | Effector Memory CD4+ T cell %T cell \|\| id:ebi-a-GCST90001544 | Inverse variance weighted (multiplicative random effects) | 20 | -0.028725254 | 0.0265147520290006 | 0.278644776596537 | -0.080694168 | 0.0232436599710082 | 0.971683393916104 | 0.922475770933899 | 1.0235158990208 | 0.885079385681701 |
| ebi-a-GCST90001544 | finngen_R11_O15_PREECLAMPS | Pre-eclampsia \|\| id:finngen_R11_O15_PREECLAMPS | Effector Memory CD4+ T cell %T cell \|\| id:ebi-a-GCST90001544 | MR Egger | 20 | -0.00187128 | 0.0426249823316083 | 0.965466649519579 | -0.085416246 | 0.0816736849591168 | 0.998130469342756 | 0.918130037098406 | 1.08510166705679 | 0.99380795269851 |
| ebi-a-GCST90001544 | finngen_R11_O15_PREECLAMPS | Pre-eclampsia \|\| id:finngen_R11_O15_PREECLAMPS | Effector Memory CD4+ T cell %T cell \|\| id:ebi-a-GCST90001544 | Weighted median | 20 | -0.021772193 | 0.0296579589328648 | 0.46288193382599 | -0.079901792 | 0.0363574066874691 | 0.978463110584954 | 0.923207007944501 | 1.03702642044192 | 0.940845681141708 |
| ebi-a-GCST90001544 | finngen_R11_O15_PREECLAMPS | Pre-eclampsia \|\| id:finngen_R11_O15_PREECLAMPS | Effector Memory CD4+ T cell %T cell \|\| id:ebi-a-GCST90001544 | Weighted mode | 20 | -0.021307452 | 0.0269497077913386 | 0.438913476928524 | -0.074128879 | 0.0315139756553539 | 0.978917948393034 | 0.92855201537946 | 1.03201579859198 | 0.933945646747087 |
| ebi-a-GCST90001545 | finngen_R11_O15_PREECLAMPS | Pre-eclampsia \|\| id:finngen_R11_O15_PREECLAMPS | Terminally Differentiated CD4+ T cell Absolute Count \|\| id:ebi-a-GCST90001545 | Inverse variance weighted | 11 | -0.044423099 | 0.0346432548107236 | 0.199736892260914 | -0.112323878 | 0.0234776808599509 | 0.95654915726421 | 0.893754743122238 | 1.02375545115035 | 0.855687495048373 |
| ebi-a-GCST90001545 | finngen_R11_O15_PREECLAMPS | Pre-eclampsia \|\| id:finngen_R11_O15_PREECLAMPS | Terminally Differentiated CD4+ T cell Absolute Count \|\| id:ebi-a-GCST90001545 | MR Egger | 11 | 0.0483548932180278 | 0.0568607035958826 | 0.417146908528044 | -0.063092086 | 0.159801872265958 | 1.04954306495352 | 0.938857014281032 | 1.17327838897339 | 0.99380795269851 |
| ebi-a-GCST90001545 | finngen_R11_O15_PREECLAMPS | Pre-eclampsia \|\| id:finngen_R11_O15_PREECLAMPS | Terminally Differentiated CD4+ T cell Absolute Count \|\| id:ebi-a-GCST90001545 | Weighted median | 11 | 0.0214367549384323 | 0.0436799707154533 | 0.623590171727154 | -0.064175988 | 0.107049497540721 | 1.02166817282754 | 0.93783993674711 | 1.11298934335128 | 0.940845681141708 |
| ebi-a-GCST90001545 | finngen_R11_O15_PREECLAMPS | Pre-eclampsia \|\| id:finngen_R11_O15_PREECLAMPS | Terminally Differentiated CD4+ T cell Absolute Count \|\| id:ebi-a-GCST90001545 | Weighted mode | 11 | 0.0243552980326986 | 0.0499384806496454 | 0.636273404930006 | -0.073524124 | 0.122234720106004 | 1.02465431088465 | 0.929113731543772 | 1.13001930890635 | 0.959313411571287 |
| ebi-a-GCST90001546 | finngen_R11_O15_PREECLAMPS | Pre-eclampsia \|\| id:finngen_R11_O15_PREECLAMPS | Terminally Differentiated CD4+ T cell %CD4+ T cell \|\| id:ebi-a-GCST90001546 | Inverse variance weighted (multiplicative random effects) | 20 | -0.032557373 | 0.0402752067983885 | 0.418876135545846 | -0.111496778 | 0.0463820327525268 | 0.967966913486448 | 0.894494273551167 | 1.04747450409573 | 0.898694924660397 |
| ebi-a-GCST90001546 | finngen_R11_O15_PREECLAMPS | Pre-eclampsia \|\| id:finngen_R11_O15_PREECLAMPS | Terminally Differentiated CD4+ T cell %CD4+ T cell \|\| id:ebi-a-GCST90001546 | MR Egger | 20 | -0.112629279 | 0.0839122356482483 | 0.196207978877542 | -0.27709726 | 0.0518387032658272 | 0.893481831557288 | 0.757980771955141 | 1.05320585014815 | 0.99380795269851 |
| ebi-a-GCST90001546 | finngen_R11_O15_PREECLAMPS | Pre-eclampsia \|\| id:finngen_R11_O15_PREECLAMPS | Terminally Differentiated CD4+ T cell %CD4+ T cell \|\| id:ebi-a-GCST90001546 | Weighted median | 20 | -0.0351777 | 0.0469529615553016 | 0.45372972826604 | -0.127205504 | 0.0568501050649117 | 0.965433843821918 | 0.880552696696581 | 1.05849713514413 | 0.940845681141708 |
| ebi-a-GCST90001546 | finngen_R11_O15_PREECLAMPS | Pre-eclampsia \|\| id:finngen_R11_O15_PREECLAMPS | Terminally Differentiated CD4+ T cell %CD4+ T cell \|\| id:ebi-a-GCST90001546 | Weighted mode | 20 | -0.02441119 | 0.0718554063023591 | 0.737789113808292 | -0.165247787 | 0.11642540610476 | 0.975884353117552 | 0.847683633416829 | 1.12347370306177 | 0.959313411571287 |
| ebi-a-GCST90001547 | finngen_R11_O15_PREECLAMPS | Pre-eclampsia \|\| id:finngen_R11_O15_PREECLAMPS | Terminally Differentiated CD4+ T cell %T cell \|\| id:ebi-a-GCST90001547 | Inverse variance weighted (multiplicative random effects) | 25 | 0.013570168803946 | 0.0178862744520702 | 0.448036975233069 | -0.021486929 | 0.0486272667300037 | 1.01366266145136 | 0.978742270406241 | 1.04982897161902 | 0.898694924660397 |
| ebi-a-GCST90001547 | finngen_R11_O15_PREECLAMPS | Pre-eclampsia \|\| id:finngen_R11_O15_PREECLAMPS | Terminally Differentiated CD4+ T cell %T cell \|\| id:ebi-a-GCST90001547 | MR Egger | 25 | 0.0292344930364122 | 0.0210834752545367 | 0.178856134679731 | -0.012089118 | 0.0705581045353042 | 1.02966601567874 | 0.987983661353626 | 1.07310691999819 | 0.99380795269851 |
| ebi-a-GCST90001547 | finngen_R11_O15_PREECLAMPS | Pre-eclampsia \|\| id:finngen_R11_O15_PREECLAMPS | Terminally Differentiated CD4+ T cell %T cell \|\| id:ebi-a-GCST90001547 | Weighted median | 25 | 0.0315395409822824 | 0.0199440207470406 | 0.113785844736891 | -0.00755074 | 0.070629821646482 | 1.03204218275053 | 0.992477695538961 | 1.07318388288622 | 0.88227178111339 |
| ebi-a-GCST90001547 | finngen_R11_O15_PREECLAMPS | Pre-eclampsia \|\| id:finngen_R11_O15_PREECLAMPS | Terminally Differentiated CD4+ T cell %T cell \|\| id:ebi-a-GCST90001547 | Weighted mode | 25 | 0.0279160973749161 | 0.0151800142742972 | 0.0783202251581372 | -0.001836731 | 0.0576689253525386 | 1.02830940294355 | 0.998164955154695 | 1.05936420901317 | 0.872755742358858 |
| ebi-a-GCST90001548 | finngen_R11_O15_PREECLAMPS | Pre-eclampsia \|\| id:finngen_R11_O15_PREECLAMPS | Central Memory CD8+ T cell Absolute Count \|\| id:ebi-a-GCST90001548 | Inverse variance weighted | 25 | 0.0328661576552373 | 0.0266875904819337 | 0.218130454788847 | -0.01944152 | 0.0851738349998273 | 1.03341221567068 | 0.98074624785689 | 1.08890634028021 | 0.857246920127122 |
| ebi-a-GCST90001548 | finngen_R11_O15_PREECLAMPS | Pre-eclampsia \|\| id:finngen_R11_O15_PREECLAMPS | Central Memory CD8+ T cell Absolute Count \|\| id:ebi-a-GCST90001548 | MR Egger | 25 | 0.0421965527832839 | 0.0464637604286047 | 0.373211388981221 | -0.048872418 | 0.133265523223349 | 1.04309948270962 | 0.952302618947977 | 1.14255333250167 | 0.99380795269851 |
| ebi-a-GCST90001548 | finngen_R11_O15_PREECLAMPS | Pre-eclampsia \|\| id:finngen_R11_O15_PREECLAMPS | Central Memory CD8+ T cell Absolute Count \|\| id:ebi-a-GCST90001548 | Weighted median | 25 | 0.0328785273522114 | 0.0404913136820809 | 0.416797574275928 | -0.046484447 | 0.11224150216909 | 1.0334249987457 | 0.954579406584472 | 1.11878301654735 | 0.940845681141708 |
| ebi-a-GCST90001548 | finngen_R11_O15_PREECLAMPS | Pre-eclampsia \|\| id:finngen_R11_O15_PREECLAMPS | Central Memory CD8+ T cell Absolute Count \|\| id:ebi-a-GCST90001548 | Weighted mode | 25 | 0.0337810699245998 | 0.0428771910331236 | 0.438493252048276 | -0.050258225 | 0.117820364349522 | 1.03435812983437 | 0.950983825469089 | 1.1250419955636 | 0.933945646747087 |
| ebi-a-GCST90001549 | finngen_R11_O15_PREECLAMPS | Pre-eclampsia \|\| id:finngen_R11_O15_PREECLAMPS | Central Memory CD8+ T cell %CD8+ T cell \|\| id:ebi-a-GCST90001549 | Inverse variance weighted | 31 | 0.024319524114119 | 0.0260206379180941 | 0.349981879814411 | -0.026680926 | 0.0753199744335833 | 1.02461765564041 | 0.973671865142533 | 1.07822910144002 | 0.885079385681701 |
| ebi-a-GCST90001549 | finngen_R11_O15_PREECLAMPS | Pre-eclampsia \|\| id:finngen_R11_O15_PREECLAMPS | Central Memory CD8+ T cell %CD8+ T cell \|\| id:ebi-a-GCST90001549 | MR Egger | 31 | 0.037360153107226 | 0.0561483215418338 | 0.511063193575858 | -0.072690557 | 0.14741086332922 | 1.03806681651175 | 0.929888532900258 | 1.15882998597901 | 0.99380795269851 |
| ebi-a-GCST90001549 | finngen_R11_O15_PREECLAMPS | Pre-eclampsia \|\| id:finngen_R11_O15_PREECLAMPS | Central Memory CD8+ T cell %CD8+ T cell \|\| id:ebi-a-GCST90001549 | Weighted median | 31 | 0.0304994018934666 | 0.0348867674672906 | 0.381987655704062 | -0.037878662 | 0.0988774661293562 | 1.03096927341914 | 0.962829761311581 | 1.10393102232994 | 0.940845681141708 |
| ebi-a-GCST90001549 | finngen_R11_O15_PREECLAMPS | Pre-eclampsia \|\| id:finngen_R11_O15_PREECLAMPS | Central Memory CD8+ T cell %CD8+ T cell \|\| id:ebi-a-GCST90001549 | Weighted mode | 31 | 0.0341894823979944 | 0.0523375921102086 | 0.518571353983624 | -0.068392198 | 0.136771162934003 | 1.03478066087415 | 0.933894130203347 | 1.14656574175703 | 0.934398527469092 |
| ebi-a-GCST90001550 | finngen_R11_O15_PREECLAMPS | Pre-eclampsia \|\| id:finngen_R11_O15_PREECLAMPS | Central Memory CD8+ T cell %T cell \|\| id:ebi-a-GCST90001550 | Inverse variance weighted | 21 | 0.0321082923358599 | 0.0281467453398092 | 0.253975561496141 | -0.023059329 | 0.0872759132018859 | 1.03262932509209 | 0.977204505946415 | 1.09119771404187 | 0.885079385681701 |
| ebi-a-GCST90001550 | finngen_R11_O15_PREECLAMPS | Pre-eclampsia \|\| id:finngen_R11_O15_PREECLAMPS | Central Memory CD8+ T cell %T cell \|\| id:ebi-a-GCST90001550 | MR Egger | 21 | 0.0595478291640278 | 0.0543982164928569 | 0.287346872983554 | -0.047072675 | 0.166168333490027 | 1.06135652356082 | 0.954018061653747 | 1.18077184844112 | 0.99380795269851 |
| ebi-a-GCST90001550 | finngen_R11_O15_PREECLAMPS | Pre-eclampsia \|\| id:finngen_R11_O15_PREECLAMPS | Central Memory CD8+ T cell %T cell \|\| id:ebi-a-GCST90001550 | Weighted median | 21 | 0.0344022248692787 | 0.0401521797482516 | 0.391557666042949 | -0.044296047 | 0.113100497175852 | 1.03500082608759 | 0.956670695637867 | 1.11974445844999 | 0.940845681141708 |
| ebi-a-GCST90001550 | finngen_R11_O15_PREECLAMPS | Pre-eclampsia \|\| id:finngen_R11_O15_PREECLAMPS | Central Memory CD8+ T cell %T cell \|\| id:ebi-a-GCST90001550 | Weighted mode | 21 | 0.0275494340675595 | 0.054267300671769 | 0.617243392421759 | -0.078814475 | 0.133913343384227 | 1.02793242873241 | 0.924211372625333 | 1.14329374138535 | 0.959313411571287 |
| ebi-a-GCST90001551 | finngen_R11_O15_PREECLAMPS | Pre-eclampsia \|\| id:finngen_R11_O15_PREECLAMPS | Naive CD8+ T cell Absolute Count \|\| id:ebi-a-GCST90001551 | Inverse variance weighted (multiplicative random effects) | 28 | -0.002202443 | 0.00527676279254369 | 0.676396664271151 | -0.012544898 | 0.00814001227742733 | 0.997799980801572 | 0.987533461350202 | 1.00817323225319 | 0.950857618427329 |
| ebi-a-GCST90001551 | finngen_R11_O15_PREECLAMPS | Pre-eclampsia \|\| id:finngen_R11_O15_PREECLAMPS | Naive CD8+ T cell Absolute Count \|\| id:ebi-a-GCST90001551 | MR Egger | 28 | -0.002130098 | 0.00551765925658314 | 0.702599846717367 | -0.012944711 | 0.00868451372163292 | 0.997872168628407 | 0.987138711853974 | 1.00872233351381 | 0.99380795269851 |
| ebi-a-GCST90001551 | finngen_R11_O15_PREECLAMPS | Pre-eclampsia \|\| id:finngen_R11_O15_PREECLAMPS | Naive CD8+ T cell Absolute Count \|\| id:ebi-a-GCST90001551 | Weighted median | 28 | -0.0022099 | 0.0045671490807753 | 0.62847907958264 | -0.011161513 | 0.00674171186748332 | 0.99779253970116 | 0.988900546048022 | 1.00676448836236 | 0.940845681141708 |
| ebi-a-GCST90001551 | finngen_R11_O15_PREECLAMPS | Pre-eclampsia \|\| id:finngen_R11_O15_PREECLAMPS | Naive CD8+ T cell Absolute Count \|\| id:ebi-a-GCST90001551 | Weighted mode | 28 | -0.001756043 | 0.00484407510430039 | 0.719790622084338 | -0.01125043 | 0.00773834447497602 | 0.998245498211463 | 0.988812619487094 | 1.00776836284345 | 0.959313411571287 |
| ebi-a-GCST90001552 | finngen_R11_O15_PREECLAMPS | Pre-eclampsia \|\| id:finngen_R11_O15_PREECLAMPS | Naive CD8+ T cell %CD8+ T cell \|\| id:ebi-a-GCST90001552 | Inverse variance weighted | 37 | -0.00577173 | 0.00361722513161523 | 0.110572865314152 | -0.012861491 | 0.00131803163459657 | 0.994244894808767 | 0.987220864642808 | 1.00131890062003 | 0.820779257935285 |
| ebi-a-GCST90001552 | finngen_R11_O15_PREECLAMPS | Pre-eclampsia \|\| id:finngen_R11_O15_PREECLAMPS | Naive CD8+ T cell %CD8+ T cell \|\| id:ebi-a-GCST90001552 | MR Egger | 37 | -0.003211916 | 0.00380031516096326 | 0.403756147340563 | -0.010660534 | 0.00423670135468288 | 0.996793236324408 | 0.98939608803118 | 1.00424568886185 | 0.99380795269851 |
| ebi-a-GCST90001552 | finngen_R11_O15_PREECLAMPS | Pre-eclampsia \|\| id:finngen_R11_O15_PREECLAMPS | Naive CD8+ T cell %CD8+ T cell \|\| id:ebi-a-GCST90001552 | Weighted median | 37 | -0.004022487 | 0.0044031311474927 | 0.360952601904129 | -0.012652624 | 0.00460765032811112 | 0.995985592642062 | 0.987427084148483 | 1.00461828187142 | 0.940845681141708 |
| ebi-a-GCST90001552 | finngen_R11_O15_PREECLAMPS | Pre-eclampsia \|\| id:finngen_R11_O15_PREECLAMPS | Naive CD8+ T cell %CD8+ T cell \|\| id:ebi-a-GCST90001552 | Weighted mode | 37 | -0.004773695 | 0.00398236014206279 | 0.2384722058018 | -0.012579121 | 0.00303173103025555 | 0.995237681124059 | 0.987499665711783 | 1.0030363313746 | 0.872755742358858 |
| ebi-a-GCST90001553 | finngen_R11_O15_PREECLAMPS | Pre-eclampsia \|\| id:finngen_R11_O15_PREECLAMPS | Naive CD8+ T cell %T cell \|\| id:ebi-a-GCST90001553 | Inverse variance weighted | 28 | -0.002773637 | 0.00414081157227534 | 0.502966556426839 | -0.010889628 | 0.00534235339556608 | 0.997230205691974 | 0.989169449392932 | 1.0053566492119 | 0.898694924660397 |
| ebi-a-GCST90001553 | finngen_R11_O15_PREECLAMPS | Pre-eclampsia \|\| id:finngen_R11_O15_PREECLAMPS | Naive CD8+ T cell %T cell \|\| id:ebi-a-GCST90001553 | MR Egger | 28 | -0.001457216 | 0.00421130169993598 | 0.732108225121315 | -0.009711368 | 0.00679693490779885 | 0.998543844800237 | 0.990335635298191 | 1.00682008649342 | 0.99380795269851 |
| ebi-a-GCST90001553 | finngen_R11_O15_PREECLAMPS | Pre-eclampsia \|\| id:finngen_R11_O15_PREECLAMPS | Naive CD8+ T cell %T cell \|\| id:ebi-a-GCST90001553 | Weighted median | 28 | -0.002238604 | 0.00439107468084573 | 0.610186073499642 | -0.010845111 | 0.00636790195664641 | 0.997763899388366 | 0.989213485403067 | 1.0063882201495 | 0.940845681141708 |
| ebi-a-GCST90001553 | finngen_R11_O15_PREECLAMPS | Pre-eclampsia \|\| id:finngen_R11_O15_PREECLAMPS | Naive CD8+ T cell %T cell \|\| id:ebi-a-GCST90001553 | Weighted mode | 28 | -0.002302554 | 0.00413145626602966 | 0.581898410902334 | -0.010400208 | 0.00579510038281202 | 0.997700094945197 | 0.989653686982946 | 1.00581192446041 | 0.948158355498791 |
| ebi-a-GCST90001554 | finngen_R11_O15_PREECLAMPS | Pre-eclampsia \|\| id:finngen_R11_O15_PREECLAMPS | Effector Memory CD8+ T cell Absolute Count \|\| id:ebi-a-GCST90001554 | Inverse variance weighted | 24 | -0.006159306 | 0.0131674235865291 | 0.639950162110408 | -0.031967456 | 0.0196488444871161 | 0.993859623896715 | 0.968538101697332 | 1.0198431535952 | 0.942541573457101 |
| ebi-a-GCST90001554 | finngen_R11_O15_PREECLAMPS | Pre-eclampsia \|\| id:finngen_R11_O15_PREECLAMPS | Effector Memory CD8+ T cell Absolute Count \|\| id:ebi-a-GCST90001554 | MR Egger | 24 | -0.030816753 | 0.0135578641938714 | 0.0331448403411234 | -0.057390166 | -0.004243339 | 0.969653243296396 | 0.944225592430751 | 0.995765651526943 | 0.839403552053506 |
| ebi-a-GCST90001554 | finngen_R11_O15_PREECLAMPS | Pre-eclampsia \|\| id:finngen_R11_O15_PREECLAMPS | Effector Memory CD8+ T cell Absolute Count \|\| id:ebi-a-GCST90001554 | Weighted median | 24 | -0.020298378 | 0.0153400073518257 | 0.185758460392786 | -0.050364793 | 0.0097680360925601 | 0.979906246905289 | 0.950882486209462 | 1.00981589907261 | 0.940845681141708 |
| ebi-a-GCST90001554 | finngen_R11_O15_PREECLAMPS | Pre-eclampsia \|\| id:finngen_R11_O15_PREECLAMPS | Effector Memory CD8+ T cell Absolute Count \|\| id:ebi-a-GCST90001554 | Weighted mode | 24 | -0.019700769 | 0.0135104717479139 | 0.158310608621479 | -0.046181293 | 0.0067797557920507 | 0.980492023186342 | 0.954868835023048 | 1.00680279036318 | 0.872755742358858 |
| ebi-a-GCST90001555 | finngen_R11_O15_PREECLAMPS | Pre-eclampsia \|\| id:finngen_R11_O15_PREECLAMPS | Effector Memory CD8+ T cell %CD8+ T cell \|\| id:ebi-a-GCST90001555 | Inverse variance weighted (multiplicative random effects) | 24 | -0.018309978 | 0.0119674627556977 | 0.12602169931804 | -0.041766205 | 0.00514624929160727 | 0.981856631512247 | 0.959093986018275 | 1.00515951397719 | 0.820779257935285 |
| ebi-a-GCST90001555 | finngen_R11_O15_PREECLAMPS | Pre-eclampsia \|\| id:finngen_R11_O15_PREECLAMPS | Effector Memory CD8+ T cell %CD8+ T cell \|\| id:ebi-a-GCST90001555 | MR Egger | 24 | -0.025780632 | 0.0136408309941013 | 0.0720113236440546 | -0.052516661 | 0.000955396599939064 | 0.974548850849313 | 0.948838512423442 | 1.00095585313665 | 0.896743733484648 |
| ebi-a-GCST90001555 | finngen_R11_O15_PREECLAMPS | Pre-eclampsia \|\| id:finngen_R11_O15_PREECLAMPS | Effector Memory CD8+ T cell %CD8+ T cell \|\| id:ebi-a-GCST90001555 | Weighted median | 24 | -0.018613352 | 0.012389002688013 | 0.132991450284377 | -0.042895797 | 0.00566909309713428 | 0.981558806463627 | 0.958011212087012 | 1.00568519281463 | 0.88227178111339 |
| ebi-a-GCST90001555 | finngen_R11_O15_PREECLAMPS | Pre-eclampsia \|\| id:finngen_R11_O15_PREECLAMPS | Effector Memory CD8+ T cell %CD8+ T cell \|\| id:ebi-a-GCST90001555 | Weighted mode | 24 | -0.021766103 | 0.0109657758767827 | 0.0592066950120969 | -0.043259023 | -0.000273182 | 0.978469069583383 | 0.957663300737164 | 0.999726855351362 | 0.838871423749761 |
| ebi-a-GCST90001556 | finngen_R11_O15_PREECLAMPS | Pre-eclampsia \|\| id:finngen_R11_O15_PREECLAMPS | Effector Memory CD8+ T cell %T cell \|\| id:ebi-a-GCST90001556 | Inverse variance weighted | 15 | 0.00916543093514063 | 0.0153157254659103 | 0.549551252535361 | -0.020853391 | 0.0391842528483247 | 1.00920756211569 | 0.979362537428418 | 1.0399620819741 | 0.905435115099076 |
| ebi-a-GCST90001556 | finngen_R11_O15_PREECLAMPS | Pre-eclampsia \|\| id:finngen_R11_O15_PREECLAMPS | Effector Memory CD8+ T cell %T cell \|\| id:ebi-a-GCST90001556 | MR Egger | 15 | -0.021303976 | 0.0194807858236963 | 0.293985521693295 | -0.059486316 | 0.01687836421695 | 0.97892135074396 | 0.94224842703079 | 1.01702160858165 | 0.99380795269851 |
| ebi-a-GCST90001556 | finngen_R11_O15_PREECLAMPS | Pre-eclampsia \|\| id:finngen_R11_O15_PREECLAMPS | Effector Memory CD8+ T cell %T cell \|\| id:ebi-a-GCST90001556 | Weighted median | 15 | -0.006871404 | 0.020359397751641 | 0.735736034508245 | -0.046775824 | 0.0330330154074416 | 0.993152149931136 | 0.954301305273287 | 1.03358466289677 | 0.956980500401294 |
| ebi-a-GCST90001556 | finngen_R11_O15_PREECLAMPS | Pre-eclampsia \|\| id:finngen_R11_O15_PREECLAMPS | Effector Memory CD8+ T cell %T cell \|\| id:ebi-a-GCST90001556 | Weighted mode | 15 | -0.006704514 | 0.0179762592612728 | 0.714760725566309 | -0.041937982 | 0.0285289543663851 | 0.993317911322372 | 0.958929249662233 | 1.02893980270326 | 0.959313411571287 |
| ebi-a-GCST90001557 | finngen_R11_O15_PREECLAMPS | Pre-eclampsia \|\| id:finngen_R11_O15_PREECLAMPS | Terminally Differentiated CD8+ T cell Absolute Count \|\| id:ebi-a-GCST90001557 | Inverse variance weighted | 17 | 0.0195966468880248 | 0.0260097051558168 | 0.451187987157782 | -0.031382375 | 0.0705756689934256 | 1.01978992162044 | 0.9691049405079 | 1.07312576870528 | 0.898694924660397 |
| ebi-a-GCST90001557 | finngen_R11_O15_PREECLAMPS | Pre-eclampsia \|\| id:finngen_R11_O15_PREECLAMPS | Terminally Differentiated CD8+ T cell Absolute Count \|\| id:ebi-a-GCST90001557 | MR Egger | 17 | 0.0431584561076509 | 0.0456639909436231 | 0.359568606251139 | -0.046342966 | 0.132659878357152 | 1.04410332629253 | 0.954714471295991 | 1.14186156044673 | 0.99380795269851 |
| ebi-a-GCST90001557 | finngen_R11_O15_PREECLAMPS | Pre-eclampsia \|\| id:finngen_R11_O15_PREECLAMPS | Terminally Differentiated CD8+ T cell Absolute Count \|\| id:ebi-a-GCST90001557 | Weighted median | 17 | 0.0430239854111167 | 0.0357001868998338 | 0.228146425799024 | -0.026948381 | 0.112996351734791 | 1.04396293443049 | 0.973411486840174 | 1.11962784824181 | 0.940845681141708 |
| ebi-a-GCST90001557 | finngen_R11_O15_PREECLAMPS | Pre-eclampsia \|\| id:finngen_R11_O15_PREECLAMPS | Terminally Differentiated CD8+ T cell Absolute Count \|\| id:ebi-a-GCST90001557 | Weighted mode | 17 | 0.044053019648432 | 0.03298350977216 | 0.200367495452032 | -0.02059466 | 0.108700698801866 | 1.04503776095431 | 0.979615962123427 | 1.11482863085769 | 0.872755742358858 |
| ebi-a-GCST90001558 | finngen_R11_O15_PREECLAMPS | Pre-eclampsia \|\| id:finngen_R11_O15_PREECLAMPS | Terminally Differentiated CD8+ T cell %CD8+ T cell \|\| id:ebi-a-GCST90001558 | Inverse variance weighted | 23 | -0.015821909 | 0.0190905817841228 | 0.407228426598357 | -0.053239449 | 0.0215956317087399 | 0.984302600286652 | 0.948152951131611 | 1.02183050506224 | 0.893945885415613 |
| ebi-a-GCST90001558 | finngen_R11_O15_PREECLAMPS | Pre-eclampsia \|\| id:finngen_R11_O15_PREECLAMPS | Terminally Differentiated CD8+ T cell %CD8+ T cell \|\| id:ebi-a-GCST90001558 | MR Egger | 23 | 0.0104511892218858 | 0.0292159370802256 | 0.72412152041842 | -0.046812047 | 0.067714425899128 | 1.01050599365741 | 0.954266737597738 | 1.07005968350957 | 0.99380795269851 |
| ebi-a-GCST90001558 | finngen_R11_O15_PREECLAMPS | Pre-eclampsia \|\| id:finngen_R11_O15_PREECLAMPS | Terminally Differentiated CD8+ T cell %CD8+ T cell \|\| id:ebi-a-GCST90001558 | Weighted median | 23 | 0.00888921823289233 | 0.0234019629280901 | 0.704057315981205 | -0.036978629 | 0.0547570655719489 | 1.00892884466225 | 0.963696730189498 | 1.05628397576003 | 0.946704357535493 |
| ebi-a-GCST90001558 | finngen_R11_O15_PREECLAMPS | Pre-eclampsia \|\| id:finngen_R11_O15_PREECLAMPS | Terminally Differentiated CD8+ T cell %CD8+ T cell \|\| id:ebi-a-GCST90001558 | Weighted mode | 23 | 0.00914499416170389 | 0.0230731012491551 | 0.695665948087799 | -0.036078284 | 0.0543682726100479 | 1.00918693738015 | 0.964564780261671 | 1.05587337980807 | 0.959313411571287 |
| ebi-a-GCST90001559 | finngen_R11_O15_PREECLAMPS | Pre-eclampsia \|\| id:finngen_R11_O15_PREECLAMPS | Terminally Differentiated CD8+ T cell %T cell \|\| id:ebi-a-GCST90001559 | Inverse variance weighted | 17 | -0.003587468 | 0.0205409508374168 | 0.861354990185891 | -0.043847732 | 0.0366727952606761 | 0.996418958895849 | 0.957099682011501 | 1.03735353830695 | 0.979238721346634 |
| ebi-a-GCST90001559 | finngen_R11_O15_PREECLAMPS | Pre-eclampsia \|\| id:finngen_R11_O15_PREECLAMPS | Terminally Differentiated CD8+ T cell %T cell \|\| id:ebi-a-GCST90001559 | MR Egger | 17 | 0.00522172010022039 | 0.0302879848818766 | 0.865425819228057 | -0.05414273 | 0.0645861704686985 | 1.00523537704118 | 0.947296888913132 | 1.0667174938308 | 0.99380795269851 |
| ebi-a-GCST90001559 | finngen_R11_O15_PREECLAMPS | Pre-eclampsia \|\| id:finngen_R11_O15_PREECLAMPS | Terminally Differentiated CD8+ T cell %T cell \|\| id:ebi-a-GCST90001559 | Weighted median | 17 | 0.00258148071469519 | 0.0297164748131689 | 0.930774563742648 | -0.05566281 | 0.0608257713485062 | 1.00258481560507 | 0.945858016066701 | 1.06271374287424 | 0.977580755884879 |
| ebi-a-GCST90001559 | finngen_R11_O15_PREECLAMPS | Pre-eclampsia \|\| id:finngen_R11_O15_PREECLAMPS | Terminally Differentiated CD8+ T cell %T cell \|\| id:ebi-a-GCST90001559 | Weighted mode | 17 | 0.00439869522292896 | 0.0267568326079588 | 0.871478926115645 | -0.048044697 | 0.0568420871345283 | 1.00440838368308 | 0.953091186105431 | 1.05848864822181 | 0.977585971930092 |
| ebi-a-GCST90001560 | finngen_R11_O15_PREECLAMPS | Pre-eclampsia \|\| id:finngen_R11_O15_PREECLAMPS | CD45RA+ CD8+ T cell Absolute Count \|\| id:ebi-a-GCST90001560 | Inverse variance weighted | 27 | -0.006032859 | 0.00442858332090221 | 0.173117467913191 | -0.014712882 | 0.00264716415639671 | 0.993985302002622 | 0.985394823126736 | 1.00265067098914 | 0.85505992597664 |
| ebi-a-GCST90001560 | finngen_R11_O15_PREECLAMPS | Pre-eclampsia \|\| id:finngen_R11_O15_PREECLAMPS | CD45RA+ CD8+ T cell Absolute Count \|\| id:ebi-a-GCST90001560 | MR Egger | 27 | -0.004799065 | 0.0048281526527756 | 0.329758754747298 | -0.014262244 | 0.00466411451794652 | 0.995212432430268 | 0.985838980120789 | 1.0046750084303 | 0.99380795269851 |
| ebi-a-GCST90001560 | finngen_R11_O15_PREECLAMPS | Pre-eclampsia \|\| id:finngen_R11_O15_PREECLAMPS | CD45RA+ CD8+ T cell Absolute Count \|\| id:ebi-a-GCST90001560 | Weighted median | 27 | -0.007158714 | 0.00601144215845582 | 0.23371327684999 | -0.01894114 | 0.00462371299510098 | 0.992866848920274 | 0.981237115898695 | 1.00463441884994 | 0.940845681141708 |
| ebi-a-GCST90001560 | finngen_R11_O15_PREECLAMPS | Pre-eclampsia \|\| id:finngen_R11_O15_PREECLAMPS | CD45RA+ CD8+ T cell Absolute Count \|\| id:ebi-a-GCST90001560 | Weighted mode | 27 | -0.005219338 | 0.00503821668636885 | 0.309765304248007 | -0.015094243 | 0.00465556676848011 | 0.994794259141246 | 0.985019104425709 | 1.00466642075673 | 0.877668362036021 |
| ebi-a-GCST90001561 | finngen_R11_O15_PREECLAMPS | Pre-eclampsia \|\| id:finngen_R11_O15_PREECLAMPS | CD45RA+ CD8+ T cell %CD8+ T cell \|\| id:ebi-a-GCST90001561 | Inverse variance weighted | 27 | 0.0111679134910842 | 0.00832273089859452 | 0.179642414037871 | -0.005144639 | 0.0274804660523295 | 1.01123050743452 | 0.994868571920451 | 1.02786153671768 | 0.855687495048373 |
| ebi-a-GCST90001561 | finngen_R11_O15_PREECLAMPS | Pre-eclampsia \|\| id:finngen_R11_O15_PREECLAMPS | CD45RA+ CD8+ T cell %CD8+ T cell \|\| id:ebi-a-GCST90001561 | MR Egger | 27 | 0.0131991521260686 | 0.00927410170472381 | 0.167030880971558 | -0.004978087 | 0.0313763914673273 | 1.01328664545663 | 0.995034282925911 | 1.03187381930358 | 0.99380795269851 |
| ebi-a-GCST90001561 | finngen_R11_O15_PREECLAMPS | Pre-eclampsia \|\| id:finngen_R11_O15_PREECLAMPS | CD45RA+ CD8+ T cell %CD8+ T cell \|\| id:ebi-a-GCST90001561 | Weighted median | 27 | 0.013591831782729 | 0.00987115413100653 | 0.168535659975261 | -0.00575563 | 0.0329392938795018 | 1.01368462064194 | 0.994260901593722 | 1.03348779830213 | 0.940845681141708 |
| ebi-a-GCST90001561 | finngen_R11_O15_PREECLAMPS | Pre-eclampsia \|\| id:finngen_R11_O15_PREECLAMPS | CD45RA+ CD8+ T cell %CD8+ T cell \|\| id:ebi-a-GCST90001561 | Weighted mode | 27 | 0.00995059865462789 | 0.00758177703131832 | 0.200841507081977 | -0.004909684 | 0.0248108816360118 | 1.01000027047951 | 0.995102348472868 | 1.02512123294069 | 0.872755742358858 |
| ebi-a-GCST90001562 | finngen_R11_O15_PREECLAMPS | Pre-eclampsia \|\| id:finngen_R11_O15_PREECLAMPS | CD45RA+ CD8+ T cell %T cell \|\| id:ebi-a-GCST90001562 | Inverse variance weighted | 34 | 0.00274839138268869 | 0.00360858693924895 | 0.446283625350689 | -0.004324439 | 0.00982122178361664 | 1.00275217167273 | 0.995684897904339 | 1.00986960825731 | 0.898694924660397 |
| ebi-a-GCST90001562 | finngen_R11_O15_PREECLAMPS | Pre-eclampsia \|\| id:finngen_R11_O15_PREECLAMPS | CD45RA+ CD8+ T cell %T cell \|\| id:ebi-a-GCST90001562 | MR Egger | 34 | 0.00122750473728723 | 0.00391022144229961 | 0.755617636374461 | -0.006436529 | 0.00889153876419447 | 1.00122825842958 | 0.993584140793386 | 1.00893118591593 | 0.99380795269851 |
| ebi-a-GCST90001562 | finngen_R11_O15_PREECLAMPS | Pre-eclampsia \|\| id:finngen_R11_O15_PREECLAMPS | CD45RA+ CD8+ T cell %T cell \|\| id:ebi-a-GCST90001562 | Weighted median | 34 | 0.00151801166249647 | 0.00425176918455752 | 0.721068905042547 | -0.006815456 | 0.00985147926422921 | 1.00151916442543 | 0.993207716606885 | 1.00990016482969 | 0.949957918887556 |
| ebi-a-GCST90001562 | finngen_R11_O15_PREECLAMPS | Pre-eclampsia \|\| id:finngen_R11_O15_PREECLAMPS | CD45RA+ CD8+ T cell %T cell \|\| id:ebi-a-GCST90001562 | Weighted mode | 34 | 0.0021800872395773 | 0.00349031939588255 | 0.536522268453691 | -0.004660939 | 0.0090211132555071 | 1.00218246535762 | 0.995349906542454 | 1.00906192613123 | 0.935508728341156 |
| ebi-a-GCST90001563 | finngen_R11_O15_PREECLAMPS | Pre-eclampsia \|\| id:finngen_R11_O15_PREECLAMPS | Central Memory CD4-CD8- T cell Absolute Count \|\| id:ebi-a-GCST90001563 | Inverse variance weighted | 4 | -0.023632738 | 0.0338832837682452 | 0.485505674532575 | -0.090043974 | 0.0427784979852528 | 0.976644328053863 | 0.913890996591907 | 1.04370668611118 | 0.898694924660397 |
| ebi-a-GCST90001563 | finngen_R11_O15_PREECLAMPS | Pre-eclampsia \|\| id:finngen_R11_O15_PREECLAMPS | Central Memory CD4-CD8- T cell Absolute Count \|\| id:ebi-a-GCST90001563 | MR Egger | 4 | -0.083854527 | 0.0328249552165878 | 0.125115751695944 | -0.148191439 | -0.019517615 | 0.919565018423721 | 0.862266027766143 | 0.980671620914137 | 0.97261503632431 |
| ebi-a-GCST90001563 | finngen_R11_O15_PREECLAMPS | Pre-eclampsia \|\| id:finngen_R11_O15_PREECLAMPS | Central Memory CD4-CD8- T cell Absolute Count \|\| id:ebi-a-GCST90001563 | Weighted median | 4 | -0.033233093 | 0.0241730120338352 | 0.169192989871601 | -0.080612197 | 0.0141460101411085 | 0.967313058975184 | 0.922551390249505 | 1.01424653840787 | 0.940845681141708 |
| ebi-a-GCST90001563 | finngen_R11_O15_PREECLAMPS | Pre-eclampsia \|\| id:finngen_R11_O15_PREECLAMPS | Central Memory CD4-CD8- T cell Absolute Count \|\| id:ebi-a-GCST90001563 | Weighted mode | 4 | -0.033484216 | 0.0215093852070293 | 0.217400395976443 | -0.075642611 | 0.00867417912756003 | 0.967070175464358 | 0.927147499780913 | 1.00871190883183 | 0.872755742358858 |
| ebi-a-GCST90001564 | finngen_R11_O15_PREECLAMPS | Pre-eclampsia \|\| id:finngen_R11_O15_PREECLAMPS | Central Memory CD4-CD8- T cell %CD4-CD8- T cell \|\| id:ebi-a-GCST90001564 | Inverse variance weighted | 16 | 0.0128821074923068 | 0.0224320324582238 | 0.565783170047349 | -0.031084676 | 0.0568488911104254 | 1.01296543928427 | 0.969393485115923 | 1.05849585017756 | 0.910672367259773 |
| ebi-a-GCST90001564 | finngen_R11_O15_PREECLAMPS | Pre-eclampsia \|\| id:finngen_R11_O15_PREECLAMPS | Central Memory CD4-CD8- T cell %CD4-CD8- T cell \|\| id:ebi-a-GCST90001564 | MR Egger | 16 | 0.00297709894690947 | 0.0313136660085592 | 0.925603876865046 | -0.058397686 | 0.0643518843236855 | 1.00298153490698 | 0.943274745269509 | 1.06646760597513 | 0.99380795269851 |
| ebi-a-GCST90001564 | finngen_R11_O15_PREECLAMPS | Pre-eclampsia \|\| id:finngen_R11_O15_PREECLAMPS | Central Memory CD4-CD8- T cell %CD4-CD8- T cell \|\| id:ebi-a-GCST90001564 | Weighted median | 16 | 0.0164384613368121 | 0.0301199348633751 | 0.585226227557393 | -0.042596611 | 0.0754735336690273 | 1.01657431623668 | 0.958297878936601 | 1.07839468618967 | 0.940845681141708 |
| ebi-a-GCST90001564 | finngen_R11_O15_PREECLAMPS | Pre-eclampsia \|\| id:finngen_R11_O15_PREECLAMPS | Central Memory CD4-CD8- T cell %CD4-CD8- T cell \|\| id:ebi-a-GCST90001564 | Weighted mode | 16 | 0.000209498653947371 | 0.0294611738377445 | 0.994419996906776 | -0.057534402 | 0.0579533993759265 | 1.00020952060032 | 0.944089411199598 | 1.05966561348078 | 0.996042942349711 |
| ebi-a-GCST90001565 | finngen_R11_O15_PREECLAMPS | Pre-eclampsia \|\| id:finngen_R11_O15_PREECLAMPS | Central Memory CD4-CD8- T cell %T cell \|\| id:ebi-a-GCST90001565 | Inverse variance weighted | 16 | -0.030146881 | 0.02428037851355 | 0.214378660666114 | -0.077736423 | 0.0174426607676108 | 0.970303003890377 | 0.925208258019875 | 1.01759567232318 | 0.855687495048373 |
| ebi-a-GCST90001565 | finngen_R11_O15_PREECLAMPS | Pre-eclampsia \|\| id:finngen_R11_O15_PREECLAMPS | Central Memory CD4-CD8- T cell %T cell \|\| id:ebi-a-GCST90001565 | MR Egger | 16 | -0.036932001 | 0.0310574739040959 | 0.25415916182212 | -0.09780465 | 0.0239406475093886 | 0.963741666260369 | 0.906826034741463 | 1.02422952551325 | 0.99380795269851 |
| ebi-a-GCST90001565 | finngen_R11_O15_PREECLAMPS | Pre-eclampsia \|\| id:finngen_R11_O15_PREECLAMPS | Central Memory CD4-CD8- T cell %T cell \|\| id:ebi-a-GCST90001565 | Weighted median | 16 | -0.028364225 | 0.0324115929768249 | 0.381505389171514 | -0.091890948 | 0.035162496755977 | 0.972034262673751 | 0.912204622121229 | 1.03578800731634 | 0.940845681141708 |
| ebi-a-GCST90001565 | finngen_R11_O15_PREECLAMPS | Pre-eclampsia \|\| id:finngen_R11_O15_PREECLAMPS | Central Memory CD4-CD8- T cell %T cell \|\| id:ebi-a-GCST90001565 | Weighted mode | 16 | -0.035078669 | 0.0318597211055384 | 0.288236555354417 | -0.097523723 | 0.0273663840014772 | 0.965529455680188 | 0.907080822865087 | 1.02774428285398 | 0.872755742358858 |
| ebi-a-GCST90001566 | finngen_R11_O15_PREECLAMPS | Pre-eclampsia \|\| id:finngen_R11_O15_PREECLAMPS | Naive CD4-CD8- T cell Absolute Count \|\| id:ebi-a-GCST90001566 | Inverse variance weighted | 18 | -0.062892866 | 0.0329375740758166 | 0.056203225721539 | -0.127450511 | 0.00166477964918269 | 0.939044072280396 | 0.880336981992521 | 1.00166616616413 | 0.789997151112252 |
| ebi-a-GCST90001566 | finngen_R11_O15_PREECLAMPS | Pre-eclampsia \|\| id:finngen_R11_O15_PREECLAMPS | Naive CD4-CD8- T cell Absolute Count \|\| id:ebi-a-GCST90001566 | MR Egger | 18 | 0.000999149459479428 | 0.0515603668742647 | 0.984778964884931 | -0.10005917 | 0.102057468533038 | 1.00099964877558 | 0.904783880739036 | 1.10744711326024 | 0.99380795269851 |
| ebi-a-GCST90001566 | finngen_R11_O15_PREECLAMPS | Pre-eclampsia \|\| id:finngen_R11_O15_PREECLAMPS | Naive CD4-CD8- T cell Absolute Count \|\| id:ebi-a-GCST90001566 | Weighted median | 18 | -0.024886659 | 0.0431156312913754 | 0.563799390315121 | -0.109393297 | 0.0596199779106077 | 0.97542046048366 | 0.896377805683073 | 1.06143310186615 | 0.940845681141708 |
| ebi-a-GCST90001566 | finngen_R11_O15_PREECLAMPS | Pre-eclampsia \|\| id:finngen_R11_O15_PREECLAMPS | Naive CD4-CD8- T cell Absolute Count \|\| id:ebi-a-GCST90001566 | Weighted mode | 18 | -0.024726034 | 0.036970409415215 | 0.512599655360815 | -0.097188036 | 0.0477359687686092 | 0.975577150696232 | 0.907385368849414 | 1.04889367807134 | 0.934398527469092 |
| ebi-a-GCST90001567 | finngen_R11_O15_PREECLAMPS | Pre-eclampsia \|\| id:finngen_R11_O15_PREECLAMPS | Naive CD4-CD8- T cell %CD4-CD8- T cell \|\| id:ebi-a-GCST90001567 | Inverse variance weighted | 24 | -0.026384653 | 0.0236487667425915 | 0.264555565549941 | -0.072736236 | 0.0199669296282767 | 0.973960380582259 | 0.929846057596311 | 1.02016760214707 | 0.885079385681701 |
| ebi-a-GCST90001567 | finngen_R11_O15_PREECLAMPS | Pre-eclampsia \|\| id:finngen_R11_O15_PREECLAMPS | Naive CD4-CD8- T cell %CD4-CD8- T cell \|\| id:ebi-a-GCST90001567 | MR Egger | 24 | -0.024852322 | 0.0329428899990461 | 0.458605076634448 | -0.089420387 | 0.039715741904275 | 0.975453953999477 | 0.914461065313909 | 1.04051495734986 | 0.99380795269851 |
| ebi-a-GCST90001567 | finngen_R11_O15_PREECLAMPS | Pre-eclampsia \|\| id:finngen_R11_O15_PREECLAMPS | Naive CD4-CD8- T cell %CD4-CD8- T cell \|\| id:ebi-a-GCST90001567 | Weighted median | 24 | -0.01610692 | 0.0369685581229007 | 0.663059916013725 | -0.088565294 | 0.0563514538753968 | 0.984022102742478 | 0.915243348916528 | 1.05796944586597 | 0.940845681141708 |
| ebi-a-GCST90001567 | finngen_R11_O15_PREECLAMPS | Pre-eclampsia \|\| id:finngen_R11_O15_PREECLAMPS | Naive CD4-CD8- T cell %CD4-CD8- T cell \|\| id:ebi-a-GCST90001567 | Weighted mode | 24 | -0.020888983 | 0.0309862928938162 | 0.506944223017062 | -0.081622117 | 0.039844151229058 | 0.979327680709728 | 0.921620157571589 | 1.04064857775183 | 0.934398527469092 |
| ebi-a-GCST90001568 | finngen_R11_O15_PREECLAMPS | Pre-eclampsia \|\| id:finngen_R11_O15_PREECLAMPS | Naive CD4-CD8- T cell %T cell \|\| id:ebi-a-GCST90001568 | Inverse variance weighted (multiplicative random effects) | 21 | -0.048311403 | 0.0350497853674961 | 0.168090572570949 | -0.117008983 | 0.020386176087565 | 0.952837024343823 | 0.889577202453137 | 1.02059539347089 | 0.85505992597664 |
| ebi-a-GCST90001568 | finngen_R11_O15_PREECLAMPS | Pre-eclampsia \|\| id:finngen_R11_O15_PREECLAMPS | Naive CD4-CD8- T cell %T cell \|\| id:ebi-a-GCST90001568 | MR Egger | 21 | -0.007476062 | 0.0534752636933467 | 0.890286128659479 | -0.112287578 | 0.0973354552040292 | 0.992551814602441 | 0.893787186582998 | 1.10223006042067 | 0.99380795269851 |
| ebi-a-GCST90001568 | finngen_R11_O15_PREECLAMPS | Pre-eclampsia \|\| id:finngen_R11_O15_PREECLAMPS | Naive CD4-CD8- T cell %T cell \|\| id:ebi-a-GCST90001568 | Weighted median | 21 | -0.039501004 | 0.0394523862365712 | 0.316714508440649 | -0.116827681 | 0.037825673136305 | 0.961268988984312 | 0.889738498881808 | 1.03855016990298 | 0.940845681141708 |
| ebi-a-GCST90001568 | finngen_R11_O15_PREECLAMPS | Pre-eclampsia \|\| id:finngen_R11_O15_PREECLAMPS | Naive CD4-CD8- T cell %T cell \|\| id:ebi-a-GCST90001568 | Weighted mode | 21 | -0.017315907 | 0.0368702756410703 | 0.643689300556121 | -0.089581648 | 0.0549498327745854 | 0.982833151241632 | 0.914313610438163 | 1.05648761229382 | 0.959313411571287 |
| ebi-a-GCST90001569 | finngen_R11_O15_PREECLAMPS | Pre-eclampsia \|\| id:finngen_R11_O15_PREECLAMPS | Effector Memory CD4-CD8- T cell Absolute Count \|\| id:ebi-a-GCST90001569 | Inverse variance weighted | 19 | -0.011333317 | 0.0292006858058221 | 0.697928569641385 | -0.068566662 | 0.0459000267899554 | 0.988730662721666 | 0.933731214041207 | 1.04696973679931 | 0.950911139356934 |
| ebi-a-GCST90001569 | finngen_R11_O15_PREECLAMPS | Pre-eclampsia \|\| id:finngen_R11_O15_PREECLAMPS | Effector Memory CD4-CD8- T cell Absolute Count \|\| id:ebi-a-GCST90001569 | MR Egger | 19 | 0.0360273347275122 | 0.0527705624467137 | 0.503983999251194 | -0.067402968 | 0.139457637123071 | 1.0366841835833 | 0.934818423827001 | 1.14965010220067 | 0.99380795269851 |
| ebi-a-GCST90001569 | finngen_R11_O15_PREECLAMPS | Pre-eclampsia \|\| id:finngen_R11_O15_PREECLAMPS | Effector Memory CD4-CD8- T cell Absolute Count \|\| id:ebi-a-GCST90001569 | Weighted median | 19 | 0.00621317355964131 | 0.0429403734540129 | 0.884953227508721 | -0.077949958 | 0.0903763055295066 | 1.00623251535972 | 0.925010714392079 | 1.09458610501887 | 0.964912412213677 |
| ebi-a-GCST90001569 | finngen_R11_O15_PREECLAMPS | Pre-eclampsia \|\| id:finngen_R11_O15_PREECLAMPS | Effector Memory CD4-CD8- T cell Absolute Count \|\| id:ebi-a-GCST90001569 | Weighted mode | 19 | 0.013177006121838 | 0.0524165175026677 | 0.804359925191638 | -0.089559368 | 0.115913380427067 | 1.01326420545478 | 0.914333981165476 | 1.12289860292317 | 0.961305639715923 |
| ebi-a-GCST90001570 | finngen_R11_O15_PREECLAMPS | Pre-eclampsia \|\| id:finngen_R11_O15_PREECLAMPS | Effector Memory CD4-CD8- T cell %CD4-CD8- T cell \|\| id:ebi-a-GCST90001570 | Inverse variance weighted | 27 | 0.00182431574840912 | 0.00571538751218839 | 0.749579625819399 | -0.009377844 | 0.0130264752722984 | 1.00182598082477 | 0.990665991069001 | 1.01311168941259 | 0.96638925304053 |
| ebi-a-GCST90001570 | finngen_R11_O15_PREECLAMPS | Pre-eclampsia \|\| id:finngen_R11_O15_PREECLAMPS | Effector Memory CD4-CD8- T cell %CD4-CD8- T cell \|\| id:ebi-a-GCST90001570 | MR Egger | 27 | -0.000386565 | 0.00600119108065714 | 0.949152383411018 | -0.012148899 | 0.0113757699687763 | 0.999613510157137 | 0.987924600858053 | 1.0114407200922 | 0.99380795269851 |
| ebi-a-GCST90001570 | finngen_R11_O15_PREECLAMPS | Pre-eclampsia \|\| id:finngen_R11_O15_PREECLAMPS | Effector Memory CD4-CD8- T cell %CD4-CD8- T cell \|\| id:ebi-a-GCST90001570 | Weighted median | 27 | 0.000799158415443348 | 0.00638910707761445 | 0.90045911356763 | -0.011723491 | 0.0133218082875677 | 1.00079947782761 | 0.988344960907917 | 1.01341093892959 | 0.967019504646225 |
| ebi-a-GCST90001570 | finngen_R11_O15_PREECLAMPS | Pre-eclampsia \|\| id:finngen_R11_O15_PREECLAMPS | Effector Memory CD4-CD8- T cell %CD4-CD8- T cell \|\| id:ebi-a-GCST90001570 | Weighted mode | 27 | 0.00155095952576556 | 0.00570551252584616 | 0.787895630832202 | -0.009631845 | 0.012733764076424 | 1.00155216288553 | 0.990414392624031 | 1.01281518367582 | 0.961305639715923 |
| ebi-a-GCST90001571 | finngen_R11_O15_PREECLAMPS | Pre-eclampsia \|\| id:finngen_R11_O15_PREECLAMPS | Effector Memory CD4-CD8- T cell %T cell \|\| id:ebi-a-GCST90001571 | Inverse variance weighted | 23 | 0.0230577712310058 | 0.0227810557398422 | 0.311467898653342 | -0.021593098 | 0.0677086404810964 | 1.02332565662247 | 0.978638363934886 | 1.07005349278489 | 0.885079385681701 |
| ebi-a-GCST90001571 | finngen_R11_O15_PREECLAMPS | Pre-eclampsia \|\| id:finngen_R11_O15_PREECLAMPS | Effector Memory CD4-CD8- T cell %T cell \|\| id:ebi-a-GCST90001571 | MR Egger | 23 | 0.00211279725208948 | 0.0387021587168559 | 0.956980184644276 | -0.073743434 | 0.0779690283371271 | 1.00211503078092 | 0.928909990146303 | 1.0810891750221 | 0.99380795269851 |
| ebi-a-GCST90001571 | finngen_R11_O15_PREECLAMPS | Pre-eclampsia \|\| id:finngen_R11_O15_PREECLAMPS | Effector Memory CD4-CD8- T cell %T cell \|\| id:ebi-a-GCST90001571 | Weighted median | 23 | 0.0122205441982901 | 0.033372422382781 | 0.714225609923636 | -0.053189404 | 0.0776304920685409 | 1.01229552015292 | 0.94820040283542 | 1.08072324906988 | 0.949957918887556 |
| ebi-a-GCST90001571 | finngen_R11_O15_PREECLAMPS | Pre-eclampsia \|\| id:finngen_R11_O15_PREECLAMPS | Effector Memory CD4-CD8- T cell %T cell \|\| id:ebi-a-GCST90001571 | Weighted mode | 23 | 0.0108363330081219 | 0.0316919581393049 | 0.735650654943005 | -0.051279905 | 0.0729525709611594 | 1.01089525871852 | 0.95001272005566 | 1.0756795172592 | 0.959313411571287 |
| ebi-a-GCST90001572 | finngen_R11_O15_PREECLAMPS | Pre-eclampsia \|\| id:finngen_R11_O15_PREECLAMPS | Terminally Differentiated CD4-CD8- T cell Absolute Count \|\| id:ebi-a-GCST90001572 | Inverse variance weighted | 21 | 0.000249152458813079 | 0.00576241868376518 | 0.965512228815283 | -0.011045188 | 0.0115434930789928 | 1.00024918349986 | 0.989015585969691 | 1.01161037630281 | 0.992671503887443 |
| ebi-a-GCST90001572 | finngen_R11_O15_PREECLAMPS | Pre-eclampsia \|\| id:finngen_R11_O15_PREECLAMPS | Terminally Differentiated CD4-CD8- T cell Absolute Count \|\| id:ebi-a-GCST90001572 | MR Egger | 21 | 0.00142906465476188 | 0.00602893947249008 | 0.815167227734445 | -0.010387657 | 0.0132457860208424 | 1.00143008625424 | 0.989666108668182 | 1.01333390006126 | 0.99380795269851 |
| ebi-a-GCST90001572 | finngen_R11_O15_PREECLAMPS | Pre-eclampsia \|\| id:finngen_R11_O15_PREECLAMPS | Terminally Differentiated CD4-CD8- T cell Absolute Count \|\| id:ebi-a-GCST90001572 | Weighted median | 21 | -5.58E-05 | 0.00631314134203444 | 0.992947825970724 | -0.012429557 | 0.0123179570396474 | 0.99994420156605 | 0.98764737086636 | 1.01239413553895 | 0.995671962667489 |
| ebi-a-GCST90001572 | finngen_R11_O15_PREECLAMPS | Pre-eclampsia \|\| id:finngen_R11_O15_PREECLAMPS | Terminally Differentiated CD4-CD8- T cell Absolute Count \|\| id:ebi-a-GCST90001572 | Weighted mode | 21 | 0.000308280827317375 | 0.00589041115774184 | 0.958780149060726 | -0.011236925 | 0.0118534866964914 | 1.00030832835074 | 0.988825973384924 | 1.01192401767376 | 0.990180645930572 |
| ebi-a-GCST90001573 | finngen_R11_O15_PREECLAMPS | Pre-eclampsia \|\| id:finngen_R11_O15_PREECLAMPS | Terminally Differentiated CD4-CD8- T cell %CD4-CD8- T cell \|\| id:ebi-a-GCST90001573 | Inverse variance weighted | 27 | 0.0010861424775661 | 0.00646215396907618 | 0.866522335023315 | -0.011579679 | 0.0137519642569554 | 1.00108673254392 | 0.988487107147422 | 1.01384695746568 | 0.981050218604997 |
| ebi-a-GCST90001573 | finngen_R11_O15_PREECLAMPS | Pre-eclampsia \|\| id:finngen_R11_O15_PREECLAMPS | Terminally Differentiated CD4-CD8- T cell %CD4-CD8- T cell \|\| id:ebi-a-GCST90001573 | MR Egger | 27 | 0.00193486815806465 | 0.00695117342266642 | 0.783032025660605 | -0.011689432 | 0.0155591680664908 | 1.00193674122331 | 0.988378624220306 | 1.01568084215207 | 0.99380795269851 |
| ebi-a-GCST90001573 | finngen_R11_O15_PREECLAMPS | Pre-eclampsia \|\| id:finngen_R11_O15_PREECLAMPS | Terminally Differentiated CD4-CD8- T cell %CD4-CD8- T cell \|\| id:ebi-a-GCST90001573 | Weighted median | 27 | -0.000768656 | 0.00691840954024762 | 0.911534647769006 | -0.014328739 | 0.0127914264513774 | 0.999231639093029 | 0.985773428872715 | 1.01287358668851 | 0.971329194634319 |
| ebi-a-GCST90001573 | finngen_R11_O15_PREECLAMPS | Pre-eclampsia \|\| id:finngen_R11_O15_PREECLAMPS | Terminally Differentiated CD4-CD8- T cell %CD4-CD8- T cell \|\| id:ebi-a-GCST90001573 | Weighted mode | 27 | -0.001519025 | 0.00604619730599254 | 0.803606424965508 | -0.013369571 | 0.0103315221775877 | 0.998482128591669 | 0.986719404492016 | 1.01038507662694 | 0.961305639715923 |
| ebi-a-GCST90001574 | finngen_R11_O15_PREECLAMPS | Pre-eclampsia \|\| id:finngen_R11_O15_PREECLAMPS | Terminally Differentiated CD4-CD8- T cell %T cell \|\| id:ebi-a-GCST90001574 | Inverse variance weighted (multiplicative random effects) | 23 | -0.000964603 | 0.00709975026122808 | 0.891928554092321 | -0.014880113 | 0.0129509078757287 | 0.999035862443294 | 0.985230048651808 | 1.01303513409237 | 0.986383922906939 |
| ebi-a-GCST90001574 | finngen_R11_O15_PREECLAMPS | Pre-eclampsia \|\| id:finngen_R11_O15_PREECLAMPS | Terminally Differentiated CD4-CD8- T cell %T cell \|\| id:ebi-a-GCST90001574 | MR Egger | 23 | 0.000638566687045012 | 0.00764939251269701 | 0.934261011098154 | -0.014354243 | 0.0156313760119311 | 1.00063877061416 | 0.985748288331937 | 1.01575418502684 | 0.99380795269851 |
| ebi-a-GCST90001574 | finngen_R11_O15_PREECLAMPS | Pre-eclampsia \|\| id:finngen_R11_O15_PREECLAMPS | Terminally Differentiated CD4-CD8- T cell %T cell \|\| id:ebi-a-GCST90001574 | Weighted median | 23 | -0.000445351 | 0.006462100823505 | 0.945055435086987 | -0.013111068 | 0.0122203669693623 | 0.999554748509171 | 0.986974507392332 | 1.01229534074488 | 0.981835244750373 |
| ebi-a-GCST90001574 | finngen_R11_O15_PREECLAMPS | Pre-eclampsia \|\| id:finngen_R11_O15_PREECLAMPS | Terminally Differentiated CD4-CD8- T cell %T cell \|\| id:ebi-a-GCST90001574 | Weighted mode | 23 | 0.000500264731186806 | 0.0059502155405336 | 0.933757121592332 | -0.011162158 | 0.0121626871906327 | 1.00050038988446 | 0.988899908010482 | 1.01223695345752 | 0.990180645930572 |
| ebi-a-GCST90001575 | finngen_R11_O15_PREECLAMPS | Pre-eclampsia \|\| id:finngen_R11_O15_PREECLAMPS | Naive CD4+ T cell %T cell \|\| id:ebi-a-GCST90001575 | Inverse variance weighted (multiplicative random effects) | 34 | 0.00210813326253217 | 0.021108528261796 | 0.920446610612938 | -0.039264582 | 0.0434808486556524 | 1.00211035693779 | 0.961496280754733 | 1.04443999169056 | 0.986667691552896 |
| ebi-a-GCST90001575 | finngen_R11_O15_PREECLAMPS | Pre-eclampsia \|\| id:finngen_R11_O15_PREECLAMPS | Naive CD4+ T cell %T cell \|\| id:ebi-a-GCST90001575 | MR Egger | 34 | 0.0579870297243346 | 0.0267260975976366 | 0.0375662403928079 | 0.00560387843296692 | 0.110370181015702 | 1.0597012510038 | 1.00561960953104 | 1.11669137190225 | 0.839403552053506 |
| ebi-a-GCST90001575 | finngen_R11_O15_PREECLAMPS | Pre-eclampsia \|\| id:finngen_R11_O15_PREECLAMPS | Naive CD4+ T cell %T cell \|\| id:ebi-a-GCST90001575 | Weighted median | 34 | 0.0280778189277359 | 0.0270356701973212 | 0.299015384049253 | -0.024912095 | 0.0810677325144853 | 1.02847571618483 | 0.975395650747107 | 1.08444434622166 | 0.940845681141708 |
| ebi-a-GCST90001575 | finngen_R11_O15_PREECLAMPS | Pre-eclampsia \|\| id:finngen_R11_O15_PREECLAMPS | Naive CD4+ T cell %T cell \|\| id:ebi-a-GCST90001575 | Weighted mode | 34 | 0.0425221036128333 | 0.0246752684900564 | 0.0941995398862453 | -0.005841423 | 0.0908856298533439 | 1.0434391198931 | 0.994175605309554 | 1.09514374634478 | 0.872755742358858 |
| ebi-a-GCST90001576 | finngen_R11_O15_PREECLAMPS | Pre-eclampsia \|\| id:finngen_R11_O15_PREECLAMPS | Transitional B cell %B cell \|\| id:ebi-a-GCST90001576 | Inverse variance weighted | 14 | 0.00775735936421224 | 0.0358355487359438 | 0.828620578018492 | -0.062480316 | 0.0779950348866621 | 1.00778752562943 | 0.939431554253544 | 1.08111729078688 | 0.977824840984872 |
| ebi-a-GCST90001576 | finngen_R11_O15_PREECLAMPS | Pre-eclampsia \|\| id:finngen_R11_O15_PREECLAMPS | Transitional B cell %B cell \|\| id:ebi-a-GCST90001576 | MR Egger | 14 | -0.022117762 | 0.0668755301509502 | 0.746551474107515 | -0.153193801 | 0.108958277210026 | 0.978125042419114 | 0.857963431543011 | 1.11511582362754 | 0.99380795269851 |
| ebi-a-GCST90001576 | finngen_R11_O15_PREECLAMPS | Pre-eclampsia \|\| id:finngen_R11_O15_PREECLAMPS | Transitional B cell %B cell \|\| id:ebi-a-GCST90001576 | Weighted median | 14 | -0.024018333 | 0.0501356387858467 | 0.631890924484986 | -0.122284185 | 0.0742475190838673 | 0.976267811738154 | 0.884896858391281 | 1.0770733687186 | 0.940845681141708 |
| ebi-a-GCST90001576 | finngen_R11_O15_PREECLAMPS | Pre-eclampsia \|\| id:finngen_R11_O15_PREECLAMPS | Transitional B cell %B cell \|\| id:ebi-a-GCST90001576 | Weighted mode | 14 | -0.009546972 | 0.0599448415559298 | 0.875909704036798 | -0.127038861 | 0.107944917597831 | 0.990498455803522 | 0.880699446805475 | 1.11398638265054 | 0.977585971930092 |
| ebi-a-GCST90001577 | finngen_R11_O15_PREECLAMPS | Pre-eclampsia \|\| id:finngen_R11_O15_PREECLAMPS | Transitional B cell Absolute Count \|\| id:ebi-a-GCST90001577 | Inverse variance weighted | 31 | 0.0177177884216203 | 0.0246419699941161 | 0.472135618973429 | -0.030580473 | 0.0660160496100879 | 1.01787567955033 | 0.969882379808092 | 1.06824386192587 | 0.898694924660397 |
| ebi-a-GCST90001577 | finngen_R11_O15_PREECLAMPS | Pre-eclampsia \|\| id:finngen_R11_O15_PREECLAMPS | Transitional B cell Absolute Count \|\| id:ebi-a-GCST90001577 | MR Egger | 31 | 0.0103951900646174 | 0.0387757977340992 | 0.790532006511323 | -0.065605373 | 0.0863957536234517 | 1.01044940775774 | 0.936500359245006 | 1.09023770846292 | 0.99380795269851 |
| ebi-a-GCST90001577 | finngen_R11_O15_PREECLAMPS | Pre-eclampsia \|\| id:finngen_R11_O15_PREECLAMPS | Transitional B cell Absolute Count \|\| id:ebi-a-GCST90001577 | Weighted median | 31 | 0.0151897481330884 | 0.035383697796424 | 0.66771465064625 | -0.0541623 | 0.0845417958140794 | 1.015305699 | 0.947278351176792 | 1.08821832625236 | 0.940845681141708 |
| ebi-a-GCST90001577 | finngen_R11_O15_PREECLAMPS | Pre-eclampsia \|\| id:finngen_R11_O15_PREECLAMPS | Transitional B cell Absolute Count \|\| id:ebi-a-GCST90001577 | Weighted mode | 31 | 0.014908303130089 | 0.0306001363931222 | 0.629659625659432 | -0.045067964 | 0.0748845704606086 | 1.01501998619265 | 0.955932510436523 | 1.07775973839415 | 0.959313411571287 |
| ebi-a-GCST90001578 | finngen_R11_O15_PREECLAMPS | Pre-eclampsia \|\| id:finngen_R11_O15_PREECLAMPS | Transitional B cell %lymphocyte \|\| id:ebi-a-GCST90001578 | Inverse variance weighted | 26 | -0.014407275 | 0.0288808556071712 | 0.617883584162061 | -0.071013752 | 0.0421992016564046 | 0.985696012828985 | 0.931449082450213 | 1.04310224575146 | 0.927459753639562 |
| ebi-a-GCST90001578 | finngen_R11_O15_PREECLAMPS | Pre-eclampsia \|\| id:finngen_R11_O15_PREECLAMPS | Transitional B cell %lymphocyte \|\| id:ebi-a-GCST90001578 | MR Egger | 26 | -0.054469533 | 0.0588483037534987 | 0.363872181189677 | -0.169812208 | 0.0608731423900354 | 0.94698736031364 | 0.843823264703697 | 1.06276408592348 | 0.99380795269851 |
| ebi-a-GCST90001578 | finngen_R11_O15_PREECLAMPS | Pre-eclampsia \|\| id:finngen_R11_O15_PREECLAMPS | Transitional B cell %lymphocyte \|\| id:ebi-a-GCST90001578 | Weighted median | 26 | -0.051680013 | 0.0391794874947146 | 0.187149752142043 | -0.128471808 | 0.0251117824958131 | 0.949632688351739 | 0.879438354769865 | 1.02542973921366 | 0.940845681141708 |
| ebi-a-GCST90001578 | finngen_R11_O15_PREECLAMPS | Pre-eclampsia \|\| id:finngen_R11_O15_PREECLAMPS | Transitional B cell %lymphocyte \|\| id:ebi-a-GCST90001578 | Weighted mode | 26 | -0.049540414 | 0.0436624230329183 | 0.267295365217464 | -0.135118763 | 0.0360379353950659 | 0.951666696939833 | 0.873612152819325 | 1.03669517318594 | 0.872755742358858 |
| ebi-a-GCST90001579 | finngen_R11_O15_PREECLAMPS | Pre-eclampsia \|\| id:finngen_R11_O15_PREECLAMPS | CD14- CD16+ monocyte Absolute Count \|\| id:ebi-a-GCST90001579 | Inverse variance weighted | 18 | 0.0253392323265537 | 0.0165376438879293 | 0.125469294269574 | -0.00707455 | 0.0577530143468951 | 1.02566299956079 | 0.992950416024467 | 1.05945329362963 | 0.820779257935285 |
| ebi-a-GCST90001579 | finngen_R11_O15_PREECLAMPS | Pre-eclampsia \|\| id:finngen_R11_O15_PREECLAMPS | CD14- CD16+ monocyte Absolute Count \|\| id:ebi-a-GCST90001579 | MR Egger | 18 | 0.0335709783755642 | 0.0208289770441507 | 0.126565741560071 | -0.007253817 | 0.0743957733820995 | 1.0341408427585 | 0.9927724287987 | 1.07723306131229 | 0.97261503632431 |
| ebi-a-GCST90001579 | finngen_R11_O15_PREECLAMPS | Pre-eclampsia \|\| id:finngen_R11_O15_PREECLAMPS | CD14- CD16+ monocyte Absolute Count \|\| id:ebi-a-GCST90001579 | Weighted median | 18 | 0.0277704341488359 | 0.0246496293468818 | 0.259908863895993 | -0.020542839 | 0.0760837076687242 | 1.0281596269872 | 0.97966672726912 | 1.07905289537924 | 0.940845681141708 |
| ebi-a-GCST90001579 | finngen_R11_O15_PREECLAMPS | Pre-eclampsia \|\| id:finngen_R11_O15_PREECLAMPS | CD14- CD16+ monocyte Absolute Count \|\| id:ebi-a-GCST90001579 | Weighted mode | 18 | 0.0306165430240304 | 0.0187659021489815 | 0.12117013862497 | -0.006164625 | 0.0673977112360342 | 1.03109004939918 | 0.99385433712868 | 1.06972083357962 | 0.872755742358858 |
| ebi-a-GCST90001580 | finngen_R11_O15_PREECLAMPS | Pre-eclampsia \|\| id:finngen_R11_O15_PREECLAMPS | CD14+ CD16+ monocyte Absolute Count \|\| id:ebi-a-GCST90001580 | Inverse variance weighted (multiplicative random effects) | 20 | 0.0325528367348815 | 0.0274020470966628 | 0.234844843222398 | -0.021155176 | 0.0862608490443405 | 1.0330884767239 | 0.979067025492917 | 1.09009064032403 | 0.884905053585426 |
| ebi-a-GCST90001580 | finngen_R11_O15_PREECLAMPS | Pre-eclampsia \|\| id:finngen_R11_O15_PREECLAMPS | CD14+ CD16+ monocyte Absolute Count \|\| id:ebi-a-GCST90001580 | MR Egger | 20 | 0.015309258134613 | 0.0417758538089221 | 0.718292558258141 | -0.066571415 | 0.0971899316001003 | 1.01542704513652 | 0.935596097565562 | 1.10206967160038 | 0.99380795269851 |
| ebi-a-GCST90001580 | finngen_R11_O15_PREECLAMPS | Pre-eclampsia \|\| id:finngen_R11_O15_PREECLAMPS | CD14+ CD16+ monocyte Absolute Count \|\| id:ebi-a-GCST90001580 | Weighted median | 20 | 0.0260186077105465 | 0.0307434473183601 | 0.397377639110984 | -0.034238549 | 0.0862757644545323 | 1.02636004650642 | 0.966340957438824 | 1.09010689959433 | 0.940845681141708 |
| ebi-a-GCST90001580 | finngen_R11_O15_PREECLAMPS | Pre-eclampsia \|\| id:finngen_R11_O15_PREECLAMPS | CD14+ CD16+ monocyte Absolute Count \|\| id:ebi-a-GCST90001580 | Weighted mode | 20 | 0.0399720486668795 | 0.0289756648140956 | 0.183760669019822 | -0.016820254 | 0.096764351702507 | 1.0407816825503 | 0.983320416300012 | 1.10160075269071 | 0.872755742358858 |
| ebi-a-GCST90001581 | finngen_R11_O15_PREECLAMPS | Pre-eclampsia \|\| id:finngen_R11_O15_PREECLAMPS | CD14- CD16- Absolute Count \|\| id:ebi-a-GCST90001581 | Inverse variance weighted | 24 | -0.002664668 | 0.00251181250864586 | 0.288756012525659 | -0.00758782 | 0.00225848481865738 | 0.997338879377391 | 0.992440894619008 | 1.00226103711658 | 0.885079385681701 |
| ebi-a-GCST90001581 | finngen_R11_O15_PREECLAMPS | Pre-eclampsia \|\| id:finngen_R11_O15_PREECLAMPS | CD14- CD16- Absolute Count \|\| id:ebi-a-GCST90001581 | MR Egger | 24 | -0.003348332 | 0.00254423701218256 | 0.201707496563589 | -0.008335036 | 0.00163837286001896 | 0.996657267727368 | 0.991699603877733 | 1.0016397157261 | 0.99380795269851 |
| ebi-a-GCST90001581 | finngen_R11_O15_PREECLAMPS | Pre-eclampsia \|\| id:finngen_R11_O15_PREECLAMPS | CD14- CD16- Absolute Count \|\| id:ebi-a-GCST90001581 | Weighted median | 24 | -0.002981339 | 0.00255245974259211 | 0.242796381047684 | -0.00798416 | 0.00202148240772845 | 0.997023101089178 | 0.992047628962089 | 1.00202352698075 | 0.940845681141708 |
| ebi-a-GCST90001581 | finngen_R11_O15_PREECLAMPS | Pre-eclampsia \|\| id:finngen_R11_O15_PREECLAMPS | CD14- CD16- Absolute Count \|\| id:ebi-a-GCST90001581 | Weighted mode | 24 | -0.003101907 | 0.00231563639704522 | 0.193479275661154 | -0.007640554 | 0.00143674053563979 | 0.996902899139858 | 0.992388560694664 | 1.00143777314179 | 0.872755742358858 |
| ebi-a-GCST90001582 | finngen_R11_O15_PREECLAMPS | Pre-eclampsia \|\| id:finngen_R11_O15_PREECLAMPS | CD14+ CD16- monocyte Absolute Count \|\| id:ebi-a-GCST90001582 | Inverse variance weighted | 27 | -0.012867718 | 0.0132450933789344 | 0.331295222329913 | -0.038828101 | 0.0130926648800383 | 0.987214716979628 | 0.961916047182761 | 1.01317874909721 | 0.885079385681701 |
| ebi-a-GCST90001582 | finngen_R11_O15_PREECLAMPS | Pre-eclampsia \|\| id:finngen_R11_O15_PREECLAMPS | CD14+ CD16- monocyte Absolute Count \|\| id:ebi-a-GCST90001582 | MR Egger | 27 | -0.011484404 | 0.0160047303039885 | 0.479678544653657 | -0.042853676 | 0.0198848671307785 | 0.988581289779472 | 0.958051566073471 | 1.02008388808071 | 0.99380795269851 |
| ebi-a-GCST90001582 | finngen_R11_O15_PREECLAMPS | Pre-eclampsia \|\| id:finngen_R11_O15_PREECLAMPS | CD14+ CD16- monocyte Absolute Count \|\| id:ebi-a-GCST90001582 | Weighted median | 27 | -0.018265908 | 0.0167791956646393 | 0.276328339829745 | -0.051153131 | 0.0146213159120249 | 0.981899903004216 | 0.950133164461647 | 1.01472873022591 | 0.940845681141708 |
| ebi-a-GCST90001582 | finngen_R11_O15_PREECLAMPS | Pre-eclampsia \|\| id:finngen_R11_O15_PREECLAMPS | CD14+ CD16- monocyte Absolute Count \|\| id:ebi-a-GCST90001582 | Weighted mode | 27 | -0.01756081 | 0.0141776291569545 | 0.226549969683341 | -0.045348963 | 0.010227343490178 | 0.982592482736023 | 0.955663932471732 | 1.01027982151864 | 0.872755742358858 |
| ebi-a-GCST90001583 | finngen_R11_O15_PREECLAMPS | Pre-eclampsia \|\| id:finngen_R11_O15_PREECLAMPS | Monocyte Absolute Count \|\| id:ebi-a-GCST90001583 | Inverse variance weighted | 27 | -0.007798289 | 0.0110284817252571 | 0.479501531225022 | -0.029414113 | 0.0138175349749627 | 0.992232038564618 | 0.97101427118586 | 1.0139134383182 | 0.898694924660397 |
| ebi-a-GCST90001583 | finngen_R11_O15_PREECLAMPS | Pre-eclampsia \|\| id:finngen_R11_O15_PREECLAMPS | Monocyte Absolute Count \|\| id:ebi-a-GCST90001583 | MR Egger | 27 | -0.010316687 | 0.0129684612619876 | 0.433799047407976 | -0.035734871 | 0.0151014968896046 | 0.989736347296597 | 0.964896081258055 | 1.01521610066321 | 0.99380795269851 |
| ebi-a-GCST90001583 | finngen_R11_O15_PREECLAMPS | Pre-eclampsia \|\| id:finngen_R11_O15_PREECLAMPS | Monocyte Absolute Count \|\| id:ebi-a-GCST90001583 | Weighted median | 27 | -0.017239086 | 0.0153787499056979 | 0.262301488078898 | -0.047381436 | 0.0129032634451071 | 0.982908656477236 | 0.953723543531023 | 1.01298686975998 | 0.940845681141708 |
| ebi-a-GCST90001583 | finngen_R11_O15_PREECLAMPS | Pre-eclampsia \|\| id:finngen_R11_O15_PREECLAMPS | Monocyte Absolute Count \|\| id:ebi-a-GCST90001583 | Weighted mode | 27 | -0.011781042 | 0.0131095496341371 | 0.377080721715392 | -0.037475759 | 0.0139136757746042 | 0.988288083241067 | 0.96321776700081 | 1.01401092145291 | 0.920254054953283 |
| ebi-a-GCST90001584 | finngen_R11_O15_PREECLAMPS | Pre-eclampsia \|\| id:finngen_R11_O15_PREECLAMPS | CD14- CD16+ monocyte %monocyte \|\| id:ebi-a-GCST90001584 | Inverse variance weighted | 23 | 0.00444899767522773 | 0.0227075769402739 | 0.844668329405303 | -0.040057853 | 0.0489558484781646 | 1.00445890915866 | 0.960733856086041 | 1.05017398293689 | 0.977824840984872 |
| ebi-a-GCST90001584 | finngen_R11_O15_PREECLAMPS | Pre-eclampsia \|\| id:finngen_R11_O15_PREECLAMPS | CD14- CD16+ monocyte %monocyte \|\| id:ebi-a-GCST90001584 | MR Egger | 23 | 0.0190801168921083 | 0.0334602013289048 | 0.574570515508926 | -0.046501878 | 0.0846621114967617 | 1.0192633055546 | 0.954562768173803 | 1.08834926385996 | 0.99380795269851 |
| ebi-a-GCST90001584 | finngen_R11_O15_PREECLAMPS | Pre-eclampsia \|\| id:finngen_R11_O15_PREECLAMPS | CD14- CD16+ monocyte %monocyte \|\| id:ebi-a-GCST90001584 | Weighted median | 23 | 0.0171233930445201 | 0.0329517218535424 | 0.603306890810109 | -0.047461982 | 0.0817087678774631 | 1.01727083872705 | 0.953646728386532 | 1.08513973625776 | 0.940845681141708 |
| ebi-a-GCST90001584 | finngen_R11_O15_PREECLAMPS | Pre-eclampsia \|\| id:finngen_R11_O15_PREECLAMPS | CD14- CD16+ monocyte %monocyte \|\| id:ebi-a-GCST90001584 | Weighted mode | 23 | 0.0130713121385972 | 0.0371638014774026 | 0.728393543898589 | -0.059769739 | 0.0859123630343063 | 1.01315711518432 | 0.941981410424467 | 1.08971082517019 | 0.959313411571287 |
| ebi-a-GCST90001585 | finngen_R11_O15_PREECLAMPS | Pre-eclampsia \|\| id:finngen_R11_O15_PREECLAMPS | CD14+ CD16+ monocyte %monocyte \|\| id:ebi-a-GCST90001585 | Inverse variance weighted | 24 | 0.0252064966326324 | 0.0255593531394338 | 0.324037617381431 | -0.024889836 | 0.0753028287859227 | 1.02552686650587 | 0.97541736245549 | 1.07821061466223 | 0.885079385681701 |
| ebi-a-GCST90001585 | finngen_R11_O15_PREECLAMPS | Pre-eclampsia \|\| id:finngen_R11_O15_PREECLAMPS | CD14+ CD16+ monocyte %monocyte \|\| id:ebi-a-GCST90001585 | MR Egger | 24 | 0.0342708362565292 | 0.0457205471684435 | 0.461453433306336 | -0.055341436 | 0.123883108706678 | 1.03486484769806 | 0.946162038830962 | 1.13188355593334 | 0.99380795269851 |
| ebi-a-GCST90001585 | finngen_R11_O15_PREECLAMPS | Pre-eclampsia \|\| id:finngen_R11_O15_PREECLAMPS | CD14+ CD16+ monocyte %monocyte \|\| id:ebi-a-GCST90001585 | Weighted median | 24 | 0.059220836651952 | 0.0309151708938658 | 0.0554171203086966 | -0.001372898 | 0.119814571603929 | 1.06100952466108 | 0.99862804369371 | 1.12728780102917 | 0.771214851704188 |
| ebi-a-GCST90001585 | finngen_R11_O15_PREECLAMPS | Pre-eclampsia \|\| id:finngen_R11_O15_PREECLAMPS | CD14+ CD16+ monocyte %monocyte \|\| id:ebi-a-GCST90001585 | Weighted mode | 24 | 0.0550019616613293 | 0.0323740931230596 | 0.102817087739014 | -0.008451261 | 0.118455184182526 | 1.05654268725239 | 0.991584350653849 | 1.12575642127716 | 0.872755742358858 |
| ebi-a-GCST90001586 | finngen_R11_O15_PREECLAMPS | Pre-eclampsia \|\| id:finngen_R11_O15_PREECLAMPS | CD14+ CD16- monocyte %monocyte \|\| id:ebi-a-GCST90001586 | Inverse variance weighted | 24 | -0.014335753 | 0.0101919613265117 | 0.159553595144374 | -0.034311997 | 0.00564049116303584 | 0.985766514592888 | 0.966269984038046 | 1.00565642868438 | 0.85505992597664 |
| ebi-a-GCST90001586 | finngen_R11_O15_PREECLAMPS | Pre-eclampsia \|\| id:finngen_R11_O15_PREECLAMPS | CD14+ CD16- monocyte %monocyte \|\| id:ebi-a-GCST90001586 | MR Egger | 24 | -0.009485992 | 0.0114932376228093 | 0.418030859390252 | -0.032012738 | 0.0130407537450476 | 0.9905588580985 | 0.968494245576257 | 1.01312615520352 | 0.99380795269851 |
| ebi-a-GCST90001586 | finngen_R11_O15_PREECLAMPS | Pre-eclampsia \|\| id:finngen_R11_O15_PREECLAMPS | CD14+ CD16- monocyte %monocyte \|\| id:ebi-a-GCST90001586 | Weighted median | 24 | -0.015137371 | 0.0137879273583257 | 0.272260642656728 | -0.042161709 | 0.0118869663381895 | 0.984976622805443 | 0.958714735325279 | 1.01195789709443 | 0.940845681141708 |
| ebi-a-GCST90001586 | finngen_R11_O15_PREECLAMPS | Pre-eclampsia \|\| id:finngen_R11_O15_PREECLAMPS | CD14+ CD16- monocyte %monocyte \|\| id:ebi-a-GCST90001586 | Weighted mode | 24 | -0.013863193 | 0.0114318999908041 | 0.237562890554227 | -0.036269717 | 0.00854333054813047 | 0.986232458110126 | 0.96438014828039 | 1.00857992894639 | 0.872755742358858 |
| ebi-a-GCST90001587 | finngen_R11_O15_PREECLAMPS | Pre-eclampsia \|\| id:finngen_R11_O15_PREECLAMPS | CD16+ monocyte %monocyte \|\| id:ebi-a-GCST90001587 | Inverse variance weighted | 22 | 0.0387176461386599 | 0.0269846872842881 | 0.151343920220061 | -0.014172341 | 0.0916076332158645 | 1.03947694188247 | 0.985927613929984 | 1.09593472932391 | 0.85505992597664 |
| ebi-a-GCST90001587 | finngen_R11_O15_PREECLAMPS | Pre-eclampsia \|\| id:finngen_R11_O15_PREECLAMPS | CD16+ monocyte %monocyte \|\| id:ebi-a-GCST90001587 | MR Egger | 22 | 0.0469384295718331 | 0.0474071490024396 | 0.333947274587039 | -0.045979582 | 0.139856441616615 | 1.04805747774542 | 0.955061461984881 | 1.15010867926265 | 0.99380795269851 |
| ebi-a-GCST90001587 | finngen_R11_O15_PREECLAMPS | Pre-eclampsia \|\| id:finngen_R11_O15_PREECLAMPS | CD16+ monocyte %monocyte \|\| id:ebi-a-GCST90001587 | Weighted median | 22 | 0.0353185998379997 | 0.037940887306778 | 0.351913095985576 | -0.039045539 | 0.109682738959284 | 1.03594970963739 | 0.96170691270561 | 1.11592397508981 | 0.940845681141708 |
| ebi-a-GCST90001587 | finngen_R11_O15_PREECLAMPS | Pre-eclampsia \|\| id:finngen_R11_O15_PREECLAMPS | CD16+ monocyte %monocyte \|\| id:ebi-a-GCST90001587 | Weighted mode | 22 | 0.0202927990931674 | 0.0486725908165053 | 0.680964645101397 | -0.075105479 | 0.115691077093518 | 1.02050009778983 | 0.927645634120232 | 1.12264900656459 | 0.959313411571287 |
| ebi-a-GCST90001588 | finngen_R11_O15_PREECLAMPS | Pre-eclampsia \|\| id:finngen_R11_O15_PREECLAMPS | T/B cell \|\| id:ebi-a-GCST90001588 | Inverse variance weighted | 28 | 0.0458883655807117 | 0.0232699952285445 | 0.0486099254418402 | 0.000279174932764505 | 0.0914975562286589 | 1.04695752793732 | 1.00027921390571 | 1.09581409877018 | 0.789641233288559 |
| ebi-a-GCST90001588 | finngen_R11_O15_PREECLAMPS | Pre-eclampsia \|\| id:finngen_R11_O15_PREECLAMPS | T/B cell \|\| id:ebi-a-GCST90001588 | MR Egger | 28 | 0.086517296351164 | 0.0427364975144023 | 0.0533113368244006 | 0.00275376122293543 | 0.170280831479392 | 1.09037022698103 | 1.00275755630616 | 1.18563776897998 | 0.862303274556491 |
| ebi-a-GCST90001588 | finngen_R11_O15_PREECLAMPS | Pre-eclampsia \|\| id:finngen_R11_O15_PREECLAMPS | T/B cell \|\| id:ebi-a-GCST90001588 | Weighted median | 28 | 0.0213668883966739 | 0.032268863455603 | 0.507873893848352 | -0.041880084 | 0.0846138607696557 | 1.02159679489898 | 0.958984771318288 | 1.08829675148352 | 0.940845681141708 |
| ebi-a-GCST90001588 | finngen_R11_O15_PREECLAMPS | Pre-eclampsia \|\| id:finngen_R11_O15_PREECLAMPS | T/B cell \|\| id:ebi-a-GCST90001588 | Weighted mode | 28 | 0.0246520215404281 | 0.0415936211377949 | 0.558322107494957 | -0.056871476 | 0.106175518970506 | 1.02495839501821 | 0.944715480280878 | 1.11201703946458 | 0.939633861977944 |
| ebi-a-GCST90001589 | finngen_R11_O15_PREECLAMPS | Pre-eclampsia \|\| id:finngen_R11_O15_PREECLAMPS | CD4+/CD8+ T cell \|\| id:ebi-a-GCST90001589 | Inverse variance weighted | 14 | -0.027530593 | 0.0296775638265764 | 0.353585631878664 | -0.085698619 | 0.0306374316572036 | 0.972844919422699 | 0.917870818783601 | 1.03111158768594 | 0.885079385681701 |
| ebi-a-GCST90001589 | finngen_R11_O15_PREECLAMPS | Pre-eclampsia \|\| id:finngen_R11_O15_PREECLAMPS | CD4+/CD8+ T cell \|\| id:ebi-a-GCST90001589 | MR Egger | 14 | -0.035545269 | 0.0557132226643981 | 0.535456385751397 | -0.144743186 | 0.0736526472161488 | 0.965079044878959 | 0.865244471795579 | 1.07643283860764 | 0.99380795269851 |
| ebi-a-GCST90001589 | finngen_R11_O15_PREECLAMPS | Pre-eclampsia \|\| id:finngen_R11_O15_PREECLAMPS | CD4+/CD8+ T cell \|\| id:ebi-a-GCST90001589 | Weighted median | 14 | -0.035073042 | 0.0400387750161743 | 0.38104259821717 | -0.113549041 | 0.0434029574824433 | 0.965534889517713 | 0.892660418752217 | 1.0443586422025 | 0.940845681141708 |
| ebi-a-GCST90001589 | finngen_R11_O15_PREECLAMPS | Pre-eclampsia \|\| id:finngen_R11_O15_PREECLAMPS | CD4+/CD8+ T cell \|\| id:ebi-a-GCST90001589 | Weighted mode | 14 | -0.035540418 | 0.0525036937125067 | 0.510327554117802 | -0.138447657 | 0.0673668221630686 | 0.965083727157204 | 0.870708825423018 | 1.06968779140506 | 0.934398527469092 |
| ebi-a-GCST90001590 | finngen_R11_O15_PREECLAMPS | Pre-eclampsia \|\| id:finngen_R11_O15_PREECLAMPS | CD4+ T cell Absolute Count \|\| id:ebi-a-GCST90001590 | Inverse variance weighted (multiplicative random effects) | 22 | -0.034641828 | 0.024055154910723 | 0.149839110422094 | -0.081789932 | 0.0125062751494417 | 0.96595133056613 | 0.921465508690104 | 1.01258480564168 | 0.85505992597664 |
| ebi-a-GCST90001590 | finngen_R11_O15_PREECLAMPS | Pre-eclampsia \|\| id:finngen_R11_O15_PREECLAMPS | CD4+ T cell Absolute Count \|\| id:ebi-a-GCST90001590 | MR Egger | 22 | -0.05389397 | 0.031098740239041 | 0.0984890314309427 | -0.114847501 | 0.00705956096488455 | 0.947532568145234 | 0.891502086920842 | 1.00708453840739 | 0.97261503632431 |
| ebi-a-GCST90001590 | finngen_R11_O15_PREECLAMPS | Pre-eclampsia \|\| id:finngen_R11_O15_PREECLAMPS | CD4+ T cell Absolute Count \|\| id:ebi-a-GCST90001590 | Weighted median | 22 | -0.045050035 | 0.0265470205940779 | 0.0896985057156288 | -0.097082195 | 0.00698212550843572 | 0.955949649833445 | 0.907481412433034 | 1.00700655737576 | 0.88227178111339 |
| ebi-a-GCST90001590 | finngen_R11_O15_PREECLAMPS | Pre-eclampsia \|\| id:finngen_R11_O15_PREECLAMPS | CD4+ T cell Absolute Count \|\| id:ebi-a-GCST90001590 | Weighted mode | 22 | -0.047086331 | 0.0236313136022631 | 0.0594698736215224 | -0.093403706 | -0.000768957 | 0.954005033519017 | 0.910825720271057 | 0.999231338909459 | 0.838871423749761 |
| ebi-a-GCST90001591 | finngen_R11_O15_PREECLAMPS | Pre-eclampsia \|\| id:finngen_R11_O15_PREECLAMPS | CD4+ T cell %T cell \|\| id:ebi-a-GCST90001591 | Inverse variance weighted | 22 | -0.01939041 | 0.0236064370830102 | 0.411416479469611 | -0.065659026 | 0.0268782070077678 | 0.980796375092751 | 0.936450114667038 | 1.02724268418411 | 0.898694924660397 |
| ebi-a-GCST90001591 | finngen_R11_O15_PREECLAMPS | Pre-eclampsia \|\| id:finngen_R11_O15_PREECLAMPS | CD4+ T cell %T cell \|\| id:ebi-a-GCST90001591 | MR Egger | 22 | 0.00867267214828291 | 0.0379814762608733 | 0.821700721738722 | -0.065771021 | 0.0831163656195945 | 1.00871038872503 | 0.936345242841518 | 1.0866682520157 | 0.99380795269851 |
| ebi-a-GCST90001591 | finngen_R11_O15_PREECLAMPS | Pre-eclampsia \|\| id:finngen_R11_O15_PREECLAMPS | CD4+ T cell %T cell \|\| id:ebi-a-GCST90001591 | Weighted median | 22 | -0.021865062 | 0.0344014150295991 | 0.525046212027883 | -0.089291836 | 0.045561711208878 | 0.978372245494728 | 0.914578627923586 | 1.04661559053441 | 0.940845681141708 |
| ebi-a-GCST90001591 | finngen_R11_O15_PREECLAMPS | Pre-eclampsia \|\| id:finngen_R11_O15_PREECLAMPS | CD4+ T cell %T cell \|\| id:ebi-a-GCST90001591 | Weighted mode | 22 | -0.012912134 | 0.0309180076426069 | 0.680460320080955 | -0.073511429 | 0.047687160960316 | 0.987170869946419 | 0.929125526756397 | 1.0488424851191 | 0.959313411571287 |
| ebi-a-GCST90001592 | finngen_R11_O15_PREECLAMPS | Pre-eclampsia \|\| id:finngen_R11_O15_PREECLAMPS | CD8+ T cell Absolute Count \|\| id:ebi-a-GCST90001592 | Inverse variance weighted (multiplicative random effects) | 21 | 0.0253280437668236 | 0.0139753569435523 | 0.0699342812476743 | -0.002063656 | 0.0527197433761862 | 1.02565152393325 | 0.997938472031194 | 1.05413417563254 | 0.789997151112252 |
| ebi-a-GCST90001592 | finngen_R11_O15_PREECLAMPS | Pre-eclampsia \|\| id:finngen_R11_O15_PREECLAMPS | CD8+ T cell Absolute Count \|\| id:ebi-a-GCST90001592 | MR Egger | 21 | 0.0214439626747181 | 0.0165348468630312 | 0.210196334080125 | -0.010964337 | 0.0538522625262592 | 1.02167553676884 | 0.989095552086171 | 1.05532867904451 | 0.99380795269851 |
| ebi-a-GCST90001592 | finngen_R11_O15_PREECLAMPS | Pre-eclampsia \|\| id:finngen_R11_O15_PREECLAMPS | CD8+ T cell Absolute Count \|\| id:ebi-a-GCST90001592 | Weighted median | 21 | 0.0371408133457954 | 0.0169186305274875 | 0.0281448656300716 | 0.00398029751191982 | 0.0703013291796709 | 1.03783915215271 | 1.00398822941635 | 1.07283140796104 | 0.709444716399391 |
| ebi-a-GCST90001592 | finngen_R11_O15_PREECLAMPS | Pre-eclampsia \|\| id:finngen_R11_O15_PREECLAMPS | CD8+ T cell Absolute Count \|\| id:ebi-a-GCST90001592 | Weighted mode | 21 | 0.0254026116543252 | 0.0139846636452656 | 0.0843191220115211 | -0.002007329 | 0.0528125523990457 | 1.02572800745228 | 0.997994684247274 | 1.05423201333538 | 0.872755742358858 |
| ebi-a-GCST90001593 | finngen_R11_O15_PREECLAMPS | Pre-eclampsia \|\| id:finngen_R11_O15_PREECLAMPS | CD8+ T cell %T cell \|\| id:ebi-a-GCST90001593 | Inverse variance weighted | 22 | -0.004674425 | 0.022775925363712 | 0.837388264486851 | -0.049315238 | 0.0399663890651541 | 0.995336483472159 | 0.951881012986823 | 1.04077579215716 | 0.977824840984872 |
| ebi-a-GCST90001593 | finngen_R11_O15_PREECLAMPS | Pre-eclampsia \|\| id:finngen_R11_O15_PREECLAMPS | CD8+ T cell %T cell \|\| id:ebi-a-GCST90001593 | MR Egger | 22 | -0.020447548 | 0.0368212251741216 | 0.584834647349553 | -0.092617149 | 0.0517220535946302 | 0.979760085751006 | 0.911542418346339 | 1.05308300119731 | 0.99380795269851 |
| ebi-a-GCST90001593 | finngen_R11_O15_PREECLAMPS | Pre-eclampsia \|\| id:finngen_R11_O15_PREECLAMPS | CD8+ T cell %T cell \|\| id:ebi-a-GCST90001593 | Weighted median | 22 | -0.00665654 | 0.0308134780253868 | 0.828966771975891 | -0.067050957 | 0.053737876499157 | 0.993365565258318 | 0.935147547472064 | 1.05520797109341 | 0.964912412213677 |
| ebi-a-GCST90001593 | finngen_R11_O15_PREECLAMPS | Pre-eclampsia \|\| id:finngen_R11_O15_PREECLAMPS | CD8+ T cell %T cell \|\| id:ebi-a-GCST90001593 | Weighted mode | 22 | 0.000627854040264886 | 0.0266615655053505 | 0.981434663092704 | -0.051628814 | 0.0528845224307518 | 1.00062805118187 | 0.949681309501964 | 1.05430788917717 | 0.994159530659367 |
| ebi-a-GCST90001594 | finngen_R11_O15_PREECLAMPS | Pre-eclampsia \|\| id:finngen_R11_O15_PREECLAMPS | CD4+CD8+ T cell Absolute Count \|\| id:ebi-a-GCST90001594 | Inverse variance weighted (multiplicative random effects) | 18 | -0.020908247 | 0.0353732154438511 | 0.554469726414638 | -0.090239749 | 0.048423255544017 | 0.979308815237473 | 0.913712097451348 | 1.04961481661119 | 0.905435115099076 |
| ebi-a-GCST90001594 | finngen_R11_O15_PREECLAMPS | Pre-eclampsia \|\| id:finngen_R11_O15_PREECLAMPS | CD4+CD8+ T cell Absolute Count \|\| id:ebi-a-GCST90001594 | MR Egger | 18 | -0.033840406 | 0.0678695348460591 | 0.62483919909397 | -0.166864694 | 0.0991838826775041 | 0.966725776326889 | 0.84631411503665 | 1.10426933689314 | 0.99380795269851 |
| ebi-a-GCST90001594 | finngen_R11_O15_PREECLAMPS | Pre-eclampsia \|\| id:finngen_R11_O15_PREECLAMPS | CD4+CD8+ T cell Absolute Count \|\| id:ebi-a-GCST90001594 | Weighted median | 18 | 0.00440179718688023 | 0.0393834385871702 | 0.911007581811422 | -0.072789742 | 0.0815933368177338 | 1.00441149932651 | 0.929796306173843 | 1.08501448465715 | 0.971329194634319 |
| ebi-a-GCST90001594 | finngen_R11_O15_PREECLAMPS | Pre-eclampsia \|\| id:finngen_R11_O15_PREECLAMPS | CD4+CD8+ T cell Absolute Count \|\| id:ebi-a-GCST90001594 | Weighted mode | 18 | -0.005685326 | 0.0467924083292235 | 0.904718820696166 | -0.097398447 | 0.0860277938975157 | 0.994330804456274 | 0.907194465421571 | 1.08983661869147 | 0.98361388732834 |
| ebi-a-GCST90001595 | finngen_R11_O15_PREECLAMPS | Pre-eclampsia \|\| id:finngen_R11_O15_PREECLAMPS | CD4+CD8+ T cell %T cell \|\| id:ebi-a-GCST90001595 | Inverse variance weighted | 29 | -0.043763855 | 0.0243906179250158 | 0.0727668078296749 | -0.091569466 | 0.00404175621503612 | 0.957179964127939 | 0.912497926322805 | 1.00404993512703 | 0.789997151112252 |
| ebi-a-GCST90001595 | finngen_R11_O15_PREECLAMPS | Pre-eclampsia \|\| id:finngen_R11_O15_PREECLAMPS | CD4+CD8+ T cell %T cell \|\| id:ebi-a-GCST90001595 | MR Egger | 29 | -0.030770966 | 0.0541982814226929 | 0.574897314349366 | -0.136999598 | 0.0754576651272086 | 0.969697640926762 | 0.871970576620832 | 1.07837757377427 | 0.99380795269851 |
| ebi-a-GCST90001595 | finngen_R11_O15_PREECLAMPS | Pre-eclampsia \|\| id:finngen_R11_O15_PREECLAMPS | CD4+CD8+ T cell %T cell \|\| id:ebi-a-GCST90001595 | Weighted median | 29 | -0.060172438 | 0.0358777978604501 | 0.0935132138913923 | -0.130492921 | 0.0101480462033158 | 0.941602151966081 | 0.87766270554151 | 1.01019971224609 | 0.88227178111339 |
| ebi-a-GCST90001595 | finngen_R11_O15_PREECLAMPS | Pre-eclampsia \|\| id:finngen_R11_O15_PREECLAMPS | CD4+CD8+ T cell %T cell \|\| id:ebi-a-GCST90001595 | Weighted mode | 29 | -0.055800381 | 0.0399490211604293 | 0.173450527739826 | -0.134100463 | 0.022499700091939 | 0.945727901945475 | 0.874502205200167 | 1.02275472743201 | 0.872755742358858 |
| ebi-a-GCST90001596 | finngen_R11_O15_PREECLAMPS | Pre-eclampsia \|\| id:finngen_R11_O15_PREECLAMPS | CD8dim T cell Absolute Count \|\| id:ebi-a-GCST90001596 | Inverse variance weighted | 27 | -0.010490817 | 0.0150383346184507 | 0.485424274306759 | -0.039965953 | 0.0189843189230728 | 0.989564019762738 | 0.960822151917413 | 1.01916566687688 | 0.898694924660397 |
| ebi-a-GCST90001596 | finngen_R11_O15_PREECLAMPS | Pre-eclampsia \|\| id:finngen_R11_O15_PREECLAMPS | CD8dim T cell Absolute Count \|\| id:ebi-a-GCST90001596 | MR Egger | 27 | -0.012804466 | 0.0204120713204919 | 0.53615119866164 | -0.052812126 | 0.0272031940609774 | 0.98727716266998 | 0.948558205626843 | 1.02757657900978 | 0.99380795269851 |
| ebi-a-GCST90001596 | finngen_R11_O15_PREECLAMPS | Pre-eclampsia \|\| id:finngen_R11_O15_PREECLAMPS | CD8dim T cell Absolute Count \|\| id:ebi-a-GCST90001596 | Weighted median | 27 | -0.001305413 | 0.0213171206465447 | 0.951169846453863 | -0.043086969 | 0.0404761434396719 | 0.998695438653392 | 0.957828084619958 | 1.04130646741563 | 0.984851498240473 |
| ebi-a-GCST90001596 | finngen_R11_O15_PREECLAMPS | Pre-eclampsia \|\| id:finngen_R11_O15_PREECLAMPS | CD8dim T cell Absolute Count \|\| id:ebi-a-GCST90001596 | Weighted mode | 27 | -0.009499796 | 0.0178664038147795 | 0.599439049538474 | -0.044517948 | 0.0255183552935223 | 0.990545184332386 | 0.956458433748405 | 1.02584673581558 | 0.955353022288269 |
| ebi-a-GCST90001597 | finngen_R11_O15_PREECLAMPS | Pre-eclampsia \|\| id:finngen_R11_O15_PREECLAMPS | CD8dim T cell %T cell \|\| id:ebi-a-GCST90001597 | Inverse variance weighted | 19 | -0.028904115 | 0.0250062036360565 | 0.24773114812968 | -0.077916275 | 0.0201080437122639 | 0.971509612797412 | 0.925041872856707 | 1.02031157232164 | 0.885079385681701 |
| ebi-a-GCST90001597 | finngen_R11_O15_PREECLAMPS | Pre-eclampsia \|\| id:finngen_R11_O15_PREECLAMPS | CD8dim T cell %T cell \|\| id:ebi-a-GCST90001597 | MR Egger | 19 | -0.016438412 | 0.0492762191049123 | 0.74276294762517 | -0.113019801 | 0.0801429779152302 | 0.983695961853907 | 0.893132975036299 | 1.08344196487467 | 0.99380795269851 |
| ebi-a-GCST90001597 | finngen_R11_O15_PREECLAMPS | Pre-eclampsia \|\| id:finngen_R11_O15_PREECLAMPS | CD8dim T cell %T cell \|\| id:ebi-a-GCST90001597 | Weighted median | 19 | -0.022620542 | 0.0368884026924151 | 0.539733834541419 | -0.094921811 | 0.0496807277089569 | 0.977633384629127 | 0.909444040349093 | 1.05093550821943 | 0.940845681141708 |
| ebi-a-GCST90001597 | finngen_R11_O15_PREECLAMPS | Pre-eclampsia \|\| id:finngen_R11_O15_PREECLAMPS | CD8dim T cell %T cell \|\| id:ebi-a-GCST90001597 | Weighted mode | 19 | -0.014776006 | 0.042936462058251 | 0.73473123713671 | -0.098931472 | 0.0693794591633499 | 0.985332623017955 | 0.905804778791511 | 1.07184285258326 | 0.959313411571287 |
| ebi-a-GCST90001598 | finngen_R11_O15_PREECLAMPS | Pre-eclampsia \|\| id:finngen_R11_O15_PREECLAMPS | CD4-CD8- T cell Absolute Count \|\| id:ebi-a-GCST90001598 | Inverse variance weighted (multiplicative random effects) | 20 | -0.014352697 | 0.0392021993387039 | 0.714275770897666 | -0.091189007 | 0.0624836139160933 | 0.98574981215218 | 0.912845160019137 | 1.06447701616525 | 0.959745573017418 |
| ebi-a-GCST90001598 | finngen_R11_O15_PREECLAMPS | Pre-eclampsia \|\| id:finngen_R11_O15_PREECLAMPS | CD4-CD8- T cell Absolute Count \|\| id:ebi-a-GCST90001598 | MR Egger | 20 | -0.070329982 | 0.0866068949331616 | 0.427366695635119 | -0.240079496 | 0.0994195324150074 | 0.932086197808702 | 0.786565330001526 | 1.1045295883354 | 0.99380795269851 |
| ebi-a-GCST90001598 | finngen_R11_O15_PREECLAMPS | Pre-eclampsia \|\| id:finngen_R11_O15_PREECLAMPS | CD4-CD8- T cell Absolute Count \|\| id:ebi-a-GCST90001598 | Weighted median | 20 | -0.029142156 | 0.0431926570699999 | 0.499865354531181 | -0.113799764 | 0.055515452121195 | 0.971278381858981 | 0.892436636297994 | 1.05708535115718 | 0.940845681141708 |
| ebi-a-GCST90001598 | finngen_R11_O15_PREECLAMPS | Pre-eclampsia \|\| id:finngen_R11_O15_PREECLAMPS | CD4-CD8- T cell Absolute Count \|\| id:ebi-a-GCST90001598 | Weighted mode | 20 | 0.0090008969534916 | 0.0713862863749405 | 0.900987587123326 | -0.130916224 | 0.148918018248375 | 1.00904152683678 | 0.877291266966238 | 1.16057783910471 | 0.98361388732834 |
| ebi-a-GCST90001599 | finngen_R11_O15_PREECLAMPS | Pre-eclampsia \|\| id:finngen_R11_O15_PREECLAMPS | CD4-CD8- T cell %T cell \|\| id:ebi-a-GCST90001599 | Inverse variance weighted | 15 | -0.028245383 | 0.0334470541207905 | 0.398400984266892 | -0.093801609 | 0.0373108428119661 | 0.972149788241954 | 0.910463371752387 | 1.03801563039255 | 0.885079385681701 |
| ebi-a-GCST90001599 | finngen_R11_O15_PREECLAMPS | Pre-eclampsia \|\| id:finngen_R11_O15_PREECLAMPS | CD4-CD8- T cell %T cell \|\| id:ebi-a-GCST90001599 | MR Egger | 15 | 0.0129112769127622 | 0.0785795236592053 | 0.872015563307809 | -0.141104589 | 0.166927143284805 | 1.01299498733002 | 0.868398481620184 | 1.18166816971078 | 0.99380795269851 |
| ebi-a-GCST90001599 | finngen_R11_O15_PREECLAMPS | Pre-eclampsia \|\| id:finngen_R11_O15_PREECLAMPS | CD4-CD8- T cell %T cell \|\| id:ebi-a-GCST90001599 | Weighted median | 15 | 0.0257783118887619 | 0.047333916101162 | 0.586024858951678 | -0.066996164 | 0.118552787447039 | 1.02611344610531 | 0.935198789061518 | 1.12586630414129 | 0.940845681141708 |
| ebi-a-GCST90001599 | finngen_R11_O15_PREECLAMPS | Pre-eclampsia \|\| id:finngen_R11_O15_PREECLAMPS | CD4-CD8- T cell %T cell \|\| id:ebi-a-GCST90001599 | Weighted mode | 15 | 0.04901000935124 | 0.0639839595556665 | 0.456413259799392 | -0.076398551 | 0.174418570080346 | 1.05023086281701 | 0.926446896282677 | 1.19055379173812 | 0.933945646747087 |
| ebi-a-GCST90001600 | finngen_R11_O15_PREECLAMPS | Pre-eclampsia \|\| id:finngen_R11_O15_PREECLAMPS | Leukocyte Absolute Count \|\| id:ebi-a-GCST90001600 | Inverse variance weighted | 28 | -0.00611046 | 0.00944311303408003 | 0.517579429934374 | -0.024618962 | 0.0123980411864438 | 0.993908170535456 | 0.97568161306878 | 1.01247521550608 | 0.898694924660397 |
| ebi-a-GCST90001600 | finngen_R11_O15_PREECLAMPS | Pre-eclampsia \|\| id:finngen_R11_O15_PREECLAMPS | Leukocyte Absolute Count \|\| id:ebi-a-GCST90001600 | MR Egger | 28 | -0.011654858 | 0.0119106305218943 | 0.336832170574486 | -0.034999694 | 0.0116899779569463 | 0.988412796899336 | 0.965605712033289 | 1.01175857277933 | 0.99380795269851 |
| ebi-a-GCST90001600 | finngen_R11_O15_PREECLAMPS | Pre-eclampsia \|\| id:finngen_R11_O15_PREECLAMPS | Leukocyte Absolute Count \|\| id:ebi-a-GCST90001600 | Weighted median | 28 | -0.009542017 | 0.0114770625209258 | 0.405748312052109 | -0.03203706 | 0.0129530252186841 | 0.990503363269459 | 0.968470690022698 | 1.01303727903744 | 0.940845681141708 |
| ebi-a-GCST90001600 | finngen_R11_O15_PREECLAMPS | Pre-eclampsia \|\| id:finngen_R11_O15_PREECLAMPS | Leukocyte Absolute Count \|\| id:ebi-a-GCST90001600 | Weighted mode | 28 | -0.010229849 | 0.00931008386550752 | 0.281560503660932 | -0.028477613 | 0.00801791585954414 | 0.989822298413604 | 0.971924052470103 | 1.00805014542731 | 0.872755742358858 |
| ebi-a-GCST90001601 | finngen_R11_O15_PREECLAMPS | Pre-eclampsia \|\| id:finngen_R11_O15_PREECLAMPS | Lymphocyte Absolute Count \|\| id:ebi-a-GCST90001601 | Inverse variance weighted | 18 | -0.007363247 | 0.0321350929844682 | 0.8187646677314 | -0.070348029 | 0.0556215357011314 | 0.992663795737727 | 0.932069376466664 | 1.05719749650382 | 0.977824840984872 |
| ebi-a-GCST90001601 | finngen_R11_O15_PREECLAMPS | Pre-eclampsia \|\| id:finngen_R11_O15_PREECLAMPS | Lymphocyte Absolute Count \|\| id:ebi-a-GCST90001601 | MR Egger | 18 | -0.067742161 | 0.0445744982500125 | 0.148086778361312 | -0.155108178 | 0.0196238552202225 | 0.934501393094501 | 0.856322537282108 | 1.01981766878087 | 0.984103954382898 |
| ebi-a-GCST90001601 | finngen_R11_O15_PREECLAMPS | Pre-eclampsia \|\| id:finngen_R11_O15_PREECLAMPS | Lymphocyte Absolute Count \|\| id:ebi-a-GCST90001601 | Weighted median | 18 | -0.035510223 | 0.035120042874838 | 0.311963839469548 | -0.104345507 | 0.0333250615024111 | 0.965112868282132 | 0.900913971948236 | 1.03388656134338 | 0.940845681141708 |
| ebi-a-GCST90001601 | finngen_R11_O15_PREECLAMPS | Pre-eclampsia \|\| id:finngen_R11_O15_PREECLAMPS | Lymphocyte Absolute Count \|\| id:ebi-a-GCST90001601 | Weighted mode | 18 | -0.038677109 | 0.0357359593203492 | 0.294231922237802 | -0.10871959 | 0.0313653709686036 | 0.962061299647454 | 0.896981905320999 | 1.03186244760213 | 0.872755742358858 |
| ebi-a-GCST90001602 | finngen_R11_O15_PREECLAMPS | Pre-eclampsia \|\| id:finngen_R11_O15_PREECLAMPS | Lymphocyte %leukocyte \|\| id:ebi-a-GCST90001602 | Inverse variance weighted | 22 | 0.0011745401257787 | 0.0163528835371717 | 0.942741456140074 | -0.030877112 | 0.0332261918586353 | 1.00117523016817 | 0.969594717691774 | 1.03378434640041 | 0.986667691552896 |
| ebi-a-GCST90001602 | finngen_R11_O15_PREECLAMPS | Pre-eclampsia \|\| id:finngen_R11_O15_PREECLAMPS | Lymphocyte %leukocyte \|\| id:ebi-a-GCST90001602 | MR Egger | 22 | 0.002952857186072 | 0.0203259369184148 | 0.88594761479308 | -0.036885979 | 0.042791693546165 | 1.0029572211632 | 0.963786020762476 | 1.0437204584972 | 0.99380795269851 |
| ebi-a-GCST90001602 | finngen_R11_O15_PREECLAMPS | Pre-eclampsia \|\| id:finngen_R11_O15_PREECLAMPS | Lymphocyte %leukocyte \|\| id:ebi-a-GCST90001602 | Weighted median | 22 | 0.000933796103324039 | 0.0220793510043712 | 0.966265338096557 | -0.042341732 | 0.0442093240718917 | 1.00093423222664 | 0.958542160196193 | 1.04520111774539 | 0.989912389390616 |
| ebi-a-GCST90001602 | finngen_R11_O15_PREECLAMPS | Pre-eclampsia \|\| id:finngen_R11_O15_PREECLAMPS | Lymphocyte %leukocyte \|\| id:ebi-a-GCST90001602 | Weighted mode | 22 | 0.000336794306081578 | 0.0181091742272848 | 0.985337375799506 | -0.035157187 | 0.0358307757915598 | 1.00033685102765 | 0.965453647394147 | 1.03648043406831 | 0.994159530659367 |
| ebi-a-GCST90001603 | finngen_R11_O15_PREECLAMPS | Pre-eclampsia \|\| id:finngen_R11_O15_PREECLAMPS | T cell Absolute Count \|\| id:ebi-a-GCST90001603 | Inverse variance weighted (multiplicative random effects) | 16 | -0.000515316 | 0.0364356039464928 | 0.988715737449746 | -0.0719291 | 0.0708984678004961 | 0.999484816817822 | 0.930596873097899 | 1.07347222833862 | 0.995223645914365 |
| ebi-a-GCST90001603 | finngen_R11_O15_PREECLAMPS | Pre-eclampsia \|\| id:finngen_R11_O15_PREECLAMPS | T cell Absolute Count \|\| id:ebi-a-GCST90001603 | MR Egger | 16 | -0.026887577 | 0.0623202684524906 | 0.672716805632743 | -0.149035304 | 0.0952601486689706 | 0.973470675381844 | 0.861538698888632 | 1.0999449671278 | 0.99380795269851 |
| ebi-a-GCST90001603 | finngen_R11_O15_PREECLAMPS | Pre-eclampsia \|\| id:finngen_R11_O15_PREECLAMPS | T cell Absolute Count \|\| id:ebi-a-GCST90001603 | Weighted median | 16 | -0.017758485 | 0.0394262646987334 | 0.652405676653351 | -0.095033964 | 0.0595169940257752 | 0.982398267839267 | 0.90934204942025 | 1.06132379699025 | 0.940845681141708 |
| ebi-a-GCST90001603 | finngen_R11_O15_PREECLAMPS | Pre-eclampsia \|\| id:finngen_R11_O15_PREECLAMPS | T cell Absolute Count \|\| id:ebi-a-GCST90001603 | Weighted mode | 16 | -0.005249608 | 0.040743185813818 | 0.899191025687528 | -0.085106252 | 0.0746070365290901 | 0.99476414744416 | 0.918414695946387 | 1.07746066500015 | 0.98361388732834 |
| ebi-a-GCST90001604 | finngen_R11_O15_PREECLAMPS | Pre-eclampsia \|\| id:finngen_R11_O15_PREECLAMPS | T cell %lymphocyte \|\| id:ebi-a-GCST90001604 | Inverse variance weighted | 17 | 0.0336310990665556 | 0.0294694541148173 | 0.253778826234858 | -0.024129031 | 0.0913912291315975 | 1.03420301788954 | 0.976159746764712 | 1.09569759023226 | 0.885079385681701 |
| ebi-a-GCST90001604 | finngen_R11_O15_PREECLAMPS | Pre-eclampsia \|\| id:finngen_R11_O15_PREECLAMPS | T cell %lymphocyte \|\| id:ebi-a-GCST90001604 | MR Egger | 17 | 0.0329912196780065 | 0.0904643025631871 | 0.720434869118662 | -0.144318813 | 0.210301252701853 | 1.03354146437461 | 0.865611735489707 | 1.23404976479125 | 0.99380795269851 |
| ebi-a-GCST90001604 | finngen_R11_O15_PREECLAMPS | Pre-eclampsia \|\| id:finngen_R11_O15_PREECLAMPS | T cell %lymphocyte \|\| id:ebi-a-GCST90001604 | Weighted median | 17 | 0.0305230534752944 | 0.0377648731441766 | 0.418952855664173 | -0.043496098 | 0.104542204837881 | 1.03099365776163 | 0.957436290107993 | 1.11020224878338 | 0.940845681141708 |
| ebi-a-GCST90001604 | finngen_R11_O15_PREECLAMPS | Pre-eclampsia \|\| id:finngen_R11_O15_PREECLAMPS | T cell %lymphocyte \|\| id:ebi-a-GCST90001604 | Weighted mode | 17 | 0.0389083854456253 | 0.0520285824306955 | 0.465414753358919 | -0.063067636 | 0.140884407009788 | 1.03967522990403 | 0.938879969344664 | 1.1512915590589 | 0.933945646747087 |
| ebi-a-GCST90001605 | finngen_R11_O15_PREECLAMPS | Pre-eclampsia \|\| id:finngen_R11_O15_PREECLAMPS | T cell %leukocyte \|\| id:ebi-a-GCST90001605 | Inverse variance weighted | 17 | -0.00940551 | 0.0178770132602549 | 0.598802652204435 | -0.044444456 | 0.0256334356473418 | 0.990638583120853 | 0.956528727731421 | 1.02596479741406 | 0.919589787313953 |
| ebi-a-GCST90001605 | finngen_R11_O15_PREECLAMPS | Pre-eclampsia \|\| id:finngen_R11_O15_PREECLAMPS | T cell %leukocyte \|\| id:ebi-a-GCST90001605 | MR Egger | 17 | -0.000314176 | 0.0223063348919308 | 0.988948111447463 | -0.044034592 | 0.0434062404813426 | 0.99968587344124 | 0.956920854811975 | 1.0443620708364 | 0.995759048854126 |
| ebi-a-GCST90001605 | finngen_R11_O15_PREECLAMPS | Pre-eclampsia \|\| id:finngen_R11_O15_PREECLAMPS | T cell %leukocyte \|\| id:ebi-a-GCST90001605 | Weighted median | 17 | -0.001329978 | 0.0227113862759112 | 0.953302609104352 | -0.045844295 | 0.0431843393968767 | 0.998670906324481 | 0.955190678763633 | 1.04413035147065 | 0.985663659484132 |
| ebi-a-GCST90001605 | finngen_R11_O15_PREECLAMPS | Pre-eclampsia \|\| id:finngen_R11_O15_PREECLAMPS | T cell %leukocyte \|\| id:ebi-a-GCST90001605 | Weighted mode | 17 | -0.002460349 | 0.0212849221011803 | 0.909414861954966 | -0.044178797 | 0.0392580981309026 | 0.997542674990964 | 0.956782872744443 | 1.04003888110351 | 0.984862613465304 |
| ebi-a-GCST90001606 | finngen_R11_O15_PREECLAMPS | Pre-eclampsia \|\| id:finngen_R11_O15_PREECLAMPS | CD4+ T cell %leukocyte \|\| id:ebi-a-GCST90001606 | Inverse variance weighted (multiplicative random effects) | 13 | 0.0545966767023684 | 0.0556790372852494 | 0.326809414324087 | -0.054534236 | 0.163727589781457 | 1.05611457315271 | 0.94692608898455 | 1.17789340118576 | 0.885079385681701 |
| ebi-a-GCST90001606 | finngen_R11_O15_PREECLAMPS | Pre-eclampsia \|\| id:finngen_R11_O15_PREECLAMPS | CD4+ T cell %leukocyte \|\| id:ebi-a-GCST90001606 | MR Egger | 13 | 0.27172753220549 | 0.112922493635407 | 0.0348407507684806 | 0.0503994446800922 | 0.493055619730888 | 1.31222941234371 | 1.05169110490231 | 1.63731158568644 | 0.839403552053506 |
| ebi-a-GCST90001606 | finngen_R11_O15_PREECLAMPS | Pre-eclampsia \|\| id:finngen_R11_O15_PREECLAMPS | CD4+ T cell %leukocyte \|\| id:ebi-a-GCST90001606 | Weighted median | 13 | 0.0484784304462531 | 0.0608239054135233 | 0.425434056020234 | -0.070736424 | 0.167693285056759 | 1.04967273060377 | 0.931707435332483 | 1.18257384194855 | 0.940845681141708 |
| ebi-a-GCST90001606 | finngen_R11_O15_PREECLAMPS | Pre-eclampsia \|\| id:finngen_R11_O15_PREECLAMPS | CD4+ T cell %leukocyte \|\| id:ebi-a-GCST90001606 | Weighted mode | 13 | 0.0383523479932093 | 0.0844313075717666 | 0.657760570075018 | -0.127133015 | 0.203837710833872 | 1.03909729223024 | 0.880616529733092 | 1.22609915469957 | 0.959313411571287 |
| ebi-a-GCST90001607 | finngen_R11_O15_PREECLAMPS | Pre-eclampsia \|\| id:finngen_R11_O15_PREECLAMPS | CD8+ T cell %leukocyte \|\| id:ebi-a-GCST90001607 | Inverse variance weighted (multiplicative random effects) | 16 | 0.0642466708997871 | 0.046843292153776 | 0.170211856916975 | -0.027566182 | 0.156059523521188 | 1.06635540516944 | 0.972810298162606 | 1.16889577781178 | 0.85505992597664 |
| ebi-a-GCST90001607 | finngen_R11_O15_PREECLAMPS | Pre-eclampsia \|\| id:finngen_R11_O15_PREECLAMPS | CD8+ T cell %leukocyte \|\| id:ebi-a-GCST90001607 | MR Egger | 16 | -0.097339799 | 0.116531980125569 | 0.417573429919913 | -0.32574248 | 0.13106288186025 | 0.907247671730117 | 0.721991090471965 | 1.14003946686054 | 0.99380795269851 |
| ebi-a-GCST90001607 | finngen_R11_O15_PREECLAMPS | Pre-eclampsia \|\| id:finngen_R11_O15_PREECLAMPS | CD8+ T cell %leukocyte \|\| id:ebi-a-GCST90001607 | Weighted median | 16 | 0.00805664678192872 | 0.0491533089495019 | 0.869803149272256 | -0.088283839 | 0.104397132322952 | 1.00808918889534 | 0.915500985177841 | 1.11004120063319 | 0.964912412213677 |
| ebi-a-GCST90001607 | finngen_R11_O15_PREECLAMPS | Pre-eclampsia \|\| id:finngen_R11_O15_PREECLAMPS | CD8+ T cell %leukocyte \|\| id:ebi-a-GCST90001607 | Weighted mode | 16 | 0.00616645510249581 | 0.0762019572386406 | 0.936573417752956 | -0.143189381 | 0.155522291290231 | 1.00618550682716 | 0.866589937611457 | 1.16826797797756 | 0.990180645930572 |
| ebi-a-GCST90001608 | finngen_R11_O15_PREECLAMPS | Pre-eclampsia \|\| id:finngen_R11_O15_PREECLAMPS | CD4+CD8+ T cell %leukocyte \|\| id:ebi-a-GCST90001608 | Inverse variance weighted | 20 | -0.02121309 | 0.0284764865783353 | 0.456311894081754 | -0.077027004 | 0.0346008235578823 | 0.979010324897781 | 0.925864851372618 | 1.03520639630667 | 0.898694924660397 |
| ebi-a-GCST90001608 | finngen_R11_O15_PREECLAMPS | Pre-eclampsia \|\| id:finngen_R11_O15_PREECLAMPS | CD4+CD8+ T cell %leukocyte \|\| id:ebi-a-GCST90001608 | MR Egger | 20 | -0.04079693 | 0.0608372447674673 | 0.510994262527433 | -0.16003793 | 0.07844406994066 | 0.96002406242957 | 0.852111468150586 | 1.08160285935842 | 0.99380795269851 |
| ebi-a-GCST90001608 | finngen_R11_O15_PREECLAMPS | Pre-eclampsia \|\| id:finngen_R11_O15_PREECLAMPS | CD4+CD8+ T cell %leukocyte \|\| id:ebi-a-GCST90001608 | Weighted median | 20 | -0.010546209 | 0.0402494061710853 | 0.793304878153322 | -0.089435045 | 0.068342626924362 | 0.989509207111317 | 0.914447660899569 | 1.07073210728635 | 0.957984984509652 |
| ebi-a-GCST90001608 | finngen_R11_O15_PREECLAMPS | Pre-eclampsia \|\| id:finngen_R11_O15_PREECLAMPS | CD4+CD8+ T cell %leukocyte \|\| id:ebi-a-GCST90001608 | Weighted mode | 20 | -0.008662359 | 0.0450377617098691 | 0.849520261896667 | -0.096936372 | 0.0796116543051537 | 0.991375051484547 | 0.907613754309285 | 1.08286645948192 | 0.977585971930092 |
| ebi-a-GCST90001609 | finngen_R11_O15_PREECLAMPS | Pre-eclampsia \|\| id:finngen_R11_O15_PREECLAMPS | CD4+ CD8dim T cell Absolute Count \|\| id:ebi-a-GCST90001609 | Inverse variance weighted | 16 | -0.028394085 | 0.0236059551805796 | 0.229039909476701 | -0.074661758 | 0.0178735866942872 | 0.972005238182426 | 0.928057341575932 | 1.01803427517714 | 0.881200914881413 |
| ebi-a-GCST90001609 | finngen_R11_O15_PREECLAMPS | Pre-eclampsia \|\| id:finngen_R11_O15_PREECLAMPS | CD4+ CD8dim T cell Absolute Count \|\| id:ebi-a-GCST90001609 | MR Egger | 16 | -0.003152554 | 0.0370507536510634 | 0.933396705599012 | -0.075772031 | 0.0694669234526071 | 0.99685241037607 | 0.927027516138453 | 1.07193660465648 | 0.99380795269851 |
| ebi-a-GCST90001609 | finngen_R11_O15_PREECLAMPS | Pre-eclampsia \|\| id:finngen_R11_O15_PREECLAMPS | CD4+ CD8dim T cell Absolute Count \|\| id:ebi-a-GCST90001609 | Weighted median | 16 | -0.025279744 | 0.0339588524782166 | 0.456620727637529 | -0.091839095 | 0.0412796066971202 | 0.975037112934829 | 0.912251923616249 | 1.04214345510134 | 0.940845681141708 |
| ebi-a-GCST90001609 | finngen_R11_O15_PREECLAMPS | Pre-eclampsia \|\| id:finngen_R11_O15_PREECLAMPS | CD4+ CD8dim T cell Absolute Count \|\| id:ebi-a-GCST90001609 | Weighted mode | 16 | -0.019870525 | 0.0298753540624689 | 0.516074262948974 | -0.078426218 | 0.0386851694618112 | 0.980325593235799 | 0.924570273630858 | 1.03944318367391 | 0.934398527469092 |
| ebi-a-GCST90001610 | finngen_R11_O15_PREECLAMPS | Pre-eclampsia \|\| id:finngen_R11_O15_PREECLAMPS | CD4+ CD8dim T cell %lymphocyte \|\| id:ebi-a-GCST90001610 | Inverse variance weighted | 20 | -0.001463954 | 0.0270960992436188 | 0.956912676913104 | -0.054572309 | 0.0516444003754871 | 0.998537116916135 | 0.946890038032995 | 1.05300122908722 | 0.992671503887443 |
| ebi-a-GCST90001610 | finngen_R11_O15_PREECLAMPS | Pre-eclampsia \|\| id:finngen_R11_O15_PREECLAMPS | CD4+ CD8dim T cell %lymphocyte \|\| id:ebi-a-GCST90001610 | MR Egger | 20 | -0.004584603 | 0.0522156882463752 | 0.931004079280299 | -0.106927352 | 0.0977581458855347 | 0.995425890173406 | 0.898590951418369 | 1.10269606127626 | 0.99380795269851 |
| ebi-a-GCST90001610 | finngen_R11_O15_PREECLAMPS | Pre-eclampsia \|\| id:finngen_R11_O15_PREECLAMPS | CD4+ CD8dim T cell %lymphocyte \|\| id:ebi-a-GCST90001610 | Weighted median | 20 | -0.005984749 | 0.0386580559340743 | 0.876969165204262 | -0.081754539 | 0.0697850407565354 | 0.994033124062506 | 0.921498123244807 | 1.07227766048414 | 0.964912412213677 |
| ebi-a-GCST90001610 | finngen_R11_O15_PREECLAMPS | Pre-eclampsia \|\| id:finngen_R11_O15_PREECLAMPS | CD4+ CD8dim T cell %lymphocyte \|\| id:ebi-a-GCST90001610 | Weighted mode | 20 | -0.009097884 | 0.0378782047007642 | 0.812757040746068 | -0.083339166 | 0.0651433968410405 | 0.990943376154925 | 0.920039048713522 | 1.06731206258951 | 0.96448928049574 |
| ebi-a-GCST90001611 | finngen_R11_O15_PREECLAMPS | Pre-eclampsia \|\| id:finngen_R11_O15_PREECLAMPS | CD4+ CD8dim T cell %leukocyte \|\| id:ebi-a-GCST90001611 | Inverse variance weighted | 16 | 0.00275049004518057 | 0.03887198572025 | 0.943590644158461 | -0.073438602 | 0.0789395820568706 | 1.00275427611331 | 0.929193194675043 | 1.08213893948629 | 0.986667691552896 |
| ebi-a-GCST90001611 | finngen_R11_O15_PREECLAMPS | Pre-eclampsia \|\| id:finngen_R11_O15_PREECLAMPS | CD4+ CD8dim T cell %leukocyte \|\| id:ebi-a-GCST90001611 | MR Egger | 16 | 0.0220700013805659 | 0.0947935158203349 | 0.819269130818079 | -0.16372529 | 0.207865292388422 | 1.02231534545128 | 0.848975212145798 | 1.23104732693384 | 0.99380795269851 |
| ebi-a-GCST90001611 | finngen_R11_O15_PREECLAMPS | Pre-eclampsia \|\| id:finngen_R11_O15_PREECLAMPS | CD4+ CD8dim T cell %leukocyte \|\| id:ebi-a-GCST90001611 | Weighted median | 16 | -0.014910327 | 0.0448468658288298 | 0.739532883415707 | -0.102810184 | 0.0729895301035236 | 0.985200281584753 | 0.902298227917197 | 1.07571927418631 | 0.957984984509652 |
| ebi-a-GCST90001611 | finngen_R11_O15_PREECLAMPS | Pre-eclampsia \|\| id:finngen_R11_O15_PREECLAMPS | CD4+ CD8dim T cell %leukocyte \|\| id:ebi-a-GCST90001611 | Weighted mode | 16 | -0.024873913 | 0.045355350917556 | 0.591471118881984 | -0.113770401 | 0.0640225745818832 | 0.975432893471035 | 0.892462840923293 | 1.0661164656233 | 0.955353022288269 |
| ebi-a-GCST90001612 | finngen_R11_O15_PREECLAMPS | Pre-eclampsia \|\| id:finngen_R11_O15_PREECLAMPS | CD8dim T cell %leukocyte \|\| id:ebi-a-GCST90001612 | Inverse variance weighted | 18 | -0.031115144 | 0.0210452615356468 | 0.139277491918741 | -0.072363857 | 0.0101335684155555 | 0.969363950018755 | 0.930192377403113 | 1.01018508689493 | 0.844911146506647 |
| ebi-a-GCST90001612 | finngen_R11_O15_PREECLAMPS | Pre-eclampsia \|\| id:finngen_R11_O15_PREECLAMPS | CD8dim T cell %leukocyte \|\| id:ebi-a-GCST90001612 | MR Egger | 18 | -0.034503189 | 0.0304241520101668 | 0.273470550874539 | -0.094134527 | 0.0251281488608971 | 0.966085258759339 | 0.910160312850915 | 1.02544652190847 | 0.99380795269851 |
| ebi-a-GCST90001612 | finngen_R11_O15_PREECLAMPS | Pre-eclampsia \|\| id:finngen_R11_O15_PREECLAMPS | CD8dim T cell %leukocyte \|\| id:ebi-a-GCST90001612 | Weighted median | 18 | -0.033777307 | 0.0315864036587201 | 0.284906473986769 | -0.095686659 | 0.0281320437221817 | 0.966786776880559 | 0.908748720039619 | 1.02853148658119 | 0.940845681141708 |
| ebi-a-GCST90001612 | finngen_R11_O15_PREECLAMPS | Pre-eclampsia \|\| id:finngen_R11_O15_PREECLAMPS | CD8dim T cell %leukocyte \|\| id:ebi-a-GCST90001612 | Weighted mode | 18 | -0.035930201 | 0.0287076435716785 | 0.227668088459387 | -0.092197182 | 0.0203367807749344 | 0.964707627122068 | 0.911925316534238 | 1.02054498208741 | 0.872755742358858 |
| ebi-a-GCST90001613 | finngen_R11_O15_PREECLAMPS | Pre-eclampsia \|\| id:finngen_R11_O15_PREECLAMPS | CD4-CD8- T cell %leukocyte \|\| id:ebi-a-GCST90001613 | Inverse variance weighted | 20 | -0.014246954 | 0.0145501655163661 | 0.327500470544291 | -0.042765278 | 0.0142713708347492 | 0.985854054013343 | 0.95813625934433 | 1.01437369302738 | 0.885079385681701 |
| ebi-a-GCST90001613 | finngen_R11_O15_PREECLAMPS | Pre-eclampsia \|\| id:finngen_R11_O15_PREECLAMPS | CD4-CD8- T cell %leukocyte \|\| id:ebi-a-GCST90001613 | MR Egger | 20 | -0.023617317 | 0.0176651301752531 | 0.197892949623766 | -0.058240972 | 0.0110063379489906 | 0.976659389008035 | 0.943422581298285 | 1.01106713051631 | 0.99380795269851 |
| ebi-a-GCST90001613 | finngen_R11_O15_PREECLAMPS | Pre-eclampsia \|\| id:finngen_R11_O15_PREECLAMPS | CD4-CD8- T cell %leukocyte \|\| id:ebi-a-GCST90001613 | Weighted median | 20 | -0.020720486 | 0.0189697430019115 | 0.274705061567744 | -0.057901182 | 0.0164602101085587 | 0.979492708063338 | 0.94374320121171 | 1.01659642571987 | 0.940845681141708 |
| ebi-a-GCST90001613 | finngen_R11_O15_PREECLAMPS | Pre-eclampsia \|\| id:finngen_R11_O15_PREECLAMPS | CD4-CD8- T cell %leukocyte \|\| id:ebi-a-GCST90001613 | Weighted mode | 20 | -0.020483095 | 0.0180778459503204 | 0.271289370432832 | -0.055915673 | 0.0149494831664621 | 0.979725258691752 | 0.945618873770053 | 1.01506178561325 | 0.872755742358858 |
| ebi-a-GCST90001614 | finngen_R11_O15_PREECLAMPS | Pre-eclampsia \|\| id:finngen_R11_O15_PREECLAMPS | CD8+ and CD8dim T cell %leukocyte \|\| id:ebi-a-GCST90001614 | Inverse variance weighted | 14 | 0.0212352234179501 | 0.0423697481929369 | 0.616238585295124 | -0.061809483 | 0.104279929876107 | 1.02146229523335 | 0.940061967479655 | 1.10991110871207 | 0.927459753639562 |
| ebi-a-GCST90001614 | finngen_R11_O15_PREECLAMPS | Pre-eclampsia \|\| id:finngen_R11_O15_PREECLAMPS | CD8+ and CD8dim T cell %leukocyte \|\| id:ebi-a-GCST90001614 | MR Egger | 14 | -0.063655916 | 0.112831874337583 | 0.583032102126219 | -0.284806389 | 0.15749455806479 | 0.938327807917077 | 0.752159866392161 | 1.17057438777438 | 0.99380795269851 |
| ebi-a-GCST90001614 | finngen_R11_O15_PREECLAMPS | Pre-eclampsia \|\| id:finngen_R11_O15_PREECLAMPS | CD8+ and CD8dim T cell %leukocyte \|\| id:ebi-a-GCST90001614 | Weighted median | 14 | 0.035663109463934 | 0.0473324178655238 | 0.451173210666512 | -0.05710843 | 0.128434648480361 | 1.03630666576823 | 0.944491653006875 | 1.13704711110649 | 0.940845681141708 |
| ebi-a-GCST90001614 | finngen_R11_O15_PREECLAMPS | Pre-eclampsia \|\| id:finngen_R11_O15_PREECLAMPS | CD8+ and CD8dim T cell %leukocyte \|\| id:ebi-a-GCST90001614 | Weighted mode | 14 | 0.0386500809998501 | 0.0657480925465445 | 0.56670432251822 | -0.09021618 | 0.167516342391077 | 1.03940671185118 | 0.913733632624178 | 1.18236461269193 | 0.939633861977944 |
| ebi-a-GCST90001615 | finngen_R11_O15_PREECLAMPS | Pre-eclampsia \|\| id:finngen_R11_O15_PREECLAMPS | TCRgd T cell Absolute Count \|\| id:ebi-a-GCST90001615 | Inverse variance weighted | 18 | -0.017186059 | 0.0235655964594538 | 0.465826748782464 | -0.063374628 | 0.0290025100403764 | 0.982960778900435 | 0.938591784978063 | 1.02942717837567 | 0.898694924660397 |
| ebi-a-GCST90001615 | finngen_R11_O15_PREECLAMPS | Pre-eclampsia \|\| id:finngen_R11_O15_PREECLAMPS | TCRgd T cell Absolute Count \|\| id:ebi-a-GCST90001615 | MR Egger | 18 | -0.03189704 | 0.033980670958898 | 0.361849410390162 | -0.098499155 | 0.0347050754547282 | 0.968606305014025 | 0.906196458618051 | 1.03531432416286 | 0.99380795269851 |
| ebi-a-GCST90001615 | finngen_R11_O15_PREECLAMPS | Pre-eclampsia \|\| id:finngen_R11_O15_PREECLAMPS | TCRgd T cell Absolute Count \|\| id:ebi-a-GCST90001615 | Weighted median | 18 | -0.009255091 | 0.0338186669971008 | 0.784339669744565 | -0.075539679 | 0.0570294958760003 | 0.990787605098803 | 0.927242937960894 | 1.05868703683653 | 0.957984984509652 |
| ebi-a-GCST90001615 | finngen_R11_O15_PREECLAMPS | Pre-eclampsia \|\| id:finngen_R11_O15_PREECLAMPS | TCRgd T cell Absolute Count \|\| id:ebi-a-GCST90001615 | Weighted mode | 18 | -0.009518319 | 0.0289216420155563 | 0.746094531496636 | -0.066204737 | 0.0471680992165834 | 0.990526836682934 | 0.935939222832162 | 1.04829821237767 | 0.959313411571287 |
| ebi-a-GCST90001616 | finngen_R11_O15_PREECLAMPS | Pre-eclampsia \|\| id:finngen_R11_O15_PREECLAMPS | TCRgd T cell %T cell \|\| id:ebi-a-GCST90001616 | Inverse variance weighted | 18 | -0.019234052 | 0.0154286755138969 | 0.212528396797822 | -0.049474256 | 0.0110061523860658 | 0.980949742494898 | 0.951729659503082 | 1.01106694289976 | 0.855687495048373 |
| ebi-a-GCST90001616 | finngen_R11_O15_PREECLAMPS | Pre-eclampsia \|\| id:finngen_R11_O15_PREECLAMPS | TCRgd T cell %T cell \|\| id:ebi-a-GCST90001616 | MR Egger | 18 | -0.005854891 | 0.0184423453752346 | 0.754992396018491 | -0.042001888 | 0.0302921058335223 | 0.994162215371175 | 0.958867970192189 | 1.03075557970053 | 0.99380795269851 |
| ebi-a-GCST90001616 | finngen_R11_O15_PREECLAMPS | Pre-eclampsia \|\| id:finngen_R11_O15_PREECLAMPS | TCRgd T cell %T cell \|\| id:ebi-a-GCST90001616 | Weighted median | 18 | -0.010590259 | 0.0182131189400276 | 0.560928289038357 | -0.046287972 | 0.02510745404478 | 0.989465620283061 | 0.954766976251701 | 1.02542530070085 | 0.940845681141708 |
| ebi-a-GCST90001616 | finngen_R11_O15_PREECLAMPS | Pre-eclampsia \|\| id:finngen_R11_O15_PREECLAMPS | TCRgd T cell %T cell \|\| id:ebi-a-GCST90001616 | Weighted mode | 18 | -0.011742135 | 0.0182256763134113 | 0.528002651957754 | -0.047464461 | 0.0239801906464296 | 0.98832653489889 | 0.953644364572232 | 1.02427002756253 | 0.935508728341156 |
| ebi-a-GCST90001617 | finngen_R11_O15_PREECLAMPS | Pre-eclampsia \|\| id:finngen_R11_O15_PREECLAMPS | TCRgd T cell %lymphocyte \|\| id:ebi-a-GCST90001617 | Inverse variance weighted | 20 | -0.014977589 | 0.0117652119528327 | 0.203003735046386 | -0.038037405 | 0.00808222625266162 | 0.98513401702184 | 0.962676931669839 | 1.00811497561305 | 0.855687495048373 |
| ebi-a-GCST90001617 | finngen_R11_O15_PREECLAMPS | Pre-eclampsia \|\| id:finngen_R11_O15_PREECLAMPS | TCRgd T cell %lymphocyte \|\| id:ebi-a-GCST90001617 | MR Egger | 20 | -0.014276138 | 0.0136307119702364 | 0.308798635004279 | -0.040992334 | 0.0124400571934489 | 0.985825282587363 | 0.959836488285386 | 1.01251775656553 | 0.99380795269851 |
| ebi-a-GCST90001617 | finngen_R11_O15_PREECLAMPS | Pre-eclampsia \|\| id:finngen_R11_O15_PREECLAMPS | TCRgd T cell %lymphocyte \|\| id:ebi-a-GCST90001617 | Weighted median | 20 | -0.011944893 | 0.0145843375017871 | 0.412773860827872 | -0.040530195 | 0.0166404082087908 | 0.988126163738905 | 0.960280168607732 | 1.01677963097061 | 0.940845681141708 |
| ebi-a-GCST90001617 | finngen_R11_O15_PREECLAMPS | Pre-eclampsia \|\| id:finngen_R11_O15_PREECLAMPS | TCRgd T cell %lymphocyte \|\| id:ebi-a-GCST90001617 | Weighted mode | 20 | -0.014262437 | 0.0128482307319077 | 0.280820914031021 | -0.039444969 | 0.0109200952303176 | 0.985838789732342 | 0.961322854863497 | 1.01097993709817 | 0.872755742358858 |
| ebi-a-GCST90001618 | finngen_R11_O15_PREECLAMPS | Pre-eclampsia \|\| id:finngen_R11_O15_PREECLAMPS | HLA DR+ T cell Absolute Count \|\| id:ebi-a-GCST90001618 | Inverse variance weighted (multiplicative random effects) | 30 | -0.018330669 | 0.0154849472121819 | 0.236502758257419 | -0.048681166 | 0.0120198272899707 | 0.981836315600254 | 0.952484766026848 | 1.01209235571576 | 0.885079385681701 |
| ebi-a-GCST90001618 | finngen_R11_O15_PREECLAMPS | Pre-eclampsia \|\| id:finngen_R11_O15_PREECLAMPS | HLA DR+ T cell Absolute Count \|\| id:ebi-a-GCST90001618 | MR Egger | 30 | -0.011881952 | 0.0226051972238677 | 0.603284174322074 | -0.056188138 | 0.032424234917264 | 0.988188359990563 | 0.945361260592153 | 1.0329556282105 | 0.99380795269851 |
| ebi-a-GCST90001618 | finngen_R11_O15_PREECLAMPS | Pre-eclampsia \|\| id:finngen_R11_O15_PREECLAMPS | HLA DR+ T cell Absolute Count \|\| id:ebi-a-GCST90001618 | Weighted median | 30 | -0.010823223 | 0.0192596972114176 | 0.574141730251214 | -0.04857223 | 0.0269257834946709 | 0.98923513730002 | 0.952588531756967 | 1.02729155794483 | 0.940845681141708 |
| ebi-a-GCST90001618 | finngen_R11_O15_PREECLAMPS | Pre-eclampsia \|\| id:finngen_R11_O15_PREECLAMPS | HLA DR+ T cell Absolute Count \|\| id:ebi-a-GCST90001618 | Weighted mode | 30 | -0.015478686 | 0.0170012623702045 | 0.37009447171086 | -0.04880116 | 0.0178437882803144 | 0.984640493189732 | 0.952370480018096 | 1.01800393982234 | 0.919136986043459 |
| ebi-a-GCST90001619 | finngen_R11_O15_PREECLAMPS | Pre-eclampsia \|\| id:finngen_R11_O15_PREECLAMPS | HLA DR+ T cell%T cell \|\| id:ebi-a-GCST90001619 | Inverse variance weighted (multiplicative random effects) | 31 | -0.002797902 | 0.0137148576389948 | 0.838349472085708 | -0.029679023 | 0.0240832193414905 | 0.997206008847934 | 0.970757074625586 | 1.02437556220331 | 0.977824840984872 |
| ebi-a-GCST90001619 | finngen_R11_O15_PREECLAMPS | Pre-eclampsia \|\| id:finngen_R11_O15_PREECLAMPS | HLA DR+ T cell%T cell \|\| id:ebi-a-GCST90001619 | MR Egger | 31 | 0.00591575897995935 | 0.0176724472400611 | 0.740226805647651 | -0.028722238 | 0.0405537555704791 | 1.00593329163806 | 0.971686324901721 | 1.0413872885657 | 0.99380795269851 |
| ebi-a-GCST90001619 | finngen_R11_O15_PREECLAMPS | Pre-eclampsia \|\| id:finngen_R11_O15_PREECLAMPS | HLA DR+ T cell%T cell \|\| id:ebi-a-GCST90001619 | Weighted median | 31 | -0.010794984 | 0.0167806928877595 | 0.5200308521538 | -0.043685142 | 0.0220951744309957 | 0.98926307311178 | 0.957255309818937 | 1.02234108057094 | 0.940845681141708 |
| ebi-a-GCST90001619 | finngen_R11_O15_PREECLAMPS | Pre-eclampsia \|\| id:finngen_R11_O15_PREECLAMPS | HLA DR+ T cell%T cell \|\| id:ebi-a-GCST90001619 | Weighted mode | 31 | -0.007576657 | 0.0130841940749665 | 0.566865298402563 | -0.033221677 | 0.0180683637466785 | 0.992451973869189 | 0.967324102288457 | 1.01823258420489 | 0.939633861977944 |
| ebi-a-GCST90001620 | finngen_R11_O15_PREECLAMPS | Pre-eclampsia \|\| id:finngen_R11_O15_PREECLAMPS | HLA DR+ T cell%lymphocyte \|\| id:ebi-a-GCST90001620 | Inverse variance weighted | 25 | 0.0131170140224414 | 0.0150753884766226 | 0.38424871153221 | -0.016430747 | 0.0426647754366218 | 1.01320341943121 | 0.983703501065048 | 1.04358799987559 | 0.885079385681701 |
| ebi-a-GCST90001620 | finngen_R11_O15_PREECLAMPS | Pre-eclampsia \|\| id:finngen_R11_O15_PREECLAMPS | HLA DR+ T cell%lymphocyte \|\| id:ebi-a-GCST90001620 | MR Egger | 25 | 0.0288947744452492 | 0.0218303209483632 | 0.198639961263297 | -0.013892655 | 0.0716822035040411 | 1.02931627840001 | 0.986203402966455 | 1.07431387662256 | 0.99380795269851 |
| ebi-a-GCST90001620 | finngen_R11_O15_PREECLAMPS | Pre-eclampsia \|\| id:finngen_R11_O15_PREECLAMPS | HLA DR+ T cell%lymphocyte \|\| id:ebi-a-GCST90001620 | Weighted median | 25 | 0.0320554606420317 | 0.022233676876421 | 0.149372189200345 | -0.011522546 | 0.0756334673198168 | 1.03257477097685 | 0.988543584257667 | 1.07856717158157 | 0.917572019373545 |
| ebi-a-GCST90001620 | finngen_R11_O15_PREECLAMPS | Pre-eclampsia \|\| id:finngen_R11_O15_PREECLAMPS | HLA DR+ T cell%lymphocyte \|\| id:ebi-a-GCST90001620 | Weighted mode | 25 | 0.0229448381372863 | 0.0227041151883901 | 0.322287537083203 | -0.021555228 | 0.0674449039065308 | 1.02321009581563 | 0.978675426050359 | 1.06977131775367 | 0.888599269773114 |
| ebi-a-GCST90001621 | finngen_R11_O15_PREECLAMPS | Pre-eclampsia \|\| id:finngen_R11_O15_PREECLAMPS | Natural Killer T Absolute Count \|\| id:ebi-a-GCST90001621 | Inverse variance weighted | 34 | -0.002908953 | 0.0211117570807864 | 0.890407723484156 | -0.044287997 | 0.0384700905486283 | 0.997095273575408 | 0.956678397087273 | 1.0392196454037 | 0.986383922906939 |
| ebi-a-GCST90001621 | finngen_R11_O15_PREECLAMPS | Pre-eclampsia \|\| id:finngen_R11_O15_PREECLAMPS | Natural Killer T Absolute Count \|\| id:ebi-a-GCST90001621 | MR Egger | 34 | 0.0174548777222549 | 0.0362472005107039 | 0.633401823396951 | -0.053589635 | 0.0884993907232345 | 1.01760810431929 | 0.94782097899843 | 1.09253358695495 | 0.99380795269851 |
| ebi-a-GCST90001621 | finngen_R11_O15_PREECLAMPS | Pre-eclampsia \|\| id:finngen_R11_O15_PREECLAMPS | Natural Killer T Absolute Count \|\| id:ebi-a-GCST90001621 | Weighted median | 34 | 0.0117277346132112 | 0.0297258959591593 | 0.693190356892503 | -0.046535021 | 0.0699904906931635 | 1.01179677412116 | 0.954531130904341 | 1.07249798249333 | 0.946704357535493 |
| ebi-a-GCST90001621 | finngen_R11_O15_PREECLAMPS | Pre-eclampsia \|\| id:finngen_R11_O15_PREECLAMPS | Natural Killer T Absolute Count \|\| id:ebi-a-GCST90001621 | Weighted mode | 34 | 0.0184279049429985 | 0.0312007227936138 | 0.558795769504835 | -0.042725512 | 0.0795813216184815 | 1.01859874658763 | 0.958174361594572 | 1.08283361373105 | 0.939633861977944 |
| ebi-a-GCST90001622 | finngen_R11_O15_PREECLAMPS | Pre-eclampsia \|\| id:finngen_R11_O15_PREECLAMPS | Natural Killer T %T cell \|\| id:ebi-a-GCST90001622 | Inverse variance weighted | 32 | -0.002752361 | 0.0168274087392528 | 0.870074245094628 | -0.035734082 | 0.0302293598171503 | 0.997251422961918 | 0.964896842384465 | 1.03069090592309 | 0.981518940068169 |
| ebi-a-GCST90001622 | finngen_R11_O15_PREECLAMPS | Pre-eclampsia \|\| id:finngen_R11_O15_PREECLAMPS | Natural Killer T %T cell \|\| id:ebi-a-GCST90001622 | MR Egger | 32 | -0.031374548 | 0.0247749380432222 | 0.215121208446145 | -0.079933427 | 0.0171843300810915 | 0.96911252546393 | 0.923177803012193 | 1.01733283008612 | 0.99380795269851 |
| ebi-a-GCST90001622 | finngen_R11_O15_PREECLAMPS | Pre-eclampsia \|\| id:finngen_R11_O15_PREECLAMPS | Natural Killer T %T cell \|\| id:ebi-a-GCST90001622 | Weighted median | 32 | -0.030222224 | 0.027530720689333 | 0.272307713416839 | -0.084182437 | 0.0237379884624882 | 0.970229901134521 | 0.919263533446174 | 1.02402197716529 | 0.940845681141708 |
| ebi-a-GCST90001622 | finngen_R11_O15_PREECLAMPS | Pre-eclampsia \|\| id:finngen_R11_O15_PREECLAMPS | Natural Killer T %T cell \|\| id:ebi-a-GCST90001622 | Weighted mode | 32 | -0.033837465 | 0.0251770236311655 | 0.188708546378669 | -0.083184431 | 0.0155095016857225 | 0.96672861946134 | 0.920181421636946 | 1.01563039821332 | 0.872755742358858 |
| ebi-a-GCST90001623 | finngen_R11_O15_PREECLAMPS | Pre-eclampsia \|\| id:finngen_R11_O15_PREECLAMPS | Natural Killer T %lymphocyte \|\| id:ebi-a-GCST90001623 | Inverse variance weighted | 33 | -0.004244646 | 0.0172813647567715 | 0.805976519102902 | -0.038116121 | 0.0296268286436987 | 0.995764349498979 | 0.962601155996932 | 1.03007006957748 | 0.977824840984872 |
| ebi-a-GCST90001623 | finngen_R11_O15_PREECLAMPS | Pre-eclampsia \|\| id:finngen_R11_O15_PREECLAMPS | Natural Killer T %lymphocyte \|\| id:ebi-a-GCST90001623 | MR Egger | 33 | -0.030568588 | 0.0256152886635364 | 0.241776710130076 | -0.080774554 | 0.0196373775503191 | 0.969893906479262 | 0.922401619751083 | 1.01983145918527 | 0.99380795269851 |
| ebi-a-GCST90001623 | finngen_R11_O15_PREECLAMPS | Pre-eclampsia \|\| id:finngen_R11_O15_PREECLAMPS | Natural Killer T %lymphocyte \|\| id:ebi-a-GCST90001623 | Weighted median | 33 | -0.029887798 | 0.0267869434529476 | 0.264524910061886 | -0.082390207 | 0.0226146110249254 | 0.970554425448456 | 0.920912541771104 | 1.02287225988471 | 0.940845681141708 |
| ebi-a-GCST90001623 | finngen_R11_O15_PREECLAMPS | Pre-eclampsia \|\| id:finngen_R11_O15_PREECLAMPS | Natural Killer T %lymphocyte \|\| id:ebi-a-GCST90001623 | Weighted mode | 33 | -0.032989252 | 0.0271307482992404 | 0.232903387924496 | -0.086165519 | 0.0201870144986763 | 0.967548958586967 | 0.917442364661331 | 1.0203921503105 | 0.872755742358858 |
| ebi-a-GCST90001624 | finngen_R11_O15_PREECLAMPS | Pre-eclampsia \|\| id:finngen_R11_O15_PREECLAMPS | HLA DR+ CD4+ T cell Absolute Count \|\| id:ebi-a-GCST90001624 | Inverse variance weighted (multiplicative random effects) | 24 | 0.0291407899166667 | 0.0144818245727481 | 0.0441954933341799 | 0.000756413754080417 | 0.057525166079253 | 1.02956953728101 | 1.00075669990711 | 1.05921192653061 | 0.781760498894217 |
| ebi-a-GCST90001624 | finngen_R11_O15_PREECLAMPS | Pre-eclampsia \|\| id:finngen_R11_O15_PREECLAMPS | HLA DR+ CD4+ T cell Absolute Count \|\| id:ebi-a-GCST90001624 | MR Egger | 24 | 0.0261861465190921 | 0.0189398481304597 | 0.180664124128982 | -0.010935956 | 0.063308248854793 | 1.02653201605113 | 0.989123624361684 | 1.06535518313804 | 0.99380795269851 |
| ebi-a-GCST90001624 | finngen_R11_O15_PREECLAMPS | Pre-eclampsia \|\| id:finngen_R11_O15_PREECLAMPS | HLA DR+ CD4+ T cell Absolute Count \|\| id:ebi-a-GCST90001624 | Weighted median | 24 | 0.026285655628145 | 0.0174093807066517 | 0.131080205605755 | -0.007836731 | 0.0604080418131823 | 1.02663417042003 | 0.992193896558314 | 1.06226990866403 | 0.88227178111339 |
| ebi-a-GCST90001624 | finngen_R11_O15_PREECLAMPS | Pre-eclampsia \|\| id:finngen_R11_O15_PREECLAMPS | HLA DR+ CD4+ T cell Absolute Count \|\| id:ebi-a-GCST90001624 | Weighted mode | 24 | 0.0265060024094662 | 0.0138794231435652 | 0.0687175494102813 | -0.000697667 | 0.0537096717708541 | 1.02686041087983 | 0.999302576361079 | 1.05517820965899 | 0.872755742358858 |
| ebi-a-GCST90001625 | finngen_R11_O15_PREECLAMPS | Pre-eclampsia \|\| id:finngen_R11_O15_PREECLAMPS | HLA DR+ CD4+ T cell %T cell \|\| id:ebi-a-GCST90001625 | Inverse variance weighted | 24 | 0.0126049710415826 | 0.0280983860375747 | 0.653719333393724 | -0.042467866 | 0.0676778076752289 | 1.01268474853433 | 0.95842126332596 | 1.0700205005419 | 0.94279131784568 |
| ebi-a-GCST90001625 | finngen_R11_O15_PREECLAMPS | Pre-eclampsia \|\| id:finngen_R11_O15_PREECLAMPS | HLA DR+ CD4+ T cell %T cell \|\| id:ebi-a-GCST90001625 | MR Egger | 24 | 0.0427619567314292 | 0.0510164400419267 | 0.410933321005333 | -0.057230266 | 0.142754179213605 | 1.04368942203675 | 0.94437658674435 | 1.15344622575473 | 0.99380795269851 |
| ebi-a-GCST90001625 | finngen_R11_O15_PREECLAMPS | Pre-eclampsia \|\| id:finngen_R11_O15_PREECLAMPS | HLA DR+ CD4+ T cell %T cell \|\| id:ebi-a-GCST90001625 | Weighted median | 24 | 0.0368533570085715 | 0.0391608170588675 | 0.34666523402119 | -0.039901844 | 0.113608558443952 | 1.03754086158621 | 0.960883750618961 | 1.1203135017817 | 0.940845681141708 |
| ebi-a-GCST90001625 | finngen_R11_O15_PREECLAMPS | Pre-eclampsia \|\| id:finngen_R11_O15_PREECLAMPS | HLA DR+ CD4+ T cell %T cell \|\| id:ebi-a-GCST90001625 | Weighted mode | 24 | 0.00850773679138783 | 0.0414984671409542 | 0.839365287849322 | -0.072829259 | 0.0898447323876581 | 1.00854403043662 | 0.929759564733385 | 1.0940044070652 | 0.977585971930092 |
| ebi-a-GCST90001626 | finngen_R11_O15_PREECLAMPS | Pre-eclampsia \|\| id:finngen_R11_O15_PREECLAMPS | HLA DR+ CD4+ T cell %lymphocyte \|\| id:ebi-a-GCST90001626 | Inverse variance weighted | 20 | 0.00621647615671993 | 0.0349366911511895 | 0.858773663354703 | -0.062259438 | 0.0746923908130513 | 1.00623583854577 | 0.93963907661337 | 1.07755263480866 | 0.977824840984872 |
| ebi-a-GCST90001626 | finngen_R11_O15_PREECLAMPS | Pre-eclampsia \|\| id:finngen_R11_O15_PREECLAMPS | HLA DR+ CD4+ T cell %lymphocyte \|\| id:ebi-a-GCST90001626 | MR Egger | 20 | -0.043678692 | 0.0789457771648143 | 0.586882393758195 | -0.198412415 | 0.111055031689922 | 0.957261484265696 | 0.82003159022919 | 1.11745640067653 | 0.99380795269851 |
| ebi-a-GCST90001626 | finngen_R11_O15_PREECLAMPS | Pre-eclampsia \|\| id:finngen_R11_O15_PREECLAMPS | HLA DR+ CD4+ T cell %lymphocyte \|\| id:ebi-a-GCST90001626 | Weighted median | 20 | -0.029681071 | 0.0455780048650935 | 0.514907838310828 | -0.119013961 | 0.0596518182431782 | 0.970755085848248 | 0.887795406316476 | 1.06146689878716 | 0.940845681141708 |
| ebi-a-GCST90001626 | finngen_R11_O15_PREECLAMPS | Pre-eclampsia \|\| id:finngen_R11_O15_PREECLAMPS | HLA DR+ CD4+ T cell %lymphocyte \|\| id:ebi-a-GCST90001626 | Weighted mode | 20 | -0.062247879 | 0.05127231290223 | 0.239598660581916 | -0.162741613 | 0.0382458538513788 | 0.93964993802307 | 0.84981074032981 | 1.03898664034782 | 0.872755742358858 |
| ebi-a-GCST90001627 | finngen_R11_O15_PREECLAMPS | Pre-eclampsia \|\| id:finngen_R11_O15_PREECLAMPS | HLA DR+ CD8+ T cell Absolute Count \|\| id:ebi-a-GCST90001627 | Inverse variance weighted (multiplicative random effects) | 34 | 0.00120056951310461 | 0.0153919128686926 | 0.937828037006778 | -0.02896758 | 0.0313687187357421 | 1.00120129048518 | 0.971447958581069 | 1.03186590204311 | 0.986667691552896 |
| ebi-a-GCST90001627 | finngen_R11_O15_PREECLAMPS | Pre-eclampsia \|\| id:finngen_R11_O15_PREECLAMPS | HLA DR+ CD8+ T cell Absolute Count \|\| id:ebi-a-GCST90001627 | MR Egger | 34 | -0.016154689 | 0.0213096524314136 | 0.453942803256925 | -0.057921607 | 0.0256122301560532 | 0.983975098542328 | 0.943723925532807 | 1.02594304155715 | 0.99380795269851 |
| ebi-a-GCST90001627 | finngen_R11_O15_PREECLAMPS | Pre-eclampsia \|\| id:finngen_R11_O15_PREECLAMPS | HLA DR+ CD8+ T cell Absolute Count \|\| id:ebi-a-GCST90001627 | Weighted median | 34 | -0.005280197 | 0.0187964070603601 | 0.778775432754214 | -0.042121155 | 0.0315607611634051 | 0.994733719060183 | 0.958753616208052 | 1.03206408310493 | 0.957984984509652 |
| ebi-a-GCST90001627 | finngen_R11_O15_PREECLAMPS | Pre-eclampsia \|\| id:finngen_R11_O15_PREECLAMPS | HLA DR+ CD8+ T cell Absolute Count \|\| id:ebi-a-GCST90001627 | Weighted mode | 34 | -0.006815198 | 0.0161302862365477 | 0.675393580420205 | -0.038430559 | 0.0248001628524649 | 0.993207972624153 | 0.962298525204188 | 1.02511024494698 | 0.959313411571287 |
| ebi-a-GCST90001628 | finngen_R11_O15_PREECLAMPS | Pre-eclampsia \|\| id:finngen_R11_O15_PREECLAMPS | HLA DR+ CD8+ T cell %T cell \|\| id:ebi-a-GCST90001628 | Inverse variance weighted | 30 | -0.00092877 | 0.0129887227227031 | 0.942995140007012 | -0.026386667 | 0.0245291264070338 | 0.999071661044015 | 0.973958419535694 | 1.0248324403593 | 0.986667691552896 |
| ebi-a-GCST90001628 | finngen_R11_O15_PREECLAMPS | Pre-eclampsia \|\| id:finngen_R11_O15_PREECLAMPS | HLA DR+ CD8+ T cell %T cell \|\| id:ebi-a-GCST90001628 | MR Egger | 30 | -0.003213025 | 0.0175843685102172 | 0.856333917741866 | -0.037678387 | 0.0312523377518557 | 0.996792131711286 | 0.963022611867377 | 1.03174581946199 | 0.99380795269851 |
| ebi-a-GCST90001628 | finngen_R11_O15_PREECLAMPS | Pre-eclampsia \|\| id:finngen_R11_O15_PREECLAMPS | HLA DR+ CD8+ T cell %T cell \|\| id:ebi-a-GCST90001628 | Weighted median | 30 | -0.002808849 | 0.0183564365067757 | 0.878384792974504 | -0.038787464 | 0.0331697669362085 | 0.997195092507334 | 0.961955137354639 | 1.0337260168445 | 0.964912412213677 |
| ebi-a-GCST90001628 | finngen_R11_O15_PREECLAMPS | Pre-eclampsia \|\| id:finngen_R11_O15_PREECLAMPS | HLA DR+ CD8+ T cell %T cell \|\| id:ebi-a-GCST90001628 | Weighted mode | 30 | -0.000568685 | 0.016013919745009 | 0.971914838777457 | -0.031955968 | 0.030818597266967 | 0.999431476237662 | 0.968549228170641 | 1.03129840656753 | 0.993663982022827 |
| ebi-a-GCST90001629 | finngen_R11_O15_PREECLAMPS | Pre-eclampsia \|\| id:finngen_R11_O15_PREECLAMPS | HLA DR+ CD8+ T cell %lymphocyte \|\| id:ebi-a-GCST90001629 | Inverse variance weighted | 30 | -0.0184051 | 0.0177465779423795 | 0.299685997669243 | -0.053188393 | 0.0163781927253701 | 0.981763239461542 | 0.948201361336801 | 1.01651305056043 | 0.885079385681701 |
| ebi-a-GCST90001629 | finngen_R11_O15_PREECLAMPS | Pre-eclampsia \|\| id:finngen_R11_O15_PREECLAMPS | HLA DR+ CD8+ T cell %lymphocyte \|\| id:ebi-a-GCST90001629 | MR Egger | 30 | -0.03481571 | 0.0280523982246797 | 0.224868200097878 | -0.089798411 | 0.0201669902123951 | 0.96578338378063 | 0.914115442473317 | 1.02037171789053 | 0.99380795269851 |
| ebi-a-GCST90001629 | finngen_R11_O15_PREECLAMPS | Pre-eclampsia \|\| id:finngen_R11_O15_PREECLAMPS | HLA DR+ CD8+ T cell %lymphocyte \|\| id:ebi-a-GCST90001629 | Weighted median | 30 | -0.036330609 | 0.0264112425574069 | 0.168953674792605 | -0.088096644 | 0.0154354265583751 | 0.964321427573886 | 0.915672377961477 | 1.01555516804861 | 0.940845681141708 |
| ebi-a-GCST90001629 | finngen_R11_O15_PREECLAMPS | Pre-eclampsia \|\| id:finngen_R11_O15_PREECLAMPS | HLA DR+ CD8+ T cell %lymphocyte \|\| id:ebi-a-GCST90001629 | Weighted mode | 30 | -0.039338866 | 0.0240407653153817 | 0.112576968716299 | -0.086458766 | 0.00778103430959116 | 0.961424860023522 | 0.91717336698227 | 1.00781138522649 | 0.872755742358858 |
| ebi-a-GCST90001630 | finngen_R11_O15_PREECLAMPS | Pre-eclampsia \|\| id:finngen_R11_O15_PREECLAMPS | CD8+ Natural Killer T Absolute Count \|\| id:ebi-a-GCST90001630 | Inverse variance weighted | 25 | 0.0427915735311902 | 0.0255090424764825 | 0.0934435639760478 | -0.00720615 | 0.0927892967850959 | 1.04372033323512 | 0.992819752318856 | 1.09723052091189 | 0.802698559103897 |
| ebi-a-GCST90001630 | finngen_R11_O15_PREECLAMPS | Pre-eclampsia \|\| id:finngen_R11_O15_PREECLAMPS | CD8+ Natural Killer T Absolute Count \|\| id:ebi-a-GCST90001630 | MR Egger | 25 | 0.137055666321877 | 0.0480059997433891 | 0.00895979382025109 | 0.0429639068248342 | 0.23114742581892 | 1.14689199000215 | 1.04390021649728 | 1.26004498892113 | 0.592319696793224 |
| ebi-a-GCST90001630 | finngen_R11_O15_PREECLAMPS | Pre-eclampsia \|\| id:finngen_R11_O15_PREECLAMPS | CD8+ Natural Killer T Absolute Count \|\| id:ebi-a-GCST90001630 | Weighted median | 25 | 0.0841211958561479 | 0.0374218094026556 | 0.0245813562690015 | 0.010774449426943 | 0.157467942285353 | 1.08776071791189 | 1.01083270283532 | 1.17054323243928 | 0.648347695240983 |
| ebi-a-GCST90001630 | finngen_R11_O15_PREECLAMPS | Pre-eclampsia \|\| id:finngen_R11_O15_PREECLAMPS | CD8+ Natural Killer T Absolute Count \|\| id:ebi-a-GCST90001630 | Weighted mode | 25 | 0.0812820296284776 | 0.0463625184694186 | 0.0923361990872274 | -0.009588507 | 0.172152565828538 | 1.08467676441772 | 0.990457316582066 | 1.18785904608966 | 0.872755742358858 |
| ebi-a-GCST90001631 | finngen_R11_O15_PREECLAMPS | Pre-eclampsia \|\| id:finngen_R11_O15_PREECLAMPS | CD8+ Natural Killer T %T cell \|\| id:ebi-a-GCST90001631 | Inverse variance weighted | 27 | 0.0578228121750278 | 0.0231628618976036 | 0.0125475642081027 | 0.0124236028557247 | 0.103222021494331 | 1.05952724374928 | 1.01250109639348 | 1.10873754531786 | 0.588591724205005 |
| ebi-a-GCST90001631 | finngen_R11_O15_PREECLAMPS | Pre-eclampsia \|\| id:finngen_R11_O15_PREECLAMPS | CD8+ Natural Killer T %T cell \|\| id:ebi-a-GCST90001631 | MR Egger | 27 | 0.0874552337397141 | 0.0426796629220814 | 0.0510845411571121 | 0.00380309441243451 | 0.171107373066994 | 1.09139340574807 | 1.00381033535241 | 1.18661815301215 | 0.862303274556491 |
| ebi-a-GCST90001631 | finngen_R11_O15_PREECLAMPS | Pre-eclampsia \|\| id:finngen_R11_O15_PREECLAMPS | CD8+ Natural Killer T %T cell \|\| id:ebi-a-GCST90001631 | Weighted median | 27 | 0.0859610353096846 | 0.0337957657703105 | 0.0109734067543836 | 0.019721334399876 | 0.152200736219493 | 1.08976386516633 | 1.01991708461603 | 1.16439394901336 | 0.598716529366782 |
| ebi-a-GCST90001631 | finngen_R11_O15_PREECLAMPS | Pre-eclampsia \|\| id:finngen_R11_O15_PREECLAMPS | CD8+ Natural Killer T %T cell \|\| id:ebi-a-GCST90001631 | Weighted mode | 27 | 0.0828110390061447 | 0.0412901789563023 | 0.0554117579970431 | 0.00188228825179217 | 0.163739789760497 | 1.08633651392518 | 1.00188406086834 | 1.17790777154822 | 0.838871423749761 |
| ebi-a-GCST90001632 | finngen_R11_O15_PREECLAMPS | Pre-eclampsia \|\| id:finngen_R11_O15_PREECLAMPS | CD8+ Natural Killer T %lymphocyte \|\| id:ebi-a-GCST90001632 | Inverse variance weighted | 27 | 0.059436663399615 | 0.0239963296731038 | 0.0132526638600489 | 0.0124038572403316 | 0.106469469558898 | 1.06123854360935 | 1.01248110413362 | 1.11234396557534 | 0.588591724205005 |
| ebi-a-GCST90001632 | finngen_R11_O15_PREECLAMPS | Pre-eclampsia \|\| id:finngen_R11_O15_PREECLAMPS | CD8+ Natural Killer T %lymphocyte \|\| id:ebi-a-GCST90001632 | MR Egger | 27 | 0.0830885542136815 | 0.0423832278186335 | 0.0611819303333216 | 1.74276891597713e-05 | 0.166159680738203 | 1.0866380306641 | 1.00001742784102 | 1.18076163155955 | 0.862303274556491 |
| ebi-a-GCST90001632 | finngen_R11_O15_PREECLAMPS | Pre-eclampsia \|\| id:finngen_R11_O15_PREECLAMPS | CD8+ Natural Killer T %lymphocyte \|\| id:ebi-a-GCST90001632 | Weighted median | 27 | 0.0802693244416465 | 0.0354075908200398 | 0.0233896804423089 | 0.0108704464343684 | 0.149668202448925 | 1.0835788626516 | 1.01092974440758 | 1.16144881291766 | 0.648347695240983 |
| ebi-a-GCST90001632 | finngen_R11_O15_PREECLAMPS | Pre-eclampsia \|\| id:finngen_R11_O15_PREECLAMPS | CD8+ Natural Killer T %lymphocyte \|\| id:ebi-a-GCST90001632 | Weighted mode | 27 | 0.0729390909759899 | 0.0470644939895202 | 0.133284952593399 | -0.019307317 | 0.16518549919545 | 1.07566501721299 | 0.980877875234303 | 1.17961191547872 | 0.872755742358858 |
| ebi-a-GCST90001633 | finngen_R11_O15_PREECLAMPS | Pre-eclampsia \|\| id:finngen_R11_O15_PREECLAMPS | CD8dim Natural Killer T Absolute Count \|\| id:ebi-a-GCST90001633 | Inverse variance weighted | 27 | -0.006271578 | 0.0174621539462875 | 0.719480949402934 | -0.040497399 | 0.0279542441063272 | 0.993748047665959 | 0.96031166193009 | 1.02834863033434 | 0.959745573017418 |
| ebi-a-GCST90001633 | finngen_R11_O15_PREECLAMPS | Pre-eclampsia \|\| id:finngen_R11_O15_PREECLAMPS | CD8dim Natural Killer T Absolute Count \|\| id:ebi-a-GCST90001633 | MR Egger | 27 | 0.0114913777466427 | 0.0245642111102851 | 0.643973174985888 | -0.036654476 | 0.0596372315228014 | 1.01155765726559 | 0.964009166085338 | 1.06145141557925 | 0.99380795269851 |
| ebi-a-GCST90001633 | finngen_R11_O15_PREECLAMPS | Pre-eclampsia \|\| id:finngen_R11_O15_PREECLAMPS | CD8dim Natural Killer T Absolute Count \|\| id:ebi-a-GCST90001633 | Weighted median | 27 | 0.00699448480056344 | 0.0255047878778476 | 0.783898630547567 | -0.042994899 | 0.0569838690411448 | 1.00701900334089 | 0.957916275963994 | 1.05863873339992 | 0.957984984509652 |
| ebi-a-GCST90001633 | finngen_R11_O15_PREECLAMPS | Pre-eclampsia \|\| id:finngen_R11_O15_PREECLAMPS | CD8dim Natural Killer T Absolute Count \|\| id:ebi-a-GCST90001633 | Weighted mode | 27 | 0.00618528565500043 | 0.0236006230579264 | 0.795323928642957 | -0.040071936 | 0.0524425068485362 | 1.00620445403457 | 0.960720326732448 | 1.05384197164069 | 0.961305639715923 |
| ebi-a-GCST90001634 | finngen_R11_O15_PREECLAMPS | Pre-eclampsia \|\| id:finngen_R11_O15_PREECLAMPS | CD8dim Natural Killer T %T cell \|\| id:ebi-a-GCST90001634 | Inverse variance weighted | 19 | -0.0246052 | 0.0255724729161725 | 0.335961580135199 | -0.074727247 | 0.0255168464331777 | 0.975695039930147 | 0.927996565290639 | 1.02584518795729 | 0.885079385681701 |
| ebi-a-GCST90001634 | finngen_R11_O15_PREECLAMPS | Pre-eclampsia \|\| id:finngen_R11_O15_PREECLAMPS | CD8dim Natural Killer T %T cell \|\| id:ebi-a-GCST90001634 | MR Egger | 19 | 0.0135392707688487 | 0.0415460439644699 | 0.748487824890021 | -0.067890975 | 0.0949695169392097 | 1.01363134175073 | 0.93436233650291 | 1.09962533466928 | 0.99380795269851 |
| ebi-a-GCST90001634 | finngen_R11_O15_PREECLAMPS | Pre-eclampsia \|\| id:finngen_R11_O15_PREECLAMPS | CD8dim Natural Killer T %T cell \|\| id:ebi-a-GCST90001634 | Weighted median | 19 | -0.008449452 | 0.0315610266210283 | 0.788916469892365 | -0.070309064 | 0.0534101603874 | 0.991586144501025 | 0.932105695099924 | 1.05486221909737 | 0.957984984509652 |
| ebi-a-GCST90001634 | finngen_R11_O15_PREECLAMPS | Pre-eclampsia \|\| id:finngen_R11_O15_PREECLAMPS | CD8dim Natural Killer T %T cell \|\| id:ebi-a-GCST90001634 | Weighted mode | 19 | -0.004070926 | 0.0326892691125691 | 0.902272679566477 | -0.068141894 | 0.0600000411418155 | 0.995937348668963 | 0.934127917232305 | 1.06183659023124 | 0.98361388732834 |
| ebi-a-GCST90001635 | finngen_R11_O15_PREECLAMPS | Pre-eclampsia \|\| id:finngen_R11_O15_PREECLAMPS | CD8dim Natural Killer T %lymphocyte \|\| id:ebi-a-GCST90001635 | Inverse variance weighted | 26 | -0.025688582 | 0.0208007720186514 | 0.216837156460051 | -0.066458095 | 0.0150809315107617 | 0.97463856268989 | 0.935702125817297 | 1.01519522257417 | 0.856799791201608 |
| ebi-a-GCST90001635 | finngen_R11_O15_PREECLAMPS | Pre-eclampsia \|\| id:finngen_R11_O15_PREECLAMPS | CD8dim Natural Killer T %lymphocyte \|\| id:ebi-a-GCST90001635 | MR Egger | 26 | 0.0139760697637249 | 0.0354309051428719 | 0.6967250293231 | -0.055468504 | 0.0834206438437537 | 1.01407419161313 | 0.946041819435121 | 1.08699895181149 | 0.99380795269851 |
| ebi-a-GCST90001635 | finngen_R11_O15_PREECLAMPS | Pre-eclampsia \|\| id:finngen_R11_O15_PREECLAMPS | CD8dim Natural Killer T %lymphocyte \|\| id:ebi-a-GCST90001635 | Weighted median | 26 | -0.017580086 | 0.0288746576394968 | 0.542629602813661 | -0.074174414 | 0.0390142434503467 | 0.982573542597917 | 0.928509734159879 | 1.03978529367489 | 0.940845681141708 |
| ebi-a-GCST90001635 | finngen_R11_O15_PREECLAMPS | Pre-eclampsia \|\| id:finngen_R11_O15_PREECLAMPS | CD8dim Natural Killer T %lymphocyte \|\| id:ebi-a-GCST90001635 | Weighted mode | 26 | -0.021634287 | 0.0350784645407839 | 0.542982533760242 | -0.090388078 | 0.0471195030616442 | 0.978598055219213 | 0.913576577553332 | 1.04824727035312 | 0.938345702550206 |
| ebi-a-GCST90001636 | finngen_R11_O15_PREECLAMPS | Pre-eclampsia \|\| id:finngen_R11_O15_PREECLAMPS | CD4-CD8- Natural Killer T Absolute Count \|\| id:ebi-a-GCST90001636 | Inverse variance weighted | 29 | -0.014773522 | 0.0197097435856321 | 0.453523221794251 | -0.053404619 | 0.023857575623383 | 0.985335071243825 | 0.947996357312088 | 1.02414444436884 | 0.898694924660397 |
| ebi-a-GCST90001636 | finngen_R11_O15_PREECLAMPS | Pre-eclampsia \|\| id:finngen_R11_O15_PREECLAMPS | CD4-CD8- Natural Killer T Absolute Count \|\| id:ebi-a-GCST90001636 | MR Egger | 29 | 9.50684926798844e-05 | 0.0326732953147442 | 0.997699814990973 | -0.06394459 | 0.0641347273095785 | 1.00009507301183 | 0.938056975523608 | 1.06623604019815 | 0.997699814990973 |
| ebi-a-GCST90001636 | finngen_R11_O15_PREECLAMPS | Pre-eclampsia \|\| id:finngen_R11_O15_PREECLAMPS | CD4-CD8- Natural Killer T Absolute Count \|\| id:ebi-a-GCST90001636 | Weighted median | 29 | -0.009602302 | 0.0300715057585803 | 0.749487176474861 | -0.068542453 | 0.0493378492785149 | 0.99044365288508 | 0.933753818335648 | 1.05057522687175 | 0.957984984509652 |
| ebi-a-GCST90001636 | finngen_R11_O15_PREECLAMPS | Pre-eclampsia \|\| id:finngen_R11_O15_PREECLAMPS | CD4-CD8- Natural Killer T Absolute Count \|\| id:ebi-a-GCST90001636 | Weighted mode | 29 | -0.015529973 | 0.0295232487431831 | 0.603012604290622 | -0.07339554 | 0.0423355947601139 | 0.984589995413566 | 0.929233208131799 | 1.04324452740715 | 0.955353022288269 |
| ebi-a-GCST90001637 | finngen_R11_O15_PREECLAMPS | Pre-eclampsia \|\| id:finngen_R11_O15_PREECLAMPS | CD4-CD8- Natural Killer T %T cell \|\| id:ebi-a-GCST90001637 | Inverse variance weighted | 31 | -0.011973339 | 0.0219666285270275 | 0.585705731529147 | -0.055027931 | 0.0310812530964925 | 0.98809805637437 | 0.946458712302068 | 1.03156931868276 | 0.915399120505076 |
| ebi-a-GCST90001637 | finngen_R11_O15_PREECLAMPS | Pre-eclampsia \|\| id:finngen_R11_O15_PREECLAMPS | CD4-CD8- Natural Killer T %T cell \|\| id:ebi-a-GCST90001637 | MR Egger | 31 | 0.0295385349844696 | 0.0411235901572538 | 0.478327863223252 | -0.051063702 | 0.110140771692687 | 1.02997912493675 | 0.950218138071105 | 1.11643522187333 | 0.99380795269851 |
| ebi-a-GCST90001637 | finngen_R11_O15_PREECLAMPS | Pre-eclampsia \|\| id:finngen_R11_O15_PREECLAMPS | CD4-CD8- Natural Killer T %T cell \|\| id:ebi-a-GCST90001637 | Weighted median | 31 | -0.01321343 | 0.0308068852178506 | 0.66798756294466 | -0.073594925 | 0.0471680648023141 | 0.986873483912035 | 0.929047951495053 | 1.04829817630125 | 0.940845681141708 |
| ebi-a-GCST90001637 | finngen_R11_O15_PREECLAMPS | Pre-eclampsia \|\| id:finngen_R11_O15_PREECLAMPS | CD4-CD8- Natural Killer T %T cell \|\| id:ebi-a-GCST90001637 | Weighted mode | 31 | -0.009994299 | 0.03405483684082 | 0.771178554290585 | -0.07674178 | 0.0567531808000825 | 0.990055477635491 | 0.92612896811089 | 1.05839454605924 | 0.961305639715923 |
| ebi-a-GCST90001638 | finngen_R11_O15_PREECLAMPS | Pre-eclampsia \|\| id:finngen_R11_O15_PREECLAMPS | CD4-CD8- Natural Killer T %lymphocyte \|\| id:ebi-a-GCST90001638 | Inverse variance weighted | 30 | -0.004368583 | 0.0242222367327734 | 0.856874464045122 | -0.051844167 | 0.0431070005190574 | 0.995640944903395 | 0.949476814686014 | 1.04404960272352 | 0.977824840984872 |
| ebi-a-GCST90001638 | finngen_R11_O15_PREECLAMPS | Pre-eclampsia \|\| id:finngen_R11_O15_PREECLAMPS | CD4-CD8- Natural Killer T %lymphocyte \|\| id:ebi-a-GCST90001638 | MR Egger | 30 | 0.00442047713631149 | 0.0470870281036915 | 0.925873946196531 | -0.087870098 | 0.0967110522195468 | 1.00443026185777 | 0.915879843668197 | 1.10154203950487 | 0.99380795269851 |
| ebi-a-GCST90001638 | finngen_R11_O15_PREECLAMPS | Pre-eclampsia \|\| id:finngen_R11_O15_PREECLAMPS | CD4-CD8- Natural Killer T %lymphocyte \|\| id:ebi-a-GCST90001638 | Weighted median | 30 | -0.00927414 | 0.0339030362465795 | 0.78443127620363 | -0.075724091 | 0.0571758113535119 | 0.990768732507096 | 0.927071959020118 | 1.05884195046872 | 0.957984984509652 |
| ebi-a-GCST90001638 | finngen_R11_O15_PREECLAMPS | Pre-eclampsia \|\| id:finngen_R11_O15_PREECLAMPS | CD4-CD8- Natural Killer T %lymphocyte \|\| id:ebi-a-GCST90001638 | Weighted mode | 30 | -0.001467434 | 0.0398195887433907 | 0.970855473314313 | -0.079513828 | 0.0765789599550946 | 0.998533642172835 | 0.923565248894858 | 1.0795874311469 | 0.993663982022827 |
| ebi-a-GCST90001639 | finngen_R11_O15_PREECLAMPS | Pre-eclampsia \|\| id:finngen_R11_O15_PREECLAMPS | CD3- lymphocyte Absolute Count \|\| id:ebi-a-GCST90001639 | Inverse variance weighted | 18 | -0.020938721 | 0.0291851651500161 | 0.47310020040258 | -0.078141644 | 0.0362642031061826 | 0.979278972370578 | 0.924833419899967 | 1.03692977036979 | 0.898694924660397 |
| ebi-a-GCST90001639 | finngen_R11_O15_PREECLAMPS | Pre-eclampsia \|\| id:finngen_R11_O15_PREECLAMPS | CD3- lymphocyte Absolute Count \|\| id:ebi-a-GCST90001639 | MR Egger | 18 | -0.021593923 | 0.0584059678653227 | 0.716438194554616 | -0.13606962 | 0.092881774162275 | 0.978637556720364 | 0.872781867413551 | 1.09733199460456 | 0.99380795269851 |
| ebi-a-GCST90001639 | finngen_R11_O15_PREECLAMPS | Pre-eclampsia \|\| id:finngen_R11_O15_PREECLAMPS | CD3- lymphocyte Absolute Count \|\| id:ebi-a-GCST90001639 | Weighted median | 18 | -0.006616739 | 0.0415896004596954 | 0.873593250027186 | -0.088132355 | 0.0748988783756271 | 0.993405103887251 | 0.915639678822803 | 1.07777515899922 | 0.964912412213677 |
| ebi-a-GCST90001639 | finngen_R11_O15_PREECLAMPS | Pre-eclampsia \|\| id:finngen_R11_O15_PREECLAMPS | CD3- lymphocyte Absolute Count \|\| id:ebi-a-GCST90001639 | Weighted mode | 18 | 0.000288527402643735 | 0.0488308086354456 | 0.995354338950191 | -0.095419858 | 0.0959969123281172 | 1.00028856903068 | 0.908991207541706 | 1.10075566521641 | 0.996042942349711 |
| ebi-a-GCST90001640 | finngen_R11_O15_PREECLAMPS | Pre-eclampsia \|\| id:finngen_R11_O15_PREECLAMPS | CD3- lymphocyte %lymphocyte \|\| id:ebi-a-GCST90001640 | Inverse variance weighted | 17 | -0.033631099 | 0.0294694541148173 | 0.253778826234858 | -0.091391229 | 0.0241290309984863 | 0.966928139545239 | 0.912660581637338 | 1.02442249162015 | 0.885079385681701 |
| ebi-a-GCST90001640 | finngen_R11_O15_PREECLAMPS | Pre-eclampsia \|\| id:finngen_R11_O15_PREECLAMPS | CD3- lymphocyte %lymphocyte \|\| id:ebi-a-GCST90001640 | MR Egger | 17 | -0.03299122 | 0.0904643025631871 | 0.720434869118662 | -0.210301253 | 0.14431881334584 | 0.967547054926422 | 0.810340092053869 | 1.15525235969018 | 0.99380795269851 |
| ebi-a-GCST90001640 | finngen_R11_O15_PREECLAMPS | Pre-eclampsia \|\| id:finngen_R11_O15_PREECLAMPS | CD3- lymphocyte %lymphocyte \|\| id:ebi-a-GCST90001640 | Weighted median | 17 | -0.030523053 | 0.0410558498869789 | 0.457208075358894 | -0.110992519 | 0.0499464123031843 | 0.9699380713661 | 0.894945443763742 | 1.05121476268865 | 0.940845681141708 |
| ebi-a-GCST90001640 | finngen_R11_O15_PREECLAMPS | Pre-eclampsia \|\| id:finngen_R11_O15_PREECLAMPS | CD3- lymphocyte %lymphocyte \|\| id:ebi-a-GCST90001640 | Weighted mode | 17 | -0.038908385 | 0.0501640910514572 | 0.449285144098818 | -0.137230004 | 0.0594132330152308 | 0.961838823545224 | 0.871769692636391 | 1.06121367867365 | 0.933945646747087 |
| ebi-a-GCST90001641 | finngen_R11_O15_PREECLAMPS | Pre-eclampsia \|\| id:finngen_R11_O15_PREECLAMPS | CD3- lymphocyte %leukocyte \|\| id:ebi-a-GCST90001641 | Inverse variance weighted | 20 | -0.006504298 | 0.0209674007645591 | 0.756401255625927 | -0.047600403 | 0.0345918078079029 | 0.993516809466387 | 0.953514732406317 | 1.0351970631867 | 0.970051434846584 |
| ebi-a-GCST90001641 | finngen_R11_O15_PREECLAMPS | Pre-eclampsia \|\| id:finngen_R11_O15_PREECLAMPS | CD3- lymphocyte %leukocyte \|\| id:ebi-a-GCST90001641 | MR Egger | 20 | -0.009781954 | 0.0313304490522872 | 0.758462154989573 | -0.071189635 | 0.0516257256978799 | 0.990265733252147 | 0.931285271483513 | 1.05298156481236 | 0.99380795269851 |
| ebi-a-GCST90001641 | finngen_R11_O15_PREECLAMPS | Pre-eclampsia \|\| id:finngen_R11_O15_PREECLAMPS | CD3- lymphocyte %leukocyte \|\| id:ebi-a-GCST90001641 | Weighted median | 20 | -0.012212231 | 0.0290783884470234 | 0.674502836354461 | -0.069205873 | 0.0447814101175443 | 0.987862035429301 | 0.93313455347027 | 1.04579923379036 | 0.941872178433165 |
| ebi-a-GCST90001641 | finngen_R11_O15_PREECLAMPS | Pre-eclampsia \|\| id:finngen_R11_O15_PREECLAMPS | CD3- lymphocyte %leukocyte \|\| id:ebi-a-GCST90001641 | Weighted mode | 20 | -0.024879301 | 0.0282198498893588 | 0.388997318850445 | -0.080190207 | 0.0304316045005039 | 0.975427637788275 | 0.922940779832543 | 1.03089937875954 | 0.920286500917063 |
| ebi-a-GCST90001642 | finngen_R11_O15_PREECLAMPS | Pre-eclampsia \|\| id:finngen_R11_O15_PREECLAMPS | B cell Absolute Count \|\| id:ebi-a-GCST90001642 | Inverse variance weighted | 30 | 0.0163007964248974 | 0.0247834653217298 | 0.510712477098549 | -0.032274796 | 0.0648763884554878 | 1.0164343792554 | 0.968240477290245 | 1.06702711936161 | 0.898694924660397 |
| ebi-a-GCST90001642 | finngen_R11_O15_PREECLAMPS | Pre-eclampsia \|\| id:finngen_R11_O15_PREECLAMPS | B cell Absolute Count \|\| id:ebi-a-GCST90001642 | MR Egger | 30 | -0.016175262 | 0.0454248832199365 | 0.724443652870446 | -0.105208033 | 0.0728575090083496 | 0.983954854945562 | 0.900137244663057 | 1.07557726592388 | 0.99380795269851 |
| ebi-a-GCST90001642 | finngen_R11_O15_PREECLAMPS | Pre-eclampsia \|\| id:finngen_R11_O15_PREECLAMPS | B cell Absolute Count \|\| id:ebi-a-GCST90001642 | Weighted median | 30 | -0.003677457 | 0.0325171195730621 | 0.909956874037739 | -0.067411011 | 0.060056097787888 | 0.996329296986938 | 0.934810904859818 | 1.06189611489753 | 0.971329194634319 |
| ebi-a-GCST90001642 | finngen_R11_O15_PREECLAMPS | Pre-eclampsia \|\| id:finngen_R11_O15_PREECLAMPS | B cell Absolute Count \|\| id:ebi-a-GCST90001642 | Weighted mode | 30 | -0.010927943 | 0.0406953878094587 | 0.790192259929014 | -0.090690903 | 0.0688350169874866 | 0.989131549941884 | 0.913299965348405 | 1.07125945495597 | 0.961305639715923 |
| ebi-a-GCST90001643 | finngen_R11_O15_PREECLAMPS | Pre-eclampsia \|\| id:finngen_R11_O15_PREECLAMPS | B cell %CD3- lymphocyte \|\| id:ebi-a-GCST90001643 | Inverse variance weighted (multiplicative random effects) | 29 | 0.0263034638244791 | 0.01488919744407 | 0.0772929040836773 | -0.002879363 | 0.0554862908148563 | 1.02665245308569 | 0.997124778224413 | 1.05705452561689 | 0.789997151112252 |
| ebi-a-GCST90001643 | finngen_R11_O15_PREECLAMPS | Pre-eclampsia \|\| id:finngen_R11_O15_PREECLAMPS | B cell %CD3- lymphocyte \|\| id:ebi-a-GCST90001643 | MR Egger | 29 | 0.0288286722309002 | 0.0190772864630412 | 0.14236635962613 | -0.008562809 | 0.0662201536984609 | 1.0292482405635 | 0.991473747198013 | 1.06846191711769 | 0.97261503632431 |
| ebi-a-GCST90001643 | finngen_R11_O15_PREECLAMPS | Pre-eclampsia \|\| id:finngen_R11_O15_PREECLAMPS | B cell %CD3- lymphocyte \|\| id:ebi-a-GCST90001643 | Weighted median | 29 | 0.0101251870683626 | 0.0162866384998077 | 0.534147877513893 | -0.021796624 | 0.0420469985279857 | 1.01017662021847 | 0.978439205486688 | 1.04294349440793 | 0.940845681141708 |
| ebi-a-GCST90001643 | finngen_R11_O15_PREECLAMPS | Pre-eclampsia \|\| id:finngen_R11_O15_PREECLAMPS | B cell %CD3- lymphocyte \|\| id:ebi-a-GCST90001643 | Weighted mode | 29 | 0.0166848544122817 | 0.0137338679643796 | 0.23456182648358 | -0.010233527 | 0.0436032356224657 | 1.01682482396932 | 0.989818657575695 | 1.04456782535559 | 0.872755742358858 |
| ebi-a-GCST90001644 | finngen_R11_O15_PREECLAMPS | Pre-eclampsia \|\| id:finngen_R11_O15_PREECLAMPS | B cell %lymphocyte \|\| id:ebi-a-GCST90001644 | Inverse variance weighted | 34 | -0.000573937 | 0.0183342841207743 | 0.975027060131266 | -0.036509134 | 0.0353612595200424 | 0.999426227313865 | 0.964149287091488 | 1.03599390386527 | 0.994537505425743 |
| ebi-a-GCST90001644 | finngen_R11_O15_PREECLAMPS | Pre-eclampsia \|\| id:finngen_R11_O15_PREECLAMPS | B cell %lymphocyte \|\| id:ebi-a-GCST90001644 | MR Egger | 34 | -0.021106866 | 0.0317933783186252 | 0.511524792452663 | -0.083421887 | 0.0412081555173207 | 0.979114324959448 | 0.919962944477849 | 1.04206899538211 | 0.99380795269851 |
| ebi-a-GCST90001644 | finngen_R11_O15_PREECLAMPS | Pre-eclampsia \|\| id:finngen_R11_O15_PREECLAMPS | B cell %lymphocyte \|\| id:ebi-a-GCST90001644 | Weighted median | 34 | -0.017493003 | 0.0261080813857074 | 0.502843418465986 | -0.068664843 | 0.0336788363818668 | 0.982659111175027 | 0.933639543801242 | 1.03425238914351 | 0.940845681141708 |
| ebi-a-GCST90001644 | finngen_R11_O15_PREECLAMPS | Pre-eclampsia \|\| id:finngen_R11_O15_PREECLAMPS | B cell %lymphocyte \|\| id:ebi-a-GCST90001644 | Weighted mode | 34 | -0.01005154 | 0.0349812618731014 | 0.775648163560951 | -0.078614813 | 0.0585117332071869 | 0.989998807832233 | 0.924395920859702 | 1.06025742584166 | 0.961305639715923 |
| ebi-a-GCST90001645 | finngen_R11_O15_PREECLAMPS | Pre-eclampsia \|\| id:finngen_R11_O15_PREECLAMPS | Natural Killer Absolute Count \|\| id:ebi-a-GCST90001645 | Inverse variance weighted | 21 | 0.0100924609708324 | 0.0269572677312774 | 0.708116127030649 | -0.042743784 | 0.062928705724136 | 1.01014356162081 | 0.958156853944692 | 1.06495091162066 | 0.956807557965628 |
| ebi-a-GCST90001645 | finngen_R11_O15_PREECLAMPS | Pre-eclampsia \|\| id:finngen_R11_O15_PREECLAMPS | Natural Killer Absolute Count \|\| id:ebi-a-GCST90001645 | MR Egger | 21 | 0.0340563617819784 | 0.0472597369637209 | 0.479915712978243 | -0.058572723 | 0.126685446230871 | 1.03464291940345 | 0.943109652456628 | 1.13505992424451 | 0.99380795269851 |
| ebi-a-GCST90001645 | finngen_R11_O15_PREECLAMPS | Pre-eclampsia \|\| id:finngen_R11_O15_PREECLAMPS | Natural Killer Absolute Count \|\| id:ebi-a-GCST90001645 | Weighted median | 21 | 0.00923073837317992 | 0.0356669226743769 | 0.795786555444962 | -0.06067643 | 0.0791379068149587 | 1.00927347302824 | 0.941127711143398 | 1.08235357571282 | 0.957984984509652 |
| ebi-a-GCST90001645 | finngen_R11_O15_PREECLAMPS | Pre-eclampsia \|\| id:finngen_R11_O15_PREECLAMPS | Natural Killer Absolute Count \|\| id:ebi-a-GCST90001645 | Weighted mode | 21 | 0.0231620971994845 | 0.0387071894911838 | 0.556294884230419 | -0.052703994 | 0.0990281886022047 | 1.02343242163175 | 0.948660780015887 | 1.10409742208326 | 0.939633861977944 |
| ebi-a-GCST90001646 | finngen_R11_O15_PREECLAMPS | Pre-eclampsia \|\| id:finngen_R11_O15_PREECLAMPS | Natural Killer %CD3- lymphocyte \|\| id:ebi-a-GCST90001646 | Inverse variance weighted | 27 | -0.024198966 | 0.011905031683301 | 0.0420861665590274 | -0.047532828 | -0.000865104 | 0.976091481107334 | 0.953579168123258 | 0.999135269875314 | 0.781760498894217 |
| ebi-a-GCST90001646 | finngen_R11_O15_PREECLAMPS | Pre-eclampsia \|\| id:finngen_R11_O15_PREECLAMPS | Natural Killer %CD3- lymphocyte \|\| id:ebi-a-GCST90001646 | MR Egger | 27 | -0.028120924 | 0.0158527040698079 | 0.0882641124528101 | -0.059192224 | 0.0029503758071254 | 0.972270788654531 | 0.942525575568428 | 1.00295473244935 | 0.936133620820556 |
| ebi-a-GCST90001646 | finngen_R11_O15_PREECLAMPS | Pre-eclampsia \|\| id:finngen_R11_O15_PREECLAMPS | Natural Killer %CD3- lymphocyte \|\| id:ebi-a-GCST90001646 | Weighted median | 27 | -0.010614731 | 0.0158003588685924 | 0.501709011596388 | -0.041583435 | 0.0203539722371902 | 0.98944140630982 | 0.959269295821863 | 1.02056252689876 | 0.940845681141708 |
| ebi-a-GCST90001646 | finngen_R11_O15_PREECLAMPS | Pre-eclampsia \|\| id:finngen_R11_O15_PREECLAMPS | Natural Killer %CD3- lymphocyte \|\| id:ebi-a-GCST90001646 | Weighted mode | 27 | -0.014393213 | 0.0142170069172714 | 0.320679014717384 | -0.042258546 | 0.01347212078362 | 0.985709874335198 | 0.958621900353413 | 1.01356327870634 | 0.888599269773114 |
| ebi-a-GCST90001647 | finngen_R11_O15_PREECLAMPS | Pre-eclampsia \|\| id:finngen_R11_O15_PREECLAMPS | Natural Killer %lymphocyte \|\| id:ebi-a-GCST90001647 | Inverse variance weighted | 27 | -0.003842897 | 0.018225335413369 | 0.833000695651461 | -0.039564554 | 0.0318787607633779 | 0.996164477831017 | 0.96120790211804 | 1.03239233125913 | 0.977824840984872 |
| ebi-a-GCST90001647 | finngen_R11_O15_PREECLAMPS | Pre-eclampsia \|\| id:finngen_R11_O15_PREECLAMPS | Natural Killer %lymphocyte \|\| id:ebi-a-GCST90001647 | MR Egger | 27 | -0.000459001 | 0.025267424194342 | 0.985650842279644 | -0.049983153 | 0.0490651500859985 | 0.999541103990085 | 0.951245450230092 | 1.05028877491509 | 0.99380795269851 |
| ebi-a-GCST90001647 | finngen_R11_O15_PREECLAMPS | Pre-eclampsia \|\| id:finngen_R11_O15_PREECLAMPS | Natural Killer %lymphocyte \|\| id:ebi-a-GCST90001647 | Weighted median | 27 | -0.013763385 | 0.0217653776943995 | 0.527156731908911 | -0.056423526 | 0.028896754843142 | 0.986330896907733 | 0.945138760538731 | 1.02931831685782 | 0.940845681141708 |
| ebi-a-GCST90001647 | finngen_R11_O15_PREECLAMPS | Pre-eclampsia \|\| id:finngen_R11_O15_PREECLAMPS | Natural Killer %lymphocyte \|\| id:ebi-a-GCST90001647 | Weighted mode | 27 | -0.015670979 | 0.0218429654995039 | 0.479500416086455 | -0.058483192 | 0.0271412331559902 | 0.984451171664792 | 0.94319409384809 | 1.02751291140747 | 0.933945646747087 |
| ebi-a-GCST90001648 | finngen_R11_O15_PREECLAMPS | Pre-eclampsia \|\| id:finngen_R11_O15_PREECLAMPS | HLA DR+ Natural Killer Absolute Count \|\| id:ebi-a-GCST90001648 | Inverse variance weighted | 18 | 0.0257491993547386 | 0.0271416745582678 | 0.342775225255855 | -0.027448483 | 0.0789468814889435 | 1.02608357377753 | 0.972924803644065 | 1.0821468385148 | 0.885079385681701 |
| ebi-a-GCST90001648 | finngen_R11_O15_PREECLAMPS | Pre-eclampsia \|\| id:finngen_R11_O15_PREECLAMPS | HLA DR+ Natural Killer Absolute Count \|\| id:ebi-a-GCST90001648 | MR Egger | 18 | 0.0865612945551232 | 0.0468556330357339 | 0.0832634163908412 | -0.005275746 | 0.178398335305162 | 1.09041820236807 | 0.994738146112541 | 1.19530135714841 | 0.936133620820556 |
| ebi-a-GCST90001648 | finngen_R11_O15_PREECLAMPS | Pre-eclampsia \|\| id:finngen_R11_O15_PREECLAMPS | HLA DR+ Natural Killer Absolute Count \|\| id:ebi-a-GCST90001648 | Weighted median | 18 | 0.0667855826959628 | 0.0330263493104053 | 0.0431566350912739 | 0.00205393804756841 | 0.131517227344357 | 1.06906622729951 | 1.00205604882321 | 1.14055755633091 | 0.743099833442067 |
| ebi-a-GCST90001648 | finngen_R11_O15_PREECLAMPS | Pre-eclampsia \|\| id:finngen_R11_O15_PREECLAMPS | HLA DR+ Natural Killer Absolute Count \|\| id:ebi-a-GCST90001648 | Weighted mode | 18 | 0.0509546923750599 | 0.0343914816898537 | 0.156742766801186 | -0.016452612 | 0.118361996487173 | 1.05227521611232 | 0.983681993267141 | 1.12565151951858 | 0.872755742358858 |
| ebi-a-GCST90001649 | finngen_R11_O15_PREECLAMPS | Pre-eclampsia \|\| id:finngen_R11_O15_PREECLAMPS | HLA DR+ Natural Killer %Natural Killer \|\| id:ebi-a-GCST90001649 | Inverse variance weighted | 22 | 0.022723292251142 | 0.0199739456951719 | 0.255268062113545 | -0.016425641 | 0.0618722258136789 | 1.02298343293728 | 0.983708523946982 | 1.06382640649004 | 0.885079385681701 |
| ebi-a-GCST90001649 | finngen_R11_O15_PREECLAMPS | Pre-eclampsia \|\| id:finngen_R11_O15_PREECLAMPS | HLA DR+ Natural Killer %Natural Killer \|\| id:ebi-a-GCST90001649 | MR Egger | 22 | 0.0136485115417749 | 0.0326471231223976 | 0.680356528547313 | -0.05033985 | 0.0776368728616742 | 1.01374207767029 | 0.950906204318117 | 1.08073014496336 | 0.99380795269851 |
| ebi-a-GCST90001649 | finngen_R11_O15_PREECLAMPS | Pre-eclampsia \|\| id:finngen_R11_O15_PREECLAMPS | HLA DR+ Natural Killer %Natural Killer \|\| id:ebi-a-GCST90001649 | Weighted median | 22 | 0.0177413808788071 | 0.028850544126687 | 0.53859372105494 | -0.038805686 | 0.0742884473671136 | 1.017899694 | 0.961937609307335 | 1.07711745238464 | 0.940845681141708 |
| ebi-a-GCST90001649 | finngen_R11_O15_PREECLAMPS | Pre-eclampsia \|\| id:finngen_R11_O15_PREECLAMPS | HLA DR+ Natural Killer %Natural Killer \|\| id:ebi-a-GCST90001649 | Weighted mode | 22 | 0.0343455094184893 | 0.0303528081241621 | 0.270583259467621 | -0.025145995 | 0.0938370133418469 | 1.03494212721376 | 0.975167532534198 | 1.09838070992604 | 0.872755742358858 |
| ebi-a-GCST90001650 | finngen_R11_O15_PREECLAMPS | Pre-eclampsia \|\| id:finngen_R11_O15_PREECLAMPS | HLA DR+ Natural Killer %CD3- lymphocyte \|\| id:ebi-a-GCST90001650 | Inverse variance weighted | 23 | 0.015061804547807 | 0.0195799898606995 | 0.441748024995798 | -0.023314976 | 0.0534385846747779 | 1.01517580515846 | 0.976954718428284 | 1.05489220323037 | 0.898694924660397 |
| ebi-a-GCST90001650 | finngen_R11_O15_PREECLAMPS | Pre-eclampsia \|\| id:finngen_R11_O15_PREECLAMPS | HLA DR+ Natural Killer %CD3- lymphocyte \|\| id:ebi-a-GCST90001650 | MR Egger | 23 | 0.0374552485005507 | 0.031890174023664 | 0.253338969007046 | -0.025049493 | 0.0999599895869321 | 1.03816553657781 | 0.975261642613299 | 1.10512670061529 | 0.99380795269851 |
| ebi-a-GCST90001650 | finngen_R11_O15_PREECLAMPS | Pre-eclampsia \|\| id:finngen_R11_O15_PREECLAMPS | HLA DR+ Natural Killer %CD3- lymphocyte \|\| id:ebi-a-GCST90001650 | Weighted median | 23 | 0.0214867210570106 | 0.028175885800281 | 0.445706419338931 | -0.033738015 | 0.0767114572255615 | 1.02171922289599 | 0.966824764939075 | 1.07973048301154 | 0.940845681141708 |
| ebi-a-GCST90001650 | finngen_R11_O15_PREECLAMPS | Pre-eclampsia \|\| id:finngen_R11_O15_PREECLAMPS | HLA DR+ Natural Killer %CD3- lymphocyte \|\| id:ebi-a-GCST90001650 | Weighted mode | 23 | 0.0389031329639157 | 0.0313180189659147 | 0.227243310818799 | -0.022480184 | 0.100286450137109 | 1.03966976904324 | 0.977770612299197 | 1.10548753978266 | 0.872755742358858 |
| ebi-a-GCST90001651 | finngen_R11_O15_PREECLAMPS | Pre-eclampsia \|\| id:finngen_R11_O15_PREECLAMPS | Granulocyte Absolute Count \|\| id:ebi-a-GCST90001651 | Inverse variance weighted | 24 | 0.00219594436518126 | 0.0245890082546397 | 0.928838777720792 | -0.045998512 | 0.050390400544275 | 1.00219835721685 | 0.955043383471933 | 1.05168159330813 | 0.986667691552896 |
| ebi-a-GCST90001651 | finngen_R11_O15_PREECLAMPS | Pre-eclampsia \|\| id:finngen_R11_O15_PREECLAMPS | Granulocyte Absolute Count \|\| id:ebi-a-GCST90001651 | MR Egger | 24 | 0.02281370521971 | 0.0397204370245377 | 0.571553048822204 | -0.055038351 | 0.100665761787804 | 1.02307592808757 | 0.946448849667883 | 1.10590694362355 | 0.99380795269851 |
| ebi-a-GCST90001651 | finngen_R11_O15_PREECLAMPS | Pre-eclampsia \|\| id:finngen_R11_O15_PREECLAMPS | Granulocyte Absolute Count \|\| id:ebi-a-GCST90001651 | Weighted median | 24 | -0.001033655 | 0.0345592339544916 | 0.97613909196865 | -0.068769754 | 0.0667024431903142 | 0.998966878677193 | 0.93354159963702 | 1.06897734935655 | 0.99075236588769 |
| ebi-a-GCST90001651 | finngen_R11_O15_PREECLAMPS | Pre-eclampsia \|\| id:finngen_R11_O15_PREECLAMPS | Granulocyte Absolute Count \|\| id:ebi-a-GCST90001651 | Weighted mode | 24 | 0.00361856923736348 | 0.0370407004822381 | 0.923023459590907 | -0.068981204 | 0.0762183421825502 | 1.00362512416313 | 0.933344223324051 | 1.07919818292135 | 0.986782054514076 |
| ebi-a-GCST90001652 | finngen_R11_O15_PREECLAMPS | Pre-eclampsia \|\| id:finngen_R11_O15_PREECLAMPS | Granulocyte %leukocyte \|\| id:ebi-a-GCST90001652 | Inverse variance weighted | 25 | 0.00141520701877061 | 0.0191683725823074 | 0.941145403593647 | -0.036154803 | 0.0389852172800931 | 1.00141620889679 | 0.96449097559554 | 1.03975511312794 | 0.986667691552896 |
| ebi-a-GCST90001652 | finngen_R11_O15_PREECLAMPS | Pre-eclampsia \|\| id:finngen_R11_O15_PREECLAMPS | Granulocyte %leukocyte \|\| id:ebi-a-GCST90001652 | MR Egger | 25 | -0.010748323 | 0.0281918339475464 | 0.706512055805651 | -0.066004318 | 0.0445076714213242 | 0.989309233711338 | 0.936126822412056 | 1.04551299725039 | 0.99380795269851 |
| ebi-a-GCST90001652 | finngen_R11_O15_PREECLAMPS | Pre-eclampsia \|\| id:finngen_R11_O15_PREECLAMPS | Granulocyte %leukocyte \|\| id:ebi-a-GCST90001652 | Weighted median | 25 | -0.004346378 | 0.0279538798390449 | 0.8764398433498 | -0.059135983 | 0.0504432261499136 | 0.99566305349798 | 0.94257858594856 | 1.05173715049264 | 0.964912412213677 |
| ebi-a-GCST90001652 | finngen_R11_O15_PREECLAMPS | Pre-eclampsia \|\| id:finngen_R11_O15_PREECLAMPS | Granulocyte %leukocyte \|\| id:ebi-a-GCST90001652 | Weighted mode | 25 | -0.007778614 | 0.0242315735264302 | 0.750980380119291 | -0.055272498 | 0.0397152705305993 | 0.992251561542519 | 0.946227268071633 | 1.04051446687862 | 0.959313411571287 |
| ebi-a-GCST90001653 | finngen_R11_O15_PREECLAMPS | Pre-eclampsia \|\| id:finngen_R11_O15_PREECLAMPS | CD28- CD4-CD8- T cell %CD4-CD8- T cell \|\| id:ebi-a-GCST90001653 | Inverse variance weighted | 27 | 0.007133330708849 | 0.0185029845382228 | 0.699849785190193 | -0.029132519 | 0.0433991804037657 | 1.00715883351627 | 0.971287741870966 | 1.04435469758519 | 0.950911139356934 |
| ebi-a-GCST90001653 | finngen_R11_O15_PREECLAMPS | Pre-eclampsia \|\| id:finngen_R11_O15_PREECLAMPS | CD28- CD4-CD8- T cell %CD4-CD8- T cell \|\| id:ebi-a-GCST90001653 | MR Egger | 27 | -0.006477776 | 0.0270557466085945 | 0.812729422633154 | -0.059507039 | 0.0465514872451498 | 0.993543159454269 | 0.942228900784717 | 1.04765201839623 | 0.99380795269851 |
| ebi-a-GCST90001653 | finngen_R11_O15_PREECLAMPS | Pre-eclampsia \|\| id:finngen_R11_O15_PREECLAMPS | CD28- CD4-CD8- T cell %CD4-CD8- T cell \|\| id:ebi-a-GCST90001653 | Weighted median | 27 | 0.0153847876746415 | 0.0295889127768246 | 0.603097659778618 | -0.042609481 | 0.0733790567172178 | 1.0155037427706 | 0.958285545365272 | 1.07613837709303 | 0.940845681141708 |
| ebi-a-GCST90001653 | finngen_R11_O15_PREECLAMPS | Pre-eclampsia \|\| id:finngen_R11_O15_PREECLAMPS | CD28- CD4-CD8- T cell %CD4-CD8- T cell \|\| id:ebi-a-GCST90001653 | Weighted mode | 27 | 0.0133898801326751 | 0.024761130527358 | 0.593274667573847 | -0.035141936 | 0.0619216959662968 | 1.01347992603007 | 0.965468372101931 | 1.0638790354465 | 0.955353022288269 |
| ebi-a-GCST90001654 | finngen_R11_O15_PREECLAMPS | Pre-eclampsia \|\| id:finngen_R11_O15_PREECLAMPS | CD28- CD4-CD8- T cell Absolute Count \|\| id:ebi-a-GCST90001654 | Inverse variance weighted | 30 | -0.014512347 | 0.0216679238360492 | 0.503009656904801 | -0.056981478 | 0.0279567833274974 | 0.985592449161758 | 0.944611565196173 | 1.02835124154227 | 0.898694924660397 |
| ebi-a-GCST90001654 | finngen_R11_O15_PREECLAMPS | Pre-eclampsia \|\| id:finngen_R11_O15_PREECLAMPS | CD28- CD4-CD8- T cell Absolute Count \|\| id:ebi-a-GCST90001654 | MR Egger | 30 | 0.00384545632953805 | 0.0341858055802441 | 0.911239918925662 | -0.063158723 | 0.0708496352668165 | 1.00385285958332 | 0.938794453959181 | 1.07341980924977 | 0.99380795269851 |
| ebi-a-GCST90001654 | finngen_R11_O15_PREECLAMPS | Pre-eclampsia \|\| id:finngen_R11_O15_PREECLAMPS | CD28- CD4-CD8- T cell Absolute Count \|\| id:ebi-a-GCST90001654 | Weighted median | 30 | 0.0160818505703269 | 0.0324063463461942 | 0.619713658423824 | -0.047434588 | 0.0795982894088675 | 1.0162118595224 | 0.953672852485272 | 1.08285198718071 | 0.940845681141708 |
| ebi-a-GCST90001654 | finngen_R11_O15_PREECLAMPS | Pre-eclampsia \|\| id:finngen_R11_O15_PREECLAMPS | CD28- CD4-CD8- T cell Absolute Count \|\| id:ebi-a-GCST90001654 | Weighted mode | 30 | 0.0171174713769362 | 0.029830456902431 | 0.570510076690852 | -0.041350224 | 0.075585166905701 | 1.01726481480513 | 0.959493033462851 | 1.07851507759862 | 0.94353589606564 |
| ebi-a-GCST90001655 | finngen_R11_O15_PREECLAMPS | Pre-eclampsia \|\| id:finngen_R11_O15_PREECLAMPS | CD28+ CD4-CD8- T cell %T cell \|\| id:ebi-a-GCST90001655 | Inverse variance weighted | 23 | -0.027615898 | 0.0241827696889209 | 0.253468198174411 | -0.075014127 | 0.0197823304711333 | 0.972761934741332 | 0.927730380458461 | 1.01997929744855 | 0.885079385681701 |
| ebi-a-GCST90001655 | finngen_R11_O15_PREECLAMPS | Pre-eclampsia \|\| id:finngen_R11_O15_PREECLAMPS | CD28+ CD4-CD8- T cell %T cell \|\| id:ebi-a-GCST90001655 | MR Egger | 23 | -0.033498535 | 0.0395346913973062 | 0.406370375047159 | -0.11098653 | 0.0439894600514618 | 0.967056327883502 | 0.894950803632929 | 1.04497134088624 | 0.99380795269851 |
| ebi-a-GCST90001655 | finngen_R11_O15_PREECLAMPS | Pre-eclampsia \|\| id:finngen_R11_O15_PREECLAMPS | CD28+ CD4-CD8- T cell %T cell \|\| id:ebi-a-GCST90001655 | Weighted median | 23 | -0.04201599 | 0.0360390221331133 | 0.24367626044395 | -0.112652474 | 0.0286204930676148 | 0.958854448066878 | 0.893461107406634 | 1.02903399482748 | 0.940845681141708 |
| ebi-a-GCST90001655 | finngen_R11_O15_PREECLAMPS | Pre-eclampsia \|\| id:finngen_R11_O15_PREECLAMPS | CD28+ CD4-CD8- T cell %T cell \|\| id:ebi-a-GCST90001655 | Weighted mode | 23 | -0.047713327 | 0.0353917629301785 | 0.191328822342749 | -0.117081182 | 0.0216545283355528 | 0.953407063961086 | 0.8895129774776 | 1.02189068920446 | 0.872755742358858 |
| ebi-a-GCST90001656 | finngen_R11_O15_PREECLAMPS | Pre-eclampsia \|\| id:finngen_R11_O15_PREECLAMPS | CD28+ CD4-CD8- T cell %CD4-CD8- T cell \|\| id:ebi-a-GCST90001656 | Inverse variance weighted | 27 | -0.007133331 | 0.0185029845382228 | 0.699849785190193 | -0.04339918 | 0.0291325189860677 | 0.992892051106499 | 0.957529086920611 | 1.02956102181803 | 0.950911139356934 |
| ebi-a-GCST90001656 | finngen_R11_O15_PREECLAMPS | Pre-eclampsia \|\| id:finngen_R11_O15_PREECLAMPS | CD28+ CD4-CD8- T cell %CD4-CD8- T cell \|\| id:ebi-a-GCST90001656 | MR Egger | 27 | 0.00647777610769535 | 0.0270557466085945 | 0.812729422633154 | -0.046551487 | 0.0595070394605405 | 1.00649880227576 | 0.954515413935652 | 1.06131323202586 | 0.99380795269851 |
| ebi-a-GCST90001656 | finngen_R11_O15_PREECLAMPS | Pre-eclampsia \|\| id:finngen_R11_O15_PREECLAMPS | CD28+ CD4-CD8- T cell %CD4-CD8- T cell \|\| id:ebi-a-GCST90001656 | Weighted median | 27 | -0.015384788 | 0.0288928607908395 | 0.594395575454103 | -0.072014795 | 0.0412452194754038 | 0.984732953589811 | 0.930517128871576 | 1.04210761929944 | 0.940845681141708 |
| ebi-a-GCST90001656 | finngen_R11_O15_PREECLAMPS | Pre-eclampsia \|\| id:finngen_R11_O15_PREECLAMPS | CD28+ CD4-CD8- T cell %CD4-CD8- T cell \|\| id:ebi-a-GCST90001656 | Weighted mode | 27 | -0.01338988 | 0.026448672305912 | 0.616940895705712 | -0.065229278 | 0.0384495175869125 | 0.986699365538627 | 0.936852639190131 | 1.03919826579764 | 0.959313411571287 |
| ebi-a-GCST90001657 | finngen_R11_O15_PREECLAMPS | Pre-eclampsia \|\| id:finngen_R11_O15_PREECLAMPS | CD28+ CD4-CD8- T cell Absolute Count \|\| id:ebi-a-GCST90001657 | Inverse variance weighted | 12 | 0.000314042092912192 | 0.0408235023738136 | 0.993862190858395 | -0.079700023 | 0.0803281067455869 | 1.00031409140929 | 0.923393302003408 | 1.08364255978576 | 0.995223645914365 |
| ebi-a-GCST90001657 | finngen_R11_O15_PREECLAMPS | Pre-eclampsia \|\| id:finngen_R11_O15_PREECLAMPS | CD28+ CD4-CD8- T cell Absolute Count \|\| id:ebi-a-GCST90001657 | MR Egger | 12 | 0.0352802950848476 | 0.0683165856059635 | 0.616785121241313 | -0.098620213 | 0.169180802872536 | 1.03591002859948 | 0.906086762928256 | 1.18433425060193 | 0.99380795269851 |
| ebi-a-GCST90001657 | finngen_R11_O15_PREECLAMPS | Pre-eclampsia \|\| id:finngen_R11_O15_PREECLAMPS | CD28+ CD4-CD8- T cell Absolute Count \|\| id:ebi-a-GCST90001657 | Weighted median | 12 | 0.00261431921940683 | 0.0597821639415925 | 0.965119022985335 | -0.114558722 | 0.119787360544928 | 1.00261773953184 | 0.891759570880424 | 1.12725712675164 | 0.989912389390616 |
| ebi-a-GCST90001657 | finngen_R11_O15_PREECLAMPS | Pre-eclampsia \|\| id:finngen_R11_O15_PREECLAMPS | CD28+ CD4-CD8- T cell Absolute Count \|\| id:ebi-a-GCST90001657 | Weighted mode | 12 | 0.0427455819344036 | 0.0594939217712529 | 0.487438419677908 | -0.073862505 | 0.159353668606059 | 1.04367233197423 | 0.928799390578486 | 1.17275263913568 | 0.933945646747087 |
| ebi-a-GCST90001658 | finngen_R11_O15_PREECLAMPS | Pre-eclampsia \|\| id:finngen_R11_O15_PREECLAMPS | CD39+ CD4+ T cell %T cell \|\| id:ebi-a-GCST90001658 | Inverse variance weighted | 31 | 0.00225471132511826 | 0.0101710380193853 | 0.824563335939192 | -0.017680523 | 0.0221899458431134 | 1.00225725509816 | 0.982474860156682 | 1.0224379738701 | 0.977824840984872 |
| ebi-a-GCST90001658 | finngen_R11_O15_PREECLAMPS | Pre-eclampsia \|\| id:finngen_R11_O15_PREECLAMPS | CD39+ CD4+ T cell %T cell \|\| id:ebi-a-GCST90001658 | MR Egger | 31 | 0.00218503569648515 | 0.0123645585175305 | 0.860958577522161 | -0.022049499 | 0.0264195703908449 | 1.00218742462663 | 0.978191814338293 | 1.02677166109715 | 0.99380795269851 |
| ebi-a-GCST90001658 | finngen_R11_O15_PREECLAMPS | Pre-eclampsia \|\| id:finngen_R11_O15_PREECLAMPS | CD39+ CD4+ T cell %T cell \|\| id:ebi-a-GCST90001658 | Weighted median | 31 | -0.006975699 | 0.0135721609740552 | 0.607272192415238 | -0.033577134 | 0.0196257369292179 | 0.993048575130585 | 0.96698032120849 | 1.01981958778276 | 0.940845681141708 |
| ebi-a-GCST90001658 | finngen_R11_O15_PREECLAMPS | Pre-eclampsia \|\| id:finngen_R11_O15_PREECLAMPS | CD39+ CD4+ T cell %T cell \|\| id:ebi-a-GCST90001658 | Weighted mode | 31 | -0.000563545 | 0.0111733375024608 | 0.960108770774285 | -0.022463287 | 0.021336196044081 | 0.999436613301176 | 0.977787134067114 | 1.02156544017112 | 0.990180645930572 |
| ebi-a-GCST90001659 | finngen_R11_O15_PREECLAMPS | Pre-eclampsia \|\| id:finngen_R11_O15_PREECLAMPS | CD39+ CD4+ T cell %CD4+ T cell \|\| id:ebi-a-GCST90001659 | Inverse variance weighted | 25 | -0.003202982 | 0.0128338382040983 | 0.80291742841177 | -0.028357305 | 0.0219513408488845 | 0.996802142043565 | 0.972040989725677 | 1.02219404416582 | 0.977824840984872 |
| ebi-a-GCST90001659 | finngen_R11_O15_PREECLAMPS | Pre-eclampsia \|\| id:finngen_R11_O15_PREECLAMPS | CD39+ CD4+ T cell %CD4+ T cell \|\| id:ebi-a-GCST90001659 | MR Egger | 25 | 0.0150458871124493 | 0.0169908693948773 | 0.385036881808934 | -0.018256217 | 0.0483479911264087 | 1.0151596462918 | 0.981909418337065 | 1.04953582093612 | 0.99380795269851 |
| ebi-a-GCST90001659 | finngen_R11_O15_PREECLAMPS | Pre-eclampsia \|\| id:finngen_R11_O15_PREECLAMPS | CD39+ CD4+ T cell %CD4+ T cell \|\| id:ebi-a-GCST90001659 | Weighted median | 25 | 0.00345540038046804 | 0.0174376039230887 | 0.84292146213549 | -0.030722303 | 0.0376331040697219 | 1.00346137715843 | 0.969744830619116 | 1.03835019652112 | 0.964912412213677 |
| ebi-a-GCST90001659 | finngen_R11_O15_PREECLAMPS | Pre-eclampsia \|\| id:finngen_R11_O15_PREECLAMPS | CD39+ CD4+ T cell %CD4+ T cell \|\| id:ebi-a-GCST90001659 | Weighted mode | 25 | 0.00283147353398816 | 0.0154708558651691 | 0.856319778521111 | -0.027491404 | 0.0331543510297197 | 1.00283548594129 | 0.972883045457388 | 1.03371008114372 | 0.977585971930092 |
| ebi-a-GCST90001660 | finngen_R11_O15_PREECLAMPS | Pre-eclampsia \|\| id:finngen_R11_O15_PREECLAMPS | CD39+ CD4+ T cell Absolute Count \|\| id:ebi-a-GCST90001660 | Inverse variance weighted | 29 | 0.0153247280239675 | 0.012307624275318 | 0.213079912736981 | -0.008798216 | 0.0394476716035908 | 1.01544275380206 | 0.991240375482471 | 1.04023606357562 | 0.855687495048373 |
| ebi-a-GCST90001660 | finngen_R11_O15_PREECLAMPS | Pre-eclampsia \|\| id:finngen_R11_O15_PREECLAMPS | CD39+ CD4+ T cell Absolute Count \|\| id:ebi-a-GCST90001660 | MR Egger | 29 | 0.0225838387908349 | 0.0170819809160485 | 0.197231510919363 | -0.010896844 | 0.05606452138629 | 1.02284078430437 | 0.989162311733304 | 1.05766592360675 | 0.99380795269851 |
| ebi-a-GCST90001660 | finngen_R11_O15_PREECLAMPS | Pre-eclampsia \|\| id:finngen_R11_O15_PREECLAMPS | CD39+ CD4+ T cell Absolute Count \|\| id:ebi-a-GCST90001660 | Weighted median | 29 | 0.0291449285731096 | 0.0174924204211649 | 0.095684312655569 | -0.005140215 | 0.0634300725985928 | 1.02957379832442 | 0.994872972848496 | 1.06548497660073 | 0.88227178111339 |
| ebi-a-GCST90001660 | finngen_R11_O15_PREECLAMPS | Pre-eclampsia \|\| id:finngen_R11_O15_PREECLAMPS | CD39+ CD4+ T cell Absolute Count \|\| id:ebi-a-GCST90001660 | Weighted mode | 29 | 0.0179228671190887 | 0.0166338923999999 | 0.290456960803538 | -0.014679562 | 0.0505252962230886 | 1.01808444557488 | 0.985427657498936 | 1.05182347017963 | 0.872755742358858 |
| ebi-a-GCST90001661 | finngen_R11_O15_PREECLAMPS | Pre-eclampsia \|\| id:finngen_R11_O15_PREECLAMPS | CD28- CD8dim T cell %T cell \|\| id:ebi-a-GCST90001661 | Inverse variance weighted | 25 | -0.028877062 | 0.0180936201081603 | 0.1104931818377 | -0.064340557 | 0.00658643336332155 | 0.971535895757801 | 0.937685609318443 | 1.00660817161519 | 0.820779257935285 |
| ebi-a-GCST90001661 | finngen_R11_O15_PREECLAMPS | Pre-eclampsia \|\| id:finngen_R11_O15_PREECLAMPS | CD28- CD8dim T cell %T cell \|\| id:ebi-a-GCST90001661 | MR Egger | 25 | -0.017576988 | 0.0265751295841327 | 0.514922951675523 | -0.069664242 | 0.0345102656309838 | 0.982576585799094 | 0.932706930835977 | 1.03511265440611 | 0.99380795269851 |
| ebi-a-GCST90001661 | finngen_R11_O15_PREECLAMPS | Pre-eclampsia \|\| id:finngen_R11_O15_PREECLAMPS | CD28- CD8dim T cell %T cell \|\| id:ebi-a-GCST90001661 | Weighted median | 25 | -0.033122709 | 0.0275704709567911 | 0.229602469032991 | -0.087160832 | 0.0209154138574697 | 0.967419840973237 | 0.916529676209002 | 1.02113567405674 | 0.940845681141708 |
| ebi-a-GCST90001661 | finngen_R11_O15_PREECLAMPS | Pre-eclampsia \|\| id:finngen_R11_O15_PREECLAMPS | CD28- CD8dim T cell %T cell \|\| id:ebi-a-GCST90001661 | Weighted mode | 25 | -0.030773508 | 0.0241774444001703 | 0.215274752332449 | -0.078161299 | 0.0166142831780291 | 0.96969517655482 | 0.92481524285806 | 1.01675306791846 | 0.872755742358858 |
| ebi-a-GCST90001662 | finngen_R11_O15_PREECLAMPS | Pre-eclampsia \|\| id:finngen_R11_O15_PREECLAMPS | CD28- CD8dim T cell %CD8dim T cell \|\| id:ebi-a-GCST90001662 | Inverse variance weighted | 14 | -0.010995504 | 0.00716321575243991 | 0.124784941151411 | -0.025035407 | 0.00304439915516229 | 0.989064725877693 | 0.975275380237191 | 1.00304903804462 | 0.820779257935285 |
| ebi-a-GCST90001662 | finngen_R11_O15_PREECLAMPS | Pre-eclampsia \|\| id:finngen_R11_O15_PREECLAMPS | CD28- CD8dim T cell %CD8dim T cell \|\| id:ebi-a-GCST90001662 | MR Egger | 14 | -0.012008018 | 0.00775141237583791 | 0.147307176662339 | -0.027200787 | 0.00318474977530931 | 0.98806379005911 | 0.973165823115148 | 1.00318982647879 | 0.984103954382898 |
| ebi-a-GCST90001662 | finngen_R11_O15_PREECLAMPS | Pre-eclampsia \|\| id:finngen_R11_O15_PREECLAMPS | CD28- CD8dim T cell %CD8dim T cell \|\| id:ebi-a-GCST90001662 | Weighted median | 14 | -0.009543325 | 0.00832954663910254 | 0.251911186545673 | -0.025869236 | 0.00678258647330943 | 0.990502068071269 | 0.974462505549973 | 1.00680564030501 | 0.940845681141708 |
| ebi-a-GCST90001662 | finngen_R11_O15_PREECLAMPS | Pre-eclampsia \|\| id:finngen_R11_O15_PREECLAMPS | CD28- CD8dim T cell %CD8dim T cell \|\| id:ebi-a-GCST90001662 | Weighted mode | 14 | -0.013140613 | 0.00807184565539223 | 0.127515720373067 | -0.028961431 | 0.00268020428164271 | 0.986945347716397 | 0.97145393205434 | 1.00268379924016 | 0.872755742358858 |
| ebi-a-GCST90001663 | finngen_R11_O15_PREECLAMPS | Pre-eclampsia \|\| id:finngen_R11_O15_PREECLAMPS | CD28- CD8dim T cell Absolute Count \|\| id:ebi-a-GCST90001663 | Inverse variance weighted | 28 | 0.000954151951407674 | 0.0152875670570014 | 0.950233472335377 | -0.029009479 | 0.0309177833831304 | 1.00095460729919 | 0.971407255986998 | 1.03140070212415 | 0.990306563277315 |
| ebi-a-GCST90001663 | finngen_R11_O15_PREECLAMPS | Pre-eclampsia \|\| id:finngen_R11_O15_PREECLAMPS | CD28- CD8dim T cell Absolute Count \|\| id:ebi-a-GCST90001663 | MR Egger | 28 | 0.00175066517662743 | 0.0219370851246858 | 0.937004302306684 | -0.041246022 | 0.0447473520210116 | 1.00175219848555 | 0.959593020229732 | 1.04576361646564 | 0.99380795269851 |
| ebi-a-GCST90001663 | finngen_R11_O15_PREECLAMPS | Pre-eclampsia \|\| id:finngen_R11_O15_PREECLAMPS | CD28- CD8dim T cell Absolute Count \|\| id:ebi-a-GCST90001663 | Weighted median | 28 | 0.00610489130793718 | 0.0243310414977107 | 0.801884020555445 | -0.04158395 | 0.0537937326434501 | 1.00612356413607 | 0.95926880131878 | 1.05526691258821 | 0.959373517227545 |
| ebi-a-GCST90001663 | finngen_R11_O15_PREECLAMPS | Pre-eclampsia \|\| id:finngen_R11_O15_PREECLAMPS | CD28- CD8dim T cell Absolute Count \|\| id:ebi-a-GCST90001663 | Weighted mode | 28 | 0.00574035929782801 | 0.0231627004402782 | 0.806142778629734 | -0.039658534 | 0.0511392521607732 | 1.00575686673135 | 0.96111757251686 | 1.05246944172328 | 0.961305639715923 |
| ebi-a-GCST90001664 | finngen_R11_O15_PREECLAMPS | Pre-eclampsia \|\| id:finngen_R11_O15_PREECLAMPS | CD28+ CD45RA+ CD8dim T cell %T cell \|\| id:ebi-a-GCST90001664 | Inverse variance weighted (multiplicative random effects) | 35 | 0.00552745253158895 | 0.00917674182846298 | 0.546952636721613 | -0.012458961 | 0.0235138665153764 | 1.00554275708273 | 0.987618330084287 | 1.02379249708261 | 0.905435115099076 |
| ebi-a-GCST90001664 | finngen_R11_O15_PREECLAMPS | Pre-eclampsia \|\| id:finngen_R11_O15_PREECLAMPS | CD28+ CD45RA+ CD8dim T cell %T cell \|\| id:ebi-a-GCST90001664 | MR Egger | 35 | 0.00212196838174307 | 0.0105940842362186 | 0.842477228643665 | -0.018642437 | 0.0228863734847316 | 1.00212422134994 | 0.981530258682718 | 1.02315027594158 | 0.99380795269851 |
| ebi-a-GCST90001664 | finngen_R11_O15_PREECLAMPS | Pre-eclampsia \|\| id:finngen_R11_O15_PREECLAMPS | CD28+ CD45RA+ CD8dim T cell %T cell \|\| id:ebi-a-GCST90001664 | Weighted median | 35 | 0.00881771066013748 | 0.00940608974571373 | 0.348528671742002 | -0.009618225 | 0.0272536465617364 | 1.00885670118892 | 0.990427881945426 | 1.02762842412576 | 0.940845681141708 |
| ebi-a-GCST90001664 | finngen_R11_O15_PREECLAMPS | Pre-eclampsia \|\| id:finngen_R11_O15_PREECLAMPS | CD28+ CD45RA+ CD8dim T cell %T cell \|\| id:ebi-a-GCST90001664 | Weighted mode | 35 | 0.00828571955349078 | 0.00757106112281986 | 0.281475213700753 | -0.00655356 | 0.0231249993542177 | 1.00832014113125 | 0.993467867493837 | 1.02339445519837 | 0.872755742358858 |
| ebi-a-GCST90001665 | finngen_R11_O15_PREECLAMPS | Pre-eclampsia \|\| id:finngen_R11_O15_PREECLAMPS | CD28+ CD45RA+ CD8dim T cell %CD8dim T cell \|\| id:ebi-a-GCST90001665 | Inverse variance weighted | 26 | 0.0103129346570354 | 0.0112511266238228 | 0.359345202811652 | -0.011739274 | 0.0323651428397282 | 1.0103662962481 | 0.988329362902657 | 1.03289459051987 | 0.885079385681701 |
| ebi-a-GCST90001665 | finngen_R11_O15_PREECLAMPS | Pre-eclampsia \|\| id:finngen_R11_O15_PREECLAMPS | CD28+ CD45RA+ CD8dim T cell %CD8dim T cell \|\| id:ebi-a-GCST90001665 | MR Egger | 26 | 0.00486770551831208 | 0.0132994676664295 | 0.717565986236491 | -0.021199251 | 0.0309346621445139 | 1.00487957204326 | 0.9790238735426 | 1.03141811103741 | 0.99380795269851 |
| ebi-a-GCST90001665 | finngen_R11_O15_PREECLAMPS | Pre-eclampsia \|\| id:finngen_R11_O15_PREECLAMPS | CD28+ CD45RA+ CD8dim T cell %CD8dim T cell \|\| id:ebi-a-GCST90001665 | Weighted median | 26 | 0.00381258401266425 | 0.0160038655035023 | 0.811703527546601 | -0.027554992 | 0.0351801603995287 | 1.00381986115639 | 0.972821183335899 | 1.03580630326808 | 0.961297867845628 |
| ebi-a-GCST90001665 | finngen_R11_O15_PREECLAMPS | Pre-eclampsia \|\| id:finngen_R11_O15_PREECLAMPS | CD28+ CD45RA+ CD8dim T cell %CD8dim T cell \|\| id:ebi-a-GCST90001665 | Weighted mode | 26 | 0.00393676237473406 | 0.0128834475385762 | 0.762463874313157 | -0.021314795 | 0.0291883195503435 | 1.00394452160247 | 0.978910760043631 | 1.02961847350691 | 0.959313411571287 |
| ebi-a-GCST90001666 | finngen_R11_O15_PREECLAMPS | Pre-eclampsia \|\| id:finngen_R11_O15_PREECLAMPS | CD28+ CD45RA+ CD8dim T cell Absolute Count \|\| id:ebi-a-GCST90001666 | Inverse variance weighted | 41 | -0.003857913 | 0.00277268004485245 | 0.164103512423202 | -0.009292366 | 0.00157653990668168 | 0.996149519204409 | 0.99075067474304 | 1.00157778329905 | 0.85505992597664 |
| ebi-a-GCST90001666 | finngen_R11_O15_PREECLAMPS | Pre-eclampsia \|\| id:finngen_R11_O15_PREECLAMPS | CD28+ CD45RA+ CD8dim T cell Absolute Count \|\| id:ebi-a-GCST90001666 | MR Egger | 41 | -0.004217487 | 0.00296441187625713 | 0.162771316286846 | -0.010027734 | 0.0015927600817599 | 0.99579139391371 | 0.990022375619389 | 1.00159402919781 | 0.99380795269851 |
| ebi-a-GCST90001666 | finngen_R11_O15_PREECLAMPS | Pre-eclampsia \|\| id:finngen_R11_O15_PREECLAMPS | CD28+ CD45RA+ CD8dim T cell Absolute Count \|\| id:ebi-a-GCST90001666 | Weighted median | 41 | -0.001968455 | 0.00345742545102202 | 0.569124476968012 | -0.008745009 | 0.00480809843990517 | 0.998033480693711 | 0.991293117046528 | 1.00481967589295 | 0.940845681141708 |
| ebi-a-GCST90001666 | finngen_R11_O15_PREECLAMPS | Pre-eclampsia \|\| id:finngen_R11_O15_PREECLAMPS | CD28+ CD45RA+ CD8dim T cell Absolute Count \|\| id:ebi-a-GCST90001666 | Weighted mode | 41 | -0.003027258 | 0.00313896440962158 | 0.340633520901998 | -0.009179628 | 0.00312511199584568 | 0.996977319278952 | 0.990862375673936 | 1.00313000024913 | 0.888599269773114 |
| ebi-a-GCST90001667 | finngen_R11_O15_PREECLAMPS | Pre-eclampsia \|\| id:finngen_R11_O15_PREECLAMPS | CD28+ CD45RA- CD8dim T cell %T cell \|\| id:ebi-a-GCST90001667 | Inverse variance weighted | 28 | 0.00319400451886457 | 0.00638547165602135 | 0.616935184928524 | -0.00932152 | 0.0157095289646664 | 1.00319911078633 | 0.99072179076165 | 1.01583357231779 | 0.927459753639562 |
| ebi-a-GCST90001667 | finngen_R11_O15_PREECLAMPS | Pre-eclampsia \|\| id:finngen_R11_O15_PREECLAMPS | CD28+ CD45RA- CD8dim T cell %T cell \|\| id:ebi-a-GCST90001667 | MR Egger | 28 | 0.00101158708282936 | 0.00722012592126655 | 0.889655544400327 | -0.01313986 | 0.0151630338885118 | 1.00101209890961 | 0.98694609136033 | 1.01527857593753 | 0.99380795269851 |
| ebi-a-GCST90001667 | finngen_R11_O15_PREECLAMPS | Pre-eclampsia \|\| id:finngen_R11_O15_PREECLAMPS | CD28+ CD45RA- CD8dim T cell %T cell \|\| id:ebi-a-GCST90001667 | Weighted median | 28 | 0.00260741045672173 | 0.00856889597182244 | 0.760908643128989 | -0.014187626 | 0.0194024465614937 | 1.00261081270775 | 0.985912544427979 | 1.01959189731348 | 0.957984984509652 |
| ebi-a-GCST90001667 | finngen_R11_O15_PREECLAMPS | Pre-eclampsia \|\| id:finngen_R11_O15_PREECLAMPS | CD28+ CD45RA- CD8dim T cell %T cell \|\| id:ebi-a-GCST90001667 | Weighted mode | 28 | -0.000412989 | 0.00649288886110685 | 0.949752282588907 | -0.013139051 | 0.0123130734738779 | 0.9995870965742 | 0.986946889663045 | 1.01238919145768 | 0.990180645930572 |
| ebi-a-GCST90001668 | finngen_R11_O15_PREECLAMPS | Pre-eclampsia \|\| id:finngen_R11_O15_PREECLAMPS | CD28+ CD45RA- CD8dim T cell %CD8dim T cell \|\| id:ebi-a-GCST90001668 | Inverse variance weighted | 27 | -0.006478301 | 0.0120238825635538 | 0.590034467380461 | -0.03004511 | 0.0170885092329307 | 0.993542638356976 | 0.970401757333979 | 1.01723535306172 | 0.91586635420875 |
| ebi-a-GCST90001668 | finngen_R11_O15_PREECLAMPS | Pre-eclampsia \|\| id:finngen_R11_O15_PREECLAMPS | CD28+ CD45RA- CD8dim T cell %CD8dim T cell \|\| id:ebi-a-GCST90001668 | MR Egger | 27 | -0.001344854 | 0.0147708378566783 | 0.928180101356881 | -0.030295697 | 0.0276059878328951 | 0.998656049545184 | 0.970158618559422 | 1.02799056382563 | 0.99380795269851 |
| ebi-a-GCST90001668 | finngen_R11_O15_PREECLAMPS | Pre-eclampsia \|\| id:finngen_R11_O15_PREECLAMPS | CD28+ CD45RA- CD8dim T cell %CD8dim T cell \|\| id:ebi-a-GCST90001668 | Weighted median | 27 | -0.018227783 | 0.0189770374384474 | 0.336794663336217 | -0.055422776 | 0.0189672106195536 | 0.98193733848555 | 0.946085081192159 | 1.01914823083046 | 0.940845681141708 |
| ebi-a-GCST90001668 | finngen_R11_O15_PREECLAMPS | Pre-eclampsia \|\| id:finngen_R11_O15_PREECLAMPS | CD28+ CD45RA- CD8dim T cell %CD8dim T cell \|\| id:ebi-a-GCST90001668 | Weighted mode | 27 | -0.005759169 | 0.0172740033731856 | 0.741505500626913 | -0.039616215 | 0.0280978777994513 | 0.994257383409786 | 0.961158246087075 | 1.02849634645419 | 0.959313411571287 |
| ebi-a-GCST90001669 | finngen_R11_O15_PREECLAMPS | Pre-eclampsia \|\| id:finngen_R11_O15_PREECLAMPS | CD28+ CD45RA- CD8dim T cell Absolute Count \|\| id:ebi-a-GCST90001669 | Inverse variance weighted | 37 | 0.0019297506487538 | 0.00346728730714349 | 0.577828636524313 | -0.004866132 | 0.00872563377075505 | 1.00193161381583 | 0.995145687968325 | 1.00876381307853 | 0.915399120505076 |
| ebi-a-GCST90001669 | finngen_R11_O15_PREECLAMPS | Pre-eclampsia \|\| id:finngen_R11_O15_PREECLAMPS | CD28+ CD45RA- CD8dim T cell Absolute Count \|\| id:ebi-a-GCST90001669 | MR Egger | 37 | -0.002194383 | 0.00383442421462702 | 0.570786946057014 | -0.009709855 | 0.00532108836003276 | 0.997808022797817 | 0.990337133869905 | 1.00533527049434 | 0.99380795269851 |
| ebi-a-GCST90001669 | finngen_R11_O15_PREECLAMPS | Pre-eclampsia \|\| id:finngen_R11_O15_PREECLAMPS | CD28+ CD45RA- CD8dim T cell Absolute Count \|\| id:ebi-a-GCST90001669 | Weighted median | 37 | -0.001252343 | 0.00466309275650908 | 0.788264483130646 | -0.010392005 | 0.00788731850364754 | 0.998748440555507 | 0.989661805222785 | 1.00791850533982 | 0.957984984509652 |
| ebi-a-GCST90001669 | finngen_R11_O15_PREECLAMPS | Pre-eclampsia \|\| id:finngen_R11_O15_PREECLAMPS | CD28+ CD45RA- CD8dim T cell Absolute Count \|\| id:ebi-a-GCST90001669 | Weighted mode | 37 | 0.000497069977681397 | 0.00374320571934517 | 0.895096907451141 | -0.006839613 | 0.00783375318759793 | 1.00049719353743 | 0.99318372368687 | 1.00786451731248 | 0.98361388732834 |
| ebi-a-GCST90001670 | finngen_R11_O15_PREECLAMPS | Pre-eclampsia \|\| id:finngen_R11_O15_PREECLAMPS | CD39+ CD8+ T cell %T cell \|\| id:ebi-a-GCST90001670 | Inverse variance weighted | 20 | 0.0259848897279301 | 0.0237096171772182 | 0.273094476615565 | -0.02048596 | 0.0724557393952778 | 1.02632544029964 | 0.979722451740531 | 1.0751452184595 | 0.885079385681701 |
| ebi-a-GCST90001670 | finngen_R11_O15_PREECLAMPS | Pre-eclampsia \|\| id:finngen_R11_O15_PREECLAMPS | CD39+ CD8+ T cell %T cell \|\| id:ebi-a-GCST90001670 | MR Egger | 20 | 0.0236288362057653 | 0.0382540822520231 | 0.544521520226507 | -0.051349165 | 0.0986068374197306 | 1.02391020895554 | 0.94994692439308 | 1.1036323073241 | 0.99380795269851 |
| ebi-a-GCST90001670 | finngen_R11_O15_PREECLAMPS | Pre-eclampsia \|\| id:finngen_R11_O15_PREECLAMPS | CD39+ CD8+ T cell %T cell \|\| id:ebi-a-GCST90001670 | Weighted median | 20 | 0.0131562422947192 | 0.0333510133761854 | 0.693228139155189 | -0.052211744 | 0.0785242285120426 | 1.01324316643041 | 0.949127873505093 | 1.08168956257339 | 0.946704357535493 |
| ebi-a-GCST90001670 | finngen_R11_O15_PREECLAMPS | Pre-eclampsia \|\| id:finngen_R11_O15_PREECLAMPS | CD39+ CD8+ T cell %T cell \|\| id:ebi-a-GCST90001670 | Weighted mode | 20 | 0.000546597231652402 | 0.0339885572693401 | 0.987336805343756 | -0.066070975 | 0.067164169479559 | 1.00054674664314 | 0.936064424746112 | 1.06947103826716 | 0.994159530659367 |
| ebi-a-GCST90001671 | finngen_R11_O15_PREECLAMPS | Pre-eclampsia \|\| id:finngen_R11_O15_PREECLAMPS | CD39+ CD8+ T cell %CD8+ T cell \|\| id:ebi-a-GCST90001671 | Inverse variance weighted | 21 | -0.014955064 | 0.0169587528306549 | 0.37785833052507 | -0.048194219 | 0.0182840919616565 | 0.98515620799522 | 0.952948688233756 | 1.01845226939801 | 0.885079385681701 |
| ebi-a-GCST90001671 | finngen_R11_O15_PREECLAMPS | Pre-eclampsia \|\| id:finngen_R11_O15_PREECLAMPS | CD39+ CD8+ T cell %CD8+ T cell \|\| id:ebi-a-GCST90001671 | MR Egger | 21 | -0.01502605 | 0.0237819908970669 | 0.535030449483985 | -0.061638752 | 0.0315866519961076 | 0.985086277611557 | 0.940222478637733 | 1.03209080444937 | 0.99380795269851 |
| ebi-a-GCST90001671 | finngen_R11_O15_PREECLAMPS | Pre-eclampsia \|\| id:finngen_R11_O15_PREECLAMPS | CD39+ CD8+ T cell %CD8+ T cell \|\| id:ebi-a-GCST90001671 | Weighted median | 21 | -0.008152933 | 0.0248917885475869 | 0.743263256363491 | -0.056940838 | 0.0406349729673344 | 0.99188021243141 | 0.944649954962494 | 1.04147187076512 | 0.957984984509652 |
| ebi-a-GCST90001671 | finngen_R11_O15_PREECLAMPS | Pre-eclampsia \|\| id:finngen_R11_O15_PREECLAMPS | CD39+ CD8+ T cell %CD8+ T cell \|\| id:ebi-a-GCST90001671 | Weighted mode | 21 | -0.014300877 | 0.0217270436832508 | 0.517911200791575 | -0.056885883 | 0.0282841286310973 | 0.985800894833528 | 0.944701870129785 | 1.02868792259412 | 0.934398527469092 |
| ebi-a-GCST90001672 | finngen_R11_O15_PREECLAMPS | Pre-eclampsia \|\| id:finngen_R11_O15_PREECLAMPS | CD39+ CD8+ T cell Absolute Count \|\| id:ebi-a-GCST90001672 | Inverse variance weighted | 24 | 0.0196420457031636 | 0.0173522725054585 | 0.257652043432366 | -0.014368408 | 0.0536524998138623 | 1.01983621992551 | 0.985734324547612 | 1.05511788478023 | 0.885079385681701 |
| ebi-a-GCST90001672 | finngen_R11_O15_PREECLAMPS | Pre-eclampsia \|\| id:finngen_R11_O15_PREECLAMPS | CD39+ CD8+ T cell Absolute Count \|\| id:ebi-a-GCST90001672 | MR Egger | 24 | 0.00907178842915769 | 0.0249606120151034 | 0.719742223745642 | -0.039851011 | 0.0579945879787603 | 1.00911306181521 | 0.960932596758532 | 1.05970926052574 | 0.99380795269851 |
| ebi-a-GCST90001672 | finngen_R11_O15_PREECLAMPS | Pre-eclampsia \|\| id:finngen_R11_O15_PREECLAMPS | CD39+ CD8+ T cell Absolute Count \|\| id:ebi-a-GCST90001672 | Weighted median | 24 | 0.0139596260421116 | 0.0256850441950954 | 0.586790829471154 | -0.036383061 | 0.0643023126644986 | 1.01405751659652 | 0.964270848576952 | 1.06641474071675 | 0.940845681141708 |
| ebi-a-GCST90001672 | finngen_R11_O15_PREECLAMPS | Pre-eclampsia \|\| id:finngen_R11_O15_PREECLAMPS | CD39+ CD8+ T cell Absolute Count \|\| id:ebi-a-GCST90001672 | Weighted mode | 24 | 0.00524855756701764 | 0.0241636036536233 | 0.82996058852105 | -0.042112106 | 0.0526092207281194 | 1.00526235537425 | 0.958762291931236 | 1.05401767637005 | 0.975403842779562 |
| ebi-a-GCST90001673 | finngen_R11_O15_PREECLAMPS | Pre-eclampsia \|\| id:finngen_R11_O15_PREECLAMPS | CD28- CD127- CD25++ CD8+ T cell %T cell \|\| id:ebi-a-GCST90001673 | Inverse variance weighted | 18 | 0.0211300629021482 | 0.0192462779557118 | 0.272257816730746 | -0.016592642 | 0.0588527676953434 | 1.02135488337936 | 0.98354425776993 | 1.06061907185358 | 0.885079385681701 |
| ebi-a-GCST90001673 | finngen_R11_O15_PREECLAMPS | Pre-eclampsia \|\| id:finngen_R11_O15_PREECLAMPS | CD28- CD127- CD25++ CD8+ T cell %T cell \|\| id:ebi-a-GCST90001673 | MR Egger | 18 | 0.0101224265499493 | 0.0249507828354171 | 0.690339742267034 | -0.038781108 | 0.059025960907367 | 1.01017383161116 | 0.961961251910004 | 1.06080277978539 | 0.99380795269851 |
| ebi-a-GCST90001673 | finngen_R11_O15_PREECLAMPS | Pre-eclampsia \|\| id:finngen_R11_O15_PREECLAMPS | CD28- CD127- CD25++ CD8+ T cell %T cell \|\| id:ebi-a-GCST90001673 | Weighted median | 18 | 0.0159273121202035 | 0.0245046181197303 | 0.515710401236937 | -0.032101739 | 0.063956363634875 | 1.01605482785066 | 0.968408051818288 | 1.06604587937931 | 0.940845681141708 |
| ebi-a-GCST90001673 | finngen_R11_O15_PREECLAMPS | Pre-eclampsia \|\| id:finngen_R11_O15_PREECLAMPS | CD28- CD127- CD25++ CD8+ T cell %T cell \|\| id:ebi-a-GCST90001673 | Weighted mode | 18 | 0.0149205834289113 | 0.0229429401225488 | 0.524168627802696 | -0.030047579 | 0.0598887460691069 | 1.01503245101793 | 0.970399361613848 | 1.06171841962675 | 0.935508728341156 |
| ebi-a-GCST90001674 | finngen_R11_O15_PREECLAMPS | Pre-eclampsia \|\| id:finngen_R11_O15_PREECLAMPS | CD28- CD127- CD25++ CD8+ T cell %CD8+ T cell \|\| id:ebi-a-GCST90001674 | Inverse variance weighted | 18 | 0.0113850603849447 | 0.0167424593217204 | 0.496497349185167 | -0.02143016 | 0.0442002806555166 | 1.01145011684107 | 0.978797834434795 | 1.04519166559923 | 0.898694924660397 |
| ebi-a-GCST90001674 | finngen_R11_O15_PREECLAMPS | Pre-eclampsia \|\| id:finngen_R11_O15_PREECLAMPS | CD28- CD127- CD25++ CD8+ T cell %CD8+ T cell \|\| id:ebi-a-GCST90001674 | MR Egger | 18 | 0.0213060081292888 | 0.022416542525413 | 0.356016461941937 | -0.022630415 | 0.0652424314790983 | 1.02153460170613 | 0.977623731864622 | 1.06741776868752 | 0.99380795269851 |
| ebi-a-GCST90001674 | finngen_R11_O15_PREECLAMPS | Pre-eclampsia \|\| id:finngen_R11_O15_PREECLAMPS | CD28- CD127- CD25++ CD8+ T cell %CD8+ T cell \|\| id:ebi-a-GCST90001674 | Weighted median | 18 | 0.0154193901441054 | 0.022205385538252 | 0.487432228719405 | -0.028103166 | 0.0589419457990792 | 1.0155388823158 | 0.97228805503307 | 1.06071366006873 | 0.940845681141708 |
| ebi-a-GCST90001674 | finngen_R11_O15_PREECLAMPS | Pre-eclampsia \|\| id:finngen_R11_O15_PREECLAMPS | CD28- CD127- CD25++ CD8+ T cell %CD8+ T cell \|\| id:ebi-a-GCST90001674 | Weighted mode | 18 | 0.0154265411991229 | 0.0194743992850162 | 0.439191024488019 | -0.022743281 | 0.0535963637977547 | 1.01554614451619 | 0.977513397436148 | 1.0550586563281 | 0.933945646747087 |
| ebi-a-GCST90001675 | finngen_R11_O15_PREECLAMPS | Pre-eclampsia \|\| id:finngen_R11_O15_PREECLAMPS | CD28- CD127- CD25++ CD8+ T cell Absolute Count \|\| id:ebi-a-GCST90001675 | Inverse variance weighted | 30 | 0.000145590454369605 | 0.0130556948865137 | 0.991102582499154 | -0.025443572 | 0.0257347524319364 | 1.00014560105317 | 0.97487738825977 | 1.02606875013443 | 0.995223645914365 |
| ebi-a-GCST90001675 | finngen_R11_O15_PREECLAMPS | Pre-eclampsia \|\| id:finngen_R11_O15_PREECLAMPS | CD28- CD127- CD25++ CD8+ T cell Absolute Count \|\| id:ebi-a-GCST90001675 | MR Egger | 30 | -0.005068247 | 0.0166107395820048 | 0.762532401346216 | -0.037625297 | 0.0274888021807457 | 0.994944574495212 | 0.963073739928881 | 1.02787010513916 | 0.99380795269851 |
| ebi-a-GCST90001675 | finngen_R11_O15_PREECLAMPS | Pre-eclampsia \|\| id:finngen_R11_O15_PREECLAMPS | CD28- CD127- CD25++ CD8+ T cell Absolute Count \|\| id:ebi-a-GCST90001675 | Weighted median | 30 | -0.007975286 | 0.0206843489005424 | 0.699814396153971 | -0.048516609 | 0.0325660382519472 | 0.992056432620416 | 0.952641516334058 | 1.03310211514908 | 0.946704357535493 |
| ebi-a-GCST90001675 | finngen_R11_O15_PREECLAMPS | Pre-eclampsia \|\| id:finngen_R11_O15_PREECLAMPS | CD28- CD127- CD25++ CD8+ T cell Absolute Count \|\| id:ebi-a-GCST90001675 | Weighted mode | 30 | 0.000225554438693099 | 0.0158163389659843 | 0.988719533227579 | -0.03077447 | 0.0312255788120223 | 1.00022557987801 | 0.969694243622855 | 1.03171821140706 | 0.994159530659367 |
| ebi-a-GCST90001676 | finngen_R11_O15_PREECLAMPS | Pre-eclampsia \|\| id:finngen_R11_O15_PREECLAMPS | CD28- CD25++ CD8+ T cell %T cell \|\| id:ebi-a-GCST90001676 | Inverse variance weighted (multiplicative random effects) | 27 | -0.022429053 | 0.0346357112006643 | 0.517263009690422 | -0.090315047 | 0.0454569412943461 | 0.977820608504644 | 0.91364329969854 | 1.04650594245246 | 0.898694924660397 |
| ebi-a-GCST90001676 | finngen_R11_O15_PREECLAMPS | Pre-eclampsia \|\| id:finngen_R11_O15_PREECLAMPS | CD28- CD25++ CD8+ T cell %T cell \|\| id:ebi-a-GCST90001676 | MR Egger | 27 | -0.055284373 | 0.0721926689835396 | 0.450971805477477 | -0.196782004 | 0.0862132586767265 | 0.946216031842824 | 0.821369669314375 | 1.09003876374415 | 0.99380795269851 |
| ebi-a-GCST90001676 | finngen_R11_O15_PREECLAMPS | Pre-eclampsia \|\| id:finngen_R11_O15_PREECLAMPS | CD28- CD25++ CD8+ T cell %T cell \|\| id:ebi-a-GCST90001676 | Weighted median | 27 | -0.01832485 | 0.0394030189487841 | 0.641886993250401 | -0.095554767 | 0.0589050667987543 | 0.981842028829165 | 0.908868583848157 | 1.06067454273062 | 0.940845681141708 |
| ebi-a-GCST90001676 | finngen_R11_O15_PREECLAMPS | Pre-eclampsia \|\| id:finngen_R11_O15_PREECLAMPS | CD28- CD25++ CD8+ T cell %T cell \|\| id:ebi-a-GCST90001676 | Weighted mode | 27 | -0.028623766 | 0.0547487753329755 | 0.60552650714271 | -0.135931366 | 0.078683833560365 | 0.971782013040639 | 0.872902541448501 | 1.08186221946638 | 0.955353022288269 |
| ebi-a-GCST90001677 | finngen_R11_O15_PREECLAMPS | Pre-eclampsia \|\| id:finngen_R11_O15_PREECLAMPS | CD28- CD25++ CD8+ T cell %CD8+ T cell \|\| id:ebi-a-GCST90001677 | Inverse variance weighted | 25 | 0.0160607407499775 | 0.0280926431967837 | 0.567520974052271 | -0.03900084 | 0.0711223214156736 | 1.01619040769903 | 0.961749901357168 | 1.07371255587582 | 0.910672367259773 |
| ebi-a-GCST90001677 | finngen_R11_O15_PREECLAMPS | Pre-eclampsia \|\| id:finngen_R11_O15_PREECLAMPS | CD28- CD25++ CD8+ T cell %CD8+ T cell \|\| id:ebi-a-GCST90001677 | MR Egger | 25 | 0.00598262388130639 | 0.0452462619652444 | 0.8959573596851 | -0.08270005 | 0.0946652973331853 | 1.00600055551714 | 0.920627248348213 | 1.09929085796296 | 0.99380795269851 |
| ebi-a-GCST90001677 | finngen_R11_O15_PREECLAMPS | Pre-eclampsia \|\| id:finngen_R11_O15_PREECLAMPS | CD28- CD25++ CD8+ T cell %CD8+ T cell \|\| id:ebi-a-GCST90001677 | Weighted median | 25 | 0.0306533981877473 | 0.0368191687974865 | 0.4051047095352 | -0.041512173 | 0.102818969030821 | 1.03112805109203 | 0.95933765758375 | 1.10829075596466 | 0.940845681141708 |
| ebi-a-GCST90001677 | finngen_R11_O15_PREECLAMPS | Pre-eclampsia \|\| id:finngen_R11_O15_PREECLAMPS | CD28- CD25++ CD8+ T cell %CD8+ T cell \|\| id:ebi-a-GCST90001677 | Weighted mode | 25 | 0.0322208867452458 | 0.0399790807243848 | 0.428189137778495 | -0.046138111 | 0.11057988496504 | 1.03274559992689 | 0.954910069045249 | 1.11692557104852 | 0.933945646747087 |
| ebi-a-GCST90001678 | finngen_R11_O15_PREECLAMPS | Pre-eclampsia \|\| id:finngen_R11_O15_PREECLAMPS | CD28- CD25++ CD8+ T cell Absolute Count \|\| id:ebi-a-GCST90001678 | Inverse variance weighted | 24 | -0.011043738 | 0.0153281190741672 | 0.471224109713024 | -0.041086851 | 0.0189993756803353 | 0.989017020494652 | 0.95974577136121 | 1.01918101232246 | 0.898694924660397 |
| ebi-a-GCST90001678 | finngen_R11_O15_PREECLAMPS | Pre-eclampsia \|\| id:finngen_R11_O15_PREECLAMPS | CD28- CD25++ CD8+ T cell Absolute Count \|\| id:ebi-a-GCST90001678 | MR Egger | 24 | -0.009840388 | 0.0197366238138426 | 0.623020019422748 | -0.048524171 | 0.028843394491643 | 0.990207870013462 | 0.952634313038257 | 1.029263394 | 0.99380795269851 |
| ebi-a-GCST90001678 | finngen_R11_O15_PREECLAMPS | Pre-eclampsia \|\| id:finngen_R11_O15_PREECLAMPS | CD28- CD25++ CD8+ T cell Absolute Count \|\| id:ebi-a-GCST90001678 | Weighted median | 24 | -0.009822711 | 0.0224739310256599 | 0.662059607626472 | -0.053871616 | 0.034226193544149 | 0.990225373990841 | 0.947553749361266 | 1.03481864955553 | 0.940845681141708 |
| ebi-a-GCST90001678 | finngen_R11_O15_PREECLAMPS | Pre-eclampsia \|\| id:finngen_R11_O15_PREECLAMPS | CD28- CD25++ CD8+ T cell Absolute Count \|\| id:ebi-a-GCST90001678 | Weighted mode | 24 | -0.009635061 | 0.0179928475321278 | 0.597448851985834 | -0.044901042 | 0.02563092066095 | 0.990411207974325 | 0.956092090433252 | 1.0259622171298 | 0.955353022288269 |
| ebi-a-GCST90001679 | finngen_R11_O15_PREECLAMPS | Pre-eclampsia \|\| id:finngen_R11_O15_PREECLAMPS | CD25++ CD8+ T cell %T cell \|\| id:ebi-a-GCST90001679 | Inverse variance weighted (multiplicative random effects) | 17 | 0.0244829699529312 | 0.0340855662173244 | 0.472584850179411 | -0.04232474 | 0.091290679738887 | 1.02478513881944 | 0.958558447913843 | 1.09558742404363 | 0.898694924660397 |
| ebi-a-GCST90001679 | finngen_R11_O15_PREECLAMPS | Pre-eclampsia \|\| id:finngen_R11_O15_PREECLAMPS | CD25++ CD8+ T cell %T cell \|\| id:ebi-a-GCST90001679 | MR Egger | 17 | -0.002125656 | 0.0578978379394694 | 0.971197263362972 | -0.115605418 | 0.111354106264433 | 0.997876601510079 | 0.890826657714146 | 1.11779065345486 | 0.99380795269851 |
| ebi-a-GCST90001679 | finngen_R11_O15_PREECLAMPS | Pre-eclampsia \|\| id:finngen_R11_O15_PREECLAMPS | CD25++ CD8+ T cell %T cell \|\| id:ebi-a-GCST90001679 | Weighted median | 17 | 0.0233768291834168 | 0.0402766395838669 | 0.561640426224711 | -0.055565384 | 0.102319042767796 | 1.0236522089029 | 0.945950171263077 | 1.10773683078109 | 0.940845681141708 |
| ebi-a-GCST90001679 | finngen_R11_O15_PREECLAMPS | Pre-eclampsia \|\| id:finngen_R11_O15_PREECLAMPS | CD25++ CD8+ T cell %T cell \|\| id:ebi-a-GCST90001679 | Weighted mode | 17 | -0.082282775 | 0.0572543180599133 | 0.169944844404097 | -0.194501239 | 0.0299356882851005 | 0.921011482744536 | 0.823245158658728 | 1.03038826578624 | 0.872755742358858 |
| ebi-a-GCST90001680 | finngen_R11_O15_PREECLAMPS | Pre-eclampsia \|\| id:finngen_R11_O15_PREECLAMPS | CD25++ CD8+ T cell %CD8+ T cell \|\| id:ebi-a-GCST90001680 | Inverse variance weighted (multiplicative random effects) | 22 | 0.00623464574997926 | 0.0313475078145823 | 0.842350291334815 | -0.05520647 | 0.0676757610665606 | 1.00625412160778 | 0.946289747747983 | 1.07001831063091 | 0.977824840984872 |
| ebi-a-GCST90001680 | finngen_R11_O15_PREECLAMPS | Pre-eclampsia \|\| id:finngen_R11_O15_PREECLAMPS | CD25++ CD8+ T cell %CD8+ T cell \|\| id:ebi-a-GCST90001680 | MR Egger | 22 | 0.0353952416550242 | 0.0509403487550683 | 0.495151320511377 | -0.064447842 | 0.135238325214958 | 1.03602910974813 | 0.937585015635138 | 1.14480958883327 | 0.99380795269851 |
| ebi-a-GCST90001680 | finngen_R11_O15_PREECLAMPS | Pre-eclampsia \|\| id:finngen_R11_O15_PREECLAMPS | CD25++ CD8+ T cell %CD8+ T cell \|\| id:ebi-a-GCST90001680 | Weighted median | 22 | 0.0308770108449792 | 0.0409719290598264 | 0.4510811599559 | -0.04942797 | 0.111181991802239 | 1.03135865015696 | 0.951773711821072 | 1.11759828207312 | 0.940845681141708 |
| ebi-a-GCST90001680 | finngen_R11_O15_PREECLAMPS | Pre-eclampsia \|\| id:finngen_R11_O15_PREECLAMPS | CD25++ CD8+ T cell %CD8+ T cell \|\| id:ebi-a-GCST90001680 | Weighted mode | 22 | 0.0531972776703972 | 0.0378294075892169 | 0.174277866073959 | -0.020948361 | 0.127342916545262 | 1.05463768106309 | 0.97926953156295 | 1.13580643782813 | 0.872755742358858 |
| ebi-a-GCST90001681 | finngen_R11_O15_PREECLAMPS | Pre-eclampsia \|\| id:finngen_R11_O15_PREECLAMPS | CD25++ CD8+ T cell Absolute Count \|\| id:ebi-a-GCST90001681 | Inverse variance weighted | 27 | 0.0118388842174202 | 0.0203905918985274 | 0.561506549314475 | -0.028126676 | 0.0518044443385338 | 1.01190924118236 | 0.972265196427665 | 1.05316976906356 | 0.90609555750305 |
| ebi-a-GCST90001681 | finngen_R11_O15_PREECLAMPS | Pre-eclampsia \|\| id:finngen_R11_O15_PREECLAMPS | CD25++ CD8+ T cell Absolute Count \|\| id:ebi-a-GCST90001681 | MR Egger | 27 | 0.0409073187638052 | 0.037466859358809 | 0.28532005121318 | -0.032527726 | 0.114342363107071 | 1.04175554987892 | 0.967995611220014 | 1.12113589475446 | 0.99380795269851 |
| ebi-a-GCST90001681 | finngen_R11_O15_PREECLAMPS | Pre-eclampsia \|\| id:finngen_R11_O15_PREECLAMPS | CD25++ CD8+ T cell Absolute Count \|\| id:ebi-a-GCST90001681 | Weighted median | 27 | -0.007752857 | 0.031707452249542 | 0.806834205683792 | -0.069899463 | 0.0543937496011732 | 0.99227711907016 | 0.932487564493372 | 1.05590028062747 | 0.961297867845628 |
| ebi-a-GCST90001681 | finngen_R11_O15_PREECLAMPS | Pre-eclampsia \|\| id:finngen_R11_O15_PREECLAMPS | CD25++ CD8+ T cell Absolute Count \|\| id:ebi-a-GCST90001681 | Weighted mode | 27 | -0.017637312 | 0.0496700391629572 | 0.725386887580921 | -0.114990589 | 0.0797159643088332 | 0.98251731454193 | 0.891374532405932 | 1.0829794191776 | 0.959313411571287 |
| ebi-a-GCST90001682 | finngen_R11_O15_PREECLAMPS | Pre-eclampsia \|\| id:finngen_R11_O15_PREECLAMPS | CD127- CD8+ T cell %T cell \|\| id:ebi-a-GCST90001682 | Inverse variance weighted | 14 | 0.0450272515973185 | 0.0329153724548635 | 0.1713213760294 | -0.019486878 | 0.109541381608851 | 1.04605636622716 | 0.98070176346656 | 1.11576624218202 | 0.85505992597664 |
| ebi-a-GCST90001682 | finngen_R11_O15_PREECLAMPS | Pre-eclampsia \|\| id:finngen_R11_O15_PREECLAMPS | CD127- CD8+ T cell %T cell \|\| id:ebi-a-GCST90001682 | MR Egger | 14 | 0.0206858171198187 | 0.0752023327815487 | 0.787942167341908 | -0.126710755 | 0.168082389371654 | 1.02090125154963 | 0.8809884571378 | 1.18303407606687 | 0.99380795269851 |
| ebi-a-GCST90001682 | finngen_R11_O15_PREECLAMPS | Pre-eclampsia \|\| id:finngen_R11_O15_PREECLAMPS | CD127- CD8+ T cell %T cell \|\| id:ebi-a-GCST90001682 | Weighted median | 14 | 0.0199096750850906 | 0.0481739354154931 | 0.6793961428226 | -0.074511238 | 0.114330588499457 | 1.0201091945891 | 0.92819704261637 | 1.12112269389693 | 0.943447857654363 |
| ebi-a-GCST90001682 | finngen_R11_O15_PREECLAMPS | Pre-eclampsia \|\| id:finngen_R11_O15_PREECLAMPS | CD127- CD8+ T cell %T cell \|\| id:ebi-a-GCST90001682 | Weighted mode | 14 | -0.001765821 | 0.0587973470478896 | 0.976497445450071 | -0.117008621 | 0.11347697921391 | 0.998235737144678 | 0.889577523892312 | 1.12016610149135 | 0.994159530659367 |
| ebi-a-GCST90001683 | finngen_R11_O15_PREECLAMPS | Pre-eclampsia \|\| id:finngen_R11_O15_PREECLAMPS | CD127- CD8+ T cell %CD8+ T cell \|\| id:ebi-a-GCST90001683 | Inverse variance weighted | 15 | -0.005021288 | 0.0216304848541387 | 0.81642973307192 | -0.047417038 | 0.0373744622391223 | 0.994991297517869 | 0.953689589475411 | 1.03808167045302 | 0.977824840984872 |
| ebi-a-GCST90001683 | finngen_R11_O15_PREECLAMPS | Pre-eclampsia \|\| id:finngen_R11_O15_PREECLAMPS | CD127- CD8+ T cell %CD8+ T cell \|\| id:ebi-a-GCST90001683 | MR Egger | 15 | 0.00163627938599193 | 0.0292265339209929 | 0.95620421543594 | -0.055647727 | 0.058920285871138 | 1.00163761882157 | 0.945872282380455 | 1.0606906853361 | 0.99380795269851 |
| ebi-a-GCST90001683 | finngen_R11_O15_PREECLAMPS | Pre-eclampsia \|\| id:finngen_R11_O15_PREECLAMPS | CD127- CD8+ T cell %CD8+ T cell \|\| id:ebi-a-GCST90001683 | Weighted median | 15 | 0.0303806805390652 | 0.0332536093622091 | 0.360924173284418 | -0.034796394 | 0.0955577548889949 | 1.030846883 | 0.965802039512756 | 1.1002723663073 | 0.940845681141708 |
| ebi-a-GCST90001683 | finngen_R11_O15_PREECLAMPS | Pre-eclampsia \|\| id:finngen_R11_O15_PREECLAMPS | CD127- CD8+ T cell %CD8+ T cell \|\| id:ebi-a-GCST90001683 | Weighted mode | 15 | 0.035153576106862 | 0.0293362043262166 | 0.250692723467592 | -0.022345384 | 0.0926525365862466 | 1.03577876745618 | 0.977902424502051 | 1.09708047370814 | 0.872755742358858 |
| ebi-a-GCST90001684 | finngen_R11_O15_PREECLAMPS | Pre-eclampsia \|\| id:finngen_R11_O15_PREECLAMPS | CD127- CD8+ T cell Absolute Count \|\| id:ebi-a-GCST90001684 | Inverse variance weighted | 13 | 0.06410746385165 | 0.0266555276379986 | 0.0161709386333822 | 0.0118626296811727 | 0.116352298022127 | 1.06620697131298 | 1.01193326972185 | 1.12339157105575 | 0.588591724205005 |
| ebi-a-GCST90001684 | finngen_R11_O15_PREECLAMPS | Pre-eclampsia \|\| id:finngen_R11_O15_PREECLAMPS | CD127- CD8+ T cell Absolute Count \|\| id:ebi-a-GCST90001684 | MR Egger | 13 | 0.063752589330944 | 0.0542935579218601 | 0.265101963843645 | -0.042662784 | 0.17016796285779 | 1.06582866875401 | 0.958234467397016 | 1.18550395523111 | 0.99380795269851 |
| ebi-a-GCST90001684 | finngen_R11_O15_PREECLAMPS | Pre-eclampsia \|\| id:finngen_R11_O15_PREECLAMPS | CD127- CD8+ T cell Absolute Count \|\| id:ebi-a-GCST90001684 | Weighted median | 13 | 0.0514521106694365 | 0.0394622555837631 | 0.192291223311371 | -0.02589391 | 0.128798131613612 | 1.05279876725669 | 0.974438462033997 | 1.13746048367551 | 0.940845681141708 |
| ebi-a-GCST90001684 | finngen_R11_O15_PREECLAMPS | Pre-eclampsia \|\| id:finngen_R11_O15_PREECLAMPS | CD127- CD8+ T cell Absolute Count \|\| id:ebi-a-GCST90001684 | Weighted mode | 13 | 0.0502984454800675 | 0.0382318981716471 | 0.212885023090982 | -0.024636075 | 0.125232965896496 | 1.05158489030592 | 0.975664916343701 | 1.13341246876419 | 0.872755742358858 |
| ebi-a-GCST90001685 | finngen_R11_O15_PREECLAMPS | Pre-eclampsia \|\| id:finngen_R11_O15_PREECLAMPS | CD28- CD8+ T cell %T cell \|\| id:ebi-a-GCST90001685 | Inverse variance weighted | 15 | -0.01429065 | 0.033796154041193 | 0.672405863374811 | -0.080531112 | 0.0519498118451081 | 0.985810976584519 | 0.922626198394287 | 1.05332287685507 | 0.950857618427329 |
| ebi-a-GCST90001685 | finngen_R11_O15_PREECLAMPS | Pre-eclampsia \|\| id:finngen_R11_O15_PREECLAMPS | CD28- CD8+ T cell %T cell \|\| id:ebi-a-GCST90001685 | MR Egger | 15 | 0.0415142089599446 | 0.0795989738255957 | 0.610759924357491 | -0.11449978 | 0.197528197658112 | 1.0423879729952 | 0.891812134850229 | 1.21838742015719 | 0.99380795269851 |
| ebi-a-GCST90001685 | finngen_R11_O15_PREECLAMPS | Pre-eclampsia \|\| id:finngen_R11_O15_PREECLAMPS | CD28- CD8+ T cell %T cell \|\| id:ebi-a-GCST90001685 | Weighted median | 15 | 0.0019755890763631 | 0.0475957489186708 | 0.966891171032694 | -0.091312079 | 0.0952632569569579 | 1.0019775418382 | 0.91273282188003 | 1.09994838607884 | 0.989912389390616 |
| ebi-a-GCST90001685 | finngen_R11_O15_PREECLAMPS | Pre-eclampsia \|\| id:finngen_R11_O15_PREECLAMPS | CD28- CD8+ T cell %T cell \|\| id:ebi-a-GCST90001685 | Weighted mode | 15 | 0.0124312091075887 | 0.0561665675420223 | 0.828033015718564 | -0.097655263 | 0.122517681489952 | 1.01250879776112 | 0.906961512808745 | 1.13033910597687 | 0.974705530580145 |
| ebi-a-GCST90001686 | finngen_R11_O15_PREECLAMPS | Pre-eclampsia \|\| id:finngen_R11_O15_PREECLAMPS | CD28- CD8+ T cell %CD8+ T cell \|\| id:ebi-a-GCST90001686 | Inverse variance weighted | 23 | -0.000271458 | 0.0273806771266252 | 0.992089721320701 | -0.053937585 | 0.0533946689331234 | 0.999728578606391 | 0.947491241940114 | 1.05484587787411 | 0.995223645914365 |
| ebi-a-GCST90001686 | finngen_R11_O15_PREECLAMPS | Pre-eclampsia \|\| id:finngen_R11_O15_PREECLAMPS | CD28- CD8+ T cell %CD8+ T cell \|\| id:ebi-a-GCST90001686 | MR Egger | 23 | 0.0528192474071043 | 0.0562731076272704 | 0.358595406000166 | -0.057476044 | 0.163114538356554 | 1.05423907145084 | 0.944144508473433 | 1.1771715132576 | 0.99380795269851 |
| ebi-a-GCST90001686 | finngen_R11_O15_PREECLAMPS | Pre-eclampsia \|\| id:finngen_R11_O15_PREECLAMPS | CD28- CD8+ T cell %CD8+ T cell \|\| id:ebi-a-GCST90001686 | Weighted median | 23 | -0.008994943 | 0.0385126194907711 | 0.815327876968932 | -0.084479677 | 0.0664897909163473 | 0.991045390193744 | 0.918990331379579 | 1.06875005306078 | 0.961297867845628 |
| ebi-a-GCST90001686 | finngen_R11_O15_PREECLAMPS | Pre-eclampsia \|\| id:finngen_R11_O15_PREECLAMPS | CD28- CD8+ T cell %CD8+ T cell \|\| id:ebi-a-GCST90001686 | Weighted mode | 23 | -0.000972325 | 0.0479608500426258 | 0.984008092970185 | -0.094975591 | 0.0930309406703817 | 0.999028147142018 | 0.909395131171386 | 1.09749569199521 | 0.994159530659367 |
| ebi-a-GCST90001687 | finngen_R11_O15_PREECLAMPS | Pre-eclampsia \|\| id:finngen_R11_O15_PREECLAMPS | CD28- CD8+ T cell Absolute Count \|\| id:ebi-a-GCST90001687 | Inverse variance weighted | 14 | 0.0745292306439606 | 0.0376148498954863 | 0.0475489151615751 | 0.000804124848807417 | 0.148254336439114 | 1.07737683548068 | 1.00080444824387 | 1.15980784025004 | 0.789641233288559 |
| ebi-a-GCST90001687 | finngen_R11_O15_PREECLAMPS | Pre-eclampsia \|\| id:finngen_R11_O15_PREECLAMPS | CD28- CD8+ T cell Absolute Count \|\| id:ebi-a-GCST90001687 | MR Egger | 14 | 0.089694195223284 | 0.11871104658257 | 0.464481837774821 | -0.142979456 | 0.322367846525122 | 1.09383973113919 | 0.866771875605903 | 1.38039245514544 | 0.99380795269851 |
| ebi-a-GCST90001687 | finngen_R11_O15_PREECLAMPS | Pre-eclampsia \|\| id:finngen_R11_O15_PREECLAMPS | CD28- CD8+ T cell Absolute Count \|\| id:ebi-a-GCST90001687 | Weighted median | 14 | 0.0481660874045664 | 0.0540976416737528 | 0.373275479290116 | -0.05786529 | 0.154197465085122 | 1.04934492382706 | 0.943777074823236 | 1.16672125074436 | 0.940845681141708 |
| ebi-a-GCST90001687 | finngen_R11_O15_PREECLAMPS | Pre-eclampsia \|\| id:finngen_R11_O15_PREECLAMPS | CD28- CD8+ T cell Absolute Count \|\| id:ebi-a-GCST90001687 | Weighted mode | 14 | 0.0381633390978603 | 0.0721771765706709 | 0.605892407355457 | -0.103303927 | 0.179630605176375 | 1.03890091215825 | 0.901852834415539 | 1.19677519889674 | 0.955353022288269 |
| ebi-a-GCST90001688 | finngen_R11_O15_PREECLAMPS | Pre-eclampsia \|\| id:finngen_R11_O15_PREECLAMPS | CD28+ CD45RA+ CD8+ T cell %T cell \|\| id:ebi-a-GCST90001688 | Inverse variance weighted | 72 | -0.002520538 | 0.00196763988933816 | 0.200195229671968 | -0.006377112 | 0.00133603624384158 | 0.997482635949295 | 0.993643178502372 | 1.00133692913787 | 0.855687495048373 |
| ebi-a-GCST90001688 | finngen_R11_O15_PREECLAMPS | Pre-eclampsia \|\| id:finngen_R11_O15_PREECLAMPS | CD28+ CD45RA+ CD8+ T cell %T cell \|\| id:ebi-a-GCST90001688 | MR Egger | 72 | -0.001820078 | 0.00220750637480461 | 0.412459065198689 | -0.00614679 | 0.00250663461925315 | 0.998181577461939 | 0.993872062497958 | 1.00250977885441 | 0.99380795269851 |
| ebi-a-GCST90001688 | finngen_R11_O15_PREECLAMPS | Pre-eclampsia \|\| id:finngen_R11_O15_PREECLAMPS | CD28+ CD45RA+ CD8+ T cell %T cell \|\| id:ebi-a-GCST90001688 | Weighted median | 72 | -0.001943302 | 0.00294415757834236 | 0.509219416260056 | -0.007713851 | 0.00382724696658372 | 0.998058585101617 | 0.992315824653228 | 1.00383458022867 | 0.940845681141708 |
| ebi-a-GCST90001688 | finngen_R11_O15_PREECLAMPS | Pre-eclampsia \|\| id:finngen_R11_O15_PREECLAMPS | CD28+ CD45RA+ CD8+ T cell %T cell \|\| id:ebi-a-GCST90001688 | Weighted mode | 72 | -0.002814939 | 0.00239645557500494 | 0.244068951385396 | -0.007511992 | 0.00188211376056582 | 0.997189019059885 | 0.992516152401317 | 1.00188388604838 | 0.872755742358858 |
| ebi-a-GCST90001689 | finngen_R11_O15_PREECLAMPS | Pre-eclampsia \|\| id:finngen_R11_O15_PREECLAMPS | CD28+ CD45RA+ CD8+ T cell %CD8+ T cell \|\| id:ebi-a-GCST90001689 | Inverse variance weighted | 36 | -0.00512807 | 0.00368605423142956 | 0.164162233251522 | -0.012352736 | 0.0020965965925921 | 0.994885056401645 | 0.987723245865586 | 1.00209879598804 | 0.85505992597664 |
| ebi-a-GCST90001689 | finngen_R11_O15_PREECLAMPS | Pre-eclampsia \|\| id:finngen_R11_O15_PREECLAMPS | CD28+ CD45RA+ CD8+ T cell %CD8+ T cell \|\| id:ebi-a-GCST90001689 | MR Egger | 36 | -0.003795853 | 0.00385834849227552 | 0.332162077647561 | -0.011358216 | 0.00376651032485813 | 0.996211342422155 | 0.988706045240919 | 1.00377361253893 | 0.99380795269851 |
| ebi-a-GCST90001689 | finngen_R11_O15_PREECLAMPS | Pre-eclampsia \|\| id:finngen_R11_O15_PREECLAMPS | CD28+ CD45RA+ CD8+ T cell %CD8+ T cell \|\| id:ebi-a-GCST90001689 | Weighted median | 36 | -0.002389969 | 0.00433736842521327 | 0.581621174908773 | -0.010891211 | 0.00611127302275303 | 0.997612884611589 | 0.989167883305164 | 1.00612998495021 | 0.940845681141708 |
| ebi-a-GCST90001689 | finngen_R11_O15_PREECLAMPS | Pre-eclampsia \|\| id:finngen_R11_O15_PREECLAMPS | CD28+ CD45RA+ CD8+ T cell %CD8+ T cell \|\| id:ebi-a-GCST90001689 | Weighted mode | 36 | -0.005189823 | 0.00389092669670127 | 0.190875753161515 | -0.01281604 | 0.00243639296157471 | 0.994823620502163 | 0.987265736027438 | 1.00243936337878 | 0.872755742358858 |
| ebi-a-GCST90001690 | finngen_R11_O15_PREECLAMPS | Pre-eclampsia \|\| id:finngen_R11_O15_PREECLAMPS | CD28+ CD45RA+ CD8+ T cell Absolute Count \|\| id:ebi-a-GCST90001690 | Inverse variance weighted (multiplicative random effects) | 38 | -0.002986773 | 0.00417980699649146 | 0.474873586804634 | -0.011179195 | 0.00520564890677254 | 0.997017683162122 | 0.988883060472731 | 1.0052192218388 | 0.898694924660397 |
| ebi-a-GCST90001690 | finngen_R11_O15_PREECLAMPS | Pre-eclampsia \|\| id:finngen_R11_O15_PREECLAMPS | CD28+ CD45RA+ CD8+ T cell Absolute Count \|\| id:ebi-a-GCST90001690 | MR Egger | 38 | -0.002850938 | 0.00440863976817664 | 0.521947007602272 | -0.011491872 | 0.00578999595042657 | 0.997153122069279 | 0.988573907402475 | 1.00580679037455 | 0.99380795269851 |
| ebi-a-GCST90001690 | finngen_R11_O15_PREECLAMPS | Pre-eclampsia \|\| id:finngen_R11_O15_PREECLAMPS | CD28+ CD45RA+ CD8+ T cell Absolute Count \|\| id:ebi-a-GCST90001690 | Weighted median | 38 | -0.002381598 | 0.00397206802911857 | 0.548781971722095 | -0.010166851 | 0.00540365571631057 | 0.997621236132785 | 0.989884656766456 | 1.00541828179676 | 0.940845681141708 |
| ebi-a-GCST90001690 | finngen_R11_O15_PREECLAMPS | Pre-eclampsia \|\| id:finngen_R11_O15_PREECLAMPS | CD28+ CD45RA+ CD8+ T cell Absolute Count \|\| id:ebi-a-GCST90001690 | Weighted mode | 38 | -0.001771954 | 0.00390156165359566 | 0.652363027826496 | -0.009419014 | 0.00587510724510632 | 0.998229615386973 | 0.990625205534404 | 1.00589239953578 | 0.959313411571287 |
| ebi-a-GCST90001691 | finngen_R11_O15_PREECLAMPS | Pre-eclampsia \|\| id:finngen_R11_O15_PREECLAMPS | CD28+ CD45RA- CD8+ T cell %T cell \|\| id:ebi-a-GCST90001691 | Inverse variance weighted | 25 | 0.00550612614538988 | 0.0080535977146513 | 0.494173925492601 | -0.010278925 | 0.0212911776661064 | 1.00552131271822 | 0.989773722236741 | 1.02151945198717 | 0.898694924660397 |
| ebi-a-GCST90001691 | finngen_R11_O15_PREECLAMPS | Pre-eclampsia \|\| id:finngen_R11_O15_PREECLAMPS | CD28+ CD45RA- CD8+ T cell %T cell \|\| id:ebi-a-GCST90001691 | MR Egger | 25 | -0.00113775 | 0.0090260665372991 | 0.900786490993731 | -0.018828841 | 0.0165533402608628 | 0.998862896840066 | 0.981347314720654 | 1.01669110590855 | 0.99380795269851 |
| ebi-a-GCST90001691 | finngen_R11_O15_PREECLAMPS | Pre-eclampsia \|\| id:finngen_R11_O15_PREECLAMPS | CD28+ CD45RA- CD8+ T cell %T cell \|\| id:ebi-a-GCST90001691 | Weighted median | 25 | 0.000645987317093581 | 0.0101781316289399 | 0.949393710372532 | -0.019303151 | 0.0205951253098159 | 1.00064619601184 | 0.980881962137028 | 1.02080866836562 | 0.984406811747973 |
| ebi-a-GCST90001691 | finngen_R11_O15_PREECLAMPS | Pre-eclampsia \|\| id:finngen_R11_O15_PREECLAMPS | CD28+ CD45RA- CD8+ T cell %T cell \|\| id:ebi-a-GCST90001691 | Weighted mode | 25 | 0.00151687761144209 | 0.00867851248716144 | 0.862714378085734 | -0.015493007 | 0.0185267620862785 | 1.00151802865221 | 0.984626392354524 | 1.01869944732728 | 0.977585971930092 |
| ebi-a-GCST90001692 | finngen_R11_O15_PREECLAMPS | Pre-eclampsia \|\| id:finngen_R11_O15_PREECLAMPS | CD28+ CD45RA- CD8+ T cell %CD8+ T cell \|\| id:ebi-a-GCST90001692 | Inverse variance weighted (multiplicative random effects) | 24 | -0.002136881 | 0.00929144364238311 | 0.818104413374845 | -0.02034811 | 0.0160743486840521 | 0.997865400649485 | 0.979857515344066 | 1.01620423604519 | 0.977824840984872 |
| ebi-a-GCST90001692 | finngen_R11_O15_PREECLAMPS | Pre-eclampsia \|\| id:finngen_R11_O15_PREECLAMPS | CD28+ CD45RA- CD8+ T cell %CD8+ T cell \|\| id:ebi-a-GCST90001692 | MR Egger | 24 | -0.004512658 | 0.0106893182515267 | 0.677001560738233 | -0.025463722 | 0.0164384057688111 | 0.99549750873819 | 0.974857744430716 | 1.01657425974768 | 0.99380795269851 |
| ebi-a-GCST90001692 | finngen_R11_O15_PREECLAMPS | Pre-eclampsia \|\| id:finngen_R11_O15_PREECLAMPS | CD28+ CD45RA- CD8+ T cell %CD8+ T cell \|\| id:ebi-a-GCST90001692 | Weighted median | 24 | -0.004213478 | 0.0100966085611772 | 0.676447186973204 | -0.02400283 | 0.0155758751016612 | 0.995795386564693 | 0.976282946425973 | 1.01569781130937 | 0.941872178433165 |
| ebi-a-GCST90001692 | finngen_R11_O15_PREECLAMPS | Pre-eclampsia \|\| id:finngen_R11_O15_PREECLAMPS | CD28+ CD45RA- CD8+ T cell %CD8+ T cell \|\| id:ebi-a-GCST90001692 | Weighted mode | 24 | -0.001330673 | 0.00782833247172338 | 0.866510903618631 | -0.016674204 | 0.014012858989336 | 0.998670212297045 | 0.983464040802521 | 1.0141114993036 | 0.977585971930092 |
| ebi-a-GCST90001693 | finngen_R11_O15_PREECLAMPS | Pre-eclampsia \|\| id:finngen_R11_O15_PREECLAMPS | CD28+ CD45RA- CD8+ T cell Absolute Count \|\| id:ebi-a-GCST90001693 | Inverse variance weighted | 30 | 0.00525893471318742 | 0.00747038307274806 | 0.481450694427841 | -0.009383016 | 0.0199008855357736 | 1.00527278718278 | 0.990660867026945 | 1.02010022832843 | 0.898694924660397 |
| ebi-a-GCST90001693 | finngen_R11_O15_PREECLAMPS | Pre-eclampsia \|\| id:finngen_R11_O15_PREECLAMPS | CD28+ CD45RA- CD8+ T cell Absolute Count \|\| id:ebi-a-GCST90001693 | MR Egger | 30 | 0.00220418257136802 | 0.00852132197978766 | 0.79778445012266 | -0.014497609 | 0.0189059736517518 | 1.00220661356756 | 0.985606975799761 | 1.01908582319391 | 0.99380795269851 |
| ebi-a-GCST90001693 | finngen_R11_O15_PREECLAMPS | Pre-eclampsia \|\| id:finngen_R11_O15_PREECLAMPS | CD28+ CD45RA- CD8+ T cell Absolute Count \|\| id:ebi-a-GCST90001693 | Weighted median | 30 | 0.00466281731405461 | 0.0092053218703014 | 0.612481156271571 | -0.013379614 | 0.0227052481798454 | 1.00467370516281 | 0.986709495619526 | 1.02296497431782 | 0.940845681141708 |
| ebi-a-GCST90001693 | finngen_R11_O15_PREECLAMPS | Pre-eclampsia \|\| id:finngen_R11_O15_PREECLAMPS | CD28+ CD45RA- CD8+ T cell Absolute Count \|\| id:ebi-a-GCST90001693 | Weighted mode | 30 | 0.00229405975079916 | 0.00788486343368327 | 0.773164294529714 | -0.013160273 | 0.0177483920808184 | 1.00229669311919 | 0.986925945177146 | 1.0179068307474 | 0.961305639715923 |
| ebi-a-GCST90001694 | finngen_R11_O15_PREECLAMPS | Pre-eclampsia \|\| id:finngen_R11_O15_PREECLAMPS | CD28- CD4-CD8- T cell %T cell \|\| id:ebi-a-GCST90001694 | Inverse variance weighted | 22 | 0.021743512320518 | 0.0205780178793822 | 0.290676904857522 | -0.018589403 | 0.0620764273641072 | 1.02198162515605 | 0.981582314537018 | 1.06404366367301 | 0.885079385681701 |
| ebi-a-GCST90001694 | finngen_R11_O15_PREECLAMPS | Pre-eclampsia \|\| id:finngen_R11_O15_PREECLAMPS | CD28- CD4-CD8- T cell %T cell \|\| id:ebi-a-GCST90001694 | MR Egger | 22 | 0.0247633555052284 | 0.0311456258931071 | 0.435899893357378 | -0.036282071 | 0.0858087822557183 | 1.02507251405263 | 0.964368234566113 | 1.08959795792002 | 0.99380795269851 |
| ebi-a-GCST90001694 | finngen_R11_O15_PREECLAMPS | Pre-eclampsia \|\| id:finngen_R11_O15_PREECLAMPS | CD28- CD4-CD8- T cell %T cell \|\| id:ebi-a-GCST90001694 | Weighted median | 22 | 0.0328526833807757 | 0.0301026029864635 | 0.275115870925945 | -0.026148418 | 0.0918537852342443 | 1.03339829128467 | 0.974190491013675 | 1.09620452907406 | 0.940845681141708 |
| ebi-a-GCST90001694 | finngen_R11_O15_PREECLAMPS | Pre-eclampsia \|\| id:finngen_R11_O15_PREECLAMPS | CD28- CD4-CD8- T cell %T cell \|\| id:ebi-a-GCST90001694 | Weighted mode | 22 | 0.0376955598553163 | 0.0280569259291568 | 0.193436637825537 | -0.017296015 | 0.0926871346764636 | 1.03841504952357 | 0.982852702460359 | 1.09711843125397 | 0.872755742358858 |
| ebi-a-GCST90001695 | finngen_R11_O15_PREECLAMPS | Pre-eclampsia \|\| id:finngen_R11_O15_PREECLAMPS | CD45RA- CD28- CD8+ T cell Absolute Count \|\| id:ebi-a-GCST90001695 | Inverse variance weighted | 338 | 2.01185702981119e-07 | 8.41779722872444e-06 | 0.980932341192833 | -1.63E-05 | 1.6700068271281e-05 | 1.00000020118572 | 0.999983702435941 | 1.00001670020772 | 0.994537505425743 |
| ebi-a-GCST90001695 | finngen_R11_O15_PREECLAMPS | Pre-eclampsia \|\| id:finngen_R11_O15_PREECLAMPS | CD45RA- CD28- CD8+ T cell Absolute Count \|\| id:ebi-a-GCST90001695 | MR Egger | 338 | -1.55E-05 | 1.10558772481627e-05 | 0.162987795341215 | -3.71E-05 | 6.21165466376511e-06 | 0.99998454225473 | 0.999962873305064 | 1.00000621167396 | 0.99380795269851 |
| ebi-a-GCST90001695 | finngen_R11_O15_PREECLAMPS | Pre-eclampsia \|\| id:finngen_R11_O15_PREECLAMPS | CD45RA- CD28- CD8+ T cell Absolute Count \|\| id:ebi-a-GCST90001695 | Weighted median | 338 | -1.15E-05 | 1.52111874428707e-05 | 0.447950500003904 | -4.14E-05 | 1.82711264943908e-05 | 0.999988457265724 | 0.999958644126896 | 1.00001827129341 | 0.940845681141708 |
| ebi-a-GCST90001695 | finngen_R11_O15_PREECLAMPS | Pre-eclampsia \|\| id:finngen_R11_O15_PREECLAMPS | CD45RA- CD28- CD8+ T cell Absolute Count \|\| id:ebi-a-GCST90001695 | Weighted mode | 338 | -1.34E-05 | 1.93188561245184e-05 | 0.487185465204505 | -5.13E-05 | 2.44275863873657e-05 | 0.999986562718664 | 0.999948698986321 | 1.00002442788474 | 0.933945646747087 |
| ebi-a-GCST90001696 | finngen_R11_O15_PREECLAMPS | Pre-eclampsia \|\| id:finngen_R11_O15_PREECLAMPS | CD45RA- CD28- CD8+ T cell %CD8+ T cell \|\| id:ebi-a-GCST90001696 | Inverse variance weighted | 50 | -0.0004219 | 0.000361225620151649 | 0.242819473675705 | -0.001129902 | 0.00028610207194028 | 0.999578188843794 | 0.998870735740264 | 1.00028614300304 | 0.885079385681701 |
| ebi-a-GCST90001696 | finngen_R11_O15_PREECLAMPS | Pre-eclampsia \|\| id:finngen_R11_O15_PREECLAMPS | CD45RA- CD28- CD8+ T cell %CD8+ T cell \|\| id:ebi-a-GCST90001696 | MR Egger | 50 | -0.00024212 | 0.000436784406145957 | 0.581930302763864 | -0.001098218 | 0.000613977061061574 | 0.999757908933788 | 0.998902385009453 | 1.00061416558356 | 0.99380795269851 |
| ebi-a-GCST90001696 | finngen_R11_O15_PREECLAMPS | Pre-eclampsia \|\| id:finngen_R11_O15_PREECLAMPS | CD45RA- CD28- CD8+ T cell %CD8+ T cell \|\| id:ebi-a-GCST90001696 | Weighted median | 50 | -0.000160246 | 0.000504623869155482 | 0.75082176174258 | -0.001149309 | 0.000828816450909542 | 0.999839766506123 | 0.998851351086592 | 1.00082916001417 | 0.957984984509652 |
| ebi-a-GCST90001696 | finngen_R11_O15_PREECLAMPS | Pre-eclampsia \|\| id:finngen_R11_O15_PREECLAMPS | CD45RA- CD28- CD8+ T cell %CD8+ T cell \|\| id:ebi-a-GCST90001696 | Weighted mode | 50 | -0.000451109 | 0.000411154908130015 | 0.277927667471033 | -0.001256972 | 0.000354754784608454 | 0.999548992898966 | 0.998743817203721 | 1.00035481771753 | 0.872755742358858 |
| ebi-a-GCST90001697 | finngen_R11_O15_PREECLAMPS | Pre-eclampsia \|\| id:finngen_R11_O15_PREECLAMPS | CD45RA- CD28- CD8+ T cell %T cell \|\| id:ebi-a-GCST90001697 | Inverse variance weighted | 176 | -2.91E-06 | 0.000263076248789243 | 0.9911831044634 | -0.000518537 | 0.000512722306511872 | 0.999997092863111 | 0.999481597828121 | 1.00051285377106 | 0.995223645914365 |
| ebi-a-GCST90001697 | finngen_R11_O15_PREECLAMPS | Pre-eclampsia \|\| id:finngen_R11_O15_PREECLAMPS | CD45RA- CD28- CD8+ T cell %T cell \|\| id:ebi-a-GCST90001697 | MR Egger | 176 | -0.000169129 | 0.000351650630437739 | 0.631149977041875 | -0.000858365 | 0.000520105756814471 | 0.99983088482274 | 0.999142003575107 | 1.00052024103527 | 0.99380795269851 |
| ebi-a-GCST90001697 | finngen_R11_O15_PREECLAMPS | Pre-eclampsia \|\| id:finngen_R11_O15_PREECLAMPS | CD45RA- CD28- CD8+ T cell %T cell \|\| id:ebi-a-GCST90001697 | Weighted median | 176 | -0.000233034 | 0.000440765197102458 | 0.597011990846682 | -0.001096933 | 0.000630866218838513 | 0.99976699358273 | 0.998903668057665 | 1.00063106525678 | 0.940845681141708 |
| ebi-a-GCST90001697 | finngen_R11_O15_PREECLAMPS | Pre-eclampsia \|\| id:finngen_R11_O15_PREECLAMPS | CD45RA- CD28- CD8+ T cell %T cell \|\| id:ebi-a-GCST90001697 | Weighted mode | 176 | -0.000128806 | 0.000347107866863655 | 0.711024717992736 | -0.000809137 | 0.000551525667820248 | 0.999871202543872 | 0.999191190092922 | 1.00055167778607 | 0.959313411571287 |
| ebi-a-GCST90001698 | finngen_R11_O15_PREECLAMPS | Pre-eclampsia \|\| id:finngen_R11_O15_PREECLAMPS | CD45RA+ CD28- CD8+ T cell Absolute Count \|\| id:ebi-a-GCST90001698 | Inverse variance weighted (multiplicative random effects) | 712 | 4.34384992696162e-07 | 4.61183098247586e-06 | 0.924958808336404 | -8.60E-06 | 9.47357371834884e-06 | 1.00000043438509 | 0.999991395233288 | 1.00000947361859 | 0.986667691552896 |
| ebi-a-GCST90001698 | finngen_R11_O15_PREECLAMPS | Pre-eclampsia \|\| id:finngen_R11_O15_PREECLAMPS | CD45RA+ CD28- CD8+ T cell Absolute Count \|\| id:ebi-a-GCST90001698 | MR Egger | 712 | 7.8247670892476e-07 | 6.10538949040068e-06 | 0.898057351248726 | -1.12E-05 | 1.27490401101101e-05 | 1.00000078247702 | 0.999988815975849 | 1.00001274912138 | 0.99380795269851 |
| ebi-a-GCST90001698 | finngen_R11_O15_PREECLAMPS | Pre-eclampsia \|\| id:finngen_R11_O15_PREECLAMPS | CD45RA+ CD28- CD8+ T cell Absolute Count \|\| id:ebi-a-GCST90001698 | Weighted median | 712 | 1.40367937575509e-07 | 7.44153642279396e-06 | 0.984950585108097 | -1.44E-05 | 1.47257793262517e-05 | 1.00000014036795 | 0.999985555060878 | 1.00001472588775 | 0.992447515164719 |
| ebi-a-GCST90001698 | finngen_R11_O15_PREECLAMPS | Pre-eclampsia \|\| id:finngen_R11_O15_PREECLAMPS | CD45RA+ CD28- CD8+ T cell Absolute Count \|\| id:ebi-a-GCST90001698 | Weighted mode | 712 | -1.32E-05 | 1.3814838620992e-05 | 0.341064381897404 | -4.02E-05 | 1.39156467919298e-05 | 0.999986838649706 | 0.999959762288956 | 1.00001391574362 | 0.888599269773114 |
| ebi-a-GCST90001699 | finngen_R11_O15_PREECLAMPS | Pre-eclampsia \|\| id:finngen_R11_O15_PREECLAMPS | CD45RA+ CD28- CD8+ T cell %CD8+ T cell \|\| id:ebi-a-GCST90001699 | Inverse variance weighted | 31 | -0.000366897 | 0.000279377627832472 | 0.189093917628015 | -0.000914477 | 0.000180683497215673 | 0.99963317064501 | 0.999085941202596 | 1.00018069982146 | 0.855687495048373 |
| ebi-a-GCST90001699 | finngen_R11_O15_PREECLAMPS | Pre-eclampsia \|\| id:finngen_R11_O15_PREECLAMPS | CD45RA+ CD28- CD8+ T cell %CD8+ T cell \|\| id:ebi-a-GCST90001699 | MR Egger | 31 | -0.000257154 | 0.000302218255412687 | 0.401802269125433 | -0.000849502 | 0.000335193531981652 | 0.999742878812692 | 0.99915085869546 | 1.00033524971561 | 0.99380795269851 |
| ebi-a-GCST90001699 | finngen_R11_O15_PREECLAMPS | Pre-eclampsia \|\| id:finngen_R11_O15_PREECLAMPS | CD45RA+ CD28- CD8+ T cell %CD8+ T cell \|\| id:ebi-a-GCST90001699 | Weighted median | 31 | -0.00048991 | 0.000389830194716708 | 0.20885256206913 | -0.001253977 | 0.000274157109014599 | 0.999510209913715 | 0.998746808646667 | 1.00027419469351 | 0.940845681141708 |
| ebi-a-GCST90001699 | finngen_R11_O15_PREECLAMPS | Pre-eclampsia \|\| id:finngen_R11_O15_PREECLAMPS | CD45RA+ CD28- CD8+ T cell %CD8+ T cell \|\| id:ebi-a-GCST90001699 | Weighted mode | 31 | -0.00043357 | 0.000281858102128225 | 0.134467718476708 | -0.000986012 | 0.000118871728182889 | 0.999566523825967 | 0.999014473917973 | 1.00011887879371 | 0.872755742358858 |
| ebi-a-GCST90001700 | finngen_R11_O15_PREECLAMPS | Pre-eclampsia \|\| id:finngen_R11_O15_PREECLAMPS | CD45RA+ CD28- CD8+ T cell %T cell \|\| id:ebi-a-GCST90001700 | Inverse variance weighted | 201 | 3.50634270882463e-05 | 0.000196694265384029 | 0.858515966930831 | -0.000350457 | 0.000420584187240942 | 1.00003506404182 | 0.999649604069933 | 1.00042067264517 | 0.977824840984872 |
| ebi-a-GCST90001700 | finngen_R11_O15_PREECLAMPS | Pre-eclampsia \|\| id:finngen_R11_O15_PREECLAMPS | CD45RA+ CD28- CD8+ T cell %T cell \|\| id:ebi-a-GCST90001700 | MR Egger | 201 | -5.70E-06 | 0.00026808400822691 | 0.983046973335031 | -0.000531148 | 0.000519740956924344 | 0.999994296317066 | 0.999468992678991 | 1.00051987604566 | 0.99380795269851 |
| ebi-a-GCST90001700 | finngen_R11_O15_PREECLAMPS | Pre-eclampsia \|\| id:finngen_R11_O15_PREECLAMPS | CD45RA+ CD28- CD8+ T cell %T cell \|\| id:ebi-a-GCST90001700 | Weighted median | 201 | -0.000352964 | 0.000309403210983526 | 0.253957530774462 | -0.000959394 | 0.000253466358904986 | 0.999647098349819 | 0.99904106584335 | 1.00025349848422 | 0.940845681141708 |
| ebi-a-GCST90001700 | finngen_R11_O15_PREECLAMPS | Pre-eclampsia \|\| id:finngen_R11_O15_PREECLAMPS | CD45RA+ CD28- CD8+ T cell %T cell \|\| id:ebi-a-GCST90001700 | Weighted mode | 201 | -0.000209471 | 0.000293086450203233 | 0.475623530757451 | -0.00078392 | 0.000364978519561055 | 0.999790551014665 | 0.999216386820059 | 1.00036504513232 | 0.933945646747087 |
| ebi-a-GCST90001701 | finngen_R11_O15_PREECLAMPS | Pre-eclampsia \|\| id:finngen_R11_O15_PREECLAMPS | BAFF-R on CD20- CD38- B cell \|\| id:ebi-a-GCST90001701 | Inverse variance weighted | 17 | 0.0340286197183812 | 0.0163268390291972 | 0.0371407435081079 | 0.00202801522115476 | 0.0660292242156077 | 1.03461421667192 | 1.00203007303488 | 1.06825793571006 | 0.781760498894217 |
| ebi-a-GCST90001701 | finngen_R11_O15_PREECLAMPS | Pre-eclampsia \|\| id:finngen_R11_O15_PREECLAMPS | BAFF-R on CD20- CD38- B cell \|\| id:ebi-a-GCST90001701 | MR Egger | 17 | 0.0463960831479375 | 0.0231722862197735 | 0.0636822685179191 | 0.000978402157181445 | 0.0918137641386934 | 1.04748922163009 | 1.00097888094871 | 1.09616065864574 | 0.878334684652809 |
| ebi-a-GCST90001701 | finngen_R11_O15_PREECLAMPS | Pre-eclampsia \|\| id:finngen_R11_O15_PREECLAMPS | BAFF-R on CD20- CD38- B cell \|\| id:ebi-a-GCST90001701 | Weighted median | 17 | 0.0377276109399001 | 0.0243478193482172 | 0.121255012461722 | -0.009994115 | 0.0854493368624059 | 1.03844833238553 | 0.990055660226805 | 1.08920637733362 | 0.88227178111339 |
| ebi-a-GCST90001701 | finngen_R11_O15_PREECLAMPS | Pre-eclampsia \|\| id:finngen_R11_O15_PREECLAMPS | BAFF-R on CD20- CD38- B cell \|\| id:ebi-a-GCST90001701 | Weighted mode | 17 | 0.0345687305348322 | 0.0231566444283507 | 0.154939780313754 | -0.010818293 | 0.0799557536143996 | 1.03517317393704 | 0.989240014730916 | 1.08323913719803 | 0.872755742358858 |
| ebi-a-GCST90001702 | finngen_R11_O15_PREECLAMPS | Pre-eclampsia \|\| id:finngen_R11_O15_PREECLAMPS | BAFF-R on CD24+ CD27+ B cell \|\| id:ebi-a-GCST90001702 | Inverse variance weighted | 19 | 0.0184377022838923 | 0.0169353200367261 | 0.276280488035248 | -0.014755525 | 0.0516309295558755 | 1.01860872619567 | 0.985352804297729 | 1.05298704439315 | 0.885079385681701 |
| ebi-a-GCST90001702 | finngen_R11_O15_PREECLAMPS | Pre-eclampsia \|\| id:finngen_R11_O15_PREECLAMPS | BAFF-R on CD24+ CD27+ B cell \|\| id:ebi-a-GCST90001702 | MR Egger | 19 | 0.0466013362703809 | 0.0211098340233021 | 0.04130497218819 | 0.00522606158470883 | 0.087976610956053 | 1.04770424412982 | 1.00523974126445 | 1.0919625817687 | 0.840804886393039 |
| ebi-a-GCST90001702 | finngen_R11_O15_PREECLAMPS | Pre-eclampsia \|\| id:finngen_R11_O15_PREECLAMPS | BAFF-R on CD24+ CD27+ B cell \|\| id:ebi-a-GCST90001702 | Weighted median | 19 | 0.0379949684358005 | 0.0178656499980919 | 0.0334445959755381 | 0.00297829443954033 | 0.0730116424320606 | 1.03872600644876 | 1.00298273396474 | 1.0757430611073 | 0.721097500353414 |
| ebi-a-GCST90001702 | finngen_R11_O15_PREECLAMPS | Pre-eclampsia \|\| id:finngen_R11_O15_PREECLAMPS | BAFF-R on CD24+ CD27+ B cell \|\| id:ebi-a-GCST90001702 | Weighted mode | 19 | 0.0394332054549096 | 0.015260762740093 | 0.0187195633337976 | 0.00952211048432734 | 0.0693443004254918 | 1.04022101547491 | 1.00956759001746 | 1.07180516860385 | 0.720210568263475 |
| ebi-a-GCST90001703 | finngen_R11_O15_PREECLAMPS | Pre-eclampsia \|\| id:finngen_R11_O15_PREECLAMPS | BAFF-R on IgD+ CD24+ B cell \|\| id:ebi-a-GCST90001703 | Inverse variance weighted (multiplicative random effects) | 18 | 0.0149050029874607 | 0.0188256495735661 | 0.428512814417414 | -0.02199327 | 0.0518032761516503 | 1.01501663648746 | 0.978246818457261 | 1.05316853876517 | 0.898694924660397 |
| ebi-a-GCST90001703 | finngen_R11_O15_PREECLAMPS | Pre-eclampsia \|\| id:finngen_R11_O15_PREECLAMPS | BAFF-R on IgD+ CD24+ B cell \|\| id:ebi-a-GCST90001703 | MR Egger | 18 | 0.0330657739022381 | 0.0256774532475889 | 0.216152640619872 | -0.017262034 | 0.0833935822675124 | 1.03361852212916 | 0.982886100856806 | 1.0869695363045 | 0.99380795269851 |
| ebi-a-GCST90001703 | finngen_R11_O15_PREECLAMPS | Pre-eclampsia \|\| id:finngen_R11_O15_PREECLAMPS | BAFF-R on IgD+ CD24+ B cell \|\| id:ebi-a-GCST90001703 | Weighted median | 18 | 0.0419144960556141 | 0.0178764783549808 | 0.01904377366211 | 0.00687659847985172 | 0.0769523936313765 | 1.0428053109714 | 1.00690029657279 | 1.07999066073525 | 0.598716529366782 |
| ebi-a-GCST90001703 | finngen_R11_O15_PREECLAMPS | Pre-eclampsia \|\| id:finngen_R11_O15_PREECLAMPS | BAFF-R on IgD+ CD24+ B cell \|\| id:ebi-a-GCST90001703 | Weighted mode | 18 | 0.0466960596283362 | 0.0166037885974948 | 0.0119889578378995 | 0.0141526339772465 | 0.079239485279426 | 1.04780349089439 | 1.01425325663299 | 1.0824635251112 | 0.720210568263475 |
| ebi-a-GCST90001704 | finngen_R11_O15_PREECLAMPS | Pre-eclampsia \|\| id:finngen_R11_O15_PREECLAMPS | BAFF-R on IgD+ CD24- B cell \|\| id:ebi-a-GCST90001704 | Inverse variance weighted (multiplicative random effects) | 22 | 0.0228462248532961 | 0.0172745815087969 | 0.185990328448851 | -0.011011955 | 0.056704404610538 | 1.02310919868285 | 0.989048454725417 | 1.05834292286525 | 0.855687495048373 |
| ebi-a-GCST90001704 | finngen_R11_O15_PREECLAMPS | Pre-eclampsia \|\| id:finngen_R11_O15_PREECLAMPS | BAFF-R on IgD+ CD24- B cell \|\| id:ebi-a-GCST90001704 | MR Egger | 22 | 0.0332047926625378 | 0.0226992735096489 | 0.159056868435138 | -0.011285783 | 0.0776953687414497 | 1.03376222448316 | 0.988777662135398 | 1.08079336507303 | 0.99380795269851 |
| ebi-a-GCST90001704 | finngen_R11_O15_PREECLAMPS | Pre-eclampsia \|\| id:finngen_R11_O15_PREECLAMPS | BAFF-R on IgD+ CD24- B cell \|\| id:ebi-a-GCST90001704 | Weighted median | 22 | 0.0512961285983551 | 0.0164156514017926 | 0.00177907526010004 | 0.0191214518508416 | 0.0834708053458687 | 1.05263456233139 | 1.01930543763203 | 1.08705347867928 | 0.507633389188193 |
| ebi-a-GCST90001704 | finngen_R11_O15_PREECLAMPS | Pre-eclampsia \|\| id:finngen_R11_O15_PREECLAMPS | BAFF-R on IgD+ CD24- B cell \|\| id:ebi-a-GCST90001704 | Weighted mode | 22 | 0.0392538425597072 | 0.0151358628788842 | 0.0169580600288374 | 0.00958755131709409 | 0.0689201338023203 | 1.04003445515342 | 1.00963365912307 | 1.07135064102931 | 0.720210568263475 |
| ebi-a-GCST90001705 | finngen_R11_O15_PREECLAMPS | Pre-eclampsia \|\| id:finngen_R11_O15_PREECLAMPS | BAFF-R on IgD+ CD38- B cell \|\| id:ebi-a-GCST90001705 | Inverse variance weighted | 22 | 0.0141078118478435 | 0.0169234807893459 | 0.404493160195642 | -0.01906221 | 0.0472778341949616 | 1.01420779666109 | 0.981118324483417 | 1.04841325367125 | 0.890615964165706 |
| ebi-a-GCST90001705 | finngen_R11_O15_PREECLAMPS | Pre-eclampsia \|\| id:finngen_R11_O15_PREECLAMPS | BAFF-R on IgD+ CD38- B cell \|\| id:ebi-a-GCST90001705 | MR Egger | 22 | 0.0407683148340451 | 0.0204884706971195 | 0.0604590876727301 | 0.000610912267690798 | 0.0809257174003993 | 1.04161075182762 | 1.0006110989126 | 1.08429034966929 | 0.862303274556491 |
| ebi-a-GCST90001705 | finngen_R11_O15_PREECLAMPS | Pre-eclampsia \|\| id:finngen_R11_O15_PREECLAMPS | BAFF-R on IgD+ CD38- B cell \|\| id:ebi-a-GCST90001705 | Weighted median | 22 | 0.0536772058539292 | 0.0184740179582985 | 0.00366606813905768 | 0.0174681306556642 | 0.0898862810521943 | 1.05514395288699 | 1.0176215907011 | 1.09404986243161 | 0.507633389188193 |
| ebi-a-GCST90001705 | finngen_R11_O15_PREECLAMPS | Pre-eclampsia \|\| id:finngen_R11_O15_PREECLAMPS | BAFF-R on IgD+ CD38- B cell \|\| id:ebi-a-GCST90001705 | Weighted mode | 22 | 0.043774003494408 | 0.0165118930623009 | 0.0149421400611957 | 0.0114106930922983 | 0.0761373138965177 | 1.04474621921176 | 1.0114760433782 | 1.07911074088498 | 0.720210568263475 |
| ebi-a-GCST90001706 | finngen_R11_O15_PREECLAMPS | Pre-eclampsia \|\| id:finngen_R11_O15_PREECLAMPS | BAFF-R on IgD+ CD38- naive B cell \|\| id:ebi-a-GCST90001706 | Inverse variance weighted | 28 | 0.0193478211957263 | 0.0137682148039548 | 0.159946144511863 | -0.00763788 | 0.0463335222114778 | 1.01953620325433 | 0.992391214663535 | 1.04742369177328 | 0.85505992597664 |
| ebi-a-GCST90001706 | finngen_R11_O15_PREECLAMPS | Pre-eclampsia \|\| id:finngen_R11_O15_PREECLAMPS | BAFF-R on IgD+ CD38- naive B cell \|\| id:ebi-a-GCST90001706 | MR Egger | 28 | 0.0637032821462538 | 0.0197789234615447 | 0.00342182237774816 | 0.0249365921616261 | 0.102469972130882 | 1.06577611703859 | 1.02525010957011 | 1.10790403341301 | 0.565056961366001 |
| ebi-a-GCST90001706 | finngen_R11_O15_PREECLAMPS | Pre-eclampsia \|\| id:finngen_R11_O15_PREECLAMPS | BAFF-R on IgD+ CD38- naive B cell \|\| id:ebi-a-GCST90001706 | Weighted median | 28 | 0.0486569898727518 | 0.017638613219853 | 0.00580584893674079 | 0.0140853079618399 | 0.0832286717836637 | 1.04986017629915 | 1.01418497330126 | 1.08679029841185 | 0.507633389188193 |
| ebi-a-GCST90001706 | finngen_R11_O15_PREECLAMPS | Pre-eclampsia \|\| id:finngen_R11_O15_PREECLAMPS | BAFF-R on IgD+ CD38- naive B cell \|\| id:ebi-a-GCST90001706 | Weighted mode | 28 | 0.0511875244020726 | 0.0175371540785674 | 0.0070043525173663 | 0.0168147024080804 | 0.0855603463960648 | 1.05252024800839 | 1.01695686520725 | 1.08932729633708 | 0.720210568263475 |
| ebi-a-GCST90001707 | finngen_R11_O15_PREECLAMPS | Pre-eclampsia \|\| id:finngen_R11_O15_PREECLAMPS | BAFF-R on IgD+ CD38- unswitched memory B cell \|\| id:ebi-a-GCST90001707 | Inverse variance weighted | 19 | 0.0279826736917484 | 0.0146056570959817 | 0.0553804818097387 | -0.000644414 | 0.0566097615998726 | 1.02837786627515 | 0.999355793373872 | 1.05824276284452 | 0.789997151112252 |
| ebi-a-GCST90001707 | finngen_R11_O15_PREECLAMPS | Pre-eclampsia \|\| id:finngen_R11_O15_PREECLAMPS | BAFF-R on IgD+ CD38- unswitched memory B cell \|\| id:ebi-a-GCST90001707 | MR Egger | 19 | 0.0571504630698925 | 0.0214465539169737 | 0.0163307146053879 | 0.0151152173926239 | 0.099185708747161 | 1.05881511098282 | 1.01523003003434 | 1.10427135336771 | 0.70222072803168 |
| ebi-a-GCST90001707 | finngen_R11_O15_PREECLAMPS | Pre-eclampsia \|\| id:finngen_R11_O15_PREECLAMPS | BAFF-R on IgD+ CD38- unswitched memory B cell \|\| id:ebi-a-GCST90001707 | Weighted median | 19 | 0.0355334014281805 | 0.0183159703484427 | 0.0523769101060965 | -0.0003659 | 0.0714327033111281 | 1.0361722571833 | 0.99963416647864 | 1.07404586853852 | 0.771214851704188 |
| ebi-a-GCST90001707 | finngen_R11_O15_PREECLAMPS | Pre-eclampsia \|\| id:finngen_R11_O15_PREECLAMPS | BAFF-R on IgD+ CD38- unswitched memory B cell \|\| id:ebi-a-GCST90001707 | Weighted mode | 19 | 0.0553125037235714 | 0.0193159744490946 | 0.010325822835402 | 0.017453193803346 | 0.0931718136437968 | 1.05687083914719 | 1.01760639075121 | 1.09765031036717 | 0.720210568263475 |
| ebi-a-GCST90001708 | finngen_R11_O15_PREECLAMPS | Pre-eclampsia \|\| id:finngen_R11_O15_PREECLAMPS | BAFF-R on IgD+ CD38+ B cell \|\| id:ebi-a-GCST90001708 | Inverse variance weighted (multiplicative random effects) | 27 | 0.00391675572654599 | 0.0166184612335475 | 0.813675537062479 | -0.028655428 | 0.036488939744299 | 1.00392443623855 | 0.971751244772314 | 1.03716283266818 | 0.977824840984872 |
| ebi-a-GCST90001708 | finngen_R11_O15_PREECLAMPS | Pre-eclampsia \|\| id:finngen_R11_O15_PREECLAMPS | BAFF-R on IgD+ CD38+ B cell \|\| id:ebi-a-GCST90001708 | MR Egger | 27 | 0.0283577914430142 | 0.0222737019762083 | 0.214676311910152 | -0.015298664 | 0.0720142473163825 | 1.02876370143009 | 0.984817765638623 | 1.07467065512757 | 0.99380795269851 |
| ebi-a-GCST90001708 | finngen_R11_O15_PREECLAMPS | Pre-eclampsia \|\| id:finngen_R11_O15_PREECLAMPS | BAFF-R on IgD+ CD38+ B cell \|\| id:ebi-a-GCST90001708 | Weighted median | 27 | 0.0521526526515311 | 0.0198343299312712 | 0.00855323168055867 | 0.0132773659862394 | 0.0910279393168227 | 1.05353655538746 | 1.01336590161606 | 1.0952996067537 | 0.568401123498944 |
| ebi-a-GCST90001708 | finngen_R11_O15_PREECLAMPS | Pre-eclampsia \|\| id:finngen_R11_O15_PREECLAMPS | BAFF-R on IgD+ CD38+ B cell \|\| id:ebi-a-GCST90001708 | Weighted mode | 27 | 0.0358936884910611 | 0.0177992065606825 | 0.054177014394265 | 0.00100724363212348 | 0.0707801333499987 | 1.03654564390164 | 1.00100775107235 | 1.073345207 | 0.838871423749761 |
| ebi-a-GCST90001709 | finngen_R11_O15_PREECLAMPS | Pre-eclampsia \|\| id:finngen_R11_O15_PREECLAMPS | BAFF-R on IgD+ CD38dim B cell \|\| id:ebi-a-GCST90001709 | Inverse variance weighted (multiplicative random effects) | 25 | 0.00967803636562623 | 0.0152067073095302 | 0.524494837023272 | -0.02012711 | 0.0394831826923054 | 1.00972502000705 | 0.980074088209738 | 1.04027300414665 | 0.904087927705763 |
| ebi-a-GCST90001709 | finngen_R11_O15_PREECLAMPS | Pre-eclampsia \|\| id:finngen_R11_O15_PREECLAMPS | BAFF-R on IgD+ CD38dim B cell \|\| id:ebi-a-GCST90001709 | MR Egger | 25 | 0.0156001160814905 | 0.0199692380362796 | 0.442647944113418 | -0.02353959 | 0.0547398226325985 | 1.01572243311796 | 0.976735304493967 | 1.05626576247653 | 0.99380795269851 |
| ebi-a-GCST90001709 | finngen_R11_O15_PREECLAMPS | Pre-eclampsia \|\| id:finngen_R11_O15_PREECLAMPS | BAFF-R on IgD+ CD38dim B cell \|\| id:ebi-a-GCST90001709 | Weighted median | 25 | 0.0266868570515613 | 0.017853046955944 | 0.134964855630217 | -0.008305115 | 0.0616788290852115 | 1.02704614014642 | 0.991729277209118 | 1.06362068583686 | 0.88227178111339 |
| ebi-a-GCST90001709 | finngen_R11_O15_PREECLAMPS | Pre-eclampsia \|\| id:finngen_R11_O15_PREECLAMPS | BAFF-R on IgD+ CD38dim B cell \|\| id:ebi-a-GCST90001709 | Weighted mode | 25 | 0.0197293667125565 | 0.0157092539644594 | 0.221238787169557 | -0.011060771 | 0.050519504482897 | 1.01992527694187 | 0.989000174362338 | 1.051817378 | 0.872755742358858 |
| ebi-a-GCST90001710 | finngen_R11_O15_PREECLAMPS | Pre-eclampsia \|\| id:finngen_R11_O15_PREECLAMPS | BAFF-R on IgD- CD24- B cell \|\| id:ebi-a-GCST90001710 | Inverse variance weighted (multiplicative random effects) | 22 | 0.0139774712945229 | 0.020392546202174 | 0.493078547246393 | -0.025991919 | 0.0539468618507839 | 1.01407561287033 | 0.974342962987399 | 1.05542851714694 | 0.898694924660397 |
| ebi-a-GCST90001710 | finngen_R11_O15_PREECLAMPS | Pre-eclampsia \|\| id:finngen_R11_O15_PREECLAMPS | BAFF-R on IgD- CD24- B cell \|\| id:ebi-a-GCST90001710 | MR Egger | 22 | 0.0403557085439483 | 0.026197437932481 | 0.139125450031649 | -0.01099127 | 0.0917026868916111 | 1.04118106533136 | 0.989068913503432 | 1.09603890689946 | 0.97261503632431 |
| ebi-a-GCST90001710 | finngen_R11_O15_PREECLAMPS | Pre-eclampsia \|\| id:finngen_R11_O15_PREECLAMPS | BAFF-R on IgD- CD24- B cell \|\| id:ebi-a-GCST90001710 | Weighted median | 22 | 0.0312546961081771 | 0.0198749792350285 | 0.115819584431473 | -0.007700263 | 0.070209655408833 | 1.03174825268913 | 0.992329307883768 | 1.07273306196834 | 0.88227178111339 |
| ebi-a-GCST90001710 | finngen_R11_O15_PREECLAMPS | Pre-eclampsia \|\| id:finngen_R11_O15_PREECLAMPS | BAFF-R on IgD- CD24- B cell \|\| id:ebi-a-GCST90001710 | Weighted mode | 22 | 0.047599950039947 | 0.0198645345967238 | 0.0259560380315987 | 0.00866546223036838 | 0.0865344378495256 | 1.04875101858915 | 1.00870311603215 | 1.09038891772068 | 0.790577658379112 |
| ebi-a-GCST90001711 | finngen_R11_O15_PREECLAMPS | Pre-eclampsia \|\| id:finngen_R11_O15_PREECLAMPS | BAFF-R on IgD- CD27- B cell \|\| id:ebi-a-GCST90001711 | Inverse variance weighted | 18 | 0.0212723423555892 | 0.0181615227892951 | 0.241483726848895 | -0.014324242 | 0.0568689270226077 | 1.02150021153229 | 0.985777861545948 | 1.05851705831992 | 0.885079385681701 |
| ebi-a-GCST90001711 | finngen_R11_O15_PREECLAMPS | Pre-eclampsia \|\| id:finngen_R11_O15_PREECLAMPS | BAFF-R on IgD- CD27- B cell \|\| id:ebi-a-GCST90001711 | MR Egger | 18 | 0.0481638048898681 | 0.0226353691391983 | 0.049247829079794 | 0.00379848137703943 | 0.0925291284026968 | 1.04934252868458 | 1.00380570475048 | 1.09694509335336 | 0.862303274556491 |
| ebi-a-GCST90001711 | finngen_R11_O15_PREECLAMPS | Pre-eclampsia \|\| id:finngen_R11_O15_PREECLAMPS | BAFF-R on IgD- CD27- B cell \|\| id:ebi-a-GCST90001711 | Weighted median | 18 | 0.0439506047762941 | 0.0189430682736186 | 0.0203332070317483 | 0.00682219096000164 | 0.0810790185925865 | 1.04493073902605 | 1.00684551511519 | 1.08445658541431 | 0.598716529366782 |
| ebi-a-GCST90001711 | finngen_R11_O15_PREECLAMPS | Pre-eclampsia \|\| id:finngen_R11_O15_PREECLAMPS | BAFF-R on IgD- CD27- B cell \|\| id:ebi-a-GCST90001711 | Weighted mode | 18 | 0.0424633212837453 | 0.0183688639988104 | 0.0335924948940593 | 0.00646034784607683 | 0.0784662947214137 | 1.04337778591407 | 1.00648126090418 | 1.08162689801196 | 0.838871423749761 |
| ebi-a-GCST90001712 | finngen_R11_O15_PREECLAMPS | Pre-eclampsia \|\| id:finngen_R11_O15_PREECLAMPS | BAFF-R on IgD- CD38- B cell \|\| id:ebi-a-GCST90001712 | Inverse variance weighted | 17 | 0.0174664850806519 | 0.0152100944231333 | 0.250824834274128 | -0.0123453 | 0.047278270149993 | 1.01761991612982 | 0.9877305906088 | 1.04841371073238 | 0.885079385681701 |
| ebi-a-GCST90001712 | finngen_R11_O15_PREECLAMPS | Pre-eclampsia \|\| id:finngen_R11_O15_PREECLAMPS | BAFF-R on IgD- CD38- B cell \|\| id:ebi-a-GCST90001712 | MR Egger | 17 | 0.0478078948459774 | 0.0186392357873539 | 0.0215511754604028 | 0.0112749927027638 | 0.0843407969891909 | 1.0489691235924 | 1.01133879499765 | 1.08799961762839 | 0.716086784616111 |
| ebi-a-GCST90001712 | finngen_R11_O15_PREECLAMPS | Pre-eclampsia \|\| id:finngen_R11_O15_PREECLAMPS | BAFF-R on IgD- CD38- B cell \|\| id:ebi-a-GCST90001712 | Weighted median | 17 | 0.0372705645058698 | 0.0186900606568861 | 0.0461369501644528 | 0.00063804561837303 | 0.0739030833933666 | 1.03797382172325 | 1.00063824921278 | 1.0767024500916 | 0.749469123782556 |
| ebi-a-GCST90001712 | finngen_R11_O15_PREECLAMPS | Pre-eclampsia \|\| id:finngen_R11_O15_PREECLAMPS | BAFF-R on IgD- CD38- B cell \|\| id:ebi-a-GCST90001712 | Weighted mode | 17 | 0.0405971564246763 | 0.0170095401743887 | 0.0296926885517438 | 0.00725845768287442 | 0.0739358551664781 | 1.04143248664439 | 1.00728486413822 | 1.07673773611819 | 0.838871423749761 |
| ebi-a-GCST90001713 | finngen_R11_O15_PREECLAMPS | Pre-eclampsia \|\| id:finngen_R11_O15_PREECLAMPS | BAFF-R on IgD- CD38+ B cell \|\| id:ebi-a-GCST90001713 | Inverse variance weighted (multiplicative random effects) | 15 | 0.0569340796823207 | 0.0364217663643579 | 0.118008307158392 | -0.014452582 | 0.128320741756462 | 1.0585860257683 | 0.985651354854062 | 1.13691760117131 | 0.820779257935285 |
| ebi-a-GCST90001713 | finngen_R11_O15_PREECLAMPS | Pre-eclampsia \|\| id:finngen_R11_O15_PREECLAMPS | BAFF-R on IgD- CD38+ B cell \|\| id:ebi-a-GCST90001713 | MR Egger | 15 | 0.080759034636548 | 0.0696202449564373 | 0.266912623992883 | -0.055696645 | 0.217214714751165 | 1.08410963221865 | 0.945826012973462 | 1.2426108803821 | 0.99380795269851 |
| ebi-a-GCST90001713 | finngen_R11_O15_PREECLAMPS | Pre-eclampsia \|\| id:finngen_R11_O15_PREECLAMPS | BAFF-R on IgD- CD38+ B cell \|\| id:ebi-a-GCST90001713 | Weighted median | 15 | 0.0124621932045656 | 0.0483728398529267 | 0.796694094387096 | -0.082348573 | 0.107272959316302 | 1.01254016991791 | 0.920950884213595 | 1.11323808171687 | 0.957984984509652 |
| ebi-a-GCST90001713 | finngen_R11_O15_PREECLAMPS | Pre-eclampsia \|\| id:finngen_R11_O15_PREECLAMPS | BAFF-R on IgD- CD38+ B cell \|\| id:ebi-a-GCST90001713 | Weighted mode | 15 | -0.032780856 | 0.070787703085228 | 0.650418683816369 | -0.171524754 | 0.105963041781635 | 0.96775061283656 | 0.842379415238674 | 1.11178078631015 | 0.959313411571287 |
| ebi-a-GCST90001714 | finngen_R11_O15_PREECLAMPS | Pre-eclampsia \|\| id:finngen_R11_O15_PREECLAMPS | BAFF-R on IgD- CD38dim B cell \|\| id:ebi-a-GCST90001714 | Inverse variance weighted (multiplicative random effects) | 16 | 0.0188365043576897 | 0.0197829567795763 | 0.341016760489738 | -0.019938091 | 0.0576110996456593 | 1.01901503048018 | 0.980259358373219 | 1.05930295230006 | 0.885079385681701 |
| ebi-a-GCST90001714 | finngen_R11_O15_PREECLAMPS | Pre-eclampsia \|\| id:finngen_R11_O15_PREECLAMPS | BAFF-R on IgD- CD38dim B cell \|\| id:ebi-a-GCST90001714 | MR Egger | 16 | 0.0510298056978257 | 0.0237784945406994 | 0.0498826854915838 | 0.0044239563980549 | 0.0976356549975966 | 1.05235425896881 | 1.00443375653963 | 1.10256099932868 | 0.862303274556491 |
| ebi-a-GCST90001714 | finngen_R11_O15_PREECLAMPS | Pre-eclampsia \|\| id:finngen_R11_O15_PREECLAMPS | BAFF-R on IgD- CD38dim B cell \|\| id:ebi-a-GCST90001714 | Weighted median | 16 | 0.0422483049795132 | 0.0181412567753617 | 0.0198668991942121 | 0.00669144169980417 | 0.0778051682592222 | 1.04315346679562 | 1.00671387941478 | 1.08091204217856 | 0.598716529366782 |
| ebi-a-GCST90001714 | finngen_R11_O15_PREECLAMPS | Pre-eclampsia \|\| id:finngen_R11_O15_PREECLAMPS | BAFF-R on IgD- CD38dim B cell \|\| id:ebi-a-GCST90001714 | Weighted mode | 16 | 0.044198990978395 | 0.0187158106791241 | 0.0321454983862694 | 0.00751600204731171 | 0.0808819799094782 | 1.04519031764032 | 1.00754431808738 | 1.08424292656705 | 0.838871423749761 |
| ebi-a-GCST90001715 | finngen_R11_O15_PREECLAMPS | Pre-eclampsia \|\| id:finngen_R11_O15_PREECLAMPS | BAFF-R on memory B cell \|\| id:ebi-a-GCST90001715 | Inverse variance weighted (multiplicative random effects) | 17 | 0.0188534722378151 | 0.0205636351902963 | 0.359228809134378 | -0.021451253 | 0.0591581972107957 | 1.01903232115175 | 0.978777189017081 | 1.0609430656989 | 0.885079385681701 |
| ebi-a-GCST90001715 | finngen_R11_O15_PREECLAMPS | Pre-eclampsia \|\| id:finngen_R11_O15_PREECLAMPS | BAFF-R on memory B cell \|\| id:ebi-a-GCST90001715 | MR Egger | 17 | 0.0554990813494303 | 0.0254514517864287 | 0.0455502273388362 | 0.00561423584803005 | 0.105383926850831 | 1.05706804599581 | 1.00563002520467 | 1.11113712385228 | 0.862303274556491 |
| ebi-a-GCST90001715 | finngen_R11_O15_PREECLAMPS | Pre-eclampsia \|\| id:finngen_R11_O15_PREECLAMPS | BAFF-R on memory B cell \|\| id:ebi-a-GCST90001715 | Weighted median | 17 | 0.0422692575503181 | 0.0175138093033554 | 0.0158009230742769 | 0.00794219131574143 | 0.0765963237848948 | 1.04317532377148 | 1.00797381418003 | 1.07960617708206 | 0.598716529366782 |
| ebi-a-GCST90001715 | finngen_R11_O15_PREECLAMPS | Pre-eclampsia \|\| id:finngen_R11_O15_PREECLAMPS | BAFF-R on memory B cell \|\| id:ebi-a-GCST90001715 | Weighted mode | 17 | 0.046523011014407 | 0.0175430372257007 | 0.0174003340527197 | 0.0121386580520337 | 0.0809073639767804 | 1.04762218564038 | 1.0122126305677 | 1.08427044941179 | 0.720210568263475 |
| ebi-a-GCST90001716 | finngen_R11_O15_PREECLAMPS | Pre-eclampsia \|\| id:finngen_R11_O15_PREECLAMPS | BAFF-R on naive-mature B cell \|\| id:ebi-a-GCST90001716 | Inverse variance weighted (multiplicative random effects) | 23 | 0.0145890397252038 | 0.0169394149419278 | 0.389101387794042 | -0.018612214 | 0.0477902930113823 | 1.01469597918038 | 0.981559924077326 | 1.04895065997388 | 0.885079385681701 |
| ebi-a-GCST90001716 | finngen_R11_O15_PREECLAMPS | Pre-eclampsia \|\| id:finngen_R11_O15_PREECLAMPS | BAFF-R on naive-mature B cell \|\| id:ebi-a-GCST90001716 | MR Egger | 23 | 0.0517559197720334 | 0.0207431331499026 | 0.0209999111987193 | 0.0110993787982244 | 0.0924124607458424 | 1.05311866569696 | 1.0111612054371 | 1.09681712280478 | 0.716086784616111 |
| ebi-a-GCST90001716 | finngen_R11_O15_PREECLAMPS | Pre-eclampsia \|\| id:finngen_R11_O15_PREECLAMPS | BAFF-R on naive-mature B cell \|\| id:ebi-a-GCST90001716 | Weighted median | 23 | 0.0515232287116616 | 0.0187152277722428 | 0.00590499045501877 | 0.0148413822780656 | 0.0882050751452575 | 1.05287364290635 | 1.01495206246314 | 1.09221208461474 | 0.507633389188193 |
| ebi-a-GCST90001716 | finngen_R11_O15_PREECLAMPS | Pre-eclampsia \|\| id:finngen_R11_O15_PREECLAMPS | BAFF-R on naive-mature B cell \|\| id:ebi-a-GCST90001716 | Weighted mode | 23 | 0.042577055352372 | 0.0159670482112115 | 0.0140956597942621 | 0.0112816408583974 | 0.0738724698463466 | 1.04349646026331 | 1.01134551855771 | 1.07666948891505 | 0.720210568263475 |
| ebi-a-GCST90001717 | finngen_R11_O15_PREECLAMPS | Pre-eclampsia \|\| id:finngen_R11_O15_PREECLAMPS | BAFF-R on unswitched memory B cell \|\| id:ebi-a-GCST90001717 | Inverse variance weighted (multiplicative random effects) | 24 | 0.0152929506555753 | 0.0150946817371879 | 0.310995673850283 | -0.014292626 | 0.0448785268604636 | 1.01541048621628 | 0.985809029142802 | 1.04590080333767 | 0.885079385681701 |
| ebi-a-GCST90001717 | finngen_R11_O15_PREECLAMPS | Pre-eclampsia \|\| id:finngen_R11_O15_PREECLAMPS | BAFF-R on unswitched memory B cell \|\| id:ebi-a-GCST90001717 | MR Egger | 24 | 0.0328246617978894 | 0.0193270482740999 | 0.103536993764894 | -0.005056353 | 0.0707056764151252 | 1.03336933423451 | 0.994956409014066 | 1.07326529208897 | 0.97261503632431 |
| ebi-a-GCST90001717 | finngen_R11_O15_PREECLAMPS | Pre-eclampsia \|\| id:finngen_R11_O15_PREECLAMPS | BAFF-R on unswitched memory B cell \|\| id:ebi-a-GCST90001717 | Weighted median | 24 | 0.0310933400447783 | 0.0174819821297605 | 0.075306428077531 | -0.003171345 | 0.0653580250191089 | 1.03158178728312 | 0.996833678473062 | 1.0675411624177 | 0.86014060819805 |
| ebi-a-GCST90001717 | finngen_R11_O15_PREECLAMPS | Pre-eclampsia \|\| id:finngen_R11_O15_PREECLAMPS | BAFF-R on unswitched memory B cell \|\| id:ebi-a-GCST90001717 | Weighted mode | 24 | 0.031967947679493 | 0.0148932004660481 | 0.0426140936496083 | 0.00277727476603861 | 0.0611586205929473 | 1.03248441125348 | 1.00278113496639 | 1.06306752521544 | 0.838871423749761 |
| ebi-a-GCST90001718 | finngen_R11_O15_PREECLAMPS | Pre-eclampsia \|\| id:finngen_R11_O15_PREECLAMPS | BAFF-R on switched memory B cell \|\| id:ebi-a-GCST90001718 | Inverse variance weighted (multiplicative random effects) | 19 | 0.0126419192679548 | 0.017277603076245 | 0.464355332457117 | -0.021222183 | 0.046506021297395 | 1.01272216613092 | 0.979001423163683 | 1.04760438698711 | 0.898694924660397 |
| ebi-a-GCST90001718 | finngen_R11_O15_PREECLAMPS | Pre-eclampsia \|\| id:finngen_R11_O15_PREECLAMPS | BAFF-R on switched memory B cell \|\| id:ebi-a-GCST90001718 | MR Egger | 19 | 0.061525566137157 | 0.0189357547315866 | 0.00472025409991285 | 0.0244114868632472 | 0.0986396454110667 | 1.06345768468628 | 1.02471188662964 | 1.10366851587724 | 0.565056961366001 |
| ebi-a-GCST90001718 | finngen_R11_O15_PREECLAMPS | Pre-eclampsia \|\| id:finngen_R11_O15_PREECLAMPS | BAFF-R on switched memory B cell \|\| id:ebi-a-GCST90001718 | Weighted median | 19 | 0.0365609683030512 | 0.0179804853557706 | 0.042015218798146 | 0.00131921700574088 | 0.0718027196003616 | 1.03723754070273 | 1.00132008755527 | 1.07444335653924 | 0.743099833442067 |
| ebi-a-GCST90001718 | finngen_R11_O15_PREECLAMPS | Pre-eclampsia \|\| id:finngen_R11_O15_PREECLAMPS | BAFF-R on switched memory B cell \|\| id:ebi-a-GCST90001718 | Weighted mode | 19 | 0.0386373898928958 | 0.0179276186113164 | 0.0449336563678236 | 0.00349925741471564 | 0.0737755223710759 | 1.03939352071313 | 1.00350538696348 | 1.07656511358594 | 0.838871423749761 |
| ebi-a-GCST90001719 | finngen_R11_O15_PREECLAMPS | Pre-eclampsia \|\| id:finngen_R11_O15_PREECLAMPS | BAFF-R on IgD+ B cell \|\| id:ebi-a-GCST90001719 | Inverse variance weighted (multiplicative random effects) | 24 | 0.016743852067504 | 0.0183991622140783 | 0.362804897963699 | -0.019318506 | 0.0528062100070975 | 1.01688481601938 | 0.980866900617431 | 1.05422532700395 | 0.885079385681701 |
| ebi-a-GCST90001719 | finngen_R11_O15_PREECLAMPS | Pre-eclampsia \|\| id:finngen_R11_O15_PREECLAMPS | BAFF-R on IgD+ B cell \|\| id:ebi-a-GCST90001719 | MR Egger | 24 | 0.0398989272025463 | 0.023823242880944 | 0.108133654586013 | -0.006794629 | 0.0865924832491965 | 1.04070558185195 | 0.993228402453898 | 1.09045221161815 | 0.97261503632431 |
| ebi-a-GCST90001719 | finngen_R11_O15_PREECLAMPS | Pre-eclampsia \|\| id:finngen_R11_O15_PREECLAMPS | BAFF-R on IgD+ B cell \|\| id:ebi-a-GCST90001719 | Weighted median | 24 | 0.0520424717839184 | 0.0186594205819148 | 0.00528592745039109 | 0.0154700074433653 | 0.0886149361244715 | 1.05342048221037 | 1.0155902874523 | 1.09265983147996 | 0.507633389188193 |
| ebi-a-GCST90001719 | finngen_R11_O15_PREECLAMPS | Pre-eclampsia \|\| id:finngen_R11_O15_PREECLAMPS | BAFF-R on IgD+ B cell \|\| id:ebi-a-GCST90001719 | Weighted mode | 24 | 0.0407726616955031 | 0.0159569782719593 | 0.0176945642072508 | 0.00949698428246282 | 0.0720483391085434 | 1.0416152795751 | 1.00954222373708 | 1.07470729320071 | 0.720210568263475 |
| ebi-a-GCST90001720 | finngen_R11_O15_PREECLAMPS | Pre-eclampsia \|\| id:finngen_R11_O15_PREECLAMPS | BAFF-R on transitional B cell \|\| id:ebi-a-GCST90001720 | Inverse variance weighted | 24 | 0.00337265298463133 | 0.0166912994745574 | 0.839869396074082 | -0.029342294 | 0.0360875999547639 | 1.00337834677797 | 0.971084011354997 | 1.03674666147382 | 0.977824840984872 |
| ebi-a-GCST90001720 | finngen_R11_O15_PREECLAMPS | Pre-eclampsia \|\| id:finngen_R11_O15_PREECLAMPS | BAFF-R on transitional B cell \|\| id:ebi-a-GCST90001720 | MR Egger | 24 | 0.0347895025729596 | 0.0207453000779866 | 0.10769927254461 | -0.005871286 | 0.0754502907258133 | 1.03540173645764 | 0.994145916734254 | 1.07836962141451 | 0.97261503632431 |
| ebi-a-GCST90001720 | finngen_R11_O15_PREECLAMPS | Pre-eclampsia \|\| id:finngen_R11_O15_PREECLAMPS | BAFF-R on transitional B cell \|\| id:ebi-a-GCST90001720 | Weighted median | 24 | 0.0492877971186263 | 0.0198639599545666 | 0.0130916106216747 | 0.0103544356076757 | 0.0882211586295769 | 1.05052264462846 | 1.01040822827999 | 1.09222965133194 | 0.598716529366782 |
| ebi-a-GCST90001720 | finngen_R11_O15_PREECLAMPS | Pre-eclampsia \|\| id:finngen_R11_O15_PREECLAMPS | BAFF-R on transitional B cell \|\| id:ebi-a-GCST90001720 | Weighted mode | 24 | 0.0411083592779281 | 0.0195016852122064 | 0.0461395739877091 | 0.00288505626200349 | 0.0793316622938526 | 1.04196500600414 | 1.00288922204203 | 1.08256330796594 | 0.838871423749761 |
| ebi-a-GCST90001721 | finngen_R11_O15_PREECLAMPS | Pre-eclampsia \|\| id:finngen_R11_O15_PREECLAMPS | CD19 on CD20- B cell \|\| id:ebi-a-GCST90001721 | Inverse variance weighted | 21 | 0.0325043872922447 | 0.0231877481150303 | 0.16097752929268 | -0.012943599 | 0.0779523735977041 | 1.0330384253755 | 0.987139809109565 | 1.08107116991353 | 0.85505992597664 |
| ebi-a-GCST90001721 | finngen_R11_O15_PREECLAMPS | Pre-eclampsia \|\| id:finngen_R11_O15_PREECLAMPS | CD19 on CD20- B cell \|\| id:ebi-a-GCST90001721 | MR Egger | 21 | 0.0128824774392413 | 0.0326554228488665 | 0.697607024167675 | -0.051122151 | 0.0768871062230197 | 1.01296581402779 | 0.950162599804379 | 1.07992015324561 | 0.99380795269851 |
| ebi-a-GCST90001721 | finngen_R11_O15_PREECLAMPS | Pre-eclampsia \|\| id:finngen_R11_O15_PREECLAMPS | CD19 on CD20- B cell \|\| id:ebi-a-GCST90001721 | Weighted median | 21 | 0.0350697382167143 | 0.0348130789475585 | 0.313755803572798 | -0.033163897 | 0.103303372953929 | 1.03569193358969 | 0.967379996379999 | 1.10882774640443 | 0.940845681141708 |
| ebi-a-GCST90001721 | finngen_R11_O15_PREECLAMPS | Pre-eclampsia \|\| id:finngen_R11_O15_PREECLAMPS | CD19 on CD20- B cell \|\| id:ebi-a-GCST90001721 | Weighted mode | 21 | 0.0420431486493318 | 0.0313367051989322 | 0.194742831281473 | -0.019376794 | 0.103463090839239 | 1.04293947920976 | 0.980809729838891 | 1.10900486 | 0.872755742358858 |
| ebi-a-GCST90001722 | finngen_R11_O15_PREECLAMPS | Pre-eclampsia \|\| id:finngen_R11_O15_PREECLAMPS | CD19 on CD20- CD38- B cell \|\| id:ebi-a-GCST90001722 | Inverse variance weighted | 19 | -0.002339787 | 0.0317064401194996 | 0.941173237184314 | -0.06448441 | 0.0598048351901333 | 0.997662947724902 | 0.93755073049059 | 1.06162933363864 | 0.986667691552896 |
| ebi-a-GCST90001722 | finngen_R11_O15_PREECLAMPS | Pre-eclampsia \|\| id:finngen_R11_O15_PREECLAMPS | CD19 on CD20- CD38- B cell \|\| id:ebi-a-GCST90001722 | MR Egger | 19 | 0.0450612874765344 | 0.0552941261349414 | 0.426377794227835 | -0.0633152 | 0.15343777470102 | 1.0460919702812 | 0.938647565580417 | 1.16583524041862 | 0.99380795269851 |
| ebi-a-GCST90001722 | finngen_R11_O15_PREECLAMPS | Pre-eclampsia \|\| id:finngen_R11_O15_PREECLAMPS | CD19 on CD20- CD38- B cell \|\| id:ebi-a-GCST90001722 | Weighted median | 19 | 0.0148453345721311 | 0.0388754372559725 | 0.702558340254134 | -0.061350522 | 0.0910411915938371 | 1.01495607386008 | 0.940493517900222 | 1.09531412206368 | 0.946704357535493 |
| ebi-a-GCST90001722 | finngen_R11_O15_PREECLAMPS | Pre-eclampsia \|\| id:finngen_R11_O15_PREECLAMPS | CD19 on CD20- CD38- B cell \|\| id:ebi-a-GCST90001722 | Weighted mode | 19 | 0.0204101309404678 | 0.0411391761096148 | 0.625810566258937 | -0.060222654 | 0.101042916115313 | 1.02061984197624 | 0.941554869065273 | 1.10632411987813 | 0.959313411571287 |
| ebi-a-GCST90001723 | finngen_R11_O15_PREECLAMPS | Pre-eclampsia \|\| id:finngen_R11_O15_PREECLAMPS | CD19 on CD24+ CD27+ B cell \|\| id:ebi-a-GCST90001723 | Inverse variance weighted (multiplicative random effects) | 29 | -0.024354964 | 0.0207269814671137 | 0.239980032184543 | -0.064979848 | 0.0162699197099962 | 0.975939225009305 | 0.93708634768753 | 1.01640299558538 | 0.885079385681701 |
| ebi-a-GCST90001723 | finngen_R11_O15_PREECLAMPS | Pre-eclampsia \|\| id:finngen_R11_O15_PREECLAMPS | CD19 on CD24+ CD27+ B cell \|\| id:ebi-a-GCST90001723 | MR Egger | 29 | -0.05718373 | 0.0283516781867242 | 0.0537456226777776 | -0.11275302 | -0.001614441 | 0.944420534768346 | 0.893371278125289 | 0.998386861466842 | 0.862303274556491 |
| ebi-a-GCST90001723 | finngen_R11_O15_PREECLAMPS | Pre-eclampsia \|\| id:finngen_R11_O15_PREECLAMPS | CD19 on CD24+ CD27+ B cell \|\| id:ebi-a-GCST90001723 | Weighted median | 29 | -0.042806065 | 0.026251799101419 | 0.102975706516014 | -0.094259591 | 0.00864746165084037 | 0.95809718102232 | 0.910046491854106 | 1.00868495895491 | 0.88227178111339 |
| ebi-a-GCST90001723 | finngen_R11_O15_PREECLAMPS | Pre-eclampsia \|\| id:finngen_R11_O15_PREECLAMPS | CD19 on CD24+ CD27+ B cell \|\| id:ebi-a-GCST90001723 | Weighted mode | 29 | -0.036002565 | 0.0262197176006114 | 0.180615166681887 | -0.087393212 | 0.0153880814160142 | 0.96463781910563 | 0.916316718442397 | 1.0155070875828 | 0.872755742358858 |
| ebi-a-GCST90001724 | finngen_R11_O15_PREECLAMPS | Pre-eclampsia \|\| id:finngen_R11_O15_PREECLAMPS | CD19 on IgD+ CD24+ B cell \|\| id:ebi-a-GCST90001724 | Inverse variance weighted (multiplicative random effects) | 34 | 0.0160545385456772 | 0.0150829677606512 | 0.28714069904308 | -0.013508078 | 0.0456171553565535 | 1.01618410509806 | 0.986582746408475 | 1.04667362085248 | 0.885079385681701 |
| ebi-a-GCST90001724 | finngen_R11_O15_PREECLAMPS | Pre-eclampsia \|\| id:finngen_R11_O15_PREECLAMPS | CD19 on IgD+ CD24+ B cell \|\| id:ebi-a-GCST90001724 | MR Egger | 34 | 0.0130395914971146 | 0.0190201392999054 | 0.497922729523587 | -0.024239882 | 0.0503190645249292 | 1.01312497770042 | 0.976051544934483 | 1.05160657320549 | 0.99380795269851 |
| ebi-a-GCST90001724 | finngen_R11_O15_PREECLAMPS | Pre-eclampsia \|\| id:finngen_R11_O15_PREECLAMPS | CD19 on IgD+ CD24+ B cell \|\| id:ebi-a-GCST90001724 | Weighted median | 34 | 0.0218356309982101 | 0.0172161326611053 | 0.204682336999636 | -0.011907989 | 0.0555792510139766 | 1.02207577308831 | 0.988162630493602 | 1.05715279418354 | 0.940845681141708 |
| ebi-a-GCST90001724 | finngen_R11_O15_PREECLAMPS | Pre-eclampsia \|\| id:finngen_R11_O15_PREECLAMPS | CD19 on IgD+ CD24+ B cell \|\| id:ebi-a-GCST90001724 | Weighted mode | 34 | 0.0173775230538111 | 0.0135629599945352 | 0.209036982211997 | -0.009205879 | 0.0439609246431002 | 1.01752939062625 | 0.990836365832766 | 1.04494152262775 | 0.872755742358858 |
| ebi-a-GCST90001725 | finngen_R11_O15_PREECLAMPS | Pre-eclampsia \|\| id:finngen_R11_O15_PREECLAMPS | CD19 on IgD+ CD24- B cell \|\| id:ebi-a-GCST90001725 | Inverse variance weighted | 24 | -0.006594016 | 0.0101421092612925 | 0.515587454433054 | -0.02647255 | 0.0132845180748137 | 0.993427676739577 | 0.973874776107782 | 1.01337314932466 | 0.898694924660397 |
| ebi-a-GCST90001725 | finngen_R11_O15_PREECLAMPS | Pre-eclampsia \|\| id:finngen_R11_O15_PREECLAMPS | CD19 on IgD+ CD24- B cell \|\| id:ebi-a-GCST90001725 | MR Egger | 24 | -0.005494017 | 0.0119179626042315 | 0.649333341407709 | -0.028853224 | 0.0178651896541797 | 0.994521047460707 | 0.971559055792419 | 1.01802572673839 | 0.99380795269851 |
| ebi-a-GCST90001725 | finngen_R11_O15_PREECLAMPS | Pre-eclampsia \|\| id:finngen_R11_O15_PREECLAMPS | CD19 on IgD+ CD24- B cell \|\| id:ebi-a-GCST90001725 | Weighted median | 24 | -0.001968551 | 0.0153051855616545 | 0.897658442 | -0.031966715 | 0.0280296127010615 | 0.998033385325944 | 0.968538819647245 | 1.02842613844631 | 0.966404007249152 |
| ebi-a-GCST90001725 | finngen_R11_O15_PREECLAMPS | Pre-eclampsia \|\| id:finngen_R11_O15_PREECLAMPS | CD19 on IgD+ CD24- B cell \|\| id:ebi-a-GCST90001725 | Weighted mode | 24 | -0.004429568 | 0.0118982173529767 | 0.713086514874657 | -0.027750074 | 0.0188909380823425 | 0.995580228137078 | 0.972631422364956 | 1.01907050077348 | 0.959313411571287 |
| ebi-a-GCST90001726 | finngen_R11_O15_PREECLAMPS | Pre-eclampsia \|\| id:finngen_R11_O15_PREECLAMPS | CD19 on IgD+ CD38- B cell \|\| id:ebi-a-GCST90001726 | Inverse variance weighted | 31 | -0.003093691 | 0.010515164666556 | 0.768595690115919 | -0.023703413 | 0.0175160320720387 | 0.996911089855486 | 0.976575305939877 | 1.01767033738414 | 0.977824840984872 |
| ebi-a-GCST90001726 | finngen_R11_O15_PREECLAMPS | Pre-eclampsia \|\| id:finngen_R11_O15_PREECLAMPS | CD19 on IgD+ CD38- B cell \|\| id:ebi-a-GCST90001726 | MR Egger | 31 | -0.015977736 | 0.0127584726890556 | 0.220457771581038 | -0.040984342 | 0.00902887055884696 | 0.984149230996748 | 0.959844158703077 | 1.00906975376104 | 0.99380795269851 |
| ebi-a-GCST90001726 | finngen_R11_O15_PREECLAMPS | Pre-eclampsia \|\| id:finngen_R11_O15_PREECLAMPS | CD19 on IgD+ CD38- B cell \|\| id:ebi-a-GCST90001726 | Weighted median | 31 | 0.00225819237333717 | 0.0162014767457046 | 0.889148386071254 | -0.029496702 | 0.0340130867949182 | 1.00226074401007 | 0.970934079729667 | 1.03459814621329 | 0.964912412213677 |
| ebi-a-GCST90001726 | finngen_R11_O15_PREECLAMPS | Pre-eclampsia \|\| id:finngen_R11_O15_PREECLAMPS | CD19 on IgD+ CD38- B cell \|\| id:ebi-a-GCST90001726 | Weighted mode | 31 | -0.00623341 | 0.0125800123389666 | 0.62385673505877 | -0.030890234 | 0.0184234140220934 | 0.993785977234729 | 0.969581994036282 | 1.0185941721515 | 0.959313411571287 |
| ebi-a-GCST90001727 | finngen_R11_O15_PREECLAMPS | Pre-eclampsia \|\| id:finngen_R11_O15_PREECLAMPS | CD19 on IgD+ CD38- naive B cell \|\| id:ebi-a-GCST90001727 | Inverse variance weighted (multiplicative random effects) | 19 | -0.010966082 | 0.0136904211020736 | 0.423129330769443 | -0.037799307 | 0.0158671433096774 | 0.989093826240996 | 0.962906169633439 | 1.01599369487941 | 0.898694924660397 |
| ebi-a-GCST90001727 | finngen_R11_O15_PREECLAMPS | Pre-eclampsia \|\| id:finngen_R11_O15_PREECLAMPS | CD19 on IgD+ CD38- naive B cell \|\| id:ebi-a-GCST90001727 | MR Egger | 19 | 0.00131640396170354 | 0.0154551504443166 | 0.933116756223889 | -0.028975691 | 0.031608498832564 | 1.00131727080173 | 0.971440079004709 | 1.03211335261468 | 0.99380795269851 |
| ebi-a-GCST90001727 | finngen_R11_O15_PREECLAMPS | Pre-eclampsia \|\| id:finngen_R11_O15_PREECLAMPS | CD19 on IgD+ CD38- naive B cell \|\| id:ebi-a-GCST90001727 | Weighted median | 19 | 0.0010135916617514 | 0.0143262792769107 | 0.943596314996924 | -0.027065916 | 0.0290930990444963 | 1.00101410551938 | 0.973297083830838 | 1.02952043738263 | 0.981835244750373 |
| ebi-a-GCST90001727 | finngen_R11_O15_PREECLAMPS | Pre-eclampsia \|\| id:finngen_R11_O15_PREECLAMPS | CD19 on IgD+ CD38- naive B cell \|\| id:ebi-a-GCST90001727 | Weighted mode | 19 | -0.00308203 | 0.0114690729646142 | 0.791196859202005 | -0.025561413 | 0.0193973526220824 | 0.996922714191532 | 0.974762513648113 | 1.01958670358736 | 0.961305639715923 |
| ebi-a-GCST90001728 | finngen_R11_O15_PREECLAMPS | Pre-eclampsia \|\| id:finngen_R11_O15_PREECLAMPS | CD19 on IgD+ CD38- unswitched memory B cell \|\| id:ebi-a-GCST90001728 | Inverse variance weighted (multiplicative random effects) | 21 | 0.0131319150682577 | 0.0134074672018874 | 0.327358738831843 | -0.013146721 | 0.039410550783957 | 1.01321851733427 | 0.986939320020853 | 1.04019744987702 | 0.885079385681701 |
| ebi-a-GCST90001728 | finngen_R11_O15_PREECLAMPS | Pre-eclampsia \|\| id:finngen_R11_O15_PREECLAMPS | CD19 on IgD+ CD38- unswitched memory B cell \|\| id:ebi-a-GCST90001728 | MR Egger | 21 | 0.000669200975177432 | 0.0199508849138675 | 0.973591910889539 | -0.038434533 | 0.0397729354063577 | 1.00066942494011 | 0.962294700786096 | 1.04057446974609 | 0.99380795269851 |
| ebi-a-GCST90001728 | finngen_R11_O15_PREECLAMPS | Pre-eclampsia \|\| id:finngen_R11_O15_PREECLAMPS | CD19 on IgD+ CD38- unswitched memory B cell \|\| id:ebi-a-GCST90001728 | Weighted median | 21 | -0.004303548 | 0.0151642090176849 | 0.776566396047975 | -0.034025398 | 0.0254183014005793 | 0.995705698720073 | 0.966546956015717 | 1.02574410099069 | 0.957984984509652 |
| ebi-a-GCST90001728 | finngen_R11_O15_PREECLAMPS | Pre-eclampsia \|\| id:finngen_R11_O15_PREECLAMPS | CD19 on IgD+ CD38- unswitched memory B cell \|\| id:ebi-a-GCST90001728 | Weighted mode | 21 | 0.00270369771407386 | 0.0139000896820127 | 0.847739252775977 | -0.024540478 | 0.0299478734908188 | 1.00270735600096 | 0.9757581913178 | 1.03040082135573 | 0.977585971930092 |
| ebi-a-GCST90001729 | finngen_R11_O15_PREECLAMPS | Pre-eclampsia \|\| id:finngen_R11_O15_PREECLAMPS | CD19 on IgD+ CD38+ B cell \|\| id:ebi-a-GCST90001729 | Inverse variance weighted (multiplicative random effects) | 21 | -0.040274335 | 0.0248728226366204 | 0.10540198712505 | -0.089025067 | 0.00847639766193317 | 0.960525897415132 | 0.914822641360532 | 1.00851242403995 | 0.820779257935285 |
| ebi-a-GCST90001729 | finngen_R11_O15_PREECLAMPS | Pre-eclampsia \|\| id:finngen_R11_O15_PREECLAMPS | CD19 on IgD+ CD38+ B cell \|\| id:ebi-a-GCST90001729 | MR Egger | 21 | -0.02685526 | 0.0354884506666317 | 0.458491810196009 | -0.096412623 | 0.0427021037535649 | 0.973502136461847 | 0.90808924037435 | 1.04362695598629 | 0.99380795269851 |
| ebi-a-GCST90001729 | finngen_R11_O15_PREECLAMPS | Pre-eclampsia \|\| id:finngen_R11_O15_PREECLAMPS | CD19 on IgD+ CD38+ B cell \|\| id:ebi-a-GCST90001729 | Weighted median | 21 | -0.027864252 | 0.0283144066752222 | 0.325065577683905 | -0.083360489 | 0.0276319851713432 | 0.972520375622514 | 0.920019430553227 | 1.02801728919163 | 0.940845681141708 |
| ebi-a-GCST90001729 | finngen_R11_O15_PREECLAMPS | Pre-eclampsia \|\| id:finngen_R11_O15_PREECLAMPS | CD19 on IgD+ CD38+ B cell \|\| id:ebi-a-GCST90001729 | Weighted mode | 21 | -0.032215633 | 0.0254284656639795 | 0.219741937535385 | -0.082055425 | 0.017624159965917 | 0.968297762870508 | 0.921220898210588 | 1.01778038188372 | 0.872755742358858 |
| ebi-a-GCST90001730 | finngen_R11_O15_PREECLAMPS | Pre-eclampsia \|\| id:finngen_R11_O15_PREECLAMPS | CD19 on IgD+ CD38dim B cell \|\| id:ebi-a-GCST90001730 | Inverse variance weighted | 24 | -0.007906412 | 0.0101268665419356 | 0.434957642580183 | -0.02775507 | 0.0119422467364686 | 0.992124761776303 | 0.972626562948476 | 1.01201384007629 | 0.898694924660397 |
| ebi-a-GCST90001730 | finngen_R11_O15_PREECLAMPS | Pre-eclampsia \|\| id:finngen_R11_O15_PREECLAMPS | CD19 on IgD+ CD38dim B cell \|\| id:ebi-a-GCST90001730 | MR Egger | 24 | -0.008613761 | 0.0121086958355133 | 0.48433016175315 | -0.032346805 | 0.0151192827607312 | 0.991423231073025 | 0.968170757473003 | 1.01523415732651 | 0.99380795269851 |
| ebi-a-GCST90001730 | finngen_R11_O15_PREECLAMPS | Pre-eclampsia \|\| id:finngen_R11_O15_PREECLAMPS | CD19 on IgD+ CD38dim B cell \|\| id:ebi-a-GCST90001730 | Weighted median | 24 | 0.00100361834526433 | 0.014700658778062 | 0.945570468268486 | -0.027809673 | 0.0298169095502658 | 1.00100412213868 | 0.972573456311545 | 1.03026588483992 | 0.981835244750373 |
| ebi-a-GCST90001730 | finngen_R11_O15_PREECLAMPS | Pre-eclampsia \|\| id:finngen_R11_O15_PREECLAMPS | CD19 on IgD+ CD38dim B cell \|\| id:ebi-a-GCST90001730 | Weighted mode | 24 | -0.005078219 | 0.0121814619823663 | 0.680630707520842 | -0.028953885 | 0.018797446165536 | 0.99493465303706 | 0.971461262558888 | 1.01897523037257 | 0.959313411571287 |
| ebi-a-GCST90001731 | finngen_R11_O15_PREECLAMPS | Pre-eclampsia \|\| id:finngen_R11_O15_PREECLAMPS | CD19 on IgD- CD24- B cell \|\| id:ebi-a-GCST90001731 | Inverse variance weighted (multiplicative random effects) | 23 | 0.0264443192796022 | 0.0313911724618678 | 0.399557041415816 | -0.035082379 | 0.087971017304863 | 1.02679707286922 | 0.965525874170908 | 1.09195647372799 | 0.885079385681701 |
| ebi-a-GCST90001731 | finngen_R11_O15_PREECLAMPS | Pre-eclampsia \|\| id:finngen_R11_O15_PREECLAMPS | CD19 on IgD- CD24- B cell \|\| id:ebi-a-GCST90001731 | MR Egger | 23 | 0.0900610094443372 | 0.0554034230259045 | 0.118959945563862 | -0.0185297 | 0.19865171857511 | 1.09424104070665 | 0.98164091973134 | 1.21975707318158 | 0.97261503632431 |
| ebi-a-GCST90001731 | finngen_R11_O15_PREECLAMPS | Pre-eclampsia \|\| id:finngen_R11_O15_PREECLAMPS | CD19 on IgD- CD24- B cell \|\| id:ebi-a-GCST90001731 | Weighted median | 23 | 0.0389913148615336 | 0.0404502583952242 | 0.335079816071588 | -0.040291192 | 0.118273821316173 | 1.03976145313875 | 0.960509706074834 | 1.12555226937912 | 0.940845681141708 |
| ebi-a-GCST90001731 | finngen_R11_O15_PREECLAMPS | Pre-eclampsia \|\| id:finngen_R11_O15_PREECLAMPS | CD19 on IgD- CD24- B cell \|\| id:ebi-a-GCST90001731 | Weighted mode | 23 | 0.0604388034813983 | 0.0610871671301284 | 0.333231795975693 | -0.059292044 | 0.18016965105645 | 1.06230258636113 | 0.942431497410821 | 1.19742048954208 | 0.888599269773114 |
| ebi-a-GCST90001732 | finngen_R11_O15_PREECLAMPS | Pre-eclampsia \|\| id:finngen_R11_O15_PREECLAMPS | CD19 on IgD- CD27- B cell \|\| id:ebi-a-GCST90001732 | Inverse variance weighted | 26 | -0.048627664 | 0.0243510021623105 | 0.0458309275569319 | -0.096355628 | -0.000899699 | 0.952535727333674 | 0.908140998348804 | 0.99910070517332 | 0.781760498894217 |
| ebi-a-GCST90001732 | finngen_R11_O15_PREECLAMPS | Pre-eclampsia \|\| id:finngen_R11_O15_PREECLAMPS | CD19 on IgD- CD27- B cell \|\| id:ebi-a-GCST90001732 | MR Egger | 26 | -0.027840076 | 0.0404375418903399 | 0.497757792645069 | -0.107097658 | 0.0514175063306399 | 0.972543887693212 | 0.898437929162919 | 1.0527623364818 | 0.99380795269851 |
| ebi-a-GCST90001732 | finngen_R11_O15_PREECLAMPS | Pre-eclampsia \|\| id:finngen_R11_O15_PREECLAMPS | CD19 on IgD- CD27- B cell \|\| id:ebi-a-GCST90001732 | Weighted median | 26 | -0.044994769 | 0.0372779033646723 | 0.227428631275101 | -0.11805946 | 0.0280699212129729 | 0.95600248230402 | 0.888643212339132 | 1.02846759360906 | 0.940845681141708 |
| ebi-a-GCST90001732 | finngen_R11_O15_PREECLAMPS | Pre-eclampsia \|\| id:finngen_R11_O15_PREECLAMPS | CD19 on IgD- CD27- B cell \|\| id:ebi-a-GCST90001732 | Weighted mode | 26 | -0.045512277 | 0.0404411980212096 | 0.271110744043572 | -0.124777025 | 0.0337524714596291 | 0.955507872052821 | 0.882693699462097 | 1.0343285492026 | 0.872755742358858 |
| ebi-a-GCST90001733 | finngen_R11_O15_PREECLAMPS | Pre-eclampsia \|\| id:finngen_R11_O15_PREECLAMPS | CD19 on IgD- CD38- B cell \|\| id:ebi-a-GCST90001733 | Inverse variance weighted (multiplicative random effects) | 22 | -0.025386641 | 0.0354879104284101 | 0.474386452467986 | -0.094942946 | 0.0441696630738586 | 0.974932889762742 | 0.909424819488614 | 1.04515966484795 | 0.898694924660397 |
| ebi-a-GCST90001733 | finngen_R11_O15_PREECLAMPS | Pre-eclampsia \|\| id:finngen_R11_O15_PREECLAMPS | CD19 on IgD- CD38- B cell \|\| id:ebi-a-GCST90001733 | MR Egger | 22 | 0.00937654190904036 | 0.067223674273801 | 0.89046374516751 | -0.12238186 | 0.14113494348569 | 1.00942063939768 | 0.884810430567386 | 1.1515800357243 | 0.99380795269851 |
| ebi-a-GCST90001733 | finngen_R11_O15_PREECLAMPS | Pre-eclampsia \|\| id:finngen_R11_O15_PREECLAMPS | CD19 on IgD- CD38- B cell \|\| id:ebi-a-GCST90001733 | Weighted median | 22 | -0.037044592 | 0.0407429356873348 | 0.363230123457869 | -0.116900746 | 0.0428115619836206 | 0.96363316409604 | 0.889673492513549 | 1.04374119579786 | 0.940845681141708 |
| ebi-a-GCST90001733 | finngen_R11_O15_PREECLAMPS | Pre-eclampsia \|\| id:finngen_R11_O15_PREECLAMPS | CD19 on IgD- CD38- B cell \|\| id:ebi-a-GCST90001733 | Weighted mode | 22 | -0.060667308 | 0.066741814161844 | 0.373672703078184 | -0.191481264 | 0.0701466479827503 | 0.941136296426356 | 0.825735096321019 | 1.07266547394853 | 0.919712949327114 |
| ebi-a-GCST90001734 | finngen_R11_O15_PREECLAMPS | Pre-eclampsia \|\| id:finngen_R11_O15_PREECLAMPS | CD19 on IgD- CD38+ B cell \|\| id:ebi-a-GCST90001734 | Inverse variance weighted (multiplicative random effects) | 18 | 0.0164418648316406 | 0.0333208024561753 | 0.62170070586248 | -0.048866908 | 0.0817506376457441 | 1.01657777614799 | 0.952307865839714 | 1.08518517175825 | 0.931277081937445 |
| ebi-a-GCST90001734 | finngen_R11_O15_PREECLAMPS | Pre-eclampsia \|\| id:finngen_R11_O15_PREECLAMPS | CD19 on IgD- CD38+ B cell \|\| id:ebi-a-GCST90001734 | MR Egger | 18 | -0.010551437 | 0.0534918015157111 | 0.846113317437116 | -0.115395368 | 0.0942924943825342 | 0.989504034547294 | 0.891013796308287 | 1.09888111546884 | 0.99380795269851 |
| ebi-a-GCST90001734 | finngen_R11_O15_PREECLAMPS | Pre-eclampsia \|\| id:finngen_R11_O15_PREECLAMPS | CD19 on IgD- CD38+ B cell \|\| id:ebi-a-GCST90001734 | Weighted median | 18 | 0.0321929673696491 | 0.033955807770508 | 0.343086553244891 | -0.034360416 | 0.0987463505998448 | 1.03271676671709 | 0.966223199707964 | 1.10378628931798 | 0.940845681141708 |
| ebi-a-GCST90001734 | finngen_R11_O15_PREECLAMPS | Pre-eclampsia \|\| id:finngen_R11_O15_PREECLAMPS | CD19 on IgD- CD38+ B cell \|\| id:ebi-a-GCST90001734 | Weighted mode | 18 | 0.0264830547675201 | 0.0334837417109743 | 0.439884633058478 | -0.039145079 | 0.0921111885210297 | 1.02683684712516 | 0.961611189449642 | 1.09648673204126 | 0.933945646747087 |
| ebi-a-GCST90001735 | finngen_R11_O15_PREECLAMPS | Pre-eclampsia \|\| id:finngen_R11_O15_PREECLAMPS | CD19 on IgD- CD38dim B cell \|\| id:ebi-a-GCST90001735 | Inverse variance weighted (multiplicative random effects) | 27 | 0.00122505595735089 | 0.0230229821905008 | 0.957564481640284 | -0.043899989 | 0.0463501010507325 | 1.00122580664491 | 0.957049668051081 | 1.04744105698624 | 0.992671503887443 |
| ebi-a-GCST90001735 | finngen_R11_O15_PREECLAMPS | Pre-eclampsia \|\| id:finngen_R11_O15_PREECLAMPS | CD19 on IgD- CD38dim B cell \|\| id:ebi-a-GCST90001735 | MR Egger | 27 | -0.022620396 | 0.0351362412401526 | 0.525573578871556 | -0.091487429 | 0.0462466369461005 | 0.977633527054268 | 0.912572788292235 | 1.04733269004127 | 0.99380795269851 |
| ebi-a-GCST90001735 | finngen_R11_O15_PREECLAMPS | Pre-eclampsia \|\| id:finngen_R11_O15_PREECLAMPS | CD19 on IgD- CD38dim B cell \|\| id:ebi-a-GCST90001735 | Weighted median | 27 | -0.025879972 | 0.0275957347009072 | 0.348334316716824 | -0.079967612 | 0.0282076682677091 | 0.974452044367096 | 0.92314624498473 | 1.02860927174862 | 0.940845681141708 |
| ebi-a-GCST90001735 | finngen_R11_O15_PREECLAMPS | Pre-eclampsia \|\| id:finngen_R11_O15_PREECLAMPS | CD19 on IgD- CD38dim B cell \|\| id:ebi-a-GCST90001735 | Weighted mode | 27 | -0.023829729 | 0.0266936128443956 | 0.38019948932951 | -0.07614921 | 0.02848975213873 | 0.97645195701971 | 0.926677926434024 | 1.0288994667615 | 0.920254054953283 |
| ebi-a-GCST90001736 | finngen_R11_O15_PREECLAMPS | Pre-eclampsia \|\| id:finngen_R11_O15_PREECLAMPS | CD19 on memory B cell \|\| id:ebi-a-GCST90001736 | Inverse variance weighted (multiplicative random effects) | 25 | -0.010449415 | 0.0277697118327277 | 0.706702654074293 | -0.06487805 | 0.0439792204335613 | 0.989604990709147 | 0.93718174576896 | 1.04496064083378 | 0.956666000237608 |
| ebi-a-GCST90001736 | finngen_R11_O15_PREECLAMPS | Pre-eclampsia \|\| id:finngen_R11_O15_PREECLAMPS | CD19 on memory B cell \|\| id:ebi-a-GCST90001736 | MR Egger | 25 | -0.04426627 | 0.0425374797506847 | 0.308858171925564 | -0.127639731 | 0.0391071899989351 | 0.956699182964781 | 0.880170420479454 | 1.03988194262073 | 0.99380795269851 |
| ebi-a-GCST90001736 | finngen_R11_O15_PREECLAMPS | Pre-eclampsia \|\| id:finngen_R11_O15_PREECLAMPS | CD19 on memory B cell \|\| id:ebi-a-GCST90001736 | Weighted median | 25 | -0.048105611 | 0.0299442086112065 | 0.108162339433647 | -0.106796259 | 0.0105850383011277 | 0.953033131383695 | 0.898708757751057 | 1.01064125800653 | 0.88227178111339 |
| ebi-a-GCST90001736 | finngen_R11_O15_PREECLAMPS | Pre-eclampsia \|\| id:finngen_R11_O15_PREECLAMPS | CD19 on memory B cell \|\| id:ebi-a-GCST90001736 | Weighted mode | 25 | -0.043837715 | 0.031754596883116 | 0.180151137987784 | -0.106076724 | 0.0184012953029965 | 0.957109269742502 | 0.899355642835793 | 1.0185716424023 | 0.872755742358858 |
| ebi-a-GCST90001737 | finngen_R11_O15_PREECLAMPS | Pre-eclampsia \|\| id:finngen_R11_O15_PREECLAMPS | CD19 on naive-mature B cell \|\| id:ebi-a-GCST90001737 | Inverse variance weighted | 28 | -0.00993144 | 0.0106235153903544 | 0.349863330814371 | -0.03075353 | 0.0108906498721008 | 0.990117713602576 | 0.969714548725316 | 1.01095016887004 | 0.885079385681701 |
| ebi-a-GCST90001737 | finngen_R11_O15_PREECLAMPS | Pre-eclampsia \|\| id:finngen_R11_O15_PREECLAMPS | CD19 on naive-mature B cell \|\| id:ebi-a-GCST90001737 | MR Egger | 28 | -0.012206403 | 0.012736543767452 | 0.346700746436542 | -0.037170029 | 0.0127572224208006 | 0.98786779258275 | 0.963512296246293 | 1.01283894292187 | 0.99380795269851 |
| ebi-a-GCST90001737 | finngen_R11_O15_PREECLAMPS | Pre-eclampsia \|\| id:finngen_R11_O15_PREECLAMPS | CD19 on naive-mature B cell \|\| id:ebi-a-GCST90001737 | Weighted median | 28 | -0.015642085 | 0.0164552312409095 | 0.341815414484941 | -0.047894339 | 0.0166101678829183 | 0.984479616683624 | 0.953234501866258 | 1.01674888368814 | 0.940845681141708 |
| ebi-a-GCST90001737 | finngen_R11_O15_PREECLAMPS | Pre-eclampsia \|\| id:finngen_R11_O15_PREECLAMPS | CD19 on naive-mature B cell \|\| id:ebi-a-GCST90001737 | Weighted mode | 28 | -0.006901039 | 0.0123985389752497 | 0.582384543938382 | -0.031202176 | 0.0174000972639618 | 0.993122718361125 | 0.969279588661158 | 1.01755236080782 | 0.948158355498791 |
| ebi-a-GCST90001738 | finngen_R11_O15_PREECLAMPS | Pre-eclampsia \|\| id:finngen_R11_O15_PREECLAMPS | CD19 on unswitched memory B cell \|\| id:ebi-a-GCST90001738 | Inverse variance weighted (multiplicative random effects) | 22 | 0.0161585619591806 | 0.0193769936399936 | 0.404334808037028 | -0.021820346 | 0.054137469493568 | 1.01628981753561 | 0.978415996025593 | 1.05562970906247 | 0.890615964165706 |
| ebi-a-GCST90001738 | finngen_R11_O15_PREECLAMPS | Pre-eclampsia \|\| id:finngen_R11_O15_PREECLAMPS | CD19 on unswitched memory B cell \|\| id:ebi-a-GCST90001738 | MR Egger | 22 | 0.015141088217282 | 0.024292124966695 | 0.540136243815402 | -0.032471477 | 0.0627536531520041 | 1.01525629521218 | 0.968050061402951 | 1.0647645055403 | 0.99380795269851 |
| ebi-a-GCST90001738 | finngen_R11_O15_PREECLAMPS | Pre-eclampsia \|\| id:finngen_R11_O15_PREECLAMPS | CD19 on unswitched memory B cell \|\| id:ebi-a-GCST90001738 | Weighted median | 22 | 0.0220033945509471 | 0.0250069084193177 | 0.378917474733864 | -0.027010146 | 0.0710169350528099 | 1.02224725453493 | 0.973351365899056 | 1.07359940717706 | 0.940845681141708 |
| ebi-a-GCST90001738 | finngen_R11_O15_PREECLAMPS | Pre-eclampsia \|\| id:finngen_R11_O15_PREECLAMPS | CD19 on unswitched memory B cell \|\| id:ebi-a-GCST90001738 | Weighted mode | 22 | 0.0211279994455653 | 0.020380344213516 | 0.311673285343416 | -0.018817475 | 0.0610734741040566 | 1.02135277586007 | 0.981358468142117 | 1.06297701260169 | 0.879664755158445 |
| ebi-a-GCST90001739 | finngen_R11_O15_PREECLAMPS | Pre-eclampsia \|\| id:finngen_R11_O15_PREECLAMPS | CD19 on Plasma Blast-Plasma Cell \|\| id:ebi-a-GCST90001739 | Inverse variance weighted | 22 | 0.0184780946422601 | 0.0305707190906847 | 0.545552688109364 | -0.041440515 | 0.078396704060002 | 1.01864987103534 | 0.959406404149435 | 1.08155162949975 | 0.905435115099076 |
| ebi-a-GCST90001739 | finngen_R11_O15_PREECLAMPS | Pre-eclampsia \|\| id:finngen_R11_O15_PREECLAMPS | CD19 on Plasma Blast-Plasma Cell \|\| id:ebi-a-GCST90001739 | MR Egger | 22 | 0.0267158251045149 | 0.0581610985103815 | 0.650942271978731 | -0.087279928 | 0.140711578184863 | 1.02707589210432 | 0.916420527981187 | 1.15109259988504 | 0.99380795269851 |
| ebi-a-GCST90001739 | finngen_R11_O15_PREECLAMPS | Pre-eclampsia \|\| id:finngen_R11_O15_PREECLAMPS | CD19 on Plasma Blast-Plasma Cell \|\| id:ebi-a-GCST90001739 | Weighted median | 22 | 0.0468888489431841 | 0.042191604096052 | 0.266425784534095 | -0.035806695 | 0.129584392971446 | 1.04800551568498 | 0.964826781216902 | 1.13835517658503 | 0.940845681141708 |
| ebi-a-GCST90001739 | finngen_R11_O15_PREECLAMPS | Pre-eclampsia \|\| id:finngen_R11_O15_PREECLAMPS | CD19 on Plasma Blast-Plasma Cell \|\| id:ebi-a-GCST90001739 | Weighted mode | 22 | 0.0433502911316355 | 0.0446636482645105 | 0.342797529515757 | -0.044190459 | 0.130891041730076 | 1.04430364109225 | 0.956771713888108 | 1.13984357916132 | 0.888599269773114 |
| ebi-a-GCST90001740 | finngen_R11_O15_PREECLAMPS | Pre-eclampsia \|\| id:finngen_R11_O15_PREECLAMPS | CD19 on switched memory B cell \|\| id:ebi-a-GCST90001740 | Inverse variance weighted (multiplicative random effects) | 27 | 0.0131297171446221 | 0.0311694491648733 | 0.673582218830828 | -0.047962403 | 0.0742218375077737 | 1.01321629035979 | 0.953169622513854 | 1.07704570813211 | 0.950857618427329 |
| ebi-a-GCST90001740 | finngen_R11_O15_PREECLAMPS | Pre-eclampsia \|\| id:finngen_R11_O15_PREECLAMPS | CD19 on switched memory B cell \|\| id:ebi-a-GCST90001740 | MR Egger | 27 | 0.0165134812507381 | 0.0593523030405008 | 0.783125476438367 | -0.099817033 | 0.13284399521012 | 1.01665058241509 | 0.905002988834014 | 1.14207181575894 | 0.99380795269851 |
| ebi-a-GCST90001740 | finngen_R11_O15_PREECLAMPS | Pre-eclampsia \|\| id:finngen_R11_O15_PREECLAMPS | CD19 on switched memory B cell \|\| id:ebi-a-GCST90001740 | Weighted median | 27 | 0.0277554350302585 | 0.0363210835383044 | 0.44476671904058 | -0.043433889 | 0.0989447587653352 | 1.02814420561469 | 0.95749585328954 | 1.10400531125789 | 0.940845681141708 |
| ebi-a-GCST90001740 | finngen_R11_O15_PREECLAMPS | Pre-eclampsia \|\| id:finngen_R11_O15_PREECLAMPS | CD19 on switched memory B cell \|\| id:ebi-a-GCST90001740 | Weighted mode | 27 | 0.0163308778138034 | 0.0386578291790231 | 0.676172084400596 | -0.059438467 | 0.0921002230046886 | 1.01646495547315 | 0.942293513598831 | 1.09647470856401 | 0.959313411571287 |
| ebi-a-GCST90001741 | finngen_R11_O15_PREECLAMPS | Pre-eclampsia \|\| id:finngen_R11_O15_PREECLAMPS | CD19 on IgD+ B cell \|\| id:ebi-a-GCST90001741 | Inverse variance weighted | 27 | -0.008748459 | 0.0099806350030812 | 0.380734750077718 | -0.028310503 | 0.0108135859061439 | 0.991289697713907 | 0.972086483868953 | 1.01087226404251 | 0.885079385681701 |
| ebi-a-GCST90001741 | finngen_R11_O15_PREECLAMPS | Pre-eclampsia \|\| id:finngen_R11_O15_PREECLAMPS | CD19 on IgD+ B cell \|\| id:ebi-a-GCST90001741 | MR Egger | 27 | -0.002367051 | 0.0118169971890995 | 0.842859454819083 | -0.025528366 | 0.0207942630092387 | 0.997635747775864 | 0.974794727573585 | 1.02101197009724 | 0.99380795269851 |
| ebi-a-GCST90001741 | finngen_R11_O15_PREECLAMPS | Pre-eclampsia \|\| id:finngen_R11_O15_PREECLAMPS | CD19 on IgD+ B cell \|\| id:ebi-a-GCST90001741 | Weighted median | 27 | 0.00550776261494292 | 0.0144102358120127 | 0.702304246967189 | -0.0227363 | 0.0337518248064878 | 1.00552295822458 | 0.977520222285403 | 1.03432788035102 | 0.946704357535493 |
| ebi-a-GCST90001741 | finngen_R11_O15_PREECLAMPS | Pre-eclampsia \|\| id:finngen_R11_O15_PREECLAMPS | CD19 on IgD+ B cell \|\| id:ebi-a-GCST90001741 | Weighted mode | 27 | -0.003388919 | 0.0105473116961482 | 0.750546935738131 | -0.02406165 | 0.0172838123116329 | 0.996616817290532 | 0.976225524050891 | 1.01743404165955 | 0.959313411571287 |
| ebi-a-GCST90001742 | finngen_R11_O15_PREECLAMPS | Pre-eclampsia \|\| id:finngen_R11_O15_PREECLAMPS | CD19 on transitional B cell \|\| id:ebi-a-GCST90001742 | Inverse variance weighted | 26 | -0.009482715 | 0.0172358747441832 | 0.582200650803307 | -0.043265029 | 0.0242995998276412 | 0.990562104486833 | 0.957657549245909 | 1.02459724106809 | 0.915399120505076 |
| ebi-a-GCST90001742 | finngen_R11_O15_PREECLAMPS | Pre-eclampsia \|\| id:finngen_R11_O15_PREECLAMPS | CD19 on transitional B cell \|\| id:ebi-a-GCST90001742 | MR Egger | 26 | -0.024345765 | 0.0237075126874233 | 0.314696649226402 | -0.07081249 | 0.022120959542122 | 0.975948202364499 | 0.931636566743696 | 1.02236744208918 | 0.99380795269851 |
| ebi-a-GCST90001742 | finngen_R11_O15_PREECLAMPS | Pre-eclampsia \|\| id:finngen_R11_O15_PREECLAMPS | CD19 on transitional B cell \|\| id:ebi-a-GCST90001742 | Weighted median | 26 | 0.000390527090214502 | 0.0256104988177205 | 0.987833760561817 | -0.049806051 | 0.0505871047729467 | 1.00039060335585 | 0.951413932776033 | 1.05188848387221 | 0.993268884416352 |
| ebi-a-GCST90001742 | finngen_R11_O15_PREECLAMPS | Pre-eclampsia \|\| id:finngen_R11_O15_PREECLAMPS | CD19 on transitional B cell \|\| id:ebi-a-GCST90001742 | Weighted mode | 26 | -0.017693388 | 0.0237374052751673 | 0.462992869285616 | -0.064218702 | 0.0288319266755472 | 0.982462221218726 | 0.937799878389426 | 1.02925159020037 | 0.933945646747087 |
| ebi-a-GCST90001743 | finngen_R11_O15_PREECLAMPS | Pre-eclampsia \|\| id:finngen_R11_O15_PREECLAMPS | CD20 on B cell \|\| id:ebi-a-GCST90001743 | Inverse variance weighted | 22 | 0.0184588302726511 | 0.0215326453917435 | 0.391307504460679 | -0.023745155 | 0.0606628152404683 | 1.01863024757674 | 0.9765345432929 | 1.062540581 | 0.885079385681701 |
| ebi-a-GCST90001743 | finngen_R11_O15_PREECLAMPS | Pre-eclampsia \|\| id:finngen_R11_O15_PREECLAMPS | CD20 on B cell \|\| id:ebi-a-GCST90001743 | MR Egger | 22 | -0.00118062 | 0.0470931406707077 | 0.980247662288604 | -0.093483176 | 0.091121935307495 | 0.998820076250991 | 0.910753339700676 | 1.09540256536409 | 0.99380795269851 |
| ebi-a-GCST90001743 | finngen_R11_O15_PREECLAMPS | Pre-eclampsia \|\| id:finngen_R11_O15_PREECLAMPS | CD20 on B cell \|\| id:ebi-a-GCST90001743 | Weighted median | 22 | -0.003746439 | 0.0285789180008486 | 0.89570332447215 | -0.059761118 | 0.0522682404789263 | 0.99626057034327 | 0.941989530972909 | 1.05365833842716 | 0.966404007249152 |
| ebi-a-GCST90001743 | finngen_R11_O15_PREECLAMPS | Pre-eclampsia \|\| id:finngen_R11_O15_PREECLAMPS | CD20 on B cell \|\| id:ebi-a-GCST90001743 | Weighted mode | 22 | -0.014281835 | 0.0389106316980603 | 0.717260399339656 | -0.090546673 | 0.0619830030766964 | 0.985819666570365 | 0.91343170014415 | 1.06394426079535 | 0.959313411571287 |
| ebi-a-GCST90001744 | finngen_R11_O15_PREECLAMPS | Pre-eclampsia \|\| id:finngen_R11_O15_PREECLAMPS | CD20 on CD20- CD38- B cell \|\| id:ebi-a-GCST90001744 | Inverse variance weighted | 16 | -0.046818055 | 0.0268030970776731 | 0.0806823606990139 | -0.099352126 | 0.00571601492006548 | 0.954261004478864 | 0.905423828952969 | 1.00573238250427 | 0.789997151112252 |
| ebi-a-GCST90001744 | finngen_R11_O15_PREECLAMPS | Pre-eclampsia \|\| id:finngen_R11_O15_PREECLAMPS | CD20 on CD20- CD38- B cell \|\| id:ebi-a-GCST90001744 | MR Egger | 16 | -0.000129104 | 0.0381478474619751 | 0.997347463607746 | -0.074898885 | 0.0746406766625441 | 0.999870903970683 | 0.9278372994937 | 1.07749691153038 | 0.997699814990973 |
| ebi-a-GCST90001744 | finngen_R11_O15_PREECLAMPS | Pre-eclampsia \|\| id:finngen_R11_O15_PREECLAMPS | CD20 on CD20- CD38- B cell \|\| id:ebi-a-GCST90001744 | Weighted median | 16 | -0.026927221 | 0.0324174815493864 | 0.40617765084128 | -0.090465485 | 0.0366110425306129 | 0.973432084061988 | 0.913505862881083 | 1.0372894808718 | 0.940845681141708 |
| ebi-a-GCST90001744 | finngen_R11_O15_PREECLAMPS | Pre-eclampsia \|\| id:finngen_R11_O15_PREECLAMPS | CD20 on CD20- CD38- B cell \|\| id:ebi-a-GCST90001744 | Weighted mode | 16 | -0.031208575 | 0.0339259688141202 | 0.372181324034014 | -0.097703474 | 0.0352863236957885 | 0.969273385620351 | 0.906917788540067 | 1.03591627371683 | 0.919136986043459 |
| ebi-a-GCST90001745 | finngen_R11_O15_PREECLAMPS | Pre-eclampsia \|\| id:finngen_R11_O15_PREECLAMPS | CD20 on CD24+ CD27+ B cell \|\| id:ebi-a-GCST90001745 | Inverse variance weighted | 29 | -0.020927342 | 0.0195292789415441 | 0.283905102568921 | -0.059204729 | 0.0173500447627104 | 0.979290115282301 | 0.942513789791827 | 1.0175014310416 | 0.885079385681701 |
| ebi-a-GCST90001745 | finngen_R11_O15_PREECLAMPS | Pre-eclampsia \|\| id:finngen_R11_O15_PREECLAMPS | CD20 on CD24+ CD27+ B cell \|\| id:ebi-a-GCST90001745 | MR Egger | 29 | -0.032938178 | 0.0332752239539857 | 0.331025753953866 | -0.098157617 | 0.0322812612254346 | 0.96759837687354 | 0.906506012030409 | 1.03280795328791 | 0.99380795269851 |
| ebi-a-GCST90001745 | finngen_R11_O15_PREECLAMPS | Pre-eclampsia \|\| id:finngen_R11_O15_PREECLAMPS | CD20 on CD24+ CD27+ B cell \|\| id:ebi-a-GCST90001745 | Weighted median | 29 | -0.022146685 | 0.0275956670908992 | 0.422239443863655 | -0.076234192 | 0.0319408227974525 | 0.978096752698699 | 0.926599178848108 | 1.03245640561545 | 0.940845681141708 |
| ebi-a-GCST90001745 | finngen_R11_O15_PREECLAMPS | Pre-eclampsia \|\| id:finngen_R11_O15_PREECLAMPS | CD20 on CD24+ CD27+ B cell \|\| id:ebi-a-GCST90001745 | Weighted mode | 29 | -0.018588785 | 0.0284314098316282 | 0.518567193676574 | -0.074314348 | 0.0371367784625323 | 0.981582921072242 | 0.928379813558152 | 1.03783496460133 | 0.934398527469092 |
| ebi-a-GCST90001746 | finngen_R11_O15_PREECLAMPS | Pre-eclampsia \|\| id:finngen_R11_O15_PREECLAMPS | CD20 on IgD+ CD24+ B cell \|\| id:ebi-a-GCST90001746 | Inverse variance weighted | 23 | -0.014327113 | 0.0246555671511131 | 0.561179540121509 | -0.062652025 | 0.0339977986672022 | 0.985775031739064 | 0.939270259806152 | 1.0345823292656 | 0.90609555750305 |
| ebi-a-GCST90001746 | finngen_R11_O15_PREECLAMPS | Pre-eclampsia \|\| id:finngen_R11_O15_PREECLAMPS | CD20 on IgD+ CD24+ B cell \|\| id:ebi-a-GCST90001746 | MR Egger | 23 | -0.035367374 | 0.0383186315683848 | 0.366500938148363 | -0.110471892 | 0.0397371441439395 | 0.96525074334674 | 0.89541149841663 | 1.04053722693866 | 0.99380795269851 |
| ebi-a-GCST90001746 | finngen_R11_O15_PREECLAMPS | Pre-eclampsia \|\| id:finngen_R11_O15_PREECLAMPS | CD20 on IgD+ CD24+ B cell \|\| id:ebi-a-GCST90001746 | Weighted median | 23 | -0.02398127 | 0.0316897760462762 | 0.449199046301292 | -0.086093231 | 0.0381306909285578 | 0.976303995641252 | 0.917508686821543 | 1.03886699449906 | 0.940845681141708 |
| ebi-a-GCST90001746 | finngen_R11_O15_PREECLAMPS | Pre-eclampsia \|\| id:finngen_R11_O15_PREECLAMPS | CD20 on IgD+ CD24+ B cell \|\| id:ebi-a-GCST90001746 | Weighted mode | 23 | -0.034539292 | 0.0312029897355975 | 0.280284326517746 | -0.095697152 | 0.0266185676880931 | 0.966050380702089 | 0.908739184175444 | 1.02697600621398 | 0.872755742358858 |
| ebi-a-GCST90001747 | finngen_R11_O15_PREECLAMPS | Pre-eclampsia \|\| id:finngen_R11_O15_PREECLAMPS | CD20 on IgD+ CD24- B cell \|\| id:ebi-a-GCST90001747 | Inverse variance weighted (multiplicative random effects) | 23 | -0.002601991 | 0.013263763945318 | 0.844474816656286 | -0.028598969 | 0.0233949859230831 | 0.997401390835748 | 0.971806110957834 | 1.0236707952583 | 0.977824840984872 |
| ebi-a-GCST90001747 | finngen_R11_O15_PREECLAMPS | Pre-eclampsia \|\| id:finngen_R11_O15_PREECLAMPS | CD20 on IgD+ CD24- B cell \|\| id:ebi-a-GCST90001747 | MR Egger | 23 | -0.006316777 | 0.0162217501342588 | 0.700899843362315 | -0.038111407 | 0.0254778533683778 | 0.99370313199833 | 0.962605693752726 | 1.02580518788927 | 0.99380795269851 |
| ebi-a-GCST90001747 | finngen_R11_O15_PREECLAMPS | Pre-eclampsia \|\| id:finngen_R11_O15_PREECLAMPS | CD20 on IgD+ CD24- B cell \|\| id:ebi-a-GCST90001747 | Weighted median | 23 | -0.004547734 | 0.0131691214895887 | 0.729843949248289 | -0.030359212 | 0.0212637442904747 | 0.995462591454228 | 0.970097000519644 | 1.02149142864472 | 0.952707012322319 |
| ebi-a-GCST90001747 | finngen_R11_O15_PREECLAMPS | Pre-eclampsia \|\| id:finngen_R11_O15_PREECLAMPS | CD20 on IgD+ CD24- B cell \|\| id:ebi-a-GCST90001747 | Weighted mode | 23 | -0.00328207 | 0.0121703614665659 | 0.789921882616233 | -0.027135979 | 0.020571838384496 | 0.996723310014495 | 0.97322889425843 | 1.02078489714718 | 0.961305639715923 |
| ebi-a-GCST90001748 | finngen_R11_O15_PREECLAMPS | Pre-eclampsia \|\| id:finngen_R11_O15_PREECLAMPS | CD20 on IgD+ CD38- B cell \|\| id:ebi-a-GCST90001748 | Inverse variance weighted | 25 | 0.023784947085973 | 0.0178651050049808 | 0.183069463548095 | -0.011230659 | 0.0588005528957354 | 1.02407006495682 | 0.988832169702401 | 1.06056369328708 | 0.855687495048373 |
| ebi-a-GCST90001748 | finngen_R11_O15_PREECLAMPS | Pre-eclampsia \|\| id:finngen_R11_O15_PREECLAMPS | CD20 on IgD+ CD38- B cell \|\| id:ebi-a-GCST90001748 | MR Egger | 25 | 0.027420787304482 | 0.0244464904199815 | 0.273579035099659 | -0.020494334 | 0.0753359085276458 | 1.02780019705856 | 0.979714247599387 | 1.07824628218082 | 0.99380795269851 |
| ebi-a-GCST90001748 | finngen_R11_O15_PREECLAMPS | Pre-eclampsia \|\| id:finngen_R11_O15_PREECLAMPS | CD20 on IgD+ CD38- B cell \|\| id:ebi-a-GCST90001748 | Weighted median | 25 | 0.0384714704363737 | 0.0271480051919694 | 0.156453344988274 | -0.01473862 | 0.0916815606126337 | 1.03922107941114 | 0.985369462072256 | 1.09601575192034 | 0.937437665462528 |
| ebi-a-GCST90001748 | finngen_R11_O15_PREECLAMPS | Pre-eclampsia \|\| id:finngen_R11_O15_PREECLAMPS | CD20 on IgD+ CD38- B cell \|\| id:ebi-a-GCST90001748 | Weighted mode | 25 | 0.0384530990275084 | 0.0267304619202076 | 0.163192225826025 | -0.013938606 | 0.0908448043911153 | 1.03920198763116 | 0.986158086262497 | 1.09509903750777 | 0.872755742358858 |
| ebi-a-GCST90001749 | finngen_R11_O15_PREECLAMPS | Pre-eclampsia \|\| id:finngen_R11_O15_PREECLAMPS | CD20 on IgD+ CD38- naive B cell \|\| id:ebi-a-GCST90001749 | Inverse variance weighted | 16 | 0.0168993557564065 | 0.0195891627465672 | 0.3883084669027 | -0.021495403 | 0.0552941147396783 | 1.01704295765494 | 0.978733976476856 | 1.05685140454504 | 0.885079385681701 |
| ebi-a-GCST90001749 | finngen_R11_O15_PREECLAMPS | Pre-eclampsia \|\| id:finngen_R11_O15_PREECLAMPS | CD20 on IgD+ CD38- naive B cell \|\| id:ebi-a-GCST90001749 | MR Egger | 16 | 0.00962690886446291 | 0.0333295808763098 | 0.776936731278398 | -0.05569907 | 0.0749528873820301 | 1.00967339660961 | 0.945823720128432 | 1.07783337013663 | 0.99380795269851 |
| ebi-a-GCST90001749 | finngen_R11_O15_PREECLAMPS | Pre-eclampsia \|\| id:finngen_R11_O15_PREECLAMPS | CD20 on IgD+ CD38- naive B cell \|\| id:ebi-a-GCST90001749 | Weighted median | 16 | 0.00603616599631375 | 0.0257923965338093 | 0.814962548422042 | -0.044516931 | 0.05658926320258 | 1.00605442035658 | 0.956459405941515 | 1.05822107078626 | 0.961297867845628 |
| ebi-a-GCST90001749 | finngen_R11_O15_PREECLAMPS | Pre-eclampsia \|\| id:finngen_R11_O15_PREECLAMPS | CD20 on IgD+ CD38- naive B cell \|\| id:ebi-a-GCST90001749 | Weighted mode | 16 | 0.00670109822553733 | 0.0261009042420326 | 0.80086900166775 | -0.044456674 | 0.0578588705399211 | 1.0067236008202 | 0.956517041168221 | 1.05956544925806 | 0.961305639715923 |
| ebi-a-GCST90001750 | finngen_R11_O15_PREECLAMPS | Pre-eclampsia \|\| id:finngen_R11_O15_PREECLAMPS | CD20 on IgD+ CD38- unswitched memory B cell \|\| id:ebi-a-GCST90001750 | Inverse variance weighted | 19 | -0.024077489 | 0.0233823167121064 | 0.303136449214527 | -0.06990683 | 0.0217518518618536 | 0.976210061389146 | 0.932480695411904 | 1.02199014804959 | 0.885079385681701 |
| ebi-a-GCST90001750 | finngen_R11_O15_PREECLAMPS | Pre-eclampsia \|\| id:finngen_R11_O15_PREECLAMPS | CD20 on IgD+ CD38- unswitched memory B cell \|\| id:ebi-a-GCST90001750 | MR Egger | 19 | -0.017965317 | 0.0659841133659892 | 0.788695765335538 | -0.147294179 | 0.111363545463935 | 0.982195097501968 | 0.863040051868731 | 1.11780120455364 | 0.99380795269851 |
| ebi-a-GCST90001750 | finngen_R11_O15_PREECLAMPS | Pre-eclampsia \|\| id:finngen_R11_O15_PREECLAMPS | CD20 on IgD+ CD38- unswitched memory B cell \|\| id:ebi-a-GCST90001750 | Weighted median | 19 | -0.032869374 | 0.030944279393915 | 0.288139614616547 | -0.093520162 | 0.0277814137035595 | 0.967664953624433 | 0.910719655748012 | 1.02817091578406 | 0.940845681141708 |
| ebi-a-GCST90001750 | finngen_R11_O15_PREECLAMPS | Pre-eclampsia \|\| id:finngen_R11_O15_PREECLAMPS | CD20 on IgD+ CD38- unswitched memory B cell \|\| id:ebi-a-GCST90001750 | Weighted mode | 19 | -0.028366678 | 0.0391401425541625 | 0.477923728280824 | -0.105081357 | 0.0483480015238679 | 0.972031878856261 | 0.900251277603931 | 1.04953583184863 | 0.933945646747087 |
| ebi-a-GCST90001751 | finngen_R11_O15_PREECLAMPS | Pre-eclampsia \|\| id:finngen_R11_O15_PREECLAMPS | CD20 on IgD+ CD38+ B cell \|\| id:ebi-a-GCST90001751 | Inverse variance weighted (multiplicative random effects) | 26 | -0.022247214 | 0.0222625499790165 | 0.317643992172828 | -0.065881812 | 0.0213873838327341 | 0.977998430136376 | 0.936241510185002 | 1.02161773318533 | 0.885079385681701 |
| ebi-a-GCST90001751 | finngen_R11_O15_PREECLAMPS | Pre-eclampsia \|\| id:finngen_R11_O15_PREECLAMPS | CD20 on IgD+ CD38+ B cell \|\| id:ebi-a-GCST90001751 | MR Egger | 26 | -0.008037656 | 0.0327497531104891 | 0.80821279454872 | -0.072227172 | 0.0561518600814787 | 0.991994559571646 | 0.930319529151874 | 1.05775830280254 | 0.99380795269851 |
| ebi-a-GCST90001751 | finngen_R11_O15_PREECLAMPS | Pre-eclampsia \|\| id:finngen_R11_O15_PREECLAMPS | CD20 on IgD+ CD38+ B cell \|\| id:ebi-a-GCST90001751 | Weighted median | 26 | -0.019692977 | 0.0248964599228263 | 0.428946870662103 | -0.068490038 | 0.0291040844868849 | 0.980499663084453 | 0.933802762216894 | 1.0295317471822 | 0.940845681141708 |
| ebi-a-GCST90001751 | finngen_R11_O15_PREECLAMPS | Pre-eclampsia \|\| id:finngen_R11_O15_PREECLAMPS | CD20 on IgD+ CD38+ B cell \|\| id:ebi-a-GCST90001751 | Weighted mode | 26 | -0.014636303 | 0.0214994108553127 | 0.502269843510453 | -0.056775148 | 0.0275025426708301 | 0.985470287409837 | 0.944806487224083 | 1.02788422867518 | 0.934398527469092 |
| ebi-a-GCST90001752 | finngen_R11_O15_PREECLAMPS | Pre-eclampsia \|\| id:finngen_R11_O15_PREECLAMPS | CD20 on IgD+ CD38dim B cell \|\| id:ebi-a-GCST90001752 | Inverse variance weighted | 27 | 0.0152100561410762 | 0.0207023315257929 | 0.462520655852885 | -0.025366514 | 0.0557866259316303 | 1.01532631774561 | 0.974952513132891 | 1.05737204388984 | 0.898694924660397 |
| ebi-a-GCST90001752 | finngen_R11_O15_PREECLAMPS | Pre-eclampsia \|\| id:finngen_R11_O15_PREECLAMPS | CD20 on IgD+ CD38dim B cell \|\| id:ebi-a-GCST90001752 | MR Egger | 27 | 0.0141551871416283 | 0.032405579217048 | 0.665996490235296 | -0.049359748 | 0.0776701224070423 | 1.01425584619159 | 0.951838645931232 | 1.08076607934675 | 0.99380795269851 |
| ebi-a-GCST90001752 | finngen_R11_O15_PREECLAMPS | Pre-eclampsia \|\| id:finngen_R11_O15_PREECLAMPS | CD20 on IgD+ CD38dim B cell \|\| id:ebi-a-GCST90001752 | Weighted median | 27 | 0.027081262734391 | 0.0267284207317119 | 0.310964160785257 | -0.025306442 | 0.0794689673685463 | 1.02745129287264 | 0.975011081995392 | 1.08271195960688 | 0.940845681141708 |
| ebi-a-GCST90001752 | finngen_R11_O15_PREECLAMPS | Pre-eclampsia \|\| id:finngen_R11_O15_PREECLAMPS | CD20 on IgD+ CD38dim B cell \|\| id:ebi-a-GCST90001752 | Weighted mode | 27 | 0.0205562430702728 | 0.0257946421258997 | 0.432715494459666 | -0.030001255 | 0.0711137416370361 | 1.02076897781008 | 0.970444315158311 | 1.07370334369929 | 0.933945646747087 |
| ebi-a-GCST90001753 | finngen_R11_O15_PREECLAMPS | Pre-eclampsia \|\| id:finngen_R11_O15_PREECLAMPS | CD20 on IgD- CD24- B cell \|\| id:ebi-a-GCST90001753 | Inverse variance weighted | 28 | 0.00498707491996263 | 0.0216576183308278 | 0.817882862449052 | -0.037461857 | 0.0474360068483851 | 1.00499953107608 | 0.963231157538027 | 1.04857909709307 | 0.977824840984872 |
| ebi-a-GCST90001753 | finngen_R11_O15_PREECLAMPS | Pre-eclampsia \|\| id:finngen_R11_O15_PREECLAMPS | CD20 on IgD- CD24- B cell \|\| id:ebi-a-GCST90001753 | MR Egger | 28 | -0.008540582 | 0.0316993790897863 | 0.789729917454933 | -0.070671365 | 0.0535902005462124 | 0.991495784698782 | 0.931768052958806 | 1.05505215375625 | 0.99380795269851 |
| ebi-a-GCST90001753 | finngen_R11_O15_PREECLAMPS | Pre-eclampsia \|\| id:finngen_R11_O15_PREECLAMPS | CD20 on IgD- CD24- B cell \|\| id:ebi-a-GCST90001753 | Weighted median | 28 | -0.008177768 | 0.0260053274407953 | 0.753167805579643 | -0.05914821 | 0.0427926734253372 | 0.991855578625824 | 0.942567060805785 | 1.04372148121764 | 0.957984984509652 |
| ebi-a-GCST90001753 | finngen_R11_O15_PREECLAMPS | Pre-eclampsia \|\| id:finngen_R11_O15_PREECLAMPS | CD20 on IgD- CD24- B cell \|\| id:ebi-a-GCST90001753 | Weighted mode | 28 | -0.003862922 | 0.0238230097931294 | 0.872394433278123 | -0.050556022 | 0.0428301767463916 | 0.996144529038851 | 0.950700667367369 | 1.04376062497344 | 0.977585971930092 |
| ebi-a-GCST90001754 | finngen_R11_O15_PREECLAMPS | Pre-eclampsia \|\| id:finngen_R11_O15_PREECLAMPS | CD20 on IgD- CD27- B cell \|\| id:ebi-a-GCST90001754 | Inverse variance weighted | 21 | -0.003009522 | 0.0280906358478717 | 0.914680987232898 | -0.058067168 | 0.0520481243441399 | 0.996995002153829 | 0.943586566316349 | 1.05342643674991 | 0.986667691552896 |
| ebi-a-GCST90001754 | finngen_R11_O15_PREECLAMPS | Pre-eclampsia \|\| id:finngen_R11_O15_PREECLAMPS | CD20 on IgD- CD27- B cell \|\| id:ebi-a-GCST90001754 | MR Egger | 21 | -0.012198643 | 0.0661268387623526 | 0.855596812823943 | -0.141807247 | 0.117409960725309 | 0.987875458579679 | 0.867788509011064 | 1.12458036898431 | 0.99380795269851 |
| ebi-a-GCST90001754 | finngen_R11_O15_PREECLAMPS | Pre-eclampsia \|\| id:finngen_R11_O15_PREECLAMPS | CD20 on IgD- CD27- B cell \|\| id:ebi-a-GCST90001754 | Weighted median | 21 | -0.012392038 | 0.0348929216888509 | 0.722481150887278 | -0.080782165 | 0.0559980884508789 | 0.987684427065548 | 0.922394599786148 | 1.05759566208862 | 0.949957918887556 |
| ebi-a-GCST90001754 | finngen_R11_O15_PREECLAMPS | Pre-eclampsia \|\| id:finngen_R11_O15_PREECLAMPS | CD20 on IgD- CD27- B cell \|\| id:ebi-a-GCST90001754 | Weighted mode | 21 | -0.012996489 | 0.0385889119459899 | 0.739781130344214 | -0.088630756 | 0.0626377788336692 | 0.987087601092881 | 0.91518343719157 | 1.06464113382697 | 0.959313411571287 |
| ebi-a-GCST90001755 | finngen_R11_O15_PREECLAMPS | Pre-eclampsia \|\| id:finngen_R11_O15_PREECLAMPS | CD20 on IgD- CD38- B cell \|\| id:ebi-a-GCST90001755 | Inverse variance weighted | 27 | 0.0188196912041887 | 0.0282300666505035 | 0.504993069330983 | -0.036511239 | 0.0741506218391756 | 1.01899789776808 | 0.964147257369047 | 1.07696900833304 | 0.898694924660397 |
| ebi-a-GCST90001755 | finngen_R11_O15_PREECLAMPS | Pre-eclampsia \|\| id:finngen_R11_O15_PREECLAMPS | CD20 on IgD- CD38- B cell \|\| id:ebi-a-GCST90001755 | MR Egger | 27 | 0.0688389762894485 | 0.053725113715368 | 0.211839813112002 | -0.036462247 | 0.17414019917157 | 1.07126369640402 | 0.964194494836696 | 1.19022242232111 | 0.99380795269851 |
| ebi-a-GCST90001755 | finngen_R11_O15_PREECLAMPS | Pre-eclampsia \|\| id:finngen_R11_O15_PREECLAMPS | CD20 on IgD- CD38- B cell \|\| id:ebi-a-GCST90001755 | Weighted median | 27 | 0.0403392060869825 | 0.0342681620070197 | 0.239130526937433 | -0.026826391 | 0.107504803620741 | 1.04116388342741 | 0.973530240030611 | 1.11349620954718 | 0.940845681141708 |
| ebi-a-GCST90001755 | finngen_R11_O15_PREECLAMPS | Pre-eclampsia \|\| id:finngen_R11_O15_PREECLAMPS | CD20 on IgD- CD38- B cell \|\| id:ebi-a-GCST90001755 | Weighted mode | 27 | 0.0482200846505088 | 0.0437706890460973 | 0.280707030124549 | -0.037570466 | 0.134010635180859 | 1.04940158709281 | 0.963126547770004 | 1.14340497989874 | 0.872755742358858 |
| ebi-a-GCST90001756 | finngen_R11_O15_PREECLAMPS | Pre-eclampsia \|\| id:finngen_R11_O15_PREECLAMPS | CD20 on IgD- CD38+ B cell \|\| id:ebi-a-GCST90001756 | Inverse variance weighted | 16 | 0.0184913546403963 | 0.0146399603146683 | 0.206562552354053 | -0.010202968 | 0.0471856768571462 | 1.01866337842028 | 0.989848906125529 | 1.0483166391488 | 0.855687495048373 |
| ebi-a-GCST90001756 | finngen_R11_O15_PREECLAMPS | Pre-eclampsia \|\| id:finngen_R11_O15_PREECLAMPS | CD20 on IgD- CD38+ B cell \|\| id:ebi-a-GCST90001756 | MR Egger | 16 | 0.017295730738314 | 0.0174976942669612 | 0.339705194392761 | -0.01699975 | 0.0515912115015579 | 1.01744616794484 | 0.983143930396352 | 1.05294522262707 | 0.99380795269851 |
| ebi-a-GCST90001756 | finngen_R11_O15_PREECLAMPS | Pre-eclampsia \|\| id:finngen_R11_O15_PREECLAMPS | CD20 on IgD- CD38+ B cell \|\| id:ebi-a-GCST90001756 | Weighted median | 16 | 0.0180117124708318 | 0.0154952938373986 | 0.245073470979803 | -0.012359063 | 0.0483824883921331 | 1.01817490166379 | 0.98771699611012 | 1.04957202767674 | 0.940845681141708 |
| ebi-a-GCST90001756 | finngen_R11_O15_PREECLAMPS | Pre-eclampsia \|\| id:finngen_R11_O15_PREECLAMPS | CD20 on IgD- CD38+ B cell \|\| id:ebi-a-GCST90001756 | Weighted mode | 16 | 0.0148383692160434 | 0.0136858692735284 | 0.295390987833559 | -0.011985935 | 0.041662672992159 | 1.01494900435423 | 0.988085610622991 | 1.04254274160531 | 0.872755742358858 |
| ebi-a-GCST90001757 | finngen_R11_O15_PREECLAMPS | Pre-eclampsia \|\| id:finngen_R11_O15_PREECLAMPS | CD20 on IgD- CD38dim B cell \|\| id:ebi-a-GCST90001757 | Inverse variance weighted | 34 | -0.007842966 | 0.00632599519823265 | 0.215049549544245 | -0.020241917 | 0.00455598467096092 | 0.99218770989079 | 0.979961575748477 | 1.00456637894852 | 0.855687495048373 |
| ebi-a-GCST90001757 | finngen_R11_O15_PREECLAMPS | Pre-eclampsia \|\| id:finngen_R11_O15_PREECLAMPS | CD20 on IgD- CD38dim B cell \|\| id:ebi-a-GCST90001757 | MR Egger | 34 | -0.001595165 | 0.00686625182952459 | 0.817769177117532 | -0.015053019 | 0.0118626883522962 | 0.998406106366262 | 0.985059711515556 | 1.01193332909311 | 0.99380795269851 |
| ebi-a-GCST90001757 | finngen_R11_O15_PREECLAMPS | Pre-eclampsia \|\| id:finngen_R11_O15_PREECLAMPS | CD20 on IgD- CD38dim B cell \|\| id:ebi-a-GCST90001757 | Weighted median | 34 | -0.00474967 | 0.00778223209171468 | 0.541648336147004 | -0.020002845 | 0.0105035052111596 | 0.995261592155406 | 0.980195885048983 | 1.01055886066101 | 0.940845681141708 |
| ebi-a-GCST90001757 | finngen_R11_O15_PREECLAMPS | Pre-eclampsia \|\| id:finngen_R11_O15_PREECLAMPS | CD20 on IgD- CD38dim B cell \|\| id:ebi-a-GCST90001757 | Weighted mode | 34 | -0.003547388 | 0.00672121862832542 | 0.601176685332591 | -0.016720976 | 0.00962620073443762 | 0.996458896769503 | 0.983418043309146 | 1.00967268162982 | 0.955353022288269 |
| ebi-a-GCST90001758 | finngen_R11_O15_PREECLAMPS | Pre-eclampsia \|\| id:finngen_R11_O15_PREECLAMPS | CD20 on memory B cell \|\| id:ebi-a-GCST90001758 | Inverse variance weighted | 28 | 0.00900626599788852 | 0.0173809232303343 | 0.604339944710105 | -0.025060344 | 0.0430728755293437 | 1.00904694444008 | 0.975251060157601 | 1.04401397514946 | 0.92242199665996 |
| ebi-a-GCST90001758 | finngen_R11_O15_PREECLAMPS | Pre-eclampsia \|\| id:finngen_R11_O15_PREECLAMPS | CD20 on memory B cell \|\| id:ebi-a-GCST90001758 | MR Egger | 28 | 0.0368889094987063 | 0.0234036816440459 | 0.127070475986479 | -0.008982307 | 0.0827601255210361 | 1.03757774940318 | 0.991057913877542 | 1.08628120615522 | 0.97261503632431 |
| ebi-a-GCST90001758 | finngen_R11_O15_PREECLAMPS | Pre-eclampsia \|\| id:finngen_R11_O15_PREECLAMPS | CD20 on memory B cell \|\| id:ebi-a-GCST90001758 | Weighted median | 28 | 0.0407741912589072 | 0.0254885824385532 | 0.109664229448323 | -0.00918343 | 0.0907318128384714 | 1.04161687279293 | 0.990858608590012 | 1.09497530755757 | 0.88227178111339 |
| ebi-a-GCST90001758 | finngen_R11_O15_PREECLAMPS | Pre-eclampsia \|\| id:finngen_R11_O15_PREECLAMPS | CD20 on memory B cell \|\| id:ebi-a-GCST90001758 | Weighted mode | 28 | 0.0412212290861884 | 0.0245626352687778 | 0.104845922648952 | -0.006921536 | 0.0893639942129929 | 1.04208261903194 | 0.993102362619696 | 1.09347860378046 | 0.872755742358858 |
| ebi-a-GCST90001759 | finngen_R11_O15_PREECLAMPS | Pre-eclampsia \|\| id:finngen_R11_O15_PREECLAMPS | CD20 on naive-mature B cell \|\| id:ebi-a-GCST90001759 | Inverse variance weighted | 23 | 0.00586066673396657 | 0.0111024098306314 | 0.59758721378725 | -0.015900057 | 0.0276213900020041 | 1.00587787404026 | 0.984225682065805 | 1.02800639723207 | 0.919589787313953 |
| ebi-a-GCST90001759 | finngen_R11_O15_PREECLAMPS | Pre-eclampsia \|\| id:finngen_R11_O15_PREECLAMPS | CD20 on naive-mature B cell \|\| id:ebi-a-GCST90001759 | MR Egger | 23 | -0.001624406 | 0.0130455054073388 | 0.902089053970734 | -0.027193596 | 0.0239447848957976 | 0.998376912930262 | 0.973172820627853 | 1.02423376315534 | 0.99380795269851 |
| ebi-a-GCST90001759 | finngen_R11_O15_PREECLAMPS | Pre-eclampsia \|\| id:finngen_R11_O15_PREECLAMPS | CD20 on naive-mature B cell \|\| id:ebi-a-GCST90001759 | Weighted median | 23 | 0.00108119694491188 | 0.0132929198456032 | 0.935174504682223 | -0.024972926 | 0.0271353198422942 | 1.00108178164904 | 0.975336317972703 | 1.02750683541926 | 0.978793627478789 |
| ebi-a-GCST90001759 | finngen_R11_O15_PREECLAMPS | Pre-eclampsia \|\| id:finngen_R11_O15_PREECLAMPS | CD20 on naive-mature B cell \|\| id:ebi-a-GCST90001759 | Weighted mode | 23 | -0.001486682 | 0.0114694891646627 | 0.89804441905614 | -0.023966881 | 0.0209935166346507 | 0.99851442243634 | 0.976318044006353 | 1.02121543070333 | 0.98361388732834 |
| ebi-a-GCST90001760 | finngen_R11_O15_PREECLAMPS | Pre-eclampsia \|\| id:finngen_R11_O15_PREECLAMPS | CD20 on unswitched memory B cell \|\| id:ebi-a-GCST90001760 | Inverse variance weighted | 21 | 0.0167032834716645 | 0.0169864964978724 | 0.325446443731143 | -0.01659025 | 0.0499968166074946 | 1.01684356326705 | 0.983546610633757 | 1.05126774977282 | 0.885079385681701 |
| ebi-a-GCST90001760 | finngen_R11_O15_PREECLAMPS | Pre-eclampsia \|\| id:finngen_R11_O15_PREECLAMPS | CD20 on unswitched memory B cell \|\| id:ebi-a-GCST90001760 | MR Egger | 21 | 0.032354547505243 | 0.0223625712018202 | 0.164245583710379 | -0.011476092 | 0.0761851870608106 | 1.03288364671417 | 0.988589507113567 | 1.07916240256736 | 0.99380795269851 |
| ebi-a-GCST90001760 | finngen_R11_O15_PREECLAMPS | Pre-eclampsia \|\| id:finngen_R11_O15_PREECLAMPS | CD20 on unswitched memory B cell \|\| id:ebi-a-GCST90001760 | Weighted median | 21 | 0.0408079014189205 | 0.024600556680522 | 0.0971520199053827 | -0.00740919 | 0.0890249925127437 | 1.04165198645622 | 0.992618190707038 | 1.09310797549995 | 0.88227178111339 |
| ebi-a-GCST90001760 | finngen_R11_O15_PREECLAMPS | Pre-eclampsia \|\| id:finngen_R11_O15_PREECLAMPS | CD20 on unswitched memory B cell \|\| id:ebi-a-GCST90001760 | Weighted mode | 21 | 0.027583776941396 | 0.0222953664022475 | 0.230343712697874 | -0.016115141 | 0.071282695089801 | 1.02796773149232 | 0.984014012971086 | 1.07388476491189 | 0.872755742358858 |
| ebi-a-GCST90001761 | finngen_R11_O15_PREECLAMPS | Pre-eclampsia \|\| id:finngen_R11_O15_PREECLAMPS | CD20 on switched memory B cell \|\| id:ebi-a-GCST90001761 | Inverse variance weighted | 31 | -0.013920044 | 0.0175399942589447 | 0.427418215054502 | -0.048298433 | 0.0204583445693508 | 0.986176391654519 | 0.952849383012711 | 1.02066905094879 | 0.898694924660397 |
| ebi-a-GCST90001761 | finngen_R11_O15_PREECLAMPS | Pre-eclampsia \|\| id:finngen_R11_O15_PREECLAMPS | CD20 on switched memory B cell \|\| id:ebi-a-GCST90001761 | MR Egger | 31 | 0.00600103138309181 | 0.029344056633692 | 0.839386745526703 | -0.05151332 | 0.0635153823851281 | 1.00601907364459 | 0.949790999023763 | 1.06557587677392 | 0.99380795269851 |
| ebi-a-GCST90001761 | finngen_R11_O15_PREECLAMPS | Pre-eclampsia \|\| id:finngen_R11_O15_PREECLAMPS | CD20 on switched memory B cell \|\| id:ebi-a-GCST90001761 | Weighted median | 31 | 0.00373885256532367 | 0.0271216975805973 | 0.890355279408681 | -0.049419675 | 0.0568973798232944 | 1.00374585079364 | 0.951781607216156 | 1.05854717652328 | 0.964912412213677 |
| ebi-a-GCST90001761 | finngen_R11_O15_PREECLAMPS | Pre-eclampsia \|\| id:finngen_R11_O15_PREECLAMPS | CD20 on switched memory B cell \|\| id:ebi-a-GCST90001761 | Weighted mode | 31 | 0.00810439164833354 | 0.0262990277417627 | 0.760087773487867 | -0.043441703 | 0.0596504860221885 | 1.00813732112802 | 0.957488371426351 | 1.06146548467962 | 0.959313411571287 |
| ebi-a-GCST90001762 | finngen_R11_O15_PREECLAMPS | Pre-eclampsia \|\| id:finngen_R11_O15_PREECLAMPS | CD20 on IgD+ B cell \|\| id:ebi-a-GCST90001762 | Inverse variance weighted | 19 | -0.003498062 | 0.0127169228975214 | 0.783261356538493 | -0.028423231 | 0.0214271066426298 | 0.996508048855456 | 0.971976908864959 | 1.02165831551835 | 0.977824840984872 |
| ebi-a-GCST90001762 | finngen_R11_O15_PREECLAMPS | Pre-eclampsia \|\| id:finngen_R11_O15_PREECLAMPS | CD20 on IgD+ B cell \|\| id:ebi-a-GCST90001762 | MR Egger | 19 | -0.01211669 | 0.0156554965041139 | 0.449585931035032 | -0.042801463 | 0.0185680829008627 | 0.987956421256339 | 0.958101589422195 | 1.01874154168794 | 0.99380795269851 |
| ebi-a-GCST90001762 | finngen_R11_O15_PREECLAMPS | Pre-eclampsia \|\| id:finngen_R11_O15_PREECLAMPS | CD20 on IgD+ B cell \|\| id:ebi-a-GCST90001762 | Weighted median | 19 | -0.008841789 | 0.0146144392989877 | 0.545176591758846 | -0.03748609 | 0.0198025123534842 | 0.991197184990717 | 0.963207816138717 | 1.01999988275846 | 0.940845681141708 |
| ebi-a-GCST90001762 | finngen_R11_O15_PREECLAMPS | Pre-eclampsia \|\| id:finngen_R11_O15_PREECLAMPS | CD20 on IgD+ B cell \|\| id:ebi-a-GCST90001762 | Weighted mode | 19 | -0.009279024 | 0.0133950539777497 | 0.497326000352886 | -0.03553333 | 0.0169752814208395 | 0.990763892924991 | 0.965090567059397 | 1.01712018024881 | 0.934398527469092 |
| ebi-a-GCST90001763 | finngen_R11_O15_PREECLAMPS | Pre-eclampsia \|\| id:finngen_R11_O15_PREECLAMPS | CD20 on transitional B cell \|\| id:ebi-a-GCST90001763 | Inverse variance weighted | 22 | 0.0159354766450161 | 0.00900326115741122 | 0.0767326104062984 | -0.001710915 | 0.0335818685135421 | 1.01606312348938 | 0.998290547557591 | 1.03415210475629 | 0.789997151112252 |
| ebi-a-GCST90001763 | finngen_R11_O15_PREECLAMPS | Pre-eclampsia \|\| id:finngen_R11_O15_PREECLAMPS | CD20 on transitional B cell \|\| id:ebi-a-GCST90001763 | MR Egger | 22 | 0.0114085828705444 | 0.0106393279919914 | 0.296353085055521 | -0.0094445 | 0.0322616657348475 | 1.01147390894171 | 0.9905999592212 | 1.03278771510767 | 0.99380795269851 |
| ebi-a-GCST90001763 | finngen_R11_O15_PREECLAMPS | Pre-eclampsia \|\| id:finngen_R11_O15_PREECLAMPS | CD20 on transitional B cell \|\| id:ebi-a-GCST90001763 | Weighted median | 22 | 0.0122616192612117 | 0.0121678488345176 | 0.313595427045614 | -0.011587364 | 0.0361106029768662 | 1.01233710110907 | 0.988479510502332 | 1.03677051005448 | 0.940845681141708 |
| ebi-a-GCST90001763 | finngen_R11_O15_PREECLAMPS | Pre-eclampsia \|\| id:finngen_R11_O15_PREECLAMPS | CD20 on transitional B cell \|\| id:ebi-a-GCST90001763 | Weighted mode | 22 | 0.0109875045538743 | 0.00916157314793498 | 0.243767593171823 | -0.006969179 | 0.0289441879238269 | 1.01104808886886 | 0.993055049593894 | 1.02936714175454 | 0.872755742358858 |
| ebi-a-GCST90001764 | finngen_R11_O15_PREECLAMPS | Pre-eclampsia \|\| id:finngen_R11_O15_PREECLAMPS | CD24 on CD24+ CD27+ B cell \|\| id:ebi-a-GCST90001764 | Inverse variance weighted | 25 | -0.010680925 | 0.0149967563787489 | 0.476331213235237 | -0.040074568 | 0.0187127170850509 | 0.989375913123858 | 0.960717797753737 | 1.01888889719578 | 0.898694924660397 |
| ebi-a-GCST90001764 | finngen_R11_O15_PREECLAMPS | Pre-eclampsia \|\| id:finngen_R11_O15_PREECLAMPS | CD24 on CD24+ CD27+ B cell \|\| id:ebi-a-GCST90001764 | MR Egger | 25 | -0.014334763 | 0.018421901580845 | 0.444419936298227 | -0.05044169 | 0.0217721639799481 | 0.9857674904218 | 0.950809368543956 | 1.022010907 | 0.99380795269851 |
| ebi-a-GCST90001764 | finngen_R11_O15_PREECLAMPS | Pre-eclampsia \|\| id:finngen_R11_O15_PREECLAMPS | CD24 on CD24+ CD27+ B cell \|\| id:ebi-a-GCST90001764 | Weighted median | 25 | -0.000797029 | 0.0211764798667088 | 0.969976744389163 | -0.042302929 | 0.0407088717331936 | 0.999203288737534 | 0.958579354770052 | 1.04154883709487 | 0.990670780705477 |
| ebi-a-GCST90001764 | finngen_R11_O15_PREECLAMPS | Pre-eclampsia \|\| id:finngen_R11_O15_PREECLAMPS | CD24 on CD24+ CD27+ B cell \|\| id:ebi-a-GCST90001764 | Weighted mode | 25 | -0.008582222 | 0.0199401593409344 | 0.67074591903096 | -0.047664934 | 0.0305004905044941 | 0.991454500334207 | 0.953453203206021 | 1.03097039574427 | 0.959313411571287 |
| ebi-a-GCST90001765 | finngen_R11_O15_PREECLAMPS | Pre-eclampsia \|\| id:finngen_R11_O15_PREECLAMPS | CD24 on IgD+ CD24+ B cell \|\| id:ebi-a-GCST90001765 | Inverse variance weighted | 25 | -0.006772487 | 0.0109331515727106 | 0.535623217308077 | -0.028201464 | 0.0146564905636331 | 0.99325039508369 | 0.97219248567104 | 1.01476442358321 | 0.904087927705763 |
| ebi-a-GCST90001765 | finngen_R11_O15_PREECLAMPS | Pre-eclampsia \|\| id:finngen_R11_O15_PREECLAMPS | CD24 on IgD+ CD24+ B cell \|\| id:ebi-a-GCST90001765 | MR Egger | 25 | -0.005647597 | 0.013189515261591 | 0.672497066503895 | -0.031499047 | 0.0202038528950714 | 0.994368320678714 | 0.968991879970037 | 1.02040933222266 | 0.99380795269851 |
| ebi-a-GCST90001765 | finngen_R11_O15_PREECLAMPS | Pre-eclampsia \|\| id:finngen_R11_O15_PREECLAMPS | CD24 on IgD+ CD24+ B cell \|\| id:ebi-a-GCST90001765 | Weighted median | 25 | -0.002397397 | 0.0152010999717868 | 0.874683707944755 | -0.032191553 | 0.0273967593664354 | 0.997605474881776 | 0.968321079967155 | 1.0277755014358 | 0.964912412213677 |
| ebi-a-GCST90001765 | finngen_R11_O15_PREECLAMPS | Pre-eclampsia \|\| id:finngen_R11_O15_PREECLAMPS | CD24 on IgD+ CD24+ B cell \|\| id:ebi-a-GCST90001765 | Weighted mode | 25 | -0.003849983 | 0.0125347709498977 | 0.761382845018026 | -0.028418134 | 0.0207181679019512 | 0.996157418523485 | 0.971981862940866 | 1.02093427903782 | 0.959313411571287 |
| ebi-a-GCST90001766 | finngen_R11_O15_PREECLAMPS | Pre-eclampsia \|\| id:finngen_R11_O15_PREECLAMPS | CD24 on IgD+ CD38- B cell \|\| id:ebi-a-GCST90001766 | Inverse variance weighted | 21 | 0.00608300185642431 | 0.0151363917371713 | 0.687772889636059 | -0.023584326 | 0.03575032966128 | 1.00610154088413 | 0.976691610749783 | 1.03639705658202 | 0.950911139356934 |
| ebi-a-GCST90001766 | finngen_R11_O15_PREECLAMPS | Pre-eclampsia \|\| id:finngen_R11_O15_PREECLAMPS | CD24 on IgD+ CD38- B cell \|\| id:ebi-a-GCST90001766 | MR Egger | 21 | 0.00934861124495862 | 0.0211107455943458 | 0.662884471010488 | -0.03202845 | 0.0507256726098764 | 1.00939244600262 | 0.968479028342712 | 1.05203425188325 | 0.99380795269851 |
| ebi-a-GCST90001766 | finngen_R11_O15_PREECLAMPS | Pre-eclampsia \|\| id:finngen_R11_O15_PREECLAMPS | CD24 on IgD+ CD38- B cell \|\| id:ebi-a-GCST90001766 | Weighted median | 21 | 0.00719528900451889 | 0.0205162672297832 | 0.725804864817367 | -0.033016595 | 0.047407172774894 | 1.00722123729426 | 0.967522503646402 | 1.04854886272221 | 0.950705688034314 |
| ebi-a-GCST90001766 | finngen_R11_O15_PREECLAMPS | Pre-eclampsia \|\| id:finngen_R11_O15_PREECLAMPS | CD24 on IgD+ CD38- B cell \|\| id:ebi-a-GCST90001766 | Weighted mode | 21 | 0.0117818107695137 | 0.0188094520359236 | 0.538151873211568 | -0.025084715 | 0.0486483367599239 | 1.01185148968105 | 0.975227291933332 | 1.0498510917799 | 0.935508728341156 |
| ebi-a-GCST90001767 | finngen_R11_O15_PREECLAMPS | Pre-eclampsia \|\| id:finngen_R11_O15_PREECLAMPS | CD24 on IgD+ CD38- unswitched memory B cell \|\| id:ebi-a-GCST90001767 | Inverse variance weighted | 21 | -0.003163185 | 0.0154840575313145 | 0.838129583065468 | -0.033511937 | 0.0271855681311249 | 0.996841812967421 | 0.967043367187105 | 1.0275584671767 | 0.977824840984872 |
| ebi-a-GCST90001767 | finngen_R11_O15_PREECLAMPS | Pre-eclampsia \|\| id:finngen_R11_O15_PREECLAMPS | CD24 on IgD+ CD38- unswitched memory B cell \|\| id:ebi-a-GCST90001767 | MR Egger | 21 | -0.017889477 | 0.0260810758988742 | 0.501049762534127 | -0.069008386 | 0.0332294316889949 | 0.982269589669506 | 0.933318853387859 | 1.03378769569175 | 0.99380795269851 |
| ebi-a-GCST90001767 | finngen_R11_O15_PREECLAMPS | Pre-eclampsia \|\| id:finngen_R11_O15_PREECLAMPS | CD24 on IgD+ CD38- unswitched memory B cell \|\| id:ebi-a-GCST90001767 | Weighted median | 21 | -0.008321115 | 0.0213673601186615 | 0.696957253873168 | -0.050201141 | 0.0335589108192068 | 0.991713409636602 | 0.95103811265054 | 1.0341283632809 | 0.946704357535493 |
| ebi-a-GCST90001767 | finngen_R11_O15_PREECLAMPS | Pre-eclampsia \|\| id:finngen_R11_O15_PREECLAMPS | CD24 on IgD+ CD38- unswitched memory B cell \|\| id:ebi-a-GCST90001767 | Weighted mode | 21 | -0.012390257 | 0.0195466930197643 | 0.533342463104203 | -0.050701775 | 0.0259212615886233 | 0.98768618645818 | 0.950562109604724 | 1.02626013919907 | 0.935508728341156 |
| ebi-a-GCST90001768 | finngen_R11_O15_PREECLAMPS | Pre-eclampsia \|\| id:finngen_R11_O15_PREECLAMPS | CD24 on IgD+ CD38+ B cell \|\| id:ebi-a-GCST90001768 | Inverse variance weighted | 25 | 0.0170843489688047 | 0.0175273942037658 | 0.329697830686922 | -0.017269344 | 0.0514380416081856 | 1.01723112110277 | 0.982878916764562 | 1.05278395547055 | 0.885079385681701 |
| ebi-a-GCST90001768 | finngen_R11_O15_PREECLAMPS | Pre-eclampsia \|\| id:finngen_R11_O15_PREECLAMPS | CD24 on IgD+ CD38+ B cell \|\| id:ebi-a-GCST90001768 | MR Egger | 25 | -0.007627114 | 0.0235456248345331 | 0.748918979971117 | -0.053776538 | 0.0385223110496203 | 0.992401898997351 | 0.947643844946129 | 1.03927391539122 | 0.99380795269851 |
| ebi-a-GCST90001768 | finngen_R11_O15_PREECLAMPS | Pre-eclampsia \|\| id:finngen_R11_O15_PREECLAMPS | CD24 on IgD+ CD38+ B cell \|\| id:ebi-a-GCST90001768 | Weighted median | 25 | -0.002145595 | 0.021962651741389 | 0.922176168957994 | -0.045192392 | 0.04090120269847 | 0.997856705428335 | 0.955813573135283 | 1.0417491784534 | 0.975775499714955 |
| ebi-a-GCST90001768 | finngen_R11_O15_PREECLAMPS | Pre-eclampsia \|\| id:finngen_R11_O15_PREECLAMPS | CD24 on IgD+ CD38+ B cell \|\| id:ebi-a-GCST90001768 | Weighted mode | 25 | 0.00569796831258718 | 0.0181233843618799 | 0.755935096287387 | -0.029823865 | 0.0412198016618718 | 1.00571423261051 | 0.970616477991151 | 1.04208113153893 | 0.959313411571287 |
| ebi-a-GCST90001769 | finngen_R11_O15_PREECLAMPS | Pre-eclampsia \|\| id:finngen_R11_O15_PREECLAMPS | CD24 on IgD- CD38- B cell \|\| id:ebi-a-GCST90001769 | Inverse variance weighted | 29 | 0.00829285648615116 | 0.0120144445239915 | 0.49004294305845 | -0.015255455 | 0.0318411677531744 | 1.00832733746988 | 0.984860320188456 | 1.03235352125319 | 0.898694924660397 |
| ebi-a-GCST90001769 | finngen_R11_O15_PREECLAMPS | Pre-eclampsia \|\| id:finngen_R11_O15_PREECLAMPS | CD24 on IgD- CD38- B cell \|\| id:ebi-a-GCST90001769 | MR Egger | 29 | 0.0084541594602772 | 0.0166015792671003 | 0.614722098405751 | -0.024084936 | 0.0409932548237938 | 1.00848999678667 | 0.976202791570747 | 1.04184507809315 | 0.99380795269851 |
| ebi-a-GCST90001769 | finngen_R11_O15_PREECLAMPS | Pre-eclampsia \|\| id:finngen_R11_O15_PREECLAMPS | CD24 on IgD- CD38- B cell \|\| id:ebi-a-GCST90001769 | Weighted median | 29 | -0.000623387 | 0.018454490727551 | 0.973052832484996 | -0.036794189 | 0.0355474148674989 | 0.999376807306779 | 0.963874491117038 | 1.03618677762209 | 0.990670780705477 |
| ebi-a-GCST90001769 | finngen_R11_O15_PREECLAMPS | Pre-eclampsia \|\| id:finngen_R11_O15_PREECLAMPS | CD24 on IgD- CD38- B cell \|\| id:ebi-a-GCST90001769 | Weighted mode | 29 | 0.0103899672039234 | 0.0160052516466102 | 0.521524759517633 | -0.020980326 | 0.0417602604312793 | 1.01044413033503 | 0.979238229889935 | 1.04264448564602 | 0.934398527469092 |
| ebi-a-GCST90001770 | finngen_R11_O15_PREECLAMPS | Pre-eclampsia \|\| id:finngen_R11_O15_PREECLAMPS | CD24 on IgD- CD38dim B cell \|\| id:ebi-a-GCST90001770 | Inverse variance weighted | 28 | -0.001231319 | 0.013473046968697 | 0.927181701153379 | -0.027638491 | 0.0251758532346143 | 0.998769438937944 | 0.972739957609236 | 1.02549544135941 | 0.986667691552896 |
| ebi-a-GCST90001770 | finngen_R11_O15_PREECLAMPS | Pre-eclampsia \|\| id:finngen_R11_O15_PREECLAMPS | CD24 on IgD- CD38dim B cell \|\| id:ebi-a-GCST90001770 | MR Egger | 28 | -0.011160958 | 0.0174666967256013 | 0.52842230451868 | -0.045395684 | 0.0230737671431887 | 0.988901093988241 | 0.955619283733668 | 1.02334202578073 | 0.99380795269851 |
| ebi-a-GCST90001770 | finngen_R11_O15_PREECLAMPS | Pre-eclampsia \|\| id:finngen_R11_O15_PREECLAMPS | CD24 on IgD- CD38dim B cell \|\| id:ebi-a-GCST90001770 | Weighted median | 28 | -0.011655289 | 0.0193907015607248 | 0.547789241904134 | -0.049661064 | 0.0263504860645748 | 0.988412370766522 | 0.951551884989716 | 1.02670072971886 | 0.940845681141708 |
| ebi-a-GCST90001770 | finngen_R11_O15_PREECLAMPS | Pre-eclampsia \|\| id:finngen_R11_O15_PREECLAMPS | CD24 on IgD- CD38dim B cell \|\| id:ebi-a-GCST90001770 | Weighted mode | 28 | -0.017010338 | 0.018940189927338 | 0.377067357545678 | -0.05413311 | 0.0201124343103467 | 0.983133520999908 | 0.947306002013134 | 1.0203160521095 | 0.920254054953283 |
| ebi-a-GCST90001771 | finngen_R11_O15_PREECLAMPS | Pre-eclampsia \|\| id:finngen_R11_O15_PREECLAMPS | CD24 on memory B cell \|\| id:ebi-a-GCST90001771 | Inverse variance weighted | 31 | -0.006872667 | 0.0130463361060625 | 0.598340090825094 | -0.032443486 | 0.01869815182128 | 0.993150895818297 | 0.968077158474316 | 1.01887405691832 | 0.919589787313953 |
| ebi-a-GCST90001771 | finngen_R11_O15_PREECLAMPS | Pre-eclampsia \|\| id:finngen_R11_O15_PREECLAMPS | CD24 on memory B cell \|\| id:ebi-a-GCST90001771 | MR Egger | 31 | -0.007299089 | 0.0173230668163836 | 0.676605648948322 | -0.0412523 | 0.0266541219889716 | 0.992727484684963 | 0.959586995670814 | 1.02701252027702 | 0.99380795269851 |
| ebi-a-GCST90001771 | finngen_R11_O15_PREECLAMPS | Pre-eclampsia \|\| id:finngen_R11_O15_PREECLAMPS | CD24 on memory B cell \|\| id:ebi-a-GCST90001771 | Weighted median | 31 | -0.007706711 | 0.0190371669322207 | 0.685606533399809 | -0.045019558 | 0.0296061359890673 | 0.992322909359456 | 0.955978784248912 | 1.03004875491382 | 0.943838749369606 |
| ebi-a-GCST90001771 | finngen_R11_O15_PREECLAMPS | Pre-eclampsia \|\| id:finngen_R11_O15_PREECLAMPS | CD24 on memory B cell \|\| id:ebi-a-GCST90001771 | Weighted mode | 31 | -0.009302256 | 0.0155725905404039 | 0.55475735272585 | -0.039824534 | 0.0212200210262148 | 0.990740875708704 | 0.960958039927085 | 1.02144676668143 | 0.939633861977944 |
| ebi-a-GCST90001772 | finngen_R11_O15_PREECLAMPS | Pre-eclampsia \|\| id:finngen_R11_O15_PREECLAMPS | CD24 on unswitched memory B cell \|\| id:ebi-a-GCST90001772 | Inverse variance weighted | 25 | -0.00576002 | 0.0125266838534029 | 0.645645396979284 | -0.030312321 | 0.0187922800705969 | 0.994256536829732 | 0.970142490709217 | 1.01896996626339 | 0.942541573457101 |
| ebi-a-GCST90001772 | finngen_R11_O15_PREECLAMPS | Pre-eclampsia \|\| id:finngen_R11_O15_PREECLAMPS | CD24 on unswitched memory B cell \|\| id:ebi-a-GCST90001772 | MR Egger | 25 | -0.018068873 | 0.0153460414053731 | 0.251065115482129 | -0.048147114 | 0.0120093680904273 | 0.982093390247945 | 0.952993577858802 | 1.01208177009521 | 0.99380795269851 |
| ebi-a-GCST90001772 | finngen_R11_O15_PREECLAMPS | Pre-eclampsia \|\| id:finngen_R11_O15_PREECLAMPS | CD24 on unswitched memory B cell \|\| id:ebi-a-GCST90001772 | Weighted median | 25 | -0.009463881 | 0.0167422305798649 | 0.571890109915502 | -0.042278653 | 0.0233508907450024 | 0.990580760393524 | 0.958602625732212 | 1.02362565730748 | 0.940845681141708 |
| ebi-a-GCST90001772 | finngen_R11_O15_PREECLAMPS | Pre-eclampsia \|\| id:finngen_R11_O15_PREECLAMPS | CD24 on unswitched memory B cell \|\| id:ebi-a-GCST90001772 | Weighted mode | 25 | -0.011135082 | 0.0141297821222276 | 0.438377822766979 | -0.038829455 | 0.0165592910873783 | 0.988926683684546 | 0.961914745070238 | 1.01669715607894 | 0.933945646747087 |
| ebi-a-GCST90001773 | finngen_R11_O15_PREECLAMPS | Pre-eclampsia \|\| id:finngen_R11_O15_PREECLAMPS | CD24 on switched memory B cell \|\| id:ebi-a-GCST90001773 | Inverse variance weighted | 38 | 0.00424690610859184 | 0.0122800716024891 | 0.7294650342062 | -0.019822034 | 0.0283158464494705 | 1.00425593699425 | 0.980373130639418 | 1.02872055084825 | 0.960612995947903 |
| ebi-a-GCST90001773 | finngen_R11_O15_PREECLAMPS | Pre-eclampsia \|\| id:finngen_R11_O15_PREECLAMPS | CD24 on switched memory B cell \|\| id:ebi-a-GCST90001773 | MR Egger | 38 | 0.000470434736355375 | 0.0168666677845545 | 0.977902825913975 | -0.032588234 | 0.0335291035940822 | 1.00047054540813 | 0.96793704098902 | 1.03409753924336 | 0.99380795269851 |
| ebi-a-GCST90001773 | finngen_R11_O15_PREECLAMPS | Pre-eclampsia \|\| id:finngen_R11_O15_PREECLAMPS | CD24 on switched memory B cell \|\| id:ebi-a-GCST90001773 | Weighted median | 38 | -0.002907873 | 0.0188113439410998 | 0.877151803542177 | -0.039778107 | 0.0339623614366105 | 0.997096351078789 | 0.961002655438238 | 1.03454566718263 | 0.964912412213677 |
| ebi-a-GCST90001773 | finngen_R11_O15_PREECLAMPS | Pre-eclampsia \|\| id:finngen_R11_O15_PREECLAMPS | CD24 on switched memory B cell \|\| id:ebi-a-GCST90001773 | Weighted mode | 38 | -0.005442663 | 0.0183737975219441 | 0.768719364185779 | -0.041455306 | 0.0305699799523324 | 0.994572121266192 | 0.959392213138718 | 1.03104203979703 | 0.961305639715923 |
| ebi-a-GCST90001774 | finngen_R11_O15_PREECLAMPS | Pre-eclampsia \|\| id:finngen_R11_O15_PREECLAMPS | CD24 on transitional B cell \|\| id:ebi-a-GCST90001774 | Inverse variance weighted | 20 | -0.020464324 | 0.0327968133605217 | 0.532645366142084 | -0.084746078 | 0.043817430618697 | 0.979743649608778 | 0.918745544716926 | 1.04479159052084 | 0.904087927705763 |
| ebi-a-GCST90001774 | finngen_R11_O15_PREECLAMPS | Pre-eclampsia \|\| id:finngen_R11_O15_PREECLAMPS | CD24 on transitional B cell \|\| id:ebi-a-GCST90001774 | MR Egger | 20 | -0.021527571 | 0.0618655527522866 | 0.731896358382766 | -0.142784055 | 0.0997289122260705 | 0.978702493125892 | 0.866941260692641 | 1.10487136035671 | 0.99380795269851 |
| ebi-a-GCST90001774 | finngen_R11_O15_PREECLAMPS | Pre-eclampsia \|\| id:finngen_R11_O15_PREECLAMPS | CD24 on transitional B cell \|\| id:ebi-a-GCST90001774 | Weighted median | 20 | -0.012730994 | 0.0422252666712617 | 0.763031881973217 | -0.095492516 | 0.0700305290095511 | 0.987349702622761 | 0.908925163713478 | 1.07254092436653 | 0.957984984509652 |
| ebi-a-GCST90001774 | finngen_R11_O15_PREECLAMPS | Pre-eclampsia \|\| id:finngen_R11_O15_PREECLAMPS | CD24 on transitional B cell \|\| id:ebi-a-GCST90001774 | Weighted mode | 20 | -0.000547138 | 0.052236815620223 | 0.991752159938777 | -0.102931297 | 0.101837020470377 | 0.99945301150752 | 0.902188954655806 | 1.10720300559714 | 0.995839050707755 |
| ebi-a-GCST90001775 | finngen_R11_O15_PREECLAMPS | Pre-eclampsia \|\| id:finngen_R11_O15_PREECLAMPS | CD25 on B cell \|\| id:ebi-a-GCST90001775 | Inverse variance weighted | 25 | -0.003554184 | 0.0142551757175851 | 0.803108752039865 | -0.031494328 | 0.0243859608781663 | 0.996452125105748 | 0.968996452649242 | 1.02468573018314 | 0.977824840984872 |
| ebi-a-GCST90001775 | finngen_R11_O15_PREECLAMPS | Pre-eclampsia \|\| id:finngen_R11_O15_PREECLAMPS | CD25 on B cell \|\| id:ebi-a-GCST90001775 | MR Egger | 25 | -0.011773637 | 0.0173571854822179 | 0.504340136271634 | -0.04579372 | 0.0222464468193212 | 0.988295401326444 | 0.955238988308433 | 1.02249574424576 | 0.99380795269851 |
| ebi-a-GCST90001775 | finngen_R11_O15_PREECLAMPS | Pre-eclampsia \|\| id:finngen_R11_O15_PREECLAMPS | CD25 on B cell \|\| id:ebi-a-GCST90001775 | Weighted median | 25 | -0.013125134 | 0.0196603592985233 | 0.504392924058522 | -0.051659438 | 0.0254091704870704 | 0.9869606252205 | 0.949652227274449 | 1.02573473505278 | 0.940845681141708 |
| ebi-a-GCST90001775 | finngen_R11_O15_PREECLAMPS | Pre-eclampsia \|\| id:finngen_R11_O15_PREECLAMPS | CD25 on B cell \|\| id:ebi-a-GCST90001775 | Weighted mode | 25 | -0.010716476 | 0.0169511600274608 | 0.533234916746321 | -0.04394075 | 0.0225077977175248 | 0.989340740921858 | 0.957010659067041 | 1.02276300935039 | 0.935508728341156 |
| ebi-a-GCST90001776 | finngen_R11_O15_PREECLAMPS | Pre-eclampsia \|\| id:finngen_R11_O15_PREECLAMPS | CD25 on CD20- CD38- B cell \|\| id:ebi-a-GCST90001776 | Inverse variance weighted | 19 | -0.038296784 | 0.026207159329219 | 0.143930388887841 | -0.089662816 | 0.0130692484872447 | 0.962427265682178 | 0.914239400127504 | 1.01315502438343 | 0.85505992597664 |
| ebi-a-GCST90001776 | finngen_R11_O15_PREECLAMPS | Pre-eclampsia \|\| id:finngen_R11_O15_PREECLAMPS | CD25 on CD20- CD38- B cell \|\| id:ebi-a-GCST90001776 | MR Egger | 19 | -0.019124151 | 0.0394786144805911 | 0.634271391191391 | -0.096502235 | 0.0582539336385069 | 0.98105755565687 | 0.908007868086023 | 1.05998412716425 | 0.99380795269851 |
| ebi-a-GCST90001776 | finngen_R11_O15_PREECLAMPS | Pre-eclampsia \|\| id:finngen_R11_O15_PREECLAMPS | CD25 on CD20- CD38- B cell \|\| id:ebi-a-GCST90001776 | Weighted median | 19 | -0.032849935 | 0.0335932972808803 | 0.3281377744323 | -0.098692798 | 0.0329929273337266 | 0.967683763831851 | 0.906020996731539 | 1.03354322930912 | 0.940845681141708 |
| ebi-a-GCST90001776 | finngen_R11_O15_PREECLAMPS | Pre-eclampsia \|\| id:finngen_R11_O15_PREECLAMPS | CD25 on CD20- CD38- B cell \|\| id:ebi-a-GCST90001776 | Weighted mode | 19 | -0.026882283 | 0.0304774828092234 | 0.389383687829083 | -0.086618149 | 0.032853583326692 | 0.973475829454013 | 0.917027196275955 | 1.03339922128766 | 0.920286500917063 |
| ebi-a-GCST90001777 | finngen_R11_O15_PREECLAMPS | Pre-eclampsia \|\| id:finngen_R11_O15_PREECLAMPS | CD25 on CD24+ CD27+ B cell \|\| id:ebi-a-GCST90001777 | Inverse variance weighted | 24 | -0.006488684 | 0.00826171787893314 | 0.432224021295013 | -0.022681651 | 0.00970428345919012 | 0.99353232246538 | 0.977573644199233 | 1.00975152270193 | 0.898694924660397 |
| ebi-a-GCST90001777 | finngen_R11_O15_PREECLAMPS | Pre-eclampsia \|\| id:finngen_R11_O15_PREECLAMPS | CD25 on CD24+ CD27+ B cell \|\| id:ebi-a-GCST90001777 | MR Egger | 24 | -0.003359872 | 0.00924155115880781 | 0.71965584864574 | -0.021473312 | 0.0147535685496955 | 0.996645766331279 | 0.978755598157012 | 1.01486293965158 | 0.99380795269851 |
| ebi-a-GCST90001777 | finngen_R11_O15_PREECLAMPS | Pre-eclampsia \|\| id:finngen_R11_O15_PREECLAMPS | CD25 on CD24+ CD27+ B cell \|\| id:ebi-a-GCST90001777 | Weighted median | 24 | -0.006526882 | 0.00856149329678786 | 0.445849203796538 | -0.023307409 | 0.0102536446720176 | 0.993494371640318 | 0.97696211061126 | 1.01030639342137 | 0.940845681141708 |
| ebi-a-GCST90001777 | finngen_R11_O15_PREECLAMPS | Pre-eclampsia \|\| id:finngen_R11_O15_PREECLAMPS | CD25 on CD24+ CD27+ B cell \|\| id:ebi-a-GCST90001777 | Weighted mode | 24 | -0.005382722 | 0.007065899392441 | 0.453927350038176 | -0.019231885 | 0.00846644048648371 | 0.994631738569145 | 0.980951867714308 | 1.0085023821548 | 0.933945646747087 |
| ebi-a-GCST90001778 | finngen_R11_O15_PREECLAMPS | Pre-eclampsia \|\| id:finngen_R11_O15_PREECLAMPS | CD25 on IgD+ CD24+ B cell \|\| id:ebi-a-GCST90001778 | Inverse variance weighted | 26 | -0.012776362 | 0.0114156015974678 | 0.263054042156979 | -0.035150941 | 0.00959821685847684 | 0.987304908958806 | 0.965459677420005 | 1.00964442747007 | 0.885079385681701 |
| ebi-a-GCST90001778 | finngen_R11_O15_PREECLAMPS | Pre-eclampsia \|\| id:finngen_R11_O15_PREECLAMPS | CD25 on IgD+ CD24+ B cell \|\| id:ebi-a-GCST90001778 | MR Egger | 26 | -0.018770437 | 0.0140770490188431 | 0.194916846242478 | -0.046361453 | 0.0088205795203997 | 0.981404631011609 | 0.954696822138059 | 1.00885959546197 | 0.99380795269851 |
| ebi-a-GCST90001778 | finngen_R11_O15_PREECLAMPS | Pre-eclampsia \|\| id:finngen_R11_O15_PREECLAMPS | CD25 on IgD+ CD24+ B cell \|\| id:ebi-a-GCST90001778 | Weighted median | 26 | -0.019687576 | 0.0152187074776579 | 0.195788733278492 | -0.049516242 | 0.0101410909777432 | 0.980504959055299 | 0.951689700348307 | 1.01019268610365 | 0.940845681141708 |
| ebi-a-GCST90001778 | finngen_R11_O15_PREECLAMPS | Pre-eclampsia \|\| id:finngen_R11_O15_PREECLAMPS | CD25 on IgD+ CD24+ B cell \|\| id:ebi-a-GCST90001778 | Weighted mode | 26 | -0.019865943 | 0.013058277989639 | 0.140725987700978 | -0.045460168 | 0.00572828165029381 | 0.980330084403129 | 0.955557663520762 | 1.00574471962776 | 0.872755742358858 |
| ebi-a-GCST90001779 | finngen_R11_O15_PREECLAMPS | Pre-eclampsia \|\| id:finngen_R11_O15_PREECLAMPS | CD25 on IgD+ CD24- B cell \|\| id:ebi-a-GCST90001779 | Inverse variance weighted | 24 | 0.00973475779917352 | 0.012037082009743 | 0.418670063489848 | -0.013857923 | 0.0333274385382698 | 1.00978229468201 | 0.986237656 | 1.03388901893173 | 0.898694924660397 |
| ebi-a-GCST90001779 | finngen_R11_O15_PREECLAMPS | Pre-eclampsia \|\| id:finngen_R11_O15_PREECLAMPS | CD25 on IgD+ CD24- B cell \|\| id:ebi-a-GCST90001779 | MR Egger | 24 | -0.001795074 | 0.0145407628711259 | 0.902870758307754 | -0.030294969 | 0.0267048210541783 | 0.998206536008806 | 0.970159324024729 | 1.02706459017169 | 0.99380795269851 |
| ebi-a-GCST90001779 | finngen_R11_O15_PREECLAMPS | Pre-eclampsia \|\| id:finngen_R11_O15_PREECLAMPS | CD25 on IgD+ CD24- B cell \|\| id:ebi-a-GCST90001779 | Weighted median | 24 | -0.008141098 | 0.0172543716656225 | 0.637049471451743 | -0.041959666 | 0.0256774705683288 | 0.99189195109535 | 0.958908456060103 | 1.02600997668757 | 0.940845681141708 |
| ebi-a-GCST90001779 | finngen_R11_O15_PREECLAMPS | Pre-eclampsia \|\| id:finngen_R11_O15_PREECLAMPS | CD25 on IgD+ CD24- B cell \|\| id:ebi-a-GCST90001779 | Weighted mode | 24 | -0.002982953 | 0.0159455307078126 | 0.853245049196899 | -0.034236193 | 0.0282702874731331 | 0.997021491868843 | 0.966343234268308 | 1.02867368446062 | 0.977585971930092 |
| ebi-a-GCST90001780 | finngen_R11_O15_PREECLAMPS | Pre-eclampsia \|\| id:finngen_R11_O15_PREECLAMPS | CD25 on IgD+ CD38- B cell \|\| id:ebi-a-GCST90001780 | Inverse variance weighted | 28 | -0.003125978 | 0.00887638976420592 | 0.724712473998123 | -0.020523702 | 0.0142717458755852 | 0.9968789027201 | 0.979685475694085 | 1.014374073 | 0.959893708083249 |
| ebi-a-GCST90001780 | finngen_R11_O15_PREECLAMPS | Pre-eclampsia \|\| id:finngen_R11_O15_PREECLAMPS | CD25 on IgD+ CD38- B cell \|\| id:ebi-a-GCST90001780 | MR Egger | 28 | -0.005374272 | 0.0104137386383614 | 0.610163274691111 | -0.0257852 | 0.015036655516012 | 0.994640143349793 | 0.974544399317319 | 1.01515027479089 | 0.99380795269851 |
| ebi-a-GCST90001780 | finngen_R11_O15_PREECLAMPS | Pre-eclampsia \|\| id:finngen_R11_O15_PREECLAMPS | CD25 on IgD+ CD38- B cell \|\| id:ebi-a-GCST90001780 | Weighted median | 28 | 0.00162211980132674 | 0.0124010336975574 | 0.895929402429409 | -0.022683906 | 0.0259281458485393 | 1.00162343614931 | 0.977571439167391 | 1.02626720426493 | 0.966404007249152 |
| ebi-a-GCST90001780 | finngen_R11_O15_PREECLAMPS | Pre-eclampsia \|\| id:finngen_R11_O15_PREECLAMPS | CD25 on IgD+ CD38- B cell \|\| id:ebi-a-GCST90001780 | Weighted mode | 28 | -0.00114705 | 0.00925282572952858 | 0.902259285421819 | -0.019282588 | 0.0169884885470479 | 0.998853607727428 | 0.980902131595285 | 1.01713361357211 | 0.98361388732834 |
| ebi-a-GCST90001781 | finngen_R11_O15_PREECLAMPS | Pre-eclampsia \|\| id:finngen_R11_O15_PREECLAMPS | CD25 on IgD+ CD38- naive B cell \|\| id:ebi-a-GCST90001781 | Inverse variance weighted | 25 | 0.0139668176779275 | 0.0133608461006611 | 0.295859258426389 | -0.012220441 | 0.0401540760352233 | 1.0140648093551 | 0.987853925667743 | 1.04097115054465 | 0.885079385681701 |
| ebi-a-GCST90001781 | finngen_R11_O15_PREECLAMPS | Pre-eclampsia \|\| id:finngen_R11_O15_PREECLAMPS | CD25 on IgD+ CD38- naive B cell \|\| id:ebi-a-GCST90001781 | MR Egger | 25 | 0.0164793501479873 | 0.0197475410017742 | 0.412582641285459 | -0.02222583 | 0.0551845305114648 | 1.01661588360175 | 0.978019343791027 | 1.05673559664501 | 0.99380795269851 |
| ebi-a-GCST90001781 | finngen_R11_O15_PREECLAMPS | Pre-eclampsia \|\| id:finngen_R11_O15_PREECLAMPS | CD25 on IgD+ CD38- naive B cell \|\| id:ebi-a-GCST90001781 | Weighted median | 25 | 0.0129248981099549 | 0.0194978093014446 | 0.507401161061054 | -0.025290808 | 0.0511406043407862 | 1.01300878562847 | 0.975026325222215 | 1.05247086485239 | 0.940845681141708 |
| ebi-a-GCST90001781 | finngen_R11_O15_PREECLAMPS | Pre-eclampsia \|\| id:finngen_R11_O15_PREECLAMPS | CD25 on IgD+ CD38- naive B cell \|\| id:ebi-a-GCST90001781 | Weighted mode | 25 | 0.0132518831250407 | 0.0181624276395829 | 0.472677046771526 | -0.022346475 | 0.0488502412986233 | 1.01334007848247 | 0.977901357927909 | 1.05006308288055 | 0.933945646747087 |
| ebi-a-GCST90001782 | finngen_R11_O15_PREECLAMPS | Pre-eclampsia \|\| id:finngen_R11_O15_PREECLAMPS | CD25 on IgD+ CD38- unswitched memory B cell \|\| id:ebi-a-GCST90001782 | Inverse variance weighted | 24 | 0.0111818079908652 | 0.0105003558072943 | 0.286922086216968 | -0.009398889 | 0.0317625053731621 | 1.0112445580742 | 0.990645142112407 | 1.03227231706209 | 0.885079385681701 |
| ebi-a-GCST90001782 | finngen_R11_O15_PREECLAMPS | Pre-eclampsia \|\| id:finngen_R11_O15_PREECLAMPS | CD25 on IgD+ CD38- unswitched memory B cell \|\| id:ebi-a-GCST90001782 | MR Egger | 24 | 0.0113308283403542 | 0.0136593236474694 | 0.415714754443248 | -0.015441446 | 0.0381031026893941 | 1.0113952653206 | 0.984677161841731 | 1.0388383343833 | 0.99380795269851 |
| ebi-a-GCST90001782 | finngen_R11_O15_PREECLAMPS | Pre-eclampsia \|\| id:finngen_R11_O15_PREECLAMPS | CD25 on IgD+ CD38- unswitched memory B cell \|\| id:ebi-a-GCST90001782 | Weighted median | 24 | 0.011983771996636 | 0.0150955158080744 | 0.427275171186181 | -0.017603439 | 0.0415709829804618 | 1.01205586508683 | 0.98255059636988 | 1.04244715523136 | 0.940845681141708 |
| ebi-a-GCST90001782 | finngen_R11_O15_PREECLAMPS | Pre-eclampsia \|\| id:finngen_R11_O15_PREECLAMPS | CD25 on IgD+ CD38- unswitched memory B cell \|\| id:ebi-a-GCST90001782 | Weighted mode | 24 | 0.012268028401349 | 0.012378339248901 | 0.331955233600219 | -0.011993517 | 0.0365295733291949 | 1.0123435893402 | 0.988078119019467 | 1.03720497716843 | 0.888599269773114 |
| ebi-a-GCST90001783 | finngen_R11_O15_PREECLAMPS | Pre-eclampsia \|\| id:finngen_R11_O15_PREECLAMPS | CD25 on IgD+ CD38+ B cell \|\| id:ebi-a-GCST90001783 | Inverse variance weighted | 13 | -0.006639369 | 0.0305853267620179 | 0.82814839685983 | -0.066586609 | 0.0533078717884659 | 0.993382623245329 | 0.935581882425008 | 1.05475432423722 | 0.977824840984872 |
| ebi-a-GCST90001783 | finngen_R11_O15_PREECLAMPS | Pre-eclampsia \|\| id:finngen_R11_O15_PREECLAMPS | CD25 on IgD+ CD38+ B cell \|\| id:ebi-a-GCST90001783 | MR Egger | 13 | -0.06061224 | 0.0736864871916779 | 0.428224313115766 | -0.205037755 | 0.0838132749448207 | 0.941188124180916 | 0.814616560107589 | 1.0874258252032 | 0.99380795269851 |
| ebi-a-GCST90001783 | finngen_R11_O15_PREECLAMPS | Pre-eclampsia \|\| id:finngen_R11_O15_PREECLAMPS | CD25 on IgD+ CD38+ B cell \|\| id:ebi-a-GCST90001783 | Weighted median | 13 | -0.044531777 | 0.0405111463891816 | 0.271660121501834 | -0.123933624 | 0.0348700701960478 | 0.956445206912736 | 0.883438478358704 | 1.03548515967505 | 0.940845681141708 |
| ebi-a-GCST90001783 | finngen_R11_O15_PREECLAMPS | Pre-eclampsia \|\| id:finngen_R11_O15_PREECLAMPS | CD25 on IgD+ CD38+ B cell \|\| id:ebi-a-GCST90001783 | Weighted mode | 13 | -0.053950571 | 0.0493291714290739 | 0.295561738222476 | -0.150635747 | 0.0427346045324261 | 0.947478937836856 | 0.860160957408432 | 1.04366087522639 | 0.872755742358858 |
| ebi-a-GCST90001784 | finngen_R11_O15_PREECLAMPS | Pre-eclampsia \|\| id:finngen_R11_O15_PREECLAMPS | CD25 on IgD+ CD38dim B cell \|\| id:ebi-a-GCST90001784 | Inverse variance weighted | 23 | 0.0262036732392898 | 0.0139274726193449 | 0.0599123909529113 | -0.001094173 | 0.0535015195732058 | 1.02655000794822 | 0.998906425294487 | 1.05495859485319 | 0.789997151112252 |
| ebi-a-GCST90001784 | finngen_R11_O15_PREECLAMPS | Pre-eclampsia \|\| id:finngen_R11_O15_PREECLAMPS | CD25 on IgD+ CD38dim B cell \|\| id:ebi-a-GCST90001784 | MR Egger | 23 | 0.00997467364341206 | 0.0175453901505925 | 0.575720581757654 | -0.024414291 | 0.0443636383385734 | 1.0100245865174 | 0.975881327096249 | 1.04536241963468 | 0.99380795269851 |
| ebi-a-GCST90001784 | finngen_R11_O15_PREECLAMPS | Pre-eclampsia \|\| id:finngen_R11_O15_PREECLAMPS | CD25 on IgD+ CD38dim B cell \|\| id:ebi-a-GCST90001784 | Weighted median | 23 | 0.0144575217563721 | 0.0192761432047858 | 0.453241758582312 | -0.023323719 | 0.0522387624377523 | 1.01456253720142 | 0.976946176612648 | 1.05362727910107 | 0.940845681141708 |
| ebi-a-GCST90001784 | finngen_R11_O15_PREECLAMPS | Pre-eclampsia \|\| id:finngen_R11_O15_PREECLAMPS | CD25 on IgD+ CD38dim B cell \|\| id:ebi-a-GCST90001784 | Weighted mode | 23 | 0.0120816407818693 | 0.0172676597033695 | 0.491466805241287 | -0.021762972 | 0.0459262538004735 | 1.01215491861198 | 0.978472132628056 | 1.0469971960457 | 0.933945646747087 |
| ebi-a-GCST90001785 | finngen_R11_O15_PREECLAMPS | Pre-eclampsia \|\| id:finngen_R11_O15_PREECLAMPS | CD25 on IgD- CD24- B cell \|\| id:ebi-a-GCST90001785 | Inverse variance weighted | 26 | -0.027923003 | 0.0259161023436011 | 0.281284467454372 | -0.078718564 | 0.0228725573757199 | 0.972463240459078 | 0.924300019318063 | 1.02313614008348 | 0.885079385681701 |
| ebi-a-GCST90001785 | finngen_R11_O15_PREECLAMPS | Pre-eclampsia \|\| id:finngen_R11_O15_PREECLAMPS | CD25 on IgD- CD24- B cell \|\| id:ebi-a-GCST90001785 | MR Egger | 26 | 0.0300576223517265 | 0.046969028868731 | 0.528270424203494 | -0.062001674 | 0.122116918934439 | 1.03051391287787 | 0.939881313211354 | 1.12988619914827 | 0.99380795269851 |
| ebi-a-GCST90001785 | finngen_R11_O15_PREECLAMPS | Pre-eclampsia \|\| id:finngen_R11_O15_PREECLAMPS | CD25 on IgD- CD24- B cell \|\| id:ebi-a-GCST90001785 | Weighted median | 26 | 0.00783405357686198 | 0.0372482002123878 | 0.833417672936196 | -0.065172419 | 0.080840525993142 | 1.00786482006421 | 0.936905909220802 | 1.08419798138307 | 0.964912412213677 |
| ebi-a-GCST90001785 | finngen_R11_O15_PREECLAMPS | Pre-eclampsia \|\| id:finngen_R11_O15_PREECLAMPS | CD25 on IgD- CD24- B cell \|\| id:ebi-a-GCST90001785 | Weighted mode | 26 | 0.0153629857860558 | 0.045102488791264 | 0.736232567882304 | -0.073037892 | 0.103763863816933 | 1.01548160311249 | 0.929565606030917 | 1.10933846903282 | 0.959313411571287 |
| ebi-a-GCST90001786 | finngen_R11_O15_PREECLAMPS | Pre-eclampsia \|\| id:finngen_R11_O15_PREECLAMPS | CD25 on IgD- CD27- B cell \|\| id:ebi-a-GCST90001786 | Inverse variance weighted | 21 | 0.0514387824205584 | 0.0264307573816859 | 0.0516341617892495 | -0.000365502 | 0.103243066888663 | 1.05278473538622 | 0.99963456474019 | 1.10876087938225 | 0.789997151112252 |
| ebi-a-GCST90001786 | finngen_R11_O15_PREECLAMPS | Pre-eclampsia \|\| id:finngen_R11_O15_PREECLAMPS | CD25 on IgD- CD27- B cell \|\| id:ebi-a-GCST90001786 | MR Egger | 21 | 0.100624834788622 | 0.051637555340463 | 0.0662570274600833 | -0.000584774 | 0.20183444325593 | 1.10586168309717 | 0.999415397268119 | 1.22364540859122 | 0.896743733484648 |
| ebi-a-GCST90001786 | finngen_R11_O15_PREECLAMPS | Pre-eclampsia \|\| id:finngen_R11_O15_PREECLAMPS | CD25 on IgD- CD27- B cell \|\| id:ebi-a-GCST90001786 | Weighted median | 21 | 0.0654877109609463 | 0.0358880880325486 | 0.0680348842922721 | -0.004852942 | 0.135828363504742 | 1.06767961647637 | 0.995158814912604 | 1.14548526964437 | 0.828891673627516 |
| ebi-a-GCST90001786 | finngen_R11_O15_PREECLAMPS | Pre-eclampsia \|\| id:finngen_R11_O15_PREECLAMPS | CD25 on IgD- CD27- B cell \|\| id:ebi-a-GCST90001786 | Weighted mode | 21 | 0.0689221465508032 | 0.0477268692254994 | 0.164195952162883 | -0.024622517 | 0.162466810232782 | 1.07135279739086 | 0.975678144308234 | 1.17640927305081 | 0.872755742358858 |
| ebi-a-GCST90001787 | finngen_R11_O15_PREECLAMPS | Pre-eclampsia \|\| id:finngen_R11_O15_PREECLAMPS | CD25 on IgD- CD38- B cell \|\| id:ebi-a-GCST90001787 | Inverse variance weighted | 22 | -0.019917602 | 0.018072942878891 | 0.270432343333586 | -0.05534057 | 0.0155053657309999 | 0.980279442739144 | 0.946162858055658 | 1.01562619762066 | 0.885079385681701 |
| ebi-a-GCST90001787 | finngen_R11_O15_PREECLAMPS | Pre-eclampsia \|\| id:finngen_R11_O15_PREECLAMPS | CD25 on IgD- CD38- B cell \|\| id:ebi-a-GCST90001787 | MR Egger | 22 | -0.018918966 | 0.0240263788382263 | 0.440265812822082 | -0.066010669 | 0.0281727362322437 | 0.981258874067562 | 0.936120876939196 | 1.02857334096063 | 0.99380795269851 |
| ebi-a-GCST90001787 | finngen_R11_O15_PREECLAMPS | Pre-eclampsia \|\| id:finngen_R11_O15_PREECLAMPS | CD25 on IgD- CD38- B cell \|\| id:ebi-a-GCST90001787 | Weighted median | 22 | -0.030476153 | 0.02267608448394 | 0.178955585829761 | -0.074921278 | 0.0139689728546633 | 0.969983563247587 | 0.927816522726932 | 1.01406699484634 | 0.940845681141708 |
| ebi-a-GCST90001787 | finngen_R11_O15_PREECLAMPS | Pre-eclampsia \|\| id:finngen_R11_O15_PREECLAMPS | CD25 on IgD- CD38- B cell \|\| id:ebi-a-GCST90001787 | Weighted mode | 22 | -0.019376666 | 0.019271109282454 | 0.32611288050163 | -0.05714804 | 0.0183947081579584 | 0.980809854896969 | 0.944454241794244 | 1.01856493294525 | 0.888599269773114 |
| ebi-a-GCST90001788 | finngen_R11_O15_PREECLAMPS | Pre-eclampsia \|\| id:finngen_R11_O15_PREECLAMPS | CD25 on IgD- CD38+ B cell \|\| id:ebi-a-GCST90001788 | Inverse variance weighted | 19 | -0.022549196 | 0.0335468896909043 | 0.501475793671278 | -0.0883011 | 0.0432027076285398 | 0.977703136764712 | 0.91548518266797 | 1.04414953047497 | 0.898694924660397 |
| ebi-a-GCST90001788 | finngen_R11_O15_PREECLAMPS | Pre-eclampsia \|\| id:finngen_R11_O15_PREECLAMPS | CD25 on IgD- CD38+ B cell \|\| id:ebi-a-GCST90001788 | MR Egger | 19 | -0.01774123 | 0.0583436724992026 | 0.764756379214606 | -0.132094828 | 0.0966123683506567 | 0.982415219302956 | 0.876257897490303 | 1.10143334043817 | 0.99380795269851 |
| ebi-a-GCST90001788 | finngen_R11_O15_PREECLAMPS | Pre-eclampsia \|\| id:finngen_R11_O15_PREECLAMPS | CD25 on IgD- CD38+ B cell \|\| id:ebi-a-GCST90001788 | Weighted median | 19 | 0.0192015815302424 | 0.0451034965369932 | 0.670309931981775 | -0.069201272 | 0.107604434742749 | 1.0193871175224 | 0.9331388467506 | 1.11360715395055 | 0.941872178433165 |
| ebi-a-GCST90001788 | finngen_R11_O15_PREECLAMPS | Pre-eclampsia \|\| id:finngen_R11_O15_PREECLAMPS | CD25 on IgD- CD38+ B cell \|\| id:ebi-a-GCST90001788 | Weighted mode | 19 | 0.015173927576745 | 0.0471206470900101 | 0.751145172233535 | -0.077182541 | 0.107530395873165 | 1.01528963612605 | 0.925720856431181 | 1.1135247067879 | 0.959313411571287 |
| ebi-a-GCST90001789 | finngen_R11_O15_PREECLAMPS | Pre-eclampsia \|\| id:finngen_R11_O15_PREECLAMPS | CD25 on IgD- CD38dim B cell \|\| id:ebi-a-GCST90001789 | Inverse variance weighted | 20 | 0.0147339209654863 | 0.0227083621539826 | 0.516446717062276 | -0.029774469 | 0.0592423107872923 | 1.01484300024239 | 0.970664423921939 | 1.06103230916785 | 0.898694924660397 |
| ebi-a-GCST90001789 | finngen_R11_O15_PREECLAMPS | Pre-eclampsia \|\| id:finngen_R11_O15_PREECLAMPS | CD25 on IgD- CD38dim B cell \|\| id:ebi-a-GCST90001789 | MR Egger | 20 | -0.009228329 | 0.0348124773996956 | 0.793954509499396 | -0.077460784 | 0.0590041271703898 | 0.990814121808484 | 0.925463316435644 | 1.06077961874936 | 0.99380795269851 |
| ebi-a-GCST90001789 | finngen_R11_O15_PREECLAMPS | Pre-eclampsia \|\| id:finngen_R11_O15_PREECLAMPS | CD25 on IgD- CD38dim B cell \|\| id:ebi-a-GCST90001789 | Weighted median | 20 | 0.0219963352450865 | 0.0336171562542928 | 0.512906476344379 | -0.043893291 | 0.0878859615035003 | 1.02224003820437 | 0.95705607850866 | 1.09186360044483 | 0.940845681141708 |
| ebi-a-GCST90001789 | finngen_R11_O15_PREECLAMPS | Pre-eclampsia \|\| id:finngen_R11_O15_PREECLAMPS | CD25 on IgD- CD38dim B cell \|\| id:ebi-a-GCST90001789 | Weighted mode | 20 | 0.0206303868064003 | 0.033216566598891 | 0.541922344443387 | -0.044474084 | 0.0857348573402267 | 1.02084466424163 | 0.956500388697196 | 1.08951741246025 | 0.938345702550206 |
| ebi-a-GCST90001790 | finngen_R11_O15_PREECLAMPS | Pre-eclampsia \|\| id:finngen_R11_O15_PREECLAMPS | CD25 on memory B cell \|\| id:ebi-a-GCST90001790 | Inverse variance weighted | 24 | -0.00429568 | 0.0198645914162046 | 0.828794365931345 | -0.043230279 | 0.0346389191238954 | 0.995713533184595 | 0.957690828368355 | 1.03524583383147 | 0.977824840984872 |
| ebi-a-GCST90001790 | finngen_R11_O15_PREECLAMPS | Pre-eclampsia \|\| id:finngen_R11_O15_PREECLAMPS | CD25 on memory B cell \|\| id:ebi-a-GCST90001790 | MR Egger | 24 | -0.00944178 | 0.0275534085227989 | 0.735097736498402 | -0.063446461 | 0.0445629005709401 | 0.990602653518082 | 0.93852436576323 | 1.04557074163872 | 0.99380795269851 |
| ebi-a-GCST90001790 | finngen_R11_O15_PREECLAMPS | Pre-eclampsia \|\| id:finngen_R11_O15_PREECLAMPS | CD25 on memory B cell \|\| id:ebi-a-GCST90001790 | Weighted median | 24 | -0.016478932 | 0.0265992788769643 | 0.535570210731531 | -0.068613519 | 0.0356556543368527 | 0.98365610258143 | 0.933687462950117 | 1.03629893999914 | 0.940845681141708 |
| ebi-a-GCST90001790 | finngen_R11_O15_PREECLAMPS | Pre-eclampsia \|\| id:finngen_R11_O15_PREECLAMPS | CD25 on memory B cell \|\| id:ebi-a-GCST90001790 | Weighted mode | 24 | -0.011711675 | 0.0240538947375816 | 0.630941973045317 | -0.058857308 | 0.0354339589347263 | 0.988356639958501 | 0.942841295057452 | 1.03606922275348 | 0.959313411571287 |
| ebi-a-GCST90001791 | finngen_R11_O15_PREECLAMPS | Pre-eclampsia \|\| id:finngen_R11_O15_PREECLAMPS | CD25 on naive-mature B cell \|\| id:ebi-a-GCST90001791 | Inverse variance weighted | 23 | -0.002497488 | 0.0225109630026058 | 0.911659625335052 | -0.046618976 | 0.041623999166029 | 0.997505627810168 | 0.954450997239507 | 1.04250242326822 | 0.986667691552896 |
| ebi-a-GCST90001791 | finngen_R11_O15_PREECLAMPS | Pre-eclampsia \|\| id:finngen_R11_O15_PREECLAMPS | CD25 on naive-mature B cell \|\| id:ebi-a-GCST90001791 | MR Egger | 23 | -0.038685401 | 0.0330258342506965 | 0.254570659070391 | -0.103416036 | 0.0260452339410661 | 0.962053322373078 | 0.901751733955843 | 1.02638737496944 | 0.99380795269851 |
| ebi-a-GCST90001791 | finngen_R11_O15_PREECLAMPS | Pre-eclampsia \|\| id:finngen_R11_O15_PREECLAMPS | CD25 on naive-mature B cell \|\| id:ebi-a-GCST90001791 | Weighted median | 23 | -0.013447499 | 0.0331418238135167 | 0.684921782833084 | -0.078405473 | 0.05151047592859 | 0.986642514927354 | 0.924589454079586 | 1.0528602159228 | 0.943838749369606 |
| ebi-a-GCST90001791 | finngen_R11_O15_PREECLAMPS | Pre-eclampsia \|\| id:finngen_R11_O15_PREECLAMPS | CD25 on naive-mature B cell \|\| id:ebi-a-GCST90001791 | Weighted mode | 23 | -0.014784359 | 0.0325360767434265 | 0.653993310659764 | -0.07855507 | 0.0489863512933889 | 0.985324392910931 | 0.924451149429426 | 1.0502060166884 | 0.959313411571287 |
| ebi-a-GCST90001792 | finngen_R11_O15_PREECLAMPS | Pre-eclampsia \|\| id:finngen_R11_O15_PREECLAMPS | CD25 on unswitched memory B cell \|\| id:ebi-a-GCST90001792 | Inverse variance weighted | 24 | -0.011082384 | 0.0113099876379469 | 0.327147404229722 | -0.03324996 | 0.0110851919983843 | 0.988978799615202 | 0.967296744316731 | 1.01114686039723 | 0.885079385681701 |
| ebi-a-GCST90001792 | finngen_R11_O15_PREECLAMPS | Pre-eclampsia \|\| id:finngen_R11_O15_PREECLAMPS | CD25 on unswitched memory B cell \|\| id:ebi-a-GCST90001792 | MR Egger | 24 | -0.016554277 | 0.0135017864513742 | 0.233130946053805 | -0.043017778 | 0.00990922475339605 | 0.983581992365685 | 0.95789436033945 | 1.00995848369252 | 0.99380795269851 |
| ebi-a-GCST90001792 | finngen_R11_O15_PREECLAMPS | Pre-eclampsia \|\| id:finngen_R11_O15_PREECLAMPS | CD25 on unswitched memory B cell \|\| id:ebi-a-GCST90001792 | Weighted median | 24 | -0.017685186 | 0.0142383041056921 | 0.214205136895017 | -0.045592263 | 0.0102218895676767 | 0.982470278605511 | 0.955431447985915 | 1.01027431154581 | 0.940845681141708 |
| ebi-a-GCST90001792 | finngen_R11_O15_PREECLAMPS | Pre-eclampsia \|\| id:finngen_R11_O15_PREECLAMPS | CD25 on unswitched memory B cell \|\| id:ebi-a-GCST90001792 | Weighted mode | 24 | -0.015597441 | 0.0124937472997555 | 0.224436574766429 | -0.040085185 | 0.00889030396990337 | 0.984523569374903 | 0.960707597362179 | 1.00892994009423 | 0.872755742358858 |
| ebi-a-GCST90001793 | finngen_R11_O15_PREECLAMPS | Pre-eclampsia \|\| id:finngen_R11_O15_PREECLAMPS | CD25 on switched memory B cell \|\| id:ebi-a-GCST90001793 | Inverse variance weighted | 20 | 0.00759731089066865 | 0.0287607495155131 | 0.791660079978754 | -0.048773758 | 0.0639683799410744 | 1.00762624368111 | 0.952396577280261 | 1.06605868938999 | 0.977824840984872 |
| ebi-a-GCST90001793 | finngen_R11_O15_PREECLAMPS | Pre-eclampsia \|\| id:finngen_R11_O15_PREECLAMPS | CD25 on switched memory B cell \|\| id:ebi-a-GCST90001793 | MR Egger | 20 | 0.0470647993728783 | 0.056477251369827 | 0.4155741273813 | -0.063630613 | 0.157760212057739 | 1.0481899289291 | 0.938351550092491 | 1.17088539684311 | 0.99380795269851 |
| ebi-a-GCST90001793 | finngen_R11_O15_PREECLAMPS | Pre-eclampsia \|\| id:finngen_R11_O15_PREECLAMPS | CD25 on switched memory B cell \|\| id:ebi-a-GCST90001793 | Weighted median | 20 | 0.0103758420184165 | 0.0389082594310509 | 0.789719761675578 | -0.065884346 | 0.0866360305032763 | 1.01042985772505 | 0.936239137394909 | 1.09049969885163 | 0.957984984509652 |
| ebi-a-GCST90001793 | finngen_R11_O15_PREECLAMPS | Pre-eclampsia \|\| id:finngen_R11_O15_PREECLAMPS | CD25 on switched memory B cell \|\| id:ebi-a-GCST90001793 | Weighted mode | 20 | 0.0152248849480974 | 0.0458845354946325 | 0.743665976133352 | -0.074708805 | 0.105158574517577 | 1.01534137393527 | 0.928013680282021 | 1.11088675472054 | 0.959313411571287 |
| ebi-a-GCST90001794 | finngen_R11_O15_PREECLAMPS | Pre-eclampsia \|\| id:finngen_R11_O15_PREECLAMPS | CD25 on IgD+ B cell \|\| id:ebi-a-GCST90001794 | Inverse variance weighted | 27 | 0.00249645251941017 | 0.0115822230276825 | 0.829344971907693 | -0.020204705 | 0.0251976096536678 | 1.00249957125172 | 0.979998042650573 | 1.02551775271067 | 0.977824840984872 |
| ebi-a-GCST90001794 | finngen_R11_O15_PREECLAMPS | Pre-eclampsia \|\| id:finngen_R11_O15_PREECLAMPS | CD25 on IgD+ B cell \|\| id:ebi-a-GCST90001794 | MR Egger | 27 | -0.004378865 | 0.0141926364740434 | 0.760234784070601 | -0.032196433 | 0.0234387020282212 | 0.995630707792032 | 0.968316354158285 | 1.0237155471366 | 0.99380795269851 |
| ebi-a-GCST90001794 | finngen_R11_O15_PREECLAMPS | Pre-eclampsia \|\| id:finngen_R11_O15_PREECLAMPS | CD25 on IgD+ B cell \|\| id:ebi-a-GCST90001794 | Weighted median | 27 | -0.007480536 | 0.0166549154286869 | 0.653324274546168 | -0.040124171 | 0.0251630979311148 | 0.992547373266399 | 0.960670144806441 | 1.02548236093724 | 0.940845681141708 |
| ebi-a-GCST90001794 | finngen_R11_O15_PREECLAMPS | Pre-eclampsia \|\| id:finngen_R11_O15_PREECLAMPS | CD25 on IgD+ B cell \|\| id:ebi-a-GCST90001794 | Weighted mode | 27 | -0.003666629 | 0.0149131175230118 | 0.80771651710387 | -0.032896339 | 0.0255630815306825 | 0.996340085060741 | 0.967638860647869 | 1.02589261910606 | 0.961305639715923 |
| ebi-a-GCST90001795 | finngen_R11_O15_PREECLAMPS | Pre-eclampsia \|\| id:finngen_R11_O15_PREECLAMPS | CD25 on transitional B cell \|\| id:ebi-a-GCST90001795 | Inverse variance weighted | 22 | -0.002297963 | 0.023084510134989 | 0.920705027273901 | -0.047543603 | 0.042947676800071 | 0.997704675231326 | 0.953568893829287 | 1.04388327410841 | 0.986667691552896 |
| ebi-a-GCST90001795 | finngen_R11_O15_PREECLAMPS | Pre-eclampsia \|\| id:finngen_R11_O15_PREECLAMPS | CD25 on transitional B cell \|\| id:ebi-a-GCST90001795 | MR Egger | 22 | -0.007756178 | 0.0354020278341755 | 0.82880146648071 | -0.077144153 | 0.0616317966070505 | 0.992273823584397 | 0.92575639388618 | 1.06357066229656 | 0.99380795269851 |
| ebi-a-GCST90001795 | finngen_R11_O15_PREECLAMPS | Pre-eclampsia \|\| id:finngen_R11_O15_PREECLAMPS | CD25 on transitional B cell \|\| id:ebi-a-GCST90001795 | Weighted median | 22 | -0.013611578 | 0.0325330913368287 | 0.675660689355853 | -0.077376437 | 0.0501532807522715 | 0.986480640375623 | 0.925541379734322 | 1.05143224835095 | 0.941872178433165 |
| ebi-a-GCST90001795 | finngen_R11_O15_PREECLAMPS | Pre-eclampsia \|\| id:finngen_R11_O15_PREECLAMPS | CD25 on transitional B cell \|\| id:ebi-a-GCST90001795 | Weighted mode | 22 | 0.00331841092379614 | 0.0330850205672501 | 0.921057950488782 | -0.061528229 | 0.0681650512356063 | 1.00332392294469 | 0.940326400525933 | 1.07054198817569 | 0.986782054514076 |
| ebi-a-GCST90001796 | finngen_R11_O15_PREECLAMPS | Pre-eclampsia \|\| id:finngen_R11_O15_PREECLAMPS | CD27 on CD20- B cell \|\| id:ebi-a-GCST90001796 | Inverse variance weighted | 15 | 0.00728185453143945 | 0.01806060653671 | 0.686808513407626 | -0.028116934 | 0.0426806433433911 | 1.00730843170535 | 0.972274667914975 | 1.04360455956406 | 0.950911139356934 |
| ebi-a-GCST90001796 | finngen_R11_O15_PREECLAMPS | Pre-eclampsia \|\| id:finngen_R11_O15_PREECLAMPS | CD27 on CD20- B cell \|\| id:ebi-a-GCST90001796 | MR Egger | 15 | 0.0361623947383432 | 0.0229938058181465 | 0.139800822123828 | -0.008905465 | 0.0812302541419103 | 1.03682420761598 | 0.991134071535436 | 1.0846206062043 | 0.97261503632431 |
| ebi-a-GCST90001796 | finngen_R11_O15_PREECLAMPS | Pre-eclampsia \|\| id:finngen_R11_O15_PREECLAMPS | CD27 on CD20- B cell \|\| id:ebi-a-GCST90001796 | Weighted median | 15 | 0.0101918149210845 | 0.0253978380572974 | 0.688209045808889 | -0.039587948 | 0.0599715775133874 | 1.01024392835982 | 0.961185416254236 | 1.06180636693922 | 0.945640624974244 |
| ebi-a-GCST90001796 | finngen_R11_O15_PREECLAMPS | Pre-eclampsia \|\| id:finngen_R11_O15_PREECLAMPS | CD27 on CD20- B cell \|\| id:ebi-a-GCST90001796 | Weighted mode | 15 | 0.0164939095283981 | 0.0229321240382125 | 0.483821293393672 | -0.028453054 | 0.0614408726432947 | 1.01663068500688 | 0.971947922544157 | 1.06336762055336 | 0.933945646747087 |
| ebi-a-GCST90001797 | finngen_R11_O15_PREECLAMPS | Pre-eclampsia \|\| id:finngen_R11_O15_PREECLAMPS | CD27 on CD20- CD38- B cell \|\| id:ebi-a-GCST90001797 | Inverse variance weighted | 20 | 0.0405159567160739 | 0.0225032515040003 | 0.0717897692847568 | -0.003590416 | 0.0846223296639145 | 1.04134792606314 | 0.996416021605449 | 1.08830596819266 | 0.789997151112252 |
| ebi-a-GCST90001797 | finngen_R11_O15_PREECLAMPS | Pre-eclampsia \|\| id:finngen_R11_O15_PREECLAMPS | CD27 on CD20- CD38- B cell \|\| id:ebi-a-GCST90001797 | MR Egger | 20 | 0.0542875788517245 | 0.0301451479405779 | 0.0885018736848574 | -0.004796911 | 0.113372068815257 | 1.0557881808543 | 0.995214575691901 | 1.12004859058325 | 0.936133620820556 |
| ebi-a-GCST90001797 | finngen_R11_O15_PREECLAMPS | Pre-eclampsia \|\| id:finngen_R11_O15_PREECLAMPS | CD27 on CD20- CD38- B cell \|\| id:ebi-a-GCST90001797 | Weighted median | 20 | 0.0489359751678201 | 0.0322472096200445 | 0.129133964385476 | -0.014268556 | 0.112140506023107 | 1.05015311271079 | 0.985832757715511 | 1.11867002948021 | 0.88227178111339 |
| ebi-a-GCST90001797 | finngen_R11_O15_PREECLAMPS | Pre-eclampsia \|\| id:finngen_R11_O15_PREECLAMPS | CD27 on CD20- CD38- B cell \|\| id:ebi-a-GCST90001797 | Weighted mode | 20 | 0.0574808114635275 | 0.0331328969440776 | 0.0989595294692097 | -0.007459667 | 0.12242128947392 | 1.05916494663453 | 0.99256808771025 | 1.1302301555627 | 0.872755742358858 |
| ebi-a-GCST90001798 | finngen_R11_O15_PREECLAMPS | Pre-eclampsia \|\| id:finngen_R11_O15_PREECLAMPS | CD27 on CD24+ CD27+ B cell \|\| id:ebi-a-GCST90001798 | Inverse variance weighted | 28 | -0.038018509 | 0.0172073662920885 | 0.0271445936885588 | -0.071744947 | -0.004292071 | 0.962695122111434 | 0.930768260751811 | 0.995717126611685 | 0.708667785226302 |
| ebi-a-GCST90001798 | finngen_R11_O15_PREECLAMPS | Pre-eclampsia \|\| id:finngen_R11_O15_PREECLAMPS | CD27 on CD24+ CD27+ B cell \|\| id:ebi-a-GCST90001798 | MR Egger | 28 | 0.00341947144597811 | 0.0297412634129624 | 0.909349086760423 | -0.054873405 | 0.0617123477353844 | 1.00342532450802 | 0.946604975973646 | 1.06365633756404 | 0.99380795269851 |
| ebi-a-GCST90001798 | finngen_R11_O15_PREECLAMPS | Pre-eclampsia \|\| id:finngen_R11_O15_PREECLAMPS | CD27 on CD24+ CD27+ B cell \|\| id:ebi-a-GCST90001798 | Weighted median | 28 | -0.027834354 | 0.0252859027858498 | 0.270990042895298 | -0.077394724 | 0.0217260154182326 | 0.972549452344998 | 0.925524455241143 | 1.02196374379985 | 0.940845681141708 |
| ebi-a-GCST90001798 | finngen_R11_O15_PREECLAMPS | Pre-eclampsia \|\| id:finngen_R11_O15_PREECLAMPS | CD27 on CD24+ CD27+ B cell \|\| id:ebi-a-GCST90001798 | Weighted mode | 28 | -0.026939514 | 0.0250825181379366 | 0.292312102427233 | -0.076101249 | 0.0222222216424287 | 0.973420118122602 | 0.926722371670986 | 1.02247097440551 | 0.872755742358858 |
| ebi-a-GCST90001799 | finngen_R11_O15_PREECLAMPS | Pre-eclampsia \|\| id:finngen_R11_O15_PREECLAMPS | CD27 on T cell \|\| id:ebi-a-GCST90001799 | Inverse variance weighted | 21 | -0.016761437 | 0.0113331648064925 | 0.139147725566496 | -0.03897444 | 0.00545156651191603 | 0.983378254803485 | 0.96177529226114 | 1.00546645334052 | 0.844911146506647 |
| ebi-a-GCST90001799 | finngen_R11_O15_PREECLAMPS | Pre-eclampsia \|\| id:finngen_R11_O15_PREECLAMPS | CD27 on T cell \|\| id:ebi-a-GCST90001799 | MR Egger | 21 | -0.006485268 | 0.013546021321959 | 0.637574031696721 | -0.03303547 | 0.020064933345182 | 0.993535715520826 | 0.967504241375757 | 1.02026758726333 | 0.99380795269851 |
| ebi-a-GCST90001799 | finngen_R11_O15_PREECLAMPS | Pre-eclampsia \|\| id:finngen_R11_O15_PREECLAMPS | CD27 on T cell \|\| id:ebi-a-GCST90001799 | Weighted median | 21 | -0.005342743 | 0.0145671624912884 | 0.713793411138153 | -0.033894382 | 0.0232088954436464 | 0.994671504028213 | 0.966673597839877 | 1.02348031759282 | 0.949957918887556 |
| ebi-a-GCST90001799 | finngen_R11_O15_PREECLAMPS | Pre-eclampsia \|\| id:finngen_R11_O15_PREECLAMPS | CD27 on T cell \|\| id:ebi-a-GCST90001799 | Weighted mode | 21 | -0.005353541 | 0.0128803440511251 | 0.682101584083041 | -0.030599016 | 0.0198919330192923 | 0.99466076334317 | 0.969864395548409 | 1.02009109590521 | 0.959313411571287 |
| ebi-a-GCST90001800 | finngen_R11_O15_PREECLAMPS | Pre-eclampsia \|\| id:finngen_R11_O15_PREECLAMPS | CD27 on IgD+ CD24+ B cell \|\| id:ebi-a-GCST90001800 | Inverse variance weighted | 31 | -0.000476488 | 0.0133537090948445 | 0.971535847230258 | -0.026649758 | 0.0256967815035112 | 0.999523625180148 | 0.973702213075729 | 1.02602979009103 | 0.994537505425743 |
| ebi-a-GCST90001800 | finngen_R11_O15_PREECLAMPS | Pre-eclampsia \|\| id:finngen_R11_O15_PREECLAMPS | CD27 on IgD+ CD24+ B cell \|\| id:ebi-a-GCST90001800 | MR Egger | 31 | 0.0193232187313449 | 0.0181978887055846 | 0.297072160502695 | -0.016344643 | 0.0549910805942907 | 1.01951112045975 | 0.983788205773868 | 1.05653119100313 | 0.99380795269851 |
| ebi-a-GCST90001800 | finngen_R11_O15_PREECLAMPS | Pre-eclampsia \|\| id:finngen_R11_O15_PREECLAMPS | CD27 on IgD+ CD24+ B cell \|\| id:ebi-a-GCST90001800 | Weighted median | 31 | 0.0148827900517156 | 0.0189995464261379 | 0.433437202294443 | -0.022356321 | 0.0521219010469458 | 1.01499409023854 | 0.977891729661245 | 1.05350415794603 | 0.940845681141708 |
| ebi-a-GCST90001800 | finngen_R11_O15_PREECLAMPS | Pre-eclampsia \|\| id:finngen_R11_O15_PREECLAMPS | CD27 on IgD+ CD24+ B cell \|\| id:ebi-a-GCST90001800 | Weighted mode | 31 | 0.00478980325128586 | 0.0172118459111887 | 0.782702499014493 | -0.028945415 | 0.0385250212372158 | 1.00480129269561 | 0.971469490939308 | 1.03927673202231 | 0.961305639715923 |
| ebi-a-GCST90001801 | finngen_R11_O15_PREECLAMPS | Pre-eclampsia \|\| id:finngen_R11_O15_PREECLAMPS | CD27 on IgD+ CD38- unswitched memory B cell \|\| id:ebi-a-GCST90001801 | Inverse variance weighted | 23 | 0.00158164542315273 | 0.0170752954979705 | 0.926199333470076 | -0.031885934 | 0.035049224599175 | 1.00158289688398 | 0.968617062291248 | 1.03567068801939 | 0.986667691552896 |
| ebi-a-GCST90001801 | finngen_R11_O15_PREECLAMPS | Pre-eclampsia \|\| id:finngen_R11_O15_PREECLAMPS | CD27 on IgD+ CD38- unswitched memory B cell \|\| id:ebi-a-GCST90001801 | MR Egger | 23 | -0.006746194 | 0.0313650816954152 | 0.831775272643864 | -0.068221754 | 0.0547293662132001 | 0.993276510571353 | 0.934053320518848 | 1.05625471777646 | 0.99380795269851 |
| ebi-a-GCST90001801 | finngen_R11_O15_PREECLAMPS | Pre-eclampsia \|\| id:finngen_R11_O15_PREECLAMPS | CD27 on IgD+ CD38- unswitched memory B cell \|\| id:ebi-a-GCST90001801 | Weighted median | 23 | -0.023830473 | 0.0217582003769344 | 0.273411074561162 | -0.066476546 | 0.0188155999323501 | 0.976451230764155 | 0.935684861577229 | 1.0189937287792 | 0.940845681141708 |
| ebi-a-GCST90001801 | finngen_R11_O15_PREECLAMPS | Pre-eclampsia \|\| id:finngen_R11_O15_PREECLAMPS | CD27 on IgD+ CD38- unswitched memory B cell \|\| id:ebi-a-GCST90001801 | Weighted mode | 23 | -0.023738838 | 0.0211657094938953 | 0.274138181149919 | -0.065223629 | 0.0177459523227615 | 0.97654071150483 | 0.936857931447184 | 1.01790434730404 | 0.872755742358858 |
| ebi-a-GCST90001802 | finngen_R11_O15_PREECLAMPS | Pre-eclampsia \|\| id:finngen_R11_O15_PREECLAMPS | CD27 on IgD- CD38- B cell \|\| id:ebi-a-GCST90001802 | Inverse variance weighted | 30 | -0.001767232 | 0.018010554452558 | 0.921835385889421 | -0.037067919 | 0.0335334543668218 | 0.998234328275445 | 0.963610685568248 | 1.03410203837653 | 0.986667691552896 |
| ebi-a-GCST90001802 | finngen_R11_O15_PREECLAMPS | Pre-eclampsia \|\| id:finngen_R11_O15_PREECLAMPS | CD27 on IgD- CD38- B cell \|\| id:ebi-a-GCST90001802 | MR Egger | 30 | -0.027770566 | 0.0338919357428735 | 0.419486576939058 | -0.09419876 | 0.0386576279785061 | 0.972611491273593 | 0.910101852296806 | 1.03941455626105 | 0.99380795269851 |
| ebi-a-GCST90001802 | finngen_R11_O15_PREECLAMPS | Pre-eclampsia \|\| id:finngen_R11_O15_PREECLAMPS | CD27 on IgD- CD38- B cell \|\| id:ebi-a-GCST90001802 | Weighted median | 30 | -0.007314953 | 0.0264367098595552 | 0.782012936944521 | -0.059130904 | 0.0445009985703883 | 0.992711736396284 | 0.942583373072354 | 1.04550602072129 | 0.957984984509652 |
| ebi-a-GCST90001802 | finngen_R11_O15_PREECLAMPS | Pre-eclampsia \|\| id:finngen_R11_O15_PREECLAMPS | CD27 on IgD- CD38- B cell \|\| id:ebi-a-GCST90001802 | Weighted mode | 30 | -0.018805082 | 0.0253703923715179 | 0.464519455206708 | -0.068531051 | 0.0309208868130166 | 0.981370630171156 | 0.933764465068387 | 1.03140390300888 | 0.933945646747087 |
| ebi-a-GCST90001803 | finngen_R11_O15_PREECLAMPS | Pre-eclampsia \|\| id:finngen_R11_O15_PREECLAMPS | CD27 on IgD- CD38+ B cell \|\| id:ebi-a-GCST90001803 | Inverse variance weighted | 15 | 0.017039955658624 | 0.0366714298736041 | 0.642170874478272 | -0.054836047 | 0.088915958210888 | 1.01718596384843 | 0.946640339855364 | 1.09298879573245 | 0.942541573457101 |
| ebi-a-GCST90001803 | finngen_R11_O15_PREECLAMPS | Pre-eclampsia \|\| id:finngen_R11_O15_PREECLAMPS | CD27 on IgD- CD38+ B cell \|\| id:ebi-a-GCST90001803 | MR Egger | 15 | 0.250100148120569 | 0.166408838818785 | 0.156756654482157 | -0.076061176 | 0.576261472205388 | 1.28415401585936 | 0.926759509418532 | 1.77937374225852 | 0.99380795269851 |
| ebi-a-GCST90001803 | finngen_R11_O15_PREECLAMPS | Pre-eclampsia \|\| id:finngen_R11_O15_PREECLAMPS | CD27 on IgD- CD38+ B cell \|\| id:ebi-a-GCST90001803 | Weighted median | 15 | 0.0290299126078554 | 0.0509098784539887 | 0.568527404226566 | -0.070753449 | 0.128813274377673 | 1.02945538770989 | 0.931691573150557 | 1.13747770810166 | 0.940845681141708 |
| ebi-a-GCST90001803 | finngen_R11_O15_PREECLAMPS | Pre-eclampsia \|\| id:finngen_R11_O15_PREECLAMPS | CD27 on IgD- CD38+ B cell \|\| id:ebi-a-GCST90001803 | Weighted mode | 15 | 0.0189547242855793 | 0.0723528046910397 | 0.797154848357089 | -0.122856773 | 0.160766221480017 | 1.01913550548473 | 0.884390322143337 | 1.17441038479759 | 0.961305639715923 |
| ebi-a-GCST90001804 | finngen_R11_O15_PREECLAMPS | Pre-eclampsia \|\| id:finngen_R11_O15_PREECLAMPS | CD27 on IgD- CD38dim B cell \|\| id:ebi-a-GCST90001804 | Inverse variance weighted | 31 | -0.006151075 | 0.0131631262608228 | 0.640288196963643 | -0.031950802 | 0.019648652677272 | 0.993867804337802 | 0.968554231581334 | 1.01984295797927 | 0.942541573457101 |
| ebi-a-GCST90001804 | finngen_R11_O15_PREECLAMPS | Pre-eclampsia \|\| id:finngen_R11_O15_PREECLAMPS | CD27 on IgD- CD38dim B cell \|\| id:ebi-a-GCST90001804 | MR Egger | 31 | -0.013574744 | 0.0172993223699228 | 0.438994138487191 | -0.047481416 | 0.0203319281471613 | 0.986516977635079 | 0.953628195630201 | 1.0205400297745 | 0.99380795269851 |
| ebi-a-GCST90001804 | finngen_R11_O15_PREECLAMPS | Pre-eclampsia \|\| id:finngen_R11_O15_PREECLAMPS | CD27 on IgD- CD38dim B cell \|\| id:ebi-a-GCST90001804 | Weighted median | 31 | -0.006964683 | 0.0158409008001457 | 0.66018006562212 | -0.038012848 | 0.0240834826982964 | 0.993059514325897 | 0.962700571612865 | 1.02437583197962 | 0.940845681141708 |
| ebi-a-GCST90001804 | finngen_R11_O15_PREECLAMPS | Pre-eclampsia \|\| id:finngen_R11_O15_PREECLAMPS | CD27 on IgD- CD38dim B cell \|\| id:ebi-a-GCST90001804 | Weighted mode | 31 | -0.009746736 | 0.0143493405510579 | 0.502189217200889 | -0.037871444 | 0.0183779710859551 | 0.99030060909486 | 0.96283671149271 | 1.01854788529329 | 0.934398527469092 |
| ebi-a-GCST90001805 | finngen_R11_O15_PREECLAMPS | Pre-eclampsia \|\| id:finngen_R11_O15_PREECLAMPS | CD27 on memory B cell \|\| id:ebi-a-GCST90001805 | Inverse variance weighted | 25 | -0.001939504 | 0.0127664705385982 | 0.879248687865907 | -0.026961786 | 0.0230827782680985 | 0.99806237563493 | 0.973398438024797 | 1.0233512472851 | 0.984283721484735 |
| ebi-a-GCST90001805 | finngen_R11_O15_PREECLAMPS | Pre-eclampsia \|\| id:finngen_R11_O15_PREECLAMPS | CD27 on memory B cell \|\| id:ebi-a-GCST90001805 | MR Egger | 25 | -0.007539272 | 0.0163808092034734 | 0.64965829159323 | -0.039645658 | 0.0245671145244647 | 0.992489077504752 | 0.961129947957898 | 1.02487137255386 | 0.99380795269851 |
| ebi-a-GCST90001805 | finngen_R11_O15_PREECLAMPS | Pre-eclampsia \|\| id:finngen_R11_O15_PREECLAMPS | CD27 on memory B cell \|\| id:ebi-a-GCST90001805 | Weighted median | 25 | -0.004488105 | 0.0147870101326693 | 0.761496117486065 | -0.033470644 | 0.0244944352425433 | 0.995521951873554 | 0.967083300050271 | 1.02479688834521 | 0.957984984509652 |
| ebi-a-GCST90001805 | finngen_R11_O15_PREECLAMPS | Pre-eclampsia \|\| id:finngen_R11_O15_PREECLAMPS | CD27 on memory B cell \|\| id:ebi-a-GCST90001805 | Weighted mode | 25 | -0.008776137 | 0.0124702926601404 | 0.488352413156258 | -0.033217911 | 0.0156656365691369 | 0.99126226083552 | 0.967327745594421 | 1.01578898592735 | 0.933945646747087 |
| ebi-a-GCST90001806 | finngen_R11_O15_PREECLAMPS | Pre-eclampsia \|\| id:finngen_R11_O15_PREECLAMPS | CD27 on unswitched memory B cell \|\| id:ebi-a-GCST90001806 | Inverse variance weighted | 30 | -0.004037601 | 0.0221823760332477 | 0.855568259563232 | -0.047515058 | 0.039439855684286 | 0.995970538812165 | 0.953596113425175 | 1.04022793320626 | 0.977824840984872 |
| ebi-a-GCST90001806 | finngen_R11_O15_PREECLAMPS | Pre-eclampsia \|\| id:finngen_R11_O15_PREECLAMPS | CD27 on unswitched memory B cell \|\| id:ebi-a-GCST90001806 | MR Egger | 30 | 0.015676119990019 | 0.0445380392248123 | 0.727494926259032 | -0.071618437 | 0.102970676870651 | 1.01579963492664 | 0.930886019819994 | 1.1084589051156 | 0.99380795269851 |
| ebi-a-GCST90001806 | finngen_R11_O15_PREECLAMPS | Pre-eclampsia \|\| id:finngen_R11_O15_PREECLAMPS | CD27 on unswitched memory B cell \|\| id:ebi-a-GCST90001806 | Weighted median | 30 | -0.026523716 | 0.0292411124654482 | 0.364370147969032 | -0.083836296 | 0.0307888648232128 | 0.973824948709897 | 0.919581782952044 | 1.0312677440015 | 0.940845681141708 |
| ebi-a-GCST90001806 | finngen_R11_O15_PREECLAMPS | Pre-eclampsia \|\| id:finngen_R11_O15_PREECLAMPS | CD27 on unswitched memory B cell \|\| id:ebi-a-GCST90001806 | Weighted mode | 30 | -0.025882313 | 0.0327283678352328 | 0.435467988392902 | -0.090029914 | 0.0382652879381291 | 0.974449762911644 | 0.913903846364611 | 1.03900683230052 | 0.933945646747087 |
| ebi-a-GCST90001807 | finngen_R11_O15_PREECLAMPS | Pre-eclampsia \|\| id:finngen_R11_O15_PREECLAMPS | CD27 on Plasma Blast-Plasma Cell \|\| id:ebi-a-GCST90001807 | Inverse variance weighted | 19 | -0.017592978 | 0.0240975896595285 | 0.465346109250856 | -0.064824254 | 0.029638297646372 | 0.982560874788044 | 0.937232163877768 | 1.03008188352161 | 0.898694924660397 |
| ebi-a-GCST90001807 | finngen_R11_O15_PREECLAMPS | Pre-eclampsia \|\| id:finngen_R11_O15_PREECLAMPS | CD27 on Plasma Blast-Plasma Cell \|\| id:ebi-a-GCST90001807 | MR Egger | 19 | -0.007930069 | 0.033372115579616 | 0.815011741553643 | -0.073339415 | 0.0574792779467255 | 0.992101291454077 | 0.929285362983697 | 1.05916332238853 | 0.99380795269851 |
| ebi-a-GCST90001807 | finngen_R11_O15_PREECLAMPS | Pre-eclampsia \|\| id:finngen_R11_O15_PREECLAMPS | CD27 on Plasma Blast-Plasma Cell \|\| id:ebi-a-GCST90001807 | Weighted median | 19 | 0.0108635989971208 | 0.0374210084744352 | 0.771581010231059 | -0.062481578 | 0.0842087756070138 | 1.01092282215329 | 0.939430369204098 | 1.08785598789637 | 0.957984984509652 |
| ebi-a-GCST90001807 | finngen_R11_O15_PREECLAMPS | Pre-eclampsia \|\| id:finngen_R11_O15_PREECLAMPS | CD27 on Plasma Blast-Plasma Cell \|\| id:ebi-a-GCST90001807 | Weighted mode | 19 | 0.00604428219420161 | 0.0324796861266386 | 0.854452010836089 | -0.057615903 | 0.0697044670024133 | 1.00606258572648 | 0.944012470532528 | 1.07219126652816 | 0.977585971930092 |
| ebi-a-GCST90001808 | finngen_R11_O15_PREECLAMPS | Pre-eclampsia \|\| id:finngen_R11_O15_PREECLAMPS | CD27 on switched memory B cell \|\| id:ebi-a-GCST90001808 | Inverse variance weighted | 30 | -0.034688947 | 0.0177716646441217 | 0.0509471737351907 | -0.06952141 | 0.000143515782228605 | 0.965905817514063 | 0.932840161414839 | 1.00014352608111 | 0.789997151112252 |
| ebi-a-GCST90001808 | finngen_R11_O15_PREECLAMPS | Pre-eclampsia \|\| id:finngen_R11_O15_PREECLAMPS | CD27 on switched memory B cell \|\| id:ebi-a-GCST90001808 | MR Egger | 30 | -0.012478319 | 0.0362106512711047 | 0.732967415050789 | -0.083451196 | 0.0584945570022633 | 0.987599211917252 | 0.91993598214933 | 1.06023921479925 | 0.99380795269851 |
| ebi-a-GCST90001808 | finngen_R11_O15_PREECLAMPS | Pre-eclampsia \|\| id:finngen_R11_O15_PREECLAMPS | CD27 on switched memory B cell \|\| id:ebi-a-GCST90001808 | Weighted median | 30 | -0.027794165 | 0.0270282591080578 | 0.303791227643912 | -0.080769553 | 0.0251812229575113 | 0.972588539064059 | 0.922406232937249 | 1.02550094800055 | 0.940845681141708 |
| ebi-a-GCST90001808 | finngen_R11_O15_PREECLAMPS | Pre-eclampsia \|\| id:finngen_R11_O15_PREECLAMPS | CD27 on switched memory B cell \|\| id:ebi-a-GCST90001808 | Weighted mode | 30 | -0.037409518 | 0.0267770805158427 | 0.172991211274103 | -0.089892596 | 0.0150735596979008 | 0.963281573312081 | 0.914029350477102 | 1.01518773877256 | 0.872755742358858 |
| ebi-a-GCST90001809 | finngen_R11_O15_PREECLAMPS | Pre-eclampsia \|\| id:finngen_R11_O15_PREECLAMPS | CD38 on CD20- B cell \|\| id:ebi-a-GCST90001809 | Inverse variance weighted | 19 | 0.0323885410303991 | 0.0416736185332884 | 0.437043546634821 | -0.049291751 | 0.114068833355644 | 1.03291875866718 | 0.951903370141313 | 1.12082927266889 | 0.898694924660397 |
| ebi-a-GCST90001809 | finngen_R11_O15_PREECLAMPS | Pre-eclampsia \|\| id:finngen_R11_O15_PREECLAMPS | CD38 on CD20- B cell \|\| id:ebi-a-GCST90001809 | MR Egger | 19 | -0.074509469 | 0.0969889256077671 | 0.452892810091719 | -0.264607763 | 0.11558882520812 | 0.928198684919707 | 0.767506935427242 | 1.12253421945572 | 0.99380795269851 |
| ebi-a-GCST90001809 | finngen_R11_O15_PREECLAMPS | Pre-eclampsia \|\| id:finngen_R11_O15_PREECLAMPS | CD38 on CD20- B cell \|\| id:ebi-a-GCST90001809 | Weighted median | 19 | 0.00456459729497793 | 0.0509631044400758 | 0.928631544313232 | -0.095323087 | 0.104452281997526 | 1.0045750309383 | 0.90907917498187 | 1.11010242073228 | 0.977431269406605 |
| ebi-a-GCST90001809 | finngen_R11_O15_PREECLAMPS | Pre-eclampsia \|\| id:finngen_R11_O15_PREECLAMPS | CD38 on CD20- B cell \|\| id:ebi-a-GCST90001809 | Weighted mode | 19 | 0.00264162323388872 | 0.0725453874403877 | 0.971353533385105 | -0.139547336 | 0.144830582617049 | 1.00264511539486 | 0.869751851526479 | 1.15584373365897 | 0.993663982022827 |
| ebi-a-GCST90001810 | finngen_R11_O15_PREECLAMPS | Pre-eclampsia \|\| id:finngen_R11_O15_PREECLAMPS | CD38 on CD3- CD19- \|\| id:ebi-a-GCST90001810 | Inverse variance weighted | 18 | -0.024229283 | 0.0192644081327741 | 0.208492068975245 | -0.061987523 | 0.0135289570186406 | 0.976061889778093 | 0.939894613912888 | 1.01362088746418 | 0.855687495048373 |
| ebi-a-GCST90001810 | finngen_R11_O15_PREECLAMPS | Pre-eclampsia \|\| id:finngen_R11_O15_PREECLAMPS | CD38 on CD3- CD19- \|\| id:ebi-a-GCST90001810 | MR Egger | 18 | -0.030893944 | 0.0356676901599604 | 0.39920477260788 | -0.100802617 | 0.0390147282894068 | 0.969578396818654 | 0.90411147138505 | 1.03978579780353 | 0.99380795269851 |
| ebi-a-GCST90001810 | finngen_R11_O15_PREECLAMPS | Pre-eclampsia \|\| id:finngen_R11_O15_PREECLAMPS | CD38 on CD3- CD19- \|\| id:ebi-a-GCST90001810 | Weighted median | 18 | -0.045771191 | 0.0267165468499025 | 0.0866729779413512 | -0.098135623 | 0.0065932408180388 | 0.955260509381448 | 0.906525949798403 | 1.00661502407806 | 0.88227178111339 |
| ebi-a-GCST90001810 | finngen_R11_O15_PREECLAMPS | Pre-eclampsia \|\| id:finngen_R11_O15_PREECLAMPS | CD38 on CD3- CD19- \|\| id:ebi-a-GCST90001810 | Weighted mode | 18 | -0.075989203 | 0.0426584816386888 | 0.0927305182010841 | -0.159599827 | 0.007621420614952 | 0.92682621308015 | 0.852484861793842 | 1.00765053756488 | 0.872755742358858 |
| ebi-a-GCST90001811 | finngen_R11_O15_PREECLAMPS | Pre-eclampsia \|\| id:finngen_R11_O15_PREECLAMPS | CD38 on IgD+ CD24- B cell \|\| id:ebi-a-GCST90001811 | Inverse variance weighted | 17 | -0.004423742 | 0.0244843610875646 | 0.856621717778607 | -0.05241309 | 0.043565605687627 | 0.99558602829038 | 0.948936789781378 | 1.04452851907593 | 0.977824840984872 |
| ebi-a-GCST90001811 | finngen_R11_O15_PREECLAMPS | Pre-eclampsia \|\| id:finngen_R11_O15_PREECLAMPS | CD38 on IgD+ CD24- B cell \|\| id:ebi-a-GCST90001811 | MR Egger | 17 | -0.021017934 | 0.0394345249031973 | 0.601853125025153 | -0.098309602 | 0.0562737353050692 | 0.979201403898532 | 0.906368246602299 | 1.05788722518826 | 0.99380795269851 |
| ebi-a-GCST90001811 | finngen_R11_O15_PREECLAMPS | Pre-eclampsia \|\| id:finngen_R11_O15_PREECLAMPS | CD38 on IgD+ CD24- B cell \|\| id:ebi-a-GCST90001811 | Weighted median | 17 | -0.042700673 | 0.0330561106815529 | 0.196439100997642 | -0.10749065 | 0.0220893036917378 | 0.958198161492893 | 0.898084919343934 | 1.02233507869064 | 0.940845681141708 |
| ebi-a-GCST90001811 | finngen_R11_O15_PREECLAMPS | Pre-eclampsia \|\| id:finngen_R11_O15_PREECLAMPS | CD38 on IgD+ CD24- B cell \|\| id:ebi-a-GCST90001811 | Weighted mode | 17 | -0.041688755 | 0.0367722651121035 | 0.27362175573446 | -0.113762395 | 0.030384884684501 | 0.959168270508803 | 0.892469986419928 | 1.03085121645532 | 0.872755742358858 |
| ebi-a-GCST90001812 | finngen_R11_O15_PREECLAMPS | Pre-eclampsia \|\| id:finngen_R11_O15_PREECLAMPS | CD38 on IgD+ CD38+ B cell \|\| id:ebi-a-GCST90001812 | Inverse variance weighted | 17 | -0.005418209 | 0.0202917790810496 | 0.789457628686376 | -0.045190096 | 0.034353678049025 | 0.994596443069768 | 0.955815767856939 | 1.03495058130815 | 0.977824840984872 |
| ebi-a-GCST90001812 | finngen_R11_O15_PREECLAMPS | Pre-eclampsia \|\| id:finngen_R11_O15_PREECLAMPS | CD38 on IgD+ CD38+ B cell \|\| id:ebi-a-GCST90001812 | MR Egger | 17 | 0.0106318807475202 | 0.0325022578769722 | 0.748103479952104 | -0.053072545 | 0.0743363061863857 | 1.01068860002428 | 0.948311215042489 | 1.0771690031877 | 0.99380795269851 |
| ebi-a-GCST90001812 | finngen_R11_O15_PREECLAMPS | Pre-eclampsia \|\| id:finngen_R11_O15_PREECLAMPS | CD38 on IgD+ CD38+ B cell \|\| id:ebi-a-GCST90001812 | Weighted median | 17 | -0.00450941 | 0.0278777478810791 | 0.871497515010105 | -0.059149796 | 0.0501309755666851 | 0.995500741844543 | 0.94256556591016 | 1.05140879622107 | 0.964912412213677 |
| ebi-a-GCST90001812 | finngen_R11_O15_PREECLAMPS | Pre-eclampsia \|\| id:finngen_R11_O15_PREECLAMPS | CD38 on IgD+ CD38+ B cell \|\| id:ebi-a-GCST90001812 | Weighted mode | 17 | 0.00152368762182398 | 0.0301959549576891 | 0.960380407612553 | -0.057660384 | 0.0607077593388946 | 1.0015248490236 | 0.943970480393432 | 1.06258833728963 | 0.990180645930572 |
| ebi-a-GCST90001813 | finngen_R11_O15_PREECLAMPS | Pre-eclampsia \|\| id:finngen_R11_O15_PREECLAMPS | CD38 on IgD+ CD38dim B cell \|\| id:ebi-a-GCST90001813 | Inverse variance weighted | 23 | 0.0103830969193452 | 0.0185839521255262 | 0.576357558887854 | -0.026041449 | 0.0468076430853765 | 1.01043718832015 | 0.974294704990227 | 1.04792041495347 | 0.915399120505076 |
| ebi-a-GCST90001813 | finngen_R11_O15_PREECLAMPS | Pre-eclampsia \|\| id:finngen_R11_O15_PREECLAMPS | CD38 on IgD+ CD38dim B cell \|\| id:ebi-a-GCST90001813 | MR Egger | 23 | 0.00852171361421398 | 0.0280536749340268 | 0.764296507135586 | -0.046463489 | 0.0635069164849066 | 1.00855812677636 | 0.954599413068057 | 1.06556685575305 | 0.99380795269851 |
| ebi-a-GCST90001813 | finngen_R11_O15_PREECLAMPS | Pre-eclampsia \|\| id:finngen_R11_O15_PREECLAMPS | CD38 on IgD+ CD38dim B cell \|\| id:ebi-a-GCST90001813 | Weighted median | 23 | 0.00993289845891868 | 0.027306845431125 | 0.716043788301802 | -0.043588519 | 0.0634543155039237 | 1.00998239343527 | 0.957347807265896 | 1.06551080736525 | 0.949957918887556 |
| ebi-a-GCST90001813 | finngen_R11_O15_PREECLAMPS | Pre-eclampsia \|\| id:finngen_R11_O15_PREECLAMPS | CD38 on IgD+ CD38dim B cell \|\| id:ebi-a-GCST90001813 | Weighted mode | 23 | 0.00118234750829244 | 0.029872788374417 | 0.968785423823343 | -0.057368318 | 0.0597330127221497 | 1.00118304675667 | 0.944246222709178 | 1.06155308753793 | 0.993663982022827 |
| ebi-a-GCST90001814 | finngen_R11_O15_PREECLAMPS | Pre-eclampsia \|\| id:finngen_R11_O15_PREECLAMPS | CD38 on IgD- CD38+ B cell \|\| id:ebi-a-GCST90001814 | Inverse variance weighted | 14 | -0.013084081 | 0.0078791149369406 | 0.0967932374106253 | -0.028527146 | 0.00235898460336207 | 0.987001143810894 | 0.97187591129369 | 1.00236176919672 | 0.804043824399626 |
| ebi-a-GCST90001814 | finngen_R11_O15_PREECLAMPS | Pre-eclampsia \|\| id:finngen_R11_O15_PREECLAMPS | CD38 on IgD- CD38+ B cell \|\| id:ebi-a-GCST90001814 | MR Egger | 14 | -0.016415523 | 0.00877082824535516 | 0.085830814683294 | -0.033606346 | 0.000775300248945097 | 0.983718477356378 | 0.966952073820839 | 1.00077560087187 | 0.936133620820556 |
| ebi-a-GCST90001814 | finngen_R11_O15_PREECLAMPS | Pre-eclampsia \|\| id:finngen_R11_O15_PREECLAMPS | CD38 on IgD- CD38+ B cell \|\| id:ebi-a-GCST90001814 | Weighted median | 14 | -0.02431815 | 0.0118405138729848 | 0.0399942106934423 | -0.047525558 | -0.001110743 | 0.975975153482589 | 0.953586101466692 | 0.998889873447505 | 0.743099833442067 |
| ebi-a-GCST90001814 | finngen_R11_O15_PREECLAMPS | Pre-eclampsia \|\| id:finngen_R11_O15_PREECLAMPS | CD38 on IgD- CD38+ B cell \|\| id:ebi-a-GCST90001814 | Weighted mode | 14 | -0.014784052 | 0.00880485587982806 | 0.116996279045135 | -0.032041569 | 0.00247346595481088 | 0.98532469595151 | 0.968466322974709 | 1.00247652749541 | 0.872755742358858 |
| ebi-a-GCST90001815 | finngen_R11_O15_PREECLAMPS | Pre-eclampsia \|\| id:finngen_R11_O15_PREECLAMPS | CD38 on IgD- CD38dim B cell \|\| id:ebi-a-GCST90001815 | Inverse variance weighted | 19 | 0.0200405181461456 | 0.0121356996777029 | 0.0986632523898939 | -0.003745453 | 0.0438264895144434 | 1.0202426775312 | 0.99626155223883 | 1.0448010552218 | 0.810368960640589 |
| ebi-a-GCST90001815 | finngen_R11_O15_PREECLAMPS | Pre-eclampsia \|\| id:finngen_R11_O15_PREECLAMPS | CD38 on IgD- CD38dim B cell \|\| id:ebi-a-GCST90001815 | MR Egger | 19 | 0.0247028630159729 | 0.0148877258535159 | 0.115393375885188 | -0.00447708 | 0.053882805688864 | 1.02501050674009 | 0.995532927524323 | 1.05536091261221 | 0.97261503632431 |
| ebi-a-GCST90001815 | finngen_R11_O15_PREECLAMPS | Pre-eclampsia \|\| id:finngen_R11_O15_PREECLAMPS | CD38 on IgD- CD38dim B cell \|\| id:ebi-a-GCST90001815 | Weighted median | 19 | 0.0212714911325697 | 0.0179094290485364 | 0.234941451575642 | -0.01383099 | 0.0563739720677011 | 1.02149934200817 | 0.98626421888797 | 1.05799326969363 | 0.940845681141708 |
| ebi-a-GCST90001815 | finngen_R11_O15_PREECLAMPS | Pre-eclampsia \|\| id:finngen_R11_O15_PREECLAMPS | CD38 on IgD- CD38dim B cell \|\| id:ebi-a-GCST90001815 | Weighted mode | 19 | 0.0221168399833287 | 0.0155720750364747 | 0.172613690468365 | -0.008404427 | 0.052638107054819 | 1.02236323039507 | 0.991630791376444 | 1.05404812350875 | 0.872755742358858 |
| ebi-a-GCST90001816 | finngen_R11_O15_PREECLAMPS | Pre-eclampsia \|\| id:finngen_R11_O15_PREECLAMPS | CD38 on naive-mature B cell \|\| id:ebi-a-GCST90001816 | Inverse variance weighted | 26 | 0.00469198300470321 | 0.0194593562795799 | 0.809464381700676 | -0.033448355 | 0.0428323213126799 | 1.00470300759261 | 0.967104855778686 | 1.04376286338969 | 0.977824840984872 |
| ebi-a-GCST90001816 | finngen_R11_O15_PREECLAMPS | Pre-eclampsia \|\| id:finngen_R11_O15_PREECLAMPS | CD38 on naive-mature B cell \|\| id:ebi-a-GCST90001816 | MR Egger | 26 | -0.001563344 | 0.0296810246516763 | 0.958429621533711 | -0.059738152 | 0.0566114643927998 | 0.998437877461063 | 0.942011164804654 | 1.05824456481434 | 0.99380795269851 |
| ebi-a-GCST90001816 | finngen_R11_O15_PREECLAMPS | Pre-eclampsia \|\| id:finngen_R11_O15_PREECLAMPS | CD38 on naive-mature B cell \|\| id:ebi-a-GCST90001816 | Weighted median | 26 | 5.18434718012953e-05 | 0.0273453868233188 | 0.998487310447797 | -0.053545115 | 0.0536488016455061 | 1.0000518448157 | 0.947863177474481 | 1.05511398278388 | 0.998487310447797 |
| ebi-a-GCST90001816 | finngen_R11_O15_PREECLAMPS | Pre-eclampsia \|\| id:finngen_R11_O15_PREECLAMPS | CD38 on naive-mature B cell \|\| id:ebi-a-GCST90001816 | Weighted mode | 26 | -0.022011233 | 0.0302871637411874 | 0.474128966264181 | -0.081374074 | 0.0373516079312279 | 0.9782292465389 | 0.921848787336171 | 1.03805794608601 | 0.933945646747087 |
| ebi-a-GCST90001817 | finngen_R11_O15_PREECLAMPS | Pre-eclampsia \|\| id:finngen_R11_O15_PREECLAMPS | CD38 on Plasma Blast-Plasma Cell \|\| id:ebi-a-GCST90001817 | Inverse variance weighted | 15 | -0.039297941 | 0.0279788500299804 | 0.160152504865981 | -0.094136487 | 0.0155406050627872 | 0.961464206864712 | 0.910158528905886 | 1.01566198823983 | 0.85505992597664 |
| ebi-a-GCST90001817 | finngen_R11_O15_PREECLAMPS | Pre-eclampsia \|\| id:finngen_R11_O15_PREECLAMPS | CD38 on Plasma Blast-Plasma Cell \|\| id:ebi-a-GCST90001817 | MR Egger | 15 | -0.017567192 | 0.0474033819739676 | 0.716911715196404 | -0.110477821 | 0.0753434367794819 | 0.982586211622808 | 0.895406189578477 | 1.07825439952093 | 0.99380795269851 |
| ebi-a-GCST90001817 | finngen_R11_O15_PREECLAMPS | Pre-eclampsia \|\| id:finngen_R11_O15_PREECLAMPS | CD38 on Plasma Blast-Plasma Cell \|\| id:ebi-a-GCST90001817 | Weighted median | 15 | -0.030610088 | 0.0379600464138982 | 0.420025815138994 | -0.105011779 | 0.0437916030778278 | 0.969853657043977 | 0.900313917848026 | 1.0447646064718 | 0.940845681141708 |
| ebi-a-GCST90001817 | finngen_R11_O15_PREECLAMPS | Pre-eclampsia \|\| id:finngen_R11_O15_PREECLAMPS | CD38 on Plasma Blast-Plasma Cell \|\| id:ebi-a-GCST90001817 | Weighted mode | 15 | -0.017247927 | 0.0368983207069198 | 0.647374366150979 | -0.089568636 | 0.0550727812722471 | 0.982899966675993 | 0.914325507417544 | 1.05661751384399 | 0.959313411571287 |
| ebi-a-GCST90001818 | finngen_R11_O15_PREECLAMPS | Pre-eclampsia \|\| id:finngen_R11_O15_PREECLAMPS | CD38 on IgD+ B cell \|\| id:ebi-a-GCST90001818 | Inverse variance weighted | 15 | 0.0548256444320418 | 0.0387823744696534 | 0.157457567067063 | -0.02118781 | 0.130839098392562 | 1.05635641699496 | 0.979035075186076 | 1.13978437341926 | 0.85505992597664 |
| ebi-a-GCST90001818 | finngen_R11_O15_PREECLAMPS | Pre-eclampsia \|\| id:finngen_R11_O15_PREECLAMPS | CD38 on IgD+ B cell \|\| id:ebi-a-GCST90001818 | MR Egger | 15 | 0.139000712627076 | 0.0728742262654615 | 0.0788048006618007 | -0.003832771 | 0.281834196107381 | 1.14912491890084 | 0.996174564837978 | 1.32555891893671 | 0.928881437593846 |
| ebi-a-GCST90001818 | finngen_R11_O15_PREECLAMPS | Pre-eclampsia \|\| id:finngen_R11_O15_PREECLAMPS | CD38 on IgD+ B cell \|\| id:ebi-a-GCST90001818 | Weighted median | 15 | 0.0238321645765649 | 0.0555090827692399 | 0.667677187877385 | -0.084965638 | 0.132629966804275 | 1.02411842011707 | 0.918543847183284 | 1.14182740610509 | 0.940845681141708 |
| ebi-a-GCST90001818 | finngen_R11_O15_PREECLAMPS | Pre-eclampsia \|\| id:finngen_R11_O15_PREECLAMPS | CD38 on IgD+ B cell \|\| id:ebi-a-GCST90001818 | Weighted mode | 15 | -0.014300742 | 0.0836393971419474 | 0.866685319702332 | -0.178233961 | 0.149632476085016 | 0.985801027596147 | 0.836746634748282 | 1.16140731931592 | 0.977585971930092 |
| ebi-a-GCST90001819 | finngen_R11_O15_PREECLAMPS | Pre-eclampsia \|\| id:finngen_R11_O15_PREECLAMPS | CD38 on transitional B cell \|\| id:ebi-a-GCST90001819 | Inverse variance weighted | 25 | -0.015461338 | 0.0171951880591423 | 0.368563940071819 | -0.049163907 | 0.0182412302051498 | 0.984657574462219 | 0.952025073348467 | 1.01840861768032 | 0.885079385681701 |
| ebi-a-GCST90001819 | finngen_R11_O15_PREECLAMPS | Pre-eclampsia \|\| id:finngen_R11_O15_PREECLAMPS | CD38 on transitional B cell \|\| id:ebi-a-GCST90001819 | MR Egger | 25 | -0.020817122 | 0.0259755876526575 | 0.43109580909544 | -0.071729274 | 0.0300950297460456 | 0.97939805849887 | 0.930782848959491 | 1.03055246243918 | 0.99380795269851 |
| ebi-a-GCST90001819 | finngen_R11_O15_PREECLAMPS | Pre-eclampsia \|\| id:finngen_R11_O15_PREECLAMPS | CD38 on transitional B cell \|\| id:ebi-a-GCST90001819 | Weighted median | 25 | -0.048307324 | 0.0254976907426251 | 0.0581485369677938 | -0.098282798 | 0.0016681493995116 | 0.952840910761196 | 0.906392541225898 | 1.00166954153471 | 0.771214851704188 |
| ebi-a-GCST90001819 | finngen_R11_O15_PREECLAMPS | Pre-eclampsia \|\| id:finngen_R11_O15_PREECLAMPS | CD38 on transitional B cell \|\| id:ebi-a-GCST90001819 | Weighted mode | 25 | -0.044927251 | 0.0281219481095528 | 0.123219212469638 | -0.10004627 | 0.010191766954539 | 0.956067031898496 | 0.904795552507535 | 1.01024387990191 | 0.872755742358858 |
| ebi-a-GCST90001820 | finngen_R11_O15_PREECLAMPS | Pre-eclampsia \|\| id:finngen_R11_O15_PREECLAMPS | IgD on IgD+ CD24+ B cell \|\| id:ebi-a-GCST90001820 | Inverse variance weighted (multiplicative random effects) | 28 | -0.00456384 | 0.0199524837580488 | 0.819074556861174 | -0.043670708 | 0.0345430282473244 | 0.995446558573917 | 0.957269126563478 | 1.03514656796042 | 0.977824840984872 |
| ebi-a-GCST90001820 | finngen_R11_O15_PREECLAMPS | Pre-eclampsia \|\| id:finngen_R11_O15_PREECLAMPS | IgD on IgD+ CD24+ B cell \|\| id:ebi-a-GCST90001820 | MR Egger | 28 | 0.0177686365356628 | 0.0311856865775683 | 0.573724998436908 | -0.043355309 | 0.0788925822276966 | 1.01792743792486 | 0.957571095837556 | 1.08208808033619 | 0.99380795269851 |
| ebi-a-GCST90001820 | finngen_R11_O15_PREECLAMPS | Pre-eclampsia \|\| id:finngen_R11_O15_PREECLAMPS | IgD on IgD+ CD24+ B cell \|\| id:ebi-a-GCST90001820 | Weighted median | 28 | -0.016926192 | 0.0215174294059098 | 0.431500219368921 | -0.059100354 | 0.0252479696082489 | 0.983216251155073 | 0.942612169826532 | 1.02556939903857 | 0.940845681141708 |
| ebi-a-GCST90001820 | finngen_R11_O15_PREECLAMPS | Pre-eclampsia \|\| id:finngen_R11_O15_PREECLAMPS | IgD on IgD+ CD24+ B cell \|\| id:ebi-a-GCST90001820 | Weighted mode | 28 | -0.003683702 | 0.022206931947142 | 0.869486704153741 | -0.047209289 | 0.0398418845084402 | 0.99632307439922 | 0.953887738749906 | 1.04064621889492 | 0.977585971930092 |
| ebi-a-GCST90001821 | finngen_R11_O15_PREECLAMPS | Pre-eclampsia \|\| id:finngen_R11_O15_PREECLAMPS | IgD on IgD+ CD24- B cell \|\| id:ebi-a-GCST90001821 | Inverse variance weighted | 29 | -0.002273173 | 0.019530042129777 | 0.907340544728882 | -0.040552056 | 0.0360057091187512 | 0.997729408246579 | 0.960259175929875 | 1.03666176489915 | 0.986667691552896 |
| ebi-a-GCST90001821 | finngen_R11_O15_PREECLAMPS | Pre-eclampsia \|\| id:finngen_R11_O15_PREECLAMPS | IgD on IgD+ CD24- B cell \|\| id:ebi-a-GCST90001821 | MR Egger | 29 | -0.016349754 | 0.0339065571733884 | 0.633549730736887 | -0.082806606 | 0.0501070981985248 | 0.983783177911099 | 0.920529154894713 | 1.05138369164587 | 0.99380795269851 |
| ebi-a-GCST90001821 | finngen_R11_O15_PREECLAMPS | Pre-eclampsia \|\| id:finngen_R11_O15_PREECLAMPS | IgD on IgD+ CD24- B cell \|\| id:ebi-a-GCST90001821 | Weighted median | 29 | -0.016071107 | 0.0228666296886814 | 0.482168410564774 | -0.060889701 | 0.028747487081774 | 0.984057344095535 | 0.940927017081449 | 1.02916468428341 | 0.940845681141708 |
| ebi-a-GCST90001821 | finngen_R11_O15_PREECLAMPS | Pre-eclampsia \|\| id:finngen_R11_O15_PREECLAMPS | IgD on IgD+ CD24- B cell \|\| id:ebi-a-GCST90001821 | Weighted mode | 29 | -0.017817175 | 0.0216135183274411 | 0.416706213243032 | -0.060179671 | 0.024545320880599 | 0.98234061232394 | 0.941595341043557 | 1.02484903711555 | 0.93252856028397 |
| ebi-a-GCST90001822 | finngen_R11_O15_PREECLAMPS | Pre-eclampsia \|\| id:finngen_R11_O15_PREECLAMPS | IgD on IgD+ CD38- B cell \|\| id:ebi-a-GCST90001822 | Inverse variance weighted | 27 | -0.014695009 | 0.0203055183379714 | 0.4692527801655 | -0.054493825 | 0.0251038064650007 | 0.985412435230149 | 0.946964355947091 | 1.02542156038708 | 0.898694924660397 |
| ebi-a-GCST90001822 | finngen_R11_O15_PREECLAMPS | Pre-eclampsia \|\| id:finngen_R11_O15_PREECLAMPS | IgD on IgD+ CD38- B cell \|\| id:ebi-a-GCST90001822 | MR Egger | 27 | -0.00470802 | 0.0315867267698002 | 0.882709559475191 | -0.066618005 | 0.0572019641737239 | 0.995303045060348 | 0.935552509689217 | 1.05886964253399 | 0.99380795269851 |
| ebi-a-GCST90001822 | finngen_R11_O15_PREECLAMPS | Pre-eclampsia \|\| id:finngen_R11_O15_PREECLAMPS | IgD on IgD+ CD38- B cell \|\| id:ebi-a-GCST90001822 | Weighted median | 27 | -0.012829662 | 0.0232781484863834 | 0.581533487453505 | -0.058454833 | 0.0327955089859928 | 0.987252287231916 | 0.943220841817282 | 1.03333920905181 | 0.940845681141708 |
| ebi-a-GCST90001822 | finngen_R11_O15_PREECLAMPS | Pre-eclampsia \|\| id:finngen_R11_O15_PREECLAMPS | IgD on IgD+ CD38- B cell \|\| id:ebi-a-GCST90001822 | Weighted mode | 27 | -0.01912721 | 0.0198715645650634 | 0.344644870850799 | -0.058075476 | 0.0198210566014152 | 0.981054554407579 | 0.943578726735327 | 1.02001879806456 | 0.890231097498001 |
| ebi-a-GCST90001823 | finngen_R11_O15_PREECLAMPS | Pre-eclampsia \|\| id:finngen_R11_O15_PREECLAMPS | IgD on IgD+ CD38- unswitched memory B cell \|\| id:ebi-a-GCST90001823 | Inverse variance weighted | 17 | -0.024454901 | 0.017546175595498 | 0.163394508892556 | -0.058845405 | 0.00993560308701719 | 0.97584169733252 | 0.942852517942622 | 1.00998512506573 | 0.85505992597664 |
| ebi-a-GCST90001823 | finngen_R11_O15_PREECLAMPS | Pre-eclampsia \|\| id:finngen_R11_O15_PREECLAMPS | IgD on IgD+ CD38- unswitched memory B cell \|\| id:ebi-a-GCST90001823 | MR Egger | 17 | -0.031228352 | 0.0343983916323513 | 0.378315605127898 | -0.098649199 | 0.0361924957073471 | 0.969254216769134 | 0.906060498763438 | 1.03685541749903 | 0.99380795269851 |
| ebi-a-GCST90001823 | finngen_R11_O15_PREECLAMPS | Pre-eclampsia \|\| id:finngen_R11_O15_PREECLAMPS | IgD on IgD+ CD38- unswitched memory B cell \|\| id:ebi-a-GCST90001823 | Weighted median | 17 | -0.017369049 | 0.0229831149715457 | 0.44981058967667 | -0.062415954 | 0.0276778565611373 | 0.982780923596867 | 0.939492019922108 | 1.028064447 | 0.940845681141708 |
| ebi-a-GCST90001823 | finngen_R11_O15_PREECLAMPS | Pre-eclampsia \|\| id:finngen_R11_O15_PREECLAMPS | IgD on IgD+ CD38- unswitched memory B cell \|\| id:ebi-a-GCST90001823 | Weighted mode | 17 | -0.018664448 | 0.0213642315692822 | 0.395242886075607 | -0.060538342 | 0.0232094460808674 | 0.981508654385643 | 0.941257678934546 | 1.02348088115933 | 0.920286500917063 |
| ebi-a-GCST90001824 | finngen_R11_O15_PREECLAMPS | Pre-eclampsia \|\| id:finngen_R11_O15_PREECLAMPS | IgD on IgD+ CD38+ B cell \|\| id:ebi-a-GCST90001824 | Inverse variance weighted | 25 | 0.00236901759418946 | 0.0202161199048745 | 0.906713783204755 | -0.037254577 | 0.0419926126077434 | 1.0023718259336 | 0.963430836390526 | 1.04288677450862 | 0.986667691552896 |
| ebi-a-GCST90001824 | finngen_R11_O15_PREECLAMPS | Pre-eclampsia \|\| id:finngen_R11_O15_PREECLAMPS | IgD on IgD+ CD38+ B cell \|\| id:ebi-a-GCST90001824 | MR Egger | 25 | 0.0102120357758892 | 0.0374440062511471 | 0.787495858477595 | -0.063178216 | 0.0836022880281374 | 1.01026435656215 | 0.938776153401811 | 1.08719641678316 | 0.99380795269851 |
| ebi-a-GCST90001824 | finngen_R11_O15_PREECLAMPS | Pre-eclampsia \|\| id:finngen_R11_O15_PREECLAMPS | IgD on IgD+ CD38+ B cell \|\| id:ebi-a-GCST90001824 | Weighted median | 25 | -0.017637965 | 0.0230318448583441 | 0.443790702051033 | -0.062780381 | 0.0275044506287216 | 0.982516673112519 | 0.939149705958493 | 1.02788618983687 | 0.940845681141708 |
| ebi-a-GCST90001824 | finngen_R11_O15_PREECLAMPS | Pre-eclampsia \|\| id:finngen_R11_O15_PREECLAMPS | IgD on IgD+ CD38+ B cell \|\| id:ebi-a-GCST90001824 | Weighted mode | 25 | -0.023802237 | 0.0240247670909249 | 0.331697965444561 | -0.07089078 | 0.0232863068806278 | 0.976478802414774 | 0.931563631843488 | 1.02355954974162 | 0.888599269773114 |
| ebi-a-GCST90001825 | finngen_R11_O15_PREECLAMPS | Pre-eclampsia \|\| id:finngen_R11_O15_PREECLAMPS | IgD on IgD+ CD38dim B cell \|\| id:ebi-a-GCST90001825 | Inverse variance weighted | 22 | -0.009159125 | 0.0139999676890485 | 0.512967048164799 | -0.036599061 | 0.0182808119580712 | 0.990882692303811 | 0.964062587792674 | 1.01844892887639 | 0.898694924660397 |
| ebi-a-GCST90001825 | finngen_R11_O15_PREECLAMPS | Pre-eclampsia \|\| id:finngen_R11_O15_PREECLAMPS | IgD on IgD+ CD38dim B cell \|\| id:ebi-a-GCST90001825 | MR Egger | 22 | 0.0239327601896608 | 0.0210573830928709 | 0.269161881894019 | -0.017339711 | 0.0652052310516879 | 1.02422144711937 | 0.982809756955379 | 1.06737806102888 | 0.99380795269851 |
| ebi-a-GCST90001825 | finngen_R11_O15_PREECLAMPS | Pre-eclampsia \|\| id:finngen_R11_O15_PREECLAMPS | IgD on IgD+ CD38dim B cell \|\| id:ebi-a-GCST90001825 | Weighted median | 22 | -0.013108597 | 0.0190869666693932 | 0.492219708283943 | -0.050519052 | 0.0243018572307866 | 0.986976946029282 | 0.950735814973372 | 1.02459955399973 | 0.940845681141708 |
| ebi-a-GCST90001825 | finngen_R11_O15_PREECLAMPS | Pre-eclampsia \|\| id:finngen_R11_O15_PREECLAMPS | IgD on IgD+ CD38dim B cell \|\| id:ebi-a-GCST90001825 | Weighted mode | 22 | -0.000390861 | 0.0165525612547921 | 0.981383981536053 | -0.032833881 | 0.0320521589624272 | 0.999609215279282 | 0.96769929932631 | 1.03257136175142 | 0.994159530659367 |
| ebi-a-GCST90001826 | finngen_R11_O15_PREECLAMPS | Pre-eclampsia \|\| id:finngen_R11_O15_PREECLAMPS | IgD on unswitched memory B cell \|\| id:ebi-a-GCST90001826 | Inverse variance weighted | 22 | -0.003133783 | 0.016679048235627 | 0.850964931234947 | -0.035824717 | 0.0295571517696115 | 0.996871122399762 | 0.964809393044424 | 1.02999830001531 | 0.977824840984872 |
| ebi-a-GCST90001826 | finngen_R11_O15_PREECLAMPS | Pre-eclampsia \|\| id:finngen_R11_O15_PREECLAMPS | IgD on unswitched memory B cell \|\| id:ebi-a-GCST90001826 | MR Egger | 22 | -0.018697591 | 0.0239248894185882 | 0.443655031446088 | -0.065590375 | 0.028195191922358 | 0.98147612425029 | 0.936514405821567 | 1.02859643854417 | 0.99380795269851 |
| ebi-a-GCST90001826 | finngen_R11_O15_PREECLAMPS | Pre-eclampsia \|\| id:finngen_R11_O15_PREECLAMPS | IgD on unswitched memory B cell \|\| id:ebi-a-GCST90001826 | Weighted median | 22 | -0.023366006 | 0.0209822153164514 | 0.265446517265993 | -0.064491148 | 0.0177591363788839 | 0.976904865640245 | 0.937544413685725 | 1.01791776750055 | 0.940845681141708 |
| ebi-a-GCST90001826 | finngen_R11_O15_PREECLAMPS | Pre-eclampsia \|\| id:finngen_R11_O15_PREECLAMPS | IgD on unswitched memory B cell \|\| id:ebi-a-GCST90001826 | Weighted mode | 22 | -0.019933771 | 0.0189184779046315 | 0.304008607802197 | -0.057013988 | 0.0171464458694394 | 0.980263593207331 | 0.944580856933593 | 1.0172942899639 | 0.875408265683677 |
| ebi-a-GCST90001827 | finngen_R11_O15_PREECLAMPS | Pre-eclampsia \|\| id:finngen_R11_O15_PREECLAMPS | IgD on IgD+ B cell \|\| id:ebi-a-GCST90001827 | Inverse variance weighted | 20 | -0.019482591 | 0.020243031295781 | 0.335831413713398 | -0.059158932 | 0.0201937505713203 | 0.980705968377375 | 0.942556954688399 | 1.0203990237693 | 0.885079385681701 |
| ebi-a-GCST90001827 | finngen_R11_O15_PREECLAMPS | Pre-eclampsia \|\| id:finngen_R11_O15_PREECLAMPS | IgD on IgD+ B cell \|\| id:ebi-a-GCST90001827 | MR Egger | 20 | -0.033125553 | 0.0382337301989419 | 0.397675114893267 | -0.108063664 | 0.0418125586670604 | 0.967417090307453 | 0.897570451943976 | 1.04269901553907 | 0.99380795269851 |
| ebi-a-GCST90001827 | finngen_R11_O15_PREECLAMPS | Pre-eclampsia \|\| id:finngen_R11_O15_PREECLAMPS | IgD on IgD+ B cell \|\| id:ebi-a-GCST90001827 | Weighted median | 20 | -0.017904732 | 0.0255768076778416 | 0.483903698819441 | -0.068035275 | 0.0322258108895221 | 0.982254605176491 | 0.934227517926302 | 1.03275068532774 | 0.940845681141708 |
| ebi-a-GCST90001827 | finngen_R11_O15_PREECLAMPS | Pre-eclampsia \|\| id:finngen_R11_O15_PREECLAMPS | IgD on IgD+ B cell \|\| id:ebi-a-GCST90001827 | Weighted mode | 20 | -0.021005404 | 0.0246580645479761 | 0.404897434870352 | -0.06933521 | 0.0273244027820056 | 0.979213673146875 | 0.93301387184331 | 1.02770113780133 | 0.930105377419646 |
| ebi-a-GCST90001828 | finngen_R11_O15_PREECLAMPS | Pre-eclampsia \|\| id:finngen_R11_O15_PREECLAMPS | IgD on transitional B cell \|\| id:ebi-a-GCST90001828 | Inverse variance weighted | 32 | -0.013012443 | 0.020832175163294 | 0.532212620999266 | -0.053843506 | 0.0278186203581677 | 0.987071852846428 | 0.947580385276795 | 1.02820917129588 | 0.904087927705763 |
| ebi-a-GCST90001828 | finngen_R11_O15_PREECLAMPS | Pre-eclampsia \|\| id:finngen_R11_O15_PREECLAMPS | IgD on transitional B cell \|\| id:ebi-a-GCST90001828 | MR Egger | 32 | -0.029815344 | 0.0367045762815652 | 0.42301540221286 | -0.101756313 | 0.0421256257386259 | 0.970624748905114 | 0.903249634788349 | 1.0430255013697 | 0.99380795269851 |
| ebi-a-GCST90001828 | finngen_R11_O15_PREECLAMPS | Pre-eclampsia \|\| id:finngen_R11_O15_PREECLAMPS | IgD on transitional B cell \|\| id:ebi-a-GCST90001828 | Weighted median | 32 | -0.018293938 | 0.0268062156460947 | 0.494953595206262 | -0.070834121 | 0.034246244603998 | 0.981872380272532 | 0.931616415163238 | 1.03483939897422 | 0.940845681141708 |
| ebi-a-GCST90001828 | finngen_R11_O15_PREECLAMPS | Pre-eclampsia \|\| id:finngen_R11_O15_PREECLAMPS | IgD on transitional B cell \|\| id:ebi-a-GCST90001828 | Weighted mode | 32 | -0.020459547 | 0.0259859708271807 | 0.437066988844156 | -0.07139205 | 0.0304729554476432 | 0.979748329065983 | 0.931096783886292 | 1.03094200830664 | 0.933945646747087 |
| ebi-a-GCST90001829 | finngen_R11_O15_PREECLAMPS | Pre-eclampsia \|\| id:finngen_R11_O15_PREECLAMPS | BAFF-R on B cell \|\| id:ebi-a-GCST90001829 | Inverse variance weighted (multiplicative random effects) | 21 | 0.0171126061522551 | 0.0177124496551124 | 0.33397695479913 | -0.017603795 | 0.0518290074762755 | 1.01725986559529 | 0.982550246400575 | 1.05319563853538 | 0.885079385681701 |
| ebi-a-GCST90001829 | finngen_R11_O15_PREECLAMPS | Pre-eclampsia \|\| id:finngen_R11_O15_PREECLAMPS | BAFF-R on B cell \|\| id:ebi-a-GCST90001829 | MR Egger | 21 | 0.0508125795485092 | 0.0216765619212286 | 0.0300943672745018 | 0.00832651818290126 | 0.0932986409141172 | 1.05212568493245 | 1.00836128005022 | 1.09778953118811 | 0.839403552053506 |
| ebi-a-GCST90001829 | finngen_R11_O15_PREECLAMPS | Pre-eclampsia \|\| id:finngen_R11_O15_PREECLAMPS | BAFF-R on B cell \|\| id:ebi-a-GCST90001829 | Weighted median | 21 | 0.052391633917943 | 0.0176998920181222 | 0.00307641663262165 | 0.0176998455624234 | 0.0870834222734626 | 1.05378836097489 | 1.01785741611418 | 1.09098768859544 | 0.507633389188193 |
| ebi-a-GCST90001829 | finngen_R11_O15_PREECLAMPS | Pre-eclampsia \|\| id:finngen_R11_O15_PREECLAMPS | BAFF-R on B cell \|\| id:ebi-a-GCST90001829 | Weighted mode | 21 | 0.042769561270489 | 0.0159871143430208 | 0.0145436313190436 | 0.0114348171581683 | 0.0741043053828098 | 1.04369735884391 | 1.01150044458723 | 1.0769191281001 | 0.720210568263475 |
| ebi-a-GCST90001830 | finngen_R11_O15_PREECLAMPS | Pre-eclampsia \|\| id:finngen_R11_O15_PREECLAMPS | BAFF-R on CD20- B cell \|\| id:ebi-a-GCST90001830 | Inverse variance weighted (multiplicative random effects) | 15 | -0.02499543 | 0.0326953772802129 | 0.444572733021978 | -0.089078369 | 0.0390875099488349 | 0.975314369672593 | 0.914773880860491 | 1.03986147789337 | 0.898694924660397 |
| ebi-a-GCST90001830 | finngen_R11_O15_PREECLAMPS | Pre-eclampsia \|\| id:finngen_R11_O15_PREECLAMPS | BAFF-R on CD20- B cell \|\| id:ebi-a-GCST90001830 | MR Egger | 15 | -0.023925697 | 0.0506902844187503 | 0.644752842322863 | -0.123278655 | 0.075427260017863 | 0.976358252977657 | 0.884017292482849 | 1.07834478608469 | 0.99380795269851 |
| ebi-a-GCST90001830 | finngen_R11_O15_PREECLAMPS | Pre-eclampsia \|\| id:finngen_R11_O15_PREECLAMPS | BAFF-R on CD20- B cell \|\| id:ebi-a-GCST90001830 | Weighted median | 15 | -0.0644776 | 0.0391031345939962 | 0.0991658104434823 | -0.141119744 | 0.0121645438134607 | 0.937557115314872 | 0.868385321717766 | 1.0122388328015 | 0.88227178111339 |
| ebi-a-GCST90001830 | finngen_R11_O15_PREECLAMPS | Pre-eclampsia \|\| id:finngen_R11_O15_PREECLAMPS | BAFF-R on CD20- B cell \|\| id:ebi-a-GCST90001830 | Weighted mode | 15 | -0.05852604 | 0.0349839616395013 | 0.116527917657355 | -0.127094604 | 0.0100425251292194 | 0.943153680655905 | 0.880650355171683 | 1.01009312051134 | 0.872755742358858 |
| ebi-a-GCST90001831 | finngen_R11_O15_PREECLAMPS | Pre-eclampsia \|\| id:finngen_R11_O15_PREECLAMPS | CD62L on CD62L+ myeloid Dendritic Cell \|\| id:ebi-a-GCST90001831 | Inverse variance weighted | 15 | 0.029069922698607 | 0.0311766820889609 | 0.351116848594245 | -0.032036374 | 0.0901762195929702 | 1.02949657713737 | 0.968471354071889 | 1.09436711564201 | 0.885079385681701 |
| ebi-a-GCST90001831 | finngen_R11_O15_PREECLAMPS | Pre-eclampsia \|\| id:finngen_R11_O15_PREECLAMPS | CD62L on CD62L+ myeloid Dendritic Cell \|\| id:ebi-a-GCST90001831 | MR Egger | 15 | -0.072584368 | 0.070868833936704 | 0.324419208312395 | -0.211487283 | 0.066318546039516 | 0.929987281740288 | 0.8093795738735 | 1.06856705075913 | 0.99380795269851 |
| ebi-a-GCST90001831 | finngen_R11_O15_PREECLAMPS | Pre-eclampsia \|\| id:finngen_R11_O15_PREECLAMPS | CD62L on CD62L+ myeloid Dendritic Cell \|\| id:ebi-a-GCST90001831 | Weighted median | 15 | 0.0489369716488969 | 0.0388942628911696 | 0.208317482094619 | -0.027295784 | 0.125169726915589 | 1.05015415916902 | 0.973073379789388 | 1.13334079518103 | 0.940845681141708 |
| ebi-a-GCST90001831 | finngen_R11_O15_PREECLAMPS | Pre-eclampsia \|\| id:finngen_R11_O15_PREECLAMPS | CD62L on CD62L+ myeloid Dendritic Cell \|\| id:ebi-a-GCST90001831 | Weighted mode | 15 | 0.0525210279575781 | 0.0540522148087397 | 0.347703314500575 | -0.053421313 | 0.158463368982708 | 1.05392472372991 | 0.94798053174917 | 1.17170900254634 | 0.891828501403228 |
| ebi-a-GCST90001832 | finngen_R11_O15_PREECLAMPS | Pre-eclampsia \|\| id:finngen_R11_O15_PREECLAMPS | CD62L on CD62L+ plasmacytoid Dendritic Cell \|\| id:ebi-a-GCST90001832 | Inverse variance weighted | 15 | -0.0164007 | 0.0253417578200507 | 0.517514919944558 | -0.066070545 | 0.0332691455958469 | 0.983733059497749 | 0.93606482721412 | 1.03382875225525 | 0.898694924660397 |
| ebi-a-GCST90001832 | finngen_R11_O15_PREECLAMPS | Pre-eclampsia \|\| id:finngen_R11_O15_PREECLAMPS | CD62L on CD62L+ plasmacytoid Dendritic Cell \|\| id:ebi-a-GCST90001832 | MR Egger | 15 | -0.074056639 | 0.0442844774916149 | 0.118347378218313 | -0.160854215 | 0.0127409365967793 | 0.928619096028455 | 0.851416185623506 | 1.0128224481394 | 0.97261503632431 |
| ebi-a-GCST90001832 | finngen_R11_O15_PREECLAMPS | Pre-eclampsia \|\| id:finngen_R11_O15_PREECLAMPS | CD62L on CD62L+ plasmacytoid Dendritic Cell \|\| id:ebi-a-GCST90001832 | Weighted median | 15 | -0.037953247 | 0.0344752957434333 | 0.270947834387237 | -0.105524827 | 0.0296183326602736 | 0.962757951664538 | 0.899852132251939 | 1.03006131815642 | 0.940845681141708 |
| ebi-a-GCST90001832 | finngen_R11_O15_PREECLAMPS | Pre-eclampsia \|\| id:finngen_R11_O15_PREECLAMPS | CD62L on CD62L+ plasmacytoid Dendritic Cell \|\| id:ebi-a-GCST90001832 | Weighted mode | 15 | -0.046207412 | 0.0390266972428918 | 0.256128480723687 | -0.122699738 | 0.0302849149446415 | 0.954843895901493 | 0.884529212983141 | 1.03074816767835 | 0.872755742358858 |
| ebi-a-GCST90001833 | finngen_R11_O15_PREECLAMPS | Pre-eclampsia \|\| id:finngen_R11_O15_PREECLAMPS | CD62L on CD62L+ Dendritic Cell \|\| id:ebi-a-GCST90001833 | Inverse variance weighted | 26 | -0.029997091 | 0.0197212512629082 | 0.128246254838205 | -0.068650743 | 0.00865656185148965 | 0.970448356943744 | 0.933652707792404 | 1.00869413823219 | 0.820779257935285 |
| ebi-a-GCST90001833 | finngen_R11_O15_PREECLAMPS | Pre-eclampsia \|\| id:finngen_R11_O15_PREECLAMPS | CD62L on CD62L+ Dendritic Cell \|\| id:ebi-a-GCST90001833 | MR Egger | 26 | -0.060034885 | 0.0364475728406898 | 0.112558516382229 | -0.131472128 | 0.0114023574397577 | 0.941731680392652 | 0.876803712986368 | 1.01146761210049 | 0.97261503632431 |
| ebi-a-GCST90001833 | finngen_R11_O15_PREECLAMPS | Pre-eclampsia \|\| id:finngen_R11_O15_PREECLAMPS | CD62L on CD62L+ Dendritic Cell \|\| id:ebi-a-GCST90001833 | Weighted median | 26 | -0.054461221 | 0.0290104118185379 | 0.0604771051931107 | -0.111321629 | 0.00239918582762221 | 0.946995231355008 | 0.894650957404144 | 1.00240206617698 | 0.771214851704188 |
| ebi-a-GCST90001833 | finngen_R11_O15_PREECLAMPS | Pre-eclampsia \|\| id:finngen_R11_O15_PREECLAMPS | CD62L on CD62L+ Dendritic Cell \|\| id:ebi-a-GCST90001833 | Weighted mode | 26 | -0.053178493 | 0.0353175584356872 | 0.144669641131458 | -0.122400908 | 0.0160439210878014 | 0.948210747972367 | 0.884793576582337 | 1.01617331586341 | 0.872755742358858 |
| ebi-a-GCST90001834 | finngen_R11_O15_PREECLAMPS | Pre-eclampsia \|\| id:finngen_R11_O15_PREECLAMPS | CD62L on monocyte \|\| id:ebi-a-GCST90001834 | Inverse variance weighted | 25 | 0.00768131390725393 | 0.0131542068776878 | 0.559258309885758 | -0.018100932 | 0.033463559387522 | 1.00771089088043 | 0.98206190630291 | 1.03402976236186 | 0.90609555750305 |
| ebi-a-GCST90001834 | finngen_R11_O15_PREECLAMPS | Pre-eclampsia \|\| id:finngen_R11_O15_PREECLAMPS | CD62L on monocyte \|\| id:ebi-a-GCST90001834 | MR Egger | 25 | 0.000564110594233611 | 0.0172553655708583 | 0.974202339159261 | -0.033256406 | 0.0343846271131158 | 1.00056426973454 | 0.967290508772236 | 1.03498261255569 | 0.99380795269851 |
| ebi-a-GCST90001834 | finngen_R11_O15_PREECLAMPS | Pre-eclampsia \|\| id:finngen_R11_O15_PREECLAMPS | CD62L on monocyte \|\| id:ebi-a-GCST90001834 | Weighted median | 25 | 0.00519253792367746 | 0.0177513415768532 | 0.769892710123336 | -0.029600092 | 0.0399851674143098 | 1.00520604251297 | 0.970833700511633 | 1.04079533639188 | 0.957984984509652 |
| ebi-a-GCST90001834 | finngen_R11_O15_PREECLAMPS | Pre-eclampsia \|\| id:finngen_R11_O15_PREECLAMPS | CD62L on monocyte \|\| id:ebi-a-GCST90001834 | Weighted mode | 25 | 0.00968974759842761 | 0.0155133946342959 | 0.538123420456527 | -0.020716506 | 0.0400960010816476 | 1.00973684520107 | 0.979496606736516 | 1.04091069794882 | 0.935508728341156 |
| ebi-a-GCST90001835 | finngen_R11_O15_PREECLAMPS | Pre-eclampsia \|\| id:finngen_R11_O15_PREECLAMPS | CD62L on granulocyte \|\| id:ebi-a-GCST90001835 | Inverse variance weighted | 16 | 0.00769437029552546 | 0.0352601664073299 | 0.82725998353222 | -0.061415556 | 0.076804296453892 | 1.00772404803098 | 0.940432356385391 | 1.0798307290097 | 0.977824840984872 |
| ebi-a-GCST90001835 | finngen_R11_O15_PREECLAMPS | Pre-eclampsia \|\| id:finngen_R11_O15_PREECLAMPS | CD62L on granulocyte \|\| id:ebi-a-GCST90001835 | MR Egger | 16 | -0.028234617 | 0.0640537089697113 | 0.666091045121976 | -0.153779887 | 0.0973106521955358 | 0.972160254345948 | 0.857460738526025 | 1.10220272213816 | 0.99380795269851 |
| ebi-a-GCST90001835 | finngen_R11_O15_PREECLAMPS | Pre-eclampsia \|\| id:finngen_R11_O15_PREECLAMPS | CD62L on granulocyte \|\| id:ebi-a-GCST90001835 | Weighted median | 16 | 0.00852112494684887 | 0.0428467394999691 | 0.842360897908658 | -0.075458484 | 0.0925007343667883 | 1.00855753307128 | 0.927318227839717 | 1.09691394709718 | 0.964912412213677 |
| ebi-a-GCST90001835 | finngen_R11_O15_PREECLAMPS | Pre-eclampsia \|\| id:finngen_R11_O15_PREECLAMPS | CD62L on granulocyte \|\| id:ebi-a-GCST90001835 | Weighted mode | 16 | -0.000638664 | 0.0426269227733756 | 0.988243486060607 | -0.084187433 | 0.0829101042147554 | 0.99936153948165 | 0.919258940433537 | 1.08644413740934 | 0.994159530659367 |
| ebi-a-GCST90001836 | finngen_R11_O15_PREECLAMPS | Pre-eclampsia \|\| id:finngen_R11_O15_PREECLAMPS | CD66b on CD66b++ myeloid cell \|\| id:ebi-a-GCST90001836 | Inverse variance weighted | 25 | 0.0512037835486687 | 0.0160100999615301 | 0.00138279893014204 | 0.0198239876240697 | 0.0825835794732676 | 1.05253736122852 | 1.02002178776714 | 1.08608944442939 | 0.252706504483457 |
| ebi-a-GCST90001836 | finngen_R11_O15_PREECLAMPS | Pre-eclampsia \|\| id:finngen_R11_O15_PREECLAMPS | CD66b on CD66b++ myeloid cell \|\| id:ebi-a-GCST90001836 | MR Egger | 25 | 0.0195899158774028 | 0.0236224891933302 | 0.415464007100249 | -0.026710163 | 0.06588999469633 | 1.01978305742675 | 0.973643398571223 | 1.06810921302474 | 0.99380795269851 |
| ebi-a-GCST90001836 | finngen_R11_O15_PREECLAMPS | Pre-eclampsia \|\| id:finngen_R11_O15_PREECLAMPS | CD66b on CD66b++ myeloid cell \|\| id:ebi-a-GCST90001836 | Weighted median | 25 | 0.0489974866225313 | 0.0239966133556653 | 0.0411664059838781 | 0.00196412444542732 | 0.0960308487996354 | 1.05021771114318 | 1.00196605460133 | 1.10079302161356 | 0.743099833442067 |
| ebi-a-GCST90001836 | finngen_R11_O15_PREECLAMPS | Pre-eclampsia \|\| id:finngen_R11_O15_PREECLAMPS | CD66b on CD66b++ myeloid cell \|\| id:ebi-a-GCST90001836 | Weighted mode | 25 | 0.0461449461459385 | 0.0233442871078048 | 0.0596734802120213 | 0.000390143414640977 | 0.0918997488772359 | 1.04722619135704 | 1.00039021953048 | 1.09625491578565 | 0.838871423749761 |
| ebi-a-GCST90001837 | finngen_R11_O15_PREECLAMPS | Pre-eclampsia \|\| id:finngen_R11_O15_PREECLAMPS | CD66b on Granulocytic Myeloid-Derived Suppressor Cells \|\| id:ebi-a-GCST90001837 | Inverse variance weighted | 21 | 0.026719320632802 | 0.0201757407416567 | 0.18539376916658 | -0.012825131 | 0.0662637724864491 | 1.02707948228343 | 0.987256760310849 | 1.06850852314797 | 0.855687495048373 |
| ebi-a-GCST90001837 | finngen_R11_O15_PREECLAMPS | Pre-eclampsia \|\| id:finngen_R11_O15_PREECLAMPS | CD66b on Granulocytic Myeloid-Derived Suppressor Cells \|\| id:ebi-a-GCST90001837 | MR Egger | 21 | 0.0128433319757327 | 0.0406781047956756 | 0.755648698207051 | -0.066885753 | 0.0925724173752568 | 1.01292616178759 | 0.935302050290063 | 1.09699258000723 | 0.99380795269851 |
| ebi-a-GCST90001837 | finngen_R11_O15_PREECLAMPS | Pre-eclampsia \|\| id:finngen_R11_O15_PREECLAMPS | CD66b on Granulocytic Myeloid-Derived Suppressor Cells \|\| id:ebi-a-GCST90001837 | Weighted median | 21 | 0.00446623053987799 | 0.0259145085146256 | 0.863166473429322 | -0.046326206 | 0.0552586672285441 | 1.00447621901223 | 0.954730472437996 | 1.05681394245708 | 0.964912412213677 |
| ebi-a-GCST90001837 | finngen_R11_O15_PREECLAMPS | Pre-eclampsia \|\| id:finngen_R11_O15_PREECLAMPS | CD66b on Granulocytic Myeloid-Derived Suppressor Cells \|\| id:ebi-a-GCST90001837 | Weighted mode | 21 | 0.00208329071442015 | 0.0277156128481467 | 0.940828875493881 | -0.05223931 | 0.0564058918967877 | 1.00208546227225 | 0.949101709689156 | 1.05802704119696 | 0.990180645930572 |
| ebi-a-GCST90001838 | finngen_R11_O15_PREECLAMPS | Pre-eclampsia \|\| id:finngen_R11_O15_PREECLAMPS | CD3 on naive CD8+ T cell \|\| id:ebi-a-GCST90001838 | Inverse variance weighted | 25 | -0.013792859 | 0.0168304817678044 | 0.412491730213141 | -0.046780604 | 0.0191948849307911 | 0.986301826321648 | 0.954296743895713 | 1.01938029111805 | 0.898694924660397 |
| ebi-a-GCST90001838 | finngen_R11_O15_PREECLAMPS | Pre-eclampsia \|\| id:finngen_R11_O15_PREECLAMPS | CD3 on naive CD8+ T cell \|\| id:ebi-a-GCST90001838 | MR Egger | 25 | -0.033208438 | 0.0300031675892792 | 0.279809282505644 | -0.092014647 | 0.0255977701232005 | 0.967336908463026 | 0.912091790196802 | 1.02592820649433 | 0.99380795269851 |
| ebi-a-GCST90001838 | finngen_R11_O15_PREECLAMPS | Pre-eclampsia \|\| id:finngen_R11_O15_PREECLAMPS | CD3 on naive CD8+ T cell \|\| id:ebi-a-GCST90001838 | Weighted median | 25 | -0.029518011 | 0.0212843989774641 | 0.16549120054113 | -0.071235433 | 0.0121994110340828 | 0.97091339039978 | 0.931242621112283 | 1.01227412737151 | 0.940845681141708 |
| ebi-a-GCST90001838 | finngen_R11_O15_PREECLAMPS | Pre-eclampsia \|\| id:finngen_R11_O15_PREECLAMPS | CD3 on naive CD8+ T cell \|\| id:ebi-a-GCST90001838 | Weighted mode | 25 | -0.03002028 | 0.0217614437893478 | 0.18045471928885 | -0.07267271 | 0.0126321495989673 | 0.970425852891241 | 0.929905128824319 | 1.01271227221891 | 0.872755742358858 |
| ebi-a-GCST90001839 | finngen_R11_O15_PREECLAMPS | Pre-eclampsia \|\| id:finngen_R11_O15_PREECLAMPS | CD3 on Effector Memory CD8+ T cell \|\| id:ebi-a-GCST90001839 | Inverse variance weighted (multiplicative random effects) | 20 | 0.010998986173411 | 0.0272770054331173 | 0.686776894232147 | -0.042463944 | 0.0644619168223208 | 1.011059697 | 0.95842502141482 | 1.0665849585267 | 0.950911139356934 |
| ebi-a-GCST90001839 | finngen_R11_O15_PREECLAMPS | Pre-eclampsia \|\| id:finngen_R11_O15_PREECLAMPS | CD3 on Effector Memory CD8+ T cell \|\| id:ebi-a-GCST90001839 | MR Egger | 20 | -0.003994267 | 0.0416223012269955 | 0.924609045600185 | -0.085573977 | 0.077585443359383 | 0.996013699428816 | 0.917985230335304 | 1.08067456497913 | 0.99380795269851 |
| ebi-a-GCST90001839 | finngen_R11_O15_PREECLAMPS | Pre-eclampsia \|\| id:finngen_R11_O15_PREECLAMPS | CD3 on Effector Memory CD8+ T cell \|\| id:ebi-a-GCST90001839 | Weighted median | 20 | 0.0170072918587092 | 0.0328017400408521 | 0.60411818624456 | -0.047284119 | 0.0812987023387794 | 1.01715273923226 | 0.953816362099248 | 1.08469484906995 | 0.940845681141708 |
| ebi-a-GCST90001839 | finngen_R11_O15_PREECLAMPS | Pre-eclampsia \|\| id:finngen_R11_O15_PREECLAMPS | CD3 on Effector Memory CD8+ T cell \|\| id:ebi-a-GCST90001839 | Weighted mode | 20 | 0.0206544942887875 | 0.0295585607976147 | 0.493164185560022 | -0.037280285 | 0.0785892734521124 | 1.02086927453303 | 0.963406069353837 | 1.08175992329444 | 0.933945646747087 |
| ebi-a-GCST90001840 | finngen_R11_O15_PREECLAMPS | Pre-eclampsia \|\| id:finngen_R11_O15_PREECLAMPS | CD3 on Terminally Differentiated CD8+ T cell \|\| id:ebi-a-GCST90001840 | Inverse variance weighted (multiplicative random effects) | 23 | 0.00125254209334823 | 0.0283680124296716 | 0.964782186406661 | -0.054348762 | 0.0568538464555046 | 1.00125332685181 | 0.947101735544604 | 1.05850109540276 | 0.992671503887443 |
| ebi-a-GCST90001840 | finngen_R11_O15_PREECLAMPS | Pre-eclampsia \|\| id:finngen_R11_O15_PREECLAMPS | CD3 on Terminally Differentiated CD8+ T cell \|\| id:ebi-a-GCST90001840 | MR Egger | 23 | 0.0484985083543972 | 0.0609549995191828 | 0.435140603692722 | -0.070973291 | 0.167970307411995 | 1.04969380604801 | 0.931486771151975 | 1.18290148671986 | 0.99380795269851 |
| ebi-a-GCST90001840 | finngen_R11_O15_PREECLAMPS | Pre-eclampsia \|\| id:finngen_R11_O15_PREECLAMPS | CD3 on Terminally Differentiated CD8+ T cell \|\| id:ebi-a-GCST90001840 | Weighted median | 23 | -0.001963551 | 0.0348012806993887 | 0.955005790077224 | -0.070174061 | 0.0662469592430585 | 0.998038375577243 | 0.932231540537051 | 1.06849055820513 | 0.986029989472388 |
| ebi-a-GCST90001840 | finngen_R11_O15_PREECLAMPS | Pre-eclampsia \|\| id:finngen_R11_O15_PREECLAMPS | CD3 on Terminally Differentiated CD8+ T cell \|\| id:ebi-a-GCST90001840 | Weighted mode | 23 | -0.016901758 | 0.047291866063508 | 0.724203690058457 | -0.109593815 | 0.0757902998666283 | 0.98324027575708 | 0.896198083503238 | 1.07873633928315 | 0.959313411571287 |
| ebi-a-GCST90001841 | finngen_R11_O15_PREECLAMPS | Pre-eclampsia \|\| id:finngen_R11_O15_PREECLAMPS | CD3 on Central Memory CD4+ T cell \|\| id:ebi-a-GCST90001841 | Inverse variance weighted | 25 | -0.024838567 | 0.0148880389813913 | 0.0952448434790749 | -0.054019123 | 0.00434198969244754 | 0.975467372224465 | 0.947413988822278 | 1.00435142978768 | 0.802698559103897 |
| ebi-a-GCST90001841 | finngen_R11_O15_PREECLAMPS | Pre-eclampsia \|\| id:finngen_R11_O15_PREECLAMPS | CD3 on Central Memory CD4+ T cell \|\| id:ebi-a-GCST90001841 | MR Egger | 25 | -0.05858615 | 0.0197198863533387 | 0.00684120775623378 | -0.097237127 | -0.019935173 | 0.94309698929635 | 0.907340825579107 | 0.980262219163525 | 0.565056961366001 |
| ebi-a-GCST90001841 | finngen_R11_O15_PREECLAMPS | Pre-eclampsia \|\| id:finngen_R11_O15_PREECLAMPS | CD3 on Central Memory CD4+ T cell \|\| id:ebi-a-GCST90001841 | Weighted median | 25 | -0.038116071 | 0.0199898374405626 | 0.0565500597902492 | -0.077296153 | 0.0010640099793403 | 0.962601203933203 | 0.925615689344538 | 1.00106457623878 | 0.771214851704188 |
| ebi-a-GCST90001841 | finngen_R11_O15_PREECLAMPS | Pre-eclampsia \|\| id:finngen_R11_O15_PREECLAMPS | CD3 on Central Memory CD4+ T cell \|\| id:ebi-a-GCST90001841 | Weighted mode | 25 | -0.039489478 | 0.0185009057161668 | 0.043221133717378 | -0.075751253 | -0.003227703 | 0.961280068720907 | 0.927046777984983 | 0.996777500838302 | 0.838871423749761 |
| ebi-a-GCST90001842 | finngen_R11_O15_PREECLAMPS | Pre-eclampsia \|\| id:finngen_R11_O15_PREECLAMPS | CD3 on Naive CD4+ T cell \|\| id:ebi-a-GCST90001842 | Inverse variance weighted | 28 | -0.016940917 | 0.014127225551163 | 0.23046261444802 | -0.044630279 | 0.0107484448013461 | 0.983201773154994 | 0.956350999181977 | 1.01080641685112 | 0.882032309746087 |
| ebi-a-GCST90001842 | finngen_R11_O15_PREECLAMPS | Pre-eclampsia \|\| id:finngen_R11_O15_PREECLAMPS | CD3 on Naive CD4+ T cell \|\| id:ebi-a-GCST90001842 | MR Egger | 28 | -0.023274773 | 0.0209127131458156 | 0.275914906843507 | -0.06426369 | 0.0177141451269275 | 0.976993995670022 | 0.937757689220932 | 1.01787197113603 | 0.99380795269851 |
| ebi-a-GCST90001842 | finngen_R11_O15_PREECLAMPS | Pre-eclampsia \|\| id:finngen_R11_O15_PREECLAMPS | CD3 on Naive CD4+ T cell \|\| id:ebi-a-GCST90001842 | Weighted median | 28 | -0.019429229 | 0.0195424558803466 | 0.320122540222711 | -0.057732442 | 0.0188739847309015 | 0.980758302179902 | 0.943902462007013 | 1.01905322425958 | 0.940845681141708 |
| ebi-a-GCST90001842 | finngen_R11_O15_PREECLAMPS | Pre-eclampsia \|\| id:finngen_R11_O15_PREECLAMPS | CD3 on Naive CD4+ T cell \|\| id:ebi-a-GCST90001842 | Weighted mode | 28 | -0.024833488 | 0.0181366551702426 | 0.182206290239596 | -0.060381332 | 0.0107143564200057 | 0.975472326633304 | 0.941405477239054 | 1.0107719606838 | 0.872755742358858 |
| ebi-a-GCST90001843 | finngen_R11_O15_PREECLAMPS | Pre-eclampsia \|\| id:finngen_R11_O15_PREECLAMPS | CD3 on Effector Memory CD4+ T cell \|\| id:ebi-a-GCST90001843 | Inverse variance weighted | 19 | 0.0106752648813969 | 0.0172818754071223 | 0.536763557625583 | -0.023197211 | 0.0445477406793566 | 1.01073244882497 | 0.977069775945798 | 1.04555489101978 | 0.904087927705763 |
| ebi-a-GCST90001843 | finngen_R11_O15_PREECLAMPS | Pre-eclampsia \|\| id:finngen_R11_O15_PREECLAMPS | CD3 on Effector Memory CD4+ T cell \|\| id:ebi-a-GCST90001843 | MR Egger | 19 | 0.00960527333020447 | 0.0272031204559298 | 0.72835792460289 | -0.043712843 | 0.0629233894238269 | 1.00965155202256 | 0.957228793185654 | 1.06494525003685 | 0.99380795269851 |
| ebi-a-GCST90001843 | finngen_R11_O15_PREECLAMPS | Pre-eclampsia \|\| id:finngen_R11_O15_PREECLAMPS | CD3 on Effector Memory CD4+ T cell \|\| id:ebi-a-GCST90001843 | Weighted median | 19 | 0.0188436064056831 | 0.026002651767838 | 0.468648256089243 | -0.032121591 | 0.0698088038706456 | 1.01902226759953 | 0.968388827497061 | 1.07230314144329 | 0.940845681141708 |
| ebi-a-GCST90001843 | finngen_R11_O15_PREECLAMPS | Pre-eclampsia \|\| id:finngen_R11_O15_PREECLAMPS | CD3 on Effector Memory CD4+ T cell \|\| id:ebi-a-GCST90001843 | Weighted mode | 19 | 0.0213178102413469 | 0.0253258321685516 | 0.410976794673797 | -0.028320821 | 0.070956441291708 | 1.02154665804312 | 0.972076454415401 | 1.07353446307538 | 0.930105377419646 |
| ebi-a-GCST90001844 | finngen_R11_O15_PREECLAMPS | Pre-eclampsia \|\| id:finngen_R11_O15_PREECLAMPS | CD3 on Terminally Differentiated CD4+ T cell \|\| id:ebi-a-GCST90001844 | Inverse variance weighted | 23 | -0.003522296 | 0.0171788394572929 | 0.837543299345304 | -0.037192821 | 0.0301482294353804 | 0.996483900106435 | 0.96349033603797 | 1.03060728896839 | 0.977824840984872 |
| ebi-a-GCST90001844 | finngen_R11_O15_PREECLAMPS | Pre-eclampsia \|\| id:finngen_R11_O15_PREECLAMPS | CD3 on Terminally Differentiated CD4+ T cell \|\| id:ebi-a-GCST90001844 | MR Egger | 23 | -0.019579647 | 0.0251030663282448 | 0.44411100514767 | -0.068781657 | 0.0296223626841656 | 0.980610789057714 | 0.933530487373568 | 1.03006546933651 | 0.99380795269851 |
| ebi-a-GCST90001844 | finngen_R11_O15_PREECLAMPS | Pre-eclampsia \|\| id:finngen_R11_O15_PREECLAMPS | CD3 on Terminally Differentiated CD4+ T cell \|\| id:ebi-a-GCST90001844 | Weighted median | 23 | -0.017625951 | 0.0243096358458778 | 0.468415052415868 | -0.065272838 | 0.0300209349599408 | 0.982528477134466 | 0.936811831055617 | 1.03047610670372 | 0.940845681141708 |
| ebi-a-GCST90001844 | finngen_R11_O15_PREECLAMPS | Pre-eclampsia \|\| id:finngen_R11_O15_PREECLAMPS | CD3 on Terminally Differentiated CD4+ T cell \|\| id:ebi-a-GCST90001844 | Weighted mode | 23 | -0.021632022 | 0.0262354645735855 | 0.418486895736211 | -0.073053532 | 0.0297894890297445 | 0.978600272630786 | 0.929551067874346 | 1.0302376348004 | 0.932664392631616 |
| ebi-a-GCST90001845 | finngen_R11_O15_PREECLAMPS | Pre-eclampsia \|\| id:finngen_R11_O15_PREECLAMPS | CD3 on CD45RA- CD4+ T cell \|\| id:ebi-a-GCST90001845 | Inverse variance weighted | 27 | -0.013444459 | 0.0175645920828189 | 0.444015772592126 | -0.047871059 | 0.0209821417886438 | 0.986645514376683 | 0.953256692857003 | 1.02120381460113 | 0.898694924660397 |
| ebi-a-GCST90001845 | finngen_R11_O15_PREECLAMPS | Pre-eclampsia \|\| id:finngen_R11_O15_PREECLAMPS | CD3 on CD45RA- CD4+ T cell \|\| id:ebi-a-GCST90001845 | MR Egger | 27 | -0.068212369 | 0.0231637129288607 | 0.0068918408363506 | -0.113613246 | -0.022811491 | 0.934062086893417 | 0.892603106854795 | 0.977446723489407 | 0.565056961366001 |
| ebi-a-GCST90001845 | finngen_R11_O15_PREECLAMPS | Pre-eclampsia \|\| id:finngen_R11_O15_PREECLAMPS | CD3 on CD45RA- CD4+ T cell \|\| id:ebi-a-GCST90001845 | Weighted median | 27 | -0.036672066 | 0.0238724447435792 | 0.124497385691586 | -0.083462058 | 0.010117925822022 | 0.963992209461815 | 0.919925990233975 | 1.01016928510381 | 0.88227178111339 |
| ebi-a-GCST90001845 | finngen_R11_O15_PREECLAMPS | Pre-eclampsia \|\| id:finngen_R11_O15_PREECLAMPS | CD3 on CD45RA- CD4+ T cell \|\| id:ebi-a-GCST90001845 | Weighted mode | 27 | -0.037808941 | 0.020967759592349 | 0.0829599471325595 | -0.078905749 | 0.0032878681902023 | 0.962896893810066 | 0.924127019855813 | 1.00329327915738 | 0.872755742358858 |
| ebi-a-GCST90001846 | finngen_R11_O15_PREECLAMPS | Pre-eclampsia \|\| id:finngen_R11_O15_PREECLAMPS | CD3 on Central Memory CD8+ T cell \|\| id:ebi-a-GCST90001846 | Inverse variance weighted | 18 | -0.040360237 | 0.0233211290595234 | 0.0835178458274891 | -0.08606965 | 0.00534917630795428 | 0.960443389918196 | 0.917530323369654 | 1.00536350869563 | 0.791351300515166 |
| ebi-a-GCST90001846 | finngen_R11_O15_PREECLAMPS | Pre-eclampsia \|\| id:finngen_R11_O15_PREECLAMPS | CD3 on Central Memory CD8+ T cell \|\| id:ebi-a-GCST90001846 | MR Egger | 18 | -0.026558591 | 0.0488822218540746 | 0.594400328921784 | -0.122367745 | 0.0692505642808475 | 0.973790987211479 | 0.884822919118112 | 1.07170470642807 | 0.99380795269851 |
| ebi-a-GCST90001846 | finngen_R11_O15_PREECLAMPS | Pre-eclampsia \|\| id:finngen_R11_O15_PREECLAMPS | CD3 on Central Memory CD8+ T cell \|\| id:ebi-a-GCST90001846 | Weighted median | 18 | -0.037743856 | 0.0329656499576806 | 0.25223194698892 | -0.10235653 | 0.026868818268785 | 0.962959565957808 | 0.902707652322073 | 1.02723303971595 | 0.940845681141708 |
| ebi-a-GCST90001846 | finngen_R11_O15_PREECLAMPS | Pre-eclampsia \|\| id:finngen_R11_O15_PREECLAMPS | CD3 on Central Memory CD8+ T cell \|\| id:ebi-a-GCST90001846 | Weighted mode | 18 | -0.041106227 | 0.0315342659951505 | 0.209767827896585 | -0.102913388 | 0.0207009346657786 | 0.959727175896647 | 0.902205111854989 | 1.02091668518788 | 0.872755742358858 |
| ebi-a-GCST90001847 | finngen_R11_O15_PREECLAMPS | Pre-eclampsia \|\| id:finngen_R11_O15_PREECLAMPS | CD3 on HLA DR+ T cell \|\| id:ebi-a-GCST90001847 | Inverse variance weighted | 27 | -0.001620276 | 0.0180388387873942 | 0.928429053308757 | -0.0369764 | 0.0337358482515598 | 0.998381036166393 | 0.963698878571748 | 1.03431135548683 | 0.986667691552896 |
| ebi-a-GCST90001847 | finngen_R11_O15_PREECLAMPS | Pre-eclampsia \|\| id:finngen_R11_O15_PREECLAMPS | CD3 on HLA DR+ T cell \|\| id:ebi-a-GCST90001847 | MR Egger | 27 | 0.0219290809448567 | 0.0245669680559231 | 0.380570228240263 | -0.026222176 | 0.070080338334466 | 1.02217129047775 | 0.974118639348507 | 1.07259434823641 | 0.99380795269851 |
| ebi-a-GCST90001847 | finngen_R11_O15_PREECLAMPS | Pre-eclampsia \|\| id:finngen_R11_O15_PREECLAMPS | CD3 on HLA DR+ T cell \|\| id:ebi-a-GCST90001847 | Weighted median | 27 | 0.0183466645158917 | 0.0239174371969878 | 0.443032043482944 | -0.028531512 | 0.0652248414219878 | 1.0185159985517 | 0.971871667664362 | 1.06739899291314 | 0.940845681141708 |
| ebi-a-GCST90001847 | finngen_R11_O15_PREECLAMPS | Pre-eclampsia \|\| id:finngen_R11_O15_PREECLAMPS | CD3 on HLA DR+ T cell \|\| id:ebi-a-GCST90001847 | Weighted mode | 27 | 0.0117674236699515 | 0.0218666156866498 | 0.595053837415763 | -0.031091143 | 0.0546259904157851 | 1.01183693217765 | 0.969387216116927 | 1.05614553224641 | 0.955353022288269 |
| ebi-a-GCST90001848 | finngen_R11_O15_PREECLAMPS | Pre-eclampsia \|\| id:finngen_R11_O15_PREECLAMPS | CD3 on Natural Killer T \|\| id:ebi-a-GCST90001848 | Inverse variance weighted | 17 | -0.01501077 | 0.0223023511703434 | 0.500910569691554 | -0.058723378 | 0.0287018386302607 | 0.985101330335982 | 0.942967578700248 | 1.02911770558148 | 0.898694924660397 |
| ebi-a-GCST90001848 | finngen_R11_O15_PREECLAMPS | Pre-eclampsia \|\| id:finngen_R11_O15_PREECLAMPS | CD3 on Natural Killer T \|\| id:ebi-a-GCST90001848 | MR Egger | 17 | 0.010193188305203 | 0.0321931742295043 | 0.755891268715087 | -0.052905433 | 0.0732918097950314 | 1.01024531581374 | 0.948469702000603 | 1.07604449142746 | 0.99380795269851 |
| ebi-a-GCST90001848 | finngen_R11_O15_PREECLAMPS | Pre-eclampsia \|\| id:finngen_R11_O15_PREECLAMPS | CD3 on Natural Killer T \|\| id:ebi-a-GCST90001848 | Weighted median | 17 | -0.004743833 | 0.0332734511969251 | 0.886628908516514 | -0.069959797 | 0.06047213119232 | 0.995267401051423 | 0.932431305222338 | 1.06233799106461 | 0.964912412213677 |
| ebi-a-GCST90001848 | finngen_R11_O15_PREECLAMPS | Pre-eclampsia \|\| id:finngen_R11_O15_PREECLAMPS | CD3 on Natural Killer T \|\| id:ebi-a-GCST90001848 | Weighted mode | 17 | -0.003476587 | 0.0317423885314779 | 0.914147965830919 | -0.065691669 | 0.0587384941921691 | 0.996529449002897 | 0.936419547099074 | 1.06049787812145 | 0.98560791006254 |
| ebi-a-GCST90001849 | finngen_R11_O15_PREECLAMPS | Pre-eclampsia \|\| id:finngen_R11_O15_PREECLAMPS | CD3 on HLA DR+ CD4+ T cell \|\| id:ebi-a-GCST90001849 | Inverse variance weighted | 26 | -0.016965625 | 0.0197328992290187 | 0.389919463640372 | -0.055642108 | 0.021710857430248 | 0.983177480722295 | 0.945877597773534 | 1.02194825300311 | 0.885079385681701 |
| ebi-a-GCST90001849 | finngen_R11_O15_PREECLAMPS | Pre-eclampsia \|\| id:finngen_R11_O15_PREECLAMPS | CD3 on HLA DR+ CD4+ T cell \|\| id:ebi-a-GCST90001849 | MR Egger | 26 | -0.000390411 | 0.0374613105333799 | 0.991770975267982 | -0.07381458 | 0.073033757635832 | 0.999609665190869 | 0.928843904432315 | 1.07576685164737 | 0.995857943572658 |
| ebi-a-GCST90001849 | finngen_R11_O15_PREECLAMPS | Pre-eclampsia \|\| id:finngen_R11_O15_PREECLAMPS | CD3 on HLA DR+ CD4+ T cell \|\| id:ebi-a-GCST90001849 | Weighted median | 26 | -0.004754641 | 0.0277020200259705 | 0.863724359312829 | -0.0590506 | 0.0495413184487011 | 0.995256644609267 | 0.942659069351336 | 1.05078900828961 | 0.964912412213677 |
| ebi-a-GCST90001849 | finngen_R11_O15_PREECLAMPS | Pre-eclampsia \|\| id:finngen_R11_O15_PREECLAMPS | CD3 on HLA DR+ CD4+ T cell \|\| id:ebi-a-GCST90001849 | Weighted mode | 26 | -0.009707388 | 0.0340222762891827 | 0.777746146870143 | -0.076391049 | 0.056976273809705 | 0.990339576880345 | 0.926453846637486 | 1.05863069282426 | 0.961305639715923 |
| ebi-a-GCST90001850 | finngen_R11_O15_PREECLAMPS | Pre-eclampsia \|\| id:finngen_R11_O15_PREECLAMPS | CD3 on HLA DR+ CD8+ T cell \|\| id:ebi-a-GCST90001850 | Inverse variance weighted | 25 | -0.006127946 | 0.0189524696400785 | 0.746443580795239 | -0.043274786 | 0.0310188945463838 | 0.993890791618799 | 0.957648205165187 | 1.03150499352134 | 0.96638925304053 |
| ebi-a-GCST90001850 | finngen_R11_O15_PREECLAMPS | Pre-eclampsia \|\| id:finngen_R11_O15_PREECLAMPS | CD3 on HLA DR+ CD8+ T cell \|\| id:ebi-a-GCST90001850 | MR Egger | 25 | 0.00845124373284914 | 0.030371468899322 | 0.783297082860788 | -0.051076835 | 0.0679793227755202 | 1.00848705630901 | 0.950205658381351 | 1.07034317652383 | 0.99380795269851 |
| ebi-a-GCST90001850 | finngen_R11_O15_PREECLAMPS | Pre-eclampsia \|\| id:finngen_R11_O15_PREECLAMPS | CD3 on HLA DR+ CD8+ T cell \|\| id:ebi-a-GCST90001850 | Weighted median | 25 | 0.0020705128321878 | 0.0261937152441847 | 0.936995900667619 | -0.049269169 | 0.0534101947107898 | 1.00207265782404 | 0.951924866502418 | 1.05486225530382 | 0.978793627478789 |
| ebi-a-GCST90001850 | finngen_R11_O15_PREECLAMPS | Pre-eclampsia \|\| id:finngen_R11_O15_PREECLAMPS | CD3 on HLA DR+ CD8+ T cell \|\| id:ebi-a-GCST90001850 | Weighted mode | 25 | -0.005130934 | 0.0285480185989349 | 0.85887344417713 | -0.06108505 | 0.0508231828580749 | 0.994882207159557 | 0.940743226115292 | 1.05213684100593 | 0.977585971930092 |
| ebi-a-GCST90001851 | finngen_R11_O15_PREECLAMPS | Pre-eclampsia \|\| id:finngen_R11_O15_PREECLAMPS | CD3 on T cell \|\| id:ebi-a-GCST90001851 | Inverse variance weighted | 30 | 0.00658787084883439 | 0.0118987510807025 | 0.57981107618226 | -0.016733681 | 0.0299094229670114 | 1.00660961860089 | 0.983405549081211 | 1.0303612026661 | 0.915399120505076 |
| ebi-a-GCST90001851 | finngen_R11_O15_PREECLAMPS | Pre-eclampsia \|\| id:finngen_R11_O15_PREECLAMPS | CD3 on T cell \|\| id:ebi-a-GCST90001851 | MR Egger | 30 | 0.000763515707673142 | 0.0161576739910897 | 0.962646180009907 | -0.030905525 | 0.0324325567302089 | 1.00076380725999 | 0.969567168302188 | 1.03296422430979 | 0.99380795269851 |
| ebi-a-GCST90001851 | finngen_R11_O15_PREECLAMPS | Pre-eclampsia \|\| id:finngen_R11_O15_PREECLAMPS | CD3 on T cell \|\| id:ebi-a-GCST90001851 | Weighted median | 30 | 0.0140430245449358 | 0.0167714593129642 | 0.402414521724414 | -0.018829036 | 0.0469150847983455 | 1.01414209100183 | 0.981347123217491 | 1.04803301136658 | 0.940845681141708 |
| ebi-a-GCST90001851 | finngen_R11_O15_PREECLAMPS | Pre-eclampsia \|\| id:finngen_R11_O15_PREECLAMPS | CD3 on T cell \|\| id:ebi-a-GCST90001851 | Weighted mode | 30 | 0.0117152298266832 | 0.0150203088597271 | 0.441734451183283 | -0.017724576 | 0.0411550351917483 | 1.01178412189759 | 0.982431580787978 | 1.04201364180802 | 0.933945646747087 |
| ebi-a-GCST90001852 | finngen_R11_O15_PREECLAMPS | Pre-eclampsia \|\| id:finngen_R11_O15_PREECLAMPS | CD3 on CD39+ resting CD4 regulatory T cell \|\| id:ebi-a-GCST90001852 | Inverse variance weighted | 20 | -0.002631161 | 0.0180306309862312 | 0.883978791368408 | -0.037971198 | 0.0327088756957317 | 0.997372297432991 | 0.962740669569785 | 1.03324969135383 | 0.986084522213934 |
| ebi-a-GCST90001852 | finngen_R11_O15_PREECLAMPS | Pre-eclampsia \|\| id:finngen_R11_O15_PREECLAMPS | CD3 on CD39+ resting CD4 regulatory T cell \|\| id:ebi-a-GCST90001852 | MR Egger | 20 | 0.0183569327227995 | 0.0267070405168359 | 0.500626963213289 | -0.033988867 | 0.0707027321357978 | 1.0185264569384 | 0.966582265837416 | 1.07326213210081 | 0.99380795269851 |
| ebi-a-GCST90001852 | finngen_R11_O15_PREECLAMPS | Pre-eclampsia \|\| id:finngen_R11_O15_PREECLAMPS | CD3 on CD39+ resting CD4 regulatory T cell \|\| id:ebi-a-GCST90001852 | Weighted median | 20 | 0.0112764674773675 | 0.0251167743291202 | 0.653459349536365 | -0.03795241 | 0.0605053451624432 | 1.01134028649552 | 0.962758757290281 | 1.06237327611288 | 0.940845681141708 |
| ebi-a-GCST90001852 | finngen_R11_O15_PREECLAMPS | Pre-eclampsia \|\| id:finngen_R11_O15_PREECLAMPS | CD3 on CD39+ resting CD4 regulatory T cell \|\| id:ebi-a-GCST90001852 | Weighted mode | 20 | 0.0149397127844565 | 0.0251517175744315 | 0.55952941090187 | -0.034357654 | 0.0642370792303422 | 1.01505186812029 | 0.966225868612519 | 1.06634517708994 | 0.939633861977944 |
| ebi-a-GCST90001853 | finngen_R11_O15_PREECLAMPS | Pre-eclampsia \|\| id:finngen_R11_O15_PREECLAMPS | CD3 on activated CD4 regulatory T cell \|\| id:ebi-a-GCST90001853 | Inverse variance weighted | 22 | -0.008923352 | 0.0194662907928768 | 0.646664934840086 | -0.047077282 | 0.0292305780043366 | 0.991116342996901 | 0.954013666749017 | 1.02966198451115 | 0.942541573457101 |
| ebi-a-GCST90001853 | finngen_R11_O15_PREECLAMPS | Pre-eclampsia \|\| id:finngen_R11_O15_PREECLAMPS | CD3 on activated CD4 regulatory T cell \|\| id:ebi-a-GCST90001853 | MR Egger | 22 | -0.004904847 | 0.0323978944697123 | 0.881181596251892 | -0.06840472 | 0.0585950264188649 | 0.995107162376691 | 0.933882436274295 | 1.06034574176588 | 0.99380795269851 |
| ebi-a-GCST90001853 | finngen_R11_O15_PREECLAMPS | Pre-eclampsia \|\| id:finngen_R11_O15_PREECLAMPS | CD3 on activated CD4 regulatory T cell \|\| id:ebi-a-GCST90001853 | Weighted median | 22 | -0.018890034 | 0.0220388334773957 | 0.391375841142269 | -0.062086147 | 0.0243060798702096 | 0.981287264794989 | 0.93980192184808 | 1.02460388052334 | 0.940845681141708 |
| ebi-a-GCST90001853 | finngen_R11_O15_PREECLAMPS | Pre-eclampsia \|\| id:finngen_R11_O15_PREECLAMPS | CD3 on activated CD4 regulatory T cell \|\| id:ebi-a-GCST90001853 | Weighted mode | 22 | -0.039495351 | 0.0235177517248345 | 0.107895496496543 | -0.085590145 | 0.00659944200270869 | 0.961274422581672 | 0.917970389105131 | 1.00662126630307 | 0.872755742358858 |
| ebi-a-GCST90001854 | finngen_R11_O15_PREECLAMPS | Pre-eclampsia \|\| id:finngen_R11_O15_PREECLAMPS | CD3 on CD39+ activated CD4 regulatory T cell \|\| id:ebi-a-GCST90001854 | Inverse variance weighted | 26 | -0.005882685 | 0.0161924839459015 | 0.716382825338149 | -0.037619954 | 0.0258545831675019 | 0.99413458374758 | 0.963078885722937 | 1.02619171207466 | 0.959745573017418 |
| ebi-a-GCST90001854 | finngen_R11_O15_PREECLAMPS | Pre-eclampsia \|\| id:finngen_R11_O15_PREECLAMPS | CD3 on CD39+ activated CD4 regulatory T cell \|\| id:ebi-a-GCST90001854 | MR Egger | 26 | -0.01944855 | 0.0280325484309551 | 0.494478190860216 | -0.074392345 | 0.0354952447029437 | 0.98073935271288 | 0.928307405477668 | 1.03613272099747 | 0.99380795269851 |
| ebi-a-GCST90001854 | finngen_R11_O15_PREECLAMPS | Pre-eclampsia \|\| id:finngen_R11_O15_PREECLAMPS | CD3 on CD39+ activated CD4 regulatory T cell \|\| id:ebi-a-GCST90001854 | Weighted median | 26 | -0.007333342 | 0.0224876192960941 | 0.744344352617969 | -0.051409076 | 0.0367423920381642 | 0.992693481560371 | 0.949890014213041 | 1.03742573728271 | 0.957984984509652 |
| ebi-a-GCST90001854 | finngen_R11_O15_PREECLAMPS | Pre-eclampsia \|\| id:finngen_R11_O15_PREECLAMPS | CD3 on CD39+ activated CD4 regulatory T cell \|\| id:ebi-a-GCST90001854 | Weighted mode | 26 | 0.00541105538933256 | 0.0220701446678723 | 0.808322074506161 | -0.037846428 | 0.0486685389383623 | 1.00542572159082 | 0.962860797842257 | 1.04987230127323 | 0.961305639715923 |
| ebi-a-GCST90001855 | finngen_R11_O15_PREECLAMPS | Pre-eclampsia \|\| id:finngen_R11_O15_PREECLAMPS | CD3 on secreting CD4 regulatory T cell \|\| id:ebi-a-GCST90001855 | Inverse variance weighted | 21 | -0.014464597 | 0.0168985581411228 | 0.392016387892645 | -0.047585771 | 0.0186565766807355 | 0.985639512438468 | 0.953528684294668 | 1.01883169796675 | 0.885079385681701 |
| ebi-a-GCST90001855 | finngen_R11_O15_PREECLAMPS | Pre-eclampsia \|\| id:finngen_R11_O15_PREECLAMPS | CD3 on secreting CD4 regulatory T cell \|\| id:ebi-a-GCST90001855 | MR Egger | 21 | 0.00329226249238291 | 0.0268319457505942 | 0.903633745557587 | -0.049298351 | 0.0558828761635474 | 1.00329768794091 | 0.951897087710284 | 1.05747382109224 | 0.99380795269851 |
| ebi-a-GCST90001855 | finngen_R11_O15_PREECLAMPS | Pre-eclampsia \|\| id:finngen_R11_O15_PREECLAMPS | CD3 on secreting CD4 regulatory T cell \|\| id:ebi-a-GCST90001855 | Weighted median | 21 | -0.025454687 | 0.0211795667314858 | 0.229421199580306 | -0.066966638 | 0.0160572639333514 | 0.97486655222913 | 0.935226402163057 | 1.01618687459747 | 0.940845681141708 |
| ebi-a-GCST90001855 | finngen_R11_O15_PREECLAMPS | Pre-eclampsia \|\| id:finngen_R11_O15_PREECLAMPS | CD3 on secreting CD4 regulatory T cell \|\| id:ebi-a-GCST90001855 | Weighted mode | 21 | -0.02740476 | 0.021322546964556 | 0.213390375346359 | -0.069196952 | 0.0143874319624971 | 0.972967343465014 | 0.933142877493334 | 1.01449142921431 | 0.872755742358858 |
| ebi-a-GCST90001856 | finngen_R11_O15_PREECLAMPS | Pre-eclampsia \|\| id:finngen_R11_O15_PREECLAMPS | CD3 on CD39+ secreting CD4 regulatory T cell \|\| id:ebi-a-GCST90001856 | Inverse variance weighted | 27 | -0.00724908 | 0.0199188146272232 | 0.715909304364867 | -0.046289957 | 0.0317917963905817 | 0.992777130929708 | 0.95476508128173 | 1.03230255381134 | 0.959745573017418 |
| ebi-a-GCST90001856 | finngen_R11_O15_PREECLAMPS | Pre-eclampsia \|\| id:finngen_R11_O15_PREECLAMPS | CD3 on CD39+ secreting CD4 regulatory T cell \|\| id:ebi-a-GCST90001856 | MR Egger | 27 | -0.008985075 | 0.0351613967500111 | 0.800398290643488 | -0.077901413 | 0.0599312625696479 | 0.991055170101084 | 0.925055620793056 | 1.06176356113814 | 0.99380795269851 |
| ebi-a-GCST90001856 | finngen_R11_O15_PREECLAMPS | Pre-eclampsia \|\| id:finngen_R11_O15_PREECLAMPS | CD3 on CD39+ secreting CD4 regulatory T cell \|\| id:ebi-a-GCST90001856 | Weighted median | 27 | -0.054996448 | 0.0260373719142992 | 0.0346682742777844 | -0.106029697 | -0.003963199 | 0.946488509855567 | 0.899397938283719 | 0.99604464404055 | 0.721097500353414 |
| ebi-a-GCST90001856 | finngen_R11_O15_PREECLAMPS | Pre-eclampsia \|\| id:finngen_R11_O15_PREECLAMPS | CD3 on CD39+ secreting CD4 regulatory T cell \|\| id:ebi-a-GCST90001856 | Weighted mode | 27 | -0.052356027 | 0.0286882571505433 | 0.0795119515626919 | -0.108585011 | 0.00387295739437864 | 0.948990940653437 | 0.897102628310518 | 1.00388046698551 | 0.872755742358858 |
| ebi-a-GCST90001857 | finngen_R11_O15_PREECLAMPS | Pre-eclampsia \|\| id:finngen_R11_O15_PREECLAMPS | CD3 on activated & secreting CD4 regulatory T cell \|\| id:ebi-a-GCST90001857 | Inverse variance weighted | 23 | 0.00425312824658771 | 0.0178303138095902 | 0.81146728852169 | -0.030694287 | 0.0392005433133845 | 1.00426218563272 | 0.969771999844677 | 1.03997902357805 | 0.977824840984872 |
| ebi-a-GCST90001857 | finngen_R11_O15_PREECLAMPS | Pre-eclampsia \|\| id:finngen_R11_O15_PREECLAMPS | CD3 on activated & secreting CD4 regulatory T cell \|\| id:ebi-a-GCST90001857 | MR Egger | 23 | -0.007218197 | 0.0282870665019966 | 0.80106922227614 | -0.062660848 | 0.0482244530862907 | 0.992807791360284 | 0.939261972627044 | 1.04940617134626 | 0.99380795269851 |
| ebi-a-GCST90001857 | finngen_R11_O15_PREECLAMPS | Pre-eclampsia \|\| id:finngen_R11_O15_PREECLAMPS | CD3 on activated & secreting CD4 regulatory T cell \|\| id:ebi-a-GCST90001857 | Weighted median | 23 | -0.003744429 | 0.0226724575450781 | 0.868823383124088 | -0.048182446 | 0.0406935873773347 | 0.996262572223021 | 0.952959907302894 | 1.04153291781346 | 0.964912412213677 |
| ebi-a-GCST90001857 | finngen_R11_O15_PREECLAMPS | Pre-eclampsia \|\| id:finngen_R11_O15_PREECLAMPS | CD3 on activated & secreting CD4 regulatory T cell \|\| id:ebi-a-GCST90001857 | Weighted mode | 23 | -0.012133017 | 0.0205362300799251 | 0.560668787481502 | -0.052384028 | 0.0281179940067366 | 0.987940291317227 | 0.948964368058842 | 1.02851703610799 | 0.939633861977944 |
| ebi-a-GCST90001858 | finngen_R11_O15_PREECLAMPS | Pre-eclampsia \|\| id:finngen_R11_O15_PREECLAMPS | CD3 on CD45RA+ CD4+ T cell \|\| id:ebi-a-GCST90001858 | Inverse variance weighted | 31 | 0.0050752184942828 | 0.0158419532999645 | 0.748691122131817 | -0.02597501 | 0.0361254469622133 | 1.00508811923112 | 0.974359438572554 | 1.03678589997496 | 0.96638925304053 |
| ebi-a-GCST90001858 | finngen_R11_O15_PREECLAMPS | Pre-eclampsia \|\| id:finngen_R11_O15_PREECLAMPS | CD3 on CD45RA+ CD4+ T cell \|\| id:ebi-a-GCST90001858 | MR Egger | 31 | 0.0139664120648335 | 0.0225821474317203 | 0.541092352746793 | -0.030294597 | 0.0582274210310054 | 1.01406439803722 | 0.970159685408463 | 1.05995602459366 | 0.99380795269851 |
| ebi-a-GCST90001858 | finngen_R11_O15_PREECLAMPS | Pre-eclampsia \|\| id:finngen_R11_O15_PREECLAMPS | CD3 on CD45RA+ CD4+ T cell \|\| id:ebi-a-GCST90001858 | Weighted median | 31 | -0.014770426 | 0.0210971349985543 | 0.483855330905738 | -0.056120811 | 0.0265799585887693 | 0.985338121644977 | 0.945424911634695 | 1.02693635636079 | 0.940845681141708 |
| ebi-a-GCST90001858 | finngen_R11_O15_PREECLAMPS | Pre-eclampsia \|\| id:finngen_R11_O15_PREECLAMPS | CD3 on CD45RA+ CD4+ T cell \|\| id:ebi-a-GCST90001858 | Weighted mode | 31 | -0.007285952 | 0.0183602776792872 | 0.694300117804536 | -0.043272096 | 0.0287001924343213 | 0.992740526384533 | 0.957650781600714 | 1.02911601145349 | 0.959313411571287 |
| ebi-a-GCST90001859 | finngen_R11_O15_PREECLAMPS | Pre-eclampsia \|\| id:finngen_R11_O15_PREECLAMPS | CD3 on CD8+ T cell \|\| id:ebi-a-GCST90001859 | Inverse variance weighted | 23 | 0.004348470007754 | 0.0170262752376994 | 0.798416016324103 | -0.029023029 | 0.0377199694736448 | 1.00435793832271 | 0.971394093529399 | 1.03844039714796 | 0.977824840984872 |
| ebi-a-GCST90001859 | finngen_R11_O15_PREECLAMPS | Pre-eclampsia \|\| id:finngen_R11_O15_PREECLAMPS | CD3 on CD8+ T cell \|\| id:ebi-a-GCST90001859 | MR Egger | 23 | -0.003650827 | 0.0241652657920443 | 0.881356464643856 | -0.051014748 | 0.0437130939385538 | 0.996355829152455 | 0.950264656 | 1.04468258612152 | 0.99380795269851 |
| ebi-a-GCST90001859 | finngen_R11_O15_PREECLAMPS | Pre-eclampsia \|\| id:finngen_R11_O15_PREECLAMPS | CD3 on CD8+ T cell \|\| id:ebi-a-GCST90001859 | Weighted median | 23 | 0.0066083368238823 | 0.0212198749973302 | 0.755479798590381 | -0.034982618 | 0.0481992918186495 | 1.00663022005904 | 0.965622200391774 | 1.0493797672889 | 0.957984984509652 |
| ebi-a-GCST90001859 | finngen_R11_O15_PREECLAMPS | Pre-eclampsia \|\| id:finngen_R11_O15_PREECLAMPS | CD3 on CD8+ T cell \|\| id:ebi-a-GCST90001859 | Weighted mode | 23 | -0.006002375 | 0.0185344357704171 | 0.749110294989375 | -0.04232987 | 0.0303251186359265 | 0.99401560279282 | 0.958553530760217 | 1.0307896083925 | 0.959313411571287 |
| ebi-a-GCST90001860 | finngen_R11_O15_PREECLAMPS | Pre-eclampsia \|\| id:finngen_R11_O15_PREECLAMPS | CD3 on CD39+ CD4+ T cell \|\| id:ebi-a-GCST90001860 | Inverse variance weighted | 28 | 0.0162355893417171 | 0.0157565513798107 | 0.302821117774831 | -0.014647251 | 0.0471184300461461 | 1.01636810269516 | 0.985459497794069 | 1.04824614556816 | 0.885079385681701 |
| ebi-a-GCST90001860 | finngen_R11_O15_PREECLAMPS | Pre-eclampsia \|\| id:finngen_R11_O15_PREECLAMPS | CD3 on CD39+ CD4+ T cell \|\| id:ebi-a-GCST90001860 | MR Egger | 28 | -0.013478363 | 0.0250734984282679 | 0.595456414850584 | -0.062622419 | 0.0356656943663685 | 0.986612063852984 | 0.939298067400904 | 1.03630934452331 | 0.99380795269851 |
| ebi-a-GCST90001860 | finngen_R11_O15_PREECLAMPS | Pre-eclampsia \|\| id:finngen_R11_O15_PREECLAMPS | CD3 on CD39+ CD4+ T cell \|\| id:ebi-a-GCST90001860 | Weighted median | 28 | 0.0129443928247352 | 0.0198639102374088 | 0.514624525035959 | -0.025988871 | 0.0518776568900566 | 1.01302853413831 | 0.974345932809886 | 1.05324687713214 | 0.940845681141708 |
| ebi-a-GCST90001860 | finngen_R11_O15_PREECLAMPS | Pre-eclampsia \|\| id:finngen_R11_O15_PREECLAMPS | CD3 on CD39+ CD4+ T cell \|\| id:ebi-a-GCST90001860 | Weighted mode | 28 | 0.0137460527033299 | 0.0209707307119632 | 0.517700606278152 | -0.027356579 | 0.0548486848987779 | 1.01384096407273 | 0.973014222740754 | 1.05638075622024 | 0.934398527469092 |
| ebi-a-GCST90001861 | finngen_R11_O15_PREECLAMPS | Pre-eclampsia \|\| id:finngen_R11_O15_PREECLAMPS | CD3 on CD28+ CD4+ T cell \|\| id:ebi-a-GCST90001861 | Inverse variance weighted | 22 | -0.036664782 | 0.0245133917518728 | 0.13473077008693 | -0.084711029 | 0.0113814662204451 | 0.963999231459371 | 0.918777745757724 | 1.0114464815295 | 0.834645702826663 |
| ebi-a-GCST90001861 | finngen_R11_O15_PREECLAMPS | Pre-eclampsia \|\| id:finngen_R11_O15_PREECLAMPS | CD3 on CD28+ CD4+ T cell \|\| id:ebi-a-GCST90001861 | MR Egger | 22 | -0.074817394 | 0.0453652794452943 | 0.114721540603276 | -0.163733342 | 0.0140985537809532 | 0.927912913387601 | 0.848968376210215 | 1.01419840710093 | 0.97261503632431 |
| ebi-a-GCST90001861 | finngen_R11_O15_PREECLAMPS | Pre-eclampsia \|\| id:finngen_R11_O15_PREECLAMPS | CD3 on CD28+ CD4+ T cell \|\| id:ebi-a-GCST90001861 | Weighted median | 22 | -0.050047918 | 0.027620501225821 | 0.0699886029982611 | -0.1041841 | 0.00408826435061777 | 0.951183844531755 | 0.901059396705919 | 1.00409663270344 | 0.83871588183162 |
| ebi-a-GCST90001861 | finngen_R11_O15_PREECLAMPS | Pre-eclampsia \|\| id:finngen_R11_O15_PREECLAMPS | CD3 on CD28+ CD4+ T cell \|\| id:ebi-a-GCST90001861 | Weighted mode | 22 | -0.046375002 | 0.025359265994059 | 0.0816809916087007 | -0.096079163 | 0.00332915959666263 | 0.954683886925575 | 0.908392102087416 | 1.00333470740328 | 0.872755742358858 |
| ebi-a-GCST90001862 | finngen_R11_O15_PREECLAMPS | Pre-eclampsia \|\| id:finngen_R11_O15_PREECLAMPS | CD3 on CD28+ CD4-CD8- T cell \|\| id:ebi-a-GCST90001862 | Inverse variance weighted | 16 | 0.00328897921432324 | 0.0181871647634221 | 0.856492632715861 | -0.032357864 | 0.0389358221506306 | 1.00329439384103 | 0.968160050718168 | 1.03970375555794 | 0.977824840984872 |
| ebi-a-GCST90001862 | finngen_R11_O15_PREECLAMPS | Pre-eclampsia \|\| id:finngen_R11_O15_PREECLAMPS | CD3 on CD28+ CD4-CD8- T cell \|\| id:ebi-a-GCST90001862 | MR Egger | 16 | 0.0362277393948121 | 0.0251674144308917 | 0.172001112761182 | -0.013100393 | 0.0855558716793599 | 1.03689196075127 | 0.986985043765672 | 1.08932242191694 | 0.99380795269851 |
| ebi-a-GCST90001862 | finngen_R11_O15_PREECLAMPS | Pre-eclampsia \|\| id:finngen_R11_O15_PREECLAMPS | CD3 on CD28+ CD4-CD8- T cell \|\| id:ebi-a-GCST90001862 | Weighted median | 16 | 0.0184397081684697 | 0.0252378121959629 | 0.46500020912565 | -0.031026404 | 0.067905820072557 | 1.01861076940926 | 0.969449975637242 | 1.07026450629853 | 0.940845681141708 |
| ebi-a-GCST90001862 | finngen_R11_O15_PREECLAMPS | Pre-eclampsia \|\| id:finngen_R11_O15_PREECLAMPS | CD3 on CD28+ CD4-CD8- T cell \|\| id:ebi-a-GCST90001862 | Weighted mode | 16 | 0.0129628145335037 | 0.0231517916712348 | 0.583809310205268 | -0.032414697 | 0.058340326209124 | 1.01304719602683 | 0.968105028434817 | 1.06007570587361 | 0.948365790577891 |
| ebi-a-GCST90001863 | finngen_R11_O15_PREECLAMPS | Pre-eclampsia \|\| id:finngen_R11_O15_PREECLAMPS | CD3 on CD28+ CD45RA- CD8+ T cell \|\| id:ebi-a-GCST90001863 | Inverse variance weighted | 18 | -0.041559415 | 0.0259977451558867 | 0.109914473821478 | -0.092514995 | 0.00939616553103578 | 0.959292337318459 | 0.911635540449413 | 1.0094404480811 | 0.820779257935285 |
| ebi-a-GCST90001863 | finngen_R11_O15_PREECLAMPS | Pre-eclampsia \|\| id:finngen_R11_O15_PREECLAMPS | CD3 on CD28+ CD45RA- CD8+ T cell \|\| id:ebi-a-GCST90001863 | MR Egger | 18 | -0.014423484 | 0.059846075005562 | 0.812608844336901 | -0.131721791 | 0.102874823394647 | 0.985680036518922 | 0.876584835275753 | 1.10835266056857 | 0.99380795269851 |
| ebi-a-GCST90001863 | finngen_R11_O15_PREECLAMPS | Pre-eclampsia \|\| id:finngen_R11_O15_PREECLAMPS | CD3 on CD28+ CD45RA- CD8+ T cell \|\| id:ebi-a-GCST90001863 | Weighted median | 18 | -0.029312355 | 0.0343943732872246 | 0.394079380603027 | -0.096725326 | 0.0381006167876344 | 0.971113085200845 | 0.907805321958137 | 1.03883575193646 | 0.940845681141708 |
| ebi-a-GCST90001863 | finngen_R11_O15_PREECLAMPS | Pre-eclampsia \|\| id:finngen_R11_O15_PREECLAMPS | CD3 on CD28+ CD45RA- CD8+ T cell \|\| id:ebi-a-GCST90001863 | Weighted mode | 18 | -0.033585653 | 0.0379428514953469 | 0.388417002093951 | -0.107953642 | 0.0407823360446065 | 0.966972083734329 | 0.897669209779231 | 1.04162535657369 | 0.920286500917063 |
| ebi-a-GCST90001864 | finngen_R11_O15_PREECLAMPS | Pre-eclampsia \|\| id:finngen_R11_O15_PREECLAMPS | CD3 on CD28+ CD45RA+ CD8+ T cell \|\| id:ebi-a-GCST90001864 | Inverse variance weighted | 24 | -0.011434736 | 0.0193668785806253 | 0.554904147146903 | -0.049393818 | 0.0265243463020984 | 0.988630392397232 | 0.951806217712034 | 1.02687924766973 | 0.905435115099076 |
| ebi-a-GCST90001864 | finngen_R11_O15_PREECLAMPS | Pre-eclampsia \|\| id:finngen_R11_O15_PREECLAMPS | CD3 on CD28+ CD45RA+ CD8+ T cell \|\| id:ebi-a-GCST90001864 | MR Egger | 24 | -0.067663767 | 0.0357827400963323 | 0.0718715467114396 | -0.137797938 | 0.00247040320368207 | 0.934574655235315 | 0.871274725496619 | 1.002473457 | 0.896743733484648 |
| ebi-a-GCST90001864 | finngen_R11_O15_PREECLAMPS | Pre-eclampsia \|\| id:finngen_R11_O15_PREECLAMPS | CD3 on CD28+ CD45RA+ CD8+ T cell \|\| id:ebi-a-GCST90001864 | Weighted median | 24 | -0.025458731 | 0.0247167884277834 | 0.303001660195647 | -0.073903637 | 0.0229861738240976 | 0.974862609258704 | 0.928761187917454 | 1.02325239178185 | 0.940845681141708 |
| ebi-a-GCST90001864 | finngen_R11_O15_PREECLAMPS | Pre-eclampsia \|\| id:finngen_R11_O15_PREECLAMPS | CD3 on CD28+ CD45RA+ CD8+ T cell \|\| id:ebi-a-GCST90001864 | Weighted mode | 24 | -0.025686426 | 0.0238970901594035 | 0.29357718449105 | -0.072524723 | 0.0211518705501322 | 0.974640663509491 | 0.930042753045569 | 1.02137715696999 | 0.872755742358858 |
| ebi-a-GCST90001865 | finngen_R11_O15_PREECLAMPS | Pre-eclampsia \|\| id:finngen_R11_O15_PREECLAMPS | CD3 on CD28- CD8+ T cell \|\| id:ebi-a-GCST90001865 | Inverse variance weighted | 20 | -0.036674716 | 0.0282916530722246 | 0.1948691526399 | -0.092126356 | 0.0187769240232445 | 0.963989654767349 | 0.911989906849643 | 1.01895431903248 | 0.855687495048373 |
| ebi-a-GCST90001865 | finngen_R11_O15_PREECLAMPS | Pre-eclampsia \|\| id:finngen_R11_O15_PREECLAMPS | CD3 on CD28- CD8+ T cell \|\| id:ebi-a-GCST90001865 | MR Egger | 20 | 0.0689623575002331 | 0.0641808426413093 | 0.296791647134368 | -0.056832094 | 0.194756809077199 | 1.07139587837017 | 0.944752685621784 | 1.21501546982438 | 0.99380795269851 |
| ebi-a-GCST90001865 | finngen_R11_O15_PREECLAMPS | Pre-eclampsia \|\| id:finngen_R11_O15_PREECLAMPS | CD3 on CD28- CD8+ T cell \|\| id:ebi-a-GCST90001865 | Weighted median | 20 | -0.016409629 | 0.0339196605472027 | 0.628542511304042 | -0.082892164 | 0.0500729052295203 | 0.983724275084512 | 0.920450399451503 | 1.0513477423305 | 0.940845681141708 |
| ebi-a-GCST90001865 | finngen_R11_O15_PREECLAMPS | Pre-eclampsia \|\| id:finngen_R11_O15_PREECLAMPS | CD3 on CD28- CD8+ T cell \|\| id:ebi-a-GCST90001865 | Weighted mode | 20 | -0.010580118 | 0.0453363396627689 | 0.817970327944917 | -0.099439344 | 0.0782791077035616 | 0.989475654546559 | 0.905344863005252 | 1.08142445044686 | 0.966106153196364 |
| ebi-a-GCST90001866 | finngen_R11_O15_PREECLAMPS | Pre-eclampsia \|\| id:finngen_R11_O15_PREECLAMPS | CD3 on CD39+ CD8+ T cell \|\| id:ebi-a-GCST90001866 | Inverse variance weighted | 16 | -0.010782372 | 0.0243868458183961 | 0.658388737580883 | -0.05858059 | 0.0370158459994815 | 0.989275549602656 | 0.943102233097144 | 1.03770946425151 | 0.945544532753684 |
| ebi-a-GCST90001866 | finngen_R11_O15_PREECLAMPS | Pre-eclampsia \|\| id:finngen_R11_O15_PREECLAMPS | CD3 on CD39+ CD8+ T cell \|\| id:ebi-a-GCST90001866 | MR Egger | 16 | 0.0566407799222537 | 0.0583699452308045 | 0.348326482206064 | -0.057764313 | 0.17104587257463 | 1.05827558826878 | 0.943872379927853 | 1.18654517765553 | 0.99380795269851 |
| ebi-a-GCST90001866 | finngen_R11_O15_PREECLAMPS | Pre-eclampsia \|\| id:finngen_R11_O15_PREECLAMPS | CD3 on CD39+ CD8+ T cell \|\| id:ebi-a-GCST90001866 | Weighted median | 16 | -0.003459579 | 0.0355067804869387 | 0.922381491522619 | -0.073052869 | 0.0661337107674372 | 0.996546398461283 | 0.929551684499082 | 1.06836956012975 | 0.975775499714955 |
| ebi-a-GCST90001866 | finngen_R11_O15_PREECLAMPS | Pre-eclampsia \|\| id:finngen_R11_O15_PREECLAMPS | CD3 on CD39+ CD8+ T cell \|\| id:ebi-a-GCST90001866 | Weighted mode | 16 | 0.0185163565767038 | 0.0498093814288828 | 0.71528209060372 | -0.079110031 | 0.116142744177314 | 1.01868884729558 | 0.92393825697946 | 1.12315618469672 | 0.959313411571287 |
| ebi-a-GCST90001867 | finngen_R11_O15_PREECLAMPS | Pre-eclampsia \|\| id:finngen_R11_O15_PREECLAMPS | CD3 on CD4+ T cell \|\| id:ebi-a-GCST90001867 | Inverse variance weighted | 26 | -0.013252085 | 0.0212967543350132 | 0.533771955398142 | -0.054993723 | 0.0284895537279099 | 0.986835337504757 | 0.94649108880826 | 1.02889926261673 | 0.904087927705763 |
| ebi-a-GCST90001867 | finngen_R11_O15_PREECLAMPS | Pre-eclampsia \|\| id:finngen_R11_O15_PREECLAMPS | CD3 on CD4+ T cell \|\| id:ebi-a-GCST90001867 | MR Egger | 26 | -0.007369486 | 0.0326564615446261 | 0.823371085777952 | -0.07137615 | 0.0566371787618172 | 0.99265760221273 | 0.931111588165183 | 1.05827177725547 | 0.99380795269851 |
| ebi-a-GCST90001867 | finngen_R11_O15_PREECLAMPS | Pre-eclampsia \|\| id:finngen_R11_O15_PREECLAMPS | CD3 on CD4+ T cell \|\| id:ebi-a-GCST90001867 | Weighted median | 26 | 0.0152827161679033 | 0.0268316791872545 | 0.568964408548265 | -0.037307375 | 0.067872807374922 | 1.01540009406336 | 0.963379970878371 | 1.0702291745632 | 0.940845681141708 |
| ebi-a-GCST90001867 | finngen_R11_O15_PREECLAMPS | Pre-eclampsia \|\| id:finngen_R11_O15_PREECLAMPS | CD3 on CD4+ T cell \|\| id:ebi-a-GCST90001867 | Weighted mode | 26 | 0.00310042084059559 | 0.0256328588709437 | 0.904693644437018 | -0.047139983 | 0.0533408242276452 | 1.00310523211633 | 0.953953851354206 | 1.0547890815376 | 0.98361388732834 |
| ebi-a-GCST90001868 | finngen_R11_O15_PREECLAMPS | Pre-eclampsia \|\| id:finngen_R11_O15_PREECLAMPS | CD3 on CD4 regulatory T cell \|\| id:ebi-a-GCST90001868 | Inverse variance weighted | 26 | -0.000750991 | 0.0173154398265469 | 0.965405631110083 | -0.034689254 | 0.033187270568224 | 0.999249290431724 | 0.965905521336871 | 1.03374411096265 | 0.992671503887443 |
| ebi-a-GCST90001868 | finngen_R11_O15_PREECLAMPS | Pre-eclampsia \|\| id:finngen_R11_O15_PREECLAMPS | CD3 on CD4 regulatory T cell \|\| id:ebi-a-GCST90001868 | MR Egger | 26 | -0.014879932 | 0.0272840965598856 | 0.590532065934958 | -0.068356761 | 0.0385968972642143 | 0.985230227131295 | 0.93392722509094 | 1.03935143378936 | 0.99380795269851 |
| ebi-a-GCST90001868 | finngen_R11_O15_PREECLAMPS | Pre-eclampsia \|\| id:finngen_R11_O15_PREECLAMPS | CD3 on CD4 regulatory T cell \|\| id:ebi-a-GCST90001868 | Weighted median | 26 | -0.003076467 | 0.02178638693939 | 0.887703619839874 | -0.045777786 | 0.0396248509380514 | 0.996928260013654 | 0.955254209596159 | 1.04042038823782 | 0.964912412213677 |
| ebi-a-GCST90001868 | finngen_R11_O15_PREECLAMPS | Pre-eclampsia \|\| id:finngen_R11_O15_PREECLAMPS | CD3 on CD4 regulatory T cell \|\| id:ebi-a-GCST90001868 | Weighted mode | 26 | -0.014087656 | 0.0192781438229648 | 0.471720714822836 | -0.051872817 | 0.0236975062938663 | 0.986011111080036 | 0.949449612547151 | 1.02398052337401 | 0.933945646747087 |
| ebi-a-GCST90001869 | finngen_R11_O15_PREECLAMPS | Pre-eclampsia \|\| id:finngen_R11_O15_PREECLAMPS | CD3 on resting CD4 regulatory T cell \|\| id:ebi-a-GCST90001869 | Inverse variance weighted | 37 | 0.0172480698105402 | 0.0187697158553318 | 0.358131774109559 | -0.019540573 | 0.0540367128869905 | 1.0173976766721 | 0.980649106244536 | 1.05552335275332 | 0.885079385681701 |
| ebi-a-GCST90001869 | finngen_R11_O15_PREECLAMPS | Pre-eclampsia \|\| id:finngen_R11_O15_PREECLAMPS | CD3 on resting CD4 regulatory T cell \|\| id:ebi-a-GCST90001869 | MR Egger | 37 | -0.009218822 | 0.0298485985642438 | 0.75926457105354 | -0.067722075 | 0.0492844316269165 | 0.990823541497368 | 0.93452016424327 | 1.05051910910916 | 0.99380795269851 |
| ebi-a-GCST90001869 | finngen_R11_O15_PREECLAMPS | Pre-eclampsia \|\| id:finngen_R11_O15_PREECLAMPS | CD3 on resting CD4 regulatory T cell \|\| id:ebi-a-GCST90001869 | Weighted median | 37 | -0.002433974 | 0.0266844444742744 | 0.927323187840133 | -0.054735485 | 0.049867537640709 | 0.997568986182927 | 0.94673554087273 | 1.05113185174888 | 0.977431269406605 |
| ebi-a-GCST90001869 | finngen_R11_O15_PREECLAMPS | Pre-eclampsia \|\| id:finngen_R11_O15_PREECLAMPS | CD3 on resting CD4 regulatory T cell \|\| id:ebi-a-GCST90001869 | Weighted mode | 37 | 0.0038472981797133 | 0.0294646675014866 | 0.896839812517827 | -0.05390345 | 0.061598046482627 | 1.00385470853159 | 0.947523585371023 | 1.06353476726011 | 0.98361388732834 |
| ebi-a-GCST90001870 | finngen_R11_O15_PREECLAMPS | Pre-eclampsia \|\| id:finngen_R11_O15_PREECLAMPS | CD34 on Hematopoietic Stem Cell \|\| id:ebi-a-GCST90001870 | Inverse variance weighted | 12 | 0.0137969857485575 | 0.012326427074876 | 0.263011624900009 | -0.010362811 | 0.0379567828153145 | 1.01389260339547 | 0.989690697617169 | 1.03868634280899 | 0.885079385681701 |
| ebi-a-GCST90001870 | finngen_R11_O15_PREECLAMPS | Pre-eclampsia \|\| id:finngen_R11_O15_PREECLAMPS | CD34 on Hematopoietic Stem Cell \|\| id:ebi-a-GCST90001870 | MR Egger | 12 | 0.0237247447287538 | 0.0174809253609096 | 0.204564468186211 | -0.010537869 | 0.0579873584361367 | 1.02400841538069 | 0.989517459842837 | 1.05970159934017 | 0.99380795269851 |
| ebi-a-GCST90001870 | finngen_R11_O15_PREECLAMPS | Pre-eclampsia \|\| id:finngen_R11_O15_PREECLAMPS | CD34 on Hematopoietic Stem Cell \|\| id:ebi-a-GCST90001870 | Weighted median | 12 | 0.019104160663264 | 0.0154807198388038 | 0.217179980716934 | -0.01123805 | 0.0494463715473194 | 1.01928781278288 | 0.988824860779395 | 1.0506892438655 | 0.940845681141708 |
| ebi-a-GCST90001870 | finngen_R11_O15_PREECLAMPS | Pre-eclampsia \|\| id:finngen_R11_O15_PREECLAMPS | CD34 on Hematopoietic Stem Cell \|\| id:ebi-a-GCST90001870 | Weighted mode | 12 | 0.0170962877838921 | 0.0145652770463622 | 0.265274811033188 | -0.011451655 | 0.045644230794762 | 1.01724326570952 | 0.988613665395889 | 1.04670196038308 | 0.872755742358858 |
| ebi-a-GCST90001871 | finngen_R11_O15_PREECLAMPS | Pre-eclampsia \|\| id:finngen_R11_O15_PREECLAMPS | HVEM on T cell \|\| id:ebi-a-GCST90001871 | Inverse variance weighted | 25 | 0.0169872133668084 | 0.0145867656465147 | 0.244195766916555 | -0.011602847 | 0.0455772740339772 | 1.01713231654425 | 0.988464206144856 | 1.04663187895654 | 0.885079385681701 |
| ebi-a-GCST90001871 | finngen_R11_O15_PREECLAMPS | Pre-eclampsia \|\| id:finngen_R11_O15_PREECLAMPS | HVEM on T cell \|\| id:ebi-a-GCST90001871 | MR Egger | 25 | 0.0183013468796971 | 0.0272683452217082 | 0.508805314247627 | -0.03514461 | 0.0717473035142452 | 1.01846984286006 | 0.965465790390913 | 1.07438381674342 | 0.99380795269851 |
| ebi-a-GCST90001871 | finngen_R11_O15_PREECLAMPS | Pre-eclampsia \|\| id:finngen_R11_O15_PREECLAMPS | HVEM on T cell \|\| id:ebi-a-GCST90001871 | Weighted median | 25 | 0.0316171987950268 | 0.0234056386562961 | 0.176747706376463 | -0.014257853 | 0.0774922505613671 | 1.03212233200117 | 0.985843308860154 | 1.08057385858531 | 0.940845681141708 |
| ebi-a-GCST90001871 | finngen_R11_O15_PREECLAMPS | Pre-eclampsia \|\| id:finngen_R11_O15_PREECLAMPS | HVEM on T cell \|\| id:ebi-a-GCST90001871 | Weighted mode | 25 | 0.0266850264654832 | 0.0248051541606527 | 0.292719358131085 | -0.021933076 | 0.0753031286203625 | 1.02704426005177 | 0.978305705295297 | 1.07821093794695 | 0.872755742358858 |
| ebi-a-GCST90001872 | finngen_R11_O15_PREECLAMPS | Pre-eclampsia \|\| id:finngen_R11_O15_PREECLAMPS | HVEM on naive CD8+ T cell \|\| id:ebi-a-GCST90001872 | Inverse variance weighted | 21 | 0.00644467619100627 | 0.0102364547630705 | 0.528968836811164 | -0.013618775 | 0.0265081275266245 | 1.00646548780062 | 0.986473540821635 | 1.02686259308083 | 0.904087927705763 |
| ebi-a-GCST90001872 | finngen_R11_O15_PREECLAMPS | Pre-eclampsia \|\| id:finngen_R11_O15_PREECLAMPS | HVEM on naive CD8+ T cell \|\| id:ebi-a-GCST90001872 | MR Egger | 21 | -0.009852978 | 0.0144751503919962 | 0.504283982136665 | -0.038224272 | 0.0185183171371623 | 0.990195403921779 | 0.962497055159462 | 1.01869084449861 | 0.99380795269851 |
| ebi-a-GCST90001872 | finngen_R11_O15_PREECLAMPS | Pre-eclampsia \|\| id:finngen_R11_O15_PREECLAMPS | HVEM on naive CD8+ T cell \|\| id:ebi-a-GCST90001872 | Weighted median | 21 | 0.00761291833489501 | 0.0135388546650428 | 0.573910614077077 | -0.018923237 | 0.0341490734783788 | 1.00764197027423 | 0.981254683592915 | 1.03473884735043 | 0.940845681141708 |
| ebi-a-GCST90001872 | finngen_R11_O15_PREECLAMPS | Pre-eclampsia \|\| id:finngen_R11_O15_PREECLAMPS | HVEM on naive CD8+ T cell \|\| id:ebi-a-GCST90001872 | Weighted mode | 21 | 0.0037114137652321 | 0.0119738287234805 | 0.75979760643318 | -0.019757291 | 0.027180118063254 | 1.00371830958975 | 0.980436605677565 | 1.02755286692857 | 0.959313411571287 |
| ebi-a-GCST90001873 | finngen_R11_O15_PREECLAMPS | Pre-eclampsia \|\| id:finngen_R11_O15_PREECLAMPS | HVEM on Effector Memory CD8+ T cell \|\| id:ebi-a-GCST90001873 | Inverse variance weighted | 18 | -0.001067406 | 0.0206098790580529 | 0.958695228183202 | -0.041462769 | 0.0393279567409871 | 0.998933163262576 | 0.95938505338167 | 1.04011153931205 | 0.992671503887443 |
| ebi-a-GCST90001873 | finngen_R11_O15_PREECLAMPS | Pre-eclampsia \|\| id:finngen_R11_O15_PREECLAMPS | HVEM on Effector Memory CD8+ T cell \|\| id:ebi-a-GCST90001873 | MR Egger | 18 | -0.01659309 | 0.0391295403522653 | 0.677171846885857 | -0.093286989 | 0.0601008092994769 | 0.983543817240638 | 0.910932035413301 | 1.06194359493942 | 0.99380795269851 |
| ebi-a-GCST90001873 | finngen_R11_O15_PREECLAMPS | Pre-eclampsia \|\| id:finngen_R11_O15_PREECLAMPS | HVEM on Effector Memory CD8+ T cell \|\| id:ebi-a-GCST90001873 | Weighted median | 18 | -0.017191693 | 0.0238641628552741 | 0.471280043892184 | -0.063965452 | 0.0295820664394008 | 0.982955241173738 | 0.938037406331075 | 1.03002396240252 | 0.940845681141708 |
| ebi-a-GCST90001873 | finngen_R11_O15_PREECLAMPS | Pre-eclampsia \|\| id:finngen_R11_O15_PREECLAMPS | HVEM on Effector Memory CD8+ T cell \|\| id:ebi-a-GCST90001873 | Weighted mode | 18 | -0.025238549 | 0.0312911030155977 | 0.431052543048323 | -0.086569111 | 0.0360920130984661 | 0.975077280755444 | 0.91707216707434 | 1.03675123679591 | 0.933945646747087 |
| ebi-a-GCST90001874 | finngen_R11_O15_PREECLAMPS | Pre-eclampsia \|\| id:finngen_R11_O15_PREECLAMPS | HVEM on Terminally Differentiated CD8+ T cell \|\| id:ebi-a-GCST90001874 | Inverse variance weighted | 25 | -0.013360656 | 0.0147312818594902 | 0.364428927985732 | -0.042233969 | 0.0155126562857818 | 0.986728201236434 | 0.958645461391909 | 1.01563360212609 | 0.885079385681701 |
| ebi-a-GCST90001874 | finngen_R11_O15_PREECLAMPS | Pre-eclampsia \|\| id:finngen_R11_O15_PREECLAMPS | HVEM on Terminally Differentiated CD8+ T cell \|\| id:ebi-a-GCST90001874 | MR Egger | 25 | -0.014164749 | 0.0306671286671211 | 0.648501488310197 | -0.074272321 | 0.0459428236818409 | 0.985935099547584 | 0.928418831753149 | 1.04701454480876 | 0.99380795269851 |
| ebi-a-GCST90001874 | finngen_R11_O15_PREECLAMPS | Pre-eclampsia \|\| id:finngen_R11_O15_PREECLAMPS | HVEM on Terminally Differentiated CD8+ T cell \|\| id:ebi-a-GCST90001874 | Weighted median | 25 | -0.014054693 | 0.0203425191547082 | 0.489626946479914 | -0.053926031 | 0.0258166444599605 | 0.98604361302261 | 0.94750219005314 | 1.02615278042592 | 0.940845681141708 |
| ebi-a-GCST90001874 | finngen_R11_O15_PREECLAMPS | Pre-eclampsia \|\| id:finngen_R11_O15_PREECLAMPS | HVEM on Terminally Differentiated CD8+ T cell \|\| id:ebi-a-GCST90001874 | Weighted mode | 25 | -0.019221744 | 0.026967923099727 | 0.482865256910749 | -0.072078873 | 0.0336353851829932 | 0.980961815636302 | 0.930457504599828 | 1.03420745061359 | 0.933945646747087 |
| ebi-a-GCST90001875 | finngen_R11_O15_PREECLAMPS | Pre-eclampsia \|\| id:finngen_R11_O15_PREECLAMPS | HVEM on CD4+ T cell \|\| id:ebi-a-GCST90001875 | Inverse variance weighted (multiplicative random effects) | 20 | -0.031702576 | 0.0247170902991966 | 0.199626063868148 | -0.080148073 | 0.0167429207390005 | 0.968794681782957 | 0.922979667683485 | 1.01688386896601 | 0.855687495048373 |
| ebi-a-GCST90001875 | finngen_R11_O15_PREECLAMPS | Pre-eclampsia \|\| id:finngen_R11_O15_PREECLAMPS | HVEM on CD4+ T cell \|\| id:ebi-a-GCST90001875 | MR Egger | 20 | -0.017133168 | 0.0417281807790414 | 0.68621958475055 | -0.098920402 | 0.0646540662284273 | 0.983012769976902 | 0.905814805815672 | 1.06678992188421 | 0.99380795269851 |
| ebi-a-GCST90001875 | finngen_R11_O15_PREECLAMPS | Pre-eclampsia \|\| id:finngen_R11_O15_PREECLAMPS | HVEM on CD4+ T cell \|\| id:ebi-a-GCST90001875 | Weighted median | 20 | -0.024435626 | 0.0261077854505023 | 0.349298056021671 | -0.075606886 | 0.0267356332814137 | 0.975860506744031 | 0.927180622901914 | 1.02709623680677 | 0.940845681141708 |
| ebi-a-GCST90001875 | finngen_R11_O15_PREECLAMPS | Pre-eclampsia \|\| id:finngen_R11_O15_PREECLAMPS | HVEM on CD4+ T cell \|\| id:ebi-a-GCST90001875 | Weighted mode | 20 | -0.020382462 | 0.0244517996548929 | 0.414876236489459 | -0.068307989 | 0.0275430653636458 | 0.979823856282223 | 0.933972775669532 | 1.02792588215597 | 0.93252856028397 |
| ebi-a-GCST90001876 | finngen_R11_O15_PREECLAMPS | Pre-eclampsia \|\| id:finngen_R11_O15_PREECLAMPS | HVEM on Central Memory CD4+ T cell \|\| id:ebi-a-GCST90001876 | Inverse variance weighted (multiplicative random effects) | 17 | 0.00879714252670152 | 0.0127269139431819 | 0.48942507195668 | -0.016147609 | 0.033741893855338 | 1.00883595110307 | 0.983982064921352 | 1.03431760854237 | 0.898694924660397 |
| ebi-a-GCST90001876 | finngen_R11_O15_PREECLAMPS | Pre-eclampsia \|\| id:finngen_R11_O15_PREECLAMPS | HVEM on Central Memory CD4+ T cell \|\| id:ebi-a-GCST90001876 | MR Egger | 17 | -0.003532102 | 0.0185363428660443 | 0.851433040341271 | -0.039863334 | 0.0327991298523981 | 0.996474128370016 | 0.960920755199478 | 1.03334295064181 | 0.99380795269851 |
| ebi-a-GCST90001876 | finngen_R11_O15_PREECLAMPS | Pre-eclampsia \|\| id:finngen_R11_O15_PREECLAMPS | HVEM on Central Memory CD4+ T cell \|\| id:ebi-a-GCST90001876 | Weighted median | 17 | 0.0100228471864989 | 0.0129201369108769 | 0.437894281234952 | -0.015300621 | 0.0353463155318177 | 1.01007324415233 | 0.984815838619552 | 1.03597842210025 | 0.940845681141708 |
| ebi-a-GCST90001876 | finngen_R11_O15_PREECLAMPS | Pre-eclampsia \|\| id:finngen_R11_O15_PREECLAMPS | HVEM on Central Memory CD4+ T cell \|\| id:ebi-a-GCST90001876 | Weighted mode | 17 | 0.0138148360392336 | 0.0130336052055452 | 0.304911365459844 | -0.01173103 | 0.039360702242102 | 1.01391070183469 | 0.988337510092972 | 1.04014559884326 | 0.875408265683677 |
| ebi-a-GCST90001877 | finngen_R11_O15_PREECLAMPS | Pre-eclampsia \|\| id:finngen_R11_O15_PREECLAMPS | HVEM on naive CD4+ T cell \|\| id:ebi-a-GCST90001877 | Inverse variance weighted | 18 | 0.00222775333305474 | 0.0132998144493302 | 0.866974611790463 | -0.023839883 | 0.028295389653742 | 1.00223023661922 | 0.976442042224383 | 1.02869950673733 | 0.981050218604997 |
| ebi-a-GCST90001877 | finngen_R11_O15_PREECLAMPS | Pre-eclampsia \|\| id:finngen_R11_O15_PREECLAMPS | HVEM on naive CD4+ T cell \|\| id:ebi-a-GCST90001877 | MR Egger | 18 | 0.00227447059074752 | 0.020754406129447 | 0.91409746567094 | -0.038404165 | 0.0429531066044636 | 1.00227705916115 | 0.962323924227085 | 1.04388894220578 | 0.99380795269851 |
| ebi-a-GCST90001877 | finngen_R11_O15_PREECLAMPS | Pre-eclampsia \|\| id:finngen_R11_O15_PREECLAMPS | HVEM on naive CD4+ T cell \|\| id:ebi-a-GCST90001877 | Weighted median | 18 | 0.00865898159870658 | 0.0150197611888583 | 0.564273253539919 | -0.02077975 | 0.0380977135288688 | 1.00869657901998 | 0.979434660974497 | 1.03883273593184 | 0.940845681141708 |
| ebi-a-GCST90001877 | finngen_R11_O15_PREECLAMPS | Pre-eclampsia \|\| id:finngen_R11_O15_PREECLAMPS | HVEM on naive CD4+ T cell \|\| id:ebi-a-GCST90001877 | Weighted mode | 18 | 0.00590114426633748 | 0.0126019908060572 | 0.645538836203263 | -0.018798758 | 0.0306010462462096 | 1.0059185903185 | 0.981376836890555 | 1.03107407094958 | 0.959313411571287 |
| ebi-a-GCST90001878 | finngen_R11_O15_PREECLAMPS | Pre-eclampsia \|\| id:finngen_R11_O15_PREECLAMPS | HVEM on Effector Memory CD4+ T cell \|\| id:ebi-a-GCST90001878 | Inverse variance weighted | 21 | 0.014729826312556 | 0.012995337773757 | 0.257016906530538 | -0.010741036 | 0.0402006883491197 | 1.01483884482103 | 0.989316443221663 | 1.04101967374956 | 0.885079385681701 |
| ebi-a-GCST90001878 | finngen_R11_O15_PREECLAMPS | Pre-eclampsia \|\| id:finngen_R11_O15_PREECLAMPS | HVEM on Effector Memory CD4+ T cell \|\| id:ebi-a-GCST90001878 | MR Egger | 21 | 0.00465089233705871 | 0.0200371692870593 | 0.818931798793011 | -0.034621959 | 0.043923744139695 | 1.00466172452342 | 0.965970523253443 | 1.04490267189815 | 0.99380795269851 |
| ebi-a-GCST90001878 | finngen_R11_O15_PREECLAMPS | Pre-eclampsia \|\| id:finngen_R11_O15_PREECLAMPS | HVEM on Effector Memory CD4+ T cell \|\| id:ebi-a-GCST90001878 | Weighted median | 21 | 0.00937153978409366 | 0.0137376141952858 | 0.495124489114795 | -0.017554184 | 0.0362972636068538 | 1.00941559016215 | 0.982598993040803 | 1.03696405235384 | 0.940845681141708 |
| ebi-a-GCST90001878 | finngen_R11_O15_PREECLAMPS | Pre-eclampsia \|\| id:finngen_R11_O15_PREECLAMPS | HVEM on Effector Memory CD4+ T cell \|\| id:ebi-a-GCST90001878 | Weighted mode | 21 | 0.00961385596322961 | 0.0143030372010021 | 0.509176321688167 | -0.018420097 | 0.0376478088771938 | 1.0096602175285 | 0.981748516157942 | 1.03836546537311 | 0.934398527469092 |
| ebi-a-GCST90001879 | finngen_R11_O15_PREECLAMPS | Pre-eclampsia \|\| id:finngen_R11_O15_PREECLAMPS | HVEM on Terminally Differentiated CD4+ T cell \|\| id:ebi-a-GCST90001879 | Inverse variance weighted | 26 | 0.00771357964692491 | 0.014334458437191 | 0.590498016395183 | -0.020381959 | 0.0358091181838194 | 1.00774340594226 | 0.97982434920231 | 1.03645798662472 | 0.91586635420875 |
| ebi-a-GCST90001879 | finngen_R11_O15_PREECLAMPS | Pre-eclampsia \|\| id:finngen_R11_O15_PREECLAMPS | HVEM on Terminally Differentiated CD4+ T cell \|\| id:ebi-a-GCST90001879 | MR Egger | 26 | 0.00122521032090859 | 0.0256075393248096 | 0.962235156013653 | -0.048965567 | 0.0514159873975355 | 1.0012259611977 | 0.952213916948427 | 1.05276073740745 | 0.99380795269851 |
| ebi-a-GCST90001879 | finngen_R11_O15_PREECLAMPS | Pre-eclampsia \|\| id:finngen_R11_O15_PREECLAMPS | HVEM on Terminally Differentiated CD4+ T cell \|\| id:ebi-a-GCST90001879 | Weighted median | 26 | 0.0113492790163044 | 0.0230746667941163 | 0.622825259720828 | -0.033877068 | 0.0565756259327724 | 1.01141392641906 | 0.966690334606073 | 1.0582066396384 | 0.940845681141708 |
| ebi-a-GCST90001879 | finngen_R11_O15_PREECLAMPS | Pre-eclampsia \|\| id:finngen_R11_O15_PREECLAMPS | HVEM on Terminally Differentiated CD4+ T cell \|\| id:ebi-a-GCST90001879 | Weighted mode | 26 | 0.0140284755316621 | 0.0229591349376948 | 0.546702712262594 | -0.030971429 | 0.0590283800095439 | 1.01412733634242 | 0.969503272410461 | 1.06080534597881 | 0.939633861977944 |
| ebi-a-GCST90001880 | finngen_R11_O15_PREECLAMPS | Pre-eclampsia \|\| id:finngen_R11_O15_PREECLAMPS | HVEM on CD45RA- CD4+ T cell \|\| id:ebi-a-GCST90001880 | Inverse variance weighted (multiplicative random effects) | 19 | -0.000411772 | 0.0160775565579633 | 0.979567113102604 | -0.031923783 | 0.0311002384920209 | 0.999588312405016 | 0.968580401350044 | 1.0315889036202 | 0.994537505425743 |
| ebi-a-GCST90001880 | finngen_R11_O15_PREECLAMPS | Pre-eclampsia \|\| id:finngen_R11_O15_PREECLAMPS | HVEM on CD45RA- CD4+ T cell \|\| id:ebi-a-GCST90001880 | MR Egger | 19 | 0.0113829080032596 | 0.0239224590541786 | 0.640256157454805 | -0.035505112 | 0.0582709277494496 | 1.01144793981671 | 0.965117800783296 | 1.06000214080516 | 0.99380795269851 |
| ebi-a-GCST90001880 | finngen_R11_O15_PREECLAMPS | Pre-eclampsia \|\| id:finngen_R11_O15_PREECLAMPS | HVEM on CD45RA- CD4+ T cell \|\| id:ebi-a-GCST90001880 | Weighted median | 19 | 0.00950688151989457 | 0.0131063058373948 | 0.468226922971095 | -0.016181478 | 0.0351952409611883 | 1.00955221546561 | 0.98394873887961 | 1.03582192392669 | 0.940845681141708 |
| ebi-a-GCST90001880 | finngen_R11_O15_PREECLAMPS | Pre-eclampsia \|\| id:finngen_R11_O15_PREECLAMPS | HVEM on CD45RA- CD4+ T cell \|\| id:ebi-a-GCST90001880 | Weighted mode | 19 | 0.0102841288020168 | 0.0117036129067452 | 0.391135834728739 | -0.012654952 | 0.0332232100992374 | 1.01033719220224 | 0.987424784704882 | 1.03378126390882 | 0.920286500917063 |
| ebi-a-GCST90001881 | finngen_R11_O15_PREECLAMPS | Pre-eclampsia \|\| id:finngen_R11_O15_PREECLAMPS | HVEM on CD8+ T cell \|\| id:ebi-a-GCST90001881 | Inverse variance weighted | 16 | -0.018061908 | 0.0210485814869603 | 0.390834094357161 | -0.059317128 | 0.0231933117412219 | 0.982100230641487 | 0.942407858138238 | 1.02346436810439 | 0.885079385681701 |
| ebi-a-GCST90001881 | finngen_R11_O15_PREECLAMPS | Pre-eclampsia \|\| id:finngen_R11_O15_PREECLAMPS | HVEM on CD8+ T cell \|\| id:ebi-a-GCST90001881 | MR Egger | 16 | -0.022000518 | 0.0479599569069814 | 0.653471003728127 | -0.116002034 | 0.0720009971934573 | 0.978239727986164 | 0.890473412177867 | 1.07465641570363 | 0.99380795269851 |
| ebi-a-GCST90001881 | finngen_R11_O15_PREECLAMPS | Pre-eclampsia \|\| id:finngen_R11_O15_PREECLAMPS | HVEM on CD8+ T cell \|\| id:ebi-a-GCST90001881 | Weighted median | 16 | -0.029098665 | 0.0292578102712476 | 0.319950029134842 | -0.086443973 | 0.0282466434540601 | 0.971320624702415 | 0.917186934752551 | 1.02864936276794 | 0.940845681141708 |
| ebi-a-GCST90001881 | finngen_R11_O15_PREECLAMPS | Pre-eclampsia \|\| id:finngen_R11_O15_PREECLAMPS | HVEM on CD8+ T cell \|\| id:ebi-a-GCST90001881 | Weighted mode | 16 | -0.040694436 | 0.0405225655542131 | 0.331188339525369 | -0.120118664 | 0.0387297928002649 | 0.960122464291427 | 0.886815197281881 | 1.03948956813381 | 0.888599269773114 |
| ebi-a-GCST90001882 | finngen_R11_O15_PREECLAMPS | Pre-eclampsia \|\| id:finngen_R11_O15_PREECLAMPS | HVEM on Central Memory CD8+ T cell \|\| id:ebi-a-GCST90001882 | Inverse variance weighted (multiplicative random effects) | 18 | 0.0115240555604849 | 0.0153126238962897 | 0.451699157321146 | -0.018488687 | 0.0415367983972128 | 1.0115907132985 | 0.981681180016982 | 1.04241152021889 | 0.898694924660397 |
| ebi-a-GCST90001882 | finngen_R11_O15_PREECLAMPS | Pre-eclampsia \|\| id:finngen_R11_O15_PREECLAMPS | HVEM on Central Memory CD8+ T cell \|\| id:ebi-a-GCST90001882 | MR Egger | 18 | 0.0189526619209424 | 0.0248715200325768 | 0.457133286848546 | -0.029795517 | 0.067700841184793 | 1.01913340365787 | 0.97064399311985 | 1.07004514715318 | 0.99380795269851 |
| ebi-a-GCST90001882 | finngen_R11_O15_PREECLAMPS | Pre-eclampsia \|\| id:finngen_R11_O15_PREECLAMPS | HVEM on Central Memory CD8+ T cell \|\| id:ebi-a-GCST90001882 | Weighted median | 18 | 0.0182174362187216 | 0.0143833293851531 | 0.205310509836186 | -0.009973889 | 0.0464087618136217 | 1.01838438596778 | 0.990075684905435 | 1.04750250247993 | 0.940845681141708 |
| ebi-a-GCST90001882 | finngen_R11_O15_PREECLAMPS | Pre-eclampsia \|\| id:finngen_R11_O15_PREECLAMPS | HVEM on Central Memory CD8+ T cell \|\| id:ebi-a-GCST90001882 | Weighted mode | 18 | 0.0191400859732496 | 0.0115628035690904 | 0.116202571505392 | -0.003523009 | 0.0418031809686668 | 1.01932443167129 | 0.996483189492839 | 1.04268923746803 | 0.872755742358858 |
| ebi-a-GCST90001883 | finngen_R11_O15_PREECLAMPS | Pre-eclampsia \|\| id:finngen_R11_O15_PREECLAMPS | CD16-CD56 on Natural Killer T \|\| id:ebi-a-GCST90001883 | Inverse variance weighted | 28 | 0.0141065146530546 | 0.0175435002579703 | 0.421346265733536 | -0.020278746 | 0.0484917751586765 | 1.01420648103688 | 0.979925485068692 | 1.04968673827796 | 0.898694924660397 |
| ebi-a-GCST90001883 | finngen_R11_O15_PREECLAMPS | Pre-eclampsia \|\| id:finngen_R11_O15_PREECLAMPS | CD16-CD56 on Natural Killer T \|\| id:ebi-a-GCST90001883 | MR Egger | 28 | -0.001856688 | 0.028223379522949 | 0.948051882749908 | -0.057174511 | 0.053461136282946 | 0.998145034996093 | 0.94442924127125 | 1.05491599301425 | 0.99380795269851 |
| ebi-a-GCST90001883 | finngen_R11_O15_PREECLAMPS | Pre-eclampsia \|\| id:finngen_R11_O15_PREECLAMPS | CD16-CD56 on Natural Killer T \|\| id:ebi-a-GCST90001883 | Weighted median | 28 | 0.00728042250529619 | 0.026000497579974 | 0.779469148850017 | -0.043680553 | 0.0582413977620453 | 1.00730698921438 | 0.95725970261387 | 1.05997083941746 | 0.957984984509652 |
| ebi-a-GCST90001883 | finngen_R11_O15_PREECLAMPS | Pre-eclampsia \|\| id:finngen_R11_O15_PREECLAMPS | CD16-CD56 on Natural Killer T \|\| id:ebi-a-GCST90001883 | Weighted mode | 28 | 0.0072235600355432 | 0.0261748920083587 | 0.784670260995561 | -0.044079228 | 0.0585263483719263 | 1.00724971287962 | 0.956878142640394 | 1.06027292179185 | 0.961305639715923 |
| ebi-a-GCST90001884 | finngen_R11_O15_PREECLAMPS | Pre-eclampsia \|\| id:finngen_R11_O15_PREECLAMPS | CD16-CD56 on Natural Killer \|\| id:ebi-a-GCST90001884 | Inverse variance weighted | 28 | 0.0348085746087878 | 0.0207353446387189 | 0.0932091007039672 | -0.005832701 | 0.0754498501006769 | 1.03542148386497 | 0.99418427629306 | 1.07836914625785 | 0.802698559103897 |
| ebi-a-GCST90001884 | finngen_R11_O15_PREECLAMPS | Pre-eclampsia \|\| id:finngen_R11_O15_PREECLAMPS | CD16-CD56 on Natural Killer \|\| id:ebi-a-GCST90001884 | MR Egger | 28 | 0.123533143254843 | 0.0384892848103725 | 0.003518459480759 | 0.0480941450265127 | 0.198972141483173 | 1.13148750509936 | 1.04926943417333 | 1.22014797391353 | 0.565056961366001 |
| ebi-a-GCST90001884 | finngen_R11_O15_PREECLAMPS | Pre-eclampsia \|\| id:finngen_R11_O15_PREECLAMPS | CD16-CD56 on Natural Killer \|\| id:ebi-a-GCST90001884 | Weighted median | 28 | 0.0372249384136291 | 0.0262050512996468 | 0.155454807800982 | -0.014136962 | 0.0885868389609368 | 1.03792646411429 | 0.98596249548768 | 1.09262913126929 | 0.937437665462528 |
| ebi-a-GCST90001884 | finngen_R11_O15_PREECLAMPS | Pre-eclampsia \|\| id:finngen_R11_O15_PREECLAMPS | CD16-CD56 on Natural Killer \|\| id:ebi-a-GCST90001884 | Weighted mode | 28 | 0.0553540995836366 | 0.0315581348823031 | 0.090770198205071 | -0.006499845 | 0.117208043952951 | 1.05691480151304 | 0.993521233512161 | 1.1243533202692 | 0.872755742358858 |
| ebi-a-GCST90001885 | finngen_R11_O15_PREECLAMPS | Pre-eclampsia \|\| id:finngen_R11_O15_PREECLAMPS | CD16-CD56 on HLA DR+ Natural Killer \|\| id:ebi-a-GCST90001885 | Inverse variance weighted | 25 | -0.011235362 | 0.0117551000620462 | 0.339180280015084 | -0.034275358 | 0.0118046341270394 | 0.988827518967886 | 0.966305387969209 | 1.01187458379291 | 0.885079385681701 |
| ebi-a-GCST90001885 | finngen_R11_O15_PREECLAMPS | Pre-eclampsia \|\| id:finngen_R11_O15_PREECLAMPS | CD16-CD56 on HLA DR+ Natural Killer \|\| id:ebi-a-GCST90001885 | MR Egger | 25 | -0.015306116 | 0.0143375576685354 | 0.296799488902988 | -0.043407729 | 0.0127954971591611 | 0.98481042735467 | 0.957520901520369 | 1.0128777098093 | 0.99380795269851 |
| ebi-a-GCST90001885 | finngen_R11_O15_PREECLAMPS | Pre-eclampsia \|\| id:finngen_R11_O15_PREECLAMPS | CD16-CD56 on HLA DR+ Natural Killer \|\| id:ebi-a-GCST90001885 | Weighted median | 25 | -0.01930505 | 0.0162164588440498 | 0.233865541811824 | -0.051089309 | 0.0124792095752987 | 0.980880099362135 | 0.950193805795559 | 1.01255739982342 | 0.940845681141708 |
| ebi-a-GCST90001885 | finngen_R11_O15_PREECLAMPS | Pre-eclampsia \|\| id:finngen_R11_O15_PREECLAMPS | CD16-CD56 on HLA DR+ Natural Killer \|\| id:ebi-a-GCST90001885 | Weighted mode | 25 | -0.019764192 | 0.0130115859881234 | 0.141835190416659 | -0.045266901 | 0.00573851653463604 | 0.980429839247783 | 0.95574235963774 | 1.00575501336132 | 0.872755742358858 |
| ebi-a-GCST90001886 | finngen_R11_O15_PREECLAMPS | Pre-eclampsia \|\| id:finngen_R11_O15_PREECLAMPS | CD28 on CD39+ activated CD4 regulatory T cell \|\| id:ebi-a-GCST90001886 | Inverse variance weighted (multiplicative random effects) | 19 | 0.000802114985738667 | 0.0226504121621371 | 0.971750561647205 | -0.043592693 | 0.0451969228235274 | 1.00080243676599 | 0.957343811049869 | 1.04623386695146 | 0.994537505425743 |
| ebi-a-GCST90001886 | finngen_R11_O15_PREECLAMPS | Pre-eclampsia \|\| id:finngen_R11_O15_PREECLAMPS | CD28 on CD39+ activated CD4 regulatory T cell \|\| id:ebi-a-GCST90001886 | MR Egger | 19 | 0.0272854731976469 | 0.0313222861080102 | 0.395824195050919 | -0.034106208 | 0.0886771539693469 | 1.02766113060193 | 0.966468852874144 | 1.09272781653478 | 0.99380795269851 |
| ebi-a-GCST90001886 | finngen_R11_O15_PREECLAMPS | Pre-eclampsia \|\| id:finngen_R11_O15_PREECLAMPS | CD28 on CD39+ activated CD4 regulatory T cell \|\| id:ebi-a-GCST90001886 | Weighted median | 19 | 0.0274268911457705 | 0.0230045391781572 | 0.23316784588508 | -0.017662006 | 0.0725157879349586 | 1.02780647060699 | 0.982493053351943 | 1.07520978129825 | 0.940845681141708 |
| ebi-a-GCST90001886 | finngen_R11_O15_PREECLAMPS | Pre-eclampsia \|\| id:finngen_R11_O15_PREECLAMPS | CD28 on CD39+ activated CD4 regulatory T cell \|\| id:ebi-a-GCST90001886 | Weighted mode | 19 | 0.00194546809492147 | 0.0243678697321309 | 0.937247489410058 | -0.045815557 | 0.0497064927698979 | 1.00194736174579 | 0.955218129642376 | 1.05096258598567 | 0.990180645930572 |
| ebi-a-GCST90001887 | finngen_R11_O15_PREECLAMPS | Pre-eclampsia \|\| id:finngen_R11_O15_PREECLAMPS | CD28 on secreting CD4 regulatory T cell \|\| id:ebi-a-GCST90001887 | Inverse variance weighted | 17 | 0.00711025907676543 | 0.012834047600007 | 0.579568356422333 | -0.018044474 | 0.0322649923727791 | 1.00713559698627 | 0.982117352484532 | 1.03279115082417 | 0.915399120505076 |
| ebi-a-GCST90001887 | finngen_R11_O15_PREECLAMPS | Pre-eclampsia \|\| id:finngen_R11_O15_PREECLAMPS | CD28 on secreting CD4 regulatory T cell \|\| id:ebi-a-GCST90001887 | MR Egger | 17 | -0.008960305 | 0.0157170577784221 | 0.577049453809488 | -0.039765739 | 0.0218451279346581 | 0.991079718593242 | 0.961014541438356 | 1.02208547972306 | 0.99380795269851 |
| ebi-a-GCST90001887 | finngen_R11_O15_PREECLAMPS | Pre-eclampsia \|\| id:finngen_R11_O15_PREECLAMPS | CD28 on secreting CD4 regulatory T cell \|\| id:ebi-a-GCST90001887 | Weighted median | 17 | 0.00637646625043611 | 0.0179684753791342 | 0.72268725966052 | -0.028841745 | 0.0415946779935392 | 1.00639683919079 | 0.97157020766556 | 1.04247185632298 | 0.949957918887556 |
| ebi-a-GCST90001887 | finngen_R11_O15_PREECLAMPS | Pre-eclampsia \|\| id:finngen_R11_O15_PREECLAMPS | CD28 on secreting CD4 regulatory T cell \|\| id:ebi-a-GCST90001887 | Weighted mode | 17 | -0.002938037 | 0.0150321724052054 | 0.847501255382855 | -0.032401095 | 0.0265250209393122 | 0.997066274831959 | 0.968118196933928 | 1.02687994044092 | 0.977585971930092 |
| ebi-a-GCST90001888 | finngen_R11_O15_PREECLAMPS | Pre-eclampsia \|\| id:finngen_R11_O15_PREECLAMPS | CD28 on CD39+ secreting CD4 regulatory T cell \|\| id:ebi-a-GCST90001888 | Inverse variance weighted | 21 | -0.00240928 | 0.0207675430359126 | 0.907643199092655 | -0.043113664 | 0.0382951045421912 | 0.997593620176974 | 0.957802516062532 | 1.03903781241772 | 0.986667691552896 |
| ebi-a-GCST90001888 | finngen_R11_O15_PREECLAMPS | Pre-eclampsia \|\| id:finngen_R11_O15_PREECLAMPS | CD28 on CD39+ secreting CD4 regulatory T cell \|\| id:ebi-a-GCST90001888 | MR Egger | 21 | 0.0375530985068817 | 0.0270131130749508 | 0.180546813207701 | -0.015392603 | 0.0904988001337852 | 1.03826712605232 | 0.984725257493272 | 1.09472019412308 | 0.99380795269851 |
| ebi-a-GCST90001888 | finngen_R11_O15_PREECLAMPS | Pre-eclampsia \|\| id:finngen_R11_O15_PREECLAMPS | CD28 on CD39+ secreting CD4 regulatory T cell \|\| id:ebi-a-GCST90001888 | Weighted median | 21 | -0.004758541 | 0.0259080841375276 | 0.854272263032238 | -0.055538385 | 0.0460213043884675 | 0.995252763395702 | 0.945975711288452 | 1.04709671847457 | 0.964912412213677 |
| ebi-a-GCST90001888 | finngen_R11_O15_PREECLAMPS | Pre-eclampsia \|\| id:finngen_R11_O15_PREECLAMPS | CD28 on CD39+ secreting CD4 regulatory T cell \|\| id:ebi-a-GCST90001888 | Weighted mode | 21 | 0.00344283284216196 | 0.0217495794313915 | 0.875812357311291 | -0.039186343 | 0.0460720085276894 | 1.00344876619838 | 0.961571510481329 | 1.04714981195838 | 0.977585971930092 |
| ebi-a-GCST90001889 | finngen_R11_O15_PREECLAMPS | Pre-eclampsia \|\| id:finngen_R11_O15_PREECLAMPS | CD28 on activated & secreting CD4 regulatory T cell \|\| id:ebi-a-GCST90001889 | Inverse variance weighted | 24 | 0.00566729545807844 | 0.0135320099487197 | 0.675357460420361 | -0.020855444 | 0.0321900349575691 | 1.00568338495727 | 0.979360526737131 | 1.03271373837041 | 0.950857618427329 |
| ebi-a-GCST90001889 | finngen_R11_O15_PREECLAMPS | Pre-eclampsia \|\| id:finngen_R11_O15_PREECLAMPS | CD28 on activated & secreting CD4 regulatory T cell \|\| id:ebi-a-GCST90001889 | MR Egger | 24 | 0.000518409745686649 | 0.0172342884135316 | 0.976274360428611 | -0.033260796 | 0.0342976150362086 | 1.00051854414324 | 0.967286262743612 | 1.03489256048687 | 0.99380795269851 |
| ebi-a-GCST90001889 | finngen_R11_O15_PREECLAMPS | Pre-eclampsia \|\| id:finngen_R11_O15_PREECLAMPS | CD28 on activated & secreting CD4 regulatory T cell \|\| id:ebi-a-GCST90001889 | Weighted median | 24 | -0.003563899 | 0.0209630352429981 | 0.865003286225039 | -0.044651448 | 0.0375236504821431 | 0.996442444554778 | 0.956330755060682 | 1.03823655158648 | 0.964912412213677 |
| ebi-a-GCST90001889 | finngen_R11_O15_PREECLAMPS | Pre-eclampsia \|\| id:finngen_R11_O15_PREECLAMPS | CD28 on activated & secreting CD4 regulatory T cell \|\| id:ebi-a-GCST90001889 | Weighted mode | 24 | -0.008899124 | 0.0179012651705789 | 0.623821430001978 | -0.043985604 | 0.0261873553663729 | 0.991140355639998 | 0.956967733783486 | 1.02653325697232 | 0.959313411571287 |
| ebi-a-GCST90001890 | finngen_R11_O15_PREECLAMPS | Pre-eclampsia \|\| id:finngen_R11_O15_PREECLAMPS | CD28 on CD45RA+ CD4+ T cell \|\| id:ebi-a-GCST90001890 | Inverse variance weighted | 13 | 0.0536800616185899 | 0.0316743629116337 | 0.0901232452034915 | -0.00840169 | 0.115761812925392 | 1.0551469661341 | 0.991633505870237 | 1.12272842088465 | 0.802698559103897 |
| ebi-a-GCST90001890 | finngen_R11_O15_PREECLAMPS | Pre-eclampsia \|\| id:finngen_R11_O15_PREECLAMPS | CD28 on CD45RA+ CD4+ T cell \|\| id:ebi-a-GCST90001890 | MR Egger | 13 | 0.105177679283501 | 0.0608740378256158 | 0.111956970511653 | -0.014135435 | 0.224490793421708 | 1.1109079781547 | 0.985964001328617 | 1.2516851875573 | 0.97261503632431 |
| ebi-a-GCST90001890 | finngen_R11_O15_PREECLAMPS | Pre-eclampsia \|\| id:finngen_R11_O15_PREECLAMPS | CD28 on CD45RA+ CD4+ T cell \|\| id:ebi-a-GCST90001890 | Weighted median | 13 | 0.0590871846950402 | 0.0370389031972676 | 0.110651064985566 | -0.013509066 | 0.131683434961685 | 1.06086772813771 | 0.986581772349491 | 1.14074714143958 | 0.88227178111339 |
| ebi-a-GCST90001890 | finngen_R11_O15_PREECLAMPS | Pre-eclampsia \|\| id:finngen_R11_O15_PREECLAMPS | CD28 on CD45RA+ CD4+ T cell \|\| id:ebi-a-GCST90001890 | Weighted mode | 13 | 0.0716589479372399 | 0.0390349249604488 | 0.0912819619554957 | -0.004849505 | 0.14816740085972 | 1.07428889313494 | 0.995162234878885 | 1.15970701606613 | 0.872755742358858 |
| ebi-a-GCST90001891 | finngen_R11_O15_PREECLAMPS | Pre-eclampsia \|\| id:finngen_R11_O15_PREECLAMPS | CD28 on CD45RA- CD4 not regulatory T cell \|\| id:ebi-a-GCST90001891 | Inverse variance weighted | 13 | 0.036909158438793 | 0.0258489043042553 | 0.153326123157069 | -0.013754694 | 0.0875730108751333 | 1.03759875946558 | 0.986339469581127 | 1.09152195450692 | 0.85505992597664 |
| ebi-a-GCST90001891 | finngen_R11_O15_PREECLAMPS | Pre-eclampsia \|\| id:finngen_R11_O15_PREECLAMPS | CD28 on CD45RA- CD4 not regulatory T cell \|\| id:ebi-a-GCST90001891 | MR Egger | 13 | -0.038293129 | 0.0511559785050058 | 0.469828081267688 | -0.138558847 | 0.0619725891226528 | 0.962430783409218 | 0.87061201718975 | 1.0639331809864 | 0.99380795269851 |
| ebi-a-GCST90001891 | finngen_R11_O15_PREECLAMPS | Pre-eclampsia \|\| id:finngen_R11_O15_PREECLAMPS | CD28 on CD45RA- CD4 not regulatory T cell \|\| id:ebi-a-GCST90001891 | Weighted median | 13 | 0.00941261683030118 | 0.0337330898363113 | 0.780220146473401 | -0.056704239 | 0.0755294729094713 | 1.0094570548246 | 0.944873484535977 | 1.07845501245661 | 0.957984984509652 |
| ebi-a-GCST90001891 | finngen_R11_O15_PREECLAMPS | Pre-eclampsia \|\| id:finngen_R11_O15_PREECLAMPS | CD28 on CD45RA- CD4 not regulatory T cell \|\| id:ebi-a-GCST90001891 | Weighted mode | 13 | 0.00617234395945743 | 0.0339624630309256 | 0.858820416633095 | -0.060394084 | 0.0727387715000715 | 1.00619143212714 | 0.941393472763538 | 1.07544956214113 | 0.977585971930092 |
| ebi-a-GCST90001892 | finngen_R11_O15_PREECLAMPS | Pre-eclampsia \|\| id:finngen_R11_O15_PREECLAMPS | CD28 on CD39+ CD4+ T cell \|\| id:ebi-a-GCST90001892 | Inverse variance weighted | 17 | 0.0377222375079921 | 0.0216329382313242 | 0.0812042935491715 | -0.004678321 | 0.0801227964413876 | 1.03844275236911 | 0.995332604874721 | 1.08342009963963 | 0.789997151112252 |
| ebi-a-GCST90001892 | finngen_R11_O15_PREECLAMPS | Pre-eclampsia \|\| id:finngen_R11_O15_PREECLAMPS | CD28 on CD39+ CD4+ T cell \|\| id:ebi-a-GCST90001892 | MR Egger | 17 | 0.104652685676962 | 0.0286097134264306 | 0.00233158115883096 | 0.0485776473611578 | 0.160727723992766 | 1.1103249116352 | 1.04977688106041 | 1.17436517381903 | 0.565056961366001 |
| ebi-a-GCST90001892 | finngen_R11_O15_PREECLAMPS | Pre-eclampsia \|\| id:finngen_R11_O15_PREECLAMPS | CD28 on CD39+ CD4+ T cell \|\| id:ebi-a-GCST90001892 | Weighted median | 17 | 0.04432835290422 | 0.0234564310262386 | 0.0587824916553985 | -0.001646252 | 0.0903029577156476 | 1.04532553421841 | 0.998355102422172 | 1.0945058224653 | 0.771214851704188 |
| ebi-a-GCST90001892 | finngen_R11_O15_PREECLAMPS | Pre-eclampsia \|\| id:finngen_R11_O15_PREECLAMPS | CD28 on CD39+ CD4+ T cell \|\| id:ebi-a-GCST90001892 | Weighted mode | 17 | 0.0549681638434009 | 0.0242758727516018 | 0.037800984784643 | 0.00738745325026132 | 0.102548874436541 | 1.05650697901845 | 1.00741480780169 | 1.10799145304445 | 0.838871423749761 |
| ebi-a-GCST90001893 | finngen_R11_O15_PREECLAMPS | Pre-eclampsia \|\| id:finngen_R11_O15_PREECLAMPS | CD28 on CD28+ CD45RA- CD8+ T cell \|\| id:ebi-a-GCST90001893 | Inverse variance weighted | 17 | 0.00422861169350635 | 0.032894936165912 | 0.897714510364634 | -0.060245463 | 0.0687026865786939 | 1.00423756488735 | 0.941533393425384 | 1.07111770413355 | 0.986667691552896 |
| ebi-a-GCST90001893 | finngen_R11_O15_PREECLAMPS | Pre-eclampsia \|\| id:finngen_R11_O15_PREECLAMPS | CD28 on CD28+ CD45RA- CD8+ T cell \|\| id:ebi-a-GCST90001893 | MR Egger | 17 | -0.033305031 | 0.0677613582856914 | 0.630183619358853 | -0.166117293 | 0.0995072309815697 | 0.96724347509195 | 0.846946886999839 | 1.10462645824464 | 0.99380795269851 |
| ebi-a-GCST90001893 | finngen_R11_O15_PREECLAMPS | Pre-eclampsia \|\| id:finngen_R11_O15_PREECLAMPS | CD28 on CD28+ CD45RA- CD8+ T cell \|\| id:ebi-a-GCST90001893 | Weighted median | 17 | -0.012059375 | 0.0456618696269013 | 0.791701592532227 | -0.10155664 | 0.0774378893598876 | 0.98801304773808 | 0.90343000799906 | 1.08051511888866 | 0.957984984509652 |
| ebi-a-GCST90001893 | finngen_R11_O15_PREECLAMPS | Pre-eclampsia \|\| id:finngen_R11_O15_PREECLAMPS | CD28 on CD28+ CD45RA- CD8+ T cell \|\| id:ebi-a-GCST90001893 | Weighted mode | 17 | -0.060372281 | 0.066714426412253 | 0.378924336133797 | -0.191132556 | 0.0703879950612266 | 0.94141399807091 | 0.826023086185345 | 1.07292438986989 | 0.920254054953283 |
| ebi-a-GCST90001894 | finngen_R11_O15_PREECLAMPS | Pre-eclampsia \|\| id:finngen_R11_O15_PREECLAMPS | CD28 on CD28+ CD4+ T cell \|\| id:ebi-a-GCST90001894 | Inverse variance weighted | 26 | 0.0010997016662322 | 0.0048516947039933 | 0.82068548113399 | -0.00840962 | 0.0106090232860591 | 1.00110030655982 | 0.991625641984555 | 1.01066549851258 | 0.977824840984872 |
| ebi-a-GCST90001894 | finngen_R11_O15_PREECLAMPS | Pre-eclampsia \|\| id:finngen_R11_O15_PREECLAMPS | CD28 on CD28+ CD4+ T cell \|\| id:ebi-a-GCST90001894 | MR Egger | 26 | 0.000594392544675666 | 0.00505026949929736 | 0.90728862532161 | -0.009304136 | 0.0104929207632985 | 1.00059456923093 | 0.990739013869609 | 1.01054816451005 | 0.99380795269851 |
| ebi-a-GCST90001894 | finngen_R11_O15_PREECLAMPS | Pre-eclampsia \|\| id:finngen_R11_O15_PREECLAMPS | CD28 on CD28+ CD4+ T cell \|\| id:ebi-a-GCST90001894 | Weighted median | 26 | 0.0017699680002862 | 0.00543188540349453 | 0.744539498719427 | -0.008876527 | 0.0124164633911355 | 1.00177153531821 | 0.991162752669265 | 1.01249386770356 | 0.957984984509652 |
| ebi-a-GCST90001894 | finngen_R11_O15_PREECLAMPS | Pre-eclampsia \|\| id:finngen_R11_O15_PREECLAMPS | CD28 on CD28+ CD4+ T cell \|\| id:ebi-a-GCST90001894 | Weighted mode | 26 | 0.000847409130822818 | 0.0052584210720689 | 0.873268318644058 | -0.009459096 | 0.0111539144320779 | 1.00084776828338 | 0.990585500354705 | 1.01121635125806 | 0.977585971930092 |
| ebi-a-GCST90001895 | finngen_R11_O15_PREECLAMPS | Pre-eclampsia \|\| id:finngen_R11_O15_PREECLAMPS | CD28 on CD28+ CD4-CD8- T cell \|\| id:ebi-a-GCST90001895 | Inverse variance weighted | 23 | 0.00191529052796128 | 0.00530787743208645 | 0.718219617194442 | -0.008488149 | 0.0123187302948507 | 1.00191712586841 | 0.991547773389094 | 1.01239491837829 | 0.959745573017418 |
| ebi-a-GCST90001895 | finngen_R11_O15_PREECLAMPS | Pre-eclampsia \|\| id:finngen_R11_O15_PREECLAMPS | CD28 on CD28+ CD4-CD8- T cell \|\| id:ebi-a-GCST90001895 | MR Egger | 23 | -0.000592225 | 0.00516119877231649 | 0.909736796756196 | -0.010708175 | 0.00952372451537988 | 0.999407950252298 | 0.989348953734818 | 1.00956921949221 | 0.99380795269851 |
| ebi-a-GCST90001895 | finngen_R11_O15_PREECLAMPS | Pre-eclampsia \|\| id:finngen_R11_O15_PREECLAMPS | CD28 on CD28+ CD4-CD8- T cell \|\| id:ebi-a-GCST90001895 | Weighted median | 23 | 0.00155424750388795 | 0.00546466154612533 | 0.776090151000886 | -0.009156489 | 0.0122649841342936 | 1.00155545597255 | 0.990885303863751 | 1.01234050750066 | 0.957984984509652 |
| ebi-a-GCST90001895 | finngen_R11_O15_PREECLAMPS | Pre-eclampsia \|\| id:finngen_R11_O15_PREECLAMPS | CD28 on CD28+ CD4-CD8- T cell \|\| id:ebi-a-GCST90001895 | Weighted mode | 23 | -0.002069353 | 0.00550369273228019 | 0.71052302394962 | -0.012856591 | 0.00871788451910208 | 0.997932786399099 | 0.987225701928202 | 1.00875599594417 | 0.959313411571287 |
| ebi-a-GCST90001896 | finngen_R11_O15_PREECLAMPS | Pre-eclampsia \|\| id:finngen_R11_O15_PREECLAMPS | CD28 on CD28+ CD45RA+ CD8+ T cell \|\| id:ebi-a-GCST90001896 | Inverse variance weighted | 15 | 0.0146859840146745 | 0.0188761559335401 | 0.436558560204156 | -0.022311282 | 0.0516832496444132 | 1.01479435292942 | 0.977935774239907 | 1.05304213820979 | 0.898694924660397 |
| ebi-a-GCST90001896 | finngen_R11_O15_PREECLAMPS | Pre-eclampsia \|\| id:finngen_R11_O15_PREECLAMPS | CD28 on CD28+ CD45RA+ CD8+ T cell \|\| id:ebi-a-GCST90001896 | MR Egger | 15 | 0.0300542232704733 | 0.0283720022672352 | 0.308752814586615 | -0.025554901 | 0.0856633477142543 | 1.0305104100833 | 0.974768861542467 | 1.08943950426323 | 0.99380795269851 |
| ebi-a-GCST90001896 | finngen_R11_O15_PREECLAMPS | Pre-eclampsia \|\| id:finngen_R11_O15_PREECLAMPS | CD28 on CD28+ CD45RA+ CD8+ T cell \|\| id:ebi-a-GCST90001896 | Weighted median | 15 | 0.00699251671270386 | 0.0259389525975496 | 0.787486536584102 | -0.04384783 | 0.0578328638039011 | 1.00701702144097 | 0.957099587874535 | 1.05953789377744 | 0.957984984509652 |
| ebi-a-GCST90001896 | finngen_R11_O15_PREECLAMPS | Pre-eclampsia \|\| id:finngen_R11_O15_PREECLAMPS | CD28 on CD28+ CD45RA+ CD8+ T cell \|\| id:ebi-a-GCST90001896 | Weighted mode | 15 | 0.00942509351511123 | 0.0263352945240625 | 0.725765227466607 | -0.042192084 | 0.0610422707822738 | 1.00946964958068 | 0.958685614955481 | 1.06294384470539 | 0.959313411571287 |
| ebi-a-GCST90001897 | finngen_R11_O15_PREECLAMPS | Pre-eclampsia \|\| id:finngen_R11_O15_PREECLAMPS | CD28 on CD39+ CD8+ T cell \|\| id:ebi-a-GCST90001897 | Inverse variance weighted | 20 | 0.00798769048516888 | 0.0121913540671362 | 0.512343466859965 | -0.015907363 | 0.0318827444567558 | 1.00801967719474 | 0.984218490401922 | 1.03239644400182 | 0.898694924660397 |
| ebi-a-GCST90001897 | finngen_R11_O15_PREECLAMPS | Pre-eclampsia \|\| id:finngen_R11_O15_PREECLAMPS | CD28 on CD39+ CD8+ T cell \|\| id:ebi-a-GCST90001897 | MR Egger | 20 | 0.00291662834299286 | 0.0145091443710775 | 0.842936958338525 | -0.025521295 | 0.0313545513103048 | 1.00292088584161 | 0.974801620710424 | 1.03185128326343 | 0.99380795269851 |
| ebi-a-GCST90001897 | finngen_R11_O15_PREECLAMPS | Pre-eclampsia \|\| id:finngen_R11_O15_PREECLAMPS | CD28 on CD39+ CD8+ T cell \|\| id:ebi-a-GCST90001897 | Weighted median | 20 | 0.00154127979936499 | 0.0169957567326761 | 0.927741973411212 | -0.031770403 | 0.0348529629954102 | 1.00154246818154 | 0.968728973429908 | 1.03546744557419 | 0.977431269406605 |
| ebi-a-GCST90001897 | finngen_R11_O15_PREECLAMPS | Pre-eclampsia \|\| id:finngen_R11_O15_PREECLAMPS | CD28 on CD39+ CD8+ T cell \|\| id:ebi-a-GCST90001897 | Weighted mode | 20 | 0.00273896522409173 | 0.0138682123593047 | 0.845534208754091 | -0.024442731 | 0.029920661448329 | 1.00274271961628 | 0.975853573476323 | 1.0303727824263 | 0.977585971930092 |
| ebi-a-GCST90001898 | finngen_R11_O15_PREECLAMPS | Pre-eclampsia \|\| id:finngen_R11_O15_PREECLAMPS | CD28 on CD4+ T cell \|\| id:ebi-a-GCST90001898 | Inverse variance weighted | 27 | 0.000811861886133287 | 0.00560895321400041 | 0.884913059606485 | -0.010181686 | 0.0118054101855741 | 1.0008121915352 | 0.989869971485638 | 1.01187536906712 | 0.986084522213934 |
| ebi-a-GCST90001898 | finngen_R11_O15_PREECLAMPS | Pre-eclampsia \|\| id:finngen_R11_O15_PREECLAMPS | CD28 on CD4+ T cell \|\| id:ebi-a-GCST90001898 | MR Egger | 27 | 0.00195811746570719 | 0.00591332939066625 | 0.743301233358281 | -0.009632008 | 0.013548243071413 | 1.00196003582964 | 0.990414231072496 | 1.01364043639862 | 0.99380795269851 |
| ebi-a-GCST90001898 | finngen_R11_O15_PREECLAMPS | Pre-eclampsia \|\| id:finngen_R11_O15_PREECLAMPS | CD28 on CD4+ T cell \|\| id:ebi-a-GCST90001898 | Weighted median | 27 | 0.00210477049743762 | 0.0059413484399291 | 0.723145504936176 | -0.009540272 | 0.0137498134396986 | 1.00210698708172 | 0.990505091578007 | 1.01384477686849 | 0.949957918887556 |
| ebi-a-GCST90001898 | finngen_R11_O15_PREECLAMPS | Pre-eclampsia \|\| id:finngen_R11_O15_PREECLAMPS | CD28 on CD4+ T cell \|\| id:ebi-a-GCST90001898 | Weighted mode | 27 | 0.002037320553964 | 0.00504406558434941 | 0.689586000384808 | -0.007849048 | 0.0119236890992888 | 1.00203939730158 | 0.99218167535028 | 1.01199505966488 | 0.959313411571287 |
| ebi-a-GCST90001899 | finngen_R11_O15_PREECLAMPS | Pre-eclampsia \|\| id:finngen_R11_O15_PREECLAMPS | CD28 on CD4 regulatory T cell \|\| id:ebi-a-GCST90001899 | Inverse variance weighted | 25 | -0.00213502 | 0.00897265489882103 | 0.811921863090834 | -0.019721424 | 0.0154513833719851 | 0.997867257304837 | 0.980471771335174 | 1.01557137320243 | 0.977824840984872 |
| ebi-a-GCST90001899 | finngen_R11_O15_PREECLAMPS | Pre-eclampsia \|\| id:finngen_R11_O15_PREECLAMPS | CD28 on CD4 regulatory T cell \|\| id:ebi-a-GCST90001899 | MR Egger | 25 | -0.008676341 | 0.0095314861332008 | 0.372115281523339 | -0.027358054 | 0.0100053715304804 | 0.991361189536646 | 0.973012787916018 | 1.010055593 | 0.99380795269851 |
| ebi-a-GCST90001899 | finngen_R11_O15_PREECLAMPS | Pre-eclampsia \|\| id:finngen_R11_O15_PREECLAMPS | CD28 on CD4 regulatory T cell \|\| id:ebi-a-GCST90001899 | Weighted median | 25 | -0.004207628 | 0.0107771589189396 | 0.6962253542789 | -0.025330859 | 0.0169156036671311 | 0.995801211849569 | 0.974987275054987 | 1.01705948261237 | 0.946704357535493 |
| ebi-a-GCST90001899 | finngen_R11_O15_PREECLAMPS | Pre-eclampsia \|\| id:finngen_R11_O15_PREECLAMPS | CD28 on CD4 regulatory T cell \|\| id:ebi-a-GCST90001899 | Weighted mode | 25 | -0.007815367 | 0.0090677482429358 | 0.397280316884269 | -0.025588154 | 0.0099574195432639 | 0.992215093562702 | 0.974736448681437 | 1.01000715960237 | 0.920286500917063 |
| ebi-a-GCST90001900 | finngen_R11_O15_PREECLAMPS | Pre-eclampsia \|\| id:finngen_R11_O15_PREECLAMPS | CD28 on resting CD4 regulatory T cell \|\| id:ebi-a-GCST90001900 | Inverse variance weighted | 21 | 0.0294334299331232 | 0.0247565093991748 | 0.234472408496504 | -0.019089328 | 0.0779561883555058 | 1.02987087461687 | 0.981091718887267 | 1.08107529394608 | 0.884905053585426 |
| ebi-a-GCST90001900 | finngen_R11_O15_PREECLAMPS | Pre-eclampsia \|\| id:finngen_R11_O15_PREECLAMPS | CD28 on resting CD4 regulatory T cell \|\| id:ebi-a-GCST90001900 | MR Egger | 21 | 0.0635214584825842 | 0.0428084465149909 | 0.154248068842246 | -0.020383097 | 0.147426013651966 | 1.06558235133646 | 0.979823234361908 | 1.1588475427603 | 0.99380795269851 |
| ebi-a-GCST90001900 | finngen_R11_O15_PREECLAMPS | Pre-eclampsia \|\| id:finngen_R11_O15_PREECLAMPS | CD28 on resting CD4 regulatory T cell \|\| id:ebi-a-GCST90001900 | Weighted median | 21 | 0.0440877980786827 | 0.0300786130998598 | 0.142715764481452 | -0.014866284 | 0.103041879754408 | 1.0450741063592 | 0.985243674035468 | 1.1085378333961 | 0.915133542420536 |
| ebi-a-GCST90001900 | finngen_R11_O15_PREECLAMPS | Pre-eclampsia \|\| id:finngen_R11_O15_PREECLAMPS | CD28 on resting CD4 regulatory T cell \|\| id:ebi-a-GCST90001900 | Weighted mode | 21 | 0.0493880887831877 | 0.0323342199459453 | 0.142316467200785 | -0.013986982 | 0.11276315987724 | 1.05062800857663 | 0.986110381057702 | 1.11936679058356 | 0.872755742358858 |
| ebi-a-GCST90001901 | finngen_R11_O15_PREECLAMPS | Pre-eclampsia \|\| id:finngen_R11_O15_PREECLAMPS | CD28 on CD39+ resting CD4 regulatory T cell \|\| id:ebi-a-GCST90001901 | Inverse variance weighted | 22 | 0.00892276885670287 | 0.0110654836312766 | 0.420035018561655 | -0.012765579 | 0.0306111167740049 | 1.00896269542222 | 0.987315555334311 | 1.03108445446196 | 0.898694924660397 |
| ebi-a-GCST90001901 | finngen_R11_O15_PREECLAMPS | Pre-eclampsia \|\| id:finngen_R11_O15_PREECLAMPS | CD28 on CD39+ resting CD4 regulatory T cell \|\| id:ebi-a-GCST90001901 | MR Egger | 22 | 0.00628003107621962 | 0.0126512701976568 | 0.625027240173398 | -0.018516459 | 0.031076520663627 | 1.00629979181574 | 0.981653917896844 | 1.03156443686177 | 0.99380795269851 |
| ebi-a-GCST90001901 | finngen_R11_O15_PREECLAMPS | Pre-eclampsia \|\| id:finngen_R11_O15_PREECLAMPS | CD28 on CD39+ resting CD4 regulatory T cell \|\| id:ebi-a-GCST90001901 | Weighted median | 22 | 0.0144888288462693 | 0.0142770963765605 | 0.310186766047024 | -0.01349428 | 0.0424719377443279 | 1.01459430069918 | 0.986596359581675 | 1.04338677617637 | 0.940845681141708 |
| ebi-a-GCST90001901 | finngen_R11_O15_PREECLAMPS | Pre-eclampsia \|\| id:finngen_R11_O15_PREECLAMPS | CD28 on CD39+ resting CD4 regulatory T cell \|\| id:ebi-a-GCST90001901 | Weighted mode | 22 | 0.0108195356200633 | 0.0135471447138627 | 0.433428873328097 | -0.015732868 | 0.0373719392592341 | 1.01087827846118 | 0.984390247052283 | 1.03807905139715 | 0.933945646747087 |
| ebi-a-GCST90001902 | finngen_R11_O15_PREECLAMPS | Pre-eclampsia \|\| id:finngen_R11_O15_PREECLAMPS | CD28 on activated CD4 regulatory T cell \|\| id:ebi-a-GCST90001902 | Inverse variance weighted | 17 | -0.00194864 | 0.0218794032102211 | 0.929032038101733 | -0.04483227 | 0.0409349903966393 | 0.998053257470699 | 0.956157844559977 | 1.04178437735485 | 0.986667691552896 |
| ebi-a-GCST90001902 | finngen_R11_O15_PREECLAMPS | Pre-eclampsia \|\| id:finngen_R11_O15_PREECLAMPS | CD28 on activated CD4 regulatory T cell \|\| id:ebi-a-GCST90001902 | MR Egger | 17 | 0.00828677694011353 | 0.0305721898764244 | 0.790042477997056 | -0.051634715 | 0.0682082690979053 | 1.00832120731604 | 0.949675705574965 | 1.0705882557117 | 0.99380795269851 |
| ebi-a-GCST90001902 | finngen_R11_O15_PREECLAMPS | Pre-eclampsia \|\| id:finngen_R11_O15_PREECLAMPS | CD28 on activated CD4 regulatory T cell \|\| id:ebi-a-GCST90001902 | Weighted median | 17 | -0.032248441 | 0.0310779987685136 | 0.299427700831038 | -0.093161319 | 0.0286644364921064 | 0.968265995131305 | 0.911046519618706 | 1.0290792150987 | 0.940845681141708 |
| ebi-a-GCST90001902 | finngen_R11_O15_PREECLAMPS | Pre-eclampsia \|\| id:finngen_R11_O15_PREECLAMPS | CD28 on activated CD4 regulatory T cell \|\| id:ebi-a-GCST90001902 | Weighted mode | 17 | -0.04106519 | 0.0286757164483434 | 0.171374574601908 | -0.097269595 | 0.0151392138877997 | 0.95976656038945 | 0.907311366922349 | 1.01525439228916 | 0.872755742358858 |
| ebi-a-GCST90001903 | finngen_R11_O15_PREECLAMPS | Pre-eclampsia \|\| id:finngen_R11_O15_PREECLAMPS | CD86 on myeloid Dendritic Cell \|\| id:ebi-a-GCST90001903 | Inverse variance weighted | 22 | -0.04814664 | 0.0270343108029363 | 0.074921316878556 | -0.10113389 | 0.00484060877311908 | 0.952994029404419 | 0.903812013778592 | 1.00485234344645 | 0.789997151112252 |
| ebi-a-GCST90001903 | finngen_R11_O15_PREECLAMPS | Pre-eclampsia \|\| id:finngen_R11_O15_PREECLAMPS | CD86 on myeloid Dendritic Cell \|\| id:ebi-a-GCST90001903 | MR Egger | 22 | -0.057112062 | 0.0537489755253199 | 0.300639411711526 | -0.162460054 | 0.0482359296715678 | 0.944488221858573 | 0.850050045316773 | 1.04941821501479 | 0.99380795269851 |
| ebi-a-GCST90001903 | finngen_R11_O15_PREECLAMPS | Pre-eclampsia \|\| id:finngen_R11_O15_PREECLAMPS | CD86 on myeloid Dendritic Cell \|\| id:ebi-a-GCST90001903 | Weighted median | 22 | -0.074889593 | 0.0348976798634868 | 0.0318748710274041 | -0.143289046 | -0.006490141 | 0.927845921094487 | 0.866503573461771 | 0.993530874722537 | 0.721097500353414 |
| ebi-a-GCST90001903 | finngen_R11_O15_PREECLAMPS | Pre-eclampsia \|\| id:finngen_R11_O15_PREECLAMPS | CD86 on myeloid Dendritic Cell \|\| id:ebi-a-GCST90001903 | Weighted mode | 22 | -0.058942798 | 0.0439676625403764 | 0.194375554110707 | -0.145119416 | 0.0272338206971138 | 0.942760695523262 | 0.864919001377162 | 1.0276080507057 | 0.872755742358858 |
| ebi-a-GCST90001904 | finngen_R11_O15_PREECLAMPS | Pre-eclampsia \|\| id:finngen_R11_O15_PREECLAMPS | CD86 on CD62L+ myeloid Dendritic Cell \|\| id:ebi-a-GCST90001904 | Inverse variance weighted (multiplicative random effects) | 22 | -0.021482947 | 0.023614409586774 | 0.362960222130024 | -0.06776719 | 0.0248012957030649 | 0.978746167800075 | 0.93447800419358 | 1.02511140624439 | 0.885079385681701 |
| ebi-a-GCST90001904 | finngen_R11_O15_PREECLAMPS | Pre-eclampsia \|\| id:finngen_R11_O15_PREECLAMPS | CD86 on CD62L+ myeloid Dendritic Cell \|\| id:ebi-a-GCST90001904 | MR Egger | 22 | -0.006601947 | 0.0351663886346808 | 0.852976330637746 | -0.075528068 | 0.0623241751448166 | 0.993419798390801 | 0.9272537037307 | 1.06430731078691 | 0.99380795269851 |
| ebi-a-GCST90001904 | finngen_R11_O15_PREECLAMPS | Pre-eclampsia \|\| id:finngen_R11_O15_PREECLAMPS | CD86 on CD62L+ myeloid Dendritic Cell \|\| id:ebi-a-GCST90001904 | Weighted median | 22 | 0.0272509235161075 | 0.0324406604810951 | 0.400895100632136 | -0.036332771 | 0.090834618059054 | 1.02762562585048 | 0.964319342546679 | 1.09508788252215 | 0.940845681141708 |
| ebi-a-GCST90001904 | finngen_R11_O15_PREECLAMPS | Pre-eclampsia \|\| id:finngen_R11_O15_PREECLAMPS | CD86 on CD62L+ myeloid Dendritic Cell \|\| id:ebi-a-GCST90001904 | Weighted mode | 22 | 0.0352464706655392 | 0.0396624954689092 | 0.384252243066346 | -0.04249202 | 0.112984961784601 | 1.03587499013688 | 0.958398113072722 | 1.11961509580901 | 0.920286500917063 |
| ebi-a-GCST90001905 | finngen_R11_O15_PREECLAMPS | Pre-eclampsia \|\| id:finngen_R11_O15_PREECLAMPS | CD86 on monocyte \|\| id:ebi-a-GCST90001905 | Inverse variance weighted (multiplicative random effects) | 16 | 0.039788959905322 | 0.0265260819680095 | 0.133615994745185 | -0.012202161 | 0.0917800805626206 | 1.0405911445642 | 0.987871983730827 | 1.09612373665664 | 0.834645702826663 |
| ebi-a-GCST90001905 | finngen_R11_O15_PREECLAMPS | Pre-eclampsia \|\| id:finngen_R11_O15_PREECLAMPS | CD86 on monocyte \|\| id:ebi-a-GCST90001905 | MR Egger | 16 | 0.0213752798874442 | 0.0374006958931911 | 0.576707700225617 | -0.051930084 | 0.0946806438380988 | 1.02160536765502 | 0.949395242380263 | 1.09930772836496 | 0.99380795269851 |
| ebi-a-GCST90001905 | finngen_R11_O15_PREECLAMPS | Pre-eclampsia \|\| id:finngen_R11_O15_PREECLAMPS | CD86 on monocyte \|\| id:ebi-a-GCST90001905 | Weighted median | 16 | 0.0423990499931293 | 0.0256859253220637 | 0.0988054705963329 | -0.007945364 | 0.0927434636243742 | 1.04331072883212 | 0.992086117332461 | 1.09718023252152 | 0.88227178111339 |
| ebi-a-GCST90001905 | finngen_R11_O15_PREECLAMPS | Pre-eclampsia \|\| id:finngen_R11_O15_PREECLAMPS | CD86 on monocyte \|\| id:ebi-a-GCST90001905 | Weighted mode | 16 | 0.0443183230179917 | 0.0255641232344873 | 0.103485971853117 | -0.005787359 | 0.0944240045575867 | 1.04531504977481 | 0.994229355977914 | 1.09902563901964 | 0.872755742358858 |
| ebi-a-GCST90001906 | finngen_R11_O15_PREECLAMPS | Pre-eclampsia \|\| id:finngen_R11_O15_PREECLAMPS | CD86 on granulocyte \|\| id:ebi-a-GCST90001906 | Inverse variance weighted | 21 | -0.002141346 | 0.0237323062616895 | 0.928105117514008 | -0.048656666 | 0.04437397436378 | 0.997860945136421 | 0.952508101808231 | 1.04537322458284 | 0.986667691552896 |
| ebi-a-GCST90001906 | finngen_R11_O15_PREECLAMPS | Pre-eclampsia \|\| id:finngen_R11_O15_PREECLAMPS | CD86 on granulocyte \|\| id:ebi-a-GCST90001906 | MR Egger | 21 | -0.037240665 | 0.0410810261622066 | 0.376015589432009 | -0.117759476 | 0.0432781460850011 | 0.963444239951718 | 0.888909830633826 | 1.04422830247505 | 0.99380795269851 |
| ebi-a-GCST90001906 | finngen_R11_O15_PREECLAMPS | Pre-eclampsia \|\| id:finngen_R11_O15_PREECLAMPS | CD86 on granulocyte \|\| id:ebi-a-GCST90001906 | Weighted median | 21 | -0.024318205 | 0.0346204504036306 | 0.482415334736991 | -0.092174287 | 0.0435378782059039 | 0.975975100589839 | 0.91194619498393 | 1.04449955733203 | 0.940845681141708 |
| ebi-a-GCST90001906 | finngen_R11_O15_PREECLAMPS | Pre-eclampsia \|\| id:finngen_R11_O15_PREECLAMPS | CD86 on granulocyte \|\| id:ebi-a-GCST90001906 | Weighted mode | 21 | -0.021036374 | 0.0429376780383803 | 0.629515867018076 | -0.105194223 | 0.0631214753897558 | 0.979183347532106 | 0.900149676268141 | 1.06515622163993 | 0.959313411571287 |
| ebi-a-GCST90001907 | finngen_R11_O15_PREECLAMPS | Pre-eclampsia \|\| id:finngen_R11_O15_PREECLAMPS | CCR7 on naive CD4+ T cell \|\| id:ebi-a-GCST90001907 | Inverse variance weighted | 24 | 0.00979956720410211 | 0.0134364144331514 | 0.465800414176297 | -0.016535805 | 0.0361349394930788 | 1.00984774019235 | 0.983600160872932 | 1.03679574174383 | 0.898694924660397 |
| ebi-a-GCST90001907 | finngen_R11_O15_PREECLAMPS | Pre-eclampsia \|\| id:finngen_R11_O15_PREECLAMPS | CCR7 on naive CD4+ T cell \|\| id:ebi-a-GCST90001907 | MR Egger | 24 | 0.00821522344716276 | 0.0172781097677394 | 0.639138960687524 | -0.025649872 | 0.042080318591932 | 1.00824906099284 | 0.974676291628393 | 1.04297824593082 | 0.99380795269851 |
| ebi-a-GCST90001907 | finngen_R11_O15_PREECLAMPS | Pre-eclampsia \|\| id:finngen_R11_O15_PREECLAMPS | CCR7 on naive CD4+ T cell \|\| id:ebi-a-GCST90001907 | Weighted median | 24 | 0.00830642333785886 | 0.0182940009612868 | 0.649791572742488 | -0.027549819 | 0.0441626652219809 | 1.00834101739014 | 0.972826216558336 | 1.04515235100102 | 0.940845681141708 |
| ebi-a-GCST90001907 | finngen_R11_O15_PREECLAMPS | Pre-eclampsia \|\| id:finngen_R11_O15_PREECLAMPS | CCR7 on naive CD4+ T cell \|\| id:ebi-a-GCST90001907 | Weighted mode | 24 | 0.00729676808827917 | 0.0164755822036301 | 0.661987282155004 | -0.024995373 | 0.0395889092073942 | 1.00732345436893 | 0.975314424767661 | 1.04038299440046 | 0.959313411571287 |
| ebi-a-GCST90001908 | finngen_R11_O15_PREECLAMPS | Pre-eclampsia \|\| id:finngen_R11_O15_PREECLAMPS | CCR7 on naive CD8+ T cell \|\| id:ebi-a-GCST90001908 | Inverse variance weighted | 24 | -0.009695178 | 0.0189551495879472 | 0.60901504641972 | -0.046847272 | 0.0274569148243138 | 0.990351668355712 | 0.954233124997886 | 1.02783732960131 | 0.92242199665996 |
| ebi-a-GCST90001908 | finngen_R11_O15_PREECLAMPS | Pre-eclampsia \|\| id:finngen_R11_O15_PREECLAMPS | CCR7 on naive CD8+ T cell \|\| id:ebi-a-GCST90001908 | MR Egger | 24 | -0.02952421 | 0.0303157104029361 | 0.340697856428111 | -0.088943003 | 0.0298945819658443 | 0.97090737127761 | 0.914897718684125 | 1.03034591118776 | 0.99380795269851 |
| ebi-a-GCST90001908 | finngen_R11_O15_PREECLAMPS | Pre-eclampsia \|\| id:finngen_R11_O15_PREECLAMPS | CCR7 on naive CD8+ T cell \|\| id:ebi-a-GCST90001908 | Weighted median | 24 | -0.004929781 | 0.0273031442446882 | 0.856715094334827 | -0.058443944 | 0.0485843814005918 | 0.995082350109646 | 0.943231112644605 | 1.04978395032313 | 0.964912412213677 |
| ebi-a-GCST90001908 | finngen_R11_O15_PREECLAMPS | Pre-eclampsia \|\| id:finngen_R11_O15_PREECLAMPS | CCR7 on naive CD8+ T cell \|\| id:ebi-a-GCST90001908 | Weighted mode | 24 | 0.00815510051652796 | 0.036729315156262 | 0.826248295218623 | -0.063834357 | 0.0801445582228014 | 1.00818844392673 | 0.93816038618387 | 1.08344367704756 | 0.97417339323357 |
| ebi-a-GCST90001909 | finngen_R11_O15_PREECLAMPS | Pre-eclampsia \|\| id:finngen_R11_O15_PREECLAMPS | CD45 on CD14+ monocyte \|\| id:ebi-a-GCST90001909 | Inverse variance weighted | 17 | -0.002122881 | 0.023541761239385 | 0.928148070272581 | -0.048264733 | 0.0440189713331222 | 0.997879371021495 | 0.952881494768996 | 1.04500217978486 | 0.986667691552896 |
| ebi-a-GCST90001909 | finngen_R11_O15_PREECLAMPS | Pre-eclampsia \|\| id:finngen_R11_O15_PREECLAMPS | CD45 on CD14+ monocyte \|\| id:ebi-a-GCST90001909 | MR Egger | 17 | -0.001454018 | 0.0336254122600178 | 0.966079292501108 | -0.067359826 | 0.0644517902810844 | 0.998547038823103 | 0.934858754530447 | 1.06657415776483 | 0.99380795269851 |
| ebi-a-GCST90001909 | finngen_R11_O15_PREECLAMPS | Pre-eclampsia \|\| id:finngen_R11_O15_PREECLAMPS | CD45 on CD14+ monocyte \|\| id:ebi-a-GCST90001909 | Weighted median | 17 | -0.013788611 | 0.0292050646740809 | 0.636833410196164 | -0.071030538 | 0.0434533154302525 | 0.986306016143821 | 0.931433447492826 | 1.04441123528473 | 0.940845681141708 |
| ebi-a-GCST90001909 | finngen_R11_O15_PREECLAMPS | Pre-eclampsia \|\| id:finngen_R11_O15_PREECLAMPS | CD45 on CD14+ monocyte \|\| id:ebi-a-GCST90001909 | Weighted mode | 17 | -0.012463766 | 0.027342852624334 | 0.654636002478538 | -0.066055757 | 0.0411282250893672 | 0.987613584982555 | 0.936078669712769 | 1.04198570569008 | 0.959313411571287 |
| ebi-a-GCST90001910 | finngen_R11_O15_PREECLAMPS | Pre-eclampsia \|\| id:finngen_R11_O15_PREECLAMPS | CD45 on B cell \|\| id:ebi-a-GCST90001910 | Inverse variance weighted | 16 | -0.013577897 | 0.012685017861813 | 0.284444472036802 | -0.038440532 | 0.0112847377266564 | 0.986513866575227 | 0.962288928155655 | 1.01134865056639 | 0.885079385681701 |
| ebi-a-GCST90001910 | finngen_R11_O15_PREECLAMPS | Pre-eclampsia \|\| id:finngen_R11_O15_PREECLAMPS | CD45 on B cell \|\| id:ebi-a-GCST90001910 | MR Egger | 16 | -0.034092627 | 0.0149751880984059 | 0.0390420256769072 | -0.063443996 | -0.004741259 | 0.966481977701002 | 0.938526678933353 | 0.995269963217714 | 0.839403552053506 |
| ebi-a-GCST90001910 | finngen_R11_O15_PREECLAMPS | Pre-eclampsia \|\| id:finngen_R11_O15_PREECLAMPS | CD45 on B cell \|\| id:ebi-a-GCST90001910 | Weighted median | 16 | -0.021415306 | 0.016770046560032 | 0.201603157554668 | -0.054284597 | 0.0114539850402497 | 0.978812373281054 | 0.947162508081817 | 1.01151983309429 | 0.940845681141708 |
| ebi-a-GCST90001910 | finngen_R11_O15_PREECLAMPS | Pre-eclampsia \|\| id:finngen_R11_O15_PREECLAMPS | CD45 on B cell \|\| id:ebi-a-GCST90001910 | Weighted mode | 16 | -0.023037245 | 0.0141499324278614 | 0.124324789519892 | -0.050771113 | 0.00469662243366304 | 0.977226086187785 | 0.950496202161367 | 1.00470766885165 | 0.872755742358858 |
| ebi-a-GCST90001911 | finngen_R11_O15_PREECLAMPS | Pre-eclampsia \|\| id:finngen_R11_O15_PREECLAMPS | CD45 on Natural Killer \|\| id:ebi-a-GCST90001911 | Inverse variance weighted | 29 | 0.0112153141696603 | 0.0180759046274193 | 0.534957224432667 | -0.024213459 | 0.0466440872394021 | 1.01127844158282 | 0.97607733512464 | 1.04774903545893 | 0.904087927705763 |
| ebi-a-GCST90001911 | finngen_R11_O15_PREECLAMPS | Pre-eclampsia \|\| id:finngen_R11_O15_PREECLAMPS | CD45 on Natural Killer \|\| id:ebi-a-GCST90001911 | MR Egger | 29 | -0.004017957 | 0.0270940048749246 | 0.883210449884069 | -0.057122207 | 0.0490862920842586 | 0.995990103720403 | 0.944478640388312 | 1.05031098035328 | 0.99380795269851 |
| ebi-a-GCST90001911 | finngen_R11_O15_PREECLAMPS | Pre-eclampsia \|\| id:finngen_R11_O15_PREECLAMPS | CD45 on Natural Killer \|\| id:ebi-a-GCST90001911 | Weighted median | 29 | -0.013684721 | 0.0267948627763926 | 0.609545872289922 | -0.066202652 | 0.0388332104457287 | 0.986408489523645 | 0.935941175059906 | 1.0395970752564 | 0.940845681141708 |
| ebi-a-GCST90001911 | finngen_R11_O15_PREECLAMPS | Pre-eclampsia \|\| id:finngen_R11_O15_PREECLAMPS | CD45 on Natural Killer \|\| id:ebi-a-GCST90001911 | Weighted mode | 29 | -0.012878088 | 0.024113735894666 | 0.597517720842484 | -0.060141011 | 0.0343848338584523 | 0.987204479268183 | 0.941631743930761 | 1.03498282653354 | 0.955353022288269 |
| ebi-a-GCST90001912 | finngen_R11_O15_PREECLAMPS | Pre-eclampsia \|\| id:finngen_R11_O15_PREECLAMPS | CD45 on HLA DR+ Natural Killer \|\| id:ebi-a-GCST90001912 | Inverse variance weighted | 12 | -0.052289815 | 0.0310908707954715 | 0.092600211826719 | -0.113227922 | 0.00864829142360751 | 0.949053776643435 | 0.89294711454388 | 1.00868579593456 | 0.802698559103897 |
| ebi-a-GCST90001912 | finngen_R11_O15_PREECLAMPS | Pre-eclampsia \|\| id:finngen_R11_O15_PREECLAMPS | CD45 on HLA DR+ Natural Killer \|\| id:ebi-a-GCST90001912 | MR Egger | 12 | -0.006435684 | 0.046294068343666 | 0.892196147993563 | -0.097172058 | 0.0843006899203519 | 0.993584980627082 | 0.907399867306783 | 1.08795598202788 | 0.99380795269851 |
| ebi-a-GCST90001912 | finngen_R11_O15_PREECLAMPS | Pre-eclampsia \|\| id:finngen_R11_O15_PREECLAMPS | CD45 on HLA DR+ Natural Killer \|\| id:ebi-a-GCST90001912 | Weighted median | 12 | -0.054067427 | 0.0425170214053786 | 0.203492016286374 | -0.137400789 | 0.0292659353931405 | 0.947368226566322 | 0.87162082050279 | 1.02969839131389 | 0.940845681141708 |
| ebi-a-GCST90001912 | finngen_R11_O15_PREECLAMPS | Pre-eclampsia \|\| id:finngen_R11_O15_PREECLAMPS | CD45 on HLA DR+ Natural Killer \|\| id:ebi-a-GCST90001912 | Weighted mode | 12 | -0.059059443 | 0.0425381010865536 | 0.192491955876875 | -0.142434121 | 0.024315235497416 | 0.94265073385078 | 0.867244685829514 | 1.02461326145744 | 0.872755742358858 |
| ebi-a-GCST90001913 | finngen_R11_O15_PREECLAMPS | Pre-eclampsia \|\| id:finngen_R11_O15_PREECLAMPS | CD45 on granulocyte \|\| id:ebi-a-GCST90001913 | Inverse variance weighted | 14 | 0.0331501665817644 | 0.0234540025869069 | 0.157534645880707 | -0.012819678 | 0.079120011652102 | 1.03370575564673 | 0.987262143572323 | 1.08233420699261 | 0.85505992597664 |
| ebi-a-GCST90001913 | finngen_R11_O15_PREECLAMPS | Pre-eclampsia \|\| id:finngen_R11_O15_PREECLAMPS | CD45 on granulocyte \|\| id:ebi-a-GCST90001913 | MR Egger | 14 | 0.0673105244424485 | 0.0294755618937914 | 0.0414076277840621 | 0.00953842313061733 | 0.12508262575428 | 1.06962757211575 | 1.00958405887079 | 1.13324208418059 | 0.840804886393039 |
| ebi-a-GCST90001913 | finngen_R11_O15_PREECLAMPS | Pre-eclampsia \|\| id:finngen_R11_O15_PREECLAMPS | CD45 on granulocyte \|\| id:ebi-a-GCST90001913 | Weighted median | 14 | 0.0517797344471396 | 0.0295969781572001 | 0.0802056723747038 | -0.006230343 | 0.109789811635252 | 1.05314374567446 | 0.993789025599687 | 1.11604346645302 | 0.875234669704391 |
| ebi-a-GCST90001913 | finngen_R11_O15_PREECLAMPS | Pre-eclampsia \|\| id:finngen_R11_O15_PREECLAMPS | CD45 on granulocyte \|\| id:ebi-a-GCST90001913 | Weighted mode | 14 | 0.0568051624826209 | 0.0258667822230936 | 0.0468346090819384 | 0.00610626932535746 | 0.107504055639884 | 1.0584495646185 | 1.00612495059282 | 1.11349537667364 | 0.838871423749761 |
| ebi-a-GCST90001914 | finngen_R11_O15_PREECLAMPS | Pre-eclampsia \|\| id:finngen_R11_O15_PREECLAMPS | CD45 on lymphocyte \|\| id:ebi-a-GCST90001914 | Inverse variance weighted | 24 | -0.004552531 | 0.0205599060426395 | 0.824759476449689 | -0.044849947 | 0.0357448844635417 | 0.99545781568322 | 0.956140942672555 | 1.03639141321048 | 0.977824840984872 |
| ebi-a-GCST90001914 | finngen_R11_O15_PREECLAMPS | Pre-eclampsia \|\| id:finngen_R11_O15_PREECLAMPS | CD45 on lymphocyte \|\| id:ebi-a-GCST90001914 | MR Egger | 24 | -0.015770404 | 0.0313002746835538 | 0.619380449975519 | -0.077118942 | 0.0455781347750645 | 0.984353298081362 | 0.925779732979023 | 1.04663277983599 | 0.99380795269851 |
| ebi-a-GCST90001914 | finngen_R11_O15_PREECLAMPS | Pre-eclampsia \|\| id:finngen_R11_O15_PREECLAMPS | CD45 on lymphocyte \|\| id:ebi-a-GCST90001914 | Weighted median | 24 | -0.027083796 | 0.0278725547967774 | 0.33119920692012 | -0.081714003 | 0.027546411746557 | 0.973279681498664 | 0.921535477341349 | 1.02792932199533 | 0.940845681141708 |
| ebi-a-GCST90001914 | finngen_R11_O15_PREECLAMPS | Pre-eclampsia \|\| id:finngen_R11_O15_PREECLAMPS | CD45 on lymphocyte \|\| id:ebi-a-GCST90001914 | Weighted mode | 24 | -0.010348701 | 0.029080607535837 | 0.725186740776509 | -0.067346692 | 0.0466492895374437 | 0.989704662335956 | 0.934871032835755 | 1.04775448617587 | 0.959313411571287 |
| ebi-a-GCST90001915 | finngen_R11_O15_PREECLAMPS | Pre-eclampsia \|\| id:finngen_R11_O15_PREECLAMPS | CD45 on T cell \|\| id:ebi-a-GCST90001915 | Inverse variance weighted | 15 | 0.00627133351229641 | 0.0229807056788119 | 0.784933467295044 | -0.03877085 | 0.0513135166427678 | 1.00629103949704 | 0.961971119941233 | 1.05265286574704 | 0.977824840984872 |
| ebi-a-GCST90001915 | finngen_R11_O15_PREECLAMPS | Pre-eclampsia \|\| id:finngen_R11_O15_PREECLAMPS | CD45 on T cell \|\| id:ebi-a-GCST90001915 | MR Egger | 15 | -0.011314807 | 0.0308293757085166 | 0.71951369550487 | -0.071740383 | 0.0491107695965501 | 0.988748964886206 | 0.930772508644531 | 1.05033668966786 | 0.99380795269851 |
| ebi-a-GCST90001915 | finngen_R11_O15_PREECLAMPS | Pre-eclampsia \|\| id:finngen_R11_O15_PREECLAMPS | CD45 on T cell \|\| id:ebi-a-GCST90001915 | Weighted median | 15 | 0.00630457406681974 | 0.0257547300498381 | 0.806616821467158 | -0.044174697 | 0.0567838449645024 | 1.00632448972515 | 0.956786795251175 | 1.05842700134123 | 0.961297867845628 |
| ebi-a-GCST90001915 | finngen_R11_O15_PREECLAMPS | Pre-eclampsia \|\| id:finngen_R11_O15_PREECLAMPS | CD45 on T cell \|\| id:ebi-a-GCST90001915 | Weighted mode | 15 | 0.00382118330778058 | 0.0237199645433456 | 0.874319643499175 | -0.042669947 | 0.050312313812738 | 1.00382849333674 | 0.958227603586887 | 1.05159947413614 | 0.977585971930092 |
| ebi-a-GCST90001916 | finngen_R11_O15_PREECLAMPS | Pre-eclampsia \|\| id:finngen_R11_O15_PREECLAMPS | CD45 on CD4+ T cell \|\| id:ebi-a-GCST90001916 | Inverse variance weighted (multiplicative random effects) | 13 | -0.030248248 | 0.056155229507824 | 0.590125514920382 | -0.140312497 | 0.0798160023194012 | 0.970204652755722 | 0.869086605696932 | 1.08308776370338 | 0.91586635420875 |
| ebi-a-GCST90001916 | finngen_R11_O15_PREECLAMPS | Pre-eclampsia \|\| id:finngen_R11_O15_PREECLAMPS | CD45 on CD4+ T cell \|\| id:ebi-a-GCST90001916 | MR Egger | 13 | -0.140327379 | 0.136550749427363 | 0.326178800684157 | -0.407966848 | 0.12731209014492 | 0.869073672583877 | 0.665000924826743 | 1.13577142554382 | 0.99380795269851 |
| ebi-a-GCST90001916 | finngen_R11_O15_PREECLAMPS | Pre-eclampsia \|\| id:finngen_R11_O15_PREECLAMPS | CD45 on CD4+ T cell \|\| id:ebi-a-GCST90001916 | Weighted median | 13 | -0.020076703 | 0.0599974640792063 | 0.737907336723233 | -0.137671732 | 0.0975183267177966 | 0.980123492131385 | 0.871384692099125 | 1.10243164533185 | 0.957984984509652 |
| ebi-a-GCST90001916 | finngen_R11_O15_PREECLAMPS | Pre-eclampsia \|\| id:finngen_R11_O15_PREECLAMPS | CD45 on CD4+ T cell \|\| id:ebi-a-GCST90001916 | Weighted mode | 13 | 0.0989419678942458 | 0.13250929582241 | 0.46963421777857 | -0.160776252 | 0.358660187706169 | 1.10400223012569 | 0.851482567386352 | 1.4314103080979 | 0.933945646747087 |
| ebi-a-GCST90001917 | finngen_R11_O15_PREECLAMPS | Pre-eclampsia \|\| id:finngen_R11_O15_PREECLAMPS | CD45 on CD8+ T cell \|\| id:ebi-a-GCST90001917 | Inverse variance weighted | 26 | 0.00335000499434403 | 0.00514451789819649 | 0.514930571585812 | -0.00673325 | 0.0134332600748092 | 1.00335562253225 | 0.993289367450592 | 1.01352389168422 | 0.898694924660397 |
| ebi-a-GCST90001917 | finngen_R11_O15_PREECLAMPS | Pre-eclampsia \|\| id:finngen_R11_O15_PREECLAMPS | CD45 on CD8+ T cell \|\| id:ebi-a-GCST90001917 | MR Egger | 26 | 0.00139035297812586 | 0.00567203458574721 | 0.808444107298773 | -0.009726835 | 0.0125075407661904 | 1.00139131996693 | 0.990320317841912 | 1.01258608718678 | 0.99380795269851 |
| ebi-a-GCST90001917 | finngen_R11_O15_PREECLAMPS | Pre-eclampsia \|\| id:finngen_R11_O15_PREECLAMPS | CD45 on CD8+ T cell \|\| id:ebi-a-GCST90001917 | Weighted median | 26 | 0.00143219432559054 | 0.00717242784281398 | 0.841730427464329 | -0.012625764 | 0.0154901528975059 | 1.00143322040567 | 0.987453606325872 | 1.01561074718595 | 0.964912412213677 |
| ebi-a-GCST90001917 | finngen_R11_O15_PREECLAMPS | Pre-eclampsia \|\| id:finngen_R11_O15_PREECLAMPS | CD45 on CD8+ T cell \|\| id:ebi-a-GCST90001917 | Weighted mode | 26 | -0.00030608 | 0.00687291293897918 | 0.964832414184467 | -0.013776989 | 0.01316482975013 | 0.999693967227316 | 0.9863174794143 | 1.01325186764829 | 0.992140112238499 |
| ebi-a-GCST90001918 | finngen_R11_O15_PREECLAMPS | Pre-eclampsia \|\| id:finngen_R11_O15_PREECLAMPS | CD45 on HLA DR+ T cell \|\| id:ebi-a-GCST90001918 | Inverse variance weighted | 18 | -0.003699853 | 0.0181465282684345 | 0.838441275335753 | -0.039267049 | 0.0318673419163728 | 0.996306982534802 | 0.961493908971995 | 1.03238054259636 | 0.977824840984872 |
| ebi-a-GCST90001918 | finngen_R11_O15_PREECLAMPS | Pre-eclampsia \|\| id:finngen_R11_O15_PREECLAMPS | CD45 on HLA DR+ T cell \|\| id:ebi-a-GCST90001918 | MR Egger | 18 | -0.030864745 | 0.0248493924270052 | 0.232108430012237 | -0.079569554 | 0.0178400640428386 | 0.96960670825219 | 0.923513783406685 | 1.01800014854098 | 0.99380795269851 |
| ebi-a-GCST90001918 | finngen_R11_O15_PREECLAMPS | Pre-eclampsia \|\| id:finngen_R11_O15_PREECLAMPS | CD45 on HLA DR+ T cell \|\| id:ebi-a-GCST90001918 | Weighted median | 18 | -0.008695055 | 0.0236486781090661 | 0.713114734466408 | -0.055046464 | 0.0376563544340676 | 0.991342638002367 | 0.946441171722563 | 1.03837433882217 | 0.949957918887556 |
| ebi-a-GCST90001918 | finngen_R11_O15_PREECLAMPS | Pre-eclampsia \|\| id:finngen_R11_O15_PREECLAMPS | CD45 on HLA DR+ T cell \|\| id:ebi-a-GCST90001918 | Weighted mode | 18 | -0.013181268 | 0.0217053007423049 | 0.551688872198865 | -0.055723657 | 0.0293611215784807 | 0.986905224591115 | 0.945800464805019 | 1.02979640904071 | 0.939633861977944 |
| ebi-a-GCST90001919 | finngen_R11_O15_PREECLAMPS | Pre-eclampsia \|\| id:finngen_R11_O15_PREECLAMPS | CD45 on Natural Killer T \|\| id:ebi-a-GCST90001919 | Inverse variance weighted | 22 | 0.00542734617126095 | 0.0179325864154159 | 0.76215452781744 | -0.029720523 | 0.0405752155454761 | 1.00544210089541 | 0.970716788460893 | 1.04140963695067 | 0.975717968186601 |
| ebi-a-GCST90001919 | finngen_R11_O15_PREECLAMPS | Pre-eclampsia \|\| id:finngen_R11_O15_PREECLAMPS | CD45 on Natural Killer T \|\| id:ebi-a-GCST90001919 | MR Egger | 22 | -0.020577106 | 0.0270789514001309 | 0.456183277396547 | -0.073651851 | 0.0324976389690938 | 0.979633158188299 | 0.928995066696908 | 1.0330314541219 | 0.99380795269851 |
| ebi-a-GCST90001919 | finngen_R11_O15_PREECLAMPS | Pre-eclampsia \|\| id:finngen_R11_O15_PREECLAMPS | CD45 on Natural Killer T \|\| id:ebi-a-GCST90001919 | Weighted median | 22 | 0.00873988596330459 | 0.0244570985319291 | 0.720825463029154 | -0.039196027 | 0.0566757990858856 | 1.00877819027709 | 0.961562198364141 | 1.05831264884369 | 0.949957918887556 |
| ebi-a-GCST90001919 | finngen_R11_O15_PREECLAMPS | Pre-eclampsia \|\| id:finngen_R11_O15_PREECLAMPS | CD45 on Natural Killer T \|\| id:ebi-a-GCST90001919 | Weighted mode | 22 | 0.0102485401663224 | 0.0251695637263752 | 0.687996484894652 | -0.039083805 | 0.0595808850700178 | 1.01030123631979 | 0.961670113257974 | 1.06139160824216 | 0.959313411571287 |
| ebi-a-GCST90001920 | finngen_R11_O15_PREECLAMPS | Pre-eclampsia \|\| id:finngen_R11_O15_PREECLAMPS | CD45 on HLA DR+ CD4+ T cell \|\| id:ebi-a-GCST90001920 | Inverse variance weighted | 17 | -0.019532516 | 0.021867318341823 | 0.371734727498509 | -0.06239246 | 0.0233274282642147 | 0.980657007935144 | 0.939514093068767 | 1.02360164079191 | 0.885079385681701 |
| ebi-a-GCST90001920 | finngen_R11_O15_PREECLAMPS | Pre-eclampsia \|\| id:finngen_R11_O15_PREECLAMPS | CD45 on HLA DR+ CD4+ T cell \|\| id:ebi-a-GCST90001920 | MR Egger | 17 | -0.013920408 | 0.0281331332053111 | 0.627906751688552 | -0.069061349 | 0.0412205331807569 | 0.986176032959083 | 0.933269423190924 | 1.04208189384123 | 0.99380795269851 |
| ebi-a-GCST90001920 | finngen_R11_O15_PREECLAMPS | Pre-eclampsia \|\| id:finngen_R11_O15_PREECLAMPS | CD45 on HLA DR+ CD4+ T cell \|\| id:ebi-a-GCST90001920 | Weighted median | 17 | -0.014786088 | 0.025462820343082 | 0.56144720257177 | -0.064693216 | 0.0351210396240152 | 0.98532268916366 | 0.937354984670022 | 1.03574506740633 | 0.940845681141708 |
| ebi-a-GCST90001920 | finngen_R11_O15_PREECLAMPS | Pre-eclampsia \|\| id:finngen_R11_O15_PREECLAMPS | CD45 on HLA DR+ CD4+ T cell \|\| id:ebi-a-GCST90001920 | Weighted mode | 17 | -0.014942027 | 0.0226719492981429 | 0.519234673173374 | -0.059379048 | 0.0294949935836259 | 0.985169051112855 | 0.942349506071529 | 1.02993427917915 | 0.934398527469092 |
| ebi-a-GCST90001921 | finngen_R11_O15_PREECLAMPS | Pre-eclampsia \|\| id:finngen_R11_O15_PREECLAMPS | CD45 on HLA DR+ CD8+ T cell \|\| id:ebi-a-GCST90001921 | Inverse variance weighted | 17 | 0.0153220421761055 | 0.0164017222687471 | 0.350214784131348 | -0.016825333 | 0.0474694178228498 | 1.01544002648097 | 0.98331542192811 | 1.04861413172777 | 0.885079385681701 |
| ebi-a-GCST90001921 | finngen_R11_O15_PREECLAMPS | Pre-eclampsia \|\| id:finngen_R11_O15_PREECLAMPS | CD45 on HLA DR+ CD8+ T cell \|\| id:ebi-a-GCST90001921 | MR Egger | 17 | 0.000589361952545589 | 0.0205191513270219 | 0.977464553650739 | -0.039628175 | 0.0408068985535086 | 1.00058953566043 | 0.961146751448101 | 1.041650942 | 0.99380795269851 |
| ebi-a-GCST90001921 | finngen_R11_O15_PREECLAMPS | Pre-eclampsia \|\| id:finngen_R11_O15_PREECLAMPS | CD45 on HLA DR+ CD8+ T cell \|\| id:ebi-a-GCST90001921 | Weighted median | 17 | -0.010398198 | 0.0223119642396459 | 0.641189117639337 | -0.054129648 | 0.0333332521816591 | 0.989655676636158 | 0.947309282129376 | 1.03389502961126 | 0.940845681141708 |
| ebi-a-GCST90001921 | finngen_R11_O15_PREECLAMPS | Pre-eclampsia \|\| id:finngen_R11_O15_PREECLAMPS | CD45 on HLA DR+ CD8+ T cell \|\| id:ebi-a-GCST90001921 | Weighted mode | 17 | -0.001997628 | 0.0196930397718473 | 0.920462866589647 | -0.040595986 | 0.0366007295646698 | 0.998004365543505 | 0.960216992372214 | 1.03727878339587 | 0.986782054514076 |
| ebi-a-GCST90001922 | finngen_R11_O15_PREECLAMPS | Pre-eclampsia \|\| id:finngen_R11_O15_PREECLAMPS | CD127 on T cell \|\| id:ebi-a-GCST90001922 | Inverse variance weighted | 19 | -0.023370127 | 0.0275725501888359 | 0.39666817256291 | -0.077412326 | 0.0306720709851573 | 0.976900839097165 | 0.925508164069108 | 1.031147305 | 0.885079385681701 |
| ebi-a-GCST90001922 | finngen_R11_O15_PREECLAMPS | Pre-eclampsia \|\| id:finngen_R11_O15_PREECLAMPS | CD127 on T cell \|\| id:ebi-a-GCST90001922 | MR Egger | 19 | 0.0225888962264978 | 0.0707168855476098 | 0.753294446155817 | -0.116016199 | 0.161193991899813 | 1.02284595726892 | 0.89046079820829 | 1.17491287028746 | 0.99380795269851 |
| ebi-a-GCST90001922 | finngen_R11_O15_PREECLAMPS | Pre-eclampsia \|\| id:finngen_R11_O15_PREECLAMPS | CD127 on T cell \|\| id:ebi-a-GCST90001922 | Weighted median | 19 | -0.049992416 | 0.0394233738888207 | 0.204765539725959 | -0.127262229 | 0.0272773965400039 | 0.951236638383696 | 0.88050274887396 | 1.02765283056832 | 0.940845681141708 |
| ebi-a-GCST90001922 | finngen_R11_O15_PREECLAMPS | Pre-eclampsia \|\| id:finngen_R11_O15_PREECLAMPS | CD127 on T cell \|\| id:ebi-a-GCST90001922 | Weighted mode | 19 | -0.068019019 | 0.0614976676447035 | 0.28327222576598 | -0.188554447 | 0.0525164098202201 | 0.93424270526728 | 0.828155411346015 | 1.05391985657201 | 0.872755742358858 |
| ebi-a-GCST90001923 | finngen_R11_O15_PREECLAMPS | Pre-eclampsia \|\| id:finngen_R11_O15_PREECLAMPS | CD127 on CD45RA- CD4 not regulatory T cell \|\| id:ebi-a-GCST90001923 | Inverse variance weighted | 16 | 0.00123030252306207 | 0.0177773243762167 | 0.94482542282767 | -0.033613253 | 0.0360738583004467 | 1.00123105965568 | 0.966945395317231 | 1.03673241495747 | 0.986667691552896 |
| ebi-a-GCST90001923 | finngen_R11_O15_PREECLAMPS | Pre-eclampsia \|\| id:finngen_R11_O15_PREECLAMPS | CD127 on CD45RA- CD4 not regulatory T cell \|\| id:ebi-a-GCST90001923 | MR Egger | 16 | 0.0042749128779403 | 0.0226532806902537 | 0.853027917092102 | -0.040125517 | 0.0486753430308375 | 1.00428406335251 | 0.960668851048217 | 1.04987944472576 | 0.99380795269851 |
| ebi-a-GCST90001923 | finngen_R11_O15_PREECLAMPS | Pre-eclampsia \|\| id:finngen_R11_O15_PREECLAMPS | CD127 on CD45RA- CD4 not regulatory T cell \|\| id:ebi-a-GCST90001923 | Weighted median | 16 | -0.012000963 | 0.0254937344585924 | 0.637825238172226 | -0.061968682 | 0.0379667569345575 | 0.988070761740325 | 0.939912322369744 | 1.03869670284209 | 0.940845681141708 |
| ebi-a-GCST90001923 | finngen_R11_O15_PREECLAMPS | Pre-eclampsia \|\| id:finngen_R11_O15_PREECLAMPS | CD127 on CD45RA- CD4 not regulatory T cell \|\| id:ebi-a-GCST90001923 | Weighted mode | 16 | 0.00283141327669989 | 0.0206116461637537 | 0.892565616443955 | -0.037567413 | 0.0432302397576571 | 1.00283542551314 | 0.963129487887389 | 1.04417827853041 | 0.98361388732834 |
| ebi-a-GCST90001924 | finngen_R11_O15_PREECLAMPS | Pre-eclampsia \|\| id:finngen_R11_O15_PREECLAMPS | CD127 on CD28+ CD4+ T cell \|\| id:ebi-a-GCST90001924 | Inverse variance weighted | 20 | 0.0122722630988777 | 0.0173358976701031 | 0.479000870624797 | -0.021706096 | 0.0462506225322797 | 1.01234787631818 | 0.978527785696032 | 1.04733686428428 | 0.898694924660397 |
| ebi-a-GCST90001924 | finngen_R11_O15_PREECLAMPS | Pre-eclampsia \|\| id:finngen_R11_O15_PREECLAMPS | CD127 on CD28+ CD4+ T cell \|\| id:ebi-a-GCST90001924 | MR Egger | 20 | 0.00993859120829412 | 0.0253184280907913 | 0.699266037991352 | -0.039685528 | 0.059562710266245 | 1.00998814302828 | 0.961091628185808 | 1.06137231783325 | 0.99380795269851 |
| ebi-a-GCST90001924 | finngen_R11_O15_PREECLAMPS | Pre-eclampsia \|\| id:finngen_R11_O15_PREECLAMPS | CD127 on CD28+ CD4+ T cell \|\| id:ebi-a-GCST90001924 | Weighted median | 20 | 0.0247709075335137 | 0.0266609611979228 | 0.352833212296586 | -0.027484576 | 0.0770263914814425 | 1.02508025545848 | 0.972889687885102 | 1.08007058067915 | 0.940845681141708 |
| ebi-a-GCST90001924 | finngen_R11_O15_PREECLAMPS | Pre-eclampsia \|\| id:finngen_R11_O15_PREECLAMPS | CD127 on CD28+ CD4+ T cell \|\| id:ebi-a-GCST90001924 | Weighted mode | 20 | 0.0298301752438423 | 0.0253873151120835 | 0.254509283508878 | -0.019928962 | 0.0795893128635259 | 1.0302795521221 | 0.980268306764979 | 1.08284226695437 | 0.872755742358858 |
| ebi-a-GCST90001925 | finngen_R11_O15_PREECLAMPS | Pre-eclampsia \|\| id:finngen_R11_O15_PREECLAMPS | CD127 on CD28+ CD4-CD8- T cell \|\| id:ebi-a-GCST90001925 | Inverse variance weighted | 24 | -0.0147795 | 0.0216567202281293 | 0.494958212853683 | -0.057226671 | 0.0276676718857849 | 0.98532918097085 | 0.94437998116313 | 1.02805397640569 | 0.898694924660397 |
| ebi-a-GCST90001925 | finngen_R11_O15_PREECLAMPS | Pre-eclampsia \|\| id:finngen_R11_O15_PREECLAMPS | CD127 on CD28+ CD4-CD8- T cell \|\| id:ebi-a-GCST90001925 | MR Egger | 24 | -0.013045261 | 0.0379056845025906 | 0.734000887086046 | -0.087340402 | 0.0612498807824412 | 0.987039459771611 | 0.916365109591124 | 1.06316454538621 | 0.99380795269851 |
| ebi-a-GCST90001925 | finngen_R11_O15_PREECLAMPS | Pre-eclampsia \|\| id:finngen_R11_O15_PREECLAMPS | CD127 on CD28+ CD4-CD8- T cell \|\| id:ebi-a-GCST90001925 | Weighted median | 24 | -0.015745532 | 0.0305848568210984 | 0.606682559116082 | -0.075691851 | 0.0442007875372349 | 0.984377780997199 | 0.927101847867644 | 1.04519219538791 | 0.940845681141708 |
| ebi-a-GCST90001925 | finngen_R11_O15_PREECLAMPS | Pre-eclampsia \|\| id:finngen_R11_O15_PREECLAMPS | CD127 on CD28+ CD4-CD8- T cell \|\| id:ebi-a-GCST90001925 | Weighted mode | 24 | -0.015147818 | 0.0348849199560741 | 0.668173183807464 | -0.083522261 | 0.0532266255995481 | 0.984966333566613 | 0.919870609545632 | 1.05466863294922 | 0.959313411571287 |
| ebi-a-GCST90001926 | finngen_R11_O15_PREECLAMPS | Pre-eclampsia \|\| id:finngen_R11_O15_PREECLAMPS | CD127 on granulocyte \|\| id:ebi-a-GCST90001926 | Inverse variance weighted | 31 | -0.002607353 | 0.0195360308455439 | 0.893826569099057 | -0.040897974 | 0.0356832669898002 | 0.997396042726253 | 0.95992706254261 | 1.03632755535719 | 0.986667691552896 |
| ebi-a-GCST90001926 | finngen_R11_O15_PREECLAMPS | Pre-eclampsia \|\| id:finngen_R11_O15_PREECLAMPS | CD127 on granulocyte \|\| id:ebi-a-GCST90001926 | MR Egger | 31 | -0.042010893 | 0.03013297252833 | 0.173853090211951 | -0.101071519 | 0.0170497330671112 | 0.958859335576075 | 0.903868386590532 | 1.01719590933973 | 0.99380795269851 |
| ebi-a-GCST90001926 | finngen_R11_O15_PREECLAMPS | Pre-eclampsia \|\| id:finngen_R11_O15_PREECLAMPS | CD127 on granulocyte \|\| id:ebi-a-GCST90001926 | Weighted median | 31 | -0.025204351 | 0.0266289645186415 | 0.343892940401574 | -0.077397121 | 0.0269884199236241 | 0.975110627290719 | 0.925522236310812 | 1.02735590583475 | 0.940845681141708 |
| ebi-a-GCST90001926 | finngen_R11_O15_PREECLAMPS | Pre-eclampsia \|\| id:finngen_R11_O15_PREECLAMPS | CD127 on granulocyte \|\| id:ebi-a-GCST90001926 | Weighted mode | 31 | -0.026724707 | 0.0275362744373399 | 0.339544042096493 | -0.080695805 | 0.0272463908243076 | 0.97362923787672 | 0.92247426085393 | 1.02762096795079 | 0.888599269773114 |
| ebi-a-GCST90001927 | finngen_R11_O15_PREECLAMPS | Pre-eclampsia \|\| id:finngen_R11_O15_PREECLAMPS | CD127 on CD8+ T cell \|\| id:ebi-a-GCST90001927 | Inverse variance weighted | 24 | -0.021271982 | 0.0233588814404172 | 0.362474921446282 | -0.06705539 | 0.0245114252858761 | 0.978952670521987 | 0.935143402346044 | 1.02481429983666 | 0.885079385681701 |
| ebi-a-GCST90001927 | finngen_R11_O15_PREECLAMPS | Pre-eclampsia \|\| id:finngen_R11_O15_PREECLAMPS | CD127 on CD8+ T cell \|\| id:ebi-a-GCST90001927 | MR Egger | 24 | -0.01062985 | 0.0442699485125637 | 0.81246483094137 | -0.09739895 | 0.0761392485847664 | 0.989426446707425 | 0.907194009255784 | 1.07911282862987 | 0.99380795269851 |
| ebi-a-GCST90001927 | finngen_R11_O15_PREECLAMPS | Pre-eclampsia \|\| id:finngen_R11_O15_PREECLAMPS | CD127 on CD8+ T cell \|\| id:ebi-a-GCST90001927 | Weighted median | 24 | -0.00912896 | 0.0358671103244523 | 0.799092496679444 | -0.079428496 | 0.061170576247935 | 0.990912582458021 | 0.923644061645627 | 1.06308023495998 | 0.959173423764653 |
| ebi-a-GCST90001927 | finngen_R11_O15_PREECLAMPS | Pre-eclampsia \|\| id:finngen_R11_O15_PREECLAMPS | CD127 on CD8+ T cell \|\| id:ebi-a-GCST90001927 | Weighted mode | 24 | -0.012673113 | 0.0378366332585327 | 0.740703678907543 | -0.086832914 | 0.0614866879181825 | 0.987406852470011 | 0.916830271921437 | 1.06341634014925 | 0.959313411571287 |
| ebi-a-GCST90001928 | finngen_R11_O15_PREECLAMPS | Pre-eclampsia \|\| id:finngen_R11_O15_PREECLAMPS | CD127 on CD28+ CD45RA- CD8+ T cell \|\| id:ebi-a-GCST90001928 | Inverse variance weighted | 19 | 0.0168848252787369 | 0.0174553158570811 | 0.333385544627425 | -0.017327594 | 0.0510972443586158 | 1.01702817964233 | 0.98282166560679 | 1.05242523072381 | 0.885079385681701 |
| ebi-a-GCST90001928 | finngen_R11_O15_PREECLAMPS | Pre-eclampsia \|\| id:finngen_R11_O15_PREECLAMPS | CD127 on CD28+ CD45RA- CD8+ T cell \|\| id:ebi-a-GCST90001928 | MR Egger | 19 | 0.00683552850239496 | 0.024759053497065 | 0.785813268530023 | -0.041692216 | 0.0553632733566424 | 1.00685894404949 | 0.959164950433546 | 1.05692449745399 | 0.99380795269851 |
| ebi-a-GCST90001928 | finngen_R11_O15_PREECLAMPS | Pre-eclampsia \|\| id:finngen_R11_O15_PREECLAMPS | CD127 on CD28+ CD45RA- CD8+ T cell \|\| id:ebi-a-GCST90001928 | Weighted median | 19 | 0.00684467634241677 | 0.0245557986478268 | 0.780444580961151 | -0.041284689 | 0.0549740416921574 | 1.00686815467616 | 0.959555916037937 | 1.05651318902493 | 0.957984984509652 |
| ebi-a-GCST90001928 | finngen_R11_O15_PREECLAMPS | Pre-eclampsia \|\| id:finngen_R11_O15_PREECLAMPS | CD127 on CD28+ CD45RA- CD8+ T cell \|\| id:ebi-a-GCST90001928 | Weighted mode | 19 | 0.00359077809797953 | 0.0225995623222245 | 0.875526977072688 | -0.040704364 | 0.0478859202495395 | 1.00359723266498 | 0.960112931889993 | 1.04905097302471 | 0.977585971930092 |
| ebi-a-GCST90001929 | finngen_R11_O15_PREECLAMPS | Pre-eclampsia \|\| id:finngen_R11_O15_PREECLAMPS | CD127 on CD28+ CD45RA+ CD8+ T cell \|\| id:ebi-a-GCST90001929 | Inverse variance weighted | 21 | 0.0247434839758366 | 0.0248920999019003 | 0.320208459646473 | -0.024045032 | 0.0735319997835612 | 1.02505214449642 | 0.976241746813826 | 1.07630297758317 | 0.885079385681701 |
| ebi-a-GCST90001929 | finngen_R11_O15_PREECLAMPS | Pre-eclampsia \|\| id:finngen_R11_O15_PREECLAMPS | CD127 on CD28+ CD45RA+ CD8+ T cell \|\| id:ebi-a-GCST90001929 | MR Egger | 21 | 0.0550042368414034 | 0.0481247616020355 | 0.26725702585611 | -0.039320296 | 0.149328769581393 | 1.056545091 | 0.961442713666242 | 1.161054646 | 0.99380795269851 |
| ebi-a-GCST90001929 | finngen_R11_O15_PREECLAMPS | Pre-eclampsia \|\| id:finngen_R11_O15_PREECLAMPS | CD127 on CD28+ CD45RA+ CD8+ T cell \|\| id:ebi-a-GCST90001929 | Weighted median | 21 | 0.0214956193913375 | 0.035620865193958 | 0.54620547945881 | -0.048321276 | 0.0913125151714952 | 1.02172831453567 | 0.952827616881591 | 1.09561134693017 | 0.940845681141708 |
| ebi-a-GCST90001929 | finngen_R11_O15_PREECLAMPS | Pre-eclampsia \|\| id:finngen_R11_O15_PREECLAMPS | CD127 on CD28+ CD45RA+ CD8+ T cell \|\| id:ebi-a-GCST90001929 | Weighted mode | 21 | 0.0714467284865313 | 0.053515496946518 | 0.196853389521701 | -0.033443646 | 0.176337102501707 | 1.07406093232585 | 0.967109410635326 | 1.19284010026418 | 0.872755742358858 |
| ebi-a-GCST90001930 | finngen_R11_O15_PREECLAMPS | Pre-eclampsia \|\| id:finngen_R11_O15_PREECLAMPS | CD127 on CD28- CD8+ T cell \|\| id:ebi-a-GCST90001930 | Inverse variance weighted | 20 | 0.0323607934590553 | 0.021919756560608 | 0.139855333416285 | -0.010601929 | 0.075323516317847 | 1.03289009807787 | 0.989454072967983 | 1.07823292040947 | 0.844911146506647 |
| ebi-a-GCST90001930 | finngen_R11_O15_PREECLAMPS | Pre-eclampsia \|\| id:finngen_R11_O15_PREECLAMPS | CD127 on CD28- CD8+ T cell \|\| id:ebi-a-GCST90001930 | MR Egger | 20 | 0.0356268377184671 | 0.0364668116854022 | 0.341534401120691 | -0.035848113 | 0.107101788621855 | 1.03626907779831 | 0.964786820752495 | 1.11304754428901 | 0.99380795269851 |
| ebi-a-GCST90001930 | finngen_R11_O15_PREECLAMPS | Pre-eclampsia \|\| id:finngen_R11_O15_PREECLAMPS | CD127 on CD28- CD8+ T cell \|\| id:ebi-a-GCST90001930 | Weighted median | 20 | 0.0626940809188983 | 0.0310852345561649 | 0.0437117549083569 | 0.00176702118881514 | 0.123621140648981 | 1.06470107703028 | 1.00176858329071 | 1.1315870774323 | 0.743099833442067 |
| ebi-a-GCST90001930 | finngen_R11_O15_PREECLAMPS | Pre-eclampsia \|\| id:finngen_R11_O15_PREECLAMPS | CD127 on CD28- CD8+ T cell \|\| id:ebi-a-GCST90001930 | Weighted mode | 20 | 0.0709539132733442 | 0.0350755845362441 | 0.0573873309548384 | 0.0022057675823057 | 0.139702058964383 | 1.07353174916398 | 1.00220820207727 | 1.14993113613953 | 0.838871423749761 |
| ebi-a-GCST90001931 | finngen_R11_O15_PREECLAMPS | Pre-eclampsia \|\| id:finngen_R11_O15_PREECLAMPS | CD127 on CD4+ T cell \|\| id:ebi-a-GCST90001931 | Inverse variance weighted | 20 | 0.00424981021521995 | 0.0182813102851384 | 0.81617493911542 | -0.031581558 | 0.0400811783740912 | 1.00425885346481 | 0.968911930766545 | 1.0408952689483 | 0.977824840984872 |
| ebi-a-GCST90001931 | finngen_R11_O15_PREECLAMPS | Pre-eclampsia \|\| id:finngen_R11_O15_PREECLAMPS | CD127 on CD4+ T cell \|\| id:ebi-a-GCST90001931 | MR Egger | 20 | 0.0131070586016118 | 0.0277175701297995 | 0.641982945089672 | -0.041219379 | 0.0674334960560188 | 1.01319333261499 | 0.95961858682959 | 1.069759114 | 0.99380795269851 |
| ebi-a-GCST90001931 | finngen_R11_O15_PREECLAMPS | Pre-eclampsia \|\| id:finngen_R11_O15_PREECLAMPS | CD127 on CD4+ T cell \|\| id:ebi-a-GCST90001931 | Weighted median | 20 | 0.0181950437728153 | 0.0285935736419795 | 0.524559188945383 | -0.037848361 | 0.0742384481110952 | 1.01836158210582 | 0.962858937206296 | 1.07706359865971 | 0.940845681141708 |
| ebi-a-GCST90001931 | finngen_R11_O15_PREECLAMPS | Pre-eclampsia \|\| id:finngen_R11_O15_PREECLAMPS | CD127 on CD4+ T cell \|\| id:ebi-a-GCST90001931 | Weighted mode | 20 | 0.0162373402034474 | 0.0275973056606771 | 0.563215523028454 | -0.037853379 | 0.0703280592983745 | 1.01636988221674 | 0.962854105278366 | 1.07286008525519 | 0.939633861977944 |
| ebi-a-GCST90001932 | finngen_R11_O15_PREECLAMPS | Pre-eclampsia \|\| id:finngen_R11_O15_PREECLAMPS | CD127 on CD45RA+ CD4+ T cell \|\| id:ebi-a-GCST90001932 | Inverse variance weighted (multiplicative random effects) | 22 | 0.0319276774068555 | 0.0310658093715135 | 0.304070594429609 | -0.028961309 | 0.0928166637750219 | 1.03244283366192 | 0.971454050305728 | 1.09726054921939 | 0.885079385681701 |
| ebi-a-GCST90001932 | finngen_R11_O15_PREECLAMPS | Pre-eclampsia \|\| id:finngen_R11_O15_PREECLAMPS | CD127 on CD45RA+ CD4+ T cell \|\| id:ebi-a-GCST90001932 | MR Egger | 22 | 0.0544984928034739 | 0.064223419520636 | 0.406159203176075 | -0.071379409 | 0.18037639506392 | 1.05601088479659 | 0.931108553711116 | 1.1976680746452 | 0.99380795269851 |
| ebi-a-GCST90001932 | finngen_R11_O15_PREECLAMPS | Pre-eclampsia \|\| id:finngen_R11_O15_PREECLAMPS | CD127 on CD45RA+ CD4+ T cell \|\| id:ebi-a-GCST90001932 | Weighted median | 22 | 0.0351454693915792 | 0.0321529768864221 | 0.274362862538606 | -0.027874365 | 0.0981653040889665 | 1.03577037072665 | 0.972510540190793 | 1.10314512443717 | 0.940845681141708 |
| ebi-a-GCST90001932 | finngen_R11_O15_PREECLAMPS | Pre-eclampsia \|\| id:finngen_R11_O15_PREECLAMPS | CD127 on CD45RA+ CD4+ T cell \|\| id:ebi-a-GCST90001932 | Weighted mode | 22 | 0.0673870096065422 | 0.0374178950946965 | 0.0860936013725209 | -0.005952065 | 0.140726083992147 | 1.06970938588485 | 0.994065613666696 | 1.15110929753357 | 0.872755742358858 |
| ebi-a-GCST90001933 | finngen_R11_O15_PREECLAMPS | Pre-eclampsia \|\| id:finngen_R11_O15_PREECLAMPS | CD25 on CD45RA- CD4 not regulatory T cell \|\| id:ebi-a-GCST90001933 | Inverse variance weighted | 25 | 0.00396325459791297 | 0.013735904551785 | 0.772939172112841 | -0.022959118 | 0.0308856275194116 | 1.0039711186771 | 0.977302436718577 | 1.03136753707696 | 0.977824840984872 |
| ebi-a-GCST90001933 | finngen_R11_O15_PREECLAMPS | Pre-eclampsia \|\| id:finngen_R11_O15_PREECLAMPS | CD25 on CD45RA- CD4 not regulatory T cell \|\| id:ebi-a-GCST90001933 | MR Egger | 25 | -0.003491245 | 0.0175970228042526 | 0.844475540751176 | -0.03798141 | 0.0309989197006341 | 0.996514842313952 | 0.962730838187609 | 1.03148438957399 | 0.99380795269851 |
| ebi-a-GCST90001933 | finngen_R11_O15_PREECLAMPS | Pre-eclampsia \|\| id:finngen_R11_O15_PREECLAMPS | CD25 on CD45RA- CD4 not regulatory T cell \|\| id:ebi-a-GCST90001933 | Weighted median | 25 | -0.015755672 | 0.0202288456383205 | 0.436055380836876 | -0.05540421 | 0.0238928649697803 | 0.984367798818045 | 0.946102646566416 | 1.02418058639461 | 0.940845681141708 |
| ebi-a-GCST90001933 | finngen_R11_O15_PREECLAMPS | Pre-eclampsia \|\| id:finngen_R11_O15_PREECLAMPS | CD25 on CD45RA- CD4 not regulatory T cell \|\| id:ebi-a-GCST90001933 | Weighted mode | 25 | -0.010226376 | 0.0171326020455388 | 0.556166598269989 | -0.043806276 | 0.023353523537588 | 0.989825735127313 | 0.957139359919109 | 1.02362835230507 | 0.939633861977944 |
| ebi-a-GCST90001934 | finngen_R11_O15_PREECLAMPS | Pre-eclampsia \|\| id:finngen_R11_O15_PREECLAMPS | CD25 on CD45RA+ CD4 not regulatory T cell \|\| id:ebi-a-GCST90001934 | Inverse variance weighted (multiplicative random effects) | 22 | -0.00679009 | 0.0166582872025486 | 0.683559763355803 | -0.039440333 | 0.0258601526141954 | 0.993232910272256 | 0.961327311584815 | 1.02619742741061 | 0.950911139356934 |
| ebi-a-GCST90001934 | finngen_R11_O15_PREECLAMPS | Pre-eclampsia \|\| id:finngen_R11_O15_PREECLAMPS | CD25 on CD45RA+ CD4 not regulatory T cell \|\| id:ebi-a-GCST90001934 | MR Egger | 22 | 0.0039219384369997 | 0.0201967332020242 | 0.84798846725908 | -0.035663659 | 0.0435075355129671 | 1.0039296393017 | 0.964964796481171 | 1.04446786488351 | 0.99380795269851 |
| ebi-a-GCST90001934 | finngen_R11_O15_PREECLAMPS | Pre-eclampsia \|\| id:finngen_R11_O15_PREECLAMPS | CD25 on CD45RA+ CD4 not regulatory T cell \|\| id:ebi-a-GCST90001934 | Weighted median | 22 | 0.0093508134326954 | 0.0189179869617795 | 0.621107296747143 | -0.027728441 | 0.0464300678777832 | 1.00939466887673 | 0.972652463458984 | 1.04752482087321 | 0.940845681141708 |
| ebi-a-GCST90001934 | finngen_R11_O15_PREECLAMPS | Pre-eclampsia \|\| id:finngen_R11_O15_PREECLAMPS | CD25 on CD45RA+ CD4 not regulatory T cell \|\| id:ebi-a-GCST90001934 | Weighted mode | 22 | -0.000930071 | 0.0155488732610209 | 0.952867804605621 | -0.031405862 | 0.0295457208409616 | 0.999070361631102 | 0.969082179286455 | 1.02998652624553 | 0.990180645930572 |
| ebi-a-GCST90001935 | finngen_R11_O15_PREECLAMPS | Pre-eclampsia \|\| id:finngen_R11_O15_PREECLAMPS | CD25 on CD39+ CD4 regulatory T cell \|\| id:ebi-a-GCST90001935 | Inverse variance weighted | 17 | -0.024573443 | 0.028601477516127 | 0.390248436276626 | -0.080632339 | 0.0314854528553569 | 0.97572602596594 | 0.922532808428372 | 1.03198636303156 | 0.885079385681701 |
| ebi-a-GCST90001935 | finngen_R11_O15_PREECLAMPS | Pre-eclampsia \|\| id:finngen_R11_O15_PREECLAMPS | CD25 on CD39+ CD4 regulatory T cell \|\| id:ebi-a-GCST90001935 | MR Egger | 17 | -0.064037344 | 0.050686971299141 | 0.22573375513478 | -0.163383808 | 0.0353091194946295 | 0.937969971090003 | 0.849265171089474 | 1.03593988852498 | 0.99380795269851 |
| ebi-a-GCST90001935 | finngen_R11_O15_PREECLAMPS | Pre-eclampsia \|\| id:finngen_R11_O15_PREECLAMPS | CD25 on CD39+ CD4 regulatory T cell \|\| id:ebi-a-GCST90001935 | Weighted median | 17 | -0.039770406 | 0.0357943909323613 | 0.266534223912467 | -0.109927412 | 0.030386599975468 | 0.961010055725859 | 0.895899164035398 | 1.03085298466662 | 0.940845681141708 |
| ebi-a-GCST90001935 | finngen_R11_O15_PREECLAMPS | Pre-eclampsia \|\| id:finngen_R11_O15_PREECLAMPS | CD25 on CD39+ CD4 regulatory T cell \|\| id:ebi-a-GCST90001935 | Weighted mode | 17 | -0.03164684 | 0.0390787482600875 | 0.429922374960549 | -0.108241186 | 0.0449475070254173 | 0.968848680689942 | 0.897411127188356 | 1.045972952 | 0.933945646747087 |
| ebi-a-GCST90001936 | finngen_R11_O15_PREECLAMPS | Pre-eclampsia \|\| id:finngen_R11_O15_PREECLAMPS | CD25 on CD4 regulatory T cell \|\| id:ebi-a-GCST90001936 | Inverse variance weighted | 22 | -0.013980197 | 0.0120819132631785 | 0.247224231104467 | -0.037660747 | 0.00970035297896111 | 0.986117072129251 | 0.963039599539139 | 1.00974755390134 | 0.885079385681701 |
| ebi-a-GCST90001936 | finngen_R11_O15_PREECLAMPS | Pre-eclampsia \|\| id:finngen_R11_O15_PREECLAMPS | CD25 on CD4 regulatory T cell \|\| id:ebi-a-GCST90001936 | MR Egger | 22 | -0.014145671 | 0.0144399543874741 | 0.338975807628904 | -0.042447981 | 0.0141566398713299 | 0.98595390917756 | 0.958440321017331 | 1.01425731963225 | 0.99380795269851 |
| ebi-a-GCST90001936 | finngen_R11_O15_PREECLAMPS | Pre-eclampsia \|\| id:finngen_R11_O15_PREECLAMPS | CD25 on CD4 regulatory T cell \|\| id:ebi-a-GCST90001936 | Weighted median | 22 | -0.015280033 | 0.0175993300455285 | 0.385275678853367 | -0.04977472 | 0.0192146535155671 | 0.984836114005163 | 0.951443741355099 | 1.01940044302294 | 0.940845681141708 |
| ebi-a-GCST90001936 | finngen_R11_O15_PREECLAMPS | Pre-eclampsia \|\| id:finngen_R11_O15_PREECLAMPS | CD25 on CD4 regulatory T cell \|\| id:ebi-a-GCST90001936 | Weighted mode | 22 | -0.014316699 | 0.0132639208534193 | 0.292663838874487 | -0.040313984 | 0.0116805855432535 | 0.985785297278638 | 0.960487813802112 | 1.01174906996888 | 0.872755742358858 |
| ebi-a-GCST90001937 | finngen_R11_O15_PREECLAMPS | Pre-eclampsia \|\| id:finngen_R11_O15_PREECLAMPS | CD25 on resting CD4 regulatory T cell \|\| id:ebi-a-GCST90001937 | Inverse variance weighted (multiplicative random effects) | 20 | -0.040162312 | 0.028057672163697 | 0.152309893352338 | -0.095155349 | 0.0148307256803075 | 0.960633504382313 | 0.909231675081651 | 1.0149412465849 | 0.85505992597664 |
| ebi-a-GCST90001937 | finngen_R11_O15_PREECLAMPS | Pre-eclampsia \|\| id:finngen_R11_O15_PREECLAMPS | CD25 on resting CD4 regulatory T cell \|\| id:ebi-a-GCST90001937 | MR Egger | 20 | -0.00800549 | 0.0382096660687625 | 0.836399391861016 | -0.082896436 | 0.0668854550008295 | 0.992026468106792 | 0.920446467412503 | 1.06917300273955 | 0.99380795269851 |
| ebi-a-GCST90001937 | finngen_R11_O15_PREECLAMPS | Pre-eclampsia \|\| id:finngen_R11_O15_PREECLAMPS | CD25 on resting CD4 regulatory T cell \|\| id:ebi-a-GCST90001937 | Weighted median | 20 | -0.020672119 | 0.0291369744909626 | 0.478026264151274 | -0.077780589 | 0.0364363508233123 | 0.979540084329155 | 0.925167396011706 | 1.03710829082808 | 0.940845681141708 |
| ebi-a-GCST90001937 | finngen_R11_O15_PREECLAMPS | Pre-eclampsia \|\| id:finngen_R11_O15_PREECLAMPS | CD25 on resting CD4 regulatory T cell \|\| id:ebi-a-GCST90001937 | Weighted mode | 20 | -0.018154735 | 0.0259258855367106 | 0.492254347147783 | -0.068969471 | 0.0326600007637788 | 0.982009069538068 | 0.933355174472587 | 1.03319919257955 | 0.933945646747087 |
| ebi-a-GCST90001938 | finngen_R11_O15_PREECLAMPS | Pre-eclampsia \|\| id:finngen_R11_O15_PREECLAMPS | CD25 on CD39+ resting CD4 regulatory T cell \|\| id:ebi-a-GCST90001938 | Inverse variance weighted | 15 | -0.044182005 | 0.0324107758339252 | 0.172823028660935 | -0.107707126 | 0.0193431153083842 | 0.956779802604983 | 0.897890526751329 | 1.0195314054431 | 0.85505992597664 |
| ebi-a-GCST90001938 | finngen_R11_O15_PREECLAMPS | Pre-eclampsia \|\| id:finngen_R11_O15_PREECLAMPS | CD25 on CD39+ resting CD4 regulatory T cell \|\| id:ebi-a-GCST90001938 | MR Egger | 15 | -0.102810616 | 0.058545057909703 | 0.102587989481097 | -0.21755893 | 0.0119376970337133 | 0.902297837651809 | 0.804480195527268 | 1.0120092357246 | 0.97261503632431 |
| ebi-a-GCST90001938 | finngen_R11_O15_PREECLAMPS | Pre-eclampsia \|\| id:finngen_R11_O15_PREECLAMPS | CD25 on CD39+ resting CD4 regulatory T cell \|\| id:ebi-a-GCST90001938 | Weighted median | 15 | -0.059379741 | 0.0424021697534904 | 0.16139539051951 | -0.142487993 | 0.0237285121146129 | 0.942348853082789 | 0.867197966399034 | 1.02401227322278 | 0.940845681141708 |
| ebi-a-GCST90001938 | finngen_R11_O15_PREECLAMPS | Pre-eclampsia \|\| id:finngen_R11_O15_PREECLAMPS | CD25 on CD39+ resting CD4 regulatory T cell \|\| id:ebi-a-GCST90001938 | Weighted mode | 15 | -0.070257609 | 0.0524551852718423 | 0.201785337309364 | -0.173069772 | 0.0325545544330752 | 0.932153658081576 | 0.841078929179139 | 1.03309025125964 | 0.872755742358858 |
| ebi-a-GCST90001939 | finngen_R11_O15_PREECLAMPS | Pre-eclampsia \|\| id:finngen_R11_O15_PREECLAMPS | CD25 on activated CD4 regulatory T cell \|\| id:ebi-a-GCST90001939 | Inverse variance weighted | 17 | -0.028492812 | 0.0329426036671599 | 0.38708111079016 | -0.093060315 | 0.0360746910791184 | 0.971909280099468 | 0.91113854304772 | 1.03673327832647 | 0.885079385681701 |
| ebi-a-GCST90001939 | finngen_R11_O15_PREECLAMPS | Pre-eclampsia \|\| id:finngen_R11_O15_PREECLAMPS | CD25 on activated CD4 regulatory T cell \|\| id:ebi-a-GCST90001939 | MR Egger | 17 | -0.060972312 | 0.0653808131787531 | 0.365809930687674 | -0.189118706 | 0.0671740817850743 | 0.940849289607902 | 0.82768824940459 | 1.06948163924337 | 0.99380795269851 |
| ebi-a-GCST90001939 | finngen_R11_O15_PREECLAMPS | Pre-eclampsia \|\| id:finngen_R11_O15_PREECLAMPS | CD25 on activated CD4 regulatory T cell \|\| id:ebi-a-GCST90001939 | Weighted median | 17 | -0.037923998 | 0.0441724148094582 | 0.390591691713696 | -0.124501931 | 0.0486539350278367 | 0.962786111781915 | 0.882936556391967 | 1.04985696914403 | 0.940845681141708 |
| ebi-a-GCST90001939 | finngen_R11_O15_PREECLAMPS | Pre-eclampsia \|\| id:finngen_R11_O15_PREECLAMPS | CD25 on activated CD4 regulatory T cell \|\| id:ebi-a-GCST90001939 | Weighted mode | 17 | -0.038276188 | 0.0557772402601749 | 0.50239028090351 | -0.147599579 | 0.0710472032181081 | 0.962447088140474 | 0.862776519964127 | 1.07363190355318 | 0.934398527469092 |
| ebi-a-GCST90001940 | finngen_R11_O15_PREECLAMPS | Pre-eclampsia \|\| id:finngen_R11_O15_PREECLAMPS | CD25 on CD39+ activated CD4 regulatory T cell \|\| id:ebi-a-GCST90001940 | Inverse variance weighted | 12 | 0.00523405145934116 | 0.0275570219871017 | 0.849359824246981 | -0.048777712 | 0.0592458145540605 | 1.00524777303605 | 0.952392812011035 | 1.06103602678411 | 0.977824840984872 |
| ebi-a-GCST90001940 | finngen_R11_O15_PREECLAMPS | Pre-eclampsia \|\| id:finngen_R11_O15_PREECLAMPS | CD25 on CD39+ activated CD4 regulatory T cell \|\| id:ebi-a-GCST90001940 | MR Egger | 12 | 0.0268878814025086 | 0.0353349972440529 | 0.464256970345652 | -0.042368713 | 0.0961444760008523 | 1.0272526221834 | 0.958516297802186 | 1.10091810875025 | 0.99380795269851 |
| ebi-a-GCST90001940 | finngen_R11_O15_PREECLAMPS | Pre-eclampsia \|\| id:finngen_R11_O15_PREECLAMPS | CD25 on CD39+ activated CD4 regulatory T cell \|\| id:ebi-a-GCST90001940 | Weighted median | 12 | 0.0273507941710463 | 0.0299424790292182 | 0.361008786126231 | -0.031336465 | 0.0860380530683139 | 1.02772826061978 | 0.969149433613042 | 1.08984779956884 | 0.940845681141708 |
| ebi-a-GCST90001940 | finngen_R11_O15_PREECLAMPS | Pre-eclampsia \|\| id:finngen_R11_O15_PREECLAMPS | CD25 on CD39+ activated CD4 regulatory T cell \|\| id:ebi-a-GCST90001940 | Weighted mode | 12 | 0.030588236893545 | 0.0267640808043526 | 0.277351815417975 | -0.021869361 | 0.0830458352700761 | 1.03106086364277 | 0.978368039252694 | 1.08659161162684 | 0.872755742358858 |
| ebi-a-GCST90001941 | finngen_R11_O15_PREECLAMPS | Pre-eclampsia \|\| id:finngen_R11_O15_PREECLAMPS | CD25 on secreting CD4 regulatory T cell \|\| id:ebi-a-GCST90001941 | Inverse variance weighted | 14 | -0.006931826 | 0.0116574309473918 | 0.552092684738149 | -0.02978039 | 0.0159167389677118 | 0.993092143997974 | 0.970658676159525 | 1.01604408500482 | 0.905435115099076 |
| ebi-a-GCST90001941 | finngen_R11_O15_PREECLAMPS | Pre-eclampsia \|\| id:finngen_R11_O15_PREECLAMPS | CD25 on secreting CD4 regulatory T cell \|\| id:ebi-a-GCST90001941 | MR Egger | 14 | -0.001475894 | 0.0134773173924159 | 0.914608416519766 | -0.027891436 | 0.0249396481431653 | 0.998525194649883 | 0.972493938868322 | 1.0252532427203 | 0.99380795269851 |
| ebi-a-GCST90001941 | finngen_R11_O15_PREECLAMPS | Pre-eclampsia \|\| id:finngen_R11_O15_PREECLAMPS | CD25 on secreting CD4 regulatory T cell \|\| id:ebi-a-GCST90001941 | Weighted median | 14 | -0.002818353 | 0.0150182774555285 | 0.851141996685585 | -0.032254177 | 0.026617471034285 | 0.997185615049187 | 0.96826044166032 | 1.02697487997745 | 0.964912412213677 |
| ebi-a-GCST90001941 | finngen_R11_O15_PREECLAMPS | Pre-eclampsia \|\| id:finngen_R11_O15_PREECLAMPS | CD25 on secreting CD4 regulatory T cell \|\| id:ebi-a-GCST90001941 | Weighted mode | 14 | -0.005011542 | 0.0122097893047477 | 0.688158336576207 | -0.028942729 | 0.0189196453418124 | 0.995000995127909 | 0.971472100311658 | 1.01909975591468 | 0.959313411571287 |
| ebi-a-GCST90001942 | finngen_R11_O15_PREECLAMPS | Pre-eclampsia \|\| id:finngen_R11_O15_PREECLAMPS | CD25 on CD39+ secreting CD4 regulatory T cell \|\| id:ebi-a-GCST90001942 | Inverse variance weighted | 16 | -0.01628232 | 0.01501688413092 | 0.278246196921578 | -0.045715413 | 0.0131507730103127 | 0.983849520558198 | 0.955313793602992 | 1.01323762473052 | 0.885079385681701 |
| ebi-a-GCST90001942 | finngen_R11_O15_PREECLAMPS | Pre-eclampsia \|\| id:finngen_R11_O15_PREECLAMPS | CD25 on CD39+ secreting CD4 regulatory T cell \|\| id:ebi-a-GCST90001942 | MR Egger | 16 | -0.010385926 | 0.0178076959333707 | 0.569017365345847 | -0.04528901 | 0.0245171579909454 | 0.989667821457937 | 0.955721228857863 | 1.02482017481163 | 0.99380795269851 |
| ebi-a-GCST90001942 | finngen_R11_O15_PREECLAMPS | Pre-eclampsia \|\| id:finngen_R11_O15_PREECLAMPS | CD25 on CD39+ secreting CD4 regulatory T cell \|\| id:ebi-a-GCST90001942 | Weighted median | 16 | -0.033019068 | 0.0192968451508047 | 0.0870603955458685 | -0.070840884 | 0.00480274861526667 | 0.967520110855472 | 0.931610114059795 | 1.00481430029827 | 0.88227178111339 |
| ebi-a-GCST90001942 | finngen_R11_O15_PREECLAMPS | Pre-eclampsia \|\| id:finngen_R11_O15_PREECLAMPS | CD25 on CD39+ secreting CD4 regulatory T cell \|\| id:ebi-a-GCST90001942 | Weighted mode | 16 | -0.019579087 | 0.0167351531631801 | 0.260280010368989 | -0.052379987 | 0.0132218133197257 | 0.980611338630484 | 0.948968202667078 | 1.01330960800171 | 0.872755742358858 |
| ebi-a-GCST90001943 | finngen_R11_O15_PREECLAMPS | Pre-eclampsia \|\| id:finngen_R11_O15_PREECLAMPS | CD25 on activated & secreting CD4 regulatory T cell \|\| id:ebi-a-GCST90001943 | Inverse variance weighted | 21 | -0.025514564 | 0.0115197843221085 | 0.0267705397597319 | -0.048093342 | -0.002935787 | 0.974808181288743 | 0.95304482405325 | 0.997068518001178 | 0.708667785226302 |
| ebi-a-GCST90001943 | finngen_R11_O15_PREECLAMPS | Pre-eclampsia \|\| id:finngen_R11_O15_PREECLAMPS | CD25 on activated & secreting CD4 regulatory T cell \|\| id:ebi-a-GCST90001943 | MR Egger | 21 | -0.018698094 | 0.0138367367866844 | 0.192459630569092 | -0.045818098 | 0.00842191059600175 | 0.981475631384683 | 0.955215702409689 | 1.00845747465404 | 0.99380795269851 |
| ebi-a-GCST90001943 | finngen_R11_O15_PREECLAMPS | Pre-eclampsia \|\| id:finngen_R11_O15_PREECLAMPS | CD25 on activated & secreting CD4 regulatory T cell \|\| id:ebi-a-GCST90001943 | Weighted median | 21 | -0.019044271 | 0.014716674308427 | 0.195644426581403 | -0.047888953 | 0.00980041057831692 | 0.981135925347812 | 0.953239635877886 | 1.00984859187226 | 0.940845681141708 |
| ebi-a-GCST90001943 | finngen_R11_O15_PREECLAMPS | Pre-eclampsia \|\| id:finngen_R11_O15_PREECLAMPS | CD25 on activated & secreting CD4 regulatory T cell \|\| id:ebi-a-GCST90001943 | Weighted mode | 21 | -0.022004992 | 0.0137613472083802 | 0.125489984122703 | -0.048977232 | 0.00496724869918039 | 0.978235351855184 | 0.95220280886469 | 1.00497960593101 | 0.872755742358858 |
| ebi-a-GCST90001944 | finngen_R11_O15_PREECLAMPS | Pre-eclampsia \|\| id:finngen_R11_O15_PREECLAMPS | CD123 on plasmacytoid Dendritic Cell \|\| id:ebi-a-GCST90001944 | Inverse variance weighted | 18 | -0.00150773 | 0.00935396525297783 | 0.871946739966348 | -0.019841502 | 0.0168260416649648 | 0.998493405823327 | 0.980354045024596 | 1.01696839680776 | 0.98174814147255 |
| ebi-a-GCST90001944 | finngen_R11_O15_PREECLAMPS | Pre-eclampsia \|\| id:finngen_R11_O15_PREECLAMPS | CD123 on plasmacytoid Dendritic Cell \|\| id:ebi-a-GCST90001944 | MR Egger | 18 | -0.000552015 | 0.0109374464834006 | 0.960372404618124 | -0.02198941 | 0.0208853800711915 | 0.999448137295995 | 0.978250594529541 | 1.02110500594668 | 0.99380795269851 |
| ebi-a-GCST90001944 | finngen_R11_O15_PREECLAMPS | Pre-eclampsia \|\| id:finngen_R11_O15_PREECLAMPS | CD123 on plasmacytoid Dendritic Cell \|\| id:ebi-a-GCST90001944 | Weighted median | 18 | -0.002152837 | 0.0130509289431529 | 0.868978154693466 | -0.027732657 | 0.0234269840706628 | 0.997849479032855 | 0.972648362400971 | 1.02370355135155 | 0.964912412213677 |
| ebi-a-GCST90001944 | finngen_R11_O15_PREECLAMPS | Pre-eclampsia \|\| id:finngen_R11_O15_PREECLAMPS | CD123 on plasmacytoid Dendritic Cell \|\| id:ebi-a-GCST90001944 | Weighted mode | 18 | 0.00105197372108223 | 0.0107910792726184 | 0.923480993926278 | -0.020098542 | 0.0222024890954143 | 1.00105252723952 | 0.980102087667907 | 1.022450799 | 0.986782054514076 |
| ebi-a-GCST90001945 | finngen_R11_O15_PREECLAMPS | Pre-eclampsia \|\| id:finngen_R11_O15_PREECLAMPS | CD123 on CD62L+ plasmacytoid Dendritic Cell \|\| id:ebi-a-GCST90001945 | Inverse variance weighted | 18 | -0.001495414 | 0.00935245182017253 | 0.872963463689682 | -0.01982622 | 0.0168353914421165 | 0.998505703449133 | 0.980369027334822 | 1.01697790528009 | 0.98174814147255 |
| ebi-a-GCST90001945 | finngen_R11_O15_PREECLAMPS | Pre-eclampsia \|\| id:finngen_R11_O15_PREECLAMPS | CD123 on CD62L+ plasmacytoid Dendritic Cell \|\| id:ebi-a-GCST90001945 | MR Egger | 18 | -0.000535934 | 0.0109338273301362 | 0.96151306772872 | -0.021966236 | 0.0208943671678697 | 0.999464209187991 | 0.978273264945116 | 1.02111418275733 | 0.99380795269851 |
| ebi-a-GCST90001945 | finngen_R11_O15_PREECLAMPS | Pre-eclampsia \|\| id:finngen_R11_O15_PREECLAMPS | CD123 on CD62L+ plasmacytoid Dendritic Cell \|\| id:ebi-a-GCST90001945 | Weighted median | 18 | -0.002151414 | 0.0123000678701115 | 0.861149746765742 | -0.026259547 | 0.0219567186717354 | 0.997850898279403 | 0.974082236 | 1.02219954135909 | 0.964912412213677 |
| ebi-a-GCST90001945 | finngen_R11_O15_PREECLAMPS | Pre-eclampsia \|\| id:finngen_R11_O15_PREECLAMPS | CD123 on CD62L+ plasmacytoid Dendritic Cell \|\| id:ebi-a-GCST90001945 | Weighted mode | 18 | 0.000753027851165211 | 0.0112707174657228 | 0.947510245798134 | -0.021337578 | 0.0228436340839819 | 1.00075331144782 | 0.978888457205327 | 1.02310654804637 | 0.990180645930572 |
| ebi-a-GCST90001946 | finngen_R11_O15_PREECLAMPS | Pre-eclampsia \|\| id:finngen_R11_O15_PREECLAMPS | CD33 on CD14+ monocyte \|\| id:ebi-a-GCST90001946 | Inverse variance weighted | 21 | -0.013790496 | 0.0109316438444355 | 0.207121259034236 | -0.035216518 | 0.00763552548930848 | 0.986304156845467 | 0.965396367568529 | 1.00766475044935 | 0.855687495048373 |
| ebi-a-GCST90001946 | finngen_R11_O15_PREECLAMPS | Pre-eclampsia \|\| id:finngen_R11_O15_PREECLAMPS | CD33 on CD14+ monocyte \|\| id:ebi-a-GCST90001946 | MR Egger | 21 | -0.021842165 | 0.0169348336041665 | 0.212606979370368 | -0.055034439 | 0.0113501086641599 | 0.978394647588571 | 0.946452552452017 | 1.0114147655368 | 0.99380795269851 |
| ebi-a-GCST90001946 | finngen_R11_O15_PREECLAMPS | Pre-eclampsia \|\| id:finngen_R11_O15_PREECLAMPS | CD33 on CD14+ monocyte \|\| id:ebi-a-GCST90001946 | Weighted median | 21 | -0.020408155 | 0.0135646321598993 | 0.132449521769419 | -0.046994834 | 0.00617852379219166 | 0.979798681715702 | 0.954092326156591 | 1.0061976502411 | 0.88227178111339 |
| ebi-a-GCST90001946 | finngen_R11_O15_PREECLAMPS | Pre-eclampsia \|\| id:finngen_R11_O15_PREECLAMPS | CD33 on CD14+ monocyte \|\| id:ebi-a-GCST90001946 | Weighted mode | 21 | -0.019800234 | 0.0124949423594654 | 0.128728906651943 | -0.044290321 | 0.00468985346687791 | 0.98039450366801 | 0.956676174367977 | 1.00470086804183 | 0.872755742358858 |
| ebi-a-GCST90001947 | finngen_R11_O15_PREECLAMPS | Pre-eclampsia \|\| id:finngen_R11_O15_PREECLAMPS | CD33 on CD33+ HLA DR+ CD14dim \|\| id:ebi-a-GCST90001947 | Inverse variance weighted | 27 | -0.003213139 | 0.00925151837363706 | 0.72835895349611 | -0.021346115 | 0.0149198375034 | 0.996792018096169 | 0.978880101312461 | 1.01503169387961 | 0.960612995947903 |
| ebi-a-GCST90001947 | finngen_R11_O15_PREECLAMPS | Pre-eclampsia \|\| id:finngen_R11_O15_PREECLAMPS | CD33 on CD33+ HLA DR+ CD14dim \|\| id:ebi-a-GCST90001947 | MR Egger | 27 | -0.001109596 | 0.0146183377697199 | 0.940099352166044 | -0.029761538 | 0.0275423463759774 | 0.998891019720956 | 0.970676975834622 | 1.02792514309021 | 0.99380795269851 |
| ebi-a-GCST90001947 | finngen_R11_O15_PREECLAMPS | Pre-eclampsia \|\| id:finngen_R11_O15_PREECLAMPS | CD33 on CD33+ HLA DR+ CD14dim \|\| id:ebi-a-GCST90001947 | Weighted median | 27 | -0.013919258 | 0.0126399084756584 | 0.2708030594867 | -0.038693478 | 0.0108549627734648 | 0.986177167124132 | 0.962045551648856 | 1.0109140916354 | 0.940845681141708 |
| ebi-a-GCST90001947 | finngen_R11_O15_PREECLAMPS | Pre-eclampsia \|\| id:finngen_R11_O15_PREECLAMPS | CD33 on CD33+ HLA DR+ CD14dim \|\| id:ebi-a-GCST90001947 | Weighted mode | 27 | -0.016152337 | 0.0124094726907307 | 0.204463994137632 | -0.040474904 | 0.008170229435581 | 0.983977412432617 | 0.960333265201176 | 1.00820369684347 | 0.872755742358858 |
| ebi-a-GCST90001948 | finngen_R11_O15_PREECLAMPS | Pre-eclampsia \|\| id:finngen_R11_O15_PREECLAMPS | CD33 on CD33dim HLA DR+ CD11b+ \|\| id:ebi-a-GCST90001948 | Inverse variance weighted | 22 | -0.011298754 | 0.0123987844895669 | 0.362148290803685 | -0.035600372 | 0.0130028633730547 | 0.988764836971266 | 0.965025867960209 | 1.01308776820387 | 0.885079385681701 |
| ebi-a-GCST90001948 | finngen_R11_O15_PREECLAMPS | Pre-eclampsia \|\| id:finngen_R11_O15_PREECLAMPS | CD33 on CD33dim HLA DR+ CD11b+ \|\| id:ebi-a-GCST90001948 | MR Egger | 22 | -0.02612929 | 0.0198616568532272 | 0.203202749980925 | -0.065058137 | 0.0127995575836882 | 0.974209126115568 | 0.937012986406591 | 1.01288182253114 | 0.99380795269851 |
| ebi-a-GCST90001948 | finngen_R11_O15_PREECLAMPS | Pre-eclampsia \|\| id:finngen_R11_O15_PREECLAMPS | CD33 on CD33dim HLA DR+ CD11b+ \|\| id:ebi-a-GCST90001948 | Weighted median | 22 | -0.020280509 | 0.0133898390772781 | 0.129868840131095 | -0.046524593 | 0.00596357581728642 | 0.9799237575384 | 0.954541084903376 | 1.00598139333665 | 0.88227178111339 |
| ebi-a-GCST90001948 | finngen_R11_O15_PREECLAMPS | Pre-eclampsia \|\| id:finngen_R11_O15_PREECLAMPS | CD33 on CD33dim HLA DR+ CD11b+ \|\| id:ebi-a-GCST90001948 | Weighted mode | 22 | -0.020356372 | 0.0141465978424928 | 0.164901403012671 | -0.048083704 | 0.00737095996127944 | 0.97984942036703 | 0.953054009704948 | 1.00739819235516 | 0.872755742358858 |
| ebi-a-GCST90001949 | finngen_R11_O15_PREECLAMPS | Pre-eclampsia \|\| id:finngen_R11_O15_PREECLAMPS | CD33 on CD33dim HLA DR+ CD11b- \|\| id:ebi-a-GCST90001949 | Inverse variance weighted | 22 | -0.011289069 | 0.011963296388336 | 0.345352610389142 | -0.03473713 | 0.0121589917187737 | 0.988774413228946 | 0.965859278198949 | 1.01223321277125 | 0.885079385681701 |
| ebi-a-GCST90001949 | finngen_R11_O15_PREECLAMPS | Pre-eclampsia \|\| id:finngen_R11_O15_PREECLAMPS | CD33 on CD33dim HLA DR+ CD11b- \|\| id:ebi-a-GCST90001949 | MR Egger | 22 | -0.021102446 | 0.019905947196683 | 0.301730987248036 | -0.060118103 | 0.0179132104765361 | 0.979118652613423 | 0.941653315373672 | 1.01807461434477 | 0.99380795269851 |
| ebi-a-GCST90001949 | finngen_R11_O15_PREECLAMPS | Pre-eclampsia \|\| id:finngen_R11_O15_PREECLAMPS | CD33 on CD33dim HLA DR+ CD11b- \|\| id:ebi-a-GCST90001949 | Weighted median | 22 | -0.019748619 | 0.0136930684995049 | 0.149236171654085 | -0.046587033 | 0.00708979557649521 | 0.98044510791386 | 0.954481485623532 | 1.00711498767758 | 0.917572019373545 |
| ebi-a-GCST90001949 | finngen_R11_O15_PREECLAMPS | Pre-eclampsia \|\| id:finngen_R11_O15_PREECLAMPS | CD33 on CD33dim HLA DR+ CD11b- \|\| id:ebi-a-GCST90001949 | Weighted mode | 22 | -0.020410078 | 0.0142774625146942 | 0.167560564113582 | -0.048393905 | 0.00757374824862758 | 0.979796797526474 | 0.952758417030067 | 1.00760250162433 | 0.872755742358858 |
| ebi-a-GCST90001950 | finngen_R11_O15_PREECLAMPS | Pre-eclampsia \|\| id:finngen_R11_O15_PREECLAMPS | CD33 on Granulocytic Myeloid-Derived Suppressor Cells \|\| id:ebi-a-GCST90001950 | Inverse variance weighted | 20 | -0.02197576 | 0.0126410993688488 | 0.0821337667367047 | -0.046752315 | 0.00280079450294131 | 0.978263947627555 | 0.954323739973572 | 1.00280472039221 | 0.789997151112252 |
| ebi-a-GCST90001950 | finngen_R11_O15_PREECLAMPS | Pre-eclampsia \|\| id:finngen_R11_O15_PREECLAMPS | CD33 on Granulocytic Myeloid-Derived Suppressor Cells \|\| id:ebi-a-GCST90001950 | MR Egger | 20 | -0.016255641 | 0.0236655372144048 | 0.500905987684642 | -0.062640094 | 0.0301288119863186 | 0.983875768963163 | 0.939281465997463 | 1.03058727739814 | 0.99380795269851 |
| ebi-a-GCST90001950 | finngen_R11_O15_PREECLAMPS | Pre-eclampsia \|\| id:finngen_R11_O15_PREECLAMPS | CD33 on Granulocytic Myeloid-Derived Suppressor Cells \|\| id:ebi-a-GCST90001950 | Weighted median | 20 | -0.029926277 | 0.0184423477026702 | 0.104654951129607 | -0.066073278 | 0.00622072457801242 | 0.970517080420265 | 0.936062268617611 | 1.00624011346862 | 0.88227178111339 |
| ebi-a-GCST90001950 | finngen_R11_O15_PREECLAMPS | Pre-eclampsia \|\| id:finngen_R11_O15_PREECLAMPS | CD33 on Granulocytic Myeloid-Derived Suppressor Cells \|\| id:ebi-a-GCST90001950 | Weighted mode | 20 | -0.030268086 | 0.0183349293640857 | 0.115203457925925 | -0.066204548 | 0.00566837546636905 | 0.970185405472458 | 0.935939400514204 | 1.00568447110425 | 0.872755742358858 |
| ebi-a-GCST90001951 | finngen_R11_O15_PREECLAMPS | Pre-eclampsia \|\| id:finngen_R11_O15_PREECLAMPS | CD33 on CD66b++ myeloid cell \|\| id:ebi-a-GCST90001951 | Inverse variance weighted | 18 | -0.016601192 | 0.0123115975801588 | 0.177523663660403 | -0.040731923 | 0.00752953944049552 | 0.983535848575681 | 0.96008647248296 | 1.00755795770329 | 0.855687495048373 |
| ebi-a-GCST90001951 | finngen_R11_O15_PREECLAMPS | Pre-eclampsia \|\| id:finngen_R11_O15_PREECLAMPS | CD33 on CD66b++ myeloid cell \|\| id:ebi-a-GCST90001951 | MR Egger | 18 | -0.013877831 | 0.0229621293085111 | 0.554065971081017 | -0.058883605 | 0.0311279419501226 | 0.986218021685187 | 0.942816501954828 | 1.03161748259604 | 0.99380795269851 |
| ebi-a-GCST90001951 | finngen_R11_O15_PREECLAMPS | Pre-eclampsia \|\| id:finngen_R11_O15_PREECLAMPS | CD33 on CD66b++ myeloid cell \|\| id:ebi-a-GCST90001951 | Weighted median | 18 | -0.023011373 | 0.0157979439162499 | 0.145225284493483 | -0.053975343 | 0.00795259669953546 | 0.977251369062497 | 0.947455467194849 | 1.00798430258899 | 0.917572019373545 |
| ebi-a-GCST90001951 | finngen_R11_O15_PREECLAMPS | Pre-eclampsia \|\| id:finngen_R11_O15_PREECLAMPS | CD33 on CD66b++ myeloid cell \|\| id:ebi-a-GCST90001951 | Weighted mode | 18 | -0.023359008 | 0.0153832792668262 | 0.147274353414353 | -0.053510235 | 0.00679221966989998 | 0.976911701993891 | 0.947896239183008 | 1.00681533910838 | 0.872755742358858 |
| ebi-a-GCST90001952 | finngen_R11_O15_PREECLAMPS | Pre-eclampsia \|\| id:finngen_R11_O15_PREECLAMPS | CD33 on Monocytic Myeloid-Derived Suppressor Cells \|\| id:ebi-a-GCST90001952 | Inverse variance weighted | 21 | -0.018069759 | 0.0114093221032929 | 0.113245723870123 | -0.040432031 | 0.00429251197847508 | 0.982092519838724 | 0.960374438303626 | 1.00430173800424 | 0.820779257935285 |
| ebi-a-GCST90001952 | finngen_R11_O15_PREECLAMPS | Pre-eclampsia \|\| id:finngen_R11_O15_PREECLAMPS | CD33 on Monocytic Myeloid-Derived Suppressor Cells \|\| id:ebi-a-GCST90001952 | MR Egger | 21 | -0.015197166 | 0.017462405996491 | 0.395010460770648 | -0.049423482 | 0.0190291499345372 | 0.984917728347632 | 0.951777983905582 | 1.01921135812876 | 0.99380795269851 |
| ebi-a-GCST90001952 | finngen_R11_O15_PREECLAMPS | Pre-eclampsia \|\| id:finngen_R11_O15_PREECLAMPS | CD33 on Monocytic Myeloid-Derived Suppressor Cells \|\| id:ebi-a-GCST90001952 | Weighted median | 21 | -0.020744682 | 0.0139834118543915 | 0.137936095268468 | -0.04815217 | 0.0066628049426232 | 0.979469008430094 | 0.952988760194963 | 1.00668505080665 | 0.892312262311947 |
| ebi-a-GCST90001952 | finngen_R11_O15_PREECLAMPS | Pre-eclampsia \|\| id:finngen_R11_O15_PREECLAMPS | CD33 on Monocytic Myeloid-Derived Suppressor Cells \|\| id:ebi-a-GCST90001952 | Weighted mode | 21 | -0.021907013 | 0.0129443649633018 | 0.106091893293083 | -0.047277969 | 0.00346394184019599 | 0.978331202427991 | 0.953822227902312 | 1.00346994821998 | 0.872755742358858 |
| ebi-a-GCST90001953 | finngen_R11_O15_PREECLAMPS | Pre-eclampsia \|\| id:finngen_R11_O15_PREECLAMPS | CD33 on CD33dim HLA DR- \|\| id:ebi-a-GCST90001953 | Inverse variance weighted | 19 | -0.014424353 | 0.0116265070620257 | 0.214737404594187 | -0.037212306 | 0.00836360121813841 | 0.985679179956268 | 0.963471562392199 | 1.00839867384043 | 0.855687495048373 |
| ebi-a-GCST90001953 | finngen_R11_O15_PREECLAMPS | Pre-eclampsia \|\| id:finngen_R11_O15_PREECLAMPS | CD33 on CD33dim HLA DR- \|\| id:ebi-a-GCST90001953 | MR Egger | 19 | -0.022782957 | 0.0186593831968531 | 0.238753643501834 | -0.059355348 | 0.013789433972063 | 0.977474614682828 | 0.94237183955347 | 1.01388494673405 | 0.99380795269851 |
| ebi-a-GCST90001953 | finngen_R11_O15_PREECLAMPS | Pre-eclampsia \|\| id:finngen_R11_O15_PREECLAMPS | CD33 on CD33dim HLA DR- \|\| id:ebi-a-GCST90001953 | Weighted median | 19 | -0.020647643 | 0.0133392918962295 | 0.121650831358117 | -0.046792655 | 0.00549736906961198 | 0.97956405997495 | 0.954285243196152 | 1.00551250733045 | 0.88227178111339 |
| ebi-a-GCST90001953 | finngen_R11_O15_PREECLAMPS | Pre-eclampsia \|\| id:finngen_R11_O15_PREECLAMPS | CD33 on CD33dim HLA DR- \|\| id:ebi-a-GCST90001953 | Weighted mode | 19 | -0.021442843 | 0.012748512974868 | 0.109838043741574 | -0.046429928 | 0.00354424265789741 | 0.978785420530976 | 0.954631450967061 | 1.00355053091275 | 0.872755742358858 |
| ebi-a-GCST90001954 | finngen_R11_O15_PREECLAMPS | Pre-eclampsia \|\| id:finngen_R11_O15_PREECLAMPS | CD33 on basophil \|\| id:ebi-a-GCST90001954 | Inverse variance weighted | 22 | 0.00177854807536278 | 0.00843509636135138 | 0.833003580335925 | -0.014754241 | 0.0183113369436115 | 1.00178013063007 | 0.985354069683888 | 1.0184800174897 | 0.977824840984872 |
| ebi-a-GCST90001954 | finngen_R11_O15_PREECLAMPS | Pre-eclampsia \|\| id:finngen_R11_O15_PREECLAMPS | CD33 on basophil \|\| id:ebi-a-GCST90001954 | MR Egger | 22 | -0.001414616 | 0.0114842200054404 | 0.903194719306936 | -0.023923687 | 0.0210944550685549 | 0.998586383955666 | 0.976360215547703 | 1.02131851579212 | 0.99380795269851 |
| ebi-a-GCST90001954 | finngen_R11_O15_PREECLAMPS | Pre-eclampsia \|\| id:finngen_R11_O15_PREECLAMPS | CD33 on basophil \|\| id:ebi-a-GCST90001954 | Weighted median | 22 | 0.00565767646192607 | 0.0125042951188491 | 0.650938605024127 | -0.018850742 | 0.0301660948948703 | 1.00567371133919 | 0.981325822070373 | 1.03062570140563 | 0.940845681141708 |
| ebi-a-GCST90001954 | finngen_R11_O15_PREECLAMPS | Pre-eclampsia \|\| id:finngen_R11_O15_PREECLAMPS | CD33 on basophil \|\| id:ebi-a-GCST90001954 | Weighted mode | 22 | -0.001590787 | 0.00867524192931638 | 0.856265932027707 | -0.018594261 | 0.0154126872085195 | 0.998410477657981 | 0.981577545598333 | 1.0155320752469 | 0.977585971930092 |
| ebi-a-GCST90001955 | finngen_R11_O15_PREECLAMPS | Pre-eclampsia \|\| id:finngen_R11_O15_PREECLAMPS | CD33 on Immature Myeloid-Derived Suppressor Cells \|\| id:ebi-a-GCST90001955 | Inverse variance weighted | 23 | -0.012239971 | 0.0119246513361903 | 0.304682987333775 | -0.035612287 | 0.0111323460069886 | 0.987834633135571 | 0.965014369354827 | 1.01119454114915 | 0.885079385681701 |
| ebi-a-GCST90001955 | finngen_R11_O15_PREECLAMPS | Pre-eclampsia \|\| id:finngen_R11_O15_PREECLAMPS | CD33 on Immature Myeloid-Derived Suppressor Cells \|\| id:ebi-a-GCST90001955 | MR Egger | 23 | -0.023611735 | 0.01875623995806 | 0.221890884500786 | -0.060373965 | 0.013150495814711 | 0.976664841411245 | 0.941412412623661 | 1.01323734386554 | 0.99380795269851 |
| ebi-a-GCST90001955 | finngen_R11_O15_PREECLAMPS | Pre-eclampsia \|\| id:finngen_R11_O15_PREECLAMPS | CD33 on Immature Myeloid-Derived Suppressor Cells \|\| id:ebi-a-GCST90001955 | Weighted median | 23 | -0.022509959 | 0.0144161364000362 | 0.118419531134138 | -0.050765586 | 0.00574566847678914 | 0.977741499947017 | 0.95050145506672 | 1.00576220648872 | 0.88227178111339 |
| ebi-a-GCST90001955 | finngen_R11_O15_PREECLAMPS | Pre-eclampsia \|\| id:finngen_R11_O15_PREECLAMPS | CD33 on Immature Myeloid-Derived Suppressor Cells \|\| id:ebi-a-GCST90001955 | Weighted mode | 23 | -0.022221958 | 0.0134095342029773 | 0.111677501356477 | -0.048504645 | 0.00406072862785084 | 0.978023130499037 | 0.952652913796154 | 1.00406898455759 | 0.872755742358858 |
| ebi-a-GCST90001956 | finngen_R11_O15_PREECLAMPS | Pre-eclampsia \|\| id:finngen_R11_O15_PREECLAMPS | CD33 on CD33+ HLA DR+ \|\| id:ebi-a-GCST90001956 | Inverse variance weighted | 23 | -0.018790702 | 0.0103017791482962 | 0.0681483095836831 | -0.038982189 | 0.00140078556292278 | 0.981384743037281 | 0.961767839330868 | 1.00140176712128 | 0.789997151112252 |
| ebi-a-GCST90001956 | finngen_R11_O15_PREECLAMPS | Pre-eclampsia \|\| id:finngen_R11_O15_PREECLAMPS | CD33 on CD33+ HLA DR+ \|\| id:ebi-a-GCST90001956 | MR Egger | 23 | -0.007980282 | 0.0160784413080924 | 0.624813906900954 | -0.039494027 | 0.0235334631896202 | 0.992051476139174 | 0.961275695924937 | 1.02381256020725 | 0.99380795269851 |
| ebi-a-GCST90001956 | finngen_R11_O15_PREECLAMPS | Pre-eclampsia \|\| id:finngen_R11_O15_PREECLAMPS | CD33 on CD33+ HLA DR+ \|\| id:ebi-a-GCST90001956 | Weighted median | 23 | -0.019389737 | 0.0128325689407907 | 0.130793910699209 | -0.044541572 | 0.00576209844561666 | 0.980797035165599 | 0.956435838505571 | 1.00577873126617 | 0.88227178111339 |
| ebi-a-GCST90001956 | finngen_R11_O15_PREECLAMPS | Pre-eclampsia \|\| id:finngen_R11_O15_PREECLAMPS | CD33 on CD33+ HLA DR+ \|\| id:ebi-a-GCST90001956 | Weighted mode | 23 | -0.020253844 | 0.0120732050555136 | 0.107579876423331 | -0.043917325 | 0.00340963838295972 | 0.979949887797124 | 0.957033076496058 | 1.00341545781208 | 0.872755742358858 |
| ebi-a-GCST90001957 | finngen_R11_O15_PREECLAMPS | Pre-eclampsia \|\| id:finngen_R11_O15_PREECLAMPS | CD33 on CD33+ HLA DR+ CD14- \|\| id:ebi-a-GCST90001957 | Inverse variance weighted | 21 | -0.019234553 | 0.0106132787784983 | 0.0699382909627573 | -0.040036579 | 0.00156747381592059 | 0.980949251069841 | 0.960754295082242 | 1.00156870294513 | 0.789997151112252 |
| ebi-a-GCST90001957 | finngen_R11_O15_PREECLAMPS | Pre-eclampsia \|\| id:finngen_R11_O15_PREECLAMPS | CD33 on CD33+ HLA DR+ CD14- \|\| id:ebi-a-GCST90001957 | MR Egger | 21 | -0.013236014 | 0.0171578114489507 | 0.449941653496141 | -0.046865325 | 0.0203932961116961 | 0.986851196510731 | 0.954215898184537 | 1.02060266016059 | 0.99380795269851 |
| ebi-a-GCST90001957 | finngen_R11_O15_PREECLAMPS | Pre-eclampsia \|\| id:finngen_R11_O15_PREECLAMPS | CD33 on CD33+ HLA DR+ CD14- \|\| id:ebi-a-GCST90001957 | Weighted median | 21 | -0.019604729 | 0.0125787299017175 | 0.119100108219084 | -0.04425904 | 0.0050495815192799 | 0.980586193912976 | 0.956706100515122 | 1.00506235214243 | 0.88227178111339 |
| ebi-a-GCST90001957 | finngen_R11_O15_PREECLAMPS | Pre-eclampsia \|\| id:finngen_R11_O15_PREECLAMPS | CD33 on CD33+ HLA DR+ CD14- \|\| id:ebi-a-GCST90001957 | Weighted mode | 21 | -0.021757046 | 0.0120215715069706 | 0.0853758039346772 | -0.045319326 | 0.00180523414112479 | 0.978477931290467 | 0.955692255558291 | 1.00180686455722 | 0.872755742358858 |
| ebi-a-GCST90001958 | finngen_R11_O15_PREECLAMPS | Pre-eclampsia \|\| id:finngen_R11_O15_PREECLAMPS | CD4 on monocyte \|\| id:ebi-a-GCST90001958 | Inverse variance weighted | 15 | -0.020247339 | 0.0271528327996159 | 0.455860411584988 | -0.073466892 | 0.0329722130062965 | 0.979956261651909 | 0.929166908541487 | 1.03352182037799 | 0.898694924660397 |
| ebi-a-GCST90001958 | finngen_R11_O15_PREECLAMPS | Pre-eclampsia \|\| id:finngen_R11_O15_PREECLAMPS | CD4 on monocyte \|\| id:ebi-a-GCST90001958 | MR Egger | 15 | -0.015157425 | 0.0408264448294794 | 0.716420601916034 | -0.095177257 | 0.0648624072227717 | 0.984956870913784 | 0.909211756482027 | 1.06701220111143 | 0.99380795269851 |
| ebi-a-GCST90001958 | finngen_R11_O15_PREECLAMPS | Pre-eclampsia \|\| id:finngen_R11_O15_PREECLAMPS | CD4 on monocyte \|\| id:ebi-a-GCST90001958 | Weighted median | 15 | -0.006824852 | 0.0335221406308382 | 0.838672059499641 | -0.072528248 | 0.0588785437226704 | 0.993198384496313 | 0.930039474952454 | 1.0606464107521 | 0.964912412213677 |
| ebi-a-GCST90001958 | finngen_R11_O15_PREECLAMPS | Pre-eclampsia \|\| id:finngen_R11_O15_PREECLAMPS | CD4 on monocyte \|\| id:ebi-a-GCST90001958 | Weighted mode | 15 | -0.000901019 | 0.0347821162872381 | 0.97969900059449 | -0.069073967 | 0.0672719286056993 | 0.999099386478732 | 0.933257647032526 | 1.06958628974126 | 0.994159530659367 |
| ebi-a-GCST90001959 | finngen_R11_O15_PREECLAMPS | Pre-eclampsia \|\| id:finngen_R11_O15_PREECLAMPS | CD4 on HLA DR+ CD4+ T cell \|\| id:ebi-a-GCST90001959 | Inverse variance weighted (multiplicative random effects) | 21 | -0.022519164 | 0.0377833437821801 | 0.551170077669901 | -0.096574518 | 0.0515361896980212 | 0.97773249963568 | 0.907942237104455 | 1.05288728927571 | 0.905435115099076 |
| ebi-a-GCST90001959 | finngen_R11_O15_PREECLAMPS | Pre-eclampsia \|\| id:finngen_R11_O15_PREECLAMPS | CD4 on HLA DR+ CD4+ T cell \|\| id:ebi-a-GCST90001959 | MR Egger | 21 | -0.122931998 | 0.0715590420296336 | 0.102067583613957 | -0.263187721 | 0.0173237240288996 | 0.884323795994196 | 0.768597602067147 | 1.01747465000973 | 0.97261503632431 |
| ebi-a-GCST90001959 | finngen_R11_O15_PREECLAMPS | Pre-eclampsia \|\| id:finngen_R11_O15_PREECLAMPS | CD4 on HLA DR+ CD4+ T cell \|\| id:ebi-a-GCST90001959 | Weighted median | 21 | -0.060553285 | 0.0380830460042944 | 0.111827455196807 | -0.135196055 | 0.0140894852239638 | 0.941243613568486 | 0.873544632007029 | 1.01418920982658 | 0.88227178111339 |
| ebi-a-GCST90001959 | finngen_R11_O15_PREECLAMPS | Pre-eclampsia \|\| id:finngen_R11_O15_PREECLAMPS | CD4 on HLA DR+ CD4+ T cell \|\| id:ebi-a-GCST90001959 | Weighted mode | 21 | -0.068519118 | 0.0437947992821914 | 0.133374382547086 | -0.154356924 | 0.0173186888439409 | 0.933775608245035 | 0.856966094358812 | 1.01746952684957 | 0.872755742358858 |
| ebi-a-GCST90001960 | finngen_R11_O15_PREECLAMPS | Pre-eclampsia \|\| id:finngen_R11_O15_PREECLAMPS | CD25 on CD4+ T cell \|\| id:ebi-a-GCST90001960 | Inverse variance weighted | 16 | -0.038366839 | 0.0111279775304698 | 0.000565210405683824 | -0.060177675 | -0.016556003 | 0.962359845351019 | 0.941597220809091 | 0.983580294712671 | 0.137722935518292 |
| ebi-a-GCST90001960 | finngen_R11_O15_PREECLAMPS | Pre-eclampsia \|\| id:finngen_R11_O15_PREECLAMPS | CD25 on CD4+ T cell \|\| id:ebi-a-GCST90001960 | MR Egger | 16 | -0.037180976 | 0.013003569937098 | 0.0126165729759958 | -0.062667974 | -0.011693979 | 0.963501748412035 | 0.939255279509616 | 0.98837412942489 | 0.658765346103783 |
| ebi-a-GCST90001960 | finngen_R11_O15_PREECLAMPS | Pre-eclampsia \|\| id:finngen_R11_O15_PREECLAMPS | CD25 on CD4+ T cell \|\| id:ebi-a-GCST90001960 | Weighted median | 16 | -0.034946766 | 0.0155736097025609 | 0.0248341114456191 | -0.065471041 | -0.004422491 | 0.965656820415685 | 0.936626169866864 | 0.995587273573489 | 0.648347695240983 |
| ebi-a-GCST90001960 | finngen_R11_O15_PREECLAMPS | Pre-eclampsia \|\| id:finngen_R11_O15_PREECLAMPS | CD25 on CD4+ T cell \|\| id:ebi-a-GCST90001960 | Weighted mode | 16 | -0.034715784 | 0.0131681567393956 | 0.0186943551326617 | -0.060525372 | -0.008906197 | 0.965879895341566 | 0.941269887142263 | 0.991133345461026 | 0.720210568263475 |
| ebi-a-GCST90001961 | finngen_R11_O15_PREECLAMPS | Pre-eclampsia \|\| id:finngen_R11_O15_PREECLAMPS | CD25 on CD39+ CD4+ T cell \|\| id:ebi-a-GCST90001961 | Inverse variance weighted | 23 | -0.0202413 | 0.0151027089834458 | 0.180166354039914 | -0.04984261 | 0.00936000951880261 | 0.97996217981399 | 0.951379150571127 | 1.0094039513997 | 0.855687495048373 |
| ebi-a-GCST90001961 | finngen_R11_O15_PREECLAMPS | Pre-eclampsia \|\| id:finngen_R11_O15_PREECLAMPS | CD25 on CD39+ CD4+ T cell \|\| id:ebi-a-GCST90001961 | MR Egger | 23 | -0.029116028 | 0.0237040430497878 | 0.232917790017967 | -0.075575952 | 0.0173438963727394 | 0.971303759490953 | 0.927209304103848 | 1.01749517506523 | 0.99380795269851 |
| ebi-a-GCST90001961 | finngen_R11_O15_PREECLAMPS | Pre-eclampsia \|\| id:finngen_R11_O15_PREECLAMPS | CD25 on CD39+ CD4+ T cell \|\| id:ebi-a-GCST90001961 | Weighted median | 23 | -0.030104386 | 0.0199820736980806 | 0.131920995788846 | -0.069269251 | 0.00906047821042101 | 0.970344237677294 | 0.933075415057435 | 1.00910164859029 | 0.88227178111339 |
| ebi-a-GCST90001961 | finngen_R11_O15_PREECLAMPS | Pre-eclampsia \|\| id:finngen_R11_O15_PREECLAMPS | CD25 on CD39+ CD4+ T cell \|\| id:ebi-a-GCST90001961 | Weighted mode | 23 | -0.026531349 | 0.01729502458448 | 0.139275043157167 | -0.060429597 | 0.00736689920797615 | 0.973817515173542 | 0.941360041102864 | 1.00739410156793 | 0.872755742358858 |
| ebi-a-GCST90001962 | finngen_R11_O15_PREECLAMPS | Pre-eclampsia \|\| id:finngen_R11_O15_PREECLAMPS | CD25 on CD28+ CD4+ T cell \|\| id:ebi-a-GCST90001962 | Inverse variance weighted | 19 | -0.046477781 | 0.0322843682694735 | 0.149970039927336 | -0.109755143 | 0.016799580926467 | 0.954585770388497 | 0.896053513690326 | 1.01694148742897 | 0.85505992597664 |
| ebi-a-GCST90001962 | finngen_R11_O15_PREECLAMPS | Pre-eclampsia \|\| id:finngen_R11_O15_PREECLAMPS | CD25 on CD28+ CD4+ T cell \|\| id:ebi-a-GCST90001962 | MR Egger | 19 | -0.115117585 | 0.0781956593475382 | 0.159245574559596 | -0.268381078 | 0.0381459068508956 | 0.891261338361534 | 0.764616347267983 | 1.03888280193883 | 0.99380795269851 |
| ebi-a-GCST90001962 | finngen_R11_O15_PREECLAMPS | Pre-eclampsia \|\| id:finngen_R11_O15_PREECLAMPS | CD25 on CD28+ CD4+ T cell \|\| id:ebi-a-GCST90001962 | Weighted median | 19 | -0.072723129 | 0.037959718108784 | 0.0553908890513723 | -0.147124177 | 0.00167791830348461 | 0.929858244994529 | 0.86318678308945 | 1.00167932679607 | 0.771214851704188 |
| ebi-a-GCST90001962 | finngen_R11_O15_PREECLAMPS | Pre-eclampsia \|\| id:finngen_R11_O15_PREECLAMPS | CD25 on CD28+ CD4+ T cell \|\| id:ebi-a-GCST90001962 | Weighted mode | 19 | -0.092325071 | 0.0495806531060282 | 0.0789938300879951 | -0.189503151 | 0.0048530094269828 | 0.911808699107659 | 0.827370110058094 | 1.0048648043498 | 0.872755742358858 |
| ebi-a-GCST90001963 | finngen_R11_O15_PREECLAMPS | Pre-eclampsia \|\| id:finngen_R11_O15_PREECLAMPS | FSC-A on myeloid Dendritic Cell \|\| id:ebi-a-GCST90001963 | Inverse variance weighted | 20 | 0.00563666825470519 | 0.0112123874982208 | 0.615162600436186 | -0.016339611 | 0.027612947751218 | 1.00565258415938 | 0.983793156100154 | 1.02799771858089 | 0.927459753639562 |
| ebi-a-GCST90001963 | finngen_R11_O15_PREECLAMPS | Pre-eclampsia \|\| id:finngen_R11_O15_PREECLAMPS | FSC-A on myeloid Dendritic Cell \|\| id:ebi-a-GCST90001963 | MR Egger | 20 | -0.011957764 | 0.0139526949869275 | 0.402699297633767 | -0.039305046 | 0.0153895184569143 | 0.988113446219286 | 0.961457375785909 | 1.01550854690907 | 0.99380795269851 |
| ebi-a-GCST90001963 | finngen_R11_O15_PREECLAMPS | Pre-eclampsia \|\| id:finngen_R11_O15_PREECLAMPS | FSC-A on myeloid Dendritic Cell \|\| id:ebi-a-GCST90001963 | Weighted median | 20 | -0.004649713 | 0.0145285687821078 | 0.74893856116558 | -0.033125707 | 0.0238262822558462 | 0.995361080621474 | 0.967416940505684 | 1.02411239594179 | 0.957984984509652 |
| ebi-a-GCST90001963 | finngen_R11_O15_PREECLAMPS | Pre-eclampsia \|\| id:finngen_R11_O15_PREECLAMPS | FSC-A on myeloid Dendritic Cell \|\| id:ebi-a-GCST90001963 | Weighted mode | 20 | -0.005803281 | 0.0124709471624315 | 0.646974520457614 | -0.030246337 | 0.0186397754063746 | 0.994213525476696 | 0.970206505892598 | 1.01881458043967 | 0.959313411571287 |
| ebi-a-GCST90001964 | finngen_R11_O15_PREECLAMPS | Pre-eclampsia \|\| id:finngen_R11_O15_PREECLAMPS | FSC-A on plasmacytoid Dendritic Cell \|\| id:ebi-a-GCST90001964 | Inverse variance weighted | 27 | -0.00077684 | 0.0102476389830014 | 0.939572842847742 | -0.020862213 | 0.019308532161445 | 0.999223461417026 | 0.979353897849613 | 1.01949614744837 | 0.986667691552896 |
| ebi-a-GCST90001964 | finngen_R11_O15_PREECLAMPS | Pre-eclampsia \|\| id:finngen_R11_O15_PREECLAMPS | FSC-A on plasmacytoid Dendritic Cell \|\| id:ebi-a-GCST90001964 | MR Egger | 27 | 0.00287789613323191 | 0.0134591086130217 | 0.832417730849592 | -0.023501957 | 0.0292577490147545 | 1.00288204125176 | 0.976772063369908 | 1.02968996184774 | 0.99380795269851 |
| ebi-a-GCST90001964 | finngen_R11_O15_PREECLAMPS | Pre-eclampsia \|\| id:finngen_R11_O15_PREECLAMPS | FSC-A on plasmacytoid Dendritic Cell \|\| id:ebi-a-GCST90001964 | Weighted median | 27 | -0.002075825 | 0.0148315308270884 | 0.888691401296697 | -0.031145625 | 0.0269939757376369 | 0.99792632835057 | 0.969334403373514 | 1.02736161364894 | 0.964912412213677 |
| ebi-a-GCST90001964 | finngen_R11_O15_PREECLAMPS | Pre-eclampsia \|\| id:finngen_R11_O15_PREECLAMPS | FSC-A on plasmacytoid Dendritic Cell \|\| id:ebi-a-GCST90001964 | Weighted mode | 27 | 0.000815259399724599 | 0.0117550729186512 | 0.945238635403632 | -0.022224684 | 0.023855202320281 | 1.000815592 | 0.978020465281227 | 1.02414201376654 | 0.990180645930572 |
| ebi-a-GCST90001965 | finngen_R11_O15_PREECLAMPS | Pre-eclampsia \|\| id:finngen_R11_O15_PREECLAMPS | FSC-A on monocyte \|\| id:ebi-a-GCST90001965 | Inverse variance weighted | 19 | -0.017756114 | 0.0183974605812135 | 0.334474987726688 | -0.053815137 | 0.0183029084101405 | 0.982400596572622 | 0.947607267768586 | 1.01847143323296 | 0.885079385681701 |
| ebi-a-GCST90001965 | finngen_R11_O15_PREECLAMPS | Pre-eclampsia \|\| id:finngen_R11_O15_PREECLAMPS | FSC-A on monocyte \|\| id:ebi-a-GCST90001965 | MR Egger | 19 | -0.020768297 | 0.0227737891756655 | 0.374552245280412 | -0.065404923 | 0.0238683301505302 | 0.979445879187248 | 0.936688099628979 | 1.0241554586173 | 0.99380795269851 |
| ebi-a-GCST90001965 | finngen_R11_O15_PREECLAMPS | Pre-eclampsia \|\| id:finngen_R11_O15_PREECLAMPS | FSC-A on monocyte \|\| id:ebi-a-GCST90001965 | Weighted median | 19 | -0.013286437 | 0.0184039935728156 | 0.470336105113517 | -0.049358265 | 0.0227853901005615 | 0.986801437793099 | 0.951840057907726 | 1.02304695998088 | 0.940845681141708 |
| ebi-a-GCST90001965 | finngen_R11_O15_PREECLAMPS | Pre-eclampsia \|\| id:finngen_R11_O15_PREECLAMPS | FSC-A on monocyte \|\| id:ebi-a-GCST90001965 | Weighted mode | 19 | -0.015164946 | 0.01792677652969 | 0.408692605720212 | -0.050301428 | 0.019971535840493 | 0.984949462574071 | 0.95094274037896 | 1.02017230126637 | 0.930105377419646 |
| ebi-a-GCST90001966 | finngen_R11_O15_PREECLAMPS | Pre-eclampsia \|\| id:finngen_R11_O15_PREECLAMPS | FSC-A on granulocyte \|\| id:ebi-a-GCST90001966 | Inverse variance weighted | 21 | -0.007158879 | 0.0209005010142749 | 0.731958192534859 | -0.048123861 | 0.0338061034124775 | 0.992866685156801 | 0.953015738700486 | 1.03438402375018 | 0.960612995947903 |
| ebi-a-GCST90001966 | finngen_R11_O15_PREECLAMPS | Pre-eclampsia \|\| id:finngen_R11_O15_PREECLAMPS | FSC-A on granulocyte \|\| id:ebi-a-GCST90001966 | MR Egger | 21 | -0.006902394 | 0.0331619204607341 | 0.837332890129547 | -0.071899758 | 0.0580949699391438 | 0.993121372644636 | 0.930624178516205 | 1.05981564155803 | 0.99380795269851 |
| ebi-a-GCST90001966 | finngen_R11_O15_PREECLAMPS | Pre-eclampsia \|\| id:finngen_R11_O15_PREECLAMPS | FSC-A on granulocyte \|\| id:ebi-a-GCST90001966 | Weighted median | 21 | -0.021829957 | 0.0268582619544168 | 0.416342089853441 | -0.074472151 | 0.0308122362446262 | 0.97840659191701 | 0.928233324424679 | 1.03129184647619 | 0.940845681141708 |
| ebi-a-GCST90001966 | finngen_R11_O15_PREECLAMPS | Pre-eclampsia \|\| id:finngen_R11_O15_PREECLAMPS | FSC-A on granulocyte \|\| id:ebi-a-GCST90001966 | Weighted mode | 21 | 0.000626949854639847 | 0.0240073106060258 | 0.979424466681669 | -0.046427379 | 0.0476812786424504 | 1.00062714642878 | 0.954633884583878 | 1.04883631551235 | 0.994159530659367 |
| ebi-a-GCST90001967 | finngen_R11_O15_PREECLAMPS | Pre-eclampsia \|\| id:finngen_R11_O15_PREECLAMPS | FSC-A on CD14+ monocyte \|\| id:ebi-a-GCST90001967 | Inverse variance weighted | 23 | 0.00526434904518671 | 0.0180340373560715 | 0.770354029804469 | -0.030082364 | 0.0406110622630869 | 1.00527823007813 | 0.970365606896566 | 1.04144696873695 | 0.977824840984872 |
| ebi-a-GCST90001967 | finngen_R11_O15_PREECLAMPS | Pre-eclampsia \|\| id:finngen_R11_O15_PREECLAMPS | FSC-A on CD14+ monocyte \|\| id:ebi-a-GCST90001967 | MR Egger | 23 | 0.0110319040865922 | 0.0250081942274523 | 0.663626044192453 | -0.037984157 | 0.0600479647723987 | 1.01109297992813 | 0.962728193658991 | 1.0618874785151 | 0.99380795269851 |
| ebi-a-GCST90001967 | finngen_R11_O15_PREECLAMPS | Pre-eclampsia \|\| id:finngen_R11_O15_PREECLAMPS | FSC-A on CD14+ monocyte \|\| id:ebi-a-GCST90001967 | Weighted median | 23 | 0.007874359648563 | 0.0263386747776224 | 0.764966236816357 | -0.043749443 | 0.059498162212703 | 1.0079054439546 | 0.957193759107303 | 1.06130381052708 | 0.957984984509652 |
| ebi-a-GCST90001967 | finngen_R11_O15_PREECLAMPS | Pre-eclampsia \|\| id:finngen_R11_O15_PREECLAMPS | FSC-A on CD14+ monocyte \|\| id:ebi-a-GCST90001967 | Weighted mode | 23 | 0.00736091791791344 | 0.0232198417979297 | 0.754224910904814 | -0.038149972 | 0.0528718078418556 | 1.00738807606962 | 0.962568571726161 | 1.05429448417101 | 0.959313411571287 |
| ebi-a-GCST90001968 | finngen_R11_O15_PREECLAMPS | Pre-eclampsia \|\| id:finngen_R11_O15_PREECLAMPS | FSC-A on B cell \|\| id:ebi-a-GCST90001968 | Inverse variance weighted | 20 | -0.026046878 | 0.022811306700138 | 0.253520256974135 | -0.070757039 | 0.0186632835824179 | 0.974289416237549 | 0.931688228830874 | 1.0188385311937 | 0.885079385681701 |
| ebi-a-GCST90001968 | finngen_R11_O15_PREECLAMPS | Pre-eclampsia \|\| id:finngen_R11_O15_PREECLAMPS | FSC-A on B cell \|\| id:ebi-a-GCST90001968 | MR Egger | 20 | -0.007521671 | 0.0419022839352357 | 0.859546381890291 | -0.089650148 | 0.0746068054684361 | 0.992506545932431 | 0.914250982266119 | 1.07746041604142 | 0.99380795269851 |
| ebi-a-GCST90001968 | finngen_R11_O15_PREECLAMPS | Pre-eclampsia \|\| id:finngen_R11_O15_PREECLAMPS | FSC-A on B cell \|\| id:ebi-a-GCST90001968 | Weighted median | 20 | -0.003454494 | 0.0328634570915424 | 0.916283308753356 | -0.06786687 | 0.0609578819108092 | 0.996551465910957 | 0.934384860058273 | 1.06285414785867 | 0.973551015550441 |
| ebi-a-GCST90001968 | finngen_R11_O15_PREECLAMPS | Pre-eclampsia \|\| id:finngen_R11_O15_PREECLAMPS | FSC-A on B cell \|\| id:ebi-a-GCST90001968 | Weighted mode | 20 | 0.00207892486295697 | 0.035247337412059 | 0.953583136318926 | -0.067005856 | 0.0711637061905926 | 1.00208108732552 | 0.93518972441515 | 1.07375699214776 | 0.990180645930572 |
| ebi-a-GCST90001969 | finngen_R11_O15_PREECLAMPS | Pre-eclampsia \|\| id:finngen_R11_O15_PREECLAMPS | FSC-A on Natural Killer \|\| id:ebi-a-GCST90001969 | Inverse variance weighted | 18 | 0.0400444846621279 | 0.0207760191053593 | 0.0539250920261916 | -0.000676513 | 0.0807654821086322 | 1.04085707533786 | 0.999323715998803 | 1.08411662200777 | 0.789997151112252 |
| ebi-a-GCST90001969 | finngen_R11_O15_PREECLAMPS | Pre-eclampsia \|\| id:finngen_R11_O15_PREECLAMPS | FSC-A on Natural Killer \|\| id:ebi-a-GCST90001969 | MR Egger | 18 | 0.0377673985263495 | 0.0320609708566119 | 0.256026407737351 | -0.025072104 | 0.100606901405309 | 1.03848965056029 | 0.975239590473807 | 1.10584185143354 | 0.99380795269851 |
| ebi-a-GCST90001969 | finngen_R11_O15_PREECLAMPS | Pre-eclampsia \|\| id:finngen_R11_O15_PREECLAMPS | FSC-A on Natural Killer \|\| id:ebi-a-GCST90001969 | Weighted median | 18 | 0.0386131358372505 | 0.0292223359238949 | 0.186382382669832 | -0.018662643 | 0.0958889142480845 | 1.03936831151056 | 0.981510426227612 | 1.10063679213713 | 0.940845681141708 |
| ebi-a-GCST90001969 | finngen_R11_O15_PREECLAMPS | Pre-eclampsia \|\| id:finngen_R11_O15_PREECLAMPS | FSC-A on Natural Killer \|\| id:ebi-a-GCST90001969 | Weighted mode | 18 | 0.0372458737772122 | 0.0266504950411536 | 0.180216096498417 | -0.014989097 | 0.0894808440578733 | 1.03794819370965 | 0.985122680826257 | 1.0936063840511 | 0.872755742358858 |
| ebi-a-GCST90001970 | finngen_R11_O15_PREECLAMPS | Pre-eclampsia \|\| id:finngen_R11_O15_PREECLAMPS | FSC-A on HLA DR+ Natural Killer \|\| id:ebi-a-GCST90001970 | Inverse variance weighted | 20 | -0.003098344 | 0.0173482970246644 | 0.858254611493191 | -0.037101006 | 0.0309043185541737 | 0.996906451299033 | 0.963578803402543 | 1.0313868145836 | 0.977824840984872 |
| ebi-a-GCST90001970 | finngen_R11_O15_PREECLAMPS | Pre-eclampsia \|\| id:finngen_R11_O15_PREECLAMPS | FSC-A on HLA DR+ Natural Killer \|\| id:ebi-a-GCST90001970 | MR Egger | 20 | -0.010895152 | 0.0246477720059563 | 0.663729696929071 | -0.059204785 | 0.0374144808648671 | 0.989163984940172 | 0.942513736341552 | 1.03812321388614 | 0.99380795269851 |
| ebi-a-GCST90001970 | finngen_R11_O15_PREECLAMPS | Pre-eclampsia \|\| id:finngen_R11_O15_PREECLAMPS | FSC-A on HLA DR+ Natural Killer \|\| id:ebi-a-GCST90001970 | Weighted median | 20 | -0.018550507 | 0.0218878875591312 | 0.396703201704792 | -0.061450766 | 0.0243497529835172 | 0.981620494994276 | 0.9403992439826 | 1.02464862914185 | 0.940845681141708 |
| ebi-a-GCST90001970 | finngen_R11_O15_PREECLAMPS | Pre-eclampsia \|\| id:finngen_R11_O15_PREECLAMPS | FSC-A on HLA DR+ Natural Killer \|\| id:ebi-a-GCST90001970 | Weighted mode | 20 | -0.016921462 | 0.0195622390561881 | 0.397825628303409 | -0.055263451 | 0.0214205265531747 | 0.98322090180881 | 0.94623582876653 | 1.02165159293736 | 0.920286500917063 |
| ebi-a-GCST90001971 | finngen_R11_O15_PREECLAMPS | Pre-eclampsia \|\| id:finngen_R11_O15_PREECLAMPS | FSC-A on lymphocyte \|\| id:ebi-a-GCST90001971 | Inverse variance weighted | 16 | -0.008425313 | 0.0196602261994943 | 0.668253578527084 | -0.046959357 | 0.0301087301053105 | 0.991610080235806 | 0.954126175737281 | 1.03056658147487 | 0.950857618427329 |
| ebi-a-GCST90001971 | finngen_R11_O15_PREECLAMPS | Pre-eclampsia \|\| id:finngen_R11_O15_PREECLAMPS | FSC-A on lymphocyte \|\| id:ebi-a-GCST90001971 | MR Egger | 16 | 0.00654980705793994 | 0.023075212466566 | 0.780682999394298 | -0.038677609 | 0.0517772234924092 | 1.00657130395206 | 0.962060818542607 | 1.05314110128151 | 0.99380795269851 |
| ebi-a-GCST90001971 | finngen_R11_O15_PREECLAMPS | Pre-eclampsia \|\| id:finngen_R11_O15_PREECLAMPS | FSC-A on lymphocyte \|\| id:ebi-a-GCST90001971 | Weighted median | 16 | -0.01618171 | 0.0250169050946522 | 0.517741331301227 | -0.065214844 | 0.0328514244240987 | 0.98394851095773 | 0.936866162104766 | 1.03339699028181 | 0.940845681141708 |
| ebi-a-GCST90001971 | finngen_R11_O15_PREECLAMPS | Pre-eclampsia \|\| id:finngen_R11_O15_PREECLAMPS | FSC-A on lymphocyte \|\| id:ebi-a-GCST90001971 | Weighted mode | 16 | -0.001751059 | 0.0230036347914685 | 0.940328875670476 | -0.046838183 | 0.043336065637187 | 0.998250473654479 | 0.954241797885706 | 1.04428878546227 | 0.990180645930572 |
| ebi-a-GCST90001972 | finngen_R11_O15_PREECLAMPS | Pre-eclampsia \|\| id:finngen_R11_O15_PREECLAMPS | FSC-A on T cell \|\| id:ebi-a-GCST90001972 | Inverse variance weighted | 22 | 0.00131445714427971 | 0.0139680993897801 | 0.925026365550608 | -0.026063018 | 0.0286919319482486 | 1.00131532142172 | 0.974273691226261 | 1.02910751049012 | 0.986667691552896 |
| ebi-a-GCST90001972 | finngen_R11_O15_PREECLAMPS | Pre-eclampsia \|\| id:finngen_R11_O15_PREECLAMPS | FSC-A on T cell \|\| id:ebi-a-GCST90001972 | MR Egger | 22 | 0.00473491747076193 | 0.0174441089227039 | 0.788839515771787 | -0.029455536 | 0.0389253709592617 | 1.00474614490582 | 0.970974050054317 | 1.0396928894718 | 0.99380795269851 |
| ebi-a-GCST90001972 | finngen_R11_O15_PREECLAMPS | Pre-eclampsia \|\| id:finngen_R11_O15_PREECLAMPS | FSC-A on T cell \|\| id:ebi-a-GCST90001972 | Weighted median | 22 | -0.008990484 | 0.0200307793744101 | 0.653551790294702 | -0.048250812 | 0.0302698433066968 | 0.991049809293244 | 0.952894759814344 | 1.03073263273222 | 0.940845681141708 |
| ebi-a-GCST90001972 | finngen_R11_O15_PREECLAMPS | Pre-eclampsia \|\| id:finngen_R11_O15_PREECLAMPS | FSC-A on T cell \|\| id:ebi-a-GCST90001972 | Weighted mode | 22 | -0.000957541 | 0.0165334030660501 | 0.95436348329376 | -0.033363011 | 0.0314479294486868 | 0.999042917734901 | 0.96718739660659 | 1.03194764011409 | 0.990180645930572 |
| ebi-a-GCST90001973 | finngen_R11_O15_PREECLAMPS | Pre-eclampsia \|\| id:finngen_R11_O15_PREECLAMPS | FSC-A on CD4+ T cell \|\| id:ebi-a-GCST90001973 | Inverse variance weighted | 25 | -0.00304117 | 0.0147776989154916 | 0.836951409156416 | -0.03200546 | 0.0259231201765719 | 0.996963449974518 | 0.968501294462064 | 1.02626204659557 | 0.977824840984872 |
| ebi-a-GCST90001973 | finngen_R11_O15_PREECLAMPS | Pre-eclampsia \|\| id:finngen_R11_O15_PREECLAMPS | FSC-A on CD4+ T cell \|\| id:ebi-a-GCST90001973 | MR Egger | 25 | 0.00932604855837794 | 0.0173553879071184 | 0.596181839554759 | -0.024690512 | 0.0433426088563299 | 1.00936967165415 | 0.975611805710248 | 1.04429561849499 | 0.99380795269851 |
| ebi-a-GCST90001973 | finngen_R11_O15_PREECLAMPS | Pre-eclampsia \|\| id:finngen_R11_O15_PREECLAMPS | FSC-A on CD4+ T cell \|\| id:ebi-a-GCST90001973 | Weighted median | 25 | 0.00651506230169577 | 0.0179635908767823 | 0.716842811937339 | -0.028693576 | 0.041723700420189 | 1.00653633148502 | 0.97171417557389 | 1.04260636724887 | 0.949957918887556 |
| ebi-a-GCST90001973 | finngen_R11_O15_PREECLAMPS | Pre-eclampsia \|\| id:finngen_R11_O15_PREECLAMPS | FSC-A on CD4+ T cell \|\| id:ebi-a-GCST90001973 | Weighted mode | 25 | 0.00674369872775638 | 0.0141102219404627 | 0.637024441662955 | -0.020912336 | 0.0343997337310633 | 1.00676648866471 | 0.979304810313669 | 1.03499824776069 | 0.959313411571287 |
| ebi-a-GCST90001974 | finngen_R11_O15_PREECLAMPS | Pre-eclampsia \|\| id:finngen_R11_O15_PREECLAMPS | FSC-A on CD8+ T cell \|\| id:ebi-a-GCST90001974 | Inverse variance weighted | 18 | 0.00100822889426117 | 0.021986230703553 | 0.963423998271186 | -0.042084783 | 0.044101241073225 | 1.00100873732787 | 0.958788487889065 | 1.04508815537914 | 0.992671503887443 |
| ebi-a-GCST90001974 | finngen_R11_O15_PREECLAMPS | Pre-eclampsia \|\| id:finngen_R11_O15_PREECLAMPS | FSC-A on CD8+ T cell \|\| id:ebi-a-GCST90001974 | MR Egger | 18 | -0.008065283 | 0.0294621776683187 | 0.787778916637907 | -0.065811151 | 0.0496805855439255 | 0.991967154442972 | 0.936307668442079 | 1.05093535881316 | 0.99380795269851 |
| ebi-a-GCST90001974 | finngen_R11_O15_PREECLAMPS | Pre-eclampsia \|\| id:finngen_R11_O15_PREECLAMPS | FSC-A on CD8+ T cell \|\| id:ebi-a-GCST90001974 | Weighted median | 18 | -0.019392325 | 0.032090533987742 | 0.545643886156273 | -0.082289772 | 0.0435051214695916 | 0.980794496407096 | 0.921005038772063 | 1.04446534349582 | 0.940845681141708 |
| ebi-a-GCST90001974 | finngen_R11_O15_PREECLAMPS | Pre-eclampsia \|\| id:finngen_R11_O15_PREECLAMPS | FSC-A on CD8+ T cell \|\| id:ebi-a-GCST90001974 | Weighted mode | 18 | -0.017688426 | 0.027538289771175 | 0.529232456039199 | -0.071663474 | 0.0362866215697636 | 0.982467095502992 | 0.930844096038359 | 1.03695301700265 | 0.935508728341156 |
| ebi-a-GCST90001975 | finngen_R11_O15_PREECLAMPS | Pre-eclampsia \|\| id:finngen_R11_O15_PREECLAMPS | FSC-A on HLA DR+ T cell \|\| id:ebi-a-GCST90001975 | Inverse variance weighted | 18 | -0.00956999 | 0.0108503993981853 | 0.377779905426065 | -0.030836773 | 0.0116967927142422 | 0.990475656520475 | 0.969633830652118 | 1.01176546769194 | 0.885079385681701 |
| ebi-a-GCST90001975 | finngen_R11_O15_PREECLAMPS | Pre-eclampsia \|\| id:finngen_R11_O15_PREECLAMPS | FSC-A on HLA DR+ T cell \|\| id:ebi-a-GCST90001975 | MR Egger | 18 | -0.001814929 | 0.0121565827843871 | 0.883185718902755 | -0.025641831 | 0.0220119733630142 | 0.998186717093127 | 0.974684128589285 | 1.02225602423963 | 0.99380795269851 |
| ebi-a-GCST90001975 | finngen_R11_O15_PREECLAMPS | Pre-eclampsia \|\| id:finngen_R11_O15_PREECLAMPS | FSC-A on HLA DR+ T cell \|\| id:ebi-a-GCST90001975 | Weighted median | 18 | -0.00127252 | 0.0127352777016256 | 0.920407177402251 | -0.026233664 | 0.023688624748746 | 0.998728289763234 | 0.974107449325367 | 1.02397142888518 | 0.975775499714955 |
| ebi-a-GCST90001975 | finngen_R11_O15_PREECLAMPS | Pre-eclampsia \|\| id:finngen_R11_O15_PREECLAMPS | FSC-A on HLA DR+ T cell \|\| id:ebi-a-GCST90001975 | Weighted mode | 18 | -0.004250467 | 0.0116997456749894 | 0.72086220162095 | -0.027181968 | 0.0186810349008299 | 0.995758553826206 | 0.973184136898865 | 1.0188566170814 | 0.959313411571287 |
| ebi-a-GCST90001976 | finngen_R11_O15_PREECLAMPS | Pre-eclampsia \|\| id:finngen_R11_O15_PREECLAMPS | FSC-A on Natural Killer T \|\| id:ebi-a-GCST90001976 | Inverse variance weighted | 20 | -0.008058538 | 0.0332057651890234 | 0.808249509628134 | -0.073141837 | 0.0570247621272528 | 0.991973845326397 | 0.929468987198615 | 1.05868202528996 | 0.977824840984872 |
| ebi-a-GCST90001976 | finngen_R11_O15_PREECLAMPS | Pre-eclampsia \|\| id:finngen_R11_O15_PREECLAMPS | FSC-A on Natural Killer T \|\| id:ebi-a-GCST90001976 | MR Egger | 20 | 0.0688860016795501 | 0.0542249781800126 | 0.220127131130708 | -0.037394956 | 0.175166958912375 | 1.07131407418176 | 0.963295601259812 | 1.19144512238914 | 0.99380795269851 |
| ebi-a-GCST90001976 | finngen_R11_O15_PREECLAMPS | Pre-eclampsia \|\| id:finngen_R11_O15_PREECLAMPS | FSC-A on Natural Killer T \|\| id:ebi-a-GCST90001976 | Weighted median | 20 | 0.0542907442088315 | 0.0419759810728219 | 0.195880900778881 | -0.027982179 | 0.136563667111562 | 1.05579152280621 | 0.972405696186396 | 1.14632785883617 | 0.940845681141708 |
| ebi-a-GCST90001976 | finngen_R11_O15_PREECLAMPS | Pre-eclampsia \|\| id:finngen_R11_O15_PREECLAMPS | FSC-A on Natural Killer T \|\| id:ebi-a-GCST90001976 | Weighted mode | 20 | 0.0451307958569998 | 0.0361177181290381 | 0.22663917774864 | -0.025659932 | 0.115921523389914 | 1.04616468496698 | 0.974666486455361 | 1.12290774668201 | 0.872755742358858 |
| ebi-a-GCST90001977 | finngen_R11_O15_PREECLAMPS | Pre-eclampsia \|\| id:finngen_R11_O15_PREECLAMPS | FSC-A on HLA DR+ CD4+ T cell \|\| id:ebi-a-GCST90001977 | Inverse variance weighted | 17 | -0.007470827 | 0.019314773586004 | 0.698909178408868 | -0.045327783 | 0.0303861296666172 | 0.992557010697227 | 0.955684173662094 | 1.03085249984745 | 0.950911139356934 |
| ebi-a-GCST90001977 | finngen_R11_O15_PREECLAMPS | Pre-eclampsia \|\| id:finngen_R11_O15_PREECLAMPS | FSC-A on HLA DR+ CD4+ T cell \|\| id:ebi-a-GCST90001977 | MR Egger | 17 | -0.00464372 | 0.024830923561389 | 0.854155984275053 | -0.05331233 | 0.0440248901546907 | 0.995367045371929 | 0.948083851010171 | 1.0450083649846 | 0.99380795269851 |
| ebi-a-GCST90001977 | finngen_R11_O15_PREECLAMPS | Pre-eclampsia \|\| id:finngen_R11_O15_PREECLAMPS | FSC-A on HLA DR+ CD4+ T cell \|\| id:ebi-a-GCST90001977 | Weighted median | 17 | -0.029403187 | 0.0267324225828721 | 0.271372513126311 | -0.081798735 | 0.0229923614788467 | 0.971024881132695 | 0.921457397115228 | 1.02325872333396 | 0.940845681141708 |
| ebi-a-GCST90001977 | finngen_R11_O15_PREECLAMPS | Pre-eclampsia \|\| id:finngen_R11_O15_PREECLAMPS | FSC-A on HLA DR+ CD4+ T cell \|\| id:ebi-a-GCST90001977 | Weighted mode | 17 | -0.028336464 | 0.0252569147804434 | 0.278448202672982 | -0.077840017 | 0.0211670890935965 | 0.972061248277174 | 0.925112417107757 | 1.02139270096092 | 0.872755742358858 |
| ebi-a-GCST90001978 | finngen_R11_O15_PREECLAMPS | Pre-eclampsia \|\| id:finngen_R11_O15_PREECLAMPS | FSC-A on HLA DR+ CD8+ T cell \|\| id:ebi-a-GCST90001978 | Inverse variance weighted | 18 | 0.0181234022533807 | 0.0136219829741471 | 0.183369203741631 | -0.008575684 | 0.044822488882709 | 1.01828862774806 | 0.991460981917596 | 1.04584219481388 | 0.855687495048373 |
| ebi-a-GCST90001978 | finngen_R11_O15_PREECLAMPS | Pre-eclampsia \|\| id:finngen_R11_O15_PREECLAMPS | FSC-A on HLA DR+ CD8+ T cell \|\| id:ebi-a-GCST90001978 | MR Egger | 18 | 0.0263233081365954 | 0.0169888683047453 | 0.140826677230232 | -0.006974874 | 0.0596214900138963 | 1.02667282649955 | 0.993049394236339 | 1.06143470686385 | 0.97261503632431 |
| ebi-a-GCST90001978 | finngen_R11_O15_PREECLAMPS | Pre-eclampsia \|\| id:finngen_R11_O15_PREECLAMPS | FSC-A on HLA DR+ CD8+ T cell \|\| id:ebi-a-GCST90001978 | Weighted median | 18 | 0.010342433216386 | 0.0165750424424106 | 0.532643167131456 | -0.02214465 | 0.0428295164035108 | 1.01039610103785 | 0.978098742863502 | 1.04375993573377 | 0.940845681141708 |
| ebi-a-GCST90001978 | finngen_R11_O15_PREECLAMPS | Pre-eclampsia \|\| id:finngen_R11_O15_PREECLAMPS | FSC-A on HLA DR+ CD8+ T cell \|\| id:ebi-a-GCST90001978 | Weighted mode | 18 | 0.0149940932749821 | 0.0152716026654284 | 0.339951990092751 | -0.014938248 | 0.0449264344992217 | 1.01510706863969 | 0.985172774163854 | 1.0459509111758 | 0.888599269773114 |
| ebi-a-GCST90001979 | finngen_R11_O15_PREECLAMPS | Pre-eclampsia \|\| id:finngen_R11_O15_PREECLAMPS | CD16 on CD14- CD16+ monocyte \|\| id:ebi-a-GCST90001979 | Inverse variance weighted | 20 | 0.0143966872286506 | 0.014364276607683 | 0.316219805686414 | -0.013757295 | 0.0425506693797093 | 1.01450081864595 | 0.986336904189615 | 1.04346892695748 | 0.885079385681701 |
| ebi-a-GCST90001979 | finngen_R11_O15_PREECLAMPS | Pre-eclampsia \|\| id:finngen_R11_O15_PREECLAMPS | CD16 on CD14- CD16+ monocyte \|\| id:ebi-a-GCST90001979 | MR Egger | 20 | 0.0225400410421753 | 0.0201743797219314 | 0.27857445476787 | -0.017001743 | 0.0620818252971608 | 1.0227959871618 | 0.983141970807738 | 1.06404940732497 | 0.99380795269851 |
| ebi-a-GCST90001979 | finngen_R11_O15_PREECLAMPS | Pre-eclampsia \|\| id:finngen_R11_O15_PREECLAMPS | CD16 on CD14- CD16+ monocyte \|\| id:ebi-a-GCST90001979 | Weighted median | 20 | 0.023436007491419 | 0.0200131865533988 | 0.241587408720247 | -0.015789838 | 0.0626618531360806 | 1.0237127887011 | 0.984334167805304 | 1.06466676462811 | 0.940845681141708 |
| ebi-a-GCST90001979 | finngen_R11_O15_PREECLAMPS | Pre-eclampsia \|\| id:finngen_R11_O15_PREECLAMPS | CD16 on CD14- CD16+ monocyte \|\| id:ebi-a-GCST90001979 | Weighted mode | 20 | 0.0232222757424093 | 0.0183003886222552 | 0.219784751884876 | -0.012646486 | 0.0590910374420296 | 1.02349401215686 | 0.987433144809728 | 1.06087181540053 | 0.872755742358858 |
| ebi-a-GCST90001980 | finngen_R11_O15_PREECLAMPS | Pre-eclampsia \|\| id:finngen_R11_O15_PREECLAMPS | CD40 on CD14+ CD16- monocyte \|\| id:ebi-a-GCST90001980 | Inverse variance weighted | 23 | 0.0190783353174684 | 0.0196896132106592 | 0.332567992917284 | -0.019513307 | 0.0576699772103605 | 1.01926148966256 | 0.980675845664738 | 1.05936532331428 | 0.885079385681701 |
| ebi-a-GCST90001980 | finngen_R11_O15_PREECLAMPS | Pre-eclampsia \|\| id:finngen_R11_O15_PREECLAMPS | CD40 on CD14+ CD16- monocyte \|\| id:ebi-a-GCST90001980 | MR Egger | 23 | 0.00275270207820475 | 0.0333163074860649 | 0.934933592597293 | -0.062547261 | 0.0680526647508919 | 1.00275649424134 | 0.939368666642766 | 1.0704216804855 | 0.99380795269851 |
| ebi-a-GCST90001980 | finngen_R11_O15_PREECLAMPS | Pre-eclampsia \|\| id:finngen_R11_O15_PREECLAMPS | CD40 on CD14+ CD16- monocyte \|\| id:ebi-a-GCST90001980 | Weighted median | 23 | 0.0132683163104565 | 0.0262489020315161 | 0.613221172137124 | -0.038179532 | 0.064716164292228 | 1.0133567310247 | 0.962540118941896 | 1.06685616952975 | 0.940845681141708 |
| ebi-a-GCST90001980 | finngen_R11_O15_PREECLAMPS | Pre-eclampsia \|\| id:finngen_R11_O15_PREECLAMPS | CD40 on CD14+ CD16- monocyte \|\| id:ebi-a-GCST90001980 | Weighted mode | 23 | 0.0292701884195645 | 0.023323218441489 | 0.222654031659871 | -0.01644332 | 0.0749836965648829 | 1.02970277065767 | 0.983691133693804 | 1.07786657781357 | 0.872755742358858 |
| ebi-a-GCST90001981 | finngen_R11_O15_PREECLAMPS | Pre-eclampsia \|\| id:finngen_R11_O15_PREECLAMPS | CD40 on CD14+ CD16+ monocyte \|\| id:ebi-a-GCST90001981 | Inverse variance weighted | 21 | 0.00469361195088642 | 0.0113163779076983 | 0.678315595526422 | -0.017486489 | 0.0268737126499751 | 1.00470464420107 | 0.982665512616553 | 1.02723806739832 | 0.950911139356934 |
| ebi-a-GCST90001981 | finngen_R11_O15_PREECLAMPS | Pre-eclampsia \|\| id:finngen_R11_O15_PREECLAMPS | CD40 on CD14+ CD16+ monocyte \|\| id:ebi-a-GCST90001981 | MR Egger | 21 | -0.011966447 | 0.0189946781956645 | 0.536205048371968 | -0.049196016 | 0.0252631224340141 | 0.988104866356789 | 0.951994505164945 | 1.02558493943072 | 0.99380795269851 |
| ebi-a-GCST90001981 | finngen_R11_O15_PREECLAMPS | Pre-eclampsia \|\| id:finngen_R11_O15_PREECLAMPS | CD40 on CD14+ CD16+ monocyte \|\| id:ebi-a-GCST90001981 | Weighted median | 21 | 0.00368751687876311 | 0.0152667417874021 | 0.809137224201398 | -0.026235297 | 0.0336103307820713 | 1.00369432413385 | 0.974105858431019 | 1.03418153949008 | 0.961297867845628 |
| ebi-a-GCST90001981 | finngen_R11_O15_PREECLAMPS | Pre-eclampsia \|\| id:finngen_R11_O15_PREECLAMPS | CD40 on CD14+ CD16+ monocyte \|\| id:ebi-a-GCST90001981 | Weighted mode | 21 | -0.000197128 | 0.0125592572007719 | 0.987632586408682 | -0.024813272 | 0.0244190162900759 | 0.999802891604976 | 0.975492046763383 | 1.02471960215185 | 0.994159530659367 |
| ebi-a-GCST90001982 | finngen_R11_O15_PREECLAMPS | Pre-eclampsia \|\| id:finngen_R11_O15_PREECLAMPS | CCR2 on CD14- CD16+ monocyte \|\| id:ebi-a-GCST90001982 | Inverse variance weighted | 28 | -0.011034142 | 0.0119044854672927 | 0.353983921840927 | -0.034366934 | 0.0122986492183498 | 0.989026510561507 | 0.96621690193119 | 1.0123745886026 | 0.885079385681701 |
| ebi-a-GCST90001982 | finngen_R11_O15_PREECLAMPS | Pre-eclampsia \|\| id:finngen_R11_O15_PREECLAMPS | CCR2 on CD14- CD16+ monocyte \|\| id:ebi-a-GCST90001982 | MR Egger | 28 | -0.02061237 | 0.0145138314008448 | 0.167433999563862 | -0.049059479 | 0.00783473972474387 | 0.979598612968897 | 0.95212449625233 | 1.00786551160876 | 0.99380795269851 |
| ebi-a-GCST90001982 | finngen_R11_O15_PREECLAMPS | Pre-eclampsia \|\| id:finngen_R11_O15_PREECLAMPS | CCR2 on CD14- CD16+ monocyte \|\| id:ebi-a-GCST90001982 | Weighted median | 28 | -0.01885902 | 0.0166848599825735 | 0.258346744525538 | -0.051561345 | 0.0138433059301051 | 0.981317699017856 | 0.949745385852959 | 1.01393956817263 | 0.940845681141708 |
| ebi-a-GCST90001982 | finngen_R11_O15_PREECLAMPS | Pre-eclampsia \|\| id:finngen_R11_O15_PREECLAMPS | CCR2 on CD14- CD16+ monocyte \|\| id:ebi-a-GCST90001982 | Weighted mode | 28 | -0.01289345 | 0.0136751471141092 | 0.354124274130626 | -0.039696739 | 0.0139098378635292 | 0.987189313964234 | 0.961080853472833 | 1.01400702977663 | 0.899262994073554 |
| ebi-a-GCST90001983 | finngen_R11_O15_PREECLAMPS | Pre-eclampsia \|\| id:finngen_R11_O15_PREECLAMPS | CD14 on CD14+ CD16+ monocyte \|\| id:ebi-a-GCST90001983 | Inverse variance weighted | 14 | 0.000139618592532501 | 0.0336332537053538 | 0.99668782567365 | -0.065781559 | 0.066060795855026 | 1.00013962833966 | 0.936335376298829 | 1.06829166289682 | 0.99668782567365 |
| ebi-a-GCST90001983 | finngen_R11_O15_PREECLAMPS | Pre-eclampsia \|\| id:finngen_R11_O15_PREECLAMPS | CD14 on CD14+ CD16+ monocyte \|\| id:ebi-a-GCST90001983 | MR Egger | 14 | 0.200433907454345 | 0.0951770853258608 | 0.0569445004973133 | 0.0138868202156579 | 0.386980994693032 | 1.22193284891854 | 1.01398368998849 | 1.47252850515093 | 0.862303274556491 |
| ebi-a-GCST90001983 | finngen_R11_O15_PREECLAMPS | Pre-eclampsia \|\| id:finngen_R11_O15_PREECLAMPS | CD14 on CD14+ CD16+ monocyte \|\| id:ebi-a-GCST90001983 | Weighted median | 14 | 0.00670418371149009 | 0.0441917779130749 | 0.879418363228466 | -0.079911701 | 0.093320068421117 | 1.00672670705652 | 0.923197860237368 | 1.097813054 | 0.964912412213677 |
| ebi-a-GCST90001983 | finngen_R11_O15_PREECLAMPS | Pre-eclampsia \|\| id:finngen_R11_O15_PREECLAMPS | CD14 on CD14+ CD16+ monocyte \|\| id:ebi-a-GCST90001983 | Weighted mode | 14 | 0.0663733104894542 | 0.0698184021103113 | 0.359119344250006 | -0.070470758 | 0.203217378625664 | 1.06862557184831 | 0.931954991684414 | 1.22533880176354 | 0.907825101194024 |
| ebi-a-GCST90001984 | finngen_R11_O15_PREECLAMPS | Pre-eclampsia \|\| id:finngen_R11_O15_PREECLAMPS | HLA DR on CD14- CD16+ monocyte \|\| id:ebi-a-GCST90001984 | Inverse variance weighted | 17 | 0.0196118130764592 | 0.0207551528425155 | 0.344703310322329 | -0.021068286 | 0.0602919126477896 | 1.01980538806384 | 0.979152099421677 | 1.06214655530872 | 0.885079385681701 |
| ebi-a-GCST90001984 | finngen_R11_O15_PREECLAMPS | Pre-eclampsia \|\| id:finngen_R11_O15_PREECLAMPS | HLA DR on CD14- CD16+ monocyte \|\| id:ebi-a-GCST90001984 | MR Egger | 17 | 0.00485905663317306 | 0.0410727040879029 | 0.907396668928792 | -0.075643443 | 0.0853615566454626 | 1.00487088099284 | 0.927146727935464 | 1.08911077075776 | 0.99380795269851 |
| ebi-a-GCST90001984 | finngen_R11_O15_PREECLAMPS | Pre-eclampsia \|\| id:finngen_R11_O15_PREECLAMPS | HLA DR on CD14- CD16+ monocyte \|\| id:ebi-a-GCST90001984 | Weighted median | 17 | 0.0106980615769884 | 0.0278672296287766 | 0.701057031511407 | -0.043921708 | 0.0653178316493906 | 1.01075549044757 | 0.957028881771127 | 1.06749825520336 | 0.946704357535493 |
| ebi-a-GCST90001984 | finngen_R11_O15_PREECLAMPS | Pre-eclampsia \|\| id:finngen_R11_O15_PREECLAMPS | HLA DR on CD14- CD16+ monocyte \|\| id:ebi-a-GCST90001984 | Weighted mode | 17 | 0.0184335017593614 | 0.0291333245767547 | 0.535849406654619 | -0.038667814 | 0.0755348179298007 | 1.01860444751372 | 0.962070241941237 | 1.07846077683598 | 0.935508728341156 |
| ebi-a-GCST90001985 | finngen_R11_O15_PREECLAMPS | Pre-eclampsia \|\| id:finngen_R11_O15_PREECLAMPS | CD40 on monocytes \|\| id:ebi-a-GCST90001985 | Inverse variance weighted | 29 | 0.00610018517497121 | 0.00892242256757886 | 0.494169942032391 | -0.011387763 | 0.0235881334074258 | 1.00611882919594 | 0.98867683208567 | 1.02386853379294 | 0.898694924660397 |
| ebi-a-GCST90001985 | finngen_R11_O15_PREECLAMPS | Pre-eclampsia \|\| id:finngen_R11_O15_PREECLAMPS | CD40 on monocytes \|\| id:ebi-a-GCST90001985 | MR Egger | 29 | 0.00783019243507606 | 0.0116817003400295 | 0.508362505200182 | -0.01506594 | 0.0307263251015338 | 1.00786092856275 | 0.985046983235471 | 1.03120325082053 | 0.99380795269851 |
| ebi-a-GCST90001985 | finngen_R11_O15_PREECLAMPS | Pre-eclampsia \|\| id:finngen_R11_O15_PREECLAMPS | CD40 on monocytes \|\| id:ebi-a-GCST90001985 | Weighted median | 29 | 0.00664325194330887 | 0.0129493142023105 | 0.607937613023356 | -0.018737404 | 0.0320239077798374 | 1.00666536728698 | 0.981437049955798 | 1.0325421908014 | 0.940845681141708 |
| ebi-a-GCST90001985 | finngen_R11_O15_PREECLAMPS | Pre-eclampsia \|\| id:finngen_R11_O15_PREECLAMPS | CD40 on monocytes \|\| id:ebi-a-GCST90001985 | Weighted mode | 29 | 0.00424204396523431 | 0.0097321184086415 | 0.666265343333051 | -0.014832908 | 0.0233169960461717 | 1.00425105416979 | 0.985276557566074 | 1.0235909624121 | 0.959313411571287 |
| ebi-a-GCST90001986 | finngen_R11_O15_PREECLAMPS | Pre-eclampsia \|\| id:finngen_R11_O15_PREECLAMPS | CD14 on CD14+ CD16- monocyte \|\| id:ebi-a-GCST90001986 | Inverse variance weighted | 16 | 0.00437767371961087 | 0.0291449456294255 | 0.880604040835864 | -0.05274642 | 0.0615017671532849 | 1.00438726973083 | 0.948620533451439 | 1.06343237577516 | 0.984283721484735 |
| ebi-a-GCST90001986 | finngen_R11_O15_PREECLAMPS | Pre-eclampsia \|\| id:finngen_R11_O15_PREECLAMPS | CD14 on CD14+ CD16- monocyte \|\| id:ebi-a-GCST90001986 | MR Egger | 16 | 0.0423917167722809 | 0.0501401606248473 | 0.412068746051494 | -0.055882998 | 0.140666431596982 | 1.04330307803218 | 0.945649772283019 | 1.15104063315489 | 0.99380795269851 |
| ebi-a-GCST90001986 | finngen_R11_O15_PREECLAMPS | Pre-eclampsia \|\| id:finngen_R11_O15_PREECLAMPS | CD14 on CD14+ CD16- monocyte \|\| id:ebi-a-GCST90001986 | Weighted median | 16 | -0.001196657 | 0.0387476955455181 | 0.975362593979859 | -0.077142141 | 0.0747488258705186 | 0.998804058310253 | 0.925758256357173 | 1.07761344826952 | 0.99075236588769 |
| ebi-a-GCST90001986 | finngen_R11_O15_PREECLAMPS | Pre-eclampsia \|\| id:finngen_R11_O15_PREECLAMPS | CD14 on CD14+ CD16- monocyte \|\| id:ebi-a-GCST90001986 | Weighted mode | 16 | 0.00818775513021586 | 0.0422497172023926 | 0.848936880964257 | -0.074621691 | 0.0909972008469053 | 1.00822136646843 | 0.928094526819543 | 1.09526593943713 | 0.977585971930092 |
| ebi-a-GCST90001987 | finngen_R11_O15_PREECLAMPS | Pre-eclampsia \|\| id:finngen_R11_O15_PREECLAMPS | CD64 on CD14+ CD16- monocyte \|\| id:ebi-a-GCST90001987 | Inverse variance weighted (multiplicative random effects) | 34 | -0.001890072 | 0.00899757862639901 | 0.833617359644864 | -0.019525326 | 0.0157451825690724 | 0.998111713521733 | 0.980664058922965 | 1.01586979109178 | 0.977824840984872 |
| ebi-a-GCST90001987 | finngen_R11_O15_PREECLAMPS | Pre-eclampsia \|\| id:finngen_R11_O15_PREECLAMPS | CD64 on CD14+ CD16- monocyte \|\| id:ebi-a-GCST90001987 | MR Egger | 34 | -0.001802561 | 0.0104371308356978 | 0.863969853234885 | -0.022259337 | 0.018654215792344 | 0.998199062991102 | 0.977986573974884 | 1.01882929262166 | 0.99380795269851 |
| ebi-a-GCST90001987 | finngen_R11_O15_PREECLAMPS | Pre-eclampsia \|\| id:finngen_R11_O15_PREECLAMPS | CD64 on CD14+ CD16- monocyte \|\| id:ebi-a-GCST90001987 | Weighted median | 34 | -0.005561031 | 0.0101029867714514 | 0.582021477948341 | -0.025362885 | 0.0142408228812872 | 0.994454402720454 | 0.974956050644017 | 1.0143427064603 | 0.940845681141708 |
| ebi-a-GCST90001987 | finngen_R11_O15_PREECLAMPS | Pre-eclampsia \|\| id:finngen_R11_O15_PREECLAMPS | CD64 on CD14+ CD16- monocyte \|\| id:ebi-a-GCST90001987 | Weighted mode | 34 | -0.000498625 | 0.00804423642437993 | 0.95094835026681 | -0.016265329 | 0.0152680779443573 | 0.999501498845582 | 0.983866237333737 | 1.01538523051858 | 0.990180645930572 |
| ebi-a-GCST90001988 | finngen_R11_O15_PREECLAMPS | Pre-eclampsia \|\| id:finngen_R11_O15_PREECLAMPS | HLA DR on CD14+ CD16- monocyte \|\| id:ebi-a-GCST90001988 | Inverse variance weighted | 22 | 0.0427338261467995 | 0.0206617702889203 | 0.038615976086382 | 0.00223675638051558 | 0.0832308959130833 | 1.04366006285608 | 1.00223925978622 | 1.08679271557682 | 0.781760498894217 |
| ebi-a-GCST90001988 | finngen_R11_O15_PREECLAMPS | Pre-eclampsia \|\| id:finngen_R11_O15_PREECLAMPS | HLA DR on CD14+ CD16- monocyte \|\| id:ebi-a-GCST90001988 | MR Egger | 22 | 0.047523812936945 | 0.0404031783593793 | 0.253310491556192 | -0.031666417 | 0.126714042521328 | 1.04867117276447 | 0.968829713644485 | 1.13509238321189 | 0.99380795269851 |
| ebi-a-GCST90001988 | finngen_R11_O15_PREECLAMPS | Pre-eclampsia \|\| id:finngen_R11_O15_PREECLAMPS | HLA DR on CD14+ CD16- monocyte \|\| id:ebi-a-GCST90001988 | Weighted median | 22 | 0.0586461046265263 | 0.0250724047259368 | 0.0193318215182671 | 0.00950419136369025 | 0.107788017889362 | 1.0603999037091 | 1.00954949961611 | 1.11381161222294 | 0.598716529366782 |
| ebi-a-GCST90001988 | finngen_R11_O15_PREECLAMPS | Pre-eclampsia \|\| id:finngen_R11_O15_PREECLAMPS | HLA DR on CD14+ CD16- monocyte \|\| id:ebi-a-GCST90001988 | Weighted mode | 22 | 0.0457003856892906 | 0.0218644282577908 | 0.0489490764750684 | 0.00284610630402066 | 0.0885546650745605 | 1.04676073947162 | 1.0028501603097 | 1.09259397770928 | 0.838871423749761 |
| ebi-a-GCST90001989 | finngen_R11_O15_PREECLAMPS | Pre-eclampsia \|\| id:finngen_R11_O15_PREECLAMPS | CD40 on CD14- CD16+ monocyte \|\| id:ebi-a-GCST90001989 | Inverse variance weighted | 27 | -0.000326176 | 0.0115947807581173 | 0.977557428318197 | -0.023051947 | 0.0223995940032428 | 0.999673876907033 | 0.977211719659168 | 1.02265234858101 | 0.994537505425743 |
| ebi-a-GCST90001989 | finngen_R11_O15_PREECLAMPS | Pre-eclampsia \|\| id:finngen_R11_O15_PREECLAMPS | CD40 on CD14- CD16+ monocyte \|\| id:ebi-a-GCST90001989 | MR Egger | 27 | -0.007467206 | 0.0165687219836722 | 0.656104107505418 | -0.039941901 | 0.0250074894915293 | 0.992560604718409 | 0.960845261982729 | 1.0253227996421 | 0.99380795269851 |
| ebi-a-GCST90001989 | finngen_R11_O15_PREECLAMPS | Pre-eclampsia \|\| id:finngen_R11_O15_PREECLAMPS | CD40 on CD14- CD16+ monocyte \|\| id:ebi-a-GCST90001989 | Weighted median | 27 | -0.021722734 | 0.0175012256243856 | 0.214527379451222 | -0.056025136 | 0.0125796679534867 | 0.978511505149007 | 0.945515368650195 | 1.01265912480711 | 0.940845681141708 |
| ebi-a-GCST90001989 | finngen_R11_O15_PREECLAMPS | Pre-eclampsia \|\| id:finngen_R11_O15_PREECLAMPS | CD40 on CD14- CD16+ monocyte \|\| id:ebi-a-GCST90001989 | Weighted mode | 27 | -0.00886537 | 0.0142326011494922 | 0.53878136064518 | -0.036761269 | 0.0190305279809711 | 0.991173811251269 | 0.963906222637677 | 1.0192127626503 | 0.935508728341156 |
| ebi-a-GCST90001990 | finngen_R11_O15_PREECLAMPS | Pre-eclampsia \|\| id:finngen_R11_O15_PREECLAMPS | CD64 on CD14- CD16+ monocyte \|\| id:ebi-a-GCST90001990 | Inverse variance weighted | 19 | -0.030341055 | 0.0296756191524579 | 0.306580427705251 | -0.088505269 | 0.0278231584334705 | 0.97011461457884 | 0.915298288342308 | 1.02821383739711 | 0.885079385681701 |
| ebi-a-GCST90001990 | finngen_R11_O15_PREECLAMPS | Pre-eclampsia \|\| id:finngen_R11_O15_PREECLAMPS | CD64 on CD14- CD16+ monocyte \|\| id:ebi-a-GCST90001990 | MR Egger | 19 | -0.07842161 | 0.0513633705439432 | 0.145201093291835 | -0.179093816 | 0.0222505961711812 | 0.924574534400848 | 0.836027462663462 | 1.02249998694918 | 0.982796288854917 |
| ebi-a-GCST90001990 | finngen_R11_O15_PREECLAMPS | Pre-eclampsia \|\| id:finngen_R11_O15_PREECLAMPS | CD64 on CD14- CD16+ monocyte \|\| id:ebi-a-GCST90001990 | Weighted median | 19 | -0.047954674 | 0.0391086888516477 | 0.220127269139557 | -0.124607704 | 0.028698355862845 | 0.953176989525659 | 0.882843170120232 | 1.02911412141011 | 0.940845681141708 |
| ebi-a-GCST90001990 | finngen_R11_O15_PREECLAMPS | Pre-eclampsia \|\| id:finngen_R11_O15_PREECLAMPS | CD64 on CD14- CD16+ monocyte \|\| id:ebi-a-GCST90001990 | Weighted mode | 19 | -0.044965019 | 0.0374423733689009 | 0.245350298284305 | -0.118352071 | 0.0284220329795706 | 0.956030924334695 | 0.888383223910717 | 1.02882979291391 | 0.872755742358858 |
| ebi-a-GCST90001991 | finngen_R11_O15_PREECLAMPS | Pre-eclampsia \|\| id:finngen_R11_O15_PREECLAMPS | HLA DR on CD14+ monocyte \|\| id:ebi-a-GCST90001991 | Inverse variance weighted (multiplicative random effects) | 21 | 0.0435824833875853 | 0.0218401351965208 | 0.0459859116996598 | 0.000775818402404503 | 0.0863891483727662 | 1.04454614846365 | 1.00077611942734 | 1.09023050719333 | 0.781760498894217 |
| ebi-a-GCST90001991 | finngen_R11_O15_PREECLAMPS | Pre-eclampsia \|\| id:finngen_R11_O15_PREECLAMPS | HLA DR on CD14+ monocyte \|\| id:ebi-a-GCST90001991 | MR Egger | 21 | 0.0496355073895749 | 0.0431773011353651 | 0.264584636024376 | -0.034992003 | 0.134263017614891 | 1.0508879856546 | 0.965613138393567 | 1.14369359164939 | 0.99380795269851 |
| ebi-a-GCST90001991 | finngen_R11_O15_PREECLAMPS | Pre-eclampsia \|\| id:finngen_R11_O15_PREECLAMPS | HLA DR on CD14+ monocyte \|\| id:ebi-a-GCST90001991 | Weighted median | 21 | 0.0602732173063663 | 0.026007743367243 | 0.020475941496812 | 0.00929804030657001 | 0.111248394306162 | 1.06212669830184 | 1.00934140137012 | 1.11767249586139 | 0.598716529366782 |
| ebi-a-GCST90001991 | finngen_R11_O15_PREECLAMPS | Pre-eclampsia \|\| id:finngen_R11_O15_PREECLAMPS | HLA DR on CD14+ monocyte \|\| id:ebi-a-GCST90001991 | Weighted mode | 21 | 0.0473741690138881 | 0.0228464612015301 | 0.05124801972702 | 0.00259510505888906 | 0.0921532329688872 | 1.0485142572372 | 1.00259847525873 | 1.09653283418966 | 0.838871423749761 |
| ebi-a-GCST90001992 | finngen_R11_O15_PREECLAMPS | Pre-eclampsia \|\| id:finngen_R11_O15_PREECLAMPS | CCR2 on CD14+ CD16+ monocyte \|\| id:ebi-a-GCST90001992 | Inverse variance weighted | 37 | -0.012296247 | 0.00980065785872212 | 0.209611359478282 | -0.031505536 | 0.00691304285809119 | 0.98777904338408 | 0.968985592184944 | 1.00693699309674 | 0.855687495048373 |
| ebi-a-GCST90001992 | finngen_R11_O15_PREECLAMPS | Pre-eclampsia \|\| id:finngen_R11_O15_PREECLAMPS | CCR2 on CD14+ CD16+ monocyte \|\| id:ebi-a-GCST90001992 | MR Egger | 37 | -0.004769815 | 0.0118229589756926 | 0.689081222909538 | -0.027942815 | 0.0184031844835576 | 0.995241542394381 | 0.972443974710556 | 1.01857356666986 | 0.99380795269851 |
| ebi-a-GCST90001992 | finngen_R11_O15_PREECLAMPS | Pre-eclampsia \|\| id:finngen_R11_O15_PREECLAMPS | CCR2 on CD14+ CD16+ monocyte \|\| id:ebi-a-GCST90001992 | Weighted median | 37 | 0.00368662202997413 | 0.0142949660842868 | 0.796486769419661 | -0.024331511 | 0.0317047555551763 | 1.0036934259796 | 0.975962113463407 | 1.03221270524497 | 0.957984984509652 |
| ebi-a-GCST90001992 | finngen_R11_O15_PREECLAMPS | Pre-eclampsia \|\| id:finngen_R11_O15_PREECLAMPS | CCR2 on CD14+ CD16+ monocyte \|\| id:ebi-a-GCST90001992 | Weighted mode | 37 | -0.004561327 | 0.0133693570143851 | 0.734953150862495 | -0.030765266 | 0.0216426131751619 | 0.995449060478108 | 0.969703168354867 | 1.02187851328554 | 0.959313411571287 |
| ebi-a-GCST90001993 | finngen_R11_O15_PREECLAMPS | Pre-eclampsia \|\| id:finngen_R11_O15_PREECLAMPS | PDL-1 on CD14+ CD16- monocyte \|\| id:ebi-a-GCST90001993 | Inverse variance weighted | 15 | 0.0187806947600962 | 0.0365816886858456 | 0.607678126211475 | -0.052919415 | 0.0904808045843536 | 1.01895816124832 | 0.948456440704195 | 1.09470049420897 | 0.92242199665996 |
| ebi-a-GCST90001993 | finngen_R11_O15_PREECLAMPS | Pre-eclampsia \|\| id:finngen_R11_O15_PREECLAMPS | PDL-1 on CD14+ CD16- monocyte \|\| id:ebi-a-GCST90001993 | MR Egger | 15 | 0.0653792354866394 | 0.056975803129264 | 0.271858346082808 | -0.046293339 | 0.177051809619997 | 1.067563806 | 0.954761852559465 | 1.19369293630251 | 0.99380795269851 |
| ebi-a-GCST90001993 | finngen_R11_O15_PREECLAMPS | Pre-eclampsia \|\| id:finngen_R11_O15_PREECLAMPS | PDL-1 on CD14+ CD16- monocyte \|\| id:ebi-a-GCST90001993 | Weighted median | 15 | 0.0205262594574419 | 0.0466383651829069 | 0.659853579807723 | -0.070884936 | 0.111937455215939 | 1.0207383719271 | 0.93156907574468 | 1.11844290568739 | 0.940845681141708 |
| ebi-a-GCST90001993 | finngen_R11_O15_PREECLAMPS | Pre-eclampsia \|\| id:finngen_R11_O15_PREECLAMPS | PDL-1 on CD14+ CD16- monocyte \|\| id:ebi-a-GCST90001993 | Weighted mode | 15 | 0.0139835119400709 | 0.053478953773913 | 0.797532227327134 | -0.090835237 | 0.11880226133694 | 1.01408173856017 | 0.913168154412689 | 1.12614721342601 | 0.961305639715923 |
| ebi-a-GCST90001994 | finngen_R11_O15_PREECLAMPS | Pre-eclampsia \|\| id:finngen_R11_O15_PREECLAMPS | CX3CR1 on CD14- CD16- \|\| id:ebi-a-GCST90001994 | Inverse variance weighted | 19 | -0.016997475 | 0.010938249639509 | 0.120196741013609 | -0.038436444 | 0.00444149448308326 | 0.983146167262297 | 0.962292862181609 | 1.00445137253873 | 0.820779257935285 |
| ebi-a-GCST90001994 | finngen_R11_O15_PREECLAMPS | Pre-eclampsia \|\| id:finngen_R11_O15_PREECLAMPS | CX3CR1 on CD14- CD16- \|\| id:ebi-a-GCST90001994 | MR Egger | 19 | -0.012364294 | 0.0140171110013659 | 0.390032708166665 | -0.039837832 | 0.0151092433395075 | 0.987711829600534 | 0.960945261294205 | 1.01522396501433 | 0.99380795269851 |
| ebi-a-GCST90001994 | finngen_R11_O15_PREECLAMPS | Pre-eclampsia \|\| id:finngen_R11_O15_PREECLAMPS | CX3CR1 on CD14- CD16- \|\| id:ebi-a-GCST90001994 | Weighted median | 19 | -0.015230098 | 0.0140477308891203 | 0.278290394889637 | -0.04276365 | 0.0123034547041231 | 0.984885293551411 | 0.95813781881601 | 1.01237945356598 | 0.940845681141708 |
| ebi-a-GCST90001994 | finngen_R11_O15_PREECLAMPS | Pre-eclampsia \|\| id:finngen_R11_O15_PREECLAMPS | CX3CR1 on CD14- CD16- \|\| id:ebi-a-GCST90001994 | Weighted mode | 19 | -0.01484082 | 0.0124616367894811 | 0.249153223208666 | -0.039265628 | 0.00958398838853283 | 0.985268762479796 | 0.961495275322786 | 1.00963006187688 | 0.872755742358858 |
| ebi-a-GCST90001995 | finngen_R11_O15_PREECLAMPS | Pre-eclampsia \|\| id:finngen_R11_O15_PREECLAMPS | CX3CR1 on monocyte \|\| id:ebi-a-GCST90001995 | Inverse variance weighted | 26 | -0.03976065 | 0.0204994602123947 | 0.0524289323401284 | -0.079939592 | 0.000418292047337551 | 0.96101943165767 | 0.923172111696824 | 1.00041837954366 | 0.789997151112252 |
| ebi-a-GCST90001995 | finngen_R11_O15_PREECLAMPS | Pre-eclampsia \|\| id:finngen_R11_O15_PREECLAMPS | CX3CR1 on monocyte \|\| id:ebi-a-GCST90001995 | MR Egger | 26 | -0.107782377 | 0.0432252770761898 | 0.0199403233216289 | -0.19250392 | -0.023060834 | 0.897822962229143 | 0.824891084687409 | 0.977203035005983 | 0.716086784616111 |
| ebi-a-GCST90001995 | finngen_R11_O15_PREECLAMPS | Pre-eclampsia \|\| id:finngen_R11_O15_PREECLAMPS | CX3CR1 on monocyte \|\| id:ebi-a-GCST90001995 | Weighted median | 26 | -0.058904261 | 0.0298612186382989 | 0.0485410738714535 | -0.117432249 | -0.000376272 | 0.942797027645425 | 0.889200753817441 | 0.999623798699048 | 0.754968617021969 |
| ebi-a-GCST90001995 | finngen_R11_O15_PREECLAMPS | Pre-eclampsia \|\| id:finngen_R11_O15_PREECLAMPS | CX3CR1 on monocyte \|\| id:ebi-a-GCST90001995 | Weighted mode | 26 | -0.062257661 | 0.0314041399430185 | 0.0585193104454175 | -0.123809776 | -0.000705547 | 0.939640746473389 | 0.883547897213356 | 0.99929470175612 | 0.838871423749761 |
| ebi-a-GCST90001996 | finngen_R11_O15_PREECLAMPS | Pre-eclampsia \|\| id:finngen_R11_O15_PREECLAMPS | CX3CR1 on CD14+ CD16+ monocyte \|\| id:ebi-a-GCST90001996 | Inverse variance weighted | 34 | -0.016432151 | 0.013214618371035 | 0.213689969873265 | -0.042332803 | 0.00946850116043987 | 0.98370212048237 | 0.958550719068114 | 1.00951346923224 | 0.855687495048373 |
| ebi-a-GCST90001996 | finngen_R11_O15_PREECLAMPS | Pre-eclampsia \|\| id:finngen_R11_O15_PREECLAMPS | CX3CR1 on CD14+ CD16+ monocyte \|\| id:ebi-a-GCST90001996 | MR Egger | 34 | -0.022911604 | 0.0191003737965409 | 0.23912579305893 | -0.060348337 | 0.0145251285694111 | 0.977348873615815 | 0.941436539551671 | 1.01463113085986 | 0.99380795269851 |
| ebi-a-GCST90001996 | finngen_R11_O15_PREECLAMPS | Pre-eclampsia \|\| id:finngen_R11_O15_PREECLAMPS | CX3CR1 on CD14+ CD16+ monocyte \|\| id:ebi-a-GCST90001996 | Weighted median | 34 | -0.010036692 | 0.0231405442595207 | 0.664486291418618 | -0.055392159 | 0.0353187749679123 | 0.990013507724176 | 0.946114048499419 | 1.03594989106319 | 0.940845681141708 |
| ebi-a-GCST90001996 | finngen_R11_O15_PREECLAMPS | Pre-eclampsia \|\| id:finngen_R11_O15_PREECLAMPS | CX3CR1 on CD14+ CD16+ monocyte \|\| id:ebi-a-GCST90001996 | Weighted mode | 34 | -0.016487374 | 0.0216221636196095 | 0.451164906905145 | -0.058866814 | 0.0258920669159707 | 0.98364779906729 | 0.942832332416471 | 1.0262301783076 | 0.933945646747087 |
| ebi-a-GCST90001997 | finngen_R11_O15_PREECLAMPS | Pre-eclampsia \|\| id:finngen_R11_O15_PREECLAMPS | CX3CR1 on CD14+ CD16- monocyte \|\| id:ebi-a-GCST90001997 | Inverse variance weighted | 27 | -0.028007397 | 0.0212067572174565 | 0.186607192461513 | -0.069572641 | 0.0135578472505039 | 0.972381174172509 | 0.932792371913658 | 1.01365017162965 | 0.855687495048373 |
| ebi-a-GCST90001997 | finngen_R11_O15_PREECLAMPS | Pre-eclampsia \|\| id:finngen_R11_O15_PREECLAMPS | CX3CR1 on CD14+ CD16- monocyte \|\| id:ebi-a-GCST90001997 | MR Egger | 27 | -0.048564373 | 0.0411029445034218 | 0.248509969613341 | -0.129126145 | 0.0319973978082234 | 0.952596015470094 | 0.878863094679598 | 1.03251481850005 | 0.99380795269851 |
| ebi-a-GCST90001997 | finngen_R11_O15_PREECLAMPS | Pre-eclampsia \|\| id:finngen_R11_O15_PREECLAMPS | CX3CR1 on CD14+ CD16- monocyte \|\| id:ebi-a-GCST90001997 | Weighted median | 27 | -0.047424776 | 0.0272671337011678 | 0.0819881288523337 | -0.100868358 | 0.00601880581327954 | 0.953682209995153 | 0.904052036003964 | 1.0060369552193 | 0.878781071149907 |
| ebi-a-GCST90001997 | finngen_R11_O15_PREECLAMPS | Pre-eclampsia \|\| id:finngen_R11_O15_PREECLAMPS | CX3CR1 on CD14+ CD16- monocyte \|\| id:ebi-a-GCST90001997 | Weighted mode | 27 | -0.051606281 | 0.0267969073595022 | 0.0651271776476762 | -0.104128219 | 0.000915657610075588 | 0.949702709420737 | 0.901109750407079 | 1.00091607695249 | 0.871935626364341 |
| ebi-a-GCST90001998 | finngen_R11_O15_PREECLAMPS | Pre-eclampsia \|\| id:finngen_R11_O15_PREECLAMPS | PDL-1 on CD14+ CD16+ monocyte \|\| id:ebi-a-GCST90001998 | Inverse variance weighted | 28 | -0.010397201 | 0.0155245407426122 | 0.503031973669975 | -0.040825301 | 0.020030899095489 | 0.989656663291706 | 0.959996826153762 | 1.02023286381241 | 0.898694924660397 |
| ebi-a-GCST90001998 | finngen_R11_O15_PREECLAMPS | Pre-eclampsia \|\| id:finngen_R11_O15_PREECLAMPS | PDL-1 on CD14+ CD16+ monocyte \|\| id:ebi-a-GCST90001998 | MR Egger | 28 | -0.032218995 | 0.0212058932396835 | 0.140744644284934 | -0.073782546 | 0.00934455551605502 | 0.968294506976457 | 0.928873659189411 | 1.0093883521888 | 0.97261503632431 |
| ebi-a-GCST90001998 | finngen_R11_O15_PREECLAMPS | Pre-eclampsia \|\| id:finngen_R11_O15_PREECLAMPS | PDL-1 on CD14+ CD16+ monocyte \|\| id:ebi-a-GCST90001998 | Weighted median | 28 | -0.021642967 | 0.0224531871430862 | 0.335088466406674 | -0.065651214 | 0.0223652794918901 | 0.978589561151914 | 0.936457430476734 | 1.02261725736746 | 0.940845681141708 |
| ebi-a-GCST90001998 | finngen_R11_O15_PREECLAMPS | Pre-eclampsia \|\| id:finngen_R11_O15_PREECLAMPS | PDL-1 on CD14+ CD16+ monocyte \|\| id:ebi-a-GCST90001998 | Weighted mode | 28 | -0.032075907 | 0.0208628238510177 | 0.135816737499894 | -0.072967042 | 0.00881522796470106 | 0.968433068650073 | 0.929631468750564 | 1.0088541965081 | 0.872755742358858 |
| ebi-a-GCST90001999 | finngen_R11_O15_PREECLAMPS | Pre-eclampsia \|\| id:finngen_R11_O15_PREECLAMPS | PDL-1 on CD14- CD16+ monocyte \|\| id:ebi-a-GCST90001999 | Inverse variance weighted | 19 | 0.0175992219761079 | 0.0204522865850611 | 0.389512692277831 | -0.02248726 | 0.0576857036828277 | 1.0177550008034 | 0.977763694086845 | 1.05938198352488 | 0.885079385681701 |
| ebi-a-GCST90001999 | finngen_R11_O15_PREECLAMPS | Pre-eclampsia \|\| id:finngen_R11_O15_PREECLAMPS | PDL-1 on CD14- CD16+ monocyte \|\| id:ebi-a-GCST90001999 | MR Egger | 19 | 0.0151037299870751 | 0.0388552451227566 | 0.702310266831578 | -0.06105255 | 0.0912600104276781 | 1.01521836774224 | 0.940773800387112 | 1.09555382364722 | 0.99380795269851 |
| ebi-a-GCST90001999 | finngen_R11_O15_PREECLAMPS | Pre-eclampsia \|\| id:finngen_R11_O15_PREECLAMPS | PDL-1 on CD14- CD16+ monocyte \|\| id:ebi-a-GCST90001999 | Weighted median | 19 | 0.0244352881279173 | 0.0286060526706772 | 0.392994223157623 | -0.031632575 | 0.0805031513624446 | 1.02473627635871 | 0.968862500889577 | 1.08383226218516 | 0.940845681141708 |
| ebi-a-GCST90001999 | finngen_R11_O15_PREECLAMPS | Pre-eclampsia \|\| id:finngen_R11_O15_PREECLAMPS | PDL-1 on CD14- CD16+ monocyte \|\| id:ebi-a-GCST90001999 | Weighted mode | 19 | 0.0143847575548082 | 0.0337629932059563 | 0.675121880762299 | -0.051790709 | 0.0805602242384825 | 1.01448871605426 | 0.949527573501443 | 1.08389412137473 | 0.959313411571287 |
| ebi-a-GCST90002000 | finngen_R11_O15_PREECLAMPS | Pre-eclampsia \|\| id:finngen_R11_O15_PREECLAMPS | PDL-1 on CD14- CD16- \|\| id:ebi-a-GCST90002000 | Inverse variance weighted (multiplicative random effects) | 27 | -0.042114073 | 0.0226628608141242 | 0.0631284374888373 | -0.08653328 | 0.00230513447531366 | 0.958760405926585 | 0.917105027098515 | 1.00230779334041 | 0.789997151112252 |
| ebi-a-GCST90002000 | finngen_R11_O15_PREECLAMPS | Pre-eclampsia \|\| id:finngen_R11_O15_PREECLAMPS | PDL-1 on CD14- CD16- \|\| id:ebi-a-GCST90002000 | MR Egger | 27 | -0.057525465 | 0.0305300468402218 | 0.0712143035085111 | -0.117364357 | 0.00231342679103633 | 0.944097848613681 | 0.889261125770637 | 1.00231610482754 | 0.896743733484648 |
| ebi-a-GCST90002000 | finngen_R11_O15_PREECLAMPS | Pre-eclampsia \|\| id:finngen_R11_O15_PREECLAMPS | PDL-1 on CD14- CD16- \|\| id:ebi-a-GCST90002000 | Weighted median | 27 | -0.028140855 | 0.0266219247438307 | 0.290486200700634 | -0.080319828 | 0.0240381171410726 | 0.972251410336611 | 0.922821155273579 | 1.02432936165328 | 0.940845681141708 |
| ebi-a-GCST90002000 | finngen_R11_O15_PREECLAMPS | Pre-eclampsia \|\| id:finngen_R11_O15_PREECLAMPS | PDL-1 on CD14- CD16- \|\| id:ebi-a-GCST90002000 | Weighted mode | 27 | -0.032977538 | 0.023801305900792 | 0.177660745934847 | -0.079628098 | 0.013673021331671 | 0.967560292457947 | 0.923459719233837 | 1.01376692458012 | 0.872755742358858 |
| ebi-a-GCST90002001 | finngen_R11_O15_PREECLAMPS | Pre-eclampsia \|\| id:finngen_R11_O15_PREECLAMPS | CD64 on CD14- CD16- \|\| id:ebi-a-GCST90002001 | Inverse variance weighted | 16 | 0.0632355417989696 | 0.0341238818495329 | 0.0638656064984437 | -0.003647267 | 0.130118350224054 | 1.0652777271151 | 0.996359376571847 | 1.13896317189492 | 0.789997151112252 |
| ebi-a-GCST90002001 | finngen_R11_O15_PREECLAMPS | Pre-eclampsia \|\| id:finngen_R11_O15_PREECLAMPS | CD64 on CD14- CD16- \|\| id:ebi-a-GCST90002001 | MR Egger | 16 | 0.040730228566608 | 0.0716898072097629 | 0.578935405418823 | -0.099781794 | 0.181242250697743 | 1.04157108151742 | 0.905034880926685 | 1.19870553137415 | 0.99380795269851 |
| ebi-a-GCST90002001 | finngen_R11_O15_PREECLAMPS | Pre-eclampsia \|\| id:finngen_R11_O15_PREECLAMPS | CD64 on CD14- CD16- \|\| id:ebi-a-GCST90002001 | Weighted median | 16 | 0.0494675104957248 | 0.0418188124639182 | 0.236848428766594 | -0.032497362 | 0.131432382925004 | 1.05071145456597 | 0.968025003542217 | 1.14046079049238 | 0.940845681141708 |
| ebi-a-GCST90002001 | finngen_R11_O15_PREECLAMPS | Pre-eclampsia \|\| id:finngen_R11_O15_PREECLAMPS | CD64 on CD14- CD16- \|\| id:ebi-a-GCST90002001 | Weighted mode | 16 | 0.0443489758042115 | 0.0444351964775793 | 0.334079594329999 | -0.042744009 | 0.131441960900267 | 1.04534709208465 | 0.958156637871365 | 1.14047171384993 | 0.888599269773114 |
| ebi-a-GCST90002002 | finngen_R11_O15_PREECLAMPS | Pre-eclampsia \|\| id:finngen_R11_O15_PREECLAMPS | PDL-1 on monocyte \|\| id:ebi-a-GCST90002002 | Inverse variance weighted | 15 | 0.0100289455441651 | 0.0283139025152879 | 0.72318473524613 | -0.045466303 | 0.0655241944741293 | 1.01007940395902 | 0.955551800889848 | 1.06771856989031 | 0.959893708083249 |
| ebi-a-GCST90002002 | finngen_R11_O15_PREECLAMPS | Pre-eclampsia \|\| id:finngen_R11_O15_PREECLAMPS | PDL-1 on monocyte \|\| id:ebi-a-GCST90002002 | MR Egger | 15 | 0.00595025520161033 | 0.0489250669404341 | 0.905060003313651 | -0.089942876 | 0.101843386404861 | 1.00596799313438 | 0.913983394165933 | 1.10721005400137 | 0.99380795269851 |
| ebi-a-GCST90002002 | finngen_R11_O15_PREECLAMPS | Pre-eclampsia \|\| id:finngen_R11_O15_PREECLAMPS | PDL-1 on monocyte \|\| id:ebi-a-GCST90002002 | Weighted median | 15 | -0.038695208 | 0.0415197202521476 | 0.351351099355182 | -0.12007386 | 0.042683443277772 | 0.962043887344854 | 0.886854931094723 | 1.04360748159244 | 0.940845681141708 |
| ebi-a-GCST90002002 | finngen_R11_O15_PREECLAMPS | Pre-eclampsia \|\| id:finngen_R11_O15_PREECLAMPS | PDL-1 on monocyte \|\| id:ebi-a-GCST90002002 | Weighted mode | 15 | -0.055289519 | 0.0562136192667719 | 0.342024817382452 | -0.165468213 | 0.0548891745970183 | 0.946211162026957 | 0.84749680221802 | 1.05642352962423 | 0.888599269773114 |
| ebi-a-GCST90002003 | finngen_R11_O15_PREECLAMPS | Pre-eclampsia \|\| id:finngen_R11_O15_PREECLAMPS | CCR2 on CD14- CD16- \|\| id:ebi-a-GCST90002003 | Inverse variance weighted | 17 | -0.006728028 | 0.0269220554135645 | 0.802658741599257 | -0.059495257 | 0.0460392001226835 | 0.993294554022125 | 0.942240002532164 | 1.04711545720681 | 0.977824840984872 |
| ebi-a-GCST90002003 | finngen_R11_O15_PREECLAMPS | Pre-eclampsia \|\| id:finngen_R11_O15_PREECLAMPS | CCR2 on CD14- CD16- \|\| id:ebi-a-GCST90002003 | MR Egger | 17 | -0.020132183 | 0.0385553139816344 | 0.609181908501108 | -0.095700598 | 0.0554362326436459 | 0.980069116503195 | 0.908736052584813 | 1.05700161272484 | 0.99380795269851 |
| ebi-a-GCST90002003 | finngen_R11_O15_PREECLAMPS | Pre-eclampsia \|\| id:finngen_R11_O15_PREECLAMPS | CCR2 on CD14- CD16- \|\| id:ebi-a-GCST90002003 | Weighted median | 17 | -0.032254416 | 0.0302180586434341 | 0.285796320396487 | -0.091481811 | 0.0269729786938757 | 0.968260209611051 | 0.91257791470883 | 1.02734004231865 | 0.940845681141708 |
| ebi-a-GCST90002003 | finngen_R11_O15_PREECLAMPS | Pre-eclampsia \|\| id:finngen_R11_O15_PREECLAMPS | CCR2 on CD14- CD16- \|\| id:ebi-a-GCST90002003 | Weighted mode | 17 | -0.028747367 | 0.0306766480248618 | 0.3626332825563 | -0.088873597 | 0.0313788631556531 | 0.971661907350611 | 0.914961220015221 | 1.0318763697772 | 0.907825101194024 |
| ebi-a-GCST90002004 | finngen_R11_O15_PREECLAMPS | Pre-eclampsia \|\| id:finngen_R11_O15_PREECLAMPS | CCR2 on CD14+ CD16- monocyte \|\| id:ebi-a-GCST90002004 | Inverse variance weighted | 28 | 0.0108293405238542 | 0.00794583456485444 | 0.172915512531672 | -0.004744495 | 0.0264031762709689 | 1.01088819007404 | 0.995266742115344 | 1.02675482821743 | 0.85505992597664 |
| ebi-a-GCST90002004 | finngen_R11_O15_PREECLAMPS | Pre-eclampsia \|\| id:finngen_R11_O15_PREECLAMPS | CCR2 on CD14+ CD16- monocyte \|\| id:ebi-a-GCST90002004 | MR Egger | 28 | 0.00629667680385015 | 0.0092669096037804 | 0.502838946442875 | -0.011866466 | 0.0244598196272597 | 1.0063165425474 | 0.988203662820415 | 1.02476141498434 | 0.99380795269851 |
| ebi-a-GCST90002004 | finngen_R11_O15_PREECLAMPS | Pre-eclampsia \|\| id:finngen_R11_O15_PREECLAMPS | CCR2 on CD14+ CD16- monocyte \|\| id:ebi-a-GCST90002004 | Weighted median | 28 | 0.00414261586099524 | 0.0107245812677489 | 0.699294516626747 | -0.016877563 | 0.025162795145783 | 1.00415120835512 | 0.983264064750891 | 1.02548205043627 | 0.946704357535493 |
| ebi-a-GCST90002004 | finngen_R11_O15_PREECLAMPS | Pre-eclampsia \|\| id:finngen_R11_O15_PREECLAMPS | CCR2 on CD14+ CD16- monocyte \|\| id:ebi-a-GCST90002004 | Weighted mode | 28 | 0.00588804265992621 | 0.00851752036983514 | 0.495287193378937 | -0.010806297 | 0.0225823825848031 | 1.00590541125539 | 0.989251881012836 | 1.02283929483854 | 0.934398527469092 |
| ebi-a-GCST90002005 | finngen_R11_O15_PREECLAMPS | Pre-eclampsia \|\| id:finngen_R11_O15_PREECLAMPS | CD16 on CD14+ CD16+ monocyte \|\| id:ebi-a-GCST90002005 | Inverse variance weighted | 22 | 0.0343797227891923 | 0.0186808900221271 | 0.0657141450932395 | -0.002234822 | 0.0709942672325615 | 1.03497753667815 | 0.997767673700499 | 1.0735750712945 | 0.789997151112252 |
| ebi-a-GCST90002005 | finngen_R11_O15_PREECLAMPS | Pre-eclampsia \|\| id:finngen_R11_O15_PREECLAMPS | CD16 on CD14+ CD16+ monocyte \|\| id:ebi-a-GCST90002005 | MR Egger | 22 | 0.0320754397845771 | 0.027779741307595 | 0.261850410649957 | -0.022372853 | 0.0865237327474633 | 1.03259540114148 | 0.977875563059202 | 1.0903772450585 | 0.99380795269851 |
| ebi-a-GCST90002005 | finngen_R11_O15_PREECLAMPS | Pre-eclampsia \|\| id:finngen_R11_O15_PREECLAMPS | CD16 on CD14+ CD16+ monocyte \|\| id:ebi-a-GCST90002005 | Weighted median | 22 | 0.0507077288027281 | 0.0282017723472885 | 0.0721717140217815 | -0.004567745 | 0.105983202603414 | 1.05201537455289 | 0.995442671283552 | 1.11180320095039 | 0.850927789514876 |
| ebi-a-GCST90002005 | finngen_R11_O15_PREECLAMPS | Pre-eclampsia \|\| id:finngen_R11_O15_PREECLAMPS | CD16 on CD14+ CD16+ monocyte \|\| id:ebi-a-GCST90002005 | Weighted mode | 22 | 0.0355462813085481 | 0.0249283663316574 | 0.168583387154177 | -0.013313317 | 0.0844058793185967 | 1.03618560304396 | 0.98677491351966 | 1.08807042948217 | 0.872755742358858 |
| ebi-a-GCST90002006 | finngen_R11_O15_PREECLAMPS | Pre-eclampsia \|\| id:finngen_R11_O15_PREECLAMPS | CD64 on monocyte \|\| id:ebi-a-GCST90002006 | Inverse variance weighted (multiplicative random effects) | 28 | -0.003325267 | 0.0186642044664767 | 0.858595137106043 | -0.039907107 | 0.0332565741482664 | 0.996680255969931 | 0.960878693564976 | 1.03381575561298 | 0.977824840984872 |
| ebi-a-GCST90002006 | finngen_R11_O15_PREECLAMPS | Pre-eclampsia \|\| id:finngen_R11_O15_PREECLAMPS | CD64 on monocyte \|\| id:ebi-a-GCST90002006 | MR Egger | 28 | -0.032219321 | 0.029008318031396 | 0.276867214571053 | -0.089075625 | 0.0246369820186047 | 0.968294191226121 | 0.914776391300887 | 1.02494298024997 | 0.99380795269851 |
| ebi-a-GCST90002006 | finngen_R11_O15_PREECLAMPS | Pre-eclampsia \|\| id:finngen_R11_O15_PREECLAMPS | CD64 on monocyte \|\| id:ebi-a-GCST90002006 | Weighted median | 28 | -0.00988762 | 0.021409401443422 | 0.644199329967701 | -0.051850047 | 0.0320748063714177 | 0.990161101348333 | 0.949471231955946 | 1.03259474708217 | 0.940845681141708 |
| ebi-a-GCST90002006 | finngen_R11_O15_PREECLAMPS | Pre-eclampsia \|\| id:finngen_R11_O15_PREECLAMPS | CD64 on monocyte \|\| id:ebi-a-GCST90002006 | Weighted mode | 28 | -0.007626517 | 0.0213792099217012 | 0.72406990312337 | -0.049529768 | 0.0342767349263761 | 0.992402491566564 | 0.951676828230678 | 1.03487095204214 | 0.959313411571287 |
| ebi-a-GCST90002007 | finngen_R11_O15_PREECLAMPS | Pre-eclampsia \|\| id:finngen_R11_O15_PREECLAMPS | HLA DR on CD14+ CD16+ monocyte \|\| id:ebi-a-GCST90002007 | Inverse variance weighted | 19 | 0.00423941139377269 | 0.0106851597415157 | 0.691546699200977 | -0.016703502 | 0.0251823244871434 | 1.00424841041061 | 0.983435228285417 | 1.02550207762085 | 0.950911139356934 |
| ebi-a-GCST90002007 | finngen_R11_O15_PREECLAMPS | Pre-eclampsia \|\| id:finngen_R11_O15_PREECLAMPS | HLA DR on CD14+ CD16+ monocyte \|\| id:ebi-a-GCST90002007 | MR Egger | 19 | -0.014351135 | 0.012654758881301 | 0.272512608580039 | -0.039154462 | 0.0104521926266188 | 0.985751351901524 | 0.96160216649985 | 1.01050700760441 | 0.99380795269851 |
| ebi-a-GCST90002007 | finngen_R11_O15_PREECLAMPS | Pre-eclampsia \|\| id:finngen_R11_O15_PREECLAMPS | HLA DR on CD14+ CD16+ monocyte \|\| id:ebi-a-GCST90002007 | Weighted median | 19 | -0.006280053 | 0.0126502283552736 | 0.619585501423129 | -0.0310745 | 0.0185143949664067 | 0.993739625705295 | 0.969403349655614 | 1.018686849 | 0.940845681141708 |
| ebi-a-GCST90002007 | finngen_R11_O15_PREECLAMPS | Pre-eclampsia \|\| id:finngen_R11_O15_PREECLAMPS | HLA DR on CD14+ CD16+ monocyte \|\| id:ebi-a-GCST90002007 | Weighted mode | 19 | -0.003916373 | 0.0113668993485595 | 0.734431583725139 | -0.026195496 | 0.0183627498390872 | 0.996091286102449 | 0.974144629996341 | 1.01853238184248 | 0.959313411571287 |
| ebi-a-GCST90002008 | finngen_R11_O15_PREECLAMPS | Pre-eclampsia \|\| id:finngen_R11_O15_PREECLAMPS | CCR2 on monocyte \|\| id:ebi-a-GCST90002008 | Inverse variance weighted | 24 | 0.000104028456315065 | 0.0127147568584298 | 0.993472012554178 | -0.024816895 | 0.0250249518988374 | 1.00010403386746 | 0.97548851251405 | 1.02534070440277 | 0.995223645914365 |
| ebi-a-GCST90002008 | finngen_R11_O15_PREECLAMPS | Pre-eclampsia \|\| id:finngen_R11_O15_PREECLAMPS | CCR2 on monocyte \|\| id:ebi-a-GCST90002008 | MR Egger | 24 | 0.0101293624329385 | 0.0150148041577026 | 0.506942214711686 | -0.019299654 | 0.0395583785820356 | 1.01018083808295 | 0.980885392247492 | 1.04035123134191 | 0.99380795269851 |
| ebi-a-GCST90002008 | finngen_R11_O15_PREECLAMPS | Pre-eclampsia \|\| id:finngen_R11_O15_PREECLAMPS | CCR2 on monocyte \|\| id:ebi-a-GCST90002008 | Weighted median | 24 | 0.0021218745323186 | 0.0141158733111702 | 0.88051347038952 | -0.025545237 | 0.0297889862222122 | 1.00212412730117 | 0.9747782817696 | 1.03023711678928 | 0.964912412213677 |
| ebi-a-GCST90002008 | finngen_R11_O15_PREECLAMPS | Pre-eclampsia \|\| id:finngen_R11_O15_PREECLAMPS | CCR2 on monocyte \|\| id:ebi-a-GCST90002008 | Weighted mode | 24 | 0.000626409466892963 | 0.0114969119198175 | 0.957019259009116 | -0.021907538 | 0.0231603568297352 | 1.00062660570228 | 0.978330689383344 | 1.02343064048247 | 0.990180645930572 |
| ebi-a-GCST90002009 | finngen_R11_O15_PREECLAMPS | Pre-eclampsia \|\| id:finngen_R11_O15_PREECLAMPS | HLA DR on CD14- CD16- \|\| id:ebi-a-GCST90002009 | Inverse variance weighted (multiplicative random effects) | 24 | -0.003255586 | 0.0029058894514988 | 0.26256859140467 | -0.00895113 | 0.00243995701360615 | 0.996749707363554 | 0.991088812460143 | 1.0024429361312 | 0.885079385681701 |
| ebi-a-GCST90002009 | finngen_R11_O15_PREECLAMPS | Pre-eclampsia \|\| id:finngen_R11_O15_PREECLAMPS | HLA DR on CD14- CD16- \|\| id:ebi-a-GCST90002009 | MR Egger | 24 | -0.00374357 | 0.00304270969308179 | 0.23156342646759 | -0.009707281 | 0.00222014144729003 | 0.996263428869593 | 0.99033968301257 | 1.00222260778618 | 0.99380795269851 |
| ebi-a-GCST90002009 | finngen_R11_O15_PREECLAMPS | Pre-eclampsia \|\| id:finngen_R11_O15_PREECLAMPS | HLA DR on CD14- CD16- \|\| id:ebi-a-GCST90002009 | Weighted median | 24 | -0.002976633 | 0.00259856098116367 | 0.252005009833918 | -0.008069813 | 0.0021165462116927 | 0.997027792469149 | 0.991962660694644 | 1.00211878767674 | 0.940845681141708 |
| ebi-a-GCST90002009 | finngen_R11_O15_PREECLAMPS | Pre-eclampsia \|\| id:finngen_R11_O15_PREECLAMPS | HLA DR on CD14- CD16- \|\| id:ebi-a-GCST90002009 | Weighted mode | 24 | -0.002444296 | 0.00249792727077848 | 0.337993570259483 | -0.007340233 | 0.00245164151117762 | 0.997558688919317 | 0.992686640329512 | 1.00245464924168 | 0.888599269773114 |
| ebi-a-GCST90002010 | finngen_R11_O15_PREECLAMPS | Pre-eclampsia \|\| id:finngen_R11_O15_PREECLAMPS | HLA DR on monocyte \|\| id:ebi-a-GCST90002010 | Inverse variance weighted (multiplicative random effects) | 17 | 0.0226526915517916 | 0.0197328531556951 | 0.250981615728523 | -0.016023701 | 0.0613290837369539 | 1.02291121214094 | 0.984103995890882 | 1.0632487544941 | 0.885079385681701 |
| ebi-a-GCST90002010 | finngen_R11_O15_PREECLAMPS | Pre-eclampsia \|\| id:finngen_R11_O15_PREECLAMPS | HLA DR on monocyte \|\| id:ebi-a-GCST90002010 | MR Egger | 17 | 0.0290099839979381 | 0.0359283086142007 | 0.432030778345901 | -0.041409501 | 0.0994294688817716 | 1.02943487229947 | 0.959436159535135 | 1.10454056351147 | 0.99380795269851 |
| ebi-a-GCST90002010 | finngen_R11_O15_PREECLAMPS | Pre-eclampsia \|\| id:finngen_R11_O15_PREECLAMPS | HLA DR on monocyte \|\| id:ebi-a-GCST90002010 | Weighted median | 17 | 0.0260025641918641 | 0.0214234594973372 | 0.224846053544132 | -0.015987416 | 0.0679925448066449 | 1.02634358021193 | 0.984139703975194 | 1.07035732872818 | 0.940845681141708 |
| ebi-a-GCST90002010 | finngen_R11_O15_PREECLAMPS | Pre-eclampsia \|\| id:finngen_R11_O15_PREECLAMPS | HLA DR on monocyte \|\| id:ebi-a-GCST90002010 | Weighted mode | 17 | 0.0247838970425518 | 0.0196294897869458 | 0.224830752619928 | -0.013689903 | 0.0632576970249655 | 1.0250935708342 | 0.98640337762891 | 1.06530132884534 | 0.872755742358858 |
| ebi-a-GCST90002011 | finngen_R11_O15_PREECLAMPS | Pre-eclampsia \|\| id:finngen_R11_O15_PREECLAMPS | CD64 on CD14+ CD16+ monocyte \|\| id:ebi-a-GCST90002011 | Inverse variance weighted (multiplicative random effects) | 25 | 0.0126352580801191 | 0.0247039878248966 | 0.609024556626244 | -0.035784558 | 0.0610550742169163 | 1.01271542022081 | 0.964848139851166 | 1.06295745412456 | 0.92242199665996 |
| ebi-a-GCST90002011 | finngen_R11_O15_PREECLAMPS | Pre-eclampsia \|\| id:finngen_R11_O15_PREECLAMPS | CD64 on CD14+ CD16+ monocyte \|\| id:ebi-a-GCST90002011 | MR Egger | 25 | -0.029110395 | 0.0449202535548415 | 0.523372478534183 | -0.117154092 | 0.0589333021155955 | 0.971309231009002 | 0.889448125923242 | 1.06070449163521 | 0.99380795269851 |
| ebi-a-GCST90002011 | finngen_R11_O15_PREECLAMPS | Pre-eclampsia \|\| id:finngen_R11_O15_PREECLAMPS | CD64 on CD14+ CD16+ monocyte \|\| id:ebi-a-GCST90002011 | Weighted median | 25 | -0.000549473 | 0.0276125907300223 | 0.984123665228204 | -0.05467015 | 0.0535712052775015 | 0.999450678379056 | 0.946797397210728 | 1.05503211294742 | 0.992447515164719 |
| ebi-a-GCST90002011 | finngen_R11_O15_PREECLAMPS | Pre-eclampsia \|\| id:finngen_R11_O15_PREECLAMPS | CD64 on CD14+ CD16+ monocyte \|\| id:ebi-a-GCST90002011 | Weighted mode | 25 | 0.0139215256127129 | 0.027450112316816 | 0.616672993984482 | -0.039880695 | 0.0677237457536723 | 1.01401888130572 | 0.960904073427729 | 1.07006965635664 | 0.959313411571287 |
| ebi-a-GCST90002012 | finngen_R11_O15_PREECLAMPS | Pre-eclampsia \|\| id:finngen_R11_O15_PREECLAMPS | CX3CR1 on CD14- CD16+ monocyte \|\| id:ebi-a-GCST90002012 | Inverse variance weighted | 17 | -0.023156025 | 0.0191466964192547 | 0.226508993515755 | -0.06068355 | 0.0143714996051952 | 0.977110017920844 | 0.941121010065321 | 1.01447526610314 | 0.876074466984215 |
| ebi-a-GCST90002012 | finngen_R11_O15_PREECLAMPS | Pre-eclampsia \|\| id:finngen_R11_O15_PREECLAMPS | CX3CR1 on CD14- CD16+ monocyte \|\| id:ebi-a-GCST90002012 | MR Egger | 17 | -0.005752938 | 0.0273525891078281 | 0.836244196903804 | -0.059364013 | 0.0478581366966544 | 0.994263578504966 | 0.942363674458521 | 1.04902182706642 | 0.99380795269851 |
| ebi-a-GCST90002012 | finngen_R11_O15_PREECLAMPS | Pre-eclampsia \|\| id:finngen_R11_O15_PREECLAMPS | CX3CR1 on CD14- CD16+ monocyte \|\| id:ebi-a-GCST90002012 | Weighted median | 17 | -0.01172019 | 0.0257885157737979 | 0.649488270897683 | -0.06226568 | 0.0388253014106649 | 0.988348224379643 | 0.9396332114769 | 1.0395888530792 | 0.940845681141708 |
| ebi-a-GCST90002012 | finngen_R11_O15_PREECLAMPS | Pre-eclampsia \|\| id:finngen_R11_O15_PREECLAMPS | CX3CR1 on CD14- CD16+ monocyte \|\| id:ebi-a-GCST90002012 | Weighted mode | 17 | -0.012465475 | 0.0237188406407614 | 0.6064073903444 | -0.058954403 | 0.0340234527087049 | 0.987611897258193 | 0.942749755111849 | 1.03460887082406 | 0.955353022288269 |
| ebi-a-GCST90002013 | finngen_R11_O15_PREECLAMPS | Pre-eclampsia \|\| id:finngen_R11_O15_PREECLAMPS | CCR2 on myeloid Dendritic Cell \|\| id:ebi-a-GCST90002013 | Inverse variance weighted | 15 | 0.022431309071798 | 0.0142970669324381 | 0.116660797629463 | -0.005590942 | 0.0504535602593766 | 1.02268478258468 | 0.994424658114222 | 1.05174801931564 | 0.820779257935285 |
| ebi-a-GCST90002013 | finngen_R11_O15_PREECLAMPS | Pre-eclampsia \|\| id:finngen_R11_O15_PREECLAMPS | CCR2 on myeloid Dendritic Cell \|\| id:ebi-a-GCST90002013 | MR Egger | 15 | 0.0170472213246805 | 0.0181169902385863 | 0.363886400072415 | -0.01846208 | 0.0525565221923096 | 1.01719335440881 | 0.981707300675504 | 1.05396213264534 | 0.99380795269851 |
| ebi-a-GCST90002013 | finngen_R11_O15_PREECLAMPS | Pre-eclampsia \|\| id:finngen_R11_O15_PREECLAMPS | CCR2 on myeloid Dendritic Cell \|\| id:ebi-a-GCST90002013 | Weighted median | 15 | 0.0206506462139485 | 0.0190254839258684 | 0.277735551430308 | -0.016639302 | 0.0579405947086506 | 1.02086534615922 | 0.983498366282234 | 1.05965204490204 | 0.940845681141708 |
| ebi-a-GCST90002013 | finngen_R11_O15_PREECLAMPS | Pre-eclampsia \|\| id:finngen_R11_O15_PREECLAMPS | CCR2 on myeloid Dendritic Cell \|\| id:ebi-a-GCST90002013 | Weighted mode | 15 | 0.0227260750503765 | 0.0173513437293338 | 0.211363595867262 | -0.011282559 | 0.0567347087598708 | 1.02298627969876 | 0.98878085070848 | 1.0583749955332 | 0.872755742358858 |
| ebi-a-GCST90002014 | finngen_R11_O15_PREECLAMPS | Pre-eclampsia \|\| id:finngen_R11_O15_PREECLAMPS | CCR2 on CD62L+ myeloid Dendritic Cell \|\| id:ebi-a-GCST90002014 | Inverse variance weighted | 14 | -0.019320808 | 0.0249842561774569 | 0.439333434404621 | -0.06828995 | 0.0296483345244145 | 0.980864642947589 | 0.933989624309612 | 1.03009222237973 | 0.898694924660397 |
| ebi-a-GCST90002014 | finngen_R11_O15_PREECLAMPS | Pre-eclampsia \|\| id:finngen_R11_O15_PREECLAMPS | CCR2 on CD62L+ myeloid Dendritic Cell \|\| id:ebi-a-GCST90002014 | MR Egger | 14 | -0.031484244 | 0.05476043667597 | 0.575949670252405 | -0.1388147 | 0.0758462116367994 | 0.969006223755115 | 0.870389296537197 | 1.07879665502759 | 0.99380795269851 |
| ebi-a-GCST90002014 | finngen_R11_O15_PREECLAMPS | Pre-eclampsia \|\| id:finngen_R11_O15_PREECLAMPS | CCR2 on CD62L+ myeloid Dendritic Cell \|\| id:ebi-a-GCST90002014 | Weighted median | 14 | -0.03981738 | 0.0322682287188801 | 0.217221546675728 | -0.103063108 | 0.0234283483583094 | 0.96096491460847 | 0.902070043650665 | 1.02370494797861 | 0.940845681141708 |
| ebi-a-GCST90002014 | finngen_R11_O15_PREECLAMPS | Pre-eclampsia \|\| id:finngen_R11_O15_PREECLAMPS | CCR2 on CD62L+ myeloid Dendritic Cell \|\| id:ebi-a-GCST90002014 | Weighted mode | 14 | -0.032126275 | 0.0381896288247187 | 0.415425221154429 | -0.106977947 | 0.0427253979133528 | 0.968384292035545 | 0.898545488323991 | 1.0436512666825 | 0.93252856028397 |
| ebi-a-GCST90002015 | finngen_R11_O15_PREECLAMPS | Pre-eclampsia \|\| id:finngen_R11_O15_PREECLAMPS | CCR2 on plasmacytoid Dendritic Cell \|\| id:ebi-a-GCST90002015 | Inverse variance weighted | 19 | -0.011844748 | 0.0195531723160588 | 0.544666708403384 | -0.050168966 | 0.0264794694282074 | 0.988225124572346 | 0.951068712599316 | 1.02683316557325 | 0.905435115099076 |
| ebi-a-GCST90002015 | finngen_R11_O15_PREECLAMPS | Pre-eclampsia \|\| id:finngen_R11_O15_PREECLAMPS | CCR2 on plasmacytoid Dendritic Cell \|\| id:ebi-a-GCST90002015 | MR Egger | 19 | -0.014231466 | 0.0296979118608607 | 0.637897232583246 | -0.072439374 | 0.0439764407718842 | 0.985869322153791 | 0.930122134793436 | 1.04495773620077 | 0.99380795269851 |
| ebi-a-GCST90002015 | finngen_R11_O15_PREECLAMPS | Pre-eclampsia \|\| id:finngen_R11_O15_PREECLAMPS | CCR2 on plasmacytoid Dendritic Cell \|\| id:ebi-a-GCST90002015 | Weighted median | 19 | -0.012312337 | 0.0251595794515367 | 0.624579937150644 | -0.061625113 | 0.037000438227045 | 0.987763149205832 | 0.940235302511164 | 1.0376934755834 | 0.940845681141708 |
| ebi-a-GCST90002015 | finngen_R11_O15_PREECLAMPS | Pre-eclampsia \|\| id:finngen_R11_O15_PREECLAMPS | CCR2 on plasmacytoid Dendritic Cell \|\| id:ebi-a-GCST90002015 | Weighted mode | 19 | -0.009780081 | 0.0218971218815274 | 0.660463694467222 | -0.05269844 | 0.0331382781578685 | 0.990267588729324 | 0.948666049447951 | 1.03369346658757 | 0.959313411571287 |
| ebi-a-GCST90002016 | finngen_R11_O15_PREECLAMPS | Pre-eclampsia \|\| id:finngen_R11_O15_PREECLAMPS | CCR2 on CD62L+ plasmacytoid Dendritic Cell \|\| id:ebi-a-GCST90002016 | Inverse variance weighted | 19 | -0.014272345 | 0.0195349704840912 | 0.465020533524264 | -0.052560887 | 0.0240161974814991 | 0.98582902242213 | 0.948796550095976 | 1.02430690894846 | 0.898694924660397 |
| ebi-a-GCST90002016 | finngen_R11_O15_PREECLAMPS | Pre-eclampsia \|\| id:finngen_R11_O15_PREECLAMPS | CCR2 on CD62L+ plasmacytoid Dendritic Cell \|\| id:ebi-a-GCST90002016 | MR Egger | 19 | -0.012224227 | 0.0295337388619794 | 0.684117109090831 | -0.070110355 | 0.0456619012638298 | 0.987850185436328 | 0.932290931193153 | 1.04672045626111 | 0.99380795269851 |
| ebi-a-GCST90002016 | finngen_R11_O15_PREECLAMPS | Pre-eclampsia \|\| id:finngen_R11_O15_PREECLAMPS | CCR2 on CD62L+ plasmacytoid Dendritic Cell \|\| id:ebi-a-GCST90002016 | Weighted median | 19 | -0.012535009 | 0.0256068998094889 | 0.624475839765017 | -0.062724532 | 0.0376545151124215 | 0.987543227467631 | 0.939202158065744 | 1.03837242891952 | 0.940845681141708 |
| ebi-a-GCST90002016 | finngen_R11_O15_PREECLAMPS | Pre-eclampsia \|\| id:finngen_R11_O15_PREECLAMPS | CCR2 on CD62L+ plasmacytoid Dendritic Cell \|\| id:ebi-a-GCST90002016 | Weighted mode | 19 | -0.010038517 | 0.0225397419028654 | 0.661362588028731 | -0.054216411 | 0.0341393769047303 | 0.990011700511471 | 0.947227093620689 | 1.03472881397763 | 0.959313411571287 |
| ebi-a-GCST90002017 | finngen_R11_O15_PREECLAMPS | Pre-eclampsia \|\| id:finngen_R11_O15_PREECLAMPS | CCR2 on monocyte \|\| id:ebi-a-GCST90002017 | Inverse variance weighted | 25 | 0.0151584464835201 | 0.0219505479293721 | 0.489834184861881 | -0.027864627 | 0.0581815204250893 | 1.01527391845421 | 0.972520010396487 | 1.05990737308647 | 0.898694924660397 |
| ebi-a-GCST90002017 | finngen_R11_O15_PREECLAMPS | Pre-eclampsia \|\| id:finngen_R11_O15_PREECLAMPS | CCR2 on monocyte \|\| id:ebi-a-GCST90002017 | MR Egger | 25 | 0.0515189221864695 | 0.0443939426386598 | 0.257751917856123 | -0.035493205 | 0.138531049758243 | 1.05286910868925 | 0.965129291889393 | 1.14858534431378 | 0.99380795269851 |
| ebi-a-GCST90002017 | finngen_R11_O15_PREECLAMPS | Pre-eclampsia \|\| id:finngen_R11_O15_PREECLAMPS | CCR2 on monocyte \|\| id:ebi-a-GCST90002017 | Weighted median | 25 | 0.0150348650074983 | 0.0285560237151015 | 0.598537969006065 | -0.040934941 | 0.0710046714890972 | 1.01514845715731 | 0.959891577047445 | 1.07358624110306 | 0.940845681141708 |
| ebi-a-GCST90002017 | finngen_R11_O15_PREECLAMPS | Pre-eclampsia \|\| id:finngen_R11_O15_PREECLAMPS | CCR2 on monocyte \|\| id:ebi-a-GCST90002017 | Weighted mode | 25 | 0.0151299350806222 | 0.0315529611546072 | 0.635917167440799 | -0.046713869 | 0.0769737389436523 | 1.01524497198312 | 0.954360430838938 | 1.08001371371919 | 0.959313411571287 |
| ebi-a-GCST90002018 | finngen_R11_O15_PREECLAMPS | Pre-eclampsia \|\| id:finngen_R11_O15_PREECLAMPS | CCR2 on granulocyte \|\| id:ebi-a-GCST90002018 | Inverse variance weighted | 16 | 0.00835385602529961 | 0.0206705098281064 | 0.68610706204455 | -0.032160343 | 0.0488680552883882 | 1.00838884684878 | 0.968351301047471 | 1.05008178886018 | 0.950911139356934 |
| ebi-a-GCST90002018 | finngen_R11_O15_PREECLAMPS | Pre-eclampsia \|\| id:finngen_R11_O15_PREECLAMPS | CCR2 on granulocyte \|\| id:ebi-a-GCST90002018 | MR Egger | 16 | 0.00788032559321663 | 0.0276322373949165 | 0.779677242125928 | -0.04627886 | 0.062039510887253 | 1.00791145708063 | 0.954775676604753 | 1.06400438365477 | 0.99380795269851 |
| ebi-a-GCST90002018 | finngen_R11_O15_PREECLAMPS | Pre-eclampsia \|\| id:finngen_R11_O15_PREECLAMPS | CCR2 on granulocyte \|\| id:ebi-a-GCST90002018 | Weighted median | 16 | 0.0126916591854535 | 0.028744853883571 | 0.658830761751286 | -0.043648254 | 0.0690315727972527 | 1.0127725401007 | 0.95729062099826 | 1.07147003792058 | 0.940845681141708 |
| ebi-a-GCST90002018 | finngen_R11_O15_PREECLAMPS | Pre-eclampsia \|\| id:finngen_R11_O15_PREECLAMPS | CCR2 on granulocyte \|\| id:ebi-a-GCST90002018 | Weighted mode | 16 | 0.0156860058749885 | 0.0256745273304863 | 0.550375411843979 | -0.034636068 | 0.0660080794427416 | 1.01580967705462 | 0.965956895217979 | 1.06823534787746 | 0.939633861977944 |
| ebi-a-GCST90002019 | finngen_R11_O15_PREECLAMPS | Pre-eclampsia \|\| id:finngen_R11_O15_PREECLAMPS | CD14 on Monocytic Myeloid-Derived Suppressor Cells \|\| id:ebi-a-GCST90002019 | Inverse variance weighted | 24 | -0.03106028 | 0.0124521083028664 | 0.012617766126995 | -0.055466412 | -0.006654148 | 0.969417134706955 | 0.946043798457634 | 0.993367941944739 | 0.588591724205005 |
| ebi-a-GCST90002019 | finngen_R11_O15_PREECLAMPS | Pre-eclampsia \|\| id:finngen_R11_O15_PREECLAMPS | CD14 on Monocytic Myeloid-Derived Suppressor Cells \|\| id:ebi-a-GCST90002019 | MR Egger | 24 | -0.019314473 | 0.0175117231532957 | 0.28197025338262 | -0.053637451 | 0.0150085039229367 | 0.980870855887384 | 0.94777565949174 | 1.0151216970957 | 0.99380795269851 |
| ebi-a-GCST90002019 | finngen_R11_O15_PREECLAMPS | Pre-eclampsia \|\| id:finngen_R11_O15_PREECLAMPS | CD14 on Monocytic Myeloid-Derived Suppressor Cells \|\| id:ebi-a-GCST90002019 | Weighted median | 24 | -0.019301571 | 0.0195832107478836 | 0.324320455170614 | -0.057684664 | 0.0190815222078269 | 0.980883511752843 | 0.943947561230174 | 1.01926473794235 | 0.940845681141708 |
| ebi-a-GCST90002019 | finngen_R11_O15_PREECLAMPS | Pre-eclampsia \|\| id:finngen_R11_O15_PREECLAMPS | CD14 on Monocytic Myeloid-Derived Suppressor Cells \|\| id:ebi-a-GCST90002019 | Weighted mode | 24 | -0.022921918 | 0.0168017685316562 | 0.185688581063418 | -0.055853384 | 0.0100095486186325 | 0.977338793651564 | 0.945677777195537 | 1.01005981171406 | 0.872755742358858 |
| ebi-a-GCST90002020 | finngen_R11_O15_PREECLAMPS | Pre-eclampsia \|\| id:finngen_R11_O15_PREECLAMPS | CD14 on CD33+ HLA DR+ CD14dim \|\| id:ebi-a-GCST90002020 | Inverse variance weighted (multiplicative random effects) | 16 | -0.031671247 | 0.0307037876144978 | 0.302301980087603 | -0.091850671 | 0.0285081767203345 | 0.968825033862744 | 0.912241363712734 | 1.02891842397833 | 0.885079385681701 |
| ebi-a-GCST90002020 | finngen_R11_O15_PREECLAMPS | Pre-eclampsia \|\| id:finngen_R11_O15_PREECLAMPS | CD14 on CD33+ HLA DR+ CD14dim \|\| id:ebi-a-GCST90002020 | MR Egger | 16 | 0.0675198823432741 | 0.0709566320878084 | 0.357457612705257 | -0.071555117 | 0.206594881235379 | 1.06985153054183 | 0.930944965707147 | 1.22948438368025 | 0.99380795269851 |
| ebi-a-GCST90002020 | finngen_R11_O15_PREECLAMPS | Pre-eclampsia \|\| id:finngen_R11_O15_PREECLAMPS | CD14 on CD33+ HLA DR+ CD14dim \|\| id:ebi-a-GCST90002020 | Weighted median | 16 | 0.00578756450833169 | 0.0333715291621105 | 0.862314854084752 | -0.059620633 | 0.0711957616660683 | 1.00580434481655 | 0.942121876077991 | 1.07379141249036 | 0.964912412213677 |
| ebi-a-GCST90002020 | finngen_R11_O15_PREECLAMPS | Pre-eclampsia \|\| id:finngen_R11_O15_PREECLAMPS | CD14 on CD33+ HLA DR+ CD14dim \|\| id:ebi-a-GCST90002020 | Weighted mode | 16 | -0.006327435 | 0.039846723756829 | 0.875949126695226 | -0.084427013 | 0.0717721438202604 | 0.993692541317477 | 0.919038730527136 | 1.07441050509767 | 0.977585971930092 |
| ebi-a-GCST90002021 | finngen_R11_O15_PREECLAMPS | Pre-eclampsia \|\| id:finngen_R11_O15_PREECLAMPS | CD14 on CD33dim HLA DR+ CD11b+ \|\| id:ebi-a-GCST90002021 | Inverse variance weighted | 19 | -0.038479498 | 0.0200286681454621 | 0.05470384878417 | -0.077735688 | 0.000776691248730609 | 0.962251432312025 | 0.925208938162942 | 1.00077699295148 | 0.789997151112252 |
| ebi-a-GCST90002021 | finngen_R11_O15_PREECLAMPS | Pre-eclampsia \|\| id:finngen_R11_O15_PREECLAMPS | CD14 on CD33dim HLA DR+ CD11b+ \|\| id:ebi-a-GCST90002021 | MR Egger | 19 | -0.020468271 | 0.0419685798345882 | 0.631989136201508 | -0.102726687 | 0.061790145756971 | 0.979739782420386 | 0.902373570032915 | 1.06373909114175 | 0.99380795269851 |
| ebi-a-GCST90002021 | finngen_R11_O15_PREECLAMPS | Pre-eclampsia \|\| id:finngen_R11_O15_PREECLAMPS | CD14 on CD33dim HLA DR+ CD11b+ \|\| id:ebi-a-GCST90002021 | Weighted median | 19 | -0.016304959 | 0.0262419417775793 | 0.534381178709377 | -0.067739165 | 0.0351292467411386 | 0.983827247188548 | 0.934504193166555 | 1.03575356792229 | 0.940845681141708 |
| ebi-a-GCST90002021 | finngen_R11_O15_PREECLAMPS | Pre-eclampsia \|\| id:finngen_R11_O15_PREECLAMPS | CD14 on CD33dim HLA DR+ CD11b+ \|\| id:ebi-a-GCST90002021 | Weighted mode | 19 | -0.024484615 | 0.0340169785951302 | 0.480905026063907 | -0.091157894 | 0.0421886625635109 | 0.975812701210073 | 0.912873562690822 | 1.04309125245793 | 0.933945646747087 |
| ebi-a-GCST90002022 | finngen_R11_O15_PREECLAMPS | Pre-eclampsia \|\| id:finngen_R11_O15_PREECLAMPS | CD4 on CD4+ T cell \|\| id:ebi-a-GCST90002022 | Inverse variance weighted | 22 | -0.01476575 | 0.0204511300932268 | 0.47029344688175 | -0.054849965 | 0.0253184647722926 | 0.985342728897732 | 0.94662716432327 | 1.02564169926997 | 0.898694924660397 |
| ebi-a-GCST90002022 | finngen_R11_O15_PREECLAMPS | Pre-eclampsia \|\| id:finngen_R11_O15_PREECLAMPS | CD4 on CD4+ T cell \|\| id:ebi-a-GCST90002022 | MR Egger | 22 | -0.037128403 | 0.0430107826188249 | 0.398242435269434 | -0.121429537 | 0.0471727307056299 | 0.963552404167146 | 0.885653456807957 | 1.0483030675706 | 0.99380795269851 |
| ebi-a-GCST90002022 | finngen_R11_O15_PREECLAMPS | Pre-eclampsia \|\| id:finngen_R11_O15_PREECLAMPS | CD4 on CD4+ T cell \|\| id:ebi-a-GCST90002022 | Weighted median | 22 | -0.024303949 | 0.0305518053905382 | 0.426322940884004 | -0.084185488 | 0.0355775894581844 | 0.975989013680526 | 0.91926072874705 | 1.03621804460571 | 0.940845681141708 |
| ebi-a-GCST90002022 | finngen_R11_O15_PREECLAMPS | Pre-eclampsia \|\| id:finngen_R11_O15_PREECLAMPS | CD4 on CD4+ T cell \|\| id:ebi-a-GCST90002022 | Weighted mode | 22 | -0.038934656 | 0.0386746249075097 | 0.325527513279344 | -0.114736921 | 0.0368676089626573 | 0.961813555976452 | 0.89160067475925 | 1.03755564867631 | 0.888599269773114 |
| ebi-a-GCST90002023 | finngen_R11_O15_PREECLAMPS | Pre-eclampsia \|\| id:finngen_R11_O15_PREECLAMPS | CD4 on Central Memory CD4+ T cell \|\| id:ebi-a-GCST90002023 | Inverse variance weighted | 15 | -0.032215215 | 0.0237470875731945 | 0.174909471872839 | -0.078759506 | 0.0143290770558742 | 0.968298167762265 | 0.924262177013286 | 1.01443223038898 | 0.855687495048373 |
| ebi-a-GCST90002023 | finngen_R11_O15_PREECLAMPS | Pre-eclampsia \|\| id:finngen_R11_O15_PREECLAMPS | CD4 on Central Memory CD4+ T cell \|\| id:ebi-a-GCST90002023 | MR Egger | 15 | -0.040354782 | 0.0481823736492713 | 0.417420131587916 | -0.134792234 | 0.0540826702836045 | 0.960448628747544 | 0.873897458638788 | 1.05557186297337 | 0.99380795269851 |
| ebi-a-GCST90002023 | finngen_R11_O15_PREECLAMPS | Pre-eclampsia \|\| id:finngen_R11_O15_PREECLAMPS | CD4 on Central Memory CD4+ T cell \|\| id:ebi-a-GCST90002023 | Weighted median | 15 | -0.03021469 | 0.0331923243812834 | 0.362668805540798 | -0.095271646 | 0.0348422653837784 | 0.970237210568572 | 0.909125940323547 | 1.03545636860484 | 0.940845681141708 |
| ebi-a-GCST90002023 | finngen_R11_O15_PREECLAMPS | Pre-eclampsia \|\| id:finngen_R11_O15_PREECLAMPS | CD4 on Central Memory CD4+ T cell \|\| id:ebi-a-GCST90002023 | Weighted mode | 15 | -0.049132069 | 0.0376334083823596 | 0.212755536688281 | -0.12289355 | 0.0246294113465302 | 0.952055384313678 | 0.884357797869225 | 1.02493522077215 | 0.872755742358858 |
| ebi-a-GCST90002024 | finngen_R11_O15_PREECLAMPS | Pre-eclampsia \|\| id:finngen_R11_O15_PREECLAMPS | CD4 on naive CD4+ T cell \|\| id:ebi-a-GCST90002024 | Inverse variance weighted | 19 | -0.010268806 | 0.0245063104396861 | 0.675196398319598 | -0.058301174 | 0.0377635626607252 | 0.98978373837611 | 0.943365787152526 | 1.03848566706118 | 0.950857618427329 |
| ebi-a-GCST90002024 | finngen_R11_O15_PREECLAMPS | Pre-eclampsia \|\| id:finngen_R11_O15_PREECLAMPS | CD4 on naive CD4+ T cell \|\| id:ebi-a-GCST90002024 | MR Egger | 19 | -0.023779394 | 0.0530986697467418 | 0.659927400524235 | -0.127852786 | 0.0802939991223475 | 0.976501108410289 | 0.879982915163854 | 1.08360559994391 | 0.99380795269851 |
| ebi-a-GCST90002024 | finngen_R11_O15_PREECLAMPS | Pre-eclampsia \|\| id:finngen_R11_O15_PREECLAMPS | CD4 on naive CD4+ T cell \|\| id:ebi-a-GCST90002024 | Weighted median | 19 | -0.012730863 | 0.0339731815660645 | 0.707859245593806 | -0.079318299 | 0.0538565726829245 | 0.987349831451724 | 0.923745850213643 | 1.05533322768626 | 0.948776258030552 |
| ebi-a-GCST90002024 | finngen_R11_O15_PREECLAMPS | Pre-eclampsia \|\| id:finngen_R11_O15_PREECLAMPS | CD4 on naive CD4+ T cell \|\| id:ebi-a-GCST90002024 | Weighted mode | 19 | -0.018670521 | 0.0440179270475619 | 0.676477167015035 | -0.104945658 | 0.0676046157546192 | 0.981502693246585 | 0.900373449106107 | 1.06994218655238 | 0.959313411571287 |
| ebi-a-GCST90002025 | finngen_R11_O15_PREECLAMPS | Pre-eclampsia \|\| id:finngen_R11_O15_PREECLAMPS | CD4 on Effector Memory CD4+ T cell \|\| id:ebi-a-GCST90002025 | Inverse variance weighted | 18 | -0.026488306 | 0.0192345346682309 | 0.168474457235298 | -0.064187993 | 0.0112113824486582 | 0.97385943256703 | 0.937828677308224 | 1.01127446552594 | 0.85505992597664 |
| ebi-a-GCST90002025 | finngen_R11_O15_PREECLAMPS | Pre-eclampsia \|\| id:finngen_R11_O15_PREECLAMPS | CD4 on Effector Memory CD4+ T cell \|\| id:ebi-a-GCST90002025 | MR Egger | 18 | -0.046742528 | 0.0293528541398433 | 0.130848190253512 | -0.104274122 | 0.0107890658643645 | 0.954333079769294 | 0.900978285269664 | 1.01084747771624 | 0.97261503632431 |
| ebi-a-GCST90002025 | finngen_R11_O15_PREECLAMPS | Pre-eclampsia \|\| id:finngen_R11_O15_PREECLAMPS | CD4 on Effector Memory CD4+ T cell \|\| id:ebi-a-GCST90002025 | Weighted median | 18 | -0.037133942 | 0.0282928630124677 | 0.18935673432907 | -0.092587954 | 0.0183200691728052 | 0.9635470669646 | 0.911569031445309 | 1.01848891112948 | 0.940845681141708 |
| ebi-a-GCST90002025 | finngen_R11_O15_PREECLAMPS | Pre-eclampsia \|\| id:finngen_R11_O15_PREECLAMPS | CD4 on Effector Memory CD4+ T cell \|\| id:ebi-a-GCST90002025 | Weighted mode | 18 | -0.04624712 | 0.0264904651452704 | 0.0988903243832959 | -0.098168432 | 0.00567419143208394 | 0.95480598113878 | 0.906496207982325 | 1.00569032014766 | 0.872755742358858 |
| ebi-a-GCST90002026 | finngen_R11_O15_PREECLAMPS | Pre-eclampsia \|\| id:finngen_R11_O15_PREECLAMPS | CD4 on Terminally Differentiated CD4+ T cell \|\| id:ebi-a-GCST90002026 | Inverse variance weighted | 24 | -0.005726723 | 0.0222854973971638 | 0.797201267412787 | -0.049406298 | 0.0379528514434214 | 0.994289642968847 | 0.951794338654915 | 1.03868225935472 | 0.977824840984872 |
| ebi-a-GCST90002026 | finngen_R11_O15_PREECLAMPS | Pre-eclampsia \|\| id:finngen_R11_O15_PREECLAMPS | CD4 on Terminally Differentiated CD4+ T cell \|\| id:ebi-a-GCST90002026 | MR Egger | 24 | 0.0161715526154643 | 0.0388721949537465 | 0.681426747665895 | -0.060017949 | 0.0923610547248074 | 1.01630301989307 | 0.941747629539228 | 1.09676074124988 | 0.99380795269851 |
| ebi-a-GCST90002026 | finngen_R11_O15_PREECLAMPS | Pre-eclampsia \|\| id:finngen_R11_O15_PREECLAMPS | CD4 on Terminally Differentiated CD4+ T cell \|\| id:ebi-a-GCST90002026 | Weighted median | 24 | -0.003755341 | 0.030150078946789 | 0.900875899677263 | -0.062849496 | 0.0553388133284427 | 0.996251701068887 | 0.939084798938188 | 1.05689864536715 | 0.967019504646225 |
| ebi-a-GCST90002026 | finngen_R11_O15_PREECLAMPS | Pre-eclampsia \|\| id:finngen_R11_O15_PREECLAMPS | CD4 on Terminally Differentiated CD4+ T cell \|\| id:ebi-a-GCST90002026 | Weighted mode | 24 | -0.011301854 | 0.0340308910207774 | 0.742817019243716 | -0.0780024 | 0.0553986928047919 | 0.988761772428501 | 0.924962206634673 | 1.05696193379941 | 0.959313411571287 |
| ebi-a-GCST90002027 | finngen_R11_O15_PREECLAMPS | Pre-eclampsia \|\| id:finngen_R11_O15_PREECLAMPS | CD4 on CD45RA+ CD4+ T cell \|\| id:ebi-a-GCST90002027 | Inverse variance weighted | 27 | -0.023853413 | 0.0196960089578592 | 0.225865315273166 | -0.062457591 | 0.0147507643027583 | 0.976428830792205 | 0.939452903403417 | 1.01486009372928 | 0.876074466984215 |
| ebi-a-GCST90002027 | finngen_R11_O15_PREECLAMPS | Pre-eclampsia \|\| id:finngen_R11_O15_PREECLAMPS | CD4 on CD45RA+ CD4+ T cell \|\| id:ebi-a-GCST90002027 | MR Egger | 27 | -0.00364384 | 0.0383629187925791 | 0.925085251601153 | -0.078835161 | 0.0715474804807176 | 0.996362790377302 | 0.924192254644763 | 1.07416915155822 | 0.99380795269851 |
| ebi-a-GCST90002027 | finngen_R11_O15_PREECLAMPS | Pre-eclampsia \|\| id:finngen_R11_O15_PREECLAMPS | CD4 on CD45RA+ CD4+ T cell \|\| id:ebi-a-GCST90002027 | Weighted median | 27 | -0.039741899 | 0.0287161299843104 | 0.166371517893327 | -0.096025514 | 0.0165417159301595 | 0.961037452026788 | 0.908440838169289 | 1.0166792876236 | 0.940845681141708 |
| ebi-a-GCST90002027 | finngen_R11_O15_PREECLAMPS | Pre-eclampsia \|\| id:finngen_R11_O15_PREECLAMPS | CD4 on CD45RA+ CD4+ T cell \|\| id:ebi-a-GCST90002027 | Weighted mode | 27 | -0.055213013 | 0.0327181013961744 | 0.103462580133713 | -0.119340491 | 0.00891446620909446 | 0.946283556231508 | 0.887505561419608 | 1.00895431839529 | 0.872755742358858 |
| ebi-a-GCST90002028 | finngen_R11_O15_PREECLAMPS | Pre-eclampsia \|\| id:finngen_R11_O15_PREECLAMPS | CD19 on B cell \|\| id:ebi-a-GCST90002028 | Inverse variance weighted | 24 | -0.021005327 | 0.0170997038528061 | 0.21929572375345 | -0.054520747 | 0.0125100921074225 | 0.979213747849082 | 0.946938862517632 | 1.01258867064271 | 0.857246920127122 |
| ebi-a-GCST90002028 | finngen_R11_O15_PREECLAMPS | Pre-eclampsia \|\| id:finngen_R11_O15_PREECLAMPS | CD19 on B cell \|\| id:ebi-a-GCST90002028 | MR Egger | 24 | -0.031749697 | 0.0225373709237376 | 0.172884130055599 | -0.075922944 | 0.0124235500832784 | 0.968749032594462 | 0.926887626118288 | 1.01250104296132 | 0.99380795269851 |
| ebi-a-GCST90002028 | finngen_R11_O15_PREECLAMPS | Pre-eclampsia \|\| id:finngen_R11_O15_PREECLAMPS | CD19 on B cell \|\| id:ebi-a-GCST90002028 | Weighted median | 24 | -0.021297484 | 0.0259605390866764 | 0.412000028549039 | -0.07218014 | 0.0295851730255762 | 0.978927706326477 | 0.930363284892115 | 1.0300271622657 | 0.940845681141708 |
| ebi-a-GCST90002028 | finngen_R11_O15_PREECLAMPS | Pre-eclampsia \|\| id:finngen_R11_O15_PREECLAMPS | CD19 on B cell \|\| id:ebi-a-GCST90002028 | Weighted mode | 24 | -0.020925564 | 0.0239494228290406 | 0.391291778611939 | -0.067866433 | 0.0260153042728772 | 0.979291855962894 | 0.934385268077203 | 1.02635665599558 | 0.920286500917063 |
| ebi-a-GCST90002029 | finngen_R11_O15_PREECLAMPS | Pre-eclampsia \|\| id:finngen_R11_O15_PREECLAMPS | CD39 on CD39+ CD8+ T cell \|\| id:ebi-a-GCST90002029 | Inverse variance weighted | 23 | -0.009566295 | 0.0233918075084362 | 0.682569611342148 | -0.055414237 | 0.0362816480959461 | 0.990479316815776 | 0.946093159659867 | 1.0369478597568 | 0.950911139356934 |
| ebi-a-GCST90002029 | finngen_R11_O15_PREECLAMPS | Pre-eclampsia \|\| id:finngen_R11_O15_PREECLAMPS | CD39 on CD39+ CD8+ T cell \|\| id:ebi-a-GCST90002029 | MR Egger | 23 | 0.0138055722888723 | 0.0495926668823336 | 0.783443851193264 | -0.083396055 | 0.111007199378246 | 1.01390130926256 | 0.919986709903459 | 1.117402951 | 0.99380795269851 |
| ebi-a-GCST90002029 | finngen_R11_O15_PREECLAMPS | Pre-eclampsia \|\| id:finngen_R11_O15_PREECLAMPS | CD39 on CD39+ CD8+ T cell \|\| id:ebi-a-GCST90002029 | Weighted median | 23 | -0.00982404 | 0.0315296427078486 | 0.755358908817492 | -0.07162214 | 0.0519740593284689 | 0.990224057870526 | 0.930882572573286 | 1.05334841759364 | 0.957984984509652 |
| ebi-a-GCST90002029 | finngen_R11_O15_PREECLAMPS | Pre-eclampsia \|\| id:finngen_R11_O15_PREECLAMPS | CD39 on CD39+ CD8+ T cell \|\| id:ebi-a-GCST90002029 | Weighted mode | 23 | -0.010133944 | 0.038193626138754 | 0.793225046618136 | -0.084993451 | 0.0647255633485167 | 0.989917231510463 | 0.918518299652109 | 1.06686619701806 | 0.961305639715923 |
| ebi-a-GCST90002030 | finngen_R11_O15_PREECLAMPS | Pre-eclampsia \|\| id:finngen_R11_O15_PREECLAMPS | CD39 on CD39+ activated CD4 regulatory T cell \|\| id:ebi-a-GCST90002030 | Inverse variance weighted | 22 | 0.000461432006589845 | 0.0187298223902202 | 0.980345129460358 | -0.03624902 | 0.0371718838914214 | 1.00046153848271 | 0.964400108781317 | 1.0378713988824 | 0.994537505425743 |
| ebi-a-GCST90002030 | finngen_R11_O15_PREECLAMPS | Pre-eclampsia \|\| id:finngen_R11_O15_PREECLAMPS | CD39 on CD39+ activated CD4 regulatory T cell \|\| id:ebi-a-GCST90002030 | MR Egger | 22 | 0.0141605961787293 | 0.0417205278288871 | 0.73783491057718 | -0.067611638 | 0.0959328307233481 | 1.01426133235393 | 0.934623374965344 | 1.10068512928698 | 0.99380795269851 |
| ebi-a-GCST90002030 | finngen_R11_O15_PREECLAMPS | Pre-eclampsia \|\| id:finngen_R11_O15_PREECLAMPS | CD39 on CD39+ activated CD4 regulatory T cell \|\| id:ebi-a-GCST90002030 | Weighted median | 22 | -0.01350912 | 0.0255283015710857 | 0.596679144909471 | -0.063544591 | 0.0365263512933622 | 0.986581718862591 | 0.93843227286062 | 1.03720163526221 | 0.940845681141708 |
| ebi-a-GCST90002030 | finngen_R11_O15_PREECLAMPS | Pre-eclampsia \|\| id:finngen_R11_O15_PREECLAMPS | CD39 on CD39+ activated CD4 regulatory T cell \|\| id:ebi-a-GCST90002030 | Weighted mode | 22 | -0.010684553 | 0.0247640775823836 | 0.670535474781294 | -0.059222145 | 0.0378530387776105 | 0.989372323806574 | 0.942497374495203 | 1.03857859088328 | 0.959313411571287 |
| ebi-a-GCST90002031 | finngen_R11_O15_PREECLAMPS | Pre-eclampsia \|\| id:finngen_R11_O15_PREECLAMPS | CD39 on CD39+ secreting CD4 regulatory T cell \|\| id:ebi-a-GCST90002031 | Inverse variance weighted | 23 | 0.010767722564101 | 0.010540747126864 | 0.307001913892442 | -0.009892142 | 0.0314275869327545 | 1.01082590312524 | 0.990156624496665 | 1.0319266479163 | 0.885079385681701 |
| ebi-a-GCST90002031 | finngen_R11_O15_PREECLAMPS | Pre-eclampsia \|\| id:finngen_R11_O15_PREECLAMPS | CD39 on CD39+ secreting CD4 regulatory T cell \|\| id:ebi-a-GCST90002031 | MR Egger | 23 | 0.0226541338171034 | 0.0143597525197918 | 0.129600343134039 | -0.005490981 | 0.0507992487558953 | 1.02291268745136 | 0.994524066760008 | 1.05211165935662 | 0.97261503632431 |
| ebi-a-GCST90002031 | finngen_R11_O15_PREECLAMPS | Pre-eclampsia \|\| id:finngen_R11_O15_PREECLAMPS | CD39 on CD39+ secreting CD4 regulatory T cell \|\| id:ebi-a-GCST90002031 | Weighted median | 23 | 0.019431043736018 | 0.01345930528321 | 0.148826628597777 | -0.006949195 | 0.0458112820911096 | 1.01962105517783 | 0.99307489519994 | 1.04687682790797 | 0.917572019373545 |
| ebi-a-GCST90002031 | finngen_R11_O15_PREECLAMPS | Pre-eclampsia \|\| id:finngen_R11_O15_PREECLAMPS | CD39 on CD39+ secreting CD4 regulatory T cell \|\| id:ebi-a-GCST90002031 | Weighted mode | 23 | 0.0128104580896981 | 0.0123128750756313 | 0.309448372782982 | -0.011322777 | 0.0369436932379354 | 1.01289286351571 | 0.988741084324962 | 1.03763459334908 | 0.877668362036021 |
| ebi-a-GCST90002032 | finngen_R11_O15_PREECLAMPS | Pre-eclampsia \|\| id:finngen_R11_O15_PREECLAMPS | CD39 on CD39+ CD4+ T cell \|\| id:ebi-a-GCST90002032 | Inverse variance weighted | 20 | 0.0124675613506942 | 0.0140477509573528 | 0.374802779672234 | -0.015066031 | 0.0400011532271057 | 1.0125456053961 | 0.985046894291312 | 1.04081197448428 | 0.885079385681701 |
| ebi-a-GCST90002032 | finngen_R11_O15_PREECLAMPS | Pre-eclampsia \|\| id:finngen_R11_O15_PREECLAMPS | CD39 on CD39+ CD4+ T cell \|\| id:ebi-a-GCST90002032 | MR Egger | 20 | 0.0268654323097628 | 0.0219135853392201 | 0.236007537043584 | -0.016085195 | 0.0698160595746342 | 1.02722956155286 | 0.984043480943817 | 1.0723109217857 | 0.99380795269851 |
| ebi-a-GCST90002032 | finngen_R11_O15_PREECLAMPS | Pre-eclampsia \|\| id:finngen_R11_O15_PREECLAMPS | CD39 on CD39+ CD4+ T cell \|\| id:ebi-a-GCST90002032 | Weighted median | 20 | 0.0146167355887065 | 0.0156853118251316 | 0.351401602396399 | -0.016126476 | 0.0453599467659645 | 1.01472408245088 | 0.984002859844027 | 1.04640444202469 | 0.940845681141708 |
| ebi-a-GCST90002032 | finngen_R11_O15_PREECLAMPS | Pre-eclampsia \|\| id:finngen_R11_O15_PREECLAMPS | CD39 on CD39+ CD4+ T cell \|\| id:ebi-a-GCST90002032 | Weighted mode | 20 | 0.016345220721308 | 0.0150756291006699 | 0.291836397268365 | -0.013203012 | 0.0458934537586209 | 1.01647953464054 | 0.986883765123412 | 1.04696285505704 | 0.872755742358858 |
| ebi-a-GCST90002033 | finngen_R11_O15_PREECLAMPS | Pre-eclampsia \|\| id:finngen_R11_O15_PREECLAMPS | CD39 on granulocyte \|\| id:ebi-a-GCST90002033 | Inverse variance weighted | 26 | 0.0254695752612701 | 0.0177817035884568 | 0.15204445043854 | -0.009382564 | 0.0603217142946453 | 1.0257966961992 | 0.990661315139901 | 1.06217820949694 | 0.85505992597664 |
| ebi-a-GCST90002033 | finngen_R11_O15_PREECLAMPS | Pre-eclampsia \|\| id:finngen_R11_O15_PREECLAMPS | CD39 on granulocyte \|\| id:ebi-a-GCST90002033 | MR Egger | 26 | 0.0179403386726908 | 0.0257274041708613 | 0.492300406647858 | -0.032485374 | 0.0683660508475789 | 1.01810223324723 | 0.968036608713088 | 1.07075718832676 | 0.99380795269851 |
| ebi-a-GCST90002033 | finngen_R11_O15_PREECLAMPS | Pre-eclampsia \|\| id:finngen_R11_O15_PREECLAMPS | CD39 on granulocyte \|\| id:ebi-a-GCST90002033 | Weighted median | 26 | 0.0215005935403236 | 0.0297186205671031 | 0.46938987436612 | -0.036747903 | 0.0797490898518457 | 1.02173339677717 | 0.963919106057071 | 1.08301529405312 | 0.940845681141708 |
| ebi-a-GCST90002033 | finngen_R11_O15_PREECLAMPS | Pre-eclampsia \|\| id:finngen_R11_O15_PREECLAMPS | CD39 on granulocyte \|\| id:ebi-a-GCST90002033 | Weighted mode | 26 | 0.0214136090445619 | 0.0259545231952362 | 0.417150258841119 | -0.029457256 | 0.0722844745072249 | 1.02164452567811 | 0.970972379591646 | 1.07496109960102 | 0.93252856028397 |
| ebi-a-GCST90002034 | finngen_R11_O15_PREECLAMPS | Pre-eclampsia \|\| id:finngen_R11_O15_PREECLAMPS | CD39 on monocyte \|\| id:ebi-a-GCST90002034 | Inverse variance weighted (multiplicative random effects) | 22 | 0.0128164123253156 | 0.0126966825824286 | 0.312768455915919 | -0.012069086 | 0.0377019101868756 | 1.01289889453643 | 0.988003453755685 | 1.03842164382437 | 0.885079385681701 |
| ebi-a-GCST90002034 | finngen_R11_O15_PREECLAMPS | Pre-eclampsia \|\| id:finngen_R11_O15_PREECLAMPS | CD39 on monocyte \|\| id:ebi-a-GCST90002034 | MR Egger | 22 | 0.0301588758048219 | 0.0140426070168513 | 0.0441768913765114 | 0.00263536605179327 | 0.0576823855578505 | 1.03061826125274 | 1.00263884168142 | 1.05937846836889 | 0.862303274556491 |
| ebi-a-GCST90002034 | finngen_R11_O15_PREECLAMPS | Pre-eclampsia \|\| id:finngen_R11_O15_PREECLAMPS | CD39 on monocyte \|\| id:ebi-a-GCST90002034 | Weighted median | 22 | 0.0291871354004829 | 0.013660903926157 | 0.0326350205996708 | 0.00241176370521514 | 0.0559625070957506 | 1.02961725428506 | 1.00241467434676 | 1.05755803207125 | 0.721097500353414 |
| ebi-a-GCST90002034 | finngen_R11_O15_PREECLAMPS | Pre-eclampsia \|\| id:finngen_R11_O15_PREECLAMPS | CD39 on monocyte \|\| id:ebi-a-GCST90002034 | Weighted mode | 22 | 0.0239526379271433 | 0.0119826736878727 | 0.0587229886069559 | 0.000466597498912879 | 0.0474386783553737 | 1.02424180652677 | 1.00046670637246 | 1.0485818983832 | 0.838871423749761 |
| ebi-a-GCST90002035 | finngen_R11_O15_PREECLAMPS | Pre-eclampsia \|\| id:finngen_R11_O15_PREECLAMPS | CD80 on myeloid Dendritic Cell \|\| id:ebi-a-GCST90002035 | Inverse variance weighted | 26 | -0.016547131 | 0.01910329662967 | 0.386384631239348 | -0.053989593 | 0.0208953300217113 | 0.983589020397749 | 0.947441966700175 | 1.02111516594152 | 0.885079385681701 |
| ebi-a-GCST90002035 | finngen_R11_O15_PREECLAMPS | Pre-eclampsia \|\| id:finngen_R11_O15_PREECLAMPS | CD80 on myeloid Dendritic Cell \|\| id:ebi-a-GCST90002035 | MR Egger | 26 | -0.008570101 | 0.0287322637802259 | 0.768060790592585 | -0.064885338 | 0.047745136271283 | 0.991466517892254 | 0.937174915804029 | 1.04890329385101 | 0.99380795269851 |
| ebi-a-GCST90002035 | finngen_R11_O15_PREECLAMPS | Pre-eclampsia \|\| id:finngen_R11_O15_PREECLAMPS | CD80 on myeloid Dendritic Cell \|\| id:ebi-a-GCST90002035 | Weighted median | 26 | -0.001053296 | 0.0241130694381509 | 0.965158263088228 | -0.048314912 | 0.0462083203629995 | 0.998947258785469 | 0.952833681223965 | 1.04729256060003 | 0.989912389390616 |
| ebi-a-GCST90002035 | finngen_R11_O15_PREECLAMPS | Pre-eclampsia \|\| id:finngen_R11_O15_PREECLAMPS | CD80 on myeloid Dendritic Cell \|\| id:ebi-a-GCST90002035 | Weighted mode | 26 | -0.005478335 | 0.0235682574851174 | 0.818084417002119 | -0.05167212 | 0.0407154493471486 | 0.99453664338999 | 0.949640183831479 | 1.04155568802357 | 0.966106153196364 |
| ebi-a-GCST90002036 | finngen_R11_O15_PREECLAMPS | Pre-eclampsia \|\| id:finngen_R11_O15_PREECLAMPS | CD80 on CD62L+ myeloid Dendritic Cell \|\| id:ebi-a-GCST90002036 | Inverse variance weighted | 26 | 0.0108593246535885 | 0.0138634319087673 | 0.433446794497204 | -0.016313002 | 0.0380316511947723 | 1.0109185011311 | 0.98381933454901 | 1.03876411048337 | 0.898694924660397 |
| ebi-a-GCST90002036 | finngen_R11_O15_PREECLAMPS | Pre-eclampsia \|\| id:finngen_R11_O15_PREECLAMPS | CD80 on CD62L+ myeloid Dendritic Cell \|\| id:ebi-a-GCST90002036 | MR Egger | 26 | 0.0102601450187272 | 0.0187810488875084 | 0.589900686018247 | -0.026550711 | 0.0470710008382436 | 1.01031296078455 | 0.973798660473531 | 1.0481964292628 | 0.99380795269851 |
| ebi-a-GCST90002036 | finngen_R11_O15_PREECLAMPS | Pre-eclampsia \|\| id:finngen_R11_O15_PREECLAMPS | CD80 on CD62L+ myeloid Dendritic Cell \|\| id:ebi-a-GCST90002036 | Weighted median | 26 | -0.001632882 | 0.0193061953900581 | 0.932596763623352 | -0.039473025 | 0.0362072605057941 | 0.998368449968511 | 0.961295884190492 | 1.03687072657331 | 0.97808928867815 |
| ebi-a-GCST90002036 | finngen_R11_O15_PREECLAMPS | Pre-eclampsia \|\| id:finngen_R11_O15_PREECLAMPS | CD80 on CD62L+ myeloid Dendritic Cell \|\| id:ebi-a-GCST90002036 | Weighted mode | 26 | -0.00139076 | 0.0198481272520825 | 0.944695378726123 | -0.040293089 | 0.0375115695853419 | 0.998610206829529 | 0.960507883365593 | 1.03822400883361 | 0.990180645930572 |
| ebi-a-GCST90002037 | finngen_R11_O15_PREECLAMPS | Pre-eclampsia \|\| id:finngen_R11_O15_PREECLAMPS | CD80 on plasmacytoid Dendritic Cell \|\| id:ebi-a-GCST90002037 | Inverse variance weighted | 21 | -0.046764176 | 0.0174901199687052 | 0.00750087543532716 | -0.081044811 | -0.012483541 | 0.954312420574331 | 0.9221523675753 | 0.987594055044351 | 0.548313994322415 |
| ebi-a-GCST90002037 | finngen_R11_O15_PREECLAMPS | Pre-eclampsia \|\| id:finngen_R11_O15_PREECLAMPS | CD80 on plasmacytoid Dendritic Cell \|\| id:ebi-a-GCST90002037 | MR Egger | 21 | -0.0466656 | 0.0243783997665742 | 0.0707755232214991 | -0.094447264 | 0.00111606329948 | 0.954406497534775 | 0.909875716761796 | 1.00111668632988 | 0.896743733484648 |
| ebi-a-GCST90002037 | finngen_R11_O15_PREECLAMPS | Pre-eclampsia \|\| id:finngen_R11_O15_PREECLAMPS | CD80 on plasmacytoid Dendritic Cell \|\| id:ebi-a-GCST90002037 | Weighted median | 21 | -0.037360927 | 0.0210956640977615 | 0.0765567121781729 | -0.078708429 | 0.00398657439242062 | 0.963328381142805 | 0.924309387091134 | 1.00399453135027 | 0.860968563111452 |
| ebi-a-GCST90002037 | finngen_R11_O15_PREECLAMPS | Pre-eclampsia \|\| id:finngen_R11_O15_PREECLAMPS | CD80 on plasmacytoid Dendritic Cell \|\| id:ebi-a-GCST90002037 | Weighted mode | 21 | -0.03886479 | 0.0185263606553537 | 0.0488309468102277 | -0.075176457 | -0.002553123 | 0.961880756184207 | 0.927579793981302 | 0.997450133261691 | 0.838871423749761 |
| ebi-a-GCST90002038 | finngen_R11_O15_PREECLAMPS | Pre-eclampsia \|\| id:finngen_R11_O15_PREECLAMPS | CD80 on CD62L+ plasmacytoid Dendritic Cell \|\| id:ebi-a-GCST90002038 | Inverse variance weighted (multiplicative random effects) | 21 | -0.031886211 | 0.0176084601187756 | 0.0701646829090723 | -0.066398793 | 0.00262637106007886 | 0.96861679396513 | 0.935757616654284 | 1.00262982299391 | 0.789997151112252 |
| ebi-a-GCST90002038 | finngen_R11_O15_PREECLAMPS | Pre-eclampsia \|\| id:finngen_R11_O15_PREECLAMPS | CD80 on CD62L+ plasmacytoid Dendritic Cell \|\| id:ebi-a-GCST90002038 | MR Egger | 21 | -0.024636857 | 0.0241751921016063 | 0.320959003461607 | -0.072020234 | 0.0227465193783278 | 0.975664153174551 | 0.930512067955948 | 1.02300719417954 | 0.99380795269851 |
| ebi-a-GCST90002038 | finngen_R11_O15_PREECLAMPS | Pre-eclampsia \|\| id:finngen_R11_O15_PREECLAMPS | CD80 on CD62L+ plasmacytoid Dendritic Cell \|\| id:ebi-a-GCST90002038 | Weighted median | 21 | -0.028528428 | 0.02084496792885 | 0.171124266483025 | -0.069384565 | 0.0123277088652546 | 0.971874665032888 | 0.932967823921622 | 1.01240400827814 | 0.940845681141708 |
| ebi-a-GCST90002038 | finngen_R11_O15_PREECLAMPS | Pre-eclampsia \|\| id:finngen_R11_O15_PREECLAMPS | CD80 on CD62L+ plasmacytoid Dendritic Cell \|\| id:ebi-a-GCST90002038 | Weighted mode | 21 | -0.020393647 | 0.0172495273121352 | 0.250963375166662 | -0.05420272 | 0.0134154267958102 | 0.979812897233129 | 0.94724006227777 | 1.01350581739105 | 0.872755742358858 |
| ebi-a-GCST90002039 | finngen_R11_O15_PREECLAMPS | Pre-eclampsia \|\| id:finngen_R11_O15_PREECLAMPS | CD80 on monocyte \|\| id:ebi-a-GCST90002039 | Inverse variance weighted | 23 | -0.022941314 | 0.0206056269033737 | 0.265557315880991 | -0.063328343 | 0.0174457144566923 | 0.977319836814594 | 0.938635228775695 | 1.01759877974872 | 0.885079385681701 |
| ebi-a-GCST90002039 | finngen_R11_O15_PREECLAMPS | Pre-eclampsia \|\| id:finngen_R11_O15_PREECLAMPS | CD80 on monocyte \|\| id:ebi-a-GCST90002039 | MR Egger | 23 | -0.036686361 | 0.032209468272839 | 0.267532694602367 | -0.099816919 | 0.0264441967929952 | 0.963978429150571 | 0.90500309188862 | 1.02679694710034 | 0.99380795269851 |
| ebi-a-GCST90002039 | finngen_R11_O15_PREECLAMPS | Pre-eclampsia \|\| id:finngen_R11_O15_PREECLAMPS | CD80 on monocyte \|\| id:ebi-a-GCST90002039 | Weighted median | 23 | -0.040500621 | 0.0293809883767099 | 0.168060217833571 | -0.098087358 | 0.017086116022978 | 0.960308567971988 | 0.906569703803325 | 1.01723291860686 | 0.940845681141708 |
| ebi-a-GCST90002039 | finngen_R11_O15_PREECLAMPS | Pre-eclampsia \|\| id:finngen_R11_O15_PREECLAMPS | CD80 on monocyte \|\| id:ebi-a-GCST90002039 | Weighted mode | 23 | -0.050903559 | 0.0381995567533168 | 0.196311712532267 | -0.125774691 | 0.0239675719718525 | 0.950370320525527 | 0.881813505361716 | 1.02425710271392 | 0.872755742358858 |
| ebi-a-GCST90002040 | finngen_R11_O15_PREECLAMPS | Pre-eclampsia \|\| id:finngen_R11_O15_PREECLAMPS | CD80 on granulocyte \|\| id:ebi-a-GCST90002040 | Inverse variance weighted | 32 | 0.015407597474424 | 0.0154815939896791 | 0.31962910373894 | -0.014936327 | 0.045751521694195 | 1.01552690647183 | 0.985174666883458 | 1.04681426800253 | 0.885079385681701 |
| ebi-a-GCST90002040 | finngen_R11_O15_PREECLAMPS | Pre-eclampsia \|\| id:finngen_R11_O15_PREECLAMPS | CD80 on granulocyte \|\| id:ebi-a-GCST90002040 | MR Egger | 32 | 0.00663438608256238 | 0.0231226457327795 | 0.776144795401964 | -0.038686 | 0.0519547717188101 | 1.00665644237158 | 0.962052746715766 | 1.05332810121646 | 0.99380795269851 |
| ebi-a-GCST90002040 | finngen_R11_O15_PREECLAMPS | Pre-eclampsia \|\| id:finngen_R11_O15_PREECLAMPS | CD80 on granulocyte \|\| id:ebi-a-GCST90002040 | Weighted median | 32 | 0.00800687498358596 | 0.0211140384455137 | 0.704524173049669 | -0.03337664 | 0.0493903903367928 | 1.00803901573214 | 0.967174214126238 | 1.05063042665609 | 0.946704357535493 |
| ebi-a-GCST90002040 | finngen_R11_O15_PREECLAMPS | Pre-eclampsia \|\| id:finngen_R11_O15_PREECLAMPS | CD80 on granulocyte \|\| id:ebi-a-GCST90002040 | Weighted mode | 32 | 0.00569058895167579 | 0.0201307733001596 | 0.779300281080369 | -0.033765727 | 0.0451469046199886 | 1.0057068111096 | 0.966797973044216 | 1.04618153752167 | 0.961305639715923 |
| ebi-a-GCST90002041 | finngen_R11_O15_PREECLAMPS | Pre-eclampsia \|\| id:finngen_R11_O15_PREECLAMPS | CD45 on lymphocyte \|\| id:ebi-a-GCST90002041 | Inverse variance weighted (multiplicative random effects) | 17 | 0.0209246362084994 | 0.0319674681375296 | 0.512750878020242 | -0.041731601 | 0.0835808737580574 | 1.0211450913718 | 0.959127174476234 | 1.08717313551474 | 0.898694924660397 |
| ebi-a-GCST90002041 | finngen_R11_O15_PREECLAMPS | Pre-eclampsia \|\| id:finngen_R11_O15_PREECLAMPS | CD45 on lymphocyte \|\| id:ebi-a-GCST90002041 | MR Egger | 17 | -0.170588119 | 0.0657217640795274 | 0.0202753516486105 | -0.299402777 | -0.041773462 | 0.843168787080793 | 0.741260786729924 | 0.95908702609723 | 0.716086784616111 |
| ebi-a-GCST90002041 | finngen_R11_O15_PREECLAMPS | Pre-eclampsia \|\| id:finngen_R11_O15_PREECLAMPS | CD45 on lymphocyte \|\| id:ebi-a-GCST90002041 | Weighted median | 17 | 0.0293363307034797 | 0.039534839196338 | 0.458064654282656 | -0.048151954 | 0.106824615528302 | 1.0297708798031 | 0.952988965473766 | 1.11273908020884 | 0.940845681141708 |
| ebi-a-GCST90002041 | finngen_R11_O15_PREECLAMPS | Pre-eclampsia \|\| id:finngen_R11_O15_PREECLAMPS | CD45 on lymphocyte \|\| id:ebi-a-GCST90002041 | Weighted mode | 17 | 0.054920103224697 | 0.0626448717986252 | 0.393628120530701 | -0.067863846 | 0.177704051950002 | 1.05645620385953 | 0.934387686004371 | 1.19447176733027 | 0.920286500917063 |
| ebi-a-GCST90002042 | finngen_R11_O15_PREECLAMPS | Pre-eclampsia \|\| id:finngen_R11_O15_PREECLAMPS | CD45 on CD33+ HLA DR+ CD14- \|\| id:ebi-a-GCST90002042 | Inverse variance weighted | 18 | -0.052018771 | 0.0219345927832791 | 0.0177141148187553 | -0.095010573 | -0.009026969 | 0.949311047410233 | 0.909363320092316 | 0.991013651885176 | 0.588591724205005 |
| ebi-a-GCST90002042 | finngen_R11_O15_PREECLAMPS | Pre-eclampsia \|\| id:finngen_R11_O15_PREECLAMPS | CD45 on CD33+ HLA DR+ CD14- \|\| id:ebi-a-GCST90002042 | MR Egger | 18 | -0.050549916 | 0.0726158599908348 | 0.496337548856175 | -0.192877002 | 0.0917771693984514 | 0.950706471849121 | 0.824583390203075 | 1.09612054566514 | 0.99380795269851 |
| ebi-a-GCST90002042 | finngen_R11_O15_PREECLAMPS | Pre-eclampsia \|\| id:finngen_R11_O15_PREECLAMPS | CD45 on CD33+ HLA DR+ CD14- \|\| id:ebi-a-GCST90002042 | Weighted median | 18 | -0.037809222 | 0.0319505071627551 | 0.236663161351448 | -0.100432216 | 0.0248137723726162 | 0.962896623182557 | 0.904446417597418 | 1.02512419630045 | 0.940845681141708 |
| ebi-a-GCST90002042 | finngen_R11_O15_PREECLAMPS | Pre-eclampsia \|\| id:finngen_R11_O15_PREECLAMPS | CD45 on CD33+ HLA DR+ CD14- \|\| id:ebi-a-GCST90002042 | Weighted mode | 18 | -0.02621098 | 0.0549846598103506 | 0.639648303998281 | -0.133980913 | 0.0815589534625567 | 0.974129546303302 | 0.874606758068443 | 1.0849771788601 | 0.959313411571287 |
| ebi-a-GCST90002043 | finngen_R11_O15_PREECLAMPS | Pre-eclampsia \|\| id:finngen_R11_O15_PREECLAMPS | CD45 on CD33+ HLA DR+ CD14dim \|\| id:ebi-a-GCST90002043 | Inverse variance weighted (multiplicative random effects) | 15 | -0.03704276 | 0.0242919925664059 | 0.127285062971713 | -0.084655065 | 0.0105695453960786 | 0.963634929405657 | 0.918829165658377 | 1.01062560035877 | 0.820779257935285 |
| ebi-a-GCST90002043 | finngen_R11_O15_PREECLAMPS | Pre-eclampsia \|\| id:finngen_R11_O15_PREECLAMPS | CD45 on CD33+ HLA DR+ CD14dim \|\| id:ebi-a-GCST90002043 | MR Egger | 15 | -0.048693228 | 0.0381822585364806 | 0.224528155060385 | -0.123530454 | 0.0261439990637792 | 0.952473277333527 | 0.883794725396828 | 1.02648875125062 | 0.99380795269851 |
| ebi-a-GCST90002043 | finngen_R11_O15_PREECLAMPS | Pre-eclampsia \|\| id:finngen_R11_O15_PREECLAMPS | CD45 on CD33+ HLA DR+ CD14dim \|\| id:ebi-a-GCST90002043 | Weighted median | 15 | -0.014087303 | 0.0295902830835448 | 0.634018313226379 | -0.072084258 | 0.0439096514317706 | 0.986011458340557 | 0.930452494204064 | 1.04488794649375 | 0.940845681141708 |
| ebi-a-GCST90002043 | finngen_R11_O15_PREECLAMPS | Pre-eclampsia \|\| id:finngen_R11_O15_PREECLAMPS | CD45 on CD33+ HLA DR+ CD14dim \|\| id:ebi-a-GCST90002043 | Weighted mode | 15 | -0.009786663 | 0.029346700818323 | 0.743710852892981 | -0.067306197 | 0.0477328706063168 | 0.990261070544442 | 0.934908891580153 | 1.04889042843353 | 0.959313411571287 |
| ebi-a-GCST90002044 | finngen_R11_O15_PREECLAMPS | Pre-eclampsia \|\| id:finngen_R11_O15_PREECLAMPS | CD45 on CD33dim HLA DR+ CD11b- \|\| id:ebi-a-GCST90002044 | Inverse variance weighted | 10 | -0.050878323 | 0.0320645639325547 | 0.112570149795395 | -0.113724869 | 0.0119682219831782 | 0.950394304316558 | 0.892503477807873 | 1.01204012772687 | 0.820779257935285 |
| ebi-a-GCST90002044 | finngen_R11_O15_PREECLAMPS | Pre-eclampsia \|\| id:finngen_R11_O15_PREECLAMPS | CD45 on CD33dim HLA DR+ CD11b- \|\| id:ebi-a-GCST90002044 | MR Egger | 10 | 0.0061935404946031 | 0.105936668064817 | 0.954812583687979 | -0.201442329 | 0.213829409901645 | 1.00621276012523 | 0.817550725240018 | 1.23841137605449 | 0.99380795269851 |
| ebi-a-GCST90002044 | finngen_R11_O15_PREECLAMPS | Pre-eclampsia \|\| id:finngen_R11_O15_PREECLAMPS | CD45 on CD33dim HLA DR+ CD11b- \|\| id:ebi-a-GCST90002044 | Weighted median | 10 | -0.017838414 | 0.0438716396960514 | 0.684298366710913 | -0.103826827 | 0.0681500002210236 | 0.98231974906309 | 0.901381378474504 | 1.07052587555387 | 0.943838749369606 |
| ebi-a-GCST90002044 | finngen_R11_O15_PREECLAMPS | Pre-eclampsia \|\| id:finngen_R11_O15_PREECLAMPS | CD45 on CD33dim HLA DR+ CD11b- \|\| id:ebi-a-GCST90002044 | Weighted mode | 10 | -0.001461021 | 0.0710605194764909 | 0.984045070135581 | -0.140739639 | 0.137817597101533 | 0.998540045699309 | 0.868715461668443 | 1.14776617530232 | 0.994159530659367 |
| ebi-a-GCST90002045 | finngen_R11_O15_PREECLAMPS | Pre-eclampsia \|\| id:finngen_R11_O15_PREECLAMPS | CD45 on CD33- HLA DR- \|\| id:ebi-a-GCST90002045 | Inverse variance weighted | 14 | 0.042229224952484 | 0.0241622809880226 | 0.0805102063901709 | -0.005128846 | 0.0895872956890084 | 1.04313356358916 | 0.994884284288535 | 1.09372280643107 | 0.789997151112252 |
| ebi-a-GCST90002045 | finngen_R11_O15_PREECLAMPS | Pre-eclampsia \|\| id:finngen_R11_O15_PREECLAMPS | CD45 on CD33- HLA DR- \|\| id:ebi-a-GCST90002045 | MR Egger | 14 | -0.005380264 | 0.0494072193997177 | 0.915084486346011 | -0.102218414 | 0.0914578857892358 | 0.994634183464978 | 0.902832338677555 | 1.09577062820561 | 0.99380795269851 |
| ebi-a-GCST90002045 | finngen_R11_O15_PREECLAMPS | Pre-eclampsia \|\| id:finngen_R11_O15_PREECLAMPS | CD45 on CD33- HLA DR- \|\| id:ebi-a-GCST90002045 | Weighted median | 14 | 0.0181984244755366 | 0.031623711394528 | 0.564975029090326 | -0.04378405 | 0.0801808988088115 | 1.01836502488942 | 0.957160634131425 | 1.08348305074112 | 0.940845681141708 |
| ebi-a-GCST90002045 | finngen_R11_O15_PREECLAMPS | Pre-eclampsia \|\| id:finngen_R11_O15_PREECLAMPS | CD45 on CD33- HLA DR- \|\| id:ebi-a-GCST90002045 | Weighted mode | 14 | 0.00953266614114934 | 0.038346595905511 | 0.807560196298093 | -0.065626662 | 0.0846919941159509 | 1.00957824672272 | 0.936480422919684 | 1.08838178707249 | 0.961305639715923 |
| ebi-a-GCST90002046 | finngen_R11_O15_PREECLAMPS | Pre-eclampsia \|\| id:finngen_R11_O15_PREECLAMPS | CD45 on CD33- HLA DR+ \|\| id:ebi-a-GCST90002046 | Inverse variance weighted (multiplicative random effects) | 25 | -0.002729688 | 0.00927757392098929 | 0.768586477460796 | -0.020913733 | 0.0154543567574364 | 0.997274034083339 | 0.979303442483076 | 1.01557439289206 | 0.977824840984872 |
| ebi-a-GCST90002046 | finngen_R11_O15_PREECLAMPS | Pre-eclampsia \|\| id:finngen_R11_O15_PREECLAMPS | CD45 on CD33- HLA DR+ \|\| id:ebi-a-GCST90002046 | MR Egger | 25 | 0.00253105334515663 | 0.0112935312276387 | 0.824646468705138 | -0.019604268 | 0.0246663745513285 | 1.0025342591648 | 0.980586646185979 | 1.0249731063628 | 0.99380795269851 |
| ebi-a-GCST90002046 | finngen_R11_O15_PREECLAMPS | Pre-eclampsia \|\| id:finngen_R11_O15_PREECLAMPS | CD45 on CD33- HLA DR+ \|\| id:ebi-a-GCST90002046 | Weighted median | 25 | 0.00656918705130773 | 0.01094343746242 | 0.548315967004901 | -0.01487995 | 0.0280183244776509 | 1.00659081148628 | 0.985230209020918 | 1.02841452940782 | 0.940845681141708 |
| ebi-a-GCST90002046 | finngen_R11_O15_PREECLAMPS | Pre-eclampsia \|\| id:finngen_R11_O15_PREECLAMPS | CD45 on CD33- HLA DR+ \|\| id:ebi-a-GCST90002046 | Weighted mode | 25 | 0.00443601221906848 | 0.00924272593782638 | 0.635610827469749 | -0.013679731 | 0.0225517550572082 | 1.00444586588621 | 0.986413411691531 | 1.02280796827954 | 0.959313411571287 |
| ebi-a-GCST90002047 | finngen_R11_O15_PREECLAMPS | Pre-eclampsia \|\| id:finngen_R11_O15_PREECLAMPS | CD45 on Granulocytic Myeloid-Derived Suppressor Cells \|\| id:ebi-a-GCST90002047 | Inverse variance weighted | 18 | 0.00655738541076704 | 0.0190922092829128 | 0.731253404642412 | -0.030863345 | 0.0439781156052761 | 1.00657893213345 | 0.969608066022841 | 1.04495948633234 | 0.960612995947903 |
| ebi-a-GCST90002047 | finngen_R11_O15_PREECLAMPS | Pre-eclampsia \|\| id:finngen_R11_O15_PREECLAMPS | CD45 on Granulocytic Myeloid-Derived Suppressor Cells \|\| id:ebi-a-GCST90002047 | MR Egger | 18 | 0.0159296019161517 | 0.0403514091635828 | 0.698223959882172 | -0.06315916 | 0.095018363876774 | 1.01605715441155 | 0.938794043296095 | 1.09967904931123 | 0.99380795269851 |
| ebi-a-GCST90002047 | finngen_R11_O15_PREECLAMPS | Pre-eclampsia \|\| id:finngen_R11_O15_PREECLAMPS | CD45 on Granulocytic Myeloid-Derived Suppressor Cells \|\| id:ebi-a-GCST90002047 | Weighted median | 18 | 0.00423041241536974 | 0.0273294015546189 | 0.876984153316342 | -0.049335215 | 0.0577960394624229 | 1.00423937324151 | 0.951861998143589 | 1.0594988777106 | 0.964912412213677 |
| ebi-a-GCST90002047 | finngen_R11_O15_PREECLAMPS | Pre-eclampsia \|\| id:finngen_R11_O15_PREECLAMPS | CD45 on Granulocytic Myeloid-Derived Suppressor Cells \|\| id:ebi-a-GCST90002047 | Weighted mode | 18 | 0.011117312885261 | 0.0276602937040599 | 0.692748601846668 | -0.043096863 | 0.0653314885452184 | 1.01117933985278 | 0.957818608605483 | 1.06751283401538 | 0.959313411571287 |
| ebi-a-GCST90002048 | finngen_R11_O15_PREECLAMPS | Pre-eclampsia \|\| id:finngen_R11_O15_PREECLAMPS | CD45 on CD66b++ myelod cell \|\| id:ebi-a-GCST90002048 | Inverse variance weighted | 16 | -0.009171016 | 0.0175891284754439 | 0.602086409543496 | -0.043645707 | 0.025303676233289 | 0.990870909920431 | 0.957293059254939 | 1.02562653163985 | 0.92242199665996 |
| ebi-a-GCST90002048 | finngen_R11_O15_PREECLAMPS | Pre-eclampsia \|\| id:finngen_R11_O15_PREECLAMPS | CD45 on CD66b++ myelod cell \|\| id:ebi-a-GCST90002048 | MR Egger | 16 | -0.015847674 | 0.0269562306401228 | 0.565959947718663 | -0.068681886 | 0.0369865378442065 | 0.984277239444351 | 0.933623631343941 | 1.03767905134707 | 0.99380795269851 |
| ebi-a-GCST90002048 | finngen_R11_O15_PREECLAMPS | Pre-eclampsia \|\| id:finngen_R11_O15_PREECLAMPS | CD45 on CD66b++ myelod cell \|\| id:ebi-a-GCST90002048 | Weighted median | 16 | -0.019610684 | 0.0258747226440751 | 0.448505495289737 | -0.07032514 | 0.0311037726556504 | 0.98058035489391 | 0.932090710556771 | 1.03159254943063 | 0.940845681141708 |
| ebi-a-GCST90002048 | finngen_R11_O15_PREECLAMPS | Pre-eclampsia \|\| id:finngen_R11_O15_PREECLAMPS | CD45 on CD66b++ myelod cell \|\| id:ebi-a-GCST90002048 | Weighted mode | 16 | -0.025171597 | 0.0242912015270495 | 0.316506205072276 | -0.072782352 | 0.022439158384829 | 0.975142566513868 | 0.929803178177543 | 1.02269280998917 | 0.888599269773114 |
| ebi-a-GCST90002049 | finngen_R11_O15_PREECLAMPS | Pre-eclampsia \|\| id:finngen_R11_O15_PREECLAMPS | CD45 on Monocytic Myeloid-Derived Suppressor Cells \|\| id:ebi-a-GCST90002049 | Inverse variance weighted | 17 | 0.0065028959837771 | 0.0219033484180798 | 0.766550112035664 | -0.036427667 | 0.0494334588832135 | 1.00652408571851 | 0.964227836947382 | 1.05067567675581 | 0.977824840984872 |
| ebi-a-GCST90002049 | finngen_R11_O15_PREECLAMPS | Pre-eclampsia \|\| id:finngen_R11_O15_PREECLAMPS | CD45 on Monocytic Myeloid-Derived Suppressor Cells \|\| id:ebi-a-GCST90002049 | MR Egger | 17 | 0.00758886115829545 | 0.0397495098802061 | 0.851150587538199 | -0.070320178 | 0.0854979005234994 | 1.00761772954499 | 0.932095335511207 | 1.08925927446742 | 0.99380795269851 |
| ebi-a-GCST90002049 | finngen_R11_O15_PREECLAMPS | Pre-eclampsia \|\| id:finngen_R11_O15_PREECLAMPS | CD45 on Monocytic Myeloid-Derived Suppressor Cells \|\| id:ebi-a-GCST90002049 | Weighted median | 17 | 0.00838659717381225 | 0.0280273996131178 | 0.764765693400893 | -0.046547106 | 0.0633203004155231 | 1.00842186319826 | 0.95451959584603 | 1.06536802240809 | 0.957984984509652 |
| ebi-a-GCST90002049 | finngen_R11_O15_PREECLAMPS | Pre-eclampsia \|\| id:finngen_R11_O15_PREECLAMPS | CD45 on Monocytic Myeloid-Derived Suppressor Cells \|\| id:ebi-a-GCST90002049 | Weighted mode | 17 | 0.0078146751802497 | 0.0271421531669995 | 0.77710667165636 | -0.045383945 | 0.0610132953875687 | 1.00784528944923 | 0.955630501808645 | 1.06291304593415 | 0.961305639715923 |
| ebi-a-GCST90002050 | finngen_R11_O15_PREECLAMPS | Pre-eclampsia \|\| id:finngen_R11_O15_PREECLAMPS | CD45 on CD33dim HLA DR- \|\| id:ebi-a-GCST90002050 | Inverse variance weighted | 18 | 0.00782844646398645 | 0.0222404299290015 | 0.724844496391181 | -0.035762796 | 0.0514196891248294 | 1.00785916886824 | 0.964869136969602 | 1.05276463444782 | 0.959893708083249 |
| ebi-a-GCST90002050 | finngen_R11_O15_PREECLAMPS | Pre-eclampsia \|\| id:finngen_R11_O15_PREECLAMPS | CD45 on CD33dim HLA DR- \|\| id:ebi-a-GCST90002050 | MR Egger | 18 | 0.0868461284640287 | 0.0380412129229607 | 0.0364457172797586 | 0.0122853511350256 | 0.161406905793032 | 1.0907288346842 | 1.01236112605048 | 1.17516305219352 | 0.839403552053506 |
| ebi-a-GCST90002050 | finngen_R11_O15_PREECLAMPS | Pre-eclampsia \|\| id:finngen_R11_O15_PREECLAMPS | CD45 on CD33dim HLA DR- \|\| id:ebi-a-GCST90002050 | Weighted median | 18 | 0.0192715317519623 | 0.025895521026369 | 0.456753502444918 | -0.031483689 | 0.0700267529636455 | 1.0194584263713 | 0.969006761348658 | 1.07253687441041 | 0.940845681141708 |
| ebi-a-GCST90002050 | finngen_R11_O15_PREECLAMPS | Pre-eclampsia \|\| id:finngen_R11_O15_PREECLAMPS | CD45 on CD33dim HLA DR- \|\| id:ebi-a-GCST90002050 | Weighted mode | 18 | 0.022905609381884 | 0.0317644115735596 | 0.48064263485438 | -0.039352637 | 0.0851638560660609 | 1.02316995734435 | 0.961411619762112 | 1.08889547421018 | 0.933945646747087 |
| ebi-a-GCST90002051 | finngen_R11_O15_PREECLAMPS | Pre-eclampsia \|\| id:finngen_R11_O15_PREECLAMPS | CD45 on basophil \|\| id:ebi-a-GCST90002051 | Inverse variance weighted | 17 | -0.025288143 | 0.0203998878515605 | 0.215115371395405 | -0.065271923 | 0.0146956376817168 | 0.975028924269048 | 0.936812688107212 | 1.0148041494635 | 0.855687495048373 |
| ebi-a-GCST90002051 | finngen_R11_O15_PREECLAMPS | Pre-eclampsia \|\| id:finngen_R11_O15_PREECLAMPS | CD45 on basophil \|\| id:ebi-a-GCST90002051 | MR Egger | 17 | 0.0182111031880798 | 0.0498365806600662 | 0.71990213719626 | -0.079468595 | 0.115890801281809 | 1.01837793652868 | 0.923607025478921 | 1.12287324911874 | 0.99380795269851 |
| ebi-a-GCST90002051 | finngen_R11_O15_PREECLAMPS | Pre-eclampsia \|\| id:finngen_R11_O15_PREECLAMPS | CD45 on basophil \|\| id:ebi-a-GCST90002051 | Weighted median | 17 | -0.037078168 | 0.0283498268946932 | 0.190914055758549 | -0.092643829 | 0.0184874925942918 | 0.963600809542009 | 0.911518098951499 | 1.01865944430295 | 0.940845681141708 |
| ebi-a-GCST90002051 | finngen_R11_O15_PREECLAMPS | Pre-eclampsia \|\| id:finngen_R11_O15_PREECLAMPS | CD45 on basophil \|\| id:ebi-a-GCST90002051 | Weighted mode | 17 | -0.043762586 | 0.0365555787133291 | 0.248683971652681 | -0.11541152 | 0.0278863483014684 | 0.957181178733934 | 0.890999404149782 | 1.02827881214662 | 0.872755742358858 |
| ebi-a-GCST90002052 | finngen_R11_O15_PREECLAMPS | Pre-eclampsia \|\| id:finngen_R11_O15_PREECLAMPS | CD45 on Immature Myeloid-Derived Suppressor Cells \|\| id:ebi-a-GCST90002052 | Inverse variance weighted | 10 | 0.00808018336322638 | 0.0144472543729804 | 0.575964836094167 | -0.020236435 | 0.036396801934268 | 1.00811291614772 | 0.979966947224914 | 1.03706727515843 | 0.915399120505076 |
| ebi-a-GCST90002052 | finngen_R11_O15_PREECLAMPS | Pre-eclampsia \|\| id:finngen_R11_O15_PREECLAMPS | CD45 on Immature Myeloid-Derived Suppressor Cells \|\| id:ebi-a-GCST90002052 | MR Egger | 10 | 0.00152512594866363 | 0.0237106485367014 | 0.950291736124767 | -0.044947745 | 0.0479979970805984 | 1.00152628954471 | 0.956047438611536 | 1.04916855392231 | 0.99380795269851 |
| ebi-a-GCST90002052 | finngen_R11_O15_PREECLAMPS | Pre-eclampsia \|\| id:finngen_R11_O15_PREECLAMPS | CD45 on Immature Myeloid-Derived Suppressor Cells \|\| id:ebi-a-GCST90002052 | Weighted median | 10 | 0.0096139641984219 | 0.0181508180368851 | 0.596339971767285 | -0.025961639 | 0.0451895675507167 | 1.00966032680928 | 0.974372466644101 | 1.04622617164424 | 0.940845681141708 |
| ebi-a-GCST90002052 | finngen_R11_O15_PREECLAMPS | Pre-eclampsia \|\| id:finngen_R11_O15_PREECLAMPS | CD45 on Immature Myeloid-Derived Suppressor Cells \|\| id:ebi-a-GCST90002052 | Weighted mode | 10 | 0.00473536539128883 | 0.017642350145819 | 0.794435430597349 | -0.029843641 | 0.039314371677094 | 1.00474659495234 | 0.970597283407481 | 1.04009740942631 | 0.961305639715923 |
| ebi-a-GCST90002053 | finngen_R11_O15_PREECLAMPS | Pre-eclampsia \|\| id:finngen_R11_O15_PREECLAMPS | CD45 on CD33+ HLA DR+ \|\| id:ebi-a-GCST90002053 | Inverse variance weighted | 16 | -0.002007454 | 0.0132356780730376 | 0.879447292418734 | -0.027949383 | 0.0239344749464161 | 0.997994559511577 | 0.972437587331761 | 1.02422320341152 | 0.984283721484735 |
| ebi-a-GCST90002053 | finngen_R11_O15_PREECLAMPS | Pre-eclampsia \|\| id:finngen_R11_O15_PREECLAMPS | CD45 on CD33+ HLA DR+ \|\| id:ebi-a-GCST90002053 | MR Egger | 16 | 0.0069713114869059 | 0.0169305107387927 | 0.686749907938817 | -0.02621249 | 0.0401551125349396 | 1.00699566764405 | 0.974128075568107 | 1.04097222951151 | 0.99380795269851 |
| ebi-a-GCST90002053 | finngen_R11_O15_PREECLAMPS | Pre-eclampsia \|\| id:finngen_R11_O15_PREECLAMPS | CD45 on CD33+ HLA DR+ \|\| id:ebi-a-GCST90002053 | Weighted median | 16 | 0.00549736609134639 | 0.0183246103885082 | 0.764177864309147 | -0.03041887 | 0.0414136024528225 | 1.00551250433576 | 0.970039127887291 | 1.04228310725735 | 0.957984984509652 |
| ebi-a-GCST90002053 | finngen_R11_O15_PREECLAMPS | Pre-eclampsia \|\| id:finngen_R11_O15_PREECLAMPS | CD45 on CD33+ HLA DR+ \|\| id:ebi-a-GCST90002053 | Weighted mode | 16 | 0.00536408283329248 | 0.0151390793406304 | 0.728030592354937 | -0.024308513 | 0.0350366783409281 | 1.00537849528395 | 0.975984559699362 | 1.03565769430899 | 0.959313411571287 |
| ebi-a-GCST90002054 | finngen_R11_O15_PREECLAMPS | Pre-eclampsia \|\| id:finngen_R11_O15_PREECLAMPS | CD8 on Central Memory CD8+ T cell \|\| id:ebi-a-GCST90002054 | Inverse variance weighted | 14 | 0.0541622415770351 | 0.0300115433093544 | 0.0711195042206668 | -0.004660383 | 0.11298486646337 | 1.05565585953363 | 0.995350459426687 | 1.11961498908593 | 0.789997151112252 |
| ebi-a-GCST90002054 | finngen_R11_O15_PREECLAMPS | Pre-eclampsia \|\| id:finngen_R11_O15_PREECLAMPS | CD8 on Central Memory CD8+ T cell \|\| id:ebi-a-GCST90002054 | MR Egger | 14 | -0.008838196 | 0.0775866237245938 | 0.911189739084828 | -0.160907978 | 0.14323158699203 | 0.991200746531512 | 0.851370412303488 | 1.15399702142151 | 0.99380795269851 |
| ebi-a-GCST90002054 | finngen_R11_O15_PREECLAMPS | Pre-eclampsia \|\| id:finngen_R11_O15_PREECLAMPS | CD8 on Central Memory CD8+ T cell \|\| id:ebi-a-GCST90002054 | Weighted median | 14 | 0.0356009847904897 | 0.040698729444662 | 0.381713007517233 | -0.044168525 | 0.115370494502027 | 1.03624228755479 | 0.956792700471211 | 1.12228916251968 | 0.940845681141708 |
| ebi-a-GCST90002054 | finngen_R11_O15_PREECLAMPS | Pre-eclampsia \|\| id:finngen_R11_O15_PREECLAMPS | CD8 on Central Memory CD8+ T cell \|\| id:ebi-a-GCST90002054 | Weighted mode | 14 | 0.0335842879260323 | 0.0472562018640258 | 0.489833881696942 | -0.059037868 | 0.126206443579523 | 1.03415460679983 | 0.942671071669988 | 1.13451635772666 | 0.933945646747087 |
| ebi-a-GCST90002055 | finngen_R11_O15_PREECLAMPS | Pre-eclampsia \|\| id:finngen_R11_O15_PREECLAMPS | CD8 on naive CD8+ T cell \|\| id:ebi-a-GCST90002055 | Inverse variance weighted | 16 | -0.034904211 | 0.0311709284510075 | 0.262812601964387 | -0.095999231 | 0.0261908088448507 | 0.965697915 | 0.908464714965697 | 1.02653680208895 | 0.885079385681701 |
| ebi-a-GCST90002055 | finngen_R11_O15_PREECLAMPS | Pre-eclampsia \|\| id:finngen_R11_O15_PREECLAMPS | CD8 on naive CD8+ T cell \|\| id:ebi-a-GCST90002055 | MR Egger | 16 | -0.022886221 | 0.0584092943818056 | 0.701087055586317 | -0.137368438 | 0.0915959962817618 | 0.977373682334096 | 0.871649018608071 | 1.09592197607789 | 0.99380795269851 |
| ebi-a-GCST90002055 | finngen_R11_O15_PREECLAMPS | Pre-eclampsia \|\| id:finngen_R11_O15_PREECLAMPS | CD8 on naive CD8+ T cell \|\| id:ebi-a-GCST90002055 | Weighted median | 16 | -0.026742187 | 0.03838854120971 | 0.486041258491902 | -0.101983728 | 0.0484993533618101 | 0.97361221865892 | 0.903044245722517 | 1.04969469304743 | 0.940845681141708 |
| ebi-a-GCST90002055 | finngen_R11_O15_PREECLAMPS | Pre-eclampsia \|\| id:finngen_R11_O15_PREECLAMPS | CD8 on naive CD8+ T cell \|\| id:ebi-a-GCST90002055 | Weighted mode | 16 | -0.036841719 | 0.0396153540427927 | 0.367104312405492 | -0.114487812 | 0.0408043754123539 | 0.96382867951431 | 0.891822807499975 | 1.04164831359095 | 0.915881407400732 |
| ebi-a-GCST90002056 | finngen_R11_O15_PREECLAMPS | Pre-eclampsia \|\| id:finngen_R11_O15_PREECLAMPS | CD8 on Effector Memory CD8+ T cell \|\| id:ebi-a-GCST90002056 | Inverse variance weighted | 22 | -0.044374257 | 0.0205658357355957 | 0.0309536124688328 | -0.084683295 | -0.004065218 | 0.95659587821007 | 0.91880322829099 | 0.995943033320172 | 0.754236357157226 |
| ebi-a-GCST90002056 | finngen_R11_O15_PREECLAMPS | Pre-eclampsia \|\| id:finngen_R11_O15_PREECLAMPS | CD8 on Effector Memory CD8+ T cell \|\| id:ebi-a-GCST90002056 | MR Egger | 22 | -0.046246634 | 0.0361713729360703 | 0.2156964730975 | -0.117142525 | 0.0246492571469312 | 0.954806445599373 | 0.889458414279843 | 1.0249555616338 | 0.99380795269851 |
| ebi-a-GCST90002056 | finngen_R11_O15_PREECLAMPS | Pre-eclampsia \|\| id:finngen_R11_O15_PREECLAMPS | CD8 on Effector Memory CD8+ T cell \|\| id:ebi-a-GCST90002056 | Weighted median | 22 | -0.022713787 | 0.0303957951114677 | 0.454902163359808 | -0.082289545 | 0.0368619714705274 | 0.977542229082882 | 0.92100524728388 | 1.03754979948099 | 0.940845681141708 |
| ebi-a-GCST90002056 | finngen_R11_O15_PREECLAMPS | Pre-eclampsia \|\| id:finngen_R11_O15_PREECLAMPS | CD8 on Effector Memory CD8+ T cell \|\| id:ebi-a-GCST90002056 | Weighted mode | 22 | -0.057556951 | 0.0345805237781216 | 0.110877397438985 | -0.125334778 | 0.0102208756608658 | 0.944068123284333 | 0.882201511882068 | 1.01027328722233 | 0.872755742358858 |
| ebi-a-GCST90002057 | finngen_R11_O15_PREECLAMPS | Pre-eclampsia \|\| id:finngen_R11_O15_PREECLAMPS | CD8 on Terminally Differentiated CD8+ T cell \|\| id:ebi-a-GCST90002057 | Inverse variance weighted | 17 | -0.026394531 | 0.0287108115168236 | 0.357926259692124 | -0.082667721 | 0.0298786597085 | 0.973950760163458 | 0.920657010989538 | 1.03032950588561 | 0.885079385681701 |
| ebi-a-GCST90002057 | finngen_R11_O15_PREECLAMPS | Pre-eclampsia \|\| id:finngen_R11_O15_PREECLAMPS | CD8 on Terminally Differentiated CD8+ T cell \|\| id:ebi-a-GCST90002057 | MR Egger | 17 | -0.051654634 | 0.0499736491156522 | 0.317683909816087 | -0.149602987 | 0.0462937178528288 | 0.949656788986689 | 0.861049756797065 | 1.04738200057475 | 0.99380795269851 |
| ebi-a-GCST90002057 | finngen_R11_O15_PREECLAMPS | Pre-eclampsia \|\| id:finngen_R11_O15_PREECLAMPS | CD8 on Terminally Differentiated CD8+ T cell \|\| id:ebi-a-GCST90002057 | Weighted median | 17 | -0.01542201 | 0.0367431276100483 | 0.674686339386225 | -0.08743854 | 0.0565945198546463 | 0.984696299964534 | 0.916275183847996 | 1.05822663350086 | 0.941872178433165 |
| ebi-a-GCST90002057 | finngen_R11_O15_PREECLAMPS | Pre-eclampsia \|\| id:finngen_R11_O15_PREECLAMPS | CD8 on Terminally Differentiated CD8+ T cell \|\| id:ebi-a-GCST90002057 | Weighted mode | 17 | -0.036376143 | 0.0343531037308808 | 0.305374976401283 | -0.103708226 | 0.030955940789965 | 0.964277519481409 | 0.901488290045118 | 1.03144005845121 | 0.875408265683677 |
| ebi-a-GCST90002058 | finngen_R11_O15_PREECLAMPS | Pre-eclampsia \|\| id:finngen_R11_O15_PREECLAMPS | CD8 on CD8+ T cell \|\| id:ebi-a-GCST90002058 | Inverse variance weighted | 26 | -0.040956187 | 0.0183504278407269 | 0.0256220511025391 | -0.076923026 | -0.004989349 | 0.959871183713234 | 0.925961126051081 | 0.99502307753708 | 0.708667785226302 |
| ebi-a-GCST90002058 | finngen_R11_O15_PREECLAMPS | Pre-eclampsia \|\| id:finngen_R11_O15_PREECLAMPS | CD8 on CD8+ T cell \|\| id:ebi-a-GCST90002058 | MR Egger | 26 | -0.017213906 | 0.0263703692737672 | 0.520106086897683 | -0.06889983 | 0.0344720176417953 | 0.982933406660064 | 0.933420176177142 | 1.03507306418562 | 0.99380795269851 |
| ebi-a-GCST90002058 | finngen_R11_O15_PREECLAMPS | Pre-eclampsia \|\| id:finngen_R11_O15_PREECLAMPS | CD8 on CD8+ T cell \|\| id:ebi-a-GCST90002058 | Weighted median | 26 | -0.043473926 | 0.0266510766490707 | 0.102842665005307 | -0.095710036 | 0.00876218409568823 | 0.957457518382158 | 0.908727475788762 | 1.00880068439751 | 0.88227178111339 |
| ebi-a-GCST90002058 | finngen_R11_O15_PREECLAMPS | Pre-eclampsia \|\| id:finngen_R11_O15_PREECLAMPS | CD8 on CD8+ T cell \|\| id:ebi-a-GCST90002058 | Weighted mode | 26 | -0.03510549 | 0.0245793738166244 | 0.165594694720884 | -0.083281063 | 0.0130700822449353 | 0.965503559494092 | 0.920092506807502 | 1.01315586910958 | 0.872755742358858 |
| ebi-a-GCST90002059 | finngen_R11_O15_PREECLAMPS | Pre-eclampsia \|\| id:finngen_R11_O15_PREECLAMPS | CD8 on Natural Killer T \|\| id:ebi-a-GCST90002059 | Inverse variance weighted | 18 | 0.0146438297822592 | 0.0275362034202289 | 0.594862590787315 | -0.039327129 | 0.0686147884859078 | 1.01475157595403 | 0.961436144128701 | 1.07102355906785 | 0.919333094853123 |
| ebi-a-GCST90002059 | finngen_R11_O15_PREECLAMPS | Pre-eclampsia \|\| id:finngen_R11_O15_PREECLAMPS | CD8 on Natural Killer T \|\| id:ebi-a-GCST90002059 | MR Egger | 18 | 0.0263393380397133 | 0.0470420100930073 | 0.583294875410807 | -0.065863002 | 0.118541677822007 | 1.0266892840974 | 0.936259121374041 | 1.1258537962583 | 0.99380795269851 |
| ebi-a-GCST90002059 | finngen_R11_O15_PREECLAMPS | Pre-eclampsia \|\| id:finngen_R11_O15_PREECLAMPS | CD8 on Natural Killer T \|\| id:ebi-a-GCST90002059 | Weighted median | 18 | 0.0133374253010355 | 0.0338059490158606 | 0.693190563696428 | -0.052922235 | 0.0795970853721222 | 1.01342676550545 | 0.948453766339753 | 1.08285068338791 | 0.946704357535493 |
| ebi-a-GCST90002059 | finngen_R11_O15_PREECLAMPS | Pre-eclampsia \|\| id:finngen_R11_O15_PREECLAMPS | CD8 on Natural Killer T \|\| id:ebi-a-GCST90002059 | Weighted mode | 18 | 0.00744905633906501 | 0.0353118132139029 | 0.835433616200442 | -0.061762098 | 0.0766602102383146 | 1.00747686957714 | 0.940106513822651 | 1.07967515149504 | 0.977585971930092 |
| ebi-a-GCST90002060 | finngen_R11_O15_PREECLAMPS | Pre-eclampsia \|\| id:finngen_R11_O15_PREECLAMPS | CD8 on HLA DR+ CD8+ T cell \|\| id:ebi-a-GCST90002060 | Inverse variance weighted | 25 | -0.0254645 | 0.0144479052751409 | 0.0779840717545631 | -0.053782395 | 0.00285339413692787 | 0.974856985577201 | 0.947638295332707 | 1.00285746894073 | 0.789997151112252 |
| ebi-a-GCST90002060 | finngen_R11_O15_PREECLAMPS | Pre-eclampsia \|\| id:finngen_R11_O15_PREECLAMPS | CD8 on HLA DR+ CD8+ T cell \|\| id:ebi-a-GCST90002060 | MR Egger | 25 | -0.034394145 | 0.0189490892124622 | 0.0825747101331699 | -0.07153436 | 0.00274606982292467 | 0.966190610348157 | 0.930964289214803 | 1.00274984372634 | 0.936133620820556 |
| ebi-a-GCST90002060 | finngen_R11_O15_PREECLAMPS | Pre-eclampsia \|\| id:finngen_R11_O15_PREECLAMPS | CD8 on HLA DR+ CD8+ T cell \|\| id:ebi-a-GCST90002060 | Weighted median | 25 | -0.013111444 | 0.0195604445907125 | 0.502664036838785 | -0.051449915 | 0.0252270277327741 | 0.986974136876008 | 0.949851222009654 | 1.02554792191681 | 0.940845681141708 |
| ebi-a-GCST90002060 | finngen_R11_O15_PREECLAMPS | Pre-eclampsia \|\| id:finngen_R11_O15_PREECLAMPS | CD8 on HLA DR+ CD8+ T cell \|\| id:ebi-a-GCST90002060 | Weighted mode | 25 | -0.023415568 | 0.0183857626971088 | 0.215012517154583 | -0.059451663 | 0.0126205268944537 | 0.976856449138699 | 0.942281079645743 | 1.01270050183181 | 0.872755742358858 |
| ebi-a-GCST90002061 | finngen_R11_O15_PREECLAMPS | Pre-eclampsia \|\| id:finngen_R11_O15_PREECLAMPS | CD4 on CD39+ CD4+ T cell \|\| id:ebi-a-GCST90002061 | Inverse variance weighted (multiplicative random effects) | 27 | 0.0148833224281121 | 0.023233047310031 | 0.521776101106096 | -0.03065345 | 0.0604200951557729 | 1.01499463059758 | 0.969811602767602 | 1.06228271264433 | 0.903834904996579 |
| ebi-a-GCST90002061 | finngen_R11_O15_PREECLAMPS | Pre-eclampsia \|\| id:finngen_R11_O15_PREECLAMPS | CD4 on CD39+ CD4+ T cell \|\| id:ebi-a-GCST90002061 | MR Egger | 27 | 0.0375106532068766 | 0.0432515220609451 | 0.394043735819865 | -0.04726233 | 0.122283636446329 | 1.03822305742793 | 0.953837144638149 | 1.13007458666742 | 0.99380795269851 |
| ebi-a-GCST90002061 | finngen_R11_O15_PREECLAMPS | Pre-eclampsia \|\| id:finngen_R11_O15_PREECLAMPS | CD4 on CD39+ CD4+ T cell \|\| id:ebi-a-GCST90002061 | Weighted median | 27 | 0.0351753938160119 | 0.0279264284442048 | 0.207823200760466 | -0.019560406 | 0.0899111935666534 | 1.0358013660226 | 0.980629657548542 | 1.09407711830414 | 0.940845681141708 |
| ebi-a-GCST90002061 | finngen_R11_O15_PREECLAMPS | Pre-eclampsia \|\| id:finngen_R11_O15_PREECLAMPS | CD4 on CD39+ CD4+ T cell \|\| id:ebi-a-GCST90002061 | Weighted mode | 27 | 0.0282308307097893 | 0.0291101897191043 | 0.341085973975528 | -0.028825141 | 0.0852868025592337 | 1.02863309712722 | 0.971586340094198 | 1.08902935832029 | 0.888599269773114 |
| ebi-a-GCST90002062 | finngen_R11_O15_PREECLAMPS | Pre-eclampsia \|\| id:finngen_R11_O15_PREECLAMPS | CD4 on CD28+ CD4+ T cell \|\| id:ebi-a-GCST90002062 | Inverse variance weighted (multiplicative random effects) | 21 | 0.0100546630019912 | 0.0302376170281401 | 0.739495377162394 | -0.049211066 | 0.0693203923771458 | 1.01010538096753 | 0.951980177488738 | 1.07177954414038 | 0.96638925304053 |
| ebi-a-GCST90002062 | finngen_R11_O15_PREECLAMPS | Pre-eclampsia \|\| id:finngen_R11_O15_PREECLAMPS | CD4 on CD28+ CD4+ T cell \|\| id:ebi-a-GCST90002062 | MR Egger | 21 | 0.072427167090946 | 0.0544355333034864 | 0.199098718094165 | -0.034266478 | 0.179120812365779 | 1.07511449952198 | 0.966313968733728 | 1.1961652469922 | 0.99380795269851 |
| ebi-a-GCST90002062 | finngen_R11_O15_PREECLAMPS | Pre-eclampsia \|\| id:finngen_R11_O15_PREECLAMPS | CD4 on CD28+ CD4+ T cell \|\| id:ebi-a-GCST90002062 | Weighted median | 21 | 0.0276981342431551 | 0.0354221065552632 | 0.434246978066603 | -0.041729195 | 0.097125463091471 | 1.02808529383031 | 0.959129482844813 | 1.1019986251024 | 0.940845681141708 |
| ebi-a-GCST90002062 | finngen_R11_O15_PREECLAMPS | Pre-eclampsia \|\| id:finngen_R11_O15_PREECLAMPS | CD4 on CD28+ CD4+ T cell \|\| id:ebi-a-GCST90002062 | Weighted mode | 21 | 0.0528367624640607 | 0.0440036893054471 | 0.243882694790579 | -0.033410469 | 0.139083993502737 | 1.05425753666993 | 0.967141496912042 | 1.14922062301544 | 0.872755742358858 |
| ebi-a-GCST90002063 | finngen_R11_O15_PREECLAMPS | Pre-eclampsia \|\| id:finngen_R11_O15_PREECLAMPS | CD4 on CD4 regulatory T cell \|\| id:ebi-a-GCST90002063 | Inverse variance weighted | 29 | 0.0404053990441739 | 0.016808821420683 | 0.0162247125976797 | 0.00746010905963519 | 0.0733506890287126 | 1.04123280342476 | 1.007488005 | 1.07610784996776 | 0.588591724205005 |
| ebi-a-GCST90002063 | finngen_R11_O15_PREECLAMPS | Pre-eclampsia \|\| id:finngen_R11_O15_PREECLAMPS | CD4 on CD4 regulatory T cell \|\| id:ebi-a-GCST90002063 | MR Egger | 29 | 0.0345381382090942 | 0.0233727510695933 | 0.151056675864689 | -0.011272454 | 0.080348730305497 | 1.03514150606651 | 0.988790842163827 | 1.08366490858347 | 0.99380795269851 |
| ebi-a-GCST90002063 | finngen_R11_O15_PREECLAMPS | Pre-eclampsia \|\| id:finngen_R11_O15_PREECLAMPS | CD4 on CD4 regulatory T cell \|\| id:ebi-a-GCST90002063 | Weighted median | 29 | 0.064468945711584 | 0.0230106821188591 | 0.00508346849618866 | 0.0193680087586202 | 0.109569882664548 | 1.06659245546062 | 1.0195567854133 | 1.11579804315102 | 0.507633389188193 |
| ebi-a-GCST90002063 | finngen_R11_O15_PREECLAMPS | Pre-eclampsia \|\| id:finngen_R11_O15_PREECLAMPS | CD4 on CD4 regulatory T cell \|\| id:ebi-a-GCST90002063 | Weighted mode | 29 | 0.0618431034880605 | 0.0247297248178731 | 0.0185185884346564 | 0.0133728428450293 | 0.110313364131092 | 1.06379542584215 | 1.01346265922813 | 1.11662792677983 | 0.720210568263475 |
| ebi-a-GCST90002064 | finngen_R11_O15_PREECLAMPS | Pre-eclampsia \|\| id:finngen_R11_O15_PREECLAMPS | CD4 on resting CD4 regulatory T cell \|\| id:ebi-a-GCST90002064 | Inverse variance weighted | 13 | 0.0488778771761985 | 0.032209349000788 | 0.129139004762841 | -0.014252447 | 0.112008201217743 | 1.05009210269635 | 0.985848638447956 | 1.11852203385018 | 0.820779257935285 |
| ebi-a-GCST90002064 | finngen_R11_O15_PREECLAMPS | Pre-eclampsia \|\| id:finngen_R11_O15_PREECLAMPS | CD4 on resting CD4 regulatory T cell \|\| id:ebi-a-GCST90002064 | MR Egger | 13 | 0.0451485014357184 | 0.0559269788263681 | 0.436615721165297 | -0.064468377 | 0.1547653799354 | 1.04618320808214 | 0.937565762375401 | 1.16738403725413 | 0.99380795269851 |
| ebi-a-GCST90002064 | finngen_R11_O15_PREECLAMPS | Pre-eclampsia \|\| id:finngen_R11_O15_PREECLAMPS | CD4 on resting CD4 regulatory T cell \|\| id:ebi-a-GCST90002064 | Weighted median | 13 | 0.0402232336268485 | 0.0467031688524813 | 0.3890988410122 | -0.051314977 | 0.131761444577712 | 1.0410431440918 | 0.949979401433678 | 1.14083613415716 | 0.940845681141708 |
| ebi-a-GCST90002064 | finngen_R11_O15_PREECLAMPS | Pre-eclampsia \|\| id:finngen_R11_O15_PREECLAMPS | CD4 on resting CD4 regulatory T cell \|\| id:ebi-a-GCST90002064 | Weighted mode | 13 | 0.0200967993501602 | 0.0625982903558636 | 0.753699538232381 | -0.10259585 | 0.142789448447653 | 1.02030009963336 | 0.902491642011263 | 1.15348690763704 | 0.959313411571287 |
| ebi-a-GCST90002065 | finngen_R11_O15_PREECLAMPS | Pre-eclampsia \|\| id:finngen_R11_O15_PREECLAMPS | CD4 on CD39+ resting CD4 regulatory T cell \|\| id:ebi-a-GCST90002065 | Inverse variance weighted | 14 | -0.007259672 | 0.022114553850055 | 0.742703154760351 | -0.050604198 | 0.0360848531200418 | 0.992766615343802 | 0.950654867202015 | 1.036743814 | 0.96638925304053 |
| ebi-a-GCST90002065 | finngen_R11_O15_PREECLAMPS | Pre-eclampsia \|\| id:finngen_R11_O15_PREECLAMPS | CD4 on CD39+ resting CD4 regulatory T cell \|\| id:ebi-a-GCST90002065 | MR Egger | 14 | -0.035797525 | 0.0299539136419442 | 0.25513801800455 | -0.094507196 | 0.0229121458237258 | 0.964835628883646 | 0.909821187845193 | 1.02317664525713 | 0.99380795269851 |
| ebi-a-GCST90002065 | finngen_R11_O15_PREECLAMPS | Pre-eclampsia \|\| id:finngen_R11_O15_PREECLAMPS | CD4 on CD39+ resting CD4 regulatory T cell \|\| id:ebi-a-GCST90002065 | Weighted median | 14 | -0.037263494 | 0.0292210243260393 | 0.202228249149949 | -0.094536701 | 0.0200097141392817 | 0.963422246363495 | 0.90979434345203 | 1.0202112504528 | 0.940845681141708 |
| ebi-a-GCST90002065 | finngen_R11_O15_PREECLAMPS | Pre-eclampsia \|\| id:finngen_R11_O15_PREECLAMPS | CD4 on CD39+ resting CD4 regulatory T cell \|\| id:ebi-a-GCST90002065 | Weighted mode | 14 | -0.033116764 | 0.0276353701108051 | 0.25217253952952 | -0.087282089 | 0.0210485613506559 | 0.967425592447677 | 0.916418547133146 | 1.02127164476382 | 0.872755742358858 |
| ebi-a-GCST90002066 | finngen_R11_O15_PREECLAMPS | Pre-eclampsia \|\| id:finngen_R11_O15_PREECLAMPS | CD4 on activated CD4 regulatory T cell \|\| id:ebi-a-GCST90002066 | Inverse variance weighted | 24 | 0.0293143985819453 | 0.0143705133717636 | 0.0413603674929865 | 0.00114819237328871 | 0.0574806047906019 | 1.02974829499068 | 1.00114885179851 | 1.05916472773383 | 0.781760498894217 |
| ebi-a-GCST90002066 | finngen_R11_O15_PREECLAMPS | Pre-eclampsia \|\| id:finngen_R11_O15_PREECLAMPS | CD4 on activated CD4 regulatory T cell \|\| id:ebi-a-GCST90002066 | MR Egger | 24 | 0.0439603277981025 | 0.0183277169016946 | 0.0253739104546595 | 0.00803800267078105 | 0.079882652925424 | 1.04494089895981 | 1.00807039414367 | 1.08315995456496 | 0.806449067058962 |
| ebi-a-GCST90002066 | finngen_R11_O15_PREECLAMPS | Pre-eclampsia \|\| id:finngen_R11_O15_PREECLAMPS | CD4 on activated CD4 regulatory T cell \|\| id:ebi-a-GCST90002066 | Weighted median | 24 | 0.0360172589912809 | 0.0192388133297422 | 0.0611907816673637 | -0.001690815 | 0.0737253331175757 | 1.03667373827954 | 0.998310613487604 | 1.07651108294244 | 0.771214851704188 |
| ebi-a-GCST90002066 | finngen_R11_O15_PREECLAMPS | Pre-eclampsia \|\| id:finngen_R11_O15_PREECLAMPS | CD4 on activated CD4 regulatory T cell \|\| id:ebi-a-GCST90002066 | Weighted mode | 24 | 0.0350998961264157 | 0.0166013139981599 | 0.045541688104873 | 0.00256132069002229 | 0.0676384715628092 | 1.03572316836449 | 1.00256460367419 | 1.06997841092303 | 0.838871423749761 |
| ebi-a-GCST90002067 | finngen_R11_O15_PREECLAMPS | Pre-eclampsia \|\| id:finngen_R11_O15_PREECLAMPS | CD4 on CD39+ activated CD4 regulatory T cell \|\| id:ebi-a-GCST90002067 | Inverse variance weighted | 23 | -0.008495764 | 0.0215612796014547 | 0.693559819164805 | -0.050755872 | 0.0337643441397292 | 0.991540223138311 | 0.950510688580571 | 1.03434082952749 | 0.950911139356934 |
| ebi-a-GCST90002067 | finngen_R11_O15_PREECLAMPS | Pre-eclampsia \|\| id:finngen_R11_O15_PREECLAMPS | CD4 on CD39+ activated CD4 regulatory T cell \|\| id:ebi-a-GCST90002067 | MR Egger | 23 | -0.015543208 | 0.0342892595985085 | 0.654984258991089 | -0.082750157 | 0.0516637410625062 | 0.984576964476763 | 0.920581119640751 | 1.05302159505137 | 0.99380795269851 |
| ebi-a-GCST90002067 | finngen_R11_O15_PREECLAMPS | Pre-eclampsia \|\| id:finngen_R11_O15_PREECLAMPS | CD4 on CD39+ activated CD4 regulatory T cell \|\| id:ebi-a-GCST90002067 | Weighted median | 23 | -0.022590138 | 0.0273226727384435 | 0.408355573139624 | -0.076142577 | 0.0309623006302427 | 0.977663108685733 | 0.926684073764118 | 1.0314466182661 | 0.940845681141708 |
| ebi-a-GCST90002067 | finngen_R11_O15_PREECLAMPS | Pre-eclampsia \|\| id:finngen_R11_O15_PREECLAMPS | CD4 on CD39+ activated CD4 regulatory T cell \|\| id:ebi-a-GCST90002067 | Weighted mode | 23 | -0.02788195 | 0.0252170646938471 | 0.280811151081528 | -0.077307397 | 0.0215434966955117 | 0.972503163922163 | 0.925605281672217 | 1.02177723330401 | 0.872755742358858 |
| ebi-a-GCST90002068 | finngen_R11_O15_PREECLAMPS | Pre-eclampsia \|\| id:finngen_R11_O15_PREECLAMPS | CD4 on secreting CD4 regulatory T cell \|\| id:ebi-a-GCST90002068 | Inverse variance weighted | 27 | 0.0105494277477928 | 0.0150643716739749 | 0.483746260799864 | -0.018976741 | 0.0400755962287836 | 1.0106052691529 | 0.981202184020781 | 1.04088945853588 | 0.898694924660397 |
| ebi-a-GCST90002068 | finngen_R11_O15_PREECLAMPS | Pre-eclampsia \|\| id:finngen_R11_O15_PREECLAMPS | CD4 on secreting CD4 regulatory T cell \|\| id:ebi-a-GCST90002068 | MR Egger | 27 | 0.00575132034727689 | 0.0219173623711214 | 0.795156149323042 | -0.03720671 | 0.0487093505946748 | 1.00576789094251 | 0.963476954538398 | 1.0499151491751 | 0.99380795269851 |
| ebi-a-GCST90002068 | finngen_R11_O15_PREECLAMPS | Pre-eclampsia \|\| id:finngen_R11_O15_PREECLAMPS | CD4 on secreting CD4 regulatory T cell \|\| id:ebi-a-GCST90002068 | Weighted median | 27 | -0.004957495 | 0.0239445076981724 | 0.8359778193306 | -0.051888731 | 0.0419737396738045 | 0.995054772684392 | 0.949434504065352 | 1.04286709236115 | 0.964912412213677 |
| ebi-a-GCST90002068 | finngen_R11_O15_PREECLAMPS | Pre-eclampsia \|\| id:finngen_R11_O15_PREECLAMPS | CD4 on secreting CD4 regulatory T cell \|\| id:ebi-a-GCST90002068 | Weighted mode | 27 | -0.030551253 | 0.0320696002222684 | 0.349538320887424 | -0.09340767 | 0.0323051632125638 | 0.969910719742774 | 0.910822110060248 | 1.03283263974534 | 0.893400393596876 |
| ebi-a-GCST90002069 | finngen_R11_O15_PREECLAMPS | Pre-eclampsia \|\| id:finngen_R11_O15_PREECLAMPS | CD4 on CD39+ secreting CD4 regulatory T cell \|\| id:ebi-a-GCST90002069 | Inverse variance weighted (multiplicative random effects) | 23 | -0.010949744 | 0.0125543540419681 | 0.383106374974708 | -0.035556278 | 0.0136567896406497 | 0.989109985959236 | 0.965068420384366 | 1.01375046956218 | 0.885079385681701 |
| ebi-a-GCST90002069 | finngen_R11_O15_PREECLAMPS | Pre-eclampsia \|\| id:finngen_R11_O15_PREECLAMPS | CD4 on CD39+ secreting CD4 regulatory T cell \|\| id:ebi-a-GCST90002069 | MR Egger | 23 | -0.010465275 | 0.0153532360693004 | 0.502919747160255 | -0.040557617 | 0.0196270678962585 | 0.989589295657898 | 0.96025383549652 | 1.01982094512992 | 0.99380795269851 |
| ebi-a-GCST90002069 | finngen_R11_O15_PREECLAMPS | Pre-eclampsia \|\| id:finngen_R11_O15_PREECLAMPS | CD4 on CD39+ secreting CD4 regulatory T cell \|\| id:ebi-a-GCST90002069 | Weighted median | 23 | -0.007806964 | 0.0133267421623326 | 0.558001912047105 | -0.033927379 | 0.0183134502152203 | 0.992223430774292 | 0.966641700516479 | 1.01848216981688 | 0.940845681141708 |
| ebi-a-GCST90002069 | finngen_R11_O15_PREECLAMPS | Pre-eclampsia \|\| id:finngen_R11_O15_PREECLAMPS | CD4 on CD39+ secreting CD4 regulatory T cell \|\| id:ebi-a-GCST90002069 | Weighted mode | 23 | -0.008352932 | 0.0109060985905199 | 0.451878212356085 | -0.029728885 | 0.013023021396624 | 0.991681856964148 | 0.970708671482132 | 1.01310819025682 | 0.933945646747087 |
| ebi-a-GCST90002070 | finngen_R11_O15_PREECLAMPS | Pre-eclampsia \|\| id:finngen_R11_O15_PREECLAMPS | CD4 on activated & secreting CD4 regulatory T cell \|\| id:ebi-a-GCST90002070 | Inverse variance weighted | 32 | 0.0419398658222304 | 0.0117519835127894 | 0.000358691438513025 | 0.0189059781371632 | 0.0649737535072977 | 1.04283176703436 | 1.01908582776493 | 1.06713101557022 | 0.137722935518292 |
| ebi-a-GCST90002070 | finngen_R11_O15_PREECLAMPS | Pre-eclampsia \|\| id:finngen_R11_O15_PREECLAMPS | CD4 on activated & secreting CD4 regulatory T cell \|\| id:ebi-a-GCST90002070 | MR Egger | 32 | 0.0429443823689788 | 0.015660892032006 | 0.0101923016394146 | 0.0122490339862471 | 0.0736397307517105 | 1.04387983511256 | 1.01232436064846 | 1.07641893499095 | 0.592319696793224 |
| ebi-a-GCST90002070 | finngen_R11_O15_PREECLAMPS | Pre-eclampsia \|\| id:finngen_R11_O15_PREECLAMPS | CD4 on activated & secreting CD4 regulatory T cell \|\| id:ebi-a-GCST90002070 | Weighted median | 32 | 0.0397320552361618 | 0.0188978195034876 | 0.0355123255988001 | 0.00269232900932604 | 0.0767717814629976 | 1.04053193175415 | 1.00269595658188 | 1.07979561889418 | 0.721097500353414 |
| ebi-a-GCST90002070 | finngen_R11_O15_PREECLAMPS | Pre-eclampsia \|\| id:finngen_R11_O15_PREECLAMPS | CD4 on activated & secreting CD4 regulatory T cell \|\| id:ebi-a-GCST90002070 | Weighted mode | 32 | 0.042400760006672 | 0.0172539792464086 | 0.0197867851381749 | 0.00858296068371126 | 0.0762185593296328 | 1.04331251290912 | 1.00861989989774 | 1.07919841726611 | 0.723206996800294 |
| ebi-a-GCST90002071 | finngen_R11_O15_PREECLAMPS | Pre-eclampsia \|\| id:finngen_R11_O15_PREECLAMPS | SSC-A on myeloid Dendritic Cell \|\| id:ebi-a-GCST90002071 | Inverse variance weighted (multiplicative random effects) | 18 | 0.00863968918365866 | 0.0112833445659858 | 0.443853126041652 | -0.013475666 | 0.0307550445329908 | 1.00867711901464 | 0.986614724144872 | 1.03123286681688 | 0.898694924660397 |
| ebi-a-GCST90002071 | finngen_R11_O15_PREECLAMPS | Pre-eclampsia \|\| id:finngen_R11_O15_PREECLAMPS | SSC-A on myeloid Dendritic Cell \|\| id:ebi-a-GCST90002071 | MR Egger | 18 | 0.0139834030119313 | 0.0132269874059743 | 0.306127536185869 | -0.011941492 | 0.0399082983276409 | 1.01408162809814 | 0.988129524352744 | 1.04071533447984 | 0.99380795269851 |
| ebi-a-GCST90002071 | finngen_R11_O15_PREECLAMPS | Pre-eclampsia \|\| id:finngen_R11_O15_PREECLAMPS | SSC-A on myeloid Dendritic Cell \|\| id:ebi-a-GCST90002071 | Weighted median | 18 | 0.0138015474853167 | 0.0104310523035529 | 0.18579504234763 | -0.006643315 | 0.0342464100002803 | 1.01389722851718 | 0.993378703003088 | 1.03483957013283 | 0.940845681141708 |
| ebi-a-GCST90002071 | finngen_R11_O15_PREECLAMPS | Pre-eclampsia \|\| id:finngen_R11_O15_PREECLAMPS | SSC-A on myeloid Dendritic Cell \|\| id:ebi-a-GCST90002071 | Weighted mode | 18 | 0.0136902533136167 | 0.00926883223709207 | 0.157955722681038 | -0.004476658 | 0.0318571644983172 | 1.01378439394398 | 0.995533347426098 | 1.03237003568145 | 0.872755742358858 |
| ebi-a-GCST90002072 | finngen_R11_O15_PREECLAMPS | Pre-eclampsia \|\| id:finngen_R11_O15_PREECLAMPS | SSC-A on plasmacytoid Dendritic Cell \|\| id:ebi-a-GCST90002072 | Inverse variance weighted | 26 | -0.013409494 | 0.0193719477216361 | 0.488804236762197 | -0.051378511 | 0.0245595238734044 | 0.986680013072456 | 0.949919047481841 | 1.02486359314242 | 0.898694924660397 |
| ebi-a-GCST90002072 | finngen_R11_O15_PREECLAMPS | Pre-eclampsia \|\| id:finngen_R11_O15_PREECLAMPS | SSC-A on plasmacytoid Dendritic Cell \|\| id:ebi-a-GCST90002072 | MR Egger | 26 | -0.009437855 | 0.0299188020996176 | 0.755147445977647 | -0.068078707 | 0.0492029969339525 | 0.990606541594022 | 0.934186943354787 | 1.05043356389128 | 0.99380795269851 |
| ebi-a-GCST90002072 | finngen_R11_O15_PREECLAMPS | Pre-eclampsia \|\| id:finngen_R11_O15_PREECLAMPS | SSC-A on plasmacytoid Dendritic Cell \|\| id:ebi-a-GCST90002072 | Weighted median | 26 | 0.000740936003892145 | 0.027563137017393 | 0.978554320343245 | -0.053282813 | 0.0547646845579825 | 1.00074121056478 | 0.948111836635946 | 1.05629202360355 | 0.99075236588769 |
| ebi-a-GCST90002072 | finngen_R11_O15_PREECLAMPS | Pre-eclampsia \|\| id:finngen_R11_O15_PREECLAMPS | SSC-A on plasmacytoid Dendritic Cell \|\| id:ebi-a-GCST90002072 | Weighted mode | 26 | -0.016581446 | 0.0235742595892328 | 0.488324111634154 | -0.062786995 | 0.0296241030608192 | 0.983555269747472 | 0.939143495088084 | 1.03006726203996 | 0.933945646747087 |
| ebi-a-GCST90002073 | finngen_R11_O15_PREECLAMPS | Pre-eclampsia \|\| id:finngen_R11_O15_PREECLAMPS | SSC-A on monocyte \|\| id:ebi-a-GCST90002073 | Inverse variance weighted | 22 | -0.013240295 | 0.0189887777866956 | 0.485634160378874 | -0.0504583 | 0.0239777091315205 | 0.986846971807675 | 0.950793576135247 | 1.02426748582433 | 0.898694924660397 |
| ebi-a-GCST90002073 | finngen_R11_O15_PREECLAMPS | Pre-eclampsia \|\| id:finngen_R11_O15_PREECLAMPS | SSC-A on monocyte \|\| id:ebi-a-GCST90002073 | MR Egger | 22 | -0.068953288 | 0.0291543269651043 | 0.028243776366239 | -0.126095769 | -0.011810807 | 0.933370278573414 | 0.881530419510231 | 0.988258666567888 | 0.839403552053506 |
| ebi-a-GCST90002073 | finngen_R11_O15_PREECLAMPS | Pre-eclampsia \|\| id:finngen_R11_O15_PREECLAMPS | SSC-A on monocyte \|\| id:ebi-a-GCST90002073 | Weighted median | 22 | -0.042624831 | 0.0245842832907948 | 0.0829492392740678 | -0.090810026 | 0.00556036424123414 | 0.958270836139268 | 0.913191176766154 | 1.00557585175859 | 0.878781071149907 |
| ebi-a-GCST90002073 | finngen_R11_O15_PREECLAMPS | Pre-eclampsia \|\| id:finngen_R11_O15_PREECLAMPS | SSC-A on monocyte \|\| id:ebi-a-GCST90002073 | Weighted mode | 22 | -0.060239486 | 0.0215824617902318 | 0.0109450395710035 | -0.102541111 | -0.01793786 | 0.94153902161435 | 0.902541044955168 | 0.982222065331712 | 0.720210568263475 |
| ebi-a-GCST90002074 | finngen_R11_O15_PREECLAMPS | Pre-eclampsia \|\| id:finngen_R11_O15_PREECLAMPS | SSC-A on CD14+ monocyte \|\| id:ebi-a-GCST90002074 | Inverse variance weighted (multiplicative random effects) | 22 | -0.019515696 | 0.0289026713048561 | 0.49953525432208 | -0.076164931 | 0.0371335400314466 | 0.980673502685205 | 0.926663357992537 | 1.03783160364976 | 0.898694924660397 |
| ebi-a-GCST90002074 | finngen_R11_O15_PREECLAMPS | Pre-eclampsia \|\| id:finngen_R11_O15_PREECLAMPS | SSC-A on CD14+ monocyte \|\| id:ebi-a-GCST90002074 | MR Egger | 22 | -0.073264302 | 0.0451226254139289 | 0.120103048889681 | -0.161704648 | 0.0151760434644576 | 0.929355166810902 | 0.850692421013182 | 1.01529178436719 | 0.97261503632431 |
| ebi-a-GCST90002074 | finngen_R11_O15_PREECLAMPS | Pre-eclampsia \|\| id:finngen_R11_O15_PREECLAMPS | SSC-A on CD14+ monocyte \|\| id:ebi-a-GCST90002074 | Weighted median | 22 | -0.072953593 | 0.0290111496666395 | 0.0119142387880453 | -0.129815446 | -0.01609174 | 0.929643971056924 | 0.878257501637211 | 0.984037040744213 | 0.598716529366782 |
| ebi-a-GCST90002074 | finngen_R11_O15_PREECLAMPS | Pre-eclampsia \|\| id:finngen_R11_O15_PREECLAMPS | SSC-A on CD14+ monocyte \|\| id:ebi-a-GCST90002074 | Weighted mode | 22 | -0.084253504 | 0.0282823751695957 | 0.0071567141536627 | -0.139686959 | -0.028820049 | 0.919198206047024 | 0.869630422398762 | 0.971591287790336 | 0.720210568263475 |
| ebi-a-GCST90002075 | finngen_R11_O15_PREECLAMPS | Pre-eclampsia \|\| id:finngen_R11_O15_PREECLAMPS | SSC-A on B cell \|\| id:ebi-a-GCST90002075 | Inverse variance weighted | 13 | -0.040992289 | 0.0258249377421924 | 0.112441517290334 | -0.091609166 | 0.00962458947189692 | 0.959836531695986 | 0.912461700485071 | 1.00967105478336 | 0.820779257935285 |
| ebi-a-GCST90002075 | finngen_R11_O15_PREECLAMPS | Pre-eclampsia \|\| id:finngen_R11_O15_PREECLAMPS | SSC-A on B cell \|\| id:ebi-a-GCST90002075 | MR Egger | 13 | -0.026650043 | 0.0450351820129055 | 0.565974344465873 | -0.114918999 | 0.0616189141124263 | 0.973701936072495 | 0.891438348043022 | 1.06355696094147 | 0.99380795269851 |
| ebi-a-GCST90002075 | finngen_R11_O15_PREECLAMPS | Pre-eclampsia \|\| id:finngen_R11_O15_PREECLAMPS | SSC-A on B cell \|\| id:ebi-a-GCST90002075 | Weighted median | 13 | -0.037504322 | 0.0358632527782164 | 0.295672285455432 | -0.107796297 | 0.0327876537835211 | 0.963190255129312 | 0.89781046439332 | 1.03333109199498 | 0.940845681141708 |
| ebi-a-GCST90002075 | finngen_R11_O15_PREECLAMPS | Pre-eclampsia \|\| id:finngen_R11_O15_PREECLAMPS | SSC-A on B cell \|\| id:ebi-a-GCST90002075 | Weighted mode | 13 | -0.031985499 | 0.0361911797320167 | 0.394170395937939 | -0.102920211 | 0.0389492131244308 | 0.968520626349383 | 0.902198955778777 | 1.03971767829691 | 0.920286500917063 |
| ebi-a-GCST90002076 | finngen_R11_O15_PREECLAMPS | Pre-eclampsia \|\| id:finngen_R11_O15_PREECLAMPS | SSC-A on Natural Killer \|\| id:ebi-a-GCST90002076 | Inverse variance weighted | 23 | 0.011932323802602 | 0.0195724468678637 | 0.542094306499008 | -0.026429672 | 0.0502943196636149 | 1.0120037979797 | 0.973916534972271 | 1.05158055166863 | 0.905435115099076 |
| ebi-a-GCST90002076 | finngen_R11_O15_PREECLAMPS | Pre-eclampsia \|\| id:finngen_R11_O15_PREECLAMPS | SSC-A on Natural Killer \|\| id:ebi-a-GCST90002076 | MR Egger | 23 | -0.014354971 | 0.0331531501974268 | 0.66943688445433 | -0.079335145 | 0.0506252033012049 | 0.985747570265918 | 0.923730288537242 | 1.05192856003875 | 0.99380795269851 |
| ebi-a-GCST90002076 | finngen_R11_O15_PREECLAMPS | Pre-eclampsia \|\| id:finngen_R11_O15_PREECLAMPS | SSC-A on Natural Killer \|\| id:ebi-a-GCST90002076 | Weighted median | 23 | -0.003728078 | 0.0288160559227447 | 0.897060914232337 | -0.060207547 | 0.0527513918531 | 0.996278862898617 | 0.941569093120017 | 1.05416753790159 | 0.966404007249152 |
| ebi-a-GCST90002076 | finngen_R11_O15_PREECLAMPS | Pre-eclampsia \|\| id:finngen_R11_O15_PREECLAMPS | SSC-A on Natural Killer \|\| id:ebi-a-GCST90002076 | Weighted mode | 23 | -0.01590671 | 0.0310662137559345 | 0.613734652762606 | -0.076796489 | 0.0449830691820647 | 0.984219133792503 | 0.926078301791128 | 1.04601014995139 | 0.959313411571287 |
| ebi-a-GCST90002077 | finngen_R11_O15_PREECLAMPS | Pre-eclampsia \|\| id:finngen_R11_O15_PREECLAMPS | SSC-A on HLA DR+ Natural Killer \|\| id:ebi-a-GCST90002077 | Inverse variance weighted | 28 | 0.0186718777927187 | 0.0147537417619988 | 0.205667440606789 | -0.010245456 | 0.0475892116462363 | 1.01884728734392 | 0.989806849839138 | 1.04873975674827 | 0.855687495048373 |
| ebi-a-GCST90002077 | finngen_R11_O15_PREECLAMPS | Pre-eclampsia \|\| id:finngen_R11_O15_PREECLAMPS | SSC-A on HLA DR+ Natural Killer \|\| id:ebi-a-GCST90002077 | MR Egger | 28 | 0.0309024782469826 | 0.0197479144636576 | 0.129709946277733 | -0.007803434 | 0.0696083905957515 | 1.03138491651678 | 0.992226933647853 | 1.07208825919238 | 0.97261503632431 |
| ebi-a-GCST90002077 | finngen_R11_O15_PREECLAMPS | Pre-eclampsia \|\| id:finngen_R11_O15_PREECLAMPS | SSC-A on HLA DR+ Natural Killer \|\| id:ebi-a-GCST90002077 | Weighted median | 28 | 0.0156804510060749 | 0.0208028869606932 | 0.450991414341338 | -0.025093207 | 0.0564541094490335 | 1.01580403438069 | 0.975219010127698 | 1.05807805790104 | 0.940845681141708 |
| ebi-a-GCST90002077 | finngen_R11_O15_PREECLAMPS | Pre-eclampsia \|\| id:finngen_R11_O15_PREECLAMPS | SSC-A on HLA DR+ Natural Killer \|\| id:ebi-a-GCST90002077 | Weighted mode | 28 | 0.0236855859937233 | 0.0186588649244022 | 0.215131165506439 | -0.012885789 | 0.0602569612455517 | 1.02396831729159 | 0.987196877069715 | 1.06210943244598 | 0.872755742358858 |
| ebi-a-GCST90002078 | finngen_R11_O15_PREECLAMPS | Pre-eclampsia \|\| id:finngen_R11_O15_PREECLAMPS | SSC-A on granulocyte \|\| id:ebi-a-GCST90002078 | Inverse variance weighted | 25 | -0.006438268 | 0.0163867417792689 | 0.69439707162293 | -0.038556282 | 0.0256797457960076 | 0.993582413149056 | 0.962177549959465 | 1.02601231109652 | 0.950911139356934 |
| ebi-a-GCST90002078 | finngen_R11_O15_PREECLAMPS | Pre-eclampsia \|\| id:finngen_R11_O15_PREECLAMPS | SSC-A on granulocyte \|\| id:ebi-a-GCST90002078 | MR Egger | 25 | -0.01912182 | 0.0251076651496246 | 0.454041942409384 | -0.068332844 | 0.0300892035985193 | 0.981059842160058 | 0.933949562527477 | 1.03054645830599 | 0.99380795269851 |
| ebi-a-GCST90002078 | finngen_R11_O15_PREECLAMPS | Pre-eclampsia \|\| id:finngen_R11_O15_PREECLAMPS | SSC-A on granulocyte \|\| id:ebi-a-GCST90002078 | Weighted median | 25 | -0.022091404 | 0.0232609198952466 | 0.342253593134153 | -0.067682807 | 0.0234999986060786 | 0.978150823686974 | 0.934556861105001 | 1.02377829931956 | 0.940845681141708 |
| ebi-a-GCST90002078 | finngen_R11_O15_PREECLAMPS | Pre-eclampsia \|\| id:finngen_R11_O15_PREECLAMPS | SSC-A on granulocyte \|\| id:ebi-a-GCST90002078 | Weighted mode | 25 | -0.024341913 | 0.0233197501494128 | 0.306963556978459 | -0.070048624 | 0.0213647968352667 | 0.975951961595043 | 0.932348484523742 | 1.02159465816878 | 0.876524844340834 |
| ebi-a-GCST90002079 | finngen_R11_O15_PREECLAMPS | Pre-eclampsia \|\| id:finngen_R11_O15_PREECLAMPS | SSC-A on lymphocyte \|\| id:ebi-a-GCST90002079 | Inverse variance weighted | 21 | -0.012776325 | 0.0281435799367237 | 0.649850824928015 | -0.067937742 | 0.0423850915454588 | 0.987304945629326 | 0.934318640757232 | 1.04329616593554 | 0.942541573457101 |
| ebi-a-GCST90002079 | finngen_R11_O15_PREECLAMPS | Pre-eclampsia \|\| id:finngen_R11_O15_PREECLAMPS | SSC-A on lymphocyte \|\| id:ebi-a-GCST90002079 | MR Egger | 21 | 0.00812537120049466 | 0.0536741564295386 | 0.881269137645191 | -0.097075975 | 0.11332671780239 | 1.00815847161939 | 0.907487056820672 | 1.119997796 | 0.99380795269851 |
| ebi-a-GCST90002079 | finngen_R11_O15_PREECLAMPS | Pre-eclampsia \|\| id:finngen_R11_O15_PREECLAMPS | SSC-A on lymphocyte \|\| id:ebi-a-GCST90002079 | Weighted median | 21 | -0.021374576 | 0.0440477420977531 | 0.627492424195636 | -0.107708151 | 0.0649589981391794 | 0.978852240969195 | 0.89788960648278 | 1.06711526977542 | 0.940845681141708 |
| ebi-a-GCST90002079 | finngen_R11_O15_PREECLAMPS | Pre-eclampsia \|\| id:finngen_R11_O15_PREECLAMPS | SSC-A on lymphocyte \|\| id:ebi-a-GCST90002079 | Weighted mode | 21 | -0.029675931 | 0.0487771908958649 | 0.549771601218556 | -0.125279226 | 0.0659273627959555 | 0.970760075476652 | 0.882250521331316 | 1.06814912698197 | 0.939633861977944 |
| ebi-a-GCST90002080 | finngen_R11_O15_PREECLAMPS | Pre-eclampsia \|\| id:finngen_R11_O15_PREECLAMPS | SSC-A on T cell \|\| id:ebi-a-GCST90002080 | Inverse variance weighted | 22 | -0.003395765 | 0.0215018897432759 | 0.874512998909149 | -0.045539469 | 0.0387479388492263 | 0.996609994041852 | 0.955481889966116 | 1.03950843093355 | 0.981980034105357 |
| ebi-a-GCST90002080 | finngen_R11_O15_PREECLAMPS | Pre-eclampsia \|\| id:finngen_R11_O15_PREECLAMPS | SSC-A on T cell \|\| id:ebi-a-GCST90002080 | MR Egger | 22 | 0.0385706982000177 | 0.0295451779639586 | 0.206547772763377 | -0.019337851 | 0.0964792470093765 | 1.03932420411112 | 0.980847926188488 | 1.10128672591354 | 0.99380795269851 |
| ebi-a-GCST90002080 | finngen_R11_O15_PREECLAMPS | Pre-eclampsia \|\| id:finngen_R11_O15_PREECLAMPS | SSC-A on T cell \|\| id:ebi-a-GCST90002080 | Weighted median | 22 | 0.0169583534038133 | 0.0298390002176163 | 0.569811949295723 | -0.041526087 | 0.0754427938303413 | 1.01710296256681 | 0.959324309100001 | 1.07836153702048 | 0.940845681141708 |
| ebi-a-GCST90002080 | finngen_R11_O15_PREECLAMPS | Pre-eclampsia \|\| id:finngen_R11_O15_PREECLAMPS | SSC-A on T cell \|\| id:ebi-a-GCST90002080 | Weighted mode | 22 | 0.0149603636333859 | 0.0265772362046436 | 0.57946277355255 | -0.037131019 | 0.0670517465944874 | 1.01507283001952 | 0.963549883420566 | 1.06935081200575 | 0.948158355498791 |
| ebi-a-GCST90002081 | finngen_R11_O15_PREECLAMPS | Pre-eclampsia \|\| id:finngen_R11_O15_PREECLAMPS | SSC-A on CD4+ T cell \|\| id:ebi-a-GCST90002081 | Inverse variance weighted (multiplicative random effects) | 29 | -0.057094496 | 0.0277318780694914 | 0.0395130363023216 | -0.111448977 | -0.002740015 | 0.944504812940192 | 0.894537031919939 | 0.997263735132912 | 0.781760498894217 |
| ebi-a-GCST90002081 | finngen_R11_O15_PREECLAMPS | Pre-eclampsia \|\| id:finngen_R11_O15_PREECLAMPS | SSC-A on CD4+ T cell \|\| id:ebi-a-GCST90002081 | MR Egger | 29 | -0.040020386 | 0.0508531889596436 | 0.43814797279413 | -0.139692637 | 0.0596518641036419 | 0.960769852451295 | 0.869625485355389 | 1.06146694746653 | 0.99380795269851 |
| ebi-a-GCST90002081 | finngen_R11_O15_PREECLAMPS | Pre-eclampsia \|\| id:finngen_R11_O15_PREECLAMPS | SSC-A on CD4+ T cell \|\| id:ebi-a-GCST90002081 | Weighted median | 29 | -0.044841808 | 0.0377547590414736 | 0.234946606823708 | -0.118841136 | 0.0291575198001426 | 0.956148725024553 | 0.887948852981649 | 1.02958676200348 | 0.940845681141708 |
| ebi-a-GCST90002081 | finngen_R11_O15_PREECLAMPS | Pre-eclampsia \|\| id:finngen_R11_O15_PREECLAMPS | SSC-A on CD4+ T cell \|\| id:ebi-a-GCST90002081 | Weighted mode | 29 | -0.034706167 | 0.0491925588963168 | 0.486317396784226 | -0.131123582 | 0.0617112487807466 | 0.965889185014296 | 0.877109372681023 | 1.06365516865462 | 0.933945646747087 |
| ebi-a-GCST90002082 | finngen_R11_O15_PREECLAMPS | Pre-eclampsia \|\| id:finngen_R11_O15_PREECLAMPS | SSC-A on CD8+ T cell \|\| id:ebi-a-GCST90002082 | Inverse variance weighted | 18 | 0.0176871735419279 | 0.0142765937420219 | 0.215385087672915 | -0.01029495 | 0.0456692972762908 | 1.01784451788587 | 0.989757861420948 | 1.04672819784727 | 0.855687495048373 |
| ebi-a-GCST90002082 | finngen_R11_O15_PREECLAMPS | Pre-eclampsia \|\| id:finngen_R11_O15_PREECLAMPS | SSC-A on CD8+ T cell \|\| id:ebi-a-GCST90002082 | MR Egger | 18 | 0.00650076841951102 | 0.0168641634808639 | 0.704959826807801 | -0.026552992 | 0.0395545288420043 | 1.00652194427611 | 0.973796439044425 | 1.04034722626784 | 0.99380795269851 |
| ebi-a-GCST90002082 | finngen_R11_O15_PREECLAMPS | Pre-eclampsia \|\| id:finngen_R11_O15_PREECLAMPS | SSC-A on CD8+ T cell \|\| id:ebi-a-GCST90002082 | Weighted median | 18 | 0.0282957815803621 | 0.0206070437093622 | 0.169717492186625 | -0.012094024 | 0.068685587250712 | 1.02869990991213 | 0.987978814685688 | 1.07109938889721 | 0.940845681141708 |
| ebi-a-GCST90002082 | finngen_R11_O15_PREECLAMPS | Pre-eclampsia \|\| id:finngen_R11_O15_PREECLAMPS | SSC-A on CD8+ T cell \|\| id:ebi-a-GCST90002082 | Weighted mode | 18 | 0.0133348847229131 | 0.017251027249893 | 0.450142751337833 | -0.020477129 | 0.0471468981327033 | 1.01342419081885 | 0.979731103955127 | 1.04827598755493 | 0.933945646747087 |
| ebi-a-GCST90002083 | finngen_R11_O15_PREECLAMPS | Pre-eclampsia \|\| id:finngen_R11_O15_PREECLAMPS | SSC-A on HLA DR+ T cell \|\| id:ebi-a-GCST90002083 | Inverse variance weighted | 19 | -0.019045187 | 0.022574034096215 | 0.398850069212564 | -0.063290294 | 0.0251999193539442 | 0.981135026226984 | 0.9386709433066 | 1.02552012135205 | 0.885079385681701 |
| ebi-a-GCST90002083 | finngen_R11_O15_PREECLAMPS | Pre-eclampsia \|\| id:finngen_R11_O15_PREECLAMPS | SSC-A on HLA DR+ T cell \|\| id:ebi-a-GCST90002083 | MR Egger | 19 | 0.0188308645426537 | 0.0435851112633742 | 0.671136621984445 | -0.066595954 | 0.104257682618867 | 1.01900928344009 | 0.935573140000558 | 1.10988641650879 | 0.99380795269851 |
| ebi-a-GCST90002083 | finngen_R11_O15_PREECLAMPS | Pre-eclampsia \|\| id:finngen_R11_O15_PREECLAMPS | SSC-A on HLA DR+ T cell \|\| id:ebi-a-GCST90002083 | Weighted median | 19 | 0.0247945190416919 | 0.0304175415712446 | 0.414992851102742 | -0.034823862 | 0.0844129005213313 | 1.02510445943506 | 0.965775510621054 | 1.08807806907206 | 0.940845681141708 |
| ebi-a-GCST90002083 | finngen_R11_O15_PREECLAMPS | Pre-eclampsia \|\| id:finngen_R11_O15_PREECLAMPS | SSC-A on HLA DR+ T cell \|\| id:ebi-a-GCST90002083 | Weighted mode | 19 | 0.0252342318476246 | 0.0402249271621307 | 0.538321207718706 | -0.053606625 | 0.104075089085401 | 1.02555531010843 | 0.947804875551184 | 1.10968377692715 | 0.935508728341156 |
| ebi-a-GCST90002084 | finngen_R11_O15_PREECLAMPS | Pre-eclampsia \|\| id:finngen_R11_O15_PREECLAMPS | SSC-A on Natural Killer T \|\| id:ebi-a-GCST90002084 | Inverse variance weighted | 20 | 0.00150432594959965 | 0.013223459499767 | 0.909426537518039 | -0.024413655 | 0.027422306569143 | 1.00150545801548 | 0.975881948129567 | 1.02780175856026 | 0.986667691552896 |
| ebi-a-GCST90002084 | finngen_R11_O15_PREECLAMPS | Pre-eclampsia \|\| id:finngen_R11_O15_PREECLAMPS | SSC-A on Natural Killer T \|\| id:ebi-a-GCST90002084 | MR Egger | 20 | -0.008563127 | 0.0151409716284633 | 0.578675443930532 | -0.038239431 | 0.0211131778272306 | 0.991473432575784 | 0.962482465203684 | 1.02133763787123 | 0.99380795269851 |
| ebi-a-GCST90002084 | finngen_R11_O15_PREECLAMPS | Pre-eclampsia \|\| id:finngen_R11_O15_PREECLAMPS | SSC-A on Natural Killer T \|\| id:ebi-a-GCST90002084 | Weighted median | 20 | -0.002606595 | 0.0159695985226101 | 0.870343364806382 | -0.033907009 | 0.028693817611204 | 0.99739679872716 | 0.966661391656705 | 1.02910945104186 | 0.964912412213677 |
| ebi-a-GCST90002084 | finngen_R11_O15_PREECLAMPS | Pre-eclampsia \|\| id:finngen_R11_O15_PREECLAMPS | SSC-A on Natural Killer T \|\| id:ebi-a-GCST90002084 | Weighted mode | 20 | 0.00529176220136762 | 0.0127582336479832 | 0.682954305703408 | -0.019714376 | 0.0302979001514148 | 1.00530578830499 | 0.980478681805669 | 1.03076155224333 | 0.959313411571287 |
| ebi-a-GCST90002085 | finngen_R11_O15_PREECLAMPS | Pre-eclampsia \|\| id:finngen_R11_O15_PREECLAMPS | SSC-A on HLA DR+ CD4+ T cell \|\| id:ebi-a-GCST90002085 | Inverse variance weighted | 22 | -0.004804044 | 0.0235782898015037 | 0.838549944386189 | -0.051017492 | 0.0414094039598537 | 0.995207476912074 | 0.950262048344344 | 1.04227873124824 | 0.977824840984872 |
| ebi-a-GCST90002085 | finngen_R11_O15_PREECLAMPS | Pre-eclampsia \|\| id:finngen_R11_O15_PREECLAMPS | SSC-A on HLA DR+ CD4+ T cell \|\| id:ebi-a-GCST90002085 | MR Egger | 22 | 0.0719936251029889 | 0.0318751337969516 | 0.0352343378268502 | 0.00951836286096386 | 0.134468887345014 | 1.07464849326851 | 1.00956380654546 | 1.14392906777832 | 0.839403552053506 |
| ebi-a-GCST90002085 | finngen_R11_O15_PREECLAMPS | Pre-eclampsia \|\| id:finngen_R11_O15_PREECLAMPS | SSC-A on HLA DR+ CD4+ T cell \|\| id:ebi-a-GCST90002085 | Weighted median | 22 | 0.0269057570917532 | 0.0329275332062988 | 0.413859660860774 | -0.037632208 | 0.0914437221760988 | 1.02727098519617 | 0.963067084137823 | 1.09575510824425 | 0.940845681141708 |
| ebi-a-GCST90002085 | finngen_R11_O15_PREECLAMPS | Pre-eclampsia \|\| id:finngen_R11_O15_PREECLAMPS | SSC-A on HLA DR+ CD4+ T cell \|\| id:ebi-a-GCST90002085 | Weighted mode | 22 | 0.0450104149349742 | 0.0343112623013569 | 0.203739453001968 | -0.022239659 | 0.112260489045634 | 1.04603875427759 | 0.978005818893969 | 1.11880425894403 | 0.872755742358858 |
| ebi-a-GCST90002086 | finngen_R11_O15_PREECLAMPS | Pre-eclampsia \|\| id:finngen_R11_O15_PREECLAMPS | SSC-A on HLA DR+ CD8+ T cell \|\| id:ebi-a-GCST90002086 | Inverse variance weighted (multiplicative random effects) | 25 | 0.0244499864663835 | 0.025002469610264 | 0.328122371927906 | -0.024554854 | 0.0734548269025009 | 1.02475133839003 | 0.975744164009553 | 1.07621991938645 | 0.885079385681701 |
| ebi-a-GCST90002086 | finngen_R11_O15_PREECLAMPS | Pre-eclampsia \|\| id:finngen_R11_O15_PREECLAMPS | SSC-A on HLA DR+ CD8+ T cell \|\| id:ebi-a-GCST90002086 | MR Egger | 25 | 0.0730522834718317 | 0.0333658339197628 | 0.0389724515068687 | 0.00765524898909666 | 0.138449317954567 | 1.07578678131224 | 1.00768462532084 | 1.14849147219813 | 0.839403552053506 |
| ebi-a-GCST90002086 | finngen_R11_O15_PREECLAMPS | Pre-eclampsia \|\| id:finngen_R11_O15_PREECLAMPS | SSC-A on HLA DR+ CD8+ T cell \|\| id:ebi-a-GCST90002086 | Weighted median | 25 | -0.005226948 | 0.030041258478544 | 0.861871509929966 | -0.064107814 | 0.0536539188184507 | 0.994786688922331 | 0.937903874519613 | 1.05511938199842 | 0.964912412213677 |
| ebi-a-GCST90002086 | finngen_R11_O15_PREECLAMPS | Pre-eclampsia \|\| id:finngen_R11_O15_PREECLAMPS | SSC-A on HLA DR+ CD8+ T cell \|\| id:ebi-a-GCST90002086 | Weighted mode | 25 | -0.008725741 | 0.0345568027044544 | 0.802801339044374 | -0.076457074 | 0.0590055924818541 | 0.99131221797112 | 0.926392679656698 | 1.06078117312303 | 0.961305639715923 |
| ebi-a-GCST90002087 | finngen_R11_O15_PREECLAMPS | Pre-eclampsia \|\| id:finngen_R11_O15_PREECLAMPS | CD11c on myeloid Dendritic Cell \|\| id:ebi-a-GCST90002087 | Inverse variance weighted (multiplicative random effects) | 30 | -0.00185007 | 0.0177601195009022 | 0.917034528618713 | -0.036659904 | 0.0329597645353406 | 0.998151640637593 | 0.964003933574739 | 1.0335089546917 | 0.986667691552896 |
| ebi-a-GCST90002087 | finngen_R11_O15_PREECLAMPS | Pre-eclampsia \|\| id:finngen_R11_O15_PREECLAMPS | CD11c on myeloid Dendritic Cell \|\| id:ebi-a-GCST90002087 | MR Egger | 30 | -0.009136619 | 0.0236066843774506 | 0.701656255651472 | -0.05540572 | 0.0371324827209995 | 0.990904993413809 | 0.94610121785205 | 1.03783050634015 | 0.99380795269851 |
| ebi-a-GCST90002087 | finngen_R11_O15_PREECLAMPS | Pre-eclampsia \|\| id:finngen_R11_O15_PREECLAMPS | CD11c on myeloid Dendritic Cell \|\| id:ebi-a-GCST90002087 | Weighted median | 30 | -0.013869936 | 0.0199425303882548 | 0.486744971043992 | -0.052957295 | 0.0252174238970747 | 0.986225808726417 | 0.948420513702247 | 1.02553807277036 | 0.940845681141708 |
| ebi-a-GCST90002087 | finngen_R11_O15_PREECLAMPS | Pre-eclampsia \|\| id:finngen_R11_O15_PREECLAMPS | CD11c on myeloid Dendritic Cell \|\| id:ebi-a-GCST90002087 | Weighted mode | 30 | -0.009629345 | 0.0163382976841965 | 0.560175239145182 | -0.041652408 | 0.0223937185833634 | 0.990416868808928 | 0.959203133644514 | 1.02264634008673 | 0.939633861977944 |
| ebi-a-GCST90002088 | finngen_R11_O15_PREECLAMPS | Pre-eclampsia \|\| id:finngen_R11_O15_PREECLAMPS | CD11c on CD62L+ myeloid Dendritic Cell \|\| id:ebi-a-GCST90002088 | Inverse variance weighted | 26 | -0.005120412 | 0.0156156915201348 | 0.742985980564991 | -0.035727167 | 0.0254863434230947 | 0.994892675006524 | 0.964903514770406 | 1.02581389706841 | 0.96638925304053 |
| ebi-a-GCST90002088 | finngen_R11_O15_PREECLAMPS | Pre-eclampsia \|\| id:finngen_R11_O15_PREECLAMPS | CD11c on CD62L+ myeloid Dendritic Cell \|\| id:ebi-a-GCST90002088 | MR Egger | 26 | -0.03635106 | 0.01833719621024 | 0.0589992463417564 | -0.072291964 | -0.000410155 | 0.964301706523744 | 0.930259253631693 | 0.999589928908958 | 0.862303274556491 |
| ebi-a-GCST90002088 | finngen_R11_O15_PREECLAMPS | Pre-eclampsia \|\| id:finngen_R11_O15_PREECLAMPS | CD11c on CD62L+ myeloid Dendritic Cell \|\| id:ebi-a-GCST90002088 | Weighted median | 26 | 0.00035420919018107 | 0.0197062498441491 | 0.985659228467286 | -0.038270041 | 0.0389784588847134 | 1.00035427192966 | 0.962453004501352 | 1.03974808607553 | 0.992447515164719 |
| ebi-a-GCST90002088 | finngen_R11_O15_PREECLAMPS | Pre-eclampsia \|\| id:finngen_R11_O15_PREECLAMPS | CD11c on CD62L+ myeloid Dendritic Cell \|\| id:ebi-a-GCST90002088 | Weighted mode | 26 | -0.00496904 | 0.0187585870740852 | 0.793262866697227 | -0.04173587 | 0.0317977908469728 | 0.995043285436778 | 0.959123079834503 | 1.03230874192253 | 0.961305639715923 |
| ebi-a-GCST90002089 | finngen_R11_O15_PREECLAMPS | Pre-eclampsia \|\| id:finngen_R11_O15_PREECLAMPS | CD11c on monocyte \|\| id:ebi-a-GCST90002089 | Inverse variance weighted | 19 | 0.00786832089570589 | 0.0185933552164281 | 0.672164532686074 | -0.028574655 | 0.0443112971199049 | 1.0078993574811 | 0.971829739169445 | 1.0453077055236 | 0.950857618427329 |
| ebi-a-GCST90002089 | finngen_R11_O15_PREECLAMPS | Pre-eclampsia \|\| id:finngen_R11_O15_PREECLAMPS | CD11c on monocyte \|\| id:ebi-a-GCST90002089 | MR Egger | 19 | -0.012669209 | 0.0287980517251747 | 0.665527671027699 | -0.06911339 | 0.043774972324631 | 0.987410707523051 | 0.933220855756947 | 1.04474723139396 | 0.99380795269851 |
| ebi-a-GCST90002089 | finngen_R11_O15_PREECLAMPS | Pre-eclampsia \|\| id:finngen_R11_O15_PREECLAMPS | CD11c on monocyte \|\| id:ebi-a-GCST90002089 | Weighted median | 19 | -0.017781125 | 0.0245709527920616 | 0.469271753663959 | -0.065940193 | 0.0303779420495358 | 0.982376025966357 | 0.936186853242442 | 1.03084405965647 | 0.940845681141708 |
| ebi-a-GCST90002089 | finngen_R11_O15_PREECLAMPS | Pre-eclampsia \|\| id:finngen_R11_O15_PREECLAMPS | CD11c on monocyte \|\| id:ebi-a-GCST90002089 | Weighted mode | 19 | -0.015242681 | 0.0236183018109616 | 0.526825418605083 | -0.061534553 | 0.0310491904682731 | 0.984872900578743 | 0.9403204546327 | 1.03153624438944 | 0.935508728341156 |
| ebi-a-GCST90002090 | finngen_R11_O15_PREECLAMPS | Pre-eclampsia \|\| id:finngen_R11_O15_PREECLAMPS | CD11c on granulocyte \|\| id:ebi-a-GCST90002090 | Inverse variance weighted | 22 | 0.00831584983975216 | 0.0220373308201956 | 0.705911393109304 | -0.034877319 | 0.0515090182473356 | 1.00835052256344 | 0.965723885379775 | 1.05285868118932 | 0.956666000237608 |
| ebi-a-GCST90002090 | finngen_R11_O15_PREECLAMPS | Pre-eclampsia \|\| id:finngen_R11_O15_PREECLAMPS | CD11c on granulocyte \|\| id:ebi-a-GCST90002090 | MR Egger | 22 | 0.038359049170169 | 0.0389858432077818 | 0.336907657502142 | -0.038053204 | 0.114771301857421 | 1.0391042554284 | 0.962661722539313 | 1.1216168965369 | 0.99380795269851 |
| ebi-a-GCST90002090 | finngen_R11_O15_PREECLAMPS | Pre-eclampsia \|\| id:finngen_R11_O15_PREECLAMPS | CD11c on granulocyte \|\| id:ebi-a-GCST90002090 | Weighted median | 22 | 0.0255978724303312 | 0.0312101451670537 | 0.412114678387738 | -0.035574012 | 0.0867697569577564 | 1.0259283114541 | 0.965051306115792 | 1.09064553726098 | 0.940845681141708 |
| ebi-a-GCST90002090 | finngen_R11_O15_PREECLAMPS | Pre-eclampsia \|\| id:finngen_R11_O15_PREECLAMPS | CD11c on granulocyte \|\| id:ebi-a-GCST90002090 | Weighted mode | 22 | 0.0418776231604315 | 0.0316524593875639 | 0.200046739573851 | -0.020161197 | 0.103916443560057 | 1.04276686042937 | 0.980040680721083 | 1.10950774452514 | 0.872755742358858 |
| ebi-a-GCST90002091 | finngen_R11_O15_PREECLAMPS | Pre-eclampsia \|\| id:finngen_R11_O15_PREECLAMPS | CD11b on CD14+ monocyte \|\| id:ebi-a-GCST90002091 | Inverse variance weighted | 20 | 0.0231641703814485 | 0.0151369905624859 | 0.125941937087086 | -0.006504331 | 0.0528326718839209 | 1.02343454339559 | 0.993516776252732 | 1.05425322415381 | 0.820779257935285 |
| ebi-a-GCST90002091 | finngen_R11_O15_PREECLAMPS | Pre-eclampsia \|\| id:finngen_R11_O15_PREECLAMPS | CD11b on CD14+ monocyte \|\| id:ebi-a-GCST90002091 | MR Egger | 20 | 0.00726508321737013 | 0.0232501124273552 | 0.758270488547176 | -0.038305137 | 0.0528353035749864 | 1.00729153796095 | 0.962419226231443 | 1.05425599862625 | 0.99380795269851 |
| ebi-a-GCST90002091 | finngen_R11_O15_PREECLAMPS | Pre-eclampsia \|\| id:finngen_R11_O15_PREECLAMPS | CD11b on CD14+ monocyte \|\| id:ebi-a-GCST90002091 | Weighted median | 20 | 0.0120629005938818 | 0.019188069019025 | 0.529567263984419 | -0.025545715 | 0.0496715158711708 | 1.01213595081627 | 0.974777816287901 | 1.05092582721659 | 0.940845681141708 |
| ebi-a-GCST90002091 | finngen_R11_O15_PREECLAMPS | Pre-eclampsia \|\| id:finngen_R11_O15_PREECLAMPS | CD11b on CD14+ monocyte \|\| id:ebi-a-GCST90002091 | Weighted mode | 20 | 0.0209212794046133 | 0.0168689568755121 | 0.22999103816549 | -0.012141876 | 0.053984434880617 | 1.02114166359374 | 0.987931539072946 | 1.0554681735391 | 0.872755742358858 |
| ebi-a-GCST90002092 | finngen_R11_O15_PREECLAMPS | Pre-eclampsia \|\| id:finngen_R11_O15_PREECLAMPS | CD11b on Granulocytic Myeloid-Derived Suppressor Cells \|\| id:ebi-a-GCST90002092 | Inverse variance weighted | 15 | 0.00300956325839064 | 0.0165221171204011 | 0.855462178657992 | -0.029373786 | 0.0353929128143769 | 1.00301409654049 | 0.971053430155781 | 1.03602669700424 | 0.977824840984872 |
| ebi-a-GCST90002092 | finngen_R11_O15_PREECLAMPS | Pre-eclampsia \|\| id:finngen_R11_O15_PREECLAMPS | CD11b on Granulocytic Myeloid-Derived Suppressor Cells \|\| id:ebi-a-GCST90002092 | MR Egger | 15 | 0.00960874803890911 | 0.02133272586437 | 0.659825703485206 | -0.032203395 | 0.0514208907330742 | 1.00965506027369 | 0.968309613048725 | 1.05276589945925 | 0.99380795269851 |
| ebi-a-GCST90002092 | finngen_R11_O15_PREECLAMPS | Pre-eclampsia \|\| id:finngen_R11_O15_PREECLAMPS | CD11b on Granulocytic Myeloid-Derived Suppressor Cells \|\| id:ebi-a-GCST90002092 | Weighted median | 15 | 0.0164860188668566 | 0.0172126655611826 | 0.338171595271229 | -0.017250806 | 0.0502228433667744 | 1.01662266314988 | 0.982897137579682 | 1.0515053912711 | 0.940845681141708 |
| ebi-a-GCST90002092 | finngen_R11_O15_PREECLAMPS | Pre-eclampsia \|\| id:finngen_R11_O15_PREECLAMPS | CD11b on Granulocytic Myeloid-Derived Suppressor Cells \|\| id:ebi-a-GCST90002092 | Weighted mode | 15 | 0.00908064226429511 | 0.0171766941430558 | 0.605321249956642 | -0.024585678 | 0.0427469627846846 | 1.00912199637545 | 0.975714087855568 | 1.04367377313046 | 0.955353022288269 |
| ebi-a-GCST90002093 | finngen_R11_O15_PREECLAMPS | Pre-eclampsia \|\| id:finngen_R11_O15_PREECLAMPS | CD11b on CD66b++ myeloid cell \|\| id:ebi-a-GCST90002093 | Inverse variance weighted | 17 | 0.000794404809306532 | 0.0203953938358954 | 0.968930087612709 | -0.039180567 | 0.0407693767276614 | 1.00079472043238 | 0.961577064278939 | 1.04161185790802 | 0.994537505425743 |
| ebi-a-GCST90002093 | finngen_R11_O15_PREECLAMPS | Pre-eclampsia \|\| id:finngen_R11_O15_PREECLAMPS | CD11b on CD66b++ myeloid cell \|\| id:ebi-a-GCST90002093 | MR Egger | 17 | 0.0268669541712759 | 0.0405862751639278 | 0.518030936446557 | -0.052682145 | 0.106416053492574 | 1.02723112485518 | 0.948681507582189 | 1.11228455012319 | 0.99380795269851 |
| ebi-a-GCST90002093 | finngen_R11_O15_PREECLAMPS | Pre-eclampsia \|\| id:finngen_R11_O15_PREECLAMPS | CD11b on CD66b++ myeloid cell \|\| id:ebi-a-GCST90002093 | Weighted median | 17 | 0.0162862218923031 | 0.0300784910098312 | 0.588192344553285 | -0.04266762 | 0.0752400642715723 | 1.01641956530737 | 0.958229833107431 | 1.07814294342033 | 0.940845681141708 |
| ebi-a-GCST90002093 | finngen_R11_O15_PREECLAMPS | Pre-eclampsia \|\| id:finngen_R11_O15_PREECLAMPS | CD11b on CD66b++ myeloid cell \|\| id:ebi-a-GCST90002093 | Weighted mode | 17 | 0.028431358090001 | 0.0370491144354342 | 0.454022113051415 | -0.044184906 | 0.101047622383452 | 1.02883938691007 | 0.956777027108159 | 1.10632932654834 | 0.933945646747087 |
| ebi-a-GCST90002094 | finngen_R11_O15_PREECLAMPS | Pre-eclampsia \|\| id:finngen_R11_O15_PREECLAMPS | CD11b on Monocytic Myeloid-Derived Suppressor Cells \|\| id:ebi-a-GCST90002094 | Inverse variance weighted | 16 | 0.0377065043476999 | 0.0162179268505321 | 0.0200725391066855 | 0.0059193677206571 | 0.0694936409747427 | 1.03842641451136 | 1.00593692179702 | 1.07196524452903 | 0.63795765595596 |
| ebi-a-GCST90002094 | finngen_R11_O15_PREECLAMPS | Pre-eclampsia \|\| id:finngen_R11_O15_PREECLAMPS | CD11b on Monocytic Myeloid-Derived Suppressor Cells \|\| id:ebi-a-GCST90002094 | MR Egger | 16 | -0.000602841 | 0.025831661148989 | 0.981710597462809 | -0.051232897 | 0.0500272149219582 | 0.999397340742025 | 0.950057379457972 | 1.05129970702619 | 0.99380795269851 |
| ebi-a-GCST90002094 | finngen_R11_O15_PREECLAMPS | Pre-eclampsia \|\| id:finngen_R11_O15_PREECLAMPS | CD11b on Monocytic Myeloid-Derived Suppressor Cells \|\| id:ebi-a-GCST90002094 | Weighted median | 16 | 0.0158989740651478 | 0.0206640249626859 | 0.441653784632946 | -0.024602515 | 0.0564004629920122 | 1.01602603524097 | 0.975697660280563 | 1.0580212972845 | 0.940845681141708 |
| ebi-a-GCST90002094 | finngen_R11_O15_PREECLAMPS | Pre-eclampsia \|\| id:finngen_R11_O15_PREECLAMPS | CD11b on Monocytic Myeloid-Derived Suppressor Cells \|\| id:ebi-a-GCST90002094 | Weighted mode | 16 | 0.0198668554048369 | 0.0210879424100926 | 0.361070356026054 | -0.021465512 | 0.0611992225286184 | 1.02006551477475 | 0.978763232748521 | 1.06311068869097 | 0.907825101194024 |
| ebi-a-GCST90002095 | finngen_R11_O15_PREECLAMPS | Pre-eclampsia \|\| id:finngen_R11_O15_PREECLAMPS | CD11b on CD33dim HLA DR- \|\| id:ebi-a-GCST90002095 | Inverse variance weighted | 23 | 0.00493707875034766 | 0.0184508099893678 | 0.789022167924981 | -0.031226509 | 0.0411006663295085 | 1.0049492862051 | 0.969256003167606 | 1.04195699025193 | 0.977824840984872 |
| ebi-a-GCST90002095 | finngen_R11_O15_PREECLAMPS | Pre-eclampsia \|\| id:finngen_R11_O15_PREECLAMPS | CD11b on CD33dim HLA DR- \|\| id:ebi-a-GCST90002095 | MR Egger | 23 | -0.019241418 | 0.0365786663298215 | 0.604379892109199 | -0.090935604 | 0.0524527677426511 | 0.980942516215327 | 0.913076507234131 | 1.05385278505705 | 0.99380795269851 |
| ebi-a-GCST90002095 | finngen_R11_O15_PREECLAMPS | Pre-eclampsia \|\| id:finngen_R11_O15_PREECLAMPS | CD11b on CD33dim HLA DR- \|\| id:ebi-a-GCST90002095 | Weighted median | 23 | -0.003903879 | 0.0229269734367557 | 0.864794279459122 | -0.048840746 | 0.0410329894351506 | 0.996103731726429 | 0.952332780011202 | 1.04188647622488 | 0.964912412213677 |
| ebi-a-GCST90002095 | finngen_R11_O15_PREECLAMPS | Pre-eclampsia \|\| id:finngen_R11_O15_PREECLAMPS | CD11b on CD33dim HLA DR- \|\| id:ebi-a-GCST90002095 | Weighted mode | 23 | 0.00465117378204644 | 0.0276018748412335 | 0.867722219218177 | -0.049448501 | 0.0587508484708641 | 1.00466200728047 | 0.951754171351185 | 1.06051097988872 | 0.977585971930092 |
| ebi-a-GCST90002096 | finngen_R11_O15_PREECLAMPS | Pre-eclampsia \|\| id:finngen_R11_O15_PREECLAMPS | CD11b on basophil \|\| id:ebi-a-GCST90002096 | Inverse variance weighted | 19 | 0.00159613592169802 | 0.0205943081879289 | 0.938222815578485 | -0.038768708 | 0.0419609799700386 | 1.00159741042464 | 0.961973179996446 | 1.04285378577088 | 0.986667691552896 |
| ebi-a-GCST90002096 | finngen_R11_O15_PREECLAMPS | Pre-eclampsia \|\| id:finngen_R11_O15_PREECLAMPS | CD11b on basophil \|\| id:ebi-a-GCST90002096 | MR Egger | 19 | -0.009306143 | 0.0499672718915914 | 0.854457170979688 | -0.107241996 | 0.0886297100228554 | 0.990737025249638 | 0.898308259865865 | 1.09267597444453 | 0.99380795269851 |
| ebi-a-GCST90002096 | finngen_R11_O15_PREECLAMPS | Pre-eclampsia \|\| id:finngen_R11_O15_PREECLAMPS | CD11b on basophil \|\| id:ebi-a-GCST90002096 | Weighted median | 19 | -0.005018792 | 0.0291354561998709 | 0.863235327962606 | -0.062124286 | 0.0520867019417249 | 0.99499378088489 | 0.939766079425532 | 1.05346707619495 | 0.964912412213677 |
| ebi-a-GCST90002096 | finngen_R11_O15_PREECLAMPS | Pre-eclampsia \|\| id:finngen_R11_O15_PREECLAMPS | CD11b on basophil \|\| id:ebi-a-GCST90002096 | Weighted mode | 19 | -0.004710795 | 0.0334796653409623 | 0.889664746899313 | -0.070330939 | 0.0609093494296368 | 0.99530028375158 | 0.932085305753287 | 1.06280256616145 | 0.98361388732834 |
| ebi-a-GCST90002097 | finngen_R11_O15_PREECLAMPS | Pre-eclampsia \|\| id:finngen_R11_O15_PREECLAMPS | CD11b on CD33+ HLA DR+ CD14dim \|\| id:ebi-a-GCST90002097 | Inverse variance weighted | 20 | -0.011661727 | 0.019715946246405 | 0.554194125182052 | -0.050304982 | 0.0269815276321809 | 0.988406007372025 | 0.950939361211995 | 1.02734882502283 | 0.905435115099076 |
| ebi-a-GCST90002097 | finngen_R11_O15_PREECLAMPS | Pre-eclampsia \|\| id:finngen_R11_O15_PREECLAMPS | CD11b on CD33+ HLA DR+ CD14dim \|\| id:ebi-a-GCST90002097 | MR Egger | 20 | 0.0175498291329469 | 0.0412405109324704 | 0.675481537748005 | -0.063281572 | 0.0983812305605889 | 1.01770473223173 | 0.938679130438272 | 1.10338334839008 | 0.99380795269851 |
| ebi-a-GCST90002097 | finngen_R11_O15_PREECLAMPS | Pre-eclampsia \|\| id:finngen_R11_O15_PREECLAMPS | CD11b on CD33+ HLA DR+ CD14dim \|\| id:ebi-a-GCST90002097 | Weighted median | 20 | 0.00125297549263974 | 0.0285961375925945 | 0.965050874486775 | -0.054795454 | 0.0573014051741249 | 1.00125376079439 | 0.946678767327159 | 1.05897494282604 | 0.989912389390616 |
| ebi-a-GCST90002097 | finngen_R11_O15_PREECLAMPS | Pre-eclampsia \|\| id:finngen_R11_O15_PREECLAMPS | CD11b on CD33+ HLA DR+ CD14dim \|\| id:ebi-a-GCST90002097 | Weighted mode | 20 | 0.0546588499948559 | 0.0302572306833178 | 0.0867103250631649 | -0.004645322 | 0.113963022134159 | 1.05618023731423 | 0.995365450676935 | 1.12071068262867 | 0.872755742358858 |
| ebi-a-GCST90002098 | finngen_R11_O15_PREECLAMPS | Pre-eclampsia \|\| id:finngen_R11_O15_PREECLAMPS | CD45RA on naive CD4+ T cell \|\| id:ebi-a-GCST90002098 | Inverse variance weighted | 32 | -0.003861373 | 0.0149295002520399 | 0.79591264352808 | -0.033123194 | 0.0254004473126509 | 0.996146072333689 | 0.967419372299549 | 1.0257257874288 | 0.977824840984872 |
| ebi-a-GCST90002098 | finngen_R11_O15_PREECLAMPS | Pre-eclampsia \|\| id:finngen_R11_O15_PREECLAMPS | CD45RA on naive CD4+ T cell \|\| id:ebi-a-GCST90002098 | MR Egger | 32 | -0.001588534 | 0.019620750691246 | 0.936009927947285 | -0.040045206 | 0.0368681369498627 | 0.998412726647968 | 0.960746006917399 | 1.03755619649256 | 0.99380795269851 |
| ebi-a-GCST90002098 | finngen_R11_O15_PREECLAMPS | Pre-eclampsia \|\| id:finngen_R11_O15_PREECLAMPS | CD45RA on naive CD4+ T cell \|\| id:ebi-a-GCST90002098 | Weighted median | 32 | -0.007476891 | 0.0183121571489925 | 0.683051959587838 | -0.043368719 | 0.0284149373353566 | 0.9925509917359 | 0.957558255343087 | 1.02882249272964 | 0.943838749369606 |
| ebi-a-GCST90002098 | finngen_R11_O15_PREECLAMPS | Pre-eclampsia \|\| id:finngen_R11_O15_PREECLAMPS | CD45RA on naive CD4+ T cell \|\| id:ebi-a-GCST90002098 | Weighted mode | 32 | -0.008365587 | 0.0156911389167573 | 0.597737863012475 | -0.03912022 | 0.0223890448570218 | 0.991669306735453 | 0.961635094717549 | 1.02264156052876 | 0.955353022288269 |
| ebi-a-GCST90002099 | finngen_R11_O15_PREECLAMPS | Pre-eclampsia \|\| id:finngen_R11_O15_PREECLAMPS | CD4RA on Terminally Differentiated CD4+ T cell \|\| id:ebi-a-GCST90002099 | Inverse variance weighted | 33 | -0.009193069 | 0.0107818872532857 | 0.393858929407554 | -0.030325568 | 0.0119394298148148 | 0.990849057867857 | 0.970129638750865 | 1.0120109893166 | 0.885079385681701 |
| ebi-a-GCST90002099 | finngen_R11_O15_PREECLAMPS | Pre-eclampsia \|\| id:finngen_R11_O15_PREECLAMPS | CD4RA on Terminally Differentiated CD4+ T cell \|\| id:ebi-a-GCST90002099 | MR Egger | 33 | -0.0191267 | 0.0138020132725003 | 0.175700171462579 | -0.046178646 | 0.0079252459002691 | 0.981055054580984 | 0.954871362881063 | 1.00795673378968 | 0.99380795269851 |
| ebi-a-GCST90002099 | finngen_R11_O15_PREECLAMPS | Pre-eclampsia \|\| id:finngen_R11_O15_PREECLAMPS | CD4RA on Terminally Differentiated CD4+ T cell \|\| id:ebi-a-GCST90002099 | Weighted median | 33 | -0.013381468 | 0.0163771899103624 | 0.413882921213114 | -0.045480761 | 0.0187178238997678 | 0.986707665499283 | 0.955537986421515 | 1.01889410048589 | 0.940845681141708 |
| ebi-a-GCST90002099 | finngen_R11_O15_PREECLAMPS | Pre-eclampsia \|\| id:finngen_R11_O15_PREECLAMPS | CD4RA on Terminally Differentiated CD4+ T cell \|\| id:ebi-a-GCST90002099 | Weighted mode | 33 | -0.015197219 | 0.0130178379979486 | 0.251668315724136 | -0.040712181 | 0.0103177438903702 | 0.984917676376457 | 0.96010542670885 | 1.01037115534706 | 0.872755742358858 |
| ebi-a-GCST90002100 | finngen_R11_O15_PREECLAMPS | Pre-eclampsia \|\| id:finngen_R11_O15_PREECLAMPS | CD45RA on naive CD8+ T cell \|\| id:ebi-a-GCST90002100 | Inverse variance weighted | 22 | -0.03464894 | 0.0219193096590388 | 0.113935303846411 | -0.077610787 | 0.00831290708373699 | 0.965944461350919 | 0.925324504995763 | 1.00834755523825 | 0.820779257935285 |
| ebi-a-GCST90002100 | finngen_R11_O15_PREECLAMPS | Pre-eclampsia \|\| id:finngen_R11_O15_PREECLAMPS | CD45RA on naive CD8+ T cell \|\| id:ebi-a-GCST90002100 | MR Egger | 22 | -0.043339019 | 0.0408988411045754 | 0.301928032347165 | -0.123500748 | 0.0368227094758422 | 0.957586694862153 | 0.883820980451458 | 1.03750906400596 | 0.99380795269851 |
| ebi-a-GCST90002100 | finngen_R11_O15_PREECLAMPS | Pre-eclampsia \|\| id:finngen_R11_O15_PREECLAMPS | CD45RA on naive CD8+ T cell \|\| id:ebi-a-GCST90002100 | Weighted median | 22 | -0.074370121 | 0.0313193321278783 | 0.0175691388197353 | -0.135756012 | -0.01298423 | 0.928328036260139 | 0.873055621355494 | 0.987099701126256 | 0.598716529366782 |
| ebi-a-GCST90002100 | finngen_R11_O15_PREECLAMPS | Pre-eclampsia \|\| id:finngen_R11_O15_PREECLAMPS | CD45RA on naive CD8+ T cell \|\| id:ebi-a-GCST90002100 | Weighted mode | 22 | -0.07526008 | 0.0471316579702309 | 0.125248599493086 | -0.16763813 | 0.0171179692519752 | 0.927502229834062 | 0.845659798240324 | 1.01726532127602 | 0.872755742358858 |
| ebi-a-GCST90002101 | finngen_R11_O15_PREECLAMPS | Pre-eclampsia \|\| id:finngen_R11_O15_PREECLAMPS | CD45RA on Terminally Differentiated CD8+ T cell \|\| id:ebi-a-GCST90002101 | Inverse variance weighted | 14 | 0.0184329865936497 | 0.0307351572663902 | 0.548682055062893 | -0.041807922 | 0.0786738948357745 | 1.01860392276377 | 0.959053976388712 | 1.08185146718917 | 0.905435115099076 |
| ebi-a-GCST90002101 | finngen_R11_O15_PREECLAMPS | Pre-eclampsia \|\| id:finngen_R11_O15_PREECLAMPS | CD45RA on Terminally Differentiated CD8+ T cell \|\| id:ebi-a-GCST90002101 | MR Egger | 14 | 0.0348360058614101 | 0.063026845570786 | 0.59060475989589 | -0.088696611 | 0.158368623180151 | 1.03544988716283 | 0.915123169347346 | 1.17159799329544 | 0.99380795269851 |
| ebi-a-GCST90002101 | finngen_R11_O15_PREECLAMPS | Pre-eclampsia \|\| id:finngen_R11_O15_PREECLAMPS | CD45RA on Terminally Differentiated CD8+ T cell \|\| id:ebi-a-GCST90002101 | Weighted median | 14 | 0.0327843782673719 | 0.0429490499505579 | 0.445265504821603 | -0.05139576 | 0.116964516170465 | 1.03332770730784 | 0.949902663001152 | 1.124079542 | 0.940845681141708 |
| ebi-a-GCST90002101 | finngen_R11_O15_PREECLAMPS | Pre-eclampsia \|\| id:finngen_R11_O15_PREECLAMPS | CD45RA on Terminally Differentiated CD8+ T cell \|\| id:ebi-a-GCST90002101 | Weighted mode | 14 | 0.0526902913132137 | 0.0539942342386648 | 0.346948896742153 | -0.053138408 | 0.158518990420997 | 1.0541031296636 | 0.948248758379862 | 1.17177417649883 | 0.891828501403228 |
| ebi-a-GCST90002102 | finngen_R11_O15_PREECLAMPS | Pre-eclampsia \|\| id:finngen_R11_O15_PREECLAMPS | CD45RA on resting CD4 regulatory T cell \|\| id:ebi-a-GCST90002102 | Inverse variance weighted | 29 | 0.0101500335843574 | 0.0104837366317315 | 0.332959746889515 | -0.01039809 | 0.0306981573825512 | 1.01020171989984 | 0.989655783038212 | 1.03117420458623 | 0.885079385681701 |
| ebi-a-GCST90002102 | finngen_R11_O15_PREECLAMPS | Pre-eclampsia \|\| id:finngen_R11_O15_PREECLAMPS | CD45RA on resting CD4 regulatory T cell \|\| id:ebi-a-GCST90002102 | MR Egger | 29 | 0.0381306579822909 | 0.0130652645849652 | 0.00700994390944475 | 0.0125227393957592 | 0.0637385765688227 | 1.03886696027227 | 1.01260147722458 | 1.06581373365505 | 0.565056961366001 |
| ebi-a-GCST90002102 | finngen_R11_O15_PREECLAMPS | Pre-eclampsia \|\| id:finngen_R11_O15_PREECLAMPS | CD45RA on resting CD4 regulatory T cell \|\| id:ebi-a-GCST90002102 | Weighted median | 29 | 0.0151143218604742 | 0.013084260423674 | 0.248027803274721 | -0.010530829 | 0.0407594722908752 | 1.01522912086361 | 0.989524426474696 | 1.04160154138031 | 0.940845681141708 |
| ebi-a-GCST90002102 | finngen_R11_O15_PREECLAMPS | Pre-eclampsia \|\| id:finngen_R11_O15_PREECLAMPS | CD45RA on resting CD4 regulatory T cell \|\| id:ebi-a-GCST90002102 | Weighted mode | 29 | 0.0172726787860867 | 0.0114033753598423 | 0.14105688379497 | -0.005077937 | 0.0396232944913776 | 1.01742271409471 | 0.99493493400734 | 1.04041876888023 | 0.872755742358858 |
| ebi-a-GCST90002103 | finngen_R11_O15_PREECLAMPS | Pre-eclampsia \|\| id:finngen_R11_O15_PREECLAMPS | CD45RA on CD39+ resting CD4 regulatory T cell \|\| id:ebi-a-GCST90002103 | Inverse variance weighted | 22 | -0.016152704 | 0.0204064093931691 | 0.4286227941279 | -0.056149267 | 0.023843857971021 | 0.983977050918065 | 0.945398008774901 | 1.02413039560774 | 0.898694924660397 |
| ebi-a-GCST90002103 | finngen_R11_O15_PREECLAMPS | Pre-eclampsia \|\| id:finngen_R11_O15_PREECLAMPS | CD45RA on CD39+ resting CD4 regulatory T cell \|\| id:ebi-a-GCST90002103 | MR Egger | 22 | -0.009564617 | 0.0289987270736176 | 0.744958889122751 | -0.066402122 | 0.0472728882466403 | 0.990480978646279 | 0.935754501263703 | 1.0484080682863 | 0.99380795269851 |
| ebi-a-GCST90002103 | finngen_R11_O15_PREECLAMPS | Pre-eclampsia \|\| id:finngen_R11_O15_PREECLAMPS | CD45RA on CD39+ resting CD4 regulatory T cell \|\| id:ebi-a-GCST90002103 | Weighted median | 22 | 0.0149614574066151 | 0.0261712835958204 | 0.56754246283542 | -0.036334258 | 0.0662571732544232 | 1.01507394027961 | 0.964317908205313 | 1.06850147183557 | 0.940845681141708 |
| ebi-a-GCST90002103 | finngen_R11_O15_PREECLAMPS | Pre-eclampsia \|\| id:finngen_R11_O15_PREECLAMPS | CD45RA on CD39+ resting CD4 regulatory T cell \|\| id:ebi-a-GCST90002103 | Weighted mode | 22 | 0.010722409674914 | 0.025481091180049 | 0.67817725223534 | -0.039220529 | 0.06066534838781 | 1.01078010072083 | 0.961538638572419 | 1.06254327286326 | 0.959313411571287 |
| ebi-a-GCST90002104 | finngen_R11_O15_PREECLAMPS | Pre-eclampsia \|\| id:finngen_R11_O15_PREECLAMPS | HLA DR on myeloid Dendritic Cell \|\| id:ebi-a-GCST90002104 | Inverse variance weighted | 15 | 0.0334626941873389 | 0.0160198639409653 | 0.036723468473178 | 0.0020637608630469 | 0.0648616275116308 | 1.0340288677195 | 1.00206589188322 | 1.06701136915045 | 0.781760498894217 |
| ebi-a-GCST90002104 | finngen_R11_O15_PREECLAMPS | Pre-eclampsia \|\| id:finngen_R11_O15_PREECLAMPS | HLA DR on myeloid Dendritic Cell \|\| id:ebi-a-GCST90002104 | MR Egger | 15 | -0.001901422 | 0.0209764903477596 | 0.929156139068706 | -0.043015344 | 0.0392124986572657 | 0.998100384134083 | 0.957896692460672 | 1.03999145695923 | 0.99380795269851 |
| ebi-a-GCST90002104 | finngen_R11_O15_PREECLAMPS | Pre-eclampsia \|\| id:finngen_R11_O15_PREECLAMPS | HLA DR on myeloid Dendritic Cell \|\| id:ebi-a-GCST90002104 | Weighted median | 15 | 0.0414548069924461 | 0.0199320345320642 | 0.0375431372970747 | 0.00238801930960032 | 0.0805215946752919 | 1.04232605493776 | 1.00239087289874 | 1.08385225182698 | 0.722211404320042 |
| ebi-a-GCST90002104 | finngen_R11_O15_PREECLAMPS | Pre-eclampsia \|\| id:finngen_R11_O15_PREECLAMPS | HLA DR on myeloid Dendritic Cell \|\| id:ebi-a-GCST90002104 | Weighted mode | 15 | 0.0360976356464536 | 0.0179340124925869 | 0.0637803338321555 | 0.000946971160983273 | 0.071248300131924 | 1.03675706599588 | 1.00094741967974 | 1.07384782932584 | 0.871935626364341 |
| ebi-a-GCST90002105 | finngen_R11_O15_PREECLAMPS | Pre-eclampsia \|\| id:finngen_R11_O15_PREECLAMPS | HLA DR on plasmacytoid Dendritic Cell \|\| id:ebi-a-GCST90002105 | Inverse variance weighted | 25 | 0.0260437137551376 | 0.0117690165915641 | 0.0269042070822815 | 0.00297644123567202 | 0.0491109862746033 | 1.02638581467098 | 1.00298087523497 | 1.05033691725279 | 0.708667785226302 |
| ebi-a-GCST90002105 | finngen_R11_O15_PREECLAMPS | Pre-eclampsia \|\| id:finngen_R11_O15_PREECLAMPS | HLA DR on plasmacytoid Dendritic Cell \|\| id:ebi-a-GCST90002105 | MR Egger | 25 | 0.0330813476541827 | 0.0163570252798598 | 0.0549080145542269 | 0.00102157810565738 | 0.0651411172027079 | 1.03363461957297 | 1.00102210009431 | 1.0673096295068 | 0.862303274556491 |
| ebi-a-GCST90002105 | finngen_R11_O15_PREECLAMPS | Pre-eclampsia \|\| id:finngen_R11_O15_PREECLAMPS | HLA DR on plasmacytoid Dendritic Cell \|\| id:ebi-a-GCST90002105 | Weighted median | 25 | 0.0280078649852612 | 0.0146307460208679 | 0.0555798455002558 | -0.000668397 | 0.0566841271861622 | 1.02840377277013 | 0.999331826112019 | 1.05832146261425 | 0.771214851704188 |
| ebi-a-GCST90002105 | finngen_R11_O15_PREECLAMPS | Pre-eclampsia \|\| id:finngen_R11_O15_PREECLAMPS | HLA DR on plasmacytoid Dendritic Cell \|\| id:ebi-a-GCST90002105 | Weighted mode | 25 | 0.0333422827352191 | 0.0120457381077896 | 0.0106943449191587 | 0.00973263604395149 | 0.0569519294264867 | 1.03390436629786 | 1.00978015217342 | 1.05860492142668 | 0.720210568263475 |
| ebi-a-GCST90002106 | finngen_R11_O15_PREECLAMPS | Pre-eclampsia \|\| id:finngen_R11_O15_PREECLAMPS | HLA DR on Dendritic Cell \|\| id:ebi-a-GCST90002106 | Inverse variance weighted | 19 | 0.0473439962677557 | 0.0165737652283567 | 0.00428255678262564 | 0.0148594164201766 | 0.0798285761153348 | 1.04848262115998 | 1.01497036641792 | 1.08310138231352 | 0.447221286871335 |
| ebi-a-GCST90002106 | finngen_R11_O15_PREECLAMPS | Pre-eclampsia \|\| id:finngen_R11_O15_PREECLAMPS | HLA DR on Dendritic Cell \|\| id:ebi-a-GCST90002106 | MR Egger | 19 | 0.0454677583876444 | 0.0285732386713944 | 0.129971503534821 | -0.010535789 | 0.101471306183578 | 1.0465172626661 | 0.989519517616137 | 1.10679815967309 | 0.97261503632431 |
| ebi-a-GCST90002106 | finngen_R11_O15_PREECLAMPS | Pre-eclampsia \|\| id:finngen_R11_O15_PREECLAMPS | HLA DR on Dendritic Cell \|\| id:ebi-a-GCST90002106 | Weighted median | 19 | 0.0560009506047287 | 0.018714601133308 | 0.00276823463068893 | 0.019320332383445 | 0.0926815688260123 | 1.05759868909445 | 1.01950817780022 | 1.09711232487385 | 0.507633389188193 |
| ebi-a-GCST90002106 | finngen_R11_O15_PREECLAMPS | Pre-eclampsia \|\| id:finngen_R11_O15_PREECLAMPS | HLA DR on Dendritic Cell \|\| id:ebi-a-GCST90002106 | Weighted mode | 19 | 0.0606376725796178 | 0.0190213041391649 | 0.00509642468093412 | 0.0233559164668546 | 0.097919428692381 | 1.06251386652637 | 1.02363080177824 | 1.10287392153463 | 0.720210568263475 |
| ebi-a-GCST90002107 | finngen_R11_O15_PREECLAMPS | Pre-eclampsia \|\| id:finngen_R11_O15_PREECLAMPS | HLA DR on Hematopoietic Stem Cell \|\| id:ebi-a-GCST90002107 | Inverse variance weighted | 24 | -0.009139564 | 0.0101008956093575 | 0.36555698213263 | -0.02893732 | 0.0106581911176101 | 0.990902074590596 | 0.971477355078427 | 1.01071519196521 | 0.885079385681701 |
| ebi-a-GCST90002107 | finngen_R11_O15_PREECLAMPS | Pre-eclampsia \|\| id:finngen_R11_O15_PREECLAMPS | HLA DR on Hematopoietic Stem Cell \|\| id:ebi-a-GCST90002107 | MR Egger | 24 | -0.004098051 | 0.0143320160657205 | 0.777601593208661 | -0.032188803 | 0.0239927000250023 | 0.995910334090368 | 0.968323742437742 | 1.0242828406242 | 0.99380795269851 |
| ebi-a-GCST90002107 | finngen_R11_O15_PREECLAMPS | Pre-eclampsia \|\| id:finngen_R11_O15_PREECLAMPS | HLA DR on Hematopoietic Stem Cell \|\| id:ebi-a-GCST90002107 | Weighted median | 24 | -0.023762782 | 0.0146224515068399 | 0.104143638851268 | -0.052422787 | 0.00489722277094818 | 0.976517329594365 | 0.948927587644103 | 1.00490923376522 | 0.88227178111339 |
| ebi-a-GCST90002107 | finngen_R11_O15_PREECLAMPS | Pre-eclampsia \|\| id:finngen_R11_O15_PREECLAMPS | HLA DR on Hematopoietic Stem Cell \|\| id:ebi-a-GCST90002107 | Weighted mode | 24 | -0.026981524 | 0.0165835617404601 | 0.117360112946702 | -0.059485305 | 0.00552225713005498 | 0.973379225628363 | 0.942249379945377 | 1.00553753289792 | 0.872755742358858 |
| ebi-a-GCST90002108 | finngen_R11_O15_PREECLAMPS | Pre-eclampsia \|\| id:finngen_R11_O15_PREECLAMPS | HLA DR on CD33+ HLA DR+ CD14- \|\| id:ebi-a-GCST90002108 | Inverse variance weighted | 25 | 0.00372262194594613 | 0.00902714597251447 | 0.680060311976437 | -0.013970584 | 0.0214158280520745 | 1.00372955950899 | 0.986126551576904 | 1.0216467927175 | 0.950911139356934 |
| ebi-a-GCST90002108 | finngen_R11_O15_PREECLAMPS | Pre-eclampsia \|\| id:finngen_R11_O15_PREECLAMPS | HLA DR on CD33+ HLA DR+ CD14- \|\| id:ebi-a-GCST90002108 | MR Egger | 25 | 0.000768916195500857 | 0.0138778345731428 | 0.956293457571502 | -0.02643164 | 0.0279694719588607 | 1.00076921188734 | 0.973914618784172 | 1.02836428999487 | 0.99380795269851 |
| ebi-a-GCST90002108 | finngen_R11_O15_PREECLAMPS | Pre-eclampsia \|\| id:finngen_R11_O15_PREECLAMPS | HLA DR on CD33+ HLA DR+ CD14- \|\| id:ebi-a-GCST90002108 | Weighted median | 25 | -0.002146172 | 0.0127576306133383 | 0.866405087390347 | -0.027151128 | 0.0228587843479397 | 0.997856129725502 | 0.973214150836142 | 1.02312204849804 | 0.964912412213677 |
| ebi-a-GCST90002108 | finngen_R11_O15_PREECLAMPS | Pre-eclampsia \|\| id:finngen_R11_O15_PREECLAMPS | HLA DR on CD33+ HLA DR+ CD14- \|\| id:ebi-a-GCST90002108 | Weighted mode | 25 | -0.002525549 | 0.0143268037244056 | 0.861551927911343 | -0.030606084 | 0.0255549862038009 | 0.997477637419952 | 0.969857539858483 | 1.02588431420358 | 0.977585971930092 |
| ebi-a-GCST90002109 | finngen_R11_O15_PREECLAMPS | Pre-eclampsia \|\| id:finngen_R11_O15_PREECLAMPS | HLA DR on CD33+ HLA DR+ CD14dim \|\| id:ebi-a-GCST90002109 | Inverse variance weighted (multiplicative random effects) | 20 | 0.0203026709832221 | 0.019979646141214 | 0.309549536748678 | -0.018857435 | 0.0594627774200016 | 1.02051017210432 | 0.9813192536051 | 1.06126625717617 | 0.885079385681701 |
| ebi-a-GCST90002109 | finngen_R11_O15_PREECLAMPS | Pre-eclampsia \|\| id:finngen_R11_O15_PREECLAMPS | HLA DR on CD33+ HLA DR+ CD14dim \|\| id:ebi-a-GCST90002109 | MR Egger | 20 | 0.00103800571332599 | 0.034184285307099 | 0.976110113662197 | -0.065963193 | 0.0680392049152401 | 1.00103854462771 | 0.936165320637063 | 1.07040727288257 | 0.99380795269851 |
| ebi-a-GCST90002109 | finngen_R11_O15_PREECLAMPS | Pre-eclampsia \|\| id:finngen_R11_O15_PREECLAMPS | HLA DR on CD33+ HLA DR+ CD14dim \|\| id:ebi-a-GCST90002109 | Weighted median | 20 | 0.000867243855826377 | 0.0245191885669046 | 0.971784701189047 | -0.047190366 | 0.0489248534469593 | 1.00086762002051 | 0.953905789327926 | 1.05014143326596 | 0.990670780705477 |
| ebi-a-GCST90002109 | finngen_R11_O15_PREECLAMPS | Pre-eclampsia \|\| id:finngen_R11_O15_PREECLAMPS | HLA DR on CD33+ HLA DR+ CD14dim \|\| id:ebi-a-GCST90002109 | Weighted mode | 20 | 0.00223484566989207 | 0.0280576904387356 | 0.937347157615205 | -0.052758228 | 0.0572279189298139 | 1.00223734479885 | 0.948609332323972 | 1.05889712559395 | 0.990180645930572 |
| ebi-a-GCST90002110 | finngen_R11_O15_PREECLAMPS | Pre-eclampsia \|\| id:finngen_R11_O15_PREECLAMPS | HLA DR on CD33dim HLA DR+ CD11b+ \|\| id:ebi-a-GCST90002110 | Inverse variance weighted (multiplicative random effects) | 22 | -0.005355606 | 0.0154034729605464 | 0.728074368317019 | -0.035546413 | 0.0248352013968219 | 0.994658710082059 | 0.965077941405847 | 1.02514616394704 | 0.960612995947903 |
| ebi-a-GCST90002110 | finngen_R11_O15_PREECLAMPS | Pre-eclampsia \|\| id:finngen_R11_O15_PREECLAMPS | HLA DR on CD33dim HLA DR+ CD11b+ \|\| id:ebi-a-GCST90002110 | MR Egger | 22 | -0.011283662 | 0.0226421949737459 | 0.623675824691034 | -0.055662364 | 0.0330950403737699 | 0.988779759969447 | 0.945858437915499 | 1.03364877293887 | 0.99380795269851 |
| ebi-a-GCST90002110 | finngen_R11_O15_PREECLAMPS | Pre-eclampsia \|\| id:finngen_R11_O15_PREECLAMPS | HLA DR on CD33dim HLA DR+ CD11b+ \|\| id:ebi-a-GCST90002110 | Weighted median | 22 | -0.026385726 | 0.0169201981920666 | 0.118896585309394 | -0.059549314 | 0.0067778629210065 | 0.973959336158117 | 0.942189069341442 | 1.00680088461714 | 0.88227178111339 |
| ebi-a-GCST90002110 | finngen_R11_O15_PREECLAMPS | Pre-eclampsia \|\| id:finngen_R11_O15_PREECLAMPS | HLA DR on CD33dim HLA DR+ CD11b+ \|\| id:ebi-a-GCST90002110 | Weighted mode | 22 | -0.019062805 | 0.0157437161547509 | 0.239415784963498 | -0.049920489 | 0.0117948784697092 | 0.981117741018088 | 0.951305060846519 | 1.01186471233936 | 0.872755742358858 |
| ebi-a-GCST90002111 | finngen_R11_O15_PREECLAMPS | Pre-eclampsia \|\| id:finngen_R11_O15_PREECLAMPS | HLA DR on CD33dim HLA DR+ CD11b- \|\| id:ebi-a-GCST90002111 | Inverse variance weighted | 17 | 0.0430096338018653 | 0.0196766385506647 | 0.0288286143033598 | 0.00444342224256247 | 0.0815758453611682 | 1.0439479519899 | 1.00445330888125 | 1.08499550633939 | 0.726679898474346 |
| ebi-a-GCST90002111 | finngen_R11_O15_PREECLAMPS | Pre-eclampsia \|\| id:finngen_R11_O15_PREECLAMPS | HLA DR on CD33dim HLA DR+ CD11b- \|\| id:ebi-a-GCST90002111 | MR Egger | 17 | -0.005761718 | 0.0400532048262107 | 0.887532455928618 | -0.084266 | 0.0727425633382225 | 0.994254848743564 | 0.919186720291336 | 1.07545364007954 | 0.99380795269851 |
| ebi-a-GCST90002111 | finngen_R11_O15_PREECLAMPS | Pre-eclampsia \|\| id:finngen_R11_O15_PREECLAMPS | HLA DR on CD33dim HLA DR+ CD11b- \|\| id:ebi-a-GCST90002111 | Weighted median | 17 | 0.0458179122052954 | 0.0237043463269541 | 0.0532496552503453 | -0.000642607 | 0.0922784310061253 | 1.04688376884388 | 0.999357599831864 | 1.09667012654245 | 0.771214851704188 |
| ebi-a-GCST90002111 | finngen_R11_O15_PREECLAMPS | Pre-eclampsia \|\| id:finngen_R11_O15_PREECLAMPS | HLA DR on CD33dim HLA DR+ CD11b- \|\| id:ebi-a-GCST90002111 | Weighted mode | 17 | 0.0358203170373581 | 0.0303413262082015 | 0.255023929751768 | -0.023648682 | 0.0952893164054331 | 1.0364695938309 | 0.976628756433667 | 1.09997705050062 | 0.872755742358858 |
| ebi-a-GCST90002112 | finngen_R11_O15_PREECLAMPS | Pre-eclampsia \|\| id:finngen_R11_O15_PREECLAMPS | HLA DR on CD33- HLA DR+ \|\| id:ebi-a-GCST90002112 | Inverse variance weighted | 12 | 0.0396347258299822 | 0.0150595118942917 | 0.00849155472268462 | 0.0101180825171704 | 0.069151369142794 | 1.04043066232745 | 1.01016944339245 | 1.07159840380421 | 0.564302409298406 |
| ebi-a-GCST90002112 | finngen_R11_O15_PREECLAMPS | Pre-eclampsia \|\| id:finngen_R11_O15_PREECLAMPS | HLA DR on CD33- HLA DR+ \|\| id:ebi-a-GCST90002112 | MR Egger | 12 | 0.0578248953457278 | 0.0230633151531882 | 0.0310600078964549 | 0.0126207976454789 | 0.103028993045977 | 1.05952945092769 | 1.01270077602155 | 1.1085235480843 | 0.839403552053506 |
| ebi-a-GCST90002112 | finngen_R11_O15_PREECLAMPS | Pre-eclampsia \|\| id:finngen_R11_O15_PREECLAMPS | HLA DR on CD33- HLA DR+ \|\| id:ebi-a-GCST90002112 | Weighted median | 12 | 0.0513887136784186 | 0.0189319351029868 | 0.00663973292910448 | 0.0142821208765646 | 0.0884953064802727 | 1.05273202509835 | 1.0143845976456 | 1.09252912479145 | 0.507633389188193 |
| ebi-a-GCST90002112 | finngen_R11_O15_PREECLAMPS | Pre-eclampsia \|\| id:finngen_R11_O15_PREECLAMPS | HLA DR on CD33- HLA DR+ \|\| id:ebi-a-GCST90002112 | Weighted mode | 12 | 0.0508517619425313 | 0.0193704937639168 | 0.0236078339789933 | 0.0128855941652543 | 0.0888179297198083 | 1.05216691054325 | 1.01296897116962 | 1.09288165694145 | 0.784423938120185 |
| ebi-a-GCST90002113 | finngen_R11_O15_PREECLAMPS | Pre-eclampsia \|\| id:finngen_R11_O15_PREECLAMPS | HLA DR on HLA DR+ T cell \|\| id:ebi-a-GCST90002113 | Inverse variance weighted | 19 | 0.00304029715113467 | 0.0185102056875237 | 0.869534517006965 | -0.033239706 | 0.0393203002986812 | 1.00304492354186 | 0.9673066625892 | 1.04010357578854 | 0.981518940068169 |
| ebi-a-GCST90002113 | finngen_R11_O15_PREECLAMPS | Pre-eclampsia \|\| id:finngen_R11_O15_PREECLAMPS | HLA DR on HLA DR+ T cell \|\| id:ebi-a-GCST90002113 | MR Egger | 19 | -0.027331485 | 0.0229573053243247 | 0.25019561925977 | -0.072327804 | 0.017664832987889 | 0.973038639909156 | 0.930225914159691 | 1.01782177892942 | 0.99380795269851 |
| ebi-a-GCST90002113 | finngen_R11_O15_PREECLAMPS | Pre-eclampsia \|\| id:finngen_R11_O15_PREECLAMPS | HLA DR on HLA DR+ T cell \|\| id:ebi-a-GCST90002113 | Weighted median | 19 | -0.024803347 | 0.0235097109559051 | 0.291413605579093 | -0.070882381 | 0.0212756859940583 | 0.975501728040719 | 0.931571456230864 | 1.02150362706541 | 0.940845681141708 |
| ebi-a-GCST90002113 | finngen_R11_O15_PREECLAMPS | Pre-eclampsia \|\| id:finngen_R11_O15_PREECLAMPS | HLA DR on HLA DR+ T cell \|\| id:ebi-a-GCST90002113 | Weighted mode | 19 | -0.022758302 | 0.0197061597915288 | 0.263240481308596 | -0.061382375 | 0.0158657714093338 | 0.977498714921236 | 0.94046356128508 | 1.01599230103826 | 0.872755742358858 |
| ebi-a-GCST90002114 | finngen_R11_O15_PREECLAMPS | Pre-eclampsia \|\| id:finngen_R11_O15_PREECLAMPS | HLA DR on HLA DR+ CD4+ T cell \|\| id:ebi-a-GCST90002114 | Inverse variance weighted | 21 | -0.007817403 | 0.0243985383400906 | 0.748661668953835 | -0.055638538 | 0.0400037321960948 | 0.99221307347675 | 0.945880974042772 | 1.04081465870954 | 0.96638925304053 |
| ebi-a-GCST90002114 | finngen_R11_O15_PREECLAMPS | Pre-eclampsia \|\| id:finngen_R11_O15_PREECLAMPS | HLA DR on HLA DR+ CD4+ T cell \|\| id:ebi-a-GCST90002114 | MR Egger | 21 | -0.060148883 | 0.0489190839228907 | 0.233871682001908 | -0.156030288 | 0.0357325209916035 | 0.941624330824089 | 0.855533277598554 | 1.03637859989353 | 0.99380795269851 |
| ebi-a-GCST90002114 | finngen_R11_O15_PREECLAMPS | Pre-eclampsia \|\| id:finngen_R11_O15_PREECLAMPS | HLA DR on HLA DR+ CD4+ T cell \|\| id:ebi-a-GCST90002114 | Weighted median | 21 | -0.015108604 | 0.0347409093196376 | 0.663639561780164 | -0.083200786 | 0.0529835784307081 | 0.985004958477133 | 0.920166372051317 | 1.05441232987205 | 0.940845681141708 |
| ebi-a-GCST90002114 | finngen_R11_O15_PREECLAMPS | Pre-eclampsia \|\| id:finngen_R11_O15_PREECLAMPS | HLA DR on HLA DR+ CD4+ T cell \|\| id:ebi-a-GCST90002114 | Weighted mode | 21 | -0.016872022 | 0.0414168852201183 | 0.688063085526566 | -0.098049117 | 0.0643050726736527 | 0.983269513097077 | 0.906604372620473 | 1.06641768403526 | 0.959313411571287 |
| ebi-a-GCST90002115 | finngen_R11_O15_PREECLAMPS | Pre-eclampsia \|\| id:finngen_R11_O15_PREECLAMPS | HLA DR on HLA DR+ CD8+ T cell \|\| id:ebi-a-GCST90002115 | Inverse variance weighted | 20 | 0.0657027564987089 | 0.0270419967845183 | 0.0151131490479552 | 0.0127004428010531 | 0.118705070196365 | 1.06790924090263 | 1.01278143594445 | 1.12603776721256 | 0.588591724205005 |
| ebi-a-GCST90002115 | finngen_R11_O15_PREECLAMPS | Pre-eclampsia \|\| id:finngen_R11_O15_PREECLAMPS | HLA DR on HLA DR+ CD8+ T cell \|\| id:ebi-a-GCST90002115 | MR Egger | 20 | 0.0989201947136676 | 0.051825980906765 | 0.0723774011978033 | -0.002658728 | 0.200499117290927 | 1.10397819274746 | 0.997344803423065 | 1.22201253355802 | 0.896743733484648 |
| ebi-a-GCST90002115 | finngen_R11_O15_PREECLAMPS | Pre-eclampsia \|\| id:finngen_R11_O15_PREECLAMPS | HLA DR on HLA DR+ CD8+ T cell \|\| id:ebi-a-GCST90002115 | Weighted median | 20 | 0.0765808892529889 | 0.0367606497512293 | 0.0372302616493904 | 0.0045300157405795 | 0.148631762765398 | 1.07958951399467 | 1.00454029177289 | 1.16024566488046 | 0.722211404320042 |
| ebi-a-GCST90002115 | finngen_R11_O15_PREECLAMPS | Pre-eclampsia \|\| id:finngen_R11_O15_PREECLAMPS | HLA DR on HLA DR+ CD8+ T cell \|\| id:ebi-a-GCST90002115 | Weighted mode | 20 | 0.0901718783255446 | 0.0433968307990836 | 0.0515217268650039 | 0.00511408995934076 | 0.175229666691749 | 1.09436236471201 | 1.00512718923817 | 1.1915198376096 | 0.838871423749761 |
| ebi-a-GCST90002116 | finngen_R11_O15_PREECLAMPS | Pre-eclampsia \|\| id:finngen_R11_O15_PREECLAMPS | HLA DR on B cell \|\| id:ebi-a-GCST90002116 | Inverse variance weighted | 22 | 0.0372638785455445 | 0.0145813352821792 | 0.0106008697034035 | 0.00868446139247338 | 0.0658432956986157 | 1.03796688189466 | 1.00872228072822 | 1.06805933455969 | 0.588591724205005 |
| ebi-a-GCST90002116 | finngen_R11_O15_PREECLAMPS | Pre-eclampsia \|\| id:finngen_R11_O15_PREECLAMPS | HLA DR on B cell \|\| id:ebi-a-GCST90002116 | MR Egger | 22 | 0.0414101509833817 | 0.0240490399280871 | 0.100518150890728 | -0.005725967 | 0.0885462692424324 | 1.04227950985526 | 0.994290394830428 | 1.09258480451217 | 0.97261503632431 |
| ebi-a-GCST90002116 | finngen_R11_O15_PREECLAMPS | Pre-eclampsia \|\| id:finngen_R11_O15_PREECLAMPS | HLA DR on B cell \|\| id:ebi-a-GCST90002116 | Weighted median | 22 | 0.0197245032726215 | 0.0212345530670398 | 0.352947578132442 | -0.021895221 | 0.0613442272840195 | 1.0199203166086 | 0.978342739710425 | 1.06326485597357 | 0.940845681141708 |
| ebi-a-GCST90002116 | finngen_R11_O15_PREECLAMPS | Pre-eclampsia \|\| id:finngen_R11_O15_PREECLAMPS | HLA DR on B cell \|\| id:ebi-a-GCST90002116 | Weighted mode | 22 | 0.0175686843052925 | 0.0196514427681144 | 0.381445935226874 | -0.020948144 | 0.0560855121307966 | 1.01772392141077 | 0.979269744734533 | 1.05768812503493 | 0.920254054953283 |
| ebi-a-GCST90002117 | finngen_R11_O15_PREECLAMPS | Pre-eclampsia \|\| id:finngen_R11_O15_PREECLAMPS | HLA DR on HLA DR+ Natural Killer \|\| id:ebi-a-GCST90002117 | Inverse variance weighted (multiplicative random effects) | 28 | -0.002034622 | 0.00358361137191098 | 0.570199774156395 | -0.0090585 | 0.00498925675363316 | 0.997967446904015 | 0.990982404780644 | 1.00500172382029 | 0.912070098267669 |
| ebi-a-GCST90002117 | finngen_R11_O15_PREECLAMPS | Pre-eclampsia \|\| id:finngen_R11_O15_PREECLAMPS | HLA DR on HLA DR+ Natural Killer \|\| id:ebi-a-GCST90002117 | MR Egger | 28 | -0.003576383 | 0.00343122708118904 | 0.306865880521745 | -0.010301588 | 0.00314882168472426 | 0.996430004247531 | 0.989751291151848 | 1.00315378443129 | 0.99380795269851 |
| ebi-a-GCST90002117 | finngen_R11_O15_PREECLAMPS | Pre-eclampsia \|\| id:finngen_R11_O15_PREECLAMPS | HLA DR on HLA DR+ Natural Killer \|\| id:ebi-a-GCST90002117 | Weighted median | 28 | -0.003119506 | 0.00335409298361456 | 0.35234034850003 | -0.009693528 | 0.00345451665770621 | 0.996885355011847 | 0.9903533029622 | 1.00346049037717 | 0.940845681141708 |
| ebi-a-GCST90002117 | finngen_R11_O15_PREECLAMPS | Pre-eclampsia \|\| id:finngen_R11_O15_PREECLAMPS | HLA DR on HLA DR+ Natural Killer \|\| id:ebi-a-GCST90002117 | Weighted mode | 28 | -0.002468557 | 0.00332473335797943 | 0.464205964665963 | -0.008985035 | 0.00404792023827588 | 0.997534487238229 | 0.991055210273873 | 1.00405612413324 | 0.933945646747087 |
| ebi-a-GCST90002118 | finngen_R11_O15_PREECLAMPS | Pre-eclampsia \|\| id:finngen_R11_O15_PREECLAMPS | CD8 on CD28+ CD45RA- CD8+ T cell \|\| id:ebi-a-GCST90002118 | Inverse variance weighted | 13 | -0.043975953 | 0.034405203760498 | 0.201186891606441 | -0.111410152 | 0.0234582463321898 | 0.956976969584813 | 0.894571762910528 | 1.02373555513995 | 0.855687495048373 |
| ebi-a-GCST90002118 | finngen_R11_O15_PREECLAMPS | Pre-eclampsia \|\| id:finngen_R11_O15_PREECLAMPS | CD8 on CD28+ CD45RA- CD8+ T cell \|\| id:ebi-a-GCST90002118 | MR Egger | 13 | -0.007519718 | 0.0741448404936044 | 0.921042665910819 | -0.152843606 | 0.137804168910334 | 0.992508483890193 | 0.85826393879673 | 1.14775076298215 | 0.99380795269851 |
| ebi-a-GCST90002118 | finngen_R11_O15_PREECLAMPS | Pre-eclampsia \|\| id:finngen_R11_O15_PREECLAMPS | CD8 on CD28+ CD45RA- CD8+ T cell \|\| id:ebi-a-GCST90002118 | Weighted median | 13 | -0.037732038 | 0.0484434388874266 | 0.436045389732985 | -0.132681178 | 0.0572171020236479 | 0.962970945754036 | 0.875744253776011 | 1.05888567166505 | 0.940845681141708 |
| ebi-a-GCST90002118 | finngen_R11_O15_PREECLAMPS | Pre-eclampsia \|\| id:finngen_R11_O15_PREECLAMPS | CD8 on CD28+ CD45RA- CD8+ T cell \|\| id:ebi-a-GCST90002118 | Weighted mode | 13 | -0.025186341 | 0.0556144540592571 | 0.658718610736696 | -0.134190671 | 0.083817988795409 | 0.975128188579054 | 0.874423321435963 | 1.08743095117815 | 0.959313411571287 |
| ebi-a-GCST90002119 | finngen_R11_O15_PREECLAMPS | Pre-eclampsia \|\| id:finngen_R11_O15_PREECLAMPS | CD8 on CD28+ CD45RA+ CD8+ T cell \|\| id:ebi-a-GCST90002119 | Inverse variance weighted (multiplicative random effects) | 22 | 0.0224901118665738 | 0.0240163439792538 | 0.349041404362382 | -0.024581922 | 0.0695621460659113 | 1.02274492107621 | 0.975717752569756 | 1.07203868212123 | 0.885079385681701 |
| ebi-a-GCST90002119 | finngen_R11_O15_PREECLAMPS | Pre-eclampsia \|\| id:finngen_R11_O15_PREECLAMPS | CD8 on CD28+ CD45RA+ CD8+ T cell \|\| id:ebi-a-GCST90002119 | MR Egger | 22 | -0.000820478 | 0.0443285167724877 | 0.985416182756504 | -0.087704371 | 0.0860634151132774 | 0.999179858739043 | 0.916031642551111 | 1.08987544068825 | 0.99380795269851 |
| ebi-a-GCST90002119 | finngen_R11_O15_PREECLAMPS | Pre-eclampsia \|\| id:finngen_R11_O15_PREECLAMPS | CD8 on CD28+ CD45RA+ CD8+ T cell \|\| id:ebi-a-GCST90002119 | Weighted median | 22 | 0.0162465544708371 | 0.025458782296992 | 0.52337520923312 | -0.033652659 | 0.0661457677729414 | 1.01637924736374 | 0.966907293026781 | 1.06838244154507 | 0.940845681141708 |
| ebi-a-GCST90002119 | finngen_R11_O15_PREECLAMPS | Pre-eclampsia \|\| id:finngen_R11_O15_PREECLAMPS | CD8 on CD28+ CD45RA+ CD8+ T cell \|\| id:ebi-a-GCST90002119 | Weighted mode | 22 | 0.0102022754786116 | 0.0232805654547857 | 0.665693397521161 | -0.035427633 | 0.0558321837699916 | 1.01025449612982 | 0.96519257997485 | 1.05742021657181 | 0.959313411571287 |
| ebi-a-GCST90002120 | finngen_R11_O15_PREECLAMPS | Pre-eclampsia \|\| id:finngen_R11_O15_PREECLAMPS | CD8 on CD28- CD8+ T cell \|\| id:ebi-a-GCST90002120 | Inverse variance weighted | 20 | -0.014403593 | 0.0293671968644407 | 0.623804620973034 | -0.071963299 | 0.0431561124424507 | 0.985699642091302 | 0.93056504760464 | 1.04410087926676 | 0.932517746280752 |
| ebi-a-GCST90002120 | finngen_R11_O15_PREECLAMPS | Pre-eclampsia \|\| id:finngen_R11_O15_PREECLAMPS | CD8 on CD28- CD8+ T cell \|\| id:ebi-a-GCST90002120 | MR Egger | 20 | 0.0272335512590615 | 0.0893324140180176 | 0.76397239541313 | -0.14785798 | 0.202325082734376 | 1.02760777382903 | 0.862553605920351 | 1.22424592464269 | 0.99380795269851 |
| ebi-a-GCST90002120 | finngen_R11_O15_PREECLAMPS | Pre-eclampsia \|\| id:finngen_R11_O15_PREECLAMPS | CD8 on CD28- CD8+ T cell \|\| id:ebi-a-GCST90002120 | Weighted median | 20 | -0.031371854 | 0.0418435201548524 | 0.453409972374332 | -0.113385153 | 0.0506414456848793 | 0.969115136901045 | 0.892806726409955 | 1.05194564600478 | 0.940845681141708 |
| ebi-a-GCST90002120 | finngen_R11_O15_PREECLAMPS | Pre-eclampsia \|\| id:finngen_R11_O15_PREECLAMPS | CD8 on CD28- CD8+ T cell \|\| id:ebi-a-GCST90002120 | Weighted mode | 20 | -0.030445323 | 0.0607818941028758 | 0.622198493207287 | -0.149577835 | 0.0886871896272959 | 0.970013468223758 | 0.861071413697408 | 1.0927387828324 | 0.959313411571287 |
| ebi-a-GCST90002121 | finngen_R11_O15_PREECLAMPS | Pre-eclampsia \|\| id:finngen_R11_O15_PREECLAMPS | CD8 on CD39+ CD8+ T cell \|\| id:ebi-a-GCST90002121 | Inverse variance weighted (multiplicative random effects) | 25 | 0.00843007429767334 | 0.0313443138463286 | 0.787967641804848 | -0.053004781 | 0.0698649294364774 | 1.00846570743362 | 0.948375478438965 | 1.0723633267528 | 0.977824840984872 |
| ebi-a-GCST90002121 | finngen_R11_O15_PREECLAMPS | Pre-eclampsia \|\| id:finngen_R11_O15_PREECLAMPS | CD8 on CD39+ CD8+ T cell \|\| id:ebi-a-GCST90002121 | MR Egger | 25 | 0.0394375532291968 | 0.066146887944809 | 0.55685667669333 | -0.090210347 | 0.169085453601022 | 1.04022553813092 | 0.913738962675293 | 1.18422133057742 | 0.99380795269851 |
| ebi-a-GCST90002121 | finngen_R11_O15_PREECLAMPS | Pre-eclampsia \|\| id:finngen_R11_O15_PREECLAMPS | CD8 on CD39+ CD8+ T cell \|\| id:ebi-a-GCST90002121 | Weighted median | 25 | 0.00176337812747675 | 0.0361837599969661 | 0.961131307114545 | -0.069156791 | 0.0726835477215304 | 1.00176493379296 | 0.933180353890886 | 1.07539017339252 | 0.989912389390616 |
| ebi-a-GCST90002121 | finngen_R11_O15_PREECLAMPS | Pre-eclampsia \|\| id:finngen_R11_O15_PREECLAMPS | CD8 on CD39+ CD8+ T cell \|\| id:ebi-a-GCST90002121 | Weighted mode | 25 | 0.0296305177686984 | 0.0381144270107434 | 0.444513236817683 | -0.045073759 | 0.104334794709756 | 1.03007386964174 | 0.955926970850511 | 1.10997200547094 | 0.933945646747087 |

| **id.exposure** | **id.outcome** | **outcome** | **exposure** | **method** | **nsnp** | **b** | **se** | **pval** | **lo_ci** | **up_ci** | **or** | **or_lci95** | **or_uci95** | **p_fdr** |
| --- | --- | --- | --- | --- | --- | --- | --- | --- | --- | --- | --- | --- | --- | --- |
| ebi-a-GCST90001391 | finngen_R11_O15_POOR_FETGRO | Poor fetal growth \|\| id:finngen_R11_O15_POOR_FETGRO | IgD+ B cell %B cell \|\| id:ebi-a-GCST90001391 | Inverse variance weighted | 17 | 0.051975415163576 | 0.0329994471826574 | 0.115247450616039 | -0.012703501 | 0.116654331641584 | 1.05334984576138 | 0.987376847561163 | 1.12373092432349 | 0.994119304454654 |
| ebi-a-GCST90001391 | finngen_R11_O15_POOR_FETGRO | Poor fetal growth \|\| id:finngen_R11_O15_POOR_FETGRO | IgD+ B cell %B cell \|\| id:ebi-a-GCST90001391 | MR Egger | 17 | 0.0188989927661923 | 0.0529628706274909 | 0.726185615159215 | -0.084908234 | 0.122706219196074 | 1.01907870909724 | 0.918596576776195 | 1.13055223761009 | 0.992899239648112 |
| ebi-a-GCST90001391 | finngen_R11_O15_POOR_FETGRO | Poor fetal growth \|\| id:finngen_R11_O15_POOR_FETGRO | IgD+ B cell %B cell \|\| id:ebi-a-GCST90001391 | Weighted median | 17 | 0.0406668562445631 | 0.0463354469988886 | 0.380127228855076 | -0.05015062 | 0.131484332362385 | 1.04150507683086 | 0.951086161234774 | 1.14052003832773 | 0.999440741871876 |
| ebi-a-GCST90001391 | finngen_R11_O15_POOR_FETGRO | Poor fetal growth \|\| id:finngen_R11_O15_POOR_FETGRO | IgD+ B cell %B cell \|\| id:ebi-a-GCST90001391 | Weighted mode | 17 | 0.0551554735486717 | 0.0493916293792206 | 0.280613615795691 | -0.04165212 | 0.151963067131944 | 1.0567048915642 | 0.959203410186737 | 1.16411724144967 | 0.999857399747617 |
| ebi-a-GCST90001392 | finngen_R11_O15_POOR_FETGRO | Poor fetal growth \|\| id:finngen_R11_O15_POOR_FETGRO | IgD+ CD38+ B cell Absolute Count \|\| id:ebi-a-GCST90001392 | Inverse variance weighted | 29 | 0.0269962539689498 | 0.0115382256414776 | 0.0192980377013469 | 0.00438133171165374 | 0.0496111762262459 | 1.02736395421901 | 1.00439094377819 | 1.05086241663844 | 0.994119304454654 |
| ebi-a-GCST90001392 | finngen_R11_O15_POOR_FETGRO | Poor fetal growth \|\| id:finngen_R11_O15_POOR_FETGRO | IgD+ CD38+ B cell Absolute Count \|\| id:ebi-a-GCST90001392 | MR Egger | 29 | 0.024211012638823 | 0.0126606031513342 | 0.0665005103404163 | -0.00060377 | 0.0490257948154379 | 1.02450647889907 | 0.999396412694358 | 1.05024744132954 | 0.992899239648112 |
| ebi-a-GCST90001392 | finngen_R11_O15_POOR_FETGRO | Poor fetal growth \|\| id:finngen_R11_O15_POOR_FETGRO | IgD+ CD38+ B cell Absolute Count \|\| id:ebi-a-GCST90001392 | Weighted median | 29 | 0.0283531163717102 | 0.0188703557554277 | 0.132962464096774 | -0.008632781 | 0.0653390136523484 | 1.02875889189767 | 0.991404374549001 | 1.06752086719405 | 0.999440741871876 |
| ebi-a-GCST90001392 | finngen_R11_O15_POOR_FETGRO | Poor fetal growth \|\| id:finngen_R11_O15_POOR_FETGRO | IgD+ CD38+ B cell Absolute Count \|\| id:ebi-a-GCST90001392 | Weighted mode | 29 | 0.0270934888409283 | 0.0137985704025707 | 0.0595890620431186 | 4.82908518898012e-05 | 0.0541386868299669 | 1.0274638546784 | 1.00004829201791 | 1.05563099411972 | 0.999857399747617 |
| ebi-a-GCST90001393 | finngen_R11_O15_POOR_FETGRO | Poor fetal growth \|\| id:finngen_R11_O15_POOR_FETGRO | IgD+ CD38dim B cell %B cell \|\| id:ebi-a-GCST90001393 | Inverse variance weighted | 15 | 0.0555539978229132 | 0.0413731480773544 | 0.179351347329753 | -0.025537372 | 0.136645368054528 | 1.05712609803912 | 0.97478594818614 | 1.14642151872918 | 0.994119304454654 |
| ebi-a-GCST90001393 | finngen_R11_O15_POOR_FETGRO | Poor fetal growth \|\| id:finngen_R11_O15_POOR_FETGRO | IgD+ CD38dim B cell %B cell \|\| id:ebi-a-GCST90001393 | MR Egger | 15 | 0.0931929951821013 | 0.0614691981728113 | 0.153429348598356 | -0.027286633 | 0.213672623600811 | 1.0976735605355 | 0.973082283822474 | 1.23821722533641 | 0.992899239648112 |
| ebi-a-GCST90001393 | finngen_R11_O15_POOR_FETGRO | Poor fetal growth \|\| id:finngen_R11_O15_POOR_FETGRO | IgD+ CD38dim B cell %B cell \|\| id:ebi-a-GCST90001393 | Weighted median | 15 | 0.0402394163383627 | 0.0530491855854828 | 0.448133561956667 | -0.063736987 | 0.144215820085909 | 1.04105999112899 | 0.938251739102239 | 1.15513338261064 | 0.999440741871876 |
| ebi-a-GCST90001393 | finngen_R11_O15_POOR_FETGRO | Poor fetal growth \|\| id:finngen_R11_O15_POOR_FETGRO | IgD+ CD38dim B cell %B cell \|\| id:ebi-a-GCST90001393 | Weighted mode | 15 | 0.0374328844929146 | 0.0495455991797196 | 0.462457554558374 | -0.05967649 | 0.134542258885165 | 1.03814231929543 | 0.942069253210591 | 1.14401300269502 | 0.999857399747617 |
| ebi-a-GCST90001394 | finngen_R11_O15_POOR_FETGRO | Poor fetal growth \|\| id:finngen_R11_O15_POOR_FETGRO | IgD+ CD38dim B cell Absolute Count \|\| id:ebi-a-GCST90001394 | Inverse variance weighted | 24 | -0.000125293 | 0.0123108405884902 | 0.991879711161886 | -0.02425454 | 0.0240039546074258 | 0.999874714902818 | 0.976037237130251 | 1.02429436856473 | 0.998984604424952 |
| ebi-a-GCST90001394 | finngen_R11_O15_POOR_FETGRO | Poor fetal growth \|\| id:finngen_R11_O15_POOR_FETGRO | IgD+ CD38dim B cell Absolute Count \|\| id:ebi-a-GCST90001394 | MR Egger | 24 | -0.009182767 | 0.0144269651662304 | 0.531017794303134 | -0.037459619 | 0.0190940847579819 | 0.99085926587909 | 0.963233313555014 | 1.01927754258717 | 0.992899239648112 |
| ebi-a-GCST90001394 | finngen_R11_O15_POOR_FETGRO | Poor fetal growth \|\| id:finngen_R11_O15_POOR_FETGRO | IgD+ CD38dim B cell Absolute Count \|\| id:ebi-a-GCST90001394 | Weighted median | 24 | -0.010465104 | 0.0165410591315243 | 0.526946332591521 | -0.042885579 | 0.0219553722975787 | 0.989589465074968 | 0.958021001039909 | 1.02219816509697 | 0.999440741871876 |
| ebi-a-GCST90001394 | finngen_R11_O15_POOR_FETGRO | Poor fetal growth \|\| id:finngen_R11_O15_POOR_FETGRO | IgD+ CD38dim B cell Absolute Count \|\| id:ebi-a-GCST90001394 | Weighted mode | 24 | -0.006793159 | 0.012808270492657 | 0.600937193374446 | -0.031897369 | 0.0183110510175743 | 0.993229862198851 | 0.968605985675302 | 1.01847972627979 | 0.999857399747617 |
| ebi-a-GCST90001395 | finngen_R11_O15_POOR_FETGRO | Poor fetal growth \|\| id:finngen_R11_O15_POOR_FETGRO | IgD+ CD38- B cell %B cell \|\| id:ebi-a-GCST90001395 | Inverse variance weighted (multiplicative random effects) | 22 | 0.00181448708233955 | 0.0217061381718911 | 0.933379817476886 | -0.040729544 | 0.0443585178992461 | 1.00181613426014 | 0.960088756857018 | 1.04535706693354 | 0.998984604424952 |
| ebi-a-GCST90001395 | finngen_R11_O15_POOR_FETGRO | Poor fetal growth \|\| id:finngen_R11_O15_POOR_FETGRO | IgD+ CD38- B cell %B cell \|\| id:ebi-a-GCST90001395 | MR Egger | 22 | 0.00515181315331905 | 0.0249425118035308 | 0.838451507469658 | -0.04373551 | 0.0540391362882394 | 1.00516510656128 | 0.957207095717659 | 1.05552591071303 | 0.992899239648112 |
| ebi-a-GCST90001395 | finngen_R11_O15_POOR_FETGRO | Poor fetal growth \|\| id:finngen_R11_O15_POOR_FETGRO | IgD+ CD38- B cell %B cell \|\| id:ebi-a-GCST90001395 | Weighted median | 22 | -0.004518728 | 0.022641858271794 | 0.841813718253081 | -0.04889677 | 0.0398593146749452 | 0.995491466550959 | 0.952279428667731 | 1.04066435768987 | 0.999440741871876 |
| ebi-a-GCST90001395 | finngen_R11_O15_POOR_FETGRO | Poor fetal growth \|\| id:finngen_R11_O15_POOR_FETGRO | IgD+ CD38- B cell %B cell \|\| id:ebi-a-GCST90001395 | Weighted mode | 22 | 0.00982292123668072 | 0.0175871593487172 | 0.582390951894294 | -0.024647911 | 0.0442937535601665 | 1.00987132448477 | 0.975653368295311 | 1.04528936726629 | 0.999857399747617 |
| ebi-a-GCST90001396 | finngen_R11_O15_POOR_FETGRO | Poor fetal growth \|\| id:finngen_R11_O15_POOR_FETGRO | IgD+ CD38- B cell Absolute Count \|\| id:ebi-a-GCST90001396 | Inverse variance weighted | 18 | -0.007767866 | 0.0383451988407671 | 0.839465442785906 | -0.082924456 | 0.0673887233053001 | 0.992262225484726 | 0.920420676714901 | 1.06971121904607 | 0.994119304454654 |
| ebi-a-GCST90001396 | finngen_R11_O15_POOR_FETGRO | Poor fetal growth \|\| id:finngen_R11_O15_POOR_FETGRO | IgD+ CD38- B cell Absolute Count \|\| id:ebi-a-GCST90001396 | MR Egger | 18 | -0.088174533 | 0.0878415020996358 | 0.330419474194919 | -0.260343877 | 0.0839948113080941 | 0.9156010603538 | 0.770786484540884 | 1.08762325045223 | 0.992899239648112 |
| ebi-a-GCST90001396 | finngen_R11_O15_POOR_FETGRO | Poor fetal growth \|\| id:finngen_R11_O15_POOR_FETGRO | IgD+ CD38- B cell Absolute Count \|\| id:ebi-a-GCST90001396 | Weighted median | 18 | 0.00621383071514418 | 0.0548761506500664 | 0.90984526233701 | -0.101343425 | 0.113771085989274 | 1.00623317661117 | 0.903622653381715 | 1.12049559838259 | 0.999440741871876 |
| ebi-a-GCST90001396 | finngen_R11_O15_POOR_FETGRO | Poor fetal growth \|\| id:finngen_R11_O15_POOR_FETGRO | IgD+ CD38- B cell Absolute Count \|\| id:ebi-a-GCST90001396 | Weighted mode | 18 | 0.0157482466282353 | 0.0737134734490636 | 0.833368018781345 | -0.128730161 | 0.1602266545884 | 1.01587290378169 | 0.879211178712918 | 1.17377688276051 | 0.999857399747617 |
| ebi-a-GCST90001397 | finngen_R11_O15_POOR_FETGRO | Poor fetal growth \|\| id:finngen_R11_O15_POOR_FETGRO | Unswitched memory B cell %B cell \|\| id:ebi-a-GCST90001397 | Inverse variance weighted (multiplicative random effects) | 31 | -0.041021357 | 0.0234822719359282 | 0.0806533842392246 | -0.08704661 | 0.00500389597617527 | 0.959808631078458 | 0.916634370297839 | 1.00501643637187 | 0.994119304454654 |
| ebi-a-GCST90001397 | finngen_R11_O15_POOR_FETGRO | Poor fetal growth \|\| id:finngen_R11_O15_POOR_FETGRO | Unswitched memory B cell %B cell \|\| id:ebi-a-GCST90001397 | MR Egger | 31 | -0.014111243 | 0.0318742405624071 | 0.661255730009048 | -0.076584755 | 0.0483622681482693 | 0.985987853565913 | 0.926274404707708 | 1.04955080528895 | 0.992899239648112 |
| ebi-a-GCST90001397 | finngen_R11_O15_POOR_FETGRO | Poor fetal growth \|\| id:finngen_R11_O15_POOR_FETGRO | Unswitched memory B cell %B cell \|\| id:ebi-a-GCST90001397 | Weighted median | 31 | -0.020195503 | 0.037092337060388 | 0.586121085051067 | -0.092896484 | 0.0525054775090333 | 0.98000706012986 | 0.911287828496364 | 1.05390833485515 | 0.999440741871876 |
| ebi-a-GCST90001397 | finngen_R11_O15_POOR_FETGRO | Poor fetal growth \|\| id:finngen_R11_O15_POOR_FETGRO | Unswitched memory B cell %B cell \|\| id:ebi-a-GCST90001397 | Weighted mode | 31 | -0.005331063 | 0.0333351762302042 | 0.87401360774735 | -0.070668009 | 0.0600058820293974 | 0.994683121518526 | 0.931771180623121 | 1.06184279231751 | 0.999857399747617 |
| ebi-a-GCST90001398 | finngen_R11_O15_POOR_FETGRO | Poor fetal growth \|\| id:finngen_R11_O15_POOR_FETGRO | Unswitched memory B cell Absolute Count \|\| id:ebi-a-GCST90001398 | Inverse variance weighted | 20 | -0.060272275 | 0.04405403300852 | 0.171266906166169 | -0.14661818 | 0.0260736296574981 | 0.941508149514024 | 0.863623663487178 | 1.02641652038809 | 0.994119304454654 |
| ebi-a-GCST90001398 | finngen_R11_O15_POOR_FETGRO | Poor fetal growth \|\| id:finngen_R11_O15_POOR_FETGRO | Unswitched memory B cell Absolute Count \|\| id:ebi-a-GCST90001398 | MR Egger | 20 | -0.089327572 | 0.0784034351860949 | 0.269500232662315 | -0.242998305 | 0.0643431608675847 | 0.914545944769035 | 0.784272843064347 | 1.06645830273236 | 0.992899239648112 |
| ebi-a-GCST90001398 | finngen_R11_O15_POOR_FETGRO | Poor fetal growth \|\| id:finngen_R11_O15_POOR_FETGRO | Unswitched memory B cell Absolute Count \|\| id:ebi-a-GCST90001398 | Weighted median | 20 | -0.036574725 | 0.0576892783659283 | 0.526083966038945 | -0.14964571 | 0.076496261027147 | 0.964086050289023 | 0.861012970535002 | 1.07949815411533 | 0.999440741871876 |
| ebi-a-GCST90001398 | finngen_R11_O15_POOR_FETGRO | Poor fetal growth \|\| id:finngen_R11_O15_POOR_FETGRO | Unswitched memory B cell Absolute Count \|\| id:ebi-a-GCST90001398 | Weighted mode | 20 | -0.030395334 | 0.0696231205008527 | 0.667344352213791 | -0.16685665 | 0.106065982047328 | 0.970061959128602 | 0.846320922478812 | 1.11189523921049 | 0.999857399747617 |
| ebi-a-GCST90001399 | finngen_R11_O15_POOR_FETGRO | Poor fetal growth \|\| id:finngen_R11_O15_POOR_FETGRO | IgD- CD27- B cell %B cell \|\| id:ebi-a-GCST90001399 | Inverse variance weighted | 15 | 0.0105999845249706 | 0.040086483961988 | 0.791450220043351 | -0.067969524 | 0.089169493090467 | 1.01065636338988 | 0.934288946495412 | 1.09326594164675 | 0.994119304454654 |
| ebi-a-GCST90001399 | finngen_R11_O15_POOR_FETGRO | Poor fetal growth \|\| id:finngen_R11_O15_POOR_FETGRO | IgD- CD27- B cell %B cell \|\| id:ebi-a-GCST90001399 | MR Egger | 15 | 0.0356875107892233 | 0.0775256846429591 | 0.652883538035087 | -0.116262831 | 0.187637852689423 | 1.0363319533328 | 0.890241209459642 | 1.20639654296667 | 0.992899239648112 |
| ebi-a-GCST90001399 | finngen_R11_O15_POOR_FETGRO | Poor fetal growth \|\| id:finngen_R11_O15_POOR_FETGRO | IgD- CD27- B cell %B cell \|\| id:ebi-a-GCST90001399 | Weighted median | 15 | 0.0195400740632819 | 0.047763767146956 | 0.682467552644206 | -0.07407691 | 0.113157057671316 | 1.01973223085581 | 0.928600272870602 | 1.11980779354247 | 0.999440741871876 |
| ebi-a-GCST90001399 | finngen_R11_O15_POOR_FETGRO | Poor fetal growth \|\| id:finngen_R11_O15_POOR_FETGRO | IgD- CD27- B cell %B cell \|\| id:ebi-a-GCST90001399 | Weighted mode | 15 | 0.0202746174640723 | 0.0438600660226912 | 0.65099923159727 | -0.065691112 | 0.106240346868547 | 1.02048154360423 | 0.936420068601399 | 1.1120891315286 | 0.999857399747617 |
| ebi-a-GCST90001400 | finngen_R11_O15_POOR_FETGRO | Poor fetal growth \|\| id:finngen_R11_O15_POOR_FETGRO | IgD+ B cell Absolute Count \|\| id:ebi-a-GCST90001400 | Inverse variance weighted | 14 | -0.005056184 | 0.0150810970225325 | 0.737424206724942 | -0.034615134 | 0.0245027664443228 | 0.994956577260717 | 0.965977116586548 | 1.02480542617043 | 0.994119304454654 |
| ebi-a-GCST90001400 | finngen_R11_O15_POOR_FETGRO | Poor fetal growth \|\| id:finngen_R11_O15_POOR_FETGRO | IgD+ B cell Absolute Count \|\| id:ebi-a-GCST90001400 | MR Egger | 14 | -0.008452556 | 0.0184005741625126 | 0.654183310539084 | -0.044517682 | 0.0276125689770286 | 0.991583066035709 | 0.956458688090241 | 1.02799732920196 | 0.992899239648112 |
| ebi-a-GCST90001400 | finngen_R11_O15_POOR_FETGRO | Poor fetal growth \|\| id:finngen_R11_O15_POOR_FETGRO | IgD+ B cell Absolute Count \|\| id:ebi-a-GCST90001400 | Weighted median | 14 | -0.012100521 | 0.0192438411070798 | 0.529480217659964 | -0.049818449 | 0.0256174076778131 | 0.987972396003724 | 0.951402136392045 | 1.02594835341333 | 0.999440741871876 |
| ebi-a-GCST90001400 | finngen_R11_O15_POOR_FETGRO | Poor fetal growth \|\| id:finngen_R11_O15_POOR_FETGRO | IgD+ B cell Absolute Count \|\| id:ebi-a-GCST90001400 | Weighted mode | 14 | -0.009035268 | 0.0182311126438653 | 0.628451503339864 | -0.044768249 | 0.0266977124946325 | 0.991005427092383 | 0.956219060813819 | 1.02705728924784 | 0.999857399747617 |
| ebi-a-GCST90001401 | finngen_R11_O15_POOR_FETGRO | Poor fetal growth \|\| id:finngen_R11_O15_POOR_FETGRO | IgD- CD27- B cell Absolute Count \|\| id:ebi-a-GCST90001401 | Inverse variance weighted | 26 | -0.016075734 | 0.0316746731888163 | 0.611785484582563 | -0.078158094 | 0.0460066251060215 | 0.984052790640485 | 0.924818206966413 | 1.0470813479589 | 0.994119304454654 |
| ebi-a-GCST90001401 | finngen_R11_O15_POOR_FETGRO | Poor fetal growth \|\| id:finngen_R11_O15_POOR_FETGRO | IgD- CD27- B cell Absolute Count \|\| id:ebi-a-GCST90001401 | MR Egger | 26 | -0.035721755 | 0.0708941824597398 | 0.61894389265148 | -0.174674352 | 0.103230842978453 | 0.96490873751123 | 0.839730432563269 | 1.10874732607165 | 0.992899239648112 |
| ebi-a-GCST90001401 | finngen_R11_O15_POOR_FETGRO | Poor fetal growth \|\| id:finngen_R11_O15_POOR_FETGRO | IgD- CD27- B cell Absolute Count \|\| id:ebi-a-GCST90001401 | Weighted median | 26 | -0.001211306 | 0.0439303925389727 | 0.978002468669979 | -0.087314876 | 0.0848922630205948 | 0.998789426979624 | 0.916388501699491 | 1.08859977792849 | 0.999440741871876 |
| ebi-a-GCST90001401 | finngen_R11_O15_POOR_FETGRO | Poor fetal growth \|\| id:finngen_R11_O15_POOR_FETGRO | IgD- CD27- B cell Absolute Count \|\| id:ebi-a-GCST90001401 | Weighted mode | 26 | -0.015311026 | 0.0554791749719566 | 0.784833583503493 | -0.124050209 | 0.0934281570649903 | 0.984805591938601 | 0.88333548853229 | 1.09793172187046 | 0.999857399747617 |
| ebi-a-GCST90001402 | finngen_R11_O15_POOR_FETGRO | Poor fetal growth \|\| id:finngen_R11_O15_POOR_FETGRO | Switched memory B cell %B cell \|\| id:ebi-a-GCST90001402 | Inverse variance weighted | 13 | -0.108668194 | 0.0376780643840143 | 0.00392504683447102 | -0.1825172 | -0.034819188 | 0.897028007267014 | 0.833170312992145 | 0.965780025132767 | 0.956403078666104 |
| ebi-a-GCST90001402 | finngen_R11_O15_POOR_FETGRO | Poor fetal growth \|\| id:finngen_R11_O15_POOR_FETGRO | Switched memory B cell %B cell \|\| id:ebi-a-GCST90001402 | MR Egger | 13 | -0.111496757 | 0.0583127490277154 | 0.0822546887563065 | -0.225789745 | 0.00279623071398324 | 0.894494291903343 | 0.797885843140078 | 1.00280014381355 | 0.992899239648112 |
| ebi-a-GCST90001402 | finngen_R11_O15_POOR_FETGRO | Poor fetal growth \|\| id:finngen_R11_O15_POOR_FETGRO | Switched memory B cell %B cell \|\| id:ebi-a-GCST90001402 | Weighted median | 13 | -0.116538804 | 0.0491734648061478 | 0.0177903433656895 | -0.212918795 | -0.020158813 | 0.889995561137889 | 0.808221766382969 | 0.980043017636102 | 0.999440741871876 |
| ebi-a-GCST90001402 | finngen_R11_O15_POOR_FETGRO | Poor fetal growth \|\| id:finngen_R11_O15_POOR_FETGRO | Switched memory B cell %B cell \|\| id:ebi-a-GCST90001402 | Weighted mode | 13 | -0.110521112 | 0.0608345343953841 | 0.0942999349640489 | -0.2297568 | 0.00871457532211474 | 0.895367426909702 | 0.794726856965377 | 1.0087526577774 | 0.999857399747617 |
| ebi-a-GCST90001403 | finngen_R11_O15_POOR_FETGRO | Poor fetal growth \|\| id:finngen_R11_O15_POOR_FETGRO | Switched memory B cell Absolute Count \|\| id:ebi-a-GCST90001403 | Inverse variance weighted | 23 | 0.0426036526273368 | 0.0302848421615807 | 0.15949711709272 | -0.016754638 | 0.101961943264035 | 1.04352421479468 | 0.983384940322733 | 1.10734132912945 | 0.994119304454654 |
| ebi-a-GCST90001403 | finngen_R11_O15_POOR_FETGRO | Poor fetal growth \|\| id:finngen_R11_O15_POOR_FETGRO | Switched memory B cell Absolute Count \|\| id:ebi-a-GCST90001403 | MR Egger | 23 | 0.0575937632810471 | 0.0436184889421164 | 0.200913235601367 | -0.027898475 | 0.143086001607595 | 1.05928458799703 | 0.972487093497469 | 1.15382902855046 | 0.992899239648112 |
| ebi-a-GCST90001403 | finngen_R11_O15_POOR_FETGRO | Poor fetal growth \|\| id:finngen_R11_O15_POOR_FETGRO | Switched memory B cell Absolute Count \|\| id:ebi-a-GCST90001403 | Weighted median | 23 | 0.0700075814158396 | 0.0426336694148724 | 0.100574954273016 | -0.013554411 | 0.153569573468989 | 1.07251631241555 | 0.986537036748476 | 1.16598890619322 | 0.999440741871876 |
| ebi-a-GCST90001403 | finngen_R11_O15_POOR_FETGRO | Poor fetal growth \|\| id:finngen_R11_O15_POOR_FETGRO | Switched memory B cell Absolute Count \|\| id:ebi-a-GCST90001403 | Weighted mode | 23 | 0.0811050754166155 | 0.0402628553904119 | 0.0563543723915438 | 0.00218987885140819 | 0.160020271981823 | 1.08448484327688 | 1.00219227838734 | 1.17353466062399 | 0.999857399747617 |
| ebi-a-GCST90001404 | finngen_R11_O15_POOR_FETGRO | Poor fetal growth \|\| id:finngen_R11_O15_POOR_FETGRO | Plasma Blast-Plasma Cell %B cell \|\| id:ebi-a-GCST90001404 | Inverse variance weighted | 26 | 0.0063239913961575 | 0.0214796974631267 | 0.768439160482704 | -0.035776216 | 0.0484241984238858 | 1.0063440300489 | 0.964856189058088 | 1.04961580627234 | 0.994119304454654 |
| ebi-a-GCST90001404 | finngen_R11_O15_POOR_FETGRO | Poor fetal growth \|\| id:finngen_R11_O15_POOR_FETGRO | Plasma Blast-Plasma Cell %B cell \|\| id:ebi-a-GCST90001404 | MR Egger | 26 | -0.021300592 | 0.0270051731434125 | 0.437975086862996 | -0.074230732 | 0.0316295470781585 | 0.978924663139997 | 0.928457444612603 | 1.03213507701864 | 0.992899239648112 |
| ebi-a-GCST90001404 | finngen_R11_O15_POOR_FETGRO | Poor fetal growth \|\| id:finngen_R11_O15_POOR_FETGRO | Plasma Blast-Plasma Cell %B cell \|\| id:ebi-a-GCST90001404 | Weighted median | 26 | 0.000421445458711409 | 0.0269583538877359 | 0.987527015573539 | -0.052416928 | 0.0532598190786738 | 1.00042153427933 | 0.948933147403036 | 1.0547036416515 | 0.999440741871876 |
| ebi-a-GCST90001404 | finngen_R11_O15_POOR_FETGRO | Poor fetal growth \|\| id:finngen_R11_O15_POOR_FETGRO | Plasma Blast-Plasma Cell %B cell \|\| id:ebi-a-GCST90001404 | Weighted mode | 26 | 0.000257495232715677 | 0.024123875779414 | 0.991568364852755 | -0.047025301 | 0.0475402917603671 | 1.00025752838746 | 0.95406325824911 | 1.04868845377394 | 0.999857399747617 |
| ebi-a-GCST90001405 | finngen_R11_O15_POOR_FETGRO | Poor fetal growth \|\| id:finngen_R11_O15_POOR_FETGRO | Plasma Blast-Plasma Cell Absolute Count \|\| id:ebi-a-GCST90001405 | Inverse variance weighted | 24 | -0.003432909 | 0.0196618927894604 | 0.861396253215348 | -0.041970219 | 0.0351044010504995 | 0.996572976877686 | 0.958898337401473 | 1.03572783422925 | 0.994119304454654 |
| ebi-a-GCST90001405 | finngen_R11_O15_POOR_FETGRO | Poor fetal growth \|\| id:finngen_R11_O15_POOR_FETGRO | Plasma Blast-Plasma Cell Absolute Count \|\| id:ebi-a-GCST90001405 | MR Egger | 24 | -0.019231556 | 0.0227186062265589 | 0.406381788277196 | -0.063760024 | 0.0252969119447139 | 0.980952190322497 | 0.938230124795026 | 1.02561959402948 | 0.992899239648112 |
| ebi-a-GCST90001405 | finngen_R11_O15_POOR_FETGRO | Poor fetal growth \|\| id:finngen_R11_O15_POOR_FETGRO | Plasma Blast-Plasma Cell Absolute Count \|\| id:ebi-a-GCST90001405 | Weighted median | 24 | -0.012763813 | 0.0220772034892387 | 0.563166589669727 | -0.056035132 | 0.0305075058600317 | 0.987317299015807 | 0.945505917965273 | 1.03097762839351 | 0.999440741871876 |
| ebi-a-GCST90001405 | finngen_R11_O15_POOR_FETGRO | Poor fetal growth \|\| id:finngen_R11_O15_POOR_FETGRO | Plasma Blast-Plasma Cell Absolute Count \|\| id:ebi-a-GCST90001405 | Weighted mode | 24 | -0.01020928 | 0.0174459519460195 | 0.564114598714266 | -0.044403346 | 0.0239847856107564 | 0.989842657598233 | 0.956568051737238 | 1.02427473405758 | 0.999857399747617 |
| ebi-a-GCST90001406 | finngen_R11_O15_POOR_FETGRO | Poor fetal growth \|\| id:finngen_R11_O15_POOR_FETGRO | Memory B cell %B cell \|\| id:ebi-a-GCST90001406 | Inverse variance weighted | 28 | -0.022997899 | 0.0219298631197 | 0.294315137072676 | -0.06598043 | 0.0199846329511914 | 0.977264537235113 | 0.936149184104386 | 1.02018566266343 | 0.994119304454654 |
| ebi-a-GCST90001406 | finngen_R11_O15_POOR_FETGRO | Poor fetal growth \|\| id:finngen_R11_O15_POOR_FETGRO | Memory B cell %B cell \|\| id:ebi-a-GCST90001406 | MR Egger | 28 | -0.053920541 | 0.0315442745616802 | 0.0992955960867679 | -0.115747319 | 0.00790623758410674 | 0.947507391920493 | 0.890700258166434 | 1.0079375744115 | 0.992899239648112 |
| ebi-a-GCST90001406 | finngen_R11_O15_POOR_FETGRO | Poor fetal growth \|\| id:finngen_R11_O15_POOR_FETGRO | Memory B cell %B cell \|\| id:ebi-a-GCST90001406 | Weighted median | 28 | -0.041514704 | 0.03025307622917 | 0.169986448113962 | -0.100810733 | 0.0177813255721258 | 0.95933522932888 | 0.904104133547955 | 1.01794035452519 | 0.999440741871876 |
| ebi-a-GCST90001406 | finngen_R11_O15_POOR_FETGRO | Poor fetal growth \|\| id:finngen_R11_O15_POOR_FETGRO | Memory B cell %B cell \|\| id:ebi-a-GCST90001406 | Weighted mode | 28 | -0.037404298 | 0.0276426754313741 | 0.187234262046104 | -0.091583942 | 0.0167753460266139 | 0.963286601938486 | 0.912484717451247 | 1.01691684225251 | 0.999857399747617 |
| ebi-a-GCST90001407 | finngen_R11_O15_POOR_FETGRO | Poor fetal growth \|\| id:finngen_R11_O15_POOR_FETGRO | Memory B cell Absolute Count \|\| id:ebi-a-GCST90001407 | Inverse variance weighted | 17 | -0.031398076 | 0.0160134760028177 | 0.0499107240773397 | -0.062784489 | -1.17E-05 | 0.96908972507898 | 0.939145848327419 | 0.999988337196204 | 0.994119304454654 |
| ebi-a-GCST90001407 | finngen_R11_O15_POOR_FETGRO | Poor fetal growth \|\| id:finngen_R11_O15_POOR_FETGRO | Memory B cell Absolute Count \|\| id:ebi-a-GCST90001407 | MR Egger | 17 | -0.044597002 | 0.0183435091119547 | 0.0280569729573034 | -0.08055028 | -0.008643724 | 0.95638282433749 | 0.922608513588804 | 0.991393525223214 | 0.992899239648112 |
| ebi-a-GCST90001407 | finngen_R11_O15_POOR_FETGRO | Poor fetal growth \|\| id:finngen_R11_O15_POOR_FETGRO | Memory B cell Absolute Count \|\| id:ebi-a-GCST90001407 | Weighted median | 17 | -0.037658317 | 0.0200749640513984 | 0.0606711207491556 | -0.077005246 | 0.00168861260993898 | 0.963041939807079 | 0.92588499596447 | 1.00169003911904 | 0.999440741871876 |
| ebi-a-GCST90001407 | finngen_R11_O15_POOR_FETGRO | Poor fetal growth \|\| id:finngen_R11_O15_POOR_FETGRO | Memory B cell Absolute Count \|\| id:ebi-a-GCST90001407 | Weighted mode | 17 | -0.036416337 | 0.0189409162761258 | 0.0725140124825088 | -0.073540533 | 0.000707858576158016 | 0.964238761315914 | 0.929098485669149 | 1.00070810916716 | 0.999857399747617 |
| ebi-a-GCST90001408 | finngen_R11_O15_POOR_FETGRO | Poor fetal growth \|\| id:finngen_R11_O15_POOR_FETGRO | Naive-mature B cell %B cell \|\| id:ebi-a-GCST90001408 | Inverse variance weighted | 28 | 0.0190982232491587 | 0.0191624804297378 | 0.318936020337918 | -0.018460238 | 0.0566566848914447 | 1.01928176086701 | 0.981709108147389 | 1.05829242024327 | 0.994119304454654 |
| ebi-a-GCST90001408 | finngen_R11_O15_POOR_FETGRO | Poor fetal growth \|\| id:finngen_R11_O15_POOR_FETGRO | Naive-mature B cell %B cell \|\| id:ebi-a-GCST90001408 | MR Egger | 28 | 0.030713763039629 | 0.0263122299961397 | 0.253691109006442 | -0.020858208 | 0.0822857338320629 | 1.03119029686282 | 0.979357820071026 | 1.08576600559223 | 0.992899239648112 |
| ebi-a-GCST90001408 | finngen_R11_O15_POOR_FETGRO | Poor fetal growth \|\| id:finngen_R11_O15_POOR_FETGRO | Naive-mature B cell %B cell \|\| id:ebi-a-GCST90001408 | Weighted median | 28 | 0.0325686756068776 | 0.0269159957140875 | 0.226274319628307 | -0.020186676 | 0.0853240272064891 | 1.03310483980963 | 0.980015710824222 | 1.08906989780853 | 0.999440741871876 |
| ebi-a-GCST90001408 | finngen_R11_O15_POOR_FETGRO | Poor fetal growth \|\| id:finngen_R11_O15_POOR_FETGRO | Naive-mature B cell %B cell \|\| id:ebi-a-GCST90001408 | Weighted mode | 28 | 0.0268871074759122 | 0.0242594485292546 | 0.277501345154179 | -0.020661412 | 0.0744356265932512 | 1.02725182716558 | 0.97955057284754 | 1.07727599336442 | 0.999857399747617 |
| ebi-a-GCST90001409 | finngen_R11_O15_POOR_FETGRO | Poor fetal growth \|\| id:finngen_R11_O15_POOR_FETGRO | Naive-mature B cell Absolute Count \|\| id:ebi-a-GCST90001409 | Inverse variance weighted | 21 | 0.0265398707788505 | 0.0223257862026662 | 0.234536429115591 | -0.01721867 | 0.0702984117360762 | 1.02689518955648 | 0.982928723933626 | 1.07282827804048 | 0.994119304454654 |
| ebi-a-GCST90001409 | finngen_R11_O15_POOR_FETGRO | Poor fetal growth \|\| id:finngen_R11_O15_POOR_FETGRO | Naive-mature B cell Absolute Count \|\| id:ebi-a-GCST90001409 | MR Egger | 21 | 0.0287763025632501 | 0.0277599405326958 | 0.312930226806413 | -0.025633181 | 0.0831857860073339 | 1.02919434058658 | 0.974692559907554 | 1.08674369156559 | 0.992899239648112 |
| ebi-a-GCST90001409 | finngen_R11_O15_POOR_FETGRO | Poor fetal growth \|\| id:finngen_R11_O15_POOR_FETGRO | Naive-mature B cell Absolute Count \|\| id:ebi-a-GCST90001409 | Weighted median | 21 | 0.0223679195030424 | 0.0249619106450069 | 0.370208955100115 | -0.026557425 | 0.0712932643672559 | 1.02261995709199 | 0.973792121865577 | 1.07389611515791 | 0.999440741871876 |
| ebi-a-GCST90001409 | finngen_R11_O15_POOR_FETGRO | Poor fetal growth \|\| id:finngen_R11_O15_POOR_FETGRO | Naive-mature B cell Absolute Count \|\| id:ebi-a-GCST90001409 | Weighted mode | 21 | 0.023983012187332 | 0.0224365655021434 | 0.297835614809295 | -0.019992656 | 0.0679586805715331 | 1.02427291758638 | 0.980205871719273 | 1.07032108250968 | 0.999857399747617 |
| ebi-a-GCST90001410 | finngen_R11_O15_POOR_FETGRO | Poor fetal growth \|\| id:finngen_R11_O15_POOR_FETGRO | IgD- CD38+ B cell %B cell \|\| id:ebi-a-GCST90001410 | Inverse variance weighted | 18 | -0.046447514 | 0.0487162352197791 | 0.340372314986873 | -0.141931335 | 0.0490363073913689 | 0.954614663504563 | 0.867680834031095 | 1.05025848219355 | 0.994119304454654 |
| ebi-a-GCST90001410 | finngen_R11_O15_POOR_FETGRO | Poor fetal growth \|\| id:finngen_R11_O15_POOR_FETGRO | IgD- CD38+ B cell %B cell \|\| id:ebi-a-GCST90001410 | MR Egger | 18 | -0.128987945 | 0.103864149321167 | 0.232173633774358 | -0.332561677 | 0.0745857881441847 | 0.878984562057857 | 0.717084439743364 | 1.07743777094445 | 0.992899239648112 |
| ebi-a-GCST90001410 | finngen_R11_O15_POOR_FETGRO | Poor fetal growth \|\| id:finngen_R11_O15_POOR_FETGRO | IgD- CD38+ B cell %B cell \|\| id:ebi-a-GCST90001410 | Weighted median | 18 | -0.064503067 | 0.061573592254763 | 0.294833631627916 | -0.185187307 | 0.0561811741637166 | 0.937533239166065 | 0.830948626385652 | 1.05778931047089 | 0.999440741871876 |
| ebi-a-GCST90001410 | finngen_R11_O15_POOR_FETGRO | Poor fetal growth \|\| id:finngen_R11_O15_POOR_FETGRO | IgD- CD38+ B cell %B cell \|\| id:ebi-a-GCST90001410 | Weighted mode | 18 | -0.061522664 | 0.0940416198641468 | 0.521731295979941 | -0.245844239 | 0.122798911132121 | 0.94033163400833 | 0.782044027605926 | 1.13065703554268 | 0.999857399747617 |
| ebi-a-GCST90001411 | finngen_R11_O15_POOR_FETGRO | Poor fetal growth \|\| id:finngen_R11_O15_POOR_FETGRO | IgD+ CD24+ B cell %B cell \|\| id:ebi-a-GCST90001411 | Inverse variance weighted | 20 | -0.124195673 | 0.0422883294547234 | 0.00331534278737066 | -0.207080799 | -0.041310548 | 0.883207003969508 | 0.812953961464326 | 0.959531103650358 | 0.956403078666104 |
| ebi-a-GCST90001411 | finngen_R11_O15_POOR_FETGRO | Poor fetal growth \|\| id:finngen_R11_O15_POOR_FETGRO | IgD+ CD24+ B cell %B cell \|\| id:ebi-a-GCST90001411 | MR Egger | 20 | -0.120251336 | 0.0874178465245689 | 0.185825781139098 | -0.291590316 | 0.0510876427929457 | 0.886697549342783 | 0.747074538083247 | 1.05241512584235 | 0.992899239648112 |
| ebi-a-GCST90001411 | finngen_R11_O15_POOR_FETGRO | Poor fetal growth \|\| id:finngen_R11_O15_POOR_FETGRO | IgD+ CD24+ B cell %B cell \|\| id:ebi-a-GCST90001411 | Weighted median | 20 | -0.195454697 | 0.060930753031411 | 0.00133746636850068 | -0.314878973 | -0.076030421 | 0.8224606026344 | 0.729877203798409 | 0.926788012237429 | 0.977687915373994 |
| ebi-a-GCST90001411 | finngen_R11_O15_POOR_FETGRO | Poor fetal growth \|\| id:finngen_R11_O15_POOR_FETGRO | IgD+ CD24+ B cell %B cell \|\| id:ebi-a-GCST90001411 | Weighted mode | 20 | -0.216033558 | 0.0793231912352844 | 0.0134882212058538 | -0.371507013 | -0.060560103 | 0.805708263622648 | 0.68969416924791 | 0.941237196158578 | 0.999857399747617 |
| ebi-a-GCST90001412 | finngen_R11_O15_POOR_FETGRO | Poor fetal growth \|\| id:finngen_R11_O15_POOR_FETGRO | IgD+ CD24+ B cell Absolute Count \|\| id:ebi-a-GCST90001412 | Inverse variance weighted | 23 | -0.060027747 | 0.037291179365412 | 0.107462572987107 | -0.133118458 | 0.0130629649111019 | 0.94173840314048 | 0.875361392230819 | 1.01314865816669 | 0.994119304454654 |
| ebi-a-GCST90001412 | finngen_R11_O15_POOR_FETGRO | Poor fetal growth \|\| id:finngen_R11_O15_POOR_FETGRO | IgD+ CD24+ B cell Absolute Count \|\| id:ebi-a-GCST90001412 | MR Egger | 23 | -0.035076645 | 0.0714362562123482 | 0.628504332451707 | -0.175091708 | 0.104938416803813 | 0.965531409907014 | 0.839380039753608 | 1.11064221135242 | 0.992899239648112 |
| ebi-a-GCST90001412 | finngen_R11_O15_POOR_FETGRO | Poor fetal growth \|\| id:finngen_R11_O15_POOR_FETGRO | IgD+ CD24+ B cell Absolute Count \|\| id:ebi-a-GCST90001412 | Weighted median | 23 | -0.047102288 | 0.053945162586983 | 0.382580619609681 | -0.152834807 | 0.0586302302559047 | 0.953989810494199 | 0.858271490470796 | 1.06038307066163 | 0.999440741871876 |
| ebi-a-GCST90001412 | finngen_R11_O15_POOR_FETGRO | Poor fetal growth \|\| id:finngen_R11_O15_POOR_FETGRO | IgD+ CD24+ B cell Absolute Count \|\| id:ebi-a-GCST90001412 | Weighted mode | 23 | -0.046082766 | 0.0632332637207169 | 0.473827092985082 | -0.170019963 | 0.0778544312827343 | 0.95496292083126 | 0.843647975103493 | 1.08096529248553 | 0.999857399747617 |
| ebi-a-GCST90001413 | finngen_R11_O15_POOR_FETGRO | Poor fetal growth \|\| id:finngen_R11_O15_POOR_FETGRO | IgD- CD24- B cell %B cell \|\| id:ebi-a-GCST90001413 | Inverse variance weighted | 17 | 0.00702588840726974 | 0.0238241757733948 | 0.768065815753801 | -0.039669496 | 0.0537212729231235 | 1.00705062786618 | 0.961107036281447 | 1.05519045101308 | 0.994119304454654 |
| ebi-a-GCST90001413 | finngen_R11_O15_POOR_FETGRO | Poor fetal growth \|\| id:finngen_R11_O15_POOR_FETGRO | IgD- CD24- B cell %B cell \|\| id:ebi-a-GCST90001413 | MR Egger | 17 | -0.018873748 | 0.0305795062044816 | 0.54635675499595 | -0.07880958 | 0.0410620846521216 | 0.981303246401916 | 0.924215897186807 | 1.04191679057897 | 0.992899239648112 |
| ebi-a-GCST90001413 | finngen_R11_O15_POOR_FETGRO | Poor fetal growth \|\| id:finngen_R11_O15_POOR_FETGRO | IgD- CD24- B cell %B cell \|\| id:ebi-a-GCST90001413 | Weighted median | 17 | -0.01295418 | 0.0326251948088082 | 0.691322763182183 | -0.076899562 | 0.0509912019468031 | 0.987129364371523 | 0.925982853076206 | 1.05231363493114 | 0.999440741871876 |
| ebi-a-GCST90001413 | finngen_R11_O15_POOR_FETGRO | Poor fetal growth \|\| id:finngen_R11_O15_POOR_FETGRO | IgD- CD24- B cell %B cell \|\| id:ebi-a-GCST90001413 | Weighted mode | 17 | -0.007343819 | 0.0310179208501973 | 0.815847156789706 | -0.068138944 | 0.0534513057335602 | 0.992683080817167 | 0.934130672708443 | 1.05490562266145 | 0.999857399747617 |
| ebi-a-GCST90001414 | finngen_R11_O15_POOR_FETGRO | Poor fetal growth \|\| id:finngen_R11_O15_POOR_FETGRO | IgD- CD24- B cell Absolute Count \|\| id:ebi-a-GCST90001414 | Inverse variance weighted | 18 | -0.011822634 | 0.0341153517804334 | 0.728930315284353 | -0.078688724 | 0.0550434552181298 | 0.988246978463669 | 0.924327600888396 | 1.05658652787594 | 0.994119304454654 |
| ebi-a-GCST90001414 | finngen_R11_O15_POOR_FETGRO | Poor fetal growth \|\| id:finngen_R11_O15_POOR_FETGRO | IgD- CD24- B cell Absolute Count \|\| id:ebi-a-GCST90001414 | MR Egger | 18 | 0.0590663092902878 | 0.0558992237806889 | 0.306362226140376 | -0.050496169 | 0.168628787900438 | 1.06084558232565 | 0.950757570713493 | 1.18368066077592 | 0.992899239648112 |
| ebi-a-GCST90001414 | finngen_R11_O15_POOR_FETGRO | Poor fetal growth \|\| id:finngen_R11_O15_POOR_FETGRO | IgD- CD24- B cell Absolute Count \|\| id:ebi-a-GCST90001414 | Weighted median | 18 | -0.001036152 | 0.0466966188661771 | 0.982297186070906 | -0.092561525 | 0.0904892213452157 | 0.998964384987256 | 0.911593123827507 | 1.09470970808002 | 0.999440741871876 |
| ebi-a-GCST90001414 | finngen_R11_O15_POOR_FETGRO | Poor fetal growth \|\| id:finngen_R11_O15_POOR_FETGRO | IgD- CD24- B cell Absolute Count \|\| id:ebi-a-GCST90001414 | Weighted mode | 18 | 0.00616748866683103 | 0.0497179844249267 | 0.902731071485965 | -0.091279761 | 0.103614738139687 | 1.00618654678516 | 0.912762320054389 | 1.10917305051671 | 0.999857399747617 |
| ebi-a-GCST90001415 | finngen_R11_O15_POOR_FETGRO | Poor fetal growth \|\| id:finngen_R11_O15_POOR_FETGRO | IgD+ CD24- B cell %B cell \|\| id:ebi-a-GCST90001415 | Inverse variance weighted | 19 | 0.0263596361621568 | 0.0221030487368825 | 0.233034216252027 | -0.016962339 | 0.0696816116864465 | 1.0267101241737 | 0.983180711150405 | 1.07216676153802 | 0.994119304454654 |
| ebi-a-GCST90001415 | finngen_R11_O15_POOR_FETGRO | Poor fetal growth \|\| id:finngen_R11_O15_POOR_FETGRO | IgD+ CD24- B cell %B cell \|\| id:ebi-a-GCST90001415 | MR Egger | 19 | -0.001865701 | 0.0302471137558032 | 0.951535519450672 | -0.061150044 | 0.0574186423747946 | 0.998136038750897 | 0.940682085908995 | 1.05909910136177 | 0.992899239648112 |
| ebi-a-GCST90001415 | finngen_R11_O15_POOR_FETGRO | Poor fetal growth \|\| id:finngen_R11_O15_POOR_FETGRO | IgD+ CD24- B cell %B cell \|\| id:ebi-a-GCST90001415 | Weighted median | 19 | -0.008489417 | 0.0298391433704058 | 0.776022586564836 | -0.066974138 | 0.0499953037335339 | 0.991546516074065 | 0.935219387407408 | 1.05126615933842 | 0.999440741871876 |
| ebi-a-GCST90001415 | finngen_R11_O15_POOR_FETGRO | Poor fetal growth \|\| id:finngen_R11_O15_POOR_FETGRO | IgD+ CD24- B cell %B cell \|\| id:ebi-a-GCST90001415 | Weighted mode | 19 | -0.001396943 | 0.0281233301536089 | 0.960930788869352 | -0.05651867 | 0.0537247844625737 | 0.998604032631683 | 0.945048840514707 | 1.05519415636248 | 0.999857399747617 |
| ebi-a-GCST90001416 | finngen_R11_O15_POOR_FETGRO | Poor fetal growth \|\| id:finngen_R11_O15_POOR_FETGRO | IgD+ CD24- B cell Absolute Count \|\| id:ebi-a-GCST90001416 | Inverse variance weighted | 22 | 0.00160393742159034 | 0.0126498021428228 | 0.899102292886506 | -0.023189675 | 0.026397549621523 | 1.00160522441721 | 0.977077139306426 | 1.0267490510442 | 0.994119304454654 |
| ebi-a-GCST90001416 | finngen_R11_O15_POOR_FETGRO | Poor fetal growth \|\| id:finngen_R11_O15_POOR_FETGRO | IgD+ CD24- B cell Absolute Count \|\| id:ebi-a-GCST90001416 | MR Egger | 22 | -0.005675563 | 0.0151852408888417 | 0.712519168185691 | -0.035438635 | 0.0240875093845154 | 0.994340512821662 | 0.965181960900552 | 1.02437995682797 | 0.992899239648112 |
| ebi-a-GCST90001416 | finngen_R11_O15_POOR_FETGRO | Poor fetal growth \|\| id:finngen_R11_O15_POOR_FETGRO | IgD+ CD24- B cell Absolute Count \|\| id:ebi-a-GCST90001416 | Weighted median | 22 | -0.009679209 | 0.0180753005858032 | 0.592308541281064 | -0.045106798 | 0.0257483803026911 | 0.990367483925325 | 0.955895388671868 | 1.02608273336202 | 0.999440741871876 |
| ebi-a-GCST90001416 | finngen_R11_O15_POOR_FETGRO | Poor fetal growth \|\| id:finngen_R11_O15_POOR_FETGRO | IgD+ CD24- B cell Absolute Count \|\| id:ebi-a-GCST90001416 | Weighted mode | 22 | -0.006437256 | 0.0145936668036765 | 0.663649060093217 | -0.035040843 | 0.022166331225496 | 0.993583419034167 | 0.965565979183784 | 1.02241382967339 | 0.999857399747617 |
| ebi-a-GCST90001417 | finngen_R11_O15_POOR_FETGRO | Poor fetal growth \|\| id:finngen_R11_O15_POOR_FETGRO | CD24+ CD27+ B cell %B cell \|\| id:ebi-a-GCST90001417 | Inverse variance weighted | 25 | -0.017157245 | 0.0252676162453449 | 0.497124482185207 | -0.066681773 | 0.032367282842563 | 0.982989102361841 | 0.935492853208445 | 1.03289680091958 | 0.994119304454654 |
| ebi-a-GCST90001417 | finngen_R11_O15_POOR_FETGRO | Poor fetal growth \|\| id:finngen_R11_O15_POOR_FETGRO | CD24+ CD27+ B cell %B cell \|\| id:ebi-a-GCST90001417 | MR Egger | 25 | -0.038366864 | 0.038095218308808 | 0.324350683679493 | -0.113033491 | 0.0362997643288643 | 0.962359821375214 | 0.893120747713694 | 1.03696664551591 | 0.992899239648112 |
| ebi-a-GCST90001417 | finngen_R11_O15_POOR_FETGRO | Poor fetal growth \|\| id:finngen_R11_O15_POOR_FETGRO | CD24+ CD27+ B cell %B cell \|\| id:ebi-a-GCST90001417 | Weighted median | 25 | -0.02756035 | 0.0342024658611871 | 0.420358129044874 | -0.094597183 | 0.039476483189695 | 0.972815971436992 | 0.909739319146199 | 1.04026603485829 | 0.999440741871876 |
| ebi-a-GCST90001417 | finngen_R11_O15_POOR_FETGRO | Poor fetal growth \|\| id:finngen_R11_O15_POOR_FETGRO | CD24+ CD27+ B cell %B cell \|\| id:ebi-a-GCST90001417 | Weighted mode | 25 | 0.017667689911631 | 0.0404592865430192 | 0.666248280405885 | -0.061632512 | 0.0969678915359485 | 1.01782468677277 | 0.940228346215689 | 1.10182499514479 | 0.999857399747617 |
| ebi-a-GCST90001418 | finngen_R11_O15_POOR_FETGRO | Poor fetal growth \|\| id:finngen_R11_O15_POOR_FETGRO | CD24+ CD27+ B cell Absolute Count \|\| id:ebi-a-GCST90001418 | Inverse variance weighted | 26 | -0.039016348 | 0.0155252604421742 | 0.0119679419767586 | -0.069445859 | -0.008586838 | 0.961734986234459 | 0.932910640900196 | 0.991449923708552 | 0.994119304454654 |
| ebi-a-GCST90001418 | finngen_R11_O15_POOR_FETGRO | Poor fetal growth \|\| id:finngen_R11_O15_POOR_FETGRO | CD24+ CD27+ B cell Absolute Count \|\| id:ebi-a-GCST90001418 | MR Egger | 26 | -0.044132455 | 0.0179086728130912 | 0.0212746751107368 | -0.079233454 | -0.009031456 | 0.956827212399912 | 0.92382422894521 | 0.991009204677705 | 0.992899239648112 |
| ebi-a-GCST90001418 | finngen_R11_O15_POOR_FETGRO | Poor fetal growth \|\| id:finngen_R11_O15_POOR_FETGRO | CD24+ CD27+ B cell Absolute Count \|\| id:ebi-a-GCST90001418 | Weighted median | 26 | -0.037472981 | 0.0201801411926225 | 0.0633220171684856 | -0.077026058 | 0.00208009527711486 | 0.963220442178885 | 0.925865726899747 | 1.0020822601761 | 0.999440741871876 |
| ebi-a-GCST90001418 | finngen_R11_O15_POOR_FETGRO | Poor fetal growth \|\| id:finngen_R11_O15_POOR_FETGRO | CD24+ CD27+ B cell Absolute Count \|\| id:ebi-a-GCST90001418 | Weighted mode | 26 | -0.035139493 | 0.018460374671593 | 0.0685480255554919 | -0.071321827 | 0.00104284182573906 | 0.965470730908526 | 0.931162170878343 | 1.00104338577434 | 0.999857399747617 |
| ebi-a-GCST90001419 | finngen_R11_O15_POOR_FETGRO | Poor fetal growth \|\| id:finngen_R11_O15_POOR_FETGRO | CD20- B cell %B cell \|\| id:ebi-a-GCST90001419 | Inverse variance weighted | 19 | -0.003731816 | 0.0306216214358923 | 0.903003034415719 | -0.063750194 | 0.0562865619708916 | 0.996275138528283 | 0.938239348023024 | 1.0579007944412 | 0.994119304454654 |
| ebi-a-GCST90001419 | finngen_R11_O15_POOR_FETGRO | Poor fetal growth \|\| id:finngen_R11_O15_POOR_FETGRO | CD20- B cell %B cell \|\| id:ebi-a-GCST90001419 | MR Egger | 19 | -0.024715306 | 0.0440063540555567 | 0.581697239794568 | -0.11096776 | 0.0615371479316588 | 0.97558761642013 | 0.894967602249727 | 1.06347000150597 | 0.992899239648112 |
| ebi-a-GCST90001419 | finngen_R11_O15_POOR_FETGRO | Poor fetal growth \|\| id:finngen_R11_O15_POOR_FETGRO | CD20- B cell %B cell \|\| id:ebi-a-GCST90001419 | Weighted median | 19 | 0.00044243527176345 | 0.0366329187612363 | 0.990363757448454 | -0.071358086 | 0.0722429560437867 | 1.00044253316068 | 0.931128408841305 | 1.07491646979439 | 0.999440741871876 |
| ebi-a-GCST90001419 | finngen_R11_O15_POOR_FETGRO | Poor fetal growth \|\| id:finngen_R11_O15_POOR_FETGRO | CD20- B cell %B cell \|\| id:ebi-a-GCST90001419 | Weighted mode | 19 | 0.00353970750715382 | 0.0341313157794523 | 0.918547457732453 | -0.063357671 | 0.0704370864348802 | 1.00354597967013 | 0.938607700494879 | 1.07297706249489 | 0.999857399747617 |
| ebi-a-GCST90001420 | finngen_R11_O15_POOR_FETGRO | Poor fetal growth \|\| id:finngen_R11_O15_POOR_FETGRO | IgD- CD38+ B cell Absolute Count \|\| id:ebi-a-GCST90001420 | Inverse variance weighted (multiplicative random effects) | 21 | 0.00412311486745399 | 0.0286472242401636 | 0.885557954196346 | -0.052025445 | 0.0602716743781746 | 1.00413162659982 | 0.949304711815839 | 1.06212505951788 | 0.994119304454654 |
| ebi-a-GCST90001420 | finngen_R11_O15_POOR_FETGRO | Poor fetal growth \|\| id:finngen_R11_O15_POOR_FETGRO | IgD- CD38+ B cell Absolute Count \|\| id:ebi-a-GCST90001420 | MR Egger | 21 | 0.00110992853966334 | 0.0368872420074079 | 0.976309286261641 | -0.071189066 | 0.0734089228741827 | 1.0011105447383 | 0.93128580119149 | 1.07617051769068 | 0.993986195344372 |
| ebi-a-GCST90001420 | finngen_R11_O15_POOR_FETGRO | Poor fetal growth \|\| id:finngen_R11_O15_POOR_FETGRO | IgD- CD38+ B cell Absolute Count \|\| id:ebi-a-GCST90001420 | Weighted median | 21 | -0.011230072 | 0.0344308891034468 | 0.74430126469865 | -0.078714614 | 0.0562544710733854 | 0.988832750299749 | 0.924303669939769 | 1.05786684599995 | 0.999440741871876 |
| ebi-a-GCST90001420 | finngen_R11_O15_POOR_FETGRO | Poor fetal growth \|\| id:finngen_R11_O15_POOR_FETGRO | IgD- CD38+ B cell Absolute Count \|\| id:ebi-a-GCST90001420 | Weighted mode | 21 | -0.002774858 | 0.027590732427292 | 0.920891515664441 | -0.056852693 | 0.0513029777500909 | 0.997228988552001 | 0.944733224589415 | 1.05264177200992 | 0.999857399747617 |
| ebi-a-GCST90001421 | finngen_R11_O15_POOR_FETGRO | Poor fetal growth \|\| id:finngen_R11_O15_POOR_FETGRO | CD20- B cell Absolute Count \|\| id:ebi-a-GCST90001421 | Inverse variance weighted | 22 | 0.00368300123799556 | 0.0168447024627929 | 0.826926999712168 | -0.029332616 | 0.0366986180650696 | 1.00368979182107 | 0.971093409936501 | 1.03738032603032 | 0.994119304454654 |
| ebi-a-GCST90001421 | finngen_R11_O15_POOR_FETGRO | Poor fetal growth \|\| id:finngen_R11_O15_POOR_FETGRO | CD20- B cell Absolute Count \|\| id:ebi-a-GCST90001421 | MR Egger | 22 | 0.00217567989457579 | 0.020180782629192 | 0.915221028343368 | -0.037378654 | 0.041730013847792 | 1.00217804840347 | 0.96331130454588 | 1.04261294968947 | 0.992899239648112 |
| ebi-a-GCST90001421 | finngen_R11_O15_POOR_FETGRO | Poor fetal growth \|\| id:finngen_R11_O15_POOR_FETGRO | CD20- B cell Absolute Count \|\| id:ebi-a-GCST90001421 | Weighted median | 22 | 0.00841927920251025 | 0.022195519846457 | 0.704447753562715 | -0.03508394 | 0.0519224981015659 | 1.0084548210091 | 0.965524367033614 | 1.05329410705705 | 0.999440741871876 |
| ebi-a-GCST90001421 | finngen_R11_O15_POOR_FETGRO | Poor fetal growth \|\| id:finngen_R11_O15_POOR_FETGRO | CD20- B cell Absolute Count \|\| id:ebi-a-GCST90001421 | Weighted mode | 22 | 0.00465768646972176 | 0.0183310391515166 | 0.801899342802026 | -0.03127115 | 0.0405865232066942 | 1.00466855035165 | 0.969212735151191 | 1.04142141292462 | 0.999857399747617 |
| ebi-a-GCST90001422 | finngen_R11_O15_POOR_FETGRO | Poor fetal growth \|\| id:finngen_R11_O15_POOR_FETGRO | CD20- CD38- B cell %B cell \|\| id:ebi-a-GCST90001422 | Inverse variance weighted | 23 | 0.0121689597959052 | 0.0229783825806349 | 0.596401113379057 | -0.03286867 | 0.0572065896539496 | 1.01224330284028 | 0.967665634712142 | 1.05887454032591 | 0.994119304454654 |
| ebi-a-GCST90001422 | finngen_R11_O15_POOR_FETGRO | Poor fetal growth \|\| id:finngen_R11_O15_POOR_FETGRO | CD20- CD38- B cell %B cell \|\| id:ebi-a-GCST90001422 | MR Egger | 23 | 0.0220374958305949 | 0.0311255310754728 | 0.486717772053308 | -0.038968545 | 0.0830435367385216 | 1.02228211506882 | 0.961780961416338 | 1.08658911406461 | 0.992899239648112 |
| ebi-a-GCST90001422 | finngen_R11_O15_POOR_FETGRO | Poor fetal growth \|\| id:finngen_R11_O15_POOR_FETGRO | CD20- CD38- B cell %B cell \|\| id:ebi-a-GCST90001422 | Weighted median | 23 | 0.00597134303397113 | 0.0344939642162059 | 0.862562848097058 | -0.061636827 | 0.0735795128977347 | 1.00598920704246 | 0.940224289029023 | 1.07635411730432 | 0.999440741871876 |
| ebi-a-GCST90001422 | finngen_R11_O15_POOR_FETGRO | Poor fetal growth \|\| id:finngen_R11_O15_POOR_FETGRO | CD20- CD38- B cell %B cell \|\| id:ebi-a-GCST90001422 | Weighted mode | 23 | -0.00674051 | 0.0303377674103251 | 0.826221722274345 | -0.066202534 | 0.0527215140521082 | 0.99328215620986 | 0.935941284978121 | 1.0541360421642 | 0.999857399747617 |
| ebi-a-GCST90001423 | finngen_R11_O15_POOR_FETGRO | Poor fetal growth \|\| id:finngen_R11_O15_POOR_FETGRO | CD20- CD38- B cell Absolute Count \|\| id:ebi-a-GCST90001423 | Inverse variance weighted | 14 | -0.000326358 | 0.0470870903505997 | 0.994469943930871 | -0.092617055 | 0.0919643386797405 | 0.999673694841677 | 0.911542503660621 | 1.09632572496091 | 0.998984604424952 |
| ebi-a-GCST90001423 | finngen_R11_O15_POOR_FETGRO | Poor fetal growth \|\| id:finngen_R11_O15_POOR_FETGRO | CD20- CD38- B cell Absolute Count \|\| id:ebi-a-GCST90001423 | MR Egger | 14 | 0.130156890415138 | 0.0879571954333343 | 0.16470159028864 | -0.042239213 | 0.302552993464473 | 1.13900706859909 | 0.958640434238785 | 1.35330939107409 | 0.992899239648112 |
| ebi-a-GCST90001423 | finngen_R11_O15_POOR_FETGRO | Poor fetal growth \|\| id:finngen_R11_O15_POOR_FETGRO | CD20- CD38- B cell Absolute Count \|\| id:ebi-a-GCST90001423 | Weighted median | 14 | -0.012452461 | 0.0716380119325569 | 0.862003178332459 | -0.152862964 | 0.127958042553974 | 0.987624750235005 | 0.858247324343442 | 1.13650531683619 | 0.999440741871876 |
| ebi-a-GCST90001423 | finngen_R11_O15_POOR_FETGRO | Poor fetal growth \|\| id:finngen_R11_O15_POOR_FETGRO | CD20- CD38- B cell Absolute Count \|\| id:ebi-a-GCST90001423 | Weighted mode | 14 | 0.0245461995473928 | 0.0863869107356821 | 0.780776480658802 | -0.144772145 | 0.19386454458933 | 1.02484993761676 | 0.865219414794225 | 1.21393183818335 | 0.999857399747617 |
| ebi-a-GCST90001424 | finngen_R11_O15_POOR_FETGRO | Poor fetal growth \|\| id:finngen_R11_O15_POOR_FETGRO | IgD+ B cell %Lymphocyte \|\| id:ebi-a-GCST90001424 | Inverse variance weighted | 18 | -0.002258955 | 0.0182428711409984 | 0.90145245424453 | -0.038014983 | 0.0334970720584813 | 0.997743594141713 | 0.962698516850104 | 1.03406441604171 | 0.994119304454654 |
| ebi-a-GCST90001424 | finngen_R11_O15_POOR_FETGRO | Poor fetal growth \|\| id:finngen_R11_O15_POOR_FETGRO | IgD+ B cell %Lymphocyte \|\| id:ebi-a-GCST90001424 | MR Egger | 18 | 0.00822986230376515 | 0.0219422200771691 | 0.712533958190664 | -0.034776889 | 0.0512366136550166 | 1.00826382071429 | 0.965820877436721 | 1.05257191670925 | 0.992899239648112 |
| ebi-a-GCST90001424 | finngen_R11_O15_POOR_FETGRO | Poor fetal growth \|\| id:finngen_R11_O15_POOR_FETGRO | IgD+ B cell %Lymphocyte \|\| id:ebi-a-GCST90001424 | Weighted median | 18 | -0.002802839 | 0.0203077878370868 | 0.890226255278408 | -0.042606103 | 0.0370004255314379 | 0.997201085655703 | 0.958288783013195 | 1.03769346240925 | 0.999440741871876 |
| ebi-a-GCST90001424 | finngen_R11_O15_POOR_FETGRO | Poor fetal growth \|\| id:finngen_R11_O15_POOR_FETGRO | IgD+ B cell %Lymphocyte \|\| id:ebi-a-GCST90001424 | Weighted mode | 18 | -0.000175866 | 0.0189736697015828 | 0.992712453433432 | -0.037364259 | 0.0370125264809015 | 0.999824149329341 | 0.963325171809909 | 1.03770601956138 | 0.999857399747617 |
| ebi-a-GCST90001425 | finngen_R11_O15_POOR_FETGRO | Poor fetal growth \|\| id:finngen_R11_O15_POOR_FETGRO | IgD- CD38+ B cell %lymphocyte \|\| id:ebi-a-GCST90001425 | Inverse variance weighted | 30 | 0.00474222570807245 | 0.0196482287501514 | 0.809278903659599 | -0.033768303 | 0.0432527540583692 | 1.00475348785592 | 0.966795482647787 | 1.04420178773882 | 0.994119304454654 |
| ebi-a-GCST90001425 | finngen_R11_O15_POOR_FETGRO | Poor fetal growth \|\| id:finngen_R11_O15_POOR_FETGRO | IgD- CD38+ B cell %lymphocyte \|\| id:ebi-a-GCST90001425 | MR Egger | 30 | -0.001672548 | 0.0246775797853043 | 0.946445606677657 | -0.050040605 | 0.0466955081219677 | 0.998328849672131 | 0.951190800959928 | 1.04780291302425 | 0.992899239648112 |
| ebi-a-GCST90001425 | finngen_R11_O15_POOR_FETGRO | Poor fetal growth \|\| id:finngen_R11_O15_POOR_FETGRO | IgD- CD38+ B cell %lymphocyte \|\| id:ebi-a-GCST90001425 | Weighted median | 30 | -0.013454912 | 0.029076973523223 | 0.643554851617629 | -0.07044578 | 0.0435359560231373 | 0.98663520064152 | 0.931978269842591 | 1.04449754961491 | 0.999440741871876 |
| ebi-a-GCST90001425 | finngen_R11_O15_POOR_FETGRO | Poor fetal growth \|\| id:finngen_R11_O15_POOR_FETGRO | IgD- CD38+ B cell %lymphocyte \|\| id:ebi-a-GCST90001425 | Weighted mode | 30 | -0.010809429 | 0.027509512297182 | 0.697241695626873 | -0.064728073 | 0.0431092152150221 | 0.989248783054234 | 0.937322311979491 | 1.04405191497852 | 0.999857399747617 |
| ebi-a-GCST90001426 | finngen_R11_O15_POOR_FETGRO | Poor fetal growth \|\| id:finngen_R11_O15_POOR_FETGRO | IgD- CD38dim B cell %lymphocyte \|\| id:ebi-a-GCST90001426 | Inverse variance weighted | 22 | -0.0030466 | 0.0201109500822371 | 0.879589508027604 | -0.042464062 | 0.0363708622212879 | 0.996958036236314 | 0.958424908679525 | 1.03704037427987 | 0.994119304454654 |
| ebi-a-GCST90001426 | finngen_R11_O15_POOR_FETGRO | Poor fetal growth \|\| id:finngen_R11_O15_POOR_FETGRO | IgD- CD38dim B cell %lymphocyte \|\| id:ebi-a-GCST90001426 | MR Egger | 22 | 0.030511861363892 | 0.0251638189549516 | 0.239438988621429 | -0.018809224 | 0.0798329465155972 | 1.03098211883033 | 0.981366565781434 | 1.08310611591043 | 0.992899239648112 |
| ebi-a-GCST90001426 | finngen_R11_O15_POOR_FETGRO | Poor fetal growth \|\| id:finngen_R11_O15_POOR_FETGRO | IgD- CD38dim B cell %lymphocyte \|\| id:ebi-a-GCST90001426 | Weighted median | 22 | 0.0119772947629585 | 0.0272533772062325 | 0.66031428772415 | -0.041439325 | 0.0653939140871742 | 1.01204930978573 | 0.959407546049264 | 1.06757947616266 | 0.999440741871876 |
| ebi-a-GCST90001426 | finngen_R11_O15_POOR_FETGRO | Poor fetal growth \|\| id:finngen_R11_O15_POOR_FETGRO | IgD- CD38dim B cell %lymphocyte \|\| id:ebi-a-GCST90001426 | Weighted mode | 22 | 0.0136977687460343 | 0.0249629473553526 | 0.588981970835411 | -0.035229608 | 0.0626251455625254 | 1.01379201300071 | 0.965383730912463 | 1.06462768401182 | 0.999857399747617 |
| ebi-a-GCST90001427 | finngen_R11_O15_POOR_FETGRO | Poor fetal growth \|\| id:finngen_R11_O15_POOR_FETGRO | IgD- CD38- B cell %lymphocyte \|\| id:ebi-a-GCST90001427 | Inverse variance weighted | 28 | 0.0195250764731367 | 0.0307563778520051 | 0.525539186388621 | -0.040757424 | 0.0798075770630667 | 1.01971693744444 | 0.960061989588648 | 1.08307863844978 | 0.994119304454654 |
| ebi-a-GCST90001427 | finngen_R11_O15_POOR_FETGRO | Poor fetal growth \|\| id:finngen_R11_O15_POOR_FETGRO | IgD- CD38- B cell %lymphocyte \|\| id:ebi-a-GCST90001427 | MR Egger | 28 | -0.009549669 | 0.053275353360514 | 0.859130085989943 | -0.113969362 | 0.0948700235541126 | 0.990495784253803 | 0.89228529364031 | 1.09951593466477 | 0.992899239648112 |
| ebi-a-GCST90001427 | finngen_R11_O15_POOR_FETGRO | Poor fetal growth \|\| id:finngen_R11_O15_POOR_FETGRO | IgD- CD38- B cell %lymphocyte \|\| id:ebi-a-GCST90001427 | Weighted median | 28 | 0.0224276331161502 | 0.0477266212439469 | 0.638413083964538 | -0.071116545 | 0.115971810754286 | 1.02268102324768 | 0.93135334167223 | 1.12296421617286 | 0.999440741871876 |
| ebi-a-GCST90001427 | finngen_R11_O15_POOR_FETGRO | Poor fetal growth \|\| id:finngen_R11_O15_POOR_FETGRO | IgD- CD38- B cell %lymphocyte \|\| id:ebi-a-GCST90001427 | Weighted mode | 28 | 0.0306483918361516 | 0.0514152182095658 | 0.556076171292105 | -0.070125436 | 0.131422219526901 | 1.03112288891539 | 0.932276871625234 | 1.14044919959425 | 0.999857399747617 |
| ebi-a-GCST90001428 | finngen_R11_O15_POOR_FETGRO | Poor fetal growth \|\| id:finngen_R11_O15_POOR_FETGRO | IgD- CD38dim B cell %B cell \|\| id:ebi-a-GCST90001428 | Inverse variance weighted | 25 | 0.0059878941243888 | 0.0179011010012935 | 0.738003309184205 | -0.029098264 | 0.0410740520869241 | 1.00600585739857 | 0.971321014046107 | 1.04192925972484 | 0.994119304454654 |
| ebi-a-GCST90001428 | finngen_R11_O15_POOR_FETGRO | Poor fetal growth \|\| id:finngen_R11_O15_POOR_FETGRO | IgD- CD38dim B cell %B cell \|\| id:ebi-a-GCST90001428 | MR Egger | 25 | -0.001299847 | 0.0217004252822833 | 0.952753034913619 | -0.04383268 | 0.0412329868851259 | 0.998700997766613 | 0.957114088193463 | 1.04209487170188 | 0.992899239648112 |
| ebi-a-GCST90001428 | finngen_R11_O15_POOR_FETGRO | Poor fetal growth \|\| id:finngen_R11_O15_POOR_FETGRO | IgD- CD38dim B cell %B cell \|\| id:ebi-a-GCST90001428 | Weighted median | 25 | -1.57E-05 | 0.0224111914726348 | 0.999440741871876 | -0.043941644 | 0.0439102266956307 | 0.999984291532646 | 0.957009803225256 | 1.0448885475802 | 0.999440741871876 |
| ebi-a-GCST90001428 | finngen_R11_O15_POOR_FETGRO | Poor fetal growth \|\| id:finngen_R11_O15_POOR_FETGRO | IgD- CD38dim B cell %B cell \|\| id:ebi-a-GCST90001428 | Weighted mode | 25 | -0.005051496 | 0.0186533546547572 | 0.788853419533596 | -0.041612071 | 0.0315090795393709 | 0.994961241763287 | 0.959241826407348 | 1.0320107457353 | 0.999857399747617 |
| ebi-a-GCST90001429 | finngen_R11_O15_POOR_FETGRO | Poor fetal growth \|\| id:finngen_R11_O15_POOR_FETGRO | IgD+ CD38+ B cell %lymphocyte \|\| id:ebi-a-GCST90001429 | Inverse variance weighted | 25 | 0.0250397643421229 | 0.0263926403294034 | 0.342752657274321 | -0.026689811 | 0.0767693393877535 | 1.02535589231629 | 0.973663214595064 | 1.07979298195525 | 0.994119304454654 |
| ebi-a-GCST90001429 | finngen_R11_O15_POOR_FETGRO | Poor fetal growth \|\| id:finngen_R11_O15_POOR_FETGRO | IgD+ CD38+ B cell %lymphocyte \|\| id:ebi-a-GCST90001429 | MR Egger | 25 | -0.031147483 | 0.038877452374645 | 0.431232008580228 | -0.10734729 | 0.0450523237355214 | 0.969332602531936 | 0.898213678572265 | 1.04608259342573 | 0.992899239648112 |
| ebi-a-GCST90001429 | finngen_R11_O15_POOR_FETGRO | Poor fetal growth \|\| id:finngen_R11_O15_POOR_FETGRO | IgD+ CD38+ B cell %lymphocyte \|\| id:ebi-a-GCST90001429 | Weighted median | 25 | -0.000762314 | 0.0383024354025076 | 0.98412115875029 | -0.075835087 | 0.0743104595486345 | 0.999237976647096 | 0.926969062991675 | 1.07714116235047 | 0.999440741871876 |
| ebi-a-GCST90001429 | finngen_R11_O15_POOR_FETGRO | Poor fetal growth \|\| id:finngen_R11_O15_POOR_FETGRO | IgD+ CD38+ B cell %lymphocyte \|\| id:ebi-a-GCST90001429 | Weighted mode | 25 | -3.39E-05 | 0.0334934808397123 | 0.999200372660642 | -0.06568114 | 0.06561330442294 | 0.999966082552314 | 0.936429406134139 | 1.0678137184767 | 0.999857399747617 |
| ebi-a-GCST90001430 | finngen_R11_O15_POOR_FETGRO | Poor fetal growth \|\| id:finngen_R11_O15_POOR_FETGRO | IgD+ CD38dim B cell %lymphocyte \|\| id:ebi-a-GCST90001430 | Inverse variance weighted | 34 | 0.0220015763307792 | 0.01296943688331 | 0.0898068909484369 | -0.00341852 | 0.0474216726220667 | 1.02224539586605 | 0.996587316526242 | 1.0485640666307 | 0.994119304454654 |
| ebi-a-GCST90001430 | finngen_R11_O15_POOR_FETGRO | Poor fetal growth \|\| id:finngen_R11_O15_POOR_FETGRO | IgD+ CD38dim B cell %lymphocyte \|\| id:ebi-a-GCST90001430 | MR Egger | 34 | 0.0146810610557705 | 0.0149465985151762 | 0.333352906323685 | -0.014614272 | 0.0439763941455159 | 1.01478935715082 | 0.985491998122721 | 1.04495768747818 | 0.992899239648112 |
| ebi-a-GCST90001430 | finngen_R11_O15_POOR_FETGRO | Poor fetal growth \|\| id:finngen_R11_O15_POOR_FETGRO | IgD+ CD38dim B cell %lymphocyte \|\| id:ebi-a-GCST90001430 | Weighted median | 34 | 0.000342875272203196 | 0.0198574445663735 | 0.986223741349925 | -0.038577716 | 0.0392634666222953 | 1.00034293406065 | 0.962156926771468 | 1.04004446455827 | 0.999440741871876 |
| ebi-a-GCST90001430 | finngen_R11_O15_POOR_FETGRO | Poor fetal growth \|\| id:finngen_R11_O15_POOR_FETGRO | IgD+ CD38dim B cell %lymphocyte \|\| id:ebi-a-GCST90001430 | Weighted mode | 34 | 0.0118253953969349 | 0.0153852769850178 | 0.447586412579153 | -0.018329747 | 0.0419805382875697 | 1.01189559181232 | 0.981837220610461 | 1.04287418243582 | 0.999857399747617 |
| ebi-a-GCST90001431 | finngen_R11_O15_POOR_FETGRO | Poor fetal growth \|\| id:finngen_R11_O15_POOR_FETGRO | IgD+ CD38- B cell %lymphocyte \|\| id:ebi-a-GCST90001431 | Inverse variance weighted | 20 | 0.0104210047626645 | 0.0272290445379726 | 0.701929975242059 | -0.042947923 | 0.0637899320570909 | 1.01047549254078 | 0.957961276966002 | 1.06586847044525 | 0.994119304454654 |
| ebi-a-GCST90001431 | finngen_R11_O15_POOR_FETGRO | Poor fetal growth \|\| id:finngen_R11_O15_POOR_FETGRO | IgD+ CD38- B cell %lymphocyte \|\| id:ebi-a-GCST90001431 | MR Egger | 20 | 0.0331200048953812 | 0.032900640979982 | 0.327436318501971 | -0.031365251 | 0.0976052612161458 | 1.03367457780811 | 0.969121535401404 | 1.10252748883989 | 0.992899239648112 |
| ebi-a-GCST90001431 | finngen_R11_O15_POOR_FETGRO | Poor fetal growth \|\| id:finngen_R11_O15_POOR_FETGRO | IgD+ CD38- B cell %lymphocyte \|\| id:ebi-a-GCST90001431 | Weighted median | 20 | 0.0341272836056807 | 0.0319755945419478 | 0.285840177996668 | -0.028544882 | 0.0967994489078984 | 1.03471630076831 | 0.971858674501176 | 1.10163941647708 | 0.999440741871876 |
| ebi-a-GCST90001431 | finngen_R11_O15_POOR_FETGRO | Poor fetal growth \|\| id:finngen_R11_O15_POOR_FETGRO | IgD+ CD38- B cell %lymphocyte \|\| id:ebi-a-GCST90001431 | Weighted mode | 20 | 0.0334890650098494 | 0.0300879926982966 | 0.279576424207065 | -0.025483401 | 0.0924615306985108 | 1.03405613627079 | 0.974838560489811 | 1.0968709448896 | 0.999857399747617 |
| ebi-a-GCST90001432 | finngen_R11_O15_POOR_FETGRO | Poor fetal growth \|\| id:finngen_R11_O15_POOR_FETGRO | Unswitched memory B cell %lymphocyte \|\| id:ebi-a-GCST90001432 | Inverse variance weighted | 22 | -0.040306065 | 0.0360814780395282 | 0.263958136951866 | -0.111025762 | 0.0304136320196917 | 0.960495420189149 | 0.89491569390769 | 1.03088085110673 | 0.994119304454654 |
| ebi-a-GCST90001432 | finngen_R11_O15_POOR_FETGRO | Poor fetal growth \|\| id:finngen_R11_O15_POOR_FETGRO | Unswitched memory B cell %lymphocyte \|\| id:ebi-a-GCST90001432 | MR Egger | 22 | -0.112115333 | 0.0615642647267695 | 0.0835848803657022 | -0.232781292 | 0.00855062557511677 | 0.89394115038127 | 0.792326842673194 | 1.00858728659102 | 0.992899239648112 |
| ebi-a-GCST90001432 | finngen_R11_O15_POOR_FETGRO | Poor fetal growth \|\| id:finngen_R11_O15_POOR_FETGRO | Unswitched memory B cell %lymphocyte \|\| id:ebi-a-GCST90001432 | Weighted median | 22 | -0.049372856 | 0.0492223885616415 | 0.315833414045133 | -0.145848737 | 0.0471030256783163 | 0.951826169522657 | 0.86428842774025 | 1.04822999812333 | 0.999440741871876 |
| ebi-a-GCST90001432 | finngen_R11_O15_POOR_FETGRO | Poor fetal growth \|\| id:finngen_R11_O15_POOR_FETGRO | Unswitched memory B cell %lymphocyte \|\| id:ebi-a-GCST90001432 | Weighted mode | 22 | -0.057430696 | 0.058894568514808 | 0.340588747760381 | -0.17286405 | 0.0580026586613076 | 0.944187324428801 | 0.841251975346879 | 1.05971781313729 | 0.999857399747617 |
| ebi-a-GCST90001433 | finngen_R11_O15_POOR_FETGRO | Poor fetal growth \|\| id:finngen_R11_O15_POOR_FETGRO | IgD- CD27- B cell %lymphocyte \|\| id:ebi-a-GCST90001433 | Inverse variance weighted | 18 | 0.0028755978959191 | 0.0366053584248139 | 0.937385191728301 | -0.068870905 | 0.0746221004085544 | 1.00287973639348 | 0.933447176021263 | 1.07747689585999 | 0.998984604424952 |
| ebi-a-GCST90001433 | finngen_R11_O15_POOR_FETGRO | Poor fetal growth \|\| id:finngen_R11_O15_POOR_FETGRO | IgD- CD27- B cell %lymphocyte \|\| id:ebi-a-GCST90001433 | MR Egger | 18 | 0.138042850645328 | 0.0936251750640279 | 0.159772501387529 | -0.045462492 | 0.321548193770822 | 1.14802474282129 | 0.955555442414527 | 1.37926147623587 | 0.992899239648112 |
| ebi-a-GCST90001433 | finngen_R11_O15_POOR_FETGRO | Poor fetal growth \|\| id:finngen_R11_O15_POOR_FETGRO | IgD- CD27- B cell %lymphocyte \|\| id:ebi-a-GCST90001433 | Weighted median | 18 | 0.0367934493902646 | 0.048160278800972 | 0.444879661212721 | -0.057600697 | 0.13118759584017 | 1.03747870684609 | 0.944026824874607 | 1.14018165458593 | 0.999440741871876 |
| ebi-a-GCST90001433 | finngen_R11_O15_POOR_FETGRO | Poor fetal growth \|\| id:finngen_R11_O15_POOR_FETGRO | IgD- CD27- B cell %lymphocyte \|\| id:ebi-a-GCST90001433 | Weighted mode | 18 | 0.0373662806203229 | 0.0585031057146835 | 0.5315258933343 | -0.077299807 | 0.152032367821103 | 1.03807317729926 | 0.925612307342783 | 1.16419791837222 | 0.999857399747617 |
| ebi-a-GCST90001434 | finngen_R11_O15_POOR_FETGRO | Poor fetal growth \|\| id:finngen_R11_O15_POOR_FETGRO | Switched memory B cell %lymphocyte \|\| id:ebi-a-GCST90001434 | Inverse variance weighted | 18 | 0.0282556032471635 | 0.0296690782759288 | 0.34091512413354 | -0.02989579 | 0.0864069966679839 | 1.02865857929469 | 0.970546668778585 | 1.09024996612293 | 0.994119304454654 |
| ebi-a-GCST90001434 | finngen_R11_O15_POOR_FETGRO | Poor fetal growth \|\| id:finngen_R11_O15_POOR_FETGRO | Switched memory B cell %lymphocyte \|\| id:ebi-a-GCST90001434 | MR Egger | 18 | 0.00207485127301789 | 0.0462238360136826 | 0.964752796253635 | -0.088523867 | 0.0926735698598357 | 1.0020770052664 | 0.91528126517013 | 1.09710354914457 | 0.992899239648112 |
| ebi-a-GCST90001434 | finngen_R11_O15_POOR_FETGRO | Poor fetal growth \|\| id:finngen_R11_O15_POOR_FETGRO | Switched memory B cell %lymphocyte \|\| id:ebi-a-GCST90001434 | Weighted median | 18 | 0.0103405393307149 | 0.0414243848992157 | 0.802877788055545 | -0.070851255 | 0.0915323337331776 | 1.01039418746497 | 0.931600452664739 | 1.09585220911264 | 0.999440741871876 |
| ebi-a-GCST90001434 | finngen_R11_O15_POOR_FETGRO | Poor fetal growth \|\| id:finngen_R11_O15_POOR_FETGRO | Switched memory B cell %lymphocyte \|\| id:ebi-a-GCST90001434 | Weighted mode | 18 | 0.00198673377498598 | 0.0425522701133183 | 0.963304994932531 | -0.081415716 | 0.0853891831970898 | 1.00198870863816 | 0.921810400772886 | 1.08914085954832 | 0.999857399747617 |
| ebi-a-GCST90001435 | finngen_R11_O15_POOR_FETGRO | Poor fetal growth \|\| id:finngen_R11_O15_POOR_FETGRO | Plasma Blast-Plasma Cell %lymphocyte \|\| id:ebi-a-GCST90001435 | Inverse variance weighted | 18 | 0.00356638956293304 | 0.0144681905391613 | 0.805296242551374 | -0.024791264 | 0.0319240430196892 | 1.00357275669717 | 0.975513515670662 | 1.03243908137176 | 0.994119304454654 |
| ebi-a-GCST90001435 | finngen_R11_O15_POOR_FETGRO | Poor fetal growth \|\| id:finngen_R11_O15_POOR_FETGRO | Plasma Blast-Plasma Cell %lymphocyte \|\| id:ebi-a-GCST90001435 | MR Egger | 18 | -0.012757885 | 0.0164949554919951 | 0.450537915868262 | -0.045087997 | 0.0195722282377024 | 0.987323152296639 | 0.955913360345859 | 1.01976502003097 | 0.992899239648112 |
| ebi-a-GCST90001435 | finngen_R11_O15_POOR_FETGRO | Poor fetal growth \|\| id:finngen_R11_O15_POOR_FETGRO | Plasma Blast-Plasma Cell %lymphocyte \|\| id:ebi-a-GCST90001435 | Weighted median | 18 | -0.003247191 | 0.0182733865969841 | 0.858958125927517 | -0.039063029 | 0.0325686462449015 | 0.996758074939178 | 0.961690092664203 | 1.03310480947564 | 0.999440741871876 |
| ebi-a-GCST90001435 | finngen_R11_O15_POOR_FETGRO | Poor fetal growth \|\| id:finngen_R11_O15_POOR_FETGRO | Plasma Blast-Plasma Cell %lymphocyte \|\| id:ebi-a-GCST90001435 | Weighted mode | 18 | -0.005546279 | 0.0149112514759362 | 0.714523903848528 | -0.034772332 | 0.023679773589768 | 0.994469072908323 | 0.965825278549144 | 1.02396236559138 | 0.999857399747617 |
| ebi-a-GCST90001436 | finngen_R11_O15_POOR_FETGRO | Poor fetal growth \|\| id:finngen_R11_O15_POOR_FETGRO | Memory B cell %lymphocyte \|\| id:ebi-a-GCST90001436 | Inverse variance weighted | 28 | -0.037402933 | 0.0149767007358269 | 0.0125104910460678 | -0.066757266 | -0.008048599 | 0.96328791687724 | 0.935422232367674 | 0.991983703929078 | 0.994119304454654 |
| ebi-a-GCST90001436 | finngen_R11_O15_POOR_FETGRO | Poor fetal growth \|\| id:finngen_R11_O15_POOR_FETGRO | Memory B cell %lymphocyte \|\| id:ebi-a-GCST90001436 | MR Egger | 28 | -0.036761989 | 0.0173212605908836 | 0.0434936814651465 | -0.07071166 | -0.002812319 | 0.963905527763744 | 0.931730508409094 | 0.997191632202686 | 0.992899239648112 |
| ebi-a-GCST90001436 | finngen_R11_O15_POOR_FETGRO | Poor fetal growth \|\| id:finngen_R11_O15_POOR_FETGRO | Memory B cell %lymphocyte \|\| id:ebi-a-GCST90001436 | Weighted median | 28 | -0.037441124 | 0.0205501345674921 | 0.0684640051766548 | -0.077719387 | 0.00283714007470524 | 0.963251128735365 | 0.925224019609391 | 1.0028411685655 | 0.999440741871876 |
| ebi-a-GCST90001436 | finngen_R11_O15_POOR_FETGRO | Poor fetal growth \|\| id:finngen_R11_O15_POOR_FETGRO | Memory B cell %lymphocyte \|\| id:ebi-a-GCST90001436 | Weighted mode | 28 | -0.032441672 | 0.0187977532577309 | 0.0958073290610562 | -0.069285268 | 0.00440192483492163 | 0.968078914726885 | 0.93306046987556 | 1.00441162753768 | 0.999857399747617 |
| ebi-a-GCST90001437 | finngen_R11_O15_POOR_FETGRO | Poor fetal growth \|\| id:finngen_R11_O15_POOR_FETGRO | Naive-mature B cell %lymphocyte \|\| id:ebi-a-GCST90001437 | Inverse variance weighted | 22 | -0.000909245 | 0.0380787490533249 | 0.980949900478623 | -0.075543594 | 0.0737251027120864 | 0.999091167805943 | 0.927239307974803 | 1.0765108349084 | 0.998984604424952 |
| ebi-a-GCST90001437 | finngen_R11_O15_POOR_FETGRO | Poor fetal growth \|\| id:finngen_R11_O15_POOR_FETGRO | Naive-mature B cell %lymphocyte \|\| id:ebi-a-GCST90001437 | MR Egger | 22 | -0.089472284 | 0.0914803527428193 | 0.339733172733591 | -0.268773776 | 0.0898292070901166 | 0.914413608399322 | 0.764316143005474 | 1.09398742245312 | 0.992899239648112 |
| ebi-a-GCST90001437 | finngen_R11_O15_POOR_FETGRO | Poor fetal growth \|\| id:finngen_R11_O15_POOR_FETGRO | Naive-mature B cell %lymphocyte \|\| id:ebi-a-GCST90001437 | Weighted median | 22 | -0.018376106 | 0.051801713320333 | 0.7227848678528 | -0.119907464 | 0.0831552519444784 | 0.981791704998107 | 0.887002512343538 | 1.086710509 | 0.999440741871876 |
| ebi-a-GCST90001437 | finngen_R11_O15_POOR_FETGRO | Poor fetal growth \|\| id:finngen_R11_O15_POOR_FETGRO | Naive-mature B cell %lymphocyte \|\| id:ebi-a-GCST90001437 | Weighted mode | 22 | -0.029192144 | 0.0704349270885966 | 0.682743553901781 | -0.167244601 | 0.108860312883956 | 0.971229830348664 | 0.845992655121279 | 1.1150065874081 | 0.999857399747617 |
| ebi-a-GCST90001438 | finngen_R11_O15_POOR_FETGRO | Poor fetal growth \|\| id:finngen_R11_O15_POOR_FETGRO | IgD- CD38dim B cell Absolute Count \|\| id:ebi-a-GCST90001438 | Inverse variance weighted (multiplicative random effects) | 17 | -0.060176516 | 0.0555508090060089 | 0.278688664767128 | -0.169056102 | 0.0487030692465 | 0.941598311365069 | 0.844461526028349 | 1.04990855431321 | 0.994119304454654 |
| ebi-a-GCST90001438 | finngen_R11_O15_POOR_FETGRO | Poor fetal growth \|\| id:finngen_R11_O15_POOR_FETGRO | IgD- CD38dim B cell Absolute Count \|\| id:ebi-a-GCST90001438 | MR Egger | 17 | -0.00397941 | 0.102175728966433 | 0.969446498670674 | -0.204243839 | 0.196285018894531 | 0.996028497479466 | 0.815263554181028 | 1.21687368790549 | 0.992899239648112 |
| ebi-a-GCST90001438 | finngen_R11_O15_POOR_FETGRO | Poor fetal growth \|\| id:finngen_R11_O15_POOR_FETGRO | IgD- CD38dim B cell Absolute Count \|\| id:ebi-a-GCST90001438 | Weighted median | 17 | -0.018242825 | 0.0677351055555892 | 0.787678829807349 | -0.151003632 | 0.114517982183877 | 0.981922568348927 | 0.859844576047104 | 1.12133280489535 | 0.999440741871876 |
| ebi-a-GCST90001438 | finngen_R11_O15_POOR_FETGRO | Poor fetal growth \|\| id:finngen_R11_O15_POOR_FETGRO | IgD- CD38dim B cell Absolute Count \|\| id:ebi-a-GCST90001438 | Weighted mode | 17 | 0.142267099153738 | 0.0876709518363355 | 0.124180894401578 | -0.029567966 | 0.314102164752955 | 1.15288454190165 | 0.970864889163161 | 1.36902959597336 | 0.999857399747617 |
| ebi-a-GCST90001439 | finngen_R11_O15_POOR_FETGRO | Poor fetal growth \|\| id:finngen_R11_O15_POOR_FETGRO | IgD+ CD24+ B cell %lymphocyte \|\| id:ebi-a-GCST90001439 | Inverse variance weighted | 15 | -0.107196217 | 0.0461216056782474 | 0.0201143341243476 | -0.197594565 | -0.01679787 | 0.89834938384072 | 0.820702527541954 | 0.983342427205753 | 0.994119304454654 |
| ebi-a-GCST90001439 | finngen_R11_O15_POOR_FETGRO | Poor fetal growth \|\| id:finngen_R11_O15_POOR_FETGRO | IgD+ CD24+ B cell %lymphocyte \|\| id:ebi-a-GCST90001439 | MR Egger | 15 | -0.154847036 | 0.10531758075342 | 0.165269182967806 | -0.361269494 | 0.0515754222073999 | 0.856546188135166 | 0.696791191875044 | 1.05292859749647 | 0.992899239648112 |
| ebi-a-GCST90001439 | finngen_R11_O15_POOR_FETGRO | Poor fetal growth \|\| id:finngen_R11_O15_POOR_FETGRO | IgD+ CD24+ B cell %lymphocyte \|\| id:ebi-a-GCST90001439 | Weighted median | 15 | -0.151021172 | 0.0625795803993387 | 0.0158102775326764 | -0.27367715 | -0.028365194 | 0.859829494203318 | 0.760577588460008 | 0.972033320885588 | 0.999440741871876 |
| ebi-a-GCST90001439 | finngen_R11_O15_POOR_FETGRO | Poor fetal growth \|\| id:finngen_R11_O15_POOR_FETGRO | IgD+ CD24+ B cell %lymphocyte \|\| id:ebi-a-GCST90001439 | Weighted mode | 15 | -0.160544965 | 0.0854411648389451 | 0.0812226784249815 | -0.328009648 | 0.00691971849245629 | 0.851679527288615 | 0.720356069909659 | 1.00694371506237 | 0.999857399747617 |
| ebi-a-GCST90001440 | finngen_R11_O15_POOR_FETGRO | Poor fetal growth \|\| id:finngen_R11_O15_POOR_FETGRO | IgD- CD24- B cell %lymphocyte \|\| id:ebi-a-GCST90001440 | Inverse variance weighted | 24 | -0.020470634 | 0.0295680904629516 | 0.488735248742432 | -0.078424092 | 0.0374828230354358 | 0.979737466756095 | 0.924572240086423 | 1.0381941639043 | 0.994119304454654 |
| ebi-a-GCST90001440 | finngen_R11_O15_POOR_FETGRO | Poor fetal growth \|\| id:finngen_R11_O15_POOR_FETGRO | IgD- CD24- B cell %lymphocyte \|\| id:ebi-a-GCST90001440 | MR Egger | 24 | 0.00917292037374592 | 0.043890559513814 | 0.836374949569662 | -0.076852576 | 0.0951984170208213 | 1.00921512054207 | 0.926026361801227 | 1.0998770678079 | 0.992899239648112 |
| ebi-a-GCST90001440 | finngen_R11_O15_POOR_FETGRO | Poor fetal growth \|\| id:finngen_R11_O15_POOR_FETGRO | IgD- CD24- B cell %lymphocyte \|\| id:ebi-a-GCST90001440 | Weighted median | 24 | -0.001737907 | 0.0432458033209042 | 0.967944256800326 | -0.086499682 | 0.0830238670758728 | 0.998263601853563 | 0.917135840486985 | 1.08656774143349 | 0.999440741871876 |
| ebi-a-GCST90001440 | finngen_R11_O15_POOR_FETGRO | Poor fetal growth \|\| id:finngen_R11_O15_POOR_FETGRO | IgD- CD24- B cell %lymphocyte \|\| id:ebi-a-GCST90001440 | Weighted mode | 24 | -0.007073385 | 0.0405462061835501 | 0.863036401635191 | -0.086543949 | 0.0723971790899102 | 0.992951572478684 | 0.917095242342987 | 1.07508225947066 | 0.999857399747617 |
| ebi-a-GCST90001441 | finngen_R11_O15_POOR_FETGRO | Poor fetal growth \|\| id:finngen_R11_O15_POOR_FETGRO | IgD+ CD24- B cell %lymphocyte \|\| id:ebi-a-GCST90001441 | Inverse variance weighted | 23 | 0.0741388436039963 | 0.0381896476245538 | 0.052217834486259 | -0.000712866 | 0.148990552948122 | 1.07695632361348 | 0.999287388288286 | 1.16066202432294 | 0.994119304454654 |
| ebi-a-GCST90001441 | finngen_R11_O15_POOR_FETGRO | Poor fetal growth \|\| id:finngen_R11_O15_POOR_FETGRO | IgD+ CD24- B cell %lymphocyte \|\| id:ebi-a-GCST90001441 | MR Egger | 23 | 0.164160408929742 | 0.0842161174086364 | 0.0647443863283944 | -0.000903181 | 0.32922399905067 | 1.17840332634915 | 0.999097226554182 | 1.38988915457213 | 0.992899239648112 |
| ebi-a-GCST90001441 | finngen_R11_O15_POOR_FETGRO | Poor fetal growth \|\| id:finngen_R11_O15_POOR_FETGRO | IgD+ CD24- B cell %lymphocyte \|\| id:ebi-a-GCST90001441 | Weighted median | 23 | 0.102226013924459 | 0.056399916228717 | 0.0699057831336468 | -0.008317822 | 0.212769849732745 | 1.10763378409823 | 0.991716675482708 | 1.23709989960451 | 0.999440741871876 |
| ebi-a-GCST90001441 | finngen_R11_O15_POOR_FETGRO | Poor fetal growth \|\| id:finngen_R11_O15_POOR_FETGRO | IgD+ CD24- B cell %lymphocyte \|\| id:ebi-a-GCST90001441 | Weighted mode | 23 | 0.0723567606078508 | 0.0717005485114527 | 0.323872761383948 | -0.068176314 | 0.212889835690298 | 1.07503880715579 | 0.934095764453407 | 1.23724834312594 | 0.999857399747617 |
| ebi-a-GCST90001442 | finngen_R11_O15_POOR_FETGRO | Poor fetal growth \|\| id:finngen_R11_O15_POOR_FETGRO | CD24+ CD27+ B cell %lymphocyte \|\| id:ebi-a-GCST90001442 | Inverse variance weighted | 24 | -0.017038464 | 0.014510380857131 | 0.240304573833826 | -0.045478811 | 0.0114018821583104 | 0.983105869407253 | 0.955539849480854 | 1.01146713136882 | 0.994119304454654 |
| ebi-a-GCST90001442 | finngen_R11_O15_POOR_FETGRO | Poor fetal growth \|\| id:finngen_R11_O15_POOR_FETGRO | CD24+ CD27+ B cell %lymphocyte \|\| id:ebi-a-GCST90001442 | MR Egger | 24 | -0.02201444 | 0.0167308514064131 | 0.201788811819549 | -0.054806909 | 0.0107780288356487 | 0.978226109441561 | 0.946667923668072 | 1.01083632102517 | 0.992899239648112 |
| ebi-a-GCST90001442 | finngen_R11_O15_POOR_FETGRO | Poor fetal growth \|\| id:finngen_R11_O15_POOR_FETGRO | CD24+ CD27+ B cell %lymphocyte \|\| id:ebi-a-GCST90001442 | Weighted median | 24 | -0.031486774 | 0.020636304407168 | 0.127060876763651 | -0.071933931 | 0.00896038245053629 | 0.969003772231181 | 0.930592377250272 | 1.00900064684898 | 0.999440741871876 |
| ebi-a-GCST90001442 | finngen_R11_O15_POOR_FETGRO | Poor fetal growth \|\| id:finngen_R11_O15_POOR_FETGRO | CD24+ CD27+ B cell %lymphocyte \|\| id:ebi-a-GCST90001442 | Weighted mode | 24 | -0.019484526 | 0.0172411625465931 | 0.270075210963573 | -0.053277205 | 0.0143081521628818 | 0.980704070065868 | 0.948117153216832 | 1.01441100372519 | 0.999857399747617 |
| ebi-a-GCST90001443 | finngen_R11_O15_POOR_FETGRO | Poor fetal growth \|\| id:finngen_R11_O15_POOR_FETGRO | CD20- B cell %lymphocyte \|\| id:ebi-a-GCST90001443 | Inverse variance weighted | 18 | 0.0089465238211836 | 0.0216900584602091 | 0.67999410668396 | -0.033565991 | 0.0514590384031935 | 1.0089866635799 | 0.96699109664766 | 1.05280606079152 | 0.994119304454654 |
| ebi-a-GCST90001443 | finngen_R11_O15_POOR_FETGRO | Poor fetal growth \|\| id:finngen_R11_O15_POOR_FETGRO | CD20- B cell %lymphocyte \|\| id:ebi-a-GCST90001443 | MR Egger | 18 | 0.0251269717692337 | 0.0270857984978789 | 0.367356967301625 | -0.027961193 | 0.0782151368250764 | 1.02544531486463 | 0.972426102730101 | 1.08135527298744 | 0.992899239648112 |
| ebi-a-GCST90001443 | finngen_R11_O15_POOR_FETGRO | Poor fetal growth \|\| id:finngen_R11_O15_POOR_FETGRO | CD20- B cell %lymphocyte \|\| id:ebi-a-GCST90001443 | Weighted median | 18 | 0.0190054209334182 | 0.025571850829297 | 0.457350628062761 | -0.031115407 | 0.0691262485588404 | 1.01918717354824 | 0.969363695562989 | 1.07157148496465 | 0.999440741871876 |
| ebi-a-GCST90001443 | finngen_R11_O15_POOR_FETGRO | Poor fetal growth \|\| id:finngen_R11_O15_POOR_FETGRO | CD20- B cell %lymphocyte \|\| id:ebi-a-GCST90001443 | Weighted mode | 18 | 0.0223730173049214 | 0.0220076489592202 | 0.32359377067254 | -0.020761975 | 0.065508009264993 | 1.02262517021921 | 0.979452071242732 | 1.06770128878181 | 0.999857399747617 |
| ebi-a-GCST90001444 | finngen_R11_O15_POOR_FETGRO | Poor fetal growth \|\| id:finngen_R11_O15_POOR_FETGRO | CD20- CD38- B cell %lymphocyte \|\| id:ebi-a-GCST90001444 | Inverse variance weighted | 23 | 0.0288987404068244 | 0.0287147966780979 | 0.314220363155359 | -0.027382261 | 0.0851797418958964 | 1.02932036063692 | 0.972989234509166 | 1.08891277235579 | 0.994119304454654 |
| ebi-a-GCST90001444 | finngen_R11_O15_POOR_FETGRO | Poor fetal growth \|\| id:finngen_R11_O15_POOR_FETGRO | CD20- CD38- B cell %lymphocyte \|\| id:ebi-a-GCST90001444 | MR Egger | 23 | 0.0620290101872781 | 0.0421676292770018 | 0.156115041571555 | -0.020619543 | 0.144677563570202 | 1.06399321092263 | 0.979591585966161 | 1.15566688108379 | 0.992899239648112 |
| ebi-a-GCST90001444 | finngen_R11_O15_POOR_FETGRO | Poor fetal growth \|\| id:finngen_R11_O15_POOR_FETGRO | CD20- CD38- B cell %lymphocyte \|\| id:ebi-a-GCST90001444 | Weighted median | 23 | 0.0489720307200666 | 0.0414190336180381 | 0.237064590830385 | -0.032209275 | 0.130153336611421 | 1.05019097724383 | 0.968303918905264 | 1.13900302079873 | 0.999440741871876 |
| ebi-a-GCST90001444 | finngen_R11_O15_POOR_FETGRO | Poor fetal growth \|\| id:finngen_R11_O15_POOR_FETGRO | CD20- CD38- B cell %lymphocyte \|\| id:ebi-a-GCST90001444 | Weighted mode | 23 | 0.0432937575125126 | 0.0372520815809225 | 0.257614066509483 | -0.029720322 | 0.116307837411121 | 1.04424460449675 | 0.970716983397209 | 1.12334162549041 | 0.999857399747617 |
| ebi-a-GCST90001445 | finngen_R11_O15_POOR_FETGRO | Poor fetal growth \|\| id:finngen_R11_O15_POOR_FETGRO | IgD- CD38- B cell %B cell \|\| id:ebi-a-GCST90001445 | Inverse variance weighted | 12 | -0.050530079 | 0.0503353480971809 | 0.315441914737528 | -0.149187362 | 0.0481272028176796 | 0.950725330944514 | 0.861407704946371 | 1.0493041212765 | 0.994119304454654 |
| ebi-a-GCST90001445 | finngen_R11_O15_POOR_FETGRO | Poor fetal growth \|\| id:finngen_R11_O15_POOR_FETGRO | IgD- CD38- B cell %B cell \|\| id:ebi-a-GCST90001445 | MR Egger | 12 | 0.0123297963482575 | 0.091901689299866 | 0.895935522628607 | -0.167797515 | 0.192457107375995 | 1.01240612165651 | 0.84552502375788 | 1.21222450710232 | 0.992899239648112 |
| ebi-a-GCST90001445 | finngen_R11_O15_POOR_FETGRO | Poor fetal growth \|\| id:finngen_R11_O15_POOR_FETGRO | IgD- CD38- B cell %B cell \|\| id:ebi-a-GCST90001445 | Weighted median | 12 | -0.113599028 | 0.0677777070119404 | 0.0937278163717216 | -0.246443333 | 0.0192452781878342 | 0.892615798473756 | 0.78157564959583 | 1.01943166230545 | 0.999440741871876 |
| ebi-a-GCST90001445 | finngen_R11_O15_POOR_FETGRO | Poor fetal growth \|\| id:finngen_R11_O15_POOR_FETGRO | IgD- CD38- B cell %B cell \|\| id:ebi-a-GCST90001445 | Weighted mode | 12 | -0.116687575 | 0.0919494579552282 | 0.230621419914745 | -0.296908512 | 0.0635333628425969 | 0.889863165460614 | 0.743111994828229 | 1.0655950364879 | 0.999857399747617 |
| ebi-a-GCST90001446 | finngen_R11_O15_POOR_FETGRO | Poor fetal growth \|\| id:finngen_R11_O15_POOR_FETGRO | IgD- CD38- B cell Absolute Count \|\| id:ebi-a-GCST90001446 | Inverse variance weighted (multiplicative random effects) | 14 | -0.00078944 | 0.0396603429372812 | 0.984119148790411 | -0.078523712 | 0.0769448326469597 | 0.999210872015276 | 0.924480138706294 | 1.07998249497356 | 0.998984604424952 |
| ebi-a-GCST90001446 | finngen_R11_O15_POOR_FETGRO | Poor fetal growth \|\| id:finngen_R11_O15_POOR_FETGRO | IgD- CD38- B cell Absolute Count \|\| id:ebi-a-GCST90001446 | MR Egger | 14 | 0.0442974016267847 | 0.0489059668404215 | 0.382885740282172 | -0.051558293 | 0.140153096634011 | 1.04529318055849 | 0.949748284310429 | 1.1504499153851 | 0.992899239648112 |
| ebi-a-GCST90001446 | finngen_R11_O15_POOR_FETGRO | Poor fetal growth \|\| id:finngen_R11_O15_POOR_FETGRO | IgD- CD38- B cell Absolute Count \|\| id:ebi-a-GCST90001446 | Weighted median | 14 | 0.0360691323114984 | 0.040737896249398 | 0.375943592951821 | -0.043777144 | 0.115915408960318 | 1.03672751538311 | 0.957167243846547 | 1.12290088076264 | 0.999440741871876 |
| ebi-a-GCST90001446 | finngen_R11_O15_POOR_FETGRO | Poor fetal growth \|\| id:finngen_R11_O15_POOR_FETGRO | IgD- CD38- B cell Absolute Count \|\| id:ebi-a-GCST90001446 | Weighted mode | 14 | 0.0319550033837979 | 0.0326571048110187 | 0.345685782187503 | -0.032052922 | 0.0959629288133945 | 1.03247104655646 | 0.968455328085749 | 1.10071825830567 | 0.999857399747617 |
| ebi-a-GCST90001447 | finngen_R11_O15_POOR_FETGRO | Poor fetal growth \|\| id:finngen_R11_O15_POOR_FETGRO | IgD+ CD38+ B cell %B cell \|\| id:ebi-a-GCST90001447 | Inverse variance weighted | 25 | 7.3696822039705e-05 | 0.0228042338348316 | 0.997421467673155 | -0.044622601 | 0.0447699951383096 | 1.00007369953772 | 0.956358341944014 | 1.04578729608196 | 0.998984604424952 |
| ebi-a-GCST90001447 | finngen_R11_O15_POOR_FETGRO | Poor fetal growth \|\| id:finngen_R11_O15_POOR_FETGRO | IgD+ CD38+ B cell %B cell \|\| id:ebi-a-GCST90001447 | MR Egger | 25 | -0.021128313 | 0.0338400966279141 | 0.538537477845338 | -0.087454903 | 0.0451982759192743 | 0.979093325645574 | 0.916260191431329 | 1.04623528260701 | 0.992899239648112 |
| ebi-a-GCST90001447 | finngen_R11_O15_POOR_FETGRO | Poor fetal growth \|\| id:finngen_R11_O15_POOR_FETGRO | IgD+ CD38+ B cell %B cell \|\| id:ebi-a-GCST90001447 | Weighted median | 25 | -0.012233398 | 0.029196754379105 | 0.675217184380954 | -0.069459037 | 0.0449922405267854 | 0.987841125755041 | 0.932898347253356 | 1.04601974331505 | 0.999440741871876 |
| ebi-a-GCST90001447 | finngen_R11_O15_POOR_FETGRO | Poor fetal growth \|\| id:finngen_R11_O15_POOR_FETGRO | IgD+ CD38+ B cell %B cell \|\| id:ebi-a-GCST90001447 | Weighted mode | 25 | -0.012659258 | 0.0284985371420999 | 0.660872336853908 | -0.068516391 | 0.043197874424827 | 0.9874205329829 | 0.933778154259555 | 1.04414448389978 | 0.999857399747617 |
| ebi-a-GCST90001448 | finngen_R11_O15_POOR_FETGRO | Poor fetal growth \|\| id:finngen_R11_O15_POOR_FETGRO | CD11c+ monocyte Absolute Count \|\| id:ebi-a-GCST90001448 | Inverse variance weighted | 15 | -0.044474422 | 0.030510758284519 | 0.144933291018046 | -0.104275508 | 0.0153266646149909 | 0.956500065500322 | 0.900977036968266 | 1.01544472030128 | 0.994119304454654 |
| ebi-a-GCST90001448 | finngen_R11_O15_POOR_FETGRO | Poor fetal growth \|\| id:finngen_R11_O15_POOR_FETGRO | CD11c+ monocyte Absolute Count \|\| id:ebi-a-GCST90001448 | MR Egger | 15 | 0.00442102866158539 | 0.0482668892192666 | 0.928415831849285 | -0.090182074 | 0.0990241315313479 | 1.0044308158266 | 0.913764797122296 | 1.10409294269087 | 0.992899239648112 |
| ebi-a-GCST90001448 | finngen_R11_O15_POOR_FETGRO | Poor fetal growth \|\| id:finngen_R11_O15_POOR_FETGRO | CD11c+ monocyte Absolute Count \|\| id:ebi-a-GCST90001448 | Weighted median | 15 | -0.028285148 | 0.046620228367455 | 0.544040683685991 | -0.119660796 | 0.0630904991432707 | 0.972111131287424 | 0.887221334656282 | 1.06512322760928 | 0.999440741871876 |
| ebi-a-GCST90001448 | finngen_R11_O15_POOR_FETGRO | Poor fetal growth \|\| id:finngen_R11_O15_POOR_FETGRO | CD11c+ monocyte Absolute Count \|\| id:ebi-a-GCST90001448 | Weighted mode | 15 | -0.02557741 | 0.0417814949641466 | 0.550238595764327 | -0.10746914 | 0.0563143200032493 | 0.974746920762744 | 0.898104237289827 | 1.05793016009325 | 0.999857399747617 |
| ebi-a-GCST90001449 | finngen_R11_O15_POOR_FETGRO | Poor fetal growth \|\| id:finngen_R11_O15_POOR_FETGRO | CD11c+ monocyte %monocyte \|\| id:ebi-a-GCST90001449 | Inverse variance weighted | 20 | 0.0222589103506396 | 0.0296780945944176 | 0.453247817043394 | -0.035910155 | 0.0804279757556981 | 1.02250848823291 | 0.964726965431256 | 1.08375078749973 | 0.994119304454654 |
| ebi-a-GCST90001449 | finngen_R11_O15_POOR_FETGRO | Poor fetal growth \|\| id:finngen_R11_O15_POOR_FETGRO | CD11c+ monocyte %monocyte \|\| id:ebi-a-GCST90001449 | MR Egger | 20 | -0.013200279 | 0.052729547063188 | 0.805159983961155 | -0.116550191 | 0.0901496332538799 | 0.986886462602189 | 0.889985426389021 | 1.09433802081355 | 0.992899239648112 |
| ebi-a-GCST90001449 | finngen_R11_O15_POOR_FETGRO | Poor fetal growth \|\| id:finngen_R11_O15_POOR_FETGRO | CD11c+ monocyte %monocyte \|\| id:ebi-a-GCST90001449 | Weighted median | 20 | 0.0147663832360805 | 0.0461860134388511 | 0.749184027508042 | -0.075758203 | 0.105290969576229 | 1.0148759448852 | 0.927040334936909 | 1.11103384037408 | 0.999440741871876 |
| ebi-a-GCST90001449 | finngen_R11_O15_POOR_FETGRO | Poor fetal growth \|\| id:finngen_R11_O15_POOR_FETGRO | CD11c+ monocyte %monocyte \|\| id:ebi-a-GCST90001449 | Weighted mode | 20 | 0.00263055580786997 | 0.0413159253445481 | 0.949898633897651 | -0.078348658 | 0.0836097694831842 | 1.00263401875563 | 0.924641986632991 | 1.08720455062471 | 0.999857399747617 |
| ebi-a-GCST90001450 | finngen_R11_O15_POOR_FETGRO | Poor fetal growth \|\| id:finngen_R11_O15_POOR_FETGRO | CD62L- monocyte Absolute Count \|\| id:ebi-a-GCST90001450 | Inverse variance weighted | 20 | 0.00947709649985411 | 0.0203560439485213 | 0.641525673057292 | -0.03042075 | 0.0493749426389559 | 1.00952214638045 | 0.970037304827424 | 1.05061419696008 | 0.994119304454654 |
| ebi-a-GCST90001450 | finngen_R11_O15_POOR_FETGRO | Poor fetal growth \|\| id:finngen_R11_O15_POOR_FETGRO | CD62L- monocyte Absolute Count \|\| id:ebi-a-GCST90001450 | MR Egger | 20 | 0.0313786084468087 | 0.0261893710291617 | 0.246399999260413 | -0.019952559 | 0.0827097756639655 | 1.0318761069492 | 0.980245176240189 | 1.08622651342865 | 0.992899239648112 |
| ebi-a-GCST90001450 | finngen_R11_O15_POOR_FETGRO | Poor fetal growth \|\| id:finngen_R11_O15_POOR_FETGRO | CD62L- monocyte Absolute Count \|\| id:ebi-a-GCST90001450 | Weighted median | 20 | 0.00953294696886583 | 0.0266304931058383 | 0.720364939580005 | -0.04266282 | 0.0617287134563089 | 1.00957853024032 | 0.958234433549612 | 1.06367374520927 | 0.999440741871876 |
| ebi-a-GCST90001450 | finngen_R11_O15_POOR_FETGRO | Poor fetal growth \|\| id:finngen_R11_O15_POOR_FETGRO | CD62L- monocyte Absolute Count \|\| id:ebi-a-GCST90001450 | Weighted mode | 20 | 0.0153355882628293 | 0.0240254672654801 | 0.530893947136546 | -0.031754328 | 0.0624255041031703 | 1.0154537818128 | 0.968744546666884 | 1.06441516140218 | 0.999857399747617 |
| ebi-a-GCST90001451 | finngen_R11_O15_POOR_FETGRO | Poor fetal growth \|\| id:finngen_R11_O15_POOR_FETGRO | CD62L- monocyte %monocyte \|\| id:ebi-a-GCST90001451 | Inverse variance weighted | 23 | 0.00110804838901258 | 0.0274620137512698 | 0.967815365421865 | -0.052717499 | 0.0549335953415015 | 1.00110866250143 | 0.94864796904547 | 1.05647045778618 | 0.998984604424952 |
| ebi-a-GCST90001451 | finngen_R11_O15_POOR_FETGRO | Poor fetal growth \|\| id:finngen_R11_O15_POOR_FETGRO | CD62L- monocyte %monocyte \|\| id:ebi-a-GCST90001451 | MR Egger | 23 | 0.00766524177276693 | 0.0653154360918779 | 0.907692136645162 | -0.120353013 | 0.135683496512848 | 1.00769469494562 | 0.886607397558713 | 1.14531933865835 | 0.992899239648112 |
| ebi-a-GCST90001451 | finngen_R11_O15_POOR_FETGRO | Poor fetal growth \|\| id:finngen_R11_O15_POOR_FETGRO | CD62L- monocyte %monocyte \|\| id:ebi-a-GCST90001451 | Weighted median | 23 | 0.00969303171355055 | 0.0365641007654779 | 0.790934774306773 | -0.061972606 | 0.0813586692138872 | 1.00974016129856 | 0.939908634495953 | 1.08475989678082 | 0.999440741871876 |
| ebi-a-GCST90001451 | finngen_R11_O15_POOR_FETGRO | Poor fetal growth \|\| id:finngen_R11_O15_POOR_FETGRO | CD62L- monocyte %monocyte \|\| id:ebi-a-GCST90001451 | Weighted mode | 23 | 0.0113502409753098 | 0.0478046826584972 | 0.814520748914253 | -0.082346937 | 0.105047418985964 | 1.01141489935826 | 0.920952390772432 | 1.11076328037532 | 0.999857399747617 |
| ebi-a-GCST90001452 | finngen_R11_O15_POOR_FETGRO | Poor fetal growth \|\| id:finngen_R11_O15_POOR_FETGRO | CD11c+ CD62L- monocyte Absolute Count \|\| id:ebi-a-GCST90001452 | Inverse variance weighted | 22 | 0.0116457937489997 | 0.0216818923724731 | 0.591184225888205 | -0.030850715 | 0.0541423027990471 | 1.01171387001574 | 0.96962031174846 | 1.05563481125565 | 0.994119304454654 |
| ebi-a-GCST90001452 | finngen_R11_O15_POOR_FETGRO | Poor fetal growth \|\| id:finngen_R11_O15_POOR_FETGRO | CD11c+ CD62L- monocyte Absolute Count \|\| id:ebi-a-GCST90001452 | MR Egger | 22 | 0.0148325456981798 | 0.0301967813834246 | 0.628634508032927 | -0.044353146 | 0.074018237209692 | 1.01494309379778 | 0.956616072854194 | 1.0768264436268 | 0.992899239648112 |
| ebi-a-GCST90001452 | finngen_R11_O15_POOR_FETGRO | Poor fetal growth \|\| id:finngen_R11_O15_POOR_FETGRO | CD11c+ CD62L- monocyte Absolute Count \|\| id:ebi-a-GCST90001452 | Weighted median | 22 | 0.00651711815895672 | 0.0295929502942316 | 0.825695646284527 | -0.051485064 | 0.0645193007356506 | 1.00653840078218 | 0.949817835938685 | 1.06664616510164 | 0.999440741871876 |
| ebi-a-GCST90001452 | finngen_R11_O15_POOR_FETGRO | Poor fetal growth \|\| id:finngen_R11_O15_POOR_FETGRO | CD11c+ CD62L- monocyte Absolute Count \|\| id:ebi-a-GCST90001452 | Weighted mode | 22 | 0.016203783547227 | 0.0272599061232475 | 0.558582832556216 | -0.037225632 | 0.069633199548792 | 1.01633577681424 | 0.963458723265981 | 1.07211485690959 | 0.999857399747617 |
| ebi-a-GCST90001453 | finngen_R11_O15_POOR_FETGRO | Poor fetal growth \|\| id:finngen_R11_O15_POOR_FETGRO | CD11c+ CD62L- monocyte %monocyte \|\| id:ebi-a-GCST90001453 | Inverse variance weighted | 18 | 0.0154884629024113 | 0.0380067959919039 | 0.683627394682349 | -0.059004857 | 0.089981783046543 | 1.01560903081022 | 0.942702190213571 | 1.09415435136476 | 0.994119304454654 |
| ebi-a-GCST90001453 | finngen_R11_O15_POOR_FETGRO | Poor fetal growth \|\| id:finngen_R11_O15_POOR_FETGRO | CD11c+ CD62L- monocyte %monocyte \|\| id:ebi-a-GCST90001453 | MR Egger | 18 | 0.0190040482040625 | 0.0760835218667705 | 0.805937030043781 | -0.130119655 | 0.168127751062933 | 1.01918577448105 | 0.877990369000585 | 1.18308774171057 | 0.992899239648112 |
| ebi-a-GCST90001453 | finngen_R11_O15_POOR_FETGRO | Poor fetal growth \|\| id:finngen_R11_O15_POOR_FETGRO | CD11c+ CD62L- monocyte %monocyte \|\| id:ebi-a-GCST90001453 | Weighted median | 18 | 0.00527598094828294 | 0.0522764846495443 | 0.919610350585386 | -0.097185929 | 0.10773789086139 | 1.0052899234451 | 0.907387280870475 | 1.11375578155642 | 0.999440741871876 |
| ebi-a-GCST90001453 | finngen_R11_O15_POOR_FETGRO | Poor fetal growth \|\| id:finngen_R11_O15_POOR_FETGRO | CD11c+ CD62L- monocyte %monocyte \|\| id:ebi-a-GCST90001453 | Weighted mode | 18 | -0.005650285 | 0.0537512357220491 | 0.917511603215159 | -0.111002707 | 0.099702137434683 | 0.994365648254911 | 0.894936326694895 | 1.10484177805256 | 0.999857399747617 |
| ebi-a-GCST90001454 | finngen_R11_O15_POOR_FETGRO | Poor fetal growth \|\| id:finngen_R11_O15_POOR_FETGRO | CD62L- HLA DR++ monocyte Absolute Count \|\| id:ebi-a-GCST90001454 | Inverse variance weighted | 18 | -0.017880257 | 0.0457516656677446 | 0.695936895702998 | -0.107553522 | 0.0717930075380626 | 0.98227864614069 | 0.898028456993678 | 1.0744329215291 | 0.994119304454654 |
| ebi-a-GCST90001454 | finngen_R11_O15_POOR_FETGRO | Poor fetal growth \|\| id:finngen_R11_O15_POOR_FETGRO | CD62L- HLA DR++ monocyte Absolute Count \|\| id:ebi-a-GCST90001454 | MR Egger | 18 | -0.054540182 | 0.113061974544287 | 0.636060672918303 | -0.276141652 | 0.167061288263173 | 0.946920459083559 | 0.758705451042123 | 1.18182669519431 | 0.992899239648112 |
| ebi-a-GCST90001454 | finngen_R11_O15_POOR_FETGRO | Poor fetal growth \|\| id:finngen_R11_O15_POOR_FETGRO | CD62L- HLA DR++ monocyte Absolute Count \|\| id:ebi-a-GCST90001454 | Weighted median | 18 | 0.00166294047345483 | 0.0606705877499151 | 0.978133252673203 | -0.117251412 | 0.120577292463288 | 1.00166432392572 | 0.889361569313034 | 1.12814793492885 | 0.999440741871876 |
| ebi-a-GCST90001454 | finngen_R11_O15_POOR_FETGRO | Poor fetal growth \|\| id:finngen_R11_O15_POOR_FETGRO | CD62L- HLA DR++ monocyte Absolute Count \|\| id:ebi-a-GCST90001454 | Weighted mode | 18 | -0.007666377 | 0.0827662840192272 | 0.927282878112447 | -0.169888293 | 0.154555540126862 | 0.992362935161183 | 0.843759064933257 | 1.16713909931103 | 0.999857399747617 |
| ebi-a-GCST90001455 | finngen_R11_O15_POOR_FETGRO | Poor fetal growth \|\| id:finngen_R11_O15_POOR_FETGRO | CD62L- HLA DR++ monocyte %monocyte \|\| id:ebi-a-GCST90001455 | Inverse variance weighted | 11 | -0.065055704 | 0.0503372445446832 | 0.196219690852362 | -0.163716704 | 0.0336052948507968 | 0.937015265997215 | 0.848982501361892 | 1.03417633143604 | 0.994119304454654 |
| ebi-a-GCST90001455 | finngen_R11_O15_POOR_FETGRO | Poor fetal growth \|\| id:finngen_R11_O15_POOR_FETGRO | CD62L- HLA DR++ monocyte %monocyte \|\| id:ebi-a-GCST90001455 | MR Egger | 11 | -0.143325833 | 0.128838483250606 | 0.294776633453398 | -0.395849261 | 0.109197593716467 | 0.866471697428362 | 0.673108152192027 | 1.11538272118595 | 0.992899239648112 |
| ebi-a-GCST90001455 | finngen_R11_O15_POOR_FETGRO | Poor fetal growth \|\| id:finngen_R11_O15_POOR_FETGRO | CD62L- HLA DR++ monocyte %monocyte \|\| id:ebi-a-GCST90001455 | Weighted median | 11 | -0.039938905 | 0.0676216085593617 | 0.55477277494356 | -0.172477258 | 0.0925994476461805 | 0.960848140251193 | 0.841577427826678 | 1.09702223241462 | 0.999440741871876 |
| ebi-a-GCST90001455 | finngen_R11_O15_POOR_FETGRO | Poor fetal growth \|\| id:finngen_R11_O15_POOR_FETGRO | CD62L- HLA DR++ monocyte %monocyte \|\| id:ebi-a-GCST90001455 | Weighted mode | 11 | -0.037517827 | 0.0735421461092003 | 0.621013895958342 | -0.181660433 | 0.106624779775026 | 0.963177247393216 | 0.833884452009532 | 1.11251673737331 | 0.999857399747617 |
| ebi-a-GCST90001456 | finngen_R11_O15_POOR_FETGRO | Poor fetal growth \|\| id:finngen_R11_O15_POOR_FETGRO | CD11c+ HLA DR++ monocyte Absolute Count \|\| id:ebi-a-GCST90001456 | Inverse variance weighted | 17 | -0.054417834 | 0.0358821402929479 | 0.129374887932642 | -0.124746829 | 0.0159111611497959 | 0.947036320013646 | 0.882720353670186 | 1.01603841771172 | 0.994119304454654 |
| ebi-a-GCST90001456 | finngen_R11_O15_POOR_FETGRO | Poor fetal growth \|\| id:finngen_R11_O15_POOR_FETGRO | CD11c+ HLA DR++ monocyte Absolute Count \|\| id:ebi-a-GCST90001456 | MR Egger | 17 | -0.030825549 | 0.0563784537342846 | 0.592584131699057 | -0.141327318 | 0.0796762201463302 | 0.969644713644561 | 0.868205085604554 | 1.08293637792291 | 0.992899239648112 |
| ebi-a-GCST90001456 | finngen_R11_O15_POOR_FETGRO | Poor fetal growth \|\| id:finngen_R11_O15_POOR_FETGRO | CD11c+ HLA DR++ monocyte Absolute Count \|\| id:ebi-a-GCST90001456 | Weighted median | 17 | -0.029931843 | 0.0519312264705017 | 0.564361969417683 | -0.131717047 | 0.0718533609576875 | 0.97051167853211 | 0.876588993646708 | 1.07449776918694 | 0.999440741871876 |
| ebi-a-GCST90001456 | finngen_R11_O15_POOR_FETGRO | Poor fetal growth \|\| id:finngen_R11_O15_POOR_FETGRO | CD11c+ HLA DR++ monocyte Absolute Count \|\| id:ebi-a-GCST90001456 | Weighted mode | 17 | -0.030175195 | 0.0541922835452483 | 0.585357446169566 | -0.136392071 | 0.0760416802575199 | 0.970275530758903 | 0.872500483073458 | 1.07900754653245 | 0.999857399747617 |
| ebi-a-GCST90001457 | finngen_R11_O15_POOR_FETGRO | Poor fetal growth \|\| id:finngen_R11_O15_POOR_FETGRO | CD11c+ HLA DR++ monocyte %monocyte \|\| id:ebi-a-GCST90001457 | Inverse variance weighted | 17 | -0.045178537 | 0.0228379410933351 | 0.0479035737156873 | -0.089940901 | -0.000416172 | 0.955826816544395 | 0.913985199170937 | 0.999583914547096 | 0.994119304454654 |
| ebi-a-GCST90001457 | finngen_R11_O15_POOR_FETGRO | Poor fetal growth \|\| id:finngen_R11_O15_POOR_FETGRO | CD11c+ HLA DR++ monocyte %monocyte \|\| id:ebi-a-GCST90001457 | MR Egger | 17 | -0.053895209 | 0.027075892235499 | 0.0650761238662199 | -0.106963958 | -0.00082646 | 0.947531393802993 | 0.898558058187056 | 0.999173880932847 | 0.992899239648112 |
| ebi-a-GCST90001457 | finngen_R11_O15_POOR_FETGRO | Poor fetal growth \|\| id:finngen_R11_O15_POOR_FETGRO | CD11c+ HLA DR++ monocyte %monocyte \|\| id:ebi-a-GCST90001457 | Weighted median | 17 | -0.048884951 | 0.032487760366455 | 0.132396329249469 | -0.112560961 | 0.0147910591588012 | 0.952290683335358 | 0.89354287375417 | 1.01490098819457 | 0.999440741871876 |
| ebi-a-GCST90001457 | finngen_R11_O15_POOR_FETGRO | Poor fetal growth \|\| id:finngen_R11_O15_POOR_FETGRO | CD11c+ HLA DR++ monocyte %monocyte \|\| id:ebi-a-GCST90001457 | Weighted mode | 17 | -0.04938423 | 0.0274908724201229 | 0.0913370054391349 | -0.10326634 | 0.00449787952783103 | 0.951815343024988 | 0.901886732653825 | 1.00450801017106 | 0.999857399747617 |
| ebi-a-GCST90001458 | finngen_R11_O15_POOR_FETGRO | Poor fetal growth \|\| id:finngen_R11_O15_POOR_FETGRO | Myeloid Dendritic Cell Absolute Count \|\| id:ebi-a-GCST90001458 | Inverse variance weighted | 23 | -0.015708154 | 0.0190437916641955 | 0.409460263796684 | -0.053033986 | 0.0216176777591114 | 0.984414575688057 | 0.948347781799873 | 1.02185303263734 | 0.994119304454654 |
| ebi-a-GCST90001458 | finngen_R11_O15_POOR_FETGRO | Poor fetal growth \|\| id:finngen_R11_O15_POOR_FETGRO | Myeloid Dendritic Cell Absolute Count \|\| id:ebi-a-GCST90001458 | MR Egger | 23 | -0.022021285 | 0.0251802208769245 | 0.39171287009956 | -0.071374518 | 0.0273319481326446 | 0.978219413638616 | 0.931113108474455 | 1.02770889219602 | 0.992899239648112 |
| ebi-a-GCST90001458 | finngen_R11_O15_POOR_FETGRO | Poor fetal growth \|\| id:finngen_R11_O15_POOR_FETGRO | Myeloid Dendritic Cell Absolute Count \|\| id:ebi-a-GCST90001458 | Weighted median | 23 | -0.016896684 | 0.0266253704372762 | 0.525683840956281 | -0.06908241 | 0.0352890420242896 | 0.983245264322924 | 0.933249767711874 | 1.03591908968139 | 0.999440741871876 |
| ebi-a-GCST90001458 | finngen_R11_O15_POOR_FETGRO | Poor fetal growth \|\| id:finngen_R11_O15_POOR_FETGRO | Myeloid Dendritic Cell Absolute Count \|\| id:ebi-a-GCST90001458 | Weighted mode | 23 | -0.020082131 | 0.0230356307445459 | 0.392740236700166 | -0.065231967 | 0.0250677053171228 | 0.980118171972056 | 0.93685011966959 | 1.0253845421599 | 0.999857399747617 |
| ebi-a-GCST90001459 | finngen_R11_O15_POOR_FETGRO | Poor fetal growth \|\| id:finngen_R11_O15_POOR_FETGRO | Myeloid Dendritic Cell %Dendritic Cell \|\| id:ebi-a-GCST90001459 | Inverse variance weighted | 26 | -0.015880124 | 0.0286944820760921 | 0.579975291325632 | -0.072121308 | 0.0403610613095558 | 0.984245300806744 | 0.930418021416801 | 1.04118663854447 | 0.994119304454654 |
| ebi-a-GCST90001459 | finngen_R11_O15_POOR_FETGRO | Poor fetal growth \|\| id:finngen_R11_O15_POOR_FETGRO | Myeloid Dendritic Cell %Dendritic Cell \|\| id:ebi-a-GCST90001459 | MR Egger | 26 | -0.044550488 | 0.048494064194713 | 0.367404617723595 | -0.139598853 | 0.0504978782315725 | 0.956427311164622 | 0.869707045446247 | 1.05179463168799 | 0.992899239648112 |
| ebi-a-GCST90001459 | finngen_R11_O15_POOR_FETGRO | Poor fetal growth \|\| id:finngen_R11_O15_POOR_FETGRO | Myeloid Dendritic Cell %Dendritic Cell \|\| id:ebi-a-GCST90001459 | Weighted median | 26 | -0.001642424 | 0.0417886461836797 | 0.968648731162436 | -0.08354817 | 0.0802633229745841 | 0.998358924494005 | 0.919846776524613 | 1.08357235960826 | 0.999440741871876 |
| ebi-a-GCST90001459 | finngen_R11_O15_POOR_FETGRO | Poor fetal growth \|\| id:finngen_R11_O15_POOR_FETGRO | Myeloid Dendritic Cell %Dendritic Cell \|\| id:ebi-a-GCST90001459 | Weighted mode | 26 | -0.001614892 | 0.0421929858242814 | 0.969773263590975 | -0.084313144 | 0.0810833601277208 | 0.998386411148734 | 0.919143386509566 | 1.0844612936309 | 0.999857399747617 |
| ebi-a-GCST90001460 | finngen_R11_O15_POOR_FETGRO | Poor fetal growth \|\| id:finngen_R11_O15_POOR_FETGRO | Plasmacytoid Dendritic Cell Absolute Count \|\| id:ebi-a-GCST90001460 | Inverse variance weighted | 31 | 0.000193591059998223 | 0.0153206878308238 | 0.989918258941938 | -0.029834957 | 0.030222139208413 | 1.00019360979996 | 0.970605711922687 | 1.03068346373419 | 0.998984604424952 |
| ebi-a-GCST90001460 | finngen_R11_O15_POOR_FETGRO | Poor fetal growth \|\| id:finngen_R11_O15_POOR_FETGRO | Plasmacytoid Dendritic Cell Absolute Count \|\| id:ebi-a-GCST90001460 | MR Egger | 31 | -0.00453561 | 0.0200520304868929 | 0.822638749850362 | -0.04383759 | 0.0347663697698251 | 0.995474660361186 | 0.957109389236756 | 1.03537778499014 | 0.992899239648112 |
| ebi-a-GCST90001460 | finngen_R11_O15_POOR_FETGRO | Poor fetal growth \|\| id:finngen_R11_O15_POOR_FETGRO | Plasmacytoid Dendritic Cell Absolute Count \|\| id:ebi-a-GCST90001460 | Weighted median | 31 | -0.011663085 | 0.0216029707065504 | 0.589277450608501 | -0.054004908 | 0.0306787374377284 | 0.988404664982822 | 0.947427456770389 | 1.03115417943452 | 0.999440741871876 |
| ebi-a-GCST90001460 | finngen_R11_O15_POOR_FETGRO | Poor fetal growth \|\| id:finngen_R11_O15_POOR_FETGRO | Plasmacytoid Dendritic Cell Absolute Count \|\| id:ebi-a-GCST90001460 | Weighted mode | 31 | -0.009760784 | 0.0201019793457578 | 0.630804323117346 | -0.049160663 | 0.0296390958562217 | 0.990286698175157 | 0.952028161539586 | 1.03008270574344 | 0.999857399747617 |
| ebi-a-GCST90001461 | finngen_R11_O15_POOR_FETGRO | Poor fetal growth \|\| id:finngen_R11_O15_POOR_FETGRO | Dendritic Cell Absolute Count \|\| id:ebi-a-GCST90001461 | Inverse variance weighted | 21 | -5.07E-05 | 0.0375535756160222 | 0.998922332423651 | -0.07365573 | 0.0735542862294254 | 0.99994927930836 | 0.92899146251336 | 1.0763269648185 | 0.998984604424952 |
| ebi-a-GCST90001461 | finngen_R11_O15_POOR_FETGRO | Poor fetal growth \|\| id:finngen_R11_O15_POOR_FETGRO | Dendritic Cell Absolute Count \|\| id:ebi-a-GCST90001461 | MR Egger | 21 | -0.011740186 | 0.0671243602593638 | 0.863006977596624 | -0.143303932 | 0.119823560344187 | 0.988328461310964 | 0.866490674737283 | 1.12729793397195 | 0.992899239648112 |
| ebi-a-GCST90001461 | finngen_R11_O15_POOR_FETGRO | Poor fetal growth \|\| id:finngen_R11_O15_POOR_FETGRO | Dendritic Cell Absolute Count \|\| id:ebi-a-GCST90001461 | Weighted median | 21 | 0.00513881694686917 | 0.0503353342258931 | 0.918683934672985 | -0.093518438 | 0.10379607202962 | 1.00515204331293 | 0.91072122526969 | 1.10937419941757 | 0.999440741871876 |
| ebi-a-GCST90001461 | finngen_R11_O15_POOR_FETGRO | Poor fetal growth \|\| id:finngen_R11_O15_POOR_FETGRO | Dendritic Cell Absolute Count \|\| id:ebi-a-GCST90001461 | Weighted mode | 21 | -0.010464487 | 0.0504826595613372 | 0.837880385291125 | -0.1094105 | 0.088481525758965 | 0.989590075274776 | 0.896362385455307 | 1.0925140690558 | 0.999857399747617 |
| ebi-a-GCST90001462 | finngen_R11_O15_POOR_FETGRO | Poor fetal growth \|\| id:finngen_R11_O15_POOR_FETGRO | CD62L- Dendritic Cell Absolute Count \|\| id:ebi-a-GCST90001462 | Inverse variance weighted | 17 | 0.0100313972078787 | 0.018550032163912 | 0.588662543944518 | -0.026326666 | 0.0463894602491463 | 1.01008188033708 | 0.974016859604967 | 1.04748228423796 | 0.994119304454654 |
| ebi-a-GCST90001462 | finngen_R11_O15_POOR_FETGRO | Poor fetal growth \|\| id:finngen_R11_O15_POOR_FETGRO | CD62L- Dendritic Cell Absolute Count \|\| id:ebi-a-GCST90001462 | MR Egger | 17 | 0.00125833266327374 | 0.0227213631132587 | 0.956565832063198 | -0.043275539 | 0.0457922043652608 | 1.001259125 | 0.95764748444326 | 1.04685685606935 | 0.992899239648112 |
| ebi-a-GCST90001462 | finngen_R11_O15_POOR_FETGRO | Poor fetal growth \|\| id:finngen_R11_O15_POOR_FETGRO | CD62L- Dendritic Cell Absolute Count \|\| id:ebi-a-GCST90001462 | Weighted median | 17 | 0.00923862295752296 | 0.0218458616725801 | 0.672367953749422 | -0.033579266 | 0.0520565118357799 | 1.00928143076143 | 0.966978259771428 | 1.0534352723924 | 0.999440741871876 |
| ebi-a-GCST90001462 | finngen_R11_O15_POOR_FETGRO | Poor fetal growth \|\| id:finngen_R11_O15_POOR_FETGRO | CD62L- Dendritic Cell Absolute Count \|\| id:ebi-a-GCST90001462 | Weighted mode | 17 | 0.00713986934020439 | 0.0210725394294546 | 0.739148660065425 | -0.034162308 | 0.0484420466219355 | 1.00716541897813 | 0.966414635137178 | 1.04963454019031 | 0.999857399747617 |
| ebi-a-GCST90001463 | finngen_R11_O15_POOR_FETGRO | Poor fetal growth \|\| id:finngen_R11_O15_POOR_FETGRO | CD62L- Dendritic Cell %Dendritic Cell \|\| id:ebi-a-GCST90001463 | Inverse variance weighted | 23 | 0.00621780256111316 | 0.0169761088305354 | 0.714165231381763 | -0.027055371 | 0.0394909758689626 | 1.0062371732223 | 0.973307347277646 | 1.04028111120954 | 0.994119304454654 |
| ebi-a-GCST90001463 | finngen_R11_O15_POOR_FETGRO | Poor fetal growth \|\| id:finngen_R11_O15_POOR_FETGRO | CD62L- Dendritic Cell %Dendritic Cell \|\| id:ebi-a-GCST90001463 | MR Egger | 23 | 0.0105696760346287 | 0.0210494285007725 | 0.62079633364845 | -0.030687204 | 0.0518265558961427 | 1.01062573238544 | 0.969778868757604 | 1.05319305654504 | 0.992899239648112 |
| ebi-a-GCST90001463 | finngen_R11_O15_POOR_FETGRO | Poor fetal growth \|\| id:finngen_R11_O15_POOR_FETGRO | CD62L- Dendritic Cell %Dendritic Cell \|\| id:ebi-a-GCST90001463 | Weighted median | 23 | 0.0165353749462996 | 0.0224754581426798 | 0.461908404131445 | -0.027516523 | 0.060587272905952 | 1.01667284089709 | 0.972858607864885 | 1.06246031752367 | 0.999440741871876 |
| ebi-a-GCST90001463 | finngen_R11_O15_POOR_FETGRO | Poor fetal growth \|\| id:finngen_R11_O15_POOR_FETGRO | CD62L- Dendritic Cell %Dendritic Cell \|\| id:ebi-a-GCST90001463 | Weighted mode | 23 | 0.00374763588398008 | 0.0181012468916469 | 0.837884933560747 | -0.031730808 | 0.039226079791608 | 1.00375466705202 | 0.968767331374372 | 1.04000558131884 | 0.999857399747617 |
| ebi-a-GCST90001464 | finngen_R11_O15_POOR_FETGRO | Poor fetal growth \|\| id:finngen_R11_O15_POOR_FETGRO | CD86+ myeloid Dendritic Cell Absolute Count \|\| id:ebi-a-GCST90001464 | Inverse variance weighted | 21 | -0.014961425 | 0.018788122910635 | 0.42584398730314 | -0.051786146 | 0.0218632957314422 | 0.985149940858253 | 0.949531906253711 | 1.02210404893303 | 0.994119304454654 |
| ebi-a-GCST90001464 | finngen_R11_O15_POOR_FETGRO | Poor fetal growth \|\| id:finngen_R11_O15_POOR_FETGRO | CD86+ myeloid Dendritic Cell Absolute Count \|\| id:ebi-a-GCST90001464 | MR Egger | 21 | -0.028921471 | 0.0236579241327763 | 0.23647286656192 | -0.075291003 | 0.0174480598824607 | 0.971492751419618 | 0.92747354973045 | 1.01760116645391 | 0.992899239648112 |
| ebi-a-GCST90001464 | finngen_R11_O15_POOR_FETGRO | Poor fetal growth \|\| id:finngen_R11_O15_POOR_FETGRO | CD86+ myeloid Dendritic Cell Absolute Count \|\| id:ebi-a-GCST90001464 | Weighted median | 21 | -0.028214782 | 0.0261688174603852 | 0.280951956672794 | -0.079505665 | 0.0230760998784756 | 0.972179537375904 | 0.923572788314557 | 1.02334441296957 | 0.999440741871876 |
| ebi-a-GCST90001464 | finngen_R11_O15_POOR_FETGRO | Poor fetal growth \|\| id:finngen_R11_O15_POOR_FETGRO | CD86+ myeloid Dendritic Cell Absolute Count \|\| id:ebi-a-GCST90001464 | Weighted mode | 21 | -0.022955678 | 0.022858356088551 | 0.327250884262629 | -0.067758056 | 0.0218467003394308 | 0.977305799357644 | 0.934486540081162 | 1.02208708685641 | 0.999857399747617 |
| ebi-a-GCST90001465 | finngen_R11_O15_POOR_FETGRO | Poor fetal growth \|\| id:finngen_R11_O15_POOR_FETGRO | CD86+ myeloid Dendritic Cell %Dendritic Cell \|\| id:ebi-a-GCST90001465 | Inverse variance weighted | 23 | -0.00468967 | 0.0118473178186707 | 0.692221357738726 | -0.027910413 | 0.018531073204464 | 0.99532130961106 | 0.972475484405588 | 1.01870383907046 | 0.994119304454654 |
| ebi-a-GCST90001465 | finngen_R11_O15_POOR_FETGRO | Poor fetal growth \|\| id:finngen_R11_O15_POOR_FETGRO | CD86+ myeloid Dendritic Cell %Dendritic Cell \|\| id:ebi-a-GCST90001465 | MR Egger | 23 | -0.001925284 | 0.0147254552534225 | 0.897221185624392 | -0.030787177 | 0.0269366079317042 | 0.998076567806096 | 0.969681922061038 | 1.02730267785779 | 0.992899239648112 |
| ebi-a-GCST90001465 | finngen_R11_O15_POOR_FETGRO | Poor fetal growth \|\| id:finngen_R11_O15_POOR_FETGRO | CD86+ myeloid Dendritic Cell %Dendritic Cell \|\| id:ebi-a-GCST90001465 | Weighted median | 23 | -0.000477396 | 0.0160585184374368 | 0.976283574421437 | -0.031952092 | 0.0309973003143874 | 0.999522718112266 | 0.968552982442385 | 1.03148271920371 | 0.999440741871876 |
| ebi-a-GCST90001465 | finngen_R11_O15_POOR_FETGRO | Poor fetal growth \|\| id:finngen_R11_O15_POOR_FETGRO | CD86+ myeloid Dendritic Cell %Dendritic Cell \|\| id:ebi-a-GCST90001465 | Weighted mode | 23 | -0.000462763 | 0.0134189791895916 | 0.972800813667202 | -0.026763962 | 0.0258384360439128 | 0.999537343890673 | 0.973591018512821 | 1.02617514216403 | 0.999857399747617 |
| ebi-a-GCST90001466 | finngen_R11_O15_POOR_FETGRO | Poor fetal growth \|\| id:finngen_R11_O15_POOR_FETGRO | CD86+ plasmacytoid Dendritic Cell Absolute Count \|\| id:ebi-a-GCST90001466 | Inverse variance weighted | 20 | -0.057229181 | 0.0346046471557862 | 0.09816806556239 | -0.125054289 | 0.0105959275645679 | 0.944377611289596 | 0.882448993758236 | 1.01065226320535 | 0.994119304454654 |
| ebi-a-GCST90001466 | finngen_R11_O15_POOR_FETGRO | Poor fetal growth \|\| id:finngen_R11_O15_POOR_FETGRO | CD86+ plasmacytoid Dendritic Cell Absolute Count \|\| id:ebi-a-GCST90001466 | MR Egger | 20 | -0.073571136 | 0.0572368393453473 | 0.214951953003285 | -0.185755341 | 0.0386130688933827 | 0.929070052905819 | 0.830476753458027 | 1.03936824193123 | 0.992899239648112 |
| ebi-a-GCST90001466 | finngen_R11_O15_POOR_FETGRO | Poor fetal growth \|\| id:finngen_R11_O15_POOR_FETGRO | CD86+ plasmacytoid Dendritic Cell Absolute Count \|\| id:ebi-a-GCST90001466 | Weighted median | 20 | -0.0740821 | 0.0492979462708039 | 0.132905558030702 | -0.170706075 | 0.0225418746634514 | 0.928595452999578 | 0.843069336450312 | 1.022797863 | 0.999440741871876 |
| ebi-a-GCST90001466 | finngen_R11_O15_POOR_FETGRO | Poor fetal growth \|\| id:finngen_R11_O15_POOR_FETGRO | CD86+ plasmacytoid Dendritic Cell Absolute Count \|\| id:ebi-a-GCST90001466 | Weighted mode | 20 | -0.06535767 | 0.0549450060850192 | 0.248891821861662 | -0.173049882 | 0.0423345419426529 | 0.936732362404137 | 0.841095658339743 | 1.04324342906167 | 0.999857399747617 |
| ebi-a-GCST90001467 | finngen_R11_O15_POOR_FETGRO | Poor fetal growth \|\| id:finngen_R11_O15_POOR_FETGRO | CD86+ plasmacytoid Dendritic Cell %Dendritic Cell \|\| id:ebi-a-GCST90001467 | Inverse variance weighted | 26 | -0.040730987 | 0.0309055867195371 | 0.187531558768115 | -0.101305937 | 0.0198439633387048 | 0.96008737154881 | 0.903656528983944 | 1.02004216363481 | 0.994119304454654 |
| ebi-a-GCST90001467 | finngen_R11_O15_POOR_FETGRO | Poor fetal growth \|\| id:finngen_R11_O15_POOR_FETGRO | CD86+ plasmacytoid Dendritic Cell %Dendritic Cell \|\| id:ebi-a-GCST90001467 | MR Egger | 26 | -0.078368145 | 0.0544782432707104 | 0.163200049986121 | -0.185145502 | 0.028409212035274 | 0.924623968395341 | 0.830983365657876 | 1.028816602 | 0.992899239648112 |
| ebi-a-GCST90001467 | finngen_R11_O15_POOR_FETGRO | Poor fetal growth \|\| id:finngen_R11_O15_POOR_FETGRO | CD86+ plasmacytoid Dendritic Cell %Dendritic Cell \|\| id:ebi-a-GCST90001467 | Weighted median | 26 | -0.065769722 | 0.043184362923871 | 0.127759070908385 | -0.150411073 | 0.0188716296834739 | 0.936346459787482 | 0.860354235345595 | 1.01905082434373 | 0.999440741871876 |
| ebi-a-GCST90001467 | finngen_R11_O15_POOR_FETGRO | Poor fetal growth \|\| id:finngen_R11_O15_POOR_FETGRO | CD86+ plasmacytoid Dendritic Cell %Dendritic Cell \|\| id:ebi-a-GCST90001467 | Weighted mode | 26 | -0.067724987 | 0.0561380750078238 | 0.238955952162511 | -0.177755614 | 0.0423056398646579 | 0.934517442545327 | 0.837146985355926 | 1.04321327759444 | 0.999857399747617 |
| ebi-a-GCST90001468 | finngen_R11_O15_POOR_FETGRO | Poor fetal growth \|\| id:finngen_R11_O15_POOR_FETGRO | CD62L- myeloid Dendritic Cell Absolute Count \|\| id:ebi-a-GCST90001468 | Inverse variance weighted | 15 | 0.00231663885297354 | 0.0336624730171067 | 0.945133194659626 | -0.063661808 | 0.0682950859665027 | 1.00231932433412 | 0.938322278720705 | 1.07068120486634 | 0.998984604424952 |
| ebi-a-GCST90001468 | finngen_R11_O15_POOR_FETGRO | Poor fetal growth \|\| id:finngen_R11_O15_POOR_FETGRO | CD62L- myeloid Dendritic Cell Absolute Count \|\| id:ebi-a-GCST90001468 | MR Egger | 15 | -0.028202365 | 0.0555202665964693 | 0.619986698385346 | -0.137022088 | 0.0806173570998442 | 0.972191608921183 | 0.871950966302808 | 1.08395604911633 | 0.992899239648112 |
| ebi-a-GCST90001468 | finngen_R11_O15_POOR_FETGRO | Poor fetal growth \|\| id:finngen_R11_O15_POOR_FETGRO | CD62L- myeloid Dendritic Cell Absolute Count \|\| id:ebi-a-GCST90001468 | Weighted median | 15 | -0.000394838 | 0.0375117946898373 | 0.991601858663229 | -0.073917956 | 0.0731282795589516 | 0.999605239905149 | 0.928747889255453 | 1.07586854000483 | 0.999440741871876 |
| ebi-a-GCST90001468 | finngen_R11_O15_POOR_FETGRO | Poor fetal growth \|\| id:finngen_R11_O15_POOR_FETGRO | CD62L- myeloid Dendritic Cell Absolute Count \|\| id:ebi-a-GCST90001468 | Weighted mode | 15 | -0.002187496 | 0.042203928610546 | 0.959395185005569 | -0.084907197 | 0.0805322036504991 | 0.99781489440051 | 0.918597529509094 | 1.08386375044964 | 0.999857399747617 |
| ebi-a-GCST90001469 | finngen_R11_O15_POOR_FETGRO | Poor fetal growth \|\| id:finngen_R11_O15_POOR_FETGRO | CD62L- myeloid Dendritic Cell %Dendritic Cell \|\| id:ebi-a-GCST90001469 | Inverse variance weighted | 24 | 0.00304128340063318 | 0.0186947031985092 | 0.870769169895909 | -0.033600335 | 0.0396829016697111 | 1.00304591279491 | 0.966957886771655 | 1.04048078715568 | 0.994119304454654 |
| ebi-a-GCST90001469 | finngen_R11_O15_POOR_FETGRO | Poor fetal growth \|\| id:finngen_R11_O15_POOR_FETGRO | CD62L- myeloid Dendritic Cell %Dendritic Cell \|\| id:ebi-a-GCST90001469 | MR Egger | 24 | -0.015417992 | 0.0262058308566737 | 0.562294748713975 | -0.06678142 | 0.0359454366737946 | 0.984700256931005 | 0.935399638380028 | 1.03659928464292 | 0.992899239648112 |
| ebi-a-GCST90001469 | finngen_R11_O15_POOR_FETGRO | Poor fetal growth \|\| id:finngen_R11_O15_POOR_FETGRO | CD62L- myeloid Dendritic Cell %Dendritic Cell \|\| id:ebi-a-GCST90001469 | Weighted median | 24 | -0.000724114 | 0.0256970599118554 | 0.977519500961679 | -0.051090351 | 0.0496421236752896 | 0.999276148355147 | 0.950192815612596 | 1.05089493865277 | 0.999440741871876 |
| ebi-a-GCST90001469 | finngen_R11_O15_POOR_FETGRO | Poor fetal growth \|\| id:finngen_R11_O15_POOR_FETGRO | CD62L- myeloid Dendritic Cell %Dendritic Cell \|\| id:ebi-a-GCST90001469 | Weighted mode | 24 | -0.003389081 | 0.0238784164290197 | 0.888370650411862 | -0.050190777 | 0.0434126153873915 | 0.99661665563863 | 0.951047969100441 | 1.04436872856771 | 0.999857399747617 |
| ebi-a-GCST90001470 | finngen_R11_O15_POOR_FETGRO | Poor fetal growth \|\| id:finngen_R11_O15_POOR_FETGRO | CD62L- plasmacytoid Dendritic Cell Absolute Count \|\| id:ebi-a-GCST90001470 | Inverse variance weighted | 25 | 0.00887997758186626 | 0.0292017447642433 | 0.761058829052174 | -0.048355442 | 0.0661153973197831 | 1.00891952154596 | 0.952795063351133 | 1.06834999477889 | 0.994119304454654 |
| ebi-a-GCST90001470 | finngen_R11_O15_POOR_FETGRO | Poor fetal growth \|\| id:finngen_R11_O15_POOR_FETGRO | CD62L- plasmacytoid Dendritic Cell Absolute Count \|\| id:ebi-a-GCST90001470 | MR Egger | 25 | -0.046288433 | 0.0467819058515925 | 0.332737969502912 | -0.137980968 | 0.0454041027407039 | 0.9547665365546 | 0.871115270481785 | 1.04645064805293 | 0.992899239648112 |
| ebi-a-GCST90001470 | finngen_R11_O15_POOR_FETGRO | Poor fetal growth \|\| id:finngen_R11_O15_POOR_FETGRO | CD62L- plasmacytoid Dendritic Cell Absolute Count \|\| id:ebi-a-GCST90001470 | Weighted median | 25 | 0.00349512213775535 | 0.0413657934790666 | 0.932664426822324 | -0.077581833 | 0.0845720773567259 | 1.00350123719935 | 0.925351296950338 | 1.08825127968095 | 0.999440741871876 |
| ebi-a-GCST90001470 | finngen_R11_O15_POOR_FETGRO | Poor fetal growth \|\| id:finngen_R11_O15_POOR_FETGRO | CD62L- plasmacytoid Dendritic Cell Absolute Count \|\| id:ebi-a-GCST90001470 | Weighted mode | 25 | -0.000158104 | 0.03631077672084 | 0.996561854349626 | -0.071327227 | 0.0710110180398592 | 0.999841908164844 | 0.931157142784803 | 1.07359305469426 | 0.999857399747617 |
| ebi-a-GCST90001471 | finngen_R11_O15_POOR_FETGRO | Poor fetal growth \|\| id:finngen_R11_O15_POOR_FETGRO | CD62L- plasmacytoid Dendritic Cell %Dendritic Cell \|\| id:ebi-a-GCST90001471 | Inverse variance weighted | 18 | 0.00142677258800167 | 0.0233064743417399 | 0.95118570260958 | -0.044253917 | 0.0471074622978118 | 1.00142779091226 | 0.95671100132513 | 1.04823464873129 | 0.998984604424952 |
| ebi-a-GCST90001471 | finngen_R11_O15_POOR_FETGRO | Poor fetal growth \|\| id:finngen_R11_O15_POOR_FETGRO | CD62L- plasmacytoid Dendritic Cell %Dendritic Cell \|\| id:ebi-a-GCST90001471 | MR Egger | 18 | -0.007756042 | 0.032204996531787 | 0.812743040084641 | -0.070877835 | 0.055365750928493 | 0.992273958210288 | 0.931575690677095 | 1.05692711606361 | 0.992899239648112 |
| ebi-a-GCST90001471 | finngen_R11_O15_POOR_FETGRO | Poor fetal growth \|\| id:finngen_R11_O15_POOR_FETGRO | CD62L- plasmacytoid Dendritic Cell %Dendritic Cell \|\| id:ebi-a-GCST90001471 | Weighted median | 18 | -0.013728766 | 0.0315468886260161 | 0.663427379051714 | -0.075560668 | 0.0481031354921193 | 0.986365043508074 | 0.927223476106198 | 1.04927886763649 | 0.999440741871876 |
| ebi-a-GCST90001471 | finngen_R11_O15_POOR_FETGRO | Poor fetal growth \|\| id:finngen_R11_O15_POOR_FETGRO | CD62L- plasmacytoid Dendritic Cell %Dendritic Cell \|\| id:ebi-a-GCST90001471 | Weighted mode | 18 | -0.003581545 | 0.0298912168429692 | 0.906030926189903 | -0.06216833 | 0.0550052401051905 | 0.996424861174759 | 0.939724689695678 | 1.05654615107396 | 0.999857399747617 |
| ebi-a-GCST90001472 | finngen_R11_O15_POOR_FETGRO | Poor fetal growth \|\| id:finngen_R11_O15_POOR_FETGRO | CD62L- CD86+ myeloid Dendritic Cell Absolute Count \|\| id:ebi-a-GCST90001472 | Inverse variance weighted | 19 | -0.003118732 | 0.0223210040828792 | 0.888879756462491 | -0.0468679 | 0.0406304358631674 | 0.996886126054022 | 0.954213440725337 | 1.04146714550948 | 0.994119304454654 |
| ebi-a-GCST90001472 | finngen_R11_O15_POOR_FETGRO | Poor fetal growth \|\| id:finngen_R11_O15_POOR_FETGRO | CD62L- CD86+ myeloid Dendritic Cell Absolute Count \|\| id:ebi-a-GCST90001472 | MR Egger | 19 | 0.00808214900852037 | 0.0304486594900179 | 0.793866126428505 | -0.051597224 | 0.0677615216089555 | 1.00811489774208 | 0.949711311128566 | 1.07011007991664 | 0.992899239648112 |
| ebi-a-GCST90001472 | finngen_R11_O15_POOR_FETGRO | Poor fetal growth \|\| id:finngen_R11_O15_POOR_FETGRO | CD62L- CD86+ myeloid Dendritic Cell Absolute Count \|\| id:ebi-a-GCST90001472 | Weighted median | 19 | 0.00764728688657278 | 0.0296004009167516 | 0.796136193486331 | -0.050369499 | 0.065664072683406 | 1.00767660206448 | 0.950878011192369 | 1.06786793089781 | 0.999440741871876 |
| ebi-a-GCST90001472 | finngen_R11_O15_POOR_FETGRO | Poor fetal growth \|\| id:finngen_R11_O15_POOR_FETGRO | CD62L- CD86+ myeloid Dendritic Cell Absolute Count \|\| id:ebi-a-GCST90001472 | Weighted mode | 19 | 0.00524062600840902 | 0.0260906562603636 | 0.84305853903832 | -0.04589706 | 0.0563783122787216 | 1.00525438210858 | 0.955140279020441 | 1.05799786161765 | 0.999857399747617 |
| ebi-a-GCST90001473 | finngen_R11_O15_POOR_FETGRO | Poor fetal growth \|\| id:finngen_R11_O15_POOR_FETGRO | CD62L- CD86+ myeloid Dendritic Cell %Dendritic Cell \|\| id:ebi-a-GCST90001473 | Inverse variance weighted | 18 | 0.00183932261925911 | 0.0230668242032641 | 0.93644493984062 | -0.043371653 | 0.0470502980576567 | 1.00184101521069 | 0.95755544574638 | 1.04817472890674 | 0.998984604424952 |
| ebi-a-GCST90001473 | finngen_R11_O15_POOR_FETGRO | Poor fetal growth \|\| id:finngen_R11_O15_POOR_FETGRO | CD62L- CD86+ myeloid Dendritic Cell %Dendritic Cell \|\| id:ebi-a-GCST90001473 | MR Egger | 18 | 0.0317127628992784 | 0.0304066032472262 | 0.31247164788408 | -0.027884179 | 0.0913097052638418 | 1.03222097056038 | 0.972500995864094 | 1.09560826836779 | 0.992899239648112 |
| ebi-a-GCST90001473 | finngen_R11_O15_POOR_FETGRO | Poor fetal growth \|\| id:finngen_R11_O15_POOR_FETGRO | CD62L- CD86+ myeloid Dendritic Cell %Dendritic Cell \|\| id:ebi-a-GCST90001473 | Weighted median | 18 | 0.00882822907431537 | 0.0310624629507705 | 0.776250279386484 | -0.052054198 | 0.0697106564578256 | 1.00886731281736 | 0.949277416217718 | 1.07219790282874 | 0.999440741871876 |
| ebi-a-GCST90001473 | finngen_R11_O15_POOR_FETGRO | Poor fetal growth \|\| id:finngen_R11_O15_POOR_FETGRO | CD62L- CD86+ myeloid Dendritic Cell %Dendritic Cell \|\| id:ebi-a-GCST90001473 | Weighted mode | 18 | 0.0109170689798224 | 0.0262417277010805 | 0.682600369567143 | -0.040516717 | 0.0623508552739401 | 1.01097687762426 | 0.960293110855477 | 1.06433570702219 | 0.999857399747617 |
| ebi-a-GCST90001474 | finngen_R11_O15_POOR_FETGRO | Poor fetal growth \|\| id:finngen_R11_O15_POOR_FETGRO | Plasmacytoid Dendritic Cell %Dendritic Cell \|\| id:ebi-a-GCST90001474 | Inverse variance weighted | 24 | -0.0064094 | 0.0224479977177917 | 0.775244159052823 | -0.050407475 | 0.0375886755984986 | 0.993611096462114 | 0.95084190081648 | 1.03830406523407 | 0.994119304454654 |
| ebi-a-GCST90001474 | finngen_R11_O15_POOR_FETGRO | Poor fetal growth \|\| id:finngen_R11_O15_POOR_FETGRO | Plasmacytoid Dendritic Cell %Dendritic Cell \|\| id:ebi-a-GCST90001474 | MR Egger | 24 | -0.018440227 | 0.033507458874452 | 0.587636578134257 | -0.084114846 | 0.0472343924486081 | 0.981728753764539 | 0.919325668844447 | 1.04836770975787 | 0.992899239648112 |
| ebi-a-GCST90001474 | finngen_R11_O15_POOR_FETGRO | Poor fetal growth \|\| id:finngen_R11_O15_POOR_FETGRO | Plasmacytoid Dendritic Cell %Dendritic Cell \|\| id:ebi-a-GCST90001474 | Weighted median | 24 | -0.020494956 | 0.031938333074213 | 0.521065390671371 | -0.083094089 | 0.042104176694026 | 0.97971363800888 | 0.920264556414394 | 1.04300312970913 | 0.999440741871876 |
| ebi-a-GCST90001474 | finngen_R11_O15_POOR_FETGRO | Poor fetal growth \|\| id:finngen_R11_O15_POOR_FETGRO | Plasmacytoid Dendritic Cell %Dendritic Cell \|\| id:ebi-a-GCST90001474 | Weighted mode | 24 | -0.019114054 | 0.0313611924929181 | 0.54817978099682 | -0.080581992 | 0.0423538829593405 | 0.981067460872735 | 0.92257925672131 | 1.04326360664537 | 0.999857399747617 |
| ebi-a-GCST90001475 | finngen_R11_O15_POOR_FETGRO | Poor fetal growth \|\| id:finngen_R11_O15_POOR_FETGRO | HLA DR++ monocyte %monocyte \|\| id:ebi-a-GCST90001475 | Inverse variance weighted | 21 | -0.010296076 | 0.0276260996219139 | 0.709376344911451 | -0.064443232 | 0.0438510789696788 | 0.989756746858377 | 0.937589338246448 | 1.04482674662645 | 0.994119304454654 |
| ebi-a-GCST90001475 | finngen_R11_O15_POOR_FETGRO | Poor fetal growth \|\| id:finngen_R11_O15_POOR_FETGRO | HLA DR++ monocyte %monocyte \|\| id:ebi-a-GCST90001475 | MR Egger | 21 | 0.0199425813777641 | 0.0499772337170816 | 0.694318022477139 | -0.078012797 | 0.117897959463244 | 1.02014276315313 | 0.924952590119859 | 1.12512929671221 | 0.992899239648112 |
| ebi-a-GCST90001475 | finngen_R11_O15_POOR_FETGRO | Poor fetal growth \|\| id:finngen_R11_O15_POOR_FETGRO | HLA DR++ monocyte %monocyte \|\| id:ebi-a-GCST90001475 | Weighted median | 21 | -0.009144101 | 0.0368258477984611 | 0.803897200264607 | -0.081322762 | 0.0630345609800132 | 0.990897579444637 | 0.921896090034611 | 1.06506364823868 | 0.999440741871876 |
| ebi-a-GCST90001475 | finngen_R11_O15_POOR_FETGRO | Poor fetal growth \|\| id:finngen_R11_O15_POOR_FETGRO | HLA DR++ monocyte %monocyte \|\| id:ebi-a-GCST90001475 | Weighted mode | 21 | -0.014770185 | 0.0448783877039259 | 0.745489741543191 | -0.102731825 | 0.0731914551443423 | 0.985338359360827 | 0.902368934136312 | 1.07593651077667 | 0.999857399747617 |
| ebi-a-GCST90001476 | finngen_R11_O15_POOR_FETGRO | Poor fetal growth \|\| id:finngen_R11_O15_POOR_FETGRO | HLA DR++ monocyte %leukocyte \|\| id:ebi-a-GCST90001476 | Inverse variance weighted | 8 | 0.0458403329893881 | 0.0417644080882883 | 0.272382136 | -0.036017907 | 0.127698572842433 | 1.04690724106197 | 0.964623019955567 | 1.1362104663835 | 0.994119304454654 |
| ebi-a-GCST90001476 | finngen_R11_O15_POOR_FETGRO | Poor fetal growth \|\| id:finngen_R11_O15_POOR_FETGRO | HLA DR++ monocyte %leukocyte \|\| id:ebi-a-GCST90001476 | MR Egger | 8 | 0.159439522341894 | 0.0810959090194069 | 0.0968777207563829 | 0.000491540663856038 | 0.318387504019931 | 1.17285332865318 | 1.00049166148976 | 1.37490894075475 | 0.992899239648112 |
| ebi-a-GCST90001476 | finngen_R11_O15_POOR_FETGRO | Poor fetal growth \|\| id:finngen_R11_O15_POOR_FETGRO | HLA DR++ monocyte %leukocyte \|\| id:ebi-a-GCST90001476 | Weighted median | 8 | 0.059168919304713 | 0.0561197611527597 | 0.291730459434404 | -0.050825813 | 0.169163651164122 | 1.06095444129107 | 0.95044421156354 | 1.18431393742041 | 0.999440741871876 |
| ebi-a-GCST90001476 | finngen_R11_O15_POOR_FETGRO | Poor fetal growth \|\| id:finngen_R11_O15_POOR_FETGRO | HLA DR++ monocyte %leukocyte \|\| id:ebi-a-GCST90001476 | Weighted mode | 8 | 0.0778742102297326 | 0.0636859474137927 | 0.260977662410557 | -0.046950247 | 0.202698667160766 | 1.0809866730522 | 0.954134867766537 | 1.22470336929599 | 0.999857399747617 |
| ebi-a-GCST90001477 | finngen_R11_O15_POOR_FETGRO | Poor fetal growth \|\| id:finngen_R11_O15_POOR_FETGRO | HLA DR++ monocyte Absolute Count \|\| id:ebi-a-GCST90001477 | Inverse variance weighted | 23 | 0.0139423717690424 | 0.0209230177382743 | 0.505177673748679 | -0.027066743 | 0.0549514865360599 | 1.01404001992217 | 0.973296278644897 | 1.05648935947378 | 0.994119304454654 |
| ebi-a-GCST90001477 | finngen_R11_O15_POOR_FETGRO | Poor fetal growth \|\| id:finngen_R11_O15_POOR_FETGRO | HLA DR++ monocyte Absolute Count \|\| id:ebi-a-GCST90001477 | MR Egger | 23 | -0.01162321 | 0.0280313194957111 | 0.682602765800097 | -0.066564596 | 0.0433181762992781 | 0.988444078636719 | 0.93560247761074 | 1.04427010399441 | 0.992899239648112 |
| ebi-a-GCST90001477 | finngen_R11_O15_POOR_FETGRO | Poor fetal growth \|\| id:finngen_R11_O15_POOR_FETGRO | HLA DR++ monocyte Absolute Count \|\| id:ebi-a-GCST90001477 | Weighted median | 23 | 0.00868504957862469 | 0.0287054262621774 | 0.76222690619491 | -0.047577586 | 0.0649476850524924 | 1.00872287404487 | 0.953536489280444 | 1.06710319747615 | 0.999440741871876 |
| ebi-a-GCST90001477 | finngen_R11_O15_POOR_FETGRO | Poor fetal growth \|\| id:finngen_R11_O15_POOR_FETGRO | HLA DR++ monocyte Absolute Count \|\| id:ebi-a-GCST90001477 | Weighted mode | 23 | 0.00235166214222693 | 0.0288121890618163 | 0.935686607065746 | -0.054120228 | 0.0588235527033869 | 1.00235442946849 | 0.947318205084818 | 1.06058808632855 | 0.999857399747617 |
| ebi-a-GCST90001478 | finngen_R11_O15_POOR_FETGRO | Poor fetal growth \|\| id:finngen_R11_O15_POOR_FETGRO | CD4 regulatory T cell %CD4+ T cell \|\| id:ebi-a-GCST90001478 | Inverse variance weighted | 15 | 0.000623507626345652 | 0.0262650006079012 | 0.981060712927218 | -0.050855894 | 0.0521029088178319 | 1.00062370204763 | 0.950415621671293 | 1.05348414974369 | 0.998984604424952 |
| ebi-a-GCST90001478 | finngen_R11_O15_POOR_FETGRO | Poor fetal growth \|\| id:finngen_R11_O15_POOR_FETGRO | CD4 regulatory T cell %CD4+ T cell \|\| id:ebi-a-GCST90001478 | MR Egger | 15 | -0.023321884 | 0.0385180579786938 | 0.555284252204989 | -0.098817278 | 0.0521735092657886 | 0.976947968873399 | 0.905908222254045 | 1.05355852882215 | 0.992899239648112 |
| ebi-a-GCST90001478 | finngen_R11_O15_POOR_FETGRO | Poor fetal growth \|\| id:finngen_R11_O15_POOR_FETGRO | CD4 regulatory T cell %CD4+ T cell \|\| id:ebi-a-GCST90001478 | Weighted median | 15 | -0.018644899 | 0.0334101413542593 | 0.576802678776935 | -0.084128776 | 0.0468389776170854 | 0.981527841455405 | 0.919312862587001 | 1.0479532515634 | 0.999440741871876 |
| ebi-a-GCST90001478 | finngen_R11_O15_POOR_FETGRO | Poor fetal growth \|\| id:finngen_R11_O15_POOR_FETGRO | CD4 regulatory T cell %CD4+ T cell \|\| id:ebi-a-GCST90001478 | Weighted mode | 15 | -0.016442997 | 0.0304603930606718 | 0.597806333006956 | -0.076145367 | 0.0432593735987543 | 0.983691451352897 | 0.926681487675464 | 1.0442086998976 | 0.999857399747617 |
| ebi-a-GCST90001479 | finngen_R11_O15_POOR_FETGRO | Poor fetal growth \|\| id:finngen_R11_O15_POOR_FETGRO | CD4 regulatory T cell %T cell \|\| id:ebi-a-GCST90001479 | Inverse variance weighted | 16 | -0.025901427 | 0.0313263774551041 | 0.408336340888336 | -0.087301127 | 0.0354982723556257 | 0.974431137030614 | 0.916401100720238 | 1.03613585805223 | 0.994119304454654 |
| ebi-a-GCST90001479 | finngen_R11_O15_POOR_FETGRO | Poor fetal growth \|\| id:finngen_R11_O15_POOR_FETGRO | CD4 regulatory T cell %T cell \|\| id:ebi-a-GCST90001479 | MR Egger | 16 | -0.07024645 | 0.0465654465995378 | 0.153646086140469 | -0.161514725 | 0.0210218254754634 | 0.932164059893236 | 0.85085400238226 | 1.02124434053759 | 0.992899239648112 |
| ebi-a-GCST90001479 | finngen_R11_O15_POOR_FETGRO | Poor fetal growth \|\| id:finngen_R11_O15_POOR_FETGRO | CD4 regulatory T cell %T cell \|\| id:ebi-a-GCST90001479 | Weighted median | 16 | -0.021134095 | 0.0474188473066648 | 0.65582135269472 | -0.114075036 | 0.0718068454067202 | 0.979087664698141 | 0.892191006894664 | 1.07444778949362 | 0.999440741871876 |
| ebi-a-GCST90001479 | finngen_R11_O15_POOR_FETGRO | Poor fetal growth \|\| id:finngen_R11_O15_POOR_FETGRO | CD4 regulatory T cell %T cell \|\| id:ebi-a-GCST90001479 | Weighted mode | 16 | -0.020542666 | 0.0524258979628004 | 0.70068571660227 | -0.123297426 | 0.0822120937147007 | 0.979666896828543 | 0.884000698400071 | 1.08568605260006 | 0.999857399747617 |
| ebi-a-GCST90001480 | finngen_R11_O15_POOR_FETGRO | Poor fetal growth \|\| id:finngen_R11_O15_POOR_FETGRO | Resting CD4 regulatory T cell Absolute Count \|\| id:ebi-a-GCST90001480 | Inverse variance weighted | 25 | -0.035879537 | 0.0276268700902025 | 0.194040432812093 | -0.090028202 | 0.0182691284367922 | 0.964756504004068 | 0.91390541065786 | 1.01843702987617 | 0.994119304454654 |
| ebi-a-GCST90001480 | finngen_R11_O15_POOR_FETGRO | Poor fetal growth \|\| id:finngen_R11_O15_POOR_FETGRO | Resting CD4 regulatory T cell Absolute Count \|\| id:ebi-a-GCST90001480 | MR Egger | 25 | 0.00379548741109366 | 0.045058472481095 | 0.933598721807064 | -0.084519119 | 0.0921100934740399 | 1.00380269939488 | 0.918954086045642 | 1.09648553133742 | 0.992899239648112 |
| ebi-a-GCST90001480 | finngen_R11_O15_POOR_FETGRO | Poor fetal growth \|\| id:finngen_R11_O15_POOR_FETGRO | Resting CD4 regulatory T cell Absolute Count \|\| id:ebi-a-GCST90001480 | Weighted median | 25 | -0.029515713 | 0.0379461729317427 | 0.436668590783106 | -0.103890212 | 0.044858785499558 | 0.970915621090507 | 0.901324246221667 | 1.04588015603624 | 0.999440741871876 |
| ebi-a-GCST90001480 | finngen_R11_O15_POOR_FETGRO | Poor fetal growth \|\| id:finngen_R11_O15_POOR_FETGRO | Resting CD4 regulatory T cell Absolute Count \|\| id:ebi-a-GCST90001480 | Weighted mode | 25 | -0.020666987 | 0.0361106425210926 | 0.572425264914647 | -0.091443846 | 0.0501098726965611 | 0.979545111865034 | 0.912612561571628 | 1.05138660871191 | 0.999857399747617 |
| ebi-a-GCST90001481 | finngen_R11_O15_POOR_FETGRO | Poor fetal growth \|\| id:finngen_R11_O15_POOR_FETGRO | Resting CD4 regulatory T cell %CD4 regulatory T cell \|\| id:ebi-a-GCST90001481 | Inverse variance weighted | 29 | -0.003809059 | 0.013815838554134 | 0.782776705676395 | -0.030888102 | 0.0232699846820303 | 0.996198186378589 | 0.969584061086926 | 1.02354284313572 | 0.994119304454654 |
| ebi-a-GCST90001481 | finngen_R11_O15_POOR_FETGRO | Poor fetal growth \|\| id:finngen_R11_O15_POOR_FETGRO | Resting CD4 regulatory T cell %CD4 regulatory T cell \|\| id:ebi-a-GCST90001481 | MR Egger | 29 | 0.0150123143646538 | 0.0167966825143153 | 0.37934471019221 | -0.017909183 | 0.0479338120927118 | 1.01512556516513 | 0.982250232970243 | 1.04910121521248 | 0.992899239648112 |
| ebi-a-GCST90001481 | finngen_R11_O15_POOR_FETGRO | Poor fetal growth \|\| id:finngen_R11_O15_POOR_FETGRO | Resting CD4 regulatory T cell %CD4 regulatory T cell \|\| id:ebi-a-GCST90001481 | Weighted median | 29 | -0.004906531 | 0.0169497655287841 | 0.772218001555008 | -0.038128072 | 0.0283150091222576 | 0.995105486048054 | 0.962589652454609 | 1.0287196894729 | 0.999440741871876 |
| ebi-a-GCST90001481 | finngen_R11_O15_POOR_FETGRO | Poor fetal growth \|\| id:finngen_R11_O15_POOR_FETGRO | Resting CD4 regulatory T cell %CD4 regulatory T cell \|\| id:ebi-a-GCST90001481 | Weighted mode | 29 | 0.00606508450630738 | 0.0133796141872445 | 0.653820673907756 | -0.020158959 | 0.0322891283133066 | 1.00608351437207 | 0.98004287399431 | 1.03281607851079 | 0.999857399747617 |
| ebi-a-GCST90001482 | finngen_R11_O15_POOR_FETGRO | Poor fetal growth \|\| id:finngen_R11_O15_POOR_FETGRO | Resting CD4 regulatory T cell %CD4+ T cell \|\| id:ebi-a-GCST90001482 | Inverse variance weighted | 31 | 0.001163536794232 | 0.0131013103019529 | 0.929232338432945 | -0.024515031 | 0.0268421049860597 | 1.00116421396578 | 0.975783021425609 | 1.02720559931585 | 0.998984604424952 |
| ebi-a-GCST90001482 | finngen_R11_O15_POOR_FETGRO | Poor fetal growth \|\| id:finngen_R11_O15_POOR_FETGRO | Resting CD4 regulatory T cell %CD4+ T cell \|\| id:ebi-a-GCST90001482 | MR Egger | 31 | 0.0127229290810973 | 0.0162177940045504 | 0.439105953498224 | -0.019063947 | 0.0445098053300161 | 1.01280420988749 | 0.981116620607562 | 1.04551522828205 | 0.992899239648112 |
| ebi-a-GCST90001482 | finngen_R11_O15_POOR_FETGRO | Poor fetal growth \|\| id:finngen_R11_O15_POOR_FETGRO | Resting CD4 regulatory T cell %CD4+ T cell \|\| id:ebi-a-GCST90001482 | Weighted median | 31 | 0.000587873295152608 | 0.017738240666863 | 0.973561686115338 | -0.034179078 | 0.0353548250022041 | 1.00058804612652 | 0.966398428045073 | 1.03598723776546 | 0.999440741871876 |
| ebi-a-GCST90001482 | finngen_R11_O15_POOR_FETGRO | Poor fetal growth \|\| id:finngen_R11_O15_POOR_FETGRO | Resting CD4 regulatory T cell %CD4+ T cell \|\| id:ebi-a-GCST90001482 | Weighted mode | 31 | 0.00706865378720656 | 0.0127622384019693 | 0.583771249371601 | -0.017945333 | 0.0320826410550664 | 1.0070936956898 | 0.982214725150963 | 1.03260283716704 | 0.999857399747617 |
| ebi-a-GCST90001483 | finngen_R11_O15_POOR_FETGRO | Poor fetal growth \|\| id:finngen_R11_O15_POOR_FETGRO | CD39+ resting CD4 regulatory T cell Absolute Count \|\| id:ebi-a-GCST90001483 | Inverse variance weighted | 28 | -0.033688777 | 0.016067286425959 | 0.0360173951890702 | -0.065180659 | -0.002196896 | 0.966872370309306 | 0.936898189150268 | 0.997805515362777 | 0.994119304454654 |
| ebi-a-GCST90001483 | finngen_R11_O15_POOR_FETGRO | Poor fetal growth \|\| id:finngen_R11_O15_POOR_FETGRO | CD39+ resting CD4 regulatory T cell Absolute Count \|\| id:ebi-a-GCST90001483 | MR Egger | 28 | -0.037536238 | 0.0213601086015892 | 0.09063685623927 | -0.079402051 | 0.00432957483108835 | 0.963159514086941 | 0.92366848804688 | 1.00433896098132 | 0.992899239648112 |
| ebi-a-GCST90001483 | finngen_R11_O15_POOR_FETGRO | Poor fetal growth \|\| id:finngen_R11_O15_POOR_FETGRO | CD39+ resting CD4 regulatory T cell Absolute Count \|\| id:ebi-a-GCST90001483 | Weighted median | 28 | -0.010267037 | 0.0219489221036494 | 0.639949402416427 | -0.053286925 | 0.0327528500354682 | 0.989785488823438 | 0.948107937950597 | 1.03329512882583 | 0.999440741871876 |
| ebi-a-GCST90001483 | finngen_R11_O15_POOR_FETGRO | Poor fetal growth \|\| id:finngen_R11_O15_POOR_FETGRO | CD39+ resting CD4 regulatory T cell Absolute Count \|\| id:ebi-a-GCST90001483 | Weighted mode | 28 | -0.016545372 | 0.0204979310355816 | 0.426620735008158 | -0.056721317 | 0.0236305725418473 | 0.98359075061552 | 0.944857348248396 | 1.02391198680932 | 0.999857399747617 |
| ebi-a-GCST90001484 | finngen_R11_O15_POOR_FETGRO | Poor fetal growth \|\| id:finngen_R11_O15_POOR_FETGRO | CD39+ resting CD4 regulatory T cell %resting CD4 regulatory T cell \|\| id:ebi-a-GCST90001484 | Inverse variance weighted | 29 | -0.003520118 | 0.0153093044423128 | 0.8181436353698 | -0.033526355 | 0.0264861185160525 | 0.996486070161784 | 0.967029424933883 | 1.02683999309987 | 0.994119304454654 |
| ebi-a-GCST90001484 | finngen_R11_O15_POOR_FETGRO | Poor fetal growth \|\| id:finngen_R11_O15_POOR_FETGRO | CD39+ resting CD4 regulatory T cell %resting CD4 regulatory T cell \|\| id:ebi-a-GCST90001484 | MR Egger | 29 | 0.00302563037692994 | 0.0218416051583263 | 0.890852737704577 | -0.039783916 | 0.0458351764872494 | 1.00303021221634 | 0.960997073066052 | 1.04690184269646 | 0.992899239648112 |
| ebi-a-GCST90001484 | finngen_R11_O15_POOR_FETGRO | Poor fetal growth \|\| id:finngen_R11_O15_POOR_FETGRO | CD39+ resting CD4 regulatory T cell %resting CD4 regulatory T cell \|\| id:ebi-a-GCST90001484 | Weighted median | 29 | 0.00550424529138412 | 0.0206004132202288 | 0.789322094731721 | -0.034872565 | 0.0458810552030325 | 1.00551942148121 | 0.965728476391403 | 1.04694987431036 | 0.999440741871876 |
| ebi-a-GCST90001484 | finngen_R11_O15_POOR_FETGRO | Poor fetal growth \|\| id:finngen_R11_O15_POOR_FETGRO | CD39+ resting CD4 regulatory T cell %resting CD4 regulatory T cell \|\| id:ebi-a-GCST90001484 | Weighted mode | 29 | -0.002467167 | 0.0171077337380662 | 0.886364832514382 | -0.035998325 | 0.0310639911671936 | 0.997535873995625 | 0.964641909173984 | 1.0315515119598 | 0.999857399747617 |
| ebi-a-GCST90001485 | finngen_R11_O15_POOR_FETGRO | Poor fetal growth \|\| id:finngen_R11_O15_POOR_FETGRO | CD39+ resting CD4 regulatory T cell %CD4 regulatory T cell \|\| id:ebi-a-GCST90001485 | Inverse variance weighted | 33 | 0.00405045587008564 | 0.0107374403211502 | 0.706004676680562 | -0.016994927 | 0.0250958388995399 | 1.00405867005311 | 0.98314867197879 | 1.02541339030622 | 0.994119304454654 |
| ebi-a-GCST90001485 | finngen_R11_O15_POOR_FETGRO | Poor fetal growth \|\| id:finngen_R11_O15_POOR_FETGRO | CD39+ resting CD4 regulatory T cell %CD4 regulatory T cell \|\| id:ebi-a-GCST90001485 | MR Egger | 33 | 0.00557527714916335 | 0.0136309053586769 | 0.685339054816142 | -0.021141297 | 0.03229185165217 | 1.0055908479305 | 0.979080613295497 | 1.03281889122279 | 0.992899239648112 |
| ebi-a-GCST90001485 | finngen_R11_O15_POOR_FETGRO | Poor fetal growth \|\| id:finngen_R11_O15_POOR_FETGRO | CD39+ resting CD4 regulatory T cell %CD4 regulatory T cell \|\| id:ebi-a-GCST90001485 | Weighted median | 33 | 0.00417366293173409 | 0.0161954078091236 | 0.796633378285437 | -0.027569336 | 0.0359166622376164 | 1.00418238479268 | 0.972807229288975 | 1.0365694575121 | 0.999440741871876 |
| ebi-a-GCST90001485 | finngen_R11_O15_POOR_FETGRO | Poor fetal growth \|\| id:finngen_R11_O15_POOR_FETGRO | CD39+ resting CD4 regulatory T cell %CD4 regulatory T cell \|\| id:ebi-a-GCST90001485 | Weighted mode | 33 | 0.00777621708604781 | 0.0130065969957004 | 0.554136572121001 | -0.017716713 | 0.0332691471976206 | 1.00780653038545 | 0.982439305199279 | 1.03382875391121 | 0.999857399747617 |
| ebi-a-GCST90001486 | finngen_R11_O15_POOR_FETGRO | Poor fetal growth \|\| id:finngen_R11_O15_POOR_FETGRO | Activated CD4 regulatory T cell Absolute Count \|\| id:ebi-a-GCST90001486 | Inverse variance weighted | 15 | 0.0357028996058146 | 0.0209665998102909 | 0.0885968115928529 | -0.005391636 | 0.0767974352339848 | 1.03634790137787 | 0.994622872760074 | 1.07982332007902 | 0.994119304454654 |
| ebi-a-GCST90001486 | finngen_R11_O15_POOR_FETGRO | Poor fetal growth \|\| id:finngen_R11_O15_POOR_FETGRO | Activated CD4 regulatory T cell Absolute Count \|\| id:ebi-a-GCST90001486 | MR Egger | 15 | 0.0565508994445963 | 0.0261056033889578 | 0.04946630541492 | 0.00538391680223896 | 0.107717882086954 | 1.05818047422793 | 1.00539843612756 | 1.11373349689115 | 0.992899239648112 |
| ebi-a-GCST90001486 | finngen_R11_O15_POOR_FETGRO | Poor fetal growth \|\| id:finngen_R11_O15_POOR_FETGRO | Activated CD4 regulatory T cell Absolute Count \|\| id:ebi-a-GCST90001486 | Weighted median | 15 | 0.0368827311182401 | 0.0281512168982332 | 0.190140118937475 | -0.018293654 | 0.0920591162387772 | 1.03757133887289 | 0.981872659183289 | 1.09642963696121 | 0.999440741871876 |
| ebi-a-GCST90001486 | finngen_R11_O15_POOR_FETGRO | Poor fetal growth \|\| id:finngen_R11_O15_POOR_FETGRO | Activated CD4 regulatory T cell Absolute Count \|\| id:ebi-a-GCST90001486 | Weighted mode | 15 | 0.0417783510947452 | 0.0257056769352982 | 0.126399485058474 | -0.008604776 | 0.0921614778879295 | 1.04266334794714 | 0.991432139425968 | 1.09654187505137 | 0.999857399747617 |
| ebi-a-GCST90001487 | finngen_R11_O15_POOR_FETGRO | Poor fetal growth \|\| id:finngen_R11_O15_POOR_FETGRO | Activated CD4 regulatory T cell %CD4 regulatory T cell \|\| id:ebi-a-GCST90001487 | Inverse variance weighted | 28 | 0.000597968566179161 | 0.0233914156129911 | 0.979605427273229 | -0.045249206 | 0.0464451431676416 | 1.00059814738502 | 0.955759271173911 | 1.04754061273256 | 0.998984604424952 |
| ebi-a-GCST90001487 | finngen_R11_O15_POOR_FETGRO | Poor fetal growth \|\| id:finngen_R11_O15_POOR_FETGRO | Activated CD4 regulatory T cell %CD4 regulatory T cell \|\| id:ebi-a-GCST90001487 | MR Egger | 28 | 0.021897324727225 | 0.0330417434705498 | 0.513344459981808 | -0.042864492 | 0.0866591419295026 | 1.0221388306992 | 0.958041203063738 | 1.09052490214621 | 0.992899239648112 |
| ebi-a-GCST90001487 | finngen_R11_O15_POOR_FETGRO | Poor fetal growth \|\| id:finngen_R11_O15_POOR_FETGRO | Activated CD4 regulatory T cell %CD4 regulatory T cell \|\| id:ebi-a-GCST90001487 | Weighted median | 28 | 0.026880382297632 | 0.0322025725621775 | 0.403870989800957 | -0.03623666 | 0.0899974245195 | 1.02724491873713 | 0.96441202879597 | 1.09417146568431 | 0.999440741871876 |
| ebi-a-GCST90001487 | finngen_R11_O15_POOR_FETGRO | Poor fetal growth \|\| id:finngen_R11_O15_POOR_FETGRO | Activated CD4 regulatory T cell %CD4 regulatory T cell \|\| id:ebi-a-GCST90001487 | Weighted mode | 28 | 0.029604769252825 | 0.030080949646186 | 0.33376952552437 | -0.029353892 | 0.0885634305593495 | 1.03004734710981 | 0.97107274872172 | 1.09260355486715 | 0.999857399747617 |
| ebi-a-GCST90001488 | finngen_R11_O15_POOR_FETGRO | Poor fetal growth \|\| id:finngen_R11_O15_POOR_FETGRO | Activated CD4 regulatory T cell %CD4+ T cell \|\| id:ebi-a-GCST90001488 | Inverse variance weighted (multiplicative random effects) | 19 | -0.007500845 | 0.0351461683307072 | 0.831000499663274 | -0.076387335 | 0.0613856450450895 | 0.992527216249316 | 0.926457287894255 | 1.06330889493532 | 0.994119304454654 |
| ebi-a-GCST90001488 | finngen_R11_O15_POOR_FETGRO | Poor fetal growth \|\| id:finngen_R11_O15_POOR_FETGRO | Activated CD4 regulatory T cell %CD4+ T cell \|\| id:ebi-a-GCST90001488 | MR Egger | 19 | 0.0273077885640526 | 0.0510937126859974 | 0.599940936410928 | -0.072835888 | 0.127451465428607 | 1.02768406349248 | 0.929753400916854 | 1.13592973504042 | 0.992899239648112 |
| ebi-a-GCST90001488 | finngen_R11_O15_POOR_FETGRO | Poor fetal growth \|\| id:finngen_R11_O15_POOR_FETGRO | Activated CD4 regulatory T cell %CD4+ T cell \|\| id:ebi-a-GCST90001488 | Weighted median | 19 | 0.0134911864612192 | 0.0400399224072174 | 0.73615958910613 | -0.064987061 | 0.0919694343793653 | 1.01358260316126 | 0.937079587743576 | 1.09633131152173 | 0.999440741871876 |
| ebi-a-GCST90001488 | finngen_R11_O15_POOR_FETGRO | Poor fetal growth \|\| id:finngen_R11_O15_POOR_FETGRO | Activated CD4 regulatory T cell %CD4+ T cell \|\| id:ebi-a-GCST90001488 | Weighted mode | 19 | 0.0104913975986521 | 0.0364370645487366 | 0.776687797383852 | -0.060925249 | 0.0819080441141758 | 1.01054662527998 | 0.940893569960812 | 1.08535600036814 | 0.999857399747617 |
| ebi-a-GCST90001489 | finngen_R11_O15_POOR_FETGRO | Poor fetal growth \|\| id:finngen_R11_O15_POOR_FETGRO | CD39+ activated CD4 regulatory T cell Absolute Count \|\| id:ebi-a-GCST90001489 | Inverse variance weighted | 30 | 0.0101779528183516 | 0.0111542007060375 | 0.361517614123101 | -0.011684281 | 0.0320401862021851 | 1.01022992435176 | 0.988383715554444 | 1.03255899909608 | 0.994119304454654 |
| ebi-a-GCST90001489 | finngen_R11_O15_POOR_FETGRO | Poor fetal growth \|\| id:finngen_R11_O15_POOR_FETGRO | CD39+ activated CD4 regulatory T cell Absolute Count \|\| id:ebi-a-GCST90001489 | MR Egger | 30 | -0.002715317 | 0.0139463691176008 | 0.847035960339503 | -0.0300502 | 0.0246195669696869 | 0.997288366636657 | 0.970396818433327 | 1.02492513097326 | 0.992899239648112 |
| ebi-a-GCST90001489 | finngen_R11_O15_POOR_FETGRO | Poor fetal growth \|\| id:finngen_R11_O15_POOR_FETGRO | CD39+ activated CD4 regulatory T cell Absolute Count \|\| id:ebi-a-GCST90001489 | Weighted median | 30 | 0.00258943594066564 | 0.0147419600620009 | 0.860568350757946 | -0.026304806 | 0.0314836776621874 | 1.00259279142556 | 0.97403815189741 | 1.03198453105804 | 0.999440741871876 |
| ebi-a-GCST90001489 | finngen_R11_O15_POOR_FETGRO | Poor fetal growth \|\| id:finngen_R11_O15_POOR_FETGRO | CD39+ activated CD4 regulatory T cell Absolute Count \|\| id:ebi-a-GCST90001489 | Weighted mode | 30 | 0.00282524050221178 | 0.0126312903696448 | 0.824582314501997 | -0.021932089 | 0.0275825696267156 | 1.00282923525532 | 0.978306670949153 | 1.02796649041253 | 0.999857399747617 |
| ebi-a-GCST90001490 | finngen_R11_O15_POOR_FETGRO | Poor fetal growth \|\| id:finngen_R11_O15_POOR_FETGRO | CD39+ activated CD4 regulatory T cell %activated CD4 regulatory T cell \|\| id:ebi-a-GCST90001490 | Inverse variance weighted | 24 | -0.017619092 | 0.025247003989875 | 0.485259364792104 | -0.067103219 | 0.0318650361615191 | 0.98253521694858 | 0.935098675957262 | 1.03237816218266 | 0.994119304454654 |
| ebi-a-GCST90001490 | finngen_R11_O15_POOR_FETGRO | Poor fetal growth \|\| id:finngen_R11_O15_POOR_FETGRO | CD39+ activated CD4 regulatory T cell %activated CD4 regulatory T cell \|\| id:ebi-a-GCST90001490 | MR Egger | 24 | 0.0281898433978647 | 0.0492447595386381 | 0.572825699624828 | -0.068329885 | 0.124709572093595 | 1.02859093708563 | 0.933952325612139 | 1.13281940291894 | 0.992899239648112 |
| ebi-a-GCST90001490 | finngen_R11_O15_POOR_FETGRO | Poor fetal growth \|\| id:finngen_R11_O15_POOR_FETGRO | CD39+ activated CD4 regulatory T cell %activated CD4 regulatory T cell \|\| id:ebi-a-GCST90001490 | Weighted median | 24 | -0.038994595 | 0.0364800191539689 | 0.285101117388433 | -0.110495432 | 0.0325062429522477 | 0.961755907817769 | 0.89539042020601 | 1.03304034234537 | 0.999440741871876 |
| ebi-a-GCST90001490 | finngen_R11_O15_POOR_FETGRO | Poor fetal growth \|\| id:finngen_R11_O15_POOR_FETGRO | CD39+ activated CD4 regulatory T cell %activated CD4 regulatory T cell \|\| id:ebi-a-GCST90001490 | Weighted mode | 24 | -0.044544518 | 0.040306943767442 | 0.280529120802508 | -0.123546128 | 0.0344570917614041 | 0.956433020638849 | 0.883780873430289 | 1.03505761492417 | 0.999857399747617 |
| ebi-a-GCST90001491 | finngen_R11_O15_POOR_FETGRO | Poor fetal growth \|\| id:finngen_R11_O15_POOR_FETGRO | CD39+ activated CD4 regulatory T cell %CD4 regulatory T cell \|\| id:ebi-a-GCST90001491 | Inverse variance weighted | 22 | 0.0158881336435392 | 0.0151704017442898 | 0.294956011739697 | -0.013845854 | 0.0456221210623472 | 1.01601502115008 | 0.986249559192489 | 1.04667881833865 | 0.994119304454654 |
| ebi-a-GCST90001491 | finngen_R11_O15_POOR_FETGRO | Poor fetal growth \|\| id:finngen_R11_O15_POOR_FETGRO | CD39+ activated CD4 regulatory T cell %CD4 regulatory T cell \|\| id:ebi-a-GCST90001491 | MR Egger | 22 | 0.0170399509545948 | 0.0233694899318195 | 0.474360765611923 | -0.028764249 | 0.062844151220961 | 1.01718595906356 | 0.971645503563661 | 1.06486086903223 | 0.992899239648112 |
| ebi-a-GCST90001491 | finngen_R11_O15_POOR_FETGRO | Poor fetal growth \|\| id:finngen_R11_O15_POOR_FETGRO | CD39+ activated CD4 regulatory T cell %CD4 regulatory T cell \|\| id:ebi-a-GCST90001491 | Weighted median | 22 | 0.0197637154080713 | 0.0214684861763125 | 0.357263510127062 | -0.022314517 | 0.0618419483136437 | 1.01996031064633 | 0.977932609759831 | 1.0637941969736 | 0.999440741871876 |
| ebi-a-GCST90001491 | finngen_R11_O15_POOR_FETGRO | Poor fetal growth \|\| id:finngen_R11_O15_POOR_FETGRO | CD39+ activated CD4 regulatory T cell %CD4 regulatory T cell \|\| id:ebi-a-GCST90001491 | Weighted mode | 22 | 0.0171099148171829 | 0.017022899164945 | 0.326284848764196 | -0.016254968 | 0.0504747971804752 | 1.01725712781182 | 0.983876431513008 | 1.05177035544252 | 0.999857399747617 |
| ebi-a-GCST90001492 | finngen_R11_O15_POOR_FETGRO | Poor fetal growth \|\| id:finngen_R11_O15_POOR_FETGRO | Secreting CD4 regulatory T cell Absolute Count \|\| id:ebi-a-GCST90001492 | Inverse variance weighted | 23 | 0.00036605520494142 | 0.0107796274083095 | 0.97291059684092 | -0.020762015 | 0.021494124925228 | 1.00036612221132 | 0.979452032201582 | 1.02172678759848 | 0.998984604424952 |
| ebi-a-GCST90001492 | finngen_R11_O15_POOR_FETGRO | Poor fetal growth \|\| id:finngen_R11_O15_POOR_FETGRO | Secreting CD4 regulatory T cell Absolute Count \|\| id:ebi-a-GCST90001492 | MR Egger | 23 | -0.012617501 | 0.0123550097469793 | 0.318759605391034 | -0.03683332 | 0.0115983182729598 | 0.987461766098731 | 0.963836774337027 | 1.01166583955829 | 0.992899239648112 |
| ebi-a-GCST90001492 | finngen_R11_O15_POOR_FETGRO | Poor fetal growth \|\| id:finngen_R11_O15_POOR_FETGRO | Secreting CD4 regulatory T cell Absolute Count \|\| id:ebi-a-GCST90001492 | Weighted median | 23 | 0.00598225682844306 | 0.0136398137847731 | 0.660960190327199 | -0.020751778 | 0.0327162918465984 | 1.00600018626182 | 0.979462058242841 | 1.03325735411784 | 0.999440741871876 |
| ebi-a-GCST90001492 | finngen_R11_O15_POOR_FETGRO | Poor fetal growth \|\| id:finngen_R11_O15_POOR_FETGRO | Secreting CD4 regulatory T cell Absolute Count \|\| id:ebi-a-GCST90001492 | Weighted mode | 23 | 0.00277559994223287 | 0.0115012736801536 | 0.811534586655888 | -0.019766896 | 0.0253180963553339 | 1.00277945548608 | 0.980427187709476 | 1.02564132140624 | 0.999857399747617 |
| ebi-a-GCST90001493 | finngen_R11_O15_POOR_FETGRO | Poor fetal growth \|\| id:finngen_R11_O15_POOR_FETGRO | Secreting CD4 regulatory T cell %CD4 regulatory T cell \|\| id:ebi-a-GCST90001493 | Inverse variance weighted | 31 | -0.006699009 | 0.0156084214286147 | 0.667783534741323 | -0.037291515 | 0.0238934970406132 | 0.993323379379923 | 0.96339525028252 | 1.02418123374949 | 0.994119304454654 |
| ebi-a-GCST90001493 | finngen_R11_O15_POOR_FETGRO | Poor fetal growth \|\| id:finngen_R11_O15_POOR_FETGRO | Secreting CD4 regulatory T cell %CD4 regulatory T cell \|\| id:ebi-a-GCST90001493 | MR Egger | 31 | 0.00823537507247652 | 0.0198531520890127 | 0.68133047531003 | -0.030676803 | 0.0471475531669415 | 1.00826937905486 | 0.969788955290865 | 1.04827667421182 | 0.992899239648112 |
| ebi-a-GCST90001493 | finngen_R11_O15_POOR_FETGRO | Poor fetal growth \|\| id:finngen_R11_O15_POOR_FETGRO | Secreting CD4 regulatory T cell %CD4 regulatory T cell \|\| id:ebi-a-GCST90001493 | Weighted median | 31 | 0.00503009221538465 | 0.0207348383654803 | 0.808321990843375 | -0.035610191 | 0.0456703754117261 | 1.00504276436769 | 0.965016392268242 | 1.04672932636264 | 0.999440741871876 |
| ebi-a-GCST90001493 | finngen_R11_O15_POOR_FETGRO | Poor fetal growth \|\| id:finngen_R11_O15_POOR_FETGRO | Secreting CD4 regulatory T cell %CD4 regulatory T cell \|\| id:ebi-a-GCST90001493 | Weighted mode | 31 | 0.00265825331881975 | 0.0180195482169265 | 0.883708183991615 | -0.032660061 | 0.0379765678239958 | 1.00266178960693 | 0.967867519409071 | 1.03870689343059 | 0.999857399747617 |
| ebi-a-GCST90001494 | finngen_R11_O15_POOR_FETGRO | Poor fetal growth \|\| id:finngen_R11_O15_POOR_FETGRO | Secreting CD4 regulatory T cell %CD4+ T cell \|\| id:ebi-a-GCST90001494 | Inverse variance weighted | 31 | 0.00530166227561103 | 0.00685330586701894 | 0.439172131794921 | -0.008130817 | 0.0187341417749681 | 1.00531574095619 | 0.991902148464117 | 1.01891072680831 | 0.994119304454654 |
| ebi-a-GCST90001494 | finngen_R11_O15_POOR_FETGRO | Poor fetal growth \|\| id:finngen_R11_O15_POOR_FETGRO | Secreting CD4 regulatory T cell %CD4+ T cell \|\| id:ebi-a-GCST90001494 | MR Egger | 31 | 0.00522457252770807 | 0.00756619379493374 | 0.495360193309764 | -0.009605167 | 0.0200543123657782 | 1.00523824440629 | 0.990440814968907 | 1.02025675107984 | 0.992899239648112 |
| ebi-a-GCST90001494 | finngen_R11_O15_POOR_FETGRO | Poor fetal growth \|\| id:finngen_R11_O15_POOR_FETGRO | Secreting CD4 regulatory T cell %CD4+ T cell \|\| id:ebi-a-GCST90001494 | Weighted median | 31 | 0.013978130832128 | 0.00946824871414841 | 0.139858974203556 | -0.004579637 | 0.0325358983118589 | 1.01407628169156 | 0.995430833898446 | 1.03307097798247 | 0.999440741871876 |
| ebi-a-GCST90001494 | finngen_R11_O15_POOR_FETGRO | Poor fetal growth \|\| id:finngen_R11_O15_POOR_FETGRO | Secreting CD4 regulatory T cell %CD4+ T cell \|\| id:ebi-a-GCST90001494 | Weighted mode | 31 | 0.00495164979311391 | 0.00650323238273046 | 0.452354307270285 | -0.007794686 | 0.0176979852632656 | 1.00496392947081 | 0.992235614108486 | 1.01785552259665 | 0.999857399747617 |
| ebi-a-GCST90001495 | finngen_R11_O15_POOR_FETGRO | Poor fetal growth \|\| id:finngen_R11_O15_POOR_FETGRO | CD39+ secreting CD4 regulatory T cell Absolute Count \|\| id:ebi-a-GCST90001495 | Inverse variance weighted | 26 | 0.0197249833970654 | 0.0166291249612283 | 0.235554832902215 | -0.012868102 | 0.0523180683210729 | 1.0199208062974 | 0.987214338497108 | 1.05371084125657 | 0.994119304454654 |
| ebi-a-GCST90001495 | finngen_R11_O15_POOR_FETGRO | Poor fetal growth \|\| id:finngen_R11_O15_POOR_FETGRO | CD39+ secreting CD4 regulatory T cell Absolute Count \|\| id:ebi-a-GCST90001495 | MR Egger | 26 | 0.00859577312647813 | 0.0269043122091398 | 0.752116232871813 | -0.044136679 | 0.0613282250563921 | 1.00863282286526 | 0.956823171089263 | 1.06324784150346 | 0.992899239648112 |
| ebi-a-GCST90001495 | finngen_R11_O15_POOR_FETGRO | Poor fetal growth \|\| id:finngen_R11_O15_POOR_FETGRO | CD39+ secreting CD4 regulatory T cell Absolute Count \|\| id:ebi-a-GCST90001495 | Weighted median | 26 | 0.0195767174052932 | 0.0210149238215134 | 0.351562610764462 | -0.021612533 | 0.0607659680954595 | 1.01976959793733 | 0.978619344023001 | 1.06265019103569 | 0.999440741871876 |
| ebi-a-GCST90001495 | finngen_R11_O15_POOR_FETGRO | Poor fetal growth \|\| id:finngen_R11_O15_POOR_FETGRO | CD39+ secreting CD4 regulatory T cell Absolute Count \|\| id:ebi-a-GCST90001495 | Weighted mode | 26 | 0.0181173566125129 | 0.0212587097175448 | 0.402175978632504 | -0.023549714 | 0.0597844276589008 | 1.01828247155933 | 0.97672541611071 | 1.06160766862592 | 0.999857399747617 |
| ebi-a-GCST90001496 | finngen_R11_O15_POOR_FETGRO | Poor fetal growth \|\| id:finngen_R11_O15_POOR_FETGRO | CD39+ secreting CD4 regulatory T cell %secreting CD4 regulatory T cell \|\| id:ebi-a-GCST90001496 | Inverse variance weighted | 28 | 0.00678642190659239 | 0.0152840224157135 | 0.657027638195169 | -0.023170262 | 0.0367431058413908 | 1.00680950184827 | 0.977096107244905 | 1.03742647780081 | 0.994119304454654 |
| ebi-a-GCST90001496 | finngen_R11_O15_POOR_FETGRO | Poor fetal growth \|\| id:finngen_R11_O15_POOR_FETGRO | CD39+ secreting CD4 regulatory T cell %secreting CD4 regulatory T cell \|\| id:ebi-a-GCST90001496 | MR Egger | 28 | 0.042327357346447 | 0.0202963629076282 | 0.0469856881848557 | 0.00254648604749579 | 0.0821082286453983 | 1.04323593380581 | 1.002549731 | 1.08557329359893 | 0.992899239648112 |
| ebi-a-GCST90001496 | finngen_R11_O15_POOR_FETGRO | Poor fetal growth \|\| id:finngen_R11_O15_POOR_FETGRO | CD39+ secreting CD4 regulatory T cell %secreting CD4 regulatory T cell \|\| id:ebi-a-GCST90001496 | Weighted median | 28 | 0.0237744291859295 | 0.0205618468106818 | 0.247583014020103 | -0.016526791 | 0.0640756489348658 | 1.02405929394688 | 0.983609027598056 | 1.06617305056651 | 0.999440741871876 |
| ebi-a-GCST90001496 | finngen_R11_O15_POOR_FETGRO | Poor fetal growth \|\| id:finngen_R11_O15_POOR_FETGRO | CD39+ secreting CD4 regulatory T cell %secreting CD4 regulatory T cell \|\| id:ebi-a-GCST90001496 | Weighted mode | 28 | 0.0243767045977318 | 0.0192517809348514 | 0.216255628059575 | -0.013356786 | 0.0621101952300406 | 1.02467624544856 | 0.986732020004555 | 1.06407959476344 | 0.999857399747617 |
| ebi-a-GCST90001497 | finngen_R11_O15_POOR_FETGRO | Poor fetal growth \|\| id:finngen_R11_O15_POOR_FETGRO | CD39+ secreting CD4 regulatory T cell %CD4 regulatory T cell \|\| id:ebi-a-GCST90001497 | Inverse variance weighted | 22 | 0.0118378645275302 | 0.01786431999821 | 0.507552126003544 | -0.023176203 | 0.0468519317240217 | 1.01190820934926 | 0.97709030268519 | 1.04796682694982 | 0.994119304454654 |
| ebi-a-GCST90001497 | finngen_R11_O15_POOR_FETGRO | Poor fetal growth \|\| id:finngen_R11_O15_POOR_FETGRO | CD39+ secreting CD4 regulatory T cell %CD4 regulatory T cell \|\| id:ebi-a-GCST90001497 | MR Egger | 22 | 0.0171660104451011 | 0.0241633741821973 | 0.485649392770454 | -0.030194203 | 0.0645262238422079 | 1.0173141930897 | 0.970257088460021 | 1.06665354963226 | 0.992899239648112 |
| ebi-a-GCST90001497 | finngen_R11_O15_POOR_FETGRO | Poor fetal growth \|\| id:finngen_R11_O15_POOR_FETGRO | CD39+ secreting CD4 regulatory T cell %CD4 regulatory T cell \|\| id:ebi-a-GCST90001497 | Weighted median | 22 | 0.0238641559743828 | 0.0222803978560832 | 0.284132099269515 | -0.019805424 | 0.0675337357723059 | 1.02415118362093 | 0.980389415173088 | 1.06986635175674 | 0.999440741871876 |
| ebi-a-GCST90001497 | finngen_R11_O15_POOR_FETGRO | Poor fetal growth \|\| id:finngen_R11_O15_POOR_FETGRO | CD39+ secreting CD4 regulatory T cell %CD4 regulatory T cell \|\| id:ebi-a-GCST90001497 | Weighted mode | 22 | 0.0174283611803699 | 0.0188560462142133 | 0.365837523114327 | -0.019529489 | 0.054386211760228 | 1.01758112122912 | 0.980659975688474 | 1.0558923214491 | 0.999857399747617 |
| ebi-a-GCST90001498 | finngen_R11_O15_POOR_FETGRO | Poor fetal growth \|\| id:finngen_R11_O15_POOR_FETGRO | Activated & resting CD4 regulatory T cell Absolute Count \|\| id:ebi-a-GCST90001498 | Inverse variance weighted | 19 | 0.0759261993001672 | 0.0451392318682241 | 0.0925601999978956 | -0.012546695 | 0.164399093761886 | 1.07888294890245 | 0.987531686465602 | 1.17868462691906 | 0.994119304454654 |
| ebi-a-GCST90001498 | finngen_R11_O15_POOR_FETGRO | Poor fetal growth \|\| id:finngen_R11_O15_POOR_FETGRO | Activated & resting CD4 regulatory T cell Absolute Count \|\| id:ebi-a-GCST90001498 | MR Egger | 19 | 0.0792013977644032 | 0.10106332262193 | 0.444009688233435 | -0.118882715 | 0.277285510103385 | 1.08242229755056 | 0.887911933784078 | 1.31954306013365 | 0.992899239648112 |
| ebi-a-GCST90001498 | finngen_R11_O15_POOR_FETGRO | Poor fetal growth \|\| id:finngen_R11_O15_POOR_FETGRO | Activated & resting CD4 regulatory T cell Absolute Count \|\| id:ebi-a-GCST90001498 | Weighted median | 19 | 0.113956315683827 | 0.0569706974299525 | 0.0454719904581891 | 0.00229374872112048 | 0.225618882646534 | 1.12070316666334 | 1.00229638137522 | 1.25309799686791 | 0.999440741871876 |
| ebi-a-GCST90001498 | finngen_R11_O15_POOR_FETGRO | Poor fetal growth \|\| id:finngen_R11_O15_POOR_FETGRO | Activated & resting CD4 regulatory T cell Absolute Count \|\| id:ebi-a-GCST90001498 | Weighted mode | 19 | 0.117002922222318 | 0.0842949851150411 | 0.182073875791783 | -0.048215249 | 0.282221093047799 | 1.12412271462222 | 0.952928648439904 | 1.32607187285054 | 0.999857399747617 |
| ebi-a-GCST90001499 | finngen_R11_O15_POOR_FETGRO | Poor fetal growth \|\| id:finngen_R11_O15_POOR_FETGRO | Activated & resting CD4 regulatory T cell %CD4 regulatory T cell \|\| id:ebi-a-GCST90001499 | Inverse variance weighted | 29 | 0.00784677159264013 | 0.0155647660016802 | 0.614165236360702 | -0.02266017 | 0.0383537129559334 | 1.0078776381864 | 0.977594643543039 | 1.03909871056028 | 0.994119304454654 |
| ebi-a-GCST90001499 | finngen_R11_O15_POOR_FETGRO | Poor fetal growth \|\| id:finngen_R11_O15_POOR_FETGRO | Activated & resting CD4 regulatory T cell %CD4 regulatory T cell \|\| id:ebi-a-GCST90001499 | MR Egger | 29 | -0.005617402 | 0.0196847909641986 | 0.777540262134479 | -0.044199592 | 0.0329647885060248 | 0.994398346315996 | 0.95676297610812 | 1.03351414702344 | 0.992899239648112 |
| ebi-a-GCST90001499 | finngen_R11_O15_POOR_FETGRO | Poor fetal growth \|\| id:finngen_R11_O15_POOR_FETGRO | Activated & resting CD4 regulatory T cell %CD4 regulatory T cell \|\| id:ebi-a-GCST90001499 | Weighted median | 29 | -0.004978822 | 0.0212249719648564 | 0.814539557263599 | -0.046579767 | 0.0366221230094133 | 0.995033551748605 | 0.954488420766827 | 1.03730097459958 | 0.999440741871876 |
| ebi-a-GCST90001499 | finngen_R11_O15_POOR_FETGRO | Poor fetal growth \|\| id:finngen_R11_O15_POOR_FETGRO | Activated & resting CD4 regulatory T cell %CD4 regulatory T cell \|\| id:ebi-a-GCST90001499 | Weighted mode | 29 | -0.003413158 | 0.0175294901114603 | 0.847026287587852 | -0.037770959 | 0.0309446424759857 | 0.996592660060412 | 0.962933467109877 | 1.03142840498341 | 0.999857399747617 |
| ebi-a-GCST90001500 | finngen_R11_O15_POOR_FETGRO | Poor fetal growth \|\| id:finngen_R11_O15_POOR_FETGRO | Activated & resting CD4 regulatory T cell %CD4+ T cell \|\| id:ebi-a-GCST90001500 | Inverse variance weighted | 26 | -0.00694787 | 0.0217068381855288 | 0.748909592572957 | -0.049493273 | 0.0355975326599444 | 0.993076210464339 | 0.951711560252586 | 1.03623871031731 | 0.994119304454654 |
| ebi-a-GCST90001500 | finngen_R11_O15_POOR_FETGRO | Poor fetal growth \|\| id:finngen_R11_O15_POOR_FETGRO | Activated & resting CD4 regulatory T cell %CD4+ T cell \|\| id:ebi-a-GCST90001500 | MR Egger | 26 | 0.000438634709200594 | 0.0302233463136691 | 0.988540595940827 | -0.058799124 | 0.059676393483992 | 1.00043873092347 | 0.942896155281149 | 1.06149298491235 | 0.994809183268002 |
| ebi-a-GCST90001500 | finngen_R11_O15_POOR_FETGRO | Poor fetal growth \|\| id:finngen_R11_O15_POOR_FETGRO | Activated & resting CD4 regulatory T cell %CD4+ T cell \|\| id:ebi-a-GCST90001500 | Weighted median | 26 | -0.005558638 | 0.0321253878406209 | 0.862628276738734 | -0.068524398 | 0.057407121886317 | 0.994456782362647 | 0.933770677269422 | 1.05908690009306 | 0.999440741871876 |
| ebi-a-GCST90001500 | finngen_R11_O15_POOR_FETGRO | Poor fetal growth \|\| id:finngen_R11_O15_POOR_FETGRO | Activated & resting CD4 regulatory T cell %CD4+ T cell \|\| id:ebi-a-GCST90001500 | Weighted mode | 26 | -0.004248585 | 0.026629800365252 | 0.874523471370177 | -0.056442993 | 0.0479458240544296 | 0.99576042780642 | 0.945120361079246 | 1.0491138170518 | 0.999857399747617 |
| ebi-a-GCST90001501 | finngen_R11_O15_POOR_FETGRO | Poor fetal growth \|\| id:finngen_R11_O15_POOR_FETGRO | Activated & secreting CD4 regulatory T cell Absolute Count \|\| id:ebi-a-GCST90001501 | Inverse variance weighted | 22 | 0.000439231073925911 | 0.00922927030117166 | 0.962042129647144 | -0.017650139 | 0.0185286008642224 | 1.00043932755002 | 0.982504712594511 | 1.01870132049108 | 0.998984604424952 |
| ebi-a-GCST90001501 | finngen_R11_O15_POOR_FETGRO | Poor fetal growth \|\| id:finngen_R11_O15_POOR_FETGRO | Activated & secreting CD4 regulatory T cell Absolute Count \|\| id:ebi-a-GCST90001501 | MR Egger | 22 | -0.006251173 | 0.0114223405823744 | 0.590244498713683 | -0.028638961 | 0.0161366143511805 | 0.993768324743378 | 0.971767247275498 | 1.01626751264985 | 0.992899239648112 |
| ebi-a-GCST90001501 | finngen_R11_O15_POOR_FETGRO | Poor fetal growth \|\| id:finngen_R11_O15_POOR_FETGRO | Activated & secreting CD4 regulatory T cell Absolute Count \|\| id:ebi-a-GCST90001501 | Weighted median | 22 | 0.00503959383640639 | 0.0137130136799299 | 0.713242757541537 | -0.021837913 | 0.031917100649069 | 1.00505231394851 | 0.978398807950373 | 1.03243191382189 | 0.999440741871876 |
| ebi-a-GCST90001501 | finngen_R11_O15_POOR_FETGRO | Poor fetal growth \|\| id:finngen_R11_O15_POOR_FETGRO | Activated & secreting CD4 regulatory T cell Absolute Count \|\| id:ebi-a-GCST90001501 | Weighted mode | 22 | 0.000873511437884944 | 0.0111915767306902 | 0.93852656970369 | -0.021061979 | 0.0228090018300376 | 1.00087389306011 | 0.979158275482779 | 1.02307111617413 | 0.999857399747617 |
| ebi-a-GCST90001502 | finngen_R11_O15_POOR_FETGRO | Poor fetal growth \|\| id:finngen_R11_O15_POOR_FETGRO | Activated & secreting CD4 regulatory T cell %CD4 regulatory T cell \|\| id:ebi-a-GCST90001502 | Inverse variance weighted | 36 | 0.00692282504472358 | 0.00839795348201317 | 0.409742603862297 | -0.009537164 | 0.0233828138694694 | 1.00694684319051 | 0.990508170731106 | 1.02365833515833 | 0.994119304454654 |
| ebi-a-GCST90001502 | finngen_R11_O15_POOR_FETGRO | Poor fetal growth \|\| id:finngen_R11_O15_POOR_FETGRO | Activated & secreting CD4 regulatory T cell %CD4 regulatory T cell \|\| id:ebi-a-GCST90001502 | MR Egger | 36 | 0.00182836455635123 | 0.00999876308817116 | 0.855994636251309 | -0.017769211 | 0.0214259402091667 | 1.00183003703397 | 0.982387730384742 | 1.0216571238226 | 0.992899239648112 |
| ebi-a-GCST90001502 | finngen_R11_O15_POOR_FETGRO | Poor fetal growth \|\| id:finngen_R11_O15_POOR_FETGRO | Activated & secreting CD4 regulatory T cell %CD4 regulatory T cell \|\| id:ebi-a-GCST90001502 | Weighted median | 36 | 0.011712461946679 | 0.0124274416049728 | 0.345953255294036 | -0.012645324 | 0.0360702474924257 | 1.01178132140443 | 0.987434292561351 | 1.03672867152251 | 0.999440741871876 |
| ebi-a-GCST90001502 | finngen_R11_O15_POOR_FETGRO | Poor fetal growth \|\| id:finngen_R11_O15_POOR_FETGRO | Activated & secreting CD4 regulatory T cell %CD4 regulatory T cell \|\| id:ebi-a-GCST90001502 | Weighted mode | 36 | 0.00548202051695224 | 0.0101933089341426 | 0.594114644551597 | -0.014496865 | 0.0254609060278718 | 1.00549707428721 | 0.985607708613651 | 1.02578780336676 | 0.999857399747617 |
| ebi-a-GCST90001503 | finngen_R11_O15_POOR_FETGRO | Poor fetal growth \|\| id:finngen_R11_O15_POOR_FETGRO | Activated & secreting CD4 regulatory T cell %CD4+ T cell \|\| id:ebi-a-GCST90001503 | Inverse variance weighted | 31 | 0.00412214691268814 | 0.00615123518737541 | 0.502772883170776 | -0.007934274 | 0.016178567879944 | 1.0041306546463 | 0.992097119215349 | 1.01631014955255 | 0.994119304454654 |
| ebi-a-GCST90001503 | finngen_R11_O15_POOR_FETGRO | Poor fetal growth \|\| id:finngen_R11_O15_POOR_FETGRO | Activated & secreting CD4 regulatory T cell %CD4+ T cell \|\| id:ebi-a-GCST90001503 | MR Egger | 31 | 0.00644086185532366 | 0.00674439525847689 | 0.347473827569708 | -0.006778153 | 0.0196598765619384 | 1.00646164881072 | 0.993244767012729 | 1.01985440464324 | 0.992899239648112 |
| ebi-a-GCST90001503 | finngen_R11_O15_POOR_FETGRO | Poor fetal growth \|\| id:finngen_R11_O15_POOR_FETGRO | Activated & secreting CD4 regulatory T cell %CD4+ T cell \|\| id:ebi-a-GCST90001503 | Weighted median | 31 | 0.0136105334176922 | 0.00872009981206536 | 0.118565490174106 | -0.003480862 | 0.0307019290493403 | 1.01370357837895 | 0.996525188963779 | 1.03117809383907 | 0.999440741871876 |
| ebi-a-GCST90001503 | finngen_R11_O15_POOR_FETGRO | Poor fetal growth \|\| id:finngen_R11_O15_POOR_FETGRO | Activated & secreting CD4 regulatory T cell %CD4+ T cell \|\| id:ebi-a-GCST90001503 | Weighted mode | 31 | 0.00627941913164809 | 0.00669147440237977 | 0.355522555271707 | -0.006835871 | 0.0193947089603124 | 1.00629917601623 | 0.99318744071889 | 1.01958400814854 | 0.999857399747617 |
| ebi-a-GCST90001504 | finngen_R11_O15_POOR_FETGRO | Poor fetal growth \|\| id:finngen_R11_O15_POOR_FETGRO | CD25++ CD4+ T cell Absolute Count \|\| id:ebi-a-GCST90001504 | Inverse variance weighted (multiplicative random effects) | 23 | 0.0153371380202342 | 0.0277390816945763 | 0.580326956217015 | -0.039031462 | 0.0697057381416038 | 1.01545535552103 | 0.961720450924285 | 1.07219262943337 | 0.994119304454654 |
| ebi-a-GCST90001504 | finngen_R11_O15_POOR_FETGRO | Poor fetal growth \|\| id:finngen_R11_O15_POOR_FETGRO | CD25++ CD4+ T cell Absolute Count \|\| id:ebi-a-GCST90001504 | MR Egger | 23 | 0.0198413160684237 | 0.0393497909492956 | 0.619349762854118 | -0.057284274 | 0.096966906329043 | 1.02003946331108 | 0.944325583814066 | 1.10182390961973 | 0.992899239648112 |
| ebi-a-GCST90001504 | finngen_R11_O15_POOR_FETGRO | Poor fetal growth \|\| id:finngen_R11_O15_POOR_FETGRO | CD25++ CD4+ T cell Absolute Count \|\| id:ebi-a-GCST90001504 | Weighted median | 23 | 0.0158161259929686 | 0.0300086672494648 | 0.598157526872921 | -0.043000862 | 0.0746331138019196 | 1.01594186292947 | 0.957910564524041 | 1.07748876260223 | 0.999440741871876 |
| ebi-a-GCST90001504 | finngen_R11_O15_POOR_FETGRO | Poor fetal growth \|\| id:finngen_R11_O15_POOR_FETGRO | CD25++ CD4+ T cell Absolute Count \|\| id:ebi-a-GCST90001504 | Weighted mode | 23 | 0.0125281978980015 | 0.0260441898254407 | 0.635240394689872 | -0.038518414 | 0.0635748099558652 | 1.01260700452709 | 0.962213986214518 | 1.06563920324136 | 0.999857399747617 |
| ebi-a-GCST90001505 | finngen_R11_O15_POOR_FETGRO | Poor fetal growth \|\| id:finngen_R11_O15_POOR_FETGRO | CD25++ CD4+ T cell %CD4+ T cell \|\| id:ebi-a-GCST90001505 | Inverse variance weighted | 26 | -0.008764353 | 0.0269169403437584 | 0.744721476419963 | -0.061521556 | 0.0439928503798044 | 0.991273942286542 | 0.940332675928214 | 1.0449748836882 | 0.994119304454654 |
| ebi-a-GCST90001505 | finngen_R11_O15_POOR_FETGRO | Poor fetal growth \|\| id:finngen_R11_O15_POOR_FETGRO | CD25++ CD4+ T cell %CD4+ T cell \|\| id:ebi-a-GCST90001505 | MR Egger | 26 | -0.004377254 | 0.0401181672688495 | 0.914023177648937 | -0.083008862 | 0.0742543539979008 | 0.995632312363582 | 0.920342991384192 | 1.07708073044764 | 0.992899239648112 |
| ebi-a-GCST90001505 | finngen_R11_O15_POOR_FETGRO | Poor fetal growth \|\| id:finngen_R11_O15_POOR_FETGRO | CD25++ CD4+ T cell %CD4+ T cell \|\| id:ebi-a-GCST90001505 | Weighted median | 26 | -0.022663461 | 0.0360542479153119 | 0.5296152979106 | -0.093329787 | 0.0480028644919384 | 0.977591425647538 | 0.910893049764134 | 1.04917366066966 | 0.999440741871876 |
| ebi-a-GCST90001505 | finngen_R11_O15_POOR_FETGRO | Poor fetal growth \|\| id:finngen_R11_O15_POOR_FETGRO | CD25++ CD4+ T cell %CD4+ T cell \|\| id:ebi-a-GCST90001505 | Weighted mode | 26 | -0.015119268 | 0.0322013159123765 | 0.642765065039966 | -0.078233848 | 0.047995310867299 | 0.984994453962367 | 0.924748151204988 | 1.04916573563558 | 0.999857399747617 |
| ebi-a-GCST90001506 | finngen_R11_O15_POOR_FETGRO | Poor fetal growth \|\| id:finngen_R11_O15_POOR_FETGRO | CD25++ CD4+ T cell %T cell \|\| id:ebi-a-GCST90001506 | Inverse variance weighted | 19 | -0.000481923 | 0.0292391302244273 | 0.986849770291489 | -0.057790618 | 0.0568267725322069 | 0.999518193398425 | 0.943847551486238 | 1.05847243801323 | 0.998984604424952 |
| ebi-a-GCST90001506 | finngen_R11_O15_POOR_FETGRO | Poor fetal growth \|\| id:finngen_R11_O15_POOR_FETGRO | CD25++ CD4+ T cell %T cell \|\| id:ebi-a-GCST90001506 | MR Egger | 19 | -0.002337922 | 0.0409790063337354 | 0.955169226734663 | -0.082656774 | 0.0779809305552968 | 0.997664808951929 | 0.920667089628454 | 1.0811020424579 | 0.992899239648112 |
| ebi-a-GCST90001506 | finngen_R11_O15_POOR_FETGRO | Poor fetal growth \|\| id:finngen_R11_O15_POOR_FETGRO | CD25++ CD4+ T cell %T cell \|\| id:ebi-a-GCST90001506 | Weighted median | 19 | -0.019204105 | 0.0420310980759347 | 0.647741276766234 | -0.101585057 | 0.0631768471976303 | 0.980979119034479 | 0.903404334976746 | 1.06521520289853 | 0.999440741871876 |
| ebi-a-GCST90001506 | finngen_R11_O15_POOR_FETGRO | Poor fetal growth \|\| id:finngen_R11_O15_POOR_FETGRO | CD25++ CD4+ T cell %T cell \|\| id:ebi-a-GCST90001506 | Weighted mode | 19 | -0.014937925 | 0.041205251594762 | 0.721181550400991 | -0.095700218 | 0.0658243677626605 | 0.985173091967032 | 0.908736397609743 | 1.06803911859233 | 0.999857399747617 |
| ebi-a-GCST90001507 | finngen_R11_O15_POOR_FETGRO | Poor fetal growth \|\| id:finngen_R11_O15_POOR_FETGRO | CD25++ CD45RA+ CD4 not regulatory T cell Absolute Count \|\| id:ebi-a-GCST90001507 | Inverse variance weighted | 26 | -0.008709499 | 0.0200382141935469 | 0.663820514277026 | -0.047984399 | 0.0305654009242343 | 0.991328318919341 | 0.953148657305863 | 1.03103731863737 | 0.994119304454654 |
| ebi-a-GCST90001507 | finngen_R11_O15_POOR_FETGRO | Poor fetal growth \|\| id:finngen_R11_O15_POOR_FETGRO | CD25++ CD45RA+ CD4 not regulatory T cell Absolute Count \|\| id:ebi-a-GCST90001507 | MR Egger | 26 | -0.059872577 | 0.0229146349217556 | 0.0152542795460177 | -0.104785262 | -0.014959893 | 0.941884543568321 | 0.90051787761295 | 0.985151450590309 | 0.992899239648112 |
| ebi-a-GCST90001507 | finngen_R11_O15_POOR_FETGRO | Poor fetal growth \|\| id:finngen_R11_O15_POOR_FETGRO | CD25++ CD45RA+ CD4 not regulatory T cell Absolute Count \|\| id:ebi-a-GCST90001507 | Weighted median | 26 | -0.008862835 | 0.0270334635685622 | 0.743027484529705 | -0.061848424 | 0.0441227535226157 | 0.991176324078566 | 0.94002536159095 | 1.04511063802702 | 0.999440741871876 |
| ebi-a-GCST90001507 | finngen_R11_O15_POOR_FETGRO | Poor fetal growth \|\| id:finngen_R11_O15_POOR_FETGRO | CD25++ CD45RA+ CD4 not regulatory T cell Absolute Count \|\| id:ebi-a-GCST90001507 | Weighted mode | 26 | -0.017761731 | 0.0252203988428425 | 0.487779524588906 | -0.067193712 | 0.0316702511583248 | 0.982395079186062 | 0.935014060063336 | 1.0321770899826 | 0.999857399747617 |
| ebi-a-GCST90001508 | finngen_R11_O15_POOR_FETGRO | Poor fetal growth \|\| id:finngen_R11_O15_POOR_FETGRO | CD25++ CD45RA+ CD4 not regulatory T cell %CD4+ T cell \|\| id:ebi-a-GCST90001508 | Inverse variance weighted | 32 | 0.00183170102651534 | 0.0117379776177673 | 0.87599430672574 | -0.021174735 | 0.0248381371573393 | 1.00183337961558 | 0.979047875589605 | 1.02514917353509 | 0.994119304454654 |
| ebi-a-GCST90001508 | finngen_R11_O15_POOR_FETGRO | Poor fetal growth \|\| id:finngen_R11_O15_POOR_FETGRO | CD25++ CD45RA+ CD4 not regulatory T cell %CD4+ T cell \|\| id:ebi-a-GCST90001508 | MR Egger | 32 | -0.017407617 | 0.0144848853348186 | 0.23885082153967 | -0.045797992 | 0.0109827580349716 | 0.982743020002517 | 0.955234907338894 | 1.01104328992139 | 0.992899239648112 |
| ebi-a-GCST90001508 | finngen_R11_O15_POOR_FETGRO | Poor fetal growth \|\| id:finngen_R11_O15_POOR_FETGRO | CD25++ CD45RA+ CD4 not regulatory T cell %CD4+ T cell \|\| id:ebi-a-GCST90001508 | Weighted median | 32 | -0.005914897 | 0.0161046855738263 | 0.713411631451759 | -0.037480081 | 0.025650286343881 | 0.994102561185857 | 0.963213603679875 | 1.02598208578118 | 0.999440741871876 |
| ebi-a-GCST90001508 | finngen_R11_O15_POOR_FETGRO | Poor fetal growth \|\| id:finngen_R11_O15_POOR_FETGRO | CD25++ CD45RA+ CD4 not regulatory T cell %CD4+ T cell \|\| id:ebi-a-GCST90001508 | Weighted mode | 32 | -0.004604143 | 0.0143065326023938 | 0.749748407114655 | -0.032644947 | 0.023436661215068 | 0.995406440131481 | 0.967882148450075 | 1.02371345792657 | 0.999857399747617 |
| ebi-a-GCST90001509 | finngen_R11_O15_POOR_FETGRO | Poor fetal growth \|\| id:finngen_R11_O15_POOR_FETGRO | CD25++ CD45RA+ CD4 not regulatory T cell %T cell \|\| id:ebi-a-GCST90001509 | Inverse variance weighted | 27 | -0.002994542 | 0.0140260754366274 | 0.830938367234513 | -0.03048565 | 0.0244965655345696 | 0.9970099368485 | 0.969974350927585 | 1.02479907146417 | 0.994119304454654 |
| ebi-a-GCST90001509 | finngen_R11_O15_POOR_FETGRO | Poor fetal growth \|\| id:finngen_R11_O15_POOR_FETGRO | CD25++ CD45RA+ CD4 not regulatory T cell %T cell \|\| id:ebi-a-GCST90001509 | MR Egger | 27 | -0.028713597 | 0.0157093888747686 | 0.0795394315009931 | -0.059504 | 0.00207680475022068 | 0.971694720469365 | 0.9422317649969 | 1.0020789628029 | 0.992899239648112 |
| ebi-a-GCST90001509 | finngen_R11_O15_POOR_FETGRO | Poor fetal growth \|\| id:finngen_R11_O15_POOR_FETGRO | CD25++ CD45RA+ CD4 not regulatory T cell %T cell \|\| id:ebi-a-GCST90001509 | Weighted median | 27 | -0.006805305 | 0.0162259026955378 | 0.674916534777038 | -0.038608075 | 0.024997463827767 | 0.993217798196839 | 0.962127717419022 | 1.02531252015199 | 0.999440741871876 |
| ebi-a-GCST90001509 | finngen_R11_O15_POOR_FETGRO | Poor fetal growth \|\| id:finngen_R11_O15_POOR_FETGRO | CD25++ CD45RA+ CD4 not regulatory T cell %T cell \|\| id:ebi-a-GCST90001509 | Weighted mode | 27 | -0.004235674 | 0.0148693966468862 | 0.778009288423831 | -0.033379691 | 0.024908343582813 | 0.995773283969454 | 0.96717126337568 | 1.02522114812065 | 0.999857399747617 |
| ebi-a-GCST90001510 | finngen_R11_O15_POOR_FETGRO | Poor fetal growth \|\| id:finngen_R11_O15_POOR_FETGRO | CD25++ CD45RA- CD4 not regulatory T cell Absolute Count \|\| id:ebi-a-GCST90001510 | Inverse variance weighted | 20 | 0.0112501440375848 | 0.0160308579242898 | 0.482816040096018 | -0.020170337 | 0.0426706255691929 | 1.01131366489078 | 0.980031722940456 | 1.0435941050216 | 0.994119304454654 |
| ebi-a-GCST90001510 | finngen_R11_O15_POOR_FETGRO | Poor fetal growth \|\| id:finngen_R11_O15_POOR_FETGRO | CD25++ CD45RA- CD4 not regulatory T cell Absolute Count \|\| id:ebi-a-GCST90001510 | MR Egger | 20 | 0.037053784410049 | 0.0211327230883239 | 0.0965486594002121 | -0.004366353 | 0.0784739216631639 | 1.03774883404602 | 0.995643165816528 | 1.08163514754876 | 0.992899239648112 |
| ebi-a-GCST90001510 | finngen_R11_O15_POOR_FETGRO | Poor fetal growth \|\| id:finngen_R11_O15_POOR_FETGRO | CD25++ CD45RA- CD4 not regulatory T cell Absolute Count \|\| id:ebi-a-GCST90001510 | Weighted median | 20 | 0.0118876606709326 | 0.0175962338545259 | 0.499307451303582 | -0.022600958 | 0.0463762790258034 | 1.01195859973018 | 0.977652530675636 | 1.04746847723102 | 0.999440741871876 |
| ebi-a-GCST90001510 | finngen_R11_O15_POOR_FETGRO | Poor fetal growth \|\| id:finngen_R11_O15_POOR_FETGRO | CD25++ CD45RA- CD4 not regulatory T cell Absolute Count \|\| id:ebi-a-GCST90001510 | Weighted mode | 20 | 0.0126748339842744 | 0.0151369367292688 | 0.412807988785562 | -0.016993562 | 0.0423432299736412 | 1.01275550014231 | 0.983150014129319 | 1.04325249283228 | 0.999857399747617 |
| ebi-a-GCST90001511 | finngen_R11_O15_POOR_FETGRO | Poor fetal growth \|\| id:finngen_R11_O15_POOR_FETGRO | CD25++ CD45RA- CD4 not regulatory T cell %CD4+ T cell \|\| id:ebi-a-GCST90001511 | Inverse variance weighted | 24 | 0.0184397172143 | 0.0195253026094267 | 0.344964720835519 | -0.019829876 | 0.0567093103287764 | 1.01861077862344 | 0.980365442909062 | 1.05834811481017 | 0.994119304454654 |
| ebi-a-GCST90001511 | finngen_R11_O15_POOR_FETGRO | Poor fetal growth \|\| id:finngen_R11_O15_POOR_FETGRO | CD25++ CD45RA- CD4 not regulatory T cell %CD4+ T cell \|\| id:ebi-a-GCST90001511 | MR Egger | 24 | 0.0296692694136294 | 0.0239407075049259 | 0.228299860112258 | -0.017254517 | 0.0765930561232842 | 1.03011378747202 | 0.982893489403549 | 1.07960264930016 | 0.992899239648112 |
| ebi-a-GCST90001511 | finngen_R11_O15_POOR_FETGRO | Poor fetal growth \|\| id:finngen_R11_O15_POOR_FETGRO | CD25++ CD45RA- CD4 not regulatory T cell %CD4+ T cell \|\| id:ebi-a-GCST90001511 | Weighted median | 24 | 0.0166673793262249 | 0.0201006213725479 | 0.406992818824253 | -0.022729839 | 0.0560645972164188 | 1.01680705502327 | 0.977526538076311 | 1.05766600380969 | 0.999440741871876 |
| ebi-a-GCST90001511 | finngen_R11_O15_POOR_FETGRO | Poor fetal growth \|\| id:finngen_R11_O15_POOR_FETGRO | CD25++ CD45RA- CD4 not regulatory T cell %CD4+ T cell \|\| id:ebi-a-GCST90001511 | Weighted mode | 24 | 0.0175894618309519 | 0.0177137164002598 | 0.331049637173707 | -0.017129422 | 0.052308345975461 | 1.01774506741534 | 0.983016452138225 | 1.05370059676539 | 0.999857399747617 |
| ebi-a-GCST90001512 | finngen_R11_O15_POOR_FETGRO | Poor fetal growth \|\| id:finngen_R11_O15_POOR_FETGRO | CD25++ CD45RA- CD4 not regulatory T cell %T cell \|\| id:ebi-a-GCST90001512 | Inverse variance weighted | 28 | 0.0134490230981864 | 0.016099133903279 | 0.403499290206343 | -0.018105279 | 0.0450033255486133 | 1.01353986801093 | 0.982057636523837 | 1.046031339 | 0.994119304454654 |
| ebi-a-GCST90001512 | finngen_R11_O15_POOR_FETGRO | Poor fetal growth \|\| id:finngen_R11_O15_POOR_FETGRO | CD25++ CD45RA- CD4 not regulatory T cell %T cell \|\| id:ebi-a-GCST90001512 | MR Egger | 28 | 0.00214862330514676 | 0.0202185886704605 | 0.916184077907099 | -0.03747981 | 0.0417770570992493 | 1.0021509332503 | 0.963213864341464 | 1.04266199874634 | 0.992899239648112 |
| ebi-a-GCST90001512 | finngen_R11_O15_POOR_FETGRO | Poor fetal growth \|\| id:finngen_R11_O15_POOR_FETGRO | CD25++ CD45RA- CD4 not regulatory T cell %T cell \|\| id:ebi-a-GCST90001512 | Weighted median | 28 | 0.0113702324595547 | 0.0190883714885475 | 0.551400526421477 | -0.026042976 | 0.0487834405771078 | 1.0114351192454 | 0.974293217816903 | 1.04999294025179 | 0.999440741871876 |
| ebi-a-GCST90001512 | finngen_R11_O15_POOR_FETGRO | Poor fetal growth \|\| id:finngen_R11_O15_POOR_FETGRO | CD25++ CD45RA- CD4 not regulatory T cell %T cell \|\| id:ebi-a-GCST90001512 | Weighted mode | 28 | 0.00247733732297462 | 0.0163712532244855 | 0.880846370820907 | -0.029610319 | 0.0345649936429662 | 1.00248040845864 | 0.970823771428634 | 1.03516930561405 | 0.999857399747617 |
| ebi-a-GCST90001513 | finngen_R11_O15_POOR_FETGRO | Poor fetal growth \|\| id:finngen_R11_O15_POOR_FETGRO | CD4 regulatory T cell Absolute Count \|\| id:ebi-a-GCST90001513 | Inverse variance weighted | 21 | 0.00970333031014782 | 0.0300922591730823 | 0.747109763247239 | -0.049277498 | 0.0686841582893891 | 1.00975056025869 | 0.951916938312401 | 1.0710978583387 | 0.994119304454654 |
| ebi-a-GCST90001513 | finngen_R11_O15_POOR_FETGRO | Poor fetal growth \|\| id:finngen_R11_O15_POOR_FETGRO | CD4 regulatory T cell Absolute Count \|\| id:ebi-a-GCST90001513 | MR Egger | 21 | -0.050783586 | 0.0456840134461762 | 0.28016744904147 | -0.140324252 | 0.0387570808450599 | 0.950484346861661 | 0.869076390067443 | 1.03951793415873 | 0.992899239648112 |
| ebi-a-GCST90001513 | finngen_R11_O15_POOR_FETGRO | Poor fetal growth \|\| id:finngen_R11_O15_POOR_FETGRO | CD4 regulatory T cell Absolute Count \|\| id:ebi-a-GCST90001513 | Weighted median | 21 | 0.0176160672737177 | 0.0405437084120863 | 0.66392851118479 | -0.061849601 | 0.0970817357614068 | 1.01777214533369 | 0.940024254666783 | 1.10195043869833 | 0.999440741871876 |
| ebi-a-GCST90001513 | finngen_R11_O15_POOR_FETGRO | Poor fetal growth \|\| id:finngen_R11_O15_POOR_FETGRO | CD4 regulatory T cell Absolute Count \|\| id:ebi-a-GCST90001513 | Weighted mode | 21 | -0.001874456 | 0.0353151874363304 | 0.958196433658891 | -0.071092223 | 0.0673433112525392 | 0.998127299573047 | 0.931375993414763 | 1.06966264236674 | 0.999857399747617 |
| ebi-a-GCST90001514 | finngen_R11_O15_POOR_FETGRO | Poor fetal growth \|\| id:finngen_R11_O15_POOR_FETGRO | Hematopoietic Stem Cell Absolute Count \|\| id:ebi-a-GCST90001514 | Inverse variance weighted | 19 | -0.011600358 | 0.0228459280966826 | 0.611618303069383 | -0.056378377 | 0.0331776611174683 | 0.98846666677964 | 0.945181433285911 | 1.03373417729726 | 0.994119304454654 |
| ebi-a-GCST90001514 | finngen_R11_O15_POOR_FETGRO | Poor fetal growth \|\| id:finngen_R11_O15_POOR_FETGRO | Hematopoietic Stem Cell Absolute Count \|\| id:ebi-a-GCST90001514 | MR Egger | 19 | 0.00448906199866733 | 0.0332681376563497 | 0.894247422912636 | -0.060716488 | 0.0696946118051128 | 1.00449915293144 | 0.941090012450078 | 1.07218069992375 | 0.992899239648112 |
| ebi-a-GCST90001514 | finngen_R11_O15_POOR_FETGRO | Poor fetal growth \|\| id:finngen_R11_O15_POOR_FETGRO | Hematopoietic Stem Cell Absolute Count \|\| id:ebi-a-GCST90001514 | Weighted median | 19 | 0.000645196189052703 | 0.0324598057080737 | 0.984141673961139 | -0.062976023 | 0.0642664153768771 | 1.00064540437288 | 0.938965987007855 | 1.06637646000717 | 0.999440741871876 |
| ebi-a-GCST90001514 | finngen_R11_O15_POOR_FETGRO | Poor fetal growth \|\| id:finngen_R11_O15_POOR_FETGRO | Hematopoietic Stem Cell Absolute Count \|\| id:ebi-a-GCST90001514 | Weighted mode | 19 | 0.0015882789714281 | 0.0362995231171287 | 0.965581582186584 | -0.069558786 | 0.0727353442810003 | 1.00158954095451 | 0.932805295565187 | 1.07544587634619 | 0.999857399747617 |
| ebi-a-GCST90001515 | finngen_R11_O15_POOR_FETGRO | Poor fetal growth \|\| id:finngen_R11_O15_POOR_FETGRO | Immature Myeloid-Derived Suppressor Cells Absolute Count \|\| id:ebi-a-GCST90001515 | Inverse variance weighted | 22 | -0.010373858 | 0.0229377038593535 | 0.651080048438084 | -0.055331758 | 0.0345840413925497 | 0.989679764709326 | 0.946171196264362 | 1.03518902344755 | 0.994119304454654 |
| ebi-a-GCST90001515 | finngen_R11_O15_POOR_FETGRO | Poor fetal growth \|\| id:finngen_R11_O15_POOR_FETGRO | Immature Myeloid-Derived Suppressor Cells Absolute Count \|\| id:ebi-a-GCST90001515 | MR Egger | 22 | -0.043165909 | 0.0438260194918749 | 0.336420820466301 | -0.129064907 | 0.0427330889931431 | 0.957752476927104 | 0.878916915469128 | 1.04365929351854 | 0.992899239648112 |
| ebi-a-GCST90001515 | finngen_R11_O15_POOR_FETGRO | Poor fetal growth \|\| id:finngen_R11_O15_POOR_FETGRO | Immature Myeloid-Derived Suppressor Cells Absolute Count \|\| id:ebi-a-GCST90001515 | Weighted median | 22 | -0.019399553 | 0.0314326753440667 | 0.537117381247624 | -0.081007597 | 0.0422084906500982 | 0.98078740736986 | 0.922186685843653 | 1.04311193516665 | 0.999440741871876 |
| ebi-a-GCST90001515 | finngen_R11_O15_POOR_FETGRO | Poor fetal growth \|\| id:finngen_R11_O15_POOR_FETGRO | Immature Myeloid-Derived Suppressor Cells Absolute Count \|\| id:ebi-a-GCST90001515 | Weighted mode | 22 | -0.026332937 | 0.039595543517007 | 0.513257982422253 | -0.103940203 | 0.0512743280733072 | 0.974010751187803 | 0.901279190040192 | 1.05261161459539 | 0.999857399747617 |
| ebi-a-GCST90001516 | finngen_R11_O15_POOR_FETGRO | Poor fetal growth \|\| id:finngen_R11_O15_POOR_FETGRO | Immature Myeloid-Derived Suppressor Cells %CD33dim HLA DR- CD66b- \|\| id:ebi-a-GCST90001516 | Inverse variance weighted | 19 | -0.011622196 | 0.0204381852449905 | 0.569592966838114 | -0.051681039 | 0.0284366473909861 | 0.988445081140065 | 0.949631714242246 | 1.02884482876565 | 0.994119304454654 |
| ebi-a-GCST90001516 | finngen_R11_O15_POOR_FETGRO | Poor fetal growth \|\| id:finngen_R11_O15_POOR_FETGRO | Immature Myeloid-Derived Suppressor Cells %CD33dim HLA DR- CD66b- \|\| id:ebi-a-GCST90001516 | MR Egger | 19 | 0.000325343438606505 | 0.0303782204859989 | 0.991579691988301 | -0.059215969 | 0.0598666555911643 | 1.00032539636852 | 0.942503195972042 | 1.06169496601848 | 0.995665872037703 |
| ebi-a-GCST90001516 | finngen_R11_O15_POOR_FETGRO | Poor fetal growth \|\| id:finngen_R11_O15_POOR_FETGRO | Immature Myeloid-Derived Suppressor Cells %CD33dim HLA DR- CD66b- \|\| id:ebi-a-GCST90001516 | Weighted median | 19 | -0.005104721 | 0.0293616788563247 | 0.861978172866735 | -0.062653612 | 0.0524441693347627 | 0.994908285724059 | 0.939268768981735 | 1.05384372363991 | 0.999440741871876 |
| ebi-a-GCST90001516 | finngen_R11_O15_POOR_FETGRO | Poor fetal growth \|\| id:finngen_R11_O15_POOR_FETGRO | Immature Myeloid-Derived Suppressor Cells %CD33dim HLA DR- CD66b- \|\| id:ebi-a-GCST90001516 | Weighted mode | 19 | -0.001822362 | 0.026595866392421 | 0.946126739555305 | -0.05395026 | 0.0503055359618985 | 0.998179297326468 | 0.947479232665976 | 1.05159234657592 | 0.999857399747617 |
| ebi-a-GCST90001517 | finngen_R11_O15_POOR_FETGRO | Poor fetal growth \|\| id:finngen_R11_O15_POOR_FETGRO | CD33+ HLA DR+ Absolute Count \|\| id:ebi-a-GCST90001517 | Inverse variance weighted | 30 | -0.012280445 | 0.011380777917921 | 0.280564787926603 | -0.03458677 | 0.0100258796879288 | 0.987794651911592 | 0.966004516079198 | 1.01007630720553 | 0.994119304454654 |
| ebi-a-GCST90001517 | finngen_R11_O15_POOR_FETGRO | Poor fetal growth \|\| id:finngen_R11_O15_POOR_FETGRO | CD33+ HLA DR+ Absolute Count \|\| id:ebi-a-GCST90001517 | MR Egger | 30 | -0.008407654 | 0.013722278379993 | 0.545018666569173 | -0.03530332 | 0.0184880117227568 | 0.991627591573617 | 0.965312573694295 | 1.0186599731182 | 0.992899239648112 |
| ebi-a-GCST90001517 | finngen_R11_O15_POOR_FETGRO | Poor fetal growth \|\| id:finngen_R11_O15_POOR_FETGRO | CD33+ HLA DR+ Absolute Count \|\| id:ebi-a-GCST90001517 | Weighted median | 30 | -0.008758165 | 0.0157344320814601 | 0.577784251698346 | -0.039597652 | 0.0220813220337721 | 0.991280076158073 | 0.961176088903989 | 1.02232691879428 | 0.999440741871876 |
| ebi-a-GCST90001517 | finngen_R11_O15_POOR_FETGRO | Poor fetal growth \|\| id:finngen_R11_O15_POOR_FETGRO | CD33+ HLA DR+ Absolute Count \|\| id:ebi-a-GCST90001517 | Weighted mode | 30 | -0.006021372 | 0.0137277591453894 | 0.664181998975127 | -0.03292778 | 0.0208850361416521 | 0.993996720344409 | 0.967608438029352 | 1.02110465475857 | 0.999857399747617 |
| ebi-a-GCST90001518 | finngen_R11_O15_POOR_FETGRO | Poor fetal growth \|\| id:finngen_R11_O15_POOR_FETGRO | CD33+ HLA DR+ CD14- Absolute Count \|\| id:ebi-a-GCST90001518 | Inverse variance weighted | 29 | -0.012743993 | 0.00929637294369507 | 0.170419712187727 | -0.030964884 | 0.0054768982368228 | 0.987336868081541 | 0.96950961806647 | 1.00549192386272 | 0.994119304454654 |
| ebi-a-GCST90001518 | finngen_R11_O15_POOR_FETGRO | Poor fetal growth \|\| id:finngen_R11_O15_POOR_FETGRO | CD33+ HLA DR+ CD14- Absolute Count \|\| id:ebi-a-GCST90001518 | MR Egger | 29 | -0.01125111 | 0.0109450450066631 | 0.313088470823297 | -0.032703398 | 0.0102011784533342 | 0.988811947266949 | 0.967825576049943 | 1.01025338785571 | 0.992899239648112 |
| ebi-a-GCST90001518 | finngen_R11_O15_POOR_FETGRO | Poor fetal growth \|\| id:finngen_R11_O15_POOR_FETGRO | CD33+ HLA DR+ CD14- Absolute Count \|\| id:ebi-a-GCST90001518 | Weighted median | 29 | -0.011965271 | 0.0129366995952521 | 0.35501318309262 | -0.037321203 | 0.0133906597885066 | 0.988106027787098 | 0.963366649751298 | 1.01348071619591 | 0.999440741871876 |
| ebi-a-GCST90001518 | finngen_R11_O15_POOR_FETGRO | Poor fetal growth \|\| id:finngen_R11_O15_POOR_FETGRO | CD33+ HLA DR+ CD14- Absolute Count \|\| id:ebi-a-GCST90001518 | Weighted mode | 29 | -0.00837646 | 0.0121475918884352 | 0.496148198598311 | -0.03218574 | 0.0154328204830127 | 0.991658525168515 | 0.968326708643498 | 1.01555252143876 | 0.999857399747617 |
| ebi-a-GCST90001519 | finngen_R11_O15_POOR_FETGRO | Poor fetal growth \|\| id:finngen_R11_O15_POOR_FETGRO | CD33+ HLA DR+ CD14- %CD33+ HLA DR+ \|\| id:ebi-a-GCST90001519 | Inverse variance weighted | 19 | 0.00172432425300943 | 0.021599068318617 | 0.936369863122214 | -0.04060985 | 0.0440584981574988 | 1.00172581175493 | 0.960203680678166 | 1.04504348621884 | 0.998984604424952 |
| ebi-a-GCST90001519 | finngen_R11_O15_POOR_FETGRO | Poor fetal growth \|\| id:finngen_R11_O15_POOR_FETGRO | CD33+ HLA DR+ CD14- %CD33+ HLA DR+ \|\| id:ebi-a-GCST90001519 | MR Egger | 19 | -0.007540013 | 0.0325552468621817 | 0.819608350841001 | -0.071348297 | 0.056268270736278 | 0.992488341475864 | 0.931137523270595 | 1.05788144430654 | 0.992899239648112 |
| ebi-a-GCST90001519 | finngen_R11_O15_POOR_FETGRO | Poor fetal growth \|\| id:finngen_R11_O15_POOR_FETGRO | CD33+ HLA DR+ CD14- %CD33+ HLA DR+ \|\| id:ebi-a-GCST90001519 | Weighted median | 19 | -0.011597376 | 0.0293167270913205 | 0.692408228498549 | -0.069058161 | 0.0458634093619873 | 0.988469614604185 | 0.933272398596781 | 1.04693140016229 | 0.999440741871876 |
| ebi-a-GCST90001519 | finngen_R11_O15_POOR_FETGRO | Poor fetal growth \|\| id:finngen_R11_O15_POOR_FETGRO | CD33+ HLA DR+ CD14- %CD33+ HLA DR+ \|\| id:ebi-a-GCST90001519 | Weighted mode | 19 | -0.016348807 | 0.0293129850218422 | 0.583897551177727 | -0.073802258 | 0.041104643402744 | 0.983784109181601 | 0.928855349505771 | 1.04196113419943 | 0.999857399747617 |
| ebi-a-GCST90001520 | finngen_R11_O15_POOR_FETGRO | Poor fetal growth \|\| id:finngen_R11_O15_POOR_FETGRO | CD33+ HLA DR+ CD14dim Absolute Count \|\| id:ebi-a-GCST90001520 | Inverse variance weighted | 28 | -0.002153342 | 0.0139300024603557 | 0.877150057935302 | -0.029456147 | 0.0251494629872327 | 0.997848974942229 | 0.97097345713927 | 1.02546837863812 | 0.994119304454654 |
| ebi-a-GCST90001520 | finngen_R11_O15_POOR_FETGRO | Poor fetal growth \|\| id:finngen_R11_O15_POOR_FETGRO | CD33+ HLA DR+ CD14dim Absolute Count \|\| id:ebi-a-GCST90001520 | MR Egger | 28 | 0.000433226439580956 | 0.0175235730501225 | 0.980465084751721 | -0.033912977 | 0.034779429617821 | 1.00043332029571 | 0.966655622502212 | 1.03539130695493 | 0.994809183268002 |
| ebi-a-GCST90001520 | finngen_R11_O15_POOR_FETGRO | Poor fetal growth \|\| id:finngen_R11_O15_POOR_FETGRO | CD33+ HLA DR+ CD14dim Absolute Count \|\| id:ebi-a-GCST90001520 | Weighted median | 28 | 0.00472989194563187 | 0.0209590053553183 | 0.821455375892493 | -0.036349759 | 0.0458095424420557 | 1.00474109554151 | 0.964302961287888 | 1.04687500671127 | 0.999440741871876 |
| ebi-a-GCST90001520 | finngen_R11_O15_POOR_FETGRO | Poor fetal growth \|\| id:finngen_R11_O15_POOR_FETGRO | CD33+ HLA DR+ CD14dim Absolute Count \|\| id:ebi-a-GCST90001520 | Weighted mode | 28 | -0.00244677 | 0.0169234263045149 | 0.886117206728169 | -0.035616685 | 0.0307231458607438 | 0.997556221205021 | 0.965010125209658 | 1.0311999723823 | 0.999857399747617 |
| ebi-a-GCST90001521 | finngen_R11_O15_POOR_FETGRO | Poor fetal growth \|\| id:finngen_R11_O15_POOR_FETGRO | CD33+ HLA DR+ CD14dim %CD33+ HLA DR+ \|\| id:ebi-a-GCST90001521 | Inverse variance weighted (multiplicative random effects) | 24 | 0.00445454300738286 | 0.0266805066235046 | 0.867402536320507 | -0.04783925 | 0.0567483359894519 | 1.00446447923239 | 0.953287015673325 | 1.05838941835052 | 0.994119304454654 |
| ebi-a-GCST90001521 | finngen_R11_O15_POOR_FETGRO | Poor fetal growth \|\| id:finngen_R11_O15_POOR_FETGRO | CD33+ HLA DR+ CD14dim %CD33+ HLA DR+ \|\| id:ebi-a-GCST90001521 | MR Egger | 24 | 0.0477999313953683 | 0.0390242812424346 | 0.233575333809578 | -0.02868766 | 0.12428752263054 | 1.04896077021185 | 0.971719924229602 | 1.13234139797616 | 0.992899239648112 |
| ebi-a-GCST90001521 | finngen_R11_O15_POOR_FETGRO | Poor fetal growth \|\| id:finngen_R11_O15_POOR_FETGRO | CD33+ HLA DR+ CD14dim %CD33+ HLA DR+ \|\| id:ebi-a-GCST90001521 | Weighted median | 24 | 0.0423024404706332 | 0.0313616761114518 | 0.177382655057495 | -0.019166445 | 0.103771325649079 | 1.04320993994945 | 0.98101606372101 | 1.10934674676115 | 0.999440741871876 |
| ebi-a-GCST90001521 | finngen_R11_O15_POOR_FETGRO | Poor fetal growth \|\| id:finngen_R11_O15_POOR_FETGRO | CD33+ HLA DR+ CD14dim %CD33+ HLA DR+ \|\| id:ebi-a-GCST90001521 | Weighted mode | 24 | 0.046693188989628 | 0.0306481454290449 | 0.141260216926078 | -0.013377176 | 0.106763554030556 | 1.04780048303344 | 0.986711900727283 | 1.11267113676838 | 0.999857399747617 |
| ebi-a-GCST90001522 | finngen_R11_O15_POOR_FETGRO | Poor fetal growth \|\| id:finngen_R11_O15_POOR_FETGRO | CD33- HLA DR- Absolute Count \|\| id:ebi-a-GCST90001522 | Inverse variance weighted | 21 | 0.00489285150448732 | 0.0247050937938756 | 0.843005700913193 | -0.043529132 | 0.0533148353404834 | 1.00490484104879 | 0.957404662254683 | 1.05476166909939 | 0.994119304454654 |
| ebi-a-GCST90001522 | finngen_R11_O15_POOR_FETGRO | Poor fetal growth \|\| id:finngen_R11_O15_POOR_FETGRO | CD33- HLA DR- Absolute Count \|\| id:ebi-a-GCST90001522 | MR Egger | 21 | 0.0498296691102486 | 0.051941450363077 | 0.349435886521871 | -0.051975574 | 0.15163491182188 | 1.051092048 | 0.949352055811421 | 1.16373529286801 | 0.992899239648112 |
| ebi-a-GCST90001522 | finngen_R11_O15_POOR_FETGRO | Poor fetal growth \|\| id:finngen_R11_O15_POOR_FETGRO | CD33- HLA DR- Absolute Count \|\| id:ebi-a-GCST90001522 | Weighted median | 21 | 0.00514194040540904 | 0.0342419051439824 | 0.880634329954647 | -0.061972194 | 0.0722560744876145 | 1.00515518286857 | 0.939909021841771 | 1.07493057111822 | 0.999440741871876 |
| ebi-a-GCST90001522 | finngen_R11_O15_POOR_FETGRO | Poor fetal growth \|\| id:finngen_R11_O15_POOR_FETGRO | CD33- HLA DR- Absolute Count \|\| id:ebi-a-GCST90001522 | Weighted mode | 21 | 0.0150254530799034 | 0.0423283754283251 | 0.726325296325059 | -0.067938163 | 0.0979890689194205 | 1.01513890269849 | 0.934318247452972 | 1.10295072859932 | 0.999857399747617 |
| ebi-a-GCST90001523 | finngen_R11_O15_POOR_FETGRO | Poor fetal growth \|\| id:finngen_R11_O15_POOR_FETGRO | CD33- HLA DR+ Absolute Count \|\| id:ebi-a-GCST90001523 | Inverse variance weighted | 36 | 0.00927498371640996 | 0.0134897025933296 | 0.491729727206803 | -0.017164833 | 0.0357148007993359 | 1.00931812966738 | 0.982981643106895 | 1.03636023522819 | 0.994119304454654 |
| ebi-a-GCST90001523 | finngen_R11_O15_POOR_FETGRO | Poor fetal growth \|\| id:finngen_R11_O15_POOR_FETGRO | CD33- HLA DR+ Absolute Count \|\| id:ebi-a-GCST90001523 | MR Egger | 36 | -0.000744269 | 0.0176747833918836 | 0.96665803607772 | -0.035386845 | 0.0338983059835535 | 0.999256007435279 | 0.965231948956299 | 1.03447940101359 | 0.992899239648112 |
| ebi-a-GCST90001523 | finngen_R11_O15_POOR_FETGRO | Poor fetal growth \|\| id:finngen_R11_O15_POOR_FETGRO | CD33- HLA DR+ Absolute Count \|\| id:ebi-a-GCST90001523 | Weighted median | 36 | 0.00347321463718363 | 0.0198005325571149 | 0.860757355717428 | -0.035335829 | 0.0422822584491287 | 1.00347925323623 | 0.96528119223247 | 1.04318888607646 | 0.999440741871876 |
| ebi-a-GCST90001523 | finngen_R11_O15_POOR_FETGRO | Poor fetal growth \|\| id:finngen_R11_O15_POOR_FETGRO | CD33- HLA DR+ Absolute Count \|\| id:ebi-a-GCST90001523 | Weighted mode | 36 | -0.001882475 | 0.0186631451797783 | 0.92023249469431 | -0.03846224 | 0.0346972890861059 | 0.998119295279378 | 0.962268039277058 | 1.03530626285527 | 0.999857399747617 |
| ebi-a-GCST90001524 | finngen_R11_O15_POOR_FETGRO | Poor fetal growth \|\| id:finngen_R11_O15_POOR_FETGRO | Granulocytic Myeloid-Derived Suppressor Cells Absolute Count \|\| id:ebi-a-GCST90001524 | Inverse variance weighted | 21 | -0.025526968 | 0.0259680662525046 | 0.325600643910592 | -0.076424377 | 0.0253704422175233 | 0.974796090663778 | 0.926422970068084 | 1.02569501089071 | 0.994119304454654 |
| ebi-a-GCST90001524 | finngen_R11_O15_POOR_FETGRO | Poor fetal growth \|\| id:finngen_R11_O15_POOR_FETGRO | Granulocytic Myeloid-Derived Suppressor Cells Absolute Count \|\| id:ebi-a-GCST90001524 | MR Egger | 21 | -0.030032232 | 0.0517716829557199 | 0.568670228100842 | -0.131504731 | 0.0714402666227423 | 0.970414254680822 | 0.876775127487413 | 1.07405399191283 | 0.992899239648112 |
| ebi-a-GCST90001524 | finngen_R11_O15_POOR_FETGRO | Poor fetal growth \|\| id:finngen_R11_O15_POOR_FETGRO | Granulocytic Myeloid-Derived Suppressor Cells Absolute Count \|\| id:ebi-a-GCST90001524 | Weighted median | 21 | -0.03940941 | 0.0383920727015938 | 0.304656611537308 | -0.114657872 | 0.0358390526661079 | 0.96135703954458 | 0.89167115710774 | 1.03648901292234 | 0.999440741871876 |
| ebi-a-GCST90001524 | finngen_R11_O15_POOR_FETGRO | Poor fetal growth \|\| id:finngen_R11_O15_POOR_FETGRO | Granulocytic Myeloid-Derived Suppressor Cells Absolute Count \|\| id:ebi-a-GCST90001524 | Weighted mode | 21 | -0.059334545 | 0.0529945576926236 | 0.276135946553476 | -0.163203878 | 0.0445347883631558 | 0.942391444338329 | 0.849417993294974 | 1.04554134874992 | 0.999857399747617 |
| ebi-a-GCST90001525 | finngen_R11_O15_POOR_FETGRO | Poor fetal growth \|\| id:finngen_R11_O15_POOR_FETGRO | CD33dim HLA DR+ CD11b+ Absolute Count \|\| id:ebi-a-GCST90001525 | Inverse variance weighted | 27 | 0.043519680674393 | 0.0213095310905615 | 0.0411253860836304 | 0.0017529997368925 | 0.0852863616118936 | 1.04448055019137 | 1.00175453713916 | 1.08902887811579 | 0.994119304454654 |
| ebi-a-GCST90001525 | finngen_R11_O15_POOR_FETGRO | Poor fetal growth \|\| id:finngen_R11_O15_POOR_FETGRO | CD33dim HLA DR+ CD11b+ Absolute Count \|\| id:ebi-a-GCST90001525 | MR Egger | 27 | 0.0741939933093272 | 0.03503624684324 | 0.0443259874587958 | 0.00552294949657671 | 0.142865037122078 | 1.07701571907519 | 1.00553822909869 | 1.15357410147874 | 0.992899239648112 |
| ebi-a-GCST90001525 | finngen_R11_O15_POOR_FETGRO | Poor fetal growth \|\| id:finngen_R11_O15_POOR_FETGRO | CD33dim HLA DR+ CD11b+ Absolute Count \|\| id:ebi-a-GCST90001525 | Weighted median | 27 | 0.0698000952983935 | 0.0299942021925866 | 0.0199589015133621 | 0.0110114590009238 | 0.128588731595863 | 1.07229380325457 | 1.01107230825685 | 1.1372223243662 | 0.999440741871876 |
| ebi-a-GCST90001525 | finngen_R11_O15_POOR_FETGRO | Poor fetal growth \|\| id:finngen_R11_O15_POOR_FETGRO | CD33dim HLA DR+ CD11b+ Absolute Count \|\| id:ebi-a-GCST90001525 | Weighted mode | 27 | 0.0691653757023097 | 0.0313512932535203 | 0.0364123704430163 | 0.00771684092540986 | 0.13061391047921 | 1.07161341331614 | 1.0077466924795 | 1.13952773665137 | 0.999857399747617 |
| ebi-a-GCST90001526 | finngen_R11_O15_POOR_FETGRO | Poor fetal growth \|\| id:finngen_R11_O15_POOR_FETGRO | CD33dim HLA DR+ CD11b+ %CD33dim HLA DR+ \|\| id:ebi-a-GCST90001526 | Inverse variance weighted | 24 | -0.005559516 | 0.0140897205232405 | 0.69315317674377 | -0.033175368 | 0.0220563360416029 | 0.994455909326787 | 0.967368898767716 | 1.02230137526101 | 0.994119304454654 |
| ebi-a-GCST90001526 | finngen_R11_O15_POOR_FETGRO | Poor fetal growth \|\| id:finngen_R11_O15_POOR_FETGRO | CD33dim HLA DR+ CD11b+ %CD33dim HLA DR+ \|\| id:ebi-a-GCST90001526 | MR Egger | 24 | -0.033152705 | 0.0205972110931503 | 0.121747949788908 | -0.073523239 | 0.00721782841516247 | 0.967390822576908 | 0.92911455378249 | 1.00724393972319 | 0.992899239648112 |
| ebi-a-GCST90001526 | finngen_R11_O15_POOR_FETGRO | Poor fetal growth \|\| id:finngen_R11_O15_POOR_FETGRO | CD33dim HLA DR+ CD11b+ %CD33dim HLA DR+ \|\| id:ebi-a-GCST90001526 | Weighted median | 24 | -0.023043528 | 0.0221712255609901 | 0.298644827266629 | -0.06649913 | 0.0204120741753357 | 0.977219946491742 | 0.935663729861204 | 1.02062182528223 | 0.999440741871876 |
| ebi-a-GCST90001526 | finngen_R11_O15_POOR_FETGRO | Poor fetal growth \|\| id:finngen_R11_O15_POOR_FETGRO | CD33dim HLA DR+ CD11b+ %CD33dim HLA DR+ \|\| id:ebi-a-GCST90001526 | Weighted mode | 24 | -0.031851415 | 0.0202942416083925 | 0.130192519442586 | -0.071628128 | 0.00792529870719516 | 0.968650498471223 | 0.930876998175249 | 1.00795678701678 | 0.999857399747617 |
| ebi-a-GCST90001527 | finngen_R11_O15_POOR_FETGRO | Poor fetal growth \|\| id:finngen_R11_O15_POOR_FETGRO | CD33dim HLA DR+ CD11b- Absolute Count \|\| id:ebi-a-GCST90001527 | Inverse variance weighted | 28 | -0.008612213 | 0.0168966383340251 | 0.610261793010853 | -0.041729624 | 0.0245051979521356 | 0.991424765692602 | 0.959129070695375 | 1.02480791799586 | 0.994119304454654 |
| ebi-a-GCST90001527 | finngen_R11_O15_POOR_FETGRO | Poor fetal growth \|\| id:finngen_R11_O15_POOR_FETGRO | CD33dim HLA DR+ CD11b- Absolute Count \|\| id:ebi-a-GCST90001527 | MR Egger | 28 | -0.013624246 | 0.0214517595865339 | 0.53090621593334 | -0.055669695 | 0.028421202411285 | 0.98646814360911 | 0.94585150362134 | 1.02882893840086 | 0.992899239648112 |
| ebi-a-GCST90001527 | finngen_R11_O15_POOR_FETGRO | Poor fetal growth \|\| id:finngen_R11_O15_POOR_FETGRO | CD33dim HLA DR+ CD11b- Absolute Count \|\| id:ebi-a-GCST90001527 | Weighted median | 28 | -0.01604846 | 0.0225228517694027 | 0.476129710782058 | -0.060193249 | 0.0280963297332186 | 0.984079630661852 | 0.941582555922956 | 1.02849475427496 | 0.999440741871876 |
| ebi-a-GCST90001527 | finngen_R11_O15_POOR_FETGRO | Poor fetal growth \|\| id:finngen_R11_O15_POOR_FETGRO | CD33dim HLA DR+ CD11b- Absolute Count \|\| id:ebi-a-GCST90001527 | Weighted mode | 28 | -0.01890406 | 0.0184161532992535 | 0.313768054605845 | -0.054999721 | 0.0171916000242403 | 0.981273500672575 | 0.946485412109165 | 1.01734022606484 | 0.999857399747617 |
| ebi-a-GCST90001528 | finngen_R11_O15_POOR_FETGRO | Poor fetal growth \|\| id:finngen_R11_O15_POOR_FETGRO | CD33dim HLA DR+ CD11b- %CD33dim HLA DR+ \|\| id:ebi-a-GCST90001528 | Inverse variance weighted | 24 | 0.00431008188967992 | 0.0117269269092597 | 0.713218348305817 | -0.018674695 | 0.0272948586318289 | 1.00431938365161 | 0.981498596861512 | 1.02767077569309 | 0.994119304454654 |
| ebi-a-GCST90001528 | finngen_R11_O15_POOR_FETGRO | Poor fetal growth \|\| id:finngen_R11_O15_POOR_FETGRO | CD33dim HLA DR+ CD11b- %CD33dim HLA DR+ \|\| id:ebi-a-GCST90001528 | MR Egger | 24 | 0.0247358222739319 | 0.0166284210512655 | 0.151059911665727 | -0.007855883 | 0.0573275275344123 | 1.02504429088254 | 0.99217489381648 | 1.05900260611234 | 0.992899239648112 |
| ebi-a-GCST90001528 | finngen_R11_O15_POOR_FETGRO | Poor fetal growth \|\| id:finngen_R11_O15_POOR_FETGRO | CD33dim HLA DR+ CD11b- %CD33dim HLA DR+ \|\| id:ebi-a-GCST90001528 | Weighted median | 24 | 0.00175320487185115 | 0.0181556094664152 | 0.92307148315937 | -0.03383179 | 0.0373381994260249 | 1.00175474263405 | 0.966734105612557 | 1.03804402737395 | 0.999440741871876 |
| ebi-a-GCST90001528 | finngen_R11_O15_POOR_FETGRO | Poor fetal growth \|\| id:finngen_R11_O15_POOR_FETGRO | CD33dim HLA DR+ CD11b- %CD33dim HLA DR+ \|\| id:ebi-a-GCST90001528 | Weighted mode | 24 | 0.0128547936444043 | 0.0158700090003231 | 0.42623865799104 | -0.018250424 | 0.0439600112850376 | 1.01293777167818 | 0.981915106461795 | 1.04494056822242 | 0.999857399747617 |
| ebi-a-GCST90001529 | finngen_R11_O15_POOR_FETGRO | Poor fetal growth \|\| id:finngen_R11_O15_POOR_FETGRO | CD66b++ myeloid cell Absolute Count \|\| id:ebi-a-GCST90001529 | Inverse variance weighted | 16 | -0.067526173 | 0.0355609000729635 | 0.0575791788670854 | -0.137225537 | 0.00217319107989102 | 0.934703256248553 | 0.871773586579084 | 1.00217555417113 | 0.994119304454654 |
| ebi-a-GCST90001529 | finngen_R11_O15_POOR_FETGRO | Poor fetal growth \|\| id:finngen_R11_O15_POOR_FETGRO | CD66b++ myeloid cell Absolute Count \|\| id:ebi-a-GCST90001529 | MR Egger | 16 | 0.00824904840197929 | 0.0586005755937769 | 0.890059068717227 | -0.10660808 | 0.123106176565782 | 1.00828316554856 | 0.898877892402521 | 1.13100450074632 | 0.992899239648112 |
| ebi-a-GCST90001529 | finngen_R11_O15_POOR_FETGRO | Poor fetal growth \|\| id:finngen_R11_O15_POOR_FETGRO | CD66b++ myeloid cell Absolute Count \|\| id:ebi-a-GCST90001529 | Weighted median | 16 | -0.072595652 | 0.0414733314052146 | 0.0800462684849882 | -0.153883382 | 0.00869207726474478 | 0.929976787996865 | 0.857372000323587 | 1.00872996305752 | 0.999440741871876 |
| ebi-a-GCST90001529 | finngen_R11_O15_POOR_FETGRO | Poor fetal growth \|\| id:finngen_R11_O15_POOR_FETGRO | CD66b++ myeloid cell Absolute Count \|\| id:ebi-a-GCST90001529 | Weighted mode | 16 | -0.033255401 | 0.0469927862080249 | 0.49000035770905 | -0.125361262 | 0.0588504600016031 | 0.967291480859565 | 0.88217814762743 | 1.06061662427241 | 0.999857399747617 |
| ebi-a-GCST90001530 | finngen_R11_O15_POOR_FETGRO | Poor fetal growth \|\| id:finngen_R11_O15_POOR_FETGRO | Monocytic Myeloid-Derived Suppressor Cells Absolute Count \|\| id:ebi-a-GCST90001530 | Inverse variance weighted | 19 | -0.018734145 | 0.0203348177763969 | 0.356902112903536 | -0.058590387 | 0.0211220982656571 | 0.981440248775609 | 0.943092992806687 | 1.02134674869138 | 0.994119304454654 |
| ebi-a-GCST90001530 | finngen_R11_O15_POOR_FETGRO | Poor fetal growth \|\| id:finngen_R11_O15_POOR_FETGRO | Monocytic Myeloid-Derived Suppressor Cells Absolute Count \|\| id:ebi-a-GCST90001530 | MR Egger | 19 | -0.014809589 | 0.0391737827030994 | 0.710073598274333 | -0.091590203 | 0.0619710254943522 | 0.985299534002461 | 0.912479004368167 | 1.06393151739167 | 0.992899239648112 |
| ebi-a-GCST90001530 | finngen_R11_O15_POOR_FETGRO | Poor fetal growth \|\| id:finngen_R11_O15_POOR_FETGRO | Monocytic Myeloid-Derived Suppressor Cells Absolute Count \|\| id:ebi-a-GCST90001530 | Weighted median | 19 | -0.013230938 | 0.0296625419435765 | 0.655562085893886 | -0.07136952 | 0.0449076446716334 | 0.986856206560199 | 0.931117762150026 | 1.04593125812315 | 0.999440741871876 |
| ebi-a-GCST90001530 | finngen_R11_O15_POOR_FETGRO | Poor fetal growth \|\| id:finngen_R11_O15_POOR_FETGRO | Monocytic Myeloid-Derived Suppressor Cells Absolute Count \|\| id:ebi-a-GCST90001530 | Weighted mode | 19 | -0.005902533 | 0.0344760459209538 | 0.86597109922761 | -0.073475583 | 0.0616705167078735 | 0.994114852429038 | 0.929158832504912 | 1.06361184465712 | 0.999857399747617 |
| ebi-a-GCST90001531 | finngen_R11_O15_POOR_FETGRO | Poor fetal growth \|\| id:finngen_R11_O15_POOR_FETGRO | CD33dim HLA DR- Absolute Count \|\| id:ebi-a-GCST90001531 | Inverse variance weighted | 24 | 0.00977724667363018 | 0.0105569346761137 | 0.35437090599227 | -0.010914345 | 0.030468838638813 | 1.00982520010665 | 0.989145000073521 | 1.03093776412421 | 0.994119304454654 |
| ebi-a-GCST90001531 | finngen_R11_O15_POOR_FETGRO | Poor fetal growth \|\| id:finngen_R11_O15_POOR_FETGRO | CD33dim HLA DR- Absolute Count \|\| id:ebi-a-GCST90001531 | MR Egger | 24 | 0.0228933979112455 | 0.0131880282313252 | 0.0965641042422834 | -0.002955137 | 0.0487419332446429 | 1.02315746301075 | 0.997049224698493 | 1.04994935875022 | 0.992899239648112 |
| ebi-a-GCST90001531 | finngen_R11_O15_POOR_FETGRO | Poor fetal growth \|\| id:finngen_R11_O15_POOR_FETGRO | CD33dim HLA DR- Absolute Count \|\| id:ebi-a-GCST90001531 | Weighted median | 24 | 0.0184832942361316 | 0.0149766089957151 | 0.217149197959755 | -0.010870859 | 0.0478374478677333 | 1.01865516761474 | 0.989188014865467 | 1.04900012425781 | 0.999440741871876 |
| ebi-a-GCST90001531 | finngen_R11_O15_POOR_FETGRO | Poor fetal growth \|\| id:finngen_R11_O15_POOR_FETGRO | CD33dim HLA DR- Absolute Count \|\| id:ebi-a-GCST90001531 | Weighted mode | 24 | 0.0220260971086301 | 0.0138603116442032 | 0.12567989485316 | -0.005140114 | 0.0491923079312684 | 1.02227046242564 | 0.994873074065251 | 1.0504223358641 | 0.999857399747617 |
| ebi-a-GCST90001532 | finngen_R11_O15_POOR_FETGRO | Poor fetal growth \|\| id:finngen_R11_O15_POOR_FETGRO | Basophil Absolute Count \|\| id:ebi-a-GCST90001532 | Inverse variance weighted | 23 | 0.00852388326300711 | 0.0107754489943575 | 0.428916746074936 | -0.012595997 | 0.0296437632919477 | 1.00856031499566 | 0.987483000769233 | 1.03008751359949 | 0.994119304454654 |
| ebi-a-GCST90001532 | finngen_R11_O15_POOR_FETGRO | Poor fetal growth \|\| id:finngen_R11_O15_POOR_FETGRO | Basophil Absolute Count \|\| id:ebi-a-GCST90001532 | MR Egger | 23 | 0.0178557890918648 | 0.013021052290574 | 0.184757725778945 | -0.007665473 | 0.0433770515813898 | 1.01801615676909 | 0.992363831417311 | 1.04433158750129 | 0.992899239648112 |
| ebi-a-GCST90001532 | finngen_R11_O15_POOR_FETGRO | Poor fetal growth \|\| id:finngen_R11_O15_POOR_FETGRO | Basophil Absolute Count \|\| id:ebi-a-GCST90001532 | Weighted median | 23 | 0.00490312724321994 | 0.0148739410234668 | 0.741667862971737 | -0.024249797 | 0.0340560516492149 | 1.00491516724144 | 0.97604186681446 | 1.03464259852683 | 0.999440741871876 |
| ebi-a-GCST90001532 | finngen_R11_O15_POOR_FETGRO | Poor fetal growth \|\| id:finngen_R11_O15_POOR_FETGRO | Basophil Absolute Count \|\| id:ebi-a-GCST90001532 | Weighted mode | 23 | 0.0188993190550175 | 0.0136958261494085 | 0.181471273149098 | -0.0079445 | 0.045743138307858 | 1.01907904161129 | 0.992086973939958 | 1.04680549219088 | 0.999857399747617 |
| ebi-a-GCST90001533 | finngen_R11_O15_POOR_FETGRO | Poor fetal growth \|\| id:finngen_R11_O15_POOR_FETGRO | Basophil %CD33dim HLA DR- CD66b- \|\| id:ebi-a-GCST90001533 | Inverse variance weighted | 20 | -0.020906672 | 0.0247527470417918 | 0.398322894563526 | -0.069422056 | 0.0276087126044244 | 0.979310357775858 | 0.9329328472557 | 1.02799336486886 | 0.994119304454654 |
| ebi-a-GCST90001533 | finngen_R11_O15_POOR_FETGRO | Poor fetal growth \|\| id:finngen_R11_O15_POOR_FETGRO | Basophil %CD33dim HLA DR- CD66b- \|\| id:ebi-a-GCST90001533 | MR Egger | 20 | -0.068218187 | 0.0493463838299766 | 0.183751888039415 | -0.1649371 | 0.0285007249069907 | 0.934056651950246 | 0.847947038528103 | 1.02891075669885 | 0.992899239648112 |
| ebi-a-GCST90001533 | finngen_R11_O15_POOR_FETGRO | Poor fetal growth \|\| id:finngen_R11_O15_POOR_FETGRO | Basophil %CD33dim HLA DR- CD66b- \|\| id:ebi-a-GCST90001533 | Weighted median | 20 | -0.068536494 | 0.0334263246062084 | 0.040327869134804 | -0.13405209 | -0.003020898 | 0.933759382820113 | 0.87454450815012 | 0.996983660498765 | 0.999440741871876 |
| ebi-a-GCST90001533 | finngen_R11_O15_POOR_FETGRO | Poor fetal growth \|\| id:finngen_R11_O15_POOR_FETGRO | Basophil %CD33dim HLA DR- CD66b- \|\| id:ebi-a-GCST90001533 | Weighted mode | 20 | -0.067358477 | 0.0347117506817838 | 0.0673040791901426 | -0.135393508 | 0.000676554336460425 | 0.934860015448546 | 0.873372164831437 | 1.00067678325097 | 0.999857399747617 |
| ebi-a-GCST90001534 | finngen_R11_O15_POOR_FETGRO | Poor fetal growth \|\| id:finngen_R11_O15_POOR_FETGRO | CD45RA- CD4+ T cell Absolute Count \|\| id:ebi-a-GCST90001534 | Inverse variance weighted (multiplicative random effects) | 15 | -0.015268036 | 0.0224440880530756 | 0.496333620555968 | -0.059258449 | 0.02872237649564 | 0.984847929435854 | 0.942463159325808 | 1.02913884167942 | 0.994119304454654 |
| ebi-a-GCST90001534 | finngen_R11_O15_POOR_FETGRO | Poor fetal growth \|\| id:finngen_R11_O15_POOR_FETGRO | CD45RA- CD4+ T cell Absolute Count \|\| id:ebi-a-GCST90001534 | MR Egger | 15 | -0.029526139 | 0.025736417531708 | 0.271953935397673 | -0.079969518 | 0.0209172390454725 | 0.970905498503212 | 0.923144485544461 | 1.02113753782302 | 0.992899239648112 |
| ebi-a-GCST90001534 | finngen_R11_O15_POOR_FETGRO | Poor fetal growth \|\| id:finngen_R11_O15_POOR_FETGRO | CD45RA- CD4+ T cell Absolute Count \|\| id:ebi-a-GCST90001534 | Weighted median | 15 | -0.028170192 | 0.0199754485170882 | 0.1584686056623 | -0.067322071 | 0.010981687502472 | 0.972222888559913 | 0.934894050876599 | 1.01104220756727 | 0.999440741871876 |
| ebi-a-GCST90001534 | finngen_R11_O15_POOR_FETGRO | Poor fetal growth \|\| id:finngen_R11_O15_POOR_FETGRO | CD45RA- CD4+ T cell Absolute Count \|\| id:ebi-a-GCST90001534 | Weighted mode | 15 | -0.019838026 | 0.0178339926815268 | 0.284715174183414 | -0.054792652 | 0.0151165994863519 | 0.980357452699286 | 0.946681420269006 | 1.01523143317837 | 0.999857399747617 |
| ebi-a-GCST90001535 | finngen_R11_O15_POOR_FETGRO | Poor fetal growth \|\| id:finngen_R11_O15_POOR_FETGRO | CD45RA- CD4+ T cell %CD4+ T cell \|\| id:ebi-a-GCST90001535 | Inverse variance weighted | 30 | -0.002591678 | 0.024023850323013 | 0.914091373645896 | -0.049678425 | 0.0444950685435276 | 0.997411677408666 | 0.951535365555593 | 1.04549982086088 | 0.995828307205886 |
| ebi-a-GCST90001535 | finngen_R11_O15_POOR_FETGRO | Poor fetal growth \|\| id:finngen_R11_O15_POOR_FETGRO | CD45RA- CD4+ T cell %CD4+ T cell \|\| id:ebi-a-GCST90001535 | MR Egger | 30 | 0.0363355630759117 | 0.0363412095964185 | 0.32594858212948 | -0.034893208 | 0.107564333884892 | 1.03700376828702 | 0.965708540955292 | 1.11356249824373 | 0.992899239648112 |
| ebi-a-GCST90001535 | finngen_R11_O15_POOR_FETGRO | Poor fetal growth \|\| id:finngen_R11_O15_POOR_FETGRO | CD45RA- CD4+ T cell %CD4+ T cell \|\| id:ebi-a-GCST90001535 | Weighted median | 30 | 0.00617844585766276 | 0.036508561085832 | 0.865613534617049 | -0.065378334 | 0.0777352255858935 | 1.00619757182356 | 0.936713006072831 | 1.08083644294453 | 0.999440741871876 |
| ebi-a-GCST90001535 | finngen_R11_O15_POOR_FETGRO | Poor fetal growth \|\| id:finngen_R11_O15_POOR_FETGRO | CD45RA- CD4+ T cell %CD4+ T cell \|\| id:ebi-a-GCST90001535 | Weighted mode | 30 | 0.00462688449712045 | 0.035103370159323 | 0.896046852535125 | -0.064175721 | 0.0734294900093935 | 1.00463760505508 | 0.937840186820946 | 1.07619265166284 | 0.999857399747617 |
| ebi-a-GCST90001536 | finngen_R11_O15_POOR_FETGRO | Poor fetal growth \|\| id:finngen_R11_O15_POOR_FETGRO | CD45RA- CD4+ T cell %T cell \|\| id:ebi-a-GCST90001536 | Inverse variance weighted | 26 | 0.0113402581693202 | 0.00906472749612746 | 0.210923044206793 | -0.006426608 | 0.02910712406173 | 1.01140480264994 | 0.993593998753446 | 1.02953487652576 | 0.994119304454654 |
| ebi-a-GCST90001536 | finngen_R11_O15_POOR_FETGRO | Poor fetal growth \|\| id:finngen_R11_O15_POOR_FETGRO | CD45RA- CD4+ T cell %T cell \|\| id:ebi-a-GCST90001536 | MR Egger | 26 | 0.0156625497303555 | 0.0103697604043798 | 0.143994915876076 | -0.004662181 | 0.03598728012294 | 1.01578585035535 | 0.995348670432231 | 1.03664266043987 | 0.992899239648112 |
| ebi-a-GCST90001536 | finngen_R11_O15_POOR_FETGRO | Poor fetal growth \|\| id:finngen_R11_O15_POOR_FETGRO | CD45RA- CD4+ T cell %T cell \|\| id:ebi-a-GCST90001536 | Weighted median | 26 | 0.013894358277053 | 0.0120393794954977 | 0.248469034144276 | -0.009702826 | 0.0374915420882284 | 1.01399133348861 | 0.990344095001004 | 1.03820321601349 | 0.999440741871876 |
| ebi-a-GCST90001536 | finngen_R11_O15_POOR_FETGRO | Poor fetal growth \|\| id:finngen_R11_O15_POOR_FETGRO | CD45RA- CD4+ T cell %T cell \|\| id:ebi-a-GCST90001536 | Weighted mode | 26 | 0.0148941957085893 | 0.00970712198428574 | 0.137501227921993 | -0.004131763 | 0.0339201547977893 | 1.01500566697888 | 0.995876760609961 | 1.03450200340877 | 0.999857399747617 |
| ebi-a-GCST90001537 | finngen_R11_O15_POOR_FETGRO | Poor fetal growth \|\| id:finngen_R11_O15_POOR_FETGRO | Central Memory CD4+ T cell Absolute Count \|\| id:ebi-a-GCST90001537 | Inverse variance weighted | 28 | 0.0154031031080601 | 0.0267865732756663 | 0.565270506015397 | -0.037098581 | 0.0679047867283661 | 1.01552234233212 | 0.963581140344397 | 1.07026340034749 | 0.994119304454654 |
| ebi-a-GCST90001537 | finngen_R11_O15_POOR_FETGRO | Poor fetal growth \|\| id:finngen_R11_O15_POOR_FETGRO | Central Memory CD4+ T cell Absolute Count \|\| id:ebi-a-GCST90001537 | MR Egger | 28 | 0.00264000870735434 | 0.0383120283657728 | 0.945590013641154 | -0.072451567 | 0.077731584304269 | 1.00264349659902 | 0.930110793728179 | 1.08083250732182 | 0.992899239648112 |
| ebi-a-GCST90001537 | finngen_R11_O15_POOR_FETGRO | Poor fetal growth \|\| id:finngen_R11_O15_POOR_FETGRO | Central Memory CD4+ T cell Absolute Count \|\| id:ebi-a-GCST90001537 | Weighted median | 28 | 0.0275205470033809 | 0.0350078235405054 | 0.431793895839613 | -0.041094787 | 0.0961358811427714 | 1.02790273521125 | 0.959738154805217 | 1.10090864655601 | 0.999440741871876 |
| ebi-a-GCST90001537 | finngen_R11_O15_POOR_FETGRO | Poor fetal growth \|\| id:finngen_R11_O15_POOR_FETGRO | Central Memory CD4+ T cell Absolute Count \|\| id:ebi-a-GCST90001537 | Weighted mode | 28 | 0.0293077969849411 | 0.0327700900007687 | 0.379040313049007 | -0.034921579 | 0.0935371733864478 | 1.02974149702986 | 0.965681142566889 | 1.09805142087244 | 0.999857399747617 |
| ebi-a-GCST90001538 | finngen_R11_O15_POOR_FETGRO | Poor fetal growth \|\| id:finngen_R11_O15_POOR_FETGRO | Central Memory CD4+ T cell %CD4+ T cell \|\| id:ebi-a-GCST90001538 | Inverse variance weighted | 32 | 0.00145602866722063 | 0.00869419371606839 | 0.866999160628856 | -0.015584591 | 0.0184966483507147 | 1.00145708919162 | 0.984536220309706 | 1.0186687709434 | 0.994119304454654 |
| ebi-a-GCST90001538 | finngen_R11_O15_POOR_FETGRO | Poor fetal growth \|\| id:finngen_R11_O15_POOR_FETGRO | Central Memory CD4+ T cell %CD4+ T cell \|\| id:ebi-a-GCST90001538 | MR Egger | 32 | -0.001049487 | 0.0095789584665819 | 0.913486573720631 | -0.019824246 | 0.0177252713447405 | 0.998951063269379 | 0.980370962436861 | 1.01788329626429 | 0.992899239648112 |
| ebi-a-GCST90001538 | finngen_R11_O15_POOR_FETGRO | Poor fetal growth \|\| id:finngen_R11_O15_POOR_FETGRO | Central Memory CD4+ T cell %CD4+ T cell \|\| id:ebi-a-GCST90001538 | Weighted median | 32 | -0.003301872 | 0.0110273016861489 | 0.764614220299571 | -0.024915384 | 0.0183116388169507 | 0.99670357269831 | 0.975392442545639 | 1.01848032494171 | 0.999440741871876 |
| ebi-a-GCST90001538 | finngen_R11_O15_POOR_FETGRO | Poor fetal growth \|\| id:finngen_R11_O15_POOR_FETGRO | Central Memory CD4+ T cell %CD4+ T cell \|\| id:ebi-a-GCST90001538 | Weighted mode | 32 | -0.003663378 | 0.0096015440050882 | 0.705404969883466 | -0.022482404 | 0.0151556481773567 | 0.996343323910372 | 0.977768441540055 | 1.01527107741092 | 0.999857399747617 |
| ebi-a-GCST90001539 | finngen_R11_O15_POOR_FETGRO | Poor fetal growth \|\| id:finngen_R11_O15_POOR_FETGRO | Central Memory CD4+ T cell %T cell \|\| id:ebi-a-GCST90001539 | Inverse variance weighted | 25 | 0.00696273745738787 | 0.0283751463056517 | 0.806161011305326 | -0.048652549 | 0.0625780242164652 | 1.00698703367049 | 0.952512023178193 | 1.06457751850424 | 0.994119304454654 |
| ebi-a-GCST90001539 | finngen_R11_O15_POOR_FETGRO | Poor fetal growth \|\| id:finngen_R11_O15_POOR_FETGRO | Central Memory CD4+ T cell %T cell \|\| id:ebi-a-GCST90001539 | MR Egger | 25 | 0.0274530443832857 | 0.0461326421711803 | 0.557593891908593 | -0.062966934 | 0.117873023038799 | 1.02783335142524 | 0.938974521051727 | 1.12510124036033 | 0.992899239648112 |
| ebi-a-GCST90001539 | finngen_R11_O15_POOR_FETGRO | Poor fetal growth \|\| id:finngen_R11_O15_POOR_FETGRO | Central Memory CD4+ T cell %T cell \|\| id:ebi-a-GCST90001539 | Weighted median | 25 | -0.012627696 | 0.0425135767919217 | 0.766445604114859 | -0.095954307 | 0.0706989140942026 | 0.987451698397862 | 0.908505527526852 | 1.07325803434917 | 0.999440741871876 |
| ebi-a-GCST90001539 | finngen_R11_O15_POOR_FETGRO | Poor fetal growth \|\| id:finngen_R11_O15_POOR_FETGRO | Central Memory CD4+ T cell %T cell \|\| id:ebi-a-GCST90001539 | Weighted mode | 25 | -0.022069055 | 0.0425981820203665 | 0.609149280919226 | -0.105561492 | 0.0614233817616587 | 0.978172685005842 | 0.899819139684593 | 1.06334902147883 | 0.999857399747617 |
| ebi-a-GCST90001540 | finngen_R11_O15_POOR_FETGRO | Poor fetal growth \|\| id:finngen_R11_O15_POOR_FETGRO | Naive CD4+ T cell Absolute Count \|\| id:ebi-a-GCST90001540 | Inverse variance weighted | 25 | 0.0719932073414823 | 0.0325982782989238 | 0.0272096306605351 | 0.00810058187559165 | 0.135885832807373 | 1.07464804432183 | 1.00813348036125 | 1.14555110177564 | 0.994119304454654 |
| ebi-a-GCST90001540 | finngen_R11_O15_POOR_FETGRO | Poor fetal growth \|\| id:finngen_R11_O15_POOR_FETGRO | Naive CD4+ T cell Absolute Count \|\| id:ebi-a-GCST90001540 | MR Egger | 25 | 0.045987959119428 | 0.0653718478716518 | 0.48881600488677 | -0.082140863 | 0.174116780947866 | 1.04706180333491 | 0.921142194972162 | 1.19019454975253 | 0.992899239648112 |
| ebi-a-GCST90001540 | finngen_R11_O15_POOR_FETGRO | Poor fetal growth \|\| id:finngen_R11_O15_POOR_FETGRO | Naive CD4+ T cell Absolute Count \|\| id:ebi-a-GCST90001540 | Weighted median | 25 | 0.0510684896787359 | 0.0474386244450241 | 0.281696064370332 | -0.041911214 | 0.144048193590983 | 1.05239496900828 | 0.95895491834037 | 1.15493976787847 | 0.999440741871876 |
| ebi-a-GCST90001540 | finngen_R11_O15_POOR_FETGRO | Poor fetal growth \|\| id:finngen_R11_O15_POOR_FETGRO | Naive CD4+ T cell Absolute Count \|\| id:ebi-a-GCST90001540 | Weighted mode | 25 | 0.0437251531843589 | 0.0681116807902473 | 0.526982195091048 | -0.089773741 | 0.177224047533244 | 1.04469518428157 | 0.914137993672143 | 1.19389855318992 | 0.999857399747617 |
| ebi-a-GCST90001541 | finngen_R11_O15_POOR_FETGRO | Poor fetal growth \|\| id:finngen_R11_O15_POOR_FETGRO | Naive CD4+ T cell %CD4+ T cell \|\| id:ebi-a-GCST90001541 | Inverse variance weighted | 35 | -0.004107125 | 0.0248143948516311 | 0.868539579811608 | -0.052743339 | 0.0445290888390672 | 0.995901297633064 | 0.94862345590417 | 1.0455353896788 | 0.994119304454654 |
| ebi-a-GCST90001541 | finngen_R11_O15_POOR_FETGRO | Poor fetal growth \|\| id:finngen_R11_O15_POOR_FETGRO | Naive CD4+ T cell %CD4+ T cell \|\| id:ebi-a-GCST90001541 | MR Egger | 35 | -0.031185191 | 0.0412925321016345 | 0.455470201823864 | -0.112118554 | 0.0497481715715678 | 0.969296051211609 | 0.893938271021586 | 1.05100638975969 | 0.992899239648112 |
| ebi-a-GCST90001541 | finngen_R11_O15_POOR_FETGRO | Poor fetal growth \|\| id:finngen_R11_O15_POOR_FETGRO | Naive CD4+ T cell %CD4+ T cell \|\| id:ebi-a-GCST90001541 | Weighted median | 35 | -0.009911857 | 0.0382599321984686 | 0.795584134912146 | -0.084901324 | 0.0650776104593641 | 0.990137103904629 | 0.918602924213256 | 1.06724185030033 | 0.999440741871876 |
| ebi-a-GCST90001541 | finngen_R11_O15_POOR_FETGRO | Poor fetal growth \|\| id:finngen_R11_O15_POOR_FETGRO | Naive CD4+ T cell %CD4+ T cell \|\| id:ebi-a-GCST90001541 | Weighted mode | 35 | -0.00549389 | 0.0369471546853633 | 0.882672045853289 | -0.077910313 | 0.0669225329363609 | 0.994521173569129 | 0.925047387150255 | 1.06921264620216 | 0.999857399747617 |
| ebi-a-GCST90001542 | finngen_R11_O15_POOR_FETGRO | Poor fetal growth \|\| id:finngen_R11_O15_POOR_FETGRO | Effector Memory CD4+ T cell Absolute Count \|\| id:ebi-a-GCST90001542 | Inverse variance weighted | 22 | -0.01079524 | 0.0147383327091275 | 0.463887815724833 | -0.039682372 | 0.0180918921393819 | 0.989262819522637 | 0.961094661174009 | 1.01825654186283 | 0.994119304454654 |
| ebi-a-GCST90001542 | finngen_R11_O15_POOR_FETGRO | Poor fetal growth \|\| id:finngen_R11_O15_POOR_FETGRO | Effector Memory CD4+ T cell Absolute Count \|\| id:ebi-a-GCST90001542 | MR Egger | 22 | -0.031527679 | 0.0189350035505424 | 0.111491681187105 | -0.068640286 | 0.00558492794679154 | 0.968964136112347 | 0.933662471169116 | 1.0056005527311 | 0.992899239648112 |
| ebi-a-GCST90001542 | finngen_R11_O15_POOR_FETGRO | Poor fetal growth \|\| id:finngen_R11_O15_POOR_FETGRO | Effector Memory CD4+ T cell Absolute Count \|\| id:ebi-a-GCST90001542 | Weighted median | 22 | -0.017839714 | 0.0205542113884692 | 0.385430066929873 | -0.058125968 | 0.0224465404808762 | 0.982318471795509 | 0.943531085074015 | 1.02270035963359 | 0.999440741871876 |
| ebi-a-GCST90001542 | finngen_R11_O15_POOR_FETGRO | Poor fetal growth \|\| id:finngen_R11_O15_POOR_FETGRO | Effector Memory CD4+ T cell Absolute Count \|\| id:ebi-a-GCST90001542 | Weighted mode | 22 | -0.021716853 | 0.0167941383886287 | 0.210014523875521 | -0.054633364 | 0.0111996586160212 | 0.978517260422861 | 0.946832227229704 | 1.01126260958286 | 0.999857399747617 |
| ebi-a-GCST90001543 | finngen_R11_O15_POOR_FETGRO | Poor fetal growth \|\| id:finngen_R11_O15_POOR_FETGRO | Effector Memory CD4+ T cell %CD4+ T cell \|\| id:ebi-a-GCST90001543 | Inverse variance weighted | 28 | -0.002516643 | 0.0217119291513703 | 0.907723376646564 | -0.045072024 | 0.0400387379059229 | 0.997486520860959 | 0.955928629198752 | 1.04085109380319 | 0.995828307205886 |
| ebi-a-GCST90001543 | finngen_R11_O15_POOR_FETGRO | Poor fetal growth \|\| id:finngen_R11_O15_POOR_FETGRO | Effector Memory CD4+ T cell %CD4+ T cell \|\| id:ebi-a-GCST90001543 | MR Egger | 28 | 0.0180561546606531 | 0.0285294417137742 | 0.532329441727512 | -0.037861551 | 0.0739738604196506 | 1.01822015259156 | 0.96284623666759 | 1.07677865858608 | 0.992899239648112 |
| ebi-a-GCST90001543 | finngen_R11_O15_POOR_FETGRO | Poor fetal growth \|\| id:finngen_R11_O15_POOR_FETGRO | Effector Memory CD4+ T cell %CD4+ T cell \|\| id:ebi-a-GCST90001543 | Weighted median | 28 | 0.00386332550133854 | 0.0298727400535859 | 0.897099605894844 | -0.054687245 | 0.0624138960063669 | 1.0038707977628 | 0.946781212207814 | 1.06440280563966 | 0.999440741871876 |
| ebi-a-GCST90001543 | finngen_R11_O15_POOR_FETGRO | Poor fetal growth \|\| id:finngen_R11_O15_POOR_FETGRO | Effector Memory CD4+ T cell %CD4+ T cell \|\| id:ebi-a-GCST90001543 | Weighted mode | 28 | -5.95E-06 | 0.0264874439207999 | 0.999822414921606 | -0.05192134 | 0.0519094399501532 | 0.999994049883087 | 0.949403543780287 | 1.05328035307292 | 0.999857399747617 |
| ebi-a-GCST90001544 | finngen_R11_O15_POOR_FETGRO | Poor fetal growth \|\| id:finngen_R11_O15_POOR_FETGRO | Effector Memory CD4+ T cell %T cell \|\| id:ebi-a-GCST90001544 | Inverse variance weighted | 20 | 0.00493417414802199 | 0.0304636517326716 | 0.871330086494426 | -0.054774583 | 0.0646429315440583 | 1.0049463672313 | 0.946698525609884 | 1.06677804358127 | 0.994119304454654 |
| ebi-a-GCST90001544 | finngen_R11_O15_POOR_FETGRO | Poor fetal growth \|\| id:finngen_R11_O15_POOR_FETGRO | Effector Memory CD4+ T cell %T cell \|\| id:ebi-a-GCST90001544 | MR Egger | 20 | 0.0994807588910867 | 0.041893974970675 | 0.0288930340436795 | 0.0173685679485636 | 0.18159294983361 | 1.10459721686012 | 1.01752027858427 | 1.1991259900911 | 0.992899239648112 |
| ebi-a-GCST90001544 | finngen_R11_O15_POOR_FETGRO | Poor fetal growth \|\| id:finngen_R11_O15_POOR_FETGRO | Effector Memory CD4+ T cell %T cell \|\| id:ebi-a-GCST90001544 | Weighted median | 20 | 0.0101482437242097 | 0.0379969262306389 | 0.78940706631116 | -0.064325732 | 0.084622219136262 | 1.01019991178166 | 0.937699511335321 | 1.08830584790476 | 0.999440741871876 |
| ebi-a-GCST90001544 | finngen_R11_O15_POOR_FETGRO | Poor fetal growth \|\| id:finngen_R11_O15_POOR_FETGRO | Effector Memory CD4+ T cell %T cell \|\| id:ebi-a-GCST90001544 | Weighted mode | 20 | 0.0183896073791564 | 0.0351870042318465 | 0.60727623670343 | -0.050576921 | 0.0873561356735755 | 1.01855973748409 | 0.950680798622595 | 1.09128525613096 | 0.999857399747617 |
| ebi-a-GCST90001545 | finngen_R11_O15_POOR_FETGRO | Poor fetal growth \|\| id:finngen_R11_O15_POOR_FETGRO | Terminally Differentiated CD4+ T cell Absolute Count \|\| id:ebi-a-GCST90001545 | Inverse variance weighted | 11 | -0.011031367 | 0.0407106392047371 | 0.786414004736381 | -0.09082422 | 0.0687614855828346 | 0.989029255152547 | 0.913178215167727 | 1.0711806866395 | 0.994119304454654 |
| ebi-a-GCST90001545 | finngen_R11_O15_POOR_FETGRO | Poor fetal growth \|\| id:finngen_R11_O15_POOR_FETGRO | Terminally Differentiated CD4+ T cell Absolute Count \|\| id:ebi-a-GCST90001545 | MR Egger | 11 | -0.111151941 | 0.0746585396730501 | 0.1707191231812 | -0.257482679 | 0.0351787967055013 | 0.894802781322335 | 0.772995015388849 | 1.03580489074618 | 0.992899239648112 |
| ebi-a-GCST90001545 | finngen_R11_O15_POOR_FETGRO | Poor fetal growth \|\| id:finngen_R11_O15_POOR_FETGRO | Terminally Differentiated CD4+ T cell Absolute Count \|\| id:ebi-a-GCST90001545 | Weighted median | 11 | -0.059147191 | 0.0550624953338432 | 0.282740715389782 | -0.167069682 | 0.0487752999907237 | 0.942568021545059 | 0.84614064874888 | 1.04998439272835 | 0.999440741871876 |
| ebi-a-GCST90001545 | finngen_R11_O15_POOR_FETGRO | Poor fetal growth \|\| id:finngen_R11_O15_POOR_FETGRO | Terminally Differentiated CD4+ T cell Absolute Count \|\| id:ebi-a-GCST90001545 | Weighted mode | 11 | -0.04896559 | 0.062510459795967 | 0.451593113567068 | -0.171486091 | 0.0735549110837062 | 0.952213894704071 | 0.842411984780228 | 1.07632763736623 | 0.999857399747617 |
| ebi-a-GCST90001546 | finngen_R11_O15_POOR_FETGRO | Poor fetal growth \|\| id:finngen_R11_O15_POOR_FETGRO | Terminally Differentiated CD4+ T cell %CD4+ T cell \|\| id:ebi-a-GCST90001546 | Inverse variance weighted | 21 | -0.041877861 | 0.0377710231081463 | 0.267546733329697 | -0.115909067 | 0.032153343860243 | 0.958986902707066 | 0.890556200808719 | 1.03267584766525 | 0.994119304454654 |
| ebi-a-GCST90001546 | finngen_R11_O15_POOR_FETGRO | Poor fetal growth \|\| id:finngen_R11_O15_POOR_FETGRO | Terminally Differentiated CD4+ T cell %CD4+ T cell \|\| id:ebi-a-GCST90001546 | MR Egger | 21 | -0.110058199 | 0.0818587902507008 | 0.194623369981431 | -0.270501428 | 0.0503850299583995 | 0.89578200022285 | 0.762996810570019 | 1.05167594517699 | 0.992899239648112 |
| ebi-a-GCST90001546 | finngen_R11_O15_POOR_FETGRO | Poor fetal growth \|\| id:finngen_R11_O15_POOR_FETGRO | Terminally Differentiated CD4+ T cell %CD4+ T cell \|\| id:ebi-a-GCST90001546 | Weighted median | 21 | -0.062208104 | 0.0534551155224254 | 0.24452751077985 | -0.166980131 | 0.0425639219600556 | 0.939687313317368 | 0.846216424739199 | 1.04348275570491 | 0.999440741871876 |
| ebi-a-GCST90001546 | finngen_R11_O15_POOR_FETGRO | Poor fetal growth \|\| id:finngen_R11_O15_POOR_FETGRO | Terminally Differentiated CD4+ T cell %CD4+ T cell \|\| id:ebi-a-GCST90001546 | Weighted mode | 21 | -0.051774434 | 0.0647014982963332 | 0.432995092858443 | -0.17858937 | 0.07504050291609 | 0.949543027553216 | 0.836449299723777 | 1.07792780922009 | 0.999857399747617 |
| ebi-a-GCST90001547 | finngen_R11_O15_POOR_FETGRO | Poor fetal growth \|\| id:finngen_R11_O15_POOR_FETGRO | Terminally Differentiated CD4+ T cell %T cell \|\| id:ebi-a-GCST90001547 | Inverse variance weighted | 26 | 0.0153276212302563 | 0.0185313322659554 | 0.408169487086378 | -0.02099379 | 0.0516490324715289 | 1.01544569169167 | 0.979225045527324 | 1.05300610670134 | 0.994119304454654 |
| ebi-a-GCST90001547 | finngen_R11_O15_POOR_FETGRO | Poor fetal growth \|\| id:finngen_R11_O15_POOR_FETGRO | Terminally Differentiated CD4+ T cell %T cell \|\| id:ebi-a-GCST90001547 | MR Egger | 26 | 0.0256773211853061 | 0.0225787188343401 | 0.266666968817735 | -0.018576968 | 0.0699316101006128 | 1.02600982341911 | 0.981594520582188 | 1.07243483503571 | 0.992899239648112 |
| ebi-a-GCST90001547 | finngen_R11_O15_POOR_FETGRO | Poor fetal growth \|\| id:finngen_R11_O15_POOR_FETGRO | Terminally Differentiated CD4+ T cell %T cell \|\| id:ebi-a-GCST90001547 | Weighted median | 26 | 0.0299847038144655 | 0.0276183649618973 | 0.277620634717116 | -0.024147292 | 0.0841166991397842 | 1.03043877205033 | 0.976141921750331 | 1.08775582657147 | 0.999440741871876 |
| ebi-a-GCST90001547 | finngen_R11_O15_POOR_FETGRO | Poor fetal growth \|\| id:finngen_R11_O15_POOR_FETGRO | Terminally Differentiated CD4+ T cell %T cell \|\| id:ebi-a-GCST90001547 | Weighted mode | 26 | 0.0268221659539912 | 0.0211925736147537 | 0.217310617056422 | -0.014715278 | 0.0683596102389085 | 1.02718511803465 | 0.985392462252275 | 1.07075029202094 | 0.999857399747617 |
| ebi-a-GCST90001548 | finngen_R11_O15_POOR_FETGRO | Poor fetal growth \|\| id:finngen_R11_O15_POOR_FETGRO | Central Memory CD8+ T cell Absolute Count \|\| id:ebi-a-GCST90001548 | Inverse variance weighted | 25 | 0.0133083855115768 | 0.0382793026702793 | 0.728091554207975 | -0.061719048 | 0.0883358187453242 | 1.01339733623286 | 0.940146986126944 | 1.0923548936902 | 0.994119304454654 |
| ebi-a-GCST90001548 | finngen_R11_O15_POOR_FETGRO | Poor fetal growth \|\| id:finngen_R11_O15_POOR_FETGRO | Central Memory CD8+ T cell Absolute Count \|\| id:ebi-a-GCST90001548 | MR Egger | 25 | 0.0248608461953234 | 0.0679287849809365 | 0.717721838137606 | -0.108279572 | 0.158001264757959 | 1.02517245395094 | 0.897376679634672 | 1.17116767595037 | 0.992899239648112 |
| ebi-a-GCST90001548 | finngen_R11_O15_POOR_FETGRO | Poor fetal growth \|\| id:finngen_R11_O15_POOR_FETGRO | Central Memory CD8+ T cell Absolute Count \|\| id:ebi-a-GCST90001548 | Weighted median | 25 | 0.0150361785007357 | 0.0559983184647092 | 0.788305816312791 | -0.094720526 | 0.124792882691566 | 1.01514979054881 | 0.909627116358539 | 1.13291378271216 | 0.999440741871876 |
| ebi-a-GCST90001548 | finngen_R11_O15_POOR_FETGRO | Poor fetal growth \|\| id:finngen_R11_O15_POOR_FETGRO | Central Memory CD8+ T cell Absolute Count \|\| id:ebi-a-GCST90001548 | Weighted mode | 25 | 0.00829449483728117 | 0.0582994700160979 | 0.888051281609245 | -0.105972466 | 0.122561456068833 | 1.00832898946546 | 0.899449412820497 | 1.13038858717822 | 0.999857399747617 |
| ebi-a-GCST90001549 | finngen_R11_O15_POOR_FETGRO | Poor fetal growth \|\| id:finngen_R11_O15_POOR_FETGRO | Central Memory CD8+ T cell %CD8+ T cell \|\| id:ebi-a-GCST90001549 | Inverse variance weighted | 31 | -0.036690543 | 0.032467016410674 | 0.258439699216218 | -0.100325895 | 0.0269448091791157 | 0.963974397835878 | 0.904542583954334 | 1.02731110305577 | 0.994119304454654 |
| ebi-a-GCST90001549 | finngen_R11_O15_POOR_FETGRO | Poor fetal growth \|\| id:finngen_R11_O15_POOR_FETGRO | Central Memory CD8+ T cell %CD8+ T cell \|\| id:ebi-a-GCST90001549 | MR Egger | 31 | 0.0193329478542993 | 0.0689500285816144 | 0.781168317725896 | -0.115809108 | 0.154475003874263 | 1.01952103945705 | 0.890645223971632 | 1.16704510608669 | 0.992899239648112 |
| ebi-a-GCST90001549 | finngen_R11_O15_POOR_FETGRO | Poor fetal growth \|\| id:finngen_R11_O15_POOR_FETGRO | Central Memory CD8+ T cell %CD8+ T cell \|\| id:ebi-a-GCST90001549 | Weighted median | 31 | -0.023955334 | 0.045716862655006 | 0.600283133382506 | -0.113560385 | 0.0656497167014095 | 0.976329317409328 | 0.892650292179525 | 1.06785260071506 | 0.999440741871876 |
| ebi-a-GCST90001549 | finngen_R11_O15_POOR_FETGRO | Poor fetal growth \|\| id:finngen_R11_O15_POOR_FETGRO | Central Memory CD8+ T cell %CD8+ T cell \|\| id:ebi-a-GCST90001549 | Weighted mode | 31 | -0.005882565 | 0.0601838486571436 | 0.922786298311959 | -0.123842909 | 0.112077778085558 | 0.994134703127266 | 0.883518623114584 | 1.11859985981729 | 0.999857399747617 |
| ebi-a-GCST90001550 | finngen_R11_O15_POOR_FETGRO | Poor fetal growth \|\| id:finngen_R11_O15_POOR_FETGRO | Central Memory CD8+ T cell %T cell \|\| id:ebi-a-GCST90001550 | Inverse variance weighted | 22 | -0.027095253 | 0.0399708967906794 | 0.497851254542232 | -0.10543821 | 0.0512477050961419 | 0.973268530737657 | 0.899930077517392 | 1.05258359131344 | 0.994119304454654 |
| ebi-a-GCST90001550 | finngen_R11_O15_POOR_FETGRO | Poor fetal growth \|\| id:finngen_R11_O15_POOR_FETGRO | Central Memory CD8+ T cell %T cell \|\| id:ebi-a-GCST90001550 | MR Egger | 22 | 0.0433239113878322 | 0.0787790031603011 | 0.588448455141992 | -0.111082935 | 0.197730757582022 | 1.0442760929931 | 0.894864530434877 | 1.21863424161754 | 0.992899239648112 |
| ebi-a-GCST90001550 | finngen_R11_O15_POOR_FETGRO | Poor fetal growth \|\| id:finngen_R11_O15_POOR_FETGRO | Central Memory CD8+ T cell %T cell \|\| id:ebi-a-GCST90001550 | Weighted median | 22 | -0.065348991 | 0.0549785806127623 | 0.234586721870127 | -0.173107009 | 0.0424090266833539 | 0.936740492027023 | 0.841047610097307 | 1.04332113767199 | 0.999440741871876 |
| ebi-a-GCST90001550 | finngen_R11_O15_POOR_FETGRO | Poor fetal growth \|\| id:finngen_R11_O15_POOR_FETGRO | Central Memory CD8+ T cell %T cell \|\| id:ebi-a-GCST90001550 | Weighted mode | 22 | -0.07880447 | 0.0771193796108766 | 0.318479953756252 | -0.229958454 | 0.0723495143588179 | 0.92422061993374 | 0.794566613107905 | 1.0750310171851 | 0.999857399747617 |
| ebi-a-GCST90001551 | finngen_R11_O15_POOR_FETGRO | Poor fetal growth \|\| id:finngen_R11_O15_POOR_FETGRO | Naive CD8+ T cell Absolute Count \|\| id:ebi-a-GCST90001551 | Inverse variance weighted | 28 | 0.00425047730113391 | 0.00646686859729099 | 0.511007464126331 | -0.008424585 | 0.0169255397518242 | 1.00425952339197 | 0.991610802223542 | 1.01706958825173 | 0.994119304454654 |
| ebi-a-GCST90001551 | finngen_R11_O15_POOR_FETGRO | Poor fetal growth \|\| id:finngen_R11_O15_POOR_FETGRO | Naive CD8+ T cell Absolute Count \|\| id:ebi-a-GCST90001551 | MR Egger | 28 | 0.00345106338950085 | 0.0067269178073349 | 0.612266954702014 | -0.009733696 | 0.0166358222918773 | 1.00345702516494 | 0.990313523571705 | 1.0167749681144 | 0.992899239648112 |
| ebi-a-GCST90001551 | finngen_R11_O15_POOR_FETGRO | Poor fetal growth \|\| id:finngen_R11_O15_POOR_FETGRO | Naive CD8+ T cell Absolute Count \|\| id:ebi-a-GCST90001551 | Weighted median | 28 | 0.00446489603103035 | 0.00570038694968031 | 0.433473319678114 | -0.006707862 | 0.0156376544524038 | 1.00447487853073 | 0.99331458509898 | 1.01576056239905 | 0.999440741871876 |
| ebi-a-GCST90001551 | finngen_R11_O15_POOR_FETGRO | Poor fetal growth \|\| id:finngen_R11_O15_POOR_FETGRO | Naive CD8+ T cell Absolute Count \|\| id:ebi-a-GCST90001551 | Weighted mode | 28 | 0.00251175230743317 | 0.00603068314522538 | 0.680339950921892 | -0.009308387 | 0.0143318912720749 | 1.00251490939999 | 0.990734802263596 | 1.01443508522461 | 0.999857399747617 |
| ebi-a-GCST90001552 | finngen_R11_O15_POOR_FETGRO | Poor fetal growth \|\| id:finngen_R11_O15_POOR_FETGRO | Naive CD8+ T cell %CD8+ T cell \|\| id:ebi-a-GCST90001552 | Inverse variance weighted | 37 | 0.00148118804383993 | 0.00476778752110381 | 0.756054775870713 | -0.007863675 | 0.0108260515852034 | 1.00148228554465 | 0.992167162312855 | 1.01088486533026 | 0.994119304454654 |
| ebi-a-GCST90001552 | finngen_R11_O15_POOR_FETGRO | Poor fetal growth \|\| id:finngen_R11_O15_POOR_FETGRO | Naive CD8+ T cell %CD8+ T cell \|\| id:ebi-a-GCST90001552 | MR Egger | 37 | 0.00419083382844427 | 0.00500748301276497 | 0.408319012106877 | -0.005623833 | 0.0140055005334636 | 1.00419962765273 | 0.994391951268543 | 1.01410403703634 | 0.992899239648112 |
| ebi-a-GCST90001552 | finngen_R11_O15_POOR_FETGRO | Poor fetal growth \|\| id:finngen_R11_O15_POOR_FETGRO | Naive CD8+ T cell %CD8+ T cell \|\| id:ebi-a-GCST90001552 | Weighted median | 37 | 0.00404358828834758 | 0.00581185919536206 | 0.486586780618278 | -0.007347656 | 0.0154348323112572 | 1.00405177462181 | 0.992679272294841 | 1.01555456455806 | 0.999440741871876 |
| ebi-a-GCST90001552 | finngen_R11_O15_POOR_FETGRO | Poor fetal growth \|\| id:finngen_R11_O15_POOR_FETGRO | Naive CD8+ T cell %CD8+ T cell \|\| id:ebi-a-GCST90001552 | Weighted mode | 37 | 0.00205128403315946 | 0.00488979656189546 | 0.67734151690344 | -0.007532717 | 0.0116352852944746 | 1.00205338935554 | 0.992495582583522 | 1.01170323852241 | 0.999857399747617 |
| ebi-a-GCST90001553 | finngen_R11_O15_POOR_FETGRO | Poor fetal growth \|\| id:finngen_R11_O15_POOR_FETGRO | Naive CD8+ T cell %T cell \|\| id:ebi-a-GCST90001553 | Inverse variance weighted | 28 | 0.00347495165539639 | 0.00550287713773183 | 0.527727339883971 | -0.007310688 | 0.0142605908453508 | 1.00348099629949 | 0.992715970539049 | 1.01436275814866 | 0.994119304454654 |
| ebi-a-GCST90001553 | finngen_R11_O15_POOR_FETGRO | Poor fetal growth \|\| id:finngen_R11_O15_POOR_FETGRO | Naive CD8+ T cell %T cell \|\| id:ebi-a-GCST90001553 | MR Egger | 28 | 0.00487111926818073 | 0.00566247016893883 | 0.397516552172438 | -0.006227322 | 0.0159695607993008 | 1.00488300245662 | 0.993792027322142 | 1.01609775573184 | 0.992899239648112 |
| ebi-a-GCST90001553 | finngen_R11_O15_POOR_FETGRO | Poor fetal growth \|\| id:finngen_R11_O15_POOR_FETGRO | Naive CD8+ T cell %T cell \|\| id:ebi-a-GCST90001553 | Weighted median | 28 | 0.00412017152116024 | 0.00566539866561966 | 0.467071703600532 | -0.00698401 | 0.0152243529057748 | 1.00412867109707 | 0.993040321656622 | 1.01534083373083 | 0.999440741871876 |
| ebi-a-GCST90001553 | finngen_R11_O15_POOR_FETGRO | Poor fetal growth \|\| id:finngen_R11_O15_POOR_FETGRO | Naive CD8+ T cell %T cell \|\| id:ebi-a-GCST90001553 | Weighted mode | 28 | 0.00513844975936295 | 0.00569314045012694 | 0.374736861682814 | -0.006020106 | 0.0162970050416118 | 1.00515167423372 | 0.993997979003916 | 1.01643052557039 | 0.999857399747617 |
| ebi-a-GCST90001554 | finngen_R11_O15_POOR_FETGRO | Poor fetal growth \|\| id:finngen_R11_O15_POOR_FETGRO | Effector Memory CD8+ T cell Absolute Count \|\| id:ebi-a-GCST90001554 | Inverse variance weighted | 24 | -0.024868916 | 0.0151021876049742 | 0.0996177611661274 | -0.054469204 | 0.00473137121230303 | 0.975437767451263 | 0.946987671652477 | 1.00474258182262 | 0.994119304454654 |
| ebi-a-GCST90001554 | finngen_R11_O15_POOR_FETGRO | Poor fetal growth \|\| id:finngen_R11_O15_POOR_FETGRO | Effector Memory CD8+ T cell Absolute Count \|\| id:ebi-a-GCST90001554 | MR Egger | 24 | -0.026801073 | 0.0179934655807993 | 0.150555853396525 | -0.062068266 | 0.00846611927811689 | 0.973554888362841 | 0.939818727125196 | 1.00850205821545 | 0.992899239648112 |
| ebi-a-GCST90001554 | finngen_R11_O15_POOR_FETGRO | Poor fetal growth \|\| id:finngen_R11_O15_POOR_FETGRO | Effector Memory CD8+ T cell Absolute Count \|\| id:ebi-a-GCST90001554 | Weighted median | 24 | -0.032122928 | 0.0214052164798769 | 0.133431732235796 | -0.074077152 | 0.00983129674024998 | 0.968387533245261 | 0.928600047855818 | 1.00987978270107 | 0.999440741871876 |
| ebi-a-GCST90001554 | finngen_R11_O15_POOR_FETGRO | Poor fetal growth \|\| id:finngen_R11_O15_POOR_FETGRO | Effector Memory CD8+ T cell Absolute Count \|\| id:ebi-a-GCST90001554 | Weighted mode | 24 | -0.029946839 | 0.0182286096356773 | 0.114017295733113 | -0.065674914 | 0.00578123602655449 | 0.970497124911304 | 0.936435237039218 | 1.00579797962223 | 0.999857399747617 |
| ebi-a-GCST90001555 | finngen_R11_O15_POOR_FETGRO | Poor fetal growth \|\| id:finngen_R11_O15_POOR_FETGRO | Effector Memory CD8+ T cell %CD8+ T cell \|\| id:ebi-a-GCST90001555 | Inverse variance weighted | 24 | -0.012272133 | 0.0141496766237618 | 0.385773067946367 | -0.040005499 | 0.0154612331129365 | 0.98780286245689 | 0.960784155543405 | 1.01558137636664 | 0.994119304454654 |
| ebi-a-GCST90001555 | finngen_R11_O15_POOR_FETGRO | Poor fetal growth \|\| id:finngen_R11_O15_POOR_FETGRO | Effector Memory CD8+ T cell %CD8+ T cell \|\| id:ebi-a-GCST90001555 | MR Egger | 24 | -0.016637624 | 0.0164753448664847 | 0.323545772007382 | -0.0489293 | 0.0156540515636956 | 0.983500016501565 | 0.952248450986183 | 1.01577721807459 | 0.992899239648112 |
| ebi-a-GCST90001555 | finngen_R11_O15_POOR_FETGRO | Poor fetal growth \|\| id:finngen_R11_O15_POOR_FETGRO | Effector Memory CD8+ T cell %CD8+ T cell \|\| id:ebi-a-GCST90001555 | Weighted median | 24 | -0.018890359 | 0.0168390106742437 | 0.261938470467425 | -0.05189482 | 0.0141141017619589 | 0.981286945470356 | 0.949428722427445 | 1.01421417596109 | 0.999440741871876 |
| ebi-a-GCST90001555 | finngen_R11_O15_POOR_FETGRO | Poor fetal growth \|\| id:finngen_R11_O15_POOR_FETGRO | Effector Memory CD8+ T cell %CD8+ T cell \|\| id:ebi-a-GCST90001555 | Weighted mode | 24 | -0.013203436 | 0.0134757772034708 | 0.337384050086047 | -0.03961596 | 0.0132090869388769 | 0.986883346621702 | 0.961158491879018 | 1.01329671231978 | 0.999857399747617 |
| ebi-a-GCST90001556 | finngen_R11_O15_POOR_FETGRO | Poor fetal growth \|\| id:finngen_R11_O15_POOR_FETGRO | Effector Memory CD8+ T cell %T cell \|\| id:ebi-a-GCST90001556 | Inverse variance weighted | 15 | -0.003900861 | 0.020369199301201 | 0.848127750820992 | -0.043824492 | 0.0360227695826507 | 0.996106737427344 | 0.95712192559577 | 1.03667945098063 | 0.994119304454654 |
| ebi-a-GCST90001556 | finngen_R11_O15_POOR_FETGRO | Poor fetal growth \|\| id:finngen_R11_O15_POOR_FETGRO | Effector Memory CD8+ T cell %T cell \|\| id:ebi-a-GCST90001556 | MR Egger | 15 | 0.0043018895371974 | 0.025933711053287 | 0.870803217780154 | -0.046528184 | 0.0551319632016399 | 1.00431115594692 | 0.954537657380055 | 1.05668004835752 | 0.992899239648112 |
| ebi-a-GCST90001556 | finngen_R11_O15_POOR_FETGRO | Poor fetal growth \|\| id:finngen_R11_O15_POOR_FETGRO | Effector Memory CD8+ T cell %T cell \|\| id:ebi-a-GCST90001556 | Weighted median | 15 | 0.00101032277061644 | 0.0285691693215519 | 0.971789416153253 | -0.054985249 | 0.0570058946408581 | 1.00101083331859 | 0.94649910956458 | 1.05866205080969 | 0.999440741871876 |
| ebi-a-GCST90001556 | finngen_R11_O15_POOR_FETGRO | Poor fetal growth \|\| id:finngen_R11_O15_POOR_FETGRO | Effector Memory CD8+ T cell %T cell \|\| id:ebi-a-GCST90001556 | Weighted mode | 15 | 0.00537228472359475 | 0.0238625875983675 | 0.825127916924404 | -0.041398387 | 0.052142956416395 | 1.0053867413219 | 0.959446822687936 | 1.05352634009882 | 0.999857399747617 |
| ebi-a-GCST90001557 | finngen_R11_O15_POOR_FETGRO | Poor fetal growth \|\| id:finngen_R11_O15_POOR_FETGRO | Terminally Differentiated CD8+ T cell Absolute Count \|\| id:ebi-a-GCST90001557 | Inverse variance weighted | 17 | -0.030953941 | 0.0428387113118325 | 0.469944482205416 | -0.114917816 | 0.0530099328382407 | 0.969520226856997 | 0.891439403394349 | 1.05444011865047 | 0.994119304454654 |
| ebi-a-GCST90001557 | finngen_R11_O15_POOR_FETGRO | Poor fetal growth \|\| id:finngen_R11_O15_POOR_FETGRO | Terminally Differentiated CD8+ T cell Absolute Count \|\| id:ebi-a-GCST90001557 | MR Egger | 17 | 0.0270512445885538 | 0.0738037011976869 | 0.719088521581214 | -0.11760401 | 0.17170649893602 | 1.0274204511528 | 0.88904803726539 | 1.18732929965618 | 0.992899239648112 |
| ebi-a-GCST90001557 | finngen_R11_O15_POOR_FETGRO | Poor fetal growth \|\| id:finngen_R11_O15_POOR_FETGRO | Terminally Differentiated CD8+ T cell Absolute Count \|\| id:ebi-a-GCST90001557 | Weighted median | 17 | -0.003386509 | 0.0525150980230759 | 0.948582942318278 | -0.106316101 | 0.0995430829380146 | 0.996619218567501 | 0.899140383694763 | 1.10466606197424 | 0.999440741871876 |
| ebi-a-GCST90001557 | finngen_R11_O15_POOR_FETGRO | Poor fetal growth \|\| id:finngen_R11_O15_POOR_FETGRO | Terminally Differentiated CD8+ T cell Absolute Count \|\| id:ebi-a-GCST90001557 | Weighted mode | 17 | 0.020744467179867 | 0.0581044133686106 | 0.725741636961336 | -0.093140183 | 0.134629117382344 | 1.02096112922523 | 0.911065775389728 | 1.14411237426076 | 0.999857399747617 |
| ebi-a-GCST90001558 | finngen_R11_O15_POOR_FETGRO | Poor fetal growth \|\| id:finngen_R11_O15_POOR_FETGRO | Terminally Differentiated CD8+ T cell %CD8+ T cell \|\| id:ebi-a-GCST90001558 | Inverse variance weighted | 23 | 0.0126755702038144 | 0.0238735045017849 | 0.595455373179673 | -0.034116499 | 0.0594676390273127 | 1.01275624575298 | 0.966458906950255 | 1.0612714166485 | 0.994119304454654 |
| ebi-a-GCST90001558 | finngen_R11_O15_POOR_FETGRO | Poor fetal growth \|\| id:finngen_R11_O15_POOR_FETGRO | Terminally Differentiated CD8+ T cell %CD8+ T cell \|\| id:ebi-a-GCST90001558 | MR Egger | 23 | 0.0294523841806793 | 0.0373864531712243 | 0.43962611433126 | -0.043825064 | 0.102729832396279 | 1.02989039522938 | 0.957121377780624 | 1.10819197105932 | 0.992899239648112 |
| ebi-a-GCST90001558 | finngen_R11_O15_POOR_FETGRO | Poor fetal growth \|\| id:finngen_R11_O15_POOR_FETGRO | Terminally Differentiated CD8+ T cell %CD8+ T cell \|\| id:ebi-a-GCST90001558 | Weighted median | 23 | -0.003575867 | 0.0312279929355756 | 0.908834790419813 | -0.064782734 | 0.0576309987442707 | 0.996430518390549 | 0.937271078804824 | 1.0593240316837 | 0.999440741871876 |
| ebi-a-GCST90001558 | finngen_R11_O15_POOR_FETGRO | Poor fetal growth \|\| id:finngen_R11_O15_POOR_FETGRO | Terminally Differentiated CD8+ T cell %CD8+ T cell \|\| id:ebi-a-GCST90001558 | Weighted mode | 23 | 0.00030519058176981 | 0.0256890117903075 | 0.990628272742626 | -0.050045273 | 0.0506556536907726 | 1.00030523715715 | 0.951186360915495 | 1.05196059216089 | 0.999857399747617 |
| ebi-a-GCST90001559 | finngen_R11_O15_POOR_FETGRO | Poor fetal growth \|\| id:finngen_R11_O15_POOR_FETGRO | Terminally Differentiated CD8+ T cell %T cell \|\| id:ebi-a-GCST90001559 | Inverse variance weighted | 17 | 0.013740667701347 | 0.028729999461544 | 0.63245876770233 | -0.042570131 | 0.0700514666459733 | 1.01383550455183 | 0.958323254762867 | 1.07256338107355 | 0.994119304454654 |
| ebi-a-GCST90001559 | finngen_R11_O15_POOR_FETGRO | Poor fetal growth \|\| id:finngen_R11_O15_POOR_FETGRO | Terminally Differentiated CD8+ T cell %T cell \|\| id:ebi-a-GCST90001559 | MR Egger | 17 | 0.0521149521649004 | 0.0416101729519785 | 0.229579246896552 | -0.029440987 | 0.133670891150778 | 1.05349683729534 | 0.970988177049588 | 1.14301658086472 | 0.992899239648112 |
| ebi-a-GCST90001559 | finngen_R11_O15_POOR_FETGRO | Poor fetal growth \|\| id:finngen_R11_O15_POOR_FETGRO | Terminally Differentiated CD8+ T cell %T cell \|\| id:ebi-a-GCST90001559 | Weighted median | 17 | 0.008260817165535 | 0.0434825108953837 | 0.84932462716932 | -0.076964904 | 0.0934865385204871 | 1.00829503186455 | 0.925922349031642 | 1.09799582259354 | 0.999440741871876 |
| ebi-a-GCST90001559 | finngen_R11_O15_POOR_FETGRO | Poor fetal growth \|\| id:finngen_R11_O15_POOR_FETGRO | Terminally Differentiated CD8+ T cell %T cell \|\| id:ebi-a-GCST90001559 | Weighted mode | 17 | 0.00327537875155393 | 0.0371478637792223 | 0.93083461127406 | -0.069534434 | 0.0760851917588297 | 1.00328074866577 | 0.932828011593218 | 1.07905449679216 | 0.999857399747617 |
| ebi-a-GCST90001560 | finngen_R11_O15_POOR_FETGRO | Poor fetal growth \|\| id:finngen_R11_O15_POOR_FETGRO | CD45RA+ CD8+ T cell Absolute Count \|\| id:ebi-a-GCST90001560 | Inverse variance weighted | 28 | -0.000453443 | 0.00606455234228183 | 0.940398216731672 | -0.012339965 | 0.0114330798630457 | 0.99954666006179 | 0.987735859839595 | 1.01149868731396 | 0.998984604424952 |
| ebi-a-GCST90001560 | finngen_R11_O15_POOR_FETGRO | Poor fetal growth \|\| id:finngen_R11_O15_POOR_FETGRO | CD45RA+ CD8+ T cell Absolute Count \|\| id:ebi-a-GCST90001560 | MR Egger | 28 | 0.000142844897895529 | 0.00664327880539856 | 0.983009244786483 | -0.012877982 | 0.0131636713564767 | 1.00014285510071 | 0.987204584834314 | 1.01325069390443 | 0.994809183268002 |
| ebi-a-GCST90001560 | finngen_R11_O15_POOR_FETGRO | Poor fetal growth \|\| id:finngen_R11_O15_POOR_FETGRO | CD45RA+ CD8+ T cell Absolute Count \|\| id:ebi-a-GCST90001560 | Weighted median | 28 | 0.00363769092373133 | 0.00807622417506819 | 0.652407802332409 | -0.012191708 | 0.019467090306865 | 1.00364431535146 | 0.98788230931179 | 1.01965780968286 | 0.999440741871876 |
| ebi-a-GCST90001560 | finngen_R11_O15_POOR_FETGRO | Poor fetal growth \|\| id:finngen_R11_O15_POOR_FETGRO | CD45RA+ CD8+ T cell Absolute Count \|\| id:ebi-a-GCST90001560 | Weighted mode | 28 | 0.000755223915492875 | 0.00610698681565867 | 0.902496110902531 | -0.01121447 | 0.0127249180741839 | 1.00075550916888 | 0.988848177522359 | 1.01280622435007 | 0.999857399747617 |
| ebi-a-GCST90001561 | finngen_R11_O15_POOR_FETGRO | Poor fetal growth \|\| id:finngen_R11_O15_POOR_FETGRO | CD45RA+ CD8+ T cell %CD8+ T cell \|\| id:ebi-a-GCST90001561 | Inverse variance weighted | 27 | 0.00738582493308198 | 0.0100431833374108 | 0.462091805202319 | -0.012298814 | 0.0270704642744071 | 1.00741316741219 | 0.987776506905843 | 1.02744019804087 | 0.994119304454654 |
| ebi-a-GCST90001561 | finngen_R11_O15_POOR_FETGRO | Poor fetal growth \|\| id:finngen_R11_O15_POOR_FETGRO | CD45RA+ CD8+ T cell %CD8+ T cell \|\| id:ebi-a-GCST90001561 | MR Egger | 27 | 0.0036930135654672 | 0.0111246348461802 | 0.74268120703484 | -0.018111271 | 0.0254972978639805 | 1.00369984114226 | 0.98205175266019 | 1.02582513434765 | 0.992899239648112 |
| ebi-a-GCST90001561 | finngen_R11_O15_POOR_FETGRO | Poor fetal growth \|\| id:finngen_R11_O15_POOR_FETGRO | CD45RA+ CD8+ T cell %CD8+ T cell \|\| id:ebi-a-GCST90001561 | Weighted median | 27 | 0.0137619630508851 | 0.0138626865657135 | 0.320839503851264 | -0.013408903 | 0.0409328287196835 | 1.01385709476316 | 0.986680596243031 | 1.04178212535601 | 0.999440741871876 |
| ebi-a-GCST90001561 | finngen_R11_O15_POOR_FETGRO | Poor fetal growth \|\| id:finngen_R11_O15_POOR_FETGRO | CD45RA+ CD8+ T cell %CD8+ T cell \|\| id:ebi-a-GCST90001561 | Weighted mode | 27 | 0.00745048769964229 | 0.011126338787391 | 0.508999726024356 | -0.014357136 | 0.0292581117229286 | 1.00747831164084 | 0.985745435890237 | 1.02969033532477 | 0.999857399747617 |
| ebi-a-GCST90001562 | finngen_R11_O15_POOR_FETGRO | Poor fetal growth \|\| id:finngen_R11_O15_POOR_FETGRO | CD45RA+ CD8+ T cell %T cell \|\| id:ebi-a-GCST90001562 | Inverse variance weighted | 35 | 0.00252415861349641 | 0.00444569986251364 | 0.570187547509148 | -0.006189413 | 0.0112377303440231 | 1.00252734698394 | 0.993829701843209 | 1.01130111083077 | 0.994119304454654 |
| ebi-a-GCST90001562 | finngen_R11_O15_POOR_FETGRO | Poor fetal growth \|\| id:finngen_R11_O15_POOR_FETGRO | CD45RA+ CD8+ T cell %T cell \|\| id:ebi-a-GCST90001562 | MR Egger | 35 | 0.00285498847662937 | 0.00479191006746502 | 0.555377905910691 | -0.006537155 | 0.0122471322088608 | 1.00285906783748 | 0.993484165459576 | 1.01232243543472 | 0.992899239648112 |
| ebi-a-GCST90001562 | finngen_R11_O15_POOR_FETGRO | Poor fetal growth \|\| id:finngen_R11_O15_POOR_FETGRO | CD45RA+ CD8+ T cell %T cell \|\| id:ebi-a-GCST90001562 | Weighted median | 35 | 0.00380473811548923 | 0.00615319076412581 | 0.536354009721627 | -0.008255516 | 0.0158649920131758 | 1.00381198531988 | 0.991778467407664 | 1.01599150917808 | 0.999440741871876 |
| ebi-a-GCST90001562 | finngen_R11_O15_POOR_FETGRO | Poor fetal growth \|\| id:finngen_R11_O15_POOR_FETGRO | CD45RA+ CD8+ T cell %T cell \|\| id:ebi-a-GCST90001562 | Weighted mode | 35 | 0.00295819138742398 | 0.00461593769730567 | 0.525906972686742 | -0.006089046 | 0.0120054292741431 | 1.00296257115323 | 0.993929454174806 | 1.0120777836989 | 0.999857399747617 |
| ebi-a-GCST90001563 | finngen_R11_O15_POOR_FETGRO | Poor fetal growth \|\| id:finngen_R11_O15_POOR_FETGRO | Central Memory CD4-CD8- T cell Absolute Count \|\| id:ebi-a-GCST90001563 | Inverse variance weighted | 4 | -0.023006512 | 0.028360478172769 | 0.417240867008659 | -0.078593049 | 0.0325800254340739 | 0.97725612007125 | 0.924416039938538 | 1.03311656543758 | 0.994119304454654 |
| ebi-a-GCST90001563 | finngen_R11_O15_POOR_FETGRO | Poor fetal growth \|\| id:finngen_R11_O15_POOR_FETGRO | Central Memory CD4-CD8- T cell Absolute Count \|\| id:ebi-a-GCST90001563 | MR Egger | 4 | -0.016843247 | 0.0434976007781556 | 0.735912882941383 | -0.102098544 | 0.0684120507805416 | 0.983297807687289 | 0.902940567665578 | 1.07080644421852 | 0.992899239648112 |
| ebi-a-GCST90001563 | finngen_R11_O15_POOR_FETGRO | Poor fetal growth \|\| id:finngen_R11_O15_POOR_FETGRO | Central Memory CD4-CD8- T cell Absolute Count \|\| id:ebi-a-GCST90001563 | Weighted median | 4 | -0.022644488 | 0.0339005826741324 | 0.504154321646277 | -0.08908963 | 0.0438006536958058 | 0.977609973740477 | 0.914763579286481 | 1.04477406227992 | 0.999440741871876 |
| ebi-a-GCST90001563 | finngen_R11_O15_POOR_FETGRO | Poor fetal growth \|\| id:finngen_R11_O15_POOR_FETGRO | Central Memory CD4-CD8- T cell Absolute Count \|\| id:ebi-a-GCST90001563 | Weighted mode | 4 | -0.022947677 | 0.0306238022380382 | 0.508058100171888 | -0.082970329 | 0.0370749755146209 | 0.977313618541123 | 0.920378455126234 | 1.03777082532309 | 0.999857399747617 |
| ebi-a-GCST90001564 | finngen_R11_O15_POOR_FETGRO | Poor fetal growth \|\| id:finngen_R11_O15_POOR_FETGRO | Central Memory CD4-CD8- T cell %CD4-CD8- T cell \|\| id:ebi-a-GCST90001564 | Inverse variance weighted | 16 | 0.0178498299303245 | 0.0306560645078914 | 0.560390909288658 | -0.042236057 | 0.0779357163657917 | 1.01801009026444 | 0.958643459836488 | 1.08105316241032 | 0.994119304454654 |
| ebi-a-GCST90001564 | finngen_R11_O15_POOR_FETGRO | Poor fetal growth \|\| id:finngen_R11_O15_POOR_FETGRO | Central Memory CD4-CD8- T cell %CD4-CD8- T cell \|\| id:ebi-a-GCST90001564 | MR Egger | 16 | 0.0624486025105981 | 0.0414655780089798 | 0.154285045322268 | -0.01882393 | 0.143721135408198 | 1.0644397479812 | 0.98135213332282 | 1.15456209714029 | 0.992899239648112 |
| ebi-a-GCST90001564 | finngen_R11_O15_POOR_FETGRO | Poor fetal growth \|\| id:finngen_R11_O15_POOR_FETGRO | Central Memory CD4-CD8- T cell %CD4-CD8- T cell \|\| id:ebi-a-GCST90001564 | Weighted median | 16 | 0.0195367712758963 | 0.0434199535247482 | 0.65274710501978 | -0.065566338 | 0.104639880184403 | 1.01972886290262 | 0.936536917056952 | 1.11031069346884 | 0.999440741871876 |
| ebi-a-GCST90001564 | finngen_R11_O15_POOR_FETGRO | Poor fetal growth \|\| id:finngen_R11_O15_POOR_FETGRO | Central Memory CD4-CD8- T cell %CD4-CD8- T cell \|\| id:ebi-a-GCST90001564 | Weighted mode | 16 | 0.0222432115726989 | 0.0422581978921079 | 0.606331212246738 | -0.060582856 | 0.10506927944123 | 1.02249243622521 | 0.941215780134394 | 1.11078756243173 | 0.999857399747617 |
| ebi-a-GCST90001565 | finngen_R11_O15_POOR_FETGRO | Poor fetal growth \|\| id:finngen_R11_O15_POOR_FETGRO | Central Memory CD4-CD8- T cell %T cell \|\| id:ebi-a-GCST90001565 | Inverse variance weighted | 16 | 0.00647655373487338 | 0.0324225273408975 | 0.841672394882141 | -0.0570716 | 0.0700247073230325 | 1.00649757195973 | 0.944526438990933 | 1.07253468038767 | 0.994119304454654 |
| ebi-a-GCST90001565 | finngen_R11_O15_POOR_FETGRO | Poor fetal growth \|\| id:finngen_R11_O15_POOR_FETGRO | Central Memory CD4-CD8- T cell %T cell \|\| id:ebi-a-GCST90001565 | MR Egger | 16 | 0.045357449807495 | 0.041558498967457 | 0.293519026897488 | -0.036097208 | 0.126812107783711 | 1.04640182919952 | 0.964546527124221 | 1.13520370180244 | 0.992899239648112 |
| ebi-a-GCST90001565 | finngen_R11_O15_POOR_FETGRO | Poor fetal growth \|\| id:finngen_R11_O15_POOR_FETGRO | Central Memory CD4-CD8- T cell %T cell \|\| id:ebi-a-GCST90001565 | Weighted median | 16 | 0.0366213457575717 | 0.0422499069879007 | 0.38606293364706 | -0.046188472 | 0.119431163453857 | 1.0373001683558 | 0.954861980541826 | 1.12685567254486 | 0.999440741871876 |
| ebi-a-GCST90001565 | finngen_R11_O15_POOR_FETGRO | Poor fetal growth \|\| id:finngen_R11_O15_POOR_FETGRO | Central Memory CD4-CD8- T cell %T cell \|\| id:ebi-a-GCST90001565 | Weighted mode | 16 | 0.0274605745190426 | 0.0378440813160412 | 0.479238396938397 | -0.046713825 | 0.101634973898483 | 1.02784109117905 | 0.954360472756367 | 1.10697932162352 | 0.999857399747617 |
| ebi-a-GCST90001566 | finngen_R11_O15_POOR_FETGRO | Poor fetal growth \|\| id:finngen_R11_O15_POOR_FETGRO | Naive CD4-CD8- T cell Absolute Count \|\| id:ebi-a-GCST90001566 | Inverse variance weighted (multiplicative random effects) | 18 | 0.00526781210000407 | 0.0503092855177772 | 0.916607081097658 | -0.093338388 | 0.103874011714847 | 1.00528171141778 | 0.910885215954765 | 1.10946066706306 | 0.99660269822999 |
| ebi-a-GCST90001566 | finngen_R11_O15_POOR_FETGRO | Poor fetal growth \|\| id:finngen_R11_O15_POOR_FETGRO | Naive CD4-CD8- T cell Absolute Count \|\| id:ebi-a-GCST90001566 | MR Egger | 18 | 0.0804638233643863 | 0.0812462877578965 | 0.336735558215599 | -0.078778901 | 0.239706547369863 | 1.08378963807022 | 0.924244251667465 | 1.27087615364579 | 0.992899239648112 |
| ebi-a-GCST90001566 | finngen_R11_O15_POOR_FETGRO | Poor fetal growth \|\| id:finngen_R11_O15_POOR_FETGRO | Naive CD4-CD8- T cell Absolute Count \|\| id:ebi-a-GCST90001566 | Weighted median | 18 | 0.10205774400235 | 0.0548620035432182 | 0.0628483547283176 | -0.005471783 | 0.209587270947058 | 1.10744741832798 | 0.994543159994668 | 1.23316899024058 | 0.999440741871876 |
| ebi-a-GCST90001566 | finngen_R11_O15_POOR_FETGRO | Poor fetal growth \|\| id:finngen_R11_O15_POOR_FETGRO | Naive CD4-CD8- T cell Absolute Count \|\| id:ebi-a-GCST90001566 | Weighted mode | 18 | 0.0920605266690551 | 0.060775339306728 | 0.148202696107053 | -0.027059138 | 0.211180191710242 | 1.09643118339986 | 0.973303680227074 | 1.23513489608007 | 0.999857399747617 |
| ebi-a-GCST90001567 | finngen_R11_O15_POOR_FETGRO | Poor fetal growth \|\| id:finngen_R11_O15_POOR_FETGRO | Naive CD4-CD8- T cell %CD4-CD8- T cell \|\| id:ebi-a-GCST90001567 | Inverse variance weighted | 23 | 0.0120891680270098 | 0.0330455218596112 | 0.714489115067482 | -0.052680055 | 0.0768583908718477 | 1.01216253737885 | 0.948683490643759 | 1.0798891434044 | 0.994119304454654 |
| ebi-a-GCST90001567 | finngen_R11_O15_POOR_FETGRO | Poor fetal growth \|\| id:finngen_R11_O15_POOR_FETGRO | Naive CD4-CD8- T cell %CD4-CD8- T cell \|\| id:ebi-a-GCST90001567 | MR Egger | 23 | 0.0678112655242162 | 0.0437855789557209 | 0.13639038867065 | -0.018008469 | 0.153631000277429 | 1.07016331270576 | 0.98215271424683 | 1.16606053137023 | 0.992899239648112 |
| ebi-a-GCST90001567 | finngen_R11_O15_POOR_FETGRO | Poor fetal growth \|\| id:finngen_R11_O15_POOR_FETGRO | Naive CD4-CD8- T cell %CD4-CD8- T cell \|\| id:ebi-a-GCST90001567 | Weighted median | 23 | 0.00380204113039968 | 0.0462192044528919 | 0.93443911251903 | -0.0867876 | 0.0943916818580678 | 1.00380927805757 | 0.916871818896357 | 1.09899011611825 | 0.999440741871876 |
| ebi-a-GCST90001567 | finngen_R11_O15_POOR_FETGRO | Poor fetal growth \|\| id:finngen_R11_O15_POOR_FETGRO | Naive CD4-CD8- T cell %CD4-CD8- T cell \|\| id:ebi-a-GCST90001567 | Weighted mode | 23 | 0.00361263006037194 | 0.0446888487085918 | 0.936300406199871 | -0.083977513 | 0.0912027735292119 | 1.00361916347358 | 0.919451931202678 | 1.09549111933877 | 0.999857399747617 |
| ebi-a-GCST90001568 | finngen_R11_O15_POOR_FETGRO | Poor fetal growth \|\| id:finngen_R11_O15_POOR_FETGRO | Naive CD4-CD8- T cell %T cell \|\| id:ebi-a-GCST90001568 | Inverse variance weighted | 21 | -0.020702221 | 0.034312545824002 | 0.546280791040006 | -0.087954811 | 0.0465503687994619 | 0.979510598817372 | 0.915802260131724 | 1.047650847 | 0.994119304454654 |
| ebi-a-GCST90001568 | finngen_R11_O15_POOR_FETGRO | Poor fetal growth \|\| id:finngen_R11_O15_POOR_FETGRO | Naive CD4-CD8- T cell %T cell \|\| id:ebi-a-GCST90001568 | MR Egger | 21 | -0.036163132 | 0.0523381812519638 | 0.49795176419136 | -0.138745967 | 0.0664197037391987 | 0.964482943085708 | 0.870449123378003 | 1.06867515001139 | 0.992899239648112 |
| ebi-a-GCST90001568 | finngen_R11_O15_POOR_FETGRO | Poor fetal growth \|\| id:finngen_R11_O15_POOR_FETGRO | Naive CD4-CD8- T cell %T cell \|\| id:ebi-a-GCST90001568 | Weighted median | 21 | 0.000271051483606396 | 0.0499880102696632 | 0.995673627875601 | -0.097705449 | 0.0982475516121462 | 1.00027108822138 | 0.90691599775161 | 1.10323585912266 | 0.999440741871876 |
| ebi-a-GCST90001568 | finngen_R11_O15_POOR_FETGRO | Poor fetal growth \|\| id:finngen_R11_O15_POOR_FETGRO | Naive CD4-CD8- T cell %T cell \|\| id:ebi-a-GCST90001568 | Weighted mode | 21 | -0.000168346 | 0.0519623317628123 | 0.997447146816711 | -0.102014516 | 0.101677824708414 | 0.999831668622619 | 0.903016443566014 | 1.10702675760036 | 0.999857399747617 |
| ebi-a-GCST90001569 | finngen_R11_O15_POOR_FETGRO | Poor fetal growth \|\| id:finngen_R11_O15_POOR_FETGRO | Effector Memory CD4-CD8- T cell Absolute Count \|\| id:ebi-a-GCST90001569 | Inverse variance weighted | 19 | -0.041553484 | 0.0386658952254816 | 0.282517791761236 | -0.117338639 | 0.0342316705706398 | 0.959298026805322 | 0.889283996179632 | 1.0348243173002 | 0.994119304454654 |
| ebi-a-GCST90001569 | finngen_R11_O15_POOR_FETGRO | Poor fetal growth \|\| id:finngen_R11_O15_POOR_FETGRO | Effector Memory CD4-CD8- T cell Absolute Count \|\| id:ebi-a-GCST90001569 | MR Egger | 19 | -0.085499706 | 0.0698032003382932 | 0.237326799644642 | -0.222313978 | 0.0513145669068323 | 0.918053413185624 | 0.800663933668558 | 1.0526539713111 | 0.992899239648112 |
| ebi-a-GCST90001569 | finngen_R11_O15_POOR_FETGRO | Poor fetal growth \|\| id:finngen_R11_O15_POOR_FETGRO | Effector Memory CD4-CD8- T cell Absolute Count \|\| id:ebi-a-GCST90001569 | Weighted median | 19 | -0.061090791 | 0.0518328910150457 | 0.23855330114792 | -0.162683257 | 0.0405016758181793 | 0.94073782577402 | 0.849860333132294 | 1.04133305478592 | 0.999440741871876 |
| ebi-a-GCST90001569 | finngen_R11_O15_POOR_FETGRO | Poor fetal growth \|\| id:finngen_R11_O15_POOR_FETGRO | Effector Memory CD4-CD8- T cell Absolute Count \|\| id:ebi-a-GCST90001569 | Weighted mode | 19 | -0.059562637 | 0.0675170192871943 | 0.38930499549548 | -0.191895995 | 0.0727707208965809 | 0.942176516720718 | 0.825392709236391 | 1.07548392265451 | 0.999857399747617 |
| ebi-a-GCST90001570 | finngen_R11_O15_POOR_FETGRO | Poor fetal growth \|\| id:finngen_R11_O15_POOR_FETGRO | Effector Memory CD4-CD8- T cell %CD4-CD8- T cell \|\| id:ebi-a-GCST90001570 | Inverse variance weighted | 28 | 0.00450331906689316 | 0.00838311874761372 | 0.591137077802751 | -0.011927594 | 0.0209342318122161 | 1.00451347424648 | 0.988143258090239 | 1.02115488992244 | 0.994119304454654 |
| ebi-a-GCST90001570 | finngen_R11_O15_POOR_FETGRO | Poor fetal growth \|\| id:finngen_R11_O15_POOR_FETGRO | Effector Memory CD4-CD8- T cell %CD4-CD8- T cell \|\| id:ebi-a-GCST90001570 | MR Egger | 28 | 0.0012772644252171 | 0.00881450249665429 | 0.885903436115835 | -0.01599916 | 0.0185536893186595 | 1.00127808047482 | 0.984128146261788 | 1.01872687845334 | 0.992899239648112 |
| ebi-a-GCST90001570 | finngen_R11_O15_POOR_FETGRO | Poor fetal growth \|\| id:finngen_R11_O15_POOR_FETGRO | Effector Memory CD4-CD8- T cell %CD4-CD8- T cell \|\| id:ebi-a-GCST90001570 | Weighted median | 28 | 0.00396523990741237 | 0.00859259320442218 | 0.644460110999174 | -0.012876243 | 0.0208067225880798 | 1.00397311187248 | 0.98720630137473 | 1.02102469155563 | 0.999440741871876 |
| ebi-a-GCST90001570 | finngen_R11_O15_POOR_FETGRO | Poor fetal growth \|\| id:finngen_R11_O15_POOR_FETGRO | Effector Memory CD4-CD8- T cell %CD4-CD8- T cell \|\| id:ebi-a-GCST90001570 | Weighted mode | 28 | 0.00256276247701726 | 0.00765567646564308 | 0.740397959818578 | -0.012442363 | 0.0175678883496777 | 1.00256604915984 | 0.987634722765227 | 1.01772311134802 | 0.999857399747617 |
| ebi-a-GCST90001571 | finngen_R11_O15_POOR_FETGRO | Poor fetal growth \|\| id:finngen_R11_O15_POOR_FETGRO | Effector Memory CD4-CD8- T cell %T cell \|\| id:ebi-a-GCST90001571 | Inverse variance weighted | 23 | 0.00663838300604388 | 0.0302016533486809 | 0.826025382665277 | -0.052556858 | 0.0658336235694584 | 1.00666046590839 | 0.948800373050496 | 1.06804900420182 | 0.994119304454654 |
| ebi-a-GCST90001571 | finngen_R11_O15_POOR_FETGRO | Poor fetal growth \|\| id:finngen_R11_O15_POOR_FETGRO | Effector Memory CD4-CD8- T cell %T cell \|\| id:ebi-a-GCST90001571 | MR Egger | 23 | -0.055089747 | 0.0512998407046651 | 0.295062662606173 | -0.155637435 | 0.045457940577882 | 0.946400207370173 | 0.855869442442092 | 1.04650698820914 | 0.992899239648112 |
| ebi-a-GCST90001571 | finngen_R11_O15_POOR_FETGRO | Poor fetal growth \|\| id:finngen_R11_O15_POOR_FETGRO | Effector Memory CD4-CD8- T cell %T cell \|\| id:ebi-a-GCST90001571 | Weighted median | 23 | 0.00863054837884442 | 0.0425854697146392 | 0.839397539252365 | -0.074836972 | 0.0920980690195372 | 1.00866789893611 | 0.927894746580187 | 1.09647234677631 | 0.999440741871876 |
| ebi-a-GCST90001571 | finngen_R11_O15_POOR_FETGRO | Poor fetal growth \|\| id:finngen_R11_O15_POOR_FETGRO | Effector Memory CD4-CD8- T cell %T cell \|\| id:ebi-a-GCST90001571 | Weighted mode | 23 | -0.019511312 | 0.048131695749604 | 0.689118186045829 | -0.113849435 | 0.0748268119604802 | 0.980677801984258 | 0.892392308478412 | 1.07769749040586 | 0.999857399747617 |
| ebi-a-GCST90001572 | finngen_R11_O15_POOR_FETGRO | Poor fetal growth \|\| id:finngen_R11_O15_POOR_FETGRO | Terminally Differentiated CD4-CD8- T cell Absolute Count \|\| id:ebi-a-GCST90001572 | Inverse variance weighted | 21 | -0.004692998 | 0.00762899332645947 | 0.538453678244362 | -0.019645825 | 0.0102598287285185 | 0.995317996718249 | 0.98054589654812 | 1.01031264123251 | 0.994119304454654 |
| ebi-a-GCST90001572 | finngen_R11_O15_POOR_FETGRO | Poor fetal growth \|\| id:finngen_R11_O15_POOR_FETGRO | Terminally Differentiated CD4-CD8- T cell Absolute Count \|\| id:ebi-a-GCST90001572 | MR Egger | 21 | -0.005039054 | 0.00798135495760693 | 0.535332135981709 | -0.020682509 | 0.0106044021328226 | 0.994973621147936 | 0.979529906841026 | 1.01066082808323 | 0.992899239648112 |
| ebi-a-GCST90001572 | finngen_R11_O15_POOR_FETGRO | Poor fetal growth \|\| id:finngen_R11_O15_POOR_FETGRO | Terminally Differentiated CD4-CD8- T cell Absolute Count \|\| id:ebi-a-GCST90001572 | Weighted median | 21 | -0.003787669 | 0.00840577835462182 | 0.652275688098094 | -0.020262995 | 0.0126876564448743 | 0.996219495040512 | 0.979940920140934 | 1.01276848624307 | 0.999440741871876 |
| ebi-a-GCST90001572 | finngen_R11_O15_POOR_FETGRO | Poor fetal growth \|\| id:finngen_R11_O15_POOR_FETGRO | Terminally Differentiated CD4-CD8- T cell Absolute Count \|\| id:ebi-a-GCST90001572 | Weighted mode | 21 | -0.005652499 | 0.00827719189223747 | 0.502504139730212 | -0.021875795 | 0.0105707968030043 | 0.994363446010643 | 0.978361744519919 | 1.01062686506344 | 0.999857399747617 |
| ebi-a-GCST90001573 | finngen_R11_O15_POOR_FETGRO | Poor fetal growth \|\| id:finngen_R11_O15_POOR_FETGRO | Terminally Differentiated CD4-CD8- T cell %CD4-CD8- T cell \|\| id:ebi-a-GCST90001573 | Inverse variance weighted | 27 | -0.001318818 | 0.00767159979495263 | 0.863508980408167 | -0.016355154 | 0.0137175176654695 | 0.998682051325559 | 0.983777865821491 | 1.01381203449519 | 0.994119304454654 |
| ebi-a-GCST90001573 | finngen_R11_O15_POOR_FETGRO | Poor fetal growth \|\| id:finngen_R11_O15_POOR_FETGRO | Terminally Differentiated CD4-CD8- T cell %CD4-CD8- T cell \|\| id:ebi-a-GCST90001573 | MR Egger | 27 | -0.002846724 | 0.00811369781183291 | 0.728637162167563 | -0.018749572 | 0.0130561233459032 | 0.997157323712352 | 0.981425107722572 | 1.0131417266678 | 0.992899239648112 |
| ebi-a-GCST90001573 | finngen_R11_O15_POOR_FETGRO | Poor fetal growth \|\| id:finngen_R11_O15_POOR_FETGRO | Terminally Differentiated CD4-CD8- T cell %CD4-CD8- T cell \|\| id:ebi-a-GCST90001573 | Weighted median | 27 | -0.003458876 | 0.00857512743621612 | 0.686682286882277 | -0.020266126 | 0.0133483732975544 | 0.996547098544874 | 0.979937851414524 | 1.01343786055889 | 0.999440741871876 |
| ebi-a-GCST90001573 | finngen_R11_O15_POOR_FETGRO | Poor fetal growth \|\| id:finngen_R11_O15_POOR_FETGRO | Terminally Differentiated CD4-CD8- T cell %CD4-CD8- T cell \|\| id:ebi-a-GCST90001573 | Weighted mode | 27 | -0.003425719 | 0.00839257646046698 | 0.686480397610996 | -0.019875169 | 0.0130237307614959 | 0.996580141979944 | 0.980321040160518 | 1.01310890892043 | 0.999857399747617 |
| ebi-a-GCST90001574 | finngen_R11_O15_POOR_FETGRO | Poor fetal growth \|\| id:finngen_R11_O15_POOR_FETGRO | Terminally Differentiated CD4-CD8- T cell %T cell \|\| id:ebi-a-GCST90001574 | Inverse variance weighted | 23 | -0.00372432 | 0.00772197516353865 | 0.629591839952023 | -0.018859391 | 0.0114107517477544 | 0.996282607103639 | 0.981317334696291 | 1.01147610270679 | 0.994119304454654 |
| ebi-a-GCST90001574 | finngen_R11_O15_POOR_FETGRO | Poor fetal growth \|\| id:finngen_R11_O15_POOR_FETGRO | Terminally Differentiated CD4-CD8- T cell %T cell \|\| id:ebi-a-GCST90001574 | MR Egger | 23 | -0.003636194 | 0.00839413315606504 | 0.669298762882701 | -0.020088695 | 0.0128163065587289 | 0.996370408522171 | 0.980111738035996 | 1.01289878740558 | 0.992899239648112 |
| ebi-a-GCST90001574 | finngen_R11_O15_POOR_FETGRO | Poor fetal growth \|\| id:finngen_R11_O15_POOR_FETGRO | Terminally Differentiated CD4-CD8- T cell %T cell \|\| id:ebi-a-GCST90001574 | Weighted median | 23 | -0.003669072 | 0.0087857816688571 | 0.676228831792711 | -0.020889204 | 0.0135510603088312 | 0.996337651056987 | 0.979327464287866 | 1.0136432920684 | 0.999440741871876 |
| ebi-a-GCST90001574 | finngen_R11_O15_POOR_FETGRO | Poor fetal growth \|\| id:finngen_R11_O15_POOR_FETGRO | Terminally Differentiated CD4-CD8- T cell %T cell \|\| id:ebi-a-GCST90001574 | Weighted mode | 23 | -0.003691134 | 0.0072031006605192 | 0.613451853610513 | -0.017809212 | 0.0104269429329747 | 0.996315669500897 | 0.982348435111512 | 1.01048149293416 | 0.999857399747617 |
| ebi-a-GCST90001575 | finngen_R11_O15_POOR_FETGRO | Poor fetal growth \|\| id:finngen_R11_O15_POOR_FETGRO | Naive CD4+ T cell %T cell \|\| id:ebi-a-GCST90001575 | Inverse variance weighted | 34 | -0.019081716 | 0.0217950263470573 | 0.381297655247797 | -0.061799968 | 0.023636535559753 | 0.981099187386909 | 0.940070912512178 | 1.02391809243304 | 0.994119304454654 |
| ebi-a-GCST90001575 | finngen_R11_O15_POOR_FETGRO | Poor fetal growth \|\| id:finngen_R11_O15_POOR_FETGRO | Naive CD4+ T cell %T cell \|\| id:ebi-a-GCST90001575 | MR Egger | 34 | -0.018562421 | 0.0305485484040531 | 0.547716713141137 | -0.078437576 | 0.0413127336127807 | 0.981608799422109 | 0.924559772728242 | 1.04217797867151 | 0.992899239648112 |
| ebi-a-GCST90001575 | finngen_R11_O15_POOR_FETGRO | Poor fetal growth \|\| id:finngen_R11_O15_POOR_FETGRO | Naive CD4+ T cell %T cell \|\| id:ebi-a-GCST90001575 | Weighted median | 34 | -0.006301273 | 0.0332418876976426 | 0.84965533562535 | -0.071455373 | 0.0588528271013875 | 0.993718538599214 | 0.931037826396931 | 1.06061913486076 | 0.999440741871876 |
| ebi-a-GCST90001575 | finngen_R11_O15_POOR_FETGRO | Poor fetal growth \|\| id:finngen_R11_O15_POOR_FETGRO | Naive CD4+ T cell %T cell \|\| id:ebi-a-GCST90001575 | Weighted mode | 34 | -0.025317216 | 0.0297681446938895 | 0.401187081893863 | -0.08366278 | 0.0330283471104903 | 0.975000576707558 | 0.919741358904446 | 1.0335798378279 | 0.999857399747617 |
| ebi-a-GCST90001576 | finngen_R11_O15_POOR_FETGRO | Poor fetal growth \|\| id:finngen_R11_O15_POOR_FETGRO | Transitional B cell %B cell \|\| id:ebi-a-GCST90001576 | Inverse variance weighted | 14 | -0.082309977 | 0.0475538233981482 | 0.0834731143255055 | -0.17551547 | 0.0108955173227586 | 0.920986430260237 | 0.839024417031412 | 1.01095508963208 | 0.994119304454654 |
| ebi-a-GCST90001576 | finngen_R11_O15_POOR_FETGRO | Poor fetal growth \|\| id:finngen_R11_O15_POOR_FETGRO | Transitional B cell %B cell \|\| id:ebi-a-GCST90001576 | MR Egger | 14 | -0.05548528 | 0.088873075134977 | 0.544104355119196 | -0.229676507 | 0.118705947617026 | 0.946025949403361 | 0.794790670209433 | 1.1260387552218 | 0.992899239648112 |
| ebi-a-GCST90001576 | finngen_R11_O15_POOR_FETGRO | Poor fetal growth \|\| id:finngen_R11_O15_POOR_FETGRO | Transitional B cell %B cell \|\| id:ebi-a-GCST90001576 | Weighted median | 14 | -0.065075822 | 0.0649203992815431 | 0.316153311216384 | -0.192319805 | 0.0621681601619825 | 0.936996415338956 | 0.825042973410458 | 1.06414127585239 | 0.999440741871876 |
| ebi-a-GCST90001576 | finngen_R11_O15_POOR_FETGRO | Poor fetal growth \|\| id:finngen_R11_O15_POOR_FETGRO | Transitional B cell %B cell \|\| id:ebi-a-GCST90001576 | Weighted mode | 14 | -0.069331579 | 0.0746019794907927 | 0.369642132379688 | -0.215551459 | 0.076888301059564 | 0.933017260092763 | 0.806096788623492 | 1.07992144357445 | 0.999857399747617 |
| ebi-a-GCST90001577 | finngen_R11_O15_POOR_FETGRO | Poor fetal growth \|\| id:finngen_R11_O15_POOR_FETGRO | Transitional B cell Absolute Count \|\| id:ebi-a-GCST90001577 | Inverse variance weighted | 31 | -0.008482663 | 0.0317083742953059 | 0.789067876024469 | -0.070631077 | 0.0536657505550948 | 0.991553213208843 | 0.93180559353479 | 1.05513186596693 | 0.994119304454654 |
| ebi-a-GCST90001577 | finngen_R11_O15_POOR_FETGRO | Poor fetal growth \|\| id:finngen_R11_O15_POOR_FETGRO | Transitional B cell Absolute Count \|\| id:ebi-a-GCST90001577 | MR Egger | 31 | -0.036957969 | 0.0495050237291406 | 0.461345139381185 | -0.133987815 | 0.0600718776956869 | 0.96371664065173 | 0.874600721266101 | 1.06191287165253 | 0.992899239648112 |
| ebi-a-GCST90001577 | finngen_R11_O15_POOR_FETGRO | Poor fetal growth \|\| id:finngen_R11_O15_POOR_FETGRO | Transitional B cell Absolute Count \|\| id:ebi-a-GCST90001577 | Weighted median | 31 | -0.029853386 | 0.046628586631089 | 0.522017981587812 | -0.121245416 | 0.0615386439266916 | 0.9705878250066 | 0.885816539657761 | 1.063471592 | 0.999440741871876 |
| ebi-a-GCST90001577 | finngen_R11_O15_POOR_FETGRO | Poor fetal growth \|\| id:finngen_R11_O15_POOR_FETGRO | Transitional B cell Absolute Count \|\| id:ebi-a-GCST90001577 | Weighted mode | 31 | -0.023034531 | 0.0422083504542903 | 0.589284158525824 | -0.105762898 | 0.0596938353941393 | 0.977228738020113 | 0.899637928394599 | 1.06151149953908 | 0.999857399747617 |
| ebi-a-GCST90001578 | finngen_R11_O15_POOR_FETGRO | Poor fetal growth \|\| id:finngen_R11_O15_POOR_FETGRO | Transitional B cell %lymphocyte \|\| id:ebi-a-GCST90001578 | Inverse variance weighted | 26 | -0.015650508 | 0.0339990448336734 | 0.645285122217968 | -0.082288636 | 0.0509876200291792 | 0.984471324943345 | 0.921006085074479 | 1.05230986563713 | 0.994119304454654 |
| ebi-a-GCST90001578 | finngen_R11_O15_POOR_FETGRO | Poor fetal growth \|\| id:finngen_R11_O15_POOR_FETGRO | Transitional B cell %lymphocyte \|\| id:ebi-a-GCST90001578 | MR Egger | 26 | -0.060162901 | 0.06881683514145 | 0.390648497441807 | -0.195043898 | 0.0747180955504166 | 0.941611131387221 | 0.822798537885097 | 1.07758033337213 | 0.992899239648112 |
| ebi-a-GCST90001578 | finngen_R11_O15_POOR_FETGRO | Poor fetal growth \|\| id:finngen_R11_O15_POOR_FETGRO | Transitional B cell %lymphocyte \|\| id:ebi-a-GCST90001578 | Weighted median | 26 | -0.024776947 | 0.051800114074032 | 0.632423617581635 | -0.12630517 | 0.0767512770228726 | 0.975527482521123 | 0.881345845298432 | 1.07977347851655 | 0.999440741871876 |
| ebi-a-GCST90001578 | finngen_R11_O15_POOR_FETGRO | Poor fetal growth \|\| id:finngen_R11_O15_POOR_FETGRO | Transitional B cell %lymphocyte \|\| id:ebi-a-GCST90001578 | Weighted mode | 26 | -0.030346603 | 0.0565088148921057 | 0.595998578972075 | -0.14110388 | 0.0804106742810223 | 0.970109232589817 | 0.86839909763037 | 1.08373203717513 | 0.999857399747617 |
| ebi-a-GCST90001579 | finngen_R11_O15_POOR_FETGRO | Poor fetal growth \|\| id:finngen_R11_O15_POOR_FETGRO | CD14- CD16+ monocyte Absolute Count \|\| id:ebi-a-GCST90001579 | Inverse variance weighted | 18 | 0.0309774876227477 | 0.0234471649082947 | 0.18644757644613 | -0.014978956 | 0.0769339308430053 | 1.03146228295711 | 0.985132670913356 | 1.07997072128031 | 0.994119304454654 |
| ebi-a-GCST90001579 | finngen_R11_O15_POOR_FETGRO | Poor fetal growth \|\| id:finngen_R11_O15_POOR_FETGRO | CD14- CD16+ monocyte Absolute Count \|\| id:ebi-a-GCST90001579 | MR Egger | 18 | 0.0255565957346027 | 0.0303368809001721 | 0.411965353051062 | -0.033903691 | 0.08501688229894 | 1.02588596539731 | 0.96666459881996 | 1.08873544690053 | 0.992899239648112 |
| ebi-a-GCST90001579 | finngen_R11_O15_POOR_FETGRO | Poor fetal growth \|\| id:finngen_R11_O15_POOR_FETGRO | CD14- CD16+ monocyte Absolute Count \|\| id:ebi-a-GCST90001579 | Weighted median | 18 | 0.0335884871649898 | 0.0312159287738462 | 0.281925138810432 | -0.027594733 | 0.0947717075617284 | 1.03415894947126 | 0.972782523356027 | 1.09940783997832 | 0.999440741871876 |
| ebi-a-GCST90001579 | finngen_R11_O15_POOR_FETGRO | Poor fetal growth \|\| id:finngen_R11_O15_POOR_FETGRO | CD14- CD16+ monocyte Absolute Count \|\| id:ebi-a-GCST90001579 | Weighted mode | 18 | 0.0277663076881557 | 0.0271704668543705 | 0.321137603937523 | -0.025487807 | 0.0810204227227219 | 1.02815538433568 | 0.974834264709778 | 1.08439304259905 | 0.999857399747617 |
| ebi-a-GCST90001580 | finngen_R11_O15_POOR_FETGRO | Poor fetal growth \|\| id:finngen_R11_O15_POOR_FETGRO | CD14+ CD16+ monocyte Absolute Count \|\| id:ebi-a-GCST90001580 | Inverse variance weighted | 20 | 0.0264420400526517 | 0.0327914468527989 | 0.420029520995979 | -0.037829196 | 0.0907132758841376 | 1.02679473256833 | 0.962877390369207 | 1.09495501023842 | 0.994119304454654 |
| ebi-a-GCST90001580 | finngen_R11_O15_POOR_FETGRO | Poor fetal growth \|\| id:finngen_R11_O15_POOR_FETGRO | CD14+ CD16+ monocyte Absolute Count \|\| id:ebi-a-GCST90001580 | MR Egger | 20 | 0.0224784518473383 | 0.0504446537603612 | 0.661194853398239 | -0.07639307 | 0.121349973217646 | 1.02273299592028 | 0.926451974944053 | 1.1290199699852 | 0.992899239648112 |
| ebi-a-GCST90001580 | finngen_R11_O15_POOR_FETGRO | Poor fetal growth \|\| id:finngen_R11_O15_POOR_FETGRO | CD14+ CD16+ monocyte Absolute Count \|\| id:ebi-a-GCST90001580 | Weighted median | 20 | 0.00868430407775889 | 0.0395263209916705 | 0.82609748717379 | -0.068787285 | 0.0861558932214331 | 1.00872212204138 | 0.933525233718348 | 1.08997623496769 | 0.999440741871876 |
| ebi-a-GCST90001580 | finngen_R11_O15_POOR_FETGRO | Poor fetal growth \|\| id:finngen_R11_O15_POOR_FETGRO | CD14+ CD16+ monocyte Absolute Count \|\| id:ebi-a-GCST90001580 | Weighted mode | 20 | 0.0344232776552769 | 0.0402404092613548 | 0.402966298570818 | -0.044447924 | 0.113294479807532 | 1.03502261596786 | 0.956525410338559 | 1.11996169049579 | 0.999857399747617 |
| ebi-a-GCST90001581 | finngen_R11_O15_POOR_FETGRO | Poor fetal growth \|\| id:finngen_R11_O15_POOR_FETGRO | CD14- CD16- Absolute Count \|\| id:ebi-a-GCST90001581 | Inverse variance weighted | 25 | -0.001052889 | 0.00299652045186904 | 0.725310454341232 | -0.006926069 | 0.00482029137008 | 0.998947665377257 | 0.993097861134607 | 1.00483192766379 | 0.994119304454654 |
| ebi-a-GCST90001581 | finngen_R11_O15_POOR_FETGRO | Poor fetal growth \|\| id:finngen_R11_O15_POOR_FETGRO | CD14- CD16- Absolute Count \|\| id:ebi-a-GCST90001581 | MR Egger | 25 | -0.001206249 | 0.0030702723843534 | 0.698025848849355 | -0.007223982 | 0.00481148526920632 | 0.998794478621287 | 0.99280204776557 | 1.00482307905143 | 0.992899239648112 |
| ebi-a-GCST90001581 | finngen_R11_O15_POOR_FETGRO | Poor fetal growth \|\| id:finngen_R11_O15_POOR_FETGRO | CD14- CD16- Absolute Count \|\| id:ebi-a-GCST90001581 | Weighted median | 25 | -0.000851947 | 0.00324324734306138 | 0.792794777729648 | -0.007208711 | 0.00550481824861925 | 0.999148416259639 | 0.992817209101667 | 1.005519998 | 0.999440741871876 |
| ebi-a-GCST90001581 | finngen_R11_O15_POOR_FETGRO | Poor fetal growth \|\| id:finngen_R11_O15_POOR_FETGRO | CD14- CD16- Absolute Count \|\| id:ebi-a-GCST90001581 | Weighted mode | 25 | -0.000281984 | 0.00297847923886665 | 0.92535983956958 | -0.006119804 | 0.00555583497147041 | 0.999718055417138 | 0.993898884212001 | 1.0055712972446 | 0.999857399747617 |
| ebi-a-GCST90001582 | finngen_R11_O15_POOR_FETGRO | Poor fetal growth \|\| id:finngen_R11_O15_POOR_FETGRO | CD14+ CD16- monocyte Absolute Count \|\| id:ebi-a-GCST90001582 | Inverse variance weighted (multiplicative random effects) | 27 | -0.024117796 | 0.0211459143613052 | 0.254060706142263 | -0.065563788 | 0.0173281958468885 | 0.976170713685506 | 0.936539304464154 | 1.01747919998134 | 0.994119304454654 |
| ebi-a-GCST90001582 | finngen_R11_O15_POOR_FETGRO | Poor fetal growth \|\| id:finngen_R11_O15_POOR_FETGRO | CD14+ CD16- monocyte Absolute Count \|\| id:ebi-a-GCST90001582 | MR Egger | 27 | -0.002506931 | 0.0242751060303825 | 0.918571309127341 | -0.050086139 | 0.0450722763632184 | 0.997496208272089 | 0.951147489815838 | 1.04610346573048 | 0.992899239648112 |
| ebi-a-GCST90001582 | finngen_R11_O15_POOR_FETGRO | Poor fetal growth \|\| id:finngen_R11_O15_POOR_FETGRO | CD14+ CD16- monocyte Absolute Count \|\| id:ebi-a-GCST90001582 | Weighted median | 27 | -0.021042212 | 0.0218197724467668 | 0.334863266964838 | -0.063808966 | 0.0217245423641956 | 0.979177631011786 | 0.938184207844492 | 1.02196223839314 | 0.999440741871876 |
| ebi-a-GCST90001582 | finngen_R11_O15_POOR_FETGRO | Poor fetal growth \|\| id:finngen_R11_O15_POOR_FETGRO | CD14+ CD16- monocyte Absolute Count \|\| id:ebi-a-GCST90001582 | Weighted mode | 27 | -0.015997713 | 0.0198264409650566 | 0.427051011133108 | -0.054857538 | 0.02286211092124 | 0.984129570392645 | 0.946619996045909 | 1.02312545199419 | 0.999857399747617 |
| ebi-a-GCST90001583 | finngen_R11_O15_POOR_FETGRO | Poor fetal growth \|\| id:finngen_R11_O15_POOR_FETGRO | Monocyte Absolute Count \|\| id:ebi-a-GCST90001583 | Inverse variance weighted | 27 | -0.011961028 | 0.0170784870097848 | 0.483704762361397 | -0.045434862 | 0.0215128069895608 | 0.988110221188112 | 0.955581845150135 | 1.02174587574235 | 0.994119304454654 |
| ebi-a-GCST90001583 | finngen_R11_O15_POOR_FETGRO | Poor fetal growth \|\| id:finngen_R11_O15_POOR_FETGRO | Monocyte Absolute Count \|\| id:ebi-a-GCST90001583 | MR Egger | 27 | -0.020741998 | 0.0202117981471669 | 0.314609878360609 | -0.060357123 | 0.0188731261279955 | 0.979471637378915 | 0.941428268224602 | 1.0190523492979 | 0.992899239648112 |
| ebi-a-GCST90001583 | finngen_R11_O15_POOR_FETGRO | Poor fetal growth \|\| id:finngen_R11_O15_POOR_FETGRO | Monocyte Absolute Count \|\| id:ebi-a-GCST90001583 | Weighted median | 27 | -0.011936994 | 0.0208403334433837 | 0.566791454100575 | -0.052784048 | 0.0289100593959915 | 0.98813396911829 | 0.948584839440932 | 1.02933201156887 | 0.999440741871876 |
| ebi-a-GCST90001583 | finngen_R11_O15_POOR_FETGRO | Poor fetal growth \|\| id:finngen_R11_O15_POOR_FETGRO | Monocyte Absolute Count \|\| id:ebi-a-GCST90001583 | Weighted mode | 27 | -0.01492227 | 0.0186212884881991 | 0.430189015966519 | -0.051419995 | 0.0215754557185935 | 0.985188515607756 | 0.949879641895676 | 1.02180988882801 | 0.999857399747617 |
| ebi-a-GCST90001584 | finngen_R11_O15_POOR_FETGRO | Poor fetal growth \|\| id:finngen_R11_O15_POOR_FETGRO | CD14- CD16+ monocyte %monocyte \|\| id:ebi-a-GCST90001584 | Inverse variance weighted | 23 | 0.0151973544599959 | 0.0301003253986822 | 0.613636070845852 | -0.043799283 | 0.074193992241413 | 1.01531342147643 | 0.957146053370724 | 1.07701571792503 | 0.994119304454654 |
| ebi-a-GCST90001584 | finngen_R11_O15_POOR_FETGRO | Poor fetal growth \|\| id:finngen_R11_O15_POOR_FETGRO | CD14- CD16+ monocyte %monocyte \|\| id:ebi-a-GCST90001584 | MR Egger | 23 | 0.0505126614062287 | 0.0440311432755247 | 0.264200399228658 | -0.035788379 | 0.136813702226257 | 1.05181018066667 | 0.964844452828901 | 1.14661451688963 | 0.992899239648112 |
| ebi-a-GCST90001584 | finngen_R11_O15_POOR_FETGRO | Poor fetal growth \|\| id:finngen_R11_O15_POOR_FETGRO | CD14- CD16+ monocyte %monocyte \|\| id:ebi-a-GCST90001584 | Weighted median | 23 | 0.00286739700554079 | 0.043147830217362 | 0.947015436610237 | -0.08170235 | 0.0874371442315703 | 1.00287151192043 | 0.921546215905999 | 1.09137366315674 | 0.999440741871876 |
| ebi-a-GCST90001584 | finngen_R11_O15_POOR_FETGRO | Poor fetal growth \|\| id:finngen_R11_O15_POOR_FETGRO | CD14- CD16+ monocyte %monocyte \|\| id:ebi-a-GCST90001584 | Weighted mode | 23 | -0.010683382 | 0.0571538461592172 | 0.853433890805883 | -0.122704921 | 0.101338156225757 | 0.989373482399398 | 0.884524628948131 | 1.10665079935554 | 0.999857399747617 |
| ebi-a-GCST90001585 | finngen_R11_O15_POOR_FETGRO | Poor fetal growth \|\| id:finngen_R11_O15_POOR_FETGRO | CD14+ CD16+ monocyte %monocyte \|\| id:ebi-a-GCST90001585 | Inverse variance weighted (multiplicative random effects) | 24 | 0.015507900603195 | 0.0360076971201769 | 0.666698930187569 | -0.055067186 | 0.0860829869587417 | 1.01562877210654 | 0.946421559772863 | 1.08989677177069 | 0.994119304454654 |
| ebi-a-GCST90001585 | finngen_R11_O15_POOR_FETGRO | Poor fetal growth \|\| id:finngen_R11_O15_POOR_FETGRO | CD14+ CD16+ monocyte %monocyte \|\| id:ebi-a-GCST90001585 | MR Egger | 24 | 0.0238398850697418 | 0.064494376578136 | 0.715182920641425 | -0.102569093 | 0.150248863162888 | 1.02412632684687 | 0.902515790054035 | 1.16212341645353 | 0.992899239648112 |
| ebi-a-GCST90001585 | finngen_R11_O15_POOR_FETGRO | Poor fetal growth \|\| id:finngen_R11_O15_POOR_FETGRO | CD14+ CD16+ monocyte %monocyte \|\| id:ebi-a-GCST90001585 | Weighted median | 24 | 0.00161253236129405 | 0.0406964278149827 | 0.968393341739573 | -0.078152466 | 0.08137753087866 | 1.00161383319072 | 0.924823411523203 | 1.08478035735132 | 0.999440741871876 |
| ebi-a-GCST90001585 | finngen_R11_O15_POOR_FETGRO | Poor fetal growth \|\| id:finngen_R11_O15_POOR_FETGRO | CD14+ CD16+ monocyte %monocyte \|\| id:ebi-a-GCST90001585 | Weighted mode | 24 | 0.0357835865845613 | 0.0431725063507919 | 0.415707714572525 | -0.048834526 | 0.120401699032113 | 1.03643152453257 | 0.952338704086102 | 1.12794985695318 | 0.999857399747617 |
| ebi-a-GCST90001586 | finngen_R11_O15_POOR_FETGRO | Poor fetal growth \|\| id:finngen_R11_O15_POOR_FETGRO | CD14+ CD16- monocyte %monocyte \|\| id:ebi-a-GCST90001586 | Inverse variance weighted | 24 | -0.004218467 | 0.013476737085217 | 0.754266641484627 | -0.030632872 | 0.022195937272205 | 0.995790417820429 | 0.969831559947839 | 1.02244409975307 | 0.994119304454654 |
| ebi-a-GCST90001586 | finngen_R11_O15_POOR_FETGRO | Poor fetal growth \|\| id:finngen_R11_O15_POOR_FETGRO | CD14+ CD16- monocyte %monocyte \|\| id:ebi-a-GCST90001586 | MR Egger | 24 | -0.008184016 | 0.0151963612253993 | 0.595604472158421 | -0.037968884 | 0.0216008520165715 | 0.991849381901895 | 0.962742897145647 | 1.02183583934595 | 0.992899239648112 |
| ebi-a-GCST90001586 | finngen_R11_O15_POOR_FETGRO | Poor fetal growth \|\| id:finngen_R11_O15_POOR_FETGRO | CD14+ CD16- monocyte %monocyte \|\| id:ebi-a-GCST90001586 | Weighted median | 24 | -0.005993254 | 0.0159557746917894 | 0.707201956733933 | -0.037266572 | 0.0252800647433555 | 0.994024670067111 | 0.96341928046429 | 1.02560231535521 | 0.999440741871876 |
| ebi-a-GCST90001586 | finngen_R11_O15_POOR_FETGRO | Poor fetal growth \|\| id:finngen_R11_O15_POOR_FETGRO | CD14+ CD16- monocyte %monocyte \|\| id:ebi-a-GCST90001586 | Weighted mode | 24 | -0.007818931 | 0.0144454455689164 | 0.59352406578364 | -0.036132004 | 0.0204941427450227 | 0.992211557753798 | 0.964512965620702 | 1.02070558969279 | 0.999857399747617 |
| ebi-a-GCST90001587 | finngen_R11_O15_POOR_FETGRO | Poor fetal growth \|\| id:finngen_R11_O15_POOR_FETGRO | CD16+ monocyte %monocyte \|\| id:ebi-a-GCST90001587 | Inverse variance weighted | 22 | -0.010038439 | 0.037128003555955 | 0.786872711041531 | -0.082809326 | 0.06273244836908 | 0.990011778350445 | 0.920526651381824 | 1.06474192767947 | 0.994119304454654 |
| ebi-a-GCST90001587 | finngen_R11_O15_POOR_FETGRO | Poor fetal growth \|\| id:finngen_R11_O15_POOR_FETGRO | CD16+ monocyte %monocyte \|\| id:ebi-a-GCST90001587 | MR Egger | 22 | 0.0398775174881351 | 0.0654495525030157 | 0.549194193810091 | -0.088403605 | 0.168158640394046 | 1.04068330088117 | 0.915391345249562 | 1.18312428706399 | 0.992899239648112 |
| ebi-a-GCST90001587 | finngen_R11_O15_POOR_FETGRO | Poor fetal growth \|\| id:finngen_R11_O15_POOR_FETGRO | CD16+ monocyte %monocyte \|\| id:ebi-a-GCST90001587 | Weighted median | 22 | 0.00328038969528172 | 0.0516037613049499 | 0.949313567544358 | -0.097862982 | 0.104423761852983 | 1.00328577606174 | 0.906773139065177 | 1.11007076090226 | 0.999440741871876 |
| ebi-a-GCST90001587 | finngen_R11_O15_POOR_FETGRO | Poor fetal growth \|\| id:finngen_R11_O15_POOR_FETGRO | CD16+ monocyte %monocyte \|\| id:ebi-a-GCST90001587 | Weighted mode | 22 | 0.0210463216548181 | 0.0632690770808164 | 0.742697952593451 | -0.102961069 | 0.145053712733218 | 1.02126935742853 | 0.902162094488232 | 1.1561016659807 | 0.999857399747617 |
| ebi-a-GCST90001588 | finngen_R11_O15_POOR_FETGRO | Poor fetal growth \|\| id:finngen_R11_O15_POOR_FETGRO | T/B cell \|\| id:ebi-a-GCST90001588 | Inverse variance weighted | 28 | -0.006242252 | 0.0272421225352741 | 0.818760328440911 | -0.059636812 | 0.0471523080468913 | 0.993777190257752 | 0.94210663300665 | 1.04828165865341 | 0.994119304454654 |
| ebi-a-GCST90001588 | finngen_R11_O15_POOR_FETGRO | Poor fetal growth \|\| id:finngen_R11_O15_POOR_FETGRO | T/B cell \|\| id:ebi-a-GCST90001588 | MR Egger | 28 | -0.03804011 | 0.050240830320353 | 0.455764221836235 | -0.136512137 | 0.0604319177428789 | 0.962674327552772 | 0.872395731829871 | 1.06229527164847 | 0.992899239648112 |
| ebi-a-GCST90001588 | finngen_R11_O15_POOR_FETGRO | Poor fetal growth \|\| id:finngen_R11_O15_POOR_FETGRO | T/B cell \|\| id:ebi-a-GCST90001588 | Weighted median | 28 | -0.003557632 | 0.0396693086230109 | 0.928539735181225 | -0.081309477 | 0.0741942127286 | 0.996448688702831 | 0.921908337797168 | 1.07701595539322 | 0.999440741871876 |
| ebi-a-GCST90001588 | finngen_R11_O15_POOR_FETGRO | Poor fetal growth \|\| id:finngen_R11_O15_POOR_FETGRO | T/B cell \|\| id:ebi-a-GCST90001588 | Weighted mode | 28 | 0.0478545907864515 | 0.065717702744908 | 0.472769450496515 | -0.080952107 | 0.176661288166471 | 1.04901810733581 | 0.922237859499552 | 1.19322686461338 | 0.999857399747617 |
| ebi-a-GCST90001589 | finngen_R11_O15_POOR_FETGRO | Poor fetal growth \|\| id:finngen_R11_O15_POOR_FETGRO | CD4+/CD8+ T cell \|\| id:ebi-a-GCST90001589 | Inverse variance weighted | 14 | -0.032306653 | 0.0487724914489929 | 0.507718131634162 | -0.127900736 | 0.0632874306213989 | 0.96820963253215 | 0.879940721369739 | 1.06533300455605 | 0.994119304454654 |
| ebi-a-GCST90001589 | finngen_R11_O15_POOR_FETGRO | Poor fetal growth \|\| id:finngen_R11_O15_POOR_FETGRO | CD4+/CD8+ T cell \|\| id:ebi-a-GCST90001589 | MR Egger | 14 | 0.0813498128075255 | 0.087360609881475 | 0.370108413313771 | -0.089876983 | 0.252576608175216 | 1.08475028974892 | 0.914043621661502 | 1.28733811300104 | 0.992899239648112 |
| ebi-a-GCST90001589 | finngen_R11_O15_POOR_FETGRO | Poor fetal growth \|\| id:finngen_R11_O15_POOR_FETGRO | CD4+/CD8+ T cell \|\| id:ebi-a-GCST90001589 | Weighted median | 14 | -0.010361226 | 0.0552434445753311 | 0.851224916787093 | -0.118638377 | 0.0979159256775628 | 0.989692266899806 | 0.88812891048785 | 1.10287005815771 | 0.999440741871876 |
| ebi-a-GCST90001589 | finngen_R11_O15_POOR_FETGRO | Poor fetal growth \|\| id:finngen_R11_O15_POOR_FETGRO | CD4+/CD8+ T cell \|\| id:ebi-a-GCST90001589 | Weighted mode | 14 | -0.008656579 | 0.0643410209827925 | 0.89503459759482 | -0.13476498 | 0.117451821975225 | 0.991380781148398 | 0.873921276290719 | 1.12462744630954 | 0.999857399747617 |
| ebi-a-GCST90001590 | finngen_R11_O15_POOR_FETGRO | Poor fetal growth \|\| id:finngen_R11_O15_POOR_FETGRO | CD4+ T cell Absolute Count \|\| id:ebi-a-GCST90001590 | Inverse variance weighted (multiplicative random effects) | 22 | -0.00798018 | 0.0292072987134017 | 0.784679856394447 | -0.065226485 | 0.0492661258134758 | 0.992051577437009 | 0.936855255550772 | 1.05049987867835 | 0.994119304454654 |
| ebi-a-GCST90001590 | finngen_R11_O15_POOR_FETGRO | Poor fetal growth \|\| id:finngen_R11_O15_POOR_FETGRO | CD4+ T cell Absolute Count \|\| id:ebi-a-GCST90001590 | MR Egger | 22 | -0.045796742 | 0.0370038913267902 | 0.230190492840642 | -0.118324369 | 0.026730884698821 | 0.955236101551233 | 0.888407833643386 | 1.02709135956704 | 0.992899239648112 |
| ebi-a-GCST90001590 | finngen_R11_O15_POOR_FETGRO | Poor fetal growth \|\| id:finngen_R11_O15_POOR_FETGRO | CD4+ T cell Absolute Count \|\| id:ebi-a-GCST90001590 | Weighted median | 22 | -0.038295822 | 0.0335467164185474 | 0.253634071492503 | -0.104047387 | 0.027455741690858 | 0.962428190872162 | 0.90118259236741 | 1.02783612381166 | 0.999440741871876 |
| ebi-a-GCST90001590 | finngen_R11_O15_POOR_FETGRO | Poor fetal growth \|\| id:finngen_R11_O15_POOR_FETGRO | CD4+ T cell Absolute Count \|\| id:ebi-a-GCST90001590 | Weighted mode | 22 | -0.035072469 | 0.0302004559880471 | 0.25854150843828 | -0.094265363 | 0.0241204242822747 | 0.965535441895516 | 0.910041238749524 | 1.02441367474443 | 0.999857399747617 |
| ebi-a-GCST90001591 | finngen_R11_O15_POOR_FETGRO | Poor fetal growth \|\| id:finngen_R11_O15_POOR_FETGRO | CD4+ T cell %T cell \|\| id:ebi-a-GCST90001591 | Inverse variance weighted | 22 | 0.0431216366147375 | 0.0313301300196914 | 0.168709229014018 | -0.018285418 | 0.104528691453333 | 1.04406488364523 | 0.981880745702265 | 1.11018724629484 | 0.994119304454654 |
| ebi-a-GCST90001591 | finngen_R11_O15_POOR_FETGRO | Poor fetal growth \|\| id:finngen_R11_O15_POOR_FETGRO | CD4+ T cell %T cell \|\| id:ebi-a-GCST90001591 | MR Egger | 22 | 0.0656843460935647 | 0.0504257530146303 | 0.207513387290568 | -0.03315013 | 0.16451882200224 | 1.06788958044182 | 0.96739331410708 | 1.17882575720384 | 0.992899239648112 |
| ebi-a-GCST90001591 | finngen_R11_O15_POOR_FETGRO | Poor fetal growth \|\| id:finngen_R11_O15_POOR_FETGRO | CD4+ T cell %T cell \|\| id:ebi-a-GCST90001591 | Weighted median | 22 | 0.0366804609584541 | 0.0454812201118422 | 0.419956420989453 | -0.05246273 | 0.125823652377665 | 1.03736149037614 | 0.948889685078152 | 1.13408215795578 | 0.999440741871876 |
| ebi-a-GCST90001591 | finngen_R11_O15_POOR_FETGRO | Poor fetal growth \|\| id:finngen_R11_O15_POOR_FETGRO | CD4+ T cell %T cell \|\| id:ebi-a-GCST90001591 | Weighted mode | 22 | 0.11175729272427 | 0.0603559790283913 | 0.078191681059761 | -0.006540426 | 0.230055011619917 | 1.11824142237712 | 0.993480915861862 | 1.25866924945932 | 0.999857399747617 |
| ebi-a-GCST90001592 | finngen_R11_O15_POOR_FETGRO | Poor fetal growth \|\| id:finngen_R11_O15_POOR_FETGRO | CD8+ T cell Absolute Count \|\| id:ebi-a-GCST90001592 | Inverse variance weighted | 21 | -0.024442033 | 0.0159452661797343 | 0.125307688020374 | -0.055694755 | 0.0068106885444611 | 0.975854254458731 | 0.945827801151894 | 1.0068339340263 | 0.994119304454654 |
| ebi-a-GCST90001592 | finngen_R11_O15_POOR_FETGRO | Poor fetal growth \|\| id:finngen_R11_O15_POOR_FETGRO | CD8+ T cell Absolute Count \|\| id:ebi-a-GCST90001592 | MR Egger | 21 | -0.025582999 | 0.0190572328439185 | 0.195277522603209 | -0.062935175 | 0.0117691775217829 | 0.974741473184683 | 0.93900434246014 | 1.01183870679126 | 0.992899239648112 |
| ebi-a-GCST90001592 | finngen_R11_O15_POOR_FETGRO | Poor fetal growth \|\| id:finngen_R11_O15_POOR_FETGRO | CD8+ T cell Absolute Count \|\| id:ebi-a-GCST90001592 | Weighted median | 21 | -0.022193173 | 0.0197209373099196 | 0.260436113954396 | -0.06084621 | 0.0164598644921459 | 0.97805128405772 | 0.940967940331757 | 1.01659607436752 | 0.999440741871876 |
| ebi-a-GCST90001592 | finngen_R11_O15_POOR_FETGRO | Poor fetal growth \|\| id:finngen_R11_O15_POOR_FETGRO | CD8+ T cell Absolute Count \|\| id:ebi-a-GCST90001592 | Weighted mode | 21 | -0.023597427 | 0.0176177530383032 | 0.195460030698795 | -0.058128223 | 0.0109333687150751 | 0.97667881491204 | 0.943528957382539 | 1.01099335641401 | 0.999857399747617 |
| ebi-a-GCST90001593 | finngen_R11_O15_POOR_FETGRO | Poor fetal growth \|\| id:finngen_R11_O15_POOR_FETGRO | CD8+ T cell %T cell \|\| id:ebi-a-GCST90001593 | Inverse variance weighted | 22 | -0.026622663 | 0.0269099557871879 | 0.322504680040917 | -0.079366177 | 0.0261208501529137 | 0.973728595854013 | 0.923701624651829 | 1.02646498942904 | 0.994119304454654 |
| ebi-a-GCST90001593 | finngen_R11_O15_POOR_FETGRO | Poor fetal growth \|\| id:finngen_R11_O15_POOR_FETGRO | CD8+ T cell %T cell \|\| id:ebi-a-GCST90001593 | MR Egger | 22 | -0.107331462 | 0.0422882826685818 | 0.0195708971803937 | -0.190216496 | -0.024446428 | 0.898227894981073 | 0.826780119725403 | 0.975849965514526 | 0.992899239648112 |
| ebi-a-GCST90001593 | finngen_R11_O15_POOR_FETGRO | Poor fetal growth \|\| id:finngen_R11_O15_POOR_FETGRO | CD8+ T cell %T cell \|\| id:ebi-a-GCST90001593 | Weighted median | 22 | -0.04903815 | 0.0409693421313784 | 0.231327032499081 | -0.129338061 | 0.0312617604216831 | 0.952144804532949 | 0.878676869183358 | 1.03175554130799 | 0.999440741871876 |
| ebi-a-GCST90001593 | finngen_R11_O15_POOR_FETGRO | Poor fetal growth \|\| id:finngen_R11_O15_POOR_FETGRO | CD8+ T cell %T cell \|\| id:ebi-a-GCST90001593 | Weighted mode | 22 | -0.04913162 | 0.0394047084416575 | 0.226182029862848 | -0.126364848 | 0.0281016088839282 | 0.952055812187622 | 0.881293249857576 | 1.0285001838681 | 0.999857399747617 |
| ebi-a-GCST90001594 | finngen_R11_O15_POOR_FETGRO | Poor fetal growth \|\| id:finngen_R11_O15_POOR_FETGRO | CD4+CD8+ T cell Absolute Count \|\| id:ebi-a-GCST90001594 | Inverse variance weighted | 18 | 0.0256854238186548 | 0.0366278639741811 | 0.483144725014618 | -0.04610519 | 0.0974760372080498 | 1.0260181368342 | 0.954941507020184 | 1.10238502502382 | 0.994119304454654 |
| ebi-a-GCST90001594 | finngen_R11_O15_POOR_FETGRO | Poor fetal growth \|\| id:finngen_R11_O15_POOR_FETGRO | CD4+CD8+ T cell Absolute Count \|\| id:ebi-a-GCST90001594 | MR Egger | 18 | 0.0293770238113668 | 0.0683803870429591 | 0.673207566158016 | -0.104648535 | 0.163402582415567 | 1.02981278523324 | 0.900641010945179 | 1.17751063935771 | 0.992899239648112 |
| ebi-a-GCST90001594 | finngen_R11_O15_POOR_FETGRO | Poor fetal growth \|\| id:finngen_R11_O15_POOR_FETGRO | CD4+CD8+ T cell Absolute Count \|\| id:ebi-a-GCST90001594 | Weighted median | 18 | 0.0136948775500663 | 0.0542595391589949 | 0.800735178159291 | -0.092653819 | 0.120043574301696 | 1.01378908193356 | 0.91150899259514 | 1.12754598253776 | 0.999440741871876 |
| ebi-a-GCST90001594 | finngen_R11_O15_POOR_FETGRO | Poor fetal growth \|\| id:finngen_R11_O15_POOR_FETGRO | CD4+CD8+ T cell Absolute Count \|\| id:ebi-a-GCST90001594 | Weighted mode | 18 | -0.005965299 | 0.0628533373163941 | 0.925497450317815 | -0.12915784 | 0.117227242014431 | 0.994052457944837 | 0.878835239009805 | 1.12437490588056 | 0.999857399747617 |
| ebi-a-GCST90001595 | finngen_R11_O15_POOR_FETGRO | Poor fetal growth \|\| id:finngen_R11_O15_POOR_FETGRO | CD4+CD8+ T cell %T cell \|\| id:ebi-a-GCST90001595 | Inverse variance weighted | 30 | -0.000256742 | 0.0358097753708341 | 0.994279520041474 | -0.070443902 | 0.0699304172513127 | 0.999743290480007 | 0.931980020085949 | 1.07243355578333 | 0.998984604424952 |
| ebi-a-GCST90001595 | finngen_R11_O15_POOR_FETGRO | Poor fetal growth \|\| id:finngen_R11_O15_POOR_FETGRO | CD4+CD8+ T cell %T cell \|\| id:ebi-a-GCST90001595 | MR Egger | 30 | -0.02463513 | 0.0799674551627855 | 0.760313427497158 | -0.181371342 | 0.132101082333758 | 0.975665838494866 | 0.834125555405657 | 1.14122367098913 | 0.992899239648112 |
| ebi-a-GCST90001595 | finngen_R11_O15_POOR_FETGRO | Poor fetal growth \|\| id:finngen_R11_O15_POOR_FETGRO | CD4+CD8+ T cell %T cell \|\| id:ebi-a-GCST90001595 | Weighted median | 30 | -0.01051193 | 0.042961421207835 | 0.806701873917878 | -0.094716316 | 0.0736924551370627 | 0.989543126822178 | 0.909630945616997 | 1.07647569001386 | 0.999440741871876 |
| ebi-a-GCST90001595 | finngen_R11_O15_POOR_FETGRO | Poor fetal growth \|\| id:finngen_R11_O15_POOR_FETGRO | CD4+CD8+ T cell %T cell \|\| id:ebi-a-GCST90001595 | Weighted mode | 30 | -0.017625043 | 0.0491284062929445 | 0.722377383779811 | -0.113916719 | 0.0786666333974796 | 0.982529369625704 | 0.892332266869846 | 1.08184361142002 | 0.999857399747617 |
| ebi-a-GCST90001596 | finngen_R11_O15_POOR_FETGRO | Poor fetal growth \|\| id:finngen_R11_O15_POOR_FETGRO | CD8dim T cell Absolute Count \|\| id:ebi-a-GCST90001596 | Inverse variance weighted | 27 | 0.0213606845907085 | 0.0232995951015426 | 0.359256095021082 | -0.024306522 | 0.067027890989732 | 1.02159045713034 | 0.975986502755801 | 1.06932530229971 | 0.994119304454654 |
| ebi-a-GCST90001596 | finngen_R11_O15_POOR_FETGRO | Poor fetal growth \|\| id:finngen_R11_O15_POOR_FETGRO | CD8dim T cell Absolute Count \|\| id:ebi-a-GCST90001596 | MR Egger | 27 | 0.00651741440130472 | 0.0319003683061314 | 0.839769048855383 | -0.056007307 | 0.0690421362813223 | 1.00653869896152 | 0.945532226408534 | 1.07148135643704 | 0.992899239648112 |
| ebi-a-GCST90001596 | finngen_R11_O15_POOR_FETGRO | Poor fetal growth \|\| id:finngen_R11_O15_POOR_FETGRO | CD8dim T cell Absolute Count \|\| id:ebi-a-GCST90001596 | Weighted median | 27 | 0.0237842700575095 | 0.0261932093985716 | 0.363861360723566 | -0.02755442 | 0.0751229604787099 | 1.02406937163247 | 0.972821739799999 | 1.07801669618457 | 0.999440741871876 |
| ebi-a-GCST90001596 | finngen_R11_O15_POOR_FETGRO | Poor fetal growth \|\| id:finngen_R11_O15_POOR_FETGRO | CD8dim T cell Absolute Count \|\| id:ebi-a-GCST90001596 | Weighted mode | 27 | 0.0210032472281525 | 0.0221749943407238 | 0.352278347053341 | -0.022459742 | 0.0644662361359712 | 1.02122536778391 | 0.977790600608197 | 1.06658956545162 | 0.999857399747617 |
| ebi-a-GCST90001597 | finngen_R11_O15_POOR_FETGRO | Poor fetal growth \|\| id:finngen_R11_O15_POOR_FETGRO | CD8dim T cell %T cell \|\| id:ebi-a-GCST90001597 | Inverse variance weighted | 19 | 0.03099069677673 | 0.0331005683409564 | 0.349139980451931 | -0.033886417 | 0.0958678107250045 | 1.03147590779122 | 0.966681296798042 | 1.10061356506828 | 0.994119304454654 |
| ebi-a-GCST90001597 | finngen_R11_O15_POOR_FETGRO | Poor fetal growth \|\| id:finngen_R11_O15_POOR_FETGRO | CD8dim T cell %T cell \|\| id:ebi-a-GCST90001597 | MR Egger | 19 | 0.0696362654430056 | 0.0652295335804732 | 0.300646575096394 | -0.05821362 | 0.197486151260733 | 1.07211814390537 | 0.94344838611099 | 1.21833619243254 | 0.992899239648112 |
| ebi-a-GCST90001597 | finngen_R11_O15_POOR_FETGRO | Poor fetal growth \|\| id:finngen_R11_O15_POOR_FETGRO | CD8dim T cell %T cell \|\| id:ebi-a-GCST90001597 | Weighted median | 19 | 0.053311755761585 | 0.0480264552946878 | 0.266977579405284 | -0.040820097 | 0.147443608139173 | 1.05475842088261 | 0.960001821989811 | 1.15886793226794 | 0.999440741871876 |
| ebi-a-GCST90001597 | finngen_R11_O15_POOR_FETGRO | Poor fetal growth \|\| id:finngen_R11_O15_POOR_FETGRO | CD8dim T cell %T cell \|\| id:ebi-a-GCST90001597 | Weighted mode | 19 | 0.0408592319163884 | 0.0577583143171188 | 0.488365987894149 | -0.072347064 | 0.154065527977941 | 1.04170545634318 | 0.93020799793768 | 1.166567327 | 0.999857399747617 |
| ebi-a-GCST90001598 | finngen_R11_O15_POOR_FETGRO | Poor fetal growth \|\| id:finngen_R11_O15_POOR_FETGRO | CD4-CD8- T cell Absolute Count \|\| id:ebi-a-GCST90001598 | Inverse variance weighted | 20 | -0.016980086 | 0.0376447875600189 | 0.651945772110727 | -0.090763869 | 0.0568036978636472 | 0.983163263395056 | 0.913233327800931 | 1.05844801439432 | 0.994119304454654 |
| ebi-a-GCST90001598 | finngen_R11_O15_POOR_FETGRO | Poor fetal growth \|\| id:finngen_R11_O15_POOR_FETGRO | CD4-CD8- T cell Absolute Count \|\| id:ebi-a-GCST90001598 | MR Egger | 20 | -0.005472145 | 0.082041954234094 | 0.947556326814828 | -0.166274375 | 0.155330085419428 | 0.994542800032726 | 0.846813857608573 | 1.16804345159186 | 0.992899239648112 |
| ebi-a-GCST90001598 | finngen_R11_O15_POOR_FETGRO | Poor fetal growth \|\| id:finngen_R11_O15_POOR_FETGRO | CD4-CD8- T cell Absolute Count \|\| id:ebi-a-GCST90001598 | Weighted median | 20 | -0.010704783 | 0.0532225737970702 | 0.8405950641669 | -0.115021028 | 0.0936114617339591 | 0.989352309378478 | 0.891347400857237 | 1.09813299632799 | 0.999440741871876 |
| ebi-a-GCST90001598 | finngen_R11_O15_POOR_FETGRO | Poor fetal growth \|\| id:finngen_R11_O15_POOR_FETGRO | CD4-CD8- T cell Absolute Count \|\| id:ebi-a-GCST90001598 | Weighted mode | 20 | -0.011182205 | 0.0648711017468279 | 0.864965012965497 | -0.138329564 | 0.115965154577923 | 0.988880083616442 | 0.870811656042621 | 1.12295674154986 | 0.999857399747617 |
| ebi-a-GCST90001599 | finngen_R11_O15_POOR_FETGRO | Poor fetal growth \|\| id:finngen_R11_O15_POOR_FETGRO | CD4-CD8- T cell %T cell \|\| id:ebi-a-GCST90001599 | Inverse variance weighted | 15 | -0.021305009 | 0.0458321028958197 | 0.642039572667123 | -0.11113593 | 0.0685259130534817 | 0.978920339885988 | 0.894817107905617 | 1.0709283756157 | 0.994119304454654 |
| ebi-a-GCST90001599 | finngen_R11_O15_POOR_FETGRO | Poor fetal growth \|\| id:finngen_R11_O15_POOR_FETGRO | CD4-CD8- T cell %T cell \|\| id:ebi-a-GCST90001599 | MR Egger | 15 | -0.184345802 | 0.104369191649807 | 0.100804242055423 | -0.388909417 | 0.0202178140805087 | 0.831648168868792 | 0.677795663871367 | 1.02042357844602 | 0.992899239648112 |
| ebi-a-GCST90001599 | finngen_R11_O15_POOR_FETGRO | Poor fetal growth \|\| id:finngen_R11_O15_POOR_FETGRO | CD4-CD8- T cell %T cell \|\| id:ebi-a-GCST90001599 | Weighted median | 15 | 0.00786735022608698 | 0.0579679496365596 | 0.892043453203337 | -0.105749831 | 0.121484531513744 | 1.00789837914429 | 0.899649684332698 | 1.12917189921006 | 0.999440741871876 |
| ebi-a-GCST90001599 | finngen_R11_O15_POOR_FETGRO | Poor fetal growth \|\| id:finngen_R11_O15_POOR_FETGRO | CD4-CD8- T cell %T cell \|\| id:ebi-a-GCST90001599 | Weighted mode | 15 | 0.00317133964929084 | 0.0764332429888923 | 0.967489897510694 | -0.146637817 | 0.15298049590752 | 1.003176374 | 0.863606704785454 | 1.16530225055823 | 0.999857399747617 |
| ebi-a-GCST90001600 | finngen_R11_O15_POOR_FETGRO | Poor fetal growth \|\| id:finngen_R11_O15_POOR_FETGRO | Leukocyte Absolute Count \|\| id:ebi-a-GCST90001600 | Inverse variance weighted | 28 | 0.00250727020980342 | 0.0110536502596082 | 0.82055799476057 | -0.019157884 | 0.0241724247186355 | 1.00251041604036 | 0.981024461655484 | 1.02446694608758 | 0.994119304454654 |
| ebi-a-GCST90001600 | finngen_R11_O15_POOR_FETGRO | Poor fetal growth \|\| id:finngen_R11_O15_POOR_FETGRO | Leukocyte Absolute Count \|\| id:ebi-a-GCST90001600 | MR Egger | 28 | -0.001861474 | 0.0138418503815269 | 0.894057409596718 | -0.028991501 | 0.0252685527258374 | 0.998140257446284 | 0.971424720793893 | 1.02559050867135 | 0.992899239648112 |
| ebi-a-GCST90001600 | finngen_R11_O15_POOR_FETGRO | Poor fetal growth \|\| id:finngen_R11_O15_POOR_FETGRO | Leukocyte Absolute Count \|\| id:ebi-a-GCST90001600 | Weighted median | 28 | -0.003141116 | 0.014919737431616 | 0.833250727139845 | -0.032383801 | 0.0261015697147794 | 0.996863812491273 | 0.968134939590818 | 1.0264451989251 | 0.999440741871876 |
| ebi-a-GCST90001600 | finngen_R11_O15_POOR_FETGRO | Poor fetal growth \|\| id:finngen_R11_O15_POOR_FETGRO | Leukocyte Absolute Count \|\| id:ebi-a-GCST90001600 | Weighted mode | 28 | -0.002439907 | 0.0120993135945712 | 0.841696764828517 | -0.026154562 | 0.0212747473267756 | 0.997563066835899 | 0.974184506101301 | 1.02150266821382 | 0.999857399747617 |
| ebi-a-GCST90001601 | finngen_R11_O15_POOR_FETGRO | Poor fetal growth \|\| id:finngen_R11_O15_POOR_FETGRO | Lymphocyte Absolute Count \|\| id:ebi-a-GCST90001601 | Inverse variance weighted | 19 | 0.044767832666246 | 0.0333045080838314 | 0.178884567146333 | -0.020509003 | 0.110044668510555 | 1.04578503459859 | 0.979699876022379 | 1.1163279340513 | 0.994119304454654 |
| ebi-a-GCST90001601 | finngen_R11_O15_POOR_FETGRO | Poor fetal growth \|\| id:finngen_R11_O15_POOR_FETGRO | Lymphocyte Absolute Count \|\| id:ebi-a-GCST90001601 | MR Egger | 19 | 0.0153837515222257 | 0.0515368244470707 | 0.7689392568674 | -0.085628424 | 0.116395927438484 | 1.01550269055449 | 0.917935250205893 | 1.12344058504355 | 0.992899239648112 |
| ebi-a-GCST90001601 | finngen_R11_O15_POOR_FETGRO | Poor fetal growth \|\| id:finngen_R11_O15_POOR_FETGRO | Lymphocyte Absolute Count \|\| id:ebi-a-GCST90001601 | Weighted median | 19 | 0.00578761115974938 | 0.0480865759242297 | 0.904199444413877 | -0.088462078 | 0.10003729997124 | 1.00580439173875 | 0.915337821837485 | 1.10521214168792 | 0.999440741871876 |
| ebi-a-GCST90001601 | finngen_R11_O15_POOR_FETGRO | Poor fetal growth \|\| id:finngen_R11_O15_POOR_FETGRO | Lymphocyte Absolute Count \|\| id:ebi-a-GCST90001601 | Weighted mode | 19 | -0.011174666 | 0.0563854672992848 | 0.84512262345518 | -0.121690181 | 0.0993408503562433 | 0.988887539103716 | 0.885422646366527 | 1.10444268509233 | 0.999857399747617 |
| ebi-a-GCST90001602 | finngen_R11_O15_POOR_FETGRO | Poor fetal growth \|\| id:finngen_R11_O15_POOR_FETGRO | Lymphocyte %leukocyte \|\| id:ebi-a-GCST90001602 | Inverse variance weighted | 21 | -0.020496189 | 0.0219026425810076 | 0.349383320624993 | -0.063425368 | 0.0224329905967237 | 0.979712430286625 | 0.938544161813711 | 1.02268650225608 | 0.994119304454654 |
| ebi-a-GCST90001602 | finngen_R11_O15_POOR_FETGRO | Poor fetal growth \|\| id:finngen_R11_O15_POOR_FETGRO | Lymphocyte %leukocyte \|\| id:ebi-a-GCST90001602 | MR Egger | 21 | 0.00382104834979169 | 0.0270949980194385 | 0.889335329642534 | -0.049285148 | 0.0569272444678912 | 1.00382835786207 | 0.951909656081234 | 1.05857879013055 | 0.992899239648112 |
| ebi-a-GCST90001602 | finngen_R11_O15_POOR_FETGRO | Poor fetal growth \|\| id:finngen_R11_O15_POOR_FETGRO | Lymphocyte %leukocyte \|\| id:ebi-a-GCST90001602 | Weighted median | 21 | -0.004717242 | 0.0304316712617004 | 0.876812699721872 | -0.064363317 | 0.0549288339765636 | 0.995293867013887 | 0.937664267922512 | 1.05646542755676 | 0.999440741871876 |
| ebi-a-GCST90001602 | finngen_R11_O15_POOR_FETGRO | Poor fetal growth \|\| id:finngen_R11_O15_POOR_FETGRO | Lymphocyte %leukocyte \|\| id:ebi-a-GCST90001602 | Weighted mode | 21 | -0.007287908 | 0.0232228769223127 | 0.756904069660171 | -0.052804747 | 0.0382289304700331 | 0.992738584108834 | 0.948565204541879 | 1.03896905732947 | 0.999857399747617 |
| ebi-a-GCST90001603 | finngen_R11_O15_POOR_FETGRO | Poor fetal growth \|\| id:finngen_R11_O15_POOR_FETGRO | T cell Absolute Count \|\| id:ebi-a-GCST90001603 | Inverse variance weighted | 17 | 0.0142010209154714 | 0.0358781516621366 | 0.69224335447178 | -0.056120156 | 0.0845221981732592 | 1.01430233443002 | 0.94542553019167 | 1.08819699994944 | 0.994119304454654 |
| ebi-a-GCST90001603 | finngen_R11_O15_POOR_FETGRO | Poor fetal growth \|\| id:finngen_R11_O15_POOR_FETGRO | T cell Absolute Count \|\| id:ebi-a-GCST90001603 | MR Egger | 17 | 0.000849821479150058 | 0.0627059785462342 | 0.989365630965578 | -0.122053896 | 0.123753539429769 | 1.00085018267973 | 0.885100663414418 | 1.13173690810019 | 0.994809183268002 |
| ebi-a-GCST90001603 | finngen_R11_O15_POOR_FETGRO | Poor fetal growth \|\| id:finngen_R11_O15_POOR_FETGRO | T cell Absolute Count \|\| id:ebi-a-GCST90001603 | Weighted median | 17 | 0.000146828157971885 | 0.0467877967524877 | 0.997496105049162 | -0.091557253 | 0.0918509097928478 | 1.00014683893775 | 0.912509070339415 | 1.09620137700671 | 0.999440741871876 |
| ebi-a-GCST90001603 | finngen_R11_O15_POOR_FETGRO | Poor fetal growth \|\| id:finngen_R11_O15_POOR_FETGRO | T cell Absolute Count \|\| id:ebi-a-GCST90001603 | Weighted mode | 17 | -0.014713541 | 0.0526854636312208 | 0.783614300715414 | -0.11797705 | 0.0885499676341154 | 0.985394174124696 | 0.888716448600569 | 1.0925888453262 | 0.999857399747617 |
| ebi-a-GCST90001604 | finngen_R11_O15_POOR_FETGRO | Poor fetal growth \|\| id:finngen_R11_O15_POOR_FETGRO | T cell %lymphocyte \|\| id:ebi-a-GCST90001604 | Inverse variance weighted | 17 | -0.000516863 | 0.0425436709429923 | 0.990306741887611 | -0.083902458 | 0.0828687321480805 | 0.999483270650434 | 0.919520943680188 | 1.08639918989984 | 0.998984604424952 |
| ebi-a-GCST90001604 | finngen_R11_O15_POOR_FETGRO | Poor fetal growth \|\| id:finngen_R11_O15_POOR_FETGRO | T cell %lymphocyte \|\| id:ebi-a-GCST90001604 | MR Egger | 17 | 0.0750046020963467 | 0.133342165827198 | 0.582086990181226 | -0.186346043 | 0.336355247117656 | 1.07788911142276 | 0.829986334383722 | 1.39983622427523 | 0.992899239648112 |
| ebi-a-GCST90001604 | finngen_R11_O15_POOR_FETGRO | Poor fetal growth \|\| id:finngen_R11_O15_POOR_FETGRO | T cell %lymphocyte \|\| id:ebi-a-GCST90001604 | Weighted median | 17 | -0.001157078 | 0.0559533466974124 | 0.98350145366434 | -0.110825638 | 0.10851148151833 | 0.998843591148047 | 0.895094806259722 | 1.1146177059685 | 0.999440741871876 |
| ebi-a-GCST90001604 | finngen_R11_O15_POOR_FETGRO | Poor fetal growth \|\| id:finngen_R11_O15_POOR_FETGRO | T cell %lymphocyte \|\| id:ebi-a-GCST90001604 | Weighted mode | 17 | -0.003748527 | 0.0789827598171018 | 0.962733919915187 | -0.158554736 | 0.151057682586574 | 0.996258490300609 | 0.853376252198359 | 1.16306374467208 | 0.999857399747617 |
| ebi-a-GCST90001605 | finngen_R11_O15_POOR_FETGRO | Poor fetal growth \|\| id:finngen_R11_O15_POOR_FETGRO | T cell %leukocyte \|\| id:ebi-a-GCST90001605 | Inverse variance weighted | 15 | -0.004877981 | 0.0248278079033249 | 0.844240364288153 | -0.053540485 | 0.0437845222968471 | 0.995133896835144 | 0.947867566107946 | 1.04475720874863 | 0.994119304454654 |
| ebi-a-GCST90001605 | finngen_R11_O15_POOR_FETGRO | Poor fetal growth \|\| id:finngen_R11_O15_POOR_FETGRO | T cell %leukocyte \|\| id:ebi-a-GCST90001605 | MR Egger | 15 | -0.00742353 | 0.0302327588882226 | 0.809867641969739 | -0.066679738 | 0.0518326771226015 | 0.99260395604544 | 0.935494757050559 | 1.05319950339797 | 0.992899239648112 |
| ebi-a-GCST90001605 | finngen_R11_O15_POOR_FETGRO | Poor fetal growth \|\| id:finngen_R11_O15_POOR_FETGRO | T cell %leukocyte \|\| id:ebi-a-GCST90001605 | Weighted median | 15 | -0.006484435 | 0.0300795765611809 | 0.829318257491324 | -0.065440405 | 0.0524715349676546 | 0.993536543487734 | 0.936654864900513 | 1.05387256313498 | 0.999440741871876 |
| ebi-a-GCST90001605 | finngen_R11_O15_POOR_FETGRO | Poor fetal growth \|\| id:finngen_R11_O15_POOR_FETGRO | T cell %leukocyte \|\| id:ebi-a-GCST90001605 | Weighted mode | 15 | -0.006034035 | 0.0292058675828175 | 0.839294112130513 | -0.063277535 | 0.0512094658100909 | 0.993984133573924 | 0.938682920062666 | 1.05254334203796 | 0.999857399747617 |
| ebi-a-GCST90001606 | finngen_R11_O15_POOR_FETGRO | Poor fetal growth \|\| id:finngen_R11_O15_POOR_FETGRO | CD4+ T cell %leukocyte \|\| id:ebi-a-GCST90001606 | Inverse variance weighted | 13 | -0.011615629 | 0.0617768804627868 | 0.850856659553555 | -0.132698315 | 0.109467056710775 | 0.988451571976682 | 0.875729246898596 | 1.11568331605157 | 0.994119304454654 |
| ebi-a-GCST90001606 | finngen_R11_O15_POOR_FETGRO | Poor fetal growth \|\| id:finngen_R11_O15_POOR_FETGRO | CD4+ T cell %leukocyte \|\| id:ebi-a-GCST90001606 | MR Egger | 13 | 0.0134954380264714 | 0.15153515090416 | 0.930636497990305 | -0.283513458 | 0.310504333798625 | 1.013586912 | 0.753132986599665 | 1.36411290892363 | 0.992899239648112 |
| ebi-a-GCST90001606 | finngen_R11_O15_POOR_FETGRO | Poor fetal growth \|\| id:finngen_R11_O15_POOR_FETGRO | CD4+ T cell %leukocyte \|\| id:ebi-a-GCST90001606 | Weighted median | 13 | 0.0683287014083866 | 0.0756385450519126 | 0.366335806534917 | -0.079922847 | 0.216580249710135 | 1.0707171968931 | 0.923187570428091 | 1.24182273726996 | 0.999440741871876 |
| ebi-a-GCST90001606 | finngen_R11_O15_POOR_FETGRO | Poor fetal growth \|\| id:finngen_R11_O15_POOR_FETGRO | CD4+ T cell %leukocyte \|\| id:ebi-a-GCST90001606 | Weighted mode | 13 | 0.0931571688054856 | 0.107235898931015 | 0.402044969244633 | -0.117025193 | 0.303339530710274 | 1.09763423557356 | 0.889562782037606 | 1.35437423803126 | 0.999857399747617 |
| ebi-a-GCST90001607 | finngen_R11_O15_POOR_FETGRO | Poor fetal growth \|\| id:finngen_R11_O15_POOR_FETGRO | CD8+ T cell %leukocyte \|\| id:ebi-a-GCST90001607 | Inverse variance weighted (multiplicative random effects) | 16 | 0.0533198320565818 | 0.0615983445671169 | 0.386706839364991 | -0.067412923 | 0.174052587408131 | 1.05476693945717 | 0.934809117169862 | 1.19011814940364 | 0.994119304454654 |
| ebi-a-GCST90001607 | finngen_R11_O15_POOR_FETGRO | Poor fetal growth \|\| id:finngen_R11_O15_POOR_FETGRO | CD8+ T cell %leukocyte \|\| id:ebi-a-GCST90001607 | MR Egger | 16 | -0.145501706 | 0.154517376545408 | 0.362336374541071 | -0.448355764 | 0.157352351755752 | 0.864588414848612 | 0.638677424980524 | 1.17040793654673 | 0.992899239648112 |
| ebi-a-GCST90001607 | finngen_R11_O15_POOR_FETGRO | Poor fetal growth \|\| id:finngen_R11_O15_POOR_FETGRO | CD8+ T cell %leukocyte \|\| id:ebi-a-GCST90001607 | Weighted median | 16 | 0.0202456303801822 | 0.0633068049635426 | 0.749118580615574 | -0.103835707 | 0.144326968108726 | 1.02045196324885 | 0.901373374278679 | 1.15526178053766 | 0.999440741871876 |
| ebi-a-GCST90001607 | finngen_R11_O15_POOR_FETGRO | Poor fetal growth \|\| id:finngen_R11_O15_POOR_FETGRO | CD8+ T cell %leukocyte \|\| id:ebi-a-GCST90001607 | Weighted mode | 16 | -0.017040627 | 0.0876186640758804 | 0.848404694705254 | -0.188773209 | 0.154691954487987 | 0.983103743168752 | 0.827974262771349 | 1.16729832470567 | 0.999857399747617 |
| ebi-a-GCST90001608 | finngen_R11_O15_POOR_FETGRO | Poor fetal growth \|\| id:finngen_R11_O15_POOR_FETGRO | CD4+CD8+ T cell %leukocyte \|\| id:ebi-a-GCST90001608 | Inverse variance weighted | 21 | 0.0216876153280171 | 0.0394024869141559 | 0.582036581427036 | -0.055541259 | 0.0989164896797626 | 1.02192450105336 | 0.945972992943093 | 1.1039741024784 | 0.994119304454654 |
| ebi-a-GCST90001608 | finngen_R11_O15_POOR_FETGRO | Poor fetal growth \|\| id:finngen_R11_O15_POOR_FETGRO | CD4+CD8+ T cell %leukocyte \|\| id:ebi-a-GCST90001608 | MR Egger | 21 | 0.0171105410265193 | 0.0875161833357527 | 0.847067162570665 | -0.154421178 | 0.188642260364595 | 1.01725776482793 | 0.856911032654673 | 1.20760886564491 | 0.992899239648112 |
| ebi-a-GCST90001608 | finngen_R11_O15_POOR_FETGRO | Poor fetal growth \|\| id:finngen_R11_O15_POOR_FETGRO | CD4+CD8+ T cell %leukocyte \|\| id:ebi-a-GCST90001608 | Weighted median | 21 | 0.0244784609127186 | 0.0524639868887152 | 0.640802937072131 | -0.078350953 | 0.1273078752146 | 1.02478051803246 | 0.924639864099665 | 1.13576663835649 | 0.999440741871876 |
| ebi-a-GCST90001608 | finngen_R11_O15_POOR_FETGRO | Poor fetal growth \|\| id:finngen_R11_O15_POOR_FETGRO | CD4+CD8+ T cell %leukocyte \|\| id:ebi-a-GCST90001608 | Weighted mode | 21 | 0.0167920116573765 | 0.0558479561671129 | 0.766766642131718 | -0.092669982 | 0.126254005744918 | 1.01693378995434 | 0.911494259785986 | 1.13457031906455 | 0.999857399747617 |
| ebi-a-GCST90001609 | finngen_R11_O15_POOR_FETGRO | Poor fetal growth \|\| id:finngen_R11_O15_POOR_FETGRO | CD4+ CD8dim T cell Absolute Count \|\| id:ebi-a-GCST90001609 | Inverse variance weighted | 16 | -0.001506747 | 0.0389818740735093 | 0.969167446731074 | -0.07791122 | 0.0748977264010414 | 0.998494387789988 | 0.925046548561089 | 1.07777391743034 | 0.998984604424952 |
| ebi-a-GCST90001609 | finngen_R11_O15_POOR_FETGRO | Poor fetal growth \|\| id:finngen_R11_O15_POOR_FETGRO | CD4+ CD8dim T cell Absolute Count \|\| id:ebi-a-GCST90001609 | MR Egger | 16 | 0.0106646491948315 | 0.0632403620732538 | 0.86849449695491 | -0.11328646 | 0.134615758858409 | 1.01072171926305 | 0.892894844401552 | 1.14409709071031 | 0.992899239648112 |
| ebi-a-GCST90001609 | finngen_R11_O15_POOR_FETGRO | Poor fetal growth \|\| id:finngen_R11_O15_POOR_FETGRO | CD4+ CD8dim T cell Absolute Count \|\| id:ebi-a-GCST90001609 | Weighted median | 16 | -0.019685086 | 0.0467614119122487 | 0.673778017871283 | -0.111337453 | 0.071967281689906 | 0.980507400535654 | 0.894636800107556 | 1.07462018373222 | 0.999440741871876 |
| ebi-a-GCST90001609 | finngen_R11_O15_POOR_FETGRO | Poor fetal growth \|\| id:finngen_R11_O15_POOR_FETGRO | CD4+ CD8dim T cell Absolute Count \|\| id:ebi-a-GCST90001609 | Weighted mode | 16 | -0.037975876 | 0.0386269623549746 | 0.341129398699659 | -0.113684722 | 0.0377329705440463 | 0.962736165974384 | 0.892539309636653 | 1.03845389807243 | 0.999857399747617 |
| ebi-a-GCST90001610 | finngen_R11_O15_POOR_FETGRO | Poor fetal growth \|\| id:finngen_R11_O15_POOR_FETGRO | CD4+ CD8dim T cell %lymphocyte \|\| id:ebi-a-GCST90001610 | Inverse variance weighted | 20 | 0.0116406271465277 | 0.0359454910666963 | 0.746058342826589 | -0.058812535 | 0.0820937896372524 | 1.01170864290586 | 0.942883509922909 | 1.08555761911046 | 0.994119304454654 |
| ebi-a-GCST90001610 | finngen_R11_O15_POOR_FETGRO | Poor fetal growth \|\| id:finngen_R11_O15_POOR_FETGRO | CD4+ CD8dim T cell %lymphocyte \|\| id:ebi-a-GCST90001610 | MR Egger | 20 | 0.0597299985079642 | 0.0694866878971341 | 0.401319253359663 | -0.07646391 | 0.195923906786347 | 1.06154988779438 | 0.926386347181469 | 1.21643433941446 | 0.992899239648112 |
| ebi-a-GCST90001610 | finngen_R11_O15_POOR_FETGRO | Poor fetal growth \|\| id:finngen_R11_O15_POOR_FETGRO | CD4+ CD8dim T cell %lymphocyte \|\| id:ebi-a-GCST90001610 | Weighted median | 20 | -0.005649807 | 0.0509818499247274 | 0.911759110086065 | -0.105574233 | 0.0942746188352146 | 0.994366123127547 | 0.899807675061605 | 1.09886147254302 | 0.999440741871876 |
| ebi-a-GCST90001610 | finngen_R11_O15_POOR_FETGRO | Poor fetal growth \|\| id:finngen_R11_O15_POOR_FETGRO | CD4+ CD8dim T cell %lymphocyte \|\| id:ebi-a-GCST90001610 | Weighted mode | 20 | -0.01510295 | 0.0547955991635325 | 0.785812646127598 | -0.122502324 | 0.0922964242684349 | 0.985010527458447 | 0.884703848488922 | 1.09668985939323 | 0.999857399747617 |
| ebi-a-GCST90001611 | finngen_R11_O15_POOR_FETGRO | Poor fetal growth \|\| id:finngen_R11_O15_POOR_FETGRO | CD4+ CD8dim T cell %leukocyte \|\| id:ebi-a-GCST90001611 | Inverse variance weighted | 16 | 0.00351170893393054 | 0.0435825963840884 | 0.935779196280579 | -0.08191018 | 0.0889335978467438 | 1.00351788220788 | 0.921354711079488 | 1.09300807582684 | 0.998984604424952 |
| ebi-a-GCST90001611 | finngen_R11_O15_POOR_FETGRO | Poor fetal growth \|\| id:finngen_R11_O15_POOR_FETGRO | CD4+ CD8dim T cell %leukocyte \|\| id:ebi-a-GCST90001611 | MR Egger | 16 | 0.0471992876534383 | 0.105841844189753 | 0.662458450334837 | -0.160250727 | 0.254649302265354 | 1.04833090767013 | 0.851930160328167 | 1.29000913825275 | 0.992899239648112 |
| ebi-a-GCST90001611 | finngen_R11_O15_POOR_FETGRO | Poor fetal growth \|\| id:finngen_R11_O15_POOR_FETGRO | CD4+ CD8dim T cell %leukocyte \|\| id:ebi-a-GCST90001611 | Weighted median | 16 | -0.005265032 | 0.0616952500525745 | 0.931991621124541 | -0.126187722 | 0.115657658518457 | 0.994748804401254 | 0.881449364089366 | 1.12261148986137 | 0.999440741871876 |
| ebi-a-GCST90001611 | finngen_R11_O15_POOR_FETGRO | Poor fetal growth \|\| id:finngen_R11_O15_POOR_FETGRO | CD4+ CD8dim T cell %leukocyte \|\| id:ebi-a-GCST90001611 | Weighted mode | 16 | 0.00379764454835063 | 0.0626397820745817 | 0.952456895368858 | -0.118976328 | 0.126571617414531 | 1.00380486473742 | 0.887828816914781 | 1.13493072907007 | 0.999857399747617 |
| ebi-a-GCST90001612 | finngen_R11_O15_POOR_FETGRO | Poor fetal growth \|\| id:finngen_R11_O15_POOR_FETGRO | CD8dim T cell %leukocyte \|\| id:ebi-a-GCST90001612 | Inverse variance weighted | 18 | 0.0475506139289638 | 0.0279388418331498 | 0.0887640567175281 | -0.007209516 | 0.102310743921938 | 1.04869927856883 | 0.992816410154352 | 1.10772763788203 | 0.994119304454654 |
| ebi-a-GCST90001612 | finngen_R11_O15_POOR_FETGRO | Poor fetal growth \|\| id:finngen_R11_O15_POOR_FETGRO | CD8dim T cell %leukocyte \|\| id:ebi-a-GCST90001612 | MR Egger | 18 | 0.0650396153787012 | 0.0404647345264777 | 0.127537130259264 | -0.014271264 | 0.144350495050598 | 1.06720130113048 | 0.985830087486873 | 1.15528896063415 | 0.992899239648112 |
| ebi-a-GCST90001612 | finngen_R11_O15_POOR_FETGRO | Poor fetal growth \|\| id:finngen_R11_O15_POOR_FETGRO | CD8dim T cell %leukocyte \|\| id:ebi-a-GCST90001612 | Weighted median | 18 | 0.0655603445656793 | 0.0387216928584018 | 0.0904332574169254 | -0.010334173 | 0.141454862568147 | 1.06775716871203 | 0.989719040667841 | 1.15194850709007 | 0.999440741871876 |
| ebi-a-GCST90001612 | finngen_R11_O15_POOR_FETGRO | Poor fetal growth \|\| id:finngen_R11_O15_POOR_FETGRO | CD8dim T cell %leukocyte \|\| id:ebi-a-GCST90001612 | Weighted mode | 18 | 0.0615249563154099 | 0.0368490467213551 | 0.113297858828186 | -0.010699175 | 0.133749087889266 | 1.06345703616685 | 0.989357857335365 | 1.14310596452809 | 0.999857399747617 |
| ebi-a-GCST90001613 | finngen_R11_O15_POOR_FETGRO | Poor fetal growth \|\| id:finngen_R11_O15_POOR_FETGRO | CD4-CD8- T cell %leukocyte \|\| id:ebi-a-GCST90001613 | Inverse variance weighted | 20 | 0.0223317422175867 | 0.019206233262596 | 0.244937404529257 | -0.015312475 | 0.0599759594122749 | 1.02258296214708 | 0.98480416486075 | 1.06181101967755 | 0.994119304454654 |
| ebi-a-GCST90001613 | finngen_R11_O15_POOR_FETGRO | Poor fetal growth \|\| id:finngen_R11_O15_POOR_FETGRO | CD4-CD8- T cell %leukocyte \|\| id:ebi-a-GCST90001613 | MR Egger | 20 | 0.0160441824136813 | 0.0233011157111543 | 0.499880288544747 | -0.029626004 | 0.0617143692075438 | 1.01617358141583 | 0.97080854380522 | 1.06365848771789 | 0.992899239648112 |
| ebi-a-GCST90001613 | finngen_R11_O15_POOR_FETGRO | Poor fetal growth \|\| id:finngen_R11_O15_POOR_FETGRO | CD4-CD8- T cell %leukocyte \|\| id:ebi-a-GCST90001613 | Weighted median | 20 | 0.0074007963718915 | 0.0239678673983632 | 0.757488938591328 | -0.039576224 | 0.0543778164726833 | 1.00742824994968 | 0.961196685202671 | 1.05588345696665 | 0.999440741871876 |
| ebi-a-GCST90001613 | finngen_R11_O15_POOR_FETGRO | Poor fetal growth \|\| id:finngen_R11_O15_POOR_FETGRO | CD4-CD8- T cell %leukocyte \|\| id:ebi-a-GCST90001613 | Weighted mode | 20 | 0.0109428481688937 | 0.0212162313614274 | 0.611959989973085 | -0.030640965 | 0.0525266616372914 | 1.01100294012427 | 0.969823710941091 | 1.05393066122097 | 0.999857399747617 |
| ebi-a-GCST90001614 | finngen_R11_O15_POOR_FETGRO | Poor fetal growth \|\| id:finngen_R11_O15_POOR_FETGRO | CD8+ and CD8dim T cell %leukocyte \|\| id:ebi-a-GCST90001614 | Inverse variance weighted | 14 | 0.0413322946964645 | 0.0494040527851622 | 0.402807610575588 | -0.055499649 | 0.138164238155383 | 1.04219836500156 | 0.946012355945435 | 1.14816410714467 | 0.994119304454654 |
| ebi-a-GCST90001614 | finngen_R11_O15_POOR_FETGRO | Poor fetal growth \|\| id:finngen_R11_O15_POOR_FETGRO | CD8+ and CD8dim T cell %leukocyte \|\| id:ebi-a-GCST90001614 | MR Egger | 14 | -0.001850642 | 0.134553632824782 | 0.989252320063881 | -0.265575762 | 0.261874478653995 | 0.998151069698861 | 0.766764349069709 | 1.29936343434559 | 0.994809183268002 |
| ebi-a-GCST90001614 | finngen_R11_O15_POOR_FETGRO | Poor fetal growth \|\| id:finngen_R11_O15_POOR_FETGRO | CD8+ and CD8dim T cell %leukocyte \|\| id:ebi-a-GCST90001614 | Weighted median | 14 | 0.00644505468079405 | 0.0694741946807728 | 0.92608704357865 | -0.129724367 | 0.142614476255109 | 1.0064658687376 | 0.878337496451076 | 1.15328509715986 | 0.999440741871876 |
| ebi-a-GCST90001614 | finngen_R11_O15_POOR_FETGRO | Poor fetal growth \|\| id:finngen_R11_O15_POOR_FETGRO | CD8+ and CD8dim T cell %leukocyte \|\| id:ebi-a-GCST90001614 | Weighted mode | 14 | 0.00184875445149291 | 0.0978569242568213 | 0.985213810303291 | -0.189950817 | 0.193648325994863 | 1.00185046445163 | 0.826999807198675 | 1.21366939192142 | 0.999857399747617 |
| ebi-a-GCST90001615 | finngen_R11_O15_POOR_FETGRO | Poor fetal growth \|\| id:finngen_R11_O15_POOR_FETGRO | TCRgd T cell Absolute Count \|\| id:ebi-a-GCST90001615 | Inverse variance weighted | 19 | 0.056777695455384 | 0.0301649699006686 | 0.0598035155943008 | -0.002345646 | 0.115901036460694 | 1.05842049255475 | 0.99765710332688 | 1.12288474198613 | 0.994119304454654 |
| ebi-a-GCST90001615 | finngen_R11_O15_POOR_FETGRO | Poor fetal growth \|\| id:finngen_R11_O15_POOR_FETGRO | TCRgd T cell Absolute Count \|\| id:ebi-a-GCST90001615 | MR Egger | 19 | 0.0505554316535448 | 0.0445402095786724 | 0.272104539022106 | -0.036743379 | 0.137854242427743 | 1.05185516781027 | 0.963923466500124 | 1.14780823633889 | 0.992899239648112 |
| ebi-a-GCST90001615 | finngen_R11_O15_POOR_FETGRO | Poor fetal growth \|\| id:finngen_R11_O15_POOR_FETGRO | TCRgd T cell Absolute Count \|\| id:ebi-a-GCST90001615 | Weighted median | 19 | 0.0451758799197418 | 0.0439371364621386 | 0.303858796112151 | -0.040940908 | 0.131292667385533 | 1.04621185138449 | 0.959885850282316 | 1.14030146152842 | 0.999440741871876 |
| ebi-a-GCST90001615 | finngen_R11_O15_POOR_FETGRO | Poor fetal growth \|\| id:finngen_R11_O15_POOR_FETGRO | TCRgd T cell Absolute Count \|\| id:ebi-a-GCST90001615 | Weighted mode | 19 | 0.0144393955069369 | 0.0417448651179124 | 0.733430555372574 | -0.06738054 | 0.0962593311380453 | 1.01454414715447 | 0.934839389743323 | 1.10104456211243 | 0.999857399747617 |
| ebi-a-GCST90001616 | finngen_R11_O15_POOR_FETGRO | Poor fetal growth \|\| id:finngen_R11_O15_POOR_FETGRO | TCRgd T cell %T cell \|\| id:ebi-a-GCST90001616 | Inverse variance weighted | 18 | -0.012437022 | 0.024603001802034 | 0.613201857537974 | -0.060658906 | 0.035784861468357 | 0.987639998064279 | 0.941144204055059 | 1.03643284586316 | 0.994119304454654 |
| ebi-a-GCST90001616 | finngen_R11_O15_POOR_FETGRO | Poor fetal growth \|\| id:finngen_R11_O15_POOR_FETGRO | TCRgd T cell %T cell \|\| id:ebi-a-GCST90001616 | MR Egger | 18 | -0.012222949 | 0.0303345732880689 | 0.692326783600044 | -0.071678712 | 0.0472328148760772 | 0.987851448045118 | 0.930829911869709 | 1.04836605588307 | 0.992899239648112 |
| ebi-a-GCST90001616 | finngen_R11_O15_POOR_FETGRO | Poor fetal growth \|\| id:finngen_R11_O15_POOR_FETGRO | TCRgd T cell %T cell \|\| id:ebi-a-GCST90001616 | Weighted median | 18 | -0.013742667 | 0.0236317737915997 | 0.56088098874354 | -0.060060943 | 0.0325756099689177 | 0.986351332687622 | 0.941707141100117 | 1.03311200375746 | 0.999440741871876 |
| ebi-a-GCST90001616 | finngen_R11_O15_POOR_FETGRO | Poor fetal growth \|\| id:finngen_R11_O15_POOR_FETGRO | TCRgd T cell %T cell \|\| id:ebi-a-GCST90001616 | Weighted mode | 18 | -0.012936549 | 0.0237694478446342 | 0.59333614289906 | -0.059524667 | 0.0336515683022968 | 0.987146768015691 | 0.942212291519678 | 1.03422418745157 | 0.999857399747617 |
| ebi-a-GCST90001617 | finngen_R11_O15_POOR_FETGRO | Poor fetal growth \|\| id:finngen_R11_O15_POOR_FETGRO | TCRgd T cell %lymphocyte \|\| id:ebi-a-GCST90001617 | Inverse variance weighted (multiplicative random effects) | 20 | 0.00463017269156185 | 0.0203195376889518 | 0.819748849985296 | -0.035196121 | 0.0444564665619075 | 1.0046409085043 | 0.965416059154166 | 1.04545946327496 | 0.994119304454654 |
| ebi-a-GCST90001617 | finngen_R11_O15_POOR_FETGRO | Poor fetal growth \|\| id:finngen_R11_O15_POOR_FETGRO | TCRgd T cell %lymphocyte \|\| id:ebi-a-GCST90001617 | MR Egger | 20 | -0.00433707 | 0.0238297529654129 | 0.857614871760081 | -0.051043386 | 0.0423692454744385 | 0.995672321169671 | 0.950237442493874 | 1.04327963392138 | 0.992899239648112 |
| ebi-a-GCST90001617 | finngen_R11_O15_POOR_FETGRO | Poor fetal growth \|\| id:finngen_R11_O15_POOR_FETGRO | TCRgd T cell %lymphocyte \|\| id:ebi-a-GCST90001617 | Weighted median | 20 | -0.013434253 | 0.0199866381233069 | 0.501480761736118 | -0.052608063 | 0.0257395581002826 | 0.986655584203506 | 0.948751790225784 | 1.02607368109239 | 0.999440741871876 |
| ebi-a-GCST90001617 | finngen_R11_O15_POOR_FETGRO | Poor fetal growth \|\| id:finngen_R11_O15_POOR_FETGRO | TCRgd T cell %lymphocyte \|\| id:ebi-a-GCST90001617 | Weighted mode | 20 | -0.007397156 | 0.0170370801411247 | 0.669048361895501 | -0.040789833 | 0.0259955214206402 | 0.992630135965018 | 0.960030875812687 | 1.02633635193435 | 0.999857399747617 |
| ebi-a-GCST90001618 | finngen_R11_O15_POOR_FETGRO | Poor fetal growth \|\| id:finngen_R11_O15_POOR_FETGRO | HLA DR+ T cell Absolute Count \|\| id:ebi-a-GCST90001618 | Inverse variance weighted | 30 | -0.007760266 | 0.0169109059376497 | 0.646312302940557 | -0.040905641 | 0.0253851098247521 | 0.992269767311147 | 0.959919702305018 | 1.0257100554926 | 0.994119304454654 |
| ebi-a-GCST90001618 | finngen_R11_O15_POOR_FETGRO | Poor fetal growth \|\| id:finngen_R11_O15_POOR_FETGRO | HLA DR+ T cell Absolute Count \|\| id:ebi-a-GCST90001618 | MR Egger | 30 | 0.0232285421357581 | 0.0243203490374636 | 0.347697328501189 | -0.024439342 | 0.0708964262491868 | 1.02350042579303 | 0.97585688067162 | 1.07347003679223 | 0.992899239648112 |
| ebi-a-GCST90001618 | finngen_R11_O15_POOR_FETGRO | Poor fetal growth \|\| id:finngen_R11_O15_POOR_FETGRO | HLA DR+ T cell Absolute Count \|\| id:ebi-a-GCST90001618 | Weighted median | 30 | -0.01111067 | 0.0257688506734064 | 0.666347172846106 | -0.061617617 | 0.0393962772932111 | 0.988950825504653 | 0.940242350425229 | 1.0401826027343 | 0.999440741871876 |
| ebi-a-GCST90001618 | finngen_R11_O15_POOR_FETGRO | Poor fetal growth \|\| id:finngen_R11_O15_POOR_FETGRO | HLA DR+ T cell Absolute Count \|\| id:ebi-a-GCST90001618 | Weighted mode | 30 | -0.002801236 | 0.0223569119618592 | 0.90115363140084 | -0.046620784 | 0.0410183113461212 | 0.997202683701768 | 0.954449271841648 | 1.04187118343466 | 0.999857399747617 |
| ebi-a-GCST90001619 | finngen_R11_O15_POOR_FETGRO | Poor fetal growth \|\| id:finngen_R11_O15_POOR_FETGRO | HLA DR+ T cell%T cell \|\| id:ebi-a-GCST90001619 | Inverse variance weighted | 31 | -0.001802775 | 0.0147865440652132 | 0.902962419953335 | -0.030784401 | 0.0271788517345287 | 0.998198849388838 | 0.969684613572683 | 1.02755156570968 | 0.994119304454654 |
| ebi-a-GCST90001619 | finngen_R11_O15_POOR_FETGRO | Poor fetal growth \|\| id:finngen_R11_O15_POOR_FETGRO | HLA DR+ T cell%T cell \|\| id:ebi-a-GCST90001619 | MR Egger | 31 | -0.012492687 | 0.018930376220101 | 0.514507530282736 | -0.049596224 | 0.0246108507558238 | 0.987585023036646 | 0.951613585639464 | 1.02491619754556 | 0.992899239648112 |
| ebi-a-GCST90001619 | finngen_R11_O15_POOR_FETGRO | Poor fetal growth \|\| id:finngen_R11_O15_POOR_FETGRO | HLA DR+ T cell%T cell \|\| id:ebi-a-GCST90001619 | Weighted median | 31 | -0.006813466 | 0.01889826419197 | 0.71844785965074 | -0.043854064 | 0.0302271316460682 | 0.99320969286282 | 0.95709362170952 | 1.03068860936997 | 0.999440741871876 |
| ebi-a-GCST90001619 | finngen_R11_O15_POOR_FETGRO | Poor fetal growth \|\| id:finngen_R11_O15_POOR_FETGRO | HLA DR+ T cell%T cell \|\| id:ebi-a-GCST90001619 | Weighted mode | 31 | -0.008082835 | 0.0174647464143564 | 0.646841190391311 | -0.042313738 | 0.0261480674961893 | 0.991949742804665 | 0.95856899344232 | 1.02649292745922 | 0.999857399747617 |
| ebi-a-GCST90001620 | finngen_R11_O15_POOR_FETGRO | Poor fetal growth \|\| id:finngen_R11_O15_POOR_FETGRO | HLA DR+ T cell%lymphocyte \|\| id:ebi-a-GCST90001620 | Inverse variance weighted | 25 | -0.000356318 | 0.0187440124868604 | 0.984833354339783 | -0.037094583 | 0.0363819461149055 | 0.999643745114507 | 0.963584992439857 | 1.03705186878876 | 0.998984604424952 |
| ebi-a-GCST90001620 | finngen_R11_O15_POOR_FETGRO | Poor fetal growth \|\| id:finngen_R11_O15_POOR_FETGRO | HLA DR+ T cell%lymphocyte \|\| id:ebi-a-GCST90001620 | MR Egger | 25 | -0.00917052 | 0.02713670673465 | 0.738475147178041 | -0.062358465 | 0.0440174249280655 | 0.990871400705585 | 0.939546031607757 | 1.04500056378945 | 0.992899239648112 |
| ebi-a-GCST90001620 | finngen_R11_O15_POOR_FETGRO | Poor fetal growth \|\| id:finngen_R11_O15_POOR_FETGRO | HLA DR+ T cell%lymphocyte \|\| id:ebi-a-GCST90001620 | Weighted median | 25 | -0.001220633 | 0.0280796576789829 | 0.965326586356092 | -0.056256762 | 0.053815496042451 | 0.998780111661095 | 0.945296388480343 | 1.055289879 | 0.999440741871876 |
| ebi-a-GCST90001620 | finngen_R11_O15_POOR_FETGRO | Poor fetal growth \|\| id:finngen_R11_O15_POOR_FETGRO | HLA DR+ T cell%lymphocyte \|\| id:ebi-a-GCST90001620 | Weighted mode | 25 | -0.002143252 | 0.0228672084129664 | 0.926104887651078 | -0.046962981 | 0.0426764763697448 | 0.997859043005185 | 0.954122717978477 | 1.04360021090042 | 0.999857399747617 |
| ebi-a-GCST90001621 | finngen_R11_O15_POOR_FETGRO | Poor fetal growth \|\| id:finngen_R11_O15_POOR_FETGRO | Natural Killer T Absolute Count \|\| id:ebi-a-GCST90001621 | Inverse variance weighted | 34 | 0.0227845029719875 | 0.0259283279664229 | 0.379537160404534 | -0.02803502 | 0.0736040257861764 | 1.0230460524071 | 0.97235431451034 | 1.0763805021761 | 0.994119304454654 |
| ebi-a-GCST90001621 | finngen_R11_O15_POOR_FETGRO | Poor fetal growth \|\| id:finngen_R11_O15_POOR_FETGRO | Natural Killer T Absolute Count \|\| id:ebi-a-GCST90001621 | MR Egger | 34 | -0.031685451 | 0.0432327072128628 | 0.468951701587415 | -0.116421557 | 0.0530506551078691 | 0.968811272745222 | 0.890099916197711 | 1.05448305871959 | 0.992899239648112 |
| ebi-a-GCST90001621 | finngen_R11_O15_POOR_FETGRO | Poor fetal growth \|\| id:finngen_R11_O15_POOR_FETGRO | Natural Killer T Absolute Count \|\| id:ebi-a-GCST90001621 | Weighted median | 34 | -0.000982664 | 0.0373807875075433 | 0.979027681642769 | -0.074249007 | 0.0722836799703344 | 0.999017819111261 | 0.928440476822338 | 1.07496024550511 | 0.999440741871876 |
| ebi-a-GCST90001621 | finngen_R11_O15_POOR_FETGRO | Poor fetal growth \|\| id:finngen_R11_O15_POOR_FETGRO | Natural Killer T Absolute Count \|\| id:ebi-a-GCST90001621 | Weighted mode | 34 | -0.022575468 | 0.0468495755792402 | 0.633077868594893 | -0.114400636 | 0.069249700458955 | 0.977677451363669 | 0.891900556990215 | 1.07170378066649 | 0.999857399747617 |
| ebi-a-GCST90001622 | finngen_R11_O15_POOR_FETGRO | Poor fetal growth \|\| id:finngen_R11_O15_POOR_FETGRO | Natural Killer T %T cell \|\| id:ebi-a-GCST90001622 | Inverse variance weighted | 32 | 0.0278956353285623 | 0.0221961693784123 | 0.208834394032108 | -0.015608857 | 0.0714001273102504 | 1.02828836184415 | 0.984512330201172 | 1.07401088098924 | 0.994119304454654 |
| ebi-a-GCST90001622 | finngen_R11_O15_POOR_FETGRO | Poor fetal growth \|\| id:finngen_R11_O15_POOR_FETGRO | Natural Killer T %T cell \|\| id:ebi-a-GCST90001622 | MR Egger | 32 | -0.038163739 | 0.032827751958185 | 0.254179023415481 | -0.102506133 | 0.0261786550642355 | 0.962555320359378 | 0.902572614608442 | 1.02652432586168 | 0.992899239648112 |
| ebi-a-GCST90001622 | finngen_R11_O15_POOR_FETGRO | Poor fetal growth \|\| id:finngen_R11_O15_POOR_FETGRO | Natural Killer T %T cell \|\| id:ebi-a-GCST90001622 | Weighted median | 32 | -0.012890299 | 0.0340671381860205 | 0.705148871247933 | -0.079661889 | 0.0538812922244707 | 0.987192425451644 | 0.923428514519323 | 1.05535931536226 | 0.999440741871876 |
| ebi-a-GCST90001622 | finngen_R11_O15_POOR_FETGRO | Poor fetal growth \|\| id:finngen_R11_O15_POOR_FETGRO | Natural Killer T %T cell \|\| id:ebi-a-GCST90001622 | Weighted mode | 32 | -0.019347202 | 0.031061984698494 | 0.537933416192636 | -0.080228692 | 0.0415342880880521 | 0.98083875401673 | 0.922905261265377 | 1.04240890344698 | 0.999857399747617 |
| ebi-a-GCST90001623 | finngen_R11_O15_POOR_FETGRO | Poor fetal growth \|\| id:finngen_R11_O15_POOR_FETGRO | Natural Killer T %lymphocyte \|\| id:ebi-a-GCST90001623 | Inverse variance weighted | 33 | 0.0322111410197606 | 0.0229119517136939 | 0.159763826921294 | -0.012696284 | 0.0771185663786007 | 1.03273553512082 | 0.98738397346125 | 1.08017014066224 | 0.994119304454654 |
| ebi-a-GCST90001623 | finngen_R11_O15_POOR_FETGRO | Poor fetal growth \|\| id:finngen_R11_O15_POOR_FETGRO | Natural Killer T %lymphocyte \|\| id:ebi-a-GCST90001623 | MR Egger | 33 | -0.025451258 | 0.0339388912978919 | 0.458960267711516 | -0.091971485 | 0.0410689692440177 | 0.974869895208745 | 0.912131158919227 | 1.04192396377555 | 0.992899239648112 |
| ebi-a-GCST90001623 | finngen_R11_O15_POOR_FETGRO | Poor fetal growth \|\| id:finngen_R11_O15_POOR_FETGRO | Natural Killer T %lymphocyte \|\| id:ebi-a-GCST90001623 | Weighted median | 33 | -0.013945959 | 0.0363331054476795 | 0.701099895670747 | -0.085158846 | 0.057266927508515 | 0.986150835233595 | 0.918366394127562 | 1.05893843247147 | 0.999440741871876 |
| ebi-a-GCST90001623 | finngen_R11_O15_POOR_FETGRO | Poor fetal growth \|\| id:finngen_R11_O15_POOR_FETGRO | Natural Killer T %lymphocyte \|\| id:ebi-a-GCST90001623 | Weighted mode | 33 | -0.023318351 | 0.0329701749722445 | 0.484526626941083 | -0.087939894 | 0.0413031921218235 | 0.976951420972699 | 0.915815921312024 | 1.04216803478719 | 0.999857399747617 |
| ebi-a-GCST90001624 | finngen_R11_O15_POOR_FETGRO | Poor fetal growth \|\| id:finngen_R11_O15_POOR_FETGRO | HLA DR+ CD4+ T cell Absolute Count \|\| id:ebi-a-GCST90001624 | Inverse variance weighted | 24 | 0.0372295092468859 | 0.0155228171438807 | 0.0164680752885751 | 0.00680478764487974 | 0.0676542308488921 | 1.03793120831394 | 1.00682799281789 | 1.06999527315177 | 0.994119304454654 |
| ebi-a-GCST90001624 | finngen_R11_O15_POOR_FETGRO | Poor fetal growth \|\| id:finngen_R11_O15_POOR_FETGRO | HLA DR+ CD4+ T cell Absolute Count \|\| id:ebi-a-GCST90001624 | MR Egger | 24 | 0.0427192610388437 | 0.0198945915380842 | 0.0430477401153911 | 0.00372586162419873 | 0.0817126604534887 | 1.0436448619453 | 1.00373281127509 | 1.0851439602549 | 0.992899239648112 |
| ebi-a-GCST90001624 | finngen_R11_O15_POOR_FETGRO | Poor fetal growth \|\| id:finngen_R11_O15_POOR_FETGRO | HLA DR+ CD4+ T cell Absolute Count \|\| id:ebi-a-GCST90001624 | Weighted median | 24 | 0.0372320974256356 | 0.0235182599623344 | 0.113395378333741 | -0.008863692 | 0.0833278869518109 | 1.03793389466891 | 0.9911754746123 | 1.08689812984322 | 0.999440741871876 |
| ebi-a-GCST90001624 | finngen_R11_O15_POOR_FETGRO | Poor fetal growth \|\| id:finngen_R11_O15_POOR_FETGRO | HLA DR+ CD4+ T cell Absolute Count \|\| id:ebi-a-GCST90001624 | Weighted mode | 24 | 0.0346835095285714 | 0.0190791174924522 | 0.0821329743640381 | -0.002711561 | 0.0720785798137777 | 1.03529199689135 | 0.997292112203665 | 1.0747397935986 | 0.999857399747617 |
| ebi-a-GCST90001625 | finngen_R11_O15_POOR_FETGRO | Poor fetal growth \|\| id:finngen_R11_O15_POOR_FETGRO | HLA DR+ CD4+ T cell %T cell \|\| id:ebi-a-GCST90001625 | Inverse variance weighted | 25 | -0.020863732 | 0.0398633479042934 | 0.600708919017014 | -0.098995894 | 0.0572684301409824 | 0.979352410114714 | 0.905746427333326 | 1.05894002366793 | 0.994119304454654 |
| ebi-a-GCST90001625 | finngen_R11_O15_POOR_FETGRO | Poor fetal growth \|\| id:finngen_R11_O15_POOR_FETGRO | HLA DR+ CD4+ T cell %T cell \|\| id:ebi-a-GCST90001625 | MR Egger | 25 | -0.005310602 | 0.0732847566702423 | 0.942858106028553 | -0.148948725 | 0.138327520789657 | 0.994703474035357 | 0.861613292679738 | 1.14835159771123 | 0.992899239648112 |
| ebi-a-GCST90001625 | finngen_R11_O15_POOR_FETGRO | Poor fetal growth \|\| id:finngen_R11_O15_POOR_FETGRO | HLA DR+ CD4+ T cell %T cell \|\| id:ebi-a-GCST90001625 | Weighted median | 25 | 0.00759900078248019 | 0.0517857543424614 | 0.883337866936694 | -0.093901078 | 0.109099079293705 | 1.00762794646189 | 0.910372813933078 | 1.11527284531328 | 0.999440741871876 |
| ebi-a-GCST90001625 | finngen_R11_O15_POOR_FETGRO | Poor fetal growth \|\| id:finngen_R11_O15_POOR_FETGRO | HLA DR+ CD4+ T cell %T cell \|\| id:ebi-a-GCST90001625 | Weighted mode | 25 | 0.0227141439111955 | 0.0582440859384075 | 0.699989427941034 | -0.091444265 | 0.136872552350474 | 1.02297407437988 | 0.912612179605056 | 1.14668199728197 | 0.999857399747617 |
| ebi-a-GCST90001626 | finngen_R11_O15_POOR_FETGRO | Poor fetal growth \|\| id:finngen_R11_O15_POOR_FETGRO | HLA DR+ CD4+ T cell %lymphocyte \|\| id:ebi-a-GCST90001626 | Inverse variance weighted | 21 | 0.00276739831943007 | 0.0467611817590226 | 0.95280752012613 | -0.088884518 | 0.0944193145671144 | 1.00277123110095 | 0.914951227937091 | 1.09902048461195 | 0.998984604424952 |
| ebi-a-GCST90001626 | finngen_R11_O15_POOR_FETGRO | Poor fetal growth \|\| id:finngen_R11_O15_POOR_FETGRO | HLA DR+ CD4+ T cell %lymphocyte \|\| id:ebi-a-GCST90001626 | MR Egger | 21 | -0.058345297 | 0.10648718366992 | 0.590134004860539 | -0.267060177 | 0.150369583371751 | 0.943324164547369 | 0.765626997238555 | 1.16226371670346 | 0.992899239648112 |
| ebi-a-GCST90001626 | finngen_R11_O15_POOR_FETGRO | Poor fetal growth \|\| id:finngen_R11_O15_POOR_FETGRO | HLA DR+ CD4+ T cell %lymphocyte \|\| id:ebi-a-GCST90001626 | Weighted median | 21 | -0.022607859 | 0.0630413703573081 | 0.719879828722718 | -0.146168945 | 0.100953227227305 | 0.977645783929477 | 0.864011720761994 | 1.1062248993476 | 0.999440741871876 |
| ebi-a-GCST90001626 | finngen_R11_O15_POOR_FETGRO | Poor fetal growth \|\| id:finngen_R11_O15_POOR_FETGRO | HLA DR+ CD4+ T cell %lymphocyte \|\| id:ebi-a-GCST90001626 | Weighted mode | 21 | -0.048109906 | 0.0680086842147421 | 0.487474803165237 | -0.181406927 | 0.085187115112631 | 0.953029037761206 | 0.834095873488474 | 1.08892080117526 | 0.999857399747617 |
| ebi-a-GCST90001627 | finngen_R11_O15_POOR_FETGRO | Poor fetal growth \|\| id:finngen_R11_O15_POOR_FETGRO | HLA DR+ CD8+ T cell Absolute Count \|\| id:ebi-a-GCST90001627 | Inverse variance weighted | 34 | 0.00526288030590204 | 0.0160362693908219 | 0.742770975998363 | -0.026168208 | 0.036693968311913 | 1.00527675358759 | 0.974171212727254 | 1.03737550247909 | 0.994119304454654 |
| ebi-a-GCST90001627 | finngen_R11_O15_POOR_FETGRO | Poor fetal growth \|\| id:finngen_R11_O15_POOR_FETGRO | HLA DR+ CD8+ T cell Absolute Count \|\| id:ebi-a-GCST90001627 | MR Egger | 34 | 0.0141989898694612 | 0.0223158203974303 | 0.529122631871444 | -0.029540018 | 0.0579379978484245 | 1.0143002743374 | 0.970892023600452 | 1.05964929313737 | 0.992899239648112 |
| ebi-a-GCST90001627 | finngen_R11_O15_POOR_FETGRO | Poor fetal growth \|\| id:finngen_R11_O15_POOR_FETGRO | HLA DR+ CD8+ T cell Absolute Count \|\| id:ebi-a-GCST90001627 | Weighted median | 34 | 0.0207172624261791 | 0.0214926998260915 | 0.335085587740939 | -0.021408429 | 0.0628429540853185 | 1.02093335460699 | 0.978819104581673 | 1.06485959425009 | 0.999440741871876 |
| ebi-a-GCST90001627 | finngen_R11_O15_POOR_FETGRO | Poor fetal growth \|\| id:finngen_R11_O15_POOR_FETGRO | HLA DR+ CD8+ T cell Absolute Count \|\| id:ebi-a-GCST90001627 | Weighted mode | 34 | 0.00994847886803241 | 0.0195257776663451 | 0.613788907928419 | -0.028322045 | 0.0482190030940687 | 1.00999812949675 | 0.97207526406089 | 1.04940045210638 | 0.999857399747617 |
| ebi-a-GCST90001628 | finngen_R11_O15_POOR_FETGRO | Poor fetal growth \|\| id:finngen_R11_O15_POOR_FETGRO | HLA DR+ CD8+ T cell %T cell \|\| id:ebi-a-GCST90001628 | Inverse variance weighted | 30 | -0.012949433 | 0.0164309268973513 | 0.430630391250401 | -0.04515405 | 0.0192551834343751 | 0.987134049884988 | 0.955850221760923 | 1.01944176007741 | 0.994119304454654 |
| ebi-a-GCST90001628 | finngen_R11_O15_POOR_FETGRO | Poor fetal growth \|\| id:finngen_R11_O15_POOR_FETGRO | HLA DR+ CD8+ T cell %T cell \|\| id:ebi-a-GCST90001628 | MR Egger | 30 | -0.003098306 | 0.0218698266316891 | 0.888354803727186 | -0.045963167 | 0.0397665538612568 | 0.996906488461029 | 0.955077140343299 | 1.04056782929437 | 0.992899239648112 |
| ebi-a-GCST90001628 | finngen_R11_O15_POOR_FETGRO | Poor fetal growth \|\| id:finngen_R11_O15_POOR_FETGRO | HLA DR+ CD8+ T cell %T cell \|\| id:ebi-a-GCST90001628 | Weighted median | 30 | -0.008781497 | 0.0234751299349415 | 0.708347334316938 | -0.054792751 | 0.0372297580982655 | 0.991256948150446 | 0.94668132614855 | 1.03793146660458 | 0.999440741871876 |
| ebi-a-GCST90001628 | finngen_R11_O15_POOR_FETGRO | Poor fetal growth \|\| id:finngen_R11_O15_POOR_FETGRO | HLA DR+ CD8+ T cell %T cell \|\| id:ebi-a-GCST90001628 | Weighted mode | 30 | -0.006489624 | 0.0198377943875103 | 0.745915944475863 | -0.045371701 | 0.03239245263774 | 0.993531387772209 | 0.955642202300736 | 1.03292279904769 | 0.999857399747617 |
| ebi-a-GCST90001629 | finngen_R11_O15_POOR_FETGRO | Poor fetal growth \|\| id:finngen_R11_O15_POOR_FETGRO | HLA DR+ CD8+ T cell %lymphocyte \|\| id:ebi-a-GCST90001629 | Inverse variance weighted | 30 | -0.007413857 | 0.021980998669241 | 0.735902195940444 | -0.050496615 | 0.0356689000396524 | 0.992613557496596 | 0.950757147223432 | 1.03631266659782 | 0.994119304454654 |
| ebi-a-GCST90001629 | finngen_R11_O15_POOR_FETGRO | Poor fetal growth \|\| id:finngen_R11_O15_POOR_FETGRO | HLA DR+ CD8+ T cell %lymphocyte \|\| id:ebi-a-GCST90001629 | MR Egger | 30 | -0.009606673 | 0.0345294326788603 | 0.782890090817462 | -0.077284361 | 0.0580710152424769 | 0.990439323863633 | 0.925626604203391 | 1.05979025429989 | 0.992899239648112 |
| ebi-a-GCST90001629 | finngen_R11_O15_POOR_FETGRO | Poor fetal growth \|\| id:finngen_R11_O15_POOR_FETGRO | HLA DR+ CD8+ T cell %lymphocyte \|\| id:ebi-a-GCST90001629 | Weighted median | 30 | -0.01583995 | 0.031155502585249 | 0.611161778837898 | -0.076904735 | 0.0452248354844458 | 0.984284842649035 | 0.925978063029294 | 1.0462630705302 | 0.999440741871876 |
| ebi-a-GCST90001629 | finngen_R11_O15_POOR_FETGRO | Poor fetal growth \|\| id:finngen_R11_O15_POOR_FETGRO | HLA DR+ CD8+ T cell %lymphocyte \|\| id:ebi-a-GCST90001629 | Weighted mode | 30 | -0.013878126 | 0.0333792379526287 | 0.680638085844336 | -0.079301432 | 0.0515451806463093 | 0.986217731494242 | 0.923761431099839 | 1.05289675577344 | 0.999857399747617 |
| ebi-a-GCST90001630 | finngen_R11_O15_POOR_FETGRO | Poor fetal growth \|\| id:finngen_R11_O15_POOR_FETGRO | CD8+ Natural Killer T Absolute Count \|\| id:ebi-a-GCST90001630 | Inverse variance weighted | 26 | 0.0164980921376229 | 0.036903099863533 | 0.654827976416998 | -0.055831984 | 0.0888281678701475 | 1.01663493718466 | 0.94569801532369 | 1.09289284608543 | 0.994119304454654 |
| ebi-a-GCST90001630 | finngen_R11_O15_POOR_FETGRO | Poor fetal growth \|\| id:finngen_R11_O15_POOR_FETGRO | CD8+ Natural Killer T Absolute Count \|\| id:ebi-a-GCST90001630 | MR Egger | 26 | -0.003563095 | 0.0734240569651992 | 0.961697339421058 | -0.147474246 | 0.140348056863779 | 0.996443245501636 | 0.862884660387271 | 1.15067422923037 | 0.992899239648112 |
| ebi-a-GCST90001630 | finngen_R11_O15_POOR_FETGRO | Poor fetal growth \|\| id:finngen_R11_O15_POOR_FETGRO | CD8+ Natural Killer T Absolute Count \|\| id:ebi-a-GCST90001630 | Weighted median | 26 | -0.013929938 | 0.0458751801176004 | 0.761395553237354 | -0.103845291 | 0.0759854147203798 | 0.986166634343458 | 0.90136473556464 | 1.0789468373012 | 0.999440741871876 |
| ebi-a-GCST90001630 | finngen_R11_O15_POOR_FETGRO | Poor fetal growth \|\| id:finngen_R11_O15_POOR_FETGRO | CD8+ Natural Killer T Absolute Count \|\| id:ebi-a-GCST90001630 | Weighted mode | 26 | -0.021550147 | 0.0578732192489426 | 0.712756810662274 | -0.134981657 | 0.091881362719041 | 0.978680398343939 | 0.873731938635945 | 1.09623476005464 | 0.999857399747617 |
| ebi-a-GCST90001631 | finngen_R11_O15_POOR_FETGRO | Poor fetal growth \|\| id:finngen_R11_O15_POOR_FETGRO | CD8+ Natural Killer T %T cell \|\| id:ebi-a-GCST90001631 | Inverse variance weighted | 27 | 0.0221999495195984 | 0.0307167703250056 | 0.469845418492396 | -0.03800492 | 0.0824048193566094 | 1.02244820205997 | 0.962708204049606 | 1.08589531230567 | 0.994119304454654 |
| ebi-a-GCST90001631 | finngen_R11_O15_POOR_FETGRO | Poor fetal growth \|\| id:finngen_R11_O15_POOR_FETGRO | CD8+ Natural Killer T %T cell \|\| id:ebi-a-GCST90001631 | MR Egger | 27 | -0.070486305 | 0.0562073064488263 | 0.221426530239996 | -0.180652626 | 0.0396800154209729 | 0.931940502159746 | 0.8347252703132 | 1.04047778407365 | 0.992899239648112 |
| ebi-a-GCST90001631 | finngen_R11_O15_POOR_FETGRO | Poor fetal growth \|\| id:finngen_R11_O15_POOR_FETGRO | CD8+ Natural Killer T %T cell \|\| id:ebi-a-GCST90001631 | Weighted median | 27 | 0.00199434987454563 | 0.0444834243209615 | 0.964239982111501 | -0.085193162 | 0.0891818615436302 | 1.00199633991298 | 0.918334880054719 | 1.09327946373897 | 0.999440741871876 |
| ebi-a-GCST90001631 | finngen_R11_O15_POOR_FETGRO | Poor fetal growth \|\| id:finngen_R11_O15_POOR_FETGRO | CD8+ Natural Killer T %T cell \|\| id:ebi-a-GCST90001631 | Weighted mode | 27 | 0.00389661711222578 | 0.0589641986504399 | 0.94781612008597 | -0.111673212 | 0.119466446467088 | 1.00390421879509 | 0.89433646796105 | 1.12689543210988 | 0.999857399747617 |
| ebi-a-GCST90001632 | finngen_R11_O15_POOR_FETGRO | Poor fetal growth \|\| id:finngen_R11_O15_POOR_FETGRO | CD8+ Natural Killer T %lymphocyte \|\| id:ebi-a-GCST90001632 | Inverse variance weighted | 27 | 0.0366893614300462 | 0.0329546149688273 | 0.265566727206651 | -0.027901684 | 0.101280406768948 | 1.03737072342371 | 0.972483972924278 | 1.1065868927183 | 0.994119304454654 |
| ebi-a-GCST90001632 | finngen_R11_O15_POOR_FETGRO | Poor fetal growth \|\| id:finngen_R11_O15_POOR_FETGRO | CD8+ Natural Killer T %lymphocyte \|\| id:ebi-a-GCST90001632 | MR Egger | 27 | -0.062124604 | 0.0561695462709756 | 0.27924821651304 | -0.172216914 | 0.047967707024996 | 0.939765781233717 | 0.841796555604254 | 1.04913677502977 | 0.992899239648112 |
| ebi-a-GCST90001632 | finngen_R11_O15_POOR_FETGRO | Poor fetal growth \|\| id:finngen_R11_O15_POOR_FETGRO | CD8+ Natural Killer T %lymphocyte \|\| id:ebi-a-GCST90001632 | Weighted median | 27 | 0.00352541594694344 | 0.046498955237438 | 0.939564611315785 | -0.087612536 | 0.0946633682123218 | 1.00353163753482 | 0.916115769553656 | 1.09928873730007 | 0.999440741871876 |
| ebi-a-GCST90001632 | finngen_R11_O15_POOR_FETGRO | Poor fetal growth \|\| id:finngen_R11_O15_POOR_FETGRO | CD8+ Natural Killer T %lymphocyte \|\| id:ebi-a-GCST90001632 | Weighted mode | 27 | -0.001377195 | 0.0562142007011543 | 0.980641602725001 | -0.111557028 | 0.10880263858663 | 0.998623753109912 | 0.894440381657895 | 1.11494228204106 | 0.999857399747617 |
| ebi-a-GCST90001633 | finngen_R11_O15_POOR_FETGRO | Poor fetal growth \|\| id:finngen_R11_O15_POOR_FETGRO | CD8dim Natural Killer T Absolute Count \|\| id:ebi-a-GCST90001633 | Inverse variance weighted | 27 | 0.0230576497363308 | 0.0230751082668097 | 0.317676794453802 | -0.022169562 | 0.0682848619392777 | 1.02332553229386 | 0.97807437628612 | 1.07067025824851 | 0.994119304454654 |
| ebi-a-GCST90001633 | finngen_R11_O15_POOR_FETGRO | Poor fetal growth \|\| id:finngen_R11_O15_POOR_FETGRO | CD8dim Natural Killer T Absolute Count \|\| id:ebi-a-GCST90001633 | MR Egger | 27 | 0.0157625046422764 | 0.0324050241203993 | 0.630905222930955 | -0.047751343 | 0.079276351918259 | 1.01588738821508 | 0.95337082028353 | 1.08250343263865 | 0.992899239648112 |
| ebi-a-GCST90001633 | finngen_R11_O15_POOR_FETGRO | Poor fetal growth \|\| id:finngen_R11_O15_POOR_FETGRO | CD8dim Natural Killer T Absolute Count \|\| id:ebi-a-GCST90001633 | Weighted median | 27 | 0.0218140066225902 | 0.0347865672703212 | 0.530605929304589 | -0.046367665 | 0.0899956784724197 | 1.02205367157685 | 0.95469089101295 | 1.09416955521108 | 0.999440741871876 |
| ebi-a-GCST90001633 | finngen_R11_O15_POOR_FETGRO | Poor fetal growth \|\| id:finngen_R11_O15_POOR_FETGRO | CD8dim Natural Killer T Absolute Count \|\| id:ebi-a-GCST90001633 | Weighted mode | 27 | 0.0100360381262287 | 0.0298293912115427 | 0.739233532564717 | -0.048429569 | 0.0685016449008523 | 1.01008656805549 | 0.952724438612778 | 1.07090238647778 | 0.999857399747617 |
| ebi-a-GCST90001634 | finngen_R11_O15_POOR_FETGRO | Poor fetal growth \|\| id:finngen_R11_O15_POOR_FETGRO | CD8dim Natural Killer T %T cell \|\| id:ebi-a-GCST90001634 | Inverse variance weighted | 19 | 0.0426700517460312 | 0.0303359667067482 | 0.15955110934989 | -0.016788443 | 0.102128546491258 | 1.0435935061833 | 0.983351697566711 | 1.10752583113741 | 0.994119304454654 |
| ebi-a-GCST90001634 | finngen_R11_O15_POOR_FETGRO | Poor fetal growth \|\| id:finngen_R11_O15_POOR_FETGRO | CD8dim Natural Killer T %T cell \|\| id:ebi-a-GCST90001634 | MR Egger | 19 | 0.0528897804535815 | 0.0510636544500652 | 0.314823033733068 | -0.047194982 | 0.152974543175709 | 1.05431343276669 | 0.953901385600307 | 1.1652953138471 | 0.992899239648112 |
| ebi-a-GCST90001634 | finngen_R11_O15_POOR_FETGRO | Poor fetal growth \|\| id:finngen_R11_O15_POOR_FETGRO | CD8dim Natural Killer T %T cell \|\| id:ebi-a-GCST90001634 | Weighted median | 19 | -0.005131798 | 0.0432651805674345 | 0.905582237202255 | -0.089931552 | 0.079667955883539 | 0.994881347151122 | 0.913993744228094 | 1.08292742828907 | 0.999440741871876 |
| ebi-a-GCST90001634 | finngen_R11_O15_POOR_FETGRO | Poor fetal growth \|\| id:finngen_R11_O15_POOR_FETGRO | CD8dim Natural Killer T %T cell \|\| id:ebi-a-GCST90001634 | Weighted mode | 19 | 0.00247495038024503 | 0.0549174020395676 | 0.964550293174065 | -0.105163158 | 0.110113058377798 | 1.00247801559817 | 0.900177639765208 | 1.1164042821812 | 0.999857399747617 |
| ebi-a-GCST90001635 | finngen_R11_O15_POOR_FETGRO | Poor fetal growth \|\| id:finngen_R11_O15_POOR_FETGRO | CD8dim Natural Killer T %lymphocyte \|\| id:ebi-a-GCST90001635 | Inverse variance weighted | 26 | 0.0250718861258848 | 0.0275889701315833 | 0.363474267796292 | -0.029002495 | 0.079146267583788 | 1.02538882910553 | 0.971414040463022 | 1.08236262505868 | 0.994119304454654 |
| ebi-a-GCST90001635 | finngen_R11_O15_POOR_FETGRO | Poor fetal growth \|\| id:finngen_R11_O15_POOR_FETGRO | CD8dim Natural Killer T %lymphocyte \|\| id:ebi-a-GCST90001635 | MR Egger | 26 | 0.0185414386595856 | 0.0487380865665491 | 0.706972415269423 | -0.076985211 | 0.114068088330022 | 1.01871439845411 | 0.925903546682747 | 1.12082843762268 | 0.992899239648112 |
| ebi-a-GCST90001635 | finngen_R11_O15_POOR_FETGRO | Poor fetal growth \|\| id:finngen_R11_O15_POOR_FETGRO | CD8dim Natural Killer T %lymphocyte \|\| id:ebi-a-GCST90001635 | Weighted median | 26 | -0.019241393 | 0.0396444040618913 | 0.627428422938512 | -0.096944425 | 0.0584616388646234 | 0.980942540902821 | 0.907606444907237 | 1.06020431427327 | 0.999440741871876 |
| ebi-a-GCST90001635 | finngen_R11_O15_POOR_FETGRO | Poor fetal growth \|\| id:finngen_R11_O15_POOR_FETGRO | CD8dim Natural Killer T %lymphocyte \|\| id:ebi-a-GCST90001635 | Weighted mode | 26 | -0.01159279 | 0.0537126070914952 | 0.830871567285709 | -0.1168695 | 0.0936839199930215 | 0.988474147568875 | 0.8897012917793 | 1.09821256801591 | 0.999857399747617 |
| ebi-a-GCST90001636 | finngen_R11_O15_POOR_FETGRO | Poor fetal growth \|\| id:finngen_R11_O15_POOR_FETGRO | CD4-CD8- Natural Killer T Absolute Count \|\| id:ebi-a-GCST90001636 | Inverse variance weighted (multiplicative random effects) | 29 | 0.0209936379744436 | 0.0327108133169461 | 0.52100626593097 | -0.043119556 | 0.0851068320756579 | 1.0212155546174 | 0.957796872737205 | 1.08883338281547 | 0.994119304454654 |
| ebi-a-GCST90001636 | finngen_R11_O15_POOR_FETGRO | Poor fetal growth \|\| id:finngen_R11_O15_POOR_FETGRO | CD4-CD8- Natural Killer T Absolute Count \|\| id:ebi-a-GCST90001636 | MR Egger | 29 | 0.0308629427576957 | 0.0552735801871424 | 0.581195156594489 | -0.077473274 | 0.139199159924495 | 1.03134414101551 | 0.925451757311195 | 1.14935298226393 | 0.992899239648112 |
| ebi-a-GCST90001636 | finngen_R11_O15_POOR_FETGRO | Poor fetal growth \|\| id:finngen_R11_O15_POOR_FETGRO | CD4-CD8- Natural Killer T Absolute Count \|\| id:ebi-a-GCST90001636 | Weighted median | 29 | -0.021279473 | 0.0420443474054741 | 0.612772612173859 | -0.103686394 | 0.061127447845099 | 0.978945337477074 | 0.90150797141774 | 1.06303438699604 | 0.999440741871876 |
| ebi-a-GCST90001636 | finngen_R11_O15_POOR_FETGRO | Poor fetal growth \|\| id:finngen_R11_O15_POOR_FETGRO | CD4-CD8- Natural Killer T Absolute Count \|\| id:ebi-a-GCST90001636 | Weighted mode | 29 | -0.026956094 | 0.0430905109319929 | 0.536663593146444 | -0.111413496 | 0.0575013072639748 | 0.97340397870281 | 0.894568772200676 | 1.05918665529038 | 0.999857399747617 |
| ebi-a-GCST90001637 | finngen_R11_O15_POOR_FETGRO | Poor fetal growth \|\| id:finngen_R11_O15_POOR_FETGRO | CD4-CD8- Natural Killer T %T cell \|\| id:ebi-a-GCST90001637 | Inverse variance weighted | 31 | 0.0453083563871106 | 0.0319959248104918 | 0.156756043220437 | -0.017403656 | 0.108020369015674 | 1.04635045901561 | 0.982746912635496 | 1.11407043767356 | 0.994119304454654 |
| ebi-a-GCST90001637 | finngen_R11_O15_POOR_FETGRO | Poor fetal growth \|\| id:finngen_R11_O15_POOR_FETGRO | CD4-CD8- Natural Killer T %T cell \|\| id:ebi-a-GCST90001637 | MR Egger | 31 | 0.118473115789987 | 0.0592001441638116 | 0.0548036963393845 | 0.00244083322891617 | 0.234505398351058 | 1.12577660808039 | 1.00244381448743 | 1.26428329746243 | 0.992899239648112 |
| ebi-a-GCST90001637 | finngen_R11_O15_POOR_FETGRO | Poor fetal growth \|\| id:finngen_R11_O15_POOR_FETGRO | CD4-CD8- Natural Killer T %T cell \|\| id:ebi-a-GCST90001637 | Weighted median | 31 | 0.00771011823159653 | 0.0432604928677059 | 0.858545970254003 | -0.077080448 | 0.0925006842523 | 1.00773991772982 | 0.925815370810844 | 1.0969138921259 | 0.999440741871876 |
| ebi-a-GCST90001637 | finngen_R11_O15_POOR_FETGRO | Poor fetal growth \|\| id:finngen_R11_O15_POOR_FETGRO | CD4-CD8- Natural Killer T %T cell \|\| id:ebi-a-GCST90001637 | Weighted mode | 31 | -0.000338652 | 0.0592293847901003 | 0.99547586357371 | -0.116428246 | 0.115750942107016 | 0.999661405254563 | 0.890093962247049 | 1.12271621597424 | 0.999857399747617 |
| ebi-a-GCST90001638 | finngen_R11_O15_POOR_FETGRO | Poor fetal growth \|\| id:finngen_R11_O15_POOR_FETGRO | CD4-CD8- Natural Killer T %lymphocyte \|\| id:ebi-a-GCST90001638 | Inverse variance weighted | 30 | 0.0478941252256629 | 0.0336969875118812 | 0.155224416353787 | -0.01815197 | 0.11394022074895 | 1.04905958049821 | 0.982011784394814 | 1.12068512916401 | 0.994119304454654 |
| ebi-a-GCST90001638 | finngen_R11_O15_POOR_FETGRO | Poor fetal growth \|\| id:finngen_R11_O15_POOR_FETGRO | CD4-CD8- Natural Killer T %lymphocyte \|\| id:ebi-a-GCST90001638 | MR Egger | 30 | 0.113117273842395 | 0.0639515164974639 | 0.0878216749776831 | -0.012227698 | 0.238462246177424 | 1.11976324418697 | 0.987846756034434 | 1.26929578436396 | 0.992899239648112 |
| ebi-a-GCST90001638 | finngen_R11_O15_POOR_FETGRO | Poor fetal growth \|\| id:finngen_R11_O15_POOR_FETGRO | CD4-CD8- Natural Killer T %lymphocyte \|\| id:ebi-a-GCST90001638 | Weighted median | 30 | 0.0416997782042955 | 0.0442121740006503 | 0.345591849762031 | -0.044956083 | 0.12835563924557 | 1.04258142609259 | 0.956039467452295 | 1.13695727743321 | 0.999440741871876 |
| ebi-a-GCST90001638 | finngen_R11_O15_POOR_FETGRO | Poor fetal growth \|\| id:finngen_R11_O15_POOR_FETGRO | CD4-CD8- Natural Killer T %lymphocyte \|\| id:ebi-a-GCST90001638 | Weighted mode | 30 | 0.0399809434145629 | 0.0645825886358842 | 0.540705408506001 | -0.08660093 | 0.166562817140896 | 1.04079094008193 | 0.917042986679071 | 1.18123773551711 | 0.999857399747617 |
| ebi-a-GCST90001639 | finngen_R11_O15_POOR_FETGRO | Poor fetal growth \|\| id:finngen_R11_O15_POOR_FETGRO | CD3- lymphocyte Absolute Count \|\| id:ebi-a-GCST90001639 | Inverse variance weighted | 18 | -0.021813925 | 0.0388818569240849 | 0.57477644325569 | -0.098022364 | 0.0543945147167095 | 0.97842227818161 | 0.90662862729861 | 1.05590108851349 | 0.994119304454654 |
| ebi-a-GCST90001639 | finngen_R11_O15_POOR_FETGRO | Poor fetal growth \|\| id:finngen_R11_O15_POOR_FETGRO | CD3- lymphocyte Absolute Count \|\| id:ebi-a-GCST90001639 | MR Egger | 18 | 0.0223135913572869 | 0.0768560945879514 | 0.775293370881567 | -0.128324354 | 0.172951536749672 | 1.02256440155503 | 0.879568041428545 | 1.18880849016449 | 0.992899239648112 |
| ebi-a-GCST90001639 | finngen_R11_O15_POOR_FETGRO | Poor fetal growth \|\| id:finngen_R11_O15_POOR_FETGRO | CD3- lymphocyte Absolute Count \|\| id:ebi-a-GCST90001639 | Weighted median | 18 | -0.006218957 | 0.0541954114626769 | 0.90864279 | -0.112441964 | 0.100004049373732 | 0.993800340595992 | 0.893649209822319 | 1.10517539333479 | 0.999440741871876 |
| ebi-a-GCST90001639 | finngen_R11_O15_POOR_FETGRO | Poor fetal growth \|\| id:finngen_R11_O15_POOR_FETGRO | CD3- lymphocyte Absolute Count \|\| id:ebi-a-GCST90001639 | Weighted mode | 18 | -0.011866581 | 0.0665296333694736 | 0.860544349306563 | -0.142264663 | 0.118531500118552 | 0.988203548914082 | 0.867391659894624 | 1.12584233771054 | 0.999857399747617 |
| ebi-a-GCST90001640 | finngen_R11_O15_POOR_FETGRO | Poor fetal growth \|\| id:finngen_R11_O15_POOR_FETGRO | CD3- lymphocyte %lymphocyte \|\| id:ebi-a-GCST90001640 | Inverse variance weighted | 17 | 0.000516862900184434 | 0.0425436709429923 | 0.990306741887611 | -0.082868732 | 0.0839024579484494 | 1.00051699649683 | 0.920471967667979 | 1.08752280942913 | 0.998984604424952 |
| ebi-a-GCST90001640 | finngen_R11_O15_POOR_FETGRO | Poor fetal growth \|\| id:finngen_R11_O15_POOR_FETGRO | CD3- lymphocyte %lymphocyte \|\| id:ebi-a-GCST90001640 | MR Egger | 17 | -0.075004602 | 0.133342165827198 | 0.582086990181226 | -0.336355247 | 0.186346042924962 | 0.927739216773468 | 0.714369283105069 | 1.20483911430002 | 0.992899239648112 |
| ebi-a-GCST90001640 | finngen_R11_O15_POOR_FETGRO | Poor fetal growth \|\| id:finngen_R11_O15_POOR_FETGRO | CD3- lymphocyte %lymphocyte \|\| id:ebi-a-GCST90001640 | Weighted median | 17 | 0.00115707800859817 | 0.0558640280016321 | 0.983475078586436 | -0.108336417 | 0.110650572891797 | 1.00115774768162 | 0.897325670149284 | 1.11700452699206 | 0.999440741871876 |
| ebi-a-GCST90001640 | finngen_R11_O15_POOR_FETGRO | Poor fetal growth \|\| id:finngen_R11_O15_POOR_FETGRO | CD3- lymphocyte %lymphocyte \|\| id:ebi-a-GCST90001640 | Weighted mode | 17 | 0.00374852665494574 | 0.0753723551824633 | 0.960950367966962 | -0.14398129 | 0.151478342812574 | 1.00375556116793 | 0.86590394940116 | 1.16355310224919 | 0.999857399747617 |
| ebi-a-GCST90001641 | finngen_R11_O15_POOR_FETGRO | Poor fetal growth \|\| id:finngen_R11_O15_POOR_FETGRO | CD3- lymphocyte %leukocyte \|\| id:ebi-a-GCST90001641 | Inverse variance weighted (multiplicative random effects) | 20 | -0.02375055 | 0.0375891935388877 | 0.527488056094609 | -0.09742537 | 0.0499242690513054 | 0.976529274327344 | 0.907170041473426 | 1.0511914856331 | 0.994119304454654 |
| ebi-a-GCST90001641 | finngen_R11_O15_POOR_FETGRO | Poor fetal growth \|\| id:finngen_R11_O15_POOR_FETGRO | CD3- lymphocyte %leukocyte \|\| id:ebi-a-GCST90001641 | MR Egger | 20 | -0.062400661 | 0.0548727176716001 | 0.270371597535468 | -0.169951188 | 0.0451498657723109 | 0.9395063879308 | 0.843705998990039 | 1.04618463542915 | 0.992899239648112 |
| ebi-a-GCST90001641 | finngen_R11_O15_POOR_FETGRO | Poor fetal growth \|\| id:finngen_R11_O15_POOR_FETGRO | CD3- lymphocyte %leukocyte \|\| id:ebi-a-GCST90001641 | Weighted median | 20 | -0.026414553 | 0.0412767381408222 | 0.522212916723019 | -0.107316959 | 0.0544878540634148 | 0.973931260083952 | 0.898240921918089 | 1.05599965023105 | 0.999440741871876 |
| ebi-a-GCST90001641 | finngen_R11_O15_POOR_FETGRO | Poor fetal growth \|\| id:finngen_R11_O15_POOR_FETGRO | CD3- lymphocyte %leukocyte \|\| id:ebi-a-GCST90001641 | Weighted mode | 20 | -0.036037169 | 0.0400760419135142 | 0.379791035892515 | -0.114586211 | 0.042511873241903 | 0.964604439522568 | 0.891735057680453 | 1.04342844517847 | 0.999857399747617 |
| ebi-a-GCST90001642 | finngen_R11_O15_POOR_FETGRO | Poor fetal growth \|\| id:finngen_R11_O15_POOR_FETGRO | B cell Absolute Count \|\| id:ebi-a-GCST90001642 | Inverse variance weighted | 30 | 0.0184241953424645 | 0.0294272292598348 | 0.531253651503093 | -0.039253174 | 0.0761015646917407 | 1.01859496800018 | 0.961507249685882 | 1.07907216422367 | 0.994119304454654 |
| ebi-a-GCST90001642 | finngen_R11_O15_POOR_FETGRO | Poor fetal growth \|\| id:finngen_R11_O15_POOR_FETGRO | B cell Absolute Count \|\| id:ebi-a-GCST90001642 | MR Egger | 30 | 0.0233626497936677 | 0.0536731369622029 | 0.666698746204125 | -0.081836699 | 0.128561998239585 | 1.02363769424217 | 0.921422415933448 | 1.137191923 | 0.992899239648112 |
| ebi-a-GCST90001642 | finngen_R11_O15_POOR_FETGRO | Poor fetal growth \|\| id:finngen_R11_O15_POOR_FETGRO | B cell Absolute Count \|\| id:ebi-a-GCST90001642 | Weighted median | 30 | 0.0329064014660535 | 0.0426732389148216 | 0.440632842086595 | -0.050733147 | 0.116545949739104 | 1.03345380495324 | 0.950532289267881 | 1.12360913882782 | 0.999440741871876 |
| ebi-a-GCST90001642 | finngen_R11_O15_POOR_FETGRO | Poor fetal growth \|\| id:finngen_R11_O15_POOR_FETGRO | B cell Absolute Count \|\| id:ebi-a-GCST90001642 | Weighted mode | 30 | 0.0652587139108652 | 0.0657941191615609 | 0.329468923650005 | -0.06369776 | 0.194215187467525 | 1.067435149 | 0.938288545341369 | 1.21435756937247 | 0.999857399747617 |
| ebi-a-GCST90001643 | finngen_R11_O15_POOR_FETGRO | Poor fetal growth \|\| id:finngen_R11_O15_POOR_FETGRO | B cell %CD3- lymphocyte \|\| id:ebi-a-GCST90001643 | Inverse variance weighted | 29 | -0.013143496 | 0.0167785528123253 | 0.433421010884619 | -0.04602946 | 0.0197424670688558 | 0.986942502119824 | 0.955013827111478 | 1.01993863841391 | 0.994119304454654 |
| ebi-a-GCST90001643 | finngen_R11_O15_POOR_FETGRO | Poor fetal growth \|\| id:finngen_R11_O15_POOR_FETGRO | B cell %CD3- lymphocyte \|\| id:ebi-a-GCST90001643 | MR Egger | 29 | -0.014558588 | 0.021496104009539 | 0.503999191851558 | -0.056690952 | 0.0275737759601675 | 0.985546875920522 | 0.944886039617919 | 1.02795745085774 | 0.992899239648112 |
| ebi-a-GCST90001643 | finngen_R11_O15_POOR_FETGRO | Poor fetal growth \|\| id:finngen_R11_O15_POOR_FETGRO | B cell %CD3- lymphocyte \|\| id:ebi-a-GCST90001643 | Weighted median | 29 | -0.007626593 | 0.0218726423551111 | 0.727328179816803 | -0.050496972 | 0.0352437858511367 | 0.992402415504153 | 0.950756807387591 | 1.03587220900853 | 0.999440741871876 |
| ebi-a-GCST90001643 | finngen_R11_O15_POOR_FETGRO | Poor fetal growth \|\| id:finngen_R11_O15_POOR_FETGRO | B cell %CD3- lymphocyte \|\| id:ebi-a-GCST90001643 | Weighted mode | 29 | -0.009714303 | 0.0175502669499885 | 0.584305421008054 | -0.044112826 | 0.0246842205065088 | 0.990332728707457 | 0.956845994336338 | 1.02499139815013 | 0.999857399747617 |
| ebi-a-GCST90001644 | finngen_R11_O15_POOR_FETGRO | Poor fetal growth \|\| id:finngen_R11_O15_POOR_FETGRO | B cell %lymphocyte \|\| id:ebi-a-GCST90001644 | Inverse variance weighted | 34 | -0.001948987 | 0.0243512571417469 | 0.936208297331714 | -0.049677451 | 0.045779476781285 | 0.998052910825759 | 0.95153629188364 | 1.0468435321956 | 0.998984604424952 |
| ebi-a-GCST90001644 | finngen_R11_O15_POOR_FETGRO | Poor fetal growth \|\| id:finngen_R11_O15_POOR_FETGRO | B cell %lymphocyte \|\| id:ebi-a-GCST90001644 | MR Egger | 34 | 0.0269452011928124 | 0.0422374952008619 | 0.528048504916934 | -0.055840289 | 0.109730691786502 | 1.02731150577587 | 0.945690160572084 | 1.11597748808242 | 0.992899239648112 |
| ebi-a-GCST90001644 | finngen_R11_O15_POOR_FETGRO | Poor fetal growth \|\| id:finngen_R11_O15_POOR_FETGRO | B cell %lymphocyte \|\| id:ebi-a-GCST90001644 | Weighted median | 34 | 0.0133157248817837 | 0.0355166635169335 | 0.707723788162833 | -0.056296936 | 0.0829283853749733 | 1.01340477395837 | 0.945258413329298 | 1.08646399905023 | 0.999440741871876 |
| ebi-a-GCST90001644 | finngen_R11_O15_POOR_FETGRO | Poor fetal growth \|\| id:finngen_R11_O15_POOR_FETGRO | B cell %lymphocyte \|\| id:ebi-a-GCST90001644 | Weighted mode | 34 | 0.00490870020283785 | 0.0607920629320787 | 0.936131884255343 | -0.114243743 | 0.124061143549712 | 1.00492076760869 | 0.892040500625641 | 1.13208508858394 | 0.999857399747617 |
| ebi-a-GCST90001645 | finngen_R11_O15_POOR_FETGRO | Poor fetal growth \|\| id:finngen_R11_O15_POOR_FETGRO | Natural Killer Absolute Count \|\| id:ebi-a-GCST90001645 | Inverse variance weighted | 22 | 0.0175679097610834 | 0.0333474561910854 | 0.598322697863963 | -0.047793104 | 0.0829289238956108 | 1.0177231331389 | 0.953331006690808 | 1.08646458413367 | 0.994119304454654 |
| ebi-a-GCST90001645 | finngen_R11_O15_POOR_FETGRO | Poor fetal growth \|\| id:finngen_R11_O15_POOR_FETGRO | Natural Killer Absolute Count \|\| id:ebi-a-GCST90001645 | MR Egger | 22 | -0.032051287 | 0.0580692985087275 | 0.587098632634146 | -0.145867112 | 0.0817645381971486 | 0.968456911672116 | 0.864272547041127 | 1.08520025653535 | 0.992899239648112 |
| ebi-a-GCST90001645 | finngen_R11_O15_POOR_FETGRO | Poor fetal growth \|\| id:finngen_R11_O15_POOR_FETGRO | Natural Killer Absolute Count \|\| id:ebi-a-GCST90001645 | Weighted median | 22 | 0.0181217782349761 | 0.0451518199329058 | 0.688160902300595 | -0.070375789 | 0.106619345303471 | 1.01828697402993 | 0.932043502546784 | 1.11251069144917 | 0.999440741871876 |
| ebi-a-GCST90001645 | finngen_R11_O15_POOR_FETGRO | Poor fetal growth \|\| id:finngen_R11_O15_POOR_FETGRO | Natural Killer Absolute Count \|\| id:ebi-a-GCST90001645 | Weighted mode | 22 | 0.0168386920278403 | 0.0604178308557467 | 0.783198014859821 | -0.101580256 | 0.135257640505104 | 1.01698126190839 | 0.903408672060273 | 1.1448317013762 | 0.999857399747617 |
| ebi-a-GCST90001646 | finngen_R11_O15_POOR_FETGRO | Poor fetal growth \|\| id:finngen_R11_O15_POOR_FETGRO | Natural Killer %CD3- lymphocyte \|\| id:ebi-a-GCST90001646 | Inverse variance weighted | 27 | 0.0114690816846415 | 0.0146763477532895 | 0.434527868953235 | -0.01729656 | 0.0402347232810888 | 1.01153510376477 | 0.982852166858881 | 1.04105510538629 | 0.994119304454654 |
| ebi-a-GCST90001646 | finngen_R11_O15_POOR_FETGRO | Poor fetal growth \|\| id:finngen_R11_O15_POOR_FETGRO | Natural Killer %CD3- lymphocyte \|\| id:ebi-a-GCST90001646 | MR Egger | 27 | 0.0202291215514694 | 0.0192092351188652 | 0.302370625371363 | -0.017420979 | 0.0578792223844452 | 1.02043511692123 | 0.98272988861882 | 1.05958701358878 | 0.992899239648112 |
| ebi-a-GCST90001646 | finngen_R11_O15_POOR_FETGRO | Poor fetal growth \|\| id:finngen_R11_O15_POOR_FETGRO | Natural Killer %CD3- lymphocyte \|\| id:ebi-a-GCST90001646 | Weighted median | 27 | 0.00559596538250249 | 0.0205874832569461 | 0.78576506949852 | -0.034755502 | 0.0459474325661168 | 1.0056116520438 | 0.965841533906672 | 1.04701937038875 | 0.999440741871876 |
| ebi-a-GCST90001646 | finngen_R11_O15_POOR_FETGRO | Poor fetal growth \|\| id:finngen_R11_O15_POOR_FETGRO | Natural Killer %CD3- lymphocyte \|\| id:ebi-a-GCST90001646 | Weighted mode | 27 | 0.00735977011311545 | 0.0206106684560006 | 0.723909885965536 | -0.03303714 | 0.0477566802868766 | 1.00738691978542 | 0.967502625815547 | 1.04891540247688 | 0.999857399747617 |
| ebi-a-GCST90001647 | finngen_R11_O15_POOR_FETGRO | Poor fetal growth \|\| id:finngen_R11_O15_POOR_FETGRO | Natural Killer %lymphocyte \|\| id:ebi-a-GCST90001647 | Inverse variance weighted | 27 | -0.007365056 | 0.020350693584252 | 0.717421613808647 | -0.047252416 | 0.0325223032774129 | 0.992661999415648 | 0.953846601464979 | 1.0330569334424 | 0.994119304454654 |
| ebi-a-GCST90001647 | finngen_R11_O15_POOR_FETGRO | Poor fetal growth \|\| id:finngen_R11_O15_POOR_FETGRO | Natural Killer %lymphocyte \|\| id:ebi-a-GCST90001647 | MR Egger | 27 | -0.00443015 | 0.0276949892021741 | 0.874196334299831 | -0.058712329 | 0.0498520289655429 | 0.995579648768067 | 0.942977997842805 | 1.05111555021285 | 0.992899239648112 |
| ebi-a-GCST90001647 | finngen_R11_O15_POOR_FETGRO | Poor fetal growth \|\| id:finngen_R11_O15_POOR_FETGRO | Natural Killer %lymphocyte \|\| id:ebi-a-GCST90001647 | Weighted median | 27 | 0.0047574021686939 | 0.0280263787731406 | 0.86520887259495 | -0.0501743 | 0.0596891045640495 | 1.00476873657337 | 0.951063639445022 | 1.06150647772042 | 0.999440741871876 |
| ebi-a-GCST90001647 | finngen_R11_O15_POOR_FETGRO | Poor fetal growth \|\| id:finngen_R11_O15_POOR_FETGRO | Natural Killer %lymphocyte \|\| id:ebi-a-GCST90001647 | Weighted mode | 27 | -0.001500068 | 0.0277105522564826 | 0.957242647268584 | -0.055812751 | 0.0528126141460562 | 0.998501056263402 | 0.945716204009747 | 1.05423207843106 | 0.999857399747617 |
| ebi-a-GCST90001648 | finngen_R11_O15_POOR_FETGRO | Poor fetal growth \|\| id:finngen_R11_O15_POOR_FETGRO | HLA DR+ Natural Killer Absolute Count \|\| id:ebi-a-GCST90001648 | Inverse variance weighted | 18 | 0.00508726419145714 | 0.0329231675182571 | 0.877200309310063 | -0.059442144 | 0.069616672527241 | 1.00510022629115 | 0.942290049011275 | 1.07209713819067 | 0.994119304454654 |
| ebi-a-GCST90001648 | finngen_R11_O15_POOR_FETGRO | Poor fetal growth \|\| id:finngen_R11_O15_POOR_FETGRO | HLA DR+ Natural Killer Absolute Count \|\| id:ebi-a-GCST90001648 | MR Egger | 18 | -0.019131934 | 0.0591519740717684 | 0.75055330181031 | -0.135069803 | 0.0968059353039312 | 0.98104991998487 | 0.873654925774625 | 1.10164656216979 | 0.992899239648112 |
| ebi-a-GCST90001648 | finngen_R11_O15_POOR_FETGRO | Poor fetal growth \|\| id:finngen_R11_O15_POOR_FETGRO | HLA DR+ Natural Killer Absolute Count \|\| id:ebi-a-GCST90001648 | Weighted median | 18 | -0.001242099 | 0.0433053718755277 | 0.977117946880312 | -0.086120628 | 0.0836364297838601 | 0.998758671993615 | 0.917483550368136 | 1.0872335362113 | 0.999440741871876 |
| ebi-a-GCST90001648 | finngen_R11_O15_POOR_FETGRO | Poor fetal growth \|\| id:finngen_R11_O15_POOR_FETGRO | HLA DR+ Natural Killer Absolute Count \|\| id:ebi-a-GCST90001648 | Weighted mode | 18 | -0.001430742 | 0.0445412808512099 | 0.974749111722117 | -0.088731653 | 0.0858701682696935 | 0.998570280824989 | 0.915091102886277 | 1.08966484604847 | 0.999857399747617 |
| ebi-a-GCST90001649 | finngen_R11_O15_POOR_FETGRO | Poor fetal growth \|\| id:finngen_R11_O15_POOR_FETGRO | HLA DR+ Natural Killer %Natural Killer \|\| id:ebi-a-GCST90001649 | Inverse variance weighted | 23 | -0.001570187 | 0.0261858349812043 | 0.952184933905968 | -0.052894423 | 0.0497540497687872 | 0.998431045303952 | 0.948480144545509 | 1.05101256780069 | 0.998984604424952 |
| ebi-a-GCST90001649 | finngen_R11_O15_POOR_FETGRO | Poor fetal growth \|\| id:finngen_R11_O15_POOR_FETGRO | HLA DR+ Natural Killer %Natural Killer \|\| id:ebi-a-GCST90001649 | MR Egger | 23 | -0.041184824 | 0.0431318675984546 | 0.350512702958923 | -0.125723284 | 0.0433536368685736 | 0.959651747241962 | 0.881858837370363 | 1.04430713506336 | 0.992899239648112 |
| ebi-a-GCST90001649 | finngen_R11_O15_POOR_FETGRO | Poor fetal growth \|\| id:finngen_R11_O15_POOR_FETGRO | HLA DR+ Natural Killer %Natural Killer \|\| id:ebi-a-GCST90001649 | Weighted median | 23 | -0.000848156 | 0.0356636395875601 | 0.981026416085152 | -0.07074889 | 0.0690525772565911 | 0.99915220324789 | 0.931695820961366 | 1.07149254380578 | 0.999440741871876 |
| ebi-a-GCST90001649 | finngen_R11_O15_POOR_FETGRO | Poor fetal growth \|\| id:finngen_R11_O15_POOR_FETGRO | HLA DR+ Natural Killer %Natural Killer \|\| id:ebi-a-GCST90001649 | Weighted mode | 23 | 0.00436077771382859 | 0.0396827517754314 | 0.913492082738192 | -0.073417416 | 0.0821389711936741 | 1.00437029974108 | 0.929212880956899 | 1.08560666740131 | 0.999857399747617 |
| ebi-a-GCST90001650 | finngen_R11_O15_POOR_FETGRO | Poor fetal growth \|\| id:finngen_R11_O15_POOR_FETGRO | HLA DR+ Natural Killer %CD3- lymphocyte \|\| id:ebi-a-GCST90001650 | Inverse variance weighted | 23 | -0.015449658 | 0.0259589794308357 | 0.551738802468965 | -0.066329257 | 0.0354299419338163 | 0.984669075960187 | 0.935822686981981 | 1.03606506087083 | 0.994119304454654 |
| ebi-a-GCST90001650 | finngen_R11_O15_POOR_FETGRO | Poor fetal growth \|\| id:finngen_R11_O15_POOR_FETGRO | HLA DR+ Natural Killer %CD3- lymphocyte \|\| id:ebi-a-GCST90001650 | MR Egger | 23 | -0.064186051 | 0.0422416218763207 | 0.14355076982455 | -0.14697963 | 0.0186075273797338 | 0.937830498529161 | 0.863311562569622 | 1.01878172620968 | 0.992899239648112 |
| ebi-a-GCST90001650 | finngen_R11_O15_POOR_FETGRO | Poor fetal growth \|\| id:finngen_R11_O15_POOR_FETGRO | HLA DR+ Natural Killer %CD3- lymphocyte \|\| id:ebi-a-GCST90001650 | Weighted median | 23 | -0.004440486 | 0.0361088924747675 | 0.902126999557821 | -0.075213916 | 0.0663329429757558 | 0.995569358107718 | 0.927545048818489 | 1.06858243496157 | 0.999440741871876 |
| ebi-a-GCST90001650 | finngen_R11_O15_POOR_FETGRO | Poor fetal growth \|\| id:finngen_R11_O15_POOR_FETGRO | HLA DR+ Natural Killer %CD3- lymphocyte \|\| id:ebi-a-GCST90001650 | Weighted mode | 23 | -0.005037418 | 0.0399121014322606 | 0.900709850476638 | -0.083265137 | 0.073190300605423 | 0.994975248311495 | 0.920107160416081 | 1.07593526856681 | 0.999857399747617 |
| ebi-a-GCST90001651 | finngen_R11_O15_POOR_FETGRO | Poor fetal growth \|\| id:finngen_R11_O15_POOR_FETGRO | Granulocyte Absolute Count \|\| id:ebi-a-GCST90001651 | Inverse variance weighted | 24 | 0.00713883684705254 | 0.0325288999364102 | 0.826290670968601 | -0.056617807 | 0.0708954807224166 | 1.00716437908727 | 0.944955155578845 | 1.07346902179805 | 0.994119304454654 |
| ebi-a-GCST90001651 | finngen_R11_O15_POOR_FETGRO | Poor fetal growth \|\| id:finngen_R11_O15_POOR_FETGRO | Granulocyte Absolute Count \|\| id:ebi-a-GCST90001651 | MR Egger | 24 | 0.0678423763974227 | 0.052391888772069 | 0.208779472641622 | -0.034845726 | 0.170530478390678 | 1.0701966069388 | 0.965754395949402 | 1.18593379673658 | 0.992899239648112 |
| ebi-a-GCST90001651 | finngen_R11_O15_POOR_FETGRO | Poor fetal growth \|\| id:finngen_R11_O15_POOR_FETGRO | Granulocyte Absolute Count \|\| id:ebi-a-GCST90001651 | Weighted median | 24 | 0.025591169468601 | 0.0470352537458736 | 0.586383078142743 | -0.066597928 | 0.117780266810513 | 1.02592143471894 | 0.935571292863141 | 1.12499688505271 | 0.999440741871876 |
| ebi-a-GCST90001651 | finngen_R11_O15_POOR_FETGRO | Poor fetal growth \|\| id:finngen_R11_O15_POOR_FETGRO | Granulocyte Absolute Count \|\| id:ebi-a-GCST90001651 | Weighted mode | 24 | 0.0384406097699999 | 0.0496221463471547 | 0.446428084363447 | -0.058818797 | 0.135700016610423 | 1.03918900885099 | 0.942877605862991 | 1.14533825960187 | 0.999857399747617 |
| ebi-a-GCST90001652 | finngen_R11_O15_POOR_FETGRO | Poor fetal growth \|\| id:finngen_R11_O15_POOR_FETGRO | Granulocyte %leukocyte \|\| id:ebi-a-GCST90001652 | Inverse variance weighted | 24 | 0.0364474870769644 | 0.0258482967988244 | 0.158523820996846 | -0.014215175 | 0.0871101488026601 | 1.03711984039338 | 0.985885383896745 | 1.09101684729941 | 0.994119304454654 |
| ebi-a-GCST90001652 | finngen_R11_O15_POOR_FETGRO | Poor fetal growth \|\| id:finngen_R11_O15_POOR_FETGRO | Granulocyte %leukocyte \|\| id:ebi-a-GCST90001652 | MR Egger | 24 | 0.053673465994323 | 0.0380265411771691 | 0.172092037930214 | -0.020858555 | 0.128205486701575 | 1.05514000680412 | 0.979357480272975 | 1.13678657322175 | 0.992899239648112 |
| ebi-a-GCST90001652 | finngen_R11_O15_POOR_FETGRO | Poor fetal growth \|\| id:finngen_R11_O15_POOR_FETGRO | Granulocyte %leukocyte \|\| id:ebi-a-GCST90001652 | Weighted median | 24 | 0.0246592295070551 | 0.0393083192339532 | 0.530443915933022 | -0.052385076 | 0.101703535205603 | 1.02496578291074 | 0.948963373274323 | 1.10705522017459 | 0.999440741871876 |
| ebi-a-GCST90001652 | finngen_R11_O15_POOR_FETGRO | Poor fetal growth \|\| id:finngen_R11_O15_POOR_FETGRO | Granulocyte %leukocyte \|\| id:ebi-a-GCST90001652 | Weighted mode | 24 | 0.0298639586425289 | 0.0346928374113679 | 0.398227499911974 | -0.038134003 | 0.09786191996881 | 1.03031435905491 | 0.962583943416634 | 1.10281049848685 | 0.999857399747617 |
| ebi-a-GCST90001653 | finngen_R11_O15_POOR_FETGRO | Poor fetal growth \|\| id:finngen_R11_O15_POOR_FETGRO | CD28- CD4-CD8- T cell %CD4-CD8- T cell \|\| id:ebi-a-GCST90001653 | Inverse variance weighted (multiplicative random effects) | 27 | 0.0305391309561474 | 0.0310498081961639 | 0.325335360466426 | -0.030318493 | 0.0913967550206286 | 1.03101023367567 | 0.970136502548794 | 1.09570364495228 | 0.994119304454654 |
| ebi-a-GCST90001653 | finngen_R11_O15_POOR_FETGRO | Poor fetal growth \|\| id:finngen_R11_O15_POOR_FETGRO | CD28- CD4-CD8- T cell %CD4-CD8- T cell \|\| id:ebi-a-GCST90001653 | MR Egger | 27 | -0.028951698 | 0.0433431802817068 | 0.510276542020578 | -0.113904331 | 0.0560009354964817 | 0.971463387098105 | 0.892343321206704 | 1.05759867311599 | 0.992899239648112 |
| ebi-a-GCST90001653 | finngen_R11_O15_POOR_FETGRO | Poor fetal growth \|\| id:finngen_R11_O15_POOR_FETGRO | CD28- CD4-CD8- T cell %CD4-CD8- T cell \|\| id:ebi-a-GCST90001653 | Weighted median | 27 | -0.041813452 | 0.0370416029863687 | 0.258972065807325 | -0.114414994 | 0.0307880896151941 | 0.959048672269419 | 0.891887750924486 | 1.03126694455479 | 0.999440741871876 |
| ebi-a-GCST90001653 | finngen_R11_O15_POOR_FETGRO | Poor fetal growth \|\| id:finngen_R11_O15_POOR_FETGRO | CD28- CD4-CD8- T cell %CD4-CD8- T cell \|\| id:ebi-a-GCST90001653 | Weighted mode | 27 | -0.030166092 | 0.0362537553929648 | 0.412940343537186 | -0.101223452 | 0.040891268726067 | 0.970284363845057 | 0.903731069432702 | 1.04173882979721 | 0.999857399747617 |
| ebi-a-GCST90001654 | finngen_R11_O15_POOR_FETGRO | Poor fetal growth \|\| id:finngen_R11_O15_POOR_FETGRO | CD28- CD4-CD8- T cell Absolute Count \|\| id:ebi-a-GCST90001654 | Inverse variance weighted | 30 | -0.017594952 | 0.0271938401761007 | 0.517619155885091 | -0.070894879 | 0.0357049748451216 | 0.982558935397811 | 0.931559813810383 | 1.036350052 | 0.994119304454654 |
| ebi-a-GCST90001654 | finngen_R11_O15_POOR_FETGRO | Poor fetal growth \|\| id:finngen_R11_O15_POOR_FETGRO | CD28- CD4-CD8- T cell Absolute Count \|\| id:ebi-a-GCST90001654 | MR Egger | 30 | -0.047026758 | 0.0426061560670847 | 0.279095256575821 | -0.130534824 | 0.0364813082508421 | 0.95406186880419 | 0.877625930381809 | 1.03715491759701 | 0.992899239648112 |
| ebi-a-GCST90001654 | finngen_R11_O15_POOR_FETGRO | Poor fetal growth \|\| id:finngen_R11_O15_POOR_FETGRO | CD28- CD4-CD8- T cell Absolute Count \|\| id:ebi-a-GCST90001654 | Weighted median | 30 | -0.038840262 | 0.042834228690021 | 0.364534999018922 | -0.12279535 | 0.0451148260578718 | 0.961904349384004 | 0.884444645277847 | 1.04614797806051 | 0.999440741871876 |
| ebi-a-GCST90001654 | finngen_R11_O15_POOR_FETGRO | Poor fetal growth \|\| id:finngen_R11_O15_POOR_FETGRO | CD28- CD4-CD8- T cell Absolute Count \|\| id:ebi-a-GCST90001654 | Weighted mode | 30 | -0.035332292 | 0.0392846950269696 | 0.37585233386549 | -0.112330294 | 0.0416657106385125 | 0.965284606979044 | 0.893749008927173 | 1.04254590848627 | 0.999857399747617 |
| ebi-a-GCST90001655 | finngen_R11_O15_POOR_FETGRO | Poor fetal growth \|\| id:finngen_R11_O15_POOR_FETGRO | CD28+ CD4-CD8- T cell %T cell \|\| id:ebi-a-GCST90001655 | Inverse variance weighted | 23 | 0.0261156934618233 | 0.032150466141007 | 0.416621833473064 | -0.03689922 | 0.0891306070981969 | 1.02645969627982 | 0.963773259355752 | 1.09322342974236 | 0.994119304454654 |
| ebi-a-GCST90001655 | finngen_R11_O15_POOR_FETGRO | Poor fetal growth \|\| id:finngen_R11_O15_POOR_FETGRO | CD28+ CD4-CD8- T cell %T cell \|\| id:ebi-a-GCST90001655 | MR Egger | 23 | 0.0561358862698988 | 0.0526271481093737 | 0.298230300071589 | -0.047013324 | 0.159285096564271 | 1.05774140650566 | 0.954074685391225 | 1.17267222384985 | 0.992899239648112 |
| ebi-a-GCST90001655 | finngen_R11_O15_POOR_FETGRO | Poor fetal growth \|\| id:finngen_R11_O15_POOR_FETGRO | CD28+ CD4-CD8- T cell %T cell \|\| id:ebi-a-GCST90001655 | Weighted median | 23 | 0.000715347807625193 | 0.0468110434493072 | 0.987807519013601 | -0.091034297 | 0.0924649929682672 | 1.00071560372989 | 0.912986397345514 | 1.09687474255927 | 0.999440741871876 |
| ebi-a-GCST90001655 | finngen_R11_O15_POOR_FETGRO | Poor fetal growth \|\| id:finngen_R11_O15_POOR_FETGRO | CD28+ CD4-CD8- T cell %T cell \|\| id:ebi-a-GCST90001655 | Weighted mode | 23 | 0.0301481527627241 | 0.0476797563908498 | 0.533704459797328 | -0.06330417 | 0.12360047528879 | 1.03060720994899 | 0.938657918905717 | 1.13156369301938 | 0.999857399747617 |
| ebi-a-GCST90001656 | finngen_R11_O15_POOR_FETGRO | Poor fetal growth \|\| id:finngen_R11_O15_POOR_FETGRO | CD28+ CD4-CD8- T cell %CD4-CD8- T cell \|\| id:ebi-a-GCST90001656 | Inverse variance weighted (multiplicative random effects) | 27 | -0.030539131 | 0.0310498081961639 | 0.325335360466426 | -0.091396755 | 0.0303184931083339 | 0.969922477330686 | 0.912655538390175 | 1.03078277889013 | 0.994119304454654 |
| ebi-a-GCST90001656 | finngen_R11_O15_POOR_FETGRO | Poor fetal growth \|\| id:finngen_R11_O15_POOR_FETGRO | CD28+ CD4-CD8- T cell %CD4-CD8- T cell \|\| id:ebi-a-GCST90001656 | MR Egger | 27 | 0.0289516978556637 | 0.0433431802817068 | 0.510276542020578 | -0.056000935 | 0.113904331207809 | 1.02937487226064 | 0.945538251342275 | 1.12064490901071 | 0.992899239648112 |
| ebi-a-GCST90001656 | finngen_R11_O15_POOR_FETGRO | Poor fetal growth \|\| id:finngen_R11_O15_POOR_FETGRO | CD28+ CD4-CD8- T cell %CD4-CD8- T cell \|\| id:ebi-a-GCST90001656 | Weighted median | 27 | 0.0418134522380886 | 0.0374754791908351 | 0.264527032721421 | -0.031638487 | 0.115265391452125 | 1.04269994726511 | 0.968856773117996 | 1.12217121270438 | 0.999440741871876 |
| ebi-a-GCST90001656 | finngen_R11_O15_POOR_FETGRO | Poor fetal growth \|\| id:finngen_R11_O15_POOR_FETGRO | CD28+ CD4-CD8- T cell %CD4-CD8- T cell \|\| id:ebi-a-GCST90001656 | Weighted mode | 27 | 0.0301660918441441 | 0.0346291741176889 | 0.391662535174666 | -0.037707089 | 0.0980392731148143 | 1.03062569826147 | 0.96299497099359 | 1.10300610274321 | 0.999857399747617 |
| ebi-a-GCST90001657 | finngen_R11_O15_POOR_FETGRO | Poor fetal growth \|\| id:finngen_R11_O15_POOR_FETGRO | CD28+ CD4-CD8- T cell Absolute Count \|\| id:ebi-a-GCST90001657 | Inverse variance weighted | 12 | -0.048746218 | 0.0526413783550057 | 0.354443001107149 | -0.15192332 | 0.0544308834475977 | 0.9524228066732 | 0.859054150742164 | 1.05593949099434 | 0.994119304454654 |
| ebi-a-GCST90001657 | finngen_R11_O15_POOR_FETGRO | Poor fetal growth \|\| id:finngen_R11_O15_POOR_FETGRO | CD28+ CD4-CD8- T cell Absolute Count \|\| id:ebi-a-GCST90001657 | MR Egger | 12 | -0.024698142 | 0.0856993200739969 | 0.779082174545568 | -0.192668809 | 0.143272525724811 | 0.975604361937009 | 0.824755080399927 | 1.15404426556425 | 0.992899239648112 |
| ebi-a-GCST90001657 | finngen_R11_O15_POOR_FETGRO | Poor fetal growth \|\| id:finngen_R11_O15_POOR_FETGRO | CD28+ CD4-CD8- T cell Absolute Count \|\| id:ebi-a-GCST90001657 | Weighted median | 12 | -0.072308823 | 0.0742995402665346 | 0.330450495093055 | -0.217935922 | 0.0733182755985975 | 0.93024357053571 | 0.804176969869509 | 1.07607297018646 | 0.999440741871876 |
| ebi-a-GCST90001657 | finngen_R11_O15_POOR_FETGRO | Poor fetal growth \|\| id:finngen_R11_O15_POOR_FETGRO | CD28+ CD4-CD8- T cell Absolute Count \|\| id:ebi-a-GCST90001657 | Weighted mode | 12 | -0.067523937 | 0.0807375407251455 | 0.420761240254523 | -0.225769517 | 0.0907216425533629 | 0.934705346055939 | 0.797901983245705 | 1.09496417140314 | 0.999857399747617 |
| ebi-a-GCST90001658 | finngen_R11_O15_POOR_FETGRO | Poor fetal growth \|\| id:finngen_R11_O15_POOR_FETGRO | CD39+ CD4+ T cell %T cell \|\| id:ebi-a-GCST90001658 | Inverse variance weighted | 31 | -0.005527117 | 0.0134560282497605 | 0.68125295251882 | -0.031900932 | 0.020846698422277 | 0.994488129461127 | 0.968602534535279 | 1.02106550868525 | 0.994119304454654 |
| ebi-a-GCST90001658 | finngen_R11_O15_POOR_FETGRO | Poor fetal growth \|\| id:finngen_R11_O15_POOR_FETGRO | CD39+ CD4+ T cell %T cell \|\| id:ebi-a-GCST90001658 | MR Egger | 31 | -0.003209207 | 0.016350879276123 | 0.845766799766268 | -0.035256931 | 0.0288385158829349 | 0.996795936503923 | 0.965357354277505 | 1.0292583721749 | 0.992899239648112 |
| ebi-a-GCST90001658 | finngen_R11_O15_POOR_FETGRO | Poor fetal growth \|\| id:finngen_R11_O15_POOR_FETGRO | CD39+ CD4+ T cell %T cell \|\| id:ebi-a-GCST90001658 | Weighted median | 31 | 0.00472642764198363 | 0.0188598307661342 | 0.80211706586429 | -0.032238841 | 0.0416916959436066 | 1.0047376148193 | 0.968275290950231 | 1.04257299971177 | 0.999440741871876 |
| ebi-a-GCST90001658 | finngen_R11_O15_POOR_FETGRO | Poor fetal growth \|\| id:finngen_R11_O15_POOR_FETGRO | CD39+ CD4+ T cell %T cell \|\| id:ebi-a-GCST90001658 | Weighted mode | 31 | 0.00719055870207974 | 0.0158199561026771 | 0.652723176390972 | -0.023816555 | 0.0381976726633268 | 1.00721647284445 | 0.976464820664889 | 1.03893658194306 | 0.999857399747617 |
| ebi-a-GCST90001659 | finngen_R11_O15_POOR_FETGRO | Poor fetal growth \|\| id:finngen_R11_O15_POOR_FETGRO | CD39+ CD4+ T cell %CD4+ T cell \|\| id:ebi-a-GCST90001659 | Inverse variance weighted | 25 | -0.01591041 | 0.0170126791013608 | 0.349680630954855 | -0.049255261 | 0.0174344414098834 | 0.984215492336848 | 0.951938106326277 | 1.0175873083747 | 0.994119304454654 |
| ebi-a-GCST90001659 | finngen_R11_O15_POOR_FETGRO | Poor fetal growth \|\| id:finngen_R11_O15_POOR_FETGRO | CD39+ CD4+ T cell %CD4+ T cell \|\| id:ebi-a-GCST90001659 | MR Egger | 25 | -0.015131876 | 0.0225263963024473 | 0.508441080003043 | -0.059283613 | 0.0290198605713762 | 0.984982035368022 | 0.942439443234529 | 1.02944503963879 | 0.992899239648112 |
| ebi-a-GCST90001659 | finngen_R11_O15_POOR_FETGRO | Poor fetal growth \|\| id:finngen_R11_O15_POOR_FETGRO | CD39+ CD4+ T cell %CD4+ T cell \|\| id:ebi-a-GCST90001659 | Weighted median | 25 | -0.014914615 | 0.0233303129280021 | 0.522640446744299 | -0.060642028 | 0.0308127983314741 | 0.985196056969855 | 0.941160088114479 | 1.03129242615194 | 0.999440741871876 |
| ebi-a-GCST90001659 | finngen_R11_O15_POOR_FETGRO | Poor fetal growth \|\| id:finngen_R11_O15_POOR_FETGRO | CD39+ CD4+ T cell %CD4+ T cell \|\| id:ebi-a-GCST90001659 | Weighted mode | 25 | -0.015239504 | 0.0203370479964731 | 0.460930489780655 | -0.055100118 | 0.0246211098728015 | 0.984876029407645 | 0.946390392238166 | 1.02492671233466 | 0.999857399747617 |
| ebi-a-GCST90001660 | finngen_R11_O15_POOR_FETGRO | Poor fetal growth \|\| id:finngen_R11_O15_POOR_FETGRO | CD39+ CD4+ T cell Absolute Count \|\| id:ebi-a-GCST90001660 | Inverse variance weighted | 29 | 0.00618585810391256 | 0.0194093056206303 | 0.749949622771381 | -0.031856381 | 0.044228097120348 | 1.00620503003538 | 0.968645688099632 | 1.0452207395408 | 0.994119304454654 |
| ebi-a-GCST90001660 | finngen_R11_O15_POOR_FETGRO | Poor fetal growth \|\| id:finngen_R11_O15_POOR_FETGRO | CD39+ CD4+ T cell Absolute Count \|\| id:ebi-a-GCST90001660 | MR Egger | 29 | -0.019402887 | 0.0264750311362391 | 0.469950026879873 | -0.071293948 | 0.0324881735779667 | 0.980784137013468 | 0.931188130561734 | 1.03302167612142 | 0.992899239648112 |
| ebi-a-GCST90001660 | finngen_R11_O15_POOR_FETGRO | Poor fetal growth \|\| id:finngen_R11_O15_POOR_FETGRO | CD39+ CD4+ T cell Absolute Count \|\| id:ebi-a-GCST90001660 | Weighted median | 29 | -0.009352805 | 0.0276848638533236 | 0.735490558351469 | -0.063615138 | 0.0449095283183237 | 0.99069079660714 | 0.938366071500316 | 1.04593322828996 | 0.999440741871876 |
| ebi-a-GCST90001660 | finngen_R11_O15_POOR_FETGRO | Poor fetal growth \|\| id:finngen_R11_O15_POOR_FETGRO | CD39+ CD4+ T cell Absolute Count \|\| id:ebi-a-GCST90001660 | Weighted mode | 29 | -0.009266672 | 0.0231087061236719 | 0.691461364851481 | -0.054559736 | 0.0360263920737195 | 0.990776131358929 | 0.946901943099131 | 1.03668320634949 | 0.999857399747617 |
| ebi-a-GCST90001661 | finngen_R11_O15_POOR_FETGRO | Poor fetal growth \|\| id:finngen_R11_O15_POOR_FETGRO | CD28- CD8dim T cell %T cell \|\| id:ebi-a-GCST90001661 | Inverse variance weighted | 25 | 0.021419467509978 | 0.025147376182399 | 0.394348671952846 | -0.02786939 | 0.0707083248274801 | 1.02165051096476 | 0.972515378927355 | 1.07326813454179 | 0.994119304454654 |
| ebi-a-GCST90001661 | finngen_R11_O15_POOR_FETGRO | Poor fetal growth \|\| id:finngen_R11_O15_POOR_FETGRO | CD28- CD8dim T cell %T cell \|\| id:ebi-a-GCST90001661 | MR Egger | 25 | 0.00445579287301065 | 0.0373968259742518 | 0.90619192836993 | -0.068841986 | 0.0777535717825441 | 1.0044657346788 | 0.933474170378597 | 1.08085627236436 | 0.992899239648112 |
| ebi-a-GCST90001661 | finngen_R11_O15_POOR_FETGRO | Poor fetal growth \|\| id:finngen_R11_O15_POOR_FETGRO | CD28- CD8dim T cell %T cell \|\| id:ebi-a-GCST90001661 | Weighted median | 25 | 0.0337973786428173 | 0.034340497977615 | 0.325024908379356 | -0.033509997 | 0.101104754678943 | 1.0343749990272 | 0.967045243251432 | 1.10639253548795 | 0.999440741871876 |
| ebi-a-GCST90001661 | finngen_R11_O15_POOR_FETGRO | Poor fetal growth \|\| id:finngen_R11_O15_POOR_FETGRO | CD28- CD8dim T cell %T cell \|\| id:ebi-a-GCST90001661 | Weighted mode | 25 | 0.00483173235367906 | 0.0308607040426843 | 0.876896618143641 | -0.055655248 | 0.0653187122773403 | 1.00484342399516 | 0.945865169002296 | 1.06749919527258 | 0.999857399747617 |
| ebi-a-GCST90001662 | finngen_R11_O15_POOR_FETGRO | Poor fetal growth \|\| id:finngen_R11_O15_POOR_FETGRO | CD28- CD8dim T cell %CD8dim T cell \|\| id:ebi-a-GCST90001662 | Inverse variance weighted | 15 | 0.00123440773600797 | 0.00950819892607648 | 0.896704399669446 | -0.017401662 | 0.0198704776311179 | 1.00123516993082 | 0.982748872315724 | 1.02006920968956 | 0.994119304454654 |
| ebi-a-GCST90001662 | finngen_R11_O15_POOR_FETGRO | Poor fetal growth \|\| id:finngen_R11_O15_POOR_FETGRO | CD28- CD8dim T cell %CD8dim T cell \|\| id:ebi-a-GCST90001662 | MR Egger | 15 | -0.00051524 | 0.0102928405692655 | 0.960837016056125 | -0.020689208 | 0.0196587271503675 | 0.99948489234813 | 0.979523345403405 | 1.01985323241146 | 0.992899239648112 |
| ebi-a-GCST90001662 | finngen_R11_O15_POOR_FETGRO | Poor fetal growth \|\| id:finngen_R11_O15_POOR_FETGRO | CD28- CD8dim T cell %CD8dim T cell \|\| id:ebi-a-GCST90001662 | Weighted median | 15 | 0.00401690144254796 | 0.0113166301560765 | 0.722622841216843 | -0.018163694 | 0.0261974965484579 | 1.00402498000445 | 0.982000271978989 | 1.02654366728578 | 0.999440741871876 |
| ebi-a-GCST90001662 | finngen_R11_O15_POOR_FETGRO | Poor fetal growth \|\| id:finngen_R11_O15_POOR_FETGRO | CD28- CD8dim T cell %CD8dim T cell \|\| id:ebi-a-GCST90001662 | Weighted mode | 15 | 0.00019199424258709 | 0.00983685266541204 | 0.984703437192808 | -0.019088237 | 0.0194722254667947 | 1.00019201267466 | 0.981092789756957 | 1.01966304580223 | 0.999857399747617 |
| ebi-a-GCST90001663 | finngen_R11_O15_POOR_FETGRO | Poor fetal growth \|\| id:finngen_R11_O15_POOR_FETGRO | CD28- CD8dim T cell Absolute Count \|\| id:ebi-a-GCST90001663 | Inverse variance weighted | 28 | 0.0102875374273991 | 0.0202042182010639 | 0.610627670019703 | -0.02931273 | 0.0498878051014844 | 1.01034063606911 | 0.971112720653452 | 1.05115315573835 | 0.994119304454654 |
| ebi-a-GCST90001663 | finngen_R11_O15_POOR_FETGRO | Poor fetal growth \|\| id:finngen_R11_O15_POOR_FETGRO | CD28- CD8dim T cell Absolute Count \|\| id:ebi-a-GCST90001663 | MR Egger | 28 | 0.00243253870615828 | 0.0287137477334072 | 0.933135452907167 | -0.053846407 | 0.0587114842636363 | 1.00243549972888 | 0.947577636758136 | 1.06046923453639 | 0.992899239648112 |
| ebi-a-GCST90001663 | finngen_R11_O15_POOR_FETGRO | Poor fetal growth \|\| id:finngen_R11_O15_POOR_FETGRO | CD28- CD8dim T cell Absolute Count \|\| id:ebi-a-GCST90001663 | Weighted median | 28 | 0.0150857440198056 | 0.0310804766978783 | 0.627409047488389 | -0.04583199 | 0.0760034783476472 | 1.01520010822212 | 0.955202431976457 | 1.07896632717074 | 0.999440741871876 |
| ebi-a-GCST90001663 | finngen_R11_O15_POOR_FETGRO | Poor fetal growth \|\| id:finngen_R11_O15_POOR_FETGRO | CD28- CD8dim T cell Absolute Count \|\| id:ebi-a-GCST90001663 | Weighted mode | 28 | 0.0093520582781178 | 0.0259387238541526 | 0.721245608396843 | -0.04148784 | 0.060191957032257 | 1.00939592541785 | 0.959361000643641 | 1.06204039310184 | 0.999857399747617 |
| ebi-a-GCST90001664 | finngen_R11_O15_POOR_FETGRO | Poor fetal growth \|\| id:finngen_R11_O15_POOR_FETGRO | CD28+ CD45RA+ CD8dim T cell %T cell \|\| id:ebi-a-GCST90001664 | Inverse variance weighted | 35 | 0.0142272461359817 | 0.00956756106755306 | 0.137007003604247 | -0.004525174 | 0.0329796658283857 | 1.01432893508121 | 0.99548504961508 | 1.03352952306094 | 0.994119304454654 |
| ebi-a-GCST90001664 | finngen_R11_O15_POOR_FETGRO | Poor fetal growth \|\| id:finngen_R11_O15_POOR_FETGRO | CD28+ CD45RA+ CD8dim T cell %T cell \|\| id:ebi-a-GCST90001664 | MR Egger | 35 | 0.00853530586390571 | 0.0109536047359076 | 0.441400649209074 | -0.012933759 | 0.0300043711462846 | 1.00857183544341 | 0.987149522213026 | 1.03045903823087 | 0.992899239648112 |
| ebi-a-GCST90001664 | finngen_R11_O15_POOR_FETGRO | Poor fetal growth \|\| id:finngen_R11_O15_POOR_FETGRO | CD28+ CD45RA+ CD8dim T cell %T cell \|\| id:ebi-a-GCST90001664 | Weighted median | 35 | 0.00538426712388377 | 0.0137543897364907 | 0.695458661950538 | -0.021574337 | 0.0323428710074055 | 1.00539878834045 | 0.97865672459537 | 1.03287158632091 | 0.999440741871876 |
| ebi-a-GCST90001664 | finngen_R11_O15_POOR_FETGRO | Poor fetal growth \|\| id:finngen_R11_O15_POOR_FETGRO | CD28+ CD45RA+ CD8dim T cell %T cell \|\| id:ebi-a-GCST90001664 | Weighted mode | 35 | 0.0135344547506226 | 0.0108187414152336 | 0.219467694558673 | -0.007670278 | 0.0347391879244805 | 1.01362646009547 | 0.992359063095178 | 1.03534964189381 | 0.999857399747617 |
| ebi-a-GCST90001665 | finngen_R11_O15_POOR_FETGRO | Poor fetal growth \|\| id:finngen_R11_O15_POOR_FETGRO | CD28+ CD45RA+ CD8dim T cell %CD8dim T cell \|\| id:ebi-a-GCST90001665 | Inverse variance weighted | 26 | -0.009455085 | 0.0149025506534489 | 0.525780100021544 | -0.038664084 | 0.0197539141239344 | 0.990589473614594 | 0.96207383044443 | 1.01995031377451 | 0.994119304454654 |
| ebi-a-GCST90001665 | finngen_R11_O15_POOR_FETGRO | Poor fetal growth \|\| id:finngen_R11_O15_POOR_FETGRO | CD28+ CD45RA+ CD8dim T cell %CD8dim T cell \|\| id:ebi-a-GCST90001665 | MR Egger | 26 | 0.000253452170127679 | 0.0176065881198506 | 0.988633607411811 | -0.034255461 | 0.0347623648850349 | 1.00025348429184 | 0.96632461529095 | 1.0353736384297 | 0.994809183268002 |
| ebi-a-GCST90001665 | finngen_R11_O15_POOR_FETGRO | Poor fetal growth \|\| id:finngen_R11_O15_POOR_FETGRO | CD28+ CD45RA+ CD8dim T cell %CD8dim T cell \|\| id:ebi-a-GCST90001665 | Weighted median | 26 | -0.000913294 | 0.0209956173748643 | 0.965303554859776 | -0.042064704 | 0.0402381162476944 | 0.999087123118814 | 0.958807740001897 | 1.04105863765749 | 0.999440741871876 |
| ebi-a-GCST90001665 | finngen_R11_O15_POOR_FETGRO | Poor fetal growth \|\| id:finngen_R11_O15_POOR_FETGRO | CD28+ CD45RA+ CD8dim T cell %CD8dim T cell \|\| id:ebi-a-GCST90001665 | Weighted mode | 26 | -0.002841263 | 0.0173680820632703 | 0.871368409341332 | -0.036882704 | 0.0312001778372244 | 0.997162769560852 | 0.963789177478384 | 1.03169200509161 | 0.999857399747617 |
| ebi-a-GCST90001666 | finngen_R11_O15_POOR_FETGRO | Poor fetal growth \|\| id:finngen_R11_O15_POOR_FETGRO | CD28+ CD45RA+ CD8dim T cell Absolute Count \|\| id:ebi-a-GCST90001666 | Inverse variance weighted | 41 | -0.000152705 | 0.00408418371546152 | 0.97017450782934 | -0.008157705 | 0.0078522948191352 | 0.999847306395686 | 0.991875478436963 | 1.00788320493825 | 0.998984604424952 |
| ebi-a-GCST90001666 | finngen_R11_O15_POOR_FETGRO | Poor fetal growth \|\| id:finngen_R11_O15_POOR_FETGRO | CD28+ CD45RA+ CD8dim T cell Absolute Count \|\| id:ebi-a-GCST90001666 | MR Egger | 41 | -0.001879705 | 0.00433481918083687 | 0.666946841136093 | -0.010375951 | 0.00661654055617745 | 0.998122060500848 | 0.989677693845262 | 1.00663847821776 | 0.992899239648112 |
| ebi-a-GCST90001666 | finngen_R11_O15_POOR_FETGRO | Poor fetal growth \|\| id:finngen_R11_O15_POOR_FETGRO | CD28+ CD45RA+ CD8dim T cell Absolute Count \|\| id:ebi-a-GCST90001666 | Weighted median | 41 | -0.001638937 | 0.00460267439087139 | 0.72177795653521 | -0.010660179 | 0.0073823049262598 | 0.998362405443772 | 0.989396439653051 | 1.00740962131721 | 0.999440741871876 |
| ebi-a-GCST90001666 | finngen_R11_O15_POOR_FETGRO | Poor fetal growth \|\| id:finngen_R11_O15_POOR_FETGRO | CD28+ CD45RA+ CD8dim T cell Absolute Count \|\| id:ebi-a-GCST90001666 | Weighted mode | 41 | -0.000318111 | 0.00396267641926241 | 0.936417363716722 | -0.008084957 | 0.00744873453395309 | 0.999681939344217 | 0.991947638332343 | 1.00747654536598 | 0.999857399747617 |
| ebi-a-GCST90001667 | finngen_R11_O15_POOR_FETGRO | Poor fetal growth \|\| id:finngen_R11_O15_POOR_FETGRO | CD28+ CD45RA- CD8dim T cell %T cell \|\| id:ebi-a-GCST90001667 | Inverse variance weighted | 28 | 0.00142164944505743 | 0.00949609096892921 | 0.880994276848028 | -0.017190689 | 0.0200339877441587 | 1.00142266046768 | 0.982956227965843 | 1.02023601495815 | 0.994119304454654 |
| ebi-a-GCST90001667 | finngen_R11_O15_POOR_FETGRO | Poor fetal growth \|\| id:finngen_R11_O15_POOR_FETGRO | CD28+ CD45RA- CD8dim T cell %T cell \|\| id:ebi-a-GCST90001667 | MR Egger | 28 | -0.001076135 | 0.0108903729676832 | 0.922042911398992 | -0.022421266 | 0.020268996494833 | 0.998924444303278 | 0.977828222941168 | 1.02047580752499 | 0.992899239648112 |
| ebi-a-GCST90001667 | finngen_R11_O15_POOR_FETGRO | Poor fetal growth \|\| id:finngen_R11_O15_POOR_FETGRO | CD28+ CD45RA- CD8dim T cell %T cell \|\| id:ebi-a-GCST90001667 | Weighted median | 28 | 0.0091262833996512 | 0.0118724172398509 | 0.442073612150258 | -0.014143654 | 0.032396221189759 | 1.00916805490015 | 0.985955897195565 | 1.03292669167832 | 0.999440741871876 |
| ebi-a-GCST90001667 | finngen_R11_O15_POOR_FETGRO | Poor fetal growth \|\| id:finngen_R11_O15_POOR_FETGRO | CD28+ CD45RA- CD8dim T cell %T cell \|\| id:ebi-a-GCST90001667 | Weighted mode | 28 | 0.00223892224509181 | 0.00980039533429176 | 0.821016117066511 | -0.016969853 | 0.0214476971003037 | 1.00224143050308 | 0.983173324297655 | 1.02167935214723 | 0.999857399747617 |
| ebi-a-GCST90001668 | finngen_R11_O15_POOR_FETGRO | Poor fetal growth \|\| id:finngen_R11_O15_POOR_FETGRO | CD28+ CD45RA- CD8dim T cell %CD8dim T cell \|\| id:ebi-a-GCST90001668 | Inverse variance weighted | 28 | 0.00969494484031607 | 0.0159603024464941 | 0.543558171385526 | -0.021587248 | 0.0409771376354445 | 1.00974209306133 | 0.97864408904896 | 1.04182828661511 | 0.994119304454654 |
| ebi-a-GCST90001668 | finngen_R11_O15_POOR_FETGRO | Poor fetal growth \|\| id:finngen_R11_O15_POOR_FETGRO | CD28+ CD45RA- CD8dim T cell %CD8dim T cell \|\| id:ebi-a-GCST90001668 | MR Egger | 28 | 0.0205827746442997 | 0.0196374864003046 | 0.30422167116713 | -0.017906699 | 0.0590722479888968 | 1.02079606077706 | 0.982252673534191 | 1.06085188238654 | 0.992899239648112 |
| ebi-a-GCST90001668 | finngen_R11_O15_POOR_FETGRO | Poor fetal growth \|\| id:finngen_R11_O15_POOR_FETGRO | CD28+ CD45RA- CD8dim T cell %CD8dim T cell \|\| id:ebi-a-GCST90001668 | Weighted median | 28 | 0.00774284669968565 | 0.0212370280444461 | 0.715416122067338 | -0.033881728 | 0.0493674216668001 | 1.00777290005329 | 0.96668582948458 | 1.05060629534967 | 0.999440741871876 |
| ebi-a-GCST90001668 | finngen_R11_O15_POOR_FETGRO | Poor fetal growth \|\| id:finngen_R11_O15_POOR_FETGRO | CD28+ CD45RA- CD8dim T cell %CD8dim T cell \|\| id:ebi-a-GCST90001668 | Weighted mode | 28 | 0.0110614386780811 | 0.0226734332653726 | 0.629587997392033 | -0.033378491 | 0.0555013678782113 | 1.01112284258724 | 0.967172424708173 | 1.05707046301509 | 0.999857399747617 |
| ebi-a-GCST90001669 | finngen_R11_O15_POOR_FETGRO | Poor fetal growth \|\| id:finngen_R11_O15_POOR_FETGRO | CD28+ CD45RA- CD8dim T cell Absolute Count \|\| id:ebi-a-GCST90001669 | Inverse variance weighted | 37 | -0.004239735 | 0.00446238875185132 | 0.342059249369283 | -0.012986017 | 0.00450654713182202 | 0.995769240165538 | 0.987097937735915 | 1.00451671688644 | 0.994119304454654 |
| ebi-a-GCST90001669 | finngen_R11_O15_POOR_FETGRO | Poor fetal growth \|\| id:finngen_R11_O15_POOR_FETGRO | CD28+ CD45RA- CD8dim T cell Absolute Count \|\| id:ebi-a-GCST90001669 | MR Egger | 37 | -0.001552041 | 0.00510459010842974 | 0.76289164756375 | -0.011557037 | 0.00845295595989192 | 0.998449163139605 | 0.988509488762132 | 1.0084887830693 | 0.992899239648112 |
| ebi-a-GCST90001669 | finngen_R11_O15_POOR_FETGRO | Poor fetal growth \|\| id:finngen_R11_O15_POOR_FETGRO | CD28+ CD45RA- CD8dim T cell Absolute Count \|\| id:ebi-a-GCST90001669 | Weighted median | 37 | -0.003132918 | 0.00609267387153891 | 0.607104743060997 | -0.015074558 | 0.00880872317479584 | 0.996871984851956 | 0.985038493969441 | 1.00884763414485 | 0.999440741871876 |
| ebi-a-GCST90001669 | finngen_R11_O15_POOR_FETGRO | Poor fetal growth \|\| id:finngen_R11_O15_POOR_FETGRO | CD28+ CD45RA- CD8dim T cell Absolute Count \|\| id:ebi-a-GCST90001669 | Weighted mode | 37 | -0.005597621 | 0.00537337398536448 | 0.304481192672382 | -0.016129434 | 0.00493419202315496 | 0.99441801650102 | 0.983999948763525 | 1.00494638519485 | 0.999857399747617 |
| ebi-a-GCST90001670 | finngen_R11_O15_POOR_FETGRO | Poor fetal growth \|\| id:finngen_R11_O15_POOR_FETGRO | CD39+ CD8+ T cell %T cell \|\| id:ebi-a-GCST90001670 | Inverse variance weighted | 20 | -0.061008556 | 0.0374437561974002 | 0.103241364178154 | -0.134398319 | 0.0123812057594027 | 0.940815189762239 | 0.874241768541588 | 1.01245817019695 | 0.994119304454654 |
| ebi-a-GCST90001670 | finngen_R11_O15_POOR_FETGRO | Poor fetal growth \|\| id:finngen_R11_O15_POOR_FETGRO | CD39+ CD8+ T cell %T cell \|\| id:ebi-a-GCST90001670 | MR Egger | 20 | 0.015361951220852 | 0.0560965623871693 | 0.787318905633591 | -0.094587311 | 0.125311213499704 | 1.0154805525311 | 0.90974830007173 | 1.13350115904317 | 0.992899239648112 |
| ebi-a-GCST90001670 | finngen_R11_O15_POOR_FETGRO | Poor fetal growth \|\| id:finngen_R11_O15_POOR_FETGRO | CD39+ CD8+ T cell %T cell \|\| id:ebi-a-GCST90001670 | Weighted median | 20 | -0.079142185 | 0.0456209525646274 | 0.082780288181211 | -0.168559252 | 0.0102748817806662 | 0.923908548941163 | 0.844881200804503 | 1.01032784963585 | 0.999440741871876 |
| ebi-a-GCST90001670 | finngen_R11_O15_POOR_FETGRO | Poor fetal growth \|\| id:finngen_R11_O15_POOR_FETGRO | CD39+ CD8+ T cell %T cell \|\| id:ebi-a-GCST90001670 | Weighted mode | 20 | -0.058361552 | 0.0415089409350313 | 0.175867862181629 | -0.139719076 | 0.0229959721169104 | 0.943308830471271 | 0.869602492996093 | 1.02326241795753 | 0.999857399747617 |
| ebi-a-GCST90001671 | finngen_R11_O15_POOR_FETGRO | Poor fetal growth \|\| id:finngen_R11_O15_POOR_FETGRO | CD39+ CD8+ T cell %CD8+ T cell \|\| id:ebi-a-GCST90001671 | Inverse variance weighted | 21 | -0.009159455 | 0.0260280189877848 | 0.724907649710708 | -0.060174372 | 0.0418554626570961 | 0.990882365464679 | 0.941600330747463 | 1.04274375244693 | 0.994119304454654 |
| ebi-a-GCST90001671 | finngen_R11_O15_POOR_FETGRO | Poor fetal growth \|\| id:finngen_R11_O15_POOR_FETGRO | CD39+ CD8+ T cell %CD8+ T cell \|\| id:ebi-a-GCST90001671 | MR Egger | 21 | -0.012480625 | 0.0373719144434295 | 0.742069539765291 | -0.085729578 | 0.060768326955727 | 0.987596934650119 | 0.917842402751069 | 1.06265269768196 | 0.992899239648112 |
| ebi-a-GCST90001671 | finngen_R11_O15_POOR_FETGRO | Poor fetal growth \|\| id:finngen_R11_O15_POOR_FETGRO | CD39+ CD8+ T cell %CD8+ T cell \|\| id:ebi-a-GCST90001671 | Weighted median | 21 | -0.044885044 | 0.0371049059228209 | 0.226401902713441 | -0.117610659 | 0.0278405719142535 | 0.956107386088675 | 0.889042125520743 | 1.0282317423349 | 0.999440741871876 |
| ebi-a-GCST90001671 | finngen_R11_O15_POOR_FETGRO | Poor fetal growth \|\| id:finngen_R11_O15_POOR_FETGRO | CD39+ CD8+ T cell %CD8+ T cell \|\| id:ebi-a-GCST90001671 | Weighted mode | 21 | -0.055586734 | 0.0332451807491168 | 0.110092599281724 | -0.120747288 | 0.0095738205276296 | 0.945929976067131 | 0.886257899293911 | 1.00961979615104 | 0.999857399747617 |
| ebi-a-GCST90001672 | finngen_R11_O15_POOR_FETGRO | Poor fetal growth \|\| id:finngen_R11_O15_POOR_FETGRO | CD39+ CD8+ T cell Absolute Count \|\| id:ebi-a-GCST90001672 | Inverse variance weighted | 24 | -0.060765381 | 0.0243643471184787 | 0.0126302326882153 | -0.108519502 | -0.013011261 | 0.941044000397299 | 0.897161398516382 | 0.987073019579522 | 0.994119304454654 |
| ebi-a-GCST90001672 | finngen_R11_O15_POOR_FETGRO | Poor fetal growth \|\| id:finngen_R11_O15_POOR_FETGRO | CD39+ CD8+ T cell Absolute Count \|\| id:ebi-a-GCST90001672 | MR Egger | 24 | -0.037160782 | 0.034629454956496 | 0.294857597204976 | -0.105034514 | 0.0307129496616319 | 0.963521205976753 | 0.900293449530946 | 1.03118945811566 | 0.992899239648112 |
| ebi-a-GCST90001672 | finngen_R11_O15_POOR_FETGRO | Poor fetal growth \|\| id:finngen_R11_O15_POOR_FETGRO | CD39+ CD8+ T cell Absolute Count \|\| id:ebi-a-GCST90001672 | Weighted median | 24 | -0.059470733 | 0.0326572461598102 | 0.0685980682691618 | -0.123478936 | 0.00453746900279078 | 0.942263109958859 | 0.883840258308994 | 1.00454777890299 | 0.999440741871876 |
| ebi-a-GCST90001672 | finngen_R11_O15_POOR_FETGRO | Poor fetal growth \|\| id:finngen_R11_O15_POOR_FETGRO | CD39+ CD8+ T cell Absolute Count \|\| id:ebi-a-GCST90001672 | Weighted mode | 24 | -0.061565246 | 0.0300692495814696 | 0.0522021910132003 | -0.120500975 | -0.002629517 | 0.940291593352268 | 0.886476222757867 | 0.997373937203101 | 0.999857399747617 |
| ebi-a-GCST90001673 | finngen_R11_O15_POOR_FETGRO | Poor fetal growth \|\| id:finngen_R11_O15_POOR_FETGRO | CD28- CD127- CD25++ CD8+ T cell %T cell \|\| id:ebi-a-GCST90001673 | Inverse variance weighted | 18 | -0.026666914 | 0.0255675859943624 | 0.296949707477514 | -0.076779383 | 0.02344555445524 | 0.973685508436988 | 0.926094143513306 | 1.02372256209671 | 0.994119304454654 |
| ebi-a-GCST90001673 | finngen_R11_O15_POOR_FETGRO | Poor fetal growth \|\| id:finngen_R11_O15_POOR_FETGRO | CD28- CD127- CD25++ CD8+ T cell %T cell \|\| id:ebi-a-GCST90001673 | MR Egger | 18 | -0.027434981 | 0.0331567025220171 | 0.420161425131737 | -0.092422118 | 0.0375521559224686 | 0.972937939928758 | 0.911720214826204 | 1.03826614739837 | 0.992899239648112 |
| ebi-a-GCST90001673 | finngen_R11_O15_POOR_FETGRO | Poor fetal growth \|\| id:finngen_R11_O15_POOR_FETGRO | CD28- CD127- CD25++ CD8+ T cell %T cell \|\| id:ebi-a-GCST90001673 | Weighted median | 18 | -0.031322425 | 0.0332883681099966 | 0.346734567702315 | -0.096567626 | 0.0339227768555815 | 0.969163040650162 | 0.907948494475535 | 1.03450471593637 | 0.999440741871876 |
| ebi-a-GCST90001673 | finngen_R11_O15_POOR_FETGRO | Poor fetal growth \|\| id:finngen_R11_O15_POOR_FETGRO | CD28- CD127- CD25++ CD8+ T cell %T cell \|\| id:ebi-a-GCST90001673 | Weighted mode | 18 | -0.029609497 | 0.0290776958435772 | 0.322814572576754 | -0.086601781 | 0.027382786915103 | 0.970824569503098 | 0.91704220675273 | 1.02776114099295 | 0.999857399747617 |
| ebi-a-GCST90001674 | finngen_R11_O15_POOR_FETGRO | Poor fetal growth \|\| id:finngen_R11_O15_POOR_FETGRO | CD28- CD127- CD25++ CD8+ T cell %CD8+ T cell \|\| id:ebi-a-GCST90001674 | Inverse variance weighted | 18 | -0.025032403 | 0.0222391760106821 | 0.260335123641849 | -0.068621188 | 0.0185563818833213 | 0.975278309478061 | 0.933680302325153 | 1.01872962144503 | 0.994119304454654 |
| ebi-a-GCST90001674 | finngen_R11_O15_POOR_FETGRO | Poor fetal growth \|\| id:finngen_R11_O15_POOR_FETGRO | CD28- CD127- CD25++ CD8+ T cell %CD8+ T cell \|\| id:ebi-a-GCST90001674 | MR Egger | 18 | -0.030961451 | 0.0301804140479468 | 0.320209404547045 | -0.090115062 | 0.0281921607964817 | 0.969512946364737 | 0.913826032322768 | 1.02859332074361 | 0.992899239648112 |
| ebi-a-GCST90001674 | finngen_R11_O15_POOR_FETGRO | Poor fetal growth \|\| id:finngen_R11_O15_POOR_FETGRO | CD28- CD127- CD25++ CD8+ T cell %CD8+ T cell \|\| id:ebi-a-GCST90001674 | Weighted median | 18 | -0.031217556 | 0.0291845396972118 | 0.28477205218533 | -0.088419254 | 0.0259841420017131 | 0.969264680978702 | 0.915377021140662 | 1.02632467288949 | 0.999440741871876 |
| ebi-a-GCST90001674 | finngen_R11_O15_POOR_FETGRO | Poor fetal growth \|\| id:finngen_R11_O15_POOR_FETGRO | CD28- CD127- CD25++ CD8+ T cell %CD8+ T cell \|\| id:ebi-a-GCST90001674 | Weighted mode | 18 | -0.03145328 | 0.0266649678860078 | 0.254414661926327 | -0.083716618 | 0.0208100565998691 | 0.969036228326214 | 0.919691843732086 | 1.02102809566966 | 0.999857399747617 |
| ebi-a-GCST90001675 | finngen_R11_O15_POOR_FETGRO | Poor fetal growth \|\| id:finngen_R11_O15_POOR_FETGRO | CD28- CD127- CD25++ CD8+ T cell Absolute Count \|\| id:ebi-a-GCST90001675 | Inverse variance weighted | 30 | -0.014312706 | 0.0182057135675992 | 0.431770466615808 | -0.049995905 | 0.021370492555487 | 0.985789233815474 | 0.951233320145613 | 1.0216004769027 | 0.994119304454654 |
| ebi-a-GCST90001675 | finngen_R11_O15_POOR_FETGRO | Poor fetal growth \|\| id:finngen_R11_O15_POOR_FETGRO | CD28- CD127- CD25++ CD8+ T cell Absolute Count \|\| id:ebi-a-GCST90001675 | MR Egger | 30 | -0.025330305 | 0.0233540341360501 | 0.287339967156035 | -0.071104212 | 0.0204436014339375 | 0.974987815024921 | 0.931364827325278 | 1.02065400319769 | 0.992899239648112 |
| ebi-a-GCST90001675 | finngen_R11_O15_POOR_FETGRO | Poor fetal growth \|\| id:finngen_R11_O15_POOR_FETGRO | CD28- CD127- CD25++ CD8+ T cell Absolute Count \|\| id:ebi-a-GCST90001675 | Weighted median | 30 | -0.02666372 | 0.0246188485036352 | 0.278781255626417 | -0.074916663 | 0.0215892232531266 | 0.973688618665821 | 0.927820805019474 | 1.02182395672778 | 0.999440741871876 |
| ebi-a-GCST90001675 | finngen_R11_O15_POOR_FETGRO | Poor fetal growth \|\| id:finngen_R11_O15_POOR_FETGRO | CD28- CD127- CD25++ CD8+ T cell Absolute Count \|\| id:ebi-a-GCST90001675 | Weighted mode | 30 | -0.028321018 | 0.0212579343186493 | 0.193151255695166 | -0.069986569 | 0.0133445332306067 | 0.972076262697707 | 0.93240634269312 | 1.01343396889713 | 0.999857399747617 |
| ebi-a-GCST90001676 | finngen_R11_O15_POOR_FETGRO | Poor fetal growth \|\| id:finngen_R11_O15_POOR_FETGRO | CD28- CD25++ CD8+ T cell %T cell \|\| id:ebi-a-GCST90001676 | Inverse variance weighted | 27 | -0.044165933 | 0.0352542171960483 | 0.210284184390723 | -0.113264199 | 0.0249323327496944 | 0.95679518044904 | 0.892914722078122 | 1.02524574261686 | 0.994119304454654 |
| ebi-a-GCST90001676 | finngen_R11_O15_POOR_FETGRO | Poor fetal growth \|\| id:finngen_R11_O15_POOR_FETGRO | CD28- CD25++ CD8+ T cell %T cell \|\| id:ebi-a-GCST90001676 | MR Egger | 27 | -0.087677689 | 0.0726252101222654 | 0.238629725026309 | -0.230023101 | 0.0546677231402762 | 0.916056084374399 | 0.794515248560853 | 1.05618960899664 | 0.992899239648112 |
| ebi-a-GCST90001676 | finngen_R11_O15_POOR_FETGRO | Poor fetal growth \|\| id:finngen_R11_O15_POOR_FETGRO | CD28- CD25++ CD8+ T cell %T cell \|\| id:ebi-a-GCST90001676 | Weighted median | 27 | -0.009393723 | 0.049096390551462 | 0.848265301657357 | -0.105622648 | 0.0868352029676938 | 0.990650260668478 | 0.899764111815721 | 1.09071691799541 | 0.999440741871876 |
| ebi-a-GCST90001676 | finngen_R11_O15_POOR_FETGRO | Poor fetal growth \|\| id:finngen_R11_O15_POOR_FETGRO | CD28- CD25++ CD8+ T cell %T cell \|\| id:ebi-a-GCST90001676 | Weighted mode | 27 | -0.018815346 | 0.0807238444707773 | 0.817522295005209 | -0.177034081 | 0.139403388913948 | 0.981360557421338 | 0.837751232291965 | 1.14958773743311 | 0.999857399747617 |
| ebi-a-GCST90001677 | finngen_R11_O15_POOR_FETGRO | Poor fetal growth \|\| id:finngen_R11_O15_POOR_FETGRO | CD28- CD25++ CD8+ T cell %CD8+ T cell \|\| id:ebi-a-GCST90001677 | Inverse variance weighted | 25 | 0.0149182896623795 | 0.0313858184618228 | 0.634559304823953 | -0.046597915 | 0.0764344938475521 | 1.01503012277314 | 0.954471099412224 | 1.07943147861817 | 0.994119304454654 |
| ebi-a-GCST90001677 | finngen_R11_O15_POOR_FETGRO | Poor fetal growth \|\| id:finngen_R11_O15_POOR_FETGRO | CD28- CD25++ CD8+ T cell %CD8+ T cell \|\| id:ebi-a-GCST90001677 | MR Egger | 25 | 0.00635086877445908 | 0.0495553343323699 | 0.899138633335769 | -0.090777587 | 0.103479324065904 | 1.00637107830159 | 0.913220800932544 | 1.10902286304439 | 0.992899239648112 |
| ebi-a-GCST90001677 | finngen_R11_O15_POOR_FETGRO | Poor fetal growth \|\| id:finngen_R11_O15_POOR_FETGRO | CD28- CD25++ CD8+ T cell %CD8+ T cell \|\| id:ebi-a-GCST90001677 | Weighted median | 25 | 0.0390297817257828 | 0.0434761706916404 | 0.369330504904459 | -0.046183513 | 0.124243076281398 | 1.0398014502707 | 0.954866715818095 | 1.13229107065348 | 0.999440741871876 |
| ebi-a-GCST90001677 | finngen_R11_O15_POOR_FETGRO | Poor fetal growth \|\| id:finngen_R11_O15_POOR_FETGRO | CD28- CD25++ CD8+ T cell %CD8+ T cell \|\| id:ebi-a-GCST90001677 | Weighted mode | 25 | 0.0423746169245095 | 0.0440843332712348 | 0.346027982614133 | -0.044030676 | 0.12877991013613 | 1.0432852378609 | 0.956924602128954 | 1.13743975765375 | 0.999857399747617 |
| ebi-a-GCST90001678 | finngen_R11_O15_POOR_FETGRO | Poor fetal growth \|\| id:finngen_R11_O15_POOR_FETGRO | CD28- CD25++ CD8+ T cell Absolute Count \|\| id:ebi-a-GCST90001678 | Inverse variance weighted | 25 | -0.027696972 | 0.0201609686017698 | 0.169506351296635 | -0.06721247 | 0.0118185266556433 | 0.97268307254384 | 0.934996521273667 | 1.01188864138715 | 0.994119304454654 |
| ebi-a-GCST90001678 | finngen_R11_O15_POOR_FETGRO | Poor fetal growth \|\| id:finngen_R11_O15_POOR_FETGRO | CD28- CD25++ CD8+ T cell Absolute Count \|\| id:ebi-a-GCST90001678 | MR Egger | 25 | -0.033597126 | 0.0258127823454478 | 0.205950426698793 | -0.084190179 | 0.0169959274932073 | 0.966960989710237 | 0.919256415927588 | 1.01714118000244 | 0.992899239648112 |
| ebi-a-GCST90001678 | finngen_R11_O15_POOR_FETGRO | Poor fetal growth \|\| id:finngen_R11_O15_POOR_FETGRO | CD28- CD25++ CD8+ T cell Absolute Count \|\| id:ebi-a-GCST90001678 | Weighted median | 25 | -0.021594027 | 0.0305821273630947 | 0.480126217889787 | -0.081534997 | 0.0383469425907871 | 0.97863745475894 | 0.921700452840515 | 1.0390916755064 | 0.999440741871876 |
| ebi-a-GCST90001678 | finngen_R11_O15_POOR_FETGRO | Poor fetal growth \|\| id:finngen_R11_O15_POOR_FETGRO | CD28- CD25++ CD8+ T cell Absolute Count \|\| id:ebi-a-GCST90001678 | Weighted mode | 25 | -0.02264087 | 0.025699945664027 | 0.387071777355351 | -0.073012764 | 0.0277310233694625 | 0.977613510948445 | 0.929588965017196 | 1.02811910721344 | 0.999857399747617 |
| ebi-a-GCST90001679 | finngen_R11_O15_POOR_FETGRO | Poor fetal growth \|\| id:finngen_R11_O15_POOR_FETGRO | CD25++ CD8+ T cell %T cell \|\| id:ebi-a-GCST90001679 | Inverse variance weighted | 17 | -0.067708568 | 0.0359748531762044 | 0.0598213202612026 | -0.13821928 | 0.00280214448512871 | 0.934532786896756 | 0.870907698195939 | 1.00280607416164 | 0.994119304454654 |
| ebi-a-GCST90001679 | finngen_R11_O15_POOR_FETGRO | Poor fetal growth \|\| id:finngen_R11_O15_POOR_FETGRO | CD25++ CD8+ T cell %T cell \|\| id:ebi-a-GCST90001679 | MR Egger | 17 | -0.005510846 | 0.0583079932123301 | 0.925952864689875 | -0.119794513 | 0.108772820213016 | 0.994504310376173 | 0.887102705904213 | 1.1149090367712 | 0.992899239648112 |
| ebi-a-GCST90001679 | finngen_R11_O15_POOR_FETGRO | Poor fetal growth \|\| id:finngen_R11_O15_POOR_FETGRO | CD25++ CD8+ T cell %T cell \|\| id:ebi-a-GCST90001679 | Weighted median | 17 | -0.029155113 | 0.0490919611517884 | 0.552587204926157 | -0.125375357 | 0.0670651303925472 | 0.971265796378511 | 0.882165713071252 | 1.06936512407685 | 0.999440741871876 |
| ebi-a-GCST90001679 | finngen_R11_O15_POOR_FETGRO | Poor fetal growth \|\| id:finngen_R11_O15_POOR_FETGRO | CD25++ CD8+ T cell %T cell \|\| id:ebi-a-GCST90001679 | Weighted mode | 17 | -0.001629854 | 0.0572370255679165 | 0.977635056485799 | -0.113814424 | 0.110554716460565 | 0.998371473837611 | 0.892423553118895 | 1.11689746005604 | 0.999857399747617 |
| ebi-a-GCST90001680 | finngen_R11_O15_POOR_FETGRO | Poor fetal growth \|\| id:finngen_R11_O15_POOR_FETGRO | CD25++ CD8+ T cell %CD8+ T cell \|\| id:ebi-a-GCST90001680 | Inverse variance weighted | 22 | -0.042391154 | 0.0315574398610175 | 0.179174392271059 | -0.104243736 | 0.0194614283286943 | 0.958494788359759 | 0.901005663205819 | 1.01965203641895 | 0.994119304454654 |
| ebi-a-GCST90001680 | finngen_R11_O15_POOR_FETGRO | Poor fetal growth \|\| id:finngen_R11_O15_POOR_FETGRO | CD25++ CD8+ T cell %CD8+ T cell \|\| id:ebi-a-GCST90001680 | MR Egger | 22 | 0.0301331587551797 | 0.0506733229596343 | 0.558742023818755 | -0.069186554 | 0.129452871756063 | 1.03059175713256 | 0.93315258026344 | 1.13820546857377 | 0.992899239648112 |
| ebi-a-GCST90001680 | finngen_R11_O15_POOR_FETGRO | Poor fetal growth \|\| id:finngen_R11_O15_POOR_FETGRO | CD25++ CD8+ T cell %CD8+ T cell \|\| id:ebi-a-GCST90001680 | Weighted median | 22 | 0.0126481016010719 | 0.0466957011416583 | 0.786496997852021 | -0.078875473 | 0.104171675838722 | 1.01272842713606 | 0.924154999865445 | 1.10979096285666 | 0.999440741871876 |
| ebi-a-GCST90001680 | finngen_R11_O15_POOR_FETGRO | Poor fetal growth \|\| id:finngen_R11_O15_POOR_FETGRO | CD25++ CD8+ T cell %CD8+ T cell \|\| id:ebi-a-GCST90001680 | Weighted mode | 22 | 0.0170338423551978 | 0.0464632925936515 | 0.717578912475187 | -0.074034211 | 0.108101895838755 | 1.017179746 | 0.928639923478219 | 1.11416126799954 | 0.999857399747617 |
| ebi-a-GCST90001681 | finngen_R11_O15_POOR_FETGRO | Poor fetal growth \|\| id:finngen_R11_O15_POOR_FETGRO | CD25++ CD8+ T cell Absolute Count \|\| id:ebi-a-GCST90001681 | Inverse variance weighted | 27 | -0.044688076 | 0.0292461196023626 | 0.126512494364993 | -0.10201047 | 0.0126343185334159 | 0.956295727011959 | 0.903020096720975 | 1.01271446872782 | 0.994119304454654 |
| ebi-a-GCST90001681 | finngen_R11_O15_POOR_FETGRO | Poor fetal growth \|\| id:finngen_R11_O15_POOR_FETGRO | CD25++ CD8+ T cell Absolute Count \|\| id:ebi-a-GCST90001681 | MR Egger | 27 | -0.086233473 | 0.0536850912201186 | 0.120770428125708 | -0.191456252 | 0.0189893056344011 | 0.917380022605068 | 0.825755749521377 | 1.01917074917453 | 0.992899239648112 |
| ebi-a-GCST90001681 | finngen_R11_O15_POOR_FETGRO | Poor fetal growth \|\| id:finngen_R11_O15_POOR_FETGRO | CD25++ CD8+ T cell Absolute Count \|\| id:ebi-a-GCST90001681 | Weighted median | 27 | -0.056535792 | 0.0411113320994647 | 0.169073310573537 | -0.137114003 | 0.0240424193087359 | 0.945032659653013 | 0.871870824994047 | 1.02433376849942 | 0.999440741871876 |
| ebi-a-GCST90001681 | finngen_R11_O15_POOR_FETGRO | Poor fetal growth \|\| id:finngen_R11_O15_POOR_FETGRO | CD25++ CD8+ T cell Absolute Count \|\| id:ebi-a-GCST90001681 | Weighted mode | 27 | -0.030468643 | 0.0579161035280374 | 0.603289960230312 | -0.143984206 | 0.0830469197491436 | 0.969990847432512 | 0.865901423928364 | 1.08659279001334 | 0.999857399747617 |
| ebi-a-GCST90001682 | finngen_R11_O15_POOR_FETGRO | Poor fetal growth \|\| id:finngen_R11_O15_POOR_FETGRO | CD127- CD8+ T cell %T cell \|\| id:ebi-a-GCST90001682 | Inverse variance weighted | 14 | 0.010759430066684 | 0.0450447023126755 | 0.811213239895123 | -0.077528186 | 0.0990470465995279 | 1.01081752088881 | 0.925400940246742 | 1.10411824334581 | 0.994119304454654 |
| ebi-a-GCST90001682 | finngen_R11_O15_POOR_FETGRO | Poor fetal growth \|\| id:finngen_R11_O15_POOR_FETGRO | CD127- CD8+ T cell %T cell \|\| id:ebi-a-GCST90001682 | MR Egger | 14 | -0.080191337 | 0.0995172419158896 | 0.436033684088102 | -0.275245131 | 0.114862456821693 | 0.922939736663009 | 0.759385950998978 | 1.12171914214506 | 0.992899239648112 |
| ebi-a-GCST90001682 | finngen_R11_O15_POOR_FETGRO | Poor fetal growth \|\| id:finngen_R11_O15_POOR_FETGRO | CD127- CD8+ T cell %T cell \|\| id:ebi-a-GCST90001682 | Weighted median | 14 | -0.014732114 | 0.0578602437981246 | 0.799020104654236 | -0.128138191 | 0.0986739643068811 | 0.985375873106313 | 0.879731799391258 | 1.103706393 | 0.999440741871876 |
| ebi-a-GCST90001682 | finngen_R11_O15_POOR_FETGRO | Poor fetal growth \|\| id:finngen_R11_O15_POOR_FETGRO | CD127- CD8+ T cell %T cell \|\| id:ebi-a-GCST90001682 | Weighted mode | 14 | -0.012515178 | 0.0615862245847454 | 0.842114074841867 | -0.133224178 | 0.108193822102537 | 0.987562811069255 | 0.875268853856268 | 1.11426369338988 | 0.999857399747617 |
| ebi-a-GCST90001683 | finngen_R11_O15_POOR_FETGRO | Poor fetal growth \|\| id:finngen_R11_O15_POOR_FETGRO | CD127- CD8+ T cell %CD8+ T cell \|\| id:ebi-a-GCST90001683 | Inverse variance weighted | 15 | 0.0148009428782684 | 0.0272482462553421 | 0.586999799661089 | -0.03860562 | 0.0682075055387389 | 1.0149110192408 | 0.962130079403652 | 1.07058743825454 | 0.994119304454654 |
| ebi-a-GCST90001683 | finngen_R11_O15_POOR_FETGRO | Poor fetal growth \|\| id:finngen_R11_O15_POOR_FETGRO | CD127- CD8+ T cell %CD8+ T cell \|\| id:ebi-a-GCST90001683 | MR Egger | 15 | 0.0144254083769137 | 0.0356566744092605 | 0.692375576760778 | -0.055461673 | 0.0843124902190643 | 1.01452995669281 | 0.946048281727965 | 1.0879688203092 | 0.992899239648112 |
| ebi-a-GCST90001683 | finngen_R11_O15_POOR_FETGRO | Poor fetal growth \|\| id:finngen_R11_O15_POOR_FETGRO | CD127- CD8+ T cell %CD8+ T cell \|\| id:ebi-a-GCST90001683 | Weighted median | 15 | 0.00676486340950504 | 0.0408375823682922 | 0.86843012455901 | -0.073276798 | 0.0868065248513578 | 1.00678779678253 | 0.929343553953571 | 1.09068563873727 | 0.999440741871876 |
| ebi-a-GCST90001683 | finngen_R11_O15_POOR_FETGRO | Poor fetal growth \|\| id:finngen_R11_O15_POOR_FETGRO | CD127- CD8+ T cell %CD8+ T cell \|\| id:ebi-a-GCST90001683 | Weighted mode | 15 | 0.0360668132614781 | 0.0361862053869752 | 0.335825831379856 | -0.034858149 | 0.10699177581995 | 1.03672511116293 | 0.965742397779923 | 1.11292510154527 | 0.999857399747617 |
| ebi-a-GCST90001684 | finngen_R11_O15_POOR_FETGRO | Poor fetal growth \|\| id:finngen_R11_O15_POOR_FETGRO | CD127- CD8+ T cell Absolute Count \|\| id:ebi-a-GCST90001684 | Inverse variance weighted | 13 | -0.028268238 | 0.0336225263849151 | 0.400486347964949 | -0.09416839 | 0.0376319133300844 | 0.97212756989621 | 0.910129492541425 | 1.03834896011712 | 0.994119304454654 |
| ebi-a-GCST90001684 | finngen_R11_O15_POOR_FETGRO | Poor fetal growth \|\| id:finngen_R11_O15_POOR_FETGRO | CD127- CD8+ T cell Absolute Count \|\| id:ebi-a-GCST90001684 | MR Egger | 13 | 0.0161398310610471 | 0.0655654740913327 | 0.810088638908396 | -0.112368498 | 0.144648160280059 | 1.01627078169284 | 0.893714864532408 | 1.15563290117474 | 0.992899239648112 |
| ebi-a-GCST90001684 | finngen_R11_O15_POOR_FETGRO | Poor fetal growth \|\| id:finngen_R11_O15_POOR_FETGRO | CD127- CD8+ T cell Absolute Count \|\| id:ebi-a-GCST90001684 | Weighted median | 13 | -0.028815085 | 0.0474480901962282 | 0.543653796268892 | -0.121813342 | 0.064183171618614 | 0.971596110389969 | 0.885313603991308 | 1.06628769451755 | 0.999440741871876 |
| ebi-a-GCST90001684 | finngen_R11_O15_POOR_FETGRO | Poor fetal growth \|\| id:finngen_R11_O15_POOR_FETGRO | CD127- CD8+ T cell Absolute Count \|\| id:ebi-a-GCST90001684 | Weighted mode | 13 | 0.025711560664493 | 0.0504888494561071 | 0.619809761270293 | -0.073246584 | 0.124669705598463 | 1.02604495406253 | 0.929371633343525 | 1.13277424227993 | 0.999857399747617 |
| ebi-a-GCST90001685 | finngen_R11_O15_POOR_FETGRO | Poor fetal growth \|\| id:finngen_R11_O15_POOR_FETGRO | CD28- CD8+ T cell %T cell \|\| id:ebi-a-GCST90001685 | Inverse variance weighted | 16 | -0.019979938 | 0.0498343444471317 | 0.68847380905947 | -0.117655253 | 0.0776953767343392 | 0.980218337875316 | 0.889002480286626 | 1.0807933737117 | 0.994119304454654 |
| ebi-a-GCST90001685 | finngen_R11_O15_POOR_FETGRO | Poor fetal growth \|\| id:finngen_R11_O15_POOR_FETGRO | CD28- CD8+ T cell %T cell \|\| id:ebi-a-GCST90001685 | MR Egger | 16 | -0.124953446 | 0.123552012415304 | 0.329013028264805 | -0.367115391 | 0.117208497992623 | 0.882537987000446 | 0.692729705883755 | 1.12435383077032 | 0.992899239648112 |
| ebi-a-GCST90001685 | finngen_R11_O15_POOR_FETGRO | Poor fetal growth \|\| id:finngen_R11_O15_POOR_FETGRO | CD28- CD8+ T cell %T cell \|\| id:ebi-a-GCST90001685 | Weighted median | 16 | -0.033215059 | 0.0582943142390292 | 0.568825469604103 | -0.147471915 | 0.0810417967135073 | 0.967330503898232 | 0.86288667206396 | 1.08441622065365 | 0.999440741871876 |
| ebi-a-GCST90001685 | finngen_R11_O15_POOR_FETGRO | Poor fetal growth \|\| id:finngen_R11_O15_POOR_FETGRO | CD28- CD8+ T cell %T cell \|\| id:ebi-a-GCST90001685 | Weighted mode | 16 | -0.018526783 | 0.0730185893018804 | 0.803151412618061 | -0.161643218 | 0.124589651793892 | 0.981643782640842 | 0.850744680559025 | 1.13268356302178 | 0.999857399747617 |
| ebi-a-GCST90001686 | finngen_R11_O15_POOR_FETGRO | Poor fetal growth \|\| id:finngen_R11_O15_POOR_FETGRO | CD28- CD8+ T cell %CD8+ T cell \|\| id:ebi-a-GCST90001686 | Inverse variance weighted | 23 | 0.0333309120444476 | 0.0363090968328451 | 0.358630961179442 | -0.037834918 | 0.104496741836824 | 1.03389261015786 | 0.962871880830301 | 1.11015177680469 | 0.994119304454654 |
| ebi-a-GCST90001686 | finngen_R11_O15_POOR_FETGRO | Poor fetal growth \|\| id:finngen_R11_O15_POOR_FETGRO | CD28- CD8+ T cell %CD8+ T cell \|\| id:ebi-a-GCST90001686 | MR Egger | 23 | 0.0548258830693212 | 0.0745048369298306 | 0.469950048764444 | -0.091203597 | 0.200855363451789 | 1.05635666908102 | 0.912831841868331 | 1.22244794838445 | 0.992899239648112 |
| ebi-a-GCST90001686 | finngen_R11_O15_POOR_FETGRO | Poor fetal growth \|\| id:finngen_R11_O15_POOR_FETGRO | CD28- CD8+ T cell %CD8+ T cell \|\| id:ebi-a-GCST90001686 | Weighted median | 23 | -0.024261632 | 0.055118465626377 | 0.659812191130024 | -0.132293824 | 0.0837705610041643 | 0.976030315953636 | 0.876083542667402 | 1.08737937795302 | 0.999440741871876 |
| ebi-a-GCST90001686 | finngen_R11_O15_POOR_FETGRO | Poor fetal growth \|\| id:finngen_R11_O15_POOR_FETGRO | CD28- CD8+ T cell %CD8+ T cell \|\| id:ebi-a-GCST90001686 | Weighted mode | 23 | -0.01707347 | 0.0625896795078128 | 0.787563305785909 | -0.139749242 | 0.105602301660784 | 0.983071455550186 | 0.869576261257362 | 1.11137979470617 | 0.999857399747617 |
| ebi-a-GCST90001687 | finngen_R11_O15_POOR_FETGRO | Poor fetal growth \|\| id:finngen_R11_O15_POOR_FETGRO | CD28- CD8+ T cell Absolute Count \|\| id:ebi-a-GCST90001687 | Inverse variance weighted | 14 | 0.0268609516277989 | 0.0555691459077889 | 0.628826739905769 | -0.082054574 | 0.135776477607065 | 1.0272249588742 | 0.921221682248592 | 1.14542583665476 | 0.994119304454654 |
| ebi-a-GCST90001687 | finngen_R11_O15_POOR_FETGRO | Poor fetal growth \|\| id:finngen_R11_O15_POOR_FETGRO | CD28- CD8+ T cell Absolute Count \|\| id:ebi-a-GCST90001687 | MR Egger | 14 | 0.248338816081218 | 0.162162704127611 | 0.151594096507299 | -0.069500084 | 0.566177716171335 | 1.28189418498825 | 0.932860055016006 | 1.76152113349787 | 0.992899239648112 |
| ebi-a-GCST90001687 | finngen_R11_O15_POOR_FETGRO | Poor fetal growth \|\| id:finngen_R11_O15_POOR_FETGRO | CD28- CD8+ T cell Absolute Count \|\| id:ebi-a-GCST90001687 | Weighted median | 14 | -0.040104175 | 0.0667511964209546 | 0.547972643325342 | -0.17093652 | 0.0907281699784516 | 0.96068935411941 | 0.842875077490307 | 1.09497131872307 | 0.999440741871876 |
| ebi-a-GCST90001687 | finngen_R11_O15_POOR_FETGRO | Poor fetal growth \|\| id:finngen_R11_O15_POOR_FETGRO | CD28- CD8+ T cell Absolute Count \|\| id:ebi-a-GCST90001687 | Weighted mode | 14 | -0.064681179 | 0.076124577137368 | 0.410880817383654 | -0.21388535 | 0.0845229922180209 | 0.937366267820152 | 0.807440952691809 | 1.08819786402691 | 0.999857399747617 |
| ebi-a-GCST90001688 | finngen_R11_O15_POOR_FETGRO | Poor fetal growth \|\| id:finngen_R11_O15_POOR_FETGRO | CD28+ CD45RA+ CD8+ T cell %T cell \|\| id:ebi-a-GCST90001688 | Inverse variance weighted (multiplicative random effects) | 72 | 0.00278446713640953 | 0.00299408782168471 | 0.352377057061776 | -0.003083945 | 0.00865287926691156 | 1.00278834736565 | 0.996920805479616 | 1.00869042363755 | 0.994119304454654 |
| ebi-a-GCST90001688 | finngen_R11_O15_POOR_FETGRO | Poor fetal growth \|\| id:finngen_R11_O15_POOR_FETGRO | CD28+ CD45RA+ CD8+ T cell %T cell \|\| id:ebi-a-GCST90001688 | MR Egger | 72 | 0.00374008788181262 | 0.00337482787021147 | 0.271555519780682 | -0.002874575 | 0.0103547505074271 | 1.00374709073821 | 0.997129552890166 | 1.01040854645734 | 0.992899239648112 |
| ebi-a-GCST90001688 | finngen_R11_O15_POOR_FETGRO | Poor fetal growth \|\| id:finngen_R11_O15_POOR_FETGRO | CD28+ CD45RA+ CD8+ T cell %T cell \|\| id:ebi-a-GCST90001688 | Weighted median | 72 | -0.000726526 | 0.00402003083642927 | 0.856582200945249 | -0.008605787 | 0.0071527340478616 | 0.999273737464856 | 0.991431136957216 | 1.00717837595015 | 0.999440741871876 |
| ebi-a-GCST90001688 | finngen_R11_O15_POOR_FETGRO | Poor fetal growth \|\| id:finngen_R11_O15_POOR_FETGRO | CD28+ CD45RA+ CD8+ T cell %T cell \|\| id:ebi-a-GCST90001688 | Weighted mode | 72 | 0.00157101462469944 | 0.00341788792293462 | 0.647175527845719 | -0.005128046 | 0.0082700749536513 | 1.00157224931466 | 0.99488508027566 | 1.00830436648953 | 0.999857399747617 |
| ebi-a-GCST90001689 | finngen_R11_O15_POOR_FETGRO | Poor fetal growth \|\| id:finngen_R11_O15_POOR_FETGRO | CD28+ CD45RA+ CD8+ T cell %CD8+ T cell \|\| id:ebi-a-GCST90001689 | Inverse variance weighted | 36 | -0.000518719 | 0.00470089084853332 | 0.912135824697971 | -0.009732466 | 0.00869502658492146 | 0.999481415033486 | 0.990314741629911 | 1.00873293812954 | 0.995828307205886 |
| ebi-a-GCST90001689 | finngen_R11_O15_POOR_FETGRO | Poor fetal growth \|\| id:finngen_R11_O15_POOR_FETGRO | CD28+ CD45RA+ CD8+ T cell %CD8+ T cell \|\| id:ebi-a-GCST90001689 | MR Egger | 36 | 9.37539237975393e-05 | 0.00493862229367963 | 0.984964981460805 | -0.009585946 | 0.00977345361940961 | 1.00009375831883 | 0.99045985294818 | 1.00982136979218 | 0.994809183268002 |
| ebi-a-GCST90001689 | finngen_R11_O15_POOR_FETGRO | Poor fetal growth \|\| id:finngen_R11_O15_POOR_FETGRO | CD28+ CD45RA+ CD8+ T cell %CD8+ T cell \|\| id:ebi-a-GCST90001689 | Weighted median | 36 | -0.001194701 | 0.0056187941309808 | 0.831618832254121 | -0.012207537 | 0.00981813580001574 | 0.998806012674054 | 0.987866672509222 | 1.00986649182107 | 0.999440741871876 |
| ebi-a-GCST90001689 | finngen_R11_O15_POOR_FETGRO | Poor fetal growth \|\| id:finngen_R11_O15_POOR_FETGRO | CD28+ CD45RA+ CD8+ T cell %CD8+ T cell \|\| id:ebi-a-GCST90001689 | Weighted mode | 36 | -0.000183884 | 0.00499361379777266 | 0.970834630959351 | -0.009971367 | 0.00960359859914361 | 0.999816132461217 | 0.990078181768625 | 1.00964986112916 | 0.999857399747617 |
| ebi-a-GCST90001690 | finngen_R11_O15_POOR_FETGRO | Poor fetal growth \|\| id:finngen_R11_O15_POOR_FETGRO | CD28+ CD45RA+ CD8+ T cell Absolute Count \|\| id:ebi-a-GCST90001690 | Inverse variance weighted | 38 | -0.001772309 | 0.0045532895410584 | 0.697100808174213 | -0.010696756 | 0.00715213857256864 | 0.998229260684146 | 0.989360250426791 | 1.00717777620049 | 0.994119304454654 |
| ebi-a-GCST90001690 | finngen_R11_O15_POOR_FETGRO | Poor fetal growth \|\| id:finngen_R11_O15_POOR_FETGRO | CD28+ CD45RA+ CD8+ T cell Absolute Count \|\| id:ebi-a-GCST90001690 | MR Egger | 38 | -0.000818342 | 0.00473813000027977 | 0.86384351254768 | -0.010105077 | 0.00846839273874334 | 0.99918199268874 | 0.989945807884516 | 1.00850435100778 | 0.992899239648112 |
| ebi-a-GCST90001690 | finngen_R11_O15_POOR_FETGRO | Poor fetal growth \|\| id:finngen_R11_O15_POOR_FETGRO | CD28+ CD45RA+ CD8+ T cell Absolute Count \|\| id:ebi-a-GCST90001690 | Weighted median | 38 | -0.00138785 | 0.00534051833174833 | 0.794962635532802 | -0.011855266 | 0.00907956561815176 | 0.998613112306794 | 0.988214730543302 | 1.00912090990872 | 0.999440741871876 |
| ebi-a-GCST90001690 | finngen_R11_O15_POOR_FETGRO | Poor fetal growth \|\| id:finngen_R11_O15_POOR_FETGRO | CD28+ CD45RA+ CD8+ T cell Absolute Count \|\| id:ebi-a-GCST90001690 | Weighted mode | 38 | 0.000340487333430839 | 0.00498820250695389 | 0.945947393274545 | -0.00943639 | 0.0101173642470605 | 1.00034054530582 | 0.990607993429122 | 1.01016871781819 | 0.999857399747617 |
| ebi-a-GCST90001691 | finngen_R11_O15_POOR_FETGRO | Poor fetal growth \|\| id:finngen_R11_O15_POOR_FETGRO | CD28+ CD45RA- CD8+ T cell %T cell \|\| id:ebi-a-GCST90001691 | Inverse variance weighted | 25 | 0.00248034847741848 | 0.0106798159506903 | 0.816346664807267 | -0.018452091 | 0.0234127877407714 | 1.00248342708652 | 0.981717106760165 | 1.02368901862138 | 0.994119304454654 |
| ebi-a-GCST90001691 | finngen_R11_O15_POOR_FETGRO | Poor fetal growth \|\| id:finngen_R11_O15_POOR_FETGRO | CD28+ CD45RA- CD8+ T cell %T cell \|\| id:ebi-a-GCST90001691 | MR Egger | 25 | 0.0109170472647119 | 0.0119719667842562 | 0.371288984796899 | -0.012548008 | 0.0343821021618541 | 1.01097685567079 | 0.987530390359872 | 1.03497999927833 | 0.992899239648112 |
| ebi-a-GCST90001691 | finngen_R11_O15_POOR_FETGRO | Poor fetal growth \|\| id:finngen_R11_O15_POOR_FETGRO | CD28+ CD45RA- CD8+ T cell %T cell \|\| id:ebi-a-GCST90001691 | Weighted median | 25 | 0.00429696401242506 | 0.014101581238271 | 0.760583187228676 | -0.023342135 | 0.0319360632394362 | 1.00430620919962 | 0.976928185054627 | 1.03245149159098 | 0.999440741871876 |
| ebi-a-GCST90001691 | finngen_R11_O15_POOR_FETGRO | Poor fetal growth \|\| id:finngen_R11_O15_POOR_FETGRO | CD28+ CD45RA- CD8+ T cell %T cell \|\| id:ebi-a-GCST90001691 | Weighted mode | 25 | 0.00652934502652514 | 0.0115676001208931 | 0.577684956479204 | -0.016143151 | 0.0292018412634755 | 1.00655070766914 | 0.983986451121226 | 1.02963239580667 | 0.999857399747617 |
| ebi-a-GCST90001692 | finngen_R11_O15_POOR_FETGRO | Poor fetal growth \|\| id:finngen_R11_O15_POOR_FETGRO | CD28+ CD45RA- CD8+ T cell %CD8+ T cell \|\| id:ebi-a-GCST90001692 | Inverse variance weighted | 24 | 0.0042138944871211 | 0.00989906481022317 | 0.670336577637251 | -0.015188273 | 0.0236160615151585 | 1.0042227854246 | 0.984926487533421 | 1.02389712890295 | 0.994119304454654 |
| ebi-a-GCST90001692 | finngen_R11_O15_POOR_FETGRO | Poor fetal growth \|\| id:finngen_R11_O15_POOR_FETGRO | CD28+ CD45RA- CD8+ T cell %CD8+ T cell \|\| id:ebi-a-GCST90001692 | MR Egger | 24 | 0.00350565907743862 | 0.0111966739989602 | 0.757154956571378 | -0.018439822 | 0.0254511401154005 | 1.00351181108707 | 0.981729151349837 | 1.02577778566178 | 0.992899239648112 |
| ebi-a-GCST90001692 | finngen_R11_O15_POOR_FETGRO | Poor fetal growth \|\| id:finngen_R11_O15_POOR_FETGRO | CD28+ CD45RA- CD8+ T cell %CD8+ T cell \|\| id:ebi-a-GCST90001692 | Weighted median | 24 | 0.00805001920132771 | 0.0151974158591657 | 0.596322319730209 | -0.021736916 | 0.0378369542852924 | 1.00808250772513 | 0.978497628376581 | 1.03856188600826 | 0.999440741871876 |
| ebi-a-GCST90001692 | finngen_R11_O15_POOR_FETGRO | Poor fetal growth \|\| id:finngen_R11_O15_POOR_FETGRO | CD28+ CD45RA- CD8+ T cell %CD8+ T cell \|\| id:ebi-a-GCST90001692 | Weighted mode | 24 | 0.00462255641980247 | 0.0101131801271132 | 0.651899780083021 | -0.015199277 | 0.0244443894689444 | 1.00463325691526 | 0.984915649374894 | 1.02474560287547 | 0.999857399747617 |
| ebi-a-GCST90001693 | finngen_R11_O15_POOR_FETGRO | Poor fetal growth \|\| id:finngen_R11_O15_POOR_FETGRO | CD28+ CD45RA- CD8+ T cell Absolute Count \|\| id:ebi-a-GCST90001693 | Inverse variance weighted | 29 | 0.00228084762067849 | 0.00992291264531272 | 0.818203150027704 | -0.017168061 | 0.0217297564054914 | 1.00228345073234 | 0.982978470246209 | 1.02196756696035 | 0.994119304454654 |
| ebi-a-GCST90001693 | finngen_R11_O15_POOR_FETGRO | Poor fetal growth \|\| id:finngen_R11_O15_POOR_FETGRO | CD28+ CD45RA- CD8+ T cell Absolute Count \|\| id:ebi-a-GCST90001693 | MR Egger | 29 | 0.00835901823184797 | 0.0113168108074698 | 0.466500033358865 | -0.013821931 | 0.0305399674144888 | 1.00839405237372 | 0.986273153349802 | 1.03101109607315 | 0.992899239648112 |
| ebi-a-GCST90001693 | finngen_R11_O15_POOR_FETGRO | Poor fetal growth \|\| id:finngen_R11_O15_POOR_FETGRO | CD28+ CD45RA- CD8+ T cell Absolute Count \|\| id:ebi-a-GCST90001693 | Weighted median | 29 | 0.0075493991471462 | 0.0128029146566488 | 0.555416915622286 | -0.017544314 | 0.0326431118741779 | 1.00757796770746 | 0.982608691791528 | 1.0331817431398 | 0.999440741871876 |
| ebi-a-GCST90001693 | finngen_R11_O15_POOR_FETGRO | Poor fetal growth \|\| id:finngen_R11_O15_POOR_FETGRO | CD28+ CD45RA- CD8+ T cell Absolute Count \|\| id:ebi-a-GCST90001693 | Weighted mode | 29 | 0.00762125514751855 | 0.00999384895337199 | 0.452082225034086 | -0.011966689 | 0.0272091990961276 | 1.00765037083155 | 0.988104627263501 | 1.02758274966178 | 0.999857399747617 |
| ebi-a-GCST90001694 | finngen_R11_O15_POOR_FETGRO | Poor fetal growth \|\| id:finngen_R11_O15_POOR_FETGRO | CD28- CD4-CD8- T cell %T cell \|\| id:ebi-a-GCST90001694 | Inverse variance weighted | 22 | 0.0021589813551306 | 0.0273675714021713 | 0.937121464483523 | -0.051481459 | 0.0557994213033864 | 1.00216131363352 | 0.949821260821389 | 1.05738557344478 | 0.998984604424952 |
| ebi-a-GCST90001694 | finngen_R11_O15_POOR_FETGRO | Poor fetal growth \|\| id:finngen_R11_O15_POOR_FETGRO | CD28- CD4-CD8- T cell %T cell \|\| id:ebi-a-GCST90001694 | MR Egger | 22 | -0.037394969 | 0.0414893800221284 | 0.378147486850395 | -0.118714154 | 0.0439242157124419 | 0.963295588180516 | 0.888061613367316 | 1.04490316464589 | 0.992899239648112 |
| ebi-a-GCST90001694 | finngen_R11_O15_POOR_FETGRO | Poor fetal growth \|\| id:finngen_R11_O15_POOR_FETGRO | CD28- CD4-CD8- T cell %T cell \|\| id:ebi-a-GCST90001694 | Weighted median | 22 | -0.020190929 | 0.0401333221064393 | 0.61489651990572 | -0.09885224 | 0.058470382727153 | 0.980011543209712 | 0.905876550517452 | 1.06021358459458 | 0.999440741871876 |
| ebi-a-GCST90001694 | finngen_R11_O15_POOR_FETGRO | Poor fetal growth \|\| id:finngen_R11_O15_POOR_FETGRO | CD28- CD4-CD8- T cell %T cell \|\| id:ebi-a-GCST90001694 | Weighted mode | 22 | -0.02870339 | 0.0382359082809931 | 0.461167540761023 | -0.10364577 | 0.046238990463377 | 0.971704639265796 | 0.901544595009062 | 1.04732468166057 | 0.999857399747617 |
| ebi-a-GCST90001695 | finngen_R11_O15_POOR_FETGRO | Poor fetal growth \|\| id:finngen_R11_O15_POOR_FETGRO | CD45RA- CD28- CD8+ T cell Absolute Count \|\| id:ebi-a-GCST90001695 | Inverse variance weighted | 338 | 8.66327140758349e-06 | 1.11714383648081e-05 | 0.438053732237951 | -1.32E-05 | 3.05592906026074e-05 | 1.00000866330893 | 0.999986767339765 | 1.00003055975754 | 0.994119304454654 |
| ebi-a-GCST90001695 | finngen_R11_O15_POOR_FETGRO | Poor fetal growth \|\| id:finngen_R11_O15_POOR_FETGRO | CD45RA- CD28- CD8+ T cell Absolute Count \|\| id:ebi-a-GCST90001695 | MR Egger | 338 | -2.10E-06 | 1.45790200417302e-05 | 0.885467358353862 | -3.07E-05 | 2.6473293520792e-05 | 0.999997898416447 | 0.999969324005475 | 1.00002647364394 | 0.992899239648112 |
| ebi-a-GCST90001695 | finngen_R11_O15_POOR_FETGRO | Poor fetal growth \|\| id:finngen_R11_O15_POOR_FETGRO | CD45RA- CD28- CD8+ T cell Absolute Count \|\| id:ebi-a-GCST90001695 | Weighted median | 338 | 6.37020277977477e-07 | 2.14946147224797e-05 | 0.97635713604157 | -4.15E-05 | 4.27664651340376e-05 | 1.00000063702048 | 0.999958508436221 | 1.00004276737963 | 0.999440741871876 |
| ebi-a-GCST90001695 | finngen_R11_O15_POOR_FETGRO | Poor fetal growth \|\| id:finngen_R11_O15_POOR_FETGRO | CD45RA- CD28- CD8+ T cell Absolute Count \|\| id:ebi-a-GCST90001695 | Weighted mode | 338 | -1.79E-06 | 2.23695310492648e-05 | 0.936382780257882 | -4.56E-05 | 4.20574778686787e-05 | 0.999998213198609 | 0.999954369957238 | 1.0000420583623 | 0.999857399747617 |
| ebi-a-GCST90001696 | finngen_R11_O15_POOR_FETGRO | Poor fetal growth \|\| id:finngen_R11_O15_POOR_FETGRO | CD45RA- CD28- CD8+ T cell %CD8+ T cell \|\| id:ebi-a-GCST90001696 | Inverse variance weighted | 51 | -0.000288527 | 0.000467522554314296 | 0.537142886348277 | -0.001204871 | 0.000627817691245754 | 0.999711515104562 | 0.998795854843628 | 1.00062801481002 | 0.994119304454654 |
| ebi-a-GCST90001696 | finngen_R11_O15_POOR_FETGRO | Poor fetal growth \|\| id:finngen_R11_O15_POOR_FETGRO | CD45RA- CD28- CD8+ T cell %CD8+ T cell \|\| id:ebi-a-GCST90001696 | MR Egger | 51 | -0.0004222 | 0.000564199553200057 | 0.457845521832644 | -0.001528032 | 0.000683630707059891 | 0.999577888696842 | 0.99847313530431 | 1.00068386443579 | 0.992899239648112 |
| ebi-a-GCST90001696 | finngen_R11_O15_POOR_FETGRO | Poor fetal growth \|\| id:finngen_R11_O15_POOR_FETGRO | CD45RA- CD28- CD8+ T cell %CD8+ T cell \|\| id:ebi-a-GCST90001696 | Weighted median | 51 | -0.000585131 | 0.000684307444661433 | 0.392511962657277 | -0.001926374 | 0.000756111288454276 | 0.999415039852854 | 0.998075480372711 | 1.00075639721265 | 0.999440741871876 |
| ebi-a-GCST90001696 | finngen_R11_O15_POOR_FETGRO | Poor fetal growth \|\| id:finngen_R11_O15_POOR_FETGRO | CD45RA- CD28- CD8+ T cell %CD8+ T cell \|\| id:ebi-a-GCST90001696 | Weighted mode | 51 | -0.000670614 | 0.000525979402060821 | 0.208208577495675 | -0.001701534 | 0.000360305447338969 | 0.999329610630733 | 0.998299912979209 | 1.00036037036514 | 0.999857399747617 |
| ebi-a-GCST90001697 | finngen_R11_O15_POOR_FETGRO | Poor fetal growth \|\| id:finngen_R11_O15_POOR_FETGRO | CD45RA- CD28- CD8+ T cell %T cell \|\| id:ebi-a-GCST90001697 | Inverse variance weighted | 175 | 0.000135061503612868 | 0.00035729070244755 | 0.705418913011167 | -0.000565228 | 0.000835351280410067 | 1.00013507062483 | 0.999434931438224 | 1.00083570028346 | 0.994119304454654 |
| ebi-a-GCST90001697 | finngen_R11_O15_POOR_FETGRO | Poor fetal growth \|\| id:finngen_R11_O15_POOR_FETGRO | CD45RA- CD28- CD8+ T cell %T cell \|\| id:ebi-a-GCST90001697 | MR Egger | 175 | 0.000298617565193071 | 0.000473277637044421 | 0.528901704515499 | -0.000629007 | 0.00122624173380014 | 1.00029866215586 | 0.999371191179769 | 1.0012269938756 | 0.992899239648112 |
| ebi-a-GCST90001697 | finngen_R11_O15_POOR_FETGRO | Poor fetal growth \|\| id:finngen_R11_O15_POOR_FETGRO | CD45RA- CD28- CD8+ T cell %T cell \|\| id:ebi-a-GCST90001697 | Weighted median | 175 | 0.000434803809205978 | 0.000572360802528666 | 0.447453454933181 | -0.000687023 | 0.00155663098216216 | 1.00043489835008 | 0.999313212582764 | 1.00155784316106 | 0.999440741871876 |
| ebi-a-GCST90001697 | finngen_R11_O15_POOR_FETGRO | Poor fetal growth \|\| id:finngen_R11_O15_POOR_FETGRO | CD45RA- CD28- CD8+ T cell %T cell \|\| id:ebi-a-GCST90001697 | Weighted mode | 175 | 7.39956708960821e-05 | 0.000484913321900706 | 0.878893948459718 | -0.000876434 | 0.00102442578182147 | 1.00007399840864 | 0.999123949516455 | 1.00102495068514 | 0.999857399747617 |
| ebi-a-GCST90001698 | finngen_R11_O15_POOR_FETGRO | Poor fetal growth \|\| id:finngen_R11_O15_POOR_FETGRO | CD45RA+ CD28- CD8+ T cell Absolute Count \|\| id:ebi-a-GCST90001698 | Inverse variance weighted | 715 | -2.19E-06 | 5.78500487874724e-06 | 0.704725207478672 | -1.35E-05 | 9.14638362914297e-06 | 0.99999780777647 | 0.999986469256046 | 1.00000914642546 | 0.994119304454654 |
| ebi-a-GCST90001698 | finngen_R11_O15_POOR_FETGRO | Poor fetal growth \|\| id:finngen_R11_O15_POOR_FETGRO | CD45RA+ CD28- CD8+ T cell Absolute Count \|\| id:ebi-a-GCST90001698 | MR Egger | 715 | -3.58E-06 | 7.66043416609833e-06 | 0.640013196563824 | -1.86E-05 | 1.14302850112885e-05 | 0.999996415840469 | 0.999981401556033 | 1.00001143035034 | 0.992899239648112 |
| ebi-a-GCST90001698 | finngen_R11_O15_POOR_FETGRO | Poor fetal growth \|\| id:finngen_R11_O15_POOR_FETGRO | CD45RA+ CD28- CD8+ T cell Absolute Count \|\| id:ebi-a-GCST90001698 | Weighted median | 715 | 8.13086593047381e-06 | 1.06852226365115e-05 | 0.446689964113482 | -1.28E-05 | 2.90739022980363e-05 | 1.00000813089899 | 0.999987187911638 | 1.00002907432495 | 0.999440741871876 |
| ebi-a-GCST90001698 | finngen_R11_O15_POOR_FETGRO | Poor fetal growth \|\| id:finngen_R11_O15_POOR_FETGRO | CD45RA+ CD28- CD8+ T cell Absolute Count \|\| id:ebi-a-GCST90001698 | Weighted mode | 715 | 1.44682498502847e-05 | 2.04167990316172e-05 | 0.478776485908412 | -2.55E-05 | 5.44851759522544e-05 | 1.00001446835452 | 0.999974451650113 | 1.0000544866603 | 0.999857399747617 |
| ebi-a-GCST90001699 | finngen_R11_O15_POOR_FETGRO | Poor fetal growth \|\| id:finngen_R11_O15_POOR_FETGRO | CD45RA+ CD28- CD8+ T cell %CD8+ T cell \|\| id:ebi-a-GCST90001699 | Inverse variance weighted | 31 | 0.000391289551695553 | 0.0003641117436372 | 0.282535311760757 | -0.000322369 | 0.00110494856922446 | 1.00039136611544 | 0.99967768248962 | 1.0011055592498 | 0.994119304454654 |
| ebi-a-GCST90001699 | finngen_R11_O15_POOR_FETGRO | Poor fetal growth \|\| id:finngen_R11_O15_POOR_FETGRO | CD45RA+ CD28- CD8+ T cell %CD8+ T cell \|\| id:ebi-a-GCST90001699 | MR Egger | 31 | 0.000202786485674733 | 0.000393353655204192 | 0.610089218951798 | -0.000568187 | 0.000973759649874949 | 1.00020280704824 | 0.999431974708958 | 1.00097423390773 | 0.992899239648112 |
| ebi-a-GCST90001699 | finngen_R11_O15_POOR_FETGRO | Poor fetal growth \|\| id:finngen_R11_O15_POOR_FETGRO | CD45RA+ CD28- CD8+ T cell %CD8+ T cell \|\| id:ebi-a-GCST90001699 | Weighted median | 31 | 0.000416238349830235 | 0.000484145812687997 | 0.389933713780068 | -0.000532687 | 0.00136516414269871 | 1.00041632498903 | 0.999467454409729 | 1.00136609640345 | 0.999440741871876 |
| ebi-a-GCST90001699 | finngen_R11_O15_POOR_FETGRO | Poor fetal growth \|\| id:finngen_R11_O15_POOR_FETGRO | CD45RA+ CD28- CD8+ T cell %CD8+ T cell \|\| id:ebi-a-GCST90001699 | Weighted mode | 31 | 0.000359261292742438 | 0.00036590940498628 | 0.334035281664675 | -0.000357921 | 0.00107644372651555 | 1.00035932583481 | 0.9996421429051 | 1.001077023 | 0.999857399747617 |
| ebi-a-GCST90001700 | finngen_R11_O15_POOR_FETGRO | Poor fetal growth \|\| id:finngen_R11_O15_POOR_FETGRO | CD45RA+ CD28- CD8+ T cell %T cell \|\| id:ebi-a-GCST90001700 | Inverse variance weighted | 204 | -0.000184342 | 0.000258199775193511 | 0.475257733863747 | -0.000690413 | 0.000321729677897706 | 0.999815675108439 | 0.999309824839658 | 1.00032178143844 | 0.994119304454654 |
| ebi-a-GCST90001700 | finngen_R11_O15_POOR_FETGRO | Poor fetal growth \|\| id:finngen_R11_O15_POOR_FETGRO | CD45RA+ CD28- CD8+ T cell %T cell \|\| id:ebi-a-GCST90001700 | MR Egger | 204 | -0.000461109 | 0.000352817634820523 | 0.192720979692368 | -0.001152632 | 0.000230413201560443 | 0.999538996931896 | 0.998848032098094 | 1.00023043974872 | 0.992899239648112 |
| ebi-a-GCST90001700 | finngen_R11_O15_POOR_FETGRO | Poor fetal growth \|\| id:finngen_R11_O15_POOR_FETGRO | CD45RA+ CD28- CD8+ T cell %T cell \|\| id:ebi-a-GCST90001700 | Weighted median | 204 | 0.000258709108026588 | 0.000418704268488167 | 0.536654263113898 | -0.000561951 | 0.00107936947426339 | 1.00025874257611 | 0.999438206606826 | 1.00107995220314 | 0.999440741871876 |
| ebi-a-GCST90001700 | finngen_R11_O15_POOR_FETGRO | Poor fetal growth \|\| id:finngen_R11_O15_POOR_FETGRO | CD45RA+ CD28- CD8+ T cell %T cell \|\| id:ebi-a-GCST90001700 | Weighted mode | 204 | -0.000176589 | 0.000359147608897363 | 0.623470594812731 | -0.000880518 | 0.000527340459488401 | 0.999823426736944 | 0.999119869374978 | 1.00052747952791 | 0.999857399747617 |
| ebi-a-GCST90001701 | finngen_R11_O15_POOR_FETGRO | Poor fetal growth \|\| id:finngen_R11_O15_POOR_FETGRO | BAFF-R on CD20- CD38- B cell \|\| id:ebi-a-GCST90001701 | Inverse variance weighted | 17 | 0.0198038618513936 | 0.0245392133059532 | 0.419649656295915 | -0.028292996 | 0.0679007199310618 | 1.0200012592471 | 0.97210350241149 | 1.07025904781203 | 0.994119304454654 |
| ebi-a-GCST90001701 | finngen_R11_O15_POOR_FETGRO | Poor fetal growth \|\| id:finngen_R11_O15_POOR_FETGRO | BAFF-R on CD20- CD38- B cell \|\| id:ebi-a-GCST90001701 | MR Egger | 17 | 0.0299136586038407 | 0.0358001737500473 | 0.416506747174175 | -0.040254682 | 0.100081999153933 | 1.0303655669112 | 0.960544774585168 | 1.10526154487149 | 0.992899239648112 |
| ebi-a-GCST90001701 | finngen_R11_O15_POOR_FETGRO | Poor fetal growth \|\| id:finngen_R11_O15_POOR_FETGRO | BAFF-R on CD20- CD38- B cell \|\| id:ebi-a-GCST90001701 | Weighted median | 17 | 0.0364744690875914 | 0.0296519093527625 | 0.218664011575049 | -0.021643273 | 0.0945922114190059 | 1.03714782434947 | 0.978589261766904 | 1.09921051822154 | 0.999440741871876 |
| ebi-a-GCST90001701 | finngen_R11_O15_POOR_FETGRO | Poor fetal growth \|\| id:finngen_R11_O15_POOR_FETGRO | BAFF-R on CD20- CD38- B cell \|\| id:ebi-a-GCST90001701 | Weighted mode | 17 | 0.0349259202168768 | 0.0276634237812662 | 0.22484975805684 | -0.01929439 | 0.0891462308281585 | 1.03554299315777 | 0.980890554976502 | 1.09324051010344 | 0.999857399747617 |
| ebi-a-GCST90001702 | finngen_R11_O15_POOR_FETGRO | Poor fetal growth \|\| id:finngen_R11_O15_POOR_FETGRO | BAFF-R on CD24+ CD27+ B cell \|\| id:ebi-a-GCST90001702 | Inverse variance weighted | 19 | -0.010437964 | 0.0216463203650721 | 0.629660323798295 | -0.052864752 | 0.0319888239584289 | 0.989616322544311 | 0.9485082877773 | 1.03250596591104 | 0.994119304454654 |
| ebi-a-GCST90001702 | finngen_R11_O15_POOR_FETGRO | Poor fetal growth \|\| id:finngen_R11_O15_POOR_FETGRO | BAFF-R on CD24+ CD27+ B cell \|\| id:ebi-a-GCST90001702 | MR Egger | 19 | -0.012279794 | 0.0299443397516673 | 0.686863564057778 | -0.0709707 | 0.0464111120020455 | 0.987795295084629 | 0.931489184524346 | 1.04750496431108 | 0.992899239648112 |
| ebi-a-GCST90001702 | finngen_R11_O15_POOR_FETGRO | Poor fetal growth \|\| id:finngen_R11_O15_POOR_FETGRO | BAFF-R on CD24+ CD27+ B cell \|\| id:ebi-a-GCST90001702 | Weighted median | 19 | 0.00298833199532258 | 0.0217330421822945 | 0.890634185493167 | -0.039608431 | 0.0455850946726199 | 1.0029928015104 | 0.961165728484649 | 1.04664006431827 | 0.999440741871876 |
| ebi-a-GCST90001702 | finngen_R11_O15_POOR_FETGRO | Poor fetal growth \|\| id:finngen_R11_O15_POOR_FETGRO | BAFF-R on CD24+ CD27+ B cell \|\| id:ebi-a-GCST90001702 | Weighted mode | 19 | 0.00678404080567152 | 0.0220027393094585 | 0.761373143402891 | -0.036341328 | 0.0499094098522103 | 1.0068071045361 | 0.964311090694979 | 1.05117586588557 | 0.999857399747617 |
| ebi-a-GCST90001703 | finngen_R11_O15_POOR_FETGRO | Poor fetal growth \|\| id:finngen_R11_O15_POOR_FETGRO | BAFF-R on IgD+ CD24+ B cell \|\| id:ebi-a-GCST90001703 | Inverse variance weighted | 18 | 0.0132575589490674 | 0.0188234779260676 | 0.481239898503218 | -0.023636458 | 0.0501515756841599 | 1.01334583003876 | 0.976640695348521 | 1.05143045558888 | 0.994119304454654 |
| ebi-a-GCST90001703 | finngen_R11_O15_POOR_FETGRO | Poor fetal growth \|\| id:finngen_R11_O15_POOR_FETGRO | BAFF-R on IgD+ CD24+ B cell \|\| id:ebi-a-GCST90001703 | MR Egger | 18 | -0.006031848 | 0.0255142402514825 | 0.816113657225038 | -0.056039759 | 0.0439760629418342 | 0.993986307122559 | 0.945501543094746 | 1.04495734138441 | 0.992899239648112 |
| ebi-a-GCST90001703 | finngen_R11_O15_POOR_FETGRO | Poor fetal growth \|\| id:finngen_R11_O15_POOR_FETGRO | BAFF-R on IgD+ CD24+ B cell \|\| id:ebi-a-GCST90001703 | Weighted median | 18 | 0.00340685046221533 | 0.0234982531751456 | 0.884724185735637 | -0.042649726 | 0.0494634266855007 | 1.00341266037321 | 0.958246980521063 | 1.05070716366856 | 0.999440741871876 |
| ebi-a-GCST90001703 | finngen_R11_O15_POOR_FETGRO | Poor fetal growth \|\| id:finngen_R11_O15_POOR_FETGRO | BAFF-R on IgD+ CD24+ B cell \|\| id:ebi-a-GCST90001703 | Weighted mode | 18 | 0.00718028573375296 | 0.0234730690194347 | 0.763399115519939 | -0.03882693 | 0.0531875010118449 | 1.00720612579468 | 0.961917174184505 | 1.05462737028099 | 0.999857399747617 |
| ebi-a-GCST90001704 | finngen_R11_O15_POOR_FETGRO | Poor fetal growth \|\| id:finngen_R11_O15_POOR_FETGRO | BAFF-R on IgD+ CD24- B cell \|\| id:ebi-a-GCST90001704 | Inverse variance weighted | 22 | -0.019701586 | 0.0204338506289793 | 0.334963652079054 | -0.059751933 | 0.0203487617127138 | 0.98049122243234 | 0.941998183498773 | 1.02055720924659 | 0.994119304454654 |
| ebi-a-GCST90001704 | finngen_R11_O15_POOR_FETGRO | Poor fetal growth \|\| id:finngen_R11_O15_POOR_FETGRO | BAFF-R on IgD+ CD24- B cell \|\| id:ebi-a-GCST90001704 | MR Egger | 22 | -0.032314197 | 0.0268091577792096 | 0.242139573408456 | -0.084860147 | 0.0202317517731022 | 0.968202327557916 | 0.918640750338848 | 1.02043780089528 | 0.992899239648112 |
| ebi-a-GCST90001704 | finngen_R11_O15_POOR_FETGRO | Poor fetal growth \|\| id:finngen_R11_O15_POOR_FETGRO | BAFF-R on IgD+ CD24- B cell \|\| id:ebi-a-GCST90001704 | Weighted median | 22 | -0.003862959 | 0.0224858037285158 | 0.863598347437712 | -0.047935134 | 0.0402092166727181 | 0.996144492991339 | 0.953195615113254 | 1.04102855194007 | 0.999440741871876 |
| ebi-a-GCST90001704 | finngen_R11_O15_POOR_FETGRO | Poor fetal growth \|\| id:finngen_R11_O15_POOR_FETGRO | BAFF-R on IgD+ CD24- B cell \|\| id:ebi-a-GCST90001704 | Weighted mode | 22 | -0.008399124 | 0.0215662649577322 | 0.7008598495761 | -0.050669003 | 0.0338707552718407 | 0.991636050050962 | 0.950593261638517 | 1.03445090076245 | 0.999857399747617 |
| ebi-a-GCST90001705 | finngen_R11_O15_POOR_FETGRO | Poor fetal growth \|\| id:finngen_R11_O15_POOR_FETGRO | BAFF-R on IgD+ CD38- B cell \|\| id:ebi-a-GCST90001705 | Inverse variance weighted | 22 | -0.028305877 | 0.0216835584467455 | 0.191754173200808 | -0.070805651 | 0.0141938977466512 | 0.972090981234523 | 0.931642938067708 | 1.01429510940899 | 0.994119304454654 |
| ebi-a-GCST90001705 | finngen_R11_O15_POOR_FETGRO | Poor fetal growth \|\| id:finngen_R11_O15_POOR_FETGRO | BAFF-R on IgD+ CD38- B cell \|\| id:ebi-a-GCST90001705 | MR Egger | 22 | -0.011754086 | 0.0282215131628261 | 0.681484490700883 | -0.067068252 | 0.0435600798479621 | 0.988314723456003 | 0.935131374805559 | 1.04452274719476 | 0.992899239648112 |
| ebi-a-GCST90001705 | finngen_R11_O15_POOR_FETGRO | Poor fetal growth \|\| id:finngen_R11_O15_POOR_FETGRO | BAFF-R on IgD+ CD38- B cell \|\| id:ebi-a-GCST90001705 | Weighted median | 22 | -0.002162248 | 0.0235758471273397 | 0.926924838906827 | -0.048370909 | 0.0440464122193266 | 0.997840087824317 | 0.95278032719002 | 1.0450308559642 | 0.999440741871876 |
| ebi-a-GCST90001705 | finngen_R11_O15_POOR_FETGRO | Poor fetal growth \|\| id:finngen_R11_O15_POOR_FETGRO | BAFF-R on IgD+ CD38- B cell \|\| id:ebi-a-GCST90001705 | Weighted mode | 22 | -0.000424895 | 0.0228176455780596 | 0.985318993388294 | -0.04514748 | 0.0442976908228814 | 0.999575195744774 | 0.955856501870571 | 1.04529348285324 | 0.999857399747617 |
| ebi-a-GCST90001706 | finngen_R11_O15_POOR_FETGRO | Poor fetal growth \|\| id:finngen_R11_O15_POOR_FETGRO | BAFF-R on IgD+ CD38- naive B cell \|\| id:ebi-a-GCST90001706 | Inverse variance weighted | 28 | -0.012041913 | 0.020385181962257 | 0.554708762515025 | -0.05199687 | 0.0279130437818835 | 0.988030300814323 | 0.94933183871191 | 1.02830626290991 | 0.994119304454654 |
| ebi-a-GCST90001706 | finngen_R11_O15_POOR_FETGRO | Poor fetal growth \|\| id:finngen_R11_O15_POOR_FETGRO | BAFF-R on IgD+ CD38- naive B cell \|\| id:ebi-a-GCST90001706 | MR Egger | 28 | -0.018511309 | 0.0316602186105468 | 0.563798218567502 | -0.080565337 | 0.0435427194960768 | 0.981658972966741 | 0.922594621627778 | 1.04450461406972 | 0.992899239648112 |
| ebi-a-GCST90001706 | finngen_R11_O15_POOR_FETGRO | Poor fetal growth \|\| id:finngen_R11_O15_POOR_FETGRO | BAFF-R on IgD+ CD38- naive B cell \|\| id:ebi-a-GCST90001706 | Weighted median | 28 | 0.00268610133783477 | 0.0233917297666772 | 0.908578886796791 | -0.043161689 | 0.0485338916805221 | 1.0026897121403 | 0.957756518848458 | 1.04973094836338 | 0.999440741871876 |
| ebi-a-GCST90001706 | finngen_R11_O15_POOR_FETGRO | Poor fetal growth \|\| id:finngen_R11_O15_POOR_FETGRO | BAFF-R on IgD+ CD38- naive B cell \|\| id:ebi-a-GCST90001706 | Weighted mode | 28 | 0.00579736050024871 | 0.0226845691210272 | 0.800224374092005 | -0.038664395 | 0.050259115977462 | 1.00581419771605 | 0.962073531682666 | 1.05154353280853 | 0.999857399747617 |
| ebi-a-GCST90001707 | finngen_R11_O15_POOR_FETGRO | Poor fetal growth \|\| id:finngen_R11_O15_POOR_FETGRO | BAFF-R on IgD+ CD38- unswitched memory B cell \|\| id:ebi-a-GCST90001707 | Inverse variance weighted | 19 | -0.001869521 | 0.0227347451938308 | 0.934462320486262 | -0.046429621 | 0.0426905796777 | 0.998132225563473 | 0.954631743773066 | 1.04361492921937 | 0.998984604424952 |
| ebi-a-GCST90001707 | finngen_R11_O15_POOR_FETGRO | Poor fetal growth \|\| id:finngen_R11_O15_POOR_FETGRO | BAFF-R on IgD+ CD38- unswitched memory B cell \|\| id:ebi-a-GCST90001707 | MR Egger | 19 | -0.009194947 | 0.0343177619441158 | 0.791972839001738 | -0.076457761 | 0.0580678661852882 | 0.990847197031736 | 0.926392043673484 | 1.05978691696502 | 0.992899239648112 |
| ebi-a-GCST90001707 | finngen_R11_O15_POOR_FETGRO | Poor fetal growth \|\| id:finngen_R11_O15_POOR_FETGRO | BAFF-R on IgD+ CD38- unswitched memory B cell \|\| id:ebi-a-GCST90001707 | Weighted median | 19 | 0.00450784055182327 | 0.0235753128881072 | 0.848360903988099 | -0.041699773 | 0.0507154538125135 | 1.00451801614929 | 0.959157702668128 | 1.05202350141334 | 0.999440741871876 |
| ebi-a-GCST90001707 | finngen_R11_O15_POOR_FETGRO | Poor fetal growth \|\| id:finngen_R11_O15_POOR_FETGRO | BAFF-R on IgD+ CD38- unswitched memory B cell \|\| id:ebi-a-GCST90001707 | Weighted mode | 19 | 0.010795910425488 | 0.0245168263907284 | 0.66492821717955 | -0.037257069 | 0.0588488901513157 | 1.01085439654727 | 0.963428435638545 | 1.0606149592644 | 0.999857399747617 |
| ebi-a-GCST90001708 | finngen_R11_O15_POOR_FETGRO | Poor fetal growth \|\| id:finngen_R11_O15_POOR_FETGRO | BAFF-R on IgD+ CD38+ B cell \|\| id:ebi-a-GCST90001708 | Inverse variance weighted (multiplicative random effects) | 26 | -0.018262808 | 0.0216723792978403 | 0.399409315507841 | -0.060740671 | 0.0242150556655943 | 0.98190294673416 | 0.941067253993376 | 1.02451062101457 | 0.994119304454654 |
| ebi-a-GCST90001708 | finngen_R11_O15_POOR_FETGRO | Poor fetal growth \|\| id:finngen_R11_O15_POOR_FETGRO | BAFF-R on IgD+ CD38+ B cell \|\| id:ebi-a-GCST90001708 | MR Egger | 26 | 0.00138089105926864 | 0.0301058130233711 | 0.963795052345501 | -0.057626502 | 0.0603882845850759 | 1.00138184492834 | 0.944002464192588 | 1.06224892136246 | 0.992899239648112 |
| ebi-a-GCST90001708 | finngen_R11_O15_POOR_FETGRO | Poor fetal growth \|\| id:finngen_R11_O15_POOR_FETGRO | BAFF-R on IgD+ CD38+ B cell \|\| id:ebi-a-GCST90001708 | Weighted median | 26 | 0.00317073854687656 | 0.0239770666078046 | 0.894794092105118 | -0.043824312 | 0.0501657890981736 | 1.00317577065544 | 0.957122097565362 | 1.05144540011146 | 0.999440741871876 |
| ebi-a-GCST90001708 | finngen_R11_O15_POOR_FETGRO | Poor fetal growth \|\| id:finngen_R11_O15_POOR_FETGRO | BAFF-R on IgD+ CD38+ B cell \|\| id:ebi-a-GCST90001708 | Weighted mode | 26 | 0.00461707068531292 | 0.021684071878473 | 0.833112559684701 | -0.03788371 | 0.0471178515671199 | 1.00462774577907 | 0.962824901099718 | 1.04824553917992 | 0.999857399747617 |
| ebi-a-GCST90001709 | finngen_R11_O15_POOR_FETGRO | Poor fetal growth \|\| id:finngen_R11_O15_POOR_FETGRO | BAFF-R on IgD+ CD38dim B cell \|\| id:ebi-a-GCST90001709 | Inverse variance weighted | 25 | -0.00339392 | 0.0170311943801122 | 0.842046323438517 | -0.036775061 | 0.0299872211874871 | 0.996611833038202 | 0.963892928286514 | 1.03044136605236 | 0.994119304454654 |
| ebi-a-GCST90001709 | finngen_R11_O15_POOR_FETGRO | Poor fetal growth \|\| id:finngen_R11_O15_POOR_FETGRO | BAFF-R on IgD+ CD38dim B cell \|\| id:ebi-a-GCST90001709 | MR Egger | 25 | -0.004001176 | 0.0224791779994212 | 0.860285499107037 | -0.048060365 | 0.0400580130070492 | 0.996006818166956 | 0.95307625313061 | 1.04087115650663 | 0.992899239648112 |
| ebi-a-GCST90001709 | finngen_R11_O15_POOR_FETGRO | Poor fetal growth \|\| id:finngen_R11_O15_POOR_FETGRO | BAFF-R on IgD+ CD38dim B cell \|\| id:ebi-a-GCST90001709 | Weighted median | 25 | 0.00342657650429285 | 0.0205771677734245 | 0.867745201103097 | -0.036904672 | 0.0437578253402048 | 1.00343245392879 | 0.963768004726888 | 1.04472931728304 | 0.999440741871876 |
| ebi-a-GCST90001709 | finngen_R11_O15_POOR_FETGRO | Poor fetal growth \|\| id:finngen_R11_O15_POOR_FETGRO | BAFF-R on IgD+ CD38dim B cell \|\| id:ebi-a-GCST90001709 | Weighted mode | 25 | 0.00673057147568357 | 0.0185788722085196 | 0.720320671358163 | -0.029684018 | 0.0431451610043819 | 1.00675327267398 | 0.970752225269614 | 1.04408944492326 | 0.999857399747617 |
| ebi-a-GCST90001710 | finngen_R11_O15_POOR_FETGRO | Poor fetal growth \|\| id:finngen_R11_O15_POOR_FETGRO | BAFF-R on IgD- CD24- B cell \|\| id:ebi-a-GCST90001710 | Inverse variance weighted (multiplicative random effects) | 22 | 0.00111397295877868 | 0.0294245094084412 | 0.969800361439886 | -0.056558065 | 0.0587860113993234 | 1.00111459365711 | 0.945011610347586 | 1.06054827121607 | 0.998984604424952 |
| ebi-a-GCST90001710 | finngen_R11_O15_POOR_FETGRO | Poor fetal growth \|\| id:finngen_R11_O15_POOR_FETGRO | BAFF-R on IgD- CD24- B cell \|\| id:ebi-a-GCST90001710 | MR Egger | 22 | -0.03139331 | 0.0382929992720742 | 0.421982723942484 | -0.106447589 | 0.0436609681148916 | 0.969094343169766 | 0.899022165548761 | 1.04462813260048 | 0.992899239648112 |
| ebi-a-GCST90001710 | finngen_R11_O15_POOR_FETGRO | Poor fetal growth \|\| id:finngen_R11_O15_POOR_FETGRO | BAFF-R on IgD- CD24- B cell \|\| id:ebi-a-GCST90001710 | Weighted median | 22 | 0.00435346569580379 | 0.0245549126025685 | 0.859276599126209 | -0.043774163 | 0.052481094396838 | 1.00436295579419 | 0.957170097484222 | 1.05388263760327 | 0.999440741871876 |
| ebi-a-GCST90001710 | finngen_R11_O15_POOR_FETGRO | Poor fetal growth \|\| id:finngen_R11_O15_POOR_FETGRO | BAFF-R on IgD- CD24- B cell \|\| id:ebi-a-GCST90001710 | Weighted mode | 22 | 0.00528969110816924 | 0.0241405285264604 | 0.828671670726737 | -0.042025745 | 0.0526051270200316 | 1.00530370622516 | 0.958845094975981 | 1.0540133615382 | 0.999857399747617 |
| ebi-a-GCST90001711 | finngen_R11_O15_POOR_FETGRO | Poor fetal growth \|\| id:finngen_R11_O15_POOR_FETGRO | BAFF-R on IgD- CD27- B cell \|\| id:ebi-a-GCST90001711 | Inverse variance weighted (multiplicative random effects) | 18 | -0.023373539 | 0.0266231002694961 | 0.379975178041028 | -0.075554815 | 0.0288077377982148 | 0.976897506557021 | 0.927228902849216 | 1.02922669406102 | 0.994119304454654 |
| ebi-a-GCST90001711 | finngen_R11_O15_POOR_FETGRO | Poor fetal growth \|\| id:finngen_R11_O15_POOR_FETGRO | BAFF-R on IgD- CD27- B cell \|\| id:ebi-a-GCST90001711 | MR Egger | 18 | -0.001387341 | 0.0353968696894563 | 0.969220683809289 | -0.070765206 | 0.0679905232090792 | 0.998613620530915 | 0.931680619492627 | 1.0703551648986 | 0.992899239648112 |
| ebi-a-GCST90001711 | finngen_R11_O15_POOR_FETGRO | Poor fetal growth \|\| id:finngen_R11_O15_POOR_FETGRO | BAFF-R on IgD- CD27- B cell \|\| id:ebi-a-GCST90001711 | Weighted median | 18 | 0.00541630226243809 | 0.0241823288387642 | 0.822774659501186 | -0.041981062 | 0.0528136667864158 | 1.00543099694584 | 0.958887939569551 | 1.05423318815888 | 0.999440741871876 |
| ebi-a-GCST90001711 | finngen_R11_O15_POOR_FETGRO | Poor fetal growth \|\| id:finngen_R11_O15_POOR_FETGRO | BAFF-R on IgD- CD27- B cell \|\| id:ebi-a-GCST90001711 | Weighted mode | 18 | 0.00987803952622635 | 0.0230750499320773 | 0.673964766017791 | -0.035349058 | 0.0551051373930978 | 1.00992698839887 | 0.965268422451921 | 1.05665170244106 | 0.999857399747617 |
| ebi-a-GCST90001712 | finngen_R11_O15_POOR_FETGRO | Poor fetal growth \|\| id:finngen_R11_O15_POOR_FETGRO | BAFF-R on IgD- CD38- B cell \|\| id:ebi-a-GCST90001712 | Inverse variance weighted (multiplicative random effects) | 17 | -0.012336104 | 0.0239087743839368 | 0.605878465456832 | -0.059197302 | 0.0345250936995114 | 0.987739673718039 | 0.942520789681706 | 1.03512800324128 | 0.994119304454654 |
| ebi-a-GCST90001712 | finngen_R11_O15_POOR_FETGRO | Poor fetal growth \|\| id:finngen_R11_O15_POOR_FETGRO | BAFF-R on IgD- CD38- B cell \|\| id:ebi-a-GCST90001712 | MR Egger | 17 | 0.0204192017639027 | 0.0316161583536864 | 0.528135833317425 | -0.041548469 | 0.082386872137128 | 1.02062909988061 | 0.95930283814017 | 1.08587582367904 | 0.992899239648112 |
| ebi-a-GCST90001712 | finngen_R11_O15_POOR_FETGRO | Poor fetal growth \|\| id:finngen_R11_O15_POOR_FETGRO | BAFF-R on IgD- CD38- B cell \|\| id:ebi-a-GCST90001712 | Weighted median | 17 | 0.00279125346329204 | 0.0223572679353913 | 0.900644124238713 | -0.041028992 | 0.0466114986166589 | 1.00279515263826 | 0.959801303282512 | 1.04771489131725 | 0.999440741871876 |
| ebi-a-GCST90001712 | finngen_R11_O15_POOR_FETGRO | Poor fetal growth \|\| id:finngen_R11_O15_POOR_FETGRO | BAFF-R on IgD- CD38- B cell \|\| id:ebi-a-GCST90001712 | Weighted mode | 17 | 0.00536399249292607 | 0.0215588552065363 | 0.806675686741501 | -0.036891364 | 0.0476193486977371 | 1.00537840445769 | 0.963780831234126 | 1.04877136314859 | 0.999857399747617 |
| ebi-a-GCST90001713 | finngen_R11_O15_POOR_FETGRO | Poor fetal growth \|\| id:finngen_R11_O15_POOR_FETGRO | BAFF-R on IgD- CD38+ B cell \|\| id:ebi-a-GCST90001713 | Inverse variance weighted | 15 | -0.009766848 | 0.0369016899793126 | 0.791262060765976 | -0.082094161 | 0.0625604640321568 | 0.990280692435466 | 0.921185215180014 | 1.06455882449094 | 0.994119304454654 |
| ebi-a-GCST90001713 | finngen_R11_O15_POOR_FETGRO | Poor fetal growth \|\| id:finngen_R11_O15_POOR_FETGRO | BAFF-R on IgD- CD38+ B cell \|\| id:ebi-a-GCST90001713 | MR Egger | 15 | -0.05318847 | 0.0683381441042901 | 0.450319029098326 | -0.187131232 | 0.0807542926787785 | 0.948201288366193 | 0.829334893792759 | 1.08410449142874 | 0.992899239648112 |
| ebi-a-GCST90001713 | finngen_R11_O15_POOR_FETGRO | Poor fetal growth \|\| id:finngen_R11_O15_POOR_FETGRO | BAFF-R on IgD- CD38+ B cell \|\| id:ebi-a-GCST90001713 | Weighted median | 15 | -0.008256598 | 0.0499038476503197 | 0.868589697626887 | -0.10606814 | 0.089554943208812 | 0.991777393903645 | 0.899363363745734 | 1.09368742235801 | 0.999440741871876 |
| ebi-a-GCST90001713 | finngen_R11_O15_POOR_FETGRO | Poor fetal growth \|\| id:finngen_R11_O15_POOR_FETGRO | BAFF-R on IgD- CD38+ B cell \|\| id:ebi-a-GCST90001713 | Weighted mode | 15 | -0.018217529 | 0.0434023793517758 | 0.681048544647942 | -0.103286193 | 0.0668511342941498 | 0.981947406855699 | 0.901868828210181 | 1.06913630859622 | 0.999857399747617 |
| ebi-a-GCST90001714 | finngen_R11_O15_POOR_FETGRO | Poor fetal growth \|\| id:finngen_R11_O15_POOR_FETGRO | BAFF-R on IgD- CD38dim B cell \|\| id:ebi-a-GCST90001714 | Inverse variance weighted | 16 | -0.007702265 | 0.023577936598066 | 0.74391555241331 | -0.053915021 | 0.0385104906961286 | 0.99232732139768 | 0.947512621975329 | 1.03926163087876 | 0.994119304454654 |
| ebi-a-GCST90001714 | finngen_R11_O15_POOR_FETGRO | Poor fetal growth \|\| id:finngen_R11_O15_POOR_FETGRO | BAFF-R on IgD- CD38dim B cell \|\| id:ebi-a-GCST90001714 | MR Egger | 16 | 0.00143689524181902 | 0.0321054371227115 | 0.964934295893909 | -0.061489762 | 0.0643635520023335 | 1.00143792807042 | 0.94036257357477 | 1.06648004924903 | 0.992899239648112 |
| ebi-a-GCST90001714 | finngen_R11_O15_POOR_FETGRO | Poor fetal growth \|\| id:finngen_R11_O15_POOR_FETGRO | BAFF-R on IgD- CD38dim B cell \|\| id:ebi-a-GCST90001714 | Weighted median | 16 | -0.000666286 | 0.022976608755551 | 0.976865830020949 | -0.045700439 | 0.0443678672136045 | 0.999333935971916 | 0.955328098268107 | 1.04536684035106 | 0.999440741871876 |
| ebi-a-GCST90001714 | finngen_R11_O15_POOR_FETGRO | Poor fetal growth \|\| id:finngen_R11_O15_POOR_FETGRO | BAFF-R on IgD- CD38dim B cell \|\| id:ebi-a-GCST90001714 | Weighted mode | 16 | 0.00362326254395307 | 0.0219903218091125 | 0.871327863221681 | -0.039477768 | 0.0467242932898136 | 1.00362983449459 | 0.961291324987685 | 1.04783307464107 | 0.999857399747617 |
| ebi-a-GCST90001715 | finngen_R11_O15_POOR_FETGRO | Poor fetal growth \|\| id:finngen_R11_O15_POOR_FETGRO | BAFF-R on memory B cell \|\| id:ebi-a-GCST90001715 | Inverse variance weighted | 17 | -0.006938069 | 0.0215438453034525 | 0.747419273519393 | -0.049164005 | 0.0352878681766575 | 0.993085944213647 | 0.952024979644273 | 1.03591787367094 | 0.994119304454654 |
| ebi-a-GCST90001715 | finngen_R11_O15_POOR_FETGRO | Poor fetal growth \|\| id:finngen_R11_O15_POOR_FETGRO | BAFF-R on memory B cell \|\| id:ebi-a-GCST90001715 | MR Egger | 17 | -0.012203352 | 0.0302735017946233 | 0.692559318580789 | -0.071539415 | 0.0471327117429809 | 0.987870807153764 | 0.930959582827956 | 1.04826111640872 | 0.992899239648112 |
| ebi-a-GCST90001715 | finngen_R11_O15_POOR_FETGRO | Poor fetal growth \|\| id:finngen_R11_O15_POOR_FETGRO | BAFF-R on memory B cell \|\| id:ebi-a-GCST90001715 | Weighted median | 17 | -3.83E-05 | 0.0232666002880648 | 0.998685780658649 | -0.04564086 | 0.04556421343939 | 0.999961677609105 | 0.955385017856119 | 1.04661820941115 | 0.999440741871876 |
| ebi-a-GCST90001715 | finngen_R11_O15_POOR_FETGRO | Poor fetal growth \|\| id:finngen_R11_O15_POOR_FETGRO | BAFF-R on memory B cell \|\| id:ebi-a-GCST90001715 | Weighted mode | 17 | 0.00524943709409065 | 0.0229746767245727 | 0.822161572454172 | -0.039780929 | 0.0502798034742531 | 1.0052632395301 | 0.960999943037469 | 1.05156528683701 | 0.999857399747617 |
| ebi-a-GCST90001716 | finngen_R11_O15_POOR_FETGRO | Poor fetal growth \|\| id:finngen_R11_O15_POOR_FETGRO | BAFF-R on naive-mature B cell \|\| id:ebi-a-GCST90001716 | Inverse variance weighted | 23 | -0.012447724 | 0.0207960421065855 | 0.54946488354067 | -0.053207967 | 0.0283125182590431 | 0.987629428193894 | 0.948182801434469 | 1.02871712707606 | 0.994119304454654 |
| ebi-a-GCST90001716 | finngen_R11_O15_POOR_FETGRO | Poor fetal growth \|\| id:finngen_R11_O15_POOR_FETGRO | BAFF-R on naive-mature B cell \|\| id:ebi-a-GCST90001716 | MR Egger | 23 | -0.011925619 | 0.029250927961251 | 0.687620044061793 | -0.069257438 | 0.0454061998744624 | 0.988145209426918 | 0.933086437498015 | 1.04645284260221 | 0.992899239648112 |
| ebi-a-GCST90001716 | finngen_R11_O15_POOR_FETGRO | Poor fetal growth \|\| id:finngen_R11_O15_POOR_FETGRO | BAFF-R on naive-mature B cell \|\| id:ebi-a-GCST90001716 | Weighted median | 23 | -0.000824037 | 0.022931020966635 | 0.971333830866275 | -0.045768838 | 0.0441207645879972 | 0.999176302918235 | 0.955262757500441 | 1.04510855937236 | 0.999440741871876 |
| ebi-a-GCST90001716 | finngen_R11_O15_POOR_FETGRO | Poor fetal growth \|\| id:finngen_R11_O15_POOR_FETGRO | BAFF-R on naive-mature B cell \|\| id:ebi-a-GCST90001716 | Weighted mode | 23 | 0.00133232768331459 | 0.024886885463494 | 0.95778855038224 | -0.047445968 | 0.0501106231917629 | 1.00133321562614 | 0.953662000172512 | 1.05138739777281 | 0.999857399747617 |
| ebi-a-GCST90001717 | finngen_R11_O15_POOR_FETGRO | Poor fetal growth \|\| id:finngen_R11_O15_POOR_FETGRO | BAFF-R on unswitched memory B cell \|\| id:ebi-a-GCST90001717 | Inverse variance weighted | 24 | -0.009242313 | 0.0177264268267236 | 0.602097810041316 | -0.04398611 | 0.0255014834576028 | 0.990800265776347 | 0.956967249939902 | 1.02582942804378 | 0.994119304454654 |
| ebi-a-GCST90001717 | finngen_R11_O15_POOR_FETGRO | Poor fetal growth \|\| id:finngen_R11_O15_POOR_FETGRO | BAFF-R on unswitched memory B cell \|\| id:ebi-a-GCST90001717 | MR Egger | 24 | -0.004289704 | 0.0236327987913751 | 0.857625440921808 | -0.05060999 | 0.0420305813152473 | 0.995719483323597 | 0.950649361048955 | 1.04292637232326 | 0.992899239648112 |
| ebi-a-GCST90001717 | finngen_R11_O15_POOR_FETGRO | Poor fetal growth \|\| id:finngen_R11_O15_POOR_FETGRO | BAFF-R on unswitched memory B cell \|\| id:ebi-a-GCST90001717 | Weighted median | 24 | 0.00257998302225272 | 0.0209257766661441 | 0.901875793631578 | -0.038434539 | 0.0435945052878952 | 1.00258331404249 | 0.962294695216924 | 1.0445587059688 | 0.999440741871876 |
| ebi-a-GCST90001717 | finngen_R11_O15_POOR_FETGRO | Poor fetal growth \|\| id:finngen_R11_O15_POOR_FETGRO | BAFF-R on unswitched memory B cell \|\| id:ebi-a-GCST90001717 | Weighted mode | 24 | 0.00446548836794769 | 0.0183039092732613 | 0.809422029055277 | -0.031410174 | 0.0403411505435399 | 1.00447547351846 | 0.969078001131172 | 1.04116590792732 | 0.999857399747617 |
| ebi-a-GCST90001718 | finngen_R11_O15_POOR_FETGRO | Poor fetal growth \|\| id:finngen_R11_O15_POOR_FETGRO | BAFF-R on switched memory B cell \|\| id:ebi-a-GCST90001718 | Inverse variance weighted (multiplicative random effects) | 19 | -0.00504098 | 0.0232955354201487 | 0.828681784892554 | -0.050700229 | 0.0406182694672852 | 0.994971704460339 | 0.950563578860392 | 1.04145447468497 | 0.994119304454654 |
| ebi-a-GCST90001718 | finngen_R11_O15_POOR_FETGRO | Poor fetal growth \|\| id:finngen_R11_O15_POOR_FETGRO | BAFF-R on switched memory B cell \|\| id:ebi-a-GCST90001718 | MR Egger | 19 | -0.013133936 | 0.0334817437808612 | 0.699731997597373 | -0.078758154 | 0.0524902816325265 | 0.986951937597123 | 0.924263426840819 | 1.05389231991593 | 0.992899239648112 |
| ebi-a-GCST90001718 | finngen_R11_O15_POOR_FETGRO | Poor fetal growth \|\| id:finngen_R11_O15_POOR_FETGRO | BAFF-R on switched memory B cell \|\| id:ebi-a-GCST90001718 | Weighted median | 19 | 0.00337426185295909 | 0.0226074550205675 | 0.881352905509896 | -0.04093635 | 0.0476848736932714 | 1.00337996108291 | 0.95989022502839 | 1.04884008613899 | 0.999440741871876 |
| ebi-a-GCST90001718 | finngen_R11_O15_POOR_FETGRO | Poor fetal growth \|\| id:finngen_R11_O15_POOR_FETGRO | BAFF-R on switched memory B cell \|\| id:ebi-a-GCST90001718 | Weighted mode | 19 | 0.00602559205485864 | 0.0212151090659181 | 0.7796336224968 | -0.035556022 | 0.0476072058240581 | 1.00604378245228 | 0.965068667914337 | 1.04875862812773 | 0.999857399747617 |
| ebi-a-GCST90001719 | finngen_R11_O15_POOR_FETGRO | Poor fetal growth \|\| id:finngen_R11_O15_POOR_FETGRO | BAFF-R on IgD+ B cell \|\| id:ebi-a-GCST90001719 | Inverse variance weighted | 24 | -0.007068848 | 0.0199156252400454 | 0.722634709385234 | -0.046103473 | 0.0319657779171413 | 0.992956077983332 | 0.954943146223478 | 1.03248217101011 | 0.994119304454654 |
| ebi-a-GCST90001719 | finngen_R11_O15_POOR_FETGRO | Poor fetal growth \|\| id:finngen_R11_O15_POOR_FETGRO | BAFF-R on IgD+ B cell \|\| id:ebi-a-GCST90001719 | MR Egger | 24 | -0.015721509 | 0.0268470622659705 | 0.564107092476354 | -0.068341751 | 0.0368987330305818 | 0.984401428812862 | 0.933941243629198 | 1.03758794213134 | 0.992899239648112 |
| ebi-a-GCST90001719 | finngen_R11_O15_POOR_FETGRO | Poor fetal growth \|\| id:finngen_R11_O15_POOR_FETGRO | BAFF-R on IgD+ B cell \|\| id:ebi-a-GCST90001719 | Weighted median | 24 | 0.000163314313250319 | 0.022810458607693 | 0.994287495131156 | -0.044545185 | 0.0448718131843286 | 1.00016332764976 | 0.956432383142934 | 1.04589378152198 | 0.999440741871876 |
| ebi-a-GCST90001719 | finngen_R11_O15_POOR_FETGRO | Poor fetal growth \|\| id:finngen_R11_O15_POOR_FETGRO | BAFF-R on IgD+ B cell \|\| id:ebi-a-GCST90001719 | Weighted mode | 24 | 0.00274730305766147 | 0.0236439387234929 | 0.908506741993723 | -0.043594817 | 0.0490894229557076 | 1.00275108035304 | 0.957341777664942 | 1.05031426874709 | 0.999857399747617 |
| ebi-a-GCST90001720 | finngen_R11_O15_POOR_FETGRO | Poor fetal growth \|\| id:finngen_R11_O15_POOR_FETGRO | BAFF-R on transitional B cell \|\| id:ebi-a-GCST90001720 | Inverse variance weighted | 24 | -0.028101563 | 0.022297604353552 | 0.207562905420378 | -0.071804867 | 0.0156017416918001 | 0.97228961329093 | 0.930712490465309 | 1.01572408428816 | 0.994119304454654 |
| ebi-a-GCST90001720 | finngen_R11_O15_POOR_FETGRO | Poor fetal growth \|\| id:finngen_R11_O15_POOR_FETGRO | BAFF-R on transitional B cell \|\| id:ebi-a-GCST90001720 | MR Egger | 24 | -0.007598278 | 0.0300060781229145 | 0.802445909021329 | -0.066410191 | 0.0512136351808173 | 0.99243051599946 | 0.935746950523534 | 1.0525477304905 | 0.992899239648112 |
| ebi-a-GCST90001720 | finngen_R11_O15_POOR_FETGRO | Poor fetal growth \|\| id:finngen_R11_O15_POOR_FETGRO | BAFF-R on transitional B cell \|\| id:ebi-a-GCST90001720 | Weighted median | 24 | 0.000668490172726176 | 0.0246542934691458 | 0.978368366579094 | -0.047653925 | 0.048990905372252 | 1.00066871366208 | 0.953463699911319 | 1.05021079942032 | 0.999440741871876 |
| ebi-a-GCST90001720 | finngen_R11_O15_POOR_FETGRO | Poor fetal growth \|\| id:finngen_R11_O15_POOR_FETGRO | BAFF-R on transitional B cell \|\| id:ebi-a-GCST90001720 | Weighted mode | 24 | 0.00580272845898489 | 0.0260082344738209 | 0.825418592770934 | -0.045173411 | 0.0567788680276739 | 1.00581959689965 | 0.955831715622168 | 1.05842173363002 | 0.999857399747617 |
| ebi-a-GCST90001721 | finngen_R11_O15_POOR_FETGRO | Poor fetal growth \|\| id:finngen_R11_O15_POOR_FETGRO | CD19 on CD20- B cell \|\| id:ebi-a-GCST90001721 | Inverse variance weighted | 21 | 0.0220024931560715 | 0.0357341797580401 | 0.538074827732066 | -0.048036499 | 0.09204148548183 | 1.02224633308691 | 0.953098999120545 | 1.09641030624718 | 0.994119304454654 |
| ebi-a-GCST90001721 | finngen_R11_O15_POOR_FETGRO | Poor fetal growth \|\| id:finngen_R11_O15_POOR_FETGRO | CD19 on CD20- B cell \|\| id:ebi-a-GCST90001721 | MR Egger | 21 | -0.017011755 | 0.0501134056156511 | 0.737982878890792 | -0.11523403 | 0.0812105202597324 | 0.983132128097609 | 0.891157562115912 | 1.08459920264024 | 0.992899239648112 |
| ebi-a-GCST90001721 | finngen_R11_O15_POOR_FETGRO | Poor fetal growth \|\| id:finngen_R11_O15_POOR_FETGRO | CD19 on CD20- B cell \|\| id:ebi-a-GCST90001721 | Weighted median | 21 | 0.0254483927251606 | 0.0465547715955575 | 0.584630623190892 | -0.06579896 | 0.116695745052453 | 1.02577496745377 | 0.936319083332214 | 1.12377746281761 | 0.999440741871876 |
| ebi-a-GCST90001721 | finngen_R11_O15_POOR_FETGRO | Poor fetal growth \|\| id:finngen_R11_O15_POOR_FETGRO | CD19 on CD20- B cell \|\| id:ebi-a-GCST90001721 | Weighted mode | 21 | 0.00570997290055564 | 0.0400239996661755 | 0.887983238065688 | -0.072737066 | 0.0841570122462595 | 1.00572630586795 | 0.929845285412978 | 1.08779967827182 | 0.999857399747617 |
| ebi-a-GCST90001722 | finngen_R11_O15_POOR_FETGRO | Poor fetal growth \|\| id:finngen_R11_O15_POOR_FETGRO | CD19 on CD20- CD38- B cell \|\| id:ebi-a-GCST90001722 | Inverse variance weighted | 19 | -0.075671089 | 0.0334721383695806 | 0.0237766826080009 | -0.14127648 | -0.010065697 | 0.92712109704306 | 0.868249225067616 | 0.989984792114716 | 0.994119304454654 |
| ebi-a-GCST90001722 | finngen_R11_O15_POOR_FETGRO | Poor fetal growth \|\| id:finngen_R11_O15_POOR_FETGRO | CD19 on CD20- CD38- B cell \|\| id:ebi-a-GCST90001722 | MR Egger | 19 | -0.048664311 | 0.0585840546252124 | 0.417671408710026 | -0.163489058 | 0.0661604363599033 | 0.952500820365522 | 0.849175790826772 | 1.06839811332077 | 0.992899239648112 |
| ebi-a-GCST90001722 | finngen_R11_O15_POOR_FETGRO | Poor fetal growth \|\| id:finngen_R11_O15_POOR_FETGRO | CD19 on CD20- CD38- B cell \|\| id:ebi-a-GCST90001722 | Weighted median | 19 | -0.044389962 | 0.0487135881247614 | 0.362166856364276 | -0.139868595 | 0.0510886706996045 | 0.956580854522747 | 0.869472481141618 | 1.05241620762742 | 0.999440741871876 |
| ebi-a-GCST90001722 | finngen_R11_O15_POOR_FETGRO | Poor fetal growth \|\| id:finngen_R11_O15_POOR_FETGRO | CD19 on CD20- CD38- B cell \|\| id:ebi-a-GCST90001722 | Weighted mode | 19 | -0.018331404 | 0.0612877666183588 | 0.768286782641604 | -0.138455427 | 0.101792618249853 | 0.981835593875988 | 0.87070206029932 | 1.10715384441656 | 0.999857399747617 |
| ebi-a-GCST90001723 | finngen_R11_O15_POOR_FETGRO | Poor fetal growth \|\| id:finngen_R11_O15_POOR_FETGRO | CD19 on CD24+ CD27+ B cell \|\| id:ebi-a-GCST90001723 | Inverse variance weighted | 29 | 0.0266042187399986 | 0.0252583751630427 | 0.292211279372018 | -0.022902197 | 0.0761106340595623 | 1.0269612702943 | 0.977358068061012 | 1.07908195077042 | 0.994119304454654 |
| ebi-a-GCST90001723 | finngen_R11_O15_POOR_FETGRO | Poor fetal growth \|\| id:finngen_R11_O15_POOR_FETGRO | CD19 on CD24+ CD27+ B cell \|\| id:ebi-a-GCST90001723 | MR Egger | 29 | 0.023820960913589 | 0.0364420022222108 | 0.518854632759217 | -0.047605363 | 0.0952472852691221 | 1.02410694630372 | 0.953510002743949 | 1.09993081818688 | 0.992899239648112 |
| ebi-a-GCST90001723 | finngen_R11_O15_POOR_FETGRO | Poor fetal growth \|\| id:finngen_R11_O15_POOR_FETGRO | CD19 on CD24+ CD27+ B cell \|\| id:ebi-a-GCST90001723 | Weighted median | 29 | 0.0437795818900359 | 0.0341880350162884 | 0.200350809113495 | -0.023228967 | 0.110788130521961 | 1.04475204723575 | 0.97703874878131 | 1.1171581900562 | 0.999440741871876 |
| ebi-a-GCST90001723 | finngen_R11_O15_POOR_FETGRO | Poor fetal growth \|\| id:finngen_R11_O15_POOR_FETGRO | CD19 on CD24+ CD27+ B cell \|\| id:ebi-a-GCST90001723 | Weighted mode | 29 | 0.0410190068941008 | 0.033327038299438 | 0.228636084258881 | -0.024301988 | 0.106340001960999 | 1.04187190810631 | 0.975990927532904 | 1.11219996239615 | 0.999857399747617 |
| ebi-a-GCST90001724 | finngen_R11_O15_POOR_FETGRO | Poor fetal growth \|\| id:finngen_R11_O15_POOR_FETGRO | CD19 on IgD+ CD24+ B cell \|\| id:ebi-a-GCST90001724 | Inverse variance weighted | 34 | 0.00666304915810853 | 0.0166458542889817 | 0.688948235196038 | -0.025962825 | 0.0392889235645127 | 1.00668529665476 | 0.974371310947038 | 1.04007094124712 | 0.994119304454654 |
| ebi-a-GCST90001724 | finngen_R11_O15_POOR_FETGRO | Poor fetal growth \|\| id:finngen_R11_O15_POOR_FETGRO | CD19 on IgD+ CD24+ B cell \|\| id:ebi-a-GCST90001724 | MR Egger | 34 | 0.00938381837968121 | 0.0209945878000809 | 0.657911899577618 | -0.031765574 | 0.0505332104678399 | 1.00942798444405 | 0.968733652100101 | 1.05183179460095 | 0.992899239648112 |
| ebi-a-GCST90001724 | finngen_R11_O15_POOR_FETGRO | Poor fetal growth \|\| id:finngen_R11_O15_POOR_FETGRO | CD19 on IgD+ CD24+ B cell \|\| id:ebi-a-GCST90001724 | Weighted median | 34 | 0.0106738702675712 | 0.0224668965764024 | 0.634720529881768 | -0.033361247 | 0.05470898755732 | 1.01073103924451 | 0.967189102289543 | 1.05623319294437 | 0.999440741871876 |
| ebi-a-GCST90001724 | finngen_R11_O15_POOR_FETGRO | Poor fetal growth \|\| id:finngen_R11_O15_POOR_FETGRO | CD19 on IgD+ CD24+ B cell \|\| id:ebi-a-GCST90001724 | Weighted mode | 34 | 0.0107666407495881 | 0.0185384041003704 | 0.565337677668569 | -0.025568631 | 0.0471019127863141 | 1.0108248095997 | 0.97475547794692 | 1.04822883155719 | 0.999857399747617 |
| ebi-a-GCST90001725 | finngen_R11_O15_POOR_FETGRO | Poor fetal growth \|\| id:finngen_R11_O15_POOR_FETGRO | CD19 on IgD+ CD24- B cell \|\| id:ebi-a-GCST90001725 | Inverse variance weighted | 24 | -0.009221056 | 0.0134653267373907 | 0.493470136060239 | -0.035613096 | 0.0171709846702983 | 0.990821327825678 | 0.965013588745952 | 1.01731925345218 | 0.994119304454654 |
| ebi-a-GCST90001725 | finngen_R11_O15_POOR_FETGRO | Poor fetal growth \|\| id:finngen_R11_O15_POOR_FETGRO | CD19 on IgD+ CD24- B cell \|\| id:ebi-a-GCST90001725 | MR Egger | 24 | -0.005568867 | 0.0157247276424934 | 0.726600432921861 | -0.036389333 | 0.0252515995232957 | 0.994446610738082 | 0.964264800443265 | 1.02557312177512 | 0.992899239648112 |
| ebi-a-GCST90001725 | finngen_R11_O15_POOR_FETGRO | Poor fetal growth \|\| id:finngen_R11_O15_POOR_FETGRO | CD19 on IgD+ CD24- B cell \|\| id:ebi-a-GCST90001725 | Weighted median | 24 | -0.016372948 | 0.0170982374441598 | 0.338274075189923 | -0.049885493 | 0.0171395977896158 | 0.983760360564838 | 0.951338353172949 | 1.01728732347525 | 0.999440741871876 |
| ebi-a-GCST90001725 | finngen_R11_O15_POOR_FETGRO | Poor fetal growth \|\| id:finngen_R11_O15_POOR_FETGRO | CD19 on IgD+ CD24- B cell \|\| id:ebi-a-GCST90001725 | Weighted mode | 24 | -0.012088106 | 0.0134290310055506 | 0.377370564360866 | -0.038409007 | 0.0142327943258282 | 0.987984661210817 | 0.962319264865162 | 1.01433456278632 | 0.999857399747617 |
| ebi-a-GCST90001726 | finngen_R11_O15_POOR_FETGRO | Poor fetal growth \|\| id:finngen_R11_O15_POOR_FETGRO | CD19 on IgD+ CD38- B cell \|\| id:ebi-a-GCST90001726 | Inverse variance weighted | 31 | -0.008085842 | 0.01592037961071 | 0.611528712084354 | -0.039289786 | 0.0231181021214386 | 0.991946760572156 | 0.961472047679046 | 1.02338739663293 | 0.994119304454654 |
| ebi-a-GCST90001726 | finngen_R11_O15_POOR_FETGRO | Poor fetal growth \|\| id:finngen_R11_O15_POOR_FETGRO | CD19 on IgD+ CD38- B cell \|\| id:ebi-a-GCST90001726 | MR Egger | 31 | -0.021552061 | 0.0191496531753001 | 0.269625023464152 | -0.059085381 | 0.0159812594584731 | 0.978678525390024 | 0.942626283357119 | 1.0161096427827 | 0.992899239648112 |
| ebi-a-GCST90001726 | finngen_R11_O15_POOR_FETGRO | Poor fetal growth \|\| id:finngen_R11_O15_POOR_FETGRO | CD19 on IgD+ CD38- B cell \|\| id:ebi-a-GCST90001726 | Weighted median | 31 | -0.014116614 | 0.0184394173591017 | 0.443933822925496 | -0.050257873 | 0.0220246435354963 | 0.985982557706961 | 0.950984160204175 | 1.02226897648184 | 0.999440741871876 |
| ebi-a-GCST90001726 | finngen_R11_O15_POOR_FETGRO | Poor fetal growth \|\| id:finngen_R11_O15_POOR_FETGRO | CD19 on IgD+ CD38- B cell \|\| id:ebi-a-GCST90001726 | Weighted mode | 31 | -0.012041923 | 0.0165655862126105 | 0.472907137446187 | -0.044510472 | 0.0204266255594431 | 0.988030290387508 | 0.956465583556744 | 1.02063667685049 | 0.999857399747617 |
| ebi-a-GCST90001727 | finngen_R11_O15_POOR_FETGRO | Poor fetal growth \|\| id:finngen_R11_O15_POOR_FETGRO | CD19 on IgD+ CD38- naive B cell \|\| id:ebi-a-GCST90001727 | Inverse variance weighted | 19 | -0.007507122 | 0.0150571703939138 | 0.618079138449785 | -0.037019176 | 0.0220049320299928 | 0.992520986116993 | 0.963657656155392 | 1.02224882621988 | 0.994119304454654 |
| ebi-a-GCST90001727 | finngen_R11_O15_POOR_FETGRO | Poor fetal growth \|\| id:finngen_R11_O15_POOR_FETGRO | CD19 on IgD+ CD38- naive B cell \|\| id:ebi-a-GCST90001727 | MR Egger | 19 | -0.011469231 | 0.0180466099355774 | 0.533542745096639 | -0.046840586 | 0.023902124728709 | 0.988596290151264 | 0.954239504393698 | 1.02419007010385 | 0.992899239648112 |
| ebi-a-GCST90001727 | finngen_R11_O15_POOR_FETGRO | Poor fetal growth \|\| id:finngen_R11_O15_POOR_FETGRO | CD19 on IgD+ CD38- naive B cell \|\| id:ebi-a-GCST90001727 | Weighted median | 19 | -0.012612119 | 0.0164635706620947 | 0.443639292650064 | -0.044880718 | 0.0196564793223573 | 0.987467080292343 | 0.956111522238655 | 1.01985093995938 | 0.999440741871876 |
| ebi-a-GCST90001727 | finngen_R11_O15_POOR_FETGRO | Poor fetal growth \|\| id:finngen_R11_O15_POOR_FETGRO | CD19 on IgD+ CD38- naive B cell \|\| id:ebi-a-GCST90001727 | Weighted mode | 19 | -0.010845817 | 0.0144686282458797 | 0.463175516966312 | -0.039204328 | 0.0175126947218443 | 0.98921278716914 | 0.961554216620687 | 1.01766694106751 | 0.999857399747617 |
| ebi-a-GCST90001728 | finngen_R11_O15_POOR_FETGRO | Poor fetal growth \|\| id:finngen_R11_O15_POOR_FETGRO | CD19 on IgD+ CD38- unswitched memory B cell \|\| id:ebi-a-GCST90001728 | Inverse variance weighted | 20 | 0.0198491067587627 | 0.0136240213243947 | 0.145138512978843 | -0.006853975 | 0.0465521885545763 | 1.02004741015363 | 0.993169459878511 | 1.04765275312472 | 0.994119304454654 |
| ebi-a-GCST90001728 | finngen_R11_O15_POOR_FETGRO | Poor fetal growth \|\| id:finngen_R11_O15_POOR_FETGRO | CD19 on IgD+ CD38- unswitched memory B cell \|\| id:ebi-a-GCST90001728 | MR Egger | 20 | 0.0290098287785467 | 0.0200360609049982 | 0.164844807448927 | -0.010260851 | 0.0682805081523431 | 1.02943471251122 | 0.989791612340776 | 1.07066559678848 | 0.992899239648112 |
| ebi-a-GCST90001728 | finngen_R11_O15_POOR_FETGRO | Poor fetal growth \|\| id:finngen_R11_O15_POOR_FETGRO | CD19 on IgD+ CD38- unswitched memory B cell \|\| id:ebi-a-GCST90001728 | Weighted median | 20 | 0.0133352310137146 | 0.0214195791003753 | 0.533565790929985 | -0.028647144 | 0.0553176060504502 | 1.01342454175839 | 0.971759295053581 | 1.05687623166143 | 0.999440741871876 |
| ebi-a-GCST90001728 | finngen_R11_O15_POOR_FETGRO | Poor fetal growth \|\| id:finngen_R11_O15_POOR_FETGRO | CD19 on IgD+ CD38- unswitched memory B cell \|\| id:ebi-a-GCST90001728 | Weighted mode | 20 | 0.0160358192540327 | 0.0190640135041143 | 0.410722175022583 | -0.021329647 | 0.0534012857220968 | 1.01616508302947 | 0.97889622096455 | 1.05485285758978 | 0.999857399747617 |
| ebi-a-GCST90001729 | finngen_R11_O15_POOR_FETGRO | Poor fetal growth \|\| id:finngen_R11_O15_POOR_FETGRO | CD19 on IgD+ CD38+ B cell \|\| id:ebi-a-GCST90001729 | Inverse variance weighted | 20 | 0.0258655760620688 | 0.0262167936276241 | 0.323837133997662 | -0.025519339 | 0.077250491572212 | 1.02620299295396 | 0.974803526621259 | 1.08031265171738 | 0.994119304454654 |
| ebi-a-GCST90001729 | finngen_R11_O15_POOR_FETGRO | Poor fetal growth \|\| id:finngen_R11_O15_POOR_FETGRO | CD19 on IgD+ CD38+ B cell \|\| id:ebi-a-GCST90001729 | MR Egger | 20 | 0.0474685316901434 | 0.0367279152550083 | 0.212551564725877 | -0.024518182 | 0.11945524558996 | 1.04861320251691 | 0.975779946921524 | 1.1268828099633 | 0.992899239648112 |
| ebi-a-GCST90001729 | finngen_R11_O15_POOR_FETGRO | Poor fetal growth \|\| id:finngen_R11_O15_POOR_FETGRO | CD19 on IgD+ CD38+ B cell \|\| id:ebi-a-GCST90001729 | Weighted median | 20 | 0.0458811191141447 | 0.0335624483006678 | 0.171613610553663 | -0.01990128 | 0.111663517783454 | 1.04694994122209 | 0.980295443732343 | 1.11813656427053 | 0.999440741871876 |
| ebi-a-GCST90001729 | finngen_R11_O15_POOR_FETGRO | Poor fetal growth \|\| id:finngen_R11_O15_POOR_FETGRO | CD19 on IgD+ CD38+ B cell \|\| id:ebi-a-GCST90001729 | Weighted mode | 20 | 0.0512614369916182 | 0.0327924987553785 | 0.134506995964924 | -0.013011861 | 0.11553473455216 | 1.05259804538054 | 0.987072427710299 | 1.12247350248558 | 0.999857399747617 |
| ebi-a-GCST90001730 | finngen_R11_O15_POOR_FETGRO | Poor fetal growth \|\| id:finngen_R11_O15_POOR_FETGRO | CD19 on IgD+ CD38dim B cell \|\| id:ebi-a-GCST90001730 | Inverse variance weighted | 23 | -0.004004198 | 0.0134795494590943 | 0.766422783403459 | -0.030424115 | 0.0224157187271067 | 0.996003807899361 | 0.970034040159471 | 1.02266883870069 | 0.994119304454654 |
| ebi-a-GCST90001730 | finngen_R11_O15_POOR_FETGRO | Poor fetal growth \|\| id:finngen_R11_O15_POOR_FETGRO | CD19 on IgD+ CD38dim B cell \|\| id:ebi-a-GCST90001730 | MR Egger | 23 | 0.00300492278641204 | 0.0160982372548846 | 0.853717465941951 | -0.028547622 | 0.0345574678059859 | 1.00300944209248 | 0.971856011090534 | 1.03516151512793 | 0.992899239648112 |
| ebi-a-GCST90001730 | finngen_R11_O15_POOR_FETGRO | Poor fetal growth \|\| id:finngen_R11_O15_POOR_FETGRO | CD19 on IgD+ CD38dim B cell \|\| id:ebi-a-GCST90001730 | Weighted median | 23 | -0.014984317 | 0.0176126585598098 | 0.394897261995095 | -0.049505128 | 0.0195364934503511 | 0.985127388912745 | 0.951700277705849 | 1.01972857959593 | 0.999440741871876 |
| ebi-a-GCST90001730 | finngen_R11_O15_POOR_FETGRO | Poor fetal growth \|\| id:finngen_R11_O15_POOR_FETGRO | CD19 on IgD+ CD38dim B cell \|\| id:ebi-a-GCST90001730 | Weighted mode | 23 | -0.010347603 | 0.0142427801132197 | 0.475182412480257 | -0.038263452 | 0.017568246155682 | 0.989705749395066 | 0.962459345755711 | 1.01772347549552 | 0.999857399747617 |
| ebi-a-GCST90001731 | finngen_R11_O15_POOR_FETGRO | Poor fetal growth \|\| id:finngen_R11_O15_POOR_FETGRO | CD19 on IgD- CD24- B cell \|\| id:ebi-a-GCST90001731 | Inverse variance weighted | 23 | -0.00633074 | 0.0334663014826317 | 0.849961461042464 | -0.071924691 | 0.0592632109916732 | 0.993689256998974 | 0.930600975968514 | 1.06105448519171 | 0.994119304454654 |
| ebi-a-GCST90001731 | finngen_R11_O15_POOR_FETGRO | Poor fetal growth \|\| id:finngen_R11_O15_POOR_FETGRO | CD19 on IgD- CD24- B cell \|\| id:ebi-a-GCST90001731 | MR Egger | 23 | 0.0232246960522372 | 0.0604488843176082 | 0.704690166934829 | -0.095255117 | 0.141704509314749 | 1.02349648933248 | 0.909140967372748 | 1.15223612318684 | 0.992899239648112 |
| ebi-a-GCST90001731 | finngen_R11_O15_POOR_FETGRO | Poor fetal growth \|\| id:finngen_R11_O15_POOR_FETGRO | CD19 on IgD- CD24- B cell \|\| id:ebi-a-GCST90001731 | Weighted median | 23 | -0.00665522 | 0.0477998195032009 | 0.889267556864038 | -0.100342866 | 0.0870324261846497 | 0.993366876888126 | 0.904527232986417 | 1.09093205390897 | 0.999440741871876 |
| ebi-a-GCST90001731 | finngen_R11_O15_POOR_FETGRO | Poor fetal growth \|\| id:finngen_R11_O15_POOR_FETGRO | CD19 on IgD- CD24- B cell \|\| id:ebi-a-GCST90001731 | Weighted mode | 23 | 0.0011372160630429 | 0.0632591063261756 | 0.985819156296531 | -0.122850632 | 0.125125064462347 | 1.00113786293842 | 0.884395752822988 | 1.13329017853109 | 0.999857399747617 |
| ebi-a-GCST90001732 | finngen_R11_O15_POOR_FETGRO | Poor fetal growth \|\| id:finngen_R11_O15_POOR_FETGRO | CD19 on IgD- CD27- B cell \|\| id:ebi-a-GCST90001732 | Inverse variance weighted | 25 | -0.002262489 | 0.0356782991085943 | 0.94943716314113 | -0.072191955 | 0.0676669771031705 | 0.997740068349767 | 0.930352292521025 | 1.07000891167052 | 0.998984604424952 |
| ebi-a-GCST90001732 | finngen_R11_O15_POOR_FETGRO | Poor fetal growth \|\| id:finngen_R11_O15_POOR_FETGRO | CD19 on IgD- CD27- B cell \|\| id:ebi-a-GCST90001732 | MR Egger | 25 | 0.0947278981945151 | 0.0538852926063777 | 0.0920580367066266 | -0.010887275 | 0.200343071703015 | 1.09935967667155 | 0.989171776568902 | 1.22182185877115 | 0.992899239648112 |
| ebi-a-GCST90001732 | finngen_R11_O15_POOR_FETGRO | Poor fetal growth \|\| id:finngen_R11_O15_POOR_FETGRO | CD19 on IgD- CD27- B cell \|\| id:ebi-a-GCST90001732 | Weighted median | 25 | 0.0576038808972035 | 0.046439962378661 | 0.214829458736453 | -0.033418445 | 0.148626207159379 | 1.05929530548611 | 0.967133782257845 | 1.16023921903056 | 0.999440741871876 |
| ebi-a-GCST90001732 | finngen_R11_O15_POOR_FETGRO | Poor fetal growth \|\| id:finngen_R11_O15_POOR_FETGRO | CD19 on IgD- CD27- B cell \|\| id:ebi-a-GCST90001732 | Weighted mode | 25 | 0.0601274873875439 | 0.0498126218604548 | 0.239166596197161 | -0.037505251 | 0.157760226234035 | 1.06197192594208 | 0.96318935955816 | 1.17088541344193 | 0.999857399747617 |
| ebi-a-GCST90001733 | finngen_R11_O15_POOR_FETGRO | Poor fetal growth \|\| id:finngen_R11_O15_POOR_FETGRO | CD19 on IgD- CD38- B cell \|\| id:ebi-a-GCST90001733 | Inverse variance weighted | 22 | 0.0184773605688563 | 0.0413623894838286 | 0.655078043626343 | -0.062592923 | 0.0995476439571604 | 1.01864912327184 | 0.939325773958679 | 1.10467110038878 | 0.994119304454654 |
| ebi-a-GCST90001733 | finngen_R11_O15_POOR_FETGRO | Poor fetal growth \|\| id:finngen_R11_O15_POOR_FETGRO | CD19 on IgD- CD38- B cell \|\| id:ebi-a-GCST90001733 | MR Egger | 22 | 0.0806743467148918 | 0.0773957239600802 | 0.309680832147363 | -0.071021272 | 0.232369965676649 | 1.08401782511458 | 0.931442078051023 | 1.26158638616042 | 0.992899239648112 |
| ebi-a-GCST90001733 | finngen_R11_O15_POOR_FETGRO | Poor fetal growth \|\| id:finngen_R11_O15_POOR_FETGRO | CD19 on IgD- CD38- B cell \|\| id:ebi-a-GCST90001733 | Weighted median | 22 | 0.0634811005322666 | 0.0487779314348601 | 0.193111049369203 | -0.032123645 | 0.159085846144592 | 1.06553934748465 | 0.968386838408329 | 1.17243859169353 | 0.999440741871876 |
| ebi-a-GCST90001733 | finngen_R11_O15_POOR_FETGRO | Poor fetal growth \|\| id:finngen_R11_O15_POOR_FETGRO | CD19 on IgD- CD38- B cell \|\| id:ebi-a-GCST90001733 | Weighted mode | 22 | 0.0752333739546002 | 0.05682189374428 | 0.19972795940176 | -0.036137538 | 0.186604285693389 | 1.07813573032643 | 0.964507628118078 | 1.20515029546682 | 0.999857399747617 |
| ebi-a-GCST90001734 | finngen_R11_O15_POOR_FETGRO | Poor fetal growth \|\| id:finngen_R11_O15_POOR_FETGRO | CD19 on IgD- CD38+ B cell \|\| id:ebi-a-GCST90001734 | Inverse variance weighted | 18 | 0.0236868394603763 | 0.035205251943699 | 0.501061055989383 | -0.045315454 | 0.0926891332700263 | 1.02396960080253 | 0.955695955830906 | 1.097120624 | 0.994119304454654 |
| ebi-a-GCST90001734 | finngen_R11_O15_POOR_FETGRO | Poor fetal growth \|\| id:finngen_R11_O15_POOR_FETGRO | CD19 on IgD- CD38+ B cell \|\| id:ebi-a-GCST90001734 | MR Egger | 18 | 0.012040016288131 | 0.0570609197761153 | 0.835549012059342 | -0.099799386 | 0.123879419049317 | 1.01211278905273 | 0.905018958870868 | 1.13187937967855 | 0.992899239648112 |
| ebi-a-GCST90001734 | finngen_R11_O15_POOR_FETGRO | Poor fetal growth \|\| id:finngen_R11_O15_POOR_FETGRO | CD19 on IgD- CD38+ B cell \|\| id:ebi-a-GCST90001734 | Weighted median | 18 | 0.000931656480342765 | 0.0490118137506115 | 0.98483407365444 | -0.095131498 | 0.0969948114315413 | 1.00093209060705 | 0.909253361179936 | 1.10185465655786 | 0.999440741871876 |
| ebi-a-GCST90001734 | finngen_R11_O15_POOR_FETGRO | Poor fetal growth \|\| id:finngen_R11_O15_POOR_FETGRO | CD19 on IgD- CD38+ B cell \|\| id:ebi-a-GCST90001734 | Weighted mode | 18 | -0.017480554 | 0.0479097480698937 | 0.719712171524128 | -0.11138366 | 0.0764225524991082 | 0.98267134478347 | 0.894595462643707 | 1.07941858882772 | 0.999857399747617 |
| ebi-a-GCST90001735 | finngen_R11_O15_POOR_FETGRO | Poor fetal growth \|\| id:finngen_R11_O15_POOR_FETGRO | CD19 on IgD- CD38dim B cell \|\| id:ebi-a-GCST90001735 | Inverse variance weighted | 27 | -0.001379441 | 0.0246189699105842 | 0.955316601726754 | -0.049632622 | 0.0468737397672711 | 0.998621509734288 | 0.951578949195634 | 1.0479896813049 | 0.998984604424952 |
| ebi-a-GCST90001735 | finngen_R11_O15_POOR_FETGRO | Poor fetal growth \|\| id:finngen_R11_O15_POOR_FETGRO | CD19 on IgD- CD38dim B cell \|\| id:ebi-a-GCST90001735 | MR Egger | 27 | 0.00627921681251969 | 0.0380575688777306 | 0.870276700112256 | -0.068313618 | 0.0808720518128716 | 1.00629897242268 | 0.933967518440958 | 1.08423216215197 | 0.992899239648112 |
| ebi-a-GCST90001735 | finngen_R11_O15_POOR_FETGRO | Poor fetal growth \|\| id:finngen_R11_O15_POOR_FETGRO | CD19 on IgD- CD38dim B cell \|\| id:ebi-a-GCST90001735 | Weighted median | 27 | 0.0378087299469748 | 0.0359961619893963 | 0.293555166623233 | -0.032743748 | 0.108361207446191 | 1.03853257369989 | 0.967786525482804 | 1.11445022041163 | 0.999440741871876 |
| ebi-a-GCST90001735 | finngen_R11_O15_POOR_FETGRO | Poor fetal growth \|\| id:finngen_R11_O15_POOR_FETGRO | CD19 on IgD- CD38dim B cell \|\| id:ebi-a-GCST90001735 | Weighted mode | 27 | 0.0208505167562787 | 0.0358817442218733 | 0.566183674106754 | -0.049477702 | 0.0911787354311504 | 1.02106940746185 | 0.951726379572152 | 1.09546478613231 | 0.999857399747617 |
| ebi-a-GCST90001736 | finngen_R11_O15_POOR_FETGRO | Poor fetal growth \|\| id:finngen_R11_O15_POOR_FETGRO | CD19 on memory B cell \|\| id:ebi-a-GCST90001736 | Inverse variance weighted | 25 | 0.0191691974918142 | 0.0277438725471152 | 0.489606731606637 | -0.035208793 | 0.07354718768416 | 1.01935410618534 | 0.965403825941084 | 1.07631932448994 | 0.994119304454654 |
| ebi-a-GCST90001736 | finngen_R11_O15_POOR_FETGRO | Poor fetal growth \|\| id:finngen_R11_O15_POOR_FETGRO | CD19 on memory B cell \|\| id:ebi-a-GCST90001736 | MR Egger | 25 | 0.0198909624876252 | 0.043164713009509 | 0.649258795283237 | -0.064711875 | 0.104493799986263 | 1.02009010587498 | 0.937337494829506 | 1.11014851090886 | 0.992899239648112 |
| ebi-a-GCST90001736 | finngen_R11_O15_POOR_FETGRO | Poor fetal growth \|\| id:finngen_R11_O15_POOR_FETGRO | CD19 on memory B cell \|\| id:ebi-a-GCST90001736 | Weighted median | 25 | 0.0506139495043875 | 0.0390070222548344 | 0.194438595018131 | -0.025839814 | 0.127067713123863 | 1.05191672191508 | 0.974491176838431 | 1.13549390301769 | 0.999440741871876 |
| ebi-a-GCST90001736 | finngen_R11_O15_POOR_FETGRO | Poor fetal growth \|\| id:finngen_R11_O15_POOR_FETGRO | CD19 on memory B cell \|\| id:ebi-a-GCST90001736 | Weighted mode | 25 | 0.0146443766032967 | 0.0397790296776089 | 0.715993664420807 | -0.063322522 | 0.0926112747714101 | 1.01475213084169 | 0.938640693 | 1.09703520711067 | 0.999857399747617 |
| ebi-a-GCST90001737 | finngen_R11_O15_POOR_FETGRO | Poor fetal growth \|\| id:finngen_R11_O15_POOR_FETGRO | CD19 on naive-mature B cell \|\| id:ebi-a-GCST90001737 | Inverse variance weighted | 27 | -7.73E-05 | 0.0141480398597367 | 0.995641095415038 | -0.02780745 | 0.0276528659131469 | 0.999922710775029 | 0.972575617880547 | 1.02803875517933 | 0.998984604424952 |
| ebi-a-GCST90001737 | finngen_R11_O15_POOR_FETGRO | Poor fetal growth \|\| id:finngen_R11_O15_POOR_FETGRO | CD19 on naive-mature B cell \|\| id:ebi-a-GCST90001737 | MR Egger | 27 | -0.013255137 | 0.0169406929312664 | 0.4413026679193 | -0.046458895 | 0.0199486214340849 | 0.986832325744665 | 0.954603798889675 | 1.02014892489148 | 0.992899239648112 |
| ebi-a-GCST90001737 | finngen_R11_O15_POOR_FETGRO | Poor fetal growth \|\| id:finngen_R11_O15_POOR_FETGRO | CD19 on naive-mature B cell \|\| id:ebi-a-GCST90001737 | Weighted median | 27 | -0.012785361 | 0.0192040715518313 | 0.505562574178252 | -0.050425341 | 0.0248546192377448 | 0.987296024507209 | 0.950824913426324 | 1.02516607026544 | 0.999440741871876 |
| ebi-a-GCST90001737 | finngen_R11_O15_POOR_FETGRO | Poor fetal growth \|\| id:finngen_R11_O15_POOR_FETGRO | CD19 on naive-mature B cell \|\| id:ebi-a-GCST90001737 | Weighted mode | 27 | -0.007771737 | 0.0149408075941919 | 0.60734743550843 | -0.037055719 | 0.0215122462765378 | 0.992258385253305 | 0.963622441299586 | 1.0217453028363 | 0.999857399747617 |
| ebi-a-GCST90001738 | finngen_R11_O15_POOR_FETGRO | Poor fetal growth \|\| id:finngen_R11_O15_POOR_FETGRO | CD19 on unswitched memory B cell \|\| id:ebi-a-GCST90001738 | Inverse variance weighted | 22 | 0.0217810160245232 | 0.0220442148538755 | 0.323123062089781 | -0.021425645 | 0.0649876771381193 | 1.02201995397115 | 0.978802253517861 | 1.06714587401196 | 0.994119304454654 |
| ebi-a-GCST90001738 | finngen_R11_O15_POOR_FETGRO | Poor fetal growth \|\| id:finngen_R11_O15_POOR_FETGRO | CD19 on unswitched memory B cell \|\| id:ebi-a-GCST90001738 | MR Egger | 22 | 0.026875464945043 | 0.027562658464759 | 0.341173608602586 | -0.027147346 | 0.0808982755359707 | 1.02723986742409 | 0.973217831549202 | 1.08426059512877 | 0.992899239648112 |
| ebi-a-GCST90001738 | finngen_R11_O15_POOR_FETGRO | Poor fetal growth \|\| id:finngen_R11_O15_POOR_FETGRO | CD19 on unswitched memory B cell \|\| id:ebi-a-GCST90001738 | Weighted median | 22 | 0.0256829519645533 | 0.027233769913732 | 0.345652600880485 | -0.027695237 | 0.0790611409954681 | 1.0260156006702 | 0.972684759895069 | 1.08227049114267 | 0.999440741871876 |
| ebi-a-GCST90001738 | finngen_R11_O15_POOR_FETGRO | Poor fetal growth \|\| id:finngen_R11_O15_POOR_FETGRO | CD19 on unswitched memory B cell \|\| id:ebi-a-GCST90001738 | Weighted mode | 22 | 0.032309346596969 | 0.0235157803455867 | 0.183948274316635 | -0.013781583 | 0.0784002760743189 | 1.03283696049034 | 0.986312948371259 | 1.08155549282455 | 0.999857399747617 |
| ebi-a-GCST90001739 | finngen_R11_O15_POOR_FETGRO | Poor fetal growth \|\| id:finngen_R11_O15_POOR_FETGRO | CD19 on Plasma Blast-Plasma Cell \|\| id:ebi-a-GCST90001739 | Inverse variance weighted | 23 | 0.0143883732692626 | 0.036719730518334 | 0.695174142557134 | -0.057582299 | 0.0863590450851973 | 1.01449238416241 | 0.944044193724167 | 1.09019768816484 | 0.994119304454654 |
| ebi-a-GCST90001739 | finngen_R11_O15_POOR_FETGRO | Poor fetal growth \|\| id:finngen_R11_O15_POOR_FETGRO | CD19 on Plasma Blast-Plasma Cell \|\| id:ebi-a-GCST90001739 | MR Egger | 23 | -0.028468436 | 0.0693928795044686 | 0.685777835819792 | -0.164478479 | 0.107541608221968 | 0.971932972136474 | 0.848336013417747 | 1.11353719208531 | 0.992899239648112 |
| ebi-a-GCST90001739 | finngen_R11_O15_POOR_FETGRO | Poor fetal growth \|\| id:finngen_R11_O15_POOR_FETGRO | CD19 on Plasma Blast-Plasma Cell \|\| id:ebi-a-GCST90001739 | Weighted median | 23 | 0.00865072636356375 | 0.0529771970283647 | 0.870288920228075 | -0.09518458 | 0.112486032539159 | 1.0086882520269 | 0.909205098073003 | 1.11905662642401 | 0.999440741871876 |
| ebi-a-GCST90001739 | finngen_R11_O15_POOR_FETGRO | Poor fetal growth \|\| id:finngen_R11_O15_POOR_FETGRO | CD19 on Plasma Blast-Plasma Cell \|\| id:ebi-a-GCST90001739 | Weighted mode | 23 | -0.002503255 | 0.0658067334528474 | 0.969999266688074 | -0.131484453 | 0.126477942540473 | 0.997499875503041 | 0.876792906886525 | 1.1348244195563 | 0.999857399747617 |
| ebi-a-GCST90001740 | finngen_R11_O15_POOR_FETGRO | Poor fetal growth \|\| id:finngen_R11_O15_POOR_FETGRO | CD19 on switched memory B cell \|\| id:ebi-a-GCST90001740 | Inverse variance weighted | 27 | 0.0109531378626677 | 0.0303277261805488 | 0.717980427016292 | -0.048489205 | 0.0703954811765434 | 1.01101334308845 | 0.952667622867469 | 1.07293242193566 | 0.994119304454654 |
| ebi-a-GCST90001740 | finngen_R11_O15_POOR_FETGRO | Poor fetal growth \|\| id:finngen_R11_O15_POOR_FETGRO | CD19 on switched memory B cell \|\| id:ebi-a-GCST90001740 | MR Egger | 27 | -0.066631507 | 0.056017963826804 | 0.245430750691172 | -0.176426716 | 0.0431637023842575 | 0.935539877989053 | 0.83826020811892 | 1.04410880396175 | 0.992899239648112 |
| ebi-a-GCST90001740 | finngen_R11_O15_POOR_FETGRO | Poor fetal growth \|\| id:finngen_R11_O15_POOR_FETGRO | CD19 on switched memory B cell \|\| id:ebi-a-GCST90001740 | Weighted median | 27 | -0.009170599 | 0.0477422104857007 | 0.847675022476377 | -0.102745332 | 0.0844041333639838 | 0.990871322509841 | 0.902356745844808 | 1.08806852976224 | 0.999440741871876 |
| ebi-a-GCST90001740 | finngen_R11_O15_POOR_FETGRO | Poor fetal growth \|\| id:finngen_R11_O15_POOR_FETGRO | CD19 on switched memory B cell \|\| id:ebi-a-GCST90001740 | Weighted mode | 27 | -0.027073475 | 0.050335437055452 | 0.595247457773956 | -0.125730931 | 0.0715839820177298 | 0.973289726813086 | 0.881852093713791 | 1.07420836109886 | 0.999857399747617 |
| ebi-a-GCST90001741 | finngen_R11_O15_POOR_FETGRO | Poor fetal growth \|\| id:finngen_R11_O15_POOR_FETGRO | CD19 on IgD+ B cell \|\| id:ebi-a-GCST90001741 | Inverse variance weighted | 26 | -0.002159114 | 0.0132845397705594 | 0.870889799595227 | -0.028196812 | 0.0238785835298261 | 0.997843214790424 | 0.97219700757302 | 1.02416595972551 | 0.994119304454654 |
| ebi-a-GCST90001741 | finngen_R11_O15_POOR_FETGRO | Poor fetal growth \|\| id:finngen_R11_O15_POOR_FETGRO | CD19 on IgD+ B cell \|\| id:ebi-a-GCST90001741 | MR Egger | 26 | -0.007688905 | 0.0157096129695865 | 0.628974201374212 | -0.038479747 | 0.0231019363099088 | 0.992340578905424 | 0.962251193467301 | 1.02337085287888 | 0.992899239648112 |
| ebi-a-GCST90001741 | finngen_R11_O15_POOR_FETGRO | Poor fetal growth \|\| id:finngen_R11_O15_POOR_FETGRO | CD19 on IgD+ B cell \|\| id:ebi-a-GCST90001741 | Weighted median | 26 | -0.016879163 | 0.0176063937623394 | 0.337712390696732 | -0.051387694 | 0.0176293691500323 | 0.983262492315901 | 0.949910324222515 | 1.01778568370292 | 0.999440741871876 |
| ebi-a-GCST90001741 | finngen_R11_O15_POOR_FETGRO | Poor fetal growth \|\| id:finngen_R11_O15_POOR_FETGRO | CD19 on IgD+ B cell \|\| id:ebi-a-GCST90001741 | Weighted mode | 26 | -0.011391308 | 0.0142311772475281 | 0.430993014288038 | -0.039284416 | 0.0165017991530588 | 0.988673327040105 | 0.961477211081723 | 1.01663870587305 | 0.999857399747617 |
| ebi-a-GCST90001742 | finngen_R11_O15_POOR_FETGRO | Poor fetal growth \|\| id:finngen_R11_O15_POOR_FETGRO | CD19 on transitional B cell \|\| id:ebi-a-GCST90001742 | Inverse variance weighted | 25 | 0.0457840688016201 | 0.0229652394264534 | 0.0461930828440931 | 0.000772199525771435 | 0.0907959380774688 | 1.04684833933342 | 1.00077249774858 | 1.09504552536221 | 0.994119304454654 |
| ebi-a-GCST90001742 | finngen_R11_O15_POOR_FETGRO | Poor fetal growth \|\| id:finngen_R11_O15_POOR_FETGRO | CD19 on transitional B cell \|\| id:ebi-a-GCST90001742 | MR Egger | 25 | 0.0620810012542285 | 0.0314140039964314 | 0.060243383529647 | 0.000509553421223018 | 0.123652449087234 | 1.06404853050294 | 1.00050968326562 | 1.13162250621105 | 0.992899239648112 |
| ebi-a-GCST90001742 | finngen_R11_O15_POOR_FETGRO | Poor fetal growth \|\| id:finngen_R11_O15_POOR_FETGRO | CD19 on transitional B cell \|\| id:ebi-a-GCST90001742 | Weighted median | 25 | 0.0513833540556905 | 0.0322192072266156 | 0.110755839758628 | -0.011766292 | 0.114533000219857 | 1.05272638286698 | 0.988302660004652 | 1.12134964523822 | 0.999440741871876 |
| ebi-a-GCST90001742 | finngen_R11_O15_POOR_FETGRO | Poor fetal growth \|\| id:finngen_R11_O15_POOR_FETGRO | CD19 on transitional B cell \|\| id:ebi-a-GCST90001742 | Weighted mode | 25 | 0.0502303356171546 | 0.0319620425381278 | 0.129143814557366 | -0.012415268 | 0.112875938991885 | 1.05151326944228 | 0.987661483720769 | 1.11949303889811 | 0.999857399747617 |
| ebi-a-GCST90001743 | finngen_R11_O15_POOR_FETGRO | Poor fetal growth \|\| id:finngen_R11_O15_POOR_FETGRO | CD20 on B cell \|\| id:ebi-a-GCST90001743 | Inverse variance weighted | 23 | -0.009101596 | 0.0278649012359465 | 0.74394548496033 | -0.063716803 | 0.0455136102836775 | 0.990939698011502 | 0.938270677762069 | 1.04656524856694 | 0.994119304454654 |
| ebi-a-GCST90001743 | finngen_R11_O15_POOR_FETGRO | Poor fetal growth \|\| id:finngen_R11_O15_POOR_FETGRO | CD20 on B cell \|\| id:ebi-a-GCST90001743 | MR Egger | 23 | 0.00568307419185365 | 0.0626754514774424 | 0.928610446765868 | -0.117160811 | 0.128526959087641 | 1.00569925349286 | 0.889442149844047 | 1.1371520774605 | 0.992899239648112 |
| ebi-a-GCST90001743 | finngen_R11_O15_POOR_FETGRO | Poor fetal growth \|\| id:finngen_R11_O15_POOR_FETGRO | CD20 on B cell \|\| id:ebi-a-GCST90001743 | Weighted median | 23 | -0.004011697 | 0.0408029492163505 | 0.921679156610231 | -0.083985477 | 0.0759620837866928 | 0.995996339428031 | 0.919444608962209 | 1.07892166475774 | 0.999440741871876 |
| ebi-a-GCST90001743 | finngen_R11_O15_POOR_FETGRO | Poor fetal growth \|\| id:finngen_R11_O15_POOR_FETGRO | CD20 on B cell \|\| id:ebi-a-GCST90001743 | Weighted mode | 23 | 0.0179054186643052 | 0.0665068382285342 | 0.790264099920337 | -0.112447984 | 0.148258821592232 | 1.01806668172944 | 0.893643829441447 | 1.15981304217746 | 0.999857399747617 |
| ebi-a-GCST90001744 | finngen_R11_O15_POOR_FETGRO | Poor fetal growth \|\| id:finngen_R11_O15_POOR_FETGRO | CD20 on CD20- CD38- B cell \|\| id:ebi-a-GCST90001744 | Inverse variance weighted | 16 | 0.0440919980252122 | 0.0334854159178471 | 0.187921670503082 | -0.021539417 | 0.109723413224192 | 1.04507849562379 | 0.978690899479585 | 1.11596936540029 | 0.994119304454654 |
| ebi-a-GCST90001744 | finngen_R11_O15_POOR_FETGRO | Poor fetal growth \|\| id:finngen_R11_O15_POOR_FETGRO | CD20 on CD20- CD38- B cell \|\| id:ebi-a-GCST90001744 | MR Egger | 16 | -0.001524039 | 0.0501609137139478 | 0.97619049646114 | -0.09983943 | 0.0967913521425589 | 0.998477122020502 | 0.904982719792793 | 1.10163049679734 | 0.993986195344372 |
| ebi-a-GCST90001744 | finngen_R11_O15_POOR_FETGRO | Poor fetal growth \|\| id:finngen_R11_O15_POOR_FETGRO | CD20 on CD20- CD38- B cell \|\| id:ebi-a-GCST90001744 | Weighted median | 16 | 0.0320915575380777 | 0.0458193558532356 | 0.483681887077638 | -0.05771438 | 0.121897495010419 | 1.03261204439374 | 0.943919511291407 | 1.129638302 | 0.999440741871876 |
| ebi-a-GCST90001744 | finngen_R11_O15_POOR_FETGRO | Poor fetal growth \|\| id:finngen_R11_O15_POOR_FETGRO | CD20 on CD20- CD38- B cell \|\| id:ebi-a-GCST90001744 | Weighted mode | 16 | 0.0294506184182972 | 0.0358619956164186 | 0.424380872495457 | -0.040838893 | 0.0997401298264776 | 1.02988857668926 | 0.959983777606224 | 1.10488375443164 | 0.999857399747617 |
| ebi-a-GCST90001745 | finngen_R11_O15_POOR_FETGRO | Poor fetal growth \|\| id:finngen_R11_O15_POOR_FETGRO | CD20 on CD24+ CD27+ B cell \|\| id:ebi-a-GCST90001745 | Inverse variance weighted (multiplicative random effects) | 29 | 0.0273117083219177 | 0.0327971859339146 | 0.404988289142538 | -0.036970776 | 0.0915941927523904 | 1.02768809177306 | 0.963704298127332 | 1.0959199995522 | 0.994119304454654 |
| ebi-a-GCST90001745 | finngen_R11_O15_POOR_FETGRO | Poor fetal growth \|\| id:finngen_R11_O15_POOR_FETGRO | CD20 on CD24+ CD27+ B cell \|\| id:ebi-a-GCST90001745 | MR Egger | 29 | 0.0745411189186557 | 0.0558037186644723 | 0.192774674709212 | -0.03483417 | 0.183916407501021 | 1.07738964370858 | 0.965765556206132 | 1.20191534778939 | 0.992899239648112 |
| ebi-a-GCST90001745 | finngen_R11_O15_POOR_FETGRO | Poor fetal growth \|\| id:finngen_R11_O15_POOR_FETGRO | CD20 on CD24+ CD27+ B cell \|\| id:ebi-a-GCST90001745 | Weighted median | 29 | 0.0473916380324656 | 0.0369184283228589 | 0.199252144023412 | -0.024968481 | 0.119751757545269 | 1.04853257391223 | 0.975340652837421 | 1.12721699373098 | 0.999440741871876 |
| ebi-a-GCST90001745 | finngen_R11_O15_POOR_FETGRO | Poor fetal growth \|\| id:finngen_R11_O15_POOR_FETGRO | CD20 on CD24+ CD27+ B cell \|\| id:ebi-a-GCST90001745 | Weighted mode | 29 | 0.0585038710400487 | 0.0374233363200411 | 0.129215287819789 | -0.014845868 | 0.131853610227329 | 1.06024908995334 | 0.985263788433568 | 1.14094128490614 | 0.999857399747617 |
| ebi-a-GCST90001746 | finngen_R11_O15_POOR_FETGRO | Poor fetal growth \|\| id:finngen_R11_O15_POOR_FETGRO | CD20 on IgD+ CD24+ B cell \|\| id:ebi-a-GCST90001746 | Inverse variance weighted | 23 | 0.00346291344484321 | 0.0290172831036441 | 0.905006243517336 | -0.053410961 | 0.0603367883279857 | 1.00346891625668 | 0.947990344942965 | 1.06219422092736 | 0.994826412046876 |
| ebi-a-GCST90001746 | finngen_R11_O15_POOR_FETGRO | Poor fetal growth \|\| id:finngen_R11_O15_POOR_FETGRO | CD20 on IgD+ CD24+ B cell \|\| id:ebi-a-GCST90001746 | MR Egger | 23 | 0.0555159161080224 | 0.0445987743025187 | 0.226923608256597 | -0.031897682 | 0.142929513740959 | 1.05708584163098 | 0.968605683265641 | 1.15364848243433 | 0.992899239648112 |
| ebi-a-GCST90001746 | finngen_R11_O15_POOR_FETGRO | Poor fetal growth \|\| id:finngen_R11_O15_POOR_FETGRO | CD20 on IgD+ CD24+ B cell \|\| id:ebi-a-GCST90001746 | Weighted median | 23 | 0.0425403113705884 | 0.0407960378329063 | 0.29706133550923 | -0.037419923 | 0.122500545523085 | 1.04345811875279 | 0.963271550738532 | 1.13031973668935 | 0.999440741871876 |
| ebi-a-GCST90001746 | finngen_R11_O15_POOR_FETGRO | Poor fetal growth \|\| id:finngen_R11_O15_POOR_FETGRO | CD20 on IgD+ CD24+ B cell \|\| id:ebi-a-GCST90001746 | Weighted mode | 23 | 0.0369323075574085 | 0.0402767423990286 | 0.369104997365746 | -0.042010108 | 0.115874722659505 | 1.03762277924036 | 0.958860088802308 | 1.12285519500902 | 0.999857399747617 |
| ebi-a-GCST90001747 | finngen_R11_O15_POOR_FETGRO | Poor fetal growth \|\| id:finngen_R11_O15_POOR_FETGRO | CD20 on IgD+ CD24- B cell \|\| id:ebi-a-GCST90001747 | Inverse variance weighted | 23 | -0.019342544 | 0.0137780258854739 | 0.160357842651171 | -0.046347475 | 0.00766238640142441 | 0.980843322369093 | 0.954710166567092 | 1.00769181760717 | 0.994119304454654 |
| ebi-a-GCST90001747 | finngen_R11_O15_POOR_FETGRO | Poor fetal growth \|\| id:finngen_R11_O15_POOR_FETGRO | CD20 on IgD+ CD24- B cell \|\| id:ebi-a-GCST90001747 | MR Egger | 23 | -0.021742339 | 0.0165374687217423 | 0.202777943789384 | -0.054155777 | 0.0106711001623166 | 0.978492322341134 | 0.947284529650414 | 1.01072823941702 | 0.992899239648112 |
| ebi-a-GCST90001747 | finngen_R11_O15_POOR_FETGRO | Poor fetal growth \|\| id:finngen_R11_O15_POOR_FETGRO | CD20 on IgD+ CD24- B cell \|\| id:ebi-a-GCST90001747 | Weighted median | 23 | -0.019189229 | 0.0176905735999945 | 0.278047952833069 | -0.053862753 | 0.0154842957344288 | 0.980993712688352 | 0.947562147850579 | 1.0156047986056 | 0.999440741871876 |
| ebi-a-GCST90001747 | finngen_R11_O15_POOR_FETGRO | Poor fetal growth \|\| id:finngen_R11_O15_POOR_FETGRO | CD20 on IgD+ CD24- B cell \|\| id:ebi-a-GCST90001747 | Weighted mode | 23 | -0.020526402 | 0.0156342931826747 | 0.202744699151338 | -0.051169616 | 0.0101168128423258 | 0.979682830747104 | 0.950117501322113 | 1.01016816080653 | 0.999857399747617 |
| ebi-a-GCST90001748 | finngen_R11_O15_POOR_FETGRO | Poor fetal growth \|\| id:finngen_R11_O15_POOR_FETGRO | CD20 on IgD+ CD38- B cell \|\| id:ebi-a-GCST90001748 | Inverse variance weighted | 25 | 0.00479026934934105 | 0.0240974273647583 | 0.842428909254895 | -0.042440688 | 0.0520212269842673 | 1.00480176103165 | 0.95844731098832 | 1.053398103 | 0.994119304454654 |
| ebi-a-GCST90001748 | finngen_R11_O15_POOR_FETGRO | Poor fetal growth \|\| id:finngen_R11_O15_POOR_FETGRO | CD20 on IgD+ CD38- B cell \|\| id:ebi-a-GCST90001748 | MR Egger | 25 | -0.011557418 | 0.0331242605858001 | 0.730330105380566 | -0.076480968 | 0.0533661330742712 | 0.98850911272455 | 0.92637054441427 | 1.05481577737051 | 0.992899239648112 |
| ebi-a-GCST90001748 | finngen_R11_O15_POOR_FETGRO | Poor fetal growth \|\| id:finngen_R11_O15_POOR_FETGRO | CD20 on IgD+ CD38- B cell \|\| id:ebi-a-GCST90001748 | Weighted median | 25 | -0.016772419 | 0.0328211839903371 | 0.60933411168323 | -0.08110194 | 0.0475571013424437 | 0.983367454645786 | 0.922099687903656 | 1.04870608193674 | 0.999440741871876 |
| ebi-a-GCST90001748 | finngen_R11_O15_POOR_FETGRO | Poor fetal growth \|\| id:finngen_R11_O15_POOR_FETGRO | CD20 on IgD+ CD38- B cell \|\| id:ebi-a-GCST90001748 | Weighted mode | 25 | -0.007536172 | 0.0299547711211826 | 0.803503603782575 | -0.066247524 | 0.0511751792152384 | 0.992492153562739 | 0.935899178503961 | 1.05250725452948 | 0.999857399747617 |
| ebi-a-GCST90001749 | finngen_R11_O15_POOR_FETGRO | Poor fetal growth \|\| id:finngen_R11_O15_POOR_FETGRO | CD20 on IgD+ CD38- naive B cell \|\| id:ebi-a-GCST90001749 | Inverse variance weighted | 16 | -0.022566206 | 0.025537413989394 | 0.376883686130094 | -0.072619538 | 0.0274871249410583 | 0.977686505870251 | 0.929954575201244 | 1.02786838115607 | 0.994119304454654 |
| ebi-a-GCST90001749 | finngen_R11_O15_POOR_FETGRO | Poor fetal growth \|\| id:finngen_R11_O15_POOR_FETGRO | CD20 on IgD+ CD38- naive B cell \|\| id:ebi-a-GCST90001749 | MR Egger | 16 | -0.010458145 | 0.0419995533195059 | 0.806972002875811 | -0.092777269 | 0.071860979849171 | 0.989596351595757 | 0.911396473790253 | 1.07450595570003 | 0.992899239648112 |
| ebi-a-GCST90001749 | finngen_R11_O15_POOR_FETGRO | Poor fetal growth \|\| id:finngen_R11_O15_POOR_FETGRO | CD20 on IgD+ CD38- naive B cell \|\| id:ebi-a-GCST90001749 | Weighted median | 16 | -0.017925892 | 0.0356647966047483 | 0.615230444859576 | -0.087828893 | 0.0519771095329205 | 0.982233821229446 | 0.915917583081633 | 1.05335163052658 | 0.999440741871876 |
| ebi-a-GCST90001749 | finngen_R11_O15_POOR_FETGRO | Poor fetal growth \|\| id:finngen_R11_O15_POOR_FETGRO | CD20 on IgD+ CD38- naive B cell \|\| id:ebi-a-GCST90001749 | Weighted mode | 16 | -0.015427207 | 0.0383032901539113 | 0.692803295559739 | -0.090501655 | 0.0596472420599624 | 0.984691183121024 | 0.913472821788522 | 1.06146204133127 | 0.999857399747617 |
| ebi-a-GCST90001750 | finngen_R11_O15_POOR_FETGRO | Poor fetal growth \|\| id:finngen_R11_O15_POOR_FETGRO | CD20 on IgD+ CD38- unswitched memory B cell \|\| id:ebi-a-GCST90001750 | Inverse variance weighted | 18 | 0.00634155553797705 | 0.0323914981334485 | 0.844783620587803 | -0.057145781 | 0.069828891879536 | 1.00636170577339 | 0.944456375720821 | 1.07232468209469 | 0.994119304454654 |
| ebi-a-GCST90001750 | finngen_R11_O15_POOR_FETGRO | Poor fetal growth \|\| id:finngen_R11_O15_POOR_FETGRO | CD20 on IgD+ CD38- unswitched memory B cell \|\| id:ebi-a-GCST90001750 | MR Egger | 18 | -0.118554388 | 0.113778153331702 | 0.312911086663935 | -0.341559568 | 0.104450792881063 | 0.888203507042474 | 0.710661133569229 | 1.11010076766172 | 0.992899239648112 |
| ebi-a-GCST90001750 | finngen_R11_O15_POOR_FETGRO | Poor fetal growth \|\| id:finngen_R11_O15_POOR_FETGRO | CD20 on IgD+ CD38- unswitched memory B cell \|\| id:ebi-a-GCST90001750 | Weighted median | 18 | 0.00552910303807791 | 0.0434466036533001 | 0.898733010470474 | -0.07962624 | 0.0906844461985461 | 1.00554441673895 | 0.923461434725351 | 1.09492344348477 | 0.999440741871876 |
| ebi-a-GCST90001750 | finngen_R11_O15_POOR_FETGRO | Poor fetal growth \|\| id:finngen_R11_O15_POOR_FETGRO | CD20 on IgD+ CD38- unswitched memory B cell \|\| id:ebi-a-GCST90001750 | Weighted mode | 18 | 0.016888323364321 | 0.0672830323349047 | 0.804819613061186 | -0.11498642 | 0.148763066740734 | 1.01703173730016 | 0.891378248730479 | 1.1603980197508 | 0.999857399747617 |
| ebi-a-GCST90001751 | finngen_R11_O15_POOR_FETGRO | Poor fetal growth \|\| id:finngen_R11_O15_POOR_FETGRO | CD20 on IgD+ CD38+ B cell \|\| id:ebi-a-GCST90001751 | Inverse variance weighted (multiplicative random effects) | 26 | 0.0172051114354634 | 0.0278909711356241 | 0.537320285059589 | -0.037461192 | 0.0718714148612867 | 1.01735397185985 | 0.963231798104395 | 1.0745171682412 | 0.994119304454654 |
| ebi-a-GCST90001751 | finngen_R11_O15_POOR_FETGRO | Poor fetal growth \|\| id:finngen_R11_O15_POOR_FETGRO | CD20 on IgD+ CD38+ B cell \|\| id:ebi-a-GCST90001751 | MR Egger | 26 | 0.0675399662976726 | 0.0388441555283788 | 0.0948943530173531 | -0.008594579 | 0.143674511133295 | 1.06987301760695 | 0.991442249270154 | 1.15450826777456 | 0.992899239648112 |
| ebi-a-GCST90001751 | finngen_R11_O15_POOR_FETGRO | Poor fetal growth \|\| id:finngen_R11_O15_POOR_FETGRO | CD20 on IgD+ CD38+ B cell \|\| id:ebi-a-GCST90001751 | Weighted median | 26 | 0.0441108655257902 | 0.0344736459619974 | 0.200702302599595 | -0.023457481 | 0.111679211611305 | 1.04509821382892 | 0.976815507434491 | 1.11815411225098 | 0.999440741871876 |
| ebi-a-GCST90001751 | finngen_R11_O15_POOR_FETGRO | Poor fetal growth \|\| id:finngen_R11_O15_POOR_FETGRO | CD20 on IgD+ CD38+ B cell \|\| id:ebi-a-GCST90001751 | Weighted mode | 26 | 0.0482883282877735 | 0.0288845351690967 | 0.107041525626552 | -0.008325361 | 0.104902017219203 | 1.04947320451775 | 0.991709199197053 | 1.11060178517303 | 0.999857399747617 |
| ebi-a-GCST90001752 | finngen_R11_O15_POOR_FETGRO | Poor fetal growth \|\| id:finngen_R11_O15_POOR_FETGRO | CD20 on IgD+ CD38dim B cell \|\| id:ebi-a-GCST90001752 | Inverse variance weighted | 27 | -0.024616247 | 0.0251395849813596 | 0.327489708661736 | -0.073889834 | 0.0246573395413208 | 0.975684261935758 | 0.928774007907628 | 1.02496384576236 | 0.994119304454654 |
| ebi-a-GCST90001752 | finngen_R11_O15_POOR_FETGRO | Poor fetal growth \|\| id:finngen_R11_O15_POOR_FETGRO | CD20 on IgD+ CD38dim B cell \|\| id:ebi-a-GCST90001752 | MR Egger | 27 | -0.07293308 | 0.0372576899922652 | 0.0615373907550889 | -0.145958152 | 9.19924557526292e-05 | 0.929663041060819 | 0.864193866941603 | 1.00009199668719 | 0.992899239648112 |
| ebi-a-GCST90001752 | finngen_R11_O15_POOR_FETGRO | Poor fetal growth \|\| id:finngen_R11_O15_POOR_FETGRO | CD20 on IgD+ CD38dim B cell \|\| id:ebi-a-GCST90001752 | Weighted median | 27 | -0.049936707 | 0.0368247929742111 | 0.175079405086078 | -0.122113301 | 0.0222398875302112 | 0.951289632856141 | 0.885048086051597 | 1.02248903742256 | 0.999440741871876 |
| ebi-a-GCST90001752 | finngen_R11_O15_POOR_FETGRO | Poor fetal growth \|\| id:finngen_R11_O15_POOR_FETGRO | CD20 on IgD+ CD38dim B cell \|\| id:ebi-a-GCST90001752 | Weighted mode | 27 | -0.043619882 | 0.0334689233496909 | 0.203897875119571 | -0.109218972 | 0.0219792074170247 | 0.957317781707706 | 0.896534080040238 | 1.0222225296027 | 0.999857399747617 |
| ebi-a-GCST90001753 | finngen_R11_O15_POOR_FETGRO | Poor fetal growth \|\| id:finngen_R11_O15_POOR_FETGRO | CD20 on IgD- CD24- B cell \|\| id:ebi-a-GCST90001753 | Inverse variance weighted | 28 | 0.0420732045353827 | 0.0257700706201388 | 0.102545089044483 | -0.008436134 | 0.0925825429508546 | 1.04297082615099 | 0.991599350443721 | 1.09700368774476 | 0.994119304454654 |
| ebi-a-GCST90001753 | finngen_R11_O15_POOR_FETGRO | Poor fetal growth \|\| id:finngen_R11_O15_POOR_FETGRO | CD20 on IgD- CD24- B cell \|\| id:ebi-a-GCST90001753 | MR Egger | 28 | 0.0310660517314184 | 0.0379442595150063 | 0.420382477231216 | -0.043304697 | 0.105436800380831 | 1.03155363754014 | 0.957619561880598 | 1.11119587514751 | 0.992899239648112 |
| ebi-a-GCST90001753 | finngen_R11_O15_POOR_FETGRO | Poor fetal growth \|\| id:finngen_R11_O15_POOR_FETGRO | CD20 on IgD- CD24- B cell \|\| id:ebi-a-GCST90001753 | Weighted median | 28 | 0.034372503987413 | 0.0362742530450888 | 0.343346859882117 | -0.036725032 | 0.105470039955787 | 1.03497006540743 | 0.963941151900854 | 1.11123281143996 | 0.999440741871876 |
| ebi-a-GCST90001753 | finngen_R11_O15_POOR_FETGRO | Poor fetal growth \|\| id:finngen_R11_O15_POOR_FETGRO | CD20 on IgD- CD24- B cell \|\| id:ebi-a-GCST90001753 | Weighted mode | 28 | 0.0459027022201111 | 0.0298300245221714 | 0.135489530293945 | -0.012564146 | 0.104369550283567 | 1.04697253789746 | 0.987514453514745 | 1.11001058385531 | 0.999857399747617 |
| ebi-a-GCST90001754 | finngen_R11_O15_POOR_FETGRO | Poor fetal growth \|\| id:finngen_R11_O15_POOR_FETGRO | CD20 on IgD- CD27- B cell \|\| id:ebi-a-GCST90001754 | Inverse variance weighted | 20 | 0.01882249724667 | 0.0411737826602245 | 0.647564935788143 | -0.061878117 | 0.09952311126071 | 1.01900075712348 | 0.939997449737134 | 1.10464400016042 | 0.994119304454654 |
| ebi-a-GCST90001754 | finngen_R11_O15_POOR_FETGRO | Poor fetal growth \|\| id:finngen_R11_O15_POOR_FETGRO | CD20 on IgD- CD27- B cell \|\| id:ebi-a-GCST90001754 | MR Egger | 20 | 0.0292842012390882 | 0.0970161094469833 | 0.766227017247676 | -0.160867373 | 0.219435775755175 | 1.02971719979786 | 0.851404982672239 | 1.24537386219141 | 0.992899239648112 |
| ebi-a-GCST90001754 | finngen_R11_O15_POOR_FETGRO | Poor fetal growth \|\| id:finngen_R11_O15_POOR_FETGRO | CD20 on IgD- CD27- B cell \|\| id:ebi-a-GCST90001754 | Weighted median | 20 | 0.03962317276735 | 0.0483166141311711 | 0.412174232763297 | -0.055077391 | 0.134323736464445 | 1.04041864223628 | 0.946411901422239 | 1.14376303751683 | 0.999440741871876 |
| ebi-a-GCST90001754 | finngen_R11_O15_POOR_FETGRO | Poor fetal growth \|\| id:finngen_R11_O15_POOR_FETGRO | CD20 on IgD- CD27- B cell \|\| id:ebi-a-GCST90001754 | Weighted mode | 20 | 0.0304281564582445 | 0.0562428633749675 | 0.594785097365386 | -0.079807856 | 0.140664168673181 | 1.03089582418105 | 0.923293734920045 | 1.15103802844059 | 0.999857399747617 |
| ebi-a-GCST90001755 | finngen_R11_O15_POOR_FETGRO | Poor fetal growth \|\| id:finngen_R11_O15_POOR_FETGRO | CD20 on IgD- CD38- B cell \|\| id:ebi-a-GCST90001755 | Inverse variance weighted | 27 | 0.0255561161962008 | 0.0328217094612655 | 0.436195034689767 | -0.038774434 | 0.0898866667402812 | 1.02588547344571 | 0.961967671540965 | 1.09405028439369 | 0.994119304454654 |
| ebi-a-GCST90001755 | finngen_R11_O15_POOR_FETGRO | Poor fetal growth \|\| id:finngen_R11_O15_POOR_FETGRO | CD20 on IgD- CD38- B cell \|\| id:ebi-a-GCST90001755 | MR Egger | 27 | 0.0166281934315177 | 0.0638208655989707 | 0.796577668078037 | -0.108460703 | 0.1417170900055 | 1.01676721130973 | 0.897214151820924 | 1.15225061920436 | 0.992899239648112 |
| ebi-a-GCST90001755 | finngen_R11_O15_POOR_FETGRO | Poor fetal growth \|\| id:finngen_R11_O15_POOR_FETGRO | CD20 on IgD- CD38- B cell \|\| id:ebi-a-GCST90001755 | Weighted median | 27 | 0.0339922580914395 | 0.0468731122431653 | 0.468330951257072 | -0.057879042 | 0.125863558088044 | 1.0345765970997 | 0.943764096440116 | 1.13412741521293 | 0.999440741871876 |
| ebi-a-GCST90001755 | finngen_R11_O15_POOR_FETGRO | Poor fetal growth \|\| id:finngen_R11_O15_POOR_FETGRO | CD20 on IgD- CD38- B cell \|\| id:ebi-a-GCST90001755 | Weighted mode | 27 | 0.0208261040451168 | 0.0538502656894406 | 0.70209522906221 | -0.084720417 | 0.12637262479642 | 1.02104448069359 | 0.918769120993275 | 1.13470490870195 | 0.999857399747617 |
| ebi-a-GCST90001756 | finngen_R11_O15_POOR_FETGRO | Poor fetal growth \|\| id:finngen_R11_O15_POOR_FETGRO | CD20 on IgD- CD38+ B cell \|\| id:ebi-a-GCST90001756 | Inverse variance weighted | 16 | -0.007785327 | 0.0160419321296431 | 0.627455696738975 | -0.039227514 | 0.0236568595601189 | 0.992244899753825 | 0.961531921911853 | 1.02393890275619 | 0.994119304454654 |
| ebi-a-GCST90001756 | finngen_R11_O15_POOR_FETGRO | Poor fetal growth \|\| id:finngen_R11_O15_POOR_FETGRO | CD20 on IgD- CD38+ B cell \|\| id:ebi-a-GCST90001756 | MR Egger | 16 | -0.015206671 | 0.0185464616330989 | 0.42599966152158 | -0.051557736 | 0.0211443939465272 | 0.984908366713894 | 0.949748814009148 | 1.0213695205664 | 0.992899239648112 |
| ebi-a-GCST90001756 | finngen_R11_O15_POOR_FETGRO | Poor fetal growth \|\| id:finngen_R11_O15_POOR_FETGRO | CD20 on IgD- CD38+ B cell \|\| id:ebi-a-GCST90001756 | Weighted median | 16 | -0.020298901 | 0.0222187992583005 | 0.360931658788891 | -0.063847747 | 0.0232499456600763 | 0.979905734836624 | 0.938147824072698 | 1.02352233254372 | 0.999440741871876 |
| ebi-a-GCST90001756 | finngen_R11_O15_POOR_FETGRO | Poor fetal growth \|\| id:finngen_R11_O15_POOR_FETGRO | CD20 on IgD- CD38+ B cell \|\| id:ebi-a-GCST90001756 | Weighted mode | 16 | -0.012712491 | 0.0182959808237524 | 0.49778795630255 | -0.048572614 | 0.023147631288604 | 0.987367971269304 | 0.952588165995029 | 1.02341761685663 | 0.999857399747617 |
| ebi-a-GCST90001757 | finngen_R11_O15_POOR_FETGRO | Poor fetal growth \|\| id:finngen_R11_O15_POOR_FETGRO | CD20 on IgD- CD38dim B cell \|\| id:ebi-a-GCST90001757 | Inverse variance weighted | 34 | -0.012502303 | 0.00907279711866327 | 0.168203661287113 | -0.030284986 | 0.00528037888822941 | 0.987575525646245 | 0.970169009739896 | 1.00529434465953 | 0.994119304454654 |
| ebi-a-GCST90001757 | finngen_R11_O15_POOR_FETGRO | Poor fetal growth \|\| id:finngen_R11_O15_POOR_FETGRO | CD20 on IgD- CD38dim B cell \|\| id:ebi-a-GCST90001757 | MR Egger | 34 | -0.011685069 | 0.0100010464721832 | 0.251282777865407 | -0.03128712 | 0.00791698212211946 | 0.988382936315127 | 0.969197257159088 | 1.00794840429327 | 0.992899239648112 |
| ebi-a-GCST90001757 | finngen_R11_O15_POOR_FETGRO | Poor fetal growth \|\| id:finngen_R11_O15_POOR_FETGRO | CD20 on IgD- CD38dim B cell \|\| id:ebi-a-GCST90001757 | Weighted median | 34 | -0.006994115 | 0.0113737975498205 | 0.538599459440948 | -0.029286758 | 0.015298528205712 | 0.993030286907285 | 0.971137942776018 | 1.01541614973497 | 0.999440741871876 |
| ebi-a-GCST90001757 | finngen_R11_O15_POOR_FETGRO | Poor fetal growth \|\| id:finngen_R11_O15_POOR_FETGRO | CD20 on IgD- CD38dim B cell \|\| id:ebi-a-GCST90001757 | Weighted mode | 34 | -0.007854337 | 0.00964337970767778 | 0.421209171892726 | -0.026755362 | 0.0110466869439636 | 0.992176427425835 | 0.973599392277791 | 1.01110792688192 | 0.999857399747617 |
| ebi-a-GCST90001758 | finngen_R11_O15_POOR_FETGRO | Poor fetal growth \|\| id:finngen_R11_O15_POOR_FETGRO | CD20 on memory B cell \|\| id:ebi-a-GCST90001758 | Inverse variance weighted | 28 | -0.032026995 | 0.0252034464271271 | 0.203821625251212 | -0.08142575 | 0.0173717604117477 | 0.968480437998412 | 0.92180115143321 | 1.01752352698548 | 0.994119304454654 |
| ebi-a-GCST90001758 | finngen_R11_O15_POOR_FETGRO | Poor fetal growth \|\| id:finngen_R11_O15_POOR_FETGRO | CD20 on memory B cell \|\| id:ebi-a-GCST90001758 | MR Egger | 28 | -0.013586129 | 0.0339694638726407 | 0.692459422605077 | -0.080166278 | 0.0529940199877787 | 0.986505745705325 | 0.922962864844718 | 1.05442333963605 | 0.992899239648112 |
| ebi-a-GCST90001758 | finngen_R11_O15_POOR_FETGRO | Poor fetal growth \|\| id:finngen_R11_O15_POOR_FETGRO | CD20 on memory B cell \|\| id:ebi-a-GCST90001758 | Weighted median | 28 | -0.017152479 | 0.0317372109009698 | 0.58888449549826 | -0.079357412 | 0.0450524546955657 | 0.982993787621467 | 0.923709720467081 | 1.04608273042076 | 0.999440741871876 |
| ebi-a-GCST90001758 | finngen_R11_O15_POOR_FETGRO | Poor fetal growth \|\| id:finngen_R11_O15_POOR_FETGRO | CD20 on memory B cell \|\| id:ebi-a-GCST90001758 | Weighted mode | 28 | -0.014445551 | 0.0302295123185907 | 0.636594274793857 | -0.073695395 | 0.0448042932779025 | 0.985658285510853 | 0.928954614959804 | 1.04582316525574 | 0.999857399747617 |
| ebi-a-GCST90001759 | finngen_R11_O15_POOR_FETGRO | Poor fetal growth \|\| id:finngen_R11_O15_POOR_FETGRO | CD20 on naive-mature B cell \|\| id:ebi-a-GCST90001759 | Inverse variance weighted | 23 | -0.020994132 | 0.0136620486506449 | 0.124372853541213 | -0.047771747 | 0.00578348325450058 | 0.979224710544533 | 0.953351367119806 | 1.00580023988209 | 0.994119304454654 |
| ebi-a-GCST90001759 | finngen_R11_O15_POOR_FETGRO | Poor fetal growth \|\| id:finngen_R11_O15_POOR_FETGRO | CD20 on naive-mature B cell \|\| id:ebi-a-GCST90001759 | MR Egger | 23 | -0.020888948 | 0.016499979325747 | 0.219378471161577 | -0.053228907 | 0.0114510116632361 | 0.979327715013221 | 0.948162946225222 | 1.01151682546894 | 0.992899239648112 |
| ebi-a-GCST90001759 | finngen_R11_O15_POOR_FETGRO | Poor fetal growth \|\| id:finngen_R11_O15_POOR_FETGRO | CD20 on naive-mature B cell \|\| id:ebi-a-GCST90001759 | Weighted median | 23 | -0.016849441 | 0.0186376098266226 | 0.365965753189217 | -0.053379157 | 0.0196802738618762 | 0.983291716516791 | 0.948020496046624 | 1.01987520713158 | 0.999440741871876 |
| ebi-a-GCST90001759 | finngen_R11_O15_POOR_FETGRO | Poor fetal growth \|\| id:finngen_R11_O15_POOR_FETGRO | CD20 on naive-mature B cell \|\| id:ebi-a-GCST90001759 | Weighted mode | 23 | -0.021358087 | 0.016008405414574 | 0.195792028052334 | -0.052734561 | 0.0100183880541789 | 0.978868382193695 | 0.948631782775632 | 1.01006874011212 | 0.999857399747617 |
| ebi-a-GCST90001760 | finngen_R11_O15_POOR_FETGRO | Poor fetal growth \|\| id:finngen_R11_O15_POOR_FETGRO | CD20 on unswitched memory B cell \|\| id:ebi-a-GCST90001760 | Inverse variance weighted | 22 | 0.00745717856993823 | 0.0219916463854362 | 0.734540862861757 | -0.035646448 | 0.0505608054853931 | 1.0074850525701 | 0.964981403951397 | 1.05186082031826 | 0.994119304454654 |
| ebi-a-GCST90001760 | finngen_R11_O15_POOR_FETGRO | Poor fetal growth \|\| id:finngen_R11_O15_POOR_FETGRO | CD20 on unswitched memory B cell \|\| id:ebi-a-GCST90001760 | MR Egger | 22 | -0.014103941 | 0.0287063695219355 | 0.628550282786921 | -0.070368425 | 0.0421605430903309 | 0.985995053454351 | 0.932050365579186 | 1.04306192169382 | 0.992899239648112 |
| ebi-a-GCST90001760 | finngen_R11_O15_POOR_FETGRO | Poor fetal growth \|\| id:finngen_R11_O15_POOR_FETGRO | CD20 on unswitched memory B cell \|\| id:ebi-a-GCST90001760 | Weighted median | 22 | -0.013233465 | 0.0307997062235332 | 0.667441492113903 | -0.073600889 | 0.0471339592421759 | 0.986853712365041 | 0.929042410760254 | 1.04826242411444 | 0.999440741871876 |
| ebi-a-GCST90001760 | finngen_R11_O15_POOR_FETGRO | Poor fetal growth \|\| id:finngen_R11_O15_POOR_FETGRO | CD20 on unswitched memory B cell \|\| id:ebi-a-GCST90001760 | Weighted mode | 22 | -0.005562143 | 0.0271690958399038 | 0.83975965896439 | -0.058813571 | 0.0476892847945324 | 0.99445329702608 | 0.942882533516913 | 1.04884471268902 | 0.999857399747617 |
| ebi-a-GCST90001761 | finngen_R11_O15_POOR_FETGRO | Poor fetal growth \|\| id:finngen_R11_O15_POOR_FETGRO | CD20 on switched memory B cell \|\| id:ebi-a-GCST90001761 | Inverse variance weighted | 31 | -0.034365136 | 0.0261477159195941 | 0.188755656050471 | -0.085614659 | 0.0168843876750332 | 0.966218639467144 | 0.917947886281329 | 1.01702773458712 | 0.994119304454654 |
| ebi-a-GCST90001761 | finngen_R11_O15_POOR_FETGRO | Poor fetal growth \|\| id:finngen_R11_O15_POOR_FETGRO | CD20 on switched memory B cell \|\| id:ebi-a-GCST90001761 | MR Egger | 31 | 0.00220352392097368 | 0.043742019503282 | 0.960168574945052 | -0.083530834 | 0.0879378821474064 | 1.002205953 | 0.919862722905772 | 1.09192029217774 | 0.992899239648112 |
| ebi-a-GCST90001761 | finngen_R11_O15_POOR_FETGRO | Poor fetal growth \|\| id:finngen_R11_O15_POOR_FETGRO | CD20 on switched memory B cell \|\| id:ebi-a-GCST90001761 | Weighted median | 31 | -0.028521332 | 0.0371706067642678 | 0.442897962147692 | -0.101375721 | 0.0443330571246776 | 0.971881561617991 | 0.90359346970372 | 1.04533045167174 | 0.999440741871876 |
| ebi-a-GCST90001761 | finngen_R11_O15_POOR_FETGRO | Poor fetal growth \|\| id:finngen_R11_O15_POOR_FETGRO | CD20 on switched memory B cell \|\| id:ebi-a-GCST90001761 | Weighted mode | 31 | -0.00989564 | 0.0339550179070265 | 0.772725218202207 | -0.076447475 | 0.0566561955966188 | 0.990153161235262 | 0.92640157262507 | 1.05829190242639 | 0.999857399747617 |
| ebi-a-GCST90001762 | finngen_R11_O15_POOR_FETGRO | Poor fetal growth \|\| id:finngen_R11_O15_POOR_FETGRO | CD20 on IgD+ B cell \|\| id:ebi-a-GCST90001762 | Inverse variance weighted | 19 | -0.016878431 | 0.0153369426448678 | 0.27111075074776 | -0.046938839 | 0.0131819761557298 | 0.983263211273708 | 0.954145752302599 | 1.01326924142473 | 0.994119304454654 |
| ebi-a-GCST90001762 | finngen_R11_O15_POOR_FETGRO | Poor fetal growth \|\| id:finngen_R11_O15_POOR_FETGRO | CD20 on IgD+ B cell \|\| id:ebi-a-GCST90001762 | MR Egger | 19 | -0.01108042 | 0.0188388862240539 | 0.564152765756005 | -0.048004637 | 0.0258437969890265 | 0.988980741735969 | 0.95312936739755 | 1.0261806434474 | 0.992899239648112 |
| ebi-a-GCST90001762 | finngen_R11_O15_POOR_FETGRO | Poor fetal growth \|\| id:finngen_R11_O15_POOR_FETGRO | CD20 on IgD+ B cell \|\| id:ebi-a-GCST90001762 | Weighted median | 19 | -0.015246155 | 0.0184913212874787 | 0.409653663031744 | -0.051489144 | 0.0209968350015091 | 0.984869479490056 | 0.949813960663538 | 1.02121881947639 | 0.999440741871876 |
| ebi-a-GCST90001762 | finngen_R11_O15_POOR_FETGRO | Poor fetal growth \|\| id:finngen_R11_O15_POOR_FETGRO | CD20 on IgD+ B cell \|\| id:ebi-a-GCST90001762 | Weighted mode | 19 | -0.014939738 | 0.0171624136156451 | 0.395492949857539 | -0.048578069 | 0.0186985922606255 | 0.985171305787803 | 0.952582969095691 | 1.01887450567064 | 0.999857399747617 |
| ebi-a-GCST90001763 | finngen_R11_O15_POOR_FETGRO | Poor fetal growth \|\| id:finngen_R11_O15_POOR_FETGRO | CD20 on transitional B cell \|\| id:ebi-a-GCST90001763 | Inverse variance weighted | 22 | 0.00168627469981633 | 0.0112901797714712 | 0.881271428559816 | -0.020442478 | 0.0238150270518999 | 1.00168769726049 | 0.979765053239704 | 1.02410086941277 | 0.994119304454654 |
| ebi-a-GCST90001763 | finngen_R11_O15_POOR_FETGRO | Poor fetal growth \|\| id:finngen_R11_O15_POOR_FETGRO | CD20 on transitional B cell \|\| id:ebi-a-GCST90001763 | MR Egger | 22 | 0.0034613252499482 | 0.0135380219345338 | 0.800813388352197 | -0.023073198 | 0.0299958482416344 | 1.00346732255373 | 0.977190952984358 | 1.03045025576417 | 0.992899239648112 |
| ebi-a-GCST90001763 | finngen_R11_O15_POOR_FETGRO | Poor fetal growth \|\| id:finngen_R11_O15_POOR_FETGRO | CD20 on transitional B cell \|\| id:ebi-a-GCST90001763 | Weighted median | 22 | -0.001000319 | 0.0135096633764804 | 0.940974792609766 | -0.02747926 | 0.0254786208251996 | 0.999000180759957 | 0.972894860562453 | 1.02580597515076 | 0.999440741871876 |
| ebi-a-GCST90001763 | finngen_R11_O15_POOR_FETGRO | Poor fetal growth \|\| id:finngen_R11_O15_POOR_FETGRO | CD20 on transitional B cell \|\| id:ebi-a-GCST90001763 | Weighted mode | 22 | 0.00124203430608949 | 0.0118975211474309 | 0.917847024001226 | -0.022077107 | 0.0245611757550541 | 1.00124280595013 | 0.978164808649634 | 1.02486528609718 | 0.999857399747617 |
| ebi-a-GCST90001764 | finngen_R11_O15_POOR_FETGRO | Poor fetal growth \|\| id:finngen_R11_O15_POOR_FETGRO | CD24 on CD24+ CD27+ B cell \|\| id:ebi-a-GCST90001764 | Inverse variance weighted | 25 | 0.0359822464968858 | 0.0227056021844497 | 0.11302771722352 | -0.008520734 | 0.0804852267784072 | 1.03663744238149 | 0.991515464781734 | 1.0838128351168 | 0.994119304454654 |
| ebi-a-GCST90001764 | finngen_R11_O15_POOR_FETGRO | Poor fetal growth \|\| id:finngen_R11_O15_POOR_FETGRO | CD24 on CD24+ CD27+ B cell \|\| id:ebi-a-GCST90001764 | MR Egger | 25 | 0.0104529289509886 | 0.0269637649758519 | 0.701827840196216 | -0.04239605 | 0.0633019083036582 | 1.01050775166562 | 0.958490095003003 | 1.06534842822044 | 0.992899239648112 |
| ebi-a-GCST90001764 | finngen_R11_O15_POOR_FETGRO | Poor fetal growth \|\| id:finngen_R11_O15_POOR_FETGRO | CD24 on CD24+ CD27+ B cell \|\| id:ebi-a-GCST90001764 | Weighted median | 25 | 0.017450826651076 | 0.0294758123755609 | 0.553824560005506 | -0.040321766 | 0.0752234189071753 | 1.01760398192478 | 0.960480339888557 | 1.07812499748752 | 0.999440741871876 |
| ebi-a-GCST90001764 | finngen_R11_O15_POOR_FETGRO | Poor fetal growth \|\| id:finngen_R11_O15_POOR_FETGRO | CD24 on CD24+ CD27+ B cell \|\| id:ebi-a-GCST90001764 | Weighted mode | 25 | 0.0270978198743174 | 0.0258946478476034 | 0.305772273305408 | -0.02365569 | 0.07785132965562 | 1.0274683046683 | 0.976621912657149 | 1.08096193973947 | 0.999857399747617 |
| ebi-a-GCST90001765 | finngen_R11_O15_POOR_FETGRO | Poor fetal growth \|\| id:finngen_R11_O15_POOR_FETGRO | CD24 on IgD+ CD24+ B cell \|\| id:ebi-a-GCST90001765 | Inverse variance weighted | 25 | 0.0226591381005136 | 0.0145001295394613 | 0.118126652799685 | -0.005761116 | 0.0510793919978578 | 1.02291780640916 | 0.99425544760762 | 1.05240644261662 | 0.994119304454654 |
| ebi-a-GCST90001765 | finngen_R11_O15_POOR_FETGRO | Poor fetal growth \|\| id:finngen_R11_O15_POOR_FETGRO | CD24 on IgD+ CD24+ B cell \|\| id:ebi-a-GCST90001765 | MR Egger | 25 | 0.0128968078992665 | 0.017492914464034 | 0.468419916031412 | -0.021389304 | 0.0471829202487732 | 1.01298033039791 | 0.978837824463377 | 1.04831374935436 | 0.992899239648112 |
| ebi-a-GCST90001765 | finngen_R11_O15_POOR_FETGRO | Poor fetal growth \|\| id:finngen_R11_O15_POOR_FETGRO | CD24 on IgD+ CD24+ B cell \|\| id:ebi-a-GCST90001765 | Weighted median | 25 | 0.0170705054595781 | 0.0218800600819884 | 0.435281700087895 | -0.025814412 | 0.0599554232202755 | 1.01721703915183 | 0.974515930996418 | 1.06178921434649 | 0.999440741871876 |
| ebi-a-GCST90001765 | finngen_R11_O15_POOR_FETGRO | Poor fetal growth \|\| id:finngen_R11_O15_POOR_FETGRO | CD24 on IgD+ CD24+ B cell \|\| id:ebi-a-GCST90001765 | Weighted mode | 25 | 0.0194594784073894 | 0.017782661127671 | 0.284687101231789 | -0.015394537 | 0.0543134942176246 | 1.01965004817966 | 0.984723352757962 | 1.05581554234586 | 0.999857399747617 |
| ebi-a-GCST90001766 | finngen_R11_O15_POOR_FETGRO | Poor fetal growth \|\| id:finngen_R11_O15_POOR_FETGRO | CD24 on IgD+ CD38- B cell \|\| id:ebi-a-GCST90001766 | Inverse variance weighted | 21 | 0.0262345150490709 | 0.0196676513880051 | 0.182239383701851 | -0.012314082 | 0.0647831117695608 | 1.02658166909654 | 0.987761426377073 | 1.06692759524983 | 0.994119304454654 |
| ebi-a-GCST90001766 | finngen_R11_O15_POOR_FETGRO | Poor fetal growth \|\| id:finngen_R11_O15_POOR_FETGRO | CD24 on IgD+ CD38- B cell \|\| id:ebi-a-GCST90001766 | MR Egger | 21 | 0.034205443739659 | 0.0267672904329152 | 0.216683703455229 | -0.018258446 | 0.0866693329881728 | 1.03479717749364 | 0.981907230048962 | 1.0905360158061 | 0.992899239648112 |
| ebi-a-GCST90001766 | finngen_R11_O15_POOR_FETGRO | Poor fetal growth \|\| id:finngen_R11_O15_POOR_FETGRO | CD24 on IgD+ CD38- B cell \|\| id:ebi-a-GCST90001766 | Weighted median | 21 | 0.0349849617846473 | 0.0258830699415573 | 0.176485943297605 | -0.015745855 | 0.0857157788700996 | 1.03560413504452 | 0.984377462581862 | 1.08949662633313 | 0.999440741871876 |
| ebi-a-GCST90001766 | finngen_R11_O15_POOR_FETGRO | Poor fetal growth \|\| id:finngen_R11_O15_POOR_FETGRO | CD24 on IgD+ CD38- B cell \|\| id:ebi-a-GCST90001766 | Weighted mode | 21 | 0.0346208814941594 | 0.0254129300190272 | 0.1882391552541 | -0.015188461 | 0.0844302243314528 | 1.03522716061885 | 0.984926301577133 | 1.0880969188932 | 0.999857399747617 |
| ebi-a-GCST90001767 | finngen_R11_O15_POOR_FETGRO | Poor fetal growth \|\| id:finngen_R11_O15_POOR_FETGRO | CD24 on IgD+ CD38- unswitched memory B cell \|\| id:ebi-a-GCST90001767 | Inverse variance weighted | 21 | -0.000203799 | 0.0178742208498022 | 0.990902863556842 | -0.035237271 | 0.034829674274924 | 0.999796222174834 | 0.965376332832793 | 1.03544333114307 | 0.998984604424952 |
| ebi-a-GCST90001767 | finngen_R11_O15_POOR_FETGRO | Poor fetal growth \|\| id:finngen_R11_O15_POOR_FETGRO | CD24 on IgD+ CD38- unswitched memory B cell \|\| id:ebi-a-GCST90001767 | MR Egger | 21 | -0.03013662 | 0.0296445400356809 | 0.322115441051317 | -0.088239918 | 0.0279666786075296 | 0.970312960469508 | 0.91554119525482 | 1.02836141741612 | 0.992899239648112 |
| ebi-a-GCST90001767 | finngen_R11_O15_POOR_FETGRO | Poor fetal growth \|\| id:finngen_R11_O15_POOR_FETGRO | CD24 on IgD+ CD38- unswitched memory B cell \|\| id:ebi-a-GCST90001767 | Weighted median | 21 | -0.018493908 | 0.0251942580857329 | 0.462916750371526 | -0.067874654 | 0.0308868380752453 | 0.981676055167147 | 0.934377587084504 | 1.0313687856057 | 0.999440741871876 |
| ebi-a-GCST90001767 | finngen_R11_O15_POOR_FETGRO | Poor fetal growth \|\| id:finngen_R11_O15_POOR_FETGRO | CD24 on IgD+ CD38- unswitched memory B cell \|\| id:ebi-a-GCST90001767 | Weighted mode | 21 | -0.020716828 | 0.0248925007018871 | 0.415094343194149 | -0.069506129 | 0.0280724734185776 | 0.979496291267813 | 0.932854415591847 | 1.02847021847316 | 0.999857399747617 |
| ebi-a-GCST90001768 | finngen_R11_O15_POOR_FETGRO | Poor fetal growth \|\| id:finngen_R11_O15_POOR_FETGRO | CD24 on IgD+ CD38+ B cell \|\| id:ebi-a-GCST90001768 | Inverse variance weighted | 25 | -0.015182039 | 0.0205972537196621 | 0.46106735739532 | -0.055552656 | 0.0251885786517008 | 0.984932627487837 | 0.945962211839584 | 1.02550849129965 | 0.994119304454654 |
| ebi-a-GCST90001768 | finngen_R11_O15_POOR_FETGRO | Poor fetal growth \|\| id:finngen_R11_O15_POOR_FETGRO | CD24 on IgD+ CD38+ B cell \|\| id:ebi-a-GCST90001768 | MR Egger | 25 | 0.0160384318803915 | 0.0284222967858889 | 0.578018447680752 | -0.03966927 | 0.0717461335807336 | 1.01616773789262 | 0.961107253769069 | 1.07438255978652 | 0.992899239648112 |
| ebi-a-GCST90001768 | finngen_R11_O15_POOR_FETGRO | Poor fetal growth \|\| id:finngen_R11_O15_POOR_FETGRO | CD24 on IgD+ CD38+ B cell \|\| id:ebi-a-GCST90001768 | Weighted median | 25 | -0.014475332 | 0.0288618609571501 | 0.615992254370741 | -0.071044579 | 0.042093915688369 | 0.985628932136298 | 0.931420369167853 | 1.04299242750302 | 0.999440741871876 |
| ebi-a-GCST90001768 | finngen_R11_O15_POOR_FETGRO | Poor fetal growth \|\| id:finngen_R11_O15_POOR_FETGRO | CD24 on IgD+ CD38+ B cell \|\| id:ebi-a-GCST90001768 | Weighted mode | 25 | -0.011015384 | 0.0240146033097221 | 0.65057999331505 | -0.058084006 | 0.0360532389683153 | 0.989045063665066 | 0.943570678503425 | 1.03671103844786 | 0.999857399747617 |
| ebi-a-GCST90001769 | finngen_R11_O15_POOR_FETGRO | Poor fetal growth \|\| id:finngen_R11_O15_POOR_FETGRO | CD24 on IgD- CD38- B cell \|\| id:ebi-a-GCST90001769 | Inverse variance weighted (multiplicative random effects) | 29 | -0.005880068 | 0.0197535591414898 | 0.765954051044133 | -0.044597044 | 0.0328369083090103 | 0.9941371861549 | 0.956382784832842 | 1.03338198948104 | 0.994119304454654 |
| ebi-a-GCST90001769 | finngen_R11_O15_POOR_FETGRO | Poor fetal growth \|\| id:finngen_R11_O15_POOR_FETGRO | CD24 on IgD- CD38- B cell \|\| id:ebi-a-GCST90001769 | MR Egger | 29 | -0.028210491 | 0.0271060994911271 | 0.307224953582969 | -0.081338446 | 0.0249174636320669 | 0.972183708981328 | 0.921881631145212 | 1.02523049823065 | 0.992899239648112 |
| ebi-a-GCST90001769 | finngen_R11_O15_POOR_FETGRO | Poor fetal growth \|\| id:finngen_R11_O15_POOR_FETGRO | CD24 on IgD- CD38- B cell \|\| id:ebi-a-GCST90001769 | Weighted median | 29 | 0.00950579951012348 | 0.0231873485395387 | 0.681838007837969 | -0.035941404 | 0.0549530026476193 | 1.00955112312084 | 0.964696819561307 | 1.05649096123072 | 0.999440741871876 |
| ebi-a-GCST90001769 | finngen_R11_O15_POOR_FETGRO | Poor fetal growth \|\| id:finngen_R11_O15_POOR_FETGRO | CD24 on IgD- CD38- B cell \|\| id:ebi-a-GCST90001769 | Weighted mode | 29 | -0.003661257 | 0.0207927621834509 | 0.861495888174527 | -0.044415071 | 0.0370925565805082 | 0.996345436931191 | 0.956556835888597 | 1.03778907060073 | 0.999857399747617 |
| ebi-a-GCST90001770 | finngen_R11_O15_POOR_FETGRO | Poor fetal growth \|\| id:finngen_R11_O15_POOR_FETGRO | CD24 on IgD- CD38dim B cell \|\| id:ebi-a-GCST90001770 | Inverse variance weighted | 28 | -0.025752864 | 0.017819081498614 | 0.148390260741526 | -0.060678263 | 0.00917253619138234 | 0.974575913084804 | 0.941125985855932 | 1.0092147328195 | 0.994119304454654 |
| ebi-a-GCST90001770 | finngen_R11_O15_POOR_FETGRO | Poor fetal growth \|\| id:finngen_R11_O15_POOR_FETGRO | CD24 on IgD- CD38dim B cell \|\| id:ebi-a-GCST90001770 | MR Egger | 28 | -0.025585067 | 0.0233332186408355 | 0.282909038351014 | -0.071318175 | 0.0201480416024486 | 0.974739457342163 | 0.93116557094619 | 1.02035238344803 | 0.992899239648112 |
| ebi-a-GCST90001770 | finngen_R11_O15_POOR_FETGRO | Poor fetal growth \|\| id:finngen_R11_O15_POOR_FETGRO | CD24 on IgD- CD38dim B cell \|\| id:ebi-a-GCST90001770 | Weighted median | 28 | -0.031539123 | 0.0257265041337923 | 0.220221656172756 | -0.081963071 | 0.0188848250655915 | 0.96895304732661 | 0.921305980848766 | 1.01906427119748 | 0.999440741871876 |
| ebi-a-GCST90001770 | finngen_R11_O15_POOR_FETGRO | Poor fetal growth \|\| id:finngen_R11_O15_POOR_FETGRO | CD24 on IgD- CD38dim B cell \|\| id:ebi-a-GCST90001770 | Weighted mode | 28 | -0.034004029 | 0.0214335643217017 | 0.124273000837849 | -0.076013815 | 0.0080057575295916 | 0.966567610772469 | 0.926803403042014 | 1.00803788929554 | 0.999857399747617 |
| ebi-a-GCST90001771 | finngen_R11_O15_POOR_FETGRO | Poor fetal growth \|\| id:finngen_R11_O15_POOR_FETGRO | CD24 on memory B cell \|\| id:ebi-a-GCST90001771 | Inverse variance weighted | 31 | 0.00183546396607897 | 0.0168677991458259 | 0.913349489422036 | -0.031225422 | 0.0348963502918977 | 1.00183714946113 | 0.969257056234849 | 1.03551237268188 | 0.995828307205886 |
| ebi-a-GCST90001771 | finngen_R11_O15_POOR_FETGRO | Poor fetal growth \|\| id:finngen_R11_O15_POOR_FETGRO | CD24 on memory B cell \|\| id:ebi-a-GCST90001771 | MR Egger | 31 | -0.025520684 | 0.0220147920232692 | 0.255810105062977 | -0.068669676 | 0.0176283085753197 | 0.974802216152609 | 0.933635031060008 | 1.01778460426574 | 0.992899239648112 |
| ebi-a-GCST90001771 | finngen_R11_O15_POOR_FETGRO | Poor fetal growth \|\| id:finngen_R11_O15_POOR_FETGRO | CD24 on memory B cell \|\| id:ebi-a-GCST90001771 | Weighted median | 31 | -0.026100901 | 0.0253312987140744 | 0.302830963896732 | -0.075750247 | 0.0235484444392422 | 0.97423678314426 | 0.927047711040678 | 1.02382789831367 | 0.999440741871876 |
| ebi-a-GCST90001771 | finngen_R11_O15_POOR_FETGRO | Poor fetal growth \|\| id:finngen_R11_O15_POOR_FETGRO | CD24 on memory B cell \|\| id:ebi-a-GCST90001771 | Weighted mode | 31 | -0.016094818 | 0.0231826493126006 | 0.492860028220128 | -0.061532811 | 0.0293431742528684 | 0.984034011101342 | 0.940322092275703 | 1.02977792711509 | 0.999857399747617 |
| ebi-a-GCST90001772 | finngen_R11_O15_POOR_FETGRO | Poor fetal growth \|\| id:finngen_R11_O15_POOR_FETGRO | CD24 on unswitched memory B cell \|\| id:ebi-a-GCST90001772 | Inverse variance weighted | 25 | 0.0233297214378426 | 0.0160571942805515 | 0.146247649487641 | -0.008142379 | 0.0548018222277235 | 1.02360398809089 | 0.991890680030524 | 1.0563312525563 | 0.994119304454654 |
| ebi-a-GCST90001772 | finngen_R11_O15_POOR_FETGRO | Poor fetal growth \|\| id:finngen_R11_O15_POOR_FETGRO | CD24 on unswitched memory B cell \|\| id:ebi-a-GCST90001772 | MR Egger | 25 | 0.0193657955085174 | 0.020001661468563 | 0.343011648752897 | -0.019837461 | 0.058569051986901 | 1.01955452888164 | 0.980358006797058 | 1.06031820024524 | 0.992899239648112 |
| ebi-a-GCST90001772 | finngen_R11_O15_POOR_FETGRO | Poor fetal growth \|\| id:finngen_R11_O15_POOR_FETGRO | CD24 on unswitched memory B cell \|\| id:ebi-a-GCST90001772 | Weighted median | 25 | 0.0316877202494278 | 0.0252450956069034 | 0.209405069463754 | -0.017792667 | 0.0811681076389584 | 1.03219512133571 | 0.982364687725514 | 1.08455320292105 | 0.999440741871876 |
| ebi-a-GCST90001772 | finngen_R11_O15_POOR_FETGRO | Poor fetal growth \|\| id:finngen_R11_O15_POOR_FETGRO | CD24 on unswitched memory B cell \|\| id:ebi-a-GCST90001772 | Weighted mode | 25 | 0.0264444855654389 | 0.0215389501520338 | 0.231446145737682 | -0.015771857 | 0.068660827863425 | 1.02679724361105 | 0.984351867691214 | 1.07107286946092 | 0.999857399747617 |
| ebi-a-GCST90001773 | finngen_R11_O15_POOR_FETGRO | Poor fetal growth \|\| id:finngen_R11_O15_POOR_FETGRO | CD24 on switched memory B cell \|\| id:ebi-a-GCST90001773 | Inverse variance weighted | 38 | -0.008138409 | 0.0165543067977279 | 0.622988808313912 | -0.040584851 | 0.0243080318908441 | 0.991894617764329 | 0.960227685009419 | 1.0246058805732 | 0.994119304454654 |
| ebi-a-GCST90001773 | finngen_R11_O15_POOR_FETGRO | Poor fetal growth \|\| id:finngen_R11_O15_POOR_FETGRO | CD24 on switched memory B cell \|\| id:ebi-a-GCST90001773 | MR Egger | 38 | -0.012932826 | 0.0230250826102585 | 0.577811550293619 | -0.058061988 | 0.0321963359372121 | 0.98715044365789 | 0.943591454375889 | 1.03272024549915 | 0.992899239648112 |
| ebi-a-GCST90001773 | finngen_R11_O15_POOR_FETGRO | Poor fetal growth \|\| id:finngen_R11_O15_POOR_FETGRO | CD24 on switched memory B cell \|\| id:ebi-a-GCST90001773 | Weighted median | 38 | -0.033379997 | 0.0249317886904785 | 0.180618577433918 | -0.082246303 | 0.0154863091626097 | 0.9671709680038 | 0.921045075048148 | 1.01560684345498 | 0.999440741871876 |
| ebi-a-GCST90001773 | finngen_R11_O15_POOR_FETGRO | Poor fetal growth \|\| id:finngen_R11_O15_POOR_FETGRO | CD24 on switched memory B cell \|\| id:ebi-a-GCST90001773 | Weighted mode | 38 | -0.035873419 | 0.0236736887286747 | 0.138186502388995 | -0.082273849 | 0.0105270110326084 | 0.964762406464556 | 0.921019704032307 | 1.01058261495634 | 0.999857399747617 |
| ebi-a-GCST90001774 | finngen_R11_O15_POOR_FETGRO | Poor fetal growth \|\| id:finngen_R11_O15_POOR_FETGRO | CD24 on transitional B cell \|\| id:ebi-a-GCST90001774 | Inverse variance weighted | 19 | 0.00471287044804116 | 0.0418211903315812 | 0.910275561621184 | -0.077256663 | 0.0866824034979403 | 1.00472399348892 | 0.925652242801843 | 1.0905502697609 | 0.995828307205886 |
| ebi-a-GCST90001774 | finngen_R11_O15_POOR_FETGRO | Poor fetal growth \|\| id:finngen_R11_O15_POOR_FETGRO | CD24 on transitional B cell \|\| id:ebi-a-GCST90001774 | MR Egger | 19 | -0.042777416 | 0.0810593330770583 | 0.604507385885955 | -0.201653709 | 0.116098877030747 | 0.958124629738195 | 0.817377929860907 | 1.12310691612039 | 0.992899239648112 |
| ebi-a-GCST90001774 | finngen_R11_O15_POOR_FETGRO | Poor fetal growth \|\| id:finngen_R11_O15_POOR_FETGRO | CD24 on transitional B cell \|\| id:ebi-a-GCST90001774 | Weighted median | 19 | 0.0389668338405804 | 0.0598228298764254 | 0.514807270817989 | -0.078285913 | 0.156219580398374 | 1.0397359990284 | 0.924700005253527 | 1.16908288259304 | 0.999440741871876 |
| ebi-a-GCST90001774 | finngen_R11_O15_POOR_FETGRO | Poor fetal growth \|\| id:finngen_R11_O15_POOR_FETGRO | CD24 on transitional B cell \|\| id:ebi-a-GCST90001774 | Weighted mode | 19 | 0.131103588615399 | 0.101715805031926 | 0.213745243377563 | -0.068259389 | 0.330466566477974 | 1.14008587511254 | 0.93401816788341 | 1.39161725898395 | 0.999857399747617 |
| ebi-a-GCST90001775 | finngen_R11_O15_POOR_FETGRO | Poor fetal growth \|\| id:finngen_R11_O15_POOR_FETGRO | CD25 on B cell \|\| id:ebi-a-GCST90001775 | Inverse variance weighted | 25 | -0.007501695 | 0.0190893044125099 | 0.694335036658645 | -0.044916731 | 0.0299133420495679 | 0.992526372883563 | 0.956077089865218 | 1.03036524074463 | 0.994119304454654 |
| ebi-a-GCST90001775 | finngen_R11_O15_POOR_FETGRO | Poor fetal growth \|\| id:finngen_R11_O15_POOR_FETGRO | CD25 on B cell \|\| id:ebi-a-GCST90001775 | MR Egger | 25 | 0.0312724374697422 | 0.0232364392172999 | 0.191472784537483 | -0.014270983 | 0.07681585833565 | 1.0317665574703 | 0.985830364403655 | 1.07984321395708 | 0.992899239648112 |
| ebi-a-GCST90001775 | finngen_R11_O15_POOR_FETGRO | Poor fetal growth \|\| id:finngen_R11_O15_POOR_FETGRO | CD25 on B cell \|\| id:ebi-a-GCST90001775 | Weighted median | 25 | 0.0275134101702046 | 0.0269304929140881 | 0.306948832683695 | -0.025270356 | 0.0802971762818173 | 1.02789539926709 | 0.975046266839529 | 1.08360904273718 | 0.999440741871876 |
| ebi-a-GCST90001775 | finngen_R11_O15_POOR_FETGRO | Poor fetal growth \|\| id:finngen_R11_O15_POOR_FETGRO | CD25 on B cell \|\| id:ebi-a-GCST90001775 | Weighted mode | 25 | 0.0159293214362326 | 0.0243327044874391 | 0.518920552605992 | -0.031762779 | 0.0636214222316133 | 1.01605686942796 | 0.968736359084114 | 1.06568887626743 | 0.999857399747617 |
| ebi-a-GCST90001776 | finngen_R11_O15_POOR_FETGRO | Poor fetal growth \|\| id:finngen_R11_O15_POOR_FETGRO | CD25 on CD20- CD38- B cell \|\| id:ebi-a-GCST90001776 | Inverse variance weighted | 19 | -0.006311613 | 0.0311075456127525 | 0.839215910542002 | -0.067282402 | 0.0546591764988887 | 0.993708263487263 | 0.934931137345965 | 1.05618058216139 | 0.994119304454654 |
| ebi-a-GCST90001776 | finngen_R11_O15_POOR_FETGRO | Poor fetal growth \|\| id:finngen_R11_O15_POOR_FETGRO | CD25 on CD20- CD38- B cell \|\| id:ebi-a-GCST90001776 | MR Egger | 19 | 0.0676880412067018 | 0.0427175352062377 | 0.131493623616276 | -0.016038328 | 0.151414410210928 | 1.0700314506864 | 0.984089601345466 | 1.16347871565009 | 0.992899239648112 |
| ebi-a-GCST90001776 | finngen_R11_O15_POOR_FETGRO | Poor fetal growth \|\| id:finngen_R11_O15_POOR_FETGRO | CD25 on CD20- CD38- B cell \|\| id:ebi-a-GCST90001776 | Weighted median | 19 | -0.025112043 | 0.0476339304373409 | 0.598062943462806 | -0.118474547 | 0.0682504604431609 | 0.975200641292753 | 0.888274424732396 | 1.07063342622332 | 0.999440741871876 |
| ebi-a-GCST90001776 | finngen_R11_O15_POOR_FETGRO | Poor fetal growth \|\| id:finngen_R11_O15_POOR_FETGRO | CD25 on CD20- CD38- B cell \|\| id:ebi-a-GCST90001776 | Weighted mode | 19 | -0.015862052 | 0.0552640428501708 | 0.777372625280569 | -0.124179576 | 0.0924554721680844 | 0.984263087993952 | 0.8832212214796 | 1.09686429948373 | 0.999857399747617 |
| ebi-a-GCST90001777 | finngen_R11_O15_POOR_FETGRO | Poor fetal growth \|\| id:finngen_R11_O15_POOR_FETGRO | CD25 on CD24+ CD27+ B cell \|\| id:ebi-a-GCST90001777 | Inverse variance weighted | 24 | 0.0049945839213649 | 0.0091916492699007 | 0.586866280319704 | -0.013021049 | 0.0230102164903703 | 1.0050070776473 | 0.987063358452798 | 1.02327699379337 | 0.994119304454654 |
| ebi-a-GCST90001777 | finngen_R11_O15_POOR_FETGRO | Poor fetal growth \|\| id:finngen_R11_O15_POOR_FETGRO | CD25 on CD24+ CD27+ B cell \|\| id:ebi-a-GCST90001777 | MR Egger | 24 | 0.00815185594238228 | 0.0101931519570102 | 0.432413237147838 | -0.011826722 | 0.0281304337781223 | 1.00818517278988 | 0.988242938891995 | 1.02852983070436 | 0.992899239648112 |
| ebi-a-GCST90001777 | finngen_R11_O15_POOR_FETGRO | Poor fetal growth \|\| id:finngen_R11_O15_POOR_FETGRO | CD25 on CD24+ CD27+ B cell \|\| id:ebi-a-GCST90001777 | Weighted median | 24 | 0.0053388275623051 | 0.0110632069147338 | 0.629397502458308 | -0.016345058 | 0.0270227131151834 | 1.00535310449823 | 0.983787797640588 | 1.02739113775173 | 0.999440741871876 |
| ebi-a-GCST90001777 | finngen_R11_O15_POOR_FETGRO | Poor fetal growth \|\| id:finngen_R11_O15_POOR_FETGRO | CD25 on CD24+ CD27+ B cell \|\| id:ebi-a-GCST90001777 | Weighted mode | 24 | 0.00636626708643945 | 0.00885494341192018 | 0.479412705169529 | -0.010989422 | 0.023721956173803 | 1.00638657483673 | 0.989070741109419 | 1.02400555988094 | 0.999857399747617 |
| ebi-a-GCST90001778 | finngen_R11_O15_POOR_FETGRO | Poor fetal growth \|\| id:finngen_R11_O15_POOR_FETGRO | CD25 on IgD+ CD24+ B cell \|\| id:ebi-a-GCST90001778 | Inverse variance weighted | 26 | -0.008450647 | 0.0137136668914133 | 0.537748791092544 | -0.035329434 | 0.0184281400920196 | 0.991584959332851 | 0.965287365276053 | 1.01859898611016 | 0.994119304454654 |
| ebi-a-GCST90001778 | finngen_R11_O15_POOR_FETGRO | Poor fetal growth \|\| id:finngen_R11_O15_POOR_FETGRO | CD25 on IgD+ CD24+ B cell \|\| id:ebi-a-GCST90001778 | MR Egger | 26 | -0.012348705 | 0.016738141472369 | 0.46781393277219 | -0.045155462 | 0.0204580525019567 | 0.987727227594154 | 0.95584887203781 | 1.02066875284469 | 0.992899239648112 |
| ebi-a-GCST90001778 | finngen_R11_O15_POOR_FETGRO | Poor fetal growth \|\| id:finngen_R11_O15_POOR_FETGRO | CD25 on IgD+ CD24+ B cell \|\| id:ebi-a-GCST90001778 | Weighted median | 26 | -0.009562755 | 0.0174656523475298 | 0.584023067253779 | -0.043795434 | 0.0246699236188999 | 0.990482822760536 | 0.957149738139354 | 1.02497674406807 | 0.999440741871876 |
| ebi-a-GCST90001778 | finngen_R11_O15_POOR_FETGRO | Poor fetal growth \|\| id:finngen_R11_O15_POOR_FETGRO | CD25 on IgD+ CD24+ B cell \|\| id:ebi-a-GCST90001778 | Weighted mode | 26 | -0.006314789 | 0.0157646092202824 | 0.692141341492188 | -0.037213423 | 0.0245838453483614 | 0.993705107652419 | 0.963470486840453 | 1.02488851963976 | 0.999857399747617 |
| ebi-a-GCST90001779 | finngen_R11_O15_POOR_FETGRO | Poor fetal growth \|\| id:finngen_R11_O15_POOR_FETGRO | CD25 on IgD+ CD24- B cell \|\| id:ebi-a-GCST90001779 | Inverse variance weighted | 24 | -0.0113768 | 0.0160911583617345 | 0.479552930685447 | -0.04291547 | 0.0201618706448487 | 0.988687671318982 | 0.957992365611664 | 1.02036649404197 | 0.994119304454654 |
| ebi-a-GCST90001779 | finngen_R11_O15_POOR_FETGRO | Poor fetal growth \|\| id:finngen_R11_O15_POOR_FETGRO | CD25 on IgD+ CD24- B cell \|\| id:ebi-a-GCST90001779 | MR Egger | 24 | 0.00145042117519469 | 0.0194703922766878 | 0.941290783281731 | -0.036711548 | 0.0396123900375029 | 1.00145147354472 | 0.963954150054233 | 1.04040742374361 | 0.992899239648112 |
| ebi-a-GCST90001779 | finngen_R11_O15_POOR_FETGRO | Poor fetal growth \|\| id:finngen_R11_O15_POOR_FETGRO | CD25 on IgD+ CD24- B cell \|\| id:ebi-a-GCST90001779 | Weighted median | 24 | -0.004595843 | 0.0242011232388956 | 0.849385897869247 | -0.052030044 | 0.0428383589983719 | 0.995414702174359 | 0.949300345541737 | 1.04376916532082 | 0.999440741871876 |
| ebi-a-GCST90001779 | finngen_R11_O15_POOR_FETGRO | Poor fetal growth \|\| id:finngen_R11_O15_POOR_FETGRO | CD25 on IgD+ CD24- B cell \|\| id:ebi-a-GCST90001779 | Weighted mode | 24 | -0.001234585 | 0.0195239003385407 | 0.95012609208651 | -0.03950143 | 0.0370322598202999 | 0.998766176943099 | 0.961268579849664 | 1.0377264971685 | 0.999857399747617 |
| ebi-a-GCST90001780 | finngen_R11_O15_POOR_FETGRO | Poor fetal growth \|\| id:finngen_R11_O15_POOR_FETGRO | CD25 on IgD+ CD38- B cell \|\| id:ebi-a-GCST90001780 | Inverse variance weighted | 27 | -0.007484816 | 0.0116465136473118 | 0.520440974782755 | -0.030311983 | 0.0153423506974838 | 0.992543125428674 | 0.970142818457131 | 1.01546064877586 | 0.994119304454654 |
| ebi-a-GCST90001780 | finngen_R11_O15_POOR_FETGRO | Poor fetal growth \|\| id:finngen_R11_O15_POOR_FETGRO | CD25 on IgD+ CD38- B cell \|\| id:ebi-a-GCST90001780 | MR Egger | 27 | 0.000550594044855391 | 0.0132583621311195 | 0.967204813780649 | -0.025435796 | 0.0265369838218495 | 1.00055074564958 | 0.974884968732121 | 1.0268922249585 | 0.992899239648112 |
| ebi-a-GCST90001780 | finngen_R11_O15_POOR_FETGRO | Poor fetal growth \|\| id:finngen_R11_O15_POOR_FETGRO | CD25 on IgD+ CD38- B cell \|\| id:ebi-a-GCST90001780 | Weighted median | 27 | -0.002109414 | 0.017938001513935 | 0.906388821270584 | -0.037267896 | 0.0330490694381686 | 0.997892809720049 | 0.963418004466398 | 1.0336012562299 | 0.999440741871876 |
| ebi-a-GCST90001780 | finngen_R11_O15_POOR_FETGRO | Poor fetal growth \|\| id:finngen_R11_O15_POOR_FETGRO | CD25 on IgD+ CD38- B cell \|\| id:ebi-a-GCST90001780 | Weighted mode | 27 | 0.00162001102643061 | 0.0125270625051999 | 0.898098980516522 | -0.022933031 | 0.0261730535366225 | 1.00162132395318 | 0.977327931783245 | 1.02651857577343 | 0.999857399747617 |
| ebi-a-GCST90001781 | finngen_R11_O15_POOR_FETGRO | Poor fetal growth \|\| id:finngen_R11_O15_POOR_FETGRO | CD25 on IgD+ CD38- naive B cell \|\| id:ebi-a-GCST90001781 | Inverse variance weighted | 25 | -0.000861557 | 0.0176831158318039 | 0.961140811815511 | -0.035520464 | 0.033797349563734 | 0.999138813567469 | 0.965102983680855 | 1.03437496894852 | 0.998984604424952 |
| ebi-a-GCST90001781 | finngen_R11_O15_POOR_FETGRO | Poor fetal growth \|\| id:finngen_R11_O15_POOR_FETGRO | CD25 on IgD+ CD38- naive B cell \|\| id:ebi-a-GCST90001781 | MR Egger | 25 | 0.00575619187436899 | 0.0261282423938496 | 0.827577121894896 | -0.045455163 | 0.0569675469663142 | 1.00577279057998 | 0.955562445956949 | 1.05862145436031 | 0.992899239648112 |
| ebi-a-GCST90001781 | finngen_R11_O15_POOR_FETGRO | Poor fetal growth \|\| id:finngen_R11_O15_POOR_FETGRO | CD25 on IgD+ CD38- naive B cell \|\| id:ebi-a-GCST90001781 | Weighted median | 25 | 0.00127498494625919 | 0.0250850516069125 | 0.959463787852221 | -0.047891716 | 0.0504416860958077 | 1.00127579808511 | 0.953237001610873 | 1.05173553076177 | 0.999440741871876 |
| ebi-a-GCST90001781 | finngen_R11_O15_POOR_FETGRO | Poor fetal growth \|\| id:finngen_R11_O15_POOR_FETGRO | CD25 on IgD+ CD38- naive B cell \|\| id:ebi-a-GCST90001781 | Weighted mode | 25 | 0.00297558570743528 | 0.0224831790236892 | 0.895813021110035 | -0.041091445 | 0.0470426165938662 | 1.00298001715688 | 0.959741362214235 | 1.04816667742144 | 0.999857399747617 |
| ebi-a-GCST90001782 | finngen_R11_O15_POOR_FETGRO | Poor fetal growth \|\| id:finngen_R11_O15_POOR_FETGRO | CD25 on IgD+ CD38- unswitched memory B cell \|\| id:ebi-a-GCST90001782 | Inverse variance weighted | 24 | -0.008024723 | 0.0140939476826063 | 0.569102546472684 | -0.035648861 | 0.019599414016255 | 0.992007388697082 | 0.964979075884416 | 1.01979274351383 | 0.994119304454654 |
| ebi-a-GCST90001782 | finngen_R11_O15_POOR_FETGRO | Poor fetal growth \|\| id:finngen_R11_O15_POOR_FETGRO | CD25 on IgD+ CD38- unswitched memory B cell \|\| id:ebi-a-GCST90001782 | MR Egger | 24 | -0.013613639 | 0.0182550586832348 | 0.463714981687278 | -0.049393554 | 0.0221662755645283 | 0.986478607056943 | 0.951806468284763 | 1.02241377276484 | 0.992899239648112 |
| ebi-a-GCST90001782 | finngen_R11_O15_POOR_FETGRO | Poor fetal growth \|\| id:finngen_R11_O15_POOR_FETGRO | CD25 on IgD+ CD38- unswitched memory B cell \|\| id:ebi-a-GCST90001782 | Weighted median | 24 | -0.009470709 | 0.0202407731989149 | 0.639854308224571 | -0.049142624 | 0.0302012068792349 | 0.990573997326414 | 0.952045335443439 | 1.03066188935445 | 0.999440741871876 |
| ebi-a-GCST90001782 | finngen_R11_O15_POOR_FETGRO | Poor fetal growth \|\| id:finngen_R11_O15_POOR_FETGRO | CD25 on IgD+ CD38- unswitched memory B cell \|\| id:ebi-a-GCST90001782 | Weighted mode | 24 | -0.015847463 | 0.0170656203753746 | 0.362730323526676 | -0.049296079 | 0.0176011528932676 | 0.984277447292197 | 0.951899250613857 | 1.01775696600589 | 0.999857399747617 |
| ebi-a-GCST90001783 | finngen_R11_O15_POOR_FETGRO | Poor fetal growth \|\| id:finngen_R11_O15_POOR_FETGRO | CD25 on IgD+ CD38+ B cell \|\| id:ebi-a-GCST90001783 | Inverse variance weighted | 13 | 0.00972236090577297 | 0.0461237996436939 | 0.833052407950547 | -0.080680286 | 0.100125008207413 | 1.00976977659614 | 0.922488576450271 | 1.10530908214664 | 0.994119304454654 |
| ebi-a-GCST90001783 | finngen_R11_O15_POOR_FETGRO | Poor fetal growth \|\| id:finngen_R11_O15_POOR_FETGRO | CD25 on IgD+ CD38+ B cell \|\| id:ebi-a-GCST90001783 | MR Egger | 13 | 0.162808709067817 | 0.102716895759408 | 0.141267870031533 | -0.038516407 | 0.364133824756257 | 1.1768115547767 | 0.962215917898792 | 1.4392668108008 | 0.992899239648112 |
| ebi-a-GCST90001783 | finngen_R11_O15_POOR_FETGRO | Poor fetal growth \|\| id:finngen_R11_O15_POOR_FETGRO | CD25 on IgD+ CD38+ B cell \|\| id:ebi-a-GCST90001783 | Weighted median | 13 | -0.05219836 | 0.0591074310231122 | 0.377176869024885 | -0.168048925 | 0.0636522043838361 | 0.949140576244074 | 0.845312476568337 | 1.06572168096954 | 0.999440741871876 |
| ebi-a-GCST90001783 | finngen_R11_O15_POOR_FETGRO | Poor fetal growth \|\| id:finngen_R11_O15_POOR_FETGRO | CD25 on IgD+ CD38+ B cell \|\| id:ebi-a-GCST90001783 | Weighted mode | 13 | -0.117383335 | 0.120051570739774 | 0.347486881452545 | -0.352684414 | 0.117917743342212 | 0.889244249101747 | 0.702798951923217 | 1.12515155635423 | 0.999857399747617 |
| ebi-a-GCST90001784 | finngen_R11_O15_POOR_FETGRO | Poor fetal growth \|\| id:finngen_R11_O15_POOR_FETGRO | CD25 on IgD+ CD38dim B cell \|\| id:ebi-a-GCST90001784 | Inverse variance weighted | 23 | -0.062125752 | 0.0175416308261858 | 0.000397681277013212 | -0.096507348 | -0.027744155 | 0.939764702485622 | 0.908003225588897 | 0.97263717919626 | 0.290705013496658 |
| ebi-a-GCST90001784 | finngen_R11_O15_POOR_FETGRO | Poor fetal growth \|\| id:finngen_R11_O15_POOR_FETGRO | CD25 on IgD+ CD38dim B cell \|\| id:ebi-a-GCST90001784 | MR Egger | 23 | -0.036482069 | 0.022666120664062 | 0.122428588362921 | -0.080907665 | 0.00794352750601995 | 0.964175382374404 | 0.922278845671958 | 1.00797516102574 | 0.992899239648112 |
| ebi-a-GCST90001784 | finngen_R11_O15_POOR_FETGRO | Poor fetal growth \|\| id:finngen_R11_O15_POOR_FETGRO | CD25 on IgD+ CD38dim B cell \|\| id:ebi-a-GCST90001784 | Weighted median | 23 | -0.062855219 | 0.0264442442649309 | 0.0174590081020896 | -0.114685938 | -0.011024501 | 0.939079424336462 | 0.891646131969477 | 0.989036046468589 | 0.999440741871876 |
| ebi-a-GCST90001784 | finngen_R11_O15_POOR_FETGRO | Poor fetal growth \|\| id:finngen_R11_O15_POOR_FETGRO | CD25 on IgD+ CD38dim B cell \|\| id:ebi-a-GCST90001784 | Weighted mode | 23 | -0.068704106 | 0.0238203645527872 | 0.0086106925217816 | -0.11539202 | -0.022016191 | 0.933602887063139 | 0.891016778910156 | 0.978224396401088 | 0.999857399747617 |
| ebi-a-GCST90001785 | finngen_R11_O15_POOR_FETGRO | Poor fetal growth \|\| id:finngen_R11_O15_POOR_FETGRO | CD25 on IgD- CD24- B cell \|\| id:ebi-a-GCST90001785 | Inverse variance weighted | 26 | -0.010646594 | 0.0390677769022167 | 0.785225305248955 | -0.087219436 | 0.0659262491906976 | 0.98940988084172 | 0.916475965502596 | 1.06814793748615 | 0.994119304454654 |
| ebi-a-GCST90001785 | finngen_R11_O15_POOR_FETGRO | Poor fetal growth \|\| id:finngen_R11_O15_POOR_FETGRO | CD25 on IgD- CD24- B cell \|\| id:ebi-a-GCST90001785 | MR Egger | 26 | -0.074855524 | 0.071797987938739 | 0.307529309457905 | -0.21557958 | 0.0658685324797671 | 0.927877532790687 | 0.8060741201336 | 1.0680862892795 | 0.992899239648112 |
| ebi-a-GCST90001785 | finngen_R11_O15_POOR_FETGRO | Poor fetal growth \|\| id:finngen_R11_O15_POOR_FETGRO | CD25 on IgD- CD24- B cell \|\| id:ebi-a-GCST90001785 | Weighted median | 26 | -0.029573802 | 0.048965166954416 | 0.545859373212244 | -0.12554553 | 0.0663979249549548 | 0.970859223377033 | 0.882015605777709 | 1.06865187581931 | 0.999440741871876 |
| ebi-a-GCST90001785 | finngen_R11_O15_POOR_FETGRO | Poor fetal growth \|\| id:finngen_R11_O15_POOR_FETGRO | CD25 on IgD- CD24- B cell \|\| id:ebi-a-GCST90001785 | Weighted mode | 26 | -0.027667051 | 0.0629891916385123 | 0.664264522603984 | -0.151125866 | 0.0957917648407412 | 0.972712176661642 | 0.859739479550826 | 1.10052987111882 | 0.999857399747617 |
| ebi-a-GCST90001786 | finngen_R11_O15_POOR_FETGRO | Poor fetal growth \|\| id:finngen_R11_O15_POOR_FETGRO | CD25 on IgD- CD27- B cell \|\| id:ebi-a-GCST90001786 | Inverse variance weighted | 21 | -0.053956793 | 0.0350402160390949 | 0.123596110412681 | -0.122635617 | 0.0147220302853277 | 0.94747304294184 | 0.884585932282664 | 1.01483093314061 | 0.994119304454654 |
| ebi-a-GCST90001786 | finngen_R11_O15_POOR_FETGRO | Poor fetal growth \|\| id:finngen_R11_O15_POOR_FETGRO | CD25 on IgD- CD27- B cell \|\| id:ebi-a-GCST90001786 | MR Egger | 21 | -0.079065702 | 0.0682833169615307 | 0.261250938808466 | -0.212901003 | 0.0547695995785181 | 0.923979215476887 | 0.808236146282054 | 1.05629721531329 | 0.992899239648112 |
| ebi-a-GCST90001786 | finngen_R11_O15_POOR_FETGRO | Poor fetal growth \|\| id:finngen_R11_O15_POOR_FETGRO | CD25 on IgD- CD27- B cell \|\| id:ebi-a-GCST90001786 | Weighted median | 21 | -0.06779681 | 0.0504605119652704 | 0.17909017026939 | -0.166699413 | 0.031105793573489 | 0.934450325363758 | 0.846454005892092 | 1.03159463419652 | 0.999440741871876 |
| ebi-a-GCST90001786 | finngen_R11_O15_POOR_FETGRO | Poor fetal growth \|\| id:finngen_R11_O15_POOR_FETGRO | CD25 on IgD- CD27- B cell \|\| id:ebi-a-GCST90001786 | Weighted mode | 21 | -0.033852696 | 0.0653698197188172 | 0.610234294700567 | -0.161977542 | 0.0942721510524305 | 0.966713895363618 | 0.850460303754625 | 1.09885876079494 | 0.999857399747617 |
| ebi-a-GCST90001787 | finngen_R11_O15_POOR_FETGRO | Poor fetal growth \|\| id:finngen_R11_O15_POOR_FETGRO | CD25 on IgD- CD38- B cell \|\| id:ebi-a-GCST90001787 | Inverse variance weighted | 22 | -0.025266788 | 0.0247231831639621 | 0.306786743389773 | -0.073724227 | 0.0231906510206493 | 0.975049745772488 | 0.928927831753292 | 1.02346164495531 | 0.994119304454654 |
| ebi-a-GCST90001787 | finngen_R11_O15_POOR_FETGRO | Poor fetal growth \|\| id:finngen_R11_O15_POOR_FETGRO | CD25 on IgD- CD38- B cell \|\| id:ebi-a-GCST90001787 | MR Egger | 22 | -0.049262935 | 0.0317443034791488 | 0.13637510302207 | -0.11148177 | 0.0129558995699252 | 0.951930800627489 | 0.89450769806884 | 1.01304019086659 | 0.992899239648112 |
| ebi-a-GCST90001787 | finngen_R11_O15_POOR_FETGRO | Poor fetal growth \|\| id:finngen_R11_O15_POOR_FETGRO | CD25 on IgD- CD38- B cell \|\| id:ebi-a-GCST90001787 | Weighted median | 22 | -0.05029642 | 0.0305680100724142 | 0.0998885854276185 | -0.11020972 | 0.00961687927915419 | 0.950947502420297 | 0.895646280477452 | 1.00966327005493 | 0.999440741871876 |
| ebi-a-GCST90001787 | finngen_R11_O15_POOR_FETGRO | Poor fetal growth \|\| id:finngen_R11_O15_POOR_FETGRO | CD25 on IgD- CD38- B cell \|\| id:ebi-a-GCST90001787 | Weighted mode | 22 | -0.053943627 | 0.0279515666755087 | 0.0672383554186102 | -0.108728698 | 0.00084144341782421 | 0.947485517345201 | 0.896973735303465 | 1.00084179753065 | 0.999857399747617 |
| ebi-a-GCST90001788 | finngen_R11_O15_POOR_FETGRO | Poor fetal growth \|\| id:finngen_R11_O15_POOR_FETGRO | CD25 on IgD- CD38+ B cell \|\| id:ebi-a-GCST90001788 | Inverse variance weighted | 19 | 0.0290521974770099 | 0.0398721698092938 | 0.466225898664566 | -0.049097255 | 0.107201650303226 | 1.02947832924413 | 0.952088529493235 | 1.11315870063827 | 0.994119304454654 |
| ebi-a-GCST90001788 | finngen_R11_O15_POOR_FETGRO | Poor fetal growth \|\| id:finngen_R11_O15_POOR_FETGRO | CD25 on IgD- CD38+ B cell \|\| id:ebi-a-GCST90001788 | MR Egger | 19 | 0.00261332571508591 | 0.0672945424449752 | 0.969475034738688 | -0.129283977 | 0.134510628907237 | 1.00261674342728 | 0.878724392174727 | 1.14397681816126 | 0.992899239648112 |
| ebi-a-GCST90001788 | finngen_R11_O15_POOR_FETGRO | Poor fetal growth \|\| id:finngen_R11_O15_POOR_FETGRO | CD25 on IgD- CD38+ B cell \|\| id:ebi-a-GCST90001788 | Weighted median | 19 | 0.00568892921360627 | 0.0586804477719714 | 0.922767998881733 | -0.109324748 | 0.12070260684667 | 1.0057051419011 | 0.896439252992639 | 1.12828931695009 | 0.999440741871876 |
| ebi-a-GCST90001788 | finngen_R11_O15_POOR_FETGRO | Poor fetal growth \|\| id:finngen_R11_O15_POOR_FETGRO | CD25 on IgD- CD38+ B cell \|\| id:ebi-a-GCST90001788 | Weighted mode | 19 | -0.003951916 | 0.0647404776541468 | 0.951998094766652 | -0.130843252 | 0.122939420330883 | 0.996055882671838 | 0.877355287235525 | 1.13081591441853 | 0.999857399747617 |
| ebi-a-GCST90001789 | finngen_R11_O15_POOR_FETGRO | Poor fetal growth \|\| id:finngen_R11_O15_POOR_FETGRO | CD25 on IgD- CD38dim B cell \|\| id:ebi-a-GCST90001789 | Inverse variance weighted | 20 | -0.000460226 | 0.0367165858076497 | 0.989999136598771 | -0.072424734 | 0.0715042822397181 | 0.999539879944439 | 0.930135751505783 | 1.07412275034257 | 0.998984604424952 |
| ebi-a-GCST90001789 | finngen_R11_O15_POOR_FETGRO | Poor fetal growth \|\| id:finngen_R11_O15_POOR_FETGRO | CD25 on IgD- CD38dim B cell \|\| id:ebi-a-GCST90001789 | MR Egger | 20 | 0.0294119876926466 | 0.0570463478118988 | 0.612421998949743 | -0.082398854 | 0.141222829403968 | 1.02984879211467 | 0.920904578943646 | 1.1516812478407 | 0.992899239648112 |
| ebi-a-GCST90001789 | finngen_R11_O15_POOR_FETGRO | Poor fetal growth \|\| id:finngen_R11_O15_POOR_FETGRO | CD25 on IgD- CD38dim B cell \|\| id:ebi-a-GCST90001789 | Weighted median | 20 | 0.0409357263238639 | 0.0466348746425893 | 0.380056531728628 | -0.050468628 | 0.132340080623339 | 1.04178514403262 | 0.950783756215638 | 1.14149645409057 | 0.999440741871876 |
| ebi-a-GCST90001789 | finngen_R11_O15_POOR_FETGRO | Poor fetal growth \|\| id:finngen_R11_O15_POOR_FETGRO | CD25 on IgD- CD38dim B cell \|\| id:ebi-a-GCST90001789 | Weighted mode | 20 | 0.000156584736203613 | 0.0577752492024483 | 0.997865798956728 | -0.113082904 | 0.113396073173002 | 1.00015659699623 | 0.893076617690341 | 1.12007547695301 | 0.999857399747617 |
| ebi-a-GCST90001790 | finngen_R11_O15_POOR_FETGRO | Poor fetal growth \|\| id:finngen_R11_O15_POOR_FETGRO | CD25 on memory B cell \|\| id:ebi-a-GCST90001790 | Inverse variance weighted | 24 | -0.000973212 | 0.0219225251533868 | 0.964590964647369 | -0.043941361 | 0.0419949377819274 | 0.999027261898029 | 0.957010074114335 | 1.04288919940485 | 0.998984604424952 |
| ebi-a-GCST90001790 | finngen_R11_O15_POOR_FETGRO | Poor fetal growth \|\| id:finngen_R11_O15_POOR_FETGRO | CD25 on memory B cell \|\| id:ebi-a-GCST90001790 | MR Egger | 24 | 0.00257112820389995 | 0.0297699915413096 | 0.931956364358239 | -0.055778055 | 0.0609203116248668 | 1.00257443638867 | 0.945749016658775 | 1.06281421687453 | 0.992899239648112 |
| ebi-a-GCST90001790 | finngen_R11_O15_POOR_FETGRO | Poor fetal growth \|\| id:finngen_R11_O15_POOR_FETGRO | CD25 on memory B cell \|\| id:ebi-a-GCST90001790 | Weighted median | 24 | 0.0127365581296828 | 0.030010020935027 | 0.671266704048214 | -0.046083083 | 0.0715561991623358 | 1.01281801353934 | 0.954962617828163 | 1.07417851693788 | 0.999440741871876 |
| ebi-a-GCST90001790 | finngen_R11_O15_POOR_FETGRO | Poor fetal growth \|\| id:finngen_R11_O15_POOR_FETGRO | CD25 on memory B cell \|\| id:ebi-a-GCST90001790 | Weighted mode | 24 | 0.0151755887509883 | 0.0281369632342473 | 0.594830664565696 | -0.039972859 | 0.070324036690113 | 1.01529132270045 | 0.960815516111605 | 1.07285576956803 | 0.999857399747617 |
| ebi-a-GCST90001791 | finngen_R11_O15_POOR_FETGRO | Poor fetal growth \|\| id:finngen_R11_O15_POOR_FETGRO | CD25 on naive-mature B cell \|\| id:ebi-a-GCST90001791 | Inverse variance weighted | 23 | -0.021514533 | 0.03274606379707 | 0.511173660539427 | -0.085696818 | 0.0426677516018151 | 0.978715253265941 | 0.917872471007888 | 1.04359110577049 | 0.994119304454654 |
| ebi-a-GCST90001791 | finngen_R11_O15_POOR_FETGRO | Poor fetal growth \|\| id:finngen_R11_O15_POOR_FETGRO | CD25 on naive-mature B cell \|\| id:ebi-a-GCST90001791 | MR Egger | 23 | 0.0126867837681498 | 0.0482806751655351 | 0.795287481829588 | -0.08194334 | 0.107316907092598 | 1.01276760242397 | 0.921324159853154 | 1.11328700713012 | 0.992899239648112 |
| ebi-a-GCST90001791 | finngen_R11_O15_POOR_FETGRO | Poor fetal growth \|\| id:finngen_R11_O15_POOR_FETGRO | CD25 on naive-mature B cell \|\| id:ebi-a-GCST90001791 | Weighted median | 23 | -0.014472206 | 0.0465820445017571 | 0.756042335562725 | -0.105773013 | 0.076828601599686 | 0.985632013378688 | 0.899628829088304 | 1.07985697477195 | 0.999440741871876 |
| ebi-a-GCST90001791 | finngen_R11_O15_POOR_FETGRO | Poor fetal growth \|\| id:finngen_R11_O15_POOR_FETGRO | CD25 on naive-mature B cell \|\| id:ebi-a-GCST90001791 | Weighted mode | 23 | -0.016494566 | 0.0424596027204806 | 0.701397286433003 | -0.099715387 | 0.0667262552440719 | 0.983640724393217 | 0.905094982799143 | 1.06900280420572 | 0.999857399747617 |
| ebi-a-GCST90001792 | finngen_R11_O15_POOR_FETGRO | Poor fetal growth \|\| id:finngen_R11_O15_POOR_FETGRO | CD25 on unswitched memory B cell \|\| id:ebi-a-GCST90001792 | Inverse variance weighted | 24 | -0.000542966 | 0.0136603695103138 | 0.968294376307634 | -0.027317291 | 0.0262313577479624 | 0.999457180887378 | 0.973052452013673 | 1.02657842787421 | 0.998984604424952 |
| ebi-a-GCST90001792 | finngen_R11_O15_POOR_FETGRO | Poor fetal growth \|\| id:finngen_R11_O15_POOR_FETGRO | CD25 on unswitched memory B cell \|\| id:ebi-a-GCST90001792 | MR Egger | 24 | 0.00141031980962197 | 0.0161402330819299 | 0.931160612871988 | -0.030224537 | 0.0330451766502046 | 1.00141131477829 | 0.970227657051282 | 1.0335972326472 | 0.992899239648112 |
| ebi-a-GCST90001792 | finngen_R11_O15_POOR_FETGRO | Poor fetal growth \|\| id:finngen_R11_O15_POOR_FETGRO | CD25 on unswitched memory B cell \|\| id:ebi-a-GCST90001792 | Weighted median | 24 | -0.005938407 | 0.0177101144451421 | 0.737390052264204 | -0.040650231 | 0.0287734176908001 | 0.994079190864017 | 0.960164907204775 | 1.02919137149647 | 0.999440741871876 |
| ebi-a-GCST90001792 | finngen_R11_O15_POOR_FETGRO | Poor fetal growth \|\| id:finngen_R11_O15_POOR_FETGRO | CD25 on unswitched memory B cell \|\| id:ebi-a-GCST90001792 | Weighted mode | 24 | -0.002275825 | 0.0155975024345392 | 0.88526404025625 | -0.03284693 | 0.0282952799496994 | 0.99772676290387 | 0.967686672445023 | 1.02869939388484 | 0.999857399747617 |
| ebi-a-GCST90001793 | finngen_R11_O15_POOR_FETGRO | Poor fetal growth \|\| id:finngen_R11_O15_POOR_FETGRO | CD25 on switched memory B cell \|\| id:ebi-a-GCST90001793 | Inverse variance weighted | 20 | -0.042733328 | 0.0347340348868248 | 0.218584295733459 | -0.110812037 | 0.0253453799207068 | 0.958166871838372 | 0.895106980258334 | 1.02566930494003 | 0.994119304454654 |
| ebi-a-GCST90001793 | finngen_R11_O15_POOR_FETGRO | Poor fetal growth \|\| id:finngen_R11_O15_POOR_FETGRO | CD25 on switched memory B cell \|\| id:ebi-a-GCST90001793 | MR Egger | 20 | -0.038989191 | 0.06760602859747 | 0.571276700219801 | -0.171497007 | 0.0935186249828755 | 0.961761104700406 | 0.84240278922738 | 1.09803105396043 | 0.992899239648112 |
| ebi-a-GCST90001793 | finngen_R11_O15_POOR_FETGRO | Poor fetal growth \|\| id:finngen_R11_O15_POOR_FETGRO | CD25 on switched memory B cell \|\| id:ebi-a-GCST90001793 | Weighted median | 20 | -0.042730661 | 0.0511273238126981 | 0.403284550075242 | -0.142940216 | 0.0574788933693576 | 0.958169427420327 | 0.866805888490159 | 1.05916291505837 | 0.999440741871876 |
| ebi-a-GCST90001793 | finngen_R11_O15_POOR_FETGRO | Poor fetal growth \|\| id:finngen_R11_O15_POOR_FETGRO | CD25 on switched memory B cell \|\| id:ebi-a-GCST90001793 | Weighted mode | 20 | -0.065559797 | 0.0650098049760094 | 0.325904872493941 | -0.192979015 | 0.0618594207612227 | 0.936543042628606 | 0.824499276285342 | 1.06381278422432 | 0.999857399747617 |
| ebi-a-GCST90001794 | finngen_R11_O15_POOR_FETGRO | Poor fetal growth \|\| id:finngen_R11_O15_POOR_FETGRO | CD25 on IgD+ B cell \|\| id:ebi-a-GCST90001794 | Inverse variance weighted | 27 | -0.033425655 | 0.0154497788386477 | 0.0305024541664441 | -0.063707221 | -0.003144088 | 0.967126809846641 | 0.938279667649606 | 0.996860849246747 | 0.994119304454654 |
| ebi-a-GCST90001794 | finngen_R11_O15_POOR_FETGRO | Poor fetal growth \|\| id:finngen_R11_O15_POOR_FETGRO | CD25 on IgD+ B cell \|\| id:ebi-a-GCST90001794 | MR Egger | 27 | -0.013578518 | 0.018959613755212 | 0.480516490584945 | -0.050739361 | 0.023582324872122 | 0.986513254142087 | 0.950526382449213 | 1.02386258663368 | 0.992899239648112 |
| ebi-a-GCST90001794 | finngen_R11_O15_POOR_FETGRO | Poor fetal growth \|\| id:finngen_R11_O15_POOR_FETGRO | CD25 on IgD+ B cell \|\| id:ebi-a-GCST90001794 | Weighted median | 27 | -0.042160404 | 0.0257075082103033 | 0.101004434452008 | -0.09254712 | 0.00822631206117525 | 0.958715986329396 | 0.911606254953367 | 1.00826024113949 | 0.999440741871876 |
| ebi-a-GCST90001794 | finngen_R11_O15_POOR_FETGRO | Poor fetal growth \|\| id:finngen_R11_O15_POOR_FETGRO | CD25 on IgD+ B cell \|\| id:ebi-a-GCST90001794 | Weighted mode | 27 | -0.019238414 | 0.022907917698878 | 0.408668420720668 | -0.064137933 | 0.0256611047676579 | 0.980945463306217 | 0.93787562697368 | 1.02599318535021 | 0.999857399747617 |
| ebi-a-GCST90001795 | finngen_R11_O15_POOR_FETGRO | Poor fetal growth \|\| id:finngen_R11_O15_POOR_FETGRO | CD25 on transitional B cell \|\| id:ebi-a-GCST90001795 | Inverse variance weighted (multiplicative random effects) | 22 | -0.004949642 | 0.0403784546897203 | 0.902438694862077 | -0.084091413 | 0.0741921293389026 | 0.995062587439095 | 0.919347211925945 | 1.07701371155161 | 0.994119304454654 |
| ebi-a-GCST90001795 | finngen_R11_O15_POOR_FETGRO | Poor fetal growth \|\| id:finngen_R11_O15_POOR_FETGRO | CD25 on transitional B cell \|\| id:ebi-a-GCST90001795 | MR Egger | 22 | -0.074308292 | 0.0602211117738624 | 0.231535206263081 | -0.192341671 | 0.0437250872355209 | 0.928385436062613 | 0.825024933303547 | 1.04469511538514 | 0.992899239648112 |
| ebi-a-GCST90001795 | finngen_R11_O15_POOR_FETGRO | Poor fetal growth \|\| id:finngen_R11_O15_POOR_FETGRO | CD25 on transitional B cell \|\| id:ebi-a-GCST90001795 | Weighted median | 22 | -0.071367744 | 0.0476350046426953 | 0.134075953207648 | -0.164732353 | 0.0219968653330921 | 0.931119415798573 | 0.848120670779657 | 1.0222405800817 | 0.999440741871876 |
| ebi-a-GCST90001795 | finngen_R11_O15_POOR_FETGRO | Poor fetal growth \|\| id:finngen_R11_O15_POOR_FETGRO | CD25 on transitional B cell \|\| id:ebi-a-GCST90001795 | Weighted mode | 22 | -0.064894148 | 0.0422236016335094 | 0.13924516167177 | -0.147652407 | 0.0178641114498003 | 0.937166659325008 | 0.862730942106536 | 1.01802462909918 | 0.999857399747617 |
| ebi-a-GCST90001796 | finngen_R11_O15_POOR_FETGRO | Poor fetal growth \|\| id:finngen_R11_O15_POOR_FETGRO | CD27 on CD20- B cell \|\| id:ebi-a-GCST90001796 | Inverse variance weighted | 15 | 0.0276202376781994 | 0.023936030285312 | 0.248533420789524 | -0.019294382 | 0.0745348570374109 | 1.02800521263651 | 0.980890563523386 | 1.0773828972437 | 0.994119304454654 |
| ebi-a-GCST90001796 | finngen_R11_O15_POOR_FETGRO | Poor fetal growth \|\| id:finngen_R11_O15_POOR_FETGRO | CD27 on CD20- B cell \|\| id:ebi-a-GCST90001796 | MR Egger | 15 | -2.24E-05 | 0.0304700545036551 | 0.999424937578259 | -0.059743694 | 0.0596989200364855 | 0.999977613459904 | 0.94200594478099 | 1.06151689695912 | 0.999424937578259 |
| ebi-a-GCST90001796 | finngen_R11_O15_POOR_FETGRO | Poor fetal growth \|\| id:finngen_R11_O15_POOR_FETGRO | CD27 on CD20- B cell \|\| id:ebi-a-GCST90001796 | Weighted median | 15 | 0.00479117888647024 | 0.0339535904505066 | 0.887783329137623 | -0.061757858 | 0.0713402161694631 | 1.00480267493658 | 0.94011049909647 | 1.0739465376996 | 0.999440741871876 |
| ebi-a-GCST90001796 | finngen_R11_O15_POOR_FETGRO | Poor fetal growth \|\| id:finngen_R11_O15_POOR_FETGRO | CD27 on CD20- B cell \|\| id:ebi-a-GCST90001796 | Weighted mode | 15 | -0.008865028 | 0.0329329710604384 | 0.791714665715914 | -0.073413651 | 0.0556835951288552 | 0.99117415035412 | 0.929216378834798 | 1.05726310761131 | 0.999857399747617 |
| ebi-a-GCST90001797 | finngen_R11_O15_POOR_FETGRO | Poor fetal growth \|\| id:finngen_R11_O15_POOR_FETGRO | CD27 on CD20- CD38- B cell \|\| id:ebi-a-GCST90001797 | Inverse variance weighted | 20 | 0.0299156189932621 | 0.0299386728099755 | 0.317683303045207 | -0.02876418 | 0.0885954177008142 | 1.03036758683094 | 0.971645571187743 | 1.09263850469059 | 0.994119304454654 |
| ebi-a-GCST90001797 | finngen_R11_O15_POOR_FETGRO | Poor fetal growth \|\| id:finngen_R11_O15_POOR_FETGRO | CD27 on CD20- CD38- B cell \|\| id:ebi-a-GCST90001797 | MR Egger | 20 | 0.0716122060693948 | 0.0401637373080625 | 0.0914578875830582 | -0.007108719 | 0.150333131193197 | 1.07423868 | 0.992916488123274 | 1.16222135043111 | 0.992899239648112 |
| ebi-a-GCST90001797 | finngen_R11_O15_POOR_FETGRO | Poor fetal growth \|\| id:finngen_R11_O15_POOR_FETGRO | CD27 on CD20- CD38- B cell \|\| id:ebi-a-GCST90001797 | Weighted median | 20 | 0.0637245976195731 | 0.0463463951799158 | 0.169142788372278 | -0.027114337 | 0.154563532172208 | 1.06579883480309 | 0.973249956747321 | 1.16714842717691 | 0.999440741871876 |
| ebi-a-GCST90001797 | finngen_R11_O15_POOR_FETGRO | Poor fetal growth \|\| id:finngen_R11_O15_POOR_FETGRO | CD27 on CD20- CD38- B cell \|\| id:ebi-a-GCST90001797 | Weighted mode | 20 | 0.0714359780599516 | 0.04297789926158 | 0.112891576488507 | -0.012800704 | 0.155672660612648 | 1.07404938577472 | 0.987280876057831 | 1.16844366285029 | 0.999857399747617 |
| ebi-a-GCST90001798 | finngen_R11_O15_POOR_FETGRO | Poor fetal growth \|\| id:finngen_R11_O15_POOR_FETGRO | CD27 on CD24+ CD27+ B cell \|\| id:ebi-a-GCST90001798 | Inverse variance weighted | 29 | -0.023559902 | 0.0215197359203151 | 0.273602324530075 | -0.065738585 | 0.0186187801511802 | 0.976715465459878 | 0.936375615252545 | 1.0187931903921 | 0.994119304454654 |
| ebi-a-GCST90001798 | finngen_R11_O15_POOR_FETGRO | Poor fetal growth \|\| id:finngen_R11_O15_POOR_FETGRO | CD27 on CD24+ CD27+ B cell \|\| id:ebi-a-GCST90001798 | MR Egger | 29 | -0.032939234 | 0.0384567290294255 | 0.39924459523086 | -0.108314423 | 0.0424359548105506 | 0.967597354739201 | 0.897345406048225 | 1.04334923273456 | 0.992899239648112 |
| ebi-a-GCST90001798 | finngen_R11_O15_POOR_FETGRO | Poor fetal growth \|\| id:finngen_R11_O15_POOR_FETGRO | CD27 on CD24+ CD27+ B cell \|\| id:ebi-a-GCST90001798 | Weighted median | 29 | -0.020039905 | 0.0310742104603923 | 0.518988934894292 | -0.080945357 | 0.0408655476067753 | 0.98015955936146 | 0.922244083884266 | 1.04171203545309 | 0.999440741871876 |
| ebi-a-GCST90001798 | finngen_R11_O15_POOR_FETGRO | Poor fetal growth \|\| id:finngen_R11_O15_POOR_FETGRO | CD27 on CD24+ CD27+ B cell \|\| id:ebi-a-GCST90001798 | Weighted mode | 29 | -0.014994532 | 0.0304149546456339 | 0.625857829050167 | -0.074607843 | 0.0446187788592211 | 0.985117325967318 | 0.928107378451361 | 1.0456291679744 | 0.999857399747617 |
| ebi-a-GCST90001799 | finngen_R11_O15_POOR_FETGRO | Poor fetal growth \|\| id:finngen_R11_O15_POOR_FETGRO | CD27 on T cell \|\| id:ebi-a-GCST90001799 | Inverse variance weighted | 21 | -0.007639854 | 0.0156873239772789 | 0.626252597930168 | -0.038387009 | 0.0231073013092179 | 0.992389255817969 | 0.962340434711287 | 1.02337634327753 | 0.994119304454654 |
| ebi-a-GCST90001799 | finngen_R11_O15_POOR_FETGRO | Poor fetal growth \|\| id:finngen_R11_O15_POOR_FETGRO | CD27 on T cell \|\| id:ebi-a-GCST90001799 | MR Egger | 21 | -0.002799489 | 0.0193953746297977 | 0.886753594288566 | -0.040814424 | 0.0352154449628226 | 0.997204425604514 | 0.960007268124413 | 1.03584285188595 | 0.992899239648112 |
| ebi-a-GCST90001799 | finngen_R11_O15_POOR_FETGRO | Poor fetal growth \|\| id:finngen_R11_O15_POOR_FETGRO | CD27 on T cell \|\| id:ebi-a-GCST90001799 | Weighted median | 21 | -0.002715245 | 0.0188079713641275 | 0.885210909435272 | -0.039578869 | 0.0341483790801779 | 0.997288438149514 | 0.961194142900105 | 1.03473812882988 | 0.999440741871876 |
| ebi-a-GCST90001799 | finngen_R11_O15_POOR_FETGRO | Poor fetal growth \|\| id:finngen_R11_O15_POOR_FETGRO | CD27 on T cell \|\| id:ebi-a-GCST90001799 | Weighted mode | 21 | -0.002757188 | 0.0167182058235092 | 0.870661467972198 | -0.035524871 | 0.0300104955588009 | 0.997246609696168 | 0.965098730701017 | 1.03046534920643 | 0.999857399747617 |
| ebi-a-GCST90001800 | finngen_R11_O15_POOR_FETGRO | Poor fetal growth \|\| id:finngen_R11_O15_POOR_FETGRO | CD27 on IgD+ CD24+ B cell \|\| id:ebi-a-GCST90001800 | Inverse variance weighted | 31 | 0.0105718942250276 | 0.01770127415285 | 0.550347713087558 | -0.024122603 | 0.0452663915646135 | 1.01062797414822 | 0.976166021426425 | 1.04630655002565 | 0.994119304454654 |
| ebi-a-GCST90001800 | finngen_R11_O15_POOR_FETGRO | Poor fetal growth \|\| id:finngen_R11_O15_POOR_FETGRO | CD27 on IgD+ CD24+ B cell \|\| id:ebi-a-GCST90001800 | MR Egger | 31 | 0.0338300991826845 | 0.0241145784073693 | 0.171265736994011 | -0.013434474 | 0.0810946728611284 | 1.03440884488932 | 0.986655365289954 | 1.0844735619218 | 0.992899239648112 |
| ebi-a-GCST90001800 | finngen_R11_O15_POOR_FETGRO | Poor fetal growth \|\| id:finngen_R11_O15_POOR_FETGRO | CD27 on IgD+ CD24+ B cell \|\| id:ebi-a-GCST90001800 | Weighted median | 31 | 0.00511675154176989 | 0.0288599756972602 | 0.859276075163965 | -0.051448801 | 0.0616823039083999 | 1.0051298644706 | 0.94985228037053 | 1.06362438173711 | 0.999440741871876 |
| ebi-a-GCST90001800 | finngen_R11_O15_POOR_FETGRO | Poor fetal growth \|\| id:finngen_R11_O15_POOR_FETGRO | CD27 on IgD+ CD24+ B cell \|\| id:ebi-a-GCST90001800 | Weighted mode | 31 | 0.000322410719222455 | 0.0245992949828956 | 0.989629598425071 | -0.047892207 | 0.0485370288856978 | 1.00032246269914 | 0.953236533339065 | 1.04973424158991 | 0.999857399747617 |
| ebi-a-GCST90001801 | finngen_R11_O15_POOR_FETGRO | Poor fetal growth \|\| id:finngen_R11_O15_POOR_FETGRO | CD27 on IgD+ CD38- unswitched memory B cell \|\| id:ebi-a-GCST90001801 | Inverse variance weighted | 23 | 0.00246945778534804 | 0.0191300915982241 | 0.897288326517224 | -0.035025522 | 0.0399644373178672 | 1.00247250940766 | 0.965580772634741 | 1.04077376082782 | 0.994119304454654 |
| ebi-a-GCST90001801 | finngen_R11_O15_POOR_FETGRO | Poor fetal growth \|\| id:finngen_R11_O15_POOR_FETGRO | CD27 on IgD+ CD38- unswitched memory B cell \|\| id:ebi-a-GCST90001801 | MR Egger | 23 | -0.003594372 | 0.0344066635069515 | 0.917789833293935 | -0.071031433 | 0.0638426882618331 | 0.996412079811367 | 0.931432614239103 | 1.0659247031038 | 0.992899239648112 |
| ebi-a-GCST90001801 | finngen_R11_O15_POOR_FETGRO | Poor fetal growth \|\| id:finngen_R11_O15_POOR_FETGRO | CD27 on IgD+ CD38- unswitched memory B cell \|\| id:ebi-a-GCST90001801 | Weighted median | 23 | -0.020903773 | 0.0263573253656345 | 0.427724470099565 | -0.07256413 | 0.0307565849859473 | 0.979313196670247 | 0.930006103040396 | 1.03123445538383 | 0.999440741871876 |
| ebi-a-GCST90001801 | finngen_R11_O15_POOR_FETGRO | Poor fetal growth \|\| id:finngen_R11_O15_POOR_FETGRO | CD27 on IgD+ CD38- unswitched memory B cell \|\| id:ebi-a-GCST90001801 | Weighted mode | 23 | -0.018569828 | 0.0260344532155309 | 0.483171500088203 | -0.069597356 | 0.0324577006191946 | 0.98160152923798 | 0.932769318287531 | 1.0329901973741 | 0.999857399747617 |
| ebi-a-GCST90001802 | finngen_R11_O15_POOR_FETGRO | Poor fetal growth \|\| id:finngen_R11_O15_POOR_FETGRO | CD27 on IgD- CD38- B cell \|\| id:ebi-a-GCST90001802 | Inverse variance weighted | 30 | -0.04488499 | 0.0225409808651525 | 0.0464521335620006 | -0.089065312 | -0.000704667 | 0.956107437551899 | 0.914785824798171 | 0.999295580846746 | 0.994119304454654 |
| ebi-a-GCST90001802 | finngen_R11_O15_POOR_FETGRO | Poor fetal growth \|\| id:finngen_R11_O15_POOR_FETGRO | CD27 on IgD- CD38- B cell \|\| id:ebi-a-GCST90001802 | MR Egger | 30 | -0.10005966 | 0.0422794296741778 | 0.0251023396472117 | -0.182927342 | -0.017191978 | 0.904783437320402 | 0.832828665322264 | 0.982954961249504 | 0.992899239648112 |
| ebi-a-GCST90001802 | finngen_R11_O15_POOR_FETGRO | Poor fetal growth \|\| id:finngen_R11_O15_POOR_FETGRO | CD27 on IgD- CD38- B cell \|\| id:ebi-a-GCST90001802 | Weighted median | 30 | -0.027861862 | 0.0336055654920775 | 0.407056446923975 | -0.093728771 | 0.038005046172616 | 0.972522699676913 | 0.910529691213644 | 1.03873647450883 | 0.999440741871876 |
| ebi-a-GCST90001802 | finngen_R11_O15_POOR_FETGRO | Poor fetal growth \|\| id:finngen_R11_O15_POOR_FETGRO | CD27 on IgD- CD38- B cell \|\| id:ebi-a-GCST90001802 | Weighted mode | 30 | -0.027507213 | 0.0362572874762186 | 0.454173972614283 | -0.098571496 | 0.0435570707556686 | 0.972867665527755 | 0.906130905426143 | 1.04451960413414 | 0.999857399747617 |
| ebi-a-GCST90001803 | finngen_R11_O15_POOR_FETGRO | Poor fetal growth \|\| id:finngen_R11_O15_POOR_FETGRO | CD27 on IgD- CD38+ B cell \|\| id:ebi-a-GCST90001803 | Inverse variance weighted | 15 | 0.0838266307817407 | 0.0576498165177593 | 0.145928044036107 | -0.02916701 | 0.196820271156549 | 1.08744034878217 | 0.971254242144894 | 1.21752519664474 | 0.994119304454654 |
| ebi-a-GCST90001803 | finngen_R11_O15_POOR_FETGRO | Poor fetal growth \|\| id:finngen_R11_O15_POOR_FETGRO | CD27 on IgD- CD38+ B cell \|\| id:ebi-a-GCST90001803 | MR Egger | 15 | 0.318472449270985 | 0.261793889080417 | 0.24542047120448 | -0.194643573 | 0.831588471868603 | 1.3750257377005 | 0.823127990548578 | 2.2969645074015 | 0.992899239648112 |
| ebi-a-GCST90001803 | finngen_R11_O15_POOR_FETGRO | Poor fetal growth \|\| id:finngen_R11_O15_POOR_FETGRO | CD27 on IgD- CD38+ B cell \|\| id:ebi-a-GCST90001803 | Weighted median | 15 | 0.0741009536306619 | 0.0698567540130649 | 0.288800915812417 | -0.062818284 | 0.211020191496269 | 1.07691551854015 | 0.939114110023984 | 1.23493729004134 | 0.999440741871876 |
| ebi-a-GCST90001803 | finngen_R11_O15_POOR_FETGRO | Poor fetal growth \|\| id:finngen_R11_O15_POOR_FETGRO | CD27 on IgD- CD38+ B cell \|\| id:ebi-a-GCST90001803 | Weighted mode | 15 | 0.0882962274173583 | 0.0881142245529808 | 0.333318001919906 | -0.084407653 | 0.261000107541201 | 1.09231164676545 | 0.91905652384065 | 1.29822780504666 | 0.999857399747617 |
| ebi-a-GCST90001804 | finngen_R11_O15_POOR_FETGRO | Poor fetal growth \|\| id:finngen_R11_O15_POOR_FETGRO | CD27 on IgD- CD38dim B cell \|\| id:ebi-a-GCST90001804 | Inverse variance weighted | 31 | -0.033918019 | 0.0155350709981492 | 0.0290123084717787 | -0.064366759 | -0.00346928 | 0.966650747958286 | 0.937661041211808 | 0.996536730715295 | 0.994119304454654 |
| ebi-a-GCST90001804 | finngen_R11_O15_POOR_FETGRO | Poor fetal growth \|\| id:finngen_R11_O15_POOR_FETGRO | CD27 on IgD- CD38dim B cell \|\| id:ebi-a-GCST90001804 | MR Egger | 31 | -0.040461746 | 0.0202146723414079 | 0.0547613621835463 | -0.080082504 | -0.000840989 | 0.960345900436225 | 0.923040188504558 | 0.999159364858043 | 0.992899239648112 |
| ebi-a-GCST90001804 | finngen_R11_O15_POOR_FETGRO | Poor fetal growth \|\| id:finngen_R11_O15_POOR_FETGRO | CD27 on IgD- CD38dim B cell \|\| id:ebi-a-GCST90001804 | Weighted median | 31 | -0.038702382 | 0.0238815239849952 | 0.105103590683288 | -0.085510169 | 0.00810540464620292 | 0.962036985716836 | 0.918043807074968 | 1.00813834236949 | 0.999440741871876 |
| ebi-a-GCST90001804 | finngen_R11_O15_POOR_FETGRO | Poor fetal growth \|\| id:finngen_R11_O15_POOR_FETGRO | CD27 on IgD- CD38dim B cell \|\| id:ebi-a-GCST90001804 | Weighted mode | 31 | -0.0331396 | 0.0200331330890147 | 0.10850606654002 | -0.07240454 | 0.00612534129651405 | 0.967403501061084 | 0.930154534590636 | 1.00614413956184 | 0.999857399747617 |
| ebi-a-GCST90001805 | finngen_R11_O15_POOR_FETGRO | Poor fetal growth \|\| id:finngen_R11_O15_POOR_FETGRO | CD27 on memory B cell \|\| id:ebi-a-GCST90001805 | Inverse variance weighted | 25 | -0.031659635 | 0.0147348813308262 | 0.0316646753738996 | -0.060540002 | -0.002779267 | 0.968836284271488 | 0.941256116087141 | 0.997224591350316 | 0.994119304454654 |
| ebi-a-GCST90001805 | finngen_R11_O15_POOR_FETGRO | Poor fetal growth \|\| id:finngen_R11_O15_POOR_FETGRO | CD27 on memory B cell \|\| id:ebi-a-GCST90001805 | MR Egger | 25 | -0.02950947 | 0.018621796248924 | 0.126695067539501 | -0.066008191 | 0.00698925055170319 | 0.970921682875929 | 0.936123196714687 | 1.00701373236662 | 0.992899239648112 |
| ebi-a-GCST90001805 | finngen_R11_O15_POOR_FETGRO | Poor fetal growth \|\| id:finngen_R11_O15_POOR_FETGRO | CD27 on memory B cell \|\| id:ebi-a-GCST90001805 | Weighted median | 25 | -0.034267981 | 0.0205091422847312 | 0.0947486066673872 | -0.0744659 | 0.00592993790234411 | 0.96631251656607 | 0.928239126609226 | 1.00594755478924 | 0.999440741871876 |
| ebi-a-GCST90001805 | finngen_R11_O15_POOR_FETGRO | Poor fetal growth \|\| id:finngen_R11_O15_POOR_FETGRO | CD27 on memory B cell \|\| id:ebi-a-GCST90001805 | Weighted mode | 25 | -0.03287608 | 0.0182150129527485 | 0.0836551686394686 | -0.068577505 | 0.00282534537708881 | 0.967658464386519 | 0.933721088874658 | 1.00282934042692 | 0.999857399747617 |
| ebi-a-GCST90001806 | finngen_R11_O15_POOR_FETGRO | Poor fetal growth \|\| id:finngen_R11_O15_POOR_FETGRO | CD27 on unswitched memory B cell \|\| id:ebi-a-GCST90001806 | Inverse variance weighted | 30 | -0.058677368 | 0.0255378429693662 | 0.0215809532582035 | -0.10873154 | -0.008623195 | 0.943010966048241 | 0.896971186295368 | 0.991413877808115 | 0.994119304454654 |
| ebi-a-GCST90001806 | finngen_R11_O15_POOR_FETGRO | Poor fetal growth \|\| id:finngen_R11_O15_POOR_FETGRO | CD27 on unswitched memory B cell \|\| id:ebi-a-GCST90001806 | MR Egger | 30 | -0.007144967 | 0.0502255346468285 | 0.887895193130921 | -0.105587015 | 0.0912970807402741 | 0.992880497426413 | 0.89979617360843 | 1.09559443692269 | 0.992899239648112 |
| ebi-a-GCST90001806 | finngen_R11_O15_POOR_FETGRO | Poor fetal growth \|\| id:finngen_R11_O15_POOR_FETGRO | CD27 on unswitched memory B cell \|\| id:ebi-a-GCST90001806 | Weighted median | 30 | -0.025377419 | 0.0398651441667154 | 0.524397383794857 | -0.103513102 | 0.0527582634881564 | 0.97494188091533 | 0.901664209379756 | 1.05417478178109 | 0.999440741871876 |
| ebi-a-GCST90001806 | finngen_R11_O15_POOR_FETGRO | Poor fetal growth \|\| id:finngen_R11_O15_POOR_FETGRO | CD27 on unswitched memory B cell \|\| id:ebi-a-GCST90001806 | Weighted mode | 30 | -0.01247758 | 0.0435543998298766 | 0.776545241434434 | -0.097844204 | 0.0728890431715393 | 0.987599941747496 | 0.906790166883855 | 1.07561118388769 | 0.999857399747617 |
| ebi-a-GCST90001807 | finngen_R11_O15_POOR_FETGRO | Poor fetal growth \|\| id:finngen_R11_O15_POOR_FETGRO | CD27 on Plasma Blast-Plasma Cell \|\| id:ebi-a-GCST90001807 | Inverse variance weighted | 19 | 0.00853731694500406 | 0.031749227526219 | 0.788007857058078 | -0.053691169 | 0.0707658028963932 | 1.00857386376521 | 0.947724748086702 | 1.07332982569452 | 0.994119304454654 |
| ebi-a-GCST90001807 | finngen_R11_O15_POOR_FETGRO | Poor fetal growth \|\| id:finngen_R11_O15_POOR_FETGRO | CD27 on Plasma Blast-Plasma Cell \|\| id:ebi-a-GCST90001807 | MR Egger | 19 | -0.00911475 | 0.0437105748195363 | 0.837297018180886 | -0.094787477 | 0.0765579766466126 | 0.990926663414282 | 0.909566217992233 | 1.07956477806847 | 0.992899239648112 |
| ebi-a-GCST90001807 | finngen_R11_O15_POOR_FETGRO | Poor fetal growth \|\| id:finngen_R11_O15_POOR_FETGRO | CD27 on Plasma Blast-Plasma Cell \|\| id:ebi-a-GCST90001807 | Weighted median | 19 | -0.010297075 | 0.04599862460007 | 0.822869240206139 | -0.100454379 | 0.0798602293939347 | 0.989755758554166 | 0.90442637227246 | 1.08313566656593 | 0.999440741871876 |
| ebi-a-GCST90001807 | finngen_R11_O15_POOR_FETGRO | Poor fetal growth \|\| id:finngen_R11_O15_POOR_FETGRO | CD27 on Plasma Blast-Plasma Cell \|\| id:ebi-a-GCST90001807 | Weighted mode | 19 | -0.004288212 | 0.0413090536071009 | 0.918469564231621 | -0.085253957 | 0.0766775326871761 | 0.995720968871567 | 0.918279050978411 | 1.07969385427463 | 0.999857399747617 |
| ebi-a-GCST90001808 | finngen_R11_O15_POOR_FETGRO | Poor fetal growth \|\| id:finngen_R11_O15_POOR_FETGRO | CD27 on switched memory B cell \|\| id:ebi-a-GCST90001808 | Inverse variance weighted (multiplicative random effects) | 30 | -0.025191933 | 0.0289370201442095 | 0.383984616489033 | -0.081908492 | 0.0315246265407343 | 0.975122735890845 | 0.921356265916919 | 1.0320267905325 | 0.994119304454654 |
| ebi-a-GCST90001808 | finngen_R11_O15_POOR_FETGRO | Poor fetal growth \|\| id:finngen_R11_O15_POOR_FETGRO | CD27 on switched memory B cell \|\| id:ebi-a-GCST90001808 | MR Egger | 30 | -0.084640492 | 0.0585967307073528 | 0.159708455151367 | -0.199490084 | 0.0302090999410989 | 0.918842556054528 | 0.81914834309398 | 1.03067002446461 | 0.992899239648112 |
| ebi-a-GCST90001808 | finngen_R11_O15_POOR_FETGRO | Poor fetal growth \|\| id:finngen_R11_O15_POOR_FETGRO | CD27 on switched memory B cell \|\| id:ebi-a-GCST90001808 | Weighted median | 30 | -0.021818698 | 0.0359805129297674 | 0.544247047583194 | -0.092340503 | 0.0487031073553578 | 0.978417608053591 | 0.911794627574853 | 1.04990859432402 | 0.999440741871876 |
| ebi-a-GCST90001808 | finngen_R11_O15_POOR_FETGRO | Poor fetal growth \|\| id:finngen_R11_O15_POOR_FETGRO | CD27 on switched memory B cell \|\| id:ebi-a-GCST90001808 | Weighted mode | 30 | -0.021766815 | 0.0350872833405738 | 0.539864458506165 | -0.09053789 | 0.0470042605473819 | 0.978468372794068 | 0.913439722879263 | 1.04812647466265 | 0.999857399747617 |
| ebi-a-GCST90001809 | finngen_R11_O15_POOR_FETGRO | Poor fetal growth \|\| id:finngen_R11_O15_POOR_FETGRO | CD38 on CD20- B cell \|\| id:ebi-a-GCST90001809 | Inverse variance weighted | 19 | 0.0232770979520404 | 0.0477888242431299 | 0.626199897600333 | -0.070388998 | 0.116943193468575 | 1.02355012389822 | 0.932031191516208 | 1.12405557417855 | 0.994119304454654 |
| ebi-a-GCST90001809 | finngen_R11_O15_POOR_FETGRO | Poor fetal growth \|\| id:finngen_R11_O15_POOR_FETGRO | CD38 on CD20- B cell \|\| id:ebi-a-GCST90001809 | MR Egger | 19 | -0.04689472 | 0.114180206069234 | 0.686417013557431 | -0.270687924 | 0.176898483588469 | 0.954187848906115 | 0.762854527696012 | 1.1935099261322 | 0.992899239648112 |
| ebi-a-GCST90001809 | finngen_R11_O15_POOR_FETGRO | Poor fetal growth \|\| id:finngen_R11_O15_POOR_FETGRO | CD38 on CD20- B cell \|\| id:ebi-a-GCST90001809 | Weighted median | 19 | 0.0412177372160041 | 0.0632016667511076 | 0.51429655035093 | -0.08265753 | 0.165093004048175 | 1.04207898022106 | 0.920666394209072 | 1.17950281214671 | 0.999440741871876 |
| ebi-a-GCST90001809 | finngen_R11_O15_POOR_FETGRO | Poor fetal growth \|\| id:finngen_R11_O15_POOR_FETGRO | CD38 on CD20- B cell \|\| id:ebi-a-GCST90001809 | Weighted mode | 19 | 0.0415346740697218 | 0.0909733725757432 | 0.653449523017456 | -0.136773136 | 0.219842484318178 | 1.04240930579779 | 0.872168067070266 | 1.24588046941907 | 0.999857399747617 |
| ebi-a-GCST90001810 | finngen_R11_O15_POOR_FETGRO | Poor fetal growth \|\| id:finngen_R11_O15_POOR_FETGRO | CD38 on CD3- CD19- \|\| id:ebi-a-GCST90001810 | Inverse variance weighted | 18 | -0.027597674 | 0.0271607748259617 | 0.309588588512876 | -0.080832793 | 0.025637444353099 | 0.972779662334811 | 0.922347901609907 | 1.02596891021329 | 0.994119304454654 |
| ebi-a-GCST90001810 | finngen_R11_O15_POOR_FETGRO | Poor fetal growth \|\| id:finngen_R11_O15_POOR_FETGRO | CD38 on CD3- CD19- \|\| id:ebi-a-GCST90001810 | MR Egger | 18 | -0.056341796 | 0.0494916451586494 | 0.271708657875974 | -0.153345421 | 0.0406618282830046 | 0.945216009405224 | 0.857833357197002 | 1.04149984019654 | 0.992899239648112 |
| ebi-a-GCST90001810 | finngen_R11_O15_POOR_FETGRO | Poor fetal growth \|\| id:finngen_R11_O15_POOR_FETGRO | CD38 on CD3- CD19- \|\| id:ebi-a-GCST90001810 | Weighted median | 18 | 0.00514724352640875 | 0.0376879351370152 | 0.891366449280433 | -0.068721109 | 0.0790155963949586 | 1.00516051334226 | 0.933587012470455 | 1.08222120068797 | 0.999440741871876 |
| ebi-a-GCST90001810 | finngen_R11_O15_POOR_FETGRO | Poor fetal growth \|\| id:finngen_R11_O15_POOR_FETGRO | CD38 on CD3- CD19- \|\| id:ebi-a-GCST90001810 | Weighted mode | 18 | 0.0273792800733028 | 0.0540176746473239 | 0.61876565761427 | -0.078495362 | 0.133253922382058 | 1.02775753680354 | 0.924506347564374 | 1.14254007799868 | 0.999857399747617 |
| ebi-a-GCST90001811 | finngen_R11_O15_POOR_FETGRO | Poor fetal growth \|\| id:finngen_R11_O15_POOR_FETGRO | CD38 on IgD+ CD24- B cell \|\| id:ebi-a-GCST90001811 | Inverse variance weighted | 17 | -0.028498444 | 0.0320446715227564 | 0.373823136022311 | -0.091306 | 0.0343091123716719 | 0.971903806609096 | 0.912738370223286 | 1.03490445906221 | 0.994119304454654 |
| ebi-a-GCST90001811 | finngen_R11_O15_POOR_FETGRO | Poor fetal growth \|\| id:finngen_R11_O15_POOR_FETGRO | CD38 on IgD+ CD24- B cell \|\| id:ebi-a-GCST90001811 | MR Egger | 17 | -0.015367092 | 0.0518981467187279 | 0.771212957522791 | -0.117087459 | 0.0863532760594745 | 0.984750379741229 | 0.889507394264829 | 1.09019139880447 | 0.992899239648112 |
| ebi-a-GCST90001811 | finngen_R11_O15_POOR_FETGRO | Poor fetal growth \|\| id:finngen_R11_O15_POOR_FETGRO | CD38 on IgD+ CD24- B cell \|\| id:ebi-a-GCST90001811 | Weighted median | 17 | -0.0335142 | 0.0440249555942404 | 0.446505016943578 | -0.119803113 | 0.0527747133796374 | 0.967041179550412 | 0.887095077412203 | 1.05419212298448 | 0.999440741871876 |
| ebi-a-GCST90001811 | finngen_R11_O15_POOR_FETGRO | Poor fetal growth \|\| id:finngen_R11_O15_POOR_FETGRO | CD38 on IgD+ CD24- B cell \|\| id:ebi-a-GCST90001811 | Weighted mode | 17 | -0.027739984 | 0.0537272883749314 | 0.612699237429696 | -0.13304547 | 0.07756550085489 | 0.972641235858323 | 0.875425285988236 | 1.08065301383666 | 0.999857399747617 |
| ebi-a-GCST90001812 | finngen_R11_O15_POOR_FETGRO | Poor fetal growth \|\| id:finngen_R11_O15_POOR_FETGRO | CD38 on IgD+ CD38+ B cell \|\| id:ebi-a-GCST90001812 | Inverse variance weighted | 17 | -0.038185607 | 0.026959200455141 | 0.15665196280802 | -0.09102564 | 0.0146544260887824 | 0.9625342714014 | 0.912994301703394 | 1.01476232862975 | 0.994119304454654 |
| ebi-a-GCST90001812 | finngen_R11_O15_POOR_FETGRO | Poor fetal growth \|\| id:finngen_R11_O15_POOR_FETGRO | CD38 on IgD+ CD38+ B cell \|\| id:ebi-a-GCST90001812 | MR Egger | 17 | -0.047118896 | 0.0437645653774988 | 0.298650866245959 | -0.132897444 | 0.0386596523035716 | 0.953973967314634 | 0.875554880931686 | 1.03941666037612 | 0.992899239648112 |
| ebi-a-GCST90001812 | finngen_R11_O15_POOR_FETGRO | Poor fetal growth \|\| id:finngen_R11_O15_POOR_FETGRO | CD38 on IgD+ CD38+ B cell \|\| id:ebi-a-GCST90001812 | Weighted median | 17 | -0.032237279 | 0.0387799950112511 | 0.405811908929667 | -0.108246069 | 0.0437715114363667 | 0.968276803275369 | 0.89740674527189 | 1.04474361564679 | 0.999440741871876 |
| ebi-a-GCST90001812 | finngen_R11_O15_POOR_FETGRO | Poor fetal growth \|\| id:finngen_R11_O15_POOR_FETGRO | CD38 on IgD+ CD38+ B cell \|\| id:ebi-a-GCST90001812 | Weighted mode | 17 | -0.037014705 | 0.0375709773878376 | 0.339193442611015 | -0.110653821 | 0.0366244103780468 | 0.963661964304537 | 0.895248611576937 | 1.03730334729201 | 0.999857399747617 |
| ebi-a-GCST90001813 | finngen_R11_O15_POOR_FETGRO | Poor fetal growth \|\| id:finngen_R11_O15_POOR_FETGRO | CD38 on IgD+ CD38dim B cell \|\| id:ebi-a-GCST90001813 | Inverse variance weighted | 23 | 0.0321109891581549 | 0.0246408632330593 | 0.19252011769403 | -0.016185103 | 0.0804070810949512 | 1.03263210991363 | 0.983945172212362 | 1.08372814313126 | 0.994119304454654 |
| ebi-a-GCST90001813 | finngen_R11_O15_POOR_FETGRO | Poor fetal growth \|\| id:finngen_R11_O15_POOR_FETGRO | CD38 on IgD+ CD38dim B cell \|\| id:ebi-a-GCST90001813 | MR Egger | 23 | 0.0533929459247186 | 0.0372173189923713 | 0.166119169211356 | -0.019552999 | 0.126338891149766 | 1.05484406036737 | 0.980636920740697 | 1.13466663161312 | 0.992899239648112 |
| ebi-a-GCST90001813 | finngen_R11_O15_POOR_FETGRO | Poor fetal growth \|\| id:finngen_R11_O15_POOR_FETGRO | CD38 on IgD+ CD38dim B cell \|\| id:ebi-a-GCST90001813 | Weighted median | 23 | 0.0529361299795512 | 0.0341152773661406 | 0.12073785338702 | -0.013929814 | 0.119802073617187 | 1.05436230082702 | 0.986166757271141 | 1.12727371228921 | 0.999440741871876 |
| ebi-a-GCST90001813 | finngen_R11_O15_POOR_FETGRO | Poor fetal growth \|\| id:finngen_R11_O15_POOR_FETGRO | CD38 on IgD+ CD38dim B cell \|\| id:ebi-a-GCST90001813 | Weighted mode | 23 | 0.0566603228421663 | 0.0448840173618728 | 0.220033266818025 | -0.031312351 | 0.144632996871437 | 1.05829627026594 | 0.969172803517584 | 1.1556153779737 | 0.999857399747617 |
| ebi-a-GCST90001814 | finngen_R11_O15_POOR_FETGRO | Poor fetal growth \|\| id:finngen_R11_O15_POOR_FETGRO | CD38 on IgD- CD38+ B cell \|\| id:ebi-a-GCST90001814 | Inverse variance weighted | 14 | -0.00766961 | 0.00991694069316142 | 0.439294743688566 | -0.027106814 | 0.0117675935832012 | 0.9923597262372 | 0.973257278533382 | 1.01183710410216 | 0.994119304454654 |
| ebi-a-GCST90001814 | finngen_R11_O15_POOR_FETGRO | Poor fetal growth \|\| id:finngen_R11_O15_POOR_FETGRO | CD38 on IgD- CD38+ B cell \|\| id:ebi-a-GCST90001814 | MR Egger | 14 | -0.002331279 | 0.0109529326221356 | 0.835020613369432 | -0.023799027 | 0.0191364687914785 | 0.997671436172859 | 0.976481936458099 | 1.01932074459621 | 0.992899239648112 |
| ebi-a-GCST90001814 | finngen_R11_O15_POOR_FETGRO | Poor fetal growth \|\| id:finngen_R11_O15_POOR_FETGRO | CD38 on IgD- CD38+ B cell \|\| id:ebi-a-GCST90001814 | Weighted median | 14 | -0.00852559 | 0.0130981014856415 | 0.51510926442239 | -0.034197869 | 0.0171466886000676 | 0.991510649471649 | 0.966380268804752 | 1.01729453689241 | 0.999440741871876 |
| ebi-a-GCST90001814 | finngen_R11_O15_POOR_FETGRO | Poor fetal growth \|\| id:finngen_R11_O15_POOR_FETGRO | CD38 on IgD- CD38+ B cell \|\| id:ebi-a-GCST90001814 | Weighted mode | 14 | -0.006937998 | 0.0104491441515605 | 0.518307966248444 | -0.02741832 | 0.0135423245879079 | 0.993086014393999 | 0.97295414972988 | 1.01363443720217 | 0.999857399747617 |
| ebi-a-GCST90001815 | finngen_R11_O15_POOR_FETGRO | Poor fetal growth \|\| id:finngen_R11_O15_POOR_FETGRO | CD38 on IgD- CD38dim B cell \|\| id:ebi-a-GCST90001815 | Inverse variance weighted | 19 | 0.00257905538639467 | 0.0176574765667353 | 0.883873828201007 | -0.032029599 | 0.0371877094571958 | 1.00258238401069 | 0.968477915982771 | 1.03788782391445 | 0.994119304454654 |
| ebi-a-GCST90001815 | finngen_R11_O15_POOR_FETGRO | Poor fetal growth \|\| id:finngen_R11_O15_POOR_FETGRO | CD38 on IgD- CD38dim B cell \|\| id:ebi-a-GCST90001815 | MR Egger | 19 | 0.0180439072717036 | 0.0213271627638581 | 0.4092748277523 | -0.023757332 | 0.0598451462888654 | 1.01820768212968 | 0.976522652055054 | 1.0616721299461 | 0.992899239648112 |
| ebi-a-GCST90001815 | finngen_R11_O15_POOR_FETGRO | Poor fetal growth \|\| id:finngen_R11_O15_POOR_FETGRO | CD38 on IgD- CD38dim B cell \|\| id:ebi-a-GCST90001815 | Weighted median | 19 | 0.0129912538141175 | 0.0228044452099171 | 0.568894237849398 | -0.031705459 | 0.057687966425555 | 1.01307600676999 | 0.968791889187973 | 1.05938438063647 | 0.999440741871876 |
| ebi-a-GCST90001815 | finngen_R11_O15_POOR_FETGRO | Poor fetal growth \|\| id:finngen_R11_O15_POOR_FETGRO | CD38 on IgD- CD38dim B cell \|\| id:ebi-a-GCST90001815 | Weighted mode | 19 | 0.0140967233000849 | 0.0193541611724404 | 0.475765297856793 | -0.023837433 | 0.0520308791980681 | 1.01419655063185 | 0.97644443489087 | 1.05340827041377 | 0.999857399747617 |
| ebi-a-GCST90001816 | finngen_R11_O15_POOR_FETGRO | Poor fetal growth \|\| id:finngen_R11_O15_POOR_FETGRO | CD38 on naive-mature B cell \|\| id:ebi-a-GCST90001816 | Inverse variance weighted | 26 | -0.022572013 | 0.0263499552628168 | 0.391652538570138 | -0.074217925 | 0.0290738997086694 | 0.977680829313443 | 0.928469335185538 | 1.02950067146376 | 0.994119304454654 |
| ebi-a-GCST90001816 | finngen_R11_O15_POOR_FETGRO | Poor fetal growth \|\| id:finngen_R11_O15_POOR_FETGRO | CD38 on naive-mature B cell \|\| id:ebi-a-GCST90001816 | MR Egger | 26 | -0.011224763 | 0.0402052108458111 | 0.782493264188218 | -0.090026976 | 0.067577450441491 | 0.988837999782584 | 0.913906531328397 | 1.06991312163264 | 0.992899239648112 |
| ebi-a-GCST90001816 | finngen_R11_O15_POOR_FETGRO | Poor fetal growth \|\| id:finngen_R11_O15_POOR_FETGRO | CD38 on naive-mature B cell \|\| id:ebi-a-GCST90001816 | Weighted median | 26 | 0.00840930414372265 | 0.0346753309183372 | 0.808380771951104 | -0.059554344 | 0.0763729527436635 | 1.00844476166315 | 0.942184329704864 | 1.07936505125743 | 0.999440741871876 |
| ebi-a-GCST90001816 | finngen_R11_O15_POOR_FETGRO | Poor fetal growth \|\| id:finngen_R11_O15_POOR_FETGRO | CD38 on naive-mature B cell \|\| id:ebi-a-GCST90001816 | Weighted mode | 26 | -0.001356405 | 0.035294410660397 | 0.969649182084594 | -0.070533449 | 0.0678206403708344 | 0.998644514977287 | 0.931896567606712 | 1.0701733453697 | 0.999857399747617 |
| ebi-a-GCST90001817 | finngen_R11_O15_POOR_FETGRO | Poor fetal growth \|\| id:finngen_R11_O15_POOR_FETGRO | CD38 on Plasma Blast-Plasma Cell \|\| id:ebi-a-GCST90001817 | Inverse variance weighted | 15 | 0.0203623522037091 | 0.0360830755010574 | 0.572537295932543 | -0.050360476 | 0.0910851801857816 | 1.0205710792144 | 0.950886591128791 | 1.09536230444938 | 0.994119304454654 |
| ebi-a-GCST90001817 | finngen_R11_O15_POOR_FETGRO | Poor fetal growth \|\| id:finngen_R11_O15_POOR_FETGRO | CD38 on Plasma Blast-Plasma Cell \|\| id:ebi-a-GCST90001817 | MR Egger | 15 | 0.00325733359270417 | 0.0617259501487724 | 0.958716751679172 | -0.117725529 | 0.124240195884298 | 1.00326264446864 | 0.888940007654267 | 1.13228780921026 | 0.992899239648112 |
| ebi-a-GCST90001817 | finngen_R11_O15_POOR_FETGRO | Poor fetal growth \|\| id:finngen_R11_O15_POOR_FETGRO | CD38 on Plasma Blast-Plasma Cell \|\| id:ebi-a-GCST90001817 | Weighted median | 15 | 0.00356823181473151 | 0.0472950299528548 | 0.939859675974331 | -0.089130027 | 0.0962664905223269 | 1.00357460553259 | 0.914726626780378 | 1.10105244494178 | 0.999440741871876 |
| ebi-a-GCST90001817 | finngen_R11_O15_POOR_FETGRO | Poor fetal growth \|\| id:finngen_R11_O15_POOR_FETGRO | CD38 on Plasma Blast-Plasma Cell \|\| id:ebi-a-GCST90001817 | Weighted mode | 15 | 0.00466853722809357 | 0.0466378386860655 | 0.921682758353577 | -0.086741627 | 0.096078701052782 | 1.00467945182648 | 0.916913971214032 | 1.10084569830024 | 0.999857399747617 |
| ebi-a-GCST90001818 | finngen_R11_O15_POOR_FETGRO | Poor fetal growth \|\| id:finngen_R11_O15_POOR_FETGRO | CD38 on IgD+ B cell \|\| id:ebi-a-GCST90001818 | Inverse variance weighted | 15 | 0.00677897930657975 | 0.0515810513531945 | 0.895440038812426 | -0.094319881 | 0.107877839958841 | 1.00680200859575 | 0.909991626332786 | 1.11391166158021 | 0.994119304454654 |
| ebi-a-GCST90001818 | finngen_R11_O15_POOR_FETGRO | Poor fetal growth \|\| id:finngen_R11_O15_POOR_FETGRO | CD38 on IgD+ B cell \|\| id:ebi-a-GCST90001818 | MR Egger | 15 | -0.022335562 | 0.0969828498933093 | 0.821439830650849 | -0.212421948 | 0.167750823877114 | 0.977912029955452 | 0.808623428773067 | 1.18264188781002 | 0.992899239648112 |
| ebi-a-GCST90001818 | finngen_R11_O15_POOR_FETGRO | Poor fetal growth \|\| id:finngen_R11_O15_POOR_FETGRO | CD38 on IgD+ B cell \|\| id:ebi-a-GCST90001818 | Weighted median | 15 | 0.00609633495753423 | 0.0754353976209044 | 0.935588833185265 | -0.141757044 | 0.153949714294507 | 1.00611495542713 | 0.867832075555498 | 1.16643223043605 | 0.999440741871876 |
| ebi-a-GCST90001818 | finngen_R11_O15_POOR_FETGRO | Poor fetal growth \|\| id:finngen_R11_O15_POOR_FETGRO | CD38 on IgD+ B cell \|\| id:ebi-a-GCST90001818 | Weighted mode | 15 | 0.0202668030096231 | 0.0929003889265847 | 0.830455219308254 | -0.161817959 | 0.202351565305729 | 1.02047356912885 | 0.850596033556077 | 1.22427834625204 | 0.999857399747617 |
| ebi-a-GCST90001819 | finngen_R11_O15_POOR_FETGRO | Poor fetal growth \|\| id:finngen_R11_O15_POOR_FETGRO | CD38 on transitional B cell \|\| id:ebi-a-GCST90001819 | Inverse variance weighted | 25 | -0.023322166 | 0.0274673896715403 | 0.395833639852212 | -0.07715825 | 0.0305139175959449 | 0.976947693581396 | 0.925743343207415 | 1.03098423877099 | 0.994119304454654 |
| ebi-a-GCST90001819 | finngen_R11_O15_POOR_FETGRO | Poor fetal growth \|\| id:finngen_R11_O15_POOR_FETGRO | CD38 on transitional B cell \|\| id:ebi-a-GCST90001819 | MR Egger | 25 | -0.079822875 | 0.0393992013387886 | 0.0545165344646436 | -0.15704531 | -0.00260044 | 0.923279867780536 | 0.854665333310287 | 0.997402937764135 | 0.992899239648112 |
| ebi-a-GCST90001819 | finngen_R11_O15_POOR_FETGRO | Poor fetal growth \|\| id:finngen_R11_O15_POOR_FETGRO | CD38 on transitional B cell \|\| id:ebi-a-GCST90001819 | Weighted median | 25 | -0.057124226 | 0.0365664772308316 | 0.118240396823579 | -0.128794521 | 0.0145460695230892 | 0.944476733654189 | 0.879154594599202 | 1.01465237842584 | 0.999440741871876 |
| ebi-a-GCST90001819 | finngen_R11_O15_POOR_FETGRO | Poor fetal growth \|\| id:finngen_R11_O15_POOR_FETGRO | CD38 on transitional B cell \|\| id:ebi-a-GCST90001819 | Weighted mode | 25 | -0.063150089 | 0.0380501528293484 | 0.109996331386637 | -0.137728389 | 0.0114282103764596 | 0.938802559018517 | 0.871335324115742 | 1.01149376184667 | 0.999857399747617 |
| ebi-a-GCST90001820 | finngen_R11_O15_POOR_FETGRO | Poor fetal growth \|\| id:finngen_R11_O15_POOR_FETGRO | IgD on IgD+ CD24+ B cell \|\| id:ebi-a-GCST90001820 | Inverse variance weighted | 28 | -0.018882027 | 0.0194807903409984 | 0.332413537380868 | -0.057064376 | 0.0193003222987053 | 0.981295121969861 | 0.944533262289004 | 1.01948777755929 | 0.994119304454654 |
| ebi-a-GCST90001820 | finngen_R11_O15_POOR_FETGRO | Poor fetal growth \|\| id:finngen_R11_O15_POOR_FETGRO | IgD on IgD+ CD24+ B cell \|\| id:ebi-a-GCST90001820 | MR Egger | 28 | -0.039382162 | 0.0304726468542653 | 0.207594999230837 | -0.09910855 | 0.0203442255940095 | 0.961383234562628 | 0.905644394920968 | 1.02055257988844 | 0.992899239648112 |
| ebi-a-GCST90001820 | finngen_R11_O15_POOR_FETGRO | Poor fetal growth \|\| id:finngen_R11_O15_POOR_FETGRO | IgD on IgD+ CD24+ B cell \|\| id:ebi-a-GCST90001820 | Weighted median | 28 | -0.046178788 | 0.028129379060102 | 0.100661134102567 | -0.101312371 | 0.00895479449511337 | 0.954871226969692 | 0.903650714136807 | 1.0089950086141 | 0.999440741871876 |
| ebi-a-GCST90001820 | finngen_R11_O15_POOR_FETGRO | Poor fetal growth \|\| id:finngen_R11_O15_POOR_FETGRO | IgD on IgD+ CD24+ B cell \|\| id:ebi-a-GCST90001820 | Weighted mode | 28 | -0.037967596 | 0.0257793072622247 | 0.152369471924511 | -0.088495038 | 0.0125598467084898 | 0.962744137603626 | 0.915307652701496 | 1.01263905284142 | 0.999857399747617 |
| ebi-a-GCST90001821 | finngen_R11_O15_POOR_FETGRO | Poor fetal growth \|\| id:finngen_R11_O15_POOR_FETGRO | IgD on IgD+ CD24- B cell \|\| id:ebi-a-GCST90001821 | Inverse variance weighted | 29 | -0.028383881 | 0.0233507572617031 | 0.22415853834708 | -0.074151366 | 0.017383602805866 | 0.972015156606145 | 0.928531135475539 | 1.01753557697146 | 0.994119304454654 |
| ebi-a-GCST90001821 | finngen_R11_O15_POOR_FETGRO | Poor fetal growth \|\| id:finngen_R11_O15_POOR_FETGRO | IgD on IgD+ CD24- B cell \|\| id:ebi-a-GCST90001821 | MR Egger | 29 | -0.048659541 | 0.0404119917823055 | 0.239006124944085 | -0.127867045 | 0.0305479632584259 | 0.952505363872538 | 0.879970368242771 | 1.03101933990993 | 0.992899239648112 |
| ebi-a-GCST90001821 | finngen_R11_O15_POOR_FETGRO | Poor fetal growth \|\| id:finngen_R11_O15_POOR_FETGRO | IgD on IgD+ CD24- B cell \|\| id:ebi-a-GCST90001821 | Weighted median | 29 | -0.050666117 | 0.0300802402018166 | 0.0921110634583592 | -0.109623388 | 0.00829115344428089 | 0.950596005065266 | 0.896171580589306 | 1.00832562024767 | 0.999440741871876 |
| ebi-a-GCST90001821 | finngen_R11_O15_POOR_FETGRO | Poor fetal growth \|\| id:finngen_R11_O15_POOR_FETGRO | IgD on IgD+ CD24- B cell \|\| id:ebi-a-GCST90001821 | Weighted mode | 29 | -0.049974279 | 0.0289720400734161 | 0.0955666912397007 | -0.106759477 | 0.00681091975728441 | 0.951253891590384 | 0.898741814776239 | 1.00683416681924 | 0.999857399747617 |
| ebi-a-GCST90001822 | finngen_R11_O15_POOR_FETGRO | Poor fetal growth \|\| id:finngen_R11_O15_POOR_FETGRO | IgD on IgD+ CD38- B cell \|\| id:ebi-a-GCST90001822 | Inverse variance weighted | 27 | -0.019366956 | 0.0244875449249406 | 0.429008146920147 | -0.067362544 | 0.0286286320766744 | 0.9808193786652 | 0.934856213353243 | 1.02904237017857 | 0.994119304454654 |
| ebi-a-GCST90001822 | finngen_R11_O15_POOR_FETGRO | Poor fetal growth \|\| id:finngen_R11_O15_POOR_FETGRO | IgD on IgD+ CD38- B cell \|\| id:ebi-a-GCST90001822 | MR Egger | 27 | -0.050399706 | 0.0373560002554125 | 0.189374929882304 | -0.123617467 | 0.0228180540876305 | 0.950849287976092 | 0.883717827541178 | 1.02308037731932 | 0.992899239648112 |
| ebi-a-GCST90001822 | finngen_R11_O15_POOR_FETGRO | Poor fetal growth \|\| id:finngen_R11_O15_POOR_FETGRO | IgD on IgD+ CD38- B cell \|\| id:ebi-a-GCST90001822 | Weighted median | 27 | -0.023750697 | 0.0324010060204779 | 0.46354412662106 | -0.087256669 | 0.0397552745626864 | 0.976529130823901 | 0.916441843197538 | 1.04055609248533 | 0.999440741871876 |
| ebi-a-GCST90001822 | finngen_R11_O15_POOR_FETGRO | Poor fetal growth \|\| id:finngen_R11_O15_POOR_FETGRO | IgD on IgD+ CD38- B cell \|\| id:ebi-a-GCST90001822 | Weighted mode | 27 | -0.036458599 | 0.0276332890108798 | 0.198546070119425 | -0.090619845 | 0.0177026475119998 | 0.964198011880746 | 0.913364864754108 | 1.01786026810334 | 0.999857399747617 |
| ebi-a-GCST90001823 | finngen_R11_O15_POOR_FETGRO | Poor fetal growth \|\| id:finngen_R11_O15_POOR_FETGRO | IgD on IgD+ CD38- unswitched memory B cell \|\| id:ebi-a-GCST90001823 | Inverse variance weighted | 17 | -0.021761298 | 0.0255774864366713 | 0.394881047806065 | -0.071893172 | 0.0283705749394402 | 0.978473770357237 | 0.930630307975863 | 1.02877685271125 | 0.994119304454654 |
| ebi-a-GCST90001823 | finngen_R11_O15_POOR_FETGRO | Poor fetal growth \|\| id:finngen_R11_O15_POOR_FETGRO | IgD on IgD+ CD38- unswitched memory B cell \|\| id:ebi-a-GCST90001823 | MR Egger | 17 | -0.094399731 | 0.0469698472709622 | 0.0627947345577332 | -0.186460631 | -0.00233883 | 0.909918966981812 | 0.829891232997422 | 0.997663902874151 | 0.992899239648112 |
| ebi-a-GCST90001823 | finngen_R11_O15_POOR_FETGRO | Poor fetal growth \|\| id:finngen_R11_O15_POOR_FETGRO | IgD on IgD+ CD38- unswitched memory B cell \|\| id:ebi-a-GCST90001823 | Weighted median | 17 | -0.04374891 | 0.0301247379820003 | 0.146429710421913 | -0.102793397 | 0.0152955762259276 | 0.957194269001476 | 0.902313375179115 | 1.01541315225145 | 0.999440741871876 |
| ebi-a-GCST90001823 | finngen_R11_O15_POOR_FETGRO | Poor fetal growth \|\| id:finngen_R11_O15_POOR_FETGRO | IgD on IgD+ CD38- unswitched memory B cell \|\| id:ebi-a-GCST90001823 | Weighted mode | 17 | -0.04566252 | 0.0267479571310618 | 0.107124267493691 | -0.098088516 | 0.00676347551571945 | 0.955364323703834 | 0.90656865397417 | 1.00678639946897 | 0.999857399747617 |
| ebi-a-GCST90001824 | finngen_R11_O15_POOR_FETGRO | Poor fetal growth \|\| id:finngen_R11_O15_POOR_FETGRO | IgD on IgD+ CD38+ B cell \|\| id:ebi-a-GCST90001824 | Inverse variance weighted | 25 | -0.029920904 | 0.0224414165641167 | 0.182436840710023 | -0.07390608 | 0.0140642729431646 | 0.97052229540757 | 0.928758918793777 | 1.01416364012578 | 0.994119304454654 |
| ebi-a-GCST90001824 | finngen_R11_O15_POOR_FETGRO | Poor fetal growth \|\| id:finngen_R11_O15_POOR_FETGRO | IgD on IgD+ CD38+ B cell \|\| id:ebi-a-GCST90001824 | MR Egger | 25 | -0.077580305 | 0.0400880138128061 | 0.0653490170910456 | -0.156152812 | 0.000992201707852872 | 0.92535271062537 | 0.855428460273835 | 1.00099269410281 | 0.992899239648112 |
| ebi-a-GCST90001824 | finngen_R11_O15_POOR_FETGRO | Poor fetal growth \|\| id:finngen_R11_O15_POOR_FETGRO | IgD on IgD+ CD38+ B cell \|\| id:ebi-a-GCST90001824 | Weighted median | 25 | -0.050150125 | 0.0289545012824116 | 0.0832678501225758 | -0.106900948 | 0.00660069731755353 | 0.951086631715611 | 0.898614678424312 | 1.00662252993048 | 0.999440741871876 |
| ebi-a-GCST90001824 | finngen_R11_O15_POOR_FETGRO | Poor fetal growth \|\| id:finngen_R11_O15_POOR_FETGRO | IgD on IgD+ CD38+ B cell \|\| id:ebi-a-GCST90001824 | Weighted mode | 25 | -0.055619132 | 0.0296393990631467 | 0.072794573008228 | -0.113712354 | 0.00247408984580278 | 0.945899329778108 | 0.892514646800783 | 1.00247715293168 | 0.999857399747617 |
| ebi-a-GCST90001825 | finngen_R11_O15_POOR_FETGRO | Poor fetal growth \|\| id:finngen_R11_O15_POOR_FETGRO | IgD on IgD+ CD38dim B cell \|\| id:ebi-a-GCST90001825 | Inverse variance weighted | 22 | -0.025291707 | 0.0186775511155489 | 0.175697041967002 | -0.061899707 | 0.0113162929276199 | 0.975025448539409 | 0.939977154774004 | 1.01138056437983 | 0.994119304454654 |
| ebi-a-GCST90001825 | finngen_R11_O15_POOR_FETGRO | Poor fetal growth \|\| id:finngen_R11_O15_POOR_FETGRO | IgD on IgD+ CD38dim B cell \|\| id:ebi-a-GCST90001825 | MR Egger | 22 | 0.00101770737542462 | 0.0278062512633813 | 0.971166834949932 | -0.053482545 | 0.0555179598516521 | 1.0010182254153 | 0.947922486750853 | 1.05708800204564 | 0.992899239648112 |
| ebi-a-GCST90001825 | finngen_R11_O15_POOR_FETGRO | Poor fetal growth \|\| id:finngen_R11_O15_POOR_FETGRO | IgD on IgD+ CD38dim B cell \|\| id:ebi-a-GCST90001825 | Weighted median | 22 | -0.019930878 | 0.0284651770595658 | 0.483812034838992 | -0.075722625 | 0.0358608687468557 | 0.980266428656954 | 0.92707331755833 | 1.03651162529699 | 0.999440741871876 |
| ebi-a-GCST90001825 | finngen_R11_O15_POOR_FETGRO | Poor fetal growth \|\| id:finngen_R11_O15_POOR_FETGRO | IgD on IgD+ CD38dim B cell \|\| id:ebi-a-GCST90001825 | Weighted mode | 22 | -0.024980321 | 0.021612468396682 | 0.260732381553813 | -0.067340759 | 0.0173801173019028 | 0.97532910557932 | 0.934876579619623 | 1.01753203035336 | 0.999857399747617 |
| ebi-a-GCST90001826 | finngen_R11_O15_POOR_FETGRO | Poor fetal growth \|\| id:finngen_R11_O15_POOR_FETGRO | IgD on unswitched memory B cell \|\| id:ebi-a-GCST90001826 | Inverse variance weighted | 22 | -0.024939509 | 0.0207661297831084 | 0.229762658490825 | -0.065641123 | 0.0157621058411225 | 0.975368911739412 | 0.936466880503961 | 1.01588698307809 | 0.994119304454654 |
| ebi-a-GCST90001826 | finngen_R11_O15_POOR_FETGRO | Poor fetal growth \|\| id:finngen_R11_O15_POOR_FETGRO | IgD on unswitched memory B cell \|\| id:ebi-a-GCST90001826 | MR Egger | 22 | -0.051714365 | 0.0292410582591493 | 0.0922192238626207 | -0.10902684 | 0.00559810875001209 | 0.949600066708223 | 0.896706349912323 | 1.00561380744146 | 0.992899239648112 |
| ebi-a-GCST90001826 | finngen_R11_O15_POOR_FETGRO | Poor fetal growth \|\| id:finngen_R11_O15_POOR_FETGRO | IgD on unswitched memory B cell \|\| id:ebi-a-GCST90001826 | Weighted median | 22 | -0.017287487 | 0.0281378802409138 | 0.538961063957569 | -0.072437732 | 0.0378627587639677 | 0.982861084713711 | 0.930123662001544 | 1.03858868590208 | 0.999440741871876 |
| ebi-a-GCST90001826 | finngen_R11_O15_POOR_FETGRO | Poor fetal growth \|\| id:finngen_R11_O15_POOR_FETGRO | IgD on unswitched memory B cell \|\| id:ebi-a-GCST90001826 | Weighted mode | 22 | -0.029562148 | 0.023573260712989 | 0.223602793383077 | -0.075765739 | 0.0166414432997312 | 0.970870538397488 | 0.927033349166316 | 1.01678068343054 | 0.999857399747617 |
| ebi-a-GCST90001827 | finngen_R11_O15_POOR_FETGRO | Poor fetal growth \|\| id:finngen_R11_O15_POOR_FETGRO | IgD on IgD+ B cell \|\| id:ebi-a-GCST90001827 | Inverse variance weighted | 21 | -0.031172986 | 0.0252009347527176 | 0.216095482523633 | -0.080566818 | 0.0182208460120198 | 0.969307881778939 | 0.922593255486317 | 1.01838785845396 | 0.994119304454654 |
| ebi-a-GCST90001827 | finngen_R11_O15_POOR_FETGRO | Poor fetal growth \|\| id:finngen_R11_O15_POOR_FETGRO | IgD on IgD+ B cell \|\| id:ebi-a-GCST90001827 | MR Egger | 21 | -0.081955125 | 0.0462477409665277 | 0.0924162031678051 | -0.172600698 | 0.00869044686974897 | 0.921313301311891 | 0.841473550078221 | 1.00872831843058 | 0.992899239648112 |
| ebi-a-GCST90001827 | finngen_R11_O15_POOR_FETGRO | Poor fetal growth \|\| id:finngen_R11_O15_POOR_FETGRO | IgD on IgD+ B cell \|\| id:ebi-a-GCST90001827 | Weighted median | 21 | -0.057170059 | 0.0337515762213268 | 0.0902940615705926 | -0.123323149 | 0.00898303021002239 | 0.944433446128201 | 0.883977960180705 | 1.00902349871173 | 0.999440741871876 |
| ebi-a-GCST90001827 | finngen_R11_O15_POOR_FETGRO | Poor fetal growth \|\| id:finngen_R11_O15_POOR_FETGRO | IgD on IgD+ B cell \|\| id:ebi-a-GCST90001827 | Weighted mode | 21 | -0.060139304 | 0.0352684515746426 | 0.103644428784027 | -0.129265469 | 0.00898686108722807 | 0.941633351155867 | 0.878740656100478 | 1.00902736416425 | 0.999857399747617 |
| ebi-a-GCST90001828 | finngen_R11_O15_POOR_FETGRO | Poor fetal growth \|\| id:finngen_R11_O15_POOR_FETGRO | IgD on transitional B cell \|\| id:ebi-a-GCST90001828 | Inverse variance weighted | 32 | -0.035287538 | 0.0241975610102 | 0.144755200284151 | -0.082714757 | 0.0121396818648512 | 0.965327808195755 | 0.920613708115795 | 1.01221366688449 | 0.994119304454654 |
| ebi-a-GCST90001828 | finngen_R11_O15_POOR_FETGRO | Poor fetal growth \|\| id:finngen_R11_O15_POOR_FETGRO | IgD on transitional B cell \|\| id:ebi-a-GCST90001828 | MR Egger | 32 | -0.066518523 | 0.0420966283186297 | 0.12456238691356 | -0.149027915 | 0.0159908683037628 | 0.935645584544821 | 0.861545064786727 | 1.01611940646997 | 0.992899239648112 |
| ebi-a-GCST90001828 | finngen_R11_O15_POOR_FETGRO | Poor fetal growth \|\| id:finngen_R11_O15_POOR_FETGRO | IgD on transitional B cell \|\| id:ebi-a-GCST90001828 | Weighted median | 32 | -0.048374634 | 0.0349232416965229 | 0.166000425362161 | -0.116824188 | 0.0200749194533529 | 0.952776777373435 | 0.889741606667328 | 1.02027777581669 | 0.999440741871876 |
| ebi-a-GCST90001828 | finngen_R11_O15_POOR_FETGRO | Poor fetal growth \|\| id:finngen_R11_O15_POOR_FETGRO | IgD on transitional B cell \|\| id:ebi-a-GCST90001828 | Weighted mode | 32 | -0.045592039 | 0.0331859151653114 | 0.179345784770592 | -0.110636432 | 0.0194523551527791 | 0.955431661959976 | 0.89526417891021 | 1.01964278497862 | 0.999857399747617 |
| ebi-a-GCST90001829 | finngen_R11_O15_POOR_FETGRO | Poor fetal growth \|\| id:finngen_R11_O15_POOR_FETGRO | BAFF-R on B cell \|\| id:ebi-a-GCST90001829 | Inverse variance weighted | 21 | -0.01061532 | 0.021770295254155 | 0.625829149963475 | -0.053285099 | 0.0320544584135365 | 0.989440823391119 | 0.948109668844657 | 1.03257373610151 | 0.994119304454654 |
| ebi-a-GCST90001829 | finngen_R11_O15_POOR_FETGRO | Poor fetal growth \|\| id:finngen_R11_O15_POOR_FETGRO | BAFF-R on B cell \|\| id:ebi-a-GCST90001829 | MR Egger | 21 | -0.013349461 | 0.0301075819107197 | 0.662489862925843 | -0.072360321 | 0.0456613996302919 | 0.986739247961342 | 0.930195665959385 | 1.04671993119115 | 0.992899239648112 |
| ebi-a-GCST90001829 | finngen_R11_O15_POOR_FETGRO | Poor fetal growth \|\| id:finngen_R11_O15_POOR_FETGRO | BAFF-R on B cell \|\| id:ebi-a-GCST90001829 | Weighted median | 21 | -0.000710649 | 0.0225087604493131 | 0.974813288047461 | -0.044827819 | 0.0434065214975124 | 0.999289603468043 | 0.956162100163762 | 1.04436236431907 | 0.999440741871876 |
| ebi-a-GCST90001829 | finngen_R11_O15_POOR_FETGRO | Poor fetal growth \|\| id:finngen_R11_O15_POOR_FETGRO | BAFF-R on B cell \|\| id:ebi-a-GCST90001829 | Weighted mode | 21 | -0.000996401 | 0.0224075511447137 | 0.964972997888391 | -0.044915201 | 0.0429223996351145 | 0.999004095633729 | 0.956078553042229 | 1.04385688803217 | 0.999857399747617 |
| ebi-a-GCST90001830 | finngen_R11_O15_POOR_FETGRO | Poor fetal growth \|\| id:finngen_R11_O15_POOR_FETGRO | BAFF-R on CD20- B cell \|\| id:ebi-a-GCST90001830 | Inverse variance weighted | 15 | -0.027162255 | 0.0383701345458549 | 0.47900677124719 | -0.102367718 | 0.0480432091064538 | 0.973203321994039 | 0.902697552210161 | 1.04921599003043 | 0.994119304454654 |
| ebi-a-GCST90001830 | finngen_R11_O15_POOR_FETGRO | Poor fetal growth \|\| id:finngen_R11_O15_POOR_FETGRO | BAFF-R on CD20- B cell \|\| id:ebi-a-GCST90001830 | MR Egger | 15 | 0.0263577204899019 | 0.0561809187329439 | 0.646727712217181 | -0.08375688 | 0.136472321206472 | 1.02670815733549 | 0.919654815188418 | 1.14622315126275 | 0.992899239648112 |
| ebi-a-GCST90001830 | finngen_R11_O15_POOR_FETGRO | Poor fetal growth \|\| id:finngen_R11_O15_POOR_FETGRO | BAFF-R on CD20- B cell \|\| id:ebi-a-GCST90001830 | Weighted median | 15 | -0.016094673 | 0.0408415978950596 | 0.693525298485971 | -0.096144205 | 0.0639548587536797 | 0.984034154061018 | 0.90833302046519 | 1.06604427510812 | 0.999440741871876 |
| ebi-a-GCST90001830 | finngen_R11_O15_POOR_FETGRO | Poor fetal growth \|\| id:finngen_R11_O15_POOR_FETGRO | BAFF-R on CD20- B cell \|\| id:ebi-a-GCST90001830 | Weighted mode | 15 | -0.01220113 | 0.0366088841804245 | 0.743859288097785 | -0.083954543 | 0.0595522828987522 | 0.987873001888623 | 0.919473051550291 | 1.06136125057175 | 0.999857399747617 |
| ebi-a-GCST90001831 | finngen_R11_O15_POOR_FETGRO | Poor fetal growth \|\| id:finngen_R11_O15_POOR_FETGRO | CD62L on CD62L+ myeloid Dendritic Cell \|\| id:ebi-a-GCST90001831 | Inverse variance weighted | 15 | 0.0296545722136023 | 0.0451983184808268 | 0.511760418473948 | -0.058934132 | 0.118243276436023 | 1.03009864779489 | 0.942768865403313 | 1.12551789004501 | 0.994119304454654 |
| ebi-a-GCST90001831 | finngen_R11_O15_POOR_FETGRO | Poor fetal growth \|\| id:finngen_R11_O15_POOR_FETGRO | CD62L on CD62L+ myeloid Dendritic Cell \|\| id:ebi-a-GCST90001831 | MR Egger | 15 | -0.163311177 | 0.0957369425159944 | 0.111800771843056 | -0.350955585 | 0.0243332299259946 | 0.849326855962244 | 0.704015022173795 | 1.02463169895348 | 0.992899239648112 |
| ebi-a-GCST90001831 | finngen_R11_O15_POOR_FETGRO | Poor fetal growth \|\| id:finngen_R11_O15_POOR_FETGRO | CD62L on CD62L+ myeloid Dendritic Cell \|\| id:ebi-a-GCST90001831 | Weighted median | 15 | 0.0818438020567984 | 0.0553148940490214 | 0.138980582525597 | -0.02657339 | 0.19026099439288 | 1.08528627710529 | 0.973776575478192 | 1.20956524621129 | 0.999440741871876 |
| ebi-a-GCST90001831 | finngen_R11_O15_POOR_FETGRO | Poor fetal growth \|\| id:finngen_R11_O15_POOR_FETGRO | CD62L on CD62L+ myeloid Dendritic Cell \|\| id:ebi-a-GCST90001831 | Weighted mode | 15 | 0.073625407065028 | 0.069040768068369 | 0.30428639412365 | -0.061694498 | 0.208945312479031 | 1.07640351681381 | 0.940170066429467 | 1.23237760101147 | 0.999857399747617 |
| ebi-a-GCST90001832 | finngen_R11_O15_POOR_FETGRO | Poor fetal growth \|\| id:finngen_R11_O15_POOR_FETGRO | CD62L on CD62L+ plasmacytoid Dendritic Cell \|\| id:ebi-a-GCST90001832 | Inverse variance weighted | 15 | 0.0047551383515883 | 0.0379905722301187 | 0.900391917126985 | -0.069706383 | 0.0792166599226209 | 1.00476646196329 | 0.932667626572708 | 1.08243881777699 | 0.994119304454654 |
| ebi-a-GCST90001832 | finngen_R11_O15_POOR_FETGRO | Poor fetal growth \|\| id:finngen_R11_O15_POOR_FETGRO | CD62L on CD62L+ plasmacytoid Dendritic Cell \|\| id:ebi-a-GCST90001832 | MR Egger | 15 | -0.049509885 | 0.0665431101375181 | 0.470095465843382 | -0.179934381 | 0.0809146104182317 | 0.951695750147967 | 0.835325022537585 | 1.08427830654259 | 0.992899239648112 |
| ebi-a-GCST90001832 | finngen_R11_O15_POOR_FETGRO | Poor fetal growth \|\| id:finngen_R11_O15_POOR_FETGRO | CD62L on CD62L+ plasmacytoid Dendritic Cell \|\| id:ebi-a-GCST90001832 | Weighted median | 15 | -0.057651273 | 0.0484046004060888 | 0.233642278714093 | -0.15252429 | 0.0372217436292582 | 0.943979080880231 | 0.858538039846236 | 1.03792314816835 | 0.999440741871876 |
| ebi-a-GCST90001832 | finngen_R11_O15_POOR_FETGRO | Poor fetal growth \|\| id:finngen_R11_O15_POOR_FETGRO | CD62L on CD62L+ plasmacytoid Dendritic Cell \|\| id:ebi-a-GCST90001832 | Weighted mode | 15 | -0.056662168 | 0.0531256390715003 | 0.304214690063772 | -0.160788421 | 0.0474640842915933 | 0.944913237107062 | 0.851472205799748 | 1.04860853892644 | 0.999857399747617 |
| ebi-a-GCST90001833 | finngen_R11_O15_POOR_FETGRO | Poor fetal growth \|\| id:finngen_R11_O15_POOR_FETGRO | CD62L on CD62L+ Dendritic Cell \|\| id:ebi-a-GCST90001833 | Inverse variance weighted | 26 | 0.0287962675429371 | 0.0298297074284383 | 0.334366859164408 | -0.029669959 | 0.0872624941026761 | 1.02921488863581 | 0.970765873206241 | 1.09118307124973 | 0.994119304454654 |
| ebi-a-GCST90001833 | finngen_R11_O15_POOR_FETGRO | Poor fetal growth \|\| id:finngen_R11_O15_POOR_FETGRO | CD62L on CD62L+ Dendritic Cell \|\| id:ebi-a-GCST90001833 | MR Egger | 26 | 0.00288008458639364 | 0.0558293294103276 | 0.959284507883417 | -0.106545401 | 0.112305570230636 | 1.00288423601454 | 0.89893423466956 | 1.1188546971027 | 0.992899239648112 |
| ebi-a-GCST90001833 | finngen_R11_O15_POOR_FETGRO | Poor fetal growth \|\| id:finngen_R11_O15_POOR_FETGRO | CD62L on CD62L+ Dendritic Cell \|\| id:ebi-a-GCST90001833 | Weighted median | 26 | 0.0617234220583665 | 0.0393844991159952 | 0.117067742555051 | -0.015470196 | 0.138917040325717 | 1.06366811690309 | 0.984648852583049 | 1.14902877299676 | 0.999440741871876 |
| ebi-a-GCST90001833 | finngen_R11_O15_POOR_FETGRO | Poor fetal growth \|\| id:finngen_R11_O15_POOR_FETGRO | CD62L on CD62L+ Dendritic Cell \|\| id:ebi-a-GCST90001833 | Weighted mode | 26 | 0.0715877618635009 | 0.0467264142879433 | 0.138065011668175 | -0.01999601 | 0.16317153386787 | 1.07421242144847 | 0.980202584169185 | 1.17723860866195 | 0.999857399747617 |
| ebi-a-GCST90001834 | finngen_R11_O15_POOR_FETGRO | Poor fetal growth \|\| id:finngen_R11_O15_POOR_FETGRO | CD62L on monocyte \|\| id:ebi-a-GCST90001834 | Inverse variance weighted | 25 | -0.020150122 | 0.0179979755999313 | 0.262894009160817 | -0.055426154 | 0.015125910447231 | 0.980051535232101 | 0.946081885544161 | 1.01524088600253 | 0.994119304454654 |
| ebi-a-GCST90001834 | finngen_R11_O15_POOR_FETGRO | Poor fetal growth \|\| id:finngen_R11_O15_POOR_FETGRO | CD62L on monocyte \|\| id:ebi-a-GCST90001834 | MR Egger | 25 | -0.061564067 | 0.0230106981147442 | 0.0135097212363456 | -0.106665035 | -0.016463098 | 0.940292702288677 | 0.89882669798418 | 0.983671677710785 | 0.992899239648112 |
| ebi-a-GCST90001834 | finngen_R11_O15_POOR_FETGRO | Poor fetal growth \|\| id:finngen_R11_O15_POOR_FETGRO | CD62L on monocyte \|\| id:ebi-a-GCST90001834 | Weighted median | 25 | -0.030293242 | 0.028800791066445 | 0.292882232660882 | -0.086742793 | 0.0261563081681062 | 0.970160999567495 | 0.916912901895128 | 1.02650138648551 | 0.999440741871876 |
| ebi-a-GCST90001834 | finngen_R11_O15_POOR_FETGRO | Poor fetal growth \|\| id:finngen_R11_O15_POOR_FETGRO | CD62L on monocyte \|\| id:ebi-a-GCST90001834 | Weighted mode | 25 | -0.039798743 | 0.0233832863234764 | 0.101672790388748 | -0.085629984 | 0.00603249840127868 | 0.960982824411052 | 0.917933818601879 | 1.00605073056315 | 0.999857399747617 |
| ebi-a-GCST90001835 | finngen_R11_O15_POOR_FETGRO | Poor fetal growth \|\| id:finngen_R11_O15_POOR_FETGRO | CD62L on granulocyte \|\| id:ebi-a-GCST90001835 | Inverse variance weighted | 16 | -0.076240204 | 0.0389768930578319 | 0.0504609199316761 | -0.152634914 | 0.000154506558507128 | 0.926593608487897 | 0.858443069959186 | 1.00015451849526 | 0.994119304454654 |
| ebi-a-GCST90001835 | finngen_R11_O15_POOR_FETGRO | Poor fetal growth \|\| id:finngen_R11_O15_POOR_FETGRO | CD62L on granulocyte \|\| id:ebi-a-GCST90001835 | MR Egger | 16 | 0.0218554754232529 | 0.0693872863473833 | 0.757423937607756 | -0.114143606 | 0.157854556664124 | 1.02209605579562 | 0.89212983164904 | 1.17099586877613 | 0.992899239648112 |
| ebi-a-GCST90001835 | finngen_R11_O15_POOR_FETGRO | Poor fetal growth \|\| id:finngen_R11_O15_POOR_FETGRO | CD62L on granulocyte \|\| id:ebi-a-GCST90001835 | Weighted median | 16 | -0.04670376 | 0.0593783251135112 | 0.431547922636643 | -0.163085277 | 0.0696777576194025 | 0.954370078688443 | 0.849518741064101 | 1.0721626293434 | 0.999440741871876 |
| ebi-a-GCST90001835 | finngen_R11_O15_POOR_FETGRO | Poor fetal growth \|\| id:finngen_R11_O15_POOR_FETGRO | CD62L on granulocyte \|\| id:ebi-a-GCST90001835 | Weighted mode | 16 | -0.042861687 | 0.0579980330898603 | 0.471302070216451 | -0.156537831 | 0.0708144582838619 | 0.958043891237992 | 0.855099167468065 | 1.07338205024356 | 0.999857399747617 |
| ebi-a-GCST90001836 | finngen_R11_O15_POOR_FETGRO | Poor fetal growth \|\| id:finngen_R11_O15_POOR_FETGRO | CD66b on CD66b++ myeloid cell \|\| id:ebi-a-GCST90001836 | Inverse variance weighted (multiplicative random effects) | 25 | 0.00504943346079298 | 0.0244203084400835 | 0.836188006083296 | -0.042814371 | 0.0529132380033566 | 1.00506220333443 | 0.95808922262705 | 1.0543381646666 | 0.994119304454654 |
| ebi-a-GCST90001836 | finngen_R11_O15_POOR_FETGRO | Poor fetal growth \|\| id:finngen_R11_O15_POOR_FETGRO | CD66b on CD66b++ myeloid cell \|\| id:ebi-a-GCST90001836 | MR Egger | 25 | 0.040168884636511 | 0.0370310091816309 | 0.289278203094622 | -0.032411893 | 0.112749662632507 | 1.04098656598551 | 0.968107742794686 | 1.119351682 | 0.992899239648112 |
| ebi-a-GCST90001836 | finngen_R11_O15_POOR_FETGRO | Poor fetal growth \|\| id:finngen_R11_O15_POOR_FETGRO | CD66b on CD66b++ myeloid cell \|\| id:ebi-a-GCST90001836 | Weighted median | 25 | 0.0315132644794416 | 0.0302899380917697 | 0.298160036882253 | -0.027855014 | 0.0908815431393101 | 1.03201506464747 | 0.972529359546278 | 1.09513927081461 | 0.999440741871876 |
| ebi-a-GCST90001836 | finngen_R11_O15_POOR_FETGRO | Poor fetal growth \|\| id:finngen_R11_O15_POOR_FETGRO | CD66b on CD66b++ myeloid cell \|\| id:ebi-a-GCST90001836 | Weighted mode | 25 | 0.0134079848233755 | 0.027206344441206 | 0.62661460778651 | -0.03991645 | 0.0667324199281391 | 1.01349827493676 | 0.960869716193125 | 1.06900939429059 | 0.999857399747617 |
| ebi-a-GCST90001837 | finngen_R11_O15_POOR_FETGRO | Poor fetal growth \|\| id:finngen_R11_O15_POOR_FETGRO | CD66b on Granulocytic Myeloid-Derived Suppressor Cells \|\| id:ebi-a-GCST90001837 | Inverse variance weighted | 21 | 0.00221657817087139 | 0.0216747459190225 | 0.918545959142252 | -0.040265924 | 0.0446990801721555 | 1.00221903659636 | 0.960533976312778 | 1.04571313674079 | 0.99660269822999 |
| ebi-a-GCST90001837 | finngen_R11_O15_POOR_FETGRO | Poor fetal growth \|\| id:finngen_R11_O15_POOR_FETGRO | CD66b on Granulocytic Myeloid-Derived Suppressor Cells \|\| id:ebi-a-GCST90001837 | MR Egger | 21 | 0.024813279619745 | 0.0428215776110663 | 0.569088207683275 | -0.059117012 | 0.108743571737435 | 1.02512369116768 | 0.942596467136694 | 1.11487642785834 | 0.992899239648112 |
| ebi-a-GCST90001837 | finngen_R11_O15_POOR_FETGRO | Poor fetal growth \|\| id:finngen_R11_O15_POOR_FETGRO | CD66b on Granulocytic Myeloid-Derived Suppressor Cells \|\| id:ebi-a-GCST90001837 | Weighted median | 21 | 0.00132288778734062 | 0.0315690538204658 | 0.966574769183767 | -0.060552458 | 0.0631982332754535 | 1.00132376318937 | 0.94124439220664 | 1.06523798391735 | 0.999440741871876 |
| ebi-a-GCST90001837 | finngen_R11_O15_POOR_FETGRO | Poor fetal growth \|\| id:finngen_R11_O15_POOR_FETGRO | CD66b on Granulocytic Myeloid-Derived Suppressor Cells \|\| id:ebi-a-GCST90001837 | Weighted mode | 21 | 0.00236786974301018 | 0.0367289062918056 | 0.949236953355186 | -0.069620787 | 0.0743565260749493 | 1.00237067536058 | 0.932747463195758 | 1.07719078364511 | 0.999857399747617 |
| ebi-a-GCST90001838 | finngen_R11_O15_POOR_FETGRO | Poor fetal growth \|\| id:finngen_R11_O15_POOR_FETGRO | CD3 on naive CD8+ T cell \|\| id:ebi-a-GCST90001838 | Inverse variance weighted | 25 | -0.00856874 | 0.0249999022680444 | 0.73178582455097 | -0.057568549 | 0.0404310680363948 | 0.991467866613815 | 0.944057174130618 | 1.04125953116452 | 0.994119304454654 |
| ebi-a-GCST90001838 | finngen_R11_O15_POOR_FETGRO | Poor fetal growth \|\| id:finngen_R11_O15_POOR_FETGRO | CD3 on naive CD8+ T cell \|\| id:ebi-a-GCST90001838 | MR Egger | 25 | 0.0183900891819505 | 0.0445908549917638 | 0.683851695282872 | -0.069007987 | 0.105788164965808 | 1.01856022822913 | 0.933319225999326 | 1.11158637862554 | 0.992899239648112 |
| ebi-a-GCST90001838 | finngen_R11_O15_POOR_FETGRO | Poor fetal growth \|\| id:finngen_R11_O15_POOR_FETGRO | CD3 on naive CD8+ T cell \|\| id:ebi-a-GCST90001838 | Weighted median | 25 | -0.005512448 | 0.0332366338517137 | 0.868271399274801 | -0.07065625 | 0.0596313546979611 | 0.994502718008725 | 0.931782137191273 | 1.06144517763353 | 0.999440741871876 |
| ebi-a-GCST90001838 | finngen_R11_O15_POOR_FETGRO | Poor fetal growth \|\| id:finngen_R11_O15_POOR_FETGRO | CD3 on naive CD8+ T cell \|\| id:ebi-a-GCST90001838 | Weighted mode | 25 | 0.0183717618592867 | 0.0354377311650383 | 0.608909686711865 | -0.051086191 | 0.0878297149427618 | 1.01854156091824 | 0.950196768380168 | 1.09180218859963 | 0.999857399747617 |
| ebi-a-GCST90001839 | finngen_R11_O15_POOR_FETGRO | Poor fetal growth \|\| id:finngen_R11_O15_POOR_FETGRO | CD3 on Effector Memory CD8+ T cell \|\| id:ebi-a-GCST90001839 | Inverse variance weighted | 20 | 0.0566870497778053 | 0.0287291998844285 | 0.0484783600329192 | 0.000377818004325403 | 0.112996281551285 | 1.05832455566023 | 1.00037788938654 | 1.1196277696624 | 0.994119304454654 |
| ebi-a-GCST90001839 | finngen_R11_O15_POOR_FETGRO | Poor fetal growth \|\| id:finngen_R11_O15_POOR_FETGRO | CD3 on Effector Memory CD8+ T cell \|\| id:ebi-a-GCST90001839 | MR Egger | 20 | 0.0507454358893739 | 0.044171881905396 | 0.265668892472779 | -0.035831453 | 0.13732232442395 | 1.05205504373567 | 0.964802894755543 | 1.14719785882287 | 0.992899239648112 |
| ebi-a-GCST90001839 | finngen_R11_O15_POOR_FETGRO | Poor fetal growth \|\| id:finngen_R11_O15_POOR_FETGRO | CD3 on Effector Memory CD8+ T cell \|\| id:ebi-a-GCST90001839 | Weighted median | 20 | 0.0378004449889606 | 0.0433792981526843 | 0.383539251315506 | -0.047222979 | 0.122823869368222 | 1.03852396953676 | 0.953874679480922 | 1.13068525510008 | 0.999440741871876 |
| ebi-a-GCST90001839 | finngen_R11_O15_POOR_FETGRO | Poor fetal growth \|\| id:finngen_R11_O15_POOR_FETGRO | CD3 on Effector Memory CD8+ T cell \|\| id:ebi-a-GCST90001839 | Weighted mode | 20 | 0.0459081447473925 | 0.0413438712183575 | 0.280683278312642 | -0.035125843 | 0.126942132335373 | 1.04697823608957 | 0.965483909374643 | 1.13535131575132 | 0.999857399747617 |
| ebi-a-GCST90001840 | finngen_R11_O15_POOR_FETGRO | Poor fetal growth \|\| id:finngen_R11_O15_POOR_FETGRO | CD3 on Terminally Differentiated CD8+ T cell \|\| id:ebi-a-GCST90001840 | Inverse variance weighted | 22 | 0.025343116863501 | 0.0299696566559238 | 0.397761491252205 | -0.03339741 | 0.0840836439091117 | 1.02566698379434 | 0.967154126307777 | 1.08771987114596 | 0.994119304454654 |
| ebi-a-GCST90001840 | finngen_R11_O15_POOR_FETGRO | Poor fetal growth \|\| id:finngen_R11_O15_POOR_FETGRO | CD3 on Terminally Differentiated CD8+ T cell \|\| id:ebi-a-GCST90001840 | MR Egger | 22 | -0.012595866 | 0.0658399075458402 | 0.850211164941837 | -0.141642084 | 0.11645035309138 | 0.987483130196136 | 0.867931847171175 | 1.12350173069481 | 0.992899239648112 |
| ebi-a-GCST90001840 | finngen_R11_O15_POOR_FETGRO | Poor fetal growth \|\| id:finngen_R11_O15_POOR_FETGRO | CD3 on Terminally Differentiated CD8+ T cell \|\| id:ebi-a-GCST90001840 | Weighted median | 22 | 0.00812078504504499 | 0.0412315426827006 | 0.843862248723308 | -0.072693039 | 0.0889346087031382 | 1.00815384805853 | 0.929886225386326 | 1.09300918070161 | 0.999440741871876 |
| ebi-a-GCST90001840 | finngen_R11_O15_POOR_FETGRO | Poor fetal growth \|\| id:finngen_R11_O15_POOR_FETGRO | CD3 on Terminally Differentiated CD8+ T cell \|\| id:ebi-a-GCST90001840 | Weighted mode | 22 | 0.0108818425651248 | 0.0595050758726347 | 0.856652053818734 | -0.105748106 | 0.127511791275489 | 1.01094126516077 | 0.899651236154469 | 1.13599826303067 | 0.999857399747617 |
| ebi-a-GCST90001841 | finngen_R11_O15_POOR_FETGRO | Poor fetal growth \|\| id:finngen_R11_O15_POOR_FETGRO | CD3 on Central Memory CD4+ T cell \|\| id:ebi-a-GCST90001841 | Inverse variance weighted (multiplicative random effects) | 25 | 0.0227783078331993 | 0.0270908137478782 | 0.400452714454973 | -0.030319687 | 0.0758763027790405 | 1.02303971451445 | 0.970135344202322 | 1.07882911773961 | 0.994119304454654 |
| ebi-a-GCST90001841 | finngen_R11_O15_POOR_FETGRO | Poor fetal growth \|\| id:finngen_R11_O15_POOR_FETGRO | CD3 on Central Memory CD4+ T cell \|\| id:ebi-a-GCST90001841 | MR Egger | 25 | 0.0625212241065794 | 0.0373789023980167 | 0.107949267647629 | -0.010741425 | 0.135783872806692 | 1.06451705210147 | 0.989316058506722 | 1.1454343073388 | 0.992899239648112 |
| ebi-a-GCST90001841 | finngen_R11_O15_POOR_FETGRO | Poor fetal growth \|\| id:finngen_R11_O15_POOR_FETGRO | CD3 on Central Memory CD4+ T cell \|\| id:ebi-a-GCST90001841 | Weighted median | 25 | 0.0409807978020192 | 0.0279926022102783 | 0.143197577782452 | -0.013884703 | 0.0958462981341647 | 1.04183209988716 | 0.986211245369363 | 1.10058988827365 | 0.999440741871876 |
| ebi-a-GCST90001841 | finngen_R11_O15_POOR_FETGRO | Poor fetal growth \|\| id:finngen_R11_O15_POOR_FETGRO | CD3 on Central Memory CD4+ T cell \|\| id:ebi-a-GCST90001841 | Weighted mode | 25 | 0.0343119767835454 | 0.0239631324997351 | 0.165077975906017 | -0.012655763 | 0.0812797164830261 | 1.03490742345908 | 0.98742398447569 | 1.0846742554055 | 0.999857399747617 |
| ebi-a-GCST90001842 | finngen_R11_O15_POOR_FETGRO | Poor fetal growth \|\| id:finngen_R11_O15_POOR_FETGRO | CD3 on Naive CD4+ T cell \|\| id:ebi-a-GCST90001842 | Inverse variance weighted (multiplicative random effects) | 28 | 0.031370304880855 | 0.0223660924612786 | 0.160741173167724 | -0.012467236 | 0.075207846104961 | 1.03186753873346 | 0.987610157684017 | 1.0781082081909 | 0.994119304454654 |
| ebi-a-GCST90001842 | finngen_R11_O15_POOR_FETGRO | Poor fetal growth \|\| id:finngen_R11_O15_POOR_FETGRO | CD3 on Naive CD4+ T cell \|\| id:ebi-a-GCST90001842 | MR Egger | 28 | 0.0112639967048429 | 0.032780968443702 | 0.733899903657402 | -0.052986701 | 0.0755146948544988 | 1.01132767437951 | 0.948392624650094 | 1.07843907510691 | 0.992899239648112 |
| ebi-a-GCST90001842 | finngen_R11_O15_POOR_FETGRO | Poor fetal growth \|\| id:finngen_R11_O15_POOR_FETGRO | CD3 on Naive CD4+ T cell \|\| id:ebi-a-GCST90001842 | Weighted median | 28 | 0.0275496186917036 | 0.0277958150710185 | 0.321615914137166 | -0.026930179 | 0.0820294162308999 | 1.02793261851357 | 0.973429205100646 | 1.08548774031792 | 0.999440741871876 |
| ebi-a-GCST90001842 | finngen_R11_O15_POOR_FETGRO | Poor fetal growth \|\| id:finngen_R11_O15_POOR_FETGRO | CD3 on Naive CD4+ T cell \|\| id:ebi-a-GCST90001842 | Weighted mode | 28 | 0.0344164298314883 | 0.0254817288415198 | 0.188025631646285 | -0.015527759 | 0.0843606183608671 | 1.03501552833964 | 0.984592175375651 | 1.08802118348692 | 0.999857399747617 |
| ebi-a-GCST90001843 | finngen_R11_O15_POOR_FETGRO | Poor fetal growth \|\| id:finngen_R11_O15_POOR_FETGRO | CD3 on Effector Memory CD4+ T cell \|\| id:ebi-a-GCST90001843 | Inverse variance weighted (multiplicative random effects) | 19 | -0.006760198 | 0.0321106622762083 | 0.833255490479983 | -0.069697096 | 0.0561767000276716 | 0.993262600701447 | 0.932676288413182 | 1.0577845777882 | 0.994119304454654 |
| ebi-a-GCST90001843 | finngen_R11_O15_POOR_FETGRO | Poor fetal growth \|\| id:finngen_R11_O15_POOR_FETGRO | CD3 on Effector Memory CD4+ T cell \|\| id:ebi-a-GCST90001843 | MR Egger | 19 | -0.088348758 | 0.0451870894270537 | 0.0672067225785025 | -0.176915453 | 0.000217937620095185 | 0.915441553792047 | 0.837850619340163 | 1.00021796137022 | 0.992899239648112 |
| ebi-a-GCST90001843 | finngen_R11_O15_POOR_FETGRO | Poor fetal growth \|\| id:finngen_R11_O15_POOR_FETGRO | CD3 on Effector Memory CD4+ T cell \|\| id:ebi-a-GCST90001843 | Weighted median | 19 | -0.046669027 | 0.0337152710750977 | 0.16629405587726 | -0.112750958 | 0.0194129042166404 | 0.954403226934815 | 0.893373119487137 | 1.01960255990969 | 0.999440741871876 |
| ebi-a-GCST90001843 | finngen_R11_O15_POOR_FETGRO | Poor fetal growth \|\| id:finngen_R11_O15_POOR_FETGRO | CD3 on Effector Memory CD4+ T cell \|\| id:ebi-a-GCST90001843 | Weighted mode | 19 | -0.055755898 | 0.039001933209338 | 0.16996855924894 | -0.132199687 | 0.020687890866153 | 0.94576997184519 | 0.876166018370418 | 1.02090336864205 | 0.999857399747617 |
| ebi-a-GCST90001844 | finngen_R11_O15_POOR_FETGRO | Poor fetal growth \|\| id:finngen_R11_O15_POOR_FETGRO | CD3 on Terminally Differentiated CD4+ T cell \|\| id:ebi-a-GCST90001844 | Inverse variance weighted | 23 | 0.00120908300355358 | 0.022210043634238 | 0.956585750397433 | -0.042322603 | 0.04474076852666 | 1.00120981423909 | 0.958560496655917 | 1.04575673170944 | 0.998984604424952 |
| ebi-a-GCST90001844 | finngen_R11_O15_POOR_FETGRO | Poor fetal growth \|\| id:finngen_R11_O15_POOR_FETGRO | CD3 on Terminally Differentiated CD4+ T cell \|\| id:ebi-a-GCST90001844 | MR Egger | 23 | 0.00601026992182275 | 0.0322898267045084 | 0.854125110080712 | -0.05727779 | 0.0692983302626592 | 1.0060283678337 | 0.944331706626811 | 1.0717558986782 | 0.992899239648112 |
| ebi-a-GCST90001844 | finngen_R11_O15_POOR_FETGRO | Poor fetal growth \|\| id:finngen_R11_O15_POOR_FETGRO | CD3 on Terminally Differentiated CD4+ T cell \|\| id:ebi-a-GCST90001844 | Weighted median | 23 | 0.000535899860818798 | 0.0332138948623795 | 0.987126843293193 | -0.064563334 | 0.0656351337910827 | 1.0005360434808 | 0.937476738164967 | 1.06783702842989 | 0.999440741871876 |
| ebi-a-GCST90001844 | finngen_R11_O15_POOR_FETGRO | Poor fetal growth \|\| id:finngen_R11_O15_POOR_FETGRO | CD3 on Terminally Differentiated CD4+ T cell \|\| id:ebi-a-GCST90001844 | Weighted mode | 23 | -0.001868416 | 0.0335706800692824 | 0.956118067077687 | -0.067666949 | 0.0639301166681448 | 0.998133328135433 | 0.934571681593293 | 1.06601789927578 | 0.999857399747617 |
| ebi-a-GCST90001845 | finngen_R11_O15_POOR_FETGRO | Poor fetal growth \|\| id:finngen_R11_O15_POOR_FETGRO | CD3 on CD45RA- CD4+ T cell \|\| id:ebi-a-GCST90001845 | Inverse variance weighted (multiplicative random effects) | 27 | 0.0337721348935583 | 0.0292725986708755 | 0.248618451135226 | -0.023602159 | 0.0911464282884743 | 1.03434888785366 | 0.976674194000235 | 1.09542939536686 | 0.994119304454654 |
| ebi-a-GCST90001845 | finngen_R11_O15_POOR_FETGRO | Poor fetal growth \|\| id:finngen_R11_O15_POOR_FETGRO | CD3 on CD45RA- CD4+ T cell \|\| id:ebi-a-GCST90001845 | MR Egger | 27 | 0.0391935258402128 | 0.0442606371333398 | 0.384316566438389 | -0.047557323 | 0.125944374621559 | 1.03997172557876 | 0.953555810942325 | 1.13421907516296 | 0.992899239648112 |
| ebi-a-GCST90001845 | finngen_R11_O15_POOR_FETGRO | Poor fetal growth \|\| id:finngen_R11_O15_POOR_FETGRO | CD3 on CD45RA- CD4+ T cell \|\| id:ebi-a-GCST90001845 | Weighted median | 27 | 0.0460567091756487 | 0.0307654267849016 | 0.134385940010908 | -0.014243527 | 0.106356945674056 | 1.04713379136732 | 0.985857431806087 | 1.11221880735283 | 0.999440741871876 |
| ebi-a-GCST90001845 | finngen_R11_O15_POOR_FETGRO | Poor fetal growth \|\| id:finngen_R11_O15_POOR_FETGRO | CD3 on CD45RA- CD4+ T cell \|\| id:ebi-a-GCST90001845 | Weighted mode | 27 | 0.0483102952998129 | 0.0264115242987517 | 0.0788716323092582 | -0.003456292 | 0.100076882925366 | 1.04949625856148 | 0.996549673777069 | 1.10525589011527 | 0.999857399747617 |
| ebi-a-GCST90001846 | finngen_R11_O15_POOR_FETGRO | Poor fetal growth \|\| id:finngen_R11_O15_POOR_FETGRO | CD3 on Central Memory CD8+ T cell \|\| id:ebi-a-GCST90001846 | Inverse variance weighted | 18 | -0.007833205 | 0.0309591652557751 | 0.800254832690908 | -0.068513169 | 0.0528467590209259 | 0.992197394719184 | 0.93378116326225 | 1.05426807566802 | 0.994119304454654 |
| ebi-a-GCST90001846 | finngen_R11_O15_POOR_FETGRO | Poor fetal growth \|\| id:finngen_R11_O15_POOR_FETGRO | CD3 on Central Memory CD8+ T cell \|\| id:ebi-a-GCST90001846 | MR Egger | 18 | -0.020348014 | 0.0648668083574961 | 0.75780974152988 | -0.147486958 | 0.106790930550076 | 0.979857609962515 | 0.862873691664347 | 1.11270159824844 | 0.992899239648112 |
| ebi-a-GCST90001846 | finngen_R11_O15_POOR_FETGRO | Poor fetal growth \|\| id:finngen_R11_O15_POOR_FETGRO | CD3 on Central Memory CD8+ T cell \|\| id:ebi-a-GCST90001846 | Weighted median | 18 | 0.0410162304896987 | 0.0454771661707242 | 0.36710561860862 | -0.048119015 | 0.130151476184318 | 1.04186901545257 | 0.95302035641464 | 1.13900090176861 | 0.999440741871876 |
| ebi-a-GCST90001846 | finngen_R11_O15_POOR_FETGRO | Poor fetal growth \|\| id:finngen_R11_O15_POOR_FETGRO | CD3 on Central Memory CD8+ T cell \|\| id:ebi-a-GCST90001846 | Weighted mode | 18 | 0.0648445168808529 | 0.0576935094334779 | 0.276656185953044 | -0.048234762 | 0.17792379537047 | 1.06699311206907 | 0.952910054119265 | 1.19473427348302 | 0.999857399747617 |
| ebi-a-GCST90001847 | finngen_R11_O15_POOR_FETGRO | Poor fetal growth \|\| id:finngen_R11_O15_POOR_FETGRO | CD3 on HLA DR+ T cell \|\| id:ebi-a-GCST90001847 | Inverse variance weighted | 27 | -0.016729305 | 0.0207956705596224 | 0.42113082108325 | -0.057488819 | 0.0240302096909735 | 0.983409853125759 | 0.944132446764114 | 1.02432126185199 | 0.994119304454654 |
| ebi-a-GCST90001847 | finngen_R11_O15_POOR_FETGRO | Poor fetal growth \|\| id:finngen_R11_O15_POOR_FETGRO | CD3 on HLA DR+ T cell \|\| id:ebi-a-GCST90001847 | MR Egger | 27 | 0.0121966332999138 | 0.0288602472137358 | 0.676192539594154 | -0.044369451 | 0.068762717838836 | 1.01227131554698 | 0.956600474949083 | 1.07118200660915 | 0.992899239648112 |
| ebi-a-GCST90001847 | finngen_R11_O15_POOR_FETGRO | Poor fetal growth \|\| id:finngen_R11_O15_POOR_FETGRO | CD3 on HLA DR+ T cell \|\| id:ebi-a-GCST90001847 | Weighted median | 27 | -0.004357045 | 0.031884953680295 | 0.891308290896273 | -0.066851554 | 0.0581374640957124 | 0.99565243303284 | 0.935334037318589 | 1.05986067848673 | 0.999440741871876 |
| ebi-a-GCST90001847 | finngen_R11_O15_POOR_FETGRO | Poor fetal growth \|\| id:finngen_R11_O15_POOR_FETGRO | CD3 on HLA DR+ T cell \|\| id:ebi-a-GCST90001847 | Weighted mode | 27 | 0.0033588676276306 | 0.0279468367808647 | 0.905258361476291 | -0.051416932 | 0.0581346677181255 | 1.00336451494459 | 0.94988255108918 | 1.05985771472023 | 0.999857399747617 |
| ebi-a-GCST90001848 | finngen_R11_O15_POOR_FETGRO | Poor fetal growth \|\| id:finngen_R11_O15_POOR_FETGRO | CD3 on Natural Killer T \|\| id:ebi-a-GCST90001848 | Inverse variance weighted | 17 | 0.0248553462944064 | 0.0306857561506449 | 0.417942351916284 | -0.035288736 | 0.0849994283496703 | 1.02516681561953 | 0.965326651689602 | 1.08871644433311 | 0.994119304454654 |
| ebi-a-GCST90001848 | finngen_R11_O15_POOR_FETGRO | Poor fetal growth \|\| id:finngen_R11_O15_POOR_FETGRO | CD3 on Natural Killer T \|\| id:ebi-a-GCST90001848 | MR Egger | 17 | 0.0504709840736954 | 0.0447471856300283 | 0.277074877398123 | -0.0372335 | 0.138175467908551 | 1.05176634493749 | 0.963451143470411 | 1.14817700081659 | 0.992899239648112 |
| ebi-a-GCST90001848 | finngen_R11_O15_POOR_FETGRO | Poor fetal growth \|\| id:finngen_R11_O15_POOR_FETGRO | CD3 on Natural Killer T \|\| id:ebi-a-GCST90001848 | Weighted median | 17 | 0.0526084353568201 | 0.0444651685915844 | 0.236754417798712 | -0.034543295 | 0.139760165796325 | 1.05401684857515 | 0.966046513717379 | 1.14999795693616 | 0.999440741871876 |
| ebi-a-GCST90001848 | finngen_R11_O15_POOR_FETGRO | Poor fetal growth \|\| id:finngen_R11_O15_POOR_FETGRO | CD3 on Natural Killer T \|\| id:ebi-a-GCST90001848 | Weighted mode | 17 | 0.0814381692628301 | 0.0489341491927469 | 0.115522828397905 | -0.014472763 | 0.177349101680614 | 1.08484613867378 | 0.985631463858246 | 1.19404786449134 | 0.999857399747617 |
| ebi-a-GCST90001849 | finngen_R11_O15_POOR_FETGRO | Poor fetal growth \|\| id:finngen_R11_O15_POOR_FETGRO | CD3 on HLA DR+ CD4+ T cell \|\| id:ebi-a-GCST90001849 | Inverse variance weighted | 26 | 0.0245344423775458 | 0.0281761398133929 | 0.38388934095227 | -0.030690792 | 0.0797596764117958 | 1.02483788835281 | 0.969775389362302 | 1.08302675952015 | 0.994119304454654 |
| ebi-a-GCST90001849 | finngen_R11_O15_POOR_FETGRO | Poor fetal growth \|\| id:finngen_R11_O15_POOR_FETGRO | CD3 on HLA DR+ CD4+ T cell \|\| id:ebi-a-GCST90001849 | MR Egger | 26 | -0.025365742 | 0.0525193658076579 | 0.633487396588359 | -0.128303699 | 0.0775722154627356 | 0.974953265922489 | 0.879586209561843 | 1.08066027002223 | 0.992899239648112 |
| ebi-a-GCST90001849 | finngen_R11_O15_POOR_FETGRO | Poor fetal growth \|\| id:finngen_R11_O15_POOR_FETGRO | CD3 on HLA DR+ CD4+ T cell \|\| id:ebi-a-GCST90001849 | Weighted median | 26 | 0.0221484991747748 | 0.0395115586838685 | 0.575099254869642 | -0.055294156 | 0.099591154195157 | 1.02239559810067 | 0.946206774758991 | 1.10471916593693 | 0.999440741871876 |
| ebi-a-GCST90001849 | finngen_R11_O15_POOR_FETGRO | Poor fetal growth \|\| id:finngen_R11_O15_POOR_FETGRO | CD3 on HLA DR+ CD4+ T cell \|\| id:ebi-a-GCST90001849 | Weighted mode | 26 | 0.0102653376497091 | 0.0489859951061916 | 0.835712087974956 | -0.085747213 | 0.106277888057845 | 1.01031820698056 | 0.917826216654989 | 1.11213088146087 | 0.999857399747617 |
| ebi-a-GCST90001850 | finngen_R11_O15_POOR_FETGRO | Poor fetal growth \|\| id:finngen_R11_O15_POOR_FETGRO | CD3 on HLA DR+ CD8+ T cell \|\| id:ebi-a-GCST90001850 | Inverse variance weighted (multiplicative random effects) | 26 | 0.0298862950891493 | 0.0308423461047586 | 0.332544190833019 | -0.030564703 | 0.0903372934544762 | 1.03033737287362 | 0.969897674479826 | 1.09454340377655 | 0.994119304454654 |
| ebi-a-GCST90001850 | finngen_R11_O15_POOR_FETGRO | Poor fetal growth \|\| id:finngen_R11_O15_POOR_FETGRO | CD3 on HLA DR+ CD8+ T cell \|\| id:ebi-a-GCST90001850 | MR Egger | 26 | -0.013707873 | 0.0495726879600731 | 0.784515622840272 | -0.110870341 | 0.0834545954496666 | 0.98638565210743 | 0.895054792998527 | 1.08703585779804 | 0.992899239648112 |
| ebi-a-GCST90001850 | finngen_R11_O15_POOR_FETGRO | Poor fetal growth \|\| id:finngen_R11_O15_POOR_FETGRO | CD3 on HLA DR+ CD8+ T cell \|\| id:ebi-a-GCST90001850 | Weighted median | 26 | 0.0140672842485648 | 0.0410421316573338 | 0.731784827757415 | -0.066375294 | 0.0945098622969391 | 1.01416669408682 | 0.935779606099074 | 1.09912000292738 | 0.999440741871876 |
| ebi-a-GCST90001850 | finngen_R11_O15_POOR_FETGRO | Poor fetal growth \|\| id:finngen_R11_O15_POOR_FETGRO | CD3 on HLA DR+ CD8+ T cell \|\| id:ebi-a-GCST90001850 | Weighted mode | 26 | -0.002986716 | 0.0405741720034964 | 0.941905533946307 | -0.082512093 | 0.0765386614660217 | 0.997017740137217 | 0.920800302746917 | 1.0795439262812 | 0.999857399747617 |
| ebi-a-GCST90001851 | finngen_R11_O15_POOR_FETGRO | Poor fetal growth \|\| id:finngen_R11_O15_POOR_FETGRO | CD3 on T cell \|\| id:ebi-a-GCST90001851 | Inverse variance weighted | 31 | 0.00922406356106298 | 0.0178003234208803 | 0.604321244495044 | -0.02566457 | 0.0441126974659884 | 1.00926673633991 | 0.974661965311657 | 1.04510012838811 | 0.994119304454654 |
| ebi-a-GCST90001851 | finngen_R11_O15_POOR_FETGRO | Poor fetal growth \|\| id:finngen_R11_O15_POOR_FETGRO | CD3 on T cell \|\| id:ebi-a-GCST90001851 | MR Egger | 31 | 0.0239933986986061 | 0.0242762522071301 | 0.331157070467153 | -0.023588056 | 0.071574853024581 | 1.02428355626384 | 0.976687968010447 | 1.07419855470286 | 0.992899239648112 |
| ebi-a-GCST90001851 | finngen_R11_O15_POOR_FETGRO | Poor fetal growth \|\| id:finngen_R11_O15_POOR_FETGRO | CD3 on T cell \|\| id:ebi-a-GCST90001851 | Weighted median | 31 | 0.0281153549436125 | 0.0240511720586321 | 0.24241142423216 | -0.019024942 | 0.0752556521785314 | 1.02851432179018 | 0.981154889686531 | 1.07815974954321 | 0.999440741871876 |
| ebi-a-GCST90001851 | finngen_R11_O15_POOR_FETGRO | Poor fetal growth \|\| id:finngen_R11_O15_POOR_FETGRO | CD3 on T cell \|\| id:ebi-a-GCST90001851 | Weighted mode | 31 | 0.0331233445211855 | 0.0212799541684389 | 0.13006483359558 | -0.008585366 | 0.0748320546913258 | 1.03367802990016 | 0.991451383359617 | 1.07770314049854 | 0.999857399747617 |
| ebi-a-GCST90001852 | finngen_R11_O15_POOR_FETGRO | Poor fetal growth \|\| id:finngen_R11_O15_POOR_FETGRO | CD3 on CD39+ resting CD4 regulatory T cell \|\| id:ebi-a-GCST90001852 | Inverse variance weighted | 20 | 0.00889372150344788 | 0.0250055238029981 | 0.722087509088401 | -0.040117105 | 0.0579045481573242 | 1.00893338815204 | 0.960676932348213 | 1.05961384878864 | 0.994119304454654 |
| ebi-a-GCST90001852 | finngen_R11_O15_POOR_FETGRO | Poor fetal growth \|\| id:finngen_R11_O15_POOR_FETGRO | CD3 on CD39+ resting CD4 regulatory T cell \|\| id:ebi-a-GCST90001852 | MR Egger | 20 | 0.0408576196693497 | 0.036675519859168 | 0.279921494513322 | -0.031026399 | 0.112741638593319 | 1.041703777 | 0.969449979981346 | 1.11934270063227 | 0.992899239648112 |
| ebi-a-GCST90001852 | finngen_R11_O15_POOR_FETGRO | Poor fetal growth \|\| id:finngen_R11_O15_POOR_FETGRO | CD3 on CD39+ resting CD4 regulatory T cell \|\| id:ebi-a-GCST90001852 | Weighted median | 20 | 0.0273875338264791 | 0.03937464505871 | 0.486702741842341 | -0.04978677 | 0.104561838141551 | 1.02776601969558 | 0.951432276312364 | 1.11022404593524 | 0.999440741871876 |
| ebi-a-GCST90001852 | finngen_R11_O15_POOR_FETGRO | Poor fetal growth \|\| id:finngen_R11_O15_POOR_FETGRO | CD3 on CD39+ resting CD4 regulatory T cell \|\| id:ebi-a-GCST90001852 | Weighted mode | 20 | 0.0476912755438572 | 0.0365199851401747 | 0.20717883798739 | -0.023887895 | 0.1192704464186 | 1.048846801 | 0.976395162079307 | 1.12667458219455 | 0.999857399747617 |
| ebi-a-GCST90001853 | finngen_R11_O15_POOR_FETGRO | Poor fetal growth \|\| id:finngen_R11_O15_POOR_FETGRO | CD3 on activated CD4 regulatory T cell \|\| id:ebi-a-GCST90001853 | Inverse variance weighted | 22 | 0.031011390670469 | 0.0211712600231668 | 0.142978985425328 | -0.010484279 | 0.072507060315876 | 1.03149725326491 | 0.98957048950808 | 1.07520039731779 | 0.994119304454654 |
| ebi-a-GCST90001853 | finngen_R11_O15_POOR_FETGRO | Poor fetal growth \|\| id:finngen_R11_O15_POOR_FETGRO | CD3 on activated CD4 regulatory T cell \|\| id:ebi-a-GCST90001853 | MR Egger | 22 | 0.0639917915712734 | 0.0344300725354611 | 0.0778641143529976 | -0.003491151 | 0.131474733740777 | 1.06608364785395 | 0.996514936382437 | 1.14050909095999 | 0.992899239648112 |
| ebi-a-GCST90001853 | finngen_R11_O15_POOR_FETGRO | Poor fetal growth \|\| id:finngen_R11_O15_POOR_FETGRO | CD3 on activated CD4 regulatory T cell \|\| id:ebi-a-GCST90001853 | Weighted median | 22 | 0.0349992902217636 | 0.0298180087659886 | 0.240489769135134 | -0.023444007 | 0.0934425874031012 | 1.03561897373955 | 0.976828668744724 | 1.09794756551074 | 0.999440741871876 |
| ebi-a-GCST90001853 | finngen_R11_O15_POOR_FETGRO | Poor fetal growth \|\| id:finngen_R11_O15_POOR_FETGRO | CD3 on activated CD4 regulatory T cell \|\| id:ebi-a-GCST90001853 | Weighted mode | 22 | 0.0516931827018522 | 0.0287979473018302 | 0.0870565478244055 | -0.004750794 | 0.108137159413439 | 1.05305259818978 | 0.995260473162394 | 1.11420055800137 | 0.999857399747617 |
| ebi-a-GCST90001854 | finngen_R11_O15_POOR_FETGRO | Poor fetal growth \|\| id:finngen_R11_O15_POOR_FETGRO | CD3 on CD39+ activated CD4 regulatory T cell \|\| id:ebi-a-GCST90001854 | Inverse variance weighted | 26 | 0.00858986899286351 | 0.0211563429644688 | 0.684728925520895 | -0.032876563 | 0.0500563012032224 | 1.00862686777989 | 0.967657996807098 | 1.05133028586987 | 0.994119304454654 |
| ebi-a-GCST90001854 | finngen_R11_O15_POOR_FETGRO | Poor fetal growth \|\| id:finngen_R11_O15_POOR_FETGRO | CD3 on CD39+ activated CD4 regulatory T cell \|\| id:ebi-a-GCST90001854 | MR Egger | 26 | 0.0412651108234404 | 0.036156930413322 | 0.26501442527349 | -0.029602473 | 0.112132694433552 | 1.04212834843096 | 0.970831388746037 | 1.11866129092323 | 0.992899239648112 |
| ebi-a-GCST90001854 | finngen_R11_O15_POOR_FETGRO | Poor fetal growth \|\| id:finngen_R11_O15_POOR_FETGRO | CD3 on CD39+ activated CD4 regulatory T cell \|\| id:ebi-a-GCST90001854 | Weighted median | 26 | 0.0326925937678574 | 0.0302854816732558 | 0.280373481013225 | -0.02666695 | 0.0920521378474387 | 1.03323286819384 | 0.973685473172034 | 1.09642198567283 | 0.999440741871876 |
| ebi-a-GCST90001854 | finngen_R11_O15_POOR_FETGRO | Poor fetal growth \|\| id:finngen_R11_O15_POOR_FETGRO | CD3 on CD39+ activated CD4 regulatory T cell \|\| id:ebi-a-GCST90001854 | Weighted mode | 26 | 0.0467658958776707 | 0.0285570691952896 | 0.11402632132246 | -0.00920596 | 0.102737751500438 | 1.04787666811541 | 0.990836285367325 | 1.10820074698171 | 0.999857399747617 |
| ebi-a-GCST90001855 | finngen_R11_O15_POOR_FETGRO | Poor fetal growth \|\| id:finngen_R11_O15_POOR_FETGRO | CD3 on secreting CD4 regulatory T cell \|\| id:ebi-a-GCST90001855 | Inverse variance weighted | 21 | 0.00819813019781928 | 0.0220642351002132 | 0.710222399114596 | -0.035047771 | 0.0514440309942372 | 1.00823182688753 | 0.965559289810575 | 1.05279026101897 | 0.994119304454654 |
| ebi-a-GCST90001855 | finngen_R11_O15_POOR_FETGRO | Poor fetal growth \|\| id:finngen_R11_O15_POOR_FETGRO | CD3 on secreting CD4 regulatory T cell \|\| id:ebi-a-GCST90001855 | MR Egger | 21 | -0.00420168 | 0.0355542178302869 | 0.90716808640031 | -0.073887947 | 0.0654845872452649 | 0.995807135004216 | 0.928775760446531 | 1.06767628135402 | 0.992899239648112 |
| ebi-a-GCST90001855 | finngen_R11_O15_POOR_FETGRO | Poor fetal growth \|\| id:finngen_R11_O15_POOR_FETGRO | CD3 on secreting CD4 regulatory T cell \|\| id:ebi-a-GCST90001855 | Weighted median | 21 | -0.007792242 | 0.0315374452187096 | 0.804847047581124 | -0.069605635 | 0.0540211506543671 | 0.992238038840332 | 0.932761596279291 | 1.05550692658118 | 0.999440741871876 |
| ebi-a-GCST90001855 | finngen_R11_O15_POOR_FETGRO | Poor fetal growth \|\| id:finngen_R11_O15_POOR_FETGRO | CD3 on secreting CD4 regulatory T cell \|\| id:ebi-a-GCST90001855 | Weighted mode | 21 | 0.0112884233192851 | 0.0342262961558499 | 0.744967514347147 | -0.055795117 | 0.0783719637847509 | 1.01135237799239 | 0.945732880493751 | 1.08152487194573 | 0.999857399747617 |
| ebi-a-GCST90001856 | finngen_R11_O15_POOR_FETGRO | Poor fetal growth \|\| id:finngen_R11_O15_POOR_FETGRO | CD3 on CD39+ secreting CD4 regulatory T cell \|\| id:ebi-a-GCST90001856 | Inverse variance weighted | 27 | 0.017909287801717 | 0.0228032448967939 | 0.432228867261417 | -0.026785072 | 0.062603647799433 | 1.01807062077695 | 0.973570466401794 | 1.06460479714409 | 0.994119304454654 |
| ebi-a-GCST90001856 | finngen_R11_O15_POOR_FETGRO | Poor fetal growth \|\| id:finngen_R11_O15_POOR_FETGRO | CD3 on CD39+ secreting CD4 regulatory T cell \|\| id:ebi-a-GCST90001856 | MR Egger | 27 | 0.0501094406649836 | 0.039523674121927 | 0.216538729551927 | -0.027356961 | 0.127575841943961 | 1.05138615447979 | 0.97301385190382 | 1.13607102680905 | 0.992899239648112 |
| ebi-a-GCST90001856 | finngen_R11_O15_POOR_FETGRO | Poor fetal growth \|\| id:finngen_R11_O15_POOR_FETGRO | CD3 on CD39+ secreting CD4 regulatory T cell \|\| id:ebi-a-GCST90001856 | Weighted median | 27 | 0.0529809271615723 | 0.0341406268851604 | 0.120699553750637 | -0.013934702 | 0.119896555856487 | 1.05440953434489 | 0.986161937022829 | 1.12738022466554 | 0.999440741871876 |
| ebi-a-GCST90001856 | finngen_R11_O15_POOR_FETGRO | Poor fetal growth \|\| id:finngen_R11_O15_POOR_FETGRO | CD3 on CD39+ secreting CD4 regulatory T cell \|\| id:ebi-a-GCST90001856 | Weighted mode | 27 | 0.0715562820147651 | 0.0399149252505127 | 0.0846613574418067 | -0.006676971 | 0.14978953550577 | 1.07417860593619 | 0.993345269968446 | 1.1615897436021 | 0.999857399747617 |
| ebi-a-GCST90001857 | finngen_R11_O15_POOR_FETGRO | Poor fetal growth \|\| id:finngen_R11_O15_POOR_FETGRO | CD3 on activated & secreting CD4 regulatory T cell \|\| id:ebi-a-GCST90001857 | Inverse variance weighted | 23 | 0.0260732551872555 | 0.0206165387846345 | 0.205987263903206 | -0.014335161 | 0.0664816712051391 | 1.02641613602572 | 0.9857670983702 | 1.06874137515423 | 0.994119304454654 |
| ebi-a-GCST90001857 | finngen_R11_O15_POOR_FETGRO | Poor fetal growth \|\| id:finngen_R11_O15_POOR_FETGRO | CD3 on activated & secreting CD4 regulatory T cell \|\| id:ebi-a-GCST90001857 | MR Egger | 23 | 0.0478773005296771 | 0.0321762470574181 | 0.151621995187729 | -0.015188144 | 0.110942744762217 | 1.04904193053818 | 0.984926614429441 | 1.11733093197483 | 0.992899239648112 |
| ebi-a-GCST90001857 | finngen_R11_O15_POOR_FETGRO | Poor fetal growth \|\| id:finngen_R11_O15_POOR_FETGRO | CD3 on activated & secreting CD4 regulatory T cell \|\| id:ebi-a-GCST90001857 | Weighted median | 23 | 0.0332137964872033 | 0.0291564683040035 | 0.254637655041504 | -0.023932881 | 0.0903604743630501 | 1.03377153233887 | 0.976351238898123 | 1.09456877658121 | 0.999440741871876 |
| ebi-a-GCST90001857 | finngen_R11_O15_POOR_FETGRO | Poor fetal growth \|\| id:finngen_R11_O15_POOR_FETGRO | CD3 on activated & secreting CD4 regulatory T cell \|\| id:ebi-a-GCST90001857 | Weighted mode | 23 | 0.0460892299108072 | 0.031282946415455 | 0.154834391463155 | -0.015225345 | 0.107403804885099 | 1.04716784548175 | 0.984889974500803 | 1.11338375351692 | 0.999857399747617 |
| ebi-a-GCST90001858 | finngen_R11_O15_POOR_FETGRO | Poor fetal growth \|\| id:finngen_R11_O15_POOR_FETGRO | CD3 on CD45RA+ CD4+ T cell \|\| id:ebi-a-GCST90001858 | Inverse variance weighted | 32 | -0.003070453 | 0.0175989259632236 | 0.861497534119219 | -0.037564348 | 0.0314234422651742 | 0.996934256396071 | 0.963132440551817 | 1.03192237093224 | 0.994119304454654 |
| ebi-a-GCST90001858 | finngen_R11_O15_POOR_FETGRO | Poor fetal growth \|\| id:finngen_R11_O15_POOR_FETGRO | CD3 on CD45RA+ CD4+ T cell \|\| id:ebi-a-GCST90001858 | MR Egger | 32 | 0.0152238160572651 | 0.024817933271918 | 0.544221972145726 | -0.033419333 | 0.0638669652702244 | 1.01534028864676 | 0.967132923645828 | 1.06595058088088 | 0.992899239648112 |
| ebi-a-GCST90001858 | finngen_R11_O15_POOR_FETGRO | Poor fetal growth \|\| id:finngen_R11_O15_POOR_FETGRO | CD3 on CD45RA+ CD4+ T cell \|\| id:ebi-a-GCST90001858 | Weighted median | 32 | -0.023205036 | 0.0289479057207575 | 0.422776496262985 | -0.079942931 | 0.0335328597006985 | 0.977062130800011 | 0.923169029470847 | 1.03410142343126 | 0.999440741871876 |
| ebi-a-GCST90001858 | finngen_R11_O15_POOR_FETGRO | Poor fetal growth \|\| id:finngen_R11_O15_POOR_FETGRO | CD3 on CD45RA+ CD4+ T cell \|\| id:ebi-a-GCST90001858 | Weighted mode | 32 | -0.013855165 | 0.0299986717266076 | 0.647407166959351 | -0.072652562 | 0.0449422313962381 | 0.986240375858629 | 0.929923865004966 | 1.04596743408513 | 0.999857399747617 |
| ebi-a-GCST90001859 | finngen_R11_O15_POOR_FETGRO | Poor fetal growth \|\| id:finngen_R11_O15_POOR_FETGRO | CD3 on CD8+ T cell \|\| id:ebi-a-GCST90001859 | Inverse variance weighted | 23 | 0.0466791582129131 | 0.0199972119540494 | 0.0195808918076013 | 0.00748462278297632 | 0.0858736936428499 | 1.04778578168196 | 1.00751270258393 | 1.08966868753044 | 0.994119304454654 |
| ebi-a-GCST90001859 | finngen_R11_O15_POOR_FETGRO | Poor fetal growth \|\| id:finngen_R11_O15_POOR_FETGRO | CD3 on CD8+ T cell \|\| id:ebi-a-GCST90001859 | MR Egger | 23 | 0.0263969604193438 | 0.0278707942174015 | 0.354350166743469 | -0.028229796 | 0.0810237170854508 | 1.0267484460816 | 0.972164941276317 | 1.08439661498896 | 0.992899239648112 |
| ebi-a-GCST90001859 | finngen_R11_O15_POOR_FETGRO | Poor fetal growth \|\| id:finngen_R11_O15_POOR_FETGRO | CD3 on CD8+ T cell \|\| id:ebi-a-GCST90001859 | Weighted median | 23 | 0.0277812581655321 | 0.0270162876799298 | 0.303801602536516 | -0.025170666 | 0.0807331820181945 | 1.0281707558644 | 0.97514347429504 | 1.08408160550835 | 0.999440741871876 |
| ebi-a-GCST90001859 | finngen_R11_O15_POOR_FETGRO | Poor fetal growth \|\| id:finngen_R11_O15_POOR_FETGRO | CD3 on CD8+ T cell \|\| id:ebi-a-GCST90001859 | Weighted mode | 23 | 0.0350364450501794 | 0.0237641924971278 | 0.154557730961489 | -0.011541372 | 0.08161426234455 | 1.03565745269966 | 0.988524973905281 | 1.08503718939439 | 0.999857399747617 |
| ebi-a-GCST90001860 | finngen_R11_O15_POOR_FETGRO | Poor fetal growth \|\| id:finngen_R11_O15_POOR_FETGRO | CD3 on CD39+ CD4+ T cell \|\| id:ebi-a-GCST90001860 | Inverse variance weighted | 28 | 0.0130498022086438 | 0.0194560110304018 | 0.502390192163424 | -0.025083979 | 0.0511835838282313 | 1.01313532248013 | 0.975228009515544 | 1.0525161004828 | 0.994119304454654 |
| ebi-a-GCST90001860 | finngen_R11_O15_POOR_FETGRO | Poor fetal growth \|\| id:finngen_R11_O15_POOR_FETGRO | CD3 on CD39+ CD4+ T cell \|\| id:ebi-a-GCST90001860 | MR Egger | 28 | 0.0405158745036916 | 0.0315545353731183 | 0.210468302345643 | -0.021331015 | 0.102362763835003 | 1.04134784045145 | 0.978894882213692 | 1.10778526327627 | 0.992899239648112 |
| ebi-a-GCST90001860 | finngen_R11_O15_POOR_FETGRO | Poor fetal growth \|\| id:finngen_R11_O15_POOR_FETGRO | CD3 on CD39+ CD4+ T cell \|\| id:ebi-a-GCST90001860 | Weighted median | 28 | 0.0472109234997802 | 0.026968960155879 | 0.0800208316985687 | -0.005648238 | 0.100070085405303 | 1.04834310595845 | 0.994367682902915 | 1.10524837714171 | 0.999440741871876 |
| ebi-a-GCST90001860 | finngen_R11_O15_POOR_FETGRO | Poor fetal growth \|\| id:finngen_R11_O15_POOR_FETGRO | CD3 on CD39+ CD4+ T cell \|\| id:ebi-a-GCST90001860 | Weighted mode | 28 | 0.0542372685712352 | 0.0261144257427806 | 0.047452156214419 | 0.0030529941153852 | 0.105421543027085 | 1.05573506519092 | 1.00305765924825 | 1.1111789213683 | 0.999857399747617 |
| ebi-a-GCST90001861 | finngen_R11_O15_POOR_FETGRO | Poor fetal growth \|\| id:finngen_R11_O15_POOR_FETGRO | CD3 on CD28+ CD4+ T cell \|\| id:ebi-a-GCST90001861 | Inverse variance weighted | 23 | -0.012414562 | 0.0297057742514881 | 0.676007452081342 | -0.07063788 | 0.0458087552295137 | 0.98766218047093 | 0.931799254339575 | 1.04687418259846 | 0.994119304454654 |
| ebi-a-GCST90001861 | finngen_R11_O15_POOR_FETGRO | Poor fetal growth \|\| id:finngen_R11_O15_POOR_FETGRO | CD3 on CD28+ CD4+ T cell \|\| id:ebi-a-GCST90001861 | MR Egger | 23 | -0.002094226 | 0.0562828422533858 | 0.970669846834353 | -0.112408597 | 0.108220145102106 | 0.99790796564614 | 0.893679028738717 | 1.11429302453864 | 0.992899239648112 |
| ebi-a-GCST90001861 | finngen_R11_O15_POOR_FETGRO | Poor fetal growth \|\| id:finngen_R11_O15_POOR_FETGRO | CD3 on CD28+ CD4+ T cell \|\| id:ebi-a-GCST90001861 | Weighted median | 23 | 0.0154864303799491 | 0.0430936995576598 | 0.719320960103404 | -0.068977221 | 0.0999500815129622 | 1.01560696656415 | 0.93334794079927 | 1.10511575099244 | 0.999440741871876 |
| ebi-a-GCST90001861 | finngen_R11_O15_POOR_FETGRO | Poor fetal growth \|\| id:finngen_R11_O15_POOR_FETGRO | CD3 on CD28+ CD4+ T cell \|\| id:ebi-a-GCST90001861 | Weighted mode | 23 | 0.0602359151290304 | 0.0490968816248049 | 0.232836655879202 | -0.035993973 | 0.156465803113648 | 1.06208707940233 | 0.964646107526999 | 1.16937077279587 | 0.999857399747617 |
| ebi-a-GCST90001862 | finngen_R11_O15_POOR_FETGRO | Poor fetal growth \|\| id:finngen_R11_O15_POOR_FETGRO | CD3 on CD28+ CD4-CD8- T cell \|\| id:ebi-a-GCST90001862 | Inverse variance weighted | 16 | -0.02189164 | 0.0244123217960017 | 0.369854724223683 | -0.069739791 | 0.025956510490112 | 0.978346242681412 | 0.9326364687842 | 1.02629631437918 | 0.994119304454654 |
| ebi-a-GCST90001862 | finngen_R11_O15_POOR_FETGRO | Poor fetal growth \|\| id:finngen_R11_O15_POOR_FETGRO | CD3 on CD28+ CD4-CD8- T cell \|\| id:ebi-a-GCST90001862 | MR Egger | 16 | 0.0152555031104941 | 0.0334678967484439 | 0.655506058012746 | -0.050341575 | 0.0808525807374442 | 1.01537246229828 | 0.950904564255151 | 1.08421105119128 | 0.992899239648112 |
| ebi-a-GCST90001862 | finngen_R11_O15_POOR_FETGRO | Poor fetal growth \|\| id:finngen_R11_O15_POOR_FETGRO | CD3 on CD28+ CD4-CD8- T cell \|\| id:ebi-a-GCST90001862 | Weighted median | 16 | 0.00722081121886814 | 0.0339075570553515 | 0.831361431901788 | -0.059238001 | 0.073679623047357 | 1.00724694413862 | 0.942482431068707 | 1.07646187666986 | 0.999440741871876 |
| ebi-a-GCST90001862 | finngen_R11_O15_POOR_FETGRO | Poor fetal growth \|\| id:finngen_R11_O15_POOR_FETGRO | CD3 on CD28+ CD4-CD8- T cell \|\| id:ebi-a-GCST90001862 | Weighted mode | 16 | -0.00238725 | 0.0300499028519625 | 0.937730546150179 | -0.06128506 | 0.0565105592299742 | 0.997615596856145 | 0.940555086972213 | 1.05813778786148 | 0.999857399747617 |
| ebi-a-GCST90001863 | finngen_R11_O15_POOR_FETGRO | Poor fetal growth \|\| id:finngen_R11_O15_POOR_FETGRO | CD3 on CD28+ CD45RA- CD8+ T cell \|\| id:ebi-a-GCST90001863 | Inverse variance weighted | 18 | 0.0250219932965977 | 0.0325782409703305 | 0.442452459775106 | -0.038831359 | 0.0888753455984455 | 1.02533767083196 | 0.961912913419419 | 1.09294440750345 | 0.994119304454654 |
| ebi-a-GCST90001863 | finngen_R11_O15_POOR_FETGRO | Poor fetal growth \|\| id:finngen_R11_O15_POOR_FETGRO | CD3 on CD28+ CD45RA- CD8+ T cell \|\| id:ebi-a-GCST90001863 | MR Egger | 18 | -0.043710714 | 0.073045045721083 | 0.557945005881805 | -0.186879004 | 0.09945757559224 | 0.95723083088128 | 0.829544102134795 | 1.10457160894958 | 0.992899239648112 |
| ebi-a-GCST90001863 | finngen_R11_O15_POOR_FETGRO | Poor fetal growth \|\| id:finngen_R11_O15_POOR_FETGRO | CD3 on CD28+ CD45RA- CD8+ T cell \|\| id:ebi-a-GCST90001863 | Weighted median | 18 | 0.00256482931092922 | 0.0443098793039394 | 0.953841095183698 | -0.084282534 | 0.0894121927466504 | 1.00256812129949 | 0.919171522083466 | 1.0935313091159 | 0.999440741871876 |
| ebi-a-GCST90001863 | finngen_R11_O15_POOR_FETGRO | Poor fetal growth \|\| id:finngen_R11_O15_POOR_FETGRO | CD3 on CD28+ CD45RA- CD8+ T cell \|\| id:ebi-a-GCST90001863 | Weighted mode | 18 | 0.0886008119609257 | 0.0664711639205249 | 0.200147269291702 | -0.041682669 | 0.218884293245154 | 1.09264439868279 | 0.959174107652423 | 1.24468724963279 | 0.999857399747617 |
| ebi-a-GCST90001864 | finngen_R11_O15_POOR_FETGRO | Poor fetal growth \|\| id:finngen_R11_O15_POOR_FETGRO | CD3 on CD28+ CD45RA+ CD8+ T cell \|\| id:ebi-a-GCST90001864 | Inverse variance weighted | 24 | 0.0399674622516499 | 0.0252789975825346 | 0.113865673115024 | -0.009579373 | 0.0895142975134176 | 1.04077690910429 | 0.990466363026158 | 1.0936429695756 | 0.994119304454654 |
| ebi-a-GCST90001864 | finngen_R11_O15_POOR_FETGRO | Poor fetal growth \|\| id:finngen_R11_O15_POOR_FETGRO | CD3 on CD28+ CD45RA+ CD8+ T cell \|\| id:ebi-a-GCST90001864 | MR Egger | 24 | 0.0488862708649206 | 0.0500891971405631 | 0.339682768774473 | -0.049288556 | 0.147061097260424 | 1.05010091687958 | 0.951906412204946 | 1.15842473744564 | 0.992899239648112 |
| ebi-a-GCST90001864 | finngen_R11_O15_POOR_FETGRO | Poor fetal growth \|\| id:finngen_R11_O15_POOR_FETGRO | CD3 on CD28+ CD45RA+ CD8+ T cell \|\| id:ebi-a-GCST90001864 | Weighted median | 24 | 0.0736922657233262 | 0.0337221164178351 | 0.0288683247303494 | 0.00759691754436948 | 0.139787613902283 | 1.07647548611459 | 1.00762584733513 | 1.15002952263514 | 0.999440741871876 |
| ebi-a-GCST90001864 | finngen_R11_O15_POOR_FETGRO | Poor fetal growth \|\| id:finngen_R11_O15_POOR_FETGRO | CD3 on CD28+ CD45RA+ CD8+ T cell \|\| id:ebi-a-GCST90001864 | Weighted mode | 24 | 0.0696036615564359 | 0.0364900430673931 | 0.0690236274015788 | -0.001916823 | 0.141124145968526 | 1.07208318925685 | 0.998085013076036 | 1.15156760158623 | 0.999857399747617 |
| ebi-a-GCST90001865 | finngen_R11_O15_POOR_FETGRO | Poor fetal growth \|\| id:finngen_R11_O15_POOR_FETGRO | CD3 on CD28- CD8+ T cell \|\| id:ebi-a-GCST90001865 | Inverse variance weighted | 20 | 0.013682085870027 | 0.0314984505723612 | 0.664017384425547 | -0.048054877 | 0.0754190489918549 | 1.01377611395094 | 0.953081483149832 | 1.07833593180395 | 0.994119304454654 |
| ebi-a-GCST90001865 | finngen_R11_O15_POOR_FETGRO | Poor fetal growth \|\| id:finngen_R11_O15_POOR_FETGRO | CD3 on CD28- CD8+ T cell \|\| id:ebi-a-GCST90001865 | MR Egger | 20 | 0.0345104919224623 | 0.0755434040208402 | 0.653258102498264 | -0.11355458 | 0.182575563803309 | 1.03511288864331 | 0.892655473982941 | 1.20030484712624 | 0.992899239648112 |
| ebi-a-GCST90001865 | finngen_R11_O15_POOR_FETGRO | Poor fetal growth \|\| id:finngen_R11_O15_POOR_FETGRO | CD3 on CD28- CD8+ T cell \|\| id:ebi-a-GCST90001865 | Weighted median | 20 | 0.0031746901924069 | 0.0430867690096137 | 0.94126395958775 | -0.081275377 | 0.0876247574512497 | 1.00317973485832 | 0.921939775414103 | 1.09157843849224 | 0.999440741871876 |
| ebi-a-GCST90001865 | finngen_R11_O15_POOR_FETGRO | Poor fetal growth \|\| id:finngen_R11_O15_POOR_FETGRO | CD3 on CD28- CD8+ T cell \|\| id:ebi-a-GCST90001865 | Weighted mode | 20 | 0.0139651997045464 | 0.0684425715597443 | 0.840489854415026 | -0.120182241 | 0.148112639961645 | 1.01406316862656 | 0.886758818573837 | 1.15964351120723 | 0.999857399747617 |
| ebi-a-GCST90001866 | finngen_R11_O15_POOR_FETGRO | Poor fetal growth \|\| id:finngen_R11_O15_POOR_FETGRO | CD3 on CD39+ CD8+ T cell \|\| id:ebi-a-GCST90001866 | Inverse variance weighted | 16 | 0.0272386507790415 | 0.0323841150054767 | 0.400285486506048 | -0.036234215 | 0.0907115161897759 | 1.02761301414876 | 0.964414387068395 | 1.09495308345396 | 0.994119304454654 |
| ebi-a-GCST90001866 | finngen_R11_O15_POOR_FETGRO | Poor fetal growth \|\| id:finngen_R11_O15_POOR_FETGRO | CD3 on CD39+ CD8+ T cell \|\| id:ebi-a-GCST90001866 | MR Egger | 16 | 0.18255989444172 | 0.0775368332650232 | 0.0336737873486789 | 0.0305877012422748 | 0.334532087641166 | 1.20028603926293 | 1.03106031135386 | 1.39728642464936 | 0.992899239648112 |
| ebi-a-GCST90001866 | finngen_R11_O15_POOR_FETGRO | Poor fetal growth \|\| id:finngen_R11_O15_POOR_FETGRO | CD3 on CD39+ CD8+ T cell \|\| id:ebi-a-GCST90001866 | Weighted median | 16 | 0.0115803953646048 | 0.0468109775597329 | 0.804609262096502 | -0.080169121 | 0.103329911381681 | 1.01164770772665 | 0.922960241548476 | 1.10885717333994 | 0.999440741871876 |
| ebi-a-GCST90001866 | finngen_R11_O15_POOR_FETGRO | Poor fetal growth \|\| id:finngen_R11_O15_POOR_FETGRO | CD3 on CD39+ CD8+ T cell \|\| id:ebi-a-GCST90001866 | Weighted mode | 16 | -0.008228947 | 0.0596638417878761 | 0.892136612447542 | -0.125170077 | 0.108712183057852 | 0.991804818256161 | 0.882346823141844 | 1.11484143390858 | 0.999857399747617 |
| ebi-a-GCST90001867 | finngen_R11_O15_POOR_FETGRO | Poor fetal growth \|\| id:finngen_R11_O15_POOR_FETGRO | CD3 on CD4+ T cell \|\| id:ebi-a-GCST90001867 | Inverse variance weighted | 28 | -0.020908661 | 0.0239339362465953 | 0.382336707662515 | -0.067819176 | 0.0260018538832582 | 0.979308409378553 | 0.934429425377856 | 1.02634285119151 | 0.994119304454654 |
| ebi-a-GCST90001867 | finngen_R11_O15_POOR_FETGRO | Poor fetal growth \|\| id:finngen_R11_O15_POOR_FETGRO | CD3 on CD4+ T cell \|\| id:ebi-a-GCST90001867 | MR Egger | 28 | 0.00138341920754322 | 0.0365389489604795 | 0.97008732907653 | -0.070232921 | 0.072999759170083 | 1.00138437657332 | 0.932176671323649 | 1.07573027784664 | 0.992899239648112 |
| ebi-a-GCST90001867 | finngen_R11_O15_POOR_FETGRO | Poor fetal growth \|\| id:finngen_R11_O15_POOR_FETGRO | CD3 on CD4+ T cell \|\| id:ebi-a-GCST90001867 | Weighted median | 28 | 0.0259761203360899 | 0.0356426844671557 | 0.466128613464618 | -0.043883541 | 0.0958357818917152 | 1.02631644008916 | 0.957065409653561 | 1.10057831426441 | 0.999440741871876 |
| ebi-a-GCST90001867 | finngen_R11_O15_POOR_FETGRO | Poor fetal growth \|\| id:finngen_R11_O15_POOR_FETGRO | CD3 on CD4+ T cell \|\| id:ebi-a-GCST90001867 | Weighted mode | 28 | 0.0130869484355614 | 0.035833932855301 | 0.717799283774156 | -0.05714756 | 0.0833214568319514 | 1.0131729573337 | 0.944454695385911 | 1.08689114098044 | 0.999857399747617 |
| ebi-a-GCST90001868 | finngen_R11_O15_POOR_FETGRO | Poor fetal growth \|\| id:finngen_R11_O15_POOR_FETGRO | CD3 on CD4 regulatory T cell \|\| id:ebi-a-GCST90001868 | Inverse variance weighted | 26 | 0.0230060884504935 | 0.0199716329695075 | 0.249346438222659 | -0.016138312 | 0.0621504890707282 | 1.02327276967385 | 0.983991212683217 | 1.06412247148094 | 0.994119304454654 |
| ebi-a-GCST90001868 | finngen_R11_O15_POOR_FETGRO | Poor fetal growth \|\| id:finngen_R11_O15_POOR_FETGRO | CD3 on CD4 regulatory T cell \|\| id:ebi-a-GCST90001868 | MR Egger | 26 | 0.0677075103517155 | 0.0305271296065315 | 0.0362751334608816 | 0.00787433632291382 | 0.127540684380517 | 1.07005228348668 | 1.00790542044455 | 1.13603108602197 | 0.992899239648112 |
| ebi-a-GCST90001868 | finngen_R11_O15_POOR_FETGRO | Poor fetal growth \|\| id:finngen_R11_O15_POOR_FETGRO | CD3 on CD4 regulatory T cell \|\| id:ebi-a-GCST90001868 | Weighted median | 26 | 0.0335748610920853 | 0.0271017076169377 | 0.215402274918721 | -0.019544486 | 0.0866942080212832 | 1.03414485804203 | 0.980645269392589 | 1.090563143 | 0.999440741871876 |
| ebi-a-GCST90001868 | finngen_R11_O15_POOR_FETGRO | Poor fetal growth \|\| id:finngen_R11_O15_POOR_FETGRO | CD3 on CD4 regulatory T cell \|\| id:ebi-a-GCST90001868 | Weighted mode | 26 | 0.0461756950538322 | 0.0286096956813486 | 0.119082880721452 | -0.009899308 | 0.102250698589276 | 1.04725839291382 | 0.990149528389327 | 1.1076611260044 | 0.999857399747617 |
| ebi-a-GCST90001869 | finngen_R11_O15_POOR_FETGRO | Poor fetal growth \|\| id:finngen_R11_O15_POOR_FETGRO | CD3 on resting CD4 regulatory T cell \|\| id:ebi-a-GCST90001869 | Inverse variance weighted | 37 | -0.006536024 | 0.0239378469904156 | 0.784821381526595 | -0.053454204 | 0.0403821565317478 | 0.993485289772468 | 0.947949352610479 | 1.04120860283963 | 0.994119304454654 |
| ebi-a-GCST90001869 | finngen_R11_O15_POOR_FETGRO | Poor fetal growth \|\| id:finngen_R11_O15_POOR_FETGRO | CD3 on resting CD4 regulatory T cell \|\| id:ebi-a-GCST90001869 | MR Egger | 37 | 0.0525713624333066 | 0.0366519687015408 | 0.160350551885573 | -0.019266496 | 0.124409221088327 | 1.05397777381345 | 0.980917916488645 | 1.1324792105636 | 0.992899239648112 |
| ebi-a-GCST90001869 | finngen_R11_O15_POOR_FETGRO | Poor fetal growth \|\| id:finngen_R11_O15_POOR_FETGRO | CD3 on resting CD4 regulatory T cell \|\| id:ebi-a-GCST90001869 | Weighted median | 37 | -0.030632939 | 0.036239919460292 | 0.397953605296802 | -0.101663181 | 0.0403973033144909 | 0.96983149523502 | 0.903333760435438 | 1.04122437391956 | 0.999440741871876 |
| ebi-a-GCST90001869 | finngen_R11_O15_POOR_FETGRO | Poor fetal growth \|\| id:finngen_R11_O15_POOR_FETGRO | CD3 on resting CD4 regulatory T cell \|\| id:ebi-a-GCST90001869 | Weighted mode | 37 | -0.01935206 | 0.0411182367764993 | 0.640734066400554 | -0.099943804 | 0.0612396841155354 | 0.980833989069101 | 0.90488826766452 | 1.06315370470675 | 0.999857399747617 |
| ebi-a-GCST90001870 | finngen_R11_O15_POOR_FETGRO | Poor fetal growth \|\| id:finngen_R11_O15_POOR_FETGRO | CD34 on Hematopoietic Stem Cell \|\| id:ebi-a-GCST90001870 | Inverse variance weighted | 12 | 0.000145873654390911 | 0.0163224062464876 | 0.992869385458736 | -0.031846043 | 0.0321377898975066 | 1.00014588429447 | 0.968655702324167 | 1.03265978558852 | 0.998984604424952 |
| ebi-a-GCST90001870 | finngen_R11_O15_POOR_FETGRO | Poor fetal growth \|\| id:finngen_R11_O15_POOR_FETGRO | CD34 on Hematopoietic Stem Cell \|\| id:ebi-a-GCST90001870 | MR Egger | 12 | -0.006673921 | 0.0231517340920267 | 0.77902756740095 | -0.05205132 | 0.0387034775919247 | 0.993348299922283 | 0.949280148489222 | 1.03946221410917 | 0.992899239648112 |
| ebi-a-GCST90001870 | finngen_R11_O15_POOR_FETGRO | Poor fetal growth \|\| id:finngen_R11_O15_POOR_FETGRO | CD34 on Hematopoietic Stem Cell \|\| id:ebi-a-GCST90001870 | Weighted median | 12 | -0.01314959 | 0.0208395546704654 | 0.528045815726796 | -0.053995118 | 0.0276959368000756 | 0.986936487798642 | 0.947436732342631 | 1.02808303467388 | 0.999440741871876 |
| ebi-a-GCST90001870 | finngen_R11_O15_POOR_FETGRO | Poor fetal growth \|\| id:finngen_R11_O15_POOR_FETGRO | CD34 on Hematopoietic Stem Cell \|\| id:ebi-a-GCST90001870 | Weighted mode | 12 | -0.009521924 | 0.0196840409726141 | 0.638057778160128 | -0.048102644 | 0.0290587963677246 | 0.990523266033581 | 0.953035958400481 | 1.02948512268153 | 0.999857399747617 |
| ebi-a-GCST90001871 | finngen_R11_O15_POOR_FETGRO | Poor fetal growth \|\| id:finngen_R11_O15_POOR_FETGRO | HVEM on T cell \|\| id:ebi-a-GCST90001871 | Inverse variance weighted | 25 | -0.003835289 | 0.0212954293911646 | 0.85707472633478 | -0.04557433 | 0.0379037527777427 | 0.996172056497777 | 0.955448581023214 | 1.03863126269366 | 0.994119304454654 |
| ebi-a-GCST90001871 | finngen_R11_O15_POOR_FETGRO | Poor fetal growth \|\| id:finngen_R11_O15_POOR_FETGRO | HVEM on T cell \|\| id:ebi-a-GCST90001871 | MR Egger | 25 | -0.008753602 | 0.039838787317816 | 0.828023297624858 | -0.086837625 | 0.0693304213161395 | 0.991284599398323 | 0.91682595318938 | 1.07179029300594 | 0.992899239648112 |
| ebi-a-GCST90001871 | finngen_R11_O15_POOR_FETGRO | Poor fetal growth \|\| id:finngen_R11_O15_POOR_FETGRO | HVEM on T cell \|\| id:ebi-a-GCST90001871 | Weighted median | 25 | -0.022074022 | 0.0280113314493483 | 0.430673888514943 | -0.076976232 | 0.0328281877554552 | 0.978167826544707 | 0.925911860856955 | 1.03337297785735 | 0.999440741871876 |
| ebi-a-GCST90001871 | finngen_R11_O15_POOR_FETGRO | Poor fetal growth \|\| id:finngen_R11_O15_POOR_FETGRO | HVEM on T cell \|\| id:ebi-a-GCST90001871 | Weighted mode | 25 | -0.025467378 | 0.0310357072949125 | 0.419959860720195 | -0.086297365 | 0.0353626079254629 | 0.974854179776897 | 0.917321411679302 | 1.035995301 | 0.999857399747617 |
| ebi-a-GCST90001872 | finngen_R11_O15_POOR_FETGRO | Poor fetal growth \|\| id:finngen_R11_O15_POOR_FETGRO | HVEM on naive CD8+ T cell \|\| id:ebi-a-GCST90001872 | Inverse variance weighted | 21 | -0.008286422 | 0.0122426369375181 | 0.498501501378985 | -0.03228199 | 0.0157091467401667 | 0.991747816099808 | 0.968233511358365 | 1.01583318404139 | 0.994119304454654 |
| ebi-a-GCST90001872 | finngen_R11_O15_POOR_FETGRO | Poor fetal growth \|\| id:finngen_R11_O15_POOR_FETGRO | HVEM on naive CD8+ T cell \|\| id:ebi-a-GCST90001872 | MR Egger | 21 | -0.005458383 | 0.0179015717549192 | 0.763748933672233 | -0.040545464 | 0.0296286975095023 | 0.994556486775542 | 0.960265506229082 | 1.03007199464201 | 0.992899239648112 |
| ebi-a-GCST90001872 | finngen_R11_O15_POOR_FETGRO | Poor fetal growth \|\| id:finngen_R11_O15_POOR_FETGRO | HVEM on naive CD8+ T cell \|\| id:ebi-a-GCST90001872 | Weighted median | 21 | -0.01057669 | 0.0163208912888269 | 0.516955121375566 | -0.042565637 | 0.0214122566593488 | 0.989479046246063 | 0.958327561525577 | 1.0216431440221 | 0.999440741871876 |
| ebi-a-GCST90001872 | finngen_R11_O15_POOR_FETGRO | Poor fetal growth \|\| id:finngen_R11_O15_POOR_FETGRO | HVEM on naive CD8+ T cell \|\| id:ebi-a-GCST90001872 | Weighted mode | 21 | -0.010812227 | 0.0153048836912066 | 0.488053872295788 | -0.040809799 | 0.0191853451320261 | 0.989246015124891 | 0.960011707830833 | 1.01937056648159 | 0.999857399747617 |
| ebi-a-GCST90001873 | finngen_R11_O15_POOR_FETGRO | Poor fetal growth \|\| id:finngen_R11_O15_POOR_FETGRO | HVEM on Effector Memory CD8+ T cell \|\| id:ebi-a-GCST90001873 | Inverse variance weighted | 18 | -0.025790199 | 0.0261338169044976 | 0.323715400461761 | -0.07701248 | 0.0254320824293867 | 0.974539527818795 | 0.925878298724844 | 1.0257582368971 | 0.994119304454654 |
| ebi-a-GCST90001873 | finngen_R11_O15_POOR_FETGRO | Poor fetal growth \|\| id:finngen_R11_O15_POOR_FETGRO | HVEM on Effector Memory CD8+ T cell \|\| id:ebi-a-GCST90001873 | MR Egger | 18 | 0.00718643839009473 | 0.0490223818236719 | 0.885282847706534 | -0.08889743 | 0.103270306764492 | 1.0072123228069 | 0.914939414111825 | 1.10879108230229 | 0.992899239648112 |
| ebi-a-GCST90001873 | finngen_R11_O15_POOR_FETGRO | Poor fetal growth \|\| id:finngen_R11_O15_POOR_FETGRO | HVEM on Effector Memory CD8+ T cell \|\| id:ebi-a-GCST90001873 | Weighted median | 18 | -0.043394689 | 0.0309356487997309 | 0.160694222598152 | -0.10402856 | 0.0172391828394533 | 0.957533387763844 | 0.901199558383771 | 1.01738863512853 | 0.999440741871876 |
| ebi-a-GCST90001873 | finngen_R11_O15_POOR_FETGRO | Poor fetal growth \|\| id:finngen_R11_O15_POOR_FETGRO | HVEM on Effector Memory CD8+ T cell \|\| id:ebi-a-GCST90001873 | Weighted mode | 18 | -0.043900513 | 0.0346608842430753 | 0.222387864850484 | -0.111835846 | 0.0240348198517703 | 0.957049166434085 | 0.894191030146997 | 1.0243259841486 | 0.999857399747617 |
| ebi-a-GCST90001874 | finngen_R11_O15_POOR_FETGRO | Poor fetal growth \|\| id:finngen_R11_O15_POOR_FETGRO | HVEM on Terminally Differentiated CD8+ T cell \|\| id:ebi-a-GCST90001874 | Inverse variance weighted | 25 | 0.0235895208151376 | 0.0195565225329486 | 0.227731645303315 | -0.014741263 | 0.0619203049797169 | 1.02386995431702 | 0.985366857143574 | 1.06387755560607 | 0.994119304454654 |
| ebi-a-GCST90001874 | finngen_R11_O15_POOR_FETGRO | Poor fetal growth \|\| id:finngen_R11_O15_POOR_FETGRO | HVEM on Terminally Differentiated CD8+ T cell \|\| id:ebi-a-GCST90001874 | MR Egger | 25 | 0.0686311790639036 | 0.0407946880984801 | 0.106028651146058 | -0.01132641 | 0.148588767736925 | 1.0710411139069 | 0.988737492679488 | 1.16019578115744 | 0.992899239648112 |
| ebi-a-GCST90001874 | finngen_R11_O15_POOR_FETGRO | Poor fetal growth \|\| id:finngen_R11_O15_POOR_FETGRO | HVEM on Terminally Differentiated CD8+ T cell \|\| id:ebi-a-GCST90001874 | Weighted median | 25 | 0.0346150604759989 | 0.0273345322735675 | 0.205388123721373 | -0.018960623 | 0.0881907437321912 | 1.03522113456028 | 0.981217999118924 | 1.09219643178436 | 0.999440741871876 |
| ebi-a-GCST90001874 | finngen_R11_O15_POOR_FETGRO | Poor fetal growth \|\| id:finngen_R11_O15_POOR_FETGRO | HVEM on Terminally Differentiated CD8+ T cell \|\| id:ebi-a-GCST90001874 | Weighted mode | 25 | 0.0499857054258508 | 0.0419009397570892 | 0.244549820423626 | -0.032140136 | 0.132111547349746 | 1.05125606901079 | 0.96837086846789 | 1.14123561397558 | 0.999857399747617 |
| ebi-a-GCST90001875 | finngen_R11_O15_POOR_FETGRO | Poor fetal growth \|\| id:finngen_R11_O15_POOR_FETGRO | HVEM on CD4+ T cell \|\| id:ebi-a-GCST90001875 | Inverse variance weighted | 20 | 0.00858951573569947 | 0.0225193517527346 | 0.70288554146004 | -0.035548414 | 0.0527274451710593 | 1.00862651147529 | 0.965076010198861 | 1.05414229438899 | 0.994119304454654 |
| ebi-a-GCST90001875 | finngen_R11_O15_POOR_FETGRO | Poor fetal growth \|\| id:finngen_R11_O15_POOR_FETGRO | HVEM on CD4+ T cell \|\| id:ebi-a-GCST90001875 | MR Egger | 20 | -0.018052664 | 0.0373396931973028 | 0.634590111106028 | -0.091238462 | 0.0551331351338278 | 0.982109309650436 | 0.912800016644609 | 1.05668128671561 | 0.992899239648112 |
| ebi-a-GCST90001875 | finngen_R11_O15_POOR_FETGRO | Poor fetal growth \|\| id:finngen_R11_O15_POOR_FETGRO | HVEM on CD4+ T cell \|\| id:ebi-a-GCST90001875 | Weighted median | 20 | -0.025379529 | 0.0306273197495395 | 0.407298786089833 | -0.085409075 | 0.0346500179801388 | 0.974939824131017 | 0.918136620429047 | 1.03525732393992 | 0.999440741871876 |
| ebi-a-GCST90001875 | finngen_R11_O15_POOR_FETGRO | Poor fetal growth \|\| id:finngen_R11_O15_POOR_FETGRO | HVEM on CD4+ T cell \|\| id:ebi-a-GCST90001875 | Weighted mode | 20 | -0.036456826 | 0.0321471262088135 | 0.270875091559241 | -0.099465193 | 0.0265515416464774 | 0.964199721623755 | 0.905321460760837 | 1.02690717438424 | 0.999857399747617 |
| ebi-a-GCST90001876 | finngen_R11_O15_POOR_FETGRO | Poor fetal growth \|\| id:finngen_R11_O15_POOR_FETGRO | HVEM on Central Memory CD4+ T cell \|\| id:ebi-a-GCST90001876 | Inverse variance weighted (multiplicative random effects) | 17 | 0.00787615168915557 | 0.016227064085281 | 0.627412965149598 | -0.023928894 | 0.0396811972963062 | 1.00790725016369 | 0.976355132077793 | 1.04047901378941 | 0.994119304454654 |
| ebi-a-GCST90001876 | finngen_R11_O15_POOR_FETGRO | Poor fetal growth \|\| id:finngen_R11_O15_POOR_FETGRO | HVEM on Central Memory CD4+ T cell \|\| id:ebi-a-GCST90001876 | MR Egger | 17 | -6.16E-05 | 0.0241312177313045 | 0.997995764342804 | -0.047358821 | 0.0472355526087903 | 0.999938367754778 | 0.953745112506864 | 1.04836892603305 | 0.999424937578259 |
| ebi-a-GCST90001876 | finngen_R11_O15_POOR_FETGRO | Poor fetal growth \|\| id:finngen_R11_O15_POOR_FETGRO | HVEM on Central Memory CD4+ T cell \|\| id:ebi-a-GCST90001876 | Weighted median | 17 | -0.00634306 | 0.0164385646405108 | 0.699596964345337 | -0.038562647 | 0.0258765268621322 | 0.993677014903244 | 0.962171426151881 | 1.02621423075929 | 0.999440741871876 |
| ebi-a-GCST90001876 | finngen_R11_O15_POOR_FETGRO | Poor fetal growth \|\| id:finngen_R11_O15_POOR_FETGRO | HVEM on Central Memory CD4+ T cell \|\| id:ebi-a-GCST90001876 | Weighted mode | 17 | 0.000505219092441278 | 0.0140247119229587 | 0.97170923940753 | -0.026983216 | 0.0279936544614402 | 1.0005053467371 | 0.97337757828732 | 1.02838915871766 | 0.999857399747617 |
| ebi-a-GCST90001877 | finngen_R11_O15_POOR_FETGRO | Poor fetal growth \|\| id:finngen_R11_O15_POOR_FETGRO | HVEM on naive CD4+ T cell \|\| id:ebi-a-GCST90001877 | Inverse variance weighted | 18 | -0.006885149 | 0.0144600153522605 | 0.633966881862611 | -0.035226779 | 0.0214564808713538 | 0.993138499115577 | 0.965386461756125 | 1.02168832638416 | 0.994119304454654 |
| ebi-a-GCST90001877 | finngen_R11_O15_POOR_FETGRO | Poor fetal growth \|\| id:finngen_R11_O15_POOR_FETGRO | HVEM on naive CD4+ T cell \|\| id:ebi-a-GCST90001877 | MR Egger | 18 | -0.029594813 | 0.0218799411928627 | 0.194990903051907 | -0.072479498 | 0.0132898713360977 | 0.970838824745656 | 0.930084814933545 | 1.01337857419043 | 0.992899239648112 |
| ebi-a-GCST90001877 | finngen_R11_O15_POOR_FETGRO | Poor fetal growth \|\| id:finngen_R11_O15_POOR_FETGRO | HVEM on naive CD4+ T cell \|\| id:ebi-a-GCST90001877 | Weighted median | 18 | -0.013200403 | 0.0198368039542678 | 0.505762647001522 | -0.052080539 | 0.0256797324893123 | 0.986886339960746 | 0.949252411913288 | 1.02601229744369 | 0.999440741871876 |
| ebi-a-GCST90001877 | finngen_R11_O15_POOR_FETGRO | Poor fetal growth \|\| id:finngen_R11_O15_POOR_FETGRO | HVEM on naive CD4+ T cell \|\| id:ebi-a-GCST90001877 | Weighted mode | 18 | -0.018161274 | 0.0161126214459436 | 0.275339612148671 | -0.049742012 | 0.0134194644665174 | 0.982002648516628 | 0.951474862315162 | 1.01350990960206 | 0.999857399747617 |
| ebi-a-GCST90001878 | finngen_R11_O15_POOR_FETGRO | Poor fetal growth \|\| id:finngen_R11_O15_POOR_FETGRO | HVEM on Effector Memory CD4+ T cell \|\| id:ebi-a-GCST90001878 | Inverse variance weighted | 21 | -0.011801245 | 0.0142325322216165 | 0.4070059621516 | -0.039697009 | 0.0160945176616752 | 0.988268116085771 | 0.961080594150877 | 1.01622473205238 | 0.994119304454654 |
| ebi-a-GCST90001878 | finngen_R11_O15_POOR_FETGRO | Poor fetal growth \|\| id:finngen_R11_O15_POOR_FETGRO | HVEM on Effector Memory CD4+ T cell \|\| id:ebi-a-GCST90001878 | MR Egger | 21 | -0.020315082 | 0.0216309204797779 | 0.35943122867094 | -0.062711686 | 0.022081522552906 | 0.979889879402936 | 0.93921422352201 | 1.02232712379041 | 0.992899239648112 |
| ebi-a-GCST90001878 | finngen_R11_O15_POOR_FETGRO | Poor fetal growth \|\| id:finngen_R11_O15_POOR_FETGRO | HVEM on Effector Memory CD4+ T cell \|\| id:ebi-a-GCST90001878 | Weighted median | 21 | -0.010907621 | 0.0181974840096807 | 0.548904387405338 | -0.046574689 | 0.0247594478964077 | 0.98915165163011 | 0.954493267357576 | 1.02506850847805 | 0.999440741871876 |
| ebi-a-GCST90001878 | finngen_R11_O15_POOR_FETGRO | Poor fetal growth \|\| id:finngen_R11_O15_POOR_FETGRO | HVEM on Effector Memory CD4+ T cell \|\| id:ebi-a-GCST90001878 | Weighted mode | 21 | -0.012591254 | 0.0161633535795341 | 0.445099815744183 | -0.044271427 | 0.0190889192038528 | 0.987487684366689 | 0.95669424974332 | 1.01927227746745 | 0.999857399747617 |
| ebi-a-GCST90001879 | finngen_R11_O15_POOR_FETGRO | Poor fetal growth \|\| id:finngen_R11_O15_POOR_FETGRO | HVEM on Terminally Differentiated CD4+ T cell \|\| id:ebi-a-GCST90001879 | Inverse variance weighted | 26 | -0.021687995 | 0.0192190782891732 | 0.259124962399063 | -0.059357388 | 0.0159813987460021 | 0.978545498807937 | 0.942369917128228 | 1.01610978431412 | 0.994119304454654 |
| ebi-a-GCST90001879 | finngen_R11_O15_POOR_FETGRO | Poor fetal growth \|\| id:finngen_R11_O15_POOR_FETGRO | HVEM on Terminally Differentiated CD4+ T cell \|\| id:ebi-a-GCST90001879 | MR Egger | 26 | 0.0275721828317209 | 0.0339637009143131 | 0.424877740649622 | -0.038996671 | 0.0941410366237745 | 1.02795581319079 | 0.961753910857957 | 1.09871469400117 | 0.992899239648112 |
| ebi-a-GCST90001879 | finngen_R11_O15_POOR_FETGRO | Poor fetal growth \|\| id:finngen_R11_O15_POOR_FETGRO | HVEM on Terminally Differentiated CD4+ T cell \|\| id:ebi-a-GCST90001879 | Weighted median | 26 | 0.0110228155570165 | 0.0299039110635029 | 0.712420063656084 | -0.04758885 | 0.0696344812414821 | 1.01108379062144 | 0.953525748484505 | 1.07211623103225 | 0.999440741871876 |
| ebi-a-GCST90001879 | finngen_R11_O15_POOR_FETGRO | Poor fetal growth \|\| id:finngen_R11_O15_POOR_FETGRO | HVEM on Terminally Differentiated CD4+ T cell \|\| id:ebi-a-GCST90001879 | Weighted mode | 26 | 0.0135402059665143 | 0.0319973417387113 | 0.675791879781905 | -0.049174584 | 0.0762549957743886 | 1.01363228969684 | 0.952014908769378 | 1.07923774013601 | 0.999857399747617 |
| ebi-a-GCST90001880 | finngen_R11_O15_POOR_FETGRO | Poor fetal growth \|\| id:finngen_R11_O15_POOR_FETGRO | HVEM on CD45RA- CD4+ T cell \|\| id:ebi-a-GCST90001880 | Inverse variance weighted (multiplicative random effects) | 19 | 0.0023795169561808 | 0.0175517798448259 | 0.892160224709795 | -0.032021972 | 0.0367810054520397 | 1.0023823502535 | 0.968485302732172 | 1.03746579660548 | 0.994119304454654 |
| ebi-a-GCST90001880 | finngen_R11_O15_POOR_FETGRO | Poor fetal growth \|\| id:finngen_R11_O15_POOR_FETGRO | HVEM on CD45RA- CD4+ T cell \|\| id:ebi-a-GCST90001880 | MR Egger | 19 | -0.010198153 | 0.0261413809790354 | 0.701295978769911 | -0.061435259 | 0.0410389539569444 | 0.989853672075758 | 0.940413826648007 | 1.04189269059801 | 0.992899239648112 |
| ebi-a-GCST90001880 | finngen_R11_O15_POOR_FETGRO | Poor fetal growth \|\| id:finngen_R11_O15_POOR_FETGRO | HVEM on CD45RA- CD4+ T cell \|\| id:ebi-a-GCST90001880 | Weighted median | 19 | -0.004598798 | 0.0171561411551489 | 0.788656596036363 | -0.038224835 | 0.0290272386133599 | 0.99541176022969 | 0.962496513932735 | 1.02945263495553 | 0.999440741871876 |
| ebi-a-GCST90001880 | finngen_R11_O15_POOR_FETGRO | Poor fetal growth \|\| id:finngen_R11_O15_POOR_FETGRO | HVEM on CD45RA- CD4+ T cell \|\| id:ebi-a-GCST90001880 | Weighted mode | 19 | -0.003364933 | 0.0161850387068767 | 0.837638171427562 | -0.035087609 | 0.0283577424070805 | 0.996640721585465 | 0.96552082392551 | 1.0287636509837 | 0.999857399747617 |
| ebi-a-GCST90001881 | finngen_R11_O15_POOR_FETGRO | Poor fetal growth \|\| id:finngen_R11_O15_POOR_FETGRO | HVEM on CD8+ T cell \|\| id:ebi-a-GCST90001881 | Inverse variance weighted | 16 | -0.02037708 | 0.027834000339314 | 0.464111736943241 | -0.074931721 | 0.0341775607026871 | 0.979829129706034 | 0.927806834234364 | 1.03476832460793 | 0.994119304454654 |
| ebi-a-GCST90001881 | finngen_R11_O15_POOR_FETGRO | Poor fetal growth \|\| id:finngen_R11_O15_POOR_FETGRO | HVEM on CD8+ T cell \|\| id:ebi-a-GCST90001881 | MR Egger | 16 | -0.022098706 | 0.0630331900481877 | 0.731116297774426 | -0.145643759 | 0.101446346002534 | 0.978143681154685 | 0.864465606441396 | 1.10677053413541 | 0.992899239648112 |
| ebi-a-GCST90001881 | finngen_R11_O15_POOR_FETGRO | Poor fetal growth \|\| id:finngen_R11_O15_POOR_FETGRO | HVEM on CD8+ T cell \|\| id:ebi-a-GCST90001881 | Weighted median | 16 | -0.027322379 | 0.0374185172689456 | 0.465278112503126 | -0.100662673 | 0.0460179151866045 | 0.973047501205393 | 0.904238005784068 | 1.04709316965843 | 0.999440741871876 |
| ebi-a-GCST90001881 | finngen_R11_O15_POOR_FETGRO | Poor fetal growth \|\| id:finngen_R11_O15_POOR_FETGRO | HVEM on CD8+ T cell \|\| id:ebi-a-GCST90001881 | Weighted mode | 16 | -0.024092114 | 0.0495099319400629 | 0.633566782362424 | -0.121131581 | 0.0729473524178724 | 0.976195784137545 | 0.885917382216849 | 1.07567390379373 | 0.999857399747617 |
| ebi-a-GCST90001882 | finngen_R11_O15_POOR_FETGRO | Poor fetal growth \|\| id:finngen_R11_O15_POOR_FETGRO | HVEM on Central Memory CD8+ T cell \|\| id:ebi-a-GCST90001882 | Inverse variance weighted | 18 | -0.011232458 | 0.0131618122231233 | 0.393430473087855 | -0.03702961 | 0.0145646940598464 | 0.988830390623182 | 0.963647601461059 | 1.01467127603234 | 0.994119304454654 |
| ebi-a-GCST90001882 | finngen_R11_O15_POOR_FETGRO | Poor fetal growth \|\| id:finngen_R11_O15_POOR_FETGRO | HVEM on Central Memory CD8+ T cell \|\| id:ebi-a-GCST90001882 | MR Egger | 18 | -0.020547922 | 0.0208298060045017 | 0.338587602026271 | -0.061374341 | 0.0202784982315944 | 0.979661748452666 | 0.940471116686888 | 1.02048550386355 | 0.992899239648112 |
| ebi-a-GCST90001882 | finngen_R11_O15_POOR_FETGRO | Poor fetal growth \|\| id:finngen_R11_O15_POOR_FETGRO | HVEM on Central Memory CD8+ T cell \|\| id:ebi-a-GCST90001882 | Weighted median | 18 | -0.01733676 | 0.0185072471623893 | 0.348884499761157 | -0.053610964 | 0.0189374446017675 | 0.982812657069923 | 0.947800763144083 | 1.01911789529759 | 0.999440741871876 |
| ebi-a-GCST90001882 | finngen_R11_O15_POOR_FETGRO | Poor fetal growth \|\| id:finngen_R11_O15_POOR_FETGRO | HVEM on Central Memory CD8+ T cell \|\| id:ebi-a-GCST90001882 | Weighted mode | 18 | -0.017946202 | 0.0155893430236571 | 0.265591166786596 | -0.048501314 | 0.0126089104480025 | 0.982213872198314 | 0.952656087319873 | 1.01268873791899 | 0.999857399747617 |
| ebi-a-GCST90001883 | finngen_R11_O15_POOR_FETGRO | Poor fetal growth \|\| id:finngen_R11_O15_POOR_FETGRO | CD16-CD56 on Natural Killer T \|\| id:ebi-a-GCST90001883 | Inverse variance weighted | 28 | 0.0132423521768178 | 0.0238228232836609 | 0.57830089321496 | -0.033450381 | 0.0599350858127932 | 1.01333042043667 | 0.967102896275477 | 1.06176762052616 | 0.994119304454654 |
| ebi-a-GCST90001883 | finngen_R11_O15_POOR_FETGRO | Poor fetal growth \|\| id:finngen_R11_O15_POOR_FETGRO | CD16-CD56 on Natural Killer T \|\| id:ebi-a-GCST90001883 | MR Egger | 28 | 0.036255097156142 | 0.0386427316881635 | 0.356769681026275 | -0.039484657 | 0.111994851264943 | 1.03692032818209 | 0.961284702914157 | 1.11850710173349 | 0.992899239648112 |
| ebi-a-GCST90001883 | finngen_R11_O15_POOR_FETGRO | Poor fetal growth \|\| id:finngen_R11_O15_POOR_FETGRO | CD16-CD56 on Natural Killer T \|\| id:ebi-a-GCST90001883 | Weighted median | 28 | 0.0343211379177865 | 0.0367346202541329 | 0.350149414012651 | -0.037678718 | 0.106320993615887 | 1.03491690442834 | 0.963022293133796 | 1.11217882151636 | 0.999440741871876 |
| ebi-a-GCST90001883 | finngen_R11_O15_POOR_FETGRO | Poor fetal growth \|\| id:finngen_R11_O15_POOR_FETGRO | CD16-CD56 on Natural Killer T \|\| id:ebi-a-GCST90001883 | Weighted mode | 28 | 0.0412986777848081 | 0.0342950474557256 | 0.23895660577491 | -0.025919615 | 0.10851697079803 | 1.04216333010008 | 0.974413414460396 | 1.11462382443364 | 0.999857399747617 |
| ebi-a-GCST90001884 | finngen_R11_O15_POOR_FETGRO | Poor fetal growth \|\| id:finngen_R11_O15_POOR_FETGRO | CD16-CD56 on Natural Killer \|\| id:ebi-a-GCST90001884 | Inverse variance weighted | 28 | -0.015160158 | 0.0229033239696653 | 0.508022787592388 | -0.060050673 | 0.0297303572904814 | 0.984954178983988 | 0.941716813069323 | 1.03017671685831 | 0.994119304454654 |
| ebi-a-GCST90001884 | finngen_R11_O15_POOR_FETGRO | Poor fetal growth \|\| id:finngen_R11_O15_POOR_FETGRO | CD16-CD56 on Natural Killer \|\| id:ebi-a-GCST90001884 | MR Egger | 28 | -0.047681246 | 0.0469876966493598 | 0.319570826413346 | -0.139777131 | 0.0444146398155789 | 0.95343765107598 | 0.869552009948343 | 1.04541573602169 | 0.992899239648112 |
| ebi-a-GCST90001884 | finngen_R11_O15_POOR_FETGRO | Poor fetal growth \|\| id:finngen_R11_O15_POOR_FETGRO | CD16-CD56 on Natural Killer \|\| id:ebi-a-GCST90001884 | Weighted median | 28 | -0.006080599 | 0.0345334075870095 | 0.860232085424461 | -0.073766078 | 0.0616048800817741 | 0.993937850638588 | 0.928888956307931 | 1.06354203505522 | 0.999440741871876 |
| ebi-a-GCST90001884 | finngen_R11_O15_POOR_FETGRO | Poor fetal growth \|\| id:finngen_R11_O15_POOR_FETGRO | CD16-CD56 on Natural Killer \|\| id:ebi-a-GCST90001884 | Weighted mode | 28 | 0.0319283277283123 | 0.0464404559635855 | 0.497624599460542 | -0.059094966 | 0.12295162141694 | 1.03244350508187 | 0.942617248354253 | 1.13082971168499 | 0.999857399747617 |
| ebi-a-GCST90001885 | finngen_R11_O15_POOR_FETGRO | Poor fetal growth \|\| id:finngen_R11_O15_POOR_FETGRO | CD16-CD56 on HLA DR+ Natural Killer \|\| id:ebi-a-GCST90001885 | Inverse variance weighted | 25 | 0.0242931218438451 | 0.0132779733721494 | 0.0673126841221429 | -0.001731706 | 0.0503179496532579 | 1.02459060376526 | 0.998269792572074 | 1.05160540079977 | 0.994119304454654 |
| ebi-a-GCST90001885 | finngen_R11_O15_POOR_FETGRO | Poor fetal growth \|\| id:finngen_R11_O15_POOR_FETGRO | CD16-CD56 on HLA DR+ Natural Killer \|\| id:ebi-a-GCST90001885 | MR Egger | 25 | 0.0277312988546053 | 0.01593298438879 | 0.0951363937004713 | -0.003497351 | 0.0589599482566338 | 1.02811939044502 | 0.996508758059614 | 1.06073275569325 | 0.992899239648112 |
| ebi-a-GCST90001885 | finngen_R11_O15_POOR_FETGRO | Poor fetal growth \|\| id:finngen_R11_O15_POOR_FETGRO | CD16-CD56 on HLA DR+ Natural Killer \|\| id:ebi-a-GCST90001885 | Weighted median | 25 | 0.0320120702549814 | 0.0184134439568056 | 0.0821199015662096 | -0.00407828 | 0.0681024204103203 | 1.03252996812989 | 0.995930024989391 | 1.07047494134709 | 0.999440741871876 |
| ebi-a-GCST90001885 | finngen_R11_O15_POOR_FETGRO | Poor fetal growth \|\| id:finngen_R11_O15_POOR_FETGRO | CD16-CD56 on HLA DR+ Natural Killer \|\| id:ebi-a-GCST90001885 | Weighted mode | 25 | 0.0298063638665106 | 0.015884076298938 | 0.0727993681018938 | -0.001326426 | 0.0609391534124292 | 1.03025502 | 0.998674453634309 | 1.06283424238288 | 0.999857399747617 |
| ebi-a-GCST90001886 | finngen_R11_O15_POOR_FETGRO | Poor fetal growth \|\| id:finngen_R11_O15_POOR_FETGRO | CD28 on CD39+ activated CD4 regulatory T cell \|\| id:ebi-a-GCST90001886 | Inverse variance weighted | 19 | -0.013363686 | 0.020099746467652 | 0.506134690735896 | -0.052759189 | 0.026031817490616 | 0.986725212019746 | 0.94860842064202 | 1.02637360458645 | 0.994119304454654 |
| ebi-a-GCST90001886 | finngen_R11_O15_POOR_FETGRO | Poor fetal growth \|\| id:finngen_R11_O15_POOR_FETGRO | CD28 on CD39+ activated CD4 regulatory T cell \|\| id:ebi-a-GCST90001886 | MR Egger | 19 | -0.013942637 | 0.0281467562616154 | 0.626691498417811 | -0.06911028 | 0.0412250049573634 | 0.986154111087673 | 0.933223758871454 | 1.04208655380909 | 0.992899239648112 |
| ebi-a-GCST90001886 | finngen_R11_O15_POOR_FETGRO | Poor fetal growth \|\| id:finngen_R11_O15_POOR_FETGRO | CD28 on CD39+ activated CD4 regulatory T cell \|\| id:ebi-a-GCST90001886 | Weighted median | 19 | -0.029996121 | 0.0300673834875159 | 0.318458852797101 | -0.088928193 | 0.0289359506098409 | 0.970449297889103 | 0.914911268559146 | 1.02935866256911 | 0.999440741871876 |
| ebi-a-GCST90001886 | finngen_R11_O15_POOR_FETGRO | Poor fetal growth \|\| id:finngen_R11_O15_POOR_FETGRO | CD28 on CD39+ activated CD4 regulatory T cell \|\| id:ebi-a-GCST90001886 | Weighted mode | 19 | -0.046430093 | 0.0306704857003711 | 0.147428781390665 | -0.106544245 | 0.013684059133459 | 0.954631293800673 | 0.898935274059142 | 1.01377811440025 | 0.999857399747617 |
| ebi-a-GCST90001887 | finngen_R11_O15_POOR_FETGRO | Poor fetal growth \|\| id:finngen_R11_O15_POOR_FETGRO | CD28 on secreting CD4 regulatory T cell \|\| id:ebi-a-GCST90001887 | Inverse variance weighted | 17 | 0.0302016332767122 | 0.0169968306928102 | 0.0755850350200935 | -0.003112155 | 0.0635154214346201 | 1.03066232882618 | 0.996892682852913 | 1.06557591838412 | 0.994119304454654 |
| ebi-a-GCST90001887 | finngen_R11_O15_POOR_FETGRO | Poor fetal growth \|\| id:finngen_R11_O15_POOR_FETGRO | CD28 on secreting CD4 regulatory T cell \|\| id:ebi-a-GCST90001887 | MR Egger | 17 | 0.028503307844805 | 0.0207999969012218 | 0.190738201141212 | -0.012264686 | 0.0692713017711997 | 1.02891341431479 | 0.987810218640391 | 1.07172693112453 | 0.992899239648112 |
| ebi-a-GCST90001887 | finngen_R11_O15_POOR_FETGRO | Poor fetal growth \|\| id:finngen_R11_O15_POOR_FETGRO | CD28 on secreting CD4 regulatory T cell \|\| id:ebi-a-GCST90001887 | Weighted median | 17 | 0.0327640092267665 | 0.0237469370916361 | 0.167674361799004 | -0.013779987 | 0.0793080059263733 | 1.03330665962818 | 0.98631452194363 | 1.08253769875342 | 0.999440741871876 |
| ebi-a-GCST90001887 | finngen_R11_O15_POOR_FETGRO | Poor fetal growth \|\| id:finngen_R11_O15_POOR_FETGRO | CD28 on secreting CD4 regulatory T cell \|\| id:ebi-a-GCST90001887 | Weighted mode | 17 | 0.0285289727502357 | 0.0209886016151816 | 0.192915501342825 | -0.012608686 | 0.0696666319159916 | 1.02893982161913 | 0.987470470035485 | 1.07215070084634 | 0.999857399747617 |
| ebi-a-GCST90001888 | finngen_R11_O15_POOR_FETGRO | Poor fetal growth \|\| id:finngen_R11_O15_POOR_FETGRO | CD28 on CD39+ secreting CD4 regulatory T cell \|\| id:ebi-a-GCST90001888 | Inverse variance weighted | 21 | -0.01838223 | 0.022408776389651 | 0.412037204364103 | -0.062303432 | 0.0255389717551661 | 0.981785692715391 | 0.939597739799723 | 1.02586788536347 | 0.994119304454654 |
| ebi-a-GCST90001888 | finngen_R11_O15_POOR_FETGRO | Poor fetal growth \|\| id:finngen_R11_O15_POOR_FETGRO | CD28 on CD39+ secreting CD4 regulatory T cell \|\| id:ebi-a-GCST90001888 | MR Egger | 21 | -0.007512786 | 0.0321590931775588 | 0.817783881447077 | -0.070544609 | 0.0555190361380067 | 0.992515363950218 | 0.93188616797852 | 1.05708913977564 | 0.992899239648112 |
| ebi-a-GCST90001888 | finngen_R11_O15_POOR_FETGRO | Poor fetal growth \|\| id:finngen_R11_O15_POOR_FETGRO | CD28 on CD39+ secreting CD4 regulatory T cell \|\| id:ebi-a-GCST90001888 | Weighted median | 21 | 0.0116978607967949 | 0.0342302919330049 | 0.732546548505819 | -0.055393511 | 0.0787892329854844 | 1.01176654834156 | 0.946112768538081 | 1.08197625313181 | 0.999440741871876 |
| ebi-a-GCST90001888 | finngen_R11_O15_POOR_FETGRO | Poor fetal growth \|\| id:finngen_R11_O15_POOR_FETGRO | CD28 on CD39+ secreting CD4 regulatory T cell \|\| id:ebi-a-GCST90001888 | Weighted mode | 21 | 0.0230739821882645 | 0.0315504491806482 | 0.473056259779884 | -0.038764898 | 0.0849128625823349 | 1.02334224584542 | 0.961976845045091 | 1.08862220283779 | 0.999857399747617 |
| ebi-a-GCST90001889 | finngen_R11_O15_POOR_FETGRO | Poor fetal growth \|\| id:finngen_R11_O15_POOR_FETGRO | CD28 on activated & secreting CD4 regulatory T cell \|\| id:ebi-a-GCST90001889 | Inverse variance weighted | 24 | 0.00773264613441839 | 0.0179244792828766 | 0.666176504655927 | -0.027399333 | 0.0428646255288566 | 1.00776262025248 | 0.972972623605776 | 1.04379658187549 | 0.994119304454654 |
| ebi-a-GCST90001889 | finngen_R11_O15_POOR_FETGRO | Poor fetal growth \|\| id:finngen_R11_O15_POOR_FETGRO | CD28 on activated & secreting CD4 regulatory T cell \|\| id:ebi-a-GCST90001889 | MR Egger | 24 | 0.0181963376375673 | 0.0228150434645519 | 0.433651089382459 | -0.026521148 | 0.062913822828089 | 1.01836289972883 | 0.973827449550219 | 1.06493506218489 | 0.992899239648112 |
| ebi-a-GCST90001889 | finngen_R11_O15_POOR_FETGRO | Poor fetal growth \|\| id:finngen_R11_O15_POOR_FETGRO | CD28 on activated & secreting CD4 regulatory T cell \|\| id:ebi-a-GCST90001889 | Weighted median | 24 | 0.00455122640508263 | 0.0261859402564537 | 0.862019343285117 | -0.046773216 | 0.0558756693077319 | 1.00456159896597 | 0.954303793408597 | 1.05746620005835 | 0.999440741871876 |
| ebi-a-GCST90001889 | finngen_R11_O15_POOR_FETGRO | Poor fetal growth \|\| id:finngen_R11_O15_POOR_FETGRO | CD28 on activated & secreting CD4 regulatory T cell \|\| id:ebi-a-GCST90001889 | Weighted mode | 24 | 0.0116858678906861 | 0.0236149202588986 | 0.625399436414807 | -0.034599376 | 0.0579711115981274 | 1.0117544143931 | 0.965992338638848 | 1.0596843826798 | 0.999857399747617 |
| ebi-a-GCST90001890 | finngen_R11_O15_POOR_FETGRO | Poor fetal growth \|\| id:finngen_R11_O15_POOR_FETGRO | CD28 on CD45RA+ CD4+ T cell \|\| id:ebi-a-GCST90001890 | Inverse variance weighted (multiplicative random effects) | 13 | 0.000243962447684349 | 0.05002522363343 | 0.996108900976147 | -0.097805476 | 0.0982934007692071 | 1.00024399220894 | 0.906825285994404 | 1.10328644271644 | 0.998984604424952 |
| ebi-a-GCST90001890 | finngen_R11_O15_POOR_FETGRO | Poor fetal growth \|\| id:finngen_R11_O15_POOR_FETGRO | CD28 on CD45RA+ CD4+ T cell \|\| id:ebi-a-GCST90001890 | MR Egger | 13 | -0.038896411 | 0.0993125063672184 | 0.702792700951054 | -0.233548923 | 0.155756101767763 | 0.961850341377902 | 0.791718861379097 | 1.16854116320692 | 0.992899239648112 |
| ebi-a-GCST90001890 | finngen_R11_O15_POOR_FETGRO | Poor fetal growth \|\| id:finngen_R11_O15_POOR_FETGRO | CD28 on CD45RA+ CD4+ T cell \|\| id:ebi-a-GCST90001890 | Weighted median | 13 | 0.0385951083483254 | 0.04929233691531 | 0.433636540920304 | -0.058017872 | 0.135208088702333 | 1.03934957447873 | 0.94363308267028 | 1.144774974 | 0.999440741871876 |
| ebi-a-GCST90001890 | finngen_R11_O15_POOR_FETGRO | Poor fetal growth \|\| id:finngen_R11_O15_POOR_FETGRO | CD28 on CD45RA+ CD4+ T cell \|\| id:ebi-a-GCST90001890 | Weighted mode | 13 | 0.0282714512476436 | 0.0519620092754075 | 0.596351540233867 | -0.073574087 | 0.130116989427442 | 1.02867488160553 | 0.929067311494816 | 1.13896162199875 | 0.999857399747617 |
| ebi-a-GCST90001891 | finngen_R11_O15_POOR_FETGRO | Poor fetal growth \|\| id:finngen_R11_O15_POOR_FETGRO | CD28 on CD45RA- CD4 not regulatory T cell \|\| id:ebi-a-GCST90001891 | Inverse variance weighted | 13 | 0.0327349960383861 | 0.0332143752657717 | 0.324345581406597 | -0.032365179 | 0.0978351715592988 | 1.0332766805423 | 0.968152967916978 | 1.10278100045454 | 0.994119304454654 |
| ebi-a-GCST90001891 | finngen_R11_O15_POOR_FETGRO | Poor fetal growth \|\| id:finngen_R11_O15_POOR_FETGRO | CD28 on CD45RA- CD4 not regulatory T cell \|\| id:ebi-a-GCST90001891 | MR Egger | 13 | -0.030437354 | 0.0678849207999392 | 0.662591591602499 | -0.163491799 | 0.10261709031714 | 0.970021197674565 | 0.849173462858951 | 1.10806703823514 | 0.992899239648112 |
| ebi-a-GCST90001891 | finngen_R11_O15_POOR_FETGRO | Poor fetal growth \|\| id:finngen_R11_O15_POOR_FETGRO | CD28 on CD45RA- CD4 not regulatory T cell \|\| id:ebi-a-GCST90001891 | Weighted median | 13 | 0.0472894709396714 | 0.046320189108632 | 0.307289647542219 | -0.0434981 | 0.13807704159259 | 1.04842545385962 | 0.957434373489095 | 1.14806399554577 | 0.999440741871876 |
| ebi-a-GCST90001891 | finngen_R11_O15_POOR_FETGRO | Poor fetal growth \|\| id:finngen_R11_O15_POOR_FETGRO | CD28 on CD45RA- CD4 not regulatory T cell \|\| id:ebi-a-GCST90001891 | Weighted mode | 13 | 0.0284902480172224 | 0.0524349168102668 | 0.596841397543392 | -0.074282189 | 0.131262684965345 | 1.02889997697074 | 0.928409669940705 | 1.14026727304338 | 0.999857399747617 |
| ebi-a-GCST90001892 | finngen_R11_O15_POOR_FETGRO | Poor fetal growth \|\| id:finngen_R11_O15_POOR_FETGRO | CD28 on CD39+ CD4+ T cell \|\| id:ebi-a-GCST90001892 | Inverse variance weighted | 17 | 0.00159823240197773 | 0.0233257274663769 | 0.945373284740678 | -0.044120193 | 0.0473166582360764 | 1.00159951025606 | 0.956838944804538 | 1.04845395810067 | 0.998984604424952 |
| ebi-a-GCST90001892 | finngen_R11_O15_POOR_FETGRO | Poor fetal growth \|\| id:finngen_R11_O15_POOR_FETGRO | CD28 on CD39+ CD4+ T cell \|\| id:ebi-a-GCST90001892 | MR Egger | 17 | 0.0377417656506168 | 0.0369650056413117 | 0.323433909247287 | -0.034709645 | 0.110193176707588 | 1.0384630314253 | 0.96588582493283 | 1.11649373021082 | 0.992899239648112 |
| ebi-a-GCST90001892 | finngen_R11_O15_POOR_FETGRO | Poor fetal growth \|\| id:finngen_R11_O15_POOR_FETGRO | CD28 on CD39+ CD4+ T cell \|\| id:ebi-a-GCST90001892 | Weighted median | 17 | 0.0344657291405534 | 0.031322348781518 | 0.271177114864933 | -0.026926074 | 0.0958575327523286 | 1.03506655514784 | 0.973433200428576 | 1.10060225305026 | 0.999440741871876 |
| ebi-a-GCST90001892 | finngen_R11_O15_POOR_FETGRO | Poor fetal growth \|\| id:finngen_R11_O15_POOR_FETGRO | CD28 on CD39+ CD4+ T cell \|\| id:ebi-a-GCST90001892 | Weighted mode | 17 | 0.0336163616260367 | 0.0321390274230539 | 0.311121688769079 | -0.029376132 | 0.0966088553752222 | 1.03418777649638 | 0.971051152236502 | 1.1014294711367 | 0.999857399747617 |
| ebi-a-GCST90001893 | finngen_R11_O15_POOR_FETGRO | Poor fetal growth \|\| id:finngen_R11_O15_POOR_FETGRO | CD28 on CD28+ CD45RA- CD8+ T cell \|\| id:ebi-a-GCST90001893 | Inverse variance weighted | 17 | 0.0324927711311036 | 0.0416212048271399 | 0.434991730799753 | -0.04908479 | 0.114070332592298 | 1.03302642550438 | 0.95210039736892 | 1.12083095305848 | 0.994119304454654 |
| ebi-a-GCST90001893 | finngen_R11_O15_POOR_FETGRO | Poor fetal growth \|\| id:finngen_R11_O15_POOR_FETGRO | CD28 on CD28+ CD45RA- CD8+ T cell \|\| id:ebi-a-GCST90001893 | MR Egger | 17 | -0.062807276 | 0.0842363125092324 | 0.467428197256541 | -0.227910448 | 0.102295896933829 | 0.939124448460075 | 0.796195557466049 | 1.10771119158505 | 0.992899239648112 |
| ebi-a-GCST90001893 | finngen_R11_O15_POOR_FETGRO | Poor fetal growth \|\| id:finngen_R11_O15_POOR_FETGRO | CD28 on CD28+ CD45RA- CD8+ T cell \|\| id:ebi-a-GCST90001893 | Weighted median | 17 | 0.0321965079360292 | 0.0554720874748928 | 0.561638726517927 | -0.076528784 | 0.140921799386819 | 1.03272042312583 | 0.926326250979774 | 1.15133460939182 | 0.999440741871876 |
| ebi-a-GCST90001893 | finngen_R11_O15_POOR_FETGRO | Poor fetal growth \|\| id:finngen_R11_O15_POOR_FETGRO | CD28 on CD28+ CD45RA- CD8+ T cell \|\| id:ebi-a-GCST90001893 | Weighted mode | 17 | 0.0293676319826999 | 0.0786475488173073 | 0.713745996817886 | -0.124781564 | 0.183516827664622 | 1.02980311345342 | 0.88268969299889 | 1.20143518258991 | 0.999857399747617 |
| ebi-a-GCST90001894 | finngen_R11_O15_POOR_FETGRO | Poor fetal growth \|\| id:finngen_R11_O15_POOR_FETGRO | CD28 on CD28+ CD4+ T cell \|\| id:ebi-a-GCST90001894 | Inverse variance weighted | 26 | 0.00108782950932594 | 0.00649897386400108 | 0.867067275952369 | -0.011650159 | 0.013825818282768 | 1.00108842141046 | 0.988417441068443 | 1.01392183691008 | 0.994119304454654 |
| ebi-a-GCST90001894 | finngen_R11_O15_POOR_FETGRO | Poor fetal growth \|\| id:finngen_R11_O15_POOR_FETGRO | CD28 on CD28+ CD4+ T cell \|\| id:ebi-a-GCST90001894 | MR Egger | 26 | -0.000376063 | 0.00676794150331697 | 0.956148124328206 | -0.013641228 | 0.0128891028394025 | 0.999624008195543 | 0.986451392066949 | 1.01297252535389 | 0.992899239648112 |
| ebi-a-GCST90001894 | finngen_R11_O15_POOR_FETGRO | Poor fetal growth \|\| id:finngen_R11_O15_POOR_FETGRO | CD28 on CD28+ CD4+ T cell \|\| id:ebi-a-GCST90001894 | Weighted median | 26 | 0.000940872052913685 | 0.0076157317132464 | 0.90167694821864 | -0.013985962 | 0.0158677062108766 | 1.00094131481187 | 0.986111387093761 | 1.01599426678364 | 0.999440741871876 |
| ebi-a-GCST90001894 | finngen_R11_O15_POOR_FETGRO | Poor fetal growth \|\| id:finngen_R11_O15_POOR_FETGRO | CD28 on CD28+ CD4+ T cell \|\| id:ebi-a-GCST90001894 | Weighted mode | 26 | -5.65E-05 | 0.00687216927968386 | 0.993509974942707 | -0.013525913 | 0.0134129909877132 | 0.999943540793414 | 0.986565151529564 | 1.01350334868838 | 0.999857399747617 |
| ebi-a-GCST90001895 | finngen_R11_O15_POOR_FETGRO | Poor fetal growth \|\| id:finngen_R11_O15_POOR_FETGRO | CD28 on CD28+ CD4-CD8- T cell \|\| id:ebi-a-GCST90001895 | Inverse variance weighted | 23 | -0.001585017 | 0.00647092936100742 | 0.806499622536534 | -0.014268038 | 0.0110980048090247 | 0.998416238737077 | 0.985833267786835 | 1.01115981611348 | 0.994119304454654 |
| ebi-a-GCST90001895 | finngen_R11_O15_POOR_FETGRO | Poor fetal growth \|\| id:finngen_R11_O15_POOR_FETGRO | CD28 on CD28+ CD4-CD8- T cell \|\| id:ebi-a-GCST90001895 | MR Egger | 23 | -0.001182285 | 0.00675517174893493 | 0.862740999516633 | -0.014422422 | 0.0120578515839549 | 0.998818413579654 | 0.985681083256857 | 1.0121308405447 | 0.992899239648112 |
| ebi-a-GCST90001895 | finngen_R11_O15_POOR_FETGRO | Poor fetal growth \|\| id:finngen_R11_O15_POOR_FETGRO | CD28 on CD28+ CD4-CD8- T cell \|\| id:ebi-a-GCST90001895 | Weighted median | 23 | 0.000748099915273286 | 0.0071140332408483 | 0.916250168472403 | -0.013195405 | 0.014691605067336 | 1.00074837981181 | 0.986891272454944 | 1.01480005715795 | 0.999440741871876 |
| ebi-a-GCST90001895 | finngen_R11_O15_POOR_FETGRO | Poor fetal growth \|\| id:finngen_R11_O15_POOR_FETGRO | CD28 on CD28+ CD4-CD8- T cell \|\| id:ebi-a-GCST90001895 | Weighted mode | 23 | 0.00446690620679346 | 0.00801860584526112 | 0.583104802669743 | -0.011249561 | 0.0201833736635053 | 1.00447689770381 | 0.988813478453132 | 1.02038843523763 | 0.999857399747617 |
| ebi-a-GCST90001896 | finngen_R11_O15_POOR_FETGRO | Poor fetal growth \|\| id:finngen_R11_O15_POOR_FETGRO | CD28 on CD28+ CD45RA+ CD8+ T cell \|\| id:ebi-a-GCST90001896 | Inverse variance weighted | 15 | -0.003191582 | 0.0250704477157139 | 0.898699364365746 | -0.052329659 | 0.0459464956207189 | 0.996813505781413 | 0.949015963213274 | 1.04701838938923 | 0.994119304454654 |
| ebi-a-GCST90001896 | finngen_R11_O15_POOR_FETGRO | Poor fetal growth \|\| id:finngen_R11_O15_POOR_FETGRO | CD28 on CD28+ CD45RA+ CD8+ T cell \|\| id:ebi-a-GCST90001896 | MR Egger | 15 | -7.35E-05 | 0.0377024456762338 | 0.998474208324498 | -0.07397029 | 0.0738232966905519 | 0.99992650586596 | 0.928699284752581 | 1.0766165469802 | 0.999424937578259 |
| ebi-a-GCST90001896 | finngen_R11_O15_POOR_FETGRO | Poor fetal growth \|\| id:finngen_R11_O15_POOR_FETGRO | CD28 on CD28+ CD45RA+ CD8+ T cell \|\| id:ebi-a-GCST90001896 | Weighted median | 15 | 8.82735943862451e-05 | 0.0335688189011797 | 0.997901860738019 | -0.065706611 | 0.0658831586406984 | 1.00008827749061 | 0.936405554660235 | 1.0681019113957 | 0.999440741871876 |
| ebi-a-GCST90001896 | finngen_R11_O15_POOR_FETGRO | Poor fetal growth \|\| id:finngen_R11_O15_POOR_FETGRO | CD28 on CD28+ CD45RA+ CD8+ T cell \|\| id:ebi-a-GCST90001896 | Weighted mode | 15 | 0.00179024358700902 | 0.0334875143195836 | 0.958120741121887 | -0.063845284 | 0.0674257716533929 | 1.00179184702977 | 0.938150134689622 | 1.06975085081381 | 0.999857399747617 |
| ebi-a-GCST90001897 | finngen_R11_O15_POOR_FETGRO | Poor fetal growth \|\| id:finngen_R11_O15_POOR_FETGRO | CD28 on CD39+ CD8+ T cell \|\| id:ebi-a-GCST90001897 | Inverse variance weighted | 20 | 0.0126575352292198 | 0.0161244668171766 | 0.432459794538791 | -0.01894642 | 0.0442614901908859 | 1.01273798088452 | 0.981231935503985 | 1.04525564325345 | 0.994119304454654 |
| ebi-a-GCST90001897 | finngen_R11_O15_POOR_FETGRO | Poor fetal growth \|\| id:finngen_R11_O15_POOR_FETGRO | CD28 on CD39+ CD8+ T cell \|\| id:ebi-a-GCST90001897 | MR Egger | 20 | 0.021271456684105 | 0.0192033155251659 | 0.282575614507245 | -0.016367042 | 0.0589099551134302 | 1.02149930681908 | 0.983766170528744 | 1.06067972765423 | 0.992899239648112 |
| ebi-a-GCST90001897 | finngen_R11_O15_POOR_FETGRO | Poor fetal growth \|\| id:finngen_R11_O15_POOR_FETGRO | CD28 on CD39+ CD8+ T cell \|\| id:ebi-a-GCST90001897 | Weighted median | 20 | 0.0194874876812456 | 0.0223918450756965 | 0.384139775014331 | -0.024400529 | 0.0633755040296107 | 1.01967860823707 | 0.975894757642843 | 1.06542683619662 | 0.999440741871876 |
| ebi-a-GCST90001897 | finngen_R11_O15_POOR_FETGRO | Poor fetal growth \|\| id:finngen_R11_O15_POOR_FETGRO | CD28 on CD39+ CD8+ T cell \|\| id:ebi-a-GCST90001897 | Weighted mode | 20 | 0.0201970373056973 | 0.0189857556137466 | 0.300753419271727 | -0.017015044 | 0.0574091183086408 | 1.02040237755536 | 0.983128894630217 | 1.0590890144799 | 0.999857399747617 |
| ebi-a-GCST90001898 | finngen_R11_O15_POOR_FETGRO | Poor fetal growth \|\| id:finngen_R11_O15_POOR_FETGRO | CD28 on CD4+ T cell \|\| id:ebi-a-GCST90001898 | Inverse variance weighted | 27 | 0.00163783336462907 | 0.0066702770245075 | 0.806036768369667 | -0.01143591 | 0.0147115763326638 | 1.00163917534624 | 0.988629231857075 | 1.01482032420152 | 0.994119304454654 |
| ebi-a-GCST90001898 | finngen_R11_O15_POOR_FETGRO | Poor fetal growth \|\| id:finngen_R11_O15_POOR_FETGRO | CD28 on CD4+ T cell \|\| id:ebi-a-GCST90001898 | MR Egger | 27 | -0.00139263 | 0.00696297083743519 | 0.843094607626497 | -0.015040053 | 0.0122547931137889 | 0.998608339531203 | 0.985072484129323 | 1.01233019077036 | 0.992899239648112 |
| ebi-a-GCST90001898 | finngen_R11_O15_POOR_FETGRO | Poor fetal growth \|\| id:finngen_R11_O15_POOR_FETGRO | CD28 on CD4+ T cell \|\| id:ebi-a-GCST90001898 | Weighted median | 27 | 0.000702059922977186 | 0.00717516230290988 | 0.922054691409323 | -0.013361258 | 0.0147653780366805 | 1.00070230642473 | 0.986727607194752 | 1.01487492473302 | 0.999440741871876 |
| ebi-a-GCST90001898 | finngen_R11_O15_POOR_FETGRO | Poor fetal growth \|\| id:finngen_R11_O15_POOR_FETGRO | CD28 on CD4+ T cell \|\| id:ebi-a-GCST90001898 | Weighted mode | 27 | -0.000697687 | 0.00690947364285271 | 0.920344605516368 | -0.014240256 | 0.0128448811567302 | 0.99930255614385 | 0.985860657339222 | 1.01292773099477 | 0.999857399747617 |
| ebi-a-GCST90001899 | finngen_R11_O15_POOR_FETGRO | Poor fetal growth \|\| id:finngen_R11_O15_POOR_FETGRO | CD28 on CD4 regulatory T cell \|\| id:ebi-a-GCST90001899 | Inverse variance weighted | 25 | 0.0078284180660243 | 0.01087372999238 | 0.471562793816959 | -0.013484093 | 0.0291409288510891 | 1.0078591402471 | 0.986606410418274 | 1.02956968032366 | 0.994119304454654 |
| ebi-a-GCST90001899 | finngen_R11_O15_POOR_FETGRO | Poor fetal growth \|\| id:finngen_R11_O15_POOR_FETGRO | CD28 on CD4 regulatory T cell \|\| id:ebi-a-GCST90001899 | MR Egger | 25 | 0.0139320515117805 | 0.0117899351246399 | 0.249402972691276 | -0.009176221 | 0.0370403243560747 | 1.01402955482229 | 0.990865751703721 | 1.03773486598471 | 0.992899239648112 |
| ebi-a-GCST90001899 | finngen_R11_O15_POOR_FETGRO | Poor fetal growth \|\| id:finngen_R11_O15_POOR_FETGRO | CD28 on CD4 regulatory T cell \|\| id:ebi-a-GCST90001899 | Weighted median | 25 | 0.00635620683335769 | 0.013827402404563 | 0.645744551578177 | -0.020745502 | 0.0334579155463011 | 1.00637645038402 | 0.979468205669767 | 1.03402392647853 | 0.999440741871876 |
| ebi-a-GCST90001899 | finngen_R11_O15_POOR_FETGRO | Poor fetal growth \|\| id:finngen_R11_O15_POOR_FETGRO | CD28 on CD4 regulatory T cell \|\| id:ebi-a-GCST90001899 | Weighted mode | 25 | 0.0106901977772207 | 0.0118006675943192 | 0.373995346456278 | -0.012439111 | 0.0338195062620864 | 1.01074754210003 | 0.987637935238061 | 1.03439788753659 | 0.999857399747617 |
| ebi-a-GCST90001900 | finngen_R11_O15_POOR_FETGRO | Poor fetal growth \|\| id:finngen_R11_O15_POOR_FETGRO | CD28 on resting CD4 regulatory T cell \|\| id:ebi-a-GCST90001900 | Inverse variance weighted | 21 | -0.013357625 | 0.0299390031322874 | 0.655480719387138 | -0.072038071 | 0.0453228209126907 | 0.98673119194727 | 0.930495469903373 | 1.04636559408805 | 0.994119304454654 |
| ebi-a-GCST90001900 | finngen_R11_O15_POOR_FETGRO | Poor fetal growth \|\| id:finngen_R11_O15_POOR_FETGRO | CD28 on resting CD4 regulatory T cell \|\| id:ebi-a-GCST90001900 | MR Egger | 21 | 0.040848838936903 | 0.0508228942266997 | 0.431483586562231 | -0.058764034 | 0.140461711621234 | 1.04169463 | 0.942929242387746 | 1.15080501626295 | 0.992899239648112 |
| ebi-a-GCST90001900 | finngen_R11_O15_POOR_FETGRO | Poor fetal growth \|\| id:finngen_R11_O15_POOR_FETGRO | CD28 on resting CD4 regulatory T cell \|\| id:ebi-a-GCST90001900 | Weighted median | 21 | 0.0122089852160567 | 0.0400828367414019 | 0.760675511147444 | -0.066353375 | 0.0907713452292045 | 1.01228381911461 | 0.935800117679599 | 1.0950185954049 | 0.999440741871876 |
| ebi-a-GCST90001900 | finngen_R11_O15_POOR_FETGRO | Poor fetal growth \|\| id:finngen_R11_O15_POOR_FETGRO | CD28 on resting CD4 regulatory T cell \|\| id:ebi-a-GCST90001900 | Weighted mode | 21 | 0.0206984182258569 | 0.0409903758963279 | 0.619107222297186 | -0.059642719 | 0.10103955498266 | 1.02091411611561 | 0.942101068715614 | 1.10632040138226 | 0.999857399747617 |
| ebi-a-GCST90001901 | finngen_R11_O15_POOR_FETGRO | Poor fetal growth \|\| id:finngen_R11_O15_POOR_FETGRO | CD28 on CD39+ resting CD4 regulatory T cell \|\| id:ebi-a-GCST90001901 | Inverse variance weighted | 22 | -0.020926719 | 0.0170779478725411 | 0.220437744090161 | -0.054399497 | 0.0125460589681792 | 0.979290725478861 | 0.947053686103066 | 1.01262509093339 | 0.994119304454654 |
| ebi-a-GCST90001901 | finngen_R11_O15_POOR_FETGRO | Poor fetal growth \|\| id:finngen_R11_O15_POOR_FETGRO | CD28 on CD39+ resting CD4 regulatory T cell \|\| id:ebi-a-GCST90001901 | MR Egger | 22 | -0.017721228 | 0.01996699225369 | 0.385344783211309 | -0.056856533 | 0.0214140766008722 | 0.982434869308347 | 0.944729597133788 | 1.02164500335457 | 0.992899239648112 |
| ebi-a-GCST90001901 | finngen_R11_O15_POOR_FETGRO | Poor fetal growth \|\| id:finngen_R11_O15_POOR_FETGRO | CD28 on CD39+ resting CD4 regulatory T cell \|\| id:ebi-a-GCST90001901 | Weighted median | 22 | -0.023412062 | 0.0221226083658113 | 0.289924754611538 | -0.066772375 | 0.0199482501950103 | 0.976859873798174 | 0.935408099749146 | 1.02014854617241 | 0.999440741871876 |
| ebi-a-GCST90001901 | finngen_R11_O15_POOR_FETGRO | Poor fetal growth \|\| id:finngen_R11_O15_POOR_FETGRO | CD28 on CD39+ resting CD4 regulatory T cell \|\| id:ebi-a-GCST90001901 | Weighted mode | 22 | -0.011242508 | 0.0165558211848742 | 0.504511752889987 | -0.043691917 | 0.0212069020191691 | 0.988820453317576 | 0.957248824114052 | 1.021433366 | 0.999857399747617 |
| ebi-a-GCST90001902 | finngen_R11_O15_POOR_FETGRO | Poor fetal growth \|\| id:finngen_R11_O15_POOR_FETGRO | CD28 on activated CD4 regulatory T cell \|\| id:ebi-a-GCST90001902 | Inverse variance weighted | 17 | 0.0176984855275183 | 0.024845715666841 | 0.476257040164631 | -0.030999117 | 0.0663960882345267 | 1.01785603179351 | 0.969476428949324 | 1.06864991300638 | 0.994119304454654 |
| ebi-a-GCST90001902 | finngen_R11_O15_POOR_FETGRO | Poor fetal growth \|\| id:finngen_R11_O15_POOR_FETGRO | CD28 on activated CD4 regulatory T cell \|\| id:ebi-a-GCST90001902 | MR Egger | 17 | 0.00542973061078038 | 0.0338637836912692 | 0.874752329965715 | -0.060943285 | 0.0718027466456681 | 1.00544449831415 | 0.940876599680171 | 1.07444338559789 | 0.992899239648112 |
| ebi-a-GCST90001902 | finngen_R11_O15_POOR_FETGRO | Poor fetal growth \|\| id:finngen_R11_O15_POOR_FETGRO | CD28 on activated CD4 regulatory T cell \|\| id:ebi-a-GCST90001902 | Weighted median | 17 | -0.027641774 | 0.0365848477399361 | 0.449917532719206 | -0.099348075 | 0.0440645277008627 | 0.972736764122101 | 0.905427496094158 | 1.04504978737286 | 0.999440741871876 |
| ebi-a-GCST90001902 | finngen_R11_O15_POOR_FETGRO | Poor fetal growth \|\| id:finngen_R11_O15_POOR_FETGRO | CD28 on activated CD4 regulatory T cell \|\| id:ebi-a-GCST90001902 | Weighted mode | 17 | -0.023635855 | 0.0379816865889578 | 0.542514651967152 | -0.09807996 | 0.0508082510517013 | 0.976641284383525 | 0.906576410664084 | 1.05212113081963 | 0.999857399747617 |
| ebi-a-GCST90001903 | finngen_R11_O15_POOR_FETGRO | Poor fetal growth \|\| id:finngen_R11_O15_POOR_FETGRO | CD86 on myeloid Dendritic Cell \|\| id:ebi-a-GCST90001903 | Inverse variance weighted | 22 | -0.058185761 | 0.0311107960442685 | 0.0614455617859825 | -0.119162922 | 0.00279139878792937 | 0.943474669926338 | 0.887663169382918 | 1.00279529836911 | 0.994119304454654 |
| ebi-a-GCST90001903 | finngen_R11_O15_POOR_FETGRO | Poor fetal growth \|\| id:finngen_R11_O15_POOR_FETGRO | CD86 on myeloid Dendritic Cell \|\| id:ebi-a-GCST90001903 | MR Egger | 22 | -0.079473727 | 0.0604031579355066 | 0.203151021389884 | -0.197863917 | 0.0389164623696109 | 0.923602285282761 | 0.820481499340259 | 1.03968362731574 | 0.992899239648112 |
| ebi-a-GCST90001903 | finngen_R11_O15_POOR_FETGRO | Poor fetal growth \|\| id:finngen_R11_O15_POOR_FETGRO | CD86 on myeloid Dendritic Cell \|\| id:ebi-a-GCST90001903 | Weighted median | 22 | -0.043750022 | 0.0441848230788481 | 0.322096172412927 | -0.130352275 | 0.0428522316086222 | 0.957193205169534 | 0.877786154453522 | 1.04378364522408 | 0.999440741871876 |
| ebi-a-GCST90001903 | finngen_R11_O15_POOR_FETGRO | Poor fetal growth \|\| id:finngen_R11_O15_POOR_FETGRO | CD86 on myeloid Dendritic Cell \|\| id:ebi-a-GCST90001903 | Weighted mode | 22 | -0.045867511 | 0.0503945529399049 | 0.373062924519969 | -0.144640834 | 0.0529058130640725 | 0.955168503416026 | 0.86533303511002 | 1.0543303362988 | 0.999857399747617 |
| ebi-a-GCST90001904 | finngen_R11_O15_POOR_FETGRO | Poor fetal growth \|\| id:finngen_R11_O15_POOR_FETGRO | CD86 on CD62L+ myeloid Dendritic Cell \|\| id:ebi-a-GCST90001904 | Inverse variance weighted | 22 | -0.02466845 | 0.0303423607125326 | 0.416215973125747 | -0.084139477 | 0.0348025773924326 | 0.97563333002748 | 0.919303025891716 | 1.03541527423693 | 0.994119304454654 |
| ebi-a-GCST90001904 | finngen_R11_O15_POOR_FETGRO | Poor fetal growth \|\| id:finngen_R11_O15_POOR_FETGRO | CD86 on CD62L+ myeloid Dendritic Cell \|\| id:ebi-a-GCST90001904 | MR Egger | 22 | -0.012094588 | 0.04533156120671 | 0.792352583444664 | -0.100944448 | 0.0767552722841965 | 0.987978257869909 | 0.903983249888426 | 1.07977779250239 | 0.992899239648112 |
| ebi-a-GCST90001904 | finngen_R11_O15_POOR_FETGRO | Poor fetal growth \|\| id:finngen_R11_O15_POOR_FETGRO | CD86 on CD62L+ myeloid Dendritic Cell \|\| id:ebi-a-GCST90001904 | Weighted median | 22 | 0.0239674029439533 | 0.0350988678811284 | 0.494699092366836 | -0.044826378 | 0.0927611839909649 | 1.0242569295859 | 0.956163478339264 | 1.09719967512972 | 0.999440741871876 |
| ebi-a-GCST90001904 | finngen_R11_O15_POOR_FETGRO | Poor fetal growth \|\| id:finngen_R11_O15_POOR_FETGRO | CD86 on CD62L+ myeloid Dendritic Cell \|\| id:ebi-a-GCST90001904 | Weighted mode | 22 | 0.0182032084168848 | 0.0316947141647472 | 0.57184634518833 | -0.043918431 | 0.0803248481797894 | 1.01836989669962 | 0.957032018102886 | 1.08363902867093 | 0.999857399747617 |
| ebi-a-GCST90001905 | finngen_R11_O15_POOR_FETGRO | Poor fetal growth \|\| id:finngen_R11_O15_POOR_FETGRO | CD86 on monocyte \|\| id:ebi-a-GCST90001905 | Inverse variance weighted | 16 | 0.0596578194551982 | 0.0284651693449404 | 0.0360983228600727 | 0.00386608753911507 | 0.115449551371281 | 1.06147326889419 | 1.00387357049569 | 1.1223778906945 | 0.994119304454654 |
| ebi-a-GCST90001905 | finngen_R11_O15_POOR_FETGRO | Poor fetal growth \|\| id:finngen_R11_O15_POOR_FETGRO | CD86 on monocyte \|\| id:ebi-a-GCST90001905 | MR Egger | 16 | 0.0587981657422872 | 0.040854806153224 | 0.172076785189178 | -0.021277254 | 0.138873585802606 | 1.06056116156183 | 0.978947509516016 | 1.14897884358423 | 0.992899239648112 |
| ebi-a-GCST90001905 | finngen_R11_O15_POOR_FETGRO | Poor fetal growth \|\| id:finngen_R11_O15_POOR_FETGRO | CD86 on monocyte \|\| id:ebi-a-GCST90001905 | Weighted median | 16 | 0.0351024174331761 | 0.0352238899077649 | 0.318982297342917 | -0.033936407 | 0.104141241652395 | 1.03572577974361 | 0.966632973980501 | 1.10975718778568 | 0.999440741871876 |
| ebi-a-GCST90001905 | finngen_R11_O15_POOR_FETGRO | Poor fetal growth \|\| id:finngen_R11_O15_POOR_FETGRO | CD86 on monocyte \|\| id:ebi-a-GCST90001905 | Weighted mode | 16 | 0.0475683992515734 | 0.0362220886789408 | 0.20883619888552 | -0.023426895 | 0.118563693062297 | 1.04871793018969 | 0.976845384771074 | 1.12587858247299 | 0.999857399747617 |
| ebi-a-GCST90001906 | finngen_R11_O15_POOR_FETGRO | Poor fetal growth \|\| id:finngen_R11_O15_POOR_FETGRO | CD86 on granulocyte \|\| id:ebi-a-GCST90001906 | Inverse variance weighted (multiplicative random effects) | 21 | -0.017535248 | 0.0396237618319769 | 0.658095772522125 | -0.095197822 | 0.0601273246999483 | 0.982617599267301 | 0.909193058577576 | 1.06197175317243 | 0.994119304454654 |
| ebi-a-GCST90001906 | finngen_R11_O15_POOR_FETGRO | Poor fetal growth \|\| id:finngen_R11_O15_POOR_FETGRO | CD86 on granulocyte \|\| id:ebi-a-GCST90001906 | MR Egger | 21 | -0.028346911 | 0.0703042304275321 | 0.691299736191051 | -0.166143203 | 0.109449380212145 | 0.972051092671978 | 0.846924943258324 | 1.11566359485127 | 0.992899239648112 |
| ebi-a-GCST90001906 | finngen_R11_O15_POOR_FETGRO | Poor fetal growth \|\| id:finngen_R11_O15_POOR_FETGRO | CD86 on granulocyte \|\| id:ebi-a-GCST90001906 | Weighted median | 21 | -0.029465491 | 0.0484209905466283 | 0.542837857576514 | -0.124370632 | 0.0654396506515024 | 0.970964384247866 | 0.88305249245495 | 1.06762830469666 | 0.999440741871876 |
| ebi-a-GCST90001906 | finngen_R11_O15_POOR_FETGRO | Poor fetal growth \|\| id:finngen_R11_O15_POOR_FETGRO | CD86 on granulocyte \|\| id:ebi-a-GCST90001906 | Weighted mode | 21 | -0.038367893 | 0.0536232786926595 | 0.482565522631952 | -0.143469519 | 0.0667337334333867 | 0.962358830868969 | 0.866347206877758 | 1.06901079844096 | 0.999857399747617 |
| ebi-a-GCST90001907 | finngen_R11_O15_POOR_FETGRO | Poor fetal growth \|\| id:finngen_R11_O15_POOR_FETGRO | CCR7 on naive CD4+ T cell \|\| id:ebi-a-GCST90001907 | Inverse variance weighted | 24 | 0.0336612428184428 | 0.0174192219281804 | 0.053307758401907 | -0.000480432 | 0.0678029177976764 | 1.03423419311857 | 0.99951968322826 | 1.07015437931236 | 0.994119304454654 |
| ebi-a-GCST90001907 | finngen_R11_O15_POOR_FETGRO | Poor fetal growth \|\| id:finngen_R11_O15_POOR_FETGRO | CCR7 on naive CD4+ T cell \|\| id:ebi-a-GCST90001907 | MR Egger | 24 | 0.0499849404008262 | 0.0222077773996789 | 0.0347261817766558 | 0.00645769669745557 | 0.0935121841041969 | 1.0512552647739 | 1.00647859257631 | 1.0980239816984 | 0.992899239648112 |
| ebi-a-GCST90001907 | finngen_R11_O15_POOR_FETGRO | Poor fetal growth \|\| id:finngen_R11_O15_POOR_FETGRO | CCR7 on naive CD4+ T cell \|\| id:ebi-a-GCST90001907 | Weighted median | 24 | 0.0561883090231444 | 0.0259886496702881 | 0.0306156599010606 | 0.00525055566937965 | 0.107126062376909 | 1.05779685767585 | 1.00526436399335 | 1.11307456246038 | 0.999440741871876 |
| ebi-a-GCST90001907 | finngen_R11_O15_POOR_FETGRO | Poor fetal growth \|\| id:finngen_R11_O15_POOR_FETGRO | CCR7 on naive CD4+ T cell \|\| id:ebi-a-GCST90001907 | Weighted mode | 24 | 0.0552337287918591 | 0.0230784172825418 | 0.0252489380834306 | 0.0100000309180771 | 0.100467426665641 | 1.05678758749812 | 1.01005019831298 | 1.10568762518478 | 0.999857399747617 |
| ebi-a-GCST90001908 | finngen_R11_O15_POOR_FETGRO | Poor fetal growth \|\| id:finngen_R11_O15_POOR_FETGRO | CCR7 on naive CD8+ T cell \|\| id:ebi-a-GCST90001908 | Inverse variance weighted | 24 | 0.00943610031599282 | 0.025133250026015 | 0.70733110359784 | -0.03982507 | 0.0586972703669822 | 1.00948076067325 | 0.960957525004749 | 1.06045416124341 | 0.994119304454654 |
| ebi-a-GCST90001908 | finngen_R11_O15_POOR_FETGRO | Poor fetal growth \|\| id:finngen_R11_O15_POOR_FETGRO | CCR7 on naive CD8+ T cell \|\| id:ebi-a-GCST90001908 | MR Egger | 24 | -0.005454914 | 0.0404399542077196 | 0.893925979424306 | -0.084717224 | 0.0738073966338483 | 0.994559937412025 | 0.918772054486064 | 1.07659942885215 | 0.992899239648112 |
| ebi-a-GCST90001908 | finngen_R11_O15_POOR_FETGRO | Poor fetal growth \|\| id:finngen_R11_O15_POOR_FETGRO | CCR7 on naive CD8+ T cell \|\| id:ebi-a-GCST90001908 | Weighted median | 24 | -0.006755275 | 0.0357258248266028 | 0.850024939874761 | -0.076777892 | 0.0632673417375498 | 0.993267490655624 | 0.926095524376202 | 1.06531160342002 | 0.999440741871876 |
| ebi-a-GCST90001908 | finngen_R11_O15_POOR_FETGRO | Poor fetal growth \|\| id:finngen_R11_O15_POOR_FETGRO | CCR7 on naive CD8+ T cell \|\| id:ebi-a-GCST90001908 | Weighted mode | 24 | -0.025549666 | 0.0392669592872466 | 0.521709876221729 | -0.102512906 | 0.0514135742293789 | 0.97477396466546 | 0.902566500995075 | 1.05275819692183 | 0.999857399747617 |
| ebi-a-GCST90001909 | finngen_R11_O15_POOR_FETGRO | Poor fetal growth \|\| id:finngen_R11_O15_POOR_FETGRO | CD45 on CD14+ monocyte \|\| id:ebi-a-GCST90001909 | Inverse variance weighted | 17 | 0.0167864578645179 | 0.0284929690758628 | 0.555764771392803 | -0.039059762 | 0.0726326772532091 | 1.0169281421304 | 0.961693235175496 | 1.0753354691822 | 0.994119304454654 |
| ebi-a-GCST90001909 | finngen_R11_O15_POOR_FETGRO | Poor fetal growth \|\| id:finngen_R11_O15_POOR_FETGRO | CD45 on CD14+ monocyte \|\| id:ebi-a-GCST90001909 | MR Egger | 17 | -0.012024556 | 0.0392864878561621 | 0.763755677775708 | -0.089026073 | 0.0649769598516342 | 0.988047449728547 | 0.914821721533448 | 1.06713443716519 | 0.992899239648112 |
| ebi-a-GCST90001909 | finngen_R11_O15_POOR_FETGRO | Poor fetal growth \|\| id:finngen_R11_O15_POOR_FETGRO | CD45 on CD14+ monocyte \|\| id:ebi-a-GCST90001909 | Weighted median | 17 | 0.0192752980834298 | 0.0394879235168787 | 0.625456722862481 | -0.058121032 | 0.0966716281765119 | 1.01946226599688 | 0.943535742498618 | 1.10149861312012 | 0.999440741871876 |
| ebi-a-GCST90001909 | finngen_R11_O15_POOR_FETGRO | Poor fetal growth \|\| id:finngen_R11_O15_POOR_FETGRO | CD45 on CD14+ monocyte \|\| id:ebi-a-GCST90001909 | Weighted mode | 17 | 0.0246241034892561 | 0.0381953445939281 | 0.528264864232024 | -0.050238772 | 0.0994869788933552 | 1.02492978057672 | 0.951002324743172 | 1.10460408747869 | 0.999857399747617 |
| ebi-a-GCST90001910 | finngen_R11_O15_POOR_FETGRO | Poor fetal growth \|\| id:finngen_R11_O15_POOR_FETGRO | CD45 on B cell \|\| id:ebi-a-GCST90001910 | Inverse variance weighted | 16 | -0.024665756 | 0.016836796829539 | 0.14292337788142 | -0.057665877 | 0.00833436624779671 | 0.975635958451635 | 0.943965294961893 | 1.00836919376603 | 0.994119304454654 |
| ebi-a-GCST90001910 | finngen_R11_O15_POOR_FETGRO | Poor fetal growth \|\| id:finngen_R11_O15_POOR_FETGRO | CD45 on B cell \|\| id:ebi-a-GCST90001910 | MR Egger | 16 | -0.012196471 | 0.0198740366297867 | 0.549263221590133 | -0.051149582 | 0.0267566412878653 | 0.987877604980891 | 0.950136536293042 | 1.02711781427779 | 0.992899239648112 |
| ebi-a-GCST90001910 | finngen_R11_O15_POOR_FETGRO | Poor fetal growth \|\| id:finngen_R11_O15_POOR_FETGRO | CD45 on B cell \|\| id:ebi-a-GCST90001910 | Weighted median | 16 | -0.015897635 | 0.0223008469245349 | 0.475925364150814 | -0.059607295 | 0.0278120249371531 | 0.984228065370329 | 0.942134441846452 | 1.02820238984586 | 0.999440741871876 |
| ebi-a-GCST90001910 | finngen_R11_O15_POOR_FETGRO | Poor fetal growth \|\| id:finngen_R11_O15_POOR_FETGRO | CD45 on B cell \|\| id:ebi-a-GCST90001910 | Weighted mode | 16 | -0.014347743 | 0.0194295719802429 | 0.471638680468433 | -0.052429704 | 0.0237342182621828 | 0.985754695537965 | 0.948921024158146 | 1.0240181164046 | 0.999857399747617 |
| ebi-a-GCST90001911 | finngen_R11_O15_POOR_FETGRO | Poor fetal growth \|\| id:finngen_R11_O15_POOR_FETGRO | CD45 on Natural Killer \|\| id:ebi-a-GCST90001911 | Inverse variance weighted (multiplicative random effects) | 29 | 0.0390340908955146 | 0.0295981179730874 | 0.187234934536687 | -0.01897822 | 0.0970464021227659 | 1.03980593096129 | 0.981200732236537 | 1.1019115034676 | 0.994119304454654 |
| ebi-a-GCST90001911 | finngen_R11_O15_POOR_FETGRO | Poor fetal growth \|\| id:finngen_R11_O15_POOR_FETGRO | CD45 on Natural Killer \|\| id:ebi-a-GCST90001911 | MR Egger | 29 | 0.0540506696830067 | 0.0449704427990123 | 0.23983247100816 | -0.034091398 | 0.142192737569071 | 1.05553808458025 | 0.966483165775925 | 1.15279881476761 | 0.992899239648112 |
| ebi-a-GCST90001911 | finngen_R11_O15_POOR_FETGRO | Poor fetal growth \|\| id:finngen_R11_O15_POOR_FETGRO | CD45 on Natural Killer \|\| id:ebi-a-GCST90001911 | Weighted median | 29 | 0.0766209685652506 | 0.0357033999073678 | 0.0318696887039472 | 0.00664230474680969 | 0.146599632383691 | 1.07963278406702 | 1.00666441377752 | 1.15789028844117 | 0.999440741871876 |
| ebi-a-GCST90001911 | finngen_R11_O15_POOR_FETGRO | Poor fetal growth \|\| id:finngen_R11_O15_POOR_FETGRO | CD45 on Natural Killer \|\| id:ebi-a-GCST90001911 | Weighted mode | 29 | 0.0498720981634174 | 0.0361599700658993 | 0.17874858423038 | -0.021001443 | 0.12074563949258 | 1.05113664547049 | 0.979217551395213 | 1.12833787126945 | 0.999857399747617 |
| ebi-a-GCST90001912 | finngen_R11_O15_POOR_FETGRO | Poor fetal growth \|\| id:finngen_R11_O15_POOR_FETGRO | CD45 on HLA DR+ Natural Killer \|\| id:ebi-a-GCST90001912 | Inverse variance weighted | 11 | -0.076219358 | 0.0421664228735622 | 0.0706712453372142 | -0.158865547 | 0.006426830754691 | 0.926612924234749 | 0.853111054676056 | 1.00644752714511 | 0.994119304454654 |
| ebi-a-GCST90001912 | finngen_R11_O15_POOR_FETGRO | Poor fetal growth \|\| id:finngen_R11_O15_POOR_FETGRO | CD45 on HLA DR+ Natural Killer \|\| id:ebi-a-GCST90001912 | MR Egger | 11 | -0.069757884 | 0.0620840471224618 | 0.290257113940004 | -0.191442616 | 0.0519268487297555 | 0.932619595043608 | 0.825767009569006 | 1.05329868955805 | 0.992899239648112 |
| ebi-a-GCST90001912 | finngen_R11_O15_POOR_FETGRO | Poor fetal growth \|\| id:finngen_R11_O15_POOR_FETGRO | CD45 on HLA DR+ Natural Killer \|\| id:ebi-a-GCST90001912 | Weighted median | 11 | -0.077987798 | 0.0571232785860928 | 0.172173159626417 | -0.189949424 | 0.0339738284004553 | 0.924975713372157 | 0.827000959569829 | 1.03455753034841 | 0.999440741871876 |
| ebi-a-GCST90001912 | finngen_R11_O15_POOR_FETGRO | Poor fetal growth \|\| id:finngen_R11_O15_POOR_FETGRO | CD45 on HLA DR+ Natural Killer \|\| id:ebi-a-GCST90001912 | Weighted mode | 11 | -0.076624977 | 0.0543199314333594 | 0.18869718813753 | -0.183092043 | 0.0298420886299269 | 0.926237148733848 | 0.832691509127562 | 1.03029182631329 | 0.999857399747617 |
| ebi-a-GCST90001913 | finngen_R11_O15_POOR_FETGRO | Poor fetal growth \|\| id:finngen_R11_O15_POOR_FETGRO | CD45 on granulocyte \|\| id:ebi-a-GCST90001913 | Inverse variance weighted | 15 | -0.027046566 | 0.0262782005008575 | 0.303367073898042 | -0.078551839 | 0.024458707058188 | 0.973315917114528 | 0.924454135999109 | 1.02476027486712 | 0.994119304454654 |
| ebi-a-GCST90001913 | finngen_R11_O15_POOR_FETGRO | Poor fetal growth \|\| id:finngen_R11_O15_POOR_FETGRO | CD45 on granulocyte \|\| id:ebi-a-GCST90001913 | MR Egger | 15 | -0.040763988 | 0.0368043655298879 | 0.288113523596484 | -0.112900544 | 0.0313725688997074 | 0.960055688317267 | 0.893239493745652 | 1.03186987490367 | 0.992899239648112 |
| ebi-a-GCST90001913 | finngen_R11_O15_POOR_FETGRO | Poor fetal growth \|\| id:finngen_R11_O15_POOR_FETGRO | CD45 on granulocyte \|\| id:ebi-a-GCST90001913 | Weighted median | 15 | -0.037503052 | 0.0374606911940864 | 0.316763570571448 | -0.110926007 | 0.035919902554155 | 0.963191477876546 | 0.895004970647456 | 1.0365728163307 | 0.999440741871876 |
| ebi-a-GCST90001913 | finngen_R11_O15_POOR_FETGRO | Poor fetal growth \|\| id:finngen_R11_O15_POOR_FETGRO | CD45 on granulocyte \|\| id:ebi-a-GCST90001913 | Weighted mode | 15 | -0.02896296 | 0.035252169072544 | 0.425079198146352 | -0.098057211 | 0.0401312917907046 | 0.971452446795686 | 0.906597034971108 | 1.04094743307361 | 0.999857399747617 |
| ebi-a-GCST90001914 | finngen_R11_O15_POOR_FETGRO | Poor fetal growth \|\| id:finngen_R11_O15_POOR_FETGRO | CD45 on lymphocyte \|\| id:ebi-a-GCST90001914 | Inverse variance weighted | 23 | 0.0184670794078499 | 0.0274271537412329 | 0.500747714593708 | -0.035290142 | 0.0722243007406664 | 1.01863865043003 | 0.965325294282865 | 1.07489641708887 | 0.994119304454654 |
| ebi-a-GCST90001914 | finngen_R11_O15_POOR_FETGRO | Poor fetal growth \|\| id:finngen_R11_O15_POOR_FETGRO | CD45 on lymphocyte \|\| id:ebi-a-GCST90001914 | MR Egger | 23 | -0.010283954 | 0.0409925355332285 | 0.804350012842279 | -0.090629323 | 0.0700614158886194 | 0.989768745289695 | 0.913356207931176 | 1.07257405231996 | 0.992899239648112 |
| ebi-a-GCST90001914 | finngen_R11_O15_POOR_FETGRO | Poor fetal growth \|\| id:finngen_R11_O15_POOR_FETGRO | CD45 on lymphocyte \|\| id:ebi-a-GCST90001914 | Weighted median | 23 | 0.0541384697301409 | 0.0437797032003074 | 0.216231473284344 | -0.031669749 | 0.139946688002743 | 1.05563076494244 | 0.968826485610961 | 1.15021247709823 | 0.999440741871876 |
| ebi-a-GCST90001914 | finngen_R11_O15_POOR_FETGRO | Poor fetal growth \|\| id:finngen_R11_O15_POOR_FETGRO | CD45 on lymphocyte \|\| id:ebi-a-GCST90001914 | Weighted mode | 23 | 0.0681054382593804 | 0.0632377770946249 | 0.293160397184042 | -0.055840605 | 0.192051481364845 | 1.07047817188376 | 0.945689862258702 | 1.21173289702255 | 0.999857399747617 |
| ebi-a-GCST90001915 | finngen_R11_O15_POOR_FETGRO | Poor fetal growth \|\| id:finngen_R11_O15_POOR_FETGRO | CD45 on T cell \|\| id:ebi-a-GCST90001915 | Inverse variance weighted | 15 | 0.0253277523252964 | 0.026806172871007 | 0.344736606119233 | -0.027212347 | 0.0778678511524702 | 1.02565122501585 | 0.973154573613017 | 1.08097979899628 | 0.994119304454654 |
| ebi-a-GCST90001915 | finngen_R11_O15_POOR_FETGRO | Poor fetal growth \|\| id:finngen_R11_O15_POOR_FETGRO | CD45 on T cell \|\| id:ebi-a-GCST90001915 | MR Egger | 15 | 0.0495166101924629 | 0.0356635663256634 | 0.188336310031137 | -0.02038398 | 0.119417200190763 | 1.05076304544629 | 0.979822369061736 | 1.12683993807249 | 0.992899239648112 |
| ebi-a-GCST90001915 | finngen_R11_O15_POOR_FETGRO | Poor fetal growth \|\| id:finngen_R11_O15_POOR_FETGRO | CD45 on T cell \|\| id:ebi-a-GCST90001915 | Weighted median | 15 | 0.0576029579893365 | 0.0384830471814091 | 0.134434938380919 | -0.017823814 | 0.133029730464898 | 1.05929432785459 | 0.982334090149086 | 1.1422839584592 | 0.999440741871876 |
| ebi-a-GCST90001915 | finngen_R11_O15_POOR_FETGRO | Poor fetal growth \|\| id:finngen_R11_O15_POOR_FETGRO | CD45 on T cell \|\| id:ebi-a-GCST90001915 | Weighted mode | 15 | 0.0675770825405931 | 0.0390881074124931 | 0.105816181150612 | -0.009035608 | 0.14418977306908 | 1.06991272801071 | 0.991005090447351 | 1.15510329522383 | 0.999857399747617 |
| ebi-a-GCST90001916 | finngen_R11_O15_POOR_FETGRO | Poor fetal growth \|\| id:finngen_R11_O15_POOR_FETGRO | CD45 on CD4+ T cell \|\| id:ebi-a-GCST90001916 | Inverse variance weighted | 12 | -0.02803977 | 0.0617776616991546 | 0.649913747672802 | -0.149123987 | 0.09304444724742 | 0.972349695993189 | 0.861462298484347 | 1.09751051550543 | 0.994119304454654 |
| ebi-a-GCST90001916 | finngen_R11_O15_POOR_FETGRO | Poor fetal growth \|\| id:finngen_R11_O15_POOR_FETGRO | CD45 on CD4+ T cell \|\| id:ebi-a-GCST90001916 | MR Egger | 12 | -0.071925069 | 0.153459144789723 | 0.649340045228757 | -0.372704993 | 0.228854854593258 | 0.930600623853035 | 0.688868423890301 | 1.25715955483186 | 0.992899239648112 |
| ebi-a-GCST90001916 | finngen_R11_O15_POOR_FETGRO | Poor fetal growth \|\| id:finngen_R11_O15_POOR_FETGRO | CD45 on CD4+ T cell \|\| id:ebi-a-GCST90001916 | Weighted median | 12 | -0.067045142 | 0.0678673065889646 | 0.323208630577606 | -0.200065062 | 0.0659747793719677 | 0.935152986135777 | 0.818677486176607 | 1.06819977615704 | 0.999440741871876 |
| ebi-a-GCST90001916 | finngen_R11_O15_POOR_FETGRO | Poor fetal growth \|\| id:finngen_R11_O15_POOR_FETGRO | CD45 on CD4+ T cell \|\| id:ebi-a-GCST90001916 | Weighted mode | 12 | -0.028588979 | 0.115404075280438 | 0.808907193722724 | -0.254780966 | 0.197603008766123 | 0.971815819309558 | 0.775086249353053 | 1.21847857247964 | 0.999857399747617 |
| ebi-a-GCST90001917 | finngen_R11_O15_POOR_FETGRO | Poor fetal growth \|\| id:finngen_R11_O15_POOR_FETGRO | CD45 on CD8+ T cell \|\| id:ebi-a-GCST90001917 | Inverse variance weighted | 26 | -0.004940542 | 0.00740095451420427 | 0.504417916168077 | -0.019446412 | 0.00956532927197269 | 0.995071642825558 | 0.980741449337768 | 1.00961122324763 | 0.994119304454654 |
| ebi-a-GCST90001917 | finngen_R11_O15_POOR_FETGRO | Poor fetal growth \|\| id:finngen_R11_O15_POOR_FETGRO | CD45 on CD8+ T cell \|\| id:ebi-a-GCST90001917 | MR Egger | 26 | 0.00307436441870966 | 0.00755204839093186 | 0.68754978833622 | -0.01172765 | 0.0178763792649361 | 1.00307909512373 | 0.988340850418597 | 1.01803711811374 | 0.992899239648112 |
| ebi-a-GCST90001917 | finngen_R11_O15_POOR_FETGRO | Poor fetal growth \|\| id:finngen_R11_O15_POOR_FETGRO | CD45 on CD8+ T cell \|\| id:ebi-a-GCST90001917 | Weighted median | 26 | -0.008525182 | 0.0100651648808853 | 0.396995805108731 | -0.028252905 | 0.0112025408577982 | 0.991511054011103 | 0.972142475554124 | 1.0112655242904 | 0.999440741871876 |
| ebi-a-GCST90001917 | finngen_R11_O15_POOR_FETGRO | Poor fetal growth \|\| id:finngen_R11_O15_POOR_FETGRO | CD45 on CD8+ T cell \|\| id:ebi-a-GCST90001917 | Weighted mode | 26 | -0.006658554 | 0.0082646101036476 | 0.42802990486269 | -0.02285719 | 0.00954008208462139 | 0.993363565329431 | 0.977402057062059 | 1.00958573372569 | 0.999857399747617 |
| ebi-a-GCST90001918 | finngen_R11_O15_POOR_FETGRO | Poor fetal growth \|\| id:finngen_R11_O15_POOR_FETGRO | CD45 on HLA DR+ T cell \|\| id:ebi-a-GCST90001918 | Inverse variance weighted | 18 | -0.008873027 | 0.0227248324401446 | 0.696200030167066 | -0.053413699 | 0.0356676442297429 | 0.99116622178226 | 0.947987749825484 | 1.03631136518692 | 0.994119304454654 |
| ebi-a-GCST90001918 | finngen_R11_O15_POOR_FETGRO | Poor fetal growth \|\| id:finngen_R11_O15_POOR_FETGRO | CD45 on HLA DR+ T cell \|\| id:ebi-a-GCST90001918 | MR Egger | 18 | -0.017561202 | 0.0323683608814579 | 0.594922137919636 | -0.081003189 | 0.0458807855642306 | 0.982592097455716 | 0.922190750489618 | 1.04694959201208 | 0.992899239648112 |
| ebi-a-GCST90001918 | finngen_R11_O15_POOR_FETGRO | Poor fetal growth \|\| id:finngen_R11_O15_POOR_FETGRO | CD45 on HLA DR+ T cell \|\| id:ebi-a-GCST90001918 | Weighted median | 18 | -0.014506077 | 0.0292220486583577 | 0.619606129492936 | -0.071781293 | 0.0427691378970661 | 0.985598628764815 | 0.930734431833863 | 1.04369691697028 | 0.999440741871876 |
| ebi-a-GCST90001918 | finngen_R11_O15_POOR_FETGRO | Poor fetal growth \|\| id:finngen_R11_O15_POOR_FETGRO | CD45 on HLA DR+ T cell \|\| id:ebi-a-GCST90001918 | Weighted mode | 18 | -0.009979759 | 0.0257795530977176 | 0.703471433432264 | -0.060507683 | 0.0405481652454448 | 0.990069873722856 | 0.94128653718254 | 1.04138146688855 | 0.999857399747617 |
| ebi-a-GCST90001919 | finngen_R11_O15_POOR_FETGRO | Poor fetal growth \|\| id:finngen_R11_O15_POOR_FETGRO | CD45 on Natural Killer T \|\| id:ebi-a-GCST90001919 | Inverse variance weighted | 22 | 0.00481082461621817 | 0.0217786501862051 | 0.825173129416059 | -0.03787533 | 0.0474969789811802 | 1.00482241521228 | 0.962832970037304 | 1.04864303314616 | 0.994119304454654 |
| ebi-a-GCST90001919 | finngen_R11_O15_POOR_FETGRO | Poor fetal growth \|\| id:finngen_R11_O15_POOR_FETGRO | CD45 on Natural Killer T \|\| id:ebi-a-GCST90001919 | MR Egger | 22 | 0.0212529777514039 | 0.033395485684505 | 0.53173060008391 | -0.044202174 | 0.0867081296930336 | 1.02148043077654 | 0.956760505637754 | 1.09057832583078 | 0.992899239648112 |
| ebi-a-GCST90001919 | finngen_R11_O15_POOR_FETGRO | Poor fetal growth \|\| id:finngen_R11_O15_POOR_FETGRO | CD45 on Natural Killer T \|\| id:ebi-a-GCST90001919 | Weighted median | 22 | -0.003805466 | 0.0337311446042699 | 0.910175189789305 | -0.069918509 | 0.0623075777146464 | 0.99620176589879 | 0.932469804581687 | 1.06428964616723 | 0.999440741871876 |
| ebi-a-GCST90001919 | finngen_R11_O15_POOR_FETGRO | Poor fetal growth \|\| id:finngen_R11_O15_POOR_FETGRO | CD45 on Natural Killer T \|\| id:ebi-a-GCST90001919 | Weighted mode | 22 | -0.027004516 | 0.0452114581677186 | 0.556697510128113 | -0.115618974 | 0.0616099421188742 | 0.973356845942107 | 0.890814582248417 | 1.06354741875808 | 0.999857399747617 |
| ebi-a-GCST90001920 | finngen_R11_O15_POOR_FETGRO | Poor fetal growth \|\| id:finngen_R11_O15_POOR_FETGRO | CD45 on HLA DR+ CD4+ T cell \|\| id:ebi-a-GCST90001920 | Inverse variance weighted | 17 | -0.026216033 | 0.0272383935924435 | 0.335815470337124 | -0.079603285 | 0.027171218078416 | 0.974124623457547 | 0.92348263331995 | 1.02754372176433 | 0.994119304454654 |
| ebi-a-GCST90001920 | finngen_R11_O15_POOR_FETGRO | Poor fetal growth \|\| id:finngen_R11_O15_POOR_FETGRO | CD45 on HLA DR+ CD4+ T cell \|\| id:ebi-a-GCST90001920 | MR Egger | 17 | -0.020585971 | 0.0351070015197834 | 0.566341900006563 | -0.089395694 | 0.0482237515527687 | 0.979624473141263 | 0.914483645910847 | 1.04940543515291 | 0.992899239648112 |
| ebi-a-GCST90001920 | finngen_R11_O15_POOR_FETGRO | Poor fetal growth \|\| id:finngen_R11_O15_POOR_FETGRO | CD45 on HLA DR+ CD4+ T cell \|\| id:ebi-a-GCST90001920 | Weighted median | 17 | -0.012974547 | 0.0338757225566294 | 0.701716584101219 | -0.079370963 | 0.0534218691851245 | 0.987109259566987 | 0.92369720317615 | 1.05487457033803 | 0.999440741871876 |
| ebi-a-GCST90001920 | finngen_R11_O15_POOR_FETGRO | Poor fetal growth \|\| id:finngen_R11_O15_POOR_FETGRO | CD45 on HLA DR+ CD4+ T cell \|\| id:ebi-a-GCST90001920 | Weighted mode | 17 | -0.010692025 | 0.0305213364085111 | 0.730672975397227 | -0.070513844 | 0.0491297946170942 | 0.989364931778717 | 0.931914837910309 | 1.05035667253504 | 0.999857399747617 |
| ebi-a-GCST90001921 | finngen_R11_O15_POOR_FETGRO | Poor fetal growth \|\| id:finngen_R11_O15_POOR_FETGRO | CD45 on HLA DR+ CD8+ T cell \|\| id:ebi-a-GCST90001921 | Inverse variance weighted | 16 | -0.001805425 | 0.0218776221005213 | 0.934230167372987 | -0.044685565 | 0.0410747140952942 | 0.998196203578015 | 0.956298128606781 | 1.04192994949096 | 0.998984604424952 |
| ebi-a-GCST90001921 | finngen_R11_O15_POOR_FETGRO | Poor fetal growth \|\| id:finngen_R11_O15_POOR_FETGRO | CD45 on HLA DR+ CD8+ T cell \|\| id:ebi-a-GCST90001921 | MR Egger | 16 | -0.002623162 | 0.0272545970433647 | 0.924688921002993 | -0.056042172 | 0.0507958485153671 | 0.99738027579265 | 0.94549926155439 | 1.052108082 | 0.992899239648112 |
| ebi-a-GCST90001921 | finngen_R11_O15_POOR_FETGRO | Poor fetal growth \|\| id:finngen_R11_O15_POOR_FETGRO | CD45 on HLA DR+ CD8+ T cell \|\| id:ebi-a-GCST90001921 | Weighted median | 16 | 0.0100227832304194 | 0.028103510384921 | 0.721362732746622 | -0.045060097 | 0.0651056635848646 | 1.01007317955201 | 0.955940030860202 | 1.06727179018984 | 0.999440741871876 |
| ebi-a-GCST90001921 | finngen_R11_O15_POOR_FETGRO | Poor fetal growth \|\| id:finngen_R11_O15_POOR_FETGRO | CD45 on HLA DR+ CD8+ T cell \|\| id:ebi-a-GCST90001921 | Weighted mode | 16 | 0.00470546353365187 | 0.0249886702643441 | 0.853162299695429 | -0.04427233 | 0.0536832572517663 | 1.00471655161188 | 0.956693385507702 | 1.05515033800214 | 0.999857399747617 |
| ebi-a-GCST90001922 | finngen_R11_O15_POOR_FETGRO | Poor fetal growth \|\| id:finngen_R11_O15_POOR_FETGRO | CD127 on T cell \|\| id:ebi-a-GCST90001922 | Inverse variance weighted | 19 | 0.0208317781103323 | 0.0365345003782062 | 0.568545657327287 | -0.050775843 | 0.0924393988516165 | 1.021050274 | 0.950491706374961 | 1.09684666937841 | 0.994119304454654 |
| ebi-a-GCST90001922 | finngen_R11_O15_POOR_FETGRO | Poor fetal growth \|\| id:finngen_R11_O15_POOR_FETGRO | CD127 on T cell \|\| id:ebi-a-GCST90001922 | MR Egger | 19 | 0.0583738712362347 | 0.0935532227111123 | 0.540934287383079 | -0.124990445 | 0.241738187750015 | 1.06011126673834 | 0.88250533463785 | 1.27346074154527 | 0.992899239648112 |
| ebi-a-GCST90001922 | finngen_R11_O15_POOR_FETGRO | Poor fetal growth \|\| id:finngen_R11_O15_POOR_FETGRO | CD127 on T cell \|\| id:ebi-a-GCST90001922 | Weighted median | 19 | 0.0147224505111806 | 0.0512938378824226 | 0.774095615771715 | -0.085813472 | 0.115258372760729 | 1.01483135959889 | 0.91776540444081 | 1.12216333655858 | 0.999440741871876 |
| ebi-a-GCST90001922 | finngen_R11_O15_POOR_FETGRO | Poor fetal growth \|\| id:finngen_R11_O15_POOR_FETGRO | CD127 on T cell \|\| id:ebi-a-GCST90001922 | Weighted mode | 19 | 0.0425379172396672 | 0.082623802625059 | 0.612930078006704 | -0.119404736 | 0.204480570384783 | 1.04345562058044 | 0.887448545774445 | 1.22688761765985 | 0.999857399747617 |
| ebi-a-GCST90001923 | finngen_R11_O15_POOR_FETGRO | Poor fetal growth \|\| id:finngen_R11_O15_POOR_FETGRO | CD127 on CD45RA- CD4 not regulatory T cell \|\| id:ebi-a-GCST90001923 | Inverse variance weighted | 16 | -0.023892422 | 0.0236323424431341 | 0.312013921628207 | -0.070211813 | 0.022426969134665 | 0.97639074221888 | 0.932196347462119 | 1.02268034420664 | 0.994119304454654 |
| ebi-a-GCST90001923 | finngen_R11_O15_POOR_FETGRO | Poor fetal growth \|\| id:finngen_R11_O15_POOR_FETGRO | CD127 on CD45RA- CD4 not regulatory T cell \|\| id:ebi-a-GCST90001923 | MR Egger | 16 | 0.00450699938164414 | 0.0301473104007636 | 0.883292014487424 | -0.054581729 | 0.0635957277671407 | 1.00451717117904 | 0.946881118044787 | 1.06566149431424 | 0.992899239648112 |
| ebi-a-GCST90001923 | finngen_R11_O15_POOR_FETGRO | Poor fetal growth \|\| id:finngen_R11_O15_POOR_FETGRO | CD127 on CD45RA- CD4 not regulatory T cell \|\| id:ebi-a-GCST90001923 | Weighted median | 16 | -0.00511143 | 0.0301021017464993 | 0.865164981106441 | -0.06411155 | 0.0538886889322055 | 0.994901610640825 | 0.937900370989394 | 1.05536712157554 | 0.999440741871876 |
| ebi-a-GCST90001923 | finngen_R11_O15_POOR_FETGRO | Poor fetal growth \|\| id:finngen_R11_O15_POOR_FETGRO | CD127 on CD45RA- CD4 not regulatory T cell \|\| id:ebi-a-GCST90001923 | Weighted mode | 16 | -0.000919336 | 0.02605987446562 | 0.972323385155235 | -0.05199669 | 0.0501580180685275 | 0.999081086575675 | 0.949332009281662 | 1.05143722932983 | 0.999857399747617 |
| ebi-a-GCST90001924 | finngen_R11_O15_POOR_FETGRO | Poor fetal growth \|\| id:finngen_R11_O15_POOR_FETGRO | CD127 on CD28+ CD4+ T cell \|\| id:ebi-a-GCST90001924 | Inverse variance weighted | 20 | -0.011682832 | 0.0268164303139636 | 0.663083817489296 | -0.064243035 | 0.0408773716534444 | 0.988385147529325 | 0.93777705901927 | 1.04172435277764 | 0.994119304454654 |
| ebi-a-GCST90001924 | finngen_R11_O15_POOR_FETGRO | Poor fetal growth \|\| id:finngen_R11_O15_POOR_FETGRO | CD127 on CD28+ CD4+ T cell \|\| id:ebi-a-GCST90001924 | MR Egger | 20 | -0.036883264 | 0.0394038640138728 | 0.361646287400009 | -0.114114838 | 0.0403483092572691 | 0.963788637410474 | 0.892155496934556 | 1.04117336136267 | 0.992899239648112 |
| ebi-a-GCST90001924 | finngen_R11_O15_POOR_FETGRO | Poor fetal growth \|\| id:finngen_R11_O15_POOR_FETGRO | CD127 on CD28+ CD4+ T cell \|\| id:ebi-a-GCST90001924 | Weighted median | 20 | 0.012481229597872 | 0.0341888704232573 | 0.715061366515182 | -0.054528956 | 0.0794914156274564 | 1.01255944521429 | 0.946931088715426 | 1.08273626487808 | 0.999440741871876 |
| ebi-a-GCST90001924 | finngen_R11_O15_POOR_FETGRO | Poor fetal growth \|\| id:finngen_R11_O15_POOR_FETGRO | CD127 on CD28+ CD4+ T cell \|\| id:ebi-a-GCST90001924 | Weighted mode | 20 | 0.0128552586523103 | 0.0357546247751652 | 0.723155410202005 | -0.057223806 | 0.0829343232116341 | 1.01293824270236 | 0.94438268728923 | 1.08647045031514 | 0.999857399747617 |
| ebi-a-GCST90001925 | finngen_R11_O15_POOR_FETGRO | Poor fetal growth \|\| id:finngen_R11_O15_POOR_FETGRO | CD127 on CD28+ CD4-CD8- T cell \|\| id:ebi-a-GCST90001925 | Inverse variance weighted | 24 | 0.00810310331211787 | 0.0287704278279406 | 0.778214234281159 | -0.048286935 | 0.0644931418548815 | 1.00813602230903 | 0.952860338647344 | 1.06661826319673 | 0.994119304454654 |
| ebi-a-GCST90001925 | finngen_R11_O15_POOR_FETGRO | Poor fetal growth \|\| id:finngen_R11_O15_POOR_FETGRO | CD127 on CD28+ CD4-CD8- T cell \|\| id:ebi-a-GCST90001925 | MR Egger | 24 | -0.018710789 | 0.050398586320222 | 0.713998013543965 | -0.117492018 | 0.0800704403741567 | 0.981463171328754 | 0.889147608893347 | 1.08336337750894 | 0.992899239648112 |
| ebi-a-GCST90001925 | finngen_R11_O15_POOR_FETGRO | Poor fetal growth \|\| id:finngen_R11_O15_POOR_FETGRO | CD127 on CD28+ CD4-CD8- T cell \|\| id:ebi-a-GCST90001925 | Weighted median | 24 | 0.0152647544757823 | 0.0417541360245078 | 0.714673542516955 | -0.066573352 | 0.0971028610838175 | 1.01538185592328 | 0.935594285503505 | 1.10197371800252 | 0.999440741871876 |
| ebi-a-GCST90001925 | finngen_R11_O15_POOR_FETGRO | Poor fetal growth \|\| id:finngen_R11_O15_POOR_FETGRO | CD127 on CD28+ CD4-CD8- T cell \|\| id:ebi-a-GCST90001925 | Weighted mode | 24 | -0.00870854 | 0.0458899289669898 | 0.851154066742106 | -0.098652801 | 0.0812357207181016 | 0.991329269442979 | 0.906057235736465 | 1.08462653538169 | 0.999857399747617 |
| ebi-a-GCST90001926 | finngen_R11_O15_POOR_FETGRO | Poor fetal growth \|\| id:finngen_R11_O15_POOR_FETGRO | CD127 on granulocyte \|\| id:ebi-a-GCST90001926 | Inverse variance weighted | 31 | 0.0071980479216039 | 0.0241270421714435 | 0.765444115688679 | -0.040090955 | 0.0544870505776331 | 1.00722401613797 | 0.960702054778118 | 1.05599880175068 | 0.994119304454654 |
| ebi-a-GCST90001926 | finngen_R11_O15_POOR_FETGRO | Poor fetal growth \|\| id:finngen_R11_O15_POOR_FETGRO | CD127 on granulocyte \|\| id:ebi-a-GCST90001926 | MR Egger | 31 | -0.005721783 | 0.0383210419256911 | 0.8823411654388 | -0.080831025 | 0.0693874595149526 | 0.994294555562894 | 0.922349532443187 | 1.07185142773725 | 0.992899239648112 |
| ebi-a-GCST90001926 | finngen_R11_O15_POOR_FETGRO | Poor fetal growth \|\| id:finngen_R11_O15_POOR_FETGRO | CD127 on granulocyte \|\| id:ebi-a-GCST90001926 | Weighted median | 31 | 0.0293764160079506 | 0.0351752730605766 | 0.403636671237671 | -0.039567119 | 0.0983199512066806 | 1.0298121593097 | 0.961205436494371 | 1.10331573584302 | 0.999440741871876 |
| ebi-a-GCST90001926 | finngen_R11_O15_POOR_FETGRO | Poor fetal growth \|\| id:finngen_R11_O15_POOR_FETGRO | CD127 on granulocyte \|\| id:ebi-a-GCST90001926 | Weighted mode | 31 | 0.0263839658938022 | 0.0394118630336776 | 0.50833326955954 | -0.050863286 | 0.10363121743981 | 1.02673510405938 | 0.950408596142237 | 1.10919132906291 | 0.999857399747617 |
| ebi-a-GCST90001927 | finngen_R11_O15_POOR_FETGRO | Poor fetal growth \|\| id:finngen_R11_O15_POOR_FETGRO | CD127 on CD8+ T cell \|\| id:ebi-a-GCST90001927 | Inverse variance weighted | 24 | -0.02689862 | 0.0308507420923349 | 0.383265447115768 | -0.087366074 | 0.0335688345943383 | 0.973459925980113 | 0.916341585023065 | 1.03413862578915 | 0.994119304454654 |
| ebi-a-GCST90001927 | finngen_R11_O15_POOR_FETGRO | Poor fetal growth \|\| id:finngen_R11_O15_POOR_FETGRO | CD127 on CD8+ T cell \|\| id:ebi-a-GCST90001927 | MR Egger | 24 | -0.015095065 | 0.058045646538745 | 0.797239652547625 | -0.128864533 | 0.0986744017571544 | 0.985018293935874 | 0.879093045863245 | 1.10370687602877 | 0.992899239648112 |
| ebi-a-GCST90001927 | finngen_R11_O15_POOR_FETGRO | Poor fetal growth \|\| id:finngen_R11_O15_POOR_FETGRO | CD127 on CD8+ T cell \|\| id:ebi-a-GCST90001927 | Weighted median | 24 | -0.003338997 | 0.0456687879425236 | 0.941715936006546 | -0.092849821 | 0.0861718273050224 | 0.996666571189185 | 0.911330352309171 | 1.0899936028785 | 0.999440741871876 |
| ebi-a-GCST90001927 | finngen_R11_O15_POOR_FETGRO | Poor fetal growth \|\| id:finngen_R11_O15_POOR_FETGRO | CD127 on CD8+ T cell \|\| id:ebi-a-GCST90001927 | Weighted mode | 24 | -0.000958889 | 0.0509493989513151 | 0.985146679986167 | -0.100819711 | 0.0989019327997658 | 0.999041570442475 | 0.904096016679272 | 1.10395803217684 | 0.999857399747617 |
| ebi-a-GCST90001928 | finngen_R11_O15_POOR_FETGRO | Poor fetal growth \|\| id:finngen_R11_O15_POOR_FETGRO | CD127 on CD28+ CD45RA- CD8+ T cell \|\| id:ebi-a-GCST90001928 | Inverse variance weighted | 19 | 0.0316148716794022 | 0.0231141287862283 | 0.171383271552688 | -0.013688821 | 0.0769185641004096 | 1.03211993013596 | 0.986404445113504 | 1.07995412577573 | 0.994119304454654 |
| ebi-a-GCST90001928 | finngen_R11_O15_POOR_FETGRO | Poor fetal growth \|\| id:finngen_R11_O15_POOR_FETGRO | CD127 on CD28+ CD45RA- CD8+ T cell \|\| id:ebi-a-GCST90001928 | MR Egger | 19 | 0.0401844971910656 | 0.0327762540751266 | 0.236902263935302 | -0.024056961 | 0.104425955178314 | 1.04100281857194 | 0.976230101330345 | 1.11007319565125 | 0.992899239648112 |
| ebi-a-GCST90001928 | finngen_R11_O15_POOR_FETGRO | Poor fetal growth \|\| id:finngen_R11_O15_POOR_FETGRO | CD127 on CD28+ CD45RA- CD8+ T cell \|\| id:ebi-a-GCST90001928 | Weighted median | 19 | 0.000664336108331662 | 0.0363531950735933 | 0.985419877912893 | -0.070587926 | 0.0719165984525745 | 1.00066455682844 | 0.931845802229794 | 1.07456571988262 | 0.999440741871876 |
| ebi-a-GCST90001928 | finngen_R11_O15_POOR_FETGRO | Poor fetal growth \|\| id:finngen_R11_O15_POOR_FETGRO | CD127 on CD28+ CD45RA- CD8+ T cell \|\| id:ebi-a-GCST90001928 | Weighted mode | 19 | 0.00967752505346231 | 0.033989825865791 | 0.779109786234116 | -0.056942534 | 0.0762975837504126 | 1.0097245037225 | 0.944648353305808 | 1.07928370366575 | 0.999857399747617 |
| ebi-a-GCST90001929 | finngen_R11_O15_POOR_FETGRO | Poor fetal growth \|\| id:finngen_R11_O15_POOR_FETGRO | CD127 on CD28+ CD45RA+ CD8+ T cell \|\| id:ebi-a-GCST90001929 | Inverse variance weighted (multiplicative random effects) | 21 | -0.069435412 | 0.046899396191696 | 0.138734996334635 | -0.161358228 | 0.0224874049691881 | 0.932920387305045 | 0.850987168979565 | 1.0227421526144 | 0.994119304454654 |
| ebi-a-GCST90001929 | finngen_R11_O15_POOR_FETGRO | Poor fetal growth \|\| id:finngen_R11_O15_POOR_FETGRO | CD127 on CD28+ CD45RA+ CD8+ T cell \|\| id:ebi-a-GCST90001929 | MR Egger | 21 | 0.0233111828529052 | 0.0888948539554275 | 0.795963733779929 | -0.150922731 | 0.197545096605543 | 1.0235850120973 | 0.85991414088418 | 1.21840800979613 | 0.992899239648112 |
| ebi-a-GCST90001929 | finngen_R11_O15_POOR_FETGRO | Poor fetal growth \|\| id:finngen_R11_O15_POOR_FETGRO | CD127 on CD28+ CD45RA+ CD8+ T cell \|\| id:ebi-a-GCST90001929 | Weighted median | 21 | 0.0145723938058827 | 0.050690698373457 | 0.77374735338766 | -0.084781375 | 0.113926162617858 | 1.01467908877355 | 0.918713116096652 | 1.12066937453629 | 0.999440741871876 |
| ebi-a-GCST90001929 | finngen_R11_O15_POOR_FETGRO | Poor fetal growth \|\| id:finngen_R11_O15_POOR_FETGRO | CD127 on CD28+ CD45RA+ CD8+ T cell \|\| id:ebi-a-GCST90001929 | Weighted mode | 21 | 0.0588875190359864 | 0.0764339645437358 | 0.45004672895734 | -0.090923051 | 0.208698089541709 | 1.06065593042867 | 0.913087968973318 | 1.23207296665891 | 0.999857399747617 |
| ebi-a-GCST90001930 | finngen_R11_O15_POOR_FETGRO | Poor fetal growth \|\| id:finngen_R11_O15_POOR_FETGRO | CD127 on CD28- CD8+ T cell \|\| id:ebi-a-GCST90001930 | Inverse variance weighted | 20 | -0.011664575 | 0.0270517248329416 | 0.666326403832824 | -0.064685956 | 0.0413568052864543 | 0.988403192024739 | 0.937361789950208 | 1.04222391021143 | 0.994119304454654 |
| ebi-a-GCST90001930 | finngen_R11_O15_POOR_FETGRO | Poor fetal growth \|\| id:finngen_R11_O15_POOR_FETGRO | CD127 on CD28- CD8+ T cell \|\| id:ebi-a-GCST90001930 | MR Egger | 20 | -0.036272 | 0.0438570639291744 | 0.419038039917854 | -0.122231845 | 0.049687845623864 | 0.964377947315222 | 0.884943175085613 | 1.05094298871557 | 0.992899239648112 |
| ebi-a-GCST90001930 | finngen_R11_O15_POOR_FETGRO | Poor fetal growth \|\| id:finngen_R11_O15_POOR_FETGRO | CD127 on CD28- CD8+ T cell \|\| id:ebi-a-GCST90001930 | Weighted median | 20 | -0.005306782 | 0.0437465240716008 | 0.903447470013096 | -0.091049969 | 0.0804364051737017 | 0.994707274085799 | 0.912972089286407 | 1.08375992292663 | 0.999440741871876 |
| ebi-a-GCST90001930 | finngen_R11_O15_POOR_FETGRO | Poor fetal growth \|\| id:finngen_R11_O15_POOR_FETGRO | CD127 on CD28- CD8+ T cell \|\| id:ebi-a-GCST90001930 | Weighted mode | 20 | -0.033194575 | 0.0467821303616922 | 0.486596303939985 | -0.124887551 | 0.0584984001407416 | 0.967350318731688 | 0.88259614416697 | 1.0602432894532 | 0.999857399747617 |
| ebi-a-GCST90001931 | finngen_R11_O15_POOR_FETGRO | Poor fetal growth \|\| id:finngen_R11_O15_POOR_FETGRO | CD127 on CD4+ T cell \|\| id:ebi-a-GCST90001931 | Inverse variance weighted | 20 | -0.031084981 | 0.0248205753046782 | 0.210428651964053 | -0.079733309 | 0.0175633461445069 | 0.969393189134108 | 0.923362565992981 | 1.01771848865134 | 0.994119304454654 |
| ebi-a-GCST90001931 | finngen_R11_O15_POOR_FETGRO | Poor fetal growth \|\| id:finngen_R11_O15_POOR_FETGRO | CD127 on CD4+ T cell \|\| id:ebi-a-GCST90001931 | MR Egger | 20 | -0.010616104 | 0.0380894649654143 | 0.78364033688033 | -0.085271455 | 0.0640392473002515 | 0.989440047919796 | 0.91826298315341 | 1.06613424083106 | 0.992899239648112 |
| ebi-a-GCST90001931 | finngen_R11_O15_POOR_FETGRO | Poor fetal growth \|\| id:finngen_R11_O15_POOR_FETGRO | CD127 on CD4+ T cell \|\| id:ebi-a-GCST90001931 | Weighted median | 20 | -0.004831993 | 0.0373610812708515 | 0.897094743081645 | -0.078059713 | 0.0683957260762752 | 0.995179662084288 | 0.924909196249153 | 1.07078896376267 | 0.999440741871876 |
| ebi-a-GCST90001931 | finngen_R11_O15_POOR_FETGRO | Poor fetal growth \|\| id:finngen_R11_O15_POOR_FETGRO | CD127 on CD4+ T cell \|\| id:ebi-a-GCST90001931 | Weighted mode | 20 | 0.0168372466359388 | 0.0369553556953001 | 0.653834158483389 | -0.055595251 | 0.089269743798727 | 1.01697979197297 | 0.945921919818062 | 1.09337554782567 | 0.999857399747617 |
| ebi-a-GCST90001932 | finngen_R11_O15_POOR_FETGRO | Poor fetal growth \|\| id:finngen_R11_O15_POOR_FETGRO | CD127 on CD45RA+ CD4+ T cell \|\| id:ebi-a-GCST90001932 | Inverse variance weighted (multiplicative random effects) | 22 | -0.019634888 | 0.0338246427560599 | 0.56158381566795 | -0.085931187 | 0.046661412219846 | 0.980556621356118 | 0.917657375452246 | 1.04776718784773 | 0.994119304454654 |
| ebi-a-GCST90001932 | finngen_R11_O15_POOR_FETGRO | Poor fetal growth \|\| id:finngen_R11_O15_POOR_FETGRO | CD127 on CD45RA+ CD4+ T cell \|\| id:ebi-a-GCST90001932 | MR Egger | 22 | 0.0489944134760343 | 0.0679966167631682 | 0.479529203663038 | -0.084278955 | 0.182267782331844 | 1.05021448367526 | 0.919174811569856 | 1.19993547238045 | 0.992899239648112 |
| ebi-a-GCST90001932 | finngen_R11_O15_POOR_FETGRO | Poor fetal growth \|\| id:finngen_R11_O15_POOR_FETGRO | CD127 on CD45RA+ CD4+ T cell \|\| id:ebi-a-GCST90001932 | Weighted median | 22 | 0.0240731596903905 | 0.0413840681043164 | 0.560768001956733 | -0.057039614 | 0.105185933174851 | 1.02436525739439 | 0.944556651152724 | 1.11091714750629 | 0.999440741871876 |
| ebi-a-GCST90001932 | finngen_R11_O15_POOR_FETGRO | Poor fetal growth \|\| id:finngen_R11_O15_POOR_FETGRO | CD127 on CD45RA+ CD4+ T cell \|\| id:ebi-a-GCST90001932 | Weighted mode | 22 | 0.0234576314611877 | 0.0416812749451837 | 0.579540052799518 | -0.058237667 | 0.105152930353748 | 1.02373492567483 | 0.94342569922698 | 1.1108804847114 | 0.999857399747617 |
| ebi-a-GCST90001933 | finngen_R11_O15_POOR_FETGRO | Poor fetal growth \|\| id:finngen_R11_O15_POOR_FETGRO | CD25 on CD45RA- CD4 not regulatory T cell \|\| id:ebi-a-GCST90001933 | Inverse variance weighted | 25 | 0.00222966272720806 | 0.0181185890900268 | 0.902060059867886 | -0.033282772 | 0.0377420973436606 | 1.0022321502736 | 0.967265005561137 | 1.03846337587632 | 0.994119304454654 |
| ebi-a-GCST90001933 | finngen_R11_O15_POOR_FETGRO | Poor fetal growth \|\| id:finngen_R11_O15_POOR_FETGRO | CD25 on CD45RA- CD4 not regulatory T cell \|\| id:ebi-a-GCST90001933 | MR Egger | 25 | -0.002034518 | 0.0233036846089112 | 0.931185080222251 | -0.04770974 | 0.0436407036361339 | 0.997967550031961 | 0.953410483816238 | 1.04460696397036 | 0.992899239648112 |
| ebi-a-GCST90001933 | finngen_R11_O15_POOR_FETGRO | Poor fetal growth \|\| id:finngen_R11_O15_POOR_FETGRO | CD25 on CD45RA- CD4 not regulatory T cell \|\| id:ebi-a-GCST90001933 | Weighted median | 25 | 0.0103274884775751 | 0.0253466701508669 | 0.683677865819507 | -0.039351985 | 0.0600069619732743 | 1.01038100104486 | 0.961412246875896 | 1.06184393904875 | 0.999440741871876 |
| ebi-a-GCST90001933 | finngen_R11_O15_POOR_FETGRO | Poor fetal growth \|\| id:finngen_R11_O15_POOR_FETGRO | CD25 on CD45RA- CD4 not regulatory T cell \|\| id:ebi-a-GCST90001933 | Weighted mode | 25 | 0.00307588674277259 | 0.0212410711840588 | 0.886071326375151 | -0.038556613 | 0.0447083862635278 | 1.00308062213633 | 0.9621772316719 | 1.04572286828807 | 0.999857399747617 |
| ebi-a-GCST90001934 | finngen_R11_O15_POOR_FETGRO | Poor fetal growth \|\| id:finngen_R11_O15_POOR_FETGRO | CD25 on CD45RA+ CD4 not regulatory T cell \|\| id:ebi-a-GCST90001934 | Inverse variance weighted | 22 | 0.00455598507883096 | 0.0194721689100295 | 0.815005010760574 | -0.033609466 | 0.0427214361424887 | 1.00456637935825 | 0.966949057406966 | 1.04364713198351 | 0.994119304454654 |
| ebi-a-GCST90001934 | finngen_R11_O15_POOR_FETGRO | Poor fetal growth \|\| id:finngen_R11_O15_POOR_FETGRO | CD25 on CD45RA+ CD4 not regulatory T cell \|\| id:ebi-a-GCST90001934 | MR Egger | 22 | -0.006267056 | 0.0237307086344511 | 0.794412278877133 | -0.052779245 | 0.0402451324959547 | 0.993752540610611 | 0.948589394889203 | 1.04106594200897 | 0.992899239648112 |
| ebi-a-GCST90001934 | finngen_R11_O15_POOR_FETGRO | Poor fetal growth \|\| id:finngen_R11_O15_POOR_FETGRO | CD25 on CD45RA+ CD4 not regulatory T cell \|\| id:ebi-a-GCST90001934 | Weighted median | 22 | 0.0122457023270205 | 0.0306893889479229 | 0.68987793104915 | -0.0479055 | 0.0723969046649494 | 1.01232098793428 | 0.953223862465981 | 1.07508196444129 | 0.999440741871876 |
| ebi-a-GCST90001934 | finngen_R11_O15_POOR_FETGRO | Poor fetal growth \|\| id:finngen_R11_O15_POOR_FETGRO | CD25 on CD45RA+ CD4 not regulatory T cell \|\| id:ebi-a-GCST90001934 | Weighted mode | 22 | -0.012189528 | 0.0237860188474775 | 0.613672985161377 | -0.058810125 | 0.0344310693379366 | 0.987884463743481 | 0.942885783029235 | 1.03503068056705 | 0.999857399747617 |
| ebi-a-GCST90001935 | finngen_R11_O15_POOR_FETGRO | Poor fetal growth \|\| id:finngen_R11_O15_POOR_FETGRO | CD25 on CD39+ CD4 regulatory T cell \|\| id:ebi-a-GCST90001935 | Inverse variance weighted | 17 | -0.02112716 | 0.0374472872100187 | 0.572628918605296 | -0.094523843 | 0.0522695228092702 | 0.979094454882603 | 0.909806041812832 | 1.05365968956609 | 0.994119304454654 |
| ebi-a-GCST90001935 | finngen_R11_O15_POOR_FETGRO | Poor fetal growth \|\| id:finngen_R11_O15_POOR_FETGRO | CD25 on CD39+ CD4 regulatory T cell \|\| id:ebi-a-GCST90001935 | MR Egger | 17 | -0.044480321 | 0.067452516015713 | 0.519615252551552 | -0.176687253 | 0.0877266102485569 | 0.956494422626108 | 0.838041839004777 | 1.09168962447188 | 0.992899239648112 |
| ebi-a-GCST90001935 | finngen_R11_O15_POOR_FETGRO | Poor fetal growth \|\| id:finngen_R11_O15_POOR_FETGRO | CD25 on CD39+ CD4 regulatory T cell \|\| id:ebi-a-GCST90001935 | Weighted median | 17 | -0.011749508 | 0.0487341992183994 | 0.80948249786261 | -0.107268539 | 0.0837695222131686 | 0.988319247670994 | 0.898284416448348 | 1.0873782483937 | 0.999440741871876 |
| ebi-a-GCST90001935 | finngen_R11_O15_POOR_FETGRO | Poor fetal growth \|\| id:finngen_R11_O15_POOR_FETGRO | CD25 on CD39+ CD4 regulatory T cell \|\| id:ebi-a-GCST90001935 | Weighted mode | 17 | -0.013150631 | 0.0478473689162102 | 0.786952365084414 | -0.106931474 | 0.0806302123162169 | 0.986935460985007 | 0.898587247618248 | 1.08396998369545 | 0.999857399747617 |
| ebi-a-GCST90001936 | finngen_R11_O15_POOR_FETGRO | Poor fetal growth \|\| id:finngen_R11_O15_POOR_FETGRO | CD25 on CD4 regulatory T cell \|\| id:ebi-a-GCST90001936 | Inverse variance weighted | 22 | -0.029000402 | 0.0149698791830464 | 0.0527147613578669 | -0.058341365 | 0.000340561122540703 | 0.971416073883213 | 0.943327873088614 | 1.00034061912006 | 0.994119304454654 |
| ebi-a-GCST90001936 | finngen_R11_O15_POOR_FETGRO | Poor fetal growth \|\| id:finngen_R11_O15_POOR_FETGRO | CD25 on CD4 regulatory T cell \|\| id:ebi-a-GCST90001936 | MR Egger | 22 | -0.030293297 | 0.0174499056464459 | 0.0979426964510673 | -0.064495112 | 0.00390851813911687 | 0.970160946591088 | 0.93754069695451 | 1.00391616635729 | 0.992899239648112 |
| ebi-a-GCST90001936 | finngen_R11_O15_POOR_FETGRO | Poor fetal growth \|\| id:finngen_R11_O15_POOR_FETGRO | CD25 on CD4 regulatory T cell \|\| id:ebi-a-GCST90001936 | Weighted median | 22 | -0.024380346 | 0.0212713399753763 | 0.25172920473858 | -0.066072173 | 0.0173114801555564 | 0.975914453809187 | 0.936063303779994 | 1.01746219225525 | 0.999440741871876 |
| ebi-a-GCST90001936 | finngen_R11_O15_POOR_FETGRO | Poor fetal growth \|\| id:finngen_R11_O15_POOR_FETGRO | CD25 on CD4 regulatory T cell \|\| id:ebi-a-GCST90001936 | Weighted mode | 22 | -0.028043998 | 0.0160409013012796 | 0.0950212845412848 | -0.059484165 | 0.00339616809089147 | 0.972345584152151 | 0.942250453999497 | 1.00340194160383 | 0.999857399747617 |
| ebi-a-GCST90001937 | finngen_R11_O15_POOR_FETGRO | Poor fetal growth \|\| id:finngen_R11_O15_POOR_FETGRO | CD25 on resting CD4 regulatory T cell \|\| id:ebi-a-GCST90001937 | Inverse variance weighted | 20 | -0.040399796 | 0.0285383304046753 | 0.156883043929795 | -0.096334924 | 0.0155353311266073 | 0.960405395704168 | 0.908159800559725 | 1.01565663171745 | 0.994119304454654 |
| ebi-a-GCST90001937 | finngen_R11_O15_POOR_FETGRO | Poor fetal growth \|\| id:finngen_R11_O15_POOR_FETGRO | CD25 on resting CD4 regulatory T cell \|\| id:ebi-a-GCST90001937 | MR Egger | 20 | -0.014033739 | 0.0394018077755833 | 0.725853776112799 | -0.091261283 | 0.0631938037567841 | 0.986064274402434 | 0.91277918630769 | 1.06523326543627 | 0.992899239648112 |
| ebi-a-GCST90001937 | finngen_R11_O15_POOR_FETGRO | Poor fetal growth \|\| id:finngen_R11_O15_POOR_FETGRO | CD25 on resting CD4 regulatory T cell \|\| id:ebi-a-GCST90001937 | Weighted median | 20 | -0.021599724 | 0.0393827920273181 | 0.58337892189516 | -0.098789996 | 0.0555905488570185 | 0.97863187999039 | 0.905932937688581 | 1.05716473779734 | 0.999440741871876 |
| ebi-a-GCST90001937 | finngen_R11_O15_POOR_FETGRO | Poor fetal growth \|\| id:finngen_R11_O15_POOR_FETGRO | CD25 on resting CD4 regulatory T cell \|\| id:ebi-a-GCST90001937 | Weighted mode | 20 | -0.017701432 | 0.0330398643820864 | 0.598340984009409 | -0.082459566 | 0.0470567020997727 | 0.982454317906531 | 0.920848670443213 | 1.04818144148334 | 0.999857399747617 |
| ebi-a-GCST90001938 | finngen_R11_O15_POOR_FETGRO | Poor fetal growth \|\| id:finngen_R11_O15_POOR_FETGRO | CD25 on CD39+ resting CD4 regulatory T cell \|\| id:ebi-a-GCST90001938 | Inverse variance weighted | 15 | -0.000957127 | 0.0430224737341511 | 0.982250808371124 | -0.085281176 | 0.0833669211866631 | 0.999043330567991 | 0.918254057233368 | 1.08694055690813 | 0.998984604424952 |
| ebi-a-GCST90001938 | finngen_R11_O15_POOR_FETGRO | Poor fetal growth \|\| id:finngen_R11_O15_POOR_FETGRO | CD25 on CD39+ resting CD4 regulatory T cell \|\| id:ebi-a-GCST90001938 | MR Egger | 15 | 0.0419340318254897 | 0.0777585914103921 | 0.598805557324695 | -0.110472807 | 0.194340870989858 | 1.04282568317498 | 0.895410678457581 | 1.21451020370077 | 0.992899239648112 |
| ebi-a-GCST90001938 | finngen_R11_O15_POOR_FETGRO | Poor fetal growth \|\| id:finngen_R11_O15_POOR_FETGRO | CD25 on CD39+ resting CD4 regulatory T cell \|\| id:ebi-a-GCST90001938 | Weighted median | 15 | 0.0019915014356726 | 0.0577325608123996 | 0.972482198039738 | -0.111164318 | 0.115147320627976 | 1.00199348579172 | 0.894791706682642 | 1.12203872484608 | 0.999440741871876 |
| ebi-a-GCST90001938 | finngen_R11_O15_POOR_FETGRO | Poor fetal growth \|\| id:finngen_R11_O15_POOR_FETGRO | CD25 on CD39+ resting CD4 regulatory T cell \|\| id:ebi-a-GCST90001938 | Weighted mode | 15 | 0.0373940977498541 | 0.076185974935346 | 0.631153181104878 | -0.111930413 | 0.186718608623132 | 1.03810205391692 | 0.894106473412438 | 1.20528807965517 | 0.999857399747617 |
| ebi-a-GCST90001939 | finngen_R11_O15_POOR_FETGRO | Poor fetal growth \|\| id:finngen_R11_O15_POOR_FETGRO | CD25 on activated CD4 regulatory T cell \|\| id:ebi-a-GCST90001939 | Inverse variance weighted | 17 | -0.022776386 | 0.0437357482480082 | 0.602525086371443 | -0.108498453 | 0.062945680564994 | 0.977481037780204 | 0.897180283142096 | 1.06496898914634 | 0.994119304454654 |
| ebi-a-GCST90001939 | finngen_R11_O15_POOR_FETGRO | Poor fetal growth \|\| id:finngen_R11_O15_POOR_FETGRO | CD25 on activated CD4 regulatory T cell \|\| id:ebi-a-GCST90001939 | MR Egger | 17 | -0.112164883 | 0.0868296708418871 | 0.215982943851356 | -0.282351038 | 0.0580212714971256 | 0.893896856637782 | 0.754008952122559 | 1.05973753767452 | 0.992899239648112 |
| ebi-a-GCST90001939 | finngen_R11_O15_POOR_FETGRO | Poor fetal growth \|\| id:finngen_R11_O15_POOR_FETGRO | CD25 on activated CD4 regulatory T cell \|\| id:ebi-a-GCST90001939 | Weighted median | 17 | -0.029633589 | 0.0600790064691331 | 0.621840430632779 | -0.147388442 | 0.0881212635330884 | 0.970801180477284 | 0.862958703048941 | 1.09212054839505 | 0.999440741871876 |
| ebi-a-GCST90001939 | finngen_R11_O15_POOR_FETGRO | Poor fetal growth \|\| id:finngen_R11_O15_POOR_FETGRO | CD25 on activated CD4 regulatory T cell \|\| id:ebi-a-GCST90001939 | Weighted mode | 17 | -0.034985157 | 0.0764251422139209 | 0.653272724519692 | -0.184778436 | 0.114808121275539 | 0.965619748397326 | 0.831288446874109 | 1.12165819457867 | 0.999857399747617 |
| ebi-a-GCST90001940 | finngen_R11_O15_POOR_FETGRO | Poor fetal growth \|\| id:finngen_R11_O15_POOR_FETGRO | CD25 on CD39+ activated CD4 regulatory T cell \|\| id:ebi-a-GCST90001940 | Inverse variance weighted | 12 | -0.034190557 | 0.0307474376714419 | 0.26614645427329 | -0.094455535 | 0.0260744209727935 | 0.966387335351411 | 0.909868191289325 | 1.0264173326075 | 0.994119304454654 |
| ebi-a-GCST90001940 | finngen_R11_O15_POOR_FETGRO | Poor fetal growth \|\| id:finngen_R11_O15_POOR_FETGRO | CD25 on CD39+ activated CD4 regulatory T cell \|\| id:ebi-a-GCST90001940 | MR Egger | 12 | -0.002821791 | 0.0393276693379627 | 0.944214995349713 | -0.079904023 | 0.0742604413038755 | 0.997182186910447 | 0.923204949036767 | 1.07708728698756 | 0.992899239648112 |
| ebi-a-GCST90001940 | finngen_R11_O15_POOR_FETGRO | Poor fetal growth \|\| id:finngen_R11_O15_POOR_FETGRO | CD25 on CD39+ activated CD4 regulatory T cell \|\| id:ebi-a-GCST90001940 | Weighted median | 12 | -0.013204026 | 0.0363279780670031 | 0.716255954732421 | -0.084406863 | 0.0579988106899863 | 0.986882764418517 | 0.919057249320094 | 1.05971373538138 | 0.999440741871876 |
| ebi-a-GCST90001940 | finngen_R11_O15_POOR_FETGRO | Poor fetal growth \|\| id:finngen_R11_O15_POOR_FETGRO | CD25 on CD39+ activated CD4 regulatory T cell \|\| id:ebi-a-GCST90001940 | Weighted mode | 12 | -0.010288958 | 0.0359956423148993 | 0.780312457356073 | -0.080840417 | 0.0602625005897535 | 0.989763791914394 | 0.922340869361179 | 1.062115316 | 0.999857399747617 |
| ebi-a-GCST90001941 | finngen_R11_O15_POOR_FETGRO | Poor fetal growth \|\| id:finngen_R11_O15_POOR_FETGRO | CD25 on secreting CD4 regulatory T cell \|\| id:ebi-a-GCST90001941 | Inverse variance weighted | 14 | -0.028412067 | 0.0155116419980958 | 0.0670019694946341 | -0.058814885 | 0.00199075142815687 | 0.97198776029691 | 0.942881294281096 | 1.00199273428936 | 0.994119304454654 |
| ebi-a-GCST90001941 | finngen_R11_O15_POOR_FETGRO | Poor fetal growth \|\| id:finngen_R11_O15_POOR_FETGRO | CD25 on secreting CD4 regulatory T cell \|\| id:ebi-a-GCST90001941 | MR Egger | 14 | -0.037301406 | 0.017933170391656 | 0.0596204826649342 | -0.07245042 | -0.002152392 | 0.963385721076724 | 0.930111860231083 | 0.997849922421087 | 0.992899239648112 |
| ebi-a-GCST90001941 | finngen_R11_O15_POOR_FETGRO | Poor fetal growth \|\| id:finngen_R11_O15_POOR_FETGRO | CD25 on secreting CD4 regulatory T cell \|\| id:ebi-a-GCST90001941 | Weighted median | 14 | -0.026650762 | 0.0193679952303347 | 0.168815231875071 | -0.064612033 | 0.0113105087476228 | 0.973701235717216 | 0.937431085578919 | 1.01137471438952 | 0.999440741871876 |
| ebi-a-GCST90001941 | finngen_R11_O15_POOR_FETGRO | Poor fetal growth \|\| id:finngen_R11_O15_POOR_FETGRO | CD25 on secreting CD4 regulatory T cell \|\| id:ebi-a-GCST90001941 | Weighted mode | 14 | -0.029037751 | 0.0174678691578044 | 0.120351384235188 | -0.063274774 | 0.00519927278078922 | 0.971379793440715 | 0.938685511578998 | 1.00521281245483 | 0.999857399747617 |
| ebi-a-GCST90001942 | finngen_R11_O15_POOR_FETGRO | Poor fetal growth \|\| id:finngen_R11_O15_POOR_FETGRO | CD25 on CD39+ secreting CD4 regulatory T cell \|\| id:ebi-a-GCST90001942 | Inverse variance weighted | 16 | -0.019097546 | 0.0167497874311108 | 0.254216942379326 | -0.05192713 | 0.0137320369007379 | 0.981083656333197 | 0.949398047120105 | 1.0138267543775 | 0.994119304454654 |
| ebi-a-GCST90001942 | finngen_R11_O15_POOR_FETGRO | Poor fetal growth \|\| id:finngen_R11_O15_POOR_FETGRO | CD25 on CD39+ secreting CD4 regulatory T cell \|\| id:ebi-a-GCST90001942 | MR Egger | 16 | -0.019042011 | 0.019465226973446 | 0.344549553560037 | -0.057193856 | 0.0191098335692059 | 0.981138142489347 | 0.944410971729023 | 1.01929359512317 | 0.992899239648112 |
| ebi-a-GCST90001942 | finngen_R11_O15_POOR_FETGRO | Poor fetal growth \|\| id:finngen_R11_O15_POOR_FETGRO | CD25 on CD39+ secreting CD4 regulatory T cell \|\| id:ebi-a-GCST90001942 | Weighted median | 16 | -0.023028755 | 0.0234952789509044 | 0.327015082529439 | -0.069079502 | 0.023021991955518 | 0.977234383201502 | 0.933252482126718 | 1.02328904342689 | 0.999440741871876 |
| ebi-a-GCST90001942 | finngen_R11_O15_POOR_FETGRO | Poor fetal growth \|\| id:finngen_R11_O15_POOR_FETGRO | CD25 on CD39+ secreting CD4 regulatory T cell \|\| id:ebi-a-GCST90001942 | Weighted mode | 16 | -0.018875447 | 0.0195197950783324 | 0.348882461186924 | -0.057134246 | 0.0193833511673055 | 0.981301578504222 | 0.944467270337122 | 1.01957242799018 | 0.999857399747617 |
| ebi-a-GCST90001943 | finngen_R11_O15_POOR_FETGRO | Poor fetal growth \|\| id:finngen_R11_O15_POOR_FETGRO | CD25 on activated & secreting CD4 regulatory T cell \|\| id:ebi-a-GCST90001943 | Inverse variance weighted | 21 | -0.033052109 | 0.0148101583912044 | 0.025633551422981 | -0.06208002 | -0.004024199 | 0.967488142980403 | 0.939807680449098 | 0.995983887214429 | 0.994119304454654 |
| ebi-a-GCST90001943 | finngen_R11_O15_POOR_FETGRO | Poor fetal growth \|\| id:finngen_R11_O15_POOR_FETGRO | CD25 on activated & secreting CD4 regulatory T cell \|\| id:ebi-a-GCST90001943 | MR Egger | 21 | -0.03351052 | 0.0176911009159187 | 0.0735240488976569 | -0.068185078 | 0.00116403732736449 | 0.96704473741483 | 0.934087578271615 | 1.00116471508177 | 0.992899239648112 |
| ebi-a-GCST90001943 | finngen_R11_O15_POOR_FETGRO | Poor fetal growth \|\| id:finngen_R11_O15_POOR_FETGRO | CD25 on activated & secreting CD4 regulatory T cell \|\| id:ebi-a-GCST90001943 | Weighted median | 21 | -0.025032004 | 0.0208428152146494 | 0.229755249705733 | -0.065883921 | 0.0158199142723282 | 0.975278699149837 | 0.936239535387766 | 1.01594571160835 | 0.999440741871876 |
| ebi-a-GCST90001943 | finngen_R11_O15_POOR_FETGRO | Poor fetal growth \|\| id:finngen_R11_O15_POOR_FETGRO | CD25 on activated & secreting CD4 regulatory T cell \|\| id:ebi-a-GCST90001943 | Weighted mode | 21 | -0.032254415 | 0.0176209022216881 | 0.0821244497524977 | -0.066791383 | 0.00228255385206223 | 0.96826021130048 | 0.935390319439652 | 1.00228516086128 | 0.999857399747617 |
| ebi-a-GCST90001944 | finngen_R11_O15_POOR_FETGRO | Poor fetal growth \|\| id:finngen_R11_O15_POOR_FETGRO | CD123 on plasmacytoid Dendritic Cell \|\| id:ebi-a-GCST90001944 | Inverse variance weighted | 18 | 0.00317491615841498 | 0.0123891648332614 | 0.797745795203705 | -0.021107847 | 0.0274576792316074 | 1.00317996154287 | 0.979113364519661 | 1.02783811528796 | 0.994119304454654 |
| ebi-a-GCST90001944 | finngen_R11_O15_POOR_FETGRO | Poor fetal growth \|\| id:finngen_R11_O15_POOR_FETGRO | CD123 on plasmacytoid Dendritic Cell \|\| id:ebi-a-GCST90001944 | MR Egger | 18 | -0.00369626 | 0.0144868348489277 | 0.801860914364111 | -0.032090457 | 0.0246979358218729 | 0.996310562279926 | 0.968418978048886 | 1.02500545632681 | 0.992899239648112 |
| ebi-a-GCST90001944 | finngen_R11_O15_POOR_FETGRO | Poor fetal growth \|\| id:finngen_R11_O15_POOR_FETGRO | CD123 on plasmacytoid Dendritic Cell \|\| id:ebi-a-GCST90001944 | Weighted median | 18 | 0.00281906429587225 | 0.0173315141324064 | 0.870789767959577 | -0.031150703 | 0.0367888319953889 | 1.00282304159417 | 0.96932948081599 | 1.03747391640828 | 0.999440741871876 |
| ebi-a-GCST90001944 | finngen_R11_O15_POOR_FETGRO | Poor fetal growth \|\| id:finngen_R11_O15_POOR_FETGRO | CD123 on plasmacytoid Dendritic Cell \|\| id:ebi-a-GCST90001944 | Weighted mode | 18 | 0.00301016126857201 | 0.0154895840166434 | 0.848217295887589 | -0.027349423 | 0.0333697459411932 | 1.00301469635331 | 0.973021185741139 | 1.03393276101633 | 0.999857399747617 |
| ebi-a-GCST90001945 | finngen_R11_O15_POOR_FETGRO | Poor fetal growth \|\| id:finngen_R11_O15_POOR_FETGRO | CD123 on CD62L+ plasmacytoid Dendritic Cell \|\| id:ebi-a-GCST90001945 | Inverse variance weighted | 18 | 0.00315902735008778 | 0.0123871931050655 | 0.798704823533997 | -0.021119871 | 0.0274379258360161 | 1.00316402233537 | 0.979101591514901 | 1.0278178121956 | 0.994119304454654 |
| ebi-a-GCST90001945 | finngen_R11_O15_POOR_FETGRO | Poor fetal growth \|\| id:finngen_R11_O15_POOR_FETGRO | CD123 on CD62L+ plasmacytoid Dendritic Cell \|\| id:ebi-a-GCST90001945 | MR Egger | 18 | -0.003711786 | 0.0144820981231803 | 0.800984186327663 | -0.032096698 | 0.024673126586304 | 0.996295094426348 | 0.968412933902814 | 1.02498002704043 | 0.992899239648112 |
| ebi-a-GCST90001945 | finngen_R11_O15_POOR_FETGRO | Poor fetal growth \|\| id:finngen_R11_O15_POOR_FETGRO | CD123 on CD62L+ plasmacytoid Dendritic Cell \|\| id:ebi-a-GCST90001945 | Weighted median | 18 | 0.00283052729896273 | 0.0161952139210271 | 0.861256006775999 | -0.028912092 | 0.0345731465841758 | 1.00283453702368 | 0.971501863512095 | 1.03517774532295 | 0.999440741871876 |
| ebi-a-GCST90001945 | finngen_R11_O15_POOR_FETGRO | Poor fetal growth \|\| id:finngen_R11_O15_POOR_FETGRO | CD123 on CD62L+ plasmacytoid Dendritic Cell \|\| id:ebi-a-GCST90001945 | Weighted mode | 18 | 0.00293880508059607 | 0.013537981997785 | 0.830731035250438 | -0.02359564 | 0.0294732497962547 | 1.00294312760156 | 0.976680560829472 | 1.02991188475064 | 0.999857399747617 |
| ebi-a-GCST90001946 | finngen_R11_O15_POOR_FETGRO | Poor fetal growth \|\| id:finngen_R11_O15_POOR_FETGRO | CD33 on CD14+ monocyte \|\| id:ebi-a-GCST90001946 | Inverse variance weighted | 21 | -0.026911861 | 0.0144740438566965 | 0.0629819700126774 | -0.055280987 | 0.00145726474442708 | 0.973447036182688 | 0.946219235127491 | 1.00145832707066 | 0.994119304454654 |
| ebi-a-GCST90001946 | finngen_R11_O15_POOR_FETGRO | Poor fetal growth \|\| id:finngen_R11_O15_POOR_FETGRO | CD33 on CD14+ monocyte \|\| id:ebi-a-GCST90001946 | MR Egger | 21 | -0.041745122 | 0.0224246389162788 | 0.078206456435478 | -0.085697415 | 0.00220716999971942 | 0.959114206267599 | 0.917871923892363 | 1.00220960759249 | 0.992899239648112 |
| ebi-a-GCST90001946 | finngen_R11_O15_POOR_FETGRO | Poor fetal growth \|\| id:finngen_R11_O15_POOR_FETGRO | CD33 on CD14+ monocyte \|\| id:ebi-a-GCST90001946 | Weighted median | 21 | -0.035602213 | 0.0180091123408705 | 0.0480529398212579 | -0.070900074 | -0.000304353 | 0.965024090802751 | 0.931554974430051 | 0.99969569310652 | 0.999440741871876 |
| ebi-a-GCST90001946 | finngen_R11_O15_POOR_FETGRO | Poor fetal growth \|\| id:finngen_R11_O15_POOR_FETGRO | CD33 on CD14+ monocyte \|\| id:ebi-a-GCST90001946 | Weighted mode | 21 | -0.036477469 | 0.0166718770686588 | 0.0407139146125022 | -0.069154348 | -0.00380059 | 0.964179817832373 | 0.933182634275173 | 0.996206623409087 | 0.999857399747617 |
| ebi-a-GCST90001947 | finngen_R11_O15_POOR_FETGRO | Poor fetal growth \|\| id:finngen_R11_O15_POOR_FETGRO | CD33 on CD33+ HLA DR+ CD14dim \|\| id:ebi-a-GCST90001947 | Inverse variance weighted | 27 | -0.016036349 | 0.0121938666161836 | 0.188470910140595 | -0.039936328 | 0.00786362911809149 | 0.984091548218984 | 0.960850616468445 | 1.00789462865255 | 0.994119304454654 |
| ebi-a-GCST90001947 | finngen_R11_O15_POOR_FETGRO | Poor fetal growth \|\| id:finngen_R11_O15_POOR_FETGRO | CD33 on CD33+ HLA DR+ CD14dim \|\| id:ebi-a-GCST90001947 | MR Egger | 27 | -0.006054545 | 0.0189160454773817 | 0.751571591129958 | -0.043129994 | 0.0310209042968557 | 0.993963746982953 | 0.957786875451486 | 1.03150706659107 | 0.992899239648112 |
| ebi-a-GCST90001947 | finngen_R11_O15_POOR_FETGRO | Poor fetal growth \|\| id:finngen_R11_O15_POOR_FETGRO | CD33 on CD33+ HLA DR+ CD14dim \|\| id:ebi-a-GCST90001947 | Weighted median | 27 | -0.03373617 | 0.0169482351853416 | 0.0465312886875415 | -0.066954711 | -0.000517629 | 0.966826548959603 | 0.935237556501154 | 0.999482505033449 | 0.999440741871876 |
| ebi-a-GCST90001947 | finngen_R11_O15_POOR_FETGRO | Poor fetal growth \|\| id:finngen_R11_O15_POOR_FETGRO | CD33 on CD33+ HLA DR+ CD14dim \|\| id:ebi-a-GCST90001947 | Weighted mode | 27 | -0.028987925 | 0.016886732847891 | 0.097943229672886 | -0.062085921 | 0.00411007179980536 | 0.97142819479723 | 0.939802134616679 | 1.0041185297285 | 0.999857399747617 |
| ebi-a-GCST90001948 | finngen_R11_O15_POOR_FETGRO | Poor fetal growth \|\| id:finngen_R11_O15_POOR_FETGRO | CD33 on CD33dim HLA DR+ CD11b+ \|\| id:ebi-a-GCST90001948 | Inverse variance weighted | 22 | -0.032293388 | 0.0171422847809221 | 0.0595861018491812 | -0.065892266 | 0.00130549061900596 | 0.968222475983005 | 0.93623172310708 | 1.00130634314283 | 0.994119304454654 |
| ebi-a-GCST90001948 | finngen_R11_O15_POOR_FETGRO | Poor fetal growth \|\| id:finngen_R11_O15_POOR_FETGRO | CD33 on CD33dim HLA DR+ CD11b+ \|\| id:ebi-a-GCST90001948 | MR Egger | 22 | -0.032271533 | 0.0280796516154411 | 0.263997365870451 | -0.08730765 | 0.0227645840958573 | 0.968243636214121 | 0.916395123084414 | 1.02302567468245 | 0.992899239648112 |
| ebi-a-GCST90001948 | finngen_R11_O15_POOR_FETGRO | Poor fetal growth \|\| id:finngen_R11_O15_POOR_FETGRO | CD33 on CD33dim HLA DR+ CD11b+ \|\| id:ebi-a-GCST90001948 | Weighted median | 22 | -0.036214342 | 0.0176303178147696 | 0.0399665304571397 | -0.070769765 | -0.001658919 | 0.964433552960685 | 0.931676372264588 | 0.99834245642136 | 0.999440741871876 |
| ebi-a-GCST90001948 | finngen_R11_O15_POOR_FETGRO | Poor fetal growth \|\| id:finngen_R11_O15_POOR_FETGRO | CD33 on CD33dim HLA DR+ CD11b+ \|\| id:ebi-a-GCST90001948 | Weighted mode | 22 | -0.035386816 | 0.0169111228044773 | 0.048718189034509 | -0.068532617 | -0.002241015 | 0.965231976875428 | 0.933763003353437 | 0.997761493908964 | 0.999857399747617 |
| ebi-a-GCST90001949 | finngen_R11_O15_POOR_FETGRO | Poor fetal growth \|\| id:finngen_R11_O15_POOR_FETGRO | CD33 on CD33dim HLA DR+ CD11b- \|\| id:ebi-a-GCST90001949 | Inverse variance weighted | 22 | -0.032777851 | 0.016500744068353 | 0.0469837290061831 | -0.065119309 | -0.000436393 | 0.967753521126253 | 0.936955669054018 | 0.999563702515221 | 0.994119304454654 |
| ebi-a-GCST90001949 | finngen_R11_O15_POOR_FETGRO | Poor fetal growth \|\| id:finngen_R11_O15_POOR_FETGRO | CD33 on CD33dim HLA DR+ CD11b- \|\| id:ebi-a-GCST90001949 | MR Egger | 22 | -0.043057225 | 0.0275584202592687 | 0.13387943082535 | -0.097071729 | 0.0109572788529169 | 0.957856575294791 | 0.907490910779359 | 1.01101752969351 | 0.992899239648112 |
| ebi-a-GCST90001949 | finngen_R11_O15_POOR_FETGRO | Poor fetal growth \|\| id:finngen_R11_O15_POOR_FETGRO | CD33 on CD33dim HLA DR+ CD11b- \|\| id:ebi-a-GCST90001949 | Weighted median | 22 | -0.036541727 | 0.0190222708514431 | 0.0547320708157968 | -0.073825378 | 0.000741923383878185 | 0.964117862843347 | 0.928833874180952 | 1.00074219867711 | 0.999440741871876 |
| ebi-a-GCST90001949 | finngen_R11_O15_POOR_FETGRO | Poor fetal growth \|\| id:finngen_R11_O15_POOR_FETGRO | CD33 on CD33dim HLA DR+ CD11b- \|\| id:ebi-a-GCST90001949 | Weighted mode | 22 | -0.03328476 | 0.0178581211012386 | 0.0763863538859352 | -0.068286678 | 0.00171715706940342 | 0.967263082253526 | 0.933992680369539 | 1.00171863222784 | 0.999857399747617 |
| ebi-a-GCST90001950 | finngen_R11_O15_POOR_FETGRO | Poor fetal growth \|\| id:finngen_R11_O15_POOR_FETGRO | CD33 on Granulocytic Myeloid-Derived Suppressor Cells \|\| id:ebi-a-GCST90001950 | Inverse variance weighted | 20 | -0.033272197 | 0.0177038550493043 | 0.060193196167113 | -0.067971753 | 0.00142735887622204 | 0.967275234315779 | 0.934286864083033 | 1.00142837803775 | 0.994119304454654 |
| ebi-a-GCST90001950 | finngen_R11_O15_POOR_FETGRO | Poor fetal growth \|\| id:finngen_R11_O15_POOR_FETGRO | CD33 on Granulocytic Myeloid-Derived Suppressor Cells \|\| id:ebi-a-GCST90001950 | MR Egger | 20 | -0.035813666 | 0.0340382942352124 | 0.306647610039408 | -0.102528723 | 0.0309013906464961 | 0.964820055462336 | 0.902552225593686 | 1.03138379478265 | 0.992899239648112 |
| ebi-a-GCST90001950 | finngen_R11_O15_POOR_FETGRO | Poor fetal growth \|\| id:finngen_R11_O15_POOR_FETGRO | CD33 on Granulocytic Myeloid-Derived Suppressor Cells \|\| id:ebi-a-GCST90001950 | Weighted median | 20 | -0.045792171 | 0.023127896418425 | 0.0477086972630705 | -0.091122848 | -0.000461494 | 0.955240468110338 | 0.912905555289039 | 0.999538612323104 | 0.999440741871876 |
| ebi-a-GCST90001950 | finngen_R11_O15_POOR_FETGRO | Poor fetal growth \|\| id:finngen_R11_O15_POOR_FETGRO | CD33 on Granulocytic Myeloid-Derived Suppressor Cells \|\| id:ebi-a-GCST90001950 | Weighted mode | 20 | -0.044083313 | 0.0228510477938537 | 0.0687837843004796 | -0.088871367 | 0.000704740217208795 | 0.95687423365007 | 0.914963260350921 | 1.00070498860494 | 0.999857399747617 |
| ebi-a-GCST90001951 | finngen_R11_O15_POOR_FETGRO | Poor fetal growth \|\| id:finngen_R11_O15_POOR_FETGRO | CD33 on CD66b++ myeloid cell \|\| id:ebi-a-GCST90001951 | Inverse variance weighted | 18 | -0.019770843 | 0.0170792149950251 | 0.247028651098242 | -0.053246105 | 0.0137044182437788 | 0.98042331828905 | 0.948146640576812 | 1.01379875423082 | 0.994119304454654 |
| ebi-a-GCST90001951 | finngen_R11_O15_POOR_FETGRO | Poor fetal growth \|\| id:finngen_R11_O15_POOR_FETGRO | CD33 on CD66b++ myeloid cell \|\| id:ebi-a-GCST90001951 | MR Egger | 18 | -0.036867189 | 0.032420361337054 | 0.272214948953424 | -0.100411097 | 0.0266767192434785 | 0.963804130661694 | 0.90446551835766 | 1.02703572820254 | 0.992899239648112 |
| ebi-a-GCST90001951 | finngen_R11_O15_POOR_FETGRO | Poor fetal growth \|\| id:finngen_R11_O15_POOR_FETGRO | CD33 on CD66b++ myeloid cell \|\| id:ebi-a-GCST90001951 | Weighted median | 18 | -0.032173451 | 0.0221304395515574 | 0.145999030936562 | -0.075549113 | 0.0112022104435188 | 0.968338608136989 | 0.927234190535119 | 1.01126519015389 | 0.999440741871876 |
| ebi-a-GCST90001951 | finngen_R11_O15_POOR_FETGRO | Poor fetal growth \|\| id:finngen_R11_O15_POOR_FETGRO | CD33 on CD66b++ myeloid cell \|\| id:ebi-a-GCST90001951 | Weighted mode | 18 | -0.027520945 | 0.0225683962703547 | 0.239324519330555 | -0.071755001 | 0.016713112076587 | 0.972854306282818 | 0.930758902597551 | 1.01685355746982 | 0.999857399747617 |
| ebi-a-GCST90001952 | finngen_R11_O15_POOR_FETGRO | Poor fetal growth \|\| id:finngen_R11_O15_POOR_FETGRO | CD33 on Monocytic Myeloid-Derived Suppressor Cells \|\| id:ebi-a-GCST90001952 | Inverse variance weighted | 20 | -0.018816356 | 0.0152264916535949 | 0.216546149877258 | -0.04866028 | 0.011027567253648 | 0.981359566111637 | 0.952504659596475 | 1.01108859499629 | 0.994119304454654 |
| ebi-a-GCST90001952 | finngen_R11_O15_POOR_FETGRO | Poor fetal growth \|\| id:finngen_R11_O15_POOR_FETGRO | CD33 on Monocytic Myeloid-Derived Suppressor Cells \|\| id:ebi-a-GCST90001952 | MR Egger | 20 | -0.045816537 | 0.0233631982138092 | 0.0655318226140318 | -0.091608405 | -2.47E-05 | 0.955217193482371 | 0.912462395187586 | 0.99997533217438 | 0.992899239648112 |
| ebi-a-GCST90001952 | finngen_R11_O15_POOR_FETGRO | Poor fetal growth \|\| id:finngen_R11_O15_POOR_FETGRO | CD33 on Monocytic Myeloid-Derived Suppressor Cells \|\| id:ebi-a-GCST90001952 | Weighted median | 20 | -0.035895179 | 0.0181959382451549 | 0.04852943038139 | -0.071559218 | -0.00023114 | 0.96474141362533 | 0.930941147798889 | 0.99976888696391 | 0.999440741871876 |
| ebi-a-GCST90001952 | finngen_R11_O15_POOR_FETGRO | Poor fetal growth \|\| id:finngen_R11_O15_POOR_FETGRO | CD33 on Monocytic Myeloid-Derived Suppressor Cells \|\| id:ebi-a-GCST90001952 | Weighted mode | 20 | -0.033974105 | 0.018336629714505 | 0.0795095298786102 | -0.069913899 | 0.0019656893670338 | 0.966596534453065 | 0.932474103296292 | 1.00196762260088 | 0.999857399747617 |
| ebi-a-GCST90001953 | finngen_R11_O15_POOR_FETGRO | Poor fetal growth \|\| id:finngen_R11_O15_POOR_FETGRO | CD33 on CD33dim HLA DR- \|\| id:ebi-a-GCST90001953 | Inverse variance weighted | 19 | -0.012026498 | 0.0160061708227483 | 0.452432230525834 | -0.043398592 | 0.019345597256566 | 0.988045531723236 | 0.957529649981544 | 1.01953393587036 | 0.994119304454654 |
| ebi-a-GCST90001953 | finngen_R11_O15_POOR_FETGRO | Poor fetal growth \|\| id:finngen_R11_O15_POOR_FETGRO | CD33 on CD33dim HLA DR- \|\| id:ebi-a-GCST90001953 | MR Egger | 19 | -0.040140764 | 0.0249629291599973 | 0.126244330018289 | -0.089068106 | 0.00878657678073571 | 0.960654203747858 | 0.914783269656685 | 1.008825292 | 0.992899239648112 |
| ebi-a-GCST90001953 | finngen_R11_O15_POOR_FETGRO | Poor fetal growth \|\| id:finngen_R11_O15_POOR_FETGRO | CD33 on CD33dim HLA DR- \|\| id:ebi-a-GCST90001953 | Weighted median | 19 | -0.032065678 | 0.018807046233706 | 0.0881977589669073 | -0.068927488 | 0.00479613304011063 | 0.968442975001457 | 0.933394359733237 | 1.00480765289574 | 0.999440741871876 |
| ebi-a-GCST90001953 | finngen_R11_O15_POOR_FETGRO | Poor fetal growth \|\| id:finngen_R11_O15_POOR_FETGRO | CD33 on CD33dim HLA DR- \|\| id:ebi-a-GCST90001953 | Weighted mode | 19 | -0.029219283 | 0.0190818637736228 | 0.143091339315491 | -0.066619736 | 0.00818117011051844 | 0.971203472814546 | 0.935550890138651 | 1.00821472733273 | 0.999857399747617 |
| ebi-a-GCST90001954 | finngen_R11_O15_POOR_FETGRO | Poor fetal growth \|\| id:finngen_R11_O15_POOR_FETGRO | CD33 on basophil \|\| id:ebi-a-GCST90001954 | Inverse variance weighted | 22 | -0.009845236 | 0.011225277599157 | 0.380454326855339 | -0.03184678 | 0.0121563078560132 | 0.990203069443036 | 0.968654987704539 | 1.01223049607987 | 0.994119304454654 |
| ebi-a-GCST90001954 | finngen_R11_O15_POOR_FETGRO | Poor fetal growth \|\| id:finngen_R11_O15_POOR_FETGRO | CD33 on basophil \|\| id:ebi-a-GCST90001954 | MR Egger | 22 | -0.020120495 | 0.0149587620890007 | 0.193659382208787 | -0.049439669 | 0.00919867843207246 | 0.980080571125961 | 0.951762577233521 | 1.00924111629881 | 0.992899239648112 |
| ebi-a-GCST90001954 | finngen_R11_O15_POOR_FETGRO | Poor fetal growth \|\| id:finngen_R11_O15_POOR_FETGRO | CD33 on basophil \|\| id:ebi-a-GCST90001954 | Weighted median | 22 | -0.012060411 | 0.0155501459025155 | 0.437995908449851 | -0.042538697 | 0.0184178747285825 | 0.98801202402716 | 0.958353379202105 | 1.01858852987504 | 0.999440741871876 |
| ebi-a-GCST90001954 | finngen_R11_O15_POOR_FETGRO | Poor fetal growth \|\| id:finngen_R11_O15_POOR_FETGRO | CD33 on basophil \|\| id:ebi-a-GCST90001954 | Weighted mode | 22 | -0.015655573 | 0.0127840723333647 | 0.234280519807347 | -0.040712355 | 0.00940120901319694 | 0.984466338692021 | 0.960105260157452 | 1.00944553918883 | 0.999857399747617 |
| ebi-a-GCST90001955 | finngen_R11_O15_POOR_FETGRO | Poor fetal growth \|\| id:finngen_R11_O15_POOR_FETGRO | CD33 on Immature Myeloid-Derived Suppressor Cells \|\| id:ebi-a-GCST90001955 | Inverse variance weighted | 22 | -0.034373427 | 0.0159257295370881 | 0.0309000016185982 | -0.065587857 | -0.003158997 | 0.966210627965505 | 0.936516763537752 | 0.99684598710962 | 0.994119304454654 |
| ebi-a-GCST90001955 | finngen_R11_O15_POOR_FETGRO | Poor fetal growth \|\| id:finngen_R11_O15_POOR_FETGRO | CD33 on Immature Myeloid-Derived Suppressor Cells \|\| id:ebi-a-GCST90001955 | MR Egger | 22 | -0.043841932 | 0.0253657920926633 | 0.0993276433709569 | -0.093558884 | 0.00587502093749753 | 0.95710523364399 | 0.910684391047792 | 1.00589231271961 | 0.992899239648112 |
| ebi-a-GCST90001955 | finngen_R11_O15_POOR_FETGRO | Poor fetal growth \|\| id:finngen_R11_O15_POOR_FETGRO | CD33 on Immature Myeloid-Derived Suppressor Cells \|\| id:ebi-a-GCST90001955 | Weighted median | 22 | -0.033267868 | 0.0187090927607842 | 0.0753766364691002 | -0.06993769 | 0.00340195386899486 | 0.967279421735043 | 0.932451919404986 | 1.00340774708161 | 0.999440741871876 |
| ebi-a-GCST90001955 | finngen_R11_O15_POOR_FETGRO | Poor fetal growth \|\| id:finngen_R11_O15_POOR_FETGRO | CD33 on Immature Myeloid-Derived Suppressor Cells \|\| id:ebi-a-GCST90001955 | Weighted mode | 22 | -0.032427063 | 0.018947520752105 | 0.101736370119378 | -0.069564204 | 0.00471007755530579 | 0.968093056944609 | 0.932800242148348 | 1.0047211874065 | 0.999857399747617 |
| ebi-a-GCST90001956 | finngen_R11_O15_POOR_FETGRO | Poor fetal growth \|\| id:finngen_R11_O15_POOR_FETGRO | CD33 on CD33+ HLA DR+ \|\| id:ebi-a-GCST90001956 | Inverse variance weighted | 23 | -0.031621818 | 0.0135893490628879 | 0.0199676027552126 | -0.058256943 | -0.004986694 | 0.968872922644437 | 0.943407514678459 | 0.99502571860848 | 0.994119304454654 |
| ebi-a-GCST90001956 | finngen_R11_O15_POOR_FETGRO | Poor fetal growth \|\| id:finngen_R11_O15_POOR_FETGRO | CD33 on CD33+ HLA DR+ \|\| id:ebi-a-GCST90001956 | MR Egger | 23 | -0.017905566 | 0.0210943467645244 | 0.40554703985328 | -0.059250486 | 0.0234393533426612 | 0.982253785822514 | 0.942470663905334 | 1.02371621389753 | 0.992899239648112 |
| ebi-a-GCST90001956 | finngen_R11_O15_POOR_FETGRO | Poor fetal growth \|\| id:finngen_R11_O15_POOR_FETGRO | CD33 on CD33+ HLA DR+ \|\| id:ebi-a-GCST90001956 | Weighted median | 23 | -0.033853496 | 0.0165584548870747 | 0.0409057569921481 | -0.066308068 | -0.001398924 | 0.966713121611013 | 0.935842517148479 | 0.998602053625907 | 0.999440741871876 |
| ebi-a-GCST90001956 | finngen_R11_O15_POOR_FETGRO | Poor fetal growth \|\| id:finngen_R11_O15_POOR_FETGRO | CD33 on CD33+ HLA DR+ \|\| id:ebi-a-GCST90001956 | Weighted mode | 23 | -0.029316306 | 0.017224749927174 | 0.102848808638673 | -0.063076816 | 0.00444420401756203 | 0.971109248355801 | 0.938871350861914 | 1.00445409413805 | 0.999857399747617 |
| ebi-a-GCST90001957 | finngen_R11_O15_POOR_FETGRO | Poor fetal growth \|\| id:finngen_R11_O15_POOR_FETGRO | CD33 on CD33+ HLA DR+ CD14- \|\| id:ebi-a-GCST90001957 | Inverse variance weighted | 21 | -0.030294614 | 0.0140660528791285 | 0.0312606057331798 | -0.057864077 | -0.00272515 | 0.970159668963583 | 0.943778219418707 | 0.997278559642158 | 0.994119304454654 |
| ebi-a-GCST90001957 | finngen_R11_O15_POOR_FETGRO | Poor fetal growth \|\| id:finngen_R11_O15_POOR_FETGRO | CD33 on CD33+ HLA DR+ CD14- \|\| id:ebi-a-GCST90001957 | MR Egger | 21 | -0.020120198 | 0.0227398211208148 | 0.387324795516877 | -0.064690247 | 0.0244498514565338 | 0.980080862525624 | 0.937357767478452 | 1.02475120003852 | 0.992899239648112 |
| ebi-a-GCST90001957 | finngen_R11_O15_POOR_FETGRO | Poor fetal growth \|\| id:finngen_R11_O15_POOR_FETGRO | CD33 on CD33+ HLA DR+ CD14- \|\| id:ebi-a-GCST90001957 | Weighted median | 21 | -0.033351015 | 0.0172534072675115 | 0.0532349219403388 | -0.067167693 | 0.000465663301898969 | 0.967198998696205 | 0.935038388621814 | 1.00046577173989 | 0.999440741871876 |
| ebi-a-GCST90001957 | finngen_R11_O15_POOR_FETGRO | Poor fetal growth \|\| id:finngen_R11_O15_POOR_FETGRO | CD33 on CD33+ HLA DR+ CD14- \|\| id:ebi-a-GCST90001957 | Weighted mode | 21 | -0.028044465 | 0.0168202938905195 | 0.111038432718155 | -0.061012241 | 0.0049233114064703 | 0.972345130884289 | 0.940811723563803 | 1.00493545081793 | 0.999857399747617 |
| ebi-a-GCST90001958 | finngen_R11_O15_POOR_FETGRO | Poor fetal growth \|\| id:finngen_R11_O15_POOR_FETGRO | CD4 on monocyte \|\| id:ebi-a-GCST90001958 | Inverse variance weighted | 15 | -0.036351952 | 0.0311320834586506 | 0.242940520472671 | -0.097370836 | 0.0246669313764001 | 0.964300845945317 | 0.907219514287938 | 1.02497367709368 | 0.994119304454654 |
| ebi-a-GCST90001958 | finngen_R11_O15_POOR_FETGRO | Poor fetal growth \|\| id:finngen_R11_O15_POOR_FETGRO | CD4 on monocyte \|\| id:ebi-a-GCST90001958 | MR Egger | 15 | 0.0350604384228057 | 0.0452265413935225 | 0.452082281795181 | -0.053583583 | 0.12370445955411 | 1.03568230191295 | 0.947826715768828 | 1.13168136395652 | 0.992899239648112 |
| ebi-a-GCST90001958 | finngen_R11_O15_POOR_FETGRO | Poor fetal growth \|\| id:finngen_R11_O15_POOR_FETGRO | CD4 on monocyte \|\| id:ebi-a-GCST90001958 | Weighted median | 15 | -0.023158186 | 0.0465032626659904 | 0.618490695785826 | -0.114304581 | 0.0679882090055017 | 0.977107906932338 | 0.891986232761441 | 1.07035268788172 | 0.999440741871876 |
| ebi-a-GCST90001958 | finngen_R11_O15_POOR_FETGRO | Poor fetal growth \|\| id:finngen_R11_O15_POOR_FETGRO | CD4 on monocyte \|\| id:ebi-a-GCST90001958 | Weighted mode | 15 | -0.007411773 | 0.0430274953072715 | 0.865701065010453 | -0.091745664 | 0.0769221176262596 | 0.992615626280173 | 0.912337160243337 | 1.07995796342745 | 0.999857399747617 |
| ebi-a-GCST90001959 | finngen_R11_O15_POOR_FETGRO | Poor fetal growth \|\| id:finngen_R11_O15_POOR_FETGRO | CD4 on HLA DR+ CD4+ T cell \|\| id:ebi-a-GCST90001959 | Inverse variance weighted | 21 | -0.054492477 | 0.0325514854118915 | 0.0941228059278647 | -0.118293389 | 0.00930843396522882 | 0.946965632434851 | 0.888435357346699 | 1.00935189217463 | 0.994119304454654 |
| ebi-a-GCST90001959 | finngen_R11_O15_POOR_FETGRO | Poor fetal growth \|\| id:finngen_R11_O15_POOR_FETGRO | CD4 on HLA DR+ CD4+ T cell \|\| id:ebi-a-GCST90001959 | MR Egger | 21 | -0.036392377 | 0.06416333334529 | 0.577230051992962 | -0.162152511 | 0.0893677559648474 | 0.964261864688931 | 0.85031151300534 | 1.09348271718336 | 0.992899239648112 |
| ebi-a-GCST90001959 | finngen_R11_O15_POOR_FETGRO | Poor fetal growth \|\| id:finngen_R11_O15_POOR_FETGRO | CD4 on HLA DR+ CD4+ T cell \|\| id:ebi-a-GCST90001959 | Weighted median | 21 | -0.044843008 | 0.0432922014042477 | 0.300285258590039 | -0.129695723 | 0.040009706289259 | 0.956147577128615 | 0.878362655627936 | 1.04082087665186 | 0.999440741871876 |
| ebi-a-GCST90001959 | finngen_R11_O15_POOR_FETGRO | Poor fetal growth \|\| id:finngen_R11_O15_POOR_FETGRO | CD4 on HLA DR+ CD4+ T cell \|\| id:ebi-a-GCST90001959 | Weighted mode | 21 | -0.030344544 | 0.0637662492567065 | 0.639322661798247 | -0.155326393 | 0.0946373042772493 | 0.970111229699105 | 0.856135695341092 | 1.09926008588318 | 0.999857399747617 |
| ebi-a-GCST90001960 | finngen_R11_O15_POOR_FETGRO | Poor fetal growth \|\| id:finngen_R11_O15_POOR_FETGRO | CD25 on CD4+ T cell \|\| id:ebi-a-GCST90001960 | Inverse variance weighted | 16 | 0.0132168732579495 | 0.0151871761969205 | 0.384155408348474 | -0.016549992 | 0.0429837386039137 | 1.01330460220202 | 0.983586206633346 | 1.04392091910104 | 0.994119304454654 |
| ebi-a-GCST90001960 | finngen_R11_O15_POOR_FETGRO | Poor fetal growth \|\| id:finngen_R11_O15_POOR_FETGRO | CD25 on CD4+ T cell \|\| id:ebi-a-GCST90001960 | MR Egger | 16 | 0.0108665992008498 | 0.0178231643591807 | 0.551834980024448 | -0.024066803 | 0.0458000013448439 | 1.01092585513226 | 0.976220493177502 | 1.04686501842271 | 0.992899239648112 |
| ebi-a-GCST90001960 | finngen_R11_O15_POOR_FETGRO | Poor fetal growth \|\| id:finngen_R11_O15_POOR_FETGRO | CD25 on CD4+ T cell \|\| id:ebi-a-GCST90001960 | Weighted median | 16 | 0.0173684367941964 | 0.0195344071166751 | 0.373938460972289 | -0.020919001 | 0.0556558747428796 | 1.01752014513205 | 0.979298283387417 | 1.0572338002761 | 0.999440741871876 |
| ebi-a-GCST90001960 | finngen_R11_O15_POOR_FETGRO | Poor fetal growth \|\| id:finngen_R11_O15_POOR_FETGRO | CD25 on CD4+ T cell \|\| id:ebi-a-GCST90001960 | Weighted mode | 16 | 0.0174198265288778 | 0.0172672117320569 | 0.329049834031281 | -0.016423908 | 0.0512635615237094 | 1.01757243656595 | 0.98371022856329 | 1.05260028166124 | 0.999857399747617 |
| ebi-a-GCST90001961 | finngen_R11_O15_POOR_FETGRO | Poor fetal growth \|\| id:finngen_R11_O15_POOR_FETGRO | CD25 on CD39+ CD4+ T cell \|\| id:ebi-a-GCST90001961 | Inverse variance weighted | 23 | -0.014353976 | 0.0224648798262055 | 0.522854283050976 | -0.05838514 | 0.0296771888722023 | 0.985748551576723 | 0.943286580030302 | 1.03012194544779 | 0.994119304454654 |
| ebi-a-GCST90001961 | finngen_R11_O15_POOR_FETGRO | Poor fetal growth \|\| id:finngen_R11_O15_POOR_FETGRO | CD25 on CD39+ CD4+ T cell \|\| id:ebi-a-GCST90001961 | MR Egger | 23 | -0.045097232 | 0.0345252187512615 | 0.205607543363231 | -0.112766661 | 0.0225721968290814 | 0.95590453287806 | 0.893359091604084 | 1.0228288765004 | 0.992899239648112 |
| ebi-a-GCST90001961 | finngen_R11_O15_POOR_FETGRO | Poor fetal growth \|\| id:finngen_R11_O15_POOR_FETGRO | CD25 on CD39+ CD4+ T cell \|\| id:ebi-a-GCST90001961 | Weighted median | 23 | -0.025334526 | 0.026583831950658 | 0.34058751164917 | -0.077438837 | 0.0267697845061644 | 0.974983699956739 | 0.925483628260978 | 1.02713131400016 | 0.999440741871876 |
| ebi-a-GCST90001961 | finngen_R11_O15_POOR_FETGRO | Poor fetal growth \|\| id:finngen_R11_O15_POOR_FETGRO | CD25 on CD39+ CD4+ T cell \|\| id:ebi-a-GCST90001961 | Weighted mode | 23 | -0.01804622 | 0.0251019521352472 | 0.479759132229509 | -0.067246046 | 0.0311536063834333 | 0.98211563811926 | 0.934965128616069 | 1.03164395881387 | 0.999857399747617 |
| ebi-a-GCST90001962 | finngen_R11_O15_POOR_FETGRO | Poor fetal growth \|\| id:finngen_R11_O15_POOR_FETGRO | CD25 on CD28+ CD4+ T cell \|\| id:ebi-a-GCST90001962 | Inverse variance weighted | 19 | -0.030633754 | 0.0364592562617538 | 0.400785693384917 | -0.102093897 | 0.0408263880396198 | 0.969830704429178 | 0.902944764329429 | 1.04167124325939 | 0.994119304454654 |
| ebi-a-GCST90001962 | finngen_R11_O15_POOR_FETGRO | Poor fetal growth \|\| id:finngen_R11_O15_POOR_FETGRO | CD25 on CD28+ CD4+ T cell \|\| id:ebi-a-GCST90001962 | MR Egger | 19 | -0.039478503 | 0.0881405297688332 | 0.659877875622455 | -0.212233941 | 0.133276935754563 | 0.961290619024782 | 0.808775469740251 | 1.14256637200165 | 0.992899239648112 |
| ebi-a-GCST90001962 | finngen_R11_O15_POOR_FETGRO | Poor fetal growth \|\| id:finngen_R11_O15_POOR_FETGRO | CD25 on CD28+ CD4+ T cell \|\| id:ebi-a-GCST90001962 | Weighted median | 19 | -0.046778866 | 0.0488774451969033 | 0.338534716639933 | -0.142578659 | 0.0490209265088415 | 0.954298402008658 | 0.867119345161302 | 1.05024232841544 | 0.999440741871876 |
| ebi-a-GCST90001962 | finngen_R11_O15_POOR_FETGRO | Poor fetal growth \|\| id:finngen_R11_O15_POOR_FETGRO | CD25 on CD28+ CD4+ T cell \|\| id:ebi-a-GCST90001962 | Weighted mode | 19 | -0.057109219 | 0.0677367852479242 | 0.410233895875026 | -0.189873318 | 0.0756548803229403 | 0.944490907604441 | 0.827063901541256 | 1.07859026719106 | 0.999857399747617 |
| ebi-a-GCST90001963 | finngen_R11_O15_POOR_FETGRO | Poor fetal growth \|\| id:finngen_R11_O15_POOR_FETGRO | FSC-A on myeloid Dendritic Cell \|\| id:ebi-a-GCST90001963 | Inverse variance weighted | 20 | 0.00751204730978234 | 0.0148664096758576 | 0.613345757280866 | -0.021626116 | 0.0366502102744632 | 1.00754033352193 | 0.978606052143225 | 1.03733010995616 | 0.994119304454654 |
| ebi-a-GCST90001963 | finngen_R11_O15_POOR_FETGRO | Poor fetal growth \|\| id:finngen_R11_O15_POOR_FETGRO | FSC-A on myeloid Dendritic Cell \|\| id:ebi-a-GCST90001963 | MR Egger | 20 | 0.0104237860145031 | 0.0185041305514728 | 0.5801659874982 | -0.02584431 | 0.0466918818953897 | 1.01047830293151 | 0.974486795778308 | 1.04779911346037 | 0.992899239648112 |
| ebi-a-GCST90001963 | finngen_R11_O15_POOR_FETGRO | Poor fetal growth \|\| id:finngen_R11_O15_POOR_FETGRO | FSC-A on myeloid Dendritic Cell \|\| id:ebi-a-GCST90001963 | Weighted median | 20 | -0.00310139 | 0.0210674223810613 | 0.882964217457263 | -0.044393538 | 0.0381907578926845 | 0.996903414367694 | 0.956577433971596 | 1.03892939795972 | 0.999440741871876 |
| ebi-a-GCST90001963 | finngen_R11_O15_POOR_FETGRO | Poor fetal growth \|\| id:finngen_R11_O15_POOR_FETGRO | FSC-A on myeloid Dendritic Cell \|\| id:ebi-a-GCST90001963 | Weighted mode | 20 | -0.005673814 | 0.017983763678934 | 0.75582386223047 | -0.04092199 | 0.0295743631836757 | 0.99434225205458 | 0.959904008718657 | 1.0300160278951 | 0.999857399747617 |
| ebi-a-GCST90001964 | finngen_R11_O15_POOR_FETGRO | Poor fetal growth \|\| id:finngen_R11_O15_POOR_FETGRO | FSC-A on plasmacytoid Dendritic Cell \|\| id:ebi-a-GCST90001964 | Inverse variance weighted | 27 | 0.00934718422506673 | 0.0135834562858282 | 0.491370899154994 | -0.01727639 | 0.03597075854529 | 1.00939100558054 | 0.982871991006804 | 1.03662553360914 | 0.994119304454654 |
| ebi-a-GCST90001964 | finngen_R11_O15_POOR_FETGRO | Poor fetal growth \|\| id:finngen_R11_O15_POOR_FETGRO | FSC-A on plasmacytoid Dendritic Cell \|\| id:ebi-a-GCST90001964 | MR Egger | 27 | 0.0197948211394723 | 0.0178422067479488 | 0.277799700224667 | -0.015175904 | 0.054765546365452 | 1.01999203775124 | 0.984938669627114 | 1.0562929339243 | 0.992899239648112 |
| ebi-a-GCST90001964 | finngen_R11_O15_POOR_FETGRO | Poor fetal growth \|\| id:finngen_R11_O15_POOR_FETGRO | FSC-A on plasmacytoid Dendritic Cell \|\| id:ebi-a-GCST90001964 | Weighted median | 27 | 0.0197461120719706 | 0.0195394441485553 | 0.312218947675629 | -0.018551198 | 0.0580434226031391 | 1.0199423561002 | 0.981619815883127 | 1.05976101229306 | 0.999440741871876 |
| ebi-a-GCST90001964 | finngen_R11_O15_POOR_FETGRO | Poor fetal growth \|\| id:finngen_R11_O15_POOR_FETGRO | FSC-A on plasmacytoid Dendritic Cell \|\| id:ebi-a-GCST90001964 | Weighted mode | 27 | 0.0229766396601055 | 0.0164121315741648 | 0.17333965464715 | -0.009191138 | 0.0551444175454686 | 1.02324263597225 | 0.990850971175819 | 1.05669320869612 | 0.999857399747617 |
| ebi-a-GCST90001965 | finngen_R11_O15_POOR_FETGRO | Poor fetal growth \|\| id:finngen_R11_O15_POOR_FETGRO | FSC-A on monocyte \|\| id:ebi-a-GCST90001965 | Inverse variance weighted | 19 | -0.006817216 | 0.0202114022661369 | 0.735894129309399 | -0.046431564 | 0.0327971328098688 | 0.99320596886823 | 0.954629889315575 | 1.03334088701405 | 0.994119304454654 |
| ebi-a-GCST90001965 | finngen_R11_O15_POOR_FETGRO | Poor fetal growth \|\| id:finngen_R11_O15_POOR_FETGRO | FSC-A on monocyte \|\| id:ebi-a-GCST90001965 | MR Egger | 19 | -0.00175245 | 0.0243615023810861 | 0.943493006815275 | -0.049500995 | 0.0459960947001055 | 0.998249084677027 | 0.951704211538861 | 1.04707032182534 | 0.992899239648112 |
| ebi-a-GCST90001965 | finngen_R11_O15_POOR_FETGRO | Poor fetal growth \|\| id:finngen_R11_O15_POOR_FETGRO | FSC-A on monocyte \|\| id:ebi-a-GCST90001965 | Weighted median | 19 | -0.000161155 | 0.0253677456105224 | 0.994931260093591 | -0.049881937 | 0.0495596260419396 | 0.999838857630142 | 0.951341736366643 | 1.05080824588345 | 0.999440741871876 |
| ebi-a-GCST90001965 | finngen_R11_O15_POOR_FETGRO | Poor fetal growth \|\| id:finngen_R11_O15_POOR_FETGRO | FSC-A on monocyte \|\| id:ebi-a-GCST90001965 | Weighted mode | 19 | 0.00089637037556578 | 0.0217174064823422 | 0.967531615483178 | -0.041669746 | 0.0434624870809564 | 1.00089677223555 | 0.959186503133254 | 1.0444208143037 | 0.999857399747617 |
| ebi-a-GCST90001966 | finngen_R11_O15_POOR_FETGRO | Poor fetal growth \|\| id:finngen_R11_O15_POOR_FETGRO | FSC-A on granulocyte \|\| id:ebi-a-GCST90001966 | Inverse variance weighted | 21 | 0.0145168311315234 | 0.0241164668648161 | 0.547209520798155 | -0.032751444 | 0.0617851061865629 | 1.014622712 | 0.967779077067052 | 1.06373373036721 | 0.994119304454654 |
| ebi-a-GCST90001966 | finngen_R11_O15_POOR_FETGRO | Poor fetal growth \|\| id:finngen_R11_O15_POOR_FETGRO | FSC-A on granulocyte \|\| id:ebi-a-GCST90001966 | MR Egger | 21 | 0.0129843001230608 | 0.0373126159705883 | 0.731675747944406 | -0.060148427 | 0.0861170274254139 | 1.01306896217692 | 0.94162476050429 | 1.0899338729969 | 0.992899239648112 |
| ebi-a-GCST90001966 | finngen_R11_O15_POOR_FETGRO | Poor fetal growth \|\| id:finngen_R11_O15_POOR_FETGRO | FSC-A on granulocyte \|\| id:ebi-a-GCST90001966 | Weighted median | 21 | 0.0189481995187624 | 0.0358702097466587 | 0.597330449715473 | -0.051357412 | 0.0892538106222135 | 1.0191288558849 | 0.949939090615409 | 1.09335812701886 | 0.999440741871876 |
| ebi-a-GCST90001966 | finngen_R11_O15_POOR_FETGRO | Poor fetal growth \|\| id:finngen_R11_O15_POOR_FETGRO | FSC-A on granulocyte \|\| id:ebi-a-GCST90001966 | Weighted mode | 21 | 0.0259679581483832 | 0.0328806922535899 | 0.438929869584441 | -0.038478199 | 0.0904141149654193 | 1.02630806313592 | 0.96225268290072 | 1.09462749148445 | 0.999857399747617 |
| ebi-a-GCST90001967 | finngen_R11_O15_POOR_FETGRO | Poor fetal growth \|\| id:finngen_R11_O15_POOR_FETGRO | FSC-A on CD14+ monocyte \|\| id:ebi-a-GCST90001967 | Inverse variance weighted | 23 | 0.0229150918805741 | 0.0238739989054867 | 0.337138429842671 | -0.023877946 | 0.0697081297353281 | 1.02317965959813 | 0.976404876631386 | 1.0721951936856 | 0.994119304454654 |
| ebi-a-GCST90001967 | finngen_R11_O15_POOR_FETGRO | Poor fetal growth \|\| id:finngen_R11_O15_POOR_FETGRO | FSC-A on CD14+ monocyte \|\| id:ebi-a-GCST90001967 | MR Egger | 23 | 0.0393470696173274 | 0.0330783935928481 | 0.247514966152635 | -0.025486582 | 0.10418072105931 | 1.04013141902525 | 0.97483545939111 | 1.10980100120613 | 0.992899239648112 |
| ebi-a-GCST90001967 | finngen_R11_O15_POOR_FETGRO | Poor fetal growth \|\| id:finngen_R11_O15_POOR_FETGRO | FSC-A on CD14+ monocyte \|\| id:ebi-a-GCST90001967 | Weighted median | 23 | 0.0432658761439888 | 0.036422119962156 | 0.234872378704221 | -0.028121479 | 0.114653231269815 | 1.04421548993398 | 0.972270249227044 | 1.1214844743886 | 0.999440741871876 |
| ebi-a-GCST90001967 | finngen_R11_O15_POOR_FETGRO | Poor fetal growth \|\| id:finngen_R11_O15_POOR_FETGRO | FSC-A on CD14+ monocyte \|\| id:ebi-a-GCST90001967 | Weighted mode | 23 | 0.043536292525044 | 0.0311968102332004 | 0.176781635402107 | -0.017609456 | 0.104682040582117 | 1.04449790109039 | 0.982544684827944 | 1.11035750559607 | 0.999857399747617 |
| ebi-a-GCST90001968 | finngen_R11_O15_POOR_FETGRO | Poor fetal growth \|\| id:finngen_R11_O15_POOR_FETGRO | FSC-A on B cell \|\| id:ebi-a-GCST90001968 | Inverse variance weighted | 20 | -0.001643001 | 0.0303072380648625 | 0.956766652137933 | -0.061045188 | 0.0577591855293663 | 0.99835834790961 | 0.940780727112441 | 1.0594598317294 | 0.998984604424952 |
| ebi-a-GCST90001968 | finngen_R11_O15_POOR_FETGRO | Poor fetal growth \|\| id:finngen_R11_O15_POOR_FETGRO | FSC-A on B cell \|\| id:ebi-a-GCST90001968 | MR Egger | 20 | 0.0253616934262392 | 0.0557279638965888 | 0.654480002848765 | -0.083865116 | 0.134588502663553 | 1.02568603733839 | 0.9195552811987 | 1.14406590740204 | 0.992899239648112 |
| ebi-a-GCST90001968 | finngen_R11_O15_POOR_FETGRO | Poor fetal growth \|\| id:finngen_R11_O15_POOR_FETGRO | FSC-A on B cell \|\| id:ebi-a-GCST90001968 | Weighted median | 20 | 0.0358698880964377 | 0.0448012771781048 | 0.423337585329568 | -0.051940615 | 0.123680391365523 | 1.03652097399985 | 0.949385244247712 | 1.13165412676381 | 0.999440741871876 |
| ebi-a-GCST90001968 | finngen_R11_O15_POOR_FETGRO | Poor fetal growth \|\| id:finngen_R11_O15_POOR_FETGRO | FSC-A on B cell \|\| id:ebi-a-GCST90001968 | Weighted mode | 20 | 0.0423081926215468 | 0.0465888876660718 | 0.375191040801912 | -0.049006027 | 0.133622412447048 | 1.04321594066772 | 0.95217539072593 | 1.14296117024566 | 0.999857399747617 |
| ebi-a-GCST90001969 | finngen_R11_O15_POOR_FETGRO | Poor fetal growth \|\| id:finngen_R11_O15_POOR_FETGRO | FSC-A on Natural Killer \|\| id:ebi-a-GCST90001969 | Inverse variance weighted | 18 | 0.0425979387842584 | 0.0276019253902364 | 0.122758993592137 | -0.011501835 | 0.0966977125491217 | 1.0435182522781 | 0.988564058250375 | 1.10152734539511 | 0.994119304454654 |
| ebi-a-GCST90001969 | finngen_R11_O15_POOR_FETGRO | Poor fetal growth \|\| id:finngen_R11_O15_POOR_FETGRO | FSC-A on Natural Killer \|\| id:ebi-a-GCST90001969 | MR Egger | 18 | 0.0458048290049601 | 0.0426198458595204 | 0.298436415293279 | -0.037730069 | 0.12933972688962 | 1.0468700723434 | 0.962972842150003 | 1.13807669375329 | 0.992899239648112 |
| ebi-a-GCST90001969 | finngen_R11_O15_POOR_FETGRO | Poor fetal growth \|\| id:finngen_R11_O15_POOR_FETGRO | FSC-A on Natural Killer \|\| id:ebi-a-GCST90001969 | Weighted median | 18 | 0.0438498617983202 | 0.0398473665460178 | 0.271137991076111 | -0.034250977 | 0.121950700228515 | 1.04482547489403 | 0.966328948216077 | 1.12969840653416 | 0.999440741871876 |
| ebi-a-GCST90001969 | finngen_R11_O15_POOR_FETGRO | Poor fetal growth \|\| id:finngen_R11_O15_POOR_FETGRO | FSC-A on Natural Killer \|\| id:ebi-a-GCST90001969 | Weighted mode | 18 | 0.0501929361548192 | 0.0360264707824823 | 0.181503153932639 | -0.020418947 | 0.120804818888485 | 1.05147394414674 | 0.979788108434363 | 1.12840464759892 | 0.999857399747617 |
| ebi-a-GCST90001970 | finngen_R11_O15_POOR_FETGRO | Poor fetal growth \|\| id:finngen_R11_O15_POOR_FETGRO | FSC-A on HLA DR+ Natural Killer \|\| id:ebi-a-GCST90001970 | Inverse variance weighted | 20 | 0.00843909457727173 | 0.0192945210768046 | 0.661833645947835 | -0.029378167 | 0.0462563558878088 | 1.00847480411729 | 0.971049176528014 | 1.0473428690561 | 0.994119304454654 |
| ebi-a-GCST90001970 | finngen_R11_O15_POOR_FETGRO | Poor fetal growth \|\| id:finngen_R11_O15_POOR_FETGRO | FSC-A on HLA DR+ Natural Killer \|\| id:ebi-a-GCST90001970 | MR Egger | 20 | 0.020017774479605 | 0.0268759415195866 | 0.465994039195738 | -0.032659071 | 0.0726946198579948 | 1.02021947373583 | 0.967868477876721 | 1.07540208032519 | 0.992899239648112 |
| ebi-a-GCST90001970 | finngen_R11_O15_POOR_FETGRO | Poor fetal growth \|\| id:finngen_R11_O15_POOR_FETGRO | FSC-A on HLA DR+ Natural Killer \|\| id:ebi-a-GCST90001970 | Weighted median | 20 | 0.0280371460912287 | 0.0254324428728843 | 0.270280616978503 | -0.021810442 | 0.077884734122082 | 1.02843388601085 | 0.978425685949049 | 1.08099804929944 | 0.999440741871876 |
| ebi-a-GCST90001970 | finngen_R11_O15_POOR_FETGRO | Poor fetal growth \|\| id:finngen_R11_O15_POOR_FETGRO | FSC-A on HLA DR+ Natural Killer \|\| id:ebi-a-GCST90001970 | Weighted mode | 20 | 0.014598896420734 | 0.0256651098589992 | 0.576139227856149 | -0.035704719 | 0.0649025117443725 | 1.01470598077899 | 0.964925175585365 | 1.06705499398338 | 0.999857399747617 |
| ebi-a-GCST90001971 | finngen_R11_O15_POOR_FETGRO | Poor fetal growth \|\| id:finngen_R11_O15_POOR_FETGRO | FSC-A on lymphocyte \|\| id:ebi-a-GCST90001971 | Inverse variance weighted | 17 | -0.01178998 | 0.0293363138937068 | 0.687764756405118 | -0.069289155 | 0.0457091950270163 | 0.988279249273472 | 0.933056842609166 | 1.04676996078111 | 0.994119304454654 |
| ebi-a-GCST90001971 | finngen_R11_O15_POOR_FETGRO | Poor fetal growth \|\| id:finngen_R11_O15_POOR_FETGRO | FSC-A on lymphocyte \|\| id:ebi-a-GCST90001971 | MR Egger | 17 | 0.00822184100152486 | 0.0352776254530304 | 0.81886440090361 | -0.060922305 | 0.0773659868894645 | 1.00825573315788 | 0.940896339984216 | 1.08043742997533 | 0.992899239648112 |
| ebi-a-GCST90001971 | finngen_R11_O15_POOR_FETGRO | Poor fetal growth \|\| id:finngen_R11_O15_POOR_FETGRO | FSC-A on lymphocyte \|\| id:ebi-a-GCST90001971 | Weighted median | 17 | 0.00111618507241155 | 0.0333292540488052 | 0.973284118839373 | -0.064209153 | 0.0664415230080698 | 1.0011168082388 | 0.937808833614385 | 1.06869846797621 | 0.999440741871876 |
| ebi-a-GCST90001971 | finngen_R11_O15_POOR_FETGRO | Poor fetal growth \|\| id:finngen_R11_O15_POOR_FETGRO | FSC-A on lymphocyte \|\| id:ebi-a-GCST90001971 | Weighted mode | 17 | -0.002152355 | 0.0285478585074098 | 0.940835394122952 | -0.058106157 | 0.0538014479720638 | 0.997849959951973 | 0.94354977735058 | 1.05527505435063 | 0.999857399747617 |
| ebi-a-GCST90001972 | finngen_R11_O15_POOR_FETGRO | Poor fetal growth \|\| id:finngen_R11_O15_POOR_FETGRO | FSC-A on T cell \|\| id:ebi-a-GCST90001972 | Inverse variance weighted | 22 | -0.012283649 | 0.0185038664972523 | 0.506791271738522 | -0.048551227 | 0.0239839296920236 | 0.987791487406459 | 0.952608538799992 | 1.02427385736202 | 0.994119304454654 |
| ebi-a-GCST90001972 | finngen_R11_O15_POOR_FETGRO | Poor fetal growth \|\| id:finngen_R11_O15_POOR_FETGRO | FSC-A on T cell \|\| id:ebi-a-GCST90001972 | MR Egger | 22 | -0.004750556 | 0.0231045740130116 | 0.839173125228512 | -0.050035521 | 0.0405344087165053 | 0.99526070969676 | 0.951195636086151 | 1.0413671411928 | 0.992899239648112 |
| ebi-a-GCST90001972 | finngen_R11_O15_POOR_FETGRO | Poor fetal growth \|\| id:finngen_R11_O15_POOR_FETGRO | FSC-A on T cell \|\| id:ebi-a-GCST90001972 | Weighted median | 22 | -0.006098115 | 0.0259077602296694 | 0.813915414932604 | -0.056877325 | 0.0446810950996933 | 0.993920440814959 | 0.944709954551368 | 1.04569432968337 | 0.999440741871876 |
| ebi-a-GCST90001972 | finngen_R11_O15_POOR_FETGRO | Poor fetal growth \|\| id:finngen_R11_O15_POOR_FETGRO | FSC-A on T cell \|\| id:ebi-a-GCST90001972 | Weighted mode | 22 | -0.007588399 | 0.0221430344151665 | 0.735231316068717 | -0.050988747 | 0.0358119479793629 | 0.992440319738726 | 0.950289364146809 | 1.03646091959306 | 0.999857399747617 |
| ebi-a-GCST90001973 | finngen_R11_O15_POOR_FETGRO | Poor fetal growth \|\| id:finngen_R11_O15_POOR_FETGRO | FSC-A on CD4+ T cell \|\| id:ebi-a-GCST90001973 | Inverse variance weighted | 25 | -0.00036933 | 0.016998297316263 | 0.982665347170791 | -0.033685993 | 0.0329473326621982 | 0.99963073811628 | 0.966875062689522 | 1.03349610631935 | 0.998984604424952 |
| ebi-a-GCST90001973 | finngen_R11_O15_POOR_FETGRO | Poor fetal growth \|\| id:finngen_R11_O15_POOR_FETGRO | FSC-A on CD4+ T cell \|\| id:ebi-a-GCST90001973 | MR Egger | 25 | -0.0122699 | 0.0202724868048732 | 0.550939509088984 | -0.052003974 | 0.0274641742102819 | 0.987805068363776 | 0.949325094155933 | 1.02784479109628 | 0.992899239648112 |
| ebi-a-GCST90001973 | finngen_R11_O15_POOR_FETGRO | Poor fetal growth \|\| id:finngen_R11_O15_POOR_FETGRO | FSC-A on CD4+ T cell \|\| id:ebi-a-GCST90001973 | Weighted median | 25 | 0.0080204263170281 | 0.0232681946342183 | 0.730323807756764 | -0.037585235 | 0.0536260878000959 | 1.00805267609752 | 0.963112323183419 | 1.05509001736016 | 0.999440741871876 |
| ebi-a-GCST90001973 | finngen_R11_O15_POOR_FETGRO | Poor fetal growth \|\| id:finngen_R11_O15_POOR_FETGRO | FSC-A on CD4+ T cell \|\| id:ebi-a-GCST90001973 | Weighted mode | 25 | 0.00119763948342555 | 0.0190670377863517 | 0.950436385768714 | -0.036173755 | 0.0385690335446748 | 1.00119835693998 | 0.964472697376894 | 1.03932247399597 | 0.999857399747617 |
| ebi-a-GCST90001974 | finngen_R11_O15_POOR_FETGRO | Poor fetal growth \|\| id:finngen_R11_O15_POOR_FETGRO | FSC-A on CD8+ T cell \|\| id:ebi-a-GCST90001974 | Inverse variance weighted | 18 | 0.00462973042056054 | 0.0289498545386409 | 0.872942197440044 | -0.052111984 | 0.0613714453162968 | 1.00464046418086 | 0.949222562700285 | 1.06329379634459 | 0.994119304454654 |
| ebi-a-GCST90001974 | finngen_R11_O15_POOR_FETGRO | Poor fetal growth \|\| id:finngen_R11_O15_POOR_FETGRO | FSC-A on CD8+ T cell \|\| id:ebi-a-GCST90001974 | MR Egger | 18 | -0.001682291 | 0.0386510486030693 | 0.965821511634513 | -0.077438347 | 0.0740737639830951 | 0.998319122979877 | 0.925484081932681 | 1.07688623798481 | 0.992899239648112 |
| ebi-a-GCST90001974 | finngen_R11_O15_POOR_FETGRO | Poor fetal growth \|\| id:finngen_R11_O15_POOR_FETGRO | FSC-A on CD8+ T cell \|\| id:ebi-a-GCST90001974 | Weighted median | 18 | -0.01401338 | 0.0375590507189116 | 0.709072065535483 | -0.08762912 | 0.0596023590176918 | 0.986084349980058 | 0.916100577290246 | 1.06141440075474 | 0.999440741871876 |
| ebi-a-GCST90001974 | finngen_R11_O15_POOR_FETGRO | Poor fetal growth \|\| id:finngen_R11_O15_POOR_FETGRO | FSC-A on CD8+ T cell \|\| id:ebi-a-GCST90001974 | Weighted mode | 18 | -0.014825951 | 0.0344784770793132 | 0.672592615170444 | -0.082403766 | 0.052751864537297 | 0.985283412727976 | 0.920900055844487 | 1.05416803619005 | 0.999857399747617 |
| ebi-a-GCST90001975 | finngen_R11_O15_POOR_FETGRO | Poor fetal growth \|\| id:finngen_R11_O15_POOR_FETGRO | FSC-A on HLA DR+ T cell \|\| id:ebi-a-GCST90001975 | Inverse variance weighted | 18 | 0.00178674074586784 | 0.0143349707366895 | 0.900806903373922 | -0.026309802 | 0.0298832833897792 | 1.00178833791822 | 0.974033285500815 | 1.03033426981188 | 0.994119304454654 |
| ebi-a-GCST90001975 | finngen_R11_O15_POOR_FETGRO | Poor fetal growth \|\| id:finngen_R11_O15_POOR_FETGRO | FSC-A on HLA DR+ T cell \|\| id:ebi-a-GCST90001975 | MR Egger | 18 | 0.00982026310188175 | 0.0160595992533599 | 0.549466577116625 | -0.021656551 | 0.0412970776384671 | 1.00986864011423 | 0.978576267958161 | 1.04216166248757 | 0.992899239648112 |
| ebi-a-GCST90001975 | finngen_R11_O15_POOR_FETGRO | Poor fetal growth \|\| id:finngen_R11_O15_POOR_FETGRO | FSC-A on HLA DR+ T cell \|\| id:ebi-a-GCST90001975 | Weighted median | 18 | 0.00591824742240389 | 0.0171516073592378 | 0.730053042845968 | -0.027698903 | 0.03953539784651 | 1.00593579484827 | 0.972681194102169 | 1.04032732358012 | 0.999440741871876 |
| ebi-a-GCST90001975 | finngen_R11_O15_POOR_FETGRO | Poor fetal growth \|\| id:finngen_R11_O15_POOR_FETGRO | FSC-A on HLA DR+ T cell \|\| id:ebi-a-GCST90001975 | Weighted mode | 18 | 0.0063114697483726 | 0.0154896282703752 | 0.688752141099261 | -0.024048202 | 0.0366711411583079 | 1.0063314290423 | 0.976238652298672 | 1.03735182241943 | 0.999857399747617 |
| ebi-a-GCST90001976 | finngen_R11_O15_POOR_FETGRO | Poor fetal growth \|\| id:finngen_R11_O15_POOR_FETGRO | FSC-A on Natural Killer T \|\| id:ebi-a-GCST90001976 | Inverse variance weighted | 20 | 0.0232148356542564 | 0.0434485990234923 | 0.593130024742852 | -0.061944418 | 0.108374089740301 | 1.02348639729952 | 0.939935128407694 | 1.11446457717961 | 0.994119304454654 |
| ebi-a-GCST90001976 | finngen_R11_O15_POOR_FETGRO | Poor fetal growth \|\| id:finngen_R11_O15_POOR_FETGRO | FSC-A on Natural Killer T \|\| id:ebi-a-GCST90001976 | MR Egger | 20 | 0.0544934702930563 | 0.076331319421664 | 0.484439775679832 | -0.095115916 | 0.204102856359518 | 1.05600558098424 | 0.909267529910362 | 1.22642429250695 | 0.992899239648112 |
| ebi-a-GCST90001976 | finngen_R11_O15_POOR_FETGRO | Poor fetal growth \|\| id:finngen_R11_O15_POOR_FETGRO | FSC-A on Natural Killer T \|\| id:ebi-a-GCST90001976 | Weighted median | 20 | 0.0615871387915432 | 0.0573311953133252 | 0.282717664755668 | -0.050782004 | 0.173956281605661 | 1.06352316661468 | 0.950485850041366 | 1.19000353963911 | 0.999440741871876 |
| ebi-a-GCST90001976 | finngen_R11_O15_POOR_FETGRO | Poor fetal growth \|\| id:finngen_R11_O15_POOR_FETGRO | FSC-A on Natural Killer T \|\| id:ebi-a-GCST90001976 | Weighted mode | 20 | 0.0519406768506701 | 0.0544682447796387 | 0.352263855465082 | -0.054817083 | 0.158698436618762 | 1.05331325480039 | 0.946658292090478 | 1.17198446578669 | 0.999857399747617 |
| ebi-a-GCST90001977 | finngen_R11_O15_POOR_FETGRO | Poor fetal growth \|\| id:finngen_R11_O15_POOR_FETGRO | FSC-A on HLA DR+ CD4+ T cell \|\| id:ebi-a-GCST90001977 | Inverse variance weighted | 17 | -0.025501213 | 0.0257681001877411 | 0.322348784217088 | -0.076006689 | 0.0250042637534558 | 0.97482119688269 | 0.92681000712274 | 1.02531949222464 | 0.994119304454654 |
| ebi-a-GCST90001977 | finngen_R11_O15_POOR_FETGRO | Poor fetal growth \|\| id:finngen_R11_O15_POOR_FETGRO | FSC-A on HLA DR+ CD4+ T cell \|\| id:ebi-a-GCST90001977 | MR Egger | 17 | -0.041494299 | 0.0331640895714114 | 0.230031225577824 | -0.106495914 | 0.0235073167013465 | 0.959354804743257 | 0.898978721004482 | 1.0237857914541 | 0.992899239648112 |
| ebi-a-GCST90001977 | finngen_R11_O15_POOR_FETGRO | Poor fetal growth \|\| id:finngen_R11_O15_POOR_FETGRO | FSC-A on HLA DR+ CD4+ T cell \|\| id:ebi-a-GCST90001977 | Weighted median | 17 | -0.031893849 | 0.0344513275515621 | 0.354567878567097 | -0.099418451 | 0.0356307527560504 | 0.968609395240848 | 0.905363778146123 | 1.03627313483864 | 0.999440741871876 |
| ebi-a-GCST90001977 | finngen_R11_O15_POOR_FETGRO | Poor fetal growth \|\| id:finngen_R11_O15_POOR_FETGRO | FSC-A on HLA DR+ CD4+ T cell \|\| id:ebi-a-GCST90001977 | Weighted mode | 17 | -0.033292205 | 0.030421902296981 | 0.290003264681238 | -0.092919134 | 0.0263347232545861 | 0.967255881046853 | 0.911267188077304 | 1.02668454615789 | 0.999857399747617 |
| ebi-a-GCST90001978 | finngen_R11_O15_POOR_FETGRO | Poor fetal growth \|\| id:finngen_R11_O15_POOR_FETGRO | FSC-A on HLA DR+ CD8+ T cell \|\| id:ebi-a-GCST90001978 | Inverse variance weighted | 18 | -0.004137769 | 0.0170894561953929 | 0.808684009893826 | -0.037633103 | 0.0293575650591448 | 0.995870779687658 | 0.963066221967615 | 1.02979274655638 | 0.994119304454654 |
| ebi-a-GCST90001978 | finngen_R11_O15_POOR_FETGRO | Poor fetal growth \|\| id:finngen_R11_O15_POOR_FETGRO | FSC-A on HLA DR+ CD8+ T cell \|\| id:ebi-a-GCST90001978 | MR Egger | 18 | -0.021114571 | 0.0211149404554679 | 0.332203193304383 | -0.062499854 | 0.0202707122358503 | 0.979106780844412 | 0.939413199639389 | 1.02047755839869 | 0.992899239648112 |
| ebi-a-GCST90001978 | finngen_R11_O15_POOR_FETGRO | Poor fetal growth \|\| id:finngen_R11_O15_POOR_FETGRO | FSC-A on HLA DR+ CD8+ T cell \|\| id:ebi-a-GCST90001978 | Weighted median | 18 | -0.002073525 | 0.0231473055127069 | 0.928621329672298 | -0.047442244 | 0.0432951934149735 | 0.997928622878753 | 0.953665551263839 | 1.04424610393122 | 0.999440741871876 |
| ebi-a-GCST90001978 | finngen_R11_O15_POOR_FETGRO | Poor fetal growth \|\| id:finngen_R11_O15_POOR_FETGRO | FSC-A on HLA DR+ CD8+ T cell \|\| id:ebi-a-GCST90001978 | Weighted mode | 18 | -0.005892231 | 0.0201025155701115 | 0.772986519118028 | -0.045293162 | 0.0335086991074342 | 0.994125093740876 | 0.955717260845803 | 1.03407643922919 | 0.999857399747617 |
| ebi-a-GCST90001979 | finngen_R11_O15_POOR_FETGRO | Poor fetal growth \|\| id:finngen_R11_O15_POOR_FETGRO | CD16 on CD14- CD16+ monocyte \|\| id:ebi-a-GCST90001979 | Inverse variance weighted | 20 | -0.008423011 | 0.0190518231550058 | 0.658409237704591 | -0.045764585 | 0.0289185620878974 | 0.991612362875043 | 0.955266820166587 | 1.02934076369904 | 0.994119304454654 |
| ebi-a-GCST90001979 | finngen_R11_O15_POOR_FETGRO | Poor fetal growth \|\| id:finngen_R11_O15_POOR_FETGRO | CD16 on CD14- CD16+ monocyte \|\| id:ebi-a-GCST90001979 | MR Egger | 20 | -0.021918282 | 0.0267861242841162 | 0.423903721838977 | -0.074419086 | 0.030582521330897 | 0.978320177892884 | 0.928282582204468 | 1.03105497056665 | 0.992899239648112 |
| ebi-a-GCST90001979 | finngen_R11_O15_POOR_FETGRO | Poor fetal growth \|\| id:finngen_R11_O15_POOR_FETGRO | CD16 on CD14- CD16+ monocyte \|\| id:ebi-a-GCST90001979 | Weighted median | 20 | -0.005994374 | 0.0243031914423302 | 0.805179376925174 | -0.053628629 | 0.041639881586301 | 0.994023556771918 | 0.947784020777387 | 1.04251898086133 | 0.999440741871876 |
| ebi-a-GCST90001979 | finngen_R11_O15_POOR_FETGRO | Poor fetal growth \|\| id:finngen_R11_O15_POOR_FETGRO | CD16 on CD14- CD16+ monocyte \|\| id:ebi-a-GCST90001979 | Weighted mode | 20 | -0.006445074 | 0.0242273090990465 | 0.793085170184741 | -0.0539306 | 0.0410404515581135 | 0.993575650666692 | 0.947497860467264 | 1.04189425093889 | 0.999857399747617 |
| ebi-a-GCST90001980 | finngen_R11_O15_POOR_FETGRO | Poor fetal growth \|\| id:finngen_R11_O15_POOR_FETGRO | CD40 on CD14+ CD16- monocyte \|\| id:ebi-a-GCST90001980 | Inverse variance weighted | 23 | -0.02690468 | 0.0217163215684524 | 0.215376923754114 | -0.06946867 | 0.0156593105543211 | 0.973454027012669 | 0.932889360355959 | 1.0157825600515 | 0.994119304454654 |
| ebi-a-GCST90001980 | finngen_R11_O15_POOR_FETGRO | Poor fetal growth \|\| id:finngen_R11_O15_POOR_FETGRO | CD40 on CD14+ CD16- monocyte \|\| id:ebi-a-GCST90001980 | MR Egger | 23 | -0.004493383 | 0.0365008700656353 | 0.903195762532767 | -0.076035088 | 0.0670483223668 | 0.995516697179745 | 0.926783686733631 | 1.06934715031136 | 0.992899239648112 |
| ebi-a-GCST90001980 | finngen_R11_O15_POOR_FETGRO | Poor fetal growth \|\| id:finngen_R11_O15_POOR_FETGRO | CD40 on CD14+ CD16- monocyte \|\| id:ebi-a-GCST90001980 | Weighted median | 23 | -0.021701303 | 0.0328872743238587 | 0.509337699978097 | -0.086160361 | 0.0427577546403231 | 0.978532476084591 | 0.917447096956064 | 1.04368503636793 | 0.999440741871876 |
| ebi-a-GCST90001980 | finngen_R11_O15_POOR_FETGRO | Poor fetal growth \|\| id:finngen_R11_O15_POOR_FETGRO | CD40 on CD14+ CD16- monocyte \|\| id:ebi-a-GCST90001980 | Weighted mode | 23 | -0.012110016 | 0.0318445510358431 | 0.707380454289202 | -0.074525336 | 0.0503053037494806 | 0.98796301486633 | 0.928183957003645 | 1.05159210238314 | 0.999857399747617 |
| ebi-a-GCST90001981 | finngen_R11_O15_POOR_FETGRO | Poor fetal growth \|\| id:finngen_R11_O15_POOR_FETGRO | CD40 on CD14+ CD16+ monocyte \|\| id:ebi-a-GCST90001981 | Inverse variance weighted | 21 | 0.0024752222796017 | 0.0149464469004259 | 0.868466972830325 | -0.026819814 | 0.0317702582044364 | 1.00247828817133 | 0.973536643740387 | 1.03228032012622 | 0.994119304454654 |
| ebi-a-GCST90001981 | finngen_R11_O15_POOR_FETGRO | Poor fetal growth \|\| id:finngen_R11_O15_POOR_FETGRO | CD40 on CD14+ CD16+ monocyte \|\| id:ebi-a-GCST90001981 | MR Egger | 21 | -0.007354283 | 0.0251973785260276 | 0.773551223004609 | -0.056741145 | 0.0420325793033311 | 0.992672693957051 | 0.944838614368527 | 1.0429284560798 | 0.992899239648112 |
| ebi-a-GCST90001981 | finngen_R11_O15_POOR_FETGRO | Poor fetal growth \|\| id:finngen_R11_O15_POOR_FETGRO | CD40 on CD14+ CD16+ monocyte \|\| id:ebi-a-GCST90001981 | Weighted median | 21 | 0.00440838996462365 | 0.0204563462195828 | 0.829375707437556 | -0.035686049 | 0.044502828555006 | 1.00441812121012 | 0.964943191173996 | 1.04550793398297 | 0.999440741871876 |
| ebi-a-GCST90001981 | finngen_R11_O15_POOR_FETGRO | Poor fetal growth \|\| id:finngen_R11_O15_POOR_FETGRO | CD40 on CD14+ CD16+ monocyte \|\| id:ebi-a-GCST90001981 | Weighted mode | 21 | -0.002134345 | 0.0171240372287267 | 0.902052572132578 | -0.035697458 | 0.0314287675525856 | 0.997867930679845 | 0.964932181468255 | 1.03192786623008 | 0.999857399747617 |
| ebi-a-GCST90001982 | finngen_R11_O15_POOR_FETGRO | Poor fetal growth \|\| id:finngen_R11_O15_POOR_FETGRO | CCR2 on CD14- CD16+ monocyte \|\| id:ebi-a-GCST90001982 | Inverse variance weighted | 28 | -0.023941601 | 0.0157720553372651 | 0.129020463389852 | -0.054854829 | 0.00697162788674597 | 0.976342725947525 | 0.94662256008934 | 1.00699598625737 | 0.994119304454654 |
| ebi-a-GCST90001982 | finngen_R11_O15_POOR_FETGRO | Poor fetal growth \|\| id:finngen_R11_O15_POOR_FETGRO | CCR2 on CD14- CD16+ monocyte \|\| id:ebi-a-GCST90001982 | MR Egger | 28 | -0.019285369 | 0.0192140694903193 | 0.324768473246707 | -0.056944945 | 0.0183742073341013 | 0.980899404147515 | 0.944646075360407 | 1.01854405173902 | 0.992899239648112 |
| ebi-a-GCST90001982 | finngen_R11_O15_POOR_FETGRO | Poor fetal growth \|\| id:finngen_R11_O15_POOR_FETGRO | CCR2 on CD14- CD16+ monocyte \|\| id:ebi-a-GCST90001982 | Weighted median | 28 | -0.034948118 | 0.0238369453070054 | 0.142612217089625 | -0.081668531 | 0.0117722946738388 | 0.965655514973876 | 0.921577382472534 | 1.01184186085128 | 0.999440741871876 |
| ebi-a-GCST90001982 | finngen_R11_O15_POOR_FETGRO | Poor fetal growth \|\| id:finngen_R11_O15_POOR_FETGRO | CCR2 on CD14- CD16+ monocyte \|\| id:ebi-a-GCST90001982 | Weighted mode | 28 | -0.03190031 | 0.0195674731465499 | 0.114656317331952 | -0.070252557 | 0.00645193723998849 | 0.968603137189827 | 0.932158366592918 | 1.00647279582235 | 0.999857399747617 |
| ebi-a-GCST90001983 | finngen_R11_O15_POOR_FETGRO | Poor fetal growth \|\| id:finngen_R11_O15_POOR_FETGRO | CD14 on CD14+ CD16+ monocyte \|\| id:ebi-a-GCST90001983 | Inverse variance weighted | 14 | 0.031179834543761 | 0.0439904192856617 | 0.478456705338793 | -0.055041387 | 0.117401056343658 | 1.03167101729187 | 0.946445976340845 | 1.12457035533609 | 0.994119304454654 |
| ebi-a-GCST90001983 | finngen_R11_O15_POOR_FETGRO | Poor fetal growth \|\| id:finngen_R11_O15_POOR_FETGRO | CD14 on CD14+ CD16+ monocyte \|\| id:ebi-a-GCST90001983 | MR Egger | 14 | 0.0644015615867876 | 0.125546674265667 | 0.617284655510423 | -0.18166992 | 0.310473043147495 | 1.06652058648293 | 0.833876540984527 | 1.36407022561029 | 0.992899239648112 |
| ebi-a-GCST90001983 | finngen_R11_O15_POOR_FETGRO | Poor fetal growth \|\| id:finngen_R11_O15_POOR_FETGRO | CD14 on CD14+ CD16+ monocyte \|\| id:ebi-a-GCST90001983 | Weighted median | 14 | 0.0569558879787575 | 0.0609043769821748 | 0.349701189199012 | -0.062416691 | 0.17632846686382 | 1.05860911197789 | 0.939491327724388 | 1.1928297993735 | 0.999440741871876 |
| ebi-a-GCST90001983 | finngen_R11_O15_POOR_FETGRO | Poor fetal growth \|\| id:finngen_R11_O15_POOR_FETGRO | CD14 on CD14+ CD16+ monocyte \|\| id:ebi-a-GCST90001983 | Weighted mode | 14 | 0.080475350431492 | 0.0863564239956101 | 0.368374050872906 | -0.088783241 | 0.249733941462888 | 1.08380213105811 | 0.915043896445557 | 1.28368383620598 | 0.999857399747617 |
| ebi-a-GCST90001984 | finngen_R11_O15_POOR_FETGRO | Poor fetal growth \|\| id:finngen_R11_O15_POOR_FETGRO | HLA DR on CD14- CD16+ monocyte \|\| id:ebi-a-GCST90001984 | Inverse variance weighted | 18 | 0.0230434847934972 | 0.0277202574533227 | 0.405811615869597 | -0.03128822 | 0.0773751894020097 | 1.02331103704886 | 0.969196191269313 | 1.08044737276008 | 0.994119304454654 |
| ebi-a-GCST90001984 | finngen_R11_O15_POOR_FETGRO | Poor fetal growth \|\| id:finngen_R11_O15_POOR_FETGRO | HLA DR on CD14- CD16+ monocyte \|\| id:ebi-a-GCST90001984 | MR Egger | 18 | -0.002937644 | 0.0565464358014441 | 0.959210809524298 | -0.113768659 | 0.107893369745201 | 0.997066666229666 | 0.892464395968391 | 1.11392896052466 | 0.992899239648112 |
| ebi-a-GCST90001984 | finngen_R11_O15_POOR_FETGRO | Poor fetal growth \|\| id:finngen_R11_O15_POOR_FETGRO | HLA DR on CD14- CD16+ monocyte \|\| id:ebi-a-GCST90001984 | Weighted median | 18 | 0.0232056963683958 | 0.0320140591733626 | 0.468538201424244 | -0.03954186 | 0.0859532523481865 | 1.0234770434075 | 0.961229716446048 | 1.08975538360913 | 0.999440741871876 |
| ebi-a-GCST90001984 | finngen_R11_O15_POOR_FETGRO | Poor fetal growth \|\| id:finngen_R11_O15_POOR_FETGRO | HLA DR on CD14- CD16+ monocyte \|\| id:ebi-a-GCST90001984 | Weighted mode | 18 | 0.0255504488845852 | 0.0290126987069663 | 0.390780258019898 | -0.031314441 | 0.0824153383502392 | 1.02587965944953 | 0.969170778535893 | 1.08590673489161 | 0.999857399747617 |
| ebi-a-GCST90001985 | finngen_R11_O15_POOR_FETGRO | Poor fetal growth \|\| id:finngen_R11_O15_POOR_FETGRO | CD40 on monocytes \|\| id:ebi-a-GCST90001985 | Inverse variance weighted | 29 | -0.009749831 | 0.0136640751060197 | 0.475513160729498 | -0.036531418 | 0.0170317559788339 | 0.990297544282769 | 0.964127802032085 | 1.01717762328344 | 0.994119304454654 |
| ebi-a-GCST90001985 | finngen_R11_O15_POOR_FETGRO | Poor fetal growth \|\| id:finngen_R11_O15_POOR_FETGRO | CD40 on monocytes \|\| id:ebi-a-GCST90001985 | MR Egger | 29 | -0.014229388 | 0.017874002044562 | 0.432921466924652 | -0.049262432 | 0.0208036558237602 | 0.985871371080069 | 0.951931279504284 | 1.02102156031834 | 0.992899239648112 |
| ebi-a-GCST90001985 | finngen_R11_O15_POOR_FETGRO | Poor fetal growth \|\| id:finngen_R11_O15_POOR_FETGRO | CD40 on monocytes \|\| id:ebi-a-GCST90001985 | Weighted median | 29 | -0.007913857 | 0.0167311982265561 | 0.636213190042936 | -0.040707005 | 0.024879292017674 | 0.992117375612883 | 0.960110396257319 | 1.02519136427432 | 0.999440741871876 |
| ebi-a-GCST90001985 | finngen_R11_O15_POOR_FETGRO | Poor fetal growth \|\| id:finngen_R11_O15_POOR_FETGRO | CD40 on monocytes \|\| id:ebi-a-GCST90001985 | Weighted mode | 29 | -0.010281998 | 0.0147376381807586 | 0.491137328003732 | -0.039167768 | 0.0186037732974654 | 0.989770681496694 | 0.961589371330551 | 1.01877790162644 | 0.999857399747617 |
| ebi-a-GCST90001986 | finngen_R11_O15_POOR_FETGRO | Poor fetal growth \|\| id:finngen_R11_O15_POOR_FETGRO | CD14 on CD14+ CD16- monocyte \|\| id:ebi-a-GCST90001986 | Inverse variance weighted | 16 | 0.0130570544444335 | 0.041277917071151 | 0.75175919606438 | -0.067847663 | 0.0939617719038895 | 1.01314267000301 | 0.934402806841977 | 1.09851775087232 | 0.994119304454654 |
| ebi-a-GCST90001986 | finngen_R11_O15_POOR_FETGRO | Poor fetal growth \|\| id:finngen_R11_O15_POOR_FETGRO | CD14 on CD14+ CD16- monocyte \|\| id:ebi-a-GCST90001986 | MR Egger | 16 | 0.0803913249285417 | 0.0700681514007329 | 0.270474642806624 | -0.056942252 | 0.217724901673978 | 1.08371106786482 | 0.944648619532873 | 1.24324500595095 | 0.992899239648112 |
| ebi-a-GCST90001986 | finngen_R11_O15_POOR_FETGRO | Poor fetal growth \|\| id:finngen_R11_O15_POOR_FETGRO | CD14 on CD14+ CD16- monocyte \|\| id:ebi-a-GCST90001986 | Weighted median | 16 | 0.00599878970263554 | 0.0568787823050077 | 0.916005879491354 | -0.105483624 | 0.117481203020451 | 1.00601681847382 | 0.899889209658108 | 1.12466048952482 | 0.999440741871876 |
| ebi-a-GCST90001986 | finngen_R11_O15_POOR_FETGRO | Poor fetal growth \|\| id:finngen_R11_O15_POOR_FETGRO | CD14 on CD14+ CD16- monocyte \|\| id:ebi-a-GCST90001986 | Weighted mode | 16 | 0.0283190743987102 | 0.0577107462160041 | 0.630734554041904 | -0.084793988 | 0.141432136982078 | 1.02872387151133 | 0.918701528277149 | 1.15192232868259 | 0.999857399747617 |
| ebi-a-GCST90001987 | finngen_R11_O15_POOR_FETGRO | Poor fetal growth \|\| id:finngen_R11_O15_POOR_FETGRO | CD64 on CD14+ CD16- monocyte \|\| id:ebi-a-GCST90001987 | Inverse variance weighted | 34 | 0.0171578580571665 | 0.00933613022969386 | 0.066093177607702 | -0.001140957 | 0.0354566733073664 | 1.01730589958355 | 0.998859693451149 | 1.03609275668316 | 0.994119304454654 |
| ebi-a-GCST90001987 | finngen_R11_O15_POOR_FETGRO | Poor fetal growth \|\| id:finngen_R11_O15_POOR_FETGRO | CD64 on CD14+ CD16- monocyte \|\| id:ebi-a-GCST90001987 | MR Egger | 34 | 0.0140629379983397 | 0.0107643839490124 | 0.200718003201229 | -0.007035255 | 0.0351611305384041 | 1.01416228627418 | 0.992989434928677 | 1.03578659220553 | 0.992899239648112 |
| ebi-a-GCST90001987 | finngen_R11_O15_POOR_FETGRO | Poor fetal growth \|\| id:finngen_R11_O15_POOR_FETGRO | CD64 on CD14+ CD16- monocyte \|\| id:ebi-a-GCST90001987 | Weighted median | 34 | 0.020500278207569 | 0.0142752398007512 | 0.150981734267737 | -0.007479192 | 0.0484797482170413 | 1.02071185221292 | 0.992548707754394 | 1.04967411383274 | 0.999440741871876 |
| ebi-a-GCST90001987 | finngen_R11_O15_POOR_FETGRO | Poor fetal growth \|\| id:finngen_R11_O15_POOR_FETGRO | CD64 on CD14+ CD16- monocyte \|\| id:ebi-a-GCST90001987 | Weighted mode | 34 | 0.0164285202926364 | 0.0109287250865796 | 0.1422872286788 | -0.004991781 | 0.0378488214623325 | 1.01656421047672 | 0.995020657356185 | 1.03857421087916 | 0.999857399747617 |
| ebi-a-GCST90001988 | finngen_R11_O15_POOR_FETGRO | Poor fetal growth \|\| id:finngen_R11_O15_POOR_FETGRO | HLA DR on CD14+ CD16- monocyte \|\| id:ebi-a-GCST90001988 | Inverse variance weighted | 22 | 0.024304103085712 | 0.0223089359391684 | 0.275962746969474 | -0.019421411 | 0.068029617526482 | 1.02460185510427 | 0.980765969228581 | 1.07039701052111 | 0.994119304454654 |
| ebi-a-GCST90001988 | finngen_R11_O15_POOR_FETGRO | Poor fetal growth \|\| id:finngen_R11_O15_POOR_FETGRO | HLA DR on CD14+ CD16- monocyte \|\| id:ebi-a-GCST90001988 | MR Egger | 22 | -0.010084316 | 0.0425361866639569 | 0.815009178549499 | -0.093455242 | 0.0732866099657264 | 0.989966360329836 | 0.910778781371961 | 1.07603889619433 | 0.992899239648112 |
| ebi-a-GCST90001988 | finngen_R11_O15_POOR_FETGRO | Poor fetal growth \|\| id:finngen_R11_O15_POOR_FETGRO | HLA DR on CD14+ CD16- monocyte \|\| id:ebi-a-GCST90001988 | Weighted median | 22 | 0.0111566307714931 | 0.0305470877054565 | 0.714941731414169 | -0.048715661 | 0.0710289226741878 | 1.01121909806863 | 0.952451910298715 | 1.07361227715741 | 0.999440741871876 |
| ebi-a-GCST90001988 | finngen_R11_O15_POOR_FETGRO | Poor fetal growth \|\| id:finngen_R11_O15_POOR_FETGRO | HLA DR on CD14+ CD16- monocyte \|\| id:ebi-a-GCST90001988 | Weighted mode | 22 | 0.0272106983431985 | 0.0272126782053303 | 0.328729071801353 | -0.026126151 | 0.0805475476256459 | 1.02758429026337 | 0.974212184074539 | 1.08388038135568 | 0.999857399747617 |
| ebi-a-GCST90001989 | finngen_R11_O15_POOR_FETGRO | Poor fetal growth \|\| id:finngen_R11_O15_POOR_FETGRO | CD40 on CD14- CD16+ monocyte \|\| id:ebi-a-GCST90001989 | Inverse variance weighted | 27 | -0.020676988 | 0.0155332458897513 | 0.183141620846204 | -0.05112215 | 0.00976817427894514 | 0.979535315463583 | 0.950162601453535 | 1.00981603861543 | 0.994119304454654 |
| ebi-a-GCST90001989 | finngen_R11_O15_POOR_FETGRO | Poor fetal growth \|\| id:finngen_R11_O15_POOR_FETGRO | CD40 on CD14- CD16+ monocyte \|\| id:ebi-a-GCST90001989 | MR Egger | 27 | -0.013467673 | 0.0225351763194409 | 0.555462857020632 | -0.057636619 | 0.0307012725643279 | 0.986622610329851 | 0.943992914578536 | 1.03117741688632 | 0.992899239648112 |
| ebi-a-GCST90001989 | finngen_R11_O15_POOR_FETGRO | Poor fetal growth \|\| id:finngen_R11_O15_POOR_FETGRO | CD40 on CD14- CD16+ monocyte \|\| id:ebi-a-GCST90001989 | Weighted median | 27 | -0.032464909 | 0.0234271343079508 | 0.165813371252375 | -0.078382093 | 0.013452273841888 | 0.968056418914237 | 0.924611071950407 | 1.01354316277463 | 0.999440741871876 |
| ebi-a-GCST90001989 | finngen_R11_O15_POOR_FETGRO | Poor fetal growth \|\| id:finngen_R11_O15_POOR_FETGRO | CD40 on CD14- CD16+ monocyte \|\| id:ebi-a-GCST90001989 | Weighted mode | 27 | -0.029189553 | 0.0185820081798056 | 0.128308883769994 | -0.065610289 | 0.00723118269450788 | 0.971232346683885 | 0.936495755536576 | 1.00725739082994 | 0.999857399747617 |
| ebi-a-GCST90001990 | finngen_R11_O15_POOR_FETGRO | Poor fetal growth \|\| id:finngen_R11_O15_POOR_FETGRO | CD64 on CD14- CD16+ monocyte \|\| id:ebi-a-GCST90001990 | Inverse variance weighted | 19 | -0.032642428 | 0.0344229123695945 | 0.342988878301632 | -0.100111336 | 0.0348264806929035 | 0.967884586581973 | 0.904736682849676 | 1.03544002437515 | 0.994119304454654 |
| ebi-a-GCST90001990 | finngen_R11_O15_POOR_FETGRO | Poor fetal growth \|\| id:finngen_R11_O15_POOR_FETGRO | CD64 on CD14- CD16+ monocyte \|\| id:ebi-a-GCST90001990 | MR Egger | 19 | -0.079165529 | 0.0601571680087968 | 0.205651596850453 | -0.197073579 | 0.0387425200184033 | 0.923886981441397 | 0.821130213497998 | 1.03950279802849 | 0.992899239648112 |
| ebi-a-GCST90001990 | finngen_R11_O15_POOR_FETGRO | Poor fetal growth \|\| id:finngen_R11_O15_POOR_FETGRO | CD64 on CD14- CD16+ monocyte \|\| id:ebi-a-GCST90001990 | Weighted median | 19 | -0.039551835 | 0.0521800516083791 | 0.448458373573644 | -0.141824736 | 0.0627210662340343 | 0.96122012793236 | 0.867773332522706 | 1.06472980871203 | 0.999440741871876 |
| ebi-a-GCST90001990 | finngen_R11_O15_POOR_FETGRO | Poor fetal growth \|\| id:finngen_R11_O15_POOR_FETGRO | CD64 on CD14- CD16+ monocyte \|\| id:ebi-a-GCST90001990 | Weighted mode | 19 | -0.039869947 | 0.0478890076677545 | 0.416009129780607 | -0.133732402 | 0.053992508508981 | 0.960914401288312 | 0.874824134867241 | 1.05547669503128 | 0.999857399747617 |
| ebi-a-GCST90001991 | finngen_R11_O15_POOR_FETGRO | Poor fetal growth \|\| id:finngen_R11_O15_POOR_FETGRO | HLA DR on CD14+ monocyte \|\| id:ebi-a-GCST90001991 | Inverse variance weighted | 21 | 0.0213038050033769 | 0.0228847195828519 | 0.351895810483943 | -0.023550245 | 0.0661578553857667 | 1.02153235113926 | 0.976724897523237 | 1.06839535581643 | 0.994119304454654 |
| ebi-a-GCST90001991 | finngen_R11_O15_POOR_FETGRO | Poor fetal growth \|\| id:finngen_R11_O15_POOR_FETGRO | HLA DR on CD14+ monocyte \|\| id:ebi-a-GCST90001991 | MR Egger | 21 | -0.01061713 | 0.0440669996291173 | 0.812188953567338 | -0.096988449 | 0.0757541892344278 | 0.989439032748217 | 0.907566489090346 | 1.07869738613528 | 0.992899239648112 |
| ebi-a-GCST90001991 | finngen_R11_O15_POOR_FETGRO | Poor fetal growth \|\| id:finngen_R11_O15_POOR_FETGRO | HLA DR on CD14+ monocyte \|\| id:ebi-a-GCST90001991 | Weighted median | 21 | 0.0112940341152766 | 0.0316819774147253 | 0.721480115655697 | -0.050802642 | 0.0733907098481382 | 1.01135805250018 | 0.950466234501739 | 1.0761509175475 | 0.999440741871876 |
| ebi-a-GCST90001991 | finngen_R11_O15_POOR_FETGRO | Poor fetal growth \|\| id:finngen_R11_O15_POOR_FETGRO | HLA DR on CD14+ monocyte \|\| id:ebi-a-GCST90001991 | Weighted mode | 21 | 0.0252344805856736 | 0.0282765977925608 | 0.38278220903708 | -0.030187651 | 0.0806566122590928 | 1.02555556520309 | 0.970263445473587 | 1.08399860081884 | 0.999857399747617 |
| ebi-a-GCST90001992 | finngen_R11_O15_POOR_FETGRO | Poor fetal growth \|\| id:finngen_R11_O15_POOR_FETGRO | CCR2 on CD14+ CD16+ monocyte \|\| id:ebi-a-GCST90001992 | Inverse variance weighted | 37 | -0.004319843 | 0.013011603463373 | 0.739889785325187 | -0.029822586 | 0.021182899507679 | 0.995689473821484 | 0.970617719379317 | 1.02140884973012 | 0.994119304454654 |
| ebi-a-GCST90001992 | finngen_R11_O15_POOR_FETGRO | Poor fetal growth \|\| id:finngen_R11_O15_POOR_FETGRO | CCR2 on CD14+ CD16+ monocyte \|\| id:ebi-a-GCST90001992 | MR Egger | 37 | 0.00513494223223851 | 0.0156884543764191 | 0.745385311446514 | -0.025614428 | 0.03588431281002 | 1.00514814864315 | 0.974710838035562 | 1.03653592562585 | 0.992899239648112 |
| ebi-a-GCST90001992 | finngen_R11_O15_POOR_FETGRO | Poor fetal growth \|\| id:finngen_R11_O15_POOR_FETGRO | CCR2 on CD14+ CD16+ monocyte \|\| id:ebi-a-GCST90001992 | Weighted median | 37 | 0.00287823834299462 | 0.0196094736895466 | 0.883307289745006 | -0.03555633 | 0.041312806774506 | 1.00288238444785 | 0.965068370312128 | 1.04217805491906 | 0.999440741871876 |
| ebi-a-GCST90001992 | finngen_R11_O15_POOR_FETGRO | Poor fetal growth \|\| id:finngen_R11_O15_POOR_FETGRO | CCR2 on CD14+ CD16+ monocyte \|\| id:ebi-a-GCST90001992 | Weighted mode | 37 | -2.97E-06 | 0.0165049862638396 | 0.999857399747617 | -0.032352743 | 0.0323468027043983 | 0.999997029631684 | 0.968165007973786 | 1.03287564726701 | 0.999857399747617 |
| ebi-a-GCST90001993 | finngen_R11_O15_POOR_FETGRO | Poor fetal growth \|\| id:finngen_R11_O15_POOR_FETGRO | PDL-1 on CD14+ CD16- monocyte \|\| id:ebi-a-GCST90001993 | Inverse variance weighted | 15 | 0.0319529039422015 | 0.0432671780220612 | 0.460209361872434 | -0.052850765 | 0.116756572865441 | 1.03246887894607 | 0.948521554552698 | 1.12384582182201 | 0.994119304454654 |
| ebi-a-GCST90001993 | finngen_R11_O15_POOR_FETGRO | Poor fetal growth \|\| id:finngen_R11_O15_POOR_FETGRO | PDL-1 on CD14+ CD16- monocyte \|\| id:ebi-a-GCST90001993 | MR Egger | 15 | 0.0539767185258027 | 0.0683833175804981 | 0.444080067953469 | -0.080054584 | 0.188008020983579 | 1.0554600292036 | 0.923065960441925 | 1.20684319538024 | 0.992899239648112 |
| ebi-a-GCST90001993 | finngen_R11_O15_POOR_FETGRO | Poor fetal growth \|\| id:finngen_R11_O15_POOR_FETGRO | PDL-1 on CD14+ CD16- monocyte \|\| id:ebi-a-GCST90001993 | Weighted median | 15 | 0.065850987920874 | 0.0626847567114777 | 0.29348351873019 | -0.057011135 | 0.18871311107537 | 1.06806755034107 | 0.944583551149444 | 1.20769442862244 | 0.999440741871876 |
| ebi-a-GCST90001993 | finngen_R11_O15_POOR_FETGRO | Poor fetal growth \|\| id:finngen_R11_O15_POOR_FETGRO | PDL-1 on CD14+ CD16- monocyte \|\| id:ebi-a-GCST90001993 | Weighted mode | 15 | 0.02283560396036 | 0.0629799822630684 | 0.722330105590764 | -0.100605161 | 0.146276369195974 | 1.02309833240729 | 0.904290011121602 | 1.15751604562823 | 0.999857399747617 |
| ebi-a-GCST90001994 | finngen_R11_O15_POOR_FETGRO | Poor fetal growth \|\| id:finngen_R11_O15_POOR_FETGRO | CX3CR1 on CD14- CD16- \|\| id:ebi-a-GCST90001994 | Inverse variance weighted | 18 | 0.0022813821344172 | 0.0146705699887997 | 0.876421323833881 | -0.026472935 | 0.0310356993124645 | 1.00228398646675 | 0.973874401347033 | 1.03152232786712 | 0.994119304454654 |
| ebi-a-GCST90001994 | finngen_R11_O15_POOR_FETGRO | Poor fetal growth \|\| id:finngen_R11_O15_POOR_FETGRO | CX3CR1 on CD14- CD16- \|\| id:ebi-a-GCST90001994 | MR Egger | 18 | 0.0157375913127548 | 0.0191682858526614 | 0.423698304993976 | -0.021832249 | 0.0533074315839713 | 1.01586207939308 | 0.978404349634326 | 1.05475385992972 | 0.992899239648112 |
| ebi-a-GCST90001994 | finngen_R11_O15_POOR_FETGRO | Poor fetal growth \|\| id:finngen_R11_O15_POOR_FETGRO | CX3CR1 on CD14- CD16- \|\| id:ebi-a-GCST90001994 | Weighted median | 18 | -0.000170003 | 0.0202798808472428 | 0.993311520916889 | -0.03991857 | 0.0395785629862047 | 0.99983001097538 | 0.960867679484331 | 1.04037223042357 | 0.999440741871876 |
| ebi-a-GCST90001994 | finngen_R11_O15_POOR_FETGRO | Poor fetal growth \|\| id:finngen_R11_O15_POOR_FETGRO | CX3CR1 on CD14- CD16- \|\| id:ebi-a-GCST90001994 | Weighted mode | 18 | 0.000279577074342119 | 0.0189147119971295 | 0.988379036818979 | -0.036793258 | 0.037352412588716 | 1.00027961615965 | 0.963875387852757 | 1.03805878136745 | 0.999857399747617 |
| ebi-a-GCST90001995 | finngen_R11_O15_POOR_FETGRO | Poor fetal growth \|\| id:finngen_R11_O15_POOR_FETGRO | CX3CR1 on monocyte \|\| id:ebi-a-GCST90001995 | Inverse variance weighted | 26 | -0.007365326 | 0.0272157631828674 | 0.786677773006797 | -0.060708222 | 0.0459775700297287 | 0.992661731733486 | 0.941097791683475 | 1.04705092537242 | 0.994119304454654 |
| ebi-a-GCST90001995 | finngen_R11_O15_POOR_FETGRO | Poor fetal growth \|\| id:finngen_R11_O15_POOR_FETGRO | CX3CR1 on monocyte \|\| id:ebi-a-GCST90001995 | MR Egger | 26 | -0.062833205 | 0.057461645950497 | 0.285037098316305 | -0.175458032 | 0.0497916205753321 | 0.939100097369581 | 0.839072611010928 | 1.05105205593234 | 0.992899239648112 |
| ebi-a-GCST90001995 | finngen_R11_O15_POOR_FETGRO | Poor fetal growth \|\| id:finngen_R11_O15_POOR_FETGRO | CX3CR1 on monocyte \|\| id:ebi-a-GCST90001995 | Weighted median | 26 | -0.025283929 | 0.0390114738353426 | 0.51691044039792 | -0.101746418 | 0.0511785593582779 | 0.975033032219204 | 0.903258572676345 | 1.05251081216057 | 0.999440741871876 |
| ebi-a-GCST90001995 | finngen_R11_O15_POOR_FETGRO | Poor fetal growth \|\| id:finngen_R11_O15_POOR_FETGRO | CX3CR1 on monocyte \|\| id:ebi-a-GCST90001995 | Weighted mode | 26 | -0.03442414 | 0.0428752431535313 | 0.429605282 | -0.118459617 | 0.0496113362492011 | 0.966161629607309 | 0.88828768673176 | 1.05086258480056 | 0.999857399747617 |
| ebi-a-GCST90001996 | finngen_R11_O15_POOR_FETGRO | Poor fetal growth \|\| id:finngen_R11_O15_POOR_FETGRO | CX3CR1 on CD14+ CD16+ monocyte \|\| id:ebi-a-GCST90001996 | Inverse variance weighted | 34 | -0.017511392 | 0.0208225789553898 | 0.400358602261364 | -0.058323647 | 0.0233008623589669 | 0.982641040967803 | 0.943344587241479 | 1.0235744482489 | 0.994119304454654 |
| ebi-a-GCST90001996 | finngen_R11_O15_POOR_FETGRO | Poor fetal growth \|\| id:finngen_R11_O15_POOR_FETGRO | CX3CR1 on CD14+ CD16+ monocyte \|\| id:ebi-a-GCST90001996 | MR Egger | 34 | -0.030116213 | 0.0304408934689454 | 0.329921892712391 | -0.089780364 | 0.0295479379435144 | 0.970332761466586 | 0.914131939090955 | 1.02998880983382 | 0.992899239648112 |
| ebi-a-GCST90001996 | finngen_R11_O15_POOR_FETGRO | Poor fetal growth \|\| id:finngen_R11_O15_POOR_FETGRO | CX3CR1 on CD14+ CD16+ monocyte \|\| id:ebi-a-GCST90001996 | Weighted median | 34 | -0.011408729 | 0.0266914704127119 | 0.669066326920971 | -0.063724011 | 0.0409065529137093 | 0.988656103667211 | 0.938263914222025 | 1.04175475205064 | 0.999440741871876 |
| ebi-a-GCST90001996 | finngen_R11_O15_POOR_FETGRO | Poor fetal growth \|\| id:finngen_R11_O15_POOR_FETGRO | CX3CR1 on CD14+ CD16+ monocyte \|\| id:ebi-a-GCST90001996 | Weighted mode | 34 | -0.009306961 | 0.0255163212193286 | 0.717628607610056 | -0.05931895 | 0.0407050289809926 | 0.990736215100301 | 0.942406140591009 | 1.04154483468848 | 0.999857399747617 |
| ebi-a-GCST90001997 | finngen_R11_O15_POOR_FETGRO | Poor fetal growth \|\| id:finngen_R11_O15_POOR_FETGRO | CX3CR1 on CD14+ CD16- monocyte \|\| id:ebi-a-GCST90001997 | Inverse variance weighted | 27 | 0.00100377100725698 | 0.0264181456099151 | 0.969691259182739 | -0.050775794 | 0.0527833364026906 | 1.00100427495398 | 0.95049175222932 | 1.05420121334665 | 0.998984604424952 |
| ebi-a-GCST90001997 | finngen_R11_O15_POOR_FETGRO | Poor fetal growth \|\| id:finngen_R11_O15_POOR_FETGRO | CX3CR1 on CD14+ CD16- monocyte \|\| id:ebi-a-GCST90001997 | MR Egger | 27 | -0.029245757 | 0.0510014498099709 | 0.571476473882612 | -0.129208599 | 0.0707170847444935 | 0.971177761516802 | 0.878790632007737 | 1.07327753632276 | 0.992899239648112 |
| ebi-a-GCST90001997 | finngen_R11_O15_POOR_FETGRO | Poor fetal growth \|\| id:finngen_R11_O15_POOR_FETGRO | CX3CR1 on CD14+ CD16- monocyte \|\| id:ebi-a-GCST90001997 | Weighted median | 27 | 0.00204698161684448 | 0.0355926460747307 | 0.954137854092782 | -0.067714605 | 0.0718085679233167 | 1.00204907811397 | 0.934527145186642 | 1.07444964024936 | 0.999440741871876 |
| ebi-a-GCST90001997 | finngen_R11_O15_POOR_FETGRO | Poor fetal growth \|\| id:finngen_R11_O15_POOR_FETGRO | CX3CR1 on CD14+ CD16- monocyte \|\| id:ebi-a-GCST90001997 | Weighted mode | 27 | -0.015379488 | 0.0400242463963198 | 0.703914810553501 | -0.093827011 | 0.0630680350110547 | 0.984738172441037 | 0.910440244891672 | 1.06509930080902 | 0.999857399747617 |
| ebi-a-GCST90001998 | finngen_R11_O15_POOR_FETGRO | Poor fetal growth \|\| id:finngen_R11_O15_POOR_FETGRO | PDL-1 on CD14+ CD16+ monocyte \|\| id:ebi-a-GCST90001998 | Inverse variance weighted | 28 | 0.0345314515838517 | 0.0206386581935222 | 0.0942989089007392 | -0.005920318 | 0.0749832216431553 | 1.03513458448632 | 0.994097172076405 | 1.07786606591143 | 0.994119304454654 |
| ebi-a-GCST90001998 | finngen_R11_O15_POOR_FETGRO | Poor fetal growth \|\| id:finngen_R11_O15_POOR_FETGRO | PDL-1 on CD14+ CD16+ monocyte \|\| id:ebi-a-GCST90001998 | MR Egger | 28 | 0.0255113620448628 | 0.028206132200635 | 0.374055554394209 | -0.029772657 | 0.0807953811581074 | 1.02583956183935 | 0.970666182561627 | 1.08414903654887 | 0.992899239648112 |
| ebi-a-GCST90001998 | finngen_R11_O15_POOR_FETGRO | Poor fetal growth \|\| id:finngen_R11_O15_POOR_FETGRO | PDL-1 on CD14+ CD16+ monocyte \|\| id:ebi-a-GCST90001998 | Weighted median | 28 | 0.0183806409565021 | 0.0308104765003409 | 0.550793747189373 | -0.042007893 | 0.0787691748971703 | 1.01855060468792 | 0.958862212258357 | 1.08195455097422 | 0.999440741871876 |
| ebi-a-GCST90001998 | finngen_R11_O15_POOR_FETGRO | Poor fetal growth \|\| id:finngen_R11_O15_POOR_FETGRO | PDL-1 on CD14+ CD16+ monocyte \|\| id:ebi-a-GCST90001998 | Weighted mode | 28 | 0.0252926349106286 | 0.027814314804143 | 0.371217624527873 | -0.029223422 | 0.0798086919267489 | 1.0256152074289 | 0.971199452798309 | 1.08307984593549 | 0.999857399747617 |
| ebi-a-GCST90001999 | finngen_R11_O15_POOR_FETGRO | Poor fetal growth \|\| id:finngen_R11_O15_POOR_FETGRO | PDL-1 on CD14- CD16+ monocyte \|\| id:ebi-a-GCST90001999 | Inverse variance weighted | 20 | 0.0354876948721547 | 0.0257505913049106 | 0.168162765310211 | -0.014983464 | 0.0859588538297795 | 1.03612489840028 | 0.985128229464582 | 1.08976148787095 | 0.994119304454654 |
| ebi-a-GCST90001999 | finngen_R11_O15_POOR_FETGRO | Poor fetal growth \|\| id:finngen_R11_O15_POOR_FETGRO | PDL-1 on CD14- CD16+ monocyte \|\| id:ebi-a-GCST90001999 | MR Egger | 20 | -0.003568387 | 0.0476887403855082 | 0.941177998361689 | -0.097038318 | 0.0899015442038102 | 0.996437972174743 | 0.907521230971038 | 1.09406656120798 | 0.992899239648112 |
| ebi-a-GCST90001999 | finngen_R11_O15_POOR_FETGRO | Poor fetal growth \|\| id:finngen_R11_O15_POOR_FETGRO | PDL-1 on CD14- CD16+ monocyte \|\| id:ebi-a-GCST90001999 | Weighted median | 20 | 0.0172212560659706 | 0.0356603824334917 | 0.629149669492755 | -0.052673094 | 0.0871156056356143 | 1.01737039679641 | 0.948690094750558 | 1.09102280081234 | 0.999440741871876 |
| ebi-a-GCST90001999 | finngen_R11_O15_POOR_FETGRO | Poor fetal growth \|\| id:finngen_R11_O15_POOR_FETGRO | PDL-1 on CD14- CD16+ monocyte \|\| id:ebi-a-GCST90001999 | Weighted mode | 20 | 0.0133295632504168 | 0.0365121220042263 | 0.719089663747365 | -0.058234196 | 0.0848933223787005 | 1.01341879792424 | 0.943428974385458 | 1.0886009311461 | 0.999857399747617 |
| ebi-a-GCST90002000 | finngen_R11_O15_POOR_FETGRO | Poor fetal growth \|\| id:finngen_R11_O15_POOR_FETGRO | PDL-1 on CD14- CD16- \|\| id:ebi-a-GCST90002000 | Inverse variance weighted | 26 | 0.00921179981821966 | 0.0229798550892882 | 0.688520153872614 | -0.035828716 | 0.0542523157932245 | 1.00925435902809 | 0.964805534931102 | 1.05575095119033 | 0.994119304454654 |
| ebi-a-GCST90002000 | finngen_R11_O15_POOR_FETGRO | Poor fetal growth \|\| id:finngen_R11_O15_POOR_FETGRO | PDL-1 on CD14- CD16- \|\| id:ebi-a-GCST90002000 | MR Egger | 26 | 0.00663016510462276 | 0.0306314003609303 | 0.830466306063126 | -0.05340738 | 0.066667709812046 | 1.00665219330591 | 0.947993740494516 | 1.06894022080672 | 0.992899239648112 |
| ebi-a-GCST90002000 | finngen_R11_O15_POOR_FETGRO | Poor fetal growth \|\| id:finngen_R11_O15_POOR_FETGRO | PDL-1 on CD14- CD16- \|\| id:ebi-a-GCST90002000 | Weighted median | 26 | 0.0123666853820438 | 0.0310932373802535 | 0.69082988307296 | -0.04857606 | 0.0733094306473406 | 1.01244346902899 | 0.952584883055369 | 1.07606345241558 | 0.999440741871876 |
| ebi-a-GCST90002000 | finngen_R11_O15_POOR_FETGRO | Poor fetal growth \|\| id:finngen_R11_O15_POOR_FETGRO | PDL-1 on CD14- CD16- \|\| id:ebi-a-GCST90002000 | Weighted mode | 26 | 0.00989097146213846 | 0.0283186377409586 | 0.729808304308095 | -0.045613559 | 0.0653955014344173 | 1.00994004879441 | 0.955411101350212 | 1.06758117078334 | 0.999857399747617 |
| ebi-a-GCST90002001 | finngen_R11_O15_POOR_FETGRO | Poor fetal growth \|\| id:finngen_R11_O15_POOR_FETGRO | CD64 on CD14- CD16- \|\| id:ebi-a-GCST90002001 | Inverse variance weighted | 16 | -0.042160564 | 0.0458736064390659 | 0.358064564882868 | -0.132072832 | 0.0477517047787122 | 0.958715833116204 | 0.876277171331025 | 1.04891018360272 | 0.994119304454654 |
| ebi-a-GCST90002001 | finngen_R11_O15_POOR_FETGRO | Poor fetal growth \|\| id:finngen_R11_O15_POOR_FETGRO | CD64 on CD14- CD16- \|\| id:ebi-a-GCST90002001 | MR Egger | 16 | -0.040965366 | 0.0966587624673119 | 0.678140696842517 | -0.23041654 | 0.148485808686742 | 0.9598623734863 | 0.794202716247958 | 1.16007633465094 | 0.992899239648112 |
| ebi-a-GCST90002001 | finngen_R11_O15_POOR_FETGRO | Poor fetal growth \|\| id:finngen_R11_O15_POOR_FETGRO | CD64 on CD14- CD16- \|\| id:ebi-a-GCST90002001 | Weighted median | 16 | 0.0171762914389352 | 0.052637960601841 | 0.744189844387615 | -0.085994111 | 0.120346694218544 | 1.01732465214441 | 0.917599634635888 | 1.12788781598788 | 0.999440741871876 |
| ebi-a-GCST90002001 | finngen_R11_O15_POOR_FETGRO | Poor fetal growth \|\| id:finngen_R11_O15_POOR_FETGRO | CD64 on CD14- CD16- \|\| id:ebi-a-GCST90002001 | Weighted mode | 16 | 0.00553290871067191 | 0.0549413931504102 | 0.921117857168955 | -0.102152222 | 0.113218039285476 | 1.00554824351906 | 0.902892101288897 | 1.11987608331146 | 0.999857399747617 |
| ebi-a-GCST90002002 | finngen_R11_O15_POOR_FETGRO | Poor fetal growth \|\| id:finngen_R11_O15_POOR_FETGRO | PDL-1 on monocyte \|\| id:ebi-a-GCST90002002 | Inverse variance weighted | 15 | -0.024968734 | 0.0374836938740046 | 0.505332770657862 | -0.098436774 | 0.0484993061615034 | 0.975340406709061 | 0.906252989713403 | 1.04969464350152 | 0.994119304454654 |
| ebi-a-GCST90002002 | finngen_R11_O15_POOR_FETGRO | Poor fetal growth \|\| id:finngen_R11_O15_POOR_FETGRO | PDL-1 on monocyte \|\| id:ebi-a-GCST90002002 | MR Egger | 15 | 0.034615727925744 | 0.0647499741453568 | 0.6019464098367 | -0.092294221 | 0.161525677250643 | 1.0352218255186 | 0.911836828166696 | 1.1753026363113 | 0.992899239648112 |
| ebi-a-GCST90002002 | finngen_R11_O15_POOR_FETGRO | Poor fetal growth \|\| id:finngen_R11_O15_POOR_FETGRO | PDL-1 on monocyte \|\| id:ebi-a-GCST90002002 | Weighted median | 15 | -0.036345582 | 0.0517080362162794 | 0.482118036183162 | -0.137693333 | 0.06500216942533 | 0.964306989182261 | 0.871365870332235 | 1.06716133950852 | 0.999440741871876 |
| ebi-a-GCST90002002 | finngen_R11_O15_POOR_FETGRO | Poor fetal growth \|\| id:finngen_R11_O15_POOR_FETGRO | PDL-1 on monocyte \|\| id:ebi-a-GCST90002002 | Weighted mode | 15 | -0.012474486 | 0.0587792227531622 | 0.834989433446574 | -0.127681762 | 0.102732791063712 | 0.987602998337043 | 0.880133426369546 | 1.10819524983566 | 0.999857399747617 |
| ebi-a-GCST90002003 | finngen_R11_O15_POOR_FETGRO | Poor fetal growth \|\| id:finngen_R11_O15_POOR_FETGRO | CCR2 on CD14- CD16- \|\| id:ebi-a-GCST90002003 | Inverse variance weighted | 17 | -0.005013081 | 0.0304821366681055 | 0.869369331822498 | -0.064758069 | 0.0547319072321154 | 0.994999463880387 | 0.937294196933881 | 1.05625740174309 | 0.994119304454654 |
| ebi-a-GCST90002003 | finngen_R11_O15_POOR_FETGRO | Poor fetal growth \|\| id:finngen_R11_O15_POOR_FETGRO | CCR2 on CD14- CD16- \|\| id:ebi-a-GCST90002003 | MR Egger | 17 | -0.004962078 | 0.043963048541247 | 0.911630883883701 | -0.091129653 | 0.0812054969253246 | 0.995050212556927 | 0.912899342762207 | 1.08459375434943 | 0.992899239648112 |
| ebi-a-GCST90002003 | finngen_R11_O15_POOR_FETGRO | Poor fetal growth \|\| id:finngen_R11_O15_POOR_FETGRO | CCR2 on CD14- CD16- \|\| id:ebi-a-GCST90002003 | Weighted median | 17 | -0.011209622 | 0.0407412215213044 | 0.783207131929226 | -0.091062416 | 0.0686431723175398 | 0.988852971844701 | 0.912960725722261 | 1.07105395925166 | 0.999440741871876 |
| ebi-a-GCST90002003 | finngen_R11_O15_POOR_FETGRO | Poor fetal growth \|\| id:finngen_R11_O15_POOR_FETGRO | CCR2 on CD14- CD16- \|\| id:ebi-a-GCST90002003 | Weighted mode | 17 | -0.002756246 | 0.0367664418384137 | 0.941170793 | -0.074818472 | 0.0693059799649714 | 0.997247548920378 | 0.927911912996148 | 1.07176409732314 | 0.999857399747617 |
| ebi-a-GCST90002004 | finngen_R11_O15_POOR_FETGRO | Poor fetal growth \|\| id:finngen_R11_O15_POOR_FETGRO | CCR2 on CD14+ CD16- monocyte \|\| id:ebi-a-GCST90002004 | Inverse variance weighted | 28 | -0.006661319 | 0.0113225481872895 | 0.556315300328888 | -0.028853514 | 0.0155308753848435 | 0.993360818341536 | 0.97155877427844 | 1.01565210622386 | 0.994119304454654 |
| ebi-a-GCST90002004 | finngen_R11_O15_POOR_FETGRO | Poor fetal growth \|\| id:finngen_R11_O15_POOR_FETGRO | CCR2 on CD14+ CD16- monocyte \|\| id:ebi-a-GCST90002004 | MR Egger | 28 | -0.006863525 | 0.0134586630644122 | 0.614373988312368 | -0.033242505 | 0.0195154543563656 | 0.993159974944084 | 0.967303955237336 | 1.0197071256562 | 0.992899239648112 |
| ebi-a-GCST90002004 | finngen_R11_O15_POOR_FETGRO | Poor fetal growth \|\| id:finngen_R11_O15_POOR_FETGRO | CCR2 on CD14+ CD16- monocyte \|\| id:ebi-a-GCST90002004 | Weighted median | 28 | -0.000257163 | 0.0148789155783436 | 0.986210254271535 | -0.029419838 | 0.0289055111693396 | 0.99974286969945 | 0.971008712621135 | 1.02932732994422 | 0.999440741871876 |
| ebi-a-GCST90002004 | finngen_R11_O15_POOR_FETGRO | Poor fetal growth \|\| id:finngen_R11_O15_POOR_FETGRO | CCR2 on CD14+ CD16- monocyte \|\| id:ebi-a-GCST90002004 | Weighted mode | 28 | -0.002669145 | 0.0126409336875451 | 0.834355117989422 | -0.027445375 | 0.0221070849995373 | 0.997334413972339 | 0.972927827230358 | 1.02235325730697 | 0.999857399747617 |
| ebi-a-GCST90002005 | finngen_R11_O15_POOR_FETGRO | Poor fetal growth \|\| id:finngen_R11_O15_POOR_FETGRO | CD16 on CD14+ CD16+ monocyte \|\| id:ebi-a-GCST90002005 | Inverse variance weighted | 22 | -0.014776476 | 0.0289574987246212 | 0.609854265833977 | -0.071533173 | 0.0419802217887705 | 0.985332160659929 | 0.930965393970469 | 1.04287385236745 | 0.994119304454654 |
| ebi-a-GCST90002005 | finngen_R11_O15_POOR_FETGRO | Poor fetal growth \|\| id:finngen_R11_O15_POOR_FETGRO | CD16 on CD14+ CD16+ monocyte \|\| id:ebi-a-GCST90002005 | MR Egger | 22 | -0.052540626 | 0.0426113733112565 | 0.231865603019735 | -0.136058918 | 0.0309776656119597 | 0.948815773609067 | 0.872791208063774 | 1.03146246654628 | 0.992899239648112 |
| ebi-a-GCST90002005 | finngen_R11_O15_POOR_FETGRO | Poor fetal growth \|\| id:finngen_R11_O15_POOR_FETGRO | CD16 on CD14+ CD16+ monocyte \|\| id:ebi-a-GCST90002005 | Weighted median | 22 | -0.008565111 | 0.0359949766285444 | 0.811917593431191 | -0.079115265 | 0.0619850436364553 | 0.991471465503434 | 0.923933421354716 | 1.06394643183941 | 0.999440741871876 |
| ebi-a-GCST90002005 | finngen_R11_O15_POOR_FETGRO | Poor fetal growth \|\| id:finngen_R11_O15_POOR_FETGRO | CD16 on CD14+ CD16+ monocyte \|\| id:ebi-a-GCST90002005 | Weighted mode | 22 | -0.033547392 | 0.0313385889448773 | 0.296552325446102 | -0.094971027 | 0.0278762419017398 | 0.96700908123501 | 0.909399282328219 | 1.02826842000243 | 0.999857399747617 |
| ebi-a-GCST90002006 | finngen_R11_O15_POOR_FETGRO | Poor fetal growth \|\| id:finngen_R11_O15_POOR_FETGRO | CD64 on monocyte \|\| id:ebi-a-GCST90002006 | Inverse variance weighted | 28 | 0.00953232299648703 | 0.0220832012146506 | 0.665992218280602 | -0.033750751 | 0.0528153973772022 | 1.0095779002914 | 0.966812451273642 | 1.0542350126067 | 0.994119304454654 |
| ebi-a-GCST90002006 | finngen_R11_O15_POOR_FETGRO | Poor fetal growth \|\| id:finngen_R11_O15_POOR_FETGRO | CD64 on monocyte \|\| id:ebi-a-GCST90002006 | MR Egger | 28 | 0.00808638285006066 | 0.0352198339486847 | 0.820201277391593 | -0.060944492 | 0.0771172573894827 | 1.00811916594985 | 0.940875464734105 | 1.08016872673221 | 0.992899239648112 |
| ebi-a-GCST90002006 | finngen_R11_O15_POOR_FETGRO | Poor fetal growth \|\| id:finngen_R11_O15_POOR_FETGRO | CD64 on monocyte \|\| id:ebi-a-GCST90002006 | Weighted median | 28 | -0.001208564 | 0.0271433804610161 | 0.964485764580824 | -0.05440959 | 0.0519924612389031 | 0.998792165555223 | 0.947044127087563 | 1.05336780139523 | 0.999440741871876 |
| ebi-a-GCST90002006 | finngen_R11_O15_POOR_FETGRO | Poor fetal growth \|\| id:finngen_R11_O15_POOR_FETGRO | CD64 on monocyte \|\| id:ebi-a-GCST90002006 | Weighted mode | 28 | 0.00697847569210253 | 0.0251842398033921 | 0.783816146944296 | -0.042382634 | 0.056339585706751 | 1.00700288199349 | 0.958502954268228 | 1.05795688978067 | 0.999857399747617 |
| ebi-a-GCST90002007 | finngen_R11_O15_POOR_FETGRO | Poor fetal growth \|\| id:finngen_R11_O15_POOR_FETGRO | HLA DR on CD14+ CD16+ monocyte \|\| id:ebi-a-GCST90002007 | Inverse variance weighted | 19 | -0.000253819 | 0.0150824078335206 | 0.986573175815973 | -0.029815339 | 0.0293077001836388 | 0.999746213039299 | 0.970624754000389 | 1.02974139734954 | 0.998984604424952 |
| ebi-a-GCST90002007 | finngen_R11_O15_POOR_FETGRO | Poor fetal growth \|\| id:finngen_R11_O15_POOR_FETGRO | HLA DR on CD14+ CD16+ monocyte \|\| id:ebi-a-GCST90002007 | MR Egger | 19 | -0.003464373 | 0.0203484559578788 | 0.866821423861275 | -0.043347346 | 0.0364186010792127 | 0.996541621416688 | 0.957578720892311 | 1.03708988258469 | 0.992899239648112 |
| ebi-a-GCST90002007 | finngen_R11_O15_POOR_FETGRO | Poor fetal growth \|\| id:finngen_R11_O15_POOR_FETGRO | HLA DR on CD14+ CD16+ monocyte \|\| id:ebi-a-GCST90002007 | Weighted median | 19 | -0.000759685 | 0.0161392301494937 | 0.962456870788249 | -0.032392576 | 0.0308732060568821 | 0.999240603451494 | 0.968126444135588 | 1.03135472606331 | 0.999440741871876 |
| ebi-a-GCST90002007 | finngen_R11_O15_POOR_FETGRO | Poor fetal growth \|\| id:finngen_R11_O15_POOR_FETGRO | HLA DR on CD14+ CD16+ monocyte \|\| id:ebi-a-GCST90002007 | Weighted mode | 19 | 0.00140152373578351 | 0.0137156098002557 | 0.919739924762116 | -0.025481071 | 0.0282841189442846 | 1.00140250632916 | 0.974840831092372 | 1.02868791262941 | 0.999857399747617 |
| ebi-a-GCST90002008 | finngen_R11_O15_POOR_FETGRO | Poor fetal growth \|\| id:finngen_R11_O15_POOR_FETGRO | CCR2 on monocyte \|\| id:ebi-a-GCST90002008 | Inverse variance weighted | 24 | -0.004660414 | 0.0169832811632973 | 0.783768137560854 | -0.037947645 | 0.0286268174798287 | 0.995350429276592 | 0.962763345354462 | 1.02904050288323 | 0.994119304454654 |
| ebi-a-GCST90002008 | finngen_R11_O15_POOR_FETGRO | Poor fetal growth \|\| id:finngen_R11_O15_POOR_FETGRO | CCR2 on monocyte \|\| id:ebi-a-GCST90002008 | MR Egger | 24 | -0.003790487 | 0.0207236562104 | 0.856546669961935 | -0.044408853 | 0.0368278794455477 | 0.996216688099752 | 0.956562784044758 | 1.03751442791026 | 0.992899239648112 |
| ebi-a-GCST90002008 | finngen_R11_O15_POOR_FETGRO | Poor fetal growth \|\| id:finngen_R11_O15_POOR_FETGRO | CCR2 on monocyte \|\| id:ebi-a-GCST90002008 | Weighted median | 24 | -0.022313775 | 0.0221708162058868 | 0.31420009324238 | -0.065768574 | 0.0211410251925971 | 0.977933336292211 | 0.936347534070409 | 1.02136607982961 | 0.999440741871876 |
| ebi-a-GCST90002008 | finngen_R11_O15_POOR_FETGRO | Poor fetal growth \|\| id:finngen_R11_O15_POOR_FETGRO | CCR2 on monocyte \|\| id:ebi-a-GCST90002008 | Weighted mode | 24 | -0.002126265 | 0.0166481626599202 | 0.899482492233295 | -0.034756664 | 0.0305041336016726 | 0.997875993688814 | 0.965840411383014 | 1.03097415167645 | 0.999857399747617 |
| ebi-a-GCST90002009 | finngen_R11_O15_POOR_FETGRO | Poor fetal growth \|\| id:finngen_R11_O15_POOR_FETGRO | HLA DR on CD14- CD16- \|\| id:ebi-a-GCST90002009 | Inverse variance weighted | 24 | -0.000854669 | 0.00321909522734643 | 0.790624565193824 | -0.007164095 | 0.00545475792986445 | 0.999145696409544 | 0.992861505597455 | 1.00546966220932 | 0.994119304454654 |
| ebi-a-GCST90002009 | finngen_R11_O15_POOR_FETGRO | Poor fetal growth \|\| id:finngen_R11_O15_POOR_FETGRO | HLA DR on CD14- CD16- \|\| id:ebi-a-GCST90002009 | MR Egger | 24 | -0.001479451 | 0.00336021844542635 | 0.66402692599668 | -0.008065479 | 0.00510657757208556 | 0.998521643266563 | 0.991966959969808 | 1.00511963836185 | 0.992899239648112 |
| ebi-a-GCST90002009 | finngen_R11_O15_POOR_FETGRO | Poor fetal growth \|\| id:finngen_R11_O15_POOR_FETGRO | HLA DR on CD14- CD16- \|\| id:ebi-a-GCST90002009 | Weighted median | 24 | -0.001038726 | 0.00323544056012069 | 0.74817530481745 | -0.00738019 | 0.00530273727983839 | 0.998961813071339 | 0.992646977011337 | 1.00531682167545 | 0.999440741871876 |
| ebi-a-GCST90002009 | finngen_R11_O15_POOR_FETGRO | Poor fetal growth \|\| id:finngen_R11_O15_POOR_FETGRO | HLA DR on CD14- CD16- \|\| id:ebi-a-GCST90002009 | Weighted mode | 24 | 0.000547718681040421 | 0.00312320138573101 | 0.862322965570793 | -0.005573756 | 0.0066691933970732 | 1.00054786870631 | 0.994441748523593 | 1.00669148198878 | 0.999857399747617 |
| ebi-a-GCST90002010 | finngen_R11_O15_POOR_FETGRO | Poor fetal growth \|\| id:finngen_R11_O15_POOR_FETGRO | HLA DR on monocyte \|\| id:ebi-a-GCST90002010 | Inverse variance weighted | 17 | 0.013818139230683 | 0.0188438138551189 | 0.463376428664881 | -0.023115736 | 0.0507520143867162 | 1.01391405098138 | 0.977149385940275 | 1.05206196469975 | 0.994119304454654 |
| ebi-a-GCST90002010 | finngen_R11_O15_POOR_FETGRO | Poor fetal growth \|\| id:finngen_R11_O15_POOR_FETGRO | HLA DR on monocyte \|\| id:ebi-a-GCST90002010 | MR Egger | 17 | 0.0364610498156476 | 0.0332470161340392 | 0.290079738902495 | -0.028703102 | 0.101625201438364 | 1.03713390667415 | 0.971704919078196 | 1.10696850376511 | 0.992899239648112 |
| ebi-a-GCST90002010 | finngen_R11_O15_POOR_FETGRO | Poor fetal growth \|\| id:finngen_R11_O15_POOR_FETGRO | HLA DR on monocyte \|\| id:ebi-a-GCST90002010 | Weighted median | 17 | 0.0168739716418118 | 0.0245585216300607 | 0.492024552562022 | -0.031260731 | 0.0650086740367309 | 1.01701714124762 | 0.969222833929605 | 1.06716828100091 | 0.999440741871876 |
| ebi-a-GCST90002010 | finngen_R11_O15_POOR_FETGRO | Poor fetal growth \|\| id:finngen_R11_O15_POOR_FETGRO | HLA DR on monocyte \|\| id:ebi-a-GCST90002010 | Weighted mode | 17 | 0.0213812063182043 | 0.0261139550543346 | 0.424947791331888 | -0.029802146 | 0.0725645582247001 | 1.02161142214644 | 0.970637559474605 | 1.07526222086955 | 0.999857399747617 |
| ebi-a-GCST90002011 | finngen_R11_O15_POOR_FETGRO | Poor fetal growth \|\| id:finngen_R11_O15_POOR_FETGRO | CD64 on CD14+ CD16+ monocyte \|\| id:ebi-a-GCST90002011 | Inverse variance weighted | 25 | 0.015215601784286 | 0.0262645883489541 | 0.562373261060184 | -0.036262991 | 0.066694194948236 | 1.01533194839872 | 0.964386634757951 | 1.06896853220896 | 0.994119304454654 |
| ebi-a-GCST90002011 | finngen_R11_O15_POOR_FETGRO | Poor fetal growth \|\| id:finngen_R11_O15_POOR_FETGRO | CD64 on CD14+ CD16+ monocyte \|\| id:ebi-a-GCST90002011 | MR Egger | 25 | -0.010148381 | 0.0485924369545017 | 0.836406332037319 | -0.105389557 | 0.085092795733725 | 0.989902940363015 | 0.899973863056444 | 1.08881809968506 | 0.992899239648112 |
| ebi-a-GCST90002011 | finngen_R11_O15_POOR_FETGRO | Poor fetal growth \|\| id:finngen_R11_O15_POOR_FETGRO | CD64 on CD14+ CD16+ monocyte \|\| id:ebi-a-GCST90002011 | Weighted median | 25 | 0.0183262904189496 | 0.034444966466236 | 0.594694462240656 | -0.049185844 | 0.0858384246927721 | 1.0184952474194 | 0.952004189128992 | 1.08963025673761 | 0.999440741871876 |
| ebi-a-GCST90002011 | finngen_R11_O15_POOR_FETGRO | Poor fetal growth \|\| id:finngen_R11_O15_POOR_FETGRO | CD64 on CD14+ CD16+ monocyte \|\| id:ebi-a-GCST90002011 | Weighted mode | 25 | 0.0225212796461955 | 0.0391021967101232 | 0.570004668065982 | -0.054119026 | 0.099161585198037 | 1.02277679826128 | 0.947319344248232 | 1.10424471474478 | 0.999857399747617 |
| ebi-a-GCST90002012 | finngen_R11_O15_POOR_FETGRO | Poor fetal growth \|\| id:finngen_R11_O15_POOR_FETGRO | CX3CR1 on CD14- CD16+ monocyte \|\| id:ebi-a-GCST90002012 | Inverse variance weighted | 18 | -0.003986012 | 0.0251827873509699 | 0.874233637463753 | -0.053344276 | 0.0453722507772625 | 0.996021921172263 | 0.948053564545183 | 1.04641731707598 | 0.994119304454654 |
| ebi-a-GCST90002012 | finngen_R11_O15_POOR_FETGRO | Poor fetal growth \|\| id:finngen_R11_O15_POOR_FETGRO | CX3CR1 on CD14- CD16+ monocyte \|\| id:ebi-a-GCST90002012 | MR Egger | 18 | -0.000683139 | 0.0360077920422925 | 0.985098033071581 | -0.071258412 | 0.0698921332767816 | 0.999317094160296 | 0.93122122273305 | 1.07239249955029 | 0.994809183268002 |
| ebi-a-GCST90002012 | finngen_R11_O15_POOR_FETGRO | Poor fetal growth \|\| id:finngen_R11_O15_POOR_FETGRO | CX3CR1 on CD14- CD16+ monocyte \|\| id:ebi-a-GCST90002012 | Weighted median | 18 | 0.0109346089938379 | 0.0362126833572672 | 0.762686195004386 | -0.06004225 | 0.0819114683740816 | 1.01099461032838 | 0.941724744509359 | 1.08535971691554 | 0.999440741871876 |
| ebi-a-GCST90002012 | finngen_R11_O15_POOR_FETGRO | Poor fetal growth \|\| id:finngen_R11_O15_POOR_FETGRO | CX3CR1 on CD14- CD16+ monocyte \|\| id:ebi-a-GCST90002012 | Weighted mode | 18 | 0.00642274249809671 | 0.0326906277997475 | 0.846571520032187 | -0.057650888 | 0.0704963729856017 | 1.00644341253778 | 0.943979444479584 | 1.07304067748967 | 0.999857399747617 |
| ebi-a-GCST90002013 | finngen_R11_O15_POOR_FETGRO | Poor fetal growth \|\| id:finngen_R11_O15_POOR_FETGRO | CCR2 on myeloid Dendritic Cell \|\| id:ebi-a-GCST90002013 | Inverse variance weighted | 15 | 0.0229149688570085 | 0.0189908603021351 | 0.227574118362432 | -0.014307117 | 0.0601370550491933 | 1.02317953372293 | 0.985794743112965 | 1.06198208657875 | 0.994119304454654 |
| ebi-a-GCST90002013 | finngen_R11_O15_POOR_FETGRO | Poor fetal growth \|\| id:finngen_R11_O15_POOR_FETGRO | CCR2 on myeloid Dendritic Cell \|\| id:ebi-a-GCST90002013 | MR Egger | 15 | 0.0204974127012327 | 0.0236675436954687 | 0.402160824637482 | -0.025890973 | 0.0668857983443513 | 1.02070892736083 | 0.974441324288309 | 1.06917336983324 | 0.992899239648112 |
| ebi-a-GCST90002013 | finngen_R11_O15_POOR_FETGRO | Poor fetal growth \|\| id:finngen_R11_O15_POOR_FETGRO | CCR2 on myeloid Dendritic Cell \|\| id:ebi-a-GCST90002013 | Weighted median | 15 | 0.0221091849742513 | 0.0241818509020754 | 0.3605653415368 | -0.025287243 | 0.069505612742319 | 1.02235540422521 | 0.975029801516154 | 1.07197807792461 | 0.999440741871876 |
| ebi-a-GCST90002013 | finngen_R11_O15_POOR_FETGRO | Poor fetal growth \|\| id:finngen_R11_O15_POOR_FETGRO | CCR2 on myeloid Dendritic Cell \|\| id:ebi-a-GCST90002013 | Weighted mode | 15 | 0.0229196642120526 | 0.0224197619015403 | 0.323981460910588 | -0.021023069 | 0.0668623975390716 | 1.02318433792539 | 0.979196375115149 | 1.06914835060813 | 0.999857399747617 |
| ebi-a-GCST90002014 | finngen_R11_O15_POOR_FETGRO | Poor fetal growth \|\| id:finngen_R11_O15_POOR_FETGRO | CCR2 on CD62L+ myeloid Dendritic Cell \|\| id:ebi-a-GCST90002014 | Inverse variance weighted | 14 | 0.00060734422542926 | 0.0331261805231448 | 0.985372191255711 | -0.06431997 | 0.065534658050793 | 1.00060752869628 | 0.937704914457904 | 1.06772974210387 | 0.998984604424952 |
| ebi-a-GCST90002014 | finngen_R11_O15_POOR_FETGRO | Poor fetal growth \|\| id:finngen_R11_O15_POOR_FETGRO | CCR2 on CD62L+ myeloid Dendritic Cell \|\| id:ebi-a-GCST90002014 | MR Egger | 14 | -0.032950209 | 0.071787757705214 | 0.654440740880217 | -0.173654214 | 0.107753796533867 | 0.967586735918463 | 0.840587511080481 | 1.11377349673199 | 0.992899239648112 |
| ebi-a-GCST90002014 | finngen_R11_O15_POOR_FETGRO | Poor fetal growth \|\| id:finngen_R11_O15_POOR_FETGRO | CCR2 on CD62L+ myeloid Dendritic Cell \|\| id:ebi-a-GCST90002014 | Weighted median | 14 | 0.0306675515952419 | 0.0417221317452583 | 0.462313442257329 | -0.051107827 | 0.112442929815948 | 1.0311426451708 | 0.95017621071418 | 1.11900839307549 | 0.999440741871876 |
| ebi-a-GCST90002014 | finngen_R11_O15_POOR_FETGRO | Poor fetal growth \|\| id:finngen_R11_O15_POOR_FETGRO | CCR2 on CD62L+ myeloid Dendritic Cell \|\| id:ebi-a-GCST90002014 | Weighted mode | 14 | 0.0311510361024124 | 0.051859564849819 | 0.558380434928141 | -0.070493711 | 0.132795783208058 | 1.03164130720239 | 0.931933600434771 | 1.1420167555175 | 0.999857399747617 |
| ebi-a-GCST90002015 | finngen_R11_O15_POOR_FETGRO | Poor fetal growth \|\| id:finngen_R11_O15_POOR_FETGRO | CCR2 on plasmacytoid Dendritic Cell \|\| id:ebi-a-GCST90002015 | Inverse variance weighted | 19 | 0.00597921043679505 | 0.0244333527447919 | 0.806677032942033 | -0.041910161 | 0.0538685818165872 | 1.00599712159592 | 0.958955928399022 | 1.05534590140015 | 0.994119304454654 |
| ebi-a-GCST90002015 | finngen_R11_O15_POOR_FETGRO | Poor fetal growth \|\| id:finngen_R11_O15_POOR_FETGRO | CCR2 on plasmacytoid Dendritic Cell \|\| id:ebi-a-GCST90002015 | MR Egger | 19 | 0.0142453826900692 | 0.0361604779434505 | 0.698516313094105 | -0.056629154 | 0.0851199194592321 | 1.01434733167962 | 0.944944433185541 | 1.08884763288885 | 0.992899239648112 |
| ebi-a-GCST90002015 | finngen_R11_O15_POOR_FETGRO | Poor fetal growth \|\| id:finngen_R11_O15_POOR_FETGRO | CCR2 on plasmacytoid Dendritic Cell \|\| id:ebi-a-GCST90002015 | Weighted median | 19 | -0.008173603 | 0.0360963616433552 | 0.820860453160771 | -0.078922472 | 0.0625752659164256 | 0.991859710163286 | 0.924111566443078 | 1.06457458208407 | 0.999440741871876 |
| ebi-a-GCST90002015 | finngen_R11_O15_POOR_FETGRO | Poor fetal growth \|\| id:finngen_R11_O15_POOR_FETGRO | CCR2 on plasmacytoid Dendritic Cell \|\| id:ebi-a-GCST90002015 | Weighted mode | 19 | -0.012106261 | 0.0350198248204599 | 0.733577584392855 | -0.080745117 | 0.0565325961179151 | 0.987966725415969 | 0.922428772732667 | 1.05816110618227 | 0.999857399747617 |
| ebi-a-GCST90002016 | finngen_R11_O15_POOR_FETGRO | Poor fetal growth \|\| id:finngen_R11_O15_POOR_FETGRO | CCR2 on CD62L+ plasmacytoid Dendritic Cell \|\| id:ebi-a-GCST90002016 | Inverse variance weighted | 19 | 0.00472188706684543 | 0.0246122180113221 | 0.847858652404983 | -0.04351806 | 0.0529618343690368 | 1.00473305274302 | 0.957415262789855 | 1.05438940291458 | 0.994119304454654 |
| ebi-a-GCST90002016 | finngen_R11_O15_POOR_FETGRO | Poor fetal growth \|\| id:finngen_R11_O15_POOR_FETGRO | CCR2 on CD62L+ plasmacytoid Dendritic Cell \|\| id:ebi-a-GCST90002016 | MR Egger | 19 | 0.0159674846734876 | 0.0362512045754433 | 0.66514793236815 | -0.055084876 | 0.0870198456413566 | 1.01609564618725 | 0.946404817210575 | 1.09091832947737 | 0.992899239648112 |
| ebi-a-GCST90002016 | finngen_R11_O15_POOR_FETGRO | Poor fetal growth \|\| id:finngen_R11_O15_POOR_FETGRO | CCR2 on CD62L+ plasmacytoid Dendritic Cell \|\| id:ebi-a-GCST90002016 | Weighted median | 19 | -0.00915124 | 0.0358869987437728 | 0.79872190083365 | -0.079489758 | 0.0611872771757143 | 0.990890504800944 | 0.923587479395602 | 1.06309798953447 | 0.999440741871876 |
| ebi-a-GCST90002016 | finngen_R11_O15_POOR_FETGRO | Poor fetal growth \|\| id:finngen_R11_O15_POOR_FETGRO | CCR2 on CD62L+ plasmacytoid Dendritic Cell \|\| id:ebi-a-GCST90002016 | Weighted mode | 19 | -0.013170877 | 0.033492404901437 | 0.698753854018152 | -0.078815991 | 0.0524742362495454 | 0.98691547910204 | 0.924209971785372 | 1.0538754099457 | 0.999857399747617 |
| ebi-a-GCST90002017 | finngen_R11_O15_POOR_FETGRO | Poor fetal growth \|\| id:finngen_R11_O15_POOR_FETGRO | CCR2 on monocyte \|\| id:ebi-a-GCST90002017 | Inverse variance weighted | 25 | 0.0397052684703431 | 0.0292639969316138 | 0.174846098684582 | -0.017652166 | 0.0970627024563062 | 1.04050405964227 | 0.982502721256715 | 1.10192946513902 | 0.994119304454654 |
| ebi-a-GCST90002017 | finngen_R11_O15_POOR_FETGRO | Poor fetal growth \|\| id:finngen_R11_O15_POOR_FETGRO | CCR2 on monocyte \|\| id:ebi-a-GCST90002017 | MR Egger | 25 | -0.055408915 | 0.0559039225297246 | 0.331928004704142 | -0.164980603 | 0.0541627734648892 | 0.946098195390115 | 0.847910150967406 | 1.05565642102431 | 0.992899239648112 |
| ebi-a-GCST90002017 | finngen_R11_O15_POOR_FETGRO | Poor fetal growth \|\| id:finngen_R11_O15_POOR_FETGRO | CCR2 on monocyte \|\| id:ebi-a-GCST90002017 | Weighted median | 25 | 0.0634885174415178 | 0.04044891040766 | 0.116508729925971 | -0.015791347 | 0.142768381840531 | 1.0655472505226 | 0.984332682638845 | 1.15346260783749 | 0.999440741871876 |
| ebi-a-GCST90002017 | finngen_R11_O15_POOR_FETGRO | Poor fetal growth \|\| id:finngen_R11_O15_POOR_FETGRO | CCR2 on monocyte \|\| id:ebi-a-GCST90002017 | Weighted mode | 25 | 0.0527480738636916 | 0.0505612383969845 | 0.30722713800069 | -0.046351953 | 0.151848101121781 | 1.05416404019067 | 0.954705891074483 | 1.16398341522794 | 0.999857399747617 |
| ebi-a-GCST90002018 | finngen_R11_O15_POOR_FETGRO | Poor fetal growth \|\| id:finngen_R11_O15_POOR_FETGRO | CCR2 on granulocyte \|\| id:ebi-a-GCST90002018 | Inverse variance weighted | 16 | 0.0128614163968425 | 0.0274207329022584 | 0.639041153373187 | -0.04088322 | 0.066606052885269 | 1.01294448013649 | 0.959941225250802 | 1.06887431526958 | 0.994119304454654 |
| ebi-a-GCST90002018 | finngen_R11_O15_POOR_FETGRO | Poor fetal growth \|\| id:finngen_R11_O15_POOR_FETGRO | CCR2 on granulocyte \|\| id:ebi-a-GCST90002018 | MR Egger | 16 | 0.0490779117520485 | 0.0366464215836584 | 0.201834695245968 | -0.022749075 | 0.120904898056019 | 1.05030217843522 | 0.977507734568454 | 1.12851758304785 | 0.992899239648112 |
| ebi-a-GCST90002018 | finngen_R11_O15_POOR_FETGRO | Poor fetal growth \|\| id:finngen_R11_O15_POOR_FETGRO | CCR2 on granulocyte \|\| id:ebi-a-GCST90002018 | Weighted median | 16 | 0.0411042165319316 | 0.0387143434678144 | 0.288357846196885 | -0.034775897 | 0.116984329728848 | 1.04196068941672 | 0.965821835900935 | 1.12410181457228 | 0.999440741871876 |
| ebi-a-GCST90002018 | finngen_R11_O15_POOR_FETGRO | Poor fetal growth \|\| id:finngen_R11_O15_POOR_FETGRO | CCR2 on granulocyte \|\| id:ebi-a-GCST90002018 | Weighted mode | 16 | 0.0379223425248088 | 0.0345944433153708 | 0.290280407514598 | -0.029882766 | 0.105727451422936 | 1.0386505707656 | 0.970559309066931 | 1.11151889232697 | 0.999857399747617 |
| ebi-a-GCST90002019 | finngen_R11_O15_POOR_FETGRO | Poor fetal growth \|\| id:finngen_R11_O15_POOR_FETGRO | CD14 on Monocytic Myeloid-Derived Suppressor Cells \|\| id:ebi-a-GCST90002019 | Inverse variance weighted | 24 | -0.011332891 | 0.0190142515494429 | 0.551161286942235 | -0.048600824 | 0.0259350423498103 | 0.988731084615461 | 0.952561293687105 | 1.02627428194241 | 0.994119304454654 |
| ebi-a-GCST90002019 | finngen_R11_O15_POOR_FETGRO | Poor fetal growth \|\| id:finngen_R11_O15_POOR_FETGRO | CD14 on Monocytic Myeloid-Derived Suppressor Cells \|\| id:ebi-a-GCST90002019 | MR Egger | 24 | -0.033500497 | 0.0264633319503757 | 0.218783150372645 | -0.085368628 | 0.0183676332264156 | 0.967054430221968 | 0.918173757436674 | 1.01853735574275 | 0.992899239648112 |
| ebi-a-GCST90002019 | finngen_R11_O15_POOR_FETGRO | Poor fetal growth \|\| id:finngen_R11_O15_POOR_FETGRO | CD14 on Monocytic Myeloid-Derived Suppressor Cells \|\| id:ebi-a-GCST90002019 | Weighted median | 24 | -0.028098617 | 0.0255189918651609 | 0.270859014250793 | -0.078115841 | 0.0219186067525261 | 0.972292477211125 | 0.924857283613341 | 1.02216058411513 | 0.999440741871876 |
| ebi-a-GCST90002019 | finngen_R11_O15_POOR_FETGRO | Poor fetal growth \|\| id:finngen_R11_O15_POOR_FETGRO | CD14 on Monocytic Myeloid-Derived Suppressor Cells \|\| id:ebi-a-GCST90002019 | Weighted mode | 24 | -0.026921658 | 0.0218936903779846 | 0.231257656466555 | -0.069833291 | 0.0159899753224747 | 0.973437499754586 | 0.932549271342509 | 1.01611849909476 | 0.999857399747617 |
| ebi-a-GCST90002020 | finngen_R11_O15_POOR_FETGRO | Poor fetal growth \|\| id:finngen_R11_O15_POOR_FETGRO | CD14 on CD33+ HLA DR+ CD14dim \|\| id:ebi-a-GCST90002020 | Inverse variance weighted | 16 | 0.014805203900998 | 0.0312105937096286 | 0.635239861506883 | -0.04636756 | 0.07597796757187 | 1.01491534380893 | 0.954690991692141 | 1.07893880225379 | 0.994119304454654 |
| ebi-a-GCST90002020 | finngen_R11_O15_POOR_FETGRO | Poor fetal growth \|\| id:finngen_R11_O15_POOR_FETGRO | CD14 on CD33+ HLA DR+ CD14dim \|\| id:ebi-a-GCST90002020 | MR Egger | 16 | -0.142508412 | 0.0704897191163793 | 0.0627525906598901 | -0.280668261 | -0.004348562 | 0.86718026 | 0.75527884911234 | 0.995660878894965 | 0.992899239648112 |
| ebi-a-GCST90002020 | finngen_R11_O15_POOR_FETGRO | Poor fetal growth \|\| id:finngen_R11_O15_POOR_FETGRO | CD14 on CD33+ HLA DR+ CD14dim \|\| id:ebi-a-GCST90002020 | Weighted median | 16 | -0.022498947 | 0.0432984732691508 | 0.603325330340992 | -0.107363954 | 0.0623660608186179 | 0.977752266972317 | 0.89819871012469 | 1.06435189094938 | 0.999440741871876 |
| ebi-a-GCST90002020 | finngen_R11_O15_POOR_FETGRO | Poor fetal growth \|\| id:finngen_R11_O15_POOR_FETGRO | CD14 on CD33+ HLA DR+ CD14dim \|\| id:ebi-a-GCST90002020 | Weighted mode | 16 | -0.053717767 | 0.0552313042989268 | 0.346177859246777 | -0.161971124 | 0.054535589095853 | 0.947699540532354 | 0.850465762442582 | 1.05605005961174 | 0.999857399747617 |
| ebi-a-GCST90002021 | finngen_R11_O15_POOR_FETGRO | Poor fetal growth \|\| id:finngen_R11_O15_POOR_FETGRO | CD14 on CD33dim HLA DR+ CD11b+ \|\| id:ebi-a-GCST90002021 | Inverse variance weighted | 19 | -0.003781522 | 0.0283786284936341 | 0.893993737768957 | -0.059403633 | 0.0518405902961602 | 0.996225619397206 | 0.942326338002237 | 1.05320783758141 | 0.994119304454654 |
| ebi-a-GCST90002021 | finngen_R11_O15_POOR_FETGRO | Poor fetal growth \|\| id:finngen_R11_O15_POOR_FETGRO | CD14 on CD33dim HLA DR+ CD11b+ \|\| id:ebi-a-GCST90002021 | MR Egger | 19 | 0.0259337620667255 | 0.0591967033203341 | 0.666833463983355 | -0.090091776 | 0.14195930057458 | 1.02627296802165 | 0.913847311768471 | 1.15252974028423 | 0.992899239648112 |
| ebi-a-GCST90002021 | finngen_R11_O15_POOR_FETGRO | Poor fetal growth \|\| id:finngen_R11_O15_POOR_FETGRO | CD14 on CD33dim HLA DR+ CD11b+ \|\| id:ebi-a-GCST90002021 | Weighted median | 19 | -0.022114253 | 0.0333757276598478 | 0.507596441300915 | -0.08753068 | 0.0433021729156075 | 0.978128474263059 | 0.916190762935113 | 1.044253392 | 0.999440741871876 |
| ebi-a-GCST90002021 | finngen_R11_O15_POOR_FETGRO | Poor fetal growth \|\| id:finngen_R11_O15_POOR_FETGRO | CD14 on CD33dim HLA DR+ CD11b+ \|\| id:ebi-a-GCST90002021 | Weighted mode | 19 | -0.023285417 | 0.0477955049939965 | 0.632004877595433 | -0.116964607 | 0.0703937727652333 | 0.976983596225989 | 0.889616678977266 | 1.07293058892734 | 0.999857399747617 |
| ebi-a-GCST90002022 | finngen_R11_O15_POOR_FETGRO | Poor fetal growth \|\| id:finngen_R11_O15_POOR_FETGRO | CD4 on CD4+ T cell \|\| id:ebi-a-GCST90002022 | Inverse variance weighted | 22 | -0.010267605 | 0.0284847202991373 | 0.718503098851429 | -0.066097657 | 0.0455624470138898 | 0.989784927135442 | 0.936039449436595 | 1.04661636063969 | 0.994119304454654 |
| ebi-a-GCST90002022 | finngen_R11_O15_POOR_FETGRO | Poor fetal growth \|\| id:finngen_R11_O15_POOR_FETGRO | CD4 on CD4+ T cell \|\| id:ebi-a-GCST90002022 | MR Egger | 22 | 0.0602183632122791 | 0.0576746132862648 | 0.308893283064645 | -0.052823879 | 0.173260605253358 | 1.06206843790193 | 0.948547056990445 | 1.18917597021104 | 0.992899239648112 |
| ebi-a-GCST90002022 | finngen_R11_O15_POOR_FETGRO | Poor fetal growth \|\| id:finngen_R11_O15_POOR_FETGRO | CD4 on CD4+ T cell \|\| id:ebi-a-GCST90002022 | Weighted median | 22 | 0.00107486624200681 | 0.0392261475890845 | 0.978139279046014 | -0.075808383 | 0.0779581155166125 | 1.00107544411775 | 0.926993817286292 | 1.08107737735435 | 0.999440741871876 |
| ebi-a-GCST90002022 | finngen_R11_O15_POOR_FETGRO | Poor fetal growth \|\| id:finngen_R11_O15_POOR_FETGRO | CD4 on CD4+ T cell \|\| id:ebi-a-GCST90002022 | Weighted mode | 22 | 0.021454088858134 | 0.0509056455759013 | 0.677710446865801 | -0.078320976 | 0.121229154186901 | 1.0216858824951 | 0.924667582368993 | 1.12888357112669 | 0.999857399747617 |
| ebi-a-GCST90002023 | finngen_R11_O15_POOR_FETGRO | Poor fetal growth \|\| id:finngen_R11_O15_POOR_FETGRO | CD4 on Central Memory CD4+ T cell \|\| id:ebi-a-GCST90002023 | Inverse variance weighted | 15 | -0.016948241 | 0.0315214628042045 | 0.590802798758622 | -0.078730308 | 0.0448338265351809 | 0.983194572917387 | 0.924289164544621 | 1.04585405227645 | 0.994119304454654 |
| ebi-a-GCST90002023 | finngen_R11_O15_POOR_FETGRO | Poor fetal growth \|\| id:finngen_R11_O15_POOR_FETGRO | CD4 on Central Memory CD4+ T cell \|\| id:ebi-a-GCST90002023 | MR Egger | 15 | 0.078227152843419 | 0.0640021547736153 | 0.243308916962265 | -0.047217071 | 0.203671376199705 | 1.0813682666503 | 0.953880315826142 | 1.22589522890565 | 0.992899239648112 |
| ebi-a-GCST90002023 | finngen_R11_O15_POOR_FETGRO | Poor fetal growth \|\| id:finngen_R11_O15_POOR_FETGRO | CD4 on Central Memory CD4+ T cell \|\| id:ebi-a-GCST90002023 | Weighted median | 15 | 0.00304780402615171 | 0.0437358520710452 | 0.944443085714398 | -0.082674466 | 0.0887700740854002 | 1.003052453 | 0.920650801551209 | 1.09282935764784 | 0.999440741871876 |
| ebi-a-GCST90002023 | finngen_R11_O15_POOR_FETGRO | Poor fetal growth \|\| id:finngen_R11_O15_POOR_FETGRO | CD4 on Central Memory CD4+ T cell \|\| id:ebi-a-GCST90002023 | Weighted mode | 15 | 0.0464572619197656 | 0.064804700873459 | 0.485236357013983 | -0.080559952 | 0.173474475631745 | 1.04755330769451 | 0.92259959042677 | 1.18943032692447 | 0.999857399747617 |
| ebi-a-GCST90002024 | finngen_R11_O15_POOR_FETGRO | Poor fetal growth \|\| id:finngen_R11_O15_POOR_FETGRO | CD4 on naive CD4+ T cell \|\| id:ebi-a-GCST90002024 | Inverse variance weighted (multiplicative random effects) | 20 | -0.015760191 | 0.0437398968967234 | 0.718610726305825 | -0.101490389 | 0.0699700068395027 | 0.98436335086696 | 0.903489862745491 | 1.07247601382661 | 0.994119304454654 |
| ebi-a-GCST90002024 | finngen_R11_O15_POOR_FETGRO | Poor fetal growth \|\| id:finngen_R11_O15_POOR_FETGRO | CD4 on naive CD4+ T cell \|\| id:ebi-a-GCST90002024 | MR Egger | 20 | 0.0432969768860377 | 0.0950096998184127 | 0.654047404605396 | -0.142922035 | 0.229515988530127 | 1.04424796631559 | 0.866821648220565 | 1.2579909804892 | 0.992899239648112 |
| ebi-a-GCST90002024 | finngen_R11_O15_POOR_FETGRO | Poor fetal growth \|\| id:finngen_R11_O15_POOR_FETGRO | CD4 on naive CD4+ T cell \|\| id:ebi-a-GCST90002024 | Weighted median | 20 | -0.045921411 | 0.0455534673337197 | 0.313417414358116 | -0.135206207 | 0.0433633853211364 | 0.955117021264308 | 0.873535764251326 | 1.04431731549155 | 0.999440741871876 |
| ebi-a-GCST90002024 | finngen_R11_O15_POOR_FETGRO | Poor fetal growth \|\| id:finngen_R11_O15_POOR_FETGRO | CD4 on naive CD4+ T cell \|\| id:ebi-a-GCST90002024 | Weighted mode | 20 | -0.046905046 | 0.057919743440246 | 0.428065317293718 | -0.160427743 | 0.0666176508990526 | 0.954177996073752 | 0.85177936804083 | 1.06888671216051 | 0.999857399747617 |
| ebi-a-GCST90002025 | finngen_R11_O15_POOR_FETGRO | Poor fetal growth \|\| id:finngen_R11_O15_POOR_FETGRO | CD4 on Effector Memory CD4+ T cell \|\| id:ebi-a-GCST90002025 | Inverse variance weighted | 19 | -0.008338226 | 0.0310871905336304 | 0.788529487689839 | -0.06926912 | 0.0525926672736734 | 0.991696440416076 | 0.933075537353676 | 1.05400022888087 | 0.994119304454654 |
| ebi-a-GCST90002025 | finngen_R11_O15_POOR_FETGRO | Poor fetal growth \|\| id:finngen_R11_O15_POOR_FETGRO | CD4 on Effector Memory CD4+ T cell \|\| id:ebi-a-GCST90002025 | MR Egger | 19 | -0.043432323 | 0.0478229447975678 | 0.376473053944639 | -0.137165295 | 0.0503006486319667 | 0.957497352282598 | 0.871826105747333 | 1.05158720710972 | 0.992899239648112 |
| ebi-a-GCST90002025 | finngen_R11_O15_POOR_FETGRO | Poor fetal growth \|\| id:finngen_R11_O15_POOR_FETGRO | CD4 on Effector Memory CD4+ T cell \|\| id:ebi-a-GCST90002025 | Weighted median | 19 | -0.012523055 | 0.0367825145843824 | 0.733508509030878 | -0.084616783 | 0.059570673954832 | 0.987555032514999 | 0.918864341177923 | 1.06138077030552 | 0.999440741871876 |
| ebi-a-GCST90002025 | finngen_R11_O15_POOR_FETGRO | Poor fetal growth \|\| id:finngen_R11_O15_POOR_FETGRO | CD4 on Effector Memory CD4+ T cell \|\| id:ebi-a-GCST90002025 | Weighted mode | 19 | -0.03028707 | 0.0329387414403952 | 0.369994412030424 | -0.094847003 | 0.0342728632356049 | 0.970166987744238 | 0.909512076251293 | 1.03486694534958 | 0.999857399747617 |
| ebi-a-GCST90002026 | finngen_R11_O15_POOR_FETGRO | Poor fetal growth \|\| id:finngen_R11_O15_POOR_FETGRO | CD4 on Terminally Differentiated CD4+ T cell \|\| id:ebi-a-GCST90002026 | Inverse variance weighted | 24 | -0.022263534 | 0.0286379145452014 | 0.436914171184221 | -0.078393847 | 0.0338667781611368 | 0.977982469115789 | 0.924600203940856 | 1.03444678664488 | 0.994119304454654 |
| ebi-a-GCST90002026 | finngen_R11_O15_POOR_FETGRO | Poor fetal growth \|\| id:finngen_R11_O15_POOR_FETGRO | CD4 on Terminally Differentiated CD4+ T cell \|\| id:ebi-a-GCST90002026 | MR Egger | 24 | -0.024692308 | 0.0505105034521476 | 0.629782353389758 | -0.123692895 | 0.0743082787786934 | 0.975610053271124 | 0.883651173127985 | 1.07713881335597 | 0.992899239648112 |
| ebi-a-GCST90002026 | finngen_R11_O15_POOR_FETGRO | Poor fetal growth \|\| id:finngen_R11_O15_POOR_FETGRO | CD4 on Terminally Differentiated CD4+ T cell \|\| id:ebi-a-GCST90002026 | Weighted median | 24 | -0.019936401 | 0.0380554764825239 | 0.600363879210777 | -0.094525134 | 0.0546523333580018 | 0.980261015387919 | 0.909804866890534 | 1.05617335459359 | 0.999440741871876 |
| ebi-a-GCST90002026 | finngen_R11_O15_POOR_FETGRO | Poor fetal growth \|\| id:finngen_R11_O15_POOR_FETGRO | CD4 on Terminally Differentiated CD4+ T cell \|\| id:ebi-a-GCST90002026 | Weighted mode | 24 | -0.018021232 | 0.0411442578070471 | 0.665471486155892 | -0.098663977 | 0.0626215134388121 | 0.982140179471191 | 0.906047109396291 | 1.06462381715938 | 0.999857399747617 |
| ebi-a-GCST90002027 | finngen_R11_O15_POOR_FETGRO | Poor fetal growth \|\| id:finngen_R11_O15_POOR_FETGRO | CD4 on CD45RA+ CD4+ T cell \|\| id:ebi-a-GCST90002027 | Inverse variance weighted | 27 | -0.018622872 | 0.0261232384458779 | 0.475916756936994 | -0.069824419 | 0.0325786755525101 | 0.981549462431404 | 0.932557544773466 | 1.03311517085352 | 0.994119304454654 |
| ebi-a-GCST90002027 | finngen_R11_O15_POOR_FETGRO | Poor fetal growth \|\| id:finngen_R11_O15_POOR_FETGRO | CD4 on CD45RA+ CD4+ T cell \|\| id:ebi-a-GCST90002027 | MR Egger | 27 | 0.0439976657116132 | 0.0508763532824335 | 0.395373370510499 | -0.055719987 | 0.143715318145183 | 1.04497991560112 | 0.945803936475465 | 1.15455538076843 | 0.992899239648112 |
| ebi-a-GCST90002027 | finngen_R11_O15_POOR_FETGRO | Poor fetal growth \|\| id:finngen_R11_O15_POOR_FETGRO | CD4 on CD45RA+ CD4+ T cell \|\| id:ebi-a-GCST90002027 | Weighted median | 27 | -0.003031269 | 0.0387106806830416 | 0.937584849751859 | -0.078904203 | 0.0728416655507735 | 0.996973321067974 | 0.92412844919037 | 1.07556022519609 | 0.999440741871876 |
| ebi-a-GCST90002027 | finngen_R11_O15_POOR_FETGRO | Poor fetal growth \|\| id:finngen_R11_O15_POOR_FETGRO | CD4 on CD45RA+ CD4+ T cell \|\| id:ebi-a-GCST90002027 | Weighted mode | 27 | 0.0281172198667072 | 0.0497833420714507 | 0.577056436939642 | -0.069458131 | 0.125692570326751 | 1.02851623989209 | 0.932899192502527 | 1.13393350988337 | 0.999857399747617 |
| ebi-a-GCST90002028 | finngen_R11_O15_POOR_FETGRO | Poor fetal growth \|\| id:finngen_R11_O15_POOR_FETGRO | CD19 on B cell \|\| id:ebi-a-GCST90002028 | Inverse variance weighted | 24 | -0.007014449 | 0.0223380149235643 | 0.753510419000047 | -0.050796958 | 0.0367680602222951 | 0.993010094799021 | 0.950471636339391 | 1.03745236645932 | 0.994119304454654 |
| ebi-a-GCST90002028 | finngen_R11_O15_POOR_FETGRO | Poor fetal growth \|\| id:finngen_R11_O15_POOR_FETGRO | CD19 on B cell \|\| id:ebi-a-GCST90002028 | MR Egger | 24 | -0.008862213 | 0.0291175929399654 | 0.763714417573597 | -0.065932696 | 0.0482082687362885 | 0.991176940239279 | 0.936193872148973 | 1.04938918752693 | 0.992899239648112 |
| ebi-a-GCST90002028 | finngen_R11_O15_POOR_FETGRO | Poor fetal growth \|\| id:finngen_R11_O15_POOR_FETGRO | CD19 on B cell \|\| id:ebi-a-GCST90002028 | Weighted median | 24 | 0.0037983572641262 | 0.0330462462463358 | 0.908492158786481 | -0.060972285 | 0.0685689999069443 | 1.00380558016524 | 0.940849314697145 | 1.07097451954378 | 0.999440741871876 |
| ebi-a-GCST90002028 | finngen_R11_O15_POOR_FETGRO | Poor fetal growth \|\| id:finngen_R11_O15_POOR_FETGRO | CD19 on B cell \|\| id:ebi-a-GCST90002028 | Weighted mode | 24 | 0.00496370896408527 | 0.0265985435159103 | 0.853598135238248 | -0.047169436 | 0.0570968542552695 | 1.00497604857572 | 0.953925754220508 | 1.05875835068125 | 0.999857399747617 |
| ebi-a-GCST90002029 | finngen_R11_O15_POOR_FETGRO | Poor fetal growth \|\| id:finngen_R11_O15_POOR_FETGRO | CD39 on CD39+ CD8+ T cell \|\| id:ebi-a-GCST90002029 | Inverse variance weighted | 23 | 0.0178895588935023 | 0.0301542799335296 | 0.553002042762319 | -0.04121283 | 0.0769919475632202 | 1.01805053555325 | 0.959624871465781 | 1.08003337945708 | 0.994119304454654 |
| ebi-a-GCST90002029 | finngen_R11_O15_POOR_FETGRO | Poor fetal growth \|\| id:finngen_R11_O15_POOR_FETGRO | CD39 on CD39+ CD8+ T cell \|\| id:ebi-a-GCST90002029 | MR Egger | 23 | 0.0488333562397136 | 0.0639769223120691 | 0.453776903670859 | -0.076561411 | 0.174228123971369 | 1.05004535265323 | 0.926296027321066 | 1.19032707699011 | 0.992899239648112 |
| ebi-a-GCST90002029 | finngen_R11_O15_POOR_FETGRO | Poor fetal growth \|\| id:finngen_R11_O15_POOR_FETGRO | CD39 on CD39+ CD8+ T cell \|\| id:ebi-a-GCST90002029 | Weighted median | 23 | 0.025779074561008 | 0.0421636496627346 | 0.54093136244032 | -0.056861679 | 0.108419827899968 | 1.02611422869385 | 0.9447247358093 | 1.11451555190412 | 0.999440741871876 |
| ebi-a-GCST90002029 | finngen_R11_O15_POOR_FETGRO | Poor fetal growth \|\| id:finngen_R11_O15_POOR_FETGRO | CD39 on CD39+ CD8+ T cell \|\| id:ebi-a-GCST90002029 | Weighted mode | 23 | 0.01216649526599 | 0.0560492731253021 | 0.830155892226237 | -0.09769008 | 0.122023070591582 | 1.01224080813946 | 0.906929935874614 | 1.12978016617645 | 0.999857399747617 |
| ebi-a-GCST90002030 | finngen_R11_O15_POOR_FETGRO | Poor fetal growth \|\| id:finngen_R11_O15_POOR_FETGRO | CD39 on CD39+ activated CD4 regulatory T cell \|\| id:ebi-a-GCST90002030 | Inverse variance weighted | 22 | 0.00504787992137148 | 0.0248196164349441 | 0.838835942114504 | -0.043598568 | 0.0536943281338618 | 1.00506064193188 | 0.957338186251163 | 1.05516201951179 | 0.994119304454654 |
| ebi-a-GCST90002030 | finngen_R11_O15_POOR_FETGRO | Poor fetal growth \|\| id:finngen_R11_O15_POOR_FETGRO | CD39 on CD39+ activated CD4 regulatory T cell \|\| id:ebi-a-GCST90002030 | MR Egger | 22 | -0.02108743 | 0.0552794346007961 | 0.706877268823962 | -0.129435122 | 0.0872602615063486 | 0.979133354893138 | 0.87859158771912 | 1.09118063508113 | 0.992899239648112 |
| ebi-a-GCST90002030 | finngen_R11_O15_POOR_FETGRO | Poor fetal growth \|\| id:finngen_R11_O15_POOR_FETGRO | CD39 on CD39+ activated CD4 regulatory T cell \|\| id:ebi-a-GCST90002030 | Weighted median | 22 | -0.019721939 | 0.0347638040429593 | 0.570501924993883 | -0.087858995 | 0.0484151173418307 | 0.980471266636502 | 0.91589001314187 | 1.04960627466835 | 0.999440741871876 |
| ebi-a-GCST90002030 | finngen_R11_O15_POOR_FETGRO | Poor fetal growth \|\| id:finngen_R11_O15_POOR_FETGRO | CD39 on CD39+ activated CD4 regulatory T cell \|\| id:ebi-a-GCST90002030 | Weighted mode | 22 | -0.039153047 | 0.0377141685522815 | 0.311005765666575 | -0.113072817 | 0.034766723338406 | 0.961603527325596 | 0.893085625587013 | 1.03537815106726 | 0.999857399747617 |
| ebi-a-GCST90002031 | finngen_R11_O15_POOR_FETGRO | Poor fetal growth \|\| id:finngen_R11_O15_POOR_FETGRO | CD39 on CD39+ secreting CD4 regulatory T cell \|\| id:ebi-a-GCST90002031 | Inverse variance weighted | 23 | 0.00930421403540252 | 0.0142620510918987 | 0.514158905568325 | -0.018649406 | 0.0372578341755239 | 1.00934763278947 | 0.981523418045792 | 1.03796060805771 | 0.994119304454654 |
| ebi-a-GCST90002031 | finngen_R11_O15_POOR_FETGRO | Poor fetal growth \|\| id:finngen_R11_O15_POOR_FETGRO | CD39 on CD39+ secreting CD4 regulatory T cell \|\| id:ebi-a-GCST90002031 | MR Egger | 23 | 0.0104892960613098 | 0.0198862067244589 | 0.603398894418153 | -0.028487669 | 0.0494662612412492 | 1.01054450158075 | 0.971914278631919 | 1.05071014196081 | 0.992899239648112 |
| ebi-a-GCST90002031 | finngen_R11_O15_POOR_FETGRO | Poor fetal growth \|\| id:finngen_R11_O15_POOR_FETGRO | CD39 on CD39+ secreting CD4 regulatory T cell \|\| id:ebi-a-GCST90002031 | Weighted median | 23 | 0.0146460201599376 | 0.0179266837510268 | 0.413930993 | -0.02049028 | 0.0497823203119502 | 1.01475379864567 | 0.979718219297093 | 1.05104228091685 | 0.999440741871876 |
| ebi-a-GCST90002031 | finngen_R11_O15_POOR_FETGRO | Poor fetal growth \|\| id:finngen_R11_O15_POOR_FETGRO | CD39 on CD39+ secreting CD4 regulatory T cell \|\| id:ebi-a-GCST90002031 | Weighted mode | 23 | 0.0122247955963795 | 0.0163252067711323 | 0.461891794928182 | -0.01977261 | 0.0442222008677988 | 1.0122998238333 | 0.980421586344778 | 1.04521457667352 | 0.999857399747617 |
| ebi-a-GCST90002032 | finngen_R11_O15_POOR_FETGRO | Poor fetal growth \|\| id:finngen_R11_O15_POOR_FETGRO | CD39 on CD39+ CD4+ T cell \|\| id:ebi-a-GCST90002032 | Inverse variance weighted | 20 | 0.0081066836901454 | 0.0206303312203882 | 0.694356588799115 | -0.032328766 | 0.0485421328821063 | 1.00813963182356 | 0.96818822286236 | 1.04973959944339 | 0.994119304454654 |
| ebi-a-GCST90002032 | finngen_R11_O15_POOR_FETGRO | Poor fetal growth \|\| id:finngen_R11_O15_POOR_FETGRO | CD39 on CD39+ CD4+ T cell \|\| id:ebi-a-GCST90002032 | MR Egger | 20 | 0.0344679683348509 | 0.0318157539884132 | 0.292947098082769 | -0.027890909 | 0.0968268461521408 | 1.03506887286556 | 0.972494450937733 | 1.10166959877469 | 0.992899239648112 |
| ebi-a-GCST90002032 | finngen_R11_O15_POOR_FETGRO | Poor fetal growth \|\| id:finngen_R11_O15_POOR_FETGRO | CD39 on CD39+ CD4+ T cell \|\| id:ebi-a-GCST90002032 | Weighted median | 20 | 0.0172344955027777 | 0.0199305992702929 | 0.387189522744026 | -0.021829479 | 0.0562984700725519 | 1.01738386629665 | 0.978407059711937 | 1.05791339210641 | 0.999440741871876 |
| ebi-a-GCST90002032 | finngen_R11_O15_POOR_FETGRO | Poor fetal growth \|\| id:finngen_R11_O15_POOR_FETGRO | CD39 on CD39+ CD4+ T cell \|\| id:ebi-a-GCST90002032 | Weighted mode | 20 | 0.0111178735181714 | 0.0199422596893917 | 0.583692899041335 | -0.027968955 | 0.0502047025093791 | 1.01117990675336 | 0.9724185546067 | 1.05148631623476 | 0.999857399747617 |
| ebi-a-GCST90002033 | finngen_R11_O15_POOR_FETGRO | Poor fetal growth \|\| id:finngen_R11_O15_POOR_FETGRO | CD39 on granulocyte \|\| id:ebi-a-GCST90002033 | Inverse variance weighted | 26 | -0.01633914 | 0.0255006698936199 | 0.521695679892025 | -0.066320453 | 0.033642173038941 | 0.983793619751406 | 0.935830926460689 | 1.03421447068861 | 0.994119304454654 |
| ebi-a-GCST90002033 | finngen_R11_O15_POOR_FETGRO | Poor fetal growth \|\| id:finngen_R11_O15_POOR_FETGRO | CD39 on granulocyte \|\| id:ebi-a-GCST90002033 | MR Egger | 26 | -0.014795979 | 0.0377577279718449 | 0.698615063446051 | -0.088801126 | 0.059209167701749 | 0.985312943508657 | 0.915027530713407 | 1.06099714386601 | 0.992899239648112 |
| ebi-a-GCST90002033 | finngen_R11_O15_POOR_FETGRO | Poor fetal growth \|\| id:finngen_R11_O15_POOR_FETGRO | CD39 on granulocyte \|\| id:ebi-a-GCST90002033 | Weighted median | 26 | -0.027257437 | 0.0385542535677549 | 0.479573244026613 | -0.102823774 | 0.0483088994975453 | 0.973110694095876 | 0.902285965278053 | 1.04949479367325 | 0.999440741871876 |
| ebi-a-GCST90002033 | finngen_R11_O15_POOR_FETGRO | Poor fetal growth \|\| id:finngen_R11_O15_POOR_FETGRO | CD39 on granulocyte \|\| id:ebi-a-GCST90002033 | Weighted mode | 26 | -0.032953317 | 0.0381132106126302 | 0.39546993292319 | -0.10765521 | 0.0417485754425761 | 0.967583727899337 | 0.897937142667877 | 1.04263230242817 | 0.999857399747617 |
| ebi-a-GCST90002034 | finngen_R11_O15_POOR_FETGRO | Poor fetal growth \|\| id:finngen_R11_O15_POOR_FETGRO | CD39 on monocyte \|\| id:ebi-a-GCST90002034 | Inverse variance weighted | 22 | 0.00249242207405346 | 0.0126378100581825 | 0.843655802871448 | -0.022277686 | 0.0272625297880911 | 1.00249553074012 | 0.977968629497708 | 1.0276375528222 | 0.994119304454654 |
[truncated: 128,072 more chars]
