# Supplementary material for: Mendelian Randomization Mediation Analysis Reveals the Impact of Dietary Preferences on Preeclampsia and Fetal Growth Restriction via Immune Modulation
Source: Food Sci Nutr. 2025 Nov 26;13(12):e71280. doi: 10.1002/fsn3.71280 (PMC12648301; doi:10.1002/fsn3.71280)
Supplement: Supplementary file 1 — Data S1: fsn371280‐sup‐0001‐Supinfo1.zip. [file FSN3-13-e71280-s001.zip › fsn371280-sup-0008-Supinfo8@Supplementary Material 3_PE - MR (Dietary Preferences to Immune Cells) MR Analysis - 1.docx]

| id.exposure | id.outcome | outcome | exposure | method | nsnp | b | se | pval | lo_ci | up_ci | or | or_lci95 | or_uci95 | p_fdr |
| --- | --- | --- | --- | --- | --- | --- | --- | --- | --- | --- | --- | --- | --- | --- |
| ebi-fl187-GCST90094707 | ebi-a-GCST90001414 | IgD- CD24- B cell Absolute Count \|\| id:ebi-a-GCST90001414 | Black olive liking \|\| id:ebi-fl187-GCST90094707 | Inverse variance weighted | 20 | -0.089624357 | 0.0852598002843569 | 0.293170755665807 | -0.256733566 | 0.0774848515183225 | 0.914274561577207 | 0.773574293116376 | 1.08056586340239 | 0.998735809224318 |
| ebi-fl187-GCST90094707 | ebi-a-GCST90001422 | CD20- CD38- B cell %B cell \|\| id:ebi-a-GCST90001422 | Black olive liking \|\| id:ebi-fl187-GCST90094707 | Inverse variance weighted | 20 | -0.007382539 | 0.0915681033366703 | 0.935741393654762 | -0.186856022 | 0.172090943507716 | 0.99264464497233 | 0.829563166988166 | 1.18778584971372 | 0.998735809224318 |
| ebi-fl187-GCST90094707 | ebi-a-GCST90001444 | CD20- CD38- B cell %lymphocyte \|\| id:ebi-a-GCST90001444 | Black olive liking \|\| id:ebi-fl187-GCST90094707 | Inverse variance weighted | 20 | -0.048133806 | 0.0980475128727598 | 0.623480718699133 | -0.240306931 | 0.144039319693442 | 0.953006261031169 | 0.786386457822139 | 1.15492951910678 | 0.998735809224318 |
| ebi-fl187-GCST90094707 | ebi-a-GCST90001459 | Myeloid Dendritic Cell %Dendritic Cell \|\| id:ebi-a-GCST90001459 | Black olive liking \|\| id:ebi-fl187-GCST90094707 | Inverse variance weighted | 20 | -0.026621744 | 0.096811758774648 | 0.783328063422447 | -0.216372791 | 0.163129303631358 | 0.973729491317659 | 0.805434987174447 | 1.1771888946468 | 0.998735809224318 |
| ebi-fl187-GCST90094707 | ebi-a-GCST90001476 | HLA DR++ monocyte %leukocyte \|\| id:ebi-a-GCST90001476 | Black olive liking \|\| id:ebi-fl187-GCST90094707 | Inverse variance weighted | 20 | 0.0702277667712262 | 0.0872456780040075 | 0.420852778304084 | -0.100773762 | 0.241229295659081 | 1.07275249080152 | 0.904137559916943 | 1.27281285231262 | 0.998735809224318 |
| ebi-fl187-GCST90094707 | ebi-a-GCST90001478 | CD4 regulatory T cell %CD4+ T cell \|\| id:ebi-a-GCST90001478 | Black olive liking \|\| id:ebi-fl187-GCST90094707 | Inverse variance weighted | 20 | 0.0175944696981981 | 0.0987622945127432 | 0.858605438657998 | -0.175979628 | 0.211168566943175 | 1.01775016416029 | 0.83863506821674 | 1.23512053800806 | 0.998735809224318 |
| ebi-fl187-GCST90094707 | ebi-a-GCST90001485 | CD39+ resting CD4 regulatory T cell %CD4 regulatory T cell \|\| id:ebi-a-GCST90001485 | Black olive liking \|\| id:ebi-fl187-GCST90094707 | Inverse variance weighted | 19 | 0.0850401980261191 | 0.0884355850613667 | 0.336247506321414 | -0.088293549 | 0.258373944746398 | 1.08876083185511 | 0.915492095765881 | 1.29482292033353 | 0.998735809224318 |
| ebi-fl187-GCST90094707 | ebi-a-GCST90001515 | Immature Myeloid-Derived Suppressor Cells Absolute Count \|\| id:ebi-a-GCST90001515 | Black olive liking \|\| id:ebi-fl187-GCST90094707 | Inverse variance weighted | 20 | -0.183957475 | 0.13838583060754 | 0.183746178480348 | -0.455193703 | 0.0872787533922101 | 0.831971182982946 | 0.634325085635727 | 1.09120081325546 | 0.998735809224318 |
| ebi-fl187-GCST90094707 | ebi-a-GCST90001517 | CD33+ HLA DR+ Absolute Count \|\| id:ebi-a-GCST90001517 | Black olive liking \|\| id:ebi-fl187-GCST90094707 | Inverse variance weighted | 20 | -0.150450082 | 0.110371331744297 | 0.172842662017973 | -0.366777892 | 0.0658777285660713 | 0.860320674721727 | 0.692963540788125 | 1.06809611153836 | 0.998735809224318 |
| ebi-fl187-GCST90094707 | ebi-a-GCST90001518 | CD33+ HLA DR+ CD14- Absolute Count \|\| id:ebi-a-GCST90001518 | Black olive liking \|\| id:ebi-fl187-GCST90094707 | Inverse variance weighted | 20 | -0.198212986 | 0.111802904940849 | 0.0762488129985804 | -0.41734668 | 0.0209207077116803 | 0.820195144472854 | 0.658792490396229 | 1.02114107981443 | 0.998735809224318 |
| ebi-fl187-GCST90094707 | ebi-a-GCST90001520 | CD33+ HLA DR+ CD14dim Absolute Count \|\| id:ebi-a-GCST90001520 | Black olive liking \|\| id:ebi-fl187-GCST90094707 | Inverse variance weighted | 20 | -0.121924046 | 0.110753882458589 | 0.270959451228195 | -0.339001656 | 0.0951535632787178 | 0.885215601314011 | 0.712481269248478 | 1.09982773531193 | 0.998735809224318 |
| ebi-fl187-GCST90094707 | ebi-a-GCST90001531 | CD33dim HLA DR- Absolute Count \|\| id:ebi-a-GCST90001531 | Black olive liking \|\| id:ebi-fl187-GCST90094707 | Inverse variance weighted | 20 | -0.114622439 | 0.112648949142412 | 0.308906629800331 | -0.335414379 | 0.106169501684696 | 0.891702752866617 | 0.715041726711736 | 1.11201034816036 | 0.998735809224318 |
| ebi-fl187-GCST90094707 | ebi-a-GCST90001532 | Basophil Absolute Count \|\| id:ebi-a-GCST90001532 | Black olive liking \|\| id:ebi-fl187-GCST90094707 | Inverse variance weighted | 19 | -0.142965868 | 0.115150945091868 | 0.214401959800428 | -0.36866172 | 0.0827299845174484 | 0.86678365356934 | 0.691659345235834 | 1.08624846504292 | 0.998735809224318 |
| ebi-fl187-GCST90094707 | ebi-a-GCST90001588 | T/B cell \|\| id:ebi-a-GCST90001588 | Black olive liking \|\| id:ebi-fl187-GCST90094707 | Inverse variance weighted | 20 | 0.171885387575809 | 0.0839221813430817 | 0.040545103347335 | 0.00739791214336874 | 0.336372863008249 | 1.18754171837866 | 1.00742534430058 | 1.3998608838542 | 0.998735809224318 |
| ebi-fl187-GCST90094707 | ebi-a-GCST90001624 | HLA DR+ CD4+ T cell Absolute Count \|\| id:ebi-a-GCST90001624 | Black olive liking \|\| id:ebi-fl187-GCST90094707 | Inverse variance weighted | 20 | 0.0207986364694078 | 0.0815510526498334 | 0.798693568746807 | -0.139041427 | 0.180638699663081 | 1.02101643546219 | 0.870191978508389 | 1.19798226969505 | 0.998735809224318 |
| ebi-fl187-GCST90094707 | ebi-a-GCST90001631 | CD8+ Natural Killer T %T cell \|\| id:ebi-a-GCST90001631 | Black olive liking \|\| id:ebi-fl187-GCST90094707 | Inverse variance weighted | 20 | 0.115899829500902 | 0.0958188644537471 | 0.226443111671673 | -0.071905145 | 0.303704803830247 | 1.12288338671021 | 0.930619165665328 | 1.35486904489912 | 0.998735809224318 |
| ebi-fl187-GCST90094707 | ebi-a-GCST90001632 | CD8+ Natural Killer T %lymphocyte \|\| id:ebi-a-GCST90001632 | Black olive liking \|\| id:ebi-fl187-GCST90094707 | Inverse variance weighted | 20 | 0.131828720991119 | 0.101114253935327 | 0.192315420502928 | -0.066355217 | 0.33001265870436 | 1.14091288810239 | 0.935798394007526 | 1.39098573642954 | 0.998735809224318 |
| ebi-fl187-GCST90094707 | ebi-a-GCST90001646 | Natural Killer %CD3- lymphocyte \|\| id:ebi-a-GCST90001646 | Black olive liking \|\| id:ebi-fl187-GCST90094707 | Inverse variance weighted | 20 | 0.111179507361027 | 0.080991096451496 | 0.169833997139566 | -0.047563042 | 0.269922056405959 | 1.11759550546934 | 0.953550357817516 | 1.30986235137494 | 0.998735809224318 |
| ebi-fl187-GCST90094707 | ebi-a-GCST90001684 | CD127- CD8+ T cell Absolute Count \|\| id:ebi-a-GCST90001684 | Black olive liking \|\| id:ebi-fl187-GCST90094707 | Inverse variance weighted | 20 | 0.0318287323230609 | 0.0899850632783677 | 0.723555264792619 | -0.144541992 | 0.208199456348662 | 1.03234068357294 | 0.865418571240883 | 1.23145876732429 | 0.998735809224318 |
| ebi-fl187-GCST90094707 | ebi-a-GCST90001687 | CD28- CD8+ T cell Absolute Count \|\| id:ebi-a-GCST90001687 | Black olive liking \|\| id:ebi-fl187-GCST90094707 | Inverse variance weighted (multiplicative random effects) | 20 | 0.0338231134273611 | 0.108827798628377 | 0.755956626425101 | -0.179479372 | 0.24712559873898 | 1.03440161878746 | 0.835705189788736 | 1.28033991175837 | 0.998735809224318 |
| ebi-fl187-GCST90094707 | ebi-a-GCST90001701 | BAFF-R on CD20- CD38- B cell \|\| id:ebi-a-GCST90001701 | Black olive liking \|\| id:ebi-fl187-GCST90094707 | Inverse variance weighted | 20 | -0.065752398 | 0.0818478589344357 | 0.42177302635459 | -0.226174202 | 0.0946694054211209 | 0.936362680779196 | 0.797579149997679 | 1.09929537395574 | 0.998735809224318 |
| ebi-fl187-GCST90094707 | ebi-a-GCST90001732 | CD19 on IgD- CD27- B cell \|\| id:ebi-a-GCST90001732 | Black olive liking \|\| id:ebi-fl187-GCST90094707 | Inverse variance weighted | 20 | -0.209399223 | 0.084788715236133 | 0.0135241861366353 | -0.375585105 | -0.043213341 | 0.81107137283119 | 0.686887260264578 | 0.957707050168442 | 0.998735809224318 |
| ebi-fl187-GCST90094707 | ebi-a-GCST90001798 | CD27 on CD24+ CD27+ B cell \|\| id:ebi-a-GCST90001798 | Black olive liking \|\| id:ebi-fl187-GCST90094707 | Inverse variance weighted | 20 | 0.0436490431548685 | 0.0917887524683335 | 0.634403528589534 | -0.136256912 | 0.223554997992802 | 1.04461567552603 | 0.872618417821873 | 1.25051441416798 | 0.998735809224318 |
| ebi-fl187-GCST90094707 | ebi-a-GCST90001836 | CD66b on CD66b++ myeloid cell \|\| id:ebi-a-GCST90001836 | Black olive liking \|\| id:ebi-fl187-GCST90094707 | Inverse variance weighted | 20 | -0.015370721 | 0.124870699430376 | 0.902033376603542 | -0.260117292 | 0.229375849623702 | 0.984746805349432 | 0.770961152814061 | 1.25781469936111 | 0.998735809224318 |
| ebi-fl187-GCST90094707 | ebi-a-GCST90001943 | CD25 on activated & secreting CD4 regulatory T cell \|\| id:ebi-a-GCST90001943 | Black olive liking \|\| id:ebi-fl187-GCST90094707 | Inverse variance weighted (multiplicative random effects) | 20 | -0.025249368 | 0.11608348210555 | 0.827810581621633 | -0.252772993 | 0.2022742571193 | 0.975066731455825 | 0.776644165682476 | 1.2241837031718 | 0.998735809224318 |
| ebi-fl187-GCST90094707 | ebi-a-GCST90001960 | CD25 on CD4+ T cell \|\| id:ebi-a-GCST90001960 | Black olive liking \|\| id:ebi-fl187-GCST90094707 | Inverse variance weighted | 20 | -0.013051831 | 0.0945691773852379 | 0.890229681563709 | -0.198407419 | 0.172303756539002 | 0.98703297465404 | 0.820035687104964 | 1.18803865291987 | 0.998735809224318 |
| ebi-fl187-GCST90094707 | ebi-a-GCST90001988 | HLA DR on CD14+ CD16- monocyte \|\| id:ebi-a-GCST90001988 | Black olive liking \|\| id:ebi-fl187-GCST90094707 | Inverse variance weighted | 19 | -0.038661193 | 0.0865538561414762 | 0.655111827264231 | -0.208306751 | 0.130984365124417 | 0.962076612308705 | 0.811957929640562 | 1.13994995819689 | 0.998735809224318 |
| ebi-fl187-GCST90094707 | ebi-a-GCST90001991 | HLA DR on CD14+ monocyte \|\| id:ebi-a-GCST90001991 | Black olive liking \|\| id:ebi-fl187-GCST90094707 | Inverse variance weighted | 19 | -0.038998041 | 0.086711602201866 | 0.652894890902991 | -0.208952781 | 0.130956699445512 | 0.961752593342739 | 0.811433549668848 | 1.13991842114363 | 0.998735809224318 |
| ebi-fl187-GCST90094707 | ebi-a-GCST90002019 | CD14 on Monocytic Myeloid-Derived Suppressor Cells \|\| id:ebi-a-GCST90002019 | Black olive liking \|\| id:ebi-fl187-GCST90094707 | Inverse variance weighted | 20 | -0.074588686 | 0.1298134568375 | 0.565572933024461 | -0.329023061 | 0.179845689524316 | 0.928125158815091 | 0.719626421050273 | 1.19703263419418 | 0.998735809224318 |
| ebi-fl187-GCST90094707 | ebi-a-GCST90002037 | CD80 on plasmacytoid Dendritic Cell \|\| id:ebi-a-GCST90002037 | Black olive liking \|\| id:ebi-fl187-GCST90094707 | Inverse variance weighted | 20 | -0.068618233 | 0.0937322351346769 | 0.46412795268702 | -0.252333414 | 0.115096947403107 | 0.933683060997569 | 0.776985636735826 | 1.12198220556063 | 0.998735809224318 |
| ebi-fl187-GCST90094707 | ebi-a-GCST90002042 | CD45 on CD33+ HLA DR+ CD14- \|\| id:ebi-a-GCST90002042 | Black olive liking \|\| id:ebi-fl187-GCST90094707 | Inverse variance weighted | 20 | 0.0102871523455802 | 0.132582598869444 | 0.938153786253178 | -0.249574741 | 0.27014904612969 | 1.01034024700537 | 0.779132045203123 | 1.31015971041565 | 0.998735809224318 |
| ebi-fl187-GCST90094707 | ebi-a-GCST90002056 | CD8 on Effector Memory CD8+ T cell \|\| id:ebi-a-GCST90002056 | Black olive liking \|\| id:ebi-fl187-GCST90094707 | Inverse variance weighted | 20 | 0.00650217715896528 | 0.0918060364627647 | 0.943536907861078 | -0.173437654 | 0.186442008625984 | 1.00652336220428 | 0.840769567888363 | 1.20495474307835 | 0.998735809224318 |
| ebi-fl187-GCST90094707 | ebi-a-GCST90002058 | CD8 on CD8+ T cell \|\| id:ebi-a-GCST90002058 | Black olive liking \|\| id:ebi-fl187-GCST90094707 | Inverse variance weighted | 20 | -0.164124528 | 0.0886674338244949 | 0.0641677549731516 | -0.337912699 | 0.00966364202962089 | 0.848636336088181 | 0.713257553590109 | 1.00971048579044 | 0.998735809224318 |
| ebi-fl187-GCST90094707 | ebi-a-GCST90002063 | CD4 on CD4 regulatory T cell \|\| id:ebi-a-GCST90002063 | Black olive liking \|\| id:ebi-fl187-GCST90094707 | Inverse variance weighted | 20 | -0.158953316 | 0.092399774870408 | 0.085381889496227 | -0.340056874 | 0.0221502430627505 | 0.853036181450877 | 0.711729842382907 | 1.02239738104562 | 0.998735809224318 |
| ebi-fl187-GCST90094707 | ebi-a-GCST90002066 | CD4 on activated CD4 regulatory T cell \|\| id:ebi-a-GCST90002066 | Black olive liking \|\| id:ebi-fl187-GCST90094707 | Inverse variance weighted | 20 | -0.130903023 | 0.0935550155910113 | 0.161750306862432 | -0.314270854 | 0.0524648071177362 | 0.877302848077618 | 0.73032119095299 | 1.05386547286239 | 0.998735809224318 |
| ebi-fl187-GCST90094707 | ebi-a-GCST90002070 | CD4 on activated & secreting CD4 regulatory T cell \|\| id:ebi-a-GCST90002070 | Black olive liking \|\| id:ebi-fl187-GCST90094707 | Inverse variance weighted | 20 | -0.17112292 | 0.0918108646201461 | 0.0623408743064749 | -0.351072215 | 0.00882637434031847 | 0.842717979945184 | 0.70393291752509 | 1.00886544163859 | 0.998735809224318 |
| ebi-fl187-GCST90094707 | ebi-a-GCST90002081 | SSC-A on CD4+ T cell \|\| id:ebi-a-GCST90002081 | Black olive liking \|\| id:ebi-fl187-GCST90094707 | Inverse variance weighted | 20 | -0.018980465 | 0.086830569451906 | 0.826967906595605 | -0.189168382 | 0.151207450675147 | 0.981198529326748 | 0.827647134432066 | 1.1632379475507 | 0.998735809224318 |
| ebi-fl187-GCST90094707 | ebi-a-GCST90002094 | CD11b on Monocytic Myeloid-Derived Suppressor Cells \|\| id:ebi-a-GCST90002094 | Black olive liking \|\| id:ebi-fl187-GCST90094707 | Inverse variance weighted | 20 | -0.169152705 | 0.12255371547355 | 0.167514860415914 | -0.409357987 | 0.0710525776436788 | 0.844379952766336 | 0.664076459017153 | 1.07363767372345 | 0.998735809224318 |
| ebi-fl187-GCST90094707 | ebi-a-GCST90002104 | HLA DR on myeloid Dendritic Cell \|\| id:ebi-a-GCST90002104 | Black olive liking \|\| id:ebi-fl187-GCST90094707 | Inverse variance weighted | 19 | -0.182593454 | 0.0955917618073038 | 0.0561154508464934 | -0.369953307 | 0.00476639896759123 | 0.833106782983311 | 0.690766583629448 | 1.0047777763163 | 0.998735809224318 |
| ebi-fl187-GCST90094707 | ebi-a-GCST90002105 | HLA DR on plasmacytoid Dendritic Cell \|\| id:ebi-a-GCST90002105 | Black olive liking \|\| id:ebi-fl187-GCST90094707 | Inverse variance weighted | 19 | -0.142080303 | 0.0942207741848813 | 0.131567087982602 | -0.326753021 | 0.0425924139579596 | 0.867551586307992 | 0.721261857673261 | 1.04351248703695 | 0.998735809224318 |
| ebi-fl187-GCST90094707 | ebi-a-GCST90002106 | HLA DR on Dendritic Cell \|\| id:ebi-a-GCST90002106 | Black olive liking \|\| id:ebi-fl187-GCST90094707 | Inverse variance weighted | 19 | -0.159277499 | 0.0953732583617517 | 0.0949107395375033 | -0.346209085 | 0.0276540875757703 | 0.852759686331507 | 0.707364572178958 | 1.02804001109662 | 0.998735809224318 |
| ebi-fl187-GCST90094707 | ebi-a-GCST90002111 | HLA DR on CD33dim HLA DR+ CD11b- \|\| id:ebi-a-GCST90002111 | Black olive liking \|\| id:ebi-fl187-GCST90094707 | Inverse variance weighted | 19 | 0.0688936615400607 | 0.123152389020961 | 0.575876488654524 | -0.172485021 | 0.310272344021143 | 1.07132228032956 | 0.841570894657427 | 1.36379648537836 | 0.998735809224318 |
| ebi-fl187-GCST90094707 | ebi-a-GCST90002112 | HLA DR on CD33- HLA DR+ \|\| id:ebi-a-GCST90002112 | Black olive liking \|\| id:ebi-fl187-GCST90094707 | Inverse variance weighted | 19 | -0.047449075 | 0.124410850897232 | 0.702913757196004 | -0.291294343 | 0.196396192716594 | 0.953659036942481 | 0.747295684538301 | 1.21700897992467 | 0.998735809224318 |
| ebi-fl187-GCST90094707 | ebi-a-GCST90002115 | HLA DR on HLA DR+ CD8+ T cell \|\| id:ebi-a-GCST90002115 | Black olive liking \|\| id:ebi-fl187-GCST90094707 | Inverse variance weighted | 20 | -0.023271369 | 0.0938949911532291 | 0.804254625535998 | -0.207305552 | 0.160762813721522 | 0.976997321070208 | 0.812771268482004 | 1.17440638269744 | 0.998735809224318 |
| ebi-fl187-GCST90094707 | ebi-a-GCST90002116 | HLA DR on B cell \|\| id:ebi-a-GCST90002116 | Black olive liking \|\| id:ebi-fl187-GCST90094707 | Inverse variance weighted (multiplicative random effects) | 20 | 0.0968355688147992 | 0.123344539961566 | 0.43240553682848 | -0.14491973 | 0.338590867139468 | 1.10167920830887 | 0.865091731661079 | 1.40296922696235 | 0.998735809224318 |
| ebi-fl187-GCST90094707 | ebi-cfmet338-GCST90026023 | Sphingomyelin (d18:1/24:1, d18:2/24:0) levels \|\| id:ebi-cfmet338-GCST90026023 | Black olive liking \|\| id:ebi-fl187-GCST90094707 | Inverse variance weighted | 19 | 0.0777124929634481 | 0.0569883192112769 | 0.17267569505472 | -0.033984613 | 0.189409598617551 | 1.08081187297702 | 0.966586377686681 | 1.20853586573796 | 0.998735809224318 |
| ebi-fl187-GCST90094707 | ebi-cfmet338-GCST90026046 | 1-arachidonoyl-gpc (20:4n6) levels \|\| id:ebi-cfmet338-GCST90026046 | Black olive liking \|\| id:ebi-fl187-GCST90094707 | Inverse variance weighted | 19 | 0.0850298132919336 | 0.0603675933124914 | 0.158972979031941 | -0.03329067 | 0.203350296184417 | 1.08874952542199 | 0.967257366411534 | 1.22550168163028 | 0.998735809224318 |
| ebi-fl187-GCST90094707 | ebi-cfmet338-GCST90026047 | 1-linoleoyl-gpc (18:2) levels \|\| id:ebi-cfmet338-GCST90026047 | Black olive liking \|\| id:ebi-fl187-GCST90094707 | Inverse variance weighted | 19 | 0.0798718189593855 | 0.0797617734293187 | 0.316643285292122 | -0.076461257 | 0.23620489488085 | 1.08314821971037 | 0.926388804710156 | 1.26643376938132 | 0.998735809224318 |
| ebi-fl187-GCST90094707 | ebi-cfmet338-GCST90026050 | 1-myristoyl-2-palmitoyl-gpc (14:0/16:0) levels \|\| id:ebi-cfmet338-GCST90026050 | Black olive liking \|\| id:ebi-fl187-GCST90094707 | Inverse variance weighted | 19 | 0.0412046527537475 | 0.0454912619135376 | 0.36505722533219 | -0.047958221 | 0.130367526104281 | 1.04206534526718 | 0.95317360927018 | 1.13924700940708 | 0.998735809224318 |
| ebi-fl187-GCST90094707 | ebi-cfmet338-GCST90026068 | 2,3-dihydroxyisovalerate levels \|\| id:ebi-cfmet338-GCST90026068 | Black olive liking \|\| id:ebi-fl187-GCST90094707 | Inverse variance weighted | 19 | 0.0130112462300035 | 0.125557884481788 | 0.917464985074539 | -0.233082207 | 0.259104699814309 | 1.01309626080931 | 0.792088455351467 | 1.29576946454848 | 0.998735809224318 |
| ebi-fl187-GCST90094707 | ebi-cfmet338-GCST90026082 | 3-hydroxyhexanoate levels \|\| id:ebi-cfmet338-GCST90026082 | Black olive liking \|\| id:ebi-fl187-GCST90094707 | Inverse variance weighted | 19 | -0.014754081 | 0.0268912757789679 | 0.583241015380058 | -0.067460981 | 0.0379528198885501 | 0.985354227492945 | 0.934764193314281 | 1.03868222657923 | 0.998735809224318 |
| ebi-fl187-GCST90094707 | ebi-cfmet338-GCST90026094 | 3-ureidopropionate levels \|\| id:ebi-cfmet338-GCST90026094 | Black olive liking \|\| id:ebi-fl187-GCST90094707 | Inverse variance weighted | 19 | -0.006555727 | 0.0612147942844694 | 0.914714535673217 | -0.126536724 | 0.113425269539527 | 0.993465714640575 | 0.881141789849254 | 1.12010817956456 | 0.998735809224318 |
| ebi-fl187-GCST90094707 | ebi-cfmet338-GCST90026173 | Glycerol levels \|\| id:ebi-cfmet338-GCST90026173 | Black olive liking \|\| id:ebi-fl187-GCST90094707 | Inverse variance weighted | 18 | 0.0156848288193804 | 0.0352393565986877 | 0.656251770648679 | -0.05338431 | 0.0847539677528083 | 1.01580848139084 | 0.948015610477714 | 1.08844924014026 | 0.998735809224318 |
| ebi-fl187-GCST90094707 | ebi-cfmet338-GCST90026179 | Histidine levels \|\| id:ebi-cfmet338-GCST90026179 | Black olive liking \|\| id:ebi-fl187-GCST90094707 | Inverse variance weighted | 19 | -0.021658755 | 0.0239518838221003 | 0.365857012803269 | -0.068604447 | 0.0252869374837176 | 0.978574111792111 | 0.933695933178919 | 1.02560936407786 | 0.998735809224318 |
| ebi-fl187-GCST90094707 | ebi-cfmet338-GCST90026188 | Isocitrate levels \|\| id:ebi-cfmet338-GCST90026188 | Black olive liking \|\| id:ebi-fl187-GCST90094707 | Inverse variance weighted | 19 | 0.0228742487230749 | 0.0470596674437949 | 0.626918196616419 | -0.069362699 | 0.115111196912913 | 1.02313787056355 | 0.932988224371584 | 1.12199819337098 | 0.998735809224318 |
| ebi-fl187-GCST90094707 | ebi-cfmet338-GCST90026207 | N-acetyl-isoputreanine levels \|\| id:ebi-cfmet338-GCST90026207 | Black olive liking \|\| id:ebi-fl187-GCST90094707 | Inverse variance weighted | 19 | 0.0300688138590585 | 0.0299542357317311 | 0.31546292088495 | -0.028641488 | 0.0887791158932513 | 1.03052544594642 | 0.971764791191879 | 1.09283923884558 | 0.998735809224318 |
| ebi-fl187-GCST90094707 | ebi-cfmet338-GCST90026213 | N-acetylglutamate levels \|\| id:ebi-cfmet338-GCST90026213 | Black olive liking \|\| id:ebi-fl187-GCST90094707 | Inverse variance weighted | 19 | -0.028302146 | 0.0619281514669339 | 0.6476596661047 | -0.149681323 | 0.0930770312173074 | 0.972094608259611 | 0.860982308372132 | 1.09754627733765 | 0.998735809224318 |
| ebi-fl187-GCST90094707 | ebi-cfmet338-GCST90026224 | N-acetyltaurine levels \|\| id:ebi-cfmet338-GCST90026224 | Black olive liking \|\| id:ebi-fl187-GCST90094707 | Inverse variance weighted | 19 | 0.0126230795414385 | 0.0409056618947512 | 0.757633403820308 | -0.067552018 | 0.0927981768551509 | 1.01270308690199 | 0.934679099426912 | 1.09724026443904 | 0.998735809224318 |
| ebi-fl187-GCST90094707 | ebi-cfmet338-GCST90026250 | Phenyllactate (pla) levels \|\| id:ebi-cfmet338-GCST90026250 | Black olive liking \|\| id:ebi-fl187-GCST90094707 | Inverse variance weighted | 19 | 0.00934894841501963 | 0.0717121335646722 | 0.896275492550543 | -0.131206833 | 0.149904730201777 | 1.00939278633959 | 0.877036355243432 | 1.16172356028684 | 0.998735809224318 |
| ebi-fl187-GCST90094707 | ebi-cfmet338-GCST90026251 | Pipecolate levels \|\| id:ebi-cfmet338-GCST90026251 | Black olive liking \|\| id:ebi-fl187-GCST90094707 | Inverse variance weighted | 19 | 0.0313213749053432 | 0.0965123817360353 | 0.745535153419526 | -0.157842893 | 0.220485643107972 | 1.03181705071526 | 0.853983938030472 | 1.2466820261305 | 0.998735809224318 |
| ebi-fl187-GCST90094707 | ebi-cfmet338-GCST90026278 | Trigonelline (n'-methylnicotinate) levels \|\| id:ebi-cfmet338-GCST90026278 | Black olive liking \|\| id:ebi-fl187-GCST90094707 | Inverse variance weighted | 19 | 0.091656154833487 | 0.108988898399328 | 0.400365969920822 | -0.121962086 | 0.305274395696169 | 1.09598790713991 | 0.885181928628223 | 1.35699730614522 | 0.998735809224318 |
| ebi-fl187-GCST90094707 | ebi-cfmet338-GCST90026299 | 1-carboxyethylvaline levels \|\| id:ebi-cfmet338-GCST90026299 | Black olive liking \|\| id:ebi-fl187-GCST90094707 | Inverse variance weighted | 19 | 0.0487673276357624 | 0.0654607306866777 | 0.456280185894249 | -0.079535705 | 0.177070359781651 | 1.04997602191344 | 0.923545044656528 | 1.19371507970483 | 0.998735809224318 |
| ebi-fl187-GCST90094707 | ebi-gm430-GCST90011306 | C_Erysipelotrichia abundance \|\| id:ebi-gm430-GCST90011306 | Black olive liking \|\| id:ebi-fl187-GCST90094707 | Inverse variance weighted | 18 | 0.0982048380889854 | 0.0606062158997801 | 0.105151601584503 | -0.020583345 | 0.216993021252554 | 1.10318873703862 | 0.97962704598277 | 1.24233543216229 | 0.998735809224318 |
| ebi-fl187-GCST90094707 | ebi-gm430-GCST90011312 | F_Erysipelotrichaceae abundance \|\| id:ebi-gm430-GCST90011312 | Black olive liking \|\| id:ebi-fl187-GCST90094707 | Inverse variance weighted | 18 | 0.0982048380889854 | 0.0606062158997801 | 0.105151601584503 | -0.020583345 | 0.216993021252554 | 1.10318873703862 | 0.97962704598277 | 1.24233543216229 | 0.998735809224318 |
| ebi-fl187-GCST90094707 | ebi-gm430-GCST90011352 | O_Erysipelotrichales abundance \|\| id:ebi-gm430-GCST90011352 | Black olive liking \|\| id:ebi-fl187-GCST90094707 | Inverse variance weighted | 18 | 0.0982048380889854 | 0.0606062158997801 | 0.105151601584503 | -0.020583345 | 0.216993021252554 | 1.10318873703862 | 0.97962704598277 | 1.24233543216229 | 0.998735809224318 |
| ebi-fl187-GCST90094707 | ebi-gm430-GCST90011362 | OTU97_11 (Parabacteroides) abundance \|\| id:ebi-gm430-GCST90011362 | Black olive liking \|\| id:ebi-fl187-GCST90094707 | Inverse variance weighted | 18 | 0.085180195104175 | 0.0703541727847102 | 0.225997334154503 | -0.052713984 | 0.223074373762207 | 1.08891326586018 | 0.948651303558067 | 1.24991353105089 | 0.998735809224318 |
| ebi-fl187-GCST90094707 | ebi-gm430-GCST90011384 | OTU97_235 (Phascolarctobacterium) abundance \|\| id:ebi-gm430-GCST90011384 | Black olive liking \|\| id:ebi-fl187-GCST90094707 | Inverse variance weighted (multiplicative random effects) | 18 | 0.13358721634431 | 0.288301014483137 | 0.643106200254661 | -0.431482772 | 0.698657204731259 | 1.14292094317481 | 0.649545252779715 | 2.01105046454801 | 0.998735809224318 |
| ebi-fl187-GCST90094707 | ebi-gm430-GCST90011390 | OTU97_3 (Bacteroides) abundance \|\| id:ebi-gm430-GCST90011390 | Black olive liking \|\| id:ebi-fl187-GCST90094707 | Inverse variance weighted | 18 | -0.021759699 | 0.0560688484616838 | 0.697950225591198 | -0.131654642 | 0.0881352437154169 | 0.978475335140544 | 0.876643698497301 | 1.09213581654616 | 0.998735809224318 |
| ebi-fl187-GCST90094707 | ebi-gm430-GCST90011398 | OTU97_4 (Alistipes) abundance \|\| id:ebi-gm430-GCST90011398 | Black olive liking \|\| id:ebi-fl187-GCST90094707 | Inverse variance weighted | 18 | 0.0527215821520875 | 0.0655490680136456 | 0.421219626340621 | -0.075754591 | 0.181197755458833 | 1.05413611395084 | 0.927043683365747 | 1.19865219587174 | 0.998735809224318 |
| ebi-fl187-GCST90094707 | ebi-gm430-GCST90011421 | OTU99_108 (Cytophagales) abundance \|\| id:ebi-gm430-GCST90011421 | Black olive liking \|\| id:ebi-fl187-GCST90094707 | Inverse variance weighted | 18 | -0.161501176 | 0.205964181051433 | 0.432968156790493 | -0.565190971 | 0.242188619123842 | 0.850865531070727 | 0.568251617043885 | 1.27403447742122 | 0.998735809224318 |
| ebi-fl187-GCST90094707 | ebi-gm430-GCST90011428 | OTU99_123 (Phascolarctobacterium) abundance \|\| id:ebi-gm430-GCST90011428 | Black olive liking \|\| id:ebi-fl187-GCST90094707 | Inverse variance weighted | 18 | 0.00779468911360053 | 0.149174112963597 | 0.95832766786409 | -0.284586572 | 0.30017595052225 | 1.00782514678739 | 0.75232522212356 | 1.35009633683425 | 0.998735809224318 |
| ebi-fl187-GCST90094707 | ebi-gm430-GCST90011450 | OTU99_298 (Phascolarctobacterium) abundance \|\| id:ebi-gm430-GCST90011450 | Black olive liking \|\| id:ebi-fl187-GCST90094707 | Inverse variance weighted (multiplicative random effects) | 18 | 0.132443694427296 | 0.289145549865714 | 0.646915090246447 | -0.434281583 | 0.699168972164096 | 1.14161473500805 | 0.647729839891981 | 2.01207991807948 | 0.998735809224318 |
| ebi-fl187-GCST90094707 | ebi-gm430-GCST90011451 | OTU99_3 (Bacteroides) abundance \|\| id:ebi-gm430-GCST90011451 | Black olive liking \|\| id:ebi-fl187-GCST90094707 | Inverse variance weighted | 18 | -0.023398752 | 0.0560678976060224 | 0.676437879088518 | -0.133291831 | 0.0864943273067151 | 0.976872876085868 | 0.875209641261142 | 1.09034518250644 | 0.998735809224318 |
| ebi-fl187-GCST90094707 | ebi-gm430-GCST90011472 | OTU99_72 (Parabacteroides) abundance \|\| id:ebi-gm430-GCST90011472 | Black olive liking \|\| id:ebi-fl187-GCST90094707 | Inverse variance weighted | 18 | 0.0913054931452054 | 0.169553360743187 | 0.590227800328814 | -0.241019094 | 0.423630080201851 | 1.0956036535455 | 0.785826621741174 | 1.52749643808531 | 0.998735809224318 |
| ebi-fl187-GCST90094707 | ebi-gm430-GCST90011507 | TestASV_3 (Bacteroides) abundance \|\| id:ebi-gm430-GCST90011507 | Black olive liking \|\| id:ebi-fl187-GCST90094707 | Inverse variance weighted | 18 | -0.040535742 | 0.0723087716177778 | 0.575075487073629 | -0.182260935 | 0.101189450209893 | 0.960274841600084 | 0.833383853416438 | 1.10648624595956 | 0.998735809224318 |
| ebi-fl187-GCST90094707 | ebi-gm430-GCST90011512 | TestASV_34 (Phascolarctobacterium) abundance \|\| id:ebi-gm430-GCST90011512 | Black olive liking \|\| id:ebi-fl187-GCST90094707 | Inverse variance weighted | 18 | -0.081828285 | 0.196476356964499 | 0.677059500929246 | -0.466921944 | 0.303265374915999 | 0.921430168738559 | 0.626929023833207 | 1.35427380705771 | 0.998735809224318 |
| ebi-fl187-GCST90094707 | ebi-gm430-GCST90011519 | TestASV_41 (Barnesiella) abundance \|\| id:ebi-gm430-GCST90011519 | Black olive liking \|\| id:ebi-fl187-GCST90094707 | Inverse variance weighted | 18 | 0.0431581710460078 | 0.267741242857712 | 0.871940932720358 | -0.481614665 | 0.567931007047123 | 1.04410302865876 | 0.617785070142988 | 1.7646123014952 | 0.998735809224318 |
| ebi-fl187-GCST90094707 | ebi-gm430-GCST90011524 | TestASV_46 (Sutterella) abundance \|\| id:ebi-gm430-GCST90011524 | Black olive liking \|\| id:ebi-fl187-GCST90094707 | Inverse variance weighted | 18 | -0.3435932 | 0.234148803073214 | 0.142263505080361 | -0.802524854 | 0.11533845396501 | 0.709217378962907 | 0.448195905054342 | 1.12225320434828 | 0.998735809224318 |
| ebi-fl187-GCST90094707 | ebi-gm430-GCST90011581 | OTU97_19 (Bacteria) prevalence \|\| id:ebi-gm430-GCST90011581 | Black olive liking \|\| id:ebi-fl187-GCST90094707 | Inverse variance weighted | 18 | -0.323691133 | 0.289032328397876 | 0.262750733807379 | -0.890194496 | 0.242812231056077 | 0.723473665301288 | 0.410575889509614 | 1.27482922830573 | 0.998735809224318 |
| ebi-fl187-GCST90094707 | ebi-gm430-GCST90011614 | OTU97_80 (Ruminococcus) prevalence \|\| id:ebi-gm430-GCST90011614 | Black olive liking \|\| id:ebi-fl187-GCST90094707 | Inverse variance weighted | 18 | 0.0385915348839201 | 0.141587077032671 | 0.785188416386555 | -0.238919136 | 0.316102205867956 | 1.03934586040665 | 0.787478558385414 | 1.37177045145113 | 0.998735809224318 |
| ebi-fl187-GCST90094707 | ebi-gm430-GCST90011623 | OTU99_11 (Parabacteroides) prevalence \|\| id:ebi-gm430-GCST90011623 | Black olive liking \|\| id:ebi-fl187-GCST90094707 | Inverse variance weighted (multiplicative random effects) | 18 | 0.251654628435331 | 0.147412017191406 | 0.0877939194358167 | -0.037272925 | 0.540582182130486 | 1.28615176032308 | 0.963413159677411 | 1.71700618158054 | 0.998735809224318 |
| ebi-fl187-GCST90094707 | ebi-gm430-GCST90011642 | OTU99_19 (Bacteria) prevalence \|\| id:ebi-gm430-GCST90011642 | Black olive liking \|\| id:ebi-fl187-GCST90094707 | Inverse variance weighted | 18 | -0.323691133 | 0.289032328397876 | 0.262750733807379 | -0.890194496 | 0.242812231056077 | 0.723473665301288 | 0.410575889509614 | 1.27482922830573 | 0.998735809224318 |
| ebi-fl187-GCST90094707 | ebi-gm430-GCST90011653 | OTU99_30 (Parasutterella) prevalence \|\| id:ebi-gm430-GCST90011653 | Black olive liking \|\| id:ebi-fl187-GCST90094707 | Inverse variance weighted | 18 | 0.0851247299730451 | 0.135179406582491 | 0.528879953579875 | -0.179826907 | 0.350076366874726 | 1.08885287081803 | 0.83541480341116 | 1.419175922 | 0.998735809224318 |
| ebi-fl187-GCST90094707 | ebi-gm430-GCST90011675 | OTU99_8 (Ruminococcaceae) prevalence \|\| id:ebi-gm430-GCST90011675 | Black olive liking \|\| id:ebi-fl187-GCST90094707 | Inverse variance weighted | 18 | 0.0554154132787275 | 0.125451510918735 | 0.658686228538526 | -0.190469548 | 0.301300374679447 | 1.05697960685162 | 0.826570927982877 | 1.35161527157335 | 0.998735809224318 |
| ebi-fl187-GCST90094707 | ebi-gm430-GCST90011679 | OTU99_92 (Ruminococcus) prevalence \|\| id:ebi-gm430-GCST90011679 | Black olive liking \|\| id:ebi-fl187-GCST90094707 | Inverse variance weighted | 18 | -0.00205518 | 0.145985914942999 | 0.988767804737618 | -0.288187573 | 0.284077213195982 | 0.997946930344285 | 0.749620970111455 | 1.3285355072651 | 0.999095833221658 |
| ebi-fl187-GCST90094707 | ebi-gm430-GCST90011684 | TestASV_11 (Lachnospiraceae) prevalence \|\| id:ebi-gm430-GCST90011684 | Black olive liking \|\| id:ebi-fl187-GCST90094707 | Inverse variance weighted | 18 | 0.0610226639997009 | 0.132079256136033 | 0.644070202773155 | -0.197852678 | 0.319898006026325 | 1.06292300400085 | 0.820490720546242 | 1.37698731276574 | 0.998735809224318 |
| ebi-fl187-GCST90094707 | ebi-gm430-GCST90011688 | TestASV_15 (Bacteroides) prevalence \|\| id:ebi-gm430-GCST90011688 | Black olive liking \|\| id:ebi-fl187-GCST90094707 | Inverse variance weighted | 18 | -0.071255308 | 0.142114040102194 | 0.616092963636984 | -0.349798827 | 0.207288210209145 | 0.931224112445355 | 0.70482986820233 | 1.23033711640425 | 0.998735809224318 |
| ebi-fl187-GCST90094707 | ebi-gm430-GCST90011692 | TestASV_19 (Prevotella) prevalence \|\| id:ebi-gm430-GCST90011692 | Black olive liking \|\| id:ebi-fl187-GCST90094707 | Inverse variance weighted | 18 | -0.207301336 | 0.256959284584301 | 0.419811803108428 | -0.710941533 | 0.296338862174531 | 0.812774695123497 | 0.491181515879401 | 1.34492582411283 | 0.998735809224318 |
| ebi-fl187-GCST90094707 | ebi-gm430-GCST90011693 | TestASV_2 (Bacteroides) prevalence \|\| id:ebi-gm430-GCST90011693 | Black olive liking \|\| id:ebi-fl187-GCST90094707 | Inverse variance weighted | 18 | -0.015027119 | 0.122252419875306 | 0.90217141264737 | -0.254641861 | 0.224587624426593 | 0.985085225178583 | 0.775194075107781 | 1.25180639536005 | 0.998735809224318 |
| ebi-fl187-GCST90094707 | ebi-gm430-GCST90011716 | TestASV_40 (Barnesiella) prevalence \|\| id:ebi-gm430-GCST90011716 | Black olive liking \|\| id:ebi-fl187-GCST90094707 | Inverse variance weighted (multiplicative random effects) | 18 | -0.124094518 | 0.314527537494353 | 0.693180468566033 | -0.740568492 | 0.49237945525494 | 0.883296349326047 | 0.476842757292077 | 1.63620486795991 | 0.998735809224318 |
| ebi-fl187-GCST90094707 | ebi-gm430-GCST90011718 | TestASV_42 (Alphaproteobacteria) prevalence \|\| id:ebi-gm430-GCST90011718 | Black olive liking \|\| id:ebi-fl187-GCST90094707 | Inverse variance weighted | 18 | -0.175662626 | 0.198868766905424 | 0.377069194245106 | -0.565445409 | 0.214120156945906 | 0.83890095881389 | 0.568107050219115 | 1.23877149285057 | 0.998735809224318 |
| ebi-fl187-GCST90094707 | ebi-hbsm44-GCST90104929 | Sulcal depth of the right inferior frontal sulcus \|\| id:ebi-hbsm44-GCST90104929 | Black olive liking \|\| id:ebi-fl187-GCST90094707 | Inverse variance weighted | 20 | 0.0465812565967003 | 0.106282989482875 | 0.661186404944144 | -0.161733403 | 0.254895915983135 | 1.0476832067817 | 0.850667960017704 | 1.29032731143363 | 0.998735809224318 |
| ebi-fl187-GCST90094707 | ebi-hbsm44-GCST90104935 | Sulcal opening distance of the left calloso-marginal posterior fissure \|\| id:ebi-hbsm44-GCST90104935 | Black olive liking \|\| id:ebi-fl187-GCST90094707 | Inverse variance weighted | 20 | 0.0331539750849982 | 0.0273917831719541 | 0.226139993852564 | -0.02053392 | 0.0868418701020283 | 1.03370969252594 | 0.979675465385723 | 1.09072418997587 | 0.998735809224318 |
| ebi-fl187-GCST90094707 | ebi-hbsm44-GCST90104936 | Sulcal opening distance of the left anterior inferior temporal sulcus \|\| id:ebi-hbsm44-GCST90104936 | Black olive liking \|\| id:ebi-fl187-GCST90094707 | Inverse variance weighted | 20 | -0.007034972 | 0.0144257697650169 | 0.625785732056609 | -0.035309481 | 0.0212395369205849 | 0.992989715669546 | 0.965306626391476 | 1.02146670132316 | 0.998735809224318 |
| ebi-fl187-GCST90094707 | ebi-hbsm44-GCST90104941 | Sulcal opening distance of the right posterior inferior temporal sulcus \|\| id:ebi-hbsm44-GCST90104941 | Black olive liking \|\| id:ebi-fl187-GCST90094707 | Inverse variance weighted | 18 | -0.00545981 | 0.0113601977810386 | 0.630794654530857 | -0.027725797 | 0.0168061781398596 | 0.99455506816024 | 0.972655035010164 | 1.01694819643111 | 0.998735809224318 |
| ebi-fl187-GCST90094707 | ebi-IPs91-GCST90274819 | Leukemia inhibitory factor levels \|\| id:ebi-IPs91-GCST90274819 | Black olive liking \|\| id:ebi-fl187-GCST90094707 | Inverse variance weighted | 22 | -0.034093278 | 0.0419795828927139 | 0.416711576692319 | -0.116373261 | 0.0481867040320612 | 0.966481348562544 | 0.890142905732064 | 1.04936655800348 | 0.998735809224318 |
| ebi-fl187-GCST90094707 | ebi-IPs91-GCST90274837 | STAM binding protein levels \|\| id:ebi-IPs91-GCST90274837 | Black olive liking \|\| id:ebi-fl187-GCST90094707 | Inverse variance weighted | 22 | -0.022504171 | 0.0371738457803291 | 0.544928886662049 | -0.095364909 | 0.0503565664694935 | 0.977747158747264 | 0.909041156647766 | 1.0516460112364 | 0.998735809224318 |
| ebi-fl187-GCST90094707 | ebi-IPs91-GCST90274840 | TNF-beta levels \|\| id:ebi-IPs91-GCST90274840 | Black olive liking \|\| id:ebi-fl187-GCST90094707 | Inverse variance weighted (multiplicative random effects) | 22 | 0.0330636109679073 | 0.0529156361343509 | 0.532078495139133 | -0.070651036 | 0.136778257791235 | 1.03361628648259 | 0.931786995651424 | 1.14657387650613 | 0.998735809224318 |
| ebi-fl187-GCST90094707 | ebi-IPs91-GCST90274842 | Tumor necrosis factor ligand superfamily member 14 levels \|\| id:ebi-IPs91-GCST90274842 | Black olive liking \|\| id:ebi-fl187-GCST90094707 | Inverse variance weighted | 22 | -0.000759773 | 0.0424099746076421 | 0.98570669634574 | -0.083883323 | 0.0823637773263959 | 0.999240515649768 | 0.919538538709731 | 1.085850746 | 0.998955228396052 |
| ebi-fl187-GCST90094707 | ebi-met1400-GCST90199628 | 3-methylhistidine levels \|\| id:ebi-met1400-GCST90199628 | Black olive liking \|\| id:ebi-fl187-GCST90094707 | Inverse variance weighted | 22 | -0.082464034 | 0.0487032279933248 | 0.0904184642054687 | -0.177922361 | 0.0129942924365144 | 0.920844555960211 | 0.837007405135259 | 1.01307908513011 | 0.998735809224318 |
| ebi-fl187-GCST90094707 | ebi-met1400-GCST90199651 | Docosahexaenoate DHA; 22:6n3 levels \|\| id:ebi-met1400-GCST90199651 | Black olive liking \|\| id:ebi-fl187-GCST90094707 | Inverse variance weighted | 22 | 0.0105934127203834 | 0.0467242582345452 | 0.820639994824608 | -0.080986133 | 0.102172958860092 | 1.01064972157558 | 0.922206479206489 | 1.1075750200754 | 0.998735809224318 |
| ebi-fl187-GCST90094707 | ebi-met1400-GCST90199652 | 1-stearoyl-2-oleoyl-GPS (18:0/18:1) levels \|\| id:ebi-met1400-GCST90199652 | Black olive liking \|\| id:ebi-fl187-GCST90094707 | Inverse variance weighted | 22 | -0.042599496 | 0.0571636625515582 | 0.456138822290794 | -0.154640275 | 0.0694412823286996 | 0.958295113985807 | 0.856723306959383 | 1.07190911934956 | 0.998735809224318 |
| ebi-fl187-GCST90094707 | ebi-met1400-GCST90199653 | 1-myristoyl-2-palmitoyl-gpc (14:0/16:0) levels \|\| id:ebi-met1400-GCST90199653 | Black olive liking \|\| id:ebi-fl187-GCST90094707 | Inverse variance weighted | 21 | 0.0127494969426671 | 0.0530693692388786 | 0.810142574718818 | -0.091266467 | 0.116765460650869 | 1.01283111828698 | 0.912774454434287 | 1.12385581036691 | 0.998735809224318 |
| ebi-fl187-GCST90094707 | ebi-met1400-GCST90199667 | Erythritol levels in elite athletes \|\| id:ebi-met1400-GCST90199667 | Black olive liking \|\| id:ebi-fl187-GCST90094707 | Inverse variance weighted | 22 | 0.0157610818234237 | 0.0482865242786749 | 0.744116092808604 | -0.078880506 | 0.110402669409626 | 1.01588594279238 | 0.924150348488407 | 1.11672765200077 | 0.998735809224318 |
| ebi-fl187-GCST90094707 | ebi-met1400-GCST90199674 | 3-hydroxymyristate levels \|\| id:ebi-met1400-GCST90199674 | Black olive liking \|\| id:ebi-fl187-GCST90094707 | Inverse variance weighted | 22 | 0.00248618171871921 | 0.0476240270022528 | 0.958365861096327 | -0.090856911 | 0.0958292746431346 | 1.0024892748313 | 0.913148362849889 | 1.10057115255104 | 0.998735809224318 |
| ebi-fl187-GCST90094707 | ebi-met1400-GCST90199685 | 2-linoleoylglycerol (18:2) levels \|\| id:ebi-met1400-GCST90199685 | Black olive liking \|\| id:ebi-fl187-GCST90094707 | Inverse variance weighted | 22 | -0.030972483 | 0.0558419363725303 | 0.579137103472019 | -0.140422679 | 0.078477711896949 | 0.969502250121191 | 0.868990853852134 | 1.08163924720661 | 0.998735809224318 |
| ebi-fl187-GCST90094707 | ebi-met1400-GCST90199703 | Gamma-glutamylvaline levels \|\| id:ebi-met1400-GCST90199703 | Black olive liking \|\| id:ebi-fl187-GCST90094707 | Inverse variance weighted | 22 | -0.066230529 | 0.0541979639761354 | 0.221703321913902 | -0.172458538 | 0.0399974804090515 | 0.935915083867197 | 0.841593181907298 | 1.04080815177829 | 0.998735809224318 |
| ebi-fl187-GCST90094707 | ebi-met1400-GCST90199779 | Gamma-glutamyl-2-aminobutyrate levels \|\| id:ebi-met1400-GCST90199779 | Black olive liking \|\| id:ebi-fl187-GCST90094707 | Inverse variance weighted | 21 | 0.0447903314131977 | 0.0542633845455469 | 0.409130584631075 | -0.061565902 | 0.15114656512247 | 1.04580856371614 | 0.940290976363169 | 1.16316712532141 | 0.998735809224318 |
| ebi-fl187-GCST90094707 | ebi-met1400-GCST90199824 | 4-vinylphenol sulfate levels \|\| id:ebi-met1400-GCST90199824 | Black olive liking \|\| id:ebi-fl187-GCST90094707 | Inverse variance weighted | 22 | -0.030351574 | 0.0488926040072623 | 0.534744073212885 | -0.126181078 | 0.065477930035302 | 0.970104410274733 | 0.881455220471149 | 1.0676691736325 | 0.998735809224318 |
| ebi-fl187-GCST90094707 | ebi-met1400-GCST90199830 | 4-ethylphenylsulfate levels \|\| id:ebi-met1400-GCST90199830 | Black olive liking \|\| id:ebi-fl187-GCST90094707 | Inverse variance weighted | 22 | -0.016336314 | 0.048962424545881 | 0.738643662528757 | -0.112302666 | 0.079630037792883 | 0.98379639959752 | 0.893773701265596 | 1.08288636652717 | 0.998735809224318 |
| ebi-fl187-GCST90094707 | ebi-met1400-GCST90199833 | Hexanoylglutamine levels \|\| id:ebi-met1400-GCST90199833 | Black olive liking \|\| id:ebi-fl187-GCST90094707 | Inverse variance weighted | 22 | 0.0103737816474133 | 0.0507447328700439 | 0.83801693565413 | -0.089085895 | 0.109833458072699 | 1.01042777586681 | 0.914766996491851 | 1.11609217883742 | 0.998735809224318 |
| ebi-fl187-GCST90094707 | ebi-met1400-GCST90199837 | Carotenoid levels (cryptoxanthin) \|\| id:ebi-met1400-GCST90199837 | Black olive liking \|\| id:ebi-fl187-GCST90094707 | Inverse variance weighted | 22 | 0.115329748047511 | 0.048627451042231 | 0.0177064388069006 | 0.0200199440047382 | 0.210639552090284 | 1.122243434 | 1.02022168713002 | 1.23446731369626 | 0.998735809224318 |
| ebi-fl187-GCST90094707 | ebi-met1400-GCST90199869 | 2-hydroxyhippurate levels \|\| id:ebi-met1400-GCST90199869 | Black olive liking \|\| id:ebi-fl187-GCST90094707 | Inverse variance weighted | 22 | 0.029491212928331 | 0.0492100666975728 | 0.548977996969031 | -0.066960518 | 0.125942943655574 | 1.02993038536002 | 0.935232125630694 | 1.13421745213521 | 0.998735809224318 |
| ebi-fl187-GCST90094707 | ebi-met1400-GCST90199902 | Hydantoin-5-propionate levels \|\| id:ebi-met1400-GCST90199902 | Black olive liking \|\| id:ebi-fl187-GCST90094707 | Inverse variance weighted | 22 | -0.006486652 | 0.0499343286559738 | 0.896642653947546 | -0.104357936 | 0.0913846324532436 | 0.993534341196998 | 0.900902774277686 | 1.09569036229152 | 0.998735809224318 |
| ebi-fl187-GCST90094707 | ebi-met1400-GCST90199904 | Pantoate levels \|\| id:ebi-met1400-GCST90199904 | Black olive liking \|\| id:ebi-fl187-GCST90094707 | Inverse variance weighted | 22 | -0.007179655 | 0.0528922385036127 | 0.892025883402219 | -0.110848443 | 0.0964891320205995 | 0.992846056708081 | 0.895074393517048 | 1.10129761219899 | 0.998735809224318 |
| ebi-fl187-GCST90094707 | ebi-met1400-GCST90199936 | 3-methyl catechol sulfate (2) levels \|\| id:ebi-met1400-GCST90199936 | Black olive liking \|\| id:ebi-fl187-GCST90094707 | Inverse variance weighted | 22 | 0.0290283466370294 | 0.0671212240415529 | 0.665395187009807 | -0.102529252 | 0.160585945758473 | 1.02945377561405 | 0.902551747485835 | 1.17419868620071 | 0.998735809224318 |
| ebi-fl187-GCST90094707 | ebi-met1400-GCST90199977 | 4-hydroxychlorothalonil levels \|\| id:ebi-met1400-GCST90199977 | Black olive liking \|\| id:ebi-fl187-GCST90094707 | Inverse variance weighted | 22 | 0.103672992021195 | 0.0484529234668453 | 0.0323819037050034 | 0.00870526202617818 | 0.198640722016212 | 1.1092376660342 | 1.00874326300911 | 1.21974366012483 | 0.998735809224318 |
| ebi-fl187-GCST90094707 | ebi-met1400-GCST90200040 | Sphingomyelin (d18:2/24:1, d18:1/24:2) levels \|\| id:ebi-met1400-GCST90200040 | Black olive liking \|\| id:ebi-fl187-GCST90094707 | Inverse variance weighted | 22 | 0.0552685730353546 | 0.0445406166303412 | 0.21465827225178 | -0.032031036 | 0.142568181630823 | 1.05682441110368 | 0.968476524401379 | 1.15323170749543 | 0.998735809224318 |
| ebi-fl187-GCST90094707 | ebi-met1400-GCST90200077 | 1-stearoyl-2-oleoyl-GPI (18:0/18:1) levels \|\| id:ebi-met1400-GCST90200077 | Black olive liking \|\| id:ebi-fl187-GCST90094707 | Inverse variance weighted | 22 | 0.00610133433370886 | 0.0582969972359528 | 0.9166460031069 | -0.10816078 | 0.120363448916176 | 1.00611998538684 | 0.897483287243533 | 1.12790671356551 | 0.998735809224318 |
| ebi-fl187-GCST90094707 | ebi-met1400-GCST90200080 | 1-palmitoyl-2-oleoyl-GPI (16:0/18:1) levels \|\| id:ebi-met1400-GCST90200080 | Black olive liking \|\| id:ebi-fl187-GCST90094707 | Inverse variance weighted | 22 | -0.00292372 | 0.051987425666633 | 0.955151422764605 | -0.104819074 | 0.0989716341246107 | 0.997080549725509 | 0.900487428997467 | 1.10403498219598 | 0.998735809224318 |
| ebi-fl187-GCST90094707 | ebi-met1400-GCST90200082 | 1-oleoyl-2-linoleoyl-GPE (18:1/18:2) levels \|\| id:ebi-met1400-GCST90200082 | Black olive liking \|\| id:ebi-fl187-GCST90094707 | Inverse variance weighted | 22 | -0.012086984 | 0.0490292930949189 | 0.805275260341914 | -0.108184399 | 0.0840104303623537 | 0.987985770067491 | 0.897462090445507 | 1.08764023823146 | 0.998735809224318 |
| ebi-fl187-GCST90094707 | ebi-met1400-GCST90200100 | Perfluorooctanesulfonate (PFOS) levels \|\| id:ebi-met1400-GCST90200100 | Black olive liking \|\| id:ebi-fl187-GCST90094707 | Inverse variance weighted | 22 | -0.032633717 | 0.045015229758839 | 0.468483710872139 | -0.120863567 | 0.0555961333112859 | 0.967893017411708 | 0.886154851806489 | 1.05717064150196 | 0.998735809224318 |
| ebi-fl187-GCST90094707 | ebi-met1400-GCST90200102 | 1-palmitoleoyl-2-linolenoyl-GPC (16:1/18:3) levels \|\| id:ebi-met1400-GCST90200102 | Black olive liking \|\| id:ebi-fl187-GCST90094707 | Inverse variance weighted | 22 | -0.014266962 | 0.0547599629594857 | 0.794450068509756 | -0.121596489 | 0.0930625657154351 | 0.985834329136457 | 0.88550560760014 | 1.09753040089475 | 0.998735809224318 |
| ebi-fl187-GCST90094707 | ebi-met1400-GCST90200114 | Glycosyl-N-tricosanoyl-sphingadienine (d18:2/23:0) levels \|\| id:ebi-met1400-GCST90200114 | Black olive liking \|\| id:ebi-fl187-GCST90094707 | Inverse variance weighted | 22 | 0.113006472426143 | 0.0510849956801061 | 0.0269579259212295 | 0.0128798808931354 | 0.213133063959151 | 1.11963917970703 | 1.01296318381877 | 1.23754931349935 | 0.998735809224318 |
| ebi-fl187-GCST90094707 | ebi-met1400-GCST90200135 | Arachidoylcarnitine (C20) levels \|\| id:ebi-met1400-GCST90200135 | Black olive liking \|\| id:ebi-fl187-GCST90094707 | Inverse variance weighted | 22 | 0.0548215215312229 | 0.0588662570822192 | 0.351703881062843 | -0.060556342 | 0.170199385412373 | 1.05635206175121 | 0.94124073580951 | 1.18554120737913 | 0.998735809224318 |
| ebi-fl187-GCST90094707 | ebi-met1400-GCST90200146 | Docosahexaenoylcarnitine (C22:6) levels \|\| id:ebi-met1400-GCST90200146 | Black olive liking \|\| id:ebi-fl187-GCST90094707 | Inverse variance weighted | 22 | -0.041766581 | 0.0589837423177761 | 0.478881131447526 | -0.157374716 | 0.0738415543537628 | 0.959093625515677 | 0.854383847929395 | 1.07663620366197 | 0.998735809224318 |
| ebi-fl187-GCST90094707 | ebi-met1400-GCST90200162 | 3-hydroxyphenylacetoylglutamine levels \|\| id:ebi-met1400-GCST90200162 | Black olive liking \|\| id:ebi-fl187-GCST90094707 | Inverse variance weighted | 22 | 0.0740830236348938 | 0.0595846823421753 | 0.213748723016304 | -0.042702954 | 0.190869001025557 | 1.07689620962257 | 0.958195976313301 | 1.2103008935202 | 0.998735809224318 |
| ebi-fl187-GCST90094707 | ebi-met1400-GCST90200172 | 3-carboxy-4-methyl-5-pentyl-2-furanpropionate (3-CMPFP) levels \|\| id:ebi-met1400-GCST90200172 | Black olive liking \|\| id:ebi-fl187-GCST90094707 | Inverse variance weighted | 22 | 0.0368912182450013 | 0.0485323461804889 | 0.447172867846298 | -0.05823218 | 0.132014616758759 | 1.03758014490973 | 0.94343087597141 | 1.14112499869416 | 0.998735809224318 |
| ebi-fl187-GCST90094707 | ebi-met1400-GCST90200222 | Dihydrocaffeate sulfate (2) levels \|\| id:ebi-met1400-GCST90200222 | Black olive liking \|\| id:ebi-fl187-GCST90094707 | Inverse variance weighted (multiplicative random effects) | 22 | 0.0463389218709085 | 0.0725113414942328 | 0.522785479368519 | -0.095783307 | 0.188461151199605 | 1.04742934751976 | 0.90866089477616 | 1.20739017641553 | 0.998735809224318 |
| ebi-fl187-GCST90094707 | ebi-met1400-GCST90200223 | 4-methylhexanoylglutamine levels \|\| id:ebi-met1400-GCST90200223 | Black olive liking \|\| id:ebi-fl187-GCST90094707 | Inverse variance weighted | 22 | 0.0488509281062967 | 0.0660765395987909 | 0.459719866090721 | -0.08065909 | 0.178360945719927 | 1.05006380407218 | 0.922508130545041 | 1.19525666616193 | 0.998735809224318 |
| ebi-fl187-GCST90094707 | ebi-met1400-GCST90200224 | 2-ketocaprylate levels \|\| id:ebi-met1400-GCST90200224 | Black olive liking \|\| id:ebi-fl187-GCST90094707 | Inverse variance weighted | 22 | -0.040346355 | 0.0494741236237315 | 0.41478376970921 | -0.137315637 | 0.0566229274107505 | 0.960456722652438 | 0.871695043327689 | 1.05825669556031 | 0.998735809224318 |
| ebi-fl187-GCST90094707 | ebi-met1400-GCST90200243 | Hydroxypalmitoyl sphingomyelin (d18:1/16:0(OH)) levels \|\| id:ebi-met1400-GCST90200243 | Black olive liking \|\| id:ebi-fl187-GCST90094707 | Inverse variance weighted | 22 | 0.0708570861065809 | 0.052614591561524 | 0.178070652807431 | -0.032267513 | 0.173981685567168 | 1.07342780715856 | 0.968247528286768 | 1.19003377082722 | 0.998735809224318 |
| ebi-fl187-GCST90094707 | ebi-met1400-GCST90200273 | 2-methoxyhydroquinone sulfate (2) levels \|\| id:ebi-met1400-GCST90200273 | Black olive liking \|\| id:ebi-fl187-GCST90094707 | Inverse variance weighted | 22 | -0.058356652 | 0.0657833411800068 | 0.375023016987613 | -0.187292001 | 0.0705786966045712 | 0.943313452702948 | 0.829201573490963 | 1.07312901771773 | 0.998735809224318 |
| ebi-fl187-GCST90094707 | ebi-met1400-GCST90200276 | 2,4-di-tert-butylphenol levels \|\| id:ebi-met1400-GCST90200276 | Black olive liking \|\| id:ebi-fl187-GCST90094707 | Inverse variance weighted | 22 | 0.027133949911139 | 0.0518667877758002 | 0.600872021101152 | -0.074524954 | 0.128792853951707 | 1.0275054278066 | 0.928184311738537 | 1.13745448055949 | 0.998735809224318 |
| ebi-fl187-GCST90094707 | ebi-met1400-GCST90200289 | N-acetylmethionine levels \|\| id:ebi-met1400-GCST90200289 | Black olive liking \|\| id:ebi-fl187-GCST90094707 | Inverse variance weighted | 22 | -0.001149755 | 0.0486271755332746 | 0.981136351447062 | -0.096459019 | 0.0941595094771169 | 0.998850906146438 | 0.908047109866973 | 1.09873499058405 | 0.998955228396052 |
| ebi-fl187-GCST90094707 | ebi-met1400-GCST90200308 | 3-hydroxyisobutyrate levels \|\| id:ebi-met1400-GCST90200308 | Black olive liking \|\| id:ebi-fl187-GCST90094707 | Inverse variance weighted | 22 | 0.066691869462976 | 0.0523459922334845 | 0.202642595750625 | -0.035906275 | 0.169290014240606 | 1.06896604634128 | 0.964730708328088 | 1.1844636004288 | 0.998735809224318 |
| ebi-fl187-GCST90094707 | ebi-met1400-GCST90200383 | Cystathionine levels \|\| id:ebi-met1400-GCST90200383 | Black olive liking \|\| id:ebi-fl187-GCST90094707 | Inverse variance weighted | 22 | -0.020588462 | 0.0588311074476638 | 0.726369645192194 | -0.135897432 | 0.0947205089893415 | 0.979622033700999 | 0.872932162623855 | 1.09935155330735 | 0.998735809224318 |
| ebi-fl187-GCST90094707 | ebi-met1400-GCST90200384 | Guanidinoacetate levels \|\| id:ebi-met1400-GCST90200384 | Black olive liking \|\| id:ebi-fl187-GCST90094707 | Inverse variance weighted | 22 | 0.0504593302423311 | 0.0577231787830027 | 0.38203078384751 | -0.0626781 | 0.163596760657016 | 1.05175408790129 | 0.939245768083129 | 1.17773930850352 | 0.998735809224318 |
| ebi-fl187-GCST90094707 | ebi-met1400-GCST90200403 | Citrulline levels \|\| id:ebi-met1400-GCST90200403 | Black olive liking \|\| id:ebi-fl187-GCST90094707 | Inverse variance weighted | 22 | 0.0627918176947843 | 0.0489274079927809 | 0.199363551917876 | -0.033105902 | 0.158689537360635 | 1.06480514256626 | 0.96743610077388 | 1.17197403604082 | 0.998735809224318 |
| ebi-fl187-GCST90094707 | ebi-met1400-GCST90200434 | 12,13-DiHOME levels \|\| id:ebi-met1400-GCST90200434 | Black olive liking \|\| id:ebi-fl187-GCST90094707 | Inverse variance weighted | 22 | 0.0478275613995051 | 0.0493080723396373 | 0.332059318282522 | -0.04881626 | 0.144471383185194 | 1.04898975340267 | 0.952356099165477 | 1.15542862980352 | 0.998735809224318 |
| ebi-fl187-GCST90094707 | ebi-met1400-GCST90200437 | Fructose levels \|\| id:ebi-met1400-GCST90200437 | Black olive liking \|\| id:ebi-fl187-GCST90094707 | Inverse variance weighted | 22 | 0.0229936168800342 | 0.0539949241563846 | 0.670218791291326 | -0.082836434 | 0.128823668226548 | 1.02326000793498 | 0.920501697258609 | 1.1374895309345 | 0.998735809224318 |
| ebi-fl187-GCST90094707 | ebi-met1400-GCST90200448 | N-stearoyl-sphinganine (d18:0/18:0) levels \|\| id:ebi-met1400-GCST90200448 | Black olive liking \|\| id:ebi-fl187-GCST90094707 | Inverse variance weighted | 22 | -0.035330305 | 0.0644023640848864 | 0.583288854505318 | -0.161558938 | 0.090898328761274 | 0.965286524778718 | 0.850816384187408 | 1.09515765356269 | 0.998735809224318 |
| ebi-fl187-GCST90094707 | ebi-met1400-GCST90200468 | X-11847 levels \|\| id:ebi-met1400-GCST90200468 | Black olive liking \|\| id:ebi-fl187-GCST90094707 | Inverse variance weighted | 22 | 0.133896321799788 | 0.0518735538470622 | 0.00984558487069091 | 0.0322241562595465 | 0.23556848734003 | 1.14327428088002 | 1.03274897650891 | 1.2656280577882 | 0.998735809224318 |
| ebi-fl187-GCST90094707 | ebi-met1400-GCST90200476 | X-11843 levels \|\| id:ebi-met1400-GCST90200476 | Black olive liking \|\| id:ebi-fl187-GCST90094707 | Inverse variance weighted | 22 | 0.0923821408498456 | 0.0588748911255177 | 0.116618509641113 | -0.023012646 | 0.20777692745586 | 1.09678386792784 | 0.977250125628333 | 1.2309385503259 | 0.998735809224318 |
| ebi-fl187-GCST90094707 | ebi-met1400-GCST90200511 | X-12906 levels \|\| id:ebi-met1400-GCST90200511 | Black olive liking \|\| id:ebi-fl187-GCST90094707 | Inverse variance weighted | 22 | -0.025355625 | 0.0560940246554723 | 0.651254528235267 | -0.135299913 | 0.0845886637030487 | 0.974963129475712 | 0.873453912265115 | 1.08826932994326 | 0.998735809224318 |
| ebi-fl187-GCST90094707 | ebi-met1400-GCST90200515 | X-12849 levels \|\| id:ebi-met1400-GCST90200515 | Black olive liking \|\| id:ebi-fl187-GCST90094707 | Inverse variance weighted | 22 | 0.00107679561779306 | 0.0526562588015421 | 0.98368477478384 | -0.102129472 | 0.104283062868816 | 1.00107737557034 | 0.90291264252634 | 1.10991458606092 | 0.998955228396052 |
| ebi-fl187-GCST90094707 | ebi-met1400-GCST90200551 | X-18886 levels \|\| id:ebi-met1400-GCST90200551 | Black olive liking \|\| id:ebi-fl187-GCST90094707 | Inverse variance weighted | 22 | -0.023318479 | 0.0600180872818583 | 0.69762815304317 | -0.14095393 | 0.0943169720637283 | 0.97695129574225 | 0.868529323851446 | 1.09890801385965 | 0.998735809224318 |
| ebi-fl187-GCST90094707 | ebi-met1400-GCST90200564 | X-21319 levels \|\| id:ebi-met1400-GCST90200564 | Black olive liking \|\| id:ebi-fl187-GCST90094707 | Inverse variance weighted | 22 | -0.085715495 | 0.0572000035610884 | 0.133997539472069 | -0.197827502 | 0.0263965120343315 | 0.917855328556998 | 0.820511377564169 | 1.02674798570309 | 0.998735809224318 |
| ebi-fl187-GCST90094707 | ebi-met1400-GCST90200579 | X-21442 levels \|\| id:ebi-met1400-GCST90200579 | Black olive liking \|\| id:ebi-fl187-GCST90094707 | Inverse variance weighted | 22 | 0.0684517880622295 | 0.0532259184935483 | 0.198421707431945 | -0.035871012 | 0.172774588309584 | 1.07084899600127 | 0.964764728351838 | 1.18859815096674 | 0.998735809224318 |
| ebi-fl187-GCST90094707 | ebi-met1400-GCST90200581 | X-21441 levels \|\| id:ebi-met1400-GCST90200581 | Black olive liking \|\| id:ebi-fl187-GCST90094707 | Inverse variance weighted | 22 | 0.00043801446112819 | 0.055125038395326 | 0.993660207618541 | -0.107607061 | 0.108483089715967 | 1.00043811040347 | 0.897980378812195 | 1.11458606041212 | 0.999107451094609 |
| ebi-fl187-GCST90094707 | ebi-met1400-GCST90200648 | X-24585 levels \|\| id:ebi-met1400-GCST90200648 | Black olive liking \|\| id:ebi-fl187-GCST90094707 | Inverse variance weighted | 22 | -0.049176277 | 0.0477488794078547 | 0.303059835560781 | -0.142764081 | 0.0444115261414322 | 0.952013296384407 | 0.866958576652283 | 1.0454124809428 | 0.998735809224318 |
| ebi-fl187-GCST90094707 | ebi-met1400-GCST90200720 | Adenosine 5'-diphosphate (ADP) to pantothenate ratio \|\| id:ebi-met1400-GCST90200720 | Black olive liking \|\| id:ebi-fl187-GCST90094707 | Inverse variance weighted | 22 | 0.00826646889285548 | 0.0649725553414847 | 0.898758231696492 | -0.11907974 | 0.135612677362165 | 1.00830073048924 | 0.887737010166388 | 1.14523823098755 | 0.998735809224318 |
| ebi-fl187-GCST90094707 | ebi-met1400-GCST90200724 | Cholate to taurocholate ratio \|\| id:ebi-met1400-GCST90200724 | Black olive liking \|\| id:ebi-fl187-GCST90094707 | Inverse variance weighted | 22 | 0.0869950801382371 | 0.0523904707137181 | 0.0968112087527219 | -0.015690242 | 0.189680402737125 | 1.09089131267062 | 0.984432208130604 | 1.20886318654696 | 0.998735809224318 |
| ebi-fl187-GCST90094707 | ebi-met1400-GCST90200726 | N-acetylputrescine to (N(1) + N(8))-acetylspermidine ratio \|\| id:ebi-met1400-GCST90200726 | Black olive liking \|\| id:ebi-fl187-GCST90094707 | Inverse variance weighted | 22 | -0.029044391 | 0.0486383333856513 | 0.550407092129373 | -0.124375525 | 0.0662867420227101 | 0.971373342874095 | 0.883048172080192 | 1.06853306657511 | 0.998735809224318 |
| ebi-fl187-GCST90094707 | ebi-met1400-GCST90200744 | Arginine to glutamate ratio \|\| id:ebi-met1400-GCST90200744 | Black olive liking \|\| id:ebi-fl187-GCST90094707 | Inverse variance weighted | 22 | 0.102072082089704 | 0.0553489275188921 | 0.0651603420235897 | -0.006411816 | 0.210555980026733 | 1.10746329711964 | 0.993608695981136 | 1.23436415102629 | 0.998735809224318 |
| ebi-fl187-GCST90094707 | ebi-met1400-GCST90200759 | Histidine to trans-urocanate ratio \|\| id:ebi-met1400-GCST90200759 | Black olive liking \|\| id:ebi-fl187-GCST90094707 | Inverse variance weighted | 22 | -0.017980565 | 0.049281529669551 | 0.71522036038475 | -0.114572364 | 0.0786112327511201 | 0.982180120449406 | 0.891747406072113 | 1.08178367824487 | 0.998735809224318 |
| ebi-fl187-GCST90094707 | ebi-met1400-GCST90200795 | Oleoyl-linoleoyl-glycerol (18:1 to 18:2) [2] to linoleoyl-arachidonoyl-glycerol (18:2 to 20:4) [2] ratio \|\| id:ebi-met1400-GCST90200795 | Black olive liking \|\| id:ebi-fl187-GCST90094707 | Inverse variance weighted | 22 | 0.101422547056533 | 0.0507507569757348 | 0.0456685426883876 | 0.00195106338409305 | 0.200894030728973 | 1.10674419447667 | 1.0019529679467 | 1.222495218 | 0.998735809224318 |
| ebi-fl187-GCST90094707 | ebi-met1400-GCST90200811 | Choline to taurocholate ratio \|\| id:ebi-met1400-GCST90200811 | Black olive liking \|\| id:ebi-fl187-GCST90094707 | Inverse variance weighted | 22 | -0.008892797 | 0.051169460357195 | 0.862029634483094 | -0.109184939 | 0.0913993451958692 | 0.99114662686626 | 0.896564592043277 | 1.09570648302041 | 0.998735809224318 |
| ebi-fl187-GCST90094707 | ebi-met1400-GCST90200831 | Cholate to phosphate ratio \|\| id:ebi-met1400-GCST90200831 | Black olive liking \|\| id:ebi-fl187-GCST90094707 | Inverse variance weighted | 22 | 0.125678590768199 | 0.0509733477726579 | 0.0136793692604732 | 0.0257708291337898 | 0.225586352402609 | 1.13391765810428 | 1.02610576797854 | 1.25305723394743 | 0.998735809224318 |
| ebi-fl187-GCST90094707 | ebi-met1400-GCST90200841 | Adenosine 5'-diphosphate (ADP) to oxalate (ethanedioate) ratio \|\| id:ebi-met1400-GCST90200841 | Black olive liking \|\| id:ebi-fl187-GCST90094707 | Inverse variance weighted | 22 | -0.009426311 | 0.0658987197136916 | 0.88625669176251 | -0.138587802 | 0.119735179333506 | 0.990617977099076 | 0.870586808698894 | 1.12719830664385 | 0.998735809224318 |
| ebi-fl187-GCST90094707 | ebi-met1400-GCST90200844 | Aspartate to phosphate ratio \|\| id:ebi-met1400-GCST90200844 | Black olive liking \|\| id:ebi-fl187-GCST90094707 | Inverse variance weighted | 22 | -0.095866192 | 0.0505830066659187 | 0.0580625648895787 | -0.195008885 | 0.00327650106084582 | 0.908585583951008 | 0.822827347145328 | 1.00328187465771 | 0.998735809224318 |
| ebi-fl187-GCST90094707 | ebi-met1400-GCST90200870 | Adenosine 5'-monophosphate (AMP) to acetoacetate ratio \|\| id:ebi-met1400-GCST90200870 | Black olive liking \|\| id:ebi-fl187-GCST90094707 | Inverse variance weighted | 22 | -0.013633263 | 0.0633327284965178 | 0.829561409570881 | -0.137765411 | 0.110498884405419 | 0.986459248597468 | 0.87130306562562 | 1.11683510311623 | 0.998735809224318 |
| ebi-fl187-GCST90094707 | ebi-met1400-GCST90200887 | Cholesterol to cortisol ratio \|\| id:ebi-met1400-GCST90200887 | Black olive liking \|\| id:ebi-fl187-GCST90094707 | Inverse variance weighted | 22 | -0.04738717 | 0.0551698346958593 | 0.390377693639946 | -0.155520046 | 0.0607457055741809 | 0.953718074662743 | 0.855969917612832 | 1.06262865928172 | 0.998735809224318 |
| ebi-fl187-GCST90094707 | ebi-met1400-GCST90200890 | Cortisol to taurocholate ratio \|\| id:ebi-met1400-GCST90200890 | Black olive liking \|\| id:ebi-fl187-GCST90094707 | Inverse variance weighted | 22 | 0.0562465232876483 | 0.050990158505289 | 0.269989877000022 | -0.043694187 | 0.156187233958015 | 1.05785843833434 | 0.957246650819778 | 1.16904506753489 | 0.998735809224318 |
| ebi-fl187-GCST90094707 | ebi-met1400-GCST90200948 | Adenosine 5'-diphosphate (ADP) to mannitol to sorbitol ratio \|\| id:ebi-met1400-GCST90200948 | Black olive liking \|\| id:ebi-fl187-GCST90094707 | Inverse variance weighted | 22 | -0.046264008 | 0.0675176567151418 | 0.493209222174469 | -0.178598615 | 0.0860705990030125 | 0.954789856601259 | 0.836441566857005 | 1.08988327026136 | 0.998735809224318 |
| ebi-fl187-GCST90094707 | ebi-met1400-GCST90200972 | Leucine to N-palmitoyl-sphingosine (d18:1 to 16:0) ratio \|\| id:ebi-met1400-GCST90200972 | Black olive liking \|\| id:ebi-fl187-GCST90094707 | Inverse variance weighted | 22 | -0.090334196 | 0.0477794867741117 | 0.0586713343766167 | -0.18398199 | 0.00331359802161556 | 0.913625804105414 | 0.831950787014855 | 1.00331909405641 | 0.998735809224318 |
| ebi-fl187-GCST90094707 | ebi-met1400-GCST90200973 | Proline to glutamate ratio \|\| id:ebi-met1400-GCST90200973 | Black olive liking \|\| id:ebi-fl187-GCST90094707 | Inverse variance weighted | 22 | 0.0568763388159959 | 0.0479043437622684 | 0.23511332928747 | -0.037016175 | 0.150768852590042 | 1.05852490385873 | 0.963660548054053 | 1.16272786548307 | 0.998735809224318 |
| ebi-fl187-GCST90094707 | ebi-met1400-GCST90200983 | Cholesterol to linoleoyl-arachidonoyl-glycerol (18:2 to 20:4) [1] ratio \|\| id:ebi-met1400-GCST90200983 | Black olive liking \|\| id:ebi-fl187-GCST90094707 | Inverse variance weighted | 22 | 0.155367015168529 | 0.0577678250239381 | 0.00715574777959769 | 0.0421420781216104 | 0.268591952215448 | 1.16808658793997 | 1.04304266176588 | 1.3081212561481 | 0.998735809224318 |
| ebi-fl187-GCST90094707 | ebi-met1400-GCST90201001 | Serine to alpha-ketobutyrate ratio \|\| id:ebi-met1400-GCST90201001 | Black olive liking \|\| id:ebi-fl187-GCST90094707 | Inverse variance weighted | 22 | -0.024145433 | 0.0526984242350623 | 0.646822182965429 | -0.127434345 | 0.0791434782010899 | 0.976143735629889 | 0.880351213571525 | 1.08235960593932 | 0.998735809224318 |
| ebi-fl187-GCST90094707 | ebi-met1400-GCST90201005 | Fructose to maltose ratio \|\| id:ebi-met1400-GCST90201005 | Black olive liking \|\| id:ebi-fl187-GCST90094707 | Inverse variance weighted | 22 | 0.030212501939274 | 0.0630794699709455 | 0.631967547551989 | -0.093423259 | 0.153848263082327 | 1.03067353080812 | 0.91080791086861 | 1.1663139004748 | 0.998735809224318 |
| ebi-fl187-GCST90094707 | ebi-met1400-GCST90201020 | Paraxanthine to 5-acetylamino-6-formylamino-3-methyluracil ratio \|\| id:ebi-met1400-GCST90201020 | Black olive liking \|\| id:ebi-fl187-GCST90094707 | Inverse variance weighted | 22 | 0.0100848499415283 | 0.0530740371105719 | 0.849297571554789 | -0.093940263 | 0.114109962678249 | 1.01013587341782 | 0.910337141612787 | 1.12087537256566 | 0.998735809224318 |
| ebi-fl187-GCST90094707 | ebi-pw11-GCST90275197 | Placental weight (fetal genotype, adjusted for sex) \|\| id:ebi-pw11-GCST90275197 | Black olive liking \|\| id:ebi-fl187-GCST90094707 | Inverse variance weighted | 19 | -0.058458712 | 0.0240117630598365 | 0.0149088198742476 | -0.105521768 | -0.011395656 | 0.943217183233145 | 0.899854885037238 | 0.988669028239428 | 0.998735809224318 |
| ebi-fl187-GCST90094707 | prot-a-1017 | FAS-associated factor 2 \|\| id:prot-a-1017 | Black olive liking \|\| id:ebi-fl187-GCST90094707 | Inverse variance weighted | 22 | -0.073759787 | 0.0781111858249845 | 0.345020326430583 | -0.226857711 | 0.0793381369931948 | 0.928894799442402 | 0.797034183067718 | 1.08257031726057 | 0.998735809224318 |
| ebi-fl187-GCST90094707 | prot-a-1019 | Fas apoptotic inhibitory molecule 3 \|\| id:prot-a-1019 | Black olive liking \|\| id:ebi-fl187-GCST90094707 | Inverse variance weighted | 22 | -0.060314976 | 0.0772533131539249 | 0.434953718094107 | -0.21173147 | 0.0911015173296969 | 0.941467946644248 | 0.809181957836811 | 1.09538019968716 | 0.998735809224318 |
| ebi-fl187-GCST90094707 | prot-a-1052 | MIP18 family protein FAM96A \|\| id:prot-a-1052 | Black olive liking \|\| id:ebi-fl187-GCST90094707 | Inverse variance weighted | 22 | -0.004728371 | 0.0772378928568042 | 0.951185368546991 | -0.156114641 | 0.146657898741875 | 0.995282789891632 | 0.855461113612099 | 1.157957756 | 0.998735809224318 |
| ebi-fl187-GCST90094707 | prot-a-1066 | F-box/LRR-repeat protein 4 \|\| id:prot-a-1066 | Black olive liking \|\| id:ebi-fl187-GCST90094707 | Inverse variance weighted | 22 | -0.066397285 | 0.0772630488201627 | 0.390138280208325 | -0.217832861 | 0.0850382906350759 | 0.935759027359624 | 0.80425985383032 | 1.08875875516443 | 0.998735809224318 |
| ebi-fl187-GCST90094707 | prot-a-1076 | Low affinity immunoglobulin gamma Fc region receptor III-B \|\| id:prot-a-1076 | Black olive liking \|\| id:ebi-fl187-GCST90094707 | Inverse variance weighted | 22 | -0.146234102 | 0.0947070152969701 | 0.122571782845516 | -0.331859852 | 0.0393916480961398 | 0.863955425914263 | 0.71758788440908 | 1.04017778753519 | 0.998735809224318 |
| ebi-fl187-GCST90094707 | prot-a-1081 | Fc receptor-like protein 3 \|\| id:prot-a-1081 | Black olive liking \|\| id:ebi-fl187-GCST90094707 | Inverse variance weighted (multiplicative random effects) | 22 | -0.043079466 | 0.0986148396283587 | 0.662223269614092 | -0.236364552 | 0.150205619181342 | 0.957835271235388 | 0.789492810154623 | 1.16207316269655 | 0.998735809224318 |
| ebi-fl187-GCST90094707 | prot-a-1105 | Fibroblast growth factor receptor 4 \|\| id:prot-a-1105 | Black olive liking \|\| id:ebi-fl187-GCST90094707 | Inverse variance weighted | 22 | -0.001546043 | 0.0949945480140456 | 0.987014948350466 | -0.187735357 | 0.184643271449113 | 0.998455151849868 | 0.828834023527594 | 1.20278929430598 | 0.998955228396052 |
| ebi-fl187-GCST90094707 | prot-a-1143 | Follistatin-related protein 1 \|\| id:prot-a-1143 | Black olive liking \|\| id:ebi-fl187-GCST90094707 | Inverse variance weighted | 22 | -0.016087342 | 0.0955915773058658 | 0.866353199930868 | -0.203446834 | 0.171272149165214 | 0.984041367811928 | 0.815913582134041 | 1.1868136954314 | 0.998735809224318 |
| ebi-fl187-GCST90094707 | prot-a-1151 | Alpha-(1,3)-fucosyltransferase 10 \|\| id:prot-a-1151 | Black olive liking \|\| id:ebi-fl187-GCST90094707 | Inverse variance weighted | 22 | -0.132073473 | 0.0772417250236925 | 0.0872895211387208 | -0.283467254 | 0.0193203081580677 | 0.876276610140571 | 0.753167785017646 | 1.01950815310225 | 0.998735809224318 |
| ebi-fl187-GCST90094707 | prot-a-1165 | Growth arrest and DNA damage-inducible proteins-interacting protein 1 \|\| id:prot-a-1165 | Black olive liking \|\| id:ebi-fl187-GCST90094707 | Inverse variance weighted | 22 | 0.0840912490732992 | 0.0772339961234008 | 0.276248521651506 | -0.067287383 | 0.235469881475165 | 1.08772814346563 | 0.934926480441759 | 1.26550326559169 | 0.998735809224318 |
| ebi-fl187-GCST90094707 | prot-a-1194 | Growth/differentiation factor 11/8 \|\| id:prot-a-1194 | Black olive liking \|\| id:ebi-fl187-GCST90094707 | Inverse variance weighted (multiplicative random effects) | 22 | -0.106132149 | 0.100242423926114 | 0.289711457555778 | -0.3026073 | 0.0903430020747466 | 0.899305798022729 | 0.738889201404106 | 1.09454965212705 | 0.998735809224318 |
| ebi-fl187-GCST90094707 | prot-a-1216 | D-glucuronyl C5-epimerase \|\| id:prot-a-1216 | Black olive liking \|\| id:ebi-fl187-GCST90094707 | Inverse variance weighted | 22 | 0.164073745311603 | 0.0772417250236925 | 0.0336568071195832 | 0.0126799642651658 | 0.31546752635804 | 1.17830120607839 | 1.01276069587583 | 1.37090009308181 | 0.998735809224318 |
| ebi-fl187-GCST90094707 | prot-a-1246 | Glypican-3 \|\| id:prot-a-1246 | Black olive liking \|\| id:ebi-fl187-GCST90094707 | Inverse variance weighted | 22 | 0.00124942155206098 | 0.0772533131539249 | 0.987096337125901 | -0.150167072 | 0.152665915333754 | 1.00125020240434 | 0.860564188036232 | 1.16493572676129 | 0.998955228396052 |
| ebi-fl187-GCST90094707 | prot-a-1275 | Glutamate receptor 4 \|\| id:prot-a-1275 | Black olive liking \|\| id:ebi-fl187-GCST90094707 | Inverse variance weighted | 21 | 0.00719874918165658 | 0.0791251188679509 | 0.927509026440189 | -0.147886484 | 0.16228398216284 | 1.00722472246419 | 0.862529020402206 | 1.17619421207415 | 0.998735809224318 |
| ebi-fl187-GCST90094707 | prot-a-1289 | Glutathione S-transferase P \|\| id:prot-a-1289 | Black olive liking \|\| id:ebi-fl187-GCST90094707 | Inverse variance weighted | 22 | -0.01982722 | 0.0822905209438985 | 0.809600282869874 | -0.181116641 | 0.141462201001361 | 0.980368046617549 | 0.834338034914827 | 1.15195696061827 | 0.998735809224318 |
| ebi-fl187-GCST90094707 | prot-a-130 | Apolipoprotein D \|\| id:prot-a-130 | Black olive liking \|\| id:ebi-fl187-GCST90094707 | Inverse variance weighted | 22 | 0.130700921333914 | 0.077238535874235 | 0.0906131006232064 | -0.020686609 | 0.282088451647414 | 1.13962689224744 | 0.97952589109147 | 1.32589599248508 | 0.998735809224318 |
| ebi-fl187-GCST90094707 | prot-a-1313 | Heparin-binding EGF-like growth factor \|\| id:prot-a-1313 | Black olive liking \|\| id:ebi-fl187-GCST90094707 | Inverse variance weighted | 22 | 0.068857328677649 | 0.0772514563089353 | 0.372746687351342 | -0.082555526 | 0.220270183043162 | 1.07128335683165 | 0.920760310587776 | 1.24641344487566 | 0.998735809224318 |
| ebi-fl187-GCST90094707 | prot-a-1347 | HLA class II histocompatibility antigen, DQ alpha 2 chain \|\| id:prot-a-1347 | Black olive liking \|\| id:ebi-fl187-GCST90094707 | Inverse variance weighted | 21 | -0.109730829 | 0.0791390095490967 | 0.165576575919977 | -0.264843287 | 0.0453816301212475 | 0.896075300685346 | 0.767326190304477 | 1.04642713182997 | 0.998735809224318 |
| ebi-fl187-GCST90094707 | prot-a-1357 | Non-histone chromosomal protein HMG-14 \|\| id:prot-a-1357 | Black olive liking \|\| id:ebi-fl187-GCST90094707 | Inverse variance weighted | 22 | -0.027752811 | 0.0772630488201627 | 0.719446213946389 | -0.179188387 | 0.123682764707515 | 0.972628760238777 | 0.835948403028526 | 1.13165681256925 | 0.998735809224318 |
| ebi-fl187-GCST90094707 | prot-a-137 | Apolipoprotein M \|\| id:prot-a-137 | Black olive liking \|\| id:ebi-fl187-GCST90094707 | Inverse variance weighted | 22 | -0.074537603 | 0.0772495856243546 | 0.334598287999925 | -0.225946791 | 0.0768715849117501 | 0.928172571411257 | 0.797760548788247 | 1.07990339159885 | 0.998735809224318 |
| ebi-fl187-GCST90094707 | prot-a-1381 | 17-beta-hydroxysteroid dehydrogenase 14 \|\| id:prot-a-1381 | Black olive liking \|\| id:ebi-fl187-GCST90094707 | Inverse variance weighted | 21 | -0.097630961 | 0.0821122921387948 | 0.234442321674755 | -0.258571054 | 0.06330913119424 | 0.906983553943672 | 0.77215416446581 | 1.06535612314336 | 0.998735809224318 |
| ebi-fl187-GCST90094707 | prot-a-1392 | Serine protease HTRA2, mitochondrial \|\| id:prot-a-1392 | Black olive liking \|\| id:ebi-fl187-GCST90094707 | Inverse variance weighted (multiplicative random effects) | 22 | -0.038572981 | 0.0981571744092087 | 0.694340463653163 | -0.230961043 | 0.153815080795996 | 0.962161482625933 | 0.793770388434758 | 1.16627520015509 | 0.998735809224318 |
| ebi-fl187-GCST90094707 | prot-a-1416 | Interferon-induced protein with tetratricopeptide repeats 2 \|\| id:prot-a-1416 | Black olive liking \|\| id:ebi-fl187-GCST90094707 | Inverse variance weighted | 22 | -0.071254437 | 0.0772630488201627 | 0.356407705274519 | -0.222690012 | 0.0801811389673568 | 0.931224924166757 | 0.800362913416679 | 1.08348331094886 | 0.998735809224318 |
| ebi-fl187-GCST90094707 | prot-a-1428 | Interferon gamma \|\| id:prot-a-1428 | Black olive liking \|\| id:ebi-fl187-GCST90094707 | Inverse variance weighted | 22 | -0.078443811 | 0.0772398550459502 | 0.309825982633587 | -0.229833927 | 0.0729463045161029 | 0.924554007891501 | 0.794665563829815 | 1.07567277659373 | 0.998735809224318 |
| ebi-fl187-GCST90094707 | prot-a-1483 | Interleukin-17C \|\| id:prot-a-1483 | Black olive liking \|\| id:ebi-fl187-GCST90094707 | Inverse variance weighted | 22 | -0.000116835 | 0.0850928048442983 | 0.998904476264918 | -0.166898733 | 0.166665062026059 | 0.999883171356232 | 0.84628530780308 | 1.18135851720828 | 0.999882738470636 |
| ebi-fl187-GCST90094707 | prot-a-1503 | Interleukin-1 receptor-like 2 \|\| id:prot-a-1503 | Black olive liking \|\| id:ebi-fl187-GCST90094707 | Inverse variance weighted | 22 | -0.09587411 | 0.0772514563089353 | 0.214581586673937 | -0.247286965 | 0.0555387441349955 | 0.908578389593349 | 0.78091656596769 | 1.05710997309052 | 0.998735809224318 |
| ebi-fl187-GCST90094707 | prot-a-1522 | Interleukin-31 receptor subunit alpha \|\| id:prot-a-1522 | Black olive liking \|\| id:ebi-fl187-GCST90094707 | Inverse variance weighted | 22 | -0.143549598 | 0.0772330280330949 | 0.0630759747790983 | -0.294926333 | 0.00782713711479602 | 0.866277833620944 | 0.744586437062214 | 1.00785784922952 | 0.998735809224318 |
| ebi-fl187-GCST90094707 | prot-a-1540 | Interleukin-6 receptor subunit alpha \|\| id:prot-a-1540 | Black olive liking \|\| id:ebi-fl187-GCST90094707 | Inverse variance weighted | 22 | 0.00447440544596192 | 0.0772630488201627 | 0.953819263091538 | -0.14696117 | 0.155909981133481 | 1.00448443054455 | 0.863327499563752 | 1.1687209914155 | 0.998735809224318 |
| ebi-fl187-GCST90094707 | prot-a-1541 | Interleukin-6 receptor subunit alpha \|\| id:prot-a-1541 | Black olive liking \|\| id:ebi-fl187-GCST90094707 | Inverse variance weighted | 22 | -0.034026484 | 0.0772514563089353 | 0.659601093615845 | -0.185439338 | 0.117386370316809 | 0.966545906249678 | 0.830739228011408 | 1.12455383998694 | 0.998735809224318 |
| ebi-fl187-GCST90094707 | prot-a-1545 | Interleukin-9 \|\| id:prot-a-1545 | Black olive liking \|\| id:ebi-fl187-GCST90094707 | Inverse variance weighted | 22 | 0.181402422141711 | 0.080536740372336 | 0.0242958548100788 | 0.023550411011932 | 0.339254433271489 | 1.19889754514715 | 1.02382991174763 | 1.40390049877167 | 0.998735809224318 |
| ebi-fl187-GCST90094707 | prot-a-1548 | Inositol monophosphatase 3 \|\| id:prot-a-1548 | Black olive liking \|\| id:ebi-fl187-GCST90094707 | Inverse variance weighted | 22 | -0.108363605 | 0.0870520054103284 | 0.213199915800456 | -0.278985536 | 0.0622583251228661 | 0.897301273446145 | 0.756550845822568 | 1.06423722843462 | 0.998735809224318 |
| ebi-fl187-GCST90094707 | prot-a-1555 | Inhibin beta A chain:Inhibin beta B chain heterodimer \|\| id:prot-a-1555 | Black olive liking \|\| id:ebi-fl187-GCST90094707 | Inverse variance weighted | 22 | 0.0154770812326946 | 0.083065354092677 | 0.852190572582538 | -0.147331013 | 0.178285175254342 | 1.01559747154945 | 0.863008263359281 | 1.19516610443884 | 0.998735809224318 |
| ebi-fl187-GCST90094707 | prot-a-1603 | Junctophilin-3 \|\| id:prot-a-1603 | Black olive liking \|\| id:ebi-fl187-GCST90094707 | Inverse variance weighted | 22 | -0.015060239 | 0.0772630488201627 | 0.845454306154331 | -0.166495815 | 0.136375336813225 | 0.98505259935608 | 0.846626360430324 | 1.1461119908964 | 0.998735809224318 |
| ebi-fl187-GCST90094707 | prot-a-1627 | UPF0258 protein KIAA1024 \|\| id:prot-a-1627 | Black olive liking \|\| id:ebi-fl187-GCST90094707 | Inverse variance weighted | 22 | 0.108087330186774 | 0.0781458065041825 | 0.166619042266915 | -0.045078451 | 0.261253110934972 | 1.11414503963245 | 0.955922486235692 | 1.29855630264097 | 0.998735809224318 |
| ebi-fl187-GCST90094707 | prot-a-1666 | Kallikrein-7 \|\| id:prot-a-1666 | Black olive liking \|\| id:ebi-fl187-GCST90094707 | Inverse variance weighted | 22 | 0.0990609954786221 | 0.0772416186939132 | 0.199673570567327 | -0.052332577 | 0.250454568118692 | 1.10413364466511 | 0.949013194238733 | 1.28460922638634 | 0.998735809224318 |
| ebi-fl187-GCST90094707 | prot-a-1678 | Importin subunit alpha-3 \|\| id:prot-a-1678 | Black olive liking \|\| id:ebi-fl187-GCST90094707 | Inverse variance weighted | 22 | 0.101740124504398 | 0.0880308588452999 | 0.247790590548496 | -0.070800359 | 0.274280607841185 | 1.10709572728988 | 0.931647868831006 | 1.31558391361043 | 0.998735809224318 |
| ebi-fl187-GCST90094707 | prot-a-1718 | Protein LDOC1 \|\| id:prot-a-1718 | Black olive liking \|\| id:ebi-fl187-GCST90094707 | Inverse variance weighted | 22 | 0.0792667837074657 | 0.0772482659540224 | 0.304830185656481 | -0.072139818 | 0.23067338497735 | 1.08249307506718 | 0.930400800344626 | 1.25944781768712 | 0.998735809224318 |
| ebi-fl187-GCST90094707 | prot-a-1723 | Leptin \|\| id:prot-a-1723 | Black olive liking \|\| id:ebi-fl187-GCST90094707 | Inverse variance weighted (multiplicative random effects) | 22 | -0.179151549 | 0.0981966886253061 | 0.0680892980111254 | -0.371617059 | 0.0133139603442297 | 0.835979197682968 | 0.689618275032244 | 1.01340298576917 | 0.998735809224318 |
| ebi-fl187-GCST90094707 | prot-a-1730 | Galectin-7 \|\| id:prot-a-1730 | Black olive liking \|\| id:ebi-fl187-GCST90094707 | Inverse variance weighted | 22 | -0.172619273 | 0.0910482802719083 | 0.0579720516690682 | -0.351073902 | 0.00583535675514449 | 0.841457919971045 | 0.703931730033437 | 1.00585241561474 | 0.998735809224318 |
| ebi-fl187-GCST90094707 | prot-a-1753 | Lipase member N \|\| id:prot-a-1753 | Black olive liking \|\| id:ebi-fl187-GCST90094707 | Inverse variance weighted | 22 | 0.198492880664291 | 0.0772425871073023 | 0.0101776712388641 | 0.0470974099339783 | 0.349888351394603 | 1.21956334490247 | 1.04822411154818 | 1.41890912052479 | 0.998735809224318 |
| ebi-fl187-GCST90094707 | prot-a-1756 | VIP36-like protein \|\| id:prot-a-1756 | Black olive liking \|\| id:ebi-fl187-GCST90094707 | Inverse variance weighted | 22 | -0.116573567 | 0.0772282729699731 | 0.131179357559582 | -0.267940982 | 0.0347938477700561 | 0.88996462231748 | 0.7649529255542 | 1.03540623549204 | 0.998735809224318 |
| ebi-fl187-GCST90094707 | prot-a-1760 | Ig Kappa chain V-I region HK102- like \|\| id:prot-a-1760 | Black olive liking \|\| id:ebi-fl187-GCST90094707 | Inverse variance weighted | 22 | -0.221912165 | 0.0846954982705291 | 0.00878994515850419 | -0.387915342 | -0.055908988 | 0.800985715884659 | 0.678469779042132 | 0.945625194915893 | 0.998735809224318 |
| ebi-fl187-GCST90094707 | prot-a-179 | Neutral ceramidase \|\| id:prot-a-179 | Black olive liking \|\| id:ebi-fl187-GCST90094707 | Inverse variance weighted | 22 | -0.071436713 | 0.0772630488201627 | 0.35517874038573 | -0.222872289 | 0.079998862659482 | 0.931055199394651 | 0.800217039514944 | 1.08328583560938 | 0.998735809224318 |
| ebi-fl187-GCST90094707 | prot-a-1809 | Lactotransferrin \|\| id:prot-a-1809 | Black olive liking \|\| id:ebi-fl187-GCST90094707 | Inverse variance weighted | 22 | -0.003289671 | 0.089467288809319 | 0.970668758871998 | -0.178645557 | 0.172066214753138 | 0.996715733726988 | 0.836402303489142 | 1.18775647761212 | 0.998919666907682 |
| ebi-fl187-GCST90094707 | prot-a-1818 | Lysophospholipase-like protein 1 \|\| id:prot-a-1818 | Black olive liking \|\| id:ebi-fl187-GCST90094707 | Inverse variance weighted | 22 | 0.0358592999262875 | 0.0852735418871369 | 0.674105021677909 | -0.131276842 | 0.202995442025076 | 1.03650999919751 | 0.876974957129205 | 1.22506688441063 | 0.998735809224318 |
| ebi-fl187-GCST90094707 | prot-a-1852 | MAP kinase-activated protein kinase 3 \|\| id:prot-a-1852 | Black olive liking \|\| id:ebi-fl187-GCST90094707 | Inverse variance weighted | 22 | 0.106669565465864 | 0.0772630488201627 | 0.167401185955856 | -0.04476601 | 0.258105141153383 | 1.1125665633197 | 0.956221201645026 | 1.29447491405499 | 0.998735809224318 |
| ebi-fl187-GCST90094707 | prot-a-1856 | Megakaryocyte-associated tyrosine-protein kinase \|\| id:prot-a-1856 | Black olive liking \|\| id:ebi-fl187-GCST90094707 | Inverse variance weighted | 22 | 0.0564671212605385 | 0.0772533131539249 | 0.464819087231214 | -0.094949373 | 0.207883615042231 | 1.05809182550286 | 0.90941897489268 | 1.23106988319448 | 0.998735809224318 |
| ebi-fl187-GCST90094707 | prot-a-1890 | Beta-1,3-N-acetylglucosaminyltransferase manic fringe \|\| id:prot-a-1890 | Black olive liking \|\| id:ebi-fl187-GCST90094707 | Inverse variance weighted | 22 | -0.042844894 | 0.0772630488201627 | 0.579214268768578 | -0.19428047 | 0.108590681360278 | 0.958059979080832 | 0.823426925316859 | 1.11470598701052 | 0.998735809224318 |
| ebi-fl187-GCST90094707 | prot-a-1919 | Matrilysin \|\| id:prot-a-1919 | Black olive liking \|\| id:ebi-fl187-GCST90094707 | Inverse variance weighted | 22 | -0.093463209 | 0.0895674222952084 | 0.296718827717814 | -0.269015357 | 0.082088938589006 | 0.91077152490516 | 0.764131520936881 | 1.08555235303085 | 0.998735809224318 |
| ebi-fl187-GCST90094707 | prot-a-1923 | Multimerin-2 \|\| id:prot-a-1923 | Black olive liking \|\| id:ebi-fl187-GCST90094707 | Inverse variance weighted | 22 | -0.058639806 | 0.083638451646702 | 0.483233987510531 | -0.222571171 | 0.105291559115097 | 0.943046387533672 | 0.800458035051928 | 1.11103449537191 | 0.998735809224318 |
| ebi-fl187-GCST90094707 | prot-a-1948 | Mesothelin \|\| id:prot-a-1948 | Black olive liking \|\| id:ebi-fl187-GCST90094707 | Inverse variance weighted | 22 | 0.0260853208331902 | 0.079748372468055 | 0.743595777922527 | -0.130221489 | 0.182392130870578 | 1.02642852047411 | 0.877900963799336 | 1.20008469188044 | 0.998735809224318 |
| ebi-fl187-GCST90094707 | prot-a-195 | ATPase family AAA domain-containing protein 1 \|\| id:prot-a-195 | Black olive liking \|\| id:ebi-fl187-GCST90094707 | Inverse variance weighted | 22 | -0.005017756 | 0.0772495856243546 | 0.94820973024176 | -0.156426944 | 0.146391431374262 | 0.994994811460729 | 0.855193992239472 | 1.15764923960849 | 0.998735809224318 |
| ebi-fl187-GCST90094707 | prot-a-1966 | Mucin-1 \|\| id:prot-a-1966 | Black olive liking \|\| id:ebi-fl187-GCST90094707 | Inverse variance weighted | 22 | 0.0236275552598068 | 0.0772630488201627 | 0.75975185377057 | -0.12780802 | 0.175063130947326 | 1.02390889738273 | 0.880022309235093 | 1.19132142348843 | 0.998735809224318 |
| ebi-fl187-GCST90094707 | prot-a-1986 | Myocardial zonula adherens protein \|\| id:prot-a-1986 | Black olive liking \|\| id:ebi-fl187-GCST90094707 | Inverse variance weighted | 22 | -0.143807973 | 0.0772514563089353 | 0.0626655882863531 | -0.295220828 | 0.00760488107787308 | 0.866054037602244 | 0.744367192454901 | 1.00763387162928 | 0.998735809224318 |
| ebi-fl187-GCST90094707 | prot-a-2071 | Epididymal secretory protein E1 \|\| id:prot-a-2071 | Black olive liking \|\| id:ebi-fl187-GCST90094707 | Inverse variance weighted | 22 | -0.052559482 | 0.0772514563089353 | 0.496270962416509 | -0.203972336 | 0.0988533726588098 | 0.948797883259897 | 0.815484930391476 | 1.10390442512078 | 0.998735809224318 |
| ebi-fl187-GCST90094707 | prot-a-2098 | Neuritin \|\| id:prot-a-2098 | Black olive liking \|\| id:ebi-fl187-GCST90094707 | Inverse variance weighted | 22 | -0.038712242 | 0.0887535840379402 | 0.662708563326403 | -0.212669266 | 0.135244783193485 | 0.962027500890401 | 0.808423465943145 | 1.14481698201289 | 0.998735809224318 |
| ebi-fl187-GCST90094707 | prot-a-2115 | Neurotrophin-4 \|\| id:prot-a-2115 | Black olive liking \|\| id:ebi-fl187-GCST90094707 | Inverse variance weighted (multiplicative random effects) | 22 | -0.019831291 | 0.0967168073514169 | 0.837537023333457 | -0.209396233 | 0.169733651623903 | 0.98036405580598 | 0.811073797604295 | 1.18498918933793 | 0.998735809224318 |
| ebi-fl187-GCST90094707 | prot-a-2118 | Netrin-1 \|\| id:prot-a-2118 | Black olive liking \|\| id:ebi-fl187-GCST90094707 | Inverse variance weighted | 22 | 0.0189638462752687 | 0.0833552410511186 | 0.820030035550781 | -0.144412426 | 0.182340118735461 | 1.01914480207071 | 0.865530706910321 | 1.20002227453654 | 0.998735809224318 |
| ebi-fl187-GCST90094707 | prot-a-2120 | Netrin-G1 \|\| id:prot-a-2120 | Black olive liking \|\| id:ebi-fl187-GCST90094707 | Inverse variance weighted | 22 | -0.103700395 | 0.0772533131539249 | 0.179485015290491 | -0.255116888 | 0.0477160992544823 | 0.90149534990511 | 0.774825924575681 | 1.04887283727063 | 0.998735809224318 |
| ebi-fl187-GCST90094707 | prot-a-2145 | Olfactomedin-like protein 3 \|\| id:prot-a-2145 | Black olive liking \|\| id:ebi-fl187-GCST90094707 | Inverse variance weighted | 22 | 0.0283822456666922 | 0.0772630488201627 | 0.713361199338763 | -0.12305333 | 0.179817821354211 | 1.02878885935536 | 0.884216506018721 | 1.19699927554993 | 0.998735809224318 |
| ebi-fl187-GCST90094707 | prot-a-2162 | Prolyl 4-hydroxylase subunit alpha-2 \|\| id:prot-a-2162 | Black olive liking \|\| id:ebi-fl187-GCST90094707 | Inverse variance weighted | 22 | 0.0120606417001047 | 0.0772416186939132 | 0.875921288302263 | -0.139332931 | 0.163454214340175 | 1.01213366451125 | 0.869938350846748 | 1.17757143806783 | 0.998735809224318 |
| ebi-fl187-GCST90094707 | prot-a-2168 | Polyadenylate-binding protein-interacting protein 1 \|\| id:prot-a-2168 | Black olive liking \|\| id:ebi-fl187-GCST90094707 | Inverse variance weighted | 22 | 0.0482128418024844 | 0.0772482659540224 | 0.532543220117648 | -0.103193759 | 0.199619443072368 | 1.04939398646412 | 0.901952194772669 | 1.22093803331186 | 0.998735809224318 |
| ebi-fl187-GCST90094707 | prot-a-219 | Beta-1,4-galactosyltransferase 5 \|\| id:prot-a-219 | Black olive liking \|\| id:ebi-fl187-GCST90094707 | Inverse variance weighted | 22 | -0.008643797 | 0.0814455970918422 | 0.915479440437223 | -0.168277168 | 0.150989572838635 | 0.991393452750873 | 0.845119562322593 | 1.16298453139122 | 0.998735809224318 |
| ebi-fl187-GCST90094707 | prot-a-2190 | Pyruvate carboxylase, mitochondrial \|\| id:prot-a-2190 | Black olive liking \|\| id:ebi-fl187-GCST90094707 | Inverse variance weighted | 22 | -0.008685755 | 0.0772630488201627 | 0.910492004242975 | -0.160121331 | 0.142749820251817 | 0.991351856762482 | 0.852040403675204 | 1.15344119793767 | 0.998735809224318 |
| ebi-fl187-GCST90094707 | prot-a-2197 | Protocadherin-9 \|\| id:prot-a-2197 | Black olive liking \|\| id:ebi-fl187-GCST90094707 | Inverse variance weighted (multiplicative random effects) | 22 | -0.09811877 | 0.107649848997729 | 0.362051885217455 | -0.309112474 | 0.112874934134587 | 0.906541227547887 | 0.734098198468608 | 1.11949191396793 | 0.998735809224318 |
| ebi-fl187-GCST90094707 | prot-a-2198 | Protocadherin alpha-4 \|\| id:prot-a-2198 | Black olive liking \|\| id:ebi-fl187-GCST90094707 | Inverse variance weighted | 22 | 0.0171942342571895 | 0.0787175897652776 | 0.827094576916282 | -0.137092242 | 0.171480710197134 | 1.01734290597951 | 0.871889797840617 | 1.18706124433404 | 0.998735809224318 |
| ebi-fl187-GCST90094707 | prot-a-2254 | Placenta growth factor \|\| id:prot-a-2254 | Black olive liking \|\| id:ebi-fl187-GCST90094707 | Inverse variance weighted | 22 | -0.055867481 | 0.0857011601363094 | 0.51447400864534 | -0.223841754 | 0.112106793291633 | 0.945664446495356 | 0.799441632446877 | 1.11863231669361 | 0.998735809224318 |
| ebi-fl187-GCST90094707 | prot-a-2261 | Membrane-associated progesterone receptor component 2 \|\| id:prot-a-2261 | Black olive liking \|\| id:ebi-fl187-GCST90094707 | Inverse variance weighted | 22 | -0.099618239 | 0.0838588526000629 | 0.234861965373997 | -0.26398159 | 0.0647451120866294 | 0.905182915609468 | 0.767987678098603 | 1.0668870531098 | 0.998735809224318 |
| ebi-fl187-GCST90094707 | prot-a-2268 | Polymeric immunoglobulin receptor \|\| id:prot-a-2268 | Black olive liking \|\| id:ebi-fl187-GCST90094707 | Inverse variance weighted | 22 | 0.0853865062787698 | 0.0813239758838668 | 0.293738852501505 | -0.074008486 | 0.244781499011149 | 1.0891379440111 | 0.92866381274548 | 1.27734218218089 | 0.998735809224318 |
| ebi-fl187-GCST90094707 | prot-a-2283 | Group 10 secretory phospholipase A2 \|\| id:prot-a-2283 | Black olive liking \|\| id:ebi-fl187-GCST90094707 | Inverse variance weighted | 22 | 0.10521520455521 | 0.0772308975169431 | 0.173088715912448 | -0.046157355 | 0.256587763688418 | 1.11094966606059 | 0.954891693788803 | 1.29251219645975 | 0.998735809224318 |
| ebi-fl187-GCST90094707 | prot-a-2298 | Inactive phospholipase D5 \|\| id:prot-a-2298 | Black olive liking \|\| id:ebi-fl187-GCST90094707 | Inverse variance weighted | 22 | 0.10733973977862 | 0.0829588134014956 | 0.195702749910086 | -0.055259534 | 0.269939014045551 | 1.11331242675301 | 0.946239534288902 | 1.30988456373694 | 0.998735809224318 |
| ebi-fl187-GCST90094707 | prot-a-2304 | Procollagen-lysine,2-oxoglutarate 5-dioxygenase 2 \|\| id:prot-a-2304 | Black olive liking \|\| id:ebi-fl187-GCST90094707 | Inverse variance weighted | 22 | -0.103138111 | 0.0834072440872852 | 0.216250260296244 | -0.266616309 | 0.0603400878471642 | 0.902002388819913 | 0.76596690971893 | 1.06219772566335 | 0.998735809224318 |
| ebi-fl187-GCST90094707 | prot-a-2331 | Periostin \|\| id:prot-a-2331 | Black olive liking \|\| id:ebi-fl187-GCST90094707 | Inverse variance weighted | 22 | -0.135035063 | 0.0824385174326807 | 0.101419742170102 | -0.296614557 | 0.0265444313500672 | 0.873685277283118 | 0.743330468688317 | 1.0268998727958 | 0.998735809224318 |
| ebi-fl187-GCST90094707 | prot-a-2333 | Periostin \|\| id:prot-a-2333 | Black olive liking \|\| id:ebi-fl187-GCST90094707 | Inverse variance weighted | 22 | -0.184960722 | 0.0872727979712158 | 0.0340617527577862 | -0.356015406 | -0.013906038 | 0.831136928528136 | 0.700461828753961 | 0.986190204242843 | 0.998735809224318 |
| ebi-fl187-GCST90094707 | prot-a-2367 | cAMP-dependent protein kinase catalytic subunit alpha \|\| id:prot-a-2367 | Black olive liking \|\| id:ebi-fl187-GCST90094707 | Inverse variance weighted | 22 | -0.01585729 | 0.0873447867118405 | 0.855937293336952 | -0.187053072 | 0.15533849201282 | 0.984267774943753 | 0.829399717400462 | 1.16805327089949 | 0.998735809224318 |
| ebi-fl187-GCST90094707 | prot-a-2385 | Phosphoribosyl pyrophosphate synthase-associated protein 1 \|\| id:prot-a-2385 | Black olive liking \|\| id:ebi-fl187-GCST90094707 | Inverse variance weighted | 22 | -0.047454754 | 0.0772533131539249 | 0.539033836297946 | -0.198871247 | 0.103961740149538 | 0.953653621519021 | 0.819655419294397 | 1.10955800258022 | 0.998735809224318 |
| ebi-fl187-GCST90094707 | prot-a-2393 | Trypsin-3 \|\| id:prot-a-2393 | Black olive liking \|\| id:ebi-fl187-GCST90094707 | Inverse variance weighted | 21 | -0.036113738 | 0.0873066239690958 | 0.679135877728364 | -0.207234721 | 0.135007244962955 | 0.964530583448752 | 0.812828839600118 | 1.14454507650796 | 0.998735809224318 |
| ebi-fl187-GCST90094707 | prot-a-2405 | Pregnancy-specific beta-1-glycoprotein 6 \|\| id:prot-a-2405 | Black olive liking \|\| id:ebi-fl187-GCST90094707 | Inverse variance weighted | 22 | -0.03687523 | 0.0897248660905388 | 0.681086343193621 | -0.212735968 | 0.138985507229288 | 0.963796380424801 | 0.808369544594435 | 1.14910744613218 | 0.998735809224318 |
| ebi-fl187-GCST90094707 | prot-a-244 | Beclin-1 \|\| id:prot-a-244 | Black olive liking \|\| id:ebi-fl187-GCST90094707 | Inverse variance weighted | 22 | -0.118167088 | 0.0780855256504867 | 0.130202621636587 | -0.271214718 | 0.0348805425613559 | 0.888547574827855 | 0.762452766503427 | 1.03549600371069 | 0.998735809224318 |
| ebi-fl187-GCST90094707 | prot-a-2454 | tRNA pseudouridine synthase A, mitochondrial \|\| id:prot-a-2454 | Black olive liking \|\| id:ebi-fl187-GCST90094707 | Inverse variance weighted | 22 | 0.111661968566443 | 0.0805544777377648 | 0.165695878339916 | -0.046224808 | 0.269548744932463 | 1.11813483203569 | 0.9548272854401 | 1.30937345599127 | 0.998735809224318 |
| ebi-fl187-GCST90094707 | prot-a-2466 | Glutaminyl-peptide cyclotransferase-like protein \|\| id:prot-a-2466 | Black olive liking \|\| id:ebi-fl187-GCST90094707 | Inverse variance weighted | 22 | 0.0514347593902826 | 0.0772630488201627 | 0.505596686001193 | -0.100000816 | 0.202870335077801 | 1.05278050000987 | 0.904836679419977 | 1.22491362961934 | 0.998735809224318 |
| ebi-fl187-GCST90094707 | prot-a-2474 | Ras-related protein Rab-27A \|\| id:prot-a-2474 | Black olive liking \|\| id:ebi-fl187-GCST90094707 | Inverse variance weighted | 22 | -0.057670664 | 0.0882704653973271 | 0.513536832504521 | -0.230680776 | 0.115339448247329 | 0.943960776581407 | 0.793992887081958 | 1.12225432018536 | 0.998735809224318 |
| ebi-fl187-GCST90094707 | prot-a-2490 | Rap1 GTPase-GDP dissociation stimulator 1 \|\| id:prot-a-2490 | Black olive liking \|\| id:ebi-fl187-GCST90094707 | Inverse variance weighted | 22 | 0.0849375780160435 | 0.0836971177567759 | 0.31019124535748 | -0.079108773 | 0.248983928819324 | 1.08864910894018 | 0.923939419513186 | 1.28272141805649 | 0.998735809224318 |
| ebi-fl187-GCST90094707 | prot-a-2545 | Receptor-interacting serine/threonine-protein kinase 2 \|\| id:prot-a-2545 | Black olive liking \|\| id:ebi-fl187-GCST90094707 | Inverse variance weighted | 22 | 0.0317175957264879 | 0.0772407742051035 | 0.681341805683447 | -0.119674322 | 0.183109513168491 | 1.03222595911803 | 0.887209334484773 | 1.20094592027246 | 0.998735809224318 |
| ebi-fl187-GCST90094707 | prot-a-2610 | RUN and FYVE domain-containing protein 1 \|\| id:prot-a-2610 | Black olive liking \|\| id:ebi-fl187-GCST90094707 | Inverse variance weighted | 22 | -0.184865759 | 0.0867379910331878 | 0.0330634116529795 | -0.354872221 | -0.014859296 | 0.831215860017743 | 0.701263044219369 | 0.985250558460775 | 0.998735809224318 |
| ebi-fl187-GCST90094707 | prot-a-2627 | Serine--tRNA ligase, mitochondrial \|\| id:prot-a-2627 | Black olive liking \|\| id:ebi-fl187-GCST90094707 | Inverse variance weighted | 22 | 0.0519158194609917 | 0.0772630488201627 | 0.501624478005999 | -0.099519756 | 0.203351395148511 | 1.05328707250778 | 0.905272064931856 | 1.22550302841336 | 0.998735809224318 |
| ebi-fl187-GCST90094707 | prot-a-264 | BolA-like protein 2 \|\| id:prot-a-264 | Black olive liking \|\| id:ebi-fl187-GCST90094707 | Inverse variance weighted | 22 | 0.0351873433887064 | 0.0772513499389633 | 0.648755587001564 | -0.116225302 | 0.186599989269074 | 1.03581374348027 | 0.890274619610005 | 1.20514511764091 | 0.998735809224318 |
| ebi-fl187-GCST90094707 | prot-a-2646 | Secretoglobin family 1C member 1 \|\| id:prot-a-2646 | Black olive liking \|\| id:ebi-fl187-GCST90094707 | Inverse variance weighted (multiplicative random effects) | 22 | -0.055281138 | 0.102102785348153 | 0.588213749984661 | -0.255402598 | 0.144840321006104 | 0.946219092151454 | 0.774604581263503 | 1.15585498976974 | 0.998735809224318 |
| ebi-fl187-GCST90094707 | prot-a-2672 | Semaphorin-3E \|\| id:prot-a-2672 | Black olive liking \|\| id:ebi-fl187-GCST90094707 | Inverse variance weighted | 22 | 0.0234557494420684 | 0.0958603268588763 | 0.806699007518723 | -0.164430491 | 0.211341990085466 | 1.02373299898794 | 0.848376724541876 | 1.23533475506744 | 0.998735809224318 |
| ebi-fl187-GCST90094707 | prot-a-2701 | Plasma protease C1 inhibitor \|\| id:prot-a-2701 | Black olive liking \|\| id:ebi-fl187-GCST90094707 | Inverse variance weighted | 22 | 0.0138389083423246 | 0.0772533131539249 | 0.857830317256081 | -0.137577585 | 0.165255402124017 | 1.01393510929418 | 0.871466734244722 | 1.17969437668829 | 0.998735809224318 |
| ebi-fl187-GCST90094707 | prot-a-2738 | Signaling threshold-regulating transmembrane adapter 1 \|\| id:prot-a-2738 | Black olive liking \|\| id:ebi-fl187-GCST90094707 | Inverse variance weighted | 22 | -0.114189065 | 0.0772417250236925 | 0.139318414478998 | -0.265582846 | 0.0372047164804608 | 0.892089277465299 | 0.766758917642167 | 1.03790547544692 | 0.998735809224318 |
| ebi-fl187-GCST90094707 | prot-a-274 | Serine/threonine-protein kinase BRSK2 \|\| id:prot-a-274 | Black olive liking \|\| id:ebi-fl187-GCST90094707 | Inverse variance weighted | 22 | 0.0747768738600453 | 0.0812260658813774 | 0.357258288777994 | -0.084426215 | 0.233979962987545 | 1.07764367358411 | 0.919039463956229 | 1.26361917280101 | 0.998735809224318 |
| ebi-fl187-GCST90094707 | prot-a-2749 | Mitochondrial glutamate carrier 2 \|\| id:prot-a-2749 | Black olive liking \|\| id:ebi-fl187-GCST90094707 | Inverse variance weighted | 22 | 0.0843109796578079 | 0.0954594398057296 | 0.377121417181076 | -0.102789522 | 0.271411481677038 | 1.08796717686689 | 0.902316871020484 | 1.31181474707553 | 0.998735809224318 |
| ebi-fl187-GCST90094707 | prot-a-2760 | Sodium-coupled monocarboxylate transporter 1 \|\| id:prot-a-2760 | Black olive liking \|\| id:ebi-fl187-GCST90094707 | Inverse variance weighted | 22 | -0.170308163 | 0.0772385358742349 | 0.0274566466146659 | -0.321695693 | -0.018920632 | 0.843404870676943 | 0.724918754656191 | 0.981257239259806 | 0.998735809224318 |
| ebi-fl187-GCST90094707 | prot-a-2777 | Small integral membrane protein 10 \|\| id:prot-a-2777 | Black olive liking \|\| id:ebi-fl187-GCST90094707 | Inverse variance weighted | 22 | -0.015755142 | 0.0883515552936791 | 0.858469073152692 | -0.18892419 | 0.157413906557456 | 0.984368321185923 | 0.827849263408166 | 1.17047998299256 | 0.998735809224318 |
| ebi-fl187-GCST90094707 | prot-a-2799 | Superoxide dismutase [Mn], mitochondrial \|\| id:prot-a-2799 | Black olive liking \|\| id:ebi-fl187-GCST90094707 | Inverse variance weighted (multiplicative random effects) | 22 | -0.007739735 | 0.0973429076380451 | 0.936626971137105 | -0.198531834 | 0.183052363550903 | 0.992290139208926 | 0.819933668236568 | 1.20087728863352 | 0.998735809224318 |
| ebi-fl187-GCST90094707 | prot-a-2805 | Protein SPACA7 \|\| id:prot-a-2805 | Black olive liking \|\| id:ebi-fl187-GCST90094707 | Inverse variance weighted | 22 | 0.00619807992161296 | 0.081891263583792 | 0.939668353401951 | -0.154308797 | 0.166704956545845 | 1.00621732776498 | 0.857007339106674 | 1.18140564787914 | 0.998735809224318 |
| ebi-fl187-GCST90094707 | prot-a-2811 | SPARC-like protein 1 \|\| id:prot-a-2811 | Black olive liking \|\| id:ebi-fl187-GCST90094707 | Inverse variance weighted | 22 | 0.0683465958170291 | 0.0772381208262667 | 0.376220465807263 | -0.083040121 | 0.219733312636512 | 1.07073635691559 | 0.920314222550221 | 1.24574446197729 | 0.998735809224318 |
| ebi-fl187-GCST90094707 | prot-a-2831 | Sepiapterin reductase \|\| id:prot-a-2831 | Black olive liking \|\| id:ebi-fl187-GCST90094707 | Inverse variance weighted | 22 | -0.042938984 | 0.0792149119068389 | 0.587779354783764 | -0.198200212 | 0.11232224306228 | 0.957969839508012 | 0.820205622007703 | 1.11887335173422 | 0.998735809224318 |
| ebi-fl187-GCST90094707 | prot-a-2834 | SPRY domain-containing SOCS box protein 1 \|\| id:prot-a-2834 | Black olive liking \|\| id:ebi-fl187-GCST90094707 | Inverse variance weighted | 22 | 0.0519523428353991 | 0.078513840273452 | 0.508165670485127 | -0.101934784 | 0.205839469771365 | 1.05332554280841 | 0.903088445473689 | 1.22855596779415 | 0.998735809224318 |
| ebi-fl187-GCST90094707 | prot-a-2835 | Spectrin alpha chain, non-erythrocytic 1 \|\| id:prot-a-2835 | Black olive liking \|\| id:ebi-fl187-GCST90094707 | Inverse variance weighted | 22 | -0.108698026 | 0.0777317325433034 | 0.162000816507395 | -0.261052222 | 0.0436561697678273 | 0.897001247643813 | 0.770240695207775 | 1.04462312012417 | 0.998735809224318 |
| ebi-fl187-GCST90094707 | prot-a-2859 | Sia-alpha-2,3-Gal-beta-1,4-GlcNAc-R:alpha 2,8-sialyltransferase \|\| id:prot-a-2859 | Black olive liking \|\| id:ebi-fl187-GCST90094707 | Inverse variance weighted | 22 | -0.078258113 | 0.0887573341069202 | 0.377934356697296 | -0.252222488 | 0.0957062614494079 | 0.924725711639464 | 0.777071829483347 | 1.10043577610535 | 0.998735809224318 |
| ebi-fl187-GCST90094707 | prot-a-2861 | Alpha-2,8-sialyltransferase 8F \|\| id:prot-a-2861 | Black olive liking \|\| id:ebi-fl187-GCST90094707 | Inverse variance weighted | 22 | -0.006934017 | 0.0772630488201627 | 0.928489394347944 | -0.158369593 | 0.144501558548303 | 0.99308996768854 | 0.853534263524226 | 1.15546349580802 | 0.998735809224318 |
| ebi-fl187-GCST90094707 | prot-a-289 | Uncharacterized protein C17orf89 \|\| id:prot-a-289 | Black olive liking \|\| id:ebi-fl187-GCST90094707 | Inverse variance weighted | 22 | 0.118595717649654 | 0.0863661301054442 | 0.169698284531341 | -0.050681897 | 0.287873332656325 | 1.12591463884735 | 0.950581004773206 | 1.33358837132792 | 0.998735809224318 |
| ebi-fl187-GCST90094707 | prot-a-2921 | Tachykinin-3 \|\| id:prot-a-2921 | Black olive liking \|\| id:ebi-fl187-GCST90094707 | Inverse variance weighted | 22 | -0.07273553 | 0.0772365737856208 | 0.346333999340402 | -0.224119215 | 0.0786481547010853 | 0.92984671414592 | 0.799219850064217 | 1.08182362054508 | 0.998735809224318 |
| ebi-fl187-GCST90094707 | prot-a-2928 | Tax1-binding protein 1 \|\| id:prot-a-2928 | Black olive liking \|\| id:ebi-fl187-GCST90094707 | Inverse variance weighted | 22 | -0.047244637 | 0.0899472074945608 | 0.599410445383972 | -0.223541164 | 0.129051889846019 | 0.953854021208568 | 0.799681973455051 | 1.13774916026277 | 0.998735809224318 |
| ebi-fl187-GCST90094707 | prot-a-296 | Uncharacterized protein C1orf185 \|\| id:prot-a-296 | Black olive liking \|\| id:ebi-fl187-GCST90094707 | Inverse variance weighted | 22 | 0.0458458058092706 | 0.0844152555447194 | 0.587061927081689 | -0.119608095 | 0.211299706676921 | 1.04691297061241 | 0.887268093338856 | 1.2352825220076 | 0.998735809224318 |
| ebi-fl187-GCST90094707 | prot-a-2964 | Transforming growth factor beta-2 \|\| id:prot-a-2964 | Black olive liking \|\| id:ebi-fl187-GCST90094707 | Inverse variance weighted | 22 | 0.0715729835117858 | 0.0789794705109214 | 0.364818027182537 | -0.083226779 | 0.226372745713192 | 1.07419654647679 | 0.920142454857352 | 1.25404301732991 | 0.998735809224318 |
| ebi-fl187-GCST90094707 | prot-a-2977 | Thymocyte nuclear protein 1 \|\| id:prot-a-2977 | Black olive liking \|\| id:ebi-fl187-GCST90094707 | Inverse variance weighted | 22 | 0.12031460401903 | 0.0772513499389633 | 0.119365252132817 | -0.031098042 | 0.271727249899398 | 1.12785162242357 | 0.969380528545567 | 1.3122290418934 | 0.998735809224318 |
| ebi-fl187-GCST90094707 | prot-a-3015 | Transmembrane protein 70, mitochondrial \|\| id:prot-a-3015 | Black olive liking \|\| id:ebi-fl187-GCST90094707 | Inverse variance weighted | 22 | -0.046406091 | 0.0772416186939132 | 0.547979008389502 | -0.197799664 | 0.104987481165217 | 0.954654206529205 | 0.820534219122103 | 1.11069670564012 | 0.998735809224318 |
| ebi-fl187-GCST90094707 | prot-a-3030 | Tumor necrosis factor alpha-induced protein 3 \|\| id:prot-a-3030 | Black olive liking \|\| id:ebi-fl187-GCST90094707 | Inverse variance weighted (multiplicative random effects) | 22 | -0.20282266 | 0.0972664780179149 | 0.0370487241317958 | -0.393464957 | -0.012180364 | 0.816423012676593 | 0.674714960951412 | 0.987893516823801 | 0.998735809224318 |
| ebi-fl187-GCST90094707 | prot-a-3032 | Tumor necrosis factor receptor superfamily member 10A \|\| id:prot-a-3032 | Black olive liking \|\| id:ebi-fl187-GCST90094707 | Inverse variance weighted | 22 | 0.144952857078948 | 0.0772533131539249 | 0.0606097171270294 | -0.006463637 | 0.296369350860641 | 1.15598507247043 | 0.993557207662653 | 1.34496682975923 | 0.998735809224318 |
| ebi-fl187-GCST90094707 | prot-a-3081 | Tumor protein p53-inducible protein 11 \|\| id:prot-a-3081 | Black olive liking \|\| id:ebi-fl187-GCST90094707 | Inverse variance weighted | 22 | -0.035295154 | 0.0772495856243546 | 0.647744612045814 | -0.186704342 | 0.116114033565911 | 0.965320455763301 | 0.829689004249874 | 1.12312393865886 | 0.998735809224318 |
| ebi-fl187-GCST90094707 | prot-a-315 | Complement C4 \|\| id:prot-a-315 | Black olive liking \|\| id:ebi-fl187-GCST90094707 | Inverse variance weighted | 21 | 0.0344191368374966 | 0.092134696479529 | 0.708721202011083 | -0.146164868 | 0.215003141937373 | 1.03501833013668 | 0.864015242749621 | 1.23986579254061 | 0.998735809224318 |
| ebi-fl187-GCST90094707 | prot-a-3157 | UDP-glucuronosyltransferase 1-8 \|\| id:prot-a-3157 | Black olive liking \|\| id:ebi-fl187-GCST90094707 | Inverse variance weighted | 22 | -0.002584951 | 0.0884484161274506 | 0.976684724468542 | -0.175943847 | 0.170773944273938 | 0.997418386773936 | 0.838665075620431 | 1.18622256630724 | 0.998955228396052 |
| ebi-fl187-GCST90094707 | prot-a-317 | Glutaredoxin-like protein C5orf63 \|\| id:prot-a-317 | Black olive liking \|\| id:ebi-fl187-GCST90094707 | Inverse variance weighted | 22 | -0.051988335 | 0.0772378928568042 | 0.50088771305643 | -0.203374605 | 0.0993979347269769 | 0.949339940570149 | 0.81597251655985 | 1.10450573330754 | 0.998735809224318 |
| ebi-fl187-GCST90094707 | prot-a-3192 | Guanine nucleotide exchange factor VAV3 \|\| id:prot-a-3192 | Black olive liking \|\| id:ebi-fl187-GCST90094707 | Inverse variance weighted | 22 | 0.0602366557225895 | 0.0772630488201627 | 0.435608197209909 | -0.09119892 | 0.211672231410108 | 1.06208786597747 | 0.912836111510705 | 1.23574278102313 | 0.998735809224318 |
| ebi-fl187-GCST90094707 | prot-a-3233 | Serine/threonine-protein kinase WNK3 \|\| id:prot-a-3233 | Black olive liking \|\| id:ebi-fl187-GCST90094707 | Inverse variance weighted | 22 | -0.022156091 | 0.0772533131539249 | 0.774267038986403 | -0.173572585 | 0.129260402572815 | 0.978087552266878 | 0.840656129930031 | 1.13798642017758 | 0.998735809224318 |
| ebi-fl187-GCST90094707 | prot-a-3250 | 14-3-3 protein theta \|\| id:prot-a-3250 | Black olive liking \|\| id:ebi-fl187-GCST90094707 | Inverse variance weighted | 22 | 0.0165993028578961 | 0.0772495856243546 | 0.829861767556513 | -0.134809885 | 0.168008490681631 | 1.01673783674609 | 0.873882034009107 | 1.1829466546286 | 0.998735809224318 |
| ebi-fl187-GCST90094707 | prot-a-3278 | E3 ubiquitin-protein ligase ZNRF3 \|\| id:prot-a-3278 | Black olive liking \|\| id:ebi-fl187-GCST90094707 | Inverse variance weighted | 22 | 0.0392049080264231 | 0.0772495856243546 | 0.611797286571148 | -0.11220428 | 0.190614095850158 | 1.03998356279796 | 0.893861640973716 | 1.20999242087596 | 0.998735809224318 |
| ebi-fl187-GCST90094707 | prot-a-355 | Calpain-3 \|\| id:prot-a-355 | Black olive liking \|\| id:ebi-fl187-GCST90094707 | Inverse variance weighted | 22 | -0.011375923 | 0.0772378928568042 | 0.882907681539579 | -0.162762193 | 0.140010346635803 | 0.988688537786074 | 0.849793250862986 | 1.15028570038287 | 0.998735809224318 |
| ebi-fl187-GCST90094707 | prot-a-385 | Coiled-coil domain-containing protein 90B, mitochondrial \|\| id:prot-a-385 | Black olive liking \|\| id:ebi-fl187-GCST90094707 | Inverse variance weighted | 22 | 0.15868667011598 | 0.0821825180235289 | 0.0534947488481237 | -0.002391065 | 0.319764405442097 | 1.17197067570934 | 0.997611791109281 | 1.37680335874474 | 0.998735809224318 |
| ebi-fl187-GCST90094707 | prot-a-390 | C-C motif chemokine 14 \|\| id:prot-a-390 | Black olive liking \|\| id:ebi-fl187-GCST90094707 | Inverse variance weighted | 22 | 0.0455902921124118 | 0.0899113296340188 | 0.612113864736494 | -0.130635914 | 0.221816498195089 | 1.04664550418112 | 0.877537215276188 | 1.24834228378312 | 0.998735809224318 |
| ebi-fl187-GCST90094707 | prot-a-40 | Pituitary adenylate cyclase-activating polypeptide 27 \|\| id:prot-a-40 | Black olive liking \|\| id:ebi-fl187-GCST90094707 | Inverse variance weighted (multiplicative random effects) | 22 | 0.138090301389447 | 0.0964815363655793 | 0.152355304559966 | -0.05101351 | 0.327194112665983 | 1.14807921874205 | 0.950265832461607 | 1.38707069904107 | 0.998735809224318 |
| ebi-fl187-GCST90094707 | prot-a-401 | C-C motif chemokine 24 \|\| id:prot-a-401 | Black olive liking \|\| id:ebi-fl187-GCST90094707 | Inverse variance weighted | 22 | 0.122147029694691 | 0.078691403805869 | 0.12060758944083 | -0.032088122 | 0.276382181154194 | 1.12992022139294 | 0.968421239330284 | 1.31835161690132 | 0.998735809224318 |
| ebi-fl187-GCST90094707 | prot-a-412 | Cerebral cavernous malformations 2 protein \|\| id:prot-a-412 | Black olive liking \|\| id:ebi-fl187-GCST90094707 | Inverse variance weighted | 22 | -0.097663664 | 0.0772425871073023 | 0.206095147127404 | -0.249059135 | 0.0537318069394702 | 0.906953893895986 | 0.779533874659299 | 1.05520156646509 | 0.998735809224318 |
| ebi-fl187-GCST90094707 | prot-a-44 | Adhesion G protein-coupled receptor F5 \|\| id:prot-a-44 | Black olive liking \|\| id:ebi-fl187-GCST90094707 | Inverse variance weighted | 22 | -0.074954093 | 0.085382483768812 | 0.380018264821335 | -0.242303761 | 0.092395575407026 | 0.927786077430639 | 0.784817744343594 | 1.0967986028324 | 0.998735809224318 |
| ebi-fl187-GCST90094707 | prot-a-48 | Adrenomedullin \|\| id:prot-a-48 | Black olive liking \|\| id:ebi-fl187-GCST90094707 | Inverse variance weighted | 22 | -0.029281208 | 0.0772365163528559 | 0.70460548560078 | -0.18066478 | 0.122102364037092 | 0.971143332776585 | 0.834715124950566 | 1.12986975389031 | 0.998735809224318 |
| ebi-fl187-GCST90094707 | prot-a-502 | Carcinoembryonic antigen-related cell adhesion molecule 1 \|\| id:prot-a-502 | Black olive liking \|\| id:ebi-fl187-GCST90094707 | Inverse variance weighted | 22 | 0.109172579825092 | 0.0773238977346523 | 0.15798333580364 | -0.04238226 | 0.260727419385011 | 1.11535482147266 | 0.958503313311732 | 1.29787384196315 | 0.998735809224318 |
| ebi-fl187-GCST90094707 | prot-a-518 | Complement factor B \|\| id:prot-a-518 | Black olive liking \|\| id:ebi-fl187-GCST90094707 | Inverse variance weighted | 22 | -0.079396681 | 0.0907263605992834 | 0.381507249489982 | -0.257220347 | 0.0984269862183421 | 0.923673448465611 | 0.773197822917057 | 1.10343383557597 | 0.998735809224318 |
| ebi-fl187-GCST90094707 | prot-a-525 | Properdin \|\| id:prot-a-525 | Black olive liking \|\| id:ebi-fl187-GCST90094707 | Inverse variance weighted (multiplicative random effects) | 22 | -0.083244643 | 0.106725382293387 | 0.435397246484116 | -0.292426392 | 0.125937106332644 | 0.92012601732837 | 0.746450187527003 | 1.13421083136099 | 0.998735809224318 |
| ebi-fl187-GCST90094707 | prot-a-533 | Chondroadherin \|\| id:prot-a-533 | Black olive liking \|\| id:ebi-fl187-GCST90094707 | Inverse variance weighted | 22 | -0.01708664 | 0.0838890475182859 | 0.838602462016505 | -0.181509174 | 0.147335892760654 | 0.983058508387115 | 0.834010594463003 | 1.15874311109266 | 0.998735809224318 |
| ebi-fl187-GCST90094707 | prot-a-535 | Coiled-coil-helix-coiled-coil-helix domain-containing protein 2 \|\| id:prot-a-535 | Black olive liking \|\| id:ebi-fl187-GCST90094707 | Inverse variance weighted | 22 | -0.071502685 | 0.0772630488201627 | 0.354734593003776 | -0.222938261 | 0.0799328902012206 | 0.930993777420472 | 0.800164248971085 | 1.08321437093719 | 0.998735809224318 |
| ebi-fl187-GCST90094707 | prot-a-591 | Caseinolytic peptidase B protein homolog \|\| id:prot-a-591 | Black olive liking \|\| id:ebi-fl187-GCST90094707 | Inverse variance weighted (multiplicative random effects) | 22 | -0.182691426 | 0.10005688839961 | 0.0678690098851444 | -0.378802927 | 0.0134200751386937 | 0.833025165885535 | 0.684680531152286 | 1.01351052852455 | 0.998735809224318 |
| ebi-fl187-GCST90094707 | prot-a-605 | Ciliary neurotrophic factor receptor subunit alpha \|\| id:prot-a-605 | Black olive liking \|\| id:ebi-fl187-GCST90094707 | Inverse variance weighted | 22 | -0.106211308 | 0.0812401258007388 | 0.191085389677291 | -0.265441955 | 0.0530193385420708 | 0.899234612506493 | 0.766866954695331 | 1.05445003644858 | 0.998735809224318 |
| ebi-fl187-GCST90094707 | prot-a-606 | Ciliary neurotrophic factor receptor subunit alpha \|\| id:prot-a-606 | Black olive liking \|\| id:ebi-fl187-GCST90094707 | Inverse variance weighted | 22 | -0.184685862 | 0.0772630488201627 | 0.0168322443436025 | -0.336121438 | -0.033250286 | 0.831365406344654 | 0.714536328944622 | 0.967296428283045 | 0.998735809224318 |
| ebi-fl187-GCST90094707 | prot-a-707 | Cancer/testis antigen 55 \|\| id:prot-a-707 | Black olive liking \|\| id:ebi-fl187-GCST90094707 | Inverse variance weighted | 22 | -0.07325727 | 0.0772278159807264 | 0.342830916910765 | -0.224623789 | 0.0781092491528698 | 0.929361702224364 | 0.798816685467843 | 1.08124077685673 | 0.998735809224318 |
| ebi-fl187-GCST90094707 | prot-a-719 | Dipeptidyl peptidase 1 \|\| id:prot-a-719 | Black olive liking \|\| id:ebi-fl187-GCST90094707 | Inverse variance weighted | 22 | -0.051826531 | 0.0772407742051035 | 0.502237263818583 | -0.203218448 | 0.0995653869346321 | 0.949493560724028 | 0.816099946592432 | 1.10469070071712 | 0.998735809224318 |
| ebi-fl187-GCST90094707 | prot-a-752 | Cytochrome b5 \|\| id:prot-a-752 | Black olive liking \|\| id:ebi-fl187-GCST90094707 | Inverse variance weighted | 22 | -0.20441272 | 0.0836530267140635 | 0.0145424138730888 | -0.368372653 | -0.040452788 | 0.815125882855664 | 0.691859310516537 | 0.96035450387329 | 0.998735809224318 |
| ebi-fl187-GCST90094707 | prot-a-755 | Cytoglobin \|\| id:prot-a-755 | Black olive liking \|\| id:ebi-fl187-GCST90094707 | Inverse variance weighted (multiplicative random effects) | 22 | 0.0682624645813762 | 0.100517164429423 | 0.497066571799741 | -0.128751178 | 0.265276106863045 | 1.07064627833209 | 0.879192701081085 | 1.30379091170439 | 0.998735809224318 |
| ebi-fl187-GCST90094707 | prot-a-807 | Desmin \|\| id:prot-a-807 | Black olive liking \|\| id:ebi-fl187-GCST90094707 | Inverse variance weighted | 22 | -0.031410919 | 0.0772156399854755 | 0.684158728130995 | -0.182753573 | 0.119931735845333 | 0.969077279441073 | 0.83297339766812 | 1.12741988658692 | 0.998735809224318 |
| ebi-fl187-GCST90094707 | prot-a-82 | Ameloblastin \|\| id:prot-a-82 | Black olive liking \|\| id:ebi-fl187-GCST90094707 | Inverse variance weighted (multiplicative random effects) | 22 | -0.042653262 | 0.0968090321021704 | 0.659508566430262 | -0.232398965 | 0.14709244070376 | 0.958243591729323 | 0.792629828547651 | 1.15846104703479 | 0.998735809224318 |
| ebi-fl187-GCST90094707 | prot-a-84 | Muellerian-inhibiting factor \|\| id:prot-a-84 | Black olive liking \|\| id:ebi-fl187-GCST90094707 | Inverse variance weighted | 22 | -0.147034197 | 0.0793890400108105 | 0.0640155230285678 | -0.302636715 | 0.00856832155772341 | 0.863264455974817 | 0.738867466877463 | 1.00860513469202 | 0.998735809224318 |
| ebi-fl187-GCST90094707 | prot-a-858 | Inactive dipeptidyl peptidase 10 \|\| id:prot-a-858 | Black olive liking \|\| id:ebi-fl187-GCST90094707 | Inverse variance weighted | 22 | 0.056168252242433 | 0.0790203614735623 | 0.47720363706404 | -0.098711656 | 0.211048160730615 | 1.057775642 | 0.906003910932669 | 1.23497183077485 | 0.998735809224318 |
| ebi-fl187-GCST90094707 | prot-a-863 | Dihydropyrimidinase-related protein 3 \|\| id:prot-a-863 | Black olive liking \|\| id:ebi-fl187-GCST90094707 | Inverse variance weighted | 21 | -0.078767495 | 0.078623995050903 | 0.31642805309909 | -0.232870525 | 0.0753355354660876 | 0.924254793479516 | 0.792256144142547 | 1.07824587992866 | 0.998735809224318 |
| ebi-fl187-GCST90094707 | prot-a-864 | Dihydropyrimidinase-related protein 5 \|\| id:prot-a-864 | Black olive liking \|\| id:ebi-fl187-GCST90094707 | Inverse variance weighted | 22 | -0.041423011 | 0.0772482659540224 | 0.591798055193946 | -0.192829612 | 0.109983590677088 | 0.959423197921383 | 0.824622468055844 | 1.11625975324184 | 0.998735809224318 |
| ebi-fl187-GCST90094707 | prot-a-884 | Endothelin-converting enzyme 1 \|\| id:prot-a-884 | Black olive liking \|\| id:ebi-fl187-GCST90094707 | Inverse variance weighted | 22 | -0.163589568 | 0.0772513499389633 | 0.0342066434978742 | -0.315002214 | -0.012176922 | 0.849090444028785 | 0.729787258404546 | 0.987896916311122 | 0.998735809224318 |
| ebi-fl187-GCST90094707 | prot-a-893 | Early endosome antigen 1 \|\| id:prot-a-893 | Black olive liking \|\| id:ebi-fl187-GCST90094707 | Inverse variance weighted | 22 | -0.06957991 | 0.0772513499389633 | 0.36775046827896 | -0.220992556 | 0.0818327354956167 | 0.932785591150767 | 0.801722648078824 | 1.08527426678477 | 0.998735809224318 |
| ebi-fl187-GCST90094707 | prot-a-897 | Ephrin-A2 \|\| id:prot-a-897 | Black olive liking \|\| id:ebi-fl187-GCST90094707 | Inverse variance weighted | 22 | -0.054504733 | 0.078826763257035 | 0.489283047076268 | -0.209005189 | 0.0999957225520743 | 0.946954026504996 | 0.811391024987322 | 1.10516619077471 | 0.998735809224318 |
| ebi-fl187-GCST90094707 | prot-a-90 | Anaphase-promoting complex subunit 10 \|\| id:prot-a-90 | Black olive liking \|\| id:ebi-fl187-GCST90094707 | Inverse variance weighted | 22 | 0.0720843084646508 | 0.0915006200083931 | 0.430812933694874 | -0.107256907 | 0.251425523681101 | 1.07474595042529 | 0.898294865327542 | 1.28585713059195 | 0.998735809224318 |
| ebi-fl187-GCST90094707 | prot-a-914 | Histone-lysine N-methyltransferase EHMT2 \|\| id:prot-a-914 | Black olive liking \|\| id:ebi-fl187-GCST90094707 | Inverse variance weighted | 22 | 0.0174021359745352 | 0.093430067832458 | 0.852242119918721 | -0.165720797 | 0.200525068926153 | 1.01755443530469 | 0.847282765077069 | 1.22204424719304 | 0.998735809224318 |
| ebi-fl187-GCST90094707 | prot-a-932 | RNA polymerase II elongation factor ELL \|\| id:prot-a-932 | Black olive liking \|\| id:ebi-fl187-GCST90094707 | Inverse variance weighted | 22 | -0.169461271 | 0.080522207957503 | 0.0353323825184557 | -0.327284799 | -0.011637743 | 0.844119445668717 | 0.72087840856051 | 0.988429713103624 | 0.998735809224318 |
| ebi-fl187-GCST90094707 | prot-a-991 | Estrogen receptor \|\| id:prot-a-991 | Black olive liking \|\| id:ebi-fl187-GCST90094707 | Inverse variance weighted | 22 | 0.00936305737712891 | 0.0945305049759872 | 0.921100159628967 | -0.175916732 | 0.194642847130064 | 1.00940702792463 | 0.838687815971511 | 1.21487701218524 | 0.998735809224318 |
| ebi-fl187-GCST90094707 | prot-a-992 | Persulfide dioxygenase ETHE1, mitochondrial \|\| id:prot-a-992 | Black olive liking \|\| id:ebi-fl187-GCST90094707 | Inverse variance weighted | 22 | -0.103260288 | 0.0886919988422937 | 0.244319843919136 | -0.277096605 | 0.070576030020159 | 0.901892191473556 | 0.757981268458234 | 1.07312615613244 | 0.998735809224318 |
| ebi-fl187-GCST90094707 | prot-a-993 | Ethanolamine kinase 1 \|\| id:prot-a-993 | Black olive liking \|\| id:ebi-fl187-GCST90094707 | Inverse variance weighted | 22 | 0.0939001407125175 | 0.0772513499389633 | 0.224169898600467 | -0.057512505 | 0.245312786592886 | 1.09845005000086 | 0.944110084057532 | 1.27802099852729 | 0.998735809224318 |
| ebi-fl187-GCST90094707 | prot-b-10 | coagulation factor III, tissue factor \|\| id:prot-b-10 | Black olive liking \|\| id:ebi-fl187-GCST90094707 | Inverse variance weighted | 16 | -0.010567672 | 0.111171514821562 | 0.924269256496267 | -0.228463841 | 0.207328496951773 | 0.989487969574831 | 0.795755070273499 | 1.23038668367745 | 0.998735809224318 |
| ebi-fl187-GCST90094707 | prot-b-28 | prolactin \|\| id:prot-b-28 | Black olive liking \|\| id:ebi-fl187-GCST90094707 | Inverse variance weighted | 16 | -0.073046748 | 0.109452231905653 | 0.504526415742608 | -0.287573123 | 0.141479626059976 | 0.929557373620302 | 0.750081716532603 | 1.15197703371072 | 0.998735809224318 |
| ebi-fl187-GCST90094707 | prot-b-54 | matrix metallopeptidase 7 \|\| id:prot-b-54 | Black olive liking \|\| id:ebi-fl187-GCST90094707 | Inverse variance weighted (multiplicative random effects) | 16 | -0.040289275 | 0.151263594613341 | 0.789968568021411 | -0.33676592 | 0.256187370774159 | 0.96051154730178 | 0.714075971165608 | 1.29199478732508 | 0.998735809224318 |
| ebi-fl187-GCST90094707 | prot-c-2421_7_3 | BDNF \|\| id:prot-c-2421_7_3 | Black olive liking \|\| id:ebi-fl187-GCST90094707 | Inverse variance weighted | 2 | 0.418048561952787 | 0.491709316263949 | 0.395216952657781 | -0.545701698 | 1.38179882183013 | 1.51899443794735 | 0.579435052281766 | 3.98205820208645 | 0.998735809224318 |
| ebi-fl187-GCST90094707 | prot-c-2443_10_4 | FGF-8B \|\| id:prot-c-2443_10_4 | Black olive liking \|\| id:ebi-fl187-GCST90094707 | Inverse variance weighted | 2 | -0.506508901 | 0.983081573051684 | 0.606395305385559 | -2.433348784 | 1.42033098212154 | 0.602595631904727 | 0.0877425093124059 | 4.13848997978583 | 0.998735809224318 |
| ebi-fl187-GCST90094707 | prot-c-2605_49_2 | CD30 \|\| id:prot-c-2605_49_2 | Black olive liking \|\| id:ebi-fl187-GCST90094707 | Inverse variance weighted | 2 | -0.241579958 | 0.913082916519002 | 0.791335998765827 | -2.031222475 | 1.54806255803515 | 0.785386003115589 | 0.13117506489098 | 4.70235081951382 | 0.998735809224318 |
| ebi-fl187-GCST90094707 | prot-c-2624_31_2 | HPV E7 Type18 \|\| id:prot-c-2624_31_2 | Black olive liking \|\| id:ebi-fl187-GCST90094707 | Inverse variance weighted | 2 | 0.0623703359771593 | 0.469876984540947 | 0.89440091766059 | -0.858588554 | 0.983329225677415 | 1.06435644123218 | 0.423759774569389 | 2.6733416005415 | 0.998735809224318 |
| ebi-fl187-GCST90094707 | prot-c-2774_10_3 | IL-16 \|\| id:prot-c-2774_10_3 | Black olive liking \|\| id:ebi-fl187-GCST90094707 | Inverse variance weighted | 2 | -0.76027895 | 0.451355664198447 | 0.092097771831012 | -1.644936052 | 0.124378151663578 | 0.467535989576371 | 0.193024905956877 | 1.13244402563258 | 0.998735809224318 |
| ebi-fl187-GCST90094707 | prot-c-2851_63_3 | C5a \|\| id:prot-c-2851_63_3 | Black olive liking \|\| id:ebi-fl187-GCST90094707 | Inverse variance weighted | 2 | -0.161843019 | 0.408096808866117 | 0.691677228099806 | -0.961712764 | 0.638026726593501 | 0.850574718313886 | 0.382237642061582 | 1.89274229385861 | 0.998735809224318 |
| ebi-fl187-GCST90094707 | prot-c-2891_1_3 | CD22 \|\| id:prot-c-2891_1_3 | Black olive liking \|\| id:ebi-fl187-GCST90094707 | Inverse variance weighted | 2 | 1.02178961368321 | 0.817404249550791 | 0.211284218277164 | -0.580322715 | 2.62390194280276 | 2.77816215478964 | 0.559717707871974 | 13.7894242932739 | 0.998735809224318 |
| ebi-fl187-GCST90094707 | prot-c-2961_1_2 | Protein C \|\| id:prot-c-2961_1_2 | Black olive liking \|\| id:ebi-fl187-GCST90094707 | Inverse variance weighted | 2 | 0.0234506744722564 | 0.45743716288212 | 0.959114081586856 | -0.873126165 | 0.920027513721212 | 1.02372780358706 | 0.417643882726721 | 2.50935943080231 | 0.998735809224318 |
| ebi-fl187-GCST90094707 | prot-c-2982_82_2 | Galectin-4 \|\| id:prot-c-2982_82_2 | Black olive liking \|\| id:ebi-fl187-GCST90094707 | Inverse variance weighted | 2 | -0.415365203 | 0.433259221342168 | 0.337710377714979 | -1.264553277 | 0.433822870373349 | 0.660099166182084 | 0.28236540701054 | 1.54314550711949 | 0.998735809224318 |
| ebi-fl187-GCST90094707 | prot-c-2994_71_2 | IL-1Rrp2 \|\| id:prot-c-2994_71_2 | Black olive liking \|\| id:ebi-fl187-GCST90094707 | Inverse variance weighted | 2 | 0.348522461856532 | 0.567098051408185 | 0.538837552305184 | -0.762989719 | 1.46003464261657 | 1.41697237039607 | 0.466270323870427 | 4.30610870063397 | 0.998735809224318 |
| ebi-fl187-GCST90094707 | prot-c-3336_50_1 | TFPI \|\| id:prot-c-3336_50_1 | Black olive liking \|\| id:ebi-fl187-GCST90094707 | Inverse variance weighted | 2 | 0.397325010708091 | 0.45374832525411 | 0.381219987594506 | -0.492021707 | 1.28667172820615 | 1.48783941521999 | 0.611389094393711 | 3.62071575332458 | 0.998735809224318 |
| ebi-fl187-GCST90094707 | prot-c-3472_40_2 | Survivin \|\| id:prot-c-3472_40_2 | Black olive liking \|\| id:ebi-fl187-GCST90094707 | Inverse variance weighted | 2 | 0.210029172971163 | 0.468575710164617 | 0.653988010884681 | -0.708379219 | 1.12843756489381 | 1.23371405053618 | 0.492441691165583 | 3.09082351432874 | 0.998735809224318 |
| ebi-fl187-GCST90094707 | prot-c-3473_78_2 | Thrombopoietin Receptor \|\| id:prot-c-3473_78_2 | Black olive liking \|\| id:ebi-fl187-GCST90094707 | Inverse variance weighted | 2 | 0.608131869325243 | 0.467398509624276 | 0.193224470550554 | -0.30796921 | 1.52423294818882 | 1.83699644190392 | 0.734937946739365 | 4.59162020758248 | 0.998735809224318 |
| ebi-fl187-GCST90094707 | prot-c-3497_13_2 | IFN-aA \|\| id:prot-c-3497_13_2 | Black olive liking \|\| id:ebi-fl187-GCST90094707 | Inverse variance weighted | 2 | -0.006909476 | 0.470223077851603 | 0.988276275345539 | -0.928546709 | 0.914727756527423 | 0.993114339485452 | 0.395127528727286 | 2.4960956136578 | 0.998955228396052 |
| ebi-fl187-GCST90094707 | prot-c-3665_64_3 | COLEC12 \|\| id:prot-c-3665_64_3 | Black olive liking \|\| id:ebi-fl187-GCST90094707 | Inverse variance weighted | 2 | -0.171778664 | 0.783923162333341 | 0.826551184831803 | -1.708268063 | 1.36471073375805 | 0.842165553746668 | 0.181179312269628 | 3.91459052930915 | 0.998735809224318 |
| ebi-fl187-GCST90094707 | prot-c-3741_4_3 | IL-5 \|\| id:prot-c-3741_4_3 | Black olive liking \|\| id:ebi-fl187-GCST90094707 | Inverse variance weighted | 2 | -0.424243888 | 1.09588995643995 | 0.698665366765754 | -2.572188203 | 1.72370042636534 | 0.654264295070447 | 0.0763682532244364 | 5.60523188275645 | 0.998735809224318 |
| ebi-fl187-GCST90094707 | prot-c-3848_14_2 | GAPDH, liver \|\| id:prot-c-3848_14_2 | Black olive liking \|\| id:ebi-fl187-GCST90094707 | Inverse variance weighted | 2 | 0.689299723823111 | 0.462423909246144 | 0.136060497201891 | -0.217051138 | 1.59565058594555 | 1.99231987048338 | 0.804888807607769 | 4.93153641696212 | 0.998735809224318 |
| ebi-fl187-GCST90094707 | prot-c-3859_50_2 | PSA1 \|\| id:prot-c-3859_50_2 | Black olive liking \|\| id:ebi-fl187-GCST90094707 | Inverse variance weighted | 2 | -0.034850608 | 0.561465392996752 | 0.950506448346465 | -1.135322778 | 1.06562156273145 | 0.965749681199764 | 0.321318390146076 | 2.90264259793359 | 0.998735809224318 |
| ebi-fl187-GCST90094707 | prot-c-3868_8_1 | SGTA \|\| id:prot-c-3868_8_1 | Black olive liking \|\| id:ebi-fl187-GCST90094707 | Inverse variance weighted | 2 | 1.04704021121833 | 0.451147054582959 | 0.0202954710834844 | 0.162791984235732 | 1.93128843820093 | 2.84920557901335 | 1.17679187296564 | 6.898392671 | 0.998735809224318 |
| ebi-fl187-GCST90094707 | prot-c-3882_10_1 | EF-1-beta \|\| id:prot-c-3882_10_1 | Black olive liking \|\| id:ebi-fl187-GCST90094707 | Inverse variance weighted | 2 | 0.652886146104725 | 0.465617552784204 | 0.160857318190895 | -0.259724257 | 1.56549654956176 | 1.92107734547051 | 0.771264226925104 | 4.78505036074802 | 0.998735809224318 |
| ebi-fl187-GCST90094707 | prot-c-4134_4_2 | HB-EGF \|\| id:prot-c-4134_4_2 | Black olive liking \|\| id:ebi-fl187-GCST90094707 | Inverse variance weighted | 2 | 0.299649236003825 | 0.469974745720488 | 0.523743325339346 | -0.621501266 | 1.22079973761598 | 1.34938540873687 | 0.537137446017237 | 3.38989767854231 | 0.998735809224318 |
| ebi-fl187-GCST90094707 | prot-c-4220_39_2 | FER \|\| id:prot-c-4220_39_2 | Black olive liking \|\| id:ebi-fl187-GCST90094707 | Inverse variance weighted | 2 | 1.19782809220521 | 0.460110243551493 | 0.00923176025765082 | 0.296012014844281 | 2.09964416956613 | 3.31291376004658 | 1.34448631052867 | 8.16326465770432 | 0.998735809224318 |
| ebi-fl187-GCST90094707 | prot-c-4228_37_2 | HSP70 protein 8 \|\| id:prot-c-4228_37_2 | Black olive liking \|\| id:ebi-fl187-GCST90094707 | Inverse variance weighted | 2 | 0.186846121606007 | 0.633619626324329 | 0.768080286462774 | -1.055048346 | 1.42874058920169 | 1.20544177933257 | 0.348175594005616 | 4.17343980559719 | 0.998735809224318 |
| ebi-fl187-GCST90094707 | prot-c-4246_40_2 | NCAM-L1 \|\| id:prot-c-4246_40_2 | Black olive liking \|\| id:ebi-fl187-GCST90094707 | Inverse variance weighted | 2 | 0.116156234462054 | 0.465617414435002 | 0.802999473037303 | -0.796453898 | 1.02876636675466 | 1.12317133649566 | 0.450925158994075 | 2.79761247728922 | 0.998735809224318 |
| ebi-fl187-GCST90094707 | prot-c-4267_81_3 | PESC \|\| id:prot-c-4267_81_3 | Black olive liking \|\| id:ebi-fl187-GCST90094707 | Inverse variance weighted | 2 | 0.312002420222703 | 0.716201481425752 | 0.663101584962362 | -1.091752483 | 1.71575732381718 | 1.36615799943208 | 0.335627795884437 | 5.56088530895966 | 0.998735809224318 |
| ebi-fl187-GCST90094707 | prot-c-4280_47_2 | PSA2 \|\| id:prot-c-4280_47_2 | Black olive liking \|\| id:ebi-fl187-GCST90094707 | Inverse variance weighted | 2 | -0.483068036 | 0.467224479073041 | 0.301178264693481 | -1.398828015 | 0.432691942875073 | 0.616887851297226 | 0.246886141284531 | 1.54140130789898 | 0.998735809224318 |
| ebi-fl187-GCST90094707 | prot-c-4324_33_2 | CYTT \|\| id:prot-c-4324_33_2 | Black olive liking \|\| id:ebi-fl187-GCST90094707 | Inverse variance weighted | 2 | -0.870063633 | 0.452538430251212 | 0.0545266251431738 | -1.757038956 | 0.0169116907202929 | 0.418924891131154 | 0.172555051229069 | 1.01705550292047 | 0.998735809224318 |
| ebi-fl187-GCST90094707 | prot-c-4462_8_2 | RASA1 \|\| id:prot-c-4462_8_2 | Black olive liking \|\| id:ebi-fl187-GCST90094707 | Inverse variance weighted | 2 | 0.24664200436719 | 0.469109971875508 | 0.599050902851885 | -0.672813541 | 1.16609754924319 | 1.27972089627133 | 0.510270888421267 | 3.20944347309396 | 0.998735809224318 |
| ebi-fl187-GCST90094707 | prot-c-4499_21_1 | PDGF-AA \|\| id:prot-c-4499_21_1 | Black olive liking \|\| id:ebi-fl187-GCST90094707 | Inverse variance weighted | 2 | 0.65308253081159 | 0.647105089658392 | 0.312860888481906 | -0.615243445 | 1.92140850654204 | 1.92145465272934 | 0.540509295049784 | 6.83057260311345 | 0.998735809224318 |
| ebi-fl187-GCST90094707 | prot-c-4535_50_2 | BST1 \|\| id:prot-c-4535_50_2 | Black olive liking \|\| id:ebi-fl187-GCST90094707 | Inverse variance weighted | 2 | -0.474596401 | 0.466193822168416 | 0.308666650701695 | -1.388336293 | 0.439143489970897 | 0.62213609901071 | 0.249490037878778 | 1.55137787858418 | 0.998735809224318 |
| ebi-fl187-GCST90094707 | prot-c-4542_24_2 | Clusterin \|\| id:prot-c-4542_24_2 | Black olive liking \|\| id:ebi-fl187-GCST90094707 | Inverse variance weighted | 2 | 0.446231107761543 | 0.466113374556036 | 0.338393366523741 | -0.467351106 | 1.35981332189137 | 1.56241251047001 | 0.626660027455494 | 3.89546603568324 | 0.998735809224318 |
| ebi-fl187-GCST90094707 | prot-c-4696_2_2 | FABP \|\| id:prot-c-4696_2_2 | Black olive liking \|\| id:ebi-fl187-GCST90094707 | Inverse variance weighted | 2 | -0.786305197 | 0.445733392852071 | 0.0777200846391284 | -1.659942647 | 0.0873322525415892 | 0.455524763854865 | 0.190149885371784 | 1.09125919313239 | 0.998735809224318 |
| ebi-fl187-GCST90094707 | prot-c-4906_35_1 | Coagulation Factor V \|\| id:prot-c-4906_35_1 | Black olive liking \|\| id:ebi-fl187-GCST90094707 | Inverse variance weighted | 2 | 0.51948198814687 | 0.469968640774346 | 0.26900597775262 | -0.401656548 | 1.44062052406459 | 1.68115656507461 | 0.669210548079527 | 4.22331567307811 | 0.998735809224318 |
| ebi-fl187-GCST90094707 | prot-c-4916_2_1 | IgD \|\| id:prot-c-4916_2_1 | Black olive liking \|\| id:ebi-fl187-GCST90094707 | Inverse variance weighted | 2 | -0.15541956 | 0.467293798602851 | 0.739440051231758 | -1.071315406 | 0.760476284806495 | 0.856055934909259 | 0.342557618676431 | 2.13929489154225 | 0.998735809224318 |
| ebi-fl187-GCST90094707 | prot-c-4922_13_1 | MIP-3b \|\| id:prot-c-4922_13_1 | Black olive liking \|\| id:ebi-fl187-GCST90094707 | Inverse variance weighted | 2 | 0.534005362532697 | 0.470451154160974 | 0.256336754392439 | -0.3880789 | 1.45608962468821 | 1.70575079457146 | 0.678358818897664 | 4.28915448893146 | 0.998735809224318 |
| ebi-fl187-GCST90094707 | prot-c-4973_18_1 | cIAP-2 \|\| id:prot-c-4973_18_1 | Black olive liking \|\| id:ebi-fl187-GCST90094707 | Inverse variance weighted | 2 | -0.835625247 | 0.46982258713103 | 0.0753057854027889 | -1.756477518 | 0.0852270237335448 | 0.433603287486765 | 0.172651957400437 | 1.08896425936989 | 0.998735809224318 |
| ebi-fl187-GCST90094707 | prot-c-4997_19_1 | IF4A3 \|\| id:prot-c-4997_19_1 | Black olive liking \|\| id:ebi-fl187-GCST90094707 | Inverse variance weighted | 2 | -0.0249986 | 0.583006929988211 | 0.965798199416808 | -1.167692183 | 1.11769498257265 | 0.975311277263966 | 0.311084038520983 | 3.05779779663658 | 0.998735809224318 |
| ebi-fl187-GCST90094707 | prot-c-5000_52_1 | LG3BP \|\| id:prot-c-5000_52_1 | Black olive liking \|\| id:ebi-fl187-GCST90094707 | Inverse variance weighted | 2 | -0.383401752 | 0.433917540808856 | 0.376921869736311 | -1.233880132 | 0.467076627668436 | 0.681539034390606 | 0.291160641260166 | 1.5953236446647 | 0.998735809224318 |
| ebi-fl187-GCST90094707 | prot-c-5006_71_1 | MK13 \|\| id:prot-c-5006_71_1 | Black olive liking \|\| id:ebi-fl187-GCST90094707 | Inverse variance weighted | 2 | -0.484589094 | 0.468806051744091 | 0.301292115894086 | -1.403448955 | 0.434270767395584 | 0.615950242407983 | 0.245747926987963 | 1.54383683220667 | 0.998735809224318 |
| ebi-fl187-GCST90094707 | prot-c-5109_24_3 | Nr-CAM \|\| id:prot-c-5109_24_3 | Black olive liking \|\| id:ebi-fl187-GCST90094707 | Inverse variance weighted | 2 | -0.344945402 | 0.46143331152471 | 0.4547304167318 | -1.249354693 | 0.559463888262381 | 0.708259021707331 | 0.286689740101813 | 1.74973419576048 | 0.998735809224318 |
| ebi-fl187-GCST90094707 | prot-c-5255_22_3 | PDE4D \|\| id:prot-c-5255_22_3 | Black olive liking \|\| id:ebi-fl187-GCST90094707 | Inverse variance weighted | 2 | -0.046834999 | 0.461649254203427 | 0.919192073280463 | -0.951667538 | 0.857997538840188 | 0.954244835573154 | 0.386096655645127 | 2.35843329100209 | 0.998735809224318 |
| ebi-fl187-GCST90094707 | prot-c-5440_26_3 | Troponin I, skeletal, fast twitch \|\| id:prot-c-5440_26_3 | Black olive liking \|\| id:ebi-fl187-GCST90094707 | Inverse variance weighted | 2 | -0.445054477 | 0.599455763741154 | 0.457826308616099 | -1.619987774 | 0.729878819667793 | 0.640789366629392 | 0.197901118568808 | 2.07482916395205 | 0.998735809224318 |
| ebi-fl187-GCST90094707 | ukb-rsfMRI191-edge-pheno716 | Net100_Pair10_33 (OccipitalPrecuneus)&(Temporal\|Frontal\|Supp_Motor_Area) (Default_mode\|Central_executive)&(Default_mode\|Salience) \|\| id:ukb-rsfMRI191-edge-pheno716 | Black olive liking \|\| id:ebi-fl187-GCST90094707 | Inverse variance weighted | 21 | 0.02006808343942 | 0.0248588613869438 | 0.419504678428249 | -0.028655285 | 0.0687914517578299 | 1.02027080120744 | 0.971751384133325 | 1.07121278630836 | 0.998735809224318 |
| ebi-fl187-GCST90094707 | ukb-rsfMRI191-edge-pheno903 | Net100_Pair27_38 (Precentral\|Frontal\|Supp_Motor_Area)&(Subcortical) (Attention\|Salience\|Motor)&(Subcortical-cerebellum) \|\| id:ukb-rsfMRI191-edge-pheno903 | Black olive liking \|\| id:ebi-fl187-GCST90094707 | Inverse variance weighted (multiplicative random effects) | 21 | 0.0227683050590672 | 0.0335692494291341 | 0.497613919612309 | -0.043027424 | 0.0885640339401701 | 1.02302948133043 | 0.957885120835747 | 1.09260421412338 | 0.998735809224318 |
| ebi-fl187-GCST90094707 | ukb-rsfMRI191-node-pheno23 | Net100_Node2 RolandicOper\|SupraMarginal\|Insula Salience\|Motor \|\| id:ukb-rsfMRI191-node-pheno23 | Black olive liking \|\| id:ebi-fl187-GCST90094707 | Inverse variance weighted (multiplicative random effects) | 21 | 0.0383399769737949 | 0.0299441091609161 | 0.200409898629835 | -0.020350477 | 0.0970304309291904 | 1.03908443761698 | 0.979855196428252 | 1.10189390476621 | 0.998735809224318 |
| ebi-fl187-GCST90094707 | ukb-rsfMRI191-node-pheno55 | Net100_Node34 Parietal\|Postcentral\|Precuneus Attention \|\| id:ukb-rsfMRI191-node-pheno55 | Black olive liking \|\| id:ebi-fl187-GCST90094707 | Inverse variance weighted | 21 | 0.0298767031334091 | 0.0288935749433884 | 0.301124082839693 | -0.026754704 | 0.0865081100224503 | 1.03032748997054 | 0.973600032667384 | 1.09036021052771 | 0.998735809224318 |
| ebi-fl187-GCST90094707 | ebi-a-GCST90001414 | IgD- CD24- B cell Absolute Count \|\| id:ebi-a-GCST90001414 | Black olive liking \|\| id:ebi-fl187-GCST90094707 | MR Egger | 20 | -0.034867544 | 0.389301537517894 | 0.92962246305874 | -0.797898558 | 0.728163469139613 | 0.965733324577625 | 0.45027419573234 | 2.07127315542228 | 0.999950574162623 |
| ebi-fl187-GCST90094707 | ebi-a-GCST90001422 | CD20- CD38- B cell %B cell \|\| id:ebi-a-GCST90001422 | Black olive liking \|\| id:ebi-fl187-GCST90094707 | MR Egger | 20 | -0.536981046 | 0.412868801041656 | 0.209798054469286 | -1.346203896 | 0.272241804438006 | 0.584510201055613 | 0.260226233973044 | 1.31290442904947 | 0.999950574162623 |
| ebi-fl187-GCST90094707 | ebi-a-GCST90001444 | CD20- CD38- B cell %lymphocyte \|\| id:ebi-a-GCST90001444 | Black olive liking \|\| id:ebi-fl187-GCST90094707 | MR Egger | 20 | -0.655811706 | 0.439702685079794 | 0.15315106751485 | -1.517628969 | 0.206005556518027 | 0.519020599299706 | 0.219231074879498 | 1.22876003160361 | 0.999950574162623 |
| ebi-fl187-GCST90094707 | ebi-a-GCST90001459 | Myeloid Dendritic Cell %Dendritic Cell \|\| id:ebi-a-GCST90001459 | Black olive liking \|\| id:ebi-fl187-GCST90094707 | MR Egger | 20 | -0.214398493 | 0.451544924204559 | 0.640631631482709 | -1.099426545 | 0.670629557971828 | 0.807026726057711 | 0.333062025058241 | 1.95546801367565 | 0.999950574162623 |
| ebi-fl187-GCST90094707 | ebi-a-GCST90001476 | HLA DR++ monocyte %leukocyte \|\| id:ebi-a-GCST90001476 | Black olive liking \|\| id:ebi-fl187-GCST90094707 | MR Egger | 20 | -0.500615344 | 0.389980767674243 | 0.215529430076099 | -1.264977649 | 0.263746960552846 | 0.606157549463948 | 0.282245604617684 | 1.30179874818542 | 0.999950574162623 |
| ebi-fl187-GCST90094707 | ebi-a-GCST90001478 | CD4 regulatory T cell %CD4+ T cell \|\| id:ebi-a-GCST90001478 | Black olive liking \|\| id:ebi-fl187-GCST90094707 | MR Egger | 20 | -0.531425752 | 0.444726159447858 | 0.247614102435293 | -1.403089024 | 0.340237520727474 | 0.587766363053547 | 0.24583639523807 | 1.40528133437133 | 0.999950574162623 |
| ebi-fl187-GCST90094707 | ebi-a-GCST90001485 | CD39+ resting CD4 regulatory T cell %CD4 regulatory T cell \|\| id:ebi-a-GCST90001485 | Black olive liking \|\| id:ebi-fl187-GCST90094707 | MR Egger | 19 | -0.1099797 | 0.417501487176435 | 0.795390527096982 | -0.928282615 | 0.708323214771406 | 0.895852320829483 | 0.395231893195014 | 2.0305835499454 | 0.999950574162623 |
| ebi-fl187-GCST90094707 | ebi-a-GCST90001515 | Immature Myeloid-Derived Suppressor Cells Absolute Count \|\| id:ebi-a-GCST90001515 | Black olive liking \|\| id:ebi-fl187-GCST90094707 | MR Egger | 20 | -0.102597983 | 0.656099478690294 | 0.877477440608367 | -1.388552961 | 1.18335699516606 | 0.902489716710221 | 0.249435987134544 | 3.26531747934337 | 0.999950574162623 |
| ebi-fl187-GCST90094707 | ebi-a-GCST90001517 | CD33+ HLA DR+ Absolute Count \|\| id:ebi-a-GCST90001517 | Black olive liking \|\| id:ebi-fl187-GCST90094707 | MR Egger | 20 | -0.440317973 | 0.510219211593146 | 0.399491756414405 | -1.440347628 | 0.559711681246863 | 0.643831667138737 | 0.236845410226403 | 1.75016782134136 | 0.999950574162623 |
| ebi-fl187-GCST90094707 | ebi-a-GCST90001518 | CD33+ HLA DR+ CD14- Absolute Count \|\| id:ebi-a-GCST90001518 | Black olive liking \|\| id:ebi-fl187-GCST90094707 | MR Egger | 20 | -0.377458206 | 0.516040635919233 | 0.473919694595291 | -1.388897852 | 0.633981440415654 | 0.685601855039016 | 0.249349973719076 | 1.88510107549684 | 0.999950574162623 |
| ebi-fl187-GCST90094707 | ebi-a-GCST90001520 | CD33+ HLA DR+ CD14dim Absolute Count \|\| id:ebi-a-GCST90001520 | Black olive liking \|\| id:ebi-fl187-GCST90094707 | MR Egger | 20 | -0.428223631 | 0.512666946456838 | 0.414506609245561 | -1.433050846 | 0.576603583889858 | 0.651665665749948 | 0.238579940094333 | 1.77998259094797 | 0.999950574162623 |
| ebi-fl187-GCST90094707 | ebi-a-GCST90001531 | CD33dim HLA DR- Absolute Count \|\| id:ebi-a-GCST90001531 | Black olive liking \|\| id:ebi-fl187-GCST90094707 | MR Egger | 20 | -0.275767556 | 0.5192391031255 | 0.601842578190159 | -1.293476198 | 0.741941086492417 | 0.758989333053241 | 0.27431554866411 | 2.10000785771708 | 0.999950574162623 |
| ebi-fl187-GCST90094707 | ebi-a-GCST90001532 | Basophil Absolute Count \|\| id:ebi-a-GCST90001532 | Black olive liking \|\| id:ebi-fl187-GCST90094707 | MR Egger | 19 | -0.252169904 | 0.518791795381066 | 0.63312271131012 | -1.269001823 | 0.764662014454741 | 0.777112691914384 | 0.281112081271575 | 2.14826816835027 | 0.999950574162623 |
| ebi-fl187-GCST90094707 | ebi-a-GCST90001588 | T/B cell \|\| id:ebi-a-GCST90001588 | Black olive liking \|\| id:ebi-fl187-GCST90094707 | MR Egger | 20 | 0.0121529224194729 | 0.383044913508496 | 0.975038802942822 | -0.738615108 | 0.762920952896126 | 1.01222706924357 | 0.47777512448425 | 2.1445311553536 | 0.999950574162623 |
| ebi-fl187-GCST90094707 | ebi-a-GCST90001624 | HLA DR+ CD4+ T cell Absolute Count \|\| id:ebi-a-GCST90001624 | Black olive liking \|\| id:ebi-fl187-GCST90094707 | MR Egger | 20 | -0.128209707 | 0.373635806055565 | 0.735467559085699 | -0.860535887 | 0.604116472611505 | 0.879668886850949 | 0.422935375955428 | 1.82963496195017 | 0.999950574162623 |
| ebi-fl187-GCST90094707 | ebi-a-GCST90001631 | CD8+ Natural Killer T %T cell \|\| id:ebi-a-GCST90001631 | Black olive liking \|\| id:ebi-fl187-GCST90094707 | MR Egger | 20 | 0.402888484373143 | 0.445627380920225 | 0.377887715129084 | -0.470541182 | 1.27631815097678 | 1.49614003936608 | 0.624664119669348 | 3.58342178926365 | 0.999950574162623 |
| ebi-fl187-GCST90094707 | ebi-a-GCST90001632 | CD8+ Natural Killer T %lymphocyte \|\| id:ebi-a-GCST90001632 | Black olive liking \|\| id:ebi-fl187-GCST90094707 | MR Egger | 20 | 0.417886539011881 | 0.470826164444538 | 0.386483619386613 | -0.504932743 | 1.34070582132317 | 1.51874834593811 | 0.603546166585828 | 3.8217400192231 | 0.999950574162623 |
| ebi-fl187-GCST90094707 | ebi-a-GCST90001646 | Natural Killer %CD3- lymphocyte \|\| id:ebi-a-GCST90001646 | Black olive liking \|\| id:ebi-fl187-GCST90094707 | MR Egger | 20 | -0.14445629 | 0.36976066482951 | 0.700622595658335 | -0.869187193 | 0.580274613410376 | 0.865492742562287 | 0.41929221454515 | 1.7865289682056 | 0.999950574162623 |
| ebi-fl187-GCST90094707 | ebi-a-GCST90001684 | CD127- CD8+ T cell Absolute Count \|\| id:ebi-a-GCST90001684 | Black olive liking \|\| id:ebi-fl187-GCST90094707 | MR Egger | 20 | -0.018306889 | 0.423029139656368 | 0.965958201863459 | -0.847444003 | 0.810830224421228 | 0.98185966388718 | 0.428508800585909 | 2.2497750297088 | 0.999950574162623 |
| ebi-fl187-GCST90094707 | ebi-a-GCST90001687 | CD28- CD8+ T cell Absolute Count \|\| id:ebi-a-GCST90001687 | Black olive liking \|\| id:ebi-fl187-GCST90094707 | MR Egger | 20 | -0.048113397 | 0.512651545746977 | 0.926263491444691 | -1.052910427 | 0.956683632823941 | 0.953025710845692 | 0.348920761662803 | 2.60304947520055 | 0.999950574162623 |
| ebi-fl187-GCST90094707 | ebi-a-GCST90001701 | BAFF-R on CD20- CD38- B cell \|\| id:ebi-a-GCST90001701 | Black olive liking \|\| id:ebi-fl187-GCST90094707 | MR Egger | 20 | 0.304645459815303 | 0.378914574044206 | 0.431890441973936 | -0.438027105 | 1.04731802494195 | 1.35614411018005 | 0.645308291338092 | 2.84999723738629 | 0.999950574162623 |
| ebi-fl187-GCST90094707 | ebi-a-GCST90001732 | CD19 on IgD- CD27- B cell \|\| id:ebi-a-GCST90001732 | Black olive liking \|\| id:ebi-fl187-GCST90094707 | MR Egger | 20 | 0.0991617400029155 | 0.395069329866469 | 0.804658309362423 | -0.675174147 | 0.873497626541194 | 1.10424488568726 | 0.509067760501092 | 2.39527399332109 | 0.999950574162623 |
| ebi-fl187-GCST90094707 | ebi-a-GCST90001798 | CD27 on CD24+ CD27+ B cell \|\| id:ebi-a-GCST90001798 | Black olive liking \|\| id:ebi-fl187-GCST90094707 | MR Egger | 20 | -0.088571943 | 0.43252344151334 | 0.840042169141921 | -0.936317888 | 0.759174002261762 | 0.91523726335742 | 0.392068821923381 | 2.13651073841744 | 0.999950574162623 |
| ebi-fl187-GCST90094707 | ebi-a-GCST90001836 | CD66b on CD66b++ myeloid cell \|\| id:ebi-a-GCST90001836 | Black olive liking \|\| id:ebi-fl187-GCST90094707 | MR Egger | 20 | -0.057855388 | 0.579526899806553 | 0.921581111925561 | -1.193728111 | 1.07801733604544 | 0.943786420811299 | 0.303089204898419 | 2.93884702494216 | 0.999950574162623 |
| ebi-fl187-GCST90094707 | ebi-a-GCST90001943 | CD25 on activated & secreting CD4 regulatory T cell \|\| id:ebi-a-GCST90001943 | Black olive liking \|\| id:ebi-fl187-GCST90094707 | MR Egger | 20 | -0.530734306 | 0.538551755897607 | 0.337448611775906 | -1.586295747 | 0.524827136035791 | 0.588172912448369 | 0.204682404587072 | 1.69016665421688 | 0.999950574162623 |
| ebi-fl187-GCST90094707 | ebi-a-GCST90001960 | CD25 on CD4+ T cell \|\| id:ebi-a-GCST90001960 | Black olive liking \|\| id:ebi-fl187-GCST90094707 | MR Egger | 20 | -0.301673255 | 0.443315108875942 | 0.50485152151533 | -1.170570869 | 0.567224358036287 | 0.739579679109896 | 0.310189813038681 | 1.76336578043615 | 0.999950574162623 |
| ebi-fl187-GCST90094707 | ebi-a-GCST90001988 | HLA DR on CD14+ CD16- monocyte \|\| id:ebi-a-GCST90001988 | Black olive liking \|\| id:ebi-fl187-GCST90094707 | MR Egger | 19 | -0.127607124 | 0.388387543673744 | 0.7465037068013 | -0.888846709 | 0.633632461951301 | 0.88019912064183 | 0.411129631440951 | 1.88444333059444 | 0.999950574162623 |
| ebi-fl187-GCST90094707 | ebi-a-GCST90001991 | HLA DR on CD14+ monocyte \|\| id:ebi-a-GCST90001991 | Black olive liking \|\| id:ebi-fl187-GCST90094707 | MR Egger | 19 | -0.149251345 | 0.389095601019338 | 0.706040729338758 | -0.911878723 | 0.613376033355838 | 0.86135259133032 | 0.401768702590653 | 1.8466552566375 | 0.999950574162623 |
| ebi-fl187-GCST90094707 | ebi-a-GCST90002019 | CD14 on Monocytic Myeloid-Derived Suppressor Cells \|\| id:ebi-a-GCST90002019 | Black olive liking \|\| id:ebi-fl187-GCST90094707 | MR Egger | 20 | -1.120265281 | 0.560249460175254 | 0.0608702529373179 | -2.218354222 | -0.022176339 | 0.326193250434316 | 0.108788002427381 | 0.978067748784438 | 0.999950574162623 |
| ebi-fl187-GCST90094707 | ebi-a-GCST90002037 | CD80 on plasmacytoid Dendritic Cell \|\| id:ebi-a-GCST90002037 | Black olive liking \|\| id:ebi-fl187-GCST90094707 | MR Egger | 20 | -0.469303231 | 0.434125502880363 | 0.29395467529312 | -1.320189217 | 0.381582754432858 | 0.625437902105661 | 0.267084760245131 | 1.46460085940999 | 0.999950574162623 |
| ebi-fl187-GCST90094707 | ebi-a-GCST90002042 | CD45 on CD33+ HLA DR+ CD14- \|\| id:ebi-a-GCST90002042 | Black olive liking \|\| id:ebi-fl187-GCST90094707 | MR Egger | 20 | -1.320731052 | 0.569168410984724 | 0.0322622465002804 | -2.436301138 | -0.205160967 | 0.266940083454919 | 0.0874838444230621 | 0.814516195817006 | 0.999950574162623 |
| ebi-fl187-GCST90094707 | ebi-a-GCST90002056 | CD8 on Effector Memory CD8+ T cell \|\| id:ebi-a-GCST90002056 | Black olive liking \|\| id:ebi-fl187-GCST90094707 | MR Egger | 20 | -0.634439931 | 0.424756766151255 | 0.152588605064414 | -1.466963193 | 0.198083330319526 | 0.530232371707804 | 0.230624785794205 | 1.21906397457973 | 0.999950574162623 |
| ebi-fl187-GCST90094707 | ebi-a-GCST90002058 | CD8 on CD8+ T cell \|\| id:ebi-a-GCST90002058 | Black olive liking \|\| id:ebi-fl187-GCST90094707 | MR Egger | 20 | -0.341439291 | 0.409695969893852 | 0.415543413765026 | -1.144443392 | 0.461564809921155 | 0.710746614976062 | 0.31840109305289 | 1.58655470009965 | 0.999950574162623 |
| ebi-fl187-GCST90094707 | ebi-a-GCST90002063 | CD4 on CD4 regulatory T cell \|\| id:ebi-a-GCST90002063 | Black olive liking \|\| id:ebi-fl187-GCST90094707 | MR Egger | 20 | -0.511766114 | 0.430215022153705 | 0.249674911621228 | -1.354987557 | 0.331455329701325 | 0.599435971300869 | 0.257950504073557 | 1.39299391943406 | 0.999950574162623 |
| ebi-fl187-GCST90094707 | ebi-a-GCST90002066 | CD4 on activated CD4 regulatory T cell \|\| id:ebi-a-GCST90002066 | Black olive liking \|\| id:ebi-fl187-GCST90094707 | MR Egger | 20 | -0.53546582 | 0.433122043004379 | 0.232239613726787 | -1.384385024 | 0.31345338427414 | 0.585396537199423 | 0.250477790190266 | 1.36814168435758 | 0.999950574162623 |
| ebi-fl187-GCST90094707 | ebi-a-GCST90002070 | CD4 on activated & secreting CD4 regulatory T cell \|\| id:ebi-a-GCST90002070 | Black olive liking \|\| id:ebi-fl187-GCST90094707 | MR Egger | 20 | -0.480076214 | 0.424113512201013 | 0.272509633133164 | -1.311338698 | 0.351186269614269 | 0.618736233460352 | 0.269459090423335 | 1.42075194418287 | 0.999950574162623 |
| ebi-fl187-GCST90094707 | ebi-a-GCST90002081 | SSC-A on CD4+ T cell \|\| id:ebi-a-GCST90002081 | Black olive liking \|\| id:ebi-fl187-GCST90094707 | MR Egger | 20 | 0.55711688177025 | 0.39987994401923 | 0.180524016339948 | -0.226647809 | 1.34088157204794 | 1.745632374 | 0.797201500440276 | 3.82241175182836 | 0.999950574162623 |
| ebi-fl187-GCST90094707 | ebi-a-GCST90002094 | CD11b on Monocytic Myeloid-Derived Suppressor Cells \|\| id:ebi-a-GCST90002094 | Black olive liking \|\| id:ebi-fl187-GCST90094707 | MR Egger | 20 | -0.701723811 | 0.567098477231006 | 0.231840781008572 | -1.813236826 | 0.409789204427345 | 0.495730021993249 | 0.16312527316723 | 1.50650018807016 | 0.999950574162623 |
| ebi-fl187-GCST90094707 | ebi-a-GCST90002104 | HLA DR on myeloid Dendritic Cell \|\| id:ebi-a-GCST90002104 | Black olive liking \|\| id:ebi-fl187-GCST90094707 | MR Egger | 19 | -0.458064715 | 0.430236307330082 | 0.301909347455232 | -1.301327877 | 0.38519844727217 | 0.63250654216202 | 0.272170144374124 | 1.46990599133397 | 0.999950574162623 |
| ebi-fl187-GCST90094707 | ebi-a-GCST90002105 | HLA DR on plasmacytoid Dendritic Cell \|\| id:ebi-a-GCST90002105 | Black olive liking \|\| id:ebi-fl187-GCST90094707 | MR Egger | 19 | -0.177280531 | 0.435893830487034 | 0.689298640130756 | -1.031632439 | 0.677071376654229 | 0.837544794200772 | 0.356424643962672 | 1.96810544437629 | 0.999950574162623 |
| ebi-fl187-GCST90094707 | ebi-a-GCST90002106 | HLA DR on Dendritic Cell \|\| id:ebi-a-GCST90002106 | Black olive liking \|\| id:ebi-fl187-GCST90094707 | MR Egger | 19 | -0.222227521 | 0.429162505764508 | 0.611259245755785 | -1.063386032 | 0.618930990635619 | 0.800733160268487 | 0.345284683738088 | 1.85694189215735 | 0.999950574162623 |
| ebi-fl187-GCST90094707 | ebi-a-GCST90002111 | HLA DR on CD33dim HLA DR+ CD11b- \|\| id:ebi-a-GCST90002111 | Black olive liking \|\| id:ebi-fl187-GCST90094707 | MR Egger | 19 | 0.137533149777459 | 0.552832534485689 | 0.806512332699533 | -0.946018618 | 1.22108491736941 | 1.14743974271373 | 0.388283856541767 | 3.39086454658547 | 0.999950574162623 |
| ebi-fl187-GCST90094707 | ebi-a-GCST90002112 | HLA DR on CD33- HLA DR+ \|\| id:ebi-a-GCST90002112 | Black olive liking \|\| id:ebi-fl187-GCST90094707 | MR Egger | 19 | 0.615722940000457 | 0.558439662761366 | 0.285575606892079 | -0.478818799 | 1.71026467901273 | 1.8509942737721 | 0.61951473120439 | 5.53042507137208 | 0.999950574162623 |
| ebi-fl187-GCST90094707 | ebi-a-GCST90002115 | HLA DR on HLA DR+ CD8+ T cell \|\| id:ebi-a-GCST90002115 | Black olive liking \|\| id:ebi-fl187-GCST90094707 | MR Egger | 20 | -0.040421021 | 0.451080888548405 | 0.929587290115737 | -0.924539563 | 0.843697520495041 | 0.960385011706584 | 0.396714039004073 | 2.32494764497403 | 0.999950574162623 |
| ebi-fl187-GCST90094707 | ebi-a-GCST90002116 | HLA DR on B cell \|\| id:ebi-a-GCST90002116 | Black olive liking \|\| id:ebi-fl187-GCST90094707 | MR Egger | 20 | -0.783167631 | 0.54262479542291 | 0.166115882169002 | -1.84671223 | 0.280376968218745 | 0.45695624767046 | 0.157754976731608 | 1.32362868424948 | 0.999950574162623 |
| ebi-fl187-GCST90094707 | ebi-cfmet338-GCST90026023 | Sphingomyelin (d18:1/24:1, d18:2/24:0) levels \|\| id:ebi-cfmet338-GCST90026023 | Black olive liking \|\| id:ebi-fl187-GCST90094707 | MR Egger | 19 | -0.129848183 | 0.269048689215482 | 0.635521768645451 | -0.657183614 | 0.397487248124169 | 0.878228751084398 | 0.518309039327203 | 1.48808081802401 | 0.999950574162623 |
| ebi-fl187-GCST90094707 | ebi-cfmet338-GCST90026046 | 1-arachidonoyl-gpc (20:4n6) levels \|\| id:ebi-cfmet338-GCST90026046 | Black olive liking \|\| id:ebi-fl187-GCST90094707 | MR Egger | 19 | -0.00850564 | 0.282122108010685 | 0.976299511944372 | -0.561464972 | 0.544453691254876 | 0.991530430173279 | 0.570372871129228 | 1.72366647104585 | 0.999950574162623 |
| ebi-fl187-GCST90094707 | ebi-cfmet338-GCST90026047 | 1-linoleoyl-gpc (18:2) levels \|\| id:ebi-cfmet338-GCST90026047 | Black olive liking \|\| id:ebi-fl187-GCST90094707 | MR Egger | 19 | -0.356863134 | 0.367331628355617 | 0.344919683963389 | -1.076833125 | 0.363106858069737 | 0.699868279696604 | 0.340672686913849 | 1.43778949044233 | 0.999950574162623 |
| ebi-fl187-GCST90094707 | ebi-cfmet338-GCST90026050 | 1-myristoyl-2-palmitoyl-gpc (14:0/16:0) levels \|\| id:ebi-cfmet338-GCST90026050 | Black olive liking \|\| id:ebi-fl187-GCST90094707 | MR Egger | 19 | -0.098962241 | 0.215693288643029 | 0.652182220970077 | -0.521721087 | 0.32379660478128 | 0.905776908645231 | 0.593498206584006 | 1.38236611186589 | 0.999950574162623 |
| ebi-fl187-GCST90094707 | ebi-cfmet338-GCST90026068 | 2,3-dihydroxyisovalerate levels \|\| id:ebi-cfmet338-GCST90026068 | Black olive liking \|\| id:ebi-fl187-GCST90094707 | MR Egger | 19 | -0.433454223 | 0.58272045345605 | 0.467127210753765 | -1.575586312 | 0.708677865681859 | 0.648265967547963 | 0.206886217270365 | 2.03130382596586 | 0.999950574162623 |
| ebi-fl187-GCST90094707 | ebi-cfmet338-GCST90026082 | 3-hydroxyhexanoate levels \|\| id:ebi-cfmet338-GCST90026082 | Black olive liking \|\| id:ebi-fl187-GCST90094707 | MR Egger | 19 | -0.046335619 | 0.125203907010253 | 0.715892254319622 | -0.291735277 | 0.199064038659877 | 0.95472148566782 | 0.746966249083155 | 1.22026010721978 | 0.999950574162623 |
| ebi-fl187-GCST90094707 | ebi-cfmet338-GCST90026094 | 3-ureidopropionate levels \|\| id:ebi-cfmet338-GCST90026094 | Black olive liking \|\| id:ebi-fl187-GCST90094707 | MR Egger | 19 | -0.019086946 | 0.285774158658755 | 0.947527859846223 | -0.579204297 | 0.541030405182428 | 0.981094056537809 | 0.560344056804879 | 1.71777595583384 | 0.999950574162623 |
| ebi-fl187-GCST90094707 | ebi-cfmet338-GCST90026173 | Glycerol levels \|\| id:ebi-cfmet338-GCST90026173 | Black olive liking \|\| id:ebi-fl187-GCST90094707 | MR Egger | 18 | 0.16008758883162 | 0.161000373715028 | 0.334862665160743 | -0.155473144 | 0.475648321313075 | 1.1736136619395 | 0.856010065926303 | 1.60905704537547 | 0.999950574162623 |
| ebi-fl187-GCST90094707 | ebi-cfmet338-GCST90026179 | Histidine levels \|\| id:ebi-cfmet338-GCST90026179 | Black olive liking \|\| id:ebi-fl187-GCST90094707 | MR Egger | 19 | -0.132103597 | 0.111761093605394 | 0.253469166325679 | -0.35115534 | 0.086948146463542 | 0.876250213481082 | 0.703874405182061 | 1.09084011433409 | 0.999950574162623 |
| ebi-fl187-GCST90094707 | ebi-cfmet338-GCST90026188 | Isocitrate levels \|\| id:ebi-cfmet338-GCST90026188 | Black olive liking \|\| id:ebi-fl187-GCST90094707 | MR Egger | 19 | -0.307235548 | 0.219619952846383 | 0.179810523829991 | -0.737690656 | 0.123219559202491 | 0.73547734000906 | 0.47821700892132 | 1.1311327442889 | 0.999950574162623 |
| ebi-fl187-GCST90094707 | ebi-cfmet338-GCST90026207 | N-acetyl-isoputreanine levels \|\| id:ebi-cfmet338-GCST90026207 | Black olive liking \|\| id:ebi-fl187-GCST90094707 | MR Egger | 19 | 0.152577646265956 | 0.139275982389343 | 0.288573230228734 | -0.120403279 | 0.425558571749068 | 1.16483290350877 | 0.886562832249831 | 1.53044504432182 | 0.999950574162623 |
| ebi-fl187-GCST90094707 | ebi-cfmet338-GCST90026213 | N-acetylglutamate levels \|\| id:ebi-cfmet338-GCST90026213 | Black olive liking \|\| id:ebi-fl187-GCST90094707 | MR Egger | 19 | 0.235172692027833 | 0.289413579624021 | 0.427690313928371 | -0.332077924 | 0.802423308090915 | 1.26512722725567 | 0.717431415525297 | 2.23094063977071 | 0.999950574162623 |
| ebi-fl187-GCST90094707 | ebi-cfmet338-GCST90026224 | N-acetyltaurine levels \|\| id:ebi-cfmet338-GCST90026224 | Black olive liking \|\| id:ebi-fl187-GCST90094707 | MR Egger | 19 | 0.102646735699117 | 0.190581455945675 | 0.59714719964139 | -0.270892918 | 0.47618638935264 | 1.10809988779266 | 0.762698163311955 | 1.60992306051206 | 0.999950574162623 |
| ebi-fl187-GCST90094707 | ebi-cfmet338-GCST90026250 | Phenyllactate (pla) levels \|\| id:ebi-cfmet338-GCST90026250 | Black olive liking \|\| id:ebi-fl187-GCST90094707 | MR Egger | 19 | 0.0817714059412332 | 0.34286451930908 | 0.814348494075869 | -0.590243052 | 0.75378586378703 | 1.08520770943859 | 0.554192570804103 | 2.12502988070771 | 0.999950574162623 |
| ebi-fl187-GCST90094707 | ebi-cfmet338-GCST90026251 | Pipecolate levels \|\| id:ebi-cfmet338-GCST90026251 | Black olive liking \|\| id:ebi-fl187-GCST90094707 | MR Egger | 19 | 0.294220375741864 | 0.447281345590306 | 0.519477155737722 | -0.582451062 | 1.17089181309886 | 1.34207963287474 | 0.558527701649031 | 3.22486733542362 | 0.999950574162623 |
| ebi-fl187-GCST90094707 | ebi-cfmet338-GCST90026278 | Trigonelline (n'-methylnicotinate) levels \|\| id:ebi-cfmet338-GCST90026278 | Black olive liking \|\| id:ebi-fl187-GCST90094707 | MR Egger | 19 | 0.0781635175934895 | 0.507683515610943 | 0.879453337324496 | -0.916896173 | 1.07322320819094 | 1.08129945569981 | 0.399757896845078 | 2.92479153538731 | 0.999950574162623 |
| ebi-fl187-GCST90094707 | ebi-cfmet338-GCST90026299 | 1-carboxyethylvaline levels \|\| id:ebi-cfmet338-GCST90026299 | Black olive liking \|\| id:ebi-fl187-GCST90094707 | MR Egger | 19 | -0.51711008 | 0.304476254811732 | 0.107666587645062 | -1.11388354 | 0.079663379209992 | 0.59624114966374 | 0.328281587752589 | 1.0829224720951 | 0.999950574162623 |
| ebi-fl187-GCST90094707 | ebi-gm430-GCST90011306 | C_Erysipelotrichia abundance \|\| id:ebi-gm430-GCST90011306 | Black olive liking \|\| id:ebi-fl187-GCST90094707 | MR Egger | 18 | -0.047463487 | 0.282260105459493 | 0.868568449491376 | -0.600693294 | 0.505766319745776 | 0.953645292990591 | 0.548431280333045 | 1.65825578783697 | 0.999950574162623 |
| ebi-fl187-GCST90094707 | ebi-gm430-GCST90011312 | F_Erysipelotrichaceae abundance \|\| id:ebi-gm430-GCST90011312 | Black olive liking \|\| id:ebi-fl187-GCST90094707 | MR Egger | 18 | -0.047463487 | 0.282260105459493 | 0.868568449491376 | -0.600693294 | 0.505766319745776 | 0.953645292990591 | 0.548431280333045 | 1.65825578783697 | 0.999950574162623 |
| ebi-fl187-GCST90094707 | ebi-gm430-GCST90011352 | O_Erysipelotrichales abundance \|\| id:ebi-gm430-GCST90011352 | Black olive liking \|\| id:ebi-fl187-GCST90094707 | MR Egger | 18 | -0.047463487 | 0.282260105459493 | 0.868568449491376 | -0.600693294 | 0.505766319745776 | 0.953645292990591 | 0.548431280333045 | 1.65825578783697 | 0.999950574162623 |
| ebi-fl187-GCST90094707 | ebi-gm430-GCST90011362 | OTU97_11 (Parabacteroides) abundance \|\| id:ebi-gm430-GCST90011362 | Black olive liking \|\| id:ebi-fl187-GCST90094707 | MR Egger | 18 | -0.027937005 | 0.319257067516719 | 0.93135491720528 | -0.653680857 | 0.597806847638208 | 0.972449624632877 | 0.520127733082897 | 1.81812699515852 | 0.999950574162623 |
| ebi-fl187-GCST90094707 | ebi-gm430-GCST90011384 | OTU97_235 (Phascolarctobacterium) abundance \|\| id:ebi-gm430-GCST90011384 | Black olive liking \|\| id:ebi-fl187-GCST90094707 | MR Egger | 18 | -1.192279849 | 1.3152887310067 | 0.378132561088434 | -3.770245761 | 1.38568606419885 | 0.303528475677942 | 0.023046398677191 | 3.99756755221611 | 0.999950574162623 |
| ebi-fl187-GCST90094707 | ebi-gm430-GCST90011390 | OTU97_3 (Bacteroides) abundance \|\| id:ebi-gm430-GCST90011390 | Black olive liking \|\| id:ebi-fl187-GCST90094707 | MR Egger | 18 | -0.021442958 | 0.255389627311745 | 0.93412839615685 | -0.522006628 | 0.479120711326038 | 0.978785307547689 | 0.593328762683619 | 1.61465403082451 | 0.999950574162623 |
| ebi-fl187-GCST90094707 | ebi-gm430-GCST90011398 | OTU97_4 (Alistipes) abundance \|\| id:ebi-gm430-GCST90011398 | Black olive liking \|\| id:ebi-fl187-GCST90094707 | MR Egger | 18 | 0.203271803854861 | 0.298628980177671 | 0.505803733539072 | -0.382040997 | 0.788584605003095 | 1.2254054929235 | 0.682467073329022 | 2.20027995601649 | 0.999950574162623 |
| ebi-fl187-GCST90094707 | ebi-gm430-GCST90011421 | OTU99_108 (Cytophagales) abundance \|\| id:ebi-gm430-GCST90011421 | Black olive liking \|\| id:ebi-fl187-GCST90094707 | MR Egger | 18 | 0.658892814109856 | 0.967407205589169 | 0.505552301961316 | -1.237225309 | 2.55501093706463 | 1.93265134508432 | 0.290188284771682 | 12.8714404325282 | 0.999950574162623 |
| ebi-fl187-GCST90094707 | ebi-gm430-GCST90011428 | OTU99_123 (Phascolarctobacterium) abundance \|\| id:ebi-gm430-GCST90011428 | Black olive liking \|\| id:ebi-fl187-GCST90094707 | MR Egger | 18 | 0.0664054388409732 | 0.702631387631437 | 0.925877795264248 | -1.310752081 | 1.44356295859859 | 1.06865990557787 | 0.269617206158806 | 4.23576080347422 | 0.999950574162623 |
| ebi-fl187-GCST90094707 | ebi-gm430-GCST90011450 | OTU99_298 (Phascolarctobacterium) abundance \|\| id:ebi-gm430-GCST90011450 | Black olive liking \|\| id:ebi-fl187-GCST90094707 | MR Egger | 18 | -1.195739016 | 1.31916664935772 | 0.378154378030091 | -3.781305649 | 1.38982761691844 | 0.302480333810083 | 0.0227929124554758 | 4.01415802041914 | 0.999950574162623 |
| ebi-fl187-GCST90094707 | ebi-gm430-GCST90011451 | OTU99_3 (Bacteroides) abundance \|\| id:ebi-gm430-GCST90011451 | Black olive liking \|\| id:ebi-fl187-GCST90094707 | MR Egger | 18 | -0.021622515 | 0.255368750202944 | 0.933572784328058 | -0.522145265 | 0.478900235585986 | 0.978609575956433 | 0.593246510784703 | 1.61429807802305 | 0.999950574162623 |
| ebi-fl187-GCST90094707 | ebi-gm430-GCST90011472 | OTU99_72 (Parabacteroides) abundance \|\| id:ebi-gm430-GCST90011472 | Black olive liking \|\| id:ebi-fl187-GCST90094707 | MR Egger | 18 | -1.167893891 | 0.773818221684173 | 0.15072551814915 | -2.684577605 | 0.348789823837085 | 0.311021296808385 | 0.0682500163630894 | 1.41735126558424 | 0.999950574162623 |
| ebi-fl187-GCST90094707 | ebi-gm430-GCST90011507 | TestASV_3 (Bacteroides) abundance \|\| id:ebi-gm430-GCST90011507 | Black olive liking \|\| id:ebi-fl187-GCST90094707 | MR Egger | 18 | 0.149848514259452 | 0.328645836624488 | 0.654548190109827 | -0.494297326 | 0.793994354043448 | 1.1616582547378 | 0.609999387737351 | 2.21221517255278 | 0.999950574162623 |
| ebi-fl187-GCST90094707 | ebi-gm430-GCST90011512 | TestASV_34 (Phascolarctobacterium) abundance \|\| id:ebi-gm430-GCST90011512 | Black olive liking \|\| id:ebi-fl187-GCST90094707 | MR Egger | 18 | -0.444812698 | 0.932496969162527 | 0.639803333414786 | -2.272506757 | 1.38288136204329 | 0.640944315252972 | 0.103053525871947 | 3.98637127433724 | 0.999950574162623 |
| ebi-fl187-GCST90094707 | ebi-gm430-GCST90011519 | TestASV_41 (Barnesiella) abundance \|\| id:ebi-gm430-GCST90011519 | Black olive liking \|\| id:ebi-fl187-GCST90094707 | MR Egger | 18 | -0.38076555 | 1.19823478714479 | 0.754767445004736 | -2.729305733 | 1.96777463234508 | 0.683338079140189 | 0.0652645849732804 | 7.15473683919348 | 0.999950574162623 |
| ebi-fl187-GCST90094707 | ebi-gm430-GCST90011524 | TestASV_46 (Sutterella) abundance \|\| id:ebi-gm430-GCST90011524 | Black olive liking \|\| id:ebi-fl187-GCST90094707 | MR Egger | 18 | -1.012115226 | 1.08383995102825 | 0.364274638126992 | -3.13644153 | 1.11221107799581 | 0.363449388325772 | 0.0434370928023731 | 3.04107502026841 | 0.999950574162623 |
| ebi-fl187-GCST90094707 | ebi-gm430-GCST90011581 | OTU97_19 (Bacteria) prevalence \|\| id:ebi-gm430-GCST90011581 | Black olive liking \|\| id:ebi-fl187-GCST90094707 | MR Egger | 18 | -2.25163564 | 1.29282404696253 | 0.100759435774994 | -4.785570772 | 0.282299491885857 | 0.105226970268688 | 0.00834935680683982 | 1.32617583941993 | 0.999950574162623 |
| ebi-fl187-GCST90094707 | ebi-gm430-GCST90011614 | OTU97_80 (Ruminococcus) prevalence \|\| id:ebi-gm430-GCST90011614 | Black olive liking \|\| id:ebi-fl187-GCST90094707 | MR Egger | 18 | 1.02757144146548 | 0.603907306517535 | 0.108190066928817 | -0.156086879 | 2.21122976223985 | 2.79427153587601 | 0.85548486320864 | 9.12693345259416 | 0.999950574162623 |
| ebi-fl187-GCST90094707 | ebi-gm430-GCST90011623 | OTU99_11 (Parabacteroides) prevalence \|\| id:ebi-gm430-GCST90011623 | Black olive liking \|\| id:ebi-fl187-GCST90094707 | MR Egger | 18 | 0.657999766808089 | 0.679444158358833 | 0.347237824699689 | -0.673710784 | 1.9897103171914 | 1.93092616646305 | 0.509813256738765 | 7.3134148848589 | 0.999950574162623 |
| ebi-fl187-GCST90094707 | ebi-gm430-GCST90011642 | OTU99_19 (Bacteria) prevalence \|\| id:ebi-gm430-GCST90011642 | Black olive liking \|\| id:ebi-fl187-GCST90094707 | MR Egger | 18 | -2.25163564 | 1.29282404696253 | 0.100759435774994 | -4.785570772 | 0.282299491885857 | 0.105226970268688 | 0.00834935680683982 | 1.32617583941993 | 0.999950574162623 |
| ebi-fl187-GCST90094707 | ebi-gm430-GCST90011653 | OTU99_30 (Parasutterella) prevalence \|\| id:ebi-gm430-GCST90011653 | Black olive liking \|\| id:ebi-fl187-GCST90094707 | MR Egger | 18 | 1.07821072696703 | 0.573309185039582 | 0.0783447535923336 | -0.045475276 | 2.20189672964461 | 2.93941542623678 | 0.955543227407234 | 9.04214775446939 | 0.999950574162623 |
| ebi-fl187-GCST90094707 | ebi-gm430-GCST90011675 | OTU99_8 (Ruminococcaceae) prevalence \|\| id:ebi-gm430-GCST90011675 | Black olive liking \|\| id:ebi-fl187-GCST90094707 | MR Egger | 18 | -0.032698242 | 0.566054991555467 | 0.954650986052158 | -1.142166026 | 1.07676954095396 | 0.967830565666293 | 0.319127035218842 | 2.93518223298004 | 0.999950574162623 |
| ebi-fl187-GCST90094707 | ebi-gm430-GCST90011679 | OTU99_92 (Ruminococcus) prevalence \|\| id:ebi-gm430-GCST90011679 | Black olive liking \|\| id:ebi-fl187-GCST90094707 | MR Egger | 18 | 1.22469355746322 | 0.595340668148917 | 0.0563560652161378 | 0.0578258478913425 | 2.3915612670351 | 3.40312306132202 | 1.0595304601783 | 10.9305461294174 | 0.999950574162623 |
| ebi-fl187-GCST90094707 | ebi-gm430-GCST90011684 | TestASV_11 (Lachnospiraceae) prevalence \|\| id:ebi-gm430-GCST90011684 | Black olive liking \|\| id:ebi-fl187-GCST90094707 | MR Egger | 18 | 0.12429866272883 | 0.595562893132666 | 0.837310133782086 | -1.043004608 | 1.29160193326886 | 1.1323540124409 | 0.352394283099555 | 3.63861070109573 | 0.999950574162623 |
| ebi-fl187-GCST90094707 | ebi-gm430-GCST90011688 | TestASV_15 (Bacteroides) prevalence \|\| id:ebi-gm430-GCST90011688 | Black olive liking \|\| id:ebi-fl187-GCST90094707 | MR Egger | 18 | -0.204178279 | 0.661171573373565 | 0.761448592121179 | -1.500074563 | 1.09171800448993 | 0.815317004066792 | 0.223113523484549 | 2.97938828063232 | 0.999950574162623 |
| ebi-fl187-GCST90094707 | ebi-gm430-GCST90011692 | TestASV_19 (Prevotella) prevalence \|\| id:ebi-gm430-GCST90011692 | Black olive liking \|\| id:ebi-fl187-GCST90094707 | MR Egger | 18 | 1.43948712747942 | 1.10685868540295 | 0.211845328445292 | -0.729955896 | 3.6089301508692 | 4.21853169310156 | 0.481930244716204 | 36.926525863057 | 0.999950574162623 |
| ebi-fl187-GCST90094707 | ebi-gm430-GCST90011693 | TestASV_2 (Bacteroides) prevalence \|\| id:ebi-gm430-GCST90011693 | Black olive liking \|\| id:ebi-fl187-GCST90094707 | MR Egger | 18 | -0.457882418 | 0.55291071012323 | 0.419778129395007 | -1.541587409 | 0.625822574241735 | 0.632621857030711 | 0.214041060805782 | 1.86978336066149 | 0.999950574162623 |
| ebi-fl187-GCST90094707 | ebi-gm430-GCST90011716 | TestASV_40 (Barnesiella) prevalence \|\| id:ebi-gm430-GCST90011716 | Black olive liking \|\| id:ebi-fl187-GCST90094707 | MR Egger | 18 | 0.511233305385824 | 1.43980267596781 | 0.727173086596861 | -2.31077994 | 3.33324655028273 | 1.66734627455547 | 0.099183863970377 | 28.0291923301587 | 0.999950574162623 |
| ebi-fl187-GCST90094707 | ebi-gm430-GCST90011718 | TestASV_42 (Alphaproteobacteria) prevalence \|\| id:ebi-gm430-GCST90011718 | Black olive liking \|\| id:ebi-fl187-GCST90094707 | MR Egger | 18 | -0.804022496 | 0.915595636834619 | 0.392863182028539 | -2.598589944 | 0.990544952153552 | 0.447525170446006 | 0.0743783819734552 | 2.69270146605507 | 0.999950574162623 |
| ebi-fl187-GCST90094707 | ebi-hbsm44-GCST90104929 | Sulcal depth of the right inferior frontal sulcus \|\| id:ebi-hbsm44-GCST90104929 | Black olive liking \|\| id:ebi-fl187-GCST90094707 | MR Egger | 20 | -0.37751844 | 0.478298058449719 | 0.440213023675508 | -1.314982634 | 0.559945754970109 | 0.685560560011176 | 0.268478985563797 | 1.75057753758964 | 0.999950574162623 |
| ebi-fl187-GCST90094707 | ebi-hbsm44-GCST90104935 | Sulcal opening distance of the left calloso-marginal posterior fissure \|\| id:ebi-hbsm44-GCST90104935 | Black olive liking \|\| id:ebi-fl187-GCST90094707 | MR Egger | 20 | -0.033356422 | 0.125046767418024 | 0.792692425841503 | -0.278448086 | 0.211735241958923 | 0.967193768835184 | 0.756957563182824 | 1.23582064830716 | 0.999950574162623 |
| ebi-fl187-GCST90094707 | ebi-hbsm44-GCST90104936 | Sulcal opening distance of the left anterior inferior temporal sulcus \|\| id:ebi-hbsm44-GCST90104936 | Black olive liking \|\| id:ebi-fl187-GCST90094707 | MR Egger | 20 | -0.015577501 | 0.0663674474307761 | 0.817078456952789 | -0.145657698 | 0.114502696239184 | 0.98454320098289 | 0.864453556995949 | 1.12131566439512 | 0.999950574162623 |
| ebi-fl187-GCST90094707 | ebi-hbsm44-GCST90104941 | Sulcal opening distance of the right posterior inferior temporal sulcus \|\| id:ebi-hbsm44-GCST90104941 | Black olive liking \|\| id:ebi-fl187-GCST90094707 | MR Egger | 18 | 0.0370325909270912 | 0.054994587725113 | 0.510312458225576 | -0.070756801 | 0.144821982868313 | 1.03772684076685 | 0.931688450263372 | 1.15583379373602 | 0.999950574162623 |
| ebi-fl187-GCST90094707 | ebi-IPs91-GCST90274819 | Leukemia inhibitory factor levels \|\| id:ebi-IPs91-GCST90274819 | Black olive liking \|\| id:ebi-fl187-GCST90094707 | MR Egger | 22 | 0.140683854091146 | 0.193863189903134 | 0.476437824606071 | -0.239287998 | 0.520655706301289 | 1.1510606873283 | 0.787188141019804 | 1.68313092750131 | 0.999950574162623 |
| ebi-fl187-GCST90094707 | ebi-IPs91-GCST90274837 | STAM binding protein levels \|\| id:ebi-IPs91-GCST90274837 | Black olive liking \|\| id:ebi-fl187-GCST90094707 | MR Egger | 22 | -0.097309106 | 0.172515102659343 | 0.57898347369861 | -0.435438707 | 0.240820495477584 | 0.907275518719557 | 0.646980769850409 | 1.2722926325278 | 0.999950574162623 |
| ebi-fl187-GCST90094707 | ebi-IPs91-GCST90274840 | TNF-beta levels \|\| id:ebi-IPs91-GCST90274840 | Black olive liking \|\| id:ebi-fl187-GCST90094707 | MR Egger | 22 | 0.415062347392252 | 0.233065530033241 | 0.0901271998368355 | -0.041746091 | 0.871870786257404 | 1.51446516070202 | 0.959113276697714 | 2.39138043305711 | 0.999950574162623 |
| ebi-fl187-GCST90094707 | ebi-IPs91-GCST90274842 | Tumor necrosis factor ligand superfamily member 14 levels \|\| id:ebi-IPs91-GCST90274842 | Black olive liking \|\| id:ebi-fl187-GCST90094707 | MR Egger | 22 | -0.357216292 | 0.194882142533967 | 0.0817343160550879 | -0.739185291 | 0.0247527077085974 | 0.699621159148095 | 0.47750278289568 | 1.02506159934707 | 0.999950574162623 |
| ebi-fl187-GCST90094707 | ebi-met1400-GCST90199628 | 3-methylhistidine levels \|\| id:ebi-met1400-GCST90199628 | Black olive liking \|\| id:ebi-fl187-GCST90094707 | MR Egger | 22 | -0.123260918 | 0.226615808222987 | 0.592508832699908 | -0.567427902 | 0.32090606643195 | 0.884032972629813 | 0.566981897936381 | 1.37837609902741 | 0.999950574162623 |
| ebi-fl187-GCST90094707 | ebi-met1400-GCST90199651 | Docosahexaenoate DHA; 22:6n3 levels \|\| id:ebi-met1400-GCST90199651 | Black olive liking \|\| id:ebi-fl187-GCST90094707 | MR Egger | 22 | 0.0136926687877393 | 0.22278198255193 | 0.951601192290776 | -0.422960017 | 0.450345354589522 | 1.0137868427169 | 0.655104825635219 | 1.56885390283813 | 0.999950574162623 |
| ebi-fl187-GCST90094707 | ebi-met1400-GCST90199652 | 1-stearoyl-2-oleoyl-GPS (18:0/18:1) levels \|\| id:ebi-met1400-GCST90199652 | Black olive liking \|\| id:ebi-fl187-GCST90094707 | MR Egger | 22 | -0.033095707 | 0.272660891088124 | 0.904600938507673 | -0.567511053 | 0.50131963956757 | 0.967445963840926 | 0.566934754390188 | 1.6508984247382 | 0.999950574162623 |
| ebi-fl187-GCST90094707 | ebi-met1400-GCST90199653 | 1-myristoyl-2-palmitoyl-gpc (14:0/16:0) levels \|\| id:ebi-met1400-GCST90199653 | Black olive liking \|\| id:ebi-fl187-GCST90094707 | MR Egger | 21 | -0.026814403 | 0.248554899674355 | 0.915221011609726 | -0.513982006 | 0.460353200369112 | 0.973541911223218 | 0.598109156130146 | 1.58463357932933 | 0.999950574162623 |
| ebi-fl187-GCST90094707 | ebi-met1400-GCST90199667 | Erythritol levels in elite athletes \|\| id:ebi-met1400-GCST90199667 | Black olive liking \|\| id:ebi-fl187-GCST90094707 | MR Egger | 22 | 0.00360332436121656 | 0.230163651436214 | 0.987664361675484 | -0.447517432 | 0.454724081176195 | 1.00360982413903 | 0.639213073100863 | 1.5757385471205 | 0.999950574162623 |
| ebi-fl187-GCST90094707 | ebi-met1400-GCST90199674 | 3-hydroxymyristate levels \|\| id:ebi-met1400-GCST90199674 | Black olive liking \|\| id:ebi-fl187-GCST90094707 | MR Egger | 22 | 0.197116466728247 | 0.221654170420635 | 0.384415222440518 | -0.237325707 | 0.631558640752691 | 1.21788587562954 | 0.788734349644089 | 1.88053938151321 | 0.999950574162623 |
| ebi-fl187-GCST90094707 | ebi-met1400-GCST90199685 | 2-linoleoylglycerol (18:2) levels \|\| id:ebi-met1400-GCST90199685 | Black olive liking \|\| id:ebi-fl187-GCST90094707 | MR Egger | 22 | -0.057767141 | 0.259083261113276 | 0.825822243347191 | -0.565570333 | 0.450036050447233 | 0.943869710089811 | 0.568036084564054 | 1.56836872486498 | 0.999950574162623 |
| ebi-fl187-GCST90094707 | ebi-met1400-GCST90199703 | Gamma-glutamylvaline levels \|\| id:ebi-met1400-GCST90199703 | Black olive liking \|\| id:ebi-fl187-GCST90094707 | MR Egger | 22 | -0.092954959 | 0.258205043061435 | 0.722617528526071 | -0.599036844 | 0.413126925019076 | 0.911234541939379 | 0.549340482073742 | 1.51153686560461 | 0.999950574162623 |
| ebi-fl187-GCST90094707 | ebi-met1400-GCST90199779 | Gamma-glutamyl-2-aminobutyrate levels \|\| id:ebi-met1400-GCST90199779 | Black olive liking \|\| id:ebi-fl187-GCST90094707 | MR Egger | 21 | 0.378342694138997 | 0.247066664581966 | 0.14216665862541 | -0.105907968 | 0.862593356719649 | 1.45986314370604 | 0.899507427336993 | 2.36929716596197 | 0.999950574162623 |
| ebi-fl187-GCST90094707 | ebi-met1400-GCST90199824 | 4-vinylphenol sulfate levels \|\| id:ebi-met1400-GCST90199824 | Black olive liking \|\| id:ebi-fl187-GCST90094707 | MR Egger | 22 | 0.172317976441962 | 0.227449637325929 | 0.457519045792046 | -0.273483313 | 0.618119265600783 | 1.18805554683434 | 0.760725030683927 | 1.85543517753697 | 0.999950574162623 |
| ebi-fl187-GCST90094707 | ebi-met1400-GCST90199830 | 4-ethylphenylsulfate levels \|\| id:ebi-met1400-GCST90199830 | Black olive liking \|\| id:ebi-fl187-GCST90094707 | MR Egger | 22 | 0.0383452645503937 | 0.227869527334602 | 0.868055242589471 | -0.408279009 | 0.484969538126213 | 1.03908993187006 | 0.664793369594071 | 1.62412553418365 | 0.999950574162623 |
| ebi-fl187-GCST90094707 | ebi-met1400-GCST90199833 | Hexanoylglutamine levels \|\| id:ebi-met1400-GCST90199833 | Black olive liking \|\| id:ebi-fl187-GCST90094707 | MR Egger | 22 | 0.160716634088409 | 0.239259543234308 | 0.50944421805178 | -0.308232071 | 0.629665338827653 | 1.17435215029379 | 0.734744785521468 | 1.87698232103936 | 0.999950574162623 |
| ebi-fl187-GCST90094707 | ebi-met1400-GCST90199837 | Carotenoid levels (cryptoxanthin) \|\| id:ebi-met1400-GCST90199837 | Black olive liking \|\| id:ebi-fl187-GCST90094707 | MR Egger | 22 | 0.111312737736721 | 0.232008687374953 | 0.636590112685003 | -0.34342429 | 0.566049764991629 | 1.11774441305769 | 0.709337183371407 | 1.76129575920952 | 0.999950574162623 |
| ebi-fl187-GCST90094707 | ebi-met1400-GCST90199869 | 2-hydroxyhippurate levels \|\| id:ebi-met1400-GCST90199869 | Black olive liking \|\| id:ebi-fl187-GCST90094707 | MR Egger | 22 | -0.255199333 | 0.229027859558032 | 0.278374459630325 | -0.704093938 | 0.193695271818296 | 0.774762046990407 | 0.494556470312723 | 1.21372636996783 | 0.999950574162623 |
| ebi-fl187-GCST90094707 | ebi-met1400-GCST90199902 | Hydantoin-5-propionate levels \|\| id:ebi-met1400-GCST90199902 | Black olive liking \|\| id:ebi-fl187-GCST90094707 | MR Egger | 22 | 0.00135778810162469 | 0.23808718942545 | 0.99550626835106 | -0.465293103 | 0.468008679375506 | 1.00135871031323 | 0.627951023776445 | 1.59681126195146 | 0.999950574162623 |
| ebi-fl187-GCST90094707 | ebi-met1400-GCST90199904 | Pantoate levels \|\| id:ebi-met1400-GCST90199904 | Black olive liking \|\| id:ebi-fl187-GCST90094707 | MR Egger | 22 | -0.337783705 | 0.240204821823049 | 0.175002736957802 | -0.808585155 | 0.133017746058885 | 0.713349565360988 | 0.445487916681957 | 1.14227026894649 | 0.999950574162623 |
| ebi-fl187-GCST90094707 | ebi-met1400-GCST90199936 | 3-methyl catechol sulfate (2) levels \|\| id:ebi-met1400-GCST90199936 | Black olive liking \|\| id:ebi-fl187-GCST90094707 | MR Egger | 22 | 0.351722011726259 | 0.310861172593311 | 0.271253091156878 | -0.257565887 | 0.961009910009148 | 1.42151330475831 | 0.772930698893522 | 2.61433538413937 | 0.999950574162623 |
| ebi-fl187-GCST90094707 | ebi-met1400-GCST90199977 | 4-hydroxychlorothalonil levels \|\| id:ebi-met1400-GCST90199977 | Black olive liking \|\| id:ebi-fl187-GCST90094707 | MR Egger | 22 | 0.496789665124421 | 0.225575436311241 | 0.03954104933471 | 0.0546618099543887 | 0.938917520294453 | 1.64343681028705 | 1.05618336356962 | 2.55721178970119 | 0.999950574162623 |
| ebi-fl187-GCST90094707 | ebi-met1400-GCST90200040 | Sphingomyelin (d18:2/24:1, d18:1/24:2) levels \|\| id:ebi-met1400-GCST90200040 | Black olive liking \|\| id:ebi-fl187-GCST90094707 | MR Egger | 22 | 0.0432373837656812 | 0.207197922842017 | 0.836811218606263 | -0.362870545 | 0.449345312536035 | 1.04418573817506 | 0.695676486466371 | 1.567285767 | 0.999950574162623 |
| ebi-fl187-GCST90094707 | ebi-met1400-GCST90200077 | 1-stearoyl-2-oleoyl-GPI (18:0/18:1) levels \|\| id:ebi-met1400-GCST90200077 | Black olive liking \|\| id:ebi-fl187-GCST90094707 | MR Egger | 22 | -0.257876889 | 0.271213304678203 | 0.353042217554381 | -0.789454966 | 0.273701188412156 | 0.772690353121307 | 0.454092223582213 | 1.31482185952615 | 0.999950574162623 |
| ebi-fl187-GCST90094707 | ebi-met1400-GCST90200080 | 1-palmitoyl-2-oleoyl-GPI (16:0/18:1) levels \|\| id:ebi-met1400-GCST90200080 | Black olive liking \|\| id:ebi-fl187-GCST90094707 | MR Egger | 22 | 0.0561396381391696 | 0.247464107668718 | 0.822835527749028 | -0.428890013 | 0.541169289169857 | 1.05774537502059 | 0.651231552317542 | 1.71801454397575 | 0.999950574162623 |
| ebi-fl187-GCST90094707 | ebi-met1400-GCST90200082 | 1-oleoyl-2-linoleoyl-GPE (18:1/18:2) levels \|\| id:ebi-met1400-GCST90200082 | Black olive liking \|\| id:ebi-fl187-GCST90094707 | MR Egger | 22 | -0.371289359 | 0.228130838152542 | 0.119277270039744 | -0.818425802 | 0.0758470835828352 | 0.689844299884024 | 0.441125527149796 | 1.07879759568047 | 0.999950574162623 |
| ebi-fl187-GCST90094707 | ebi-met1400-GCST90200100 | Perfluorooctanesulfonate (PFOS) levels \|\| id:ebi-met1400-GCST90200100 | Black olive liking \|\| id:ebi-fl187-GCST90094707 | MR Egger | 22 | -0.18635078 | 0.209561139131441 | 0.384443715695925 | -0.597090613 | 0.224389052693475 | 0.829982402682043 | 0.550410666668898 | 1.25155784667278 | 0.999950574162623 |
| ebi-fl187-GCST90094707 | ebi-met1400-GCST90200102 | 1-palmitoleoyl-2-linolenoyl-GPC (16:1/18:3) levels \|\| id:ebi-met1400-GCST90200102 | Black olive liking \|\| id:ebi-fl187-GCST90094707 | MR Egger | 22 | -0.400535769 | 0.245510621843053 | 0.11844472178634 | -0.881736588 | 0.0806650498111136 | 0.66996100552402 | 0.414063229808093 | 1.08400774715201 | 0.999950574162623 |
| ebi-fl187-GCST90094707 | ebi-met1400-GCST90200114 | Glycosyl-N-tricosanoyl-sphingadienine (d18:2/23:0) levels \|\| id:ebi-met1400-GCST90200114 | Black olive liking \|\| id:ebi-fl187-GCST90094707 | MR Egger | 22 | 0.0268915707086747 | 0.237430402934567 | 0.910952939309256 | -0.438472019 | 0.492255160460426 | 1.02725641203982 | 0.645021248677418 | 1.63600150885056 | 0.999950574162623 |
| ebi-fl187-GCST90094707 | ebi-met1400-GCST90200135 | Arachidoylcarnitine (C20) levels \|\| id:ebi-met1400-GCST90200135 | Black olive liking \|\| id:ebi-fl187-GCST90094707 | MR Egger | 22 | 0.0378907902486897 | 0.280877038910362 | 0.894038014856427 | -0.512628206 | 0.588409786512999 | 1.03861779949301 | 0.598919424855603 | 1.80112196842467 | 0.999950574162623 |
| ebi-fl187-GCST90094707 | ebi-met1400-GCST90200146 | Docosahexaenoylcarnitine (C22:6) levels \|\| id:ebi-met1400-GCST90200146 | Black olive liking \|\| id:ebi-fl187-GCST90094707 | MR Egger | 22 | -0.04183743 | 0.281203615229924 | 0.883217283829003 | -0.592996516 | 0.509321655433011 | 0.959025676303859 | 0.552668720206674 | 1.66416193676772 | 0.999950574162623 |
| ebi-fl187-GCST90094707 | ebi-met1400-GCST90200162 | 3-hydroxyphenylacetoylglutamine levels \|\| id:ebi-met1400-GCST90200162 | Black olive liking \|\| id:ebi-fl187-GCST90094707 | MR Egger | 22 | 0.215450620898286 | 0.277523764661227 | 0.446637415806893 | -0.328495958 | 0.759397199634291 | 1.24042073054941 | 0.720005838600382 | 2.13698765522194 | 0.999950574162623 |
| ebi-fl187-GCST90094707 | ebi-met1400-GCST90200172 | 3-carboxy-4-methyl-5-pentyl-2-furanpropionate (3-CMPFP) levels \|\| id:ebi-met1400-GCST90200172 | Black olive liking \|\| id:ebi-fl187-GCST90094707 | MR Egger | 22 | -0.089584189 | 0.225806603386686 | 0.695768271580309 | -0.532165132 | 0.352996753435969 | 0.914311286746424 | 0.587331940952239 | 1.42332652250541 | 0.999950574162623 |
| ebi-fl187-GCST90094707 | ebi-met1400-GCST90200222 | Dihydrocaffeate sulfate (2) levels \|\| id:ebi-met1400-GCST90200222 | Black olive liking \|\| id:ebi-fl187-GCST90094707 | MR Egger | 22 | -0.210362102 | 0.340613873199589 | 0.543809698382383 | -0.877965293 | 0.457241089709307 | 0.810290785121115 | 0.415627732468602 | 1.57970969009339 | 0.999950574162623 |
| ebi-fl187-GCST90094707 | ebi-met1400-GCST90200223 | 4-methylhexanoylglutamine levels \|\| id:ebi-met1400-GCST90200223 | Black olive liking \|\| id:ebi-fl187-GCST90094707 | MR Egger | 22 | 0.205010358811542 | 0.312298548214095 | 0.519012931810662 | -0.407094796 | 0.817115513311168 | 1.22753778072985 | 0.665581093093201 | 2.26396004747713 | 0.999950574162623 |
| ebi-fl187-GCST90094707 | ebi-met1400-GCST90200224 | 2-ketocaprylate levels \|\| id:ebi-met1400-GCST90200224 | Black olive liking \|\| id:ebi-fl187-GCST90094707 | MR Egger | 22 | 0.185052428370023 | 0.230183740710873 | 0.430887754152991 | -0.266107703 | 0.636212560163334 | 1.20328152456299 | 0.766356583828444 | 1.88931165714203 | 0.999950574162623 |
| ebi-fl187-GCST90094707 | ebi-met1400-GCST90200243 | Hydroxypalmitoyl sphingomyelin (d18:1/16:0(OH)) levels \|\| id:ebi-met1400-GCST90200243 | Black olive liking \|\| id:ebi-fl187-GCST90094707 | MR Egger | 22 | -0.324937547 | 0.234099769514287 | 0.180397546608216 | -0.783773095 | 0.133898001125872 | 0.722572478963735 | 0.456679660597321 | 1.14327620081195 | 0.999950574162623 |
| ebi-fl187-GCST90094707 | ebi-met1400-GCST90200273 | 2-methoxyhydroquinone sulfate (2) levels \|\| id:ebi-met1400-GCST90200273 | Black olive liking \|\| id:ebi-fl187-GCST90094707 | MR Egger | 22 | 0.347116078256121 | 0.30121690207281 | 0.262753976253567 | -0.24326905 | 0.937501206318828 | 1.41498096436074 | 0.784060534055764 | 2.55359253850783 | 0.999950574162623 |
| ebi-fl187-GCST90094707 | ebi-met1400-GCST90200276 | 2,4-di-tert-butylphenol levels \|\| id:ebi-met1400-GCST90200276 | Black olive liking \|\| id:ebi-fl187-GCST90094707 | MR Egger | 22 | -0.077952962 | 0.246119959612159 | 0.754732614121455 | -0.560348082 | 0.404442159189776 | 0.925007936367229 | 0.571010270575861 | 1.49846636117324 | 0.999950574162623 |
| ebi-fl187-GCST90094707 | ebi-met1400-GCST90200289 | N-acetylmethionine levels \|\| id:ebi-met1400-GCST90200289 | Black olive liking \|\| id:ebi-fl187-GCST90094707 | MR Egger | 22 | -0.149979778 | 0.226084826957761 | 0.514662650208432 | -0.593106039 | 0.293146482575194 | 0.860725381612217 | 0.552608193678444 | 1.34063915632526 | 0.999950574162623 |
| ebi-fl187-GCST90094707 | ebi-met1400-GCST90200308 | 3-hydroxyisobutyrate levels \|\| id:ebi-met1400-GCST90200308 | Black olive liking \|\| id:ebi-fl187-GCST90094707 | MR Egger | 22 | -0.055406939 | 0.24786880655069 | 0.825387868454136 | -0.5412298 | 0.430415921738068 | 0.94610006449607 | 0.582032029103751 | 1.53789703535355 | 0.999950574162623 |
| ebi-fl187-GCST90094707 | ebi-met1400-GCST90200383 | Cystathionine levels \|\| id:ebi-met1400-GCST90200383 | Black olive liking \|\| id:ebi-fl187-GCST90094707 | MR Egger | 22 | -0.003944982 | 0.280388820749263 | 0.988913793892545 | -0.553507071 | 0.54561710643601 | 0.996062788987396 | 0.57492995045537 | 1.72567297775934 | 0.999950574162623 |
| ebi-fl187-GCST90094707 | ebi-met1400-GCST90200384 | Guanidinoacetate levels \|\| id:ebi-met1400-GCST90200384 | Black olive liking \|\| id:ebi-fl187-GCST90094707 | MR Egger | 22 | -0.02882167 | 0.274534083909473 | 0.91743430663245 | -0.566908475 | 0.509265134302795 | 0.971589712456691 | 0.56727648024504 | 1.66406787911234 | 0.999950574162623 |
| ebi-fl187-GCST90094707 | ebi-met1400-GCST90200403 | Citrulline levels \|\| id:ebi-met1400-GCST90200403 | Black olive liking \|\| id:ebi-fl187-GCST90094707 | MR Egger | 22 | 0.144681236117354 | 0.227760860531898 | 0.532477571141899 | -0.301730051 | 0.591092522759874 | 1.1556711253327 | 0.739537675753085 | 1.8059603908181 | 0.999950574162623 |
| ebi-fl187-GCST90094707 | ebi-met1400-GCST90200434 | 12,13-DiHOME levels \|\| id:ebi-met1400-GCST90200434 | Black olive liking \|\| id:ebi-fl187-GCST90094707 | MR Egger | 22 | 0.135610644728511 | 0.229341335312144 | 0.560940578267084 | -0.313898372 | 0.585119661940313 | 1.14523590314014 | 0.730593272766749 | 1.79520579059582 | 0.999950574162623 |
| ebi-fl187-GCST90094707 | ebi-met1400-GCST90200437 | Fructose levels \|\| id:ebi-met1400-GCST90200437 | Black olive liking \|\| id:ebi-fl187-GCST90094707 | MR Egger | 22 | -0.233427143 | 0.250681780255718 | 0.362870878992005 | -0.724763432 | 0.257909146452798 | 0.791815283044863 | 0.484439158131051 | 1.2942212286931 | 0.999950574162623 |
| ebi-fl187-GCST90094707 | ebi-met1400-GCST90200448 | N-stearoyl-sphinganine (d18:0/18:0) levels \|\| id:ebi-met1400-GCST90200448 | Black olive liking \|\| id:ebi-fl187-GCST90094707 | MR Egger | 22 | 0.055488144724584 | 0.306119463443719 | 0.857985487732822 | -0.544506004 | 0.655482293074273 | 1.05705648530238 | 0.580128293840577 | 1.92607122421592 | 0.999950574162623 |
| ebi-fl187-GCST90094707 | ebi-met1400-GCST90200468 | X-11847 levels \|\| id:ebi-met1400-GCST90200468 | Black olive liking \|\| id:ebi-fl187-GCST90094707 | MR Egger | 22 | -0.031436308 | 0.241110132571792 | 0.897567070572768 | -0.504012168 | 0.44113955216594 | 0.96905267570638 | 0.604102032390312 | 1.55447761792492 | 0.999950574162623 |
| ebi-fl187-GCST90094707 | ebi-met1400-GCST90200476 | X-11843 levels \|\| id:ebi-met1400-GCST90200476 | Black olive liking \|\| id:ebi-fl187-GCST90094707 | MR Egger | 22 | 0.439914865889229 | 0.268905804448022 | 0.117492058171835 | -0.087140511 | 0.966970242607351 | 1.55257503578971 | 0.916548301623306 | 2.62996422282184 | 0.999950574162623 |
| ebi-fl187-GCST90094707 | ebi-met1400-GCST90200511 | X-12906 levels \|\| id:ebi-met1400-GCST90200511 | Black olive liking \|\| id:ebi-fl187-GCST90094707 | MR Egger | 22 | -0.630647508 | 0.229131160524405 | 0.0122828569269389 | -1.079744583 | -0.181550433 | 0.532247055174094 | 0.339682275316211 | 0.833976184002484 | 0.999950574162623 |
| ebi-fl187-GCST90094707 | ebi-met1400-GCST90200515 | X-12849 levels \|\| id:ebi-met1400-GCST90200515 | Black olive liking \|\| id:ebi-fl187-GCST90094707 | MR Egger | 22 | 0.281581100009045 | 0.242233258176596 | 0.258741476053189 | -0.193196086 | 0.756358286035173 | 1.32522346759871 | 0.82432032060216 | 2.13050339192348 | 0.999950574162623 |
| ebi-fl187-GCST90094707 | ebi-met1400-GCST90200551 | X-18886 levels \|\| id:ebi-met1400-GCST90200551 | Black olive liking \|\| id:ebi-fl187-GCST90094707 | MR Egger | 22 | 0.210362656046321 | 0.281048805922904 | 0.462875844141454 | -0.340493004 | 0.761218315655214 | 1.23412554190051 | 0.711419503942325 | 2.14088290345032 | 0.999950574162623 |
| ebi-fl187-GCST90094707 | ebi-met1400-GCST90200564 | X-21319 levels \|\| id:ebi-met1400-GCST90200564 | Black olive liking \|\| id:ebi-fl187-GCST90094707 | MR Egger | 22 | -0.260811912 | 0.269777252113215 | 0.345206208083389 | -0.789575326 | 0.267951502053643 | 0.770425813770697 | 0.454037572193243 | 1.30728373790092 | 0.999950574162623 |
| ebi-fl187-GCST90094707 | ebi-met1400-GCST90200579 | X-21442 levels \|\| id:ebi-met1400-GCST90200579 | Black olive liking \|\| id:ebi-fl187-GCST90094707 | MR Egger | 22 | 0.234516185753616 | 0.247319656188386 | 0.354329594549845 | -0.25023034 | 0.719262711882853 | 1.26429693586887 | 0.778621414465207 | 2.05291905970155 | 0.999950574162623 |
| ebi-fl187-GCST90094707 | ebi-met1400-GCST90200581 | X-21441 levels \|\| id:ebi-met1400-GCST90200581 | Black olive liking \|\| id:ebi-fl187-GCST90094707 | MR Egger | 22 | -0.042849582 | 0.262412272022779 | 0.871928006442518 | -0.557177636 | 0.471478470753681 | 0.958055487625964 | 0.572823501183075 | 1.60236148739448 | 0.999950574162623 |
| ebi-fl187-GCST90094707 | ebi-met1400-GCST90200648 | X-24585 levels \|\| id:ebi-met1400-GCST90200648 | Black olive liking \|\| id:ebi-fl187-GCST90094707 | MR Egger | 22 | 0.10615168907167 | 0.222128074154501 | 0.637913670163626 | -0.329219336 | 0.541522714414492 | 1.11199054052676 | 0.719485190240393 | 1.71862184099697 | 0.999950574162623 |
| ebi-fl187-GCST90094707 | ebi-met1400-GCST90200720 | Adenosine 5'-diphosphate (ADP) to pantothenate ratio \|\| id:ebi-met1400-GCST90200720 | Black olive liking \|\| id:ebi-fl187-GCST90094707 | MR Egger | 22 | 0.346325063945687 | 0.303818192467402 | 0.267792442212585 | -0.249158593 | 0.941808721181794 | 1.41386213673161 | 0.779456347034989 | 2.56461590092556 | 0.999950574162623 |
| ebi-fl187-GCST90094707 | ebi-met1400-GCST90200724 | Cholate to taurocholate ratio \|\| id:ebi-met1400-GCST90200724 | Black olive liking \|\| id:ebi-fl187-GCST90094707 | MR Egger | 22 | -0.056482373 | 0.244486940019136 | 0.819643050670696 | -0.535676776 | 0.422712029069245 | 0.945083142980854 | 0.585273057434523 | 1.52609476175399 | 0.999950574162623 |
| ebi-fl187-GCST90094707 | ebi-met1400-GCST90200726 | N-acetylputrescine to (N(1) + N(8))-acetylspermidine ratio \|\| id:ebi-met1400-GCST90200726 | Black olive liking \|\| id:ebi-fl187-GCST90094707 | MR Egger | 22 | -0.035036492 | 0.226215359884192 | 0.878467416355937 | -0.478418597 | 0.408345613811902 | 0.965570180451415 | 0.619762711904862 | 1.50432698751986 | 0.999950574162623 |
| ebi-fl187-GCST90094707 | ebi-met1400-GCST90200744 | Arginine to glutamate ratio \|\| id:ebi-met1400-GCST90200744 | Black olive liking \|\| id:ebi-fl187-GCST90094707 | MR Egger | 22 | 0.00585036182278394 | 0.263038576609906 | 0.982475754798205 | -0.509705248 | 0.521405971978199 | 1.00586750861151 | 0.600672601973023 | 1.68439419670046 | 0.999950574162623 |
| ebi-fl187-GCST90094707 | ebi-met1400-GCST90200759 | Histidine to trans-urocanate ratio \|\| id:ebi-met1400-GCST90200759 | Black olive liking \|\| id:ebi-fl187-GCST90094707 | MR Egger | 22 | 0.259408219183715 | 0.229008885905804 | 0.270719889330132 | -0.189449197 | 0.708265635559091 | 1.29616281537103 | 0.827414750822729 | 2.03046663391005 | 0.999950574162623 |
| ebi-fl187-GCST90094707 | ebi-met1400-GCST90200795 | Oleoyl-linoleoyl-glycerol (18:1 to 18:2) [2] to linoleoyl-arachidonoyl-glycerol (18:2 to 20:4) [2] ratio \|\| id:ebi-met1400-GCST90200795 | Black olive liking \|\| id:ebi-fl187-GCST90094707 | MR Egger | 22 | 0.442826433963802 | 0.236220934925485 | 0.0755251561708567 | -0.020166598 | 0.905819466417753 | 1.55710205085552 | 0.980035387289716 | 2.47395841846445 | 0.999950574162623 |
| ebi-fl187-GCST90094707 | ebi-met1400-GCST90200811 | Choline to taurocholate ratio \|\| id:ebi-met1400-GCST90200811 | Black olive liking \|\| id:ebi-fl187-GCST90094707 | MR Egger | 22 | -0.253193726 | 0.238647936230693 | 0.301356906504687 | -0.720943681 | 0.214556229267429 | 0.776317474574421 | 0.486293133889054 | 1.23931180461023 | 0.999950574162623 |
| ebi-fl187-GCST90094707 | ebi-met1400-GCST90200831 | Cholate to phosphate ratio \|\| id:ebi-met1400-GCST90200831 | Black olive liking \|\| id:ebi-fl187-GCST90094707 | MR Egger | 22 | 0.115421527345129 | 0.237344736801399 | 0.632038136627059 | -0.349774157 | 0.580617211475871 | 1.12234643758787 | 0.704847256714752 | 1.78714113443156 | 0.999950574162623 |
| ebi-fl187-GCST90094707 | ebi-met1400-GCST90200841 | Adenosine 5'-diphosphate (ADP) to oxalate (ethanedioate) ratio \|\| id:ebi-met1400-GCST90200841 | Black olive liking \|\| id:ebi-fl187-GCST90094707 | MR Egger | 22 | 0.233175821287593 | 0.308111058179484 | 0.457998033626554 | -0.370721853 | 0.837073495319383 | 1.26260345237761 | 0.690235902082927 | 2.30959802749342 | 0.999950574162623 |
| ebi-fl187-GCST90094707 | ebi-met1400-GCST90200844 | Aspartate to phosphate ratio \|\| id:ebi-met1400-GCST90200844 | Black olive liking \|\| id:ebi-fl187-GCST90094707 | MR Egger | 22 | 0.232494676677046 | 0.229127833709334 | 0.322370529204881 | -0.216595877 | 0.681585230747341 | 1.26174372967175 | 0.805255325439906 | 1.97700926534858 | 0.999950574162623 |
| ebi-fl187-GCST90094707 | ebi-met1400-GCST90200870 | Adenosine 5'-monophosphate (AMP) to acetoacetate ratio \|\| id:ebi-met1400-GCST90200870 | Black olive liking \|\| id:ebi-fl187-GCST90094707 | MR Egger | 22 | -0.093693771 | 0.300656154858637 | 0.758546233983046 | -0.682979834 | 0.495592292696791 | 0.910561560064864 | 0.50510960464816 | 1.64147018199207 | 0.999950574162623 |
| ebi-fl187-GCST90094707 | ebi-met1400-GCST90200887 | Cholesterol to cortisol ratio \|\| id:ebi-met1400-GCST90200887 | Black olive liking \|\| id:ebi-fl187-GCST90094707 | MR Egger | 22 | -0.230958205 | 0.25952793706928 | 0.384090601464148 | -0.739632962 | 0.277716551198896 | 0.793772640706828 | 0.477289066546163 | 1.32011196002067 | 0.999950574162623 |
| ebi-fl187-GCST90094707 | ebi-met1400-GCST90200890 | Cortisol to taurocholate ratio \|\| id:ebi-met1400-GCST90200890 | Black olive liking \|\| id:ebi-fl187-GCST90094707 | MR Egger | 22 | -0.035336121 | 0.237604031247023 | 0.883265046077411 | -0.501040022 | 0.430367780445203 | 0.965280910733155 | 0.605900182368696 | 1.53782300078404 | 0.999950574162623 |
| ebi-fl187-GCST90094707 | ebi-met1400-GCST90200948 | Adenosine 5'-diphosphate (ADP) to mannitol to sorbitol ratio \|\| id:ebi-met1400-GCST90200948 | Black olive liking \|\| id:ebi-fl187-GCST90094707 | MR Egger | 22 | 0.0125704211361745 | 0.323203956713345 | 0.96936119352065 | -0.620909334 | 0.64605017629433 | 1.01264976097647 | 0.537455488758233 | 1.90798970306358 | 0.999950574162623 |
| ebi-fl187-GCST90094707 | ebi-met1400-GCST90200972 | Leucine to N-palmitoyl-sphingosine (d18:1 to 16:0) ratio \|\| id:ebi-met1400-GCST90200972 | Black olive liking \|\| id:ebi-fl187-GCST90094707 | MR Egger | 22 | -0.006979941 | 0.226805090121046 | 0.975753971882952 | -0.451517917 | 0.437558035800867 | 0.993044362372794 | 0.636661018908264 | 1.54892018884932 | 0.999950574162623 |
| ebi-fl187-GCST90094707 | ebi-met1400-GCST90200973 | Proline to glutamate ratio \|\| id:ebi-met1400-GCST90200973 | Black olive liking \|\| id:ebi-fl187-GCST90094707 | MR Egger | 22 | 0.0959267457793832 | 0.222802775113793 | 0.671402970360512 | -0.340766693 | 0.532620185002418 | 1.10067843170002 | 0.71122482226531 | 1.70338966256959 | 0.999950574162623 |
| ebi-fl187-GCST90094707 | ebi-met1400-GCST90200983 | Cholesterol to linoleoyl-arachidonoyl-glycerol (18:2 to 20:4) [1] ratio \|\| id:ebi-met1400-GCST90200983 | Black olive liking \|\| id:ebi-fl187-GCST90094707 | MR Egger | 22 | -0.25053537 | 0.259723820487431 | 0.346253817634567 | -0.759594058 | 0.258523317998929 | 0.778383947964833 | 0.467856310847078 | 1.29501634669059 | 0.999950574162623 |
| ebi-fl187-GCST90094707 | ebi-met1400-GCST90201001 | Serine to alpha-ketobutyrate ratio \|\| id:ebi-met1400-GCST90201001 | Black olive liking \|\| id:ebi-fl187-GCST90094707 | MR Egger | 22 | 0.111495587495972 | 0.249244375831798 | 0.659440756039448 | -0.377023389 | 0.600014564126296 | 1.11794881104102 | 0.685900031100428 | 1.82214533815209 | 0.999950574162623 |
| ebi-fl187-GCST90094707 | ebi-met1400-GCST90201005 | Fructose to maltose ratio \|\| id:ebi-met1400-GCST90201005 | Black olive liking \|\| id:ebi-fl187-GCST90094707 | MR Egger | 22 | -0.496450733 | 0.277003011448074 | 0.0882396612881568 | -1.039376636 | 0.046475169066344 | 0.608687223588275 | 0.35367508163776 | 1.04757206655309 | 0.999950574162623 |
| ebi-fl187-GCST90094707 | ebi-met1400-GCST90201020 | Paraxanthine to 5-acetylamino-6-formylamino-3-methyluracil ratio \|\| id:ebi-met1400-GCST90201020 | Black olive liking \|\| id:ebi-fl187-GCST90094707 | MR Egger | 22 | 0.12975456927459 | 0.25157934602202 | 0.611676807451828 | -0.363340949 | 0.622850087477749 | 1.13854891414495 | 0.695349314474902 | 1.86423370659328 | 0.999950574162623 |
| ebi-fl187-GCST90094707 | ebi-pw11-GCST90275197 | Placental weight (fetal genotype, adjusted for sex) \|\| id:ebi-pw11-GCST90275197 | Black olive liking \|\| id:ebi-fl187-GCST90094707 | MR Egger | 19 | 0.0651706942012174 | 0.108710157619744 | 0.556752184532496 | -0.147901215 | 0.278242603135916 | 1.06734119778896 | 0.862516314637757 | 1.32080659016407 | 0.999950574162623 |
| ebi-fl187-GCST90094707 | prot-a-1017 | FAS-associated factor 2 \|\| id:prot-a-1017 | Black olive liking \|\| id:ebi-fl187-GCST90094707 | MR Egger | 22 | 0.25887976487172 | 0.354452444937489 | 0.473636292197619 | -0.435847027 | 0.953606556949197 | 1.2954780334961 | 0.646716648421814 | 2.59505200518094 | 0.999950574162623 |
| ebi-fl187-GCST90094707 | prot-a-1019 | Fas apoptotic inhibitory molecule 3 \|\| id:prot-a-1019 | Black olive liking \|\| id:ebi-fl187-GCST90094707 | MR Egger | 22 | -0.724479854 | 0.349939965389584 | 0.051584658526007 | -1.410362186 | -0.038597522 | 0.484576553987118 | 0.244054873853531 | 0.962137870743583 | 0.999950574162623 |
| ebi-fl187-GCST90094707 | prot-a-1052 | MIP18 family protein FAM96A \|\| id:prot-a-1052 | Black olive liking \|\| id:ebi-fl187-GCST90094707 | MR Egger | 22 | -0.288556355 | 0.349902427117658 | 0.419282646059612 | -0.974365112 | 0.397252402159815 | 0.749344574650958 | 0.377431904444716 | 1.48773138928183 | 0.999950574162623 |
| ebi-fl187-GCST90094707 | prot-a-1066 | F-box/LRR-repeat protein 4 \|\| id:prot-a-1066 | Black olive liking \|\| id:ebi-fl187-GCST90094707 | MR Egger | 22 | 0.079313245342779 | 0.34994497061527 | 0.823000292985158 | -0.606578897 | 0.765205387748708 | 1.08254337063406 | 0.545212911631958 | 2.14943579710165 | 0.999950574162623 |
| ebi-fl187-GCST90094707 | prot-a-1076 | Low affinity immunoglobulin gamma Fc region receptor III-B \|\| id:prot-a-1076 | Black olive liking \|\| id:ebi-fl187-GCST90094707 | MR Egger | 22 | -0.622093246 | 0.425922408274379 | 0.159663318431144 | -1.456901166 | 0.212714674096975 | 0.536819565215578 | 0.232957052523749 | 1.23703164371409 | 0.999950574162623 |
| ebi-fl187-GCST90094707 | prot-a-1081 | Fc receptor-like protein 3 \|\| id:prot-a-1081 | Black olive liking \|\| id:ebi-fl187-GCST90094707 | MR Egger | 22 | -0.583049552 | 0.440704262427363 | 0.200764814873653 | -1.446829906 | 0.280730802573152 | 0.558193528319988 | 0.235315077826275 | 1.32409711241855 | 0.999950574162623 |
| ebi-fl187-GCST90094707 | prot-a-1105 | Fibroblast growth factor receptor 4 \|\| id:prot-a-1105 | Black olive liking \|\| id:ebi-fl187-GCST90094707 | MR Egger | 22 | -0.306263423 | 0.435358970382999 | 0.489870189310991 | -1.159567005 | 0.547040159295759 | 0.736192664084997 | 0.313621948346359 | 1.72813044976691 | 0.999950574162623 |
| ebi-fl187-GCST90094707 | prot-a-1143 | Follistatin-related protein 1 \|\| id:prot-a-1143 | Black olive liking \|\| id:ebi-fl187-GCST90094707 | MR Egger | 22 | -1.161804397 | 0.357533404794988 | 0.00401525407696522 | -1.862569871 | -0.461038924 | 0.312921037313173 | 0.15527308545781 | 0.630628130460889 | 0.999950574162623 |
| ebi-fl187-GCST90094707 | prot-a-1151 | Alpha-(1,3)-fucosyltransferase 10 \|\| id:prot-a-1151 | Black olive liking \|\| id:ebi-fl187-GCST90094707 | MR Egger | 22 | 0.266034456244146 | 0.349927979438374 | 0.455971819313126 | -0.419824383 | 0.951893295943359 | 1.30478001573118 | 0.657162218240051 | 2.59060981017866 | 0.999950574162623 |
| ebi-fl187-GCST90094707 | prot-a-1165 | Growth arrest and DNA damage-inducible proteins-interacting protein 1 \|\| id:prot-a-1165 | Black olive liking \|\| id:ebi-fl187-GCST90094707 | MR Egger | 22 | 0.358162481888348 | 0.349771559645323 | 0.318066968067432 | -0.327389775 | 1.04371473879318 | 1.43069806411856 | 0.720802737366832 | 2.83974636132782 | 0.999950574162623 |
| ebi-fl187-GCST90094707 | prot-a-1194 | Growth/differentiation factor 11/8 \|\| id:prot-a-1194 | Black olive liking \|\| id:ebi-fl187-GCST90094707 | MR Egger | 22 | -0.343304942 | 0.462335480524676 | 0.466389637268331 | -1.249482483 | 0.562872600296072 | 0.709421846387642 | 0.286653106232791 | 1.75570871268822 | 0.999950574162623 |
| ebi-fl187-GCST90094707 | prot-a-1216 | D-glucuronyl C5-epimerase \|\| id:prot-a-1216 | Black olive liking \|\| id:ebi-fl187-GCST90094707 | MR Egger | 22 | 0.0973927892160896 | 0.349927979438374 | 0.783622050466261 | -0.58846605 | 0.783251628915303 | 1.1022932575039 | 0.555178247305069 | 2.1885771487565 | 0.999950574162623 |
| ebi-fl187-GCST90094707 | prot-a-1246 | Glypican-3 \|\| id:prot-a-1246 | Black olive liking \|\| id:ebi-fl187-GCST90094707 | MR Egger | 22 | 0.499679508200397 | 0.349939965389584 | 0.168750178384899 | -0.186202824 | 1.18556184036398 | 1.64819295371819 | 0.830105212677428 | 3.2725249416569 | 0.999950574162623 |
| ebi-fl187-GCST90094707 | prot-a-1275 | Glutamate receptor 4 \|\| id:prot-a-1275 | Black olive liking \|\| id:ebi-fl187-GCST90094707 | MR Egger | 21 | 0.418759842059093 | 0.35009827002921 | 0.246363544640571 | -0.267432767 | 1.10495245131635 | 1.52007525280814 | 0.765341784965666 | 3.01908091207041 | 0.999950574162623 |
| ebi-fl187-GCST90094707 | prot-a-1289 | Glutathione S-transferase P \|\| id:prot-a-1289 | Black olive liking \|\| id:ebi-fl187-GCST90094707 | MR Egger | 22 | 0.440816615884075 | 0.367118754898705 | 0.243878210116318 | -0.278736144 | 1.16036937548554 | 1.55397570175058 | 0.75673954735908 | 3.19111177691013 | 0.999950574162623 |
| ebi-fl187-GCST90094707 | prot-a-130 | Apolipoprotein D \|\| id:prot-a-130 | Black olive liking \|\| id:ebi-fl187-GCST90094707 | MR Egger | 22 | 0.180593940305884 | 0.349934486573461 | 0.611457842204318 | -0.505277653 | 0.866465533989868 | 1.19792864997873 | 0.603338033325712 | 2.37848928987567 | 0.999950574162623 |
| ebi-fl187-GCST90094707 | prot-a-1313 | Heparin-binding EGF-like growth factor \|\| id:prot-a-1313 | Black olive liking \|\| id:ebi-fl187-GCST90094707 | MR Egger | 22 | 0.0146286742414935 | 0.349932978832408 | 0.967069302398423 | -0.671239964 | 0.700497312753013 | 1.01473619696169 | 0.511074470649764 | 2.01475442143524 | 0.999950574162623 |
| ebi-fl187-GCST90094707 | prot-a-1347 | HLA class II histocompatibility antigen, DQ alpha 2 chain \|\| id:prot-a-1347 | Black olive liking \|\| id:ebi-fl187-GCST90094707 | MR Egger | 21 | -0.147489775 | 0.350096644095003 | 0.67827592637204 | -0.833679198 | 0.538699647177885 | 0.862871260920644 | 0.434447922474796 | 1.71377689800318 | 0.999950574162623 |
| ebi-fl187-GCST90094707 | prot-a-1357 | Non-histone chromosomal protein HMG-14 \|\| id:prot-a-1357 | Black olive liking \|\| id:ebi-fl187-GCST90094707 | MR Egger | 22 | 0.324440726902269 | 0.34994497061527 | 0.36491849249004 | -0.361451416 | 1.0103328693082 | 1.38325681128665 | 0.696664442344336 | 2.74651509345326 | 0.999950574162623 |
| ebi-fl187-GCST90094707 | prot-a-137 | Apolipoprotein M \|\| id:prot-a-137 | Black olive liking \|\| id:ebi-fl187-GCST90094707 | MR Egger | 22 | -0.179166695 | 0.349943820698913 | 0.614267322094941 | -0.865056583 | 0.50672319377025 | 0.835966536507532 | 0.421027728785768 | 1.65984328912453 | 0.999950574162623 |
| ebi-fl187-GCST90094707 | prot-a-1381 | 17-beta-hydroxysteroid dehydrogenase 14 \|\| id:prot-a-1381 | Black olive liking \|\| id:ebi-fl187-GCST90094707 | MR Egger | 21 | 0.100972308241086 | 0.369753703861936 | 0.787737908133582 | -0.623744951 | 0.825689567810481 | 1.10624600744153 | 0.535933629398036 | 2.2834548194986 | 0.999950574162623 |
| ebi-fl187-GCST90094707 | prot-a-1392 | Serine protease HTRA2, mitochondrial \|\| id:prot-a-1392 | Black olive liking \|\| id:ebi-fl187-GCST90094707 | MR Egger | 22 | 0.404584686600915 | 0.444201459346682 | 0.373241096460548 | -0.466050174 | 1.27521954692041 | 1.49867994892507 | 0.627475800463294 | 3.57948718922978 | 0.999950574162623 |
| ebi-fl187-GCST90094707 | prot-a-1416 | Interferon-induced protein with tetratricopeptide repeats 2 \|\| id:prot-a-1416 | Black olive liking \|\| id:ebi-fl187-GCST90094707 | MR Egger | 22 | -0.169460548 | 0.34994497061527 | 0.633469560717386 | -0.855352691 | 0.516431593919972 | 0.844120055637697 | 0.42513322402187 | 1.67603618834818 | 0.999950574162623 |
| ebi-fl187-GCST90094707 | prot-a-1428 | Interferon gamma \|\| id:prot-a-1428 | Black olive liking \|\| id:ebi-fl187-GCST90094707 | MR Egger | 22 | -0.157393758 | 0.35734784141929 | 0.664335063567633 | -0.857795528 | 0.543008010764529 | 0.854367578150638 | 0.424095960425391 | 1.72117640041375 | 0.999950574162623 |
| ebi-fl187-GCST90094707 | prot-a-1483 | Interleukin-17C \|\| id:prot-a-1483 | Black olive liking \|\| id:ebi-fl187-GCST90094707 | MR Egger | 22 | -0.240533356 | 0.390975309533344 | 0.545350958608014 | -1.006844963 | 0.525778250763477 | 0.786208420304091 | 0.365369918744563 | 1.691774961 | 0.999950574162623 |
| ebi-fl187-GCST90094707 | prot-a-1503 | Interleukin-1 receptor-like 2 \|\| id:prot-a-1503 | Black olive liking \|\| id:ebi-fl187-GCST90094707 | MR Egger | 22 | 0.16107907939029 | 0.349932978832408 | 0.650256246609677 | -0.524789559 | 0.846947717901809 | 1.17477786585806 | 0.591679864897075 | 2.33251647721711 | 0.999950574162623 |
| ebi-fl187-GCST90094707 | prot-a-1522 | Interleukin-31 receptor subunit alpha \|\| id:prot-a-1522 | Black olive liking \|\| id:ebi-fl187-GCST90094707 | MR Egger | 22 | -0.254040196 | 0.34989235959484 | 0.476218236927912 | -0.939829221 | 0.431748828601188 | 0.775660622806665 | 0.390694552082226 | 1.53994827561916 | 0.999950574162623 |
| ebi-fl187-GCST90094707 | prot-a-1540 | Interleukin-6 receptor subunit alpha \|\| id:prot-a-1540 | Black olive liking \|\| id:ebi-fl187-GCST90094707 | MR Egger | 22 | -0.048355316 | 0.34994497061527 | 0.891480119574765 | -0.734247458 | 0.637536826867753 | 0.952795183992026 | 0.479866442809526 | 1.89181526702158 | 0.999950574162623 |
| ebi-fl187-GCST90094707 | prot-a-1541 | Interleukin-6 receptor subunit alpha \|\| id:prot-a-1541 | Black olive liking \|\| id:ebi-fl187-GCST90094707 | MR Egger | 22 | 0.401977692211246 | 0.349932978832408 | 0.264221977480828 | -0.283890946 | 1.08784633072277 | 1.49477798711254 | 0.752848741170212 | 2.96787536269659 | 0.999950574162623 |
| ebi-fl187-GCST90094707 | prot-a-1545 | Interleukin-9 \|\| id:prot-a-1545 | Black olive liking \|\| id:ebi-fl187-GCST90094707 | MR Egger | 22 | -0.262717705 | 0.359561643593511 | 0.473459511216944 | -0.967458527 | 0.442023116039351 | 0.768958939628038 | 0.380047692770152 | 1.55585170514767 | 0.999950574162623 |
| ebi-fl187-GCST90094707 | prot-a-1548 | Inositol monophosphatase 3 \|\| id:prot-a-1548 | Black olive liking \|\| id:ebi-fl187-GCST90094707 | MR Egger | 22 | -0.089378908 | 0.404156121834793 | 0.827218234475347 | -0.881524907 | 0.702767090617642 | 0.914498996769053 | 0.414150888337429 | 2.01933266025069 | 0.999950574162623 |
| ebi-fl187-GCST90094707 | prot-a-1555 | Inhibin beta A chain:Inhibin beta B chain heterodimer \|\| id:prot-a-1555 | Black olive liking \|\| id:ebi-fl187-GCST90094707 | MR Egger | 22 | -0.41538088 | 0.372651249726877 | 0.278210441868174 | -1.14577733 | 0.315015568979912 | 0.66008881787044 | 0.317976648925689 | 1.37028064466275 | 0.999950574162623 |
| ebi-fl187-GCST90094707 | prot-a-1603 | Junctophilin-3 \|\| id:prot-a-1603 | Black olive liking \|\| id:ebi-fl187-GCST90094707 | MR Egger | 22 | 0.0517243904394655 | 0.34994497061527 | 0.883974904060992 | -0.634167752 | 0.737616532845395 | 1.05308546209176 | 0.530376709662953 | 2.09094586972675 | 0.999950574162623 |
| ebi-fl187-GCST90094707 | prot-a-1627 | UPF0258 protein KIAA1024 \|\| id:prot-a-1627 | Black olive liking \|\| id:ebi-fl187-GCST90094707 | MR Egger | 22 | -0.401025856 | 0.349796220046128 | 0.265138451774869 | -1.086626448 | 0.284574734849145 | 0.669632746494309 | 0.337352652990551 | 1.32919664689897 | 0.999950574162623 |
| ebi-fl187-GCST90094707 | prot-a-1666 | Kallikrein-7 \|\| id:prot-a-1666 | Black olive liking \|\| id:ebi-fl187-GCST90094707 | MR Egger | 22 | 0.0762989071226188 | 0.34989858936457 | 0.829591821564469 | -0.609502328 | 0.762100142277175 | 1.07928513196075 | 0.543621346869886 | 2.14277162362856 | 0.999950574162623 |
| ebi-fl187-GCST90094707 | prot-a-1678 | Importin subunit alpha-3 \|\| id:prot-a-1678 | Black olive liking \|\| id:ebi-fl187-GCST90094707 | MR Egger | 22 | -0.306677062 | 0.397758936169158 | 0.449713036412837 | -1.086284577 | 0.472930453131868 | 0.735888208982101 | 0.337468003822954 | 1.60468977794703 | 0.999950574162623 |
| ebi-fl187-GCST90094707 | prot-a-1718 | Protein LDOC1 \|\| id:prot-a-1718 | Black olive liking \|\| id:ebi-fl187-GCST90094707 | MR Egger | 22 | -0.046186946 | 0.349939487659149 | 0.896314523326956 | -0.732068342 | 0.639694449793591 | 0.954863437586287 | 0.480913267669759 | 1.89590149770042 | 0.999950574162623 |
| ebi-fl187-GCST90094707 | prot-a-1723 | Leptin \|\| id:prot-a-1723 | Black olive liking \|\| id:ebi-fl187-GCST90094707 | MR Egger | 22 | 1.01003377796924 | 0.365383674082053 | 0.0119634042429013 | 0.293881776768421 | 1.72618577917007 | 2.74569375740988 | 1.34162528301431 | 5.61918018758683 | 0.999950574162623 |
| ebi-fl187-GCST90094707 | prot-a-1730 | Galectin-7 \|\| id:prot-a-1730 | Black olive liking \|\| id:ebi-fl187-GCST90094707 | MR Egger | 22 | -0.201777191 | 0.422422844966322 | 0.638066631308074 | -1.029725967 | 0.626171585162045 | 0.817277004352341 | 0.357104805625523 | 1.87043604936409 | 0.999950574162623 |
| ebi-fl187-GCST90094707 | prot-a-1753 | Lipase member N \|\| id:prot-a-1753 | Black olive liking \|\| id:ebi-fl187-GCST90094707 | MR Egger | 22 | -0.147403627 | 0.349777755810135 | 0.677942486357141 | -0.832968029 | 0.538160774210507 | 0.86294559881725 | 0.434756998306428 | 1.7128536387428 | 0.999950574162623 |
| ebi-fl187-GCST90094707 | prot-a-1756 | VIP36-like protein \|\| id:prot-a-1756 | Black olive liking \|\| id:ebi-fl187-GCST90094707 | MR Egger | 22 | -0.151254448 | 0.35260971766514 | 0.672539469397055 | -0.842369495 | 0.539860598276325 | 0.859628939665921 | 0.430688798478981 | 1.7157676645431 | 0.999950574162623 |
| ebi-fl187-GCST90094707 | prot-a-1760 | Ig Kappa chain V-I region HK102- like \|\| id:prot-a-1760 | Black olive liking \|\| id:ebi-fl187-GCST90094707 | MR Egger | 22 | 0.502551600425153 | 0.356366463639065 | 0.173841077311765 | -0.195926668 | 1.20102986915772 | 1.65293352029896 | 0.822072516436936 | 3.3235379700685 | 0.999950574162623 |
| ebi-fl187-GCST90094707 | prot-a-179 | Neutral ceramidase \|\| id:prot-a-179 | Black olive liking \|\| id:ebi-fl187-GCST90094707 | MR Egger | 22 | 0.405746874060348 | 0.34994497061527 | 0.259924933564163 | -0.280145268 | 1.09163901646628 | 1.50042270847791 | 0.755673957976213 | 2.97915295393446 | 0.999950574162623 |
| ebi-fl187-GCST90094707 | prot-a-1809 | Lactotransferrin \|\| id:prot-a-1809 | Black olive liking \|\| id:ebi-fl187-GCST90094707 | MR Egger | 22 | 0.845595056174723 | 0.366902137321431 | 0.0320345020803663 | 0.126466867024718 | 1.56472324532473 | 2.32936350438416 | 1.13481185086016 | 4.78135149138963 | 0.999950574162623 |
| ebi-fl187-GCST90094707 | prot-a-1818 | Lysophospholipase-like protein 1 \|\| id:prot-a-1818 | Black olive liking \|\| id:ebi-fl187-GCST90094707 | MR Egger | 22 | -0.419687981 | 0.381735082403708 | 0.284642466449716 | -1.167888743 | 0.328512780451473 | 0.657251862854433 | 0.311022897979015 | 1.38890099099639 | 0.999950574162623 |
| ebi-fl187-GCST90094707 | prot-a-1852 | MAP kinase-activated protein kinase 3 \|\| id:prot-a-1852 | Black olive liking \|\| id:ebi-fl187-GCST90094707 | MR Egger | 22 | -0.201840644 | 0.34994497061527 | 0.570524825205019 | -0.887732786 | 0.484051498384657 | 0.817225147279531 | 0.411587853284953 | 1.62263520659264 | 0.999950574162623 |
| ebi-fl187-GCST90094707 | prot-a-1856 | Megakaryocyte-associated tyrosine-protein kinase \|\| id:prot-a-1856 | Black olive liking \|\| id:ebi-fl187-GCST90094707 | MR Egger | 22 | -0.352481465 | 0.349939965389584 | 0.325840380261123 | -1.038363797 | 0.333400867408141 | 0.702941598884413 | 0.354033478983995 | 1.39570667966267 | 0.999950574162623 |
| ebi-fl187-GCST90094707 | prot-a-1890 | Beta-1,3-N-acetylglucosaminyltransferase manic fringe \|\| id:prot-a-1890 | Black olive liking \|\| id:ebi-fl187-GCST90094707 | MR Egger | 22 | -0.203731144 | 0.34994497061527 | 0.566950757540216 | -0.889623287 | 0.482160998273386 | 0.815681642505346 | 0.410810481444822 | 1.61957051236918 | 0.999950574162623 |
| ebi-fl187-GCST90094707 | prot-a-1919 | Matrilysin \|\| id:prot-a-1919 | Black olive liking \|\| id:ebi-fl187-GCST90094707 | MR Egger | 22 | -0.77183472 | 0.38563003186152 | 0.0590926408420851 | -1.527669582 | -0.015999857 | 0.462164347898777 | 0.217040874327371 | 0.984127460464094 | 0.999950574162623 |
| ebi-fl187-GCST90094707 | prot-a-1923 | Multimerin-2 \|\| id:prot-a-1923 | Black olive liking \|\| id:ebi-fl187-GCST90094707 | MR Egger | 22 | -0.408090348 | 0.379862588123627 | 0.295474476667657 | -1.152621021 | 0.336440324494557 | 0.664918801873172 | 0.315807944431212 | 1.39995532373554 | 0.999950574162623 |
| ebi-fl187-GCST90094707 | prot-a-1948 | Mesothelin \|\| id:prot-a-1948 | Black olive liking \|\| id:ebi-fl187-GCST90094707 | MR Egger | 22 | 0.154472541933166 | 0.369069860994044 | 0.680008596555398 | -0.568904386 | 0.877849469481491 | 1.16704223289392 | 0.566145376049296 | 2.40572056396948 | 0.999950574162623 |
| ebi-fl187-GCST90094707 | prot-a-195 | ATPase family AAA domain-containing protein 1 \|\| id:prot-a-195 | Black olive liking \|\| id:ebi-fl187-GCST90094707 | MR Egger | 22 | -0.090209992 | 0.349943820698913 | 0.799207334014903 | -0.776099881 | 0.595679896304297 | 0.913739286940343 | 0.460197340302692 | 1.81426403713912 | 0.999950574162623 |
| ebi-fl187-GCST90094707 | prot-a-1966 | Mucin-1 \|\| id:prot-a-1966 | Black olive liking \|\| id:ebi-fl187-GCST90094707 | MR Egger | 22 | -0.292869764 | 0.34994497061527 | 0.412536274739615 | -0.978761907 | 0.39302237811017 | 0.746119305743136 | 0.375776056778923 | 1.48145154104412 | 0.999950574162623 |
| ebi-fl187-GCST90094707 | prot-a-1986 | Myocardial zonula adherens protein \|\| id:prot-a-1986 | Black olive liking \|\| id:ebi-fl187-GCST90094707 | MR Egger | 22 | -0.407975768 | 0.349932978832408 | 0.257383523439239 | -1.093844406 | 0.277892870878107 | 0.664994993029608 | 0.334926422320362 | 1.32034474225942 | 0.999950574162623 |
| ebi-fl187-GCST90094707 | prot-a-2071 | Epididymal secretory protein E1 \|\| id:prot-a-2071 | Black olive liking \|\| id:ebi-fl187-GCST90094707 | MR Egger | 22 | 0.429216957060072 | 0.349932978832408 | 0.234229583102042 | -0.256651681 | 1.11508559557159 | 1.53605425606415 | 0.773637639179375 | 3.04982921988589 | 0.999950574162623 |
| ebi-fl187-GCST90094707 | prot-a-2098 | Neuritin \|\| id:prot-a-2098 | Black olive liking \|\| id:ebi-fl187-GCST90094707 | MR Egger | 22 | -0.091868589 | 0.411851992184353 | 0.825749568245218 | -0.899098493 | 0.715361315928053 | 0.912225018295811 | 0.406936350220246 | 2.04492541291631 | 0.999950574162623 |
| ebi-fl187-GCST90094707 | prot-a-2115 | Neurotrophin-4 \|\| id:prot-a-2115 | Black olive liking \|\| id:ebi-fl187-GCST90094707 | MR Egger | 22 | -0.339235627 | 0.442991608103531 | 0.452748712581599 | -1.207499179 | 0.529027924427845 | 0.712314588439992 | 0.298943950603842 | 1.6972816204494 | 0.999950574162623 |
| ebi-fl187-GCST90094707 | prot-a-2118 | Netrin-1 \|\| id:prot-a-2118 | Black olive liking \|\| id:ebi-fl187-GCST90094707 | MR Egger | 22 | -0.150904409 | 0.384941267421587 | 0.699190012183946 | -0.905389293 | 0.603580475324693 | 0.859929896442579 | 0.404384430441901 | 1.8286545453485 | 0.999950574162623 |
| ebi-fl187-GCST90094707 | prot-a-2120 | Netrin-G1 \|\| id:prot-a-2120 | Black olive liking \|\| id:ebi-fl187-GCST90094707 | MR Egger | 22 | 0.0587242916617806 | 0.349939965389584 | 0.868416113288188 | -0.627158041 | 0.744606623825366 | 1.06048281647507 | 0.534107558174472 | 2.10561297406602 | 0.999950574162623 |
| ebi-fl187-GCST90094707 | prot-a-2145 | Olfactomedin-like protein 3 \|\| id:prot-a-2145 | Black olive liking \|\| id:ebi-fl187-GCST90094707 | MR Egger | 22 | -0.029327096 | 0.34994497061527 | 0.934044733768587 | -0.715219238 | 0.656565046585431 | 0.971098770162187 | 0.489084873941107 | 1.92815781402405 | 0.999950574162623 |
| ebi-fl187-GCST90094707 | prot-a-2162 | Prolyl 4-hydroxylase subunit alpha-2 \|\| id:prot-a-2162 | Black olive liking \|\| id:ebi-fl187-GCST90094707 | MR Egger | 22 | 0.152676977972411 | 0.34989858936457 | 0.667259493575864 | -0.533124257 | 0.838478213126967 | 1.16494861409558 | 0.586768886066595 | 2.31284464072485 | 0.999950574162623 |
| ebi-fl187-GCST90094707 | prot-a-2168 | Polyadenylate-binding protein-interacting protein 1 \|\| id:prot-a-2168 | Black olive liking \|\| id:ebi-fl187-GCST90094707 | MR Egger | 22 | 0.10417917255452 | 0.349939487659149 | 0.768997812868468 | -0.581702223 | 0.790060568366452 | 1.10979928267529 | 0.558946105254659 | 2.20352988641981 | 0.999950574162623 |
| ebi-fl187-GCST90094707 | prot-a-219 | Beta-1,4-galactosyltransferase 5 \|\| id:prot-a-219 | Black olive liking \|\| id:ebi-fl187-GCST90094707 | MR Egger | 22 | -0.155279344 | 0.37646712006123 | 0.684386756987802 | -0.893154899 | 0.58259621172827 | 0.856175976803059 | 0.409362216925191 | 1.79068138911469 | 0.999950574162623 |
| ebi-fl187-GCST90094707 | prot-a-2190 | Pyruvate carboxylase, mitochondrial \|\| id:prot-a-2190 | Black olive liking \|\| id:ebi-fl187-GCST90094707 | MR Egger | 22 | 0.0116007305717772 | 0.34994497061527 | 0.973883455953844 | -0.674291412 | 0.697492872977707 | 1.01166828000154 | 0.509517330674958 | 2.00871029726405 | 0.999950574162623 |
| ebi-fl187-GCST90094707 | prot-a-2197 | Protocadherin-9 \|\| id:prot-a-2197 | Black olive liking \|\| id:ebi-fl187-GCST90094707 | MR Egger | 22 | -0.006804933 | 0.499341157835052 | 0.989261935083836 | -0.985513603 | 0.971903736130961 | 0.993218167902116 | 0.37324747683121 | 2.64297119280177 | 0.999950574162623 |
| ebi-fl187-GCST90094707 | prot-a-2198 | Protocadherin alpha-4 \|\| id:prot-a-2198 | Black olive liking \|\| id:ebi-fl187-GCST90094707 | MR Egger | 22 | -0.537758037 | 0.349777755810135 | 0.139859565916996 | -1.223322438 | 0.147806364499869 | 0.584056218117008 | 0.294250910577422 | 1.15928839523977 | 0.999950574162623 |
| ebi-fl187-GCST90094707 | prot-a-2254 | Placenta growth factor \|\| id:prot-a-2254 | Black olive liking \|\| id:ebi-fl187-GCST90094707 | MR Egger | 22 | -0.280317075 | 0.394476963413863 | 0.485534177996466 | -1.053491923 | 0.49285777310864 | 0.755544139175925 | 0.348717924277681 | 1.63698768116241 | 0.999950574162623 |
| ebi-fl187-GCST90094707 | prot-a-2261 | Membrane-associated progesterone receptor component 2 \|\| id:prot-a-2261 | Black olive liking \|\| id:ebi-fl187-GCST90094707 | MR Egger | 22 | 0.306932787855196 | 0.377943652382124 | 0.426288062901867 | -0.433836771 | 1.04770234652416 | 1.35924960692032 | 0.648018022307446 | 2.85109276333751 | 0.999950574162623 |
| ebi-fl187-GCST90094707 | prot-a-2268 | Polymeric immunoglobulin receptor \|\| id:prot-a-2268 | Black olive liking \|\| id:ebi-fl187-GCST90094707 | MR Egger | 22 | 0.497792888485605 | 0.365510053358775 | 0.188369642027129 | -0.218606816 | 1.2141925930688 | 1.64508637178873 | 0.803637633423986 | 3.36757396379569 | 0.999950574162623 |
| ebi-fl187-GCST90094707 | prot-a-2283 | Group 10 secretory phospholipase A2 \|\| id:prot-a-2283 | Black olive liking \|\| id:ebi-fl187-GCST90094707 | MR Egger | 22 | -0.205197514 | 0.349736367751358 | 0.563956097404542 | -0.890680795 | 0.480285766921135 | 0.814486428155375 | 0.410376275651107 | 1.61653628879195 | 0.999950574162623 |
| ebi-fl187-GCST90094707 | prot-a-2298 | Inactive phospholipase D5 \|\| id:prot-a-2298 | Black olive liking \|\| id:ebi-fl187-GCST90094707 | MR Egger | 22 | 0.406997856676115 | 0.378824888992093 | 0.295448718898051 | -0.335498926 | 1.14949463910062 | 1.50230088574113 | 0.714981274781118 | 3.15659728569745 | 0.999950574162623 |
| ebi-fl187-GCST90094707 | prot-a-2304 | Procollagen-lysine,2-oxoglutarate 5-dioxygenase 2 \|\| id:prot-a-2304 | Black olive liking \|\| id:ebi-fl187-GCST90094707 | MR Egger | 22 | 0.367291525830459 | 0.371880091112609 | 0.335117581866568 | -0.361593453 | 1.09617650441117 | 1.44381876823345 | 0.696565497072269 | 2.9927015395752 | 0.999950574162623 |
| ebi-fl187-GCST90094707 | prot-a-2331 | Periostin \|\| id:prot-a-2331 | Black olive liking \|\| id:ebi-fl187-GCST90094707 | MR Egger | 22 | -0.939926102 | 0.349635459803368 | 0.0141349686795637 | -1.625211603 | -0.254640601 | 0.390656702968877 | 0.196870012432564 | 0.775195052252002 | 0.999950574162623 |
| ebi-fl187-GCST90094707 | prot-a-2333 | Periostin \|\| id:prot-a-2333 | Black olive liking \|\| id:ebi-fl187-GCST90094707 | MR Egger | 22 | -0.840783483 | 0.3761249338294 | 0.0369566724155336 | -1.577988353 | -0.103578613 | 0.431372418096472 | 0.20638986439883 | 0.901605142463829 | 0.999950574162623 |
| ebi-fl187-GCST90094707 | prot-a-2367 | cAMP-dependent protein kinase catalytic subunit alpha \|\| id:prot-a-2367 | Black olive liking \|\| id:ebi-fl187-GCST90094707 | MR Egger | 22 | 0.71805295576809 | 0.368762688765057 | 0.065690679812904 | -0.004721914 | 1.4408278257476 | 2.05043703 | 0.995289216499176 | 4.22419126427767 | 0.999950574162623 |
| ebi-fl187-GCST90094707 | prot-a-2385 | Phosphoribosyl pyrophosphate synthase-associated protein 1 \|\| id:prot-a-2385 | Black olive liking \|\| id:ebi-fl187-GCST90094707 | MR Egger | 22 | 0.0974082196893868 | 0.350095310020374 | 0.7836893547704 | -0.588778588 | 0.78359502732932 | 1.1023102665418 | 0.555004760413677 | 2.18932883173432 | 0.999950574162623 |
| ebi-fl187-GCST90094707 | prot-a-2393 | Trypsin-3 \|\| id:prot-a-2393 | Black olive liking \|\| id:ebi-fl187-GCST90094707 | MR Egger | 21 | 0.470275154743943 | 0.377933409942303 | 0.228508917987284 | -0.270474329 | 1.21102463823086 | 1.60043449978335 | 0.763017487362838 | 3.35692252211614 | 0.999950574162623 |
| ebi-fl187-GCST90094707 | prot-a-2405 | Pregnancy-specific beta-1-glycoprotein 6 \|\| id:prot-a-2405 | Black olive liking \|\| id:ebi-fl187-GCST90094707 | MR Egger | 22 | -0.028378352 | 0.416447991112239 | 0.946347833667534 | -0.844616415 | 0.787859710146937 | 0.972020530886992 | 0.429722161570414 | 2.19868556234797 | 0.999950574162623 |
| ebi-fl187-GCST90094707 | prot-a-244 | Beclin-1 \|\| id:prot-a-244 | Black olive liking \|\| id:ebi-fl187-GCST90094707 | MR Egger | 22 | 0.398645466375513 | 0.349939965389584 | 0.268088149343438 | -0.287236866 | 1.0845277985391 | 1.4898053388799 | 0.750333979337178 | 2.9580426968211 | 0.999950574162623 |
| ebi-fl187-GCST90094707 | prot-a-2454 | tRNA pseudouridine synthase A, mitochondrial \|\| id:prot-a-2454 | Black olive liking \|\| id:ebi-fl187-GCST90094707 | MR Egger | 22 | 0.562178063914069 | 0.359422390211825 | 0.133476126098725 | -0.142289821 | 1.26664594872925 | 1.75448973247311 | 0.867369838146444 | 3.54892928710976 | 0.999950574162623 |
| ebi-fl187-GCST90094707 | prot-a-2466 | Glutaminyl-peptide cyclotransferase-like protein \|\| id:prot-a-2466 | Black olive liking \|\| id:ebi-fl187-GCST90094707 | MR Egger | 22 | 0.0947798170476181 | 0.34994497061527 | 0.78928802745179 | -0.591112325 | 0.780671959453548 | 1.09941675564684 | 0.553711035237403 | 2.18293861901953 | 0.999950574162623 |
| ebi-fl187-GCST90094707 | prot-a-2474 | Ras-related protein Rab-27A \|\| id:prot-a-2474 | Black olive liking \|\| id:ebi-fl187-GCST90094707 | MR Egger | 22 | -0.458114754 | 0.399275863515295 | 0.264771763503881 | -1.240695446 | 0.324465938764222 | 0.632474893192417 | 0.289183036743251 | 1.38329168620605 | 0.999950574162623 |
| ebi-fl187-GCST90094707 | prot-a-2490 | Rap1 GTPase-GDP dissociation stimulator 1 \|\| id:prot-a-2490 | Black olive liking \|\| id:ebi-fl187-GCST90094707 | MR Egger | 22 | -0.133696697 | 0.385269412239852 | 0.732200154403192 | -0.888824745 | 0.621431351147847 | 0.874855370763719 | 0.411138661762956 | 1.86159072579607 | 0.999950574162623 |
| ebi-fl187-GCST90094707 | prot-a-2545 | Receptor-interacting serine/threonine-protein kinase 2 \|\| id:prot-a-2545 | Black olive liking \|\| id:ebi-fl187-GCST90094707 | MR Egger | 22 | 0.33187290862484 | 0.349802377527351 | 0.354074956160846 | -0.353739751 | 1.01748556857845 | 1.39357572580199 | 0.702057653153856 | 2.76623051514394 | 0.999950574162623 |
| ebi-fl187-GCST90094707 | prot-a-2610 | RUN and FYVE domain-containing protein 1 \|\| id:prot-a-2610 | Black olive liking \|\| id:ebi-fl187-GCST90094707 | MR Egger | 22 | -0.011508021 | 0.400678931925078 | 0.977371534995272 | -0.796838728 | 0.773822685281308 | 0.988557942704347 | 0.450751662835014 | 2.16803816082992 | 0.999950574162623 |
| ebi-fl187-GCST90094707 | prot-a-2627 | Serine--tRNA ligase, mitochondrial \|\| id:prot-a-2627 | Black olive liking \|\| id:ebi-fl187-GCST90094707 | MR Egger | 22 | 0.29569212696774 | 0.34994497061527 | 0.408123891821155 | -0.390200015 | 0.981584269373669 | 1.34405629443989 | 0.676921466213058 | 2.66868080388945 | 0.999950574162623 |
| ebi-fl187-GCST90094707 | prot-a-264 | BolA-like protein 2 \|\| id:prot-a-264 | Black olive liking \|\| id:ebi-fl187-GCST90094707 | MR Egger | 22 | 0.598034058729706 | 0.349903582091341 | 0.102897856171342 | -0.087776962 | 1.28384507962873 | 1.81854014071143 | 0.915965148822047 | 3.61049571332679 | 0.999950574162623 |
| ebi-fl187-GCST90094707 | prot-a-2646 | Secretoglobin family 1C member 1 \|\| id:prot-a-2646 | Black olive liking \|\| id:ebi-fl187-GCST90094707 | MR Egger | 22 | -0.21069047 | 0.472481410169424 | 0.660442645605807 | -1.136754034 | 0.715373093902506 | 0.810024755020003 | 0.32085883017788 | 2.04494949813742 | 0.999950574162623 |
| ebi-fl187-GCST90094707 | prot-a-2672 | Semaphorin-3E \|\| id:prot-a-2672 | Black olive liking \|\| id:ebi-fl187-GCST90094707 | MR Egger | 22 | -0.214923 | 0.441594537254524 | 0.631763539205958 | -1.080448293 | 0.65060229257565 | 0.806603545901774 | 0.339443321309693 | 1.91669489254063 | 0.999950574162623 |
| ebi-fl187-GCST90094707 | prot-a-2701 | Plasma protease C1 inhibitor \|\| id:prot-a-2701 | Black olive liking \|\| id:ebi-fl187-GCST90094707 | MR Egger | 22 | 0.0389967282252463 | 0.349939965389584 | 0.912379559318121 | -0.646885604 | 0.724879060388831 | 1.0397670817609 | 0.523674168484342 | 2.06448140728926 | 0.999950574162623 |
| ebi-fl187-GCST90094707 | prot-a-2738 | Signaling threshold-regulating transmembrane adapter 1 \|\| id:prot-a-2738 | Black olive liking \|\| id:ebi-fl187-GCST90094707 | MR Egger | 22 | -0.100000128 | 0.349927979438374 | 0.777990480895289 | -0.785858968 | 0.585858711693626 | 0.904837302211722 | 0.455728077912491 | 1.7965330273791 | 0.999950574162623 |
| ebi-fl187-GCST90094707 | prot-a-274 | Serine/threonine-protein kinase BRSK2 \|\| id:prot-a-274 | Black olive liking \|\| id:ebi-fl187-GCST90094707 | MR Egger | 22 | -0.001425017 | 0.376574683099037 | 0.997018180484378 | -0.739511395 | 0.736661362269859 | 0.998575998249789 | 0.477347092498516 | 2.08894961329143 | 0.999950574162623 |
| ebi-fl187-GCST90094707 | prot-a-2749 | Mitochondrial glutamate carrier 2 \|\| id:prot-a-2749 | Black olive liking \|\| id:ebi-fl187-GCST90094707 | MR Egger | 22 | -0.280233951 | 0.435029794166288 | 0.52678343128929 | -1.132892347 | 0.572424445979998 | 0.755606946088074 | 0.322100281996461 | 1.77255932046287 | 0.999950574162623 |
| ebi-fl187-GCST90094707 | prot-a-2760 | Sodium-coupled monocarboxylate transporter 1 \|\| id:prot-a-2760 | Black olive liking \|\| id:ebi-fl187-GCST90094707 | MR Egger | 22 | 0.275601612445083 | 0.349934486573461 | 0.440176717220137 | -0.410269981 | 0.961473206129067 | 1.31732295440467 | 0.663471101204104 | 2.61554687619712 | 0.999950574162623 |
| ebi-fl187-GCST90094707 | prot-a-2777 | Small integral membrane protein 10 \|\| id:prot-a-2777 | Black olive liking \|\| id:ebi-fl187-GCST90094707 | MR Egger | 22 | 0.256453209031451 | 0.405349211670801 | 0.534115014826073 | -0.538031246 | 1.05093766390622 | 1.29233829462444 | 0.583896670523714 | 2.86033189100875 | 0.999950574162623 |
| ebi-fl187-GCST90094707 | prot-a-2799 | Superoxide dismutase [Mn], mitochondrial \|\| id:prot-a-2799 | Black olive liking \|\| id:ebi-fl187-GCST90094707 | MR Egger | 22 | 0.330911108036148 | 0.44505490933024 | 0.465807306412489 | -0.541396514 | 1.20321873032342 | 1.39223602821277 | 0.581935004123289 | 3.33082070079944 | 0.999950574162623 |
| ebi-fl187-GCST90094707 | prot-a-2805 | Protein SPACA7 \|\| id:prot-a-2805 | Black olive liking \|\| id:ebi-fl187-GCST90094707 | MR Egger | 22 | 0.191429255811646 | 0.37772974993032 | 0.617844792249543 | -0.548921054 | 0.931779565675074 | 1.21097916037179 | 0.577572643982196 | 2.53902351874541 | 0.999950574162623 |
| ebi-fl187-GCST90094707 | prot-a-2811 | SPARC-like protein 1 \|\| id:prot-a-2811 | Black olive liking \|\| id:ebi-fl187-GCST90094707 | MR Egger | 22 | -0.43134999 | 0.349925121929478 | 0.231984860194403 | -1.117203229 | 0.254503248628843 | 0.649631506222232 | 0.327193601640003 | 1.28982074148533 | 0.999950574162623 |
| ebi-fl187-GCST90094707 | prot-a-2831 | Sepiapterin reductase \|\| id:prot-a-2831 | Black olive liking \|\| id:ebi-fl187-GCST90094707 | MR Egger | 22 | 0.371655611869886 | 0.355232650124889 | 0.307934654956509 | -0.324600382 | 1.06791160611467 | 1.45013348652822 | 0.722816146006623 | 2.90929739238452 | 0.999950574162623 |
| ebi-fl187-GCST90094707 | prot-a-2834 | SPRY domain-containing SOCS box protein 1 \|\| id:prot-a-2834 | Black olive liking \|\| id:ebi-fl187-GCST90094707 | MR Egger | 22 | -0.398368761 | 0.349777755810135 | 0.2681951786059 | -1.083933163 | 0.287195639893908 | 0.671414390231636 | 0.338262464420615 | 1.33268491430835 | 0.999950574162623 |
| ebi-fl187-GCST90094707 | prot-a-2835 | Spectrin alpha chain, non-erythrocytic 1 \|\| id:prot-a-2835 | Black olive liking \|\| id:ebi-fl187-GCST90094707 | MR Egger | 22 | -0.399785535 | 0.354476563600198 | 0.272744972098109 | -1.0945596 | 0.294988529770045 | 0.670463821717381 | 0.334686970791652 | 1.34311095280644 | 0.999950574162623 |
| ebi-fl187-GCST90094707 | prot-a-2859 | Sia-alpha-2,3-Gal-beta-1,4-GlcNAc-R:alpha 2,8-sialyltransferase \|\| id:prot-a-2859 | Black olive liking \|\| id:ebi-fl187-GCST90094707 | MR Egger | 22 | -0.475383542 | 0.401841846372107 | 0.250677107332213 | -1.262993561 | 0.312226477203191 | 0.621646583356991 | 0.282806160690969 | 1.36646413096248 | 0.999950574162623 |
| ebi-fl187-GCST90094707 | prot-a-2861 | Alpha-2,8-sialyltransferase 8F \|\| id:prot-a-2861 | Black olive liking \|\| id:ebi-fl187-GCST90094707 | MR Egger | 22 | -0.49122616 | 0.34994497061527 | 0.175738289077404 | -1.177118302 | 0.194665982381968 | 0.611875676534286 | 0.308165500071039 | 1.21490511899608 | 0.999950574162623 |
| ebi-fl187-GCST90094707 | prot-a-289 | Uncharacterized protein C17orf89 \|\| id:prot-a-289 | Black olive liking \|\| id:ebi-fl187-GCST90094707 | MR Egger | 22 | 0.0600651841055535 | 0.400727582008479 | 0.882352437460381 | -0.725360877 | 0.845491244842172 | 1.06190576366679 | 0.484149819070048 | 2.32912170260588 | 0.999950574162623 |
| ebi-fl187-GCST90094707 | prot-a-2921 | Tachykinin-3 \|\| id:prot-a-2921 | Black olive liking \|\| id:ebi-fl187-GCST90094707 | MR Egger | 22 | 0.175365136018817 | 0.349898114764179 | 0.621709664704045 | -0.510435169 | 0.861165440956608 | 1.19168126293403 | 0.600234318650659 | 2.36591642347354 | 0.999950574162623 |
| ebi-fl187-GCST90094707 | prot-a-2928 | Tax1-binding protein 1 \|\| id:prot-a-2928 | Black olive liking \|\| id:ebi-fl187-GCST90094707 | MR Egger | 22 | -0.334803305 | 0.412173952007271 | 0.426190622930908 | -1.142664251 | 0.47305764053742 | 0.715478803332269 | 0.318968077629666 | 1.6048938872564 | 0.999950574162623 |
| ebi-fl187-GCST90094707 | prot-a-296 | Uncharacterized protein C1orf185 \|\| id:prot-a-296 | Black olive liking \|\| id:ebi-fl187-GCST90094707 | MR Egger | 22 | 0.255301662543403 | 0.388827016740176 | 0.5189259257745 | -0.50679929 | 1.01740261535415 | 1.2908509635297 | 0.602420670041432 | 2.76600105692084 | 0.999950574162623 |
| ebi-fl187-GCST90094707 | prot-a-2964 | Transforming growth factor beta-2 \|\| id:prot-a-2964 | Black olive liking \|\| id:ebi-fl187-GCST90094707 | MR Egger | 22 | -0.494824581 | 0.34989858936457 | 0.1726838914067 | -1.180625816 | 0.190976653898904 | 0.609677846838784 | 0.307086498683728 | 1.21043119290243 | 0.999950574162623 |
| ebi-fl187-GCST90094707 | prot-a-2977 | Thymocyte nuclear protein 1 \|\| id:prot-a-2977 | Black olive liking \|\| id:ebi-fl187-GCST90094707 | MR Egger | 22 | 0.474794249694213 | 0.349903582091341 | 0.18992236815832 | -0.211016771 | 1.16060527059324 | 1.60768338210679 | 0.80976048610843 | 3.19186463336053 | 0.999950574162623 |
| ebi-fl187-GCST90094707 | prot-a-3015 | Transmembrane protein 70, mitochondrial \|\| id:prot-a-3015 | Black olive liking \|\| id:ebi-fl187-GCST90094707 | MR Egger | 22 | -0.688203711 | 0.34989858936457 | 0.063228191711685 | -1.374004946 | -0.002402476 | 0.502477854370734 | 0.253091310049885 | 0.997600407865649 | 0.999950574162623 |
| ebi-fl187-GCST90094707 | prot-a-3030 | Tumor necrosis factor alpha-induced protein 3 \|\| id:prot-a-3030 | Black olive liking \|\| id:ebi-fl187-GCST90094707 | MR Egger | 22 | -0.33539854 | 0.450406634951502 | 0.465139764242978 | -1.218195544 | 0.547398464906479 | 0.71505305260125 | 0.295763377672531 | 1.72874975954757 | 0.999950574162623 |
| ebi-fl187-GCST90094707 | prot-a-3032 | Tumor necrosis factor receptor superfamily member 10A \|\| id:prot-a-3032 | Black olive liking \|\| id:ebi-fl187-GCST90094707 | MR Egger | 22 | -0.097084205 | 0.349939965389584 | 0.784296565526893 | -0.782966537 | 0.588798126941996 | 0.907479588396103 | 0.457048147807319 | 1.8018215527322 | 0.999950574162623 |
| ebi-fl187-GCST90094707 | prot-a-3081 | Tumor protein p53-inducible protein 11 \|\| id:prot-a-3081 | Black olive liking \|\| id:ebi-fl187-GCST90094707 | MR Egger | 22 | 0.468212113508714 | 0.349943820698913 | 0.195923498109417 | -0.217677775 | 1.15410200207858 | 1.59713614091105 | 0.804384592688042 | 3.17117443047981 | 0.999950574162623 |
| ebi-fl187-GCST90094707 | prot-a-315 | Complement C4 \|\| id:prot-a-315 | Black olive liking \|\| id:ebi-fl187-GCST90094707 | MR Egger | 21 | 0.53758816564088 | 0.40099809061827 | 0.195852410510086 | -0.248368092 | 1.32354442325269 | 1.71187312482287 | 0.78007275190814 | 3.75671318902304 | 0.999950574162623 |
| ebi-fl187-GCST90094707 | prot-a-3157 | UDP-glucuronosyltransferase 1-8 \|\| id:prot-a-3157 | Black olive liking \|\| id:ebi-fl187-GCST90094707 | MR Egger | 22 | 0.152604505987455 | 0.408912863927962 | 0.712929044751988 | -0.648864707 | 0.954073719286261 | 1.16486419101634 | 0.522638788071521 | 2.5962645989575 | 0.999950574162623 |
| ebi-fl187-GCST90094707 | prot-a-317 | Glutaredoxin-like protein C5orf63 \|\| id:prot-a-317 | Black olive liking \|\| id:ebi-fl187-GCST90094707 | MR Egger | 22 | -0.027609185 | 0.349902427117658 | 0.93789203008172 | -0.713417943 | 0.658199571752683 | 0.972768464642935 | 0.489966654345852 | 1.93131201360455 | 0.999950574162623 |
| ebi-fl187-GCST90094707 | prot-a-3192 | Guanine nucleotide exchange factor VAV3 \|\| id:prot-a-3192 | Black olive liking \|\| id:ebi-fl187-GCST90094707 | MR Egger | 22 | 0.171705257356559 | 0.34994497061527 | 0.629004620761191 | -0.514186885 | 0.857597399762489 | 1.18732782549342 | 0.597986628858807 | 2.35748977846092 | 0.999950574162623 |
| ebi-fl187-GCST90094707 | prot-a-3233 | Serine/threonine-protein kinase WNK3 \|\| id:prot-a-3233 | Black olive liking \|\| id:ebi-fl187-GCST90094707 | MR Egger | 22 | -0.179403 | 0.349939965389584 | 0.613799427343729 | -0.865285332 | 0.506479332489189 | 0.83576901687815 | 0.420931430352095 | 1.65943856696372 | 0.999950574162623 |
| ebi-fl187-GCST90094707 | prot-a-3250 | 14-3-3 protein theta \|\| id:prot-a-3250 | Black olive liking \|\| id:ebi-fl187-GCST90094707 | MR Egger | 22 | 0.0642680387150965 | 0.349943820698913 | 0.856135285220255 | -0.62162185 | 0.750157927284966 | 1.06637819109824 | 0.537072679608057 | 2.11733437507904 | 0.999950574162623 |
| ebi-fl187-GCST90094707 | prot-a-3278 | E3 ubiquitin-protein ligase ZNRF3 \|\| id:prot-a-3278 | Black olive liking \|\| id:ebi-fl187-GCST90094707 | MR Egger | 22 | -0.053069236 | 0.349943820698913 | 0.880981718620145 | -0.738959125 | 0.632820652533247 | 0.948314352682061 | 0.477610790203055 | 1.88291414253949 | 0.999950574162623 |
| ebi-fl187-GCST90094707 | prot-a-355 | Calpain-3 \|\| id:prot-a-355 | Black olive liking \|\| id:ebi-fl187-GCST90094707 | MR Egger | 22 | 0.168681397182114 | 0.349902427117658 | 0.634981838333722 | -0.51712736 | 0.854490154332724 | 1.1837429350033 | 0.596230846856245 | 2.35017584809408 | 0.999950574162623 |
| ebi-fl187-GCST90094707 | prot-a-385 | Coiled-coil domain-containing protein 90B, mitochondrial \|\| id:prot-a-385 | Black olive liking \|\| id:ebi-fl187-GCST90094707 | MR Egger | 22 | 0.10507265983587 | 0.381284681571793 | 0.785701376830055 | -0.642245316 | 0.852390635716585 | 1.1107913173384 | 0.526109814066436 | 2.34524678628892 | 0.999950574162623 |
| ebi-fl187-GCST90094707 | prot-a-390 | C-C motif chemokine 14 \|\| id:prot-a-390 | Black olive liking \|\| id:ebi-fl187-GCST90094707 | MR Egger | 22 | -0.627894979 | 0.387721584845169 | 0.121012694284559 | -1.387829286 | 0.13203932681362 | 0.533714098490446 | 0.249616563183706 | 1.14115319630386 | 0.999950574162623 |
| ebi-fl187-GCST90094707 | prot-a-40 | Pituitary adenylate cyclase-activating polypeptide 27 \|\| id:prot-a-40 | Black olive liking \|\| id:ebi-fl187-GCST90094707 | MR Egger | 22 | -0.59132541 | 0.415279688251623 | 0.169884853518686 | -1.405273599 | 0.222622778863626 | 0.553593060428962 | 0.245299933434164 | 1.24934920390982 | 0.999950574162623 |
| ebi-fl187-GCST90094707 | prot-a-401 | C-C motif chemokine 24 \|\| id:prot-a-401 | Black olive liking \|\| id:ebi-fl187-GCST90094707 | MR Egger | 22 | 0.00491479108514667 | 0.364188560822844 | 0.989366443184344 | -0.708894788 | 0.71872437029792 | 1.00492688848146 | 0.492187868845676 | 2.05181418542778 | 0.999950574162623 |
| ebi-fl187-GCST90094707 | prot-a-412 | Cerebral cavernous malformations 2 protein \|\| id:prot-a-412 | Black olive liking \|\| id:ebi-fl187-GCST90094707 | MR Egger | 22 | 0.0249593972083573 | 0.349777755810135 | 0.943821684478954 | -0.660605004 | 0.710523798596222 | 1.02527349071337 | 0.516538731846636 | 2.03505693948946 | 0.999950574162623 |
| ebi-fl187-GCST90094707 | prot-a-44 | Adhesion G protein-coupled receptor F5 \|\| id:prot-a-44 | Black olive liking \|\| id:ebi-fl187-GCST90094707 | MR Egger | 22 | 0.0939299984367062 | 0.394464629528172 | 0.814210488171579 | -0.679220675 | 0.867080672311923 | 1.09848284770912 | 0.507011965317079 | 2.37995283988317 | 0.999950574162623 |
| ebi-fl187-GCST90094707 | prot-a-48 | Adrenomedullin \|\| id:prot-a-48 | Black olive liking \|\| id:ebi-fl187-GCST90094707 | MR Egger | 22 | 0.097178884008326 | 0.349934582959342 | 0.784088575311716 | -0.588692899 | 0.783050666608637 | 1.10205749645179 | 0.555052320453261 | 2.18813737143518 | 0.999950574162623 |
| ebi-fl187-GCST90094707 | prot-a-502 | Carcinoembryonic antigen-related cell adhesion molecule 1 \|\| id:prot-a-502 | Black olive liking \|\| id:ebi-fl187-GCST90094707 | MR Egger | 22 | -0.096038009 | 0.355824468739407 | 0.790000351834948 | -0.793453968 | 0.601377949831494 | 0.908429487009025 | 0.452279934097432 | 1.82463131934945 | 0.999950574162623 |
| ebi-fl187-GCST90094707 | prot-a-518 | Complement factor B \|\| id:prot-a-518 | Black olive liking \|\| id:ebi-fl187-GCST90094707 | MR Egger | 22 | 0.617052994884793 | 0.389704051149255 | 0.129018478883384 | -0.146766945 | 1.38087293513733 | 1.85345783571992 | 0.863495195523247 | 3.97837297370244 | 0.999950574162623 |
| ebi-fl187-GCST90094707 | prot-a-525 | Properdin \|\| id:prot-a-525 | Black olive liking \|\| id:ebi-fl187-GCST90094707 | MR Egger | 22 | 0.801553609384834 | 0.452126393815969 | 0.0914849273226056 | -0.084614122 | 1.68772134126413 | 2.22900123705322 | 0.918866786023268 | 5.40714561714387 | 0.999950574162623 |
| ebi-fl187-GCST90094707 | prot-a-533 | Chondroadherin \|\| id:prot-a-533 | Black olive liking \|\| id:ebi-fl187-GCST90094707 | MR Egger | 22 | -0.258738903 | 0.385457297388324 | 0.509739315283438 | -1.014235206 | 0.496757400188553 | 0.772024570267413 | 0.362679699204433 | 1.64338378575919 | 0.999950574162623 |
| ebi-fl187-GCST90094707 | prot-a-535 | Coiled-coil-helix-coiled-coil-helix domain-containing protein 2 \|\| id:prot-a-535 | Black olive liking \|\| id:ebi-fl187-GCST90094707 | MR Egger | 22 | -0.420798774 | 0.34994497061527 | 0.243224568168786 | -1.106690916 | 0.265093368600174 | 0.656522197581377 | 0.330651305623628 | 1.30355268098563 | 0.999950574162623 |
| ebi-fl187-GCST90094707 | prot-a-591 | Caseinolytic peptidase B protein homolog \|\| id:prot-a-591 | Black olive liking \|\| id:ebi-fl187-GCST90094707 | MR Egger | 22 | 0.689903498393439 | 0.419253840737235 | 0.115481895700987 | -0.131834029 | 1.51164102623842 | 1.9935231457741 | 0.876486453945573 | 4.53416537682605 | 0.999950574162623 |
| ebi-fl187-GCST90094707 | prot-a-605 | Ciliary neurotrophic factor receptor subunit alpha \|\| id:prot-a-605 | Black olive liking \|\| id:ebi-fl187-GCST90094707 | MR Egger | 22 | -0.642139008 | 0.356463272858944 | 0.0867341725860016 | -1.340807023 | 0.0565290067508653 | 0.526165746717426 | 0.261634438386247 | 1.05815730806048 | 0.999950574162623 |
| ebi-fl187-GCST90094707 | prot-a-606 | Ciliary neurotrophic factor receptor subunit alpha \|\| id:prot-a-606 | Black olive liking \|\| id:ebi-fl187-GCST90094707 | MR Egger | 22 | -0.357334312 | 0.34994497061527 | 0.319393195934258 | -1.043226454 | 0.328557830377758 | 0.69953859447215 | 0.352316114288982 | 1.38896356229305 | 0.999950574162623 |
| ebi-fl187-GCST90094707 | prot-a-707 | Cancer/testis antigen 55 \|\| id:prot-a-707 | Black olive liking \|\| id:ebi-fl187-GCST90094707 | MR Egger | 22 | 0.40499341917641 | 0.349772259280521 | 0.260554946913164 | -0.280560209 | 1.09054704736623 | 1.49929263344397 | 0.755360463164799 | 2.97590158648393 | 0.999950574162623 |
| ebi-fl187-GCST90094707 | prot-a-719 | Dipeptidyl peptidase 1 \|\| id:prot-a-719 | Black olive liking \|\| id:ebi-fl187-GCST90094707 | MR Egger | 22 | 0.103278024677434 | 0.349802377527351 | 0.770848607348718 | -0.582334635 | 0.788890684631042 | 1.10879963988835 | 0.558592732770137 | 2.20095351996365 | 0.999950574162623 |
| ebi-fl187-GCST90094707 | prot-a-752 | Cytochrome b5 \|\| id:prot-a-752 | Black olive liking \|\| id:ebi-fl187-GCST90094707 | MR Egger | 22 | 0.294750886787732 | 0.371169912288984 | 0.43645130969334 | -0.432742141 | 1.02224391487414 | 1.34279180983658 | 0.648727750334729 | 2.77942456390042 | 0.999950574162623 |
| ebi-fl187-GCST90094707 | prot-a-755 | Cytoglobin \|\| id:prot-a-755 | Black olive liking \|\| id:ebi-fl187-GCST90094707 | MR Egger | 22 | -1.049054838 | 0.390119300203019 | 0.0141115564305779 | -1.813688667 | -0.28442101 | 0.350268653158043 | 0.163051583200776 | 0.752449789058939 | 0.999950574162623 |
| ebi-fl187-GCST90094707 | prot-a-807 | Desmin \|\| id:prot-a-807 | Black olive liking \|\| id:ebi-fl187-GCST90094707 | MR Egger | 22 | 0.39816454868183 | 0.349759835189643 | 0.268409302696406 | -0.287364728 | 1.08369382565353 | 1.48908903738712 | 0.750238045890671 | 2.95557679780698 | 0.999950574162623 |
| ebi-fl187-GCST90094707 | prot-a-82 | Ameloblastin \|\| id:prot-a-82 | Black olive liking \|\| id:ebi-fl187-GCST90094707 | MR Egger | 22 | -0.354211886 | 0.443541659705917 | 0.433904457832846 | -1.223553539 | 0.515129767195462 | 0.701726265751126 | 0.294182916879531 | 1.67385569926437 | 0.999950574162623 |
| ebi-fl187-GCST90094707 | prot-a-84 | Muellerian-inhibiting factor \|\| id:prot-a-84 | Black olive liking \|\| id:ebi-fl187-GCST90094707 | MR Egger | 22 | 0.135773359647544 | 0.362758480937139 | 0.712134589151039 | -0.575233263 | 0.846779982284336 | 1.14542226526893 | 0.562573625894846 | 2.33212526393665 | 0.999950574162623 |
| ebi-fl187-GCST90094707 | prot-a-858 | Inactive dipeptidyl peptidase 10 \|\| id:prot-a-858 | Black olive liking \|\| id:ebi-fl187-GCST90094707 | MR Egger | 22 | -0.022431503 | 0.366342321575861 | 0.951783012962012 | -0.740462454 | 0.695599446810707 | 0.97781821204623 | 0.476893323404431 | 2.00491055102996 | 0.999950574162623 |
| ebi-fl187-GCST90094707 | prot-a-863 | Dihydropyrimidinase-related protein 3 \|\| id:prot-a-863 | Black olive liking \|\| id:ebi-fl187-GCST90094707 | MR Egger | 21 | -0.013932672 | 0.357942232016779 | 0.969356784463051 | -0.715499447 | 0.687634102796729 | 0.986163938516627 | 0.488947847409603 | 1.98900418272201 | 0.999950574162623 |
| ebi-fl187-GCST90094707 | prot-a-864 | Dihydropyrimidinase-related protein 5 \|\| id:prot-a-864 | Black olive liking \|\| id:ebi-fl187-GCST90094707 | MR Egger | 22 | -0.259762911 | 0.349939487659149 | 0.466530014432351 | -0.945644307 | 0.426118484495117 | 0.771234415081232 | 0.3884292225427 | 1.53130219995305 | 0.999950574162623 |
| ebi-fl187-GCST90094707 | prot-a-884 | Endothelin-converting enzyme 1 \|\| id:prot-a-884 | Black olive liking \|\| id:ebi-fl187-GCST90094707 | MR Egger | 22 | -0.14153091 | 0.349903582091341 | 0.690148864660293 | -0.827341931 | 0.544280110692575 | 0.868028344234886 | 0.437209877147842 | 1.72336730201631 | 0.999950574162623 |
| ebi-fl187-GCST90094707 | prot-a-893 | Early endosome antigen 1 \|\| id:prot-a-893 | Black olive liking \|\| id:ebi-fl187-GCST90094707 | MR Egger | 22 | -0.173648439 | 0.349903582091341 | 0.625110523996533 | -0.859459459 | 0.512162582307093 | 0.840592365553476 | 0.423390880396874 | 1.66889642111433 | 0.999950574162623 |
| ebi-fl187-GCST90094707 | prot-a-897 | Ephrin-A2 \|\| id:prot-a-897 | Black olive liking \|\| id:ebi-fl187-GCST90094707 | MR Egger | 22 | 0.0724932947563679 | 0.36468380920457 | 0.844438598730737 | -0.642286971 | 0.787273560797326 | 1.07518559668461 | 0.526087899292434 | 2.19739718186418 | 0.999950574162623 |
| ebi-fl187-GCST90094707 | prot-a-90 | Anaphase-promoting complex subunit 10 \|\| id:prot-a-90 | Black olive liking \|\| id:ebi-fl187-GCST90094707 | MR Egger | 22 | 0.108471023697622 | 0.424705257857967 | 0.801019979997691 | -0.723951282 | 0.940893329099237 | 1.1145726118774 | 0.484832755417677 | 2.5622693460078 | 0.999950574162623 |
| ebi-fl187-GCST90094707 | prot-a-914 | Histone-lysine N-methyltransferase EHMT2 \|\| id:prot-a-914 | Black olive liking \|\| id:ebi-fl187-GCST90094707 | MR Egger | 22 | -0.471917341 | 0.419115437576919 | 0.273502637626916 | -1.293383598 | 0.349548916940801 | 0.623805074071265 | 0.27434095129512 | 1.41842757561356 | 0.999950574162623 |
| ebi-fl187-GCST90094707 | prot-a-932 | RNA polymerase II elongation factor ELL \|\| id:prot-a-932 | Black olive liking \|\| id:ebi-fl187-GCST90094707 | MR Egger | 22 | 0.103358314347739 | 0.368563251188635 | 0.782023493459847 | -0.619025658 | 0.825742286677464 | 1.10888866861987 | 0.538468834893255 | 2.28357520382273 | 0.999950574162623 |
| ebi-fl187-GCST90094707 | prot-a-991 | Estrogen receptor \|\| id:prot-a-991 | Black olive liking \|\| id:ebi-fl187-GCST90094707 | MR Egger | 22 | 0.0524253574326593 | 0.43876821277707 | 0.906084736466218 | -0.80756034 | 0.912411054475717 | 1.05382389902142 | 0.445944693788253 | 2.4903195970665 | 0.999950574162623 |
| ebi-fl187-GCST90094707 | prot-a-992 | Persulfide dioxygenase ETHE1, mitochondrial \|\| id:prot-a-992 | Black olive liking \|\| id:ebi-fl187-GCST90094707 | MR Egger | 22 | 0.198578464792824 | 0.405891372026678 | 0.629994005069782 | -0.596968624 | 0.994125553965113 | 1.2196677246351 | 0.550477814438246 | 2.70236023959407 | 0.999950574162623 |
| ebi-fl187-GCST90094707 | prot-a-993 | Ethanolamine kinase 1 \|\| id:prot-a-993 | Black olive liking \|\| id:ebi-fl187-GCST90094707 | MR Egger | 22 | 0.331748865283445 | 0.349903582091341 | 0.354387198258394 | -0.354062156 | 1.01755988618247 | 1.39340287273333 | 0.701831343240492 | 2.76643610240729 | 0.999950574162623 |
| ebi-fl187-GCST90094707 | prot-b-10 | coagulation factor III, tissue factor \|\| id:prot-b-10 | Black olive liking \|\| id:ebi-fl187-GCST90094707 | MR Egger | 16 | 0.703075245002926 | 0.506482918256672 | 0.186785537349441 | -0.289631275 | 1.695781765 | 2.01995502235217 | 0.748539522099595 | 5.45090562603971 | 0.999950574162623 |
| ebi-fl187-GCST90094707 | prot-b-28 | prolactin \|\| id:prot-b-28 | Black olive liking \|\| id:ebi-fl187-GCST90094707 | MR Egger | 16 | 0.0469049244457683 | 0.506480368252695 | 0.927525941972182 | -0.945796597 | 1.03960644622105 | 1.04802236303577 | 0.388370073082471 | 2.82810378437639 | 0.999950574162623 |
| ebi-fl187-GCST90094707 | prot-b-54 | matrix metallopeptidase 7 \|\| id:prot-b-54 | Black olive liking \|\| id:ebi-fl187-GCST90094707 | MR Egger | 16 | 0.960864250202319 | 0.670660772285341 | 0.173890648241002 | -0.353630863 | 2.27535936388159 | 2.61395460828487 | 0.702134102865708 | 9.73141550351466 | 0.999950574162623 |
| ebi-fl187-GCST90094707 | ukb-rsfMRI191-edge-pheno716 | Net100_Pair10_33 (OccipitalPrecuneus)&(Temporal\|Frontal\|Supp_Motor_Area) (Default_mode\|Central_executive)&(Default_mode\|Salience) \|\| id:ukb-rsfMRI191-edge-pheno716 | Black olive liking \|\| id:ebi-fl187-GCST90094707 | MR Egger | 21 | -0.096089872 | 0.112997184480487 | 0.405706376374304 | -0.317564354 | 0.125384609114446 | 0.90838237383487 | 0.727919834678204 | 1.13358435611067 | 0.999950574162623 |
| ebi-fl187-GCST90094707 | ukb-rsfMRI191-edge-pheno903 | Net100_Pair27_38 (Precentral\|Frontal\|Supp_Motor_Area)&(Subcortical) (Attention\|Salience\|Motor)&(Subcortical-cerebellum) \|\| id:ukb-rsfMRI191-edge-pheno903 | Black olive liking \|\| id:ebi-fl187-GCST90094707 | MR Egger | 21 | 0.145031580161314 | 0.153888115173148 | 0.357793266886547 | -0.156589126 | 0.446652285900683 | 1.1560760787606 | 0.855055307008292 | 1.56307070306218 | 0.999950574162623 |
| ebi-fl187-GCST90094707 | ukb-rsfMRI191-node-pheno23 | Net100_Node2 RolandicOper\|SupraMarginal\|Insula Salience\|Motor \|\| id:ukb-rsfMRI191-node-pheno23 | Black olive liking \|\| id:ebi-fl187-GCST90094707 | MR Egger | 21 | -0.060709062 | 0.137687931531969 | 0.664248601767682 | -0.330577408 | 0.209159283674917 | 0.941097000709341 | 0.718508740988383 | 1.23264132253394 | 0.999950574162623 |
| ebi-fl187-GCST90094707 | ukb-rsfMRI191-node-pheno55 | Net100_Node34 Parietal\|Postcentral\|Precuneus Attention \|\| id:ukb-rsfMRI191-node-pheno55 | Black olive liking \|\| id:ebi-fl187-GCST90094707 | MR Egger | 21 | 0.098032122377646 | 0.13378966323207 | 0.472664818170735 | -0.164195618 | 0.360259862312503 | 1.10299821546467 | 0.848576009277204 | 1.43370193125601 | 0.999950574162623 |
| ebi-fl187-GCST90094707 | ebi-a-GCST90001414 | IgD- CD24- B cell Absolute Count \|\| id:ebi-a-GCST90001414 | Black olive liking \|\| id:ebi-fl187-GCST90094707 | Weighted median | 20 | -0.113185394 | 0.109725105612878 | 0.302289544611098 | -0.328246601 | 0.101875812649651 | 0.892985090376832 | 0.720185399111752 | 1.10724595724772 | 0.999332001504091 |
| ebi-fl187-GCST90094707 | ebi-a-GCST90001422 | CD20- CD38- B cell %B cell \|\| id:ebi-a-GCST90001422 | Black olive liking \|\| id:ebi-fl187-GCST90094707 | Weighted median | 20 | -0.120014457 | 0.118739962929743 | 0.312144002462779 | -0.352744785 | 0.112715869940268 | 0.886907614244527 | 0.70275652467835 | 1.1193138570502 | 0.999332001504091 |
| ebi-fl187-GCST90094707 | ebi-a-GCST90001444 | CD20- CD38- B cell %lymphocyte \|\| id:ebi-a-GCST90001444 | Black olive liking \|\| id:ebi-fl187-GCST90094707 | Weighted median | 20 | -0.111183701 | 0.125904601059703 | 0.377193623821151 | -0.357956719 | 0.135589316871813 | 0.894774362701705 | 0.69910333204576 | 1.14521147797338 | 0.999332001504091 |
| ebi-fl187-GCST90094707 | ebi-a-GCST90001459 | Myeloid Dendritic Cell %Dendritic Cell \|\| id:ebi-a-GCST90001459 | Black olive liking \|\| id:ebi-fl187-GCST90094707 | Weighted median | 20 | -0.066615884 | 0.123520026881915 | 0.589671302629788 | -0.308715137 | 0.175483368479209 | 0.93555449358145 | 0.734389940829024 | 1.19182216667128 | 0.999332001504091 |
| ebi-fl187-GCST90094707 | ebi-a-GCST90001476 | HLA DR++ monocyte %leukocyte \|\| id:ebi-a-GCST90001476 | Black olive liking \|\| id:ebi-fl187-GCST90094707 | Weighted median | 20 | 0.0238112204564709 | 0.117910498393086 | 0.839961148276542 | -0.207293356 | 0.254915797306919 | 1.0240969710825 | 0.812781180454809 | 1.29035296510371 | 0.999332001504091 |
| ebi-fl187-GCST90094707 | ebi-a-GCST90001478 | CD4 regulatory T cell %CD4+ T cell \|\| id:ebi-a-GCST90001478 | Black olive liking \|\| id:ebi-fl187-GCST90094707 | Weighted median | 20 | 0.100314067080412 | 0.123250323469914 | 0.415699317737672 | -0.141256567 | 0.341884701081442 | 1.10551807039095 | 0.868266514651773 | 1.40759799363112 | 0.999332001504091 |
| ebi-fl187-GCST90094707 | ebi-a-GCST90001485 | CD39+ resting CD4 regulatory T cell %CD4 regulatory T cell \|\| id:ebi-a-GCST90001485 | Black olive liking \|\| id:ebi-fl187-GCST90094707 | Weighted median | 19 | -0.020244123 | 0.118924879003576 | 0.864832297205259 | -0.253336886 | 0.212848640081689 | 0.979959413701612 | 0.776206345022383 | 1.23719737497729 | 0.999332001504091 |
| ebi-fl187-GCST90094707 | ebi-a-GCST90001515 | Immature Myeloid-Derived Suppressor Cells Absolute Count \|\| id:ebi-a-GCST90001515 | Black olive liking \|\| id:ebi-fl187-GCST90094707 | Weighted median | 20 | -0.212064289 | 0.169508838401364 | 0.210915683024972 | -0.544301612 | 0.12017303408443 | 0.80891269155936 | 0.580246879063369 | 1.12769196384484 | 0.999332001504091 |
| ebi-fl187-GCST90094707 | ebi-a-GCST90001517 | CD33+ HLA DR+ Absolute Count \|\| id:ebi-a-GCST90001517 | Black olive liking \|\| id:ebi-fl187-GCST90094707 | Weighted median | 20 | -0.071540514 | 0.152041534163474 | 0.637974475002261 | -0.369541921 | 0.226460892876169 | 0.930958559897298 | 0.691050813979604 | 1.25415356253619 | 0.999332001504091 |
| ebi-fl187-GCST90094707 | ebi-a-GCST90001518 | CD33+ HLA DR+ CD14- Absolute Count \|\| id:ebi-a-GCST90001518 | Black olive liking \|\| id:ebi-fl187-GCST90094707 | Weighted median | 20 | -0.14850368 | 0.15043827082977 | 0.32357386292337 | -0.443362691 | 0.146355330769967 | 0.861996834967707 | 0.641874362885834 | 1.15760744852572 | 0.999332001504091 |
| ebi-fl187-GCST90094707 | ebi-a-GCST90001520 | CD33+ HLA DR+ CD14dim Absolute Count \|\| id:ebi-a-GCST90001520 | Black olive liking \|\| id:ebi-fl187-GCST90094707 | Weighted median | 20 | -0.14829276 | 0.159641332212497 | 0.352934657374712 | -0.461189771 | 0.164604250808114 | 0.862178666280909 | 0.630533008870831 | 1.17892646718232 | 0.999332001504091 |
| ebi-fl187-GCST90094707 | ebi-a-GCST90001531 | CD33dim HLA DR- Absolute Count \|\| id:ebi-a-GCST90001531 | Black olive liking \|\| id:ebi-fl187-GCST90094707 | Weighted median | 20 | -0.106005219 | 0.154875416285906 | 0.493688034855846 | -0.409561035 | 0.197550596975629 | 0.899419954040631 | 0.663941633406751 | 1.21841471150953 | 0.999332001504091 |
| ebi-fl187-GCST90094707 | ebi-a-GCST90001532 | Basophil Absolute Count \|\| id:ebi-a-GCST90001532 | Black olive liking \|\| id:ebi-fl187-GCST90094707 | Weighted median | 19 | -0.062013469 | 0.154122662952527 | 0.687415885873527 | -0.364093888 | 0.240066950783797 | 0.939870227966723 | 0.694825955867914 | 1.27133426429761 | 0.999332001504091 |
| ebi-fl187-GCST90094707 | ebi-a-GCST90001588 | T/B cell \|\| id:ebi-a-GCST90001588 | Black olive liking \|\| id:ebi-fl187-GCST90094707 | Weighted median | 20 | 0.122726281360636 | 0.112999615253366 | 0.277444934064806 | -0.098752965 | 0.344205527257234 | 1.13057491916265 | 0.905966486233164 | 1.41086857766023 | 0.999332001504091 |
| ebi-fl187-GCST90094707 | ebi-a-GCST90001624 | HLA DR+ CD4+ T cell Absolute Count \|\| id:ebi-a-GCST90001624 | Black olive liking \|\| id:ebi-fl187-GCST90094707 | Weighted median | 20 | 0.0024893047512699 | 0.10781157877001 | 0.981578960383676 | -0.20882139 | 0.21379999914049 | 1.00249240564283 | 0.811540172183414 | 1.2383749539689 | 0.999332001504091 |
| ebi-fl187-GCST90094707 | ebi-a-GCST90001631 | CD8+ Natural Killer T %T cell \|\| id:ebi-a-GCST90001631 | Black olive liking \|\| id:ebi-fl187-GCST90094707 | Weighted median | 20 | 0.0726391547878064 | 0.113775524828934 | 0.523185533900218 | -0.150360874 | 0.295639183452518 | 1.07534243472747 | 0.860397425438962 | 1.34398513725868 | 0.999332001504091 |
| ebi-fl187-GCST90094707 | ebi-a-GCST90001632 | CD8+ Natural Killer T %lymphocyte \|\| id:ebi-a-GCST90001632 | Black olive liking \|\| id:ebi-fl187-GCST90094707 | Weighted median | 20 | 0.0385660639675818 | 0.120231727424299 | 0.748388901411195 | -0.197088122 | 0.274220249719207 | 1.03931938765234 | 0.821118271717374 | 1.31550450983245 | 0.999332001504091 |
| ebi-fl187-GCST90094707 | ebi-a-GCST90001646 | Natural Killer %CD3- lymphocyte \|\| id:ebi-a-GCST90001646 | Black olive liking \|\| id:ebi-fl187-GCST90094707 | Weighted median | 20 | 0.0507753427067177 | 0.109864937159443 | 0.643965519488802 | -0.164559934 | 0.266110619539226 | 1.05208650782419 | 0.848266915284775 | 1.30487939586104 | 0.999332001504091 |
| ebi-fl187-GCST90094707 | ebi-a-GCST90001684 | CD127- CD8+ T cell Absolute Count \|\| id:ebi-a-GCST90001684 | Black olive liking \|\| id:ebi-fl187-GCST90094707 | Weighted median | 20 | 0.0293983039184873 | 0.12150768985688 | 0.808821977939289 | -0.208756768 | 0.267553376037972 | 1.02983469999279 | 0.811592616769989 | 1.30676337782631 | 0.999332001504091 |
| ebi-fl187-GCST90094707 | ebi-a-GCST90001687 | CD28- CD8+ T cell Absolute Count \|\| id:ebi-a-GCST90001687 | Black olive liking \|\| id:ebi-fl187-GCST90094707 | Weighted median | 20 | -0.044533684 | 0.122796996999975 | 0.716858121842471 | -0.285215798 | 0.196148429979793 | 0.956443382578062 | 0.751851988460697 | 1.21670748779989 | 0.999332001504091 |
| ebi-fl187-GCST90094707 | ebi-a-GCST90001701 | BAFF-R on CD20- CD38- B cell \|\| id:ebi-a-GCST90001701 | Black olive liking \|\| id:ebi-fl187-GCST90094707 | Weighted median | 20 | -0.030428398 | 0.113988556191948 | 0.789513380164031 | -0.253845968 | 0.192989172110958 | 0.970029885636043 | 0.775811292483301 | 1.21286966062991 | 0.999332001504091 |
| ebi-fl187-GCST90094707 | ebi-a-GCST90001732 | CD19 on IgD- CD27- B cell \|\| id:ebi-a-GCST90001732 | Black olive liking \|\| id:ebi-fl187-GCST90094707 | Weighted median | 20 | -0.197217607 | 0.115262443257324 | 0.0870754011705984 | -0.423131996 | 0.0286967820171207 | 0.821011956115048 | 0.654992171352703 | 1.02911250174452 | 0.999332001504091 |
| ebi-fl187-GCST90094707 | ebi-a-GCST90001798 | CD27 on CD24+ CD27+ B cell \|\| id:ebi-a-GCST90001798 | Black olive liking \|\| id:ebi-fl187-GCST90094707 | Weighted median | 20 | -0.021011361 | 0.115377342503905 | 0.855496446526256 | -0.247150952 | 0.20512823014373 | 0.979207839565483 | 0.78102278731238 | 1.2276824807712 | 0.999332001504091 |
| ebi-fl187-GCST90094707 | ebi-a-GCST90001836 | CD66b on CD66b++ myeloid cell \|\| id:ebi-a-GCST90001836 | Black olive liking \|\| id:ebi-fl187-GCST90094707 | Weighted median | 20 | -0.052864013 | 0.174644750458225 | 0.762122656120488 | -0.395167724 | 0.289439697798151 | 0.948508988509571 | 0.673567056414481 | 1.3356788945002 | 0.999332001504091 |
| ebi-fl187-GCST90094707 | ebi-a-GCST90001943 | CD25 on activated & secreting CD4 regulatory T cell \|\| id:ebi-a-GCST90001943 | Black olive liking \|\| id:ebi-fl187-GCST90094707 | Weighted median | 20 | 0.029398807780159 | 0.129514967770076 | 0.820430285806124 | -0.224450529 | 0.283248144609508 | 1.02983521888716 | 0.798955100790758 | 1.32743451667144 | 0.999332001504091 |
| ebi-fl187-GCST90094707 | ebi-a-GCST90001960 | CD25 on CD4+ T cell \|\| id:ebi-a-GCST90001960 | Black olive liking \|\| id:ebi-fl187-GCST90094707 | Weighted median | 20 | -0.089360913 | 0.127837844631466 | 0.484540986773938 | -0.339923088 | 0.161201262908172 | 0.914515453883552 | 0.71182506851337 | 1.17492141311982 | 0.999332001504091 |
| ebi-fl187-GCST90094707 | ebi-a-GCST90001988 | HLA DR on CD14+ CD16- monocyte \|\| id:ebi-a-GCST90001988 | Black olive liking \|\| id:ebi-fl187-GCST90094707 | Weighted median | 19 | -0.052457016 | 0.118346997825645 | 0.657586692666694 | -0.284417132 | 0.179503099524838 | 0.948895107283994 | 0.752452707167763 | 1.19662261302324 | 0.999332001504091 |
| ebi-fl187-GCST90094707 | ebi-a-GCST90001991 | HLA DR on CD14+ monocyte \|\| id:ebi-a-GCST90001991 | Black olive liking \|\| id:ebi-fl187-GCST90094707 | Weighted median | 19 | -0.089284791 | 0.118209808199774 | 0.450064317889539 | -0.320976015 | 0.142406433552951 | 0.914585071325155 | 0.725440650772791 | 1.15304518956827 | 0.999332001504091 |
| ebi-fl187-GCST90094707 | ebi-a-GCST90002019 | CD14 on Monocytic Myeloid-Derived Suppressor Cells \|\| id:ebi-a-GCST90002019 | Black olive liking \|\| id:ebi-fl187-GCST90094707 | Weighted median | 20 | -0.097986649 | 0.179245994321211 | 0.584612208794482 | -0.449308798 | 0.253335499463424 | 0.906661008 | 0.638069033651279 | 1.28831543347307 | 0.999332001504091 |
| ebi-fl187-GCST90094707 | ebi-a-GCST90002037 | CD80 on plasmacytoid Dendritic Cell \|\| id:ebi-a-GCST90002037 | Black olive liking \|\| id:ebi-fl187-GCST90094707 | Weighted median | 20 | -0.094174117 | 0.130397158949793 | 0.470165446977265 | -0.349752549 | 0.161404314412374 | 0.910124280217046 | 0.704862487299744 | 1.17516000690266 | 0.999332001504091 |
| ebi-fl187-GCST90094707 | ebi-a-GCST90002042 | CD45 on CD33+ HLA DR+ CD14- \|\| id:ebi-a-GCST90002042 | Black olive liking \|\| id:ebi-fl187-GCST90094707 | Weighted median | 20 | -0.036332509 | 0.171150466315716 | 0.831885463050823 | -0.371787423 | 0.299122405298894 | 0.964319595532931 | 0.689500799193066 | 1.34867469829924 | 0.999332001504091 |
| ebi-fl187-GCST90094707 | ebi-a-GCST90002056 | CD8 on Effector Memory CD8+ T cell \|\| id:ebi-a-GCST90002056 | Black olive liking \|\| id:ebi-fl187-GCST90094707 | Weighted median | 20 | 0.049238009358248 | 0.131107479005475 | 0.707248090296166 | -0.207732649 | 0.306208668208978 | 1.05047034276077 | 0.812424209704775 | 1.3582657038506 | 0.999332001504091 |
| ebi-fl187-GCST90094707 | ebi-a-GCST90002058 | CD8 on CD8+ T cell \|\| id:ebi-a-GCST90002058 | Black olive liking \|\| id:ebi-fl187-GCST90094707 | Weighted median | 20 | -0.164577127 | 0.119642937417408 | 0.168955079643548 | -0.399077285 | 0.0699230299328389 | 0.848252330919995 | 0.670938846013106 | 1.07242563340437 | 0.999332001504091 |
| ebi-fl187-GCST90094707 | ebi-a-GCST90002063 | CD4 on CD4 regulatory T cell \|\| id:ebi-a-GCST90002063 | Black olive liking \|\| id:ebi-fl187-GCST90094707 | Weighted median | 20 | -0.121328527 | 0.128479128154101 | 0.344993757056831 | -0.373147618 | 0.130490564404386 | 0.885742921520758 | 0.688563580990378 | 1.13938718904608 | 0.999332001504091 |
| ebi-fl187-GCST90094707 | ebi-a-GCST90002066 | CD4 on activated CD4 regulatory T cell \|\| id:ebi-a-GCST90002066 | Black olive liking \|\| id:ebi-fl187-GCST90094707 | Weighted median | 20 | -0.093780296 | 0.128205811435868 | 0.464484545842256 | -0.345063686 | 0.157503094710668 | 0.910482777245814 | 0.708175251099059 | 1.17058438059606 | 0.999332001504091 |
| ebi-fl187-GCST90094707 | ebi-a-GCST90002070 | CD4 on activated & secreting CD4 regulatory T cell \|\| id:ebi-a-GCST90002070 | Black olive liking \|\| id:ebi-fl187-GCST90094707 | Weighted median | 20 | -0.065078116 | 0.132424385550524 | 0.623118078318487 | -0.324629911 | 0.194473679881724 | 0.936994266464329 | 0.722794802210462 | 1.21467151216644 | 0.999332001504091 |
| ebi-fl187-GCST90094707 | ebi-a-GCST90002081 | SSC-A on CD4+ T cell \|\| id:ebi-a-GCST90002081 | Black olive liking \|\| id:ebi-fl187-GCST90094707 | Weighted median | 20 | 0.0919427821006825 | 0.113958756331086 | 0.419777383298147 | -0.13141638 | 0.315301944509611 | 1.09630209218347 | 0.876852594215937 | 1.37067311570257 | 0.999332001504091 |
| ebi-fl187-GCST90094707 | ebi-a-GCST90002094 | CD11b on Monocytic Myeloid-Derived Suppressor Cells \|\| id:ebi-a-GCST90002094 | Black olive liking \|\| id:ebi-fl187-GCST90094707 | Weighted median | 20 | -0.279544606 | 0.165890226718904 | 0.0919658083972137 | -0.604689451 | 0.045600238091575 | 0.756127999007585 | 0.546244036034532 | 1.04665591414727 | 0.999332001504091 |
| ebi-fl187-GCST90094707 | ebi-a-GCST90002104 | HLA DR on myeloid Dendritic Cell \|\| id:ebi-a-GCST90002104 | Black olive liking \|\| id:ebi-fl187-GCST90094707 | Weighted median | 19 | -0.199013577 | 0.127682969370856 | 0.119078283074078 | -0.449272197 | 0.051245042750875 | 0.819538766202537 | 0.638092388102616 | 1.05258078897623 | 0.999332001504091 |
| ebi-fl187-GCST90094707 | ebi-a-GCST90002105 | HLA DR on plasmacytoid Dendritic Cell \|\| id:ebi-a-GCST90002105 | Black olive liking \|\| id:ebi-fl187-GCST90094707 | Weighted median | 19 | -0.236158284 | 0.137819547867168 | 0.0866146065008993 | -0.506284598 | 0.0339680296069074 | 0.789655674013609 | 0.602730811089225 | 1.03455153118028 | 0.999332001504091 |
| ebi-fl187-GCST90094707 | ebi-a-GCST90002106 | HLA DR on Dendritic Cell \|\| id:ebi-a-GCST90002106 | Black olive liking \|\| id:ebi-fl187-GCST90094707 | Weighted median | 19 | -0.206853068 | 0.130653970105657 | 0.113372916592931 | -0.46293485 | 0.0492287129501154 | 0.813139116995776 | 0.629433638859499 | 1.05046057720512 | 0.999332001504091 |
| ebi-fl187-GCST90094707 | ebi-a-GCST90002111 | HLA DR on CD33dim HLA DR+ CD11b- \|\| id:ebi-a-GCST90002111 | Black olive liking \|\| id:ebi-fl187-GCST90094707 | Weighted median | 19 | -0.086435744 | 0.161906703822073 | 0.593437410144253 | -0.403772884 | 0.23090139535883 | 0.917194482018451 | 0.667795771402874 | 1.25973501760553 | 0.999332001504091 |
| ebi-fl187-GCST90094707 | ebi-a-GCST90002112 | HLA DR on CD33- HLA DR+ \|\| id:ebi-a-GCST90002112 | Black olive liking \|\| id:ebi-fl187-GCST90094707 | Weighted median | 19 | -0.123137731 | 0.176176441902068 | 0.484586093150956 | -0.468443557 | 0.222168095466216 | 0.884141880728078 | 0.62597580625122 | 1.24878127469301 | 0.999332001504091 |
| ebi-fl187-GCST90094707 | ebi-a-GCST90002115 | HLA DR on HLA DR+ CD8+ T cell \|\| id:ebi-a-GCST90002115 | Black olive liking \|\| id:ebi-fl187-GCST90094707 | Weighted median | 20 | -0.020901077 | 0.120722859014373 | 0.862547103349096 | -0.257517881 | 0.21571572624412 | 0.97931583622317 | 0.77296780468131 | 1.24074961630892 | 0.999332001504091 |
| ebi-fl187-GCST90094707 | ebi-a-GCST90002116 | HLA DR on B cell \|\| id:ebi-a-GCST90002116 | Black olive liking \|\| id:ebi-fl187-GCST90094707 | Weighted median | 20 | -0.032574643 | 0.139594338511185 | 0.815487944035938 | -0.306179547 | 0.241030260462134 | 0.967950196409068 | 0.736254415683388 | 1.27255954296546 | 0.999332001504091 |
| ebi-fl187-GCST90094707 | ebi-cfmet338-GCST90026023 | Sphingomyelin (d18:1/24:1, d18:2/24:0) levels \|\| id:ebi-cfmet338-GCST90026023 | Black olive liking \|\| id:ebi-fl187-GCST90094707 | Weighted median | 19 | 0.0979400827069104 | 0.0740596848690899 | 0.186019011590811 | -0.0472169 | 0.243097065050327 | 1.10289670054387 | 0.953880478821753 | 1.27519239472543 | 0.999332001504091 |
| ebi-fl187-GCST90094707 | ebi-cfmet338-GCST90026046 | 1-arachidonoyl-gpc (20:4n6) levels \|\| id:ebi-cfmet338-GCST90026046 | Black olive liking \|\| id:ebi-fl187-GCST90094707 | Weighted median | 19 | 0.0531216742151205 | 0.0854802192885913 | 0.534303644801077 | -0.114419556 | 0.220662904020759 | 1.05455794982435 | 0.89188368258855 | 1.24690303371183 | 0.999332001504091 |
| ebi-fl187-GCST90094707 | ebi-cfmet338-GCST90026047 | 1-linoleoyl-gpc (18:2) levels \|\| id:ebi-cfmet338-GCST90026047 | Black olive liking \|\| id:ebi-fl187-GCST90094707 | Weighted median | 19 | 0.0209540324976833 | 0.109505942081706 | 0.848250886171058 | -0.193677614 | 0.235585678977827 | 1.02117510968941 | 0.823923482867262 | 1.26564981619438 | 0.999332001504091 |
| ebi-fl187-GCST90094707 | ebi-cfmet338-GCST90026050 | 1-myristoyl-2-palmitoyl-gpc (14:0/16:0) levels \|\| id:ebi-cfmet338-GCST90026050 | Black olive liking \|\| id:ebi-fl187-GCST90094707 | Weighted median | 19 | 0.0350631690984411 | 0.0582556283011526 | 0.547250915225869 | -0.079117862 | 0.1492442005687 | 1.03568513002923 | 0.923931021325853 | 1.16095646082368 | 0.999332001504091 |
| ebi-fl187-GCST90094707 | ebi-cfmet338-GCST90026068 | 2,3-dihydroxyisovalerate levels \|\| id:ebi-cfmet338-GCST90026068 | Black olive liking \|\| id:ebi-fl187-GCST90094707 | Weighted median | 19 | 0.109054003550277 | 0.175924135246558 | 0.535328745206611 | -0.235757302 | 0.453865308633531 | 1.11522257469365 | 0.789972375753578 | 1.57438592700145 | 0.999332001504091 |
| ebi-fl187-GCST90094707 | ebi-cfmet338-GCST90026082 | 3-hydroxyhexanoate levels \|\| id:ebi-cfmet338-GCST90026082 | Black olive liking \|\| id:ebi-fl187-GCST90094707 | Weighted median | 19 | -0.006106946 | 0.0364985791370551 | 0.867118188311641 | -0.077644161 | 0.0654302691395733 | 0.99391166352382 | 0.925293623455326 | 1.06761828877596 | 0.999332001504091 |
| ebi-fl187-GCST90094707 | ebi-cfmet338-GCST90026094 | 3-ureidopropionate levels \|\| id:ebi-cfmet338-GCST90026094 | Black olive liking \|\| id:ebi-fl187-GCST90094707 | Weighted median | 19 | 0.0274457030176397 | 0.0806850211047143 | 0.733737101048896 | -0.130696938 | 0.18558834438288 | 1.02782580575248 | 0.877483665747967 | 1.20392655522567 | 0.999332001504091 |
| ebi-fl187-GCST90094707 | ebi-cfmet338-GCST90026173 | Glycerol levels \|\| id:ebi-cfmet338-GCST90026173 | Black olive liking \|\| id:ebi-fl187-GCST90094707 | Weighted median | 18 | 0.0721928527769303 | 0.0460591193387943 | 0.117022293461442 | -0.018083021 | 0.162468726680967 | 1.07486261431684 | 0.982079495627077 | 1.17641152758039 | 0.999332001504091 |
| ebi-fl187-GCST90094707 | ebi-cfmet338-GCST90026179 | Histidine levels \|\| id:ebi-cfmet338-GCST90026179 | Black olive liking \|\| id:ebi-fl187-GCST90094707 | Weighted median | 19 | -0.045455361 | 0.0305819916726865 | 0.137188141641448 | -0.105396065 | 0.0144853424172309 | 0.955562256713885 | 0.899968006214995 | 1.01459076339432 | 0.999332001504091 |
| ebi-fl187-GCST90094707 | ebi-cfmet338-GCST90026188 | Isocitrate levels \|\| id:ebi-cfmet338-GCST90026188 | Black olive liking \|\| id:ebi-fl187-GCST90094707 | Weighted median | 19 | -0.008378568 | 0.0636539913797821 | 0.895279522512827 | -0.133140391 | 0.116383254800073 | 0.991656434074292 | 0.875342192958441 | 1.12342634817745 | 0.999332001504091 |
| ebi-fl187-GCST90094707 | ebi-cfmet338-GCST90026207 | N-acetyl-isoputreanine levels \|\| id:ebi-cfmet338-GCST90026207 | Black olive liking \|\| id:ebi-fl187-GCST90094707 | Weighted median | 19 | 0.025770843176543 | 0.042242653703574 | 0.541817517289059 | -0.057024758 | 0.108566444435548 | 1.02610578238789 | 0.944570683318159 | 1.11467897029282 | 0.999332001504091 |
| ebi-fl187-GCST90094707 | ebi-cfmet338-GCST90026213 | N-acetylglutamate levels \|\| id:ebi-cfmet338-GCST90026213 | Black olive liking \|\| id:ebi-fl187-GCST90094707 | Weighted median | 19 | -0.041922032 | 0.076577905253684 | 0.584074474972546 | -0.192014726 | 0.108170662200789 | 0.958944544553628 | 0.825294714787343 | 1.11423788745105 | 0.999332001504091 |
| ebi-fl187-GCST90094707 | ebi-cfmet338-GCST90026224 | N-acetyltaurine levels \|\| id:ebi-cfmet338-GCST90026224 | Black olive liking \|\| id:ebi-fl187-GCST90094707 | Weighted median | 19 | 0.0249564650616494 | 0.0553390310810183 | 0.652008276865379 | -0.083508036 | 0.133420965980445 | 1.02527048446549 | 0.919883694587575 | 1.14273094794597 | 0.999332001504091 |
| ebi-fl187-GCST90094707 | ebi-cfmet338-GCST90026250 | Phenyllactate (pla) levels \|\| id:ebi-cfmet338-GCST90026250 | Black olive liking \|\| id:ebi-fl187-GCST90094707 | Weighted median | 19 | 0.0230814220403101 | 0.0925229338394687 | 0.802999536669343 | -0.158263528 | 0.204426372365669 | 1.02334985938864 | 0.853624798045866 | 1.22682112458321 | 0.999332001504091 |
| ebi-fl187-GCST90094707 | ebi-cfmet338-GCST90026251 | Pipecolate levels \|\| id:ebi-cfmet338-GCST90026251 | Black olive liking \|\| id:ebi-fl187-GCST90094707 | Weighted median | 19 | -0.035521181 | 0.131417388132613 | 0.786934960557138 | -0.293099262 | 0.222056899501658 | 0.965102291951911 | 0.745948092738901 | 1.24864242297466 | 0.999332001504091 |
| ebi-fl187-GCST90094707 | ebi-cfmet338-GCST90026278 | Trigonelline (n'-methylnicotinate) levels \|\| id:ebi-cfmet338-GCST90026278 | Black olive liking \|\| id:ebi-fl187-GCST90094707 | Weighted median | 19 | 0.0517304492627512 | 0.140664255234914 | 0.713053455013681 | -0.223971491 | 0.327432389523183 | 1.05309184256981 | 0.799337922371215 | 1.38740124526714 | 0.999332001504091 |
| ebi-fl187-GCST90094707 | ebi-cfmet338-GCST90026299 | 1-carboxyethylvaline levels \|\| id:ebi-cfmet338-GCST90026299 | Black olive liking \|\| id:ebi-fl187-GCST90094707 | Weighted median | 19 | -0.034939545 | 0.0889336205024358 | 0.694413889696664 | -0.209249442 | 0.139370350786523 | 0.965663793313016 | 0.811192865172228 | 1.14954975783435 | 0.999332001504091 |
| ebi-fl187-GCST90094707 | ebi-gm430-GCST90011306 | C_Erysipelotrichia abundance \|\| id:ebi-gm430-GCST90011306 | Black olive liking \|\| id:ebi-fl187-GCST90094707 | Weighted median | 18 | 0.12976970930133 | 0.076886044157409 | 0.0914460212929144 | -0.020926937 | 0.280466355849852 | 1.13856615193645 | 0.979290511616294 | 1.32374700557019 | 0.999332001504091 |
| ebi-fl187-GCST90094707 | ebi-gm430-GCST90011312 | F_Erysipelotrichaceae abundance \|\| id:ebi-gm430-GCST90011312 | Black olive liking \|\| id:ebi-fl187-GCST90094707 | Weighted median | 18 | 0.12976970930133 | 0.0782597939866519 | 0.097278860832424 | -0.023619487 | 0.283158905515168 | 1.13856615193645 | 0.976657269934876 | 1.32731606290281 | 0.999332001504091 |
| ebi-fl187-GCST90094707 | ebi-gm430-GCST90011352 | O_Erysipelotrichales abundance \|\| id:ebi-gm430-GCST90011352 | Black olive liking \|\| id:ebi-fl187-GCST90094707 | Weighted median | 18 | 0.12976970930133 | 0.0774948142024875 | 0.0940206100881327 | -0.022120127 | 0.281659545138206 | 1.13856615193645 | 0.978122729498751 | 1.32532742900237 | 0.999332001504091 |
| ebi-fl187-GCST90094707 | ebi-gm430-GCST90011362 | OTU97_11 (Parabacteroides) abundance \|\| id:ebi-gm430-GCST90011362 | Black olive liking \|\| id:ebi-fl187-GCST90094707 | Weighted median | 18 | 0.0933825612406905 | 0.101695822144116 | 0.358486079891654 | -0.10594125 | 0.292706372643158 | 1.09788166190984 | 0.899477490680725 | 1.34004925753697 | 0.999332001504091 |
| ebi-fl187-GCST90094707 | ebi-gm430-GCST90011384 | OTU97_235 (Phascolarctobacterium) abundance \|\| id:ebi-gm430-GCST90011384 | Black olive liking \|\| id:ebi-fl187-GCST90094707 | Weighted median | 18 | -0.005536082 | 0.33021376079621 | 0.986623975629772 | -0.652755053 | 0.641682889323996 | 0.994479214025078 | 0.520609492407348 | 1.89967513376439 | 0.999332001504091 |
| ebi-fl187-GCST90094707 | ebi-gm430-GCST90011390 | OTU97_3 (Bacteroides) abundance \|\| id:ebi-gm430-GCST90011390 | Black olive liking \|\| id:ebi-fl187-GCST90094707 | Weighted median | 18 | -0.043352119 | 0.0716612043918206 | 0.545205972290475 | -0.18380808 | 0.0971038412676631 | 0.957574150318093 | 0.832095484311629 | 1.10197479813989 | 0.999332001504091 |
| ebi-fl187-GCST90094707 | ebi-gm430-GCST90011398 | OTU97_4 (Alistipes) abundance \|\| id:ebi-gm430-GCST90011398 | Black olive liking \|\| id:ebi-fl187-GCST90094707 | Weighted median | 18 | 0.110843525625823 | 0.0882232098655425 | 0.208971455041401 | -0.062073966 | 0.283761016962287 | 1.11722007686426 | 0.939813370256401 | 1.32811549574791 | 0.999332001504091 |
| ebi-fl187-GCST90094707 | ebi-gm430-GCST90011421 | OTU99_108 (Cytophagales) abundance \|\| id:ebi-gm430-GCST90011421 | Black olive liking \|\| id:ebi-fl187-GCST90094707 | Weighted median | 18 | -0.086041989 | 0.299127229656538 | 0.773619680689066 | -0.672331359 | 0.50024738147382 | 0.917555703483256 | 0.51051699104883 | 1.64912918425104 | 0.999332001504091 |
| ebi-fl187-GCST90094707 | ebi-gm430-GCST90011428 | OTU99_123 (Phascolarctobacterium) abundance \|\| id:ebi-gm430-GCST90011428 | Black olive liking \|\| id:ebi-fl187-GCST90094707 | Weighted median | 18 | 0.0652219145813241 | 0.178150800643971 | 0.714286660877106 | -0.283953655 | 0.414397483843507 | 1.06739586881094 | 0.752801532725026 | 1.51345858267643 | 0.999332001504091 |
| ebi-fl187-GCST90094707 | ebi-gm430-GCST90011450 | OTU99_298 (Phascolarctobacterium) abundance \|\| id:ebi-gm430-GCST90011450 | Black olive liking \|\| id:ebi-fl187-GCST90094707 | Weighted median | 18 | -0.011767422 | 0.320054365542732 | 0.970670827185958 | -0.639073979 | 0.615539133963806 | 0.988301542836385 | 0.527780934070365 | 1.85065408111646 | 0.999332001504091 |
| ebi-fl187-GCST90094707 | ebi-gm430-GCST90011451 | OTU99_3 (Bacteroides) abundance \|\| id:ebi-gm430-GCST90011451 | Black olive liking \|\| id:ebi-fl187-GCST90094707 | Weighted median | 18 | -0.045856202 | 0.0734255289571908 | 0.532281755138134 | -0.189770238 | 0.0980578350186297 | 0.95517930544015 | 0.827149159149146 | 1.10302657682641 | 0.999332001504091 |
| ebi-fl187-GCST90094707 | ebi-gm430-GCST90011472 | OTU99_72 (Parabacteroides) abundance \|\| id:ebi-gm430-GCST90011472 | Black olive liking \|\| id:ebi-fl187-GCST90094707 | Weighted median | 18 | 0.0252202051506254 | 0.237963985965991 | 0.915595613436509 | -0.441189207 | 0.491629617643967 | 1.02554092505573 | 0.643270983464439 | 1.63497843987907 | 0.999332001504091 |
| ebi-fl187-GCST90094707 | ebi-gm430-GCST90011507 | TestASV_3 (Bacteroides) abundance \|\| id:ebi-gm430-GCST90011507 | Black olive liking \|\| id:ebi-fl187-GCST90094707 | Weighted median | 18 | -0.007221455 | 0.0983873195369313 | 0.941489225898474 | -0.200060601 | 0.185617691164753 | 0.992804556926824 | 0.818681138335111 | 1.20396188711411 | 0.999332001504091 |
| ebi-fl187-GCST90094707 | ebi-gm430-GCST90011512 | TestASV_34 (Phascolarctobacterium) abundance \|\| id:ebi-gm430-GCST90011512 | Black olive liking \|\| id:ebi-fl187-GCST90094707 | Weighted median | 18 | -0.258831834 | 0.260254360595969 | 0.319962917681006 | -0.76893038 | 0.251266713239043 | 0.771952828711856 | 0.463508581169684 | 1.2856529392669 | 0.999332001504091 |
| ebi-fl187-GCST90094707 | ebi-gm430-GCST90011519 | TestASV_41 (Barnesiella) abundance \|\| id:ebi-gm430-GCST90011519 | Black olive liking \|\| id:ebi-fl187-GCST90094707 | Weighted median | 18 | 0.114914435677531 | 0.335933602595369 | 0.732294566126479 | -0.543515425 | 0.773344296764455 | 1.121777449 | 0.58070324100883 | 2.16700124431393 | 0.999332001504091 |
| ebi-fl187-GCST90094707 | ebi-gm430-GCST90011524 | TestASV_46 (Sutterella) abundance \|\| id:ebi-gm430-GCST90011524 | Black olive liking \|\| id:ebi-fl187-GCST90094707 | Weighted median | 18 | -0.180713518 | 0.331181515630437 | 0.585297571241924 | -0.829829289 | 0.468402252650417 | 0.834674443663673 | 0.436123731250679 | 1.59743984787844 | 0.999332001504091 |
| ebi-fl187-GCST90094707 | ebi-gm430-GCST90011581 | OTU97_19 (Bacteria) prevalence \|\| id:ebi-gm430-GCST90011581 | Black olive liking \|\| id:ebi-fl187-GCST90094707 | Weighted median | 18 | -0.28321844 | 0.3890815086749 | 0.466665347035644 | -1.045818197 | 0.479381316894541 | 0.753355206891876 | 0.351404183834798 | 1.61507487349074 | 0.999332001504091 |
| ebi-fl187-GCST90094707 | ebi-gm430-GCST90011614 | OTU97_80 (Ruminococcus) prevalence \|\| id:ebi-gm430-GCST90011614 | Black olive liking \|\| id:ebi-fl187-GCST90094707 | Weighted median | 18 | 0.0604222359367226 | 0.186145809250928 | 0.745486648260727 | -0.30442355 | 0.425268022068542 | 1.06228498676136 | 0.737548411511498 | 1.53000043859629 | 0.999332001504091 |
| ebi-fl187-GCST90094707 | ebi-gm430-GCST90011623 | OTU99_11 (Parabacteroides) prevalence \|\| id:ebi-gm430-GCST90011623 | Black olive liking \|\| id:ebi-fl187-GCST90094707 | Weighted median | 18 | 0.127101181510624 | 0.172913331255127 | 0.462304685681106 | -0.211808948 | 0.466011310770674 | 1.13553190680276 | 0.809119266857541 | 1.59362502437374 | 0.999332001504091 |
| ebi-fl187-GCST90094707 | ebi-gm430-GCST90011642 | OTU99_19 (Bacteria) prevalence \|\| id:ebi-gm430-GCST90011642 | Black olive liking \|\| id:ebi-fl187-GCST90094707 | Weighted median | 18 | -0.28321844 | 0.392293873490483 | 0.470322275259451 | -1.052114432 | 0.485677551933084 | 0.753355206891876 | 0.349198611187946 | 1.62527584465574 | 0.999332001504091 |
| ebi-fl187-GCST90094707 | ebi-gm430-GCST90011653 | OTU99_30 (Parasutterella) prevalence \|\| id:ebi-gm430-GCST90011653 | Black olive liking \|\| id:ebi-fl187-GCST90094707 | Weighted median | 18 | 0.0888606174978704 | 0.172485494701164 | 0.606429105548349 | -0.249210952 | 0.426932187112152 | 1.09292831062683 | 0.779415536684148 | 1.5325487316449 | 0.999332001504091 |
| ebi-fl187-GCST90094707 | ebi-gm430-GCST90011675 | OTU99_8 (Ruminococcaceae) prevalence \|\| id:ebi-gm430-GCST90011675 | Black olive liking \|\| id:ebi-fl187-GCST90094707 | Weighted median | 18 | -0.023669167 | 0.167403154436104 | 0.887561690067905 | -0.351779349 | 0.304441016001175 | 0.976608751020728 | 0.703435318286865 | 1.35586688324529 | 0.999332001504091 |
| ebi-fl187-GCST90094707 | ebi-gm430-GCST90011679 | OTU99_92 (Ruminococcus) prevalence \|\| id:ebi-gm430-GCST90011679 | Black olive liking \|\| id:ebi-fl187-GCST90094707 | Weighted median | 18 | -0.01938984 | 0.194664410040774 | 0.920656734753823 | -0.400932084 | 0.362152403492527 | 0.980796933644229 | 0.669695542624789 | 1.43641784037509 | 0.999332001504091 |
| ebi-fl187-GCST90094707 | ebi-gm430-GCST90011684 | TestASV_11 (Lachnospiraceae) prevalence \|\| id:ebi-gm430-GCST90011684 | Black olive liking \|\| id:ebi-fl187-GCST90094707 | Weighted median | 18 | 0.00377508818652149 | 0.180091990460116 | 0.983275969137634 | -0.349205213 | 0.356755389488349 | 1.00378222280705 | 0.705248389200067 | 1.42868635540778 | 0.999332001504091 |
| ebi-fl187-GCST90094707 | ebi-gm430-GCST90011688 | TestASV_15 (Bacteroides) prevalence \|\| id:ebi-gm430-GCST90011688 | Black olive liking \|\| id:ebi-fl187-GCST90094707 | Weighted median | 18 | -0.09880933 | 0.177520925473649 | 0.57779620200366 | -0.446750343 | 0.249131684409776 | 0.905915422887023 | 0.639703594535009 | 1.2829109613197 | 0.999332001504091 |
| ebi-fl187-GCST90094707 | ebi-gm430-GCST90011692 | TestASV_19 (Prevotella) prevalence \|\| id:ebi-gm430-GCST90011692 | Black olive liking \|\| id:ebi-fl187-GCST90094707 | Weighted median | 18 | -0.024947948 | 0.350919578572679 | 0.943323688269429 | -0.712750322 | 0.662854425917129 | 0.975360680097935 | 0.490293875326943 | 1.94032294539011 | 0.999332001504091 |
| ebi-fl187-GCST90094707 | ebi-gm430-GCST90011693 | TestASV_2 (Bacteroides) prevalence \|\| id:ebi-gm430-GCST90011693 | Black olive liking \|\| id:ebi-fl187-GCST90094707 | Weighted median | 18 | -0.102205949 | 0.158401334739386 | 0.518775464932943 | -0.412672565 | 0.208260667020356 | 0.902843592723268 | 0.661878969586319 | 1.23153414804963 | 0.999332001504091 |
| ebi-fl187-GCST90094707 | ebi-gm430-GCST90011716 | TestASV_40 (Barnesiella) prevalence \|\| id:ebi-gm430-GCST90011716 | Black olive liking \|\| id:ebi-fl187-GCST90094707 | Weighted median | 18 | -0.044503109 | 0.328585309728258 | 0.892265168165095 | -0.688530316 | 0.599524098202469 | 0.956472626544807 | 0.502313769383919 | 1.82125185708439 | 0.999332001504091 |
| ebi-fl187-GCST90094707 | ebi-gm430-GCST90011718 | TestASV_42 (Alphaproteobacteria) prevalence \|\| id:ebi-gm430-GCST90011718 | Black olive liking \|\| id:ebi-fl187-GCST90094707 | Weighted median | 18 | -0.198449898 | 0.265389676339704 | 0.454600129082504 | -0.718613663 | 0.321713867877389 | 0.820000853600278 | 0.487427526 | 1.37949000307864 | 0.999332001504091 |
| ebi-fl187-GCST90094707 | ebi-hbsm44-GCST90104929 | Sulcal depth of the right inferior frontal sulcus \|\| id:ebi-hbsm44-GCST90104929 | Black olive liking \|\| id:ebi-fl187-GCST90094707 | Weighted median | 20 | 0.110581918556831 | 0.136028577059815 | 0.416257318887519 | -0.156034092 | 0.377197929594069 | 1.1169278424215 | 0.855530022733286 | 1.45819290033878 | 0.999332001504091 |
| ebi-fl187-GCST90094707 | ebi-hbsm44-GCST90104935 | Sulcal opening distance of the left calloso-marginal posterior fissure \|\| id:ebi-hbsm44-GCST90104935 | Black olive liking \|\| id:ebi-fl187-GCST90094707 | Weighted median | 20 | 0.0196031110547312 | 0.0342745766216542 | 0.567360455373717 | -0.047575059 | 0.0867812812331735 | 1.01979651373381 | 0.953538898652344 | 1.09065810623296 | 0.999332001504091 |
| ebi-fl187-GCST90094707 | ebi-hbsm44-GCST90104936 | Sulcal opening distance of the left anterior inferior temporal sulcus \|\| id:ebi-hbsm44-GCST90104936 | Black olive liking \|\| id:ebi-fl187-GCST90094707 | Weighted median | 20 | -0.005702826 | 0.0180037469356153 | 0.751427460948443 | -0.04099017 | 0.0295845184721845 | 0.99431340472044 | 0.959838565579631 | 1.03002648805814 | 0.999332001504091 |
| ebi-fl187-GCST90094707 | ebi-hbsm44-GCST90104941 | Sulcal opening distance of the right posterior inferior temporal sulcus \|\| id:ebi-hbsm44-GCST90104941 | Black olive liking \|\| id:ebi-fl187-GCST90094707 | Weighted median | 18 | -0.006748394 | 0.015096797819414 | 0.654869087409868 | -0.036338117 | 0.0228413302220176 | 0.993274325768484 | 0.96431418711326 | 1.02310419095282 | 0.999332001504091 |
| ebi-fl187-GCST90094707 | ebi-IPs91-GCST90274819 | Leukemia inhibitory factor levels \|\| id:ebi-IPs91-GCST90274819 | Black olive liking \|\| id:ebi-fl187-GCST90094707 | Weighted median | 22 | -0.014757036 | 0.0574287494057812 | 0.797207706298167 | -0.127317385 | 0.09780331265509 | 0.985351315241431 | 0.880454185281654 | 1.10274586761993 | 0.999332001504091 |
| ebi-fl187-GCST90094707 | ebi-IPs91-GCST90274837 | STAM binding protein levels \|\| id:ebi-IPs91-GCST90274837 | Black olive liking \|\| id:ebi-fl187-GCST90094707 | Weighted median | 22 | -0.036004611 | 0.0511770458079421 | 0.481725225995227 | -0.136311621 | 0.0643023987840914 | 0.964635845537491 | 0.872570678959498 | 1.06641483255596 | 0.999332001504091 |
| ebi-fl187-GCST90094707 | ebi-IPs91-GCST90274840 | TNF-beta levels \|\| id:ebi-IPs91-GCST90274840 | Black olive liking \|\| id:ebi-fl187-GCST90094707 | Weighted median | 22 | 0.0349623154312105 | 0.0641002754955846 | 0.585456716117619 | -0.090674225 | 0.160598855402556 | 1.03558068265281 | 0.913315198118286 | 1.17421384478568 | 0.999332001504091 |
| ebi-fl187-GCST90094707 | ebi-IPs91-GCST90274842 | Tumor necrosis factor ligand superfamily member 14 levels \|\| id:ebi-IPs91-GCST90274842 | Black olive liking \|\| id:ebi-fl187-GCST90094707 | Weighted median | 22 | 0.00608894295616395 | 0.0592522912362879 | 0.91815107479535 | -0.110045548 | 0.122223433779288 | 1.00610751825149 | 0.895793332891754 | 1.13000655521121 | 0.999332001504091 |
| ebi-fl187-GCST90094707 | ebi-met1400-GCST90199628 | 3-methylhistidine levels \|\| id:ebi-met1400-GCST90199628 | Black olive liking \|\| id:ebi-fl187-GCST90094707 | Weighted median | 22 | -0.09138364 | 0.0700538357700214 | 0.192070887024849 | -0.228689158 | 0.045921878071826 | 0.91266750793073 | 0.795575793327824 | 1.04699261468009 | 0.999332001504091 |
| ebi-fl187-GCST90094707 | ebi-met1400-GCST90199651 | Docosahexaenoate DHA; 22:6n3 levels \|\| id:ebi-met1400-GCST90199651 | Black olive liking \|\| id:ebi-fl187-GCST90094707 | Weighted median | 22 | 0.0495526294339389 | 0.0673130502380584 | 0.461638264363434 | -0.082380949 | 0.181486207900533 | 1.05080089381579 | 0.920921067874873 | 1.198998 | 0.999332001504091 |
| ebi-fl187-GCST90094707 | ebi-met1400-GCST90199652 | 1-stearoyl-2-oleoyl-GPS (18:0/18:1) levels \|\| id:ebi-met1400-GCST90199652 | Black olive liking \|\| id:ebi-fl187-GCST90094707 | Weighted median | 22 | -0.02605479 | 0.0730652836026379 | 0.721394250771621 | -0.169262746 | 0.117153165877823 | 0.974281707267837 | 0.844287041328954 | 1.12429161961624 | 0.999332001504091 |
| ebi-fl187-GCST90094707 | ebi-met1400-GCST90199653 | 1-myristoyl-2-palmitoyl-gpc (14:0/16:0) levels \|\| id:ebi-met1400-GCST90199653 | Black olive liking \|\| id:ebi-fl187-GCST90094707 | Weighted median | 21 | 0.049684293931337 | 0.0699889578440229 | 0.477773835011011 | -0.087494063 | 0.186862651305622 | 1.05093925609584 | 0.916224310852627 | 1.20546170508777 | 0.999332001504091 |
| ebi-fl187-GCST90094707 | ebi-met1400-GCST90199667 | Erythritol levels in elite athletes \|\| id:ebi-met1400-GCST90199667 | Black olive liking \|\| id:ebi-fl187-GCST90094707 | Weighted median | 22 | 0.0447665536170692 | 0.0639189171953087 | 0.483699572070929 | -0.080514524 | 0.170047631319874 | 1.04578369698896 | 0.922641502962149 | 1.18536131029948 | 0.999332001504091 |
| ebi-fl187-GCST90094707 | ebi-met1400-GCST90199674 | 3-hydroxymyristate levels \|\| id:ebi-met1400-GCST90199674 | Black olive liking \|\| id:ebi-fl187-GCST90094707 | Weighted median | 22 | -0.004343067 | 0.0673778046549146 | 0.948605209201897 | -0.136403564 | 0.127717429864563 | 0.995666350219026 | 0.872490455358396 | 1.13623189213142 | 0.999332001504091 |
| ebi-fl187-GCST90094707 | ebi-met1400-GCST90199685 | 2-linoleoylglycerol (18:2) levels \|\| id:ebi-met1400-GCST90199685 | Black olive liking \|\| id:ebi-fl187-GCST90094707 | Weighted median | 22 | 0.00333678731374455 | 0.0757401698581198 | 0.964859993043964 | -0.145113946 | 0.151787520235659 | 1.00334236058575 | 0.864923733234811 | 1.16391290221711 | 0.999332001504091 |
| ebi-fl187-GCST90094707 | ebi-met1400-GCST90199703 | Gamma-glutamylvaline levels \|\| id:ebi-met1400-GCST90199703 | Black olive liking \|\| id:ebi-fl187-GCST90094707 | Weighted median | 22 | -0.056414947 | 0.0686814467857267 | 0.411418410527807 | -0.191030582 | 0.0782006889132237 | 0.945146868854766 | 0.826107323348533 | 1.08133964977464 | 0.999332001504091 |
| ebi-fl187-GCST90094707 | ebi-met1400-GCST90199779 | Gamma-glutamyl-2-aminobutyrate levels \|\| id:ebi-met1400-GCST90199779 | Black olive liking \|\| id:ebi-fl187-GCST90094707 | Weighted median | 21 | 0.0349511269447265 | 0.0727882294504507 | 0.631102582202946 | -0.107713803 | 0.17761605666761 | 1.03556909613716 | 0.897884531720112 | 1.19436666407416 | 0.999332001504091 |
| ebi-fl187-GCST90094707 | ebi-met1400-GCST90199824 | 4-vinylphenol sulfate levels \|\| id:ebi-met1400-GCST90199824 | Black olive liking \|\| id:ebi-fl187-GCST90094707 | Weighted median | 22 | -2.83E-05 | 0.0704573510749356 | 0.999678975992922 | -0.138124756 | 0.138068060018828 | 0.999971652313757 | 0.870990023566272 | 1.14805368417061 | 0.99994023009904 |
| ebi-fl187-GCST90094707 | ebi-met1400-GCST90199830 | 4-ethylphenylsulfate levels \|\| id:ebi-met1400-GCST90199830 | Black olive liking \|\| id:ebi-fl187-GCST90094707 | Weighted median | 22 | 0.0268617355490823 | 0.0648662509796022 | 0.678793861666737 | -0.100276116 | 0.153999587469103 | 1.02722576413802 | 0.904587612101144 | 1.16649040556501 | 0.999332001504091 |
| ebi-fl187-GCST90094707 | ebi-met1400-GCST90199833 | Hexanoylglutamine levels \|\| id:ebi-met1400-GCST90199833 | Black olive liking \|\| id:ebi-fl187-GCST90094707 | Weighted median | 22 | 0.0216167611729782 | 0.06793048953138 | 0.750318947268659 | -0.111526998 | 0.154760520654483 | 1.02185209602145 | 0.894467241974707 | 1.16737836462094 | 0.999332001504091 |
| ebi-fl187-GCST90094707 | ebi-met1400-GCST90199837 | Carotenoid levels (cryptoxanthin) \|\| id:ebi-met1400-GCST90199837 | Black olive liking \|\| id:ebi-fl187-GCST90094707 | Weighted median | 22 | 0.185556037517502 | 0.071056894435804 | 0.0090179542755303 | 0.0462845244233258 | 0.324827550611678 | 1.2038876607608 | 1.04737237158642 | 1.38379199132095 | 0.999332001504091 |
| ebi-fl187-GCST90094707 | ebi-met1400-GCST90199869 | 2-hydroxyhippurate levels \|\| id:ebi-met1400-GCST90199869 | Black olive liking \|\| id:ebi-fl187-GCST90094707 | Weighted median | 22 | -0.05445573 | 0.0687655920403245 | 0.428416799981672 | -0.189236291 | 0.0803248301603249 | 0.947000431412907 | 0.82759093160034 | 1.08363900914434 | 0.999332001504091 |
| ebi-fl187-GCST90094707 | ebi-met1400-GCST90199902 | Hydantoin-5-propionate levels \|\| id:ebi-met1400-GCST90199902 | Black olive liking \|\| id:ebi-fl187-GCST90094707 | Weighted median | 22 | -0.02531062 | 0.0714717558150886 | 0.723238029445624 | -0.165395261 | 0.114774021302832 | 0.975007008217497 | 0.847558630582303 | 1.12161994671697 | 0.999332001504091 |
| ebi-fl187-GCST90094707 | ebi-met1400-GCST90199904 | Pantoate levels \|\| id:ebi-met1400-GCST90199904 | Black olive liking \|\| id:ebi-fl187-GCST90094707 | Weighted median | 22 | 0.00854867853160087 | 0.0717762864238495 | 0.905194775109413 | -0.132132843 | 0.149230199922346 | 1.0085853228296 | 0.876224587168153 | 1.16094020679662 | 0.999332001504091 |
| ebi-fl187-GCST90094707 | ebi-met1400-GCST90199936 | 3-methyl catechol sulfate (2) levels \|\| id:ebi-met1400-GCST90199936 | Black olive liking \|\| id:ebi-fl187-GCST90094707 | Weighted median | 22 | -0.028571293 | 0.0804226819546213 | 0.722391735521681 | -0.18619975 | 0.129057163612106 | 0.971833006767343 | 0.830107764684746 | 1.13775516050152 | 0.999332001504091 |
| ebi-fl187-GCST90094707 | ebi-met1400-GCST90199977 | 4-hydroxychlorothalonil levels \|\| id:ebi-met1400-GCST90199977 | Black olive liking \|\| id:ebi-fl187-GCST90094707 | Weighted median | 22 | 0.0770662692335811 | 0.0698453775138533 | 0.269860459294475 | -0.059830671 | 0.213963209160734 | 1.08011365232485 | 0.941924015421633 | 1.23857708566472 | 0.999332001504091 |
| ebi-fl187-GCST90094707 | ebi-met1400-GCST90200040 | Sphingomyelin (d18:2/24:1, d18:1/24:2) levels \|\| id:ebi-met1400-GCST90200040 | Black olive liking \|\| id:ebi-fl187-GCST90094707 | Weighted median | 22 | -0.000892879 | 0.0642010611831274 | 0.988903748683046 | -0.126726959 | 0.124941201287307 | 0.99910751986589 | 0.880974182228755 | 1.13308182735527 | 0.999332001504091 |
| ebi-fl187-GCST90094707 | ebi-met1400-GCST90200077 | 1-stearoyl-2-oleoyl-GPI (18:0/18:1) levels \|\| id:ebi-met1400-GCST90200077 | Black olive liking \|\| id:ebi-fl187-GCST90094707 | Weighted median | 22 | -0.029172726 | 0.0739647635201767 | 0.693275534680564 | -0.174143662 | 0.11579821082442 | 0.971248690391813 | 0.840176187449696 | 1.12276928658406 | 0.999332001504091 |
| ebi-fl187-GCST90094707 | ebi-met1400-GCST90200080 | 1-palmitoyl-2-oleoyl-GPI (16:0/18:1) levels \|\| id:ebi-met1400-GCST90200080 | Black olive liking \|\| id:ebi-fl187-GCST90094707 | Weighted median | 22 | 0.0137405656456761 | 0.0710424897108305 | 0.846635264149138 | -0.125502714 | 0.152983845478904 | 1.01383540108417 | 0.882053370365506 | 1.16530615382784 | 0.999332001504091 |
| ebi-fl187-GCST90094707 | ebi-met1400-GCST90200082 | 1-oleoyl-2-linoleoyl-GPE (18:1/18:2) levels \|\| id:ebi-met1400-GCST90200082 | Black olive liking \|\| id:ebi-fl187-GCST90094707 | Weighted median | 22 | -0.070058585 | 0.0674286278795262 | 0.298803073969485 | -0.202218696 | 0.0621015252705587 | 0.932339196866006 | 0.816916252074397 | 1.06407036927646 | 0.999332001504091 |
| ebi-fl187-GCST90094707 | ebi-met1400-GCST90200100 | Perfluorooctanesulfonate (PFOS) levels \|\| id:ebi-met1400-GCST90200100 | Black olive liking \|\| id:ebi-fl187-GCST90094707 | Weighted median | 22 | -0.079450813 | 0.0646013922946757 | 0.218748696171815 | -0.206069542 | 0.0471679158365859 | 0.923623449061606 | 0.813776482704706 | 1.04829802014076 | 0.999332001504091 |
| ebi-fl187-GCST90094707 | ebi-met1400-GCST90200102 | 1-palmitoleoyl-2-linolenoyl-GPC (16:1/18:3) levels \|\| id:ebi-met1400-GCST90200102 | Black olive liking \|\| id:ebi-fl187-GCST90094707 | Weighted median | 22 | 0.0204516082178082 | 0.0718507657909621 | 0.775919896171201 | -0.120375893 | 0.161279109168094 | 1.02066217538642 | 0.886587112421725 | 1.17501287991766 | 0.999332001504091 |
| ebi-fl187-GCST90094707 | ebi-met1400-GCST90200114 | Glycosyl-N-tricosanoyl-sphingadienine (d18:2/23:0) levels \|\| id:ebi-met1400-GCST90200114 | Black olive liking \|\| id:ebi-fl187-GCST90094707 | Weighted median | 22 | 0.159506147193682 | 0.0703956209302147 | 0.0234603691529375 | 0.0215307301704613 | 0.297481564216903 | 1.1729314724355 | 1.02176418884263 | 1.34646355201396 | 0.999332001504091 |
| ebi-fl187-GCST90094707 | ebi-met1400-GCST90200135 | Arachidoylcarnitine (C20) levels \|\| id:ebi-met1400-GCST90200135 | Black olive liking \|\| id:ebi-fl187-GCST90094707 | Weighted median | 22 | 0.0914897732879419 | 0.0730208482746068 | 0.210232369838355 | -0.051631089 | 0.234610635906171 | 1.0958055701472 | 0.949679148998374 | 1.2644163545468 | 0.999332001504091 |
| ebi-fl187-GCST90094707 | ebi-met1400-GCST90200146 | Docosahexaenoylcarnitine (C22:6) levels \|\| id:ebi-met1400-GCST90200146 | Black olive liking \|\| id:ebi-fl187-GCST90094707 | Weighted median | 22 | -0.118802053 | 0.0798676204433135 | 0.136886213705098 | -0.275342589 | 0.0377384827437935 | 0.887983556758644 | 0.759311946441025 | 1.03845962225352 | 0.999332001504091 |
| ebi-fl187-GCST90094707 | ebi-met1400-GCST90200162 | 3-hydroxyphenylacetoylglutamine levels \|\| id:ebi-met1400-GCST90200162 | Black olive liking \|\| id:ebi-fl187-GCST90094707 | Weighted median | 22 | 0.0458146497826208 | 0.0818648692418481 | 0.575726729135689 | -0.114640494 | 0.206269793496643 | 1.04688035347211 | 0.891686653053949 | 1.22908475834232 | 0.999332001504091 |
| ebi-fl187-GCST90094707 | ebi-met1400-GCST90200172 | 3-carboxy-4-methyl-5-pentyl-2-furanpropionate (3-CMPFP) levels \|\| id:ebi-met1400-GCST90200172 | Black olive liking \|\| id:ebi-fl187-GCST90094707 | Weighted median | 22 | -0.014227764 | 0.0658797433176223 | 0.82901449929582 | -0.14335206 | 0.114896533308315 | 0.985872972717507 | 0.866448972736725 | 1.12175736704368 | 0.999332001504091 |
| ebi-fl187-GCST90094707 | ebi-met1400-GCST90200222 | Dihydrocaffeate sulfate (2) levels \|\| id:ebi-met1400-GCST90200222 | Black olive liking \|\| id:ebi-fl187-GCST90094707 | Weighted median | 22 | -0.036601962 | 0.0815669763273305 | 0.653622789570559 | -0.196473236 | 0.12326931154525 | 0.964059791366104 | 0.821623321208637 | 1.13118902219287 | 0.999332001504091 |
| ebi-fl187-GCST90094707 | ebi-met1400-GCST90200223 | 4-methylhexanoylglutamine levels \|\| id:ebi-met1400-GCST90200223 | Black olive liking \|\| id:ebi-fl187-GCST90094707 | Weighted median | 22 | -0.004613533 | 0.0895299489566426 | 0.958902709218095 | -0.180092233 | 0.170865166872629 | 0.995397092913952 | 0.8351931754553 | 1.18633078154812 | 0.999332001504091 |
| ebi-fl187-GCST90094707 | ebi-met1400-GCST90200224 | 2-ketocaprylate levels \|\| id:ebi-met1400-GCST90200224 | Black olive liking \|\| id:ebi-fl187-GCST90094707 | Weighted median | 22 | -0.0097267 | 0.0703589763933617 | 0.890047653526852 | -0.147630294 | 0.128176893771323 | 0.990320451386886 | 0.86275002011361 | 1.13675406962724 | 0.999332001504091 |
| ebi-fl187-GCST90094707 | ebi-met1400-GCST90200243 | Hydroxypalmitoyl sphingomyelin (d18:1/16:0(OH)) levels \|\| id:ebi-met1400-GCST90200243 | Black olive liking \|\| id:ebi-fl187-GCST90094707 | Weighted median | 22 | 0.10257742918246 | 0.0718353737508533 | 0.153305820862382 | -0.038219903 | 0.243374761734133 | 1.10802309191059 | 0.962501260347399 | 1.27554656059773 | 0.999332001504091 |
| ebi-fl187-GCST90094707 | ebi-met1400-GCST90200273 | 2-methoxyhydroquinone sulfate (2) levels \|\| id:ebi-met1400-GCST90200273 | Black olive liking \|\| id:ebi-fl187-GCST90094707 | Weighted median | 22 | 0.0196640818574159 | 0.0806756168749797 | 0.807430204605681 | -0.138460127 | 0.177788290932376 | 1.01985869344138 | 0.870697967727643 | 1.19457239265465 | 0.999332001504091 |
| ebi-fl187-GCST90094707 | ebi-met1400-GCST90200276 | 2,4-di-tert-butylphenol levels \|\| id:ebi-met1400-GCST90200276 | Black olive liking \|\| id:ebi-fl187-GCST90094707 | Weighted median | 22 | 0.00988744456035764 | 0.0727196564401391 | 0.89184768373627 | -0.132643082 | 0.15241797118303 | 1.00993648684134 | 0.875777617073527 | 1.16464692356688 | 0.999332001504091 |
| ebi-fl187-GCST90094707 | ebi-met1400-GCST90200289 | N-acetylmethionine levels \|\| id:ebi-met1400-GCST90200289 | Black olive liking \|\| id:ebi-fl187-GCST90094707 | Weighted median | 22 | -0.00862485 | 0.0686823576162945 | 0.900067624677553 | -0.143242271 | 0.125992570646294 | 0.991412237038762 | 0.866544104773967 | 1.1342737413309 | 0.999332001504091 |
| ebi-fl187-GCST90094707 | ebi-met1400-GCST90200308 | 3-hydroxyisobutyrate levels \|\| id:ebi-met1400-GCST90200308 | Black olive liking \|\| id:ebi-fl187-GCST90094707 | Weighted median | 22 | 0.050878888465297 | 0.0667276293803563 | 0.445769948770115 | -0.079907265 | 0.181665042050795 | 1.05219545256002 | 0.923201955439469 | 1.19921244085859 | 0.999332001504091 |
| ebi-fl187-GCST90094707 | ebi-met1400-GCST90200383 | Cystathionine levels \|\| id:ebi-met1400-GCST90200383 | Black olive liking \|\| id:ebi-fl187-GCST90094707 | Weighted median | 22 | -0.054007489 | 0.0714974656078947 | 0.450023248298822 | -0.194142521 | 0.086127543816199 | 0.947425011422231 | 0.82354052378314 | 1.08994533522771 | 0.999332001504091 |
| ebi-fl187-GCST90094707 | ebi-met1400-GCST90200384 | Guanidinoacetate levels \|\| id:ebi-met1400-GCST90200384 | Black olive liking \|\| id:ebi-fl187-GCST90094707 | Weighted median | 22 | 0.115736566854678 | 0.0719641039828957 | 0.107779534118071 | -0.025313077 | 0.256786210661153 | 1.12270007676134 | 0.975004612767591 | 1.29276871704438 | 0.999332001504091 |
| ebi-fl187-GCST90094707 | ebi-met1400-GCST90200403 | Citrulline levels \|\| id:ebi-met1400-GCST90200403 | Black olive liking \|\| id:ebi-fl187-GCST90094707 | Weighted median | 22 | 0.0905777242285892 | 0.0699887429304891 | 0.19560476865506 | -0.046600212 | 0.227755660372348 | 1.09480659733307 | 0.954468906620115 | 1.25577845150389 | 0.999332001504091 |
| ebi-fl187-GCST90094707 | ebi-met1400-GCST90200434 | 12,13-DiHOME levels \|\| id:ebi-met1400-GCST90200434 | Black olive liking \|\| id:ebi-fl187-GCST90094707 | Weighted median | 22 | 0.0499493414732683 | 0.0644981168237466 | 0.438675954038451 | -0.076466968 | 0.176365650447812 | 1.05121784187999 | 0.926383514545681 | 1.19287415388515 | 0.999332001504091 |
| ebi-fl187-GCST90094707 | ebi-met1400-GCST90200437 | Fructose levels \|\| id:ebi-met1400-GCST90200437 | Black olive liking \|\| id:ebi-fl187-GCST90094707 | Weighted median | 22 | -0.024709009 | 0.0724252913133954 | 0.732979016236893 | -0.16666258 | 0.11724456240126 | 0.975593760148088 | 0.846485184569578 | 1.12439438065753 | 0.999332001504091 |
| ebi-fl187-GCST90094707 | ebi-met1400-GCST90200448 | N-stearoyl-sphinganine (d18:0/18:0) levels \|\| id:ebi-met1400-GCST90200448 | Black olive liking \|\| id:ebi-fl187-GCST90094707 | Weighted median | 22 | -0.012833406 | 0.0784561534509287 | 0.870066318351732 | -0.166607467 | 0.140940654424272 | 0.987248590677769 | 0.846531837722382 | 1.15135631805366 | 0.999332001504091 |
| ebi-fl187-GCST90094707 | ebi-met1400-GCST90200468 | X-11847 levels \|\| id:ebi-met1400-GCST90200468 | Black olive liking \|\| id:ebi-fl187-GCST90094707 | Weighted median | 22 | 0.101034500972158 | 0.0738310305466731 | 0.171169452465228 | -0.043674319 | 0.245743320843637 | 1.10631481004146 | 0.957265670047907 | 1.27857134880416 | 0.999332001504091 |
| ebi-fl187-GCST90094707 | ebi-met1400-GCST90200476 | X-11843 levels \|\| id:ebi-met1400-GCST90200476 | Black olive liking \|\| id:ebi-fl187-GCST90094707 | Weighted median | 22 | 0.101021863167544 | 0.0780427608726863 | 0.195512633229135 | -0.051941948 | 0.253985674478009 | 1.10630082873939 | 0.949383978746247 | 1.28915333634105 | 0.999332001504091 |
| ebi-fl187-GCST90094707 | ebi-met1400-GCST90200511 | X-12906 levels \|\| id:ebi-met1400-GCST90200511 | Black olive liking \|\| id:ebi-fl187-GCST90094707 | Weighted median | 22 | -0.097009246 | 0.0742709448036112 | 0.191500399622285 | -0.242580298 | 0.0485618054537084 | 0.907547614581273 | 0.784600743040276 | 1.04976025072395 | 0.999332001504091 |
| ebi-fl187-GCST90094707 | ebi-met1400-GCST90200515 | X-12849 levels \|\| id:ebi-met1400-GCST90200515 | Black olive liking \|\| id:ebi-fl187-GCST90094707 | Weighted median | 22 | 0.0628899609101446 | 0.071366644724707 | 0.378196889088443 | -0.076988663 | 0.20276858457057 | 1.064909651 | 0.925900350710482 | 1.22478900037686 | 0.999332001504091 |
| ebi-fl187-GCST90094707 | ebi-met1400-GCST90200551 | X-18886 levels \|\| id:ebi-met1400-GCST90200551 | Black olive liking \|\| id:ebi-fl187-GCST90094707 | Weighted median | 22 | -0.005624394 | 0.0681865287113348 | 0.934260715093869 | -0.13926999 | 0.128021202082306 | 0.994391393101249 | 0.869993106901929 | 1.13657710024283 | 0.999332001504091 |
| ebi-fl187-GCST90094707 | ebi-met1400-GCST90200564 | X-21319 levels \|\| id:ebi-met1400-GCST90200564 | Black olive liking \|\| id:ebi-fl187-GCST90094707 | Weighted median | 22 | -0.033157059 | 0.0710449689188813 | 0.64071078382108 | -0.172405198 | 0.106091080165288 | 0.967386610964702 | 0.841638074005262 | 1.11192314603857 | 0.999332001504091 |
| ebi-fl187-GCST90094707 | ebi-met1400-GCST90200579 | X-21442 levels \|\| id:ebi-met1400-GCST90200579 | Black olive liking \|\| id:ebi-fl187-GCST90094707 | Weighted median | 22 | 0.0548418104393926 | 0.0737719659622079 | 0.457241751742934 | -0.089751243 | 0.19943486372532 | 1.0563734941986 | 0.914158560470771 | 1.220712694 | 0.999332001504091 |
| ebi-fl187-GCST90094707 | ebi-met1400-GCST90200581 | X-21441 levels \|\| id:ebi-met1400-GCST90200581 | Black olive liking \|\| id:ebi-fl187-GCST90094707 | Weighted median | 22 | 0.0398703722844037 | 0.0737234044302815 | 0.588638381787094 | -0.1046275 | 0.184368244967755 | 1.04067586501353 | 0.900659955582196 | 1.20245854088361 | 0.999332001504091 |
| ebi-fl187-GCST90094707 | ebi-met1400-GCST90200648 | X-24585 levels \|\| id:ebi-met1400-GCST90200648 | Black olive liking \|\| id:ebi-fl187-GCST90094707 | Weighted median | 22 | -0.00643056 | 0.0655108214171947 | 0.921805028152466 | -0.13483177 | 0.121970649906769 | 0.993590071732108 | 0.873862909237714 | 1.1297209438787 | 0.999332001504091 |
| ebi-fl187-GCST90094707 | ebi-met1400-GCST90200720 | Adenosine 5'-diphosphate (ADP) to pantothenate ratio \|\| id:ebi-met1400-GCST90200720 | Black olive liking \|\| id:ebi-fl187-GCST90094707 | Weighted median | 22 | 0.0804164083724665 | 0.0892232285405971 | 0.367431738153453 | -0.09446112 | 0.255293936312037 | 1.08373825141155 | 0.90986310980991 | 1.29084099015503 | 0.999332001504091 |
| ebi-fl187-GCST90094707 | ebi-met1400-GCST90200724 | Cholate to taurocholate ratio \|\| id:ebi-met1400-GCST90200724 | Black olive liking \|\| id:ebi-fl187-GCST90094707 | Weighted median | 22 | 0.132730396951416 | 0.0732919144387586 | 0.070143513082805 | -0.010921755 | 0.276382549251383 | 1.14194208575805 | 0.989137670479848 | 1.31835210218294 | 0.999332001504091 |
| ebi-fl187-GCST90094707 | ebi-met1400-GCST90200726 | N-acetylputrescine to (N(1) + N(8))-acetylspermidine ratio \|\| id:ebi-met1400-GCST90200726 | Black olive liking \|\| id:ebi-fl187-GCST90094707 | Weighted median | 22 | -0.027737719 | 0.0675120402532684 | 0.681178209125666 | -0.160061318 | 0.104585879592579 | 0.972643438947829 | 0.852091538644696 | 1.11025073765313 | 0.999332001504091 |
| ebi-fl187-GCST90094707 | ebi-met1400-GCST90200744 | Arginine to glutamate ratio \|\| id:ebi-met1400-GCST90200744 | Black olive liking \|\| id:ebi-fl187-GCST90094707 | Weighted median | 22 | 0.0658336462679539 | 0.0715578003519247 | 0.357569327328773 | -0.074419642 | 0.206086934957726 | 1.06804902844492 | 0.928282065560605 | 1.22886003024656 | 0.999332001504091 |
| ebi-fl187-GCST90094707 | ebi-met1400-GCST90200759 | Histidine to trans-urocanate ratio \|\| id:ebi-met1400-GCST90200759 | Black olive liking \|\| id:ebi-fl187-GCST90094707 | Weighted median | 22 | 0.046855591925274 | 0.073963693422207 | 0.526410780707798 | -0.098113247 | 0.1918244310328 | 1.04797066272633 | 0.906546234133911 | 1.2114578038971 | 0.999332001504091 |
| ebi-fl187-GCST90094707 | ebi-met1400-GCST90200795 | Oleoyl-linoleoyl-glycerol (18:1 to 18:2) [2] to linoleoyl-arachidonoyl-glycerol (18:2 to 20:4) [2] ratio \|\| id:ebi-met1400-GCST90200795 | Black olive liking \|\| id:ebi-fl187-GCST90094707 | Weighted median | 22 | 0.0731022810422229 | 0.0704815164355834 | 0.299650246181266 | -0.065041491 | 0.211246053255966 | 1.07584056938219 | 0.937028584157132 | 1.23521624665241 | 0.999332001504091 |
| ebi-fl187-GCST90094707 | ebi-met1400-GCST90200811 | Choline to taurocholate ratio \|\| id:ebi-met1400-GCST90200811 | Black olive liking \|\| id:ebi-fl187-GCST90094707 | Weighted median | 22 | 0.00722231531418702 | 0.0673870966947025 | 0.914648933057231 | -0.124856394 | 0.139301024835804 | 1.00724845913518 | 0.882623643351868 | 1.14947006696684 | 0.999332001504091 |
| ebi-fl187-GCST90094707 | ebi-met1400-GCST90200831 | Cholate to phosphate ratio \|\| id:ebi-met1400-GCST90200831 | Black olive liking \|\| id:ebi-fl187-GCST90094707 | Weighted median | 22 | 0.13428691433606 | 0.0712034354581282 | 0.0593001876321364 | -0.005271819 | 0.273845647833992 | 1.14372092250281 | 0.994742052489782 | 1.31501181165165 | 0.999332001504091 |
| ebi-fl187-GCST90094707 | ebi-met1400-GCST90200841 | Adenosine 5'-diphosphate (ADP) to oxalate (ethanedioate) ratio \|\| id:ebi-met1400-GCST90200841 | Black olive liking \|\| id:ebi-fl187-GCST90094707 | Weighted median | 22 | -0.013692602 | 0.0915631382544826 | 0.881125388400488 | -0.193156353 | 0.165771148580332 | 0.986400714877431 | 0.824353073675252 | 1.18030295680563 | 0.999332001504091 |
| ebi-fl187-GCST90094707 | ebi-met1400-GCST90200844 | Aspartate to phosphate ratio \|\| id:ebi-met1400-GCST90200844 | Black olive liking \|\| id:ebi-fl187-GCST90094707 | Weighted median | 22 | -0.077940857 | 0.0684409091540947 | 0.25478445335797 | -0.212085038 | 0.0562033254146231 | 0.925019133769526 | 0.808895907371745 | 1.05781274208683 | 0.999332001504091 |
| ebi-fl187-GCST90094707 | ebi-met1400-GCST90200870 | Adenosine 5'-monophosphate (AMP) to acetoacetate ratio \|\| id:ebi-met1400-GCST90200870 | Black olive liking \|\| id:ebi-fl187-GCST90094707 | Weighted median | 22 | -0.035725802 | 0.0802420183541481 | 0.656156715801022 | -0.193000158 | 0.121548553834394 | 0.964904832053818 | 0.824481843777085 | 1.12924419372967 | 0.999332001504091 |
| ebi-fl187-GCST90094707 | ebi-met1400-GCST90200887 | Cholesterol to cortisol ratio \|\| id:ebi-met1400-GCST90200887 | Black olive liking \|\| id:ebi-fl187-GCST90094707 | Weighted median | 22 | -0.051855193 | 0.0695734142779451 | 0.456071901530287 | -0.188219085 | 0.0845086990664238 | 0.949466346339387 | 0.828433190144375 | 1.08818231036103 | 0.999332001504091 |
| ebi-fl187-GCST90094707 | ebi-met1400-GCST90200890 | Cortisol to taurocholate ratio \|\| id:ebi-met1400-GCST90200890 | Black olive liking \|\| id:ebi-fl187-GCST90094707 | Weighted median | 22 | 0.0529692648684389 | 0.0742786479652665 | 0.475774208191809 | -0.092616885 | 0.198555414880361 | 1.05439723758352 | 0.911542658942927 | 1.21963961172481 | 0.999332001504091 |
| ebi-fl187-GCST90094707 | ebi-met1400-GCST90200948 | Adenosine 5'-diphosphate (ADP) to mannitol to sorbitol ratio \|\| id:ebi-met1400-GCST90200948 | Black olive liking \|\| id:ebi-fl187-GCST90094707 | Weighted median | 22 | -0.10415664 | 0.0945635491687709 | 0.270702609040225 | -0.289501196 | 0.0811879165729135 | 0.901084140728441 | 0.748636897414355 | 1.08457468697661 | 0.999332001504091 |
| ebi-fl187-GCST90094707 | ebi-met1400-GCST90200972 | Leucine to N-palmitoyl-sphingosine (d18:1 to 16:0) ratio \|\| id:ebi-met1400-GCST90200972 | Black olive liking \|\| id:ebi-fl187-GCST90094707 | Weighted median | 22 | -0.126835284 | 0.068593225331665 | 0.0644443186884182 | -0.261278006 | 0.00760743733986619 | 0.880878755199934 | 0.770066806692065 | 1.00763644740874 | 0.999332001504091 |
| ebi-fl187-GCST90094707 | ebi-met1400-GCST90200973 | Proline to glutamate ratio \|\| id:ebi-met1400-GCST90200973 | Black olive liking \|\| id:ebi-fl187-GCST90094707 | Weighted median | 22 | 0.0758522969000347 | 0.0678469272149281 | 0.26357056719181 | -0.05712768 | 0.208832274241294 | 1.07880321980919 | 0.944473470878167 | 1.23223830309237 | 0.999332001504091 |
| ebi-fl187-GCST90094707 | ebi-met1400-GCST90200983 | Cholesterol to linoleoyl-arachidonoyl-glycerol (18:2 to 20:4) [1] ratio \|\| id:ebi-met1400-GCST90200983 | Black olive liking \|\| id:ebi-fl187-GCST90094707 | Weighted median | 22 | 0.0690541050328023 | 0.0746155585781183 | 0.354723962195053 | -0.07719239 | 0.215300599845914 | 1.07149418080785 | 0.925711738995234 | 1.24023465528398 | 0.999332001504091 |
| ebi-fl187-GCST90094707 | ebi-met1400-GCST90201001 | Serine to alpha-ketobutyrate ratio \|\| id:ebi-met1400-GCST90201001 | Black olive liking \|\| id:ebi-fl187-GCST90094707 | Weighted median | 22 | -0.05419624 | 0.0707105758150668 | 0.443407588985709 | -0.192788969 | 0.0843964885534989 | 0.947246200625186 | 0.824655984050246 | 1.08806021171633 | 0.999332001504091 |
| ebi-fl187-GCST90094707 | ebi-met1400-GCST90201005 | Fructose to maltose ratio \|\| id:ebi-met1400-GCST90201005 | Black olive liking \|\| id:ebi-fl187-GCST90094707 | Weighted median | 22 | 0.0305270753972014 | 0.0783652307512758 | 0.696870277084074 | -0.123068777 | 0.184122927669702 | 1.03099780434595 | 0.884202847760519 | 1.20216359318271 | 0.999332001504091 |
| ebi-fl187-GCST90094707 | ebi-met1400-GCST90201020 | Paraxanthine to 5-acetylamino-6-formylamino-3-methyluracil ratio \|\| id:ebi-met1400-GCST90201020 | Black olive liking \|\| id:ebi-fl187-GCST90094707 | Weighted median | 22 | 0.0183545051434401 | 0.0750186542438844 | 0.806715148105274 | -0.128682057 | 0.165391067461453 | 1.0185239843876 | 0.879253473443076 | 1.17985443118065 | 0.999332001504091 |
| ebi-fl187-GCST90094707 | ebi-pw11-GCST90275197 | Placental weight (fetal genotype, adjusted for sex) \|\| id:ebi-pw11-GCST90275197 | Black olive liking \|\| id:ebi-fl187-GCST90094707 | Weighted median | 19 | -0.059647685 | 0.0288363575843388 | 0.0385940758488336 | -0.116166946 | -0.003128424 | 0.942096389997776 | 0.890326574725386 | 0.996876464482258 | 0.999332001504091 |
| ebi-fl187-GCST90094707 | prot-a-1017 | FAS-associated factor 2 \|\| id:prot-a-1017 | Black olive liking \|\| id:ebi-fl187-GCST90094707 | Weighted median | 22 | -0.069870356 | 0.116655963683726 | 0.549210421455167 | -0.298516045 | 0.158775333020605 | 0.932514707193282 | 0.741918377956461 | 1.17207459064022 | 0.999332001504091 |
| ebi-fl187-GCST90094707 | prot-a-1019 | Fas apoptotic inhibitory molecule 3 \|\| id:prot-a-1019 | Black olive liking \|\| id:ebi-fl187-GCST90094707 | Weighted median | 22 | -0.084259193 | 0.106039535335526 | 0.426845242317887 | -0.292096682 | 0.123578296612814 | 0.919192977157538 | 0.746696340460667 | 1.13153859671319 | 0.999332001504091 |
| ebi-fl187-GCST90094707 | prot-a-1052 | MIP18 family protein FAM96A \|\| id:prot-a-1052 | Black olive liking \|\| id:ebi-fl187-GCST90094707 | Weighted median | 22 | 0.0741383537827074 | 0.10504132091814 | 0.4803114001462 | -0.131742635 | 0.280019342782262 | 1.07695579609747 | 0.876566563414968 | 1.32315540559682 | 0.999332001504091 |
| ebi-fl187-GCST90094707 | prot-a-1066 | F-box/LRR-repeat protein 4 \|\| id:prot-a-1066 | Black olive liking \|\| id:ebi-fl187-GCST90094707 | Weighted median | 22 | -0.085336789 | 0.109782699428948 | 0.436967307424434 | -0.30051088 | 0.12983730215095 | 0.918202991901936 | 0.740439848417232 | 1.13864311346813 | 0.999332001504091 |
| ebi-fl187-GCST90094707 | prot-a-1076 | Low affinity immunoglobulin gamma Fc region receptor III-B \|\| id:prot-a-1076 | Black olive liking \|\| id:ebi-fl187-GCST90094707 | Weighted median | 22 | -0.248399403 | 0.116656087133345 | 0.0332268984544522 | -0.477045334 | -0.019753473 | 0.780048327153656 | 0.620614393654584 | 0.980440348977591 | 0.999332001504091 |
| ebi-fl187-GCST90094707 | prot-a-1081 | Fc receptor-like protein 3 \|\| id:prot-a-1081 | Black olive liking \|\| id:ebi-fl187-GCST90094707 | Weighted median | 22 | -0.111753722 | 0.117878863537892 | 0.343109764795205 | -0.342796294 | 0.119288851 | 0.894264468206338 | 0.709782783593289 | 1.12669531803494 | 0.999332001504091 |
| ebi-fl187-GCST90094707 | prot-a-1105 | Fibroblast growth factor receptor 4 \|\| id:prot-a-1105 | Black olive liking \|\| id:ebi-fl187-GCST90094707 | Weighted median | 22 | 0.026621907873465 | 0.114780035318359 | 0.816585790208584 | -0.198346961 | 0.251590777097448 | 1.02697943650995 | 0.820085265878906 | 1.28606964043422 | 0.999332001504091 |
| ebi-fl187-GCST90094707 | prot-a-1143 | Follistatin-related protein 1 \|\| id:prot-a-1143 | Black olive liking \|\| id:ebi-fl187-GCST90094707 | Weighted median | 22 | -0.088375192 | 0.118941487980728 | 0.457473197529659 | -0.321500509 | 0.144750124331895 | 0.915417354914793 | 0.725060261296095 | 1.15575074019535 | 0.999332001504091 |
| ebi-fl187-GCST90094707 | prot-a-1151 | Alpha-(1,3)-fucosyltransferase 10 \|\| id:prot-a-1151 | Black olive liking \|\| id:ebi-fl187-GCST90094707 | Weighted median | 22 | 0.00648832032440236 | 0.106218072153991 | 0.951291594681838 | -0.201699101 | 0.214675741746225 | 1.0065094150732 | 0.817340827903017 | 1.23945992668704 | 0.999332001504091 |
| ebi-fl187-GCST90094707 | prot-a-1165 | Growth arrest and DNA damage-inducible proteins-interacting protein 1 \|\| id:prot-a-1165 | Black olive liking \|\| id:ebi-fl187-GCST90094707 | Weighted median | 22 | 0.117769233382509 | 0.107044393413206 | 0.27124909362326 | -0.092037778 | 0.327576244472393 | 1.12498447254905 | 0.912070692954505 | 1.38760084415912 | 0.999332001504091 |
| ebi-fl187-GCST90094707 | prot-a-1194 | Growth/differentiation factor 11/8 \|\| id:prot-a-1194 | Black olive liking \|\| id:ebi-fl187-GCST90094707 | Weighted median | 22 | -0.237944568 | 0.116501374723267 | 0.0411100316160601 | -0.466287263 | -0.009601874 | 0.78824638364885 | 0.627327050406442 | 0.990444076870162 | 0.999332001504091 |
| ebi-fl187-GCST90094707 | prot-a-1216 | D-glucuronyl C5-epimerase \|\| id:prot-a-1216 | Black olive liking \|\| id:ebi-fl187-GCST90094707 | Weighted median | 22 | 0.170700685668193 | 0.105512733294483 | 0.105701240673836 | -0.036104272 | 0.37750564292538 | 1.18613566847897 | 0.964539714150789 | 1.45864167477723 | 0.999332001504091 |
| ebi-fl187-GCST90094707 | prot-a-1246 | Glypican-3 \|\| id:prot-a-1246 | Black olive liking \|\| id:ebi-fl187-GCST90094707 | Weighted median | 22 | 0.00379671165121955 | 0.109917166703894 | 0.972445293005728 | -0.211640935 | 0.219234358390851 | 1.00380392829118 | 0.809255220559295 | 1.24512304753055 | 0.999332001504091 |
| ebi-fl187-GCST90094707 | prot-a-1275 | Glutamate receptor 4 \|\| id:prot-a-1275 | Black olive liking \|\| id:ebi-fl187-GCST90094707 | Weighted median | 21 | 0.00823794702258631 | 0.106895273880787 | 0.938571369200132 | -0.20127679 | 0.217752683828929 | 1.00827197227673 | 0.8176860730771 | 1.24327954645615 | 0.999332001504091 |
| ebi-fl187-GCST90094707 | prot-a-1289 | Glutathione S-transferase P \|\| id:prot-a-1289 | Black olive liking \|\| id:ebi-fl187-GCST90094707 | Weighted median | 22 | 0.0999254069784379 | 0.108255603778271 | 0.355980803178819 | -0.112255576 | 0.31210639038385 | 1.1050884831121 | 0.893815790060158 | 1.36630004648364 | 0.999332001504091 |
| ebi-fl187-GCST90094707 | prot-a-130 | Apolipoprotein D \|\| id:prot-a-130 | Black olive liking \|\| id:ebi-fl187-GCST90094707 | Weighted median | 22 | 0.0562661203163967 | 0.103940898531753 | 0.588281513810838 | -0.147458041 | 0.259990281438633 | 1.0578791694197 | 0.862898644093536 | 1.29691748243238 | 0.999332001504091 |
| ebi-fl187-GCST90094707 | prot-a-1313 | Heparin-binding EGF-like growth factor \|\| id:prot-a-1313 | Black olive liking \|\| id:ebi-fl187-GCST90094707 | Weighted median | 22 | 0.0792503089086251 | 0.11087985027243 | 0.474769466904404 | -0.138074198 | 0.296574815442589 | 1.08247524135842 | 0.871034060689201 | 1.34524320119793 | 0.999332001504091 |
| ebi-fl187-GCST90094707 | prot-a-1347 | HLA class II histocompatibility antigen, DQ alpha 2 chain \|\| id:prot-a-1347 | Black olive liking \|\| id:ebi-fl187-GCST90094707 | Weighted median | 21 | -0.075760587 | 0.104121475406142 | 0.466847888359378 | -0.279838678 | 0.128317505142214 | 0.927038125292778 | 0.755905675495496 | 1.13691392141356 | 0.999332001504091 |
| ebi-fl187-GCST90094707 | prot-a-1357 | Non-histone chromosomal protein HMG-14 \|\| id:prot-a-1357 | Black olive liking \|\| id:ebi-fl187-GCST90094707 | Weighted median | 22 | 0.0331713512129748 | 0.110512242238014 | 0.764055138035597 | -0.183432644 | 0.249775345999482 | 1.0337276545539 | 0.832407941873995 | 1.2837369876407 | 0.999332001504091 |
| ebi-fl187-GCST90094707 | prot-a-137 | Apolipoprotein M \|\| id:prot-a-137 | Black olive liking \|\| id:ebi-fl187-GCST90094707 | Weighted median | 22 | -0.116560063 | 0.102059397457764 | 0.253420496347623 | -0.316596482 | 0.0834763556814858 | 0.889976640405557 | 0.728624708742032 | 1.08705951220766 | 0.999332001504091 |
| ebi-fl187-GCST90094707 | prot-a-1381 | 17-beta-hydroxysteroid dehydrogenase 14 \|\| id:prot-a-1381 | Black olive liking \|\| id:ebi-fl187-GCST90094707 | Weighted median | 21 | -0.182843662 | 0.108964895820617 | 0.0933460830785089 | -0.396414858 | 0.0307275335565567 | 0.832898359012866 | 0.67272755159161 | 1.03120449698403 | 0.999332001504091 |
| ebi-fl187-GCST90094707 | prot-a-1392 | Serine protease HTRA2, mitochondrial \|\| id:prot-a-1392 | Black olive liking \|\| id:ebi-fl187-GCST90094707 | Weighted median | 22 | 0.00929282773830139 | 0.115786351828139 | 0.936031740689419 | -0.217648422 | 0.236234077321454 | 1.00933614012287 | 0.804408204309521 | 1.26647072754884 | 0.999332001504091 |
| ebi-fl187-GCST90094707 | prot-a-1416 | Interferon-induced protein with tetratricopeptide repeats 2 \|\| id:prot-a-1416 | Black olive liking \|\| id:ebi-fl187-GCST90094707 | Weighted median | 22 | -0.173585208 | 0.105231952396286 | 0.0990353176840126 | -0.379839835 | 0.0326694184909128 | 0.840645518213741 | 0.683970948713589 | 1.03320892301344 | 0.999332001504091 |
| ebi-fl187-GCST90094707 | prot-a-1428 | Interferon gamma \|\| id:prot-a-1428 | Black olive liking \|\| id:ebi-fl187-GCST90094707 | Weighted median | 22 | -0.101129297 | 0.1090679189064 | 0.353815457466879 | -0.314902418 | 0.112643824339725 | 0.903816164867976 | 0.729860092167126 | 1.11923321831604 | 0.999332001504091 |
| ebi-fl187-GCST90094707 | prot-a-1483 | Interleukin-17C \|\| id:prot-a-1483 | Black olive liking \|\| id:ebi-fl187-GCST90094707 | Weighted median | 22 | 0.0885782628718616 | 0.112007129679773 | 0.429044949113082 | -0.130955711 | 0.308112237044217 | 1.09261976082471 | 0.877256626085794 | 1.36085371856501 | 0.999332001504091 |
| ebi-fl187-GCST90094707 | prot-a-1503 | Interleukin-1 receptor-like 2 \|\| id:prot-a-1503 | Black olive liking \|\| id:ebi-fl187-GCST90094707 | Weighted median | 22 | -0.125185287 | 0.106813802338342 | 0.241198896793273 | -0.334540339 | 0.084169765937167 | 0.882333402841051 | 0.715666974791527 | 1.08781355182115 | 0.999332001504091 |
| ebi-fl187-GCST90094707 | prot-a-1522 | Interleukin-31 receptor subunit alpha \|\| id:prot-a-1522 | Black olive liking \|\| id:ebi-fl187-GCST90094707 | Weighted median | 22 | -0.1649094 | 0.107918558582548 | 0.126489895749167 | -0.376429775 | 0.0466109749984293 | 0.847970526887301 | 0.686307312169039 | 1.04771434271477 | 0.999332001504091 |
| ebi-fl187-GCST90094707 | prot-a-1540 | Interleukin-6 receptor subunit alpha \|\| id:prot-a-1540 | Black olive liking \|\| id:ebi-fl187-GCST90094707 | Weighted median | 22 | 0.000233530690697996 | 0.105031631443392 | 0.99822595939437 | -0.205628467 | 0.206095528319745 | 1.00023355796111 | 0.814135498353845 | 1.22887059033104 | 0.999381480690152 |
| ebi-fl187-GCST90094707 | prot-a-1541 | Interleukin-6 receptor subunit alpha \|\| id:prot-a-1541 | Black olive liking \|\| id:ebi-fl187-GCST90094707 | Weighted median | 22 | -0.05138813 | 0.102288854144922 | 0.61539848941573 | -0.251874284 | 0.149098024269858 | 0.949909910578597 | 0.7773424563276 | 1.16078676890789 | 0.999332001504091 |
| ebi-fl187-GCST90094707 | prot-a-1545 | Interleukin-9 \|\| id:prot-a-1545 | Black olive liking \|\| id:ebi-fl187-GCST90094707 | Weighted median | 22 | 0.166055439472677 | 0.104370207559472 | 0.111604316197601 | -0.038510167 | 0.370621046289242 | 1.18063855388779 | 0.962221921448911 | 1.44863400412591 | 0.999332001504091 |
| ebi-fl187-GCST90094707 | prot-a-1548 | Inositol monophosphatase 3 \|\| id:prot-a-1548 | Black olive liking \|\| id:ebi-fl187-GCST90094707 | Weighted median | 22 | -0.100887591 | 0.110679965630378 | 0.362018649506032 | -0.317820324 | 0.116045141268401 | 0.904034648473415 | 0.727733532916728 | 1.12304656673553 | 0.999332001504091 |
| ebi-fl187-GCST90094707 | prot-a-1555 | Inhibin beta A chain:Inhibin beta B chain heterodimer \|\| id:prot-a-1555 | Black olive liking \|\| id:ebi-fl187-GCST90094707 | Weighted median | 22 | 0.00115983744465082 | 0.105835936505889 | 0.991256298211133 | -0.206278598 | 0.208598272996194 | 1.00116051031622 | 0.813606375509212 | 1.23194999152914 | 0.999332001504091 |
| ebi-fl187-GCST90094707 | prot-a-1603 | Junctophilin-3 \|\| id:prot-a-1603 | Black olive liking \|\| id:ebi-fl187-GCST90094707 | Weighted median | 22 | -0.074621058 | 0.107452376957109 | 0.487395640589589 | -0.285227717 | 0.135985600466864 | 0.928095113577242 | 0.751843027231684 | 1.14566539642925 | 0.999332001504091 |
| ebi-fl187-GCST90094707 | prot-a-1627 | UPF0258 protein KIAA1024 \|\| id:prot-a-1627 | Black olive liking \|\| id:ebi-fl187-GCST90094707 | Weighted median | 22 | 0.125675256350455 | 0.111978781465214 | 0.261729326838278 | -0.093803155 | 0.345153668022273 | 1.13391387715542 | 0.910461964195465 | 1.41220691403821 | 0.999332001504091 |
| ebi-fl187-GCST90094707 | prot-a-1666 | Kallikrein-7 \|\| id:prot-a-1666 | Black olive liking \|\| id:ebi-fl187-GCST90094707 | Weighted median | 22 | 0.0621378087382593 | 0.103595489614274 | 0.548631593425579 | -0.140909351 | 0.265184968382237 | 1.06410897813977 | 0.868568043035575 | 1.30367209159603 | 0.999332001504091 |
| ebi-fl187-GCST90094707 | prot-a-1678 | Importin subunit alpha-3 \|\| id:prot-a-1678 | Black olive liking \|\| id:ebi-fl187-GCST90094707 | Weighted median | 22 | 0.0859080353674582 | 0.114073885222684 | 0.45139513184812 | -0.13767678 | 0.309492850403918 | 1.08970610927498 | 0.871380294060621 | 1.36273382894358 | 0.999332001504091 |
| ebi-fl187-GCST90094707 | prot-a-1718 | Protein LDOC1 \|\| id:prot-a-1718 | Black olive liking \|\| id:ebi-fl187-GCST90094707 | Weighted median | 22 | 0.131295895601442 | 0.11192008861626 | 0.240746966287055 | -0.088067478 | 0.350659269289312 | 1.14030514267368 | 0.915699085016555 | 1.4200034047042 | 0.999332001504091 |
| ebi-fl187-GCST90094707 | prot-a-1723 | Leptin \|\| id:prot-a-1723 | Black olive liking \|\| id:ebi-fl187-GCST90094707 | Weighted median | 22 | -0.2214118 | 0.111749447982026 | 0.0475550141437859 | -0.440440718 | -0.002382882 | 0.801386601397568 | 0.643752645203001 | 0.997619954939409 | 0.999332001504091 |
| ebi-fl187-GCST90094707 | prot-a-1730 | Galectin-7 \|\| id:prot-a-1730 | Black olive liking \|\| id:ebi-fl187-GCST90094707 | Weighted median | 22 | -0.262218111 | 0.11470055767271 | 0.022247814277927 | -0.487031204 | -0.037405018 | 0.769343203041064 | 0.61444785927099 | 0.963285908047151 | 0.999332001504091 |
| ebi-fl187-GCST90094707 | prot-a-1753 | Lipase member N \|\| id:prot-a-1753 | Black olive liking \|\| id:ebi-fl187-GCST90094707 | Weighted median | 22 | 0.1415853219734 | 0.106259106820937 | 0.182711201986516 | -0.066682527 | 0.349853171342436 | 1.1520987994105 | 0.935492147326547 | 1.41885920410595 | 0.999332001504091 |
| ebi-fl187-GCST90094707 | prot-a-1756 | VIP36-like protein \|\| id:prot-a-1756 | Black olive liking \|\| id:ebi-fl187-GCST90094707 | Weighted median | 22 | -0.173923743 | 0.112286841205269 | 0.121400077118546 | -0.394005952 | 0.046158465847345 | 0.840360978693681 | 0.674350042714652 | 1.04724034963816 | 0.999332001504091 |
| ebi-fl187-GCST90094707 | prot-a-1760 | Ig Kappa chain V-I region HK102- like \|\| id:prot-a-1760 | Black olive liking \|\| id:ebi-fl187-GCST90094707 | Weighted median | 22 | -0.158930365 | 0.111335948597209 | 0.153440226895742 | -0.377148824 | 0.059288094025981 | 0.853055759247188 | 0.685814000392081 | 1.06108088777535 | 0.999332001504091 |
| ebi-fl187-GCST90094707 | prot-a-179 | Neutral ceramidase \|\| id:prot-a-179 | Black olive liking \|\| id:ebi-fl187-GCST90094707 | Weighted median | 22 | -0.106931148 | 0.109642420636568 | 0.329425511812513 | -0.321830293 | 0.107967996112025 | 0.898587540108156 | 0.724821187279155 | 1.11401209209775 | 0.999332001504091 |
| ebi-fl187-GCST90094707 | prot-a-1809 | Lactotransferrin \|\| id:prot-a-1809 | Black olive liking \|\| id:ebi-fl187-GCST90094707 | Weighted median | 22 | 0.0646234421079036 | 0.115620837705751 | 0.576212520742755 | -0.1619934 | 0.291240284011175 | 1.0667572528813 | 0.850446817644734 | 1.33808606601223 | 0.999332001504091 |
| ebi-fl187-GCST90094707 | prot-a-1818 | Lysophospholipase-like protein 1 \|\| id:prot-a-1818 | Black olive liking \|\| id:ebi-fl187-GCST90094707 | Weighted median | 22 | 0.0777244602850248 | 0.106107441569752 | 0.463859076405164 | -0.130246125 | 0.285695045761739 | 1.08082480747766 | 0.877879336108584 | 1.33068659485411 | 0.999332001504091 |
| ebi-fl187-GCST90094707 | prot-a-1852 | MAP kinase-activated protein kinase 3 \|\| id:prot-a-1852 | Black olive liking \|\| id:ebi-fl187-GCST90094707 | Weighted median | 22 | 0.100064367940919 | 0.109256921534301 | 0.359738774370408 | -0.114079198 | 0.314207934148149 | 1.10524205794155 | 0.89218729339741 | 1.36917440506378 | 0.999332001504091 |
| ebi-fl187-GCST90094707 | prot-a-1856 | Megakaryocyte-associated tyrosine-protein kinase \|\| id:prot-a-1856 | Black olive liking \|\| id:ebi-fl187-GCST90094707 | Weighted median | 22 | -0.073839767 | 0.102146592439296 | 0.469753384753983 | -0.274047088 | 0.126367554667134 | 0.9288205100666 | 0.760296273819006 | 1.13469915561595 | 0.999332001504091 |
| ebi-fl187-GCST90094707 | prot-a-1890 | Beta-1,3-N-acetylglucosaminyltransferase manic fringe \|\| id:prot-a-1890 | Black olive liking \|\| id:ebi-fl187-GCST90094707 | Weighted median | 22 | -0.024913314 | 0.103762414105735 | 0.810253042265058 | -0.228287646 | 0.178461017253358 | 0.975394461023746 | 0.79589529077647 | 1.19537628331439 | 0.999332001504091 |
| ebi-fl187-GCST90094707 | prot-a-1919 | Matrilysin \|\| id:prot-a-1919 | Black olive liking \|\| id:ebi-fl187-GCST90094707 | Weighted median | 22 | -0.093248791 | 0.114267843522903 | 0.414468951248381 | -0.317213764 | 0.130716182521543 | 0.910966831949088 | 0.72817508080528 | 1.13964428443998 | 0.999332001504091 |
| ebi-fl187-GCST90094707 | prot-a-1923 | Multimerin-2 \|\| id:prot-a-1923 | Black olive liking \|\| id:ebi-fl187-GCST90094707 | Weighted median | 22 | -0.079071231 | 0.111201091634025 | 0.477043807838582 | -0.297025371 | 0.138882908517306 | 0.923974106422503 | 0.743025161063213 | 1.14898955523611 | 0.999332001504091 |
| ebi-fl187-GCST90094707 | prot-a-1948 | Mesothelin \|\| id:prot-a-1948 | Black olive liking \|\| id:ebi-fl187-GCST90094707 | Weighted median | 22 | 0.172824178358327 | 0.10936744517855 | 0.114057426408392 | -0.041536014 | 0.387184370908285 | 1.18865709506849 | 0.95931478577281 | 1.47282801288051 | 0.999332001504091 |
| ebi-fl187-GCST90094707 | prot-a-195 | ATPase family AAA domain-containing protein 1 \|\| id:prot-a-195 | Black olive liking \|\| id:ebi-fl187-GCST90094707 | Weighted median | 22 | -0.034664116 | 0.0987008855166883 | 0.725435535470446 | -0.228117852 | 0.15878961955127 | 0.96592980208282 | 0.796030440787077 | 1.17209133563941 | 0.999332001504091 |
| ebi-fl187-GCST90094707 | prot-a-1966 | Mucin-1 \|\| id:prot-a-1966 | Black olive liking \|\| id:ebi-fl187-GCST90094707 | Weighted median | 22 | 0.0170957193441194 | 0.105956907271911 | 0.871820923440311 | -0.190579819 | 0.224771257597065 | 1.01724268746816 | 0.826479786381495 | 1.25203628964471 | 0.999332001504091 |
| ebi-fl187-GCST90094707 | prot-a-1986 | Myocardial zonula adherens protein \|\| id:prot-a-1986 | Black olive liking \|\| id:ebi-fl187-GCST90094707 | Weighted median | 22 | -0.168874676 | 0.102146525487304 | 0.0982781363252709 | -0.369081866 | 0.0313325137663778 | 0.844614747092681 | 0.691368808435118 | 1.03182854404601 | 0.999332001504091 |
| ebi-fl187-GCST90094707 | prot-a-2071 | Epididymal secretory protein E1 \|\| id:prot-a-2071 | Black olive liking \|\| id:ebi-fl187-GCST90094707 | Weighted median | 22 | -0.02757211 | 0.113370282630329 | 0.807847344005989 | -0.249777864 | 0.194633644179184 | 0.972804531307091 | 0.778973802187299 | 1.21486583178322 | 0.999332001504091 |
| ebi-fl187-GCST90094707 | prot-a-2098 | Neuritin \|\| id:prot-a-2098 | Black olive liking \|\| id:ebi-fl187-GCST90094707 | Weighted median | 22 | -0.067128708 | 0.115275371626304 | 0.56034208258397 | -0.293068436 | 0.158811020802373 | 0.93507484236646 | 0.745971087693289 | 1.17211642012882 | 0.999332001504091 |
| ebi-fl187-GCST90094707 | prot-a-2115 | Neurotrophin-4 \|\| id:prot-a-2115 | Black olive liking \|\| id:ebi-fl187-GCST90094707 | Weighted median | 22 | -0.047525952 | 0.109890916119059 | 0.665390409265805 | -0.262912148 | 0.16786024313312 | 0.953585724915883 | 0.768809435749774 | 1.1827712986854 | 0.999332001504091 |
| ebi-fl187-GCST90094707 | prot-a-2118 | Netrin-1 \|\| id:prot-a-2118 | Black olive liking \|\| id:ebi-fl187-GCST90094707 | Weighted median | 22 | 0.0100494460814771 | 0.111887146890107 | 0.928432083267596 | -0.209249362 | 0.229348253986087 | 1.01010011134179 | 0.811192929872886 | 1.2577799896414 | 0.999332001504091 |
| ebi-fl187-GCST90094707 | prot-a-2120 | Netrin-G1 \|\| id:prot-a-2120 | Black olive liking \|\| id:ebi-fl187-GCST90094707 | Weighted median | 22 | -0.111387521 | 0.103507919043894 | 0.281871089343316 | -0.314263042 | 0.0914880001639574 | 0.894592008414043 | 0.730326895887231 | 1.09580362714978 | 0.999332001504091 |
| ebi-fl187-GCST90094707 | prot-a-2145 | Olfactomedin-like protein 3 \|\| id:prot-a-2145 | Black olive liking \|\| id:ebi-fl187-GCST90094707 | Weighted median | 22 | 0.0530113601113863 | 0.107028153331907 | 0.620386288661392 | -0.15676382 | 0.262786540641923 | 1.05444162362561 | 0.854905946303959 | 1.30054907494924 | 0.999332001504091 |
| ebi-fl187-GCST90094707 | prot-a-2162 | Prolyl 4-hydroxylase subunit alpha-2 \|\| id:prot-a-2162 | Black olive liking \|\| id:ebi-fl187-GCST90094707 | Weighted median | 22 | 0.0607280464896568 | 0.110230143408999 | 0.581688169048105 | -0.155323035 | 0.276779127571294 | 1.0626098943981 | 0.856138570435417 | 1.31887503572987 | 0.999332001504091 |
| ebi-fl187-GCST90094707 | prot-a-2168 | Polyadenylate-binding protein-interacting protein 1 \|\| id:prot-a-2168 | Black olive liking \|\| id:ebi-fl187-GCST90094707 | Weighted median | 22 | 0.0293139793446124 | 0.104474703784051 | 0.779029129195228 | -0.17545644 | 0.234084398761352 | 1.02974786328184 | 0.839073946378002 | 1.2637511467384 | 0.999332001504091 |
| ebi-fl187-GCST90094707 | prot-a-219 | Beta-1,4-galactosyltransferase 5 \|\| id:prot-a-219 | Black olive liking \|\| id:ebi-fl187-GCST90094707 | Weighted median | 22 | -0.045096404 | 0.112316799047922 | 0.688044116095423 | -0.26523733 | 0.175044522534098 | 0.955905324676636 | 0.767023890796895 | 1.19129925509335 | 0.999332001504091 |
| ebi-fl187-GCST90094707 | prot-a-2190 | Pyruvate carboxylase, mitochondrial \|\| id:prot-a-2190 | Black olive liking \|\| id:ebi-fl187-GCST90094707 | Weighted median | 22 | -0.019167115 | 0.106878757922473 | 0.857674571123982 | -0.228649481 | 0.190315250523099 | 0.981015406148974 | 0.795607360503044 | 1.20963087432115 | 0.999332001504091 |
| ebi-fl187-GCST90094707 | prot-a-2197 | Protocadherin-9 \|\| id:prot-a-2197 | Black olive liking \|\| id:ebi-fl187-GCST90094707 | Weighted median | 22 | -0.036274019 | 0.116407490637457 | 0.75533509910806 | -0.264432701 | 0.191884662548367 | 0.964375999829477 | 0.767641308813213 | 1.21153077403421 | 0.999332001504091 |
| ebi-fl187-GCST90094707 | prot-a-2198 | Protocadherin alpha-4 \|\| id:prot-a-2198 | Black olive liking \|\| id:ebi-fl187-GCST90094707 | Weighted median | 22 | -0.004965874 | 0.112450227380788 | 0.964776354416987 | -0.22536832 | 0.215436571411627 | 0.995046435314486 | 0.798222163485249 | 1.24040330339733 | 0.999332001504091 |
| ebi-fl187-GCST90094707 | prot-a-2254 | Placenta growth factor \|\| id:prot-a-2254 | Black olive liking \|\| id:ebi-fl187-GCST90094707 | Weighted median | 22 | -0.192223711 | 0.113017769884491 | 0.0889754161777103 | -0.41373854 | 0.0292911174818717 | 0.825122258511453 | 0.661173798862386 | 1.02972432159664 | 0.999332001504091 |
| ebi-fl187-GCST90094707 | prot-a-2261 | Membrane-associated progesterone receptor component 2 \|\| id:prot-a-2261 | Black olive liking \|\| id:ebi-fl187-GCST90094707 | Weighted median | 22 | -0.032054209 | 0.112524816030655 | 0.775748746761073 | -0.252602849 | 0.18849443032251 | 0.968454081634403 | 0.776776318437971 | 1.2074303579702 | 0.999332001504091 |
| ebi-fl187-GCST90094707 | prot-a-2268 | Polymeric immunoglobulin receptor \|\| id:prot-a-2268 | Black olive liking \|\| id:ebi-fl187-GCST90094707 | Weighted median | 22 | -0.009320523 | 0.116479118611884 | 0.936222278733753 | -0.237619595 | 0.218978549529818 | 0.99072277848944 | 0.788502584037227 | 1.24480457475773 | 0.999332001504091 |
| ebi-fl187-GCST90094707 | prot-a-2283 | Group 10 secretory phospholipase A2 \|\| id:prot-a-2283 | Black olive liking \|\| id:ebi-fl187-GCST90094707 | Weighted median | 22 | 0.15663025210494 | 0.110206104761211 | 0.155244553194419 | -0.059373713 | 0.372634217436913 | 1.16956309045269 | 0.942354532990009 | 1.45155328983147 | 0.999332001504091 |
| ebi-fl187-GCST90094707 | prot-a-2298 | Inactive phospholipase D5 \|\| id:prot-a-2298 | Black olive liking \|\| id:ebi-fl187-GCST90094707 | Weighted median | 22 | 0.179750558290423 | 0.114929854846977 | 0.117817026297815 | -0.045511957 | 0.405013073790498 | 1.19691876441905 | 0.955508177292048 | 1.49932210175168 | 0.999332001504091 |
| ebi-fl187-GCST90094707 | prot-a-2304 | Procollagen-lysine,2-oxoglutarate 5-dioxygenase 2 \|\| id:prot-a-2304 | Black olive liking \|\| id:ebi-fl187-GCST90094707 | Weighted median | 22 | -0.021997276 | 0.109711860568028 | 0.841089213204207 | -0.237032523 | 0.193037970296857 | 0.978242899373824 | 0.788965627968804 | 1.21292884791319 | 0.999332001504091 |
| ebi-fl187-GCST90094707 | prot-a-2331 | Periostin \|\| id:prot-a-2331 | Black olive liking \|\| id:ebi-fl187-GCST90094707 | Weighted median | 22 | -0.068967206 | 0.111797213708777 | 0.537303606243672 | -0.288089745 | 0.150155332527939 | 0.933357287776704 | 0.749694307746421 | 1.16201472739546 | 0.999332001504091 |
| ebi-fl187-GCST90094707 | prot-a-2333 | Periostin \|\| id:prot-a-2333 | Black olive liking \|\| id:ebi-fl187-GCST90094707 | Weighted median | 22 | -0.146400637 | 0.113221272220006 | 0.195994160342287 | -0.368314331 | 0.0755130564218326 | 0.86381155886687 | 0.691899662212274 | 1.07843730815855 | 0.999332001504091 |
| ebi-fl187-GCST90094707 | prot-a-2367 | cAMP-dependent protein kinase catalytic subunit alpha \|\| id:prot-a-2367 | Black olive liking \|\| id:ebi-fl187-GCST90094707 | Weighted median | 22 | 0.0659391305571124 | 0.115842902690633 | 0.569212510951327 | -0.161112959 | 0.292991219830753 | 1.06816169677974 | 0.851195915678307 | 1.34043102116878 | 0.999332001504091 |
| ebi-fl187-GCST90094707 | prot-a-2385 | Phosphoribosyl pyrophosphate synthase-associated protein 1 \|\| id:prot-a-2385 | Black olive liking \|\| id:ebi-fl187-GCST90094707 | Weighted median | 22 | -0.036952549 | 0.112694312160912 | 0.742986707993082 | -0.257833401 | 0.183928302558601 | 0.963721863563529 | 0.772723956335696 | 1.20192964472669 | 0.999332001504091 |
| ebi-fl187-GCST90094707 | prot-a-2393 | Trypsin-3 \|\| id:prot-a-2393 | Black olive liking \|\| id:ebi-fl187-GCST90094707 | Weighted median | 21 | -0.027239806 | 0.121892145850946 | 0.823166099032447 | -0.266148412 | 0.211668799418405 | 0.973127851206346 | 0.766325386934494 | 1.23573853997144 | 0.999332001504091 |
| ebi-fl187-GCST90094707 | prot-a-2405 | Pregnancy-specific beta-1-glycoprotein 6 \|\| id:prot-a-2405 | Black olive liking \|\| id:ebi-fl187-GCST90094707 | Weighted median | 22 | -0.154280972 | 0.116033514907789 | 0.18364211380559 | -0.381706661 | 0.0731447173895864 | 0.857031185558754 | 0.682695284954847 | 1.07588622509503 | 0.999332001504091 |
| ebi-fl187-GCST90094707 | prot-a-244 | Beclin-1 \|\| id:prot-a-244 | Black olive liking \|\| id:ebi-fl187-GCST90094707 | Weighted median | 22 | -0.095014392 | 0.107731978613147 | 0.377802974110186 | -0.30616907 | 0.116140285739967 | 0.909359846556316 | 0.736262128782579 | 1.123153423 | 0.999332001504091 |
| ebi-fl187-GCST90094707 | prot-a-2454 | tRNA pseudouridine synthase A, mitochondrial \|\| id:prot-a-2454 | Black olive liking \|\| id:ebi-fl187-GCST90094707 | Weighted median | 22 | 0.183938415646825 | 0.112245557605089 | 0.10127274608871 | -0.036062877 | 0.403939708552799 | 1.20194180000869 | 0.964579641452241 | 1.4977136449128 | 0.999332001504091 |
| ebi-fl187-GCST90094707 | prot-a-2466 | Glutaminyl-peptide cyclotransferase-like protein \|\| id:prot-a-2466 | Black olive liking \|\| id:ebi-fl187-GCST90094707 | Weighted median | 22 | 0.068387483636753 | 0.103392464521067 | 0.50833283544161 | -0.134261747 | 0.271036714098043 | 1.07078013788577 | 0.874361173388233 | 1.31132321354985 | 0.999332001504091 |
| ebi-fl187-GCST90094707 | prot-a-2474 | Ras-related protein Rab-27A \|\| id:prot-a-2474 | Black olive liking \|\| id:ebi-fl187-GCST90094707 | Weighted median | 22 | 0.0122113040135167 | 0.110605105088397 | 0.912088565185884 | -0.204574702 | 0.228997309986775 | 1.01228616639848 | 0.814993858005093 | 1.25733865674768 | 0.999332001504091 |
| ebi-fl187-GCST90094707 | prot-a-2490 | Rap1 GTPase-GDP dissociation stimulator 1 \|\| id:prot-a-2490 | Black olive liking \|\| id:ebi-fl187-GCST90094707 | Weighted median | 22 | 0.131332384391116 | 0.113198201498478 | 0.245967994653649 | -0.090536091 | 0.353200859328133 | 1.14034675178732 | 0.913441366708482 | 1.42361706148447 | 0.999332001504091 |
| ebi-fl187-GCST90094707 | prot-a-2545 | Receptor-interacting serine/threonine-protein kinase 2 \|\| id:prot-a-2545 | Black olive liking \|\| id:ebi-fl187-GCST90094707 | Weighted median | 22 | -0.032954195 | 0.107473772644422 | 0.759128473131763 | -0.243602789 | 0.177694399527557 | 0.967582878847571 | 0.783798905798744 | 1.19446023783982 | 0.999332001504091 |
| ebi-fl187-GCST90094707 | prot-a-2610 | RUN and FYVE domain-containing protein 1 \|\| id:prot-a-2610 | Black olive liking \|\| id:ebi-fl187-GCST90094707 | Weighted median | 22 | -0.189026297 | 0.114424348253368 | 0.0985394082552987 | -0.413298019 | 0.0352454256759316 | 0.827764738761407 | 0.661465123960241 | 1.03587390765885 | 0.999332001504091 |
| ebi-fl187-GCST90094707 | prot-a-2627 | Serine--tRNA ligase, mitochondrial \|\| id:prot-a-2627 | Black olive liking \|\| id:ebi-fl187-GCST90094707 | Weighted median | 22 | 0.0287499790838598 | 0.100833710106882 | 0.775550143963537 | -0.168884093 | 0.226384050893348 | 1.02916724896714 | 0.844606793784203 | 1.25405719459228 | 0.999332001504091 |
| ebi-fl187-GCST90094707 | prot-a-264 | BolA-like protein 2 \|\| id:prot-a-264 | Black olive liking \|\| id:ebi-fl187-GCST90094707 | Weighted median | 22 | 0.0409416875571322 | 0.104401681359281 | 0.694943364318811 | -0.163685608 | 0.245568983021322 | 1.04179135437539 | 0.849008901611074 | 1.27834846488864 | 0.999332001504091 |
| ebi-fl187-GCST90094707 | prot-a-2646 | Secretoglobin family 1C member 1 \|\| id:prot-a-2646 | Black olive liking \|\| id:ebi-fl187-GCST90094707 | Weighted median | 22 | -0.003895913 | 0.123974409131746 | 0.97493049292722 | -0.246885755 | 0.239093928843428 | 0.996111666168611 | 0.781229940084501 | 1.27009782980141 | 0.999332001504091 |
| ebi-fl187-GCST90094707 | prot-a-2672 | Semaphorin-3E \|\| id:prot-a-2672 | Black olive liking \|\| id:ebi-fl187-GCST90094707 | Weighted median | 22 | -0.007524339 | 0.113368466581617 | 0.947082733126728 | -0.229726534 | 0.214677855192879 | 0.992503897668002 | 0.794750910294587 | 1.23946254622224 | 0.999332001504091 |
| ebi-fl187-GCST90094707 | prot-a-2701 | Plasma protease C1 inhibitor \|\| id:prot-a-2701 | Black olive liking \|\| id:ebi-fl187-GCST90094707 | Weighted median | 22 | 0.0813904941024692 | 0.10258250666522 | 0.427536105032799 | -0.119671219 | 0.282452207166301 | 1.08479441969302 | 0.887212087281491 | 1.32637838220046 | 0.999332001504091 |
| ebi-fl187-GCST90094707 | prot-a-2738 | Signaling threshold-regulating transmembrane adapter 1 \|\| id:prot-a-2738 | Black olive liking \|\| id:ebi-fl187-GCST90094707 | Weighted median | 22 | -0.168026054 | 0.110910379321668 | 0.129779644867673 | -0.385410397 | 0.0493582898849538 | 0.845331810473142 | 0.68017143856161 | 1.05059670148598 | 0.999332001504091 |
| ebi-fl187-GCST90094707 | prot-a-274 | Serine/threonine-protein kinase BRSK2 \|\| id:prot-a-274 | Black olive liking \|\| id:ebi-fl187-GCST90094707 | Weighted median | 22 | 0.0595704942936988 | 0.115568438368841 | 0.606234110637664 | -0.166943645 | 0.286084633496628 | 1.06138057961667 | 0.846247300336841 | 1.33120511502844 | 0.999332001504091 |
| ebi-fl187-GCST90094707 | prot-a-2749 | Mitochondrial glutamate carrier 2 \|\| id:prot-a-2749 | Black olive liking \|\| id:ebi-fl187-GCST90094707 | Weighted median | 22 | 0.0684488217518365 | 0.1151798961549 | 0.552326519085517 | -0.157303775 | 0.29420141821544 | 1.07084581953547 | 0.854444460770229 | 1.3420541906058 | 0.999332001504091 |
| ebi-fl187-GCST90094707 | prot-a-2760 | Sodium-coupled monocarboxylate transporter 1 \|\| id:prot-a-2760 | Black olive liking \|\| id:ebi-fl187-GCST90094707 | Weighted median | 22 | -0.117051786 | 0.110684005540271 | 0.290269234512924 | -0.333992437 | 0.0998888644209717 | 0.889539125907836 | 0.716059197549852 | 1.10504810109054 | 0.999332001504091 |
| ebi-fl187-GCST90094707 | prot-a-2777 | Small integral membrane protein 10 \|\| id:prot-a-2777 | Black olive liking \|\| id:ebi-fl187-GCST90094707 | Weighted median | 22 | -0.129113589 | 0.109263243492944 | 0.23733488043322 | -0.343269546 | 0.085042368695198 | 0.878874129836699 | 0.709446957339835 | 1.08876319519715 | 0.999332001504091 |
| ebi-fl187-GCST90094707 | prot-a-2799 | Superoxide dismutase [Mn], mitochondrial \|\| id:prot-a-2799 | Black olive liking \|\| id:ebi-fl187-GCST90094707 | Weighted median | 22 | 0.0182234400903691 | 0.114408502924877 | 0.873445152754523 | -0.206017226 | 0.242464105823128 | 1.01839050023528 | 0.813819057606125 | 1.27438550532372 | 0.999332001504091 |
| ebi-fl187-GCST90094707 | prot-a-2805 | Protein SPACA7 \|\| id:prot-a-2805 | Black olive liking \|\| id:ebi-fl187-GCST90094707 | Weighted median | 22 | -0.014993553 | 0.119117787427915 | 0.899833517420751 | -0.248464417 | 0.218477309893758 | 0.985118290182173 | 0.779997615134013 | 1.24418078571267 | 0.999332001504091 |
| ebi-fl187-GCST90094707 | prot-a-2811 | SPARC-like protein 1 \|\| id:prot-a-2811 | Black olive liking \|\| id:ebi-fl187-GCST90094707 | Weighted median | 22 | 0.0982901953128622 | 0.105305477306606 | 0.350622975115558 | -0.10810854 | 0.30468893083381 | 1.10328290618558 | 0.897530173031609 | 1.35620306442715 | 0.999332001504091 |
| ebi-fl187-GCST90094707 | prot-a-2831 | Sepiapterin reductase \|\| id:prot-a-2831 | Black olive liking \|\| id:ebi-fl187-GCST90094707 | Weighted median | 22 | -0.080701938 | 0.106400100040036 | 0.448165985362199 | -0.289246134 | 0.127842257796391 | 0.922468603048812 | 0.74882787044896 | 1.13637373446135 | 0.999332001504091 |
| ebi-fl187-GCST90094707 | prot-a-2834 | SPRY domain-containing SOCS box protein 1 \|\| id:prot-a-2834 | Black olive liking \|\| id:ebi-fl187-GCST90094707 | Weighted median | 22 | 0.117930494560599 | 0.110554672508751 | 0.286099868913846 | -0.098756664 | 0.334617652677752 | 1.12516590349892 | 0.905963135050673 | 1.39740598862858 | 0.999332001504091 |
| ebi-fl187-GCST90094707 | prot-a-2835 | Spectrin alpha chain, non-erythrocytic 1 \|\| id:prot-a-2835 | Black olive liking \|\| id:ebi-fl187-GCST90094707 | Weighted median | 22 | -0.039269671 | 0.10663387571432 | 0.712674622258988 | -0.248272067 | 0.169732725535396 | 0.961491387968288 | 0.78014766176149 | 1.18498809193357 | 0.999332001504091 |
| ebi-fl187-GCST90094707 | prot-a-2859 | Sia-alpha-2,3-Gal-beta-1,4-GlcNAc-R:alpha 2,8-sialyltransferase \|\| id:prot-a-2859 | Black olive liking \|\| id:ebi-fl187-GCST90094707 | Weighted median | 22 | -0.055311393 | 0.117713557869055 | 0.638439865907888 | -0.286029966 | 0.175407180527155 | 0.946190465085517 | 0.751240103708585 | 1.19173136764013 | 0.999332001504091 |
| ebi-fl187-GCST90094707 | prot-a-2861 | Alpha-2,8-sialyltransferase 8F \|\| id:prot-a-2861 | Black olive liking \|\| id:ebi-fl187-GCST90094707 | Weighted median | 22 | 0.0105990396814434 | 0.107820606302404 | 0.921692059722046 | -0.200729349 | 0.221927428034155 | 1.01065540847821 | 0.818133830599867 | 1.24848076987273 | 0.999332001504091 |
| ebi-fl187-GCST90094707 | prot-a-289 | Uncharacterized protein C17orf89 \|\| id:prot-a-289 | Black olive liking \|\| id:ebi-fl187-GCST90094707 | Weighted median | 22 | 0.171594553043026 | 0.109931984254934 | 0.118543657701922 | -0.043872136 | 0.387061242182697 | 1.18719639045691 | 0.957076325164418 | 1.47264667660835 | 0.999332001504091 |
| ebi-fl187-GCST90094707 | prot-a-2921 | Tachykinin-3 \|\| id:prot-a-2921 | Black olive liking \|\| id:ebi-fl187-GCST90094707 | Weighted median | 22 | -0.093650456 | 0.113542793124623 | 0.409483343760339 | -0.316194331 | 0.128893418241653 | 0.910601001477382 | 0.728917785221795 | 1.13756887361351 | 0.999332001504091 |
| ebi-fl187-GCST90094707 | prot-a-2928 | Tax1-binding protein 1 \|\| id:prot-a-2928 | Black olive liking \|\| id:ebi-fl187-GCST90094707 | Weighted median | 22 | 0.00219565953937357 | 0.112310307223718 | 0.984402399904449 | -0.217932543 | 0.222323861697861 | 1.00219807176493 | 0.804179687692385 | 1.24897580779677 | 0.999332001504091 |
| ebi-fl187-GCST90094707 | prot-a-296 | Uncharacterized protein C1orf185 \|\| id:prot-a-296 | Black olive liking \|\| id:ebi-fl187-GCST90094707 | Weighted median | 22 | 0.00338523424356202 | 0.110831450904208 | 0.975633214272277 | -0.21384441 | 0.220614878015809 | 1.00339097062017 | 0.807474010511233 | 1.24684315137853 | 0.999332001504091 |
| ebi-fl187-GCST90094707 | prot-a-2964 | Transforming growth factor beta-2 \|\| id:prot-a-2964 | Black olive liking \|\| id:ebi-fl187-GCST90094707 | Weighted median | 22 | -0.053600762 | 0.109403838181864 | 0.624179940539019 | -0.268032285 | 0.160830760390138 | 0.947810432494227 | 0.764883086237501 | 1.17448618240978 | 0.999332001504091 |
| ebi-fl187-GCST90094707 | prot-a-2977 | Thymocyte nuclear protein 1 \|\| id:prot-a-2977 | Black olive liking \|\| id:ebi-fl187-GCST90094707 | Weighted median | 22 | 0.153324284448784 | 0.111995792065067 | 0.170993566947857 | -0.066187468 | 0.372836036896316 | 1.16570293699083 | 0.935955386160704 | 1.45184627109538 | 0.999332001504091 |
| ebi-fl187-GCST90094707 | prot-a-3015 | Transmembrane protein 70, mitochondrial \|\| id:prot-a-3015 | Black olive liking \|\| id:ebi-fl187-GCST90094707 | Weighted median | 22 | -0.118202485 | 0.100480432123104 | 0.239445785303133 | -0.315144132 | 0.0787391617543908 | 0.888516123027693 | 0.72968369579628 | 1.0819220786051 | 0.999332001504091 |
| ebi-fl187-GCST90094707 | prot-a-3030 | Tumor necrosis factor alpha-induced protein 3 \|\| id:prot-a-3030 | Black olive liking \|\| id:ebi-fl187-GCST90094707 | Weighted median | 22 | -0.239727586 | 0.115630581693632 | 0.0381518486494808 | -0.466363527 | -0.013091646 | 0.786842178366827 | 0.627279209952551 | 0.986993676554155 | 0.999332001504091 |
| ebi-fl187-GCST90094707 | prot-a-3032 | Tumor necrosis factor receptor superfamily member 10A \|\| id:prot-a-3032 | Black olive liking \|\| id:ebi-fl187-GCST90094707 | Weighted median | 22 | 0.249194130513453 | 0.111966787985653 | 0.026040478949397 | 0.0297392260615726 | 0.468649034965333 | 1.28299107661201 | 1.03018585330031 | 1.59783411642929 | 0.999332001504091 |
| ebi-fl187-GCST90094707 | prot-a-3081 | Tumor protein p53-inducible protein 11 \|\| id:prot-a-3081 | Black olive liking \|\| id:ebi-fl187-GCST90094707 | Weighted median | 22 | -0.121586511 | 0.110442406018459 | 0.27093834548065 | -0.338053627 | 0.0948806050093156 | 0.885514443483841 | 0.71315704269744 | 1.09952756920494 | 0.999332001504091 |
| ebi-fl187-GCST90094707 | prot-a-315 | Complement C4 \|\| id:prot-a-315 | Black olive liking \|\| id:ebi-fl187-GCST90094707 | Weighted median | 21 | 0.0704448925936093 | 0.112649027009104 | 0.531742398345152 | -0.1503472 | 0.291236985531453 | 1.07298543835684 | 0.860409190191701 | 1.33808165236975 | 0.999332001504091 |
| ebi-fl187-GCST90094707 | prot-a-3157 | UDP-glucuronosyltransferase 1-8 \|\| id:prot-a-3157 | Black olive liking \|\| id:ebi-fl187-GCST90094707 | Weighted median | 22 | 0.111361871646982 | 0.113122723080252 | 0.324902094364595 | -0.110358666 | 0.333082408884275 | 1.1177993335606 | 0.895512888031185 | 1.39526227573958 | 0.999332001504091 |
| ebi-fl187-GCST90094707 | prot-a-317 | Glutaredoxin-like protein C5orf63 \|\| id:prot-a-317 | Black olive liking \|\| id:ebi-fl187-GCST90094707 | Weighted median | 22 | 0.0459704272133666 | 0.110177602650826 | 0.676503372772289 | -0.169977674 | 0.261918528408985 | 1.04704344650665 | 0.843683652482317 | 1.29942067224717 | 0.999332001504091 |
| ebi-fl187-GCST90094707 | prot-a-3192 | Guanine nucleotide exchange factor VAV3 \|\| id:prot-a-3192 | Black olive liking \|\| id:ebi-fl187-GCST90094707 | Weighted median | 22 | 0.00983897216398439 | 0.101390263882032 | 0.922694151479818 | -0.188885945 | 0.208563889372768 | 1.00988753398607 | 0.827880925232019 | 1.23190763335277 | 0.999332001504091 |
| ebi-fl187-GCST90094707 | prot-a-3233 | Serine/threonine-protein kinase WNK3 \|\| id:prot-a-3233 | Black olive liking \|\| id:ebi-fl187-GCST90094707 | Weighted median | 22 | -0.104907312 | 0.104105799013832 | 0.313598971718118 | -0.308954679 | 0.0991400535788555 | 0.90040797529348 | 0.734214044913412 | 1.10422093882407 | 0.999332001504091 |
| ebi-fl187-GCST90094707 | prot-a-3250 | 14-3-3 protein theta \|\| id:prot-a-3250 | Black olive liking \|\| id:ebi-fl187-GCST90094707 | Weighted median | 22 | 0.0947766851885222 | 0.104544008036846 | 0.364633099593449 | -0.110129571 | 0.299682940940741 | 1.09941331243386 | 0.895718069082184 | 1.34943089045343 | 0.999332001504091 |
| ebi-fl187-GCST90094707 | prot-a-3278 | E3 ubiquitin-protein ligase ZNRF3 \|\| id:prot-a-3278 | Black olive liking \|\| id:ebi-fl187-GCST90094707 | Weighted median | 22 | 0.0194205637408819 | 0.103224661112826 | 0.850767847138601 | -0.182899772 | 0.22174089952202 | 1.01961036961012 | 0.832851626573266 | 1.24824791433007 | 0.999332001504091 |
| ebi-fl187-GCST90094707 | prot-a-355 | Calpain-3 \|\| id:prot-a-355 | Black olive liking \|\| id:ebi-fl187-GCST90094707 | Weighted median | 22 | 0.00492663394128151 | 0.111278085116531 | 0.964686655345609 | -0.213178413 | 0.223031680769683 | 1.0049387897565 | 0.808011964608312 | 1.24986016964094 | 0.999332001504091 |
| ebi-fl187-GCST90094707 | prot-a-385 | Coiled-coil domain-containing protein 90B, mitochondrial \|\| id:prot-a-385 | Black olive liking \|\| id:ebi-fl187-GCST90094707 | Weighted median | 22 | 0.0442840345325472 | 0.108945823261087 | 0.684391727447355 | -0.169249779 | 0.257817848124278 | 1.04527920811942 | 0.844297989088758 | 1.29410307385193 | 0.999332001504091 |
| ebi-fl187-GCST90094707 | prot-a-390 | C-C motif chemokine 14 \|\| id:prot-a-390 | Black olive liking \|\| id:ebi-fl187-GCST90094707 | Weighted median | 22 | -0.014522529 | 0.122393862441991 | 0.905549433180384 | -0.254414499 | 0.225369441539533 | 0.985582414447072 | 0.775370345015877 | 1.25278546169757 | 0.999332001504091 |
| ebi-fl187-GCST90094707 | prot-a-40 | Pituitary adenylate cyclase-activating polypeptide 27 \|\| id:prot-a-40 | Black olive liking \|\| id:ebi-fl187-GCST90094707 | Weighted median | 22 | 0.102556913786056 | 0.110396677873185 | 0.352896597173201 | -0.113820575 | 0.318934402417499 | 1.10800036061081 | 0.892418063767359 | 1.3756610819049 | 0.999332001504091 |
| ebi-fl187-GCST90094707 | prot-a-401 | C-C motif chemokine 24 \|\| id:prot-a-401 | Black olive liking \|\| id:ebi-fl187-GCST90094707 | Weighted median | 22 | 0.171899685111739 | 0.105010865371803 | 0.101636640969784 | -0.033921611 | 0.377720981240472 | 1.18755869742042 | 0.966647276164545 | 1.45895581003927 | 0.999332001504091 |
| ebi-fl187-GCST90094707 | prot-a-412 | Cerebral cavernous malformations 2 protein \|\| id:prot-a-412 | Black olive liking \|\| id:ebi-fl187-GCST90094707 | Weighted median | 22 | -0.065168006 | 0.107567844716842 | 0.544626540599386 | -0.276000982 | 0.145664969198304 | 0.936910043226727 | 0.758812185537713 | 1.15680855662205 | 0.999332001504091 |
| ebi-fl187-GCST90094707 | prot-a-44 | Adhesion G protein-coupled receptor F5 \|\| id:prot-a-44 | Black olive liking \|\| id:ebi-fl187-GCST90094707 | Weighted median | 22 | -0.029590874 | 0.110231731220728 | 0.788358780561567 | -0.245645067 | 0.186463319028246 | 0.970842649117925 | 0.782199803905392 | 1.20498042142224 | 0.999332001504091 |
| ebi-fl187-GCST90094707 | prot-a-48 | Adrenomedullin \|\| id:prot-a-48 | Black olive liking \|\| id:ebi-fl187-GCST90094707 | Weighted median | 22 | 0.0998655751725136 | 0.11175497921702 | 0.371529596948862 | -0.119174184 | 0.318905334437873 | 1.10502236565043 | 0.887653172232859 | 1.37562109479777 | 0.999332001504091 |
| ebi-fl187-GCST90094707 | prot-a-502 | Carcinoembryonic antigen-related cell adhesion molecule 1 \|\| id:prot-a-502 | Black olive liking \|\| id:ebi-fl187-GCST90094707 | Weighted median | 22 | 0.183044480119345 | 0.109077370574133 | 0.0933244570310352 | -0.030747166 | 0.396836126444644 | 1.20086782163692 | 0.96972072025284 | 1.48711221171701 | 0.999332001504091 |
| ebi-fl187-GCST90094707 | prot-a-518 | Complement factor B \|\| id:prot-a-518 | Black olive liking \|\| id:ebi-fl187-GCST90094707 | Weighted median | 22 | -0.183303832 | 0.115136050345619 | 0.111370575834961 | -0.408970491 | 0.0423628263766306 | 0.832515172306529 | 0.664333835874622 | 1.04327293702887 | 0.999332001504091 |
| ebi-fl187-GCST90094707 | prot-a-525 | Properdin \|\| id:prot-a-525 | Black olive liking \|\| id:ebi-fl187-GCST90094707 | Weighted median | 22 | -0.030010243 | 0.119788504642745 | 0.802179904872024 | -0.264795712 | 0.204775226413896 | 0.970435593630646 | 0.767362697119657 | 1.2272491807592 | 0.999332001504091 |
| ebi-fl187-GCST90094707 | prot-a-533 | Chondroadherin \|\| id:prot-a-533 | Black olive liking \|\| id:ebi-fl187-GCST90094707 | Weighted median | 22 | -0.057154934 | 0.10855569012103 | 0.598538526073816 | -0.269924087 | 0.155614218288453 | 0.944447730636279 | 0.763437446974758 | 1.16837537828231 | 0.999332001504091 |
| ebi-fl187-GCST90094707 | prot-a-535 | Coiled-coil-helix-coiled-coil-helix domain-containing protein 2 \|\| id:prot-a-535 | Black olive liking \|\| id:ebi-fl187-GCST90094707 | Weighted median | 22 | -0.013080444 | 0.111462950688024 | 0.906580808979727 | -0.231547827 | 0.205386939464113 | 0.987004733331956 | 0.793304753024574 | 1.2280001347597 | 0.999332001504091 |
| ebi-fl187-GCST90094707 | prot-a-591 | Caseinolytic peptidase B protein homolog \|\| id:prot-a-591 | Black olive liking \|\| id:ebi-fl187-GCST90094707 | Weighted median | 22 | -0.145954361 | 0.113860844578618 | 0.199889609305451 | -0.369121616 | 0.0772128945674078 | 0.864197143545121 | 0.691341327045655 | 1.08027203596094 | 0.999332001504091 |
| ebi-fl187-GCST90094707 | prot-a-605 | Ciliary neurotrophic factor receptor subunit alpha \|\| id:prot-a-605 | Black olive liking \|\| id:ebi-fl187-GCST90094707 | Weighted median | 22 | -0.162099666 | 0.107899105841462 | 0.133012855394155 | -0.373581914 | 0.0493825812171917 | 0.850356448493505 | 0.688264605699616 | 1.05062222218947 | 0.999332001504091 |
| ebi-fl187-GCST90094707 | prot-a-606 | Ciliary neurotrophic factor receptor subunit alpha \|\| id:prot-a-606 | Black olive liking \|\| id:ebi-fl187-GCST90094707 | Weighted median | 22 | -0.229909773 | 0.106234951107899 | 0.0304518259331286 | -0.438130277 | -0.021689268 | 0.794605294514278 | 0.645241717435098 | 0.978544252501207 | 0.999332001504091 |
| ebi-fl187-GCST90094707 | prot-a-707 | Cancer/testis antigen 55 \|\| id:prot-a-707 | Black olive liking \|\| id:ebi-fl187-GCST90094707 | Weighted median | 22 | -0.05454034 | 0.111738580923712 | 0.625474328744387 | -0.273547959 | 0.164467278574692 | 0.946920309288184 | 0.760675854496839 | 1.17876499804971 | 0.999332001504091 |
| ebi-fl187-GCST90094707 | prot-a-719 | Dipeptidyl peptidase 1 \|\| id:prot-a-719 | Black olive liking \|\| id:ebi-fl187-GCST90094707 | Weighted median | 22 | -0.074707548 | 0.103659235874711 | 0.471092110748947 | -0.277879651 | 0.128464553849135 | 0.928014846012783 | 0.757387967080855 | 1.13708111542811 | 0.999332001504091 |
| ebi-fl187-GCST90094707 | prot-a-752 | Cytochrome b5 \|\| id:prot-a-752 | Black olive liking \|\| id:ebi-fl187-GCST90094707 | Weighted median | 22 | -0.218044093 | 0.117797654820529 | 0.0641687785771647 | -0.448927497 | 0.0128393101764765 | 0.804089985926617 | 0.638312376757075 | 1.0129220880101 | 0.999332001504091 |
| ebi-fl187-GCST90094707 | prot-a-755 | Cytoglobin \|\| id:prot-a-755 | Black olive liking \|\| id:ebi-fl187-GCST90094707 | Weighted median | 22 | -0.104320012 | 0.113153766945246 | 0.356564352607051 | -0.326101395 | 0.117461370984961 | 0.900936940447438 | 0.721732003387177 | 1.12463818543925 | 0.999332001504091 |
| ebi-fl187-GCST90094707 | prot-a-807 | Desmin \|\| id:prot-a-807 | Black olive liking \|\| id:ebi-fl187-GCST90094707 | Weighted median | 22 | 0.05511114417449 | 0.110348598117642 | 0.617478124265423 | -0.161172108 | 0.271394396485068 | 1.05665804953591 | 0.851145569422954 | 1.31179233466021 | 0.999332001504091 |
| ebi-fl187-GCST90094707 | prot-a-82 | Ameloblastin \|\| id:prot-a-82 | Black olive liking \|\| id:ebi-fl187-GCST90094707 | Weighted median | 22 | -0.099425181 | 0.119070231883564 | 0.403710439519492 | -0.332802835 | 0.13395247366172 | 0.905357685445036 | 0.716911529853083 | 1.14333847966201 | 0.999332001504091 |
| ebi-fl187-GCST90094707 | prot-a-84 | Muellerian-inhibiting factor \|\| id:prot-a-84 | Black olive liking \|\| id:ebi-fl187-GCST90094707 | Weighted median | 22 | -0.107200777 | 0.108223371812435 | 0.321904837178573 | -0.319318586 | 0.10491703135416 | 0.898345287452731 | 0.726644013707107 | 1.11061846002329 | 0.999332001504091 |
| ebi-fl187-GCST90094707 | prot-a-858 | Inactive dipeptidyl peptidase 10 \|\| id:prot-a-858 | Black olive liking \|\| id:ebi-fl187-GCST90094707 | Weighted median | 22 | 0.0570351633887477 | 0.114201551534175 | 0.617479680717914 | -0.166799878 | 0.280870204395731 | 1.05869303697581 | 0.846368971764698 | 1.32428170683539 | 0.999332001504091 |
| ebi-fl187-GCST90094707 | prot-a-863 | Dihydropyrimidinase-related protein 3 \|\| id:prot-a-863 | Black olive liking \|\| id:ebi-fl187-GCST90094707 | Weighted median | 21 | -0.100385821 | 0.106629664993823 | 0.34647755507302 | -0.309379964 | 0.108608322687406 | 0.904488380366815 | 0.733901860690396 | 1.11472565207694 | 0.999332001504091 |
| ebi-fl187-GCST90094707 | prot-a-864 | Dihydropyrimidinase-related protein 5 \|\| id:prot-a-864 | Black olive liking \|\| id:ebi-fl187-GCST90094707 | Weighted median | 22 | -0.159981831 | 0.109344990124253 | 0.143442079856609 | -0.374298012 | 0.0543343496911847 | 0.852159271747971 | 0.687771917278447 | 1.0558375621086 | 0.999332001504091 |
| ebi-fl187-GCST90094707 | prot-a-884 | Endothelin-converting enzyme 1 \|\| id:prot-a-884 | Black olive liking \|\| id:ebi-fl187-GCST90094707 | Weighted median | 22 | -0.071007057 | 0.106715966214243 | 0.505805154255579 | -0.28017035 | 0.138156237084788 | 0.931455319108188 | 0.755655004301867 | 1.14815492063935 | 0.999332001504091 |
| ebi-fl187-GCST90094707 | prot-a-893 | Early endosome antigen 1 \|\| id:prot-a-893 | Black olive liking \|\| id:ebi-fl187-GCST90094707 | Weighted median | 22 | -0.006599712 | 0.104544438117577 | 0.949664347585838 | -0.21150681 | 0.198307387172289 | 0.993422018727258 | 0.809363769065471 | 1.21933714481888 | 0.999332001504091 |
| ebi-fl187-GCST90094707 | prot-a-897 | Ephrin-A2 \|\| id:prot-a-897 | Black olive liking \|\| id:ebi-fl187-GCST90094707 | Weighted median | 22 | 0.0684116230117337 | 0.112809184808522 | 0.544224844039771 | -0.152694379 | 0.289517625236437 | 1.07080598616102 | 0.858392024172833 | 1.33578298459051 | 0.999332001504091 |
| ebi-fl187-GCST90094707 | prot-a-90 | Anaphase-promoting complex subunit 10 \|\| id:prot-a-90 | Black olive liking \|\| id:ebi-fl187-GCST90094707 | Weighted median | 22 | 0.087242999156837 | 0.115626812226742 | 0.450535707621603 | -0.139385553 | 0.313871551121251 | 1.09116179890221 | 0.869892574270455 | 1.36871391548784 | 0.999332001504091 |
| ebi-fl187-GCST90094707 | prot-a-914 | Histone-lysine N-methyltransferase EHMT2 \|\| id:prot-a-914 | Black olive liking \|\| id:ebi-fl187-GCST90094707 | Weighted median | 22 | 0.033625583319867 | 0.118163370196301 | 0.775974680349085 | -0.197974622 | 0.265225788904618 | 1.0341973135034 | 0.820390672530835 | 1.303725309 | 0.999332001504091 |
| ebi-fl187-GCST90094707 | prot-a-932 | RNA polymerase II elongation factor ELL \|\| id:prot-a-932 | Black olive liking \|\| id:ebi-fl187-GCST90094707 | Weighted median | 22 | -0.205850477 | 0.108168488950796 | 0.0570334978430938 | -0.417860715 | 0.00615976148357747 | 0.813954772257892 | 0.658453934660302 | 1.00617877182736 | 0.999332001504091 |
| ebi-fl187-GCST90094707 | prot-a-991 | Estrogen receptor \|\| id:prot-a-991 | Black olive liking \|\| id:ebi-fl187-GCST90094707 | Weighted median | 22 | 0.0589155163790895 | 0.116660993411188 | 0.613548512534266 | -0.169740031 | 0.287571063465018 | 1.06068562639237 | 0.843884172054026 | 1.33318532956601 | 0.999332001504091 |
| ebi-fl187-GCST90094707 | prot-a-992 | Persulfide dioxygenase ETHE1, mitochondrial \|\| id:prot-a-992 | Black olive liking \|\| id:ebi-fl187-GCST90094707 | Weighted median | 22 | -0.159495322 | 0.120207816826495 | 0.184564966294185 | -0.395102643 | 0.0761119986764205 | 0.852573955469294 | 0.673610894066355 | 1.07908342330484 | 0.999332001504091 |
| ebi-fl187-GCST90094707 | prot-a-993 | Ethanolamine kinase 1 \|\| id:prot-a-993 | Black olive liking \|\| id:ebi-fl187-GCST90094707 | Weighted median | 22 | 0.13724806493506 | 0.107013670402591 | 0.199657370379085 | -0.072498729 | 0.346994858924138 | 1.14711267165923 | 0.930066928724353 | 1.41480945170901 | 0.999332001504091 |
| ebi-fl187-GCST90094707 | prot-b-10 | coagulation factor III, tissue factor \|\| id:prot-b-10 | Black olive liking \|\| id:ebi-fl187-GCST90094707 | Weighted median | 16 | -0.05778048 | 0.147400546900183 | 0.695060891604425 | -0.346685552 | 0.231124591740781 | 0.94385712003844 | 0.707027616650545 | 1.26001621728388 | 0.999332001504091 |
| ebi-fl187-GCST90094707 | prot-b-28 | prolactin \|\| id:prot-b-28 | Black olive liking \|\| id:ebi-fl187-GCST90094707 | Weighted median | 16 | 0.0583380848227368 | 0.151703538469475 | 0.70056845555628 | -0.239000851 | 0.355677020222908 | 1.06007332983702 | 0.787414212615642 | 1.42714653434923 | 0.999332001504091 |
| ebi-fl187-GCST90094707 | prot-b-54 | matrix metallopeptidase 7 \|\| id:prot-b-54 | Black olive liking \|\| id:ebi-fl187-GCST90094707 | Weighted median | 16 | 0.194016866805517 | 0.163910209598069 | 0.236540653015517 | -0.127247144 | 0.515280877617733 | 1.21411676105482 | 0.880516031443938 | 1.67410865541758 | 0.999332001504091 |
| ebi-fl187-GCST90094707 | ukb-rsfMRI191-edge-pheno716 | Net100_Pair10_33 (OccipitalPrecuneus)&(Temporal\|Frontal\|Supp_Motor_Area) (Default_mode\|Central_executive)&(Default_mode\|Salience) \|\| id:ukb-rsfMRI191-edge-pheno716 | Black olive liking \|\| id:ebi-fl187-GCST90094707 | Weighted median | 21 | 0.0320079054890955 | 0.0343575999846723 | 0.351537785711576 | -0.03533299 | 0.0993488014590532 | 1.03252566789326 | 0.965283932374191 | 1.10445146666458 | 0.999332001504091 |
| ebi-fl187-GCST90094707 | ukb-rsfMRI191-edge-pheno903 | Net100_Pair27_38 (Precentral\|Frontal\|Supp_Motor_Area)&(Subcortical) (Attention\|Salience\|Motor)&(Subcortical-cerebellum) \|\| id:ukb-rsfMRI191-edge-pheno903 | Black olive liking \|\| id:ebi-fl187-GCST90094707 | Weighted median | 21 | 0.0586021108157665 | 0.0384071743948103 | 0.127056800032236 | -0.016675951 | 0.133880172629595 | 1.06035325370257 | 0.98346232298911 | 1.14325581809816 | 0.999332001504091 |
| ebi-fl187-GCST90094707 | ukb-rsfMRI191-node-pheno23 | Net100_Node2 RolandicOper\|SupraMarginal\|Insula Salience\|Motor \|\| id:ukb-rsfMRI191-node-pheno23 | Black olive liking \|\| id:ebi-fl187-GCST90094707 | Weighted median | 21 | 0.051034292428759 | 0.0347889953456986 | 0.142385155441233 | -0.017152138 | 0.119220723306328 | 1.05235898060981 | 0.98299412205717 | 1.12661856182057 | 0.999332001504091 |
| ebi-fl187-GCST90094707 | ukb-rsfMRI191-node-pheno55 | Net100_Node34 Parietal\|Postcentral\|Precuneus Attention \|\| id:ukb-rsfMRI191-node-pheno55 | Black olive liking \|\| id:ebi-fl187-GCST90094707 | Weighted median | 21 | 0.0486703298177358 | 0.0358782513020432 | 0.174927002720757 | -0.021651043 | 0.118991702369741 | 1.04987418146956 | 0.978581658656521 | 1.12636057212601 | 0.999332001504091 |
| ebi-fl187-GCST90094707 | ebi-a-GCST90001414 | IgD- CD24- B cell Absolute Count \|\| id:ebi-a-GCST90001414 | Black olive liking \|\| id:ebi-fl187-GCST90094707 | Weighted mode | 20 | -0.15842436 | 0.199234018371671 | 0.436337664466479 | -0.548923036 | 0.232074315529251 | 0.853487518736356 | 0.577571498982577 | 1.26121345309097 | 0.999714303421225 |
| ebi-fl187-GCST90094707 | ebi-a-GCST90001422 | CD20- CD38- B cell %B cell \|\| id:ebi-a-GCST90001422 | Black olive liking \|\| id:ebi-fl187-GCST90094707 | Weighted mode | 20 | -0.188502221 | 0.229786087773431 | 0.422196511336537 | -0.638882953 | 0.261878510721203 | 0.828198663746714 | 0.527881763377038 | 1.29936867347685 | 0.999714303421225 |
| ebi-fl187-GCST90094707 | ebi-a-GCST90001444 | CD20- CD38- B cell %lymphocyte \|\| id:ebi-a-GCST90001444 | Black olive liking \|\| id:ebi-fl187-GCST90094707 | Weighted mode | 20 | -0.212553176 | 0.233440867899372 | 0.37395419152076 | -0.670097277 | 0.244990925325602 | 0.808517321657564 | 0.511658802814142 | 1.27760971926007 | 0.999714303421225 |
| ebi-fl187-GCST90094707 | ebi-a-GCST90001459 | Myeloid Dendritic Cell %Dendritic Cell \|\| id:ebi-a-GCST90001459 | Black olive liking \|\| id:ebi-fl187-GCST90094707 | Weighted mode | 20 | -0.09537428 | 0.219493124078466 | 0.668805241152815 | -0.525580803 | 0.334832243480743 | 0.909032638314209 | 0.5912118870476 | 1.39770589127881 | 0.999714303421225 |
| ebi-fl187-GCST90094707 | ebi-a-GCST90001476 | HLA DR++ monocyte %leukocyte \|\| id:ebi-a-GCST90001476 | Black olive liking \|\| id:ebi-fl187-GCST90094707 | Weighted mode | 20 | -0.016286755 | 0.192187121000688 | 0.933351294063409 | -0.392973512 | 0.360400002488372 | 0.983845157405118 | 0.675046628096244 | 1.43390286457589 | 0.999714303421225 |
| ebi-fl187-GCST90094707 | ebi-a-GCST90001478 | CD4 regulatory T cell %CD4+ T cell \|\| id:ebi-a-GCST90001478 | Black olive liking \|\| id:ebi-fl187-GCST90094707 | Weighted mode | 20 | 0.215340969240061 | 0.242693366538638 | 0.3860135583707 | -0.260338029 | 0.691019967655791 | 1.24028472381623 | 0.770790991918268 | 1.99575009601957 | 0.999714303421225 |
| ebi-fl187-GCST90094707 | ebi-a-GCST90001485 | CD39+ resting CD4 regulatory T cell %CD4 regulatory T cell \|\| id:ebi-a-GCST90001485 | Black olive liking \|\| id:ebi-fl187-GCST90094707 | Weighted mode | 19 | -0.048150378 | 0.208752479685853 | 0.820181989960642 | -0.457305238 | 0.361004482683899 | 0.952990467977336 | 0.632987099055515 | 1.43476989248404 | 0.999714303421225 |
| ebi-fl187-GCST90094707 | ebi-a-GCST90001515 | Immature Myeloid-Derived Suppressor Cells Absolute Count \|\| id:ebi-a-GCST90001515 | Black olive liking \|\| id:ebi-fl187-GCST90094707 | Weighted mode | 20 | -0.26234576 | 0.380369355419112 | 0.498712762966289 | -1.007869696 | 0.483178177097873 | 0.769245003934709 | 0.364995703703214 | 1.62121874332983 | 0.999714303421225 |
| ebi-fl187-GCST90094707 | ebi-a-GCST90001517 | CD33+ HLA DR+ Absolute Count \|\| id:ebi-a-GCST90001517 | Black olive liking \|\| id:ebi-fl187-GCST90094707 | Weighted mode | 20 | -0.039422731 | 0.249964823689416 | 0.876346905331745 | -0.529353786 | 0.450508323310226 | 0.961344233112026 | 0.588985457638926 | 1.56910959778622 | 0.999714303421225 |
| ebi-fl187-GCST90094707 | ebi-a-GCST90001518 | CD33+ HLA DR+ CD14- Absolute Count \|\| id:ebi-a-GCST90001518 | Black olive liking \|\| id:ebi-fl187-GCST90094707 | Weighted mode | 20 | -0.008880536 | 0.27987105961798 | 0.975017679263894 | -0.557427813 | 0.539666740654763 | 0.991158779298123 | 0.572680211572251 | 1.71543508214236 | 0.999714303421225 |
| ebi-fl187-GCST90094707 | ebi-a-GCST90001520 | CD33+ HLA DR+ CD14dim Absolute Count \|\| id:ebi-a-GCST90001520 | Black olive liking \|\| id:ebi-fl187-GCST90094707 | Weighted mode | 20 | 0.24100241960547 | 0.280358475922325 | 0.400713718555764 | -0.308500193 | 0.790505032413228 | 1.27252411431081 | 0.734547810282839 | 2.20450949391435 | 0.999714303421225 |
| ebi-fl187-GCST90094707 | ebi-a-GCST90001531 | CD33dim HLA DR- Absolute Count \|\| id:ebi-a-GCST90001531 | Black olive liking \|\| id:ebi-fl187-GCST90094707 | Weighted mode | 20 | -0.083909595 | 0.250054478626744 | 0.740875691202165 | -0.574016373 | 0.406197183576534 | 0.91951438146567 | 0.563258633015515 | 1.50109851525153 | 0.999714303421225 |
| ebi-fl187-GCST90094707 | ebi-a-GCST90001532 | Basophil Absolute Count \|\| id:ebi-a-GCST90001532 | Black olive liking \|\| id:ebi-fl187-GCST90094707 | Weighted mode | 19 | -0.0861556 | 0.244998496994255 | 0.729178041831557 | -0.566352654 | 0.394041453891669 | 0.917451464466147 | 0.567591871668863 | 1.4829620219476 | 0.999714303421225 |
| ebi-fl187-GCST90094707 | ebi-a-GCST90001588 | T/B cell \|\| id:ebi-a-GCST90001588 | Black olive liking \|\| id:ebi-fl187-GCST90094707 | Weighted mode | 20 | -0.041111832 | 0.189031586914688 | 0.830147698890266 | -0.411613743 | 0.329390078147864 | 0.959721796141645 | 0.662580153146989 | 1.39012000527733 | 0.999714303421225 |
| ebi-fl187-GCST90094707 | ebi-a-GCST90001624 | HLA DR+ CD4+ T cell Absolute Count \|\| id:ebi-a-GCST90001624 | Black olive liking \|\| id:ebi-fl187-GCST90094707 | Weighted mode | 20 | -0.01344422 | 0.190552034657368 | 0.944490002839919 | -0.386926208 | 0.360037767708612 | 0.986645749665867 | 0.679141208164976 | 1.43338354915028 | 0.999714303421225 |
| ebi-fl187-GCST90094707 | ebi-a-GCST90001631 | CD8+ Natural Killer T %T cell \|\| id:ebi-a-GCST90001631 | Black olive liking \|\| id:ebi-fl187-GCST90094707 | Weighted mode | 20 | 0.0411404026981757 | 0.165053949771824 | 0.805838800460993 | -0.282365339 | 0.364646144250951 | 1.04199839466165 | 0.753998169380387 | 1.44000436416139 | 0.999714303421225 |
| ebi-fl187-GCST90094707 | ebi-a-GCST90001632 | CD8+ Natural Killer T %lymphocyte \|\| id:ebi-a-GCST90001632 | Black olive liking \|\| id:ebi-fl187-GCST90094707 | Weighted mode | 20 | 0.0418378963071038 | 0.176840257445468 | 0.815510035066424 | -0.304769008 | 0.388444800900221 | 1.0427254354061 | 0.737293663450221 | 1.47468557990157 | 0.999714303421225 |
| ebi-fl187-GCST90094707 | ebi-a-GCST90001646 | Natural Killer %CD3- lymphocyte \|\| id:ebi-a-GCST90001646 | Black olive liking \|\| id:ebi-fl187-GCST90094707 | Weighted mode | 20 | -0.057028185 | 0.189995226707061 | 0.767317577341013 | -0.429418829 | 0.315362459259705 | 0.944567446276505 | 0.650887261342413 | 1.37075606415341 | 0.999714303421225 |
| ebi-fl187-GCST90094707 | ebi-a-GCST90001684 | CD127- CD8+ T cell Absolute Count \|\| id:ebi-a-GCST90001684 | Black olive liking \|\| id:ebi-fl187-GCST90094707 | Weighted mode | 20 | 0.063234824321343 | 0.21816715104334 | 0.775073682515828 | -0.364372792 | 0.49084244036629 | 1.06527696280244 | 0.694632193336495 | 1.63369192842443 | 0.999714303421225 |
| ebi-fl187-GCST90094707 | ebi-a-GCST90001687 | CD28- CD8+ T cell Absolute Count \|\| id:ebi-a-GCST90001687 | Black olive liking \|\| id:ebi-fl187-GCST90094707 | Weighted mode | 20 | -0.081348372 | 0.199574340709023 | 0.68811660293543 | -0.47251408 | 0.309817335326029 | 0.921872480510123 | 0.623432935962011 | 1.36317608727312 | 0.999714303421225 |
| ebi-fl187-GCST90094707 | ebi-a-GCST90001701 | BAFF-R on CD20- CD38- B cell \|\| id:ebi-a-GCST90001701 | Black olive liking \|\| id:ebi-fl187-GCST90094707 | Weighted mode | 20 | 0.0254462061492251 | 0.209241712087882 | 0.90448366152654 | -0.38466755 | 0.435559961841474 | 1.02577272452137 | 0.680676889936538 | 1.54582842157325 | 0.999714303421225 |
| ebi-fl187-GCST90094707 | ebi-a-GCST90001732 | CD19 on IgD- CD27- B cell \|\| id:ebi-a-GCST90001732 | Black olive liking \|\| id:ebi-fl187-GCST90094707 | Weighted mode | 20 | -0.177909016 | 0.195306334718857 | 0.373748546341453 | -0.560709433 | 0.204891399578948 | 0.837018574929065 | 0.570803973272993 | 1.22739176246276 | 0.999714303421225 |
| ebi-fl187-GCST90094707 | ebi-a-GCST90001798 | CD27 on CD24+ CD27+ B cell \|\| id:ebi-a-GCST90001798 | Black olive liking \|\| id:ebi-fl187-GCST90094707 | Weighted mode | 20 | -0.072607932 | 0.166050872825489 | 0.666850110583943 | -0.398067643 | 0.252851778736392 | 0.929965368219774 | 0.671616596138998 | 1.28769239929436 | 0.999714303421225 |
| ebi-fl187-GCST90094707 | ebi-a-GCST90001836 | CD66b on CD66b++ myeloid cell \|\| id:ebi-a-GCST90001836 | Black olive liking \|\| id:ebi-fl187-GCST90094707 | Weighted mode | 20 | -0.069729795 | 0.306138682386853 | 0.822256720646278 | -0.669761612 | 0.530302022843238 | 0.932645791758926 | 0.51183057745409 | 1.6994455024791 | 0.999714303421225 |
| ebi-fl187-GCST90094707 | ebi-a-GCST90001943 | CD25 on activated & secreting CD4 regulatory T cell \|\| id:ebi-a-GCST90001943 | Black olive liking \|\| id:ebi-fl187-GCST90094707 | Weighted mode | 20 | 0.027444838919044 | 0.212396093474506 | 0.898545310312554 | -0.388851504 | 0.443741182129076 | 1.02782491761003 | 0.67783491811764 | 1.558527059 | 0.999714303421225 |
| ebi-fl187-GCST90094707 | ebi-a-GCST90001960 | CD25 on CD4+ T cell \|\| id:ebi-a-GCST90001960 | Black olive liking \|\| id:ebi-fl187-GCST90094707 | Weighted mode | 20 | -0.150880168 | 0.286392209336979 | 0.604408299804577 | -0.712208898 | 0.410448562523397 | 0.859950742294157 | 0.490559404078438 | 1.50749383871566 | 0.999714303421225 |
| ebi-fl187-GCST90094707 | ebi-a-GCST90001988 | HLA DR on CD14+ CD16- monocyte \|\| id:ebi-a-GCST90001988 | Black olive liking \|\| id:ebi-fl187-GCST90094707 | Weighted mode | 19 | -0.059244922 | 0.194635260922435 | 0.764322247093568 | -0.440730034 | 0.322240189078698 | 0.942475907492117 | 0.643566424345346 | 1.3802162490168 | 0.999714303421225 |
| ebi-fl187-GCST90094707 | ebi-a-GCST90001991 | HLA DR on CD14+ monocyte \|\| id:ebi-a-GCST90001991 | Black olive liking \|\| id:ebi-fl187-GCST90094707 | Weighted mode | 19 | -0.182477754 | 0.214539553315477 | 0.40618901454022 | -0.602975279 | 0.238019770049526 | 0.833203178786137 | 0.547181195098353 | 1.26873427551643 | 0.999714303421225 |
| ebi-fl187-GCST90094707 | ebi-a-GCST90002019 | CD14 on Monocytic Myeloid-Derived Suppressor Cells \|\| id:ebi-a-GCST90002019 | Black olive liking \|\| id:ebi-fl187-GCST90094707 | Weighted mode | 20 | -0.22155463 | 0.399608305737196 | 0.585752610023849 | -1.004786909 | 0.561677649332558 | 0.801272147525059 | 0.366122643874944 | 1.75361197986629 | 0.999714303421225 |
| ebi-fl187-GCST90094707 | ebi-a-GCST90002037 | CD80 on plasmacytoid Dendritic Cell \|\| id:ebi-a-GCST90002037 | Black olive liking \|\| id:ebi-fl187-GCST90094707 | Weighted mode | 20 | -0.169084152 | 0.220514183632296 | 0.452639800910735 | -0.601291952 | 0.263123648050886 | 0.844437839374049 | 0.548103055744042 | 1.30098757358454 | 0.999714303421225 |
| ebi-fl187-GCST90094707 | ebi-a-GCST90002042 | CD45 on CD33+ HLA DR+ CD14- \|\| id:ebi-a-GCST90002042 | Black olive liking \|\| id:ebi-fl187-GCST90094707 | Weighted mode | 20 | -0.032515123 | 0.308863728162763 | 0.917262349681881 | -0.63788803 | 0.572857784651968 | 0.968007810976955 | 0.528407226758468 | 1.77332760541656 | 0.999714303421225 |
| ebi-fl187-GCST90094707 | ebi-a-GCST90002056 | CD8 on Effector Memory CD8+ T cell \|\| id:ebi-a-GCST90002056 | Black olive liking \|\| id:ebi-fl187-GCST90094707 | Weighted mode | 20 | 0.183159215293231 | 0.23894315983406 | 0.452775311582504 | -0.285169378 | 0.65148780856799 | 1.20100561131977 | 0.751886890449528 | 1.91839290821947 | 0.999714303421225 |
| ebi-fl187-GCST90094707 | ebi-a-GCST90002058 | CD8 on CD8+ T cell \|\| id:ebi-a-GCST90002058 | Black olive liking \|\| id:ebi-fl187-GCST90094707 | Weighted mode | 20 | -0.274714489 | 0.217439021568157 | 0.221725491709731 | -0.700894972 | 0.15146599284523 | 0.75978902005777 | 0.496141072813643 | 1.1635387324951 | 0.999714303421225 |
| ebi-fl187-GCST90094707 | ebi-a-GCST90002063 | CD4 on CD4 regulatory T cell \|\| id:ebi-a-GCST90002063 | Black olive liking \|\| id:ebi-fl187-GCST90094707 | Weighted mode | 20 | -0.079593171 | 0.246657918565226 | 0.750456689673734 | -0.563042691 | 0.403856349560347 | 0.923491973448803 | 0.569473692486554 | 1.49758880221583 | 0.999714303421225 |
| ebi-fl187-GCST90094707 | ebi-a-GCST90002066 | CD4 on activated CD4 regulatory T cell \|\| id:ebi-a-GCST90002066 | Black olive liking \|\| id:ebi-fl187-GCST90094707 | Weighted mode | 20 | -0.131284925 | 0.249235236107526 | 0.604462451357933 | -0.619785988 | 0.357216137654213 | 0.876967868618353 | 0.538059576540411 | 1.42934477169603 | 0.999714303421225 |
| ebi-fl187-GCST90094707 | ebi-a-GCST90002070 | CD4 on activated & secreting CD4 regulatory T cell \|\| id:ebi-a-GCST90002070 | Black olive liking \|\| id:ebi-fl187-GCST90094707 | Weighted mode | 20 | -0.053904136 | 0.255964226459032 | 0.835447342544486 | -0.55559402 | 0.447785747362574 | 0.947522935015761 | 0.573731351850564 | 1.5648433879115 | 0.999714303421225 |
| ebi-fl187-GCST90094707 | ebi-a-GCST90002081 | SSC-A on CD4+ T cell \|\| id:ebi-a-GCST90002081 | Black olive liking \|\| id:ebi-fl187-GCST90094707 | Weighted mode | 20 | 0.132355348607009 | 0.189249788686801 | 0.492795646035663 | -0.238574237 | 0.50328493443314 | 1.14151388257284 | 0.787750205701743 | 1.65414611722728 | 0.999714303421225 |
| ebi-fl187-GCST90094707 | ebi-a-GCST90002094 | CD11b on Monocytic Myeloid-Derived Suppressor Cells \|\| id:ebi-a-GCST90002094 | Black olive liking \|\| id:ebi-fl187-GCST90094707 | Weighted mode | 20 | -0.445685071 | 0.292078665846583 | 0.143507452863969 | -1.018159256 | 0.126789114241177 | 0.640385416363402 | 0.361259314473781 | 1.13517759974793 | 0.999714303421225 |
| ebi-fl187-GCST90094707 | ebi-a-GCST90002104 | HLA DR on myeloid Dendritic Cell \|\| id:ebi-a-GCST90002104 | Black olive liking \|\| id:ebi-fl187-GCST90094707 | Weighted mode | 19 | -0.157627384 | 0.202391091554119 | 0.446201113588456 | -0.554313924 | 0.239059155076358 | 0.8541679990258 | 0.574466253641821 | 1.27005366448322 | 0.999714303421225 |
| ebi-fl187-GCST90094707 | ebi-a-GCST90002105 | HLA DR on plasmacytoid Dendritic Cell \|\| id:ebi-a-GCST90002105 | Black olive liking \|\| id:ebi-fl187-GCST90094707 | Weighted mode | 19 | 0.0922513435344499 | 0.241066091859043 | 0.706437816879189 | -0.380238197 | 0.564740883578175 | 1.09664042092379 | 0.683698535210633 | 1.75899193996869 | 0.999714303421225 |
| ebi-fl187-GCST90094707 | ebi-a-GCST90002106 | HLA DR on Dendritic Cell \|\| id:ebi-a-GCST90002106 | Black olive liking \|\| id:ebi-fl187-GCST90094707 | Weighted mode | 19 | -0.270057761 | 0.237536360176083 | 0.270484748084761 | -0.735629027 | 0.195513504635932 | 0.763335401811284 | 0.479203931816948 | 1.21593521457368 | 0.999714303421225 |
| ebi-fl187-GCST90094707 | ebi-a-GCST90002111 | HLA DR on CD33dim HLA DR+ CD11b- \|\| id:ebi-a-GCST90002111 | Black olive liking \|\| id:ebi-fl187-GCST90094707 | Weighted mode | 19 | -0.127703766 | 0.262802961245135 | 0.632879724170523 | -0.64279757 | 0.387390038222235 | 0.880114060399936 | 0.525819348127958 | 1.47313095661357 | 0.999714303421225 |
| ebi-fl187-GCST90094707 | ebi-a-GCST90002112 | HLA DR on CD33- HLA DR+ \|\| id:ebi-a-GCST90002112 | Black olive liking \|\| id:ebi-fl187-GCST90094707 | Weighted mode | 19 | -0.379060427 | 0.385650932141907 | 0.338679442406053 | -1.134936254 | 0.376815400137346 | 0.684504248973588 | 0.321442611407356 | 1.45763520527501 | 0.999714303421225 |
| ebi-fl187-GCST90094707 | ebi-a-GCST90002115 | HLA DR on HLA DR+ CD8+ T cell \|\| id:ebi-a-GCST90002115 | Black olive liking \|\| id:ebi-fl187-GCST90094707 | Weighted mode | 20 | 0.116770392069371 | 0.220811757939581 | 0.60305170683174 | -0.316020653 | 0.549561437630949 | 1.12386135258391 | 0.729044392699446 | 1.73249304497765 | 0.999714303421225 |
| ebi-fl187-GCST90094707 | ebi-a-GCST90002116 | HLA DR on B cell \|\| id:ebi-a-GCST90002116 | Black olive liking \|\| id:ebi-fl187-GCST90094707 | Weighted mode | 20 | -0.073564763 | 0.24672910545067 | 0.768817067606729 | -0.557153809 | 0.410024283893626 | 0.929075974291177 | 0.572837149497187 | 1.50685437695996 | 0.999714303421225 |
| ebi-fl187-GCST90094707 | ebi-cfmet338-GCST90026023 | Sphingomyelin (d18:1/24:1, d18:2/24:0) levels \|\| id:ebi-cfmet338-GCST90026023 | Black olive liking \|\| id:ebi-fl187-GCST90094707 | Weighted mode | 19 | 0.169117435369938 | 0.141994940703315 | 0.249118882705908 | -0.109192648 | 0.447427519148434 | 1.18425920467599 | 0.896557680449691 | 1.56428291725344 | 0.999714303421225 |
| ebi-fl187-GCST90094707 | ebi-cfmet338-GCST90026046 | 1-arachidonoyl-gpc (20:4n6) levels \|\| id:ebi-cfmet338-GCST90026046 | Black olive liking \|\| id:ebi-fl187-GCST90094707 | Weighted mode | 19 | 0.0632529012904722 | 0.130371722451543 | 0.633405595469444 | -0.192275675 | 0.318781477295496 | 1.06529621995527 | 0.825079383613649 | 1.37545072485096 | 0.999714303421225 |
| ebi-fl187-GCST90094707 | ebi-cfmet338-GCST90026047 | 1-linoleoyl-gpc (18:2) levels \|\| id:ebi-cfmet338-GCST90026047 | Black olive liking \|\| id:ebi-fl187-GCST90094707 | Weighted mode | 19 | -0.05397204 | 0.178515148667698 | 0.7658596335573 | -0.403861732 | 0.295917650966425 | 0.947458596673755 | 0.66773644159484 | 1.34435944557251 | 0.999714303421225 |
| ebi-fl187-GCST90094707 | ebi-cfmet338-GCST90026050 | 1-myristoyl-2-palmitoyl-gpc (14:0/16:0) levels \|\| id:ebi-cfmet338-GCST90026050 | Black olive liking \|\| id:ebi-fl187-GCST90094707 | Weighted mode | 19 | 0.0264385705513517 | 0.0995962499213755 | 0.793673439486577 | -0.168770079 | 0.221647220397248 | 1.02679117010885 | 0.844703095792437 | 1.24813098503496 | 0.999714303421225 |
| ebi-fl187-GCST90094707 | ebi-cfmet338-GCST90026068 | 2,3-dihydroxyisovalerate levels \|\| id:ebi-cfmet338-GCST90026068 | Black olive liking \|\| id:ebi-fl187-GCST90094707 | Weighted mode | 19 | 0.13012477009212 | 0.29744379494284 | 0.666969453630013 | -0.452865068 | 0.713114608180086 | 1.13897048391169 | 0.63580391813567 | 2.04033622036473 | 0.999714303421225 |
| ebi-fl187-GCST90094707 | ebi-cfmet338-GCST90026082 | 3-hydroxyhexanoate levels \|\| id:ebi-cfmet338-GCST90026082 | Black olive liking \|\| id:ebi-fl187-GCST90094707 | Weighted mode | 19 | 0.0028045351983802 | 0.0641729775435244 | 0.965622516026836 | -0.122974501 | 0.128583571183688 | 1.00280847158627 | 0.884286210875846 | 1.13721645584541 | 0.999714303421225 |
| ebi-fl187-GCST90094707 | ebi-cfmet338-GCST90026094 | 3-ureidopropionate levels \|\| id:ebi-cfmet338-GCST90026094 | Black olive liking \|\| id:ebi-fl187-GCST90094707 | Weighted mode | 19 | 0.0688543485584429 | 0.126741858743941 | 0.593612426377209 | -0.179559695 | 0.317268391696568 | 1.0712801642843 | 0.835638066391115 | 1.37337112387106 | 0.999714303421225 |
| ebi-fl187-GCST90094707 | ebi-cfmet338-GCST90026173 | Glycerol levels \|\| id:ebi-cfmet338-GCST90026173 | Black olive liking \|\| id:ebi-fl187-GCST90094707 | Weighted mode | 18 | 0.0863519352694809 | 0.0790222739144496 | 0.289745779933527 | -0.068531722 | 0.241235592141802 | 1.09018993708773 | 0.933763839148061 | 1.27282086658198 | 0.999714303421225 |
| ebi-fl187-GCST90094707 | ebi-cfmet338-GCST90026179 | Histidine levels \|\| id:ebi-cfmet338-GCST90026179 | Black olive liking \|\| id:ebi-fl187-GCST90094707 | Weighted mode | 19 | -0.056029998 | 0.0487607114952363 | 0.265563158646132 | -0.151600993 | 0.0395409961674336 | 0.945510771689392 | 0.85933109155663 | 1.04033314768264 | 0.999714303421225 |
| ebi-fl187-GCST90094707 | ebi-cfmet338-GCST90026188 | Isocitrate levels \|\| id:ebi-cfmet338-GCST90026188 | Black olive liking \|\| id:ebi-fl187-GCST90094707 | Weighted mode | 19 | -0.041647539 | 0.11345146308913 | 0.717827911610917 | -0.264012407 | 0.1807173284698 | 0.959207804163461 | 0.767964011591298 | 1.19807646931474 | 0.999714303421225 |
| ebi-fl187-GCST90094707 | ebi-cfmet338-GCST90026207 | N-acetyl-isoputreanine levels \|\| id:ebi-cfmet338-GCST90026207 | Black olive liking \|\| id:ebi-fl187-GCST90094707 | Weighted mode | 19 | 0.0230931207603979 | 0.0677757751690889 | 0.737252751157121 | -0.109747399 | 0.155933640091812 | 1.02336183134222 | 0.896060452862103 | 1.16874864246383 | 0.999714303421225 |
| ebi-fl187-GCST90094707 | ebi-cfmet338-GCST90026213 | N-acetylglutamate levels \|\| id:ebi-cfmet338-GCST90026213 | Black olive liking \|\| id:ebi-fl187-GCST90094707 | Weighted mode | 19 | -0.155886591 | 0.176401963959967 | 0.388508643586975 | -0.50163444 | 0.189861258633074 | 0.855656224217721 | 0.605540131386698 | 1.2090818363531 | 0.999714303421225 |
| ebi-fl187-GCST90094707 | ebi-cfmet338-GCST90026224 | N-acetyltaurine levels \|\| id:ebi-cfmet338-GCST90026224 | Black olive liking \|\| id:ebi-fl187-GCST90094707 | Weighted mode | 19 | 0.0528122366566456 | 0.099468293397716 | 0.601947069232861 | -0.142145618 | 0.247770091716169 | 1.05423168046969 | 0.867494924062628 | 1.28116534780521 | 0.999714303421225 |
| ebi-fl187-GCST90094707 | ebi-cfmet338-GCST90026250 | Phenyllactate (pla) levels \|\| id:ebi-cfmet338-GCST90026250 | Black olive liking \|\| id:ebi-fl187-GCST90094707 | Weighted mode | 19 | 0.0308792238890241 | 0.160216610580439 | 0.849325018613906 | -0.283145333 | 0.344903780626684 | 1.0313609326016 | 0.753410284639844 | 1.41185406541847 | 0.999714303421225 |
| ebi-fl187-GCST90094707 | ebi-cfmet338-GCST90026251 | Pipecolate levels \|\| id:ebi-cfmet338-GCST90026251 | Black olive liking \|\| id:ebi-fl187-GCST90094707 | Weighted mode | 19 | -0.090213261 | 0.248538914843767 | 0.720851422061898 | -0.577349534 | 0.396923011824939 | 0.913736299928508 | 0.561384326319137 | 1.48724142564039 | 0.999714303421225 |
| ebi-fl187-GCST90094707 | ebi-cfmet338-GCST90026278 | Trigonelline (n'-methylnicotinate) levels \|\| id:ebi-cfmet338-GCST90026278 | Black olive liking \|\| id:ebi-fl187-GCST90094707 | Weighted mode | 19 | -0.000260438 | 0.228011712678655 | 0.999101210443371 | -0.447163395 | 0.446642519067678 | 0.99973959612849 | 0.639439418769591 | 1.56305543688619 | 0.999714303421225 |
| ebi-fl187-GCST90094707 | ebi-cfmet338-GCST90026299 | 1-carboxyethylvaline levels \|\| id:ebi-cfmet338-GCST90026299 | Black olive liking \|\| id:ebi-fl187-GCST90094707 | Weighted mode | 19 | -0.054125414 | 0.147897080384918 | 0.71865564415725 | -0.344003691 | 0.235752863824579 | 0.947313292958114 | 0.70892631119612 | 1.26586143135388 | 0.999714303421225 |
| ebi-fl187-GCST90094707 | ebi-gm430-GCST90011306 | C_Erysipelotrichia abundance \|\| id:ebi-gm430-GCST90011306 | Black olive liking \|\| id:ebi-fl187-GCST90094707 | Weighted mode | 18 | 0.132490123571092 | 0.118189703290639 | 0.277876092158048 | -0.099161695 | 0.364141942020745 | 1.14166774043323 | 0.905596265906157 | 1.43927849375759 | 0.999714303421225 |
| ebi-fl187-GCST90094707 | ebi-gm430-GCST90011312 | F_Erysipelotrichaceae abundance \|\| id:ebi-gm430-GCST90011312 | Black olive liking \|\| id:ebi-fl187-GCST90094707 | Weighted mode | 18 | 0.132490123571092 | 0.120833604948587 | 0.28816420415367 | -0.104343742 | 0.369323989270323 | 1.14166774043323 | 0.900915561557219 | 1.44675626125605 | 0.999714303421225 |
| ebi-fl187-GCST90094707 | ebi-gm430-GCST90011352 | O_Erysipelotrichales abundance \|\| id:ebi-gm430-GCST90011352 | Black olive liking \|\| id:ebi-fl187-GCST90094707 | Weighted mode | 18 | 0.132490123571092 | 0.120771958813227 | 0.287926368302436 | -0.104222916 | 0.369203162845016 | 1.14166774043323 | 0.901024422540535 | 1.4465814654289 | 0.999714303421225 |
| ebi-fl187-GCST90094707 | ebi-gm430-GCST90011362 | OTU97_11 (Parabacteroides) abundance \|\| id:ebi-gm430-GCST90011362 | Black olive liking \|\| id:ebi-fl187-GCST90094707 | Weighted mode | 18 | 0.128385683572831 | 0.181102873829702 | 0.487988902326172 | -0.226575949 | 0.483347316279047 | 1.13699143706288 | 0.797258788899426 | 1.62149297813185 | 0.999714303421225 |
| ebi-fl187-GCST90094707 | ebi-gm430-GCST90011384 | OTU97_235 (Phascolarctobacterium) abundance \|\| id:ebi-gm430-GCST90011384 | Black olive liking \|\| id:ebi-fl187-GCST90094707 | Weighted mode | 18 | 0.00819909474766156 | 0.548297119511953 | 0.988243180536829 | -1.066463259 | 1.08286144899109 | 1.00823279937785 | 0.344223797333628 | 2.95311766825944 | 0.999714303421225 |
| ebi-fl187-GCST90094707 | ebi-gm430-GCST90011390 | OTU97_3 (Bacteroides) abundance \|\| id:ebi-gm430-GCST90011390 | Black olive liking \|\| id:ebi-fl187-GCST90094707 | Weighted mode | 18 | -0.059679605 | 0.113829961045554 | 0.606847894029989 | -0.282786328 | 0.163427118950044 | 0.94206631885794 | 0.753680810879621 | 1.17753953174257 | 0.999714303421225 |
| ebi-fl187-GCST90094707 | ebi-gm430-GCST90011398 | OTU97_4 (Alistipes) abundance \|\| id:ebi-gm430-GCST90011398 | Black olive liking \|\| id:ebi-fl187-GCST90094707 | Weighted mode | 18 | 0.143857057082599 | 0.144969901745901 | 0.334954905657513 | -0.14028395 | 0.427998064504566 | 1.15471903781931 | 0.869111415876734 | 1.5341831115603 | 0.999714303421225 |
| ebi-fl187-GCST90094707 | ebi-gm430-GCST90011421 | OTU99_108 (Cytophagales) abundance \|\| id:ebi-gm430-GCST90011421 | Black olive liking \|\| id:ebi-fl187-GCST90094707 | Weighted mode | 18 | 0.390907760925761 | 0.495668454122626 | 0.441177995956344 | -0.580602409 | 1.36241793100611 | 1.47832214805777 | 0.559561180236008 | 3.90562542690397 | 0.999714303421225 |
| ebi-fl187-GCST90094707 | ebi-gm430-GCST90011428 | OTU99_123 (Phascolarctobacterium) abundance \|\| id:ebi-gm430-GCST90011428 | Black olive liking \|\| id:ebi-fl187-GCST90094707 | Weighted mode | 18 | 0.130650741566237 | 0.348506956761184 | 0.712380288477834 | -0.552422894 | 0.813724376818157 | 1.13956970746952 | 0.575553614428159 | 2.25629565279059 | 0.999714303421225 |
| ebi-fl187-GCST90094707 | ebi-gm430-GCST90011450 | OTU99_298 (Phascolarctobacterium) abundance \|\| id:ebi-gm430-GCST90011450 | Black olive liking \|\| id:ebi-fl187-GCST90094707 | Weighted mode | 18 | 0.0138453338459099 | 0.512273186512411 | 0.978752742870284 | -0.990210112 | 1.01790077941023 | 1.01394162435879 | 0.371498626606384 | 2.76737932249917 | 0.999714303421225 |
| ebi-fl187-GCST90094707 | ebi-gm430-GCST90011451 | OTU99_3 (Bacteroides) abundance \|\| id:ebi-gm430-GCST90011451 | Black olive liking \|\| id:ebi-fl187-GCST90094707 | Weighted mode | 18 | -0.057042564 | 0.115946986812121 | 0.629032204990398 | -0.284298658 | 0.170213530372193 | 0.944553864728414 | 0.752541858546077 | 1.18555797693047 | 0.999714303421225 |
| ebi-fl187-GCST90094707 | ebi-gm430-GCST90011472 | OTU99_72 (Parabacteroides) abundance \|\| id:ebi-gm430-GCST90011472 | Black olive liking \|\| id:ebi-fl187-GCST90094707 | Weighted mode | 18 | -0.315225239 | 0.421688204338902 | 0.464958160100849 | -1.14173412 | 0.511283641322015 | 0.729624515730499 | 0.319264897951874 | 1.66743020410347 | 0.999714303421225 |
| ebi-fl187-GCST90094707 | ebi-gm430-GCST90011507 | TestASV_3 (Bacteroides) abundance \|\| id:ebi-gm430-GCST90011507 | Black olive liking \|\| id:ebi-fl187-GCST90094707 | Weighted mode | 18 | 0.0761898803639437 | 0.162508709209781 | 0.645143143275293 | -0.24232719 | 0.394706950415114 | 1.07916746741553 | 0.784799357283404 | 1.48394925648174 | 0.999714303421225 |
| ebi-fl187-GCST90094707 | ebi-gm430-GCST90011512 | TestASV_34 (Phascolarctobacterium) abundance \|\| id:ebi-gm430-GCST90011512 | Black olive liking \|\| id:ebi-fl187-GCST90094707 | Weighted mode | 18 | -0.391428613 | 0.471379085340826 | 0.417827916278489 | -1.31533162 | 0.532474394595475 | 0.67609031305258 | 0.26838530656079 | 1.70314134279926 | 0.999714303421225 |
| ebi-fl187-GCST90094707 | ebi-gm430-GCST90011519 | TestASV_41 (Barnesiella) abundance \|\| id:ebi-gm430-GCST90011519 | Black olive liking \|\| id:ebi-fl187-GCST90094707 | Weighted mode | 18 | 0.551925722786468 | 0.631987855599017 | 0.39465940790175 | -0.686770474 | 1.79062191976054 | 1.73659399856747 | 0.503198540424374 | 5.9931785837797 | 0.999714303421225 |
| ebi-fl187-GCST90094707 | ebi-gm430-GCST90011524 | TestASV_46 (Sutterella) abundance \|\| id:ebi-gm430-GCST90011524 | Black olive liking \|\| id:ebi-fl187-GCST90094707 | Weighted mode | 18 | -0.295797338 | 0.556996007033876 | 0.602248438015394 | -1.387509512 | 0.795914835610836 | 0.74393818061272 | 0.249696396788691 | 2.21646777322832 | 0.999714303421225 |
| ebi-fl187-GCST90094707 | ebi-gm430-GCST90011581 | OTU97_19 (Bacteria) prevalence \|\| id:ebi-gm430-GCST90011581 | Black olive liking \|\| id:ebi-fl187-GCST90094707 | Weighted mode | 18 | -0.448141174 | 0.614850253217141 | 0.476007998165178 | -1.65324767 | 0.756965322422087 | 0.638814493742886 | 0.191427205572093 | 2.13179707762224 | 0.999714303421225 |
| ebi-fl187-GCST90094707 | ebi-gm430-GCST90011614 | OTU97_80 (Ruminococcus) prevalence \|\| id:ebi-gm430-GCST90011614 | Black olive liking \|\| id:ebi-fl187-GCST90094707 | Weighted mode | 18 | 0.0999159341516185 | 0.329324797166907 | 0.765269457585369 | -0.545560668 | 0.745392536598756 | 1.10507801484986 | 0.579516775554805 | 2.10726845264382 | 0.999714303421225 |
| ebi-fl187-GCST90094707 | ebi-gm430-GCST90011623 | OTU99_11 (Parabacteroides) prevalence \|\| id:ebi-gm430-GCST90011623 | Black olive liking \|\| id:ebi-fl187-GCST90094707 | Weighted mode | 18 | 0.0588426022773978 | 0.329181427897695 | 0.860243637712815 | -0.586352996 | 0.704038200956881 | 1.06060829027223 | 0.556352609274457 | 2.02190108690451 | 0.999714303421225 |
| ebi-fl187-GCST90094707 | ebi-gm430-GCST90011642 | OTU99_19 (Bacteria) prevalence \|\| id:ebi-gm430-GCST90011642 | Black olive liking \|\| id:ebi-fl187-GCST90094707 | Weighted mode | 18 | -0.448141174 | 0.675685439007551 | 0.516070802364194 | -1.772484634 | 0.876202286571292 | 0.638814493742886 | 0.169910298964751 | 2.401761164 | 0.999714303421225 |
| ebi-fl187-GCST90094707 | ebi-gm430-GCST90011653 | OTU99_30 (Parasutterella) prevalence \|\| id:ebi-gm430-GCST90011653 | Black olive liking \|\| id:ebi-fl187-GCST90094707 | Weighted mode | 18 | 0.2868262183806 | 0.299684134460428 | 0.351933438206356 | -0.300554685 | 0.87420712192304 | 1.33219268275661 | 0.740407413751873 | 2.39697403217115 | 0.999714303421225 |
| ebi-fl187-GCST90094707 | ebi-gm430-GCST90011675 | OTU99_8 (Ruminococcaceae) prevalence \|\| id:ebi-gm430-GCST90011675 | Black olive liking \|\| id:ebi-fl187-GCST90094707 | Weighted mode | 18 | -0.08478573 | 0.302215950071373 | 0.782443533312785 | -0.677128992 | 0.507557531912196 | 0.918709114906156 | 0.50807358355908 | 1.66122873757617 | 0.999714303421225 |
| ebi-fl187-GCST90094707 | ebi-gm430-GCST90011679 | OTU99_92 (Ruminococcus) prevalence \|\| id:ebi-gm430-GCST90011679 | Black olive liking \|\| id:ebi-fl187-GCST90094707 | Weighted mode | 18 | -0.004907648 | 0.407896354954645 | 0.990540470802639 | -0.804384504 | 0.794569207833471 | 0.995104374950236 | 0.447363192277513 | 2.21348723841998 | 0.999714303421225 |
| ebi-fl187-GCST90094707 | ebi-gm430-GCST90011684 | TestASV_11 (Lachnospiraceae) prevalence \|\| id:ebi-gm430-GCST90011684 | Black olive liking \|\| id:ebi-fl187-GCST90094707 | Weighted mode | 18 | -0.02414954 | 0.29095950498271 | 0.934821297942299 | -0.59443017 | 0.54613108990241 | 0.976139727041329 | 0.551876952533949 | 1.72656017312067 | 0.999714303421225 |
| ebi-fl187-GCST90094707 | ebi-gm430-GCST90011688 | TestASV_15 (Bacteroides) prevalence \|\| id:ebi-gm430-GCST90011688 | Black olive liking \|\| id:ebi-fl187-GCST90094707 | Weighted mode | 18 | -0.101090621 | 0.377135512699617 | 0.791887288091407 | -0.840276226 | 0.638094984108547 | 0.903851121478407 | 0.431591290367168 | 1.89287149215354 | 0.999714303421225 |
| ebi-fl187-GCST90094707 | ebi-gm430-GCST90011692 | TestASV_19 (Prevotella) prevalence \|\| id:ebi-gm430-GCST90011692 | Black olive liking \|\| id:ebi-fl187-GCST90094707 | Weighted mode | 18 | 0.46008787927836 | 0.633906694632394 | 0.477836887716365 | -0.782369242 | 1.70254500075785 | 1.58421319839005 | 0.457321222009713 | 5.48789633449357 | 0.999714303421225 |
| ebi-fl187-GCST90094707 | ebi-gm430-GCST90011693 | TestASV_2 (Bacteroides) prevalence \|\| id:ebi-gm430-GCST90011693 | Black olive liking \|\| id:ebi-fl187-GCST90094707 | Weighted mode | 18 | -0.094166479 | 0.264211490839262 | 0.725921755671226 | -0.612021001 | 0.423688042951219 | 0.910131231805142 | 0.542253865245774 | 1.52758497854451 | 0.999714303421225 |
| ebi-fl187-GCST90094707 | ebi-gm430-GCST90011716 | TestASV_40 (Barnesiella) prevalence \|\| id:ebi-gm430-GCST90011716 | Black olive liking \|\| id:ebi-fl187-GCST90094707 | Weighted mode | 18 | 0.0853186616469914 | 0.507783475665476 | 0.868548894743005 | -0.909936951 | 1.08057427395132 | 1.08906405435487 | 0.402549603721444 | 2.94637108948343 | 0.999714303421225 |
| ebi-fl187-GCST90094707 | ebi-gm430-GCST90011718 | TestASV_42 (Alphaproteobacteria) prevalence \|\| id:ebi-gm430-GCST90011718 | Black olive liking \|\| id:ebi-fl187-GCST90094707 | Weighted mode | 18 | -0.321805233 | 0.39675732936454 | 0.428525096615202 | -1.099449598 | 0.455839132865818 | 0.724839351594306 | 0.333054346956926 | 1.57749655700366 | 0.999714303421225 |
| ebi-fl187-GCST90094707 | ebi-hbsm44-GCST90104929 | Sulcal depth of the right inferior frontal sulcus \|\| id:ebi-hbsm44-GCST90104929 | Black olive liking \|\| id:ebi-fl187-GCST90094707 | Weighted mode | 20 | 0.101121667016154 | 0.220942929604969 | 0.652371379205787 | -0.331926475 | 0.534169809041893 | 1.10641124732983 | 0.717540078042368 | 1.70603132240046 | 0.999714303421225 |
| ebi-fl187-GCST90094707 | ebi-hbsm44-GCST90104935 | Sulcal opening distance of the left calloso-marginal posterior fissure \|\| id:ebi-hbsm44-GCST90104935 | Black olive liking \|\| id:ebi-fl187-GCST90094707 | Weighted mode | 20 | 0.0146029847404566 | 0.0615282382111598 | 0.814934995734971 | -0.105992362 | 0.13519833163433 | 1.01471012922995 | 0.899431517769634 | 1.14476380471422 | 0.999714303421225 |
| ebi-fl187-GCST90094707 | ebi-hbsm44-GCST90104936 | Sulcal opening distance of the left anterior inferior temporal sulcus \|\| id:ebi-hbsm44-GCST90104936 | Black olive liking \|\| id:ebi-fl187-GCST90094707 | Weighted mode | 20 | -0.004444084 | 0.0288618587965098 | 0.879251013380021 | -0.061013327 | 0.0521251590867731 | 0.99556577617548 | 0.940810701136109 | 1.05350759031013 | 0.999714303421225 |
| ebi-fl187-GCST90094707 | ebi-hbsm44-GCST90104941 | Sulcal opening distance of the right posterior inferior temporal sulcus \|\| id:ebi-hbsm44-GCST90104941 | Black olive liking \|\| id:ebi-fl187-GCST90094707 | Weighted mode | 18 | -0.011922647 | 0.0254636836863721 | 0.645573298436777 | -0.061831467 | 0.0379861731494569 | 0.988148146251965 | 0.94004130151527 | 1.03871687059628 | 0.999714303421225 |
| ebi-fl187-GCST90094707 | ebi-IPs91-GCST90274819 | Leukemia inhibitory factor levels \|\| id:ebi-IPs91-GCST90274819 | Black olive liking \|\| id:ebi-fl187-GCST90094707 | Weighted mode | 22 | -0.010000087 | 0.107373843477245 | 0.926680439730599 | -0.22045282 | 0.200452646585779 | 0.99004974798153 | 0.802155483788902 | 1.22195574709335 | 0.999714303421225 |
| ebi-fl187-GCST90094707 | ebi-IPs91-GCST90274837 | STAM binding protein levels \|\| id:ebi-IPs91-GCST90274837 | Black olive liking \|\| id:ebi-fl187-GCST90094707 | Weighted mode | 22 | -0.067421145 | 0.0964772943791452 | 0.492326529316464 | -0.256516642 | 0.121674352378206 | 0.934801431845956 | 0.773742118154749 | 1.12938625994052 | 0.999714303421225 |
| ebi-fl187-GCST90094707 | ebi-IPs91-GCST90274840 | TNF-beta levels \|\| id:ebi-IPs91-GCST90274840 | Black olive liking \|\| id:ebi-fl187-GCST90094707 | Weighted mode | 22 | 0.0879416041023302 | 0.120717068008674 | 0.474357658421873 | -0.148663849 | 0.324547057399332 | 1.09192435626341 | 0.861858780733752 | 1.38340390149091 | 0.999714303421225 |
| ebi-fl187-GCST90094707 | ebi-IPs91-GCST90274842 | Tumor necrosis factor ligand superfamily member 14 levels \|\| id:ebi-IPs91-GCST90274842 | Black olive liking \|\| id:ebi-fl187-GCST90094707 | Weighted mode | 22 | 0.0442482630640958 | 0.124653351073628 | 0.72615187338377 | -0.200072305 | 0.288568831168406 | 1.04524181761597 | 0.818671556858074 | 1.33451620267117 | 0.999714303421225 |
| ebi-fl187-GCST90094707 | ebi-met1400-GCST90199628 | 3-methylhistidine levels \|\| id:ebi-met1400-GCST90199628 | Black olive liking \|\| id:ebi-fl187-GCST90094707 | Weighted mode | 22 | -0.202629478 | 0.130715953858066 | 0.136045522262912 | -0.458832748 | 0.0535737910727695 | 0.816580746111773 | 0.632020942794747 | 1.055034841 | 0.999714303421225 |
| ebi-fl187-GCST90094707 | ebi-met1400-GCST90199651 | Docosahexaenoate DHA; 22:6n3 levels \|\| id:ebi-met1400-GCST90199651 | Black olive liking \|\| id:ebi-fl187-GCST90094707 | Weighted mode | 22 | 0.0382303775208014 | 0.117120899868538 | 0.74733886833723 | -0.191326586 | 0.267787341263137 | 1.03897056077153 | 0.825862828683004 | 1.30706915078297 | 0.999714303421225 |
| ebi-fl187-GCST90094707 | ebi-met1400-GCST90199652 | 1-stearoyl-2-oleoyl-GPS (18:0/18:1) levels \|\| id:ebi-met1400-GCST90199652 | Black olive liking \|\| id:ebi-fl187-GCST90094707 | Weighted mode | 22 | -0.024743327 | 0.137211316793879 | 0.858622320165557 | -0.293677508 | 0.244190853743998 | 0.975560279711542 | 0.745516875842907 | 1.27658794883056 | 0.999714303421225 |
| ebi-fl187-GCST90094707 | ebi-met1400-GCST90199653 | 1-myristoyl-2-palmitoyl-gpc (14:0/16:0) levels \|\| id:ebi-met1400-GCST90199653 | Black olive liking \|\| id:ebi-fl187-GCST90094707 | Weighted mode | 21 | 0.0602819608945103 | 0.121373665298915 | 0.624840893895324 | -0.177610423 | 0.298174344880383 | 1.06213598514085 | 0.837268540450513 | 1.34739667911577 | 0.999714303421225 |
| ebi-fl187-GCST90094707 | ebi-met1400-GCST90199667 | Erythritol levels in elite athletes \|\| id:ebi-met1400-GCST90199667 | Black olive liking \|\| id:ebi-fl187-GCST90094707 | Weighted mode | 22 | 0.0306834027198121 | 0.109792822144784 | 0.782620916524863 | -0.184510529 | 0.245877334123588 | 1.03115899007086 | 0.831511185134835 | 1.27874270582606 | 0.999714303421225 |
| ebi-fl187-GCST90094707 | ebi-met1400-GCST90199674 | 3-hydroxymyristate levels \|\| id:ebi-met1400-GCST90199674 | Black olive liking \|\| id:ebi-fl187-GCST90094707 | Weighted mode | 22 | -0.167413079 | 0.125558950337936 | 0.196703502564604 | -0.413508622 | 0.078682463702275 | 0.845850136267086 | 0.661325832654222 | 1.08186073746968 | 0.999714303421225 |
| ebi-fl187-GCST90094707 | ebi-met1400-GCST90199685 | 2-linoleoylglycerol (18:2) levels \|\| id:ebi-met1400-GCST90199685 | Black olive liking \|\| id:ebi-fl187-GCST90094707 | Weighted mode | 22 | 0.0389371060329466 | 0.132642512269969 | 0.771983863170185 | -0.221042218 | 0.298916430082086 | 1.03970509041606 | 0.801682834116869 | 1.34839693334319 | 0.999714303421225 |
| ebi-fl187-GCST90094707 | ebi-met1400-GCST90199703 | Gamma-glutamylvaline levels \|\| id:ebi-met1400-GCST90199703 | Black olive liking \|\| id:ebi-fl187-GCST90094707 | Weighted mode | 22 | -0.052135367 | 0.13538367933235 | 0.70404092535594 | -0.317487378 | 0.213216644605595 | 0.949200367848025 | 0.727975868953139 | 1.23765275299369 | 0.999714303421225 |
| ebi-fl187-GCST90094707 | ebi-met1400-GCST90199779 | Gamma-glutamyl-2-aminobutyrate levels \|\| id:ebi-met1400-GCST90199779 | Black olive liking \|\| id:ebi-fl187-GCST90094707 | Weighted mode | 21 | 0.023678083626856 | 0.156501664887711 | 0.881257854752811 | -0.28306518 | 0.33042134680677 | 1.02396063513443 | 0.753470675377306 | 1.39155433193184 | 0.999714303421225 |
| ebi-fl187-GCST90094707 | ebi-met1400-GCST90199824 | 4-vinylphenol sulfate levels \|\| id:ebi-met1400-GCST90199824 | Black olive liking \|\| id:ebi-fl187-GCST90094707 | Weighted mode | 22 | 0.0833745085018146 | 0.119292108408615 | 0.492276708574423 | -0.150438024 | 0.3171870409827 | 1.08694880389997 | 0.860331048250223 | 1.37325940369405 | 0.999714303421225 |
| ebi-fl187-GCST90094707 | ebi-met1400-GCST90199830 | 4-ethylphenylsulfate levels \|\| id:ebi-met1400-GCST90199830 | Black olive liking \|\| id:ebi-fl187-GCST90094707 | Weighted mode | 22 | 0.0274129808370166 | 0.100803543495121 | 0.788320325097546 | -0.170161964 | 0.224987926087454 | 1.02779217360108 | 0.843528183984349 | 1.25230759584819 | 0.999714303421225 |
| ebi-fl187-GCST90094707 | ebi-met1400-GCST90199833 | Hexanoylglutamine levels \|\| id:ebi-met1400-GCST90199833 | Black olive liking \|\| id:ebi-fl187-GCST90094707 | Weighted mode | 22 | 0.0387445273404115 | 0.119534847401647 | 0.749047751222736 | -0.195543774 | 0.273032828247639 | 1.03950488464742 | 0.822387343948923 | 1.31394337857535 | 0.999714303421225 |
| ebi-fl187-GCST90094707 | ebi-met1400-GCST90199837 | Carotenoid levels (cryptoxanthin) \|\| id:ebi-met1400-GCST90199837 | Black olive liking \|\| id:ebi-fl187-GCST90094707 | Weighted mode | 22 | 0.234140225071388 | 0.127966232268429 | 0.081528573639309 | -0.01667359 | 0.484954040317508 | 1.26382169927105 | 0.983464644772647 | 1.62410036399185 | 0.999714303421225 |
| ebi-fl187-GCST90094707 | ebi-met1400-GCST90199869 | 2-hydroxyhippurate levels \|\| id:ebi-met1400-GCST90199869 | Black olive liking \|\| id:ebi-fl187-GCST90094707 | Weighted mode | 22 | -0.079958743 | 0.116653065608022 | 0.500563187499333 | -0.308598752 | 0.148681265659387 | 0.92315443224585 | 0.734475418050884 | 1.16030310182024 | 0.999714303421225 |
| ebi-fl187-GCST90094707 | ebi-met1400-GCST90199902 | Hydantoin-5-propionate levels \|\| id:ebi-met1400-GCST90199902 | Black olive liking \|\| id:ebi-fl187-GCST90094707 | Weighted mode | 22 | 0.0287195172130925 | 0.128174704717908 | 0.824871976620407 | -0.222502904 | 0.279941938460191 | 1.0291358990849 | 0.800512682030802 | 1.32305299161335 | 0.999714303421225 |
| ebi-fl187-GCST90094707 | ebi-met1400-GCST90199904 | Pantoate levels \|\| id:ebi-met1400-GCST90199904 | Black olive liking \|\| id:ebi-fl187-GCST90094707 | Weighted mode | 22 | -0.064169357 | 0.126318938592405 | 0.616751555120179 | -0.311754476 | 0.183415762789171 | 0.937846155407952 | 0.732161268964525 | 1.2013137658284 | 0.999714303421225 |
| ebi-fl187-GCST90094707 | ebi-met1400-GCST90199936 | 3-methyl catechol sulfate (2) levels \|\| id:ebi-met1400-GCST90199936 | Black olive liking \|\| id:ebi-fl187-GCST90094707 | Weighted mode | 22 | -0.046402663 | 0.189491992618986 | 0.808926893868944 | -0.417806968 | 0.325001642849272 | 0.954657479844484 | 0.658489325526033 | 1.38403291973636 | 0.999714303421225 |
| ebi-fl187-GCST90094707 | ebi-met1400-GCST90199977 | 4-hydroxychlorothalonil levels \|\| id:ebi-met1400-GCST90199977 | Black olive liking \|\| id:ebi-fl187-GCST90094707 | Weighted mode | 22 | 0.0262460671443907 | 0.138218758854749 | 0.851220479504972 | -0.2446627 | 0.297154834499698 | 1.02659352833433 | 0.782968588847135 | 1.34602369421961 | 0.999714303421225 |
| ebi-fl187-GCST90094707 | ebi-met1400-GCST90200040 | Sphingomyelin (d18:2/24:1, d18:1/24:2) levels \|\| id:ebi-met1400-GCST90200040 | Black olive liking \|\| id:ebi-fl187-GCST90094707 | Weighted mode | 22 | -0.070893468 | 0.132542227910318 | 0.59835512872483 | -0.330676235 | 0.188889298796435 | 0.931561127997585 | 0.718437736662423 | 1.20790722829736 | 0.999714303421225 |
| ebi-fl187-GCST90094707 | ebi-met1400-GCST90200077 | 1-stearoyl-2-oleoyl-GPI (18:0/18:1) levels \|\| id:ebi-met1400-GCST90200077 | Black olive liking \|\| id:ebi-fl187-GCST90094707 | Weighted mode | 22 | -0.086609317 | 0.109545516868355 | 0.438001568076921 | -0.30131853 | 0.12809989651496 | 0.917035296173101 | 0.739842073603701 | 1.1366665460523 | 0.999714303421225 |
| ebi-fl187-GCST90094707 | ebi-met1400-GCST90200080 | 1-palmitoyl-2-oleoyl-GPI (16:0/18:1) levels \|\| id:ebi-met1400-GCST90200080 | Black olive liking \|\| id:ebi-fl187-GCST90094707 | Weighted mode | 22 | 0.0679631973489338 | 0.159005463696606 | 0.673419331509128 | -0.243687511 | 0.379613906194282 | 1.07032591692267 | 0.783732503398673 | 1.46172011938825 | 0.999714303421225 |
| ebi-fl187-GCST90094707 | ebi-met1400-GCST90200082 | 1-oleoyl-2-linoleoyl-GPE (18:1/18:2) levels \|\| id:ebi-met1400-GCST90200082 | Black olive liking \|\| id:ebi-fl187-GCST90094707 | Weighted mode | 22 | -0.085048106 | 0.121719663870001 | 0.492392637540529 | -0.323618647 | 0.153522434940987 | 0.918468099287987 | 0.723526108316696 | 1.16593394448793 | 0.999714303421225 |
| ebi-fl187-GCST90094707 | ebi-met1400-GCST90200100 | Perfluorooctanesulfonate (PFOS) levels \|\| id:ebi-met1400-GCST90200100 | Black olive liking \|\| id:ebi-fl187-GCST90094707 | Weighted mode | 22 | -0.111956672 | 0.113307810406411 | 0.334364342833918 | -0.334039981 | 0.110126635933783 | 0.894082995075393 | 0.716025154353942 | 1.11641944032575 | 0.999714303421225 |
| ebi-fl187-GCST90094707 | ebi-met1400-GCST90200102 | 1-palmitoleoyl-2-linolenoyl-GPC (16:1/18:3) levels \|\| id:ebi-met1400-GCST90200102 | Black olive liking \|\| id:ebi-fl187-GCST90094707 | Weighted mode | 22 | 0.0627700337889329 | 0.137678685315511 | 0.653129372744608 | -0.207080189 | 0.332620257007335 | 1.06478194720393 | 0.81295445701962 | 1.39461760164017 | 0.999714303421225 |
| ebi-fl187-GCST90094707 | ebi-met1400-GCST90200114 | Glycosyl-N-tricosanoyl-sphingadienine (d18:2/23:0) levels \|\| id:ebi-met1400-GCST90200114 | Black olive liking \|\| id:ebi-fl187-GCST90094707 | Weighted mode | 22 | 0.271952926022541 | 0.16405833030999 | 0.11225086400853 | -0.049601401 | 0.593507253430122 | 1.31252521407449 | 0.951608658807943 | 1.81032656821272 | 0.999714303421225 |
| ebi-fl187-GCST90094707 | ebi-met1400-GCST90200135 | Arachidoylcarnitine (C20) levels \|\| id:ebi-met1400-GCST90200135 | Black olive liking \|\| id:ebi-fl187-GCST90094707 | Weighted mode | 22 | 0.12100785484023 | 0.120108309550422 | 0.325168088220844 | -0.114404432 | 0.356420141559056 | 1.12863377757053 | 0.891897171282432 | 1.42820747154242 | 0.999714303421225 |
| ebi-fl187-GCST90094707 | ebi-met1400-GCST90200146 | Docosahexaenoylcarnitine (C22:6) levels \|\| id:ebi-met1400-GCST90200146 | Black olive liking \|\| id:ebi-fl187-GCST90094707 | Weighted mode | 22 | -0.221613682 | 0.169423353341061 | 0.204995404676276 | -0.553683455 | 0.110456090448808 | 0.801224832049159 | 0.574828551099278 | 1.11678731034592 | 0.999714303421225 |
| ebi-fl187-GCST90094707 | ebi-met1400-GCST90200162 | 3-hydroxyphenylacetoylglutamine levels \|\| id:ebi-met1400-GCST90200162 | Black olive liking \|\| id:ebi-fl187-GCST90094707 | Weighted mode | 22 | 0.0209547726182049 | 0.145558895563832 | 0.886904002050778 | -0.264340663 | 0.306250207923315 | 1.02117586548235 | 0.767711964284229 | 1.35832212699182 | 0.999714303421225 |
| ebi-fl187-GCST90094707 | ebi-met1400-GCST90200172 | 3-carboxy-4-methyl-5-pentyl-2-furanpropionate (3-CMPFP) levels \|\| id:ebi-met1400-GCST90200172 | Black olive liking \|\| id:ebi-fl187-GCST90094707 | Weighted mode | 22 | -0.048335035 | 0.108074191724301 | 0.659281115596052 | -0.26016045 | 0.163490381185484 | 0.952814507773777 | 0.770927880212994 | 1.17761402788204 | 0.999714303421225 |
| ebi-fl187-GCST90094707 | ebi-met1400-GCST90200222 | Dihydrocaffeate sulfate (2) levels \|\| id:ebi-met1400-GCST90200222 | Black olive liking \|\| id:ebi-fl187-GCST90094707 | Weighted mode | 22 | -0.080456789 | 0.133877894247375 | 0.554290985639791 | -0.342857461 | 0.181943884165522 | 0.922694773692795 | 0.709739369688878 | 1.19954687841708 | 0.999714303421225 |
| ebi-fl187-GCST90094707 | ebi-met1400-GCST90200223 | 4-methylhexanoylglutamine levels \|\| id:ebi-met1400-GCST90200223 | Black olive liking \|\| id:ebi-fl187-GCST90094707 | Weighted mode | 22 | -0.094973802 | 0.15691349077835 | 0.551490291192474 | -0.402524244 | 0.212576640078485 | 0.909396758671503 | 0.668630128612838 | 1.23686090304959 | 0.999714303421225 |
| ebi-fl187-GCST90094707 | ebi-met1400-GCST90200224 | 2-ketocaprylate levels \|\| id:ebi-met1400-GCST90200224 | Black olive liking \|\| id:ebi-fl187-GCST90094707 | Weighted mode | 22 | 0.0757551262696201 | 0.130171606107597 | 0.566787091094439 | -0.179381222 | 0.33089147424051 | 1.07869839691316 | 0.835787218431536 | 1.39220869360345 | 0.999714303421225 |
| ebi-fl187-GCST90094707 | ebi-met1400-GCST90200243 | Hydroxypalmitoyl sphingomyelin (d18:1/16:0(OH)) levels \|\| id:ebi-met1400-GCST90200243 | Black olive liking \|\| id:ebi-fl187-GCST90094707 | Weighted mode | 22 | 0.212737217048925 | 0.164604869812156 | 0.210255474903305 | -0.109888328 | 0.535362761880752 | 1.23705953037331 | 0.89593418066661 | 1.70806775175028 | 0.999714303421225 |
| ebi-fl187-GCST90094707 | ebi-met1400-GCST90200273 | 2-methoxyhydroquinone sulfate (2) levels \|\| id:ebi-met1400-GCST90200273 | Black olive liking \|\| id:ebi-fl187-GCST90094707 | Weighted mode | 22 | 0.137694339609686 | 0.145507655518937 | 0.354756427323237 | -0.147500665 | 0.422889344426802 | 1.14762471324063 | 0.862861864339061 | 1.52636538578453 | 0.999714303421225 |
| ebi-fl187-GCST90094707 | ebi-met1400-GCST90200276 | 2,4-di-tert-butylphenol levels \|\| id:ebi-met1400-GCST90200276 | Black olive liking \|\| id:ebi-fl187-GCST90094707 | Weighted mode | 22 | 0.0213048701571135 | 0.131512836758751 | 0.872855684653719 | -0.23646029 | 0.279070030204265 | 1.02153343922884 | 0.789417229524554 | 1.32189991354913 | 0.999714303421225 |
| ebi-fl187-GCST90094707 | ebi-met1400-GCST90200289 | N-acetylmethionine levels \|\| id:ebi-met1400-GCST90200289 | Black olive liking \|\| id:ebi-fl187-GCST90094707 | Weighted mode | 22 | -0.063651234 | 0.115903250220894 | 0.588677588557897 | -0.290821604 | 0.16351913645495 | 0.938332200858066 | 0.747649043413338 | 1.17764789097761 | 0.999714303421225 |
| ebi-fl187-GCST90094707 | ebi-met1400-GCST90200308 | 3-hydroxyisobutyrate levels \|\| id:ebi-met1400-GCST90200308 | Black olive liking \|\| id:ebi-fl187-GCST90094707 | Weighted mode | 22 | 0.0735938616196697 | 0.128565881252864 | 0.573114456025557 | -0.178395266 | 0.325582988875283 | 1.07636956172106 | 0.836611674280704 | 1.38483775569554 | 0.999714303421225 |
| ebi-fl187-GCST90094707 | ebi-met1400-GCST90200383 | Cystathionine levels \|\| id:ebi-met1400-GCST90200383 | Black olive liking \|\| id:ebi-fl187-GCST90094707 | Weighted mode | 22 | -0.05021537 | 0.137578244823798 | 0.718765295223081 | -0.31986873 | 0.219437989715301 | 0.951024580146483 | 0.726244364918861 | 1.24537661940254 | 0.999714303421225 |
| ebi-fl187-GCST90094707 | ebi-met1400-GCST90200384 | Guanidinoacetate levels \|\| id:ebi-met1400-GCST90200384 | Black olive liking \|\| id:ebi-fl187-GCST90094707 | Weighted mode | 22 | 0.131306250785241 | 0.135929560349044 | 0.345044141446273 | -0.135115687 | 0.397728189069367 | 1.14031695080415 | 0.873614839525981 | 1.48843940081974 | 0.999714303421225 |
| ebi-fl187-GCST90094707 | ebi-met1400-GCST90200403 | Citrulline levels \|\| id:ebi-met1400-GCST90200403 | Black olive liking \|\| id:ebi-fl187-GCST90094707 | Weighted mode | 22 | 0.198042804748999 | 0.135617297841526 | 0.159006007296187 | -0.067767099 | 0.46385270851839 | 1.21901457231769 | 0.934478089097169 | 1.59018873193543 | 0.999714303421225 |
| ebi-fl187-GCST90094707 | ebi-met1400-GCST90200434 | 12,13-DiHOME levels \|\| id:ebi-met1400-GCST90200434 | Black olive liking \|\| id:ebi-fl187-GCST90094707 | Weighted mode | 22 | 0.0228866086403865 | 0.10807583197054 | 0.834332625481715 | -0.188942022 | 0.234715239302645 | 1.02315051654118 | 0.82783450147402 | 1.26454862370982 | 0.999714303421225 |
| ebi-fl187-GCST90094707 | ebi-met1400-GCST90200437 | Fructose levels \|\| id:ebi-met1400-GCST90200437 | Black olive liking \|\| id:ebi-fl187-GCST90094707 | Weighted mode | 22 | -0.064905134 | 0.149020179105579 | 0.667610168565628 | -0.356984685 | 0.227174416927794 | 0.93715636332446 | 0.699783214716262 | 1.25504875059861 | 0.999714303421225 |
| ebi-fl187-GCST90094707 | ebi-met1400-GCST90200448 | N-stearoyl-sphinganine (d18:0/18:0) levels \|\| id:ebi-met1400-GCST90200448 | Black olive liking \|\| id:ebi-fl187-GCST90094707 | Weighted mode | 22 | 0.0428401390216038 | 0.143120842159462 | 0.767631833762438 | -0.237676712 | 0.32335698965415 | 1.04377102325584 | 0.788457549066117 | 1.38175853637136 | 0.999714303421225 |
| ebi-fl187-GCST90094707 | ebi-met1400-GCST90200468 | X-11847 levels \|\| id:ebi-met1400-GCST90200468 | Black olive liking \|\| id:ebi-fl187-GCST90094707 | Weighted mode | 22 | 0.0770547038960296 | 0.143446863740789 | 0.596799659945347 | -0.204101149 | 0.358210556827975 | 1.08010116051811 | 0.81537989212603 | 1.43076684649496 | 0.999714303421225 |
| ebi-fl187-GCST90094707 | ebi-met1400-GCST90200476 | X-11843 levels \|\| id:ebi-met1400-GCST90200476 | Black olive liking \|\| id:ebi-fl187-GCST90094707 | Weighted mode | 22 | 0.180999919682566 | 0.137539964723892 | 0.202365863263848 | -0.088578411 | 0.450578250541394 | 1.19841508303957 | 0.915231343556159 | 1.56921932511222 | 0.999714303421225 |
| ebi-fl187-GCST90094707 | ebi-met1400-GCST90200511 | X-12906 levels \|\| id:ebi-met1400-GCST90200511 | Black olive liking \|\| id:ebi-fl187-GCST90094707 | Weighted mode | 22 | -0.204896066 | 0.153402747893592 | 0.195952656483734 | -0.505565452 | 0.0957733198792317 | 0.814731990372121 | 0.603164418537681 | 1.10050957207491 | 0.999714303421225 |
| ebi-fl187-GCST90094707 | ebi-met1400-GCST90200515 | X-12849 levels \|\| id:ebi-met1400-GCST90200515 | Black olive liking \|\| id:ebi-fl187-GCST90094707 | Weighted mode | 22 | 0.0676179021978874 | 0.128138886113097 | 0.603244471236587 | -0.183534315 | 0.318770118979557 | 1.06995640237298 | 0.832323314419756 | 1.37543510213579 | 0.999714303421225 |
| ebi-fl187-GCST90094707 | ebi-met1400-GCST90200551 | X-18886 levels \|\| id:ebi-met1400-GCST90200551 | Black olive liking \|\| id:ebi-fl187-GCST90094707 | Weighted mode | 22 | 0.0316912106616432 | 0.132773591702647 | 0.813662817883137 | -0.228545029 | 0.291927450398831 | 1.03219872412846 | 0.795690467191638 | 1.3390058697735 | 0.999714303421225 |
| ebi-fl187-GCST90094707 | ebi-met1400-GCST90200564 | X-21319 levels \|\| id:ebi-met1400-GCST90200564 | Black olive liking \|\| id:ebi-fl187-GCST90094707 | Weighted mode | 22 | -0.053156249 | 0.105571717083295 | 0.619847729427379 | -0.260076814 | 0.153764316506216 | 0.94823184065165 | 0.77099236016686 | 1.1662159965256 | 0.999714303421225 |
| ebi-fl187-GCST90094707 | ebi-met1400-GCST90200579 | X-21442 levels \|\| id:ebi-met1400-GCST90200579 | Black olive liking \|\| id:ebi-fl187-GCST90094707 | Weighted mode | 22 | 0.0302788716713173 | 0.135004114898764 | 0.824706324798447 | -0.234329194 | 0.294886936872895 | 1.03074193860426 | 0.791101347580291 | 1.34297450920452 | 0.999714303421225 |
| ebi-fl187-GCST90094707 | ebi-met1400-GCST90200581 | X-21441 levels \|\| id:ebi-met1400-GCST90200581 | Black olive liking \|\| id:ebi-fl187-GCST90094707 | Weighted mode | 22 | 0.0870652009477336 | 0.136428535199383 | 0.53025854831249 | -0.180334728 | 0.354465129938525 | 1.09096780953452 | 0.834990669835794 | 1.42541803691603 | 0.999714303421225 |
| ebi-fl187-GCST90094707 | ebi-met1400-GCST90200648 | X-24585 levels \|\| id:ebi-met1400-GCST90200648 | Black olive liking \|\| id:ebi-fl187-GCST90094707 | Weighted mode | 22 | 0.0230720136253039 | 0.126916032972694 | 0.857491149010053 | -0.225683411 | 0.271827438251784 | 1.02334023133376 | 0.797970690422125 | 1.31236051854517 | 0.999714303421225 |
| ebi-fl187-GCST90094707 | ebi-met1400-GCST90200720 | Adenosine 5'-diphosphate (ADP) to pantothenate ratio \|\| id:ebi-met1400-GCST90200720 | Black olive liking \|\| id:ebi-fl187-GCST90094707 | Weighted mode | 22 | 0.113298064338086 | 0.163009258038526 | 0.494649259748012 | -0.206200081 | 0.432796210093597 | 1.11996570503989 | 0.813670259696301 | 1.54156203390507 | 0.999714303421225 |
| ebi-fl187-GCST90094707 | ebi-met1400-GCST90200724 | Cholate to taurocholate ratio \|\| id:ebi-met1400-GCST90200724 | Black olive liking \|\| id:ebi-fl187-GCST90094707 | Weighted mode | 22 | 0.184885279647281 | 0.144009082391247 | 0.213181654859229 | -0.097372522 | 0.467143081134126 | 1.20308041440118 | 0.907217984664459 | 1.59542966297239 | 0.999714303421225 |
| ebi-fl187-GCST90094707 | ebi-met1400-GCST90200726 | N-acetylputrescine to (N(1) + N(8))-acetylspermidine ratio \|\| id:ebi-met1400-GCST90200726 | Black olive liking \|\| id:ebi-fl187-GCST90094707 | Weighted mode | 22 | -0.045883347 | 0.124422193235203 | 0.715989875060794 | -0.289750846 | 0.197984151391118 | 0.955153376864863 | 0.748450023598641 | 1.21894307511651 | 0.999714303421225 |
| ebi-fl187-GCST90094707 | ebi-met1400-GCST90200744 | Arginine to glutamate ratio \|\| id:ebi-met1400-GCST90200744 | Black olive liking \|\| id:ebi-fl187-GCST90094707 | Weighted mode | 22 | -0.008700546 | 0.146217141622223 | 0.953113085835311 | -0.295286144 | 0.2778850514136 | 0.991337194053019 | 0.744318574886382 | 1.32033441791094 | 0.999714303421225 |
| ebi-fl187-GCST90094707 | ebi-met1400-GCST90200759 | Histidine to trans-urocanate ratio \|\| id:ebi-met1400-GCST90200759 | Black olive liking \|\| id:ebi-fl187-GCST90094707 | Weighted mode | 22 | 0.0685947100945923 | 0.109527813509516 | 0.537882185201037 | -0.146079804 | 0.283269224573243 | 1.07100205485361 | 0.864088742363135 | 1.32746249923785 | 0.999714303421225 |
| ebi-fl187-GCST90094707 | ebi-met1400-GCST90200795 | Oleoyl-linoleoyl-glycerol (18:1 to 18:2) [2] to linoleoyl-arachidonoyl-glycerol (18:2 to 20:4) [2] ratio \|\| id:ebi-met1400-GCST90200795 | Black olive liking \|\| id:ebi-fl187-GCST90094707 | Weighted mode | 22 | 0.0325570181324876 | 0.132436745707636 | 0.808200001647063 | -0.227019003 | 0.292133039719453 | 1.03309279648662 | 0.796905638186226 | 1.33928118338038 | 0.999714303421225 |
| ebi-fl187-GCST90094707 | ebi-met1400-GCST90200811 | Choline to taurocholate ratio \|\| id:ebi-met1400-GCST90200811 | Black olive liking \|\| id:ebi-fl187-GCST90094707 | Weighted mode | 22 | -0.026455415 | 0.118850431388767 | 0.826002074624121 | -0.259402261 | 0.20649143030615 | 0.973891463608304 | 0.771512611382388 | 1.22935719895709 | 0.999714303421225 |
| ebi-fl187-GCST90094707 | ebi-met1400-GCST90200831 | Cholate to phosphate ratio \|\| id:ebi-met1400-GCST90200831 | Black olive liking \|\| id:ebi-fl187-GCST90094707 | Weighted mode | 22 | 0.117297714981764 | 0.143671047968303 | 0.423417969978854 | -0.164297539 | 0.398892968999638 | 1.12445414670871 | 0.848489525562729 | 1.49017411524538 | 0.999714303421225 |
| ebi-fl187-GCST90094707 | ebi-met1400-GCST90200841 | Adenosine 5'-diphosphate (ADP) to oxalate (ethanedioate) ratio \|\| id:ebi-met1400-GCST90200841 | Black olive liking \|\| id:ebi-fl187-GCST90094707 | Weighted mode | 22 | -0.018971804 | 0.182941231810413 | 0.918387986895688 | -0.377536618 | 0.339593010453431 | 0.981207028069182 | 0.685548097557574 | 1.40437590792308 | 0.999714303421225 |
| ebi-fl187-GCST90094707 | ebi-met1400-GCST90200844 | Aspartate to phosphate ratio \|\| id:ebi-met1400-GCST90200844 | Black olive liking \|\| id:ebi-fl187-GCST90094707 | Weighted mode | 22 | -0.067829728 | 0.111440948705373 | 0.549277279577908 | -0.286253988 | 0.150594531227824 | 0.934419565301323 | 0.751071828714871 | 1.16252519484309 | 0.999714303421225 |
| ebi-fl187-GCST90094707 | ebi-met1400-GCST90200870 | Adenosine 5'-monophosphate (AMP) to acetoacetate ratio \|\| id:ebi-met1400-GCST90200870 | Black olive liking \|\| id:ebi-fl187-GCST90094707 | Weighted mode | 22 | -0.044679666 | 0.12990489592001 | 0.734310399823368 | -0.299293262 | 0.209933930037228 | 0.956303769417506 | 0.741341970147206 | 1.23359655358584 | 0.999714303421225 |
| ebi-fl187-GCST90094707 | ebi-met1400-GCST90200887 | Cholesterol to cortisol ratio \|\| id:ebi-met1400-GCST90200887 | Black olive liking \|\| id:ebi-fl187-GCST90094707 | Weighted mode | 22 | -0.037308688 | 0.129323764640053 | 0.775799588575763 | -0.290783267 | 0.216165890362102 | 0.963378705678754 | 0.747677706871286 | 1.241308283 | 0.999714303421225 |
| ebi-fl187-GCST90094707 | ebi-met1400-GCST90200890 | Cortisol to taurocholate ratio \|\| id:ebi-met1400-GCST90200890 | Black olive liking \|\| id:ebi-fl187-GCST90094707 | Weighted mode | 22 | 0.147814565967752 | 0.132503076739649 | 0.277215769233849 | -0.111891464 | 0.407520596377463 | 1.1592979031453 | 0.894141298358855 | 1.50308640335021 | 0.999714303421225 |
| ebi-fl187-GCST90094707 | ebi-met1400-GCST90200948 | Adenosine 5'-diphosphate (ADP) to mannitol to sorbitol ratio \|\| id:ebi-met1400-GCST90200948 | Black olive liking \|\| id:ebi-fl187-GCST90094707 | Weighted mode | 22 | -0.154904989 | 0.183504080013011 | 0.408099446357537 | -0.514572986 | 0.204763008118447 | 0.85649655046254 | 0.597755790498735 | 1.22723418595772 | 0.999714303421225 |
| ebi-fl187-GCST90094707 | ebi-met1400-GCST90200972 | Leucine to N-palmitoyl-sphingosine (d18:1 to 16:0) ratio \|\| id:ebi-met1400-GCST90200972 | Black olive liking \|\| id:ebi-fl187-GCST90094707 | Weighted mode | 22 | -0.175864655 | 0.137940798242423 | 0.216260497289472 | -0.446228619 | 0.0944993100388336 | 0.838731494175071 | 0.640037430570976 | 1.09910840479061 | 0.999714303421225 |
| ebi-fl187-GCST90094707 | ebi-met1400-GCST90200973 | Proline to glutamate ratio \|\| id:ebi-met1400-GCST90200973 | Black olive liking \|\| id:ebi-fl187-GCST90094707 | Weighted mode | 22 | 0.0739449993772773 | 0.117172174222966 | 0.534797369808111 | -0.155712462 | 0.303602460854291 | 1.07674758208004 | 0.85580523143554 | 1.3547303906643 | 0.999714303421225 |
| ebi-fl187-GCST90094707 | ebi-met1400-GCST90200983 | Cholesterol to linoleoyl-arachidonoyl-glycerol (18:2 to 20:4) [1] ratio \|\| id:ebi-met1400-GCST90200983 | Black olive liking \|\| id:ebi-fl187-GCST90094707 | Weighted mode | 22 | -0.005102294 | 0.125432920726679 | 0.967937280997014 | -0.250950819 | 0.240746230384189 | 0.994910700353015 | 0.778060636523669 | 1.27219814910507 | 0.999714303421225 |
| ebi-fl187-GCST90094707 | ebi-met1400-GCST90201001 | Serine to alpha-ketobutyrate ratio \|\| id:ebi-met1400-GCST90201001 | Black olive liking \|\| id:ebi-fl187-GCST90094707 | Weighted mode | 22 | -0.12416301 | 0.1297402713133 | 0.349449822989462 | -0.378453942 | 0.130127921810151 | 0.883235852902821 | 0.684919516531158 | 1.13897407363115 | 0.999714303421225 |
| ebi-fl187-GCST90094707 | ebi-met1400-GCST90201005 | Fructose to maltose ratio \|\| id:ebi-met1400-GCST90201005 | Black olive liking \|\| id:ebi-fl187-GCST90094707 | Weighted mode | 22 | 0.0286520951373672 | 0.136307405298818 | 0.835536062164394 | -0.238510419 | 0.295814609523051 | 1.02906651494542 | 0.787800479925555 | 1.34422092797148 | 0.999714303421225 |
| ebi-fl187-GCST90094707 | ebi-met1400-GCST90201020 | Paraxanthine to 5-acetylamino-6-formylamino-3-methyluracil ratio \|\| id:ebi-met1400-GCST90201020 | Black olive liking \|\| id:ebi-fl187-GCST90094707 | Weighted mode | 22 | -0.030004416 | 0.123496104845353 | 0.810394999056786 | -0.272056781 | 0.212047949873829 | 0.97044124843629 | 0.761811003379773 | 1.23620715963472 | 0.999714303421225 |
| ebi-fl187-GCST90094707 | ebi-pw11-GCST90275197 | Placental weight (fetal genotype, adjusted for sex) \|\| id:ebi-pw11-GCST90275197 | Black olive liking \|\| id:ebi-fl187-GCST90094707 | Weighted mode | 19 | -0.015405275 | 0.0546910286439992 | 0.781402994929638 | -0.122599691 | 0.091789141188549 | 0.984712779297662 | 0.884617712038343 | 1.09613366826878 | 0.999714303421225 |
| ebi-fl187-GCST90094707 | prot-a-1017 | FAS-associated factor 2 \|\| id:prot-a-1017 | Black olive liking \|\| id:ebi-fl187-GCST90094707 | Weighted mode | 22 | -0.067748796 | 0.207463150029882 | 0.747234352153136 | -0.47437657 | 0.338878977665678 | 0.934495192658061 | 0.622272878862426 | 1.40337349539878 | 0.999714303421225 |
| ebi-fl187-GCST90094707 | prot-a-1019 | Fas apoptotic inhibitory molecule 3 \|\| id:prot-a-1019 | Black olive liking \|\| id:ebi-fl187-GCST90094707 | Weighted mode | 22 | -0.018738342 | 0.207326655471398 | 0.928841162063233 | -0.425098586 | 0.387621903184963 | 0.981436129715945 | 0.653705335586896 | 1.47347256366974 | 0.999714303421225 |
| ebi-fl187-GCST90094707 | prot-a-1052 | MIP18 family protein FAM96A \|\| id:prot-a-1052 | Black olive liking \|\| id:ebi-fl187-GCST90094707 | Weighted mode | 22 | 0.228296749648924 | 0.227809462493606 | 0.327684772250511 | -0.218209797 | 0.674803296136392 | 1.25645812362282 | 0.803956756386543 | 1.96364668108931 | 0.999714303421225 |
| ebi-fl187-GCST90094707 | prot-a-1066 | F-box/LRR-repeat protein 4 \|\| id:prot-a-1066 | Black olive liking \|\| id:ebi-fl187-GCST90094707 | Weighted mode | 22 | -0.04437193 | 0.255950014158673 | 0.864027287303919 | -0.546033958 | 0.457290097348408 | 0.956598103382962 | 0.579242561038946 | 1.57978710983283 | 0.999714303421225 |
| ebi-fl187-GCST90094707 | prot-a-1076 | Low affinity immunoglobulin gamma Fc region receptor III-B \|\| id:prot-a-1076 | Black olive liking \|\| id:ebi-fl187-GCST90094707 | Weighted mode | 22 | -0.241740956 | 0.19663617773221 | 0.23252584913184 | -0.627147864 | 0.143665952833434 | 0.785259568362277 | 0.534112993614446 | 1.15449838718889 | 0.999714303421225 |
| ebi-fl187-GCST90094707 | prot-a-1081 | Fc receptor-like protein 3 \|\| id:prot-a-1081 | Black olive liking \|\| id:ebi-fl187-GCST90094707 | Weighted mode | 22 | -0.261535407 | 0.194934175725372 | 0.194035249649606 | -0.643606392 | 0.120535577128832 | 0.769868615978175 | 0.525394225894173 | 1.128100875 | 0.999714303421225 |
| ebi-fl187-GCST90094707 | prot-a-1105 | Fibroblast growth factor receptor 4 \|\| id:prot-a-1105 | Black olive liking \|\| id:ebi-fl187-GCST90094707 | Weighted mode | 22 | -0.00850036 | 0.224664175201158 | 0.970175939914992 | -0.448842143 | 0.431841423886666 | 0.991535666398291 | 0.638366861480792 | 1.54009087417124 | 0.999714303421225 |
| ebi-fl187-GCST90094707 | prot-a-1143 | Follistatin-related protein 1 \|\| id:prot-a-1143 | Black olive liking \|\| id:ebi-fl187-GCST90094707 | Weighted mode | 22 | -0.073267022 | 0.267685432495232 | 0.786984826395167 | -0.59793047 | 0.451396425341103 | 0.929352638965769 | 0.549948594295943 | 1.57050374618807 | 0.999714303421225 |
| ebi-fl187-GCST90094707 | prot-a-1151 | Alpha-(1,3)-fucosyltransferase 10 \|\| id:prot-a-1151 | Black olive liking \|\| id:ebi-fl187-GCST90094707 | Weighted mode | 22 | 0.0790418967025091 | 0.184515002742386 | 0.67273872286594 | -0.282607509 | 0.440691302077587 | 1.08224966381268 | 0.753815595888746 | 1.55378097934116 | 0.999714303421225 |
| ebi-fl187-GCST90094707 | prot-a-1165 | Growth arrest and DNA damage-inducible proteins-interacting protein 1 \|\| id:prot-a-1165 | Black olive liking \|\| id:ebi-fl187-GCST90094707 | Weighted mode | 22 | 0.149993988692639 | 0.204843009030814 | 0.472116803380123 | -0.251498309 | 0.551486286393034 | 1.16182725860654 | 0.777634772583025 | 1.73583104361125 | 0.999714303421225 |
| ebi-fl187-GCST90094707 | prot-a-1194 | Growth/differentiation factor 11/8 \|\| id:prot-a-1194 | Black olive liking \|\| id:ebi-fl187-GCST90094707 | Weighted mode | 22 | -0.325932785 | 0.210084328220576 | 0.13573769657985 | -0.737698069 | 0.085832497855548 | 0.721853704868203 | 0.478213464000826 | 1.08962379869558 | 0.999714303421225 |
| ebi-fl187-GCST90094707 | prot-a-1216 | D-glucuronyl C5-epimerase \|\| id:prot-a-1216 | Black olive liking \|\| id:ebi-fl187-GCST90094707 | Weighted mode | 22 | 0.188683980217826 | 0.179985259133495 | 0.306402672020777 | -0.164087128 | 0.541455088119476 | 1.20765924796051 | 0.848668076175083 | 1.71850562089914 | 0.999714303421225 |
| ebi-fl187-GCST90094707 | prot-a-1246 | Glypican-3 \|\| id:prot-a-1246 | Black olive liking \|\| id:ebi-fl187-GCST90094707 | Weighted mode | 22 | 0.0678024645460547 | 0.208946178036471 | 0.748771772278258 | -0.341732044 | 0.477336973497537 | 1.07015389426327 | 0.710538571988573 | 1.6117764785122 | 0.999714303421225 |
| ebi-fl187-GCST90094707 | prot-a-1275 | Glutamate receptor 4 \|\| id:prot-a-1275 | Black olive liking \|\| id:ebi-fl187-GCST90094707 | Weighted mode | 21 | 0.221395693343812 | 0.199698486913343 | 0.280735538491382 | -0.170013341 | 0.612804727693965 | 1.24781708580473 | 0.843653561333795 | 1.84560055334152 | 0.999714303421225 |
| ebi-fl187-GCST90094707 | prot-a-1289 | Glutathione S-transferase P \|\| id:prot-a-1289 | Black olive liking \|\| id:ebi-fl187-GCST90094707 | Weighted mode | 22 | 0.217989126195914 | 0.21111602100747 | 0.31355742925593 | -0.195798275 | 0.631776527370555 | 1.24357354517045 | 0.822178071839904 | 1.88094917052096 | 0.999714303421225 |
| ebi-fl187-GCST90094707 | prot-a-130 | Apolipoprotein D \|\| id:prot-a-130 | Black olive liking \|\| id:ebi-fl187-GCST90094707 | Weighted mode | 22 | 0.0207849158217832 | 0.184897233176924 | 0.911563310319528 | -0.341613661 | 0.383183492848554 | 1.02100242655156 | 0.710622692797908 | 1.46694717969081 | 0.999714303421225 |
| ebi-fl187-GCST90094707 | prot-a-1313 | Heparin-binding EGF-like growth factor \|\| id:prot-a-1313 | Black olive liking \|\| id:ebi-fl187-GCST90094707 | Weighted mode | 22 | 0.0850765770743094 | 0.190712460097337 | 0.660091647891737 | -0.288719845 | 0.45887299886509 | 1.08880044065834 | 0.74922207452604 | 1.58228973742898 | 0.999714303421225 |
| ebi-fl187-GCST90094707 | prot-a-1347 | HLA class II histocompatibility antigen, DQ alpha 2 chain \|\| id:prot-a-1347 | Black olive liking \|\| id:ebi-fl187-GCST90094707 | Weighted mode | 21 | -0.093260537 | 0.173858862116081 | 0.5975882867496 | -0.434023907 | 0.247502832669234 | 0.910956131526847 | 0.647896766145074 | 1.28082299052646 | 0.999714303421225 |
| ebi-fl187-GCST90094707 | prot-a-1357 | Non-histone chromosomal protein HMG-14 \|\| id:prot-a-1357 | Black olive liking \|\| id:ebi-fl187-GCST90094707 | Weighted mode | 22 | 0.155920204604696 | 0.216522198986427 | 0.479395275002902 | -0.268463305 | 0.580303714618094 | 1.16873293986199 | 0.764553477272499 | 1.7865809591127 | 0.999714303421225 |
| ebi-fl187-GCST90094707 | prot-a-137 | Apolipoprotein M \|\| id:prot-a-137 | Black olive liking \|\| id:ebi-fl187-GCST90094707 | Weighted mode | 22 | -0.104748874 | 0.15944769399904 | 0.518349798552993 | -0.417266354 | 0.207768606506194 | 0.90055064611527 | 0.658845410480611 | 1.2309283077908 | 0.999714303421225 |
| ebi-fl187-GCST90094707 | prot-a-1381 | 17-beta-hydroxysteroid dehydrogenase 14 \|\| id:prot-a-1381 | Black olive liking \|\| id:ebi-fl187-GCST90094707 | Weighted mode | 21 | -0.178097033 | 0.207465283256614 | 0.400819598053352 | -0.584728988 | 0.228534922576726 | 0.836861216724063 | 0.557256864764331 | 1.25675741357275 | 0.999714303421225 |
| ebi-fl187-GCST90094707 | prot-a-1392 | Serine protease HTRA2, mitochondrial \|\| id:prot-a-1392 | Black olive liking \|\| id:ebi-fl187-GCST90094707 | Weighted mode | 22 | -0.071569768 | 0.295066433115507 | 0.810703583505694 | -0.649899977 | 0.50676044072866 | 0.930931325946901 | 0.522097995913338 | 1.65990511438988 | 0.999714303421225 |
| ebi-fl187-GCST90094707 | prot-a-1416 | Interferon-induced protein with tetratricopeptide repeats 2 \|\| id:prot-a-1416 | Black olive liking \|\| id:ebi-fl187-GCST90094707 | Weighted mode | 22 | -0.249471012 | 0.211753825626347 | 0.251928027615488 | -0.66450851 | 0.165566486488059 | 0.77921286852743 | 0.514526350144387 | 1.18006141825071 | 0.999714303421225 |
| ebi-fl187-GCST90094707 | prot-a-1428 | Interferon gamma \|\| id:prot-a-1428 | Black olive liking \|\| id:ebi-fl187-GCST90094707 | Weighted mode | 22 | -0.115671996 | 0.183039244124416 | 0.534238349535419 | -0.474428914 | 0.243084922681486 | 0.890767350815508 | 0.622240307566138 | 1.27517691096304 | 0.999714303421225 |
| ebi-fl187-GCST90094707 | prot-a-1483 | Interleukin-17C \|\| id:prot-a-1483 | Black olive liking \|\| id:ebi-fl187-GCST90094707 | Weighted mode | 22 | 0.152558825519992 | 0.222497752805981 | 0.500424737340001 | -0.28353677 | 0.588654421019715 | 1.16481098069091 | 0.75311542959186 | 1.80156263890835 | 0.999714303421225 |
| ebi-fl187-GCST90094707 | prot-a-1503 | Interleukin-1 receptor-like 2 \|\| id:prot-a-1503 | Black olive liking \|\| id:ebi-fl187-GCST90094707 | Weighted mode | 22 | -0.023859507 | 0.190419563440476 | 0.901478096236832 | -0.397081852 | 0.349362837077535 | 0.976422880442151 | 0.672278996262937 | 1.41816365935975 | 0.999714303421225 |
| ebi-fl187-GCST90094707 | prot-a-1522 | Interleukin-31 receptor subunit alpha \|\| id:prot-a-1522 | Black olive liking \|\| id:ebi-fl187-GCST90094707 | Weighted mode | 22 | -0.127748605 | 0.181487462153129 | 0.489228809585519 | -0.483464031 | 0.227966820955867 | 0.880074597809804 | 0.616643615415155 | 1.25604365041327 | 0.999714303421225 |
| ebi-fl187-GCST90094707 | prot-a-1540 | Interleukin-6 receptor subunit alpha \|\| id:prot-a-1540 | Black olive liking \|\| id:ebi-fl187-GCST90094707 | Weighted mode | 22 | -0.020913836 | 0.189461671619955 | 0.913151735256514 | -0.392258713 | 0.350431040136158 | 0.979303341393395 | 0.675529323394207 | 1.41967935550982 | 0.999714303421225 |
| ebi-fl187-GCST90094707 | prot-a-1541 | Interleukin-6 receptor subunit alpha \|\| id:prot-a-1541 | Black olive liking \|\| id:ebi-fl187-GCST90094707 | Weighted mode | 22 | -0.105960316 | 0.208618493296063 | 0.616807007103507 | -0.514852563 | 0.302931930509813 | 0.899460341236649 | 0.597588694681917 | 1.35382230731152 | 0.999714303421225 |
| ebi-fl187-GCST90094707 | prot-a-1545 | Interleukin-9 \|\| id:prot-a-1545 | Black olive liking \|\| id:ebi-fl187-GCST90094707 | Weighted mode | 22 | 0.153615880431335 | 0.1751219824299 | 0.390306609802716 | -0.189623205 | 0.496854965993938 | 1.16604290084775 | 0.827270786612559 | 1.64354413164381 | 0.999714303421225 |
| ebi-fl187-GCST90094707 | prot-a-1548 | Inositol monophosphatase 3 \|\| id:prot-a-1548 | Black olive liking \|\| id:ebi-fl187-GCST90094707 | Weighted mode | 22 | -0.152323892 | 0.233986576642939 | 0.5221105033756 | -0.610937582 | 0.306289798169938 | 0.858710106317431 | 0.542841671677586 | 1.35837590436435 | 0.999714303421225 |
| ebi-fl187-GCST90094707 | prot-a-1555 | Inhibin beta A chain:Inhibin beta B chain heterodimer \|\| id:prot-a-1555 | Black olive liking \|\| id:ebi-fl187-GCST90094707 | Weighted mode | 22 | -0.179187838 | 0.217974265142031 | 0.420279174192233 | -0.606417398 | 0.248041721672003 | 0.835948861681061 | 0.545300970288797 | 1.28151339796033 | 0.999714303421225 |
| ebi-fl187-GCST90094707 | prot-a-1603 | Junctophilin-3 \|\| id:prot-a-1603 | Black olive liking \|\| id:ebi-fl187-GCST90094707 | Weighted mode | 22 | -0.059874624 | 0.215000228941764 | 0.783362822922484 | -0.481275073 | 0.361525824275196 | 0.941882615232115 | 0.617994900500058 | 1.43551809271993 | 0.999714303421225 |
| ebi-fl187-GCST90094707 | prot-a-1627 | UPF0258 protein KIAA1024 \|\| id:prot-a-1627 | Black olive liking \|\| id:ebi-fl187-GCST90094707 | Weighted mode | 22 | 0.305237072904168 | 0.231640220026911 | 0.201792709693619 | -0.148777758 | 0.759251904156914 | 1.35694666016215 | 0.861760612720475 | 2.13667718313607 | 0.999714303421225 |
| ebi-fl187-GCST90094707 | prot-a-1666 | Kallikrein-7 \|\| id:prot-a-1666 | Black olive liking \|\| id:ebi-fl187-GCST90094707 | Weighted mode | 22 | 0.0560991798710185 | 0.180424196390208 | 0.758919616041184 | -0.297532245 | 0.409730604795826 | 1.05770258134025 | 0.742648636089479 | 1.5064119103008 | 0.999714303421225 |
| ebi-fl187-GCST90094707 | prot-a-1678 | Importin subunit alpha-3 \|\| id:prot-a-1678 | Black olive liking \|\| id:ebi-fl187-GCST90094707 | Weighted mode | 22 | 0.100421281111219 | 0.182788979419305 | 0.588537340252676 | -0.257845119 | 0.458687680773056 | 1.10563660379351 | 0.772714902043349 | 1.58199653768224 | 0.999714303421225 |
| ebi-fl187-GCST90094707 | prot-a-1718 | Protein LDOC1 \|\| id:prot-a-1718 | Black olive liking \|\| id:ebi-fl187-GCST90094707 | Weighted mode | 22 | 0.150651069369963 | 0.188434185235036 | 0.432957561357516 | -0.218679934 | 0.519982072430634 | 1.16259092371588 | 0.803578875522639 | 1.68199749530182 | 0.999714303421225 |
| ebi-fl187-GCST90094707 | prot-a-1723 | Leptin \|\| id:prot-a-1723 | Black olive liking \|\| id:ebi-fl187-GCST90094707 | Weighted mode | 22 | -0.205250545 | 0.190565268045446 | 0.293670687966776 | -0.57875847 | 0.168257380288477 | 0.814443236100593 | 0.560593928623755 | 1.18324111439886 | 0.999714303421225 |
| ebi-fl187-GCST90094707 | prot-a-1730 | Galectin-7 \|\| id:prot-a-1730 | Black olive liking \|\| id:ebi-fl187-GCST90094707 | Weighted mode | 22 | -0.329922905 | 0.194112480866712 | 0.10396322819569 | -0.710383368 | 0.050537557155498 | 0.718979160746927 | 0.491455753010025 | 1.05183636659516 | 0.999714303421225 |
| ebi-fl187-GCST90094707 | prot-a-1753 | Lipase member N \|\| id:prot-a-1753 | Black olive liking \|\| id:ebi-fl187-GCST90094707 | Weighted mode | 22 | 0.0207186779936804 | 0.178741123753109 | 0.908821657715695 | -0.329613925 | 0.371051280549774 | 1.02093479980809 | 0.719201345813115 | 1.44925739019686 | 0.999714303421225 |
| ebi-fl187-GCST90094707 | prot-a-1756 | VIP36-like protein \|\| id:prot-a-1756 | Black olive liking \|\| id:ebi-fl187-GCST90094707 | Weighted mode | 22 | 0.193992178993238 | 0.223247098069351 | 0.394693687565221 | -0.243572133 | 0.631556491209167 | 1.21408678753813 | 0.783822934318759 | 1.88053533921631 | 0.999714303421225 |
| ebi-fl187-GCST90094707 | prot-a-1760 | Ig Kappa chain V-I region HK102- like \|\| id:prot-a-1760 | Black olive liking \|\| id:ebi-fl187-GCST90094707 | Weighted mode | 22 | -0.104091109 | 0.1841505226817 | 0.577893255612537 | -0.465026133 | 0.256843915546357 | 0.901143191507242 | 0.62811869011959 | 1.2928433182672 | 0.999714303421225 |
| ebi-fl187-GCST90094707 | prot-a-179 | Neutral ceramidase \|\| id:prot-a-179 | Black olive liking \|\| id:ebi-fl187-GCST90094707 | Weighted mode | 22 | -0.164932889 | 0.21957591952387 | 0.46090191435913 | -0.595301692 | 0.265435912770622 | 0.84795060857102 | 0.551396189083471 | 1.30399928184327 | 0.999714303421225 |
| ebi-fl187-GCST90094707 | prot-a-1809 | Lactotransferrin \|\| id:prot-a-1809 | Black olive liking \|\| id:ebi-fl187-GCST90094707 | Weighted mode | 22 | 0.135217136272211 | 0.204866505212949 | 0.516411945198677 | -0.266321214 | 0.53675548648959 | 1.14478533178543 | 0.76619297610089 | 1.71044827706501 | 0.999714303421225 |
| ebi-fl187-GCST90094707 | prot-a-1818 | Lysophospholipase-like protein 1 \|\| id:prot-a-1818 | Black olive liking \|\| id:ebi-fl187-GCST90094707 | Weighted mode | 22 | 0.078983273467085 | 0.175731596476436 | 0.657708708664011 | -0.265450656 | 0.423417202560899 | 1.08218622069549 | 0.766860282192055 | 1.52717130285525 | 0.999714303421225 |
| ebi-fl187-GCST90094707 | prot-a-1852 | MAP kinase-activated protein kinase 3 \|\| id:prot-a-1852 | Black olive liking \|\| id:ebi-fl187-GCST90094707 | Weighted mode | 22 | -0.162676442 | 0.237770485109419 | 0.501346266944631 | -0.628706593 | 0.30335370896947 | 0.849866125048622 | 0.533281104831053 | 1.35439344083639 | 0.999714303421225 |
| ebi-fl187-GCST90094707 | prot-a-1856 | Megakaryocyte-associated tyrosine-protein kinase \|\| id:prot-a-1856 | Black olive liking \|\| id:ebi-fl187-GCST90094707 | Weighted mode | 22 | -0.112662115 | 0.17183008387264 | 0.519162163114744 | -0.44944908 | 0.224124849169362 | 0.893452493118928 | 0.637979530752932 | 1.25122722435678 | 0.999714303421225 |
| ebi-fl187-GCST90094707 | prot-a-1890 | Beta-1,3-N-acetylglucosaminyltransferase manic fringe \|\| id:prot-a-1890 | Black olive liking \|\| id:ebi-fl187-GCST90094707 | Weighted mode | 22 | 0.00265141404005398 | 0.181079468749247 | 0.988455797916584 | -0.352264345 | 0.357567172788578 | 1.00265493214689 | 0.703094238167307 | 1.4298466100063 | 0.999714303421225 |
| ebi-fl187-GCST90094707 | prot-a-1919 | Matrilysin \|\| id:prot-a-1919 | Black olive liking \|\| id:ebi-fl187-GCST90094707 | Weighted mode | 22 | -0.158127331 | 0.208642114461654 | 0.456939401858715 | -0.567065875 | 0.250811213551951 | 0.85374106752023 | 0.567187197661428 | 1.28506745810873 | 0.999714303421225 |
| ebi-fl187-GCST90094707 | prot-a-1923 | Multimerin-2 \|\| id:prot-a-1923 | Black olive liking \|\| id:ebi-fl187-GCST90094707 | Weighted mode | 22 | -0.219525922 | 0.233683911757844 | 0.358198873833931 | -0.677546389 | 0.238494545217269 | 0.802899344804476 | 0.507861559672659 | 1.26933678206116 | 0.999714303421225 |
| ebi-fl187-GCST90094707 | prot-a-1948 | Mesothelin \|\| id:prot-a-1948 | Black olive liking \|\| id:ebi-fl187-GCST90094707 | Weighted mode | 22 | 0.210666968481733 | 0.16703645323874 | 0.221066844895125 | -0.11672448 | 0.538058416829663 | 1.23450115879932 | 0.889830325562766 | 1.71267832450308 | 0.999714303421225 |
| ebi-fl187-GCST90094707 | prot-a-195 | ATPase family AAA domain-containing protein 1 \|\| id:prot-a-195 | Black olive liking \|\| id:ebi-fl187-GCST90094707 | Weighted mode | 22 | -0.051954766 | 0.187516758369241 | 0.784437395287263 | -0.419487612 | 0.31557808042486 | 0.949371809776162 | 0.657383568735227 | 1.37105166004032 | 0.999714303421225 |
| ebi-fl187-GCST90094707 | prot-a-1966 | Mucin-1 \|\| id:prot-a-1966 | Black olive liking \|\| id:ebi-fl187-GCST90094707 | Weighted mode | 22 | 0.0741097887206537 | 0.207286480969228 | 0.724268059344939 | -0.332171714 | 0.480391291420341 | 1.0769250332277 | 0.717364130828529 | 1.61670688197499 | 0.999714303421225 |
| ebi-fl187-GCST90094707 | prot-a-1986 | Myocardial zonula adherens protein \|\| id:prot-a-1986 | Black olive liking \|\| id:ebi-fl187-GCST90094707 | Weighted mode | 22 | -0.262426302 | 0.18717670738939 | 0.175516912622301 | -0.629292648 | 0.104440044529108 | 0.769183049567446 | 0.532968663921193 | 1.11008883597209 | 0.999714303421225 |
| ebi-fl187-GCST90094707 | prot-a-2071 | Epididymal secretory protein E1 \|\| id:prot-a-2071 | Black olive liking \|\| id:ebi-fl187-GCST90094707 | Weighted mode | 22 | 0.246062615671539 | 0.21814330798755 | 0.272048729863193 | -0.181498268 | 0.673623499327138 | 1.27897965520452 | 0.834019689837626 | 1.96133134308322 | 0.999714303421225 |
| ebi-fl187-GCST90094707 | prot-a-2098 | Neuritin \|\| id:prot-a-2098 | Black olive liking \|\| id:ebi-fl187-GCST90094707 | Weighted mode | 22 | -0.146180828 | 0.208939475415518 | 0.491834847798259 | -0.5557022 | 0.263340543376199 | 0.864001453024465 | 0.573669289009739 | 1.30126978231131 | 0.999714303421225 |
| ebi-fl187-GCST90094707 | prot-a-2115 | Neurotrophin-4 \|\| id:prot-a-2115 | Black olive liking \|\| id:ebi-fl187-GCST90094707 | Weighted mode | 22 | -0.10381257 | 0.174582726305555 | 0.558442166465179 | -0.445994713 | 0.238369574020248 | 0.901394230325581 | 0.640187156659761 | 1.2691781614358 | 0.999714303421225 |
| ebi-fl187-GCST90094707 | prot-a-2118 | Netrin-1 \|\| id:prot-a-2118 | Black olive liking \|\| id:ebi-fl187-GCST90094707 | Weighted mode | 22 | 0.0394142859453766 | 0.229209214599635 | 0.865117139537758 | -0.409835775 | 0.488664346560661 | 1.04020133518966 | 0.663759247267513 | 1.63013746653576 | 0.999714303421225 |
| ebi-fl187-GCST90094707 | prot-a-2120 | Netrin-G1 \|\| id:prot-a-2120 | Black olive liking \|\| id:ebi-fl187-GCST90094707 | Weighted mode | 22 | -0.05647348 | 0.199624845330896 | 0.780024795070163 | -0.447738177 | 0.334791217024714 | 0.945091548157141 | 0.639071986083144 | 1.39764854953579 | 0.999714303421225 |
| ebi-fl187-GCST90094707 | prot-a-2145 | Olfactomedin-like protein 3 \|\| id:prot-a-2145 | Black olive liking \|\| id:ebi-fl187-GCST90094707 | Weighted mode | 22 | 0.0970887998336196 | 0.194093785849279 | 0.622124629230589 | -0.28333502 | 0.477512620098207 | 1.1019582229833 | 0.753267385617961 | 1.61205960643619 | 0.999714303421225 |
| ebi-fl187-GCST90094707 | prot-a-2162 | Prolyl 4-hydroxylase subunit alpha-2 \|\| id:prot-a-2162 | Black olive liking \|\| id:ebi-fl187-GCST90094707 | Weighted mode | 22 | 0.14757282781095 | 0.196958163059806 | 0.462011865171294 | -0.238465172 | 0.53361082740817 | 1.15901769047742 | 0.787836126704328 | 1.7050779487087 | 0.999714303421225 |
| ebi-fl187-GCST90094707 | prot-a-2168 | Polyadenylate-binding protein-interacting protein 1 \|\| id:prot-a-2168 | Black olive liking \|\| id:ebi-fl187-GCST90094707 | Weighted mode | 22 | -0.023366221 | 0.208505243404367 | 0.911835848006839 | -0.432036498 | 0.385304055808918 | 0.976904654997812 | 0.649185682055462 | 1.47006123415221 | 0.999714303421225 |
| ebi-fl187-GCST90094707 | prot-a-219 | Beta-1,4-galactosyltransferase 5 \|\| id:prot-a-219 | Black olive liking \|\| id:ebi-fl187-GCST90094707 | Weighted mode | 22 | -0.050604469 | 0.247535088169371 | 0.839983075687247 | -0.535773242 | 0.434564303808556 | 0.950654609544884 | 0.585216601201236 | 1.54429007105041 | 0.999714303421225 |
| ebi-fl187-GCST90094707 | prot-a-2190 | Pyruvate carboxylase, mitochondrial \|\| id:prot-a-2190 | Black olive liking \|\| id:ebi-fl187-GCST90094707 | Weighted mode | 22 | -0.13231898 | 0.20733804952938 | 0.53025503497889 | -0.538701557 | 0.274063596874273 | 0.876061504228987 | 0.583505409055268 | 1.31529844844894 | 0.999714303421225 |
| ebi-fl187-GCST90094707 | prot-a-2197 | Protocadherin-9 \|\| id:prot-a-2197 | Black olive liking \|\| id:ebi-fl187-GCST90094707 | Weighted mode | 22 | 0.0928971325231998 | 0.217592059199134 | 0.673773647335983 | -0.333583304 | 0.519377568553502 | 1.09734884795481 | 0.71635222150197 | 1.68098102855458 | 0.999714303421225 |
| ebi-fl187-GCST90094707 | prot-a-2198 | Protocadherin alpha-4 \|\| id:prot-a-2198 | Black olive liking \|\| id:ebi-fl187-GCST90094707 | Weighted mode | 22 | -0.031442191 | 0.184706311453442 | 0.866460143620126 | -0.393466562 | 0.330582179357459 | 0.969046974382638 | 0.674713878592687 | 1.39177815716317 | 0.999714303421225 |
| ebi-fl187-GCST90094707 | prot-a-2254 | Placenta growth factor \|\| id:prot-a-2254 | Black olive liking \|\| id:ebi-fl187-GCST90094707 | Weighted mode | 22 | -0.288322675 | 0.214419968816819 | 0.193078856630881 | -0.708585814 | 0.131940464253624 | 0.749519702224655 | 0.492339965900941 | 1.14104038455404 | 0.999714303421225 |
| ebi-fl187-GCST90094707 | prot-a-2261 | Membrane-associated progesterone receptor component 2 \|\| id:prot-a-2261 | Black olive liking \|\| id:ebi-fl187-GCST90094707 | Weighted mode | 22 | -0.018856224 | 0.227650104899417 | 0.934771292920752 | -0.46505043 | 0.427337981332477 | 0.981320442163191 | 0.628103429214624 | 1.53317075726123 | 0.999714303421225 |
| ebi-fl187-GCST90094707 | prot-a-2268 | Polymeric immunoglobulin receptor \|\| id:prot-a-2268 | Black olive liking \|\| id:ebi-fl187-GCST90094707 | Weighted mode | 22 | -0.132052475 | 0.224924887327356 | 0.563398746561782 | -0.572905255 | 0.308800303692228 | 0.876295009880869 | 0.563884827650192 | 1.36179039883385 | 0.999714303421225 |
| ebi-fl187-GCST90094707 | prot-a-2283 | Group 10 secretory phospholipase A2 \|\| id:prot-a-2283 | Black olive liking \|\| id:ebi-fl187-GCST90094707 | Weighted mode | 22 | 0.311880553573552 | 0.196517356188775 | 0.127447745495846 | -0.073293465 | 0.697054571703551 | 1.36599152047881 | 0.929328065155904 | 2.00783006989783 | 0.999714303421225 |
| ebi-fl187-GCST90094707 | prot-a-2298 | Inactive phospholipase D5 \|\| id:prot-a-2298 | Black olive liking \|\| id:ebi-fl187-GCST90094707 | Weighted mode | 22 | 0.210236716835409 | 0.236236694858794 | 0.383579680298103 | -0.252787205 | 0.673260638758645 | 1.23397012689023 | 0.776633127819586 | 1.96061978238353 | 0.999714303421225 |
| ebi-fl187-GCST90094707 | prot-a-2304 | Procollagen-lysine,2-oxoglutarate 5-dioxygenase 2 \|\| id:prot-a-2304 | Black olive liking \|\| id:ebi-fl187-GCST90094707 | Weighted mode | 22 | 0.112523140910434 | 0.201997144151344 | 0.5833801247679 | -0.283391262 | 0.508437543447069 | 1.11909815356328 | 0.75322502215116 | 1.66269128146066 | 0.999714303421225 |
| ebi-fl187-GCST90094707 | prot-a-2331 | Periostin \|\| id:prot-a-2331 | Black olive liking \|\| id:ebi-fl187-GCST90094707 | Weighted mode | 22 | 0.161978532873255 | 0.25749148457059 | 0.536091090532768 | -0.342704777 | 0.666661842631611 | 1.1758349992511 | 0.709847744091378 | 1.94772464514005 | 0.999714303421225 |
| ebi-fl187-GCST90094707 | prot-a-2333 | Periostin \|\| id:prot-a-2333 | Black olive liking \|\| id:ebi-fl187-GCST90094707 | Weighted mode | 22 | -0.047512092 | 0.27048022748864 | 0.862245195240674 | -0.577653338 | 0.482629153970558 | 0.953598942233018 | 0.56121380174387 | 1.62032890103965 | 0.999714303421225 |
| ebi-fl187-GCST90094707 | prot-a-2367 | cAMP-dependent protein kinase catalytic subunit alpha \|\| id:prot-a-2367 | Black olive liking \|\| id:ebi-fl187-GCST90094707 | Weighted mode | 22 | 0.264722995596836 | 0.213453643302376 | 0.228584252724117 | -0.153646145 | 0.683092136469492 | 1.30306996966131 | 0.857575424443203 | 1.97999067771278 | 0.999714303421225 |
| ebi-fl187-GCST90094707 | prot-a-2385 | Phosphoribosyl pyrophosphate synthase-associated protein 1 \|\| id:prot-a-2385 | Black olive liking \|\| id:ebi-fl187-GCST90094707 | Weighted mode | 22 | -0.063719036 | 0.201509203176463 | 0.754965356833196 | -0.458677074 | 0.331239002111841 | 0.938268582087317 | 0.632119339499005 | 1.39269260900936 | 0.999714303421225 |
| ebi-fl187-GCST90094707 | prot-a-2393 | Trypsin-3 \|\| id:prot-a-2393 | Black olive liking \|\| id:ebi-fl187-GCST90094707 | Weighted mode | 21 | -0.137063833 | 0.226736769365908 | 0.552303885166347 | -0.581467901 | 0.307340234877757 | 0.871914567363864 | 0.559077094093553 | 1.35980354196751 | 0.999714303421225 |
| ebi-fl187-GCST90094707 | prot-a-2405 | Pregnancy-specific beta-1-glycoprotein 6 \|\| id:prot-a-2405 | Black olive liking \|\| id:ebi-fl187-GCST90094707 | Weighted mode | 22 | -0.19399377 | 0.221578184120536 | 0.391201127092862 | -0.62828701 | 0.240299471296452 | 0.82366303601941 | 0.533504907040481 | 1.27162990996301 | 0.999714303421225 |
| ebi-fl187-GCST90094707 | prot-a-244 | Beclin-1 \|\| id:prot-a-244 | Black olive liking \|\| id:ebi-fl187-GCST90094707 | Weighted mode | 22 | -0.01277494 | 0.191861665538898 | 0.947542640256331 | -0.388823804 | 0.363273924728939 | 0.987306313445722 | 0.677853694477755 | 1.43802971719552 | 0.999714303421225 |
| ebi-fl187-GCST90094707 | prot-a-2454 | tRNA pseudouridine synthase A, mitochondrial \|\| id:prot-a-2454 | Black olive liking \|\| id:ebi-fl187-GCST90094707 | Weighted mode | 22 | 0.228043197774836 | 0.188107261466939 | 0.238859271295147 | -0.140647035 | 0.596733430250036 | 1.25613958669538 | 0.868795912394322 | 1.81617643309892 | 0.999714303421225 |
| ebi-fl187-GCST90094707 | prot-a-2466 | Glutaminyl-peptide cyclotransferase-like protein \|\| id:prot-a-2466 | Black olive liking \|\| id:ebi-fl187-GCST90094707 | Weighted mode | 22 | 0.109874990772536 | 0.182963151576895 | 0.554579074893487 | -0.248732786 | 0.468482767863249 | 1.11613853412149 | 0.779788315654376 | 1.59756847126578 | 0.999714303421225 |
| ebi-fl187-GCST90094707 | prot-a-2474 | Ras-related protein Rab-27A \|\| id:prot-a-2474 | Black olive liking \|\| id:ebi-fl187-GCST90094707 | Weighted mode | 22 | 0.112041353577546 | 0.204792800683555 | 0.590079914873417 | -0.289352536 | 0.513435242917314 | 1.11855911610977 | 0.748748198352621 | 1.67102171195213 | 0.999714303421225 |
| ebi-fl187-GCST90094707 | prot-a-2490 | Rap1 GTPase-GDP dissociation stimulator 1 \|\| id:prot-a-2490 | Black olive liking \|\| id:ebi-fl187-GCST90094707 | Weighted mode | 22 | 0.0982095274381216 | 0.181550578859591 | 0.594235364204211 | -0.257629607 | 0.45404866200292 | 1.1031939102879 | 0.772881448877917 | 1.57467462243172 | 0.999714303421225 |
| ebi-fl187-GCST90094707 | prot-a-2545 | Receptor-interacting serine/threonine-protein kinase 2 \|\| id:prot-a-2545 | Black olive liking \|\| id:ebi-fl187-GCST90094707 | Weighted mode | 22 | -0.044265673 | 0.212374635242842 | 0.83690000259141 | -0.460519958 | 0.371988611946701 | 0.956699754289572 | 0.630955489716653 | 1.45061646150152 | 0.999714303421225 |
| ebi-fl187-GCST90094707 | prot-a-2610 | RUN and FYVE domain-containing protein 1 \|\| id:prot-a-2610 | Black olive liking \|\| id:ebi-fl187-GCST90094707 | Weighted mode | 22 | -0.174623429 | 0.222172226411305 | 0.440657025429865 | -0.610080993 | 0.260834134575379 | 0.839773195306138 | 0.543306863263063 | 1.29801235220771 | 0.999714303421225 |
| ebi-fl187-GCST90094707 | prot-a-2627 | Serine--tRNA ligase, mitochondrial \|\| id:prot-a-2627 | Black olive liking \|\| id:ebi-fl187-GCST90094707 | Weighted mode | 22 | 0.0204293008831354 | 0.186254132383992 | 0.913700543476262 | -0.344628799 | 0.385487400355759 | 1.02063940738763 | 0.708483294661012 | 1.47033078657274 | 0.999714303421225 |
| ebi-fl187-GCST90094707 | prot-a-264 | BolA-like protein 2 \|\| id:prot-a-264 | Black olive liking \|\| id:ebi-fl187-GCST90094707 | Weighted mode | 22 | 0.0592176628506911 | 0.189897471729283 | 0.758236961984214 | -0.312981382 | 0.431416707440085 | 1.06100615723306 | 0.731263527297568 | 1.53943691113201 | 0.999714303421225 |
| ebi-fl187-GCST90094707 | prot-a-2646 | Secretoglobin family 1C member 1 \|\| id:prot-a-2646 | Black olive liking \|\| id:ebi-fl187-GCST90094707 | Weighted mode | 22 | 0.0135951118524575 | 0.242644006679574 | 0.955848336624459 | -0.461987141 | 0.489177364944422 | 1.01368794560363 | 0.630030441300374 | 1.63097397157703 | 0.999714303421225 |
| ebi-fl187-GCST90094707 | prot-a-2672 | Semaphorin-3E \|\| id:prot-a-2672 | Black olive liking \|\| id:ebi-fl187-GCST90094707 | Weighted mode | 22 | -0.004737772 | 0.194919266983234 | 0.980837753453391 | -0.386779535 | 0.377303991332008 | 0.995273433583006 | 0.679240827085082 | 1.458347567 | 0.999714303421225 |
| ebi-fl187-GCST90094707 | prot-a-2701 | Plasma protease C1 inhibitor \|\| id:prot-a-2701 | Black olive liking \|\| id:ebi-fl187-GCST90094707 | Weighted mode | 22 | 0.101875874774602 | 0.179656451792333 | 0.576685720155815 | -0.250250771 | 0.454002520287575 | 1.10724602603532 | 0.778605507109762 | 1.57460196591979 | 0.999714303421225 |
| ebi-fl187-GCST90094707 | prot-a-2738 | Signaling threshold-regulating transmembrane adapter 1 \|\| id:prot-a-2738 | Black olive liking \|\| id:ebi-fl187-GCST90094707 | Weighted mode | 22 | -0.255881024 | 0.195248645140252 | 0.204166448381769 | -0.638568369 | 0.126806320086295 | 0.77423407828558 | 0.528047852914075 | 1.13519713160592 | 0.999714303421225 |
| ebi-fl187-GCST90094707 | prot-a-274 | Serine/threonine-protein kinase BRSK2 \|\| id:prot-a-274 | Black olive liking \|\| id:ebi-fl187-GCST90094707 | Weighted mode | 22 | -0.13855669 | 0.198613211452385 | 0.49306673992627 | -0.527838585 | 0.250725204164943 | 0.870613894523146 | 0.589878565337484 | 1.28495693499747 | 0.999714303421225 |
| ebi-fl187-GCST90094707 | prot-a-2749 | Mitochondrial glutamate carrier 2 \|\| id:prot-a-2749 | Black olive liking \|\| id:ebi-fl187-GCST90094707 | Weighted mode | 22 | 0.0363903045663938 | 0.217336862925692 | 0.868627868116198 | -0.389589947 | 0.46237055590075 | 1.03706053697272 | 0.677334560787015 | 1.58783357532576 | 0.999714303421225 |
| ebi-fl187-GCST90094707 | prot-a-2760 | Sodium-coupled monocarboxylate transporter 1 \|\| id:prot-a-2760 | Black olive liking \|\| id:ebi-fl187-GCST90094707 | Weighted mode | 22 | -0.088787759 | 0.174327400491793 | 0.615840998996304 | -0.430469464 | 0.252893946154017 | 0.915039762094419 | 0.65020377594247 | 1.28774669910237 | 0.999714303421225 |
| ebi-fl187-GCST90094707 | prot-a-2777 | Small integral membrane protein 10 \|\| id:prot-a-2777 | Black olive liking \|\| id:ebi-fl187-GCST90094707 | Weighted mode | 22 | -0.173018577 | 0.215373472041636 | 0.430776552535725 | -0.595150583 | 0.249113427902683 | 0.84112198892486 | 0.551479516450514 | 1.28288754006043 | 0.999714303421225 |
| ebi-fl187-GCST90094707 | prot-a-2799 | Superoxide dismutase [Mn], mitochondrial \|\| id:prot-a-2799 | Black olive liking \|\| id:ebi-fl187-GCST90094707 | Weighted mode | 22 | 0.0747688115447902 | 0.236159975310903 | 0.754671049204361 | -0.38810474 | 0.53764236315416 | 1.0776349853161 | 0.678341290032584 | 1.71196590660353 | 0.999714303421225 |
| ebi-fl187-GCST90094707 | prot-a-2805 | Protein SPACA7 \|\| id:prot-a-2805 | Black olive liking \|\| id:ebi-fl187-GCST90094707 | Weighted mode | 22 | -0.213698306 | 0.223585456936699 | 0.350058042571797 | -0.651925802 | 0.224529189551135 | 0.807591993896001 | 0.521041387585173 | 1.25173324834643 | 0.999714303421225 |
| ebi-fl187-GCST90094707 | prot-a-2811 | SPARC-like protein 1 \|\| id:prot-a-2811 | Black olive liking \|\| id:ebi-fl187-GCST90094707 | Weighted mode | 22 | -0.1170524 | 0.191548725625952 | 0.547702065943569 | -0.492487903 | 0.258383101763889 | 0.889538579708726 | 0.611104133733581 | 1.29483477710394 | 0.999714303421225 |
| ebi-fl187-GCST90094707 | prot-a-2831 | Sepiapterin reductase \|\| id:prot-a-2831 | Black olive liking \|\| id:ebi-fl187-GCST90094707 | Weighted mode | 22 | -0.083410804 | 0.18976592872877 | 0.66475616645892 | -0.455352024 | 0.28853041673847 | 0.919973141331667 | 0.634224666420006 | 1.33446493897664 | 0.999714303421225 |
| ebi-fl187-GCST90094707 | prot-a-2834 | SPRY domain-containing SOCS box protein 1 \|\| id:prot-a-2834 | Black olive liking \|\| id:ebi-fl187-GCST90094707 | Weighted mode | 22 | 0.186086500011729 | 0.234858889952038 | 0.437026259076892 | -0.274236924 | 0.646409924317724 | 1.2045264474233 | 0.760151955458765 | 1.90867622206743 | 0.999714303421225 |
| ebi-fl187-GCST90094707 | prot-a-2835 | Spectrin alpha chain, non-erythrocytic 1 \|\| id:prot-a-2835 | Black olive liking \|\| id:ebi-fl187-GCST90094707 | Weighted mode | 22 | 0.0117979669269856 | 0.212462311627461 | 0.956241407184883 | -0.404628164 | 0.42822409771681 | 1.01186783744512 | 0.667224863053927 | 1.53452992709161 | 0.999714303421225 |
| ebi-fl187-GCST90094707 | prot-a-2859 | Sia-alpha-2,3-Gal-beta-1,4-GlcNAc-R:alpha 2,8-sialyltransferase \|\| id:prot-a-2859 | Black olive liking \|\| id:ebi-fl187-GCST90094707 | Weighted mode | 22 | -0.216502072 | 0.216142680359809 | 0.327909446673939 | -0.640141725 | 0.207137581771724 | 0.805330866490008 | 0.527217698693732 | 1.23015180660371 | 0.999714303421225 |
| ebi-fl187-GCST90094707 | prot-a-2861 | Alpha-2,8-sialyltransferase 8F \|\| id:prot-a-2861 | Black olive liking \|\| id:ebi-fl187-GCST90094707 | Weighted mode | 22 | -0.074871742 | 0.192581710532871 | 0.701353435486499 | -0.452331894 | 0.302588410919161 | 0.927862484738606 | 0.636143002403584 | 1.35335732269693 | 0.999714303421225 |
| ebi-fl187-GCST90094707 | prot-a-289 | Uncharacterized protein C17orf89 \|\| id:prot-a-289 | Black olive liking \|\| id:ebi-fl187-GCST90094707 | Weighted mode | 22 | 0.167069743088041 | 0.168541412521054 | 0.332839741746595 | -0.163271425 | 0.497410911629307 | 1.18183668737428 | 0.849360619033833 | 1.64445810686706 | 0.999714303421225 |
| ebi-fl187-GCST90094707 | prot-a-2921 | Tachykinin-3 \|\| id:prot-a-2921 | Black olive liking \|\| id:ebi-fl187-GCST90094707 | Weighted mode | 22 | -0.163138764 | 0.20686834171026 | 0.439151646747256 | -0.568600713 | 0.242323186032888 | 0.849473304160687 | 0.566317324736052 | 1.27420593183865 | 0.999714303421225 |
| ebi-fl187-GCST90094707 | prot-a-2928 | Tax1-binding protein 1 \|\| id:prot-a-2928 | Black olive liking \|\| id:ebi-fl187-GCST90094707 | Weighted mode | 22 | 0.124328653189527 | 0.222797008723534 | 0.582721138581289 | -0.312353484 | 0.561010790287653 | 1.13238797276864 | 0.73172283026217 | 1.75244295768609 | 0.999714303421225 |
| ebi-fl187-GCST90094707 | prot-a-296 | Uncharacterized protein C1orf185 \|\| id:prot-a-296 | Black olive liking \|\| id:ebi-fl187-GCST90094707 | Weighted mode | 22 | -0.073855366 | 0.211141136566091 | 0.729978859541116 | -0.487691994 | 0.339981261824286 | 0.928806021200692 | 0.614041972829164 | 1.40492126465542 | 0.999714303421225 |
| ebi-fl187-GCST90094707 | prot-a-2964 | Transforming growth factor beta-2 \|\| id:prot-a-2964 | Black olive liking \|\| id:ebi-fl187-GCST90094707 | Weighted mode | 22 | -0.112355388 | 0.183517742878938 | 0.546957025877408 | -0.472050164 | 0.247339388504906 | 0.89372658176533 | 0.623722223992964 | 1.28061366459014 | 0.999714303421225 |
| ebi-fl187-GCST90094707 | prot-a-2977 | Thymocyte nuclear protein 1 \|\| id:prot-a-2977 | Black olive liking \|\| id:ebi-fl187-GCST90094707 | Weighted mode | 22 | 0.0842223869797159 | 0.182700518226007 | 0.649547307917808 | -0.273870629 | 0.44231540270269 | 1.08787079521044 | 0.760430446740083 | 1.55630652631706 | 0.999714303421225 |
| ebi-fl187-GCST90094707 | prot-a-3015 | Transmembrane protein 70, mitochondrial \|\| id:prot-a-3015 | Black olive liking \|\| id:ebi-fl187-GCST90094707 | Weighted mode | 22 | -0.179612528 | 0.160755449767888 | 0.276485208061069 | -0.49469321 | 0.135468153542042 | 0.835593917937664 | 0.609757946519894 | 1.14507272874325 | 0.999714303421225 |
| ebi-fl187-GCST90094707 | prot-a-3030 | Tumor necrosis factor alpha-induced protein 3 \|\| id:prot-a-3030 | Black olive liking \|\| id:ebi-fl187-GCST90094707 | Weighted mode | 22 | -0.271100992 | 0.198432079679407 | 0.186324122657822 | -0.660027868 | 0.117825884274787 | 0.762539482207495 | 0.516836931044011 | 1.12504820572847 | 0.999714303421225 |
| ebi-fl187-GCST90094707 | prot-a-3032 | Tumor necrosis factor receptor superfamily member 10A \|\| id:prot-a-3032 | Black olive liking \|\| id:ebi-fl187-GCST90094707 | Weighted mode | 22 | 0.327288854395959 | 0.232408589425767 | 0.173690834904943 | -0.128231981 | 0.782809689670463 | 1.38720211874406 | 0.87964929365764 | 2.18761014431845 | 0.999714303421225 |
| ebi-fl187-GCST90094707 | prot-a-3081 | Tumor protein p53-inducible protein 11 \|\| id:prot-a-3081 | Black olive liking \|\| id:ebi-fl187-GCST90094707 | Weighted mode | 22 | -0.211611373 | 0.219809501913044 | 0.346651703717542 | -0.642437996 | 0.219215251130922 | 0.80927914449591 | 0.526008452822808 | 1.24509925686813 | 0.999714303421225 |
| ebi-fl187-GCST90094707 | prot-a-315 | Complement C4 \|\| id:prot-a-315 | Black olive liking \|\| id:ebi-fl187-GCST90094707 | Weighted mode | 21 | 0.149868953607484 | 0.214307635402561 | 0.492407780326419 | -0.270174012 | 0.569911918996503 | 1.16168199851782 | 0.76324666886799 | 1.76811130755653 | 0.999714303421225 |
| ebi-fl187-GCST90094707 | prot-a-3157 | UDP-glucuronosyltransferase 1-8 \|\| id:prot-a-3157 | Black olive liking \|\| id:ebi-fl187-GCST90094707 | Weighted mode | 22 | 0.243938036049842 | 0.183162243398878 | 0.197196948992211 | -0.115059961 | 0.602936033111643 | 1.27626524560321 | 0.891312698293159 | 1.82747646281022 | 0.999714303421225 |
| ebi-fl187-GCST90094707 | prot-a-317 | Glutaredoxin-like protein C5orf63 \|\| id:prot-a-317 | Black olive liking \|\| id:ebi-fl187-GCST90094707 | Weighted mode | 22 | 0.111927537823838 | 0.23333826485559 | 0.636413447099278 | -0.345415461 | 0.569270536940795 | 1.11843181370555 | 0.707926176437752 | 1.76697763628844 | 0.999714303421225 |
| ebi-fl187-GCST90094707 | prot-a-3192 | Guanine nucleotide exchange factor VAV3 \|\| id:prot-a-3192 | Black olive liking \|\| id:ebi-fl187-GCST90094707 | Weighted mode | 22 | -0.064244867 | 0.181718256848219 | 0.727207406931109 | -0.420412651 | 0.2919229162787 | 0.937775341044672 | 0.656775745006196 | 1.3389997985738 | 0.999714303421225 |
| ebi-fl187-GCST90094707 | prot-a-3233 | Serine/threonine-protein kinase WNK3 \|\| id:prot-a-3233 | Black olive liking \|\| id:ebi-fl187-GCST90094707 | Weighted mode | 22 | -0.145233675 | 0.179499068212133 | 0.427527003832178 | -0.497051849 | 0.206584498529493 | 0.864820182497591 | 0.608321442218934 | 1.22947161837178 | 0.999714303421225 |
| ebi-fl187-GCST90094707 | prot-a-3250 | 14-3-3 protein theta \|\| id:prot-a-3250 | Black olive liking \|\| id:ebi-fl187-GCST90094707 | Weighted mode | 22 | 0.106254784898182 | 0.16020434397593 | 0.51438877502288 | -0.207745729 | 0.420255299091005 | 1.11210518802035 | 0.812413583426338 | 1.52235016062347 | 0.999714303421225 |
| ebi-fl187-GCST90094707 | prot-a-3278 | E3 ubiquitin-protein ligase ZNRF3 \|\| id:prot-a-3278 | Black olive liking \|\| id:ebi-fl187-GCST90094707 | Weighted mode | 22 | -0.063577135 | 0.218380929789607 | 0.773808309452057 | -0.491603758 | 0.364449487067026 | 0.938401732590416 | 0.611644677310895 | 1.43972120480187 | 0.999714303421225 |
| ebi-fl187-GCST90094707 | prot-a-355 | Calpain-3 \|\| id:prot-a-355 | Black olive liking \|\| id:ebi-fl187-GCST90094707 | Weighted mode | 22 | 0.0279339752896153 | 0.179544139639837 | 0.877847725339046 | -0.323972538 | 0.379840488983696 | 1.02832778713567 | 0.723270104258187 | 1.46205135753526 | 0.999714303421225 |
| ebi-fl187-GCST90094707 | prot-a-385 | Coiled-coil domain-containing protein 90B, mitochondrial \|\| id:prot-a-385 | Black olive liking \|\| id:ebi-fl187-GCST90094707 | Weighted mode | 22 | 0.0723885247208114 | 0.185959174252343 | 0.7009950031928 | -0.292091457 | 0.436868506255404 | 1.07507295535224 | 0.746700242025446 | 1.54785253074875 | 0.999714303421225 |
| ebi-fl187-GCST90094707 | prot-a-390 | C-C motif chemokine 14 \|\| id:prot-a-390 | Black olive liking \|\| id:ebi-fl187-GCST90094707 | Weighted mode | 22 | -0.082679419 | 0.239226966551684 | 0.733073389514779 | -0.551564273 | 0.386205435913539 | 0.920646242044315 | 0.576048008903328 | 1.47138691548288 | 0.999714303421225 |
| ebi-fl187-GCST90094707 | prot-a-40 | Pituitary adenylate cyclase-activating polypeptide 27 \|\| id:prot-a-40 | Black olive liking \|\| id:ebi-fl187-GCST90094707 | Weighted mode | 22 | 0.0676849097670358 | 0.185403961526995 | 0.718712169848891 | -0.295706855 | 0.431076674359945 | 1.07002809995272 | 0.744005497676752 | 1.53891354064412 | 0.999714303421225 |
| ebi-fl187-GCST90094707 | prot-a-401 | C-C motif chemokine 24 \|\| id:prot-a-401 | Black olive liking \|\| id:ebi-fl187-GCST90094707 | Weighted mode | 22 | 0.303258659449972 | 0.197536520695972 | 0.139663916097121 | -0.083912921 | 0.690430240014078 | 1.3542647125085 | 0.919511322630538 | 1.99457349399346 | 0.999714303421225 |
| ebi-fl187-GCST90094707 | prot-a-412 | Cerebral cavernous malformations 2 protein \|\| id:prot-a-412 | Black olive liking \|\| id:ebi-fl187-GCST90094707 | Weighted mode | 22 | 0.117241501203635 | 0.216887187787796 | 0.594495497808908 | -0.307857387 | 0.542340389267715 | 1.12439093866939 | 0.735020134063773 | 1.72002768954373 | 0.999714303421225 |
| ebi-fl187-GCST90094707 | prot-a-44 | Adhesion G protein-coupled receptor F5 \|\| id:prot-a-44 | Black olive liking \|\| id:ebi-fl187-GCST90094707 | Weighted mode | 22 | -0.040123544 | 0.22849906318913 | 0.862293437842746 | -0.487981708 | 0.40773461983591 | 0.960670746699669 | 0.613864101824845 | 1.50340813352827 | 0.999714303421225 |
| ebi-fl187-GCST90094707 | prot-a-48 | Adrenomedullin \|\| id:prot-a-48 | Black olive liking \|\| id:ebi-fl187-GCST90094707 | Weighted mode | 22 | 0.129643665919932 | 0.181203951579775 | 0.482206353072349 | -0.225516079 | 0.484803411016291 | 1.13842265225249 | 0.798104227486039 | 1.62385574531275 | 0.999714303421225 |
| ebi-fl187-GCST90094707 | prot-a-502 | Carcinoembryonic antigen-related cell adhesion molecule 1 \|\| id:prot-a-502 | Black olive liking \|\| id:ebi-fl187-GCST90094707 | Weighted mode | 22 | 0.212341184746054 | 0.193964914085864 | 0.286028269014148 | -0.167830047 | 0.592512416354347 | 1.23656971183709 | 0.845497517430702 | 1.80852648376724 | 0.999714303421225 |
| ebi-fl187-GCST90094707 | prot-a-518 | Complement factor B \|\| id:prot-a-518 | Black olive liking \|\| id:ebi-fl187-GCST90094707 | Weighted mode | 22 | -0.299060941 | 0.228320764849684 | 0.204402736555896 | -0.74656964 | 0.14844775807727 | 0.741514219419171 | 0.473989722442215 | 1.16003219387917 | 0.999714303421225 |
| ebi-fl187-GCST90094707 | prot-a-525 | Properdin \|\| id:prot-a-525 | Black olive liking \|\| id:ebi-fl187-GCST90094707 | Weighted mode | 22 | -0.032722169 | 0.2208774196969 | 0.88364061269952 | -0.465641912 | 0.400197573284156 | 0.967807408828813 | 0.627732027157557 | 1.49211947146493 | 0.999714303421225 |
| ebi-fl187-GCST90094707 | prot-a-533 | Chondroadherin \|\| id:prot-a-533 | Black olive liking \|\| id:ebi-fl187-GCST90094707 | Weighted mode | 22 | -0.102492615 | 0.171512155246767 | 0.556508473687313 | -0.438656439 | 0.233671209207251 | 0.902584815248206 | 0.644902304622453 | 1.26322908582807 | 0.999714303421225 |
| ebi-fl187-GCST90094707 | prot-a-535 | Coiled-coil-helix-coiled-coil-helix domain-containing protein 2 \|\| id:prot-a-535 | Black olive liking \|\| id:ebi-fl187-GCST90094707 | Weighted mode | 22 | 0.031680396193206 | 0.19783353412018 | 0.874303791928485 | -0.356073331 | 0.419434123068759 | 1.0321875615083 | 0.700421255983356 | 1.52110055631687 | 0.999714303421225 |
| ebi-fl187-GCST90094707 | prot-a-591 | Caseinolytic peptidase B protein homolog \|\| id:prot-a-591 | Black olive liking \|\| id:ebi-fl187-GCST90094707 | Weighted mode | 22 | -0.00701467 | 0.198548561499925 | 0.972150482001033 | -0.39616985 | 0.382140510784838 | 0.993009875614782 | 0.672892395258909 | 1.46541797769772 | 0.999714303421225 |
| ebi-fl187-GCST90094707 | prot-a-605 | Ciliary neurotrophic factor receptor subunit alpha \|\| id:prot-a-605 | Black olive liking \|\| id:ebi-fl187-GCST90094707 | Weighted mode | 22 | -0.191019173 | 0.178900948545562 | 0.297760423554703 | -0.541665032 | 0.159626686412197 | 0.826116749080107 | 0.581778765289472 | 1.17307286520008 | 0.999714303421225 |
| ebi-fl187-GCST90094707 | prot-a-606 | Ciliary neurotrophic factor receptor subunit alpha \|\| id:prot-a-606 | Black olive liking \|\| id:ebi-fl187-GCST90094707 | Weighted mode | 22 | -0.209625517 | 0.185224355871747 | 0.27050294640614 | -0.572665255 | 0.153414220401906 | 0.810887852748091 | 0.564020176258847 | 1.16580778031005 | 0.999714303421225 |
| ebi-fl187-GCST90094707 | prot-a-707 | Cancer/testis antigen 55 \|\| id:prot-a-707 | Black olive liking \|\| id:ebi-fl187-GCST90094707 | Weighted mode | 22 | -0.040822277 | 0.223640141777141 | 0.856913125955419 | -0.479156954 | 0.397512401323576 | 0.959999729242247 | 0.619305274349931 | 1.4881182484883 | 0.999714303421225 |
| ebi-fl187-GCST90094707 | prot-a-719 | Dipeptidyl peptidase 1 \|\| id:prot-a-719 | Black olive liking \|\| id:ebi-fl187-GCST90094707 | Weighted mode | 22 | -0.092452341 | 0.166906633014591 | 0.585486560352995 | -0.419589342 | 0.234684659282309 | 0.91169265990093 | 0.657316696669095 | 1.26450995437845 | 0.999714303421225 |
| ebi-fl187-GCST90094707 | prot-a-752 | Cytochrome b5 \|\| id:prot-a-752 | Black olive liking \|\| id:ebi-fl187-GCST90094707 | Weighted mode | 22 | -0.205204235 | 0.238279710117903 | 0.398859780575256 | -0.672232467 | 0.26182399709947 | 0.814480954124439 | 0.510567479702368 | 1.29929784211515 | 0.999714303421225 |
| ebi-fl187-GCST90094707 | prot-a-755 | Cytoglobin \|\| id:prot-a-755 | Black olive liking \|\| id:ebi-fl187-GCST90094707 | Weighted mode | 22 | -0.187235421 | 0.16889119092588 | 0.280131626255336 | -0.518262155 | 0.143791313487961 | 0.829248491121468 | 0.595554630844322 | 1.15464312493438 | 0.999714303421225 |
| ebi-fl187-GCST90094707 | prot-a-807 | Desmin \|\| id:prot-a-807 | Black olive liking \|\| id:ebi-fl187-GCST90094707 | Weighted mode | 22 | 0.163727373601337 | 0.218679042206641 | 0.462335706265486 | -0.264883549 | 0.592338296326353 | 1.17789314654865 | 0.767295296983217 | 1.80821161049892 | 0.999714303421225 |
| ebi-fl187-GCST90094707 | prot-a-82 | Ameloblastin \|\| id:prot-a-82 | Black olive liking \|\| id:ebi-fl187-GCST90094707 | Weighted mode | 22 | -0.130086525 | 0.259190205046008 | 0.620962303355771 | -0.638099326 | 0.377926277356492 | 0.878019457409705 | 0.528295587861809 | 1.45925535874755 | 0.999714303421225 |
| ebi-fl187-GCST90094707 | prot-a-84 | Muellerian-inhibiting factor \|\| id:prot-a-84 | Black olive liking \|\| id:ebi-fl187-GCST90094707 | Weighted mode | 22 | 0.0157153661445124 | 0.180951963198342 | 0.931614905076181 | -0.338950482 | 0.370381214013264 | 1.01583950193835 | 0.71251773086512 | 1.44828661659467 | 0.999714303421225 |
| ebi-fl187-GCST90094707 | prot-a-858 | Inactive dipeptidyl peptidase 10 \|\| id:prot-a-858 | Black olive liking \|\| id:ebi-fl187-GCST90094707 | Weighted mode | 22 | 0.10762181069734 | 0.174638245892083 | 0.544348743483352 | -0.234669151 | 0.449912772645822 | 1.113626504 | 0.790832452278287 | 1.56817539173387 | 0.999714303421225 |
| ebi-fl187-GCST90094707 | prot-a-863 | Dihydropyrimidinase-related protein 3 \|\| id:prot-a-863 | Black olive liking \|\| id:ebi-fl187-GCST90094707 | Weighted mode | 21 | -0.100905007 | 0.194205672429353 | 0.609060682345375 | -0.481548125 | 0.279738111181453 | 0.904018904473795 | 0.617826179061132 | 1.32278334480407 | 0.999714303421225 |
| ebi-fl187-GCST90094707 | prot-a-864 | Dihydropyrimidinase-related protein 5 \|\| id:prot-a-864 | Black olive liking \|\| id:ebi-fl187-GCST90094707 | Weighted mode | 22 | -0.193130575 | 0.166858758966293 | 0.2600847487486 | -0.520173743 | 0.133912592493329 | 0.824374324367219 | 0.594417263364742 | 1.14329288289681 | 0.999714303421225 |
| ebi-fl187-GCST90094707 | prot-a-884 | Endothelin-converting enzyme 1 \|\| id:prot-a-884 | Black olive liking \|\| id:ebi-fl187-GCST90094707 | Weighted mode | 22 | 0.0412132530827316 | 0.193863447727845 | 0.833697507144839 | -0.338759104 | 0.421185610629307 | 1.04207430741051 | 0.71265410360534 | 1.52376707952904 | 0.999714303421225 |
| ebi-fl187-GCST90094707 | prot-a-893 | Early endosome antigen 1 \|\| id:prot-a-893 | Black olive liking \|\| id:ebi-fl187-GCST90094707 | Weighted mode | 22 | 0.0395021625503422 | 0.20968325685556 | 0.852379815617333 | -0.371477021 | 0.450481345987239 | 1.04029274856797 | 0.689714854682922 | 1.56906726798078 | 0.999714303421225 |
| ebi-fl187-GCST90094707 | prot-a-897 | Ephrin-A2 \|\| id:prot-a-897 | Black olive liking \|\| id:ebi-fl187-GCST90094707 | Weighted mode | 22 | 0.170757080454552 | 0.204978591220483 | 0.41419204325793 | -0.231000958 | 0.572515119246699 | 1.1862025622328 | 0.793738705365237 | 1.77272005149377 | 0.999714303421225 |
| ebi-fl187-GCST90094707 | prot-a-90 | Anaphase-promoting complex subunit 10 \|\| id:prot-a-90 | Black olive liking \|\| id:ebi-fl187-GCST90094707 | Weighted mode | 22 | 0.116668287317251 | 0.207628685950989 | 0.580127034299979 | -0.290283937 | 0.52362051178119 | 1.12374660685723 | 0.74805113791598 | 1.68812848803524 | 0.999714303421225 |
| ebi-fl187-GCST90094707 | prot-a-914 | Histone-lysine N-methyltransferase EHMT2 \|\| id:prot-a-914 | Black olive liking \|\| id:ebi-fl187-GCST90094707 | Weighted mode | 22 | -0.000762608 | 0.215768379337462 | 0.997213336793337 | -0.423668632 | 0.42214341543879 | 0.999237682648989 | 0.654640773260538 | 1.52522725013393 | 0.999714303421225 |
| ebi-fl187-GCST90094707 | prot-a-932 | RNA polymerase II elongation factor ELL \|\| id:prot-a-932 | Black olive liking \|\| id:ebi-fl187-GCST90094707 | Weighted mode | 22 | -0.176856623 | 0.187320981446672 | 0.355836492419799 | -0.544005747 | 0.190292500574234 | 0.83789991143536 | 0.580418579641611 | 1.20960335559364 | 0.999714303421225 |
| ebi-fl187-GCST90094707 | prot-a-991 | Estrogen receptor \|\| id:prot-a-991 | Black olive liking \|\| id:ebi-fl187-GCST90094707 | Weighted mode | 22 | 0.0481906429400811 | 0.211770871338065 | 0.82218898224158 | -0.366880265 | 0.463261550762688 | 1.04937069136997 | 0.692892603655058 | 1.5892489573384 | 0.999714303421225 |
| ebi-fl187-GCST90094707 | prot-a-992 | Persulfide dioxygenase ETHE1, mitochondrial \|\| id:prot-a-992 | Black olive liking \|\| id:ebi-fl187-GCST90094707 | Weighted mode | 22 | -0.32985716 | 0.234547418793042 | 0.174244292781511 | -0.789570101 | 0.129855780402868 | 0.719026431522299 | 0.454039944529547 | 1.13866415379682 | 0.999714303421225 |
| ebi-fl187-GCST90094707 | prot-a-993 | Ethanolamine kinase 1 \|\| id:prot-a-993 | Black olive liking \|\| id:ebi-fl187-GCST90094707 | Weighted mode | 22 | 0.284345233718961 | 0.189572738822073 | 0.148518416446244 | -0.087217334 | 0.655907801810225 | 1.3288916297685 | 0.916477891839669 | 1.92689095873762 | 0.999714303421225 |
| ebi-fl187-GCST90094707 | prot-b-10 | coagulation factor III, tissue factor \|\| id:prot-b-10 | Black olive liking \|\| id:ebi-fl187-GCST90094707 | Weighted mode | 16 | -0.082292131 | 0.223606944574791 | 0.717998871752089 | -0.520561743 | 0.355977480209077 | 0.9210028657598 | 0.59418667428125 | 1.42757539920233 | 0.999714303421225 |
| ebi-fl187-GCST90094707 | prot-b-28 | prolactin \|\| id:prot-b-28 | Black olive liking \|\| id:ebi-fl187-GCST90094707 | Weighted mode | 16 | 0.178303527729697 | 0.243888345638071 | 0.475992315979536 | -0.29971763 | 0.656324685180318 | 1.19518803889659 | 0.741027435266038 | 1.92769441499644 | 0.999714303421225 |
| ebi-fl187-GCST90094707 | prot-b-54 | matrix metallopeptidase 7 \|\| id:prot-b-54 | Black olive liking \|\| id:ebi-fl187-GCST90094707 | Weighted mode | 16 | 0.316265627024074 | 0.238108296596539 | 0.203954387097662 | -0.150426634 | 0.782957888353291 | 1.37199464608281 | 0.860340847196135 | 2.18793436928464 | 0.999714303421225 |
| ebi-fl187-GCST90094707 | ukb-rsfMRI191-edge-pheno716 | Net100_Pair10_33 (OccipitalPrecuneus)&(Temporal\|Frontal\|Supp_Motor_Area) (Default_mode\|Central_executive)&(Default_mode\|Salience) \|\| id:ukb-rsfMRI191-edge-pheno716 | Black olive liking \|\| id:ebi-fl187-GCST90094707 | Weighted mode | 21 | 0.0555907818719498 | 0.0617385659380616 | 0.378610361520422 | -0.065416807 | 0.176598371110551 | 1.05716498413254 | 0.936676968141917 | 1.1931517926537 | 0.999714303421225 |
| ebi-fl187-GCST90094707 | ukb-rsfMRI191-edge-pheno903 | Net100_Pair27_38 (Precentral\|Frontal\|Supp_Motor_Area)&(Subcortical) (Attention\|Salience\|Motor)&(Subcortical-cerebellum) \|\| id:ukb-rsfMRI191-edge-pheno903 | Black olive liking \|\| id:ebi-fl187-GCST90094707 | Weighted mode | 21 | 0.0675986741341201 | 0.080840598040038 | 0.412923543946894 | -0.090848898 | 0.226046246292595 | 1.06993582938084 | 0.913155680102629 | 1.25363363984574 | 0.999714303421225 |
| ebi-fl187-GCST90094707 | ukb-rsfMRI191-node-pheno23 | Net100_Node2 RolandicOper\|SupraMarginal\|Insula Salience\|Motor \|\| id:ukb-rsfMRI191-node-pheno23 | Black olive liking \|\| id:ebi-fl187-GCST90094707 | Weighted mode | 21 | 0.0935791227642012 | 0.0703281995924985 | 0.19829157796631 | -0.044264148 | 0.231422393965498 | 1.09809748441245 | 0.956701212963311 | 1.26039150879512 | 0.999714303421225 |
| ebi-fl187-GCST90094707 | ukb-rsfMRI191-node-pheno55 | Net100_Node34 Parietal\|Postcentral\|Precuneus Attention \|\| id:ukb-rsfMRI191-node-pheno55 | Black olive liking \|\| id:ebi-fl187-GCST90094707 | Weighted mode | 21 | 0.0716508834998186 | 0.0624701307954528 | 0.264933889728461 | -0.050790573 | 0.194092339858906 | 1.07428022963432 | 0.950477705518231 | 1.21420839761195 | 0.999714303421225 |
| ebi-fl187-GCST90094716 | ebi-a-GCST90001414 | IgD- CD24- B cell Absolute Count \|\| id:ebi-a-GCST90001414 | F-burn/spicy liking (derived food-liking factor) \|\| id:ebi-fl187-GCST90094716 | Inverse variance weighted | 15 | -0.031916037 | 0.151550873746993 | 0.833202381669755 | -0.32895575 | 0.265123675182394 | 0.968587903860976 | 0.71967486172291 | 1.3035921878108 | 0.998735809224318 |
| ebi-fl187-GCST90094716 | ebi-a-GCST90001422 | CD20- CD38- B cell %B cell \|\| id:ebi-a-GCST90001422 | F-burn/spicy liking (derived food-liking factor) \|\| id:ebi-fl187-GCST90094716 | Inverse variance weighted | 15 | 0.0844361930476287 | 0.132976657733011 | 0.525447958199856 | -0.176198056 | 0.345070442204331 | 1.08810341345427 | 0.838451906369117 | 1.4120893868534 | 0.998735809224318 |
| ebi-fl187-GCST90094716 | ebi-a-GCST90001444 | CD20- CD38- B cell %lymphocyte \|\| id:ebi-a-GCST90001444 | F-burn/spicy liking (derived food-liking factor) \|\| id:ebi-fl187-GCST90094716 | Inverse variance weighted | 15 | 0.0633808219734039 | 0.126242475865842 | 0.615627965280338 | -0.184054431 | 0.310816074670455 | 1.06543250209171 | 0.831890522191209 | 1.36453822496188 | 0.998735809224318 |
| ebi-fl187-GCST90094716 | ebi-a-GCST90001459 | Myeloid Dendritic Cell %Dendritic Cell \|\| id:ebi-a-GCST90001459 | F-burn/spicy liking (derived food-liking factor) \|\| id:ebi-fl187-GCST90094716 | Inverse variance weighted | 15 | 0.0916923449776692 | 0.121328801608989 | 0.449808475116731 | -0.146112106 | 0.329496796131289 | 1.09602757181803 | 0.864060831199196 | 1.39026836399714 | 0.998735809224318 |
| ebi-fl187-GCST90094716 | ebi-a-GCST90001476 | HLA DR++ monocyte %leukocyte \|\| id:ebi-a-GCST90001476 | F-burn/spicy liking (derived food-liking factor) \|\| id:ebi-fl187-GCST90094716 | Inverse variance weighted | 15 | 0.0572111093198711 | 0.118534949046253 | 0.629342975696843 | -0.175117391 | 0.289539609450527 | 1.0588793260959 | 0.839358482012806 | 1.33581235105242 | 0.998735809224318 |
| ebi-fl187-GCST90094716 | ebi-a-GCST90001478 | CD4 regulatory T cell %CD4+ T cell \|\| id:ebi-a-GCST90001478 | F-burn/spicy liking (derived food-liking factor) \|\| id:ebi-fl187-GCST90094716 | Inverse variance weighted | 15 | 0.0714303488478481 | 0.119402507092286 | 0.549685431553716 | -0.162598565 | 0.305459262748729 | 1.07404333973994 | 0.849932312473234 | 1.35724819342722 | 0.998735809224318 |
| ebi-fl187-GCST90094716 | ebi-a-GCST90001485 | CD39+ resting CD4 regulatory T cell %CD4 regulatory T cell \|\| id:ebi-a-GCST90001485 | F-burn/spicy liking (derived food-liking factor) \|\| id:ebi-fl187-GCST90094716 | Inverse variance weighted | 15 | 0.167001137707612 | 0.12851068763067 | 0.193768392719853 | -0.08487981 | 0.418882085463725 | 1.18175560979995 | 0.918622686982879 | 1.52026108334039 | 0.998735809224318 |
| ebi-fl187-GCST90094716 | ebi-a-GCST90001515 | Immature Myeloid-Derived Suppressor Cells Absolute Count \|\| id:ebi-a-GCST90001515 | F-burn/spicy liking (derived food-liking factor) \|\| id:ebi-fl187-GCST90094716 | Inverse variance weighted | 15 | -0.026052964 | 0.161398624106024 | 0.871762624334349 | -0.342394267 | 0.290288339153469 | 0.974283486199722 | 0.710068192813908 | 1.33681288795632 | 0.998735809224318 |
| ebi-fl187-GCST90094716 | ebi-a-GCST90001517 | CD33+ HLA DR+ Absolute Count \|\| id:ebi-a-GCST90001517 | F-burn/spicy liking (derived food-liking factor) \|\| id:ebi-fl187-GCST90094716 | Inverse variance weighted | 15 | 0.328165262492814 | 0.152436898950038 | 0.0313348610505015 | 0.0293889405507388 | 0.626941584434889 | 1.38841840681741 | 1.02982505731692 | 1.87187683839407 | 0.998735809224318 |
| ebi-fl187-GCST90094716 | ebi-a-GCST90001518 | CD33+ HLA DR+ CD14- Absolute Count \|\| id:ebi-a-GCST90001518 | F-burn/spicy liking (derived food-liking factor) \|\| id:ebi-fl187-GCST90094716 | Inverse variance weighted | 14 | 0.173040085346151 | 0.160492403399971 | 0.280952362816273 | -0.141525025 | 0.487605196010094 | 1.18891376214855 | 0.868033452500076 | 1.62841181956187 | 0.998735809224318 |
| ebi-fl187-GCST90094716 | ebi-a-GCST90001520 | CD33+ HLA DR+ CD14dim Absolute Count \|\| id:ebi-a-GCST90001520 | F-burn/spicy liking (derived food-liking factor) \|\| id:ebi-fl187-GCST90094716 | Inverse variance weighted | 15 | 0.422935256393357 | 0.152843644651391 | 0.00565555792288225 | 0.12336171287663 | 0.722508799910083 | 1.52643546582982 | 1.13129355039376 | 2.0595938432886 | 0.998735809224318 |
| ebi-fl187-GCST90094716 | ebi-a-GCST90001531 | CD33dim HLA DR- Absolute Count \|\| id:ebi-a-GCST90001531 | F-burn/spicy liking (derived food-liking factor) \|\| id:ebi-fl187-GCST90094716 | Inverse variance weighted | 15 | -0.02404914 | 0.163824703727563 | 0.883291459392366 | -0.345145559 | 0.297047279626559 | 0.97623773656977 | 0.708117273133964 | 1.34587893059709 | 0.998735809224318 |
| ebi-fl187-GCST90094716 | ebi-a-GCST90001532 | Basophil Absolute Count \|\| id:ebi-a-GCST90001532 | F-burn/spicy liking (derived food-liking factor) \|\| id:ebi-fl187-GCST90094716 | Inverse variance weighted | 15 | -0.036487213 | 0.163065100823322 | 0.822945171006509 | -0.356094811 | 0.283120384274512 | 0.964170422337649 | 0.700406210906857 | 1.3272649340261 | 0.998735809224318 |
| ebi-fl187-GCST90094716 | ebi-a-GCST90001588 | T/B cell \|\| id:ebi-a-GCST90001588 | F-burn/spicy liking (derived food-liking factor) \|\| id:ebi-fl187-GCST90094716 | Inverse variance weighted | 15 | 0.0523301538550654 | 0.126717308879593 | 0.679630288837148 | -0.196035772 | 0.300696079259068 | 1.05372357599171 | 0.821982830553226 | 1.35079874339159 | 0.998735809224318 |
| ebi-fl187-GCST90094716 | ebi-a-GCST90001624 | HLA DR+ CD4+ T cell Absolute Count \|\| id:ebi-a-GCST90001624 | F-burn/spicy liking (derived food-liking factor) \|\| id:ebi-fl187-GCST90094716 | Inverse variance weighted | 15 | 0.0278199456750221 | 0.116217646192183 | 0.810812501213047 | -0.199966641 | 0.255606532211701 | 1.02821053399972 | 0.818758065685999 | 1.29124456483028 | 0.998735809224318 |
| ebi-fl187-GCST90094716 | ebi-a-GCST90001631 | CD8+ Natural Killer T %T cell \|\| id:ebi-a-GCST90001631 | F-burn/spicy liking (derived food-liking factor) \|\| id:ebi-fl187-GCST90094716 | Inverse variance weighted | 15 | 0.110024232295448 | 0.114707070469043 | 0.337470250108772 | -0.114801626 | 0.334850090414771 | 1.11630512076662 | 0.891542985471073 | 1.39773083626624 | 0.998735809224318 |
| ebi-fl187-GCST90094716 | ebi-a-GCST90001632 | CD8+ Natural Killer T %lymphocyte \|\| id:ebi-a-GCST90001632 | F-burn/spicy liking (derived food-liking factor) \|\| id:ebi-fl187-GCST90094716 | Inverse variance weighted | 15 | 0.107155892036998 | 0.11541393140375 | 0.353174967873248 | -0.119055414 | 0.333367197588348 | 1.11310776559145 | 0.887758605574693 | 1.3956596872614 | 0.998735809224318 |
| ebi-fl187-GCST90094716 | ebi-a-GCST90001646 | Natural Killer %CD3- lymphocyte \|\| id:ebi-a-GCST90001646 | F-burn/spicy liking (derived food-liking factor) \|\| id:ebi-fl187-GCST90094716 | Inverse variance weighted | 15 | 0.0303200879249854 | 0.121913636743398 | 0.803591792545915 | -0.20863064 | 0.269270815942045 | 1.03078442280098 | 0.811694987867759 | 1.30900959371485 | 0.998735809224318 |
| ebi-fl187-GCST90094716 | ebi-a-GCST90001684 | CD127- CD8+ T cell Absolute Count \|\| id:ebi-a-GCST90001684 | F-burn/spicy liking (derived food-liking factor) \|\| id:ebi-fl187-GCST90094716 | Inverse variance weighted | 15 | -0.054762822 | 0.121536834471194 | 0.652287803380224 | -0.292975018 | 0.183449373278627 | 0.946709659761785 | 0.746040778168198 | 1.20135414325061 | 0.998735809224318 |
| ebi-fl187-GCST90094716 | ebi-a-GCST90001687 | CD28- CD8+ T cell Absolute Count \|\| id:ebi-a-GCST90001687 | F-burn/spicy liking (derived food-liking factor) \|\| id:ebi-fl187-GCST90094716 | Inverse variance weighted | 15 | -0.030473522 | 0.118183187896298 | 0.79652275684872 | -0.262112571 | 0.201165525898906 | 0.969986114653049 | 0.769424404223427 | 1.22282716463787 | 0.998735809224318 |
| ebi-fl187-GCST90094716 | ebi-a-GCST90001701 | BAFF-R on CD20- CD38- B cell \|\| id:ebi-a-GCST90001701 | F-burn/spicy liking (derived food-liking factor) \|\| id:ebi-fl187-GCST90094716 | Inverse variance weighted (multiplicative random effects) | 15 | 0.0723783142985799 | 0.15439987500457 | 0.639232726664654 | -0.230245441 | 0.375002069307538 | 1.07506197845947 | 0.794338615541364 | 1.45499442544602 | 0.998735809224318 |
| ebi-fl187-GCST90094716 | ebi-a-GCST90001732 | CD19 on IgD- CD27- B cell \|\| id:ebi-a-GCST90001732 | F-burn/spicy liking (derived food-liking factor) \|\| id:ebi-fl187-GCST90094716 | Inverse variance weighted (multiplicative random effects) | 15 | 0.152704887512827 | 0.156333202172733 | 0.328672560911605 | -0.153708189 | 0.459117963771383 | 1.16498112772973 | 0.857522219138699 | 1.58267739036498 | 0.998735809224318 |
| ebi-fl187-GCST90094716 | ebi-a-GCST90001798 | CD27 on CD24+ CD27+ B cell \|\| id:ebi-a-GCST90001798 | F-burn/spicy liking (derived food-liking factor) \|\| id:ebi-fl187-GCST90094716 | Inverse variance weighted | 15 | -0.088452288 | 0.116733721890004 | 0.448614459475092 | -0.317250382 | 0.140345807398114 | 0.915346783171759 | 0.728148416743559 | 1.15067164083111 | 0.998735809224318 |
| ebi-fl187-GCST90094716 | ebi-a-GCST90001836 | CD66b on CD66b++ myeloid cell \|\| id:ebi-a-GCST90001836 | F-burn/spicy liking (derived food-liking factor) \|\| id:ebi-fl187-GCST90094716 | Inverse variance weighted | 15 | -0.027120419 | 0.171633548222552 | 0.874446156880798 | -0.363522173 | 0.30928133596897 | 0.973244037834591 | 0.695223311814664 | 1.36244562154885 | 0.998735809224318 |
| ebi-fl187-GCST90094716 | ebi-a-GCST90001943 | CD25 on activated & secreting CD4 regulatory T cell \|\| id:ebi-a-GCST90001943 | F-burn/spicy liking (derived food-liking factor) \|\| id:ebi-fl187-GCST90094716 | Inverse variance weighted | 15 | 0.178011780925667 | 0.142984930664267 | 0.213143210388031 | -0.102238683 | 0.458262245027631 | 1.19483939746601 | 0.902814039427792 | 1.58132364295286 | 0.998735809224318 |
| ebi-fl187-GCST90094716 | ebi-a-GCST90001960 | CD25 on CD4+ T cell \|\| id:ebi-a-GCST90001960 | F-burn/spicy liking (derived food-liking factor) \|\| id:ebi-fl187-GCST90094716 | Inverse variance weighted | 15 | 0.266486195448728 | 0.136906237050429 | 0.0515962813402643 | -0.001850029 | 0.534822420067569 | 1.30536956916981 | 0.99815168107902 | 1.70714506063101 | 0.998735809224318 |
| ebi-fl187-GCST90094716 | ebi-a-GCST90001988 | HLA DR on CD14+ CD16- monocyte \|\| id:ebi-a-GCST90001988 | F-burn/spicy liking (derived food-liking factor) \|\| id:ebi-fl187-GCST90094716 | Inverse variance weighted | 15 | 0.138528291609843 | 0.116669324480961 | 0.235086318145497 | -0.090143584 | 0.367200167592527 | 1.14858217634932 | 0.913799968455739 | 1.44368686951999 | 0.998735809224318 |
| ebi-fl187-GCST90094716 | ebi-a-GCST90001991 | HLA DR on CD14+ monocyte \|\| id:ebi-a-GCST90001991 | F-burn/spicy liking (derived food-liking factor) \|\| id:ebi-fl187-GCST90094716 | Inverse variance weighted | 15 | 0.142089723261715 | 0.116864738217434 | 0.224042943909101 | -0.086965164 | 0.371144610167886 | 1.1526800661127 | 0.916709029878608 | 1.44939265514765 | 0.998735809224318 |
| ebi-fl187-GCST90094716 | ebi-a-GCST90002019 | CD14 on Monocytic Myeloid-Derived Suppressor Cells \|\| id:ebi-a-GCST90002019 | F-burn/spicy liking (derived food-liking factor) \|\| id:ebi-fl187-GCST90094716 | Inverse variance weighted | 15 | 0.186510311170706 | 0.166834473882302 | 0.263594208544211 | -0.140485258 | 0.513505879980017 | 1.20503704736434 | 0.868936475013895 | 1.67113975218654 | 0.998735809224318 |
| ebi-fl187-GCST90094716 | ebi-a-GCST90002037 | CD80 on plasmacytoid Dendritic Cell \|\| id:ebi-a-GCST90002037 | F-burn/spicy liking (derived food-liking factor) \|\| id:ebi-fl187-GCST90094716 | Inverse variance weighted | 15 | 0.0419683732372748 | 0.129207299322323 | 0.745321651795869 | -0.211277933 | 0.295214679909027 | 1.04286149589611 | 0.809549034867126 | 1.34341473188363 | 0.998735809224318 |
| ebi-fl187-GCST90094716 | ebi-a-GCST90002042 | CD45 on CD33+ HLA DR+ CD14- \|\| id:ebi-a-GCST90002042 | F-burn/spicy liking (derived food-liking factor) \|\| id:ebi-fl187-GCST90094716 | Inverse variance weighted | 15 | 0.0202127339829818 | 0.184748292393408 | 0.912879778229706 | -0.341893919 | 0.382319387074061 | 1.02041839460789 | 0.710423563077265 | 1.46568012967342 | 0.998735809224318 |
| ebi-fl187-GCST90094716 | ebi-a-GCST90002056 | CD8 on Effector Memory CD8+ T cell \|\| id:ebi-a-GCST90002056 | F-burn/spicy liking (derived food-liking factor) \|\| id:ebi-fl187-GCST90094716 | Inverse variance weighted | 15 | 0.0885124133212223 | 0.126585688975731 | 0.484408788723235 | -0.159595537 | 0.336620363713655 | 1.09254781467328 | 0.852488519249457 | 1.40020739328932 | 0.998735809224318 |
| ebi-fl187-GCST90094716 | ebi-a-GCST90002058 | CD8 on CD8+ T cell \|\| id:ebi-a-GCST90002058 | F-burn/spicy liking (derived food-liking factor) \|\| id:ebi-fl187-GCST90094716 | Inverse variance weighted (multiplicative random effects) | 15 | -0.016746727 | 0.172790140053052 | 0.922790353376723 | -0.355415402 | 0.32192194714576 | 0.9833927195687 | 0.70088223494295 | 1.37977707621515 | 0.998735809224318 |
| ebi-fl187-GCST90094716 | ebi-a-GCST90002063 | CD4 on CD4 regulatory T cell \|\| id:ebi-a-GCST90002063 | F-burn/spicy liking (derived food-liking factor) \|\| id:ebi-fl187-GCST90094716 | Inverse variance weighted | 15 | -0.056729968 | 0.126031763789162 | 0.652620767186027 | -0.303752225 | 0.19029228902686 | 0.944849174434075 | 0.738043712558517 | 1.20960309970525 | 0.998735809224318 |
| ebi-fl187-GCST90094716 | ebi-a-GCST90002066 | CD4 on activated CD4 regulatory T cell \|\| id:ebi-a-GCST90002066 | F-burn/spicy liking (derived food-liking factor) \|\| id:ebi-fl187-GCST90094716 | Inverse variance weighted | 15 | -0.032658075 | 0.126054550279397 | 0.795574361965175 | -0.279724993 | 0.214408843909779 | 0.967869442126766 | 0.755991615717055 | 1.23912916165645 | 0.998735809224318 |
| ebi-fl187-GCST90094716 | ebi-a-GCST90002070 | CD4 on activated & secreting CD4 regulatory T cell \|\| id:ebi-a-GCST90002070 | F-burn/spicy liking (derived food-liking factor) \|\| id:ebi-fl187-GCST90094716 | Inverse variance weighted | 15 | -0.02060357 | 0.126627174203865 | 0.870746384818878 | -0.268792831 | 0.227585691488536 | 0.979607233347148 | 0.764301578543044 | 1.2555650263805 | 0.998735809224318 |
| ebi-fl187-GCST90094716 | ebi-a-GCST90002081 | SSC-A on CD4+ T cell \|\| id:ebi-a-GCST90002081 | F-burn/spicy liking (derived food-liking factor) \|\| id:ebi-fl187-GCST90094716 | Inverse variance weighted | 15 | 0.0578424914060675 | 0.119967325759392 | 0.629697940545669 | -0.177293467 | 0.292978449894476 | 1.05954809463587 | 0.837533959806481 | 1.34041390405935 | 0.998735809224318 |
| ebi-fl187-GCST90094716 | ebi-a-GCST90002094 | CD11b on Monocytic Myeloid-Derived Suppressor Cells \|\| id:ebi-a-GCST90002094 | F-burn/spicy liking (derived food-liking factor) \|\| id:ebi-fl187-GCST90094716 | Inverse variance weighted | 15 | 0.311130048658774 | 0.168645077992488 | 0.0650548955315928 | -0.019414304 | 0.64167440152405 | 1.36496672173549 | 0.980772939702793 | 1.89965900977032 | 0.998735809224318 |
| ebi-fl187-GCST90094716 | ebi-a-GCST90002104 | HLA DR on myeloid Dendritic Cell \|\| id:ebi-a-GCST90002104 | F-burn/spicy liking (derived food-liking factor) \|\| id:ebi-fl187-GCST90094716 | Inverse variance weighted | 15 | 0.17280786082837 | 0.128512364295669 | 0.178728961054152 | -0.079076373 | 0.424692094847881 | 1.18863769927898 | 0.923969355262147 | 1.52911952339195 | 0.998735809224318 |
| ebi-fl187-GCST90094716 | ebi-a-GCST90002105 | HLA DR on plasmacytoid Dendritic Cell \|\| id:ebi-a-GCST90002105 | F-burn/spicy liking (derived food-liking factor) \|\| id:ebi-fl187-GCST90094716 | Inverse variance weighted | 15 | 0.123091151817747 | 0.126042557002161 | 0.328775114588711 | -0.12395226 | 0.370134563541983 | 1.13098750781633 | 0.883422014525639 | 1.44792944006883 | 0.998735809224318 |
| ebi-fl187-GCST90094716 | ebi-a-GCST90002106 | HLA DR on Dendritic Cell \|\| id:ebi-a-GCST90002106 | F-burn/spicy liking (derived food-liking factor) \|\| id:ebi-fl187-GCST90094716 | Inverse variance weighted | 15 | 0.151884239605774 | 0.128325707157711 | 0.23657782081622 | -0.099634146 | 0.403402625634887 | 1.16402548058405 | 0.905168516604749 | 1.49690946447331 | 0.998735809224318 |
| ebi-fl187-GCST90094716 | ebi-a-GCST90002111 | HLA DR on CD33dim HLA DR+ CD11b- \|\| id:ebi-a-GCST90002111 | F-burn/spicy liking (derived food-liking factor) \|\| id:ebi-fl187-GCST90094716 | Inverse variance weighted | 15 | -0.107415176 | 0.174258132882857 | 0.537621227266294 | -0.448961117 | 0.234130764370291 | 0.898152704052765 | 0.638290917176657 | 1.26380974268828 | 0.998735809224318 |
| ebi-fl187-GCST90094716 | ebi-a-GCST90002112 | HLA DR on CD33- HLA DR+ \|\| id:ebi-a-GCST90002112 | F-burn/spicy liking (derived food-liking factor) \|\| id:ebi-fl187-GCST90094716 | Inverse variance weighted | 15 | -0.078099622 | 0.16704337308058 | 0.64011288116331 | -0.405504633 | 0.249305389191528 | 0.924872284284287 | 0.666640317069951 | 1.28313382844422 | 0.998735809224318 |
| ebi-fl187-GCST90094716 | ebi-a-GCST90002115 | HLA DR on HLA DR+ CD8+ T cell \|\| id:ebi-a-GCST90002115 | F-burn/spicy liking (derived food-liking factor) \|\| id:ebi-fl187-GCST90094716 | Inverse variance weighted | 15 | -0.225244546 | 0.128963387546857 | 0.0807106593216141 | -0.478012786 | 0.0275236933343088 | 0.798320968481337 | 0.620014269522257 | 1.0279059693385 | 0.998735809224318 |
| ebi-fl187-GCST90094716 | ebi-a-GCST90002116 | HLA DR on B cell \|\| id:ebi-a-GCST90002116 | F-burn/spicy liking (derived food-liking factor) \|\| id:ebi-fl187-GCST90094716 | Inverse variance weighted | 15 | 0.15322991910527 | 0.124902443470416 | 0.219898970427765 | -0.09157887 | 0.398038708307285 | 1.16559294022277 | 0.912489345190935 | 1.48890166165489 | 0.998735809224318 |
| ebi-fl187-GCST90094716 | ebi-cfmet338-GCST90026023 | Sphingomyelin (d18:1/24:1, d18:2/24:0) levels \|\| id:ebi-cfmet338-GCST90026023 | F-burn/spicy liking (derived food-liking factor) \|\| id:ebi-fl187-GCST90094716 | Inverse variance weighted | 12 | -0.085256249 | 0.0834466839992015 | 0.306929876024106 | -0.24881175 | 0.0782992511916947 | 0.918276946290673 | 0.779726743062545 | 1.08144623432684 | 0.998735809224318 |
| ebi-fl187-GCST90094716 | ebi-cfmet338-GCST90026046 | 1-arachidonoyl-gpc (20:4n6) levels \|\| id:ebi-cfmet338-GCST90026046 | F-burn/spicy liking (derived food-liking factor) \|\| id:ebi-fl187-GCST90094716 | Inverse variance weighted (multiplicative random effects) | 12 | -0.037922542 | 0.110556167201874 | 0.731586371747778 | -0.25461263 | 0.178767545885294 | 0.962787513761573 | 0.77521673586456 | 1.19574275653556 | 0.998735809224318 |
| ebi-fl187-GCST90094716 | ebi-cfmet338-GCST90026047 | 1-linoleoyl-gpc (18:2) levels \|\| id:ebi-cfmet338-GCST90026047 | F-burn/spicy liking (derived food-liking factor) \|\| id:ebi-fl187-GCST90094716 | Inverse variance weighted (multiplicative random effects) | 12 | 0.0317991062773172 | 0.134692684842102 | 0.813365654525987 | -0.232198556 | 0.295796768567838 | 1.03231009985367 | 0.792788694715469 | 1.34419694600004 | 0.998735809224318 |
| ebi-fl187-GCST90094716 | ebi-cfmet338-GCST90026050 | 1-myristoyl-2-palmitoyl-gpc (14:0/16:0) levels \|\| id:ebi-cfmet338-GCST90026050 | F-burn/spicy liking (derived food-liking factor) \|\| id:ebi-fl187-GCST90094716 | Inverse variance weighted | 12 | -0.114423405 | 0.0549005338714259 | 0.0371424554626481 | -0.222028451 | -0.006818358 | 0.891880249494103 | 0.800892577616791 | 0.993204833792664 | 0.998735809224318 |
| ebi-fl187-GCST90094716 | ebi-cfmet338-GCST90026068 | 2,3-dihydroxyisovalerate levels \|\| id:ebi-cfmet338-GCST90026068 | F-burn/spicy liking (derived food-liking factor) \|\| id:ebi-fl187-GCST90094716 | Inverse variance weighted | 12 | -0.193039955 | 0.188662471537641 | 0.306212004927821 | -0.562818399 | 0.176738489286133 | 0.82444903267958 | 0.569601435247393 | 1.19331898661926 | 0.998735809224318 |
| ebi-fl187-GCST90094716 | ebi-cfmet338-GCST90026082 | 3-hydroxyhexanoate levels \|\| id:ebi-cfmet338-GCST90026082 | F-burn/spicy liking (derived food-liking factor) \|\| id:ebi-fl187-GCST90094716 | Inverse variance weighted (multiplicative random effects) | 12 | 0.016001665021525 | 0.0602239454439125 | 0.790468182373357 | -0.102037268 | 0.134040598091593 | 1.01613037728364 | 0.902995898146813 | 1.14343924015335 | 0.998735809224318 |
| ebi-fl187-GCST90094716 | ebi-cfmet338-GCST90026094 | 3-ureidopropionate levels \|\| id:ebi-cfmet338-GCST90026094 | F-burn/spicy liking (derived food-liking factor) \|\| id:ebi-fl187-GCST90094716 | Inverse variance weighted | 12 | 0.0258708927085477 | 0.103018186938163 | 0.801714086929361 | -0.176044754 | 0.227786539107347 | 1.02620844892699 | 0.838580452927427 | 1.2558172289526 | 0.998735809224318 |
| ebi-fl187-GCST90094716 | ebi-cfmet338-GCST90026173 | Glycerol levels \|\| id:ebi-cfmet338-GCST90026173 | F-burn/spicy liking (derived food-liking factor) \|\| id:ebi-fl187-GCST90094716 | Inverse variance weighted | 12 | -0.043349566 | 0.0465708311889565 | 0.351941071804839 | -0.134628395 | 0.0479292630117905 | 0.957576595220353 | 0.874040649005141 | 1.04909644277701 | 0.998735809224318 |
| ebi-fl187-GCST90094716 | ebi-cfmet338-GCST90026179 | Histidine levels \|\| id:ebi-cfmet338-GCST90026179 | F-burn/spicy liking (derived food-liking factor) \|\| id:ebi-fl187-GCST90094716 | Inverse variance weighted (multiplicative random effects) | 12 | 0.00262758966810154 | 0.0460424299695897 | 0.95449033036894 | -0.087615573 | 0.0928707524084974 | 1.0026310448074 | 0.916112987539781 | 1.09731990014815 | 0.998735809224318 |
| ebi-fl187-GCST90094716 | ebi-cfmet338-GCST90026188 | Isocitrate levels \|\| id:ebi-cfmet338-GCST90026188 | F-burn/spicy liking (derived food-liking factor) \|\| id:ebi-fl187-GCST90094716 | Inverse variance weighted | 12 | -0.019413759 | 0.0686953613664213 | 0.777478456441325 | -0.154056668 | 0.115229148993165 | 0.980773474147182 | 0.857223442871437 | 1.12213054319724 | 0.998735809224318 |
| ebi-fl187-GCST90094716 | ebi-cfmet338-GCST90026207 | N-acetyl-isoputreanine levels \|\| id:ebi-cfmet338-GCST90026207 | F-burn/spicy liking (derived food-liking factor) \|\| id:ebi-fl187-GCST90094716 | Inverse variance weighted | 12 | -0.000340915 | 0.0403571061967448 | 0.993259977726302 | -0.079440843 | 0.0787590128765451 | 0.999659142835933 | 0.923632657306594 | 1.08194355618562 | 0.999107451094609 |
| ebi-fl187-GCST90094716 | ebi-cfmet338-GCST90026213 | N-acetylglutamate levels \|\| id:ebi-cfmet338-GCST90026213 | F-burn/spicy liking (derived food-liking factor) \|\| id:ebi-fl187-GCST90094716 | Inverse variance weighted | 12 | 0.0231450552453718 | 0.076786274893015 | 0.763092892201247 | -0.127356044 | 0.173646154035681 | 1.02341498 | 0.88042014887558 | 1.18963454395392 | 0.998735809224318 |
| ebi-fl187-GCST90094716 | ebi-cfmet338-GCST90026224 | N-acetyltaurine levels \|\| id:ebi-cfmet338-GCST90026224 | F-burn/spicy liking (derived food-liking factor) \|\| id:ebi-fl187-GCST90094716 | Inverse variance weighted | 12 | 0.0438138756941541 | 0.0638061656768304 | 0.492289758544178 | -0.081246209 | 0.168873960420742 | 1.04478787637217 | 0.921966666977005 | 1.18397090232492 | 0.998735809224318 |
| ebi-fl187-GCST90094716 | ebi-cfmet338-GCST90026250 | Phenyllactate (pla) levels \|\| id:ebi-cfmet338-GCST90026250 | F-burn/spicy liking (derived food-liking factor) \|\| id:ebi-fl187-GCST90094716 | Inverse variance weighted | 12 | 0.212114357342531 | 0.0883970634416733 | 0.0164146373864795 | 0.0388561129968513 | 0.385372601688211 | 1.23628925574885 | 1.03962088495422 | 1.47016200424576 | 0.998735809224318 |
| ebi-fl187-GCST90094716 | ebi-cfmet338-GCST90026251 | Pipecolate levels \|\| id:ebi-cfmet338-GCST90026251 | F-burn/spicy liking (derived food-liking factor) \|\| id:ebi-fl187-GCST90094716 | Inverse variance weighted | 12 | -0.050183496 | 0.165305843850889 | 0.761448128376649 | -0.37418295 | 0.273815957463204 | 0.951054893258753 | 0.687851057668449 | 1.31497276904297 | 0.998735809224318 |
| ebi-fl187-GCST90094716 | ebi-cfmet338-GCST90026278 | Trigonelline (n'-methylnicotinate) levels \|\| id:ebi-cfmet338-GCST90026278 | F-burn/spicy liking (derived food-liking factor) \|\| id:ebi-fl187-GCST90094716 | Inverse variance weighted | 12 | 0.0731437981300022 | 0.173147060103362 | 0.672705766188724 | -0.26622444 | 0.412512035932591 | 1.07588523607676 | 0.766267127456724 | 1.51060772377099 | 0.998735809224318 |
| ebi-fl187-GCST90094716 | ebi-cfmet338-GCST90026299 | 1-carboxyethylvaline levels \|\| id:ebi-cfmet338-GCST90026299 | F-burn/spicy liking (derived food-liking factor) \|\| id:ebi-fl187-GCST90094716 | Inverse variance weighted | 12 | -0.015019288 | 0.113361086633821 | 0.894596193329924 | -0.237207017 | 0.207168442206362 | 0.985092939345263 | 0.788827969999367 | 1.23018977020892 | 0.998735809224318 |
| ebi-fl187-GCST90094716 | ebi-gm430-GCST90011306 | C_Erysipelotrichia abundance \|\| id:ebi-gm430-GCST90011306 | F-burn/spicy liking (derived food-liking factor) \|\| id:ebi-fl187-GCST90094716 | Inverse variance weighted | 12 | -0.010647223 | 0.0772480083012231 | 0.890373442321943 | -0.162053319 | 0.140758873122111 | 0.989409257898929 | 0.850395860718446 | 1.15114704202473 | 0.998735809224318 |
| ebi-fl187-GCST90094716 | ebi-gm430-GCST90011312 | F_Erysipelotrichaceae abundance \|\| id:ebi-gm430-GCST90011312 | F-burn/spicy liking (derived food-liking factor) \|\| id:ebi-fl187-GCST90094716 | Inverse variance weighted | 12 | -0.010647223 | 0.0772480083012231 | 0.890373442321943 | -0.162053319 | 0.140758873122111 | 0.989409257898929 | 0.850395860718446 | 1.15114704202473 | 0.998735809224318 |
| ebi-fl187-GCST90094716 | ebi-gm430-GCST90011352 | O_Erysipelotrichales abundance \|\| id:ebi-gm430-GCST90011352 | F-burn/spicy liking (derived food-liking factor) \|\| id:ebi-fl187-GCST90094716 | Inverse variance weighted | 12 | -0.010647223 | 0.0772480083012231 | 0.890373442321943 | -0.162053319 | 0.140758873122111 | 0.989409257898929 | 0.850395860718446 | 1.15114704202473 | 0.998735809224318 |
| ebi-fl187-GCST90094716 | ebi-gm430-GCST90011362 | OTU97_11 (Parabacteroides) abundance \|\| id:ebi-gm430-GCST90011362 | F-burn/spicy liking (derived food-liking factor) \|\| id:ebi-fl187-GCST90094716 | Inverse variance weighted | 12 | -0.135637339 | 0.100819732705941 | 0.178513798981452 | -0.333244015 | 0.0619693375580169 | 0.87315923627378 | 0.716595313065804 | 1.06392972154452 | 0.998735809224318 |
| ebi-fl187-GCST90094716 | ebi-gm430-GCST90011384 | OTU97_235 (Phascolarctobacterium) abundance \|\| id:ebi-gm430-GCST90011384 | F-burn/spicy liking (derived food-liking factor) \|\| id:ebi-fl187-GCST90094716 | Inverse variance weighted | 12 | -0.011487543 | 0.378015316921605 | 0.975756742739989 | -0.752397564 | 0.72942247824877 | 0.988578186971169 | 0.47123538023087 | 2.07388254947327 | 0.998955228396052 |
| ebi-fl187-GCST90094716 | ebi-gm430-GCST90011390 | OTU97_3 (Bacteroides) abundance \|\| id:ebi-gm430-GCST90011390 | F-burn/spicy liking (derived food-liking factor) \|\| id:ebi-fl187-GCST90094716 | Inverse variance weighted | 12 | -0.080005004 | 0.0800428357375435 | 0.317539292969696 | -0.236888962 | 0.0768789539137586 | 0.923111727002305 | 0.789078900756346 | 1.07991134943843 | 0.998735809224318 |
| ebi-fl187-GCST90094716 | ebi-gm430-GCST90011398 | OTU97_4 (Alistipes) abundance \|\| id:ebi-gm430-GCST90011398 | F-burn/spicy liking (derived food-liking factor) \|\| id:ebi-fl187-GCST90094716 | Inverse variance weighted | 12 | 0.00527440211867961 | 0.0934984822565943 | 0.955013889480323 | -0.177982623 | 0.188531427341604 | 1.00528833626486 | 0.836956967076375 | 1.20747503012057 | 0.998735809224318 |
| ebi-fl187-GCST90094716 | ebi-gm430-GCST90011421 | OTU99_108 (Cytophagales) abundance \|\| id:ebi-gm430-GCST90011421 | F-burn/spicy liking (derived food-liking factor) \|\| id:ebi-fl187-GCST90094716 | Inverse variance weighted | 12 | -0.164357573 | 0.29464743772306 | 0.57697347152796 | -0.741866551 | 0.413151405129762 | 0.848438589065549 | 0.476224188805094 | 1.5115738686473 | 0.998735809224318 |
| ebi-fl187-GCST90094716 | ebi-gm430-GCST90011428 | OTU99_123 (Phascolarctobacterium) abundance \|\| id:ebi-gm430-GCST90011428 | F-burn/spicy liking (derived food-liking factor) \|\| id:ebi-fl187-GCST90094716 | Inverse variance weighted | 13 | -0.040094719 | 0.175337267646315 | 0.81912387838297 | -0.383755764 | 0.303566325303607 | 0.960698438175211 | 0.681297804400216 | 1.35468143762008 | 0.998735809224318 |
| ebi-fl187-GCST90094716 | ebi-gm430-GCST90011450 | OTU99_298 (Phascolarctobacterium) abundance \|\| id:ebi-gm430-GCST90011450 | F-burn/spicy liking (derived food-liking factor) \|\| id:ebi-fl187-GCST90094716 | Inverse variance weighted | 12 | -0.016169722 | 0.376116782054094 | 0.965708527068204 | -0.753358615 | 0.721019170794249 | 0.983960306140372 | 0.470782716655201 | 2.05652809631273 | 0.998735809224318 |
| ebi-fl187-GCST90094716 | ebi-gm430-GCST90011451 | OTU99_3 (Bacteroides) abundance \|\| id:ebi-gm430-GCST90011451 | F-burn/spicy liking (derived food-liking factor) \|\| id:ebi-fl187-GCST90094716 | Inverse variance weighted | 12 | -0.086672897 | 0.0798544530154871 | 0.277750775464049 | -0.243187625 | 0.0698418306981536 | 0.916976992312491 | 0.784124378536233 | 1.07233855679901 | 0.998735809224318 |
| ebi-fl187-GCST90094716 | ebi-gm430-GCST90011472 | OTU99_72 (Parabacteroides) abundance \|\| id:ebi-gm430-GCST90011472 | F-burn/spicy liking (derived food-liking factor) \|\| id:ebi-fl187-GCST90094716 | Inverse variance weighted | 13 | -0.11779575 | 0.232587547203603 | 0.612535389851863 | -0.573667343 | 0.338075842421029 | 0.888877587234826 | 0.563455261501895 | 1.40224684916843 | 0.998735809224318 |
| ebi-fl187-GCST90094716 | ebi-gm430-GCST90011507 | TestASV_3 (Bacteroides) abundance \|\| id:ebi-gm430-GCST90011507 | F-burn/spicy liking (derived food-liking factor) \|\| id:ebi-fl187-GCST90094716 | Inverse variance weighted | 12 | 0.0982708490056119 | 0.108182444521046 | 0.363677097899661 | -0.113766742 | 0.310308440266861 | 1.10326156194196 | 0.892466106235984 | 1.36384571419989 | 0.998735809224318 |
| ebi-fl187-GCST90094716 | ebi-gm430-GCST90011512 | TestASV_34 (Phascolarctobacterium) abundance \|\| id:ebi-gm430-GCST90011512 | F-burn/spicy liking (derived food-liking factor) \|\| id:ebi-fl187-GCST90094716 | Inverse variance weighted | 12 | 0.161420135276406 | 0.260408148302113 | 0.535341010637024 | -0.348979835 | 0.671820105948547 | 1.17517859909642 | 0.705407354386689 | 1.95779747855757 | 0.998735809224318 |
| ebi-fl187-GCST90094716 | ebi-gm430-GCST90011519 | TestASV_41 (Barnesiella) abundance \|\| id:ebi-gm430-GCST90011519 | F-burn/spicy liking (derived food-liking factor) \|\| id:ebi-fl187-GCST90094716 | Inverse variance weighted | 12 | -0.213665662 | 0.398088287623641 | 0.591454589532736 | -0.993918706 | 0.566587381723014 | 0.807618357379924 | 0.370123440592162 | 1.76224291585941 | 0.998735809224318 |
| ebi-fl187-GCST90094716 | ebi-gm430-GCST90011524 | TestASV_46 (Sutterella) abundance \|\| id:ebi-gm430-GCST90011524 | F-burn/spicy liking (derived food-liking factor) \|\| id:ebi-fl187-GCST90094716 | Inverse variance weighted | 12 | -0.584931999 | 0.332028952242961 | 0.0781217318495661 | -1.235708746 | 0.0658447469842152 | 0.557143746626443 | 0.290628707478042 | 1.06806088461995 | 0.998735809224318 |
| ebi-fl187-GCST90094716 | ebi-gm430-GCST90011581 | OTU97_19 (Bacteria) prevalence \|\| id:ebi-gm430-GCST90011581 | F-burn/spicy liking (derived food-liking factor) \|\| id:ebi-fl187-GCST90094716 | Inverse variance weighted | 12 | -0.02620992 | 0.386934177800632 | 0.945994667386948 | -0.784600908 | 0.732181068750898 | 0.974130578907851 | 0.456301771734754 | 2.07961144037581 | 0.998735809224318 |
| ebi-fl187-GCST90094716 | ebi-gm430-GCST90011614 | OTU97_80 (Ruminococcus) prevalence \|\| id:ebi-gm430-GCST90011614 | F-burn/spicy liking (derived food-liking factor) \|\| id:ebi-fl187-GCST90094716 | Inverse variance weighted | 12 | 0.400979945192324 | 0.180793037401951 | 0.026562003262815 | 0.0466255918845004 | 0.755334298500148 | 1.49328732060964 | 1.04772965714788 | 2.12832289959583 | 0.998735809224318 |
| ebi-fl187-GCST90094716 | ebi-gm430-GCST90011623 | OTU99_11 (Parabacteroides) prevalence \|\| id:ebi-gm430-GCST90011623 | F-burn/spicy liking (derived food-liking factor) \|\| id:ebi-fl187-GCST90094716 | Inverse variance weighted | 12 | -0.018251386 | 0.157644009416578 | 0.907830107798143 | -0.327233644 | 0.290730872780678 | 0.981914162174535 | 0.720915285721442 | 1.33740460352989 | 0.998735809224318 |
| ebi-fl187-GCST90094716 | ebi-gm430-GCST90011642 | OTU99_19 (Bacteria) prevalence \|\| id:ebi-gm430-GCST90011642 | F-burn/spicy liking (derived food-liking factor) \|\| id:ebi-fl187-GCST90094716 | Inverse variance weighted | 12 | -0.02620992 | 0.386934177800632 | 0.945994667386948 | -0.784600908 | 0.732181068750898 | 0.974130578907851 | 0.456301771734754 | 2.07961144037581 | 0.998735809224318 |
| ebi-fl187-GCST90094716 | ebi-gm430-GCST90011653 | OTU99_30 (Parasutterella) prevalence \|\| id:ebi-gm430-GCST90011653 | F-burn/spicy liking (derived food-liking factor) \|\| id:ebi-fl187-GCST90094716 | Inverse variance weighted | 12 | -0.109581814 | 0.202053917335306 | 0.587584671066779 | -0.505607492 | 0.2864438638988 | 0.896208838862416 | 0.603139061922752 | 1.33168341028124 | 0.998735809224318 |
| ebi-fl187-GCST90094716 | ebi-gm430-GCST90011675 | OTU99_8 (Ruminococcaceae) prevalence \|\| id:ebi-gm430-GCST90011675 | F-burn/spicy liking (derived food-liking factor) \|\| id:ebi-fl187-GCST90094716 | Inverse variance weighted | 12 | -0.349467474 | 0.239440480740118 | 0.144422839460242 | -0.818770817 | 0.119835867783625 | 0.705063454055961 | 0.440973358591339 | 1.12731180820837 | 0.998735809224318 |
| ebi-fl187-GCST90094716 | ebi-gm430-GCST90011679 | OTU99_92 (Ruminococcus) prevalence \|\| id:ebi-gm430-GCST90011679 | F-burn/spicy liking (derived food-liking factor) \|\| id:ebi-fl187-GCST90094716 | Inverse variance weighted | 12 | 0.457115938225007 | 0.189034853766205 | 0.0155993483879518 | 0.0866076248432446 | 0.82762425160677 | 1.5795119994518 | 1.09046872292787 | 2.28787685878201 | 0.998735809224318 |
| ebi-fl187-GCST90094716 | ebi-gm430-GCST90011684 | TestASV_11 (Lachnospiraceae) prevalence \|\| id:ebi-gm430-GCST90011684 | F-burn/spicy liking (derived food-liking factor) \|\| id:ebi-fl187-GCST90094716 | Inverse variance weighted | 12 | 0.0804805122010706 | 0.234151381084814 | 0.731063294610014 | -0.378456195 | 0.539417219127307 | 1.08380772540942 | 0.684917973418005 | 1.71500709755831 | 0.998735809224318 |
| ebi-fl187-GCST90094716 | ebi-gm430-GCST90011688 | TestASV_15 (Bacteroides) prevalence \|\| id:ebi-gm430-GCST90011688 | F-burn/spicy liking (derived food-liking factor) \|\| id:ebi-fl187-GCST90094716 | Inverse variance weighted | 12 | -0.186415877 | 0.180727141189596 | 0.302317219707304 | -0.540641074 | 0.167809319352309 | 0.829928374764766 | 0.582374787278409 | 1.18271106903261 | 0.998735809224318 |
| ebi-fl187-GCST90094716 | ebi-gm430-GCST90011692 | TestASV_19 (Prevotella) prevalence \|\| id:ebi-gm430-GCST90011692 | F-burn/spicy liking (derived food-liking factor) \|\| id:ebi-fl187-GCST90094716 | Inverse variance weighted | 12 | -0.069140847 | 0.35113747918821 | 0.843901609725941 | -0.757370306 | 0.619088612217063 | 0.933195233080093 | 0.468897864955868 | 1.85723460934371 | 0.998735809224318 |
| ebi-fl187-GCST90094716 | ebi-gm430-GCST90011693 | TestASV_2 (Bacteroides) prevalence \|\| id:ebi-gm430-GCST90011693 | F-burn/spicy liking (derived food-liking factor) \|\| id:ebi-fl187-GCST90094716 | Inverse variance weighted | 12 | -0.223871858 | 0.219008171256836 | 0.306682565643829 | -0.653127874 | 0.205384157421682 | 0.799417566578887 | 0.520415434480365 | 1.22799671841597 | 0.998735809224318 |
| ebi-fl187-GCST90094716 | ebi-gm430-GCST90011716 | TestASV_40 (Barnesiella) prevalence \|\| id:ebi-gm430-GCST90011716 | F-burn/spicy liking (derived food-liking factor) \|\| id:ebi-fl187-GCST90094716 | Inverse variance weighted | 12 | -0.112834551 | 0.376061453144491 | 0.76414446988121 | -0.849914999 | 0.624245897486075 | 0.893298443512949 | 0.427451264257725 | 1.86683763953386 | 0.998735809224318 |
| ebi-fl187-GCST90094716 | ebi-gm430-GCST90011718 | TestASV_42 (Alphaproteobacteria) prevalence \|\| id:ebi-gm430-GCST90011718 | F-burn/spicy liking (derived food-liking factor) \|\| id:ebi-fl187-GCST90094716 | Inverse variance weighted | 12 | 0.207788354101642 | 0.283570963790661 | 0.4637071996405 | -0.348010735 | 0.763587443131338 | 1.23095261590506 | 0.706091296334434 | 2.14596094084386 | 0.998735809224318 |
| ebi-fl187-GCST90094716 | ebi-hbsm44-GCST90104929 | Sulcal depth of the right inferior frontal sulcus \|\| id:ebi-hbsm44-GCST90104929 | F-burn/spicy liking (derived food-liking factor) \|\| id:ebi-fl187-GCST90094716 | Inverse variance weighted | 11 | -0.004660216 | 0.151421735879053 | 0.975447861955983 | -0.301446818 | 0.292126386483305 | 0.995350626117705 | 0.739747166422076 | 1.33927227285604 | 0.998955228396052 |
| ebi-fl187-GCST90094716 | ebi-hbsm44-GCST90104935 | Sulcal opening distance of the left calloso-marginal posterior fissure \|\| id:ebi-hbsm44-GCST90104935 | F-burn/spicy liking (derived food-liking factor) \|\| id:ebi-fl187-GCST90094716 | Inverse variance weighted | 11 | -0.000623522 | 0.0355972723031775 | 0.986024955121457 | -0.070394176 | 0.0691471312888825 | 0.999376671924366 | 0.932026364935204 | 1.07159386253634 | 0.998955228396052 |
| ebi-fl187-GCST90094716 | ebi-hbsm44-GCST90104936 | Sulcal opening distance of the left anterior inferior temporal sulcus \|\| id:ebi-hbsm44-GCST90104936 | F-burn/spicy liking (derived food-liking factor) \|\| id:ebi-fl187-GCST90094716 | Inverse variance weighted | 12 | -0.008678857 | 0.0206397767704989 | 0.674126228366653 | -0.049132819 | 0.0317751058646296 | 0.991358695954156 | 0.952054670279232 | 1.03228532428256 | 0.998735809224318 |
| ebi-fl187-GCST90094716 | ebi-hbsm44-GCST90104941 | Sulcal opening distance of the right posterior inferior temporal sulcus \|\| id:ebi-hbsm44-GCST90104941 | F-burn/spicy liking (derived food-liking factor) \|\| id:ebi-fl187-GCST90094716 | Inverse variance weighted | 12 | 0.0152489610817169 | 0.015365734894514 | 0.321002255787395 | -0.014867879 | 0.0453658014749644 | 1.01536581972414 | 0.985242101869117 | 1.04641056843613 | 0.998735809224318 |
| ebi-fl187-GCST90094716 | ebi-IPs91-GCST90274819 | Leukemia inhibitory factor levels \|\| id:ebi-IPs91-GCST90274819 | F-burn/spicy liking (derived food-liking factor) \|\| id:ebi-fl187-GCST90094716 | Inverse variance weighted | 15 | -0.05464632 | 0.0574892976936586 | 0.341834022673909 | -0.167325344 | 0.0580327030968399 | 0.946819959662911 | 0.845924350267035 | 1.0597496522391 | 0.998735809224318 |
| ebi-fl187-GCST90094716 | ebi-IPs91-GCST90274837 | STAM binding protein levels \|\| id:ebi-IPs91-GCST90274837 | F-burn/spicy liking (derived food-liking factor) \|\| id:ebi-fl187-GCST90094716 | Inverse variance weighted | 15 | -0.052831239 | 0.0510412696852402 | 0.300636660533068 | -0.152872128 | 0.0472096495398269 | 0.948540075506388 | 0.858239459911938 | 1.04834177041217 | 0.998735809224318 |
| ebi-fl187-GCST90094716 | ebi-IPs91-GCST90274840 | TNF-beta levels \|\| id:ebi-IPs91-GCST90274840 | F-burn/spicy liking (derived food-liking factor) \|\| id:ebi-fl187-GCST90094716 | Inverse variance weighted | 15 | -0.03677059 | 0.0703870733787049 | 0.601388065313733 | -0.174729254 | 0.101188073395196 | 0.963897237240186 | 0.839684330960698 | 1.10648472253408 | 0.998735809224318 |
| ebi-fl187-GCST90094716 | ebi-IPs91-GCST90274842 | Tumor necrosis factor ligand superfamily member 14 levels \|\| id:ebi-IPs91-GCST90274842 | F-burn/spicy liking (derived food-liking factor) \|\| id:ebi-fl187-GCST90094716 | Inverse variance weighted | 15 | -0.027274036 | 0.0581378277338602 | 0.638978726232061 | -0.141224178 | 0.0866761066947264 | 0.973094542374763 | 0.868294637303314 | 1.09054340280207 | 0.998735809224318 |
| ebi-fl187-GCST90094716 | ebi-met1400-GCST90199628 | 3-methylhistidine levels \|\| id:ebi-met1400-GCST90199628 | F-burn/spicy liking (derived food-liking factor) \|\| id:ebi-fl187-GCST90094716 | Inverse variance weighted (multiplicative random effects) | 14 | 0.0643108050416129 | 0.0965999016996635 | 0.505574743841501 | -0.125025002 | 0.253646612372953 | 1.06642379715134 | 0.882474838417182 | 1.28871630739126 | 0.998735809224318 |
| ebi-fl187-GCST90094716 | ebi-met1400-GCST90199651 | Docosahexaenoate DHA; 22:6n3 levels \|\| id:ebi-met1400-GCST90199651 | F-burn/spicy liking (derived food-liking factor) \|\| id:ebi-fl187-GCST90094716 | Inverse variance weighted | 14 | -0.006624896 | 0.0839983731895054 | 0.937136548297279 | -0.171261708 | 0.158011914976166 | 0.993396999771251 | 0.842601029245398 | 1.1711801492081 | 0.998735809224318 |
| ebi-fl187-GCST90094716 | ebi-met1400-GCST90199652 | 1-stearoyl-2-oleoyl-GPS (18:0/18:1) levels \|\| id:ebi-met1400-GCST90199652 | F-burn/spicy liking (derived food-liking factor) \|\| id:ebi-fl187-GCST90094716 | Inverse variance weighted | 14 | -0.065719123 | 0.078228062085791 | 0.400854267271245 | -0.219046125 | 0.0876078784229445 | 0.936393838602075 | 0.803284665831371 | 1.09156001386437 | 0.998735809224318 |
| ebi-fl187-GCST90094716 | ebi-met1400-GCST90199653 | 1-myristoyl-2-palmitoyl-gpc (14:0/16:0) levels \|\| id:ebi-met1400-GCST90199653 | F-burn/spicy liking (derived food-liking factor) \|\| id:ebi-fl187-GCST90094716 | Inverse variance weighted | 14 | -0.093920962 | 0.0683844524251298 | 0.169620053911668 | -0.227954489 | 0.0401125643272593 | 0.910354711625268 | 0.796160492928431 | 1.04092793895115 | 0.998735809224318 |
| ebi-fl187-GCST90094716 | ebi-met1400-GCST90199667 | Erythritol levels in elite athletes \|\| id:ebi-met1400-GCST90199667 | F-burn/spicy liking (derived food-liking factor) \|\| id:ebi-fl187-GCST90094716 | Inverse variance weighted (multiplicative random effects) | 14 | -0.006330988 | 0.0998747489114831 | 0.949456530346427 | -0.202085495 | 0.189423520313433 | 0.993689010923001 | 0.817025073054714 | 1.20855269072386 | 0.998735809224318 |
| ebi-fl187-GCST90094716 | ebi-met1400-GCST90199674 | 3-hydroxymyristate levels \|\| id:ebi-met1400-GCST90199674 | F-burn/spicy liking (derived food-liking factor) \|\| id:ebi-fl187-GCST90094716 | Inverse variance weighted (multiplicative random effects) | 14 | -0.043316071 | 0.0942014281210713 | 0.645642601347048 | -0.227950871 | 0.141318727715512 | 0.957608669514358 | 0.796163373967902 | 1.15179169742369 | 0.998735809224318 |
| ebi-fl187-GCST90094716 | ebi-met1400-GCST90199685 | 2-linoleoylglycerol (18:2) levels \|\| id:ebi-met1400-GCST90199685 | F-burn/spicy liking (derived food-liking factor) \|\| id:ebi-fl187-GCST90094716 | Inverse variance weighted | 14 | -0.045022269 | 0.0787344550073376 | 0.567441059223265 | -0.199341801 | 0.109297262576359 | 0.955976192734674 | 0.819269818184603 | 1.11549389565033 | 0.998735809224318 |
| ebi-fl187-GCST90094716 | ebi-met1400-GCST90199703 | Gamma-glutamylvaline levels \|\| id:ebi-met1400-GCST90199703 | F-burn/spicy liking (derived food-liking factor) \|\| id:ebi-fl187-GCST90094716 | Inverse variance weighted | 14 | 0.0257312067762754 | 0.067200762538077 | 0.701793527379334 | -0.105982288 | 0.157444701350906 | 1.0260651120544 | 0.899440579007741 | 1.17051602823687 | 0.998735809224318 |
| ebi-fl187-GCST90094716 | ebi-met1400-GCST90199779 | Gamma-glutamyl-2-aminobutyrate levels \|\| id:ebi-met1400-GCST90199779 | F-burn/spicy liking (derived food-liking factor) \|\| id:ebi-fl187-GCST90094716 | Inverse variance weighted | 14 | 0.0988151416968089 | 0.0713381206826993 | 0.166001704069505 | -0.041007575 | 0.2386378582349 | 1.10386222259938 | 0.959821859422019 | 1.26951870758163 | 0.998735809224318 |
| ebi-fl187-GCST90094716 | ebi-met1400-GCST90199824 | 4-vinylphenol sulfate levels \|\| id:ebi-met1400-GCST90199824 | F-burn/spicy liking (derived food-liking factor) \|\| id:ebi-fl187-GCST90094716 | Inverse variance weighted | 14 | -0.118693103 | 0.0738205486398829 | 0.107865856347596 | -0.263381379 | 0.0259951721330466 | 0.888080307947674 | 0.768448771551834 | 1.02633599344786 | 0.998735809224318 |
| ebi-fl187-GCST90094716 | ebi-met1400-GCST90199830 | 4-ethylphenylsulfate levels \|\| id:ebi-met1400-GCST90199830 | F-burn/spicy liking (derived food-liking factor) \|\| id:ebi-fl187-GCST90094716 | Inverse variance weighted | 14 | -0.055372155 | 0.0689789394498969 | 0.422125015510001 | -0.190570877 | 0.0798265659678665 | 0.946132973974037 | 0.826487176989439 | 1.0830992051222 | 0.998735809224318 |
| ebi-fl187-GCST90094716 | ebi-met1400-GCST90199833 | Hexanoylglutamine levels \|\| id:ebi-met1400-GCST90199833 | F-burn/spicy liking (derived food-liking factor) \|\| id:ebi-fl187-GCST90094716 | Inverse variance weighted (multiplicative random effects) | 14 | -0.104617989 | 0.105484509992015 | 0.321302257351685 | -0.311367629 | 0.102131650462407 | 0.900668522049261 | 0.732444558722455 | 1.10752926887097 | 0.998735809224318 |
| ebi-fl187-GCST90094716 | ebi-met1400-GCST90199837 | Carotenoid levels (cryptoxanthin) \|\| id:ebi-met1400-GCST90199837 | F-burn/spicy liking (derived food-liking factor) \|\| id:ebi-fl187-GCST90094716 | Inverse variance weighted | 14 | 0.162921183059552 | 0.0713550177778358 | 0.0224157794736557 | 0.0230653482149937 | 0.30277701790411 | 1.17694392291364 | 1.02333341037396 | 1.35361259941385 | 0.998735809224318 |
| ebi-fl187-GCST90094716 | ebi-met1400-GCST90199869 | 2-hydroxyhippurate levels \|\| id:ebi-met1400-GCST90199869 | F-burn/spicy liking (derived food-liking factor) \|\| id:ebi-fl187-GCST90094716 | Inverse variance weighted | 14 | 0.187975538022541 | 0.0693808889442903 | 0.00674197897619379 | 0.0519889956917316 | 0.323962080353349 | 1.20680399417643 | 1.05336415090575 | 1.38259487861619 | 0.998735809224318 |
| ebi-fl187-GCST90094716 | ebi-met1400-GCST90199902 | Hydantoin-5-propionate levels \|\| id:ebi-met1400-GCST90199902 | F-burn/spicy liking (derived food-liking factor) \|\| id:ebi-fl187-GCST90094716 | Inverse variance weighted | 14 | -0.063508461 | 0.0688144372371477 | 0.356062223740983 | -0.198384758 | 0.0713678361739053 | 0.938466179082192 | 0.820054270156867 | 1.07397620051739 | 0.998735809224318 |
| ebi-fl187-GCST90094716 | ebi-met1400-GCST90199904 | Pantoate levels \|\| id:ebi-met1400-GCST90199904 | F-burn/spicy liking (derived food-liking factor) \|\| id:ebi-fl187-GCST90094716 | Inverse variance weighted | 14 | 0.0251326524019048 | 0.0806627395228638 | 0.755362034772461 | -0.132966317 | 0.183231621866718 | 1.02545114005934 | 0.875494580841026 | 1.20109257516916 | 0.998735809224318 |
| ebi-fl187-GCST90094716 | ebi-met1400-GCST90199936 | 3-methyl catechol sulfate (2) levels \|\| id:ebi-met1400-GCST90199936 | F-burn/spicy liking (derived food-liking factor) \|\| id:ebi-fl187-GCST90094716 | Inverse variance weighted | 14 | 0.0369149752160795 | 0.0784342026675268 | 0.637891444760004 | -0.116816062 | 0.190646012444432 | 1.03760479496403 | 0.889748836723426 | 1.21003104032943 | 0.998735809224318 |
| ebi-fl187-GCST90094716 | ebi-met1400-GCST90199977 | 4-hydroxychlorothalonil levels \|\| id:ebi-met1400-GCST90199977 | F-burn/spicy liking (derived food-liking factor) \|\| id:ebi-fl187-GCST90094716 | Inverse variance weighted | 14 | 0.00984763503099879 | 0.0683365542539522 | 0.885417604111006 | -0.124092011 | 0.143787281368745 | 1.00989628254537 | 0.883298563688505 | 1.15463846928504 | 0.998735809224318 |
| ebi-fl187-GCST90094716 | ebi-met1400-GCST90200040 | Sphingomyelin (d18:2/24:1, d18:1/24:2) levels \|\| id:ebi-met1400-GCST90200040 | F-burn/spicy liking (derived food-liking factor) \|\| id:ebi-fl187-GCST90094716 | Inverse variance weighted | 14 | 0.123957264626471 | 0.0627807076333865 | 0.0483307848668799 | 0.000907077665032924 | 0.247007451587908 | 1.13196749491178 | 1.0009074891844 | 1.28018865218101 | 0.998735809224318 |
| ebi-fl187-GCST90094716 | ebi-met1400-GCST90200077 | 1-stearoyl-2-oleoyl-GPI (18:0/18:1) levels \|\| id:ebi-met1400-GCST90200077 | F-burn/spicy liking (derived food-liking factor) \|\| id:ebi-fl187-GCST90094716 | Inverse variance weighted | 14 | 0.0183284235802996 | 0.0828168788948317 | 0.824848994820538 | -0.143992659 | 0.18064950621417 | 1.01849742003641 | 0.865894104518109 | 1.19799521582161 | 0.998735809224318 |
| ebi-fl187-GCST90094716 | ebi-met1400-GCST90200080 | 1-palmitoyl-2-oleoyl-GPI (16:0/18:1) levels \|\| id:ebi-met1400-GCST90200080 | F-burn/spicy liking (derived food-liking factor) \|\| id:ebi-fl187-GCST90094716 | Inverse variance weighted | 14 | -0.015590919 | 0.0687009693047318 | 0.820471288102995 | -0.150244819 | 0.119062980513713 | 0.984529989881682 | 0.860497284412318 | 1.12644085987838 | 0.998735809224318 |
| ebi-fl187-GCST90094716 | ebi-met1400-GCST90200082 | 1-oleoyl-2-linoleoyl-GPE (18:1/18:2) levels \|\| id:ebi-met1400-GCST90200082 | F-burn/spicy liking (derived food-liking factor) \|\| id:ebi-fl187-GCST90094716 | Inverse variance weighted (multiplicative random effects) | 14 | -0.120028209 | 0.092773697356809 | 0.195743452034965 | -0.301864656 | 0.061808237500766 | 0.88689541764889 | 0.739438136530501 | 1.06375833621093 | 0.998735809224318 |
| ebi-fl187-GCST90094716 | ebi-met1400-GCST90200100 | Perfluorooctanesulfonate (PFOS) levels \|\| id:ebi-met1400-GCST90200100 | F-burn/spicy liking (derived food-liking factor) \|\| id:ebi-fl187-GCST90094716 | Inverse variance weighted (multiplicative random effects) | 14 | 0.00303174807805787 | 0.103611851406113 | 0.976656725921531 | -0.200047481 | 0.20611097683404 | 1.00303634847417 | 0.818691880109653 | 1.22888957470256 | 0.998955228396052 |
| ebi-fl187-GCST90094716 | ebi-met1400-GCST90200102 | 1-palmitoleoyl-2-linolenoyl-GPC (16:1/18:3) levels \|\| id:ebi-met1400-GCST90200102 | F-burn/spicy liking (derived food-liking factor) \|\| id:ebi-fl187-GCST90094716 | Inverse variance weighted | 14 | -0.073324073 | 0.0674941219795651 | 0.277312161008338 | -0.205612552 | 0.0589644063383788 | 0.929299620545769 | 0.814148455518526 | 1.06073748453713 | 0.998735809224318 |
| ebi-fl187-GCST90094716 | ebi-met1400-GCST90200114 | Glycosyl-N-tricosanoyl-sphingadienine (d18:2/23:0) levels \|\| id:ebi-met1400-GCST90200114 | F-burn/spicy liking (derived food-liking factor) \|\| id:ebi-fl187-GCST90094716 | Inverse variance weighted | 14 | -0.004909174 | 0.0732976098156632 | 0.946600877781154 | -0.14857249 | 0.138754140809086 | 0.995102855872841 | 0.861937523340636 | 1.14884161200968 | 0.998735809224318 |
| ebi-fl187-GCST90094716 | ebi-met1400-GCST90200135 | Arachidoylcarnitine (C20) levels \|\| id:ebi-met1400-GCST90200135 | F-burn/spicy liking (derived food-liking factor) \|\| id:ebi-fl187-GCST90094716 | Inverse variance weighted | 14 | -0.017809682 | 0.0878179897770213 | 0.839289634336418 | -0.189932942 | 0.154313577989188 | 0.982347973095947 | 0.827014590080653 | 1.16685673000231 | 0.998735809224318 |
| ebi-fl187-GCST90094716 | ebi-met1400-GCST90200146 | Docosahexaenoylcarnitine (C22:6) levels \|\| id:ebi-met1400-GCST90200146 | F-burn/spicy liking (derived food-liking factor) \|\| id:ebi-fl187-GCST90094716 | Inverse variance weighted | 14 | 0.0801526341408682 | 0.0910681807034359 | 0.378784152888817 | -0.098341 | 0.258646268319603 | 1.08345242688526 | 0.906339789150451 | 1.29517557915433 | 0.998735809224318 |
| ebi-fl187-GCST90094716 | ebi-met1400-GCST90200162 | 3-hydroxyphenylacetoylglutamine levels \|\| id:ebi-met1400-GCST90200162 | F-burn/spicy liking (derived food-liking factor) \|\| id:ebi-fl187-GCST90094716 | Inverse variance weighted | 14 | 0.132899200842309 | 0.0839766852035671 | 0.113518913308327 | -0.031695102 | 0.2974935038413 | 1.14213486629589 | 0.968801922669379 | 1.34647962837901 | 0.998735809224318 |
| ebi-fl187-GCST90094716 | ebi-met1400-GCST90200172 | 3-carboxy-4-methyl-5-pentyl-2-furanpropionate (3-CMPFP) levels \|\| id:ebi-met1400-GCST90200172 | F-burn/spicy liking (derived food-liking factor) \|\| id:ebi-fl187-GCST90094716 | Inverse variance weighted | 14 | 0.0453385283151205 | 0.068418287236372 | 0.507543969407664 | -0.088761315 | 0.17943837129841 | 1.0463820299026 | 0.915063959855519 | 1.19654516027052 | 0.998735809224318 |
| ebi-fl187-GCST90094716 | ebi-met1400-GCST90200222 | Dihydrocaffeate sulfate (2) levels \|\| id:ebi-met1400-GCST90200222 | F-burn/spicy liking (derived food-liking factor) \|\| id:ebi-fl187-GCST90094716 | Inverse variance weighted | 14 | 0.145979267690963 | 0.0782603178267075 | 0.0621388337855871 | -0.007410955 | 0.299369490631309 | 1.15717219695051 | 0.992616438167238 | 1.34900797720786 | 0.998735809224318 |
| ebi-fl187-GCST90094716 | ebi-met1400-GCST90200223 | 4-methylhexanoylglutamine levels \|\| id:ebi-met1400-GCST90200223 | F-burn/spicy liking (derived food-liking factor) \|\| id:ebi-fl187-GCST90094716 | Inverse variance weighted (multiplicative random effects) | 14 | -0.065377004 | 0.128586951865236 | 0.611154328241775 | -0.317407429 | 0.186653422029663 | 0.936714252130861 | 0.728034072291779 | 1.20520951359186 | 0.998735809224318 |
| ebi-fl187-GCST90094716 | ebi-met1400-GCST90200224 | 2-ketocaprylate levels \|\| id:ebi-met1400-GCST90200224 | F-burn/spicy liking (derived food-liking factor) \|\| id:ebi-fl187-GCST90094716 | Inverse variance weighted | 14 | -0.001766884 | 0.0708797864605063 | 0.980112472330272 | -0.140691266 | 0.137157497159631 | 0.998234675718181 | 0.868757485475523 | 1.14700878492086 | 0.998955228396052 |
| ebi-fl187-GCST90094716 | ebi-met1400-GCST90200243 | Hydroxypalmitoyl sphingomyelin (d18:1/16:0(OH)) levels \|\| id:ebi-met1400-GCST90200243 | F-burn/spicy liking (derived food-liking factor) \|\| id:ebi-fl187-GCST90094716 | Inverse variance weighted | 14 | -0.025920544 | 0.0692114010123761 | 0.708022957878674 | -0.16157489 | 0.109733801800371 | 0.974412509274143 | 0.850802812313763 | 1.11598095879328 | 0.998735809224318 |
| ebi-fl187-GCST90094716 | ebi-met1400-GCST90200273 | 2-methoxyhydroquinone sulfate (2) levels \|\| id:ebi-met1400-GCST90200273 | F-burn/spicy liking (derived food-liking factor) \|\| id:ebi-fl187-GCST90094716 | Inverse variance weighted (multiplicative random effects) | 14 | 0.127535574860979 | 0.111248276028966 | 0.251627633486108 | -0.090511046 | 0.345582195877752 | 1.13602528146377 | 0.913464243576877 | 1.4128122137232 | 0.998735809224318 |
| ebi-fl187-GCST90094716 | ebi-met1400-GCST90200276 | 2,4-di-tert-butylphenol levels \|\| id:ebi-met1400-GCST90200276 | F-burn/spicy liking (derived food-liking factor) \|\| id:ebi-fl187-GCST90094716 | Inverse variance weighted | 14 | 0.000377627256855727 | 0.077091756597862 | 0.996091647585273 | -0.150722216 | 0.151477470188665 | 1.000377699 | 0.860086584049592 | 1.16355208690537 | 0.999337126751992 |
| ebi-fl187-GCST90094716 | ebi-met1400-GCST90200289 | N-acetylmethionine levels \|\| id:ebi-met1400-GCST90200289 | F-burn/spicy liking (derived food-liking factor) \|\| id:ebi-fl187-GCST90094716 | Inverse variance weighted | 14 | -0.039627744 | 0.0790136976302476 | 0.615998128567569 | -0.194494592 | 0.115239102936421 | 0.961147164961928 | 0.823250630569825 | 1.12214171287658 | 0.998735809224318 |
| ebi-fl187-GCST90094716 | ebi-met1400-GCST90200308 | 3-hydroxyisobutyrate levels \|\| id:ebi-met1400-GCST90200308 | F-burn/spicy liking (derived food-liking factor) \|\| id:ebi-fl187-GCST90094716 | Inverse variance weighted | 14 | -0.116795403 | 0.069570213941366 | 0.0931884282936969 | -0.253153022 | 0.0195622162257745 | 0.889767218157078 | 0.776349073916377 | 1.01975481018254 | 0.998735809224318 |
| ebi-fl187-GCST90094716 | ebi-met1400-GCST90200383 | Cystathionine levels \|\| id:ebi-met1400-GCST90200383 | F-burn/spicy liking (derived food-liking factor) \|\| id:ebi-fl187-GCST90094716 | Inverse variance weighted | 14 | -0.030897385 | 0.0686020400326084 | 0.65243227371185 | -0.165357384 | 0.103562612981641 | 0.969575060448743 | 0.847590734631439 | 1.10911523620296 | 0.998735809224318 |
| ebi-fl187-GCST90094716 | ebi-met1400-GCST90200384 | Guanidinoacetate levels \|\| id:ebi-met1400-GCST90200384 | F-burn/spicy liking (derived food-liking factor) \|\| id:ebi-fl187-GCST90094716 | Inverse variance weighted | 14 | 0.115282386622518 | 0.0826516649050132 | 0.163076726735158 | -0.046714877 | 0.277279649836343 | 1.12219028435741 | 0.954359469026486 | 1.3195353272816 | 0.998735809224318 |
| ebi-fl187-GCST90094716 | ebi-met1400-GCST90200403 | Citrulline levels \|\| id:ebi-met1400-GCST90200403 | F-burn/spicy liking (derived food-liking factor) \|\| id:ebi-fl187-GCST90094716 | Inverse variance weighted | 14 | -0.067832486 | 0.0690017354709736 | 0.325580479504339 | -0.203075887 | 0.0674109157895894 | 0.934416988644034 | 0.816216298659587 | 1.06973495885891 | 0.998735809224318 |
| ebi-fl187-GCST90094716 | ebi-met1400-GCST90200434 | 12,13-DiHOME levels \|\| id:ebi-met1400-GCST90200434 | F-burn/spicy liking (derived food-liking factor) \|\| id:ebi-fl187-GCST90094716 | Inverse variance weighted | 14 | 0.0347673664101427 | 0.0695083997654253 | 0.616941704844931 | -0.101469097 | 0.171003829950376 | 1.0353788168899 | 0.903509099934982 | 1.1864952932311 | 0.998735809224318 |
| ebi-fl187-GCST90094716 | ebi-met1400-GCST90200437 | Fructose levels \|\| id:ebi-met1400-GCST90200437 | F-burn/spicy liking (derived food-liking factor) \|\| id:ebi-fl187-GCST90094716 | Inverse variance weighted | 14 | 0.109230004000932 | 0.0692837959597142 | 0.114896993351807 | -0.026566236 | 0.245026244081972 | 1.11541887164305 | 0.973783542094686 | 1.27765484364335 | 0.998735809224318 |
| ebi-fl187-GCST90094716 | ebi-met1400-GCST90200448 | N-stearoyl-sphinganine (d18:0/18:0) levels \|\| id:ebi-met1400-GCST90200448 | F-burn/spicy liking (derived food-liking factor) \|\| id:ebi-fl187-GCST90094716 | Inverse variance weighted | 14 | -0.018953076 | 0.0767213996218135 | 0.804879067859403 | -0.169327019 | 0.131420867154835 | 0.98122540408146 | 0.844232777774349 | 1.14044765728365 | 0.998735809224318 |
| ebi-fl187-GCST90094716 | ebi-met1400-GCST90200468 | X-11847 levels \|\| id:ebi-met1400-GCST90200468 | F-burn/spicy liking (derived food-liking factor) \|\| id:ebi-fl187-GCST90094716 | Inverse variance weighted | 14 | 0.00893968006713425 | 0.0730004759523423 | 0.902534269623976 | -0.134141253 | 0.152020612933725 | 1.00897975834696 | 0.874466535032986 | 1.16418423343723 | 0.998735809224318 |
| ebi-fl187-GCST90094716 | ebi-met1400-GCST90200476 | X-11843 levels \|\| id:ebi-met1400-GCST90200476 | F-burn/spicy liking (derived food-liking factor) \|\| id:ebi-fl187-GCST90094716 | Inverse variance weighted | 14 | -0.002728583 | 0.0726944748290092 | 0.970058480315032 | -0.145209754 | 0.139752587789848 | 0.99727513632376 | 0.864840870650363 | 1.14998924227721 | 0.998735809224318 |
| ebi-fl187-GCST90094716 | ebi-met1400-GCST90200511 | X-12906 levels \|\| id:ebi-met1400-GCST90200511 | F-burn/spicy liking (derived food-liking factor) \|\| id:ebi-fl187-GCST90094716 | Inverse variance weighted | 14 | 0.0721503010399119 | 0.0753648803551133 | 0.338392414999369 | -0.075564864 | 0.219865466535934 | 1.07481687801863 | 0.927219584989291 | 1.24590910284435 | 0.998735809224318 |
| ebi-fl187-GCST90094716 | ebi-met1400-GCST90200515 | X-12849 levels \|\| id:ebi-met1400-GCST90200515 | F-burn/spicy liking (derived food-liking factor) \|\| id:ebi-fl187-GCST90094716 | Inverse variance weighted | 14 | -0.012423664 | 0.0725278147084813 | 0.863991679132139 | -0.154578181 | 0.129730852571956 | 0.987653190856834 | 0.856776505806523 | 1.13852191183912 | 0.998735809224318 |
| ebi-fl187-GCST90094716 | ebi-met1400-GCST90200551 | X-18886 levels \|\| id:ebi-met1400-GCST90200551 | F-burn/spicy liking (derived food-liking factor) \|\| id:ebi-fl187-GCST90094716 | Inverse variance weighted | 14 | -0.151654363 | 0.0679183445461332 | 0.0255560333468499 | -0.284774318 | -0.018534408 | 0.859285230079698 | 0.7521839892236 | 0.981636298048914 | 0.998735809224318 |
| ebi-fl187-GCST90094716 | ebi-met1400-GCST90200564 | X-21319 levels \|\| id:ebi-met1400-GCST90200564 | F-burn/spicy liking (derived food-liking factor) \|\| id:ebi-fl187-GCST90094716 | Inverse variance weighted | 14 | -0.100814427 | 0.076884485646469 | 0.189774871730687 | -0.251508019 | 0.0498791644810763 | 0.904100793667077 | 0.77762722159527 | 1.05114407316218 | 0.998735809224318 |
| ebi-fl187-GCST90094716 | ebi-met1400-GCST90200579 | X-21442 levels \|\| id:ebi-met1400-GCST90200579 | F-burn/spicy liking (derived food-liking factor) \|\| id:ebi-fl187-GCST90094716 | Inverse variance weighted | 14 | -0.002599617 | 0.0849628711157046 | 0.975590861873488 | -0.169126844 | 0.163927610662344 | 0.997403759352984 | 0.844401789198314 | 1.17812902802581 | 0.998955228396052 |
| ebi-fl187-GCST90094716 | ebi-met1400-GCST90200581 | X-21441 levels \|\| id:ebi-met1400-GCST90200581 | F-burn/spicy liking (derived food-liking factor) \|\| id:ebi-fl187-GCST90094716 | Inverse variance weighted | 14 | 0.0083736173034242 | 0.069013852770405 | 0.90342774761283 | -0.126893534 | 0.143640768733418 | 1.00840877409813 | 0.880827445668597 | 1.15446931255215 | 0.998735809224318 |
| ebi-fl187-GCST90094716 | ebi-met1400-GCST90200648 | X-24585 levels \|\| id:ebi-met1400-GCST90200648 | F-burn/spicy liking (derived food-liking factor) \|\| id:ebi-fl187-GCST90094716 | Inverse variance weighted | 14 | 0.0271883475884532 | 0.0696994012479919 | 0.696477336789617 | -0.109422479 | 0.163799174034517 | 1.02756132323558 | 0.896351647872579 | 1.17797772282308 | 0.998735809224318 |
| ebi-fl187-GCST90094716 | ebi-met1400-GCST90200720 | Adenosine 5'-diphosphate (ADP) to pantothenate ratio \|\| id:ebi-met1400-GCST90200720 | F-burn/spicy liking (derived food-liking factor) \|\| id:ebi-fl187-GCST90094716 | Inverse variance weighted | 14 | -0.152573336 | 0.103102315993477 | 0.138920080593692 | -0.354653876 | 0.0495072030636967 | 0.858495932746629 | 0.701416178430214 | 1.05075316082951 | 0.998735809224318 |
| ebi-fl187-GCST90094716 | ebi-met1400-GCST90200724 | Cholate to taurocholate ratio \|\| id:ebi-met1400-GCST90200724 | F-burn/spicy liking (derived food-liking factor) \|\| id:ebi-fl187-GCST90094716 | Inverse variance weighted | 14 | -0.050777937 | 0.0767981393471142 | 0.508492350321364 | -0.20130229 | 0.0997464161511661 | 0.950489715725931 | 0.817665222098071 | 1.1048907001115 | 0.998735809224318 |
| ebi-fl187-GCST90094716 | ebi-met1400-GCST90200726 | N-acetylputrescine to (N(1) + N(8))-acetylspermidine ratio \|\| id:ebi-met1400-GCST90200726 | F-burn/spicy liking (derived food-liking factor) \|\| id:ebi-fl187-GCST90094716 | Inverse variance weighted | 14 | -0.038061201 | 0.0768936738239367 | 0.620610835847979 | -0.188772802 | 0.112650399395223 | 0.962654023410918 | 0.827974599504291 | 1.11924057736076 | 0.998735809224318 |
| ebi-fl187-GCST90094716 | ebi-met1400-GCST90200744 | Arginine to glutamate ratio \|\| id:ebi-met1400-GCST90200744 | F-burn/spicy liking (derived food-liking factor) \|\| id:ebi-fl187-GCST90094716 | Inverse variance weighted | 14 | 0.138493134413408 | 0.0664095744604907 | 0.0370294454462517 | 0.00833036847084656 | 0.26865590035597 | 1.14854179612995 | 1.00836516253897 | 1.30820491074476 | 0.998735809224318 |
| ebi-fl187-GCST90094716 | ebi-met1400-GCST90200759 | Histidine to trans-urocanate ratio \|\| id:ebi-met1400-GCST90200759 | F-burn/spicy liking (derived food-liking factor) \|\| id:ebi-fl187-GCST90094716 | Inverse variance weighted | 14 | -0.053724952 | 0.0785920021595958 | 0.494232133213408 | -0.207765276 | 0.100315372628235 | 0.94769273202314 | 0.812397703704705 | 1.1055195136986 | 0.998735809224318 |
| ebi-fl187-GCST90094716 | ebi-met1400-GCST90200795 | Oleoyl-linoleoyl-glycerol (18:1 to 18:2) [2] to linoleoyl-arachidonoyl-glycerol (18:2 to 20:4) [2] ratio \|\| id:ebi-met1400-GCST90200795 | F-burn/spicy liking (derived food-liking factor) \|\| id:ebi-fl187-GCST90094716 | Inverse variance weighted | 14 | -0.023494619 | 0.0853366747946238 | 0.783072867732285 | -0.190754502 | 0.143765263396343 | 0.976779230507293 | 0.826335427112999 | 1.15461304676696 | 0.998735809224318 |
| ebi-fl187-GCST90094716 | ebi-met1400-GCST90200811 | Choline to taurocholate ratio \|\| id:ebi-met1400-GCST90200811 | F-burn/spicy liking (derived food-liking factor) \|\| id:ebi-fl187-GCST90094716 | Inverse variance weighted | 14 | 0.0210778271000392 | 0.0720875070684139 | 0.769986746734856 | -0.120213687 | 0.16236934095413 | 1.02130153348118 | 0.886730933815784 | 1.17629461487549 | 0.998735809224318 |
| ebi-fl187-GCST90094716 | ebi-met1400-GCST90200831 | Cholate to phosphate ratio \|\| id:ebi-met1400-GCST90200831 | F-burn/spicy liking (derived food-liking factor) \|\| id:ebi-fl187-GCST90094716 | Inverse variance weighted | 14 | -0.056599313 | 0.0717737752600993 | 0.430357934920173 | -0.197275913 | 0.0840772862472261 | 0.944972631519727 | 0.820964087583249 | 1.08771295581278 | 0.998735809224318 |
| ebi-fl187-GCST90094716 | ebi-met1400-GCST90200841 | Adenosine 5'-diphosphate (ADP) to oxalate (ethanedioate) ratio \|\| id:ebi-met1400-GCST90200841 | F-burn/spicy liking (derived food-liking factor) \|\| id:ebi-fl187-GCST90094716 | Inverse variance weighted | 14 | -0.139937935 | 0.0941273695305245 | 0.137097503667419 | -0.324427579 | 0.0445517097615605 | 0.869412194210957 | 0.722941062014678 | 1.04555904092132 | 0.998735809224318 |
| ebi-fl187-GCST90094716 | ebi-met1400-GCST90200844 | Aspartate to phosphate ratio \|\| id:ebi-met1400-GCST90200844 | F-burn/spicy liking (derived food-liking factor) \|\| id:ebi-fl187-GCST90094716 | Inverse variance weighted | 14 | -0.095098713 | 0.0647458537865571 | 0.141887030894646 | -0.222000587 | 0.0318031602347132 | 0.909283171798207 | 0.800914894510964 | 1.03231428480331 | 0.998735809224318 |
| ebi-fl187-GCST90094716 | ebi-met1400-GCST90200870 | Adenosine 5'-monophosphate (AMP) to acetoacetate ratio \|\| id:ebi-met1400-GCST90200870 | F-burn/spicy liking (derived food-liking factor) \|\| id:ebi-fl187-GCST90094716 | Inverse variance weighted | 14 | -0.007678163 | 0.0797094384344988 | 0.923260948496856 | -0.163908662 | 0.148552336345413 | 0.992351238808502 | 0.848819547549706 | 1.16015351438063 | 0.998735809224318 |
| ebi-fl187-GCST90094716 | ebi-met1400-GCST90200887 | Cholesterol to cortisol ratio \|\| id:ebi-met1400-GCST90200887 | F-burn/spicy liking (derived food-liking factor) \|\| id:ebi-fl187-GCST90094716 | Inverse variance weighted | 14 | -0.017093632 | 0.0669381174257824 | 0.798441433036224 | -0.148292342 | 0.114105077929824 | 0.983051635013981 | 0.862179026627814 | 1.12086989738482 | 0.998735809224318 |
| ebi-fl187-GCST90094716 | ebi-met1400-GCST90200890 | Cortisol to taurocholate ratio \|\| id:ebi-met1400-GCST90200890 | F-burn/spicy liking (derived food-liking factor) \|\| id:ebi-fl187-GCST90094716 | Inverse variance weighted | 14 | 0.0446656811296492 | 0.0718316793173728 | 0.534066584694491 | -0.09612441 | 0.1854557725917 | 1.04567821150652 | 0.908351000788772 | 1.203766959 | 0.998735809224318 |
| ebi-fl187-GCST90094716 | ebi-met1400-GCST90200948 | Adenosine 5'-diphosphate (ADP) to mannitol to sorbitol ratio \|\| id:ebi-met1400-GCST90200948 | F-burn/spicy liking (derived food-liking factor) \|\| id:ebi-fl187-GCST90094716 | Inverse variance weighted (multiplicative random effects) | 14 | -0.083947053 | 0.145883150796231 | 0.564993514541488 | -0.369878028 | 0.201983922677325 | 0.919479938617939 | 0.690818585716772 | 1.2238283320702 | 0.998735809224318 |
| ebi-fl187-GCST90094716 | ebi-met1400-GCST90200972 | Leucine to N-palmitoyl-sphingosine (d18:1 to 16:0) ratio \|\| id:ebi-met1400-GCST90200972 | F-burn/spicy liking (derived food-liking factor) \|\| id:ebi-fl187-GCST90094716 | Inverse variance weighted | 14 | -0.026757134 | 0.0741458277989115 | 0.718195324448548 | -0.172082957 | 0.118568688017335 | 0.973597666128102 | 0.841909328037737 | 1.12588420619993 | 0.998735809224318 |
| ebi-fl187-GCST90094716 | ebi-met1400-GCST90200973 | Proline to glutamate ratio \|\| id:ebi-met1400-GCST90200973 | F-burn/spicy liking (derived food-liking factor) \|\| id:ebi-fl187-GCST90094716 | Inverse variance weighted | 14 | 0.0892452388549268 | 0.067504660366701 | 0.186148036128352 | -0.043063895 | 0.221554373173661 | 1.0933487550476 | 0.957850185829834 | 1.24801510491799 | 0.998735809224318 |
| ebi-fl187-GCST90094716 | ebi-met1400-GCST90200983 | Cholesterol to linoleoyl-arachidonoyl-glycerol (18:2 to 20:4) [1] ratio \|\| id:ebi-met1400-GCST90200983 | F-burn/spicy liking (derived food-liking factor) \|\| id:ebi-fl187-GCST90094716 | Inverse variance weighted | 14 | -0.076472559 | 0.0709651049177108 | 0.281208768740995 | -0.215564164 | 0.0626190469294182 | 0.926378334957225 | 0.806086546581028 | 1.06462119125797 | 0.998735809224318 |
| ebi-fl187-GCST90094716 | ebi-met1400-GCST90201001 | Serine to alpha-ketobutyrate ratio \|\| id:ebi-met1400-GCST90201001 | F-burn/spicy liking (derived food-liking factor) \|\| id:ebi-fl187-GCST90094716 | Inverse variance weighted | 14 | 0.0940302539013427 | 0.0700941349792428 | 0.179762929052818 | -0.043354251 | 0.231414758460659 | 1.09859298213811 | 0.957572109425563 | 1.26038188510639 | 0.998735809224318 |
| ebi-fl187-GCST90094716 | ebi-met1400-GCST90201005 | Fructose to maltose ratio \|\| id:ebi-met1400-GCST90201005 | F-burn/spicy liking (derived food-liking factor) \|\| id:ebi-fl187-GCST90094716 | Inverse variance weighted | 14 | 0.11634330204867 | 0.0785411528736552 | 0.138525657241583 | -0.037597358 | 0.270283961681035 | 1.12338146510045 | 0.963100648004356 | 1.31033648325976 | 0.998735809224318 |
| ebi-fl187-GCST90094716 | ebi-met1400-GCST90201020 | Paraxanthine to 5-acetylamino-6-formylamino-3-methyluracil ratio \|\| id:ebi-met1400-GCST90201020 | F-burn/spicy liking (derived food-liking factor) \|\| id:ebi-fl187-GCST90094716 | Inverse variance weighted | 14 | 0.0217485800245878 | 0.0728012152114871 | 0.765139010894232 | -0.120941802 | 0.164438961839102 | 1.02198680426961 | 0.88608552668393 | 1.17873161974553 | 0.998735809224318 |
| ebi-fl187-GCST90094716 | ebi-pw11-GCST90275197 | Placental weight (fetal genotype, adjusted for sex) \|\| id:ebi-pw11-GCST90275197 | F-burn/spicy liking (derived food-liking factor) \|\| id:ebi-fl187-GCST90094716 | Inverse variance weighted | 14 | -0.007246982 | 0.0291650448490545 | 0.803761675757572 | -0.06441047 | 0.0499165060305434 | 0.99277921418056 | 0.937620055836324 | 1.05118332524344 | 0.998735809224318 |
| ebi-fl187-GCST90094716 | prot-a-1017 | FAS-associated factor 2 \|\| id:prot-a-1017 | F-burn/spicy liking (derived food-liking factor) \|\| id:ebi-fl187-GCST90094716 | Inverse variance weighted | 15 | -0.085588798 | 0.11261791305492 | 0.44725891643092 | -0.306319907 | 0.135142311748976 | 0.917971625538618 | 0.736151081585067 | 1.14469967697336 | 0.998735809224318 |
| ebi-fl187-GCST90094716 | prot-a-1019 | Fas apoptotic inhibitory molecule 3 \|\| id:prot-a-1019 | F-burn/spicy liking (derived food-liking factor) \|\| id:ebi-fl187-GCST90094716 | Inverse variance weighted | 15 | 0.0460563090809541 | 0.11054081074459 | 0.676937870914728 | -0.17060368 | 0.26271629814035 | 1.04713337241473 | 0.843155666735339 | 1.30045772433718 | 0.998735809224318 |
| ebi-fl187-GCST90094716 | prot-a-1052 | MIP18 family protein FAM96A \|\| id:prot-a-1052 | F-burn/spicy liking (derived food-liking factor) \|\| id:ebi-fl187-GCST90094716 | Inverse variance weighted | 15 | -0.058255198 | 0.107264358153653 | 0.587061433380646 | -0.26849334 | 0.151982943966363 | 0.943409160569021 | 0.764530514569213 | 1.16414038064529 | 0.998735809224318 |
| ebi-fl187-GCST90094716 | prot-a-1066 | F-box/LRR-repeat protein 4 \|\| id:prot-a-1066 | F-burn/spicy liking (derived food-liking factor) \|\| id:ebi-fl187-GCST90094716 | Inverse variance weighted | 15 | -0.03973462 | 0.113209588642713 | 0.725601243552414 | -0.261625413 | 0.182156174028817 | 0.961044447567056 | 0.769799326180401 | 1.19980155709181 | 0.998735809224318 |
| ebi-fl187-GCST90094716 | prot-a-1076 | Low affinity immunoglobulin gamma Fc region receptor III-B \|\| id:prot-a-1076 | F-burn/spicy liking (derived food-liking factor) \|\| id:ebi-fl187-GCST90094716 | Inverse variance weighted | 15 | 0.0887609487628568 | 0.12000674022178 | 0.459522694738428 | -0.146452262 | 0.323974159597546 | 1.09281938527297 | 0.863766965795921 | 1.38261157941822 | 0.998735809224318 |
| ebi-fl187-GCST90094716 | prot-a-1081 | Fc receptor-like protein 3 \|\| id:prot-a-1081 | F-burn/spicy liking (derived food-liking factor) \|\| id:ebi-fl187-GCST90094716 | Inverse variance weighted | 15 | -0.139625279 | 0.107259637560949 | 0.193001713571727 | -0.349854169 | 0.0706036101349637 | 0.869684062808226 | 0.704790862507809 | 1.07315575348317 | 0.998735809224318 |
| ebi-fl187-GCST90094716 | prot-a-1105 | Fibroblast growth factor receptor 4 \|\| id:prot-a-1105 | F-burn/spicy liking (derived food-liking factor) \|\| id:ebi-fl187-GCST90094716 | Inverse variance weighted | 15 | 0.122243369967382 | 0.123694323025534 | 0.32302050756694 | -0.120197503 | 0.364684243097429 | 1.130029083 | 0.886745284423009 | 1.4400592277117 | 0.998735809224318 |
| ebi-fl187-GCST90094716 | prot-a-1143 | Follistatin-related protein 1 \|\| id:prot-a-1143 | F-burn/spicy liking (derived food-liking factor) \|\| id:ebi-fl187-GCST90094716 | Inverse variance weighted | 15 | -0.031885875 | 0.114193514675696 | 0.780070877996942 | -0.255705164 | 0.191933413988369 | 0.968617119417256 | 0.774370247720408 | 1.2115898393438 | 0.998735809224318 |
| ebi-fl187-GCST90094716 | prot-a-1151 | Alpha-(1,3)-fucosyltransferase 10 \|\| id:prot-a-1151 | F-burn/spicy liking (derived food-liking factor) \|\| id:ebi-fl187-GCST90094716 | Inverse variance weighted | 15 | 0.0122868669222848 | 0.10729218355228 | 0.908827321312951 | -0.198005813 | 0.222579546684754 | 1.01236266057574 | 0.820365084472843 | 1.24929519298907 | 0.998735809224318 |
| ebi-fl187-GCST90094716 | prot-a-1165 | Growth arrest and DNA damage-inducible proteins-interacting protein 1 \|\| id:prot-a-1165 | F-burn/spicy liking (derived food-liking factor) \|\| id:ebi-fl187-GCST90094716 | Inverse variance weighted | 15 | -0.009190845 | 0.107230630436261 | 0.931696154997952 | -0.219362881 | 0.200981190742127 | 0.990851261804652 | 0.803030261197475 | 1.22260177537499 | 0.998735809224318 |
| ebi-fl187-GCST90094716 | prot-a-1194 | Growth/differentiation factor 11/8 \|\| id:prot-a-1194 | F-burn/spicy liking (derived food-liking factor) \|\| id:ebi-fl187-GCST90094716 | Inverse variance weighted | 15 | 0.0957517659515211 | 0.108564713054023 | 0.377788318565813 | -0.117035072 | 0.308538603537406 | 1.10048585202678 | 0.889553994503879 | 1.36143406470402 | 0.998735809224318 |
| ebi-fl187-GCST90094716 | prot-a-1216 | D-glucuronyl C5-epimerase \|\| id:prot-a-1216 | F-burn/spicy liking (derived food-liking factor) \|\| id:ebi-fl187-GCST90094716 | Inverse variance weighted | 15 | -0.022090011 | 0.118377825994244 | 0.851969571307712 | -0.25411055 | 0.20993052774636 | 0.978152186434198 | 0.775606053940391 | 1.23359235653869 | 0.998735809224318 |
| ebi-fl187-GCST90094716 | prot-a-1246 | Glypican-3 \|\| id:prot-a-1246 | F-burn/spicy liking (derived food-liking factor) \|\| id:ebi-fl187-GCST90094716 | Inverse variance weighted | 15 | -0.088635085 | 0.120378678339591 | 0.461546810195258 | -0.324577295 | 0.14730712435341 | 0.915179475190228 | 0.722832834316146 | 1.15870977637845 | 0.998735809224318 |
| ebi-fl187-GCST90094716 | prot-a-1275 | Glutamate receptor 4 \|\| id:prot-a-1275 | F-burn/spicy liking (derived food-liking factor) \|\| id:ebi-fl187-GCST90094716 | Inverse variance weighted | 15 | -0.166367156 | 0.11552628086197 | 0.149844596056608 | -0.392798667 | 0.060064354226117 | 0.846735292944681 | 0.675164666997923 | 1.0619048824134 | 0.998735809224318 |
| ebi-fl187-GCST90094716 | prot-a-1289 | Glutathione S-transferase P \|\| id:prot-a-1289 | F-burn/spicy liking (derived food-liking factor) \|\| id:ebi-fl187-GCST90094716 | Inverse variance weighted | 15 | 0.0770953093134064 | 0.107264358153653 | 0.472300417052773 | -0.133142833 | 0.287333451294567 | 1.08014501936699 | 0.875340056023971 | 1.33286858613877 | 0.998735809224318 |
| ebi-fl187-GCST90094716 | prot-a-130 | Apolipoprotein D \|\| id:prot-a-130 | F-burn/spicy liking (derived food-liking factor) \|\| id:ebi-fl187-GCST90094716 | Inverse variance weighted | 15 | 0.147429975203561 | 0.11318800255177 | 0.192737665087708 | -0.07441851 | 0.369278460205031 | 1.15885213360373 | 0.928283116955662 | 1.44669039329523 | 0.998735809224318 |
| ebi-fl187-GCST90094716 | prot-a-1313 | Heparin-binding EGF-like growth factor \|\| id:prot-a-1313 | F-burn/spicy liking (derived food-liking factor) \|\| id:ebi-fl187-GCST90094716 | Inverse variance weighted | 15 | -0.081215786 | 0.108900300576154 | 0.455799660901935 | -0.294660375 | 0.132228803544771 | 0.921994716808588 | 0.744784492163408 | 1.14136943876705 | 0.998735809224318 |
| ebi-fl187-GCST90094716 | prot-a-1347 | HLA class II histocompatibility antigen, DQ alpha 2 chain \|\| id:prot-a-1347 | F-burn/spicy liking (derived food-liking factor) \|\| id:ebi-fl187-GCST90094716 | Inverse variance weighted | 15 | 0.013342244156008 | 0.107245694045902 | 0.900992078160477 | -0.196859316 | 0.223543804485975 | 1.01343164907383 | 0.821306169679719 | 1.25050041660469 | 0.998735809224318 |
| ebi-fl187-GCST90094716 | prot-a-1357 | Non-histone chromosomal protein HMG-14 \|\| id:prot-a-1357 | F-burn/spicy liking (derived food-liking factor) \|\| id:ebi-fl187-GCST90094716 | Inverse variance weighted | 15 | -0.05275872 | 0.127967725162604 | 0.680133140095509 | -0.303575461 | 0.198058021452099 | 0.948608865345933 | 0.738174183531166 | 1.21903312184164 | 0.998735809224318 |
| ebi-fl187-GCST90094716 | prot-a-137 | Apolipoprotein M \|\| id:prot-a-137 | F-burn/spicy liking (derived food-liking factor) \|\| id:ebi-fl187-GCST90094716 | Inverse variance weighted | 15 | -0.024842691 | 0.117351535102922 | 0.832345266914699 | -0.2548517 | 0.205166317361211 | 0.975463348693778 | 0.77503142641173 | 1.22772924067131 | 0.998735809224318 |
| ebi-fl187-GCST90094716 | prot-a-1381 | 17-beta-hydroxysteroid dehydrogenase 14 \|\| id:prot-a-1381 | F-burn/spicy liking (derived food-liking factor) \|\| id:ebi-fl187-GCST90094716 | Inverse variance weighted | 15 | -0.028352884 | 0.11249120696404 | 0.801006021509113 | -0.24883565 | 0.192129881576248 | 0.972045286970834 | 0.779708108098634 | 1.21182790086195 | 0.998735809224318 |
| ebi-fl187-GCST90094716 | prot-a-1392 | Serine protease HTRA2, mitochondrial \|\| id:prot-a-1392 | F-burn/spicy liking (derived food-liking factor) \|\| id:ebi-fl187-GCST90094716 | Inverse variance weighted | 15 | -0.075133487 | 0.128913907803452 | 0.560015089784631 | -0.327804746 | 0.177537772349625 | 0.927619652949942 | 0.720503687025995 | 1.19427316755414 | 0.998735809224318 |
| ebi-fl187-GCST90094716 | prot-a-1416 | Interferon-induced protein with tetratricopeptide repeats 2 \|\| id:prot-a-1416 | F-burn/spicy liking (derived food-liking factor) \|\| id:ebi-fl187-GCST90094716 | Inverse variance weighted | 15 | 0.0279336444813411 | 0.126355833596522 | 0.825036940803726 | -0.219723789 | 0.275591078330525 | 1.02832744695639 | 0.802740492802809 | 1.31730907764685 | 0.998735809224318 |
| ebi-fl187-GCST90094716 | prot-a-1428 | Interferon gamma \|\| id:prot-a-1428 | F-burn/spicy liking (derived food-liking factor) \|\| id:ebi-fl187-GCST90094716 | Inverse variance weighted | 15 | 0.00198768797364511 | 0.107304375500778 | 0.985220967505279 | -0.208328888 | 0.21230426395517 | 1.0019896647349 | 0.811939955479964 | 1.23652405754815 | 0.998955228396052 |
| ebi-fl187-GCST90094716 | prot-a-1483 | Interleukin-17C \|\| id:prot-a-1483 | F-burn/spicy liking (derived food-liking factor) \|\| id:ebi-fl187-GCST90094716 | Inverse variance weighted | 15 | 0.141285056839854 | 0.137641316167953 | 0.304668813736429 | -0.128491923 | 0.411062036529042 | 1.15175291624156 | 0.879420665602943 | 1.50841893073032 | 0.998735809224318 |
| ebi-fl187-GCST90094716 | prot-a-1503 | Interleukin-1 receptor-like 2 \|\| id:prot-a-1503 | F-burn/spicy liking (derived food-liking factor) \|\| id:ebi-fl187-GCST90094716 | Inverse variance weighted | 15 | 0.0401066589873174 | 0.107258429588462 | 0.708459764410977 | -0.170119863 | 0.250333180980703 | 1.04092179193597 | 0.843563698455636 | 1.28445330081279 | 0.998735809224318 |
| ebi-fl187-GCST90094716 | prot-a-1522 | Interleukin-31 receptor subunit alpha \|\| id:prot-a-1522 | F-burn/spicy liking (derived food-liking factor) \|\| id:ebi-fl187-GCST90094716 | Inverse variance weighted | 15 | -0.03459003 | 0.107230630436261 | 0.747016359349393 | -0.244762065 | 0.17558200601037 | 0.966001367011637 | 0.782890792968894 | 1.19193973086541 | 0.998735809224318 |
| ebi-fl187-GCST90094716 | prot-a-1540 | Interleukin-6 receptor subunit alpha \|\| id:prot-a-1540 | F-burn/spicy liking (derived food-liking factor) \|\| id:ebi-fl187-GCST90094716 | Inverse variance weighted | 15 | -0.035788662 | 0.129090204974974 | 0.781597650300872 | -0.288805464 | 0.217228139815758 | 0.964844180239743 | 0.749157929650015 | 1.24262756262542 | 0.998735809224318 |
| ebi-fl187-GCST90094716 | prot-a-1541 | Interleukin-6 receptor subunit alpha \|\| id:prot-a-1541 | F-burn/spicy liking (derived food-liking factor) \|\| id:ebi-fl187-GCST90094716 | Inverse variance weighted (multiplicative random effects) | 15 | -0.034654535 | 0.144003272369767 | 0.809825600756362 | -0.316900949 | 0.247591878913498 | 0.965939056826346 | 0.728402900751479 | 1.28093704808146 | 0.998735809224318 |
| ebi-fl187-GCST90094716 | prot-a-1545 | Interleukin-9 \|\| id:prot-a-1545 | F-burn/spicy liking (derived food-liking factor) \|\| id:ebi-fl187-GCST90094716 | Inverse variance weighted | 15 | -0.060465289 | 0.107219670227091 | 0.572796054236735 | -0.270615843 | 0.149685264191124 | 0.941326442406152 | 0.762909517074274 | 1.16146862942694 | 0.998735809224318 |
| ebi-fl187-GCST90094716 | prot-a-1548 | Inositol monophosphatase 3 \|\| id:prot-a-1548 | F-burn/spicy liking (derived food-liking factor) \|\| id:ebi-fl187-GCST90094716 | Inverse variance weighted | 15 | -0.054376569 | 0.107276540617871 | 0.61223747395024 | -0.264638589 | 0.155885450411641 | 0.947075399918551 | 0.767483276902402 | 1.16869232219759 | 0.998735809224318 |
| ebi-fl187-GCST90094716 | prot-a-1555 | Inhibin beta A chain:Inhibin beta B chain heterodimer \|\| id:prot-a-1555 | F-burn/spicy liking (derived food-liking factor) \|\| id:ebi-fl187-GCST90094716 | Inverse variance weighted | 15 | -0.177665155 | 0.107264358153653 | 0.0976550452989806 | -0.387903297 | 0.032572987230291 | 0.83722271660786 | 0.678477951126925 | 1.03310929417825 | 0.998735809224318 |
| ebi-fl187-GCST90094716 | prot-a-1603 | Junctophilin-3 \|\| id:prot-a-1603 | F-burn/spicy liking (derived food-liking factor) \|\| id:ebi-fl187-GCST90094716 | Inverse variance weighted (multiplicative random effects) | 15 | 0.000497963951680341 | 0.147420079044495 | 0.997304865145249 | -0.288445391 | 0.289441318878891 | 1.00049808795631 | 0.749427729547293 | 1.33568105974529 | 0.999604360968858 |
| ebi-fl187-GCST90094716 | prot-a-1627 | UPF0258 protein KIAA1024 \|\| id:prot-a-1627 | F-burn/spicy liking (derived food-liking factor) \|\| id:ebi-fl187-GCST90094716 | Inverse variance weighted | 15 | -0.075258543 | 0.123603546777107 | 0.542610335639554 | -0.317521494 | 0.16700440888159 | 0.927503655933035 | 0.727951033673817 | 1.18175947553447 | 0.998735809224318 |
| ebi-fl187-GCST90094716 | prot-a-1666 | Kallikrein-7 \|\| id:prot-a-1666 | F-burn/spicy liking (derived food-liking factor) \|\| id:ebi-fl187-GCST90094716 | Inverse variance weighted | 15 | 0.0331046990001036 | 0.126153094064039 | 0.793000074537019 | -0.214155365 | 0.280364763365619 | 1.03365875661435 | 0.807222960789371 | 1.32361252965438 | 0.998735809224318 |
| ebi-fl187-GCST90094716 | prot-a-1678 | Importin subunit alpha-3 \|\| id:prot-a-1678 | F-burn/spicy liking (derived food-liking factor) \|\| id:ebi-fl187-GCST90094716 | Inverse variance weighted | 15 | -0.01915387 | 0.107264358153653 | 0.858277741443691 | -0.229392012 | 0.19108427188697 | 0.981028399696528 | 0.795016816218551 | 1.21056146408174 | 0.998735809224318 |
| ebi-fl187-GCST90094716 | prot-a-1718 | Protein LDOC1 \|\| id:prot-a-1718 | F-burn/spicy liking (derived food-liking factor) \|\| id:ebi-fl187-GCST90094716 | Inverse variance weighted | 15 | -0.039019237 | 0.107264358153653 | 0.71603198115663 | -0.249257379 | 0.171218904499707 | 0.961732207662899 | 0.779379351329192 | 1.18675050561544 | 0.998735809224318 |
| ebi-fl187-GCST90094716 | prot-a-1723 | Leptin \|\| id:prot-a-1723 | F-burn/spicy liking (derived food-liking factor) \|\| id:ebi-fl187-GCST90094716 | Inverse variance weighted (multiplicative random effects) | 15 | 0.0185277296784287 | 0.139740431987153 | 0.894520212105275 | -0.255363517 | 0.292418976373249 | 1.01870043301334 | 0.774634853822109 | 1.33966418771532 | 0.998735809224318 |
| ebi-fl187-GCST90094716 | prot-a-1730 | Galectin-7 \|\| id:prot-a-1730 | F-burn/spicy liking (derived food-liking factor) \|\| id:ebi-fl187-GCST90094716 | Inverse variance weighted | 15 | 0.10533993251725 | 0.107259637560949 | 0.326049471919822 | -0.104888957 | 0.315568822136711 | 1.11108824119028 | 0.900424502781147 | 1.37103896650775 | 0.998735809224318 |
| ebi-fl187-GCST90094716 | prot-a-1753 | Lipase member N \|\| id:prot-a-1753 | F-burn/spicy liking (derived food-liking factor) \|\| id:ebi-fl187-GCST90094716 | Inverse variance weighted | 15 | 0.050337506936688 | 0.107792458896704 | 0.640510307815914 | -0.160935713 | 0.261610726374228 | 1.05162596754576 | 0.85134680030463 | 1.29902076946885 | 0.998735809224318 |
| ebi-fl187-GCST90094716 | prot-a-1756 | VIP36-like protein \|\| id:prot-a-1756 | F-burn/spicy liking (derived food-liking factor) \|\| id:ebi-fl187-GCST90094716 | Inverse variance weighted | 15 | 0.134986254230258 | 0.107304375500778 | 0.208400688282645 | -0.075330322 | 0.345302830211782 | 1.14452105192034 | 0.927437083084036 | 1.41241757762467 | 0.998735809224318 |
| ebi-fl187-GCST90094716 | prot-a-1760 | Ig Kappa chain V-I region HK102- like \|\| id:prot-a-1760 | F-burn/spicy liking (derived food-liking factor) \|\| id:ebi-fl187-GCST90094716 | Inverse variance weighted | 15 | 0.145875706371489 | 0.129522550691515 | 0.260056664770163 | -0.107988493 | 0.399739905726857 | 1.15705236487602 | 0.897637925505085 | 1.49143673303675 | 0.998735809224318 |
| ebi-fl187-GCST90094716 | prot-a-179 | Neutral ceramidase \|\| id:prot-a-179 | F-burn/spicy liking (derived food-liking factor) \|\| id:ebi-fl187-GCST90094716 | Inverse variance weighted | 15 | 0.173682739432681 | 0.130355781357787 | 0.182737105728909 | -0.081814592 | 0.429180070893943 | 1.18967806800216 | 0.92144278569719 | 1.53599759795664 | 0.998735809224318 |
| ebi-fl187-GCST90094716 | prot-a-1809 | Lactotransferrin \|\| id:prot-a-1809 | F-burn/spicy liking (derived food-liking factor) \|\| id:ebi-fl187-GCST90094716 | Inverse variance weighted | 15 | 0.119235112034989 | 0.114887052039102 | 0.299341576262723 | -0.10594351 | 0.344413734031629 | 1.12663477254598 | 0.899475458043902 | 1.41116236063863 | 0.998735809224318 |
| ebi-fl187-GCST90094716 | prot-a-1818 | Lysophospholipase-like protein 1 \|\| id:prot-a-1818 | F-burn/spicy liking (derived food-liking factor) \|\| id:ebi-fl187-GCST90094716 | Inverse variance weighted | 15 | -0.106923702 | 0.107219670227091 | 0.318648219127532 | -0.317074256 | 0.103226851605986 | 0.898594231282356 | 0.728276674435073 | 1.10874290065693 | 0.998735809224318 |
| ebi-fl187-GCST90094716 | prot-a-1852 | MAP kinase-activated protein kinase 3 \|\| id:prot-a-1852 | F-burn/spicy liking (derived food-liking factor) \|\| id:ebi-fl187-GCST90094716 | Inverse variance weighted | 15 | -0.069816745 | 0.115564817233501 | 0.54575392400921 | -0.296323787 | 0.156690296556204 | 0.932564701185856 | 0.74354663830527 | 1.16963331833505 | 0.998735809224318 |
| ebi-fl187-GCST90094716 | prot-a-1856 | Megakaryocyte-associated tyrosine-protein kinase \|\| id:prot-a-1856 | F-burn/spicy liking (derived food-liking factor) \|\| id:ebi-fl187-GCST90094716 | Inverse variance weighted | 15 | 0.102531453389542 | 0.116850450671627 | 0.380237500704697 | -0.12649543 | 0.33155833670593 | 1.10797215084141 | 0.881178176583042 | 1.39313741495553 | 0.998735809224318 |
| ebi-fl187-GCST90094716 | prot-a-1890 | Beta-1,3-N-acetylglucosaminyltransferase manic fringe \|\| id:prot-a-1890 | F-burn/spicy liking (derived food-liking factor) \|\| id:ebi-fl187-GCST90094716 | Inverse variance weighted | 15 | 0.127426324641256 | 0.108031321071074 | 0.238186680538812 | -0.084315065 | 0.339167713940562 | 1.13590117723149 | 0.919141621430042 | 1.40377875873841 | 0.998735809224318 |
| ebi-fl187-GCST90094716 | prot-a-1919 | Matrilysin \|\| id:prot-a-1919 | F-burn/spicy liking (derived food-liking factor) \|\| id:ebi-fl187-GCST90094716 | Inverse variance weighted | 15 | -0.100308141 | 0.107264358153653 | 0.349711597055131 | -0.310546283 | 0.109930001335018 | 0.904558643802354 | 0.733046396313477 | 1.1161999352189 | 0.998735809224318 |
| ebi-fl187-GCST90094716 | prot-a-1923 | Multimerin-2 \|\| id:prot-a-1923 | F-burn/spicy liking (derived food-liking factor) \|\| id:ebi-fl187-GCST90094716 | Inverse variance weighted | 15 | -0.129736737 | 0.107304375500778 | 0.226642269805235 | -0.340053313 | 0.080579838972506 | 0.878326631 | 0.711732377169628 | 1.08391538187814 | 0.998735809224318 |
| ebi-fl187-GCST90094716 | prot-a-1948 | Mesothelin \|\| id:prot-a-1948 | F-burn/spicy liking (derived food-liking factor) \|\| id:ebi-fl187-GCST90094716 | Inverse variance weighted | 15 | 0.0668245470235432 | 0.107219670227091 | 0.533120938313356 | -0.143326007 | 0.276975100668641 | 1.06910788355774 | 0.866471547384214 | 1.31913352508329 | 0.998735809224318 |
| ebi-fl187-GCST90094716 | prot-a-195 | ATPase family AAA domain-containing protein 1 \|\| id:prot-a-195 | F-burn/spicy liking (derived food-liking factor) \|\| id:ebi-fl187-GCST90094716 | Inverse variance weighted | 15 | -0.195696561 | 0.110911775960344 | 0.0776588580031382 | -0.413083642 | 0.0216905195802276 | 0.822261702847613 | 0.661606942263664 | 1.02192746898416 | 0.998735809224318 |
| ebi-fl187-GCST90094716 | prot-a-1966 | Mucin-1 \|\| id:prot-a-1966 | F-burn/spicy liking (derived food-liking factor) \|\| id:ebi-fl187-GCST90094716 | Inverse variance weighted | 15 | 0.0891056623452739 | 0.112946823984302 | 0.430160556251535 | -0.132270113 | 0.310481437354505 | 1.09319615989413 | 0.876104316245072 | 1.3640816759462 | 0.998735809224318 |
| ebi-fl187-GCST90094716 | prot-a-1986 | Myocardial zonula adherens protein \|\| id:prot-a-1986 | F-burn/spicy liking (derived food-liking factor) \|\| id:ebi-fl187-GCST90094716 | Inverse variance weighted | 15 | -0.042783101 | 0.120791653621362 | 0.723197086934304 | -0.279534742 | 0.193968540341536 | 0.958119182857271 | 0.756135457811005 | 1.21405808850262 | 0.998735809224318 |
| ebi-fl187-GCST90094716 | prot-a-2071 | Epididymal secretory protein E1 \|\| id:prot-a-2071 | F-burn/spicy liking (derived food-liking factor) \|\| id:ebi-fl187-GCST90094716 | Inverse variance weighted | 14 | -0.190894277 | 0.116586529436141 | 0.101554988791754 | -0.419403875 | 0.0376153203562634 | 0.826219933704226 | 0.657438618597484 | 1.03833173096295 | 0.998735809224318 |
| ebi-fl187-GCST90094716 | prot-a-2098 | Neuritin \|\| id:prot-a-2098 | F-burn/spicy liking (derived food-liking factor) \|\| id:ebi-fl187-GCST90094716 | Inverse variance weighted | 15 | -0.044235539 | 0.107264358153653 | 0.680048189453486 | -0.254473681 | 0.166002602626153 | 0.95672858369837 | 0.775324458326028 | 1.1805761743177 | 0.998735809224318 |
| ebi-fl187-GCST90094716 | prot-a-2115 | Neurotrophin-4 \|\| id:prot-a-2115 | F-burn/spicy liking (derived food-liking factor) \|\| id:ebi-fl187-GCST90094716 | Inverse variance weighted | 15 | 0.0560456154189593 | 0.107264358153653 | 0.60132232248495 | -0.154192527 | 0.26628375740012 | 1.05764592759837 | 0.857106989263388 | 1.30510533944749 | 0.998735809224318 |
| ebi-fl187-GCST90094716 | prot-a-2118 | Netrin-1 \|\| id:prot-a-2118 | F-burn/spicy liking (derived food-liking factor) \|\| id:ebi-fl187-GCST90094716 | Inverse variance weighted | 15 | 0.141312507795234 | 0.107304375500778 | 0.187861600329712 | -0.069004068 | 0.351629083776759 | 1.15178453339343 | 0.93332288313912 | 1.42138121257934 | 0.998735809224318 |
| ebi-fl187-GCST90094716 | prot-a-2120 | Netrin-G1 \|\| id:prot-a-2120 | F-burn/spicy liking (derived food-liking factor) \|\| id:ebi-fl187-GCST90094716 | Inverse variance weighted | 15 | 0.0276301640100054 | 0.10729218355228 | 0.796775357215529 | -0.182662516 | 0.237922843772474 | 1.02801541700799 | 0.833049249301159 | 1.26861130778603 | 0.998735809224318 |
| ebi-fl187-GCST90094716 | prot-a-2145 | Olfactomedin-like protein 3 \|\| id:prot-a-2145 | F-burn/spicy liking (derived food-liking factor) \|\| id:ebi-fl187-GCST90094716 | Inverse variance weighted | 15 | -0.08994086 | 0.126420744095612 | 0.476811201039668 | -0.337725518 | 0.157843798912417 | 0.91398523720311 | 0.713391074077055 | 1.17098327156108 | 0.998735809224318 |
| ebi-fl187-GCST90094716 | prot-a-2162 | Prolyl 4-hydroxylase subunit alpha-2 \|\| id:prot-a-2162 | F-burn/spicy liking (derived food-liking factor) \|\| id:ebi-fl187-GCST90094716 | Inverse variance weighted | 15 | 0.0559166846903907 | 0.126016220596716 | 0.657240631118513 | -0.191075108 | 0.302908477059954 | 1.05750957332867 | 0.826070541579925 | 1.35379055588026 | 0.998735809224318 |
| ebi-fl187-GCST90094716 | prot-a-2168 | Polyadenylate-binding protein-interacting protein 1 \|\| id:prot-a-2168 | F-burn/spicy liking (derived food-liking factor) \|\| id:ebi-fl187-GCST90094716 | Inverse variance weighted | 15 | -0.226394646 | 0.136616316732344 | 0.0974877964636435 | -0.494162627 | 0.0413733350188093 | 0.797403347698193 | 0.610081559550049 | 1.04224113803611 | 0.998735809224318 |
| ebi-fl187-GCST90094716 | prot-a-219 | Beta-1,4-galactosyltransferase 5 \|\| id:prot-a-219 | F-burn/spicy liking (derived food-liking factor) \|\| id:ebi-fl187-GCST90094716 | Inverse variance weighted | 15 | -0.044858986 | 0.10729218355228 | 0.675873162784976 | -0.255151665 | 0.165433694225244 | 0.956132300809904 | 0.774798978930185 | 1.17990472562873 | 0.998735809224318 |
| ebi-fl187-GCST90094716 | prot-a-2190 | Pyruvate carboxylase, mitochondrial \|\| id:prot-a-2190 | F-burn/spicy liking (derived food-liking factor) \|\| id:ebi-fl187-GCST90094716 | Inverse variance weighted | 15 | -0.128995541 | 0.113021360786035 | 0.253729416019932 | -0.350517408 | 0.0925263262642543 | 0.878977885007898 | 0.704323572761737 | 1.09694201956566 | 0.998735809224318 |
| ebi-fl187-GCST90094716 | prot-a-2197 | Protocadherin-9 \|\| id:prot-a-2197 | F-burn/spicy liking (derived food-liking factor) \|\| id:ebi-fl187-GCST90094716 | Inverse variance weighted | 15 | -0.095270662 | 0.107304375500778 | 0.374619077639883 | -0.305587238 | 0.115045914356509 | 0.90912683541833 | 0.736690634898413 | 1.12192494885145 | 0.998735809224318 |
| ebi-fl187-GCST90094716 | prot-a-2198 | Protocadherin alpha-4 \|\| id:prot-a-2198 | F-burn/spicy liking (derived food-liking factor) \|\| id:ebi-fl187-GCST90094716 | Inverse variance weighted | 15 | -0.010072902 | 0.135513873040837 | 0.940746891778011 | -0.275680093 | 0.255534289188995 | 0.989977659795697 | 0.759055719062764 | 1.29115128478932 | 0.998735809224318 |
| ebi-fl187-GCST90094716 | prot-a-2254 | Placenta growth factor \|\| id:prot-a-2254 | F-burn/spicy liking (derived food-liking factor) \|\| id:ebi-fl187-GCST90094716 | Inverse variance weighted | 15 | -0.275593249 | 0.133160108204926 | 0.0384862366850848 | -0.536587061 | -0.014599436 | 0.759121641816099 | 0.584740534673301 | 0.985506618581752 | 0.998735809224318 |
| ebi-fl187-GCST90094716 | prot-a-2261 | Membrane-associated progesterone receptor component 2 \|\| id:prot-a-2261 | F-burn/spicy liking (derived food-liking factor) \|\| id:ebi-fl187-GCST90094716 | Inverse variance weighted | 15 | 0.104650249498922 | 0.107264358153653 | 0.329248204727215 | -0.105587892 | 0.314888391480083 | 1.11032220668933 | 0.899795384121365 | 1.37010638687737 | 0.998735809224318 |
| ebi-fl187-GCST90094716 | prot-a-2268 | Polymeric immunoglobulin receptor \|\| id:prot-a-2268 | F-burn/spicy liking (derived food-liking factor) \|\| id:ebi-fl187-GCST90094716 | Inverse variance weighted | 15 | 0.0514965232142039 | 0.12790240107093 | 0.687225156760097 | -0.199192183 | 0.302185229313227 | 1.0528455257674 | 0.819392405003937 | 1.35281178390115 | 0.998735809224318 |
| ebi-fl187-GCST90094716 | prot-a-2283 | Group 10 secretory phospholipase A2 \|\| id:prot-a-2283 | F-burn/spicy liking (derived food-liking factor) \|\| id:ebi-fl187-GCST90094716 | Inverse variance weighted | 15 | 0.0537928620192607 | 0.129992168708349 | 0.679008737275898 | -0.200991789 | 0.308577512687625 | 1.05526599384771 | 0.817919147747412 | 1.36148703797713 | 0.998735809224318 |
| ebi-fl187-GCST90094716 | prot-a-2298 | Inactive phospholipase D5 \|\| id:prot-a-2298 | F-burn/spicy liking (derived food-liking factor) \|\| id:ebi-fl187-GCST90094716 | Inverse variance weighted (multiplicative random effects) | 15 | 0.0835776809592884 | 0.141020716050835 | 0.553407086119476 | -0.192822923 | 0.359978284418925 | 1.08716966439523 | 0.82462798427277 | 1.43329828931718 | 0.998735809224318 |
| ebi-fl187-GCST90094716 | prot-a-2304 | Procollagen-lysine,2-oxoglutarate 5-dioxygenase 2 \|\| id:prot-a-2304 | F-burn/spicy liking (derived food-liking factor) \|\| id:ebi-fl187-GCST90094716 | Inverse variance weighted | 15 | -0.126645196 | 0.119602214838455 | 0.289651374805728 | -0.361065538 | 0.107775144591668 | 0.881046215436438 | 0.696933321650225 | 1.11379727388677 | 0.998735809224318 |
| ebi-fl187-GCST90094716 | prot-a-2331 | Periostin \|\| id:prot-a-2331 | F-burn/spicy liking (derived food-liking factor) \|\| id:ebi-fl187-GCST90094716 | Inverse variance weighted | 15 | 0.034115117226595 | 0.116506501712845 | 0.769661817444698 | -0.194237626 | 0.26246786058377 | 1.03470371209413 | 0.823462204880429 | 1.3001346819273 | 0.998735809224318 |
| ebi-fl187-GCST90094716 | prot-a-2333 | Periostin \|\| id:prot-a-2333 | F-burn/spicy liking (derived food-liking factor) \|\| id:ebi-fl187-GCST90094716 | Inverse variance weighted | 15 | 0.0778562743593453 | 0.119154235540026 | 0.513493587654084 | -0.155686027 | 0.311398576017796 | 1.08096728478921 | 0.855827854775231 | 1.36533330186062 | 0.998735809224318 |
| ebi-fl187-GCST90094716 | prot-a-2367 | cAMP-dependent protein kinase catalytic subunit alpha \|\| id:prot-a-2367 | F-burn/spicy liking (derived food-liking factor) \|\| id:ebi-fl187-GCST90094716 | Inverse variance weighted | 15 | -0.057527989 | 0.117114536886773 | 0.623276978108832 | -0.287072481 | 0.172016503514623 | 0.944095465933076 | 0.750457332906626 | 1.18769743423414 | 0.998735809224318 |
| ebi-fl187-GCST90094716 | prot-a-2385 | Phosphoribosyl pyrophosphate synthase-associated protein 1 \|\| id:prot-a-2385 | F-burn/spicy liking (derived food-liking factor) \|\| id:ebi-fl187-GCST90094716 | Inverse variance weighted | 15 | 0.265831823353217 | 0.10718598463331 | 0.0131346011965068 | 0.0557472934719293 | 0.475916353234504 | 1.30451565116994 | 1.05733045566442 | 1.60948838183042 | 0.998735809224318 |
| ebi-fl187-GCST90094716 | prot-a-2393 | Trypsin-3 \|\| id:prot-a-2393 | F-burn/spicy liking (derived food-liking factor) \|\| id:ebi-fl187-GCST90094716 | Inverse variance weighted | 15 | 0.16994700825888 | 0.1182964527852 | 0.150826097879452 | -0.061914039 | 0.401808055717872 | 1.18524204161675 | 0.939963683348457 | 1.49452443972249 | 0.998735809224318 |
| ebi-fl187-GCST90094716 | prot-a-2405 | Pregnancy-specific beta-1-glycoprotein 6 \|\| id:prot-a-2405 | F-burn/spicy liking (derived food-liking factor) \|\| id:ebi-fl187-GCST90094716 | Inverse variance weighted | 15 | 0.0772913070005343 | 0.112128809527168 | 0.490629343645477 | -0.14248116 | 0.297063773673784 | 1.08035674604084 | 0.867203892543504 | 1.34590112977081 | 0.998735809224318 |
| ebi-fl187-GCST90094716 | prot-a-244 | Beclin-1 \|\| id:prot-a-244 | F-burn/spicy liking (derived food-liking factor) \|\| id:ebi-fl187-GCST90094716 | Inverse variance weighted | 15 | 0.0451281241353562 | 0.107276540617871 | 0.673995377618369 | -0.165133895 | 0.255390143746384 | 1.04616188990988 | 0.847780182557267 | 1.29096518462896 | 0.998735809224318 |
| ebi-fl187-GCST90094716 | prot-a-2454 | tRNA pseudouridine synthase A, mitochondrial \|\| id:prot-a-2454 | F-burn/spicy liking (derived food-liking factor) \|\| id:ebi-fl187-GCST90094716 | Inverse variance weighted | 15 | -0.234448698 | 0.107259637560949 | 0.028829843647742 | -0.444677587 | -0.024219808 | 0.791006813279627 | 0.64103091918316 | 0.976071137804219 | 0.998735809224318 |
| ebi-fl187-GCST90094716 | prot-a-2466 | Glutaminyl-peptide cyclotransferase-like protein \|\| id:prot-a-2466 | F-burn/spicy liking (derived food-liking factor) \|\| id:ebi-fl187-GCST90094716 | Inverse variance weighted | 15 | -0.15561124 | 0.109020288253405 | 0.15347604426763 | -0.369291005 | 0.0580685247115126 | 0.855891861995483 | 0.691224231305043 | 1.05978761486273 | 0.998735809224318 |
| ebi-fl187-GCST90094716 | prot-a-2474 | Ras-related protein Rab-27A \|\| id:prot-a-2474 | F-burn/spicy liking (derived food-liking factor) \|\| id:ebi-fl187-GCST90094716 | Inverse variance weighted | 15 | 0.167830682064306 | 0.120753778916672 | 0.164571809379124 | -0.068846725 | 0.404508088740983 | 1.18273633521843 | 0.933469747050918 | 1.4985651576447 | 0.998735809224318 |
| ebi-fl187-GCST90094716 | prot-a-2490 | Rap1 GTPase-GDP dissociation stimulator 1 \|\| id:prot-a-2490 | F-burn/spicy liking (derived food-liking factor) \|\| id:ebi-fl187-GCST90094716 | Inverse variance weighted | 15 | -0.082654431 | 0.10970565042483 | 0.451197566454993 | -0.297677506 | 0.132368643382581 | 0.920669246590885 | 0.742540765870783 | 1.14152905884461 | 0.998735809224318 |
| ebi-fl187-GCST90094716 | prot-a-2545 | Receptor-interacting serine/threonine-protein kinase 2 \|\| id:prot-a-2545 | F-burn/spicy liking (derived food-liking factor) \|\| id:ebi-fl187-GCST90094716 | Inverse variance weighted | 15 | 0.0754652320769505 | 0.111544495827218 | 0.498692630521002 | -0.14316198 | 0.294092443898298 | 1.07838573383405 | 0.866613683663043 | 1.34190794913524 | 0.998735809224318 |
| ebi-fl187-GCST90094716 | prot-a-2610 | RUN and FYVE domain-containing protein 1 \|\| id:prot-a-2610 | F-burn/spicy liking (derived food-liking factor) \|\| id:ebi-fl187-GCST90094716 | Inverse variance weighted | 15 | 0.106585341168036 | 0.107304375500778 | 0.320564208952207 | -0.103731235 | 0.316901917149561 | 1.11247286212813 | 0.901467547959152 | 1.37286791052364 | 0.998735809224318 |
| ebi-fl187-GCST90094716 | prot-a-2627 | Serine--tRNA ligase, mitochondrial \|\| id:prot-a-2627 | F-burn/spicy liking (derived food-liking factor) \|\| id:ebi-fl187-GCST90094716 | Inverse variance weighted | 15 | -0.184218045 | 0.107264358153653 | 0.0859023046426962 | -0.394456187 | 0.0260200967428265 | 0.831754423961274 | 0.67404649469673 | 1.0263615747908 | 0.998735809224318 |
| ebi-fl187-GCST90094716 | prot-a-264 | BolA-like protein 2 \|\| id:prot-a-264 | F-burn/spicy liking (derived food-liking factor) \|\| id:ebi-fl187-GCST90094716 | Inverse variance weighted | 15 | 0.00804647361387003 | 0.129459129119368 | 0.950439758159342 | -0.245693419 | 0.261786366687831 | 1.00807893348677 | 0.782161983814189 | 1.29924894992243 | 0.998735809224318 |
| ebi-fl187-GCST90094716 | prot-a-2646 | Secretoglobin family 1C member 1 \|\| id:prot-a-2646 | F-burn/spicy liking (derived food-liking factor) \|\| id:ebi-fl187-GCST90094716 | Inverse variance weighted | 15 | -0.262426636 | 0.107245694045902 | 0.014406417297413 | -0.472628196 | -0.052225075 | 0.769182792988256 | 0.623361796594842 | 0.949115220504532 | 0.998735809224318 |
| ebi-fl187-GCST90094716 | prot-a-2672 | Semaphorin-3E \|\| id:prot-a-2672 | F-burn/spicy liking (derived food-liking factor) \|\| id:ebi-fl187-GCST90094716 | Inverse variance weighted (multiplicative random effects) | 15 | 0.0282501425543241 | 0.142797393849699 | 0.843175146783717 | -0.251632749 | 0.308133034499733 | 1.02865296212149 | 0.777530234093363 | 1.360882021 | 0.998735809224318 |
| ebi-fl187-GCST90094716 | prot-a-2701 | Plasma protease C1 inhibitor \|\| id:prot-a-2701 | F-burn/spicy liking (derived food-liking factor) \|\| id:ebi-fl187-GCST90094716 | Inverse variance weighted | 15 | -0.153672566 | 0.133067313021492 | 0.248152535998755 | -0.4144845 | 0.107139367457791 | 0.857552766923596 | 0.660680774147481 | 1.11308937210598 | 0.998735809224318 |
| ebi-fl187-GCST90094716 | prot-a-2738 | Signaling threshold-regulating transmembrane adapter 1 \|\| id:prot-a-2738 | F-burn/spicy liking (derived food-liking factor) \|\| id:ebi-fl187-GCST90094716 | Inverse variance weighted | 15 | 0.0783882673536453 | 0.107276540617871 | 0.464955017057317 | -0.131873752 | 0.288650286964673 | 1.08154250480473 | 0.876451638135836 | 1.3346249111785 | 0.998735809224318 |
| ebi-fl187-GCST90094716 | prot-a-274 | Serine/threonine-protein kinase BRSK2 \|\| id:prot-a-274 | F-burn/spicy liking (derived food-liking factor) \|\| id:ebi-fl187-GCST90094716 | Inverse variance weighted | 15 | -0.00526113 | 0.107218463604437 | 0.96086410269721 | -0.215409319 | 0.204887058245575 | 0.994752685088507 | 0.806211374929582 | 1.2273864339575 | 0.998735809224318 |
| ebi-fl187-GCST90094716 | prot-a-2749 | Mitochondrial glutamate carrier 2 \|\| id:prot-a-2749 | F-burn/spicy liking (derived food-liking factor) \|\| id:ebi-fl187-GCST90094716 | Inverse variance weighted | 15 | 0.0311267946291045 | 0.11611279246209 | 0.788642531590076 | -0.196454279 | 0.2587078678548 | 1.0316162990003 | 0.821638896919933 | 1.29525536382533 | 0.998735809224318 |
| ebi-fl187-GCST90094716 | prot-a-2760 | Sodium-coupled monocarboxylate transporter 1 \|\| id:prot-a-2760 | F-burn/spicy liking (derived food-liking factor) \|\| id:ebi-fl187-GCST90094716 | Inverse variance weighted | 15 | -0.178638919 | 0.138535240080144 | 0.197230016123637 | -0.45016799 | 0.0928901512086536 | 0.836407855571958 | 0.637521045525454 | 1.09734118704407 | 0.998735809224318 |
| ebi-fl187-GCST90094716 | prot-a-2777 | Small integral membrane protein 10 \|\| id:prot-a-2777 | F-burn/spicy liking (derived food-liking factor) \|\| id:ebi-fl187-GCST90094716 | Inverse variance weighted | 15 | 0.220972559100916 | 0.107231837469737 | 0.0393317483177302 | 0.0107981576602313 | 0.431146960541601 | 1.2472892033573 | 1.01085666817694 | 1.53902170880211 | 0.998735809224318 |
| ebi-fl187-GCST90094716 | prot-a-2799 | Superoxide dismutase [Mn], mitochondrial \|\| id:prot-a-2799 | F-burn/spicy liking (derived food-liking factor) \|\| id:ebi-fl187-GCST90094716 | Inverse variance weighted | 15 | 0.181370899268081 | 0.107264358153653 | 0.0908601844610747 | -0.028867243 | 0.391609041249242 | 1.198859753 | 0.97154543564164 | 1.47935922989201 | 0.998735809224318 |
| ebi-fl187-GCST90094716 | prot-a-2805 | Protein SPACA7 \|\| id:prot-a-2805 | F-burn/spicy liking (derived food-liking factor) \|\| id:ebi-fl187-GCST90094716 | Inverse variance weighted | 15 | -0.28957374 | 0.10732321082826 | 0.00697267593467615 | -0.499927233 | -0.079220247 | 0.748582590458188 | 0.606574796650376 | 0.923836430118091 | 0.998735809224318 |
| ebi-fl187-GCST90094716 | prot-a-2811 | SPARC-like protein 1 \|\| id:prot-a-2811 | F-burn/spicy liking (derived food-liking factor) \|\| id:ebi-fl187-GCST90094716 | Inverse variance weighted (multiplicative random effects) | 15 | 0.00223514945406984 | 0.155406073333764 | 0.988524710015091 | -0.302360754 | 0.306831053188247 | 1.00223764926275 | 0.739071393622469 | 1.3591113311481 | 0.999069694613173 |
| ebi-fl187-GCST90094716 | prot-a-2831 | Sepiapterin reductase \|\| id:prot-a-2831 | F-burn/spicy liking (derived food-liking factor) \|\| id:ebi-fl187-GCST90094716 | Inverse variance weighted (multiplicative random effects) | 15 | 0.126503972437739 | 0.143379364092662 | 0.377613240918718 | -0.154519581 | 0.407527526059356 | 1.13485395930381 | 0.856826714296388 | 1.50309681929693 | 0.998735809224318 |
| ebi-fl187-GCST90094716 | prot-a-2834 | SPRY domain-containing SOCS box protein 1 \|\| id:prot-a-2834 | F-burn/spicy liking (derived food-liking factor) \|\| id:ebi-fl187-GCST90094716 | Inverse variance weighted | 15 | -0.018357813 | 0.107276540617871 | 0.864124635647605 | -0.228619833 | 0.1919042065831 | 0.98180966521102 | 0.795630948935435 | 1.21155445246512 | 0.998735809224318 |
| ebi-fl187-GCST90094716 | prot-a-2835 | Spectrin alpha chain, non-erythrocytic 1 \|\| id:prot-a-2835 | F-burn/spicy liking (derived food-liking factor) \|\| id:ebi-fl187-GCST90094716 | Inverse variance weighted | 15 | 0.00444561360556818 | 0.107264358153653 | 0.966940824540054 | -0.205792528 | 0.214683755586729 | 1.00445551000549 | 0.814001941069974 | 1.23946985956101 | 0.998735809224318 |
| ebi-fl187-GCST90094716 | prot-a-2859 | Sia-alpha-2,3-Gal-beta-1,4-GlcNAc-R:alpha 2,8-sialyltransferase \|\| id:prot-a-2859 | F-burn/spicy liking (derived food-liking factor) \|\| id:ebi-fl187-GCST90094716 | Inverse variance weighted | 15 | -0.099404377 | 0.117996202040149 | 0.399543338936378 | -0.330676933 | 0.131868178826204 | 0.905376520392226 | 0.718437234791329 | 1.14095790694314 | 0.998735809224318 |
| ebi-fl187-GCST90094716 | prot-a-2861 | Alpha-2,8-sialyltransferase 8F \|\| id:prot-a-2861 | F-burn/spicy liking (derived food-liking factor) \|\| id:ebi-fl187-GCST90094716 | Inverse variance weighted | 15 | -0.135931322 | 0.124714871656425 | 0.275741004801483 | -0.380372471 | 0.10850982600609 | 0.872902579249018 | 0.683606738178333 | 1.11461586070677 | 0.998735809224318 |
| ebi-fl187-GCST90094716 | prot-a-289 | Uncharacterized protein C17orf89 \|\| id:prot-a-289 | F-burn/spicy liking (derived food-liking factor) \|\| id:ebi-fl187-GCST90094716 | Inverse variance weighted | 15 | -0.123251326 | 0.11318083490087 | 0.27616402958926 | -0.345085762 | 0.0985831106653533 | 0.884041452265915 | 0.708159617574864 | 1.10360612202207 | 0.998735809224318 |
| ebi-fl187-GCST90094716 | prot-a-2921 | Tachykinin-3 \|\| id:prot-a-2921 | F-burn/spicy liking (derived food-liking factor) \|\| id:ebi-fl187-GCST90094716 | Inverse variance weighted | 15 | -0.003101101 | 0.125375280753502 | 0.980266697910798 | -0.248836651 | 0.242634449333974 | 0.996903702504032 | 0.779707327223722 | 1.27460260711529 | 0.998955228396052 |
| ebi-fl187-GCST90094716 | prot-a-2928 | Tax1-binding protein 1 \|\| id:prot-a-2928 | F-burn/spicy liking (derived food-liking factor) \|\| id:ebi-fl187-GCST90094716 | Inverse variance weighted (multiplicative random effects) | 15 | -0.134405126 | 0.150005154477999 | 0.370250956322074 | -0.428415229 | 0.159604976869061 | 0.874235817271675 | 0.651540820185506 | 1.17304739860054 | 0.998735809224318 |
| ebi-fl187-GCST90094716 | prot-a-296 | Uncharacterized protein C1orf185 \|\| id:prot-a-296 | F-burn/spicy liking (derived food-liking factor) \|\| id:ebi-fl187-GCST90094716 | Inverse variance weighted | 15 | 0.0757096493596518 | 0.107276540617871 | 0.480348080703326 | -0.13455237 | 0.28597166897068 | 1.07864934215872 | 0.874107100469288 | 1.33105474456711 | 0.998735809224318 |
| ebi-fl187-GCST90094716 | prot-a-2964 | Transforming growth factor beta-2 \|\| id:prot-a-2964 | F-burn/spicy liking (derived food-liking factor) \|\| id:ebi-fl187-GCST90094716 | Inverse variance weighted | 15 | -0.020373543 | 0.107304375500778 | 0.849413478048775 | -0.230690119 | 0.189943033346231 | 0.979832595688273 | 0.793985469232801 | 1.20918071271619 | 0.998735809224318 |
| ebi-fl187-GCST90094716 | prot-a-2977 | Thymocyte nuclear protein 1 \|\| id:prot-a-2977 | F-burn/spicy liking (derived food-liking factor) \|\| id:ebi-fl187-GCST90094716 | Inverse variance weighted | 15 | -0.081046884 | 0.110987816089961 | 0.46524822782602 | -0.298583003 | 0.13648923598434 | 0.922150456742242 | 0.741868701901101 | 1.14624253953672 | 0.998735809224318 |
| ebi-fl187-GCST90094716 | prot-a-3015 | Transmembrane protein 70, mitochondrial \|\| id:prot-a-3015 | F-burn/spicy liking (derived food-liking factor) \|\| id:ebi-fl187-GCST90094716 | Inverse variance weighted | 15 | 0.0239742229923653 | 0.107304375500778 | 0.823206613490003 | -0.186342353 | 0.23429079897389 | 1.02426391509157 | 0.829989396985663 | 1.2640120121641 | 0.998735809224318 |
| ebi-fl187-GCST90094716 | prot-a-3030 | Tumor necrosis factor alpha-induced protein 3 \|\| id:prot-a-3030 | F-burn/spicy liking (derived food-liking factor) \|\| id:ebi-fl187-GCST90094716 | Inverse variance weighted | 15 | -0.079660522 | 0.138636509028634 | 0.565561929078174 | -0.35138808 | 0.192067035391303 | 0.923429776994632 | 0.703710604845025 | 1.21175174449467 | 0.998735809224318 |
| ebi-fl187-GCST90094716 | prot-a-3032 | Tumor necrosis factor receptor superfamily member 10A \|\| id:prot-a-3032 | F-burn/spicy liking (derived food-liking factor) \|\| id:ebi-fl187-GCST90094716 | Inverse variance weighted | 15 | -0.002675762 | 0.126000202822819 | 0.983057256649247 | -0.24963616 | 0.244284635071835 | 0.997327814200674 | 0.779084193508192 | 1.27670767455743 | 0.998955228396052 |
| ebi-fl187-GCST90094716 | prot-a-3081 | Tumor protein p53-inducible protein 11 \|\| id:prot-a-3081 | F-burn/spicy liking (derived food-liking factor) \|\| id:ebi-fl187-GCST90094716 | Inverse variance weighted | 15 | 0.0321978056203257 | 0.126792833044275 | 0.799541707166644 | -0.216316147 | 0.280711758387104 | 1.03272176327177 | 0.805480611218979 | 1.32407189630691 | 0.998735809224318 |
| ebi-fl187-GCST90094716 | prot-a-315 | Complement C4 \|\| id:prot-a-315 | F-burn/spicy liking (derived food-liking factor) \|\| id:ebi-fl187-GCST90094716 | Inverse variance weighted | 15 | -0.132202204 | 0.126509411308986 | 0.296023447415295 | -0.38016065 | 0.115756242158349 | 0.876163813332472 | 0.68375155558298 | 1.12272216644359 | 0.998735809224318 |
| ebi-fl187-GCST90094716 | prot-a-3157 | UDP-glucuronosyltransferase 1-8 \|\| id:prot-a-3157 | F-burn/spicy liking (derived food-liking factor) \|\| id:ebi-fl187-GCST90094716 | Inverse variance weighted | 15 | 0.126028876062327 | 0.12689326861539 | 0.320618328310544 | -0.12268193 | 0.374739682548491 | 1.13431492235852 | 0.884544964663637 | 1.45461270425578 | 0.998735809224318 |
| ebi-fl187-GCST90094716 | prot-a-317 | Glutaredoxin-like protein C5orf63 \|\| id:prot-a-317 | F-burn/spicy liking (derived food-liking factor) \|\| id:ebi-fl187-GCST90094716 | Inverse variance weighted | 15 | -0.041449158 | 0.107259637560949 | 0.699172599652473 | -0.251678048 | 0.168779731505776 | 0.959398111711247 | 0.777495014060568 | 1.18385934328757 | 0.998735809224318 |
| ebi-fl187-GCST90094716 | prot-a-3192 | Guanine nucleotide exchange factor VAV3 \|\| id:prot-a-3192 | F-burn/spicy liking (derived food-liking factor) \|\| id:ebi-fl187-GCST90094716 | Inverse variance weighted | 15 | -0.031244135 | 0.128362378923943 | 0.807691128988791 | -0.282834397 | 0.220346128057838 | 0.969238919401558 | 0.75364458308592 | 1.24650810735755 | 0.998735809224318 |
| ebi-fl187-GCST90094716 | prot-a-3233 | Serine/threonine-protein kinase WNK3 \|\| id:prot-a-3233 | F-burn/spicy liking (derived food-liking factor) \|\| id:ebi-fl187-GCST90094716 | Inverse variance weighted | 15 | 0.202675285034791 | 0.107276540617871 | 0.0588544918014164 | -0.007586735 | 0.412937304645819 | 1.22467473346231 | 0.992441972052131 | 1.51125027459257 | 0.998735809224318 |
| ebi-fl187-GCST90094716 | prot-a-3250 | 14-3-3 protein theta \|\| id:prot-a-3250 | F-burn/spicy liking (derived food-liking factor) \|\| id:ebi-fl187-GCST90094716 | Inverse variance weighted | 15 | 0.0432228262324666 | 0.10724746085377 | 0.68693382317204 | -0.166982197 | 0.253427849505856 | 1.04417053751713 | 0.84621467632834 | 1.2884344149519 | 0.998735809224318 |
| ebi-fl187-GCST90094716 | prot-a-3278 | E3 ubiquitin-protein ligase ZNRF3 \|\| id:prot-a-3278 | F-burn/spicy liking (derived food-liking factor) \|\| id:ebi-fl187-GCST90094716 | Inverse variance weighted | 15 | 0.10694243405776 | 0.107245694045902 | 0.318680889926085 | -0.103259126 | 0.317143994387727 | 1.11287018921432 | 0.901893238966505 | 1.37320029082502 | 0.998735809224318 |
| ebi-fl187-GCST90094716 | prot-a-355 | Calpain-3 \|\| id:prot-a-355 | F-burn/spicy liking (derived food-liking factor) \|\| id:ebi-fl187-GCST90094716 | Inverse variance weighted | 15 | 0.0666527458040891 | 0.107201029432803 | 0.534103165796744 | -0.143461272 | 0.276766763492384 | 1.06892422529643 | 0.866354351809201 | 1.31885872915567 | 0.998735809224318 |
| ebi-fl187-GCST90094716 | prot-a-385 | Coiled-coil domain-containing protein 90B, mitochondrial \|\| id:prot-a-385 | F-burn/spicy liking (derived food-liking factor) \|\| id:ebi-fl187-GCST90094716 | Inverse variance weighted | 15 | -0.22830178 | 0.108434031472381 | 0.0352526782105327 | -0.440832481 | -0.015771078 | 0.795884042017192 | 0.643500495915254 | 0.984352634315674 | 0.998735809224318 |
| ebi-fl187-GCST90094716 | prot-a-390 | C-C motif chemokine 14 \|\| id:prot-a-390 | F-burn/spicy liking (derived food-liking factor) \|\| id:ebi-fl187-GCST90094716 | Inverse variance weighted | 15 | -0.132117286 | 0.126351057643983 | 0.295728861465364 | -0.379765359 | 0.115530787307303 | 0.876238218861527 | 0.684021890198765 | 1.12246907181657 | 0.998735809224318 |
| ebi-fl187-GCST90094716 | prot-a-40 | Pituitary adenylate cyclase-activating polypeptide 27 \|\| id:prot-a-40 | F-burn/spicy liking (derived food-liking factor) \|\| id:ebi-fl187-GCST90094716 | Inverse variance weighted | 15 | 0.124654939160547 | 0.107276540617871 | 0.245236983448669 | -0.08560708 | 0.334916958771575 | 1.13275751536291 | 0.917954842773148 | 1.39782430335547 | 0.998735809224318 |
| ebi-fl187-GCST90094716 | prot-a-401 | C-C motif chemokine 24 \|\| id:prot-a-401 | F-burn/spicy liking (derived food-liking factor) \|\| id:ebi-fl187-GCST90094716 | Inverse variance weighted | 15 | 0.0584139713373114 | 0.107264358153653 | 0.586042751976349 | -0.151824171 | 0.268652113318472 | 1.06015377815964 | 0.85913932937647 | 1.30819995653309 | 0.998735809224318 |
| ebi-fl187-GCST90094716 | prot-a-412 | Cerebral cavernous malformations 2 protein \|\| id:prot-a-412 | F-burn/spicy liking (derived food-liking factor) \|\| id:ebi-fl187-GCST90094716 | Inverse variance weighted (multiplicative random effects) | 15 | 0.0814138742310218 | 0.168459120267699 | 0.628892932752748 | -0.248766001 | 0.411593749955712 | 1.0848197826225 | 0.779762415278771 | 1.50922119059611 | 0.998735809224318 |
| ebi-fl187-GCST90094716 | prot-a-44 | Adhesion G protein-coupled receptor F5 \|\| id:prot-a-44 | F-burn/spicy liking (derived food-liking factor) \|\| id:ebi-fl187-GCST90094716 | Inverse variance weighted | 15 | 0.0451538178611687 | 0.112493242461044 | 0.688131994630521 | -0.175332937 | 0.265640573084816 | 1.04618877005196 | 0.839177580683403 | 1.30426618605742 | 0.998735809224318 |
| ebi-fl187-GCST90094716 | prot-a-48 | Adrenomedullin \|\| id:prot-a-48 | F-burn/spicy liking (derived food-liking factor) \|\| id:ebi-fl187-GCST90094716 | Inverse variance weighted | 15 | 0.0176987260207902 | 0.11857062043515 | 0.88134265277547 | -0.21469969 | 0.250097142073685 | 1.01785627658107 | 0.806783688984378 | 1.28415015563798 | 0.998735809224318 |
| ebi-fl187-GCST90094716 | prot-a-502 | Carcinoembryonic antigen-related cell adhesion molecule 1 \|\| id:prot-a-502 | F-burn/spicy liking (derived food-liking factor) \|\| id:ebi-fl187-GCST90094716 | Inverse variance weighted | 15 | -0.070842158 | 0.107304375500778 | 0.509126630554833 | -0.281158734 | 0.139474417631737 | 0.93160892721358 | 0.754908496073819 | 1.14966939407603 | 0.998735809224318 |
| ebi-fl187-GCST90094716 | prot-a-518 | Complement factor B \|\| id:prot-a-518 | F-burn/spicy liking (derived food-liking factor) \|\| id:ebi-fl187-GCST90094716 | Inverse variance weighted | 15 | 0.129829806619818 | 0.107264358153653 | 0.226136070522178 | -0.080408335 | 0.340067948600978 | 1.13863457876521 | 0.922739482288478 | 1.40504305803022 | 0.998735809224318 |
| ebi-fl187-GCST90094716 | prot-a-525 | Properdin \|\| id:prot-a-525 | F-burn/spicy liking (derived food-liking factor) \|\| id:ebi-fl187-GCST90094716 | Inverse variance weighted | 15 | 0.0808682560075694 | 0.107395442314996 | 0.451452779172635 | -0.129626811 | 0.291363322944961 | 1.08422804662559 | 0.878423187691773 | 1.33825071282388 | 0.998735809224318 |
| ebi-fl187-GCST90094716 | prot-a-533 | Chondroadherin \|\| id:prot-a-533 | F-burn/spicy liking (derived food-liking factor) \|\| id:ebi-fl187-GCST90094716 | Inverse variance weighted | 15 | 0.0896720030436594 | 0.128499303833203 | 0.485277022895982 | -0.162186632 | 0.341530638556737 | 1.09381545672075 | 0.850282499408361 | 1.40709970414975 | 0.998735809224318 |
| ebi-fl187-GCST90094716 | prot-a-535 | Coiled-coil-helix-coiled-coil-helix domain-containing protein 2 \|\| id:prot-a-535 | F-burn/spicy liking (derived food-liking factor) \|\| id:ebi-fl187-GCST90094716 | Inverse variance weighted (multiplicative random effects) | 15 | -0.046323568 | 0.150403163232204 | 0.758085398857552 | -0.341113768 | 0.248466631783955 | 0.954732991018036 | 0.710978016996695 | 1.28205804166584 | 0.998735809224318 |
| ebi-fl187-GCST90094716 | prot-a-591 | Caseinolytic peptidase B protein homolog \|\| id:prot-a-591 | F-burn/spicy liking (derived food-liking factor) \|\| id:ebi-fl187-GCST90094716 | Inverse variance weighted (multiplicative random effects) | 15 | 0.277700821797525 | 0.14344881760116 | 0.052881439258482 | -0.003458861 | 0.558860504295799 | 1.3200911956131 | 0.996547114267078 | 1.74867875265172 | 0.998735809224318 |
| ebi-fl187-GCST90094716 | prot-a-605 | Ciliary neurotrophic factor receptor subunit alpha \|\| id:prot-a-605 | F-burn/spicy liking (derived food-liking factor) \|\| id:ebi-fl187-GCST90094716 | Inverse variance weighted | 15 | 0.0192392077483585 | 0.107264358153653 | 0.857653042980115 | -0.190998934 | 0.229477349729519 | 1.01942547392603 | 0.826133468616677 | 1.25794237416559 | 0.998735809224318 |
| ebi-fl187-GCST90094716 | prot-a-606 | Ciliary neurotrophic factor receptor subunit alpha \|\| id:prot-a-606 | F-burn/spicy liking (derived food-liking factor) \|\| id:ebi-fl187-GCST90094716 | Inverse variance weighted | 15 | 0.0864249891070755 | 0.110650004786174 | 0.434763505177914 | -0.13044902 | 0.303298998487977 | 1.09026958255551 | 0.877701236776929 | 1.35431934334608 | 0.998735809224318 |
| ebi-fl187-GCST90094716 | prot-a-707 | Cancer/testis antigen 55 \|\| id:prot-a-707 | F-burn/spicy liking (derived food-liking factor) \|\| id:ebi-fl187-GCST90094716 | Inverse variance weighted (multiplicative random effects) | 15 | 0.0963976918313783 | 0.155359153322896 | 0.534939934782718 | -0.208106249 | 0.400901632344255 | 1.10119691394095 | 0.812120745369436 | 1.49317038160555 | 0.998735809224318 |
| ebi-fl187-GCST90094716 | prot-a-719 | Dipeptidyl peptidase 1 \|\| id:prot-a-719 | F-burn/spicy liking (derived food-liking factor) \|\| id:ebi-fl187-GCST90094716 | Inverse variance weighted | 15 | -0.163774646 | 0.107213270814927 | 0.126621954599537 | -0.373912656 | 0.0463633651302221 | 0.848933311125527 | 0.688037004789271 | 1.04745495041983 | 0.998735809224318 |
| ebi-fl187-GCST90094716 | prot-a-752 | Cytochrome b5 \|\| id:prot-a-752 | F-burn/spicy liking (derived food-liking factor) \|\| id:ebi-fl187-GCST90094716 | Inverse variance weighted | 15 | 0.0284770391826128 | 0.128206978790354 | 0.824222259817584 | -0.222808639 | 0.279762717611707 | 1.02888638649088 | 0.800267974525629 | 1.32281589418064 | 0.998735809224318 |
| ebi-fl187-GCST90094716 | prot-a-755 | Cytoglobin \|\| id:prot-a-755 | F-burn/spicy liking (derived food-liking factor) \|\| id:ebi-fl187-GCST90094716 | Inverse variance weighted | 15 | 0.0608381298412862 | 0.107264358153653 | 0.570592330544889 | -0.149400012 | 0.271076271822447 | 1.06272687649552 | 0.861224545714181 | 1.31137508753813 | 0.998735809224318 |
| ebi-fl187-GCST90094716 | prot-a-807 | Desmin \|\| id:prot-a-807 | F-burn/spicy liking (derived food-liking factor) \|\| id:ebi-fl187-GCST90094716 | Inverse variance weighted | 15 | 0.132533691816696 | 0.137251236021124 | 0.334230157601028 | -0.136478731 | 0.4015461144181 | 1.14171748197731 | 0.872424875854801 | 1.49413301331581 | 0.998735809224318 |
| ebi-fl187-GCST90094716 | prot-a-82 | Ameloblastin \|\| id:prot-a-82 | F-burn/spicy liking (derived food-liking factor) \|\| id:ebi-fl187-GCST90094716 | Inverse variance weighted | 15 | -0.228858791 | 0.109901387186278 | 0.0373058301490482 | -0.44426551 | -0.013452072 | 0.795440848871182 | 0.641295127936188 | 0.986638002520215 | 0.998735809224318 |
| ebi-fl187-GCST90094716 | prot-a-84 | Muellerian-inhibiting factor \|\| id:prot-a-84 | F-burn/spicy liking (derived food-liking factor) \|\| id:ebi-fl187-GCST90094716 | Inverse variance weighted | 15 | 0.00950896034444734 | 0.107264358153653 | 0.929360255500111 | -0.200729182 | 0.219747102325608 | 1.00955431414972 | 0.818133967256499 | 1.24576164052455 | 0.998735809224318 |
| ebi-fl187-GCST90094716 | prot-a-858 | Inactive dipeptidyl peptidase 10 \|\| id:prot-a-858 | F-burn/spicy liking (derived food-liking factor) \|\| id:ebi-fl187-GCST90094716 | Inverse variance weighted | 15 | -0.11529535 | 0.138182529110296 | 0.40407254360336 | -0.386133107 | 0.155542406733399 | 0.891102917502427 | 0.679680049229027 | 1.16829147844204 | 0.998735809224318 |
| ebi-fl187-GCST90094716 | prot-a-863 | Dihydropyrimidinase-related protein 3 \|\| id:prot-a-863 | F-burn/spicy liking (derived food-liking factor) \|\| id:ebi-fl187-GCST90094716 | Inverse variance weighted | 15 | -0.015736935 | 0.107304375500778 | 0.883402777829359 | -0.226053511 | 0.19457964053315 | 0.984386243122724 | 0.797675415771499 | 1.21480022637034 | 0.998735809224318 |
| ebi-fl187-GCST90094716 | prot-a-864 | Dihydropyrimidinase-related protein 5 \|\| id:prot-a-864 | F-burn/spicy liking (derived food-liking factor) \|\| id:ebi-fl187-GCST90094716 | Inverse variance weighted | 15 | -0.042290177 | 0.123037346622152 | 0.731058211500046 | -0.283443377 | 0.198863022251136 | 0.958591578859001 | 0.753185768941235 | 1.22001483956754 | 0.998735809224318 |
| ebi-fl187-GCST90094716 | prot-a-884 | Endothelin-converting enzyme 1 \|\| id:prot-a-884 | F-burn/spicy liking (derived food-liking factor) \|\| id:ebi-fl187-GCST90094716 | Inverse variance weighted | 15 | -0.041764111 | 0.107259158693337 | 0.696998235756014 | -0.251992062 | 0.168463839687936 | 0.959095993749098 | 0.77725090755908 | 1.1834854308686 | 0.998735809224318 |
| ebi-fl187-GCST90094716 | prot-a-893 | Early endosome antigen 1 \|\| id:prot-a-893 | F-burn/spicy liking (derived food-liking factor) \|\| id:ebi-fl187-GCST90094716 | Inverse variance weighted | 15 | -0.099193225 | 0.10729218355228 | 0.355218282326472 | -0.309485905 | 0.111099454296475 | 0.905567712374073 | 0.733824114408903 | 1.11750604210515 | 0.998735809224318 |
| ebi-fl187-GCST90094716 | prot-a-897 | Ephrin-A2 \|\| id:prot-a-897 | F-burn/spicy liking (derived food-liking factor) \|\| id:ebi-fl187-GCST90094716 | Inverse variance weighted | 15 | 0.134231507905022 | 0.107301711650642 | 0.210944451501122 | -0.076079847 | 0.344542862740281 | 1.14365755476389 | 0.92674220608479 | 1.41134459397748 | 0.998735809224318 |
| ebi-fl187-GCST90094716 | prot-a-90 | Anaphase-promoting complex subunit 10 \|\| id:prot-a-90 | F-burn/spicy liking (derived food-liking factor) \|\| id:ebi-fl187-GCST90094716 | Inverse variance weighted | 15 | 0.141195810569467 | 0.107433904992092 | 0.188759579360638 | -0.069374643 | 0.351766264353966 | 1.15165013117604 | 0.93297708106164 | 1.42157621184926 | 0.998735809224318 |
| ebi-fl187-GCST90094716 | prot-a-914 | Histone-lysine N-methyltransferase EHMT2 \|\| id:prot-a-914 | F-burn/spicy liking (derived food-liking factor) \|\| id:ebi-fl187-GCST90094716 | Inverse variance weighted | 15 | -0.082233877 | 0.125521032752152 | 0.512377480617396 | -0.328255101 | 0.163787347526158 | 0.92105651987431 | 0.720179277915161 | 1.17796379154207 | 0.998735809224318 |
| ebi-fl187-GCST90094716 | prot-a-932 | RNA polymerase II elongation factor ELL \|\| id:prot-a-932 | F-burn/spicy liking (derived food-liking factor) \|\| id:ebi-fl187-GCST90094716 | Inverse variance weighted | 15 | 0.0294852336331359 | 0.107259158693337 | 0.783395281175604 | -0.180742717 | 0.239713184672076 | 1.02992422712063 | 0.834650072009378 | 1.27088458886289 | 0.998735809224318 |
| ebi-fl187-GCST90094716 | prot-a-991 | Estrogen receptor \|\| id:prot-a-991 | F-burn/spicy liking (derived food-liking factor) \|\| id:ebi-fl187-GCST90094716 | Inverse variance weighted | 15 | 0.119222591455327 | 0.10729218355228 | 0.266484208175989 | -0.091070088 | 0.329515271217795 | 1.12662066651387 | 0.912953721276006 | 1.39029404956271 | 0.998735809224318 |
| ebi-fl187-GCST90094716 | prot-a-992 | Persulfide dioxygenase ETHE1, mitochondrial \|\| id:prot-a-992 | F-burn/spicy liking (derived food-liking factor) \|\| id:ebi-fl187-GCST90094716 | Inverse variance weighted (multiplicative random effects) | 15 | 0.0605615729962196 | 0.161674667900535 | 0.70796605929268 | -0.256320776 | 0.377443922081269 | 1.06243301274028 | 0.773893682383601 | 1.45855164896007 | 0.998735809224318 |
| ebi-fl187-GCST90094716 | prot-a-993 | Ethanolamine kinase 1 \|\| id:prot-a-993 | F-burn/spicy liking (derived food-liking factor) \|\| id:ebi-fl187-GCST90094716 | Inverse variance weighted | 15 | -0.053144307 | 0.107264358153653 | 0.620281318282104 | -0.263382449 | 0.1570938349172 | 0.948243164421486 | 0.768447948920313 | 1.17010540549353 | 0.998735809224318 |
| ebi-fl187-GCST90094716 | prot-b-10 | coagulation factor III, tissue factor \|\| id:prot-b-10 | F-burn/spicy liking (derived food-liking factor) \|\| id:ebi-fl187-GCST90094716 | Inverse variance weighted | 10 | 0.203580677096747 | 0.139297934315878 | 0.143884681525746 | -0.069443274 | 0.476604628355867 | 1.22578404635022 | 0.932913052157927 | 1.61059653395478 | 0.998735809224318 |
| ebi-fl187-GCST90094716 | prot-b-28 | prolactin \|\| id:prot-b-28 | F-burn/spicy liking (derived food-liking factor) \|\| id:ebi-fl187-GCST90094716 | Inverse variance weighted | 10 | 0.123954892987913 | 0.161121086569948 | 0.441698878111556 | -0.191842437 | 0.439752222665012 | 1.13196481029721 | 0.825436916819474 | 1.55232254051393 | 0.998735809224318 |
| ebi-fl187-GCST90094716 | prot-b-54 | matrix metallopeptidase 7 \|\| id:prot-b-54 | F-burn/spicy liking (derived food-liking factor) \|\| id:ebi-fl187-GCST90094716 | Inverse variance weighted | 10 | 0.0723522112898621 | 0.17430974422609 | 0.67808450466954 | -0.269294887 | 0.413999309972999 | 1.07503391647353 | 0.763917952656591 | 1.51285608297249 | 0.998735809224318 |
| ebi-fl187-GCST90094716 | ukb-rsfMRI191-edge-pheno716 | Net100_Pair10_33 (OccipitalPrecuneus)&(Temporal\|Frontal\|Supp_Motor_Area) (Default_mode\|Central_executive)&(Default_mode\|Salience) \|\| id:ukb-rsfMRI191-edge-pheno716 | F-burn/spicy liking (derived food-liking factor) \|\| id:ebi-fl187-GCST90094716 | Inverse variance weighted | 13 | -0.011737582 | 0.036715826446495 | 0.749205415333449 | -0.083700602 | 0.0602254377300313 | 0.988331034584673 | 0.919706573241945 | 1.06207595155052 | 0.998735809224318 |
| ebi-fl187-GCST90094716 | ukb-rsfMRI191-edge-pheno903 | Net100_Pair27_38 (Precentral\|Frontal\|Supp_Motor_Area)&(Subcortical) (Attention\|Salience\|Motor)&(Subcortical-cerebellum) \|\| id:ukb-rsfMRI191-edge-pheno903 | F-burn/spicy liking (derived food-liking factor) \|\| id:ebi-fl187-GCST90094716 | Inverse variance weighted (multiplicative random effects) | 13 | -0.002615775 | 0.0513685826887149 | 0.959387922248577 | -0.103298197 | 0.0980666470548188 | 0.997387643143375 | 0.901858001953031 | 1.10303629677935 | 0.998735809224318 |
| ebi-fl187-GCST90094716 | ukb-rsfMRI191-node-pheno23 | Net100_Node2 RolandicOper\|SupraMarginal\|Insula Salience\|Motor \|\| id:ukb-rsfMRI191-node-pheno23 | F-burn/spicy liking (derived food-liking factor) \|\| id:ebi-fl187-GCST90094716 | Inverse variance weighted (multiplicative random effects) | 13 | -0.028637517 | 0.0621417926067254 | 0.644912320854665 | -0.150435431 | 0.0931603962238721 | 0.971768649970462 | 0.860333279250322 | 1.09763777810419 | 0.998735809224318 |
| ebi-fl187-GCST90094716 | ukb-rsfMRI191-node-pheno55 | Net100_Node34 Parietal\|Postcentral\|Precuneus Attention \|\| id:ukb-rsfMRI191-node-pheno55 | F-burn/spicy liking (derived food-liking factor) \|\| id:ebi-fl187-GCST90094716 | Inverse variance weighted (multiplicative random effects) | 13 | -0.037561614 | 0.0508735893557312 | 0.460312551559043 | -0.137273849 | 0.0621506216038662 | 0.963135073737642 | 0.871731470937769 | 1.06412261251244 | 0.998735809224318 |
| ebi-fl187-GCST90094716 | ebi-a-GCST90001414 | IgD- CD24- B cell Absolute Count \|\| id:ebi-a-GCST90001414 | F-burn/spicy liking (derived food-liking factor) \|\| id:ebi-fl187-GCST90094716 | MR Egger | 15 | 1.73588301966227 | 0.862604037123532 | 0.0653741743205184 | 0.0451791069001504 | 3.4265869324244 | 5.67393578926916 | 1.04621522749509 | 30.7714383185085 | 0.999950574162623 |
| ebi-fl187-GCST90094716 | ebi-a-GCST90001422 | CD20- CD38- B cell %B cell \|\| id:ebi-a-GCST90001422 | F-burn/spicy liking (derived food-liking factor) \|\| id:ebi-fl187-GCST90094716 | MR Egger | 15 | 0.323536433760298 | 0.875652319919351 | 0.717718308680943 | -1.392742113 | 2.03981498080223 | 1.3820065070446 | 0.248393247497052 | 7.68918642015938 | 0.999950574162623 |
| ebi-fl187-GCST90094716 | ebi-a-GCST90001444 | CD20- CD38- B cell %lymphocyte \|\| id:ebi-a-GCST90001444 | F-burn/spicy liking (derived food-liking factor) \|\| id:ebi-fl187-GCST90094716 | MR Egger | 15 | 0.607207173040762 | 0.820764353105006 | 0.472566429120012 | -1.001490959 | 2.21590530512657 | 1.83529856325151 | 0.367331356678437 | 9.16970673761916 | 0.999950574162623 |
| ebi-fl187-GCST90094716 | ebi-a-GCST90001459 | Myeloid Dendritic Cell %Dendritic Cell \|\| id:ebi-a-GCST90001459 | F-burn/spicy liking (derived food-liking factor) \|\| id:ebi-fl187-GCST90094716 | MR Egger | 15 | 0.430716020995418 | 0.761949830987374 | 0.581507466729107 | -1.062705648 | 1.92413768973067 | 1.53835862636983 | 0.345519689926987 | 6.84923994874666 | 0.999950574162623 |
| ebi-fl187-GCST90094716 | ebi-a-GCST90001476 | HLA DR++ monocyte %leukocyte \|\| id:ebi-a-GCST90001476 | F-burn/spicy liking (derived food-liking factor) \|\| id:ebi-fl187-GCST90094716 | MR Egger | 15 | -0.425100362 | 0.748338876139862 | 0.579674991352916 | -1.891844559 | 1.04164383553113 | 0.653704174973273 | 0.150793404826473 | 2.83387160644884 | 0.999950574162623 |
| ebi-fl187-GCST90094716 | ebi-a-GCST90001478 | CD4 regulatory T cell %CD4+ T cell \|\| id:ebi-a-GCST90001478 | F-burn/spicy liking (derived food-liking factor) \|\| id:ebi-fl187-GCST90094716 | MR Egger | 15 | 1.03750468446874 | 0.752655227085355 | 0.191322220063796 | -0.437699561 | 2.51270892955603 | 2.82216602607325 | 0.645519693264027 | 12.3383084386621 | 0.999950574162623 |
| ebi-fl187-GCST90094716 | ebi-a-GCST90001485 | CD39+ resting CD4 regulatory T cell %CD4 regulatory T cell \|\| id:ebi-a-GCST90001485 | F-burn/spicy liking (derived food-liking factor) \|\| id:ebi-fl187-GCST90094716 | MR Egger | 15 | -0.775073222 | 0.795752458311057 | 0.347826434045799 | -2.33474804 | 0.784601596212803 | 0.460670048546295 | 0.0968348779333526 | 2.19153365147738 | 0.999950574162623 |
| ebi-fl187-GCST90094716 | ebi-a-GCST90001515 | Immature Myeloid-Derived Suppressor Cells Absolute Count \|\| id:ebi-a-GCST90001515 | F-burn/spicy liking (derived food-liking factor) \|\| id:ebi-fl187-GCST90094716 | MR Egger | 15 | 0.589954449009237 | 1.05389961608939 | 0.585144619865288 | -1.475688799 | 2.65559769654445 | 1.80390624380972 | 0.228621198854081 | 14.2334908257246 | 0.999950574162623 |
| ebi-fl187-GCST90094716 | ebi-a-GCST90001517 | CD33+ HLA DR+ Absolute Count \|\| id:ebi-a-GCST90001517 | F-burn/spicy liking (derived food-liking factor) \|\| id:ebi-fl187-GCST90094716 | MR Egger | 15 | -0.482520858 | 0.972666584304308 | 0.628119009454322 | -2.388947363 | 1.42390564712559 | 0.617225491122332 | 0.0917261874239036 | 4.15331016790876 | 0.999950574162623 |
| ebi-fl187-GCST90094716 | ebi-a-GCST90001518 | CD33+ HLA DR+ CD14- Absolute Count \|\| id:ebi-a-GCST90001518 | F-burn/spicy liking (derived food-liking factor) \|\| id:ebi-fl187-GCST90094716 | MR Egger | 14 | -0.927968511 | 0.994533774439696 | 0.369179068309145 | -2.877254709 | 1.02131768679915 | 0.395356056556375 | 0.0562890808200483 | 2.77685137470102 | 0.999950574162623 |
| ebi-fl187-GCST90094716 | ebi-a-GCST90001520 | CD33+ HLA DR+ CD14dim Absolute Count \|\| id:ebi-a-GCST90001520 | F-burn/spicy liking (derived food-liking factor) \|\| id:ebi-fl187-GCST90094716 | MR Egger | 15 | 0.0544152675423215 | 0.976227013816369 | 0.956396208752115 | -1.85898968 | 1.9678202146224 | 1.05592300167202 | 0.155829989089852 | 7.15506297582524 | 0.999950574162623 |
| ebi-fl187-GCST90094716 | ebi-a-GCST90001531 | CD33dim HLA DR- Absolute Count \|\| id:ebi-a-GCST90001531 | F-burn/spicy liking (derived food-liking factor) \|\| id:ebi-fl187-GCST90094716 | MR Egger | 15 | 0.489072284236651 | 1.0726400339127 | 0.65594784079263 | -1.613302182 | 2.59144675070554 | 1.630802597 | 0.199228637376887 | 13.3490704165871 | 0.999950574162623 |
| ebi-fl187-GCST90094716 | ebi-a-GCST90001532 | Basophil Absolute Count \|\| id:ebi-a-GCST90001532 | F-burn/spicy liking (derived food-liking factor) \|\| id:ebi-fl187-GCST90094716 | MR Egger | 15 | 0.00348038954583889 | 1.07775639444874 | 0.997472431944621 | -2.108922144 | 2.11588292266537 | 1.00348645313404 | 0.121368714005511 | 8.2969080613132 | 0.999950574162623 |
| ebi-fl187-GCST90094716 | ebi-a-GCST90001588 | T/B cell \|\| id:ebi-a-GCST90001588 | F-burn/spicy liking (derived food-liking factor) \|\| id:ebi-fl187-GCST90094716 | MR Egger | 15 | -0.224511736 | 0.826019090043031 | 0.790044666164541 | -1.843509152 | 1.39448568057252 | 0.798906200752183 | 0.158261088263586 | 4.03289984040341 | 0.999950574162623 |
| ebi-fl187-GCST90094716 | ebi-a-GCST90001624 | HLA DR+ CD4+ T cell Absolute Count \|\| id:ebi-a-GCST90001624 | F-burn/spicy liking (derived food-liking factor) \|\| id:ebi-fl187-GCST90094716 | MR Egger | 15 | 0.592033561018283 | 0.745651327986676 | 0.441459054303876 | -0.869443042 | 2.05351016387217 | 1.80766066852674 | 0.419184952725432 | 7.79521562330233 | 0.999950574162623 |
| ebi-fl187-GCST90094716 | ebi-a-GCST90001631 | CD8+ Natural Killer T %T cell \|\| id:ebi-a-GCST90001631 | F-burn/spicy liking (derived food-liking factor) \|\| id:ebi-fl187-GCST90094716 | MR Egger | 15 | -0.276169194 | 0.725687133342309 | 0.709673884313498 | -1.698515975 | 1.14617758782879 | 0.758684555389292 | 0.18295483225701 | 3.14614403722147 | 0.999950574162623 |
| ebi-fl187-GCST90094716 | ebi-a-GCST90001632 | CD8+ Natural Killer T %lymphocyte \|\| id:ebi-a-GCST90001632 | F-burn/spicy liking (derived food-liking factor) \|\| id:ebi-fl187-GCST90094716 | MR Egger | 15 | -0.306213588 | 0.730142240767696 | 0.681779340326591 | -1.73729238 | 1.1248652040925 | 0.736229353044819 | 0.175996287183135 | 3.07980167627499 | 0.999950574162623 |
| ebi-fl187-GCST90094716 | ebi-a-GCST90001646 | Natural Killer %CD3- lymphocyte \|\| id:ebi-a-GCST90001646 | F-burn/spicy liking (derived food-liking factor) \|\| id:ebi-fl187-GCST90094716 | MR Egger | 15 | 0.0754752856652848 | 0.798215257169906 | 0.926110142646748 | -1.489026618 | 1.6399771897183 | 1.07839657553478 | 0.225592135940013 | 5.15505192270703 | 0.999950574162623 |
| ebi-fl187-GCST90094716 | ebi-a-GCST90001684 | CD127- CD8+ T cell Absolute Count \|\| id:ebi-a-GCST90001684 | F-burn/spicy liking (derived food-liking factor) \|\| id:ebi-fl187-GCST90094716 | MR Egger | 15 | 0.458578386626559 | 0.766484951939318 | 0.559928028197634 | -1.043732119 | 1.96088889242762 | 1.58182364416919 | 0.352138005487975 | 7.10564040875202 | 0.999950574162623 |
| ebi-fl187-GCST90094716 | ebi-a-GCST90001687 | CD28- CD8+ T cell Absolute Count \|\| id:ebi-a-GCST90001687 | F-burn/spicy liking (derived food-liking factor) \|\| id:ebi-fl187-GCST90094716 | MR Egger | 15 | -0.084406272 | 0.746759761692595 | 0.91173356961584 | -1.548055405 | 1.37924286096503 | 0.919057792832522 | 0.212661111765835 | 3.97189321335001 | 0.999950574162623 |
| ebi-fl187-GCST90094716 | ebi-a-GCST90001701 | BAFF-R on CD20- CD38- B cell \|\| id:ebi-a-GCST90001701 | F-burn/spicy liking (derived food-liking factor) \|\| id:ebi-fl187-GCST90094716 | MR Egger | 15 | -0.664257289 | 1.00642182396315 | 0.520764785406718 | -2.636844064 | 1.30832948635332 | 0.514655626387905 | 0.071586836936734 | 3.69998766682218 | 0.999950574162623 |
| ebi-fl187-GCST90094716 | ebi-a-GCST90001732 | CD19 on IgD- CD27- B cell \|\| id:ebi-a-GCST90001732 | F-burn/spicy liking (derived food-liking factor) \|\| id:ebi-fl187-GCST90094716 | MR Egger | 15 | 0.545285278498423 | 1.03205160504367 | 0.606158456601684 | -1.477535867 | 2.56810642438401 | 1.72510044625041 | 0.228199309505953 | 13.041106724189 | 0.999950574162623 |
| ebi-fl187-GCST90094716 | ebi-a-GCST90001798 | CD27 on CD24+ CD27+ B cell \|\| id:ebi-a-GCST90001798 | F-burn/spicy liking (derived food-liking factor) \|\| id:ebi-fl187-GCST90094716 | MR Egger | 15 | 1.30301320719936 | 0.731601212266202 | 0.0982703786668324 | -0.130925169 | 2.73695158324112 | 3.68036969303662 | 0.877283420068717 | 15.439846197437 | 0.999950574162623 |
| ebi-fl187-GCST90094716 | ebi-a-GCST90001836 | CD66b on CD66b++ myeloid cell \|\| id:ebi-a-GCST90001836 | F-burn/spicy liking (derived food-liking factor) \|\| id:ebi-fl187-GCST90094716 | MR Egger | 15 | -1.098930742 | 1.09029540432574 | 0.331885779939054 | -3.235909734 | 1.03804825067905 | 0.333227199189843 | 0.0393244138951279 | 2.82370047716502 | 0.999950574162623 |
| ebi-fl187-GCST90094716 | ebi-a-GCST90001943 | CD25 on activated & secreting CD4 regulatory T cell \|\| id:ebi-a-GCST90001943 | F-burn/spicy liking (derived food-liking factor) \|\| id:ebi-fl187-GCST90094716 | MR Egger | 15 | 0.423336528217655 | 0.942912997912528 | 0.660848739083599 | -1.424772948 | 2.27144600412621 | 1.52704810428292 | 0.240563077410177 | 9.69340739193336 | 0.999950574162623 |
| ebi-fl187-GCST90094716 | ebi-a-GCST90001960 | CD25 on CD4+ T cell \|\| id:ebi-a-GCST90001960 | F-burn/spicy liking (derived food-liking factor) \|\| id:ebi-fl187-GCST90094716 | MR Egger | 15 | -0.578426322 | 0.873787188809786 | 0.519549188437498 | -2.291049212 | 1.13419656783331 | 0.560780159823404 | 0.101160267556346 | 3.10867492987205 | 0.999950574162623 |
| ebi-fl187-GCST90094716 | ebi-a-GCST90001988 | HLA DR on CD14+ CD16- monocyte \|\| id:ebi-a-GCST90001988 | F-burn/spicy liking (derived food-liking factor) \|\| id:ebi-fl187-GCST90094716 | MR Egger | 15 | 0.314445767107129 | 0.744870296874417 | 0.679814677691897 | -1.145500015 | 1.77439154898099 | 1.36950007859031 | 0.318064840906108 | 5.89669219620701 | 0.999950574162623 |
| ebi-fl187-GCST90094716 | ebi-a-GCST90001991 | HLA DR on CD14+ monocyte \|\| id:ebi-a-GCST90001991 | F-burn/spicy liking (derived food-liking factor) \|\| id:ebi-fl187-GCST90094716 | MR Egger | 15 | 0.343118702267652 | 0.74618032616041 | 0.653231626855913 | -1.119394737 | 1.80563214154206 | 1.40933604338214 | 0.326477339485436 | 6.08381606609067 | 0.999950574162623 |
| ebi-fl187-GCST90094716 | ebi-a-GCST90002019 | CD14 on Monocytic Myeloid-Derived Suppressor Cells \|\| id:ebi-a-GCST90002019 | F-burn/spicy liking (derived food-liking factor) \|\| id:ebi-fl187-GCST90094716 | MR Egger | 15 | -0.715777043 | 1.05859455880723 | 0.510791275946647 | -2.790622378 | 1.35906829236132 | 0.488812136186499 | 0.061382998586139 | 3.89256487931111 | 0.999950574162623 |
| ebi-fl187-GCST90094716 | ebi-a-GCST90002037 | CD80 on plasmacytoid Dendritic Cell \|\| id:ebi-a-GCST90002037 | F-burn/spicy liking (derived food-liking factor) \|\| id:ebi-fl187-GCST90094716 | MR Egger | 15 | -1.153336358 | 0.824236964477209 | 0.185135713381933 | -2.768840808 | 0.462168092542013 | 0.315582116141817 | 0.0627346841402223 | 1.58751212974856 | 0.999950574162623 |
| ebi-fl187-GCST90094716 | ebi-a-GCST90002042 | CD45 on CD33+ HLA DR+ CD14- \|\| id:ebi-a-GCST90002042 | F-burn/spicy liking (derived food-liking factor) \|\| id:ebi-fl187-GCST90094716 | MR Egger | 15 | 0.0502179270760365 | 1.21930703053262 | 0.967773738643161 | -2.339623853 | 2.44005970691997 | 1.05150022177759 | 0.0963638784203846 | 11.4737257831709 | 0.999950574162623 |
| ebi-fl187-GCST90094716 | ebi-a-GCST90002056 | CD8 on Effector Memory CD8+ T cell \|\| id:ebi-a-GCST90002056 | F-burn/spicy liking (derived food-liking factor) \|\| id:ebi-fl187-GCST90094716 | MR Egger | 15 | -1.097175808 | 0.80693668014733 | 0.197047774767863 | -2.678771701 | 0.484420085033139 | 0.333812504281653 | 0.0686474219404803 | 1.62323339850115 | 0.999950574162623 |
| ebi-fl187-GCST90094716 | ebi-a-GCST90002058 | CD8 on CD8+ T cell \|\| id:ebi-a-GCST90002058 | F-burn/spicy liking (derived food-liking factor) \|\| id:ebi-fl187-GCST90094716 | MR Egger | 15 | -1.251426382 | 1.09305988604352 | 0.272898671348312 | -3.393823759 | 0.890970994434683 | 0.286096422832655 | 0.0335800291634644 | 2.43749529695753 | 0.999950574162623 |
| ebi-fl187-GCST90094716 | ebi-a-GCST90002063 | CD4 on CD4 regulatory T cell \|\| id:ebi-a-GCST90002063 | F-burn/spicy liking (derived food-liking factor) \|\| id:ebi-fl187-GCST90094716 | MR Egger | 15 | -1.153344346 | 0.803171337276482 | 0.174630302264653 | -2.727560167 | 0.42087147494269 | 0.315579595191718 | 0.0653786081076302 | 1.523288485 | 0.999950574162623 |
| ebi-fl187-GCST90094716 | ebi-a-GCST90002066 | CD4 on activated CD4 regulatory T cell \|\| id:ebi-a-GCST90002066 | F-burn/spicy liking (derived food-liking factor) \|\| id:ebi-fl187-GCST90094716 | MR Egger | 15 | -0.934478696 | 0.803319633719404 | 0.265627496497822 | -2.508985178 | 0.640027785743438 | 0.392790575330843 | 0.0813507538642188 | 1.89653357516819 | 0.999950574162623 |
| ebi-fl187-GCST90094716 | ebi-a-GCST90002070 | CD4 on activated & secreting CD4 regulatory T cell \|\| id:ebi-a-GCST90002070 | F-burn/spicy liking (derived food-liking factor) \|\| id:ebi-fl187-GCST90094716 | MR Egger | 15 | -1.331575293 | 0.806935002651498 | 0.122848769346623 | -2.913167898 | 0.250017311993843 | 0.264060960052078 | 0.0543034293510594 | 1.28404764592027 | 0.999950574162623 |
| ebi-fl187-GCST90094716 | ebi-a-GCST90002081 | SSC-A on CD4+ T cell \|\| id:ebi-a-GCST90002081 | F-burn/spicy liking (derived food-liking factor) \|\| id:ebi-fl187-GCST90094716 | MR Egger | 15 | 0.800230212566696 | 0.765935320016873 | 0.315165883567639 | -0.701003015 | 2.30146343979977 | 2.22605333496084 | 0.496087471157054 | 9.98878975623539 | 0.999950574162623 |
| ebi-fl187-GCST90094716 | ebi-a-GCST90002094 | CD11b on Monocytic Myeloid-Derived Suppressor Cells \|\| id:ebi-a-GCST90002094 | F-burn/spicy liking (derived food-liking factor) \|\| id:ebi-fl187-GCST90094716 | MR Egger | 15 | 0.0146045184735351 | 1.10977711594472 | 0.989700066960329 | -2.160558629 | 2.18976766572518 | 1.01471168552563 | 0.115260715097793 | 8.93313739957859 | 0.999950574162623 |
| ebi-fl187-GCST90094716 | ebi-a-GCST90002104 | HLA DR on myeloid Dendritic Cell \|\| id:ebi-a-GCST90002104 | F-burn/spicy liking (derived food-liking factor) \|\| id:ebi-fl187-GCST90094716 | MR Egger | 15 | -1.233728812 | 0.818366383316205 | 0.155581024016826 | -2.837726923 | 0.370269299459391 | 0.291204703156541 | 0.0585586230474135 | 1.44812454131356 | 0.999950574162623 |
| ebi-fl187-GCST90094716 | ebi-a-GCST90002105 | HLA DR on plasmacytoid Dendritic Cell \|\| id:ebi-a-GCST90002105 | F-burn/spicy liking (derived food-liking factor) \|\| id:ebi-fl187-GCST90094716 | MR Egger | 15 | -1.092965534 | 0.801318910017431 | 0.195731243739522 | -2.663550598 | 0.477619529206241 | 0.3352209090693 | 0.0697003041124673 | 1.61223195950371 | 0.999950574162623 |
| ebi-fl187-GCST90094716 | ebi-a-GCST90002106 | HLA DR on Dendritic Cell \|\| id:ebi-a-GCST90002106 | F-burn/spicy liking (derived food-liking factor) \|\| id:ebi-fl187-GCST90094716 | MR Egger | 15 | -1.465104003 | 0.816839828966218 | 0.0961551525161756 | -3.066110068 | 0.135902061372156 | 0.231053959829883 | 0.0466020816533535 | 1.14556969257675 | 0.999950574162623 |
| ebi-fl187-GCST90094716 | ebi-a-GCST90002111 | HLA DR on CD33dim HLA DR+ CD11b- \|\| id:ebi-a-GCST90002111 | F-burn/spicy liking (derived food-liking factor) \|\| id:ebi-fl187-GCST90094716 | MR Egger | 15 | 0.0703446769418032 | 1.14913723240323 | 0.952118938777239 | -2.181964299 | 2.32265365245214 | 1.07287791380967 | 0.112819701264517 | 10.2027128687551 | 0.999950574162623 |
| ebi-fl187-GCST90094716 | ebi-a-GCST90002112 | HLA DR on CD33- HLA DR+ \|\| id:ebi-a-GCST90002112 | F-burn/spicy liking (derived food-liking factor) \|\| id:ebi-fl187-GCST90094716 | MR Egger | 15 | -0.727368562 | 1.06244273241586 | 0.505607397895729 | -2.809756318 | 1.35501919307245 | 0.483178773441388 | 0.0602196650531249 | 3.87683536430114 | 0.999950574162623 |
| ebi-fl187-GCST90094716 | ebi-a-GCST90002115 | HLA DR on HLA DR+ CD8+ T cell \|\| id:ebi-a-GCST90002115 | F-burn/spicy liking (derived food-liking factor) \|\| id:ebi-fl187-GCST90094716 | MR Egger | 15 | 0.349574186314947 | 0.843811444526612 | 0.685423244254696 | -1.304296245 | 2.00344461758711 | 1.41846341884354 | 0.271363441252327 | 7.4145524589158 | 0.999950574162623 |
| ebi-fl187-GCST90094716 | ebi-a-GCST90002116 | HLA DR on B cell \|\| id:ebi-a-GCST90002116 | F-burn/spicy liking (derived food-liking factor) \|\| id:ebi-fl187-GCST90094716 | MR Egger | 15 | -0.690274374 | 0.79613261552057 | 0.401643804628664 | -2.250694301 | 0.870145552060986 | 0.501438468331369 | 0.105326071196235 | 2.38725829860346 | 0.999950574162623 |
| ebi-fl187-GCST90094716 | ebi-cfmet338-GCST90026023 | Sphingomyelin (d18:1/24:1, d18:2/24:0) levels \|\| id:ebi-cfmet338-GCST90026023 | F-burn/spicy liking (derived food-liking factor) \|\| id:ebi-fl187-GCST90094716 | MR Egger | 12 | 0.0384819022890462 | 0.588295975638729 | 0.949134805513901 | -1.11457821 | 1.19154201454095 | 1.03923192046888 | 0.32805361947054 | 3.2921538444371 | 0.999950574162623 |
| ebi-fl187-GCST90094716 | ebi-cfmet338-GCST90026046 | 1-arachidonoyl-gpc (20:4n6) levels \|\| id:ebi-cfmet338-GCST90026046 | F-burn/spicy liking (derived food-liking factor) \|\| id:ebi-fl187-GCST90094716 | MR Egger | 12 | 0.580182372166379 | 0.754101474258322 | 0.459460992876104 | -0.897856517 | 2.05822126171269 | 1.7863641841512 | 0.40744206940355 | 7.83202628803058 | 0.999950574162623 |
| ebi-fl187-GCST90094716 | ebi-cfmet338-GCST90026047 | 1-linoleoyl-gpc (18:2) levels \|\| id:ebi-cfmet338-GCST90026047 | F-burn/spicy liking (derived food-liking factor) \|\| id:ebi-fl187-GCST90094716 | MR Egger | 12 | 0.363481043316531 | 0.944398424621964 | 0.708384548865304 | -1.487539869 | 2.21450195557558 | 1.43832759072597 | 0.2259277843737 | 9.15684745892807 | 0.999950574162623 |
| ebi-fl187-GCST90094716 | ebi-cfmet338-GCST90026050 | 1-myristoyl-2-palmitoyl-gpc (14:0/16:0) levels \|\| id:ebi-cfmet338-GCST90026050 | F-burn/spicy liking (derived food-liking factor) \|\| id:ebi-fl187-GCST90094716 | MR Egger | 12 | -0.493631734 | 0.366065605952321 | 0.207250937216815 | -1.211120322 | 0.223856853211157 | 0.610405533030363 | 0.297863389488106 | 1.25089194544656 | 0.999950574162623 |
| ebi-fl187-GCST90094716 | ebi-cfmet338-GCST90026068 | 2,3-dihydroxyisovalerate levels \|\| id:ebi-cfmet338-GCST90026068 | F-burn/spicy liking (derived food-liking factor) \|\| id:ebi-fl187-GCST90094716 | MR Egger | 12 | 1.56087507792173 | 1.20528345071326 | 0.224410286481657 | -0.801480485 | 3.92323064131972 | 4.76298740713528 | 0.448664231296841 | 50.5635338367767 | 0.999950574162623 |
| ebi-fl187-GCST90094716 | ebi-cfmet338-GCST90026082 | 3-hydroxyhexanoate levels \|\| id:ebi-cfmet338-GCST90026082 | F-burn/spicy liking (derived food-liking factor) \|\| id:ebi-fl187-GCST90094716 | MR Egger | 12 | 0.151630991288829 | 0.421164115649227 | 0.726313603855427 | -0.673850675 | 0.977112657961314 | 1.16373073041428 | 0.509741943028535 | 2.65677414117506 | 0.999950574162623 |
| ebi-fl187-GCST90094716 | ebi-cfmet338-GCST90026094 | 3-ureidopropionate levels \|\| id:ebi-cfmet338-GCST90026094 | F-burn/spicy liking (derived food-liking factor) \|\| id:ebi-fl187-GCST90094716 | MR Egger | 12 | -0.370739239 | 0.716558246800441 | 0.616135344970094 | -1.775193402 | 1.03371492505678 | 0.690223901795629 | 0.169450674162933 | 2.8114909366008 | 0.999950574162623 |
| ebi-fl187-GCST90094716 | ebi-cfmet338-GCST90026173 | Glycerol levels \|\| id:ebi-cfmet338-GCST90026173 | F-burn/spicy liking (derived food-liking factor) \|\| id:ebi-fl187-GCST90094716 | MR Egger | 12 | 0.0938280890953763 | 0.313831258144889 | 0.771079133746743 | -0.521281177 | 0.70893735505936 | 1.09837090774961 | 0.593759349715142 | 2.03183099612575 | 0.999950574162623 |
| ebi-fl187-GCST90094716 | ebi-cfmet338-GCST90026179 | Histidine levels \|\| id:ebi-cfmet338-GCST90026179 | F-burn/spicy liking (derived food-liking factor) \|\| id:ebi-fl187-GCST90094716 | MR Egger | 12 | 0.0553186248947834 | 0.324242069433924 | 0.867933704929001 | -0.580195831 | 0.690833080985274 | 1.05687730845434 | 0.559788731734114 | 1.99537715177918 | 0.999950574162623 |
| ebi-fl187-GCST90094716 | ebi-cfmet338-GCST90026188 | Isocitrate levels \|\| id:ebi-cfmet338-GCST90026188 | F-burn/spicy liking (derived food-liking factor) \|\| id:ebi-fl187-GCST90094716 | MR Egger | 12 | -0.087303956 | 0.484289078010254 | 0.860539579321197 | -1.036510549 | 0.861902636898995 | 0.916398508470126 | 0.3546901991719 | 2.36766121050689 | 0.999950574162623 |
| ebi-fl187-GCST90094716 | ebi-cfmet338-GCST90026207 | N-acetyl-isoputreanine levels \|\| id:ebi-cfmet338-GCST90026207 | F-burn/spicy liking (derived food-liking factor) \|\| id:ebi-fl187-GCST90094716 | MR Egger | 12 | -0.201257399 | 0.276681416246407 | 0.483664349392848 | -0.743552975 | 0.341038176436966 | 0.817701928472671 | 0.475421749480396 | 1.40640693144287 | 0.999950574162623 |
| ebi-fl187-GCST90094716 | ebi-cfmet338-GCST90026213 | N-acetylglutamate levels \|\| id:ebi-cfmet338-GCST90026213 | F-burn/spicy liking (derived food-liking factor) \|\| id:ebi-fl187-GCST90094716 | MR Egger | 12 | -0.234862383 | 0.535881387929885 | 0.67050453922881 | -1.285189904 | 0.815465136856184 | 0.790679652719202 | 0.27659805161503 | 2.26022674264632 | 0.999950574162623 |
| ebi-fl187-GCST90094716 | ebi-cfmet338-GCST90026224 | N-acetyltaurine levels \|\| id:ebi-cfmet338-GCST90026224 | F-burn/spicy liking (derived food-liking factor) \|\| id:ebi-fl187-GCST90094716 | MR Egger | 12 | -0.129497593 | 0.446677854520419 | 0.777805434764809 | -1.004986188 | 0.745991001423738 | 0.878536702668255 | 0.366049690538964 | 2.10852995613461 | 0.999950574162623 |
| ebi-fl187-GCST90094716 | ebi-cfmet338-GCST90026250 | Phenyllactate (pla) levels \|\| id:ebi-cfmet338-GCST90026250 | F-burn/spicy liking (derived food-liking factor) \|\| id:ebi-fl187-GCST90094716 | MR Egger | 12 | 0.208288758541146 | 0.621143723386526 | 0.744303903535573 | -1.009152939 | 1.42573045637874 | 1.23156874420269 | 0.364527625859223 | 4.16089608605614 | 0.999950574162623 |
| ebi-fl187-GCST90094716 | ebi-cfmet338-GCST90026251 | Pipecolate levels \|\| id:ebi-cfmet338-GCST90026251 | F-burn/spicy liking (derived food-liking factor) \|\| id:ebi-fl187-GCST90094716 | MR Egger | 12 | -1.67328524 | 1.04079847876378 | 0.138981729179812 | -3.713250258 | 0.366679778633651 | 0.187629643642091 | 0.0243980941553633 | 1.44293578625769 | 0.999950574162623 |
| ebi-fl187-GCST90094716 | ebi-cfmet338-GCST90026278 | Trigonelline (n'-methylnicotinate) levels \|\| id:ebi-cfmet338-GCST90026278 | F-burn/spicy liking (derived food-liking factor) \|\| id:ebi-fl187-GCST90094716 | MR Egger | 12 | 0.633438600605644 | 1.20579202476016 | 0.610802996187028 | -1.729913768 | 2.99679096913555 | 1.88407804528305 | 0.177299698231706 | 20.0211851239509 | 0.999950574162623 |
| ebi-fl187-GCST90094716 | ebi-cfmet338-GCST90026299 | 1-carboxyethylvaline levels \|\| id:ebi-cfmet338-GCST90026299 | F-burn/spicy liking (derived food-liking factor) \|\| id:ebi-fl187-GCST90094716 | MR Egger | 12 | -0.515375411 | 0.782147179452789 | 0.524826145956911 | -2.048383883 | 1.01763306065132 | 0.597276328375661 | 0.12894312248702 | 2.7666385423063 | 0.999950574162623 |
| ebi-fl187-GCST90094716 | ebi-gm430-GCST90011306 | C_Erysipelotrichia abundance \|\| id:ebi-gm430-GCST90011306 | F-burn/spicy liking (derived food-liking factor) \|\| id:ebi-fl187-GCST90094716 | MR Egger | 12 | 0.0307773036337015 | 0.542625681522195 | 0.955886171662314 | -1.032769032 | 1.0943236394172 | 1.0312558213886 | 0.356019764237599 | 2.98716160161861 | 0.999950574162623 |
| ebi-fl187-GCST90094716 | ebi-gm430-GCST90011312 | F_Erysipelotrichaceae abundance \|\| id:ebi-gm430-GCST90011312 | F-burn/spicy liking (derived food-liking factor) \|\| id:ebi-fl187-GCST90094716 | MR Egger | 12 | 0.0307773036337015 | 0.542625681522195 | 0.955886171662314 | -1.032769032 | 1.0943236394172 | 1.0312558213886 | 0.356019764237599 | 2.98716160161861 | 0.999950574162623 |
| ebi-fl187-GCST90094716 | ebi-gm430-GCST90011352 | O_Erysipelotrichales abundance \|\| id:ebi-gm430-GCST90011352 | F-burn/spicy liking (derived food-liking factor) \|\| id:ebi-fl187-GCST90094716 | MR Egger | 12 | 0.0307773036337015 | 0.542625681522195 | 0.955886171662314 | -1.032769032 | 1.0943236394172 | 1.0312558213886 | 0.356019764237599 | 2.98716160161861 | 0.999950574162623 |
| ebi-fl187-GCST90094716 | ebi-gm430-GCST90011362 | OTU97_11 (Parabacteroides) abundance \|\| id:ebi-gm430-GCST90011362 | F-burn/spicy liking (derived food-liking factor) \|\| id:ebi-fl187-GCST90094716 | MR Egger | 12 | 0.381692625860543 | 0.707712476756491 | 0.601458134772032 | -1.005423829 | 1.76880908030327 | 1.46476178603786 | 0.365889527497209 | 5.86386580811116 | 0.999950574162623 |
| ebi-fl187-GCST90094716 | ebi-gm430-GCST90011384 | OTU97_235 (Phascolarctobacterium) abundance \|\| id:ebi-gm430-GCST90011384 | F-burn/spicy liking (derived food-liking factor) \|\| id:ebi-fl187-GCST90094716 | MR Egger | 12 | -2.102843536 | 2.69006698020895 | 0.452497589980137 | -7.375374817 | 3.1696877455081 | 0.122108713631137 | 0.000626491834701522 | 23.8000515230257 | 0.999950574162623 |
| ebi-fl187-GCST90094716 | ebi-gm430-GCST90011390 | OTU97_3 (Bacteroides) abundance \|\| id:ebi-gm430-GCST90011390 | F-burn/spicy liking (derived food-liking factor) \|\| id:ebi-fl187-GCST90094716 | MR Egger | 12 | 0.342491061696092 | 0.573964630170174 | 0.563964430933731 | -0.782479613 | 1.46746173682963 | 1.40845176443606 | 0.457270749686444 | 4.33820963642067 | 0.999950574162623 |
| ebi-fl187-GCST90094716 | ebi-gm430-GCST90011398 | OTU97_4 (Alistipes) abundance \|\| id:ebi-gm430-GCST90011398 | F-burn/spicy liking (derived food-liking factor) \|\| id:ebi-fl187-GCST90094716 | MR Egger | 12 | 0.0282586644240832 | 0.658088755028405 | 0.966594270293237 | -1.261595295 | 1.31811262427976 | 1.02866172820541 | 0.283201875280423 | 3.73636279783383 | 0.999950574162623 |
| ebi-fl187-GCST90094716 | ebi-gm430-GCST90011421 | OTU99_108 (Cytophagales) abundance \|\| id:ebi-gm430-GCST90011421 | F-burn/spicy liking (derived food-liking factor) \|\| id:ebi-fl187-GCST90094716 | MR Egger | 12 | 1.76314085794421 | 2.07393899426473 | 0.41513733811463 | -2.301779571 | 5.82806128670308 | 5.83072213442193 | 0.100080584669951 | 339.699460399391 | 0.999950574162623 |
| ebi-fl187-GCST90094716 | ebi-gm430-GCST90011428 | OTU99_123 (Phascolarctobacterium) abundance \|\| id:ebi-gm430-GCST90011428 | F-burn/spicy liking (derived food-liking factor) \|\| id:ebi-fl187-GCST90094716 | MR Egger | 13 | -1.051958826 | 1.06177810756046 | 0.343100041482058 | -3.133043917 | 1.02912626499815 | 0.349252952929868 | 0.0435849262490018 | 2.79861951431032 | 0.999950574162623 |
| ebi-fl187-GCST90094716 | ebi-gm430-GCST90011450 | OTU99_298 (Phascolarctobacterium) abundance \|\| id:ebi-gm430-GCST90011450 | F-burn/spicy liking (derived food-liking factor) \|\| id:ebi-fl187-GCST90094716 | MR Egger | 12 | -2.138372929 | 2.67320203453154 | 0.442339473672065 | -7.377848917 | 3.10110305875169 | 0.117846431634967 | 0.000624943747291588 | 22.2224504353912 | 0.999950574162623 |
| ebi-fl187-GCST90094716 | ebi-gm430-GCST90011451 | OTU99_3 (Bacteroides) abundance \|\| id:ebi-gm430-GCST90011451 | F-burn/spicy liking (derived food-liking factor) \|\| id:ebi-fl187-GCST90094716 | MR Egger | 12 | 0.314436662911457 | 0.573485860603384 | 0.59551985351615 | -0.809595624 | 1.43846894969409 | 1.36948761045038 | 0.445037992582147 | 4.21423866374937 | 0.999950574162623 |
| ebi-fl187-GCST90094716 | ebi-gm430-GCST90011472 | OTU99_72 (Parabacteroides) abundance \|\| id:ebi-gm430-GCST90011472 | F-burn/spicy liking (derived food-liking factor) \|\| id:ebi-fl187-GCST90094716 | MR Egger | 13 | 0.235940364812284 | 1.33875774490019 | 0.863309457202207 | -2.388024815 | 2.85990554481666 | 1.26609880387565 | 0.0918108482947563 | 17.4598776827434 | 0.999950574162623 |
| ebi-fl187-GCST90094716 | ebi-gm430-GCST90011507 | TestASV_3 (Bacteroides) abundance \|\| id:ebi-gm430-GCST90011507 | F-burn/spicy liking (derived food-liking factor) \|\| id:ebi-fl187-GCST90094716 | MR Egger | 12 | 0.153104350605289 | 0.798391854954034 | 0.851763510853385 | -1.411743685 | 1.7179523863152 | 1.16544658765451 | 0.243717945082807 | 5.57310520657004 | 0.999950574162623 |
| ebi-fl187-GCST90094716 | ebi-gm430-GCST90011512 | TestASV_34 (Phascolarctobacterium) abundance \|\| id:ebi-gm430-GCST90011512 | F-burn/spicy liking (derived food-liking factor) \|\| id:ebi-fl187-GCST90094716 | MR Egger | 12 | -2.384564408 | 1.83524519121851 | 0.22299125334253 | -5.981644983 | 1.21251616632325 | 0.0921291014970003 | 0.00252466983491667 | 3.36193320221801 | 0.999950574162623 |
| ebi-fl187-GCST90094716 | ebi-gm430-GCST90011519 | TestASV_41 (Barnesiella) abundance \|\| id:ebi-gm430-GCST90011519 | F-burn/spicy liking (derived food-liking factor) \|\| id:ebi-fl187-GCST90094716 | MR Egger | 12 | -4.569977645 | 2.57343395400265 | 0.1061436534107 | -9.613908195 | 0.473952904827946 | 0.0103581912722128 | 6.67932724137561e-05 | 1.60633133479546 | 0.999950574162623 |
| ebi-fl187-GCST90094716 | ebi-gm430-GCST90011524 | TestASV_46 (Sutterella) abundance \|\| id:ebi-gm430-GCST90011524 | F-burn/spicy liking (derived food-liking factor) \|\| id:ebi-fl187-GCST90094716 | MR Egger | 12 | -1.109054988 | 2.33308244791184 | 0.644742916607349 | -5.681896586 | 3.46378660957652 | 0.329870545341326 | 0.003407090470902 | 31.9376833732788 | 0.999950574162623 |
| ebi-fl187-GCST90094716 | ebi-gm430-GCST90011581 | OTU97_19 (Bacteria) prevalence \|\| id:ebi-gm430-GCST90011581 | F-burn/spicy liking (derived food-liking factor) \|\| id:ebi-fl187-GCST90094716 | MR Egger | 12 | -2.770785673 | 2.70727552659057 | 0.330211562798604 | -8.077045705 | 2.53547435929823 | 0.0626127922432187 | 0.000310587247744178 | 12.6224169889987 | 0.999950574162623 |
| ebi-fl187-GCST90094716 | ebi-gm430-GCST90011614 | OTU97_80 (Ruminococcus) prevalence \|\| id:ebi-gm430-GCST90011614 | F-burn/spicy liking (derived food-liking factor) \|\| id:ebi-fl187-GCST90094716 | MR Egger | 12 | 0.27387067560512 | 1.25876633155427 | 0.832138791723851 | -2.193311334 | 2.74105268545149 | 1.31504472387817 | 0.111546767758674 | 15.5032966041754 | 0.999950574162623 |
| ebi-fl187-GCST90094716 | ebi-gm430-GCST90011623 | OTU99_11 (Parabacteroides) prevalence \|\| id:ebi-gm430-GCST90011623 | F-burn/spicy liking (derived food-liking factor) \|\| id:ebi-fl187-GCST90094716 | MR Egger | 12 | 1.05900375057821 | 1.09853710128996 | 0.357772255888586 | -1.094128968 | 3.21213646910653 | 2.88349687574351 | 0.33483112861214 | 24.8320825691626 | 0.999950574162623 |
| ebi-fl187-GCST90094716 | ebi-gm430-GCST90011642 | OTU99_19 (Bacteria) prevalence \|\| id:ebi-gm430-GCST90011642 | F-burn/spicy liking (derived food-liking factor) \|\| id:ebi-fl187-GCST90094716 | MR Egger | 12 | -2.770785673 | 2.70727552659057 | 0.330211562798604 | -8.077045705 | 2.53547435929823 | 0.0626127922432187 | 0.000310587247744178 | 12.6224169889987 | 0.999950574162623 |
| ebi-fl187-GCST90094716 | ebi-gm430-GCST90011653 | OTU99_30 (Parasutterella) prevalence \|\| id:ebi-gm430-GCST90011653 | F-burn/spicy liking (derived food-liking factor) \|\| id:ebi-fl187-GCST90094716 | MR Egger | 12 | -0.152712666 | 1.47900009765468 | 0.919802744411865 | -3.051552857 | 2.74612752588015 | 0.858376327493639 | 0.0472854398276551 | 15.5821733346877 | 0.999950574162623 |
| ebi-fl187-GCST90094716 | ebi-gm430-GCST90011675 | OTU99_8 (Ruminococcaceae) prevalence \|\| id:ebi-gm430-GCST90011675 | F-burn/spicy liking (derived food-liking factor) \|\| id:ebi-fl187-GCST90094716 | MR Egger | 12 | -0.677694649 | 1.75117344111833 | 0.706868202274746 | -4.109994593 | 2.75460529584198 | 0.507786269761936 | 0.0164078632377601 | 15.714836967031 | 0.999950574162623 |
| ebi-fl187-GCST90094716 | ebi-gm430-GCST90011679 | OTU99_92 (Ruminococcus) prevalence \|\| id:ebi-gm430-GCST90011679 | F-burn/spicy liking (derived food-liking factor) \|\| id:ebi-fl187-GCST90094716 | MR Egger | 12 | 1.19274379317549 | 1.31591306246869 | 0.386043515480152 | -1.386445809 | 3.77193339561413 | 3.29611266292423 | 0.249962140831108 | 43.4640167929677 | 0.999950574162623 |
| ebi-fl187-GCST90094716 | ebi-gm430-GCST90011684 | TestASV_11 (Lachnospiraceae) prevalence \|\| id:ebi-gm430-GCST90011684 | F-burn/spicy liking (derived food-liking factor) \|\| id:ebi-fl187-GCST90094716 | MR Egger | 12 | -1.402823508 | 1.64982947637531 | 0.41506163289444 | -4.636489282 | 1.83084226536051 | 0.2459016773941 | 0.00969166266609092 | 6.23913945713315 | 0.999950574162623 |
| ebi-fl187-GCST90094716 | ebi-gm430-GCST90011688 | TestASV_15 (Bacteroides) prevalence \|\| id:ebi-gm430-GCST90011688 | F-burn/spicy liking (derived food-liking factor) \|\| id:ebi-fl187-GCST90094716 | MR Egger | 12 | -0.97783029 | 1.26055038425963 | 0.455869621149829 | -3.448509043 | 1.49284846360905 | 0.376126299323089 | 0.0317930030688106 | 4.4497524419537 | 0.999950574162623 |
| ebi-fl187-GCST90094716 | ebi-gm430-GCST90011692 | TestASV_19 (Prevotella) prevalence \|\| id:ebi-gm430-GCST90011692 | F-burn/spicy liking (derived food-liking factor) \|\| id:ebi-fl187-GCST90094716 | MR Egger | 12 | -1.743652461 | 2.42936716280688 | 0.489345458244272 | -6.5052121 | 3.01790717817483 | 0.174880488550215 | 0.00149562350328915 | 20.4484519053786 | 0.999950574162623 |
| ebi-fl187-GCST90094716 | ebi-gm430-GCST90011693 | TestASV_2 (Bacteroides) prevalence \|\| id:ebi-gm430-GCST90011693 | F-burn/spicy liking (derived food-liking factor) \|\| id:ebi-fl187-GCST90094716 | MR Egger | 12 | -1.935230009 | 1.50338480629528 | 0.227002849372626 | -4.88186423 | 1.01140421100723 | 0.144391053714054 | 0.00758286464157239 | 2.74945912635092 | 0.999950574162623 |
| ebi-fl187-GCST90094716 | ebi-gm430-GCST90011716 | TestASV_40 (Barnesiella) prevalence \|\| id:ebi-gm430-GCST90011716 | F-burn/spicy liking (derived food-liking factor) \|\| id:ebi-fl187-GCST90094716 | MR Egger | 12 | 0.130812454639394 | 2.74696192990026 | 0.962956115439057 | -5.253232928 | 5.5148578372439 | 1.1397540056903 | 0.0052305809437402 | 248.354667953613 | 0.999950574162623 |
| ebi-fl187-GCST90094716 | ebi-gm430-GCST90011718 | TestASV_42 (Alphaproteobacteria) prevalence \|\| id:ebi-gm430-GCST90011718 | F-burn/spicy liking (derived food-liking factor) \|\| id:ebi-fl187-GCST90094716 | MR Egger | 12 | -0.796044551 | 2.05162626289042 | 0.706142615594647 | -4.817232026 | 3.22514292416937 | 0.451109781520238 | 0.00808914672434679 | 25.1571694664334 | 0.999950574162623 |
| ebi-fl187-GCST90094716 | ebi-hbsm44-GCST90104929 | Sulcal depth of the right inferior frontal sulcus \|\| id:ebi-hbsm44-GCST90104929 | F-burn/spicy liking (derived food-liking factor) \|\| id:ebi-fl187-GCST90094716 | MR Egger | 11 | 1.13440722611763 | 1.02840297344674 | 0.298614141791932 | -0.881262602 | 3.15007705407323 | 3.10932986698083 | 0.414259536491701 | 23.3378627890506 | 0.999950574162623 |
| ebi-fl187-GCST90094716 | ebi-hbsm44-GCST90104935 | Sulcal opening distance of the left calloso-marginal posterior fissure \|\| id:ebi-hbsm44-GCST90104935 | F-burn/spicy liking (derived food-liking factor) \|\| id:ebi-fl187-GCST90094716 | MR Egger | 11 | 0.344842599456103 | 0.281358158026704 | 0.251439785904271 | -0.20661939 | 0.896304589188442 | 1.41176768917638 | 0.813329152067981 | 2.45053064080487 | 0.999950574162623 |
| ebi-fl187-GCST90094716 | ebi-hbsm44-GCST90104936 | Sulcal opening distance of the left anterior inferior temporal sulcus \|\| id:ebi-hbsm44-GCST90104936 | F-burn/spicy liking (derived food-liking factor) \|\| id:ebi-fl187-GCST90094716 | MR Egger | 12 | -0.213769579 | 0.135644086948608 | 0.146113208811446 | -0.479631989 | 0.0520928319138827 | 0.80753443687858 | 0.619011152854284 | 1.05347353393859 | 0.999950574162623 |
| ebi-fl187-GCST90094716 | ebi-hbsm44-GCST90104941 | Sulcal opening distance of the right posterior inferior temporal sulcus \|\| id:ebi-hbsm44-GCST90104941 | F-burn/spicy liking (derived food-liking factor) \|\| id:ebi-fl187-GCST90094716 | MR Egger | 12 | 0.0379087395820921 | 0.107020257851899 | 0.730529204628702 | -0.171850966 | 0.247668444971813 | 1.03863644215748 | 0.842104666205711 | 1.28103512813693 | 0.999950574162623 |
| ebi-fl187-GCST90094716 | ebi-IPs91-GCST90274819 | Leukemia inhibitory factor levels \|\| id:ebi-IPs91-GCST90274819 | F-burn/spicy liking (derived food-liking factor) \|\| id:ebi-fl187-GCST90094716 | MR Egger | 15 | 0.0282179153257246 | 0.307601650944231 | 0.928306921187775 | -0.574681321 | 0.631117151176417 | 1.0286198120215 | 0.562884219875379 | 1.87970932622236 | 0.999950574162623 |
| ebi-fl187-GCST90094716 | ebi-IPs91-GCST90274837 | STAM binding protein levels \|\| id:ebi-IPs91-GCST90274837 | F-burn/spicy liking (derived food-liking factor) \|\| id:ebi-fl187-GCST90094716 | MR Egger | 15 | -0.28638858 | 0.2777957382912 | 0.321372119413971 | -0.830868227 | 0.258091067164419 | 0.750970747115827 | 0.435670860888605 | 1.29445669575753 | 0.999950574162623 |
| ebi-fl187-GCST90094716 | ebi-IPs91-GCST90274840 | TNF-beta levels \|\| id:ebi-IPs91-GCST90274840 | F-burn/spicy liking (derived food-liking factor) \|\| id:ebi-fl187-GCST90094716 | MR Egger | 15 | -0.196488481 | 0.388273898836781 | 0.621289399297414 | -0.957505322 | 0.564528361135239 | 0.821610795716588 | 0.383849272759741 | 1.75861815442482 | 0.999950574162623 |
| ebi-fl187-GCST90094716 | ebi-IPs91-GCST90274842 | Tumor necrosis factor ligand superfamily member 14 levels \|\| id:ebi-IPs91-GCST90274842 | F-burn/spicy liking (derived food-liking factor) \|\| id:ebi-fl187-GCST90094716 | MR Egger | 15 | -0.09190995 | 0.311255306399195 | 0.772437274377624 | -0.70197035 | 0.518150450719149 | 0.912187288473257 | 0.495607820065432 | 1.6789195318636 | 0.999950574162623 |
| ebi-fl187-GCST90094716 | ebi-met1400-GCST90199628 | 3-methylhistidine levels \|\| id:ebi-met1400-GCST90199628 | F-burn/spicy liking (derived food-liking factor) \|\| id:ebi-fl187-GCST90094716 | MR Egger | 14 | 1.14849464923286 | 0.429069209249776 | 0.0201591034395017 | 0.307518999103302 | 1.98947029936242 | 3.15344229813588 | 1.36004664792326 | 7.31165974553661 | 0.999950574162623 |
| ebi-fl187-GCST90094716 | ebi-met1400-GCST90199651 | Docosahexaenoate DHA; 22:6n3 levels \|\| id:ebi-met1400-GCST90199651 | F-burn/spicy liking (derived food-liking factor) \|\| id:ebi-fl187-GCST90094716 | MR Egger | 14 | -0.729711602 | 0.413454066464048 | 0.102989243475792 | -1.540081572 | 0.0806583685428333 | 0.482047991853802 | 0.214363614645805 | 1.08400050462963 | 0.999950574162623 |
| ebi-fl187-GCST90094716 | ebi-met1400-GCST90199652 | 1-stearoyl-2-oleoyl-GPS (18:0/18:1) levels \|\| id:ebi-met1400-GCST90199652 | F-burn/spicy liking (derived food-liking factor) \|\| id:ebi-fl187-GCST90094716 | MR Egger | 14 | -0.135039163 | 0.43116704099865 | 0.759509555596238 | -0.980126563 | 0.710048237586123 | 0.873681695221674 | 0.375263601310318 | 2.03408937584171 | 0.999950574162623 |
| ebi-fl187-GCST90094716 | ebi-met1400-GCST90199653 | 1-myristoyl-2-palmitoyl-gpc (14:0/16:0) levels \|\| id:ebi-met1400-GCST90199653 | F-burn/spicy liking (derived food-liking factor) \|\| id:ebi-fl187-GCST90094716 | MR Egger | 14 | 0.397374262340799 | 0.363471047468744 | 0.295733988113023 | -0.315028991 | 1.10977751537954 | 1.48791269554497 | 0.72976771748692 | 3.03368337145384 | 0.999950574162623 |
| ebi-fl187-GCST90094716 | ebi-met1400-GCST90199667 | Erythritol levels in elite athletes \|\| id:ebi-met1400-GCST90199667 | F-burn/spicy liking (derived food-liking factor) \|\| id:ebi-fl187-GCST90094716 | MR Egger | 14 | 1.30794247381285 | 0.395413981437703 | 0.0062500302882744 | 0.532931070194951 | 2.08295387743075 | 3.69855600224947 | 1.70391930351729 | 8.02814808984103 | 0.999950574162623 |
| ebi-fl187-GCST90094716 | ebi-met1400-GCST90199674 | 3-hydroxymyristate levels \|\| id:ebi-met1400-GCST90199674 | F-burn/spicy liking (derived food-liking factor) \|\| id:ebi-fl187-GCST90094716 | MR Egger | 14 | -1.160330109 | 0.404903207891539 | 0.0142028077589439 | -1.953940397 | -0.366719822 | 0.31338271326024 | 0.141714558371913 | 0.693003782382138 | 0.999950574162623 |
| ebi-fl187-GCST90094716 | ebi-met1400-GCST90199685 | 2-linoleoylglycerol (18:2) levels \|\| id:ebi-met1400-GCST90199685 | F-burn/spicy liking (derived food-liking factor) \|\| id:ebi-fl187-GCST90094716 | MR Egger | 14 | 0.204229841234598 | 0.416765619393276 | 0.632945644916216 | -0.612630773 | 1.02109045524542 | 1.22658003973109 | 0.541923315008726 | 2.77622045813345 | 0.999950574162623 |
| ebi-fl187-GCST90094716 | ebi-met1400-GCST90199703 | Gamma-glutamylvaline levels \|\| id:ebi-met1400-GCST90199703 | F-burn/spicy liking (derived food-liking factor) \|\| id:ebi-fl187-GCST90094716 | MR Egger | 14 | -0.065124228 | 0.357597504269928 | 0.858531936492458 | -0.766015336 | 0.635766880537703 | 0.936951060748966 | 0.464861700365898 | 1.8884698170394 | 0.999950574162623 |
| ebi-fl187-GCST90094716 | ebi-met1400-GCST90199779 | Gamma-glutamyl-2-aminobutyrate levels \|\| id:ebi-met1400-GCST90199779 | F-burn/spicy liking (derived food-liking factor) \|\| id:ebi-fl187-GCST90094716 | MR Egger | 14 | 0.742225285519547 | 0.379331665200256 | 0.0740620087791295 | -0.001264778 | 1.48571534931205 | 2.1006047627231 | 0.998736021221987 | 4.41812478514203 | 0.999950574162623 |
| ebi-fl187-GCST90094716 | ebi-met1400-GCST90199824 | 4-vinylphenol sulfate levels \|\| id:ebi-met1400-GCST90199824 | F-burn/spicy liking (derived food-liking factor) \|\| id:ebi-fl187-GCST90094716 | MR Egger | 14 | 0.377866716298465 | 0.381554096181037 | 0.341559355577694 | -0.369979312 | 1.1257127448133 | 1.45916844654277 | 0.690748620547547 | 3.08241304006988 | 0.999950574162623 |
| ebi-fl187-GCST90094716 | ebi-met1400-GCST90199830 | 4-ethylphenylsulfate levels \|\| id:ebi-met1400-GCST90199830 | F-burn/spicy liking (derived food-liking factor) \|\| id:ebi-fl187-GCST90094716 | MR Egger | 14 | 0.359675003380815 | 0.367207468733261 | 0.346674275273845 | -0.360051635 | 1.07940164209801 | 1.43286366303437 | 0.697640302249313 | 2.9429181058272 | 0.999950574162623 |
| ebi-fl187-GCST90094716 | ebi-met1400-GCST90199833 | Hexanoylglutamine levels \|\| id:ebi-met1400-GCST90199833 | F-burn/spicy liking (derived food-liking factor) \|\| id:ebi-fl187-GCST90094716 | MR Egger | 14 | -1.016452717 | 0.518913200960695 | 0.0737853947836781 | -2.033522591 | 0.000617156716431921 | 0.361876343821091 | 0.130873693709371 | 1.00061734719682 | 0.999950574162623 |
| ebi-fl187-GCST90094716 | ebi-met1400-GCST90199837 | Carotenoid levels (cryptoxanthin) \|\| id:ebi-met1400-GCST90199837 | F-burn/spicy liking (derived food-liking factor) \|\| id:ebi-fl187-GCST90094716 | MR Egger | 14 | 0.413932952818577 | 0.388053381762377 | 0.307102444930023 | -0.346651675 | 1.17451758107284 | 1.51275569747846 | 0.707051568799097 | 3.23658117913571 | 0.999950574162623 |
| ebi-fl187-GCST90094716 | ebi-met1400-GCST90199869 | 2-hydroxyhippurate levels \|\| id:ebi-met1400-GCST90199869 | F-burn/spicy liking (derived food-liking factor) \|\| id:ebi-fl187-GCST90094716 | MR Egger | 14 | 0.3240749627308 | 0.369490625305484 | 0.39765954193596 | -0.400126663 | 1.04827658832955 | 1.38275095802228 | 0.670235146753072 | 2.85273045016238 | 0.999950574162623 |
| ebi-fl187-GCST90094716 | ebi-met1400-GCST90199902 | Hydantoin-5-propionate levels \|\| id:ebi-met1400-GCST90199902 | F-burn/spicy liking (derived food-liking factor) \|\| id:ebi-fl187-GCST90094716 | MR Egger | 14 | 0.389964605309306 | 0.365640745215349 | 0.307174798201127 | -0.326691255 | 1.10662046593139 | 1.47692851752944 | 0.72130640817288 | 3.0241209854452 | 0.999950574162623 |
| ebi-fl187-GCST90094716 | ebi-met1400-GCST90199904 | Pantoate levels \|\| id:ebi-met1400-GCST90199904 | F-burn/spicy liking (derived food-liking factor) \|\| id:ebi-fl187-GCST90094716 | MR Egger | 14 | 0.611205993885176 | 0.410445113537257 | 0.162258134249681 | -0.193266429 | 1.4156784164182 | 1.84265228671411 | 0.824262337781571 | 4.11928010549539 | 0.999950574162623 |
| ebi-fl187-GCST90094716 | ebi-met1400-GCST90199936 | 3-methyl catechol sulfate (2) levels \|\| id:ebi-met1400-GCST90199936 | F-burn/spicy liking (derived food-liking factor) \|\| id:ebi-fl187-GCST90094716 | MR Egger | 14 | -0.023192502 | 0.418199068289915 | 0.956686220117853 | -0.842862676 | 0.79647767208852 | 0.97707437713146 | 0.430476443472026 | 2.21771563328033 | 0.999950574162623 |
| ebi-fl187-GCST90094716 | ebi-met1400-GCST90199977 | 4-hydroxychlorothalonil levels \|\| id:ebi-met1400-GCST90199977 | F-burn/spicy liking (derived food-liking factor) \|\| id:ebi-fl187-GCST90094716 | MR Egger | 14 | -0.372568824 | 0.363646178034797 | 0.325792388622523 | -1.085315333 | 0.340177684555234 | 0.688962232518675 | 0.337795250993465 | 1.40519725023103 | 0.999950574162623 |
| ebi-fl187-GCST90094716 | ebi-met1400-GCST90200040 | Sphingomyelin (d18:2/24:1, d18:1/24:2) levels \|\| id:ebi-met1400-GCST90200040 | F-burn/spicy liking (derived food-liking factor) \|\| id:ebi-fl187-GCST90094716 | MR Egger | 14 | -0.430555487 | 0.333896995078507 | 0.221529084258114 | -1.084993597 | 0.22388262379562 | 0.650147846008868 | 0.337903949516467 | 1.25092418207847 | 0.999950574162623 |
| ebi-fl187-GCST90094716 | ebi-met1400-GCST90200077 | 1-stearoyl-2-oleoyl-GPI (18:0/18:1) levels \|\| id:ebi-met1400-GCST90200077 | F-burn/spicy liking (derived food-liking factor) \|\| id:ebi-fl187-GCST90094716 | MR Egger | 14 | 0.202076656489875 | 0.454506455550777 | 0.664516689776097 | -0.688755996 | 1.0929093093694 | 1.22394182759969 | 0.502200419773589 | 2.98293975545349 | 0.999950574162623 |
| ebi-fl187-GCST90094716 | ebi-met1400-GCST90200080 | 1-palmitoyl-2-oleoyl-GPI (16:0/18:1) levels \|\| id:ebi-met1400-GCST90200080 | F-burn/spicy liking (derived food-liking factor) \|\| id:ebi-fl187-GCST90094716 | MR Egger | 14 | 0.536761690976651 | 0.364678597844986 | 0.166789261431946 | -0.178008361 | 1.25153174275282 | 1.71045888955214 | 0.836935426010206 | 3.49569336166713 | 0.999950574162623 |
| ebi-fl187-GCST90094716 | ebi-met1400-GCST90200082 | 1-oleoyl-2-linoleoyl-GPE (18:1/18:2) levels \|\| id:ebi-met1400-GCST90200082 | F-burn/spicy liking (derived food-liking factor) \|\| id:ebi-fl187-GCST90094716 | MR Egger | 14 | 0.635715838575918 | 0.462296192364466 | 0.194222066554233 | -0.270384698 | 1.54181637561027 | 1.88837342829512 | 0.763085879902312 | 4.67307061840482 | 0.999950574162623 |
| ebi-fl187-GCST90094716 | ebi-met1400-GCST90200100 | Perfluorooctanesulfonate (PFOS) levels \|\| id:ebi-met1400-GCST90200100 | F-burn/spicy liking (derived food-liking factor) \|\| id:ebi-fl187-GCST90094716 | MR Egger | 14 | -0.975051314 | 0.496389620012521 | 0.0730835559495209 | -1.947974969 | -0.002127658 | 0.377172998924854 | 0.142562472915126 | 0.997874603386419 | 0.999950574162623 |
| ebi-fl187-GCST90094716 | ebi-met1400-GCST90200102 | 1-palmitoleoyl-2-linolenoyl-GPC (16:1/18:3) levels \|\| id:ebi-met1400-GCST90200102 | F-burn/spicy liking (derived food-liking factor) \|\| id:ebi-fl187-GCST90094716 | MR Egger | 14 | -0.193929391 | 0.359532241648314 | 0.599480964099198 | -0.898612585 | 0.510753802585363 | 0.823716063945475 | 0.407134132205113 | 1.66654696899742 | 0.999950574162623 |
| ebi-fl187-GCST90094716 | ebi-met1400-GCST90200114 | Glycosyl-N-tricosanoyl-sphingadienine (d18:2/23:0) levels \|\| id:ebi-met1400-GCST90200114 | F-burn/spicy liking (derived food-liking factor) \|\| id:ebi-fl187-GCST90094716 | MR Egger | 14 | -0.646863124 | 0.382719645693588 | 0.116782128482714 | -1.39699363 | 0.103267381527317 | 0.523685940762861 | 0.247339441278537 | 1.10878783883014 | 0.999950574162623 |
| ebi-fl187-GCST90094716 | ebi-met1400-GCST90200135 | Arachidoylcarnitine (C20) levels \|\| id:ebi-met1400-GCST90200135 | F-burn/spicy liking (derived food-liking factor) \|\| id:ebi-fl187-GCST90094716 | MR Egger | 14 | -0.467240258 | 0.467481367724464 | 0.337288671355276 | -1.383503739 | 0.449023222597453 | 0.626729495457844 | 0.25069862991206 | 1.56678104150241 | 0.999950574162623 |
| ebi-fl187-GCST90094716 | ebi-met1400-GCST90200146 | Docosahexaenoylcarnitine (C22:6) levels \|\| id:ebi-met1400-GCST90200146 | F-burn/spicy liking (derived food-liking factor) \|\| id:ebi-fl187-GCST90094716 | MR Egger | 14 | 1.66159594097132e-05 | 0.506746841034908 | 0.999974376584515 | -0.993207192 | 0.993240424387828 | 1.00001661609746 | 0.370386882050001 | 2.69996935889324 | 0.999974376584515 |
| ebi-fl187-GCST90094716 | ebi-met1400-GCST90200162 | 3-hydroxyphenylacetoylglutamine levels \|\| id:ebi-met1400-GCST90200162 | F-burn/spicy liking (derived food-liking factor) \|\| id:ebi-fl187-GCST90094716 | MR Egger | 14 | 0.522109973905913 | 0.447755573811849 | 0.266243018573422 | -0.355490951 | 1.39971089857714 | 1.68558043096858 | 0.700829286059034 | 4.054027772 | 0.999950574162623 |
| ebi-fl187-GCST90094716 | ebi-met1400-GCST90200172 | 3-carboxy-4-methyl-5-pentyl-2-furanpropionate (3-CMPFP) levels \|\| id:ebi-met1400-GCST90200172 | F-burn/spicy liking (derived food-liking factor) \|\| id:ebi-fl187-GCST90094716 | MR Egger | 14 | 0.250492940064751 | 0.372794189155138 | 0.514354808615726 | -0.480183671 | 0.981169550808821 | 1.28465852028803 | 0.618669749876881 | 2.66757428187989 | 0.999950574162623 |
| ebi-fl187-GCST90094716 | ebi-met1400-GCST90200222 | Dihydrocaffeate sulfate (2) levels \|\| id:ebi-met1400-GCST90200222 | F-burn/spicy liking (derived food-liking factor) \|\| id:ebi-fl187-GCST90094716 | MR Egger | 14 | -0.075983664 | 0.416359786350686 | 0.85824088814144 | -0.892048845 | 0.740081517267513 | 0.926831347171292 | 0.409815244003496 | 2.09610637639439 | 0.999950574162623 |
| ebi-fl187-GCST90094716 | ebi-met1400-GCST90200223 | 4-methylhexanoylglutamine levels \|\| id:ebi-met1400-GCST90200223 | F-burn/spicy liking (derived food-liking factor) \|\| id:ebi-fl187-GCST90094716 | MR Egger | 14 | -1.023528479 | 0.657482454199737 | 0.145501126312111 | -2.312194089 | 0.265137131272113 | 0.359324830609014 | 0.0990437022690753 | 1.30360972918225 | 0.999950574162623 |
| ebi-fl187-GCST90094716 | ebi-met1400-GCST90200224 | 2-ketocaprylate levels \|\| id:ebi-met1400-GCST90200224 | F-burn/spicy liking (derived food-liking factor) \|\| id:ebi-fl187-GCST90094716 | MR Egger | 14 | -0.316466794 | 0.381112869877623 | 0.422539877733013 | -1.063448019 | 0.430514431139659 | 0.728719209138905 | 0.345263281302107 | 1.53804854013241 | 0.999950574162623 |
| ebi-fl187-GCST90094716 | ebi-met1400-GCST90200243 | Hydroxypalmitoyl sphingomyelin (d18:1/16:0(OH)) levels \|\| id:ebi-met1400-GCST90200243 | F-burn/spicy liking (derived food-liking factor) \|\| id:ebi-fl187-GCST90094716 | MR Egger | 14 | -0.671586826 | 0.357996211513002 | 0.0851888622218598 | -1.3732594 | 0.0300857490275219 | 0.510897229451694 | 0.253280071613914 | 1.03054289821623 | 0.999950574162623 |
| ebi-fl187-GCST90094716 | ebi-met1400-GCST90200273 | 2-methoxyhydroquinone sulfate (2) levels \|\| id:ebi-met1400-GCST90200273 | F-burn/spicy liking (derived food-liking factor) \|\| id:ebi-fl187-GCST90094716 | MR Egger | 14 | 0.455321931484472 | 0.606834714123649 | 0.467517836415229 | -0.734074108 | 1.64471797116682 | 1.57668088455695 | 0.479949634746052 | 5.17954891879985 | 0.999950574162623 |
| ebi-fl187-GCST90094716 | ebi-met1400-GCST90200276 | 2,4-di-tert-butylphenol levels \|\| id:ebi-met1400-GCST90200276 | F-burn/spicy liking (derived food-liking factor) \|\| id:ebi-fl187-GCST90094716 | MR Egger | 14 | -0.248473761 | 0.420251063522398 | 0.565323735808439 | -1.072165845 | 0.575218323704234 | 0.779990326889071 | 0.34226641795858 | 1.77751855899034 | 0.999950574162623 |
| ebi-fl187-GCST90094716 | ebi-met1400-GCST90200289 | N-acetylmethionine levels \|\| id:ebi-met1400-GCST90200289 | F-burn/spicy liking (derived food-liking factor) \|\| id:ebi-fl187-GCST90094716 | MR Egger | 14 | 0.534858489590763 | 0.402576435595084 | 0.208696022205697 | -0.254191324 | 1.32390830335713 | 1.70720663764989 | 0.775543407648099 | 3.75808043095159 | 0.999950574162623 |
| ebi-fl187-GCST90094716 | ebi-met1400-GCST90200308 | 3-hydroxyisobutyrate levels \|\| id:ebi-met1400-GCST90200308 | F-burn/spicy liking (derived food-liking factor) \|\| id:ebi-fl187-GCST90094716 | MR Egger | 14 | -0.160072749 | 0.369860064597641 | 0.672841695623194 | -0.884998476 | 0.564852977401916 | 0.852081798434126 | 0.412714802329873 | 1.75918912315247 | 0.999950574162623 |
| ebi-fl187-GCST90094716 | ebi-met1400-GCST90200383 | Cystathionine levels \|\| id:ebi-met1400-GCST90200383 | F-burn/spicy liking (derived food-liking factor) \|\| id:ebi-fl187-GCST90094716 | MR Egger | 14 | 0.39549408175814 | 0.364067523981166 | 0.29867720858547 | -0.318078265 | 1.10906642876122 | 1.48511777928311 | 0.727545844632526 | 3.03152692660461 | 0.999950574162623 |
| ebi-fl187-GCST90094716 | ebi-met1400-GCST90200384 | Guanidinoacetate levels \|\| id:ebi-met1400-GCST90200384 | F-burn/spicy liking (derived food-liking factor) \|\| id:ebi-fl187-GCST90094716 | MR Egger | 14 | 0.267036919744674 | 0.45523225850957 | 0.568346958673502 | -0.625218307 | 1.15929214642343 | 1.30608866590059 | 0.535144589991391 | 3.18767606941784 | 0.999950574162623 |
| ebi-fl187-GCST90094716 | ebi-met1400-GCST90200403 | Citrulline levels \|\| id:ebi-met1400-GCST90200403 | F-burn/spicy liking (derived food-liking factor) \|\| id:ebi-fl187-GCST90094716 | MR Egger | 14 | 0.114310260039895 | 0.367439370548865 | 0.7610627823661 | -0.605870906 | 0.834491426315672 | 1.12109990343124 | 0.545599054048817 | 2.3036421785311 | 0.999950574162623 |
| ebi-fl187-GCST90094716 | ebi-met1400-GCST90200434 | 12,13-DiHOME levels \|\| id:ebi-met1400-GCST90200434 | F-burn/spicy liking (derived food-liking factor) \|\| id:ebi-fl187-GCST90094716 | MR Egger | 14 | 0.478386587924642 | 0.369755741372801 | 0.220085670110785 | -0.246334665 | 1.20310784101533 | 1.6134691105074 | 0.781660586577298 | 3.33045136887443 | 0.999950574162623 |
| ebi-fl187-GCST90094716 | ebi-met1400-GCST90200437 | Fructose levels \|\| id:ebi-met1400-GCST90200437 | F-burn/spicy liking (derived food-liking factor) \|\| id:ebi-fl187-GCST90094716 | MR Egger | 14 | 0.216272220616348 | 0.368405861343631 | 0.568051835821481 | -0.505803268 | 0.938347708849864 | 1.24144027864477 | 0.603020993592023 | 2.5557550762226 | 0.999950574162623 |
| ebi-fl187-GCST90094716 | ebi-met1400-GCST90200448 | N-stearoyl-sphinganine (d18:0/18:0) levels \|\| id:ebi-met1400-GCST90200448 | F-burn/spicy liking (derived food-liking factor) \|\| id:ebi-fl187-GCST90094716 | MR Egger | 14 | -0.498555243 | 0.408478858506732 | 0.245710563707719 | -1.299173806 | 0.302063319837162 | 0.607407582546157 | 0.272757050340337 | 1.35264687337765 | 0.999950574162623 |
| ebi-fl187-GCST90094716 | ebi-met1400-GCST90200468 | X-11847 levels \|\| id:ebi-met1400-GCST90200468 | F-burn/spicy liking (derived food-liking factor) \|\| id:ebi-fl187-GCST90094716 | MR Egger | 14 | -0.090820917 | 0.386551821339493 | 0.818207784778602 | -0.848462487 | 0.666820653012515 | 0.913181231662293 | 0.428072593569797 | 1.94803398859566 | 0.999950574162623 |
| ebi-fl187-GCST90094716 | ebi-met1400-GCST90200476 | X-11843 levels \|\| id:ebi-met1400-GCST90200476 | F-burn/spicy liking (derived food-liking factor) \|\| id:ebi-fl187-GCST90094716 | MR Egger | 14 | -0.382105135 | 0.388419482628194 | 0.344660991483016 | -1.143407321 | 0.379197050854166 | 0.682423302793515 | 0.318731150148123 | 1.46111092053345 | 0.999950574162623 |
| ebi-fl187-GCST90094716 | ebi-met1400-GCST90200511 | X-12906 levels \|\| id:ebi-met1400-GCST90200511 | F-burn/spicy liking (derived food-liking factor) \|\| id:ebi-fl187-GCST90094716 | MR Egger | 14 | 0.19007583482068 | 0.415422336430139 | 0.655450486563969 | -0.624151945 | 1.00430361422375 | 1.20934130436088 | 0.535715552407177 | 2.73000547372888 | 0.999950574162623 |
| ebi-fl187-GCST90094716 | ebi-met1400-GCST90200515 | X-12849 levels \|\| id:ebi-met1400-GCST90200515 | F-burn/spicy liking (derived food-liking factor) \|\| id:ebi-fl187-GCST90094716 | MR Egger | 14 | 0.206323634217467 | 0.392028182457588 | 0.608271469647547 | -0.562051603 | 0.97469887183434 | 1.22915093493315 | 0.5700383707013 | 2.65036899005294 | 0.999950574162623 |
| ebi-fl187-GCST90094716 | ebi-met1400-GCST90200551 | X-18886 levels \|\| id:ebi-met1400-GCST90200551 | F-burn/spicy liking (derived food-liking factor) \|\| id:ebi-fl187-GCST90094716 | MR Egger | 14 | -0.718391602 | 0.360962797189602 | 0.0698436816319438 | -1.425878685 | -0.01090452 | 0.487535777063582 | 0.240297224877689 | 0.989154718861091 | 0.999950574162623 |
| ebi-fl187-GCST90094716 | ebi-met1400-GCST90200564 | X-21319 levels \|\| id:ebi-met1400-GCST90200564 | F-burn/spicy liking (derived food-liking factor) \|\| id:ebi-fl187-GCST90094716 | MR Egger | 14 | -0.897266583 | 0.370123603108599 | 0.0320684729355172 | -1.622708845 | -0.171824321 | 0.407682504476075 | 0.197363347619284 | 0.842127104453537 | 0.999950574162623 |
| ebi-fl187-GCST90094716 | ebi-met1400-GCST90200579 | X-21442 levels \|\| id:ebi-met1400-GCST90200579 | F-burn/spicy liking (derived food-liking factor) \|\| id:ebi-fl187-GCST90094716 | MR Egger | 14 | -0.439379751 | 0.451720864474644 | 0.349911782885793 | -1.324752646 | 0.445993143185492 | 0.644436007819046 | 0.265868714764465 | 1.56204075587331 | 0.999950574162623 |
| ebi-fl187-GCST90094716 | ebi-met1400-GCST90200581 | X-21441 levels \|\| id:ebi-met1400-GCST90200581 | F-burn/spicy liking (derived food-liking factor) \|\| id:ebi-fl187-GCST90094716 | MR Egger | 14 | -0.222264309 | 0.366705204014121 | 0.55573270656944 | -0.941006509 | 0.496477890680533 | 0.800703703018988 | 0.39023486270361 | 1.6429245085549 | 0.999950574162623 |
| ebi-fl187-GCST90094716 | ebi-met1400-GCST90200648 | X-24585 levels \|\| id:ebi-met1400-GCST90200648 | F-burn/spicy liking (derived food-liking factor) \|\| id:ebi-fl187-GCST90094716 | MR Egger | 14 | 0.794931581821433 | 0.358000790120666 | 0.0463988956397529 | 0.0932500331849275 | 1.49661313045794 | 2.21428949396736 | 1.09773617142873 | 4.4665358496047 | 0.999950574162623 |
| ebi-fl187-GCST90094716 | ebi-met1400-GCST90200720 | Adenosine 5'-diphosphate (ADP) to pantothenate ratio \|\| id:ebi-met1400-GCST90200720 | F-burn/spicy liking (derived food-liking factor) \|\| id:ebi-fl187-GCST90094716 | MR Egger | 14 | -0.813044429 | 0.534471196108022 | 0.154110948262806 | -1.860607973 | 0.234519115627027 | 0.443505787042098 | 0.155578014358681 | 1.26430064010427 | 0.999950574162623 |
| ebi-fl187-GCST90094716 | ebi-met1400-GCST90200724 | Cholate to taurocholate ratio \|\| id:ebi-met1400-GCST90200724 | F-burn/spicy liking (derived food-liking factor) \|\| id:ebi-fl187-GCST90094716 | MR Egger | 14 | 0.0584906884132204 | 0.422109569451671 | 0.89209008333773 | -0.768844068 | 0.885825444538495 | 1.06023511317737 | 0.463548589520129 | 2.42498525640626 | 0.999950574162623 |
| ebi-fl187-GCST90094716 | ebi-met1400-GCST90200726 | N-acetylputrescine to (N(1) + N(8))-acetylspermidine ratio \|\| id:ebi-met1400-GCST90200726 | F-burn/spicy liking (derived food-liking factor) \|\| id:ebi-fl187-GCST90094716 | MR Egger | 14 | 0.468111182764782 | 0.398823256777643 | 0.263272420746027 | -0.313582401 | 1.24980476604896 | 1.59697494890694 | 0.730824156232382 | 3.48966159053093 | 0.999950574162623 |
| ebi-fl187-GCST90094716 | ebi-met1400-GCST90200744 | Arginine to glutamate ratio \|\| id:ebi-met1400-GCST90200744 | F-burn/spicy liking (derived food-liking factor) \|\| id:ebi-fl187-GCST90094716 | MR Egger | 14 | 0.0284511612703338 | 0.353309254958536 | 0.937145054101703 | -0.664034978 | 0.720937300989064 | 1.02885976140372 | 0.514770052284214 | 2.05635973565004 | 0.999950574162623 |
| ebi-fl187-GCST90094716 | ebi-met1400-GCST90200759 | Histidine to trans-urocanate ratio \|\| id:ebi-met1400-GCST90200759 | F-burn/spicy liking (derived food-liking factor) \|\| id:ebi-fl187-GCST90094716 | MR Egger | 14 | -0.471700405 | 0.416813959963117 | 0.279877267565442 | -1.288655767 | 0.345254956435375 | 0.623940414289813 | 0.275641060078496 | 1.4123499614799 | 0.999950574162623 |
| ebi-fl187-GCST90094716 | ebi-met1400-GCST90200795 | Oleoyl-linoleoyl-glycerol (18:1 to 18:2) [2] to linoleoyl-arachidonoyl-glycerol (18:2 to 20:4) [2] ratio \|\| id:ebi-met1400-GCST90200795 | F-burn/spicy liking (derived food-liking factor) \|\| id:ebi-fl187-GCST90094716 | MR Egger | 14 | -0.88104084 | 0.398537223659977 | 0.0472221679150113 | -1.662173798 | -0.099907881 | 0.414351413733683 | 0.189726105256998 | 0.904920774242105 | 0.999950574162623 |
| ebi-fl187-GCST90094716 | ebi-met1400-GCST90200811 | Choline to taurocholate ratio \|\| id:ebi-met1400-GCST90200811 | F-burn/spicy liking (derived food-liking factor) \|\| id:ebi-fl187-GCST90094716 | MR Egger | 14 | 0.0827204811707079 | 0.385064791308551 | 0.833513375327452 | -0.67200651 | 0.837447472135468 | 1.08623814209616 | 0.51068285891513 | 2.31046192513895 | 0.999950574162623 |
| ebi-fl187-GCST90094716 | ebi-met1400-GCST90200831 | Cholate to phosphate ratio \|\| id:ebi-met1400-GCST90200831 | F-burn/spicy liking (derived food-liking factor) \|\| id:ebi-fl187-GCST90094716 | MR Egger | 14 | -0.22527346 | 0.380750689877988 | 0.565060962067186 | -0.971544812 | 0.520997892641566 | 0.798297886751892 | 0.378497878295614 | 1.68370697 | 0.999950574162623 |
| ebi-fl187-GCST90094716 | ebi-met1400-GCST90200841 | Adenosine 5'-diphosphate (ADP) to oxalate (ethanedioate) ratio \|\| id:ebi-met1400-GCST90200841 | F-burn/spicy liking (derived food-liking factor) \|\| id:ebi-fl187-GCST90094716 | MR Egger | 14 | -0.182291917 | 0.518445447791644 | 0.731225672207785 | -1.198444995 | 0.833861160590132 | 0.83335803345977 | 0.301662934830907 | 2.30219072926947 | 0.999950574162623 |
| ebi-fl187-GCST90094716 | ebi-met1400-GCST90200844 | Aspartate to phosphate ratio \|\| id:ebi-met1400-GCST90200844 | F-burn/spicy liking (derived food-liking factor) \|\| id:ebi-fl187-GCST90094716 | MR Egger | 14 | -0.04460838 | 0.344218285021304 | 0.899035106992345 | -0.719276219 | 0.630059458296586 | 0.956371942555258 | 0.487104685526436 | 1.87772222211005 | 0.999950574162623 |
| ebi-fl187-GCST90094716 | ebi-met1400-GCST90200870 | Adenosine 5'-monophosphate (AMP) to acetoacetate ratio \|\| id:ebi-met1400-GCST90200870 | F-burn/spicy liking (derived food-liking factor) \|\| id:ebi-fl187-GCST90094716 | MR Egger | 14 | 0.886266551414401 | 0.40340190781233 | 0.0483981821732467 | 0.0955988121022345 | 1.67693429072657 | 2.42605517003261 | 1.10031754135184 | 5.34913192496304 | 0.999950574162623 |
| ebi-fl187-GCST90094716 | ebi-met1400-GCST90200887 | Cholesterol to cortisol ratio \|\| id:ebi-met1400-GCST90200887 | F-burn/spicy liking (derived food-liking factor) \|\| id:ebi-fl187-GCST90094716 | MR Egger | 14 | 0.0404166372753649 | 0.355956919047473 | 0.911477082134821 | -0.657258924 | 0.738092198608413 | 1.04124450510548 | 0.518270006706302 | 2.09194069767334 | 0.999950574162623 |
| ebi-fl187-GCST90094716 | ebi-met1400-GCST90200890 | Cortisol to taurocholate ratio \|\| id:ebi-met1400-GCST90200890 | F-burn/spicy liking (derived food-liking factor) \|\| id:ebi-fl187-GCST90094716 | MR Egger | 14 | -0.25312063 | 0.381347333625064 | 0.519397253063298 | -1.000561404 | 0.494320143958309 | 0.776374222193641 | 0.367672970198341 | 1.63938331545456 | 0.999950574162623 |
| ebi-fl187-GCST90094716 | ebi-met1400-GCST90200948 | Adenosine 5'-diphosphate (ADP) to mannitol to sorbitol ratio \|\| id:ebi-met1400-GCST90200948 | F-burn/spicy liking (derived food-liking factor) \|\| id:ebi-fl187-GCST90094716 | MR Egger | 14 | -0.734843484 | 0.781661006730307 | 0.36570305882134 | -2.266899057 | 0.797212088977918 | 0.47958051502252 | 0.103633042453697 | 2.21934495932635 | 0.999950574162623 |
| ebi-fl187-GCST90094716 | ebi-met1400-GCST90200972 | Leucine to N-palmitoyl-sphingosine (d18:1 to 16:0) ratio \|\| id:ebi-met1400-GCST90200972 | F-burn/spicy liking (derived food-liking factor) \|\| id:ebi-fl187-GCST90094716 | MR Egger | 14 | 0.518875483169293 | 0.377330385469914 | 0.194223351015556 | -0.220692072 | 1.25844303869032 | 1.68013724439258 | 0.801963589035193 | 3.51993681332985 | 0.999950574162623 |
| ebi-fl187-GCST90094716 | ebi-met1400-GCST90200973 | Proline to glutamate ratio \|\| id:ebi-met1400-GCST90200973 | F-burn/spicy liking (derived food-liking factor) \|\| id:ebi-fl187-GCST90094716 | MR Egger | 14 | 0.379493857054776 | 0.359019374375707 | 0.311314053988434 | -0.324184117 | 1.08317183083116 | 1.46154465167831 | 0.723117092174277 | 2.95403440461706 | 0.999950574162623 |
| ebi-fl187-GCST90094716 | ebi-met1400-GCST90200983 | Cholesterol to linoleoyl-arachidonoyl-glycerol (18:2 to 20:4) [1] ratio \|\| id:ebi-met1400-GCST90200983 | F-burn/spicy liking (derived food-liking factor) \|\| id:ebi-fl187-GCST90094716 | MR Egger | 14 | -0.385299957 | 0.376562441691312 | 0.326396879809219 | -1.123362343 | 0.352762428502176 | 0.68024656073679 | 0.325184572351367 | 1.42299304068533 | 0.999950574162623 |
| ebi-fl187-GCST90094716 | ebi-met1400-GCST90201001 | Serine to alpha-ketobutyrate ratio \|\| id:ebi-met1400-GCST90201001 | F-burn/spicy liking (derived food-liking factor) \|\| id:ebi-fl187-GCST90094716 | MR Egger | 14 | 0.683965489370545 | 0.372506368866888 | 0.0912268358867328 | -0.046146994 | 1.41407797234965 | 1.98172066364938 | 0.954901587443718 | 4.11269270087625 | 0.999950574162623 |
| ebi-fl187-GCST90094716 | ebi-met1400-GCST90201005 | Fructose to maltose ratio \|\| id:ebi-met1400-GCST90201005 | F-burn/spicy liking (derived food-liking factor) \|\| id:ebi-fl187-GCST90094716 | MR Egger | 14 | 0.194784793017609 | 0.416910489518222 | 0.648719121681138 | -0.622359766 | 1.01192935247333 | 1.21504947122063 | 0.536676510959003 | 2.75090336052793 | 0.999950574162623 |
| ebi-fl187-GCST90094716 | ebi-met1400-GCST90201020 | Paraxanthine to 5-acetylamino-6-formylamino-3-methyluracil ratio \|\| id:ebi-met1400-GCST90201020 | F-burn/spicy liking (derived food-liking factor) \|\| id:ebi-fl187-GCST90094716 | MR Egger | 14 | 0.219065930646277 | 0.387085207771218 | 0.581865205930841 | -0.539621077 | 0.977752937877864 | 1.24491335192372 | 0.582969111173326 | 2.65847576500011 | 0.999950574162623 |
| ebi-fl187-GCST90094716 | ebi-pw11-GCST90275197 | Placental weight (fetal genotype, adjusted for sex) \|\| id:ebi-pw11-GCST90275197 | F-burn/spicy liking (derived food-liking factor) \|\| id:ebi-fl187-GCST90094716 | MR Egger | 14 | 0.00516439557906621 | 0.167097020580171 | 0.975852145797451 | -0.322345765 | 0.332674555916201 | 1.00517775405617 | 0.724447658550477 | 1.39469332991019 | 0.999950574162623 |
| ebi-fl187-GCST90094716 | prot-a-1017 | FAS-associated factor 2 \|\| id:prot-a-1017 | F-burn/spicy liking (derived food-liking factor) \|\| id:ebi-fl187-GCST90094716 | MR Egger | 15 | -0.325633353 | 0.606516919507603 | 0.600411638901669 | -1.514406515 | 0.863139809175483 | 0.722069883617442 | 0.219938676362181 | 2.37059222802961 | 0.999950574162623 |
| ebi-fl187-GCST90094716 | prot-a-1019 | Fas apoptotic inhibitory molecule 3 \|\| id:prot-a-1019 | F-burn/spicy liking (derived food-liking factor) \|\| id:ebi-fl187-GCST90094716 | MR Egger | 15 | 0.0751286675383368 | 0.599258803970383 | 0.902149211366832 | -1.099418588 | 1.24967592332029 | 1.07802284850769 | 0.333064675132217 | 3.48921200197318 | 0.999950574162623 |
| ebi-fl187-GCST90094716 | prot-a-1052 | MIP18 family protein FAM96A \|\| id:prot-a-1052 | F-burn/spicy liking (derived food-liking factor) \|\| id:ebi-fl187-GCST90094716 | MR Egger | 15 | -0.565578391 | 0.560141464840443 | 0.331060915380546 | -1.663455662 | 0.53229888047091 | 0.568031507631998 | 0.189483058084643 | 1.70284244366874 | 0.999950574162623 |
| ebi-fl187-GCST90094716 | prot-a-1066 | F-box/LRR-repeat protein 4 \|\| id:prot-a-1066 | F-burn/spicy liking (derived food-liking factor) \|\| id:ebi-fl187-GCST90094716 | MR Egger | 15 | -0.904670993 | 0.562575228956304 | 0.131820759513048 | -2.007318442 | 0.197976455898094 | 0.40467500417052 | 0.134348455294231 | 1.21893369478467 | 0.999950574162623 |
| ebi-fl187-GCST90094716 | prot-a-1076 | Low affinity immunoglobulin gamma Fc region receptor III-B \|\| id:prot-a-1076 | F-burn/spicy liking (derived food-liking factor) \|\| id:ebi-fl187-GCST90094716 | MR Egger | 15 | -0.851501635 | 0.593708616729368 | 0.175127962461232 | -2.015170524 | 0.312167253463568 | 0.426773592237752 | 0.133297670960347 | 1.36638320624294 | 0.999950574162623 |
| ebi-fl187-GCST90094716 | prot-a-1081 | Fc receptor-like protein 3 \|\| id:prot-a-1081 | F-burn/spicy liking (derived food-liking factor) \|\| id:ebi-fl187-GCST90094716 | MR Egger | 15 | -1.155377507 | 0.560387929691838 | 0.0598152450459889 | -2.253737849 | -0.057017164 | 0.314938623071693 | 0.105005993581076 | 0.94457785617457 | 0.999950574162623 |
| ebi-fl187-GCST90094716 | prot-a-1105 | Fibroblast growth factor receptor 4 \|\| id:prot-a-1105 | F-burn/spicy liking (derived food-liking factor) \|\| id:ebi-fl187-GCST90094716 | MR Egger | 15 | -0.970979765 | 0.595402451622914 | 0.126910574744919 | -2.13796857 | 0.19600904027414 | 0.378711807734853 | 0.117894093517253 | 1.21653790312075 | 0.999950574162623 |
| ebi-fl187-GCST90094716 | prot-a-1143 | Follistatin-related protein 1 \|\| id:prot-a-1143 | F-burn/spicy liking (derived food-liking factor) \|\| id:ebi-fl187-GCST90094716 | MR Egger | 15 | -0.379268786 | 0.611287523042291 | 0.545690141884359 | -1.577392331 | 0.81885475922649 | 0.684361641158354 | 0.20651291398077 | 2.267901057 | 0.999950574162623 |
| ebi-fl187-GCST90094716 | prot-a-1151 | Alpha-(1,3)-fucosyltransferase 10 \|\| id:prot-a-1151 | F-burn/spicy liking (derived food-liking factor) \|\| id:ebi-fl187-GCST90094716 | MR Egger | 15 | 0.0622351273996816 | 0.560169039636447 | 0.913233543257555 | -1.03569619 | 1.16016644508712 | 1.06421254084034 | 0.354979161834348 | 3.19046426902758 | 0.999950574162623 |
| ebi-fl187-GCST90094716 | prot-a-1165 | Growth arrest and DNA damage-inducible proteins-interacting protein 1 \|\| id:prot-a-1165 | F-burn/spicy liking (derived food-liking factor) \|\| id:ebi-fl187-GCST90094716 | MR Egger | 15 | -0.059310254 | 0.568427584823078 | 0.918491427332583 | -1.17342832 | 1.05480781265438 | 0.942414336355816 | 0.309304725966597 | 2.87142324965603 | 0.999950574162623 |
| ebi-fl187-GCST90094716 | prot-a-1194 | Growth/differentiation factor 11/8 \|\| id:prot-a-1194 | F-burn/spicy liking (derived food-liking factor) \|\| id:ebi-fl187-GCST90094716 | MR Egger | 15 | -0.199077322 | 0.582880047348556 | 0.738154112710388 | -1.341522215 | 0.943367570507067 | 0.819486526303276 | 0.261447386362443 | 2.56861686833477 | 0.999950574162623 |
| ebi-fl187-GCST90094716 | prot-a-1216 | D-glucuronyl C5-epimerase \|\| id:prot-a-1216 | F-burn/spicy liking (derived food-liking factor) \|\| id:ebi-fl187-GCST90094716 | MR Egger | 15 | -0.376546882 | 0.634077755057669 | 0.562804071594505 | -1.619339282 | 0.866245517841602 | 0.686226945191957 | 0.198029497525036 | 2.37796604138709 | 0.999950574162623 |
| ebi-fl187-GCST90094716 | prot-a-1246 | Glypican-3 \|\| id:prot-a-1246 | F-burn/spicy liking (derived food-liking factor) \|\| id:ebi-fl187-GCST90094716 | MR Egger | 15 | 0.59417500454943 | 0.623168052600697 | 0.357743128479642 | -0.627234379 | 1.8155843876468 | 1.81153581949947 | 0.534066787003273 | 6.14466599532152 | 0.999950574162623 |
| ebi-fl187-GCST90094716 | prot-a-1275 | Glutamate receptor 4 \|\| id:prot-a-1275 | F-burn/spicy liking (derived food-liking factor) \|\| id:ebi-fl187-GCST90094716 | MR Egger | 15 | 0.222487162471848 | 0.616631106358973 | 0.724036272502305 | -0.986109806 | 1.43108413093544 | 1.24917978316709 | 0.373025011736826 | 4.18323190557067 | 0.999950574162623 |
| ebi-fl187-GCST90094716 | prot-a-1289 | Glutathione S-transferase P \|\| id:prot-a-1289 | F-burn/spicy liking (derived food-liking factor) \|\| id:ebi-fl187-GCST90094716 | MR Egger | 15 | 0.18834966466421 | 0.560141464840443 | 0.742045194728695 | -0.909527606 | 1.28622693575148 | 1.20725557617108 | 0.402714418811491 | 3.61910564438571 | 0.999950574162623 |
| ebi-fl187-GCST90094716 | prot-a-130 | Apolipoprotein D \|\| id:prot-a-130 | F-burn/spicy liking (derived food-liking factor) \|\| id:ebi-fl187-GCST90094716 | MR Egger | 15 | 1.16713634550663 | 0.560118686657315 | 0.0574835286157082 | 0.06930371965829 | 2.26496897135496 | 3.2127791632328 | 1.07176167481033 | 9.630825765 | 0.999950574162623 |
| ebi-fl187-GCST90094716 | prot-a-1313 | Heparin-binding EGF-like growth factor \|\| id:prot-a-1313 | F-burn/spicy liking (derived food-liking factor) \|\| id:ebi-fl187-GCST90094716 | MR Egger | 15 | -0.861733183 | 0.560141464840443 | 0.147924199122019 | -1.959610454 | 0.236144087757636 | 0.422429300057377 | 0.140913302385479 | 1.26635676352834 | 0.999950574162623 |
| ebi-fl187-GCST90094716 | prot-a-1347 | HLA class II histocompatibility antigen, DQ alpha 2 chain \|\| id:prot-a-1347 | F-burn/spicy liking (derived food-liking factor) \|\| id:ebi-fl187-GCST90094716 | MR Egger | 15 | 0.0202426719206832 | 0.560118686657315 | 0.971719804975023 | -1.077589954 | 1.11807529776902 | 1.02044894428751 | 0.340414953540572 | 3.05896094477349 | 0.999950574162623 |
| ebi-fl187-GCST90094716 | prot-a-1357 | Non-histone chromosomal protein HMG-14 \|\| id:prot-a-1357 | F-burn/spicy liking (derived food-liking factor) \|\| id:ebi-fl187-GCST90094716 | MR Egger | 15 | 0.966024782944192 | 0.630902390482965 | 0.149690805568088 | -0.270543902 | 2.2025934682908 | 2.62747887281784 | 0.762964403290667 | 9.04844996349587 | 0.999950574162623 |
| ebi-fl187-GCST90094716 | prot-a-137 | Apolipoprotein M \|\| id:prot-a-137 | F-burn/spicy liking (derived food-liking factor) \|\| id:ebi-fl187-GCST90094716 | MR Egger | 15 | 0.680055418185494 | 0.603950829587519 | 0.280518863288647 | -0.503688208 | 1.86379904417703 | 1.97398712398387 | 0.604297768812844 | 6.4481872460147 | 0.999950574162623 |
| ebi-fl187-GCST90094716 | prot-a-1381 | 17-beta-hydroxysteroid dehydrogenase 14 \|\| id:prot-a-1381 | F-burn/spicy liking (derived food-liking factor) \|\| id:ebi-fl187-GCST90094716 | MR Egger | 15 | -0.630064001 | 0.585447095283011 | 0.301400997933555 | -1.777540307 | 0.517412306117233 | 0.532557715882868 | 0.16905345582914 | 1.67768070374749 | 0.999950574162623 |
| ebi-fl187-GCST90094716 | prot-a-1392 | Serine protease HTRA2, mitochondrial \|\| id:prot-a-1392 | F-burn/spicy liking (derived food-liking factor) \|\| id:ebi-fl187-GCST90094716 | MR Egger | 15 | -0.117845846 | 0.69835826977043 | 0.868592931573696 | -1.486628055 | 1.25093636284729 | 0.888833059312135 | 0.226133882503268 | 3.49361271553259 | 0.999950574162623 |
| ebi-fl187-GCST90094716 | prot-a-1416 | Interferon-induced protein with tetratricopeptide repeats 2 \|\| id:prot-a-1416 | F-burn/spicy liking (derived food-liking factor) \|\| id:ebi-fl187-GCST90094716 | MR Egger | 15 | -0.828762218 | 0.641047249504536 | 0.218576278272067 | -2.085214827 | 0.427690391382358 | 0.43658935460793 | 0.124280418548635 | 1.53371115725988 | 0.999950574162623 |
| ebi-fl187-GCST90094716 | prot-a-1428 | Interferon gamma \|\| id:prot-a-1428 | F-burn/spicy liking (derived food-liking factor) \|\| id:ebi-fl187-GCST90094716 | MR Egger | 15 | 0.270956092659431 | 0.560605910397702 | 0.636902056302173 | -0.827831492 | 1.36974367703893 | 1.31121749704745 | 0.436995888796091 | 3.93434210399545 | 0.999950574162623 |
| ebi-fl187-GCST90094716 | prot-a-1483 | Interleukin-17C \|\| id:prot-a-1483 | F-burn/spicy liking (derived food-liking factor) \|\| id:ebi-fl187-GCST90094716 | MR Egger | 15 | -0.668579657 | 0.710488321665049 | 0.363855750058098 | -2.061136768 | 0.723977453339591 | 0.512435895817858 | 0.127309166672357 | 2.06262089515089 | 0.999950574162623 |
| ebi-fl187-GCST90094716 | prot-a-1503 | Interleukin-1 receptor-like 2 \|\| id:prot-a-1503 | F-burn/spicy liking (derived food-liking factor) \|\| id:ebi-fl187-GCST90094716 | MR Egger | 15 | -0.144010781 | 0.560390235042523 | 0.801214325366025 | -1.242375641 | 0.954354079987916 | 0.865878413237435 | 0.288697560765087 | 2.59699259156764 | 0.999950574162623 |
| ebi-fl187-GCST90094716 | prot-a-1522 | Interleukin-31 receptor subunit alpha \|\| id:prot-a-1522 | F-burn/spicy liking (derived food-liking factor) \|\| id:ebi-fl187-GCST90094716 | MR Egger | 15 | -0.174844856 | 0.560362575161617 | 0.759972683457723 | -1.273155503 | 0.923465791603725 | 0.839587267833407 | 0.279946853423754 | 2.51800215536252 | 0.999950574162623 |
| ebi-fl187-GCST90094716 | prot-a-1540 | Interleukin-6 receptor subunit alpha \|\| id:prot-a-1540 | F-burn/spicy liking (derived food-liking factor) \|\| id:ebi-fl187-GCST90094716 | MR Egger | 15 | -1.197514357 | 0.617791490982824 | 0.0746007436328375 | -2.408385679 | 0.0133569657898556 | 0.301943804560383 | 0.0899604023945174 | 1.01344656855331 | 0.999950574162623 |
| ebi-fl187-GCST90094716 | prot-a-1541 | Interleukin-6 receptor subunit alpha \|\| id:prot-a-1541 | F-burn/spicy liking (derived food-liking factor) \|\| id:ebi-fl187-GCST90094716 | MR Egger | 15 | -1.092015384 | 0.720953800852284 | 0.153783042204572 | -2.505084834 | 0.321054065562521 | 0.335539570687558 | 0.0816686694296026 | 1.3785801125882 | 0.999950574162623 |
| ebi-fl187-GCST90094716 | prot-a-1545 | Interleukin-9 \|\| id:prot-a-1545 | F-burn/spicy liking (derived food-liking factor) \|\| id:ebi-fl187-GCST90094716 | MR Egger | 15 | 0.102405544896347 | 0.559924044890671 | 0.857704345796979 | -0.995045583 | 1.19985667288206 | 1.10783265651934 | 0.369706591791348 | 3.31964109404719 | 0.999950574162623 |
| ebi-fl187-GCST90094716 | prot-a-1548 | Inositol monophosphatase 3 \|\| id:prot-a-1548 | F-burn/spicy liking (derived food-liking factor) \|\| id:ebi-fl187-GCST90094716 | MR Egger | 15 | -0.283868206 | 0.56057831334044 | 0.62106532969161 | -1.3826017 | 0.814865288526418 | 0.752865861656759 | 0.250924871906375 | 2.25887135596371 | 0.999950574162623 |
| ebi-fl187-GCST90094716 | prot-a-1555 | Inhibin beta A chain:Inhibin beta B chain heterodimer \|\| id:prot-a-1555 | F-burn/spicy liking (derived food-liking factor) \|\| id:ebi-fl187-GCST90094716 | MR Egger | 15 | -0.038093458 | 0.562523655351244 | 0.947039909737132 | -1.140639822 | 1.06445290672228 | 0.962622972094497 | 0.319614459937301 | 2.89925238859913 | 0.999950574162623 |
| ebi-fl187-GCST90094716 | prot-a-1603 | Junctophilin-3 \|\| id:prot-a-1603 | F-burn/spicy liking (derived food-liking factor) \|\| id:ebi-fl187-GCST90094716 | MR Egger | 15 | -0.423991461 | 0.789841322934875 | 0.600468651466605 | -1.972080454 | 1.12409753181764 | 0.654429469970081 | 0.139167024224183 | 3.07743830517934 | 0.999950574162623 |
| ebi-fl187-GCST90094716 | prot-a-1627 | UPF0258 protein KIAA1024 \|\| id:prot-a-1627 | F-burn/spicy liking (derived food-liking factor) \|\| id:ebi-fl187-GCST90094716 | MR Egger | 15 | 0.18646643408109 | 0.666275214289365 | 0.783985386343385 | -1.119432986 | 1.49236585408825 | 1.20498417500577 | 0.326464852318833 | 4.44760546717691 | 0.999950574162623 |
| ebi-fl187-GCST90094716 | prot-a-1666 | Kallikrein-7 \|\| id:prot-a-1666 | F-burn/spicy liking (derived food-liking factor) \|\| id:ebi-fl187-GCST90094716 | MR Egger | 15 | 0.313130457024458 | 0.679603769397201 | 0.652587919399409 | -1.018892931 | 1.64515384504297 | 1.36769994545488 | 0.360994364709511 | 5.18180704095627 | 0.999950574162623 |
| ebi-fl187-GCST90094716 | prot-a-1678 | Importin subunit alpha-3 \|\| id:prot-a-1678 | F-burn/spicy liking (derived food-liking factor) \|\| id:ebi-fl187-GCST90094716 | MR Egger | 15 | -0.473563042 | 0.560141464840443 | 0.413158641514314 | -1.571440314 | 0.624314228615329 | 0.622779321234865 | 0.20774574775157 | 1.86696520701627 | 0.999950574162623 |
| ebi-fl187-GCST90094716 | prot-a-1718 | Protein LDOC1 \|\| id:prot-a-1718 | F-burn/spicy liking (derived food-liking factor) \|\| id:ebi-fl187-GCST90094716 | MR Egger | 15 | 0.0673911104641148 | 0.560141464840443 | 0.906076438264019 | -1.030486161 | 1.16526838155138 | 1.06971377261968 | 0.356833440025572 | 3.20678340922932 | 0.999950574162623 |
| ebi-fl187-GCST90094716 | prot-a-1723 | Leptin \|\| id:prot-a-1723 | F-burn/spicy liking (derived food-liking factor) \|\| id:ebi-fl187-GCST90094716 | MR Egger | 15 | -0.316197294 | 0.752111117380546 | 0.681049942259607 | -1.790335085 | 1.15794049561581 | 0.728915624972763 | 0.166904233271641 | 3.18337035505085 | 0.999950574162623 |
| ebi-fl187-GCST90094716 | prot-a-1730 | Galectin-7 \|\| id:prot-a-1730 | F-burn/spicy liking (derived food-liking factor) \|\| id:ebi-fl187-GCST90094716 | MR Egger | 15 | 0.7749688485297 | 0.560387929691838 | 0.189984047525513 | -0.323391494 | 1.8733291907257 | 2.17052451110043 | 0.723690478662878 | 6.50993317197202 | 0.999950574162623 |
| ebi-fl187-GCST90094716 | prot-a-1753 | Lipase member N \|\| id:prot-a-1753 | F-burn/spicy liking (derived food-liking factor) \|\| id:ebi-fl187-GCST90094716 | MR Egger | 15 | -0.468199551 | 0.565955740797713 | 0.423008737584201 | -1.577472803 | 0.641073701234396 | 0.626128566778328 | 0.206496296226173 | 1.89851822672156 | 0.999950574162623 |
| ebi-fl187-GCST90094716 | prot-a-1756 | VIP36-like protein \|\| id:prot-a-1756 | F-burn/spicy liking (derived food-liking factor) \|\| id:ebi-fl187-GCST90094716 | MR Egger | 15 | -0.047506397 | 0.572153029979325 | 0.935091844235661 | -1.168926336 | 1.07391354141395 | 0.953604372576445 | 0.310700349996952 | 2.92681131323423 | 0.999950574162623 |
| ebi-fl187-GCST90094716 | prot-a-1760 | Ig Kappa chain V-I region HK102- like \|\| id:prot-a-1760 | F-burn/spicy liking (derived food-liking factor) \|\| id:ebi-fl187-GCST90094716 | MR Egger | 15 | -0.201652444 | 0.695561795654488 | 0.776455300314276 | -1.564953563 | 1.1616486756312 | 0.817378963664598 | 0.209097722748873 | 3.19519677908599 | 0.999950574162623 |
| ebi-fl187-GCST90094716 | prot-a-179 | Neutral ceramidase \|\| id:prot-a-179 | F-burn/spicy liking (derived food-liking factor) \|\| id:ebi-fl187-GCST90094716 | MR Egger | 15 | -0.558052198 | 0.676009627124544 | 0.423973082247822 | -1.883031067 | 0.766926671131754 | 0.572322750255501 | 0.152128295135901 | 2.15313877124178 | 0.999950574162623 |
| ebi-fl187-GCST90094716 | prot-a-1809 | Lactotransferrin \|\| id:prot-a-1809 | F-burn/spicy liking (derived food-liking factor) \|\| id:ebi-fl187-GCST90094716 | MR Egger | 15 | 0.687385680778518 | 0.601489350304219 | 0.273729711969174 | -0.491533446 | 1.86630480737479 | 1.98851013165783 | 0.611687684716409 | 6.46436513682482 | 0.999950574162623 |
| ebi-fl187-GCST90094716 | prot-a-1818 | Lysophospholipase-like protein 1 \|\| id:prot-a-1818 | F-burn/spicy liking (derived food-liking factor) \|\| id:ebi-fl187-GCST90094716 | MR Egger | 15 | -0.098005373 | 0.560407669634452 | 0.86386646587518 | -1.196404405 | 1.00039365997836 | 0.906644032791088 | 0.302279133618677 | 2.71935211787605 | 0.999950574162623 |
| ebi-fl187-GCST90094716 | prot-a-1852 | MAP kinase-activated protein kinase 3 \|\| id:prot-a-1852 | F-burn/spicy liking (derived food-liking factor) \|\| id:ebi-fl187-GCST90094716 | MR Egger | 15 | 0.90891928955262 | 0.561920702349063 | 0.129761188759736 | -0.192445287 | 2.01028386615678 | 2.48163915030956 | 0.824939451838603 | 7.46543623125693 | 0.999950574162623 |
| ebi-fl187-GCST90094716 | prot-a-1856 | Megakaryocyte-associated tyrosine-protein kinase \|\| id:prot-a-1856 | F-burn/spicy liking (derived food-liking factor) \|\| id:ebi-fl187-GCST90094716 | MR Egger | 15 | -0.294402927 | 0.623515675913255 | 0.644635861062726 | -1.516493651 | 0.927687798127207 | 0.744976260163405 | 0.219480113106548 | 2.52865565062667 | 0.999950574162623 |
| ebi-fl187-GCST90094716 | prot-a-1890 | Beta-1,3-N-acetylglucosaminyltransferase manic fringe \|\| id:prot-a-1890 | F-burn/spicy liking (derived food-liking factor) \|\| id:ebi-fl187-GCST90094716 | MR Egger | 15 | 0.27265227473354 | 0.584364844263562 | 0.64852363940744 | -0.87270282 | 1.41800736949012 | 1.31344344793652 | 0.417820727503884 | 4.1288848957627 | 0.999950574162623 |
| ebi-fl187-GCST90094716 | prot-a-1919 | Matrilysin \|\| id:prot-a-1919 | F-burn/spicy liking (derived food-liking factor) \|\| id:ebi-fl187-GCST90094716 | MR Egger | 15 | -0.126512932 | 0.560141464840443 | 0.82482329018311 | -1.224390203 | 0.971364338791233 | 0.881162754012239 | 0.293936887403603 | 2.64154596558784 | 0.999950574162623 |
| ebi-fl187-GCST90094716 | prot-a-1923 | Multimerin-2 \|\| id:prot-a-1923 | F-burn/spicy liking (derived food-liking factor) \|\| id:ebi-fl187-GCST90094716 | MR Egger | 15 | -0.608045987 | 0.560605910397702 | 0.297794081589877 | -1.706833571 | 0.490741597406917 | 0.544413621729251 | 0.181439398906728 | 1.63352719040214 | 0.999950574162623 |
| ebi-fl187-GCST90094716 | prot-a-1948 | Mesothelin \|\| id:prot-a-1948 | F-burn/spicy liking (derived food-liking factor) \|\| id:ebi-fl187-GCST90094716 | MR Egger | 15 | 0.563639465248579 | 0.559924044890671 | 0.332479937647931 | -0.533811663 | 1.66109059323429 | 1.757055621 | 0.58636567647495 | 5.26504974210356 | 0.999950574162623 |
| ebi-fl187-GCST90094716 | prot-a-195 | ATPase family AAA domain-containing protein 1 \|\| id:prot-a-195 | F-burn/spicy liking (derived food-liking factor) \|\| id:ebi-fl187-GCST90094716 | MR Egger | 15 | -0.109566934 | 0.600833629039459 | 0.858114617488145 | -1.287200847 | 1.06806697923352 | 0.896222174902782 | 0.276042387653797 | 2.90974945411223 | 0.999950574162623 |
| ebi-fl187-GCST90094716 | prot-a-1966 | Mucin-1 \|\| id:prot-a-1966 | F-burn/spicy liking (derived food-liking factor) \|\| id:ebi-fl187-GCST90094716 | MR Egger | 15 | -0.983309628 | 0.560118686657314 | 0.102686080494724 | -2.081142253 | 0.114522998319479 | 0.37407101215125 | 0.124787591711658 | 1.12133842966686 | 0.999950574162623 |
| ebi-fl187-GCST90094716 | prot-a-1986 | Myocardial zonula adherens protein \|\| id:prot-a-1986 | F-burn/spicy liking (derived food-liking factor) \|\| id:ebi-fl187-GCST90094716 | MR Egger | 15 | 0.841081843199858 | 0.605415902652882 | 0.18809084187656 | -0.345533326 | 2.02769701239951 | 2.31887427865249 | 0.707842741843713 | 7.59657138842765 | 0.999950574162623 |
| ebi-fl187-GCST90094716 | prot-a-2071 | Epididymal secretory protein E1 \|\| id:prot-a-2071 | F-burn/spicy liking (derived food-liking factor) \|\| id:ebi-fl187-GCST90094716 | MR Egger | 14 | -0.066494255 | 0.618721656790151 | 0.916191745991872 | -1.279188702 | 1.14620019234821 | 0.935668291292199 | 0.278262963011519 | 3.14621515509925 | 0.999950574162623 |
| ebi-fl187-GCST90094716 | prot-a-2098 | Neuritin \|\| id:prot-a-2098 | F-burn/spicy liking (derived food-liking factor) \|\| id:ebi-fl187-GCST90094716 | MR Egger | 15 | 0.0104146362987238 | 0.560141464840443 | 0.985448228629557 | -1.087462635 | 1.10829190738599 | 1.01046905738453 | 0.337070680975625 | 3.02917985324694 | 0.999950574162623 |
| ebi-fl187-GCST90094716 | prot-a-2115 | Neurotrophin-4 \|\| id:prot-a-2115 | F-burn/spicy liking (derived food-liking factor) \|\| id:ebi-fl187-GCST90094716 | MR Egger | 15 | -0.555162205 | 0.560141464840443 | 0.339721317835919 | -1.653039476 | 0.542715066082811 | 0.573979151354395 | 0.191467063735344 | 1.72067226478648 | 0.999950574162623 |
| ebi-fl187-GCST90094716 | prot-a-2118 | Netrin-1 \|\| id:prot-a-2118 | F-burn/spicy liking (derived food-liking factor) \|\| id:ebi-fl187-GCST90094716 | MR Egger | 15 | -0.091368724 | 0.560605910397702 | 0.873038831053223 | -1.190156309 | 1.00741886017988 | 0.912681121232772 | 0.304173715389413 | 2.73852337302812 | 0.999950574162623 |
| ebi-fl187-GCST90094716 | prot-a-2120 | Netrin-G1 \|\| id:prot-a-2120 | F-burn/spicy liking (derived food-liking factor) \|\| id:ebi-fl187-GCST90094716 | MR Egger | 15 | 0.255068412608855 | 0.560169039636447 | 0.656375209252891 | -0.842862905 | 1.35299973029629 | 1.29054990773893 | 0.430476344690334 | 3.86901413958779 | 0.999950574162623 |
| ebi-fl187-GCST90094716 | prot-a-2145 | Olfactomedin-like protein 3 \|\| id:prot-a-2145 | F-burn/spicy liking (derived food-liking factor) \|\| id:ebi-fl187-GCST90094716 | MR Egger | 15 | 0.102360891001952 | 0.683397925155435 | 0.883235251899933 | -1.237099042 | 1.44182082430661 | 1.10778318858136 | 0.290224928156359 | 4.22838796342931 | 0.999950574162623 |
| ebi-fl187-GCST90094716 | prot-a-2162 | Prolyl 4-hydroxylase subunit alpha-2 \|\| id:prot-a-2162 | F-burn/spicy liking (derived food-liking factor) \|\| id:ebi-fl187-GCST90094716 | MR Egger | 15 | -0.837806773 | 0.634336987697432 | 0.209360452591376 | -2.081107268 | 0.405493723358628 | 0.432658401885444 | 0.124791957477166 | 1.50004292348983 | 0.999950574162623 |
| ebi-fl187-GCST90094716 | prot-a-2168 | Polyadenylate-binding protein-interacting protein 1 \|\| id:prot-a-2168 | F-burn/spicy liking (derived food-liking factor) \|\| id:ebi-fl187-GCST90094716 | MR Egger | 15 | -1.296597994 | 0.675767688074934 | 0.077246954396534 | -2.621102663 | 0.0279066741737406 | 0.273460526588229 | 0.0727226300414724 | 1.02829971302283 | 0.999950574162623 |
| ebi-fl187-GCST90094716 | prot-a-219 | Beta-1,4-galactosyltransferase 5 \|\| id:prot-a-219 | F-burn/spicy liking (derived food-liking factor) \|\| id:ebi-fl187-GCST90094716 | MR Egger | 15 | -0.941452885 | 0.560169039636447 | 0.116683801678514 | -2.039384203 | 0.156478432396688 | 0.390060710006174 | 0.130108806830229 | 1.16938554120359 | 0.999950574162623 |
| ebi-fl187-GCST90094716 | prot-a-2190 | Pyruvate carboxylase, mitochondrial \|\| id:prot-a-2190 | F-burn/spicy liking (derived food-liking factor) \|\| id:ebi-fl187-GCST90094716 | MR Egger | 15 | -0.728288863 | 0.588473013257541 | 0.237755195725918 | -1.881695969 | 0.425118242521435 | 0.482734308084999 | 0.152331536923832 | 1.52977129298461 | 0.999950574162623 |
| ebi-fl187-GCST90094716 | prot-a-2197 | Protocadherin-9 \|\| id:prot-a-2197 | F-burn/spicy liking (derived food-liking factor) \|\| id:ebi-fl187-GCST90094716 | MR Egger | 15 | -0.649112368 | 0.560605910397702 | 0.267745225359799 | -1.747899953 | 0.449675216080932 | 0.522509366860266 | 0.174139260412205 | 1.56780290561968 | 0.999950574162623 |
| ebi-fl187-GCST90094716 | prot-a-2198 | Protocadherin alpha-4 \|\| id:prot-a-2198 | F-burn/spicy liking (derived food-liking factor) \|\| id:ebi-fl187-GCST90094716 | MR Egger | 15 | -0.32657183 | 0.728909498314522 | 0.661508614649588 | -1.755234447 | 1.10209078667362 | 0.721392555543794 | 0.172866709507592 | 3.01045366500223 | 0.999950574162623 |
| ebi-fl187-GCST90094716 | prot-a-2254 | Placenta growth factor \|\| id:prot-a-2254 | F-burn/spicy liking (derived food-liking factor) \|\| id:ebi-fl187-GCST90094716 | MR Egger | 15 | -0.542444658 | 0.717613937098105 | 0.463186712931328 | -1.948967975 | 0.864078658376343 | 0.581325371937964 | 0.142420977770766 | 2.37281890174032 | 0.999950574162623 |
| ebi-fl187-GCST90094716 | prot-a-2261 | Membrane-associated progesterone receptor component 2 \|\| id:prot-a-2261 | F-burn/spicy liking (derived food-liking factor) \|\| id:ebi-fl187-GCST90094716 | MR Egger | 15 | -0.213915477 | 0.560141464840443 | 0.708708579891127 | -1.311792748 | 0.883961794063752 | 0.807416627395226 | 0.269336770322876 | 2.42047014008807 | 0.999950574162623 |
| ebi-fl187-GCST90094716 | prot-a-2268 | Polymeric immunoglobulin receptor \|\| id:prot-a-2268 | F-burn/spicy liking (derived food-liking factor) \|\| id:ebi-fl187-GCST90094716 | MR Egger | 15 | -0.543484577 | 0.672458795406911 | 0.433513811086143 | -1.861503816 | 0.774534661950685 | 0.58072115502924 | 0.155438703304495 | 2.16958230305014 | 0.999950574162623 |
| ebi-fl187-GCST90094716 | prot-a-2283 | Group 10 secretory phospholipase A2 \|\| id:prot-a-2283 | F-burn/spicy liking (derived food-liking factor) \|\| id:ebi-fl187-GCST90094716 | MR Egger | 15 | -0.157462756 | 0.7024842637067 | 0.826123959234784 | -1.534331913 | 1.21940640090587 | 0.854308630921425 | 0.215599682447931 | 3.38517769868748 | 0.999950574162623 |
| ebi-fl187-GCST90094716 | prot-a-2298 | Inactive phospholipase D5 \|\| id:prot-a-2298 | F-burn/spicy liking (derived food-liking factor) \|\| id:ebi-fl187-GCST90094716 | MR Egger | 15 | 0.104373532545546 | 0.76422939879566 | 0.893460248532346 | -1.393516089 | 1.60226315418504 | 1.11001500421705 | 0.248201071510922 | 4.96425459441567 | 0.999950574162623 |
| ebi-fl187-GCST90094716 | prot-a-2304 | Procollagen-lysine,2-oxoglutarate 5-dioxygenase 2 \|\| id:prot-a-2304 | F-burn/spicy liking (derived food-liking factor) \|\| id:ebi-fl187-GCST90094716 | MR Egger | 15 | -0.173177983 | 0.64844601888273 | 0.79360797307433 | -1.44413218 | 1.09777621352812 | 0.840987919565104 | 0.235950750397121 | 2.99749282282033 | 0.999950574162623 |
| ebi-fl187-GCST90094716 | prot-a-2331 | Periostin \|\| id:prot-a-2331 | F-burn/spicy liking (derived food-liking factor) \|\| id:ebi-fl187-GCST90094716 | MR Egger | 15 | 0.700609485427245 | 0.603086979016919 | 0.266240801804478 | -0.481440993 | 1.88265996430041 | 2.01498043450262 | 0.617892371125767 | 6.5709601561045 | 0.999950574162623 |
| ebi-fl187-GCST90094716 | prot-a-2333 | Periostin \|\| id:prot-a-2333 | F-burn/spicy liking (derived food-liking factor) \|\| id:ebi-fl187-GCST90094716 | MR Egger | 15 | 0.370576926117434 | 0.640852011007566 | 0.572972405210624 | -0.885493015 | 1.62664686769226 | 1.44857009155468 | 0.412510748961924 | 5.08678941197827 | 0.999950574162623 |
| ebi-fl187-GCST90094716 | prot-a-2367 | cAMP-dependent protein kinase catalytic subunit alpha \|\| id:prot-a-2367 | F-burn/spicy liking (derived food-liking factor) \|\| id:ebi-fl187-GCST90094716 | MR Egger | 15 | 0.443403032512815 | 0.619113876729296 | 0.486541157235199 | -0.770060166 | 1.65686623090224 | 1.55800013253012 | 0.462985211562115 | 5.24285517624619 | 0.999950574162623 |
| ebi-fl187-GCST90094716 | prot-a-2385 | Phosphoribosyl pyrophosphate synthase-associated protein 1 \|\| id:prot-a-2385 | F-burn/spicy liking (derived food-liking factor) \|\| id:ebi-fl187-GCST90094716 | MR Egger | 15 | 0.429703999048695 | 0.564240227493134 | 0.459914655305353 | -0.676206847 | 1.53561484493524 | 1.53680256119688 | 0.508542317430143 | 4.64418010291879 | 0.999950574162623 |
| ebi-fl187-GCST90094716 | prot-a-2393 | Trypsin-3 \|\| id:prot-a-2393 | F-burn/spicy liking (derived food-liking factor) \|\| id:ebi-fl187-GCST90094716 | MR Egger | 15 | -0.063539187 | 0.637578899046332 | 0.922137076643441 | -1.313193829 | 1.18611545526825 | 0.938437344164891 | 0.268959671957343 | 3.27433716182896 | 0.999950574162623 |
| ebi-fl187-GCST90094716 | prot-a-2405 | Pregnancy-specific beta-1-glycoprotein 6 \|\| id:prot-a-2405 | F-burn/spicy liking (derived food-liking factor) \|\| id:ebi-fl187-GCST90094716 | MR Egger | 15 | -0.373348306 | 0.594154705155635 | 0.540645475070876 | -1.537891528 | 0.791194915952586 | 0.688425408274983 | 0.21483359478856 | 2.20603087345357 | 0.999950574162623 |
| ebi-fl187-GCST90094716 | prot-a-244 | Beclin-1 \|\| id:prot-a-244 | F-burn/spicy liking (derived food-liking factor) \|\| id:ebi-fl187-GCST90094716 | MR Egger | 15 | -0.00053734 | 0.56057831334044 | 0.999249744668593 | -1.099270834 | 1.09819615432065 | 0.999462804514575 | 0.333113890495867 | 2.99875185667328 | 0.999974376584515 |
| ebi-fl187-GCST90094716 | prot-a-2454 | tRNA pseudouridine synthase A, mitochondrial \|\| id:prot-a-2454 | F-burn/spicy liking (derived food-liking factor) \|\| id:ebi-fl187-GCST90094716 | MR Egger | 15 | -0.148502939 | 0.560387929691838 | 0.79516466146982 | -1.246863281 | 0.949857403598378 | 0.861997474103047 | 0.287404892895476 | 2.5853409727101 | 0.999950574162623 |
| ebi-fl187-GCST90094716 | prot-a-2466 | Glutaminyl-peptide cyclotransferase-like protein \|\| id:prot-a-2466 | F-burn/spicy liking (derived food-liking factor) \|\| id:ebi-fl187-GCST90094716 | MR Egger | 15 | 0.140900746228836 | 0.58472204121522 | 0.813336784338285 | -1.005154455 | 1.28695594701067 | 1.15131037041755 | 0.365988101909564 | 3.62174497508266 | 0.999950574162623 |
| ebi-fl187-GCST90094716 | prot-a-2474 | Ras-related protein Rab-27A \|\| id:prot-a-2474 | F-burn/spicy liking (derived food-liking factor) \|\| id:ebi-fl187-GCST90094716 | MR Egger | 15 | 0.787281425869996 | 0.631175327304738 | 0.23428129636207 | -0.449822216 | 2.02438506738728 | 2.19741446462066 | 0.637741522007421 | 7.57145357906918 | 0.999950574162623 |
| ebi-fl187-GCST90094716 | prot-a-2490 | Rap1 GTPase-GDP dissociation stimulator 1 \|\| id:prot-a-2490 | F-burn/spicy liking (derived food-liking factor) \|\| id:ebi-fl187-GCST90094716 | MR Egger | 15 | 0.271124729005279 | 0.586452782623692 | 0.651499236602682 | -0.878322725 | 1.42057218294772 | 1.31143863462015 | 0.415479200486526 | 4.13948830738195 | 0.999950574162623 |
| ebi-fl187-GCST90094716 | prot-a-2545 | Receptor-interacting serine/threonine-protein kinase 2 \|\| id:prot-a-2545 | F-burn/spicy liking (derived food-liking factor) \|\| id:ebi-fl187-GCST90094716 | MR Egger | 15 | -0.279175506 | 0.596395995626739 | 0.647461128929005 | -1.448111657 | 0.889760645728707 | 0.756407137800867 | 0.235013655708567 | 2.43454686235598 | 0.999950574162623 |
| ebi-fl187-GCST90094716 | prot-a-2610 | RUN and FYVE domain-containing protein 1 \|\| id:prot-a-2610 | F-burn/spicy liking (derived food-liking factor) \|\| id:ebi-fl187-GCST90094716 | MR Egger | 15 | 0.00496769601331204 | 0.560605910397702 | 0.993064342428681 | -1.093819888 | 1.10375528039281 | 1.00498005547269 | 0.334934634072947 | 3.01546871882446 | 0.999950574162623 |
| ebi-fl187-GCST90094716 | prot-a-2627 | Serine--tRNA ligase, mitochondrial \|\| id:prot-a-2627 | F-burn/spicy liking (derived food-liking factor) \|\| id:ebi-fl187-GCST90094716 | MR Egger | 15 | -0.467036063 | 0.569318112782203 | 0.426807099844518 | -1.582899564 | 0.648827437643575 | 0.626857483386588 | 0.205378725158813 | 1.91329605427197 | 0.999950574162623 |
| ebi-fl187-GCST90094716 | prot-a-264 | BolA-like protein 2 \|\| id:prot-a-264 | F-burn/spicy liking (derived food-liking factor) \|\| id:ebi-fl187-GCST90094716 | MR Egger | 15 | -0.20808256 | 0.698994566029806 | 0.770645047315607 | -1.57811191 | 1.16194678899667 | 0.812139983324455 | 0.206364365130859 | 3.19614945194632 | 0.999950574162623 |
| ebi-fl187-GCST90094716 | prot-a-2646 | Secretoglobin family 1C member 1 \|\| id:prot-a-2646 | F-burn/spicy liking (derived food-liking factor) \|\| id:ebi-fl187-GCST90094716 | MR Egger | 15 | 0.0782855871815073 | 0.560118686657314 | 0.890987005816744 | -1.019547039 | 1.17611821302984 | 1.08143145753544 | 0.360758312735847 | 3.24176590271266 | 0.999950574162623 |
| ebi-fl187-GCST90094716 | prot-a-2672 | Semaphorin-3E \|\| id:prot-a-2672 | F-burn/spicy liking (derived food-liking factor) \|\| id:ebi-fl187-GCST90094716 | MR Egger | 15 | -0.236773072 | 0.770766071621813 | 0.763562938936133 | -1.747474572 | 1.27392842869087 | 0.789170352795543 | 0.17421335163464 | 3.5748686302618 | 0.999950574162623 |
| ebi-fl187-GCST90094716 | prot-a-2701 | Plasma protease C1 inhibitor \|\| id:prot-a-2701 | F-burn/spicy liking (derived food-liking factor) \|\| id:ebi-fl187-GCST90094716 | MR Egger | 15 | 0.234804628470428 | 0.712810290795974 | 0.747095187066525 | -1.162303541 | 1.63191279843054 | 1.26466166571121 | 0.312764883546623 | 5.11364674506701 | 0.999950574162623 |
| ebi-fl187-GCST90094716 | prot-a-2738 | Signaling threshold-regulating transmembrane adapter 1 \|\| id:prot-a-2738 | F-burn/spicy liking (derived food-liking factor) \|\| id:ebi-fl187-GCST90094716 | MR Egger | 15 | -0.037458433 | 0.56057831334044 | 0.947740910962587 | -1.136191927 | 1.06127506087079 | 0.963234455388995 | 0.321039237723445 | 2.89005363527493 | 0.999950574162623 |
| ebi-fl187-GCST90094716 | prot-a-274 | Serine/threonine-protein kinase BRSK2 \|\| id:prot-a-274 | F-burn/spicy liking (derived food-liking factor) \|\| id:ebi-fl187-GCST90094716 | MR Egger | 15 | -0.902461301 | 0.559926344760663 | 0.13101810105227 | -1.999916937 | 0.194994334696207 | 0.405570199906727 | 0.135346525089854 | 1.21530410140329 | 0.999950574162623 |
| ebi-fl187-GCST90094716 | prot-a-2749 | Mitochondrial glutamate carrier 2 \|\| id:prot-a-2749 | F-burn/spicy liking (derived food-liking factor) \|\| id:ebi-fl187-GCST90094716 | MR Egger | 15 | -0.399190001 | 0.617376507617547 | 0.52914774728723 | -1.609247956 | 0.810867953635462 | 0.670863224362082 | 0.20003799485032 | 2.24985991355417 | 0.999950574162623 |
| ebi-fl187-GCST90094716 | prot-a-2760 | Sodium-coupled monocarboxylate transporter 1 \|\| id:prot-a-2760 | F-burn/spicy liking (derived food-liking factor) \|\| id:ebi-fl187-GCST90094716 | MR Egger | 15 | 0.333426757910434 | 0.73654543444893 | 0.65823438393699 | -1.110202294 | 1.77705580943034 | 1.39574281567745 | 0.329492300146188 | 5.91242346680298 | 0.999950574162623 |
| ebi-fl187-GCST90094716 | prot-a-2777 | Small integral membrane protein 10 \|\| id:prot-a-2777 | F-burn/spicy liking (derived food-liking factor) \|\| id:ebi-fl187-GCST90094716 | MR Egger | 15 | 0.0957714950686522 | 0.560360271299497 | 0.866925547759572 | -1.002534637 | 1.19407762681567 | 1.10050756385524 | 0.366948181148104 | 3.300512062 | 0.999950574162623 |
| ebi-fl187-GCST90094716 | prot-a-2799 | Superoxide dismutase [Mn], mitochondrial \|\| id:prot-a-2799 | F-burn/spicy liking (derived food-liking factor) \|\| id:ebi-fl187-GCST90094716 | MR Egger | 15 | 0.0457082743545996 | 0.560141464840444 | 0.936206739355004 | -1.052168997 | 1.14358554544187 | 1.04676899704932 | 0.349179557831146 | 3.13799965836918 | 0.999950574162623 |
| ebi-fl187-GCST90094716 | prot-a-2805 | Protein SPACA7 \|\| id:prot-a-2805 | F-burn/spicy liking (derived food-liking factor) \|\| id:ebi-fl187-GCST90094716 | MR Egger | 15 | -0.68878604 | 0.570700153653144 | 0.248968265390507 | -1.807358341 | 0.429786260995739 | 0.502185332003934 | 0.164087026694888 | 1.53692898676772 | 0.999950574162623 |
| ebi-fl187-GCST90094716 | prot-a-2811 | SPARC-like protein 1 \|\| id:prot-a-2811 | F-burn/spicy liking (derived food-liking factor) \|\| id:ebi-fl187-GCST90094716 | MR Egger | 15 | -0.016414936 | 0.8427564808257 | 0.984755824721472 | -1.668217638 | 1.63538776683594 | 0.983719055320195 | 0.188582889245716 | 5.13144741641528 | 0.999950574162623 |
| ebi-fl187-GCST90094716 | prot-a-2831 | Sepiapterin reductase \|\| id:prot-a-2831 | F-burn/spicy liking (derived food-liking factor) \|\| id:ebi-fl187-GCST90094716 | MR Egger | 15 | 0.320821343702785 | 0.775598506619136 | 0.685877721731352 | -1.199351729 | 1.84099441667629 | 1.37825932418929 | 0.301389530606469 | 6.30280275792019 | 0.999950574162623 |
| ebi-fl187-GCST90094716 | prot-a-2834 | SPRY domain-containing SOCS box protein 1 \|\| id:prot-a-2834 | F-burn/spicy liking (derived food-liking factor) \|\| id:ebi-fl187-GCST90094716 | MR Egger | 15 | -0.10054551 | 0.56057831334044 | 0.860420135207055 | -1.199279004 | 0.998187984375689 | 0.90434395498943 | 0.301411450063178 | 2.71336071922451 | 0.999950574162623 |
| ebi-fl187-GCST90094716 | prot-a-2835 | Spectrin alpha chain, non-erythrocytic 1 \|\| id:prot-a-2835 | F-burn/spicy liking (derived food-liking factor) \|\| id:ebi-fl187-GCST90094716 | MR Egger | 15 | -0.767159888 | 0.560141464840443 | 0.194011448190541 | -1.865037159 | 0.330717383037449 | 0.464329946415061 | 0.154890454182348 | 1.3919663434132 | 0.999950574162623 |
| ebi-fl187-GCST90094716 | prot-a-2859 | Sia-alpha-2,3-Gal-beta-1,4-GlcNAc-R:alpha 2,8-sialyltransferase \|\| id:prot-a-2859 | F-burn/spicy liking (derived food-liking factor) \|\| id:ebi-fl187-GCST90094716 | MR Egger | 15 | -0.613007237 | 0.622823691823509 | 0.342962521125987 | -1.833741673 | 0.607727198812872 | 0.541719338578572 | 0.159814474143316 | 1.8362532140039 | 0.999950574162623 |
| ebi-fl187-GCST90094716 | prot-a-2861 | Alpha-2,8-sialyltransferase 8F \|\| id:prot-a-2861 | F-burn/spicy liking (derived food-liking factor) \|\| id:ebi-fl187-GCST90094716 | MR Egger | 15 | -0.34991544 | 0.673686403715833 | 0.612208365480624 | -1.670340792 | 0.970509911006302 | 0.704747680468025 | 0.188182923557788 | 2.63928991927125 | 0.999950574162623 |
| ebi-fl187-GCST90094716 | prot-a-289 | Uncharacterized protein C17orf89 \|\| id:prot-a-289 | F-burn/spicy liking (derived food-liking factor) \|\| id:ebi-fl187-GCST90094716 | MR Egger | 15 | -0.318524082 | 0.611224704329462 | 0.611042744438609 | -1.516524502 | 0.879476338763253 | 0.727221564996792 | 0.21947334208369 | 2.40963754219736 | 0.999950574162623 |
| ebi-fl187-GCST90094716 | prot-a-2921 | Tachykinin-3 \|\| id:prot-a-2921 | F-burn/spicy liking (derived food-liking factor) \|\| id:ebi-fl187-GCST90094716 | MR Egger | 15 | 0.705114077678041 | 0.649741752441163 | 0.297538422209504 | -0.568379757 | 1.97860791246272 | 2.02407757382226 | 0.566442469978697 | 7.23266746754402 | 0.999950574162623 |
| ebi-fl187-GCST90094716 | prot-a-2928 | Tax1-binding protein 1 \|\| id:prot-a-2928 | F-burn/spicy liking (derived food-liking factor) \|\| id:ebi-fl187-GCST90094716 | MR Egger | 15 | -1.565411156 | 0.705122379094299 | 0.0448141822982162 | -2.947451019 | -0.183371293 | 0.209002063039863 | 0.0524732890379458 | 0.832459012114344 | 0.999950574162623 |
| ebi-fl187-GCST90094716 | prot-a-296 | Uncharacterized protein C1orf185 \|\| id:prot-a-296 | F-burn/spicy liking (derived food-liking factor) \|\| id:ebi-fl187-GCST90094716 | MR Egger | 15 | -0.509650815 | 0.56057831334044 | 0.379816816856926 | -1.608384309 | 0.589082679499642 | 0.600705299686154 | 0.200210831774903 | 1.80233433861726 | 0.999950574162623 |
| ebi-fl187-GCST90094716 | prot-a-2964 | Transforming growth factor beta-2 \|\| id:prot-a-2964 | F-burn/spicy liking (derived food-liking factor) \|\| id:ebi-fl187-GCST90094716 | MR Egger | 15 | -0.222590434 | 0.560605910397702 | 0.697769942369533 | -1.321378018 | 0.876197150406468 | 0.800442616271028 | 0.2667674381369 | 2.40174882818348 | 0.999950574162623 |
| ebi-fl187-GCST90094716 | prot-a-2977 | Thymocyte nuclear protein 1 \|\| id:prot-a-2977 | F-burn/spicy liking (derived food-liking factor) \|\| id:ebi-fl187-GCST90094716 | MR Egger | 15 | 0.249006352848925 | 0.594296218914955 | 0.682060974850174 | -0.915814236 | 1.41382694192224 | 1.28275018216204 | 0.400190643676741 | 4.1116604194422 | 0.999950574162623 |
| ebi-fl187-GCST90094716 | prot-a-3015 | Transmembrane protein 70, mitochondrial \|\| id:prot-a-3015 | F-burn/spicy liking (derived food-liking factor) \|\| id:ebi-fl187-GCST90094716 | MR Egger | 15 | -0.488032389 | 0.560605910397702 | 0.399793094863739 | -1.586819973 | 0.610755195320053 | 0.613832991247601 | 0.204575132795762 | 1.84182180891041 | 0.999950574162623 |
| ebi-fl187-GCST90094716 | prot-a-3030 | Tumor necrosis factor alpha-induced protein 3 \|\| id:prot-a-3030 | F-burn/spicy liking (derived food-liking factor) \|\| id:ebi-fl187-GCST90094716 | MR Egger | 15 | 0.148750803918041 | 0.748383593165451 | 0.845523138705831 | -1.318081039 | 1.61558264652232 | 1.16038379008288 | 0.267648416441818 | 5.03081825847385 | 0.999950574162623 |
| ebi-fl187-GCST90094716 | prot-a-3032 | Tumor necrosis factor receptor superfamily member 10A \|\| id:prot-a-3032 | F-burn/spicy liking (derived food-liking factor) \|\| id:ebi-fl187-GCST90094716 | MR Egger | 15 | -0.177146066 | 0.681037231013585 | 0.798851520910401 | -1.511979038 | 1.15768690711579 | 0.837657422593409 | 0.220473220939379 | 3.18256319128555 | 0.999950574162623 |
| ebi-fl187-GCST90094716 | prot-a-3081 | Tumor protein p53-inducible protein 11 \|\| id:prot-a-3081 | F-burn/spicy liking (derived food-liking factor) \|\| id:ebi-fl187-GCST90094716 | MR Egger | 15 | 0.171928765963846 | 0.685926564906497 | 0.80600119714403 | -1.172487301 | 1.51634483318058 | 1.18759323314143 | 0.30959592445666 | 4.55554345516187 | 0.999950574162623 |
| ebi-fl187-GCST90094716 | prot-a-315 | Complement C4 \|\| id:prot-a-315 | F-burn/spicy liking (derived food-liking factor) \|\| id:ebi-fl187-GCST90094716 | MR Egger | 15 | 0.281324423281813 | 0.675400501306741 | 0.683817586731318 | -1.042460559 | 1.60510940584303 | 1.32488335722727 | 0.352586054853729 | 4.97840423945866 | 0.999950574162623 |
| ebi-fl187-GCST90094716 | prot-a-3157 | UDP-glucuronosyltransferase 1-8 \|\| id:prot-a-3157 | F-burn/spicy liking (derived food-liking factor) \|\| id:ebi-fl187-GCST90094716 | MR Egger | 15 | 0.478351930620658 | 0.680433660311069 | 0.494447258240438 | -0.855298044 | 1.81200190483035 | 1.61341319298695 | 0.425156457040719 | 6.12269221882015 | 0.999950574162623 |
| ebi-fl187-GCST90094716 | prot-a-317 | Glutaredoxin-like protein C5orf63 \|\| id:prot-a-317 | F-burn/spicy liking (derived food-liking factor) \|\| id:ebi-fl187-GCST90094716 | MR Egger | 15 | -0.286758238 | 0.560387929691838 | 0.617432383079532 | -1.38511858 | 0.811602104438955 | 0.750693196171085 | 0.250294118167127 | 2.25151225647768 | 0.999950574162623 |
| ebi-fl187-GCST90094716 | prot-a-3192 | Guanine nucleotide exchange factor VAV3 \|\| id:prot-a-3192 | F-burn/spicy liking (derived food-liking factor) \|\| id:ebi-fl187-GCST90094716 | MR Egger | 15 | -0.012161213 | 0.695599616372542 | 0.986316722141239 | -1.375536461 | 1.35121403546536 | 0.987912436066644 | 0.25270399367026 | 3.86211142594218 | 0.999950574162623 |
| ebi-fl187-GCST90094716 | prot-a-3233 | Serine/threonine-protein kinase WNK3 \|\| id:prot-a-3233 | F-burn/spicy liking (derived food-liking factor) \|\| id:ebi-fl187-GCST90094716 | MR Egger | 15 | 0.846169611178959 | 0.56057831334044 | 0.155098285195175 | -0.252563883 | 1.94490310532622 | 2.33070223639305 | 0.776806586543647 | 6.99295424218491 | 0.999950574162623 |
| ebi-fl187-GCST90094716 | prot-a-3250 | 14-3-3 protein theta \|\| id:prot-a-3250 | F-burn/spicy liking (derived food-liking factor) \|\| id:ebi-fl187-GCST90094716 | MR Egger | 15 | 0.728885180353769 | 0.559951680937867 | 0.215616058888845 | -0.368620114 | 1.82639047499199 | 2.07276855604492 | 0.691688122984317 | 6.21142584954572 | 0.999950574162623 |
| ebi-fl187-GCST90094716 | prot-a-3278 | E3 ubiquitin-protein ligase ZNRF3 \|\| id:prot-a-3278 | F-burn/spicy liking (derived food-liking factor) \|\| id:ebi-fl187-GCST90094716 | MR Egger | 15 | -0.438331061 | 0.560118686657314 | 0.447901849364769 | -1.536163687 | 0.65950156482688 | 0.645112176004852 | 0.215205114035843 | 1.93382820614756 | 0.999950574162623 |
| ebi-fl187-GCST90094716 | prot-a-355 | Calpain-3 \|\| id:prot-a-355 | F-burn/spicy liking (derived food-liking factor) \|\| id:ebi-fl187-GCST90094716 | MR Egger | 15 | -0.376021962 | 0.559901223166098 | 0.513605033931724 | -1.473428359 | 0.721384435723131 | 0.686587254265491 | 0.229138567748565 | 2.05727941110769 | 0.999950574162623 |
| ebi-fl187-GCST90094716 | prot-a-385 | Coiled-coil domain-containing protein 90B, mitochondrial \|\| id:prot-a-385 | F-burn/spicy liking (derived food-liking factor) \|\| id:ebi-fl187-GCST90094716 | MR Egger | 15 | 0.0178263819804462 | 0.583764396200416 | 0.976102671949715 | -1.126351835 | 1.16200459853326 | 1.01798622029472 | 0.324213887439509 | 3.19633422520579 | 0.999950574162623 |
| ebi-fl187-GCST90094716 | prot-a-390 | C-C motif chemokine 14 \|\| id:prot-a-390 | F-burn/spicy liking (derived food-liking factor) \|\| id:ebi-fl187-GCST90094716 | MR Egger | 15 | -0.346484606 | 0.682036005888508 | 0.619952672108372 | -1.683275177 | 0.990305965957051 | 0.707169705667807 | 0.185764566762634 | 2.69205802446328 | 0.999950574162623 |
| ebi-fl187-GCST90094716 | prot-a-40 | Pituitary adenylate cyclase-activating polypeptide 27 \|\| id:prot-a-40 | F-burn/spicy liking (derived food-liking factor) \|\| id:ebi-fl187-GCST90094716 | MR Egger | 15 | -0.046251803 | 0.56057831334044 | 0.935500180582485 | -1.144985297 | 1.0524816910335 | 0.95480150992545 | 0.318228596588015 | 2.86475173234087 | 0.999950574162623 |
| ebi-fl187-GCST90094716 | prot-a-401 | C-C motif chemokine 24 \|\| id:prot-a-401 | F-burn/spicy liking (derived food-liking factor) \|\| id:ebi-fl187-GCST90094716 | MR Egger | 15 | -0.300871868 | 0.560141464840443 | 0.600247376297146 | -1.398749139 | 0.797005403182815 | 0.740172606538093 | 0.246905615468396 | 2.21888629964967 | 0.999950574162623 |
| ebi-fl187-GCST90094716 | prot-a-412 | Cerebral cavernous malformations 2 protein \|\| id:prot-a-412 | F-burn/spicy liking (derived food-liking factor) \|\| id:ebi-fl187-GCST90094716 | MR Egger | 15 | -0.662685599 | 0.888490390017916 | 0.469027393542274 | -2.404126764 | 1.07875556509503 | 0.515465141102044 | 0.0903443531360132 | 2.94101736819492 | 0.999950574162623 |
| ebi-fl187-GCST90094716 | prot-a-44 | Adhesion G protein-coupled receptor F5 \|\| id:prot-a-44 | F-burn/spicy liking (derived food-liking factor) \|\| id:ebi-fl187-GCST90094716 | MR Egger | 15 | -0.994914692 | 0.560110046044998 | 0.0990811732714902 | -2.092730382 | 0.102900998270386 | 0.369754986265232 | 0.123349883283913 | 1.10838167194142 | 0.999950574162623 |
| ebi-fl187-GCST90094716 | prot-a-48 | Adrenomedullin \|\| id:prot-a-48 | F-burn/spicy liking (derived food-liking factor) \|\| id:ebi-fl187-GCST90094716 | MR Egger | 15 | 0.815240531555188 | 0.601737808816894 | 0.198555267401046 | -0.364165574 | 1.9946466368363 | 2.25971914074564 | 0.694776148543262 | 7.34960548913298 | 0.999950574162623 |
| ebi-fl187-GCST90094716 | prot-a-502 | Carcinoembryonic antigen-related cell adhesion molecule 1 \|\| id:prot-a-502 | F-burn/spicy liking (derived food-liking factor) \|\| id:ebi-fl187-GCST90094716 | MR Egger | 15 | -0.013545785 | 0.560605910397702 | 0.981089743199102 | -1.11233337 | 1.08524179922599 | 0.986545546144409 | 0.328790874699257 | 2.96015549551859 | 0.999950574162623 |
| ebi-fl187-GCST90094716 | prot-a-518 | Complement factor B \|\| id:prot-a-518 | F-burn/spicy liking (derived food-liking factor) \|\| id:ebi-fl187-GCST90094716 | MR Egger | 15 | 0.22802444962515 | 0.560141464840443 | 0.690569805340838 | -0.869852821 | 1.32590172071242 | 1.25611603662314 | 0.419013214461887 | 3.76557932543522 | 0.999950574162623 |
| ebi-fl187-GCST90094716 | prot-a-525 | Properdin \|\| id:prot-a-525 | F-burn/spicy liking (derived food-liking factor) \|\| id:ebi-fl187-GCST90094716 | MR Egger | 15 | 0.677230651985185 | 0.560108312436351 | 0.248153341962926 | -0.42058164 | 1.77504294436043 | 1.96841893998769 | 0.656664765966236 | 5.90053452555956 | 0.999950574162623 |
| ebi-fl187-GCST90094716 | prot-a-533 | Chondroadherin \|\| id:prot-a-533 | F-burn/spicy liking (derived food-liking factor) \|\| id:ebi-fl187-GCST90094716 | MR Egger | 15 | 0.956061352117969 | 0.652504067744199 | 0.166625182184965 | -0.322846621 | 2.2349693248966 | 2.6014301516246 | 0.724084905515655 | 9.34619515229668 | 0.999950574162623 |
| ebi-fl187-GCST90094716 | prot-a-535 | Coiled-coil-helix-coiled-coil-helix domain-containing protein 2 \|\| id:prot-a-535 | F-burn/spicy liking (derived food-liking factor) \|\| id:ebi-fl187-GCST90094716 | MR Egger | 15 | -1.173482036 | 0.750816094748641 | 0.142073541709817 | -2.645081582 | 0.298117509576796 | 0.309288111714041 | 0.0709995612286649 | 1.34732010159263 | 0.999950574162623 |
| ebi-fl187-GCST90094716 | prot-a-591 | Caseinolytic peptidase B protein homolog \|\| id:prot-a-591 | F-burn/spicy liking (derived food-liking factor) \|\| id:ebi-fl187-GCST90094716 | MR Egger | 15 | 0.275582364479006 | 0.77791612697626 | 0.728826216513108 | -1.249133244 | 1.80029797335248 | 1.31729759886116 | 0.286753234150535 | 6.0514503667443 | 0.999950574162623 |
| ebi-fl187-GCST90094716 | prot-a-605 | Ciliary neurotrophic factor receptor subunit alpha \|\| id:prot-a-605 | F-burn/spicy liking (derived food-liking factor) \|\| id:ebi-fl187-GCST90094716 | MR Egger | 15 | 0.386030378321445 | 0.560141464840443 | 0.502834585593723 | -0.711846893 | 1.48390764940871 | 1.47112936057654 | 0.490737021335693 | 4.41014535577477 | 0.999950574162623 |
| ebi-fl187-GCST90094716 | prot-a-606 | Ciliary neurotrophic factor receptor subunit alpha \|\| id:prot-a-606 | F-burn/spicy liking (derived food-liking factor) \|\| id:ebi-fl187-GCST90094716 | MR Egger | 15 | 0.515073200984818 | 0.587272349278612 | 0.396372891602521 | -0.635980604 | 1.6661270055709 | 1.6737610182682 | 0.529416086374616 | 5.29163359099721 | 0.999950574162623 |
| ebi-fl187-GCST90094716 | prot-a-707 | Cancer/testis antigen 55 \|\| id:prot-a-707 | F-burn/spicy liking (derived food-liking factor) \|\| id:ebi-fl187-GCST90094716 | MR Egger | 15 | -0.399733822 | 0.829986817041472 | 0.638085876956923 | -2.026507983 | 1.22704033975388 | 0.670498494469707 | 0.131794948712211 | 3.41111882874833 | 0.999950574162623 |
| ebi-fl187-GCST90094716 | prot-a-719 | Dipeptidyl peptidase 1 \|\| id:prot-a-719 | F-burn/spicy liking (derived food-liking factor) \|\| id:ebi-fl187-GCST90094716 | MR Egger | 15 | -0.825811322 | 0.560329420512625 | 0.164335588686084 | -1.924056986 | 0.27243434263106 | 0.437879587154712 | 0.14601338465181 | 1.31315723763276 | 0.999950574162623 |
| ebi-fl187-GCST90094716 | prot-a-752 | Cytochrome b5 \|\| id:prot-a-752 | F-burn/spicy liking (derived food-liking factor) \|\| id:ebi-fl187-GCST90094716 | MR Egger | 15 | -0.004072968 | 0.694571481854668 | 0.995410262727913 | -1.365433073 | 1.3572871361271 | 0.995935314977702 | 0.25527010152329 | 3.88563778406003 | 0.999950574162623 |
| ebi-fl187-GCST90094716 | prot-a-755 | Cytoglobin \|\| id:prot-a-755 | F-burn/spicy liking (derived food-liking factor) \|\| id:ebi-fl187-GCST90094716 | MR Egger | 15 | 0.483484912558854 | 0.560141464840443 | 0.403699704238382 | -0.614392359 | 1.58136218364612 | 1.62171610488373 | 0.540969510968682 | 4.86157365898483 | 0.999950574162623 |
| ebi-fl187-GCST90094716 | prot-a-807 | Desmin \|\| id:prot-a-807 | F-burn/spicy liking (derived food-liking factor) \|\| id:ebi-fl187-GCST90094716 | MR Egger | 15 | 0.565448718522677 | 0.733661790189201 | 0.454652150885031 | -0.87252839 | 2.00342582729351 | 1.76023745668902 | 0.417893614235948 | 7.41441313860715 | 0.999950574162623 |
| ebi-fl187-GCST90094716 | prot-a-82 | Ameloblastin \|\| id:prot-a-82 | F-burn/spicy liking (derived food-liking factor) \|\| id:ebi-fl187-GCST90094716 | MR Egger | 15 | -0.114350818 | 0.594776748270884 | 0.850510569026487 | -1.280113244 | 1.05141160907062 | 0.891944991040988 | 0.278005816137831 | 2.86168785277744 | 0.999950574162623 |
| ebi-fl187-GCST90094716 | prot-a-84 | Muellerian-inhibiting factor \|\| id:prot-a-84 | F-burn/spicy liking (derived food-liking factor) \|\| id:ebi-fl187-GCST90094716 | MR Egger | 15 | -0.335057468 | 0.560141464840443 | 0.560006809678651 | -1.432934739 | 0.762819803318036 | 0.715296978649685 | 0.238607642590587 | 2.14431424790227 | 0.999950574162623 |
| ebi-fl187-GCST90094716 | prot-a-858 | Inactive dipeptidyl peptidase 10 \|\| id:prot-a-858 | F-burn/spicy liking (derived food-liking factor) \|\| id:ebi-fl187-GCST90094716 | MR Egger | 15 | -0.944635597 | 0.711160706969655 | 0.206927095930695 | -2.338510583 | 0.449239388517794 | 0.388821232657359 | 0.0964712171693232 | 1.56711976277689 | 0.999950574162623 |
| ebi-fl187-GCST90094716 | prot-a-863 | Dihydropyrimidinase-related protein 3 \|\| id:prot-a-863 | F-burn/spicy liking (derived food-liking factor) \|\| id:ebi-fl187-GCST90094716 | MR Egger | 15 | 0.0685351194969616 | 0.560605910397702 | 0.904569199251051 | -1.030252465 | 1.16732270387646 | 1.07093823510264 | 0.356916840225373 | 3.21337794731277 | 0.999950574162623 |
| ebi-fl187-GCST90094716 | prot-a-864 | Dihydropyrimidinase-related protein 5 \|\| id:prot-a-864 | F-burn/spicy liking (derived food-liking factor) \|\| id:ebi-fl187-GCST90094716 | MR Egger | 15 | -1.151099693 | 0.588556946494797 | 0.0723271714584517 | -2.304671308 | 0.00247192226119797 | 0.316288757569953 | 0.0997915959629647 | 1.00247497997999 | 0.999950574162623 |
| ebi-fl187-GCST90094716 | prot-a-884 | Endothelin-converting enzyme 1 \|\| id:prot-a-884 | F-burn/spicy liking (derived food-liking factor) \|\| id:ebi-fl187-GCST90094716 | MR Egger | 15 | -0.017757269 | 0.560545053387743 | 0.975209582183895 | -1.116425573 | 1.08091103597979 | 0.982399462557664 | 0.32744814467942 | 2.94736348247889 | 0.999950574162623 |
| ebi-fl187-GCST90094716 | prot-a-893 | Early endosome antigen 1 \|\| id:prot-a-893 | F-burn/spicy liking (derived food-liking factor) \|\| id:ebi-fl187-GCST90094716 | MR Egger | 15 | -0.716233233 | 0.560169039636447 | 0.223392555700591 | -1.81416455 | 0.381698085105843 | 0.488589195989528 | 0.162974008121279 | 1.46476978255359 | 0.999950574162623 |
| ebi-fl187-GCST90094716 | prot-a-897 | Ephrin-A2 \|\| id:prot-a-897 | F-burn/spicy liking (derived food-liking factor) \|\| id:ebi-fl187-GCST90094716 | MR Egger | 15 | 0.979870348633352 | 0.560141464840443 | 0.103787841200992 | -0.118006922 | 2.07774761972062 | 2.66411081392937 | 0.88868990067844 | 7.98646009533479 | 0.999950574162623 |
| ebi-fl187-GCST90094716 | prot-a-90 | Anaphase-promoting complex subunit 10 \|\| id:prot-a-90 | F-burn/spicy liking (derived food-liking factor) \|\| id:ebi-fl187-GCST90094716 | MR Egger | 15 | -0.091728076 | 0.578863169365624 | 0.876527544270499 | -1.226299888 | 1.04284373551832 | 0.912353206150683 | 0.293376096173098 | 2.83727400981676 | 0.999950574162623 |
| ebi-fl187-GCST90094716 | prot-a-914 | Histone-lysine N-methyltransferase EHMT2 \|\| id:prot-a-914 | F-burn/spicy liking (derived food-liking factor) \|\| id:ebi-fl187-GCST90094716 | MR Egger | 15 | -0.443419855 | 0.673072742243281 | 0.52152291650619 | -1.762642429 | 0.875802720283182 | 0.64183767206536 | 0.1715908475493 | 2.4008016928987 | 0.999950574162623 |
| ebi-fl187-GCST90094716 | prot-a-932 | RNA polymerase II elongation factor ELL \|\| id:prot-a-932 | F-burn/spicy liking (derived food-liking factor) \|\| id:ebi-fl187-GCST90094716 | MR Egger | 15 | -0.191960225 | 0.560545053387743 | 0.737483672939617 | -1.29062853 | 0.90670807968664 | 0.825339695763737 | 0.275097821617802 | 2.47615778779143 | 0.999950574162623 |
| ebi-fl187-GCST90094716 | prot-a-991 | Estrogen receptor \|\| id:prot-a-991 | F-burn/spicy liking (derived food-liking factor) \|\| id:ebi-fl187-GCST90094716 | MR Egger | 15 | 0.188548399399064 | 0.560169039636447 | 0.741796052091791 | -0.909382918 | 1.2864797170865 | 1.20749552363007 | 0.402772691025108 | 3.62002060237935 | 0.999950574162623 |
| ebi-fl187-GCST90094716 | prot-a-992 | Persulfide dioxygenase ETHE1, mitochondrial \|\| id:prot-a-992 | F-burn/spicy liking (derived food-liking factor) \|\| id:ebi-fl187-GCST90094716 | MR Egger | 15 | 0.825380788181094 | 0.849225546884666 | 0.34882714029784 | -0.839101284 | 2.48986286007504 | 2.28274984401235 | 0.432098683104404 | 12.0596221513578 | 0.999950574162623 |
| ebi-fl187-GCST90094716 | prot-a-993 | Ethanolamine kinase 1 \|\| id:prot-a-993 | F-burn/spicy liking (derived food-liking factor) \|\| id:ebi-fl187-GCST90094716 | MR Egger | 15 | -0.115818419 | 0.560141464840443 | 0.839395671484322 | -1.213695691 | 0.982058851610661 | 0.890636930935621 | 0.297097268460212 | 2.66994761297394 | 0.999950574162623 |
| ebi-fl187-GCST90094716 | prot-b-10 | coagulation factor III, tissue factor \|\| id:prot-b-10 | F-burn/spicy liking (derived food-liking factor) \|\| id:ebi-fl187-GCST90094716 | MR Egger | 10 | 0.114564173254835 | 0.911880968381964 | 0.903120762302923 | -1.672722525 | 1.90185087128348 | 1.12138460165478 | 0.187735255363159 | 6.69828063139179 | 0.999950574162623 |
| ebi-fl187-GCST90094716 | prot-b-28 | prolactin \|\| id:prot-b-28 | F-burn/spicy liking (derived food-liking factor) \|\| id:ebi-fl187-GCST90094716 | MR Egger | 10 | -1.009702225 | 1.0426183108119 | 0.361192274338448 | -3.053234114 | 1.0338296644206 | 0.364327451110939 | 0.047206007640924 | 2.81181354378977 | 0.999950574162623 |
| ebi-fl187-GCST90094716 | prot-b-54 | matrix metallopeptidase 7 \|\| id:prot-b-54 | F-burn/spicy liking (derived food-liking factor) \|\| id:ebi-fl187-GCST90094716 | MR Egger | 10 | 2.56705585574469 | 0.911288304294791 | 0.0225997055353022 | 0.780930779326901 | 4.35318093216248 | 13.0274133406216 | 2.1835036800378 | 77.7253090521324 | 0.999950574162623 |
| ebi-fl187-GCST90094716 | ukb-rsfMRI191-edge-pheno716 | Net100_Pair10_33 (OccipitalPrecuneus)&(Temporal\|Frontal\|Supp_Motor_Area) (Default_mode\|Central_executive)&(Default_mode\|Salience) \|\| id:ukb-rsfMRI191-edge-pheno716 | F-burn/spicy liking (derived food-liking factor) \|\| id:ebi-fl187-GCST90094716 | MR Egger | 13 | -0.053831376 | 0.252866536718641 | 0.835310885482145 | -0.549449788 | 0.441787035838544 | 0.947591879640569 | 0.577267342379641 | 1.55548444271809 | 0.999950574162623 |
| ebi-fl187-GCST90094716 | ukb-rsfMRI191-edge-pheno903 | Net100_Pair27_38 (Precentral\|Frontal\|Supp_Motor_Area)&(Subcortical) (Attention\|Salience\|Motor)&(Subcortical-cerebellum) \|\| id:ukb-rsfMRI191-edge-pheno903 | F-burn/spicy liking (derived food-liking factor) \|\| id:ebi-fl187-GCST90094716 | MR Egger | 13 | 0.341840537953453 | 0.338293406037285 | 0.33397395344153 | -0.321214538 | 1.00489561378653 | 1.40753583107343 | 0.725267636917197 | 2.73162211425374 | 0.999950574162623 |
| ebi-fl187-GCST90094716 | ukb-rsfMRI191-node-pheno23 | Net100_Node2 RolandicOper\|SupraMarginal\|Insula Salience\|Motor \|\| id:ukb-rsfMRI191-node-pheno23 | F-burn/spicy liking (derived food-liking factor) \|\| id:ebi-fl187-GCST90094716 | MR Egger | 13 | -0.009571303 | 0.428487741956232 | 0.982578864819777 | -0.849407277 | 0.830264671477746 | 0.990474356373193 | 0.427668345708082 | 2.29392579665582 | 0.999950574162623 |
| ebi-fl187-GCST90094716 | ukb-rsfMRI191-node-pheno55 | Net100_Node34 Parietal\|Postcentral\|Precuneus Attention \|\| id:ukb-rsfMRI191-node-pheno55 | F-burn/spicy liking (derived food-liking factor) \|\| id:ebi-fl187-GCST90094716 | MR Egger | 13 | -0.06688366 | 0.350701098862951 | 0.852223800418256 | -0.754257814 | 0.620490493368695 | 0.935304007899041 | 0.470359579429135 | 1.85984005737425 | 0.999950574162623 |
| ebi-fl187-GCST90094716 | ebi-a-GCST90001414 | IgD- CD24- B cell Absolute Count \|\| id:ebi-a-GCST90001414 | F-burn/spicy liking (derived food-liking factor) \|\| id:ebi-fl187-GCST90094716 | Weighted median | 15 | 0.0843051216468495 | 0.179606297335783 | 0.638792002113196 | -0.267723221 | 0.436333464424985 | 1.08796080356192 | 0.765119520714362 | 1.54702458640966 | 0.999332001504091 |
| ebi-fl187-GCST90094716 | ebi-a-GCST90001422 | CD20- CD38- B cell %B cell \|\| id:ebi-a-GCST90001422 | F-burn/spicy liking (derived food-liking factor) \|\| id:ebi-fl187-GCST90094716 | Weighted median | 15 | -0.04935365 | 0.16013946922241 | 0.7579364932906 | -0.36322701 | 0.264519709796245 | 0.951844450493343 | 0.695428546653532 | 1.30280510096223 | 0.999332001504091 |
| ebi-fl187-GCST90094716 | ebi-a-GCST90001444 | CD20- CD38- B cell %lymphocyte \|\| id:ebi-a-GCST90001444 | F-burn/spicy liking (derived food-liking factor) \|\| id:ebi-fl187-GCST90094716 | Weighted median | 15 | 0.0506181090588905 | 0.16356863455246 | 0.756970893928153 | -0.269976415 | 0.371212632781712 | 1.05192109742912 | 0.763397499111099 | 1.44949124997781 | 0.999332001504091 |
| ebi-fl187-GCST90094716 | ebi-a-GCST90001459 | Myeloid Dendritic Cell %Dendritic Cell \|\| id:ebi-a-GCST90001459 | F-burn/spicy liking (derived food-liking factor) \|\| id:ebi-fl187-GCST90094716 | Weighted median | 15 | 0.117186718937263 | 0.16319116143582 | 0.472698856975209 | -0.202667957 | 0.437041395351471 | 1.12432934367265 | 0.81654932551564 | 1.54812016070812 | 0.999332001504091 |
| ebi-fl187-GCST90094716 | ebi-a-GCST90001476 | HLA DR++ monocyte %leukocyte \|\| id:ebi-a-GCST90001476 | F-burn/spicy liking (derived food-liking factor) \|\| id:ebi-fl187-GCST90094716 | Weighted median | 15 | 0.0325067547639391 | 0.159935871693232 | 0.838940665865683 | -0.280967554 | 0.345981063282674 | 1.03304087106763 | 0.755052833712153 | 1.41337585086529 | 0.999332001504091 |
| ebi-fl187-GCST90094716 | ebi-a-GCST90001478 | CD4 regulatory T cell %CD4+ T cell \|\| id:ebi-a-GCST90001478 | F-burn/spicy liking (derived food-liking factor) \|\| id:ebi-fl187-GCST90094716 | Weighted median | 15 | 0.0291868958589307 | 0.169391410120781 | 0.863198176320748 | -0.302820268 | 0.361194059695661 | 1.02961700764897 | 0.738731858210088 | 1.43504191765686 | 0.999332001504091 |
| ebi-fl187-GCST90094716 | ebi-a-GCST90001485 | CD39+ resting CD4 regulatory T cell %CD4 regulatory T cell \|\| id:ebi-a-GCST90001485 | F-burn/spicy liking (derived food-liking factor) \|\| id:ebi-fl187-GCST90094716 | Weighted median | 15 | 0.121926995381711 | 0.171759200711033 | 0.477783702780801 | -0.214721038 | 0.458575028775336 | 1.1296716275239 | 0.806766465966314 | 1.58181833264965 | 0.999332001504091 |
| ebi-fl187-GCST90094716 | ebi-a-GCST90001515 | Immature Myeloid-Derived Suppressor Cells Absolute Count \|\| id:ebi-a-GCST90001515 | F-burn/spicy liking (derived food-liking factor) \|\| id:ebi-fl187-GCST90094716 | Weighted median | 15 | 0.00217152693791651 | 0.214419417629997 | 0.991919583502736 | -0.418090532 | 0.422433585492711 | 1.00217388641011 | 0.658302628525675 | 1.5256698896246 | 0.999332001504091 |
| ebi-fl187-GCST90094716 | ebi-a-GCST90001517 | CD33+ HLA DR+ Absolute Count \|\| id:ebi-a-GCST90001517 | F-burn/spicy liking (derived food-liking factor) \|\| id:ebi-fl187-GCST90094716 | Weighted median | 15 | 0.270843210270139 | 0.200950091981605 | 0.177718433228933 | -0.12301897 | 0.664705390554085 | 1.31106949203724 | 0.884246888226039 | 1.94391774043923 | 0.999332001504091 |
| ebi-fl187-GCST90094716 | ebi-a-GCST90001518 | CD33+ HLA DR+ CD14- Absolute Count \|\| id:ebi-a-GCST90001518 | F-burn/spicy liking (derived food-liking factor) \|\| id:ebi-fl187-GCST90094716 | Weighted median | 14 | 0.18135526544321 | 0.211698062488938 | 0.391627584598675 | -0.233572937 | 0.596283467921529 | 1.19884101043009 | 0.791699849394639 | 1.81535940595161 | 0.999332001504091 |
| ebi-fl187-GCST90094716 | ebi-a-GCST90001520 | CD33+ HLA DR+ CD14dim Absolute Count \|\| id:ebi-a-GCST90001520 | F-burn/spicy liking (derived food-liking factor) \|\| id:ebi-fl187-GCST90094716 | Weighted median | 15 | 0.255687001388454 | 0.209567330154069 | 0.222437998337625 | -0.155064966 | 0.666438968490429 | 1.2913484743981 | 0.856359541667768 | 1.94729059605346 | 0.999332001504091 |
| ebi-fl187-GCST90094716 | ebi-a-GCST90001531 | CD33dim HLA DR- Absolute Count \|\| id:ebi-a-GCST90001531 | F-burn/spicy liking (derived food-liking factor) \|\| id:ebi-fl187-GCST90094716 | Weighted median | 15 | 0.0759056419895151 | 0.215289807196337 | 0.724407646012118 | -0.34606238 | 0.497873664094336 | 1.07886077019848 | 0.707468353772511 | 1.64521926000882 | 0.999332001504091 |
| ebi-fl187-GCST90094716 | ebi-a-GCST90001532 | Basophil Absolute Count \|\| id:ebi-a-GCST90001532 | F-burn/spicy liking (derived food-liking factor) \|\| id:ebi-fl187-GCST90094716 | Weighted median | 15 | 0.0685847390379362 | 0.222915693812351 | 0.758332536343075 | -0.368330021 | 0.505499498910144 | 1.07099137588468 | 0.691888806285409 | 1.65781338966512 | 0.999332001504091 |
| ebi-fl187-GCST90094716 | ebi-a-GCST90001588 | T/B cell \|\| id:ebi-a-GCST90001588 | F-burn/spicy liking (derived food-liking factor) \|\| id:ebi-fl187-GCST90094716 | Weighted median | 15 | -0.090629817 | 0.163811823425406 | 0.580088365438221 | -0.411700991 | 0.230441357118456 | 0.913355757287085 | 0.662522346775244 | 1.25915562460638 | 0.999332001504091 |
| ebi-fl187-GCST90094716 | ebi-a-GCST90001624 | HLA DR+ CD4+ T cell Absolute Count \|\| id:ebi-a-GCST90001624 | F-burn/spicy liking (derived food-liking factor) \|\| id:ebi-fl187-GCST90094716 | Weighted median | 15 | 0.0987537125001371 | 0.152613392081259 | 0.517577459930094 | -0.200368536 | 0.397875960979405 | 1.1037944153125 | 0.818429076930923 | 1.48865936660495 | 0.999332001504091 |
| ebi-fl187-GCST90094716 | ebi-a-GCST90001631 | CD8+ Natural Killer T %T cell \|\| id:ebi-a-GCST90001631 | F-burn/spicy liking (derived food-liking factor) \|\| id:ebi-fl187-GCST90094716 | Weighted median | 15 | 0.0953907586738219 | 0.157180524981784 | 0.543926153397774 | -0.21268307 | 0.403464587638118 | 1.10008864032767 | 0.808412306497992 | 1.49700221885598 | 0.999332001504091 |
| ebi-fl187-GCST90094716 | ebi-a-GCST90001632 | CD8+ Natural Killer T %lymphocyte \|\| id:ebi-a-GCST90001632 | F-burn/spicy liking (derived food-liking factor) \|\| id:ebi-fl187-GCST90094716 | Weighted median | 15 | 0.0676485851263892 | 0.150429892993557 | 0.652925514156402 | -0.227194005 | 0.362491175393762 | 1.06998923227243 | 0.796766190557699 | 1.4369045408134 | 0.999332001504091 |
| ebi-fl187-GCST90094716 | ebi-a-GCST90001646 | Natural Killer %CD3- lymphocyte \|\| id:ebi-a-GCST90001646 | F-burn/spicy liking (derived food-liking factor) \|\| id:ebi-fl187-GCST90094716 | Weighted median | 15 | -0.100228494 | 0.153525871931352 | 0.513856645528164 | -0.401139203 | 0.20068221494813 | 0.904630691699961 | 0.669556850213004 | 1.22223630167507 | 0.999332001504091 |
| ebi-fl187-GCST90094716 | ebi-a-GCST90001684 | CD127- CD8+ T cell Absolute Count \|\| id:ebi-a-GCST90001684 | F-burn/spicy liking (derived food-liking factor) \|\| id:ebi-fl187-GCST90094716 | Weighted median | 15 | -0.147902124 | 0.170695486078274 | 0.386233736534974 | -0.482465277 | 0.186661028924569 | 0.862515530563029 | 0.617259798461412 | 1.20521868152884 | 0.999332001504091 |
| ebi-fl187-GCST90094716 | ebi-a-GCST90001687 | CD28- CD8+ T cell Absolute Count \|\| id:ebi-a-GCST90001687 | F-burn/spicy liking (derived food-liking factor) \|\| id:ebi-fl187-GCST90094716 | Weighted median | 15 | 0.036359758157698 | 0.155107278906165 | 0.814661386513672 | -0.267650508 | 0.340370024813781 | 1.03702885898154 | 0.765175156591887 | 1.40546755222759 | 0.999332001504091 |
| ebi-fl187-GCST90094716 | ebi-a-GCST90001701 | BAFF-R on CD20- CD38- B cell \|\| id:ebi-a-GCST90001701 | F-burn/spicy liking (derived food-liking factor) \|\| id:ebi-fl187-GCST90094716 | Weighted median | 15 | 0.14774646726931 | 0.168272453330952 | 0.37993322704503 | -0.182067541 | 0.477560475797975 | 1.15921895915501 | 0.833545039832731 | 1.6121367545227 | 0.999332001504091 |
| ebi-fl187-GCST90094716 | ebi-a-GCST90001732 | CD19 on IgD- CD27- B cell \|\| id:ebi-a-GCST90001732 | F-burn/spicy liking (derived food-liking factor) \|\| id:ebi-fl187-GCST90094716 | Weighted median | 15 | 0.0012280721186812 | 0.167642094356694 | 0.994155101539132 | -0.327350433 | 0.329806577057802 | 1.00122882650803 | 0.720831095887276 | 1.39069910933394 | 0.999332001504091 |
| ebi-fl187-GCST90094716 | ebi-a-GCST90001798 | CD27 on CD24+ CD27+ B cell \|\| id:ebi-a-GCST90001798 | F-burn/spicy liking (derived food-liking factor) \|\| id:ebi-fl187-GCST90094716 | Weighted median | 15 | -0.000334171 | 0.164651523277129 | 0.99838064282725 | -0.323051157 | 0.322382814715039 | 0.999665884920744 | 0.723936819324068 | 1.3804131172765 | 0.999391734334742 |
| ebi-fl187-GCST90094716 | ebi-a-GCST90001836 | CD66b on CD66b++ myeloid cell \|\| id:ebi-a-GCST90001836 | F-burn/spicy liking (derived food-liking factor) \|\| id:ebi-fl187-GCST90094716 | Weighted median | 15 | -0.01958488 | 0.24235509080339 | 0.935592512377411 | -0.494600858 | 0.455431097657102 | 0.980605657536502 | 0.609814261032364 | 1.57685301416977 | 0.999332001504091 |
| ebi-fl187-GCST90094716 | ebi-a-GCST90001943 | CD25 on activated & secreting CD4 regulatory T cell \|\| id:ebi-a-GCST90001943 | F-burn/spicy liking (derived food-liking factor) \|\| id:ebi-fl187-GCST90094716 | Weighted median | 15 | 0.20414739092199 | 0.169058442219623 | 0.227218423265655 | -0.127207156 | 0.535501937672452 | 1.22647891199244 | 0.880551242379942 | 1.70830548997523 | 0.999332001504091 |
| ebi-fl187-GCST90094716 | ebi-a-GCST90001960 | CD25 on CD4+ T cell \|\| id:ebi-a-GCST90001960 | F-burn/spicy liking (derived food-liking factor) \|\| id:ebi-fl187-GCST90094716 | Weighted median | 15 | 0.317239927136066 | 0.168840081593308 | 0.060253054928286 | -0.013686633 | 0.64816648705895 | 1.37333203202198 | 0.986406603324192 | 1.9120318779514 | 0.999332001504091 |
| ebi-fl187-GCST90094716 | ebi-a-GCST90001988 | HLA DR on CD14+ CD16- monocyte \|\| id:ebi-a-GCST90001988 | F-burn/spicy liking (derived food-liking factor) \|\| id:ebi-fl187-GCST90094716 | Weighted median | 15 | 0.156984246986035 | 0.148525489538393 | 0.290533698055612 | -0.134125713 | 0.448094206481285 | 1.16997718308884 | 0.87448012460234 | 1.56532615257661 | 0.999332001504091 |
| ebi-fl187-GCST90094716 | ebi-a-GCST90001991 | HLA DR on CD14+ monocyte \|\| id:ebi-a-GCST90001991 | F-burn/spicy liking (derived food-liking factor) \|\| id:ebi-fl187-GCST90094716 | Weighted median | 15 | 0.135012684957812 | 0.156120411705305 | 0.387149439977731 | -0.170983322 | 0.44100869190021 | 1.14455130284422 | 0.842835630180032 | 1.55427421187994 | 0.999332001504091 |
| ebi-fl187-GCST90094716 | ebi-a-GCST90002019 | CD14 on Monocytic Myeloid-Derived Suppressor Cells \|\| id:ebi-a-GCST90002019 | F-burn/spicy liking (derived food-liking factor) \|\| id:ebi-fl187-GCST90094716 | Weighted median | 15 | 0.149099463488815 | 0.221945706536403 | 0.501721534603922 | -0.285914121 | 0.584113048300165 | 1.16078843953542 | 0.751327136157188 | 1.79339962117003 | 0.999332001504091 |
| ebi-fl187-GCST90094716 | ebi-a-GCST90002037 | CD80 on plasmacytoid Dendritic Cell \|\| id:ebi-a-GCST90002037 | F-burn/spicy liking (derived food-liking factor) \|\| id:ebi-fl187-GCST90094716 | Weighted median | 15 | -0.071148986 | 0.168023322810812 | 0.671969098356451 | -0.400474699 | 0.2581767265993 | 0.931323127581024 | 0.670001921414044 | 1.29456758293577 | 0.999332001504091 |
| ebi-fl187-GCST90094716 | ebi-a-GCST90002042 | CD45 on CD33+ HLA DR+ CD14- \|\| id:ebi-a-GCST90002042 | F-burn/spicy liking (derived food-liking factor) \|\| id:ebi-fl187-GCST90094716 | Weighted median | 15 | 0.142691641410153 | 0.239795999395282 | 0.551807172743732 | -0.327308517 | 0.612691800224906 | 1.15337409401688 | 0.720861310455633 | 1.84539214610981 | 0.999332001504091 |
| ebi-fl187-GCST90094716 | ebi-a-GCST90002056 | CD8 on Effector Memory CD8+ T cell \|\| id:ebi-a-GCST90002056 | F-burn/spicy liking (derived food-liking factor) \|\| id:ebi-fl187-GCST90094716 | Weighted median | 15 | 0.097569430362239 | 0.17594938254537 | 0.579215327695373 | -0.247291359 | 0.442430220151165 | 1.10248798504624 | 0.780913133979167 | 1.55648522772028 | 0.999332001504091 |
| ebi-fl187-GCST90094716 | ebi-a-GCST90002058 | CD8 on CD8+ T cell \|\| id:ebi-a-GCST90002058 | F-burn/spicy liking (derived food-liking factor) \|\| id:ebi-fl187-GCST90094716 | Weighted median | 15 | -0.168704957 | 0.192905102384645 | 0.381819368785122 | -0.546798958 | 0.209389043750016 | 0.844758106651736 | 0.578799610251849 | 1.23292456683465 | 0.999332001504091 |
| ebi-fl187-GCST90094716 | ebi-a-GCST90002063 | CD4 on CD4 regulatory T cell \|\| id:ebi-a-GCST90002063 | F-burn/spicy liking (derived food-liking factor) \|\| id:ebi-fl187-GCST90094716 | Weighted median | 15 | 0.0946514282697084 | 0.185885337404018 | 0.610617267183033 | -0.269683833 | 0.458986689581584 | 1.09927561193399 | 0.763620887867568 | 1.58246963930932 | 0.999332001504091 |
| ebi-fl187-GCST90094716 | ebi-a-GCST90002066 | CD4 on activated CD4 regulatory T cell \|\| id:ebi-a-GCST90002066 | F-burn/spicy liking (derived food-liking factor) \|\| id:ebi-fl187-GCST90094716 | Weighted median | 15 | 0.0335623600949053 | 0.170367215115307 | 0.843827451808274 | -0.300357382 | 0.367482101720907 | 1.03413193028088 | 0.74055351323534 | 1.44409395150166 | 0.999332001504091 |
| ebi-fl187-GCST90094716 | ebi-a-GCST90002070 | CD4 on activated & secreting CD4 regulatory T cell \|\| id:ebi-a-GCST90002070 | F-burn/spicy liking (derived food-liking factor) \|\| id:ebi-fl187-GCST90094716 | Weighted median | 15 | 0.0953364522560798 | 0.178253629314713 | 0.592763289555883 | -0.254040661 | 0.444713565712917 | 1.10002890007657 | 0.775660262127617 | 1.56004328194471 | 0.999332001504091 |
| ebi-fl187-GCST90094716 | ebi-a-GCST90002081 | SSC-A on CD4+ T cell \|\| id:ebi-a-GCST90002081 | F-burn/spicy liking (derived food-liking factor) \|\| id:ebi-fl187-GCST90094716 | Weighted median | 15 | -0.025676271 | 0.166900218285759 | 0.87773427977642 | -0.352800699 | 0.301448156621573 | 0.974650560980894 | 0.702717231627701 | 1.35181503066322 | 0.999332001504091 |
| ebi-fl187-GCST90094716 | ebi-a-GCST90002094 | CD11b on Monocytic Myeloid-Derived Suppressor Cells \|\| id:ebi-a-GCST90002094 | F-burn/spicy liking (derived food-liking factor) \|\| id:ebi-fl187-GCST90094716 | Weighted median | 15 | 0.339794805983944 | 0.237709321077244 | 0.152873465649491 | -0.126115463 | 0.805705075295343 | 1.40465933330048 | 0.881513058476195 | 2.23827409436095 | 0.999332001504091 |
| ebi-fl187-GCST90094716 | ebi-a-GCST90002104 | HLA DR on myeloid Dendritic Cell \|\| id:ebi-a-GCST90002104 | F-burn/spicy liking (derived food-liking factor) \|\| id:ebi-fl187-GCST90094716 | Weighted median | 15 | 0.0830417106901465 | 0.175639931049036 | 0.636359321829544 | -0.261212554 | 0.427295975546257 | 1.08658712990213 | 0.770117210595786 | 1.53310635657077 | 0.999332001504091 |
| ebi-fl187-GCST90094716 | ebi-a-GCST90002105 | HLA DR on plasmacytoid Dendritic Cell \|\| id:ebi-a-GCST90002105 | F-burn/spicy liking (derived food-liking factor) \|\| id:ebi-fl187-GCST90094716 | Weighted median | 15 | 0.151416029184692 | 0.173557691349933 | 0.382976691703606 | -0.188757046 | 0.491589104230561 | 1.16348059929314 | 0.827987645285335 | 1.63491220266338 | 0.999332001504091 |
| ebi-fl187-GCST90094716 | ebi-a-GCST90002106 | HLA DR on Dendritic Cell \|\| id:ebi-a-GCST90002106 | F-burn/spicy liking (derived food-liking factor) \|\| id:ebi-fl187-GCST90094716 | Weighted median | 15 | -0.086661292 | 0.184173191921676 | 0.637967565162344 | -0.447640748 | 0.274318164606469 | 0.916987634490281 | 0.639134253226363 | 1.31563332361452 | 0.999332001504091 |
| ebi-fl187-GCST90094716 | ebi-a-GCST90002111 | HLA DR on CD33dim HLA DR+ CD11b- \|\| id:ebi-a-GCST90002111 | F-burn/spicy liking (derived food-liking factor) \|\| id:ebi-fl187-GCST90094716 | Weighted median | 15 | -0.083060182 | 0.224170909009771 | 0.710993825822494 | -0.522435163 | 0.356314799818311 | 0.920295760460522 | 0.593074554561532 | 1.42805702960527 | 0.999332001504091 |
| ebi-fl187-GCST90094716 | ebi-a-GCST90002112 | HLA DR on CD33- HLA DR+ \|\| id:ebi-a-GCST90002112 | F-burn/spicy liking (derived food-liking factor) \|\| id:ebi-fl187-GCST90094716 | Weighted median | 15 | -0.11916689 | 0.22184092894533 | 0.591148329308501 | -0.553975111 | 0.315641330738778 | 0.88765964688639 | 0.574660923304123 | 1.37113838223079 | 0.999332001504091 |
| ebi-fl187-GCST90094716 | ebi-a-GCST90002115 | HLA DR on HLA DR+ CD8+ T cell \|\| id:ebi-a-GCST90002115 | F-burn/spicy liking (derived food-liking factor) \|\| id:ebi-fl187-GCST90094716 | Weighted median | 15 | -0.187033542 | 0.170273206530996 | 0.272015886677363 | -0.520769027 | 0.146701942540404 | 0.829415915434246 | 0.594063521335874 | 1.15800875843828 | 0.999332001504091 |
| ebi-fl187-GCST90094716 | ebi-a-GCST90002116 | HLA DR on B cell \|\| id:ebi-a-GCST90002116 | F-burn/spicy liking (derived food-liking factor) \|\| id:ebi-fl187-GCST90094716 | Weighted median | 15 | 0.0843403792632176 | 0.160392011526973 | 0.599000056032932 | -0.230027963 | 0.398708721856084 | 1.08799916314278 | 0.794511385008929 | 1.48989958021318 | 0.999332001504091 |
| ebi-fl187-GCST90094716 | ebi-cfmet338-GCST90026023 | Sphingomyelin (d18:1/24:1, d18:2/24:0) levels \|\| id:ebi-cfmet338-GCST90026023 | F-burn/spicy liking (derived food-liking factor) \|\| id:ebi-fl187-GCST90094716 | Weighted median | 12 | -0.0707369 | 0.102853055379489 | 0.491611993636651 | -0.272328889 | 0.130855088112939 | 0.931706991591449 | 0.761603736822662 | 1.13980259839838 | 0.999332001504091 |
| ebi-fl187-GCST90094716 | ebi-cfmet338-GCST90026046 | 1-arachidonoyl-gpc (20:4n6) levels \|\| id:ebi-cfmet338-GCST90026046 | F-burn/spicy liking (derived food-liking factor) \|\| id:ebi-fl187-GCST90094716 | Weighted median | 12 | 0.031816230818482 | 0.117825958319467 | 0.78713921568801 | -0.199122647 | 0.262755109124637 | 1.03232777784183 | 0.819449383761235 | 1.30050819736093 | 0.999332001504091 |
| ebi-fl187-GCST90094716 | ebi-cfmet338-GCST90026047 | 1-linoleoyl-gpc (18:2) levels \|\| id:ebi-cfmet338-GCST90026047 | F-burn/spicy liking (derived food-liking factor) \|\| id:ebi-fl187-GCST90094716 | Weighted median | 12 | -0.051112933 | 0.14994468301669 | 0.733194979167682 | -0.345004512 | 0.242778645773625 | 0.950171358828839 | 0.708217158231376 | 1.274786414 | 0.999332001504091 |
| ebi-fl187-GCST90094716 | ebi-cfmet338-GCST90026050 | 1-myristoyl-2-palmitoyl-gpc (14:0/16:0) levels \|\| id:ebi-cfmet338-GCST90026050 | F-burn/spicy liking (derived food-liking factor) \|\| id:ebi-fl187-GCST90094716 | Weighted median | 12 | -0.0826774 | 0.0705891606173943 | 0.241499180107936 | -0.221032155 | 0.0556773545529109 | 0.920648100159416 | 0.801690901450868 | 1.05725650970119 | 0.999332001504091 |
| ebi-fl187-GCST90094716 | ebi-cfmet338-GCST90026068 | 2,3-dihydroxyisovalerate levels \|\| id:ebi-cfmet338-GCST90026068 | F-burn/spicy liking (derived food-liking factor) \|\| id:ebi-fl187-GCST90094716 | Weighted median | 12 | -0.268642035 | 0.249434794856318 | 0.281478902304707 | -0.757534233 | 0.220250162634118 | 0.764416840935506 | 0.468821006234437 | 1.24638849141845 | 0.999332001504091 |
| ebi-fl187-GCST90094716 | ebi-cfmet338-GCST90026082 | 3-hydroxyhexanoate levels \|\| id:ebi-cfmet338-GCST90026082 | F-burn/spicy liking (derived food-liking factor) \|\| id:ebi-fl187-GCST90094716 | Weighted median | 12 | -0.076912532 | 0.0545922669983536 | 0.158878350789234 | -0.183913376 | 0.0300883108726474 | 0.925970842470897 | 0.832007872754072 | 1.03054553831091 | 0.999332001504091 |
| ebi-fl187-GCST90094716 | ebi-cfmet338-GCST90026094 | 3-ureidopropionate levels \|\| id:ebi-cfmet338-GCST90026094 | F-burn/spicy liking (derived food-liking factor) \|\| id:ebi-fl187-GCST90094716 | Weighted median | 12 | -0.102227505 | 0.120523279502738 | 0.396328171475135 | -0.338453133 | 0.133998122889345 | 0.902824131356452 | 0.712872188956953 | 1.14339067337182 | 0.999332001504091 |
| ebi-fl187-GCST90094716 | ebi-cfmet338-GCST90026173 | Glycerol levels \|\| id:ebi-cfmet338-GCST90026173 | F-burn/spicy liking (derived food-liking factor) \|\| id:ebi-fl187-GCST90094716 | Weighted median | 12 | -0.046921531 | 0.0634163023989748 | 0.459362500224527 | -0.171217484 | 0.0773744213619405 | 0.954162266487328 | 0.842638293160096 | 1.08044654293353 | 0.999332001504091 |
| ebi-fl187-GCST90094716 | ebi-cfmet338-GCST90026179 | Histidine levels \|\| id:ebi-cfmet338-GCST90026179 | F-burn/spicy liking (derived food-liking factor) \|\| id:ebi-fl187-GCST90094716 | Weighted median | 12 | -0.007164539 | 0.0495744100962218 | 0.885089142923845 | -0.104330383 | 0.0900013047366137 | 0.992861065074188 | 0.90092759722767 | 1.09417571131539 | 0.999332001504091 |
| ebi-fl187-GCST90094716 | ebi-cfmet338-GCST90026188 | Isocitrate levels \|\| id:ebi-cfmet338-GCST90026188 | F-burn/spicy liking (derived food-liking factor) \|\| id:ebi-fl187-GCST90094716 | Weighted median | 12 | 0.00517154492060847 | 0.0875491268059348 | 0.952896189675181 | -0.166424744 | 0.176767833460241 | 1.00518494044093 | 0.846686533102188 | 1.19335400409314 | 0.999332001504091 |
| ebi-fl187-GCST90094716 | ebi-cfmet338-GCST90026207 | N-acetyl-isoputreanine levels \|\| id:ebi-cfmet338-GCST90026207 | F-burn/spicy liking (derived food-liking factor) \|\| id:ebi-fl187-GCST90094716 | Weighted median | 12 | 0.0322962953719432 | 0.0544209682941253 | 0.552877924074754 | -0.074368802 | 0.138961393228429 | 1.03282348079071 | 0.928329260562278 | 1.14907973688833 | 0.999332001504091 |
| ebi-fl187-GCST90094716 | ebi-cfmet338-GCST90026213 | N-acetylglutamate levels \|\| id:ebi-cfmet338-GCST90026213 | F-burn/spicy liking (derived food-liking factor) \|\| id:ebi-fl187-GCST90094716 | Weighted median | 12 | 0.071470586879742 | 0.0993215818358196 | 0.471778898259014 | -0.123199714 | 0.266140887277948 | 1.0740865579996 | 0.884087080786991 | 1.30491889220739 | 0.999332001504091 |
| ebi-fl187-GCST90094716 | ebi-cfmet338-GCST90026224 | N-acetyltaurine levels \|\| id:ebi-cfmet338-GCST90026224 | F-burn/spicy liking (derived food-liking factor) \|\| id:ebi-fl187-GCST90094716 | Weighted median | 12 | -0.033096368 | 0.0757906068043408 | 0.662342231538553 | -0.181645957 | 0.115453221334449 | 0.96744532432365 | 0.833896523103419 | 1.12238200978758 | 0.999332001504091 |
| ebi-fl187-GCST90094716 | ebi-cfmet338-GCST90026250 | Phenyllactate (pla) levels \|\| id:ebi-cfmet338-GCST90026250 | F-burn/spicy liking (derived food-liking factor) \|\| id:ebi-fl187-GCST90094716 | Weighted median | 12 | 0.21910862250117 | 0.116311950092949 | 0.0595918347278644 | -0.0088628 | 0.44708004468335 | 1.24496650071839 | 0.991176359157047 | 1.56373946330718 | 0.999332001504091 |
| ebi-fl187-GCST90094716 | ebi-cfmet338-GCST90026251 | Pipecolate levels \|\| id:ebi-cfmet338-GCST90026251 | F-burn/spicy liking (derived food-liking factor) \|\| id:ebi-fl187-GCST90094716 | Weighted median | 12 | 0.194653609516983 | 0.195928670182022 | 0.320470139984115 | -0.189366584 | 0.578673803073747 | 1.21489008723206 | 0.82748310898681 | 1.78367136201961 | 0.999332001504091 |
| ebi-fl187-GCST90094716 | ebi-cfmet338-GCST90026278 | Trigonelline (n'-methylnicotinate) levels \|\| id:ebi-cfmet338-GCST90026278 | F-burn/spicy liking (derived food-liking factor) \|\| id:ebi-fl187-GCST90094716 | Weighted median | 12 | 0.229287555216327 | 0.215255204459316 | 0.286790243878132 | -0.192612646 | 0.651187755956586 | 1.2577036462604 | 0.824801402784291 | 1.91781737576699 | 0.999332001504091 |
| ebi-fl187-GCST90094716 | ebi-cfmet338-GCST90026299 | 1-carboxyethylvaline levels \|\| id:ebi-cfmet338-GCST90026299 | F-burn/spicy liking (derived food-liking factor) \|\| id:ebi-fl187-GCST90094716 | Weighted median | 12 | -0.0862285 | 0.119147554474104 | 0.469242642615276 | -0.319757707 | 0.147300706989921 | 0.917384585093768 | 0.726324999546555 | 1.15870234054049 | 0.999332001504091 |
| ebi-fl187-GCST90094716 | ebi-gm430-GCST90011306 | C_Erysipelotrichia abundance \|\| id:ebi-gm430-GCST90011306 | F-burn/spicy liking (derived food-liking factor) \|\| id:ebi-fl187-GCST90094716 | Weighted median | 12 | -0.093871158 | 0.106717178883971 | 0.379062190526216 | -0.303036828 | 0.115294512954768 | 0.910400052759735 | 0.738571895544128 | 1.12220389249215 | 0.999332001504091 |
| ebi-fl187-GCST90094716 | ebi-gm430-GCST90011312 | F_Erysipelotrichaceae abundance \|\| id:ebi-gm430-GCST90011312 | F-burn/spicy liking (derived food-liking factor) \|\| id:ebi-fl187-GCST90094716 | Weighted median | 12 | -0.093871158 | 0.108861232034919 | 0.388521860093048 | -0.307239172 | 0.119496857130627 | 0.910400052759735 | 0.735474674592646 | 1.12692970226879 | 0.999332001504091 |
| ebi-fl187-GCST90094716 | ebi-gm430-GCST90011352 | O_Erysipelotrichales abundance \|\| id:ebi-gm430-GCST90011352 | F-burn/spicy liking (derived food-liking factor) \|\| id:ebi-fl187-GCST90094716 | Weighted median | 12 | -0.093871158 | 0.104759932625106 | 0.370220859512972 | -0.299200626 | 0.111458310287392 | 0.910400052759735 | 0.741410648554435 | 1.11790713780674 | 0.999332001504091 |
| ebi-fl187-GCST90094716 | ebi-gm430-GCST90011362 | OTU97_11 (Parabacteroides) abundance \|\| id:ebi-gm430-GCST90011362 | F-burn/spicy liking (derived food-liking factor) \|\| id:ebi-fl187-GCST90094716 | Weighted median | 12 | -0.18087273 | 0.139612854000187 | 0.195137238247275 | -0.454513924 | 0.0927684636043886 | 0.834541563845191 | 0.634756432792853 | 1.09720766234828 | 0.999332001504091 |
| ebi-fl187-GCST90094716 | ebi-gm430-GCST90011384 | OTU97_235 (Phascolarctobacterium) abundance \|\| id:ebi-gm430-GCST90011384 | F-burn/spicy liking (derived food-liking factor) \|\| id:ebi-fl187-GCST90094716 | Weighted median | 12 | -0.242863348 | 0.425285948912443 | 0.5679596826314 | -1.076423808 | 0.590697111848164 | 0.784378693349579 | 0.34081215864484 | 1.80524643553561 | 0.999332001504091 |
| ebi-fl187-GCST90094716 | ebi-gm430-GCST90011390 | OTU97_3 (Bacteroides) abundance \|\| id:ebi-gm430-GCST90011390 | F-burn/spicy liking (derived food-liking factor) \|\| id:ebi-fl187-GCST90094716 | Weighted median | 12 | -0.141364825 | 0.110569384984656 | 0.201067238669931 | -0.358080819 | 0.0753511700366584 | 0.868172523279487 | 0.699016578830553 | 1.07826273797174 | 0.999332001504091 |
| ebi-fl187-GCST90094716 | ebi-gm430-GCST90011398 | OTU97_4 (Alistipes) abundance \|\| id:ebi-gm430-GCST90011398 | F-burn/spicy liking (derived food-liking factor) \|\| id:ebi-fl187-GCST90094716 | Weighted median | 12 | 0.00572019494114091 | 0.121137490566171 | 0.962337343917608 | -0.231709287 | 0.243149676450837 | 1.00573658649561 | 0.793176676906031 | 1.27525948614811 | 0.999332001504091 |
| ebi-fl187-GCST90094716 | ebi-gm430-GCST90011421 | OTU99_108 (Cytophagales) abundance \|\| id:ebi-gm430-GCST90011421 | F-burn/spicy liking (derived food-liking factor) \|\| id:ebi-fl187-GCST90094716 | Weighted median | 12 | 0.0826457006247687 | 0.381784507547676 | 0.828619757231554 | -0.665651934 | 0.830943335418213 | 1.08615691565199 | 0.513938364485924 | 2.29548312976926 | 0.999332001504091 |
| ebi-fl187-GCST90094716 | ebi-gm430-GCST90011428 | OTU99_123 (Phascolarctobacterium) abundance \|\| id:ebi-gm430-GCST90011428 | F-burn/spicy liking (derived food-liking factor) \|\| id:ebi-fl187-GCST90094716 | Weighted median | 13 | -0.071020021 | 0.246231284959932 | 0.773019062864654 | -0.553633339 | 0.411593297585402 | 0.931443243575284 | 0.574857359463552 | 1.5092205078694 | 0.999332001504091 |
| ebi-fl187-GCST90094716 | ebi-gm430-GCST90011450 | OTU99_298 (Phascolarctobacterium) abundance \|\| id:ebi-gm430-GCST90011450 | F-burn/spicy liking (derived food-liking factor) \|\| id:ebi-fl187-GCST90094716 | Weighted median | 12 | -0.241118296 | 0.427829548661058 | 0.573036629986596 | -1.079664211 | 0.597427619565826 | 0.785748670115018 | 0.33970957717144 | 1.81743764108228 | 0.999332001504091 |
| ebi-fl187-GCST90094716 | ebi-gm430-GCST90011451 | OTU99_3 (Bacteroides) abundance \|\| id:ebi-gm430-GCST90011451 | F-burn/spicy liking (derived food-liking factor) \|\| id:ebi-fl187-GCST90094716 | Weighted median | 12 | -0.150403914 | 0.107292302953079 | 0.160970626383162 | -0.360696827 | 0.0598890002356788 | 0.860360395009907 | 0.697190335477713 | 1.06171868948012 | 0.999332001504091 |
| ebi-fl187-GCST90094716 | ebi-gm430-GCST90011472 | OTU99_72 (Parabacteroides) abundance \|\| id:ebi-gm430-GCST90011472 | F-burn/spicy liking (derived food-liking factor) \|\| id:ebi-fl187-GCST90094716 | Weighted median | 13 | 0.0868190163124421 | 0.32861582538924 | 0.791628823483355 | -0.557268001 | 0.730906034075353 | 1.09069926307957 | 0.572771739825019 | 2.07696155338556 | 0.999332001504091 |
| ebi-fl187-GCST90094716 | ebi-gm430-GCST90011507 | TestASV_3 (Bacteroides) abundance \|\| id:ebi-gm430-GCST90011507 | F-burn/spicy liking (derived food-liking factor) \|\| id:ebi-fl187-GCST90094716 | Weighted median | 12 | 0.117028081911931 | 0.142645025378015 | 0.411979713404843 | -0.162556168 | 0.396612331652841 | 1.1241509975566 | 0.849968348007815 | 1.48677944098677 | 0.999332001504091 |
| ebi-fl187-GCST90094716 | ebi-gm430-GCST90011512 | TestASV_34 (Phascolarctobacterium) abundance \|\| id:ebi-gm430-GCST90011512 | F-burn/spicy liking (derived food-liking factor) \|\| id:ebi-fl187-GCST90094716 | Weighted median | 12 | 0.331684367147469 | 0.365836915797867 | 0.364594330450231 | -0.385355988 | 1.04872472211129 | 1.3933130037436 | 0.680208447179199 | 2.8540091415383 | 0.999332001504091 |
| ebi-fl187-GCST90094716 | ebi-gm430-GCST90011519 | TestASV_41 (Barnesiella) abundance \|\| id:ebi-gm430-GCST90011519 | F-burn/spicy liking (derived food-liking factor) \|\| id:ebi-fl187-GCST90094716 | Weighted median | 12 | 0.075456044750586 | 0.445495369341295 | 0.86550121587322 | -0.797714879 | 0.948626968659524 | 1.07837582639788 | 0.450356909139872 | 2.58216183511051 | 0.999332001504091 |
| ebi-fl187-GCST90094716 | ebi-gm430-GCST90011524 | TestASV_46 (Sutterella) abundance \|\| id:ebi-gm430-GCST90011524 | F-burn/spicy liking (derived food-liking factor) \|\| id:ebi-fl187-GCST90094716 | Weighted median | 12 | -0.359601705 | 0.414283101579008 | 0.385389082353108 | -1.171596585 | 0.452393173671043 | 0.697954262114174 | 0.309871809576926 | 1.57206992358693 | 0.999332001504091 |
| ebi-fl187-GCST90094716 | ebi-gm430-GCST90011581 | OTU97_19 (Bacteria) prevalence \|\| id:ebi-gm430-GCST90011581 | F-burn/spicy liking (derived food-liking factor) \|\| id:ebi-fl187-GCST90094716 | Weighted median | 12 | 0.00609124718410637 | 0.488619307203521 | 0.990053634531497 | -0.951602595 | 0.963785089303007 | 1.00610983655522 | 0.386121730619561 | 2.62160071019295 | 0.999332001504091 |
| ebi-fl187-GCST90094716 | ebi-gm430-GCST90011614 | OTU97_80 (Ruminococcus) prevalence \|\| id:ebi-gm430-GCST90011614 | F-burn/spicy liking (derived food-liking factor) \|\| id:ebi-fl187-GCST90094716 | Weighted median | 12 | 0.415558832417923 | 0.253088467287651 | 0.100600183481636 | -0.080494563 | 0.911612228301719 | 1.51521725666295 | 0.922659919642263 | 2.48833105894464 | 0.999332001504091 |
| ebi-fl187-GCST90094716 | ebi-gm430-GCST90011623 | OTU99_11 (Parabacteroides) prevalence \|\| id:ebi-gm430-GCST90011623 | F-burn/spicy liking (derived food-liking factor) \|\| id:ebi-fl187-GCST90094716 | Weighted median | 12 | 0.183845640102593 | 0.224393975600692 | 0.412616147296403 | -0.255966552 | 0.62365783227995 | 1.20183029437663 | 0.774167862667988 | 1.86574014000484 | 0.999332001504091 |
| ebi-fl187-GCST90094716 | ebi-gm430-GCST90011642 | OTU99_19 (Bacteria) prevalence \|\| id:ebi-gm430-GCST90011642 | F-burn/spicy liking (derived food-liking factor) \|\| id:ebi-fl187-GCST90094716 | Weighted median | 12 | 0.00609124718410637 | 0.487815766537214 | 0.990037251514283 | -0.950027655 | 0.962210149597045 | 1.00610983655522 | 0.38673032819089 | 2.61747509679024 | 0.999332001504091 |
| ebi-fl187-GCST90094716 | ebi-gm430-GCST90011653 | OTU99_30 (Parasutterella) prevalence \|\| id:ebi-gm430-GCST90011653 | F-burn/spicy liking (derived food-liking factor) \|\| id:ebi-fl187-GCST90094716 | Weighted median | 12 | -0.036636019 | 0.250074956559973 | 0.883526570974756 | -0.526782934 | 0.453510896039652 | 0.964026959170725 | 0.590501600061876 | 1.57382804366757 | 0.999332001504091 |
| ebi-fl187-GCST90094716 | ebi-gm430-GCST90011675 | OTU99_8 (Ruminococcaceae) prevalence \|\| id:ebi-gm430-GCST90011675 | F-burn/spicy liking (derived food-liking factor) \|\| id:ebi-fl187-GCST90094716 | Weighted median | 12 | -0.480508074 | 0.274494963188793 | 0.0800291591062982 | -1.018518202 | 0.0575020534952934 | 0.618469083686333 | 0.361129665039617 | 1.05918744568893 | 0.999332001504091 |
| ebi-fl187-GCST90094716 | ebi-gm430-GCST90011679 | OTU99_92 (Ruminococcus) prevalence \|\| id:ebi-gm430-GCST90011679 | F-burn/spicy liking (derived food-liking factor) \|\| id:ebi-fl187-GCST90094716 | Weighted median | 12 | 0.475552576218397 | 0.26212471240357 | 0.0696434627219786 | -0.03821186 | 0.989317012529393 | 1.60890299343129 | 0.96250900204241 | 2.68939702047391 | 0.999332001504091 |
| ebi-fl187-GCST90094716 | ebi-gm430-GCST90011684 | TestASV_11 (Lachnospiraceae) prevalence \|\| id:ebi-gm430-GCST90011684 | F-burn/spicy liking (derived food-liking factor) \|\| id:ebi-fl187-GCST90094716 | Weighted median | 12 | 0.102818365680446 | 0.281974060231275 | 0.71538188792468 | -0.449850792 | 0.655487523733745 | 1.10829008727722 | 0.637723297703363 | 1.92608129886496 | 0.999332001504091 |
| ebi-fl187-GCST90094716 | ebi-gm430-GCST90011688 | TestASV_15 (Bacteroides) prevalence \|\| id:ebi-gm430-GCST90011688 | F-burn/spicy liking (derived food-liking factor) \|\| id:ebi-fl187-GCST90094716 | Weighted median | 12 | -0.244801033 | 0.243142241613374 | 0.314020172689831 | -0.721359826 | 0.231757760828132 | 0.782860286317734 | 0.486090807272121 | 1.26081427322774 | 0.999332001504091 |
| ebi-fl187-GCST90094716 | ebi-gm430-GCST90011692 | TestASV_19 (Prevotella) prevalence \|\| id:ebi-gm430-GCST90011692 | F-burn/spicy liking (derived food-liking factor) \|\| id:ebi-fl187-GCST90094716 | Weighted median | 12 | 0.0697827292171046 | 0.466486209171968 | 0.881086310140241 | -0.844530241 | 0.984095699194162 | 1.07227518187491 | 0.429759194152326 | 2.67539143154977 | 0.999332001504091 |
| ebi-fl187-GCST90094716 | ebi-gm430-GCST90011693 | TestASV_2 (Bacteroides) prevalence \|\| id:ebi-gm430-GCST90011693 | F-burn/spicy liking (derived food-liking factor) \|\| id:ebi-fl187-GCST90094716 | Weighted median | 12 | -0.443056893 | 0.246669821647566 | 0.072470088731693 | -0.926529743 | 0.0404159574260763 | 0.64207067671961 | 0.395925291468993 | 1.04124379721638 | 0.999332001504091 |
| ebi-fl187-GCST90094716 | ebi-gm430-GCST90011716 | TestASV_40 (Barnesiella) prevalence \|\| id:ebi-gm430-GCST90011716 | F-burn/spicy liking (derived food-liking factor) \|\| id:ebi-fl187-GCST90094716 | Weighted median | 12 | 0.29008568602051 | 0.488080790618188 | 0.552285074091514 | -0.666552664 | 1.24672403563216 | 1.33654200608485 | 0.513475653499682 | 3.47892742694649 | 0.999332001504091 |
| ebi-fl187-GCST90094716 | ebi-gm430-GCST90011718 | TestASV_42 (Alphaproteobacteria) prevalence \|\| id:ebi-gm430-GCST90011718 | F-burn/spicy liking (derived food-liking factor) \|\| id:ebi-fl187-GCST90094716 | Weighted median | 12 | -0.035670373 | 0.391965387938414 | 0.927489504465126 | -0.803922533 | 0.732581787459034 | 0.964958317477142 | 0.447569908543431 | 2.08044494657523 | 0.999332001504091 |
| ebi-fl187-GCST90094716 | ebi-hbsm44-GCST90104929 | Sulcal depth of the right inferior frontal sulcus \|\| id:ebi-hbsm44-GCST90104929 | F-burn/spicy liking (derived food-liking factor) \|\| id:ebi-fl187-GCST90094716 | Weighted median | 11 | 0.0325023449871614 | 0.206679847861409 | 0.875040430967711 | -0.372590157 | 0.437594846795523 | 1.03303631559803 | 0.688947535438058 | 1.54897720719156 | 0.999332001504091 |
| ebi-fl187-GCST90094716 | ebi-hbsm44-GCST90104935 | Sulcal opening distance of the left calloso-marginal posterior fissure \|\| id:ebi-hbsm44-GCST90104935 | F-burn/spicy liking (derived food-liking factor) \|\| id:ebi-fl187-GCST90094716 | Weighted median | 11 | 0.00304723845069049 | 0.0448460663097057 | 0.945826355136146 | -0.084851052 | 0.0909455284177137 | 1.00305188600131 | 0.918649105602851 | 1.09520934584761 | 0.999332001504091 |
| ebi-fl187-GCST90094716 | ebi-hbsm44-GCST90104936 | Sulcal opening distance of the left anterior inferior temporal sulcus \|\| id:ebi-hbsm44-GCST90104936 | F-burn/spicy liking (derived food-liking factor) \|\| id:ebi-fl187-GCST90094716 | Weighted median | 12 | -0.015969516 | 0.0263802696367873 | 0.544940920218368 | -0.067674845 | 0.0357358120442042 | 0.98415732021291 | 0.934564302498107 | 1.03638201067563 | 0.999332001504091 |
| ebi-fl187-GCST90094716 | ebi-hbsm44-GCST90104941 | Sulcal opening distance of the right posterior inferior temporal sulcus \|\| id:ebi-hbsm44-GCST90104941 | F-burn/spicy liking (derived food-liking factor) \|\| id:ebi-fl187-GCST90094716 | Weighted median | 12 | 0.0199011318339119 | 0.0203417885475152 | 0.327907494803486 | -0.019968774 | 0.0597710373870417 | 1.02010047957725 | 0.980229281743642 | 1.0615934535058 | 0.999332001504091 |
| ebi-fl187-GCST90094716 | ebi-IPs91-GCST90274819 | Leukemia inhibitory factor levels \|\| id:ebi-IPs91-GCST90274819 | F-burn/spicy liking (derived food-liking factor) \|\| id:ebi-fl187-GCST90094716 | Weighted median | 15 | -0.067219439 | 0.0749397537456717 | 0.369729740094328 | -0.214101356 | 0.0796624782097073 | 0.934990005428553 | 0.80726655918449 | 1.08292149638209 | 0.999332001504091 |
| ebi-fl187-GCST90094716 | ebi-IPs91-GCST90274837 | STAM binding protein levels \|\| id:ebi-IPs91-GCST90274837 | F-burn/spicy liking (derived food-liking factor) \|\| id:ebi-fl187-GCST90094716 | Weighted median | 15 | -0.022889839 | 0.0680244722997592 | 0.736498241864905 | -0.156217805 | 0.110438126879756 | 0.977370146084058 | 0.855372865991022 | 1.11676724904014 | 0.999332001504091 |
| ebi-fl187-GCST90094716 | ebi-IPs91-GCST90274840 | TNF-beta levels \|\| id:ebi-IPs91-GCST90274840 | F-burn/spicy liking (derived food-liking factor) \|\| id:ebi-fl187-GCST90094716 | Weighted median | 15 | -0.109149903 | 0.0843596465722793 | 0.195712428655499 | -0.27449481 | 0.0561950041965863 | 0.896596004916848 | 0.759955948131347 | 1.05780393983299 | 0.999332001504091 |
| ebi-fl187-GCST90094716 | ebi-IPs91-GCST90274842 | Tumor necrosis factor ligand superfamily member 14 levels \|\| id:ebi-IPs91-GCST90274842 | F-burn/spicy liking (derived food-liking factor) \|\| id:ebi-fl187-GCST90094716 | Weighted median | 15 | -0.061294367 | 0.078380068384099 | 0.434205928073827 | -0.214919301 | 0.0923305675021092 | 0.940546333660837 | 0.806606530243346 | 1.09672730457062 | 0.999332001504091 |
| ebi-fl187-GCST90094716 | ebi-met1400-GCST90199628 | 3-methylhistidine levels \|\| id:ebi-met1400-GCST90199628 | F-burn/spicy liking (derived food-liking factor) \|\| id:ebi-fl187-GCST90094716 | Weighted median | 14 | 0.115833921110509 | 0.102261611636493 | 0.257331246543026 | -0.084598838 | 0.316266679918036 | 1.1228093817124 | 0.918880830823308 | 1.37199609064845 | 0.999332001504091 |
| ebi-fl187-GCST90094716 | ebi-met1400-GCST90199651 | Docosahexaenoate DHA; 22:6n3 levels \|\| id:ebi-met1400-GCST90199651 | F-burn/spicy liking (derived food-liking factor) \|\| id:ebi-fl187-GCST90094716 | Weighted median | 14 | 0.0516008710497409 | 0.0882591302118917 | 0.558781780632456 | -0.121387024 | 0.224588766265049 | 1.05295539365131 | 0.885691109388937 | 1.25180782472155 | 0.999332001504091 |
| ebi-fl187-GCST90094716 | ebi-met1400-GCST90199652 | 1-stearoyl-2-oleoyl-GPS (18:0/18:1) levels \|\| id:ebi-met1400-GCST90199652 | F-burn/spicy liking (derived food-liking factor) \|\| id:ebi-fl187-GCST90094716 | Weighted median | 14 | -0.038012332 | 0.0974738404179177 | 0.696555252820761 | -0.229061059 | 0.153036395225245 | 0.962701068794314 | 0.79527997285332 | 1.16536739197964 | 0.999332001504091 |
| ebi-fl187-GCST90094716 | ebi-met1400-GCST90199653 | 1-myristoyl-2-palmitoyl-gpc (14:0/16:0) levels \|\| id:ebi-met1400-GCST90199653 | F-burn/spicy liking (derived food-liking factor) \|\| id:ebi-fl187-GCST90094716 | Weighted median | 14 | -0.106149545 | 0.0939213750197312 | 0.258393722741432 | -0.29023544 | 0.0779363500334997 | 0.899290153669008 | 0.748087417108657 | 1.08105384743902 | 0.999332001504091 |
| ebi-fl187-GCST90094716 | ebi-met1400-GCST90199667 | Erythritol levels in elite athletes \|\| id:ebi-met1400-GCST90199667 | F-burn/spicy liking (derived food-liking factor) \|\| id:ebi-fl187-GCST90094716 | Weighted median | 14 | 0.0986524163440844 | 0.0964837226635209 | 0.306555050618132 | -0.09045568 | 0.287760512764585 | 1.10368261084393 | 0.913514819910788 | 1.3334379245191 | 0.999332001504091 |
| ebi-fl187-GCST90094716 | ebi-met1400-GCST90199674 | 3-hydroxymyristate levels \|\| id:ebi-met1400-GCST90199674 | F-burn/spicy liking (derived food-liking factor) \|\| id:ebi-fl187-GCST90094716 | Weighted median | 14 | 0.0113791045012324 | 0.0875078731233907 | 0.896538571048946 | -0.160136327 | 0.182894535823078 | 1.01144409277974 | 0.852027626830943 | 1.20068777185556 | 0.999332001504091 |
| ebi-fl187-GCST90094716 | ebi-met1400-GCST90199685 | 2-linoleoylglycerol (18:2) levels \|\| id:ebi-met1400-GCST90199685 | F-burn/spicy liking (derived food-liking factor) \|\| id:ebi-fl187-GCST90094716 | Weighted median | 14 | -0.074494935 | 0.102948032933609 | 0.469300972681561 | -0.276273079 | 0.127283209704165 | 0.92821217558496 | 0.758605742875407 | 1.13573862443811 | 0.999332001504091 |
| ebi-fl187-GCST90094716 | ebi-met1400-GCST90199703 | Gamma-glutamylvaline levels \|\| id:ebi-met1400-GCST90199703 | F-burn/spicy liking (derived food-liking factor) \|\| id:ebi-fl187-GCST90094716 | Weighted median | 14 | 0.00787012484961956 | 0.0887847056864296 | 0.929365792998613 | -0.166147898 | 0.181888147995021 | 1.00790117568673 | 0.846920966758562 | 1.19948002213091 | 0.999332001504091 |
| ebi-fl187-GCST90094716 | ebi-met1400-GCST90199779 | Gamma-glutamyl-2-aminobutyrate levels \|\| id:ebi-met1400-GCST90199779 | F-burn/spicy liking (derived food-liking factor) \|\| id:ebi-fl187-GCST90094716 | Weighted median | 14 | -0.008210244 | 0.0996214627843223 | 0.934317178096805 | -0.203468311 | 0.187047823052319 | 0.991823367997874 | 0.81589605879246 | 1.20568494320534 | 0.999332001504091 |
| ebi-fl187-GCST90094716 | ebi-met1400-GCST90199824 | 4-vinylphenol sulfate levels \|\| id:ebi-met1400-GCST90199824 | F-burn/spicy liking (derived food-liking factor) \|\| id:ebi-fl187-GCST90094716 | Weighted median | 14 | -0.034335956 | 0.0908800385277054 | 0.705567099411979 | -0.212460831 | 0.143788919939687 | 0.966246834092753 | 0.808591987368844 | 1.15464036124363 | 0.999332001504091 |
| ebi-fl187-GCST90094716 | ebi-met1400-GCST90199830 | 4-ethylphenylsulfate levels \|\| id:ebi-met1400-GCST90199830 | F-burn/spicy liking (derived food-liking factor) \|\| id:ebi-fl187-GCST90094716 | Weighted median | 14 | -0.009279276 | 0.0971850524501098 | 0.92393318601359 | -0.199761979 | 0.18120342635338 | 0.990763643179913 | 0.818925651178994 | 1.19865899332122 | 0.999332001504091 |
| ebi-fl187-GCST90094716 | ebi-met1400-GCST90199833 | Hexanoylglutamine levels \|\| id:ebi-met1400-GCST90199833 | F-burn/spicy liking (derived food-liking factor) \|\| id:ebi-fl187-GCST90094716 | Weighted median | 14 | -0.132148324 | 0.104422086042802 | 0.205684690618444 | -0.336815612 | 0.0725189649226516 | 0.87621102256115 | 0.71404048800199 | 1.07521319723192 | 0.999332001504091 |
| ebi-fl187-GCST90094716 | ebi-met1400-GCST90199837 | Carotenoid levels (cryptoxanthin) \|\| id:ebi-met1400-GCST90199837 | F-burn/spicy liking (derived food-liking factor) \|\| id:ebi-fl187-GCST90094716 | Weighted median | 14 | 0.14640107625402 | 0.0921040418659784 | 0.111943386171543 | -0.034122846 | 0.326924998311337 | 1.15766040505005 | 0.966452772677585 | 1.38669746862813 | 0.999332001504091 |
| ebi-fl187-GCST90094716 | ebi-met1400-GCST90199869 | 2-hydroxyhippurate levels \|\| id:ebi-met1400-GCST90199869 | F-burn/spicy liking (derived food-liking factor) \|\| id:ebi-fl187-GCST90094716 | Weighted median | 14 | 0.2199488950021 | 0.0987378846340297 | 0.025906941648651 | 0.0264226411194021 | 0.413475148884799 | 1.24601305146586 | 1.02677481403905 | 1.51206331047015 | 0.999332001504091 |
| ebi-fl187-GCST90094716 | ebi-met1400-GCST90199902 | Hydantoin-5-propionate levels \|\| id:ebi-met1400-GCST90199902 | F-burn/spicy liking (derived food-liking factor) \|\| id:ebi-fl187-GCST90094716 | Weighted median | 14 | -0.074488004 | 0.0877934048108481 | 0.396189374697874 | -0.246563077 | 0.097587069431988 | 0.928218608905159 | 0.781482066104692 | 1.10250743208022 | 0.999332001504091 |
| ebi-fl187-GCST90094716 | ebi-met1400-GCST90199904 | Pantoate levels \|\| id:ebi-met1400-GCST90199904 | F-burn/spicy liking (derived food-liking factor) \|\| id:ebi-fl187-GCST90094716 | Weighted median | 14 | -0.027600312 | 0.0958879424114661 | 0.773469801635715 | -0.215540679 | 0.160340055024497 | 0.972777096343708 | 0.806105477842249 | 1.17390999711837 | 0.999332001504091 |
| ebi-fl187-GCST90094716 | ebi-met1400-GCST90199936 | 3-methyl catechol sulfate (2) levels \|\| id:ebi-met1400-GCST90199936 | F-burn/spicy liking (derived food-liking factor) \|\| id:ebi-fl187-GCST90094716 | Weighted median | 14 | 0.0819345776494976 | 0.106044584698687 | 0.439733847446191 | -0.125912808 | 0.289781963658924 | 1.08538479908197 | 0.881691719579102 | 1.33613613003034 | 0.999332001504091 |
| ebi-fl187-GCST90094716 | ebi-met1400-GCST90199977 | 4-hydroxychlorothalonil levels \|\| id:ebi-met1400-GCST90199977 | F-burn/spicy liking (derived food-liking factor) \|\| id:ebi-fl187-GCST90094716 | Weighted median | 14 | -0.022639615 | 0.0930018996312921 | 0.807670954222306 | -0.204923338 | 0.159644108547707 | 0.977614738247568 | 0.814709771292245 | 1.17309330281253 | 0.999332001504091 |
| ebi-fl187-GCST90094716 | ebi-met1400-GCST90200040 | Sphingomyelin (d18:2/24:1, d18:1/24:2) levels \|\| id:ebi-met1400-GCST90200040 | F-burn/spicy liking (derived food-liking factor) \|\| id:ebi-fl187-GCST90094716 | Weighted median | 14 | 0.131443127107817 | 0.0868169053144006 | 0.130019025563934 | -0.038718007 | 0.301604261524042 | 1.14047304387743 | 0.962021954060224 | 1.35202607208861 | 0.999332001504091 |
| ebi-fl187-GCST90094716 | ebi-met1400-GCST90200077 | 1-stearoyl-2-oleoyl-GPI (18:0/18:1) levels \|\| id:ebi-met1400-GCST90200077 | F-burn/spicy liking (derived food-liking factor) \|\| id:ebi-fl187-GCST90094716 | Weighted median | 14 | -0.04154638 | 0.0899414632202285 | 0.644133749038921 | -0.217831648 | 0.134738887414914 | 0.959304841274595 | 0.80426082886053 | 1.14423797040657 | 0.999332001504091 |
| ebi-fl187-GCST90094716 | ebi-met1400-GCST90200080 | 1-palmitoyl-2-oleoyl-GPI (16:0/18:1) levels \|\| id:ebi-met1400-GCST90200080 | F-burn/spicy liking (derived food-liking factor) \|\| id:ebi-fl187-GCST90094716 | Weighted median | 14 | -0.024079676 | 0.0957409468450803 | 0.80142087706666 | -0.211731932 | 0.163572579737411 | 0.976207926239408 | 0.809181584268647 | 1.1777108300283 | 0.999332001504091 |
| ebi-fl187-GCST90094716 | ebi-met1400-GCST90200082 | 1-oleoyl-2-linoleoyl-GPE (18:1/18:2) levels \|\| id:ebi-met1400-GCST90200082 | F-burn/spicy liking (derived food-liking factor) \|\| id:ebi-fl187-GCST90094716 | Weighted median | 14 | -0.176887992 | 0.104567171717413 | 0.0907183170214003 | -0.381839649 | 0.0280636643167943 | 0.837873627607689 | 0.682604500870407 | 1.02846115861423 | 0.999332001504091 |
| ebi-fl187-GCST90094716 | ebi-met1400-GCST90200100 | Perfluorooctanesulfonate (PFOS) levels \|\| id:ebi-met1400-GCST90200100 | F-burn/spicy liking (derived food-liking factor) \|\| id:ebi-fl187-GCST90094716 | Weighted median | 14 | -0.008068186 | 0.096101707016371 | 0.933092480997288 | -0.196427532 | 0.180291159671788 | 0.991964274375351 | 0.821660873395669 | 1.19756599528767 | 0.999332001504091 |
| ebi-fl187-GCST90094716 | ebi-met1400-GCST90200102 | 1-palmitoleoyl-2-linolenoyl-GPC (16:1/18:3) levels \|\| id:ebi-met1400-GCST90200102 | F-burn/spicy liking (derived food-liking factor) \|\| id:ebi-fl187-GCST90094716 | Weighted median | 14 | -0.122114375 | 0.0920508709398526 | 0.184642152359632 | -0.302534082 | 0.0583053321772813 | 0.885047135566987 | 0.738943303232874 | 1.06003861019967 | 0.999332001504091 |
| ebi-fl187-GCST90094716 | ebi-met1400-GCST90200114 | Glycosyl-N-tricosanoyl-sphingadienine (d18:2/23:0) levels \|\| id:ebi-met1400-GCST90200114 | F-burn/spicy liking (derived food-liking factor) \|\| id:ebi-fl187-GCST90094716 | Weighted median | 14 | 0.0569661366090194 | 0.0986976923420232 | 0.563819217555299 | -0.13648134 | 0.250413613599385 | 1.05861996132687 | 0.872422599180748 | 1.28455661691028 | 0.999332001504091 |
| ebi-fl187-GCST90094716 | ebi-met1400-GCST90200135 | Arachidoylcarnitine (C20) levels \|\| id:ebi-met1400-GCST90200135 | F-burn/spicy liking (derived food-liking factor) \|\| id:ebi-fl187-GCST90094716 | Weighted median | 14 | -0.116668906 | 0.101181781493936 | 0.248884779525078 | -0.314985198 | 0.0816473854799494 | 0.889879778027505 | 0.729799676701233 | 1.08507312982336 | 0.999332001504091 |
| ebi-fl187-GCST90094716 | ebi-met1400-GCST90200146 | Docosahexaenoylcarnitine (C22:6) levels \|\| id:ebi-met1400-GCST90200146 | F-burn/spicy liking (derived food-liking factor) \|\| id:ebi-fl187-GCST90094716 | Weighted median | 14 | 0.111684122321102 | 0.112808450256857 | 0.322157842695793 | -0.10942044 | 0.332788684824542 | 1.11815960319482 | 0.896353475244382 | 1.39485251382097 | 0.999332001504091 |
| ebi-fl187-GCST90094716 | ebi-met1400-GCST90200162 | 3-hydroxyphenylacetoylglutamine levels \|\| id:ebi-met1400-GCST90200162 | F-burn/spicy liking (derived food-liking factor) \|\| id:ebi-fl187-GCST90094716 | Weighted median | 14 | 0.163467255322456 | 0.113923083230255 | 0.151318039406098 | -0.059821988 | 0.386756498453756 | 1.17758679485623 | 0.941932194074789 | 1.47219796514318 | 0.999332001504091 |
| ebi-fl187-GCST90094716 | ebi-met1400-GCST90200172 | 3-carboxy-4-methyl-5-pentyl-2-furanpropionate (3-CMPFP) levels \|\| id:ebi-met1400-GCST90200172 | F-burn/spicy liking (derived food-liking factor) \|\| id:ebi-fl187-GCST90094716 | Weighted median | 14 | 0.011261336703104 | 0.0975232034475541 | 0.908069896463745 | -0.179884142 | 0.20240681546031 | 1.01132498424971 | 0.835366989708395 | 1.22434598968856 | 0.999332001504091 |
| ebi-fl187-GCST90094716 | ebi-met1400-GCST90200222 | Dihydrocaffeate sulfate (2) levels \|\| id:ebi-met1400-GCST90200222 | F-burn/spicy liking (derived food-liking factor) \|\| id:ebi-fl187-GCST90094716 | Weighted median | 14 | 0.165397319830377 | 0.103191452173538 | 0.108974407517764 | -0.036857926 | 0.367652566090512 | 1.17986180808889 | 0.963813057984474 | 1.44434013904926 | 0.999332001504091 |
| ebi-fl187-GCST90094716 | ebi-met1400-GCST90200223 | 4-methylhexanoylglutamine levels \|\| id:ebi-met1400-GCST90200223 | F-burn/spicy liking (derived food-liking factor) \|\| id:ebi-fl187-GCST90094716 | Weighted median | 14 | -0.042163318 | 0.121351655209542 | 0.728255221667352 | -0.280012562 | 0.195685926005114 | 0.958713192467721 | 0.755774247045376 | 1.2161448858636 | 0.999332001504091 |
| ebi-fl187-GCST90094716 | ebi-met1400-GCST90200224 | 2-ketocaprylate levels \|\| id:ebi-met1400-GCST90200224 | F-burn/spicy liking (derived food-liking factor) \|\| id:ebi-fl187-GCST90094716 | Weighted median | 14 | 0.00839676521570099 | 0.0990541541561225 | 0.932444689105312 | -0.185749377 | 0.202542907361701 | 1.00843211692614 | 0.830481706776718 | 1.22451262460083 | 0.999332001504091 |
| ebi-fl187-GCST90094716 | ebi-met1400-GCST90200243 | Hydroxypalmitoyl sphingomyelin (d18:1/16:0(OH)) levels \|\| id:ebi-met1400-GCST90200243 | F-burn/spicy liking (derived food-liking factor) \|\| id:ebi-fl187-GCST90094716 | Weighted median | 14 | 0.0597211022641896 | 0.0948330832754056 | 0.528858383913324 | -0.126151741 | 0.245593945483985 | 1.06154044402981 | 0.881481079853307 | 1.27838037601276 | 0.999332001504091 |
| ebi-fl187-GCST90094716 | ebi-met1400-GCST90200273 | 2-methoxyhydroquinone sulfate (2) levels \|\| id:ebi-met1400-GCST90200273 | F-burn/spicy liking (derived food-liking factor) \|\| id:ebi-fl187-GCST90094716 | Weighted median | 14 | 0.171630997157181 | 0.130186681910093 | 0.187388090832961 | -0.083534899 | 0.426796893700963 | 1.1872396575661 | 0.919858983596765 | 1.53234140192467 | 0.999332001504091 |
| ebi-fl187-GCST90094716 | ebi-met1400-GCST90200276 | 2,4-di-tert-butylphenol levels \|\| id:ebi-met1400-GCST90200276 | F-burn/spicy liking (derived food-liking factor) \|\| id:ebi-fl187-GCST90094716 | Weighted median | 14 | 0.0402334624737288 | 0.0996006238623728 | 0.686251034435243 | -0.15498376 | 0.23545068524398 | 1.04105379281718 | 0.856429085525081 | 1.2654789729316 | 0.999332001504091 |
| ebi-fl187-GCST90094716 | ebi-met1400-GCST90200289 | N-acetylmethionine levels \|\| id:ebi-met1400-GCST90200289 | F-burn/spicy liking (derived food-liking factor) \|\| id:ebi-fl187-GCST90094716 | Weighted median | 14 | 0.0471852324905685 | 0.101565440531862 | 0.642232538647614 | -0.151883031 | 0.246253495933018 | 1.04831617331202 | 0.859088761659131 | 1.27922381047701 | 0.999332001504091 |
| ebi-fl187-GCST90094716 | ebi-met1400-GCST90200308 | 3-hydroxyisobutyrate levels \|\| id:ebi-met1400-GCST90200308 | F-burn/spicy liking (derived food-liking factor) \|\| id:ebi-fl187-GCST90094716 | Weighted median | 14 | -0.125779995 | 0.0896363205629841 | 0.160549523995976 | -0.301467184 | 0.0499071929610269 | 0.881808827493446 | 0.739732101266926 | 1.05117353554565 | 0.999332001504091 |
| ebi-fl187-GCST90094716 | ebi-met1400-GCST90200383 | Cystathionine levels \|\| id:ebi-met1400-GCST90200383 | F-burn/spicy liking (derived food-liking factor) \|\| id:ebi-fl187-GCST90094716 | Weighted median | 14 | -0.042469298 | 0.0967751472991822 | 0.660773789797908 | -0.232148586 | 0.147209990974671 | 0.958419890733839 | 0.792828311019469 | 1.15859723244886 | 0.999332001504091 |
| ebi-fl187-GCST90094716 | ebi-met1400-GCST90200384 | Guanidinoacetate levels \|\| id:ebi-met1400-GCST90200384 | F-burn/spicy liking (derived food-liking factor) \|\| id:ebi-fl187-GCST90094716 | Weighted median | 14 | 0.218872636079516 | 0.0921675290099354 | 0.017561977001755 | 0.0382242792200421 | 0.399520992938989 | 1.24467274019192 | 1.03896422483589 | 1.49111027419791 | 0.999332001504091 |
| ebi-fl187-GCST90094716 | ebi-met1400-GCST90200403 | Citrulline levels \|\| id:ebi-met1400-GCST90200403 | F-burn/spicy liking (derived food-liking factor) \|\| id:ebi-fl187-GCST90094716 | Weighted median | 14 | -0.045944959 | 0.0929506294083205 | 0.621098403319543 | -0.228128193 | 0.136238274351047 | 0.955094529825766 | 0.79602220887553 | 1.145954913 | 0.999332001504091 |
| ebi-fl187-GCST90094716 | ebi-met1400-GCST90200434 | 12,13-DiHOME levels \|\| id:ebi-met1400-GCST90200434 | F-burn/spicy liking (derived food-liking factor) \|\| id:ebi-fl187-GCST90094716 | Weighted median | 14 | 0.0750014309951102 | 0.0953848890290725 | 0.43168995483856 | -0.111952952 | 0.261955813492092 | 1.07788569333268 | 0.894086321929459 | 1.29946912215815 | 0.999332001504091 |
| ebi-fl187-GCST90094716 | ebi-met1400-GCST90200437 | Fructose levels \|\| id:ebi-met1400-GCST90200437 | F-burn/spicy liking (derived food-liking factor) \|\| id:ebi-fl187-GCST90094716 | Weighted median | 14 | 0.0758515709329845 | 0.0931329283949091 | 0.415390541234637 | -0.106688969 | 0.258392110587006 | 1.07880243663388 | 0.898805186045955 | 1.29484644209396 | 0.999332001504091 |
| ebi-fl187-GCST90094716 | ebi-met1400-GCST90200448 | N-stearoyl-sphinganine (d18:0/18:0) levels \|\| id:ebi-met1400-GCST90200448 | F-burn/spicy liking (derived food-liking factor) \|\| id:ebi-fl187-GCST90094716 | Weighted median | 14 | -0.044371291 | 0.104441029525632 | 0.670949379220609 | -0.249075709 | 0.160333126889542 | 0.956598715052929 | 0.779520954514857 | 1.17390186413966 | 0.999332001504091 |
| ebi-fl187-GCST90094716 | ebi-met1400-GCST90200468 | X-11847 levels \|\| id:ebi-met1400-GCST90200468 | F-burn/spicy liking (derived food-liking factor) \|\| id:ebi-fl187-GCST90094716 | Weighted median | 14 | 0.0921821648959227 | 0.0982137786567983 | 0.347942887246849 | -0.100316841 | 0.284681171063247 | 1.09656455945654 | 0.904550773610807 | 1.32933812908714 | 0.999332001504091 |
| ebi-fl187-GCST90094716 | ebi-met1400-GCST90200476 | X-11843 levels \|\| id:ebi-met1400-GCST90200476 | F-burn/spicy liking (derived food-liking factor) \|\| id:ebi-fl187-GCST90094716 | Weighted median | 14 | -0.017082112 | 0.0963870307369654 | 0.859332304827737 | -0.206000693 | 0.171836467936299 | 0.983062959752017 | 0.813832512660899 | 1.18748362568684 | 0.999332001504091 |
| ebi-fl187-GCST90094716 | ebi-met1400-GCST90200511 | X-12906 levels \|\| id:ebi-met1400-GCST90200511 | F-burn/spicy liking (derived food-liking factor) \|\| id:ebi-fl187-GCST90094716 | Weighted median | 14 | -0.038073346 | 0.0983759816369338 | 0.698742351948561 | -0.23089027 | 0.154743577874397 | 0.962642332208308 | 0.793826567732576 | 1.16735858615358 | 0.999332001504091 |
| ebi-fl187-GCST90094716 | ebi-met1400-GCST90200515 | X-12849 levels \|\| id:ebi-met1400-GCST90200515 | F-burn/spicy liking (derived food-liking factor) \|\| id:ebi-fl187-GCST90094716 | Weighted median | 14 | -0.049136678 | 0.101585861833337 | 0.628601610306972 | -0.248244967 | 0.14997161100522 | 0.952050996200344 | 0.780168803958538 | 1.16180125989021 | 0.999332001504091 |
| ebi-fl187-GCST90094716 | ebi-met1400-GCST90200551 | X-18886 levels \|\| id:ebi-met1400-GCST90200551 | F-burn/spicy liking (derived food-liking factor) \|\| id:ebi-fl187-GCST90094716 | Weighted median | 14 | -0.148571103 | 0.0950824771300362 | 0.118158530209416 | -0.334932758 | 0.0377905521817412 | 0.861938718568838 | 0.715386188613209 | 1.03851369567016 | 0.999332001504091 |
| ebi-fl187-GCST90094716 | ebi-met1400-GCST90200564 | X-21319 levels \|\| id:ebi-met1400-GCST90200564 | F-burn/spicy liking (derived food-liking factor) \|\| id:ebi-fl187-GCST90094716 | Weighted median | 14 | -0.070940317 | 0.0935903848785306 | 0.448458626318718 | -0.254377471 | 0.112496837578018 | 0.931517486427994 | 0.775399056028216 | 1.11906871793967 | 0.999332001504091 |
| ebi-fl187-GCST90094716 | ebi-met1400-GCST90200579 | X-21442 levels \|\| id:ebi-met1400-GCST90200579 | F-burn/spicy liking (derived food-liking factor) \|\| id:ebi-fl187-GCST90094716 | Weighted median | 14 | 0.0831692480769004 | 0.109000550101108 | 0.445453392371751 | -0.13047183 | 0.296810326275072 | 1.08672571922263 | 0.877681216773942 | 1.34556005785426 | 0.999332001504091 |
| ebi-fl187-GCST90094716 | ebi-met1400-GCST90200581 | X-21441 levels \|\| id:ebi-met1400-GCST90200581 | F-burn/spicy liking (derived food-liking factor) \|\| id:ebi-fl187-GCST90094716 | Weighted median | 14 | 0.0469579193709532 | 0.0961714268835077 | 0.625356422604714 | -0.141538077 | 0.235453916062628 | 1.04807790437418 | 0.868022122998848 | 1.26548306147127 | 0.999332001504091 |
| ebi-fl187-GCST90094716 | ebi-met1400-GCST90200648 | X-24585 levels \|\| id:ebi-met1400-GCST90200648 | F-burn/spicy liking (derived food-liking factor) \|\| id:ebi-fl187-GCST90094716 | Weighted median | 14 | 0.0215181015346819 | 0.0934846172809535 | 0.817953462204972 | -0.161711748 | 0.204747951405351 | 1.02175128543632 | 0.850686380967146 | 1.22721570798379 | 0.999332001504091 |
| ebi-fl187-GCST90094716 | ebi-met1400-GCST90200720 | Adenosine 5'-diphosphate (ADP) to pantothenate ratio \|\| id:ebi-met1400-GCST90200720 | F-burn/spicy liking (derived food-liking factor) \|\| id:ebi-fl187-GCST90094716 | Weighted median | 14 | -0.035233041 | 0.127693553994814 | 0.78261049865033 | -0.285512407 | 0.215046324407351 | 0.965380416415964 | 0.751629015469642 | 1.239919334 | 0.999332001504091 |
| ebi-fl187-GCST90094716 | ebi-met1400-GCST90200724 | Cholate to taurocholate ratio \|\| id:ebi-met1400-GCST90200724 | F-burn/spicy liking (derived food-liking factor) \|\| id:ebi-fl187-GCST90094716 | Weighted median | 14 | -0.02152078 | 0.0959481195152326 | 0.822526986125297 | -0.209579094 | 0.166537534546584 | 0.978709139972326 | 0.810925497593213 | 1.18120787114018 | 0.999332001504091 |
| ebi-fl187-GCST90094716 | ebi-met1400-GCST90200726 | N-acetylputrescine to (N(1) + N(8))-acetylspermidine ratio \|\| id:ebi-met1400-GCST90200726 | F-burn/spicy liking (derived food-liking factor) \|\| id:ebi-fl187-GCST90094716 | Weighted median | 14 | -0.054514231 | 0.0928573379812752 | 0.557153270712535 | -0.236514613 | 0.127486151583518 | 0.946945032919954 | 0.789374346851109 | 1.13596913675852 | 0.999332001504091 |
| ebi-fl187-GCST90094716 | ebi-met1400-GCST90200744 | Arginine to glutamate ratio \|\| id:ebi-met1400-GCST90200744 | F-burn/spicy liking (derived food-liking factor) \|\| id:ebi-fl187-GCST90094716 | Weighted median | 14 | 0.0497772991268938 | 0.0896888010984384 | 0.578894549731509 | -0.126012751 | 0.225567349279833 | 1.05103700345231 | 0.881603605361213 | 1.25303342217321 | 0.999332001504091 |
| ebi-fl187-GCST90094716 | ebi-met1400-GCST90200759 | Histidine to trans-urocanate ratio \|\| id:ebi-met1400-GCST90200759 | F-burn/spicy liking (derived food-liking factor) \|\| id:ebi-fl187-GCST90094716 | Weighted median | 14 | -0.061498649 | 0.107101261062881 | 0.56582549051744 | -0.27141712 | 0.148419822912421 | 0.940354216372622 | 0.7622984598002 | 1.15999978864123 | 0.999332001504091 |
| ebi-fl187-GCST90094716 | ebi-met1400-GCST90200795 | Oleoyl-linoleoyl-glycerol (18:1 to 18:2) [2] to linoleoyl-arachidonoyl-glycerol (18:2 to 20:4) [2] ratio \|\| id:ebi-met1400-GCST90200795 | F-burn/spicy liking (derived food-liking factor) \|\| id:ebi-fl187-GCST90094716 | Weighted median | 14 | 0.0231151115655349 | 0.107963604622058 | 0.830468325948562 | -0.188493553 | 0.234723776624769 | 1.02338433614029 | 0.828205842455615 | 1.26455941961485 | 0.999332001504091 |
| ebi-fl187-GCST90094716 | ebi-met1400-GCST90200811 | Choline to taurocholate ratio \|\| id:ebi-met1400-GCST90200811 | F-burn/spicy liking (derived food-liking factor) \|\| id:ebi-fl187-GCST90094716 | Weighted median | 14 | -0.062217768 | 0.0985414033290674 | 0.527787465263646 | -0.255358919 | 0.130923382153667 | 0.93967823231006 | 0.774638415694401 | 1.13988044278158 | 0.999332001504091 |
| ebi-fl187-GCST90094716 | ebi-met1400-GCST90200831 | Cholate to phosphate ratio \|\| id:ebi-met1400-GCST90200831 | F-burn/spicy liking (derived food-liking factor) \|\| id:ebi-fl187-GCST90094716 | Weighted median | 14 | -0.117491869 | 0.0972894492534175 | 0.227180867895934 | -0.308179189 | 0.0731954516413301 | 0.889147741470374 | 0.734783640748557 | 1.07594081076229 | 0.999332001504091 |
| ebi-fl187-GCST90094716 | ebi-met1400-GCST90200841 | Adenosine 5'-diphosphate (ADP) to oxalate (ethanedioate) ratio \|\| id:ebi-met1400-GCST90200841 | F-burn/spicy liking (derived food-liking factor) \|\| id:ebi-fl187-GCST90094716 | Weighted median | 14 | -0.099802114 | 0.130555631445649 | 0.444604318542521 | -0.355691151 | 0.156086923989522 | 0.905016490732869 | 0.70068899372083 | 1.16892780654232 | 0.999332001504091 |
| ebi-fl187-GCST90094716 | ebi-met1400-GCST90200844 | Aspartate to phosphate ratio \|\| id:ebi-met1400-GCST90200844 | F-burn/spicy liking (derived food-liking factor) \|\| id:ebi-fl187-GCST90094716 | Weighted median | 14 | -0.042205524 | 0.0900663686987222 | 0.639352218635604 | -0.218735606 | 0.134324559149262 | 0.958672730548812 | 0.803534139555915 | 1.1437639784737 | 0.999332001504091 |
| ebi-fl187-GCST90094716 | ebi-met1400-GCST90200870 | Adenosine 5'-monophosphate (AMP) to acetoacetate ratio \|\| id:ebi-met1400-GCST90200870 | F-burn/spicy liking (derived food-liking factor) \|\| id:ebi-fl187-GCST90094716 | Weighted median | 14 | -0.053527212 | 0.102754204201673 | 0.602419164500791 | -0.254925453 | 0.147871027862905 | 0.947880146585326 | 0.774974268118571 | 1.15936336114989 | 0.999332001504091 |
| ebi-fl187-GCST90094716 | ebi-met1400-GCST90200887 | Cholesterol to cortisol ratio \|\| id:ebi-met1400-GCST90200887 | F-burn/spicy liking (derived food-liking factor) \|\| id:ebi-fl187-GCST90094716 | Weighted median | 14 | -0.010128891 | 0.0858786509431139 | 0.906111847389288 | -0.178451047 | 0.158193264495862 | 0.9899222331104 | 0.836565008073616 | 1.17139256142548 | 0.999332001504091 |
| ebi-fl187-GCST90094716 | ebi-met1400-GCST90200890 | Cortisol to taurocholate ratio \|\| id:ebi-met1400-GCST90200890 | F-burn/spicy liking (derived food-liking factor) \|\| id:ebi-fl187-GCST90094716 | Weighted median | 14 | 0.0302132630417199 | 0.0928037817291888 | 0.744756759353536 | -0.151682149 | 0.21210867523093 | 1.03067431525656 | 0.859261354294485 | 1.23628223103528 | 0.999332001504091 |
| ebi-fl187-GCST90094716 | ebi-met1400-GCST90200948 | Adenosine 5'-diphosphate (ADP) to mannitol to sorbitol ratio \|\| id:ebi-met1400-GCST90200948 | F-burn/spicy liking (derived food-liking factor) \|\| id:ebi-fl187-GCST90094716 | Weighted median | 14 | 0.0086188668235078 | 0.133524968504432 | 0.948533308941467 | -0.253090071 | 0.270327805092194 | 1.00865611619505 | 0.776397947389083 | 1.31039393414037 | 0.999332001504091 |
| ebi-fl187-GCST90094716 | ebi-met1400-GCST90200972 | Leucine to N-palmitoyl-sphingosine (d18:1 to 16:0) ratio \|\| id:ebi-met1400-GCST90200972 | F-burn/spicy liking (derived food-liking factor) \|\| id:ebi-fl187-GCST90094716 | Weighted median | 14 | -0.02263149 | 0.096029608835624 | 0.813687196877689 | -0.210849524 | 0.165586543117931 | 0.977622680939842 | 0.809895928002692 | 1.18008508654315 | 0.999332001504091 |
| ebi-fl187-GCST90094716 | ebi-met1400-GCST90200973 | Proline to glutamate ratio \|\| id:ebi-met1400-GCST90200973 | F-burn/spicy liking (derived food-liking factor) \|\| id:ebi-fl187-GCST90094716 | Weighted median | 14 | 0.011811854558396 | 0.0867817535802171 | 0.891734412621861 | -0.158280382 | 0.181904091575622 | 1.01188188999026 | 0.853610411026417 | 1.19949914628978 | 0.999332001504091 |
| ebi-fl187-GCST90094716 | ebi-met1400-GCST90200983 | Cholesterol to linoleoyl-arachidonoyl-glycerol (18:2 to 20:4) [1] ratio \|\| id:ebi-met1400-GCST90200983 | F-burn/spicy liking (derived food-liking factor) \|\| id:ebi-fl187-GCST90094716 | Weighted median | 14 | -0.134709547 | 0.0960473112360895 | 0.160756069883614 | -0.322962277 | 0.0535431828286404 | 0.873969721784291 | 0.724001165191761 | 1.05500254877822 | 0.999332001504091 |
| ebi-fl187-GCST90094716 | ebi-met1400-GCST90201001 | Serine to alpha-ketobutyrate ratio \|\| id:ebi-met1400-GCST90201001 | F-burn/spicy liking (derived food-liking factor) \|\| id:ebi-fl187-GCST90094716 | Weighted median | 14 | 0.0419186615757689 | 0.0914615568133167 | 0.646722550648211 | -0.13734599 | 0.22118331292987 | 1.04280965480699 | 0.871668585532149 | 1.24755210203519 | 0.999332001504091 |
| ebi-fl187-GCST90094716 | ebi-met1400-GCST90201005 | Fructose to maltose ratio \|\| id:ebi-met1400-GCST90201005 | F-burn/spicy liking (derived food-liking factor) \|\| id:ebi-fl187-GCST90094716 | Weighted median | 14 | 0.131728044732521 | 0.107638921627282 | 0.221029323365599 | -0.079244242 | 0.342700331121995 | 1.14079803104322 | 0.92381426296194 | 1.40874654117101 | 0.999332001504091 |
| ebi-fl187-GCST90094716 | ebi-met1400-GCST90201020 | Paraxanthine to 5-acetylamino-6-formylamino-3-methyluracil ratio \|\| id:ebi-met1400-GCST90201020 | F-burn/spicy liking (derived food-liking factor) \|\| id:ebi-fl187-GCST90094716 | Weighted median | 14 | 0.0683088291151502 | 0.0952516846115686 | 0.473287483377311 | -0.118384473 | 0.255002130953825 | 1.07069591949841 | 0.88835443889767 | 1.29046437078995 | 0.999332001504091 |
| ebi-fl187-GCST90094716 | ebi-pw11-GCST90275197 | Placental weight (fetal genotype, adjusted for sex) \|\| id:ebi-pw11-GCST90275197 | F-burn/spicy liking (derived food-liking factor) \|\| id:ebi-fl187-GCST90094716 | Weighted median | 14 | -0.021345439 | 0.0369687819266584 | 0.563675408909726 | -0.093804251 | 0.0511133736930641 | 0.978880762681348 | 0.910460966203989 | 1.05244220577923 | 0.999332001504091 |
| ebi-fl187-GCST90094716 | prot-a-1017 | FAS-associated factor 2 \|\| id:prot-a-1017 | F-burn/spicy liking (derived food-liking factor) \|\| id:ebi-fl187-GCST90094716 | Weighted median | 15 | -0.195693877 | 0.151148437358879 | 0.195419184330806 | -0.491944814 | 0.10055706007319 | 0.822263909925832 | 0.611436107385955 | 1.10578673617607 | 0.999332001504091 |
| ebi-fl187-GCST90094716 | prot-a-1019 | Fas apoptotic inhibitory molecule 3 \|\| id:prot-a-1019 | F-burn/spicy liking (derived food-liking factor) \|\| id:ebi-fl187-GCST90094716 | Weighted median | 15 | 0.0882111955560575 | 0.153645687058641 | 0.565886011422452 | -0.212934351 | 0.389356742190993 | 1.09221876942183 | 0.808209193536521 | 1.47603101996072 | 0.999332001504091 |
| ebi-fl187-GCST90094716 | prot-a-1052 | MIP18 family protein FAM96A \|\| id:prot-a-1052 | F-burn/spicy liking (derived food-liking factor) \|\| id:ebi-fl187-GCST90094716 | Weighted median | 15 | -0.077621741 | 0.146758754218937 | 0.59686992490366 | -0.365268899 | 0.210025417196185 | 0.925314368775313 | 0.694010006950644 | 1.23370941699252 | 0.999332001504091 |
| ebi-fl187-GCST90094716 | prot-a-1066 | F-box/LRR-repeat protein 4 \|\| id:prot-a-1066 | F-burn/spicy liking (derived food-liking factor) \|\| id:ebi-fl187-GCST90094716 | Weighted median | 15 | -0.029830886 | 0.152363024961122 | 0.844775926380557 | -0.328462415 | 0.268800642630053 | 0.970609663067289 | 0.720029989887795 | 1.30839427700283 | 0.999332001504091 |
| ebi-fl187-GCST90094716 | prot-a-1076 | Low affinity immunoglobulin gamma Fc region receptor III-B \|\| id:prot-a-1076 | F-burn/spicy liking (derived food-liking factor) \|\| id:ebi-fl187-GCST90094716 | Weighted median | 15 | -0.008555769 | 0.143519411872687 | 0.952463052141969 | -0.289853816 | 0.272742278227228 | 0.991480727389536 | 0.748372959500655 | 1.31356166775561 | 0.999332001504091 |
| ebi-fl187-GCST90094716 | prot-a-1081 | Fc receptor-like protein 3 \|\| id:prot-a-1081 | F-burn/spicy liking (derived food-liking factor) \|\| id:ebi-fl187-GCST90094716 | Weighted median | 15 | -0.1295008 | 0.15289212250564 | 0.396990849044035 | -0.42916936 | 0.170167760417302 | 0.878533885857906 | 0.651049658200528 | 1.18550371523713 | 0.999332001504091 |
| ebi-fl187-GCST90094716 | prot-a-1105 | Fibroblast growth factor receptor 4 \|\| id:prot-a-1105 | F-burn/spicy liking (derived food-liking factor) \|\| id:ebi-fl187-GCST90094716 | Weighted median | 15 | 0.197192949177921 | 0.152059333790396 | 0.194694567127965 | -0.100843345 | 0.495229243407097 | 1.21797902608687 | 0.904074649560986 | 1.64087435557217 | 0.999332001504091 |
| ebi-fl187-GCST90094716 | prot-a-1143 | Follistatin-related protein 1 \|\| id:prot-a-1143 | F-burn/spicy liking (derived food-liking factor) \|\| id:ebi-fl187-GCST90094716 | Weighted median | 15 | -0.036376731 | 0.144704291598736 | 0.801515165246791 | -0.319997143 | 0.247243680142866 | 0.96427695164931 | 0.72615111173951 | 1.28049110501898 | 0.999332001504091 |
| ebi-fl187-GCST90094716 | prot-a-1151 | Alpha-(1,3)-fucosyltransferase 10 \|\| id:prot-a-1151 | F-burn/spicy liking (derived food-liking factor) \|\| id:ebi-fl187-GCST90094716 | Weighted median | 15 | 0.0220525980711721 | 0.134386500182045 | 0.869653749248991 | -0.241344942 | 0.285450138427981 | 1.02229755393584 | 0.78557060312797 | 1.33036073985187 | 0.999332001504091 |
| ebi-fl187-GCST90094716 | prot-a-1165 | Growth arrest and DNA damage-inducible proteins-interacting protein 1 \|\| id:prot-a-1165 | F-burn/spicy liking (derived food-liking factor) \|\| id:ebi-fl187-GCST90094716 | Weighted median | 15 | 0.109429083781804 | 0.145091417867795 | 0.450724435195722 | -0.174950095 | 0.393808262802682 | 1.11564095109262 | 0.839498914716481 | 1.48261624873596 | 0.999332001504091 |
| ebi-fl187-GCST90094716 | prot-a-1194 | Growth/differentiation factor 11/8 \|\| id:prot-a-1194 | F-burn/spicy liking (derived food-liking factor) \|\| id:ebi-fl187-GCST90094716 | Weighted median | 15 | 0.0148001892422874 | 0.151842229018895 | 0.922352492175678 | -0.28281058 | 0.312410958119321 | 1.01491025436762 | 0.753662533372187 | 1.36671624077123 | 0.999332001504091 |
| ebi-fl187-GCST90094716 | prot-a-1216 | D-glucuronyl C5-epimerase \|\| id:prot-a-1216 | F-burn/spicy liking (derived food-liking factor) \|\| id:ebi-fl187-GCST90094716 | Weighted median | 15 | -0.008080636 | 0.154123577400163 | 0.958186395820081 | -0.310162848 | 0.294001575479777 | 0.991951924353931 | 0.733327525631314 | 1.34178601762204 | 0.999332001504091 |
| ebi-fl187-GCST90094716 | prot-a-1246 | Glypican-3 \|\| id:prot-a-1246 | F-burn/spicy liking (derived food-liking factor) \|\| id:ebi-fl187-GCST90094716 | Weighted median | 15 | 0.0358504332092907 | 0.154659524986206 | 0.816691572573076 | -0.267282236 | 0.338983102182254 | 1.03650080879743 | 0.765457001634076 | 1.40351962859346 | 0.999332001504091 |
| ebi-fl187-GCST90094716 | prot-a-1275 | Glutamate receptor 4 \|\| id:prot-a-1275 | F-burn/spicy liking (derived food-liking factor) \|\| id:ebi-fl187-GCST90094716 | Weighted median | 15 | -0.250867783 | 0.151211161500445 | 0.0971045101892857 | -0.54724166 | 0.0455060930974775 | 0.778125245798575 | 0.578543430992867 | 1.04655738137066 | 0.999332001504091 |
| ebi-fl187-GCST90094716 | prot-a-1289 | Glutathione S-transferase P \|\| id:prot-a-1289 | F-burn/spicy liking (derived food-liking factor) \|\| id:ebi-fl187-GCST90094716 | Weighted median | 15 | 0.0584113743046045 | 0.143902365982861 | 0.684808909235159 | -0.223637263 | 0.340460011631011 | 1.06015102490918 | 0.799605128118382 | 1.40559403146998 | 0.999332001504091 |
| ebi-fl187-GCST90094716 | prot-a-130 | Apolipoprotein D \|\| id:prot-a-130 | F-burn/spicy liking (derived food-liking factor) \|\| id:ebi-fl187-GCST90094716 | Weighted median | 15 | 0.145502373886667 | 0.148375670281619 | 0.326772773252495 | -0.14531394 | 0.43631868763864 | 1.15662048026487 | 0.864750770751632 | 1.54700172652677 | 0.999332001504091 |
| ebi-fl187-GCST90094716 | prot-a-1313 | Heparin-binding EGF-like growth factor \|\| id:prot-a-1313 | F-burn/spicy liking (derived food-liking factor) \|\| id:ebi-fl187-GCST90094716 | Weighted median | 15 | -0.051334552 | 0.151035230208788 | 0.733943418775514 | -0.347363603 | 0.244694499268376 | 0.949960806132895 | 0.70654837833064 | 1.27723105857354 | 0.999332001504091 |
| ebi-fl187-GCST90094716 | prot-a-1347 | HLA class II histocompatibility antigen, DQ alpha 2 chain \|\| id:prot-a-1347 | F-burn/spicy liking (derived food-liking factor) \|\| id:ebi-fl187-GCST90094716 | Weighted median | 15 | -0.009641906 | 0.142707806266648 | 0.946132738362615 | -0.289349206 | 0.27006539447683 | 0.990404428331944 | 0.748750691444025 | 1.31005011797422 | 0.999332001504091 |
| ebi-fl187-GCST90094716 | prot-a-1357 | Non-histone chromosomal protein HMG-14 \|\| id:prot-a-1357 | F-burn/spicy liking (derived food-liking factor) \|\| id:ebi-fl187-GCST90094716 | Weighted median | 15 | -0.035810589 | 0.152831056677515 | 0.814740541664113 | -0.33535946 | 0.263738281720167 | 0.964823023915991 | 0.715080996807598 | 1.30178745014093 | 0.999332001504091 |
| ebi-fl187-GCST90094716 | prot-a-137 | Apolipoprotein M \|\| id:prot-a-137 | F-burn/spicy liking (derived food-liking factor) \|\| id:ebi-fl187-GCST90094716 | Weighted median | 15 | -0.028028485 | 0.150301929333129 | 0.852067416417312 | -0.322620266 | 0.266563296566592 | 0.972360668784733 | 0.724248823756236 | 1.30547021850286 | 0.999332001504091 |
| ebi-fl187-GCST90094716 | prot-a-1381 | 17-beta-hydroxysteroid dehydrogenase 14 \|\| id:prot-a-1381 | F-burn/spicy liking (derived food-liking factor) \|\| id:ebi-fl187-GCST90094716 | Weighted median | 15 | -0.169771671 | 0.145735226065005 | 0.244046400163032 | -0.455412714 | 0.115869371870064 | 0.843857471550425 | 0.634186176222545 | 1.12284918686337 | 0.999332001504091 |
| ebi-fl187-GCST90094716 | prot-a-1392 | Serine protease HTRA2, mitochondrial \|\| id:prot-a-1392 | F-burn/spicy liking (derived food-liking factor) \|\| id:ebi-fl187-GCST90094716 | Weighted median | 15 | -0.06123228 | 0.142519262725158 | 0.667455983604822 | -0.340570035 | 0.218105474949435 | 0.94060473074014 | 0.711364704433523 | 1.24371824181981 | 0.999332001504091 |
| ebi-fl187-GCST90094716 | prot-a-1416 | Interferon-induced protein with tetratricopeptide repeats 2 \|\| id:prot-a-1416 | F-burn/spicy liking (derived food-liking factor) \|\| id:ebi-fl187-GCST90094716 | Weighted median | 15 | -0.01658345 | 0.158288556231466 | 0.916560529442125 | -0.326829021 | 0.293662119800595 | 0.983553298036853 | 0.721207044013712 | 1.34133061803647 | 0.999332001504091 |
| ebi-fl187-GCST90094716 | prot-a-1428 | Interferon gamma \|\| id:prot-a-1428 | F-burn/spicy liking (derived food-liking factor) \|\| id:ebi-fl187-GCST90094716 | Weighted median | 15 | -0.075236635 | 0.150031295490841 | 0.616038316124668 | -0.369297974 | 0.218824704039715 | 0.927523975608168 | 0.691219414150749 | 1.24461308191838 | 0.999332001504091 |
| ebi-fl187-GCST90094716 | prot-a-1483 | Interleukin-17C \|\| id:prot-a-1483 | F-burn/spicy liking (derived food-liking factor) \|\| id:ebi-fl187-GCST90094716 | Weighted median | 15 | 0.19054907397847 | 0.161673129430952 | 0.238554722885881 | -0.12633026 | 0.507428407663136 | 1.2099137474618 | 0.881323732997362 | 1.66101424651121 | 0.999332001504091 |
| ebi-fl187-GCST90094716 | prot-a-1503 | Interleukin-1 receptor-like 2 \|\| id:prot-a-1503 | F-burn/spicy liking (derived food-liking factor) \|\| id:ebi-fl187-GCST90094716 | Weighted median | 15 | 0.0356177538799644 | 0.142961390626154 | 0.803250579178658 | -0.244586572 | 0.315822079507226 | 1.03625966454012 | 0.783028197311788 | 1.37138623620373 | 0.999332001504091 |
| ebi-fl187-GCST90094716 | prot-a-1522 | Interleukin-31 receptor subunit alpha \|\| id:prot-a-1522 | F-burn/spicy liking (derived food-liking factor) \|\| id:ebi-fl187-GCST90094716 | Weighted median | 15 | 0.0347818306607991 | 0.144745446148267 | 0.810100373 | -0.248919244 | 0.318482905111402 | 1.03539379297694 | 0.779642931851005 | 1.37504011482535 | 0.999332001504091 |
| ebi-fl187-GCST90094716 | prot-a-1540 | Interleukin-6 receptor subunit alpha \|\| id:prot-a-1540 | F-burn/spicy liking (derived food-liking factor) \|\| id:ebi-fl187-GCST90094716 | Weighted median | 15 | -0.067263126 | 0.15087655154366 | 0.655730426316322 | -0.362981167 | 0.228454915261297 | 0.934949159756035 | 0.695599533747933 | 1.25665686780819 | 0.999332001504091 |
| ebi-fl187-GCST90094716 | prot-a-1541 | Interleukin-6 receptor subunit alpha \|\| id:prot-a-1541 | F-burn/spicy liking (derived food-liking factor) \|\| id:ebi-fl187-GCST90094716 | Weighted median | 15 | -0.12337321 | 0.162163598379748 | 0.446779685805999 | -0.441213863 | 0.194467442642716 | 0.883933707933785 | 0.643255123387257 | 1.21466393599353 | 0.999332001504091 |
| ebi-fl187-GCST90094716 | prot-a-1545 | Interleukin-9 \|\| id:prot-a-1545 | F-burn/spicy liking (derived food-liking factor) \|\| id:ebi-fl187-GCST90094716 | Weighted median | 15 | -0.03325833 | 0.151124482593807 | 0.825814755929614 | -0.329462316 | 0.262945655669616 | 0.967288647426963 | 0.719310391090564 | 1.30075602831557 | 0.999332001504091 |
| ebi-fl187-GCST90094716 | prot-a-1548 | Inositol monophosphatase 3 \|\| id:prot-a-1548 | F-burn/spicy liking (derived food-liking factor) \|\| id:ebi-fl187-GCST90094716 | Weighted median | 15 | 0.0127761582168175 | 0.153742537664353 | 0.933771232811257 | -0.288559216 | 0.314111532038949 | 1.01285812201507 | 0.749342431068053 | 1.36904242012519 | 0.999332001504091 |
| ebi-fl187-GCST90094716 | prot-a-1555 | Inhibin beta A chain:Inhibin beta B chain heterodimer \|\| id:prot-a-1555 | F-burn/spicy liking (derived food-liking factor) \|\| id:ebi-fl187-GCST90094716 | Weighted median | 15 | -0.139819207 | 0.148359626946581 | 0.345970312356797 | -0.430604076 | 0.150965661577483 | 0.8695154232843 | 0.650116256420913 | 1.16295672329684 | 0.999332001504091 |
| ebi-fl187-GCST90094716 | prot-a-1603 | Junctophilin-3 \|\| id:prot-a-1603 | F-burn/spicy liking (derived food-liking factor) \|\| id:ebi-fl187-GCST90094716 | Weighted median | 15 | 0.228198268101294 | 0.164858738348174 | 0.166295733308134 | -0.094924859 | 0.551321395263716 | 1.25633439177502 | 0.90944126817161 | 1.73554484406669 | 0.999332001504091 |
| ebi-fl187-GCST90094716 | prot-a-1627 | UPF0258 protein KIAA1024 \|\| id:prot-a-1627 | F-burn/spicy liking (derived food-liking factor) \|\| id:ebi-fl187-GCST90094716 | Weighted median | 15 | -0.150332411 | 0.147152113208004 | 0.306964425889054 | -0.438750553 | 0.138085730606321 | 0.8604219149314 | 0.644841613266139 | 1.14807397113293 | 0.999332001504091 |
| ebi-fl187-GCST90094716 | prot-a-1666 | Kallikrein-7 \|\| id:prot-a-1666 | F-burn/spicy liking (derived food-liking factor) \|\| id:ebi-fl187-GCST90094716 | Weighted median | 15 | -0.085100927 | 0.150149297011226 | 0.570866787708968 | -0.37939355 | 0.209191694646165 | 0.918419585934686 | 0.684276262993037 | 1.23268127428384 | 0.999332001504091 |
| ebi-fl187-GCST90094716 | prot-a-1678 | Importin subunit alpha-3 \|\| id:prot-a-1678 | F-burn/spicy liking (derived food-liking factor) \|\| id:ebi-fl187-GCST90094716 | Weighted median | 15 | -0.112160245 | 0.150320712479412 | 0.455583055194179 | -0.406788841 | 0.182468351536091 | 0.893901002924874 | 0.665784761648927 | 1.20017616662041 | 0.999332001504091 |
| ebi-fl187-GCST90094716 | prot-a-1718 | Protein LDOC1 \|\| id:prot-a-1718 | F-burn/spicy liking (derived food-liking factor) \|\| id:ebi-fl187-GCST90094716 | Weighted median | 15 | -0.032731379 | 0.145429703236071 | 0.821927408792194 | -0.317773597 | 0.252310839223821 | 0.96779849555998 | 0.727767538182036 | 1.28699602396099 | 0.999332001504091 |
| ebi-fl187-GCST90094716 | prot-a-1723 | Leptin \|\| id:prot-a-1723 | F-burn/spicy liking (derived food-liking factor) \|\| id:ebi-fl187-GCST90094716 | Weighted median | 15 | -0.250926958 | 0.167183530975981 | 0.133379576603537 | -0.578606679 | 0.076752762419989 | 0.778079201716573 | 0.560679028444123 | 1.07977508241016 | 0.999332001504091 |
| ebi-fl187-GCST90094716 | prot-a-1730 | Galectin-7 \|\| id:prot-a-1730 | F-burn/spicy liking (derived food-liking factor) \|\| id:ebi-fl187-GCST90094716 | Weighted median | 15 | 0.235597217013833 | 0.143769187310599 | 0.101271913792385 | -0.04619039 | 0.517384824142607 | 1.26566441939178 | 0.95486014895003 | 1.6776345984025 | 0.999332001504091 |
| ebi-fl187-GCST90094716 | prot-a-1753 | Lipase member N \|\| id:prot-a-1753 | F-burn/spicy liking (derived food-liking factor) \|\| id:ebi-fl187-GCST90094716 | Weighted median | 15 | -0.07391746 | 0.151761066094998 | 0.626212474298186 | -0.371369149 | 0.223534229937951 | 0.928748349930276 | 0.689789259431973 | 1.25048844368572 | 0.999332001504091 |
| ebi-fl187-GCST90094716 | prot-a-1756 | VIP36-like protein \|\| id:prot-a-1756 | F-burn/spicy liking (derived food-liking factor) \|\| id:ebi-fl187-GCST90094716 | Weighted median | 15 | 0.213462635097913 | 0.143809997731686 | 0.1377192911249 | -0.06840496 | 0.495330230652017 | 1.23795724125279 | 0.933882211625368 | 1.64104007132005 | 0.999332001504091 |
| ebi-fl187-GCST90094716 | prot-a-1760 | Ig Kappa chain V-I region HK102- like \|\| id:prot-a-1760 | F-burn/spicy liking (derived food-liking factor) \|\| id:ebi-fl187-GCST90094716 | Weighted median | 15 | 0.135404543791511 | 0.157362714048515 | 0.38953474350779 | -0.173026376 | 0.4438354633266 | 1.14499989326918 | 0.841115429507158 | 1.55867400548652 | 0.999332001504091 |
| ebi-fl187-GCST90094716 | prot-a-179 | Neutral ceramidase \|\| id:prot-a-179 | F-burn/spicy liking (derived food-liking factor) \|\| id:ebi-fl187-GCST90094716 | Weighted median | 15 | 0.21900538722309 | 0.153516202016131 | 0.153696777088525 | -0.081886369 | 0.519897143174706 | 1.24483798288939 | 0.921376649948376 | 1.681854651 | 0.999332001504091 |
| ebi-fl187-GCST90094716 | prot-a-1809 | Lactotransferrin \|\| id:prot-a-1809 | F-burn/spicy liking (derived food-liking factor) \|\| id:ebi-fl187-GCST90094716 | Weighted median | 15 | 0.0799142793365112 | 0.150789719536531 | 0.596131573797469 | -0.215633571 | 0.375462129628112 | 1.08319421156867 | 0.806030600790342 | 1.45566396465022 | 0.999332001504091 |
| ebi-fl187-GCST90094716 | prot-a-1818 | Lysophospholipase-like protein 1 \|\| id:prot-a-1818 | F-burn/spicy liking (derived food-liking factor) \|\| id:ebi-fl187-GCST90094716 | Weighted median | 15 | -0.17419419 | 0.151730751007028 | 0.250948735519807 | -0.471586462 | 0.123198081781887 | 0.840133736085334 | 0.624011511937146 | 1.13110845073608 | 0.999332001504091 |
| ebi-fl187-GCST90094716 | prot-a-1852 | MAP kinase-activated protein kinase 3 \|\| id:prot-a-1852 | F-burn/spicy liking (derived food-liking factor) \|\| id:ebi-fl187-GCST90094716 | Weighted median | 15 | -0.079212014 | 0.156768711625532 | 0.613362547535626 | -0.386478689 | 0.228054660428398 | 0.923844035480437 | 0.679445204779483 | 1.25615398547081 | 0.999332001504091 |
| ebi-fl187-GCST90094716 | prot-a-1856 | Megakaryocyte-associated tyrosine-protein kinase \|\| id:prot-a-1856 | F-burn/spicy liking (derived food-liking factor) \|\| id:ebi-fl187-GCST90094716 | Weighted median | 15 | -0.073983832 | 0.143498335577146 | 0.606152845969542 | -0.355240569 | 0.207272906105357 | 0.928686709074083 | 0.701004782652251 | 1.2303182873414 | 0.999332001504091 |
| ebi-fl187-GCST90094716 | prot-a-1890 | Beta-1,3-N-acetylglucosaminyltransferase manic fringe \|\| id:prot-a-1890 | F-burn/spicy liking (derived food-liking factor) \|\| id:ebi-fl187-GCST90094716 | Weighted median | 15 | 0.0514243525375292 | 0.151577263305322 | 0.734412629675265 | -0.245667084 | 0.34851578861596 | 1.05276954393523 | 0.782182583040236 | 1.41696291463011 | 0.999332001504091 |
| ebi-fl187-GCST90094716 | prot-a-1919 | Matrilysin \|\| id:prot-a-1919 | F-burn/spicy liking (derived food-liking factor) \|\| id:ebi-fl187-GCST90094716 | Weighted median | 15 | 0.00274922830441109 | 0.141676084138284 | 0.98451799982425 | -0.274935897 | 0.280434353215447 | 1.00275301089816 | 0.759620815936248 | 1.32370464285658 | 0.999332001504091 |
| ebi-fl187-GCST90094716 | prot-a-1923 | Multimerin-2 \|\| id:prot-a-1923 | F-burn/spicy liking (derived food-liking factor) \|\| id:ebi-fl187-GCST90094716 | Weighted median | 15 | -0.179946492 | 0.146276887572836 | 0.218630756647784 | -0.466649191 | 0.106756207975246 | 0.835314906523231 | 0.627100043974067 | 1.11266296305469 | 0.999332001504091 |
| ebi-fl187-GCST90094716 | prot-a-1948 | Mesothelin \|\| id:prot-a-1948 | F-burn/spicy liking (derived food-liking factor) \|\| id:ebi-fl187-GCST90094716 | Weighted median | 15 | 0.141676115183526 | 0.146194539226087 | 0.332498753852003 | -0.144865182 | 0.428217412066657 | 1.15220340690763 | 0.865138921807767 | 1.53451966779567 | 0.999332001504091 |
| ebi-fl187-GCST90094716 | prot-a-195 | ATPase family AAA domain-containing protein 1 \|\| id:prot-a-195 | F-burn/spicy liking (derived food-liking factor) \|\| id:ebi-fl187-GCST90094716 | Weighted median | 15 | -0.181711918 | 0.153990091978343 | 0.237990891669464 | -0.483532499 | 0.120108661957164 | 0.833841520284058 | 0.616601396558744 | 1.12761937425062 | 0.999332001504091 |
| ebi-fl187-GCST90094716 | prot-a-1966 | Mucin-1 \|\| id:prot-a-1966 | F-burn/spicy liking (derived food-liking factor) \|\| id:ebi-fl187-GCST90094716 | Weighted median | 15 | 0.0315302335608011 | 0.152127221466387 | 0.83580503691231 | -0.266639121 | 0.329699587634919 | 1.03203257714365 | 0.765949437034704 | 1.39055032719803 | 0.999332001504091 |
| ebi-fl187-GCST90094716 | prot-a-1986 | Myocardial zonula adherens protein \|\| id:prot-a-1986 | F-burn/spicy liking (derived food-liking factor) \|\| id:ebi-fl187-GCST90094716 | Weighted median | 15 | 0.0446047725479653 | 0.154315683816397 | 0.772543987372178 | -0.257853968 | 0.347063512828104 | 1.04561452266938 | 0.772708064179146 | 1.41490658723557 | 0.999332001504091 |
| ebi-fl187-GCST90094716 | prot-a-2071 | Epididymal secretory protein E1 \|\| id:prot-a-2071 | F-burn/spicy liking (derived food-liking factor) \|\| id:ebi-fl187-GCST90094716 | Weighted median | 14 | -0.283182064 | 0.163074796751784 | 0.0824725613443847 | -0.602808666 | 0.0364445373100741 | 0.753382611277215 | 0.54727237018873 | 1.03711678113613 | 0.999332001504091 |
| ebi-fl187-GCST90094716 | prot-a-2098 | Neuritin \|\| id:prot-a-2098 | F-burn/spicy liking (derived food-liking factor) \|\| id:ebi-fl187-GCST90094716 | Weighted median | 15 | 0.044018300960906 | 0.144851661959276 | 0.761215223713765 | -0.239890956 | 0.327927558401086 | 1.04500147924466 | 0.78671364241489 | 1.38808841330304 | 0.999332001504091 |
| ebi-fl187-GCST90094716 | prot-a-2115 | Neurotrophin-4 \|\| id:prot-a-2115 | F-burn/spicy liking (derived food-liking factor) \|\| id:ebi-fl187-GCST90094716 | Weighted median | 15 | 0.0865801292694781 | 0.140245741102401 | 0.537006616435708 | -0.188301523 | 0.361461781830183 | 1.09043874027685 | 0.828364898262501 | 1.43542616157519 | 0.999332001504091 |
| ebi-fl187-GCST90094716 | prot-a-2118 | Netrin-1 \|\| id:prot-a-2118 | F-burn/spicy liking (derived food-liking factor) \|\| id:ebi-fl187-GCST90094716 | Weighted median | 15 | 0.186803349478565 | 0.144504297117054 | 0.19610850572928 | -0.096425073 | 0.470031771827991 | 1.2053902211258 | 0.908077934723487 | 1.60004502876522 | 0.999332001504091 |
| ebi-fl187-GCST90094716 | prot-a-2120 | Netrin-G1 \|\| id:prot-a-2120 | F-burn/spicy liking (derived food-liking factor) \|\| id:ebi-fl187-GCST90094716 | Weighted median | 15 | 0.173557273952044 | 0.147409663171845 | 0.239043639603568 | -0.115365666 | 0.462480213768861 | 1.18952881383488 | 0.891040261320692 | 1.58800770331762 | 0.999332001504091 |
| ebi-fl187-GCST90094716 | prot-a-2145 | Olfactomedin-like protein 3 \|\| id:prot-a-2145 | F-burn/spicy liking (derived food-liking factor) \|\| id:ebi-fl187-GCST90094716 | Weighted median | 15 | -0.286282789 | 0.157523051945636 | 0.0691555044488876 | -0.595027971 | 0.022462392592956 | 0.751050197013647 | 0.551547138308345 | 1.02271657172281 | 0.999332001504091 |
| ebi-fl187-GCST90094716 | prot-a-2162 | Prolyl 4-hydroxylase subunit alpha-2 \|\| id:prot-a-2162 | F-burn/spicy liking (derived food-liking factor) \|\| id:ebi-fl187-GCST90094716 | Weighted median | 15 | -0.024454581 | 0.152639584609962 | 0.872714654704982 | -0.323628167 | 0.274719004677652 | 0.975842009526075 | 0.7235192206964 | 1.31616078787695 | 0.999332001504091 |
| ebi-fl187-GCST90094716 | prot-a-2168 | Polyadenylate-binding protein-interacting protein 1 \|\| id:prot-a-2168 | F-burn/spicy liking (derived food-liking factor) \|\| id:ebi-fl187-GCST90094716 | Weighted median | 15 | -0.178008678 | 0.158572371040391 | 0.261619918221934 | -0.488810525 | 0.132793169503858 | 0.836935160755461 | 0.613355531539645 | 1.14201377062741 | 0.999332001504091 |
| ebi-fl187-GCST90094716 | prot-a-219 | Beta-1,4-galactosyltransferase 5 \|\| id:prot-a-219 | F-burn/spicy liking (derived food-liking factor) \|\| id:ebi-fl187-GCST90094716 | Weighted median | 15 | -0.040495303 | 0.148829006146993 | 0.785550652994976 | -0.332200155 | 0.251209548616009 | 0.960313674679204 | 0.717343728205906 | 1.28557944750186 | 0.999332001504091 |
| ebi-fl187-GCST90094716 | prot-a-2190 | Pyruvate carboxylase, mitochondrial \|\| id:prot-a-2190 | F-burn/spicy liking (derived food-liking factor) \|\| id:ebi-fl187-GCST90094716 | Weighted median | 15 | -0.115650173 | 0.142756438194033 | 0.417869894426821 | -0.395452792 | 0.164152446210332 | 0.890786790379269 | 0.673375071695021 | 1.17839394309147 | 0.999332001504091 |
| ebi-fl187-GCST90094716 | prot-a-2197 | Protocadherin-9 \|\| id:prot-a-2197 | F-burn/spicy liking (derived food-liking factor) \|\| id:ebi-fl187-GCST90094716 | Weighted median | 15 | -0.111244079 | 0.145625584505351 | 0.444923994385113 | -0.396670224 | 0.174182066924705 | 0.894720340092996 | 0.672555781595059 | 1.19027225530286 | 0.999332001504091 |
| ebi-fl187-GCST90094716 | prot-a-2198 | Protocadherin alpha-4 \|\| id:prot-a-2198 | F-burn/spicy liking (derived food-liking factor) \|\| id:ebi-fl187-GCST90094716 | Weighted median | 15 | -0.073234849 | 0.153419903228639 | 0.633113570987538 | -0.373937859 | 0.227468161490093 | 0.929382539984605 | 0.688019664616268 | 1.25541746849631 | 0.999332001504091 |
| ebi-fl187-GCST90094716 | prot-a-2254 | Placenta growth factor \|\| id:prot-a-2254 | F-burn/spicy liking (derived food-liking factor) \|\| id:ebi-fl187-GCST90094716 | Weighted median | 15 | -0.233577777 | 0.155858155474709 | 0.133962868704897 | -0.539059762 | 0.0719042079522744 | 0.79169601778007 | 0.583296432381263 | 1.07455240555823 | 0.999332001504091 |
| ebi-fl187-GCST90094716 | prot-a-2261 | Membrane-associated progesterone receptor component 2 \|\| id:prot-a-2261 | F-burn/spicy liking (derived food-liking factor) \|\| id:ebi-fl187-GCST90094716 | Weighted median | 15 | 0.156968127885069 | 0.146739456818188 | 0.284751509843785 | -0.130641207 | 0.444577463248717 | 1.16995832426049 | 0.877532570037938 | 1.55983097065815 | 0.999332001504091 |
| ebi-fl187-GCST90094716 | prot-a-2268 | Polymeric immunoglobulin receptor \|\| id:prot-a-2268 | F-burn/spicy liking (derived food-liking factor) \|\| id:ebi-fl187-GCST90094716 | Weighted median | 15 | 0.0905062390715972 | 0.147027973379814 | 0.538177325793876 | -0.197668589 | 0.378681066896032 | 1.09472833770882 | 0.820641777990982 | 1.46035720520663 | 0.999332001504091 |
| ebi-fl187-GCST90094716 | prot-a-2283 | Group 10 secretory phospholipase A2 \|\| id:prot-a-2283 | F-burn/spicy liking (derived food-liking factor) \|\| id:ebi-fl187-GCST90094716 | Weighted median | 15 | 0.00615152495116957 | 0.149124471114473 | 0.967095842136904 | -0.286132438 | 0.298435488335537 | 1.00617048443743 | 0.751163126491369 | 1.34774858888748 | 0.999332001504091 |
| ebi-fl187-GCST90094716 | prot-a-2298 | Inactive phospholipase D5 \|\| id:prot-a-2298 | F-burn/spicy liking (derived food-liking factor) \|\| id:ebi-fl187-GCST90094716 | Weighted median | 15 | 0.0570221153370183 | 0.161147392153163 | 0.723450785445639 | -0.258826773 | 0.372871003957217 | 1.05867922318442 | 0.771956734992858 | 1.45189703877996 | 0.999332001504091 |
| ebi-fl187-GCST90094716 | prot-a-2304 | Procollagen-lysine,2-oxoglutarate 5-dioxygenase 2 \|\| id:prot-a-2304 | F-burn/spicy liking (derived food-liking factor) \|\| id:ebi-fl187-GCST90094716 | Weighted median | 15 | -0.037361075 | 0.154471498568447 | 0.808885644609218 | -0.340125212 | 0.265403062346446 | 0.96332823894734 | 0.711681206126511 | 1.30395644561734 | 0.999332001504091 |
| ebi-fl187-GCST90094716 | prot-a-2331 | Periostin \|\| id:prot-a-2331 | F-burn/spicy liking (derived food-liking factor) \|\| id:ebi-fl187-GCST90094716 | Weighted median | 15 | 0.00796643104402392 | 0.155963992559125 | 0.959262844241977 | -0.297722994 | 0.313655856459909 | 1.00799824748753 | 0.742506989878467 | 1.36841872304022 | 0.999332001504091 |
| ebi-fl187-GCST90094716 | prot-a-2333 | Periostin \|\| id:prot-a-2333 | F-burn/spicy liking (derived food-liking factor) \|\| id:ebi-fl187-GCST90094716 | Weighted median | 15 | -0.090469123 | 0.151512635901518 | 0.550436530470428 | -0.387433889 | 0.206495643828528 | 0.913502540105044 | 0.678796508748236 | 1.22936237889208 | 0.999332001504091 |
| ebi-fl187-GCST90094716 | prot-a-2367 | cAMP-dependent protein kinase catalytic subunit alpha \|\| id:prot-a-2367 | F-burn/spicy liking (derived food-liking factor) \|\| id:ebi-fl187-GCST90094716 | Weighted median | 15 | -0.012154426 | 0.147571927353885 | 0.934358279278683 | -0.301395403 | 0.277086552068684 | 0.987919141130031 | 0.739785201503404 | 1.31928055255456 | 0.999332001504091 |
| ebi-fl187-GCST90094716 | prot-a-2385 | Phosphoribosyl pyrophosphate synthase-associated protein 1 \|\| id:prot-a-2385 | F-burn/spicy liking (derived food-liking factor) \|\| id:ebi-fl187-GCST90094716 | Weighted median | 15 | 0.349511248531663 | 0.143479391028452 | 0.0148519859440135 | 0.0682916421158977 | 0.630730854947429 | 1.41837414670961 | 1.07067751760658 | 1.87898334182959 | 0.999332001504091 |
| ebi-fl187-GCST90094716 | prot-a-2393 | Trypsin-3 \|\| id:prot-a-2393 | F-burn/spicy liking (derived food-liking factor) \|\| id:ebi-fl187-GCST90094716 | Weighted median | 15 | 0.213937479342694 | 0.156085959890175 | 0.170487568317383 | -0.091991002 | 0.519865960727436 | 1.23854521771165 | 0.912113356665797 | 1.68180220704571 | 0.999332001504091 |
| ebi-fl187-GCST90094716 | prot-a-2405 | Pregnancy-specific beta-1-glycoprotein 6 \|\| id:prot-a-2405 | F-burn/spicy liking (derived food-liking factor) \|\| id:ebi-fl187-GCST90094716 | Weighted median | 15 | 0.196949078805392 | 0.146378742125502 | 0.178471579077764 | -0.089953256 | 0.483851413371375 | 1.21768203330339 | 0.913973907287853 | 1.62231057408391 | 0.999332001504091 |
| ebi-fl187-GCST90094716 | prot-a-244 | Beclin-1 \|\| id:prot-a-244 | F-burn/spicy liking (derived food-liking factor) \|\| id:ebi-fl187-GCST90094716 | Weighted median | 15 | -0.025939681 | 0.142193935018791 | 0.855249471443572 | -0.304639793 | 0.252760431812556 | 0.974393862470783 | 0.737388938877217 | 1.2875747779271 | 0.999332001504091 |
| ebi-fl187-GCST90094716 | prot-a-2454 | tRNA pseudouridine synthase A, mitochondrial \|\| id:prot-a-2454 | F-burn/spicy liking (derived food-liking factor) \|\| id:ebi-fl187-GCST90094716 | Weighted median | 15 | -0.234302725 | 0.15244131123684 | 0.124292619406316 | -0.533087695 | 0.064482245143386 | 0.791122287241652 | 0.586790340065413 | 1.06660664062856 | 0.999332001504091 |
| ebi-fl187-GCST90094716 | prot-a-2466 | Glutaminyl-peptide cyclotransferase-like protein \|\| id:prot-a-2466 | F-burn/spicy liking (derived food-liking factor) \|\| id:ebi-fl187-GCST90094716 | Weighted median | 15 | -0.160337377 | 0.153164634171371 | 0.295177861481842 | -0.46054006 | 0.139865305870583 | 0.851856343652848 | 0.630942806455146 | 1.15011887416326 | 0.999332001504091 |
| ebi-fl187-GCST90094716 | prot-a-2474 | Ras-related protein Rab-27A \|\| id:prot-a-2474 | F-burn/spicy liking (derived food-liking factor) \|\| id:ebi-fl187-GCST90094716 | Weighted median | 15 | 0.157408600903096 | 0.154579881029411 | 0.308535691012172 | -0.145567966 | 0.460385167720741 | 1.17047377284681 | 0.864531129428248 | 1.58468423667785 | 0.999332001504091 |
| ebi-fl187-GCST90094716 | prot-a-2490 | Rap1 GTPase-GDP dissociation stimulator 1 \|\| id:prot-a-2490 | F-burn/spicy liking (derived food-liking factor) \|\| id:ebi-fl187-GCST90094716 | Weighted median | 15 | -0.088366772 | 0.15145690147184 | 0.559593773567435 | -0.385222298 | 0.208488755360499 | 0.915425063297834 | 0.680299389922205 | 1.23181507866657 | 0.999332001504091 |
| ebi-fl187-GCST90094716 | prot-a-2545 | Receptor-interacting serine/threonine-protein kinase 2 \|\| id:prot-a-2545 | F-burn/spicy liking (derived food-liking factor) \|\| id:ebi-fl187-GCST90094716 | Weighted median | 15 | 0.0085772966242983 | 0.146940617133875 | 0.953451889685348 | -0.279426313 | 0.296580906206694 | 1.00861418703088 | 0.756217449189038 | 1.34525139478189 | 0.999332001504091 |
| ebi-fl187-GCST90094716 | prot-a-2610 | RUN and FYVE domain-containing protein 1 \|\| id:prot-a-2610 | F-burn/spicy liking (derived food-liking factor) \|\| id:ebi-fl187-GCST90094716 | Weighted median | 15 | 0.0215275499323618 | 0.151907100570441 | 0.887304943330156 | -0.276210367 | 0.319265467050426 | 1.0217609393944 | 0.758653318209733 | 1.37611659003316 | 0.999332001504091 |
| ebi-fl187-GCST90094716 | prot-a-2627 | Serine--tRNA ligase, mitochondrial \|\| id:prot-a-2627 | F-burn/spicy liking (derived food-liking factor) \|\| id:ebi-fl187-GCST90094716 | Weighted median | 15 | -0.166389508 | 0.146685071424543 | 0.256655489183228 | -0.453892248 | 0.12111323187561 | 0.846716367053271 | 0.635151168298569 | 1.12875271591861 | 0.999332001504091 |
| ebi-fl187-GCST90094716 | prot-a-264 | BolA-like protein 2 \|\| id:prot-a-264 | F-burn/spicy liking (derived food-liking factor) \|\| id:ebi-fl187-GCST90094716 | Weighted median | 15 | -0.015116659 | 0.149101757400222 | 0.919244964101221 | -0.307356104 | 0.277122785505646 | 0.984997024133584 | 0.735388679789402 | 1.31932835548933 | 0.999332001504091 |
| ebi-fl187-GCST90094716 | prot-a-2646 | Secretoglobin family 1C member 1 \|\| id:prot-a-2646 | F-burn/spicy liking (derived food-liking factor) \|\| id:ebi-fl187-GCST90094716 | Weighted median | 15 | -0.126630844 | 0.140990814449513 | 0.369105798367724 | -0.40297284 | 0.149711152593934 | 0.881058860976113 | 0.668330250894492 | 1.16149869838388 | 0.999332001504091 |
| ebi-fl187-GCST90094716 | prot-a-2672 | Semaphorin-3E \|\| id:prot-a-2672 | F-burn/spicy liking (derived food-liking factor) \|\| id:ebi-fl187-GCST90094716 | Weighted median | 15 | 0.0767045778243621 | 0.157857415738455 | 0.627030614434794 | -0.232695957 | 0.386105112671734 | 1.07972305513791 | 0.792394458872936 | 1.47123930858189 | 0.999332001504091 |
| ebi-fl187-GCST90094716 | prot-a-2701 | Plasma protease C1 inhibitor \|\| id:prot-a-2701 | F-burn/spicy liking (derived food-liking factor) \|\| id:ebi-fl187-GCST90094716 | Weighted median | 15 | 0.00984216757831954 | 0.15293549137907 | 0.948687539522952 | -0.289911396 | 0.309595730681296 | 1.00989076100033 | 0.748329870016692 | 1.36287403458996 | 0.999332001504091 |
| ebi-fl187-GCST90094716 | prot-a-2738 | Signaling threshold-regulating transmembrane adapter 1 \|\| id:prot-a-2738 | F-burn/spicy liking (derived food-liking factor) \|\| id:ebi-fl187-GCST90094716 | Weighted median | 15 | 0.0883271045644625 | 0.149629352765149 | 0.554985518216997 | -0.204946427 | 0.381600635984154 | 1.09234537475356 | 0.814690960799107 | 1.46462704897954 | 0.999332001504091 |
| ebi-fl187-GCST90094716 | prot-a-274 | Serine/threonine-protein kinase BRSK2 \|\| id:prot-a-274 | F-burn/spicy liking (derived food-liking factor) \|\| id:ebi-fl187-GCST90094716 | Weighted median | 15 | -0.029671984 | 0.148127218494816 | 0.841234864203966 | -0.320001332 | 0.260657364226353 | 0.970763907400848 | 0.726148069645342 | 1.29778292238996 | 0.999332001504091 |
| ebi-fl187-GCST90094716 | prot-a-2749 | Mitochondrial glutamate carrier 2 \|\| id:prot-a-2749 | F-burn/spicy liking (derived food-liking factor) \|\| id:ebi-fl187-GCST90094716 | Weighted median | 15 | 0.056257084862807 | 0.152830359055322 | 0.712797550270444 | -0.243290419 | 0.355804588611239 | 1.05786961104474 | 0.784043779583255 | 1.42732860474549 | 0.999332001504091 |
| ebi-fl187-GCST90094716 | prot-a-2760 | Sodium-coupled monocarboxylate transporter 1 \|\| id:prot-a-2760 | F-burn/spicy liking (derived food-liking factor) \|\| id:ebi-fl187-GCST90094716 | Weighted median | 15 | -0.081824845 | 0.159386758400932 | 0.607690485686117 | -0.394222892 | 0.230573201154372 | 0.921433337932091 | 0.674203765016292 | 1.25932164771011 | 0.999332001504091 |
| ebi-fl187-GCST90094716 | prot-a-2777 | Small integral membrane protein 10 \|\| id:prot-a-2777 | F-burn/spicy liking (derived food-liking factor) \|\| id:ebi-fl187-GCST90094716 | Weighted median | 15 | 0.208593528647576 | 0.140150574773848 | 0.136657824112868 | -0.066101598 | 0.483288655204318 | 1.23194414674276 | 0.936035760184372 | 1.62139786240091 | 0.999332001504091 |
| ebi-fl187-GCST90094716 | prot-a-2799 | Superoxide dismutase [Mn], mitochondrial \|\| id:prot-a-2799 | F-burn/spicy liking (derived food-liking factor) \|\| id:ebi-fl187-GCST90094716 | Weighted median | 15 | 0.206416593572786 | 0.142125005118607 | 0.146401686505235 | -0.072148416 | 0.484981603605255 | 1.22926520132263 | 0.930392799958126 | 1.62414513015446 | 0.999332001504091 |
| ebi-fl187-GCST90094716 | prot-a-2805 | Protein SPACA7 \|\| id:prot-a-2805 | F-burn/spicy liking (derived food-liking factor) \|\| id:ebi-fl187-GCST90094716 | Weighted median | 15 | -0.325427987 | 0.14559587585981 | 0.0254077890735304 | -0.610795903 | -0.04006007 | 0.722218187671932 | 0.542918586301417 | 0.960731726201299 | 0.999332001504091 |
| ebi-fl187-GCST90094716 | prot-a-2811 | SPARC-like protein 1 \|\| id:prot-a-2811 | F-burn/spicy liking (derived food-liking factor) \|\| id:ebi-fl187-GCST90094716 | Weighted median | 15 | 0.195125818545287 | 0.151842373679284 | 0.198773025928092 | -0.102485234 | 0.492736870956682 | 1.2154639047699 | 0.902591477441131 | 1.63678977779274 | 0.999332001504091 |
| ebi-fl187-GCST90094716 | prot-a-2831 | Sepiapterin reductase \|\| id:prot-a-2831 | F-burn/spicy liking (derived food-liking factor) \|\| id:ebi-fl187-GCST90094716 | Weighted median | 15 | -0.07251776 | 0.164547821796239 | 0.659423443940019 | -0.39503149 | 0.249995971034035 | 0.93004922913078 | 0.673658825124116 | 1.28402024340346 | 0.999332001504091 |
| ebi-fl187-GCST90094716 | prot-a-2834 | SPRY domain-containing SOCS box protein 1 \|\| id:prot-a-2834 | F-burn/spicy liking (derived food-liking factor) \|\| id:ebi-fl187-GCST90094716 | Weighted median | 15 | -0.165415233 | 0.145895123220116 | 0.256880343409605 | -0.451369674 | 0.120539208849382 | 0.847541704014057 | 0.636755406634791 | 1.12810497179656 | 0.999332001504091 |
| ebi-fl187-GCST90094716 | prot-a-2835 | Spectrin alpha chain, non-erythrocytic 1 \|\| id:prot-a-2835 | F-burn/spicy liking (derived food-liking factor) \|\| id:ebi-fl187-GCST90094716 | Weighted median | 15 | 0.0653933995326583 | 0.155647021117504 | 0.674383839826114 | -0.239674762 | 0.370461560922967 | 1.06757892683496 | 0.786883744060007 | 1.4484029866236 | 0.999332001504091 |
| ebi-fl187-GCST90094716 | prot-a-2859 | Sia-alpha-2,3-Gal-beta-1,4-GlcNAc-R:alpha 2,8-sialyltransferase \|\| id:prot-a-2859 | F-burn/spicy liking (derived food-liking factor) \|\| id:ebi-fl187-GCST90094716 | Weighted median | 15 | -0.099718946 | 0.145835984781648 | 0.494117493198299 | -0.385557476 | 0.186119584585238 | 0.905091762326152 | 0.680071407185142 | 1.20456629932634 | 0.999332001504091 |
| ebi-fl187-GCST90094716 | prot-a-2861 | Alpha-2,8-sialyltransferase 8F \|\| id:prot-a-2861 | F-burn/spicy liking (derived food-liking factor) \|\| id:ebi-fl187-GCST90094716 | Weighted median | 15 | -0.13864529 | 0.151253109711045 | 0.359329101521148 | -0.435101385 | 0.157810805421939 | 0.87053676213241 | 0.647199047705764 | 1.17094463737301 | 0.999332001504091 |
| ebi-fl187-GCST90094716 | prot-a-289 | Uncharacterized protein C17orf89 \|\| id:prot-a-289 | F-burn/spicy liking (derived food-liking factor) \|\| id:ebi-fl187-GCST90094716 | Weighted median | 15 | -0.12202886 | 0.152107058191555 | 0.422404662216838 | -0.420158694 | 0.176100974387068 | 0.885122823782854 | 0.656942558881489 | 1.19255847043191 | 0.999332001504091 |
| ebi-fl187-GCST90094716 | prot-a-2921 | Tachykinin-3 \|\| id:prot-a-2921 | F-burn/spicy liking (derived food-liking factor) \|\| id:ebi-fl187-GCST90094716 | Weighted median | 15 | 0.0393886206830091 | 0.150928128958782 | 0.794111008651673 | -0.256430512 | 0.335207753442221 | 1.04017463849207 | 0.773808763055681 | 1.3982308423202 | 0.999332001504091 |
| ebi-fl187-GCST90094716 | prot-a-2928 | Tax1-binding protein 1 \|\| id:prot-a-2928 | F-burn/spicy liking (derived food-liking factor) \|\| id:ebi-fl187-GCST90094716 | Weighted median | 15 | -0.028885169 | 0.160648517920725 | 0.857306821634207 | -0.343756264 | 0.28598592643435 | 0.971528019896418 | 0.709101740741445 | 1.33107372216706 | 0.999332001504091 |
| ebi-fl187-GCST90094716 | prot-a-296 | Uncharacterized protein C1orf185 \|\| id:prot-a-296 | F-burn/spicy liking (derived food-liking factor) \|\| id:ebi-fl187-GCST90094716 | Weighted median | 15 | -0.053402561 | 0.149227047137041 | 0.720447234427477 | -0.345887573 | 0.239082451432034 | 0.947998308551988 | 0.707592034840238 | 1.27008325244976 | 0.999332001504091 |
| ebi-fl187-GCST90094716 | prot-a-2964 | Transforming growth factor beta-2 \|\| id:prot-a-2964 | F-burn/spicy liking (derived food-liking factor) \|\| id:ebi-fl187-GCST90094716 | Weighted median | 15 | -0.021930068 | 0.144927818454164 | 0.879725519654481 | -0.305988593 | 0.262128455747497 | 0.97830864732592 | 0.736395019766017 | 1.29969348480491 | 0.999332001504091 |
| ebi-fl187-GCST90094716 | prot-a-2977 | Thymocyte nuclear protein 1 \|\| id:prot-a-2977 | F-burn/spicy liking (derived food-liking factor) \|\| id:ebi-fl187-GCST90094716 | Weighted median | 15 | -0.068283371 | 0.152124866293372 | 0.653529788992152 | -0.366448109 | 0.229881366575815 | 0.933995768423675 | 0.693192105776932 | 1.25845070675697 | 0.999332001504091 |
| ebi-fl187-GCST90094716 | prot-a-3015 | Transmembrane protein 70, mitochondrial \|\| id:prot-a-3015 | F-burn/spicy liking (derived food-liking factor) \|\| id:ebi-fl187-GCST90094716 | Weighted median | 15 | -0.033487935 | 0.152667344260903 | 0.826375616705925 | -0.332715929 | 0.265740060176455 | 0.967066579230377 | 0.716973836470687 | 1.30439595016181 | 0.999332001504091 |
| ebi-fl187-GCST90094716 | prot-a-3030 | Tumor necrosis factor alpha-induced protein 3 \|\| id:prot-a-3030 | F-burn/spicy liking (derived food-liking factor) \|\| id:ebi-fl187-GCST90094716 | Weighted median | 15 | -0.106994886 | 0.160218561444255 | 0.504257558743414 | -0.421023266 | 0.207033494564699 | 0.898530268182713 | 0.656374829819925 | 1.23002377020154 | 0.999332001504091 |
| ebi-fl187-GCST90094716 | prot-a-3032 | Tumor necrosis factor receptor superfamily member 10A \|\| id:prot-a-3032 | F-burn/spicy liking (derived food-liking factor) \|\| id:ebi-fl187-GCST90094716 | Weighted median | 15 | 0.0889038660584053 | 0.156533518477628 | 0.570066081389077 | -0.21790183 | 0.395709562274556 | 1.09297557922518 | 0.804204386409254 | 1.48543782770999 | 0.999332001504091 |
| ebi-fl187-GCST90094716 | prot-a-3081 | Tumor protein p53-inducible protein 11 \|\| id:prot-a-3081 | F-burn/spicy liking (derived food-liking factor) \|\| id:ebi-fl187-GCST90094716 | Weighted median | 15 | 0.13798977731074 | 0.15234518539748 | 0.365057329372203 | -0.160606786 | 0.436586340689801 | 1.14796381493683 | 0.851626876830262 | 1.54741584167615 | 0.999332001504091 |
| ebi-fl187-GCST90094716 | prot-a-315 | Complement C4 \|\| id:prot-a-315 | F-burn/spicy liking (derived food-liking factor) \|\| id:ebi-fl187-GCST90094716 | Weighted median | 15 | -0.126931367 | 0.151881197356865 | 0.403307947640979 | -0.424618514 | 0.170755779699304 | 0.880794121959871 | 0.654019236767717 | 1.1862010192746 | 0.999332001504091 |
| ebi-fl187-GCST90094716 | prot-a-3157 | UDP-glucuronosyltransferase 1-8 \|\| id:prot-a-3157 | F-burn/spicy liking (derived food-liking factor) \|\| id:ebi-fl187-GCST90094716 | Weighted median | 15 | 0.196225834903761 | 0.153505085260628 | 0.201143014810337 | -0.104644132 | 0.497095802014592 | 1.21680167059553 | 0.9006449761032 | 1.64394000394049 | 0.999332001504091 |
| ebi-fl187-GCST90094716 | prot-a-317 | Glutaredoxin-like protein C5orf63 \|\| id:prot-a-317 | F-burn/spicy liking (derived food-liking factor) \|\| id:ebi-fl187-GCST90094716 | Weighted median | 15 | 0.000187044043384071 | 0.1464551250802 | 0.998980988109812 | -0.286865001 | 0.287239089200577 | 1.00018706153721 | 0.750613053923768 | 1.33274281980185 | 0.999758154020451 |
| ebi-fl187-GCST90094716 | prot-a-3192 | Guanine nucleotide exchange factor VAV3 \|\| id:prot-a-3192 | F-burn/spicy liking (derived food-liking factor) \|\| id:ebi-fl187-GCST90094716 | Weighted median | 15 | -0.197938655 | 0.151030502612218 | 0.189997241026219 | -0.49395844 | 0.0980811304159009 | 0.820420180512921 | 0.610206142838394 | 1.10305227256802 | 0.999332001504091 |
| ebi-fl187-GCST90094716 | prot-a-3233 | Serine/threonine-protein kinase WNK3 \|\| id:prot-a-3233 | F-burn/spicy liking (derived food-liking factor) \|\| id:ebi-fl187-GCST90094716 | Weighted median | 15 | 0.198422680904049 | 0.14445253805901 | 0.169560145656402 | -0.084704294 | 0.481549655499708 | 1.219477735 | 0.918783934440624 | 1.61858070222745 | 0.999332001504091 |
| ebi-fl187-GCST90094716 | prot-a-3250 | 14-3-3 protein theta \|\| id:prot-a-3250 | F-burn/spicy liking (derived food-liking factor) \|\| id:ebi-fl187-GCST90094716 | Weighted median | 15 | 0.112236581913666 | 0.144538148738974 | 0.437442757095478 | -0.17105819 | 0.395531353442055 | 1.11877751186269 | 0.842772531435861 | 1.48517313315512 | 0.999332001504091 |
| ebi-fl187-GCST90094716 | prot-a-3278 | E3 ubiquitin-protein ligase ZNRF3 \|\| id:prot-a-3278 | F-burn/spicy liking (derived food-liking factor) \|\| id:ebi-fl187-GCST90094716 | Weighted median | 15 | 0.053434310916772 | 0.139765342596339 | 0.702228133348492 | -0.220505761 | 0.327374382405596 | 1.05488769488601 | 0.802113018218486 | 1.38732076845411 | 0.999332001504091 |
| ebi-fl187-GCST90094716 | prot-a-355 | Calpain-3 \|\| id:prot-a-355 | F-burn/spicy liking (derived food-liking factor) \|\| id:ebi-fl187-GCST90094716 | Weighted median | 15 | 0.106253458840481 | 0.153891268567243 | 0.489914378320454 | -0.195373428 | 0.407880345232278 | 1.11210371330568 | 0.822527446288762 | 1.50362723423832 | 0.999332001504091 |
| ebi-fl187-GCST90094716 | prot-a-385 | Coiled-coil domain-containing protein 90B, mitochondrial \|\| id:prot-a-385 | F-burn/spicy liking (derived food-liking factor) \|\| id:ebi-fl187-GCST90094716 | Weighted median | 15 | -0.133515855 | 0.153236654063971 | 0.383587667909203 | -0.433859697 | 0.166827986610985 | 0.875013595216975 | 0.648003165688665 | 1.18155100523443 | 0.999332001504091 |
| ebi-fl187-GCST90094716 | prot-a-390 | C-C motif chemokine 14 \|\| id:prot-a-390 | F-burn/spicy liking (derived food-liking factor) \|\| id:ebi-fl187-GCST90094716 | Weighted median | 15 | -0.410799884 | 0.154132629551373 | 0.00769342349913598 | -0.712899838 | -0.10869993 | 0.66311961891214 | 0.490220573919493 | 0.89699953935103 | 0.999332001504091 |
| ebi-fl187-GCST90094716 | prot-a-40 | Pituitary adenylate cyclase-activating polypeptide 27 \|\| id:prot-a-40 | F-burn/spicy liking (derived food-liking factor) \|\| id:ebi-fl187-GCST90094716 | Weighted median | 15 | 0.190814479555426 | 0.147713211502908 | 0.196429808869941 | -0.098703415 | 0.480332374101126 | 1.21023490793502 | 0.906011377573128 | 1.61661163274549 | 0.999332001504091 |
| ebi-fl187-GCST90094716 | prot-a-401 | C-C motif chemokine 24 \|\| id:prot-a-401 | F-burn/spicy liking (derived food-liking factor) \|\| id:ebi-fl187-GCST90094716 | Weighted median | 15 | 0.0322712234493471 | 0.142520141372418 | 0.820864880815605 | -0.247068254 | 0.311610700539285 | 1.03279758624496 | 0.781087379654683 | 1.36562295325395 | 0.999332001504091 |
| ebi-fl187-GCST90094716 | prot-a-412 | Cerebral cavernous malformations 2 protein \|\| id:prot-a-412 | F-burn/spicy liking (derived food-liking factor) \|\| id:ebi-fl187-GCST90094716 | Weighted median | 15 | -0.130590397 | 0.159122178263612 | 0.411821376202673 | -0.442469867 | 0.181289072201878 | 0.877577158849621 | 0.642447699815435 | 1.19876165788409 | 0.999332001504091 |
| ebi-fl187-GCST90094716 | prot-a-44 | Adhesion G protein-coupled receptor F5 \|\| id:prot-a-44 | F-burn/spicy liking (derived food-liking factor) \|\| id:ebi-fl187-GCST90094716 | Weighted median | 15 | 0.0214439898061561 | 0.142977648395383 | 0.880779279255099 | -0.258792201 | 0.301680180661106 | 1.02167556448837 | 0.771983423723378 | 1.35212872063773 | 0.999332001504091 |
| ebi-fl187-GCST90094716 | prot-a-48 | Adrenomedullin \|\| id:prot-a-48 | F-burn/spicy liking (derived food-liking factor) \|\| id:ebi-fl187-GCST90094716 | Weighted median | 15 | -0.057066661 | 0.156429194854891 | 0.715254546748421 | -0.363667883 | 0.249534561184823 | 0.944531104134261 | 0.695122018495748 | 1.28342792047889 | 0.999332001504091 |
| ebi-fl187-GCST90094716 | prot-a-502 | Carcinoembryonic antigen-related cell adhesion molecule 1 \|\| id:prot-a-502 | F-burn/spicy liking (derived food-liking factor) \|\| id:ebi-fl187-GCST90094716 | Weighted median | 15 | -0.010604927 | 0.1428305084428 | 0.940812786565205 | -0.290552724 | 0.269342869176154 | 0.989451106616825 | 0.747850098684182 | 1.30910391548761 | 0.999332001504091 |
| ebi-fl187-GCST90094716 | prot-a-518 | Complement factor B \|\| id:prot-a-518 | F-burn/spicy liking (derived food-liking factor) \|\| id:ebi-fl187-GCST90094716 | Weighted median | 15 | 0.111647369747346 | 0.147590026784729 | 0.449367687386745 | -0.177629083 | 0.400923822245416 | 1.1181185087067 | 0.837252917450515 | 1.49320351527635 | 0.999332001504091 |
| ebi-fl187-GCST90094716 | prot-a-525 | Properdin \|\| id:prot-a-525 | F-burn/spicy liking (derived food-liking factor) \|\| id:ebi-fl187-GCST90094716 | Weighted median | 15 | -0.097377319 | 0.150355812182016 | 0.517214030366719 | -0.392074711 | 0.197320073014941 | 0.907213632730043 | 0.675653633492964 | 1.21813387009605 | 0.999332001504091 |
| ebi-fl187-GCST90094716 | prot-a-533 | Chondroadherin \|\| id:prot-a-533 | F-burn/spicy liking (derived food-liking factor) \|\| id:ebi-fl187-GCST90094716 | Weighted median | 15 | 0.215560390883399 | 0.153308637276352 | 0.159707764591106 | -0.084924538 | 0.516045319945048 | 1.24055689898801 | 0.918581599627031 | 1.67538890421015 | 0.999332001504091 |
| ebi-fl187-GCST90094716 | prot-a-535 | Coiled-coil-helix-coiled-coil-helix domain-containing protein 2 \|\| id:prot-a-535 | F-burn/spicy liking (derived food-liking factor) \|\| id:ebi-fl187-GCST90094716 | Weighted median | 15 | -0.070563072 | 0.16379691506218 | 0.666616636899956 | -0.391605025 | 0.250478881621372 | 0.931868962925735 | 0.675971052621161 | 1.28464046011591 | 0.999332001504091 |
| ebi-fl187-GCST90094716 | prot-a-591 | Caseinolytic peptidase B protein homolog \|\| id:prot-a-591 | F-burn/spicy liking (derived food-liking factor) \|\| id:ebi-fl187-GCST90094716 | Weighted median | 15 | 0.24177851550977 | 0.16285303823144 | 0.137638593712518 | -0.077413439 | 0.560970470443392 | 1.27351209839966 | 0.92550713336014 | 1.7523723008834 | 0.999332001504091 |
| ebi-fl187-GCST90094716 | prot-a-605 | Ciliary neurotrophic factor receptor subunit alpha \|\| id:prot-a-605 | F-burn/spicy liking (derived food-liking factor) \|\| id:ebi-fl187-GCST90094716 | Weighted median | 15 | 0.138954611149431 | 0.146309296159997 | 0.342248477951353 | -0.147811609 | 0.425720831623025 | 1.14907194376521 | 0.862593604227904 | 1.53069339428988 | 0.999332001504091 |
| ebi-fl187-GCST90094716 | prot-a-606 | Ciliary neurotrophic factor receptor subunit alpha \|\| id:prot-a-606 | F-burn/spicy liking (derived food-liking factor) \|\| id:ebi-fl187-GCST90094716 | Weighted median | 15 | 0.141097566772899 | 0.149807021198589 | 0.346263208026751 | -0.152524195 | 0.434719328322134 | 1.15153699425243 | 0.858538121567277 | 1.54452949242511 | 0.999332001504091 |
| ebi-fl187-GCST90094716 | prot-a-707 | Cancer/testis antigen 55 \|\| id:prot-a-707 | F-burn/spicy liking (derived food-liking factor) \|\| id:ebi-fl187-GCST90094716 | Weighted median | 15 | 0.0443115829442704 | 0.160606083304202 | 0.782623130427575 | -0.27047634 | 0.359099506220507 | 1.04530800429806 | 0.763015952486801 | 1.43203929129973 | 0.999332001504091 |
| ebi-fl187-GCST90094716 | prot-a-719 | Dipeptidyl peptidase 1 \|\| id:prot-a-719 | F-burn/spicy liking (derived food-liking factor) \|\| id:ebi-fl187-GCST90094716 | Weighted median | 15 | -0.184972679 | 0.147610491579534 | 0.210164455786742 | -0.474289242 | 0.104343884899145 | 0.831126991097611 | 0.622327223304554 | 1.10998209537255 | 0.999332001504091 |
| ebi-fl187-GCST90094716 | prot-a-752 | Cytochrome b5 \|\| id:prot-a-752 | F-burn/spicy liking (derived food-liking factor) \|\| id:ebi-fl187-GCST90094716 | Weighted median | 15 | 0.0824240491122828 | 0.146106847402865 | 0.57266196776878 | -0.203945372 | 0.368793470021899 | 1.08591619400798 | 0.815506919647763 | 1.44598893277093 | 0.999332001504091 |
| ebi-fl187-GCST90094716 | prot-a-755 | Cytoglobin \|\| id:prot-a-755 | F-burn/spicy liking (derived food-liking factor) \|\| id:ebi-fl187-GCST90094716 | Weighted median | 15 | 0.148276520541882 | 0.145830635233595 | 0.309261859725477 | -0.137551525 | 0.434104565599729 | 1.15983356983175 | 0.871489445768472 | 1.5435802650742 | 0.999332001504091 |
| ebi-fl187-GCST90094716 | prot-a-807 | Desmin \|\| id:prot-a-807 | F-burn/spicy liking (derived food-liking factor) \|\| id:ebi-fl187-GCST90094716 | Weighted median | 15 | 0.010485083696919 | 0.162508823915915 | 0.948556112146248 | -0.308032211 | 0.329002378572113 | 1.01054024480804 | 0.734891645901999 | 1.38958116080268 | 0.999332001504091 |
| ebi-fl187-GCST90094716 | prot-a-82 | Ameloblastin \|\| id:prot-a-82 | F-burn/spicy liking (derived food-liking factor) \|\| id:ebi-fl187-GCST90094716 | Weighted median | 15 | -0.213552072 | 0.148583474168305 | 0.150646064767298 | -0.504775681 | 0.0776715373646585 | 0.807710099970952 | 0.603640968152829 | 1.08076760858602 | 0.999332001504091 |
| ebi-fl187-GCST90094716 | prot-a-84 | Muellerian-inhibiting factor \|\| id:prot-a-84 | F-burn/spicy liking (derived food-liking factor) \|\| id:ebi-fl187-GCST90094716 | Weighted median | 15 | -0.060455495 | 0.142334318680884 | 0.671024087411103 | -0.33943076 | 0.218519769563209 | 0.941335662181505 | 0.712175606479948 | 1.24423361433911 | 0.999332001504091 |
| ebi-fl187-GCST90094716 | prot-a-858 | Inactive dipeptidyl peptidase 10 \|\| id:prot-a-858 | F-burn/spicy liking (derived food-liking factor) \|\| id:ebi-fl187-GCST90094716 | Weighted median | 15 | -0.102423435 | 0.157988247550979 | 0.516792349795712 | -0.4120804 | 0.207233530204624 | 0.902647258298822 | 0.662271027191671 | 1.23026984340442 | 0.999332001504091 |
| ebi-fl187-GCST90094716 | prot-a-863 | Dihydropyrimidinase-related protein 3 \|\| id:prot-a-863 | F-burn/spicy liking (derived food-liking factor) \|\| id:ebi-fl187-GCST90094716 | Weighted median | 15 | -0.014171219 | 0.143554906082805 | 0.921363472417035 | -0.295538835 | 0.267196396959372 | 0.98592872011735 | 0.744130515938801 | 1.30629697389292 | 0.999332001504091 |
| ebi-fl187-GCST90094716 | prot-a-864 | Dihydropyrimidinase-related protein 5 \|\| id:prot-a-864 | F-burn/spicy liking (derived food-liking factor) \|\| id:ebi-fl187-GCST90094716 | Weighted median | 15 | 0.00418797484128088 | 0.165913881593555 | 0.979862048755235 | -0.321003233 | 0.329379182764648 | 1.00419675666299 | 0.725420905641015 | 1.39010485946967 | 0.999332001504091 |
| ebi-fl187-GCST90094716 | prot-a-884 | Endothelin-converting enzyme 1 \|\| id:prot-a-884 | F-burn/spicy liking (derived food-liking factor) \|\| id:ebi-fl187-GCST90094716 | Weighted median | 15 | -0.078168438 | 0.143104514522895 | 0.584905644190897 | -0.358653286 | 0.202316410892657 | 0.924808640901591 | 0.698616529471366 | 1.22423530822184 | 0.999332001504091 |
| ebi-fl187-GCST90094716 | prot-a-893 | Early endosome antigen 1 \|\| id:prot-a-893 | F-burn/spicy liking (derived food-liking factor) \|\| id:ebi-fl187-GCST90094716 | Weighted median | 15 | -0.115283896 | 0.149510454555986 | 0.440661991335117 | -0.408324387 | 0.177756594437171 | 0.891113124102405 | 0.664763203021171 | 1.1945345294966 | 0.999332001504091 |
| ebi-fl187-GCST90094716 | prot-a-897 | Ephrin-A2 \|\| id:prot-a-897 | F-burn/spicy liking (derived food-liking factor) \|\| id:ebi-fl187-GCST90094716 | Weighted median | 15 | 0.184614070698061 | 0.145842020168766 | 0.20556691426943 | -0.101236289 | 0.470464430228842 | 1.20275417246803 | 0.90371946883706 | 1.6007374514691 | 0.999332001504091 |
| ebi-fl187-GCST90094716 | prot-a-90 | Anaphase-promoting complex subunit 10 \|\| id:prot-a-90 | F-burn/spicy liking (derived food-liking factor) \|\| id:ebi-fl187-GCST90094716 | Weighted median | 15 | 0.150603765998314 | 0.150378205386128 | 0.316585161639432 | -0.144137517 | 0.445345048555126 | 1.16253593054603 | 0.865768682342988 | 1.56102873362553 | 0.999332001504091 |
| ebi-fl187-GCST90094716 | prot-a-914 | Histone-lysine N-methyltransferase EHMT2 \|\| id:prot-a-914 | F-burn/spicy liking (derived food-liking factor) \|\| id:ebi-fl187-GCST90094716 | Weighted median | 15 | -0.034994591 | 0.142142575346142 | 0.805532379536181 | -0.313594038 | 0.243604857168487 | 0.965610639704584 | 0.73081565119225 | 1.27584009180643 | 0.999332001504091 |
| ebi-fl187-GCST90094716 | prot-a-932 | RNA polymerase II elongation factor ELL \|\| id:prot-a-932 | F-burn/spicy liking (derived food-liking factor) \|\| id:ebi-fl187-GCST90094716 | Weighted median | 15 | 0.0202330658930637 | 0.14447030616298 | 0.888620488005156 | -0.262928734 | 0.303394865972504 | 1.02043914187385 | 0.768796684280134 | 1.35444918475846 | 0.999332001504091 |
| ebi-fl187-GCST90094716 | prot-a-991 | Estrogen receptor \|\| id:prot-a-991 | F-burn/spicy liking (derived food-liking factor) \|\| id:ebi-fl187-GCST90094716 | Weighted median | 15 | 0.0682351218084313 | 0.137900126028701 | 0.620730351894029 | -0.202049125 | 0.338519368824685 | 1.07061700429421 | 0.817054788970017 | 1.40286892061284 | 0.999332001504091 |
| ebi-fl187-GCST90094716 | prot-a-992 | Persulfide dioxygenase ETHE1, mitochondrial \|\| id:prot-a-992 | F-burn/spicy liking (derived food-liking factor) \|\| id:ebi-fl187-GCST90094716 | Weighted median | 15 | 0.0597919777825297 | 0.171397844506147 | 0.727202626022732 | -0.276147797 | 0.395731753014577 | 1.06161568392532 | 0.758700788432793 | 1.48547079104038 | 0.999332001504091 |
| ebi-fl187-GCST90094716 | prot-a-993 | Ethanolamine kinase 1 \|\| id:prot-a-993 | F-burn/spicy liking (derived food-liking factor) \|\| id:ebi-fl187-GCST90094716 | Weighted median | 15 | -0.154463809 | 0.160337323018228 | 0.335362995326376 | -0.468724962 | 0.159797344021453 | 0.85687450264529 | 0.62579967804936 | 1.17327307609401 | 0.999332001504091 |
| ebi-fl187-GCST90094716 | prot-b-10 | coagulation factor III, tissue factor \|\| id:prot-b-10 | F-burn/spicy liking (derived food-liking factor) \|\| id:ebi-fl187-GCST90094716 | Weighted median | 10 | 0.112422241130333 | 0.180980476167107 | 0.53447832917569 | -0.242299492 | 0.467143974417863 | 1.11898524250212 | 0.784821094588203 | 1.5954310881444 | 0.999332001504091 |
| ebi-fl187-GCST90094716 | prot-b-28 | prolactin \|\| id:prot-b-28 | F-burn/spicy liking (derived food-liking factor) \|\| id:ebi-fl187-GCST90094716 | Weighted median | 10 | 0.033172407859069 | 0.193958540493533 | 0.86420147916153 | -0.346986332 | 0.413331147226394 | 1.03372874683877 | 0.706814989286569 | 1.51184558652251 | 0.999332001504091 |
| ebi-fl187-GCST90094716 | prot-b-54 | matrix metallopeptidase 7 \|\| id:prot-b-54 | F-burn/spicy liking (derived food-liking factor) \|\| id:ebi-fl187-GCST90094716 | Weighted median | 10 | 0.344567882722155 | 0.192792298758164 | 0.0738972197251117 | -0.033305023 | 0.722440788288156 | 1.41137990623539 | 0.967243483230858 | 2.0594537717341 | 0.999332001504091 |
| ebi-fl187-GCST90094716 | ukb-rsfMRI191-edge-pheno716 | Net100_Pair10_33 (OccipitalPrecuneus)&(Temporal\|Frontal\|Supp_Motor_Area) (Default_mode\|Central_executive)&(Default_mode\|Salience) \|\| id:ukb-rsfMRI191-edge-pheno716 | F-burn/spicy liking (derived food-liking factor) \|\| id:ebi-fl187-GCST90094716 | Weighted median | 13 | -0.031904097 | 0.0520330058342542 | 0.539776406519475 | -0.133888788 | 0.0700805944517808 | 0.968599469236065 | 0.874687334556259 | 1.07259462294643 | 0.999332001504091 |
| ebi-fl187-GCST90094716 | ukb-rsfMRI191-edge-pheno903 | Net100_Pair27_38 (Precentral\|Frontal\|Supp_Motor_Area)&(Subcortical) (Attention\|Salience\|Motor)&(Subcortical-cerebellum) \|\| id:ukb-rsfMRI191-edge-pheno903 | F-burn/spicy liking (derived food-liking factor) \|\| id:ebi-fl187-GCST90094716 | Weighted median | 13 | 0.014771380477586 | 0.0497121364651842 | 0.766360913269688 | -0.082664407 | 0.112207167949347 | 1.01488101647807 | 0.920660062460032 | 1.11874460466484 | 0.999332001504091 |
| ebi-fl187-GCST90094716 | ukb-rsfMRI191-node-pheno23 | Net100_Node2 RolandicOper\|SupraMarginal\|Insula Salience\|Motor \|\| id:ukb-rsfMRI191-node-pheno23 | F-burn/spicy liking (derived food-liking factor) \|\| id:ebi-fl187-GCST90094716 | Weighted median | 13 | -0.094701251 | 0.0516159207839606 | 0.066545783392073 | -0.195868456 | 0.00646595372131292 | 0.909644649294406 | 0.822120372771914 | 1.00648690312837 | 0.999332001504091 |
| ebi-fl187-GCST90094716 | ukb-rsfMRI191-node-pheno55 | Net100_Node34 Parietal\|Postcentral\|Precuneus Attention \|\| id:ukb-rsfMRI191-node-pheno55 | F-burn/spicy liking (derived food-liking factor) \|\| id:ebi-fl187-GCST90094716 | Weighted median | 13 | -0.036254621 | 0.0551233900264224 | 0.510731303387853 | -0.144296465 | 0.0717872235108348 | 0.964394707130956 | 0.865631080356333 | 1.0744267069978 | 0.999332001504091 |
| ebi-fl187-GCST90094716 | ebi-a-GCST90001414 | IgD- CD24- B cell Absolute Count \|\| id:ebi-a-GCST90001414 | F-burn/spicy liking (derived food-liking factor) \|\| id:ebi-fl187-GCST90094716 | Weighted mode | 15 | 0.0721783990913738 | 0.258540923659682 | 0.784191577438292 | -0.434561811 | 0.578918609464351 | 1.07484707870287 | 0.647548353302477 | 1.78410807 | 0.999714303421225 |
| ebi-fl187-GCST90094716 | ebi-a-GCST90001422 | CD20- CD38- B cell %B cell \|\| id:ebi-a-GCST90001422 | F-burn/spicy liking (derived food-liking factor) \|\| id:ebi-fl187-GCST90094716 | Weighted mode | 15 | -0.008606852 | 0.243977819506242 | 0.972356765944864 | -0.486803378 | 0.469589674128557 | 0.991430080813218 | 0.614587862353626 | 1.59933780888067 | 0.999714303421225 |
| ebi-fl187-GCST90094716 | ebi-a-GCST90001444 | CD20- CD38- B cell %lymphocyte \|\| id:ebi-a-GCST90001444 | F-burn/spicy liking (derived food-liking factor) \|\| id:ebi-fl187-GCST90094716 | Weighted mode | 15 | -0.009838121 | 0.222808982343782 | 0.965404495443721 | -0.446543727 | 0.426867483931531 | 0.990210114541183 | 0.639835781566402 | 1.53244957407544 | 0.999714303421225 |
| ebi-fl187-GCST90094716 | ebi-a-GCST90001459 | Myeloid Dendritic Cell %Dendritic Cell \|\| id:ebi-a-GCST90001459 | F-burn/spicy liking (derived food-liking factor) \|\| id:ebi-fl187-GCST90094716 | Weighted mode | 15 | 0.0177136649436505 | 0.253892815219886 | 0.945364861692519 | -0.479916253 | 0.515343582774626 | 1.01787148237105 | 0.618835215298579 | 1.67421363395479 | 0.999714303421225 |
| ebi-fl187-GCST90094716 | ebi-a-GCST90001476 | HLA DR++ monocyte %leukocyte \|\| id:ebi-a-GCST90001476 | F-burn/spicy liking (derived food-liking factor) \|\| id:ebi-fl187-GCST90094716 | Weighted mode | 15 | 0.0720967850295295 | 0.210256032836129 | 0.73676796293403 | -0.340005039 | 0.484198609388343 | 1.07475935964652 | 0.711766735926616 | 1.62287393164561 | 0.999714303421225 |
| ebi-fl187-GCST90094716 | ebi-a-GCST90001478 | CD4 regulatory T cell %CD4+ T cell \|\| id:ebi-a-GCST90001478 | F-burn/spicy liking (derived food-liking factor) \|\| id:ebi-fl187-GCST90094716 | Weighted mode | 15 | 0.0190042339527599 | 0.286075575296544 | 0.947974190973125 | -0.541703894 | 0.579712361533986 | 1.0191859637935 | 0.581756156792465 | 1.78552477127326 | 0.999714303421225 |
| ebi-fl187-GCST90094716 | ebi-a-GCST90001485 | CD39+ resting CD4 regulatory T cell %CD4 regulatory T cell \|\| id:ebi-a-GCST90001485 | F-burn/spicy liking (derived food-liking factor) \|\| id:ebi-fl187-GCST90094716 | Weighted mode | 15 | 0.244213477906755 | 0.291518470696997 | 0.41625585463106 | -0.327162725 | 0.81558968047287 | 1.2766168308908 | 0.720966414466537 | 2.26050825698942 | 0.999714303421225 |
| ebi-fl187-GCST90094716 | ebi-a-GCST90001515 | Immature Myeloid-Derived Suppressor Cells Absolute Count \|\| id:ebi-a-GCST90001515 | F-burn/spicy liking (derived food-liking factor) \|\| id:ebi-fl187-GCST90094716 | Weighted mode | 15 | -0.002845075 | 0.298146722014042 | 0.992520922170247 | -0.587212651 | 0.581522499648418 | 0.997158967892699 | 0.555874543907334 | 1.78875974470705 | 0.999714303421225 |
| ebi-fl187-GCST90094716 | ebi-a-GCST90001517 | CD33+ HLA DR+ Absolute Count \|\| id:ebi-a-GCST90001517 | F-burn/spicy liking (derived food-liking factor) \|\| id:ebi-fl187-GCST90094716 | Weighted mode | 15 | -0.051182594 | 0.322316031755691 | 0.876097313587873 | -0.682922016 | 0.580556828631489 | 0.95010517156017 | 0.505138810171252 | 1.78703322502452 | 0.999714303421225 |
| ebi-fl187-GCST90094716 | ebi-a-GCST90001518 | CD33+ HLA DR+ CD14- Absolute Count \|\| id:ebi-a-GCST90001518 | F-burn/spicy liking (derived food-liking factor) \|\| id:ebi-fl187-GCST90094716 | Weighted mode | 14 | -0.035599526 | 0.319463205889372 | 0.91297321273994 | -0.661747409 | 0.590548358016493 | 0.965026684661341 | 0.515948972409499 | 1.8049779181832 | 0.999714303421225 |
| ebi-fl187-GCST90094716 | ebi-a-GCST90001520 | CD33+ HLA DR+ CD14dim Absolute Count \|\| id:ebi-a-GCST90001520 | F-burn/spicy liking (derived food-liking factor) \|\| id:ebi-fl187-GCST90094716 | Weighted mode | 15 | 0.135367916496459 | 0.315748554634967 | 0.674649742759228 | -0.483499251 | 0.754235083580994 | 1.14495795578829 | 0.616621897668867 | 2.12598470063884 | 0.999714303421225 |
| ebi-fl187-GCST90094716 | ebi-a-GCST90001531 | CD33dim HLA DR- Absolute Count \|\| id:ebi-a-GCST90001531 | F-burn/spicy liking (derived food-liking factor) \|\| id:ebi-fl187-GCST90094716 | Weighted mode | 15 | 0.335579438613944 | 0.351846537128629 | 0.356380599927735 | -0.354039774 | 1.02519865138606 | 1.39875064058561 | 0.701847051424598 | 2.78764917593851 | 0.999714303421225 |
| ebi-fl187-GCST90094716 | ebi-a-GCST90001532 | Basophil Absolute Count \|\| id:ebi-a-GCST90001532 | F-burn/spicy liking (derived food-liking factor) \|\| id:ebi-fl187-GCST90094716 | Weighted mode | 15 | 0.262919729756988 | 0.334542074366941 | 0.445019305995319 | -0.392782736 | 0.918622195516193 | 1.30072230546558 | 0.675175422963515 | 2.50583545904206 | 0.999714303421225 |
| ebi-fl187-GCST90094716 | ebi-a-GCST90001588 | T/B cell \|\| id:ebi-a-GCST90001588 | F-burn/spicy liking (derived food-liking factor) \|\| id:ebi-fl187-GCST90094716 | Weighted mode | 15 | -0.117092613 | 0.198657779465258 | 0.564971624144887 | -0.50646186 | 0.272276635155006 | 0.88950281018342 | 0.6026239790984 | 1.31295015924849 | 0.999714303421225 |
| ebi-fl187-GCST90094716 | ebi-a-GCST90001624 | HLA DR+ CD4+ T cell Absolute Count \|\| id:ebi-a-GCST90001624 | F-burn/spicy liking (derived food-liking factor) \|\| id:ebi-fl187-GCST90094716 | Weighted mode | 15 | 0.106381242262268 | 0.228217580588003 | 0.648285474929456 | -0.340925216 | 0.553687700214753 | 1.11224583080348 | 0.711112086244492 | 1.73965653526299 | 0.999714303421225 |
| ebi-fl187-GCST90094716 | ebi-a-GCST90001631 | CD8+ Natural Killer T %T cell \|\| id:ebi-a-GCST90001631 | F-burn/spicy liking (derived food-liking factor) \|\| id:ebi-fl187-GCST90094716 | Weighted mode | 15 | 0.0353074337729713 | 0.234953366583042 | 0.88269185013571 | -0.425201165 | 0.495816032275734 | 1.03593814222015 | 0.653638282934921 | 1.64183748492801 | 0.999714303421225 |
| ebi-fl187-GCST90094716 | ebi-a-GCST90001632 | CD8+ Natural Killer T %lymphocyte \|\| id:ebi-a-GCST90001632 | F-burn/spicy liking (derived food-liking factor) \|\| id:ebi-fl187-GCST90094716 | Weighted mode | 15 | 0.0225141913195119 | 0.227117489620624 | 0.922440369230433 | -0.422636088 | 0.467664470975934 | 1.02276954851091 | 0.655317067248487 | 1.59626172068635 | 0.999714303421225 |
| ebi-fl187-GCST90094716 | ebi-a-GCST90001646 | Natural Killer %CD3- lymphocyte \|\| id:ebi-a-GCST90001646 | F-burn/spicy liking (derived food-liking factor) \|\| id:ebi-fl187-GCST90094716 | Weighted mode | 15 | -0.17372809 | 0.194949826288643 | 0.387911713936361 | -0.555829749 | 0.208373569756472 | 0.840525414048321 | 0.573596122707529 | 1.23167319947406 | 0.999714303421225 |
| ebi-fl187-GCST90094716 | ebi-a-GCST90001684 | CD127- CD8+ T cell Absolute Count \|\| id:ebi-a-GCST90001684 | F-burn/spicy liking (derived food-liking factor) \|\| id:ebi-fl187-GCST90094716 | Weighted mode | 15 | -0.216432235 | 0.247215265549912 | 0.396084669171277 | -0.700974156 | 0.268109685030201 | 0.805387109770511 | 0.496101787823437 | 1.30749054428998 | 0.999714303421225 |
| ebi-fl187-GCST90094716 | ebi-a-GCST90001687 | CD28- CD8+ T cell Absolute Count \|\| id:ebi-a-GCST90001687 | F-burn/spicy liking (derived food-liking factor) \|\| id:ebi-fl187-GCST90094716 | Weighted mode | 15 | 0.0754878144043143 | 0.230555657227161 | 0.7481971204383 | -0.376401274 | 0.52737690256955 | 1.07841008656869 | 0.686326872813041 | 1.69448168340901 | 0.999714303421225 |
| ebi-fl187-GCST90094716 | ebi-a-GCST90001701 | BAFF-R on CD20- CD38- B cell \|\| id:ebi-a-GCST90001701 | F-burn/spicy liking (derived food-liking factor) \|\| id:ebi-fl187-GCST90094716 | Weighted mode | 15 | 0.180119480507673 | 0.245907007196037 | 0.475966816113415 | -0.301858254 | 0.662097214611906 | 1.19736041580598 | 0.739442870828914 | 1.93885426704018 | 0.999714303421225 |
| ebi-fl187-GCST90094716 | ebi-a-GCST90001732 | CD19 on IgD- CD27- B cell \|\| id:ebi-a-GCST90001732 | F-burn/spicy liking (derived food-liking factor) \|\| id:ebi-fl187-GCST90094716 | Weighted mode | 15 | -0.195088198 | 0.29433731942032 | 0.518219777294634 | -0.771989344 | 0.381812948500323 | 0.822762089244255 | 0.462092891791526 | 1.46493804064611 | 0.999714303421225 |
| ebi-fl187-GCST90094716 | ebi-a-GCST90001798 | CD27 on CD24+ CD27+ B cell \|\| id:ebi-a-GCST90001798 | F-burn/spicy liking (derived food-liking factor) \|\| id:ebi-fl187-GCST90094716 | Weighted mode | 15 | 0.119926224325812 | 0.222887891937895 | 0.598988475073793 | -0.316934044 | 0.556786492524085 | 1.12741367280733 | 0.728378794586108 | 1.74505573072754 | 0.999714303421225 |
| ebi-fl187-GCST90094716 | ebi-a-GCST90001836 | CD66b on CD66b++ myeloid cell \|\| id:ebi-a-GCST90001836 | F-burn/spicy liking (derived food-liking factor) \|\| id:ebi-fl187-GCST90094716 | Weighted mode | 15 | -0.139330529 | 0.388187457102804 | 0.725005824102401 | -0.900177945 | 0.62151688646637 | 0.869940439993018 | 0.406497318985916 | 1.86174996436192 | 0.999714303421225 |
| ebi-fl187-GCST90094716 | ebi-a-GCST90001943 | CD25 on activated & secreting CD4 regulatory T cell \|\| id:ebi-a-GCST90001943 | F-burn/spicy liking (derived food-liking factor) \|\| id:ebi-fl187-GCST90094716 | Weighted mode | 15 | 0.25048423865866 | 0.239276115945166 | 0.312910517345095 | -0.218496949 | 0.719465425911186 | 1.28464734200119 | 0.803725931935154 | 2.05333525737711 | 0.999714303421225 |
| ebi-fl187-GCST90094716 | ebi-a-GCST90001960 | CD25 on CD4+ T cell \|\| id:ebi-a-GCST90001960 | F-burn/spicy liking (derived food-liking factor) \|\| id:ebi-fl187-GCST90094716 | Weighted mode | 15 | 0.354521699712023 | 0.260175861345032 | 0.194519716429182 | -0.155422989 | 0.864466387948285 | 1.42549867477233 | 0.856053000295351 | 2.37373909217838 | 0.999714303421225 |
| ebi-fl187-GCST90094716 | ebi-a-GCST90001988 | HLA DR on CD14+ CD16- monocyte \|\| id:ebi-a-GCST90001988 | F-burn/spicy liking (derived food-liking factor) \|\| id:ebi-fl187-GCST90094716 | Weighted mode | 15 | 0.205738618403018 | 0.222225655831961 | 0.370229646784237 | -0.229823667 | 0.641300903833663 | 1.22843207249132 | 0.794673717328183 | 1.89894962400286 | 0.999714303421225 |
| ebi-fl187-GCST90094716 | ebi-a-GCST90001991 | HLA DR on CD14+ monocyte \|\| id:ebi-a-GCST90001991 | F-burn/spicy liking (derived food-liking factor) \|\| id:ebi-fl187-GCST90094716 | Weighted mode | 15 | 0.211121599391128 | 0.219898130722044 | 0.353299852882189 | -0.219878737 | 0.642121935606335 | 1.2350625287822 | 0.802616119841318 | 1.90050936218856 | 0.999714303421225 |
| ebi-fl187-GCST90094716 | ebi-a-GCST90002019 | CD14 on Monocytic Myeloid-Derived Suppressor Cells \|\| id:ebi-a-GCST90002019 | F-burn/spicy liking (derived food-liking factor) \|\| id:ebi-fl187-GCST90094716 | Weighted mode | 15 | 0.0111047147161156 | 0.284593347576286 | 0.969425739600452 | -0.546698247 | 0.568907675965637 | 1.01116660092467 | 0.578857904712062 | 1.76633658537347 | 0.999714303421225 |
| ebi-fl187-GCST90094716 | ebi-a-GCST90002037 | CD80 on plasmacytoid Dendritic Cell \|\| id:ebi-a-GCST90002037 | F-burn/spicy liking (derived food-liking factor) \|\| id:ebi-fl187-GCST90094716 | Weighted mode | 15 | -0.157540711 | 0.250937972082936 | 0.540236142432681 | -0.649379136 | 0.334297714380928 | 0.854242035937078 | 0.522369996731817 | 1.39695897644858 | 0.999714303421225 |
| ebi-fl187-GCST90094716 | ebi-a-GCST90002042 | CD45 on CD33+ HLA DR+ CD14- \|\| id:ebi-a-GCST90002042 | F-burn/spicy liking (derived food-liking factor) \|\| id:ebi-fl187-GCST90094716 | Weighted mode | 15 | 0.304470596364032 | 0.360849891802684 | 0.412989064290757 | -0.402795192 | 1.01173638429729 | 1.35590699087287 | 0.66844898929352 | 2.75037257493802 | 0.999714303421225 |
| ebi-fl187-GCST90094716 | ebi-a-GCST90002056 | CD8 on Effector Memory CD8+ T cell \|\| id:ebi-a-GCST90002056 | F-burn/spicy liking (derived food-liking factor) \|\| id:ebi-fl187-GCST90094716 | Weighted mode | 15 | 0.163457157224429 | 0.270738539131637 | 0.555669850764066 | -0.367190379 | 0.694104693922437 | 1.17757490352938 | 0.692677760863297 | 2.00191594384952 | 0.999714303421225 |
| ebi-fl187-GCST90094716 | ebi-a-GCST90002058 | CD8 on CD8+ T cell \|\| id:ebi-a-GCST90002058 | F-burn/spicy liking (derived food-liking factor) \|\| id:ebi-fl187-GCST90094716 | Weighted mode | 15 | -0.532224709 | 0.364476907970784 | 0.166299013748796 | -1.246599448 | 0.18215003100723 | 0.587296950651265 | 0.287480729666463 | 1.19979418670758 | 0.999714303421225 |
| ebi-fl187-GCST90094716 | ebi-a-GCST90002063 | CD4 on CD4 regulatory T cell \|\| id:ebi-a-GCST90002063 | F-burn/spicy liking (derived food-liking factor) \|\| id:ebi-fl187-GCST90094716 | Weighted mode | 15 | 0.28790772501748 | 0.3618172477672 | 0.439474957665441 | -0.421254081 | 0.997069530641192 | 1.33363423736954 | 0.656223346599759 | 2.71032764728655 | 0.999714303421225 |
| ebi-fl187-GCST90094716 | ebi-a-GCST90002066 | CD4 on activated CD4 regulatory T cell \|\| id:ebi-a-GCST90002066 | F-burn/spicy liking (derived food-liking factor) \|\| id:ebi-fl187-GCST90094716 | Weighted mode | 15 | 0.320935699788633 | 0.325529495453224 | 0.34092096206912 | -0.317102111 | 0.958973510876952 | 1.3784169455432 | 0.728256388122616 | 2.60901697087583 | 0.999714303421225 |
| ebi-fl187-GCST90094716 | ebi-a-GCST90002070 | CD4 on activated & secreting CD4 regulatory T cell \|\| id:ebi-a-GCST90002070 | F-burn/spicy liking (derived food-liking factor) \|\| id:ebi-fl187-GCST90094716 | Weighted mode | 15 | 0.286035142554332 | 0.323538645009708 | 0.391581295991175 | -0.348100602 | 0.920170886773358 | 1.33113923406318 | 0.706027845064984 | 2.50971923111505 | 0.999714303421225 |
| ebi-fl187-GCST90094716 | ebi-a-GCST90002081 | SSC-A on CD4+ T cell \|\| id:ebi-a-GCST90002081 | F-burn/spicy liking (derived food-liking factor) \|\| id:ebi-fl187-GCST90094716 | Weighted mode | 15 | -0.112220958 | 0.288542369651967 | 0.703183685155745 | -0.677764002 | 0.453322086602201 | 0.893846733167803 | 0.507751054134844 | 1.57353091813094 | 0.999714303421225 |
| ebi-fl187-GCST90094716 | ebi-a-GCST90002094 | CD11b on Monocytic Myeloid-Derived Suppressor Cells \|\| id:ebi-a-GCST90002094 | F-burn/spicy liking (derived food-liking factor) \|\| id:ebi-fl187-GCST90094716 | Weighted mode | 15 | 0.241996026613285 | 0.330120618368246 | 0.475621980222637 | -0.405040385 | 0.889032438615046 | 1.27378913154933 | 0.666949875284768 | 2.43277465335819 | 0.999714303421225 |
| ebi-fl187-GCST90094716 | ebi-a-GCST90002104 | HLA DR on myeloid Dendritic Cell \|\| id:ebi-a-GCST90002104 | F-burn/spicy liking (derived food-liking factor) \|\| id:ebi-fl187-GCST90094716 | Weighted mode | 15 | -0.041842227 | 0.244019420179788 | 0.866307283532534 | -0.520120291 | 0.436435836487635 | 0.959021076207153 | 0.594449037027714 | 1.54718296661425 | 0.999714303421225 |
| ebi-fl187-GCST90094716 | ebi-a-GCST90002105 | HLA DR on plasmacytoid Dendritic Cell \|\| id:ebi-a-GCST90002105 | F-burn/spicy liking (derived food-liking factor) \|\| id:ebi-fl187-GCST90094716 | Weighted mode | 15 | -0.044859282 | 0.237948870558787 | 0.853170691086216 | -0.511239068 | 0.421520504161961 | 0.956132017224896 | 0.599751984476385 | 1.52427746472683 | 0.999714303421225 |
| ebi-fl187-GCST90094716 | ebi-a-GCST90002106 | HLA DR on Dendritic Cell \|\| id:ebi-a-GCST90002106 | F-burn/spicy liking (derived food-liking factor) \|\| id:ebi-fl187-GCST90094716 | Weighted mode | 15 | -0.1404393 | 0.231423482776257 | 0.553665278586608 | -0.594029327 | 0.313150725752179 | 0.868976409774865 | 0.552098212833877 | 1.36772766727362 | 0.999714303421225 |
| ebi-fl187-GCST90094716 | ebi-a-GCST90002111 | HLA DR on CD33dim HLA DR+ CD11b- \|\| id:ebi-a-GCST90002111 | F-burn/spicy liking (derived food-liking factor) \|\| id:ebi-fl187-GCST90094716 | Weighted mode | 15 | -0.024017816 | 0.343574441733037 | 0.945257425664296 | -0.697423722 | 0.649388089358742 | 0.976268315979028 | 0.497866294853989 | 1.91436904774613 | 0.999714303421225 |
| ebi-fl187-GCST90094716 | ebi-a-GCST90002112 | HLA DR on CD33- HLA DR+ \|\| id:ebi-a-GCST90002112 | F-burn/spicy liking (derived food-liking factor) \|\| id:ebi-fl187-GCST90094716 | Weighted mode | 15 | -0.199939478 | 0.345532398734545 | 0.572023090340673 | -0.87718298 | 0.477304023215936 | 0.818780305551397 | 0.41595301083534 | 1.61172337089832 | 0.999714303421225 |
| ebi-fl187-GCST90094716 | ebi-a-GCST90002115 | HLA DR on HLA DR+ CD8+ T cell \|\| id:ebi-a-GCST90002115 | F-burn/spicy liking (derived food-liking factor) \|\| id:ebi-fl187-GCST90094716 | Weighted mode | 15 | -0.16463392 | 0.25977436240029 | 0.536456700388113 | -0.67379167 | 0.34452383079894 | 0.848204158256423 | 0.509772021531591 | 1.41131773360556 | 0.999714303421225 |
| ebi-fl187-GCST90094716 | ebi-a-GCST90002116 | HLA DR on B cell \|\| id:ebi-a-GCST90002116 | F-burn/spicy liking (derived food-liking factor) \|\| id:ebi-fl187-GCST90094716 | Weighted mode | 15 | 0.0731806598302425 | 0.239044346325561 | 0.764004516296587 | -0.395346259 | 0.541707578628342 | 1.07592489576678 | 0.673446811874677 | 1.71893958204108 | 0.999714303421225 |
| ebi-fl187-GCST90094716 | ebi-cfmet338-GCST90026023 | Sphingomyelin (d18:1/24:1, d18:2/24:0) levels \|\| id:ebi-cfmet338-GCST90026023 | F-burn/spicy liking (derived food-liking factor) \|\| id:ebi-fl187-GCST90094716 | Weighted mode | 12 | -0.293761124 | 0.188965426990339 | 0.148329724806603 | -0.664133361 | 0.0766111127194097 | 0.745454541240124 | 0.514719410341584 | 1.07962214342518 | 0.999714303421225 |
| ebi-fl187-GCST90094716 | ebi-cfmet338-GCST90026046 | 1-arachidonoyl-gpc (20:4n6) levels \|\| id:ebi-cfmet338-GCST90026046 | F-burn/spicy liking (derived food-liking factor) \|\| id:ebi-fl187-GCST90094716 | Weighted mode | 12 | 0.0172553116179548 | 0.154911311858841 | 0.91331491734202 | -0.28637086 | 0.320881482861284 | 1.01740504449681 | 0.750984054631343 | 1.37834221403768 | 0.999714303421225 |
| ebi-fl187-GCST90094716 | ebi-cfmet338-GCST90026047 | 1-linoleoyl-gpc (18:2) levels \|\| id:ebi-cfmet338-GCST90026047 | F-burn/spicy liking (derived food-liking factor) \|\| id:ebi-fl187-GCST90094716 | Weighted mode | 12 | -0.267577852 | 0.268726808812974 | 0.340783466294598 | -0.794282397 | 0.25912669371756 | 0.765230753898995 | 0.451905407348271 | 1.29579796389012 | 0.999714303421225 |
| ebi-fl187-GCST90094716 | ebi-cfmet338-GCST90026050 | 1-myristoyl-2-palmitoyl-gpc (14:0/16:0) levels \|\| id:ebi-cfmet338-GCST90026050 | F-burn/spicy liking (derived food-liking factor) \|\| id:ebi-fl187-GCST90094716 | Weighted mode | 12 | -0.093616251 | 0.109851343353007 | 0.412268571610519 | -0.308924884 | 0.121692382238086 | 0.910632149617091 | 0.734235921036396 | 1.12940662280011 | 0.999714303421225 |
| ebi-fl187-GCST90094716 | ebi-cfmet338-GCST90026068 | 2,3-dihydroxyisovalerate levels \|\| id:ebi-cfmet338-GCST90026068 | F-burn/spicy liking (derived food-liking factor) \|\| id:ebi-fl187-GCST90094716 | Weighted mode | 12 | -0.385148764 | 0.536630080724583 | 0.487894131604131 | -1.436943722 | 0.666646194556526 | 0.680349417404035 | 0.237652983485925 | 1.94769416723701 | 0.999714303421225 |
| ebi-fl187-GCST90094716 | ebi-cfmet338-GCST90026082 | 3-hydroxyhexanoate levels \|\| id:ebi-cfmet338-GCST90026082 | F-burn/spicy liking (derived food-liking factor) \|\| id:ebi-fl187-GCST90094716 | Weighted mode | 12 | -0.118819044 | 0.0730139152521083 | 0.131941557827428 | -0.261926318 | 0.0242882295458262 | 0.887968469137603 | 0.769567724721155 | 1.02458559117494 | 0.999714303421225 |
| ebi-fl187-GCST90094716 | ebi-cfmet338-GCST90026094 | 3-ureidopropionate levels \|\| id:ebi-cfmet338-GCST90026094 | F-burn/spicy liking (derived food-liking factor) \|\| id:ebi-fl187-GCST90094716 | Weighted mode | 12 | -0.207586671 | 0.210243089947905 | 0.344684573495521 | -0.619663127 | 0.204489785738076 | 0.812542815180518 | 0.538125687155084 | 1.22689892391479 | 0.999714303421225 |
| ebi-fl187-GCST90094716 | ebi-cfmet338-GCST90026173 | Glycerol levels \|\| id:ebi-cfmet338-GCST90026173 | F-burn/spicy liking (derived food-liking factor) \|\| id:ebi-fl187-GCST90094716 | Weighted mode | 12 | -0.046231089 | 0.0868879259211744 | 0.605252359411523 | -0.216531424 | 0.12406924609494 | 0.954821288273747 | 0.805307228940427 | 1.13209426139173 | 0.999714303421225 |
| ebi-fl187-GCST90094716 | ebi-cfmet338-GCST90026179 | Histidine levels \|\| id:ebi-cfmet338-GCST90026179 | F-burn/spicy liking (derived food-liking factor) \|\| id:ebi-fl187-GCST90094716 | Weighted mode | 12 | -0.027083002 | 0.0723676468493252 | 0.715338218462396 | -0.16892359 | 0.114757585867947 | 0.973280453989492 | 0.844573434961349 | 1.12160151255686 | 0.999714303421225 |
| ebi-fl187-GCST90094716 | ebi-cfmet338-GCST90026188 | Isocitrate levels \|\| id:ebi-cfmet338-GCST90026188 | F-burn/spicy liking (derived food-liking factor) \|\| id:ebi-fl187-GCST90094716 | Weighted mode | 12 | 0.0234549106784931 | 0.117850431651973 | 0.845875942932837 | -0.207531935 | 0.25444175671636 | 1.02373214031835 | 0.812587291091564 | 1.2897414303797 | 0.999714303421225 |
| ebi-fl187-GCST90094716 | ebi-cfmet338-GCST90026207 | N-acetyl-isoputreanine levels \|\| id:ebi-cfmet338-GCST90026207 | F-burn/spicy liking (derived food-liking factor) \|\| id:ebi-fl187-GCST90094716 | Weighted mode | 12 | 0.0393075707806277 | 0.0850094869884507 | 0.652813155804777 | -0.127311024 | 0.205926165277991 | 1.04009033585556 | 0.880459786131653 | 1.22866248269331 | 0.999714303421225 |
| ebi-fl187-GCST90094716 | ebi-cfmet338-GCST90026213 | N-acetylglutamate levels \|\| id:ebi-cfmet338-GCST90026213 | F-burn/spicy liking (derived food-liking factor) \|\| id:ebi-fl187-GCST90094716 | Weighted mode | 12 | 0.196111938023202 | 0.17015165061209 | 0.27351857026062 | -0.137385297 | 0.529609173222898 | 1.21666308857317 | 0.871634323181379 | 1.69826845011515 | 0.999714303421225 |
| ebi-fl187-GCST90094716 | ebi-cfmet338-GCST90026224 | N-acetyltaurine levels \|\| id:ebi-cfmet338-GCST90026224 | F-burn/spicy liking (derived food-liking factor) \|\| id:ebi-fl187-GCST90094716 | Weighted mode | 12 | -0.088898284 | 0.109781576482621 | 0.43523757943535 | -0.304070174 | 0.126273605683764 | 0.914938632536262 | 0.737809089524343 | 1.13459255679135 | 0.999714303421225 |
| ebi-fl187-GCST90094716 | ebi-cfmet338-GCST90026250 | Phenyllactate (pla) levels \|\| id:ebi-cfmet338-GCST90026250 | F-burn/spicy liking (derived food-liking factor) \|\| id:ebi-fl187-GCST90094716 | Weighted mode | 12 | 0.256605636922301 | 0.16448468904333 | 0.147036017528198 | -0.065784354 | 0.578995627447228 | 1.29253529803898 | 0.936332759308159 | 1.78424548331635 | 0.999714303421225 |
| ebi-fl187-GCST90094716 | ebi-cfmet338-GCST90026251 | Pipecolate levels \|\| id:ebi-cfmet338-GCST90026251 | F-burn/spicy liking (derived food-liking factor) \|\| id:ebi-fl187-GCST90094716 | Weighted mode | 12 | 0.323298017130365 | 0.386577080711381 | 0.420777118211901 | -0.434393061 | 1.08098909532467 | 1.38167705298587 | 0.647657636448561 | 2.94759356072124 | 0.999714303421225 |
| ebi-fl187-GCST90094716 | ebi-cfmet338-GCST90026278 | Trigonelline (n'-methylnicotinate) levels \|\| id:ebi-cfmet338-GCST90026278 | F-burn/spicy liking (derived food-liking factor) \|\| id:ebi-fl187-GCST90094716 | Weighted mode | 12 | 0.356629877352109 | 0.301930353920993 | 0.262445041439579 | -0.235153616 | 0.948413371037256 | 1.42850704918409 | 0.790449414361268 | 2.58161035038223 | 0.999714303421225 |
| ebi-fl187-GCST90094716 | ebi-cfmet338-GCST90026299 | 1-carboxyethylvaline levels \|\| id:ebi-cfmet338-GCST90026299 | F-burn/spicy liking (derived food-liking factor) \|\| id:ebi-fl187-GCST90094716 | Weighted mode | 12 | -0.058687916 | 0.165611348211452 | 0.729760507396009 | -0.383286159 | 0.265910326077809 | 0.943001018376466 | 0.681617820362285 | 1.30461806322259 | 0.999714303421225 |
| ebi-fl187-GCST90094716 | ebi-gm430-GCST90011306 | C_Erysipelotrichia abundance \|\| id:ebi-gm430-GCST90011306 | F-burn/spicy liking (derived food-liking factor) \|\| id:ebi-fl187-GCST90094716 | Weighted mode | 12 | -0.163824175 | 0.182747153853558 | 0.389208466432728 | -0.522008596 | 0.194360246832893 | 0.848891265303781 | 0.593327594695114 | 1.21453373608782 | 0.999714303421225 |
| ebi-fl187-GCST90094716 | ebi-gm430-GCST90011312 | F_Erysipelotrichaceae abundance \|\| id:ebi-gm430-GCST90011312 | F-burn/spicy liking (derived food-liking factor) \|\| id:ebi-fl187-GCST90094716 | Weighted mode | 12 | -0.163824175 | 0.173580188103254 | 0.365542535370068 | -0.504041343 | 0.176392993962295 | 0.848891265303781 | 0.604084407434808 | 1.1929067717028 | 0.999714303421225 |
| ebi-fl187-GCST90094716 | ebi-gm430-GCST90011352 | O_Erysipelotrichales abundance \|\| id:ebi-gm430-GCST90011352 | F-burn/spicy liking (derived food-liking factor) \|\| id:ebi-fl187-GCST90094716 | Weighted mode | 12 | -0.163824175 | 0.185565756277066 | 0.396208423177194 | -0.527533057 | 0.199884707582967 | 0.848891265303781 | 0.590058817116498 | 1.22126194780135 | 0.999714303421225 |
| ebi-fl187-GCST90094716 | ebi-gm430-GCST90011362 | OTU97_11 (Parabacteroides) abundance \|\| id:ebi-gm430-GCST90011362 | F-burn/spicy liking (derived food-liking factor) \|\| id:ebi-fl187-GCST90094716 | Weighted mode | 12 | -0.278483979 | 0.250653970169458 | 0.290250379980249 | -0.76976576 | 0.212797802992411 | 0.756930394783009 | 0.463121537162447 | 1.23713448106257 | 0.999714303421225 |
| ebi-fl187-GCST90094716 | ebi-gm430-GCST90011384 | OTU97_235 (Phascolarctobacterium) abundance \|\| id:ebi-gm430-GCST90011384 | F-burn/spicy liking (derived food-liking factor) \|\| id:ebi-fl187-GCST90094716 | Weighted mode | 12 | -0.335194898 | 0.644686027186339 | 0.613411505466858 | -1.598779511 | 0.928389715229029 | 0.715198681935273 | 0.202143080838045 | 2.5304311803369 | 0.999714303421225 |
| ebi-fl187-GCST90094716 | ebi-gm430-GCST90011390 | OTU97_3 (Bacteroides) abundance \|\| id:ebi-gm430-GCST90011390 | F-burn/spicy liking (derived food-liking factor) \|\| id:ebi-fl187-GCST90094716 | Weighted mode | 12 | -0.182688267 | 0.169235460171306 | 0.303469624539626 | -0.514389769 | 0.149013235212818 | 0.833027797750735 | 0.597865319479472 | 1.16068835106477 | 0.999714303421225 |
| ebi-fl187-GCST90094716 | ebi-gm430-GCST90011398 | OTU97_4 (Alistipes) abundance \|\| id:ebi-gm430-GCST90011398 | F-burn/spicy liking (derived food-liking factor) \|\| id:ebi-fl187-GCST90094716 | Weighted mode | 12 | -0.058592012 | 0.175506195977429 | 0.744773596083356 | -0.402584156 | 0.285400131903865 | 0.943091460476023 | 0.668590070473481 | 1.33029421479881 | 0.999714303421225 |
| ebi-fl187-GCST90094716 | ebi-gm430-GCST90011421 | OTU99_108 (Cytophagales) abundance \|\| id:ebi-gm430-GCST90011421 | F-burn/spicy liking (derived food-liking factor) \|\| id:ebi-fl187-GCST90094716 | Weighted mode | 12 | 0.306802771109153 | 0.553230838255294 | 0.590289076671737 | -0.777529672 | 1.39113521408953 | 1.35907289319749 | 0.459539824437996 | 4.01941035531168 | 0.999714303421225 |
| ebi-fl187-GCST90094716 | ebi-gm430-GCST90011428 | OTU99_123 (Phascolarctobacterium) abundance \|\| id:ebi-gm430-GCST90011428 | F-burn/spicy liking (derived food-liking factor) \|\| id:ebi-fl187-GCST90094716 | Weighted mode | 13 | -0.210087785 | 0.373195110886508 | 0.583838251829311 | -0.941550203 | 0.521374632169506 | 0.810513091819121 | 0.390022752231253 | 1.68434140893576 | 0.999714303421225 |
| ebi-fl187-GCST90094716 | ebi-gm430-GCST90011450 | OTU99_298 (Phascolarctobacterium) abundance \|\| id:ebi-gm430-GCST90011450 | F-burn/spicy liking (derived food-liking factor) \|\| id:ebi-fl187-GCST90094716 | Weighted mode | 12 | -0.351215928 | 0.621744503788416 | 0.583486391354216 | -1.569835156 | 0.867403299277029 | 0.703831760357669 | 0.208079480272612 | 2.38072080072078 | 0.999714303421225 |
| ebi-fl187-GCST90094716 | ebi-gm430-GCST90011451 | OTU99_3 (Bacteroides) abundance \|\| id:ebi-gm430-GCST90011451 | F-burn/spicy liking (derived food-liking factor) \|\| id:ebi-fl187-GCST90094716 | Weighted mode | 12 | -0.197658978 | 0.165376299382558 | 0.257138091742175 | -0.521796524 | 0.12647856918188 | 0.820649665335928 | 0.593453436134037 | 1.1348251306845 | 0.999714303421225 |
| ebi-fl187-GCST90094716 | ebi-gm430-GCST90011472 | OTU99_72 (Parabacteroides) abundance \|\| id:ebi-gm430-GCST90011472 | F-burn/spicy liking (derived food-liking factor) \|\| id:ebi-fl187-GCST90094716 | Weighted mode | 13 | 0.283931422787886 | 0.543618570068887 | 0.610968689631209 | -0.781560975 | 1.34942382012291 | 1.32834183364955 | 0.457691009384407 | 3.85520359990617 | 0.999714303421225 |
| ebi-fl187-GCST90094716 | ebi-gm430-GCST90011507 | TestASV_3 (Bacteroides) abundance \|\| id:ebi-gm430-GCST90011507 | F-burn/spicy liking (derived food-liking factor) \|\| id:ebi-fl187-GCST90094716 | Weighted mode | 12 | 0.153986131848025 | 0.233624841214318 | 0.523378846356588 | -0.303918557 | 0.611890820628088 | 1.16647470981772 | 0.737920962550456 | 1.84391461646719 | 0.999714303421225 |
| ebi-fl187-GCST90094716 | ebi-gm430-GCST90011512 | TestASV_34 (Phascolarctobacterium) abundance \|\| id:ebi-gm430-GCST90011512 | F-burn/spicy liking (derived food-liking factor) \|\| id:ebi-fl187-GCST90094716 | Weighted mode | 12 | 0.501330440149328 | 0.601402486913709 | 0.422237497965098 | -0.677418434 | 1.6800793145002 | 1.6509162554979 | 0.50792654708955 | 5.36598155438935 | 0.999714303421225 |
| ebi-fl187-GCST90094716 | ebi-gm430-GCST90011519 | TestASV_41 (Barnesiella) abundance \|\| id:ebi-gm430-GCST90011519 | F-burn/spicy liking (derived food-liking factor) \|\| id:ebi-fl187-GCST90094716 | Weighted mode | 12 | 0.444021214851612 | 0.725069524898048 | 0.552732076210733 | -0.977115054 | 1.86515748365179 | 1.55896355845166 | 0.376395414468065 | 6.45695267041161 | 0.999714303421225 |
| ebi-fl187-GCST90094716 | ebi-gm430-GCST90011524 | TestASV_46 (Sutterella) abundance \|\| id:ebi-gm430-GCST90011524 | F-burn/spicy liking (derived food-liking factor) \|\| id:ebi-fl187-GCST90094716 | Weighted mode | 12 | -0.167831333 | 0.666788717867412 | 0.805912772989063 | -1.47473722 | 1.13907455414017 | 0.845496430106613 | 0.228838853441638 | 3.12387604889544 | 0.999714303421225 |
| ebi-fl187-GCST90094716 | ebi-gm430-GCST90011581 | OTU97_19 (Bacteria) prevalence \|\| id:ebi-gm430-GCST90011581 | F-burn/spicy liking (derived food-liking factor) \|\| id:ebi-fl187-GCST90094716 | Weighted mode | 12 | -0.052326604 | 0.736954802331689 | 0.944669193945012 | -1.496758016 | 1.39210480893773 | 0.949018863213568 | 0.223854718380772 | 4.0233094448481 | 0.999714303421225 |
| ebi-fl187-GCST90094716 | ebi-gm430-GCST90011614 | OTU97_80 (Ruminococcus) prevalence \|\| id:ebi-gm430-GCST90011614 | F-burn/spicy liking (derived food-liking factor) \|\| id:ebi-fl187-GCST90094716 | Weighted mode | 12 | 0.880193964472555 | 0.421995631245742 | 0.0610838300678394 | 0.0530825272309008 | 1.70730540171421 | 2.41136738066178 | 1.05451666786897 | 5.51408320199462 | 0.999714303421225 |
| ebi-fl187-GCST90094716 | ebi-gm430-GCST90011623 | OTU99_11 (Parabacteroides) prevalence \|\| id:ebi-gm430-GCST90011623 | F-burn/spicy liking (derived food-liking factor) \|\| id:ebi-fl187-GCST90094716 | Weighted mode | 12 | 0.206739305549177 | 0.310450863095614 | 0.519175338854287 | -0.401744386 | 0.815222997216581 | 1.22966196394184 | 0.669151768312531 | 2.25967951841245 | 0.999714303421225 |
| ebi-fl187-GCST90094716 | ebi-gm430-GCST90011642 | OTU99_19 (Bacteria) prevalence \|\| id:ebi-gm430-GCST90011642 | F-burn/spicy liking (derived food-liking factor) \|\| id:ebi-fl187-GCST90094716 | Weighted mode | 12 | -0.052326604 | 0.707242923056458 | 0.942349228351154 | -1.438522733 | 1.33386952555828 | 0.949018863213568 | 0.237278022936578 | 3.79570257535356 | 0.999714303421225 |
| ebi-fl187-GCST90094716 | ebi-gm430-GCST90011653 | OTU99_30 (Parasutterella) prevalence \|\| id:ebi-gm430-GCST90011653 | F-burn/spicy liking (derived food-liking factor) \|\| id:ebi-fl187-GCST90094716 | Weighted mode | 12 | 0.291954790014592 | 0.412373558783214 | 0.493682704577732 | -0.516297385 | 1.10020696522969 | 1.33904247817991 | 0.596725908831086 | 3.00478784620319 | 0.999714303421225 |
| ebi-fl187-GCST90094716 | ebi-gm430-GCST90011675 | OTU99_8 (Ruminococcaceae) prevalence \|\| id:ebi-gm430-GCST90011675 | F-burn/spicy liking (derived food-liking factor) \|\| id:ebi-fl187-GCST90094716 | Weighted mode | 12 | -0.687440927 | 0.460231213982274 | 0.163377252146009 | -1.589494106 | 0.21461225238471 | 0.502861282607777 | 0.204028802490374 | 1.23938123666569 | 0.999714303421225 |
| ebi-fl187-GCST90094716 | ebi-gm430-GCST90011679 | OTU99_92 (Ruminococcus) prevalence \|\| id:ebi-gm430-GCST90011679 | F-burn/spicy liking (derived food-liking factor) \|\| id:ebi-fl187-GCST90094716 | Weighted mode | 12 | 0.264514881580149 | 0.436334865441989 | 0.556671949659386 | -0.590701455 | 1.11973121784645 | 1.30279881075286 | 0.553938585606321 | 3.06403053587842 | 0.999714303421225 |
| ebi-fl187-GCST90094716 | ebi-gm430-GCST90011684 | TestASV_11 (Lachnospiraceae) prevalence \|\| id:ebi-gm430-GCST90011684 | F-burn/spicy liking (derived food-liking factor) \|\| id:ebi-fl187-GCST90094716 | Weighted mode | 12 | 0.250681057630346 | 0.529368652543182 | 0.645081819883185 | -0.786881501 | 1.28824361661498 | 1.2849002098538 | 0.455262318785581 | 3.62641158988587 | 0.999714303421225 |
| ebi-fl187-GCST90094716 | ebi-gm430-GCST90011688 | TestASV_15 (Bacteroides) prevalence \|\| id:ebi-gm430-GCST90011688 | F-burn/spicy liking (derived food-liking factor) \|\| id:ebi-fl187-GCST90094716 | Weighted mode | 12 | -0.317066641 | 0.347887646337495 | 0.381621055 | -0.998926428 | 0.364793145514137 | 0.72828221982924 | 0.368274598268279 | 1.44021606218148 | 0.999714303421225 |
| ebi-fl187-GCST90094716 | ebi-gm430-GCST90011692 | TestASV_19 (Prevotella) prevalence \|\| id:ebi-gm430-GCST90011692 | F-burn/spicy liking (derived food-liking factor) \|\| id:ebi-fl187-GCST90094716 | Weighted mode | 12 | 0.237604317609872 | 0.719259773895004 | 0.747345066312568 | -1.172144839 | 1.64735347444408 | 1.26820728624346 | 0.309701967461796 | 5.19321764101938 | 0.999714303421225 |
| ebi-fl187-GCST90094716 | ebi-gm430-GCST90011693 | TestASV_2 (Bacteroides) prevalence \|\| id:ebi-gm430-GCST90011693 | F-burn/spicy liking (derived food-liking factor) \|\| id:ebi-fl187-GCST90094716 | Weighted mode | 12 | -0.523618082 | 0.323994985501351 | 0.134359051958818 | -1.158648254 | 0.111412089089464 | 0.59237341018682 | 0.313910221082635 | 1.11785546799379 | 0.999714303421225 |
| ebi-fl187-GCST90094716 | ebi-gm430-GCST90011716 | TestASV_40 (Barnesiella) prevalence \|\| id:ebi-gm430-GCST90011716 | F-burn/spicy liking (derived food-liking factor) \|\| id:ebi-fl187-GCST90094716 | Weighted mode | 12 | 0.727026131440459 | 0.914204174812962 | 0.44328649668711 | -1.064814051 | 2.51886631407386 | 2.06891875750392 | 0.344791962460762 | 12.4145145223294 | 0.999714303421225 |
| ebi-fl187-GCST90094716 | ebi-gm430-GCST90011718 | TestASV_42 (Alphaproteobacteria) prevalence \|\| id:ebi-gm430-GCST90011718 | F-burn/spicy liking (derived food-liking factor) \|\| id:ebi-fl187-GCST90094716 | Weighted mode | 12 | 0.075124486401609 | 0.586138550411556 | 0.900328860810669 | -1.073707072 | 1.22395604520826 | 1.07801834115619 | 0.341739313977107 | 3.40061414165238 | 0.999714303421225 |
| ebi-fl187-GCST90094716 | ebi-hbsm44-GCST90104929 | Sulcal depth of the right inferior frontal sulcus \|\| id:ebi-hbsm44-GCST90104929 | F-burn/spicy liking (derived food-liking factor) \|\| id:ebi-fl187-GCST90094716 | Weighted mode | 11 | 0.0084673115226499 | 0.290456237436178 | 0.97731714286199 | -0.560826914 | 0.577761536897559 | 1.00850326059723 | 0.570736918400092 | 1.78204492095299 | 0.999714303421225 |
| ebi-fl187-GCST90094716 | ebi-hbsm44-GCST90104935 | Sulcal opening distance of the left calloso-marginal posterior fissure \|\| id:ebi-hbsm44-GCST90104935 | F-burn/spicy liking (derived food-liking factor) \|\| id:ebi-fl187-GCST90094716 | Weighted mode | 11 | 0.0290772771447206 | 0.0745691353604961 | 0.704759324766443 | -0.117078228 | 0.175232782451293 | 1.02950414854231 | 0.889515605270842 | 1.19152355010469 | 0.999714303421225 |
| ebi-fl187-GCST90094716 | ebi-hbsm44-GCST90104936 | Sulcal opening distance of the left anterior inferior temporal sulcus \|\| id:ebi-hbsm44-GCST90104936 | F-burn/spicy liking (derived food-liking factor) \|\| id:ebi-fl187-GCST90094716 | Weighted mode | 12 | -0.023975831 | 0.0427102780453519 | 0.585806823104295 | -0.107687976 | 0.0597363136873771 | 0.976309305617531 | 0.897907721259342 | 1.06155659169355 | 0.999714303421225 |
| ebi-fl187-GCST90094716 | ebi-hbsm44-GCST90104941 | Sulcal opening distance of the right posterior inferior temporal sulcus \|\| id:ebi-hbsm44-GCST90104941 | F-burn/spicy liking (derived food-liking factor) \|\| id:ebi-fl187-GCST90094716 | Weighted mode | 12 | 0.0236825093887408 | 0.0316919911373062 | 0.470572457305001 | -0.038433793 | 0.0857988120178609 | 1.0239651669504 | 0.962295413091931 | 1.08958709442337 | 0.999714303421225 |
| ebi-fl187-GCST90094716 | ebi-IPs91-GCST90274819 | Leukemia inhibitory factor levels \|\| id:ebi-IPs91-GCST90274819 | F-burn/spicy liking (derived food-liking factor) \|\| id:ebi-fl187-GCST90094716 | Weighted mode | 15 | -0.084774982 | 0.1085743052046 | 0.447921290732519 | -0.297580621 | 0.128030655896275 | 0.918718989174005 | 0.742612710995009 | 1.13658784528208 | 0.999714303421225 |
| ebi-fl187-GCST90094716 | ebi-IPs91-GCST90274837 | STAM binding protein levels \|\| id:ebi-IPs91-GCST90274837 | F-burn/spicy liking (derived food-liking factor) \|\| id:ebi-fl187-GCST90094716 | Weighted mode | 15 | -0.001574055 | 0.0999709471694144 | 0.98765991142326 | -0.197517112 | 0.194369001276355 | 0.998427182999414 | 0.820766095863659 | 1.21454436870128 | 0.999714303421225 |
| ebi-fl187-GCST90094716 | ebi-IPs91-GCST90274840 | TNF-beta levels \|\| id:ebi-IPs91-GCST90274840 | F-burn/spicy liking (derived food-liking factor) \|\| id:ebi-fl187-GCST90094716 | Weighted mode | 15 | -0.151212963 | 0.154192605711313 | 0.34339674376814 | -0.45343047 | 0.151004544031832 | 0.859664602271251 | 0.635444534707125 | 1.16300194278766 | 0.999714303421225 |
| ebi-fl187-GCST90094716 | ebi-IPs91-GCST90274842 | Tumor necrosis factor ligand superfamily member 14 levels \|\| id:ebi-IPs91-GCST90274842 | F-burn/spicy liking (derived food-liking factor) \|\| id:ebi-fl187-GCST90094716 | Weighted mode | 15 | -0.058849512 | 0.113950192930779 | 0.613601239704926 | -0.28219189 | 0.164492866377889 | 0.942848646108695 | 0.754128960909017 | 1.17879516044239 | 0.999714303421225 |
| ebi-fl187-GCST90094716 | ebi-met1400-GCST90199628 | 3-methylhistidine levels \|\| id:ebi-met1400-GCST90199628 | F-burn/spicy liking (derived food-liking factor) \|\| id:ebi-fl187-GCST90094716 | Weighted mode | 14 | 0.124669195422448 | 0.180869238105531 | 0.502766286948346 | -0.229834511 | 0.47917290210929 | 1.13277366436583 | 0.794665099744965 | 1.61473830308215 | 0.999714303421225 |
| ebi-fl187-GCST90094716 | ebi-met1400-GCST90199651 | Docosahexaenoate DHA; 22:6n3 levels \|\| id:ebi-met1400-GCST90199651 | F-burn/spicy liking (derived food-liking factor) \|\| id:ebi-fl187-GCST90094716 | Weighted mode | 14 | 0.177175365627176 | 0.140165777280375 | 0.228410466058876 | -0.097549558 | 0.45190028909671 | 1.19384043334741 | 0.907057388634851 | 1.57129526549602 | 0.999714303421225 |
| ebi-fl187-GCST90094716 | ebi-met1400-GCST90199652 | 1-stearoyl-2-oleoyl-GPS (18:0/18:1) levels \|\| id:ebi-met1400-GCST90199652 | F-burn/spicy liking (derived food-liking factor) \|\| id:ebi-fl187-GCST90094716 | Weighted mode | 14 | -0.040248117 | 0.136278613289591 | 0.772400659119682 | -0.307354199 | 0.226857965333238 | 0.960551080805057 | 0.735390080515902 | 1.25465165125492 | 0.999714303421225 |
| ebi-fl187-GCST90094716 | ebi-met1400-GCST90199653 | 1-myristoyl-2-palmitoyl-gpc (14:0/16:0) levels \|\| id:ebi-met1400-GCST90199653 | F-burn/spicy liking (derived food-liking factor) \|\| id:ebi-fl187-GCST90094716 | Weighted mode | 14 | -0.003242057 | 0.166280283415618 | 0.984740274282397 | -0.329151413 | 0.322667298150954 | 0.996763192449344 | 0.719534061804167 | 1.38080587780737 | 0.999714303421225 |
| ebi-fl187-GCST90094716 | ebi-met1400-GCST90199667 | Erythritol levels in elite athletes \|\| id:ebi-met1400-GCST90199667 | F-burn/spicy liking (derived food-liking factor) \|\| id:ebi-fl187-GCST90094716 | Weighted mode | 14 | 0.0928098655826941 | 0.145049099065631 | 0.533383960705333 | -0.191486369 | 0.377106099751331 | 1.0972530898565 | 0.825730880909304 | 1.45805900086213 | 0.999714303421225 |
| ebi-fl187-GCST90094716 | ebi-met1400-GCST90199674 | 3-hydroxymyristate levels \|\| id:ebi-met1400-GCST90199674 | F-burn/spicy liking (derived food-liking factor) \|\| id:ebi-fl187-GCST90094716 | Weighted mode | 14 | 0.00260234789753966 | 0.122303010650367 | 0.983347120459928 | -0.237111553 | 0.24231624877226 | 1.00260573694402 | 0.78890327859941 | 1.27419709237067 | 0.999714303421225 |
| ebi-fl187-GCST90094716 | ebi-met1400-GCST90199685 | 2-linoleoylglycerol (18:2) levels \|\| id:ebi-met1400-GCST90199685 | F-burn/spicy liking (derived food-liking factor) \|\| id:ebi-fl187-GCST90094716 | Weighted mode | 14 | -0.075404216 | 0.163466387712791 | 0.652218827128657 | -0.395798336 | 0.244989904319381 | 0.927368553722634 | 0.673142431172315 | 1.27760841481327 | 0.999714303421225 |
| ebi-fl187-GCST90094716 | ebi-met1400-GCST90199703 | Gamma-glutamylvaline levels \|\| id:ebi-met1400-GCST90199703 | F-burn/spicy liking (derived food-liking factor) \|\| id:ebi-fl187-GCST90094716 | Weighted mode | 14 | -0.017536451 | 0.125032923260666 | 0.890608591800377 | -0.262600981 | 0.227528078562342 | 0.982616417633168 | 0.769048701433137 | 1.25549269168903 | 0.999714303421225 |
| ebi-fl187-GCST90094716 | ebi-met1400-GCST90199779 | Gamma-glutamyl-2-aminobutyrate levels \|\| id:ebi-met1400-GCST90199779 | F-burn/spicy liking (derived food-liking factor) \|\| id:ebi-fl187-GCST90094716 | Weighted mode | 14 | -0.030809044 | 0.153650061378741 | 0.844181238717409 | -0.331963164 | 0.27034507661339 | 0.969660718231876 | 0.71751375271051 | 1.31041656683243 | 0.999714303421225 |
| ebi-fl187-GCST90094716 | ebi-met1400-GCST90199824 | 4-vinylphenol sulfate levels \|\| id:ebi-met1400-GCST90199824 | F-burn/spicy liking (derived food-liking factor) \|\| id:ebi-fl187-GCST90094716 | Weighted mode | 14 | -0.009145861 | 0.13960735429843 | 0.948763635730215 | -0.282776275 | 0.264484553701159 | 0.99089583544781 | 0.753688387821515 | 1.30275930022732 | 0.999714303421225 |
| ebi-fl187-GCST90094716 | ebi-met1400-GCST90199830 | 4-ethylphenylsulfate levels \|\| id:ebi-met1400-GCST90199830 | F-burn/spicy liking (derived food-liking factor) \|\| id:ebi-fl187-GCST90094716 | Weighted mode | 14 | -0.01363524 | 0.133997354149654 | 0.920501921274356 | -0.276270054 | 0.248999574215168 | 0.986457298891891 | 0.758608037922293 | 1.28274148689783 | 0.999714303421225 |
| ebi-fl187-GCST90094716 | ebi-met1400-GCST90199833 | Hexanoylglutamine levels \|\| id:ebi-met1400-GCST90199833 | F-burn/spicy liking (derived food-liking factor) \|\| id:ebi-fl187-GCST90094716 | Weighted mode | 14 | -0.110043606 | 0.142152539366426 | 0.452707083243709 | -0.388662584 | 0.168575370702508 | 0.895795071996718 | 0.67796298724641 | 1.1836174335605 | 0.999714303421225 |
| ebi-fl187-GCST90094716 | ebi-met1400-GCST90199837 | Carotenoid levels (cryptoxanthin) \|\| id:ebi-met1400-GCST90199837 | F-burn/spicy liking (derived food-liking factor) \|\| id:ebi-fl187-GCST90094716 | Weighted mode | 14 | 0.0836835471947389 | 0.138382417376264 | 0.555769166060752 | -0.187545991 | 0.354913085252216 | 1.08728476504743 | 0.828990991292771 | 1.42605670353627 | 0.999714303421225 |
| ebi-fl187-GCST90094716 | ebi-met1400-GCST90199869 | 2-hydroxyhippurate levels \|\| id:ebi-met1400-GCST90199869 | F-burn/spicy liking (derived food-liking factor) \|\| id:ebi-fl187-GCST90094716 | Weighted mode | 14 | 0.264887197264925 | 0.168367137605302 | 0.139669105538424 | -0.065112392 | 0.594886786971318 | 1.30328395349173 | 0.9369621499957 | 1.81282569785433 | 0.999714303421225 |
| ebi-fl187-GCST90094716 | ebi-met1400-GCST90199902 | Hydantoin-5-propionate levels \|\| id:ebi-met1400-GCST90199902 | F-burn/spicy liking (derived food-liking factor) \|\| id:ebi-fl187-GCST90094716 | Weighted mode | 14 | -0.085873211 | 0.124737620854825 | 0.503282293007311 | -0.330358948 | 0.158612525877413 | 0.917710579452611 | 0.718665723595414 | 1.17188378405727 | 0.999714303421225 |
| ebi-fl187-GCST90094716 | ebi-met1400-GCST90199904 | Pantoate levels \|\| id:ebi-met1400-GCST90199904 | F-burn/spicy liking (derived food-liking factor) \|\| id:ebi-fl187-GCST90094716 | Weighted mode | 14 | -0.033659281 | 0.143319497213904 | 0.817980091422623 | -0.314565496 | 0.247246933147338 | 0.966900889645798 | 0.730106039404099 | 1.28049527046905 | 0.999714303421225 |
| ebi-fl187-GCST90094716 | ebi-met1400-GCST90199936 | 3-methyl catechol sulfate (2) levels \|\| id:ebi-met1400-GCST90199936 | F-burn/spicy liking (derived food-liking factor) \|\| id:ebi-fl187-GCST90094716 | Weighted mode | 14 | 0.141479732817745 | 0.181950878056624 | 0.450741388373225 | -0.215143988 | 0.498103453808728 | 1.15197715669323 | 0.806425316109161 | 1.64559735791253 | 0.999714303421225 |
| ebi-fl187-GCST90094716 | ebi-met1400-GCST90199977 | 4-hydroxychlorothalonil levels \|\| id:ebi-met1400-GCST90199977 | F-burn/spicy liking (derived food-liking factor) \|\| id:ebi-fl187-GCST90094716 | Weighted mode | 14 | -0.080394504 | 0.145103526981486 | 0.588949215385249 | -0.364797417 | 0.204008408894758 | 0.922752245130138 | 0.694337297652347 | 1.22630846531168 | 0.999714303421225 |
| ebi-fl187-GCST90094716 | ebi-met1400-GCST90200040 | Sphingomyelin (d18:2/24:1, d18:1/24:2) levels \|\| id:ebi-met1400-GCST90200040 | F-burn/spicy liking (derived food-liking factor) \|\| id:ebi-fl187-GCST90094716 | Weighted mode | 14 | 0.143262242833155 | 0.135330473414138 | 0.309051589233331 | -0.121985485 | 0.408509970724865 | 1.15403239871311 | 0.88516121647261 | 1.50457425437907 | 0.999714303421225 |
| ebi-fl187-GCST90094716 | ebi-met1400-GCST90200077 | 1-stearoyl-2-oleoyl-GPI (18:0/18:1) levels \|\| id:ebi-met1400-GCST90200077 | F-burn/spicy liking (derived food-liking factor) \|\| id:ebi-fl187-GCST90094716 | Weighted mode | 14 | -0.06597819 | 0.120510278253578 | 0.593317578676962 | -0.302178336 | 0.170221954950823 | 0.936151281129439 | 0.739206226198019 | 1.18556796479894 | 0.999714303421225 |
| ebi-fl187-GCST90094716 | ebi-met1400-GCST90200080 | 1-palmitoyl-2-oleoyl-GPI (16:0/18:1) levels \|\| id:ebi-met1400-GCST90200080 | F-burn/spicy liking (derived food-liking factor) \|\| id:ebi-fl187-GCST90094716 | Weighted mode | 14 | -0.035852968 | 0.148912092303573 | 0.813491920001886 | -0.327720668 | 0.256014733414301 | 0.964782137383972 | 0.720564267955049 | 1.29177176000802 | 0.999714303421225 |
| ebi-fl187-GCST90094716 | ebi-met1400-GCST90200082 | 1-oleoyl-2-linoleoyl-GPE (18:1/18:2) levels \|\| id:ebi-met1400-GCST90200082 | F-burn/spicy liking (derived food-liking factor) \|\| id:ebi-fl187-GCST90094716 | Weighted mode | 14 | -0.222489007 | 0.171308904006942 | 0.216591863029722 | -0.558254459 | 0.113276445027459 | 0.800523806999242 | 0.572207003591405 | 1.11994149241515 | 0.999714303421225 |
| ebi-fl187-GCST90094716 | ebi-met1400-GCST90200100 | Perfluorooctanesulfonate (PFOS) levels \|\| id:ebi-met1400-GCST90200100 | F-burn/spicy liking (derived food-liking factor) \|\| id:ebi-fl187-GCST90094716 | Weighted mode | 14 | 0.0161695752980362 | 0.127138771162473 | 0.900743690763662 | -0.233022416 | 0.265361566776483 | 1.01630101034138 | 0.792135816665918 | 1.30390233832403 | 0.999714303421225 |
| ebi-fl187-GCST90094716 | ebi-met1400-GCST90200102 | 1-palmitoleoyl-2-linolenoyl-GPC (16:1/18:3) levels \|\| id:ebi-met1400-GCST90200102 | F-burn/spicy liking (derived food-liking factor) \|\| id:ebi-fl187-GCST90094716 | Weighted mode | 14 | -0.172428232 | 0.130063143219983 | 0.207755073871666 | -0.427351992 | 0.0824955289229302 | 0.841618688112605 | 0.652233930366339 | 1.08599381786614 | 0.999714303421225 |
| ebi-fl187-GCST90094716 | ebi-met1400-GCST90200114 | Glycosyl-N-tricosanoyl-sphingadienine (d18:2/23:0) levels \|\| id:ebi-met1400-GCST90200114 | F-burn/spicy liking (derived food-liking factor) \|\| id:ebi-fl187-GCST90094716 | Weighted mode | 14 | 0.0475933864423794 | 0.173680080201692 | 0.788368154131883 | -0.292819571 | 0.388006343637695 | 1.0487441350321 | 0.746156757054359 | 1.4740391350287 | 0.999714303421225 |
| ebi-fl187-GCST90094716 | ebi-met1400-GCST90200135 | Arachidoylcarnitine (C20) levels \|\| id:ebi-met1400-GCST90200135 | F-burn/spicy liking (derived food-liking factor) \|\| id:ebi-fl187-GCST90094716 | Weighted mode | 14 | -0.140443447 | 0.140810053698191 | 0.336775884937288 | -0.416431152 | 0.135544258566695 | 0.868972806838883 | 0.659395909368274 | 1.1451598778477 | 0.999714303421225 |
| ebi-fl187-GCST90094716 | ebi-met1400-GCST90200146 | Docosahexaenoylcarnitine (C22:6) levels \|\| id:ebi-met1400-GCST90200146 | F-burn/spicy liking (derived food-liking factor) \|\| id:ebi-fl187-GCST90094716 | Weighted mode | 14 | 0.162044531666716 | 0.208809881003124 | 0.451614438013583 | -0.247222835 | 0.571311898432839 | 1.17591260550329 | 0.780966647359801 | 1.77058836053531 | 0.999714303421225 |
| ebi-fl187-GCST90094716 | ebi-met1400-GCST90200162 | 3-hydroxyphenylacetoylglutamine levels \|\| id:ebi-met1400-GCST90200162 | F-burn/spicy liking (derived food-liking factor) \|\| id:ebi-fl187-GCST90094716 | Weighted mode | 14 | 0.257947504618844 | 0.180280173716225 | 0.17608000235183 | -0.095401636 | 0.611296645102645 | 1.29427087359803 | 0.909007771019406 | 1.84281933295863 | 0.999714303421225 |
| ebi-fl187-GCST90094716 | ebi-met1400-GCST90200172 | 3-carboxy-4-methyl-5-pentyl-2-furanpropionate (3-CMPFP) levels \|\| id:ebi-met1400-GCST90200172 | F-burn/spicy liking (derived food-liking factor) \|\| id:ebi-fl187-GCST90094716 | Weighted mode | 14 | 0.213277738244086 | 0.176306811997888 | 0.247934429797751 | -0.132283613 | 0.558839089759946 | 1.23772836801333 | 0.876092488384133 | 1.74864130590883 | 0.999714303421225 |
| ebi-fl187-GCST90094716 | ebi-met1400-GCST90200222 | Dihydrocaffeate sulfate (2) levels \|\| id:ebi-met1400-GCST90200222 | F-burn/spicy liking (derived food-liking factor) \|\| id:ebi-fl187-GCST90094716 | Weighted mode | 14 | 0.244980627705259 | 0.181137541918218 | 0.199287263286251 | -0.110048954 | 0.600010209864966 | 1.27759656298797 | 0.895790281298591 | 1.82213740407238 | 0.999714303421225 |
| ebi-fl187-GCST90094716 | ebi-met1400-GCST90200223 | 4-methylhexanoylglutamine levels \|\| id:ebi-met1400-GCST90200223 | F-burn/spicy liking (derived food-liking factor) \|\| id:ebi-fl187-GCST90094716 | Weighted mode | 14 | -0.086588707 | 0.206717320317779 | 0.68214556952153 | -0.491754655 | 0.318577240406186 | 0.917054195667812 | 0.611552388602317 | 1.37516983575845 | 0.999714303421225 |
| ebi-fl187-GCST90094716 | ebi-met1400-GCST90200224 | 2-ketocaprylate levels \|\| id:ebi-met1400-GCST90200224 | F-burn/spicy liking (derived food-liking factor) \|\| id:ebi-fl187-GCST90094716 | Weighted mode | 14 | 0.0160687959266769 | 0.151366864048716 | 0.917077684248394 | -0.280610258 | 0.312747849462159 | 1.01619859332529 | 0.755322659380622 | 1.36717675320783 | 0.999714303421225 |
| ebi-fl187-GCST90094716 | ebi-met1400-GCST90200243 | Hydroxypalmitoyl sphingomyelin (d18:1/16:0(OH)) levels \|\| id:ebi-met1400-GCST90200243 | F-burn/spicy liking (derived food-liking factor) \|\| id:ebi-fl187-GCST90094716 | Weighted mode | 14 | 0.0863959812114256 | 0.174487595687279 | 0.62876393121754 | -0.255599706 | 0.428391668758492 | 1.09023795658794 | 0.774451914948273 | 1.53478709141603 | 0.999714303421225 |
| ebi-fl187-GCST90094716 | ebi-met1400-GCST90200273 | 2-methoxyhydroquinone sulfate (2) levels \|\| id:ebi-met1400-GCST90200273 | F-burn/spicy liking (derived food-liking factor) \|\| id:ebi-fl187-GCST90094716 | Weighted mode | 14 | 0.368031181729797 | 0.294792682526854 | 0.233886120476801 | -0.209762476 | 0.94582483948243 | 1.44488709235035 | 0.810776802031552 | 2.57493641210447 | 0.999714303421225 |
| ebi-fl187-GCST90094716 | ebi-met1400-GCST90200276 | 2,4-di-tert-butylphenol levels \|\| id:ebi-met1400-GCST90200276 | F-burn/spicy liking (derived food-liking factor) \|\| id:ebi-fl187-GCST90094716 | Weighted mode | 14 | 0.175615906745551 | 0.215515934626361 | 0.429826710291507 | -0.246795325 | 0.598027138613218 | 1.19198013918351 | 0.781300589770248 | 1.81852755624536 | 0.999714303421225 |
| ebi-fl187-GCST90094716 | ebi-met1400-GCST90200289 | N-acetylmethionine levels \|\| id:ebi-met1400-GCST90200289 | F-burn/spicy liking (derived food-liking factor) \|\| id:ebi-fl187-GCST90094716 | Weighted mode | 14 | 0.0894331514404301 | 0.165789718077938 | 0.59870339807514 | -0.235514696 | 0.414380998873188 | 1.09355422834392 | 0.790164050678656 | 1.51343363356226 | 0.999714303421225 |
| ebi-fl187-GCST90094716 | ebi-met1400-GCST90200308 | 3-hydroxyisobutyrate levels \|\| id:ebi-met1400-GCST90200308 | F-burn/spicy liking (derived food-liking factor) \|\| id:ebi-fl187-GCST90094716 | Weighted mode | 14 | -0.14847763 | 0.137025897487229 | 0.298242217298358 | -0.417048389 | 0.120093128892069 | 0.862019290168677 | 0.658989031182427 | 1.12760185900151 | 0.999714303421225 |
| ebi-fl187-GCST90094716 | ebi-met1400-GCST90200383 | Cystathionine levels \|\| id:ebi-met1400-GCST90200383 | F-burn/spicy liking (derived food-liking factor) \|\| id:ebi-fl187-GCST90094716 | Weighted mode | 14 | -0.006177189 | 0.166843115838551 | 0.971028420050761 | -0.333189696 | 0.320835318087988 | 0.993841850652199 | 0.716634238613088 | 1.37827858465057 | 0.999714303421225 |
| ebi-fl187-GCST90094716 | ebi-met1400-GCST90200384 | Guanidinoacetate levels \|\| id:ebi-met1400-GCST90200384 | F-burn/spicy liking (derived food-liking factor) \|\| id:ebi-fl187-GCST90094716 | Weighted mode | 14 | 0.250575987663018 | 0.136359356267515 | 0.0890762057271101 | -0.016688351 | 0.517840325947348 | 1.28476521252294 | 0.983450128502427 | 1.6783989380556 | 0.999714303421225 |
| ebi-fl187-GCST90094716 | ebi-met1400-GCST90200403 | Citrulline levels \|\| id:ebi-met1400-GCST90200403 | F-burn/spicy liking (derived food-liking factor) \|\| id:ebi-fl187-GCST90094716 | Weighted mode | 14 | -0.01179901 | 0.159715637574915 | 0.942234392399451 | -0.324841659 | 0.301243639959432 | 0.988270325663324 | 0.722641768162278 | 1.35153859023461 | 0.999714303421225 |
| ebi-fl187-GCST90094716 | ebi-met1400-GCST90200434 | 12,13-DiHOME levels \|\| id:ebi-met1400-GCST90200434 | F-burn/spicy liking (derived food-liking factor) \|\| id:ebi-fl187-GCST90094716 | Weighted mode | 14 | 0.0744588441485191 | 0.146765806150897 | 0.620419380370186 | -0.213202136 | 0.362119824204277 | 1.0773010053697 | 0.807992796351585 | 1.43637104366652 | 0.999714303421225 |
| ebi-fl187-GCST90094716 | ebi-met1400-GCST90200437 | Fructose levels \|\| id:ebi-met1400-GCST90200437 | F-burn/spicy liking (derived food-liking factor) \|\| id:ebi-fl187-GCST90094716 | Weighted mode | 14 | 0.0581116956096455 | 0.149921155684247 | 0.704572981678775 | -0.23573377 | 0.351957160750769 | 1.05983336783348 | 0.789990965603436 | 1.42184761152967 | 0.999714303421225 |
| ebi-fl187-GCST90094716 | ebi-met1400-GCST90200448 | N-stearoyl-sphinganine (d18:0/18:0) levels \|\| id:ebi-met1400-GCST90200448 | F-burn/spicy liking (derived food-liking factor) \|\| id:ebi-fl187-GCST90094716 | Weighted mode | 14 | -0.127616162 | 0.166194788239385 | 0.456285708373051 | -0.453357947 | 0.198125623383197 | 0.880191165511395 | 0.635490621256898 | 1.21911553362031 | 0.999714303421225 |
| ebi-fl187-GCST90094716 | ebi-met1400-GCST90200468 | X-11847 levels \|\| id:ebi-met1400-GCST90200468 | F-burn/spicy liking (derived food-liking factor) \|\| id:ebi-fl187-GCST90094716 | Weighted mode | 14 | 0.114316373532175 | 0.164833456209934 | 0.500183752573627 | -0.208757201 | 0.437389947703646 | 1.12110675728779 | 0.811592265806335 | 1.5486598556821 | 0.999714303421225 |
| ebi-fl187-GCST90094716 | ebi-met1400-GCST90200476 | X-11843 levels \|\| id:ebi-met1400-GCST90200476 | F-burn/spicy liking (derived food-liking factor) \|\| id:ebi-fl187-GCST90094716 | Weighted mode | 14 | -0.054119819 | 0.148221158543326 | 0.720886867262605 | -0.34463329 | 0.236393651336816 | 0.947318592548302 | 0.708480112470428 | 1.26667283949365 | 0.999714303421225 |
| ebi-fl187-GCST90094716 | ebi-met1400-GCST90200511 | X-12906 levels \|\| id:ebi-met1400-GCST90200511 | F-burn/spicy liking (derived food-liking factor) \|\| id:ebi-fl187-GCST90094716 | Weighted mode | 14 | -0.135186632 | 0.181883366042517 | 0.470544074398611 | -0.49167803 | 0.221304765313169 | 0.873552863441772 | 0.611599251006801 | 1.24770362941277 | 0.999714303421225 |
| ebi-fl187-GCST90094716 | ebi-met1400-GCST90200515 | X-12849 levels \|\| id:ebi-met1400-GCST90200515 | F-burn/spicy liking (derived food-liking factor) \|\| id:ebi-fl187-GCST90094716 | Weighted mode | 14 | -0.068801278 | 0.18278401040463 | 0.712685718684571 | -0.427057938 | 0.289455382314413 | 0.933512170979248 | 0.652425750581917 | 1.33569984414184 | 0.999714303421225 |
| ebi-fl187-GCST90094716 | ebi-met1400-GCST90200551 | X-18886 levels \|\| id:ebi-met1400-GCST90200551 | F-burn/spicy liking (derived food-liking factor) \|\| id:ebi-fl187-GCST90094716 | Weighted mode | 14 | -0.192034183 | 0.148114375380334 | 0.217336895234287 | -0.482338359 | 0.0982699929003758 | 0.825278657637021 | 0.617338144757699 | 1.10326061743436 | 0.999714303421225 |
| ebi-fl187-GCST90094716 | ebi-met1400-GCST90200564 | X-21319 levels \|\| id:ebi-met1400-GCST90200564 | F-burn/spicy liking (derived food-liking factor) \|\| id:ebi-fl187-GCST90094716 | Weighted mode | 14 | -0.084814297 | 0.12259485365742 | 0.501216557272215 | -0.325100211 | 0.155471615734533 | 0.918682870328196 | 0.72245495236835 | 1.16820877684861 | 0.999714303421225 |
| ebi-fl187-GCST90094716 | ebi-met1400-GCST90200579 | X-21442 levels \|\| id:ebi-met1400-GCST90200579 | F-burn/spicy liking (derived food-liking factor) \|\| id:ebi-fl187-GCST90094716 | Weighted mode | 14 | 0.182518673162615 | 0.189791338645677 | 0.353757280583403 | -0.189472351 | 0.554509696908142 | 1.20023656295684 | 0.827395593587054 | 1.74108711506799 | 0.999714303421225 |
| ebi-fl187-GCST90094716 | ebi-met1400-GCST90200581 | X-21441 levels \|\| id:ebi-met1400-GCST90200581 | F-burn/spicy liking (derived food-liking factor) \|\| id:ebi-fl187-GCST90094716 | Weighted mode | 14 | 0.0506377248562458 | 0.13010659877664 | 0.703427375644685 | -0.204371209 | 0.30564665845846 | 1.05194173190258 | 0.815159720599848 | 1.35750255974854 | 0.999714303421225 |
| ebi-fl187-GCST90094716 | ebi-met1400-GCST90200648 | X-24585 levels \|\| id:ebi-met1400-GCST90200648 | F-burn/spicy liking (derived food-liking factor) \|\| id:ebi-fl187-GCST90094716 | Weighted mode | 14 | -0.022145509 | 0.175127418181946 | 0.901307676510242 | -0.365395249 | 0.321104230274999 | 0.978097902294726 | 0.693922324564316 | 1.37864927039781 | 0.999714303421225 |
| ebi-fl187-GCST90094716 | ebi-met1400-GCST90200720 | Adenosine 5'-diphosphate (ADP) to pantothenate ratio \|\| id:ebi-met1400-GCST90200720 | F-burn/spicy liking (derived food-liking factor) \|\| id:ebi-fl187-GCST90094716 | Weighted mode | 14 | 0.0687313558383604 | 0.216302343339476 | 0.755716399699957 | -0.355221237 | 0.492683948783734 | 1.07114841272533 | 0.701018334782985 | 1.63670315761312 | 0.999714303421225 |
| ebi-fl187-GCST90094716 | ebi-met1400-GCST90200724 | Cholate to taurocholate ratio \|\| id:ebi-met1400-GCST90200724 | F-burn/spicy liking (derived food-liking factor) \|\| id:ebi-fl187-GCST90094716 | Weighted mode | 14 | 0.00494942287257327 | 0.153785474717638 | 0.974814247369647 | -0.296470108 | 0.306368953319143 | 1.00496169149848 | 0.743437850092878 | 1.35848343106732 | 0.999714303421225 |
| ebi-fl187-GCST90094716 | ebi-met1400-GCST90200726 | N-acetylputrescine to (N(1) + N(8))-acetylspermidine ratio \|\| id:ebi-met1400-GCST90200726 | F-burn/spicy liking (derived food-liking factor) \|\| id:ebi-fl187-GCST90094716 | Weighted mode | 14 | -0.044953351 | 0.141230096756451 | 0.755314043580945 | -0.321764341 | 0.231857638381699 | 0.956042078950358 | 0.72486899217532 | 1.26094020656171 | 0.999714303421225 |
| ebi-fl187-GCST90094716 | ebi-met1400-GCST90200744 | Arginine to glutamate ratio \|\| id:ebi-met1400-GCST90200744 | F-burn/spicy liking (derived food-liking factor) \|\| id:ebi-fl187-GCST90094716 | Weighted mode | 14 | -0.003716115 | 0.141064856298197 | 0.979383607117952 | -0.280203234 | 0.272771003041859 | 0.996290780908847 | 0.755630156376908 | 1.31359940011295 | 0.999714303421225 |
| ebi-fl187-GCST90094716 | ebi-met1400-GCST90200759 | Histidine to trans-urocanate ratio \|\| id:ebi-met1400-GCST90200759 | F-burn/spicy liking (derived food-liking factor) \|\| id:ebi-fl187-GCST90094716 | Weighted mode | 14 | 0.0346327193517544 | 0.167568189595135 | 0.839462992123131 | -0.293800932 | 0.36306637095822 | 1.03523941556309 | 0.745424866721933 | 1.4377312796773 | 0.999714303421225 |
| ebi-fl187-GCST90094716 | ebi-met1400-GCST90200795 | Oleoyl-linoleoyl-glycerol (18:1 to 18:2) [2] to linoleoyl-arachidonoyl-glycerol (18:2 to 20:4) [2] ratio \|\| id:ebi-met1400-GCST90200795 | F-burn/spicy liking (derived food-liking factor) \|\| id:ebi-fl187-GCST90094716 | Weighted mode | 14 | 0.0120927613370641 | 0.205819125440014 | 0.954041359317179 | -0.391312725 | 0.415498247199492 | 1.01216617439921 | 0.676168668446429 | 1.51512545967529 | 0.999714303421225 |
| ebi-fl187-GCST90094716 | ebi-met1400-GCST90200811 | Choline to taurocholate ratio \|\| id:ebi-met1400-GCST90200811 | F-burn/spicy liking (derived food-liking factor) \|\| id:ebi-fl187-GCST90094716 | Weighted mode | 14 | -0.121885166 | 0.164773812192218 | 0.472622116286808 | -0.444841838 | 0.201071506282623 | 0.885250019808354 | 0.640925638410562 | 1.22271220030161 | 0.999714303421225 |
| ebi-fl187-GCST90094716 | ebi-met1400-GCST90200831 | Cholate to phosphate ratio \|\| id:ebi-met1400-GCST90200831 | F-burn/spicy liking (derived food-liking factor) \|\| id:ebi-fl187-GCST90094716 | Weighted mode | 14 | -0.115676307 | 0.153892906786863 | 0.46564242118447 | -0.417306404 | 0.18595379029148 | 0.890763510540099 | 0.658819024024347 | 1.20436660566195 | 0.999714303421225 |
| ebi-fl187-GCST90094716 | ebi-met1400-GCST90200841 | Adenosine 5'-diphosphate (ADP) to oxalate (ethanedioate) ratio \|\| id:ebi-met1400-GCST90200841 | F-burn/spicy liking (derived food-liking factor) \|\| id:ebi-fl187-GCST90094716 | Weighted mode | 14 | -0.119260693 | 0.212304149580996 | 0.583845457316743 | -0.535376826 | 0.296855440166691 | 0.887576385637701 | 0.585448636193816 | 1.34562076267419 | 0.999714303421225 |
| ebi-fl187-GCST90094716 | ebi-met1400-GCST90200844 | Aspartate to phosphate ratio \|\| id:ebi-met1400-GCST90200844 | F-burn/spicy liking (derived food-liking factor) \|\| id:ebi-fl187-GCST90094716 | Weighted mode | 14 | 0.0211538925964325 | 0.133442430776414 | 0.876479641727611 | -0.240393272 | 0.282701056918203 | 1.02137922224398 | 0.786318563393434 | 1.32670849220399 | 0.999714303421225 |
| ebi-fl187-GCST90094716 | ebi-met1400-GCST90200870 | Adenosine 5'-monophosphate (AMP) to acetoacetate ratio \|\| id:ebi-met1400-GCST90200870 | F-burn/spicy liking (derived food-liking factor) \|\| id:ebi-fl187-GCST90094716 | Weighted mode | 14 | -0.020895206 | 0.163466919660413 | 0.900243429345921 | -0.341290368 | 0.299499956964239 | 0.979321586639547 | 0.710852469352069 | 1.34918398881329 | 0.999714303421225 |
| ebi-fl187-GCST90094716 | ebi-met1400-GCST90200887 | Cholesterol to cortisol ratio \|\| id:ebi-met1400-GCST90200887 | F-burn/spicy liking (derived food-liking factor) \|\| id:ebi-fl187-GCST90094716 | Weighted mode | 14 | 0.0243777684917217 | 0.135308906685777 | 0.859802080529795 | -0.240827689 | 0.289583225595845 | 1.02467733559604 | 0.785977047516387 | 1.33587061530866 | 0.999714303421225 |
| ebi-fl187-GCST90094716 | ebi-met1400-GCST90200890 | Cortisol to taurocholate ratio \|\| id:ebi-met1400-GCST90200890 | F-burn/spicy liking (derived food-liking factor) \|\| id:ebi-fl187-GCST90094716 | Weighted mode | 14 | 0.0521304495166329 | 0.127588539055682 | 0.689496537273308 | -0.197943087 | 0.30220398606577 | 1.05351316383289 | 0.820416544149267 | 1.35283715849499 | 0.999714303421225 |
| ebi-fl187-GCST90094716 | ebi-met1400-GCST90200948 | Adenosine 5'-diphosphate (ADP) to mannitol to sorbitol ratio \|\| id:ebi-met1400-GCST90200948 | F-burn/spicy liking (derived food-liking factor) \|\| id:ebi-fl187-GCST90094716 | Weighted mode | 14 | 0.0806173773347894 | 0.205526415023315 | 0.701230046648921 | -0.322214396 | 0.483449150780486 | 1.08395607105012 | 0.724542834510752 | 1.62165811046881 | 0.999714303421225 |
| ebi-fl187-GCST90094716 | ebi-met1400-GCST90200972 | Leucine to N-palmitoyl-sphingosine (d18:1 to 16:0) ratio \|\| id:ebi-met1400-GCST90200972 | F-burn/spicy liking (derived food-liking factor) \|\| id:ebi-fl187-GCST90094716 | Weighted mode | 14 | 0.0815310115899042 | 0.17209248505982 | 0.643526786905005 | -0.255770259 | 0.418832282307151 | 1.08494686298948 | 0.774319841275227 | 1.52018537142498 | 0.999714303421225 |
| ebi-fl187-GCST90094716 | ebi-met1400-GCST90200973 | Proline to glutamate ratio \|\| id:ebi-met1400-GCST90200973 | F-burn/spicy liking (derived food-liking factor) \|\| id:ebi-fl187-GCST90094716 | Weighted mode | 14 | -0.015114271 | 0.121096468286663 | 0.902581906719149 | -0.252463349 | 0.222234807168314 | 0.984999376629652 | 0.776884686294624 | 1.24886458579627 | 0.999714303421225 |
| ebi-fl187-GCST90094716 | ebi-met1400-GCST90200983 | Cholesterol to linoleoyl-arachidonoyl-glycerol (18:2 to 20:4) [1] ratio \|\| id:ebi-met1400-GCST90200983 | F-burn/spicy liking (derived food-liking factor) \|\| id:ebi-fl187-GCST90094716 | Weighted mode | 14 | -0.188135459 | 0.125428410184712 | 0.157518899008076 | -0.433975143 | 0.0577042246306638 | 0.828502471240352 | 0.647928360650342 | 1.05940160446503 | 0.999714303421225 |
| ebi-fl187-GCST90094716 | ebi-met1400-GCST90201001 | Serine to alpha-ketobutyrate ratio \|\| id:ebi-met1400-GCST90201001 | F-burn/spicy liking (derived food-liking factor) \|\| id:ebi-fl187-GCST90094716 | Weighted mode | 14 | 0.0202854531799875 | 0.126079390351993 | 0.8746500728591 | -0.226830152 | 0.267401058269894 | 1.02049260131225 | 0.797056149258543 | 1.30656434970334 | 0.999714303421225 |
| ebi-fl187-GCST90094716 | ebi-met1400-GCST90201005 | Fructose to maltose ratio \|\| id:ebi-met1400-GCST90201005 | F-burn/spicy liking (derived food-liking factor) \|\| id:ebi-fl187-GCST90094716 | Weighted mode | 14 | 0.275745023084506 | 0.18903003150306 | 0.168373345771759 | -0.094753839 | 0.646243884830503 | 1.31751188607897 | 0.909596814481155 | 1.90835933275505 | 0.999714303421225 |
| ebi-fl187-GCST90094716 | ebi-met1400-GCST90201020 | Paraxanthine to 5-acetylamino-6-formylamino-3-methyluracil ratio \|\| id:ebi-met1400-GCST90201020 | F-burn/spicy liking (derived food-liking factor) \|\| id:ebi-fl187-GCST90094716 | Weighted mode | 14 | 0.129164318964883 | 0.146138051442841 | 0.392829169169823 | -0.157266262 | 0.41559489979285 | 1.13787708358936 | 0.854476514017193 | 1.5152719075574 | 0.999714303421225 |
| ebi-fl187-GCST90094716 | ebi-pw11-GCST90275197 | Placental weight (fetal genotype, adjusted for sex) \|\| id:ebi-pw11-GCST90275197 | F-burn/spicy liking (derived food-liking factor) \|\| id:ebi-fl187-GCST90094716 | Weighted mode | 14 | -0.048757939 | 0.0601792306235823 | 0.432400140581509 | -0.166709231 | 0.0691933527044559 | 0.952411643210374 | 0.846445695439319 | 1.07164339426629 | 0.999714303421225 |
| ebi-fl187-GCST90094716 | prot-a-1017 | FAS-associated factor 2 \|\| id:prot-a-1017 | F-burn/spicy liking (derived food-liking factor) \|\| id:ebi-fl187-GCST90094716 | Weighted mode | 15 | -0.213102679 | 0.267539891256617 | 0.439025215513398 | -0.737480866 | 0.311275507813643 | 0.808073160772656 | 0.478317344612548 | 1.36516528308219 | 0.999714303421225 |
| ebi-fl187-GCST90094716 | prot-a-1019 | Fas apoptotic inhibitory molecule 3 \|\| id:prot-a-1019 | F-burn/spicy liking (derived food-liking factor) \|\| id:ebi-fl187-GCST90094716 | Weighted mode | 15 | 0.110141841484745 | 0.228640283085987 | 0.637444297698339 | -0.337993113 | 0.558276796333281 | 1.11643641622751 | 0.713200199431689 | 1.74765833278249 | 0.999714303421225 |
| ebi-fl187-GCST90094716 | prot-a-1052 | MIP18 family protein FAM96A \|\| id:prot-a-1052 | F-burn/spicy liking (derived food-liking factor) \|\| id:ebi-fl187-GCST90094716 | Weighted mode | 15 | -0.053980105 | 0.242145396576901 | 0.826814080516166 | -0.528585082 | 0.420624872616135 | 0.947450956159369 | 0.589438386934768 | 1.52291288491641 | 0.999714303421225 |
| ebi-fl187-GCST90094716 | prot-a-1066 | F-box/LRR-repeat protein 4 \|\| id:prot-a-1066 | F-burn/spicy liking (derived food-liking factor) \|\| id:ebi-fl187-GCST90094716 | Weighted mode | 15 | 0.0401255419375195 | 0.284491678287739 | 0.889845362770246 | -0.517478148 | 0.597729231381489 | 1.04094144779591 | 0.596021733184803 | 1.81798588442323 | 0.999714303421225 |
| ebi-fl187-GCST90094716 | prot-a-1076 | Low affinity immunoglobulin gamma Fc region receptor III-B \|\| id:prot-a-1076 | F-burn/spicy liking (derived food-liking factor) \|\| id:ebi-fl187-GCST90094716 | Weighted mode | 15 | -0.172109591 | 0.209220208319427 | 0.424512116561968 | -0.582181199 | 0.237962017616303 | 0.841886905146087 | 0.558678447736992 | 1.2686610051407 | 0.999714303421225 |
| ebi-fl187-GCST90094716 | prot-a-1081 | Fc receptor-like protein 3 \|\| id:prot-a-1081 | F-burn/spicy liking (derived food-liking factor) \|\| id:ebi-fl187-GCST90094716 | Weighted mode | 15 | 0.0967020846948934 | 0.255192195768858 | 0.710418725111155 | -0.403474619 | 0.596878788401856 | 1.10153216144376 | 0.667994980956382 | 1.81644044833654 | 0.999714303421225 |
| ebi-fl187-GCST90094716 | prot-a-1105 | Fibroblast growth factor receptor 4 \|\| id:prot-a-1105 | F-burn/spicy liking (derived food-liking factor) \|\| id:ebi-fl187-GCST90094716 | Weighted mode | 15 | 0.290983353468613 | 0.304505364076077 | 0.355488311149008 | -0.30584716 | 0.887813867057724 | 1.33774231499525 | 0.736499177299729 | 2.42981194886056 | 0.999714303421225 |
| ebi-fl187-GCST90094716 | prot-a-1143 | Follistatin-related protein 1 \|\| id:prot-a-1143 | F-burn/spicy liking (derived food-liking factor) \|\| id:ebi-fl187-GCST90094716 | Weighted mode | 15 | -0.036361527 | 0.206881312499097 | 0.862999126823209 | -0.4418489 | 0.369125845265239 | 0.96429161277957 | 0.642846762435977 | 1.44646962357477 | 0.999714303421225 |
| ebi-fl187-GCST90094716 | prot-a-1151 | Alpha-(1,3)-fucosyltransferase 10 \|\| id:prot-a-1151 | F-burn/spicy liking (derived food-liking factor) \|\| id:ebi-fl187-GCST90094716 | Weighted mode | 15 | -0.134011419 | 0.219274931764475 | 0.550890542728663 | -0.563790285 | 0.295767447434007 | 0.874580077870014 | 0.569048116545211 | 1.3441575331993 | 0.999714303421225 |
| ebi-fl187-GCST90094716 | prot-a-1165 | Growth arrest and DNA damage-inducible proteins-interacting protein 1 \|\| id:prot-a-1165 | F-burn/spicy liking (derived food-liking factor) \|\| id:ebi-fl187-GCST90094716 | Weighted mode | 15 | 0.239681537865827 | 0.308528093638372 | 0.450171756513303 | -0.365033526 | 0.844396601397035 | 1.27084437006094 | 0.694173377863477 | 2.32657353972054 | 0.999714303421225 |
| ebi-fl187-GCST90094716 | prot-a-1194 | Growth/differentiation factor 11/8 \|\| id:prot-a-1194 | F-burn/spicy liking (derived food-liking factor) \|\| id:ebi-fl187-GCST90094716 | Weighted mode | 15 | 0.0473336773671305 | 0.212395055494856 | 0.826865653092143 | -0.368960631 | 0.463627986137049 | 1.04847180202783 | 0.691452631434617 | 1.58983142108618 | 0.999714303421225 |
| ebi-fl187-GCST90094716 | prot-a-1216 | D-glucuronyl C5-epimerase \|\| id:prot-a-1216 | F-burn/spicy liking (derived food-liking factor) \|\| id:ebi-fl187-GCST90094716 | Weighted mode | 15 | 0.00416360396702742 | 0.272059373163617 | 0.988005589315889 | -0.529072767 | 0.537399975367717 | 1.00417228380832 | 0.589150996482746 | 1.71155099726347 | 0.999714303421225 |
| ebi-fl187-GCST90094716 | prot-a-1246 | Glypican-3 \|\| id:prot-a-1246 | F-burn/spicy liking (derived food-liking factor) \|\| id:ebi-fl187-GCST90094716 | Weighted mode | 15 | 0.146514287452805 | 0.265800649468574 | 0.590173250578307 | -0.374454986 | 0.66748356041121 | 1.15779147259131 | 0.687663963504784 | 1.94932578286229 | 0.999714303421225 |
| ebi-fl187-GCST90094716 | prot-a-1275 | Glutamate receptor 4 \|\| id:prot-a-1275 | F-burn/spicy liking (derived food-liking factor) \|\| id:ebi-fl187-GCST90094716 | Weighted mode | 15 | -0.386998724 | 0.269402636947806 | 0.172827613172309 | -0.915027892 | 0.141030444436973 | 0.679091961460515 | 0.400505454877304 | 1.15145970299348 | 0.999714303421225 |
| ebi-fl187-GCST90094716 | prot-a-1289 | Glutathione S-transferase P \|\| id:prot-a-1289 | F-burn/spicy liking (derived food-liking factor) \|\| id:ebi-fl187-GCST90094716 | Weighted mode | 15 | 0.223011006388218 | 0.234347095056573 | 0.357427517819184 | -0.2363093 | 0.682331312699101 | 1.24983432982212 | 0.789536432605291 | 1.9784848266563 | 0.999714303421225 |
| ebi-fl187-GCST90094716 | prot-a-130 | Apolipoprotein D \|\| id:prot-a-130 | F-burn/spicy liking (derived food-liking factor) \|\| id:ebi-fl187-GCST90094716 | Weighted mode | 15 | 0.181084957412287 | 0.231492905564619 | 0.44709803053963 | -0.272641137 | 0.634811052318939 | 1.19851699787074 | 0.761365964307225 | 1.88666562668338 | 0.999714303421225 |
| ebi-fl187-GCST90094716 | prot-a-1313 | Heparin-binding EGF-like growth factor \|\| id:prot-a-1313 | F-burn/spicy liking (derived food-liking factor) \|\| id:ebi-fl187-GCST90094716 | Weighted mode | 15 | -0.215004263 | 0.23990460485663 | 0.385293275072058 | -0.685217288 | 0.255208762817445 | 0.806538002139207 | 0.503980708605086 | 1.29073104939902 | 0.999714303421225 |
| ebi-fl187-GCST90094716 | prot-a-1347 | HLA class II histocompatibility antigen, DQ alpha 2 chain \|\| id:prot-a-1347 | F-burn/spicy liking (derived food-liking factor) \|\| id:ebi-fl187-GCST90094716 | Weighted mode | 15 | -0.076716094 | 0.216994424842786 | 0.728951301442238 | -0.502025167 | 0.34859297853319 | 0.926152756462425 | 0.605303576869888 | 1.41707229410165 | 0.999714303421225 |
| ebi-fl187-GCST90094716 | prot-a-1357 | Non-histone chromosomal protein HMG-14 \|\| id:prot-a-1357 | F-burn/spicy liking (derived food-liking factor) \|\| id:ebi-fl187-GCST90094716 | Weighted mode | 15 | -0.036099981 | 0.222048735899954 | 0.873174894140968 | -0.471315503 | 0.399115541574809 | 0.96454385280657 | 0.624180616389457 | 1.49050582404894 | 0.999714303421225 |
| ebi-fl187-GCST90094716 | prot-a-137 | Apolipoprotein M \|\| id:prot-a-137 | F-burn/spicy liking (derived food-liking factor) \|\| id:ebi-fl187-GCST90094716 | Weighted mode | 15 | 0.0822904039338397 | 0.217205095745644 | 0.710475267469572 | -0.343431584 | 0.508012391595301 | 1.08577107624182 | 0.709332009336302 | 1.66198453543127 | 0.999714303421225 |
| ebi-fl187-GCST90094716 | prot-a-1381 | 17-beta-hydroxysteroid dehydrogenase 14 \|\| id:prot-a-1381 | F-burn/spicy liking (derived food-liking factor) \|\| id:ebi-fl187-GCST90094716 | Weighted mode | 15 | -0.202234327 | 0.238083482672574 | 0.409935007872506 | -0.668877953 | 0.264409299119836 | 0.816903483036947 | 0.512283061121693 | 1.30266126531045 | 0.999714303421225 |
| ebi-fl187-GCST90094716 | prot-a-1392 | Serine protease HTRA2, mitochondrial \|\| id:prot-a-1392 | F-burn/spicy liking (derived food-liking factor) \|\| id:ebi-fl187-GCST90094716 | Weighted mode | 15 | -0.096603291 | 0.212433018306324 | 0.656262717792819 | -0.512972007 | 0.319765424759346 | 0.90791611308314 | 0.598713551158726 | 1.37680476214487 | 0.999714303421225 |
| ebi-fl187-GCST90094716 | prot-a-1416 | Interferon-induced protein with tetratricopeptide repeats 2 \|\| id:prot-a-1416 | F-burn/spicy liking (derived food-liking factor) \|\| id:ebi-fl187-GCST90094716 | Weighted mode | 15 | -0.058401385 | 0.270346096374571 | 0.832084452413198 | -0.588279734 | 0.471476963888202 | 0.94327125650254 | 0.555281695855858 | 1.60235907285309 | 0.999714303421225 |
| ebi-fl187-GCST90094716 | prot-a-1428 | Interferon gamma \|\| id:prot-a-1428 | F-burn/spicy liking (derived food-liking factor) \|\| id:ebi-fl187-GCST90094716 | Weighted mode | 15 | -0.122991192 | 0.269540949951241 | 0.655173621591729 | -0.651291454 | 0.405309069897247 | 0.884271451183107 | 0.521372013861085 | 1.49976596094362 | 0.999714303421225 |
| ebi-fl187-GCST90094716 | prot-a-1483 | Interleukin-17C \|\| id:prot-a-1483 | F-burn/spicy liking (derived food-liking factor) \|\| id:ebi-fl187-GCST90094716 | Weighted mode | 15 | 0.234798799672616 | 0.250660160414401 | 0.364780457889008 | -0.256495115 | 0.726092714084841 | 1.26465429427554 | 0.773758774563333 | 2.06698849383925 | 0.999714303421225 |
| ebi-fl187-GCST90094716 | prot-a-1503 | Interleukin-1 receptor-like 2 \|\| id:prot-a-1503 | F-burn/spicy liking (derived food-liking factor) \|\| id:ebi-fl187-GCST90094716 | Weighted mode | 15 | 0.0346940225863863 | 0.2531371023818 | 0.892937848362176 | -0.461454698 | 0.530842743254714 | 1.03530288103318 | 0.63036598601922 | 1.70036467583599 | 0.999714303421225 |
| ebi-fl187-GCST90094716 | prot-a-1522 | Interleukin-31 receptor subunit alpha \|\| id:prot-a-1522 | F-burn/spicy liking (derived food-liking factor) \|\| id:ebi-fl187-GCST90094716 | Weighted mode | 15 | 0.0830247391797783 | 0.252459345017217 | 0.747126392853128 | -0.411795577 | 0.577845055413524 | 1.08656868903388 | 0.662459684171654 | 1.78219376091553 | 0.999714303421225 |
| ebi-fl187-GCST90094716 | prot-a-1540 | Interleukin-6 receptor subunit alpha \|\| id:prot-a-1540 | F-burn/spicy liking (derived food-liking factor) \|\| id:ebi-fl187-GCST90094716 | Weighted mode | 15 | 0.0420664673123241 | 0.259064536731622 | 0.873328293499698 | -0.465700025 | 0.549832959306304 | 1.04296379944756 | 0.627695548980641 | 1.73296351826072 | 0.999714303421225 |
| ebi-fl187-GCST90094716 | prot-a-1541 | Interleukin-6 receptor subunit alpha \|\| id:prot-a-1541 | F-burn/spicy liking (derived food-liking factor) \|\| id:ebi-fl187-GCST90094716 | Weighted mode | 15 | -0.226738026 | 0.303711969196314 | 0.46768413989899 | -0.822013486 | 0.368537433491145 | 0.797129582057514 | 0.439545743825669 | 1.44561875417272 | 0.999714303421225 |
| ebi-fl187-GCST90094716 | prot-a-1545 | Interleukin-9 \|\| id:prot-a-1545 | F-burn/spicy liking (derived food-liking factor) \|\| id:ebi-fl187-GCST90094716 | Weighted mode | 15 | 0.16325988063057 | 0.297516431042761 | 0.591826617382353 | -0.419872324 | 0.746392085474382 | 1.17734261847635 | 0.657130714140236 | 2.10937582349093 | 0.999714303421225 |
| ebi-fl187-GCST90094716 | prot-a-1548 | Inositol monophosphatase 3 \|\| id:prot-a-1548 | F-burn/spicy liking (derived food-liking factor) \|\| id:ebi-fl187-GCST90094716 | Weighted mode | 15 | 0.133048714738058 | 0.239122776794732 | 0.586719239281176 | -0.335631928 | 0.601729357255733 | 1.14230564409573 | 0.714886187142846 | 1.82527262101406 | 0.999714303421225 |
| ebi-fl187-GCST90094716 | prot-a-1555 | Inhibin beta A chain:Inhibin beta B chain heterodimer \|\| id:prot-a-1555 | F-burn/spicy liking (derived food-liking factor) \|\| id:ebi-fl187-GCST90094716 | Weighted mode | 15 | -0.112710187 | 0.225526659067891 | 0.625003654560797 | -0.554742439 | 0.329322064955251 | 0.89340954446323 | 0.574220139099968 | 1.39002546199279 | 0.999714303421225 |
| ebi-fl187-GCST90094716 | prot-a-1603 | Junctophilin-3 \|\| id:prot-a-1603 | F-burn/spicy liking (derived food-liking factor) \|\| id:ebi-fl187-GCST90094716 | Weighted mode | 15 | 0.306296922189542 | 0.279080255639029 | 0.290931604810314 | -0.240700379 | 0.853294223242039 | 1.35838558149539 | 0.786077116427116 | 2.34736687972986 | 0.999714303421225 |
| ebi-fl187-GCST90094716 | prot-a-1627 | UPF0258 protein KIAA1024 \|\| id:prot-a-1627 | F-burn/spicy liking (derived food-liking factor) \|\| id:ebi-fl187-GCST90094716 | Weighted mode | 15 | -0.15182807 | 0.2269661291516 | 0.514410178577515 | -0.596681683 | 0.293025543305657 | 0.859135979437556 | 0.550635791983027 | 1.34047703020889 | 0.999714303421225 |
| ebi-fl187-GCST90094716 | prot-a-1666 | Kallikrein-7 \|\| id:prot-a-1666 | F-burn/spicy liking (derived food-liking factor) \|\| id:ebi-fl187-GCST90094716 | Weighted mode | 15 | -0.144139384 | 0.229398557343982 | 0.539899730837281 | -0.593760557 | 0.305481788011443 | 0.865767065240746 | 0.552246620187847 | 1.35727876614367 | 0.999714303421225 |
| ebi-fl187-GCST90094716 | prot-a-1678 | Importin subunit alpha-3 \|\| id:prot-a-1678 | F-burn/spicy liking (derived food-liking factor) \|\| id:ebi-fl187-GCST90094716 | Weighted mode | 15 | -0.131191474 | 0.263839305540374 | 0.626737546129793 | -0.648316513 | 0.385933564829354 | 0.877049826048173 | 0.522925374285398 | 1.47098694229994 | 0.999714303421225 |
| ebi-fl187-GCST90094716 | prot-a-1718 | Protein LDOC1 \|\| id:prot-a-1718 | F-burn/spicy liking (derived food-liking factor) \|\| id:ebi-fl187-GCST90094716 | Weighted mode | 15 | 0.0841923883803075 | 0.217990121486028 | 0.705138941655043 | -0.34306825 | 0.511453026492923 | 1.08783816109973 | 0.709589780594953 | 1.66771266597532 | 0.999714303421225 |
| ebi-fl187-GCST90094716 | prot-a-1723 | Leptin \|\| id:prot-a-1723 | F-burn/spicy liking (derived food-liking factor) \|\| id:ebi-fl187-GCST90094716 | Weighted mode | 15 | -0.352768976 | 0.341541789369093 | 0.319177359213674 | -1.022190884 | 0.316652930692427 | 0.702739523990227 | 0.359805783410978 | 1.37252612755792 | 0.999714303421225 |
| ebi-fl187-GCST90094716 | prot-a-1730 | Galectin-7 \|\| id:prot-a-1730 | F-burn/spicy liking (derived food-liking factor) \|\| id:ebi-fl187-GCST90094716 | Weighted mode | 15 | 0.331694715574415 | 0.29423112025315 | 0.278555410591738 | -0.24499828 | 0.90838771127059 | 1.39332742241604 | 0.78270588439953 | 2.48032031539647 | 0.999714303421225 |
| ebi-fl187-GCST90094716 | prot-a-1753 | Lipase member N \|\| id:prot-a-1753 | F-burn/spicy liking (derived food-liking factor) \|\| id:ebi-fl187-GCST90094716 | Weighted mode | 15 | -0.07289263 | 0.227344965989122 | 0.753229842232061 | -0.518488763 | 0.372703503460526 | 0.929700646738752 | 0.595419688526653 | 1.45165386567119 | 0.999714303421225 |
| ebi-fl187-GCST90094716 | prot-a-1756 | VIP36-like protein \|\| id:prot-a-1756 | F-burn/spicy liking (derived food-liking factor) \|\| id:ebi-fl187-GCST90094716 | Weighted mode | 15 | 0.26921108807915 | 0.215070579675304 | 0.231177634263808 | -0.152327248 | 0.690749424242746 | 1.30893141170416 | 0.858707224461762 | 1.99521023200862 | 0.999714303421225 |
| ebi-fl187-GCST90094716 | prot-a-1760 | Ig Kappa chain V-I region HK102- like \|\| id:prot-a-1760 | F-burn/spicy liking (derived food-liking factor) \|\| id:ebi-fl187-GCST90094716 | Weighted mode | 15 | 0.260600708274597 | 0.294861907513555 | 0.391725994992256 | -0.31732863 | 0.838530047001166 | 1.29770939734616 | 0.728091442785276 | 2.31296452753007 | 0.999714303421225 |
| ebi-fl187-GCST90094716 | prot-a-179 | Neutral ceramidase \|\| id:prot-a-179 | F-burn/spicy liking (derived food-liking factor) \|\| id:ebi-fl187-GCST90094716 | Weighted mode | 15 | 0.500877231342797 | 0.296392953757794 | 0.113180107118392 | -0.080052958 | 1.08180742070807 | 1.65016821523408 | 0.923067461264853 | 2.95000663855869 | 0.999714303421225 |
| ebi-fl187-GCST90094716 | prot-a-1809 | Lactotransferrin \|\| id:prot-a-1809 | F-burn/spicy liking (derived food-liking factor) \|\| id:ebi-fl187-GCST90094716 | Weighted mode | 15 | 0.040352513026561 | 0.220656985674303 | 0.857518427104789 | -0.392135179 | 0.472840204948195 | 1.04117773822448 | 0.67561277919851 | 1.60454496414391 | 0.999714303421225 |
| ebi-fl187-GCST90094716 | prot-a-1818 | Lysophospholipase-like protein 1 \|\| id:prot-a-1818 | F-burn/spicy liking (derived food-liking factor) \|\| id:ebi-fl187-GCST90094716 | Weighted mode | 15 | -0.143079126 | 0.274157567369841 | 0.609907717977027 | -0.680427958 | 0.394269705848915 | 0.866685488656456 | 0.506400227835139 | 1.48330054956495 | 0.999714303421225 |
| ebi-fl187-GCST90094716 | prot-a-1852 | MAP kinase-activated protein kinase 3 \|\| id:prot-a-1852 | F-burn/spicy liking (derived food-liking factor) \|\| id:ebi-fl187-GCST90094716 | Weighted mode | 15 | -0.434110004 | 0.337302160355373 | 0.218964910128485 | -1.095222238 | 0.2270022307922 | 0.647840986786724 | 0.334465267862842 | 1.25483266720808 | 0.999714303421225 |
| ebi-fl187-GCST90094716 | prot-a-1856 | Megakaryocyte-associated tyrosine-protein kinase \|\| id:prot-a-1856 | F-burn/spicy liking (derived food-liking factor) \|\| id:ebi-fl187-GCST90094716 | Weighted mode | 15 | -0.091685151 | 0.20149744017939 | 0.656071833832998 | -0.486620134 | 0.30324983183569 | 0.912392370229227 | 0.614700492621795 | 1.35425275763475 | 0.999714303421225 |
| ebi-fl187-GCST90094716 | prot-a-1890 | Beta-1,3-N-acetylglucosaminyltransferase manic fringe \|\| id:prot-a-1890 | F-burn/spicy liking (derived food-liking factor) \|\| id:ebi-fl187-GCST90094716 | Weighted mode | 15 | 0.013836020197316 | 0.256630326631274 | 0.957765320406601 | -0.48915942 | 0.516831460394614 | 1.01393218090679 | 0.613141572172673 | 1.67670651304144 | 0.999714303421225 |
| ebi-fl187-GCST90094716 | prot-a-1919 | Matrilysin \|\| id:prot-a-1919 | F-burn/spicy liking (derived food-liking factor) \|\| id:ebi-fl187-GCST90094716 | Weighted mode | 15 | 0.0383224821944714 | 0.228068603029838 | 0.868962507224007 | -0.40869198 | 0.485336944132954 | 1.03906625922305 | 0.664518886079247 | 1.6247223572921 | 0.999714303421225 |
| ebi-fl187-GCST90094716 | prot-a-1923 | Multimerin-2 \|\| id:prot-a-1923 | F-burn/spicy liking (derived food-liking factor) \|\| id:ebi-fl187-GCST90094716 | Weighted mode | 15 | -0.209427311 | 0.23064341919556 | 0.379236278802809 | -0.661488413 | 0.242633790499715 | 0.811048591501323 | 0.51608261860238 | 1.2746017673637 | 0.999714303421225 |
| ebi-fl187-GCST90094716 | prot-a-1948 | Mesothelin \|\| id:prot-a-1948 | F-burn/spicy liking (derived food-liking factor) \|\| id:ebi-fl187-GCST90094716 | Weighted mode | 15 | 0.162358921312065 | 0.236189097731379 | 0.503053593314855 | -0.30057171 | 0.625289552865567 | 1.17628235837049 | 0.740394808364023 | 1.86878699172802 | 0.999714303421225 |
| ebi-fl187-GCST90094716 | prot-a-195 | ATPase family AAA domain-containing protein 1 \|\| id:prot-a-195 | F-burn/spicy liking (derived food-liking factor) \|\| id:ebi-fl187-GCST90094716 | Weighted mode | 15 | -0.115331731 | 0.246283563080021 | 0.646785801503458 | -0.598047514 | 0.367384053098361 | 0.891070499575767 | 0.549884229805182 | 1.44395236702008 | 0.999714303421225 |
| ebi-fl187-GCST90094716 | prot-a-1966 | Mucin-1 \|\| id:prot-a-1966 | F-burn/spicy liking (derived food-liking factor) \|\| id:ebi-fl187-GCST90094716 | Weighted mode | 15 | -0.17889231 | 0.245468855249001 | 0.478152315961312 | -0.660011266 | 0.302226646425854 | 0.836195944604971 | 0.51684551159972 | 1.35286781461948 | 0.999714303421225 |
| ebi-fl187-GCST90094716 | prot-a-1986 | Myocardial zonula adherens protein \|\| id:prot-a-1986 | F-burn/spicy liking (derived food-liking factor) \|\| id:ebi-fl187-GCST90094716 | Weighted mode | 15 | 0.0744432135053821 | 0.295150474625464 | 0.804535259459612 | -0.504051717 | 0.652938143771292 | 1.07728416659373 | 0.604078141083448 | 1.92117723960688 | 0.999714303421225 |
| ebi-fl187-GCST90094716 | prot-a-2071 | Epididymal secretory protein E1 \|\| id:prot-a-2071 | F-burn/spicy liking (derived food-liking factor) \|\| id:ebi-fl187-GCST90094716 | Weighted mode | 14 | -0.45048361 | 0.321374440551193 | 0.184414854144636 | -1.080377513 | 0.17941029375666 | 0.637319862999254 | 0.339467348045873 | 1.19651156469546 | 0.999714303421225 |
| ebi-fl187-GCST90094716 | prot-a-2098 | Neuritin \|\| id:prot-a-2098 | F-burn/spicy liking (derived food-liking factor) \|\| id:ebi-fl187-GCST90094716 | Weighted mode | 15 | 0.196635187957558 | 0.256531457796729 | 0.456101365431024 | -0.306166469 | 0.699436845239146 | 1.21729987403885 | 0.736264043876154 | 2.01261897231022 | 0.999714303421225 |
| ebi-fl187-GCST90094716 | prot-a-2115 | Neurotrophin-4 \|\| id:prot-a-2115 | F-burn/spicy liking (derived food-liking factor) \|\| id:ebi-fl187-GCST90094716 | Weighted mode | 15 | 0.119484644249269 | 0.214202349599583 | 0.585782589513568 | -0.300351961 | 0.539321249464452 | 1.12691593929408 | 0.74055752746481 | 1.71484251680287 | 0.999714303421225 |
| ebi-fl187-GCST90094716 | prot-a-2118 | Netrin-1 \|\| id:prot-a-2118 | F-burn/spicy liking (derived food-liking factor) \|\| id:ebi-fl187-GCST90094716 | Weighted mode | 15 | 0.186960523306745 | 0.244757247840949 | 0.45763111608547 | -0.292763682 | 0.666684729075006 | 1.20557969181083 | 0.746198459645975 | 1.94776922213999 | 0.999714303421225 |
| ebi-fl187-GCST90094716 | prot-a-2120 | Netrin-G1 \|\| id:prot-a-2120 | F-burn/spicy liking (derived food-liking factor) \|\| id:ebi-fl187-GCST90094716 | Weighted mode | 15 | 0.209176799100055 | 0.224616950974825 | 0.367500809192123 | -0.231072425 | 0.649426023010711 | 1.23266291295983 | 0.79368198168642 | 1.9144416681327 | 0.999714303421225 |
| ebi-fl187-GCST90094716 | prot-a-2145 | Olfactomedin-like protein 3 \|\| id:prot-a-2145 | F-burn/spicy liking (derived food-liking factor) \|\| id:ebi-fl187-GCST90094716 | Weighted mode | 15 | -0.454635564 | 0.286614775299652 | 0.135010406728122 | -1.016400523 | 0.10712939591572 | 0.634679225973083 | 0.361895232054469 | 1.11307827294382 | 0.999714303421225 |
| ebi-fl187-GCST90094716 | prot-a-2162 | Prolyl 4-hydroxylase subunit alpha-2 \|\| id:prot-a-2162 | F-burn/spicy liking (derived food-liking factor) \|\| id:ebi-fl187-GCST90094716 | Weighted mode | 15 | -0.064979089 | 0.211336102340027 | 0.76301350649569 | -0.47919785 | 0.349239671651733 | 0.93708705866118 | 0.619279948330069 | 1.41798900138492 | 0.999714303421225 |
| ebi-fl187-GCST90094716 | prot-a-2168 | Polyadenylate-binding protein-interacting protein 1 \|\| id:prot-a-2168 | F-burn/spicy liking (derived food-liking factor) \|\| id:ebi-fl187-GCST90094716 | Weighted mode | 15 | -0.19090904 | 0.280750736778779 | 0.507596957552671 | -0.741180484 | 0.35936240430768 | 0.826207736771929 | 0.476551022550334 | 1.43241582118249 | 0.999714303421225 |
| ebi-fl187-GCST90094716 | prot-a-219 | Beta-1,4-galactosyltransferase 5 \|\| id:prot-a-219 | F-burn/spicy liking (derived food-liking factor) \|\| id:ebi-fl187-GCST90094716 | Weighted mode | 15 | -0.083985177 | 0.231606695741225 | 0.722304749888019 | -0.5379343 | 0.369963947008631 | 0.919444885252809 | 0.583953279449395 | 1.44768242044064 | 0.999714303421225 |
| ebi-fl187-GCST90094716 | prot-a-2190 | Pyruvate carboxylase, mitochondrial \|\| id:prot-a-2190 | F-burn/spicy liking (derived food-liking factor) \|\| id:ebi-fl187-GCST90094716 | Weighted mode | 15 | -0.094985903 | 0.211257330976802 | 0.659865838012167 | -0.509050272 | 0.319078465540081 | 0.909385753830204 | 0.601066157248308 | 1.37585927821202 | 0.999714303421225 |
| ebi-fl187-GCST90094716 | prot-a-2197 | Protocadherin-9 \|\| id:prot-a-2197 | F-burn/spicy liking (derived food-liking factor) \|\| id:ebi-fl187-GCST90094716 | Weighted mode | 15 | -0.146787492 | 0.216685084224844 | 0.509178042931399 | -0.571490257 | 0.27791527324333 | 0.863477453927713 | 0.564683288168364 | 1.32037432143588 | 0.999714303421225 |
| ebi-fl187-GCST90094716 | prot-a-2198 | Protocadherin alpha-4 \|\| id:prot-a-2198 | F-burn/spicy liking (derived food-liking factor) \|\| id:ebi-fl187-GCST90094716 | Weighted mode | 15 | 0.0133729100421907 | 0.237630165346194 | 0.955917098315606 | -0.452382214 | 0.479128034120731 | 1.01346272732995 | 0.636110992705124 | 1.61466585464777 | 0.999714303421225 |
| ebi-fl187-GCST90094716 | prot-a-2254 | Placenta growth factor \|\| id:prot-a-2254 | F-burn/spicy liking (derived food-liking factor) \|\| id:ebi-fl187-GCST90094716 | Weighted mode | 15 | -0.248243311 | 0.228701838094613 | 0.296062114281704 | -0.696498914 | 0.200012291239848 | 0.780170095884717 | 0.498326938628685 | 1.22141777080668 | 0.999714303421225 |
| ebi-fl187-GCST90094716 | prot-a-2261 | Membrane-associated progesterone receptor component 2 \|\| id:prot-a-2261 | F-burn/spicy liking (derived food-liking factor) \|\| id:ebi-fl187-GCST90094716 | Weighted mode | 15 | 0.263956950910722 | 0.28558669145222 | 0.371006887836762 | -0.295792964 | 0.823706866157073 | 1.30207214207447 | 0.743941434486355 | 2.27893189513898 | 0.999714303421225 |
| ebi-fl187-GCST90094716 | prot-a-2268 | Polymeric immunoglobulin receptor \|\| id:prot-a-2268 | F-burn/spicy liking (derived food-liking factor) \|\| id:ebi-fl187-GCST90094716 | Weighted mode | 15 | 0.123816198419028 | 0.292916023203112 | 0.678933170544519 | -0.450299207 | 0.697931603897127 | 1.13180782381269 | 0.637437397316686 | 2.00959177393102 | 0.999714303421225 |
| ebi-fl187-GCST90094716 | prot-a-2283 | Group 10 secretory phospholipase A2 \|\| id:prot-a-2283 | F-burn/spicy liking (derived food-liking factor) \|\| id:ebi-fl187-GCST90094716 | Weighted mode | 15 | 0.00621818886174586 | 0.241122856741262 | 0.979790046555831 | -0.46638261 | 0.478818988074619 | 1.00623756193243 | 0.627267239189809 | 1.61416692564958 | 0.999714303421225 |
| ebi-fl187-GCST90094716 | prot-a-2298 | Inactive phospholipase D5 \|\| id:prot-a-2298 | F-burn/spicy liking (derived food-liking factor) \|\| id:ebi-fl187-GCST90094716 | Weighted mode | 15 | -0.12862746 | 0.265770987930942 | 0.635884104195649 | -0.649538596 | 0.392283676572292 | 0.879301479709115 | 0.522286706288153 | 1.48035759461216 | 0.999714303421225 |
| ebi-fl187-GCST90094716 | prot-a-2304 | Procollagen-lysine,2-oxoglutarate 5-dioxygenase 2 \|\| id:prot-a-2304 | F-burn/spicy liking (derived food-liking factor) \|\| id:ebi-fl187-GCST90094716 | Weighted mode | 15 | 0.0268334067714167 | 0.24219250201713 | 0.913352590119415 | -0.447863897 | 0.501530710724991 | 1.02719666449992 | 0.638991646677542 | 1.65124691855669 | 0.999714303421225 |
| ebi-fl187-GCST90094716 | prot-a-2331 | Periostin \|\| id:prot-a-2331 | F-burn/spicy liking (derived food-liking factor) \|\| id:ebi-fl187-GCST90094716 | Weighted mode | 15 | 0.0146992694855688 | 0.244333168644008 | 0.952877959366624 | -0.464193741 | 0.493592280027824 | 1.01480783503982 | 0.628641748951396 | 1.63819050162674 | 0.999714303421225 |
| ebi-fl187-GCST90094716 | prot-a-2333 | Periostin \|\| id:prot-a-2333 | F-burn/spicy liking (derived food-liking factor) \|\| id:ebi-fl187-GCST90094716 | Weighted mode | 15 | -0.102666925 | 0.250470716489525 | 0.688085528074867 | -0.593589529 | 0.388255679403456 | 0.902427499545069 | 0.552341077646774 | 1.47440671152828 | 0.999714303421225 |
| ebi-fl187-GCST90094716 | prot-a-2367 | cAMP-dependent protein kinase catalytic subunit alpha \|\| id:prot-a-2367 | F-burn/spicy liking (derived food-liking factor) \|\| id:ebi-fl187-GCST90094716 | Weighted mode | 15 | 0.0649713259103966 | 0.235112828070153 | 0.786323755114942 | -0.395849817 | 0.525792468927896 | 1.06712842500941 | 0.673107777620101 | 1.69179901544056 | 0.999714303421225 |
| ebi-fl187-GCST90094716 | prot-a-2385 | Phosphoribosyl pyrophosphate synthase-associated protein 1 \|\| id:prot-a-2385 | F-burn/spicy liking (derived food-liking factor) \|\| id:ebi-fl187-GCST90094716 | Weighted mode | 15 | 0.374414314113813 | 0.228374717810614 | 0.123383261363952 | -0.073200133 | 0.822028761022616 | 1.45413949618479 | 0.929414805028926 | 2.27511081480862 | 0.999714303421225 |
| ebi-fl187-GCST90094716 | prot-a-2393 | Trypsin-3 \|\| id:prot-a-2393 | F-burn/spicy liking (derived food-liking factor) \|\| id:ebi-fl187-GCST90094716 | Weighted mode | 15 | 0.21588558133639 | 0.284638931911733 | 0.460757619281598 | -0.342006725 | 0.773777887883388 | 1.24096038184878 | 0.71034342748396 | 2.16794104053711 | 0.999714303421225 |
| ebi-fl187-GCST90094716 | prot-a-2405 | Pregnancy-specific beta-1-glycoprotein 6 \|\| id:prot-a-2405 | F-burn/spicy liking (derived food-liking factor) \|\| id:ebi-fl187-GCST90094716 | Weighted mode | 15 | 0.272755444333492 | 0.209978567686613 | 0.214941886409645 | -0.138802548 | 0.684313436999254 | 1.31357896236197 | 0.870399873398752 | 1.98241031863001 | 0.999714303421225 |
| ebi-fl187-GCST90094716 | prot-a-244 | Beclin-1 \|\| id:prot-a-244 | F-burn/spicy liking (derived food-liking factor) \|\| id:ebi-fl187-GCST90094716 | Weighted mode | 15 | -0.105989296 | 0.223906471160781 | 0.643249721895765 | -0.54484598 | 0.332867387286671 | 0.899434275399377 | 0.579931097644314 | 1.39496229646814 | 0.999714303421225 |
| ebi-fl187-GCST90094716 | prot-a-2454 | tRNA pseudouridine synthase A, mitochondrial \|\| id:prot-a-2454 | F-burn/spicy liking (derived food-liking factor) \|\| id:ebi-fl187-GCST90094716 | Weighted mode | 15 | -0.201440912 | 0.260084903615357 | 0.451506556235977 | -0.711207323 | 0.30832549906129 | 0.817551883618346 | 0.49105098223231 | 1.36114396792246 | 0.999714303421225 |
| ebi-fl187-GCST90094716 | prot-a-2466 | Glutaminyl-peptide cyclotransferase-like protein \|\| id:prot-a-2466 | F-burn/spicy liking (derived food-liking factor) \|\| id:ebi-fl187-GCST90094716 | Weighted mode | 15 | -0.212426616 | 0.240254199055117 | 0.391533831135778 | -0.683324846 | 0.258471614436745 | 0.808619654122353 | 0.504935366079503 | 1.29494939146331 | 0.999714303421225 |
| ebi-fl187-GCST90094716 | prot-a-2474 | Ras-related protein Rab-27A \|\| id:prot-a-2474 | F-burn/spicy liking (derived food-liking factor) \|\| id:ebi-fl187-GCST90094716 | Weighted mode | 15 | 0.165538538417014 | 0.231037504961861 | 0.485463860431668 | -0.287294971 | 0.618372048142262 | 1.18002843827122 | 0.750290382057648 | 1.85590425844193 | 0.999714303421225 |
| ebi-fl187-GCST90094716 | prot-a-2490 | Rap1 GTPase-GDP dissociation stimulator 1 \|\| id:prot-a-2490 | F-burn/spicy liking (derived food-liking factor) \|\| id:ebi-fl187-GCST90094716 | Weighted mode | 15 | -0.099940179 | 0.262217488919755 | 0.708824761986195 | -0.613886457 | 0.414006099305534 | 0.904891547954824 | 0.541243257368992 | 1.51286635429038 | 0.999714303421225 |
| ebi-fl187-GCST90094716 | prot-a-2545 | Receptor-interacting serine/threonine-protein kinase 2 \|\| id:prot-a-2545 | F-burn/spicy liking (derived food-liking factor) \|\| id:ebi-fl187-GCST90094716 | Weighted mode | 15 | 0.0451202357983287 | 0.247581709081916 | 0.858003923398766 | -0.440139914 | 0.530380385598885 | 1.04615363746486 | 0.643946317673399 | 1.69957868092979 | 0.999714303421225 |
| ebi-fl187-GCST90094716 | prot-a-2610 | RUN and FYVE domain-containing protein 1 \|\| id:prot-a-2610 | F-burn/spicy liking (derived food-liking factor) \|\| id:ebi-fl187-GCST90094716 | Weighted mode | 15 | -0.119937106 | 0.258015034307232 | 0.649189428220887 | -0.625646574 | 0.385772360853602 | 0.886976220100662 | 0.534915454454661 | 1.4707498324686 | 0.999714303421225 |
| ebi-fl187-GCST90094716 | prot-a-2627 | Serine--tRNA ligase, mitochondrial \|\| id:prot-a-2627 | F-burn/spicy liking (derived food-liking factor) \|\| id:ebi-fl187-GCST90094716 | Weighted mode | 15 | -0.093480665 | 0.203265679187929 | 0.652653272234576 | -0.491881396 | 0.304920066617237 | 0.910755627088409 | 0.611474885021682 | 1.35651656771445 | 0.999714303421225 |
| ebi-fl187-GCST90094716 | prot-a-264 | BolA-like protein 2 \|\| id:prot-a-264 | F-burn/spicy liking (derived food-liking factor) \|\| id:ebi-fl187-GCST90094716 | Weighted mode | 15 | 0.196402557315119 | 0.214665837401891 | 0.375722035753206 | -0.224342484 | 0.617147598622825 | 1.21701672572287 | 0.799041428603408 | 1.85363318805383 | 0.999714303421225 |
| ebi-fl187-GCST90094716 | prot-a-2646 | Secretoglobin family 1C member 1 \|\| id:prot-a-2646 | F-burn/spicy liking (derived food-liking factor) \|\| id:ebi-fl187-GCST90094716 | Weighted mode | 15 | -0.118682901 | 0.208183943513841 | 0.577653641760279 | -0.526723431 | 0.289357628044631 | 0.888089368152448 | 0.590536737810082 | 1.33556928016062 | 0.999714303421225 |
| ebi-fl187-GCST90094716 | prot-a-2672 | Semaphorin-3E \|\| id:prot-a-2672 | F-burn/spicy liking (derived food-liking factor) \|\| id:ebi-fl187-GCST90094716 | Weighted mode | 15 | 0.0460842842806664 | 0.253829699998748 | 0.858533619923651 | -0.451421928 | 0.543590496278213 | 1.0471626665897 | 0.636722134777805 | 1.72217925277862 | 0.999714303421225 |
| ebi-fl187-GCST90094716 | prot-a-2701 | Plasma protease C1 inhibitor \|\| id:prot-a-2701 | F-burn/spicy liking (derived food-liking factor) \|\| id:ebi-fl187-GCST90094716 | Weighted mode | 15 | 0.141261470720906 | 0.247858253142962 | 0.577758077722833 | -0.344540705 | 0.627063646881111 | 1.15172575118064 | 0.708545709935434 | 1.87210533820533 | 0.999714303421225 |
| ebi-fl187-GCST90094716 | prot-a-2738 | Signaling threshold-regulating transmembrane adapter 1 \|\| id:prot-a-2738 | F-burn/spicy liking (derived food-liking factor) \|\| id:ebi-fl187-GCST90094716 | Weighted mode | 15 | 0.149320512635748 | 0.243334363516454 | 0.549292314516653 | -0.32761484 | 0.626255865127998 | 1.16104505919148 | 0.720640528268529 | 1.8705936962938 | 0.999714303421225 |
| ebi-fl187-GCST90094716 | prot-a-274 | Serine/threonine-protein kinase BRSK2 \|\| id:prot-a-274 | F-burn/spicy liking (derived food-liking factor) \|\| id:ebi-fl187-GCST90094716 | Weighted mode | 15 | -0.143942875 | 0.225528458992672 | 0.53361465903116 | -0.585978655 | 0.298092904202248 | 0.865937212943022 | 0.556560914049104 | 1.34728695068474 | 0.999714303421225 |
| ebi-fl187-GCST90094716 | prot-a-2749 | Mitochondrial glutamate carrier 2 \|\| id:prot-a-2749 | F-burn/spicy liking (derived food-liking factor) \|\| id:ebi-fl187-GCST90094716 | Weighted mode | 15 | 0.102031446535277 | 0.24543430275836 | 0.683918198123243 | -0.379019787 | 0.583082679941663 | 1.10741829564889 | 0.684532067784478 | 1.79155271060873 | 0.999714303421225 |
| ebi-fl187-GCST90094716 | prot-a-2760 | Sodium-coupled monocarboxylate transporter 1 \|\| id:prot-a-2760 | F-burn/spicy liking (derived food-liking factor) \|\| id:ebi-fl187-GCST90094716 | Weighted mode | 15 | -0.036249308 | 0.269327119462748 | 0.894850191195951 | -0.564130462 | 0.491631845815152 | 0.96439983059668 | 0.568854572160256 | 1.63498208289498 | 0.999714303421225 |
| ebi-fl187-GCST90094716 | prot-a-2777 | Small integral membrane protein 10 \|\| id:prot-a-2777 | F-burn/spicy liking (derived food-liking factor) \|\| id:ebi-fl187-GCST90094716 | Weighted mode | 15 | 0.26970099420509 | 0.243143895319918 | 0.286024585803492 | -0.206861041 | 0.74626302903213 | 1.30957282232385 | 0.813132634542427 | 2.10910361251745 | 0.999714303421225 |
| ebi-fl187-GCST90094716 | prot-a-2799 | Superoxide dismutase [Mn], mitochondrial \|\| id:prot-a-2799 | F-burn/spicy liking (derived food-liking factor) \|\| id:ebi-fl187-GCST90094716 | Weighted mode | 15 | 0.20715884710218 | 0.264472748780385 | 0.446505999894099 | -0.311207741 | 0.725525434711735 | 1.23017796646654 | 0.732561677326448 | 2.0658162664239 | 0.999714303421225 |
| ebi-fl187-GCST90094716 | prot-a-2805 | Protein SPACA7 \|\| id:prot-a-2805 | F-burn/spicy liking (derived food-liking factor) \|\| id:ebi-fl187-GCST90094716 | Weighted mode | 15 | -0.318189443 | 0.221136299252254 | 0.172164363223791 | -0.751616589 | 0.115237703720864 | 0.7274649623507 | 0.471603546903238 | 1.12214014275954 | 0.999714303421225 |
| ebi-fl187-GCST90094716 | prot-a-2811 | SPARC-like protein 1 \|\| id:prot-a-2811 | F-burn/spicy liking (derived food-liking factor) \|\| id:ebi-fl187-GCST90094716 | Weighted mode | 15 | 0.315380604329123 | 0.232311757368479 | 0.196079694392171 | -0.13995044 | 0.770711648771342 | 1.37078093684301 | 0.8694013217623 | 2.16130379581587 | 0.999714303421225 |
| ebi-fl187-GCST90094716 | prot-a-2831 | Sepiapterin reductase \|\| id:prot-a-2831 | F-burn/spicy liking (derived food-liking factor) \|\| id:ebi-fl187-GCST90094716 | Weighted mode | 15 | -0.190046151 | 0.297418281624287 | 0.53314763733276 | -0.772985983 | 0.392893681188671 | 0.826920970002609 | 0.461632581343882 | 1.48126089505948 | 0.999714303421225 |
| ebi-fl187-GCST90094716 | prot-a-2834 | SPRY domain-containing SOCS box protein 1 \|\| id:prot-a-2834 | F-burn/spicy liking (derived food-liking factor) \|\| id:ebi-fl187-GCST90094716 | Weighted mode | 15 | -0.265447007 | 0.262357561094576 | 0.328813157133643 | -0.779667827 | 0.248773812940292 | 0.766863080333562 | 0.458558306904172 | 1.28245192623143 | 0.999714303421225 |
| ebi-fl187-GCST90094716 | prot-a-2835 | Spectrin alpha chain, non-erythrocytic 1 \|\| id:prot-a-2835 | F-burn/spicy liking (derived food-liking factor) \|\| id:ebi-fl187-GCST90094716 | Weighted mode | 15 | 0.256407281830778 | 0.243158205210338 | 0.309518949005917 | -0.2201828 | 0.732997364043041 | 1.29227894250719 | 0.802372110627721 | 2.0813097104547 | 0.999714303421225 |
| ebi-fl187-GCST90094716 | prot-a-2859 | Sia-alpha-2,3-Gal-beta-1,4-GlcNAc-R:alpha 2,8-sialyltransferase \|\| id:prot-a-2859 | F-burn/spicy liking (derived food-liking factor) \|\| id:ebi-fl187-GCST90094716 | Weighted mode | 15 | -0.087866634 | 0.211712025275034 | 0.684410775447806 | -0.502822204 | 0.327088935450479 | 0.915883016151723 | 0.60482131987161 | 1.38692481847903 | 0.999714303421225 |
| ebi-fl187-GCST90094716 | prot-a-2861 | Alpha-2,8-sialyltransferase 8F \|\| id:prot-a-2861 | F-burn/spicy liking (derived food-liking factor) \|\| id:ebi-fl187-GCST90094716 | Weighted mode | 15 | -0.212587412 | 0.270038404618584 | 0.444260244297404 | -0.741862685 | 0.316687861337852 | 0.808489641766805 | 0.476226029880717 | 1.37257407161877 | 0.999714303421225 |
| ebi-fl187-GCST90094716 | prot-a-289 | Uncharacterized protein C17orf89 \|\| id:prot-a-289 | F-burn/spicy liking (derived food-liking factor) \|\| id:ebi-fl187-GCST90094716 | Weighted mode | 15 | -0.116306659 | 0.234788894304582 | 0.628025638184896 | -0.576492891 | 0.343879574298548 | 0.890202193332611 | 0.561865438306593 | 1.41040877581399 | 0.999714303421225 |
| ebi-fl187-GCST90094716 | prot-a-2921 | Tachykinin-3 \|\| id:prot-a-2921 | F-burn/spicy liking (derived food-liking factor) \|\| id:ebi-fl187-GCST90094716 | Weighted mode | 15 | 0.0817951535627581 | 0.228983912429008 | 0.726262424681277 | -0.367013315 | 0.530603621923614 | 1.08523348084655 | 0.692800420485259 | 1.69995813098006 | 0.999714303421225 |
| ebi-fl187-GCST90094716 | prot-a-2928 | Tax1-binding protein 1 \|\| id:prot-a-2928 | F-burn/spicy liking (derived food-liking factor) \|\| id:ebi-fl187-GCST90094716 | Weighted mode | 15 | 0.0216566690736695 | 0.226504025335437 | 0.925183269584024 | -0.422291221 | 0.465604558731126 | 1.02189287680715 | 0.655543103947196 | 1.59297694595734 | 0.999714303421225 |
| ebi-fl187-GCST90094716 | prot-a-296 | Uncharacterized protein C1orf185 \|\| id:prot-a-296 | F-burn/spicy liking (derived food-liking factor) \|\| id:ebi-fl187-GCST90094716 | Weighted mode | 15 | -0.192807162 | 0.261236830061349 | 0.47267211019436 | -0.704831349 | 0.31921702471961 | 0.824640980759369 | 0.494191913128961 | 1.37604992935268 | 0.999714303421225 |
| ebi-fl187-GCST90094716 | prot-a-2964 | Transforming growth factor beta-2 \|\| id:prot-a-2964 | F-burn/spicy liking (derived food-liking factor) \|\| id:ebi-fl187-GCST90094716 | Weighted mode | 15 | -0.075159527 | 0.221328731405015 | 0.739210946 | -0.50896384 | 0.358644786821085 | 0.927595498245694 | 0.601118110604605 | 1.43138826328199 | 0.999714303421225 |
| ebi-fl187-GCST90094716 | prot-a-2977 | Thymocyte nuclear protein 1 \|\| id:prot-a-2977 | F-burn/spicy liking (derived food-liking factor) \|\| id:ebi-fl187-GCST90094716 | Weighted mode | 15 | -0.081581935 | 0.246685106040861 | 0.745758868799234 | -0.565084743 | 0.401920872496047 | 0.921657190461088 | 0.568311984149728 | 1.49469305666591 | 0.999714303421225 |
| ebi-fl187-GCST90094716 | prot-a-3015 | Transmembrane protein 70, mitochondrial \|\| id:prot-a-3015 | F-burn/spicy liking (derived food-liking factor) \|\| id:ebi-fl187-GCST90094716 | Weighted mode | 15 | -0.16067117 | 0.270365046917921 | 0.561809372443915 | -0.690586662 | 0.36924432155075 | 0.85157204716093 | 0.501281899559422 | 1.44664100607505 | 0.999714303421225 |
| ebi-fl187-GCST90094716 | prot-a-3030 | Tumor necrosis factor alpha-induced protein 3 \|\| id:prot-a-3030 | F-burn/spicy liking (derived food-liking factor) \|\| id:ebi-fl187-GCST90094716 | Weighted mode | 15 | -0.193068569 | 0.261608400897099 | 0.472700987140503 | -0.705821035 | 0.319683897015397 | 0.824425442384762 | 0.49370306056247 | 1.37669251893428 | 0.999714303421225 |
| ebi-fl187-GCST90094716 | prot-a-3032 | Tumor necrosis factor receptor superfamily member 10A \|\| id:prot-a-3032 | F-burn/spicy liking (derived food-liking factor) \|\| id:ebi-fl187-GCST90094716 | Weighted mode | 15 | 0.235307871291597 | 0.277355088131396 | 0.410487309262234 | -0.308308101 | 0.778923844029133 | 1.26529825778245 | 0.734688924414835 | 2.17912592383564 | 0.999714303421225 |
| ebi-fl187-GCST90094716 | prot-a-3081 | Tumor protein p53-inducible protein 11 \|\| id:prot-a-3081 | F-burn/spicy liking (derived food-liking factor) \|\| id:ebi-fl187-GCST90094716 | Weighted mode | 15 | 0.112120418203079 | 0.220267485628455 | 0.618666300142777 | -0.319603854 | 0.543844690034852 | 1.11864755806369 | 0.726436755365271 | 1.72261707563605 | 0.999714303421225 |
| ebi-fl187-GCST90094716 | prot-a-315 | Complement C4 \|\| id:prot-a-315 | F-burn/spicy liking (derived food-liking factor) \|\| id:ebi-fl187-GCST90094716 | Weighted mode | 15 | -0.161021738 | 0.214043772443603 | 0.464341553904672 | -0.580547532 | 0.258504056144316 | 0.851273566053067 | 0.559591888296524 | 1.29499140251423 | 0.999714303421225 |
| ebi-fl187-GCST90094716 | prot-a-3157 | UDP-glucuronosyltransferase 1-8 \|\| id:prot-a-3157 | F-burn/spicy liking (derived food-liking factor) \|\| id:ebi-fl187-GCST90094716 | Weighted mode | 15 | 0.296815807092911 | 0.20275220411322 | 0.165302443390183 | -0.100578513 | 0.694210127154822 | 1.34556743264404 | 0.904314109239846 | 2.00212702344565 | 0.999714303421225 |
| ebi-fl187-GCST90094716 | prot-a-317 | Glutaredoxin-like protein C5orf63 \|\| id:prot-a-317 | F-burn/spicy liking (derived food-liking factor) \|\| id:ebi-fl187-GCST90094716 | Weighted mode | 15 | 0.101511676515602 | 0.260424193228362 | 0.702554788389722 | -0.408919742 | 0.611943095243192 | 1.1068428423842 | 0.664367550852633 | 1.84401100891347 | 0.999714303421225 |
| ebi-fl187-GCST90094716 | prot-a-3192 | Guanine nucleotide exchange factor VAV3 \|\| id:prot-a-3192 | F-burn/spicy liking (derived food-liking factor) \|\| id:ebi-fl187-GCST90094716 | Weighted mode | 15 | -0.317973724 | 0.285529729384053 | 0.284194031539487 | -0.877611994 | 0.241664545385489 | 0.727621907005842 | 0.415774599453341 | 1.27336696433817 | 0.999714303421225 |
| ebi-fl187-GCST90094716 | prot-a-3233 | Serine/threonine-protein kinase WNK3 \|\| id:prot-a-3233 | F-burn/spicy liking (derived food-liking factor) \|\| id:ebi-fl187-GCST90094716 | Weighted mode | 15 | 0.214605049349464 | 0.219285031773327 | 0.344358594622304 | -0.215193613 | 0.644403711625184 | 1.23937230939111 | 0.806385298444963 | 1.90485085014269 | 0.999714303421225 |
| ebi-fl187-GCST90094716 | prot-a-3250 | 14-3-3 protein theta \|\| id:prot-a-3250 | F-burn/spicy liking (derived food-liking factor) \|\| id:ebi-fl187-GCST90094716 | Weighted mode | 15 | 0.120890713681921 | 0.220745441918974 | 0.592558336697902 | -0.311770352 | 0.553551779843111 | 1.1285015758458 | 0.732149645274584 | 1.73942009656897 | 0.999714303421225 |
| ebi-fl187-GCST90094716 | prot-a-3278 | E3 ubiquitin-protein ligase ZNRF3 \|\| id:prot-a-3278 | F-burn/spicy liking (derived food-liking factor) \|\| id:ebi-fl187-GCST90094716 | Weighted mode | 15 | 0.155569155664597 | 0.241473960276984 | 0.529829083976538 | -0.317719806 | 0.628858117807486 | 1.16832272940838 | 0.727806686566423 | 1.87546779281708 | 0.999714303421225 |
| ebi-fl187-GCST90094716 | prot-a-355 | Calpain-3 \|\| id:prot-a-355 | F-burn/spicy liking (derived food-liking factor) \|\| id:ebi-fl187-GCST90094716 | Weighted mode | 15 | 0.12578029201742 | 0.252253254513676 | 0.625784580044826 | -0.368636087 | 0.620196670864226 | 1.13403298481096 | 0.691677075052924 | 1.85929367478436 | 0.999714303421225 |
| ebi-fl187-GCST90094716 | prot-a-385 | Coiled-coil domain-containing protein 90B, mitochondrial \|\| id:prot-a-385 | F-burn/spicy liking (derived food-liking factor) \|\| id:ebi-fl187-GCST90094716 | Weighted mode | 15 | -0.084202905 | 0.28366207050275 | 0.7709453536622 | -0.640180563 | 0.471774753212542 | 0.91924471784648 | 0.527197223052803 | 1.60283630933318 | 0.999714303421225 |
| ebi-fl187-GCST90094716 | prot-a-390 | C-C motif chemokine 14 \|\| id:prot-a-390 | F-burn/spicy liking (derived food-liking factor) \|\| id:ebi-fl187-GCST90094716 | Weighted mode | 15 | -0.461021358 | 0.29725594369862 | 0.143226489801887 | -1.043643008 | 0.121600291267973 | 0.630639207821533 | 0.35216938630662 | 1.12930261943752 | 0.999714303421225 |
| ebi-fl187-GCST90094716 | prot-a-40 | Pituitary adenylate cyclase-activating polypeptide 27 \|\| id:prot-a-40 | F-burn/spicy liking (derived food-liking factor) \|\| id:ebi-fl187-GCST90094716 | Weighted mode | 15 | 0.324429621061171 | 0.250837131561051 | 0.216811328723386 | -0.167211157 | 0.816070398920831 | 1.38324144914161 | 0.846020949399881 | 2.26159518624287 | 0.999714303421225 |
| ebi-fl187-GCST90094716 | prot-a-401 | C-C motif chemokine 24 \|\| id:prot-a-401 | F-burn/spicy liking (derived food-liking factor) \|\| id:ebi-fl187-GCST90094716 | Weighted mode | 15 | -0.18404187 | 0.255920184338533 | 0.483887764317248 | -0.685645432 | 0.317561691043655 | 0.831900971187601 | 0.503764978804706 | 1.37377399180256 | 0.999714303421225 |
| ebi-fl187-GCST90094716 | prot-a-412 | Cerebral cavernous malformations 2 protein \|\| id:prot-a-412 | F-burn/spicy liking (derived food-liking factor) \|\| id:ebi-fl187-GCST90094716 | Weighted mode | 15 | -0.150818255 | 0.214359625914719 | 0.493233190647239 | -0.570963122 | 0.269326611952174 | 0.860003986017999 | 0.564981031122658 | 1.30908263326504 | 0.999714303421225 |
| ebi-fl187-GCST90094716 | prot-a-44 | Adhesion G protein-coupled receptor F5 \|\| id:prot-a-44 | F-burn/spicy liking (derived food-liking factor) \|\| id:ebi-fl187-GCST90094716 | Weighted mode | 15 | 0.0210116890801719 | 0.197802076315827 | 0.9169102799331 | -0.36668038 | 0.408703758659192 | 1.02123398885373 | 0.693031115908852 | 1.5048658509689 | 0.999714303421225 |
| ebi-fl187-GCST90094716 | prot-a-48 | Adrenomedullin \|\| id:prot-a-48 | F-burn/spicy liking (derived food-liking factor) \|\| id:ebi-fl187-GCST90094716 | Weighted mode | 15 | -0.069621696 | 0.277069717532115 | 0.805249440539249 | -0.612678342 | 0.473434950437413 | 0.932746615014732 | 0.541897536594088 | 1.60549954386143 | 0.999714303421225 |
| ebi-fl187-GCST90094716 | prot-a-502 | Carcinoembryonic antigen-related cell adhesion molecule 1 \|\| id:prot-a-502 | F-burn/spicy liking (derived food-liking factor) \|\| id:ebi-fl187-GCST90094716 | Weighted mode | 15 | 0.205828065813752 | 0.273575202588386 | 0.464295207813862 | -0.330379331 | 0.742035462886988 | 1.22854195747385 | 0.718651074903871 | 2.10020605823984 | 0.999714303421225 |
| ebi-fl187-GCST90094716 | prot-a-518 | Complement factor B \|\| id:prot-a-518 | F-burn/spicy liking (derived food-liking factor) \|\| id:ebi-fl187-GCST90094716 | Weighted mode | 15 | 0.406241652189134 | 0.277166013721188 | 0.164827922578758 | -0.137003735 | 0.949487039082661 | 1.50116526850408 | 0.871966969587156 | 2.58438363144635 | 0.999714303421225 |
| ebi-fl187-GCST90094716 | prot-a-525 | Properdin \|\| id:prot-a-525 | F-burn/spicy liking (derived food-liking factor) \|\| id:ebi-fl187-GCST90094716 | Weighted mode | 15 | -0.132328896 | 0.27831044531259 | 0.641783714974505 | -0.677817369 | 0.41315957681983 | 0.87605281743056 | 0.507723958026217 | 1.51158622081094 | 0.999714303421225 |
| ebi-fl187-GCST90094716 | prot-a-533 | Chondroadherin \|\| id:prot-a-533 | F-burn/spicy liking (derived food-liking factor) \|\| id:ebi-fl187-GCST90094716 | Weighted mode | 15 | 0.274217487457155 | 0.238142792168598 | 0.268819516856115 | -0.192542385 | 0.740977360107608 | 1.31550087606928 | 0.824859355639429 | 2.09798499963371 | 0.999714303421225 |
| ebi-fl187-GCST90094716 | prot-a-535 | Coiled-coil-helix-coiled-coil-helix domain-containing protein 2 \|\| id:prot-a-535 | F-burn/spicy liking (derived food-liking factor) \|\| id:ebi-fl187-GCST90094716 | Weighted mode | 15 | 0.00546376630936551 | 0.356888267240098 | 0.988001332279761 | -0.694037237 | 0.704964770099957 | 1.00547871990242 | 0.499555169530945 | 2.02377538626186 | 0.999714303421225 |
| ebi-fl187-GCST90094716 | prot-a-591 | Caseinolytic peptidase B protein homolog \|\| id:prot-a-591 | F-burn/spicy liking (derived food-liking factor) \|\| id:ebi-fl187-GCST90094716 | Weighted mode | 15 | 0.275322490826186 | 0.316503235441028 | 0.399030851873274 | -0.345023851 | 0.8956688322906 | 1.31695531239983 | 0.708203462161799 | 2.44897319417778 | 0.999714303421225 |
| ebi-fl187-GCST90094716 | prot-a-605 | Ciliary neurotrophic factor receptor subunit alpha \|\| id:prot-a-605 | F-burn/spicy liking (derived food-liking factor) \|\| id:ebi-fl187-GCST90094716 | Weighted mode | 15 | 0.211827141557789 | 0.24438509037818 | 0.400676801412561 | -0.267167636 | 0.690821918699022 | 1.23593422494776 | 0.765544728171063 | 1.99535487893254 | 0.999714303421225 |
| ebi-fl187-GCST90094716 | prot-a-606 | Ciliary neurotrophic factor receptor subunit alpha \|\| id:prot-a-606 | F-burn/spicy liking (derived food-liking factor) \|\| id:ebi-fl187-GCST90094716 | Weighted mode | 15 | 0.188412936853104 | 0.233677076014129 | 0.433555556789172 | -0.269594132 | 0.646420005840798 | 1.20733196429054 | 0.763689388426491 | 1.9086954645278 | 0.999714303421225 |
| ebi-fl187-GCST90094716 | prot-a-707 | Cancer/testis antigen 55 \|\| id:prot-a-707 | F-burn/spicy liking (derived food-liking factor) \|\| id:ebi-fl187-GCST90094716 | Weighted mode | 15 | -0.016251586 | 0.274265161957237 | 0.953586506066185 | -0.553811304 | 0.521308131035639 | 0.983879758148064 | 0.574755064433153 | 1.68422940204658 | 0.999714303421225 |
| ebi-fl187-GCST90094716 | prot-a-719 | Dipeptidyl peptidase 1 \|\| id:prot-a-719 | F-burn/spicy liking (derived food-liking factor) \|\| id:ebi-fl187-GCST90094716 | Weighted mode | 15 | -0.167355387 | 0.234375200106802 | 0.486931928527276 | -0.626730779 | 0.292020005352343 | 0.845898936548029 | 0.534335810494716 | 1.33912980713495 | 0.999714303421225 |
| ebi-fl187-GCST90094716 | prot-a-752 | Cytochrome b5 \|\| id:prot-a-752 | F-burn/spicy liking (derived food-liking factor) \|\| id:ebi-fl187-GCST90094716 | Weighted mode | 15 | 0.120255303591208 | 0.228979029937194 | 0.607675402300228 | -0.328543595 | 0.569054202268108 | 1.12778474232287 | 0.719971540320649 | 1.76659541910475 | 0.999714303421225 |
| ebi-fl187-GCST90094716 | prot-a-755 | Cytoglobin \|\| id:prot-a-755 | F-burn/spicy liking (derived food-liking factor) \|\| id:ebi-fl187-GCST90094716 | Weighted mode | 15 | 0.173659107967705 | 0.236324348860007 | 0.474570665268254 | -0.289536616 | 0.636854831733318 | 1.18964995449875 | 0.748610381442609 | 1.89052549807229 | 0.999714303421225 |
| ebi-fl187-GCST90094716 | prot-a-807 | Desmin \|\| id:prot-a-807 | F-burn/spicy liking (derived food-liking factor) \|\| id:ebi-fl187-GCST90094716 | Weighted mode | 15 | -0.045870358 | 0.27949144262332 | 0.871981934598887 | -0.593673586 | 0.5019328692533 | 0.955165783491366 | 0.552294651687944 | 1.65191111513454 | 0.999714303421225 |
| ebi-fl187-GCST90094716 | prot-a-82 | Ameloblastin \|\| id:prot-a-82 | F-burn/spicy liking (derived food-liking factor) \|\| id:ebi-fl187-GCST90094716 | Weighted mode | 15 | -0.16753409 | 0.204792113979287 | 0.427022627190101 | -0.568926634 | 0.233858453132281 | 0.845747785029489 | 0.566132780558004 | 1.26346563994628 | 0.999714303421225 |
| ebi-fl187-GCST90094716 | prot-a-84 | Muellerian-inhibiting factor \|\| id:prot-a-84 | F-burn/spicy liking (derived food-liking factor) \|\| id:ebi-fl187-GCST90094716 | Weighted mode | 15 | -0.023134133 | 0.230666325203061 | 0.92153408619119 | -0.47524013 | 0.428971864861683 | 0.977131409868516 | 0.621735741175512 | 1.53567782728145 | 0.999714303421225 |
| ebi-fl187-GCST90094716 | prot-a-858 | Inactive dipeptidyl peptidase 10 \|\| id:prot-a-858 | F-burn/spicy liking (derived food-liking factor) \|\| id:ebi-fl187-GCST90094716 | Weighted mode | 15 | -0.035383099 | 0.218522106144539 | 0.873682505120794 | -0.463686427 | 0.392920229264389 | 0.965235564851027 | 0.628960748768762 | 1.48130022020788 | 0.999714303421225 |
| ebi-fl187-GCST90094716 | prot-a-863 | Dihydropyrimidinase-related protein 3 \|\| id:prot-a-863 | F-burn/spicy liking (derived food-liking factor) \|\| id:ebi-fl187-GCST90094716 | Weighted mode | 15 | -0.111792244 | 0.227125892001797 | 0.630203656761424 | -0.556958992 | 0.333374504259686 | 0.894230019797989 | 0.572948758832549 | 1.39566988492529 | 0.999714303421225 |
| ebi-fl187-GCST90094716 | prot-a-864 | Dihydropyrimidinase-related protein 5 \|\| id:prot-a-864 | F-burn/spicy liking (derived food-liking factor) \|\| id:ebi-fl187-GCST90094716 | Weighted mode | 15 | 0.351854662768417 | 0.344100775426273 | 0.3238735077173 | -0.322582857 | 1.02629218260391 | 1.42170188248687 | 0.724275917942348 | 2.79069922469465 | 0.999714303421225 |
| ebi-fl187-GCST90094716 | prot-a-884 | Endothelin-converting enzyme 1 \|\| id:prot-a-884 | F-burn/spicy liking (derived food-liking factor) \|\| id:ebi-fl187-GCST90094716 | Weighted mode | 15 | -0.061100542 | 0.206930100231073 | 0.772120285012186 | -0.466683538 | 0.344482454550573 | 0.940728652372804 | 0.627078505310557 | 1.41125933978055 | 0.999714303421225 |
| ebi-fl187-GCST90094716 | prot-a-893 | Early endosome antigen 1 \|\| id:prot-a-893 | F-burn/spicy liking (derived food-liking factor) \|\| id:ebi-fl187-GCST90094716 | Weighted mode | 15 | -0.178691276 | 0.229416738787736 | 0.449008212976453 | -0.628348084 | 0.270965532193979 | 0.836364065345856 | 0.533472325077967 | 1.31122987438873 | 0.999714303421225 |
| ebi-fl187-GCST90094716 | prot-a-897 | Ephrin-A2 \|\| id:prot-a-897 | F-burn/spicy liking (derived food-liking factor) \|\| id:ebi-fl187-GCST90094716 | Weighted mode | 15 | 0.29127153233478 | 0.22394164697918 | 0.21437824198473 | -0.147654096 | 0.730197160413973 | 1.33812787961193 | 0.86272948513564 | 2.07548977175991 | 0.999714303421225 |
| ebi-fl187-GCST90094716 | prot-a-90 | Anaphase-promoting complex subunit 10 \|\| id:prot-a-90 | F-burn/spicy liking (derived food-liking factor) \|\| id:ebi-fl187-GCST90094716 | Weighted mode | 15 | 0.1254086114984 | 0.254013927676597 | 0.62916776729491 | -0.372458687 | 0.623275909744529 | 1.13361156516416 | 0.689038117375436 | 1.86502770785574 | 0.999714303421225 |
| ebi-fl187-GCST90094716 | prot-a-914 | Histone-lysine N-methyltransferase EHMT2 \|\| id:prot-a-914 | F-burn/spicy liking (derived food-liking factor) \|\| id:ebi-fl187-GCST90094716 | Weighted mode | 15 | -0.004936512 | 0.221126808688325 | 0.982504292927695 | -0.438345057 | 0.428472033133744 | 0.995075652654428 | 0.645103147140576 | 1.53491043857807 | 0.999714303421225 |
| ebi-fl187-GCST90094716 | prot-a-932 | RNA polymerase II elongation factor ELL \|\| id:prot-a-932 | F-burn/spicy liking (derived food-liking factor) \|\| id:ebi-fl187-GCST90094716 | Weighted mode | 15 | 0.04955364606071 | 0.22460842509663 | 0.828571466181755 | -0.390678867 | 0.489786159250106 | 1.05080196208865 | 0.67659739882093 | 1.63196720154935 | 0.999714303421225 |
| ebi-fl187-GCST90094716 | prot-a-991 | Estrogen receptor \|\| id:prot-a-991 | F-burn/spicy liking (derived food-liking factor) \|\| id:ebi-fl187-GCST90094716 | Weighted mode | 15 | 0.00160412474458804 | 0.202193011105224 | 0.993781888939972 | -0.394694177 | 0.397902426510826 | 1.00160541204092 | 0.673886097592008 | 1.48869876528755 | 0.999714303421225 |
| ebi-fl187-GCST90094716 | prot-a-992 | Persulfide dioxygenase ETHE1, mitochondrial \|\| id:prot-a-992 | F-burn/spicy liking (derived food-liking factor) \|\| id:ebi-fl187-GCST90094716 | Weighted mode | 15 | 0.163716836539363 | 0.27461774497297 | 0.560582720474588 | -0.374533944 | 0.701967616686384 | 1.17788073508095 | 0.687609669006895 | 2.01771890159522 | 0.999714303421225 |
| ebi-fl187-GCST90094716 | prot-a-993 | Ethanolamine kinase 1 \|\| id:prot-a-993 | F-burn/spicy liking (derived food-liking factor) \|\| id:ebi-fl187-GCST90094716 | Weighted mode | 15 | -0.220104532 | 0.249634033572445 | 0.392820908251139 | -0.709387237 | 0.269178174290132 | 0.802434913843575 | 0.491945550999703 | 1.30888833052082 | 0.999714303421225 |
| ebi-fl187-GCST90094716 | prot-b-10 | coagulation factor III, tissue factor \|\| id:prot-b-10 | F-burn/spicy liking (derived food-liking factor) \|\| id:ebi-fl187-GCST90094716 | Weighted mode | 10 | -0.048687952 | 0.264441545126221 | 0.858003970433043 | -0.56699338 | 0.469617476515875 | 0.952478302344535 | 0.567228317250987 | 1.59938227490801 | 0.999714303421225 |
| ebi-fl187-GCST90094716 | prot-b-28 | prolactin \|\| id:prot-b-28 | F-burn/spicy liking (derived food-liking factor) \|\| id:ebi-fl187-GCST90094716 | Weighted mode | 10 | -0.080134093 | 0.265858114553563 | 0.769944802809048 | -0.601215998 | 0.440947811260143 | 0.922992571000829 | 0.548144687943427 | 1.55417958955145 | 0.999714303421225 |
| ebi-fl187-GCST90094716 | prot-b-54 | matrix metallopeptidase 7 \|\| id:prot-b-54 | F-burn/spicy liking (derived food-liking factor) \|\| id:ebi-fl187-GCST90094716 | Weighted mode | 10 | 0.449006548523978 | 0.28696005972345 | 0.152092526016331 | -0.113435169 | 1.01144826558194 | 1.566754917 | 0.892762073609069 | 2.74958025527153 | 0.999714303421225 |
| ebi-fl187-GCST90094716 | ukb-rsfMRI191-edge-pheno716 | Net100_Pair10_33 (OccipitalPrecuneus)&(Temporal\|Frontal\|Supp_Motor_Area) (Default_mode\|Central_executive)&(Default_mode\|Salience) \|\| id:ukb-rsfMRI191-edge-pheno716 | F-burn/spicy liking (derived food-liking factor) \|\| id:ebi-fl187-GCST90094716 | Weighted mode | 13 | 0.0401538366990368 | 0.0882129130961688 | 0.657096867242224 | -0.132743473 | 0.213051146367528 | 1.04097090140262 | 0.875689701377139 | 1.23744794059224 | 0.999714303421225 |
| ebi-fl187-GCST90094716 | ukb-rsfMRI191-edge-pheno903 | Net100_Pair27_38 (Precentral\|Frontal\|Supp_Motor_Area)&(Subcortical) (Attention\|Salience\|Motor)&(Subcortical-cerebellum) \|\| id:ukb-rsfMRI191-edge-pheno903 | F-burn/spicy liking (derived food-liking factor) \|\| id:ebi-fl187-GCST90094716 | Weighted mode | 13 | 0.019773195529097 | 0.0903006106912214 | 0.830352896763813 | -0.157216001 | 0.196762392483891 | 1.01996998003935 | 0.854519461459796 | 1.21745472994171 | 0.999714303421225 |
| ebi-fl187-GCST90094716 | ukb-rsfMRI191-node-pheno23 | Net100_Node2 RolandicOper\|SupraMarginal\|Insula Salience\|Motor \|\| id:ukb-rsfMRI191-node-pheno23 | F-burn/spicy liking (derived food-liking factor) \|\| id:ebi-fl187-GCST90094716 | Weighted mode | 13 | -0.109364045 | 0.0840573725558305 | 0.21766420930818 | -0.274116495 | 0.0553884055612701 | 0.896404027002866 | 0.76024350564311 | 1.05695106063053 | 0.999714303421225 |
| ebi-fl187-GCST90094716 | ukb-rsfMRI191-node-pheno55 | Net100_Node34 Parietal\|Postcentral\|Precuneus Attention \|\| id:ukb-rsfMRI191-node-pheno55 | F-burn/spicy liking (derived food-liking factor) \|\| id:ebi-fl187-GCST90094716 | Weighted mode | 13 | -0.067938662 | 0.115764984306302 | 0.568169960243577 | -0.294838031 | 0.158960707388955 | 0.934317781142527 | 0.74465218820081 | 1.17229188336674 | 0.999714303421225 |
| ebi-fl187-GCST90094726 | ebi-a-GCST90001414 | IgD- CD24- B cell Absolute Count \|\| id:ebi-a-GCST90001414 | F-capsicum liking (derived food-liking factor) \|\| id:ebi-fl187-GCST90094726 | Inverse variance weighted | 20 | 0.00103505872510808 | 0.0968701997165673 | 0.991474760407522 | -0.188830533 | 0.19090065016958 | 1.00103559458326 | 0.827926801310308 | 1.21033919911366 | 0.999107451094609 |
| ebi-fl187-GCST90094726 | ebi-a-GCST90001422 | CD20- CD38- B cell %B cell \|\| id:ebi-a-GCST90001422 | F-capsicum liking (derived food-liking factor) \|\| id:ebi-fl187-GCST90094726 | Inverse variance weighted | 20 | 0.0980952582419068 | 0.0783535952047442 | 0.210585875948561 | -0.055477788 | 0.251668304843205 | 1.10306785640868 | 0.946033036382878 | 1.28616935037942 | 0.998735809224318 |
| ebi-fl187-GCST90094726 | ebi-a-GCST90001444 | CD20- CD38- B cell %lymphocyte \|\| id:ebi-a-GCST90001444 | F-capsicum liking (derived food-liking factor) \|\| id:ebi-fl187-GCST90094726 | Inverse variance weighted | 20 | 0.0864113337396486 | 0.0783558333678427 | 0.270111141641618 | -0.0671661 | 0.23998876714062 | 1.09025469462542 | 0.935039878630445 | 1.27123487063866 | 0.998735809224318 |
| ebi-fl187-GCST90094726 | ebi-a-GCST90001459 | Myeloid Dendritic Cell %Dendritic Cell \|\| id:ebi-a-GCST90001459 | F-capsicum liking (derived food-liking factor) \|\| id:ebi-fl187-GCST90094726 | Inverse variance weighted | 20 | 0.0103562281851261 | 0.0844627915274842 | 0.902413635300132 | -0.155190843 | 0.175903299578995 | 1.01041003951662 | 0.856251752057939 | 1.19232275496342 | 0.998735809224318 |
| ebi-fl187-GCST90094726 | ebi-a-GCST90001476 | HLA DR++ monocyte %leukocyte \|\| id:ebi-a-GCST90001476 | F-capsicum liking (derived food-liking factor) \|\| id:ebi-fl187-GCST90094726 | Inverse variance weighted | 20 | -0.053789309 | 0.0825572268082401 | 0.514698169345785 | -0.215601474 | 0.108022855152579 | 0.947631742578747 | 0.806056472385345 | 1.11407320740864 | 0.998735809224318 |
| ebi-fl187-GCST90094726 | ebi-a-GCST90001478 | CD4 regulatory T cell %CD4+ T cell \|\| id:ebi-a-GCST90001478 | F-capsicum liking (derived food-liking factor) \|\| id:ebi-fl187-GCST90094726 | Inverse variance weighted | 20 | -0.059941306 | 0.0831951933487929 | 0.471223342652375 | -0.223003885 | 0.103121272499841 | 0.941819810697188 | 0.800111740512413 | 1.10862584675187 | 0.998735809224318 |
| ebi-fl187-GCST90094726 | ebi-a-GCST90001485 | CD39+ resting CD4 regulatory T cell %CD4 regulatory T cell \|\| id:ebi-a-GCST90001485 | F-capsicum liking (derived food-liking factor) \|\| id:ebi-fl187-GCST90094726 | Inverse variance weighted | 20 | 0.0477757197582246 | 0.0891555700394109 | 0.592048985161663 | -0.126969198 | 0.22252063703547 | 1.04893537346175 | 0.880760801797176 | 1.2492215996151 | 0.998735809224318 |
| ebi-fl187-GCST90094726 | ebi-a-GCST90001515 | Immature Myeloid-Derived Suppressor Cells Absolute Count \|\| id:ebi-a-GCST90001515 | F-capsicum liking (derived food-liking factor) \|\| id:ebi-fl187-GCST90094726 | Inverse variance weighted | 20 | 0.116747142243456 | 0.109069606534428 | 0.284443278383427 | -0.097029287 | 0.330523571050936 | 1.12383522330687 | 0.907529427325397 | 1.39169658979261 | 0.998735809224318 |
| ebi-fl187-GCST90094726 | ebi-a-GCST90001517 | CD33+ HLA DR+ Absolute Count \|\| id:ebi-a-GCST90001517 | F-capsicum liking (derived food-liking factor) \|\| id:ebi-fl187-GCST90094726 | Inverse variance weighted | 20 | 0.290704635892889 | 0.106595077943777 | 0.00638769634652157 | 0.0817782831230873 | 0.499630988662691 | 1.33736951465569 | 1.08521517263502 | 1.64811298609804 | 0.998735809224318 |
| ebi-fl187-GCST90094726 | ebi-a-GCST90001518 | CD33+ HLA DR+ CD14- Absolute Count \|\| id:ebi-a-GCST90001518 | F-capsicum liking (derived food-liking factor) \|\| id:ebi-fl187-GCST90094726 | Inverse variance weighted | 20 | 0.192508951101805 | 0.108077556634297 | 0.074877959563149 | -0.01932306 | 0.404340962105027 | 1.21228735496641 | 0.980862433730971 | 1.49831472841842 | 0.998735809224318 |
| ebi-fl187-GCST90094726 | ebi-a-GCST90001520 | CD33+ HLA DR+ CD14dim Absolute Count \|\| id:ebi-a-GCST90001520 | F-capsicum liking (derived food-liking factor) \|\| id:ebi-fl187-GCST90094726 | Inverse variance weighted | 20 | 0.334590605425078 | 0.106874596259313 | 0.00174399614663795 | 0.125116396756825 | 0.544064814093331 | 1.39736819314685 | 1.13328035554813 | 1.72299630683537 | 0.998735809224318 |
| ebi-fl187-GCST90094726 | ebi-a-GCST90001531 | CD33dim HLA DR- Absolute Count \|\| id:ebi-a-GCST90001531 | F-capsicum liking (derived food-liking factor) \|\| id:ebi-fl187-GCST90094726 | Inverse variance weighted | 20 | 0.0958424856120746 | 0.108982024516725 | 0.379166064285453 | -0.117762282 | 0.309447253664855 | 1.10058569225839 | 0.888907336383135 | 1.36267169414135 | 0.998735809224318 |
| ebi-fl187-GCST90094726 | ebi-a-GCST90001532 | Basophil Absolute Count \|\| id:ebi-a-GCST90001532 | F-capsicum liking (derived food-liking factor) \|\| id:ebi-fl187-GCST90094726 | Inverse variance weighted | 20 | 0.059979514015202 | 0.108619275072405 | 0.580811647707231 | -0.152914265 | 0.272873293157116 | 1.06181479400082 | 0.858203296608276 | 1.31373377521949 | 0.998735809224318 |
| ebi-fl187-GCST90094726 | ebi-a-GCST90001588 | T/B cell \|\| id:ebi-a-GCST90001588 | F-capsicum liking (derived food-liking factor) \|\| id:ebi-fl187-GCST90094726 | Inverse variance weighted | 20 | 0.0200682343453908 | 0.0813217814079071 | 0.805081866924115 | -0.139322457 | 0.179458925904889 | 1.0202709551724 | 0.869947462390264 | 1.19656975503819 | 0.998735809224318 |
| ebi-fl187-GCST90094726 | ebi-a-GCST90001624 | HLA DR+ CD4+ T cell Absolute Count \|\| id:ebi-a-GCST90001624 | F-capsicum liking (derived food-liking factor) \|\| id:ebi-fl187-GCST90094726 | Inverse variance weighted | 20 | 0.0403120980351523 | 0.0789119331757124 | 0.609456654048121 | -0.114355291 | 0.194979487059549 | 1.04113565988544 | 0.891941000979547 | 1.21528605714352 | 0.998735809224318 |
| ebi-fl187-GCST90094726 | ebi-a-GCST90001631 | CD8+ Natural Killer T %T cell \|\| id:ebi-a-GCST90001631 | F-capsicum liking (derived food-liking factor) \|\| id:ebi-fl187-GCST90094726 | Inverse variance weighted | 20 | 0.151805743892172 | 0.0799467268738784 | 0.0575860079992663 | -0.004889841 | 0.308501328564973 | 1.16393411315932 | 0.99512209502814 | 1.36138331823258 | 0.998735809224318 |
| ebi-fl187-GCST90094726 | ebi-a-GCST90001632 | CD8+ Natural Killer T %lymphocyte \|\| id:ebi-a-GCST90001632 | F-capsicum liking (derived food-liking factor) \|\| id:ebi-fl187-GCST90094726 | Inverse variance weighted | 20 | 0.158204444176484 | 0.0804339478272102 | 0.0491963000338124 | 0.000553906435152574 | 0.315854981917816 | 1.1714056572934 | 1.00055405986965 | 1.37143135885907 | 0.998735809224318 |
| ebi-fl187-GCST90094726 | ebi-a-GCST90001646 | Natural Killer %CD3- lymphocyte \|\| id:ebi-a-GCST90001646 | F-capsicum liking (derived food-liking factor) \|\| id:ebi-fl187-GCST90094726 | Inverse variance weighted | 20 | -0.043163952 | 0.0784712390879695 | 0.582277674169174 | -0.196967581 | 0.110639676276419 | 0.95775435113075 | 0.821217255965694 | 1.11699235548967 | 0.998735809224318 |
| ebi-fl187-GCST90094726 | ebi-a-GCST90001684 | CD127- CD8+ T cell Absolute Count \|\| id:ebi-a-GCST90001684 | F-capsicum liking (derived food-liking factor) \|\| id:ebi-fl187-GCST90094726 | Inverse variance weighted | 20 | 0.0187340217459501 | 0.0847339047879119 | 0.825020493507055 | -0.147344432 | 0.184812475130257 | 1.01891060450947 | 0.862996682859059 | 1.20299282790105 | 0.998735809224318 |
| ebi-fl187-GCST90094726 | ebi-a-GCST90001687 | CD28- CD8+ T cell Absolute Count \|\| id:ebi-a-GCST90001687 | F-capsicum liking (derived food-liking factor) \|\| id:ebi-fl187-GCST90094726 | Inverse variance weighted | 20 | 0.0562961912908122 | 0.0824083013753983 | 0.494520035680935 | -0.105224079 | 0.217816461986593 | 1.05791098135544 | 0.900122801004636 | 1.24335884306375 | 0.998735809224318 |
| ebi-fl187-GCST90094726 | ebi-a-GCST90001701 | BAFF-R on CD20- CD38- B cell \|\| id:ebi-a-GCST90001701 | F-capsicum liking (derived food-liking factor) \|\| id:ebi-fl187-GCST90094726 | Inverse variance weighted | 20 | 0.0370506219378594 | 0.0873074883191749 | 0.67129647100901 | -0.134072055 | 0.208173299043442 | 1.03774555219938 | 0.874527048139898 | 1.23142655610273 | 0.998735809224318 |
| ebi-fl187-GCST90094726 | ebi-a-GCST90001732 | CD19 on IgD- CD27- B cell \|\| id:ebi-a-GCST90001732 | F-capsicum liking (derived food-liking factor) \|\| id:ebi-fl187-GCST90094726 | Inverse variance weighted | 20 | 0.0795340005674989 | 0.0975726297181358 | 0.415000113015762 | -0.111708354 | 0.270776354815045 | 1.08278237411877 | 0.894305040244247 | 1.31098184281963 | 0.998735809224318 |
| ebi-fl187-GCST90094726 | ebi-a-GCST90001798 | CD27 on CD24+ CD27+ B cell \|\| id:ebi-a-GCST90001798 | F-capsicum liking (derived food-liking factor) \|\| id:ebi-fl187-GCST90094726 | Inverse variance weighted | 20 | -0.053593594 | 0.079819322497607 | 0.501942596846553 | -0.210039466 | 0.102852277798361 | 0.947817226565329 | 0.810552255765651 | 1.10832767237863 | 0.998735809224318 |
| ebi-fl187-GCST90094726 | ebi-a-GCST90001836 | CD66b on CD66b++ myeloid cell \|\| id:ebi-a-GCST90001836 | F-capsicum liking (derived food-liking factor) \|\| id:ebi-fl187-GCST90094726 | Inverse variance weighted | 20 | -0.102019514 | 0.126457735377286 | 0.419811733849399 | -0.349876675 | 0.145837647275582 | 0.903011930064438 | 0.704775000452126 | 1.15700832934708 | 0.998735809224318 |
| ebi-fl187-GCST90094726 | ebi-a-GCST90001943 | CD25 on activated & secreting CD4 regulatory T cell \|\| id:ebi-a-GCST90001943 | F-capsicum liking (derived food-liking factor) \|\| id:ebi-fl187-GCST90094726 | Inverse variance weighted | 20 | 0.0878808269250542 | 0.0881166237671159 | 0.318607250065857 | -0.084827756 | 0.260589409508601 | 1.09185799419991 | 0.918670506571096 | 1.29769473491418 | 0.998735809224318 |
| ebi-fl187-GCST90094726 | ebi-a-GCST90001960 | CD25 on CD4+ T cell \|\| id:ebi-a-GCST90001960 | F-capsicum liking (derived food-liking factor) \|\| id:ebi-fl187-GCST90094726 | Inverse variance weighted | 20 | 0.111930790921803 | 0.0975183022285283 | 0.25105417064667 | -0.079205081 | 0.303066663289719 | 1.11843545207973 | 0.923850440431608 | 1.35400472384295 | 0.998735809224318 |
| ebi-fl187-GCST90094726 | ebi-a-GCST90001988 | HLA DR on CD14+ CD16- monocyte \|\| id:ebi-a-GCST90001988 | F-capsicum liking (derived food-liking factor) \|\| id:ebi-fl187-GCST90094726 | Inverse variance weighted | 20 | 0.0854456811515788 | 0.0812563136833645 | 0.293002643203991 | -0.073816694 | 0.244708055970973 | 1.08920239551735 | 0.928841940846489 | 1.27724837373251 | 0.998735809224318 |
| ebi-fl187-GCST90094726 | ebi-a-GCST90001991 | HLA DR on CD14+ monocyte \|\| id:ebi-a-GCST90001991 | F-capsicum liking (derived food-liking factor) \|\| id:ebi-fl187-GCST90094726 | Inverse variance weighted | 20 | 0.0861948364226095 | 0.0813937907578412 | 0.289606426914616 | -0.073336993 | 0.245726666307978 | 1.09001868295801 | 0.92928761340204 | 1.27855005496935 | 0.998735809224318 |
| ebi-fl187-GCST90094726 | ebi-a-GCST90002019 | CD14 on Monocytic Myeloid-Derived Suppressor Cells \|\| id:ebi-a-GCST90002019 | F-capsicum liking (derived food-liking factor) \|\| id:ebi-fl187-GCST90094726 | Inverse variance weighted | 20 | 0.164309351039868 | 0.116545117462512 | 0.158588497059277 | -0.064119079 | 0.392737781266391 | 1.17857885329851 | 0.937893309308429 | 1.48102998460099 | 0.998735809224318 |
| ebi-fl187-GCST90094726 | ebi-a-GCST90002037 | CD80 on plasmacytoid Dendritic Cell \|\| id:ebi-a-GCST90002037 | F-capsicum liking (derived food-liking factor) \|\| id:ebi-fl187-GCST90094726 | Inverse variance weighted | 20 | 0.0586452272139209 | 0.0900700504989544 | 0.514977512436272 | -0.117892072 | 0.235182526191871 | 1.06039897330127 | 0.888791973188066 | 1.26513966878553 | 0.998735809224318 |
| ebi-fl187-GCST90094726 | ebi-a-GCST90002042 | CD45 on CD33+ HLA DR+ CD14- \|\| id:ebi-a-GCST90002042 | F-capsicum liking (derived food-liking factor) \|\| id:ebi-fl187-GCST90094726 | Inverse variance weighted | 20 | 0.102683988790555 | 0.121294648083807 | 0.39723677876139 | -0.135053521 | 0.340421499034817 | 1.10814116870802 | 0.873669150393691 | 1.40553989943703 | 0.998735809224318 |
| ebi-fl187-GCST90094726 | ebi-a-GCST90002056 | CD8 on Effector Memory CD8+ T cell \|\| id:ebi-a-GCST90002056 | F-capsicum liking (derived food-liking factor) \|\| id:ebi-fl187-GCST90094726 | Inverse variance weighted | 20 | -0.010967676 | 0.0882220517835541 | 0.901062717457342 | -0.183882897 | 0.161947545720093 | 0.98909224989841 | 0.832033231483698 | 1.1757985640364 | 0.998735809224318 |
| ebi-fl187-GCST90094726 | ebi-a-GCST90002058 | CD8 on CD8+ T cell \|\| id:ebi-a-GCST90002058 | F-capsicum liking (derived food-liking factor) \|\| id:ebi-fl187-GCST90094726 | Inverse variance weighted (multiplicative random effects) | 20 | -0.164114689 | 0.130687800326869 | 0.20919695626614 | -0.420262778 | 0.0920333993123156 | 0.848644685809587 | 0.656874185069497 | 1.09640144052343 | 0.998735809224318 |
| ebi-fl187-GCST90094726 | ebi-a-GCST90002063 | CD4 on CD4 regulatory T cell \|\| id:ebi-a-GCST90002063 | F-capsicum liking (derived food-liking factor) \|\| id:ebi-fl187-GCST90094726 | Inverse variance weighted | 20 | -0.136110867 | 0.0881480237350858 | 0.122559978133521 | -0.308880993 | 0.0366592597984979 | 0.872745868651041 | 0.734268147697891 | 1.03733949734239 | 0.998735809224318 |
| ebi-fl187-GCST90094726 | ebi-a-GCST90002066 | CD4 on activated CD4 regulatory T cell \|\| id:ebi-a-GCST90002066 | F-capsicum liking (derived food-liking factor) \|\| id:ebi-fl187-GCST90094726 | Inverse variance weighted | 20 | -0.107951172 | 0.0878496449778873 | 0.219140278282001 | -0.280136476 | 0.0642341323119364 | 0.897671427000169 | 0.755680602150962 | 1.06634203466234 | 0.998735809224318 |
| ebi-fl187-GCST90094726 | ebi-a-GCST90002070 | CD4 on activated & secreting CD4 regulatory T cell \|\| id:ebi-a-GCST90002070 | F-capsicum liking (derived food-liking factor) \|\| id:ebi-fl187-GCST90094726 | Inverse variance weighted | 20 | -0.096806696 | 0.0882516371656201 | 0.272667893217789 | -0.269779905 | 0.0761665128199424 | 0.907731457274245 | 0.763547528937562 | 1.07914225021689 | 0.998735809224318 |
| ebi-fl187-GCST90094726 | ebi-a-GCST90002081 | SSC-A on CD4+ T cell \|\| id:ebi-a-GCST90002081 | F-capsicum liking (derived food-liking factor) \|\| id:ebi-fl187-GCST90094726 | Inverse variance weighted | 20 | 0.0565911944942297 | 0.0836268579025373 | 0.498589488721612 | -0.107317447 | 0.220499835983203 | 1.05822311452175 | 0.898240483984306 | 1.24669972025852 | 0.998735809224318 |
| ebi-fl187-GCST90094726 | ebi-a-GCST90002094 | CD11b on Monocytic Myeloid-Derived Suppressor Cells \|\| id:ebi-a-GCST90002094 | F-capsicum liking (derived food-liking factor) \|\| id:ebi-fl187-GCST90094726 | Inverse variance weighted | 20 | 0.151884591059197 | 0.117679315176776 | 0.196819813848988 | -0.078766867 | 0.382536048805677 | 1.16402588968486 | 0.924255374047019 | 1.465997721 | 0.998735809224318 |
| ebi-fl187-GCST90094726 | ebi-a-GCST90002104 | HLA DR on myeloid Dendritic Cell \|\| id:ebi-a-GCST90002104 | F-capsicum liking (derived food-liking factor) \|\| id:ebi-fl187-GCST90094726 | Inverse variance weighted (multiplicative random effects) | 20 | 0.0258325049992083 | 0.114507243281192 | 0.821514776293014 | -0.198601692 | 0.250266701830344 | 1.02616905589144 | 0.819876391768763 | 1.28436791428692 | 0.998735809224318 |
| ebi-fl187-GCST90094726 | ebi-a-GCST90002105 | HLA DR on plasmacytoid Dendritic Cell \|\| id:ebi-a-GCST90002105 | F-capsicum liking (derived food-liking factor) \|\| id:ebi-fl187-GCST90094726 | Inverse variance weighted | 20 | 0.100813224700772 | 0.0878793624158869 | 0.251308280805827 | -0.071430326 | 0.27305677503591 | 1.10607003590767 | 0.931061146429732 | 1.31397484367598 | 0.998735809224318 |
| ebi-fl187-GCST90094726 | ebi-a-GCST90002106 | HLA DR on Dendritic Cell \|\| id:ebi-a-GCST90002106 | F-capsicum liking (derived food-liking factor) \|\| id:ebi-fl187-GCST90094726 | Inverse variance weighted | 20 | 0.0532743209247109 | 0.0961798849094912 | 0.579645273631866 | -0.135238253 | 0.241786895347314 | 1.05471893691223 | 0.873507770612065 | 1.27352277026887 | 0.998735809224318 |
| ebi-fl187-GCST90094726 | ebi-a-GCST90002111 | HLA DR on CD33dim HLA DR+ CD11b- \|\| id:ebi-a-GCST90002111 | F-capsicum liking (derived food-liking factor) \|\| id:ebi-fl187-GCST90094726 | Inverse variance weighted (multiplicative random effects) | 20 | 0.0678580429768625 | 0.145962145690347 | 0.642001885882998 | -0.218227763 | 0.353943848529942 | 1.07021337339029 | 0.803942312840102 | 1.42467518662777 | 0.998735809224318 |
| ebi-fl187-GCST90094726 | ebi-a-GCST90002112 | HLA DR on CD33- HLA DR+ \|\| id:ebi-a-GCST90002112 | F-capsicum liking (derived food-liking factor) \|\| id:ebi-fl187-GCST90094726 | Inverse variance weighted | 20 | 0.071151736670061 | 0.124830236399025 | 0.568685837726378 | -0.173515527 | 0.31581900001215 | 1.07374413986831 | 0.840704097723745 | 1.37138201303307 | 0.998735809224318 |
| ebi-fl187-GCST90094726 | ebi-a-GCST90002115 | HLA DR on HLA DR+ CD8+ T cell \|\| id:ebi-a-GCST90002115 | F-capsicum liking (derived food-liking factor) \|\| id:ebi-fl187-GCST90094726 | Inverse variance weighted | 20 | -0.25855732 | 0.0889319280581387 | 0.00364490975029131 | -0.432863899 | -0.084250741 | 0.772164769421755 | 0.648648767653039 | 0.919200746026976 | 0.998735809224318 |
| ebi-fl187-GCST90094726 | ebi-a-GCST90002116 | HLA DR on B cell \|\| id:ebi-a-GCST90002116 | F-capsicum liking (derived food-liking factor) \|\| id:ebi-fl187-GCST90094726 | Inverse variance weighted | 20 | 0.0351380341286536 | 0.0870517769325375 | 0.686473623748095 | -0.135483449 | 0.205759516916427 | 1.03576266953025 | 0.873293616989205 | 1.22845774516371 | 0.998735809224318 |
| ebi-fl187-GCST90094726 | ebi-cfmet338-GCST90026023 | Sphingomyelin (d18:1/24:1, d18:2/24:0) levels \|\| id:ebi-cfmet338-GCST90026023 | F-capsicum liking (derived food-liking factor) \|\| id:ebi-fl187-GCST90094726 | Inverse variance weighted | 18 | -0.086654435 | 0.0533629224344671 | 0.104403612891636 | -0.191245763 | 0.0179368932238779 | 0.916993922123963 | 0.82592958050676 | 1.01809872543414 | 0.998735809224318 |
| ebi-fl187-GCST90094726 | ebi-cfmet338-GCST90026046 | 1-arachidonoyl-gpc (20:4n6) levels \|\| id:ebi-cfmet338-GCST90026046 | F-capsicum liking (derived food-liking factor) \|\| id:ebi-fl187-GCST90094726 | Inverse variance weighted | 18 | -0.094053135 | 0.0558491041437756 | 0.0921704451836685 | -0.203517379 | 0.0154111091526908 | 0.910234395679304 | 0.815856025363431 | 1.01553047268186 | 0.998735809224318 |
| ebi-fl187-GCST90094726 | ebi-cfmet338-GCST90026047 | 1-linoleoyl-gpc (18:2) levels \|\| id:ebi-cfmet338-GCST90026047 | F-capsicum liking (derived food-liking factor) \|\| id:ebi-fl187-GCST90094726 | Inverse variance weighted | 18 | -0.024850017 | 0.0681282746374476 | 0.715295528873611 | -0.158381435 | 0.108681401726222 | 0.97545620333127 | 0.853524156009271 | 1.11480711813284 | 0.998735809224318 |
| ebi-fl187-GCST90094726 | ebi-cfmet338-GCST90026050 | 1-myristoyl-2-palmitoyl-gpc (14:0/16:0) levels \|\| id:ebi-cfmet338-GCST90026050 | F-capsicum liking (derived food-liking factor) \|\| id:ebi-fl187-GCST90094726 | Inverse variance weighted | 18 | -0.075826349 | 0.0357532922355117 | 0.0339368309956869 | -0.145902802 | -0.005749896 | 0.926977163061161 | 0.86424170182427 | 0.994266602760671 | 0.998735809224318 |
| ebi-fl187-GCST90094726 | ebi-cfmet338-GCST90026068 | 2,3-dihydroxyisovalerate levels \|\| id:ebi-cfmet338-GCST90026068 | F-capsicum liking (derived food-liking factor) \|\| id:ebi-fl187-GCST90094726 | Inverse variance weighted | 18 | -0.132166741 | 0.116017432252146 | 0.254620888878482 | -0.359560908 | 0.0952274264826358 | 0.876194885522295 | 0.697982737468711 | 1.0999089751125 | 0.998735809224318 |
| ebi-fl187-GCST90094726 | ebi-cfmet338-GCST90026082 | 3-hydroxyhexanoate levels \|\| id:ebi-cfmet338-GCST90026082 | F-capsicum liking (derived food-liking factor) \|\| id:ebi-fl187-GCST90094726 | Inverse variance weighted (multiplicative random effects) | 18 | 0.00749570126256505 | 0.0330031740581974 | 0.820329952699644 | -0.05719052 | 0.072181922416632 | 1.00752386435467 | 0.944414122549183 | 1.0748508657454 | 0.998735809224318 |
| ebi-fl187-GCST90094726 | ebi-cfmet338-GCST90026094 | 3-ureidopropionate levels \|\| id:ebi-cfmet338-GCST90026094 | F-capsicum liking (derived food-liking factor) \|\| id:ebi-fl187-GCST90094726 | Inverse variance weighted | 18 | -0.028361891 | 0.0718057847206338 | 0.692857219759217 | -0.169101229 | 0.112377447523315 | 0.972036532327289 | 0.844423419274412 | 1.1189351202395 | 0.998735809224318 |
| ebi-fl187-GCST90094726 | ebi-cfmet338-GCST90026173 | Glycerol levels \|\| id:ebi-cfmet338-GCST90026173 | F-capsicum liking (derived food-liking factor) \|\| id:ebi-fl187-GCST90094726 | Inverse variance weighted | 18 | -0.02431158 | 0.0319641002905506 | 0.446901900854316 | -0.086961217 | 0.0383380565188641 | 0.975981565992074 | 0.916712648158622 | 1.03908244210406 | 0.998735809224318 |
| ebi-fl187-GCST90094726 | ebi-cfmet338-GCST90026179 | Histidine levels \|\| id:ebi-cfmet338-GCST90026179 | F-capsicum liking (derived food-liking factor) \|\| id:ebi-fl187-GCST90094726 | Inverse variance weighted (multiplicative random effects) | 18 | 0.0154091465831313 | 0.0297625070750712 | 0.60464188576148 | -0.042925367 | 0.0737436604502709 | 1.01552847963462 | 0.957982884263621 | 1.076530813 | 0.998735809224318 |
| ebi-fl187-GCST90094726 | ebi-cfmet338-GCST90026188 | Isocitrate levels \|\| id:ebi-cfmet338-GCST90026188 | F-capsicum liking (derived food-liking factor) \|\| id:ebi-fl187-GCST90094726 | Inverse variance weighted | 18 | -0.051509389 | 0.0482421615302551 | 0.285644324663481 | -0.146064026 | 0.0430452476035146 | 0.949794732301597 | 0.864102376744622 | 1.04398513160724 | 0.998735809224318 |
| ebi-fl187-GCST90094726 | ebi-cfmet338-GCST90026207 | N-acetyl-isoputreanine levels \|\| id:ebi-cfmet338-GCST90026207 | F-capsicum liking (derived food-liking factor) \|\| id:ebi-fl187-GCST90094726 | Inverse variance weighted | 17 | -0.00309884 | 0.0299990754864223 | 0.917726581761603 | -0.061897028 | 0.05569934794676 | 0.996905956442311 | 0.939979673432362 | 1.05727976261571 | 0.998735809224318 |
| ebi-fl187-GCST90094726 | ebi-cfmet338-GCST90026213 | N-acetylglutamate levels \|\| id:ebi-cfmet338-GCST90026213 | F-capsicum liking (derived food-liking factor) \|\| id:ebi-fl187-GCST90094726 | Inverse variance weighted | 18 | -0.016118947 | 0.0471053161379371 | 0.732208243355964 | -0.108445367 | 0.0762074723474532 | 0.984010267746199 | 0.897227911808315 | 1.07918645227882 | 0.998735809224318 |
| ebi-fl187-GCST90094726 | ebi-cfmet338-GCST90026224 | N-acetyltaurine levels \|\| id:ebi-cfmet338-GCST90026224 | F-capsicum liking (derived food-liking factor) \|\| id:ebi-fl187-GCST90094726 | Inverse variance weighted | 18 | 0.035656024479908 | 0.0437088780486857 | 0.414636497251434 | -0.050013376 | 0.121325425455332 | 1.03629932357806 | 0.951216700469684 | 1.1289922554115 | 0.998735809224318 |
| ebi-fl187-GCST90094726 | ebi-cfmet338-GCST90026250 | Phenyllactate (pla) levels \|\| id:ebi-cfmet338-GCST90026250 | F-capsicum liking (derived food-liking factor) \|\| id:ebi-fl187-GCST90094726 | Inverse variance weighted | 18 | 0.143758785500679 | 0.0606996381036207 | 0.0178669893858667 | 0.0247874948175829 | 0.262730076183776 | 1.15460556732834 | 1.02509725889689 | 1.30047564222362 | 0.998735809224318 |
| ebi-fl187-GCST90094726 | ebi-cfmet338-GCST90026251 | Pipecolate levels \|\| id:ebi-cfmet338-GCST90026251 | F-capsicum liking (derived food-liking factor) \|\| id:ebi-fl187-GCST90094726 | Inverse variance weighted | 17 | -0.055667002 | 0.102461509731178 | 0.586925525529689 | -0.256491561 | 0.145157557398318 | 0.945854051269304 | 0.773761524500488 | 1.15622172720478 | 0.998735809224318 |
| ebi-fl187-GCST90094726 | ebi-cfmet338-GCST90026278 | Trigonelline (n'-methylnicotinate) levels \|\| id:ebi-cfmet338-GCST90026278 | F-capsicum liking (derived food-liking factor) \|\| id:ebi-fl187-GCST90094726 | Inverse variance weighted | 18 | -0.00078888 | 0.100548328182309 | 0.993740035994213 | -0.197863603 | 0.196285842986041 | 0.999211430832933 | 0.82048175635528 | 1.21687469072117 | 0.999107451094609 |
| ebi-fl187-GCST90094726 | ebi-cfmet338-GCST90026299 | 1-carboxyethylvaline levels \|\| id:ebi-cfmet338-GCST90026299 | F-capsicum liking (derived food-liking factor) \|\| id:ebi-fl187-GCST90094726 | Inverse variance weighted | 18 | -0.031311985 | 0.0635430089112382 | 0.622176262029557 | -0.155856282 | 0.0932323125155908 | 0.969173158464268 | 0.855682158106533 | 1.09771671898137 | 0.998735809224318 |
| ebi-fl187-GCST90094726 | ebi-gm430-GCST90011306 | C_Erysipelotrichia abundance \|\| id:ebi-gm430-GCST90011306 | F-capsicum liking (derived food-liking factor) \|\| id:ebi-fl187-GCST90094726 | Inverse variance weighted | 18 | -0.045898529 | 0.0497774849789081 | 0.356489934803802 | -0.143462399 | 0.051665341773363 | 0.955138876375628 | 0.86635337503009 | 1.05302328063582 | 0.998735809224318 |
| ebi-fl187-GCST90094726 | ebi-gm430-GCST90011312 | F_Erysipelotrichaceae abundance \|\| id:ebi-gm430-GCST90011312 | F-capsicum liking (derived food-liking factor) \|\| id:ebi-fl187-GCST90094726 | Inverse variance weighted | 18 | -0.045898529 | 0.0497774849789081 | 0.356489934803802 | -0.143462399 | 0.051665341773363 | 0.955138876375628 | 0.86635337503009 | 1.05302328063582 | 0.998735809224318 |
| ebi-fl187-GCST90094726 | ebi-gm430-GCST90011352 | O_Erysipelotrichales abundance \|\| id:ebi-gm430-GCST90011352 | F-capsicum liking (derived food-liking factor) \|\| id:ebi-fl187-GCST90094726 | Inverse variance weighted | 18 | -0.045898529 | 0.0497774849789081 | 0.356489934803802 | -0.143462399 | 0.051665341773363 | 0.955138876375628 | 0.86635337503009 | 1.05302328063582 | 0.998735809224318 |
| ebi-fl187-GCST90094726 | ebi-gm430-GCST90011362 | OTU97_11 (Parabacteroides) abundance \|\| id:ebi-gm430-GCST90011362 | F-capsicum liking (derived food-liking factor) \|\| id:ebi-fl187-GCST90094726 | Inverse variance weighted | 18 | -0.112400332 | 0.0649251342592367 | 0.0834107742661307 | -0.239653595 | 0.014852931555612 | 0.893686414971608 | 0.786900400296607 | 1.01496378449389 | 0.998735809224318 |
| ebi-fl187-GCST90094726 | ebi-gm430-GCST90011384 | OTU97_235 (Phascolarctobacterium) abundance \|\| id:ebi-gm430-GCST90011384 | F-capsicum liking (derived food-liking factor) \|\| id:ebi-fl187-GCST90094726 | Inverse variance weighted | 18 | 0.0243370158972932 | 0.198911123871396 | 0.902620880041595 | -0.365528787 | 0.414202818685229 | 1.02463557818703 | 0.69382966582646 | 1.51316399369592 | 0.998735809224318 |
| ebi-fl187-GCST90094726 | ebi-gm430-GCST90011390 | OTU97_3 (Bacteroides) abundance \|\| id:ebi-gm430-GCST90011390 | F-capsicum liking (derived food-liking factor) \|\| id:ebi-fl187-GCST90094726 | Inverse variance weighted | 18 | -0.075608826 | 0.0575301295064053 | 0.188762945591119 | -0.18836788 | 0.0371502278978372 | 0.927178823941306 | 0.828309932710654 | 1.03784892298941 | 0.998735809224318 |
| ebi-fl187-GCST90094726 | ebi-gm430-GCST90011398 | OTU97_4 (Alistipes) abundance \|\| id:ebi-gm430-GCST90011398 | F-capsicum liking (derived food-liking factor) \|\| id:ebi-fl187-GCST90094726 | Inverse variance weighted | 18 | -0.049031634 | 0.060234305758113 | 0.41563630808434 | -0.167090873 | 0.0690276056456782 | 0.952151009219633 | 0.846122718196226 | 1.07146578724496 | 0.998735809224318 |
| ebi-fl187-GCST90094726 | ebi-gm430-GCST90011421 | OTU99_108 (Cytophagales) abundance \|\| id:ebi-gm430-GCST90011421 | F-capsicum liking (derived food-liking factor) \|\| id:ebi-fl187-GCST90094726 | Inverse variance weighted | 18 | -0.01746919 | 0.189521213150329 | 0.926558697869445 | -0.388930768 | 0.353992387743825 | 0.982682511616567 | 0.677781192669056 | 1.4247443409197 | 0.998735809224318 |
| ebi-fl187-GCST90094726 | ebi-gm430-GCST90011428 | OTU99_123 (Phascolarctobacterium) abundance \|\| id:ebi-gm430-GCST90011428 | F-capsicum liking (derived food-liking factor) \|\| id:ebi-fl187-GCST90094726 | Inverse variance weighted | 19 | -0.158031817 | 0.113701121506661 | 0.164562909201176 | -0.380886015 | 0.0648223809346482 | 0.853822615275669 | 0.683255765836135 | 1.06696949342835 | 0.998735809224318 |
| ebi-fl187-GCST90094726 | ebi-gm430-GCST90011450 | OTU99_298 (Phascolarctobacterium) abundance \|\| id:ebi-gm430-GCST90011450 | F-capsicum liking (derived food-liking factor) \|\| id:ebi-fl187-GCST90094726 | Inverse variance weighted | 18 | 0.0168976920277962 | 0.198908319524607 | 0.932299421375375 | -0.372962614 | 0.406757998296027 | 1.01704126557289 | 0.68869097959805 | 1.50194058949603 | 0.998735809224318 |
| ebi-fl187-GCST90094726 | ebi-gm430-GCST90011451 | OTU99_3 (Bacteroides) abundance \|\| id:ebi-gm430-GCST90011451 | F-capsicum liking (derived food-liking factor) \|\| id:ebi-fl187-GCST90094726 | Inverse variance weighted | 18 | -0.07977947 | 0.0574026587237427 | 0.164582978485335 | -0.192288681 | 0.03272974121143 | 0.923319943787573 | 0.825068652477301 | 1.03327125086641 | 0.998735809224318 |
| ebi-fl187-GCST90094726 | ebi-gm430-GCST90011472 | OTU99_72 (Parabacteroides) abundance \|\| id:ebi-gm430-GCST90011472 | F-capsicum liking (derived food-liking factor) \|\| id:ebi-fl187-GCST90094726 | Inverse variance weighted | 19 | -0.163143454 | 0.151897999755689 | 0.282807922335434 | -0.460863533 | 0.134576625629466 | 0.84946931999373 | 0.630738746289255 | 1.14405231937295 | 0.998735809224318 |
| ebi-fl187-GCST90094726 | ebi-gm430-GCST90011507 | TestASV_3 (Bacteroides) abundance \|\| id:ebi-gm430-GCST90011507 | F-capsicum liking (derived food-liking factor) \|\| id:ebi-fl187-GCST90094726 | Inverse variance weighted | 18 | -0.043316003 | 0.0824785047754187 | 0.599458557910561 | -0.204973872 | 0.118341866486595 | 0.957608735137906 | 0.814668601604713 | 1.12562886038091 | 0.998735809224318 |
| ebi-fl187-GCST90094726 | ebi-gm430-GCST90011512 | TestASV_34 (Phascolarctobacterium) abundance \|\| id:ebi-gm430-GCST90011512 | F-capsicum liking (derived food-liking factor) \|\| id:ebi-fl187-GCST90094726 | Inverse variance weighted | 18 | -0.019542565 | 0.16708162244169 | 0.906888439323531 | -0.347022545 | 0.307937415130571 | 0.98064715319628 | 0.706789393623731 | 1.36061583230821 | 0.998735809224318 |
| ebi-fl187-GCST90094726 | ebi-gm430-GCST90011519 | TestASV_41 (Barnesiella) abundance \|\| id:ebi-gm430-GCST90011519 | F-capsicum liking (derived food-liking factor) \|\| id:ebi-fl187-GCST90094726 | Inverse variance weighted | 18 | -0.20822038 | 0.236521198481061 | 0.37867215343514 | -0.671801929 | 0.255361168612973 | 0.812028061914253 | 0.510787345286464 | 1.29092777928242 | 0.998735809224318 |
| ebi-fl187-GCST90094726 | ebi-gm430-GCST90011524 | TestASV_46 (Sutterella) abundance \|\| id:ebi-gm430-GCST90011524 | F-capsicum liking (derived food-liking factor) \|\| id:ebi-fl187-GCST90094726 | Inverse variance weighted | 18 | -0.642390913 | 0.253757208572307 | 0.011357002395061 | -1.139755042 | -0.145026784 | 0.52603321961676 | 0.319897373680167 | 0.864999124428666 | 0.998735809224318 |
| ebi-fl187-GCST90094726 | ebi-gm430-GCST90011581 | OTU97_19 (Bacteria) prevalence \|\| id:ebi-gm430-GCST90011581 | F-capsicum liking (derived food-liking factor) \|\| id:ebi-fl187-GCST90094726 | Inverse variance weighted | 18 | -0.01957604 | 0.247169273971851 | 0.93687279386429 | -0.504027817 | 0.464875737194109 | 0.98061432664544 | 0.604092578714476 | 1.5918163730278 | 0.998735809224318 |
| ebi-fl187-GCST90094726 | ebi-gm430-GCST90011614 | OTU97_80 (Ruminococcus) prevalence \|\| id:ebi-gm430-GCST90011614 | F-capsicum liking (derived food-liking factor) \|\| id:ebi-fl187-GCST90094726 | Inverse variance weighted | 18 | 0.234440554562257 | 0.142914307659099 | 0.100916319893498 | -0.045671488 | 0.514552597574091 | 1.26420131920122 | 0.955355756046064 | 1.67288987935197 | 0.998735809224318 |
| ebi-fl187-GCST90094726 | ebi-gm430-GCST90011623 | OTU99_11 (Parabacteroides) prevalence \|\| id:ebi-gm430-GCST90011623 | F-capsicum liking (derived food-liking factor) \|\| id:ebi-fl187-GCST90094726 | Inverse variance weighted | 18 | -0.007895062 | 0.104828644619485 | 0.939964904029686 | -0.213359205 | 0.197569081719713 | 0.992136022407884 | 0.807865895470036 | 1.21843723380182 | 0.998735809224318 |
| ebi-fl187-GCST90094726 | ebi-gm430-GCST90011642 | OTU99_19 (Bacteria) prevalence \|\| id:ebi-gm430-GCST90011642 | F-capsicum liking (derived food-liking factor) \|\| id:ebi-fl187-GCST90094726 | Inverse variance weighted | 18 | -0.01957604 | 0.247169273971851 | 0.93687279386429 | -0.504027817 | 0.464875737194109 | 0.98061432664544 | 0.604092578714476 | 1.5918163730278 | 0.998735809224318 |
| ebi-fl187-GCST90094726 | ebi-gm430-GCST90011653 | OTU99_30 (Parasutterella) prevalence \|\| id:ebi-gm430-GCST90011653 | F-capsicum liking (derived food-liking factor) \|\| id:ebi-fl187-GCST90094726 | Inverse variance weighted | 18 | -0.120608799 | 0.123549055199855 | 0.328964516659343 | -0.362764947 | 0.121547349397177 | 0.886380644953391 | 0.695749952403661 | 1.12924283362675 | 0.998735809224318 |
| ebi-fl187-GCST90094726 | ebi-gm430-GCST90011675 | OTU99_8 (Ruminococcaceae) prevalence \|\| id:ebi-gm430-GCST90011675 | F-capsicum liking (derived food-liking factor) \|\| id:ebi-fl187-GCST90094726 | Inverse variance weighted | 18 | 0.00167196866113351 | 0.145124928005574 | 0.990807861279047 | -0.28277289 | 0.286116827552059 | 1.00167336718005 | 0.753690938999898 | 1.33124797260958 | 0.999107451094609 |
| ebi-fl187-GCST90094726 | ebi-gm430-GCST90011679 | OTU99_92 (Ruminococcus) prevalence \|\| id:ebi-gm430-GCST90011679 | F-capsicum liking (derived food-liking factor) \|\| id:ebi-fl187-GCST90094726 | Inverse variance weighted (multiplicative random effects) | 18 | 0.160189658401809 | 0.155561982586289 | 0.303128262463017 | -0.144711827 | 0.465091144270934 | 1.17373345829522 | 0.865271604696425 | 1.59215929847252 | 0.998735809224318 |
| ebi-fl187-GCST90094726 | ebi-gm430-GCST90011684 | TestASV_11 (Lachnospiraceae) prevalence \|\| id:ebi-gm430-GCST90011684 | F-capsicum liking (derived food-liking factor) \|\| id:ebi-fl187-GCST90094726 | Inverse variance weighted | 18 | 0.0327289140664742 | 0.125163246243952 | 0.793714803064722 | -0.212591049 | 0.278048876704621 | 1.03327039620166 | 0.808486701410857 | 1.32055073980021 | 0.998735809224318 |
| ebi-fl187-GCST90094726 | ebi-gm430-GCST90011688 | TestASV_15 (Bacteroides) prevalence \|\| id:ebi-gm430-GCST90011688 | F-capsicum liking (derived food-liking factor) \|\| id:ebi-fl187-GCST90094726 | Inverse variance weighted | 18 | -0.176212872 | 0.13658154013473 | 0.196992994904404 | -0.443912691 | 0.0914869462052053 | 0.838439483664418 | 0.641521428897852 | 1.09580247221857 | 0.998735809224318 |
| ebi-fl187-GCST90094726 | ebi-gm430-GCST90011692 | TestASV_19 (Prevotella) prevalence \|\| id:ebi-gm430-GCST90011692 | F-capsicum liking (derived food-liking factor) \|\| id:ebi-fl187-GCST90094726 | Inverse variance weighted | 18 | -0.030084919 | 0.225727822558229 | 0.893972241162535 | -0.472511452 | 0.412341612795891 | 0.970363127377359 | 0.623434574733056 | 1.51035030320022 | 0.998735809224318 |
| ebi-fl187-GCST90094726 | ebi-gm430-GCST90011693 | TestASV_2 (Bacteroides) prevalence \|\| id:ebi-gm430-GCST90011693 | F-capsicum liking (derived food-liking factor) \|\| id:ebi-fl187-GCST90094726 | Inverse variance weighted | 18 | -0.151167107 | 0.113567675530975 | 0.183164296336622 | -0.373759751 | 0.0714255373112535 | 0.859704024327259 | 0.688142217608686 | 1.07403817195353 | 0.998735809224318 |
| ebi-fl187-GCST90094726 | ebi-gm430-GCST90011716 | TestASV_40 (Barnesiella) prevalence \|\| id:ebi-gm430-GCST90011716 | F-capsicum liking (derived food-liking factor) \|\| id:ebi-fl187-GCST90094726 | Inverse variance weighted | 18 | -0.106843747 | 0.232535808437488 | 0.645895086447381 | -0.562613932 | 0.348926437081803 | 0.898666080882112 | 0.569717911935931 | 1.41754490776628 | 0.998735809224318 |
| ebi-fl187-GCST90094726 | ebi-gm430-GCST90011718 | TestASV_42 (Alphaproteobacteria) prevalence \|\| id:ebi-gm430-GCST90011718 | F-capsicum liking (derived food-liking factor) \|\| id:ebi-fl187-GCST90094726 | Inverse variance weighted | 18 | 0.199710793016087 | 0.181217912456295 | 0.2704409122358 | -0.155476315 | 0.554897901430426 | 1.22104957102689 | 0.856007350882038 | 1.74176314417004 | 0.998735809224318 |
| ebi-fl187-GCST90094726 | ebi-hbsm44-GCST90104929 | Sulcal depth of the right inferior frontal sulcus \|\| id:ebi-hbsm44-GCST90104929 | F-capsicum liking (derived food-liking factor) \|\| id:ebi-fl187-GCST90094726 | Inverse variance weighted | 18 | 0.0236580994161852 | 0.0964343318452076 | 0.806202010317277 | -0.165353191 | 0.212669389832792 | 1.02394017229384 | 0.847594288540869 | 1.23697562691468 | 0.998735809224318 |
| ebi-fl187-GCST90094726 | ebi-hbsm44-GCST90104935 | Sulcal opening distance of the left calloso-marginal posterior fissure \|\| id:ebi-hbsm44-GCST90104935 | F-capsicum liking (derived food-liking factor) \|\| id:ebi-fl187-GCST90094726 | Inverse variance weighted | 18 | -0.015609719 | 0.0223814043992713 | 0.48552658021081 | -0.059477271 | 0.0282578338861376 | 0.984511481469891 | 0.942256949567782 | 1.02866087386317 | 0.998735809224318 |
| ebi-fl187-GCST90094726 | ebi-hbsm44-GCST90104936 | Sulcal opening distance of the left anterior inferior temporal sulcus \|\| id:ebi-hbsm44-GCST90104936 | F-capsicum liking (derived food-liking factor) \|\| id:ebi-fl187-GCST90094726 | Inverse variance weighted | 20 | -0.01520201 | 0.0120977579097517 | 0.208899489712535 | -0.038913616 | 0.00850959547010606 | 0.984912957206525 | 0.96183379305429 | 1.00854590499769 | 0.998735809224318 |
| ebi-fl187-GCST90094726 | ebi-hbsm44-GCST90104941 | Sulcal opening distance of the right posterior inferior temporal sulcus \|\| id:ebi-hbsm44-GCST90104941 | F-capsicum liking (derived food-liking factor) \|\| id:ebi-fl187-GCST90094726 | Inverse variance weighted | 20 | 0.00310777961821234 | 0.00968370635180601 | 0.748264398322021 | -0.015872285 | 0.0220878440677521 | 1.00311261377182 | 0.984253016068434 | 1.02233358646693 | 0.998735809224318 |
| ebi-fl187-GCST90094726 | ebi-IPs91-GCST90274819 | Leukemia inhibitory factor levels \|\| id:ebi-IPs91-GCST90274819 | F-capsicum liking (derived food-liking factor) \|\| id:ebi-fl187-GCST90094726 | Inverse variance weighted | 21 | -0.018083878 | 0.0411673086123139 | 0.660460373666026 | -0.098771803 | 0.0626040466924423 | 0.982078653925809 | 0.905949419315401 | 1.06460522180759 | 0.998735809224318 |
| ebi-fl187-GCST90094726 | ebi-IPs91-GCST90274837 | STAM binding protein levels \|\| id:ebi-IPs91-GCST90274837 | F-capsicum liking (derived food-liking factor) \|\| id:ebi-fl187-GCST90094726 | Inverse variance weighted | 21 | -0.022598865 | 0.0358172180159988 | 0.528073369775355 | -0.092800613 | 0.0476028819381624 | 0.977654576230669 | 0.911375198835607 | 1.04875409342489 | 0.998735809224318 |
| ebi-fl187-GCST90094726 | ebi-IPs91-GCST90274840 | TNF-beta levels \|\| id:ebi-IPs91-GCST90274840 | F-capsicum liking (derived food-liking factor) \|\| id:ebi-fl187-GCST90094726 | Inverse variance weighted | 21 | -0.010777958 | 0.0405951577754972 | 0.790625371756204 | -0.090344468 | 0.0687885508166178 | 0.989279915662421 | 0.913616419747777 | 1.07120967878755 | 0.998735809224318 |
| ebi-fl187-GCST90094726 | ebi-IPs91-GCST90274842 | Tumor necrosis factor ligand superfamily member 14 levels \|\| id:ebi-IPs91-GCST90274842 | F-capsicum liking (derived food-liking factor) \|\| id:ebi-fl187-GCST90094726 | Inverse variance weighted | 21 | -0.023659428 | 0.0406020307964841 | 0.560084909652194 | -0.103239409 | 0.0559205520600666 | 0.97661826166642 | 0.901911022321025 | 1.05751366311704 | 0.998735809224318 |
| ebi-fl187-GCST90094726 | ebi-met1400-GCST90199628 | 3-methylhistidine levels \|\| id:ebi-met1400-GCST90199628 | F-capsicum liking (derived food-liking factor) \|\| id:ebi-fl187-GCST90094726 | Inverse variance weighted | 18 | 0.0380150719389031 | 0.053714689992063 | 0.479117865107841 | -0.06726572 | 0.143295864323347 | 1.03874688869016 | 0.934946733864116 | 1.15407119965436 | 0.998735809224318 |
| ebi-fl187-GCST90094726 | ebi-met1400-GCST90199651 | Docosahexaenoate DHA; 22:6n3 levels \|\| id:ebi-met1400-GCST90199651 | F-capsicum liking (derived food-liking factor) \|\| id:ebi-fl187-GCST90094726 | Inverse variance weighted (multiplicative random effects) | 18 | 0.0651545280534092 | 0.0614714142231131 | 0.289182870878227 | -0.055329444 | 0.185638499930711 | 1.06732394313287 | 0.946173385623991 | 1.2039869403359 | 0.998735809224318 |
| ebi-fl187-GCST90094726 | ebi-met1400-GCST90199652 | 1-stearoyl-2-oleoyl-GPS (18:0/18:1) levels \|\| id:ebi-met1400-GCST90199652 | F-capsicum liking (derived food-liking factor) \|\| id:ebi-fl187-GCST90094726 | Inverse variance weighted | 18 | -0.01308514 | 0.0508659858638187 | 0.796987830068565 | -0.112782473 | 0.0866121919944407 | 0.987000097959766 | 0.893344965996968 | 1.09047370327477 | 0.998735809224318 |
| ebi-fl187-GCST90094726 | ebi-met1400-GCST90199653 | 1-myristoyl-2-palmitoyl-gpc (14:0/16:0) levels \|\| id:ebi-met1400-GCST90199653 | F-capsicum liking (derived food-liking factor) \|\| id:ebi-fl187-GCST90094726 | Inverse variance weighted | 18 | -0.060473222 | 0.0498915795585606 | 0.225476877719172 | -0.158260718 | 0.0373142739423826 | 0.941318975327596 | 0.853627197040409 | 1.03801919196566 | 0.998735809224318 |
| ebi-fl187-GCST90094726 | ebi-met1400-GCST90199667 | Erythritol levels in elite athletes \|\| id:ebi-met1400-GCST90199667 | F-capsicum liking (derived food-liking factor) \|\| id:ebi-fl187-GCST90094726 | Inverse variance weighted (multiplicative random effects) | 18 | -0.003586571 | 0.0691919494791884 | 0.958660098074304 | -0.139202792 | 0.132029650287562 | 0.996419853370603 | 0.870051571354955 | 1.14114215395864 | 0.998735809224318 |
| ebi-fl187-GCST90094726 | ebi-met1400-GCST90199674 | 3-hydroxymyristate levels \|\| id:ebi-met1400-GCST90199674 | F-capsicum liking (derived food-liking factor) \|\| id:ebi-fl187-GCST90094726 | Inverse variance weighted (multiplicative random effects) | 18 | 0.00418636911511965 | 0.0656643317556774 | 0.949166042011247 | -0.124515721 | 0.132888459356247 | 1.00419514419928 | 0.882924380691834 | 1.14212259813604 | 0.998735809224318 |
| ebi-fl187-GCST90094726 | ebi-met1400-GCST90199685 | 2-linoleoylglycerol (18:2) levels \|\| id:ebi-met1400-GCST90199685 | F-capsicum liking (derived food-liking factor) \|\| id:ebi-fl187-GCST90094726 | Inverse variance weighted | 18 | -0.08823511 | 0.0574211053439393 | 0.124383441512926 | -0.200780477 | 0.0243102561389894 | 0.915545597184913 | 0.818092002009805 | 1.02460815955347 | 0.998735809224318 |
| ebi-fl187-GCST90094726 | ebi-met1400-GCST90199703 | Gamma-glutamylvaline levels \|\| id:ebi-met1400-GCST90199703 | F-capsicum liking (derived food-liking factor) \|\| id:ebi-fl187-GCST90094726 | Inverse variance weighted | 18 | 0.000965875624697142 | 0.050134951380725 | 0.984629294391037 | -0.097298629 | 0.0992303803309181 | 1.00096634223277 | 0.907285023980319 | 1.10432068401979 | 0.998955228396052 |
| ebi-fl187-GCST90094726 | ebi-met1400-GCST90199779 | Gamma-glutamyl-2-aminobutyrate levels \|\| id:ebi-met1400-GCST90199779 | F-capsicum liking (derived food-liking factor) \|\| id:ebi-fl187-GCST90094726 | Inverse variance weighted | 18 | 0.0663743270089153 | 0.0520145203621777 | 0.201929701626582 | -0.035574133 | 0.168322786918784 | 1.06862665812756 | 0.965051189533877 | 1.18331850874402 | 0.998735809224318 |
| ebi-fl187-GCST90094726 | ebi-met1400-GCST90199824 | 4-vinylphenol sulfate levels \|\| id:ebi-met1400-GCST90199824 | F-capsicum liking (derived food-liking factor) \|\| id:ebi-fl187-GCST90094726 | Inverse variance weighted | 18 | -0.026198793 | 0.0502698596682007 | 0.602253201940727 | -0.124727718 | 0.0723301322849843 | 0.974141418190852 | 0.882737223662663 | 1.07501018105647 | 0.998735809224318 |
| ebi-fl187-GCST90094726 | ebi-met1400-GCST90199830 | 4-ethylphenylsulfate levels \|\| id:ebi-met1400-GCST90199830 | F-capsicum liking (derived food-liking factor) \|\| id:ebi-fl187-GCST90094726 | Inverse variance weighted | 18 | 0.078649442596366 | 0.0503182227619404 | 0.118042963796914 | -0.019974274 | 0.177273159209769 | 1.08182501382151 | 0.980223890205488 | 1.19395718898931 | 0.998735809224318 |
| ebi-fl187-GCST90094726 | ebi-met1400-GCST90199833 | Hexanoylglutamine levels \|\| id:ebi-met1400-GCST90199833 | F-capsicum liking (derived food-liking factor) \|\| id:ebi-fl187-GCST90094726 | Inverse variance weighted | 18 | 0.00388911781224728 | 0.0546416709661629 | 0.943258523479296 | -0.103208557 | 0.110986792905927 | 1.00389669024444 | 0.901938847950577 | 1.11738014941226 | 0.998735809224318 |
| ebi-fl187-GCST90094726 | ebi-met1400-GCST90199837 | Carotenoid levels (cryptoxanthin) \|\| id:ebi-met1400-GCST90199837 | F-capsicum liking (derived food-liking factor) \|\| id:ebi-fl187-GCST90094726 | Inverse variance weighted | 18 | 0.0831210099015833 | 0.0542794368395124 | 0.125682030158074 | -0.023266686 | 0.189508706107028 | 1.08667329882121 | 0.977001896002717 | 1.20865564662905 | 0.998735809224318 |
| ebi-fl187-GCST90094726 | ebi-met1400-GCST90199869 | 2-hydroxyhippurate levels \|\| id:ebi-met1400-GCST90199869 | F-capsicum liking (derived food-liking factor) \|\| id:ebi-fl187-GCST90094726 | Inverse variance weighted | 18 | 0.102250425946346 | 0.057545422290151 | 0.0755905137638254 | -0.010538602 | 0.215039453635042 | 1.10766082400846 | 0.989516734760607 | 1.23991081498984 | 0.998735809224318 |
| ebi-fl187-GCST90094726 | ebi-met1400-GCST90199902 | Hydantoin-5-propionate levels \|\| id:ebi-met1400-GCST90199902 | F-capsicum liking (derived food-liking factor) \|\| id:ebi-fl187-GCST90094726 | Inverse variance weighted | 18 | 0.0424775020773955 | 0.0501853995874816 | 0.397323194940138 | -0.055885881 | 0.140840885268859 | 1.04339258194406 | 0.945647045920358 | 1.15124145393627 | 0.998735809224318 |
| ebi-fl187-GCST90094726 | ebi-met1400-GCST90199904 | Pantoate levels \|\| id:ebi-met1400-GCST90199904 | F-capsicum liking (derived food-liking factor) \|\| id:ebi-fl187-GCST90094726 | Inverse variance weighted | 18 | 0.0572895913976144 | 0.0590346828594322 | 0.331827440027782 | -0.058418387 | 0.172997569802101 | 1.05896243240663 | 0.943255219140108 | 1.18886321590763 | 0.998735809224318 |
| ebi-fl187-GCST90094726 | ebi-met1400-GCST90199936 | 3-methyl catechol sulfate (2) levels \|\| id:ebi-met1400-GCST90199936 | F-capsicum liking (derived food-liking factor) \|\| id:ebi-fl187-GCST90094726 | Inverse variance weighted | 18 | 0.029334596718796 | 0.0572627416959992 | 0.608454579026255 | -0.082900377 | 0.141569570442954 | 1.02976909419771 | 0.920442839924804 | 1.15208065223411 | 0.998735809224318 |
| ebi-fl187-GCST90094726 | ebi-met1400-GCST90199977 | 4-hydroxychlorothalonil levels \|\| id:ebi-met1400-GCST90199977 | F-capsicum liking (derived food-liking factor) \|\| id:ebi-fl187-GCST90094726 | Inverse variance weighted | 18 | 0.0748439077678361 | 0.0498569946057815 | 0.133311165837462 | -0.022875802 | 0.172563617195168 | 1.07771591467203 | 0.977383865689559 | 1.18834741753992 | 0.998735809224318 |
| ebi-fl187-GCST90094726 | ebi-met1400-GCST90200040 | Sphingomyelin (d18:2/24:1, d18:1/24:2) levels \|\| id:ebi-met1400-GCST90200040 | F-capsicum liking (derived food-liking factor) \|\| id:ebi-fl187-GCST90094726 | Inverse variance weighted | 18 | 0.053865073397773 | 0.0458070689555465 | 0.239630118753489 | -0.035916782 | 0.143646928550644 | 1.05534219881122 | 0.964720572495601 | 1.15447642389402 | 0.998735809224318 |
| ebi-fl187-GCST90094726 | ebi-met1400-GCST90200077 | 1-stearoyl-2-oleoyl-GPI (18:0/18:1) levels \|\| id:ebi-met1400-GCST90200077 | F-capsicum liking (derived food-liking factor) \|\| id:ebi-fl187-GCST90094726 | Inverse variance weighted | 18 | -0.039557496 | 0.0497123354210047 | 0.426190161501716 | -0.136993674 | 0.0578786812918383 | 0.961214686274012 | 0.871975742618185 | 1.05958644025424 | 0.998735809224318 |
| ebi-fl187-GCST90094726 | ebi-met1400-GCST90200080 | 1-palmitoyl-2-oleoyl-GPI (16:0/18:1) levels \|\| id:ebi-met1400-GCST90200080 | F-capsicum liking (derived food-liking factor) \|\| id:ebi-fl187-GCST90094726 | Inverse variance weighted | 18 | -0.041364926 | 0.0516586866380507 | 0.423284986542005 | -0.142615952 | 0.0598860994026713 | 0.959478926854267 | 0.867087007800897 | 1.06171560961597 | 0.998735809224318 |
| ebi-fl187-GCST90094726 | ebi-met1400-GCST90200082 | 1-oleoyl-2-linoleoyl-GPE (18:1/18:2) levels \|\| id:ebi-met1400-GCST90200082 | F-capsicum liking (derived food-liking factor) \|\| id:ebi-fl187-GCST90094726 | Inverse variance weighted | 18 | -0.123851068 | 0.053205787727906 | 0.0199240890158685 | -0.228134412 | -0.019567724 | 0.883511414473123 | 0.796017258655204 | 0.980622481506288 | 0.998735809224318 |
| ebi-fl187-GCST90094726 | ebi-met1400-GCST90200100 | Perfluorooctanesulfonate (PFOS) levels \|\| id:ebi-met1400-GCST90200100 | F-capsicum liking (derived food-liking factor) \|\| id:ebi-fl187-GCST90094726 | Inverse variance weighted (multiplicative random effects) | 18 | 0.0746700502542671 | 0.0661298956913258 | 0.258837887856475 | -0.054944545 | 0.204284645809266 | 1.07752856194958 | 0.946537636458079 | 1.22664726377054 | 0.998735809224318 |
| ebi-fl187-GCST90094726 | ebi-met1400-GCST90200102 | 1-palmitoleoyl-2-linolenoyl-GPC (16:1/18:3) levels \|\| id:ebi-met1400-GCST90200102 | F-capsicum liking (derived food-liking factor) \|\| id:ebi-fl187-GCST90094726 | Inverse variance weighted | 18 | -0.080989723 | 0.0492179633362828 | 0.0998601955635942 | -0.177456931 | 0.0154774855761538 | 0.922203169280914 | 0.837397064662754 | 1.01559788219973 | 0.998735809224318 |
| ebi-fl187-GCST90094726 | ebi-met1400-GCST90200114 | Glycosyl-N-tricosanoyl-sphingadienine (d18:2/23:0) levels \|\| id:ebi-met1400-GCST90200114 | F-capsicum liking (derived food-liking factor) \|\| id:ebi-fl187-GCST90094726 | Inverse variance weighted | 18 | -0.001750307 | 0.0525798904362468 | 0.973444505325999 | -0.104806892 | 0.101306278317945 | 0.99825122395678 | 0.900498399069095 | 1.10661552220566 | 0.998955228396052 |
| ebi-fl187-GCST90094726 | ebi-met1400-GCST90200135 | Arachidoylcarnitine (C20) levels \|\| id:ebi-met1400-GCST90200135 | F-capsicum liking (derived food-liking factor) \|\| id:ebi-fl187-GCST90094726 | Inverse variance weighted | 18 | 0.00136652570942669 | 0.0514626604507587 | 0.978815676503182 | -0.099500289 | 0.102233340192914 | 1.00136745983114 | 0.905289688444291 | 1.10764189895041 | 0.998955228396052 |
| ebi-fl187-GCST90094726 | ebi-met1400-GCST90200146 | Docosahexaenoylcarnitine (C22:6) levels \|\| id:ebi-met1400-GCST90200146 | F-capsicum liking (derived food-liking factor) \|\| id:ebi-fl187-GCST90094726 | Inverse variance weighted | 18 | 0.0722576593167471 | 0.0634766206218891 | 0.254980621978718 | -0.052156517 | 0.19667183573565 | 1.07493227470085 | 0.949180292267193 | 1.21734448619197 | 0.998735809224318 |
| ebi-fl187-GCST90094726 | ebi-met1400-GCST90200162 | 3-hydroxyphenylacetoylglutamine levels \|\| id:ebi-met1400-GCST90200162 | F-capsicum liking (derived food-liking factor) \|\| id:ebi-fl187-GCST90094726 | Inverse variance weighted | 18 | 0.0377626754565786 | 0.0690279256159275 | 0.584334939373052 | -0.097532059 | 0.173057409663796 | 1.0384847457128 | 0.907073261454168 | 1.18893435944664 | 0.998735809224318 |
| ebi-fl187-GCST90094726 | ebi-met1400-GCST90200172 | 3-carboxy-4-methyl-5-pentyl-2-furanpropionate (3-CMPFP) levels \|\| id:ebi-met1400-GCST90200172 | F-capsicum liking (derived food-liking factor) \|\| id:ebi-fl187-GCST90094726 | Inverse variance weighted | 18 | 0.0310716642713348 | 0.0533087153218643 | 0.559985700261459 | -0.073413418 | 0.135556746302189 | 1.03155942719235 | 0.929216595963462 | 1.14517417839064 | 0.998735809224318 |
| ebi-fl187-GCST90094726 | ebi-met1400-GCST90200222 | Dihydrocaffeate sulfate (2) levels \|\| id:ebi-met1400-GCST90200222 | F-capsicum liking (derived food-liking factor) \|\| id:ebi-fl187-GCST90094726 | Inverse variance weighted | 18 | 0.0955069015508493 | 0.057111360257776 | 0.0944666505184561 | -0.016431365 | 0.20744516765609 | 1.10021641520727 | 0.983702893960173 | 1.23053024213278 | 0.998735809224318 |
| ebi-fl187-GCST90094726 | ebi-met1400-GCST90200223 | 4-methylhexanoylglutamine levels \|\| id:ebi-met1400-GCST90200223 | F-capsicum liking (derived food-liking factor) \|\| id:ebi-fl187-GCST90094726 | Inverse variance weighted | 18 | 0.0464696667344738 | 0.070429891896328 | 0.509381964069339 | -0.091572921 | 0.184512254851277 | 1.04756630247978 | 0.912494773345604 | 1.20263171926743 | 0.998735809224318 |
| ebi-fl187-GCST90094726 | ebi-met1400-GCST90200224 | 2-ketocaprylate levels \|\| id:ebi-met1400-GCST90200224 | F-capsicum liking (derived food-liking factor) \|\| id:ebi-fl187-GCST90094726 | Inverse variance weighted | 18 | -0.007211458 | 0.055487916214667 | 0.896594527373535 | -0.115967774 | 0.101544857479452 | 0.992814481871175 | 0.89050392014135 | 1.10687956910585 | 0.998735809224318 |
| ebi-fl187-GCST90094726 | ebi-met1400-GCST90200243 | Hydroxypalmitoyl sphingomyelin (d18:1/16:0(OH)) levels \|\| id:ebi-met1400-GCST90200243 | F-capsicum liking (derived food-liking factor) \|\| id:ebi-fl187-GCST90094726 | Inverse variance weighted | 18 | -0.012232227 | 0.0501448826478916 | 0.807279075122077 | -0.110516197 | 0.0860517429436445 | 0.987842282527592 | 0.89537182770223 | 1.08986271955147 | 0.998735809224318 |
| ebi-fl187-GCST90094726 | ebi-met1400-GCST90200273 | 2-methoxyhydroquinone sulfate (2) levels \|\| id:ebi-met1400-GCST90200273 | F-capsicum liking (derived food-liking factor) \|\| id:ebi-fl187-GCST90094726 | Inverse variance weighted (multiplicative random effects) | 18 | 0.0503675074210339 | 0.0786506121081766 | 0.521915430374134 | -0.103787692 | 0.20452270715306 | 1.05165751730739 | 0.901416654793978 | 1.22693931582829 | 0.998735809224318 |
| ebi-fl187-GCST90094726 | ebi-met1400-GCST90200276 | 2,4-di-tert-butylphenol levels \|\| id:ebi-met1400-GCST90200276 | F-capsicum liking (derived food-liking factor) \|\| id:ebi-fl187-GCST90094726 | Inverse variance weighted | 18 | 0.0597102396650155 | 0.0499860730755478 | 0.232267773841611 | -0.038262464 | 0.157682942893089 | 1.06152891300409 | 0.962460296978892 | 1.17079492700191 | 0.998735809224318 |
| ebi-fl187-GCST90094726 | ebi-met1400-GCST90200289 | N-acetylmethionine levels \|\| id:ebi-met1400-GCST90200289 | F-capsicum liking (derived food-liking factor) \|\| id:ebi-fl187-GCST90094726 | Inverse variance weighted | 18 | 0.00334947738394866 | 0.0584000464755521 | 0.95426319007374 | -0.111114614 | 0.117813568476031 | 1.00335509315153 | 0.894836182558332 | 1.1250343499465 | 0.998735809224318 |
| ebi-fl187-GCST90094726 | ebi-met1400-GCST90200308 | 3-hydroxyisobutyrate levels \|\| id:ebi-met1400-GCST90200308 | F-capsicum liking (derived food-liking factor) \|\| id:ebi-fl187-GCST90094726 | Inverse variance weighted | 18 | -0.003559261 | 0.0507452306824022 | 0.944082383079108 | -0.103019913 | 0.0959013914372137 | 0.996447065959769 | 0.902109009752271 | 1.10065052507623 | 0.998735809224318 |
| ebi-fl187-GCST90094726 | ebi-met1400-GCST90200383 | Cystathionine levels \|\| id:ebi-met1400-GCST90200383 | F-capsicum liking (derived food-liking factor) \|\| id:ebi-fl187-GCST90094726 | Inverse variance weighted | 18 | 0.0106944209744721 | 0.0500514069136817 | 0.830805391413209 | -0.087406337 | 0.108795178525288 | 1.01075181069528 | 0.916304691866259 | 1.11493396453427 | 0.998735809224318 |
| ebi-fl187-GCST90094726 | ebi-met1400-GCST90200384 | Guanidinoacetate levels \|\| id:ebi-met1400-GCST90200384 | F-capsicum liking (derived food-liking factor) \|\| id:ebi-fl187-GCST90094726 | Inverse variance weighted | 18 | 0.0704588402003305 | 0.054957862376015 | 0.199824387462758 | -0.03725857 | 0.17817625045732 | 1.07300040404012 | 0.963426989768317 | 1.19503592830334 | 0.998735809224318 |
| ebi-fl187-GCST90094726 | ebi-met1400-GCST90200403 | Citrulline levels \|\| id:ebi-met1400-GCST90200403 | F-capsicum liking (derived food-liking factor) \|\| id:ebi-fl187-GCST90094726 | Inverse variance weighted | 18 | -0.025019995 | 0.050324161179099 | 0.619064523093684 | -0.123655351 | 0.07361536069221 | 0.975290410688188 | 0.88368434921803 | 1.0763927029171 | 0.998735809224318 |
| ebi-fl187-GCST90094726 | ebi-met1400-GCST90200434 | 12,13-DiHOME levels \|\| id:ebi-met1400-GCST90200434 | F-capsicum liking (derived food-liking factor) \|\| id:ebi-fl187-GCST90094726 | Inverse variance weighted | 18 | -0.018761584 | 0.0507045837193631 | 0.711369445874093 | -0.118142568 | 0.0806194004177378 | 0.981413319311736 | 0.888569362238347 | 1.08395826398538 | 0.998735809224318 |
| ebi-fl187-GCST90094726 | ebi-met1400-GCST90200437 | Fructose levels \|\| id:ebi-met1400-GCST90200437 | F-capsicum liking (derived food-liking factor) \|\| id:ebi-fl187-GCST90094726 | Inverse variance weighted | 18 | 0.0515353403015086 | 0.0505456495392872 | 0.30792762906716 | -0.047534133 | 0.150604813398512 | 1.0528863949573 | 0.95357792429686 | 1.16253714818703 | 0.998735809224318 |
| ebi-fl187-GCST90094726 | ebi-met1400-GCST90200448 | N-stearoyl-sphinganine (d18:0/18:0) levels \|\| id:ebi-met1400-GCST90200448 | F-capsicum liking (derived food-liking factor) \|\| id:ebi-fl187-GCST90094726 | Inverse variance weighted | 18 | -0.031529051 | 0.0559473217600025 | 0.573061179993345 | -0.141185802 | 0.0781276996883704 | 0.968962806743917 | 0.868327959974727 | 1.08126072651211 | 0.998735809224318 |
| ebi-fl187-GCST90094726 | ebi-met1400-GCST90200468 | X-11847 levels \|\| id:ebi-met1400-GCST90200468 | F-capsicum liking (derived food-liking factor) \|\| id:ebi-fl187-GCST90094726 | Inverse variance weighted | 18 | 0.00649415562741007 | 0.0532453636020832 | 0.90292547509196 | -0.097866757 | 0.110855068287493 | 1.00651528837776 | 0.906769716392719 | 1.11723297263205 | 0.998735809224318 |
| ebi-fl187-GCST90094726 | ebi-met1400-GCST90200476 | X-11843 levels \|\| id:ebi-met1400-GCST90200476 | F-capsicum liking (derived food-liking factor) \|\| id:ebi-fl187-GCST90094726 | Inverse variance weighted | 18 | 0.0780119278515588 | 0.0530596395808227 | 0.141488991919956 | -0.025984966 | 0.182008821429971 | 1.08113555421758 | 0.974349738138737 | 1.19962477623915 | 0.998735809224318 |
| ebi-fl187-GCST90094726 | ebi-met1400-GCST90200511 | X-12906 levels \|\| id:ebi-met1400-GCST90200511 | F-capsicum liking (derived food-liking factor) \|\| id:ebi-fl187-GCST90094726 | Inverse variance weighted | 18 | 0.0434786557585213 | 0.0544950085398078 | 0.42495953127725 | -0.063331561 | 0.150288872496545 | 1.04443770134361 | 0.938632208275884 | 1.1621699131672 | 0.998735809224318 |
| ebi-fl187-GCST90094726 | ebi-met1400-GCST90200515 | X-12849 levels \|\| id:ebi-met1400-GCST90200515 | F-capsicum liking (derived food-liking factor) \|\| id:ebi-fl187-GCST90094726 | Inverse variance weighted (multiplicative random effects) | 18 | -0.015422626 | 0.0771149894141323 | 0.841484357161733 | -0.166568005 | 0.135722753692846 | 0.984695694083248 | 0.846565244468789 | 1.14536430154834 | 0.998735809224318 |
| ebi-fl187-GCST90094726 | ebi-met1400-GCST90200551 | X-18886 levels \|\| id:ebi-met1400-GCST90200551 | F-capsicum liking (derived food-liking factor) \|\| id:ebi-fl187-GCST90094726 | Inverse variance weighted | 18 | -0.012627797 | 0.0513926239548185 | 0.805904985540152 | -0.11335734 | 0.0881017454736418 | 0.987451598606158 | 0.892831558293135 | 1.09209923252928 | 0.998735809224318 |
| ebi-fl187-GCST90094726 | ebi-met1400-GCST90200564 | X-21319 levels \|\| id:ebi-met1400-GCST90200564 | F-capsicum liking (derived food-liking factor) \|\| id:ebi-fl187-GCST90094726 | Inverse variance weighted | 18 | -0.007326136 | 0.0621529422548597 | 0.906168511721042 | -0.129145903 | 0.114493630687611 | 0.992700634588192 | 0.878845730004971 | 1.12130549909621 | 0.998735809224318 |
| ebi-fl187-GCST90094726 | ebi-met1400-GCST90200579 | X-21442 levels \|\| id:ebi-met1400-GCST90200579 | F-capsicum liking (derived food-liking factor) \|\| id:ebi-fl187-GCST90094726 | Inverse variance weighted | 18 | -0.051928653 | 0.0686278831857168 | 0.449247655786899 | -0.186439304 | 0.0825819979439898 | 0.949396600930885 | 0.829908932455731 | 1.0860877267485 | 0.998735809224318 |
| ebi-fl187-GCST90094726 | ebi-met1400-GCST90200581 | X-21441 levels \|\| id:ebi-met1400-GCST90200581 | F-capsicum liking (derived food-liking factor) \|\| id:ebi-fl187-GCST90094726 | Inverse variance weighted | 18 | -0.000885021 | 0.0503450389953265 | 0.985974627460499 | -0.099561297 | 0.0977912558028104 | 0.999115370887218 | 0.905234459957858 | 1.10273257205606 | 0.998955228396052 |
| ebi-fl187-GCST90094726 | ebi-met1400-GCST90200648 | X-24585 levels \|\| id:ebi-met1400-GCST90200648 | F-capsicum liking (derived food-liking factor) \|\| id:ebi-fl187-GCST90094726 | Inverse variance weighted | 18 | -0.015836475 | 0.0580081375262351 | 0.784849853929782 | -0.129532425 | 0.0978594743276959 | 0.984288262413796 | 0.878506102591585 | 1.10280780141145 | 0.998735809224318 |
| ebi-fl187-GCST90094726 | ebi-met1400-GCST90200720 | Adenosine 5'-diphosphate (ADP) to pantothenate ratio \|\| id:ebi-met1400-GCST90200720 | F-capsicum liking (derived food-liking factor) \|\| id:ebi-fl187-GCST90094726 | Inverse variance weighted | 18 | -0.135392695 | 0.079541944623118 | 0.08872637239581 | -0.291294906 | 0.0205095166883539 | 0.873372875375157 | 0.747295263486837 | 1.02072128208331 | 0.998735809224318 |
| ebi-fl187-GCST90094726 | ebi-met1400-GCST90200724 | Cholate to taurocholate ratio \|\| id:ebi-met1400-GCST90200724 | F-capsicum liking (derived food-liking factor) \|\| id:ebi-fl187-GCST90094726 | Inverse variance weighted | 18 | -0.045817284 | 0.066678948664934 | 0.491999129225149 | -0.176508023 | 0.0848734554380703 | 0.955216479633298 | 0.838192054038034 | 1.08857930419086 | 0.998735809224318 |
| ebi-fl187-GCST90094726 | ebi-met1400-GCST90200726 | N-acetylputrescine to (N(1) + N(8))-acetylspermidine ratio \|\| id:ebi-met1400-GCST90200726 | F-capsicum liking (derived food-liking factor) \|\| id:ebi-fl187-GCST90094726 | Inverse variance weighted | 18 | 0.0197743936514579 | 0.0500030347895051 | 0.692500580656329 | -0.078231555 | 0.117780341838888 | 1.01997120208892 | 0.924750271630188 | 1.1249969694594 | 0.998735809224318 |
| ebi-fl187-GCST90094726 | ebi-met1400-GCST90200744 | Arginine to glutamate ratio \|\| id:ebi-met1400-GCST90200744 | F-capsicum liking (derived food-liking factor) \|\| id:ebi-fl187-GCST90094726 | Inverse variance weighted | 18 | 0.0731673204093695 | 0.0484433439633203 | 0.130949318242818 | -0.021781634 | 0.168116274577477 | 1.07591054364749 | 0.9784538730192 | 1.18307416409923 | 0.998735809224318 |
| ebi-fl187-GCST90094726 | ebi-met1400-GCST90200759 | Histidine to trans-urocanate ratio \|\| id:ebi-met1400-GCST90200759 | F-capsicum liking (derived food-liking factor) \|\| id:ebi-fl187-GCST90094726 | Inverse variance weighted | 18 | -0.026250357 | 0.0577536786709227 | 0.649452667750923 | -0.139447567 | 0.0869468530636954 | 0.974091188403254 | 0.869838629973784 | 1.09083870344257 | 0.998735809224318 |
| ebi-fl187-GCST90094726 | ebi-met1400-GCST90200795 | Oleoyl-linoleoyl-glycerol (18:1 to 18:2) [2] to linoleoyl-arachidonoyl-glycerol (18:2 to 20:4) [2] ratio \|\| id:ebi-met1400-GCST90200795 | F-capsicum liking (derived food-liking factor) \|\| id:ebi-fl187-GCST90094726 | Inverse variance weighted | 18 | 0.0305193130588819 | 0.0620457468746412 | 0.622801316703848 | -0.091090351 | 0.152128976933179 | 1.03098980142325 | 0.912935222731091 | 1.16431039593248 | 0.998735809224318 |
| ebi-fl187-GCST90094726 | ebi-met1400-GCST90200811 | Choline to taurocholate ratio \|\| id:ebi-met1400-GCST90200811 | F-capsicum liking (derived food-liking factor) \|\| id:ebi-fl187-GCST90094726 | Inverse variance weighted | 18 | 0.0695379358426022 | 0.054871049033474 | 0.205048982443747 | -0.03800932 | 0.177085191948211 | 1.07201272813952 | 0.962703968195204 | 1.19373278521702 | 0.998735809224318 |
| ebi-fl187-GCST90094726 | ebi-met1400-GCST90200831 | Cholate to phosphate ratio \|\| id:ebi-met1400-GCST90200831 | F-capsicum liking (derived food-liking factor) \|\| id:ebi-fl187-GCST90094726 | Inverse variance weighted | 18 | -0.065703604 | 0.0523951295099862 | 0.209841230836684 | -0.168398058 | 0.0369908497748095 | 0.936408370798513 | 0.845017401873113 | 1.03768352575678 | 0.998735809224318 |
| ebi-fl187-GCST90094726 | ebi-met1400-GCST90200841 | Adenosine 5'-diphosphate (ADP) to oxalate (ethanedioate) ratio \|\| id:ebi-met1400-GCST90200841 | F-capsicum liking (derived food-liking factor) \|\| id:ebi-fl187-GCST90094726 | Inverse variance weighted | 18 | -0.073039231 | 0.0699183821270014 | 0.296191420463949 | -0.21007926 | 0.0640007980539714 | 0.92956436165004 | 0.810520001703011 | 1.06609324956112 | 0.998735809224318 |
| ebi-fl187-GCST90094726 | ebi-met1400-GCST90200844 | Aspartate to phosphate ratio \|\| id:ebi-met1400-GCST90200844 | F-capsicum liking (derived food-liking factor) \|\| id:ebi-fl187-GCST90094726 | Inverse variance weighted | 18 | -0.06614483 | 0.0472319473834232 | 0.161385898012105 | -0.158719447 | 0.026429786976214 | 0.935995294374086 | 0.853235703428405 | 1.02678215125106 | 0.998735809224318 |
| ebi-fl187-GCST90094726 | ebi-met1400-GCST90200870 | Adenosine 5'-monophosphate (AMP) to acetoacetate ratio \|\| id:ebi-met1400-GCST90200870 | F-capsicum liking (derived food-liking factor) \|\| id:ebi-fl187-GCST90094726 | Inverse variance weighted | 18 | -0.000511407 | 0.0553795080970875 | 0.992631968966737 | -0.109055243 | 0.108032428890212 | 0.999488723766181 | 0.896680880922242 | 1.11408387330428 | 0.999107451094609 |
| ebi-fl187-GCST90094726 | ebi-met1400-GCST90200887 | Cholesterol to cortisol ratio \|\| id:ebi-met1400-GCST90200887 | F-capsicum liking (derived food-liking factor) \|\| id:ebi-fl187-GCST90094726 | Inverse variance weighted | 18 | -0.04668393 | 0.0498177690578528 | 0.348710235825049 | -0.144326757 | 0.0509588976193955 | 0.954389003909802 | 0.865604859321112 | 1.05227964119601 | 0.998735809224318 |
| ebi-fl187-GCST90094726 | ebi-met1400-GCST90200890 | Cortisol to taurocholate ratio \|\| id:ebi-met1400-GCST90200890 | F-capsicum liking (derived food-liking factor) \|\| id:ebi-fl187-GCST90094726 | Inverse variance weighted | 18 | 0.0699312676700851 | 0.0524054894156883 | 0.182064158477452 | -0.032783492 | 0.172646026924834 | 1.07243446780134 | 0.967748062508099 | 1.18844535296471 | 0.998735809224318 |
| ebi-fl187-GCST90094726 | ebi-met1400-GCST90200948 | Adenosine 5'-diphosphate (ADP) to mannitol to sorbitol ratio \|\| id:ebi-met1400-GCST90200948 | F-capsicum liking (derived food-liking factor) \|\| id:ebi-fl187-GCST90094726 | Inverse variance weighted (multiplicative random effects) | 18 | -0.086396908 | 0.0998806226737588 | 0.38703786066962 | -0.282162928 | 0.10936911293176 | 0.917230103447046 | 0.75415080227915 | 1.11557404716261 | 0.998735809224318 |
| ebi-fl187-GCST90094726 | ebi-met1400-GCST90200972 | Leucine to N-palmitoyl-sphingosine (d18:1 to 16:0) ratio \|\| id:ebi-met1400-GCST90200972 | F-capsicum liking (derived food-liking factor) \|\| id:ebi-fl187-GCST90094726 | Inverse variance weighted (multiplicative random effects) | 18 | 0.0356876907938871 | 0.0620223895254052 | 0.565020718444622 | -0.085876193 | 0.157251574263681 | 1.0363321398774 | 0.91770784313937 | 1.17028999171338 | 0.998735809224318 |
| ebi-fl187-GCST90094726 | ebi-met1400-GCST90200973 | Proline to glutamate ratio \|\| id:ebi-met1400-GCST90200973 | F-capsicum liking (derived food-liking factor) \|\| id:ebi-fl187-GCST90094726 | Inverse variance weighted | 18 | 0.0647757001008654 | 0.0492438239881626 | 0.188372954219221 | -0.031742195 | 0.161293595117664 | 1.06691968756528 | 0.968756300187925 | 1.17502990121827 | 0.998735809224318 |
| ebi-fl187-GCST90094726 | ebi-met1400-GCST90200983 | Cholesterol to linoleoyl-arachidonoyl-glycerol (18:2 to 20:4) [1] ratio \|\| id:ebi-met1400-GCST90200983 | F-capsicum liking (derived food-liking factor) \|\| id:ebi-fl187-GCST90094726 | Inverse variance weighted | 18 | 0.0128878024408714 | 0.0517725164299288 | 0.803413884153549 | -0.08858633 | 0.114361934643532 | 1.01297120808677 | 0.915224096247183 | 1.12115783732122 | 0.998735809224318 |
| ebi-fl187-GCST90094726 | ebi-met1400-GCST90201001 | Serine to alpha-ketobutyrate ratio \|\| id:ebi-met1400-GCST90201001 | F-capsicum liking (derived food-liking factor) \|\| id:ebi-fl187-GCST90094726 | Inverse variance weighted | 18 | 0.00265436427607111 | 0.0511424688174562 | 0.958607281411917 | -0.097584875 | 0.102893603158285 | 1.00265789021995 | 0.907025354868984 | 1.10837347536501 | 0.998735809224318 |
| ebi-fl187-GCST90094726 | ebi-met1400-GCST90201005 | Fructose to maltose ratio \|\| id:ebi-met1400-GCST90201005 | F-capsicum liking (derived food-liking factor) \|\| id:ebi-fl187-GCST90094726 | Inverse variance weighted | 18 | 0.0344280060723204 | 0.0572456220851841 | 0.547567911156473 | -0.077773413 | 0.146629425359281 | 1.03502750999801 | 0.925174035005879 | 1.15792478595216 | 0.998735809224318 |
| ebi-fl187-GCST90094726 | ebi-met1400-GCST90201020 | Paraxanthine to 5-acetylamino-6-formylamino-3-methyluracil ratio \|\| id:ebi-met1400-GCST90201020 | F-capsicum liking (derived food-liking factor) \|\| id:ebi-fl187-GCST90094726 | Inverse variance weighted | 18 | -0.001903807 | 0.0531158833680933 | 0.971407936624895 | -0.106010938 | 0.102203324812356 | 0.998098004501153 | 0.899414810231409 | 1.10760865315624 | 0.998955228396052 |
| ebi-fl187-GCST90094726 | ebi-pw11-GCST90275197 | Placental weight (fetal genotype, adjusted for sex) \|\| id:ebi-pw11-GCST90275197 | F-capsicum liking (derived food-liking factor) \|\| id:ebi-fl187-GCST90094726 | Inverse variance weighted | 20 | -0.01791384 | 0.0192408981212105 | 0.351838401839332 | -0.055626 | 0.0197983201761349 | 0.982245658859586 | 0.945892833230406 | 1.01999560674702 | 0.998735809224318 |
| ebi-fl187-GCST90094726 | prot-a-1017 | FAS-associated factor 2 \|\| id:prot-a-1017 | F-capsicum liking (derived food-liking factor) \|\| id:ebi-fl187-GCST90094726 | Inverse variance weighted | 21 | -0.115089405 | 0.0871579213745046 | 0.18667820547168 | -0.285918931 | 0.0557401210330975 | 0.891286455003037 | 0.751323522708787 | 1.0573228720536 | 0.998735809224318 |
| ebi-fl187-GCST90094726 | prot-a-1019 | Fas apoptotic inhibitory molecule 3 \|\| id:prot-a-1019 | F-capsicum liking (derived food-liking factor) \|\| id:ebi-fl187-GCST90094726 | Inverse variance weighted | 21 | 0.00775043044440188 | 0.0752368376006189 | 0.9179520405265 | -0.139713771 | 0.155214632141615 | 1.00778054277467 | 0.869607106332691 | 1.16790860493114 | 0.998735809224318 |
| ebi-fl187-GCST90094726 | prot-a-1052 | MIP18 family protein FAM96A \|\| id:prot-a-1052 | F-capsicum liking (derived food-liking factor) \|\| id:ebi-fl187-GCST90094726 | Inverse variance weighted | 21 | -0.05340106 | 0.0752228911145964 | 0.477763432891855 | -0.200837927 | 0.0940358065569305 | 0.947999731431104 | 0.818045004135447 | 1.0985990822635 | 0.998735809224318 |
| ebi-fl187-GCST90094726 | prot-a-1066 | F-box/LRR-repeat protein 4 \|\| id:prot-a-1066 | F-capsicum liking (derived food-liking factor) \|\| id:ebi-fl187-GCST90094726 | Inverse variance weighted | 21 | -0.040202516 | 0.0784295142414994 | 0.608235190957622 | -0.193924364 | 0.113519331534889 | 0.960594883255696 | 0.823720204573524 | 1.12021354413028 | 0.998735809224318 |
| ebi-fl187-GCST90094726 | prot-a-1076 | Low affinity immunoglobulin gamma Fc region receptor III-B \|\| id:prot-a-1076 | F-capsicum liking (derived food-liking factor) \|\| id:ebi-fl187-GCST90094726 | Inverse variance weighted (multiplicative random effects) | 21 | -0.001616852 | 0.0954249256027103 | 0.986481528391925 | -0.188649706 | 0.185416002652609 | 0.99838445487155 | 0.828076526374509 | 1.20371908631831 | 0.998955228396052 |
| ebi-fl187-GCST90094726 | prot-a-1081 | Fc receptor-like protein 3 \|\| id:prot-a-1081 | F-capsicum liking (derived food-liking factor) \|\| id:ebi-fl187-GCST90094726 | Inverse variance weighted | 21 | 0.0753295755367448 | 0.083756746401471 | 0.368447536012071 | -0.088833647 | 0.239492798483628 | 1.07823945367855 | 0.914997773163987 | 1.2706045343136 | 0.998735809224318 |
| ebi-fl187-GCST90094726 | prot-a-1105 | Fibroblast growth factor receptor 4 \|\| id:prot-a-1105 | F-capsicum liking (derived food-liking factor) \|\| id:ebi-fl187-GCST90094726 | Inverse variance weighted | 21 | 0.00239410576460181 | 0.0929359043022409 | 0.979448106895778 | -0.179760267 | 0.184548478196994 | 1.00239697392424 | 0.835470477526622 | 1.20267528340098 | 0.998955228396052 |
| ebi-fl187-GCST90094726 | prot-a-1143 | Follistatin-related protein 1 \|\| id:prot-a-1143 | F-capsicum liking (derived food-liking factor) \|\| id:ebi-fl187-GCST90094726 | Inverse variance weighted | 21 | -0.029897494 | 0.0857603526713739 | 0.72737706128853 | -0.197987785 | 0.138192796975886 | 0.970545014884664 | 0.820379873609963 | 1.14819689782555 | 0.998735809224318 |
| ebi-fl187-GCST90094726 | prot-a-1151 | Alpha-(1,3)-fucosyltransferase 10 \|\| id:prot-a-1151 | F-capsicum liking (derived food-liking factor) \|\| id:ebi-fl187-GCST90094726 | Inverse variance weighted | 21 | -0.027398405 | 0.0752677979151261 | 0.715848812803088 | -0.174923289 | 0.120126478498634 | 0.972973526373986 | 0.839521418550798 | 1.12763946470694 | 0.998735809224318 |
| ebi-fl187-GCST90094726 | prot-a-1165 | Growth arrest and DNA damage-inducible proteins-interacting protein 1 \|\| id:prot-a-1165 | F-capsicum liking (derived food-liking factor) \|\| id:ebi-fl187-GCST90094726 | Inverse variance weighted | 21 | 0.0403235609472423 | 0.0752545102364945 | 0.592076627626732 | -0.107175279 | 0.187822401010771 | 1.04114759440038 | 0.898368194006175 | 1.2066192019686 | 0.998735809224318 |
| ebi-fl187-GCST90094726 | prot-a-1194 | Growth/differentiation factor 11/8 \|\| id:prot-a-1194 | F-capsicum liking (derived food-liking factor) \|\| id:ebi-fl187-GCST90094726 | Inverse variance weighted | 21 | 0.0190017849042276 | 0.0932058308105623 | 0.83845582558412 | -0.163681643 | 0.20168521329293 | 1.01918346776066 | 0.849012267447809 | 1.22346281765653 | 0.998735809224318 |
| ebi-fl187-GCST90094726 | prot-a-1216 | D-glucuronyl C5-epimerase \|\| id:prot-a-1216 | F-capsicum liking (derived food-liking factor) \|\| id:ebi-fl187-GCST90094726 | Inverse variance weighted | 21 | -0.046030118 | 0.0807280789607911 | 0.568550729120136 | -0.204257153 | 0.112196916553942 | 0.955013198470253 | 0.81525269957469 | 1.11873313603033 | 0.998735809224318 |
| ebi-fl187-GCST90094726 | prot-a-1246 | Glypican-3 \|\| id:prot-a-1246 | F-capsicum liking (derived food-liking factor) \|\| id:ebi-fl187-GCST90094726 | Inverse variance weighted (multiplicative random effects) | 21 | -0.039479228 | 0.0964646353715423 | 0.682348021295634 | -0.228549913 | 0.149591457516239 | 0.961289921878204 | 0.795686580997434 | 1.16135968102695 | 0.998735809224318 |
| ebi-fl187-GCST90094726 | prot-a-1275 | Glutamate receptor 4 \|\| id:prot-a-1275 | F-capsicum liking (derived food-liking factor) \|\| id:ebi-fl187-GCST90094726 | Inverse variance weighted (multiplicative random effects) | 20 | 0.0199917812883355 | 0.103368079543506 | 0.846642725211362 | -0.182609655 | 0.222593217193607 | 1.02019295532056 | 0.833093286394343 | 1.2493122716068 | 0.998735809224318 |
| ebi-fl187-GCST90094726 | prot-a-1289 | Glutathione S-transferase P \|\| id:prot-a-1289 | F-capsicum liking (derived food-liking factor) \|\| id:ebi-fl187-GCST90094726 | Inverse variance weighted | 21 | 0.00771299893246276 | 0.0752441139006306 | 0.918354778552737 | -0.139765464 | 0.155191462177699 | 1.00774282073126 | 0.86956215484225 | 1.1678815448444 | 0.998735809224318 |
| ebi-fl187-GCST90094726 | prot-a-130 | Apolipoprotein D \|\| id:prot-a-130 | F-capsicum liking (derived food-liking factor) \|\| id:ebi-fl187-GCST90094726 | Inverse variance weighted | 21 | 0.00574416277135777 | 0.0759352259001079 | 0.939701087756332 | -0.14308888 | 0.154577205535569 | 1.00576069210824 | 0.866677035223466 | 1.16716438613056 | 0.998735809224318 |
| ebi-fl187-GCST90094726 | prot-a-1313 | Heparin-binding EGF-like growth factor \|\| id:prot-a-1313 | F-capsicum liking (derived food-liking factor) \|\| id:ebi-fl187-GCST90094726 | Inverse variance weighted (multiplicative random effects) | 21 | -0.139624355 | 0.0977882021363183 | 0.153342484320589 | -0.331289231 | 0.0520405211007616 | 0.869684866742871 | 0.717997471682552 | 1.05341842732278 | 0.998735809224318 |
| ebi-fl187-GCST90094726 | prot-a-1347 | HLA class II histocompatibility antigen, DQ alpha 2 chain \|\| id:prot-a-1347 | F-capsicum liking (derived food-liking factor) \|\| id:ebi-fl187-GCST90094726 | Inverse variance weighted | 20 | 0.027718487969594 | 0.0770885007366874 | 0.719170897216119 | -0.123374973 | 0.178811949413501 | 1.0281062194101 | 0.883932149301285 | 1.19579585291161 | 0.998735809224318 |
| ebi-fl187-GCST90094726 | prot-a-1357 | Non-histone chromosomal protein HMG-14 \|\| id:prot-a-1357 | F-capsicum liking (derived food-liking factor) \|\| id:ebi-fl187-GCST90094726 | Inverse variance weighted | 21 | -0.05906558 | 0.0883410543201472 | 0.503745247350567 | -0.232214047 | 0.114082886423438 | 0.942644948432777 | 0.792776414118629 | 1.12084502386932 | 0.998735809224318 |
| ebi-fl187-GCST90094726 | prot-a-137 | Apolipoprotein M \|\| id:prot-a-137 | F-capsicum liking (derived food-liking factor) \|\| id:ebi-fl187-GCST90094726 | Inverse variance weighted | 21 | -0.084276049 | 0.0899121832309525 | 0.348596489186007 | -0.260503928 | 0.0919518305872157 | 0.919177483462632 | 0.770663129453397 | 1.09631201210306 | 0.998735809224318 |
| ebi-fl187-GCST90094726 | prot-a-1381 | 17-beta-hydroxysteroid dehydrogenase 14 \|\| id:prot-a-1381 | F-capsicum liking (derived food-liking factor) \|\| id:ebi-fl187-GCST90094726 | Inverse variance weighted | 21 | -0.017133224 | 0.0800133742116289 | 0.830446068313612 | -0.173959438 | 0.139692989303651 | 0.983012714876435 | 0.840330982803609 | 1.14992070670156 | 0.998735809224318 |
| ebi-fl187-GCST90094726 | prot-a-1392 | Serine protease HTRA2, mitochondrial \|\| id:prot-a-1392 | F-capsicum liking (derived food-liking factor) \|\| id:ebi-fl187-GCST90094726 | Inverse variance weighted | 21 | -0.0532316 | 0.0925577837632608 | 0.56521183858355 | -0.234644857 | 0.128181655700606 | 0.948160392653572 | 0.790851665469585 | 1.13675948278262 | 0.998735809224318 |
| ebi-fl187-GCST90094726 | prot-a-1416 | Interferon-induced protein with tetratricopeptide repeats 2 \|\| id:prot-a-1416 | F-capsicum liking (derived food-liking factor) \|\| id:ebi-fl187-GCST90094726 | Inverse variance weighted | 21 | 0.0366470565940276 | 0.0777813386296131 | 0.637530143061386 | -0.115804367 | 0.189098480308069 | 1.03732683855364 | 0.890649446571307 | 1.20815992658627 | 0.998735809224318 |
| ebi-fl187-GCST90094726 | prot-a-1428 | Interferon gamma \|\| id:prot-a-1428 | F-capsicum liking (derived food-liking factor) \|\| id:ebi-fl187-GCST90094726 | Inverse variance weighted | 21 | 0.0224301058422462 | 0.0752451401614897 | 0.765631613231914 | -0.125050369 | 0.169910580558766 | 1.02268355206086 | 0.882452453328501 | 1.18519886676148 | 0.998735809224318 |
| ebi-fl187-GCST90094726 | prot-a-1483 | Interleukin-17C \|\| id:prot-a-1483 | F-capsicum liking (derived food-liking factor) \|\| id:ebi-fl187-GCST90094726 | Inverse variance weighted | 21 | 0.0787932078108478 | 0.0853710351764165 | 0.356033195820085 | -0.088534021 | 0.246120436756624 | 1.08198055380701 | 0.915271971615064 | 1.27905360933402 | 0.998735809224318 |
| ebi-fl187-GCST90094726 | prot-a-1503 | Interleukin-1 receptor-like 2 \|\| id:prot-a-1503 | F-capsicum liking (derived food-liking factor) \|\| id:ebi-fl187-GCST90094726 | Inverse variance weighted | 21 | 0.0130475555873847 | 0.0752239165073103 | 0.86229806830834 | -0.134391321 | 0.160486431941713 | 1.01313304635133 | 0.874247886303596 | 1.17408184302167 | 0.998735809224318 |
| ebi-fl187-GCST90094726 | prot-a-1522 | Interleukin-31 receptor subunit alpha \|\| id:prot-a-1522 | F-capsicum liking (derived food-liking factor) \|\| id:ebi-fl187-GCST90094726 | Inverse variance weighted | 21 | -0.023081923 | 0.0875967349706261 | 0.792163609490588 | -0.194771524 | 0.148607677416412 | 0.977182426654979 | 0.82302267777838 | 1.16021772029524 | 0.998735809224318 |
| ebi-fl187-GCST90094726 | prot-a-1540 | Interleukin-6 receptor subunit alpha \|\| id:prot-a-1540 | F-capsicum liking (derived food-liking factor) \|\| id:ebi-fl187-GCST90094726 | Inverse variance weighted | 21 | -0.022708719 | 0.0940638625170121 | 0.809231089282358 | -0.207073889 | 0.161656451989994 | 0.977547183674969 | 0.812959578935625 | 1.17545634625769 | 0.998735809224318 |
| ebi-fl187-GCST90094726 | prot-a-1541 | Interleukin-6 receptor subunit alpha \|\| id:prot-a-1541 | F-capsicum liking (derived food-liking factor) \|\| id:ebi-fl187-GCST90094726 | Inverse variance weighted (multiplicative random effects) | 21 | -0.016656259 | 0.0970390467975843 | 0.86371669671333 | -0.20685279 | 0.173540273072375 | 0.983481689861293 | 0.813139343115824 | 1.18950859097055 | 0.998735809224318 |
| ebi-fl187-GCST90094726 | prot-a-1545 | Interleukin-9 \|\| id:prot-a-1545 | F-capsicum liking (derived food-liking factor) \|\| id:ebi-fl187-GCST90094726 | Inverse variance weighted | 21 | -0.107528415 | 0.0752228911145964 | 0.152870912818753 | -0.254965282 | 0.039908451211175 | 0.89805100363361 | 0.774943402011596 | 1.04071549358809 | 0.998735809224318 |
| ebi-fl187-GCST90094726 | prot-a-1548 | Inositol monophosphatase 3 \|\| id:prot-a-1548 | F-capsicum liking (derived food-liking factor) \|\| id:ebi-fl187-GCST90094726 | Inverse variance weighted | 21 | -0.071518526 | 0.089216805509932 | 0.422769232726037 | -0.246383465 | 0.10334641238742 | 0.93097902973398 | 0.78162244243586 | 1.10887547074948 | 0.998735809224318 |
| ebi-fl187-GCST90094726 | prot-a-1555 | Inhibin beta A chain:Inhibin beta B chain heterodimer \|\| id:prot-a-1555 | F-capsicum liking (derived food-liking factor) \|\| id:ebi-fl187-GCST90094726 | Inverse variance weighted | 21 | -0.143988123 | 0.0806790271951125 | 0.0743094622644944 | -0.302119016 | 0.014142770643483 | 0.865898032564387 | 0.73925007709505 | 1.01424325276394 | 0.998735809224318 |
| ebi-fl187-GCST90094726 | prot-a-1603 | Junctophilin-3 \|\| id:prot-a-1603 | F-capsicum liking (derived food-liking factor) \|\| id:ebi-fl187-GCST90094726 | Inverse variance weighted (multiplicative random effects) | 21 | -0.075948921 | 0.0951326153516601 | 0.424668644009712 | -0.262408847 | 0.110511004962473 | 0.926863548495971 | 0.769196475573271 | 1.11684863986186 | 0.998735809224318 |
| ebi-fl187-GCST90094726 | prot-a-1627 | UPF0258 protein KIAA1024 \|\| id:prot-a-1627 | F-capsicum liking (derived food-liking factor) \|\| id:ebi-fl187-GCST90094726 | Inverse variance weighted | 21 | -0.033031727 | 0.0835643192720357 | 0.692632520911622 | -0.196817793 | 0.130754338472966 | 0.967507862689641 | 0.821340273566426 | 1.13968776948154 | 0.998735809224318 |
| ebi-fl187-GCST90094726 | prot-a-1666 | Kallikrein-7 \|\| id:prot-a-1666 | F-capsicum liking (derived food-liking factor) \|\| id:ebi-fl187-GCST90094726 | Inverse variance weighted | 21 | 0.0433226148479186 | 0.0858533713882124 | 0.613831597420266 | -0.124949993 | 0.211595222768815 | 1.04427473904834 | 0.882541034646246 | 1.23564762181467 | 0.998735809224318 |
| ebi-fl187-GCST90094726 | prot-a-1678 | Importin subunit alpha-3 \|\| id:prot-a-1678 | F-capsicum liking (derived food-liking factor) \|\| id:ebi-fl187-GCST90094726 | Inverse variance weighted (multiplicative random effects) | 21 | 0.000542617728479605 | 0.096532441431212 | 0.995515041144513 | -0.188660967 | 0.189746202933655 | 1.00054276497211 | 0.828067200822386 | 1.20894273259926 | 0.999107451094609 |
| ebi-fl187-GCST90094726 | prot-a-1718 | Protein LDOC1 \|\| id:prot-a-1718 | F-capsicum liking (derived food-liking factor) \|\| id:ebi-fl187-GCST90094726 | Inverse variance weighted | 21 | -0.062820838 | 0.0765241192801947 | 0.411686942577723 | -0.212808111 | 0.0871664361338291 | 0.939111712073912 | 0.808311228009848 | 1.09107825945435 | 0.998735809224318 |
| ebi-fl187-GCST90094726 | prot-a-1723 | Leptin \|\| id:prot-a-1723 | F-capsicum liking (derived food-liking factor) \|\| id:ebi-fl187-GCST90094726 | Inverse variance weighted (multiplicative random effects) | 21 | -0.007616146 | 0.0956698398295059 | 0.93654851978269 | -0.195129032 | 0.179896739804521 | 0.992412783090645 | 0.822728492634769 | 1.19709374460541 | 0.998735809224318 |
| ebi-fl187-GCST90094726 | prot-a-1730 | Galectin-7 \|\| id:prot-a-1730 | F-capsicum liking (derived food-liking factor) \|\| id:ebi-fl187-GCST90094726 | Inverse variance weighted | 21 | 0.0365896894792277 | 0.0759336792778745 | 0.629902719902912 | -0.112240322 | 0.185419700863862 | 1.03726733181269 | 0.893829424896402 | 1.20372353793401 | 0.998735809224318 |
| ebi-fl187-GCST90094726 | prot-a-1753 | Lipase member N \|\| id:prot-a-1753 | F-capsicum liking (derived food-liking factor) \|\| id:ebi-fl187-GCST90094726 | Inverse variance weighted | 21 | 0.0371691073175591 | 0.0866755797736806 | 0.668046790086151 | -0.132715029 | 0.207053243673973 | 1.0378685171598 | 0.875714609788476 | 1.23004806231526 | 0.998735809224318 |
| ebi-fl187-GCST90094726 | prot-a-1756 | VIP36-like protein \|\| id:prot-a-1756 | F-capsicum liking (derived food-liking factor) \|\| id:ebi-fl187-GCST90094726 | Inverse variance weighted | 21 | 0.168101959859957 | 0.0752757598036418 | 0.0255391446220521 | 0.020561470644819 | 0.315642449075095 | 1.18305722884797 | 1.02077431396996 | 1.3711399156255 | 0.998735809224318 |
| ebi-fl187-GCST90094726 | prot-a-1760 | Ig Kappa chain V-I region HK102- like \|\| id:prot-a-1760 | F-capsicum liking (derived food-liking factor) \|\| id:ebi-fl187-GCST90094726 | Inverse variance weighted | 21 | 0.177498722763055 | 0.0843026764579081 | 0.0352483875456115 | 0.0122654769055547 | 0.342731968620554 | 1.19422653259124 | 1.01234100635309 | 1.40879111109272 | 0.998735809224318 |
| ebi-fl187-GCST90094726 | prot-a-179 | Neutral ceramidase \|\| id:prot-a-179 | F-capsicum liking (derived food-liking factor) \|\| id:ebi-fl187-GCST90094726 | Inverse variance weighted | 21 | 0.156677611714977 | 0.0752239165073103 | 0.0372679563688225 | 0.00923873536064915 | 0.304116488069306 | 1.16961848181621 | 1.00928154420782 | 1.3554269379609 | 0.998735809224318 |
| ebi-fl187-GCST90094726 | prot-a-1809 | Lactotransferrin \|\| id:prot-a-1809 | F-capsicum liking (derived food-liking factor) \|\| id:ebi-fl187-GCST90094726 | Inverse variance weighted | 21 | 0.117350573406374 | 0.0796198218541247 | 0.14051217736381 | -0.038704277 | 0.273405424240459 | 1.12451358515435 | 0.962035162597557 | 1.31443303983024 | 0.998735809224318 |
| ebi-fl187-GCST90094726 | prot-a-1818 | Lysophospholipase-like protein 1 \|\| id:prot-a-1818 | F-capsicum liking (derived food-liking factor) \|\| id:ebi-fl187-GCST90094726 | Inverse variance weighted | 21 | -0.138976705 | 0.0752194308465952 | 0.064657757499504 | -0.286406789 | 0.0084533795016305 | 0.870248300693109 | 0.750957072415465 | 1.00848921020648 | 0.998735809224318 |
| ebi-fl187-GCST90094726 | prot-a-1852 | MAP kinase-activated protein kinase 3 \|\| id:prot-a-1852 | F-capsicum liking (derived food-liking factor) \|\| id:ebi-fl187-GCST90094726 | Inverse variance weighted | 21 | -0.067104558 | 0.0752309793952497 | 0.372403447755685 | -0.214557277 | 0.0803481618817025 | 0.935097424558373 | 0.806898593397008 | 1.08366429260272 | 0.998735809224318 |
| ebi-fl187-GCST90094726 | prot-a-1856 | Megakaryocyte-associated tyrosine-protein kinase \|\| id:prot-a-1856 | F-capsicum liking (derived food-liking factor) \|\| id:ebi-fl187-GCST90094726 | Inverse variance weighted | 21 | 0.00844334225778509 | 0.0824090951262401 | 0.918394389112443 | -0.153078484 | 0.169965168705216 | 1.00847908780516 | 0.858062374838438 | 1.18526356633669 | 0.998735809224318 |
| ebi-fl187-GCST90094726 | prot-a-1890 | Beta-1,3-N-acetylglucosaminyltransferase manic fringe \|\| id:prot-a-1890 | F-capsicum liking (derived food-liking factor) \|\| id:ebi-fl187-GCST90094726 | Inverse variance weighted | 21 | 0.0584804658865227 | 0.0793511246443035 | 0.461132411703021 | -0.097047738 | 0.214008670189357 | 1.06022427495102 | 0.907512681880949 | 1.23863339393297 | 0.998735809224318 |
| ebi-fl187-GCST90094726 | prot-a-1919 | Matrilysin \|\| id:prot-a-1919 | F-capsicum liking (derived food-liking factor) \|\| id:ebi-fl187-GCST90094726 | Inverse variance weighted | 21 | -0.089258485 | 0.0752581161363925 | 0.235609917561471 | -0.236764393 | 0.0582474222303795 | 0.914609129913152 | 0.789177201769278 | 1.05997722519746 | 0.998735809224318 |
| ebi-fl187-GCST90094726 | prot-a-1923 | Multimerin-2 \|\| id:prot-a-1923 | F-capsicum liking (derived food-liking factor) \|\| id:ebi-fl187-GCST90094726 | Inverse variance weighted | 21 | -0.073509174 | 0.0763113639708623 | 0.3354072654767 | -0.223079448 | 0.0760610989274779 | 0.929127621512481 | 0.800051284424463 | 1.07902849962732 | 0.998735809224318 |
| ebi-fl187-GCST90094726 | prot-a-1948 | Mesothelin \|\| id:prot-a-1948 | F-capsicum liking (derived food-liking factor) \|\| id:ebi-fl187-GCST90094726 | Inverse variance weighted | 21 | 0.0157147798108771 | 0.0833651483525264 | 0.850480490572842 | -0.147680911 | 0.179110470581829 | 1.01583890631766 | 0.86270635116861 | 1.19615287657361 | 0.998735809224318 |
| ebi-fl187-GCST90094726 | prot-a-195 | ATPase family AAA domain-containing protein 1 \|\| id:prot-a-195 | F-capsicum liking (derived food-liking factor) \|\| id:ebi-fl187-GCST90094726 | Inverse variance weighted | 21 | -0.220834254 | 0.0752510456034022 | 0.00333941651795981 | -0.368326304 | -0.073342205 | 0.801849572380941 | 0.691891378133286 | 0.929282770457703 | 0.998735809224318 |
| ebi-fl187-GCST90094726 | prot-a-1966 | Mucin-1 \|\| id:prot-a-1966 | F-capsicum liking (derived food-liking factor) \|\| id:ebi-fl187-GCST90094726 | Inverse variance weighted | 21 | -0.073829387 | 0.0785037743101367 | 0.346983437822664 | -0.227696785 | 0.0800380105567708 | 0.928830150737401 | 0.796365693462046 | 1.08332824480212 | 0.998735809224318 |
| ebi-fl187-GCST90094726 | prot-a-1986 | Myocardial zonula adherens protein \|\| id:prot-a-1986 | F-capsicum liking (derived food-liking factor) \|\| id:ebi-fl187-GCST90094726 | Inverse variance weighted | 21 | -0.013601155 | 0.0752486039789311 | 0.856564129453286 | -0.161088418 | 0.133886109179496 | 0.986490923156864 | 0.851216804536566 | 1.14326260511352 | 0.998735809224318 |
| ebi-fl187-GCST90094726 | prot-a-2071 | Epididymal secretory protein E1 \|\| id:prot-a-2071 | F-capsicum liking (derived food-liking factor) \|\| id:ebi-fl187-GCST90094726 | Inverse variance weighted | 20 | -0.151561233 | 0.0862427822908743 | 0.0788532735629947 | -0.320597086 | 0.0174746206835527 | 0.859365259487002 | 0.725715593139278 | 1.01762819511504 | 0.998735809224318 |
| ebi-fl187-GCST90094726 | prot-a-2098 | Neuritin \|\| id:prot-a-2098 | F-capsicum liking (derived food-liking factor) \|\| id:ebi-fl187-GCST90094726 | Inverse variance weighted | 21 | -0.007030244 | 0.0752441139006306 | 0.925559985336507 | -0.154508708 | 0.140448218820297 | 0.992994409934176 | 0.85683603106406 | 1.1507894887847 | 0.998735809224318 |
| ebi-fl187-GCST90094726 | prot-a-2115 | Neurotrophin-4 \|\| id:prot-a-2115 | F-capsicum liking (derived food-liking factor) \|\| id:ebi-fl187-GCST90094726 | Inverse variance weighted | 21 | 0.124700688122207 | 0.0757000875113448 | 0.0994965771221149 | -0.023671483 | 0.273072859644443 | 1.13280933902848 | 0.976606488507567 | 1.31399597861693 | 0.998735809224318 |
| ebi-fl187-GCST90094726 | prot-a-2118 | Netrin-1 \|\| id:prot-a-2118 | F-capsicum liking (derived food-liking factor) \|\| id:ebi-fl187-GCST90094726 | Inverse variance weighted | 21 | 0.102470285581865 | 0.0752660749525394 | 0.173374512634198 | -0.045051221 | 0.249991792488842 | 1.10790438068667 | 0.955948515629344 | 1.28401487807805 | 0.998735809224318 |
| ebi-fl187-GCST90094726 | prot-a-2120 | Netrin-G1 \|\| id:prot-a-2120 | F-capsicum liking (derived food-liking factor) \|\| id:ebi-fl187-GCST90094726 | Inverse variance weighted | 21 | 0.0148557154280863 | 0.0752581161363925 | 0.843516990659088 | -0.132650192 | 0.162361623055415 | 1.01496661002757 | 0.875771390196888 | 1.17628553638782 | 0.998735809224318 |
| ebi-fl187-GCST90094726 | prot-a-2145 | Olfactomedin-like protein 3 \|\| id:prot-a-2145 | F-capsicum liking (derived food-liking factor) \|\| id:ebi-fl187-GCST90094726 | Inverse variance weighted | 21 | -0.004347174 | 0.0781093191241962 | 0.95561665528839 | -0.157441439 | 0.148747091973505 | 0.995662261771622 | 0.8543268423835 | 1.1603794828106 | 0.998735809224318 |
| ebi-fl187-GCST90094726 | prot-a-2162 | Prolyl 4-hydroxylase subunit alpha-2 \|\| id:prot-a-2162 | F-capsicum liking (derived food-liking factor) \|\| id:ebi-fl187-GCST90094726 | Inverse variance weighted | 21 | 0.0724872498436015 | 0.0904691542205178 | 0.42299435179712 | -0.104832292 | 0.249806792115816 | 1.07517909730111 | 0.900475526487303 | 1.28377735681808 | 0.998735809224318 |
| ebi-fl187-GCST90094726 | prot-a-2168 | Polyadenylate-binding protein-interacting protein 1 \|\| id:prot-a-2168 | F-capsicum liking (derived food-liking factor) \|\| id:ebi-fl187-GCST90094726 | Inverse variance weighted (multiplicative random effects) | 21 | -0.144976063 | 0.0963644525014555 | 0.132464037823575 | -0.333850389 | 0.0438982643810305 | 0.865042999810838 | 0.716160919459763 | 1.04487604836943 | 0.998735809224318 |
| ebi-fl187-GCST90094726 | prot-a-219 | Beta-1,4-galactosyltransferase 5 \|\| id:prot-a-219 | F-capsicum liking (derived food-liking factor) \|\| id:ebi-fl187-GCST90094726 | Inverse variance weighted | 21 | -0.013919897 | 0.0752581161363925 | 0.853258785519967 | -0.161425805 | 0.133586010640565 | 0.98617653681123 | 0.850929664178079 | 1.14291956515158 | 0.998735809224318 |
| ebi-fl187-GCST90094726 | prot-a-2190 | Pyruvate carboxylase, mitochondrial \|\| id:prot-a-2190 | F-capsicum liking (derived food-liking factor) \|\| id:ebi-fl187-GCST90094726 | Inverse variance weighted | 21 | -0.104846677 | 0.0752712648627195 | 0.163644680129477 | -0.252378356 | 0.0426850021966919 | 0.900462573685188 | 0.776950718433741 | 1.04360910849315 | 0.998735809224318 |
| ebi-fl187-GCST90094726 | prot-a-2197 | Protocadherin-9 \|\| id:prot-a-2197 | F-capsicum liking (derived food-liking factor) \|\| id:ebi-fl187-GCST90094726 | Inverse variance weighted | 21 | -0.043266158 | 0.0752486039789311 | 0.565307375669846 | -0.190753422 | 0.104221105468194 | 0.957656467897008 | 0.826336319282528 | 1.10984582076854 | 0.998735809224318 |
| ebi-fl187-GCST90094726 | prot-a-2198 | Protocadherin alpha-4 \|\| id:prot-a-2198 | F-capsicum liking (derived food-liking factor) \|\| id:ebi-fl187-GCST90094726 | Inverse variance weighted (multiplicative random effects) | 21 | 0.0141839534554509 | 0.101839728273503 | 0.889231096310443 | -0.185421914 | 0.213789820871516 | 1.01428502301321 | 0.830753703314654 | 1.23836234951967 | 0.998735809224318 |
| ebi-fl187-GCST90094726 | prot-a-2254 | Placenta growth factor \|\| id:prot-a-2254 | F-capsicum liking (derived food-liking factor) \|\| id:ebi-fl187-GCST90094726 | Inverse variance weighted | 21 | -0.194552875 | 0.0752439236775403 | 0.00972014761260716 | -0.342030966 | -0.047074785 | 0.823202649901852 | 0.710326208513872 | 0.954016048799918 | 0.998735809224318 |
| ebi-fl187-GCST90094726 | prot-a-2261 | Membrane-associated progesterone receptor component 2 \|\| id:prot-a-2261 | F-capsicum liking (derived food-liking factor) \|\| id:ebi-fl187-GCST90094726 | Inverse variance weighted | 21 | 0.0761900204092897 | 0.0752562324049797 | 0.311342959772702 | -0.071312195 | 0.22369223592305 | 1.07916761854793 | 0.931171139672977 | 1.25068604395472 | 0.998735809224318 |
| ebi-fl187-GCST90094726 | prot-a-2268 | Polymeric immunoglobulin receptor \|\| id:prot-a-2268 | F-capsicum liking (derived food-liking factor) \|\| id:ebi-fl187-GCST90094726 | Inverse variance weighted | 21 | 0.153831233106555 | 0.0772214454568608 | 0.0463623718433529 | 0.00247720001110771 | 0.305185266202002 | 1.16629403834646 | 1.00248027080619 | 1.35687636305161 | 0.998735809224318 |
| ebi-fl187-GCST90094726 | prot-a-2283 | Group 10 secretory phospholipase A2 \|\| id:prot-a-2283 | F-capsicum liking (derived food-liking factor) \|\| id:ebi-fl187-GCST90094726 | Inverse variance weighted | 21 | 0.0408024001160177 | 0.0888019817547279 | 0.645892215102435 | -0.133249484 | 0.214854284355284 | 1.04164625602889 | 0.875246704711058 | 1.23968124285277 | 0.998735809224318 |
| ebi-fl187-GCST90094726 | prot-a-2298 | Inactive phospholipase D5 \|\| id:prot-a-2298 | F-capsicum liking (derived food-liking factor) \|\| id:ebi-fl187-GCST90094726 | Inverse variance weighted | 21 | -0.019678609 | 0.0777044391287025 | 0.800075429455458 | -0.17197931 | 0.132622091545656 | 0.980513750823674 | 0.841996594033491 | 1.14181841395438 | 0.998735809224318 |
| ebi-fl187-GCST90094726 | prot-a-2304 | Procollagen-lysine,2-oxoglutarate 5-dioxygenase 2 \|\| id:prot-a-2304 | F-capsicum liking (derived food-liking factor) \|\| id:ebi-fl187-GCST90094726 | Inverse variance weighted | 21 | -0.021904713 | 0.0904803457624583 | 0.808707631154174 | -0.199246191 | 0.155436764355055 | 0.97833345273764 | 0.819348152331883 | 1.16816806387068 | 0.998735809224318 |
| ebi-fl187-GCST90094726 | prot-a-2331 | Periostin \|\| id:prot-a-2331 | F-capsicum liking (derived food-liking factor) \|\| id:ebi-fl187-GCST90094726 | Inverse variance weighted | 21 | 0.0525195552334132 | 0.0767821343877132 | 0.493970434405817 | -0.097973428 | 0.203012538633331 | 1.05392317159064 | 0.906672995397708 | 1.22508782907828 | 0.998735809224318 |
| ebi-fl187-GCST90094726 | prot-a-2333 | Periostin \|\| id:prot-a-2333 | F-capsicum liking (derived food-liking factor) \|\| id:ebi-fl187-GCST90094726 | Inverse variance weighted | 21 | 0.080354474524849 | 0.0847074599839317 | 0.342818216798231 | -0.085672147 | 0.246381096093355 | 1.08367113341029 | 0.917895116521952 | 1.27938705005481 | 0.998735809224318 |
| ebi-fl187-GCST90094726 | prot-a-2367 | cAMP-dependent protein kinase catalytic subunit alpha \|\| id:prot-a-2367 | F-capsicum liking (derived food-liking factor) \|\| id:ebi-fl187-GCST90094726 | Inverse variance weighted | 21 | -0.057320355 | 0.0769378216004838 | 0.456258537384983 | -0.208118485 | 0.0934777755100452 | 0.944291512562292 | 0.812110807929067 | 1.09798620088684 | 0.998735809224318 |
| ebi-fl187-GCST90094726 | prot-a-2385 | Phosphoribosyl pyrophosphate synthase-associated protein 1 \|\| id:prot-a-2385 | F-capsicum liking (derived food-liking factor) \|\| id:ebi-fl187-GCST90094726 | Inverse variance weighted | 21 | 0.09906322477686 | 0.0892947157269392 | 0.267259269716027 | -0.075954418 | 0.274080867601661 | 1.10413610611104 | 0.926858453614122 | 1.31532116480604 | 0.998735809224318 |
| ebi-fl187-GCST90094726 | prot-a-2393 | Trypsin-3 \|\| id:prot-a-2393 | F-capsicum liking (derived food-liking factor) \|\| id:ebi-fl187-GCST90094726 | Inverse variance weighted | 20 | 0.185808510636054 | 0.0770640506446157 | 0.0159048389293739 | 0.0347629713726076 | 0.336854049899501 | 1.20419164840564 | 1.03537426637114 | 1.40053464064956 | 0.998735809224318 |
| ebi-fl187-GCST90094726 | prot-a-2405 | Pregnancy-specific beta-1-glycoprotein 6 \|\| id:prot-a-2405 | F-capsicum liking (derived food-liking factor) \|\| id:ebi-fl187-GCST90094726 | Inverse variance weighted | 21 | 0.0453221031457761 | 0.0830417239331872 | 0.585220558218326 | -0.117439676 | 0.208083882054823 | 1.04636484304171 | 0.889194150086994 | 1.23131645057111 | 0.998735809224318 |
| ebi-fl187-GCST90094726 | prot-a-244 | Beclin-1 \|\| id:prot-a-244 | F-capsicum liking (derived food-liking factor) \|\| id:ebi-fl187-GCST90094726 | Inverse variance weighted | 21 | 0.056831714883544 | 0.0752626087219957 | 0.450181656959483 | -0.090682998 | 0.204346427978655 | 1.05847766936882 | 0.913307185025975 | 1.2267230510407 | 0.998735809224318 |
| ebi-fl187-GCST90094726 | prot-a-2454 | tRNA pseudouridine synthase A, mitochondrial \|\| id:prot-a-2454 | F-capsicum liking (derived food-liking factor) \|\| id:ebi-fl187-GCST90094726 | Inverse variance weighted | 21 | -0.15067312 | 0.0752486039789311 | 0.0452484189408757 | -0.298160384 | -0.003185856 | 0.860128811511104 | 0.742182296093864 | 0.996819213130273 | 0.998735809224318 |
| ebi-fl187-GCST90094726 | prot-a-2466 | Glutaminyl-peptide cyclotransferase-like protein \|\| id:prot-a-2466 | F-capsicum liking (derived food-liking factor) \|\| id:ebi-fl187-GCST90094726 | Inverse variance weighted | 21 | -0.131094714 | 0.0859671089837662 | 0.127273974844146 | -0.299590247 | 0.0374008197979963 | 0.877134693687776 | 0.741121835059302 | 1.03810903211236 | 0.998735809224318 |
| ebi-fl187-GCST90094726 | prot-a-2474 | Ras-related protein Rab-27A \|\| id:prot-a-2474 | F-capsicum liking (derived food-liking factor) \|\| id:ebi-fl187-GCST90094726 | Inverse variance weighted | 21 | 0.0882774052895499 | 0.0818927045548781 | 0.281049724489905 | -0.072232296 | 0.248787106217111 | 1.09229108732952 | 0.930314762647442 | 1.28246897433321 | 0.998735809224318 |
| ebi-fl187-GCST90094726 | prot-a-2490 | Rap1 GTPase-GDP dissociation stimulator 1 \|\| id:prot-a-2490 | F-capsicum liking (derived food-liking factor) \|\| id:ebi-fl187-GCST90094726 | Inverse variance weighted | 21 | -0.141516828 | 0.0905357203806056 | 0.118027922948635 | -0.31896684 | 0.0359331836591637 | 0.868040567826331 | 0.726899652729959 | 1.03658658325454 | 0.998735809224318 |
| ebi-fl187-GCST90094726 | prot-a-2545 | Receptor-interacting serine/threonine-protein kinase 2 \|\| id:prot-a-2545 | F-capsicum liking (derived food-liking factor) \|\| id:ebi-fl187-GCST90094726 | Inverse variance weighted | 21 | 0.101431328284159 | 0.0752389296041676 | 0.177618838717696 | -0.046036974 | 0.248899630308328 | 1.10675391309203 | 0.955006651370297 | 1.28261329110846 | 0.998735809224318 |
| ebi-fl187-GCST90094726 | prot-a-2610 | RUN and FYVE domain-containing protein 1 \|\| id:prot-a-2610 | F-capsicum liking (derived food-liking factor) \|\| id:ebi-fl187-GCST90094726 | Inverse variance weighted | 21 | 0.125172402503811 | 0.0752352905080094 | 0.0961632290193552 | -0.022288767 | 0.272633571899509 | 1.13334382753836 | 0.977957792441015 | 1.31341888305142 | 0.998735809224318 |
| ebi-fl187-GCST90094726 | prot-a-2627 | Serine--tRNA ligase, mitochondrial \|\| id:prot-a-2627 | F-capsicum liking (derived food-liking factor) \|\| id:ebi-fl187-GCST90094726 | Inverse variance weighted | 21 | -0.1338308 | 0.0752596977545709 | 0.0753622747038197 | -0.281339808 | 0.0136782072826692 | 0.874738057485463 | 0.75477181446198 | 1.01377218193935 | 0.998735809224318 |
| ebi-fl187-GCST90094726 | prot-a-264 | BolA-like protein 2 \|\| id:prot-a-264 | F-capsicum liking (derived food-liking factor) \|\| id:ebi-fl187-GCST90094726 | Inverse variance weighted | 21 | 0.0468104986418318 | 0.0776630888317986 | 0.546682965268482 | -0.105409155 | 0.199030152752157 | 1.04792340735365 | 0.899956225235006 | 1.22021875829897 | 0.998735809224318 |
| ebi-fl187-GCST90094726 | prot-a-2646 | Secretoglobin family 1C member 1 \|\| id:prot-a-2646 | F-capsicum liking (derived food-liking factor) \|\| id:ebi-fl187-GCST90094726 | Inverse variance weighted | 21 | -0.217104819 | 0.076065400654853 | 0.00431472298437638 | -0.366193005 | -0.068016634 | 0.804845601550254 | 0.693368964923101 | 0.934244933224885 | 0.998735809224318 |
| ebi-fl187-GCST90094726 | prot-a-2672 | Semaphorin-3E \|\| id:prot-a-2672 | F-capsicum liking (derived food-liking factor) \|\| id:ebi-fl187-GCST90094726 | Inverse variance weighted (multiplicative random effects) | 21 | 0.0507419243920773 | 0.103201822979206 | 0.622947962700757 | -0.151533649 | 0.25301749743132 | 1.05205134945372 | 0.859388964510363 | 1.28790581168097 | 0.998735809224318 |
| ebi-fl187-GCST90094726 | prot-a-2701 | Plasma protease C1 inhibitor \|\| id:prot-a-2701 | F-capsicum liking (derived food-liking factor) \|\| id:ebi-fl187-GCST90094726 | Inverse variance weighted (multiplicative random effects) | 21 | 0.024674872264486 | 0.100779800569831 | 0.806580736521422 | -0.172853537 | 0.222203281381355 | 1.02498181632726 | 0.841260819529525 | 1.248825215 | 0.998735809224318 |
| ebi-fl187-GCST90094726 | prot-a-2738 | Signaling threshold-regulating transmembrane adapter 1 \|\| id:prot-a-2738 | F-capsicum liking (derived food-liking factor) \|\| id:ebi-fl187-GCST90094726 | Inverse variance weighted | 21 | 0.0204356103054785 | 0.0752389296041676 | 0.785922270989044 | -0.127032692 | 0.167903912329647 | 1.02064584705303 | 0.880704880370328 | 1.18282295048548 | 0.998735809224318 |
| ebi-fl187-GCST90094726 | prot-a-274 | Serine/threonine-protein kinase BRSK2 \|\| id:prot-a-274 | F-capsicum liking (derived food-liking factor) \|\| id:ebi-fl187-GCST90094726 | Inverse variance weighted | 21 | -0.053701323 | 0.0754276706229502 | 0.476490873971735 | -0.201539557 | 0.0941369118019259 | 0.947715125305544 | 0.817471240177939 | 1.09871016200814 | 0.998735809224318 |
| ebi-fl187-GCST90094726 | prot-a-2749 | Mitochondrial glutamate carrier 2 \|\| id:prot-a-2749 | F-capsicum liking (derived food-liking factor) \|\| id:ebi-fl187-GCST90094726 | Inverse variance weighted (multiplicative random effects) | 21 | 0.026193990334059 | 0.0959607055118773 | 0.784879508522006 | -0.161888992 | 0.214276973137339 | 1.0265400680099 | 0.85053561515847 | 1.23896576751044 | 0.998735809224318 |
| ebi-fl187-GCST90094726 | prot-a-2760 | Sodium-coupled monocarboxylate transporter 1 \|\| id:prot-a-2760 | F-capsicum liking (derived food-liking factor) \|\| id:ebi-fl187-GCST90094726 | Inverse variance weighted | 21 | -0.00417358 | 0.075246555088039 | 0.955767694428332 | -0.151656828 | 0.143309667708208 | 0.995835117017954 | 0.859283111849866 | 1.15408712985323 | 0.998735809224318 |
| ebi-fl187-GCST90094726 | prot-a-2777 | Small integral membrane protein 10 \|\| id:prot-a-2777 | F-capsicum liking (derived food-liking factor) \|\| id:ebi-fl187-GCST90094726 | Inverse variance weighted | 21 | 0.0947217553555953 | 0.0752389296041676 | 0.208049808911958 | -0.052746547 | 0.242190057379764 | 1.09935292350288 | 0.948620413019792 | 1.27403630981017 | 0.998735809224318 |
| ebi-fl187-GCST90094726 | prot-a-2799 | Superoxide dismutase [Mn], mitochondrial \|\| id:prot-a-2799 | F-capsicum liking (derived food-liking factor) \|\| id:ebi-fl187-GCST90094726 | Inverse variance weighted | 21 | 0.163996650320437 | 0.0752677979151261 | 0.0293433968339444 | 0.0164717664067896 | 0.311521534234084 | 1.17821036845892 | 1.01660817387923 | 1.36550119112953 | 0.998735809224318 |
| ebi-fl187-GCST90094726 | prot-a-2805 | Protein SPACA7 \|\| id:prot-a-2805 | F-capsicum liking (derived food-liking factor) \|\| id:ebi-fl187-GCST90094726 | Inverse variance weighted | 21 | -0.196785412 | 0.0752036820699847 | 0.00887845511110251 | -0.344184629 | -0.049386195 | 0.821366869630984 | 0.70879805118182 | 0.951813472684538 | 0.998735809224318 |
| ebi-fl187-GCST90094726 | prot-a-2811 | SPARC-like protein 1 \|\| id:prot-a-2811 | F-capsicum liking (derived food-liking factor) \|\| id:ebi-fl187-GCST90094726 | Inverse variance weighted (multiplicative random effects) | 21 | -0.001314941 | 0.10302159924241 | 0.989816281607879 | -0.203237276 | 0.200607393114894 | 0.9986859227564 | 0.81608458123505 | 1.22214485513547 | 0.999107451094609 |
| ebi-fl187-GCST90094726 | prot-a-2831 | Sepiapterin reductase \|\| id:prot-a-2831 | F-capsicum liking (derived food-liking factor) \|\| id:ebi-fl187-GCST90094726 | Inverse variance weighted | 21 | 0.117606257864942 | 0.0836436166752203 | 0.159712293200568 | -0.046335231 | 0.281547746548373 | 1.12480114256194 | 0.954721856349706 | 1.32517926754702 | 0.998735809224318 |
| ebi-fl187-GCST90094726 | prot-a-2834 | SPRY domain-containing SOCS box protein 1 \|\| id:prot-a-2834 | F-capsicum liking (derived food-liking factor) \|\| id:ebi-fl187-GCST90094726 | Inverse variance weighted | 21 | 0.0576495369366964 | 0.0771125559346114 | 0.454699977162932 | -0.093491073 | 0.208790146568535 | 1.05934366981847 | 0.910746147898418 | 1.23218639285381 | 0.998735809224318 |
| ebi-fl187-GCST90094726 | prot-a-2835 | Spectrin alpha chain, non-erythrocytic 1 \|\| id:prot-a-2835 | F-capsicum liking (derived food-liking factor) \|\| id:ebi-fl187-GCST90094726 | Inverse variance weighted | 21 | 0.0255050223067322 | 0.0752441139006306 | 0.734637085665502 | -0.121973441 | 0.172983485551968 | 1.02583305830578 | 0.885171877524767 | 1.18884647177864 | 0.998735809224318 |
| ebi-fl187-GCST90094726 | prot-a-2859 | Sia-alpha-2,3-Gal-beta-1,4-GlcNAc-R:alpha 2,8-sialyltransferase \|\| id:prot-a-2859 | F-capsicum liking (derived food-liking factor) \|\| id:ebi-fl187-GCST90094726 | Inverse variance weighted | 21 | -0.088564475 | 0.0902206066483734 | 0.326275494859345 | -0.265396864 | 0.0882679143237595 | 0.91524409873848 | 0.766901534164631 | 1.09228072048137 | 0.998735809224318 |
| ebi-fl187-GCST90094726 | prot-a-2861 | Alpha-2,8-sialyltransferase 8F \|\| id:prot-a-2861 | F-capsicum liking (derived food-liking factor) \|\| id:ebi-fl187-GCST90094726 | Inverse variance weighted | 21 | -0.13532884 | 0.0790359615708235 | 0.0868517366855417 | -0.290239325 | 0.0195816443158632 | 0.873428645865406 | 0.748084510796244 | 1.01977462226332 | 0.998735809224318 |
| ebi-fl187-GCST90094726 | prot-a-289 | Uncharacterized protein C17orf89 \|\| id:prot-a-289 | F-capsicum liking (derived food-liking factor) \|\| id:ebi-fl187-GCST90094726 | Inverse variance weighted | 21 | -0.149242545 | 0.0779216650760999 | 0.0554555968567061 | -0.301969009 | 0.00348391840425599 | 0.861360170833353 | 0.739360978296754 | 1.00348999430191 | 0.998735809224318 |
| ebi-fl187-GCST90094726 | prot-a-2921 | Tachykinin-3 \|\| id:prot-a-2921 | F-capsicum liking (derived food-liking factor) \|\| id:ebi-fl187-GCST90094726 | Inverse variance weighted | 21 | -0.038291461 | 0.0752212874710623 | 0.610716574864517 | -0.185725185 | 0.109142262380563 | 0.962432388441398 | 0.830501798385571 | 1.11532100727731 | 0.998735809224318 |
| ebi-fl187-GCST90094726 | prot-a-2928 | Tax1-binding protein 1 \|\| id:prot-a-2928 | F-capsicum liking (derived food-liking factor) \|\| id:ebi-fl187-GCST90094726 | Inverse variance weighted (multiplicative random effects) | 21 | -0.085818139 | 0.0957539077419318 | 0.370126510747755 | -0.273495798 | 0.101859520171144 | 0.917761120996754 | 0.760715532740997 | 1.10722791761371 | 0.998735809224318 |
| ebi-fl187-GCST90094726 | prot-a-296 | Uncharacterized protein C1orf185 \|\| id:prot-a-296 | F-capsicum liking (derived food-liking factor) \|\| id:ebi-fl187-GCST90094726 | Inverse variance weighted | 21 | 0.0871715387890549 | 0.0924887313193928 | 0.345931678805125 | -0.094106375 | 0.268449452175065 | 1.09108382686474 | 0.910185936428813 | 1.30793486209717 | 0.998735809224318 |
| ebi-fl187-GCST90094726 | prot-a-2964 | Transforming growth factor beta-2 \|\| id:prot-a-2964 | F-capsicum liking (derived food-liking factor) \|\| id:ebi-fl187-GCST90094726 | Inverse variance weighted | 21 | 0.0367995290108835 | 0.0752389296041676 | 0.624769315628248 | -0.110668773 | 0.184267831035052 | 1.0374850143422 | 0.895235225892008 | 1.20233780335458 | 0.998735809224318 |
| ebi-fl187-GCST90094726 | prot-a-2977 | Thymocyte nuclear protein 1 \|\| id:prot-a-2977 | F-capsicum liking (derived food-liking factor) \|\| id:ebi-fl187-GCST90094726 | Inverse variance weighted | 21 | -0.084307583 | 0.0752194308465952 | 0.262363696289061 | -0.231737667 | 0.0631225019006249 | 0.919148498564696 | 0.793154166514858 | 1.06515731503493 | 0.998735809224318 |
| ebi-fl187-GCST90094726 | prot-a-3015 | Transmembrane protein 70, mitochondrial \|\| id:prot-a-3015 | F-capsicum liking (derived food-liking factor) \|\| id:ebi-fl187-GCST90094726 | Inverse variance weighted | 21 | -0.077567249 | 0.0798281653145881 | 0.331210908891312 | -0.234030453 | 0.0788959551072904 | 0.925364792531143 | 0.791337716979792 | 1.08209173009515 | 0.998735809224318 |
| ebi-fl187-GCST90094726 | prot-a-3030 | Tumor necrosis factor alpha-induced protein 3 \|\| id:prot-a-3030 | F-capsicum liking (derived food-liking factor) \|\| id:ebi-fl187-GCST90094726 | Inverse variance weighted | 21 | -0.141230613 | 0.0881780367663821 | 0.109232372925434 | -0.314059566 | 0.0315983385823755 | 0.868289049447861 | 0.730475515693621 | 1.03210286613807 | 0.998735809224318 |
| ebi-fl187-GCST90094726 | prot-a-3032 | Tumor necrosis factor receptor superfamily member 10A \|\| id:prot-a-3032 | F-capsicum liking (derived food-liking factor) \|\| id:ebi-fl187-GCST90094726 | Inverse variance weighted | 21 | 0.000540583898457572 | 0.0813705478750545 | 0.994699306015805 | -0.15894569 | 0.160026857733564 | 1.00054073004027 | 0.85304268651344 | 1.17354238925737 | 0.999107451094609 |
| ebi-fl187-GCST90094726 | prot-a-3081 | Tumor protein p53-inducible protein 11 \|\| id:prot-a-3081 | F-capsicum liking (derived food-liking factor) \|\| id:ebi-fl187-GCST90094726 | Inverse variance weighted | 21 | 0.0329916852783605 | 0.0810653622538919 | 0.684025357990972 | -0.125896425 | 0.191879795295989 | 1.033541946 | 0.88170616500011 | 1.21152487722252 | 0.998735809224318 |
| ebi-fl187-GCST90094726 | prot-a-315 | Complement C4 \|\| id:prot-a-315 | F-capsicum liking (derived food-liking factor) \|\| id:ebi-fl187-GCST90094726 | Inverse variance weighted | 20 | 0.0540731418993655 | 0.0954121322712981 | 0.570896038819992 | -0.132934637 | 0.24108092115111 | 1.05556180512698 | 0.875522316695246 | 1.27262401334171 | 0.998735809224318 |
| ebi-fl187-GCST90094726 | prot-a-3157 | UDP-glucuronosyltransferase 1-8 \|\| id:prot-a-3157 | F-capsicum liking (derived food-liking factor) \|\| id:ebi-fl187-GCST90094726 | Inverse variance weighted | 21 | 0.0622280069782959 | 0.0809769977992169 | 0.442210841860642 | -0.096486909 | 0.220942922664761 | 1.06420496322558 | 0.908021784700113 | 1.24725223869821 | 0.998735809224318 |
| ebi-fl187-GCST90094726 | prot-a-317 | Glutaredoxin-like protein C5orf63 \|\| id:prot-a-317 | F-capsicum liking (derived food-liking factor) \|\| id:ebi-fl187-GCST90094726 | Inverse variance weighted | 21 | 0.0199601160574468 | 0.0894938752237415 | 0.82350957443526 | -0.155447879 | 0.19536811149598 | 1.02016065118654 | 0.85603169266785 | 1.21575843878616 | 0.998735809224318 |
| ebi-fl187-GCST90094726 | prot-a-3192 | Guanine nucleotide exchange factor VAV3 \|\| id:prot-a-3192 | F-capsicum liking (derived food-liking factor) \|\| id:ebi-fl187-GCST90094726 | Inverse variance weighted | 21 | 0.0774207639864537 | 0.0878960745327813 | 0.378414368415424 | -0.094855542 | 0.249697070070705 | 1.0804966148222 | 0.909504310063557 | 1.28363650586839 | 0.998735809224318 |
| ebi-fl187-GCST90094726 | prot-a-3233 | Serine/threonine-protein kinase WNK3 \|\| id:prot-a-3233 | F-capsicum liking (derived food-liking factor) \|\| id:ebi-fl187-GCST90094726 | Inverse variance weighted | 21 | 0.111585846000092 | 0.0785848723918482 | 0.155624992788264 | -0.042440504 | 0.265612195888114 | 1.11804971998226 | 0.958447487723772 | 1.30422917516444 | 0.998735809224318 |
| ebi-fl187-GCST90094726 | prot-a-3250 | 14-3-3 protein theta \|\| id:prot-a-3250 | F-capsicum liking (derived food-liking factor) \|\| id:ebi-fl187-GCST90094726 | Inverse variance weighted | 21 | 0.0181936470152104 | 0.0759027932197919 | 0.810565414238168 | -0.130575828 | 0.166963121726002 | 1.01836015970253 | 0.877589944802494 | 1.18171068505435 | 0.998735809224318 |
| ebi-fl187-GCST90094726 | prot-a-3278 | E3 ubiquitin-protein ligase ZNRF3 \|\| id:prot-a-3278 | F-capsicum liking (derived food-liking factor) \|\| id:ebi-fl187-GCST90094726 | Inverse variance weighted | 21 | 0.01360171648291 | 0.0752036820699847 | 0.856473515322549 | -0.1337975 | 0.16100093334008 | 1.01369464066001 | 0.874767186697066 | 1.17468606519495 | 0.998735809224318 |
| ebi-fl187-GCST90094726 | prot-a-355 | Calpain-3 \|\| id:prot-a-355 | F-capsicum liking (derived food-liking factor) \|\| id:ebi-fl187-GCST90094726 | Inverse variance weighted | 21 | 0.133120355080384 | 0.0752071401649004 | 0.0767189078299469 | -0.01428564 | 0.280526349803589 | 1.14238748219454 | 0.985815915936554 | 1.32382642476911 | 0.998735809224318 |
| ebi-fl187-GCST90094726 | prot-a-385 | Coiled-coil domain-containing protein 90B, mitochondrial \|\| id:prot-a-385 | F-capsicum liking (derived food-liking factor) \|\| id:ebi-fl187-GCST90094726 | Inverse variance weighted | 21 | -0.157844923 | 0.0752370473130841 | 0.0359079660847414 | -0.305309536 | -0.01038031 | 0.853982204753753 | 0.736895243723766 | 0.989673379286271 | 0.998735809224318 |
| ebi-fl187-GCST90094726 | prot-a-390 | C-C motif chemokine 14 \|\| id:prot-a-390 | F-capsicum liking (derived food-liking factor) \|\| id:ebi-fl187-GCST90094726 | Inverse variance weighted | 21 | -0.166821092 | 0.0867490800750367 | 0.0544762693525874 | -0.336849289 | 0.00320710450905098 | 0.846351016389951 | 0.714016441651129 | 1.00321225277092 | 0.998735809224318 |
| ebi-fl187-GCST90094726 | prot-a-40 | Pituitary adenylate cyclase-activating polypeptide 27 \|\| id:prot-a-40 | F-capsicum liking (derived food-liking factor) \|\| id:ebi-fl187-GCST90094726 | Inverse variance weighted | 21 | 0.0625707872796597 | 0.0752757598036418 | 0.40584883155709 | -0.084969702 | 0.210111276494798 | 1.06456981425189 | 0.918540113967503 | 1.23381534696523 | 0.998735809224318 |
| ebi-fl187-GCST90094726 | prot-a-401 | C-C motif chemokine 24 \|\| id:prot-a-401 | F-capsicum liking (derived food-liking factor) \|\| id:ebi-fl187-GCST90094726 | Inverse variance weighted | 21 | 0.124626072572342 | 0.0752309793952497 | 0.0976045887970334 | -0.022826647 | 0.272078792187031 | 1.13272481699013 | 0.977431909800069 | 1.31269042698612 | 0.998735809224318 |
| ebi-fl187-GCST90094726 | prot-a-412 | Cerebral cavernous malformations 2 protein \|\| id:prot-a-412 | F-capsicum liking (derived food-liking factor) \|\| id:ebi-fl187-GCST90094726 | Inverse variance weighted (multiplicative random effects) | 21 | 0.0260637843598243 | 0.101474189015797 | 0.797293513271329 | -0.172825626 | 0.224953194830786 | 1.02640641506165 | 0.841284300070258 | 1.25226410238695 | 0.998735809224318 |
| ebi-fl187-GCST90094726 | prot-a-44 | Adhesion G protein-coupled receptor F5 \|\| id:prot-a-44 | F-capsicum liking (derived food-liking factor) \|\| id:ebi-fl187-GCST90094726 | Inverse variance weighted | 21 | 0.018181091849342 | 0.0752290977007716 | 0.809030975913135 | -0.12926794 | 0.165630123342854 | 1.01834737410208 | 0.878738485122699 | 1.1801365160373 | 0.998735809224318 |
| ebi-fl187-GCST90094726 | prot-a-48 | Adrenomedullin \|\| id:prot-a-48 | F-capsicum liking (derived food-liking factor) \|\| id:ebi-fl187-GCST90094726 | Inverse variance weighted | 21 | 0.133072728896544 | 0.0777431999013921 | 0.0869526952490523 | -0.019303943 | 0.285449400703273 | 1.14233307593388 | 0.98088118504875 | 1.33035975841224 | 0.998735809224318 |
| ebi-fl187-GCST90094726 | prot-a-502 | Carcinoembryonic antigen-related cell adhesion molecule 1 \|\| id:prot-a-502 | F-capsicum liking (derived food-liking factor) \|\| id:ebi-fl187-GCST90094726 | Inverse variance weighted | 21 | -0.090739806 | 0.0775557459888265 | 0.242003130677674 | -0.242749068 | 0.0612694559588174 | 0.913255303374554 | 0.7844683370357 | 1.0631853572234 | 0.998735809224318 |
| ebi-fl187-GCST90094726 | prot-a-518 | Complement factor B \|\| id:prot-a-518 | F-capsicum liking (derived food-liking factor) \|\| id:ebi-fl187-GCST90094726 | Inverse variance weighted | 21 | 0.16423539949886 | 0.0752406507031808 | 0.0290504367737788 | 0.016763724120626 | 0.311707074877095 | 1.17849169879876 | 1.01690502380925 | 1.36575457060393 | 0.998735809224318 |
| ebi-fl187-GCST90094726 | prot-a-525 | Properdin \|\| id:prot-a-525 | F-capsicum liking (derived food-liking factor) \|\| id:ebi-fl187-GCST90094726 | Inverse variance weighted | 21 | 0.105850331235052 | 0.085352483998279 | 0.214917976199769 | -0.061440537 | 0.273141199871679 | 1.11165548395163 | 0.940408863231503 | 1.3140857804692 | 0.998735809224318 |
| ebi-fl187-GCST90094726 | prot-a-533 | Chondroadherin \|\| id:prot-a-533 | F-capsicum liking (derived food-liking factor) \|\| id:ebi-fl187-GCST90094726 | Inverse variance weighted (multiplicative random effects) | 21 | 0.00132893455901369 | 0.0985010864801159 | 0.989235609086114 | -0.191733195 | 0.194391064060041 | 1.00132981798384 | 0.825527093915909 | 1.21457116522657 | 0.999107451094609 |
| ebi-fl187-GCST90094726 | prot-a-535 | Coiled-coil-helix-coiled-coil-helix domain-containing protein 2 \|\| id:prot-a-535 | F-capsicum liking (derived food-liking factor) \|\| id:ebi-fl187-GCST90094726 | Inverse variance weighted | 21 | -0.081260942 | 0.0921776637774867 | 0.378010095492426 | -0.261929163 | 0.0994072791286983 | 0.921953083887146 | 0.769565535583744 | 1.10451605430103 | 0.998735809224318 |
| ebi-fl187-GCST90094726 | prot-a-591 | Caseinolytic peptidase B protein homolog \|\| id:prot-a-591 | F-capsicum liking (derived food-liking factor) \|\| id:ebi-fl187-GCST90094726 | Inverse variance weighted | 21 | 0.0918781419214532 | 0.0923189942646598 | 0.319626998857259 | -0.089067087 | 0.272823370680186 | 1.09623122931006 | 0.914784201536972 | 1.31366819201246 | 0.998735809224318 |
| ebi-fl187-GCST90094726 | prot-a-605 | Ciliary neurotrophic factor receptor subunit alpha \|\| id:prot-a-605 | F-capsicum liking (derived food-liking factor) \|\| id:ebi-fl187-GCST90094726 | Inverse variance weighted | 21 | -0.033772464 | 0.0752228911145964 | 0.653456722076657 | -0.181209331 | 0.113664402193262 | 0.966791459095908 | 0.834260703808758 | 1.12037606603494 | 0.998735809224318 |
| ebi-fl187-GCST90094726 | prot-a-606 | Ciliary neurotrophic factor receptor subunit alpha \|\| id:prot-a-606 | F-capsicum liking (derived food-liking factor) \|\| id:ebi-fl187-GCST90094726 | Inverse variance weighted | 21 | 0.0540585512515636 | 0.0797425536886069 | 0.497826559056675 | -0.102236854 | 0.210353956481233 | 1.05554640390881 | 0.902815690855109 | 1.23411480559173 | 0.998735809224318 |
| ebi-fl187-GCST90094726 | prot-a-707 | Cancer/testis antigen 55 \|\| id:prot-a-707 | F-capsicum liking (derived food-liking factor) \|\| id:ebi-fl187-GCST90094726 | Inverse variance weighted (multiplicative random effects) | 21 | 0.0891965271923812 | 0.0977926288467747 | 0.36171677580939 | -0.102477025 | 0.28087007973206 | 1.09329549750914 | 0.902598886410661 | 1.32428154174558 | 0.998735809224318 |
| ebi-fl187-GCST90094726 | prot-a-719 | Dipeptidyl peptidase 1 \|\| id:prot-a-719 | F-capsicum liking (derived food-liking factor) \|\| id:ebi-fl187-GCST90094726 | Inverse variance weighted | 21 | -0.016835006 | 0.0752757598036418 | 0.823033976521572 | -0.164375495 | 0.130705483610148 | 0.983305911215234 | 0.848423383474645 | 1.13963209155199 | 0.998735809224318 |
| ebi-fl187-GCST90094726 | prot-a-752 | Cytochrome b5 \|\| id:prot-a-752 | F-capsicum liking (derived food-liking factor) \|\| id:ebi-fl187-GCST90094726 | Inverse variance weighted | 21 | -0.001919806 | 0.0800577595165987 | 0.980868353051489 | -0.158833015 | 0.154993402627208 | 0.998082035623538 | 0.853138808734098 | 1.16765025765567 | 0.998955228396052 |
| ebi-fl187-GCST90094726 | prot-a-755 | Cytoglobin \|\| id:prot-a-755 | F-capsicum liking (derived food-liking factor) \|\| id:ebi-fl187-GCST90094726 | Inverse variance weighted | 21 | -0.050531487 | 0.0786414264173564 | 0.520512519266611 | -0.204668682 | 0.103605709062698 | 0.950723993025325 | 0.814917268046478 | 1.10916303575306 | 0.998735809224318 |
| ebi-fl187-GCST90094726 | prot-a-807 | Desmin \|\| id:prot-a-807 | F-capsicum liking (derived food-liking factor) \|\| id:ebi-fl187-GCST90094726 | Inverse variance weighted | 21 | 0.0707474840057597 | 0.08365580127845 | 0.397721638194856 | -0.093217887 | 0.234712854511522 | 1.07331016366292 | 0.910994985161206 | 1.26454560802909 | 0.998735809224318 |
| ebi-fl187-GCST90094726 | prot-a-82 | Ameloblastin \|\| id:prot-a-82 | F-capsicum liking (derived food-liking factor) \|\| id:ebi-fl187-GCST90094726 | Inverse variance weighted | 21 | -0.187779514 | 0.086332763227523 | 0.0296251221941933 | -0.35699173 | -0.018567298 | 0.828797425251602 | 0.699778284873136 | 0.981604012231123 | 0.998735809224318 |
| ebi-fl187-GCST90094726 | prot-a-84 | Muellerian-inhibiting factor \|\| id:prot-a-84 | F-capsicum liking (derived food-liking factor) \|\| id:ebi-fl187-GCST90094726 | Inverse variance weighted | 21 | 0.0396824762983858 | 0.0752344412573488 | 0.597880481178389 | -0.107777029 | 0.187141981162789 | 1.04048034456508 | 0.897827764057795 | 1.20579847356623 | 0.998735809224318 |
| ebi-fl187-GCST90094726 | prot-a-858 | Inactive dipeptidyl peptidase 10 \|\| id:prot-a-858 | F-capsicum liking (derived food-liking factor) \|\| id:ebi-fl187-GCST90094726 | Inverse variance weighted | 21 | -0.0711909 | 0.0796930889000894 | 0.371689394735962 | -0.227389354 | 0.0850075546198494 | 0.931284093373709 | 0.796610558498002 | 1.08872529157303 | 0.998735809224318 |
| ebi-fl187-GCST90094726 | prot-a-863 | Dihydropyrimidinase-related protein 3 \|\| id:prot-a-863 | F-capsicum liking (derived food-liking factor) \|\| id:ebi-fl187-GCST90094726 | Inverse variance weighted | 21 | -0.018936125 | 0.0752545102364945 | 0.801328785812708 | -0.166434965 | 0.128562714890666 | 0.981242036906605 | 0.846677878640647 | 1.13719273797311 | 0.998735809224318 |
| ebi-fl187-GCST90094726 | prot-a-864 | Dihydropyrimidinase-related protein 5 \|\| id:prot-a-864 | F-capsicum liking (derived food-liking factor) \|\| id:ebi-fl187-GCST90094726 | Inverse variance weighted | 21 | 0.0116537466055672 | 0.0887242208878696 | 0.895500043197781 | -0.162245726 | 0.185553219545792 | 1.01172191606303 | 0.850232254413526 | 1.20388426824421 | 0.998735809224318 |
| ebi-fl187-GCST90094726 | prot-a-884 | Endothelin-converting enzyme 1 \|\| id:prot-a-884 | F-capsicum liking (derived food-liking factor) \|\| id:ebi-fl187-GCST90094726 | Inverse variance weighted | 21 | 0.0193121376811453 | 0.0752660749525394 | 0.797499239399015 | -0.128209369 | 0.166833644588122 | 1.01949982326844 | 0.879669184206855 | 1.18155769044192 | 0.998735809224318 |
| ebi-fl187-GCST90094726 | prot-a-893 | Early endosome antigen 1 \|\| id:prot-a-893 | F-capsicum liking (derived food-liking factor) \|\| id:ebi-fl187-GCST90094726 | Inverse variance weighted | 21 | -0.07717741 | 0.0752596977545709 | 0.305136161954914 | -0.224686418 | 0.0703315976959761 | 0.925725606147388 | 0.79876665873474 | 1.07286388146746 | 0.998735809224318 |
| ebi-fl187-GCST90094726 | prot-a-897 | Ephrin-A2 \|\| id:prot-a-897 | F-capsicum liking (derived food-liking factor) \|\| id:ebi-fl187-GCST90094726 | Inverse variance weighted | 21 | 0.0551317718456602 | 0.075246555088039 | 0.463752604614701 | -0.092351476 | 0.202615019818217 | 1.0566798461555 | 0.911784622691874 | 1.22460093039816 | 0.998735809224318 |
| ebi-fl187-GCST90094726 | prot-a-90 | Anaphase-promoting complex subunit 10 \|\| id:prot-a-90 | F-capsicum liking (derived food-liking factor) \|\| id:ebi-fl187-GCST90094726 | Inverse variance weighted | 21 | 0.0189716946058806 | 0.0752660749525394 | 0.800993375316806 | -0.128549812 | 0.166493201512858 | 1.01915280068744 | 0.879369757896267 | 1.18115550577256 | 0.998735809224318 |
| ebi-fl187-GCST90094726 | prot-a-914 | Histone-lysine N-methyltransferase EHMT2 \|\| id:prot-a-914 | F-capsicum liking (derived food-liking factor) \|\| id:ebi-fl187-GCST90094726 | Inverse variance weighted | 21 | -0.110524944 | 0.0928739117323678 | 0.234025278132606 | -0.292557811 | 0.0715079227466953 | 0.895363995728702 | 0.746352096245366 | 1.07412666070105 | 0.998735809224318 |
| ebi-fl187-GCST90094726 | prot-a-932 | RNA polymerase II elongation factor ELL \|\| id:prot-a-932 | F-capsicum liking (derived food-liking factor) \|\| id:ebi-fl187-GCST90094726 | Inverse variance weighted | 21 | 0.0883097912763663 | 0.0752486039789311 | 0.240565774404254 | -0.059177473 | 0.235797055075071 | 1.09232646282711 | 0.942539479454054 | 1.26591737258953 | 0.998735809224318 |
| ebi-fl187-GCST90094726 | prot-a-991 | Estrogen receptor \|\| id:prot-a-991 | F-capsicum liking (derived food-liking factor) \|\| id:ebi-fl187-GCST90094726 | Inverse variance weighted | 21 | 0.0881497011113405 | 0.0752228911145964 | 0.241258585982095 | -0.059287165 | 0.235586567695949 | 1.0921516061002 | 0.942436095187551 | 1.26565094100081 | 0.998735809224318 |
| ebi-fl187-GCST90094726 | prot-a-992 | Persulfide dioxygenase ETHE1, mitochondrial \|\| id:prot-a-992 | F-capsicum liking (derived food-liking factor) \|\| id:ebi-fl187-GCST90094726 | Inverse variance weighted | 21 | 0.104344692638584 | 0.0888899847046223 | 0.240449776144784 | -0.069879677 | 0.278569062659644 | 1.10998299194922 | 0.932506014720575 | 1.32123785044511 | 0.998735809224318 |
| ebi-fl187-GCST90094726 | prot-a-993 | Ethanolamine kinase 1 \|\| id:prot-a-993 | F-capsicum liking (derived food-liking factor) \|\| id:ebi-fl187-GCST90094726 | Inverse variance weighted | 21 | -0.064272402 | 0.0868867673915791 | 0.459466264270999 | -0.234570466 | 0.10602566258493 | 0.937749520357479 | 0.790910499952675 | 1.11185040909597 | 0.998735809224318 |
| ebi-fl187-GCST90094726 | prot-b-10 | coagulation factor III, tissue factor \|\| id:prot-b-10 | F-capsicum liking (derived food-liking factor) \|\| id:ebi-fl187-GCST90094726 | Inverse variance weighted | 14 | 0.222323358315966 | 0.111007036245161 | 0.0452002436355222 | 0.00474956727545031 | 0.439897149356482 | 1.24897517908512 | 1.00476086434842 | 1.5525475297869 | 0.998735809224318 |
| ebi-fl187-GCST90094726 | prot-b-28 | prolactin \|\| id:prot-b-28 | F-capsicum liking (derived food-liking factor) \|\| id:ebi-fl187-GCST90094726 | Inverse variance weighted | 14 | 0.0525315888708091 | 0.102914196745816 | 0.609742788412526 | -0.149180237 | 0.254243414492608 | 1.05393585419624 | 0.861413842474255 | 1.28948564556364 | 0.998735809224318 |
| ebi-fl187-GCST90094726 | prot-b-54 | matrix metallopeptidase 7 \|\| id:prot-b-54 | F-capsicum liking (derived food-liking factor) \|\| id:ebi-fl187-GCST90094726 | Inverse variance weighted | 14 | 0.11652870637328 | 0.108581937188123 | 0.283187292716657 | -0.096291891 | 0.329349303 | 1.12358976419144 | 0.908198882735632 | 1.39006332444836 | 0.998735809224318 |
| ebi-fl187-GCST90094726 | prot-c-2421_7_3 | BDNF \|\| id:prot-c-2421_7_3 | F-capsicum liking (derived food-liking factor) \|\| id:ebi-fl187-GCST90094726 | Inverse variance weighted | 2 | 0.0708671315929486 | 0.494219814624713 | 0.885980658391768 | -0.897803705 | 1.03953796825739 | 1.07343859031713 | 0.407463587928008 | 2.82791012821887 | 0.998735809224318 |
| ebi-fl187-GCST90094726 | prot-c-2443_10_4 | FGF-8B \|\| id:prot-c-2443_10_4 | F-capsicum liking (derived food-liking factor) \|\| id:ebi-fl187-GCST90094726 | Inverse variance weighted | 2 | 0.747120498234191 | 0.50127151854708 | 0.136105763510905 | -0.235371678 | 1.72961267458647 | 2.11091287949364 | 0.790277066342923 | 5.63846956287423 | 0.998735809224318 |
| ebi-fl187-GCST90094726 | prot-c-2605_49_2 | CD30 \|\| id:prot-c-2605_49_2 | F-capsicum liking (derived food-liking factor) \|\| id:ebi-fl187-GCST90094726 | Inverse variance weighted | 2 | 0.202655370224984 | 0.49339700608229 | 0.681266932711247 | -0.764402762 | 1.16971350214627 | 1.22465034454077 | 0.465611929229241 | 3.22106967677243 | 0.998735809224318 |
| ebi-fl187-GCST90094726 | prot-c-2624_31_2 | HPV E7 Type18 \|\| id:prot-c-2624_31_2 | F-capsicum liking (derived food-liking factor) \|\| id:ebi-fl187-GCST90094726 | Inverse variance weighted | 2 | 0.543692540545528 | 0.55834484925917 | 0.330176909999019 | -0.550663364 | 1.6380484450935 | 1.72235500026551 | 0.576567209560899 | 5.14511872639937 | 0.998735809224318 |
| ebi-fl187-GCST90094726 | prot-c-2774_10_3 | IL-16 \|\| id:prot-c-2774_10_3 | F-capsicum liking (derived food-liking factor) \|\| id:ebi-fl187-GCST90094726 | Inverse variance weighted | 2 | -0.240226703 | 0.480279201293028 | 0.616947383285279 | -1.181573938 | 0.701120531255538 | 0.786449550163854 | 0.306795481383444 | 2.01601044501663 | 0.998735809224318 |
| ebi-fl187-GCST90094726 | prot-c-2851_63_3 | C5a \|\| id:prot-c-2851_63_3 | F-capsicum liking (derived food-liking factor) \|\| id:ebi-fl187-GCST90094726 | Inverse variance weighted | 2 | -0.15302825 | 0.433625372882163 | 0.724160201766182 | -1.002933981 | 0.696877480679104 | 0.858105479843347 | 0.366801671724466 | 2.00747453269599 | 0.998735809224318 |
| ebi-fl187-GCST90094726 | prot-c-2891_1_3 | CD22 \|\| id:prot-c-2891_1_3 | F-capsicum liking (derived food-liking factor) \|\| id:ebi-fl187-GCST90094726 | Inverse variance weighted | 2 | -0.108253848 | 0.545149330694739 | 0.842594034892361 | -1.176746536 | 0.960238840076772 | 0.897399764302711 | 0.308280086881252 | 2.61232032570683 | 0.998735809224318 |
| ebi-fl187-GCST90094726 | prot-c-2961_1_2 | Protein C \|\| id:prot-c-2961_1_2 | F-capsicum liking (derived food-liking factor) \|\| id:ebi-fl187-GCST90094726 | Inverse variance weighted | 2 | 0.164735284332938 | 1.2529807330122 | 0.895399787717467 | -2.291106952 | 2.62057752103685 | 1.17908095619422 | 0.101154426724051 | 13.7436585454874 | 0.998735809224318 |
| ebi-fl187-GCST90094726 | prot-c-2982_82_2 | Galectin-4 \|\| id:prot-c-2982_82_2 | F-capsicum liking (derived food-liking factor) \|\| id:ebi-fl187-GCST90094726 | Inverse variance weighted | 2 | 0.0470363698061467 | 0.46004705955878 | 0.918564180014001 | -0.854655867 | 0.948728606541355 | 1.04816012976716 | 0.425429570278356 | 2.58242429390763 | 0.998735809224318 |
| ebi-fl187-GCST90094726 | prot-c-2994_71_2 | IL-1Rrp2 \|\| id:prot-c-2994_71_2 | F-capsicum liking (derived food-liking factor) \|\| id:ebi-fl187-GCST90094726 | Inverse variance weighted | 2 | -0.349407295 | 0.765438476741575 | 0.648044907038299 | -1.849666709 | 1.15085211975112 | 0.70510588591364 | 0.157289580768244 | 3.16088521516638 | 0.998735809224318 |
| ebi-fl187-GCST90094726 | prot-c-3336_50_1 | TFPI \|\| id:prot-c-3336_50_1 | F-capsicum liking (derived food-liking factor) \|\| id:ebi-fl187-GCST90094726 | Inverse variance weighted | 2 | -0.453823816 | 0.766586335056972 | 0.55384647761922 | -1.956333033 | 1.04868540040269 | 0.635194634323261 | 0.141375892294693 | 2.8538969192289 | 0.998735809224318 |
| ebi-fl187-GCST90094726 | prot-c-3472_40_2 | Survivin \|\| id:prot-c-3472_40_2 | F-capsicum liking (derived food-liking factor) \|\| id:ebi-fl187-GCST90094726 | Inverse variance weighted | 2 | 0.315421310435584 | 0.499242267798293 | 0.527517513896031 | -0.663093534 | 1.29393615532024 | 1.37083673713346 | 0.51525490765725 | 3.64711394680155 | 0.998735809224318 |
| ebi-fl187-GCST90094726 | prot-c-3473_78_2 | Thrombopoietin Receptor \|\| id:prot-c-3473_78_2 | F-capsicum liking (derived food-liking factor) \|\| id:ebi-fl187-GCST90094726 | Inverse variance weighted | 2 | -0.489327685 | 0.497830048317706 | 0.325646246226071 | -1.46507458 | 0.486419209736194 | 0.613038410606037 | 0.23106075839979 | 1.62648169027527 | 0.998735809224318 |
| ebi-fl187-GCST90094726 | prot-c-3497_13_2 | IFN-aA \|\| id:prot-c-3497_13_2 | F-capsicum liking (derived food-liking factor) \|\| id:ebi-fl187-GCST90094726 | Inverse variance weighted | 2 | 0.413434042476997 | 0.500404711529104 | 0.408691022495278 | -0.567359192 | 1.39422727707404 | 1.51200115625656 | 0.567020856420749 | 4.03185785960715 | 0.998735809224318 |
| ebi-fl187-GCST90094726 | prot-c-3665_64_3 | COLEC12 \|\| id:prot-c-3665_64_3 | F-capsicum liking (derived food-liking factor) \|\| id:ebi-fl187-GCST90094726 | Inverse variance weighted | 2 | -0.261112305 | 0.494219133291143 | 0.59726819185892 | -1.229781807 | 0.707557195909368 | 0.770194417810924 | 0.292356360952834 | 2.02902868025098 | 0.998735809224318 |
| ebi-fl187-GCST90094726 | prot-c-3741_4_3 | IL-5 \|\| id:prot-c-3741_4_3 | F-capsicum liking (derived food-liking factor) \|\| id:ebi-fl187-GCST90094726 | Inverse variance weighted | 2 | -0.825274014 | 0.486482966264891 | 0.089808476962262 | -1.778780628 | 0.128232599646167 | 0.438114926290278 | 0.168843905306874 | 1.13681739527095 | 0.998735809224318 |
| ebi-fl187-GCST90094726 | prot-c-3848_14_2 | GAPDH, liver \|\| id:prot-c-3848_14_2 | F-capsicum liking (derived food-liking factor) \|\| id:ebi-fl187-GCST90094726 | Inverse variance weighted | 2 | -0.546408786 | 0.493022020637301 | 0.267738860423015 | -1.512731946 | 0.419914374710864 | 0.579025485634328 | 0.220307287421252 | 1.5218312427995 | 0.998735809224318 |
| ebi-fl187-GCST90094726 | prot-c-3859_50_2 | PSA1 \|\| id:prot-c-3859_50_2 | F-capsicum liking (derived food-liking factor) \|\| id:ebi-fl187-GCST90094726 | Inverse variance weighted | 2 | -0.209439207 | 0.492001679725033 | 0.670335213322839 | -1.173762499 | 0.754884085298258 | 0.811038943455061 | 0.309201379976593 | 2.12736491619312 | 0.998735809224318 |
| ebi-fl187-GCST90094726 | prot-c-3868_8_1 | SGTA \|\| id:prot-c-3868_8_1 | F-capsicum liking (derived food-liking factor) \|\| id:ebi-fl187-GCST90094726 | Inverse variance weighted | 2 | -0.253500897 | 0.794250978957817 | 0.749597784047493 | -1.810232816 | 1.30323102164376 | 0.776079048693787 | 0.163616039957268 | 3.68117141802696 | 0.998735809224318 |
| ebi-fl187-GCST90094726 | prot-c-3882_10_1 | EF-1-beta \|\| id:prot-c-3882_10_1 | F-capsicum liking (derived food-liking factor) \|\| id:ebi-fl187-GCST90094726 | Inverse variance weighted | 2 | -0.344477559 | 0.496432758505082 | 0.487741300932008 | -1.317485765 | 0.628530648029746 | 0.70859045374172 | 0.267807787848344 | 1.87485373434409 | 0.998735809224318 |
| ebi-fl187-GCST90094726 | prot-c-4134_4_2 | HB-EGF \|\| id:prot-c-4134_4_2 | F-capsicum liking (derived food-liking factor) \|\| id:ebi-fl187-GCST90094726 | Inverse variance weighted | 2 | -0.077137617 | 0.559256867656844 | 0.890296704950523 | -1.173281078 | 1.01900584336494 | 0.925762443965093 | 0.309350271966798 | 2.77043914397531 | 0.998735809224318 |
| ebi-fl187-GCST90094726 | prot-c-4220_39_2 | FER \|\| id:prot-c-4220_39_2 | F-capsicum liking (derived food-liking factor) \|\| id:ebi-fl187-GCST90094726 | Inverse variance weighted | 2 | -0.679896503 | 0.576925872244182 | 0.238604678417756 | -1.810671213 | 0.450878206142528 | 0.506669428186813 | 0.163544326866686 | 1.56969009183922 | 0.998735809224318 |
| ebi-fl187-GCST90094726 | prot-c-4228_37_2 | HSP70 protein 8 \|\| id:prot-c-4228_37_2 | F-capsicum liking (derived food-liking factor) \|\| id:ebi-fl187-GCST90094726 | Inverse variance weighted | 2 | 0.703674046260442 | 0.879500499353929 | 0.423662282959761 | -1.020146932 | 2.42749502499414 | 2.02116493617248 | 0.360541960958953 | 11.3304639724812 | 0.998735809224318 |
| ebi-fl187-GCST90094726 | prot-c-4246_40_2 | NCAM-L1 \|\| id:prot-c-4246_40_2 | F-capsicum liking (derived food-liking factor) \|\| id:ebi-fl187-GCST90094726 | Inverse variance weighted | 2 | 0.323606951012617 | 0.494157552025559 | 0.512554069580629 | -0.644941851 | 1.29215575298271 | 1.38210396578238 | 0.524693051618104 | 3.64062639354663 | 0.998735809224318 |
| ebi-fl187-GCST90094726 | prot-c-4267_81_3 | PESC \|\| id:prot-c-4267_81_3 | F-capsicum liking (derived food-liking factor) \|\| id:ebi-fl187-GCST90094726 | Inverse variance weighted | 2 | 0.867066888581693 | 0.913648732647471 | 0.342612684432912 | -0.923684627 | 2.65781840457074 | 2.37992003548135 | 0.39705334882656 | 14.2651343755816 | 0.998735809224318 |
| ebi-fl187-GCST90094726 | prot-c-4280_47_2 | PSA2 \|\| id:prot-c-4280_47_2 | F-capsicum liking (derived food-liking factor) \|\| id:ebi-fl187-GCST90094726 | Inverse variance weighted | 2 | -0.347255676 | 0.497072983855357 | 0.484801428427079 | -1.321518724 | 0.627007372570923 | 0.706624638350199 | 0.266729905054514 | 1.87199998973306 | 0.998735809224318 |
| ebi-fl187-GCST90094726 | prot-c-4324_33_2 | CYTT \|\| id:prot-c-4324_33_2 | F-capsicum liking (derived food-liking factor) \|\| id:ebi-fl187-GCST90094726 | Inverse variance weighted | 2 | 0.507883484789367 | 0.482187329540974 | 0.292207727748399 | -0.437203681 | 1.45297065068968 | 1.66177030812127 | 0.645839872630312 | 4.27579756837677 | 0.998735809224318 |
| ebi-fl187-GCST90094726 | prot-c-4462_8_2 | RASA1 \|\| id:prot-c-4462_8_2 | F-capsicum liking (derived food-liking factor) \|\| id:ebi-fl187-GCST90094726 | Inverse variance weighted | 2 | 1.51427443871181 | 0.497846399016061 | 0.00235285397219689 | 0.538495496640326 | 2.49005338078329 | 4.54612144019952 | 1.71342706523869 | 12.0619199779961 | 0.998735809224318 |
| ebi-fl187-GCST90094726 | prot-c-4499_21_1 | PDGF-AA \|\| id:prot-c-4499_21_1 | F-capsicum liking (derived food-liking factor) \|\| id:ebi-fl187-GCST90094726 | Inverse variance weighted | 2 | -0.560453289 | 0.498904814225042 | 0.261281587060435 | -1.538306725 | 0.417400146761367 | 0.57095019966968 | 0.214744415094494 | 1.51800981813383 | 0.998735809224318 |
| ebi-fl187-GCST90094726 | prot-c-4535_50_2 | BST1 \|\| id:prot-c-4535_50_2 | F-capsicum liking (derived food-liking factor) \|\| id:ebi-fl187-GCST90094726 | Inverse variance weighted | 2 | 0.339812184550895 | 0.496168814773827 | 0.493424624916845 | -0.632678692 | 1.31230306150759 | 1.40468374447886 | 0.531167060466901 | 3.71471909472089 | 0.998735809224318 |
| ebi-fl187-GCST90094726 | prot-c-4542_24_2 | Clusterin \|\| id:prot-c-4542_24_2 | F-capsicum liking (derived food-liking factor) \|\| id:ebi-fl187-GCST90094726 | Inverse variance weighted | 2 | 0.954051800503203 | 0.658378480093766 | 0.147311547458895 | -0.33637002 | 2.24447362148698 | 2.59620769262066 | 0.714358729546346 | 9.43544763217649 | 0.998735809224318 |
| ebi-fl187-GCST90094726 | prot-c-4696_2_2 | FABP \|\| id:prot-c-4696_2_2 | F-capsicum liking (derived food-liking factor) \|\| id:ebi-fl187-GCST90094726 | Inverse variance weighted | 2 | -0.509339037 | 0.474657576994708 | 0.28324138193675 | -1.439667888 | 0.420989813607171 | 0.600892615188563 | 0.237006458251468 | 1.52346875967847 | 0.998735809224318 |
| ebi-fl187-GCST90094726 | prot-c-4906_35_1 | Coagulation Factor V \|\| id:prot-c-4906_35_1 | F-capsicum liking (derived food-liking factor) \|\| id:ebi-fl187-GCST90094726 | Inverse variance weighted | 2 | 0.615814059928271 | 1.04396864525823 | 0.555272493666065 | -1.430364485 | 2.66199260463439 | 1.85116294392119 | 0.239221713678456 | 14.3248043509673 | 0.998735809224318 |
| ebi-fl187-GCST90094726 | prot-c-4916_2_1 | IgD \|\| id:prot-c-4916_2_1 | F-capsicum liking (derived food-liking factor) \|\| id:ebi-fl187-GCST90094726 | Inverse variance weighted | 2 | -0.215010932 | 0.497320034700841 | 0.665494920351446 | -1.1897582 | 0.759736335633588 | 0.806532622807966 | 0.304294833542758 | 2.1377125075707 | 0.998735809224318 |
| ebi-fl187-GCST90094726 | prot-c-4922_13_1 | MIP-3b \|\| id:prot-c-4922_13_1 | F-capsicum liking (derived food-liking factor) \|\| id:ebi-fl187-GCST90094726 | Inverse variance weighted | 2 | 0.388648450278926 | 0.590829589482439 | 0.510665842617058 | -0.769377545 | 1.54667444566451 | 1.47498592928565 | 0.463301362777123 | 4.69582795645098 | 0.998735809224318 |
| ebi-fl187-GCST90094726 | prot-c-4973_18_1 | cIAP-2 \|\| id:prot-c-4973_18_1 | F-capsicum liking (derived food-liking factor) \|\| id:ebi-fl187-GCST90094726 | Inverse variance weighted | 2 | 0.0861314071716689 | 0.500867692439774 | 0.863465524612421 | -0.89556927 | 1.06783208435363 | 1.08994954608211 | 0.408375056784799 | 2.90906604913101 | 0.998735809224318 |
| ebi-fl187-GCST90094726 | prot-c-4997_19_1 | IF4A3 \|\| id:prot-c-4997_19_1 | F-capsicum liking (derived food-liking factor) \|\| id:ebi-fl187-GCST90094726 | Inverse variance weighted | 2 | -0.510941805 | 0.500463886719455 | 0.307284566207284 | -1.491851023 | 0.469967412898437 | 0.59993029526585 | 0.224955871418545 | 1.59994205489361 | 0.998735809224318 |
| ebi-fl187-GCST90094726 | prot-c-5000_52_1 | LG3BP \|\| id:prot-c-5000_52_1 | F-capsicum liking (derived food-liking factor) \|\| id:ebi-fl187-GCST90094726 | Inverse variance weighted | 2 | -0.160900927 | 0.461996066774852 | 0.727634891171546 | -1.066413218 | 0.744611363817906 | 0.851376415292794 | 0.344241023259148 | 2.10562295467946 | 0.998735809224318 |
| ebi-fl187-GCST90094726 | prot-c-5006_71_1 | MK13 \|\| id:prot-c-5006_71_1 | F-capsicum liking (derived food-liking factor) \|\| id:ebi-fl187-GCST90094726 | Inverse variance weighted | 2 | -0.328911229 | 0.499634414832214 | 0.510342942887389 | -1.308194682 | 0.65037222398967 | 0.719706902954263 | 0.270307607307836 | 1.91625397197987 | 0.998735809224318 |
| ebi-fl187-GCST90094726 | prot-c-5109_24_3 | Nr-CAM \|\| id:prot-c-5109_24_3 | F-capsicum liking (derived food-liking factor) \|\| id:ebi-fl187-GCST90094726 | Inverse variance weighted | 2 | -0.009395016 | 0.543037321458198 | 0.986196594395686 | -1.073748166 | 1.05495813389262 | 0.990648979112338 | 0.341725270892315 | 2.87185491799807 | 0.998955228396052 |
| ebi-fl187-GCST90094726 | prot-c-5255_22_3 | PDE4D \|\| id:prot-c-5255_22_3 | F-capsicum liking (derived food-liking factor) \|\| id:ebi-fl187-GCST90094726 | Inverse variance weighted | 2 | 0.141649544990729 | 0.645821347985788 | 0.826391126235925 | -1.124160297 | 1.40745938704287 | 1.15217279304768 | 0.324925193477647 | 4.08556237462275 | 0.998735809224318 |
| ebi-fl187-GCST90094726 | prot-c-5440_26_3 | Troponin I, skeletal, fast twitch \|\| id:prot-c-5440_26_3 | F-capsicum liking (derived food-liking factor) \|\| id:ebi-fl187-GCST90094726 | Inverse variance weighted | 2 | 0.0823350647347303 | 0.475149499985209 | 0.862429425723782 | -0.848957955 | 1.01362808470574 | 1.08581956873052 | 0.427860549576935 | 2.75558038011197 | 0.998735809224318 |
| ebi-fl187-GCST90094726 | ukb-rsfMRI191-edge-pheno716 | Net100_Pair10_33 (OccipitalPrecuneus)&(Temporal\|Frontal\|Supp_Motor_Area) (Default_mode\|Central_executive)&(Default_mode\|Salience) \|\| id:ukb-rsfMRI191-edge-pheno716 | F-capsicum liking (derived food-liking factor) \|\| id:ebi-fl187-GCST90094726 | Inverse variance weighted | 20 | -0.015663919 | 0.0243659814949564 | 0.520314842928781 | -0.063421242 | 0.0320934049807239 | 0.984458122380884 | 0.938548034105984 | 1.03261395208703 | 0.998735809224318 |
| ebi-fl187-GCST90094726 | ukb-rsfMRI191-edge-pheno903 | Net100_Pair27_38 (Precentral\|Frontal\|Supp_Motor_Area)&(Subcortical) (Attention\|Salience\|Motor)&(Subcortical-cerebellum) \|\| id:ukb-rsfMRI191-edge-pheno903 | F-capsicum liking (derived food-liking factor) \|\| id:ebi-fl187-GCST90094726 | Inverse variance weighted (multiplicative random effects) | 20 | 0.000917842180822718 | 0.0335632662708912 | 0.978183266431269 | -0.06486616 | 0.0667018440717695 | 1.00091826352686 | 0.937192889151659 | 1.06897670891259 | 0.998955228396052 |
| ebi-fl187-GCST90094726 | ukb-rsfMRI191-node-pheno23 | Net100_Node2 RolandicOper\|SupraMarginal\|Insula Salience\|Motor \|\| id:ukb-rsfMRI191-node-pheno23 | F-capsicum liking (derived food-liking factor) \|\| id:ebi-fl187-GCST90094726 | Inverse variance weighted (multiplicative random effects) | 20 | -0.006510049 | 0.0383068094899195 | 0.865053426445875 | -0.081591396 | 0.0685712975907449 | 0.99351109545085 | 0.921648471380425 | 1.07097698030741 | 0.998735809224318 |
| ebi-fl187-GCST90094726 | ukb-rsfMRI191-node-pheno55 | Net100_Node34 Parietal\|Postcentral\|Precuneus Attention \|\| id:ukb-rsfMRI191-node-pheno55 | F-capsicum liking (derived food-liking factor) \|\| id:ebi-fl187-GCST90094726 | Inverse variance weighted (multiplicative random effects) | 20 | -0.002077281 | 0.0348814646209155 | 0.952511988614454 | -0.070444952 | 0.0662903895166545 | 0.997924874914959 | 0.931979041884914 | 1.06853696405011 | 0.998735809224318 |
| ebi-fl187-GCST90094726 | ebi-a-GCST90001414 | IgD- CD24- B cell Absolute Count \|\| id:ebi-a-GCST90001414 | F-capsicum liking (derived food-liking factor) \|\| id:ebi-fl187-GCST90094726 | MR Egger | 20 | 0.724115117115871 | 0.490590041446131 | 0.157221498891944 | -0.237441364 | 1.68567159835029 | 2.06290486287793 | 0.788643132410581 | 5.39607370988922 | 0.999950574162623 |
| ebi-fl187-GCST90094726 | ebi-a-GCST90001422 | CD20- CD38- B cell %B cell \|\| id:ebi-a-GCST90001422 | F-capsicum liking (derived food-liking factor) \|\| id:ebi-fl187-GCST90094726 | MR Egger | 20 | 0.312004284033748 | 0.413500412873833 | 0.460281259847294 | -0.498456525 | 1.12246509326646 | 1.36616054569483 | 0.607467547347186 | 3.07241867448515 | 0.999950574162623 |
| ebi-fl187-GCST90094726 | ebi-a-GCST90001444 | CD20- CD38- B cell %lymphocyte \|\| id:ebi-a-GCST90001444 | F-capsicum liking (derived food-liking factor) \|\| id:ebi-fl187-GCST90094726 | MR Egger | 20 | 0.561035621281399 | 0.410303329618517 | 0.188343229392686 | -0.243158905 | 1.36523014733369 | 1.75248647312648 | 0.78414689918753 | 3.91662434892418 | 0.999950574162623 |
| ebi-fl187-GCST90094726 | ebi-a-GCST90001459 | Myeloid Dendritic Cell %Dendritic Cell \|\| id:ebi-a-GCST90001459 | F-capsicum liking (derived food-liking factor) \|\| id:ebi-fl187-GCST90094726 | MR Egger | 20 | 0.124597721691747 | 0.43957750830618 | 0.78006715631728 | -0.736974195 | 0.98616963797186 | 1.13269270369932 | 0.478559755700989 | 2.68094578729119 | 0.999950574162623 |
| ebi-fl187-GCST90094726 | ebi-a-GCST90001476 | HLA DR++ monocyte %leukocyte \|\| id:ebi-a-GCST90001476 | F-capsicum liking (derived food-liking factor) \|\| id:ebi-fl187-GCST90094726 | MR Egger | 20 | -0.344888775 | 0.430367501644376 | 0.433362630971883 | -1.188409078 | 0.498631528061906 | 0.708299129543388 | 0.304705641452803 | 1.64646658499636 | 0.999950574162623 |
| ebi-fl187-GCST90094726 | ebi-a-GCST90001478 | CD4 regulatory T cell %CD4+ T cell \|\| id:ebi-a-GCST90001478 | F-capsicum liking (derived food-liking factor) \|\| id:ebi-fl187-GCST90094726 | MR Egger | 20 | 0.333146560055777 | 0.433634356092789 | 0.452291381682331 | -0.516776778 | 1.18306989799764 | 1.39535178632019 | 0.596439911353122 | 3.26438015049974 | 0.999950574162623 |
| ebi-fl187-GCST90094726 | ebi-a-GCST90001485 | CD39+ resting CD4 regulatory T cell %CD4 regulatory T cell \|\| id:ebi-a-GCST90001485 | F-capsicum liking (derived food-liking factor) \|\| id:ebi-fl187-GCST90094726 | MR Egger | 20 | 0.235224306599345 | 0.474927784065895 | 0.626391382545993 | -0.69563415 | 1.1660827633685 | 1.26519252794063 | 0.498758060168495 | 3.20939601901578 | 0.999950574162623 |
| ebi-fl187-GCST90094726 | ebi-a-GCST90001515 | Immature Myeloid-Derived Suppressor Cells Absolute Count \|\| id:ebi-a-GCST90001515 | F-capsicum liking (derived food-liking factor) \|\| id:ebi-fl187-GCST90094726 | MR Egger | 20 | 0.0325502734214027 | 0.587325748961408 | 0.956413472209771 | -1.118608195 | 1.18370874138576 | 1.03308582859768 | 0.326734228790338 | 3.2664662444479 | 0.999950574162623 |
| ebi-fl187-GCST90094726 | ebi-a-GCST90001517 | CD33+ HLA DR+ Absolute Count \|\| id:ebi-a-GCST90001517 | F-capsicum liking (derived food-liking factor) \|\| id:ebi-fl187-GCST90094726 | MR Egger | 20 | -0.532846655 | 0.563893463703145 | 0.357201079634976 | -1.638077844 | 0.572384533963055 | 0.586931797062516 | 0.194353260818173 | 1.77248857545704 | 0.999950574162623 |
| ebi-fl187-GCST90094726 | ebi-a-GCST90001518 | CD33+ HLA DR+ CD14- Absolute Count \|\| id:ebi-a-GCST90001518 | F-capsicum liking (derived food-liking factor) \|\| id:ebi-fl187-GCST90094726 | MR Egger | 20 | -0.874675485 | 0.571590593358657 | 0.143340132000457 | -1.994993048 | 0.245642077924001 | 0.416997319584476 | 0.136014599731356 | 1.27844190906038 | 0.999950574162623 |
| ebi-fl187-GCST90094726 | ebi-a-GCST90001520 | CD33+ HLA DR+ CD14dim Absolute Count \|\| id:ebi-a-GCST90001520 | F-capsicum liking (derived food-liking factor) \|\| id:ebi-fl187-GCST90094726 | MR Egger | 20 | -0.162788784 | 0.565434641958781 | 0.776711716457249 | -1.271040682 | 0.945463114062851 | 0.849770654469577 | 0.280539517268235 | 2.57400516058924 | 0.999950574162623 |
| ebi-fl187-GCST90094726 | ebi-a-GCST90001531 | CD33dim HLA DR- Absolute Count \|\| id:ebi-a-GCST90001531 | F-capsicum liking (derived food-liking factor) \|\| id:ebi-fl187-GCST90094726 | MR Egger | 20 | 0.2400757171296 | 0.576076866346702 | 0.681794362555909 | -0.889034941 | 1.36918637516914 | 1.27134540930225 | 0.411052251110826 | 3.93215009864557 | 0.999950574162623 |
| ebi-fl187-GCST90094726 | ebi-a-GCST90001532 | Basophil Absolute Count \|\| id:ebi-a-GCST90001532 | F-capsicum liking (derived food-liking factor) \|\| id:ebi-fl187-GCST90094726 | MR Egger | 20 | 0.123099515975527 | 0.574384844856996 | 0.832710247620721 | -1.00269478 | 1.24889381189524 | 1.13099696761385 | 0.366889421573088 | 3.48648411629635 | 0.999950574162623 |
| ebi-fl187-GCST90094726 | ebi-a-GCST90001588 | T/B cell \|\| id:ebi-a-GCST90001588 | F-capsicum liking (derived food-liking factor) \|\| id:ebi-fl187-GCST90094726 | MR Egger | 20 | -0.430582014 | 0.423687950573818 | 0.322966491131453 | -1.261010397 | 0.399846369157025 | 0.650130599499537 | 0.283367568038933 | 1.49159552495985 | 0.999950574162623 |
| ebi-fl187-GCST90094726 | ebi-a-GCST90001624 | HLA DR+ CD4+ T cell Absolute Count \|\| id:ebi-a-GCST90001624 | F-capsicum liking (derived food-liking factor) \|\| id:ebi-fl187-GCST90094726 | MR Egger | 20 | 0.325366432927196 | 0.411422999245274 | 0.439338430539157 | -0.481022646 | 1.13175551144793 | 1.38453789331207 | 0.618150919149962 | 3.10109573347086 | 0.999950574162623 |
| ebi-fl187-GCST90094726 | ebi-a-GCST90001631 | CD8+ Natural Killer T %T cell \|\| id:ebi-a-GCST90001631 | F-capsicum liking (derived food-liking factor) \|\| id:ebi-fl187-GCST90094726 | MR Egger | 20 | 0.0730105434411671 | 0.416895272620907 | 0.862933028003996 | -0.744104191 | 0.890125277778145 | 1.07574187887612 | 0.475159761785715 | 2.43543473803198 | 0.999950574162623 |
| ebi-fl187-GCST90094726 | ebi-a-GCST90001632 | CD8+ Natural Killer T %lymphocyte \|\| id:ebi-a-GCST90001632 | F-capsicum liking (derived food-liking factor) \|\| id:ebi-fl187-GCST90094726 | MR Egger | 20 | 0.027433462366366 | 0.419471419533627 | 0.948576354314915 | -0.79473052 | 0.849597444652275 | 1.02781322457222 | 0.45170294346839 | 2.33870520411936 | 0.999950574162623 |
| ebi-fl187-GCST90094726 | ebi-a-GCST90001646 | Natural Killer %CD3- lymphocyte \|\| id:ebi-a-GCST90001646 | F-capsicum liking (derived food-liking factor) \|\| id:ebi-fl187-GCST90094726 | MR Egger | 20 | -0.251359011 | 0.408917030046347 | 0.546449162762524 | -1.05283639 | 0.550118368192426 | 0.777743103337102 | 0.348946595635884 | 1.73345819203695 | 0.999950574162623 |
| ebi-fl187-GCST90094726 | ebi-a-GCST90001684 | CD127- CD8+ T cell Absolute Count \|\| id:ebi-a-GCST90001684 | F-capsicum liking (derived food-liking factor) \|\| id:ebi-fl187-GCST90094726 | MR Egger | 20 | 0.414568172913148 | 0.441695921479659 | 0.360369774470673 | -0.451155833 | 1.28029217901328 | 1.51371693556222 | 0.636891585598902 | 3.59769074175038 | 0.999950574162623 |
| ebi-fl187-GCST90094726 | ebi-a-GCST90001687 | CD28- CD8+ T cell Absolute Count \|\| id:ebi-a-GCST90001687 | F-capsicum liking (derived food-liking factor) \|\| id:ebi-fl187-GCST90094726 | MR Egger | 20 | 0.251455004861483 | 0.429871203044941 | 0.565839702621231 | -0.591092553 | 1.09400256282957 | 1.28589503973676 | 0.553721983459602 | 2.98620264792187 | 0.999950574162623 |
| ebi-fl187-GCST90094726 | ebi-a-GCST90001701 | BAFF-R on CD20- CD38- B cell \|\| id:ebi-a-GCST90001701 | F-capsicum liking (derived food-liking factor) \|\| id:ebi-fl187-GCST90094726 | MR Egger | 20 | -0.284050939 | 0.463330463580885 | 0.547503274478843 | -1.192178648 | 0.624076769230341 | 0.752728300210494 | 0.303559194486304 | 1.8665219312385 | 0.999950574162623 |
| ebi-fl187-GCST90094726 | ebi-a-GCST90001732 | CD19 on IgD- CD27- B cell \|\| id:ebi-a-GCST90001732 | F-capsicum liking (derived food-liking factor) \|\| id:ebi-fl187-GCST90094726 | MR Egger | 20 | 0.252960612166591 | 0.523312446075478 | 0.634651096523215 | -0.772731782 | 1.27865300647453 | 1.28783255090167 | 0.461749943556349 | 3.59179833653734 | 0.999950574162623 |
| ebi-fl187-GCST90094726 | ebi-a-GCST90001798 | CD27 on CD24+ CD27+ B cell \|\| id:ebi-a-GCST90001798 | F-capsicum liking (derived food-liking factor) \|\| id:ebi-fl187-GCST90094726 | MR Egger | 20 | 0.264141595654703 | 0.417850828931478 | 0.535240838680506 | -0.554846029 | 1.0831292203604 | 1.30231258504937 | 0.57416065845242 | 2.95390853450214 | 0.999950574162623 |
| ebi-fl187-GCST90094726 | ebi-a-GCST90001836 | CD66b on CD66b++ myeloid cell \|\| id:ebi-a-GCST90001836 | F-capsicum liking (derived food-liking factor) \|\| id:ebi-fl187-GCST90094726 | MR Egger | 20 | -0.120331692 | 0.686870605821169 | 0.862887285557375 | -1.46659808 | 1.22593469500206 | 0.886626300726337 | 0.230709005316212 | 3.40734942731093 | 0.999950574162623 |
| ebi-fl187-GCST90094726 | ebi-a-GCST90001943 | CD25 on activated & secreting CD4 regulatory T cell \|\| id:ebi-a-GCST90001943 | F-capsicum liking (derived food-liking factor) \|\| id:ebi-fl187-GCST90094726 | MR Egger | 20 | -0.120906954 | 0.470081967859269 | 0.799938490563266 | -1.042267611 | 0.800453702887253 | 0.886116405240645 | 0.352654092247296 | 2.22655089193176 | 0.999950574162623 |
| ebi-fl187-GCST90094726 | ebi-a-GCST90001960 | CD25 on CD4+ T cell \|\| id:ebi-a-GCST90001960 | F-capsicum liking (derived food-liking factor) \|\| id:ebi-fl187-GCST90094726 | MR Egger | 20 | -0.383760373 | 0.51132930493526 | 0.462643374946251 | -1.38596581 | 0.618445065142265 | 0.681294664536901 | 0.250082151223675 | 1.85603977595066 | 0.999950574162623 |
| ebi-fl187-GCST90094726 | ebi-a-GCST90001988 | HLA DR on CD14+ CD16- monocyte \|\| id:ebi-a-GCST90001988 | F-capsicum liking (derived food-liking factor) \|\| id:ebi-fl187-GCST90094726 | MR Egger | 20 | 0.126888294185749 | 0.425812939320891 | 0.769122521257619 | -0.707705067 | 0.961481655254696 | 1.13529019218271 | 0.492773783678043 | 2.61556897537462 | 0.999950574162623 |
| ebi-fl187-GCST90094726 | ebi-a-GCST90001991 | HLA DR on CD14+ monocyte \|\| id:ebi-a-GCST90001991 | F-capsicum liking (derived food-liking factor) \|\| id:ebi-fl187-GCST90094726 | MR Egger | 20 | 0.0955377091735746 | 0.426539413065921 | 0.825292897604204 | -0.74047954 | 0.93155495878278 | 1.10025031078162 | 0.476885174955699 | 2.53845330060326 | 0.999950574162623 |
| ebi-fl187-GCST90094726 | ebi-a-GCST90002019 | CD14 on Monocytic Myeloid-Derived Suppressor Cells \|\| id:ebi-a-GCST90002019 | F-capsicum liking (derived food-liking factor) \|\| id:ebi-fl187-GCST90094726 | MR Egger | 20 | -0.4048535 | 0.615716089592987 | 0.519159090146116 | -1.611657035 | 0.801950035986612 | 0.667074530375422 | 0.199556667529767 | 2.22988504761042 | 0.999950574162623 |
| ebi-fl187-GCST90094726 | ebi-a-GCST90002037 | CD80 on plasmacytoid Dendritic Cell \|\| id:ebi-a-GCST90002037 | F-capsicum liking (derived food-liking factor) \|\| id:ebi-fl187-GCST90094726 | MR Egger | 20 | -0.847924792 | 0.471940792921636 | 0.0891886151642187 | -1.772928746 | 0.0770791622024513 | 0.428302827833041 | 0.169834856564649 | 1.08012757828632 | 0.999950574162623 |
| ebi-fl187-GCST90094726 | ebi-a-GCST90002042 | CD45 on CD33+ HLA DR+ CD14- \|\| id:ebi-a-GCST90002042 | F-capsicum liking (derived food-liking factor) \|\| id:ebi-fl187-GCST90094726 | MR Egger | 20 | -0.502910313 | 0.642033397862162 | 0.443630965257981 | -1.761295773 | 0.75547514648171 | 0.604768031593917 | 0.171822077081693 | 2.12862269069234 | 0.999950574162623 |
| ebi-fl187-GCST90094726 | ebi-a-GCST90002056 | CD8 on Effector Memory CD8+ T cell \|\| id:ebi-a-GCST90002056 | F-capsicum liking (derived food-liking factor) \|\| id:ebi-fl187-GCST90094726 | MR Egger | 20 | -0.542954561 | 0.462171485363427 | 0.255386413373526 | -1.448810672 | 0.36290155076432 | 0.581029028404422 | 0.234849435103955 | 1.43749433205642 | 0.999950574162623 |
| ebi-fl187-GCST90094726 | ebi-a-GCST90002058 | CD8 on CD8+ T cell \|\| id:ebi-a-GCST90002058 | F-capsicum liking (derived food-liking factor) \|\| id:ebi-fl187-GCST90094726 | MR Egger | 20 | -1.493854385 | 0.626370868975859 | 0.0282872427216703 | -2.721541288 | -0.266167481 | 0.224505654578355 | 0.0657733006707268 | 0.766310773880444 | 0.999950574162623 |
| ebi-fl187-GCST90094726 | ebi-a-GCST90002063 | CD4 on CD4 regulatory T cell \|\| id:ebi-a-GCST90002063 | F-capsicum liking (derived food-liking factor) \|\| id:ebi-fl187-GCST90094726 | MR Egger | 20 | -0.872079106 | 0.460247784517277 | 0.0742977909081312 | -1.774164763 | 0.0300065521352161 | 0.418081409639028 | 0.169625067452174 | 1.03046128565308 | 0.999950574162623 |
| ebi-fl187-GCST90094726 | ebi-a-GCST90002066 | CD4 on activated CD4 regulatory T cell \|\| id:ebi-a-GCST90002066 | F-capsicum liking (derived food-liking factor) \|\| id:ebi-fl187-GCST90094726 | MR Egger | 20 | -0.865046771 | 0.460298720054261 | 0.0764912804019279 | -1.767232262 | 0.0371387201543192 | 0.421031860021666 | 0.170805078842423 | 1.03783697975891 | 0.999950574162623 |
| ebi-fl187-GCST90094726 | ebi-a-GCST90002070 | CD4 on activated & secreting CD4 regulatory T cell \|\| id:ebi-a-GCST90002070 | F-capsicum liking (derived food-liking factor) \|\| id:ebi-fl187-GCST90094726 | MR Egger | 20 | -0.776884127 | 0.462374484795818 | 0.110188966780391 | -1.683138117 | 0.129369863436091 | 0.459836573895643 | 0.185790029429239 | 1.13811099197127 | 0.999950574162623 |
| ebi-fl187-GCST90094726 | ebi-a-GCST90002081 | SSC-A on CD4+ T cell \|\| id:ebi-a-GCST90002081 | F-capsicum liking (derived food-liking factor) \|\| id:ebi-fl187-GCST90094726 | MR Egger | 20 | 0.481431966428469 | 0.437867078773685 | 0.28604137115793 | -0.376787508 | 1.33965144082489 | 1.61839022417669 | 0.686061841083011 | 3.81771257467993 | 0.999950574162623 |
| ebi-fl187-GCST90094726 | ebi-a-GCST90002094 | CD11b on Monocytic Myeloid-Derived Suppressor Cells \|\| id:ebi-a-GCST90002094 | F-capsicum liking (derived food-liking factor) \|\| id:ebi-fl187-GCST90094726 | MR Egger | 20 | 0.0206925986095288 | 0.623670625641333 | 0.973897209982658 | -1.201701828 | 1.24308702486654 | 1.02090817480443 | 0.300682067189739 | 3.46629751193253 | 0.999950574162623 |
| ebi-fl187-GCST90094726 | ebi-a-GCST90002104 | HLA DR on myeloid Dendritic Cell \|\| id:ebi-a-GCST90002104 | F-capsicum liking (derived food-liking factor) \|\| id:ebi-fl187-GCST90094726 | MR Egger | 20 | -1.01912535 | 0.562924692953799 | 0.0869581377088227 | -2.122457748 | 0.0842070481895649 | 0.360910472507606 | 0.119736983224099 | 1.08785410871657 | 0.999950574162623 |
| ebi-fl187-GCST90094726 | ebi-a-GCST90002105 | HLA DR on plasmacytoid Dendritic Cell \|\| id:ebi-a-GCST90002105 | F-capsicum liking (derived food-liking factor) \|\| id:ebi-fl187-GCST90094726 | MR Egger | 20 | -0.590533807 | 0.460216890158531 | 0.215710352490167 | -1.492558912 | 0.311491297890422 | 0.554031460012889 | 0.224796684096675 | 1.36545990399043 | 0.999950574162623 |
| ebi-fl187-GCST90094726 | ebi-a-GCST90002106 | HLA DR on Dendritic Cell \|\| id:ebi-a-GCST90002106 | F-capsicum liking (derived food-liking factor) \|\| id:ebi-fl187-GCST90094726 | MR Egger | 20 | -1.016658479 | 0.468772784146278 | 0.0437415933029356 | -1.935453136 | -0.097863822 | 0.361801891116315 | 0.144358839813988 | 0.906772377666742 | 0.999950574162623 |
| ebi-fl187-GCST90094726 | ebi-a-GCST90002111 | HLA DR on CD33dim HLA DR+ CD11b- \|\| id:ebi-a-GCST90002111 | F-capsicum liking (derived food-liking factor) \|\| id:ebi-fl187-GCST90094726 | MR Egger | 20 | 0.939632632010649 | 0.763732189740215 | 0.234415572452069 | -0.55728246 | 2.43654772390147 | 2.55904113582827 | 0.57276345850495 | 11.4335009289085 | 0.999950574162623 |
| ebi-fl187-GCST90094726 | ebi-a-GCST90002112 | HLA DR on CD33- HLA DR+ \|\| id:ebi-a-GCST90002112 | F-capsicum liking (derived food-liking factor) \|\| id:ebi-fl187-GCST90094726 | MR Egger | 20 | -0.233215341 | 0.673154818588378 | 0.733020557329785 | -1.552598785 | 1.08616810371771 | 0.791983008972404 | 0.211697103051943 | 2.96289877120842 | 0.999950574162623 |
| ebi-fl187-GCST90094726 | ebi-a-GCST90002115 | HLA DR on HLA DR+ CD8+ T cell \|\| id:ebi-a-GCST90002115 | F-capsicum liking (derived food-liking factor) \|\| id:ebi-fl187-GCST90094726 | MR Egger | 20 | -0.14324891 | 0.477468943423231 | 0.767601189984589 | -1.079088039 | 0.792590218824977 | 0.866538351741782 | 0.339905364645278 | 2.20911110309478 | 0.999950574162623 |
| ebi-fl187-GCST90094726 | ebi-a-GCST90002116 | HLA DR on B cell \|\| id:ebi-a-GCST90002116 | F-capsicum liking (derived food-liking factor) \|\| id:ebi-fl187-GCST90094726 | MR Egger | 20 | 0.163141651641101 | 0.455699316142396 | 0.724505038286879 | -0.730029008 | 1.0563123112802 | 1.17720343067649 | 0.481895011077937 | 2.87574655337604 | 0.999950574162623 |
| ebi-fl187-GCST90094726 | ebi-cfmet338-GCST90026023 | Sphingomyelin (d18:1/24:1, d18:2/24:0) levels \|\| id:ebi-cfmet338-GCST90026023 | F-capsicum liking (derived food-liking factor) \|\| id:ebi-fl187-GCST90094726 | MR Egger | 18 | -0.191587146 | 0.303462788420384 | 0.536736430484675 | -0.786374212 | 0.403199918890475 | 0.825647669737989 | 0.455493327231094 | 1.49660606158104 | 0.999950574162623 |
| ebi-fl187-GCST90094726 | ebi-cfmet338-GCST90026046 | 1-arachidonoyl-gpc (20:4n6) levels \|\| id:ebi-cfmet338-GCST90026046 | F-capsicum liking (derived food-liking factor) \|\| id:ebi-fl187-GCST90094726 | MR Egger | 18 | 0.0857350145959223 | 0.309564502771986 | 0.785361803729197 | -0.521011411 | 0.692481440029016 | 1.08951758379309 | 0.593919547425521 | 1.99866896205026 | 0.999950574162623 |
| ebi-fl187-GCST90094726 | ebi-cfmet338-GCST90026047 | 1-linoleoyl-gpc (18:2) levels \|\| id:ebi-cfmet338-GCST90026047 | F-capsicum liking (derived food-liking factor) \|\| id:ebi-fl187-GCST90094726 | MR Egger | 18 | -0.187806771 | 0.377351982425648 | 0.625468528076398 | -0.927416656 | 0.551803114883834 | 0.828774835367254 | 0.395574295937406 | 1.73638109147189 | 0.999950574162623 |
| ebi-fl187-GCST90094726 | ebi-cfmet338-GCST90026050 | 1-myristoyl-2-palmitoyl-gpc (14:0/16:0) levels \|\| id:ebi-cfmet338-GCST90026050 | F-capsicum liking (derived food-liking factor) \|\| id:ebi-fl187-GCST90094726 | MR Egger | 18 | -0.292152056 | 0.197431170895127 | 0.158355643096253 | -0.679117151 | 0.0948130388839441 | 0.746654993916799 | 0.507064456150449 | 1.09945328089706 | 0.999950574162623 |
| ebi-fl187-GCST90094726 | ebi-cfmet338-GCST90026068 | 2,3-dihydroxyisovalerate levels \|\| id:ebi-cfmet338-GCST90026068 | F-capsicum liking (derived food-liking factor) \|\| id:ebi-fl187-GCST90094726 | MR Egger | 18 | 0.417509353896666 | 0.640692130943072 | 0.523875510433947 | -0.838247223 | 1.67326593054509 | 1.51817560468979 | 0.432467879356596 | 5.32954532971158 | 0.999950574162623 |
| ebi-fl187-GCST90094726 | ebi-cfmet338-GCST90026082 | 3-hydroxyhexanoate levels \|\| id:ebi-cfmet338-GCST90026082 | F-capsicum liking (derived food-liking factor) \|\| id:ebi-fl187-GCST90094726 | MR Egger | 18 | 0.0598399680851617 | 0.187791861497375 | 0.754113456869159 | -0.30823208 | 0.427912016620017 | 1.06166663240578 | 0.734744778321808 | 1.5340511040286 | 0.999950574162623 |
| ebi-fl187-GCST90094726 | ebi-cfmet338-GCST90026094 | 3-ureidopropionate levels \|\| id:ebi-cfmet338-GCST90026094 | F-capsicum liking (derived food-liking factor) \|\| id:ebi-fl187-GCST90094726 | MR Egger | 18 | 0.370105750468269 | 0.396910309912309 | 0.36495379335594 | -0.407838457 | 1.14804995789639 | 1.44788772137217 | 0.665086310209282 | 3.15204030141684 | 0.999950574162623 |
| ebi-fl187-GCST90094726 | ebi-cfmet338-GCST90026173 | Glycerol levels \|\| id:ebi-cfmet338-GCST90026173 | F-capsicum liking (derived food-liking factor) \|\| id:ebi-fl187-GCST90094726 | MR Egger | 18 | -0.106936535 | 0.177317499764285 | 0.554909016998346 | -0.454478834 | 0.240605764962302 | 0.898582700112995 | 0.634778706763165 | 1.27201946180532 | 0.999950574162623 |
| ebi-fl187-GCST90094726 | ebi-cfmet338-GCST90026179 | Histidine levels \|\| id:ebi-cfmet338-GCST90026179 | F-capsicum liking (derived food-liking factor) \|\| id:ebi-fl187-GCST90094726 | MR Egger | 18 | -0.07297098 | 0.167897141190955 | 0.669645026702645 | -0.402049377 | 0.256107416954642 | 0.929627807638184 | 0.668947714568746 | 1.29189149153654 | 0.999950574162623 |
| ebi-fl187-GCST90094726 | ebi-cfmet338-GCST90026188 | Isocitrate levels \|\| id:ebi-cfmet338-GCST90026188 | F-capsicum liking (derived food-liking factor) \|\| id:ebi-fl187-GCST90094726 | MR Egger | 18 | 0.114319674178498 | 0.271941945818999 | 0.679797507414808 | -0.41868654 | 0.647325887983736 | 1.12111045767079 | 0.657910391785891 | 1.91042530106114 | 0.999950574162623 |
| ebi-fl187-GCST90094726 | ebi-cfmet338-GCST90026207 | N-acetyl-isoputreanine levels \|\| id:ebi-cfmet338-GCST90026207 | F-capsicum liking (derived food-liking factor) \|\| id:ebi-fl187-GCST90094726 | MR Egger | 17 | -0.290271905 | 0.154339001166514 | 0.0795656824061636 | -0.592776347 | 0.0122325374650495 | 0.748060138764815 | 0.552790414211035 | 1.01230766095593 | 0.999950574162623 |
| ebi-fl187-GCST90094726 | ebi-cfmet338-GCST90026213 | N-acetylglutamate levels \|\| id:ebi-cfmet338-GCST90026213 | F-capsicum liking (derived food-liking factor) \|\| id:ebi-fl187-GCST90094726 | MR Egger | 18 | -0.173313779 | 0.266138672586404 | 0.524150659765112 | -0.694945578 | 0.348318018980872 | 0.840873724686121 | 0.499101609574512 | 1.41668271010044 | 0.999950574162623 |
| ebi-fl187-GCST90094726 | ebi-cfmet338-GCST90026224 | N-acetyltaurine levels \|\| id:ebi-cfmet338-GCST90026224 | F-capsicum liking (derived food-liking factor) \|\| id:ebi-fl187-GCST90094726 | MR Egger | 18 | 0.169952267465069 | 0.2466555290512 | 0.50067700256211 | -0.313492569 | 0.65339710440542 | 1.18524827506542 | 0.730889809878225 | 1.92205918670506 | 0.999950574162623 |
| ebi-fl187-GCST90094726 | ebi-cfmet338-GCST90026250 | Phenyllactate (pla) levels \|\| id:ebi-cfmet338-GCST90026250 | F-capsicum liking (derived food-liking factor) \|\| id:ebi-fl187-GCST90094726 | MR Egger | 18 | 0.285959031785579 | 0.344323507792026 | 0.41847941870695 | -0.388915043 | 0.960833107057951 | 1.33103792388821 | 0.677791850400313 | 2.61387320278675 | 0.999950574162623 |
| ebi-fl187-GCST90094726 | ebi-cfmet338-GCST90026251 | Pipecolate levels \|\| id:ebi-cfmet338-GCST90026251 | F-capsicum liking (derived food-liking factor) \|\| id:ebi-fl187-GCST90094726 | MR Egger | 17 | -0.787212342 | 0.548334712982163 | 0.171629160150539 | -1.861948379 | 0.287523695431544 | 0.455111724412667 | 0.155369616301782 | 1.33312218069432 | 0.999950574162623 |
| ebi-fl187-GCST90094726 | ebi-cfmet338-GCST90026278 | Trigonelline (n'-methylnicotinate) levels \|\| id:ebi-cfmet338-GCST90026278 | F-capsicum liking (derived food-liking factor) \|\| id:ebi-fl187-GCST90094726 | MR Egger | 18 | -0.107247017 | 0.559115580685984 | 0.850299962417492 | -1.203113556 | 0.988619520725949 | 0.898303748908721 | 0.30025788540684 | 2.68752184213271 | 0.999950574162623 |
| ebi-fl187-GCST90094726 | ebi-cfmet338-GCST90026299 | 1-carboxyethylvaline levels \|\| id:ebi-cfmet338-GCST90026299 | F-capsicum liking (derived food-liking factor) \|\| id:ebi-fl187-GCST90094726 | MR Egger | 18 | -0.253282666 | 0.358175931035456 | 0.489647963059378 | -0.955307491 | 0.448742158911533 | 0.776248431834133 | 0.384693836569258 | 1.56634073812739 | 0.999950574162623 |
| ebi-fl187-GCST90094726 | ebi-gm430-GCST90011306 | C_Erysipelotrichia abundance \|\| id:ebi-gm430-GCST90011306 | F-capsicum liking (derived food-liking factor) \|\| id:ebi-fl187-GCST90094726 | MR Egger | 18 | -0.187390482 | 0.277233241559986 | 0.508737592941068 | -0.730767636 | 0.355986671286003 | 0.829119916621063 | 0.481539201529002 | 1.42758852021794 | 0.999950574162623 |
| ebi-fl187-GCST90094726 | ebi-gm430-GCST90011312 | F_Erysipelotrichaceae abundance \|\| id:ebi-gm430-GCST90011312 | F-capsicum liking (derived food-liking factor) \|\| id:ebi-fl187-GCST90094726 | MR Egger | 18 | -0.187390482 | 0.277233241559986 | 0.508737592941068 | -0.730767636 | 0.355986671286003 | 0.829119916621063 | 0.481539201529002 | 1.42758852021794 | 0.999950574162623 |
| ebi-fl187-GCST90094726 | ebi-gm430-GCST90011352 | O_Erysipelotrichales abundance \|\| id:ebi-gm430-GCST90011352 | F-capsicum liking (derived food-liking factor) \|\| id:ebi-fl187-GCST90094726 | MR Egger | 18 | -0.187390482 | 0.277233241559986 | 0.508737592941068 | -0.730767636 | 0.355986671286003 | 0.829119916621063 | 0.481539201529002 | 1.42758852021794 | 0.999950574162623 |
| ebi-fl187-GCST90094726 | ebi-gm430-GCST90011362 | OTU97_11 (Parabacteroides) abundance \|\| id:ebi-gm430-GCST90011362 | F-capsicum liking (derived food-liking factor) \|\| id:ebi-fl187-GCST90094726 | MR Egger | 18 | 0.0663065219317017 | 0.361343735829667 | 0.856711111973488 | -0.6419272 | 0.774540244157849 | 1.06855420227095 | 0.526277204508086 | 2.16959441414182 | 0.999950574162623 |
| ebi-fl187-GCST90094726 | ebi-gm430-GCST90011384 | OTU97_235 (Phascolarctobacterium) abundance \|\| id:ebi-gm430-GCST90011384 | F-capsicum liking (derived food-liking factor) \|\| id:ebi-fl187-GCST90094726 | MR Egger | 18 | 0.133787663378003 | 1.11637877613651 | 0.906101206708226 | -2.054314738 | 2.32189006460557 | 1.14315006124987 | 0.128180642831553 | 10.194925174879 | 0.999950574162623 |
| ebi-fl187-GCST90094726 | ebi-gm430-GCST90011390 | OTU97_3 (Bacteroides) abundance \|\| id:ebi-gm430-GCST90011390 | F-capsicum liking (derived food-liking factor) \|\| id:ebi-fl187-GCST90094726 | MR Egger | 18 | 0.346437282419341 | 0.31220254939675 | 0.283547494259859 | -0.265479714 | 0.958354279236971 | 1.41402080708526 | 0.766837998498066 | 2.60740188512593 | 0.999950574162623 |
| ebi-fl187-GCST90094726 | ebi-gm430-GCST90011398 | OTU97_4 (Alistipes) abundance \|\| id:ebi-gm430-GCST90011398 | F-capsicum liking (derived food-liking factor) \|\| id:ebi-fl187-GCST90094726 | MR Egger | 18 | -0.185395263 | 0.335877149314687 | 0.588594139731294 | -0.843714475 | 0.472923949850884 | 0.830775844155167 | 0.430109919824563 | 1.60467934223245 | 0.999950574162623 |
| ebi-fl187-GCST90094726 | ebi-gm430-GCST90011421 | OTU99_108 (Cytophagales) abundance \|\| id:ebi-gm430-GCST90011421 | F-capsicum liking (derived food-liking factor) \|\| id:ebi-fl187-GCST90094726 | MR Egger | 18 | -0.316108233 | 1.06420286741739 | 0.770259414513541 | -2.401945853 | 1.76972938700251 | 0.728980546047411 | 0.0905416010964148 | 5.86926484710268 | 0.999950574162623 |
| ebi-fl187-GCST90094726 | ebi-gm430-GCST90011428 | OTU99_123 (Phascolarctobacterium) abundance \|\| id:ebi-gm430-GCST90011428 | F-capsicum liking (derived food-liking factor) \|\| id:ebi-fl187-GCST90094726 | MR Egger | 19 | 0.218282192149925 | 0.614800623374499 | 0.726922151433774 | -0.98672703 | 1.42329141396394 | 1.24393804764687 | 0.372794842909334 | 4.15075985039802 | 0.999950574162623 |
| ebi-fl187-GCST90094726 | ebi-gm430-GCST90011450 | OTU99_298 (Phascolarctobacterium) abundance \|\| id:ebi-gm430-GCST90011450 | F-capsicum liking (derived food-liking factor) \|\| id:ebi-fl187-GCST90094726 | MR Egger | 18 | 0.132138575127189 | 1.11634982429055 | 0.907250460642136 | -2.05590708 | 2.32018423073666 | 1.14126645945461 | 0.127976697747602 | 10.1775491507435 | 0.999950574162623 |
| ebi-fl187-GCST90094726 | ebi-gm430-GCST90011451 | OTU99_3 (Bacteroides) abundance \|\| id:ebi-gm430-GCST90011451 | F-capsicum liking (derived food-liking factor) \|\| id:ebi-fl187-GCST90094726 | MR Egger | 18 | 0.337076147497835 | 0.311882496282665 | 0.29581733270377 | -0.274213545 | 0.948365840211858 | 1.40084573057451 | 0.760169727318425 | 2.58148764722754 | 0.999950574162623 |
| ebi-fl187-GCST90094726 | ebi-gm430-GCST90011472 | OTU99_72 (Parabacteroides) abundance \|\| id:ebi-gm430-GCST90011472 | F-capsicum liking (derived food-liking factor) \|\| id:ebi-fl187-GCST90094726 | MR Egger | 19 | 0.261501347948857 | 0.829175514508578 | 0.756315348570663 | -1.36368266 | 1.88668535638567 | 1.29887869239278 | 0.255717320732197 | 6.59746415581606 | 0.999950574162623 |
| ebi-fl187-GCST90094726 | ebi-gm430-GCST90011507 | TestASV_3 (Bacteroides) abundance \|\| id:ebi-gm430-GCST90011507 | F-capsicum liking (derived food-liking factor) \|\| id:ebi-fl187-GCST90094726 | MR Egger | 18 | 0.517655232470271 | 0.451969350317706 | 0.268914732464574 | -0.368204694 | 1.40351515909297 | 1.67808830610909 | 0.691975523847602 | 4.06947972298548 | 0.999950574162623 |
| ebi-fl187-GCST90094726 | ebi-gm430-GCST90011512 | TestASV_34 (Phascolarctobacterium) abundance \|\| id:ebi-gm430-GCST90011512 | F-capsicum liking (derived food-liking factor) \|\| id:ebi-fl187-GCST90094726 | MR Egger | 18 | -0.900586862 | 0.932453831412687 | 0.348504566324845 | -2.728196371 | 0.927022647942984 | 0.406331129607986 | 0.0653370272031184 | 2.52697427410077 | 0.999950574162623 |
| ebi-fl187-GCST90094726 | ebi-gm430-GCST90011519 | TestASV_41 (Barnesiella) abundance \|\| id:ebi-gm430-GCST90011519 | F-capsicum liking (derived food-liking factor) \|\| id:ebi-fl187-GCST90094726 | MR Egger | 18 | -1.755650702 | 1.30227459166257 | 0.196390257813218 | -4.308108901 | 0.796807498097277 | 0.172794767876794 | 0.0134589777995399 | 2.2184472142168 | 0.999950574162623 |
| ebi-fl187-GCST90094726 | ebi-gm430-GCST90011524 | TestASV_46 (Sutterella) abundance \|\| id:ebi-gm430-GCST90011524 | F-capsicum liking (derived food-liking factor) \|\| id:ebi-fl187-GCST90094726 | MR Egger | 18 | 1.42069949482276 | 1.36472771208086 | 0.313345287041984 | -1.254166821 | 4.09556581050125 | 4.14001534694855 | 0.285313466451257 | 60.0733196583895 | 0.999950574162623 |
| ebi-fl187-GCST90094726 | ebi-gm430-GCST90011581 | OTU97_19 (Bacteria) prevalence \|\| id:ebi-gm430-GCST90011581 | F-capsicum liking (derived food-liking factor) \|\| id:ebi-fl187-GCST90094726 | MR Egger | 18 | 0.510065601948799 | 1.38328259648407 | 0.71715848188547 | -2.201168287 | 3.22129949105758 | 1.66540044487706 | 0.110673784043027 | 25.0606651410637 | 0.999950574162623 |
| ebi-fl187-GCST90094726 | ebi-gm430-GCST90011614 | OTU97_80 (Ruminococcus) prevalence \|\| id:ebi-gm430-GCST90011614 | F-capsicum liking (derived food-liking factor) \|\| id:ebi-fl187-GCST90094726 | MR Egger | 18 | 0.359266904678768 | 0.821834954959182 | 0.667843861101174 | -1.251529607 | 1.97006341639877 | 1.43227903253486 | 0.286066892102048 | 7.17113124124546 | 0.999950574162623 |
| ebi-fl187-GCST90094726 | ebi-gm430-GCST90011623 | OTU99_11 (Parabacteroides) prevalence \|\| id:ebi-gm430-GCST90011623 | F-capsicum liking (derived food-liking factor) \|\| id:ebi-fl187-GCST90094726 | MR Egger | 18 | -0.093769168 | 0.603166841978419 | 0.878401970148614 | -1.275976179 | 1.08843784197302 | 0.910492908607279 | 0.279158326789988 | 2.96963141367373 | 0.999950574162623 |
| ebi-fl187-GCST90094726 | ebi-gm430-GCST90011642 | OTU99_19 (Bacteria) prevalence \|\| id:ebi-gm430-GCST90011642 | F-capsicum liking (derived food-liking factor) \|\| id:ebi-fl187-GCST90094726 | MR Egger | 18 | 0.510065601948799 | 1.38328259648407 | 0.71715848188547 | -2.201168287 | 3.22129949105758 | 1.66540044487706 | 0.110673784043027 | 25.0606651410637 | 0.999950574162623 |
| ebi-fl187-GCST90094726 | ebi-gm430-GCST90011653 | OTU99_30 (Parasutterella) prevalence \|\| id:ebi-gm430-GCST90011653 | F-capsicum liking (derived food-liking factor) \|\| id:ebi-fl187-GCST90094726 | MR Egger | 18 | -0.955563832 | 0.679614831726529 | 0.178836125447189 | -2.287608902 | 0.376481238179167 | 0.384595236305685 | 0.101508889587876 | 1.45714820041428 | 0.999950574162623 |
| ebi-fl187-GCST90094726 | ebi-gm430-GCST90011675 | OTU99_8 (Ruminococcaceae) prevalence \|\| id:ebi-gm430-GCST90011675 | F-capsicum liking (derived food-liking factor) \|\| id:ebi-fl187-GCST90094726 | MR Egger | 18 | -0.75620356 | 0.813562994562057 | 0.3664442232631 | -2.35078703 | 0.838379908912553 | 0.46944526883562 | 0.0952941333745168 | 2.31261728952435 | 0.999950574162623 |
| ebi-fl187-GCST90094726 | ebi-gm430-GCST90011679 | OTU99_92 (Ruminococcus) prevalence \|\| id:ebi-gm430-GCST90011679 | F-capsicum liking (derived food-liking factor) \|\| id:ebi-fl187-GCST90094726 | MR Egger | 18 | 0.939202957070243 | 0.872716069667268 | 0.297805425348039 | -0.771320539 | 2.64972645361809 | 2.5579418161727 | 0.462402044805002 | 14.1501673888232 | 0.999950574162623 |
| ebi-fl187-GCST90094726 | ebi-gm430-GCST90011684 | TestASV_11 (Lachnospiraceae) prevalence \|\| id:ebi-gm430-GCST90011684 | F-capsicum liking (derived food-liking factor) \|\| id:ebi-fl187-GCST90094726 | MR Egger | 18 | -0.415796737 | 0.712324691255145 | 0.567548568960879 | -1.811953131 | 0.980359658239578 | 0.659814372954152 | 0.163334810700629 | 2.66541470792059 | 0.999950574162623 |
| ebi-fl187-GCST90094726 | ebi-gm430-GCST90011688 | TestASV_15 (Bacteroides) prevalence \|\| id:ebi-gm430-GCST90011688 | F-capsicum liking (derived food-liking factor) \|\| id:ebi-fl187-GCST90094726 | MR Egger | 18 | -0.269325509 | 0.786496881954545 | 0.736479302184604 | -1.810859398 | 1.27220837932678 | 0.763894560387308 | 0.163513553192761 | 3.5687249649661 | 0.999950574162623 |
| ebi-fl187-GCST90094726 | ebi-gm430-GCST90011692 | TestASV_19 (Prevotella) prevalence \|\| id:ebi-gm430-GCST90011692 | F-capsicum liking (derived food-liking factor) \|\| id:ebi-fl187-GCST90094726 | MR Egger | 18 | 0.153311437597688 | 1.25415898158532 | 0.904229251785402 | -2.304840166 | 2.61146304150491 | 1.16568796147498 | 0.0997747467452108 | 13.6189613890742 | 0.999950574162623 |
| ebi-fl187-GCST90094726 | ebi-gm430-GCST90011693 | TestASV_2 (Bacteroides) prevalence \|\| id:ebi-gm430-GCST90011693 | F-capsicum liking (derived food-liking factor) \|\| id:ebi-fl187-GCST90094726 | MR Egger | 18 | -0.329153435 | 0.634137872912197 | 0.610826446553474 | -1.572063666 | 0.913756795999392 | 0.71953260685722 | 0.207616289324076 | 2.49367318 | 0.999950574162623 |
| ebi-fl187-GCST90094726 | ebi-gm430-GCST90011716 | TestASV_40 (Barnesiella) prevalence \|\| id:ebi-gm430-GCST90011716 | F-capsicum liking (derived food-liking factor) \|\| id:ebi-fl187-GCST90094726 | MR Egger | 18 | -0.092703836 | 1.33671570766436 | 0.945568848282947 | -2.712666623 | 2.52725895129247 | 0.911463403220113 | 0.0663596145097799 | 12.5191434812683 | 0.999950574162623 |
| ebi-fl187-GCST90094726 | ebi-gm430-GCST90011718 | TestASV_42 (Alphaproteobacteria) prevalence \|\| id:ebi-gm430-GCST90011718 | F-capsicum liking (derived food-liking factor) \|\| id:ebi-fl187-GCST90094726 | MR Egger | 18 | 0.777232779353483 | 1.01154447848934 | 0.453464227637822 | -1.205394398 | 2.75985995719259 | 2.17544399502936 | 0.29957382474799 | 15.7976304488233 | 0.999950574162623 |
| ebi-fl187-GCST90094726 | ebi-hbsm44-GCST90104929 | Sulcal depth of the right inferior frontal sulcus \|\| id:ebi-hbsm44-GCST90104929 | F-capsicum liking (derived food-liking factor) \|\| id:ebi-fl187-GCST90094726 | MR Egger | 18 | 0.400592626470036 | 0.457287550905635 | 0.393981665954731 | -0.495690973 | 1.29687622624508 | 1.49270905446649 | 0.609149855556366 | 3.65785249879302 | 0.999950574162623 |
| ebi-fl187-GCST90094726 | ebi-hbsm44-GCST90104935 | Sulcal opening distance of the left calloso-marginal posterior fissure \|\| id:ebi-hbsm44-GCST90104935 | F-capsicum liking (derived food-liking factor) \|\| id:ebi-fl187-GCST90094726 | MR Egger | 18 | 0.142363859856689 | 0.114642145663845 | 0.232202406986981 | -0.082334746 | 0.367062465357825 | 1.15299610121753 | 0.920963618531467 | 1.44348808429875 | 0.999950574162623 |
| ebi-fl187-GCST90094726 | ebi-hbsm44-GCST90104936 | Sulcal opening distance of the left anterior inferior temporal sulcus \|\| id:ebi-hbsm44-GCST90104936 | F-capsicum liking (derived food-liking factor) \|\| id:ebi-fl187-GCST90094726 | MR Egger | 20 | -0.063183125 | 0.0600441861695051 | 0.3065944628402 | -0.18086973 | 0.0545034795582704 | 0.938771545094666 | 0.834544067481807 | 1.05601615087706 | 0.999950574162623 |
| ebi-fl187-GCST90094726 | ebi-hbsm44-GCST90104941 | Sulcal opening distance of the right posterior inferior temporal sulcus \|\| id:ebi-hbsm44-GCST90104941 | F-capsicum liking (derived food-liking factor) \|\| id:ebi-fl187-GCST90094726 | MR Egger | 20 | 0.00158051719438759 | 0.04764858411094 | 0.973903849187694 | -0.091810708 | 0.0949717420518301 | 1.00158176686998 | 0.912277820402507 | 1.09962778146221 | 0.999950574162623 |
| ebi-fl187-GCST90094726 | ebi-IPs91-GCST90274819 | Leukemia inhibitory factor levels \|\| id:ebi-IPs91-GCST90274819 | F-capsicum liking (derived food-liking factor) \|\| id:ebi-fl187-GCST90094726 | MR Egger | 21 | -0.003526877 | 0.200958475071668 | 0.986180610766544 | -0.397405488 | 0.39035173382065 | 0.996479334806713 | 0.672061457209213 | 1.4775003893546 | 0.999950574162623 |
| ebi-fl187-GCST90094726 | ebi-IPs91-GCST90274837 | STAM binding protein levels \|\| id:ebi-IPs91-GCST90274837 | F-capsicum liking (derived food-liking factor) \|\| id:ebi-fl187-GCST90094726 | MR Egger | 21 | -0.032220006 | 0.172881716676885 | 0.854129897825249 | -0.371068171 | 0.306628158197411 | 0.968293527783749 | 0.689996902055204 | 1.35883560223997 | 0.999950574162623 |
| ebi-fl187-GCST90094726 | ebi-IPs91-GCST90274840 | TNF-beta levels \|\| id:ebi-IPs91-GCST90274840 | F-capsicum liking (derived food-liking factor) \|\| id:ebi-fl187-GCST90094726 | MR Egger | 21 | -0.113303177 | 0.193285760175392 | 0.564644090160326 | -0.492143267 | 0.265536913137753 | 0.89287991859494 | 0.611314778476974 | 1.3041309929008 | 0.999950574162623 |
| ebi-fl187-GCST90094726 | ebi-IPs91-GCST90274842 | Tumor necrosis factor ligand superfamily member 14 levels \|\| id:ebi-IPs91-GCST90274842 | F-capsicum liking (derived food-liking factor) \|\| id:ebi-fl187-GCST90094726 | MR Egger | 21 | 0.108143873037661 | 0.193445400623936 | 0.58266390438358 | -0.271009112 | 0.487296858260575 | 1.11420803835034 | 0.762609547333934 | 1.6279097961265 | 0.999950574162623 |
| ebi-fl187-GCST90094726 | ebi-met1400-GCST90199628 | 3-methylhistidine levels \|\| id:ebi-met1400-GCST90199628 | F-capsicum liking (derived food-liking factor) \|\| id:ebi-fl187-GCST90094726 | MR Egger | 18 | 0.606455574892718 | 0.235209863908698 | 0.020209029146544 | 0.145444241631671 | 1.06746690815377 | 1.83391967450029 | 1.15655324526246 | 2.90800392138973 | 0.999950574162623 |
| ebi-fl187-GCST90094726 | ebi-met1400-GCST90199651 | Docosahexaenoate DHA; 22:6n3 levels \|\| id:ebi-met1400-GCST90199651 | F-capsicum liking (derived food-liking factor) \|\| id:ebi-fl187-GCST90094726 | MR Egger | 18 | -0.369042239 | 0.276106036654396 | 0.20005146300979 | -0.910210071 | 0.172125592446316 | 0.691396205675185 | 0.40243967415226 | 1.1878270059457 | 0.999950574162623 |
| ebi-fl187-GCST90094726 | ebi-met1400-GCST90199652 | 1-stearoyl-2-oleoyl-GPS (18:0/18:1) levels \|\| id:ebi-met1400-GCST90199652 | F-capsicum liking (derived food-liking factor) \|\| id:ebi-fl187-GCST90094726 | MR Egger | 18 | -0.239113948 | 0.23890892226356 | 0.331792498257766 | -0.707375435 | 0.229147540132838 | 0.78732516352415 | 0.492936244333826 | 1.25752756110694 | 0.999950574162623 |
| ebi-fl187-GCST90094726 | ebi-met1400-GCST90199653 | 1-myristoyl-2-palmitoyl-gpc (14:0/16:0) levels \|\| id:ebi-met1400-GCST90199653 | F-capsicum liking (derived food-liking factor) \|\| id:ebi-fl187-GCST90094726 | MR Egger | 18 | -0.256051685 | 0.234264006542353 | 0.290575750687766 | -0.715209138 | 0.203105767875912 | 0.774101958339522 | 0.48908981394646 | 1.22520204841289 | 0.999950574162623 |
| ebi-fl187-GCST90094726 | ebi-met1400-GCST90199667 | Erythritol levels in elite athletes \|\| id:ebi-met1400-GCST90199667 | F-capsicum liking (derived food-liking factor) \|\| id:ebi-fl187-GCST90094726 | MR Egger | 18 | 0.21342865189769 | 0.330542264762402 | 0.527630964766483 | -0.434434187 | 0.861291490831998 | 1.23791517221882 | 0.647631001 | 2.36621466574021 | 0.999950574162623 |
| ebi-fl187-GCST90094726 | ebi-met1400-GCST90199674 | 3-hydroxymyristate levels \|\| id:ebi-met1400-GCST90199674 | F-capsicum liking (derived food-liking factor) \|\| id:ebi-fl187-GCST90094726 | MR Egger | 18 | -0.575626282 | 0.281254469108848 | 0.0574876451671709 | -1.126885041 | -0.024367522 | 0.562352567448302 | 0.32404106058271 | 0.975926969091552 | 0.999950574162623 |
| ebi-fl187-GCST90094726 | ebi-met1400-GCST90199685 | 2-linoleoylglycerol (18:2) levels \|\| id:ebi-met1400-GCST90199685 | F-capsicum liking (derived food-liking factor) \|\| id:ebi-fl187-GCST90094726 | MR Egger | 18 | -0.180020655 | 0.268513054407394 | 0.51214209998305 | -0.706306241 | 0.34626493212382 | 0.835252959488601 | 0.493463570659151 | 1.41377712118156 | 0.999950574162623 |
| ebi-fl187-GCST90094726 | ebi-met1400-GCST90199703 | Gamma-glutamylvaline levels \|\| id:ebi-met1400-GCST90199703 | F-capsicum liking (derived food-liking factor) \|\| id:ebi-fl187-GCST90094726 | MR Egger | 18 | 0.0478333580502595 | 0.2424481005733 | 0.846082658790609 | -0.427364919 | 0.523031635173927 | 1.04899583404754 | 0.652225499270663 | 1.68713468130207 | 0.999950574162623 |
| ebi-fl187-GCST90094726 | ebi-met1400-GCST90199779 | Gamma-glutamyl-2-aminobutyrate levels \|\| id:ebi-met1400-GCST90199779 | F-capsicum liking (derived food-liking factor) \|\| id:ebi-fl187-GCST90094726 | MR Egger | 18 | 0.390894255434801 | 0.244284459162168 | 0.129120373686623 | -0.087903285 | 0.869691795392651 | 1.47830218272619 | 0.915849449256389 | 2.38617530995667 | 0.999950574162623 |
| ebi-fl187-GCST90094726 | ebi-met1400-GCST90199824 | 4-vinylphenol sulfate levels \|\| id:ebi-met1400-GCST90199824 | F-capsicum liking (derived food-liking factor) \|\| id:ebi-fl187-GCST90094726 | MR Egger | 18 | 0.137009308019993 | 0.236123430204595 | 0.569832075865231 | -0.325792615 | 0.599811231220999 | 1.14683882326941 | 0.721954894392794 | 1.82177487371169 | 0.999950574162623 |
| ebi-fl187-GCST90094726 | ebi-met1400-GCST90199830 | 4-ethylphenylsulfate levels \|\| id:ebi-met1400-GCST90199830 | F-capsicum liking (derived food-liking factor) \|\| id:ebi-fl187-GCST90094726 | MR Egger | 18 | -0.026387743 | 0.236377483978957 | 0.912502144737606 | -0.489687612 | 0.436912125129638 | 0.973957370774759 | 0.612817801171921 | 1.54792004780612 | 0.999950574162623 |
| ebi-fl187-GCST90094726 | ebi-met1400-GCST90199833 | Hexanoylglutamine levels \|\| id:ebi-met1400-GCST90199833 | F-capsicum liking (derived food-liking factor) \|\| id:ebi-fl187-GCST90094726 | MR Egger | 18 | -0.322327319 | 0.251106796795816 | 0.217552210936137 | -0.814496641 | 0.169842002908715 | 0.724461021796828 | 0.442862190143523 | 1.18511759139522 | 0.999950574162623 |
| ebi-fl187-GCST90094726 | ebi-met1400-GCST90199837 | Carotenoid levels (cryptoxanthin) \|\| id:ebi-met1400-GCST90199837 | F-capsicum liking (derived food-liking factor) \|\| id:ebi-fl187-GCST90094726 | MR Egger | 18 | 0.39862019040593 | 0.250179910466154 | 0.130646192079316 | -0.091732434 | 0.888972814919592 | 1.48976768308137 | 0.912349230425674 | 2.4326296066673 | 0.999950574162623 |
| ebi-fl187-GCST90094726 | ebi-met1400-GCST90199869 | 2-hydroxyhippurate levels \|\| id:ebi-met1400-GCST90199869 | F-capsicum liking (derived food-liking factor) \|\| id:ebi-fl187-GCST90094726 | MR Egger | 18 | 0.437463415001168 | 0.265202109576805 | 0.118526850882614 | -0.08233272 | 0.957259549771706 | 1.54877363571598 | 0.920965484290598 | 2.60454904728223 | 0.999950574162623 |
| ebi-fl187-GCST90094726 | ebi-met1400-GCST90199902 | Hydantoin-5-propionate levels \|\| id:ebi-met1400-GCST90199902 | F-capsicum liking (derived food-liking factor) \|\| id:ebi-fl187-GCST90094726 | MR Egger | 18 | 0.0920643119919698 | 0.235604847101761 | 0.701130517300115 | -0.369721188 | 0.553849812311421 | 1.0964353337538 | 0.690926942281286 | 1.73993857749201 | 0.999950574162623 |
| ebi-fl187-GCST90094726 | ebi-met1400-GCST90199904 | Pantoate levels \|\| id:ebi-met1400-GCST90199904 | F-capsicum liking (derived food-liking factor) \|\| id:ebi-fl187-GCST90094726 | MR Egger | 18 | 0.332731417664453 | 0.276770217817548 | 0.246779501270595 | -0.209738209 | 0.875201044586848 | 1.39477263686594 | 0.810796477200313 | 2.3993576233427 | 0.999950574162623 |
| ebi-fl187-GCST90094726 | ebi-met1400-GCST90199936 | 3-methyl catechol sulfate (2) levels \|\| id:ebi-met1400-GCST90199936 | F-capsicum liking (derived food-liking factor) \|\| id:ebi-fl187-GCST90094726 | MR Egger | 18 | 0.503542427220835 | 0.269213406415016 | 0.0798327931660795 | -0.024115849 | 1.03120070379427 | 1.65457210276401 | 0.976172614241633 | 2.8044311050169 | 0.999950574162623 |
| ebi-fl187-GCST90094726 | ebi-met1400-GCST90199977 | 4-hydroxychlorothalonil levels \|\| id:ebi-met1400-GCST90199977 | F-capsicum liking (derived food-liking factor) \|\| id:ebi-fl187-GCST90094726 | MR Egger | 18 | -0.243608624 | 0.23423268092583 | 0.313788699645503 | -0.702704679 | 0.21548743030668 | 0.783794332291099 | 0.495244014682836 | 1.24046639054302 | 0.999950574162623 |
| ebi-fl187-GCST90094726 | ebi-met1400-GCST90200040 | Sphingomyelin (d18:2/24:1, d18:1/24:2) levels \|\| id:ebi-met1400-GCST90200040 | F-capsicum liking (derived food-liking factor) \|\| id:ebi-fl187-GCST90094726 | MR Egger | 18 | -0.026706284 | 0.215219145618326 | 0.902790526829522 | -0.448535809 | 0.395123241671735 | 0.973647175537326 | 0.638562444750547 | 1.48456714018336 | 0.999950574162623 |
| ebi-fl187-GCST90094726 | ebi-met1400-GCST90200077 | 1-stearoyl-2-oleoyl-GPI (18:0/18:1) levels \|\| id:ebi-met1400-GCST90200077 | F-capsicum liking (derived food-liking factor) \|\| id:ebi-fl187-GCST90094726 | MR Egger | 18 | -0.294714218 | 0.233494652951014 | 0.224969747394271 | -0.752363737 | 0.16293530209566 | 0.744744391830502 | 0.471251320796659 | 1.17696054034469 | 0.999950574162623 |
| ebi-fl187-GCST90094726 | ebi-met1400-GCST90200080 | 1-palmitoyl-2-oleoyl-GPI (16:0/18:1) levels \|\| id:ebi-met1400-GCST90200080 | F-capsicum liking (derived food-liking factor) \|\| id:ebi-fl187-GCST90094726 | MR Egger | 18 | -0.165655838 | 0.248147041987989 | 0.513921627041038 | -0.65202404 | 0.320712364215806 | 0.847337805418113 | 0.520990203651816 | 1.37810913037939 | 0.999950574162623 |
| ebi-fl187-GCST90094726 | ebi-met1400-GCST90200082 | 1-oleoyl-2-linoleoyl-GPE (18:1/18:2) levels \|\| id:ebi-met1400-GCST90200082 | F-capsicum liking (derived food-liking factor) \|\| id:ebi-fl187-GCST90094726 | MR Egger | 18 | -0.104101214 | 0.257457899369985 | 0.691314345057999 | -0.608718697 | 0.40051626850856 | 0.901134085188755 | 0.54404751237152 | 1.49259507859756 | 0.999950574162623 |
| ebi-fl187-GCST90094726 | ebi-met1400-GCST90200100 | Perfluorooctanesulfonate (PFOS) levels \|\| id:ebi-met1400-GCST90200100 | F-capsicum liking (derived food-liking factor) \|\| id:ebi-fl187-GCST90094726 | MR Egger | 18 | -0.419049909 | 0.294309736919229 | 0.17370246876019 | -0.995896993 | 0.157797175860011 | 0.657671371055994 | 0.369391953947459 | 1.17092867801932 | 0.999950574162623 |
| ebi-fl187-GCST90094726 | ebi-met1400-GCST90200102 | 1-palmitoleoyl-2-linolenoyl-GPC (16:1/18:3) levels \|\| id:ebi-met1400-GCST90200102 | F-capsicum liking (derived food-liking factor) \|\| id:ebi-fl187-GCST90094726 | MR Egger | 18 | -0.433342311 | 0.231175137180004 | 0.0792352858373038 | -0.88644558 | 0.0197609581372368 | 0.648338520579698 | 0.41211799310284 | 1.01995749834339 | 0.999950574162623 |
| ebi-fl187-GCST90094726 | ebi-met1400-GCST90200114 | Glycosyl-N-tricosanoyl-sphingadienine (d18:2/23:0) levels \|\| id:ebi-met1400-GCST90200114 | F-capsicum liking (derived food-liking factor) \|\| id:ebi-fl187-GCST90094726 | MR Egger | 18 | -0.282681725 | 0.247201359082899 | 0.26964091656031 | -0.767196389 | 0.201832938895403 | 0.753759652609679 | 0.464312998374988 | 1.22364356778875 | 0.999950574162623 |
| ebi-fl187-GCST90094726 | ebi-met1400-GCST90200135 | Arachidoylcarnitine (C20) levels \|\| id:ebi-met1400-GCST90200135 | F-capsicum liking (derived food-liking factor) \|\| id:ebi-fl187-GCST90094726 | MR Egger | 18 | -0.479548329 | 0.241614621036143 | 0.06459117499295 | -0.953112986 | -0.005983672 | 0.61906294132481 | 0.385538975846929 | 0.994034194545047 | 0.999950574162623 |
| ebi-fl187-GCST90094726 | ebi-met1400-GCST90200146 | Docosahexaenoylcarnitine (C22:6) levels \|\| id:ebi-met1400-GCST90200146 | F-capsicum liking (derived food-liking factor) \|\| id:ebi-fl187-GCST90094726 | MR Egger | 18 | 0.18887712924457 | 0.305348928580785 | 0.544911420671958 | -0.409606771 | 0.787361029262909 | 1.20789252869714 | 0.663911268127256 | 2.19758939323004 | 0.999950574162623 |
| ebi-fl187-GCST90094726 | ebi-met1400-GCST90200162 | 3-hydroxyphenylacetoylglutamine levels \|\| id:ebi-met1400-GCST90200162 | F-capsicum liking (derived food-liking factor) \|\| id:ebi-fl187-GCST90094726 | MR Egger | 18 | 0.412963184915281 | 0.320236885917582 | 0.215535180615852 | -0.214701111 | 1.04062748131374 | 1.51128938666301 | 0.806782542181652 | 2.83099285225487 | 0.999950574162623 |
| ebi-fl187-GCST90094726 | ebi-met1400-GCST90200172 | 3-carboxy-4-methyl-5-pentyl-2-furanpropionate (3-CMPFP) levels \|\| id:ebi-met1400-GCST90200172 | F-capsicum liking (derived food-liking factor) \|\| id:ebi-fl187-GCST90094726 | MR Egger | 18 | 0.308456307287568 | 0.248293805875747 | 0.232025374422266 | -0.178199552 | 0.795112166804032 | 1.36132202839624 | 0.836775426425575 | 2.2146893975043 | 0.999950574162623 |
| ebi-fl187-GCST90094726 | ebi-met1400-GCST90200222 | Dihydrocaffeate sulfate (2) levels \|\| id:ebi-met1400-GCST90200222 | F-capsicum liking (derived food-liking factor) \|\| id:ebi-fl187-GCST90094726 | MR Egger | 18 | 0.109101494994438 | 0.267671706565055 | 0.68897310576434 | -0.41553505 | 0.633738039861946 | 1.11527553948196 | 0.659987060225321 | 1.88464229668704 | 0.999950574162623 |
| ebi-fl187-GCST90094726 | ebi-met1400-GCST90200223 | 4-methylhexanoylglutamine levels \|\| id:ebi-met1400-GCST90200223 | F-capsicum liking (derived food-liking factor) \|\| id:ebi-fl187-GCST90094726 | MR Egger | 18 | -0.285978269 | 0.330442004116774 | 0.399587408128305 | -0.933644597 | 0.361688058868056 | 0.751278941661309 | 0.393118338261517 | 1.43575100230573 | 0.999950574162623 |
| ebi-fl187-GCST90094726 | ebi-met1400-GCST90200224 | 2-ketocaprylate levels \|\| id:ebi-met1400-GCST90200224 | F-capsicum liking (derived food-liking factor) \|\| id:ebi-fl187-GCST90094726 | MR Egger | 18 | 0.0822783592050841 | 0.267632668298352 | 0.762481445169068 | -0.442281671 | 0.606838389069855 | 1.08575799850248 | 0.642568617236657 | 1.834621859 | 0.999950574162623 |
| ebi-fl187-GCST90094726 | ebi-met1400-GCST90200243 | Hydroxypalmitoyl sphingomyelin (d18:1/16:0(OH)) levels \|\| id:ebi-met1400-GCST90200243 | F-capsicum liking (derived food-liking factor) \|\| id:ebi-fl187-GCST90094726 | MR Egger | 18 | -0.368116633 | 0.230616330877902 | 0.129997087783207 | -0.820124641 | 0.0838913756642446 | 0.692036462792166 | 0.440376761919064 | 1.08751075725906 | 0.999950574162623 |
| ebi-fl187-GCST90094726 | ebi-met1400-GCST90200273 | 2-methoxyhydroquinone sulfate (2) levels \|\| id:ebi-met1400-GCST90200273 | F-capsicum liking (derived food-liking factor) \|\| id:ebi-fl187-GCST90094726 | MR Egger | 18 | 0.206751528845945 | 0.378788081935645 | 0.592716485494275 | -0.535673112 | 0.949176169439809 | 1.22967699455681 | 0.58527520191279 | 2.58358034989424 | 0.999950574162623 |
| ebi-fl187-GCST90094726 | ebi-met1400-GCST90200276 | 2,4-di-tert-butylphenol levels \|\| id:ebi-met1400-GCST90200276 | F-capsicum liking (derived food-liking factor) \|\| id:ebi-fl187-GCST90094726 | MR Egger | 18 | -0.240734255 | 0.234796815239279 | 0.320479787273608 | -0.700936013 | 0.219467502775227 | 0.786050487548336 | 0.496120710976321 | 1.24541337481972 | 0.999950574162623 |
| ebi-fl187-GCST90094726 | ebi-met1400-GCST90200289 | N-acetylmethionine levels \|\| id:ebi-met1400-GCST90200289 | F-capsicum liking (derived food-liking factor) \|\| id:ebi-fl187-GCST90094726 | MR Egger | 18 | 0.300641007075418 | 0.271870703139419 | 0.285153725122813 | -0.232225571 | 0.833507585228679 | 1.35072435400353 | 0.792767277766928 | 2.30137687523808 | 0.999950574162623 |
| ebi-fl187-GCST90094726 | ebi-met1400-GCST90200308 | 3-hydroxyisobutyrate levels \|\| id:ebi-met1400-GCST90200308 | F-capsicum liking (derived food-liking factor) \|\| id:ebi-fl187-GCST90094726 | MR Egger | 18 | -0.548467334 | 0.238274705193866 | 0.0351190659987868 | -1.015485756 | -0.081448912 | 0.57783475966854 | 0.362226433322416 | 0.921779800603351 | 0.999950574162623 |
| ebi-fl187-GCST90094726 | ebi-met1400-GCST90200383 | Cystathionine levels \|\| id:ebi-met1400-GCST90200383 | F-capsicum liking (derived food-liking factor) \|\| id:ebi-fl187-GCST90094726 | MR Egger | 18 | 0.0393809974650233 | 0.23526833871812 | 0.869162443843047 | -0.421744946 | 0.500506941352538 | 1.04016670904428 | 0.65590130803641 | 1.64955728757891 | 0.999950574162623 |
| ebi-fl187-GCST90094726 | ebi-met1400-GCST90200384 | Guanidinoacetate levels \|\| id:ebi-met1400-GCST90200384 | F-capsicum liking (derived food-liking factor) \|\| id:ebi-fl187-GCST90094726 | MR Egger | 18 | 0.315597230170634 | 0.258634193878854 | 0.240052263922509 | -0.19132579 | 0.822520250173187 | 1.37107791558245 | 0.825863486391846 | 2.27622928192516 | 0.999950574162623 |
| ebi-fl187-GCST90094726 | ebi-met1400-GCST90200403 | Citrulline levels \|\| id:ebi-met1400-GCST90200403 | F-capsicum liking (derived food-liking factor) \|\| id:ebi-fl187-GCST90094726 | MR Egger | 18 | 0.236632105633016 | 0.236333614987119 | 0.331602750395846 | -0.22658178 | 0.699845991007769 | 1.26697491908876 | 0.797254140409225 | 2.01344259532604 | 0.999950574162623 |
| ebi-fl187-GCST90094726 | ebi-met1400-GCST90200434 | 12,13-DiHOME levels \|\| id:ebi-met1400-GCST90200434 | F-capsicum liking (derived food-liking factor) \|\| id:ebi-fl187-GCST90094726 | MR Egger | 18 | 0.316138161568545 | 0.237963293618373 | 0.20264587335735 | -0.150269894 | 0.782546217060557 | 1.37181977530549 | 0.86047570791768 | 2.18703384488716 | 0.999950574162623 |
| ebi-fl187-GCST90094726 | ebi-met1400-GCST90200437 | Fructose levels \|\| id:ebi-met1400-GCST90200437 | F-capsicum liking (derived food-liking factor) \|\| id:ebi-fl187-GCST90094726 | MR Egger | 18 | -0.093796638 | 0.237442632337895 | 0.698038241699809 | -0.559184197 | 0.371590921373469 | 0.910467897979995 | 0.571675247824539 | 1.45003967970734 | 0.999950574162623 |
| ebi-fl187-GCST90094726 | ebi-met1400-GCST90200448 | N-stearoyl-sphinganine (d18:0/18:0) levels \|\| id:ebi-met1400-GCST90200448 | F-capsicum liking (derived food-liking factor) \|\| id:ebi-fl187-GCST90094726 | MR Egger | 18 | -0.460657288 | 0.261931507515734 | 0.097737166705178 | -0.974043042 | 0.0527284671422888 | 0.630868846937789 | 0.377553483448416 | 1.05414337169264 | 0.999950574162623 |
| ebi-fl187-GCST90094726 | ebi-met1400-GCST90200468 | X-11847 levels \|\| id:ebi-met1400-GCST90200468 | F-capsicum liking (derived food-liking factor) \|\| id:ebi-fl187-GCST90094726 | MR Egger | 18 | 0.0572741607887141 | 0.249600444949763 | 0.821416376915116 | -0.431942711 | 0.546491032890251 | 1.05894609209757 | 0.649246570103412 | 1.72718174820715 | 0.999950574162623 |
| ebi-fl187-GCST90094726 | ebi-met1400-GCST90200476 | X-11843 levels \|\| id:ebi-met1400-GCST90200476 | F-capsicum liking (derived food-liking factor) \|\| id:ebi-fl187-GCST90094726 | MR Egger | 18 | -0.159226645 | 0.249527434603355 | 0.53242772394753 | -0.648300416 | 0.329847127155033 | 0.852803053799559 | 0.522933791568506 | 1.39075550346143 | 0.999950574162623 |
| ebi-fl187-GCST90094726 | ebi-met1400-GCST90200511 | X-12906 levels \|\| id:ebi-met1400-GCST90200511 | F-capsicum liking (derived food-liking factor) \|\| id:ebi-fl187-GCST90094726 | MR Egger | 18 | 0.5462721459757 | 0.23627559982968 | 0.034422250719268 | 0.0831719703095267 | 1.00937232164187 | 1.72680373209632 | 1.08672867754687 | 2.74387820142271 | 0.999950574162623 |
| ebi-fl187-GCST90094726 | ebi-met1400-GCST90200515 | X-12849 levels \|\| id:ebi-met1400-GCST90200515 | F-capsicum liking (derived food-liking factor) \|\| id:ebi-fl187-GCST90094726 | MR Egger | 18 | -0.17308855 | 0.370981134082647 | 0.647097456973577 | -0.900211573 | 0.554034472746388 | 0.841063135359692 | 0.406483649734892 | 1.74025990497387 | 0.999950574162623 |
| ebi-fl187-GCST90094726 | ebi-met1400-GCST90200551 | X-18886 levels \|\| id:ebi-met1400-GCST90200551 | F-capsicum liking (derived food-liking factor) \|\| id:ebi-fl187-GCST90094726 | MR Egger | 18 | -0.559502695 | 0.232665052479624 | 0.0286467748557011 | -1.015526198 | -0.103479192 | 0.571493199819612 | 0.362211784740466 | 0.901694785204407 | 0.999950574162623 |
| ebi-fl187-GCST90094726 | ebi-met1400-GCST90200564 | X-21319 levels \|\| id:ebi-met1400-GCST90200564 | F-capsicum liking (derived food-liking factor) \|\| id:ebi-fl187-GCST90094726 | MR Egger | 18 | -0.322638206 | 0.28981915865922 | 0.28205122400994 | -0.890683757 | 0.245407344990077 | 0.72423583116558 | 0.410375059999421 | 1.27814185185824 | 0.999950574162623 |
| ebi-fl187-GCST90094726 | ebi-met1400-GCST90200579 | X-21442 levels \|\| id:ebi-met1400-GCST90200579 | F-capsicum liking (derived food-liking factor) \|\| id:ebi-fl187-GCST90094726 | MR Egger | 18 | 0.399875393373559 | 0.311825907811667 | 0.217982425189722 | -0.211303386 | 1.01105417268443 | 1.49163881797961 | 0.809528430080248 | 2.7484968787113 | 0.999950574162623 |
| ebi-fl187-GCST90094726 | ebi-met1400-GCST90200581 | X-21441 levels \|\| id:ebi-met1400-GCST90200581 | F-capsicum liking (derived food-liking factor) \|\| id:ebi-fl187-GCST90094726 | MR Egger | 18 | -0.110495671 | 0.23609230426691 | 0.646082926794935 | -0.573236587 | 0.352245245639389 | 0.895390206572646 | 0.563698025253964 | 1.42225728334777 | 0.999950574162623 |
| ebi-fl187-GCST90094726 | ebi-met1400-GCST90200648 | X-24585 levels \|\| id:ebi-met1400-GCST90200648 | F-capsicum liking (derived food-liking factor) \|\| id:ebi-fl187-GCST90094726 | MR Egger | 18 | -0.152025117 | 0.278796614018671 | 0.593074686726756 | -0.698466481 | 0.394416246335476 | 0.85896670568221 | 0.497347411183608 | 1.48351792907626 | 0.999950574162623 |
| ebi-fl187-GCST90094726 | ebi-met1400-GCST90200720 | Adenosine 5'-diphosphate (ADP) to pantothenate ratio \|\| id:ebi-met1400-GCST90200720 | F-capsicum liking (derived food-liking factor) \|\| id:ebi-fl187-GCST90094726 | MR Egger | 18 | -0.568608133 | 0.369492417995793 | 0.143372293593734 | -1.292813272 | 0.155597006193996 | 0.566313122899977 | 0.274497459701749 | 1.16835526826801 | 0.999950574162623 |
| ebi-fl187-GCST90094726 | ebi-met1400-GCST90200724 | Cholate to taurocholate ratio \|\| id:ebi-met1400-GCST90200724 | F-capsicum liking (derived food-liking factor) \|\| id:ebi-fl187-GCST90094726 | MR Egger | 18 | -0.141527068 | 0.321186319517394 | 0.665370101223175 | -0.771052255 | 0.487998117979156 | 0.868031679146744 | 0.462526116956413 | 1.62905178406028 | 0.999950574162623 |
| ebi-fl187-GCST90094726 | ebi-met1400-GCST90200726 | N-acetylputrescine to (N(1) + N(8))-acetylspermidine ratio \|\| id:ebi-met1400-GCST90200726 | F-capsicum liking (derived food-liking factor) \|\| id:ebi-fl187-GCST90094726 | MR Egger | 18 | -0.043694034 | 0.234928696151869 | 0.854791317183981 | -0.504154279 | 0.416766210091618 | 0.957246797294486 | 0.604016188760008 | 1.51704780100626 | 0.999950574162623 |
| ebi-fl187-GCST90094726 | ebi-met1400-GCST90200744 | Arginine to glutamate ratio \|\| id:ebi-met1400-GCST90200744 | F-capsicum liking (derived food-liking factor) \|\| id:ebi-fl187-GCST90094726 | MR Egger | 18 | 0.282930551793477 | 0.227478497869896 | 0.231499863439605 | -0.162927304 | 0.728788407618474 | 1.32701299994408 | 0.849652952513818 | 2.0725679782675 | 0.999950574162623 |
| ebi-fl187-GCST90094726 | ebi-met1400-GCST90200759 | Histidine to trans-urocanate ratio \|\| id:ebi-met1400-GCST90200759 | F-capsicum liking (derived food-liking factor) \|\| id:ebi-fl187-GCST90094726 | MR Egger | 18 | -0.243107744 | 0.273970372077337 | 0.388034514450166 | -0.780089673 | 0.293874185229034 | 0.784187017740262 | 0.45836490636201 | 1.34161509805179 | 0.999950574162623 |
| ebi-fl187-GCST90094726 | ebi-met1400-GCST90200795 | Oleoyl-linoleoyl-glycerol (18:1 to 18:2) [2] to linoleoyl-arachidonoyl-glycerol (18:2 to 20:4) [2] ratio \|\| id:ebi-met1400-GCST90200795 | F-capsicum liking (derived food-liking factor) \|\| id:ebi-fl187-GCST90094726 | MR Egger | 18 | -0.791608647 | 0.244882342515061 | 0.0052081301087137 | -1.271578038 | -0.311639255 | 0.453115306229793 | 0.280388808265443 | 0.732245634231413 | 0.999950574162623 |
| ebi-fl187-GCST90094726 | ebi-met1400-GCST90200811 | Choline to taurocholate ratio \|\| id:ebi-met1400-GCST90200811 | F-capsicum liking (derived food-liking factor) \|\| id:ebi-fl187-GCST90094726 | MR Egger | 18 | 0.0339522187853228 | 0.265403421409983 | 0.899800973715762 | -0.486238487 | 0.554142924748889 | 1.0345351741999 | 0.614935135679261 | 1.74044864988013 | 0.999950574162623 |
| ebi-fl187-GCST90094726 | ebi-met1400-GCST90200831 | Cholate to phosphate ratio \|\| id:ebi-met1400-GCST90200831 | F-capsicum liking (derived food-liking factor) \|\| id:ebi-fl187-GCST90094726 | MR Egger | 18 | -0.282902865 | 0.245996258107646 | 0.267031508252869 | -0.76505553 | 0.199249801367976 | 0.75359298491869 | 0.465308091505925 | 1.22048680709742 | 0.999950574162623 |
| ebi-fl187-GCST90094726 | ebi-met1400-GCST90200841 | Adenosine 5'-diphosphate (ADP) to oxalate (ethanedioate) ratio \|\| id:ebi-met1400-GCST90200841 | F-capsicum liking (derived food-liking factor) \|\| id:ebi-fl187-GCST90094726 | MR Egger | 18 | -0.427285336 | 0.326750315925933 | 0.209460112498634 | -1.067715955 | 0.213145283549604 | 0.652277407664296 | 0.343792859744665 | 1.23756443593752 | 0.999950574162623 |
| ebi-fl187-GCST90094726 | ebi-met1400-GCST90200844 | Aspartate to phosphate ratio \|\| id:ebi-met1400-GCST90200844 | F-capsicum liking (derived food-liking factor) \|\| id:ebi-fl187-GCST90094726 | MR Egger | 18 | -0.393423823 | 0.221822215753005 | 0.0951605985058468 | -0.828195366 | 0.0413477201771715 | 0.674742715698304 | 0.436836906344149 | 1.04221444153632 | 0.999950574162623 |
| ebi-fl187-GCST90094726 | ebi-met1400-GCST90200870 | Adenosine 5'-monophosphate (AMP) to acetoacetate ratio \|\| id:ebi-met1400-GCST90200870 | F-capsicum liking (derived food-liking factor) \|\| id:ebi-fl187-GCST90094726 | MR Egger | 18 | -0.090223811 | 0.261390165414208 | 0.734463206941013 | -0.602548535 | 0.422100912994497 | 0.913726660108445 | 0.547414750957184 | 1.52516242562531 | 0.999950574162623 |
| ebi-fl187-GCST90094726 | ebi-met1400-GCST90200887 | Cholesterol to cortisol ratio \|\| id:ebi-met1400-GCST90200887 | F-capsicum liking (derived food-liking factor) \|\| id:ebi-fl187-GCST90094726 | MR Egger | 18 | -0.38651417 | 0.229461635403529 | 0.111500177670966 | -0.836258975 | 0.0632306354362426 | 0.679421097940264 | 0.433328587863163 | 1.06527250048899 | 0.999950574162623 |
| ebi-fl187-GCST90094726 | ebi-met1400-GCST90200890 | Cortisol to taurocholate ratio \|\| id:ebi-met1400-GCST90200890 | F-capsicum liking (derived food-liking factor) \|\| id:ebi-fl187-GCST90094726 | MR Egger | 18 | -0.085490951 | 0.246016116969947 | 0.732743816535382 | -0.567682541 | 0.396700637794341 | 0.918061450126128 | 0.566837540655277 | 1.48691073853957 | 0.999950574162623 |
| ebi-fl187-GCST90094726 | ebi-met1400-GCST90200948 | Adenosine 5'-diphosphate (ADP) to mannitol to sorbitol ratio \|\| id:ebi-met1400-GCST90200948 | F-capsicum liking (derived food-liking factor) \|\| id:ebi-fl187-GCST90094726 | MR Egger | 18 | -0.043036718 | 0.483699016078982 | 0.930206631010055 | -0.99108679 | 0.90501335307635 | 0.957876217702352 | 0.371173084564825 | 2.47196493117355 | 0.999950574162623 |
| ebi-fl187-GCST90094726 | ebi-met1400-GCST90200972 | Leucine to N-palmitoyl-sphingosine (d18:1 to 16:0) ratio \|\| id:ebi-met1400-GCST90200972 | F-capsicum liking (derived food-liking factor) \|\| id:ebi-fl187-GCST90094726 | MR Egger | 18 | 0.357629284796783 | 0.288664109564106 | 0.23324603018547 | -0.20815237 | 0.92341093954243 | 1.42993542340884 | 0.812083290195152 | 2.5178640415419 | 0.999950574162623 |
| ebi-fl187-GCST90094726 | ebi-met1400-GCST90200973 | Proline to glutamate ratio \|\| id:ebi-met1400-GCST90200973 | F-capsicum liking (derived food-liking factor) \|\| id:ebi-fl187-GCST90094726 | MR Egger | 18 | 0.213363907112579 | 0.231321042426648 | 0.370035358185645 | -0.240025336 | 0.666753150268809 | 1.23783502626155 | 0.7866079312812 | 1.94790249539473 | 0.999950574162623 |
| ebi-fl187-GCST90094726 | ebi-met1400-GCST90200983 | Cholesterol to linoleoyl-arachidonoyl-glycerol (18:2 to 20:4) [1] ratio \|\| id:ebi-met1400-GCST90200983 | F-capsicum liking (derived food-liking factor) \|\| id:ebi-fl187-GCST90094726 | MR Egger | 18 | -0.286269308 | 0.242777178863543 | 0.255578326960921 | -0.762112579 | 0.189573962161653 | 0.751060321846598 | 0.46667948760573 | 1.20873452130144 | 0.999950574162623 |
| ebi-fl187-GCST90094726 | ebi-met1400-GCST90201001 | Serine to alpha-ketobutyrate ratio \|\| id:ebi-met1400-GCST90201001 | F-capsicum liking (derived food-liking factor) \|\| id:ebi-fl187-GCST90094726 | MR Egger | 18 | 0.478712726278392 | 0.240191080871952 | 0.0635961068505408 | 0.00793820776936577 | 0.949487244787418 | 1.61399541048557 | 1.00796979887759 | 2.5843841630664 | 0.999950574162623 |
| ebi-fl187-GCST90094726 | ebi-met1400-GCST90201005 | Fructose to maltose ratio \|\| id:ebi-met1400-GCST90201005 | F-capsicum liking (derived food-liking factor) \|\| id:ebi-fl187-GCST90094726 | MR Egger | 18 | 0.196974768844731 | 0.268223570431084 | 0.473350513736592 | -0.328743429 | 0.722692966889656 | 1.21771331600455 | 0.71982767982002 | 2.05997318739612 | 0.999950574162623 |
| ebi-fl187-GCST90094726 | ebi-met1400-GCST90201020 | Paraxanthine to 5-acetylamino-6-formylamino-3-methyluracil ratio \|\| id:ebi-met1400-GCST90201020 | F-capsicum liking (derived food-liking factor) \|\| id:ebi-fl187-GCST90094726 | MR Egger | 18 | 0.316417902074623 | 0.249099888218343 | 0.222159211064231 | -0.171817879 | 0.804653682982575 | 1.37220358254439 | 0.842132529362111 | 2.23592202687378 | 0.999950574162623 |
| ebi-fl187-GCST90094726 | ebi-pw11-GCST90275197 | Placental weight (fetal genotype, adjusted for sex) \|\| id:ebi-pw11-GCST90275197 | F-capsicum liking (derived food-liking factor) \|\| id:ebi-fl187-GCST90094726 | MR Egger | 20 | 0.00695325709639463 | 0.0950555701271463 | 0.942493972344503 | -0.17935566 | 0.193262174545601 | 1.00697748711515 | 0.835808582552909 | 1.21320082220207 | 0.999950574162623 |
| ebi-fl187-GCST90094726 | prot-a-1017 | FAS-associated factor 2 \|\| id:prot-a-1017 | F-capsicum liking (derived food-liking factor) \|\| id:ebi-fl187-GCST90094726 | MR Egger | 21 | -0.508785065 | 0.40885501063824 | 0.228479768686623 | -1.310140886 | 0.292570755968255 | 0.601225585344242 | 0.269782045265766 | 1.33986753683491 | 0.999950574162623 |
| ebi-fl187-GCST90094726 | prot-a-1019 | Fas apoptotic inhibitory molecule 3 \|\| id:prot-a-1019 | F-capsicum liking (derived food-liking factor) \|\| id:ebi-fl187-GCST90094726 | MR Egger | 21 | 0.390040892477116 | 0.352739589543465 | 0.282641054877524 | -0.301328703 | 1.08141048798231 | 1.47704119252087 | 0.739834546918518 | 2.94883591674688 | 0.999950574162623 |
| ebi-fl187-GCST90094726 | prot-a-1052 | MIP18 family protein FAM96A \|\| id:prot-a-1052 | F-capsicum liking (derived food-liking factor) \|\| id:ebi-fl187-GCST90094726 | MR Egger | 21 | 0.193110685382583 | 0.352694831195281 | 0.590389843331988 | -0.498171184 | 0.884392554525335 | 1.21301704934509 | 0.607640907743445 | 2.42151300751977 | 0.999950574162623 |
| ebi-fl187-GCST90094726 | prot-a-1066 | F-box/LRR-repeat protein 4 \|\| id:prot-a-1066 | F-capsicum liking (derived food-liking factor) \|\| id:ebi-fl187-GCST90094726 | MR Egger | 21 | -0.704109517 | 0.352719898825255 | 0.0604430212862639 | -1.395440519 | -0.012778515 | 0.494548765472303 | 0.247723885308279 | 0.987302783200798 | 0.999950574162623 |
| ebi-fl187-GCST90094726 | prot-a-1076 | Low affinity immunoglobulin gamma Fc region receptor III-B \|\| id:prot-a-1076 | F-capsicum liking (derived food-liking factor) \|\| id:ebi-fl187-GCST90094726 | MR Egger | 21 | -0.529679346 | 0.441946044641973 | 0.245452312535546 | -1.395893594 | 0.336534901322085 | 0.588793738375686 | 0.247611673252681 | 1.40008773333008 | 0.999950574162623 |
| ebi-fl187-GCST90094726 | prot-a-1081 | Fc receptor-like protein 3 \|\| id:prot-a-1081 | F-capsicum liking (derived food-liking factor) \|\| id:ebi-fl187-GCST90094726 | MR Egger | 21 | -0.907415919 | 0.352708381477243 | 0.0186379806365195 | -1.598724347 | -0.216107491 | 0.403565724255302 | 0.202154232267726 | 0.805648696872264 | 0.999950574162623 |
| ebi-fl187-GCST90094726 | prot-a-1105 | Fibroblast growth factor receptor 4 \|\| id:prot-a-1105 | F-capsicum liking (derived food-liking factor) \|\| id:ebi-fl187-GCST90094726 | MR Egger | 21 | 0.207935098600459 | 0.444515381208691 | 0.645263321377861 | -0.663315049 | 1.07918524576949 | 1.23113326468403 | 0.515140784060519 | 2.94228133805357 | 0.999950574162623 |
| ebi-fl187-GCST90094726 | prot-a-1143 | Follistatin-related protein 1 \|\| id:prot-a-1143 | F-capsicum liking (derived food-liking factor) \|\| id:ebi-fl187-GCST90094726 | MR Egger | 21 | -0.343371006 | 0.40605496738072 | 0.40828461933387 | -1.139238742 | 0.452496730017174 | 0.709374980324302 | 0.320062579261606 | 1.57223272983374 | 0.999950574162623 |
| ebi-fl187-GCST90094726 | prot-a-1151 | Alpha-(1,3)-fucosyltransferase 10 \|\| id:prot-a-1151 | F-capsicum liking (derived food-liking factor) \|\| id:ebi-fl187-GCST90094726 | MR Egger | 21 | -0.492236466 | 0.352806196449286 | 0.179050347330931 | -1.183736611 | 0.199263679241818 | 0.611257807175857 | 0.306132700019296 | 1.22050374497689 | 0.999950574162623 |
| ebi-fl187-GCST90094726 | prot-a-1165 | Growth arrest and DNA damage-inducible proteins-interacting protein 1 \|\| id:prot-a-1165 | F-capsicum liking (derived food-liking factor) \|\| id:ebi-fl187-GCST90094726 | MR Egger | 21 | 0.0892510774022852 | 0.352950342671947 | 0.803083189824861 | -0.602531594 | 0.781033749039302 | 1.09335513863472 | 0.547424024895489 | 2.18372852635975 | 0.999950574162623 |
| ebi-fl187-GCST90094726 | prot-a-1194 | Growth/differentiation factor 11/8 \|\| id:prot-a-1194 | F-capsicum liking (derived food-liking factor) \|\| id:ebi-fl187-GCST90094726 | MR Egger | 21 | 0.284622058576353 | 0.444142145773008 | 0.529283891095908 | -0.585896547 | 1.15514066429145 | 1.32925955092691 | 0.556606613978839 | 3.17446992068546 | 0.999950574162623 |
| ebi-fl187-GCST90094726 | prot-a-1216 | D-glucuronyl C5-epimerase \|\| id:prot-a-1216 | F-capsicum liking (derived food-liking factor) \|\| id:ebi-fl187-GCST90094726 | MR Egger | 21 | -0.497309171 | 0.373497725187028 | 0.198782545400805 | -1.229364712 | 0.23474637032622 | 0.608164927758509 | 0.292478326524727 | 1.26458799101561 | 0.999950574162623 |
| ebi-fl187-GCST90094726 | prot-a-1246 | Glypican-3 \|\| id:prot-a-1246 | F-capsicum liking (derived food-liking factor) \|\| id:ebi-fl187-GCST90094726 | MR Egger | 21 | 0.683959568268556 | 0.431786304460429 | 0.129691856796733 | -0.162341588 | 1.530260725 | 1.98170892971395 | 0.850150753237278 | 4.61938105348233 | 0.999950574162623 |
| ebi-fl187-GCST90094726 | prot-a-1275 | Glutamate receptor 4 \|\| id:prot-a-1275 | F-capsicum liking (derived food-liking factor) \|\| id:ebi-fl187-GCST90094726 | MR Egger | 20 | -0.397675202 | 0.490409264589309 | 0.428012778304304 | -1.358877361 | 0.56352695660833 | 0.671880217586101 | 0.256949076256681 | 1.75685794773045 | 0.999950574162623 |
| ebi-fl187-GCST90094726 | prot-a-1289 | Glutathione S-transferase P \|\| id:prot-a-1289 | F-capsicum liking (derived food-liking factor) \|\| id:ebi-fl187-GCST90094726 | MR Egger | 21 | 0.018957828236328 | 0.352724196350407 | 0.957697934044289 | -0.672381597 | 0.710297253083125 | 1.01913866883606 | 0.510491344426906 | 2.03459595868946 | 0.999950574162623 |
| ebi-fl187-GCST90094726 | prot-a-130 | Apolipoprotein D \|\| id:prot-a-130 | F-capsicum liking (derived food-liking factor) \|\| id:ebi-fl187-GCST90094726 | MR Egger | 21 | 0.245379973288915 | 0.360880536228751 | 0.504738528327646 | -0.461945878 | 0.952705824297267 | 1.27810686742023 | 0.630056439110509 | 2.59271560949832 | 0.999950574162623 |
| ebi-fl187-GCST90094726 | prot-a-1313 | Heparin-binding EGF-like growth factor \|\| id:prot-a-1313 | F-capsicum liking (derived food-liking factor) \|\| id:ebi-fl187-GCST90094726 | MR Egger | 21 | -0.64643417 | 0.455067187822669 | 0.171658156070298 | -1.538365858 | 0.245497517977184 | 0.523910626063782 | 0.214731716926835 | 1.27825711092352 | 0.999950574162623 |
| ebi-fl187-GCST90094726 | prot-a-1347 | HLA class II histocompatibility antigen, DQ alpha 2 chain \|\| id:prot-a-1347 | F-capsicum liking (derived food-liking factor) \|\| id:ebi-fl187-GCST90094726 | MR Egger | 20 | 0.300214015856584 | 0.363376756232909 | 0.419519649783391 | -0.412004426 | 1.01243245807308 | 1.35014772968083 | 0.662321344372983 | 2.75228770361919 | 0.999950574162623 |
| ebi-fl187-GCST90094726 | prot-a-1357 | Non-histone chromosomal protein HMG-14 \|\| id:prot-a-1357 | F-capsicum liking (derived food-liking factor) \|\| id:ebi-fl187-GCST90094726 | MR Egger | 21 | 0.259440098623004 | 0.418389173542964 | 0.542563091096544 | -0.560602682 | 1.07948287876721 | 1.29620413697346 | 0.570864910419162 | 2.94315718840287 | 0.999950574162623 |
| ebi-fl187-GCST90094726 | prot-a-137 | Apolipoprotein M \|\| id:prot-a-137 | F-capsicum liking (derived food-liking factor) \|\| id:ebi-fl187-GCST90094726 | MR Egger | 21 | 0.098741014326337 | 0.430282800615997 | 0.820948627011816 | -0.744613275 | 0.942095303533692 | 1.10378039922817 | 0.474917927122875 | 2.56535097990746 | 0.999950574162623 |
| ebi-fl187-GCST90094726 | prot-a-1381 | 17-beta-hydroxysteroid dehydrogenase 14 \|\| id:prot-a-1381 | F-capsicum liking (derived food-liking factor) \|\| id:ebi-fl187-GCST90094726 | MR Egger | 21 | 0.0844471639043248 | 0.38416138619554 | 0.8283542083728 | -0.668509153 | 0.837403480847583 | 1.08811535094637 | 0.512472025915444 | 2.31036028717886 | 0.999950574162623 |
| ebi-fl187-GCST90094726 | prot-a-1392 | Serine protease HTRA2, mitochondrial \|\| id:prot-a-1392 | F-capsicum liking (derived food-liking factor) \|\| id:ebi-fl187-GCST90094726 | MR Egger | 21 | -0.491443194 | 0.43300278924191 | 0.27050562160621 | -1.340128661 | 0.357242272610917 | 0.611742892947646 | 0.261811981365009 | 1.42938212804791 | 0.999950574162623 |
| ebi-fl187-GCST90094726 | prot-a-1416 | Interferon-induced protein with tetratricopeptide repeats 2 \|\| id:prot-a-1416 | F-capsicum liking (derived food-liking factor) \|\| id:ebi-fl187-GCST90094726 | MR Egger | 21 | 0.262452013344543 | 0.370344697763563 | 0.487134316421266 | -0.463423594 | 0.988327620961126 | 1.30011407854523 | 0.629126081852819 | 2.68673746962368 | 0.999950574162623 |
| ebi-fl187-GCST90094726 | prot-a-1428 | Interferon gamma \|\| id:prot-a-1428 | F-capsicum liking (derived food-liking factor) \|\| id:ebi-fl187-GCST90094726 | MR Egger | 21 | 0.178846709770715 | 0.352708381477243 | 0.617942563470221 | -0.512461718 | 0.87015513746611 | 1.19583741992505 | 0.599019146108244 | 2.38728118155106 | 0.999950574162623 |
| ebi-fl187-GCST90094726 | prot-a-1483 | Interleukin-17C \|\| id:prot-a-1483 | F-capsicum liking (derived food-liking factor) \|\| id:ebi-fl187-GCST90094726 | MR Egger | 21 | -0.068114448 | 0.409266869919725 | 0.869576603211911 | -0.870277513 | 0.734048617311043 | 0.934153555703643 | 0.418835300971856 | 2.08349884455512 | 0.999950574162623 |
| ebi-fl187-GCST90094726 | prot-a-1503 | Interleukin-1 receptor-like 2 \|\| id:prot-a-1503 | F-capsicum liking (derived food-liking factor) \|\| id:ebi-fl187-GCST90094726 | MR Egger | 21 | 0.0506613675612438 | 0.352679023484205 | 0.887291630294652 | -0.640589518 | 0.741912253590285 | 1.05196660294463 | 0.526981667028645 | 2.09994730926894 | 0.999950574162623 |
| ebi-fl187-GCST90094726 | prot-a-1522 | Interleukin-31 receptor subunit alpha \|\| id:prot-a-1522 | F-capsicum liking (derived food-liking factor) \|\| id:ebi-fl187-GCST90094726 | MR Egger | 21 | -0.469075161 | 0.408133519414439 | 0.264687638632337 | -1.269016859 | 0.330866536731059 | 0.625580561927667 | 0.28110785452051 | 1.39217397581889 | 0.999950574162623 |
| ebi-fl187-GCST90094726 | prot-a-1540 | Interleukin-6 receptor subunit alpha \|\| id:prot-a-1540 | F-capsicum liking (derived food-liking factor) \|\| id:ebi-fl187-GCST90094726 | MR Egger | 21 | -0.076588158 | 0.452224226645056 | 0.867304529875644 | -0.962947642 | 0.809771326177142 | 0.926271252424538 | 0.381765916486397 | 2.24739400773273 | 0.999950574162623 |
| ebi-fl187-GCST90094726 | prot-a-1541 | Interleukin-6 receptor subunit alpha \|\| id:prot-a-1541 | F-capsicum liking (derived food-liking factor) \|\| id:ebi-fl187-GCST90094726 | MR Egger | 21 | 0.0105749584411548 | 0.46666655042211 | 0.982157275769258 | -0.90409148 | 0.92524139726849 | 1.01063107093551 | 0.404909586346851 | 2.52247710595156 | 0.999950574162623 |
| ebi-fl187-GCST90094726 | prot-a-1545 | Interleukin-9 \|\| id:prot-a-1545 | F-capsicum liking (derived food-liking factor) \|\| id:ebi-fl187-GCST90094726 | MR Egger | 21 | -0.2052311 | 0.352694831195281 | 0.567478802623309 | -0.896512969 | 0.486050768863352 | 0.814459072941377 | 0.407989855269701 | 1.62588253832445 | 0.999950574162623 |
| ebi-fl187-GCST90094726 | prot-a-1548 | Inositol monophosphatase 3 \|\| id:prot-a-1548 | F-capsicum liking (derived food-liking factor) \|\| id:ebi-fl187-GCST90094726 | MR Egger | 21 | -0.002147069 | 0.428995839963897 | 0.996058883001151 | -0.842978915 | 0.83868477720225 | 0.997855234177184 | 0.430426407863671 | 2.31332244068579 | 0.999950574162623 |
| ebi-fl187-GCST90094726 | prot-a-1555 | Inhibin beta A chain:Inhibin beta B chain heterodimer \|\| id:prot-a-1555 | F-capsicum liking (derived food-liking factor) \|\| id:ebi-fl187-GCST90094726 | MR Egger | 21 | -0.092615769 | 0.38794836419148 | 0.813869270534026 | -0.852994563 | 0.667763024432531 | 0.911543676006982 | 0.426136925413909 | 1.94987062541284 | 0.999950574162623 |
| ebi-fl187-GCST90094726 | prot-a-1603 | Junctophilin-3 \|\| id:prot-a-1603 | F-capsicum liking (derived food-liking factor) \|\| id:ebi-fl187-GCST90094726 | MR Egger | 21 | -0.376858416 | 0.45214449106143 | 0.414923554890048 | -1.263061618 | 0.50934478651559 | 0.686013195536836 | 0.282786914161067 | 1.66420043108011 | 0.999950574162623 |
| ebi-fl187-GCST90094726 | prot-a-1627 | UPF0258 protein KIAA1024 \|\| id:prot-a-1627 | F-capsicum liking (derived food-liking factor) \|\| id:ebi-fl187-GCST90094726 | MR Egger | 21 | -0.168791224 | 0.400746227604676 | 0.678341034026582 | -0.95425383 | 0.616671382481209 | 0.844685235300749 | 0.38509938708771 | 1.85275066816083 | 0.999950574162623 |
| ebi-fl187-GCST90094726 | prot-a-1666 | Kallikrein-7 \|\| id:prot-a-1666 | F-capsicum liking (derived food-liking factor) \|\| id:ebi-fl187-GCST90094726 | MR Egger | 21 | 0.273272202368681 | 0.409620972143775 | 0.51270786410106 | -0.529584903 | 1.07612930777048 | 1.31425794026416 | 0.588849348532912 | 2.93330363335002 | 0.999950574162623 |
| ebi-fl187-GCST90094726 | prot-a-1678 | Importin subunit alpha-3 \|\| id:prot-a-1678 | F-capsicum liking (derived food-liking factor) \|\| id:ebi-fl187-GCST90094726 | MR Egger | 21 | -0.422063718 | 0.45353315401783 | 0.363728301095362 | -1.3109887 | 0.466861263488911 | 0.655692258409906 | 0.269553417059427 | 1.59498010609117 | 0.999950574162623 |
| ebi-fl187-GCST90094726 | prot-a-1718 | Protein LDOC1 \|\| id:prot-a-1718 | F-capsicum liking (derived food-liking factor) \|\| id:ebi-fl187-GCST90094726 | MR Egger | 21 | -0.112643933 | 0.367878749907936 | 0.762783428857799 | -0.833686283 | 0.608398416889169 | 0.893468738279507 | 0.434444844389408 | 1.83748615409334 | 0.999950574162623 |
| ebi-fl187-GCST90094726 | prot-a-1723 | Leptin \|\| id:prot-a-1723 | F-capsicum liking (derived food-liking factor) \|\| id:ebi-fl187-GCST90094726 | MR Egger | 21 | 0.254135512401392 | 0.456089554594683 | 0.583892368717273 | -0.639800015 | 1.14807103940697 | 1.28934651487227 | 0.527397885372192 | 3.15210675188823 | 0.999950574162623 |
| ebi-fl187-GCST90094726 | prot-a-1730 | Galectin-7 \|\| id:prot-a-1730 | F-capsicum liking (derived food-liking factor) \|\| id:ebi-fl187-GCST90094726 | MR Egger | 21 | 0.855829072246546 | 0.35277353822061 | 0.0253914389595633 | 0.164392937334151 | 1.54726520715894 | 2.35332464815973 | 1.17867737045466 | 4.69860289037349 | 0.999950574162623 |
| ebi-fl187-GCST90094726 | prot-a-1753 | Lipase member N \|\| id:prot-a-1753 | F-capsicum liking (derived food-liking factor) \|\| id:ebi-fl187-GCST90094726 | MR Egger | 21 | 0.355097719470869 | 0.410148193296492 | 0.397412271376103 | -0.448792739 | 1.15898817833199 | 1.42632002670997 | 0.638398399824497 | 3.18670726485719 | 0.999950574162623 |
| ebi-fl187-GCST90094726 | prot-a-1756 | VIP36-like protein \|\| id:prot-a-1756 | F-capsicum liking (derived food-liking factor) \|\| id:ebi-fl187-GCST90094726 | MR Egger | 21 | 0.217465975567272 | 0.352979682255062 | 0.545148460705522 | -0.474374202 | 0.909306152787194 | 1.24292313903355 | 0.622274352903426 | 2.48259939098722 | 0.999950574162623 |
| ebi-fl187-GCST90094726 | prot-a-1760 | Ig Kappa chain V-I region HK102- like \|\| id:prot-a-1760 | F-capsicum liking (derived food-liking factor) \|\| id:ebi-fl187-GCST90094726 | MR Egger | 21 | -0.074816897 | 0.40129474931891 | 0.85407691277087 | -0.861354606 | 0.711720811753709 | 0.927913374579427 | 0.422589252668024 | 2.03749438796017 | 0.999950574162623 |
| ebi-fl187-GCST90094726 | prot-a-179 | Neutral ceramidase \|\| id:prot-a-179 | F-capsicum liking (derived food-liking factor) \|\| id:ebi-fl187-GCST90094726 | MR Egger | 21 | 0.496297934046054 | 0.352679023484205 | 0.175510180942994 | -0.194952952 | 1.1875488200751 | 1.6426288799906 | 0.822873371705703 | 3.27903384670975 | 0.999950574162623 |
| ebi-fl187-GCST90094726 | prot-a-1809 | Lactotransferrin \|\| id:prot-a-1809 | F-capsicum liking (derived food-liking factor) \|\| id:ebi-fl187-GCST90094726 | MR Egger | 21 | 0.866100382925199 | 0.352740600905155 | 0.0238797973046655 | 0.174728805151095 | 1.5574719606993 | 2.37762094052817 | 1.19092320057676 | 4.74680595197095 | 0.999950574162623 |
| ebi-fl187-GCST90094726 | prot-a-1818 | Lysophospholipase-like protein 1 \|\| id:prot-a-1818 | F-capsicum liking (derived food-liking factor) \|\| id:ebi-fl187-GCST90094726 | MR Egger | 21 | -0.560620267 | 0.352600385080782 | 0.128345588030794 | -1.251717022 | 0.130476487614137 | 0.570854871492339 | 0.286013283938782 | 1.13937115024449 | 0.999950574162623 |
| ebi-fl187-GCST90094726 | prot-a-1852 | MAP kinase-activated protein kinase 3 \|\| id:prot-a-1852 | F-capsicum liking (derived food-liking factor) \|\| id:ebi-fl187-GCST90094726 | MR Egger | 21 | 0.134224713917462 | 0.352604674388972 | 0.707673530906836 | -0.556880448 | 0.825329875719846 | 1.14364978479509 | 0.572993762575107 | 2.28263362655786 | 0.999950574162623 |
| ebi-fl187-GCST90094726 | prot-a-1856 | Megakaryocyte-associated tyrosine-protein kinase \|\| id:prot-a-1856 | F-capsicum liking (derived food-liking factor) \|\| id:ebi-fl187-GCST90094726 | MR Egger | 21 | -0.463311371 | 0.380586013678798 | 0.238370464640895 | -1.209259958 | 0.282637215499632 | 0.629196688206464 | 0.298418039580597 | 1.32662379595541 | 0.999950574162623 |
| ebi-fl187-GCST90094726 | prot-a-1890 | Beta-1,3-N-acetylglucosaminyltransferase manic fringe \|\| id:prot-a-1890 | F-capsicum liking (derived food-liking factor) \|\| id:ebi-fl187-GCST90094726 | MR Egger | 21 | 0.423725865210428 | 0.372062056683482 | 0.268918405804018 | -0.305515766 | 1.15296749631005 | 1.52764275635217 | 0.736743289324869 | 3.16757875483846 | 0.999950574162623 |
| ebi-fl187-GCST90094726 | prot-a-1919 | Matrilysin \|\| id:prot-a-1919 | F-capsicum liking (derived food-liking factor) \|\| id:ebi-fl187-GCST90094726 | MR Egger | 21 | -0.36709974 | 0.352781146175736 | 0.311128161941411 | -1.058550786 | 0.324351306786109 | 0.69274054785146 | 0.34695826285621 | 1.38313312583197 | 0.999950574162623 |
| ebi-fl187-GCST90094726 | prot-a-1923 | Multimerin-2 \|\| id:prot-a-1923 | F-capsicum liking (derived food-liking factor) \|\| id:ebi-fl187-GCST90094726 | MR Egger | 21 | -0.590834981 | 0.352704085471135 | 0.110283573765246 | -1.282134989 | 0.100465026500875 | 0.553864625154338 | 0.277444327222142 | 1.10568497135549 | 0.999950574162623 |
| ebi-fl187-GCST90094726 | prot-a-1948 | Mesothelin \|\| id:prot-a-1948 | F-capsicum liking (derived food-liking factor) \|\| id:ebi-fl187-GCST90094726 | MR Egger | 21 | 0.10114049179258 | 0.400411182785572 | 0.803296332270575 | -0.683665426 | 0.8859464100523 | 1.10643207547024 | 0.504763424167314 | 2.42527861373648 | 0.999950574162623 |
| ebi-fl187-GCST90094726 | prot-a-195 | ATPase family AAA domain-containing protein 1 \|\| id:prot-a-195 | F-capsicum liking (derived food-liking factor) \|\| id:ebi-fl187-GCST90094726 | MR Egger | 21 | -0.422672982 | 0.352855660426851 | 0.245700194105502 | -1.114270077 | 0.268924112009735 | 0.655292890367636 | 0.328154719224348 | 1.30855583360603 | 0.999950574162623 |
| ebi-fl187-GCST90094726 | prot-a-1966 | Mucin-1 \|\| id:prot-a-1966 | F-capsicum liking (derived food-liking factor) \|\| id:ebi-fl187-GCST90094726 | MR Egger | 21 | 0.108376985878435 | 0.375217704466013 | 0.7758334130744 | -0.627049715 | 0.843803686631821 | 1.11446780482764 | 0.534165418844359 | 2.32519448878671 | 0.999950574162623 |
| ebi-fl187-GCST90094726 | prot-a-1986 | Myocardial zonula adherens protein \|\| id:prot-a-1986 | F-capsicum liking (derived food-liking factor) \|\| id:ebi-fl187-GCST90094726 | MR Egger | 21 | 0.359820939675475 | 0.352802907345278 | 0.320591209652058 | -0.331672759 | 1.05131463807222 | 1.43307278510698 | 0.717722152744367 | 2.86141036550358 | 0.999950574162623 |
| ebi-fl187-GCST90094726 | prot-a-2071 | Epididymal secretory protein E1 \|\| id:prot-a-2071 | F-capsicum liking (derived food-liking factor) \|\| id:ebi-fl187-GCST90094726 | MR Egger | 20 | -0.216390134 | 0.406927464970324 | 0.601390222375033 | -1.013967965 | 0.581187697718691 | 0.805421018751058 | 0.362776634900011 | 1.78816096473465 | 0.999950574162623 |
| ebi-fl187-GCST90094726 | prot-a-2098 | Neuritin \|\| id:prot-a-2098 | F-capsicum liking (derived food-liking factor) \|\| id:ebi-fl187-GCST90094726 | MR Egger | 21 | 0.0292304153278862 | 0.352724196350407 | 0.934821391775577 | -0.66210901 | 0.720569840174683 | 1.02966181700941 | 0.51576243875674 | 2.05560424284242 | 0.999950574162623 |
| ebi-fl187-GCST90094726 | prot-a-2115 | Neurotrophin-4 \|\| id:prot-a-2115 | F-capsicum liking (derived food-liking factor) \|\| id:ebi-fl187-GCST90094726 | MR Egger | 21 | 0.117904228190049 | 0.3641488077047 | 0.749641262638105 | -0.595827435 | 0.831635891291261 | 1.1251363498625 | 0.551106372506324 | 2.29707343071483 | 0.999950574162623 |
| ebi-fl187-GCST90094726 | prot-a-2118 | Netrin-1 \|\| id:prot-a-2118 | F-capsicum liking (derived food-liking factor) \|\| id:ebi-fl187-GCST90094726 | MR Egger | 21 | 0.139053843195753 | 0.352954614964722 | 0.697989149311807 | -0.552737202 | 0.830844888526609 | 1.1491859741832 | 0.575372741490486 | 2.29525715771371 | 0.999950574162623 |
| ebi-fl187-GCST90094726 | prot-a-2120 | Netrin-G1 \|\| id:prot-a-2120 | F-capsicum liking (derived food-liking factor) \|\| id:ebi-fl187-GCST90094726 | MR Egger | 21 | 0.241869407105536 | 0.352781146175736 | 0.501241166431861 | -0.449581639 | 0.933320453609979 | 1.27362785520709 | 0.637894965926965 | 2.54293888524768 | 0.999950574162623 |
| ebi-fl187-GCST90094726 | prot-a-2145 | Olfactomedin-like protein 3 \|\| id:prot-a-2145 | F-capsicum liking (derived food-liking factor) \|\| id:ebi-fl187-GCST90094726 | MR Egger | 21 | -0.049056552 | 0.375657094080059 | 0.897473464109059 | -0.785344456 | 0.68723135256012 | 0.952127283629219 | 0.455962615567257 | 1.9882032721111 | 0.999950574162623 |
| ebi-fl187-GCST90094726 | prot-a-2162 | Prolyl 4-hydroxylase subunit alpha-2 \|\| id:prot-a-2162 | F-capsicum liking (derived food-liking factor) \|\| id:ebi-fl187-GCST90094726 | MR Egger | 21 | -0.473052445 | 0.415891680502895 | 0.269495211391784 | -1.288200139 | 0.342095248733849 | 0.623097391945721 | 0.275766678418908 | 1.40789439128603 | 0.999950574162623 |
| ebi-fl187-GCST90094726 | prot-a-2168 | Polyadenylate-binding protein-interacting protein 1 \|\| id:prot-a-2168 | F-capsicum liking (derived food-liking factor) \|\| id:ebi-fl187-GCST90094726 | MR Egger | 21 | -0.982670463 | 0.419681089197593 | 0.0302596864105819 | -1.805245398 | -0.160095528 | 0.374310181486094 | 0.164434099848849 | 0.852062389084395 | 0.999950574162623 |
| ebi-fl187-GCST90094726 | prot-a-219 | Beta-1,4-galactosyltransferase 5 \|\| id:prot-a-219 | F-capsicum liking (derived food-liking factor) \|\| id:ebi-fl187-GCST90094726 | MR Egger | 21 | -0.139996926 | 0.352781146175736 | 0.695909137282168 | -0.831447973 | 0.551454120489429 | 0.869360907797076 | 0.435418355833053 | 1.7357752099352 | 0.999950574162623 |
| ebi-fl187-GCST90094726 | prot-a-2190 | Pyruvate carboxylase, mitochondrial \|\| id:prot-a-2190 | F-capsicum liking (derived food-liking factor) \|\| id:ebi-fl187-GCST90094726 | MR Egger | 21 | -0.35024447 | 0.352900820979844 | 0.333439088530636 | -1.041930079 | 0.34144113921546 | 0.704515835744655 | 0.35277314441289 | 1.40697377528847 | 0.999950574162623 |
| ebi-fl187-GCST90094726 | prot-a-2197 | Protocadherin-9 \|\| id:prot-a-2197 | F-capsicum liking (derived food-liking factor) \|\| id:ebi-fl187-GCST90094726 | MR Egger | 21 | -0.316073183 | 0.352802907345278 | 0.381521168847069 | -1.007566881 | 0.375420515702039 | 0.729006097584726 | 0.365106246632979 | 1.45560339001799 | 0.999950574162623 |
| ebi-fl187-GCST90094726 | prot-a-2198 | Protocadherin alpha-4 \|\| id:prot-a-2198 | F-capsicum liking (derived food-liking factor) \|\| id:ebi-fl187-GCST90094726 | MR Egger | 21 | -0.1750003 | 0.487831516916158 | 0.723751613826063 | -1.131150073 | 0.781149473052263 | 0.839456768845334 | 0.322661958063332 | 2.1839812508109 | 0.999950574162623 |
| ebi-fl187-GCST90094726 | prot-a-2254 | Placenta growth factor \|\| id:prot-a-2254 | F-capsicum liking (derived food-liking factor) \|\| id:ebi-fl187-GCST90094726 | MR Egger | 21 | -0.735367013 | 0.352860241083659 | 0.0508979011908385 | -1.426973086 | -0.043760941 | 0.479329506426669 | 0.240034387240279 | 0.957182753574568 | 0.999950574162623 |
| ebi-fl187-GCST90094726 | prot-a-2261 | Membrane-associated progesterone receptor component 2 \|\| id:prot-a-2261 | F-capsicum liking (derived food-liking factor) \|\| id:ebi-fl187-GCST90094726 | MR Egger | 21 | 0.276314594849211 | 0.35663992901012 | 0.448009756947516 | -0.422699666 | 0.975328855709046 | 1.31826251739786 | 0.655275405038214 | 2.65203920582796 | 0.999950574162623 |
| ebi-fl187-GCST90094726 | prot-a-2268 | Polymeric immunoglobulin receptor \|\| id:prot-a-2268 | F-capsicum liking (derived food-liking factor) \|\| id:ebi-fl187-GCST90094726 | MR Egger | 21 | -0.05444317 | 0.36823771755038 | 0.884020543372907 | -0.776189097 | 0.66730275619844 | 0.947012325849393 | 0.460156285276972 | 1.94897336840866 | 0.999950574162623 |
| ebi-fl187-GCST90094726 | prot-a-2283 | Group 10 secretory phospholipase A2 \|\| id:prot-a-2283 | F-capsicum liking (derived food-liking factor) \|\| id:ebi-fl187-GCST90094726 | MR Egger | 21 | -0.496755986 | 0.408061472008109 | 0.238373191226102 | -1.296556471 | 0.303044498870685 | 0.608501448407791 | 0.27347188173965 | 1.35397471344746 | 0.999950574162623 |
| ebi-fl187-GCST90094726 | prot-a-2298 | Inactive phospholipase D5 \|\| id:prot-a-2298 | F-capsicum liking (derived food-liking factor) \|\| id:ebi-fl187-GCST90094726 | MR Egger | 21 | -0.153011732 | 0.372397287773302 | 0.685755318151712 | -0.882910416 | 0.576886951924721 | 0.858119654197344 | 0.413577475808983 | 1.78048705258764 | 0.999950574162623 |
| ebi-fl187-GCST90094726 | prot-a-2304 | Procollagen-lysine,2-oxoglutarate 5-dioxygenase 2 \|\| id:prot-a-2304 | F-capsicum liking (derived food-liking factor) \|\| id:ebi-fl187-GCST90094726 | MR Egger | 21 | -0.578554726 | 0.415236800336448 | 0.179612467991774 | -1.392418854 | 0.235309403154549 | 0.560708158439265 | 0.248473555858487 | 1.26530019604746 | 0.999950574162623 |
| ebi-fl187-GCST90094726 | prot-a-2331 | Periostin \|\| id:prot-a-2331 | F-capsicum liking (derived food-liking factor) \|\| id:ebi-fl187-GCST90094726 | MR Egger | 21 | 0.629045729939898 | 0.352683311075054 | 0.090470810656477 | -0.06221356 | 1.32030502 | 1.87581968633767 | 0.939682187052042 | 3.74456336848396 | 0.999950574162623 |
| ebi-fl187-GCST90094726 | prot-a-2333 | Periostin \|\| id:prot-a-2333 | F-capsicum liking (derived food-liking factor) \|\| id:ebi-fl187-GCST90094726 | MR Egger | 21 | 0.541412364720496 | 0.392846046867903 | 0.184162591129175 | -0.228565887 | 1.31139061658159 | 1.71843220206621 | 0.795673870801198 | 3.71133116401651 | 0.999950574162623 |
| ebi-fl187-GCST90094726 | prot-a-2367 | cAMP-dependent protein kinase catalytic subunit alpha \|\| id:prot-a-2367 | F-capsicum liking (derived food-liking factor) \|\| id:ebi-fl187-GCST90094726 | MR Egger | 21 | -0.361116051 | 0.363069619596265 | 0.332420526441492 | -1.072732505 | 0.350500403904514 | 0.696898118395885 | 0.342072524344938 | 1.41977783323513 | 0.999950574162623 |
| ebi-fl187-GCST90094726 | prot-a-2385 | Phosphoribosyl pyrophosphate synthase-associated protein 1 \|\| id:prot-a-2385 | F-capsicum liking (derived food-liking factor) \|\| id:ebi-fl187-GCST90094726 | MR Egger | 21 | 0.633301887773347 | 0.410796119919433 | 0.139650648873907 | -0.171858507 | 1.43846228281544 | 1.8838204852435 | 0.842098315530065 | 4.21421056802524 | 0.999950574162623 |
| ebi-fl187-GCST90094726 | prot-a-2393 | Trypsin-3 \|\| id:prot-a-2393 | F-capsicum liking (derived food-liking factor) \|\| id:ebi-fl187-GCST90094726 | MR Egger | 20 | 0.43327215434575 | 0.363283633599092 | 0.24848980426275 | -0.278763768 | 1.14530807619997 | 1.54229590612172 | 0.756718643632641 | 3.14340961737236 | 0.999950574162623 |
| ebi-fl187-GCST90094726 | prot-a-2405 | Pregnancy-specific beta-1-glycoprotein 6 \|\| id:prot-a-2405 | F-capsicum liking (derived food-liking factor) \|\| id:ebi-fl187-GCST90094726 | MR Egger | 21 | 0.000702979109415271 | 0.399242784432483 | 0.998613456826148 | -0.781812878 | 0.783218836597083 | 1.00070322625714 | 0.45757572978597 | 2.1885053814149 | 0.999974376584515 |
| ebi-fl187-GCST90094726 | prot-a-244 | Beclin-1 \|\| id:prot-a-244 | F-capsicum liking (derived food-liking factor) \|\| id:ebi-fl187-GCST90094726 | MR Egger | 21 | 0.0369713426630578 | 0.352859932926938 | 0.917651533991274 | -0.654634126 | 0.728576811199856 | 1.03766328374571 | 0.519632147769344 | 2.07212947670028 | 0.999950574162623 |
| ebi-fl187-GCST90094726 | prot-a-2454 | tRNA pseudouridine synthase A, mitochondrial \|\| id:prot-a-2454 | F-capsicum liking (derived food-liking factor) \|\| id:ebi-fl187-GCST90094726 | MR Egger | 21 | -0.472556713 | 0.352802907345278 | 0.196232175316354 | -1.164050412 | 0.218936984993999 | 0.623406357619071 | 0.31221900078961 | 1.24475283610865 | 0.999950574162623 |
| ebi-fl187-GCST90094726 | prot-a-2466 | Glutaminyl-peptide cyclotransferase-like protein \|\| id:prot-a-2466 | F-capsicum liking (derived food-liking factor) \|\| id:ebi-fl187-GCST90094726 | MR Egger | 21 | -0.12202731 | 0.413444767093674 | 0.771081981361511 | -0.932379053 | 0.688324433987612 | 0.885124195859179 | 0.393616161856566 | 1.9903777283943 | 0.999950574162623 |
| ebi-fl187-GCST90094726 | prot-a-2474 | Ras-related protein Rab-27A \|\| id:prot-a-2474 | F-capsicum liking (derived food-liking factor) \|\| id:ebi-fl187-GCST90094726 | MR Egger | 21 | -0.175129953 | 0.389084669336679 | 0.657726344522275 | -0.937735905 | 0.587475998500398 | 0.839347937563651 | 0.391513255730024 | 1.79944088733016 | 0.999950574162623 |
| ebi-fl187-GCST90094726 | prot-a-2490 | Rap1 GTPase-GDP dissociation stimulator 1 \|\| id:prot-a-2490 | F-capsicum liking (derived food-liking factor) \|\| id:ebi-fl187-GCST90094726 | MR Egger | 21 | -0.131464894 | 0.435558913657075 | 0.766060266579389 | -0.985160365 | 0.72223057658808 | 0.876810055733682 | 0.373379345176177 | 2.05902089595489 | 0.999950574162623 |
| ebi-fl187-GCST90094726 | prot-a-2545 | Receptor-interacting serine/threonine-protein kinase 2 \|\| id:prot-a-2545 | F-capsicum liking (derived food-liking factor) \|\| id:ebi-fl187-GCST90094726 | MR Egger | 21 | -0.693801638 | 0.352777825610259 | 0.0639993456622118 | -1.385246176 | -0.0023571 | 0.499672878295546 | 0.250262183595511 | 0.997645675895216 | 0.999950574162623 |
| ebi-fl187-GCST90094726 | prot-a-2610 | RUN and FYVE domain-containing protein 1 \|\| id:prot-a-2610 | F-capsicum liking (derived food-liking factor) \|\| id:ebi-fl187-GCST90094726 | MR Egger | 21 | 0.70740020585477 | 0.352615457755405 | 0.0592888886425066 | 0.0162739086541767 | 1.39852650305536 | 2.02871016792998 | 1.01640704996828 | 4.04922904223356 | 0.999950574162623 |
| ebi-fl187-GCST90094726 | prot-a-2627 | Serine--tRNA ligase, mitochondrial \|\| id:prot-a-2627 | F-capsicum liking (derived food-liking factor) \|\| id:ebi-fl187-GCST90094726 | MR Egger | 21 | -0.136505341 | 0.352896538563651 | 0.703192113953459 | -0.828182557 | 0.555171874097498 | 0.872401660324825 | 0.436842501606631 | 1.74224040503929 | 0.999950574162623 |
| ebi-fl187-GCST90094726 | prot-a-264 | BolA-like protein 2 \|\| id:prot-a-264 | F-capsicum liking (derived food-liking factor) \|\| id:ebi-fl187-GCST90094726 | MR Egger | 21 | -0.461303253 | 0.353961806606615 | 0.208059790631398 | -1.155068394 | 0.232461887997044 | 0.630461459107217 | 0.31503598962632 | 1.26170235943859 | 0.999950574162623 |
| ebi-fl187-GCST90094726 | prot-a-2646 | Secretoglobin family 1C member 1 \|\| id:prot-a-2646 | F-capsicum liking (derived food-liking factor) \|\| id:ebi-fl187-GCST90094726 | MR Egger | 21 | -0.194580872 | 0.365829825140672 | 0.600967470042849 | -0.911607329 | 0.522445585515416 | 0.823179603580968 | 0.401877754844175 | 1.68614622626839 | 0.999950574162623 |
| ebi-fl187-GCST90094726 | prot-a-2672 | Semaphorin-3E \|\| id:prot-a-2672 | F-capsicum liking (derived food-liking factor) \|\| id:ebi-fl187-GCST90094726 | MR Egger | 21 | -0.109744009 | 0.495097486095645 | 0.826942451158521 | -1.080135082 | 0.860647063430585 | 0.896063489843847 | 0.339549655478482 | 2.36469030339516 | 0.999950574162623 |
| ebi-fl187-GCST90094726 | prot-a-2701 | Plasma protease C1 inhibitor \|\| id:prot-a-2701 | F-capsicum liking (derived food-liking factor) \|\| id:ebi-fl187-GCST90094726 | MR Egger | 21 | 0.0807300529688679 | 0.484738942364673 | 0.86948893566794 | -0.869358274 | 1.03081838000363 | 1.08407821336884 | 0.419220487605057 | 2.80335910922405 | 0.999950574162623 |
| ebi-fl187-GCST90094726 | prot-a-2738 | Signaling threshold-regulating transmembrane adapter 1 \|\| id:prot-a-2738 | F-capsicum liking (derived food-liking factor) \|\| id:ebi-fl187-GCST90094726 | MR Egger | 21 | 0.00116457688470779 | 0.352777825610259 | 0.99740047291831 | -0.690279961 | 0.692609115080815 | 1.00116525526769 | 0.501435666826508 | 1.99892415850428 | 0.999950574162623 |
| ebi-fl187-GCST90094726 | prot-a-274 | Serine/threonine-protein kinase BRSK2 \|\| id:prot-a-274 | F-capsicum liking (derived food-liking factor) \|\| id:ebi-fl187-GCST90094726 | MR Egger | 21 | -0.387055649 | 0.354297605588035 | 0.28829036800436 | -1.081478956 | 0.307367658111823 | 0.67905330534594 | 0.339093650092275 | 1.35984083268964 | 0.999950574162623 |
| ebi-fl187-GCST90094726 | prot-a-2749 | Mitochondrial glutamate carrier 2 \|\| id:prot-a-2749 | F-capsicum liking (derived food-liking factor) \|\| id:ebi-fl187-GCST90094726 | MR Egger | 21 | 0.017161360120185 | 0.461610967897163 | 0.970731627054047 | -0.887596137 | 0.921918857198624 | 1.01730946225916 | 0.411644100389511 | 2.51410998244053 | 0.999950574162623 |
| ebi-fl187-GCST90094726 | prot-a-2760 | Sodium-coupled monocarboxylate transporter 1 \|\| id:prot-a-2760 | F-capsicum liking (derived food-liking factor) \|\| id:ebi-fl187-GCST90094726 | MR Egger | 21 | 0.138998565379621 | 0.353129661079997 | 0.698244570077462 | -0.55313557 | 0.831132701096416 | 1.14912245144794 | 0.575143576934904 | 2.29591785664881 | 0.999950574162623 |
| ebi-fl187-GCST90094726 | prot-a-2777 | Small integral membrane protein 10 \|\| id:prot-a-2777 | F-capsicum liking (derived food-liking factor) \|\| id:ebi-fl187-GCST90094726 | MR Egger | 21 | 0.047354993765541 | 0.352777825610259 | 0.894628777181087 | -0.644089544 | 0.738799531961649 | 1.04849415190869 | 0.525140441560224 | 2.09342092054559 | 0.999950574162623 |
| ebi-fl187-GCST90094726 | prot-a-2799 | Superoxide dismutase [Mn], mitochondrial \|\| id:prot-a-2799 | F-capsicum liking (derived food-liking factor) \|\| id:ebi-fl187-GCST90094726 | MR Egger | 21 | -0.092208578 | 0.352806196449286 | 0.796628861319682 | -0.783708723 | 0.599291567062243 | 0.911914924336033 | 0.456709059087022 | 1.82082840854782 | 0.999950574162623 |
| ebi-fl187-GCST90094726 | prot-a-2805 | Protein SPACA7 \|\| id:prot-a-2805 | F-capsicum liking (derived food-liking factor) \|\| id:ebi-fl187-GCST90094726 | MR Egger | 21 | -0.574530917 | 0.35256422700624 | 0.119657809011292 | -1.265556802 | 0.116494967894091 | 0.562968885991609 | 0.282082188487301 | 1.12355185662104 | 0.999950574162623 |
| ebi-fl187-GCST90094726 | prot-a-2811 | SPARC-like protein 1 \|\| id:prot-a-2811 | F-capsicum liking (derived food-liking factor) \|\| id:ebi-fl187-GCST90094726 | MR Egger | 21 | 0.16244795735492 | 0.494098836581399 | 0.745920987878939 | -0.805985762 | 1.13088167705446 | 1.17638709455954 | 0.446647421269054 | 3.09838707299421 | 0.999950574162623 |
| ebi-fl187-GCST90094726 | prot-a-2831 | Sepiapterin reductase \|\| id:prot-a-2831 | F-capsicum liking (derived food-liking factor) \|\| id:ebi-fl187-GCST90094726 | MR Egger | 21 | 0.551999335944351 | 0.389264960017309 | 0.172367965303397 | -0.210959986 | 1.31495865757828 | 1.73672183944099 | 0.809806470080454 | 3.72459699820829 | 0.999950574162623 |
| ebi-fl187-GCST90094726 | prot-a-2834 | SPRY domain-containing SOCS box protein 1 \|\| id:prot-a-2834 | F-capsicum liking (derived food-liking factor) \|\| id:ebi-fl187-GCST90094726 | MR Egger | 21 | 0.0692347653584424 | 0.370998112231348 | 0.853938664279129 | -0.657921535 | 0.796391065331885 | 1.07168777478233 | 0.517926709277128 | 2.2175235724392 | 0.999950574162623 |
| ebi-fl187-GCST90094726 | prot-a-2835 | Spectrin alpha chain, non-erythrocytic 1 \|\| id:prot-a-2835 | F-capsicum liking (derived food-liking factor) \|\| id:ebi-fl187-GCST90094726 | MR Egger | 21 | -0.388029562 | 0.352724196350407 | 0.2850347914745 | -1.079368987 | 0.303309862726308 | 0.678392288253107 | 0.339809882471335 | 1.35433405707413 | 0.999950574162623 |
| ebi-fl187-GCST90094726 | prot-a-2859 | Sia-alpha-2,3-Gal-beta-1,4-GlcNAc-R:alpha 2,8-sialyltransferase \|\| id:prot-a-2859 | F-capsicum liking (derived food-liking factor) \|\| id:ebi-fl187-GCST90094726 | MR Egger | 21 | 0.165814926254037 | 0.429779080869017 | 0.70392011520096 | -0.676552072 | 1.00818192475731 | 1.18035462885438 | 0.508366786000099 | 2.74061384068802 | 0.999950574162623 |
| ebi-fl187-GCST90094726 | prot-a-2861 | Alpha-2,8-sialyltransferase 8F \|\| id:prot-a-2861 | F-capsicum liking (derived food-liking factor) \|\| id:ebi-fl187-GCST90094726 | MR Egger | 21 | -0.530009873 | 0.368781847168018 | 0.166928721660741 | -1.252822293 | 0.192802547854401 | 0.588599158648611 | 0.285697336345945 | 1.21264333085117 | 0.999950574162623 |
| ebi-fl187-GCST90094726 | prot-a-289 | Uncharacterized protein C17orf89 \|\| id:prot-a-289 | F-capsicum liking (derived food-liking factor) \|\| id:ebi-fl187-GCST90094726 | MR Egger | 21 | -0.863520693 | 0.352823632179536 | 0.024277706069571 | -1.555055012 | -0.171986374 | 0.421674877993193 | 0.211177764957737 | 0.841990645966723 | 0.999950574162623 |
| ebi-fl187-GCST90094726 | prot-a-2921 | Tachykinin-3 \|\| id:prot-a-2921 | F-capsicum liking (derived food-liking factor) \|\| id:ebi-fl187-GCST90094726 | MR Egger | 21 | -0.168847568 | 0.352762390444257 | 0.637653848746524 | -0.860261853 | 0.522566717290819 | 0.844637643395951 | 0.423051290457173 | 1.6863504845252 | 0.999950574162623 |
| ebi-fl187-GCST90094726 | prot-a-2928 | Tax1-binding protein 1 \|\| id:prot-a-2928 | F-capsicum liking (derived food-liking factor) \|\| id:ebi-fl187-GCST90094726 | MR Egger | 21 | -0.866345519 | 0.422682228248433 | 0.0544610072141544 | -1.694802686 | -0.037888351 | 0.42048540087391 | 0.183635459294508 | 0.962820432542575 | 0.999950574162623 |
| ebi-fl187-GCST90094726 | prot-a-296 | Uncharacterized protein C1orf185 \|\| id:prot-a-296 | F-capsicum liking (derived food-liking factor) \|\| id:ebi-fl187-GCST90094726 | MR Egger | 21 | 0.439954522993636 | 0.437043233915317 | 0.326746567658071 | -0.416650215 | 1.29655926146766 | 1.55263660764088 | 0.659251475580065 | 3.6566932721166 | 0.999950574162623 |
| ebi-fl187-GCST90094726 | prot-a-2964 | Transforming growth factor beta-2 \|\| id:prot-a-2964 | F-capsicum liking (derived food-liking factor) \|\| id:ebi-fl187-GCST90094726 | MR Egger | 21 | 0.197114731062657 | 0.352777825610259 | 0.58285821808444 | -0.494329807 | 0.888559269258765 | 1.21788376178876 | 0.609979574297594 | 2.43162381123456 | 0.999950574162623 |
| ebi-fl187-GCST90094726 | prot-a-2977 | Thymocyte nuclear protein 1 \|\| id:prot-a-2977 | F-capsicum liking (derived food-liking factor) \|\| id:ebi-fl187-GCST90094726 | MR Egger | 21 | -0.274074745 | 0.352600385080782 | 0.446555114546555 | -0.9651715 | 0.417022009591079 | 0.760275246236622 | 0.380917866751393 | 1.51743591071143 | 0.999950574162623 |
| ebi-fl187-GCST90094726 | prot-a-3015 | Transmembrane protein 70, mitochondrial \|\| id:prot-a-3015 | F-capsicum liking (derived food-liking factor) \|\| id:ebi-fl187-GCST90094726 | MR Egger | 21 | 0.148867681886934 | 0.380298128007646 | 0.699820122422961 | -0.596516649 | 0.89425201278192 | 1.1605194213094 | 0.550726673087634 | 2.44550589802646 | 0.999950574162623 |
| ebi-fl187-GCST90094726 | prot-a-3030 | Tumor necrosis factor alpha-induced protein 3 \|\| id:prot-a-3030 | F-capsicum liking (derived food-liking factor) \|\| id:ebi-fl187-GCST90094726 | MR Egger | 21 | -0.032755424 | 0.423233334000022 | 0.939119878486887 | -0.862292758 | 0.79678191102842 | 0.967775225615832 | 0.422192985338794 | 2.21839045138137 | 0.999950574162623 |
| ebi-fl187-GCST90094726 | prot-a-3032 | Tumor necrosis factor receptor superfamily member 10A \|\| id:prot-a-3032 | F-capsicum liking (derived food-liking factor) \|\| id:ebi-fl187-GCST90094726 | MR Egger | 21 | -0.097343205 | 0.390732410423429 | 0.805933443800313 | -0.863178729 | 0.668492319490964 | 0.907244581873816 | 0.421819100198069 | 1.95129317509071 | 0.999950574162623 |
| ebi-fl187-GCST90094726 | prot-a-3081 | Tumor protein p53-inducible protein 11 \|\| id:prot-a-3081 | F-capsicum liking (derived food-liking factor) \|\| id:ebi-fl187-GCST90094726 | MR Egger | 21 | 0.0787307406980846 | 0.389768835808652 | 0.842068902934609 | -0.685216177 | 0.842677658883043 | 1.08191296771672 | 0.50398126839374 | 2.32257772881969 | 0.999950574162623 |
| ebi-fl187-GCST90094726 | prot-a-315 | Complement C4 \|\| id:prot-a-315 | F-capsicum liking (derived food-liking factor) \|\| id:ebi-fl187-GCST90094726 | MR Egger | 20 | -0.102493275 | 0.460556781418252 | 0.826397555698233 | -1.005184566 | 0.80019801700235 | 0.902584219992804 | 0.365977081586618 | 2.22598166707116 | 0.999950574162623 |
| ebi-fl187-GCST90094726 | prot-a-3157 | UDP-glucuronosyltransferase 1-8 \|\| id:prot-a-3157 | F-capsicum liking (derived food-liking factor) \|\| id:ebi-fl187-GCST90094726 | MR Egger | 21 | 0.160767025647071 | 0.388794192882946 | 0.683868940435052 | -0.601269592 | 0.922803643697645 | 1.17441132922011 | 0.548115311127863 | 2.51633541738227 | 0.999950574162623 |
| ebi-fl187-GCST90094726 | prot-a-317 | Glutaredoxin-like protein C5orf63 \|\| id:prot-a-317 | F-capsicum liking (derived food-liking factor) \|\| id:ebi-fl187-GCST90094726 | MR Egger | 21 | 0.30421711842293 | 0.425214109887496 | 0.483034519107264 | -0.529202537 | 1.13763677380242 | 1.35556334191606 | 0.589074547599491 | 3.11938782864537 | 0.999950574162623 |
| ebi-fl187-GCST90094726 | prot-a-3192 | Guanine nucleotide exchange factor VAV3 \|\| id:prot-a-3192 | F-capsicum liking (derived food-liking factor) \|\| id:ebi-fl187-GCST90094726 | MR Egger | 21 | 0.497456084959957 | 0.41105956461315 | 0.241050915589434 | -0.308220662 | 1.30313283160173 | 1.64453239419475 | 0.734753168249782 | 3.68080998139573 | 0.999950574162623 |
| ebi-fl187-GCST90094726 | prot-a-3233 | Serine/threonine-protein kinase WNK3 \|\| id:prot-a-3233 | F-capsicum liking (derived food-liking factor) \|\| id:ebi-fl187-GCST90094726 | MR Egger | 21 | 0.748265332650387 | 0.352798611538555 | 0.0473070855998835 | 0.0567800540348193 | 1.43975061126595 | 2.11333090906531 | 1.0584229889265 | 4.21964335425159 | 0.999950574162623 |
| ebi-fl187-GCST90094726 | prot-a-3250 | 14-3-3 protein theta \|\| id:prot-a-3250 | F-capsicum liking (derived food-liking factor) \|\| id:ebi-fl187-GCST90094726 | MR Egger | 21 | 0.309697842524071 | 0.358580264234372 | 0.398537009007694 | -0.393119475 | 1.01251516042344 | 1.36301320727458 | 0.67494810309079 | 2.75251533369378 | 0.999950574162623 |
| ebi-fl187-GCST90094726 | prot-a-3278 | E3 ubiquitin-protein ligase ZNRF3 \|\| id:prot-a-3278 | F-capsicum liking (derived food-liking factor) \|\| id:ebi-fl187-GCST90094726 | MR Egger | 21 | 0.147801275482334 | 0.35256422700624 | 0.679758338862528 | -0.543224609 | 0.838827160414564 | 1.15928249561581 | 0.580872143337603 | 2.31365184241609 | 0.999950574162623 |
| ebi-fl187-GCST90094726 | prot-a-355 | Calpain-3 \|\| id:prot-a-355 | F-capsicum liking (derived food-liking factor) \|\| id:ebi-fl187-GCST90094726 | MR Egger | 21 | 0.142422057323309 | 0.352658634133722 | 0.690830503023704 | -0.548788866 | 0.833632980225403 | 1.15306320462225 | 0.577648997474463 | 2.30166547447789 | 0.999950574162623 |
| ebi-fl187-GCST90094726 | prot-a-385 | Coiled-coil domain-containing protein 90B, mitochondrial \|\| id:prot-a-385 | F-capsicum liking (derived food-liking factor) \|\| id:ebi-fl187-GCST90094726 | MR Egger | 21 | -0.586029219 | 0.352798611538555 | 0.113108330393739 | -1.277514497 | 0.105456060115459 | 0.556532773120009 | 0.278729222496329 | 1.11121727669128 | 0.999950574162623 |
| ebi-fl187-GCST90094726 | prot-a-390 | C-C motif chemokine 14 \|\| id:prot-a-390 | F-capsicum liking (derived food-liking factor) \|\| id:ebi-fl187-GCST90094726 | MR Egger | 21 | -0.465332198 | 0.411270640203534 | 0.271944957352577 | -1.271422653 | 0.340758256454159 | 0.627926474402726 | 0.280432379847539 | 1.40601330513331 | 0.999950574162623 |
| ebi-fl187-GCST90094726 | prot-a-40 | Pituitary adenylate cyclase-activating polypeptide 27 \|\| id:prot-a-40 | F-capsicum liking (derived food-liking factor) \|\| id:ebi-fl187-GCST90094726 | MR Egger | 21 | -0.245941912 | 0.352979682255062 | 0.494390593725564 | -0.937782089 | 0.44589826530938 | 0.781967646612844 | 0.391495174565487 | 1.56189255979436 | 0.999950574162623 |
| ebi-fl187-GCST90094726 | prot-a-401 | C-C motif chemokine 24 \|\| id:prot-a-401 | F-capsicum liking (derived food-liking factor) \|\| id:ebi-fl187-GCST90094726 | MR Egger | 21 | -0.678663184 | 0.352604674388972 | 0.0693711454091934 | -1.369768346 | 0.0124419773151063 | 0.507294698703239 | 0.254165831191457 | 1.01251970072467 | 0.999950574162623 |
| ebi-fl187-GCST90094726 | prot-a-412 | Cerebral cavernous malformations 2 protein \|\| id:prot-a-412 | F-capsicum liking (derived food-liking factor) \|\| id:ebi-fl187-GCST90094726 | MR Egger | 21 | -0.438630103 | 0.47567438787283 | 0.368026517690205 | -1.370951903 | 0.493691697447075 | 0.644919289364931 | 0.253865189461113 | 1.63835337439471 | 0.999950574162623 |
| ebi-fl187-GCST90094726 | prot-a-44 | Adhesion G protein-coupled receptor F5 \|\| id:prot-a-44 | F-capsicum liking (derived food-liking factor) \|\| id:ebi-fl187-GCST90094726 | MR Egger | 21 | 0.151120644807764 | 0.352625441389864 | 0.673063802964797 | -0.54002522 | 0.842266509931897 | 1.16313697605421 | 0.582733555464032 | 2.32162299970392 | 0.999950574162623 |
| ebi-fl187-GCST90094726 | prot-a-48 | Adrenomedullin \|\| id:prot-a-48 | F-capsicum liking (derived food-liking factor) \|\| id:ebi-fl187-GCST90094726 | MR Egger | 21 | 0.440591512605009 | 0.366921428128706 | 0.244593638428659 | -0.278574487 | 1.15975751173727 | 1.5536259360927 | 0.756861889636456 | 3.18915984851519 | 0.999950574162623 |
| ebi-fl187-GCST90094726 | prot-a-502 | Carcinoembryonic antigen-related cell adhesion molecule 1 \|\| id:prot-a-502 | F-capsicum liking (derived food-liking factor) \|\| id:ebi-fl187-GCST90094726 | MR Egger | 21 | -0.154606409 | 0.37270322322617 | 0.682916796332192 | -0.885104727 | 0.575891908114679 | 0.856752320783654 | 0.412670953253117 | 1.77871627111578 | 0.999950574162623 |
| ebi-fl187-GCST90094726 | prot-a-518 | Complement factor B \|\| id:prot-a-518 | F-capsicum liking (derived food-liking factor) \|\| id:ebi-fl187-GCST90094726 | MR Egger | 21 | 0.173181738959239 | 0.352629739114003 | 0.628969866299553 | -0.51797255 | 0.864336027622684 | 1.18908218800739 | 0.595727131561851 | 2.373429671 | 0.999950574162623 |
| ebi-fl187-GCST90094726 | prot-a-525 | Properdin \|\| id:prot-a-525 | F-capsicum liking (derived food-liking factor) \|\| id:ebi-fl187-GCST90094726 | MR Egger | 21 | 0.167345514639454 | 0.410215851535543 | 0.687874350981304 | -0.636677554 | 0.971368583649117 | 1.18216264925452 | 0.529047237975642 | 2.64155717859886 | 0.999950574162623 |
| ebi-fl187-GCST90094726 | prot-a-533 | Chondroadherin \|\| id:prot-a-533 | F-capsicum liking (derived food-liking factor) \|\| id:ebi-fl187-GCST90094726 | MR Egger | 21 | 0.565944701762134 | 0.455043463133367 | 0.228731202215109 | -0.325940486 | 1.45782988950353 | 1.76111072150946 | 0.72184814623892 | 4.29662525224387 | 0.999950574162623 |
| ebi-fl187-GCST90094726 | prot-a-535 | Coiled-coil-helix-coiled-coil-helix domain-containing protein 2 \|\| id:prot-a-535 | F-capsicum liking (derived food-liking factor) \|\| id:ebi-fl187-GCST90094726 | MR Egger | 21 | -0.686059787 | 0.420093252487666 | 0.118907364837459 | -1.509442562 | 0.137322987717486 | 0.503556284207075 | 0.221033155896964 | 1.14719861975205 | 0.999950574162623 |
| ebi-fl187-GCST90094726 | prot-a-591 | Caseinolytic peptidase B protein homolog \|\| id:prot-a-591 | F-capsicum liking (derived food-liking factor) \|\| id:ebi-fl187-GCST90094726 | MR Egger | 21 | 0.543851257861726 | 0.431304219145496 | 0.222592656188597 | -0.301505012 | 1.3892075273869 | 1.72262838952393 | 0.739704119197255 | 4.01166965463724 | 0.999950574162623 |
| ebi-fl187-GCST90094726 | prot-a-605 | Ciliary neurotrophic factor receptor subunit alpha \|\| id:prot-a-605 | F-capsicum liking (derived food-liking factor) \|\| id:ebi-fl187-GCST90094726 | MR Egger | 21 | 0.35099305799485 | 0.352694831195281 | 0.332157214289409 | -0.340288811 | 1.0422749271376 | 1.42047746491609 | 0.711564785240843 | 2.83566060348461 | 0.999950574162623 |
| ebi-fl187-GCST90094726 | prot-a-606 | Ciliary neurotrophic factor receptor subunit alpha \|\| id:prot-a-606 | F-capsicum liking (derived food-liking factor) \|\| id:ebi-fl187-GCST90094726 | MR Egger | 21 | 0.42701502443279 | 0.373467533553157 | 0.267083569775361 | -0.304981341 | 1.15901139019698 | 1.53267568913391 | 0.737137128260704 | 3.18678123513445 | 0.999950574162623 |
| ebi-fl187-GCST90094726 | prot-a-707 | Cancer/testis antigen 55 \|\| id:prot-a-707 | F-capsicum liking (derived food-liking factor) \|\| id:ebi-fl187-GCST90094726 | MR Egger | 21 | 0.15442749461544 | 0.470112269235377 | 0.746133470068904 | -0.766992553 | 1.07584754231678 | 1.16698966195575 | 0.464407651551103 | 2.93247724615007 | 0.999950574162623 |
| ebi-fl187-GCST90094726 | prot-a-719 | Dipeptidyl peptidase 1 \|\| id:prot-a-719 | F-capsicum liking (derived food-liking factor) \|\| id:ebi-fl187-GCST90094726 | MR Egger | 21 | -0.882437482 | 0.352979682255062 | 0.0217419333722611 | -1.574277659 | -0.190597305 | 0.413773116927059 | 0.20715713667124 | 0.82646533468573 | 0.999950574162623 |
| ebi-fl187-GCST90094726 | prot-a-752 | Cytochrome b5 \|\| id:prot-a-752 | F-capsicum liking (derived food-liking factor) \|\| id:ebi-fl187-GCST90094726 | MR Egger | 21 | 0.195780511672492 | 0.382198524263247 | 0.614380466061394 | -0.553328596 | 0.944889619228456 | 1.21625992117952 | 0.575032570245674 | 2.57252940513545 | 0.999950574162623 |
| ebi-fl187-GCST90094726 | prot-a-755 | Cytoglobin \|\| id:prot-a-755 | F-capsicum liking (derived food-liking factor) \|\| id:ebi-fl187-GCST90094726 | MR Egger | 21 | -0.173842152 | 0.377032630295708 | 0.649975720526146 | -0.912826107 | 0.565141803827036 | 0.840429547688148 | 0.401388253476945 | 1.75969729684149 | 0.999950574162623 |
| ebi-fl187-GCST90094726 | prot-a-807 | Desmin \|\| id:prot-a-807 | F-capsicum liking (derived food-liking factor) \|\| id:ebi-fl187-GCST90094726 | MR Egger | 21 | 0.665667480058511 | 0.377397738214929 | 0.0938334883081082 | -0.074032087 | 1.40536704695977 | 1.94578886324425 | 0.92864189617674 | 4.07702292553552 | 0.999950574162623 |
| ebi-fl187-GCST90094726 | prot-a-82 | Ameloblastin \|\| id:prot-a-82 | F-capsicum liking (derived food-liking factor) \|\| id:ebi-fl187-GCST90094726 | MR Egger | 21 | -0.311762883 | 0.414323681133493 | 0.460994674329433 | -1.123837298 | 0.500311532141868 | 0.732155114159626 | 0.325030160993496 | 1.64923498038332 | 0.999950574162623 |
| ebi-fl187-GCST90094726 | prot-a-84 | Muellerian-inhibiting factor \|\| id:prot-a-84 | F-capsicum liking (derived food-liking factor) \|\| id:ebi-fl187-GCST90094726 | MR Egger | 21 | 0.197558251254515 | 0.352699120302668 | 0.581933860610532 | -0.493732025 | 0.888848527047744 | 1.21842403763122 | 0.610344318478253 | 2.4323272790987 | 0.999950574162623 |
| ebi-fl187-GCST90094726 | prot-a-858 | Inactive dipeptidyl peptidase 10 \|\| id:prot-a-858 | F-capsicum liking (derived food-liking factor) \|\| id:ebi-fl187-GCST90094726 | MR Egger | 21 | -0.758481404 | 0.352864530635291 | 0.0446895177415169 | -1.450095884 | -0.066866924 | 0.46837716286719 | 0.234547797607999 | 0.935319661633168 | 0.999950574162623 |
| ebi-fl187-GCST90094726 | prot-a-863 | Dihydropyrimidinase-related protein 3 \|\| id:prot-a-863 | F-capsicum liking (derived food-liking factor) \|\| id:ebi-fl187-GCST90094726 | MR Egger | 21 | 0.104953719382814 | 0.352950342671947 | 0.769417554341867 | -0.586828952 | 0.796736391019831 | 1.11065920717264 | 0.556087873000619 | 2.21828947252727 | 0.999950574162623 |
| ebi-fl187-GCST90094726 | prot-a-864 | Dihydropyrimidinase-related protein 5 \|\| id:prot-a-864 | F-capsicum liking (derived food-liking factor) \|\| id:ebi-fl187-GCST90094726 | MR Egger | 21 | -0.536825826 | 0.406809087829448 | 0.202648922568388 | -1.334171639 | 0.260519985714458 | 0.584600935286931 | 0.263376255848951 | 1.2976046471492 | 0.999950574162623 |
| ebi-fl187-GCST90094726 | prot-a-884 | Endothelin-converting enzyme 1 \|\| id:prot-a-884 | F-capsicum liking (derived food-liking factor) \|\| id:ebi-fl187-GCST90094726 | MR Egger | 21 | -0.016477123 | 0.352954614964722 | 0.96325275456159 | -0.708268168 | 0.675313922390025 | 0.983657882332847 | 0.492496380186719 | 1.96464962668092 | 0.999950574162623 |
| ebi-fl187-GCST90094726 | prot-a-893 | Early endosome antigen 1 \|\| id:prot-a-893 | F-capsicum liking (derived food-liking factor) \|\| id:ebi-fl187-GCST90094726 | MR Egger | 21 | -0.184317414 | 0.356408223865432 | 0.611017898898272 | -0.882877533 | 0.514242704518668 | 0.831671777446233 | 0.413591075747374 | 1.67237154271448 | 0.999950574162623 |
| ebi-fl187-GCST90094726 | prot-a-897 | Ephrin-A2 \|\| id:prot-a-897 | F-capsicum liking (derived food-liking factor) \|\| id:ebi-fl187-GCST90094726 | MR Egger | 21 | 0.791563007575705 | 0.352776871954005 | 0.0369555230997299 | 0.100120338545856 | 1.48300567660555 | 2.20684304440592 | 1.10530392073936 | 4.4061693180144 | 0.999950574162623 |
| ebi-fl187-GCST90094726 | prot-a-90 | Anaphase-promoting complex subunit 10 \|\| id:prot-a-90 | F-capsicum liking (derived food-liking factor) \|\| id:ebi-fl187-GCST90094726 | MR Egger | 21 | -0.048226316 | 0.352954614964722 | 0.892755918821371 | -0.740017361 | 0.643564729384469 | 0.952918102109844 | 0.477105632285975 | 1.90325338432463 | 0.999950574162623 |
| ebi-fl187-GCST90094726 | prot-a-914 | Histone-lysine N-methyltransferase EHMT2 \|\| id:prot-a-914 | F-capsicum liking (derived food-liking factor) \|\| id:ebi-fl187-GCST90094726 | MR Egger | 21 | -0.587136348 | 0.432478890194573 | 0.190495442237535 | -1.434794973 | 0.260522276419229 | 0.555916960023128 | 0.238164189064308 | 1.29760761958176 | 0.999950574162623 |
| ebi-fl187-GCST90094726 | prot-a-932 | RNA polymerase II elongation factor ELL \|\| id:prot-a-932 | F-capsicum liking (derived food-liking factor) \|\| id:ebi-fl187-GCST90094726 | MR Egger | 21 | -0.189572542 | 0.352802907345278 | 0.597275333761305 | -0.881066241 | 0.501921156202703 | 0.827312699642199 | 0.414340888997922 | 1.65189176632938 | 0.999950574162623 |
| ebi-fl187-GCST90094726 | prot-a-991 | Estrogen receptor \|\| id:prot-a-991 | F-capsicum liking (derived food-liking factor) \|\| id:ebi-fl187-GCST90094726 | MR Egger | 21 | 0.194740604948314 | 0.352694831195281 | 0.587282171482163 | -0.496541264 | 0.886022474091065 | 1.21499578171662 | 0.608632121127517 | 2.42546309723916 | 0.999950574162623 |
| ebi-fl187-GCST90094726 | prot-a-992 | Persulfide dioxygenase ETHE1, mitochondrial \|\| id:prot-a-992 | F-capsicum liking (derived food-liking factor) \|\| id:ebi-fl187-GCST90094726 | MR Egger | 21 | 0.703597840486904 | 0.403684685246183 | 0.0975060100593717 | -0.087624143 | 1.49481982356942 | 2.0210109176037 | 0.916105136921809 | 4.45853315788358 | 0.999950574162623 |
| ebi-fl187-GCST90094726 | prot-a-993 | Ethanolamine kinase 1 \|\| id:prot-a-993 | F-capsicum liking (derived food-liking factor) \|\| id:ebi-fl187-GCST90094726 | MR Egger | 21 | -0.349473018 | 0.412415600357798 | 0.407330883161247 | -1.157807595 | 0.45886155834445 | 0.705059545272686 | 0.314174223493196 | 1.58227163531413 | 0.999950574162623 |
| ebi-fl187-GCST90094726 | prot-b-10 | coagulation factor III, tissue factor \|\| id:prot-b-10 | F-capsicum liking (derived food-liking factor) \|\| id:ebi-fl187-GCST90094726 | MR Egger | 14 | 0.693574785864431 | 0.628442626434878 | 0.291389096555705 | -0.538172762 | 1.92532233367679 | 2.00085539348133 | 0.583814045587923 | 6.85735866733342 | 0.999950574162623 |
| ebi-fl187-GCST90094726 | prot-b-28 | prolactin \|\| id:prot-b-28 | F-capsicum liking (derived food-liking factor) \|\| id:ebi-fl187-GCST90094726 | MR Egger | 14 | -0.034611218 | 0.573167644570589 | 0.95284236314038 | -1.158019802 | 1.088797365 | 0.965980898962985 | 0.314107560724195 | 2.97069925668128 | 0.999950574162623 |
| ebi-fl187-GCST90094726 | prot-b-54 | matrix metallopeptidase 7 \|\| id:prot-b-54 | F-capsicum liking (derived food-liking factor) \|\| id:ebi-fl187-GCST90094726 | MR Egger | 14 | 1.44293126497711 | 0.573108454297796 | 0.0270216989748973 | 0.319638694553428 | 2.56622383540079 | 4.2330859453269 | 1.3766302904495 | 13.0165787756081 | 0.999950574162623 |
| ebi-fl187-GCST90094726 | ukb-rsfMRI191-edge-pheno716 | Net100_Pair10_33 (OccipitalPrecuneus)&(Temporal\|Frontal\|Supp_Motor_Area) (Default_mode\|Central_executive)&(Default_mode\|Salience) \|\| id:ukb-rsfMRI191-edge-pheno716 | F-capsicum liking (derived food-liking factor) \|\| id:ebi-fl187-GCST90094726 | MR Egger | 20 | 0.0153384550822887 | 0.123374827860133 | 0.902436583334012 | -0.226476208 | 0.257153117688149 | 1.01545669293963 | 0.797338312740321 | 1.29324312999835 | 0.999950574162623 |
| ebi-fl187-GCST90094726 | ukb-rsfMRI191-edge-pheno903 | Net100_Pair27_38 (Precentral\|Frontal\|Supp_Motor_Area)&(Subcortical) (Attention\|Salience\|Motor)&(Subcortical-cerebellum) \|\| id:ukb-rsfMRI191-edge-pheno903 | F-capsicum liking (derived food-liking factor) \|\| id:ebi-fl187-GCST90094726 | MR Egger | 20 | -0.043742321 | 0.169922897591745 | 0.79977090031595 | -0.3767912 | 0.2893065586822 | 0.95720057656988 | 0.686059308209467 | 1.33550107523061 | 0.999950574162623 |
| ebi-fl187-GCST90094726 | ukb-rsfMRI191-node-pheno23 | Net100_Node2 RolandicOper\|SupraMarginal\|Insula Salience\|Motor \|\| id:ukb-rsfMRI191-node-pheno23 | F-capsicum liking (derived food-liking factor) \|\| id:ebi-fl187-GCST90094726 | MR Egger | 20 | -0.285421044 | 0.182356043639542 | 0.134950203837659 | -0.642838889 | 0.0719968019544537 | 0.751697690194572 | 0.525797622113685 | 1.07465190727257 | 0.999950574162623 |
| ebi-fl187-GCST90094726 | ukb-rsfMRI191-node-pheno55 | Net100_Node34 Parietal\|Postcentral\|Precuneus Attention \|\| id:ukb-rsfMRI191-node-pheno55 | F-capsicum liking (derived food-liking factor) \|\| id:ebi-fl187-GCST90094726 | MR Egger | 20 | -0.301003456 | 0.161645210483102 | 0.0789930485466337 | -0.617828068 | 0.0158211569152633 | 0.740075215314976 | 0.539114085975358 | 1.0159469740669 | 0.999950574162623 |
| ebi-fl187-GCST90094726 | ebi-a-GCST90001414 | IgD- CD24- B cell Absolute Count \|\| id:ebi-a-GCST90001414 | F-capsicum liking (derived food-liking factor) \|\| id:ebi-fl187-GCST90094726 | Weighted median | 20 | 0.00687873309778432 | 0.119311061467465 | 0.954024414583122 | -0.226970947 | 0.240728413574016 | 1.00690244592252 | 0.79694393526436 | 1.27217548279407 | 0.999332001504091 |
| ebi-fl187-GCST90094726 | ebi-a-GCST90001422 | CD20- CD38- B cell %B cell \|\| id:ebi-a-GCST90001422 | F-capsicum liking (derived food-liking factor) \|\| id:ebi-fl187-GCST90094726 | Weighted median | 20 | 0.04873103433978 | 0.101768259598632 | 0.632050214489337 | -0.150734754 | 0.248196823153099 | 1.04993791551441 | 0.860075799664761 | 1.28171217800154 | 0.999332001504091 |
| ebi-fl187-GCST90094726 | ebi-a-GCST90001444 | CD20- CD38- B cell %lymphocyte \|\| id:ebi-a-GCST90001444 | F-capsicum liking (derived food-liking factor) \|\| id:ebi-fl187-GCST90094726 | Weighted median | 20 | 0.0897393312744387 | 0.107668110026603 | 0.404573471055448 | -0.121290164 | 0.30076882692658 | 1.09388910385948 | 0.885776901396736 | 1.3508970144239 | 0.999332001504091 |
| ebi-fl187-GCST90094726 | ebi-a-GCST90001459 | Myeloid Dendritic Cell %Dendritic Cell \|\| id:ebi-a-GCST90001459 | F-capsicum liking (derived food-liking factor) \|\| id:ebi-fl187-GCST90094726 | Weighted median | 20 | -0.090619463 | 0.112385161818888 | 0.420052930430826 | -0.31089438 | 0.12965545465178 | 0.913365214479215 | 0.732791269431739 | 1.13843607289098 | 0.999332001504091 |
| ebi-fl187-GCST90094726 | ebi-a-GCST90001476 | HLA DR++ monocyte %leukocyte \|\| id:ebi-a-GCST90001476 | F-capsicum liking (derived food-liking factor) \|\| id:ebi-fl187-GCST90094726 | Weighted median | 20 | -0.008376049 | 0.111337567558763 | 0.940030834286917 | -0.226597681 | 0.209845583903525 | 0.991658932846034 | 0.797241463224368 | 1.23348757491378 | 0.999332001504091 |
| ebi-fl187-GCST90094726 | ebi-a-GCST90001478 | CD4 regulatory T cell %CD4+ T cell \|\| id:ebi-a-GCST90001478 | F-capsicum liking (derived food-liking factor) \|\| id:ebi-fl187-GCST90094726 | Weighted median | 20 | -0.026975794 | 0.118583542866236 | 0.820047831896777 | -0.259399538 | 0.205447949808476 | 0.973384802787938 | 0.771514711836547 | 1.22807505775631 | 0.999332001504091 |
| ebi-fl187-GCST90094726 | ebi-a-GCST90001485 | CD39+ resting CD4 regulatory T cell %CD4 regulatory T cell \|\| id:ebi-a-GCST90001485 | F-capsicum liking (derived food-liking factor) \|\| id:ebi-fl187-GCST90094726 | Weighted median | 20 | -0.004546926 | 0.122183116353535 | 0.970314353020112 | -0.244025834 | 0.234931982045781 | 0.995463395611105 | 0.783467393857696 | 1.26482273515212 | 0.999332001504091 |
| ebi-fl187-GCST90094726 | ebi-a-GCST90001515 | Immature Myeloid-Derived Suppressor Cells Absolute Count \|\| id:ebi-a-GCST90001515 | F-capsicum liking (derived food-liking factor) \|\| id:ebi-fl187-GCST90094726 | Weighted median | 20 | 0.0777923074549806 | 0.150751728840185 | 0.605833953009402 | -0.217681081 | 0.373265695981744 | 1.08089814086977 | 0.804381933388427 | 1.45247020406604 | 0.999332001504091 |
| ebi-fl187-GCST90094726 | ebi-a-GCST90001517 | CD33+ HLA DR+ Absolute Count \|\| id:ebi-a-GCST90001517 | F-capsicum liking (derived food-liking factor) \|\| id:ebi-fl187-GCST90094726 | Weighted median | 20 | 0.300134954732651 | 0.150247360291064 | 0.0457594454544862 | 0.00565012856216446 | 0.594619780903137 | 1.35004098970339 | 1.00566612064347 | 1.8123417270069 | 0.999332001504091 |
| ebi-fl187-GCST90094726 | ebi-a-GCST90001518 | CD33+ HLA DR+ CD14- Absolute Count \|\| id:ebi-a-GCST90001518 | F-capsicum liking (derived food-liking factor) \|\| id:ebi-fl187-GCST90094726 | Weighted median | 20 | 0.155427351716606 | 0.155402398906085 | 0.317232808098466 | -0.14916135 | 0.460016053572532 | 1.16815706837879 | 0.86143011181662 | 1.58409941524522 | 0.999332001504091 |
| ebi-fl187-GCST90094726 | ebi-a-GCST90001520 | CD33+ HLA DR+ CD14dim Absolute Count \|\| id:ebi-a-GCST90001520 | F-capsicum liking (derived food-liking factor) \|\| id:ebi-fl187-GCST90094726 | Weighted median | 20 | 0.178004981497625 | 0.150708481292668 | 0.237554581249166 | -0.117383642 | 0.473393604831255 | 1.19483127326913 | 0.889243976523297 | 1.60543316488185 | 0.999332001504091 |
| ebi-fl187-GCST90094726 | ebi-a-GCST90001531 | CD33dim HLA DR- Absolute Count \|\| id:ebi-a-GCST90001531 | F-capsicum liking (derived food-liking factor) \|\| id:ebi-fl187-GCST90094726 | Weighted median | 20 | 0.167374286553045 | 0.152773806156922 | 0.273267318069923 | -0.132062374 | 0.466810946620612 | 1.18219666282543 | 0.876286336316238 | 1.59489985370627 | 0.999332001504091 |
| ebi-fl187-GCST90094726 | ebi-a-GCST90001532 | Basophil Absolute Count \|\| id:ebi-a-GCST90001532 | F-capsicum liking (derived food-liking factor) \|\| id:ebi-fl187-GCST90094726 | Weighted median | 20 | 0.121872266907627 | 0.150813956340474 | 0.419034892462187 | -0.173723088 | 0.417467621334957 | 1.12960980401127 | 0.840529618576725 | 1.5181122486547 | 0.999332001504091 |
| ebi-fl187-GCST90094726 | ebi-a-GCST90001588 | T/B cell \|\| id:ebi-a-GCST90001588 | F-capsicum liking (derived food-liking factor) \|\| id:ebi-fl187-GCST90094726 | Weighted median | 20 | 0.00425270441408552 | 0.1117631924496 | 0.969646996652589 | -0.214803153 | 0.223308561615301 | 1.00426175999385 | 0.806700221239182 | 1.25020627989505 | 0.999332001504091 |
| ebi-fl187-GCST90094726 | ebi-a-GCST90001624 | HLA DR+ CD4+ T cell Absolute Count \|\| id:ebi-a-GCST90001624 | F-capsicum liking (derived food-liking factor) \|\| id:ebi-fl187-GCST90094726 | Weighted median | 20 | 0.119910864355984 | 0.106361266579816 | 0.259576687369528 | -0.088557218 | 0.328378946852422 | 1.12739635590032 | 0.915250740292332 | 1.38871512181609 | 0.999332001504091 |
| ebi-fl187-GCST90094726 | ebi-a-GCST90001631 | CD8+ Natural Killer T %T cell \|\| id:ebi-a-GCST90001631 | F-capsicum liking (derived food-liking factor) \|\| id:ebi-fl187-GCST90094726 | Weighted median | 20 | 0.0914505141333556 | 0.10590183333018 | 0.38784040334236 | -0.116117079 | 0.299018107460509 | 1.09576255059139 | 0.890370973279105 | 1.34853404177874 | 0.999332001504091 |
| ebi-fl187-GCST90094726 | ebi-a-GCST90001632 | CD8+ Natural Killer T %lymphocyte \|\| id:ebi-a-GCST90001632 | F-capsicum liking (derived food-liking factor) \|\| id:ebi-fl187-GCST90094726 | Weighted median | 20 | 0.109734142566652 | 0.108571633803784 | 0.312156543385062 | -0.10306626 | 0.322534544822069 | 1.11598133908203 | 0.902067200809308 | 1.38062258339729 | 0.999332001504091 |
| ebi-fl187-GCST90094726 | ebi-a-GCST90001646 | Natural Killer %CD3- lymphocyte \|\| id:ebi-a-GCST90001646 | F-capsicum liking (derived food-liking factor) \|\| id:ebi-fl187-GCST90094726 | Weighted median | 20 | -0.105088784 | 0.105790312125014 | 0.320530313594452 | -0.312437796 | 0.102260227897569 | 0.900244591841515 | 0.731661140049583 | 1.10767168129904 | 0.999332001504091 |
| ebi-fl187-GCST90094726 | ebi-a-GCST90001684 | CD127- CD8+ T cell Absolute Count \|\| id:ebi-a-GCST90001684 | F-capsicum liking (derived food-liking factor) \|\| id:ebi-fl187-GCST90094726 | Weighted median | 20 | 0.0358908888125396 | 0.113600586574877 | 0.75204858806018 | -0.186766261 | 0.258548038499298 | 1.03654274191113 | 0.829637632498903 | 1.29504836053836 | 0.999332001504091 |
| ebi-fl187-GCST90094726 | ebi-a-GCST90001687 | CD28- CD8+ T cell Absolute Count \|\| id:ebi-a-GCST90001687 | F-capsicum liking (derived food-liking factor) \|\| id:ebi-fl187-GCST90094726 | Weighted median | 20 | 0.0731996483496765 | 0.111646094637833 | 0.512055765863337 | -0.145626697 | 0.292025993839829 | 1.07594532618154 | 0.864480355946173 | 1.33913782652106 | 0.999332001504091 |
| ebi-fl187-GCST90094726 | ebi-a-GCST90001701 | BAFF-R on CD20- CD38- B cell \|\| id:ebi-a-GCST90001701 | F-capsicum liking (derived food-liking factor) \|\| id:ebi-fl187-GCST90094726 | Weighted median | 20 | 0.126041780720204 | 0.113919106471872 | 0.268547040298449 | -0.097239668 | 0.349323229405074 | 1.13432956039896 | 0.907338520095722 | 1.41810749031041 | 0.999332001504091 |
| ebi-fl187-GCST90094726 | ebi-a-GCST90001732 | CD19 on IgD- CD27- B cell \|\| id:ebi-a-GCST90001732 | F-capsicum liking (derived food-liking factor) \|\| id:ebi-fl187-GCST90094726 | Weighted median | 20 | -0.07538996 | 0.117791801681776 | 0.522154902241898 | -0.306261891 | 0.155481971762529 | 0.927381774442265 | 0.736193791804103 | 1.16822087491405 | 0.999332001504091 |
| ebi-fl187-GCST90094726 | ebi-a-GCST90001798 | CD27 on CD24+ CD27+ B cell \|\| id:ebi-a-GCST90001798 | F-capsicum liking (derived food-liking factor) \|\| id:ebi-fl187-GCST90094726 | Weighted median | 20 | -0.011675568 | 0.114708093309018 | 0.918927297078418 | -0.236503431 | 0.213152294550882 | 0.988392326618894 | 0.78938317374962 | 1.23757311253378 | 0.999332001504091 |
| ebi-fl187-GCST90094726 | ebi-a-GCST90001836 | CD66b on CD66b++ myeloid cell \|\| id:ebi-a-GCST90001836 | F-capsicum liking (derived food-liking factor) \|\| id:ebi-fl187-GCST90094726 | Weighted median | 20 | -0.01580706 | 0.172488111045968 | 0.926982922676818 | -0.353883757 | 0.322269638136082 | 0.984317216377442 | 0.701956560034228 | 1.38025689568282 | 0.999332001504091 |
| ebi-fl187-GCST90094726 | ebi-a-GCST90001943 | CD25 on activated & secreting CD4 regulatory T cell \|\| id:ebi-a-GCST90001943 | F-capsicum liking (derived food-liking factor) \|\| id:ebi-fl187-GCST90094726 | Weighted median | 20 | 0.0477093357604043 | 0.117180549189714 | 0.683902365014792 | -0.181964541 | 0.277383212172244 | 1.0488657432494 | 0.833630899900306 | 1.31967198851875 | 0.999332001504091 |
| ebi-fl187-GCST90094726 | ebi-a-GCST90001960 | CD25 on CD4+ T cell \|\| id:ebi-a-GCST90001960 | F-capsicum liking (derived food-liking factor) \|\| id:ebi-fl187-GCST90094726 | Weighted median | 20 | 0.0914946851165552 | 0.119118449884428 | 0.442428755591678 | -0.141977477 | 0.324966846890034 | 1.09581095256957 | 0.867640798437208 | 1.38398476182115 | 0.999332001504091 |
| ebi-fl187-GCST90094726 | ebi-a-GCST90001988 | HLA DR on CD14+ CD16- monocyte \|\| id:ebi-a-GCST90001988 | F-capsicum liking (derived food-liking factor) \|\| id:ebi-fl187-GCST90094726 | Weighted median | 20 | 0.0431490850607017 | 0.112212536198585 | 0.700585674865261 | -0.176787486 | 0.263085656009929 | 1.04409354199708 | 0.837957843468862 | 1.30093814735027 | 0.999332001504091 |
| ebi-fl187-GCST90094726 | ebi-a-GCST90001991 | HLA DR on CD14+ monocyte \|\| id:ebi-a-GCST90001991 | F-capsicum liking (derived food-liking factor) \|\| id:ebi-fl187-GCST90094726 | Weighted median | 20 | 0.071109564002754 | 0.111040757574505 | 0.521918068314898 | -0.146530321 | 0.288749448848784 | 1.07369885816876 | 0.86369954383923 | 1.33475726166123 | 0.999332001504091 |
| ebi-fl187-GCST90094726 | ebi-a-GCST90002019 | CD14 on Monocytic Myeloid-Derived Suppressor Cells \|\| id:ebi-a-GCST90002019 | F-capsicum liking (derived food-liking factor) \|\| id:ebi-fl187-GCST90094726 | Weighted median | 20 | 0.0891667345673113 | 0.152976934017521 | 0.559976112815459 | -0.210668056 | 0.389001525241653 | 1.09326292585149 | 0.810042911055585 | 1.47550680183577 | 0.999332001504091 |
| ebi-fl187-GCST90094726 | ebi-a-GCST90002037 | CD80 on plasmacytoid Dendritic Cell \|\| id:ebi-a-GCST90002037 | F-capsicum liking (derived food-liking factor) \|\| id:ebi-fl187-GCST90094726 | Weighted median | 20 | -0.048979481 | 0.124540144450836 | 0.694110716285634 | -0.293078164 | 0.195119202557689 | 0.952200668116857 | 0.745963831133065 | 1.21545586330238 | 0.999332001504091 |
| ebi-fl187-GCST90094726 | ebi-a-GCST90002042 | CD45 on CD33+ HLA DR+ CD14- \|\| id:ebi-a-GCST90002042 | F-capsicum liking (derived food-liking factor) \|\| id:ebi-fl187-GCST90094726 | Weighted median | 20 | 0.0756880056534655 | 0.169784424298118 | 0.65574976651898 | -0.257089466 | 0.408465477277777 | 1.07862599644193 | 0.773299026722372 | 1.50450731217334 | 0.999332001504091 |
| ebi-fl187-GCST90094726 | ebi-a-GCST90002056 | CD8 on Effector Memory CD8+ T cell \|\| id:ebi-a-GCST90002056 | F-capsicum liking (derived food-liking factor) \|\| id:ebi-fl187-GCST90094726 | Weighted median | 20 | -0.055274604 | 0.121755834608657 | 0.649843873715128 | -0.293916039 | 0.183366832301736 | 0.946225275472181 | 0.745339067958303 | 1.20125498639834 | 0.999332001504091 |
| ebi-fl187-GCST90094726 | ebi-a-GCST90002058 | CD8 on CD8+ T cell \|\| id:ebi-a-GCST90002058 | F-capsicum liking (derived food-liking factor) \|\| id:ebi-fl187-GCST90094726 | Weighted median | 20 | -0.406190464 | 0.130278930125679 | 0.00182174065696152 | -0.661537167 | -0.150843761 | 0.666183271576411 | 0.51605745809803 | 0.859982051153585 | 0.999332001504091 |
| ebi-fl187-GCST90094726 | ebi-a-GCST90002063 | CD4 on CD4 regulatory T cell \|\| id:ebi-a-GCST90002063 | F-capsicum liking (derived food-liking factor) \|\| id:ebi-fl187-GCST90094726 | Weighted median | 20 | -0.242471398 | 0.133481040615114 | 0.0692901628321272 | -0.504094238 | 0.0191514416454061 | 0.784686190883407 | 0.604052455740501 | 1.01933600685108 | 0.999332001504091 |
| ebi-fl187-GCST90094726 | ebi-a-GCST90002066 | CD4 on activated CD4 regulatory T cell \|\| id:ebi-a-GCST90002066 | F-capsicum liking (derived food-liking factor) \|\| id:ebi-fl187-GCST90094726 | Weighted median | 20 | -0.199291931 | 0.121671097249679 | 0.101430825735137 | -0.437767282 | 0.039183419435518 | 0.819310676089718 | 0.645475979398778 | 1.03996121525674 | 0.999332001504091 |
| ebi-fl187-GCST90094726 | ebi-a-GCST90002070 | CD4 on activated & secreting CD4 regulatory T cell \|\| id:ebi-a-GCST90002070 | F-capsicum liking (derived food-liking factor) \|\| id:ebi-fl187-GCST90094726 | Weighted median | 20 | -0.144657768 | 0.124411653094819 | 0.244937085563436 | -0.388504608 | 0.0991890716360416 | 0.86531838171091 | 0.67807009698959 | 1.10427506691581 | 0.999332001504091 |
| ebi-fl187-GCST90094726 | ebi-a-GCST90002081 | SSC-A on CD4+ T cell \|\| id:ebi-a-GCST90002081 | F-capsicum liking (derived food-liking factor) \|\| id:ebi-fl187-GCST90094726 | Weighted median | 20 | -0.001082697 | 0.122155889056302 | 0.992928249805161 | -0.24050824 | 0.238342845522468 | 0.998917888877071 | 0.786228167232933 | 1.26914423866348 | 0.999332001504091 |
| ebi-fl187-GCST90094726 | ebi-a-GCST90002094 | CD11b on Monocytic Myeloid-Derived Suppressor Cells \|\| id:ebi-a-GCST90002094 | F-capsicum liking (derived food-liking factor) \|\| id:ebi-fl187-GCST90094726 | Weighted median | 20 | 0.210551889438292 | 0.161979162068411 | 0.193645452498172 | -0.106927268 | 0.528031047092377 | 1.23435910176089 | 0.898591026742277 | 1.69559048193895 | 0.999332001504091 |
| ebi-fl187-GCST90094726 | ebi-a-GCST90002104 | HLA DR on myeloid Dendritic Cell \|\| id:ebi-a-GCST90002104 | F-capsicum liking (derived food-liking factor) \|\| id:ebi-fl187-GCST90094726 | Weighted median | 20 | -0.000924108 | 0.134239675054954 | 0.994507393655327 | -0.264033871 | 0.262185655096671 | 0.999076318845272 | 0.767947527974274 | 1.29976782855255 | 0.999332001504091 |
| ebi-fl187-GCST90094726 | ebi-a-GCST90002105 | HLA DR on plasmacytoid Dendritic Cell \|\| id:ebi-a-GCST90002105 | F-capsicum liking (derived food-liking factor) \|\| id:ebi-fl187-GCST90094726 | Weighted median | 20 | 0.0931990200255634 | 0.121998954501635 | 0.444907206220472 | -0.145918931 | 0.332316970848768 | 1.0976801738668 | 0.864227762600144 | 1.39419469755882 | 0.999332001504091 |
| ebi-fl187-GCST90094726 | ebi-a-GCST90002106 | HLA DR on Dendritic Cell \|\| id:ebi-a-GCST90002106 | F-capsicum liking (derived food-liking factor) \|\| id:ebi-fl187-GCST90094726 | Weighted median | 20 | -0.039843939 | 0.120750942178523 | 0.741424188183954 | -0.276515786 | 0.196827907243173 | 0.960939392203563 | 0.758421646519768 | 1.2175344938081 | 0.999332001504091 |
| ebi-fl187-GCST90094726 | ebi-a-GCST90002111 | HLA DR on CD33dim HLA DR+ CD11b- \|\| id:ebi-a-GCST90002111 | F-capsicum liking (derived food-liking factor) \|\| id:ebi-fl187-GCST90094726 | Weighted median | 20 | 0.141328875337585 | 0.176530622351146 | 0.423368305704516 | -0.204671144 | 0.487328895145831 | 1.15180338542984 | 0.814915261741115 | 1.62796195012127 | 0.999332001504091 |
| ebi-fl187-GCST90094726 | ebi-a-GCST90002112 | HLA DR on CD33- HLA DR+ \|\| id:ebi-a-GCST90002112 | F-capsicum liking (derived food-liking factor) \|\| id:ebi-fl187-GCST90094726 | Weighted median | 20 | -0.0084228 | 0.163416201461179 | 0.958893622620796 | -0.328718555 | 0.31187295443903 | 0.991612571977389 | 0.719845584972638 | 1.36598114016493 | 0.999332001504091 |
| ebi-fl187-GCST90094726 | ebi-a-GCST90002115 | HLA DR on HLA DR+ CD8+ T cell \|\| id:ebi-a-GCST90002115 | F-capsicum liking (derived food-liking factor) \|\| id:ebi-fl187-GCST90094726 | Weighted median | 20 | -0.256837373 | 0.114722766878105 | 0.0251711777135633 | -0.481693996 | -0.03198075 | 0.773493994283342 | 0.617736062292457 | 0.968525225760815 | 0.999332001504091 |
| ebi-fl187-GCST90094726 | ebi-a-GCST90002116 | HLA DR on B cell \|\| id:ebi-a-GCST90002116 | F-capsicum liking (derived food-liking factor) \|\| id:ebi-fl187-GCST90094726 | Weighted median | 20 | 0.0520828027684217 | 0.117111449270592 | 0.656515948406817 | -0.177455638 | 0.281621243338781 | 1.05346296855226 | 0.837398147334233 | 1.32527666754915 | 0.999332001504091 |
| ebi-fl187-GCST90094726 | ebi-cfmet338-GCST90026023 | Sphingomyelin (d18:1/24:1, d18:2/24:0) levels \|\| id:ebi-cfmet338-GCST90026023 | F-capsicum liking (derived food-liking factor) \|\| id:ebi-fl187-GCST90094726 | Weighted median | 18 | -0.073448063 | 0.0702128778516225 | 0.295525575307981 | -0.211065303 | 0.064169177964811 | 0.929184403937725 | 0.809721187759067 | 1.0662727733611 | 0.999332001504091 |
| ebi-fl187-GCST90094726 | ebi-cfmet338-GCST90026046 | 1-arachidonoyl-gpc (20:4n6) levels \|\| id:ebi-cfmet338-GCST90026046 | F-capsicum liking (derived food-liking factor) \|\| id:ebi-fl187-GCST90094726 | Weighted median | 18 | -0.059651447 | 0.0742401159630493 | 0.421689577677503 | -0.205162075 | 0.0858591799450972 | 0.94209284532883 | 0.814515293448057 | 1.08965287252322 | 0.999332001504091 |
| ebi-fl187-GCST90094726 | ebi-cfmet338-GCST90026047 | 1-linoleoyl-gpc (18:2) levels \|\| id:ebi-cfmet338-GCST90026047 | F-capsicum liking (derived food-liking factor) \|\| id:ebi-fl187-GCST90094726 | Weighted median | 18 | -0.032680885 | 0.0969220174870035 | 0.735975622644198 | -0.222648039 | 0.157286269600195 | 0.96784736524126 | 0.800396508121507 | 1.17033059602285 | 0.999332001504091 |
| ebi-fl187-GCST90094726 | ebi-cfmet338-GCST90026050 | 1-myristoyl-2-palmitoyl-gpc (14:0/16:0) levels \|\| id:ebi-cfmet338-GCST90026050 | F-capsicum liking (derived food-liking factor) \|\| id:ebi-fl187-GCST90094726 | Weighted median | 18 | -0.060125599 | 0.046505019854629 | 0.196051162092571 | -0.151275438 | 0.0310242401377205 | 0.941646256538158 | 0.859610896806825 | 1.03151050754024 | 0.999332001504091 |
| ebi-fl187-GCST90094726 | ebi-cfmet338-GCST90026068 | 2,3-dihydroxyisovalerate levels \|\| id:ebi-cfmet338-GCST90026068 | F-capsicum liking (derived food-liking factor) \|\| id:ebi-fl187-GCST90094726 | Weighted median | 18 | -0.185716111 | 0.162732366568145 | 0.253771531967129 | -0.50467155 | 0.133239327087467 | 0.830509333662153 | 0.603703829474488 | 1.14252340241135 | 0.999332001504091 |
| ebi-fl187-GCST90094726 | ebi-cfmet338-GCST90026082 | 3-hydroxyhexanoate levels \|\| id:ebi-cfmet338-GCST90026082 | F-capsicum liking (derived food-liking factor) \|\| id:ebi-fl187-GCST90094726 | Weighted median | 18 | -0.017499319 | 0.0373101144922165 | 0.639052876340591 | -0.090627143 | 0.0556285058882951 | 0.982652905326636 | 0.913358199488696 | 1.05720486539392 | 0.999332001504091 |
| ebi-fl187-GCST90094726 | ebi-cfmet338-GCST90026094 | 3-ureidopropionate levels \|\| id:ebi-cfmet338-GCST90026094 | F-capsicum liking (derived food-liking factor) \|\| id:ebi-fl187-GCST90094726 | Weighted median | 18 | -0.08968498 | 0.0788033843133613 | 0.255084830226882 | -0.244139613 | 0.0647696532800906 | 0.914219137249824 | 0.783378256660379 | 1.06691323611265 | 0.999332001504091 |
| ebi-fl187-GCST90094726 | ebi-cfmet338-GCST90026173 | Glycerol levels \|\| id:ebi-cfmet338-GCST90026173 | F-capsicum liking (derived food-liking factor) \|\| id:ebi-fl187-GCST90094726 | Weighted median | 18 | -0.008081698 | 0.0436853414865126 | 0.853230658473642 | -0.093704967 | 0.0775415715283377 | 0.991950871337326 | 0.910551365226442 | 1.0806271548472 | 0.999332001504091 |
| ebi-fl187-GCST90094726 | ebi-cfmet338-GCST90026179 | Histidine levels \|\| id:ebi-cfmet338-GCST90026179 | F-capsicum liking (derived food-liking factor) \|\| id:ebi-fl187-GCST90094726 | Weighted median | 18 | -0.003058607 | 0.0373139257559321 | 0.934670909340402 | -0.076193901 | 0.07007668792527 | 0.996946066215406 | 0.926636513356823 | 1.07259043283528 | 0.999332001504091 |
| ebi-fl187-GCST90094726 | ebi-cfmet338-GCST90026188 | Isocitrate levels \|\| id:ebi-cfmet338-GCST90026188 | F-capsicum liking (derived food-liking factor) \|\| id:ebi-fl187-GCST90094726 | Weighted median | 18 | -0.073362302 | 0.0610368733285404 | 0.229389046639621 | -0.192994573 | 0.0462699699848206 | 0.929264095031909 | 0.824486448233139 | 1.04735712778058 | 0.999332001504091 |
| ebi-fl187-GCST90094726 | ebi-cfmet338-GCST90026207 | N-acetyl-isoputreanine levels \|\| id:ebi-cfmet338-GCST90026207 | F-capsicum liking (derived food-liking factor) \|\| id:ebi-fl187-GCST90094726 | Weighted median | 17 | -0.014500232 | 0.0401526540042954 | 0.718004048507226 | -0.093199434 | 0.064198969566987 | 0.985604389794757 | 0.911011795332971 | 1.06630453980856 | 0.999332001504091 |
| ebi-fl187-GCST90094726 | ebi-cfmet338-GCST90026213 | N-acetylglutamate levels \|\| id:ebi-cfmet338-GCST90026213 | F-capsicum liking (derived food-liking factor) \|\| id:ebi-fl187-GCST90094726 | Weighted median | 18 | -0.047724247 | 0.0676663614825948 | 0.480631261739567 | -0.180350316 | 0.0849018213061149 | 0.953396652629575 | 0.8349776543844 | 1.08861018312571 | 0.999332001504091 |
| ebi-fl187-GCST90094726 | ebi-cfmet338-GCST90026224 | N-acetyltaurine levels \|\| id:ebi-cfmet338-GCST90026224 | F-capsicum liking (derived food-liking factor) \|\| id:ebi-fl187-GCST90094726 | Weighted median | 18 | -0.020796406 | 0.0549490498789335 | 0.705083656666392 | -0.128496543 | 0.0869037321990896 | 0.979418348398674 | 0.879416602269376 | 1.09079166654868 | 0.999332001504091 |
| ebi-fl187-GCST90094726 | ebi-cfmet338-GCST90026250 | Phenyllactate (pla) levels \|\| id:ebi-cfmet338-GCST90026250 | F-capsicum liking (derived food-liking factor) \|\| id:ebi-fl187-GCST90094726 | Weighted median | 18 | 0.145431755863517 | 0.0871857183055951 | 0.0953021169155169 | -0.025452252 | 0.316315763742483 | 1.15653880489693 | 0.974868925880883 | 1.37206343511645 | 0.999332001504091 |
| ebi-fl187-GCST90094726 | ebi-cfmet338-GCST90026251 | Pipecolate levels \|\| id:ebi-cfmet338-GCST90026251 | F-capsicum liking (derived food-liking factor) \|\| id:ebi-fl187-GCST90094726 | Weighted median | 17 | -0.00564227 | 0.134668140984299 | 0.966580341993345 | -0.269591826 | 0.258307286511983 | 0.994373617892144 | 0.763691149487178 | 1.29473661260037 | 0.999332001504091 |
| ebi-fl187-GCST90094726 | ebi-cfmet338-GCST90026278 | Trigonelline (n'-methylnicotinate) levels \|\| id:ebi-cfmet338-GCST90026278 | F-capsicum liking (derived food-liking factor) \|\| id:ebi-fl187-GCST90094726 | Weighted median | 18 | -0.017260963 | 0.138100316768038 | 0.90053258806122 | -0.287937584 | 0.253415657813656 | 0.982887153932684 | 0.749808390881253 | 1.28841870685185 | 0.999332001504091 |
| ebi-fl187-GCST90094726 | ebi-cfmet338-GCST90026299 | 1-carboxyethylvaline levels \|\| id:ebi-cfmet338-GCST90026299 | F-capsicum liking (derived food-liking factor) \|\| id:ebi-fl187-GCST90094726 | Weighted median | 18 | -0.070817475 | 0.0820977334563088 | 0.388357627684757 | -0.231729033 | 0.0900940821474983 | 0.931631922328715 | 0.793161014650817 | 1.09427723081423 | 0.999332001504091 |
| ebi-fl187-GCST90094726 | ebi-gm430-GCST90011306 | C_Erysipelotrichia abundance \|\| id:ebi-gm430-GCST90011306 | F-capsicum liking (derived food-liking factor) \|\| id:ebi-fl187-GCST90094726 | Weighted median | 18 | -0.090210659 | 0.0677630486321776 | 0.183101422305477 | -0.223026234 | 0.0426049163188934 | 0.913738677718947 | 0.8000938591015 | 1.04352553348825 | 0.999332001504091 |
| ebi-fl187-GCST90094726 | ebi-gm430-GCST90011312 | F_Erysipelotrichaceae abundance \|\| id:ebi-gm430-GCST90011312 | F-capsicum liking (derived food-liking factor) \|\| id:ebi-fl187-GCST90094726 | Weighted median | 18 | -0.090210659 | 0.0698967239601706 | 0.196832751876085 | -0.227208238 | 0.0467869199617596 | 0.913738677718947 | 0.796754850407731 | 1.04789869899419 | 0.999332001504091 |
| ebi-fl187-GCST90094726 | ebi-gm430-GCST90011352 | O_Erysipelotrichales abundance \|\| id:ebi-gm430-GCST90011352 | F-capsicum liking (derived food-liking factor) \|\| id:ebi-fl187-GCST90094726 | Weighted median | 18 | -0.090210659 | 0.0670492691164554 | 0.178483712914391 | -0.221627226 | 0.0412059084680778 | 0.913738677718947 | 0.80121398003807 | 1.04206665380439 | 0.999332001504091 |
| ebi-fl187-GCST90094726 | ebi-gm430-GCST90011362 | OTU97_11 (Parabacteroides) abundance \|\| id:ebi-gm430-GCST90011362 | F-capsicum liking (derived food-liking factor) \|\| id:ebi-fl187-GCST90094726 | Weighted median | 18 | -0.128281987 | 0.0900831117711063 | 0.154434790431432 | -0.304844886 | 0.0482809125654537 | 0.879605307342636 | 0.737237721726549 | 1.04946542194474 | 0.999332001504091 |
| ebi-fl187-GCST90094726 | ebi-gm430-GCST90011384 | OTU97_235 (Phascolarctobacterium) abundance \|\| id:ebi-gm430-GCST90011384 | F-capsicum liking (derived food-liking factor) \|\| id:ebi-fl187-GCST90094726 | Weighted median | 18 | 0.00683121594076279 | 0.268385765808815 | 0.979693657568646 | -0.519204885 | 0.53286731692604 | 1.0068546019176 | 0.594993448129309 | 1.70381067655443 | 0.999332001504091 |
| ebi-fl187-GCST90094726 | ebi-gm430-GCST90011390 | OTU97_3 (Bacteroides) abundance \|\| id:ebi-gm430-GCST90011390 | F-capsicum liking (derived food-liking factor) \|\| id:ebi-fl187-GCST90094726 | Weighted median | 18 | -0.111973484 | 0.0712816544794599 | 0.116215223244633 | -0.251685527 | 0.0277385588804705 | 0.894067964382251 | 0.77748919923919 | 1.02812685464548 | 0.999332001504091 |
| ebi-fl187-GCST90094726 | ebi-gm430-GCST90011398 | OTU97_4 (Alistipes) abundance \|\| id:ebi-gm430-GCST90011398 | F-capsicum liking (derived food-liking factor) \|\| id:ebi-fl187-GCST90094726 | Weighted median | 18 | -0.084043108 | 0.0829407532651586 | 0.310921245736694 | -0.246606985 | 0.0785207681148238 | 0.919391621844891 | 0.781447754123223 | 1.08168581950431 | 0.999332001504091 |
| ebi-fl187-GCST90094726 | ebi-gm430-GCST90011421 | OTU99_108 (Cytophagales) abundance \|\| id:ebi-gm430-GCST90011421 | F-capsicum liking (derived food-liking factor) \|\| id:ebi-fl187-GCST90094726 | Weighted median | 18 | 0.135865935183277 | 0.250756395550633 | 0.587939476316927 | -0.3556166 | 0.627348470462517 | 1.1455283082572 | 0.700741232860368 | 1.87263863389652 | 0.999332001504091 |
| ebi-fl187-GCST90094726 | ebi-gm430-GCST90011428 | OTU99_123 (Phascolarctobacterium) abundance \|\| id:ebi-gm430-GCST90011428 | F-capsicum liking (derived food-liking factor) \|\| id:ebi-fl187-GCST90094726 | Weighted median | 19 | -0.056200337 | 0.159279143262957 | 0.724207055300946 | -0.368387458 | 0.255986783423107 | 0.945349728037885 | 0.691849067178624 | 1.29173565550327 | 0.999332001504091 |
| ebi-fl187-GCST90094726 | ebi-gm430-GCST90011450 | OTU99_298 (Phascolarctobacterium) abundance \|\| id:ebi-gm430-GCST90011450 | F-capsicum liking (derived food-liking factor) \|\| id:ebi-fl187-GCST90094726 | Weighted median | 18 | 0.0120386255156031 | 0.27474288155218 | 0.965049647413934 | -0.526457422 | 0.550534673357875 | 1.01211138143504 | 0.590693846321638 | 1.73417998987004 | 0.999332001504091 |
| ebi-fl187-GCST90094726 | ebi-gm430-GCST90011451 | OTU99_3 (Bacteroides) abundance \|\| id:ebi-gm430-GCST90011451 | F-capsicum liking (derived food-liking factor) \|\| id:ebi-fl187-GCST90094726 | Weighted median | 18 | -0.12220486 | 0.0721803493196963 | 0.0904461627799581 | -0.263678345 | 0.0192686248129924 | 0.884967055709921 | 0.768220602286459 | 1.01945546287218 | 0.999332001504091 |
| ebi-fl187-GCST90094726 | ebi-gm430-GCST90011472 | OTU99_72 (Parabacteroides) abundance \|\| id:ebi-gm430-GCST90011472 | F-capsicum liking (derived food-liking factor) \|\| id:ebi-fl187-GCST90094726 | Weighted median | 19 | 0.139582525944283 | 0.215602178320725 | 0.517368067065605 | -0.282997744 | 0.562162795452905 | 1.14979368961278 | 0.753521488130556 | 1.75446294431927 | 0.999332001504091 |
| ebi-fl187-GCST90094726 | ebi-gm430-GCST90011507 | TestASV_3 (Bacteroides) abundance \|\| id:ebi-gm430-GCST90011507 | F-capsicum liking (derived food-liking factor) \|\| id:ebi-fl187-GCST90094726 | Weighted median | 18 | -0.077575693 | 0.0951945377841455 | 0.41511954848878 | -0.264156987 | 0.109005601505077 | 0.925356979114597 | 0.767852987557868 | 1.11516859694651 | 0.999332001504091 |
| ebi-fl187-GCST90094726 | ebi-gm430-GCST90011512 | TestASV_34 (Phascolarctobacterium) abundance \|\| id:ebi-gm430-GCST90011512 | F-capsicum liking (derived food-liking factor) \|\| id:ebi-fl187-GCST90094726 | Weighted median | 18 | 0.24178509259328 | 0.227749086922912 | 0.288404029017431 | -0.204603118 | 0.688173302962188 | 1.27352047442263 | 0.81497069961871 | 1.99007694329678 | 0.999332001504091 |
| ebi-fl187-GCST90094726 | ebi-gm430-GCST90011519 | TestASV_41 (Barnesiella) abundance \|\| id:ebi-gm430-GCST90011519 | F-capsicum liking (derived food-liking factor) \|\| id:ebi-fl187-GCST90094726 | Weighted median | 18 | -0.028312494 | 0.298725651815543 | 0.924491495510545 | -0.613814772 | 0.557189783289002 | 0.972084548482143 | 0.541282058016406 | 1.74575963751803 | 0.999332001504091 |
| ebi-fl187-GCST90094726 | ebi-gm430-GCST90011524 | TestASV_46 (Sutterella) abundance \|\| id:ebi-gm430-GCST90011524 | F-capsicum liking (derived food-liking factor) \|\| id:ebi-fl187-GCST90094726 | Weighted median | 18 | -0.256535135 | 0.311275288606516 | 0.409858702644543 | -0.866634701 | 0.353564430644847 | 0.77372780913681 | 0.420363821632897 | 1.42413474191519 | 0.999332001504091 |
| ebi-fl187-GCST90094726 | ebi-gm430-GCST90011581 | OTU97_19 (Bacteria) prevalence \|\| id:ebi-gm430-GCST90011581 | F-capsicum liking (derived food-liking factor) \|\| id:ebi-fl187-GCST90094726 | Weighted median | 18 | -0.116772406 | 0.339288289759044 | 0.730719426744905 | -0.781777454 | 0.548232641444097 | 0.88978768002711 | 0.457591939220574 | 1.73019244367937 | 0.999332001504091 |
| ebi-fl187-GCST90094726 | ebi-gm430-GCST90011614 | OTU97_80 (Ruminococcus) prevalence \|\| id:ebi-gm430-GCST90011614 | F-capsicum liking (derived food-liking factor) \|\| id:ebi-fl187-GCST90094726 | Weighted median | 18 | 0.200271724861378 | 0.169401170918038 | 0.237113097237161 | -0.13175457 | 0.532298019860732 | 1.22173468875017 | 0.8765561017246 | 1.70284097818584 | 0.999332001504091 |
| ebi-fl187-GCST90094726 | ebi-gm430-GCST90011623 | OTU99_11 (Parabacteroides) prevalence \|\| id:ebi-gm430-GCST90011623 | F-capsicum liking (derived food-liking factor) \|\| id:ebi-fl187-GCST90094726 | Weighted median | 18 | 0.0257301455229421 | 0.136141665737926 | 0.850096439647563 | -0.241107519 | 0.292567810369276 | 1.02606402313996 | 0.785757137770544 | 1.33986359012828 | 0.999332001504091 |
| ebi-fl187-GCST90094726 | ebi-gm430-GCST90011642 | OTU99_19 (Bacteria) prevalence \|\| id:ebi-gm430-GCST90011642 | F-capsicum liking (derived food-liking factor) \|\| id:ebi-fl187-GCST90094726 | Weighted median | 18 | -0.116772406 | 0.334722566653008 | 0.727191967080884 | -0.772828637 | 0.539283824156266 | 0.88978768002711 | 0.461705222939538 | 1.71477833949413 | 0.999332001504091 |
| ebi-fl187-GCST90094726 | ebi-gm430-GCST90011653 | OTU99_30 (Parasutterella) prevalence \|\| id:ebi-gm430-GCST90011653 | F-capsicum liking (derived food-liking factor) \|\| id:ebi-fl187-GCST90094726 | Weighted median | 18 | -0.192597996 | 0.164674396921922 | 0.242174639787219 | -0.515359814 | 0.130163822318047 | 0.824813486110262 | 0.597285644442113 | 1.13901496411287 | 0.999332001504091 |
| ebi-fl187-GCST90094726 | ebi-gm430-GCST90011675 | OTU99_8 (Ruminococcaceae) prevalence \|\| id:ebi-gm430-GCST90011675 | F-capsicum liking (derived food-liking factor) \|\| id:ebi-fl187-GCST90094726 | Weighted median | 18 | 0.058099063078344 | 0.181728403074453 | 0.749193704691912 | -0.298088607 | 0.414286733104273 | 1.05981997953985 | 0.74223556960576 | 1.51329097530109 | 0.999332001504091 |
| ebi-fl187-GCST90094726 | ebi-gm430-GCST90011679 | OTU99_92 (Ruminococcus) prevalence \|\| id:ebi-gm430-GCST90011679 | F-capsicum liking (derived food-liking factor) \|\| id:ebi-fl187-GCST90094726 | Weighted median | 18 | 0.164896093977213 | 0.173835774806379 | 0.342837326469518 | -0.175822025 | 0.505614212597716 | 1.17927057902954 | 0.838767249954298 | 1.65800357446054 | 0.999332001504091 |
| ebi-fl187-GCST90094726 | ebi-gm430-GCST90011684 | TestASV_11 (Lachnospiraceae) prevalence \|\| id:ebi-gm430-GCST90011684 | F-capsicum liking (derived food-liking factor) \|\| id:ebi-fl187-GCST90094726 | Weighted median | 18 | -0.003844594 | 0.173831619101935 | 0.982354808521833 | -0.344554568 | 0.336865379348692 | 0.996162786898762 | 0.70853588807798 | 1.40055050802549 | 0.999332001504091 |
| ebi-fl187-GCST90094726 | ebi-gm430-GCST90011688 | TestASV_15 (Bacteroides) prevalence \|\| id:ebi-gm430-GCST90011688 | F-capsicum liking (derived food-liking factor) \|\| id:ebi-fl187-GCST90094726 | Weighted median | 18 | -0.167586019 | 0.164609419206893 | 0.308638606696975 | -0.490220481 | 0.155048442583456 | 0.845703867506497 | 0.612491336772878 | 1.16771452684344 | 0.999332001504091 |
| ebi-fl187-GCST90094726 | ebi-gm430-GCST90011692 | TestASV_19 (Prevotella) prevalence \|\| id:ebi-gm430-GCST90011692 | F-capsicum liking (derived food-liking factor) \|\| id:ebi-fl187-GCST90094726 | Weighted median | 18 | 0.135111050066427 | 0.30148906995331 | 0.654048000776298 | -0.455807527 | 0.726029627174914 | 1.14466389229479 | 0.633935840863395 | 2.0668580980355 | 0.999332001504091 |
| ebi-fl187-GCST90094726 | ebi-gm430-GCST90011693 | TestASV_2 (Bacteroides) prevalence \|\| id:ebi-gm430-GCST90011693 | F-capsicum liking (derived food-liking factor) \|\| id:ebi-fl187-GCST90094726 | Weighted median | 18 | -0.259459008 | 0.150913595947973 | 0.0855678857739658 | -0.555249656 | 0.036331640427002 | 0.771468831680814 | 0.573928958679391 | 1.0369997004933 | 0.999332001504091 |
| ebi-fl187-GCST90094726 | ebi-gm430-GCST90011716 | TestASV_40 (Barnesiella) prevalence \|\| id:ebi-gm430-GCST90011716 | F-capsicum liking (derived food-liking factor) \|\| id:ebi-fl187-GCST90094726 | Weighted median | 18 | -0.027051234 | 0.324906440236637 | 0.933645970808309 | -0.663867857 | 0.609765388947091 | 0.973311373693026 | 0.514856088702997 | 1.83999966388023 | 0.999332001504091 |
| ebi-fl187-GCST90094726 | ebi-gm430-GCST90011718 | TestASV_42 (Alphaproteobacteria) prevalence \|\| id:ebi-gm430-GCST90011718 | F-capsicum liking (derived food-liking factor) \|\| id:ebi-fl187-GCST90094726 | Weighted median | 18 | 0.0767921387308934 | 0.256834919763524 | 0.764944509 | -0.426604304 | 0.5801885814674 | 1.07981760080661 | 0.652721780528584 | 1.78637527625859 | 0.999332001504091 |
| ebi-fl187-GCST90094726 | ebi-hbsm44-GCST90104929 | Sulcal depth of the right inferior frontal sulcus \|\| id:ebi-hbsm44-GCST90104929 | F-capsicum liking (derived food-liking factor) \|\| id:ebi-fl187-GCST90094726 | Weighted median | 18 | 0.0565702911965182 | 0.135781793519011 | 0.67695163483927 | -0.209562024 | 0.322702606493779 | 1.05820099440013 | 0.810939340089897 | 1.3808546326354 | 0.999332001504091 |
| ebi-fl187-GCST90094726 | ebi-hbsm44-GCST90104935 | Sulcal opening distance of the left calloso-marginal posterior fissure \|\| id:ebi-hbsm44-GCST90104935 | F-capsicum liking (derived food-liking factor) \|\| id:ebi-fl187-GCST90094726 | Weighted median | 18 | 0.00765248115986667 | 0.0297532064626741 | 0.79702551368266 | -0.050663804 | 0.0659687658267078 | 1.00768183622574 | 0.950598204598963 | 1.06819335250866 | 0.999332001504091 |
| ebi-fl187-GCST90094726 | ebi-hbsm44-GCST90104936 | Sulcal opening distance of the left anterior inferior temporal sulcus \|\| id:ebi-hbsm44-GCST90104936 | F-capsicum liking (derived food-liking factor) \|\| id:ebi-fl187-GCST90094726 | Weighted median | 20 | -0.019983598 | 0.0159964439072718 | 0.211572717977193 | -0.051336628 | 0.0113694317580781 | 0.980214750363009 | 0.949958833619625 | 1.01143430938813 | 0.999332001504091 |
| ebi-fl187-GCST90094726 | ebi-hbsm44-GCST90104941 | Sulcal opening distance of the right posterior inferior temporal sulcus \|\| id:ebi-hbsm44-GCST90104941 | F-capsicum liking (derived food-liking factor) \|\| id:ebi-fl187-GCST90094726 | Weighted median | 20 | 0.014309963128238 | 0.0128506536359319 | 0.265468353153297 | -0.010877318 | 0.0394972442546644 | 1.01441284079004 | 0.989181626113696 | 1.04028763211322 | 0.999332001504091 |
| ebi-fl187-GCST90094726 | ebi-IPs91-GCST90274819 | Leukemia inhibitory factor levels \|\| id:ebi-IPs91-GCST90274819 | F-capsicum liking (derived food-liking factor) \|\| id:ebi-fl187-GCST90094726 | Weighted median | 21 | -0.014203068 | 0.0568363154359717 | 0.802669133852376 | -0.125602246 | 0.0971961101173934 | 0.985897319601851 | 0.881965582018408 | 1.10207648077794 | 0.999332001504091 |
| ebi-fl187-GCST90094726 | ebi-IPs91-GCST90274837 | STAM binding protein levels \|\| id:ebi-IPs91-GCST90274837 | F-capsicum liking (derived food-liking factor) \|\| id:ebi-fl187-GCST90094726 | Weighted median | 21 | -0.002973959 | 0.048082085271961 | 0.950680930146815 | -0.097214846 | 0.0912669283304113 | 0.997030459032276 | 0.907361042358294 | 1.09556140260822 | 0.999332001504091 |
| ebi-fl187-GCST90094726 | ebi-IPs91-GCST90274840 | TNF-beta levels \|\| id:ebi-IPs91-GCST90274840 | F-capsicum liking (derived food-liking factor) \|\| id:ebi-fl187-GCST90094726 | Weighted median | 21 | -0.000234072 | 0.0582081158366088 | 0.996791485455391 | -0.114321979 | 0.113853835472515 | 0.999765955825374 | 0.891970714154024 | 1.12058832265072 | 0.999364451047625 |
| ebi-fl187-GCST90094726 | ebi-IPs91-GCST90274842 | Tumor necrosis factor ligand superfamily member 14 levels \|\| id:ebi-IPs91-GCST90274842 | F-capsicum liking (derived food-liking factor) \|\| id:ebi-fl187-GCST90094726 | Weighted median | 21 | -0.034060111 | 0.0585704035074492 | 0.560887163996179 | -0.148858102 | 0.0807378795667687 | 0.966513404506505 | 0.861691378350299 | 1.08408669804632 | 0.999332001504091 |
| ebi-fl187-GCST90094726 | ebi-met1400-GCST90199628 | 3-methylhistidine levels \|\| id:ebi-met1400-GCST90199628 | F-capsicum liking (derived food-liking factor) \|\| id:ebi-fl187-GCST90094726 | Weighted median | 18 | 0.0982488360808488 | 0.0709202890977311 | 0.165947999014638 | -0.040754931 | 0.237252602712402 | 1.1032372761955 | 0.960064383569654 | 1.26776131727937 | 0.999332001504091 |
| ebi-fl187-GCST90094726 | ebi-met1400-GCST90199651 | Docosahexaenoate DHA; 22:6n3 levels \|\| id:ebi-met1400-GCST90199651 | F-capsicum liking (derived food-liking factor) \|\| id:ebi-fl187-GCST90094726 | Weighted median | 18 | 0.149481416 | 0.0668241118023885 | 0.0252903715891746 | 0.0185061572663187 | 0.280456675531682 | 1.16123189074135 | 1.01867845742482 | 1.32373419134002 | 0.999332001504091 |
| ebi-fl187-GCST90094726 | ebi-met1400-GCST90199652 | 1-stearoyl-2-oleoyl-GPS (18:0/18:1) levels \|\| id:ebi-met1400-GCST90199652 | F-capsicum liking (derived food-liking factor) \|\| id:ebi-fl187-GCST90094726 | Weighted median | 18 | 0.0036925306868083 | 0.0708503303783549 | 0.958435197214703 | -0.135174117 | 0.142559178228384 | 1.00369935647714 | 0.873563796264845 | 1.153221325 | 0.999332001504091 |
| ebi-fl187-GCST90094726 | ebi-met1400-GCST90199653 | 1-myristoyl-2-palmitoyl-gpc (14:0/16:0) levels \|\| id:ebi-met1400-GCST90199653 | F-capsicum liking (derived food-liking factor) \|\| id:ebi-fl187-GCST90094726 | Weighted median | 18 | -0.065192406 | 0.0668011598923625 | 0.329105461461925 | -0.196122679 | 0.0657378675658641 | 0.936887183484757 | 0.821911397050287 | 1.06794673699396 | 0.999332001504091 |
| ebi-fl187-GCST90094726 | ebi-met1400-GCST90199667 | Erythritol levels in elite athletes \|\| id:ebi-met1400-GCST90199667 | F-capsicum liking (derived food-liking factor) \|\| id:ebi-fl187-GCST90094726 | Weighted median | 18 | 0.0225869186130357 | 0.0724687655601164 | 0.7552852627618 | -0.119451862 | 0.164625699110864 | 1.02284393447698 | 0.887406724878041 | 1.17895175342528 | 0.999332001504091 |
| ebi-fl187-GCST90094726 | ebi-met1400-GCST90199674 | 3-hydroxymyristate levels \|\| id:ebi-met1400-GCST90199674 | F-capsicum liking (derived food-liking factor) \|\| id:ebi-fl187-GCST90094726 | Weighted median | 18 | 0.0492403951703216 | 0.0687420935040132 | 0.47380223291092 | -0.085494108 | 0.183974898438188 | 1.05047284898858 | 0.918058552149662 | 1.2019856510005 | 0.999332001504091 |
| ebi-fl187-GCST90094726 | ebi-met1400-GCST90199685 | 2-linoleoylglycerol (18:2) levels \|\| id:ebi-met1400-GCST90199685 | F-capsicum liking (derived food-liking factor) \|\| id:ebi-fl187-GCST90094726 | Weighted median | 18 | -0.08659305 | 0.0784071437931142 | 0.269418552913944 | -0.240271052 | 0.0670849518898883 | 0.917050213342977 | 0.786414673079103 | 1.06938632070489 | 0.999332001504091 |
| ebi-fl187-GCST90094726 | ebi-met1400-GCST90199703 | Gamma-glutamylvaline levels \|\| id:ebi-met1400-GCST90199703 | F-capsicum liking (derived food-liking factor) \|\| id:ebi-fl187-GCST90094726 | Weighted median | 18 | 0.0310267442921224 | 0.0654941789624236 | 0.635690422215931 | -0.097341846 | 0.159395335058473 | 1.03151309060505 | 0.907245814334419 | 1.17280150459573 | 0.999332001504091 |
| ebi-fl187-GCST90094726 | ebi-met1400-GCST90199779 | Gamma-glutamyl-2-aminobutyrate levels \|\| id:ebi-met1400-GCST90199779 | F-capsicum liking (derived food-liking factor) \|\| id:ebi-fl187-GCST90094726 | Weighted median | 18 | 0.0403149411358778 | 0.0737640048686193 | 0.584695154619729 | -0.104262508 | 0.184892390678372 | 1.04113861994319 | 0.900988749253676 | 1.20308896957383 | 0.999332001504091 |
| ebi-fl187-GCST90094726 | ebi-met1400-GCST90199824 | 4-vinylphenol sulfate levels \|\| id:ebi-met1400-GCST90199824 | F-capsicum liking (derived food-liking factor) \|\| id:ebi-fl187-GCST90094726 | Weighted median | 18 | 0.0131386839259041 | 0.0692761318579767 | 0.849578137792768 | -0.122642535 | 0.148919902367538 | 1.01322537568939 | 0.884579812802213 | 1.16058002577371 | 0.999332001504091 |
| ebi-fl187-GCST90094726 | ebi-met1400-GCST90199830 | 4-ethylphenylsulfate levels \|\| id:ebi-met1400-GCST90199830 | F-capsicum liking (derived food-liking factor) \|\| id:ebi-fl187-GCST90094726 | Weighted median | 18 | 0.0389794812450199 | 0.0662066774011536 | 0.55602595203254 | -0.090785606 | 0.168744568951281 | 1.03974914907325 | 0.913213476981997 | 1.18381771650076 | 0.999332001504091 |
| ebi-fl187-GCST90094726 | ebi-met1400-GCST90199833 | Hexanoylglutamine levels \|\| id:ebi-met1400-GCST90199833 | F-capsicum liking (derived food-liking factor) \|\| id:ebi-fl187-GCST90094726 | Weighted median | 18 | -0.016748474 | 0.0694574239691814 | 0.809452068104001 | -0.152885025 | 0.119388076757601 | 0.983391001717086 | 0.858228390775282 | 1.12680712110274 | 0.999332001504091 |
| ebi-fl187-GCST90094726 | ebi-met1400-GCST90199837 | Carotenoid levels (cryptoxanthin) \|\| id:ebi-met1400-GCST90199837 | F-capsicum liking (derived food-liking factor) \|\| id:ebi-fl187-GCST90094726 | Weighted median | 18 | 0.1105380403818 | 0.0693572877833429 | 0.11099248312572 | -0.025402244 | 0.246478324437152 | 1.11687883474132 | 0.974917678678447 | 1.2795114487862 | 0.999332001504091 |
| ebi-fl187-GCST90094726 | ebi-met1400-GCST90199869 | 2-hydroxyhippurate levels \|\| id:ebi-met1400-GCST90199869 | F-capsicum liking (derived food-liking factor) \|\| id:ebi-fl187-GCST90094726 | Weighted median | 18 | 0.116841943085624 | 0.0715140088311336 | 0.102293934347418 | -0.023325514 | 0.257009400394646 | 1.12394176888272 | 0.976944422704325 | 1.29305728195097 | 0.999332001504091 |
| ebi-fl187-GCST90094726 | ebi-met1400-GCST90199902 | Hydantoin-5-propionate levels \|\| id:ebi-met1400-GCST90199902 | F-capsicum liking (derived food-liking factor) \|\| id:ebi-fl187-GCST90094726 | Weighted median | 18 | -0.032421559 | 0.0687152855582924 | 0.637052026876101 | -0.167103518 | 0.102260401080717 | 0.968098385832622 | 0.846112018719169 | 1.10767187312913 | 0.999332001504091 |
| ebi-fl187-GCST90094726 | ebi-met1400-GCST90199904 | Pantoate levels \|\| id:ebi-met1400-GCST90199904 | F-capsicum liking (derived food-liking factor) \|\| id:ebi-fl187-GCST90094726 | Weighted median | 18 | 0.0231704548892995 | 0.0721903062230537 | 0.748238035284023 | -0.118322545 | 0.164663455086485 | 1.02344097519822 | 0.888409454095698 | 1.17899626673926 | 0.999332001504091 |
| ebi-fl187-GCST90094726 | ebi-met1400-GCST90199936 | 3-methyl catechol sulfate (2) levels \|\| id:ebi-met1400-GCST90199936 | F-capsicum liking (derived food-liking factor) \|\| id:ebi-fl187-GCST90094726 | Weighted median | 18 | 0.0417929717982879 | 0.0785204924256583 | 0.5945489791949 | -0.112107193 | 0.195693136952578 | 1.04267859253029 | 0.893948427032268 | 1.2161536554521 | 0.999332001504091 |
| ebi-fl187-GCST90094726 | ebi-met1400-GCST90199977 | 4-hydroxychlorothalonil levels \|\| id:ebi-met1400-GCST90199977 | F-capsicum liking (derived food-liking factor) \|\| id:ebi-fl187-GCST90094726 | Weighted median | 18 | 0.0631176625774711 | 0.0709383909516478 | 0.373598404225401 | -0.075921584 | 0.202156908842701 | 1.06515216040695 | 0.926888886918052 | 1.22404005575255 | 0.999332001504091 |
| ebi-fl187-GCST90094726 | ebi-met1400-GCST90200040 | Sphingomyelin (d18:2/24:1, d18:1/24:2) levels \|\| id:ebi-met1400-GCST90200040 | F-capsicum liking (derived food-liking factor) \|\| id:ebi-fl187-GCST90094726 | Weighted median | 18 | 0.0804806461657243 | 0.0621877616592751 | 0.195611645456113 | -0.041407367 | 0.202368659017903 | 1.08380787060135 | 0.959438207165435 | 1.22429927389258 | 0.999332001504091 |
| ebi-fl187-GCST90094726 | ebi-met1400-GCST90200077 | 1-stearoyl-2-oleoyl-GPI (18:0/18:1) levels \|\| id:ebi-met1400-GCST90200077 | F-capsicum liking (derived food-liking factor) \|\| id:ebi-fl187-GCST90094726 | Weighted median | 18 | -0.028879238 | 0.0683856286865627 | 0.672806193095694 | -0.162915071 | 0.105156593874915 | 0.971533781404515 | 0.849663346768649 | 1.11088455445302 | 0.999332001504091 |
| ebi-fl187-GCST90094726 | ebi-met1400-GCST90200080 | 1-palmitoyl-2-oleoyl-GPI (16:0/18:1) levels \|\| id:ebi-met1400-GCST90200080 | F-capsicum liking (derived food-liking factor) \|\| id:ebi-fl187-GCST90094726 | Weighted median | 18 | -0.041769792 | 0.0702379841383726 | 0.552051039807612 | -0.179436241 | 0.0958966570369623 | 0.959090545597487 | 0.835741235449183 | 1.10064531416844 | 0.999332001504091 |
| ebi-fl187-GCST90094726 | ebi-met1400-GCST90200082 | 1-oleoyl-2-linoleoyl-GPE (18:1/18:2) levels \|\| id:ebi-met1400-GCST90200082 | F-capsicum liking (derived food-liking factor) \|\| id:ebi-fl187-GCST90094726 | Weighted median | 18 | -0.176812151 | 0.0735309441240138 | 0.0161903913767502 | -0.320932801 | -0.0326915 | 0.837937175627936 | 0.725472000191896 | 0.967837091043622 | 0.999332001504091 |
| ebi-fl187-GCST90094726 | ebi-met1400-GCST90200100 | Perfluorooctanesulfonate (PFOS) levels \|\| id:ebi-met1400-GCST90200100 | F-capsicum liking (derived food-liking factor) \|\| id:ebi-fl187-GCST90094726 | Weighted median | 18 | 0.0217607102034323 | 0.0707053855822504 | 0.758260432314982 | -0.116821846 | 0.160343265944643 | 1.02199920122751 | 0.889743690853216 | 1.17391376645568 | 0.999332001504091 |
| ebi-fl187-GCST90094726 | ebi-met1400-GCST90200102 | 1-palmitoleoyl-2-linolenoyl-GPC (16:1/18:3) levels \|\| id:ebi-met1400-GCST90200102 | F-capsicum liking (derived food-liking factor) \|\| id:ebi-fl187-GCST90094726 | Weighted median | 18 | -0.101577789 | 0.0705251054052477 | 0.149780616279518 | -0.239806996 | 0.0366514171780093 | 0.903410900802162 | 0.786779698034041 | 1.0373313619143 | 0.999332001504091 |
| ebi-fl187-GCST90094726 | ebi-met1400-GCST90200114 | Glycosyl-N-tricosanoyl-sphingadienine (d18:2/23:0) levels \|\| id:ebi-met1400-GCST90200114 | F-capsicum liking (derived food-liking factor) \|\| id:ebi-fl187-GCST90094726 | Weighted median | 18 | 0.0344022164059015 | 0.0729131024865429 | 0.637051784923183 | -0.108507464 | 0.177311897279526 | 1.03500081732799 | 0.897172197879064 | 1.19400344148204 | 0.999332001504091 |
| ebi-fl187-GCST90094726 | ebi-met1400-GCST90200135 | Arachidoylcarnitine (C20) levels \|\| id:ebi-met1400-GCST90200135 | F-capsicum liking (derived food-liking factor) \|\| id:ebi-fl187-GCST90094726 | Weighted median | 18 | -0.065744729 | 0.0756684921315587 | 0.3849280244303 | -0.214054974 | 0.082565515241305 | 0.936369861541618 | 0.807304003141602 | 1.08606982523494 | 0.999332001504091 |
| ebi-fl187-GCST90094726 | ebi-met1400-GCST90200146 | Docosahexaenoylcarnitine (C22:6) levels \|\| id:ebi-met1400-GCST90200146 | F-capsicum liking (derived food-liking factor) \|\| id:ebi-fl187-GCST90094726 | Weighted median | 18 | 0.0842460643313358 | 0.0879718429333233 | 0.338240258178283 | -0.088178748 | 0.25667087648065 | 1.08789655341471 | 0.915597201093588 | 1.29261962522168 | 0.999332001504091 |
| ebi-fl187-GCST90094726 | ebi-met1400-GCST90200162 | 3-hydroxyphenylacetoylglutamine levels \|\| id:ebi-met1400-GCST90200162 | F-capsicum liking (derived food-liking factor) \|\| id:ebi-fl187-GCST90094726 | Weighted median | 18 | 0.013193698422891 | 0.0891272585750224 | 0.882317407122744 | -0.161495728 | 0.187883125229935 | 1.0132811193071 | 0.850870166048096 | 1.20669247520217 | 0.999332001504091 |
| ebi-fl187-GCST90094726 | ebi-met1400-GCST90200172 | 3-carboxy-4-methyl-5-pentyl-2-furanpropionate (3-CMPFP) levels \|\| id:ebi-met1400-GCST90200172 | F-capsicum liking (derived food-liking factor) \|\| id:ebi-fl187-GCST90094726 | Weighted median | 18 | -0.026293854 | 0.0721411662778036 | 0.715501243215346 | -0.16769054 | 0.115102831823981 | 0.974048819329785 | 0.845615478377056 | 1.12198880779556 | 0.999332001504091 |
| ebi-fl187-GCST90094726 | ebi-met1400-GCST90200222 | Dihydrocaffeate sulfate (2) levels \|\| id:ebi-met1400-GCST90200222 | F-capsicum liking (derived food-liking factor) \|\| id:ebi-fl187-GCST90094726 | Weighted median | 18 | 0.0831680030707549 | 0.080900420310058 | 0.303936041791145 | -0.075396821 | 0.241732826878469 | 1.08672436624328 | 0.92737541150929 | 1.27345391470412 | 0.999332001504091 |
| ebi-fl187-GCST90094726 | ebi-met1400-GCST90200223 | 4-methylhexanoylglutamine levels \|\| id:ebi-met1400-GCST90200223 | F-capsicum liking (derived food-liking factor) \|\| id:ebi-fl187-GCST90094726 | Weighted median | 18 | 0.0271914848040755 | 0.0851471901042063 | 0.74946352891546 | -0.139697008 | 0.19407997740832 | 1.02756454692207 | 0.869621684072422 | 1.21419338711341 | 0.999332001504091 |
| ebi-fl187-GCST90094726 | ebi-met1400-GCST90200224 | 2-ketocaprylate levels \|\| id:ebi-met1400-GCST90200224 | F-capsicum liking (derived food-liking factor) \|\| id:ebi-fl187-GCST90094726 | Weighted median | 18 | -0.014578025 | 0.0735686574864441 | 0.842923501639882 | -0.158772593 | 0.129616543848348 | 0.985527720104445 | 0.853190357944384 | 1.13839177629054 | 0.999332001504091 |
| ebi-fl187-GCST90094726 | ebi-met1400-GCST90200243 | Hydroxypalmitoyl sphingomyelin (d18:1/16:0(OH)) levels \|\| id:ebi-met1400-GCST90200243 | F-capsicum liking (derived food-liking factor) \|\| id:ebi-fl187-GCST90094726 | Weighted median | 18 | 0.0162038472617691 | 0.0694697655390704 | 0.815567008722937 | -0.119956893 | 0.152364587718347 | 1.01633584156961 | 0.886958669847691 | 1.16458475233844 | 0.999332001504091 |
| ebi-fl187-GCST90094726 | ebi-met1400-GCST90200273 | 2-methoxyhydroquinone sulfate (2) levels \|\| id:ebi-met1400-GCST90200273 | F-capsicum liking (derived food-liking factor) \|\| id:ebi-fl187-GCST90094726 | Weighted median | 18 | 0.079814278450732 | 0.0937695890312205 | 0.394672453030574 | -0.103974116 | 0.263602672951924 | 1.08308589660393 | 0.901248624993231 | 1.30161092831753 | 0.999332001504091 |
| ebi-fl187-GCST90094726 | ebi-met1400-GCST90200276 | 2,4-di-tert-butylphenol levels \|\| id:ebi-met1400-GCST90200276 | F-capsicum liking (derived food-liking factor) \|\| id:ebi-fl187-GCST90094726 | Weighted median | 18 | 0.128668943635405 | 0.0692447361762967 | 0.0631439496878495 | -0.007050739 | 0.264388626540947 | 1.13731354694677 | 0.992974058876009 | 1.30263433622102 | 0.999332001504091 |
| ebi-fl187-GCST90094726 | ebi-met1400-GCST90200289 | N-acetylmethionine levels \|\| id:ebi-met1400-GCST90200289 | F-capsicum liking (derived food-liking factor) \|\| id:ebi-fl187-GCST90094726 | Weighted median | 18 | -0.003558248 | 0.0716246435635016 | 0.960378108345967 | -0.14394255 | 0.136826053255245 | 0.996448074933758 | 0.865937495160413 | 1.14662867884622 | 0.999332001504091 |
| ebi-fl187-GCST90094726 | ebi-met1400-GCST90200308 | 3-hydroxyisobutyrate levels \|\| id:ebi-met1400-GCST90200308 | F-capsicum liking (derived food-liking factor) \|\| id:ebi-fl187-GCST90094726 | Weighted median | 18 | -0.074193434 | 0.0675417270150839 | 0.271993666718354 | -0.206575219 | 0.0581883506435165 | 0.928492074249498 | 0.813365078440178 | 1.05991461251006 | 0.999332001504091 |
| ebi-fl187-GCST90094726 | ebi-met1400-GCST90200383 | Cystathionine levels \|\| id:ebi-met1400-GCST90200383 | F-capsicum liking (derived food-liking factor) \|\| id:ebi-fl187-GCST90094726 | Weighted median | 18 | 0.00801551203719565 | 0.0711875963258087 | 0.910350038478157 | -0.131512177 | 0.147543200835781 | 1.00804772225676 | 0.876768598870774 | 1.15898335279775 | 0.999332001504091 |
| ebi-fl187-GCST90094726 | ebi-met1400-GCST90200384 | Guanidinoacetate levels \|\| id:ebi-met1400-GCST90200384 | F-capsicum liking (derived food-liking factor) \|\| id:ebi-fl187-GCST90094726 | Weighted median | 18 | 0.133245325199075 | 0.0632938546298931 | 0.0352751361832762 | 0.00918937012448491 | 0.257301280273666 | 1.14253025541479 | 1.00923172201579 | 1.29343475443963 | 0.999332001504091 |
| ebi-fl187-GCST90094726 | ebi-met1400-GCST90200403 | Citrulline levels \|\| id:ebi-met1400-GCST90200403 | F-capsicum liking (derived food-liking factor) \|\| id:ebi-fl187-GCST90094726 | Weighted median | 18 | -0.004992873 | 0.0694136127512492 | 0.942658165997713 | -0.141043554 | 0.131057807857675 | 0.99501957053779 | 0.868451486227429 | 1.14003368231203 | 0.999332001504091 |
| ebi-fl187-GCST90094726 | ebi-met1400-GCST90200434 | 12,13-DiHOME levels \|\| id:ebi-met1400-GCST90200434 | F-capsicum liking (derived food-liking factor) \|\| id:ebi-fl187-GCST90094726 | Weighted median | 18 | 0.0222330429559058 | 0.071204072312381 | 0.754855110242028 | -0.117326939 | 0.161793024688173 | 1.02248203894431 | 0.889294400807129 | 1.17561689246536 | 0.999332001504091 |
| ebi-fl187-GCST90094726 | ebi-met1400-GCST90200437 | Fructose levels \|\| id:ebi-met1400-GCST90200437 | F-capsicum liking (derived food-liking factor) \|\| id:ebi-fl187-GCST90094726 | Weighted median | 18 | 0.0543177213042327 | 0.0677457872746943 | 0.422675925653647 | -0.078464022 | 0.187099464362634 | 1.05582000537903 | 0.924535322492298 | 1.20574720796334 | 0.999332001504091 |
| ebi-fl187-GCST90094726 | ebi-met1400-GCST90200448 | N-stearoyl-sphinganine (d18:0/18:0) levels \|\| id:ebi-met1400-GCST90200448 | F-capsicum liking (derived food-liking factor) \|\| id:ebi-fl187-GCST90094726 | Weighted median | 18 | 0.0134200696916425 | 0.0746149058776593 | 0.857264238479074 | -0.132825146 | 0.159665285211855 | 1.01351052300391 | 0.875618184216075 | 1.17311814527846 | 0.999332001504091 |
| ebi-fl187-GCST90094726 | ebi-met1400-GCST90200468 | X-11847 levels \|\| id:ebi-met1400-GCST90200468 | F-capsicum liking (derived food-liking factor) \|\| id:ebi-fl187-GCST90094726 | Weighted median | 18 | 0.0700118959588746 | 0.0694817639938675 | 0.313632217291148 | -0.066172361 | 0.206196153386855 | 1.07252093984332 | 0.935969525305286 | 1.22899425173828 | 0.999332001504091 |
| ebi-fl187-GCST90094726 | ebi-met1400-GCST90200476 | X-11843 levels \|\| id:ebi-met1400-GCST90200476 | F-capsicum liking (derived food-liking factor) \|\| id:ebi-fl187-GCST90094726 | Weighted median | 18 | 0.0304035042160113 | 0.0703235500100679 | 0.665496172968977 | -0.107430654 | 0.168237662235744 | 1.03087041060072 | 0.898138802801029 | 1.18321778341819 | 0.999332001504091 |
| ebi-fl187-GCST90094726 | ebi-met1400-GCST90200511 | X-12906 levels \|\| id:ebi-met1400-GCST90200511 | F-capsicum liking (derived food-liking factor) \|\| id:ebi-fl187-GCST90094726 | Weighted median | 18 | 0.0672776443567245 | 0.0734167408648735 | 0.359467540338242 | -0.076619168 | 0.211174456451876 | 1.06959240324766 | 0.926242529484325 | 1.23512781228264 | 0.999332001504091 |
| ebi-fl187-GCST90094726 | ebi-met1400-GCST90200515 | X-12849 levels \|\| id:ebi-met1400-GCST90200515 | F-capsicum liking (derived food-liking factor) \|\| id:ebi-fl187-GCST90094726 | Weighted median | 18 | -0.050674745 | 0.0794850253531011 | 0.523774297754046 | -0.206465394 | 0.105115905056038 | 0.950587804038215 | 0.813454411106331 | 1.11083935479216 | 0.999332001504091 |
| ebi-fl187-GCST90094726 | ebi-met1400-GCST90200551 | X-18886 levels \|\| id:ebi-met1400-GCST90200551 | F-capsicum liking (derived food-liking factor) \|\| id:ebi-fl187-GCST90094726 | Weighted median | 18 | -0.017952028 | 0.0680487140637289 | 0.791925232650283 | -0.151327508 | 0.115423451529342 | 0.982208149682559 | 0.85956613811177 | 1.12234859719124 | 0.999332001504091 |
| ebi-fl187-GCST90094726 | ebi-met1400-GCST90200564 | X-21319 levels \|\| id:ebi-met1400-GCST90200564 | F-capsicum liking (derived food-liking factor) \|\| id:ebi-fl187-GCST90094726 | Weighted median | 18 | -0.043280355 | 0.0686237675561859 | 0.528242946668728 | -0.177782939 | 0.091222229789191 | 0.957642872824164 | 0.837124110740052 | 1.09551243370614 | 0.999332001504091 |
| ebi-fl187-GCST90094726 | ebi-met1400-GCST90200579 | X-21442 levels \|\| id:ebi-met1400-GCST90200579 | F-capsicum liking (derived food-liking factor) \|\| id:ebi-fl187-GCST90094726 | Weighted median | 18 | 0.0236645628343345 | 0.0847553685516689 | 0.780083521491204 | -0.14245596 | 0.189785085195606 | 1.02394679046872 | 0.867225746483376 | 1.20898973994114 | 0.999332001504091 |
| ebi-fl187-GCST90094726 | ebi-met1400-GCST90200581 | X-21441 levels \|\| id:ebi-met1400-GCST90200581 | F-capsicum liking (derived food-liking factor) \|\| id:ebi-fl187-GCST90094726 | Weighted median | 18 | -0.042209781 | 0.0663110423240535 | 0.524423041736064 | -0.172179424 | 0.0877598618141237 | 0.958668648873381 | 0.841828115368473 | 1.09172592546454 | 0.999332001504091 |
| ebi-fl187-GCST90094726 | ebi-met1400-GCST90200648 | X-24585 levels \|\| id:ebi-met1400-GCST90200648 | F-capsicum liking (derived food-liking factor) \|\| id:ebi-fl187-GCST90094726 | Weighted median | 18 | 0.0245490928905364 | 0.0724587218483314 | 0.734759591026585 | -0.117470002 | 0.166568187713266 | 1.02485290286359 | 0.889167184643875 | 1.18124407945689 | 0.999332001504091 |
| ebi-fl187-GCST90094726 | ebi-met1400-GCST90200720 | Adenosine 5'-diphosphate (ADP) to pantothenate ratio \|\| id:ebi-met1400-GCST90200720 | F-capsicum liking (derived food-liking factor) \|\| id:ebi-fl187-GCST90094726 | Weighted median | 18 | -0.067363887 | 0.0973758604092554 | 0.489067664325415 | -0.258220574 | 0.123492799133911 | 0.934854957618634 | 0.772424836733929 | 1.13144185715144 | 0.999332001504091 |
| ebi-fl187-GCST90094726 | ebi-met1400-GCST90200724 | Cholate to taurocholate ratio \|\| id:ebi-met1400-GCST90200724 | F-capsicum liking (derived food-liking factor) \|\| id:ebi-fl187-GCST90094726 | Weighted median | 18 | -0.010901735 | 0.0765531083549489 | 0.88675815619688 | -0.160945827 | 0.139142357301148 | 0.989157473485263 | 0.851338189018374 | 1.14928769785356 | 0.999332001504091 |
| ebi-fl187-GCST90094726 | ebi-met1400-GCST90200726 | N-acetylputrescine to (N(1) + N(8))-acetylspermidine ratio \|\| id:ebi-met1400-GCST90200726 | F-capsicum liking (derived food-liking factor) \|\| id:ebi-fl187-GCST90094726 | Weighted median | 18 | -0.040281909 | 0.0640065802360774 | 0.529126409266675 | -0.165734806 | 0.0851709882093868 | 0.960518622085772 | 0.847270895288767 | 1.08890324039646 | 0.999332001504091 |
| ebi-fl187-GCST90094726 | ebi-met1400-GCST90200744 | Arginine to glutamate ratio \|\| id:ebi-met1400-GCST90200744 | F-capsicum liking (derived food-liking factor) \|\| id:ebi-fl187-GCST90094726 | Weighted median | 18 | 0.0744001366358963 | 0.0666166685724401 | 0.264062893408722 | -0.056168534 | 0.204968807037879 | 1.07723776156379 | 0.94537979404646 | 1.22748677541751 | 0.999332001504091 |
| ebi-fl187-GCST90094726 | ebi-met1400-GCST90200759 | Histidine to trans-urocanate ratio \|\| id:ebi-met1400-GCST90200759 | F-capsicum liking (derived food-liking factor) \|\| id:ebi-fl187-GCST90094726 | Weighted median | 18 | -0.070469232 | 0.0740075155362994 | 0.341000526414863 | -0.215523962 | 0.0745854988799589 | 0.931956413919243 | 0.806118953786312 | 1.0774374592803 | 0.999332001504091 |
| ebi-fl187-GCST90094726 | ebi-met1400-GCST90200795 | Oleoyl-linoleoyl-glycerol (18:1 to 18:2) [2] to linoleoyl-arachidonoyl-glycerol (18:2 to 20:4) [2] ratio \|\| id:ebi-met1400-GCST90200795 | F-capsicum liking (derived food-liking factor) \|\| id:ebi-fl187-GCST90094726 | Weighted median | 18 | 0.0319856784147239 | 0.0776725165121896 | 0.680484736824029 | -0.120252454 | 0.184223810778616 | 1.03250271812351 | 0.886696558410981 | 1.20228487730107 | 0.999332001504091 |
| ebi-fl187-GCST90094726 | ebi-met1400-GCST90200811 | Choline to taurocholate ratio \|\| id:ebi-met1400-GCST90200811 | F-capsicum liking (derived food-liking factor) \|\| id:ebi-fl187-GCST90094726 | Weighted median | 18 | 0.0544872661624712 | 0.076491860354786 | 0.476261913740772 | -0.09543678 | 0.204411312457852 | 1.05599902940804 | 0.90897582516809 | 1.22680264890929 | 0.999332001504091 |
| ebi-fl187-GCST90094726 | ebi-met1400-GCST90200831 | Cholate to phosphate ratio \|\| id:ebi-met1400-GCST90200831 | F-capsicum liking (derived food-liking factor) \|\| id:ebi-fl187-GCST90094726 | Weighted median | 18 | -0.089055254 | 0.0680877804279507 | 0.190891059778303 | -0.222507303 | 0.0443967959886357 | 0.914795026413661 | 0.80050916037919 | 1.04539708197064 | 0.999332001504091 |
| ebi-fl187-GCST90094726 | ebi-met1400-GCST90200841 | Adenosine 5'-diphosphate (ADP) to oxalate (ethanedioate) ratio \|\| id:ebi-met1400-GCST90200841 | F-capsicum liking (derived food-liking factor) \|\| id:ebi-fl187-GCST90094726 | Weighted median | 18 | -0.073648474 | 0.0969369544847052 | 0.447400191982348 | -0.263644905 | 0.116347956466189 | 0.928998203171246 | 0.768246291556869 | 1.12338669379899 | 0.999332001504091 |
| ebi-fl187-GCST90094726 | ebi-met1400-GCST90200844 | Aspartate to phosphate ratio \|\| id:ebi-met1400-GCST90200844 | F-capsicum liking (derived food-liking factor) \|\| id:ebi-fl187-GCST90094726 | Weighted median | 18 | -0.0895918 | 0.0690028291021561 | 0.194157251627979 | -0.224837345 | 0.0456537448879347 | 0.914304327995093 | 0.798646111824791 | 1.04671191885042 | 0.999332001504091 |
| ebi-fl187-GCST90094726 | ebi-met1400-GCST90200870 | Adenosine 5'-monophosphate (AMP) to acetoacetate ratio \|\| id:ebi-met1400-GCST90200870 | F-capsicum liking (derived food-liking factor) \|\| id:ebi-fl187-GCST90094726 | Weighted median | 18 | 0.0441346268914538 | 0.0764118078008375 | 0.563541571294139 | -0.105632516 | 0.193901770181095 | 1.04512304708477 | 0.899755232623638 | 1.2139770283555 | 0.999332001504091 |
| ebi-fl187-GCST90094726 | ebi-met1400-GCST90200887 | Cholesterol to cortisol ratio \|\| id:ebi-met1400-GCST90200887 | F-capsicum liking (derived food-liking factor) \|\| id:ebi-fl187-GCST90094726 | Weighted median | 18 | -0.037748593 | 0.0670291746535332 | 0.573321435597513 | -0.169125776 | 0.0936285890970221 | 0.962955003874838 | 0.844402691498168 | 1.09815180461158 | 0.999332001504091 |
| ebi-fl187-GCST90094726 | ebi-met1400-GCST90200890 | Cortisol to taurocholate ratio \|\| id:ebi-met1400-GCST90200890 | F-capsicum liking (derived food-liking factor) \|\| id:ebi-fl187-GCST90094726 | Weighted median | 18 | 0.0598502333589552 | 0.0728771608437694 | 0.411504936352989 | -0.082989002 | 0.202689468612743 | 1.06167753076037 | 0.920361269394495 | 1.22469210385504 | 0.999332001504091 |
| ebi-fl187-GCST90094726 | ebi-met1400-GCST90200948 | Adenosine 5'-diphosphate (ADP) to mannitol to sorbitol ratio \|\| id:ebi-met1400-GCST90200948 | F-capsicum liking (derived food-liking factor) \|\| id:ebi-fl187-GCST90094726 | Weighted median | 18 | 0.00496831003989615 | 0.0984831784772966 | 0.95976513856745 | -0.18805872 | 0.197995339855398 | 1.00498067255735 | 0.828566052591518 | 1.21895671329386 | 0.999332001504091 |
| ebi-fl187-GCST90094726 | ebi-met1400-GCST90200972 | Leucine to N-palmitoyl-sphingosine (d18:1 to 16:0) ratio \|\| id:ebi-met1400-GCST90200972 | F-capsicum liking (derived food-liking factor) \|\| id:ebi-fl187-GCST90094726 | Weighted median | 18 | 0.126499242122751 | 0.0735264644709421 | 0.0853491030457111 | -0.017612628 | 0.270611112485798 | 1.13484859109982 | 0.982541567505245 | 1.31076523102354 | 0.999332001504091 |
| ebi-fl187-GCST90094726 | ebi-met1400-GCST90200973 | Proline to glutamate ratio \|\| id:ebi-met1400-GCST90200973 | F-capsicum liking (derived food-liking factor) \|\| id:ebi-fl187-GCST90094726 | Weighted median | 18 | 0.0115601978494315 | 0.0650252595839445 | 0.858895667848245 | -0.115889311 | 0.139009706633963 | 1.01162727516307 | 0.890573794622539 | 1.14913525418476 | 0.999332001504091 |
| ebi-fl187-GCST90094726 | ebi-met1400-GCST90200983 | Cholesterol to linoleoyl-arachidonoyl-glycerol (18:2 to 20:4) [1] ratio \|\| id:ebi-met1400-GCST90200983 | F-capsicum liking (derived food-liking factor) \|\| id:ebi-fl187-GCST90094726 | Weighted median | 18 | -0.049207552 | 0.0697634636635288 | 0.480593408965982 | -0.185943941 | 0.0875288370247482 | 0.951983523340709 | 0.830320140979064 | 1.09147373884435 | 0.999332001504091 |
| ebi-fl187-GCST90094726 | ebi-met1400-GCST90201001 | Serine to alpha-ketobutyrate ratio \|\| id:ebi-met1400-GCST90201001 | F-capsicum liking (derived food-liking factor) \|\| id:ebi-fl187-GCST90094726 | Weighted median | 18 | -0.003928312 | 0.0692023425523367 | 0.954731925335857 | -0.139564903 | 0.131708279634218 | 0.996079393954849 | 0.869736572711057 | 1.14077548328082 | 0.999332001504091 |
| ebi-fl187-GCST90094726 | ebi-met1400-GCST90201005 | Fructose to maltose ratio \|\| id:ebi-met1400-GCST90201005 | F-capsicum liking (derived food-liking factor) \|\| id:ebi-fl187-GCST90094726 | Weighted median | 18 | 0.0016775370790751 | 0.0804595112165578 | 0.983365745717972 | -0.156023105 | 0.159378179063528 | 1.00167894493153 | 0.855539422985242 | 1.17278138419164 | 0.999332001504091 |
| ebi-fl187-GCST90094726 | ebi-met1400-GCST90201020 | Paraxanthine to 5-acetylamino-6-formylamino-3-methyluracil ratio \|\| id:ebi-met1400-GCST90201020 | F-capsicum liking (derived food-liking factor) \|\| id:ebi-fl187-GCST90094726 | Weighted median | 18 | 0.0438029450910917 | 0.07685304369841 | 0.568706731960101 | -0.106829021 | 0.194434910739975 | 1.04477645627302 | 0.898679315543168 | 1.21462442130725 | 0.999332001504091 |
| ebi-fl187-GCST90094726 | ebi-pw11-GCST90275197 | Placental weight (fetal genotype, adjusted for sex) \|\| id:ebi-pw11-GCST90275197 | F-capsicum liking (derived food-liking factor) \|\| id:ebi-fl187-GCST90094726 | Weighted median | 20 | -0.014603212 | 0.02593822357898 | 0.573435097890659 | -0.06544213 | 0.036235706489539 | 0.985502898028732 | 0.93665324937094 | 1.03690022180065 | 0.999332001504091 |
| ebi-fl187-GCST90094726 | prot-a-1017 | FAS-associated factor 2 \|\| id:prot-a-1017 | F-capsicum liking (derived food-liking factor) \|\| id:ebi-fl187-GCST90094726 | Weighted median | 21 | -0.133660681 | 0.111464206202705 | 0.230475282387188 | -0.352130525 | 0.0848091632785227 | 0.874886880090221 | 0.703188332303601 | 1.08850931932632 | 0.999332001504091 |
| ebi-fl187-GCST90094726 | prot-a-1019 | Fas apoptotic inhibitory molecule 3 \|\| id:prot-a-1019 | F-capsicum liking (derived food-liking factor) \|\| id:ebi-fl187-GCST90094726 | Weighted median | 21 | 0.0174379912661147 | 0.101987827149973 | 0.864238623730832 | -0.18245815 | 0.217334132480062 | 1.01759092066976 | 0.833219513478785 | 1.24275927901188 | 0.999332001504091 |
| ebi-fl187-GCST90094726 | prot-a-1052 | MIP18 family protein FAM96A \|\| id:prot-a-1052 | F-capsicum liking (derived food-liking factor) \|\| id:ebi-fl187-GCST90094726 | Weighted median | 21 | -0.050471238 | 0.106373019051296 | 0.635161895724813 | -0.258962355 | 0.158019879733916 | 0.950781275024097 | 0.771852078909086 | 1.17118947741142 | 0.999332001504091 |
| ebi-fl187-GCST90094726 | prot-a-1066 | F-box/LRR-repeat protein 4 \|\| id:prot-a-1066 | F-capsicum liking (derived food-liking factor) \|\| id:ebi-fl187-GCST90094726 | Weighted median | 21 | -0.02287355 | 0.104570209289396 | 0.826853610957436 | -0.22783116 | 0.182084059949927 | 0.977386066176228 | 0.796258688433792 | 1.19971503762734 | 0.999332001504091 |
| ebi-fl187-GCST90094726 | prot-a-1076 | Low affinity immunoglobulin gamma Fc region receptor III-B \|\| id:prot-a-1076 | F-capsicum liking (derived food-liking factor) \|\| id:ebi-fl187-GCST90094726 | Weighted median | 21 | -0.001599979 | 0.107938118106888 | 0.988173299722922 | -0.21315869 | 0.209958732516937 | 0.998401300311426 | 0.808027900720878 | 1.23362715021876 | 0.999332001504091 |
| ebi-fl187-GCST90094726 | prot-a-1081 | Fc receptor-like protein 3 \|\| id:prot-a-1081 | F-capsicum liking (derived food-liking factor) \|\| id:ebi-fl187-GCST90094726 | Weighted median | 21 | 0.129289523403661 | 0.10753977425356 | 0.229267295531383 | -0.081488434 | 0.340067480940639 | 1.13801955977015 | 0.921743370553177 | 1.40504240094746 | 0.999332001504091 |
| ebi-fl187-GCST90094726 | prot-a-1105 | Fibroblast growth factor receptor 4 \|\| id:prot-a-1105 | F-capsicum liking (derived food-liking factor) \|\| id:ebi-fl187-GCST90094726 | Weighted median | 21 | 0.118008720708169 | 0.11242284356914 | 0.293862356351489 | -0.102340053 | 0.338357494103683 | 1.12525392433564 | 0.902722526248448 | 1.4026418499767 | 0.999332001504091 |
| ebi-fl187-GCST90094726 | prot-a-1143 | Follistatin-related protein 1 \|\| id:prot-a-1143 | F-capsicum liking (derived food-liking factor) \|\| id:ebi-fl187-GCST90094726 | Weighted median | 21 | -0.081517004 | 0.103801521649158 | 0.43226826972862 | -0.284967987 | 0.121933978267536 | 0.921717036692229 | 0.752038329240003 | 1.12967951591943 | 0.999332001504091 |
| ebi-fl187-GCST90094726 | prot-a-1151 | Alpha-(1,3)-fucosyltransferase 10 \|\| id:prot-a-1151 | F-capsicum liking (derived food-liking factor) \|\| id:ebi-fl187-GCST90094726 | Weighted median | 21 | 0.0246351806791828 | 0.102573803366047 | 0.810198240464992 | -0.176409474 | 0.225679835276636 | 1.02494113398144 | 0.838274661440983 | 1.25317437881441 | 0.999332001504091 |
| ebi-fl187-GCST90094726 | prot-a-1165 | Growth arrest and DNA damage-inducible proteins-interacting protein 1 \|\| id:prot-a-1165 | F-capsicum liking (derived food-liking factor) \|\| id:ebi-fl187-GCST90094726 | Weighted median | 21 | 0.109469952843196 | 0.108496475800916 | 0.312987856523675 | -0.10318314 | 0.322123045412992 | 1.11568654722288 | 0.901961773322053 | 1.38005457489566 | 0.999332001504091 |
| ebi-fl187-GCST90094726 | prot-a-1194 | Growth/differentiation factor 11/8 \|\| id:prot-a-1194 | F-capsicum liking (derived food-liking factor) \|\| id:ebi-fl187-GCST90094726 | Weighted median | 21 | 0.0107954819807903 | 0.108269897944183 | 0.920575364523904 | -0.201413518 | 0.223004481951388 | 1.01085396345216 | 0.817574279970025 | 1.24982617538359 | 0.999332001504091 |
| ebi-fl187-GCST90094726 | prot-a-1216 | D-glucuronyl C5-epimerase \|\| id:prot-a-1216 | F-capsicum liking (derived food-liking factor) \|\| id:ebi-fl187-GCST90094726 | Weighted median | 21 | -0.113982349 | 0.109867042567504 | 0.299522854559475 | -0.329321753 | 0.10135705428442 | 0.892273705134627 | 0.719411506996044 | 1.10667171310488 | 0.999332001504091 |
| ebi-fl187-GCST90094726 | prot-a-1246 | Glypican-3 \|\| id:prot-a-1246 | F-capsicum liking (derived food-liking factor) \|\| id:ebi-fl187-GCST90094726 | Weighted median | 21 | 0.0945076385898206 | 0.11377618511615 | 0.406174109520518 | -0.128493684 | 0.317508961417475 | 1.09911755880912 | 0.879419116602862 | 1.37370155512331 | 0.999332001504091 |
| ebi-fl187-GCST90094726 | prot-a-1275 | Glutamate receptor 4 \|\| id:prot-a-1275 | F-capsicum liking (derived food-liking factor) \|\| id:ebi-fl187-GCST90094726 | Weighted median | 20 | 0.0685363312132112 | 0.117588575638567 | 0.559995256193045 | -0.161937277 | 0.299009939464802 | 1.07093953277669 | 0.850494548404191 | 1.34852302700346 | 0.999332001504091 |
| ebi-fl187-GCST90094726 | prot-a-1289 | Glutathione S-transferase P \|\| id:prot-a-1289 | F-capsicum liking (derived food-liking factor) \|\| id:ebi-fl187-GCST90094726 | Weighted median | 21 | 0.0238868234654835 | 0.0992897806862661 | 0.809883088121054 | -0.170721147 | 0.218494793610565 | 1.02417439882189 | 0.84305662983749 | 1.24420253880735 | 0.999332001504091 |
| ebi-fl187-GCST90094726 | prot-a-130 | Apolipoprotein D \|\| id:prot-a-130 | F-capsicum liking (derived food-liking factor) \|\| id:ebi-fl187-GCST90094726 | Weighted median | 21 | 0.0831914395470371 | 0.104843995818649 | 0.427499185195408 | -0.122302792 | 0.28868567135159 | 1.08674983553156 | 0.884880393002393 | 1.33467213689827 | 0.999332001504091 |
| ebi-fl187-GCST90094726 | prot-a-1313 | Heparin-binding EGF-like growth factor \|\| id:prot-a-1313 | F-capsicum liking (derived food-liking factor) \|\| id:ebi-fl187-GCST90094726 | Weighted median | 21 | -0.160098348 | 0.112097660088405 | 0.15323309511838 | -0.379809762 | 0.0596130660035981 | 0.852059986646076 | 0.683991518327166 | 1.06142576536461 | 0.999332001504091 |
| ebi-fl187-GCST90094726 | prot-a-1347 | HLA class II histocompatibility antigen, DQ alpha 2 chain \|\| id:prot-a-1347 | F-capsicum liking (derived food-liking factor) \|\| id:ebi-fl187-GCST90094726 | Weighted median | 20 | 0.0315613151153922 | 0.10461372969631 | 0.762885484354995 | -0.173481595 | 0.236604225320161 | 1.03206465481904 | 0.840732624628302 | 1.26693959592405 | 0.999332001504091 |
| ebi-fl187-GCST90094726 | prot-a-1357 | Non-histone chromosomal protein HMG-14 \|\| id:prot-a-1357 | F-capsicum liking (derived food-liking factor) \|\| id:ebi-fl187-GCST90094726 | Weighted median | 21 | -0.00124923 | 0.108288698021479 | 0.990795720557071 | -0.213495078 | 0.210996617852891 | 0.998751549694106 | 0.807756135600398 | 1.23490817841321 | 0.999332001504091 |
| ebi-fl187-GCST90094726 | prot-a-137 | Apolipoprotein M \|\| id:prot-a-137 | F-capsicum liking (derived food-liking factor) \|\| id:ebi-fl187-GCST90094726 | Weighted median | 21 | -0.024883603 | 0.109189214454374 | 0.81972839122777 | -0.238894463 | 0.189127257525197 | 0.975423441973137 | 0.787497988055359 | 1.20819469456706 | 0.999332001504091 |
| ebi-fl187-GCST90094726 | prot-a-1381 | 17-beta-hydroxysteroid dehydrogenase 14 \|\| id:prot-a-1381 | F-capsicum liking (derived food-liking factor) \|\| id:ebi-fl187-GCST90094726 | Weighted median | 21 | -0.113317836 | 0.104362917152375 | 0.277565024701987 | -0.317869154 | 0.0912334816077856 | 0.892866829781239 | 0.72769799882744 | 1.09552476028266 | 0.999332001504091 |
| ebi-fl187-GCST90094726 | prot-a-1392 | Serine protease HTRA2, mitochondrial \|\| id:prot-a-1392 | F-capsicum liking (derived food-liking factor) \|\| id:ebi-fl187-GCST90094726 | Weighted median | 21 | -0.033604306 | 0.105590510232972 | 0.750294399129721 | -0.240561706 | 0.173353093615915 | 0.966954046436157 | 0.786186131158902 | 1.18928596023564 | 0.999332001504091 |
| ebi-fl187-GCST90094726 | prot-a-1416 | Interferon-induced protein with tetratricopeptide repeats 2 \|\| id:prot-a-1416 | F-capsicum liking (derived food-liking factor) \|\| id:ebi-fl187-GCST90094726 | Weighted median | 21 | -0.024800179 | 0.106349810556918 | 0.815610464297315 | -0.233245808 | 0.183645449818623 | 0.97550481902681 | 0.791958880113236 | 1.20158972370948 | 0.999332001504091 |
| ebi-fl187-GCST90094726 | prot-a-1428 | Interferon gamma \|\| id:prot-a-1428 | F-capsicum liking (derived food-liking factor) \|\| id:ebi-fl187-GCST90094726 | Weighted median | 21 | -0.051725925 | 0.103374695872681 | 0.61681233996821 | -0.254340328 | 0.150888479408914 | 0.949589090284077 | 0.775427857003834 | 1.16286696723881 | 0.999332001504091 |
| ebi-fl187-GCST90094726 | prot-a-1483 | Interleukin-17C \|\| id:prot-a-1483 | F-capsicum liking (derived food-liking factor) \|\| id:ebi-fl187-GCST90094726 | Weighted median | 21 | 0.150761484488607 | 0.110562435560067 | 0.172697629540255 | -0.065940889 | 0.367463858186338 | 1.16271929841778 | 0.936186201362863 | 1.44406760636405 | 0.999332001504091 |
| ebi-fl187-GCST90094726 | prot-a-1503 | Interleukin-1 receptor-like 2 \|\| id:prot-a-1503 | F-capsicum liking (derived food-liking factor) \|\| id:ebi-fl187-GCST90094726 | Weighted median | 21 | -0.016507343 | 0.106325119516221 | 0.87662150190582 | -0.224904577 | 0.19188989125407 | 0.98362815658484 | 0.798592419008837 | 1.21153710878864 | 0.999332001504091 |
| ebi-fl187-GCST90094726 | prot-a-1522 | Interleukin-31 receptor subunit alpha \|\| id:prot-a-1522 | F-capsicum liking (derived food-liking factor) \|\| id:ebi-fl187-GCST90094726 | Weighted median | 21 | 0.0391439283860279 | 0.103195181276868 | 0.704450737264893 | -0.163118627 | 0.24140648368869 | 1.03992014690785 | 0.849490410009143 | 1.27303839949552 | 0.999332001504091 |
| ebi-fl187-GCST90094726 | prot-a-1540 | Interleukin-6 receptor subunit alpha \|\| id:prot-a-1540 | F-capsicum liking (derived food-liking factor) \|\| id:ebi-fl187-GCST90094726 | Weighted median | 21 | 0.0578755871255601 | 0.109077960731998 | 0.595703486411901 | -0.155917216 | 0.271668390160277 | 1.05958316172268 | 0.855630019992498 | 1.31215180670738 | 0.999332001504091 |
| ebi-fl187-GCST90094726 | prot-a-1541 | Interleukin-6 receptor subunit alpha \|\| id:prot-a-1541 | F-capsicum liking (derived food-liking factor) \|\| id:ebi-fl187-GCST90094726 | Weighted median | 21 | 0.0723749806889456 | 0.110277430220729 | 0.51163168202575 | -0.143768783 | 0.288518743921575 | 1.07505839462848 | 0.866087979569385 | 1.33444936210267 | 0.999332001504091 |
| ebi-fl187-GCST90094726 | prot-a-1545 | Interleukin-9 \|\| id:prot-a-1545 | F-capsicum liking (derived food-liking factor) \|\| id:ebi-fl187-GCST90094726 | Weighted median | 21 | -0.11168574 | 0.105986938129313 | 0.291988731488132 | -0.319420139 | 0.0960486586262063 | 0.894325263905044 | 0.726570224799552 | 1.10081262672095 | 0.999332001504091 |
| ebi-fl187-GCST90094726 | prot-a-1548 | Inositol monophosphatase 3 \|\| id:prot-a-1548 | F-capsicum liking (derived food-liking factor) \|\| id:ebi-fl187-GCST90094726 | Weighted median | 21 | 0.00236212067641777 | 0.104911321317171 | 0.982036825315088 | -0.203264069 | 0.207988310458073 | 1.00236491268138 | 0.816062716018825 | 1.23119877731502 | 0.999332001504091 |
| ebi-fl187-GCST90094726 | prot-a-1555 | Inhibin beta A chain:Inhibin beta B chain heterodimer \|\| id:prot-a-1555 | F-capsicum liking (derived food-liking factor) \|\| id:ebi-fl187-GCST90094726 | Weighted median | 21 | -0.119487619 | 0.104589949040643 | 0.253271658098083 | -0.324483919 | 0.0855086809567469 | 0.887374994196277 | 0.72290033231237 | 1.08927101721761 | 0.999332001504091 |
| ebi-fl187-GCST90094726 | prot-a-1603 | Junctophilin-3 \|\| id:prot-a-1603 | F-capsicum liking (derived food-liking factor) \|\| id:ebi-fl187-GCST90094726 | Weighted median | 21 | -0.039693131 | 0.117822698682869 | 0.736200833882258 | -0.270625621 | 0.191239358297699 | 0.961084320773418 | 0.762902057808654 | 1.21074922027301 | 0.999332001504091 |
| ebi-fl187-GCST90094726 | prot-a-1627 | UPF0258 protein KIAA1024 \|\| id:prot-a-1627 | F-capsicum liking (derived food-liking factor) \|\| id:ebi-fl187-GCST90094726 | Weighted median | 21 | -0.083883602 | 0.108133046298193 | 0.437899933778719 | -0.295824373 | 0.128057168423201 | 0.91953828198776 | 0.743918068597589 | 1.1366179794974 | 0.999332001504091 |
| ebi-fl187-GCST90094726 | prot-a-1666 | Kallikrein-7 \|\| id:prot-a-1666 | F-capsicum liking (derived food-liking factor) \|\| id:ebi-fl187-GCST90094726 | Weighted median | 21 | -0.03060053 | 0.111258989941575 | 0.783286491909346 | -0.24866815 | 0.187467090271647 | 0.969862926832735 | 0.779838719695588 | 1.20619055336434 | 0.999332001504091 |
| ebi-fl187-GCST90094726 | prot-a-1678 | Importin subunit alpha-3 \|\| id:prot-a-1678 | F-capsicum liking (derived food-liking factor) \|\| id:ebi-fl187-GCST90094726 | Weighted median | 21 | -0.058631939 | 0.113218560112801 | 0.60455240611015 | -0.280540317 | 0.16327643890512 | 0.943053806694067 | 0.755375489153328 | 1.17736211340007 | 0.999332001504091 |
| ebi-fl187-GCST90094726 | prot-a-1718 | Protein LDOC1 \|\| id:prot-a-1718 | F-capsicum liking (derived food-liking factor) \|\| id:ebi-fl187-GCST90094726 | Weighted median | 21 | -0.058339602 | 0.105130455881081 | 0.578945413462182 | -0.264395296 | 0.147716091116183 | 0.943329536049078 | 0.767670022829691 | 1.15918374707721 | 0.999332001504091 |
| ebi-fl187-GCST90094726 | prot-a-1723 | Leptin \|\| id:prot-a-1723 | F-capsicum liking (derived food-liking factor) \|\| id:ebi-fl187-GCST90094726 | Weighted median | 21 | -0.160334835 | 0.118034298595491 | 0.174344737168227 | -0.39168206 | 0.0710123902102795 | 0.851858509132711 | 0.675918981290308 | 1.0735945278479 | 0.999332001504091 |
| ebi-fl187-GCST90094726 | prot-a-1730 | Galectin-7 \|\| id:prot-a-1730 | F-capsicum liking (derived food-liking factor) \|\| id:ebi-fl187-GCST90094726 | Weighted median | 21 | 0.0889796227040833 | 0.104714370943127 | 0.395471602602079 | -0.116260544 | 0.294219789752613 | 1.09305838252528 | 0.890243245235771 | 1.34207884643073 | 0.999332001504091 |
| ebi-fl187-GCST90094726 | prot-a-1753 | Lipase member N \|\| id:prot-a-1753 | F-capsicum liking (derived food-liking factor) \|\| id:ebi-fl187-GCST90094726 | Weighted median | 21 | -0.01342841 | 0.111228235211957 | 0.903906142522155 | -0.231435751 | 0.204578931187269 | 0.986661349044917 | 0.793393668739519 | 1.22700830124565 | 0.999332001504091 |
| ebi-fl187-GCST90094726 | prot-a-1756 | VIP36-like protein \|\| id:prot-a-1756 | F-capsicum liking (derived food-liking factor) \|\| id:ebi-fl187-GCST90094726 | Weighted median | 21 | 0.177029014700352 | 0.104062436845949 | 0.088909050814881 | -0.026933362 | 0.380991390918413 | 1.19366572647807 | 0.973426107001438 | 1.46373500394141 | 0.999332001504091 |
| ebi-fl187-GCST90094726 | prot-a-1760 | Ig Kappa chain V-I region HK102- like \|\| id:prot-a-1760 | F-capsicum liking (derived food-liking factor) \|\| id:ebi-fl187-GCST90094726 | Weighted median | 21 | 0.12023780025477 | 0.111426482702631 | 0.280553221069468 | -0.098158106 | 0.338633706351927 | 1.12776500249985 | 0.906505568596606 | 1.40302933034652 | 0.999332001504091 |
| ebi-fl187-GCST90094726 | prot-a-179 | Neutral ceramidase \|\| id:prot-a-179 | F-capsicum liking (derived food-liking factor) \|\| id:ebi-fl187-GCST90094726 | Weighted median | 21 | 0.136614113222508 | 0.111235696782632 | 0.219390690555576 | -0.081407852 | 0.354636078916468 | 1.14638568807714 | 0.921817649158496 | 1.42566173150166 | 0.999332001504091 |
| ebi-fl187-GCST90094726 | prot-a-1809 | Lactotransferrin \|\| id:prot-a-1809 | F-capsicum liking (derived food-liking factor) \|\| id:ebi-fl187-GCST90094726 | Weighted median | 21 | 0.0858326943737935 | 0.103311877563714 | 0.406080133753639 | -0.116658586 | 0.288323974398673 | 1.08962401282656 | 0.889888962165537 | 1.33418947734671 | 0.999332001504091 |
| ebi-fl187-GCST90094726 | prot-a-1818 | Lysophospholipase-like protein 1 \|\| id:prot-a-1818 | F-capsicum liking (derived food-liking factor) \|\| id:ebi-fl187-GCST90094726 | Weighted median | 21 | -0.169914751 | 0.104093668833332 | 0.102610932028682 | -0.373938342 | 0.0341088398507143 | 0.843736741191211 | 0.688019332433729 | 1.03469721689039 | 0.999332001504091 |
| ebi-fl187-GCST90094726 | prot-a-1852 | MAP kinase-activated protein kinase 3 \|\| id:prot-a-1852 | F-capsicum liking (derived food-liking factor) \|\| id:ebi-fl187-GCST90094726 | Weighted median | 21 | -0.057829364 | 0.105272544113178 | 0.582779019373123 | -0.264163551 | 0.148504822317821 | 0.943810981692055 | 0.767847947389655 | 1.16009839212409 | 0.999332001504091 |
| ebi-fl187-GCST90094726 | prot-a-1856 | Megakaryocyte-associated tyrosine-protein kinase \|\| id:prot-a-1856 | F-capsicum liking (derived food-liking factor) \|\| id:ebi-fl187-GCST90094726 | Weighted median | 21 | -0.048033705 | 0.109665051224825 | 0.661383557021042 | -0.262977205 | 0.166909795508054 | 0.953101662346958 | 0.768759420756988 | 1.18164767057299 | 0.999332001504091 |
| ebi-fl187-GCST90094726 | prot-a-1890 | Beta-1,3-N-acetylglucosaminyltransferase manic fringe \|\| id:prot-a-1890 | F-capsicum liking (derived food-liking factor) \|\| id:ebi-fl187-GCST90094726 | Weighted median | 21 | -0.005694148 | 0.109829742832542 | 0.958652020027493 | -0.220960444 | 0.209572148275663 | 0.994322033255985 | 0.801748393920752 | 1.23315034157217 | 0.999332001504091 |
| ebi-fl187-GCST90094726 | prot-a-1919 | Matrilysin \|\| id:prot-a-1919 | F-capsicum liking (derived food-liking factor) \|\| id:ebi-fl187-GCST90094726 | Weighted median | 21 | 0.0130675237161527 | 0.104157618335268 | 0.900160082387685 | -0.191081408 | 0.217216455653278 | 1.01315327692444 | 0.826065336904345 | 1.24261304364789 | 0.999332001504091 |
| ebi-fl187-GCST90094726 | prot-a-1923 | Multimerin-2 \|\| id:prot-a-1923 | F-capsicum liking (derived food-liking factor) \|\| id:ebi-fl187-GCST90094726 | Weighted median | 21 | -0.100258073 | 0.109475386401484 | 0.359769340260024 | -0.31482983 | 0.114313684429248 | 0.90460393413271 | 0.729913072815829 | 1.12110374252038 | 0.999332001504091 |
| ebi-fl187-GCST90094726 | prot-a-1948 | Mesothelin \|\| id:prot-a-1948 | F-capsicum liking (derived food-liking factor) \|\| id:ebi-fl187-GCST90094726 | Weighted median | 21 | 0.0897347632582645 | 0.110188008540481 | 0.415428057224269 | -0.126233733 | 0.305703259997608 | 1.09388410696778 | 0.881408807956331 | 1.3575793986574 | 0.999332001504091 |
| ebi-fl187-GCST90094726 | prot-a-195 | ATPase family AAA domain-containing protein 1 \|\| id:prot-a-195 | F-capsicum liking (derived food-liking factor) \|\| id:ebi-fl187-GCST90094726 | Weighted median | 21 | -0.200606915 | 0.104288383805211 | 0.0544074118538146 | -0.405012147 | 0.00379831752715004 | 0.818234004080554 | 0.666968709147511 | 1.00380554027704 | 0.999332001504091 |
| ebi-fl187-GCST90094726 | prot-a-1966 | Mucin-1 \|\| id:prot-a-1966 | F-capsicum liking (derived food-liking factor) \|\| id:ebi-fl187-GCST90094726 | Weighted median | 21 | -0.066219255 | 0.103543237374783 | 0.522476641271773 | -0.269164001 | 0.136725489861992 | 0.935925635051091 | 0.76401794593547 | 1.14651337577321 | 0.999332001504091 |
| ebi-fl187-GCST90094726 | prot-a-1986 | Myocardial zonula adherens protein \|\| id:prot-a-1986 | F-capsicum liking (derived food-liking factor) \|\| id:ebi-fl187-GCST90094726 | Weighted median | 21 | 0.0625815488734018 | 0.111046469641702 | 0.573052386937619 | -0.155069532 | 0.280232629371139 | 1.06458127078138 | 0.856355631615403 | 1.32343764699792 | 0.999332001504091 |
| ebi-fl187-GCST90094726 | prot-a-2071 | Epididymal secretory protein E1 \|\| id:prot-a-2071 | F-capsicum liking (derived food-liking factor) \|\| id:ebi-fl187-GCST90094726 | Weighted median | 20 | -0.197083343 | 0.112217815628286 | 0.0790445068034303 | -0.417030262 | 0.0228635752696923 | 0.821122195376605 | 0.659000976959289 | 1.02312695020746 | 0.999332001504091 |
| ebi-fl187-GCST90094726 | prot-a-2098 | Neuritin \|\| id:prot-a-2098 | F-capsicum liking (derived food-liking factor) \|\| id:ebi-fl187-GCST90094726 | Weighted median | 21 | 0.0553209103771326 | 0.101526220747362 | 0.585827223548677 | -0.143670482 | 0.254312303041962 | 1.05687972393154 | 0.866173120424099 | 1.28957447941895 | 0.999332001504091 |
| ebi-fl187-GCST90094726 | prot-a-2115 | Neurotrophin-4 \|\| id:prot-a-2115 | F-capsicum liking (derived food-liking factor) \|\| id:ebi-fl187-GCST90094726 | Weighted median | 21 | 0.0891723683351548 | 0.10085015941498 | 0.376584740236923 | -0.108493944 | 0.286838680788515 | 1.09326908505836 | 0.897184328042758 | 1.33220928518869 | 0.999332001504091 |
| ebi-fl187-GCST90094726 | prot-a-2118 | Netrin-1 \|\| id:prot-a-2118 | F-capsicum liking (derived food-liking factor) \|\| id:ebi-fl187-GCST90094726 | Weighted median | 21 | 0.0953830673072086 | 0.103721782389869 | 0.357779224944464 | -0.107911626 | 0.298677760791351 | 1.10008017917517 | 0.89770692671813 | 1.34807515080481 | 0.999332001504091 |
| ebi-fl187-GCST90094726 | prot-a-2120 | Netrin-G1 \|\| id:prot-a-2120 | F-capsicum liking (derived food-liking factor) \|\| id:ebi-fl187-GCST90094726 | Weighted median | 21 | 0.0634782109427253 | 0.10231828062675 | 0.53499479637812 | -0.137065619 | 0.264022040971155 | 1.06553626851774 | 0.871913010120361 | 1.3021568967872 | 0.999332001504091 |
| ebi-fl187-GCST90094726 | prot-a-2145 | Olfactomedin-like protein 3 \|\| id:prot-a-2145 | F-capsicum liking (derived food-liking factor) \|\| id:ebi-fl187-GCST90094726 | Weighted median | 21 | -0.045892532 | 0.11438925210662 | 0.688275689393917 | -0.270095466 | 0.178310402593824 | 0.955144604599569 | 0.763306621284937 | 1.19519625568021 | 0.999332001504091 |
| ebi-fl187-GCST90094726 | prot-a-2162 | Prolyl 4-hydroxylase subunit alpha-2 \|\| id:prot-a-2162 | F-capsicum liking (derived food-liking factor) \|\| id:ebi-fl187-GCST90094726 | Weighted median | 21 | 0.046968688333481 | 0.110561882908882 | 0.67096928659882 | -0.169732602 | 0.263669978834889 | 1.04808919114663 | 0.843890440903719 | 1.3016985373386 | 0.999332001504091 |
| ebi-fl187-GCST90094726 | prot-a-2168 | Polyadenylate-binding protein-interacting protein 1 \|\| id:prot-a-2168 | F-capsicum liking (derived food-liking factor) \|\| id:ebi-fl187-GCST90094726 | Weighted median | 21 | -0.123875926 | 0.115390387304325 | 0.283029951802447 | -0.350041085 | 0.102289232959694 | 0.883489452014425 | 0.704659138010731 | 1.10770380985092 | 0.999332001504091 |
| ebi-fl187-GCST90094726 | prot-a-219 | Beta-1,4-galactosyltransferase 5 \|\| id:prot-a-219 | F-capsicum liking (derived food-liking factor) \|\| id:ebi-fl187-GCST90094726 | Weighted median | 21 | -0.025959266 | 0.100002279182285 | 0.795182564978571 | -0.221963733 | 0.170045201454097 | 0.974374779232874 | 0.800944411706591 | 1.1853584300341 | 0.999332001504091 |
| ebi-fl187-GCST90094726 | prot-a-2190 | Pyruvate carboxylase, mitochondrial \|\| id:prot-a-2190 | F-capsicum liking (derived food-liking factor) \|\| id:ebi-fl187-GCST90094726 | Weighted median | 21 | -0.168631099 | 0.102056378237148 | 0.0984667309270472 | -0.3686616 | 0.0313994024271813 | 0.8448205011054 | 0.69165942822129 | 1.0318975639838 | 0.999332001504091 |
| ebi-fl187-GCST90094726 | prot-a-2197 | Protocadherin-9 \|\| id:prot-a-2197 | F-capsicum liking (derived food-liking factor) \|\| id:ebi-fl187-GCST90094726 | Weighted median | 21 | -0.073910634 | 0.102077013975341 | 0.469024383087463 | -0.273981581 | 0.126160313552941 | 0.928754689374089 | 0.760346079771171 | 1.13446402366401 | 0.999332001504091 |
| ebi-fl187-GCST90094726 | prot-a-2198 | Protocadherin alpha-4 \|\| id:prot-a-2198 | F-capsicum liking (derived food-liking factor) \|\| id:ebi-fl187-GCST90094726 | Weighted median | 21 | -0.062493835 | 0.109806221620985 | 0.569269029975152 | -0.27771403 | 0.152726358971922 | 0.939418853932201 | 0.757513416819646 | 1.1650061418429 | 0.999332001504091 |
| ebi-fl187-GCST90094726 | prot-a-2254 | Placenta growth factor \|\| id:prot-a-2254 | F-capsicum liking (derived food-liking factor) \|\| id:ebi-fl187-GCST90094726 | Weighted median | 21 | -0.242754894 | 0.1066163695975 | 0.0227921519408083 | -0.451722978 | -0.03378681 | 0.78446376701172 | 0.636530478009068 | 0.966777590413276 | 0.999332001504091 |
| ebi-fl187-GCST90094726 | prot-a-2261 | Membrane-associated progesterone receptor component 2 \|\| id:prot-a-2261 | F-capsicum liking (derived food-liking factor) \|\| id:ebi-fl187-GCST90094726 | Weighted median | 21 | 0.066076508609793 | 0.106790753226842 | 0.53608273114004 | -0.143233368 | 0.275386384934404 | 1.06830844883362 | 0.866551820079211 | 1.31703946077335 | 0.999332001504091 |
| ebi-fl187-GCST90094726 | prot-a-2268 | Polymeric immunoglobulin receptor \|\| id:prot-a-2268 | F-capsicum liking (derived food-liking factor) \|\| id:ebi-fl187-GCST90094726 | Weighted median | 21 | 0.204997636346081 | 0.108159467791793 | 0.0580489993435044 | -0.006994921 | 0.416990193217996 | 1.22752216352218 | 0.99302948698805 | 1.51738763217239 | 0.999332001504091 |
| ebi-fl187-GCST90094726 | prot-a-2283 | Group 10 secretory phospholipase A2 \|\| id:prot-a-2283 | F-capsicum liking (derived food-liking factor) \|\| id:ebi-fl187-GCST90094726 | Weighted median | 21 | 0.0361837663054411 | 0.114722472409958 | 0.752455991478659 | -0.18867228 | 0.261039812228959 | 1.03684636641088 | 0.828057833662119 | 1.29827935179962 | 0.999332001504091 |
| ebi-fl187-GCST90094726 | prot-a-2298 | Inactive phospholipase D5 \|\| id:prot-a-2298 | F-capsicum liking (derived food-liking factor) \|\| id:ebi-fl187-GCST90094726 | Weighted median | 21 | -0.101104199 | 0.108948702156072 | 0.353408504076043 | -0.314643656 | 0.112435256890876 | 0.903838848571987 | 0.730048976816396 | 1.11899980704087 | 0.999332001504091 |
| ebi-fl187-GCST90094726 | prot-a-2304 | Procollagen-lysine,2-oxoglutarate 5-dioxygenase 2 \|\| id:prot-a-2304 | F-capsicum liking (derived food-liking factor) \|\| id:ebi-fl187-GCST90094726 | Weighted median | 21 | -0.014139819 | 0.113084215141275 | 0.90049350055112 | -0.235784881 | 0.20750524241272 | 0.985959678468186 | 0.789950589083473 | 1.23060416815813 | 0.999332001504091 |
| ebi-fl187-GCST90094726 | prot-a-2331 | Periostin \|\| id:prot-a-2331 | F-capsicum liking (derived food-liking factor) \|\| id:ebi-fl187-GCST90094726 | Weighted median | 21 | 0.00490935020358254 | 0.105353924920298 | 0.962833111562115 | -0.201584343 | 0.211403043047366 | 1.00492142080815 | 0.817434630057642 | 1.23541017821555 | 0.999332001504091 |
| ebi-fl187-GCST90094726 | prot-a-2333 | Periostin \|\| id:prot-a-2333 | F-capsicum liking (derived food-liking factor) \|\| id:ebi-fl187-GCST90094726 | Weighted median | 21 | -0.032902839 | 0.106613589147942 | 0.757612365020139 | -0.241865474 | 0.176059795454953 | 0.967632570903965 | 0.785161795119258 | 1.19250936315871 | 0.999332001504091 |
| ebi-fl187-GCST90094726 | prot-a-2367 | cAMP-dependent protein kinase catalytic subunit alpha \|\| id:prot-a-2367 | F-capsicum liking (derived food-liking factor) \|\| id:ebi-fl187-GCST90094726 | Weighted median | 21 | -0.007769295 | 0.110571648064111 | 0.943982898041655 | -0.224489725 | 0.208951135368265 | 0.992260808123701 | 0.798923785565251 | 1.23238477703063 | 0.999332001504091 |
| ebi-fl187-GCST90094726 | prot-a-2385 | Phosphoribosyl pyrophosphate synthase-associated protein 1 \|\| id:prot-a-2385 | F-capsicum liking (derived food-liking factor) \|\| id:ebi-fl187-GCST90094726 | Weighted median | 21 | 0.239927637896604 | 0.105043233753852 | 0.0223665564711367 | 0.0340428997390541 | 0.445812376054155 | 1.27115716338717 | 1.03462899108981 | 1.56175841576651 | 0.999332001504091 |
| ebi-fl187-GCST90094726 | prot-a-2393 | Trypsin-3 \|\| id:prot-a-2393 | F-capsicum liking (derived food-liking factor) \|\| id:ebi-fl187-GCST90094726 | Weighted median | 20 | 0.194921729019443 | 0.107723566251248 | 0.0703792162300327 | -0.016216461 | 0.406059918871889 | 1.21521586662975 | 0.983914318089927 | 1.50089248154809 | 0.999332001504091 |
| ebi-fl187-GCST90094726 | prot-a-2405 | Pregnancy-specific beta-1-glycoprotein 6 \|\| id:prot-a-2405 | F-capsicum liking (derived food-liking factor) \|\| id:ebi-fl187-GCST90094726 | Weighted median | 21 | 0.14617745077434 | 0.105911221320537 | 0.16752861071581 | -0.061408543 | 0.353763444562593 | 1.15740155163085 | 0.940438951518559 | 1.42441819275397 | 0.999332001504091 |
| ebi-fl187-GCST90094726 | prot-a-244 | Beclin-1 \|\| id:prot-a-244 | F-capsicum liking (derived food-liking factor) \|\| id:ebi-fl187-GCST90094726 | Weighted median | 21 | 0.0190358459923784 | 0.100487982103123 | 0.849752635114282 | -0.177920599 | 0.2159922909145 | 1.01921818284982 | 0.83700888025127 | 1.2410928112732 | 0.999332001504091 |
| ebi-fl187-GCST90094726 | prot-a-2454 | tRNA pseudouridine synthase A, mitochondrial \|\| id:prot-a-2454 | F-capsicum liking (derived food-liking factor) \|\| id:ebi-fl187-GCST90094726 | Weighted median | 21 | -0.173680135 | 0.104892841244583 | 0.0977650925672017 | -0.379270104 | 0.0319098338567056 | 0.840565722231653 | 0.684360739248554 | 1.0324244113808 | 0.999332001504091 |
| ebi-fl187-GCST90094726 | prot-a-2466 | Glutaminyl-peptide cyclotransferase-like protein \|\| id:prot-a-2466 | F-capsicum liking (derived food-liking factor) \|\| id:ebi-fl187-GCST90094726 | Weighted median | 21 | -0.060277767 | 0.110161003488042 | 0.584255888626703 | -0.276193334 | 0.155637799406696 | 0.941502978397654 | 0.758666240400511 | 1.16840293020511 | 0.999332001504091 |
| ebi-fl187-GCST90094726 | prot-a-2474 | Ras-related protein Rab-27A \|\| id:prot-a-2474 | F-capsicum liking (derived food-liking factor) \|\| id:ebi-fl187-GCST90094726 | Weighted median | 21 | 0.0796690846131934 | 0.108019894303125 | 0.460793511126486 | -0.132049908 | 0.291388077447319 | 1.08292865062207 | 0.876297259550769 | 1.33828384096434 | 0.999332001504091 |
| ebi-fl187-GCST90094726 | prot-a-2490 | Rap1 GTPase-GDP dissociation stimulator 1 \|\| id:prot-a-2490 | F-capsicum liking (derived food-liking factor) \|\| id:ebi-fl187-GCST90094726 | Weighted median | 21 | -0.065611487 | 0.11708712962554 | 0.575230744151395 | -0.295102261 | 0.163879286640821 | 0.936494633563912 | 0.744455454448263 | 1.17807209746887 | 0.999332001504091 |
| ebi-fl187-GCST90094726 | prot-a-2545 | Receptor-interacting serine/threonine-protein kinase 2 \|\| id:prot-a-2545 | F-capsicum liking (derived food-liking factor) \|\| id:ebi-fl187-GCST90094726 | Weighted median | 21 | 0.0789306471748433 | 0.10682634557997 | 0.459986706937809 | -0.13044899 | 0.288310284511584 | 1.08212927074572 | 0.877701263206206 | 1.33417121256843 | 0.999332001504091 |
| ebi-fl187-GCST90094726 | prot-a-2610 | RUN and FYVE domain-containing protein 1 \|\| id:prot-a-2610 | F-capsicum liking (derived food-liking factor) \|\| id:ebi-fl187-GCST90094726 | Weighted median | 21 | 0.0860698543706272 | 0.106472995941664 | 0.418876110567736 | -0.122617218 | 0.294756926416288 | 1.08988245869929 | 0.884602207851902 | 1.34279991982483 | 0.999332001504091 |
| ebi-fl187-GCST90094726 | prot-a-2627 | Serine--tRNA ligase, mitochondrial \|\| id:prot-a-2627 | F-capsicum liking (derived food-liking factor) \|\| id:ebi-fl187-GCST90094726 | Weighted median | 21 | -0.085115364 | 0.0986731831044909 | 0.388358118802535 | -0.278514803 | 0.108284074646611 | 0.91840632704346 | 0.756907063078625 | 1.11436426306124 | 0.999332001504091 |
| ebi-fl187-GCST90094726 | prot-a-264 | BolA-like protein 2 \|\| id:prot-a-264 | F-capsicum liking (derived food-liking factor) \|\| id:ebi-fl187-GCST90094726 | Weighted median | 21 | 0.00410307254471089 | 0.104844532682025 | 0.968782895085552 | -0.201392212 | 0.20959835660148 | 1.00411150167136 | 0.817591699783806 | 1.23318266080162 | 0.999332001504091 |
| ebi-fl187-GCST90094726 | prot-a-2646 | Secretoglobin family 1C member 1 \|\| id:prot-a-2646 | F-capsicum liking (derived food-liking factor) \|\| id:ebi-fl187-GCST90094726 | Weighted median | 21 | -0.149703878 | 0.106968007860303 | 0.161657073104265 | -0.359361173 | 0.0599534176112229 | 0.860962888909683 | 0.698122162796099 | 1.06178708481456 | 0.999332001504091 |
| ebi-fl187-GCST90094726 | prot-a-2672 | Semaphorin-3E \|\| id:prot-a-2672 | F-capsicum liking (derived food-liking factor) \|\| id:ebi-fl187-GCST90094726 | Weighted median | 21 | 0.0689108505318195 | 0.119707558073702 | 0.564845030434755 | -0.165715963 | 0.303537664356276 | 1.07134069543767 | 0.84728686058449 | 1.35464261172314 | 0.999332001504091 |
| ebi-fl187-GCST90094726 | prot-a-2701 | Plasma protease C1 inhibitor \|\| id:prot-a-2701 | F-capsicum liking (derived food-liking factor) \|\| id:ebi-fl187-GCST90094726 | Weighted median | 21 | 0.140535659871072 | 0.109953831126367 | 0.201202407322055 | -0.074973849 | 0.356045168878752 | 1.15089011942638 | 0.927767747938957 | 1.42767203315244 | 0.999332001504091 |
| ebi-fl187-GCST90094726 | prot-a-2738 | Signaling threshold-regulating transmembrane adapter 1 \|\| id:prot-a-2738 | F-capsicum liking (derived food-liking factor) \|\| id:ebi-fl187-GCST90094726 | Weighted median | 21 | 0.0329475853851605 | 0.102268964933813 | 0.747326925479461 | -0.167499586 | 0.233394756655435 | 1.03349636750758 | 0.845776967537604 | 1.2628799111911 | 0.999332001504091 |
| ebi-fl187-GCST90094726 | prot-a-274 | Serine/threonine-protein kinase BRSK2 \|\| id:prot-a-274 | F-capsicum liking (derived food-liking factor) \|\| id:ebi-fl187-GCST90094726 | Weighted median | 21 | -0.051392297 | 0.11111774473564 | 0.643720610912041 | -0.269183076 | 0.166398483102664 | 0.949905952573469 | 0.764003371963134 | 1.18104363389911 | 0.999332001504091 |
| ebi-fl187-GCST90094726 | prot-a-2749 | Mitochondrial glutamate carrier 2 \|\| id:prot-a-2749 | F-capsicum liking (derived food-liking factor) \|\| id:ebi-fl187-GCST90094726 | Weighted median | 21 | 0.0385244671074339 | 0.112606696160359 | 0.732264167346366 | -0.182184657 | 0.259233591581737 | 1.03927615612828 | 0.833447423998533 | 1.29593648932877 | 0.999332001504091 |
| ebi-fl187-GCST90094726 | prot-a-2760 | Sodium-coupled monocarboxylate transporter 1 \|\| id:prot-a-2760 | F-capsicum liking (derived food-liking factor) \|\| id:ebi-fl187-GCST90094726 | Weighted median | 21 | -0.051070688 | 0.105819171415399 | 0.629363971431718 | -0.258476264 | 0.156334888425027 | 0.950211500036279 | 0.77222736078837 | 1.16921769500555 | 0.999332001504091 |
| ebi-fl187-GCST90094726 | prot-a-2777 | Small integral membrane protein 10 \|\| id:prot-a-2777 | F-capsicum liking (derived food-liking factor) \|\| id:ebi-fl187-GCST90094726 | Weighted median | 21 | 0.120703357528201 | 0.102825333365063 | 0.240447944112565 | -0.080834296 | 0.322241010923725 | 1.12829016393636 | 0.922346515411841 | 1.38021738334113 | 0.999332001504091 |
| ebi-fl187-GCST90094726 | prot-a-2799 | Superoxide dismutase [Mn], mitochondrial \|\| id:prot-a-2799 | F-capsicum liking (derived food-liking factor) \|\| id:ebi-fl187-GCST90094726 | Weighted median | 21 | 0.231141947474052 | 0.105172381439847 | 0.0279673695779608 | 0.0250040798519513 | 0.437279815096153 | 1.26003808597904 | 1.02531930366686 | 1.54848930712572 | 0.999332001504091 |
| ebi-fl187-GCST90094726 | prot-a-2805 | Protein SPACA7 \|\| id:prot-a-2805 | F-capsicum liking (derived food-liking factor) \|\| id:ebi-fl187-GCST90094726 | Weighted median | 21 | -0.216684985 | 0.110457249553065 | 0.0497963692273435 | -0.433181194 | -0.000188775 | 0.805183574633298 | 0.648442987399825 | 0.999811242402264 | 0.999332001504091 |
| ebi-fl187-GCST90094726 | prot-a-2811 | SPARC-like protein 1 \|\| id:prot-a-2811 | F-capsicum liking (derived food-liking factor) \|\| id:ebi-fl187-GCST90094726 | Weighted median | 21 | 0.164379802276613 | 0.110362431672695 | 0.136367769708035 | -0.051930564 | 0.380690168355095 | 1.17866188856126 | 0.949394786918772 | 1.46329416033086 | 0.999332001504091 |
| ebi-fl187-GCST90094726 | prot-a-2831 | Sepiapterin reductase \|\| id:prot-a-2831 | F-capsicum liking (derived food-liking factor) \|\| id:ebi-fl187-GCST90094726 | Weighted median | 21 | 0.11769473974642 | 0.110292935357145 | 0.285921980446001 | -0.098479414 | 0.333868893046424 | 1.12490067148651 | 0.906214348155364 | 1.3963600590595 | 0.999332001504091 |
| ebi-fl187-GCST90094726 | prot-a-2834 | SPRY domain-containing SOCS box protein 1 \|\| id:prot-a-2834 | F-capsicum liking (derived food-liking factor) \|\| id:ebi-fl187-GCST90094726 | Weighted median | 21 | 0.026011241016121 | 0.10648015761219 | 0.807012022044627 | -0.182689868 | 0.234712349936013 | 1.02635248565343 | 0.833026463923662 | 1.2645449699705 | 0.999332001504091 |
| ebi-fl187-GCST90094726 | prot-a-2835 | Spectrin alpha chain, non-erythrocytic 1 \|\| id:prot-a-2835 | F-capsicum liking (derived food-liking factor) \|\| id:ebi-fl187-GCST90094726 | Weighted median | 21 | -0.022602575 | 0.100925167569722 | 0.822793250303909 | -0.220415904 | 0.175210753373157 | 0.977650949441695 | 0.80218509698398 | 1.19149730222841 | 0.999332001504091 |
| ebi-fl187-GCST90094726 | prot-a-2859 | Sia-alpha-2,3-Gal-beta-1,4-GlcNAc-R:alpha 2,8-sialyltransferase \|\| id:prot-a-2859 | F-capsicum liking (derived food-liking factor) \|\| id:ebi-fl187-GCST90094726 | Weighted median | 21 | -0.031229737 | 0.105429697556387 | 0.767066769800526 | -0.237871944 | 0.17541247022817 | 0.969252874265463 | 0.788303631488427 | 1.19173767155943 | 0.999332001504091 |
| ebi-fl187-GCST90094726 | prot-a-2861 | Alpha-2,8-sialyltransferase 8F \|\| id:prot-a-2861 | F-capsicum liking (derived food-liking factor) \|\| id:ebi-fl187-GCST90094726 | Weighted median | 21 | -0.182561132 | 0.106735231066093 | 0.087189865979331 | -0.391762185 | 0.0266399208927075 | 0.833133711244139 | 0.675864825720348 | 1.02699793567691 | 0.999332001504091 |
| ebi-fl187-GCST90094726 | prot-a-289 | Uncharacterized protein C17orf89 \|\| id:prot-a-289 | F-capsicum liking (derived food-liking factor) \|\| id:ebi-fl187-GCST90094726 | Weighted median | 21 | -0.188006564 | 0.104640817238196 | 0.0723856762217917 | -0.393102566 | 0.0170894378714347 | 0.828609268293669 | 0.674959516339006 | 1.01723629770607 | 0.999332001504091 |
| ebi-fl187-GCST90094726 | prot-a-2921 | Tachykinin-3 \|\| id:prot-a-2921 | F-capsicum liking (derived food-liking factor) \|\| id:ebi-fl187-GCST90094726 | Weighted median | 21 | -0.018302686 | 0.101908327164888 | 0.857466987230697 | -0.218043007 | 0.181437635339165 | 0.98186379104598 | 0.804090859268977 | 1.19893976290644 | 0.999332001504091 |
| ebi-fl187-GCST90094726 | prot-a-2928 | Tax1-binding protein 1 \|\| id:prot-a-2928 | F-capsicum liking (derived food-liking factor) \|\| id:ebi-fl187-GCST90094726 | Weighted median | 21 | -0.030150565 | 0.109068944619505 | 0.782213333237899 | -0.243925697 | 0.183624566248749 | 0.970299429216732 | 0.78354585217403 | 1.20156463048854 | 0.999332001504091 |
| ebi-fl187-GCST90094726 | prot-a-296 | Uncharacterized protein C1orf185 \|\| id:prot-a-296 | F-capsicum liking (derived food-liking factor) \|\| id:ebi-fl187-GCST90094726 | Weighted median | 21 | 0.0397797719966978 | 0.109777894190988 | 0.71707857355775 | -0.175384901 | 0.254944444611035 | 1.04058158375178 | 0.839133975417597 | 1.290389931 | 0.999332001504091 |
| ebi-fl187-GCST90094726 | prot-a-2964 | Transforming growth factor beta-2 \|\| id:prot-a-2964 | F-capsicum liking (derived food-liking factor) \|\| id:ebi-fl187-GCST90094726 | Weighted median | 21 | 0.0392324262782457 | 0.104793491205347 | 0.708122763127566 | -0.166162816 | 0.244627669040726 | 1.04001218172131 | 0.846908332326218 | 1.2771457037833 | 0.999332001504091 |
| ebi-fl187-GCST90094726 | prot-a-2977 | Thymocyte nuclear protein 1 \|\| id:prot-a-2977 | F-capsicum liking (derived food-liking factor) \|\| id:ebi-fl187-GCST90094726 | Weighted median | 21 | -0.107267325 | 0.105650757561509 | 0.309962343219247 | -0.31434281 | 0.0998081595504188 | 0.898285506474766 | 0.730268641785065 | 1.10495892192523 | 0.999332001504091 |
| ebi-fl187-GCST90094726 | prot-a-3015 | Transmembrane protein 70, mitochondrial \|\| id:prot-a-3015 | F-capsicum liking (derived food-liking factor) \|\| id:ebi-fl187-GCST90094726 | Weighted median | 21 | -0.055306482 | 0.106698469269421 | 0.604218468313164 | -0.264435482 | 0.153822517389743 | 0.946195111382112 | 0.767639173701773 | 1.1662838733022 | 0.999332001504091 |
| ebi-fl187-GCST90094726 | prot-a-3030 | Tumor necrosis factor alpha-induced protein 3 \|\| id:prot-a-3030 | F-capsicum liking (derived food-liking factor) \|\| id:ebi-fl187-GCST90094726 | Weighted median | 21 | -0.164687035 | 0.109266347700343 | 0.131757534838227 | -0.378849076 | 0.0494750067906402 | 0.848159106922434 | 0.684648934691722 | 1.05071933103843 | 0.999332001504091 |
| ebi-fl187-GCST90094726 | prot-a-3032 | Tumor necrosis factor receptor superfamily member 10A \|\| id:prot-a-3032 | F-capsicum liking (derived food-liking factor) \|\| id:ebi-fl187-GCST90094726 | Weighted median | 21 | 0.0418485082277171 | 0.10630781668301 | 0.693836431218427 | -0.166514812 | 0.250211828926416 | 1.04273650078436 | 0.846610276452409 | 1.28429743922335 | 0.999332001504091 |
| ebi-fl187-GCST90094726 | prot-a-3081 | Tumor protein p53-inducible protein 11 \|\| id:prot-a-3081 | F-capsicum liking (derived food-liking factor) \|\| id:ebi-fl187-GCST90094726 | Weighted median | 21 | 0.0855641578226025 | 0.104532455112083 | 0.413048026845513 | -0.119319454 | 0.290447769842285 | 1.08933144823596 | 0.887524232129803 | 1.33702603394642 | 0.999332001504091 |
| ebi-fl187-GCST90094726 | prot-a-315 | Complement C4 \|\| id:prot-a-315 | F-capsicum liking (derived food-liking factor) \|\| id:ebi-fl187-GCST90094726 | Weighted median | 20 | 0.0410243427053546 | 0.114250843219989 | 0.719540808902586 | -0.18290731 | 0.264955995416534 | 1.04187746735299 | 0.832845348590043 | 1.30337362010317 | 0.999332001504091 |
| ebi-fl187-GCST90094726 | prot-a-3157 | UDP-glucuronosyltransferase 1-8 \|\| id:prot-a-3157 | F-capsicum liking (derived food-liking factor) \|\| id:ebi-fl187-GCST90094726 | Weighted median | 21 | 0.157715884948438 | 0.10485593745698 | 0.132550539503195 | -0.047801752 | 0.363233522364119 | 1.17083349602846 | 0.95332276223049 | 1.43797161856793 | 0.999332001504091 |
| ebi-fl187-GCST90094726 | prot-a-317 | Glutaredoxin-like protein C5orf63 \|\| id:prot-a-317 | F-capsicum liking (derived food-liking factor) \|\| id:ebi-fl187-GCST90094726 | Weighted median | 21 | -0.006100415 | 0.110315256081886 | 0.95589959499757 | -0.222318317 | 0.210117486652252 | 0.993918154484722 | 0.800660459779869 | 1.2338230091766 | 0.999332001504091 |
| ebi-fl187-GCST90094726 | prot-a-3192 | Guanine nucleotide exchange factor VAV3 \|\| id:prot-a-3192 | F-capsicum liking (derived food-liking factor) \|\| id:ebi-fl187-GCST90094726 | Weighted median | 21 | 0.036998786327584 | 0.108632393537434 | 0.733414480632813 | -0.175920705 | 0.249918277660954 | 1.03769176141953 | 0.838684484181768 | 1.28392048741489 | 0.999332001504091 |
| ebi-fl187-GCST90094726 | prot-a-3233 | Serine/threonine-protein kinase WNK3 \|\| id:prot-a-3233 | F-capsicum liking (derived food-liking factor) \|\| id:ebi-fl187-GCST90094726 | Weighted median | 21 | 0.133972710327359 | 0.107907609980983 | 0.214402580710508 | -0.077526205 | 0.345471625890085 | 1.14336161725465 | 0.925402773681397 | 1.41265600773015 | 0.999332001504091 |
| ebi-fl187-GCST90094726 | prot-a-3250 | 14-3-3 protein theta \|\| id:prot-a-3250 | F-capsicum liking (derived food-liking factor) \|\| id:ebi-fl187-GCST90094726 | Weighted median | 21 | 0.103603376591777 | 0.10843869852592 | 0.339370587109107 | -0.108936473 | 0.31614322570258 | 1.10916044866555 | 0.896787386332295 | 1.3718267224023 | 0.999332001504091 |
| ebi-fl187-GCST90094726 | prot-a-3278 | E3 ubiquitin-protein ligase ZNRF3 \|\| id:prot-a-3278 | F-capsicum liking (derived food-liking factor) \|\| id:ebi-fl187-GCST90094726 | Weighted median | 21 | 0.0287698527858959 | 0.104640798042103 | 0.783362983140882 | -0.176326111 | 0.233865816948419 | 1.02918770253364 | 0.838344545060154 | 1.26347494390921 | 0.999332001504091 |
| ebi-fl187-GCST90094726 | prot-a-355 | Calpain-3 \|\| id:prot-a-355 | F-capsicum liking (derived food-liking factor) \|\| id:ebi-fl187-GCST90094726 | Weighted median | 21 | 0.153515686770006 | 0.103687450099472 | 0.138723357900395 | -0.049711715 | 0.356743088964972 | 1.16592607659287 | 0.951503688802307 | 1.42866878192595 | 0.999332001504091 |
| ebi-fl187-GCST90094726 | prot-a-385 | Coiled-coil domain-containing protein 90B, mitochondrial \|\| id:prot-a-385 | F-capsicum liking (derived food-liking factor) \|\| id:ebi-fl187-GCST90094726 | Weighted median | 21 | -0.089035349 | 0.113343301901822 | 0.432138829914549 | -0.311188221 | 0.133117522947669 | 0.914813235471187 | 0.732575977069882 | 1.14238424680616 | 0.999332001504091 |
| ebi-fl187-GCST90094726 | prot-a-390 | C-C motif chemokine 14 \|\| id:prot-a-390 | F-capsicum liking (derived food-liking factor) \|\| id:ebi-fl187-GCST90094726 | Weighted median | 21 | -0.352152699 | 0.110243580306098 | 0.00140163191692005 | -0.568230116 | -0.136075281 | 0.7031727402476 | 0.566527239368957 | 0.872776926274682 | 0.999332001504091 |
| ebi-fl187-GCST90094726 | prot-a-40 | Pituitary adenylate cyclase-activating polypeptide 27 \|\| id:prot-a-40 | F-capsicum liking (derived food-liking factor) \|\| id:ebi-fl187-GCST90094726 | Weighted median | 21 | 0.10698614713762 | 0.100767944187704 | 0.28836820039978 | -0.090519023 | 0.30449131774552 | 1.11291883726105 | 0.913456956614399 | 1.35593508742999 | 0.999332001504091 |
| ebi-fl187-GCST90094726 | prot-a-401 | C-C motif chemokine 24 \|\| id:prot-a-401 | F-capsicum liking (derived food-liking factor) \|\| id:ebi-fl187-GCST90094726 | Weighted median | 21 | 0.21629125896195 | 0.102037293824482 | 0.034029031456384 | 0.0162981630659663 | 0.416284354857934 | 1.24146391383883 | 1.01643170262238 | 1.51631697967278 | 0.999332001504091 |
| ebi-fl187-GCST90094726 | prot-a-412 | Cerebral cavernous malformations 2 protein \|\| id:prot-a-412 | F-capsicum liking (derived food-liking factor) \|\| id:ebi-fl187-GCST90094726 | Weighted median | 21 | -0.130583034 | 0.110599342722801 | 0.237727707897235 | -0.347357745 | 0.0861916781713132 | 0.877583621026402 | 0.70655251719577 | 1.09001524041053 | 0.999332001504091 |
| ebi-fl187-GCST90094726 | prot-a-44 | Adhesion G protein-coupled receptor F5 \|\| id:prot-a-44 | F-capsicum liking (derived food-liking factor) \|\| id:ebi-fl187-GCST90094726 | Weighted median | 21 | 0.0263153067752063 | 0.102298182045443 | 0.796992583352161 | -0.17418913 | 0.226819743584274 | 1.0266646117521 | 0.840137987305557 | 1.25460369719092 | 0.999332001504091 |
| ebi-fl187-GCST90094726 | prot-a-48 | Adrenomedullin \|\| id:prot-a-48 | F-capsicum liking (derived food-liking factor) \|\| id:ebi-fl187-GCST90094726 | Weighted median | 21 | 0.0691878291733028 | 0.107610200561623 | 0.520257444287032 | -0.141728164 | 0.280103822274084 | 1.07163747502697 | 0.86785713929999 | 1.32326718981476 | 0.999332001504091 |
| ebi-fl187-GCST90094726 | prot-a-502 | Carcinoembryonic antigen-related cell adhesion molecule 1 \|\| id:prot-a-502 | F-capsicum liking (derived food-liking factor) \|\| id:ebi-fl187-GCST90094726 | Weighted median | 21 | -0.065184665 | 0.107338658817286 | 0.543664832990079 | -0.275568437 | 0.14519910593619 | 0.936894435466965 | 0.759140477302145 | 1.15626976752499 | 0.999332001504091 |
| ebi-fl187-GCST90094726 | prot-a-518 | Complement factor B \|\| id:prot-a-518 | F-capsicum liking (derived food-liking factor) \|\| id:ebi-fl187-GCST90094726 | Weighted median | 21 | 0.259198930383804 | 0.105685768425301 | 0.0141850279540041 | 0.0520548242702142 | 0.466343036497394 | 1.29589157139606 | 1.0534334946528 | 1.59415375848557 | 0.999332001504091 |
| ebi-fl187-GCST90094726 | prot-a-525 | Properdin \|\| id:prot-a-525 | F-capsicum liking (derived food-liking factor) \|\| id:ebi-fl187-GCST90094726 | Weighted median | 21 | 0.0520878156480008 | 0.111219320032599 | 0.639545586789649 | -0.165902052 | 0.270077682911894 | 1.0534682494485 | 0.847129205062668 | 1.31006621653894 | 0.999332001504091 |
| ebi-fl187-GCST90094726 | prot-a-533 | Chondroadherin \|\| id:prot-a-533 | F-capsicum liking (derived food-liking factor) \|\| id:ebi-fl187-GCST90094726 | Weighted median | 21 | 0.0175290530724705 | 0.114202689291303 | 0.878011307497527 | -0.206308218 | 0.241366324083424 | 1.01768358855631 | 0.813582276982287 | 1.27298727580247 | 0.999332001504091 |
| ebi-fl187-GCST90094726 | prot-a-535 | Coiled-coil-helix-coiled-coil-helix domain-containing protein 2 \|\| id:prot-a-535 | F-capsicum liking (derived food-liking factor) \|\| id:ebi-fl187-GCST90094726 | Weighted median | 21 | -0.127236975 | 0.112232918670635 | 0.256925962706799 | -0.347213495 | 0.0927395459602482 | 0.880524985784986 | 0.70665444479961 | 1.0971759341463 | 0.999332001504091 |
| ebi-fl187-GCST90094726 | prot-a-591 | Caseinolytic peptidase B protein homolog \|\| id:prot-a-591 | F-capsicum liking (derived food-liking factor) \|\| id:ebi-fl187-GCST90094726 | Weighted median | 21 | 0.0794334864127851 | 0.113247161560923 | 0.483042804303552 | -0.14253095 | 0.301397923072193 | 1.08267354463324 | 0.867160715038924 | 1.35174712590168 | 0.999332001504091 |
| ebi-fl187-GCST90094726 | prot-a-605 | Ciliary neurotrophic factor receptor subunit alpha \|\| id:prot-a-605 | F-capsicum liking (derived food-liking factor) \|\| id:ebi-fl187-GCST90094726 | Weighted median | 21 | -0.002889961 | 0.11133964450095 | 0.979292221450624 | -0.221115665 | 0.215335741805112 | 0.997114210501883 | 0.8016239553827 | 1.24027824032546 | 0.999332001504091 |
| ebi-fl187-GCST90094726 | prot-a-606 | Ciliary neurotrophic factor receptor subunit alpha \|\| id:prot-a-606 | F-capsicum liking (derived food-liking factor) \|\| id:ebi-fl187-GCST90094726 | Weighted median | 21 | 0.155345937632679 | 0.108454853669267 | 0.152042024052802 | -0.057225576 | 0.367917450824441 | 1.1680619678125 | 0.944381016061931 | 1.44472277337746 | 0.999332001504091 |
| ebi-fl187-GCST90094726 | prot-a-707 | Cancer/testis antigen 55 \|\| id:prot-a-707 | F-capsicum liking (derived food-liking factor) \|\| id:ebi-fl187-GCST90094726 | Weighted median | 21 | 0.0784316792063637 | 0.115687606704386 | 0.497796456000145 | -0.14831603 | 0.30517938834696 | 1.0815894575878 | 0.862158603956593 | 1.35686838755248 | 0.999332001504091 |
| ebi-fl187-GCST90094726 | prot-a-719 | Dipeptidyl peptidase 1 \|\| id:prot-a-719 | F-capsicum liking (derived food-liking factor) \|\| id:ebi-fl187-GCST90094726 | Weighted median | 21 | -0.040137083 | 0.111823840258986 | 0.719646437983731 | -0.25931181 | 0.179037643719344 | 0.960657740099818 | 0.771582398349439 | 1.19606576768453 | 0.999332001504091 |
| ebi-fl187-GCST90094726 | prot-a-752 | Cytochrome b5 \|\| id:prot-a-752 | F-capsicum liking (derived food-liking factor) \|\| id:ebi-fl187-GCST90094726 | Weighted median | 21 | -0.007169153 | 0.107095703367918 | 0.946628232087468 | -0.217076731 | 0.202738425815734 | 0.992856484288495 | 0.804868208281953 | 1.22475206282267 | 0.999332001504091 |
| ebi-fl187-GCST90094726 | prot-a-755 | Cytoglobin \|\| id:prot-a-755 | F-capsicum liking (derived food-liking factor) \|\| id:ebi-fl187-GCST90094726 | Weighted median | 21 | -0.08161055 | 0.109841281366972 | 0.457490071567673 | -0.296899461 | 0.133678361792818 | 0.921630818223973 | 0.74311872089626 | 1.14302511996433 | 0.999332001504091 |
| ebi-fl187-GCST90094726 | prot-a-807 | Desmin \|\| id:prot-a-807 | F-capsicum liking (derived food-liking factor) \|\| id:ebi-fl187-GCST90094726 | Weighted median | 21 | 0.0537786022155069 | 0.105482709280355 | 0.610168224512287 | -0.152967508 | 0.260524712405002 | 1.05525094606902 | 0.858157604637618 | 1.29761078053931 | 0.999332001504091 |
| ebi-fl187-GCST90094726 | prot-a-82 | Ameloblastin \|\| id:prot-a-82 | F-capsicum liking (derived food-liking factor) \|\| id:ebi-fl187-GCST90094726 | Weighted median | 21 | -0.212431392 | 0.10818869960345 | 0.0495850135499292 | -0.424481244 | -0.000381541 | 0.808615791527122 | 0.654109020292962 | 0.999618531501345 | 0.999332001504091 |
| ebi-fl187-GCST90094726 | prot-a-84 | Muellerian-inhibiting factor \|\| id:prot-a-84 | F-capsicum liking (derived food-liking factor) \|\| id:ebi-fl187-GCST90094726 | Weighted median | 21 | -0.002063286 | 0.0975795493007674 | 0.983130265800115 | -0.193319202 | 0.189192631029346 | 0.997938841510379 | 0.824218839653439 | 1.20827368106973 | 0.999332001504091 |
| ebi-fl187-GCST90094726 | prot-a-858 | Inactive dipeptidyl peptidase 10 \|\| id:prot-a-858 | F-capsicum liking (derived food-liking factor) \|\| id:ebi-fl187-GCST90094726 | Weighted median | 21 | -0.021008094 | 0.109074113914322 | 0.847269228361513 | -0.234793358 | 0.192777168835093 | 0.979211038375342 | 0.790734231880522 | 1.21261255554318 | 0.999332001504091 |
| ebi-fl187-GCST90094726 | prot-a-863 | Dihydropyrimidinase-related protein 3 \|\| id:prot-a-863 | F-capsicum liking (derived food-liking factor) \|\| id:ebi-fl187-GCST90094726 | Weighted median | 21 | -0.109321304 | 0.108143037572667 | 0.31206647412899 | -0.321281658 | 0.102639049496407 | 0.896442340579863 | 0.725218958653193 | 1.10809137074503 | 0.999332001504091 |
| ebi-fl187-GCST90094726 | prot-a-864 | Dihydropyrimidinase-related protein 5 \|\| id:prot-a-864 | F-capsicum liking (derived food-liking factor) \|\| id:ebi-fl187-GCST90094726 | Weighted median | 21 | -0.036927783 | 0.109329984104248 | 0.735540697431321 | -0.251214551 | 0.177358986259943 | 0.963745732062056 | 0.777855463653376 | 1.19405966721051 | 0.999332001504091 |
| ebi-fl187-GCST90094726 | prot-a-884 | Endothelin-converting enzyme 1 \|\| id:prot-a-884 | F-capsicum liking (derived food-liking factor) \|\| id:ebi-fl187-GCST90094726 | Weighted median | 21 | 0.0334363059617186 | 0.100618246012949 | 0.739656265482268 | -0.163775456 | 0.230648068147098 | 1.03400158189246 | 0.848932623017286 | 1.25941593286413 | 0.999332001504091 |
| ebi-fl187-GCST90094726 | prot-a-893 | Early endosome antigen 1 \|\| id:prot-a-893 | F-capsicum liking (derived food-liking factor) \|\| id:ebi-fl187-GCST90094726 | Weighted median | 21 | -0.133471094 | 0.107393925566649 | 0.213934940726141 | -0.343963188 | 0.0770209998709014 | 0.87505276267743 | 0.708955025373168 | 1.08006475737492 | 0.999332001504091 |
| ebi-fl187-GCST90094726 | prot-a-897 | Ephrin-A2 \|\| id:prot-a-897 | F-capsicum liking (derived food-liking factor) \|\| id:ebi-fl187-GCST90094726 | Weighted median | 21 | 0.125509268884907 | 0.102896611996713 | 0.222555509858788 | -0.076168091 | 0.327186628398465 | 1.13372567728465 | 0.92666043053319 | 1.38706031787174 | 0.999332001504091 |
| ebi-fl187-GCST90094726 | prot-a-90 | Anaphase-promoting complex subunit 10 \|\| id:prot-a-90 | F-capsicum liking (derived food-liking factor) \|\| id:ebi-fl187-GCST90094726 | Weighted median | 21 | 0.0170524299749639 | 0.103272242854948 | 0.86884863786231 | -0.185361166 | 0.219466025970661 | 1.01719865262707 | 0.83080417142358 | 1.24541153558892 | 0.999332001504091 |
| ebi-fl187-GCST90094726 | prot-a-914 | Histone-lysine N-methyltransferase EHMT2 \|\| id:prot-a-914 | F-capsicum liking (derived food-liking factor) \|\| id:ebi-fl187-GCST90094726 | Weighted median | 21 | -0.08364757 | 0.109324604661873 | 0.44419394591446 | -0.297923795 | 0.130628655223981 | 0.919755348439026 | 0.742357908939237 | 1.13954453882088 | 0.999332001504091 |
| ebi-fl187-GCST90094726 | prot-a-932 | RNA polymerase II elongation factor ELL \|\| id:prot-a-932 | F-capsicum liking (derived food-liking factor) \|\| id:ebi-fl187-GCST90094726 | Weighted median | 21 | 0.0299241484218604 | 0.101006083839495 | 0.767030880478673 | -0.168047776 | 0.227896072747271 | 1.03037637531518 | 0.845313448106147 | 1.25595479071845 | 0.999332001504091 |
| ebi-fl187-GCST90094726 | prot-a-991 | Estrogen receptor \|\| id:prot-a-991 | F-capsicum liking (derived food-liking factor) \|\| id:ebi-fl187-GCST90094726 | Weighted median | 21 | 0.0738303910848639 | 0.100358744032592 | 0.461934742339552 | -0.122872747 | 0.270533529388743 | 1.0766241849496 | 0.884376194730869 | 1.31066354174214 | 0.999332001504091 |
| ebi-fl187-GCST90094726 | prot-a-992 | Persulfide dioxygenase ETHE1, mitochondrial \|\| id:prot-a-992 | F-capsicum liking (derived food-liking factor) \|\| id:ebi-fl187-GCST90094726 | Weighted median | 21 | 0.0454672723949609 | 0.107379305501161 | 0.671983917516145 | -0.164996166 | 0.255930711177236 | 1.04651675406649 | 0.847896954590207 | 1.29166322701463 | 0.999332001504091 |
| ebi-fl187-GCST90094726 | prot-a-993 | Ethanolamine kinase 1 \|\| id:prot-a-993 | F-capsicum liking (derived food-liking factor) \|\| id:ebi-fl187-GCST90094726 | Weighted median | 21 | -0.140023521 | 0.111927546055584 | 0.210927433123852 | -0.359401511 | 0.0793544690444949 | 0.869337787269106 | 0.698094002308261 | 1.08258799799891 | 0.999332001504091 |
| ebi-fl187-GCST90094726 | prot-b-10 | coagulation factor III, tissue factor \|\| id:prot-b-10 | F-capsicum liking (derived food-liking factor) \|\| id:ebi-fl187-GCST90094726 | Weighted median | 14 | 0.236315629117924 | 0.138651500236829 | 0.0883090606578863 | -0.035441311 | 0.50807256958211 | 1.26657401472342 | 0.965179377646111 | 1.66208455332412 | 0.999332001504091 |
| ebi-fl187-GCST90094726 | prot-b-28 | prolactin \|\| id:prot-b-28 | F-capsicum liking (derived food-liking factor) \|\| id:ebi-fl187-GCST90094726 | Weighted median | 14 | 0.010273687536794 | 0.13524838082046 | 0.939449624346436 | -0.254813139 | 0.275360513944895 | 1.01032664305872 | 0.775061313262287 | 1.31700538810002 | 0.999332001504091 |
| ebi-fl187-GCST90094726 | prot-b-54 | matrix metallopeptidase 7 \|\| id:prot-b-54 | F-capsicum liking (derived food-liking factor) \|\| id:ebi-fl187-GCST90094726 | Weighted median | 14 | 0.213577667224719 | 0.141600750530572 | 0.131475495559734 | -0.063959804 | 0.49111513826464 | 1.23809965429803 | 0.938042704510824 | 1.63413749352944 | 0.999332001504091 |
| ebi-fl187-GCST90094726 | ukb-rsfMRI191-edge-pheno716 | Net100_Pair10_33 (OccipitalPrecuneus)&(Temporal\|Frontal\|Supp_Motor_Area) (Default_mode\|Central_executive)&(Default_mode\|Salience) \|\| id:ukb-rsfMRI191-edge-pheno716 | F-capsicum liking (derived food-liking factor) \|\| id:ebi-fl187-GCST90094726 | Weighted median | 20 | -0.024177687 | 0.0345859340273325 | 0.484513742315248 | -0.091966118 | 0.0436107437852676 | 0.976112251979566 | 0.91213605437846 | 1.04457566817032 | 0.999332001504091 |
| ebi-fl187-GCST90094726 | ukb-rsfMRI191-edge-pheno903 | Net100_Pair27_38 (Precentral\|Frontal\|Supp_Motor_Area)&(Subcortical) (Attention\|Salience\|Motor)&(Subcortical-cerebellum) \|\| id:ukb-rsfMRI191-edge-pheno903 | F-capsicum liking (derived food-liking factor) \|\| id:ebi-fl187-GCST90094726 | Weighted median | 20 | 0.035094299828521 | 0.0371227056017429 | 0.344475406734771 | -0.037666203 | 0.107854802807937 | 1.03571737216532 | 0.963034345076329 | 1.11388600052475 | 0.999332001504091 |
| ebi-fl187-GCST90094726 | ukb-rsfMRI191-node-pheno23 | Net100_Node2 RolandicOper\|SupraMarginal\|Insula Salience\|Motor \|\| id:ukb-rsfMRI191-node-pheno23 | F-capsicum liking (derived food-liking factor) \|\| id:ebi-fl187-GCST90094726 | Weighted median | 20 | 0.0051734213929424 | 0.0379487718201201 | 0.891563213982662 | -0.069206171 | 0.0795530141603777 | 1.00518682664443 | 0.933134274668645 | 1.08280296189774 | 0.999332001504091 |
| ebi-fl187-GCST90094726 | ukb-rsfMRI191-node-pheno55 | Net100_Node34 Parietal\|Postcentral\|Precuneus Attention \|\| id:ukb-rsfMRI191-node-pheno55 | F-capsicum liking (derived food-liking factor) \|\| id:ebi-fl187-GCST90094726 | Weighted median | 20 | 0.00717941110465613 | 0.035982297720844 | 0.841851151305754 | -0.063345892 | 0.0777047146375104 | 1.00720524486328 | 0.938618756412939 | 1.08080346610269 | 0.999332001504091 |
| ebi-fl187-GCST90094726 | ebi-a-GCST90001414 | IgD- CD24- B cell Absolute Count \|\| id:ebi-a-GCST90001414 | F-capsicum liking (derived food-liking factor) \|\| id:ebi-fl187-GCST90094726 | Weighted mode | 20 | -0.097265287 | 0.203206918111283 | 0.637648573859642 | -0.495550847 | 0.301020272220817 | 0.907315275004276 | 0.609235219592374 | 1.35123673382981 | 0.999714303421225 |
| ebi-fl187-GCST90094726 | ebi-a-GCST90001422 | CD20- CD38- B cell %B cell \|\| id:ebi-a-GCST90001422 | F-capsicum liking (derived food-liking factor) \|\| id:ebi-fl187-GCST90094726 | Weighted mode | 20 | -0.011916888 | 0.145016341700601 | 0.935366157971202 | -0.296148917 | 0.272315142002913 | 0.988153837157369 | 0.743676673329918 | 1.313000718 | 0.999714303421225 |
| ebi-fl187-GCST90094726 | ebi-a-GCST90001444 | CD20- CD38- B cell %lymphocyte \|\| id:ebi-a-GCST90001444 | F-capsicum liking (derived food-liking factor) \|\| id:ebi-fl187-GCST90094726 | Weighted mode | 20 | 0.0798487252231408 | 0.14705637269291 | 0.593456658435487 | -0.208381765 | 0.368079215701244 | 1.0831232060599 | 0.811897023465395 | 1.44495649768259 | 0.999714303421225 |
| ebi-fl187-GCST90094726 | ebi-a-GCST90001459 | Myeloid Dendritic Cell %Dendritic Cell \|\| id:ebi-a-GCST90001459 | F-capsicum liking (derived food-liking factor) \|\| id:ebi-fl187-GCST90094726 | Weighted mode | 20 | -0.12375797 | 0.193835255253809 | 0.530789274764609 | -0.50367507 | 0.256159130790333 | 0.883593671616764 | 0.604305708129958 | 1.29195830192836 | 0.999714303421225 |
| ebi-fl187-GCST90094726 | ebi-a-GCST90001476 | HLA DR++ monocyte %leukocyte \|\| id:ebi-a-GCST90001476 | F-capsicum liking (derived food-liking factor) \|\| id:ebi-fl187-GCST90094726 | Weighted mode | 20 | 0.038270473124558 | 0.17216039759204 | 0.826455939291387 | -0.299163906 | 0.375704852404956 | 1.03901221975862 | 0.74143787324342 | 1.45601733033309 | 0.999714303421225 |
| ebi-fl187-GCST90094726 | ebi-a-GCST90001478 | CD4 regulatory T cell %CD4+ T cell \|\| id:ebi-a-GCST90001478 | F-capsicum liking (derived food-liking factor) \|\| id:ebi-fl187-GCST90094726 | Weighted mode | 20 | 0.0206994369587141 | 0.196669081534573 | 0.917280573868129 | -0.364771963 | 0.406170836766476 | 1.02091515615489 | 0.694354971555257 | 1.50105896661506 | 0.999714303421225 |
| ebi-fl187-GCST90094726 | ebi-a-GCST90001485 | CD39+ resting CD4 regulatory T cell %CD4 regulatory T cell \|\| id:ebi-a-GCST90001485 | F-capsicum liking (derived food-liking factor) \|\| id:ebi-fl187-GCST90094726 | Weighted mode | 20 | -0.04467622 | 0.188416924078824 | 0.815106479363595 | -0.413973391 | 0.324620951148894 | 0.956307064769842 | 0.661018539915525 | 1.38350613016933 | 0.999714303421225 |
| ebi-fl187-GCST90094726 | ebi-a-GCST90001515 | Immature Myeloid-Derived Suppressor Cells Absolute Count \|\| id:ebi-a-GCST90001515 | F-capsicum liking (derived food-liking factor) \|\| id:ebi-fl187-GCST90094726 | Weighted mode | 20 | 0.0334318995590543 | 0.215157379660749 | 0.878157913659753 | -0.388276565 | 0.455140363694122 | 1.03399702567517 | 0.678224744384825 | 1.5763946360802 | 0.999714303421225 |
| ebi-fl187-GCST90094726 | ebi-a-GCST90001517 | CD33+ HLA DR+ Absolute Count \|\| id:ebi-a-GCST90001517 | F-capsicum liking (derived food-liking factor) \|\| id:ebi-fl187-GCST90094726 | Weighted mode | 20 | 0.0137972401692066 | 0.236736534258005 | 0.954133404556663 | -0.450206367 | 0.477800847314896 | 1.01389286135072 | 0.637496579804509 | 1.612524313 | 0.999714303421225 |
| ebi-fl187-GCST90094726 | ebi-a-GCST90001518 | CD33+ HLA DR+ CD14- Absolute Count \|\| id:ebi-a-GCST90001518 | F-capsicum liking (derived food-liking factor) \|\| id:ebi-fl187-GCST90094726 | Weighted mode | 20 | 0.0113968741788013 | 0.251055866619977 | 0.964265584531182 | -0.480672624 | 0.503466372753955 | 1.01146206597483 | 0.618367322945474 | 1.65444626994995 | 0.999714303421225 |
| ebi-fl187-GCST90094726 | ebi-a-GCST90001520 | CD33+ HLA DR+ CD14dim Absolute Count \|\| id:ebi-a-GCST90001520 | F-capsicum liking (derived food-liking factor) \|\| id:ebi-fl187-GCST90094726 | Weighted mode | 20 | 0.110417718254307 | 0.219854547350808 | 0.621275999214543 | -0.320497195 | 0.541332631061891 | 1.11674445758821 | 0.725788089465619 | 1.71829519064196 | 0.999714303421225 |
| ebi-fl187-GCST90094726 | ebi-a-GCST90001531 | CD33dim HLA DR- Absolute Count \|\| id:ebi-a-GCST90001531 | F-capsicum liking (derived food-liking factor) \|\| id:ebi-fl187-GCST90094726 | Weighted mode | 20 | 0.282455540396044 | 0.254687511651693 | 0.281258994593934 | -0.216731982 | 0.781643063233361 | 1.3263828033317 | 0.805145733583336 | 2.18505950859884 | 0.999714303421225 |
| ebi-fl187-GCST90094726 | ebi-a-GCST90001532 | Basophil Absolute Count \|\| id:ebi-a-GCST90001532 | F-capsicum liking (derived food-liking factor) \|\| id:ebi-fl187-GCST90094726 | Weighted mode | 20 | 0.226713865247242 | 0.247243444062986 | 0.370653652060223 | -0.257883285 | 0.711311015610695 | 1.25447086886977 | 0.772685410732152 | 2.03665960167635 | 0.999714303421225 |
| ebi-fl187-GCST90094726 | ebi-a-GCST90001588 | T/B cell \|\| id:ebi-a-GCST90001588 | F-capsicum liking (derived food-liking factor) \|\| id:ebi-fl187-GCST90094726 | Weighted mode | 20 | -0.041569224 | 0.157643595373641 | 0.794856610514567 | -0.350550671 | 0.267412223313125 | 0.959282928006897 | 0.704300145564194 | 1.30657893763222 | 0.999714303421225 |
| ebi-fl187-GCST90094726 | ebi-a-GCST90001624 | HLA DR+ CD4+ T cell Absolute Count \|\| id:ebi-a-GCST90001624 | F-capsicum liking (derived food-liking factor) \|\| id:ebi-fl187-GCST90094726 | Weighted mode | 20 | 0.180286540622212 | 0.153122467392496 | 0.253574303317988 | -0.119833495 | 0.480406576711504 | 1.19756046368374 | 0.887068125285277 | 1.61673159399927 | 0.999714303421225 |
| ebi-fl187-GCST90094726 | ebi-a-GCST90001631 | CD8+ Natural Killer T %T cell \|\| id:ebi-a-GCST90001631 | F-capsicum liking (derived food-liking factor) \|\| id:ebi-fl187-GCST90094726 | Weighted mode | 20 | 0.0897083120677442 | 0.164367386296811 | 0.591568674244539 | -0.232451765 | 0.411868389209494 | 1.09385517281353 | 0.792587978847276 | 1.5096357389007 | 0.999714303421225 |
| ebi-fl187-GCST90094726 | ebi-a-GCST90001632 | CD8+ Natural Killer T %lymphocyte \|\| id:ebi-a-GCST90001632 | F-capsicum liking (derived food-liking factor) \|\| id:ebi-fl187-GCST90094726 | Weighted mode | 20 | 0.10572838207489 | 0.14893350218851 | 0.486386235172015 | -0.186181282 | 0.397638046364369 | 1.11151992676468 | 0.830123094787836 | 1.48830523491305 | 0.999714303421225 |
| ebi-fl187-GCST90094726 | ebi-a-GCST90001646 | Natural Killer %CD3- lymphocyte \|\| id:ebi-a-GCST90001646 | F-capsicum liking (derived food-liking factor) \|\| id:ebi-fl187-GCST90094726 | Weighted mode | 20 | -0.131111763 | 0.160303479910104 | 0.423554879987006 | -0.445306583 | 0.183083057758887 | 0.877119739497855 | 0.640627840003677 | 1.20091414917648 | 0.999714303421225 |
| ebi-fl187-GCST90094726 | ebi-a-GCST90001684 | CD127- CD8+ T cell Absolute Count \|\| id:ebi-a-GCST90001684 | F-capsicum liking (derived food-liking factor) \|\| id:ebi-fl187-GCST90094726 | Weighted mode | 20 | 0.0879456687858464 | 0.171103014682104 | 0.613183504319245 | -0.24741624 | 0.42330757756277 | 1.09192879459937 | 0.780815619195234 | 1.52700389588019 | 0.999714303421225 |
| ebi-fl187-GCST90094726 | ebi-a-GCST90001687 | CD28- CD8+ T cell Absolute Count \|\| id:ebi-a-GCST90001687 | F-capsicum liking (derived food-liking factor) \|\| id:ebi-fl187-GCST90094726 | Weighted mode | 20 | 0.088102586599913 | 0.158154607548763 | 0.583985509403609 | -0.221880444 | 0.398085617395489 | 1.09210015112303 | 0.801011124139454 | 1.48897150631243 | 0.999714303421225 |
| ebi-fl187-GCST90094726 | ebi-a-GCST90001701 | BAFF-R on CD20- CD38- B cell \|\| id:ebi-a-GCST90001701 | F-capsicum liking (derived food-liking factor) \|\| id:ebi-fl187-GCST90094726 | Weighted mode | 20 | 0.224189910426187 | 0.193522400657564 | 0.261026627310952 | -0.155113995 | 0.603493815715012 | 1.25130863342083 | 0.856317556117367 | 1.82849608172565 | 0.999714303421225 |
| ebi-fl187-GCST90094726 | ebi-a-GCST90001732 | CD19 on IgD- CD27- B cell \|\| id:ebi-a-GCST90001732 | F-capsicum liking (derived food-liking factor) \|\| id:ebi-fl187-GCST90094726 | Weighted mode | 20 | -0.166055055 | 0.23022142923713 | 0.47951710059363 | -0.617289056 | 0.285178946743494 | 0.846999601714268 | 0.539404753435141 | 1.33000000599808 | 0.999714303421225 |
| ebi-fl187-GCST90094726 | ebi-a-GCST90001798 | CD27 on CD24+ CD27+ B cell \|\| id:ebi-a-GCST90001798 | F-capsicum liking (derived food-liking factor) \|\| id:ebi-fl187-GCST90094726 | Weighted mode | 20 | 0.0553794703688861 | 0.185067464820249 | 0.768006052231991 | -0.307352761 | 0.418111701416574 | 1.05694161661165 | 0.73539113806875 | 1.51909034946953 | 0.999714303421225 |
| ebi-fl187-GCST90094726 | ebi-a-GCST90001836 | CD66b on CD66b++ myeloid cell \|\| id:ebi-a-GCST90001836 | F-capsicum liking (derived food-liking factor) \|\| id:ebi-fl187-GCST90094726 | Weighted mode | 20 | -0.005371391 | 0.300367809832545 | 0.985918875437111 | -0.594092299 | 0.58334951582706 | 0.994643008683852 | 0.552063447207717 | 1.7920308249487 | 0.999714303421225 |
| ebi-fl187-GCST90094726 | ebi-a-GCST90001943 | CD25 on activated & secreting CD4 regulatory T cell \|\| id:ebi-a-GCST90001943 | F-capsicum liking (derived food-liking factor) \|\| id:ebi-fl187-GCST90094726 | Weighted mode | 20 | 0.0908069648282037 | 0.184774238688222 | 0.628738578039265 | -0.271350543 | 0.45296447265712 | 1.09505760022283 | 0.762349213379857 | 1.57296830213726 | 0.999714303421225 |
| ebi-fl187-GCST90094726 | ebi-a-GCST90001960 | CD25 on CD4+ T cell \|\| id:ebi-a-GCST90001960 | F-capsicum liking (derived food-liking factor) \|\| id:ebi-fl187-GCST90094726 | Weighted mode | 20 | 0.140719208955131 | 0.160144520723092 | 0.39054067325368 | -0.173164052 | 0.454602469572391 | 1.15110138364174 | 0.840999636138956 | 1.57554693068023 | 0.999714303421225 |
| ebi-fl187-GCST90094726 | ebi-a-GCST90001988 | HLA DR on CD14+ CD16- monocyte \|\| id:ebi-a-GCST90001988 | F-capsicum liking (derived food-liking factor) \|\| id:ebi-fl187-GCST90094726 | Weighted mode | 20 | 0.0340711313679801 | 0.182942374533447 | 0.854230837932845 | -0.324495923 | 0.392638185453537 | 1.03465820076388 | 0.722891655077297 | 1.48088248756098 | 0.999714303421225 |
| ebi-fl187-GCST90094726 | ebi-a-GCST90001991 | HLA DR on CD14+ monocyte \|\| id:ebi-a-GCST90001991 | F-capsicum liking (derived food-liking factor) \|\| id:ebi-fl187-GCST90094726 | Weighted mode | 20 | 0.0802031054582887 | 0.177497569125363 | 0.656489527881841 | -0.26769213 | 0.428098341 | 1.0835071115366 | 0.765143309494656 | 1.53433696169381 | 0.999714303421225 |
| ebi-fl187-GCST90094726 | ebi-a-GCST90002019 | CD14 on Monocytic Myeloid-Derived Suppressor Cells \|\| id:ebi-a-GCST90002019 | F-capsicum liking (derived food-liking factor) \|\| id:ebi-fl187-GCST90094726 | Weighted mode | 20 | 0.014613746223314 | 0.210221024607093 | 0.945305247856279 | -0.397419462 | 0.426646954453217 | 1.01472104907436 | 0.672052066192938 | 1.53211166103159 | 0.999714303421225 |
| ebi-fl187-GCST90094726 | ebi-a-GCST90002037 | CD80 on plasmacytoid Dendritic Cell \|\| id:ebi-a-GCST90002037 | F-capsicum liking (derived food-liking factor) \|\| id:ebi-fl187-GCST90094726 | Weighted mode | 20 | -0.159816258 | 0.204586891225088 | 0.444330151618908 | -0.560806565 | 0.241174048780751 | 0.852300377938425 | 0.570748532461314 | 1.27274253531825 | 0.999714303421225 |
| ebi-fl187-GCST90094726 | ebi-a-GCST90002042 | CD45 on CD33+ HLA DR+ CD14- \|\| id:ebi-a-GCST90002042 | F-capsicum liking (derived food-liking factor) \|\| id:ebi-fl187-GCST90094726 | Weighted mode | 20 | 0.11579923186595 | 0.249293251720156 | 0.647560983540161 | -0.372815542 | 0.604414005237456 | 1.12277043297872 | 0.688792274712598 | 1.83017941903778 | 0.999714303421225 |
| ebi-fl187-GCST90094726 | ebi-a-GCST90002056 | CD8 on Effector Memory CD8+ T cell \|\| id:ebi-a-GCST90002056 | F-capsicum liking (derived food-liking factor) \|\| id:ebi-fl187-GCST90094726 | Weighted mode | 20 | -0.040714745 | 0.196503013581267 | 0.838060841799598 | -0.425860652 | 0.344431161455998 | 0.960102964904052 | 0.653207359060103 | 1.41118695377824 | 0.999714303421225 |
| ebi-fl187-GCST90094726 | ebi-a-GCST90002058 | CD8 on CD8+ T cell \|\| id:ebi-a-GCST90002058 | F-capsicum liking (derived food-liking factor) \|\| id:ebi-fl187-GCST90094726 | Weighted mode | 20 | -0.428551028 | 0.225208208249496 | 0.0723169417796956 | -0.869959116 | 0.0128570598618628 | 0.651452347195547 | 0.418968677813425 | 1.01294006721805 | 0.999714303421225 |
| ebi-fl187-GCST90094726 | ebi-a-GCST90002063 | CD4 on CD4 regulatory T cell \|\| id:ebi-a-GCST90002063 | F-capsicum liking (derived food-liking factor) \|\| id:ebi-fl187-GCST90094726 | Weighted mode | 20 | -0.321891012 | 0.221442251073572 | 0.162372917509584 | -0.755917824 | 0.112135800101435 | 0.724777178038544 | 0.469579425565982 | 1.11866476511906 | 0.999714303421225 |
| ebi-fl187-GCST90094726 | ebi-a-GCST90002066 | CD4 on activated CD4 regulatory T cell \|\| id:ebi-a-GCST90002066 | F-capsicum liking (derived food-liking factor) \|\| id:ebi-fl187-GCST90094726 | Weighted mode | 20 | -0.310607442 | 0.21547847434445 | 0.165729417235188 | -0.732945251 | 0.1117303680948 | 0.733001565304967 | 0.480491735104146 | 1.11821131454649 | 0.999714303421225 |
| ebi-fl187-GCST90094726 | ebi-a-GCST90002070 | CD4 on activated & secreting CD4 regulatory T cell \|\| id:ebi-a-GCST90002070 | F-capsicum liking (derived food-liking factor) \|\| id:ebi-fl187-GCST90094726 | Weighted mode | 20 | -0.175596522 | 0.213875088312281 | 0.421815270211353 | -0.594791695 | 0.243598651471753 | 0.83895641583267 | 0.551677471234031 | 1.27583217435431 | 0.999714303421225 |
| ebi-fl187-GCST90094726 | ebi-a-GCST90002081 | SSC-A on CD4+ T cell \|\| id:ebi-a-GCST90002081 | F-capsicum liking (derived food-liking factor) \|\| id:ebi-fl187-GCST90094726 | Weighted mode | 20 | -0.204612321 | 0.217185553552607 | 0.357962781247736 | -0.630296006 | 0.221071364286756 | 0.814963199558778 | 0.532434174160637 | 1.24741244808734 | 0.999714303421225 |
| ebi-fl187-GCST90094726 | ebi-a-GCST90002094 | CD11b on Monocytic Myeloid-Derived Suppressor Cells \|\| id:ebi-a-GCST90002094 | F-capsicum liking (derived food-liking factor) \|\| id:ebi-fl187-GCST90094726 | Weighted mode | 20 | 0.207802497950288 | 0.282215881771898 | 0.470527387067475 | -0.34534063 | 0.760945626223209 | 1.23097002643567 | 0.707979153222694 | 2.14029918689768 | 0.999714303421225 |
| ebi-fl187-GCST90094726 | ebi-a-GCST90002104 | HLA DR on myeloid Dendritic Cell \|\| id:ebi-a-GCST90002104 | F-capsicum liking (derived food-liking factor) \|\| id:ebi-fl187-GCST90094726 | Weighted mode | 20 | 0.0573421726148458 | 0.174026898234411 | 0.745381231562818 | -0.283750548 | 0.39843489315429 | 1.05901811540426 | 0.752954447330914 | 1.48949165879817 | 0.999714303421225 |
| ebi-fl187-GCST90094726 | ebi-a-GCST90002105 | HLA DR on plasmacytoid Dendritic Cell \|\| id:ebi-a-GCST90002105 | F-capsicum liking (derived food-liking factor) \|\| id:ebi-fl187-GCST90094726 | Weighted mode | 20 | -0.087169876 | 0.195811224180251 | 0.661225083548833 | -0.470959875 | 0.296620123319872 | 0.916521387353482 | 0.624402631772964 | 1.34530415269259 | 0.999714303421225 |
| ebi-fl187-GCST90094726 | ebi-a-GCST90002106 | HLA DR on Dendritic Cell \|\| id:ebi-a-GCST90002106 | F-capsicum liking (derived food-liking factor) \|\| id:ebi-fl187-GCST90094726 | Weighted mode | 20 | -0.06039577 | 0.184712231786866 | 0.747263644233348 | -0.422431744 | 0.301640204634226 | 0.941391885493699 | 0.655450991282376 | 1.35207466898404 | 0.999714303421225 |
| ebi-fl187-GCST90094726 | ebi-a-GCST90002111 | HLA DR on CD33dim HLA DR+ CD11b- \|\| id:ebi-a-GCST90002111 | F-capsicum liking (derived food-liking factor) \|\| id:ebi-fl187-GCST90094726 | Weighted mode | 20 | 0.116293768769984 | 0.304087303305361 | 0.706382761474225 | -0.479717346 | 0.712304883248491 | 1.12332582171138 | 0.618958318308028 | 2.03868477795554 | 0.999714303421225 |
| ebi-fl187-GCST90094726 | ebi-a-GCST90002112 | HLA DR on CD33- HLA DR+ \|\| id:ebi-a-GCST90002112 | F-capsicum liking (derived food-liking factor) \|\| id:ebi-fl187-GCST90094726 | Weighted mode | 20 | -0.155778852 | 0.250751339809758 | 0.541818614278891 | -0.647251478 | 0.335693773745082 | 0.855748416256211 | 0.523482604573727 | 1.39891057606648 | 0.999714303421225 |
| ebi-fl187-GCST90094726 | ebi-a-GCST90002115 | HLA DR on HLA DR+ CD8+ T cell \|\| id:ebi-a-GCST90002115 | F-capsicum liking (derived food-liking factor) \|\| id:ebi-fl187-GCST90094726 | Weighted mode | 20 | -0.345552053 | 0.195038408167569 | 0.0924804330369397 | -0.727827333 | 0.0367232271770585 | 0.707829486316016 | 0.482957156172864 | 1.03740585535307 | 0.999714303421225 |
| ebi-fl187-GCST90094726 | ebi-a-GCST90002116 | HLA DR on B cell \|\| id:ebi-a-GCST90002116 | F-capsicum liking (derived food-liking factor) \|\| id:ebi-fl187-GCST90094726 | Weighted mode | 20 | 0.0349565567449592 | 0.179519124787808 | 0.847676600888874 | -0.316900928 | 0.386814041329063 | 1.03557471908575 | 0.728402916001938 | 1.47228268208452 | 0.999714303421225 |
| ebi-fl187-GCST90094726 | ebi-cfmet338-GCST90026023 | Sphingomyelin (d18:1/24:1, d18:2/24:0) levels \|\| id:ebi-cfmet338-GCST90026023 | F-capsicum liking (derived food-liking factor) \|\| id:ebi-fl187-GCST90094726 | Weighted mode | 18 | 0.00127235146737259 | 0.126653951957878 | 0.992101626607042 | -0.246969394 | 0.249514097304813 | 1.00127316124991 | 0.781164601200221 | 1.28340165683265 | 0.999714303421225 |
| ebi-fl187-GCST90094726 | ebi-cfmet338-GCST90026046 | 1-arachidonoyl-gpc (20:4n6) levels \|\| id:ebi-cfmet338-GCST90026046 | F-capsicum liking (derived food-liking factor) \|\| id:ebi-fl187-GCST90094726 | Weighted mode | 18 | -0.008167404 | 0.130719415209782 | 0.950908998408633 | -0.264377458 | 0.248042649998196 | 0.991865858811516 | 0.767683716890365 | 1.28151458762334 | 0.999714303421225 |
| ebi-fl187-GCST90094726 | ebi-cfmet338-GCST90026047 | 1-linoleoyl-gpc (18:2) levels \|\| id:ebi-cfmet338-GCST90026047 | F-capsicum liking (derived food-liking factor) \|\| id:ebi-fl187-GCST90094726 | Weighted mode | 18 | -0.17576667 | 0.18285400762594 | 0.349905213680695 | -0.534160525 | 0.182627185058151 | 0.838813680994662 | 0.586161151392227 | 1.20036680996793 | 0.999714303421225 |
| ebi-fl187-GCST90094726 | ebi-cfmet338-GCST90026050 | 1-myristoyl-2-palmitoyl-gpc (14:0/16:0) levels \|\| id:ebi-cfmet338-GCST90026050 | F-capsicum liking (derived food-liking factor) \|\| id:ebi-fl187-GCST90094726 | Weighted mode | 18 | -0.060382747 | 0.0738671412357748 | 0.42497998252988 | -0.205162344 | 0.0843968495734231 | 0.941404144773413 | 0.814515073984468 | 1.08806060452782 | 0.999714303421225 |
| ebi-fl187-GCST90094726 | ebi-cfmet338-GCST90026068 | 2,3-dihydroxyisovalerate levels \|\| id:ebi-cfmet338-GCST90026068 | F-capsicum liking (derived food-liking factor) \|\| id:ebi-fl187-GCST90094726 | Weighted mode | 18 | -0.299169647 | 0.324981197460319 | 0.370151823438139 | -0.936132794 | 0.337793500193046 | 0.741433616903006 | 0.392141398469203 | 1.40185099155514 | 0.999714303421225 |
| ebi-fl187-GCST90094726 | ebi-cfmet338-GCST90026082 | 3-hydroxyhexanoate levels \|\| id:ebi-cfmet338-GCST90026082 | F-capsicum liking (derived food-liking factor) \|\| id:ebi-fl187-GCST90094726 | Weighted mode | 18 | -0.077509786 | 0.0584237287033719 | 0.202159987303749 | -0.192020295 | 0.0370007218572729 | 0.925417967840684 | 0.825290119339396 | 1.03769376990468 | 0.999714303421225 |
| ebi-fl187-GCST90094726 | ebi-cfmet338-GCST90026094 | 3-ureidopropionate levels \|\| id:ebi-cfmet338-GCST90026094 | F-capsicum liking (derived food-liking factor) \|\| id:ebi-fl187-GCST90094726 | Weighted mode | 18 | -0.106025296 | 0.138622863500755 | 0.454849804681484 | -0.377726109 | 0.165675516381156 | 0.899401896445545 | 0.685418205151052 | 1.18019008723524 | 0.999714303421225 |
| ebi-fl187-GCST90094726 | ebi-cfmet338-GCST90026173 | Glycerol levels \|\| id:ebi-cfmet338-GCST90026173 | F-capsicum liking (derived food-liking factor) \|\| id:ebi-fl187-GCST90094726 | Weighted mode | 18 | 2.3802493911651e-05 | 0.0655050011948059 | 0.999714303421225 | -0.128366 | 0.128413604835731 | 1.00002380277719 | 0.879531411865278 | 1.13702318374292 | 0.999714303421225 |
| ebi-fl187-GCST90094726 | ebi-cfmet338-GCST90026179 | Histidine levels \|\| id:ebi-cfmet338-GCST90026179 | F-capsicum liking (derived food-liking factor) \|\| id:ebi-fl187-GCST90094726 | Weighted mode | 18 | -0.041274272 | 0.0639173507574235 | 0.527065884222109 | -0.166552279 | 0.0840037357923036 | 0.959565912086315 | 0.846578557348451 | 1.08763295697207 | 0.999714303421225 |
| ebi-fl187-GCST90094726 | ebi-cfmet338-GCST90026188 | Isocitrate levels \|\| id:ebi-cfmet338-GCST90026188 | F-capsicum liking (derived food-liking factor) \|\| id:ebi-fl187-GCST90094726 | Weighted mode | 18 | -0.126866314 | 0.10574359248315 | 0.246689021327314 | -0.334123755 | 0.0803911276005756 | 0.880851422523329 | 0.715965172522156 | 1.08371085401834 | 0.999714303421225 |
| ebi-fl187-GCST90094726 | ebi-cfmet338-GCST90026207 | N-acetyl-isoputreanine levels \|\| id:ebi-cfmet338-GCST90026207 | F-capsicum liking (derived food-liking factor) \|\| id:ebi-fl187-GCST90094726 | Weighted mode | 17 | -0.000274338 | 0.0628760494805354 | 0.996572658277016 | -0.123511395 | 0.122962719270386 | 0.999725699915686 | 0.883811570424911 | 1.13084226153704 | 0.999714303421225 |
| ebi-fl187-GCST90094726 | ebi-cfmet338-GCST90026213 | N-acetylglutamate levels \|\| id:ebi-cfmet338-GCST90026213 | F-capsicum liking (derived food-liking factor) \|\| id:ebi-fl187-GCST90094726 | Weighted mode | 18 | -0.096525032 | 0.10557254266723 | 0.373345322736202 | -0.303447216 | 0.110397151360394 | 0.907987168337695 | 0.738268856964103 | 1.11672148985961 | 0.999714303421225 |
| ebi-fl187-GCST90094726 | ebi-cfmet338-GCST90026224 | N-acetyltaurine levels \|\| id:ebi-cfmet338-GCST90026224 | F-capsicum liking (derived food-liking factor) \|\| id:ebi-fl187-GCST90094726 | Weighted mode | 18 | -0.054480127 | 0.0879514211668858 | 0.54384355346734 | -0.226864913 | 0.117904658241112 | 0.946977327718326 | 0.797028443412231 | 1.12513683372869 | 0.999714303421225 |
| ebi-fl187-GCST90094726 | ebi-cfmet338-GCST90026250 | Phenyllactate (pla) levels \|\| id:ebi-cfmet338-GCST90026250 | F-capsicum liking (derived food-liking factor) \|\| id:ebi-fl187-GCST90094726 | Weighted mode | 18 | 0.154667269811841 | 0.130010239239736 | 0.250532673415695 | -0.100152799 | 0.409487338721724 | 1.1672695106802 | 0.904699170256946 | 1.5060454959593 | 0.999714303421225 |
| ebi-fl187-GCST90094726 | ebi-cfmet338-GCST90026251 | Pipecolate levels \|\| id:ebi-cfmet338-GCST90026251 | F-capsicum liking (derived food-liking factor) \|\| id:ebi-fl187-GCST90094726 | Weighted mode | 17 | 0.296217405710822 | 0.344184898479761 | 0.40215233848874 | -0.378384995 | 0.970819806731153 | 1.34476248409783 | 0.684966740913573 | 2.64010795067954 | 0.999714303421225 |
| ebi-fl187-GCST90094726 | ebi-cfmet338-GCST90026278 | Trigonelline (n'-methylnicotinate) levels \|\| id:ebi-cfmet338-GCST90026278 | F-capsicum liking (derived food-liking factor) \|\| id:ebi-fl187-GCST90094726 | Weighted mode | 18 | 0.185476000909108 | 0.248039244883846 | 0.464818766010715 | -0.300680919 | 0.671632920881447 | 1.20379130953141 | 0.740313955134363 | 1.95743104240194 | 0.999714303421225 |
| ebi-fl187-GCST90094726 | ebi-cfmet338-GCST90026299 | 1-carboxyethylvaline levels \|\| id:ebi-cfmet338-GCST90026299 | F-capsicum liking (derived food-liking factor) \|\| id:ebi-fl187-GCST90094726 | Weighted mode | 18 | -0.070201199 | 0.138220738215421 | 0.618055442763184 | -0.341113846 | 0.200711447807014 | 0.93220624198389 | 0.710977961603581 | 1.22227203165864 | 0.999714303421225 |
| ebi-fl187-GCST90094726 | ebi-gm430-GCST90011306 | C_Erysipelotrichia abundance \|\| id:ebi-gm430-GCST90011306 | F-capsicum liking (derived food-liking factor) \|\| id:ebi-fl187-GCST90094726 | Weighted mode | 18 | -0.155895097 | 0.124837887860737 | 0.228669090389783 | -0.400577357 | 0.0887871633680047 | 0.855648945942217 | 0.669933143735062 | 1.09284803347715 | 0.999714303421225 |
| ebi-fl187-GCST90094726 | ebi-gm430-GCST90011312 | F_Erysipelotrichaceae abundance \|\| id:ebi-gm430-GCST90011312 | F-capsicum liking (derived food-liking factor) \|\| id:ebi-fl187-GCST90094726 | Weighted mode | 18 | -0.155895097 | 0.121358086110043 | 0.216165755318648 | -0.393756946 | 0.0819667519366447 | 0.855648945942217 | 0.674517980871536 | 1.08541972112596 | 0.999714303421225 |
| ebi-fl187-GCST90094726 | ebi-gm430-GCST90011352 | O_Erysipelotrichales abundance \|\| id:ebi-gm430-GCST90011352 | F-capsicum liking (derived food-liking factor) \|\| id:ebi-fl187-GCST90094726 | Weighted mode | 18 | -0.155895097 | 0.129329895797955 | 0.244557622754308 | -0.409381693 | 0.0975914989249521 | 0.855648945942217 | 0.664060716879214 | 1.10251231563995 | 0.999714303421225 |
| ebi-fl187-GCST90094726 | ebi-gm430-GCST90011362 | OTU97_11 (Parabacteroides) abundance \|\| id:ebi-gm430-GCST90011362 | F-capsicum liking (derived food-liking factor) \|\| id:ebi-fl187-GCST90094726 | Weighted mode | 18 | -0.21512417 | 0.159576820059205 | 0.195318738914656 | -0.527894738 | 0.0976463970625494 | 0.80644129793972 | 0.589845442910088 | 1.10257284317416 | 0.999714303421225 |
| ebi-fl187-GCST90094726 | ebi-gm430-GCST90011384 | OTU97_235 (Phascolarctobacterium) abundance \|\| id:ebi-gm430-GCST90011384 | F-capsicum liking (derived food-liking factor) \|\| id:ebi-fl187-GCST90094726 | Weighted mode | 18 | -0.189728903 | 0.467898926695927 | 0.690174100307975 | -1.106810799 | 0.727352993379684 | 0.827183350520585 | 0.330611668510376 | 2.06959511883346 | 0.999714303421225 |
| ebi-fl187-GCST90094726 | ebi-gm430-GCST90011390 | OTU97_3 (Bacteroides) abundance \|\| id:ebi-gm430-GCST90011390 | F-capsicum liking (derived food-liking factor) \|\| id:ebi-fl187-GCST90094726 | Weighted mode | 18 | -0.147703242 | 0.103643248988223 | 0.172226392760726 | -0.35084401 | 0.0554375260950372 | 0.862687086321135 | 0.704093576889898 | 1.05700297990593 | 0.999714303421225 |
| ebi-fl187-GCST90094726 | ebi-gm430-GCST90011398 | OTU97_4 (Alistipes) abundance \|\| id:ebi-gm430-GCST90011398 | F-capsicum liking (derived food-liking factor) \|\| id:ebi-fl187-GCST90094726 | Weighted mode | 18 | -0.076594849 | 0.142799551440804 | 0.598645373715653 | -0.35648197 | 0.203292272102066 | 0.926265055065598 | 0.700135095109522 | 1.22543057508275 | 0.999714303421225 |
| ebi-fl187-GCST90094726 | ebi-gm430-GCST90011421 | OTU99_108 (Cytophagales) abundance \|\| id:ebi-gm430-GCST90011421 | F-capsicum liking (derived food-liking factor) \|\| id:ebi-fl187-GCST90094726 | Weighted mode | 18 | 0.213928432119896 | 0.393431003931614 | 0.593672933512145 | -0.557196336 | 0.98505319982586 | 1.23853401236781 | 0.57281278947777 | 2.67795434733645 | 0.999714303421225 |
| ebi-fl187-GCST90094726 | ebi-gm430-GCST90011428 | OTU99_123 (Phascolarctobacterium) abundance \|\| id:ebi-gm430-GCST90011428 | F-capsicum liking (derived food-liking factor) \|\| id:ebi-fl187-GCST90094726 | Weighted mode | 19 | -0.074360247 | 0.258653512997679 | 0.777020931640419 | -0.581321132 | 0.432600638664385 | 0.928337203078287 | 0.559159155161708 | 1.54126057789393 | 0.999714303421225 |
| ebi-fl187-GCST90094726 | ebi-gm430-GCST90011450 | OTU99_298 (Phascolarctobacterium) abundance \|\| id:ebi-gm430-GCST90011450 | F-capsicum liking (derived food-liking factor) \|\| id:ebi-fl187-GCST90094726 | Weighted mode | 18 | -0.194337844 | 0.441786519965547 | 0.665557502294857 | -1.060239424 | 0.671563734663994 | 0.823379683002106 | 0.346372870561644 | 1.95729561983693 | 0.999714303421225 |
| ebi-fl187-GCST90094726 | ebi-gm430-GCST90011451 | OTU99_3 (Bacteroides) abundance \|\| id:ebi-gm430-GCST90011451 | F-capsicum liking (derived food-liking factor) \|\| id:ebi-fl187-GCST90094726 | Weighted mode | 18 | -0.154002331 | 0.0997897374974827 | 0.141175451218971 | -0.349590216 | 0.0415855549345308 | 0.857270023089445 | 0.704976918758399 | 1.0424623458341 | 0.999714303421225 |
| ebi-fl187-GCST90094726 | ebi-gm430-GCST90011472 | OTU99_72 (Parabacteroides) abundance \|\| id:ebi-gm430-GCST90011472 | F-capsicum liking (derived food-liking factor) \|\| id:ebi-fl187-GCST90094726 | Weighted mode | 19 | 0.222706367943553 | 0.40813800988931 | 0.591995428473567 | -0.577244131 | 1.0226568673266 | 1.24945364022509 | 0.561443500986668 | 2.78057257111039 | 0.999714303421225 |
| ebi-fl187-GCST90094726 | ebi-gm430-GCST90011507 | TestASV_3 (Bacteroides) abundance \|\| id:ebi-gm430-GCST90011507 | F-capsicum liking (derived food-liking factor) \|\| id:ebi-fl187-GCST90094726 | Weighted mode | 18 | -0.027840987 | 0.145028941466048 | 0.85004124631552 | -0.312097713 | 0.256415738011654 | 0.972543001232142 | 0.731910007951701 | 1.29228987029788 | 0.999714303421225 |
| ebi-fl187-GCST90094726 | ebi-gm430-GCST90011512 | TestASV_34 (Phascolarctobacterium) abundance \|\| id:ebi-gm430-GCST90011512 | F-capsicum liking (derived food-liking factor) \|\| id:ebi-fl187-GCST90094726 | Weighted mode | 18 | 0.301079046586245 | 0.416320398764296 | 0.47939572434571 | -0.514908935 | 1.11706702816427 | 1.3513161542453 | 0.597555008492363 | 3.05587824178976 | 0.999714303421225 |
| ebi-fl187-GCST90094726 | ebi-gm430-GCST90011519 | TestASV_41 (Barnesiella) abundance \|\| id:ebi-gm430-GCST90011519 | F-capsicum liking (derived food-liking factor) \|\| id:ebi-fl187-GCST90094726 | Weighted mode | 18 | 0.290208120862425 | 0.586761554372806 | 0.627218287537145 | -0.859844526 | 1.44026076743313 | 1.33670565541206 | 0.423227878257438 | 4.22179658052616 | 0.999714303421225 |
| ebi-fl187-GCST90094726 | ebi-gm430-GCST90011524 | TestASV_46 (Sutterella) abundance \|\| id:ebi-gm430-GCST90011524 | F-capsicum liking (derived food-liking factor) \|\| id:ebi-fl187-GCST90094726 | Weighted mode | 18 | -0.211987941 | 0.394409970985993 | 0.597901292398385 | -0.985031484 | 0.561055602444533 | 0.808974453182998 | 0.373427469828131 | 1.75252149019443 | 0.999714303421225 |
| ebi-fl187-GCST90094726 | ebi-gm430-GCST90011581 | OTU97_19 (Bacteria) prevalence \|\| id:ebi-gm430-GCST90011581 | F-capsicum liking (derived food-liking factor) \|\| id:ebi-fl187-GCST90094726 | Weighted mode | 18 | -0.380433964 | 0.549892083626731 | 0.498382508546878 | -1.458222448 | 0.697354520211837 | 0.683564702571894 | 0.232649453991222 | 2.00843240586256 | 0.999714303421225 |
| ebi-fl187-GCST90094726 | ebi-gm430-GCST90011614 | OTU97_80 (Ruminococcus) prevalence \|\| id:ebi-gm430-GCST90011614 | F-capsicum liking (derived food-liking factor) \|\| id:ebi-fl187-GCST90094726 | Weighted mode | 18 | 0.0472081220769476 | 0.27931432058921 | 0.867780112713371 | -0.500247946 | 0.594664190431799 | 1.04834016911025 | 0.606380291335533 | 1.81242221403595 | 0.999714303421225 |
| ebi-fl187-GCST90094726 | ebi-gm430-GCST90011623 | OTU99_11 (Parabacteroides) prevalence \|\| id:ebi-gm430-GCST90011623 | F-capsicum liking (derived food-liking factor) \|\| id:ebi-fl187-GCST90094726 | Weighted mode | 18 | 0.0655903862168232 | 0.200957778139545 | 0.74811395560101 | -0.328286859 | 0.459467631370331 | 1.06778924638223 | 0.720156406771242 | 1.58323089813421 | 0.999714303421225 |
| ebi-fl187-GCST90094726 | ebi-gm430-GCST90011642 | OTU99_19 (Bacteria) prevalence \|\| id:ebi-gm430-GCST90011642 | F-capsicum liking (derived food-liking factor) \|\| id:ebi-fl187-GCST90094726 | Weighted mode | 18 | -0.380433964 | 0.592272675844019 | 0.52922744049254 | -1.541288408 | 0.780420480957722 | 0.683564702571894 | 0.214105068885139 | 2.18238972591944 | 0.999714303421225 |
| ebi-fl187-GCST90094726 | ebi-gm430-GCST90011653 | OTU99_30 (Parasutterella) prevalence \|\| id:ebi-gm430-GCST90011653 | F-capsicum liking (derived food-liking factor) \|\| id:ebi-fl187-GCST90094726 | Weighted mode | 18 | -0.463695636 | 0.346703199487236 | 0.198698280008921 | -1.143233907 | 0.215842634566795 | 0.628954956314759 | 0.318786427265093 | 1.2409070877536 | 0.999714303421225 |
| ebi-fl187-GCST90094726 | ebi-gm430-GCST90011675 | OTU99_8 (Ruminococcaceae) prevalence \|\| id:ebi-gm430-GCST90011675 | F-capsicum liking (derived food-liking factor) \|\| id:ebi-fl187-GCST90094726 | Weighted mode | 18 | 0.365724439509209 | 0.380029367656117 | 0.349359635804962 | -0.379133121 | 1.1105820001152 | 1.44155795150154 | 0.684454491268749 | 3.03612490537571 | 0.999714303421225 |
| ebi-fl187-GCST90094726 | ebi-gm430-GCST90011679 | OTU99_92 (Ruminococcus) prevalence \|\| id:ebi-gm430-GCST90011679 | F-capsicum liking (derived food-liking factor) \|\| id:ebi-fl187-GCST90094726 | Weighted mode | 18 | 0.198183313755077 | 0.284868778699487 | 0.496017953758533 | -0.360159492 | 0.756526120006071 | 1.21918586687757 | 0.697565060805667 | 2.1308609927757 | 0.999714303421225 |
| ebi-fl187-GCST90094726 | ebi-gm430-GCST90011684 | TestASV_11 (Lachnospiraceae) prevalence \|\| id:ebi-gm430-GCST90011684 | F-capsicum liking (derived food-liking factor) \|\| id:ebi-fl187-GCST90094726 | Weighted mode | 18 | 0.0853990208352533 | 0.290827568525117 | 0.772586624767343 | -0.484623013 | 0.655421055144484 | 1.08915157417471 | 0.61592935006816 | 1.92595327921291 | 0.999714303421225 |
| ebi-fl187-GCST90094726 | ebi-gm430-GCST90011688 | TestASV_15 (Bacteroides) prevalence \|\| id:ebi-gm430-GCST90011688 | F-capsicum liking (derived food-liking factor) \|\| id:ebi-fl187-GCST90094726 | Weighted mode | 18 | -0.258168146 | 0.262436016430622 | 0.339039163547662 | -0.772542738 | 0.256206446330689 | 0.772465334328088 | 0.46183724289368 | 1.29201943307975 | 0.999714303421225 |
| ebi-fl187-GCST90094726 | ebi-gm430-GCST90011692 | TestASV_19 (Prevotella) prevalence \|\| id:ebi-gm430-GCST90011692 | F-capsicum liking (derived food-liking factor) \|\| id:ebi-fl187-GCST90094726 | Weighted mode | 18 | 0.22429331818318 | 0.516248121024516 | 0.669412999844713 | -0.787552999 | 1.23613963539123 | 1.25143803513037 | 0.454956713817008 | 3.44229925223363 | 0.999714303421225 |
| ebi-fl187-GCST90094726 | ebi-gm430-GCST90011693 | TestASV_2 (Bacteroides) prevalence \|\| id:ebi-gm430-GCST90011693 | F-capsicum liking (derived food-liking factor) \|\| id:ebi-fl187-GCST90094726 | Weighted mode | 18 | -0.285724719 | 0.247296782312571 | 0.263911605486718 | -0.770426412 | 0.198976974429946 | 0.751469452812003 | 0.462815675962028 | 1.22015387083799 | 0.999714303421225 |
| ebi-fl187-GCST90094726 | ebi-gm430-GCST90011716 | TestASV_40 (Barnesiella) prevalence \|\| id:ebi-gm430-GCST90011716 | F-capsicum liking (derived food-liking factor) \|\| id:ebi-fl187-GCST90094726 | Weighted mode | 18 | 0.492662008806457 | 0.616389150213962 | 0.435159228975152 | -0.715460726 | 1.70078474322582 | 1.63666724877696 | 0.488966780372759 | 5.47824472079077 | 0.999714303421225 |
| ebi-fl187-GCST90094726 | ebi-gm430-GCST90011718 | TestASV_42 (Alphaproteobacteria) prevalence \|\| id:ebi-gm430-GCST90011718 | F-capsicum liking (derived food-liking factor) \|\| id:ebi-fl187-GCST90094726 | Weighted mode | 18 | -0.030322004 | 0.471648661347342 | 0.94948972925389 | -0.954753381 | 0.894109371963544 | 0.970133096241645 | 0.384907058428045 | 2.44515709394127 | 0.999714303421225 |
| ebi-fl187-GCST90094726 | ebi-hbsm44-GCST90104929 | Sulcal depth of the right inferior frontal sulcus \|\| id:ebi-hbsm44-GCST90104929 | F-capsicum liking (derived food-liking factor) \|\| id:ebi-fl187-GCST90094726 | Weighted mode | 18 | 0.0691620251450018 | 0.208285153406286 | 0.743907049078524 | -0.339076876 | 0.477400925821322 | 1.071609823 | 0.71242767872764 | 1.6118795586595 | 0.999714303421225 |
| ebi-fl187-GCST90094726 | ebi-hbsm44-GCST90104935 | Sulcal opening distance of the left calloso-marginal posterior fissure \|\| id:ebi-hbsm44-GCST90104935 | F-capsicum liking (derived food-liking factor) \|\| id:ebi-fl187-GCST90094726 | Weighted mode | 18 | 0.0255665831475461 | 0.0470369475724162 | 0.593813146513909 | -0.066625834 | 0.117759000389482 | 1.02589621139524 | 0.935545184968095 | 1.12497296064969 | 0.999714303421225 |
| ebi-fl187-GCST90094726 | ebi-hbsm44-GCST90104936 | Sulcal opening distance of the left anterior inferior temporal sulcus \|\| id:ebi-hbsm44-GCST90104936 | F-capsicum liking (derived food-liking factor) \|\| id:ebi-fl187-GCST90094726 | Weighted mode | 20 | -0.010832842 | 0.0309958750636036 | 0.730563016813778 | -0.071584757 | 0.049919072925415 | 0.989225621735178 | 0.930917372185755 | 1.05118602352399 | 0.999714303421225 |
| ebi-fl187-GCST90094726 | ebi-hbsm44-GCST90104941 | Sulcal opening distance of the right posterior inferior temporal sulcus \|\| id:ebi-hbsm44-GCST90104941 | F-capsicum liking (derived food-liking factor) \|\| id:ebi-fl187-GCST90094726 | Weighted mode | 20 | 0.0204284268364209 | 0.0220673754198555 | 0.366196175800335 | -0.022823629 | 0.0636804826593377 | 1.0206385153015 | 0.977434859748616 | 1.06575181816695 | 0.999714303421225 |
| ebi-fl187-GCST90094726 | ebi-IPs91-GCST90274819 | Leukemia inhibitory factor levels \|\| id:ebi-IPs91-GCST90274819 | F-capsicum liking (derived food-liking factor) \|\| id:ebi-fl187-GCST90094726 | Weighted mode | 21 | -0.030389736 | 0.0857506518178243 | 0.726750968235082 | -0.198461014 | 0.137681541248789 | 0.970067389376228 | 0.819991738415959 | 1.14761002561937 | 0.999714303421225 |
| ebi-fl187-GCST90094726 | ebi-IPs91-GCST90274837 | STAM binding protein levels \|\| id:ebi-IPs91-GCST90274837 | F-capsicum liking (derived food-liking factor) \|\| id:ebi-fl187-GCST90094726 | Weighted mode | 21 | 0.0405534333860119 | 0.0739531223570577 | 0.589509460586909 | -0.104394686 | 0.185501553205845 | 1.04138695304695 | 0.90086966620852 | 1.20382206955716 | 0.999714303421225 |
| ebi-fl187-GCST90094726 | ebi-IPs91-GCST90274840 | TNF-beta levels \|\| id:ebi-IPs91-GCST90274840 | F-capsicum liking (derived food-liking factor) \|\| id:ebi-fl187-GCST90094726 | Weighted mode | 21 | -0.048204886 | 0.10779468988435 | 0.659542083641571 | -0.259482478 | 0.163072705929412 | 0.95293852308006 | 0.771450724913286 | 1.17712227034602 | 0.999714303421225 |
| ebi-fl187-GCST90094726 | ebi-IPs91-GCST90274842 | Tumor necrosis factor ligand superfamily member 14 levels \|\| id:ebi-IPs91-GCST90274842 | F-capsicum liking (derived food-liking factor) \|\| id:ebi-fl187-GCST90094726 | Weighted mode | 21 | -0.029116229 | 0.0912342537504454 | 0.752931850404889 | -0.207935367 | 0.149702907918467 | 0.971303563843625 | 0.81225953396188 | 1.16148912224354 | 0.999714303421225 |
| ebi-fl187-GCST90094726 | ebi-met1400-GCST90199628 | 3-methylhistidine levels \|\| id:ebi-met1400-GCST90199628 | F-capsicum liking (derived food-liking factor) \|\| id:ebi-fl187-GCST90094726 | Weighted mode | 18 | 0.114695570006263 | 0.119699162730445 | 0.35139273345084 | -0.119914789 | 0.349305928957936 | 1.12153195762937 | 0.88699601536293 | 1.41808295662896 | 0.999714303421225 |
| ebi-fl187-GCST90094726 | ebi-met1400-GCST90199651 | Docosahexaenoate DHA; 22:6n3 levels \|\| id:ebi-met1400-GCST90199651 | F-capsicum liking (derived food-liking factor) \|\| id:ebi-fl187-GCST90094726 | Weighted mode | 18 | 0.161546896551653 | 0.0967793075176708 | 0.113381839083122 | -0.028140546 | 0.351234339286287 | 1.17532757567632 | 0.972251710931372 | 1.42082024090434 | 0.999714303421225 |
| ebi-fl187-GCST90094726 | ebi-met1400-GCST90199652 | 1-stearoyl-2-oleoyl-GPS (18:0/18:1) levels \|\| id:ebi-met1400-GCST90199652 | F-capsicum liking (derived food-liking factor) \|\| id:ebi-fl187-GCST90094726 | Weighted mode | 18 | 0.0128823790572723 | 0.0969925988848532 | 0.895896665331035 | -0.177223115 | 0.202987872871585 | 1.01296571437023 | 0.837592884340787 | 1.22505761172644 | 0.999714303421225 |
| ebi-fl187-GCST90094726 | ebi-met1400-GCST90199653 | 1-myristoyl-2-palmitoyl-gpc (14:0/16:0) levels \|\| id:ebi-met1400-GCST90199653 | F-capsicum liking (derived food-liking factor) \|\| id:ebi-fl187-GCST90094726 | Weighted mode | 18 | -0.014094502 | 0.10857281685779 | 0.898235560681263 | -0.226897223 | 0.19870821878368 | 0.986004360221847 | 0.797002691388317 | 1.21982599165756 | 0.999714303421225 |
| ebi-fl187-GCST90094726 | ebi-met1400-GCST90199667 | Erythritol levels in elite athletes \|\| id:ebi-met1400-GCST90199667 | F-capsicum liking (derived food-liking factor) \|\| id:ebi-fl187-GCST90094726 | Weighted mode | 18 | 0.0631297041563739 | 0.118213093352603 | 0.600233094258447 | -0.168567959 | 0.294827367127475 | 1.06516498659795 | 0.84487384484282 | 1.34289451093766 | 0.999714303421225 |
| ebi-fl187-GCST90094726 | ebi-met1400-GCST90199674 | 3-hydroxymyristate levels \|\| id:ebi-met1400-GCST90199674 | F-capsicum liking (derived food-liking factor) \|\| id:ebi-fl187-GCST90094726 | Weighted mode | 18 | 0.0422844103554478 | 0.096008625723896 | 0.6651796399996 | -0.145892496 | 0.230461316774284 | 1.04319113092363 | 0.864250608533344 | 1.2591807571701 | 0.999714303421225 |
| ebi-fl187-GCST90094726 | ebi-met1400-GCST90199685 | 2-linoleoylglycerol (18:2) levels \|\| id:ebi-met1400-GCST90199685 | F-capsicum liking (derived food-liking factor) \|\| id:ebi-fl187-GCST90094726 | Weighted mode | 18 | -0.105220735 | 0.125043810877303 | 0.41176511853948 | -0.350306605 | 0.139865133897775 | 0.900125811005219 | 0.704472062128535 | 1.1501186763741 | 0.999714303421225 |
| ebi-fl187-GCST90094726 | ebi-met1400-GCST90199703 | Gamma-glutamylvaline levels \|\| id:ebi-met1400-GCST90199703 | F-capsicum liking (derived food-liking factor) \|\| id:ebi-fl187-GCST90094726 | Weighted mode | 18 | 0.0378921504370319 | 0.0956099470753499 | 0.696799223820578 | -0.149503346 | 0.225287646704718 | 1.03861921220979 | 0.861135556801138 | 1.25268299450837 | 0.999714303421225 |
| ebi-fl187-GCST90094726 | ebi-met1400-GCST90199779 | Gamma-glutamyl-2-aminobutyrate levels \|\| id:ebi-met1400-GCST90199779 | F-capsicum liking (derived food-liking factor) \|\| id:ebi-fl187-GCST90094726 | Weighted mode | 18 | -0.000533804 | 0.107648911739614 | 0.996101232827808 | -0.211525671 | 0.210458063059925 | 0.999466338498263 | 0.809348504033488 | 1.23424329174987 | 0.999714303421225 |
| ebi-fl187-GCST90094726 | ebi-met1400-GCST90199824 | 4-vinylphenol sulfate levels \|\| id:ebi-met1400-GCST90199824 | F-capsicum liking (derived food-liking factor) \|\| id:ebi-fl187-GCST90094726 | Weighted mode | 18 | 0.0356150163733624 | 0.112173495746603 | 0.754731161602455 | -0.184245035 | 0.255475068036704 | 1.03625682777633 | 0.831731975169364 | 1.29107482358653 | 0.999714303421225 |
| ebi-fl187-GCST90094726 | ebi-met1400-GCST90199830 | 4-ethylphenylsulfate levels \|\| id:ebi-met1400-GCST90199830 | F-capsicum liking (derived food-liking factor) \|\| id:ebi-fl187-GCST90094726 | Weighted mode | 18 | 0.000520882708646586 | 0.105263466330545 | 0.996109392292353 | -0.205795511 | 0.206837276716515 | 1.0005210183916 | 0.813999512967971 | 1.22978244126143 | 0.999714303421225 |
| ebi-fl187-GCST90094726 | ebi-met1400-GCST90199833 | Hexanoylglutamine levels \|\| id:ebi-met1400-GCST90199833 | F-capsicum liking (derived food-liking factor) \|\| id:ebi-fl187-GCST90094726 | Weighted mode | 18 | -0.049693889 | 0.108308869724437 | 0.652177705615758 | -0.261979274 | 0.162591495668768 | 0.951520650871042 | 0.769526973026879 | 1.1765559632988 | 0.999714303421225 |
| ebi-fl187-GCST90094726 | ebi-met1400-GCST90199837 | Carotenoid levels (cryptoxanthin) \|\| id:ebi-met1400-GCST90199837 | F-capsicum liking (derived food-liking factor) \|\| id:ebi-fl187-GCST90094726 | Weighted mode | 18 | 0.0647677703363506 | 0.105100607954388 | 0.545896591581212 | -0.141229421 | 0.27076496192695 | 1.06691122717694 | 0.868290084644811 | 1.31096690703529 | 0.999714303421225 |
| ebi-fl187-GCST90094726 | ebi-met1400-GCST90199869 | 2-hydroxyhippurate levels \|\| id:ebi-met1400-GCST90199869 | F-capsicum liking (derived food-liking factor) \|\| id:ebi-fl187-GCST90094726 | Weighted mode | 18 | 0.114351984790392 | 0.114254131348168 | 0.330930357401578 | -0.109586113 | 0.338290082232802 | 1.12114668202089 | 0.896204986451024 | 1.40254729845241 | 0.999714303421225 |
| ebi-fl187-GCST90094726 | ebi-met1400-GCST90199902 | Hydantoin-5-propionate levels \|\| id:ebi-met1400-GCST90199902 | F-capsicum liking (derived food-liking factor) \|\| id:ebi-fl187-GCST90094726 | Weighted mode | 18 | -0.043488947 | 0.0999374455805444 | 0.668919314120863 | -0.23936634 | 0.152388446634043 | 0.957443136935043 | 0.787126473602917 | 1.16461253839934 | 0.999714303421225 |
| ebi-fl187-GCST90094726 | ebi-met1400-GCST90199904 | Pantoate levels \|\| id:ebi-met1400-GCST90199904 | F-capsicum liking (derived food-liking factor) \|\| id:ebi-fl187-GCST90094726 | Weighted mode | 18 | 0.0207007070894087 | 0.099065684988216 | 0.836963287036194 | -0.173468035 | 0.214869449666312 | 1.02091645285139 | 0.840744024705271 | 1.23970004314695 | 0.999714303421225 |
| ebi-fl187-GCST90094726 | ebi-met1400-GCST90199936 | 3-methyl catechol sulfate (2) levels \|\| id:ebi-met1400-GCST90199936 | F-capsicum liking (derived food-liking factor) \|\| id:ebi-fl187-GCST90094726 | Weighted mode | 18 | -0.019976047 | 0.131861636150697 | 0.881370474689155 | -0.278424853 | 0.238472760298778 | 0.980222152721415 | 0.756975149712374 | 1.26930912996403 | 0.999714303421225 |
| ebi-fl187-GCST90094726 | ebi-met1400-GCST90199977 | 4-hydroxychlorothalonil levels \|\| id:ebi-met1400-GCST90199977 | F-capsicum liking (derived food-liking factor) \|\| id:ebi-fl187-GCST90094726 | Weighted mode | 18 | 0.0620813101936344 | 0.10916941162654 | 0.577016469862193 | -0.151890737 | 0.276053356981652 | 1.06404885922951 | 0.859082141853761 | 1.31791818228757 | 0.999714303421225 |
| ebi-fl187-GCST90094726 | ebi-met1400-GCST90200040 | Sphingomyelin (d18:2/24:1, d18:1/24:2) levels \|\| id:ebi-met1400-GCST90200040 | F-capsicum liking (derived food-liking factor) \|\| id:ebi-fl187-GCST90094726 | Weighted mode | 18 | 0.102012343217302 | 0.0968686494470307 | 0.307034283764786 | -0.08785021 | 0.291874896133482 | 1.10739714048712 | 0.915898059094843 | 1.33893550115282 | 0.999714303421225 |
| ebi-fl187-GCST90094726 | ebi-met1400-GCST90200077 | 1-stearoyl-2-oleoyl-GPI (18:0/18:1) levels \|\| id:ebi-met1400-GCST90200077 | F-capsicum liking (derived food-liking factor) \|\| id:ebi-fl187-GCST90094726 | Weighted mode | 18 | -0.044131141 | 0.0952580234072026 | 0.649039560455516 | -0.230836867 | 0.14257458476609 | 0.956828469695388 | 0.793868961705601 | 1.15323909181769 | 0.999714303421225 |
| ebi-fl187-GCST90094726 | ebi-met1400-GCST90200080 | 1-palmitoyl-2-oleoyl-GPI (16:0/18:1) levels \|\| id:ebi-met1400-GCST90200080 | F-capsicum liking (derived food-liking factor) \|\| id:ebi-fl187-GCST90094726 | Weighted mode | 18 | -0.032111946 | 0.100353247726179 | 0.752875847912072 | -0.228804312 | 0.16458041930879 | 0.96839816748264 | 0.795484185160955 | 1.17889837193179 | 0.999714303421225 |
| ebi-fl187-GCST90094726 | ebi-met1400-GCST90200082 | 1-oleoyl-2-linoleoyl-GPE (18:1/18:2) levels \|\| id:ebi-met1400-GCST90200082 | F-capsicum liking (derived food-liking factor) \|\| id:ebi-fl187-GCST90094726 | Weighted mode | 18 | -0.236383457 | 0.122141561032173 | 0.0697646273422952 | -0.475780917 | 0.00301400262929533 | 0.789477885066809 | 0.621399605663643 | 1.00301854930197 | 0.999714303421225 |
| ebi-fl187-GCST90094726 | ebi-met1400-GCST90200100 | Perfluorooctanesulfonate (PFOS) levels \|\| id:ebi-met1400-GCST90200100 | F-capsicum liking (derived food-liking factor) \|\| id:ebi-fl187-GCST90094726 | Weighted mode | 18 | 0.0494991554697473 | 0.103693216200623 | 0.639184189996781 | -0.153739548 | 0.252737859222968 | 1.05074470482876 | 0.85749532805995 | 1.28754571435809 | 0.999714303421225 |
| ebi-fl187-GCST90094726 | ebi-met1400-GCST90200102 | 1-palmitoleoyl-2-linolenoyl-GPC (16:1/18:3) levels \|\| id:ebi-met1400-GCST90200102 | F-capsicum liking (derived food-liking factor) \|\| id:ebi-fl187-GCST90094726 | Weighted mode | 18 | -0.109253382 | 0.114045150249713 | 0.351498044288079 | -0.332781876 | 0.114275112701213 | 0.896503231125155 | 0.716926555790981 | 1.12106050044569 | 0.999714303421225 |
| ebi-fl187-GCST90094726 | ebi-met1400-GCST90200114 | Glycosyl-N-tricosanoyl-sphingadienine (d18:2/23:0) levels \|\| id:ebi-met1400-GCST90200114 | F-capsicum liking (derived food-liking factor) \|\| id:ebi-fl187-GCST90094726 | Weighted mode | 18 | 0.0237671971824949 | 0.122236100487898 | 0.848138483395227 | -0.21581556 | 0.263349954138774 | 1.02405188797333 | 0.805883925580501 | 1.3012820283099 | 0.999714303421225 |
| ebi-fl187-GCST90094726 | ebi-met1400-GCST90200135 | Arachidoylcarnitine (C20) levels \|\| id:ebi-met1400-GCST90200135 | F-capsicum liking (derived food-liking factor) \|\| id:ebi-fl187-GCST90094726 | Weighted mode | 18 | -0.111853533 | 0.123436530502466 | 0.377514866332117 | -0.353789133 | 0.130082066935623 | 0.8941752152057 | 0.702022985486539 | 1.13892184731534 | 0.999714303421225 |
| ebi-fl187-GCST90094726 | ebi-met1400-GCST90200146 | Docosahexaenoylcarnitine (C22:6) levels \|\| id:ebi-met1400-GCST90200146 | F-capsicum liking (derived food-liking factor) \|\| id:ebi-fl187-GCST90094726 | Weighted mode | 18 | 0.143197034423328 | 0.157761327167376 | 0.37673417254319 | -0.166015167 | 0.452409235671385 | 1.153957149 | 0.847033387285052 | 1.57209517437737 | 0.999714303421225 |
| ebi-fl187-GCST90094726 | ebi-met1400-GCST90200162 | 3-hydroxyphenylacetoylglutamine levels \|\| id:ebi-met1400-GCST90200162 | F-capsicum liking (derived food-liking factor) \|\| id:ebi-fl187-GCST90094726 | Weighted mode | 18 | -0.001901641 | 0.137461865462156 | 0.989123511364182 | -0.271326897 | 0.267523615163928 | 0.998100165832032 | 0.762367239761699 | 1.30672448798472 | 0.999714303421225 |
| ebi-fl187-GCST90094726 | ebi-met1400-GCST90200172 | 3-carboxy-4-methyl-5-pentyl-2-furanpropionate (3-CMPFP) levels \|\| id:ebi-met1400-GCST90200172 | F-capsicum liking (derived food-liking factor) \|\| id:ebi-fl187-GCST90094726 | Weighted mode | 18 | 0.220148635698784 | 0.134652315654744 | 0.120441056112714 | -0.043769903 | 0.484067174382083 | 1.24626195583817 | 0.95717417505735 | 1.62266064321734 | 0.999714303421225 |
| ebi-fl187-GCST90094726 | ebi-met1400-GCST90200222 | Dihydrocaffeate sulfate (2) levels \|\| id:ebi-met1400-GCST90200222 | F-capsicum liking (derived food-liking factor) \|\| id:ebi-fl187-GCST90094726 | Weighted mode | 18 | 0.1082129144587 | 0.144046091521855 | 0.462782972761494 | -0.174117425 | 0.390543253841535 | 1.11428496751226 | 0.84019823165201 | 1.47778338735905 | 0.999714303421225 |
| ebi-fl187-GCST90094726 | ebi-met1400-GCST90200223 | 4-methylhexanoylglutamine levels \|\| id:ebi-met1400-GCST90200223 | F-capsicum liking (derived food-liking factor) \|\| id:ebi-fl187-GCST90094726 | Weighted mode | 18 | 0.0244241974609514 | 0.134923127688235 | 0.85849012637113 | -0.240025133 | 0.288873527729893 | 1.02472491141297 | 0.786608091147999 | 1.3349228871239 | 0.999714303421225 |
| ebi-fl187-GCST90094726 | ebi-met1400-GCST90200224 | 2-ketocaprylate levels \|\| id:ebi-met1400-GCST90200224 | F-capsicum liking (derived food-liking factor) \|\| id:ebi-fl187-GCST90094726 | Weighted mode | 18 | -0.023941166 | 0.109839138879519 | 0.830050716091002 | -0.239225879 | 0.191343545804554 | 0.976343149851211 | 0.787237042284841 | 1.21087537178729 | 0.999714303421225 |
| ebi-fl187-GCST90094726 | ebi-met1400-GCST90200243 | Hydroxypalmitoyl sphingomyelin (d18:1/16:0(OH)) levels \|\| id:ebi-met1400-GCST90200243 | F-capsicum liking (derived food-liking factor) \|\| id:ebi-fl187-GCST90094726 | Weighted mode | 18 | 0.0363412553163992 | 0.139472747702824 | 0.797560459128296 | -0.23702533 | 0.309707840813934 | 1.03700967117866 | 0.788971302978484 | 1.36302683514384 | 0.999714303421225 |
| ebi-fl187-GCST90094726 | ebi-met1400-GCST90200273 | 2-methoxyhydroquinone sulfate (2) levels \|\| id:ebi-met1400-GCST90200273 | F-capsicum liking (derived food-liking factor) \|\| id:ebi-fl187-GCST90094726 | Weighted mode | 18 | -0.27491047 | 0.247620472458412 | 0.282366406420556 | -0.760246596 | 0.210425655641102 | 0.759640130474795 | 0.467551116372716 | 1.2342032937587 | 0.999714303421225 |
| ebi-fl187-GCST90094726 | ebi-met1400-GCST90200276 | 2,4-di-tert-butylphenol levels \|\| id:ebi-met1400-GCST90200276 | F-capsicum liking (derived food-liking factor) \|\| id:ebi-fl187-GCST90094726 | Weighted mode | 18 | 0.186378710219186 | 0.158709705600636 | 0.256448771403024 | -0.124692313 | 0.497449733196433 | 1.20487847377674 | 0.882768477400482 | 1.64452194854705 | 0.999714303421225 |
| ebi-fl187-GCST90094726 | ebi-met1400-GCST90200289 | N-acetylmethionine levels \|\| id:ebi-met1400-GCST90200289 | F-capsicum liking (derived food-liking factor) \|\| id:ebi-fl187-GCST90094726 | Weighted mode | 18 | 0.000517666742481437 | 0.1241276201016 | 0.996721029202194 | -0.242772469 | 0.243807802141618 | 1.00051780075503 | 0.784449980425241 | 1.27609904341516 | 0.999714303421225 |
| ebi-fl187-GCST90094726 | ebi-met1400-GCST90200308 | 3-hydroxyisobutyrate levels \|\| id:ebi-met1400-GCST90200308 | F-capsicum liking (derived food-liking factor) \|\| id:ebi-fl187-GCST90094726 | Weighted mode | 18 | -0.095372597 | 0.0984241450002104 | 0.346132387966961 | -0.288283921 | 0.0975387274867999 | 0.909034168216916 | 0.74954874945916 | 1.1024541360146 | 0.999714303421225 |
| ebi-fl187-GCST90094726 | ebi-met1400-GCST90200383 | Cystathionine levels \|\| id:ebi-met1400-GCST90200383 | F-capsicum liking (derived food-liking factor) \|\| id:ebi-fl187-GCST90094726 | Weighted mode | 18 | -0.034313005 | 0.134262962143185 | 0.801352721548996 | -0.297468411 | 0.228842400989551 | 0.966269010449829 | 0.742696044163924 | 1.25714389876246 | 0.999714303421225 |
| ebi-fl187-GCST90094726 | ebi-met1400-GCST90200384 | Guanidinoacetate levels \|\| id:ebi-met1400-GCST90200384 | F-capsicum liking (derived food-liking factor) \|\| id:ebi-fl187-GCST90094726 | Weighted mode | 18 | 0.193166232666674 | 0.106715462624455 | 0.0879925338471765 | -0.015996074 | 0.402328539410606 | 1.21308443101915 | 0.984131183670696 | 1.49530251779266 | 0.999714303421225 |
| ebi-fl187-GCST90094726 | ebi-met1400-GCST90200403 | Citrulline levels \|\| id:ebi-met1400-GCST90200403 | F-capsicum liking (derived food-liking factor) \|\| id:ebi-fl187-GCST90094726 | Weighted mode | 18 | 0.00404096155365785 | 0.113897310620789 | 0.972111071157996 | -0.219197767 | 0.227279690370405 | 1.00404913724764 | 0.803162863124727 | 1.25518088085602 | 0.999714303421225 |
| ebi-fl187-GCST90094726 | ebi-met1400-GCST90200434 | 12,13-DiHOME levels \|\| id:ebi-met1400-GCST90200434 | F-capsicum liking (derived food-liking factor) \|\| id:ebi-fl187-GCST90094726 | Weighted mode | 18 | 0.0415137204500018 | 0.129372032965914 | 0.752207607086273 | -0.212055464 | 0.295082905063194 | 1.04238746377844 | 0.808919830260774 | 1.34323771527787 | 0.999714303421225 |
| ebi-fl187-GCST90094726 | ebi-met1400-GCST90200437 | Fructose levels \|\| id:ebi-met1400-GCST90200437 | F-capsicum liking (derived food-liking factor) \|\| id:ebi-fl187-GCST90094726 | Weighted mode | 18 | -0.029483797 | 0.110205612586446 | 0.792276464600398 | -0.245486797 | 0.186519204036334 | 0.970946610117899 | 0.782323612508304 | 1.20504776364453 | 0.999714303421225 |
| ebi-fl187-GCST90094726 | ebi-met1400-GCST90200448 | N-stearoyl-sphinganine (d18:0/18:0) levels \|\| id:ebi-met1400-GCST90200448 | F-capsicum liking (derived food-liking factor) \|\| id:ebi-fl187-GCST90094726 | Weighted mode | 18 | -0.004648252 | 0.113086449553719 | 0.967692253533899 | -0.226297693 | 0.217001189244834 | 0.995362534523206 | 0.797480661910085 | 1.24234557958995 | 0.999714303421225 |
| ebi-fl187-GCST90094726 | ebi-met1400-GCST90200468 | X-11847 levels \|\| id:ebi-met1400-GCST90200468 | F-capsicum liking (derived food-liking factor) \|\| id:ebi-fl187-GCST90094726 | Weighted mode | 18 | 0.0904978979149371 | 0.114215702460198 | 0.439079059660017 | -0.133364879 | 0.314360674736925 | 1.09471920644634 | 0.875145711633944 | 1.36938354954057 | 0.999714303421225 |
| ebi-fl187-GCST90094726 | ebi-met1400-GCST90200476 | X-11843 levels \|\| id:ebi-met1400-GCST90200476 | F-capsicum liking (derived food-liking factor) \|\| id:ebi-fl187-GCST90094726 | Weighted mode | 18 | -0.006374888 | 0.109338947644167 | 0.954186414604301 | -0.220679226 | 0.207929449174121 | 0.993645388281722 | 0.801973891735708 | 1.23112630950698 | 0.999714303421225 |
| ebi-fl187-GCST90094726 | ebi-met1400-GCST90200511 | X-12906 levels \|\| id:ebi-met1400-GCST90200511 | F-capsicum liking (derived food-liking factor) \|\| id:ebi-fl187-GCST90094726 | Weighted mode | 18 | -0.029728161 | 0.128462633862563 | 0.819754972399125 | -0.281514924 | 0.222058600952949 | 0.970709373945947 | 0.754639653508815 | 1.24864454748073 | 0.999714303421225 |
| ebi-fl187-GCST90094726 | ebi-met1400-GCST90200515 | X-12849 levels \|\| id:ebi-met1400-GCST90200515 | F-capsicum liking (derived food-liking factor) \|\| id:ebi-fl187-GCST90094726 | Weighted mode | 18 | -0.082261007 | 0.13831952144603 | 0.559865569378767 | -0.353367269 | 0.188845254854188 | 0.921031531478349 | 0.702319205781832 | 1.20785402847273 | 0.999714303421225 |
| ebi-fl187-GCST90094726 | ebi-met1400-GCST90200551 | X-18886 levels \|\| id:ebi-met1400-GCST90200551 | F-capsicum liking (derived food-liking factor) \|\| id:ebi-fl187-GCST90094726 | Weighted mode | 18 | -0.038896067 | 0.110398336755338 | 0.728924723872714 | -0.255276807 | 0.177484673320268 | 0.96185067224658 | 0.77470202552058 | 1.19420975449176 | 0.999714303421225 |
| ebi-fl187-GCST90094726 | ebi-met1400-GCST90200564 | X-21319 levels \|\| id:ebi-met1400-GCST90200564 | F-capsicum liking (derived food-liking factor) \|\| id:ebi-fl187-GCST90094726 | Weighted mode | 18 | -0.064536148 | 0.0915867089331356 | 0.490572758475143 | -0.244046097 | 0.114973801679753 | 0.937502224979245 | 0.783451518400887 | 1.12184404675734 | 0.999714303421225 |
| ebi-fl187-GCST90094726 | ebi-met1400-GCST90200579 | X-21442 levels \|\| id:ebi-met1400-GCST90200579 | F-capsicum liking (derived food-liking factor) \|\| id:ebi-fl187-GCST90094726 | Weighted mode | 18 | 0.145680519313145 | 0.164232719857957 | 0.387436158834415 | -0.176215612 | 0.467576650234741 | 1.15682654526782 | 0.8384371870564 | 1.59612154195429 | 0.999714303421225 |
| ebi-fl187-GCST90094726 | ebi-met1400-GCST90200581 | X-21441 levels \|\| id:ebi-met1400-GCST90200581 | F-capsicum liking (derived food-liking factor) \|\| id:ebi-fl187-GCST90094726 | Weighted mode | 18 | -0.055204384 | 0.10067423103754 | 0.590582879826723 | -0.252525877 | 0.142117108534554 | 0.94629172101737 | 0.776836110288179 | 1.15271163300304 | 0.999714303421225 |
| ebi-fl187-GCST90094726 | ebi-met1400-GCST90200648 | X-24585 levels \|\| id:ebi-met1400-GCST90200648 | F-capsicum liking (derived food-liking factor) \|\| id:ebi-fl187-GCST90094726 | Weighted mode | 18 | -0.004199222 | 0.165804213906126 | 0.980089545990263 | -0.329175481 | 0.320777037234011 | 0.995809582382604 | 0.71951674395037 | 1.37819825973837 | 0.999714303421225 |
| ebi-fl187-GCST90094726 | ebi-met1400-GCST90200720 | Adenosine 5'-diphosphate (ADP) to pantothenate ratio \|\| id:ebi-met1400-GCST90200720 | F-capsicum liking (derived food-liking factor) \|\| id:ebi-fl187-GCST90094726 | Weighted mode | 18 | 0.0326486621312949 | 0.147850609442926 | 0.827861433011457 | -0.257138532 | 0.32243585663943 | 1.03318747758004 | 0.773261084649221 | 1.38048633898659 | 0.999714303421225 |
| ebi-fl187-GCST90094726 | ebi-met1400-GCST90200724 | Cholate to taurocholate ratio \|\| id:ebi-met1400-GCST90200724 | F-capsicum liking (derived food-liking factor) \|\| id:ebi-fl187-GCST90094726 | Weighted mode | 18 | 0.00722041353335912 | 0.113707762446408 | 0.95010924410376 | -0.215646801 | 0.230087627928319 | 1.00724654357119 | 0.806019937151388 | 1.25871030327325 | 0.999714303421225 |
| ebi-fl187-GCST90094726 | ebi-met1400-GCST90200726 | N-acetylputrescine to (N(1) + N(8))-acetylspermidine ratio \|\| id:ebi-met1400-GCST90200726 | F-capsicum liking (derived food-liking factor) \|\| id:ebi-fl187-GCST90094726 | Weighted mode | 18 | -0.059697836 | 0.106665419691996 | 0.583001778203848 | -0.268762059 | 0.149366386629774 | 0.94204914395163 | 0.764325098625767 | 1.16109832218727 | 0.999714303421225 |
| ebi-fl187-GCST90094726 | ebi-met1400-GCST90200744 | Arginine to glutamate ratio \|\| id:ebi-met1400-GCST90200744 | F-capsicum liking (derived food-liking factor) \|\| id:ebi-fl187-GCST90094726 | Weighted mode | 18 | 0.0541199992302106 | 0.109147318796729 | 0.626353890722649 | -0.159808746 | 0.268048744071799 | 1.05561126709453 | 0.852306780791542 | 1.30741086699094 | 0.999714303421225 |
| ebi-fl187-GCST90094726 | ebi-met1400-GCST90200759 | Histidine to trans-urocanate ratio \|\| id:ebi-met1400-GCST90200759 | F-capsicum liking (derived food-liking factor) \|\| id:ebi-fl187-GCST90094726 | Weighted mode | 18 | -0.086298838 | 0.112897038257424 | 0.455107349980382 | -0.307577033 | 0.13497935710809 | 0.917320060277012 | 0.735226228786412 | 1.14451315804605 | 0.999714303421225 |
| ebi-fl187-GCST90094726 | ebi-met1400-GCST90200795 | Oleoyl-linoleoyl-glycerol (18:1 to 18:2) [2] to linoleoyl-arachidonoyl-glycerol (18:2 to 20:4) [2] ratio \|\| id:ebi-met1400-GCST90200795 | F-capsicum liking (derived food-liking factor) \|\| id:ebi-fl187-GCST90094726 | Weighted mode | 18 | 0.13764124024375 | 0.171737269861609 | 0.43392325916389 | -0.198963809 | 0.474246289172503 | 1.14756377671389 | 0.819579554458005 | 1.606802676 | 0.999714303421225 |
| ebi-fl187-GCST90094726 | ebi-met1400-GCST90200811 | Choline to taurocholate ratio \|\| id:ebi-met1400-GCST90200811 | F-capsicum liking (derived food-liking factor) \|\| id:ebi-fl187-GCST90094726 | Weighted mode | 18 | -0.057389703 | 0.129636921005096 | 0.6635670928276 | -0.311478068 | 0.196698661981731 | 0.944226029763844 | 0.732363672266934 | 1.21737714341275 | 0.999714303421225 |
| ebi-fl187-GCST90094726 | ebi-met1400-GCST90200831 | Cholate to phosphate ratio \|\| id:ebi-met1400-GCST90200831 | F-capsicum liking (derived food-liking factor) \|\| id:ebi-fl187-GCST90094726 | Weighted mode | 18 | -0.062522616 | 0.106609863885703 | 0.565272023081195 | -0.271477949 | 0.14643271762079 | 0.939391817668169 | 0.762252091847533 | 1.15769703558704 | 0.999714303421225 |
| ebi-fl187-GCST90094726 | ebi-met1400-GCST90200841 | Adenosine 5'-diphosphate (ADP) to oxalate (ethanedioate) ratio \|\| id:ebi-met1400-GCST90200841 | F-capsicum liking (derived food-liking factor) \|\| id:ebi-fl187-GCST90094726 | Weighted mode | 18 | -0.114788634 | 0.155417868148645 | 0.470236453721854 | -0.419407656 | 0.189830387574519 | 0.89155456831859 | 0.657436133132631 | 1.20904451129307 | 0.999714303421225 |
| ebi-fl187-GCST90094726 | ebi-met1400-GCST90200844 | Aspartate to phosphate ratio \|\| id:ebi-met1400-GCST90200844 | F-capsicum liking (derived food-liking factor) \|\| id:ebi-fl187-GCST90094726 | Weighted mode | 18 | -0.1277049 | 0.10202656074883 | 0.227635067673314 | -0.327676959 | 0.0722671591899156 | 0.880113062298736 | 0.720595764125788 | 1.07494248646963 | 0.999714303421225 |
| ebi-fl187-GCST90094726 | ebi-met1400-GCST90200870 | Adenosine 5'-monophosphate (AMP) to acetoacetate ratio \|\| id:ebi-met1400-GCST90200870 | F-capsicum liking (derived food-liking factor) \|\| id:ebi-fl187-GCST90094726 | Weighted mode | 18 | 0.111082044525334 | 0.138403749215544 | 0.433287140950544 | -0.160189304 | 0.3823533929878 | 1.11748658675007 | 0.851982490059699 | 1.46572997231295 | 0.999714303421225 |
| ebi-fl187-GCST90094726 | ebi-met1400-GCST90200887 | Cholesterol to cortisol ratio \|\| id:ebi-met1400-GCST90200887 | F-capsicum liking (derived food-liking factor) \|\| id:ebi-fl187-GCST90094726 | Weighted mode | 18 | -0.02355093 | 0.0869990127544635 | 0.789878410552044 | -0.194068995 | 0.14696713537027 | 0.976724229199977 | 0.823601078258877 | 1.15831589478132 | 0.999714303421225 |
| ebi-fl187-GCST90094726 | ebi-met1400-GCST90200890 | Cortisol to taurocholate ratio \|\| id:ebi-met1400-GCST90200890 | F-capsicum liking (derived food-liking factor) \|\| id:ebi-fl187-GCST90094726 | Weighted mode | 18 | 0.0878968845553776 | 0.115023644459482 | 0.455246232510472 | -0.137549459 | 0.313343227695962 | 1.09187552699272 | 0.871491246207183 | 1.3679909828517 | 0.999714303421225 |
| ebi-fl187-GCST90094726 | ebi-met1400-GCST90200948 | Adenosine 5'-diphosphate (ADP) to mannitol to sorbitol ratio \|\| id:ebi-met1400-GCST90200948 | F-capsicum liking (derived food-liking factor) \|\| id:ebi-fl187-GCST90094726 | Weighted mode | 18 | 0.0899117027716976 | 0.148379192758732 | 0.552547620916275 | -0.200911515 | 0.380734920578813 | 1.09407767541386 | 0.81798480770845 | 1.46335964746383 | 0.999714303421225 |
| ebi-fl187-GCST90094726 | ebi-met1400-GCST90200972 | Leucine to N-palmitoyl-sphingosine (d18:1 to 16:0) ratio \|\| id:ebi-met1400-GCST90200972 | F-capsicum liking (derived food-liking factor) \|\| id:ebi-fl187-GCST90094726 | Weighted mode | 18 | 0.139679297033462 | 0.14162313147923 | 0.337828695066237 | -0.137902041 | 0.417260634732753 | 1.14990496178434 | 0.87118402817332 | 1.51779805227693 | 0.999714303421225 |
| ebi-fl187-GCST90094726 | ebi-met1400-GCST90200973 | Proline to glutamate ratio \|\| id:ebi-met1400-GCST90200973 | F-capsicum liking (derived food-liking factor) \|\| id:ebi-fl187-GCST90094726 | Weighted mode | 18 | -0.023462883 | 0.0994711278739559 | 0.816346945878255 | -0.218426294 | 0.171500527227092 | 0.976810229864857 | 0.80378272083943 | 1.18708476859537 | 0.999714303421225 |
| ebi-fl187-GCST90094726 | ebi-met1400-GCST90200983 | Cholesterol to linoleoyl-arachidonoyl-glycerol (18:2 to 20:4) [1] ratio \|\| id:ebi-met1400-GCST90200983 | F-capsicum liking (derived food-liking factor) \|\| id:ebi-fl187-GCST90094726 | Weighted mode | 18 | -0.08770979 | 0.104258438831656 | 0.411873032831329 | -0.29205633 | 0.116636750087024 | 0.916026678233536 | 0.746726471587112 | 1.12371116756061 | 0.999714303421225 |
| ebi-fl187-GCST90094726 | ebi-met1400-GCST90201001 | Serine to alpha-ketobutyrate ratio \|\| id:ebi-met1400-GCST90201001 | F-capsicum liking (derived food-liking factor) \|\| id:ebi-fl187-GCST90094726 | Weighted mode | 18 | -0.003711296 | 0.100993241349488 | 0.971114033914245 | -0.201658049 | 0.194235456600031 | 0.996295581903858 | 0.81737438174656 | 1.2143821835964 | 0.999714303421225 |
| ebi-fl187-GCST90094726 | ebi-met1400-GCST90201005 | Fructose to maltose ratio \|\| id:ebi-met1400-GCST90201005 | F-capsicum liking (derived food-liking factor) \|\| id:ebi-fl187-GCST90094726 | Weighted mode | 18 | -0.062561742 | 0.132572202086078 | 0.642993665609485 | -0.322403259 | 0.197279773606854 | 0.939355062910035 | 0.72440600848995 | 1.21808478101125 | 0.999714303421225 |
| ebi-fl187-GCST90094726 | ebi-met1400-GCST90201020 | Paraxanthine to 5-acetylamino-6-formylamino-3-methyluracil ratio \|\| id:ebi-met1400-GCST90201020 | F-capsicum liking (derived food-liking factor) \|\| id:ebi-fl187-GCST90094726 | Weighted mode | 18 | 0.126219786417502 | 0.13058931214777 | 0.347324093863214 | -0.129735265 | 0.382174838227131 | 1.13453149549559 | 0.878327923943258 | 1.4654682826122 | 0.999714303421225 |
| ebi-fl187-GCST90094726 | ebi-pw11-GCST90275197 | Placental weight (fetal genotype, adjusted for sex) \|\| id:ebi-pw11-GCST90275197 | F-capsicum liking (derived food-liking factor) \|\| id:ebi-fl187-GCST90094726 | Weighted mode | 20 | -0.038622699 | 0.0499189319538217 | 0.448623587256712 | -0.136463805 | 0.0592184078919062 | 0.962113647367276 | 0.872437897257601 | 1.06100694772667 | 0.999714303421225 |
| ebi-fl187-GCST90094726 | prot-a-1017 | FAS-associated factor 2 \|\| id:prot-a-1017 | F-capsicum liking (derived food-liking factor) \|\| id:ebi-fl187-GCST90094726 | Weighted mode | 21 | -0.140108611 | 0.206834773652274 | 0.505917108023441 | -0.545504767 | 0.265287545442453 | 0.869263818731957 | 0.579549172039769 | 1.30380582530556 | 0.999714303421225 |
| ebi-fl187-GCST90094726 | prot-a-1019 | Fas apoptotic inhibitory molecule 3 \|\| id:prot-a-1019 | F-capsicum liking (derived food-liking factor) \|\| id:ebi-fl187-GCST90094726 | Weighted mode | 21 | 0.0618364896483494 | 0.186155710899369 | 0.743212376550379 | -0.303028704 | 0.426701683011113 | 1.06378839009298 | 0.738577896137218 | 1.53219551358788 | 0.999714303421225 |
| ebi-fl187-GCST90094726 | prot-a-1052 | MIP18 family protein FAM96A \|\| id:prot-a-1052 | F-capsicum liking (derived food-liking factor) \|\| id:ebi-fl187-GCST90094726 | Weighted mode | 21 | -0.050534464 | 0.18009741393027 | 0.781902954723085 | -0.403525395 | 0.302456467123313 | 0.950721162282415 | 0.667961063389533 | 1.35317876737453 | 0.999714303421225 |
| ebi-fl187-GCST90094726 | prot-a-1066 | F-box/LRR-repeat protein 4 \|\| id:prot-a-1066 | F-capsicum liking (derived food-liking factor) \|\| id:ebi-fl187-GCST90094726 | Weighted mode | 21 | 0.00727064660803611 | 0.174337958437647 | 0.967147944358822 | -0.334431752 | 0.348973045145824 | 1.00729714193288 | 0.715744691355017 | 1.41761097832979 | 0.999714303421225 |
| ebi-fl187-GCST90094726 | prot-a-1076 | Low affinity immunoglobulin gamma Fc region receptor III-B \|\| id:prot-a-1076 | F-capsicum liking (derived food-liking factor) \|\| id:ebi-fl187-GCST90094726 | Weighted mode | 21 | -0.076781168 | 0.144810655732979 | 0.601798835245684 | -0.360610053 | 0.207047717383472 | 0.926092490241787 | 0.697250836271507 | 1.23004126473112 | 0.999714303421225 |
| ebi-fl187-GCST90094726 | prot-a-1081 | Fc receptor-like protein 3 \|\| id:prot-a-1081 | F-capsicum liking (derived food-liking factor) \|\| id:ebi-fl187-GCST90094726 | Weighted mode | 21 | 0.205985538067511 | 0.181635631757368 | 0.27018048419493 | -0.1500203 | 0.561991376311952 | 1.22873543397793 | 0.860690504078198 | 1.75416222156408 | 0.999714303421225 |
| ebi-fl187-GCST90094726 | prot-a-1105 | Fibroblast growth factor receptor 4 \|\| id:prot-a-1105 | F-capsicum liking (derived food-liking factor) \|\| id:ebi-fl187-GCST90094726 | Weighted mode | 21 | 0.196668778915956 | 0.203929954211238 | 0.346365499282241 | -0.203033931 | 0.596371489169982 | 1.21734076499505 | 0.816250544482541 | 1.81551920318559 | 0.999714303421225 |
| ebi-fl187-GCST90094726 | prot-a-1143 | Follistatin-related protein 1 \|\| id:prot-a-1143 | F-capsicum liking (derived food-liking factor) \|\| id:ebi-fl187-GCST90094726 | Weighted mode | 21 | -0.145926683 | 0.175357170508323 | 0.415140337042339 | -0.489626738 | 0.197773370792291 | 0.864221062608448 | 0.612855107264747 | 1.21868617264118 | 0.999714303421225 |
| ebi-fl187-GCST90094726 | prot-a-1151 | Alpha-(1,3)-fucosyltransferase 10 \|\| id:prot-a-1151 | F-capsicum liking (derived food-liking factor) \|\| id:ebi-fl187-GCST90094726 | Weighted mode | 21 | 0.0667493136696677 | 0.176258093670424 | 0.708899338775103 | -0.27871655 | 0.412215177263698 | 1.06902745401154 | 0.756754374902408 | 1.51015935332751 | 0.999714303421225 |
| ebi-fl187-GCST90094726 | prot-a-1165 | Growth arrest and DNA damage-inducible proteins-interacting protein 1 \|\| id:prot-a-1165 | F-capsicum liking (derived food-liking factor) \|\| id:ebi-fl187-GCST90094726 | Weighted mode | 21 | 0.216563290809598 | 0.206459502298302 | 0.306717427234605 | -0.188097334 | 0.621223915314269 | 1.24180167750025 | 0.828534059026391 | 1.86120460522109 | 0.999714303421225 |
| ebi-fl187-GCST90094726 | prot-a-1194 | Growth/differentiation factor 11/8 \|\| id:prot-a-1194 | F-capsicum liking (derived food-liking factor) \|\| id:ebi-fl187-GCST90094726 | Weighted mode | 21 | -0.022515664 | 0.165696886087481 | 0.893270886222449 | -0.347281561 | 0.302250232914412 | 0.977735921996783 | 0.706606347775674 | 1.35289972439703 | 0.999714303421225 |
| ebi-fl187-GCST90094726 | prot-a-1216 | D-glucuronyl C5-epimerase \|\| id:prot-a-1216 | F-capsicum liking (derived food-liking factor) \|\| id:ebi-fl187-GCST90094726 | Weighted mode | 21 | -0.213362194 | 0.196638630495067 | 0.290807262868085 | -0.59877391 | 0.172049521365696 | 0.807863480588013 | 0.549484941138786 | 1.18773665009855 | 0.999714303421225 |
| ebi-fl187-GCST90094726 | prot-a-1246 | Glypican-3 \|\| id:prot-a-1246 | F-capsicum liking (derived food-liking factor) \|\| id:ebi-fl187-GCST90094726 | Weighted mode | 21 | 0.120652089418387 | 0.192809928393598 | 0.538550681335052 | -0.25725537 | 0.498559549069838 | 1.12823232011512 | 0.773170743759509 | 1.64634807825615 | 0.999714303421225 |
| ebi-fl187-GCST90094726 | prot-a-1275 | Glutamate receptor 4 \|\| id:prot-a-1275 | F-capsicum liking (derived food-liking factor) \|\| id:ebi-fl187-GCST90094726 | Weighted mode | 20 | -0.007713337 | 0.195079522200996 | 0.968872819321709 | -0.3900692 | 0.374642526982089 | 0.992316334910745 | 0.677010023752486 | 1.45447138739957 | 0.999714303421225 |
| ebi-fl187-GCST90094726 | prot-a-1289 | Glutathione S-transferase P \|\| id:prot-a-1289 | F-capsicum liking (derived food-liking factor) \|\| id:ebi-fl187-GCST90094726 | Weighted mode | 21 | 0.0587031414222956 | 0.183419772886688 | 0.752251377025774 | -0.300799613 | 0.418205896280205 | 1.06046038724672 | 0.740226089248788 | 1.51923344671725 | 0.999714303421225 |
| ebi-fl187-GCST90094726 | prot-a-130 | Apolipoprotein D \|\| id:prot-a-130 | F-capsicum liking (derived food-liking factor) \|\| id:ebi-fl187-GCST90094726 | Weighted mode | 21 | 0.0988013196039872 | 0.155045343693204 | 0.53119529930976 | -0.205087554 | 0.402690193242668 | 1.10384696501872 | 0.814575993874344 | 1.49584339747803 | 0.999714303421225 |
| ebi-fl187-GCST90094726 | prot-a-1313 | Heparin-binding EGF-like growth factor \|\| id:prot-a-1313 | F-capsicum liking (derived food-liking factor) \|\| id:ebi-fl187-GCST90094726 | Weighted mode | 21 | -0.241053743 | 0.183600939114658 | 0.204075243243013 | -0.600911584 | 0.118804097334969 | 0.78579939377755 | 0.548311576148524 | 1.12614928103197 | 0.999714303421225 |
| ebi-fl187-GCST90094726 | prot-a-1347 | HLA class II histocompatibility antigen, DQ alpha 2 chain \|\| id:prot-a-1347 | F-capsicum liking (derived food-liking factor) \|\| id:ebi-fl187-GCST90094726 | Weighted mode | 20 | -0.009730853 | 0.170041600848832 | 0.954962468467956 | -0.343012391 | 0.323550684449943 | 0.990316338342949 | 0.70962941857455 | 1.38202620173075 | 0.999714303421225 |
| ebi-fl187-GCST90094726 | prot-a-1357 | Non-histone chromosomal protein HMG-14 \|\| id:prot-a-1357 | F-capsicum liking (derived food-liking factor) \|\| id:ebi-fl187-GCST90094726 | Weighted mode | 21 | 0.0230592414385284 | 0.170476416489585 | 0.893755620730983 | -0.311074535 | 0.357193017758116 | 1.02332716112466 | 0.732659265162971 | 1.42931172577543 | 0.999714303421225 |
| ebi-fl187-GCST90094726 | prot-a-137 | Apolipoprotein M \|\| id:prot-a-137 | F-capsicum liking (derived food-liking factor) \|\| id:ebi-fl187-GCST90094726 | Weighted mode | 21 | 0.120411711742612 | 0.183957053923759 | 0.520205594589516 | -0.240144114 | 0.480967537433179 | 1.1279611508451 | 0.786514505188201 | 1.61763877134264 | 0.999714303421225 |
| ebi-fl187-GCST90094726 | prot-a-1381 | 17-beta-hydroxysteroid dehydrogenase 14 \|\| id:prot-a-1381 | F-capsicum liking (derived food-liking factor) \|\| id:ebi-fl187-GCST90094726 | Weighted mode | 21 | -0.137323527 | 0.154038155871747 | 0.383266337134941 | -0.439238312 | 0.164591258899356 | 0.871688166190874 | 0.644527162692959 | 1.17891115077672 | 0.999714303421225 |
| ebi-fl187-GCST90094726 | prot-a-1392 | Serine protease HTRA2, mitochondrial \|\| id:prot-a-1392 | F-capsicum liking (derived food-liking factor) \|\| id:ebi-fl187-GCST90094726 | Weighted mode | 21 | 0.0303096703226247 | 0.14811324408705 | 0.839922776940685 | -0.259992288 | 0.320611628733243 | 1.03077368455468 | 0.771057532108477 | 1.37797031288316 | 0.999714303421225 |
| ebi-fl187-GCST90094726 | prot-a-1416 | Interferon-induced protein with tetratricopeptide repeats 2 \|\| id:prot-a-1416 | F-capsicum liking (derived food-liking factor) \|\| id:ebi-fl187-GCST90094726 | Weighted mode | 21 | -0.055176945 | 0.185710550856274 | 0.769444413920186 | -0.419169625 | 0.308815734764248 | 0.946317687036446 | 0.657592641923173 | 1.36181141288171 | 0.999714303421225 |
| ebi-fl187-GCST90094726 | prot-a-1428 | Interferon gamma \|\| id:prot-a-1428 | F-capsicum liking (derived food-liking factor) \|\| id:ebi-fl187-GCST90094726 | Weighted mode | 21 | -0.192605586 | 0.233558910937408 | 0.419294495736576 | -0.650381052 | 0.265169879144498 | 0.824807225268566 | 0.521846888210388 | 1.30365242032632 | 0.999714303421225 |
| ebi-fl187-GCST90094726 | prot-a-1483 | Interleukin-17C \|\| id:prot-a-1483 | F-capsicum liking (derived food-liking factor) \|\| id:ebi-fl187-GCST90094726 | Weighted mode | 21 | 0.202302126546336 | 0.202368791871314 | 0.32941212143044 | -0.194340706 | 0.598944958614112 | 1.22421782094561 | 0.823377327272595 | 1.82019740340099 | 0.999714303421225 |
| ebi-fl187-GCST90094726 | prot-a-1503 | Interleukin-1 receptor-like 2 \|\| id:prot-a-1503 | F-capsicum liking (derived food-liking factor) \|\| id:ebi-fl187-GCST90094726 | Weighted mode | 21 | -0.001491247 | 0.183160385961835 | 0.993584555685817 | -0.360485604 | 0.35750310911094 | 0.998509863982604 | 0.697337614001042 | 1.4297550117081 | 0.999714303421225 |
| ebi-fl187-GCST90094726 | prot-a-1522 | Interleukin-31 receptor subunit alpha \|\| id:prot-a-1522 | F-capsicum liking (derived food-liking factor) \|\| id:ebi-fl187-GCST90094726 | Weighted mode | 21 | 0.0715175632005756 | 0.151730087067959 | 0.642493723917422 | -0.225873407 | 0.368908533853776 | 1.0741370158195 | 0.797819093224506 | 1.44615532337098 | 0.999714303421225 |
| ebi-fl187-GCST90094726 | prot-a-1540 | Interleukin-6 receptor subunit alpha \|\| id:prot-a-1540 | F-capsicum liking (derived food-liking factor) \|\| id:ebi-fl187-GCST90094726 | Weighted mode | 21 | 0.140515571707075 | 0.188002427475721 | 0.463511626659234 | -0.227969186 | 0.509000329559487 | 1.15086700038913 | 0.796148791870651 | 1.66362728438312 | 0.999714303421225 |
| ebi-fl187-GCST90094726 | prot-a-1541 | Interleukin-6 receptor subunit alpha \|\| id:prot-a-1541 | F-capsicum liking (derived food-liking factor) \|\| id:ebi-fl187-GCST90094726 | Weighted mode | 21 | 0.175762189034344 | 0.191747230775502 | 0.370255424931603 | -0.200062383 | 0.551586761354328 | 1.19215451752039 | 0.818679679556635 | 1.73600545993026 | 0.999714303421225 |
| ebi-fl187-GCST90094726 | prot-a-1545 | Interleukin-9 \|\| id:prot-a-1545 | F-capsicum liking (derived food-liking factor) \|\| id:ebi-fl187-GCST90094726 | Weighted mode | 21 | -0.143837707 | 0.204949104050306 | 0.49087770271503 | -0.545537951 | 0.257862536854237 | 0.866028286910373 | 0.579529940744884 | 1.29416090696697 | 0.999714303421225 |
| ebi-fl187-GCST90094726 | prot-a-1548 | Inositol monophosphatase 3 \|\| id:prot-a-1548 | F-capsicum liking (derived food-liking factor) \|\| id:ebi-fl187-GCST90094726 | Weighted mode | 21 | 0.0384735572825987 | 0.178411221157546 | 0.831448180316097 | -0.311212436 | 0.388159550751388 | 1.03922324810799 | 0.732558237460174 | 1.47426498561057 | 0.999714303421225 |
| ebi-fl187-GCST90094726 | prot-a-1555 | Inhibin beta A chain:Inhibin beta B chain heterodimer \|\| id:prot-a-1555 | F-capsicum liking (derived food-liking factor) \|\| id:ebi-fl187-GCST90094726 | Weighted mode | 21 | -0.113744555 | 0.176452540524347 | 0.52649985428899 | -0.459591534 | 0.232102424549646 | 0.892485907938119 | 0.631541555889614 | 1.2612489050639 | 0.999714303421225 |
| ebi-fl187-GCST90094726 | prot-a-1603 | Junctophilin-3 \|\| id:prot-a-1603 | F-capsicum liking (derived food-liking factor) \|\| id:ebi-fl187-GCST90094726 | Weighted mode | 21 | 0.185439937436573 | 0.235773277799663 | 0.440784531718865 | -0.276675687 | 0.647555561923913 | 1.20374789741938 | 0.75830038387012 | 1.91086412635889 | 0.999714303421225 |
| ebi-fl187-GCST90094726 | prot-a-1627 | UPF0258 protein KIAA1024 \|\| id:prot-a-1627 | F-capsicum liking (derived food-liking factor) \|\| id:ebi-fl187-GCST90094726 | Weighted mode | 21 | -0.156912548 | 0.194298688874427 | 0.428830896696433 | -0.537737978 | 0.223912882133512 | 0.854778807613858 | 0.584067933604277 | 1.2509620335377 | 0.999714303421225 |
| ebi-fl187-GCST90094726 | prot-a-1666 | Kallikrein-7 \|\| id:prot-a-1666 | F-capsicum liking (derived food-liking factor) \|\| id:ebi-fl187-GCST90094726 | Weighted mode | 21 | -0.078100317 | 0.209540288545516 | 0.713275934039895 | -0.488799283 | 0.33259864811895 | 0.924871641143259 | 0.613362426918223 | 1.39458746582968 | 0.999714303421225 |
| ebi-fl187-GCST90094726 | prot-a-1678 | Importin subunit alpha-3 \|\| id:prot-a-1678 | F-capsicum liking (derived food-liking factor) \|\| id:ebi-fl187-GCST90094726 | Weighted mode | 21 | -0.114397661 | 0.197688144166399 | 0.56926705300545 | -0.501866424 | 0.273071101604843 | 0.891903210264861 | 0.605399672398217 | 1.313993669 | 0.999714303421225 |
| ebi-fl187-GCST90094726 | prot-a-1718 | Protein LDOC1 \|\| id:prot-a-1718 | F-capsicum liking (derived food-liking factor) \|\| id:ebi-fl187-GCST90094726 | Weighted mode | 21 | 0.00815787442289673 | 0.163242136326114 | 0.96063877417193 | -0.311796713 | 0.32811246162208 | 1.00819124055096 | 0.732130345846844 | 1.38834509905196 | 0.999714303421225 |
| ebi-fl187-GCST90094726 | prot-a-1723 | Leptin \|\| id:prot-a-1723 | F-capsicum liking (derived food-liking factor) \|\| id:ebi-fl187-GCST90094726 | Weighted mode | 21 | -0.270033225 | 0.243241977929177 | 0.280107312949543 | -0.746787502 | 0.206721051885547 | 0.763354131584695 | 0.473886469595495 | 1.22963951831083 | 0.999714303421225 |
| ebi-fl187-GCST90094726 | prot-a-1730 | Galectin-7 \|\| id:prot-a-1730 | F-capsicum liking (derived food-liking factor) \|\| id:ebi-fl187-GCST90094726 | Weighted mode | 21 | 0.229186876900049 | 0.206305100889499 | 0.279783635630686 | -0.175171121 | 0.633544874643468 | 1.25757702914882 | 0.839313384465774 | 1.88427828450443 | 0.999714303421225 |
| ebi-fl187-GCST90094726 | prot-a-1753 | Lipase member N \|\| id:prot-a-1753 | F-capsicum liking (derived food-liking factor) \|\| id:ebi-fl187-GCST90094726 | Weighted mode | 21 | -0.177342459 | 0.2045731508727 | 0.396283850775205 | -0.578305835 | 0.223620916848162 | 0.837492928532095 | 0.560847730983886 | 1.25059684936376 | 0.999714303421225 |
| ebi-fl187-GCST90094726 | prot-a-1756 | VIP36-like protein \|\| id:prot-a-1756 | F-capsicum liking (derived food-liking factor) \|\| id:ebi-fl187-GCST90094726 | Weighted mode | 21 | 0.218601625334451 | 0.170679162194246 | 0.214926483534904 | -0.115929533 | 0.553132783235174 | 1.24433546620974 | 0.890537975012207 | 1.7386914381121 | 0.999714303421225 |
| ebi-fl187-GCST90094726 | prot-a-1760 | Ig Kappa chain V-I region HK102- like \|\| id:prot-a-1760 | F-capsicum liking (derived food-liking factor) \|\| id:ebi-fl187-GCST90094726 | Weighted mode | 21 | 0.143423991734763 | 0.215153651007924 | 0.512637443860073 | -0.278277164 | 0.565125147710295 | 1.15421907728315 | 0.757086955000854 | 1.75966798736197 | 0.999714303421225 |
| ebi-fl187-GCST90094726 | prot-a-179 | Neutral ceramidase \|\| id:prot-a-179 | F-capsicum liking (derived food-liking factor) \|\| id:ebi-fl187-GCST90094726 | Weighted mode | 21 | 0.37994581748697 | 0.200866793488693 | 0.0731253490759376 | -0.013753098 | 0.773644732724808 | 1.46220536132681 | 0.986341044023486 | 2.16765238722233 | 0.999714303421225 |
| ebi-fl187-GCST90094726 | prot-a-1809 | Lactotransferrin \|\| id:prot-a-1809 | F-capsicum liking (derived food-liking factor) \|\| id:ebi-fl187-GCST90094726 | Weighted mode | 21 | 0.0449908317663521 | 0.186086193741866 | 0.811416868573245 | -0.319738108 | 0.40971977150041 | 1.04601826972486 | 0.726339234625283 | 1.50639559098395 | 0.999714303421225 |
| ebi-fl187-GCST90094726 | prot-a-1818 | Lysophospholipase-like protein 1 \|\| id:prot-a-1818 | F-capsicum liking (derived food-liking factor) \|\| id:ebi-fl187-GCST90094726 | Weighted mode | 21 | -0.151190244 | 0.188518483130733 | 0.431984376255123 | -0.520686471 | 0.218305982457916 | 0.85968413294157 | 0.594112566658422 | 1.24396764166817 | 0.999714303421225 |
| ebi-fl187-GCST90094726 | prot-a-1852 | MAP kinase-activated protein kinase 3 \|\| id:prot-a-1852 | F-capsicum liking (derived food-liking factor) \|\| id:ebi-fl187-GCST90094726 | Weighted mode | 21 | -0.022014355 | 0.203297540648383 | 0.914847545368928 | -0.420477535 | 0.376448824816792 | 0.97822619265621 | 0.656733132178439 | 1.45710097010708 | 0.999714303421225 |
| ebi-fl187-GCST90094726 | prot-a-1856 | Megakaryocyte-associated tyrosine-protein kinase \|\| id:prot-a-1856 | F-capsicum liking (derived food-liking factor) \|\| id:ebi-fl187-GCST90094726 | Weighted mode | 21 | -0.045596386 | 0.166858373596879 | 0.787452200802055 | -0.372638798 | 0.281446026538412 | 0.955427508573581 | 0.688914025059427 | 1.32504447715428 | 0.999714303421225 |
| ebi-fl187-GCST90094726 | prot-a-1890 | Beta-1,3-N-acetylglucosaminyltransferase manic fringe \|\| id:prot-a-1890 | F-capsicum liking (derived food-liking factor) \|\| id:ebi-fl187-GCST90094726 | Weighted mode | 21 | -0.010847838 | 0.196925099640717 | 0.956616438139867 | -0.396821034 | 0.375125356836132 | 0.989210787161367 | 0.672454361495997 | 1.45517381917113 | 0.999714303421225 |
| ebi-fl187-GCST90094726 | prot-a-1919 | Matrilysin \|\| id:prot-a-1919 | F-capsicum liking (derived food-liking factor) \|\| id:ebi-fl187-GCST90094726 | Weighted mode | 21 | 0.0328845049454394 | 0.166838873287116 | 0.845735931624933 | -0.294119687 | 0.359888696588186 | 1.03343117615844 | 0.745187297099242 | 1.43316988898428 | 0.999714303421225 |
| ebi-fl187-GCST90094726 | prot-a-1923 | Multimerin-2 \|\| id:prot-a-1923 | F-capsicum liking (derived food-liking factor) \|\| id:ebi-fl187-GCST90094726 | Weighted mode | 21 | -0.126632019 | 0.177141592391689 | 0.482955313365421 | -0.47382954 | 0.220565502172771 | 0.881057825567072 | 0.6226133741968 | 1.24678158896666 | 0.999714303421225 |
| ebi-fl187-GCST90094726 | prot-a-1948 | Mesothelin \|\| id:prot-a-1948 | F-capsicum liking (derived food-liking factor) \|\| id:ebi-fl187-GCST90094726 | Weighted mode | 21 | 0.114792962757796 | 0.163772007980495 | 0.491421121295889 | -0.206200173 | 0.435786098399567 | 1.12164119203189 | 0.813670185272692 | 1.54617802941997 | 0.999714303421225 |
| ebi-fl187-GCST90094726 | prot-a-195 | ATPase family AAA domain-containing protein 1 \|\| id:prot-a-195 | F-capsicum liking (derived food-liking factor) \|\| id:ebi-fl187-GCST90094726 | Weighted mode | 21 | -0.160576401 | 0.16911939848359 | 0.353707109024545 | -0.492050422 | 0.170897620216649 | 0.851652754125039 | 0.611371538577931 | 1.18636928257384 | 0.999714303421225 |
| ebi-fl187-GCST90094726 | prot-a-1966 | Mucin-1 \|\| id:prot-a-1966 | F-capsicum liking (derived food-liking factor) \|\| id:ebi-fl187-GCST90094726 | Weighted mode | 21 | -0.06114747 | 0.158909689377512 | 0.704451526859719 | -0.372610462 | 0.250315520731624 | 0.940684506380859 | 0.688933546633323 | 1.28443061724787 | 0.999714303421225 |
| ebi-fl187-GCST90094726 | prot-a-1986 | Myocardial zonula adherens protein \|\| id:prot-a-1986 | F-capsicum liking (derived food-liking factor) \|\| id:ebi-fl187-GCST90094726 | Weighted mode | 21 | 0.0711357502293033 | 0.218139541195144 | 0.74773471041625 | -0.356417751 | 0.498689250971786 | 1.07372697465844 | 0.700180058551832 | 1.64656162658168 | 0.999714303421225 |
| ebi-fl187-GCST90094726 | prot-a-2071 | Epididymal secretory protein E1 \|\| id:prot-a-2071 | F-capsicum liking (derived food-liking factor) \|\| id:ebi-fl187-GCST90094726 | Weighted mode | 20 | -0.259692661 | 0.203842114270282 | 0.218031019286458 | -0.659223205 | 0.139837883271254 | 0.771288596678881 | 0.517252978172812 | 1.15008733534663 | 0.999714303421225 |
| ebi-fl187-GCST90094726 | prot-a-2098 | Neuritin \|\| id:prot-a-2098 | F-capsicum liking (derived food-liking factor) \|\| id:ebi-fl187-GCST90094726 | Weighted mode | 21 | 0.109589743369769 | 0.192830191864609 | 0.576142097219068 | -0.268357433 | 0.487536919424402 | 1.1158202039071 | 0.764634426916761 | 1.62830064095822 | 0.999714303421225 |
| ebi-fl187-GCST90094726 | prot-a-2115 | Neurotrophin-4 \|\| id:prot-a-2115 | F-capsicum liking (derived food-liking factor) \|\| id:ebi-fl187-GCST90094726 | Weighted mode | 21 | 0.0826902242092666 | 0.151033412185397 | 0.590096066262503 | -0.213335264 | 0.378715712092644 | 1.08620527632779 | 0.807885237234669 | 1.46040780044557 | 0.999714303421225 |
| ebi-fl187-GCST90094726 | prot-a-2118 | Netrin-1 \|\| id:prot-a-2118 | F-capsicum liking (derived food-liking factor) \|\| id:ebi-fl187-GCST90094726 | Weighted mode | 21 | 0.0904198952936838 | 0.175737431165921 | 0.612529685111936 | -0.25402547 | 0.434865260378889 | 1.09463381880897 | 0.775672045589591 | 1.54475490523774 | 0.999714303421225 |
| ebi-fl187-GCST90094726 | prot-a-2120 | Netrin-G1 \|\| id:prot-a-2120 | F-capsicum liking (derived food-liking factor) \|\| id:ebi-fl187-GCST90094726 | Weighted mode | 21 | 0.109884138291598 | 0.173957243268171 | 0.534754553218219 | -0.231072059 | 0.450840335097213 | 1.11614874406671 | 0.793682272409472 | 1.56963064716024 | 0.999714303421225 |
| ebi-fl187-GCST90094726 | prot-a-2145 | Olfactomedin-like protein 3 \|\| id:prot-a-2145 | F-capsicum liking (derived food-liking factor) \|\| id:ebi-fl187-GCST90094726 | Weighted mode | 21 | -0.32345239 | 0.24727841087942 | 0.205691921761499 | -0.808118075 | 0.161213295632463 | 0.723646410131283 | 0.445696043990937 | 1.1749355507103 | 0.999714303421225 |
| ebi-fl187-GCST90094726 | prot-a-2162 | Prolyl 4-hydroxylase subunit alpha-2 \|\| id:prot-a-2162 | F-capsicum liking (derived food-liking factor) \|\| id:ebi-fl187-GCST90094726 | Weighted mode | 21 | -0.017338711 | 0.166716460178683 | 0.918204264966457 | -0.344102973 | 0.309425551245442 | 0.982810739733774 | 0.708855931513974 | 1.36264212118962 | 0.999714303421225 |
| ebi-fl187-GCST90094726 | prot-a-2168 | Polyadenylate-binding protein-interacting protein 1 \|\| id:prot-a-2168 | F-capsicum liking (derived food-liking factor) \|\| id:ebi-fl187-GCST90094726 | Weighted mode | 21 | -0.159559148 | 0.191414462165105 | 0.414363062870072 | -0.534731494 | 0.215613198036861 | 0.852519541244069 | 0.58582656718199 | 1.24062241099629 | 0.999714303421225 |
| ebi-fl187-GCST90094726 | prot-a-219 | Beta-1,4-galactosyltransferase 5 \|\| id:prot-a-219 | F-capsicum liking (derived food-liking factor) \|\| id:ebi-fl187-GCST90094726 | Weighted mode | 21 | -0.00695873 | 0.172238423801584 | 0.968173450469448 | -0.344546041 | 0.330628580362774 | 0.993065425611342 | 0.708541929499788 | 1.39184273856715 | 0.999714303421225 |
| ebi-fl187-GCST90094726 | prot-a-2190 | Pyruvate carboxylase, mitochondrial \|\| id:prot-a-2190 | F-capsicum liking (derived food-liking factor) \|\| id:ebi-fl187-GCST90094726 | Weighted mode | 21 | -0.188827941 | 0.160206123894286 | 0.252368353409285 | -0.502831943 | 0.125176062299064 | 0.827928947453045 | 0.604815429098571 | 1.13334797535232 | 0.999714303421225 |
| ebi-fl187-GCST90094726 | prot-a-2197 | Protocadherin-9 \|\| id:prot-a-2197 | F-capsicum liking (derived food-liking factor) \|\| id:ebi-fl187-GCST90094726 | Weighted mode | 21 | -0.068877336 | 0.153329099886186 | 0.658108399063102 | -0.369402371 | 0.231647700193345 | 0.933441173072708 | 0.691147256631537 | 1.26067551484454 | 0.999714303421225 |
| ebi-fl187-GCST90094726 | prot-a-2198 | Protocadherin alpha-4 \|\| id:prot-a-2198 | F-capsicum liking (derived food-liking factor) \|\| id:ebi-fl187-GCST90094726 | Weighted mode | 21 | -0.045824223 | 0.160804004223526 | 0.778597580894141 | -0.361000072 | 0.269351624888487 | 0.955209850984625 | 0.696978948515968 | 1.30911537767509 | 0.999714303421225 |
| ebi-fl187-GCST90094726 | prot-a-2254 | Placenta growth factor \|\| id:prot-a-2254 | F-capsicum liking (derived food-liking factor) \|\| id:ebi-fl187-GCST90094726 | Weighted mode | 21 | -0.296549601 | 0.177322590444805 | 0.110022865054909 | -0.644101878 | 0.0510026763195172 | 0.743378754055518 | 0.525133964626376 | 1.05232570963927 | 0.999714303421225 |
| ebi-fl187-GCST90094726 | prot-a-2261 | Membrane-associated progesterone receptor component 2 \|\| id:prot-a-2261 | F-capsicum liking (derived food-liking factor) \|\| id:ebi-fl187-GCST90094726 | Weighted mode | 21 | 0.105290916849229 | 0.18563779080078 | 0.576900113091071 | -0.258559153 | 0.469140986818758 | 1.1110337817926 | 0.772163353826463 | 1.59862036726726 | 0.999714303421225 |
| ebi-fl187-GCST90094726 | prot-a-2268 | Polymeric immunoglobulin receptor \|\| id:prot-a-2268 | F-capsicum liking (derived food-liking factor) \|\| id:ebi-fl187-GCST90094726 | Weighted mode | 21 | 0.309120061706653 | 0.209340227213756 | 0.155345411228977 | -0.101186784 | 0.719426907045616 | 1.36222591185352 | 0.903764208757901 | 2.05325616675561 | 0.999714303421225 |
| ebi-fl187-GCST90094726 | prot-a-2283 | Group 10 secretory phospholipase A2 \|\| id:prot-a-2283 | F-capsicum liking (derived food-liking factor) \|\| id:ebi-fl187-GCST90094726 | Weighted mode | 21 | -0.077740236 | 0.190898465891547 | 0.688162214983243 | -0.451901229 | 0.296420757559019 | 0.925204730593409 | 0.636417026335044 | 1.34503597184054 | 0.999714303421225 |
| ebi-fl187-GCST90094726 | prot-a-2298 | Inactive phospholipase D5 \|\| id:prot-a-2298 | F-capsicum liking (derived food-liking factor) \|\| id:ebi-fl187-GCST90094726 | Weighted mode | 21 | -0.178873614 | 0.183764397664895 | 0.34198833391397 | -0.539051834 | 0.181304605189614 | 0.836211577959932 | 0.583301056687272 | 1.19878027837884 | 0.999714303421225 |
| ebi-fl187-GCST90094726 | prot-a-2304 | Procollagen-lysine,2-oxoglutarate 5-dioxygenase 2 \|\| id:prot-a-2304 | F-capsicum liking (derived food-liking factor) \|\| id:ebi-fl187-GCST90094726 | Weighted mode | 21 | 0.0205777083840504 | 0.221105463604599 | 0.926776014418554 | -0.412789 | 0.453944417049064 | 1.02079088917165 | 0.661801908113552 | 1.57451047910407 | 0.999714303421225 |
| ebi-fl187-GCST90094726 | prot-a-2331 | Periostin \|\| id:prot-a-2331 | F-capsicum liking (derived food-liking factor) \|\| id:ebi-fl187-GCST90094726 | Weighted mode | 21 | 0.00163481815036048 | 0.198640490440252 | 0.993514997321306 | -0.387700543 | 0.390970179413254 | 1.00163615519406 | 0.678615528933993 | 1.47841442557016 | 0.999714303421225 |
| ebi-fl187-GCST90094726 | prot-a-2333 | Periostin \|\| id:prot-a-2333 | F-capsicum liking (derived food-liking factor) \|\| id:ebi-fl187-GCST90094726 | Weighted mode | 21 | -0.055898157 | 0.209655277189026 | 0.792491839656872 | -0.4668225 | 0.355026186187099 | 0.945635437238572 | 0.626991371257368 | 1.42621800100391 | 0.999714303421225 |
| ebi-fl187-GCST90094726 | prot-a-2367 | cAMP-dependent protein kinase catalytic subunit alpha \|\| id:prot-a-2367 | F-capsicum liking (derived food-liking factor) \|\| id:ebi-fl187-GCST90094726 | Weighted mode | 21 | 0.0212300358364614 | 0.185930548913579 | 0.910231529465637 | -0.34319384 | 0.385653911707075 | 1.0214569963282 | 0.709500668596141 | 1.47057563372324 | 0.999714303421225 |
| ebi-fl187-GCST90094726 | prot-a-2385 | Phosphoribosyl pyrophosphate synthase-associated protein 1 \|\| id:prot-a-2385 | F-capsicum liking (derived food-liking factor) \|\| id:ebi-fl187-GCST90094726 | Weighted mode | 21 | 0.266974405117653 | 0.156123897713862 | 0.102732889564807 | -0.039028434 | 0.572977244636824 | 1.30600701880688 | 0.961723362729335 | 1.77353945975925 | 0.999714303421225 |
| ebi-fl187-GCST90094726 | prot-a-2393 | Trypsin-3 \|\| id:prot-a-2393 | F-capsicum liking (derived food-liking factor) \|\| id:ebi-fl187-GCST90094726 | Weighted mode | 20 | 0.0892853535152616 | 0.207799849721462 | 0.672269073030616 | -0.318002352 | 0.496573058969327 | 1.09339261524125 | 0.727601077139376 | 1.64308087030922 | 0.999714303421225 |
| ebi-fl187-GCST90094726 | prot-a-2405 | Pregnancy-specific beta-1-glycoprotein 6 \|\| id:prot-a-2405 | F-capsicum liking (derived food-liking factor) \|\| id:ebi-fl187-GCST90094726 | Weighted mode | 21 | 0.166372533702564 | 0.148971993073785 | 0.277315847124408 | -0.125612573 | 0.458357640127183 | 1.18101298692295 | 0.881956474597395 | 1.58147450067463 | 0.999714303421225 |
| ebi-fl187-GCST90094726 | prot-a-244 | Beclin-1 \|\| id:prot-a-244 | F-capsicum liking (derived food-liking factor) \|\| id:ebi-fl187-GCST90094726 | Weighted mode | 21 | -0.025517636 | 0.173530245350008 | 0.884564745339874 | -0.365636916 | 0.314601645334021 | 0.974805187586581 | 0.693754646394725 | 1.36971357047323 | 0.999714303421225 |
| ebi-fl187-GCST90094726 | prot-a-2454 | tRNA pseudouridine synthase A, mitochondrial \|\| id:prot-a-2454 | F-capsicum liking (derived food-liking factor) \|\| id:ebi-fl187-GCST90094726 | Weighted mode | 21 | -0.19584694 | 0.185307100720999 | 0.303166960096816 | -0.559048858 | 0.167354977227359 | 0.822138061347354 | 0.571752623469623 | 1.18217383562543 | 0.999714303421225 |
| ebi-fl187-GCST90094726 | prot-a-2466 | Glutaminyl-peptide cyclotransferase-like protein \|\| id:prot-a-2466 | F-capsicum liking (derived food-liking factor) \|\| id:ebi-fl187-GCST90094726 | Weighted mode | 21 | -0.114757356 | 0.209562426511037 | 0.590023004238956 | -0.525499712 | 0.295985000334987 | 0.891582455128539 | 0.591259831142971 | 1.34444999038133 | 0.999714303421225 |
| ebi-fl187-GCST90094726 | prot-a-2474 | Ras-related protein Rab-27A \|\| id:prot-a-2474 | F-capsicum liking (derived food-liking factor) \|\| id:ebi-fl187-GCST90094726 | Weighted mode | 21 | 0.0784287778239745 | 0.178928998990108 | 0.665849752473869 | -0.27227206 | 0.429129615844587 | 1.08158631948775 | 0.761647019062188 | 1.53592010107709 | 0.999714303421225 |
| ebi-fl187-GCST90094726 | prot-a-2490 | Rap1 GTPase-GDP dissociation stimulator 1 \|\| id:prot-a-2490 | F-capsicum liking (derived food-liking factor) \|\| id:ebi-fl187-GCST90094726 | Weighted mode | 21 | 0.160763545774915 | 0.270853059454384 | 0.559469927829541 | -0.370108451 | 0.691635542305508 | 1.17440724242594 | 0.690659424039137 | 1.99697900739038 | 0.999714303421225 |
| ebi-fl187-GCST90094726 | prot-a-2545 | Receptor-interacting serine/threonine-protein kinase 2 \|\| id:prot-a-2545 | F-capsicum liking (derived food-liking factor) \|\| id:ebi-fl187-GCST90094726 | Weighted mode | 21 | 0.000304388100746067 | 0.180100606674142 | 0.998668240162129 | -0.352692801 | 0.353301577182065 | 1.0003044344315 | 0.702793057557054 | 1.42376045236062 | 0.999714303421225 |
| ebi-fl187-GCST90094726 | prot-a-2610 | RUN and FYVE domain-containing protein 1 \|\| id:prot-a-2610 | F-capsicum liking (derived food-liking factor) \|\| id:ebi-fl187-GCST90094726 | Weighted mode | 21 | -0.09403867 | 0.193146485596108 | 0.631638299731589 | -0.472605782 | 0.28452844158398 | 0.910247562119104 | 0.623375768723513 | 1.32913511547039 | 0.999714303421225 |
| ebi-fl187-GCST90094726 | prot-a-2627 | Serine--tRNA ligase, mitochondrial \|\| id:prot-a-2627 | F-capsicum liking (derived food-liking factor) \|\| id:ebi-fl187-GCST90094726 | Weighted mode | 21 | -0.01500058 | 0.15061110034496 | 0.921654975507912 | -0.310198337 | 0.28019717673259 | 0.985111368293931 | 0.733301501259297 | 1.32339072847308 | 0.999714303421225 |
| ebi-fl187-GCST90094726 | prot-a-264 | BolA-like protein 2 \|\| id:prot-a-264 | F-capsicum liking (derived food-liking factor) \|\| id:ebi-fl187-GCST90094726 | Weighted mode | 21 | -0.016769333 | 0.156535027601105 | 0.915754538317398 | -0.323577987 | 0.290039320953193 | 0.983370489453855 | 0.723555527621131 | 1.33648003866134 | 0.999714303421225 |
| ebi-fl187-GCST90094726 | prot-a-2646 | Secretoglobin family 1C member 1 \|\| id:prot-a-2646 | F-capsicum liking (derived food-liking factor) \|\| id:ebi-fl187-GCST90094726 | Weighted mode | 21 | -0.133506602 | 0.176889089771964 | 0.459196360123348 | -0.480209218 | 0.213196013469331 | 0.875021691642072 | 0.618653944454067 | 1.23762721907449 | 0.999714303421225 |
| ebi-fl187-GCST90094726 | prot-a-2672 | Semaphorin-3E \|\| id:prot-a-2672 | F-capsicum liking (derived food-liking factor) \|\| id:ebi-fl187-GCST90094726 | Weighted mode | 21 | 0.114040705886507 | 0.209097072124291 | 0.591512519323499 | -0.295789555 | 0.523870967250118 | 1.12079774702149 | 0.74394397048178 | 1.68855134199816 | 0.999714303421225 |
| ebi-fl187-GCST90094726 | prot-a-2701 | Plasma protease C1 inhibitor \|\| id:prot-a-2701 | F-capsicum liking (derived food-liking factor) \|\| id:ebi-fl187-GCST90094726 | Weighted mode | 21 | 0.232635937519377 | 0.175204218430348 | 0.199201013115791 | -0.110764331 | 0.576036205642859 | 1.26192197724321 | 0.895149683457785 | 1.77897295399594 | 0.999714303421225 |
| ebi-fl187-GCST90094726 | prot-a-2738 | Signaling threshold-regulating transmembrane adapter 1 \|\| id:prot-a-2738 | F-capsicum liking (derived food-liking factor) \|\| id:ebi-fl187-GCST90094726 | Weighted mode | 21 | 0.0173346802198329 | 0.16350050856307 | 0.916620964455633 | -0.303126317 | 0.33779567700345 | 1.01748579771733 | 0.738505804962864 | 1.40185404312229 | 0.999714303421225 |
| ebi-fl187-GCST90094726 | prot-a-274 | Serine/threonine-protein kinase BRSK2 \|\| id:prot-a-274 | F-capsicum liking (derived food-liking factor) \|\| id:ebi-fl187-GCST90094726 | Weighted mode | 21 | -0.057159552 | 0.177750795750038 | 0.751114081228379 | -0.405551111 | 0.291232007910522 | 0.944443369743208 | 0.666609333584479 | 1.33807499192309 | 0.999714303421225 |
| ebi-fl187-GCST90094726 | prot-a-2749 | Mitochondrial glutamate carrier 2 \|\| id:prot-a-2749 | F-capsicum liking (derived food-liking factor) \|\| id:ebi-fl187-GCST90094726 | Weighted mode | 21 | 0.188530868174835 | 0.167758750573106 | 0.274397525510131 | -0.140276283 | 0.517338019298123 | 1.20747435494085 | 0.869118079719255 | 1.67755607881358 | 0.999714303421225 |
| ebi-fl187-GCST90094726 | prot-a-2760 | Sodium-coupled monocarboxylate transporter 1 \|\| id:prot-a-2760 | F-capsicum liking (derived food-liking factor) \|\| id:ebi-fl187-GCST90094726 | Weighted mode | 21 | -0.07457577 | 0.185900136537742 | 0.692555810411811 | -0.438940038 | 0.289788497277234 | 0.928137146130528 | 0.644719437169464 | 1.33614485986234 | 0.999714303421225 |
| ebi-fl187-GCST90094726 | prot-a-2777 | Small integral membrane protein 10 \|\| id:prot-a-2777 | F-capsicum liking (derived food-liking factor) \|\| id:ebi-fl187-GCST90094726 | Weighted mode | 21 | 0.2342996297614 | 0.179558080901394 | 0.206752795102127 | -0.117634209 | 0.586233468328133 | 1.26402317443487 | 0.889021189267836 | 1.79720641622081 | 0.999714303421225 |
| ebi-fl187-GCST90094726 | prot-a-2799 | Superoxide dismutase [Mn], mitochondrial \|\| id:prot-a-2799 | F-capsicum liking (derived food-liking factor) \|\| id:ebi-fl187-GCST90094726 | Weighted mode | 21 | 0.293859998636257 | 0.203510265759709 | 0.164234514484263 | -0.105020122 | 0.692740119525287 | 1.34159606523973 | 0.900306406210916 | 1.99918604360689 | 0.999714303421225 |
| ebi-fl187-GCST90094726 | prot-a-2805 | Protein SPACA7 \|\| id:prot-a-2805 | F-capsicum liking (derived food-liking factor) \|\| id:ebi-fl187-GCST90094726 | Weighted mode | 21 | -0.264654481 | 0.199597671640813 | 0.199805057365581 | -0.655865917 | 0.126556955820486 | 0.767471080320016 | 0.518992463554384 | 1.13491408929843 | 0.999714303421225 |
| ebi-fl187-GCST90094726 | prot-a-2811 | SPARC-like protein 1 \|\| id:prot-a-2811 | F-capsicum liking (derived food-liking factor) \|\| id:ebi-fl187-GCST90094726 | Weighted mode | 21 | 0.251885144522731 | 0.17630233665986 | 0.168519804186929 | -0.093667435 | 0.597437724376056 | 1.28644827316885 | 0.910585540470538 | 1.81745600603753 | 0.999714303421225 |
| ebi-fl187-GCST90094726 | prot-a-2831 | Sepiapterin reductase \|\| id:prot-a-2831 | F-capsicum liking (derived food-liking factor) \|\| id:ebi-fl187-GCST90094726 | Weighted mode | 21 | 0.044883869066618 | 0.241553988921755 | 0.85446303046382 | -0.428561949 | 0.518329687353258 | 1.0459063907703 | 0.651445232780071 | 1.67922048271933 | 0.999714303421225 |
| ebi-fl187-GCST90094726 | prot-a-2834 | SPRY domain-containing SOCS box protein 1 \|\| id:prot-a-2834 | F-capsicum liking (derived food-liking factor) \|\| id:ebi-fl187-GCST90094726 | Weighted mode | 21 | -0.137253649 | 0.178720551815555 | 0.451472489001844 | -0.487545931 | 0.213038632159978 | 0.871749079456779 | 0.614131667920696 | 1.23743245500878 | 0.999714303421225 |
| ebi-fl187-GCST90094726 | prot-a-2835 | Spectrin alpha chain, non-erythrocytic 1 \|\| id:prot-a-2835 | F-capsicum liking (derived food-liking factor) \|\| id:ebi-fl187-GCST90094726 | Weighted mode | 21 | -0.047241554 | 0.171584992010587 | 0.785891329137432 | -0.383548138 | 0.28906503044673 | 0.953856961896686 | 0.681439273975445 | 1.33517855296313 | 0.999714303421225 |
| ebi-fl187-GCST90094726 | prot-a-2859 | Sia-alpha-2,3-Gal-beta-1,4-GlcNAc-R:alpha 2,8-sialyltransferase \|\| id:prot-a-2859 | F-capsicum liking (derived food-liking factor) \|\| id:ebi-fl187-GCST90094726 | Weighted mode | 21 | 0.0222116736352245 | 0.175917722222679 | 0.900785454296352 | -0.322587062 | 0.367010409191675 | 1.02246018943119 | 0.724272872474153 | 1.44341294379898 | 0.999714303421225 |
| ebi-fl187-GCST90094726 | prot-a-2861 | Alpha-2,8-sialyltransferase 8F \|\| id:prot-a-2861 | F-capsicum liking (derived food-liking factor) \|\| id:ebi-fl187-GCST90094726 | Weighted mode | 21 | -0.200833935 | 0.22509378820151 | 0.382882816578578 | -0.64201776 | 0.240349889842868 | 0.818048269434201 | 0.526229547206268 | 1.27169402531094 | 0.999714303421225 |
| ebi-fl187-GCST90094726 | prot-a-289 | Uncharacterized protein C17orf89 \|\| id:prot-a-289 | F-capsicum liking (derived food-liking factor) \|\| id:ebi-fl187-GCST90094726 | Weighted mode | 21 | -0.15843576 | 0.178641664421317 | 0.38567827724733 | -0.508573422 | 0.191701902717773 | 0.853477789828874 | 0.601352844038464 | 1.21130937510722 | 0.999714303421225 |
| ebi-fl187-GCST90094726 | prot-a-2921 | Tachykinin-3 \|\| id:prot-a-2921 | F-capsicum liking (derived food-liking factor) \|\| id:ebi-fl187-GCST90094726 | Weighted mode | 21 | -0.059745999 | 0.17773272953277 | 0.74025412470029 | -0.408102149 | 0.288610151177523 | 0.94200377337608 | 0.664910955636117 | 1.33457134603209 | 0.999714303421225 |
| ebi-fl187-GCST90094726 | prot-a-2928 | Tax1-binding protein 1 \|\| id:prot-a-2928 | F-capsicum liking (derived food-liking factor) \|\| id:ebi-fl187-GCST90094726 | Weighted mode | 21 | -0.005541664 | 0.170478550668367 | 0.974390341039391 | -0.339679624 | 0.328596295114832 | 0.994473662500988 | 0.711998393776088 | 1.38901698943881 | 0.999714303421225 |
| ebi-fl187-GCST90094726 | prot-a-296 | Uncharacterized protein C1orf185 \|\| id:prot-a-296 | F-capsicum liking (derived food-liking factor) \|\| id:ebi-fl187-GCST90094726 | Weighted mode | 21 | 0.0107110931937613 | 0.189287582362259 | 0.955436220415844 | -0.360292568 | 0.381714754623788 | 1.0107686623116 | 0.697472237995128 | 1.46479419976327 | 0.999714303421225 |
| ebi-fl187-GCST90094726 | prot-a-2964 | Transforming growth factor beta-2 \|\| id:prot-a-2964 | F-capsicum liking (derived food-liking factor) \|\| id:ebi-fl187-GCST90094726 | Weighted mode | 21 | 0.0739100503591832 | 0.168387463191421 | 0.665418616160598 | -0.256129377 | 0.403949478214367 | 1.07670995146689 | 0.774041818721499 | 1.49772827713971 | 0.999714303421225 |
| ebi-fl187-GCST90094726 | prot-a-2977 | Thymocyte nuclear protein 1 \|\| id:prot-a-2977 | F-capsicum liking (derived food-liking factor) \|\| id:ebi-fl187-GCST90094726 | Weighted mode | 21 | -0.148783007 | 0.17932008811656 | 0.416498762586639 | -0.500250379 | 0.202684365985696 | 0.861756089890192 | 0.606378815921112 | 1.22468585472393 | 0.999714303421225 |
| ebi-fl187-GCST90094726 | prot-a-3015 | Transmembrane protein 70, mitochondrial \|\| id:prot-a-3015 | F-capsicum liking (derived food-liking factor) \|\| id:ebi-fl187-GCST90094726 | Weighted mode | 21 | 0.0201716140268189 | 0.192693701563278 | 0.917670649829886 | -0.357508041 | 0.397851269090844 | 1.02037643591091 | 0.699417074881396 | 1.48862260924757 | 0.999714303421225 |
| ebi-fl187-GCST90094726 | prot-a-3030 | Tumor necrosis factor alpha-induced protein 3 \|\| id:prot-a-3030 | F-capsicum liking (derived food-liking factor) \|\| id:ebi-fl187-GCST90094726 | Weighted mode | 21 | -0.172828959 | 0.184211930108963 | 0.359330915 | -0.533884342 | 0.188226424032558 | 0.841281496183832 | 0.58632306140163 | 1.20710680239899 | 0.999714303421225 |
| ebi-fl187-GCST90094726 | prot-a-3032 | Tumor necrosis factor receptor superfamily member 10A \|\| id:prot-a-3032 | F-capsicum liking (derived food-liking factor) \|\| id:ebi-fl187-GCST90094726 | Weighted mode | 21 | 0.127693497873618 | 0.208939732288673 | 0.547984317727432 | -0.281828377 | 0.537215373159417 | 1.13620470016545 | 0.754403146043694 | 1.71123507033106 | 0.999714303421225 |
| ebi-fl187-GCST90094726 | prot-a-3081 | Tumor protein p53-inducible protein 11 \|\| id:prot-a-3081 | F-capsicum liking (derived food-liking factor) \|\| id:ebi-fl187-GCST90094726 | Weighted mode | 21 | 0.0804073249586215 | 0.157966693484651 | 0.616311946660604 | -0.229207394 | 0.390022044188537 | 1.08372840741322 | 0.795163604026754 | 1.4770133530846 | 0.999714303421225 |
| ebi-fl187-GCST90094726 | prot-a-315 | Complement C4 \|\| id:prot-a-315 | F-capsicum liking (derived food-liking factor) \|\| id:ebi-fl187-GCST90094726 | Weighted mode | 20 | -0.048973466 | 0.168469378767805 | 0.774432723666592 | -0.379173449 | 0.281226515996073 | 0.952206394837554 | 0.684426889365707 | 1.32475364784369 | 0.999714303421225 |
| ebi-fl187-GCST90094726 | prot-a-3157 | UDP-glucuronosyltransferase 1-8 \|\| id:prot-a-3157 | F-capsicum liking (derived food-liking factor) \|\| id:ebi-fl187-GCST90094726 | Weighted mode | 21 | 0.221324547205626 | 0.176524149900758 | 0.224375774951084 | -0.124662787 | 0.567311881011112 | 1.24772831159592 | 0.882794542547001 | 1.76352012220908 | 0.999714303421225 |
| ebi-fl187-GCST90094726 | prot-a-317 | Glutaredoxin-like protein C5orf63 \|\| id:prot-a-317 | F-capsicum liking (derived food-liking factor) \|\| id:ebi-fl187-GCST90094726 | Weighted mode | 21 | 0.0213828294924958 | 0.176646710213392 | 0.904860468442905 | -0.324844723 | 0.367610381510745 | 1.02161308040118 | 0.722639554575533 | 1.44427921145257 | 0.999714303421225 |
| ebi-fl187-GCST90094726 | prot-a-3192 | Guanine nucleotide exchange factor VAV3 \|\| id:prot-a-3192 | F-capsicum liking (derived food-liking factor) \|\| id:ebi-fl187-GCST90094726 | Weighted mode | 21 | -0.129455281 | 0.223588138842211 | 0.569060206650225 | -0.567688033 | 0.308777471263325 | 0.878573876599452 | 0.56683442743884 | 1.36175930620635 | 0.999714303421225 |
| ebi-fl187-GCST90094726 | prot-a-3233 | Serine/threonine-protein kinase WNK3 \|\| id:prot-a-3233 | F-capsicum liking (derived food-liking factor) \|\| id:ebi-fl187-GCST90094726 | Weighted mode | 21 | 0.138138483589041 | 0.17485369970947 | 0.438781421986413 | -0.204574768 | 0.480851735019602 | 1.14813453705679 | 0.814993804311849 | 1.61745145571463 | 0.999714303421225 |
| ebi-fl187-GCST90094726 | prot-a-3250 | 14-3-3 protein theta \|\| id:prot-a-3250 | F-capsicum liking (derived food-liking factor) \|\| id:ebi-fl187-GCST90094726 | Weighted mode | 21 | 0.123339764724066 | 0.182476377020728 | 0.506831689517701 | -0.234313934 | 0.480993463684692 | 1.1312687208628 | 0.791113419320202 | 1.61768071119595 | 0.999714303421225 |
| ebi-fl187-GCST90094726 | prot-a-3278 | E3 ubiquitin-protein ligase ZNRF3 \|\| id:prot-a-3278 | F-capsicum liking (derived food-liking factor) \|\| id:ebi-fl187-GCST90094726 | Weighted mode | 21 | 0.0468633188415195 | 0.157712960315166 | 0.769421463702345 | -0.262254083 | 0.355980721059244 | 1.04797876033915 | 0.769315528585727 | 1.4275800257678 | 0.999714303421225 |
| ebi-fl187-GCST90094726 | prot-a-355 | Calpain-3 \|\| id:prot-a-355 | F-capsicum liking (derived food-liking factor) \|\| id:ebi-fl187-GCST90094726 | Weighted mode | 21 | 0.178229722349045 | 0.207665803638901 | 0.400921970096801 | -0.228795253 | 0.585254697481291 | 1.19509983084358 | 0.795491391480559 | 1.79544822354908 | 0.999714303421225 |
| ebi-fl187-GCST90094726 | prot-a-385 | Coiled-coil domain-containing protein 90B, mitochondrial \|\| id:prot-a-385 | F-capsicum liking (derived food-liking factor) \|\| id:ebi-fl187-GCST90094726 | Weighted mode | 21 | 0.105211507585808 | 0.227207917214833 | 0.648318571205634 | -0.34011601 | 0.55053902532688 | 1.11094555892126 | 0.711687754966397 | 1.73418753698403 | 0.999714303421225 |
| ebi-fl187-GCST90094726 | prot-a-390 | C-C motif chemokine 14 \|\| id:prot-a-390 | F-capsicum liking (derived food-liking factor) \|\| id:ebi-fl187-GCST90094726 | Weighted mode | 21 | -0.426260075 | 0.217602779891506 | 0.0642165296913153 | -0.852761523 | 0.000241374085730051 | 0.652946505299568 | 0.426236243981653 | 1.0002414032188 | 0.999714303421225 |
| ebi-fl187-GCST90094726 | prot-a-40 | Pituitary adenylate cyclase-activating polypeptide 27 \|\| id:prot-a-40 | F-capsicum liking (derived food-liking factor) \|\| id:ebi-fl187-GCST90094726 | Weighted mode | 21 | 0.220288108114246 | 0.176146977349323 | 0.225517023213692 | -0.124959967 | 0.56553618371892 | 1.2464357871255 | 0.88253223185745 | 1.76039142293685 | 0.999714303421225 |
| ebi-fl187-GCST90094726 | prot-a-401 | C-C motif chemokine 24 \|\| id:prot-a-401 | F-capsicum liking (derived food-liking factor) \|\| id:ebi-fl187-GCST90094726 | Weighted mode | 21 | 0.310061171328803 | 0.19851257366207 | 0.133991424436206 | -0.079023473 | 0.69914581570646 | 1.36350851920911 | 0.92401823466536 | 2.01203332597556 | 0.999714303421225 |
| ebi-fl187-GCST90094726 | prot-a-412 | Cerebral cavernous malformations 2 protein \|\| id:prot-a-412 | F-capsicum liking (derived food-liking factor) \|\| id:ebi-fl187-GCST90094726 | Weighted mode | 21 | -0.145808602 | 0.187428361058894 | 0.445708587894324 | -0.51316819 | 0.221550985241667 | 0.864323116695242 | 0.59859610519444 | 1.24801087673485 | 0.999714303421225 |
| ebi-fl187-GCST90094726 | prot-a-44 | Adhesion G protein-coupled receptor F5 \|\| id:prot-a-44 | F-capsicum liking (derived food-liking factor) \|\| id:ebi-fl187-GCST90094726 | Weighted mode | 21 | 0.0302427329618112 | 0.154739286433492 | 0.847017986014402 | -0.273046268 | 0.333531734371455 | 1.03070468959384 | 0.761057573861644 | 1.39588934350959 | 0.999714303421225 |
| ebi-fl187-GCST90094726 | prot-a-48 | Adrenomedullin \|\| id:prot-a-48 | F-capsicum liking (derived food-liking factor) \|\| id:ebi-fl187-GCST90094726 | Weighted mode | 21 | 0.0337381520230693 | 0.16624877409076 | 0.841234046798446 | -0.292109445 | 0.35958574924096 | 1.0343137383066 | 0.746686810217778 | 1.43273577972773 | 0.999714303421225 |
| ebi-fl187-GCST90094726 | prot-a-502 | Carcinoembryonic antigen-related cell adhesion molecule 1 \|\| id:prot-a-502 | F-capsicum liking (derived food-liking factor) \|\| id:ebi-fl187-GCST90094726 | Weighted mode | 21 | 0.0590520571024638 | 0.212595096115971 | 0.784041754315066 | -0.357634331 | 0.475738445489766 | 1.0608304630629 | 0.699328750903157 | 1.60920206685181 | 0.999714303421225 |
| ebi-fl187-GCST90094726 | prot-a-518 | Complement factor B \|\| id:prot-a-518 | F-capsicum liking (derived food-liking factor) \|\| id:ebi-fl187-GCST90094726 | Weighted mode | 21 | 0.343112591247163 | 0.202357818862889 | 0.105479551384878 | -0.053508734 | 0.739733916218426 | 1.40932743092702 | 0.947897662290993 | 2.09537789423688 | 0.999714303421225 |
| ebi-fl187-GCST90094726 | prot-a-525 | Properdin \|\| id:prot-a-525 | F-capsicum liking (derived food-liking factor) \|\| id:ebi-fl187-GCST90094726 | Weighted mode | 21 | -0.07049407 | 0.205011571198961 | 0.734544728929008 | -0.472316749 | 0.331328609901868 | 0.931933266201633 | 0.623555970780058 | 1.39281741070776 | 0.999714303421225 |
| ebi-fl187-GCST90094726 | prot-a-533 | Chondroadherin \|\| id:prot-a-533 | F-capsicum liking (derived food-liking factor) \|\| id:ebi-fl187-GCST90094726 | Weighted mode | 21 | 0.113008782052453 | 0.233688710991749 | 0.633931398501096 | -0.345021091 | 0.571038655596281 | 1.11964176565812 | 0.708205416201848 | 1.7701046260408 | 0.999714303421225 |
| ebi-fl187-GCST90094726 | prot-a-535 | Coiled-coil-helix-coiled-coil-helix domain-containing protein 2 \|\| id:prot-a-535 | F-capsicum liking (derived food-liking factor) \|\| id:ebi-fl187-GCST90094726 | Weighted mode | 21 | 0.055156479687589 | 0.262918544981323 | 0.83595741317075 | -0.460163868 | 0.570476827850983 | 1.05670595475665 | 0.631180206493565 | 1.76911041146463 | 0.999714303421225 |
| ebi-fl187-GCST90094726 | prot-a-591 | Caseinolytic peptidase B protein homolog \|\| id:prot-a-591 | F-capsicum liking (derived food-liking factor) \|\| id:ebi-fl187-GCST90094726 | Weighted mode | 21 | 0.110471209366227 | 0.192797106041317 | 0.573036581552245 | -0.267411118 | 0.488353537207208 | 1.11680419508868 | 0.765358353817625 | 1.62963088329323 | 0.999714303421225 |
| ebi-fl187-GCST90094726 | prot-a-605 | Ciliary neurotrophic factor receptor subunit alpha \|\| id:prot-a-605 | F-capsicum liking (derived food-liking factor) \|\| id:ebi-fl187-GCST90094726 | Weighted mode | 21 | 0.143346784172363 | 0.172102370137405 | 0.414728474549325 | -0.193973861 | 0.480667429641677 | 1.15412996628178 | 0.823679433899328 | 1.6171533781823 | 0.999714303421225 |
| ebi-fl187-GCST90094726 | prot-a-606 | Ciliary neurotrophic factor receptor subunit alpha \|\| id:prot-a-606 | F-capsicum liking (derived food-liking factor) \|\| id:ebi-fl187-GCST90094726 | Weighted mode | 21 | 0.245974312230677 | 0.187143372073128 | 0.203599133571711 | -0.120826697 | 0.612775321494008 | 1.27886672188645 | 0.886187525213547 | 1.84554628204057 | 0.999714303421225 |
| ebi-fl187-GCST90094726 | prot-a-707 | Cancer/testis antigen 55 \|\| id:prot-a-707 | F-capsicum liking (derived food-liking factor) \|\| id:ebi-fl187-GCST90094726 | Weighted mode | 21 | 0.0325528083404865 | 0.214300096824159 | 0.880785394226512 | -0.387475381 | 0.452580998115838 | 1.03308844738998 | 0.678768344348421 | 1.572365224 | 0.999714303421225 |
| ebi-fl187-GCST90094726 | prot-a-719 | Dipeptidyl peptidase 1 \|\| id:prot-a-719 | F-capsicum liking (derived food-liking factor) \|\| id:ebi-fl187-GCST90094726 | Weighted mode | 21 | -0.061961949 | 0.193470529135885 | 0.752088936354003 | -0.441164187 | 0.317240287688719 | 0.939918650562716 | 0.643287078832492 | 1.37333252718058 | 0.999714303421225 |
| ebi-fl187-GCST90094726 | prot-a-752 | Cytochrome b5 \|\| id:prot-a-752 | F-capsicum liking (derived food-liking factor) \|\| id:ebi-fl187-GCST90094726 | Weighted mode | 21 | 0.0894025808572851 | 0.178495094744531 | 0.621931267380432 | -0.260447805 | 0.439252966556566 | 1.09352079826445 | 0.770706382467646 | 1.55154772743447 | 0.999714303421225 |
| ebi-fl187-GCST90094726 | prot-a-755 | Cytoglobin \|\| id:prot-a-755 | F-capsicum liking (derived food-liking factor) \|\| id:ebi-fl187-GCST90094726 | Weighted mode | 21 | 0.059733137734179 | 0.199717220410572 | 0.767958215101137 | -0.331712614 | 0.4511788897389 | 1.06155322024485 | 0.717693548103777 | 1.57016214286667 | 0.999714303421225 |
| ebi-fl187-GCST90094726 | prot-a-807 | Desmin \|\| id:prot-a-807 | F-capsicum liking (derived food-liking factor) \|\| id:ebi-fl187-GCST90094726 | Weighted mode | 21 | 0.061442202450954 | 0.188924337070378 | 0.74839129077518 | -0.308849498 | 0.431733903108895 | 1.06336903462871 | 0.734291273863853 | 1.53992529130457 | 0.999714303421225 |
| ebi-fl187-GCST90094726 | prot-a-82 | Ameloblastin \|\| id:prot-a-82 | F-capsicum liking (derived food-liking factor) \|\| id:ebi-fl187-GCST90094726 | Weighted mode | 21 | -0.19212589 | 0.169851994116947 | 0.271379185786914 | -0.525035798 | 0.140784018794644 | 0.82520297741813 | 0.59153418816186 | 1.15117598875522 | 0.999714303421225 |
| ebi-fl187-GCST90094726 | prot-a-84 | Muellerian-inhibiting factor \|\| id:prot-a-84 | F-capsicum liking (derived food-liking factor) \|\| id:ebi-fl187-GCST90094726 | Weighted mode | 21 | -0.044405719 | 0.174591715661319 | 0.80182933625225 | -0.386605482 | 0.297794043445436 | 0.95656578158096 | 0.679359061478607 | 1.34688435964934 | 0.999714303421225 |
| ebi-fl187-GCST90094726 | prot-a-858 | Inactive dipeptidyl peptidase 10 \|\| id:prot-a-858 | F-capsicum liking (derived food-liking factor) \|\| id:ebi-fl187-GCST90094726 | Weighted mode | 21 | -0.004647435 | 0.165422960952738 | 0.97786542388329 | -0.328876439 | 0.319581568225027 | 0.995363347374521 | 0.719731942260538 | 1.37655165086166 | 0.999714303421225 |
| ebi-fl187-GCST90094726 | prot-a-863 | Dihydropyrimidinase-related protein 3 \|\| id:prot-a-863 | F-capsicum liking (derived food-liking factor) \|\| id:ebi-fl187-GCST90094726 | Weighted mode | 21 | -0.124070993 | 0.175164256572969 | 0.486925457857584 | -0.467392936 | 0.21925094950464 | 0.883317128989546 | 0.626633814881768 | 1.24514370568009 | 0.999714303421225 |
| ebi-fl187-GCST90094726 | prot-a-864 | Dihydropyrimidinase-related protein 5 \|\| id:prot-a-864 | F-capsicum liking (derived food-liking factor) \|\| id:ebi-fl187-GCST90094726 | Weighted mode | 21 | 0.218095223024762 | 0.227973446537461 | 0.350156226397263 | -0.228732732 | 0.664923178238185 | 1.24370549137944 | 0.795541127630057 | 1.94434114788679 | 0.999714303421225 |
| ebi-fl187-GCST90094726 | prot-a-884 | Endothelin-converting enzyme 1 \|\| id:prot-a-884 | F-capsicum liking (derived food-liking factor) \|\| id:ebi-fl187-GCST90094726 | Weighted mode | 21 | 0.0521915283662141 | 0.158494851991481 | 0.745356456347387 | -0.258458382 | 0.362841438269516 | 1.05357751317012 | 0.772241169870883 | 1.437407923 | 0.999714303421225 |
| ebi-fl187-GCST90094726 | prot-a-893 | Early endosome antigen 1 \|\| id:prot-a-893 | F-capsicum liking (derived food-liking factor) \|\| id:ebi-fl187-GCST90094726 | Weighted mode | 21 | -0.228377984 | 0.174433652283346 | 0.205291294943596 | -0.570267942 | 0.11351197492116 | 0.795823394822734 | 0.565373930964278 | 1.12020530318225 | 0.999714303421225 |
| ebi-fl187-GCST90094726 | prot-a-897 | Ephrin-A2 \|\| id:prot-a-897 | F-capsicum liking (derived food-liking factor) \|\| id:ebi-fl187-GCST90094726 | Weighted mode | 21 | 0.176554088917942 | 0.164519569151967 | 0.295982366152573 | -0.145904267 | 0.499012444455797 | 1.19309895844602 | 0.864240435882575 | 1.64709387057467 | 0.999714303421225 |
| ebi-fl187-GCST90094726 | prot-a-90 | Anaphase-promoting complex subunit 10 \|\| id:prot-a-90 | F-capsicum liking (derived food-liking factor) \|\| id:ebi-fl187-GCST90094726 | Weighted mode | 21 | -0.128405936 | 0.181758376839798 | 0.488048382202344 | -0.484652355 | 0.227840482427825 | 0.879496287309707 | 0.615911278159162 | 1.25588497373102 | 0.999714303421225 |
| ebi-fl187-GCST90094726 | prot-a-914 | Histone-lysine N-methyltransferase EHMT2 \|\| id:prot-a-914 | F-capsicum liking (derived food-liking factor) \|\| id:ebi-fl187-GCST90094726 | Weighted mode | 21 | -0.062693164 | 0.180940784776581 | 0.73259740493995 | -0.417337102 | 0.291950774557612 | 0.939231619924806 | 0.658798800268362 | 1.33903710132324 | 0.999714303421225 |
| ebi-fl187-GCST90094726 | prot-a-932 | RNA polymerase II elongation factor ELL \|\| id:prot-a-932 | F-capsicum liking (derived food-liking factor) \|\| id:ebi-fl187-GCST90094726 | Weighted mode | 21 | 0.0223127956223583 | 0.167077876080979 | 0.895095226160068 | -0.305159841 | 0.349785432741076 | 1.02256358786515 | 0.737005560904456 | 1.41876309582309 | 0.999714303421225 |
| ebi-fl187-GCST90094726 | prot-a-991 | Estrogen receptor \|\| id:prot-a-991 | F-capsicum liking (derived food-liking factor) \|\| id:ebi-fl187-GCST90094726 | Weighted mode | 21 | 0.075585303876893 | 0.166062105055156 | 0.653894552576318 | -0.249896422 | 0.401067029784999 | 1.07851522532413 | 0.778881453852391 | 1.49341736859021 | 0.999714303421225 |
| ebi-fl187-GCST90094726 | prot-a-992 | Persulfide dioxygenase ETHE1, mitochondrial \|\| id:prot-a-992 | F-capsicum liking (derived food-liking factor) \|\| id:ebi-fl187-GCST90094726 | Weighted mode | 21 | -0.008848376 | 0.18210995418924 | 0.96172950143662 | -0.365783886 | 0.348087134436167 | 0.991190655895052 | 0.69365269308037 | 1.41635565771505 | 0.999714303421225 |
| ebi-fl187-GCST90094726 | prot-a-993 | Ethanolamine kinase 1 \|\| id:prot-a-993 | F-capsicum liking (derived food-liking factor) \|\| id:ebi-fl187-GCST90094726 | Weighted mode | 21 | -0.236452284 | 0.217123151655665 | 0.289092254349786 | -0.662013661 | 0.189109093419308 | 0.789423549674908 | 0.515811618249375 | 1.20817274899001 | 0.999714303421225 |
| ebi-fl187-GCST90094726 | prot-b-10 | coagulation factor III, tissue factor \|\| id:prot-b-10 | F-capsicum liking (derived food-liking factor) \|\| id:ebi-fl187-GCST90094726 | Weighted mode | 14 | 0.301217624668934 | 0.198663096914374 | 0.153396607347766 | -0.088162045 | 0.690597294621107 | 1.35150343002292 | 0.915612494015362 | 1.99490672451776 | 0.999714303421225 |
| ebi-fl187-GCST90094726 | prot-b-28 | prolactin \|\| id:prot-b-28 | F-capsicum liking (derived food-liking factor) \|\| id:ebi-fl187-GCST90094726 | Weighted mode | 14 | -0.100945028 | 0.20054720496727 | 0.623140023757471 | -0.49401755 | 0.292127493561253 | 0.903982725100547 | 0.610170074566547 | 1.33927375553566 | 0.999714303421225 |
| ebi-fl187-GCST90094726 | prot-b-54 | matrix metallopeptidase 7 \|\| id:prot-b-54 | F-capsicum liking (derived food-liking factor) \|\| id:ebi-fl187-GCST90094726 | Weighted mode | 14 | 0.246862529537382 | 0.194975284525987 | 0.227687974130195 | -0.135289028 | 0.629014087208317 | 1.28000313805934 | 0.873463419699226 | 1.87576033121793 | 0.999714303421225 |
| ebi-fl187-GCST90094726 | ukb-rsfMRI191-edge-pheno716 | Net100_Pair10_33 (OccipitalPrecuneus)&(Temporal\|Frontal\|Supp_Motor_Area) (Default_mode\|Central_executive)&(Default_mode\|Salience) \|\| id:ukb-rsfMRI191-edge-pheno716 | F-capsicum liking (derived food-liking factor) \|\| id:ebi-fl187-GCST90094726 | Weighted mode | 20 | -0.019547554 | 0.0673906400938437 | 0.774909746063885 | -0.151633209 | 0.112538100137411 | 0.980642260179904 | 0.859303407674767 | 1.11911489453179 | 0.999714303421225 |
| ebi-fl187-GCST90094726 | ukb-rsfMRI191-edge-pheno903 | Net100_Pair27_38 (Precentral\|Frontal\|Supp_Motor_Area)&(Subcortical) (Attention\|Salience\|Motor)&(Subcortical-cerebellum) \|\| id:ukb-rsfMRI191-edge-pheno903 | F-capsicum liking (derived food-liking factor) \|\| id:ebi-fl187-GCST90094726 | Weighted mode | 20 | 0.0371341093519784 | 0.0658452307720079 | 0.579377724779627 | -0.091922543 | 0.166190761665114 | 1.03783219450877 | 0.912175801245288 | 1.18079833129585 | 0.999714303421225 |
| ebi-fl187-GCST90094726 | ukb-rsfMRI191-node-pheno23 | Net100_Node2 RolandicOper\|SupraMarginal\|Insula Salience\|Motor \|\| id:ukb-rsfMRI191-node-pheno23 | F-capsicum liking (derived food-liking factor) \|\| id:ebi-fl187-GCST90094726 | Weighted mode | 20 | 0.0794779507316221 | 0.0822144177756673 | 0.345831231643656 | -0.081662308 | 0.24061820957193 | 1.08272168604521 | 0.921583117301408 | 1.27203529168946 | 0.999714303421225 |
| ebi-fl187-GCST90094726 | ukb-rsfMRI191-node-pheno55 | Net100_Node34 Parietal\|Postcentral\|Precuneus Attention \|\| id:ukb-rsfMRI191-node-pheno55 | F-capsicum liking (derived food-liking factor) \|\| id:ebi-fl187-GCST90094726 | Weighted mode | 20 | 0.0537150835622262 | 0.0815389396135301 | 0.517950479418606 | -0.106101238 | 0.213531405204745 | 1.05518392007878 | 0.899333596660198 | 1.23804237863194 | 0.999714303421225 |
| ebi-fl187-GCST90094729 | ebi-a-GCST90001414 | IgD- CD24- B cell Absolute Count \|\| id:ebi-a-GCST90001414 | F-cheese liking (derived food-liking factor) \|\| id:ebi-fl187-GCST90094729 | Inverse variance weighted | 14 | 0.0679166442764196 | 0.0908099253557175 | 0.454521134517176 | -0.110070809 | 0.245904097973626 | 1.07027609112243 | 0.895770704046079 | 1.27877693036207 | 0.998735809224318 |
| ebi-fl187-GCST90094729 | ebi-a-GCST90001422 | CD20- CD38- B cell %B cell \|\| id:ebi-a-GCST90001422 | F-cheese liking (derived food-liking factor) \|\| id:ebi-fl187-GCST90094729 | Inverse variance weighted | 14 | -0.062415637 | 0.0862223571407752 | 0.469132309611427 | -0.231411457 | 0.106580183053808 | 0.93949231791513 | 0.79341294360452 | 1.11246712388083 | 0.998735809224318 |
| ebi-fl187-GCST90094729 | ebi-a-GCST90001444 | CD20- CD38- B cell %lymphocyte \|\| id:ebi-a-GCST90001444 | F-cheese liking (derived food-liking factor) \|\| id:ebi-fl187-GCST90094729 | Inverse variance weighted | 14 | -0.058033866 | 0.086284907267216 | 0.501211854036413 | -0.227152284 | 0.111084552609816 | 0.943617990674244 | 0.796799433343194 | 1.11748938950435 | 0.998735809224318 |
| ebi-fl187-GCST90094729 | ebi-a-GCST90001459 | Myeloid Dendritic Cell %Dendritic Cell \|\| id:ebi-a-GCST90001459 | F-cheese liking (derived food-liking factor) \|\| id:ebi-fl187-GCST90094729 | Inverse variance weighted | 14 | 0.01540493586528 | 0.100603486493815 | 0.878299489604361 | -0.181777898 | 0.212587769393158 | 1.01552420353973 | 0.833786505783973 | 1.23687466854038 | 0.998735809224318 |
| ebi-fl187-GCST90094729 | ebi-a-GCST90001476 | HLA DR++ monocyte %leukocyte \|\| id:ebi-a-GCST90001476 | F-cheese liking (derived food-liking factor) \|\| id:ebi-fl187-GCST90094729 | Inverse variance weighted | 14 | 0.0256389680387213 | 0.0908611674286407 | 0.777807486215478 | -0.15244892 | 0.203726856198857 | 1.02597047346856 | 0.858602750160532 | 1.22596324345862 | 0.998735809224318 |
| ebi-fl187-GCST90094729 | ebi-a-GCST90001478 | CD4 regulatory T cell %CD4+ T cell \|\| id:ebi-a-GCST90001478 | F-cheese liking (derived food-liking factor) \|\| id:ebi-fl187-GCST90094729 | Inverse variance weighted | 14 | -0.029353116 | 0.0923894909224974 | 0.750704508997252 | -0.210436518 | 0.151730286376502 | 0.971073502490147 | 0.810230488540502 | 1.16384628889628 | 0.998735809224318 |
| ebi-fl187-GCST90094729 | ebi-a-GCST90001485 | CD39+ resting CD4 regulatory T cell %CD4 regulatory T cell \|\| id:ebi-a-GCST90001485 | F-cheese liking (derived food-liking factor) \|\| id:ebi-fl187-GCST90094729 | Inverse variance weighted | 13 | -0.045360522 | 0.095962072111676 | 0.636432799548974 | -0.233446183 | 0.142725139339928 | 0.955652885830904 | 0.791800206637866 | 1.1534127303084 | 0.998735809224318 |
| ebi-fl187-GCST90094729 | ebi-a-GCST90001515 | Immature Myeloid-Derived Suppressor Cells Absolute Count \|\| id:ebi-a-GCST90001515 | F-cheese liking (derived food-liking factor) \|\| id:ebi-fl187-GCST90094729 | Inverse variance weighted | 14 | -0.10403798 | 0.136962889220464 | 0.447489485871167 | -0.372485243 | 0.164409282532023 | 0.901191069227917 | 0.689019819202124 | 1.17869663632696 | 0.998735809224318 |
| ebi-fl187-GCST90094729 | ebi-a-GCST90001517 | CD33+ HLA DR+ Absolute Count \|\| id:ebi-a-GCST90001517 | F-cheese liking (derived food-liking factor) \|\| id:ebi-fl187-GCST90094729 | Inverse variance weighted | 14 | 0.110356959406232 | 0.136995214359929 | 0.420500409762493 | -0.158153661 | 0.378867579551693 | 1.11667660754264 | 0.853718588859667 | 1.46062960570947 | 0.998735809224318 |
| ebi-fl187-GCST90094729 | ebi-a-GCST90001518 | CD33+ HLA DR+ CD14- Absolute Count \|\| id:ebi-a-GCST90001518 | F-cheese liking (derived food-liking factor) \|\| id:ebi-fl187-GCST90094729 | Inverse variance weighted | 14 | 0.0635105045043092 | 0.137159293697557 | 0.643334310231891 | -0.205321711 | 0.332342720151521 | 1.06557067903447 | 0.814385277444877 | 1.39423059756238 | 0.998735809224318 |
| ebi-fl187-GCST90094729 | ebi-a-GCST90001520 | CD33+ HLA DR+ CD14dim Absolute Count \|\| id:ebi-a-GCST90001520 | F-cheese liking (derived food-liking factor) \|\| id:ebi-fl187-GCST90094729 | Inverse variance weighted | 14 | 0.137863745351496 | 0.13664144293661 | 0.313000855228353 | -0.129953483 | 0.405680973507251 | 1.14781914392487 | 0.878136278407648 | 1.50032383304955 | 0.998735809224318 |
| ebi-fl187-GCST90094729 | ebi-a-GCST90001531 | CD33dim HLA DR- Absolute Count \|\| id:ebi-a-GCST90001531 | F-cheese liking (derived food-liking factor) \|\| id:ebi-fl187-GCST90094729 | Inverse variance weighted | 14 | 0.0146108336868207 | 0.119754082934521 | 0.902893492412628 | -0.220107169 | 0.249328836238481 | 1.01471809366658 | 0.802432797542254 | 1.28316391449606 | 0.998735809224318 |
| ebi-fl187-GCST90094729 | ebi-a-GCST90001532 | Basophil Absolute Count \|\| id:ebi-a-GCST90001532 | F-cheese liking (derived food-liking factor) \|\| id:ebi-fl187-GCST90094729 | Inverse variance weighted | 14 | 0.00855673873948773 | 0.119340447319027 | 0.942840464996763 | -0.225350538 | 0.242464015484781 | 1.00859345226973 | 0.798236357530314 | 1.27438539019785 | 0.998735809224318 |
| ebi-fl187-GCST90094729 | ebi-a-GCST90001588 | T/B cell \|\| id:ebi-a-GCST90001588 | F-cheese liking (derived food-liking factor) \|\| id:ebi-fl187-GCST90094729 | Inverse variance weighted | 14 | -0.046930184 | 0.0895379385613558 | 0.600182965062687 | -0.222424543 | 0.128564175684413 | 0.954154010580797 | 0.800575413108965 | 1.13719439917836 | 0.998735809224318 |
| ebi-fl187-GCST90094729 | ebi-a-GCST90001624 | HLA DR+ CD4+ T cell Absolute Count \|\| id:ebi-a-GCST90001624 | F-cheese liking (derived food-liking factor) \|\| id:ebi-fl187-GCST90094729 | Inverse variance weighted | 14 | 0.0232809203277797 | 0.0869361440248366 | 0.788858397886926 | -0.147113922 | 0.19367576261646 | 1.02355403629886 | 0.863195634875373 | 1.21370269136608 | 0.998735809224318 |
| ebi-fl187-GCST90094729 | ebi-a-GCST90001631 | CD8+ Natural Killer T %T cell \|\| id:ebi-a-GCST90001631 | F-cheese liking (derived food-liking factor) \|\| id:ebi-fl187-GCST90094729 | Inverse variance weighted | 14 | -0.08333003 | 0.0952836079157682 | 0.381820226607305 | -0.270085902 | 0.1034258413423 | 0.92004745368892 | 0.763313921566524 | 1.10896355106723 | 0.998735809224318 |
| ebi-fl187-GCST90094729 | ebi-a-GCST90001632 | CD8+ Natural Killer T %lymphocyte \|\| id:ebi-a-GCST90001632 | F-cheese liking (derived food-liking factor) \|\| id:ebi-fl187-GCST90094729 | Inverse variance weighted | 14 | -0.088256934 | 0.0974645016891499 | 0.365185194371854 | -0.279287358 | 0.102773488867067 | 0.915525616436463 | 0.756322536839798 | 1.10824035186579 | 0.998735809224318 |
| ebi-fl187-GCST90094729 | ebi-a-GCST90001646 | Natural Killer %CD3- lymphocyte \|\| id:ebi-a-GCST90001646 | F-cheese liking (derived food-liking factor) \|\| id:ebi-fl187-GCST90094729 | Inverse variance weighted | 14 | 0.0539164181506572 | 0.0863968139676542 | 0.532591051856215 | -0.115421337 | 0.223254173527259 | 1.05539638648674 | 0.890990657277259 | 1.25013828541489 | 0.998735809224318 |
| ebi-fl187-GCST90094729 | ebi-a-GCST90001684 | CD127- CD8+ T cell Absolute Count \|\| id:ebi-a-GCST90001684 | F-cheese liking (derived food-liking factor) \|\| id:ebi-fl187-GCST90094729 | Inverse variance weighted | 14 | -0.030464024 | 0.0935296772288856 | 0.744639952758065 | -0.213782191 | 0.15285414342143 | 0.969995328042648 | 0.807524251664058 | 1.16515502102343 | 0.998735809224318 |
| ebi-fl187-GCST90094729 | ebi-a-GCST90001687 | CD28- CD8+ T cell Absolute Count \|\| id:ebi-a-GCST90001687 | F-cheese liking (derived food-liking factor) \|\| id:ebi-fl187-GCST90094729 | Inverse variance weighted | 14 | -0.061491188 | 0.0909957239172777 | 0.499193609266559 | -0.239842807 | 0.116860430817986 | 0.940361232109789 | 0.786751523228053 | 1.12396254820941 | 0.998735809224318 |
| ebi-fl187-GCST90094729 | ebi-a-GCST90001701 | BAFF-R on CD20- CD38- B cell \|\| id:ebi-a-GCST90001701 | F-cheese liking (derived food-liking factor) \|\| id:ebi-fl187-GCST90094729 | Inverse variance weighted | 14 | 0.0204453332954027 | 0.102491445144872 | 0.841884693323571 | -0.180437899 | 0.221328565779351 | 1.02065577083056 | 0.834904527335702 | 1.2477333256942 | 0.998735809224318 |
| ebi-fl187-GCST90094729 | ebi-a-GCST90001732 | CD19 on IgD- CD27- B cell \|\| id:ebi-a-GCST90001732 | F-cheese liking (derived food-liking factor) \|\| id:ebi-fl187-GCST90094729 | Inverse variance weighted | 14 | 0.00853239880446754 | 0.109623755604144 | 0.937960511020952 | -0.206330162 | 0.223394959788589 | 1.0085689034694 | 0.813564423732477 | 1.25031430010017 | 0.998735809224318 |
| ebi-fl187-GCST90094729 | ebi-a-GCST90001798 | CD27 on CD24+ CD27+ B cell \|\| id:ebi-a-GCST90001798 | F-cheese liking (derived food-liking factor) \|\| id:ebi-fl187-GCST90094729 | Inverse variance weighted | 14 | -0.048585757 | 0.0877915474209107 | 0.579974611935393 | -0.220657189 | 0.123485676393684 | 0.952575646200754 | 0.801991564303073 | 1.13143379821371 | 0.998735809224318 |
| ebi-fl187-GCST90094729 | ebi-a-GCST90001836 | CD66b on CD66b++ myeloid cell \|\| id:ebi-a-GCST90001836 | F-cheese liking (derived food-liking factor) \|\| id:ebi-fl187-GCST90094729 | Inverse variance weighted | 14 | -0.11790974 | 0.132867190895217 | 0.374849833265667 | -0.378329434 | 0.142509954330649 | 0.888776270096926 | 0.685004799634613 | 1.15316455988156 | 0.998735809224318 |
| ebi-fl187-GCST90094729 | ebi-a-GCST90001943 | CD25 on activated & secreting CD4 regulatory T cell \|\| id:ebi-a-GCST90001943 | F-cheese liking (derived food-liking factor) \|\| id:ebi-fl187-GCST90094729 | Inverse variance weighted | 14 | 0.121908673036445 | 0.0897956968055837 | 0.17458344791727 | -0.054090893 | 0.297908238775389 | 1.12965092947992 | 0.947345995750683 | 1.34703817633561 | 0.998735809224318 |
| ebi-fl187-GCST90094729 | ebi-a-GCST90001960 | CD25 on CD4+ T cell \|\| id:ebi-a-GCST90001960 | F-cheese liking (derived food-liking factor) \|\| id:ebi-fl187-GCST90094729 | Inverse variance weighted | 14 | -0.058458066 | 0.118059501485082 | 0.62048883940356 | -0.289854689 | 0.172938557130466 | 0.943217792672512 | 0.748372306637322 | 1.18879305998312 | 0.998735809224318 |
| ebi-fl187-GCST90094729 | ebi-a-GCST90001988 | HLA DR on CD14+ CD16- monocyte \|\| id:ebi-a-GCST90001988 | F-cheese liking (derived food-liking factor) \|\| id:ebi-fl187-GCST90094729 | Inverse variance weighted | 14 | -0.016088581 | 0.089386069488277 | 0.857160570103346 | -0.191285278 | 0.159108114772988 | 0.984040148516789 | 0.825896944625278 | 1.17246470058355 | 0.998735809224318 |
| ebi-fl187-GCST90094729 | ebi-a-GCST90001991 | HLA DR on CD14+ monocyte \|\| id:ebi-a-GCST90001991 | F-cheese liking (derived food-liking factor) \|\| id:ebi-fl187-GCST90094729 | Inverse variance weighted | 14 | -0.017346778 | 0.0895539339674586 | 0.846409209030578 | -0.192872489 | 0.158178932548825 | 0.982802811114446 | 0.824587111689902 | 1.17137577320963 | 0.998735809224318 |
| ebi-fl187-GCST90094729 | ebi-a-GCST90002019 | CD14 on Monocytic Myeloid-Derived Suppressor Cells \|\| id:ebi-a-GCST90002019 | F-cheese liking (derived food-liking factor) \|\| id:ebi-fl187-GCST90094729 | Inverse variance weighted | 14 | -0.086492837 | 0.128545712620215 | 0.501038497725488 | -0.338442433 | 0.165456760053135 | 0.917142118541376 | 0.712879816262023 | 1.17993194142194 | 0.998735809224318 |
| ebi-fl187-GCST90094729 | ebi-a-GCST90002037 | CD80 on plasmacytoid Dendritic Cell \|\| id:ebi-a-GCST90002037 | F-cheese liking (derived food-liking factor) \|\| id:ebi-fl187-GCST90094729 | Inverse variance weighted | 14 | -0.07087722 | 0.0998771230235628 | 0.47792481240141 | -0.266636381 | 0.124881941582493 | 0.931576264464943 | 0.765951535619123 | 1.13301468325014 | 0.998735809224318 |
| ebi-fl187-GCST90094729 | ebi-a-GCST90002042 | CD45 on CD33+ HLA DR+ CD14- \|\| id:ebi-a-GCST90002042 | F-cheese liking (derived food-liking factor) \|\| id:ebi-fl187-GCST90094729 | Inverse variance weighted | 14 | -0.266368218 | 0.145257057240682 | 0.0666879542351949 | -0.55107205 | 0.0183356139649458 | 0.766156962596866 | 0.576331622518284 | 1.01850474345095 | 0.998735809224318 |
| ebi-fl187-GCST90094729 | ebi-a-GCST90002056 | CD8 on Effector Memory CD8+ T cell \|\| id:ebi-a-GCST90002056 | F-cheese liking (derived food-liking factor) \|\| id:ebi-fl187-GCST90094729 | Inverse variance weighted | 14 | -0.136591206 | 0.0979081138287689 | 0.162986928476926 | -0.328491109 | 0.0553086968979503 | 0.872326755017157 | 0.720009329576517 | 1.05686681583186 | 0.998735809224318 |
| ebi-fl187-GCST90094729 | ebi-a-GCST90002058 | CD8 on CD8+ T cell \|\| id:ebi-a-GCST90002058 | F-cheese liking (derived food-liking factor) \|\| id:ebi-fl187-GCST90094729 | Inverse variance weighted | 14 | -0.145167569 | 0.0943225584251645 | 0.123790961983258 | -0.330039784 | 0.0397046450414731 | 0.864877353925977 | 0.718895132349712 | 1.0405034109622 | 0.998735809224318 |
| ebi-fl187-GCST90094729 | ebi-a-GCST90002063 | CD4 on CD4 regulatory T cell \|\| id:ebi-a-GCST90002063 | F-cheese liking (derived food-liking factor) \|\| id:ebi-fl187-GCST90094729 | Inverse variance weighted | 14 | -0.261301718 | 0.107156345547063 | 0.0147479893905361 | -0.471328156 | -0.051275281 | 0.770048546728743 | 0.62417271896833 | 0.950017112729875 | 0.998735809224318 |
| ebi-fl187-GCST90094729 | ebi-a-GCST90002066 | CD4 on activated CD4 regulatory T cell \|\| id:ebi-a-GCST90002066 | F-cheese liking (derived food-liking factor) \|\| id:ebi-fl187-GCST90094729 | Inverse variance weighted | 14 | -0.288008662 | 0.105746393480718 | 0.00645787585257776 | -0.495271593 | -0.080745731 | 0.749755097741294 | 0.609405374375315 | 0.922428206619091 | 0.998735809224318 |
| ebi-fl187-GCST90094729 | ebi-a-GCST90002070 | CD4 on activated & secreting CD4 regulatory T cell \|\| id:ebi-a-GCST90002070 | F-cheese liking (derived food-liking factor) \|\| id:ebi-fl187-GCST90094729 | Inverse variance weighted | 14 | -0.258995661 | 0.108821208250086 | 0.0173121264849422 | -0.472285229 | -0.045706092 | 0.77182637233298 | 0.623575625867647 | 0.955322697547399 | 0.998735809224318 |
| ebi-fl187-GCST90094729 | ebi-a-GCST90002081 | SSC-A on CD4+ T cell \|\| id:ebi-a-GCST90002081 | F-cheese liking (derived food-liking factor) \|\| id:ebi-fl187-GCST90094729 | Inverse variance weighted | 14 | -0.088938626 | 0.0923021666415946 | 0.335266837998884 | -0.269850873 | 0.0919736203973375 | 0.914901722828275 | 0.763493343443377 | 1.09633590079391 | 0.998735809224318 |
| ebi-fl187-GCST90094729 | ebi-a-GCST90002094 | CD11b on Monocytic Myeloid-Derived Suppressor Cells \|\| id:ebi-a-GCST90002094 | F-cheese liking (derived food-liking factor) \|\| id:ebi-fl187-GCST90094729 | Inverse variance weighted | 14 | -0.071793046 | 0.13015456717201 | 0.581223557993643 | -0.326895998 | 0.183309905421007 | 0.930723492611118 | 0.721158741154924 | 1.20118660464541 | 0.998735809224318 |
| ebi-fl187-GCST90094729 | ebi-a-GCST90002104 | HLA DR on myeloid Dendritic Cell \|\| id:ebi-a-GCST90002104 | F-cheese liking (derived food-liking factor) \|\| id:ebi-fl187-GCST90094729 | Inverse variance weighted | 14 | -0.118627811 | 0.099138158208747 | 0.231466075442282 | -0.312938601 | 0.0756829788267164 | 0.888138294325698 | 0.731294811703536 | 1.07862057438954 | 0.998735809224318 |
| ebi-fl187-GCST90094729 | ebi-a-GCST90002105 | HLA DR on plasmacytoid Dendritic Cell \|\| id:ebi-a-GCST90002105 | F-cheese liking (derived food-liking factor) \|\| id:ebi-fl187-GCST90094729 | Inverse variance weighted | 14 | -0.11024386 | 0.121767954814011 | 0.365274594343683 | -0.348909051 | 0.128421331699976 | 0.895615704055631 | 0.705457287866659 | 1.13703196940064 | 0.998735809224318 |
| ebi-fl187-GCST90094729 | ebi-a-GCST90002106 | HLA DR on Dendritic Cell \|\| id:ebi-a-GCST90002106 | F-cheese liking (derived food-liking factor) \|\| id:ebi-fl187-GCST90094729 | Inverse variance weighted | 14 | -0.08869232 | 0.1191079906565 | 0.45649066789398 | -0.322143982 | 0.144759341225125 | 0.915127096145369 | 0.724593854238997 | 1.15576139267562 | 0.998735809224318 |
| ebi-fl187-GCST90094729 | ebi-a-GCST90002111 | HLA DR on CD33dim HLA DR+ CD11b- \|\| id:ebi-a-GCST90002111 | F-cheese liking (derived food-liking factor) \|\| id:ebi-fl187-GCST90094729 | Inverse variance weighted | 14 | -0.22967572 | 0.127293025053726 | 0.0711831642896621 | -0.479170049 | 0.0198186095960915 | 0.794791296030135 | 0.619297165113428 | 1.02001630207618 | 0.998735809224318 |
| ebi-fl187-GCST90094729 | ebi-a-GCST90002112 | HLA DR on CD33- HLA DR+ \|\| id:ebi-a-GCST90002112 | F-cheese liking (derived food-liking factor) \|\| id:ebi-fl187-GCST90094729 | Inverse variance weighted | 14 | -0.09230748 | 0.12864659024376 | 0.473048606442343 | -0.344454797 | 0.15983983644056 | 0.911824738167643 | 0.70860658238305 | 1.17332293236453 | 0.998735809224318 |
| ebi-fl187-GCST90094729 | ebi-a-GCST90002115 | HLA DR on HLA DR+ CD8+ T cell \|\| id:ebi-a-GCST90002115 | F-cheese liking (derived food-liking factor) \|\| id:ebi-fl187-GCST90094729 | Inverse variance weighted | 14 | -0.02039692 | 0.092485886944572 | 0.82544992719241 | -0.201669259 | 0.160875418188842 | 0.979809689834043 | 0.817365219730828 | 1.17453863354846 | 0.998735809224318 |
| ebi-fl187-GCST90094729 | ebi-a-GCST90002116 | HLA DR on B cell \|\| id:ebi-a-GCST90002116 | F-cheese liking (derived food-liking factor) \|\| id:ebi-fl187-GCST90094729 | Inverse variance weighted | 14 | 0.105039754838571 | 0.0959517106656432 | 0.273641615942765 | -0.083025598 | 0.293105107743232 | 1.11075476735444 | 0.920327588312165 | 1.34058368875294 | 0.998735809224318 |
| ebi-fl187-GCST90094729 | ebi-cfmet338-GCST90026023 | Sphingomyelin (d18:1/24:1, d18:2/24:0) levels \|\| id:ebi-cfmet338-GCST90026023 | F-cheese liking (derived food-liking factor) \|\| id:ebi-fl187-GCST90094729 | Inverse variance weighted | 15 | -0.039925701 | 0.0556726637206595 | 0.47328098824566 | -0.149044122 | 0.069192719687776 | 0.960860827302167 | 0.86153110150134 | 1.07164271589836 | 0.998735809224318 |
| ebi-fl187-GCST90094729 | ebi-cfmet338-GCST90026046 | 1-arachidonoyl-gpc (20:4n6) levels \|\| id:ebi-cfmet338-GCST90026046 | F-cheese liking (derived food-liking factor) \|\| id:ebi-fl187-GCST90094729 | Inverse variance weighted | 15 | -0.023082444 | 0.0788054819083733 | 0.769595497826098 | -0.177541189 | 0.131376300213986 | 0.977181917347229 | 0.837326510095348 | 1.14039683215293 | 0.998735809224318 |
| ebi-fl187-GCST90094729 | ebi-cfmet338-GCST90026047 | 1-linoleoyl-gpc (18:2) levels \|\| id:ebi-cfmet338-GCST90026047 | F-cheese liking (derived food-liking factor) \|\| id:ebi-fl187-GCST90094729 | Inverse variance weighted | 15 | -0.032862729 | 0.0916978205040162 | 0.720058431608022 | -0.212590457 | 0.1468649989228 | 0.967671383434384 | 0.808487179322631 | 1.15819759455226 | 0.998735809224318 |
| ebi-fl187-GCST90094729 | ebi-cfmet338-GCST90026050 | 1-myristoyl-2-palmitoyl-gpc (14:0/16:0) levels \|\| id:ebi-cfmet338-GCST90026050 | F-cheese liking (derived food-liking factor) \|\| id:ebi-fl187-GCST90094729 | Inverse variance weighted | 15 | -0.044605101 | 0.0395229838939584 | 0.259072358982038 | -0.12207015 | 0.0328599471197985 | 0.956375078535377 | 0.885086277748567 | 1.03340579764743 | 0.998735809224318 |
| ebi-fl187-GCST90094729 | ebi-cfmet338-GCST90026068 | 2,3-dihydroxyisovalerate levels \|\| id:ebi-cfmet338-GCST90026068 | F-cheese liking (derived food-liking factor) \|\| id:ebi-fl187-GCST90094729 | Inverse variance weighted | 15 | -0.119064393 | 0.128435681118119 | 0.353908275429568 | -0.370798328 | 0.13266954183381 | 0.887750633854824 | 0.690183118031122 | 1.14187259485258 | 0.998735809224318 |
| ebi-fl187-GCST90094729 | ebi-cfmet338-GCST90026082 | 3-hydroxyhexanoate levels \|\| id:ebi-cfmet338-GCST90026082 | F-cheese liking (derived food-liking factor) \|\| id:ebi-fl187-GCST90094729 | Inverse variance weighted (multiplicative random effects) | 15 | 0.0313917234745548 | 0.0356732633103537 | 0.378870672278271 | -0.038527873 | 0.101311319562848 | 1.03188964012172 | 0.962204885200951 | 1.10662110093958 | 0.998735809224318 |
| ebi-fl187-GCST90094729 | ebi-cfmet338-GCST90026094 | 3-ureidopropionate levels \|\| id:ebi-cfmet338-GCST90026094 | F-cheese liking (derived food-liking factor) \|\| id:ebi-fl187-GCST90094729 | Inverse variance weighted | 15 | -0.099088826 | 0.0663637493398656 | 0.135406629275551 | -0.229161774 | 0.0309841231105329 | 0.905662258461088 | 0.795199880193613 | 1.0314691272352 | 0.998735809224318 |
| ebi-fl187-GCST90094729 | ebi-cfmet338-GCST90026173 | Glycerol levels \|\| id:ebi-cfmet338-GCST90026173 | F-cheese liking (derived food-liking factor) \|\| id:ebi-fl187-GCST90094729 | Inverse variance weighted | 15 | -0.007695362 | 0.035358808136635 | 0.827712457462677 | -0.076998626 | 0.0616079018858704 | 0.992334171431167 | 0.925891125771011 | 1.06354524887575 | 0.998735809224318 |
| ebi-fl187-GCST90094729 | ebi-cfmet338-GCST90026179 | Histidine levels \|\| id:ebi-cfmet338-GCST90026179 | F-cheese liking (derived food-liking factor) \|\| id:ebi-fl187-GCST90094729 | Inverse variance weighted | 15 | 0.0159201128350275 | 0.0244940936745346 | 0.515719860682411 | -0.032088311 | 0.0639285364371154 | 1.01604751300853 | 0.96842105629651 | 1.06601621472255 | 0.998735809224318 |
| ebi-fl187-GCST90094729 | ebi-cfmet338-GCST90026188 | Isocitrate levels \|\| id:ebi-cfmet338-GCST90026188 | F-cheese liking (derived food-liking factor) \|\| id:ebi-fl187-GCST90094729 | Inverse variance weighted | 15 | -0.006426244 | 0.048006437095358 | 0.893511599287381 | -0.10051886 | 0.0876663729475187 | 0.993594360385666 | 0.904368055448701 | 1.09162386601591 | 0.998735809224318 |
| ebi-fl187-GCST90094729 | ebi-cfmet338-GCST90026207 | N-acetyl-isoputreanine levels \|\| id:ebi-cfmet338-GCST90026207 | F-cheese liking (derived food-liking factor) \|\| id:ebi-fl187-GCST90094729 | Inverse variance weighted | 15 | -0.055565202 | 0.03056343562979 | 0.0690597511939788 | -0.115469536 | 0.00433913165943438 | 0.945950343639817 | 0.890947713646483 | 1.00434855932224 | 0.998735809224318 |
| ebi-fl187-GCST90094729 | ebi-cfmet338-GCST90026213 | N-acetylglutamate levels \|\| id:ebi-cfmet338-GCST90026213 | F-cheese liking (derived food-liking factor) \|\| id:ebi-fl187-GCST90094729 | Inverse variance weighted | 15 | -0.052358952 | 0.0533756243163696 | 0.326616170134464 | -0.156975175 | 0.052257271993231 | 0.948988164815183 | 0.854725276829874 | 1.05364678145413 | 0.998735809224318 |
| ebi-fl187-GCST90094729 | ebi-cfmet338-GCST90026224 | N-acetyltaurine levels \|\| id:ebi-cfmet338-GCST90026224 | F-cheese liking (derived food-liking factor) \|\| id:ebi-fl187-GCST90094729 | Inverse variance weighted | 15 | -0.019665137 | 0.0417982841658044 | 0.638014159987939 | -0.101589774 | 0.0622594996082839 | 0.980526960187904 | 0.903400073572866 | 1.06423847836646 | 0.998735809224318 |
| ebi-fl187-GCST90094729 | ebi-cfmet338-GCST90026250 | Phenyllactate (pla) levels \|\| id:ebi-cfmet338-GCST90026250 | F-cheese liking (derived food-liking factor) \|\| id:ebi-fl187-GCST90094729 | Inverse variance weighted | 15 | -0.036270943 | 0.0659378531480479 | 0.582266072559135 | -0.165509136 | 0.0929672487329476 | 0.964378965930411 | 0.847462121088333 | 1.0974257925943 | 0.998735809224318 |
| ebi-fl187-GCST90094729 | ebi-cfmet338-GCST90026251 | Pipecolate levels \|\| id:ebi-cfmet338-GCST90026251 | F-cheese liking (derived food-liking factor) \|\| id:ebi-fl187-GCST90094729 | Inverse variance weighted (multiplicative random effects) | 15 | -0.065288244 | 0.132656001123068 | 0.622604818150639 | -0.325294006 | 0.194717518013364 | 0.936797398051713 | 0.722314957208314 | 1.21496773151184 | 0.998735809224318 |
| ebi-fl187-GCST90094729 | ebi-cfmet338-GCST90026278 | Trigonelline (n'-methylnicotinate) levels \|\| id:ebi-cfmet338-GCST90026278 | F-cheese liking (derived food-liking factor) \|\| id:ebi-fl187-GCST90094729 | Inverse variance weighted | 15 | -0.034441259 | 0.11104205475637 | 0.756436170297498 | -0.252083686 | 0.183201168501112 | 0.966145090521012 | 0.777179696175574 | 1.20105599841479 | 0.998735809224318 |
| ebi-fl187-GCST90094729 | ebi-cfmet338-GCST90026299 | 1-carboxyethylvaline levels \|\| id:ebi-cfmet338-GCST90026299 | F-cheese liking (derived food-liking factor) \|\| id:ebi-fl187-GCST90094729 | Inverse variance weighted | 15 | -0.121305229 | 0.0668457718292911 | 0.0695695486442583 | -0.252322942 | 0.00971248352631612 | 0.885763557373291 | 0.776993773589826 | 1.00975980276615 | 0.998735809224318 |
| ebi-fl187-GCST90094729 | ebi-gm430-GCST90011306 | C_Erysipelotrichia abundance \|\| id:ebi-gm430-GCST90011306 | F-cheese liking (derived food-liking factor) \|\| id:ebi-fl187-GCST90094729 | Inverse variance weighted | 14 | 0.0294201585807629 | 0.0562467276009554 | 0.600935651998468 | -0.080823428 | 0.139663744678636 | 1.0298572069283 | 0.922356539851263 | 1.14988707819342 | 0.998735809224318 |
| ebi-fl187-GCST90094729 | ebi-gm430-GCST90011312 | F_Erysipelotrichaceae abundance \|\| id:ebi-gm430-GCST90011312 | F-cheese liking (derived food-liking factor) \|\| id:ebi-fl187-GCST90094729 | Inverse variance weighted | 14 | 0.0294201585807629 | 0.0562467276009554 | 0.600935651998468 | -0.080823428 | 0.139663744678636 | 1.0298572069283 | 0.922356539851263 | 1.14988707819342 | 0.998735809224318 |
| ebi-fl187-GCST90094729 | ebi-gm430-GCST90011352 | O_Erysipelotrichales abundance \|\| id:ebi-gm430-GCST90011352 | F-cheese liking (derived food-liking factor) \|\| id:ebi-fl187-GCST90094729 | Inverse variance weighted | 14 | 0.0294201585807629 | 0.0562467276009554 | 0.600935651998468 | -0.080823428 | 0.139663744678636 | 1.0298572069283 | 0.922356539851263 | 1.14988707819342 | 0.998735809224318 |
| ebi-fl187-GCST90094729 | ebi-gm430-GCST90011362 | OTU97_11 (Parabacteroides) abundance \|\| id:ebi-gm430-GCST90011362 | F-cheese liking (derived food-liking factor) \|\| id:ebi-fl187-GCST90094729 | Inverse variance weighted | 14 | -0.114463178 | 0.0735493086217757 | 0.11964197919242 | -0.258619822 | 0.0296934673772817 | 0.891844777748753 | 0.772116508637518 | 1.0301387144296 | 0.998735809224318 |
| ebi-fl187-GCST90094729 | ebi-gm430-GCST90011384 | OTU97_235 (Phascolarctobacterium) abundance \|\| id:ebi-gm430-GCST90011384 | F-cheese liking (derived food-liking factor) \|\| id:ebi-fl187-GCST90094729 | Inverse variance weighted | 14 | 0.303035352175419 | 0.281508055827907 | 0.281716514870686 | -0.248720437 | 0.854791141598118 | 1.35396232910999 | 0.779797945375031 | 2.35088332756161 | 0.998735809224318 |
| ebi-fl187-GCST90094729 | ebi-gm430-GCST90011390 | OTU97_3 (Bacteroides) abundance \|\| id:ebi-gm430-GCST90011390 | F-cheese liking (derived food-liking factor) \|\| id:ebi-fl187-GCST90094729 | Inverse variance weighted | 14 | -0.081401534 | 0.0587648617278779 | 0.16598899329881 | -0.196580663 | 0.0337775951043483 | 0.921823473763902 | 0.821535061247554 | 1.03435453563203 | 0.998735809224318 |
| ebi-fl187-GCST90094729 | ebi-gm430-GCST90011398 | OTU97_4 (Alistipes) abundance \|\| id:ebi-gm430-GCST90011398 | F-cheese liking (derived food-liking factor) \|\| id:ebi-fl187-GCST90094729 | Inverse variance weighted | 14 | -0.027842461 | 0.0682817689130034 | 0.68345109822419 | -0.161674728 | 0.105989806008641 | 0.972541567900251 | 0.850717874134855 | 1.1118105426617 | 0.998735809224318 |
| ebi-fl187-GCST90094729 | ebi-gm430-GCST90011421 | OTU99_108 (Cytophagales) abundance \|\| id:ebi-gm430-GCST90011421 | F-cheese liking (derived food-liking factor) \|\| id:ebi-fl187-GCST90094729 | Inverse variance weighted | 14 | 0.154862591789183 | 0.212161528070799 | 0.465434334613473 | -0.260974003 | 0.570699186807948 | 1.16749752633662 | 0.770300944691596 | 1.76950383274923 | 0.998735809224318 |
| ebi-fl187-GCST90094729 | ebi-gm430-GCST90011428 | OTU99_123 (Phascolarctobacterium) abundance \|\| id:ebi-gm430-GCST90011428 | F-cheese liking (derived food-liking factor) \|\| id:ebi-fl187-GCST90094729 | Inverse variance weighted | 14 | -0.118980688 | 0.129182264448673 | 0.357034979913782 | -0.372177926 | 0.134216550599575 | 0.887824946520501 | 0.689231599365256 | 1.14364044885645 | 0.998735809224318 |
| ebi-fl187-GCST90094729 | ebi-gm430-GCST90011450 | OTU99_298 (Phascolarctobacterium) abundance \|\| id:ebi-gm430-GCST90011450 | F-cheese liking (derived food-liking factor) \|\| id:ebi-fl187-GCST90094729 | Inverse variance weighted | 14 | 0.300750655014044 | 0.281951187311192 | 0.286118023573608 | -0.251873672 | 0.85337498214398 | 1.35087246626455 | 0.777342931954323 | 2.34755645815649 | 0.998735809224318 |
| ebi-fl187-GCST90094729 | ebi-gm430-GCST90011451 | OTU99_3 (Bacteroides) abundance \|\| id:ebi-gm430-GCST90011451 | F-cheese liking (derived food-liking factor) \|\| id:ebi-fl187-GCST90094729 | Inverse variance weighted | 14 | -0.082911153 | 0.0585778559338516 | 0.156950972757111 | -0.19772375 | 0.0319014449927896 | 0.920432921624741 | 0.820596511395676 | 1.03241575054924 | 0.998735809224318 |
| ebi-fl187-GCST90094729 | ebi-gm430-GCST90011472 | OTU99_72 (Parabacteroides) abundance \|\| id:ebi-gm430-GCST90011472 | F-cheese liking (derived food-liking factor) \|\| id:ebi-fl187-GCST90094729 | Inverse variance weighted | 14 | -0.294630969 | 0.173830898055688 | 0.0900889576489544 | -0.635339529 | 0.0460775915992515 | 0.744806393710447 | 0.529755590510216 | 1.04715565828703 | 0.998735809224318 |
| ebi-fl187-GCST90094729 | ebi-gm430-GCST90011507 | TestASV_3 (Bacteroides) abundance \|\| id:ebi-gm430-GCST90011507 | F-cheese liking (derived food-liking factor) \|\| id:ebi-fl187-GCST90094729 | Inverse variance weighted | 14 | -0.12177455 | 0.0748150488286311 | 0.103593913576832 | -0.268412046 | 0.0248629452317098 | 0.885347947280781 | 0.764592668701156 | 1.02517460582749 | 0.998735809224318 |
| ebi-fl187-GCST90094729 | ebi-gm430-GCST90011512 | TestASV_34 (Phascolarctobacterium) abundance \|\| id:ebi-gm430-GCST90011512 | F-cheese liking (derived food-liking factor) \|\| id:ebi-fl187-GCST90094729 | Inverse variance weighted | 14 | -0.371899506 | 0.186568184723259 | 0.0462208971705444 | -0.737573148 | -0.006225864 | 0.689423521731276 | 0.478273206332514 | 0.993793476245679 | 0.998735809224318 |
| ebi-fl187-GCST90094729 | ebi-gm430-GCST90011519 | TestASV_41 (Barnesiella) abundance \|\| id:ebi-gm430-GCST90011519 | F-cheese liking (derived food-liking factor) \|\| id:ebi-fl187-GCST90094729 | Inverse variance weighted (multiplicative random effects) | 14 | -0.101042543 | 0.364923962765259 | 0.7818672078975 | -0.81629351 | 0.614208423952116 | 0.903894577619575 | 0.44206713907164 | 1.84819303503504 | 0.998735809224318 |
| ebi-fl187-GCST90094729 | ebi-gm430-GCST90011524 | TestASV_46 (Sutterella) abundance \|\| id:ebi-gm430-GCST90011524 | F-cheese liking (derived food-liking factor) \|\| id:ebi-fl187-GCST90094729 | Inverse variance weighted | 14 | 0.071253222778409 | 0.264158918131566 | 0.78736320883344 | -0.446498257 | 0.589004702316279 | 1.07385311551209 | 0.639864875622671 | 1.80219380314141 | 0.998735809224318 |
| ebi-fl187-GCST90094729 | ebi-gm430-GCST90011581 | OTU97_19 (Bacteria) prevalence \|\| id:ebi-gm430-GCST90011581 | F-cheese liking (derived food-liking factor) \|\| id:ebi-fl187-GCST90094729 | Inverse variance weighted | 14 | -0.157607879 | 0.273824708958729 | 0.564899464651251 | -0.694304309 | 0.379088550185799 | 0.854184659732035 | 0.499421770421207 | 1.46095239762209 | 0.998735809224318 |
| ebi-fl187-GCST90094729 | ebi-gm430-GCST90011614 | OTU97_80 (Ruminococcus) prevalence \|\| id:ebi-gm430-GCST90011614 | F-cheese liking (derived food-liking factor) \|\| id:ebi-fl187-GCST90094729 | Inverse variance weighted | 14 | 0.158952791178104 | 0.146388265503021 | 0.277554148043361 | -0.127968209 | 0.445873791564024 | 1.17228260329375 | 0.87988135082519 | 1.56185433490134 | 0.998735809224318 |
| ebi-fl187-GCST90094729 | ebi-gm430-GCST90011623 | OTU99_11 (Parabacteroides) prevalence \|\| id:ebi-gm430-GCST90011623 | F-cheese liking (derived food-liking factor) \|\| id:ebi-fl187-GCST90094729 | Inverse variance weighted (multiplicative random effects) | 14 | 0.27915606879915 | 0.157758545237831 | 0.0768080156459625 | -0.03005068 | 0.588362817465298 | 1.32201365285318 | 0.970396352744188 | 1.80103737342771 | 0.998735809224318 |
| ebi-fl187-GCST90094729 | ebi-gm430-GCST90011642 | OTU99_19 (Bacteria) prevalence \|\| id:ebi-gm430-GCST90011642 | F-cheese liking (derived food-liking factor) \|\| id:ebi-fl187-GCST90094729 | Inverse variance weighted | 14 | -0.157607879 | 0.273824708958729 | 0.564899464651251 | -0.694304309 | 0.379088550185799 | 0.854184659732035 | 0.499421770421207 | 1.46095239762209 | 0.998735809224318 |
| ebi-fl187-GCST90094729 | ebi-gm430-GCST90011653 | OTU99_30 (Parasutterella) prevalence \|\| id:ebi-gm430-GCST90011653 | F-cheese liking (derived food-liking factor) \|\| id:ebi-fl187-GCST90094729 | Inverse variance weighted (multiplicative random effects) | 14 | -0.089339773 | 0.172009598366699 | 0.603489986114062 | -0.426478586 | 0.247799039300747 | 0.914534786095469 | 0.652803844373712 | 1.28120243498426 | 0.998735809224318 |
| ebi-fl187-GCST90094729 | ebi-gm430-GCST90011675 | OTU99_8 (Ruminococcaceae) prevalence \|\| id:ebi-gm430-GCST90011675 | F-cheese liking (derived food-liking factor) \|\| id:ebi-fl187-GCST90094729 | Inverse variance weighted | 14 | 0.209874409942403 | 0.128961380101301 | 0.103647793798878 | -0.042889895 | 0.462638714940953 | 1.23352313198705 | 0.95801686665349 | 1.5882594243484 | 0.998735809224318 |
| ebi-fl187-GCST90094729 | ebi-gm430-GCST90011679 | OTU99_92 (Ruminococcus) prevalence \|\| id:ebi-gm430-GCST90011679 | F-cheese liking (derived food-liking factor) \|\| id:ebi-fl187-GCST90094729 | Inverse variance weighted | 14 | 0.193158388433096 | 0.145333486384341 | 0.18382485018633 | -0.091695245 | 0.478012021746403 | 1.21307491533885 | 0.912383160619697 | 1.61286487271956 | 0.998735809224318 |
| ebi-fl187-GCST90094729 | ebi-gm430-GCST90011684 | TestASV_11 (Lachnospiraceae) prevalence \|\| id:ebi-gm430-GCST90011684 | F-cheese liking (derived food-liking factor) \|\| id:ebi-fl187-GCST90094729 | Inverse variance weighted | 14 | 0.0844635307624734 | 0.135815592737676 | 0.534008527452395 | -0.181735031 | 0.350662092528318 | 1.08813316012171 | 0.833822248192057 | 1.42000741371886 | 0.998735809224318 |
| ebi-fl187-GCST90094729 | ebi-gm430-GCST90011688 | TestASV_15 (Bacteroides) prevalence \|\| id:ebi-gm430-GCST90011688 | F-cheese liking (derived food-liking factor) \|\| id:ebi-fl187-GCST90094729 | Inverse variance weighted | 14 | -0.023517014 | 0.13112681561026 | 0.857666362824893 | -0.280525573 | 0.233491544380864 | 0.976757355767571 | 0.755386626435521 | 1.26300214838063 | 0.998735809224318 |
| ebi-fl187-GCST90094729 | ebi-gm430-GCST90011692 | TestASV_19 (Prevotella) prevalence \|\| id:ebi-gm430-GCST90011692 | F-cheese liking (derived food-liking factor) \|\| id:ebi-fl187-GCST90094729 | Inverse variance weighted | 13 | -0.041901588 | 0.254882627582508 | 0.869419710877617 | -0.541471538 | 0.457668362303882 | 0.95896415 | 0.581891346920366 | 1.58038480096926 | 0.998735809224318 |
| ebi-fl187-GCST90094729 | ebi-gm430-GCST90011693 | TestASV_2 (Bacteroides) prevalence \|\| id:ebi-gm430-GCST90011693 | F-cheese liking (derived food-liking factor) \|\| id:ebi-fl187-GCST90094729 | Inverse variance weighted | 14 | 0.069565861268033 | 0.125905924972794 | 0.580590396975939 | -0.177209752 | 0.31634147421471 | 1.07204266496902 | 0.83760407723495 | 1.37209871196878 | 0.998735809224318 |
| ebi-fl187-GCST90094729 | ebi-gm430-GCST90011716 | TestASV_40 (Barnesiella) prevalence \|\| id:ebi-gm430-GCST90011716 | F-cheese liking (derived food-liking factor) \|\| id:ebi-fl187-GCST90094729 | Inverse variance weighted | 14 | 0.523658349243341 | 0.276728120600912 | 0.0584482653468745 | -0.018728767 | 1.06604546562113 | 1.68819236374145 | 0.981445526427455 | 2.90387329734863 | 0.998735809224318 |
| ebi-fl187-GCST90094729 | ebi-gm430-GCST90011718 | TestASV_42 (Alphaproteobacteria) prevalence \|\| id:ebi-gm430-GCST90011718 | F-cheese liking (derived food-liking factor) \|\| id:ebi-fl187-GCST90094729 | Inverse variance weighted | 14 | 0.182703728375872 | 0.213757614918876 | 0.392704052142581 | -0.236261197 | 0.601668653616869 | 1.20045869354254 | 0.78957441263521 | 1.82516182368698 | 0.998735809224318 |
| ebi-fl187-GCST90094729 | ebi-hbsm44-GCST90104929 | Sulcal depth of the right inferior frontal sulcus \|\| id:ebi-hbsm44-GCST90104929 | F-cheese liking (derived food-liking factor) \|\| id:ebi-fl187-GCST90094729 | Inverse variance weighted | 13 | -0.027794139 | 0.123555276877223 | 0.822015779418169 | -0.269962482 | 0.214374203 | 0.972588563803722 | 0.763408135241628 | 1.23908623811349 | 0.998735809224318 |
| ebi-fl187-GCST90094729 | ebi-hbsm44-GCST90104935 | Sulcal opening distance of the left calloso-marginal posterior fissure \|\| id:ebi-hbsm44-GCST90104935 | F-cheese liking (derived food-liking factor) \|\| id:ebi-fl187-GCST90094729 | Inverse variance weighted (multiplicative random effects) | 13 | 0.0243136407137576 | 0.0357135331444324 | 0.496000400633776 | -0.045684884 | 0.094312165676845 | 1.02461162742226 | 0.955342958377381 | 1.09890273209528 | 0.998735809224318 |
| ebi-fl187-GCST90094729 | ebi-hbsm44-GCST90104936 | Sulcal opening distance of the left anterior inferior temporal sulcus \|\| id:ebi-hbsm44-GCST90104936 | F-cheese liking (derived food-liking factor) \|\| id:ebi-fl187-GCST90094729 | Inverse variance weighted | 13 | 0.00913111838389909 | 0.0142997285030343 | 0.523114499945852 | -0.018896349 | 0.0371585862498463 | 1.00917293422359 | 0.981281067262704 | 1.03785759773229 | 0.998735809224318 |
| ebi-fl187-GCST90094729 | ebi-hbsm44-GCST90104941 | Sulcal opening distance of the right posterior inferior temporal sulcus \|\| id:ebi-hbsm44-GCST90104941 | F-cheese liking (derived food-liking factor) \|\| id:ebi-fl187-GCST90094729 | Inverse variance weighted | 12 | -0.008559305 | 0.0147767836093607 | 0.562427218586289 | -0.037521801 | 0.0204031911746429 | 0.99147722186049 | 0.963173419758477 | 1.02061275913812 | 0.998735809224318 |
| ebi-fl187-GCST90094729 | ebi-IPs91-GCST90274819 | Leukemia inhibitory factor levels \|\| id:ebi-IPs91-GCST90274819 | F-cheese liking (derived food-liking factor) \|\| id:ebi-fl187-GCST90094729 | Inverse variance weighted | 16 | -0.025194646 | 0.0455990690763081 | 0.580588408304043 | -0.114568821 | 0.0641795295993255 | 0.975120090534355 | 0.891750564980282 | 1.06628381108427 | 0.998735809224318 |
| ebi-fl187-GCST90094729 | ebi-IPs91-GCST90274837 | STAM binding protein levels \|\| id:ebi-IPs91-GCST90274837 | F-cheese liking (derived food-liking factor) \|\| id:ebi-fl187-GCST90094729 | Inverse variance weighted | 16 | 0.0629897874907826 | 0.0391109417566459 | 0.107279733986891 | -0.013667658 | 0.139647233333809 | 1.06501596269043 | 0.98642532000932 | 1.1498680921681 | 0.998735809224318 |
| ebi-fl187-GCST90094729 | ebi-IPs91-GCST90274840 | TNF-beta levels \|\| id:ebi-IPs91-GCST90274840 | F-cheese liking (derived food-liking factor) \|\| id:ebi-fl187-GCST90094729 | Inverse variance weighted | 16 | 0.0113594892672912 | 0.0448265287618523 | 0.799951424814234 | -0.076500507 | 0.0992194856405217 | 1.01142425326182 | 0.926352444529876 | 1.10430865285338 | 0.998735809224318 |
| ebi-fl187-GCST90094729 | ebi-IPs91-GCST90274842 | Tumor necrosis factor ligand superfamily member 14 levels \|\| id:ebi-IPs91-GCST90274842 | F-cheese liking (derived food-liking factor) \|\| id:ebi-fl187-GCST90094729 | Inverse variance weighted | 16 | 0.0370914736160623 | 0.0446257005455084 | 0.405878484633287 | -0.050374899 | 0.124557846685259 | 1.03778794671268 | 0.950872875948768 | 1.13264753847089 | 0.998735809224318 |
| ebi-fl187-GCST90094729 | ebi-met1400-GCST90199628 | 3-methylhistidine levels \|\| id:ebi-met1400-GCST90199628 | F-cheese liking (derived food-liking factor) \|\| id:ebi-fl187-GCST90094729 | Inverse variance weighted | 15 | 0.0443165979118701 | 0.0660154236959568 | 0.502025151096725 | -0.085073633 | 0.173706828355945 | 1.04531324649697 | 0.918444654506006 | 1.18970672641102 | 0.998735809224318 |
| ebi-fl187-GCST90094729 | ebi-met1400-GCST90199651 | Docosahexaenoate DHA; 22:6n3 levels \|\| id:ebi-met1400-GCST90199651 | F-cheese liking (derived food-liking factor) \|\| id:ebi-fl187-GCST90094729 | Inverse variance weighted | 15 | 0.051916540159671 | 0.0569984649491609 | 0.362379143769413 | -0.059800451 | 0.163633531460026 | 1.05328783161065 | 0.94195248037433 | 1.17778261571985 | 0.998735809224318 |
| ebi-fl187-GCST90094729 | ebi-met1400-GCST90199652 | 1-stearoyl-2-oleoyl-GPS (18:0/18:1) levels \|\| id:ebi-met1400-GCST90199652 | F-cheese liking (derived food-liking factor) \|\| id:ebi-fl187-GCST90094729 | Inverse variance weighted | 15 | 0.00922590655064533 | 0.0540967481956614 | 0.864581887518148 | -0.09680372 | 0.115255533014142 | 1.00926859640971 | 0.907734158788586 | 1.1221601499036 | 0.998735809224318 |
| ebi-fl187-GCST90094729 | ebi-met1400-GCST90199653 | 1-myristoyl-2-palmitoyl-gpc (14:0/16:0) levels \|\| id:ebi-met1400-GCST90199653 | F-cheese liking (derived food-liking factor) \|\| id:ebi-fl187-GCST90094729 | Inverse variance weighted | 15 | -0.024379503 | 0.0530376950456084 | 0.645757632785686 | -0.128333385 | 0.079574379441336 | 0.975915276845158 | 0.879560097995407 | 1.08282609653438 | 0.998735809224318 |
| ebi-fl187-GCST90094729 | ebi-met1400-GCST90199667 | Erythritol levels in elite athletes \|\| id:ebi-met1400-GCST90199667 | F-cheese liking (derived food-liking factor) \|\| id:ebi-fl187-GCST90094729 | Inverse variance weighted | 15 | 0.0176114978623537 | 0.0613690778538317 | 0.774130130740074 | -0.102671895 | 0.137894890455864 | 1.0177674947247 | 0.902423014658361 | 1.14785489342861 | 0.998735809224318 |
| ebi-fl187-GCST90094729 | ebi-met1400-GCST90199674 | 3-hydroxymyristate levels \|\| id:ebi-met1400-GCST90199674 | F-cheese liking (derived food-liking factor) \|\| id:ebi-fl187-GCST90094729 | Inverse variance weighted | 15 | 0.0761457313868898 | 0.0520524083385404 | 0.14350387549457 | -0.025876989 | 0.178168451730429 | 1.07911982432748 | 0.974454950956679 | 1.19502660858085 | 0.998735809224318 |
| ebi-fl187-GCST90094729 | ebi-met1400-GCST90199685 | 2-linoleoylglycerol (18:2) levels \|\| id:ebi-met1400-GCST90199685 | F-cheese liking (derived food-liking factor) \|\| id:ebi-fl187-GCST90094729 | Inverse variance weighted | 15 | -0.018168908 | 0.0611928236286208 | 0.766533388969131 | -0.138106842 | 0.101769026182095 | 0.981995151384681 | 0.871005626406016 | 1.10712772467616 | 0.998735809224318 |
| ebi-fl187-GCST90094729 | ebi-met1400-GCST90199703 | Gamma-glutamylvaline levels \|\| id:ebi-met1400-GCST90199703 | F-cheese liking (derived food-liking factor) \|\| id:ebi-fl187-GCST90094729 | Inverse variance weighted | 15 | 0.0274839555408262 | 0.0670117721953453 | 0.681706017802986 | -0.103859118 | 0.158827029043703 | 1.02786512343494 | 0.901352272821824 | 1.17213518380153 | 0.998735809224318 |
| ebi-fl187-GCST90094729 | ebi-met1400-GCST90199779 | Gamma-glutamyl-2-aminobutyrate levels \|\| id:ebi-met1400-GCST90199779 | F-cheese liking (derived food-liking factor) \|\| id:ebi-fl187-GCST90094729 | Inverse variance weighted | 15 | 0.0359295284376011 | 0.0655233685452946 | 0.583453809073361 | -0.092496274 | 0.164355330786378 | 1.03658279430783 | 0.911652607856727 | 1.17863304530129 | 0.998735809224318 |
| ebi-fl187-GCST90094729 | ebi-met1400-GCST90199824 | 4-vinylphenol sulfate levels \|\| id:ebi-met1400-GCST90199824 | F-cheese liking (derived food-liking factor) \|\| id:ebi-fl187-GCST90094729 | Inverse variance weighted | 15 | -0.011172724 | 0.0639842904880619 | 0.861380841425774 | -0.136581934 | 0.114236484996968 | 0.988889458724895 | 0.872334843695947 | 1.1210171972886 | 0.998735809224318 |
| ebi-fl187-GCST90094729 | ebi-met1400-GCST90199830 | 4-ethylphenylsulfate levels \|\| id:ebi-met1400-GCST90199830 | F-cheese liking (derived food-liking factor) \|\| id:ebi-fl187-GCST90094729 | Inverse variance weighted | 15 | 0.0535612689556636 | 0.0552274441924479 | 0.332130904733122 | -0.054684522 | 0.161807059572861 | 1.05502162986088 | 0.946783790620512 | 1.17563339222867 | 0.998735809224318 |
| ebi-fl187-GCST90094729 | ebi-met1400-GCST90199833 | Hexanoylglutamine levels \|\| id:ebi-met1400-GCST90199833 | F-cheese liking (derived food-liking factor) \|\| id:ebi-fl187-GCST90094729 | Inverse variance weighted | 15 | 0.103116689264401 | 0.0527138260948935 | 0.0504462838639631 | -0.00020241 | 0.206435788410392 | 1.10862076567024 | 0.999797610601908 | 1.229288797 | 0.998735809224318 |
| ebi-fl187-GCST90094729 | ebi-met1400-GCST90199837 | Carotenoid levels (cryptoxanthin) \|\| id:ebi-met1400-GCST90199837 | F-cheese liking (derived food-liking factor) \|\| id:ebi-fl187-GCST90094729 | Inverse variance weighted (multiplicative random effects) | 15 | 0.00245339469866949 | 0.0743357649605699 | 0.973671214270769 | -0.143244705 | 0.148151494021386 | 1.00245640673418 | 0.866541996115572 | 1.15968856894083 | 0.998955228396052 |
| ebi-fl187-GCST90094729 | ebi-met1400-GCST90199869 | 2-hydroxyhippurate levels \|\| id:ebi-met1400-GCST90199869 | F-cheese liking (derived food-liking factor) \|\| id:ebi-fl187-GCST90094729 | Inverse variance weighted | 15 | 0.0354745848906231 | 0.0609717755837165 | 0.560688081071384 | -0.084030095 | 0.154979265034707 | 1.03611131491104 | 0.919403585994804 | 1.16763375000884 | 0.998735809224318 |
| ebi-fl187-GCST90094729 | ebi-met1400-GCST90199902 | Hydantoin-5-propionate levels \|\| id:ebi-met1400-GCST90199902 | F-cheese liking (derived food-liking factor) \|\| id:ebi-fl187-GCST90094729 | Inverse variance weighted | 15 | 0.00875591292335741 | 0.0533453906739954 | 0.869623877389876 | -0.095801053 | 0.113312878644388 | 1.0087943580544 | 0.908644770422807 | 1.11998229667779 | 0.998735809224318 |
| ebi-fl187-GCST90094729 | ebi-met1400-GCST90199904 | Pantoate levels \|\| id:ebi-met1400-GCST90199904 | F-cheese liking (derived food-liking factor) \|\| id:ebi-fl187-GCST90094729 | Inverse variance weighted | 15 | 0.0664188306737284 | 0.0541449956447498 | 0.219941169513141 | -0.039705361 | 0.172543022137438 | 1.06867421698842 | 0.961072567101919 | 1.18832294370827 | 0.998735809224318 |
| ebi-fl187-GCST90094729 | ebi-met1400-GCST90199936 | 3-methyl catechol sulfate (2) levels \|\| id:ebi-met1400-GCST90199936 | F-cheese liking (derived food-liking factor) \|\| id:ebi-fl187-GCST90094729 | Inverse variance weighted | 15 | -0.012305158 | 0.0724972444389371 | 0.865220302810511 | -0.154399757 | 0.129789441127648 | 0.987770240901808 | 0.856929388946944 | 1.13858861814765 | 0.998735809224318 |
| ebi-fl187-GCST90094729 | ebi-met1400-GCST90199977 | 4-hydroxychlorothalonil levels \|\| id:ebi-met1400-GCST90199977 | F-cheese liking (derived food-liking factor) \|\| id:ebi-fl187-GCST90094729 | Inverse variance weighted (multiplicative random effects) | 15 | 0.065693291286186 | 0.0702936612908841 | 0.350017688904325 | -0.072082285 | 0.203468867416319 | 1.06789913296254 | 0.930454330371787 | 1.22564699948945 | 0.998735809224318 |
| ebi-fl187-GCST90094729 | ebi-met1400-GCST90200040 | Sphingomyelin (d18:2/24:1, d18:1/24:2) levels \|\| id:ebi-met1400-GCST90200040 | F-cheese liking (derived food-liking factor) \|\| id:ebi-fl187-GCST90094729 | Inverse variance weighted | 15 | 0.00410573174546726 | 0.0486834027701503 | 0.932789808351969 | -0.091313738 | 0.0995252011749618 | 1.00411417180898 | 0.912731307767249 | 1.10464630877407 | 0.998735809224318 |
| ebi-fl187-GCST90094729 | ebi-met1400-GCST90200077 | 1-stearoyl-2-oleoyl-GPI (18:0/18:1) levels \|\| id:ebi-met1400-GCST90200077 | F-cheese liking (derived food-liking factor) \|\| id:ebi-fl187-GCST90094729 | Inverse variance weighted | 15 | -0.000515538 | 0.052827007901289 | 0.992213581176586 | -0.104056473 | 0.10302539757791 | 0.999484594958218 | 0.901174403605958 | 1.10851956243044 | 0.999107451094609 |
| ebi-fl187-GCST90094729 | ebi-met1400-GCST90200080 | 1-palmitoyl-2-oleoyl-GPI (16:0/18:1) levels \|\| id:ebi-met1400-GCST90200080 | F-cheese liking (derived food-liking factor) \|\| id:ebi-fl187-GCST90094729 | Inverse variance weighted | 15 | -0.011329591 | 0.053273936732449 | 0.831586965163087 | -0.115746507 | 0.0930873252472204 | 0.988734347372833 | 0.890700981374098 | 1.09755757557001 | 0.998735809224318 |
| ebi-fl187-GCST90094729 | ebi-met1400-GCST90200082 | 1-oleoyl-2-linoleoyl-GPE (18:1/18:2) levels \|\| id:ebi-met1400-GCST90200082 | F-cheese liking (derived food-liking factor) \|\| id:ebi-fl187-GCST90094729 | Inverse variance weighted | 15 | -0.026491138 | 0.0567727429187168 | 0.640773778704984 | -0.137765714 | 0.0847834377667059 | 0.973856673770415 | 0.871302801469457 | 1.08848131722715 | 0.998735809224318 |
| ebi-fl187-GCST90094729 | ebi-met1400-GCST90200100 | Perfluorooctanesulfonate (PFOS) levels \|\| id:ebi-met1400-GCST90200100 | F-cheese liking (derived food-liking factor) \|\| id:ebi-fl187-GCST90094729 | Inverse variance weighted | 15 | -0.004566684 | 0.0492174664930029 | 0.926073706999669 | -0.101032918 | 0.091899550756426 | 0.995443727874933 | 0.903903277801905 | 1.09625469859476 | 0.998735809224318 |
| ebi-fl187-GCST90094729 | ebi-met1400-GCST90200102 | 1-palmitoleoyl-2-linolenoyl-GPC (16:1/18:3) levels \|\| id:ebi-met1400-GCST90200102 | F-cheese liking (derived food-liking factor) \|\| id:ebi-fl187-GCST90094729 | Inverse variance weighted | 15 | -0.009842072 | 0.0522581180861568 | 0.850613457537857 | -0.112267984 | 0.0925838392672204 | 0.990206202506636 | 0.89380470037452 | 1.09700510980952 | 0.998735809224318 |
| ebi-fl187-GCST90094729 | ebi-met1400-GCST90200114 | Glycosyl-N-tricosanoyl-sphingadienine (d18:2/23:0) levels \|\| id:ebi-met1400-GCST90200114 | F-cheese liking (derived food-liking factor) \|\| id:ebi-fl187-GCST90094729 | Inverse variance weighted | 15 | -0.079185596 | 0.0569775131196595 | 0.164598958559812 | -0.190861521 | 0.0324903300491675 | 0.923868442554126 | 0.826246997773483 | 1.03302390380532 | 0.998735809224318 |
| ebi-fl187-GCST90094729 | ebi-met1400-GCST90200135 | Arachidoylcarnitine (C20) levels \|\| id:ebi-met1400-GCST90200135 | F-cheese liking (derived food-liking factor) \|\| id:ebi-fl187-GCST90094729 | Inverse variance weighted | 15 | -0.043010874 | 0.0547118962408752 | 0.431789069532027 | -0.150246191 | 0.0642244424820049 | 0.957900973651418 | 0.860496104136646 | 1.06633170203944 | 0.998735809224318 |
| ebi-fl187-GCST90094729 | ebi-met1400-GCST90200146 | Docosahexaenoylcarnitine (C22:6) levels \|\| id:ebi-met1400-GCST90200146 | F-cheese liking (derived food-liking factor) \|\| id:ebi-fl187-GCST90094729 | Inverse variance weighted (multiplicative random effects) | 15 | 0.0337151074111519 | 0.0787699067772792 | 0.668636449316947 | -0.12067391 | 0.188104124694619 | 1.03428990322254 | 0.886322933633122 | 1.20695918306329 | 0.998735809224318 |
| ebi-fl187-GCST90094729 | ebi-met1400-GCST90200162 | 3-hydroxyphenylacetoylglutamine levels \|\| id:ebi-met1400-GCST90200162 | F-cheese liking (derived food-liking factor) \|\| id:ebi-fl187-GCST90094729 | Inverse variance weighted | 15 | 0.0938271300242999 | 0.0653228677363412 | 0.150900034728987 | -0.034205691 | 0.221859950787529 | 1.09836985433434 | 0.966372710276271 | 1.24839652867013 | 0.998735809224318 |
| ebi-fl187-GCST90094729 | ebi-met1400-GCST90200172 | 3-carboxy-4-methyl-5-pentyl-2-furanpropionate (3-CMPFP) levels \|\| id:ebi-met1400-GCST90200172 | F-cheese liking (derived food-liking factor) \|\| id:ebi-fl187-GCST90094729 | Inverse variance weighted | 15 | 0.0149167412402984 | 0.0530397529416859 | 0.778528623996987 | -0.089041175 | 0.118874657006003 | 1.0150285510793 | 0.914807906017618 | 1.1262287445582 | 0.998735809224318 |
| ebi-fl187-GCST90094729 | ebi-met1400-GCST90200222 | Dihydrocaffeate sulfate (2) levels \|\| id:ebi-met1400-GCST90200222 | F-cheese liking (derived food-liking factor) \|\| id:ebi-fl187-GCST90094729 | Inverse variance weighted | 15 | -0.045097018 | 0.0653591839964864 | 0.490202068195632 | -0.173201019 | 0.083006982746219 | 0.95590473747654 | 0.840968547599557 | 1.08654939562043 | 0.998735809224318 |
| ebi-fl187-GCST90094729 | ebi-met1400-GCST90200223 | 4-methylhexanoylglutamine levels \|\| id:ebi-met1400-GCST90200223 | F-cheese liking (derived food-liking factor) \|\| id:ebi-fl187-GCST90094729 | Inverse variance weighted | 15 | 0.0791289282330365 | 0.068094263662674 | 0.245215220354148 | -0.054335829 | 0.212593685011877 | 1.0823438577562 | 0.947113985175324 | 1.23688198544097 | 0.998735809224318 |
| ebi-fl187-GCST90094729 | ebi-met1400-GCST90200224 | 2-ketocaprylate levels \|\| id:ebi-met1400-GCST90200224 | F-cheese liking (derived food-liking factor) \|\| id:ebi-fl187-GCST90094729 | Inverse variance weighted | 15 | 0.00533678956296237 | 0.0540724239201167 | 0.921378798917904 | -0.100645161 | 0.111318740446391 | 1.00535105559136 | 0.904253840203696 | 1.11775112257303 | 0.998735809224318 |
| ebi-fl187-GCST90094729 | ebi-met1400-GCST90200243 | Hydroxypalmitoyl sphingomyelin (d18:1/16:0(OH)) levels \|\| id:ebi-met1400-GCST90200243 | F-cheese liking (derived food-liking factor) \|\| id:ebi-fl187-GCST90094729 | Inverse variance weighted (multiplicative random effects) | 15 | -0.090528052 | 0.0698268462194129 | 0.19481537918719 | -0.227388671 | 0.0463325662286673 | 0.913448709148244 | 0.796611102517136 | 1.04742269045471 | 0.998735809224318 |
| ebi-fl187-GCST90094729 | ebi-met1400-GCST90200273 | 2-methoxyhydroquinone sulfate (2) levels \|\| id:ebi-met1400-GCST90200273 | F-cheese liking (derived food-liking factor) \|\| id:ebi-fl187-GCST90094729 | Inverse variance weighted | 15 | 0.059153474898512 | 0.0634879145642202 | 0.351477053354784 | -0.065282838 | 0.183589787444383 | 1.06093805560625 | 0.936802462898467 | 1.201522842 | 0.998735809224318 |
| ebi-fl187-GCST90094729 | ebi-met1400-GCST90200276 | 2,4-di-tert-butylphenol levels \|\| id:ebi-met1400-GCST90200276 | F-cheese liking (derived food-liking factor) \|\| id:ebi-fl187-GCST90094729 | Inverse variance weighted | 15 | 0.0101789121112547 | 0.053129700221307 | 0.848066430646319 | -0.0939553 | 0.114313124545016 | 1.01023089345862 | 0.91032345249608 | 1.12110311483225 | 0.998735809224318 |
| ebi-fl187-GCST90094729 | ebi-met1400-GCST90200289 | N-acetylmethionine levels \|\| id:ebi-met1400-GCST90200289 | F-cheese liking (derived food-liking factor) \|\| id:ebi-fl187-GCST90094729 | Inverse variance weighted | 15 | 0.00494680484459367 | 0.0552384623165583 | 0.928641933766381 | -0.103320581 | 0.113214190985048 | 1.0049590604841 | 0.901837814799236 | 1.11987177370014 | 0.998735809224318 |
| ebi-fl187-GCST90094729 | ebi-met1400-GCST90200308 | 3-hydroxyisobutyrate levels \|\| id:ebi-met1400-GCST90200308 | F-cheese liking (derived food-liking factor) \|\| id:ebi-fl187-GCST90094729 | Inverse variance weighted | 15 | -0.021308941 | 0.0539403517356654 | 0.692808651975887 | -0.127032031 | 0.0844141481565792 | 0.978916490168915 | 0.880705462579382 | 1.08807942659746 | 0.998735809224318 |
| ebi-fl187-GCST90094729 | ebi-met1400-GCST90200383 | Cystathionine levels \|\| id:ebi-met1400-GCST90200383 | F-cheese liking (derived food-liking factor) \|\| id:ebi-fl187-GCST90094729 | Inverse variance weighted | 15 | -0.054950807 | 0.064865715054417 | 0.396913097904529 | -0.182087608 | 0.0721859947961591 | 0.946531709816633 | 0.833528313287418 | 1.07485524295497 | 0.998735809224318 |
| ebi-fl187-GCST90094729 | ebi-met1400-GCST90200384 | Guanidinoacetate levels \|\| id:ebi-met1400-GCST90200384 | F-cheese liking (derived food-liking factor) \|\| id:ebi-fl187-GCST90094729 | Inverse variance weighted | 15 | -0.045363606 | 0.0520323422538621 | 0.38329851653006 | -0.147346997 | 0.0566197850362422 | 0.955649938809926 | 0.86299446930946 | 1.05825337012667 | 0.998735809224318 |
| ebi-fl187-GCST90094729 | ebi-met1400-GCST90200403 | Citrulline levels \|\| id:ebi-met1400-GCST90200403 | F-cheese liking (derived food-liking factor) \|\| id:ebi-fl187-GCST90094729 | Inverse variance weighted | 15 | -0.021707875 | 0.0534796843911921 | 0.684809605777682 | -0.126528056 | 0.08311230644548 | 0.978526045261902 | 0.881149427344119 | 1.08666384104901 | 0.998735809224318 |
| ebi-fl187-GCST90094729 | ebi-met1400-GCST90200434 | 12,13-DiHOME levels \|\| id:ebi-met1400-GCST90200434 | F-cheese liking (derived food-liking factor) \|\| id:ebi-fl187-GCST90094729 | Inverse variance weighted | 15 | -0.038637668 | 0.053933646406537 | 0.473748928904664 | -0.144347615 | 0.0670722788057547 | 0.962099245198076 | 0.865586804705519 | 1.06937276836795 | 0.998735809224318 |
| ebi-fl187-GCST90094729 | ebi-met1400-GCST90200437 | Fructose levels \|\| id:ebi-met1400-GCST90200437 | F-cheese liking (derived food-liking factor) \|\| id:ebi-fl187-GCST90094729 | Inverse variance weighted | 15 | -0.034215382 | 0.0537420087706928 | 0.524346395653662 | -0.139549719 | 0.0711189549945775 | 0.966363344762036 | 0.869749778703924 | 1.0737089413133 | 0.998735809224318 |
| ebi-fl187-GCST90094729 | ebi-met1400-GCST90200448 | N-stearoyl-sphinganine (d18:0/18:0) levels \|\| id:ebi-met1400-GCST90200448 | F-cheese liking (derived food-liking factor) \|\| id:ebi-fl187-GCST90094729 | Inverse variance weighted | 15 | -0.036794942 | 0.0620149920690983 | 0.552964788961484 | -0.158344326 | 0.0847544429077626 | 0.963873765548094 | 0.853555829896382 | 1.08844975732243 | 0.998735809224318 |
| ebi-fl187-GCST90094729 | ebi-met1400-GCST90200468 | X-11847 levels \|\| id:ebi-met1400-GCST90200468 | F-cheese liking (derived food-liking factor) \|\| id:ebi-fl187-GCST90094729 | Inverse variance weighted | 15 | 0.117999856231065 | 0.0628394155241818 | 0.0604085264458992 | -0.005165398 | 0.241165110658461 | 1.12524394959221 | 0.994847919532607 | 1.27273115944066 | 0.998735809224318 |
| ebi-fl187-GCST90094729 | ebi-met1400-GCST90200476 | X-11843 levels \|\| id:ebi-met1400-GCST90200476 | F-cheese liking (derived food-liking factor) \|\| id:ebi-fl187-GCST90094729 | Inverse variance weighted (multiplicative random effects) | 15 | 0.018661711771138 | 0.0868929885585014 | 0.829949294094974 | -0.151648546 | 0.188971969345801 | 1.01883692977306 | 0.859290228834467 | 1.20800709077929 | 0.998735809224318 |
| ebi-fl187-GCST90094729 | ebi-met1400-GCST90200511 | X-12906 levels \|\| id:ebi-met1400-GCST90200511 | F-cheese liking (derived food-liking factor) \|\| id:ebi-fl187-GCST90094729 | Inverse variance weighted | 15 | -0.114411726 | 0.0535348023300453 | 0.0325860613461997 | -0.219339939 | -0.009483514 | 0.891890665424797 | 0.803048684282255 | 0.990561312958078 | 0.998735809224318 |
| ebi-fl187-GCST90094729 | ebi-met1400-GCST90200515 | X-12849 levels \|\| id:ebi-met1400-GCST90200515 | F-cheese liking (derived food-liking factor) \|\| id:ebi-fl187-GCST90094729 | Inverse variance weighted | 15 | 0.0835092355300418 | 0.0716899623934581 | 0.244073035860676 | -0.057003091 | 0.22402156182122 | 1.0870952551474 | 0.944591149856396 | 1.25109799508884 | 0.998735809224318 |
| ebi-fl187-GCST90094729 | ebi-met1400-GCST90200551 | X-18886 levels \|\| id:ebi-met1400-GCST90200551 | F-cheese liking (derived food-liking factor) \|\| id:ebi-fl187-GCST90094729 | Inverse variance weighted | 15 | 0.0400605248432883 | 0.0552491768002226 | 0.468397937608417 | -0.068227862 | 0.148348911371725 | 1.04087377100781 | 0.93404761566334 | 1.15991753418544 | 0.998735809224318 |
| ebi-fl187-GCST90094729 | ebi-met1400-GCST90200564 | X-21319 levels \|\| id:ebi-met1400-GCST90200564 | F-cheese liking (derived food-liking factor) \|\| id:ebi-fl187-GCST90094729 | Inverse variance weighted | 15 | 0.0565098115021958 | 0.0539729217907425 | 0.295098190551392 | -0.049277115 | 0.162296738212051 | 1.05813699666276 | 0.951917302383988 | 1.1762092157611 | 0.998735809224318 |
| ebi-fl187-GCST90094729 | ebi-met1400-GCST90200579 | X-21442 levels \|\| id:ebi-met1400-GCST90200579 | F-cheese liking (derived food-liking factor) \|\| id:ebi-fl187-GCST90094729 | Inverse variance weighted | 15 | 0.0571896422348777 | 0.0582325715938712 | 0.32605537620623 | -0.056946198 | 0.171325482558865 | 1.0588565952874 | 0.944644891699628 | 1.18687699392135 | 0.998735809224318 |
| ebi-fl187-GCST90094729 | ebi-met1400-GCST90200581 | X-21441 levels \|\| id:ebi-met1400-GCST90200581 | F-cheese liking (derived food-liking factor) \|\| id:ebi-fl187-GCST90094729 | Inverse variance weighted | 15 | 0.0650544817691542 | 0.0536290651999028 | 0.225112528004039 | -0.040058486 | 0.170167449560964 | 1.06721716667964 | 0.960733248042631 | 1.18550334671585 | 0.998735809224318 |
| ebi-fl187-GCST90094729 | ebi-met1400-GCST90200648 | X-24585 levels \|\| id:ebi-met1400-GCST90200648 | F-cheese liking (derived food-liking factor) \|\| id:ebi-fl187-GCST90094729 | Inverse variance weighted | 15 | 0.0944653899956637 | 0.0608110515314951 | 0.120321703760941 | -0.024724271 | 0.213655050997394 | 1.09907112361837 | 0.975578870327246 | 1.23819546682734 | 0.998735809224318 |
| ebi-fl187-GCST90094729 | ebi-met1400-GCST90200720 | Adenosine 5'-diphosphate (ADP) to pantothenate ratio \|\| id:ebi-met1400-GCST90200720 | F-cheese liking (derived food-liking factor) \|\| id:ebi-fl187-GCST90094729 | Inverse variance weighted | 15 | -0.087192843 | 0.0881375427490544 | 0.322525420068991 | -0.259942427 | 0.0855567409865063 | 0.916500338097588 | 0.771095979150737 | 1.08932336887311 | 0.998735809224318 |
| ebi-fl187-GCST90094729 | ebi-met1400-GCST90200724 | Cholate to taurocholate ratio \|\| id:ebi-met1400-GCST90200724 | F-cheese liking (derived food-liking factor) \|\| id:ebi-fl187-GCST90094729 | Inverse variance weighted | 15 | -0.028553169 | 0.0573093140349232 | 0.61832256694932 | -0.140879424 | 0.0837730869826106 | 0.971850620907605 | 0.868594036948684 | 1.08738212465336 | 0.998735809224318 |
| ebi-fl187-GCST90094729 | ebi-met1400-GCST90200726 | N-acetylputrescine to (N(1) + N(8))-acetylspermidine ratio \|\| id:ebi-met1400-GCST90200726 | F-cheese liking (derived food-liking factor) \|\| id:ebi-fl187-GCST90094729 | Inverse variance weighted | 15 | 0.0240537883245126 | 0.0531552925995222 | 0.650895021920952 | -0.080130585 | 0.128238161819576 | 1.02434541423239 | 0.922995808951477 | 1.13682371846403 | 0.998735809224318 |
| ebi-fl187-GCST90094729 | ebi-met1400-GCST90200744 | Arginine to glutamate ratio \|\| id:ebi-met1400-GCST90200744 | F-cheese liking (derived food-liking factor) \|\| id:ebi-fl187-GCST90094729 | Inverse variance weighted | 15 | -0.022052394 | 0.0540993205355253 | 0.683546851204407 | -0.128087063 | 0.0839822738600812 | 0.978188982093958 | 0.879776780121871 | 1.08760961451775 | 0.998735809224318 |
| ebi-fl187-GCST90094729 | ebi-met1400-GCST90200759 | Histidine to trans-urocanate ratio \|\| id:ebi-met1400-GCST90200759 | F-cheese liking (derived food-liking factor) \|\| id:ebi-fl187-GCST90094729 | Inverse variance weighted | 15 | -0.07489354 | 0.0538613446107384 | 0.164380818214334 | -0.180461775 | 0.0306746958417588 | 0.9278422595332 | 0.834884593523597 | 1.03115001193437 | 0.998735809224318 |
| ebi-fl187-GCST90094729 | ebi-met1400-GCST90200795 | Oleoyl-linoleoyl-glycerol (18:1 to 18:2) [2] to linoleoyl-arachidonoyl-glycerol (18:2 to 20:4) [2] ratio \|\| id:ebi-met1400-GCST90200795 | F-cheese liking (derived food-liking factor) \|\| id:ebi-fl187-GCST90094729 | Inverse variance weighted | 15 | 0.0103950256052876 | 0.0694694246314862 | 0.881052953643623 | -0.125765047 | 0.146555098 | 1.01044924157992 | 0.881822009461134 | 1.15783872352354 | 0.998735809224318 |
| ebi-fl187-GCST90094729 | ebi-met1400-GCST90200811 | Choline to taurocholate ratio \|\| id:ebi-met1400-GCST90200811 | F-cheese liking (derived food-liking factor) \|\| id:ebi-fl187-GCST90094729 | Inverse variance weighted | 15 | -0.008166833 | 0.0558915633274357 | 0.883827247831056 | -0.117714298 | 0.101380630638417 | 0.991866424502155 | 0.888949991478904 | 1.10669780469652 | 0.998735809224318 |
| ebi-fl187-GCST90094729 | ebi-met1400-GCST90200831 | Cholate to phosphate ratio \|\| id:ebi-met1400-GCST90200831 | F-cheese liking (derived food-liking factor) \|\| id:ebi-fl187-GCST90094729 | Inverse variance weighted | 15 | -0.053400007 | 0.0695365266211902 | 0.442521750272794 | -0.189691599 | 0.0828915853348714 | 0.948000729850743 | 0.827214208281096 | 1.08642401786715 | 0.998735809224318 |
| ebi-fl187-GCST90094729 | ebi-met1400-GCST90200841 | Adenosine 5'-diphosphate (ADP) to oxalate (ethanedioate) ratio \|\| id:ebi-met1400-GCST90200841 | F-cheese liking (derived food-liking factor) \|\| id:ebi-fl187-GCST90094729 | Inverse variance weighted | 15 | -0.005294072 | 0.0823884762629545 | 0.948765249977687 | -0.166775485 | 0.156187341798692 | 0.994719917223793 | 0.846389617042983 | 1.16904519360551 | 0.998735809224318 |
| ebi-fl187-GCST90094729 | ebi-met1400-GCST90200844 | Aspartate to phosphate ratio \|\| id:ebi-met1400-GCST90200844 | F-cheese liking (derived food-liking factor) \|\| id:ebi-fl187-GCST90094729 | Inverse variance weighted | 15 | 0.0320621650715291 | 0.0502138159295145 | 0.523139913315444 | -0.066356914 | 0.130481244293378 | 1.03258169382481 | 0.935796805558293 | 1.13937656988048 | 0.998735809224318 |
| ebi-fl187-GCST90094729 | ebi-met1400-GCST90200870 | Adenosine 5'-monophosphate (AMP) to acetoacetate ratio \|\| id:ebi-met1400-GCST90200870 | F-cheese liking (derived food-liking factor) \|\| id:ebi-fl187-GCST90094729 | Inverse variance weighted | 15 | 0.0323586720171403 | 0.0588262107886098 | 0.582269750505985 | -0.082940701 | 0.147658045162816 | 1.03288790686385 | 0.92040572462268 | 1.15911646310428 | 0.998735809224318 |
| ebi-fl187-GCST90094729 | ebi-met1400-GCST90200887 | Cholesterol to cortisol ratio \|\| id:ebi-met1400-GCST90200887 | F-cheese liking (derived food-liking factor) \|\| id:ebi-fl187-GCST90094729 | Inverse variance weighted | 14 | -0.086048011 | 0.0660994102875713 | 0.192985806113873 | -0.215602855 | 0.043506833337355 | 0.917550177820445 | 0.806055359178435 | 1.04446713148391 | 0.998735809224318 |
| ebi-fl187-GCST90094729 | ebi-met1400-GCST90200890 | Cortisol to taurocholate ratio \|\| id:ebi-met1400-GCST90200890 | F-cheese liking (derived food-liking factor) \|\| id:ebi-fl187-GCST90094729 | Inverse variance weighted | 15 | 0.0184851484180708 | 0.055702935736468 | 0.740000775601707 | -0.090692606 | 0.127662902461548 | 1.0186570563885 | 0.913298410548037 | 1.13616993804623 | 0.998735809224318 |
| ebi-fl187-GCST90094729 | ebi-met1400-GCST90200948 | Adenosine 5'-diphosphate (ADP) to mannitol to sorbitol ratio \|\| id:ebi-met1400-GCST90200948 | F-cheese liking (derived food-liking factor) \|\| id:ebi-fl187-GCST90094729 | Inverse variance weighted | 15 | -0.081201064 | 0.0804662541567335 | 0.312911380824577 | -0.238914922 | 0.0765127941764054 | 0.922008290158551 | 0.787481876812976 | 1.07951600176697 | 0.998735809224318 |
| ebi-fl187-GCST90094729 | ebi-met1400-GCST90200972 | Leucine to N-palmitoyl-sphingosine (d18:1 to 16:0) ratio \|\| id:ebi-met1400-GCST90200972 | F-cheese liking (derived food-liking factor) \|\| id:ebi-fl187-GCST90094729 | Inverse variance weighted | 15 | 0.00137858199813967 | 0.0514705743569907 | 0.978632105683006 | -0.099503744 | 0.102260907737841 | 1.00137953267912 | 0.905286560703241 | 1.10767243433912 | 0.998955228396052 |
| ebi-fl187-GCST90094729 | ebi-met1400-GCST90200973 | Proline to glutamate ratio \|\| id:ebi-met1400-GCST90200973 | F-cheese liking (derived food-liking factor) \|\| id:ebi-fl187-GCST90094729 | Inverse variance weighted | 15 | -0.05470222 | 0.0523638966115604 | 0.296182329098073 | -0.157335457 | 0.0479310172635936 | 0.946767034178808 | 0.854417390055702 | 1.04909828315795 | 0.998735809224318 |
| ebi-fl187-GCST90094729 | ebi-met1400-GCST90200983 | Cholesterol to linoleoyl-arachidonoyl-glycerol (18:2 to 20:4) [1] ratio \|\| id:ebi-met1400-GCST90200983 | F-cheese liking (derived food-liking factor) \|\| id:ebi-fl187-GCST90094729 | Inverse variance weighted | 15 | -0.052878198 | 0.0603870802194717 | 0.381218076320231 | -0.171236875 | 0.0654804796408157 | 0.948495534489318 | 0.842621953906879 | 1.06767189577119 | 0.998735809224318 |
| ebi-fl187-GCST90094729 | ebi-met1400-GCST90201001 | Serine to alpha-ketobutyrate ratio \|\| id:ebi-met1400-GCST90201001 | F-cheese liking (derived food-liking factor) \|\| id:ebi-fl187-GCST90094729 | Inverse variance weighted | 15 | -0.082968873 | 0.054352072697427 | 0.126883403851367 | -0.189498935 | 0.0235611896184099 | 0.920379795557135 | 0.827373597755735 | 1.02384094726683 | 0.998735809224318 |
| ebi-fl187-GCST90094729 | ebi-met1400-GCST90201005 | Fructose to maltose ratio \|\| id:ebi-met1400-GCST90201005 | F-cheese liking (derived food-liking factor) \|\| id:ebi-fl187-GCST90094729 | Inverse variance weighted | 15 | -0.091816494 | 0.0609716824280192 | 0.132096057439599 | -0.211320992 | 0.0276880034886763 | 0.912272541606844 | 0.809514177897623 | 1.02807487860338 | 0.998735809224318 |
| ebi-fl187-GCST90094729 | ebi-met1400-GCST90201020 | Paraxanthine to 5-acetylamino-6-formylamino-3-methyluracil ratio \|\| id:ebi-met1400-GCST90201020 | F-cheese liking (derived food-liking factor) \|\| id:ebi-fl187-GCST90094729 | Inverse variance weighted | 15 | -0.006590226 | 0.0564553120521118 | 0.90707124861154 | -0.117242637 | 0.104062185881824 | 0.993431442172398 | 0.889369372742572 | 1.10966945854385 | 0.998735809224318 |
| ebi-fl187-GCST90094729 | ebi-pw11-GCST90275197 | Placental weight (fetal genotype, adjusted for sex) \|\| id:ebi-pw11-GCST90275197 | F-cheese liking (derived food-liking factor) \|\| id:ebi-fl187-GCST90094729 | Inverse variance weighted | 15 | 0.0308763853997082 | 0.0226247080533643 | 0.172341156958276 | -0.013468042 | 0.0752208131843023 | 1.03135800509877 | 0.986622245907922 | 1.07812218819622 | 0.998735809224318 |
| ebi-fl187-GCST90094729 | prot-a-1017 | FAS-associated factor 2 \|\| id:prot-a-1017 | F-cheese liking (derived food-liking factor) \|\| id:ebi-fl187-GCST90094729 | Inverse variance weighted | 16 | 0.0979809253001078 | 0.0826642118152172 | 0.235903467305435 | -0.06404093 | 0.260002780457934 | 1.10294174662504 | 0.937966607905247 | 1.29693369273033 | 0.998735809224318 |
| ebi-fl187-GCST90094729 | prot-a-1019 | Fas apoptotic inhibitory molecule 3 \|\| id:prot-a-1019 | F-cheese liking (derived food-liking factor) \|\| id:ebi-fl187-GCST90094729 | Inverse variance weighted | 16 | -0.068474379 | 0.0826642118152172 | 0.407475878401898 | -0.230496235 | 0.093547475713344 | 0.93381738471719 | 0.794139425247362 | 1.0980627334154 | 0.998735809224318 |
| ebi-fl187-GCST90094729 | prot-a-1052 | MIP18 family protein FAM96A \|\| id:prot-a-1052 | F-cheese liking (derived food-liking factor) \|\| id:ebi-fl187-GCST90094729 | Inverse variance weighted | 16 | -0.104638732 | 0.08627485049986 | 0.225185937182138 | -0.273737439 | 0.0644599752881901 | 0.900649840063519 | 0.760531735271197 | 1.06658288771761 | 0.998735809224318 |
| ebi-fl187-GCST90094729 | prot-a-1066 | F-box/LRR-repeat protein 4 \|\| id:prot-a-1066 | F-cheese liking (derived food-liking factor) \|\| id:ebi-fl187-GCST90094729 | Inverse variance weighted | 16 | 0.0278779747332437 | 0.103985684574465 | 0.788626693132524 | -0.175933967 | 0.231689916499195 | 1.02827020181988 | 0.838673361599314 | 1.26072873703107 | 0.998735809224318 |
| ebi-fl187-GCST90094729 | prot-a-1076 | Low affinity immunoglobulin gamma Fc region receptor III-B \|\| id:prot-a-1076 | F-cheese liking (derived food-liking factor) \|\| id:ebi-fl187-GCST90094729 | Inverse variance weighted | 16 | -0.036347681 | 0.0826642118152172 | 0.660152413881043 | -0.198369536 | 0.125674174272229 | 0.964304964788669 | 0.820066752914837 | 1.13391265017257 | 0.998735809224318 |
| ebi-fl187-GCST90094729 | prot-a-1081 | Fc receptor-like protein 3 \|\| id:prot-a-1081 | F-cheese liking (derived food-liking factor) \|\| id:ebi-fl187-GCST90094729 | Inverse variance weighted | 16 | -0.079145832 | 0.0956515771196996 | 0.407988361477301 | -0.266622923 | 0.108331259046702 | 0.923905179580416 | 0.765961843410308 | 1.11441684491099 | 0.998735809224318 |
| ebi-fl187-GCST90094729 | prot-a-1105 | Fibroblast growth factor receptor 4 \|\| id:prot-a-1105 | F-cheese liking (derived food-liking factor) \|\| id:ebi-fl187-GCST90094729 | Inverse variance weighted | 16 | -0.04391638 | 0.100173714584809 | 0.661094731460566 | -0.24025686 | 0.152424100899293 | 0.957033981608336 | 0.786425833566815 | 1.16465406254395 | 0.998735809224318 |
| ebi-fl187-GCST90094729 | prot-a-1143 | Follistatin-related protein 1 \|\| id:prot-a-1143 | F-cheese liking (derived food-liking factor) \|\| id:ebi-fl187-GCST90094729 | Inverse variance weighted | 16 | -0.23521636 | 0.0826642118152172 | 0.00443495995445674 | -0.397238215 | -0.073194505 | 0.790399820082373 | 0.672173884432374 | 0.929420035581732 | 0.998735809224318 |
| ebi-fl187-GCST90094729 | prot-a-1151 | Alpha-(1,3)-fucosyltransferase 10 \|\| id:prot-a-1151 | F-cheese liking (derived food-liking factor) \|\| id:ebi-fl187-GCST90094729 | Inverse variance weighted | 16 | -0.03071376 | 0.0918000595423987 | 0.73794771589747 | -0.210641877 | 0.149214356320246 | 0.969753115112762 | 0.810064117463604 | 1.16092181346764 | 0.998735809224318 |
| ebi-fl187-GCST90094729 | prot-a-1165 | Growth arrest and DNA damage-inducible proteins-interacting protein 1 \|\| id:prot-a-1165 | F-cheese liking (derived food-liking factor) \|\| id:ebi-fl187-GCST90094729 | Inverse variance weighted | 16 | 0.0186852465514545 | 0.0915333513036324 | 0.838247270916164 | -0.160720122 | 0.198090615106574 | 1.01886090815854 | 0.851530362370994 | 1.21907285523353 | 0.998735809224318 |
| ebi-fl187-GCST90094729 | prot-a-1194 | Growth/differentiation factor 11/8 \|\| id:prot-a-1194 | F-cheese liking (derived food-liking factor) \|\| id:ebi-fl187-GCST90094729 | Inverse variance weighted | 16 | -0.261091875 | 0.0978486132523781 | 0.00762305386966328 | -0.452875157 | -0.069308593 | 0.770210153091097 | 0.635797503377983 | 0.933038706149085 | 0.998735809224318 |
| ebi-fl187-GCST90094729 | prot-a-1216 | D-glucuronyl C5-epimerase \|\| id:prot-a-1216 | F-cheese liking (derived food-liking factor) \|\| id:ebi-fl187-GCST90094729 | Inverse variance weighted | 16 | -0.104651294 | 0.10241176936921 | 0.30684345335788 | -0.305378362 | 0.096075773946022 | 0.900638525877598 | 0.736844527687067 | 1.10084247601207 | 0.998735809224318 |
| ebi-fl187-GCST90094729 | prot-a-1246 | Glypican-3 \|\| id:prot-a-1246 | F-cheese liking (derived food-liking factor) \|\| id:ebi-fl187-GCST90094729 | Inverse variance weighted | 16 | -0.03199231 | 0.0826642118152172 | 0.698744925443937 | -0.194014165 | 0.130029545371563 | 0.968514030130458 | 0.823646237283046 | 1.13886203092942 | 0.998735809224318 |
| ebi-fl187-GCST90094729 | prot-a-1275 | Glutamate receptor 4 \|\| id:prot-a-1275 | F-cheese liking (derived food-liking factor) \|\| id:ebi-fl187-GCST90094729 | Inverse variance weighted | 16 | -0.073103903 | 0.0956146353317354 | 0.444528752323786 | -0.260508588 | 0.114300782231339 | 0.929504246710952 | 0.770659537716163 | 1.12108927791133 | 0.998735809224318 |
| ebi-fl187-GCST90094729 | prot-a-1289 | Glutathione S-transferase P \|\| id:prot-a-1289 | F-cheese liking (derived food-liking factor) \|\| id:ebi-fl187-GCST90094729 | Inverse variance weighted | 16 | 0.0474600494832131 | 0.0826576086395749 | 0.565848426868976 | -0.114548863 | 0.20946896241678 | 1.04860430800046 | 0.891768362474392 | 1.2330231044597 | 0.998735809224318 |
| ebi-fl187-GCST90094729 | prot-a-130 | Apolipoprotein D \|\| id:prot-a-130 | F-cheese liking (derived food-liking factor) \|\| id:ebi-fl187-GCST90094729 | Inverse variance weighted | 16 | 0.0889466734881645 | 0.0826322264282783 | 0.281741126225806 | -0.07301249 | 0.25090583728759 | 1.09302236770195 | 0.929589219094589 | 1.28518906174536 | 0.998735809224318 |
| ebi-fl187-GCST90094729 | prot-a-1313 | Heparin-binding EGF-like growth factor \|\| id:prot-a-1313 | F-cheese liking (derived food-liking factor) \|\| id:ebi-fl187-GCST90094729 | Inverse variance weighted | 16 | 0.0172349368194362 | 0.0826576086395749 | 0.834830786295499 | -0.144773976 | 0.179243849753003 | 1.0173843152852 | 0.865217830908068 | 1.19631242909314 | 0.998735809224318 |
| ebi-fl187-GCST90094729 | prot-a-1347 | HLA class II histocompatibility antigen, DQ alpha 2 chain \|\| id:prot-a-1347 | F-cheese liking (derived food-liking factor) \|\| id:ebi-fl187-GCST90094729 | Inverse variance weighted | 16 | -0.178714634 | 0.0826642118152172 | 0.0306232046529803 | -0.340736489 | -0.016692778 | 0.83634453001042 | 0.711246304942585 | 0.983445774013286 | 0.998735809224318 |
| ebi-fl187-GCST90094729 | prot-a-1357 | Non-histone chromosomal protein HMG-14 \|\| id:prot-a-1357 | F-cheese liking (derived food-liking factor) \|\| id:ebi-fl187-GCST90094729 | Inverse variance weighted | 16 | 0.170995963781985 | 0.0826576086395749 | 0.038571783580981 | 0.00898705084841819 | 0.333004876715552 | 1.18648596009604 | 1.0090275556385 | 1.39515410222292 | 0.998735809224318 |
| ebi-fl187-GCST90094729 | prot-a-137 | Apolipoprotein M \|\| id:prot-a-137 | F-cheese liking (derived food-liking factor) \|\| id:ebi-fl187-GCST90094729 | Inverse variance weighted | 16 | -0.017936765 | 0.0826642118152172 | 0.82822124983087 | -0.17995862 | 0.144085090045289 | 0.982223141164354 | 0.835304775381914 | 1.15498238184699 | 0.998735809224318 |
| ebi-fl187-GCST90094729 | prot-a-1381 | 17-beta-hydroxysteroid dehydrogenase 14 \|\| id:prot-a-1381 | F-cheese liking (derived food-liking factor) \|\| id:ebi-fl187-GCST90094729 | Inverse variance weighted | 16 | -0.032145948 | 0.0826642118152172 | 0.697369492418058 | -0.194167803 | 0.129875907657617 | 0.968365241279027 | 0.82351970387845 | 1.13868707221096 | 0.998735809224318 |
| ebi-fl187-GCST90094729 | prot-a-1392 | Serine protease HTRA2, mitochondrial \|\| id:prot-a-1392 | F-cheese liking (derived food-liking factor) \|\| id:ebi-fl187-GCST90094729 | Inverse variance weighted | 16 | 0.114914250514626 | 0.083107409711523 | 0.166750836803467 | -0.047976273 | 0.277804773549211 | 1.12177724162645 | 0.95315640280872 | 1.32022842853795 | 0.998735809224318 |
| ebi-fl187-GCST90094729 | prot-a-1416 | Interferon-induced protein with tetratricopeptide repeats 2 \|\| id:prot-a-1416 | F-cheese liking (derived food-liking factor) \|\| id:ebi-fl187-GCST90094729 | Inverse variance weighted | 16 | 0.0303475920573522 | 0.0943325946096492 | 0.747673581669913 | -0.154544293 | 0.215239477492265 | 1.03081277402208 | 0.856805540490299 | 1.24015885153945 | 0.998735809224318 |
| ebi-fl187-GCST90094729 | prot-a-1428 | Interferon gamma \|\| id:prot-a-1428 | F-cheese liking (derived food-liking factor) \|\| id:ebi-fl187-GCST90094729 | Inverse variance weighted | 16 | 0.0213237037487687 | 0.0826642118152172 | 0.796441104840914 | -0.140698151 | 0.183345558906595 | 1.02155267855367 | 0.868751503541781 | 1.20122943189817 | 0.998735809224318 |
| ebi-fl187-GCST90094729 | prot-a-1483 | Interleukin-17C \|\| id:prot-a-1483 | F-cheese liking (derived food-liking factor) \|\| id:ebi-fl187-GCST90094729 | Inverse variance weighted | 16 | -0.123614097 | 0.0878050170507336 | 0.159182898017121 | -0.295711931 | 0.0484837359843299 | 0.883720805214395 | 0.744001721093098 | 1.04967829969718 | 0.998735809224318 |
| ebi-fl187-GCST90094729 | prot-a-1503 | Interleukin-1 receptor-like 2 \|\| id:prot-a-1503 | F-cheese liking (derived food-liking factor) \|\| id:ebi-fl187-GCST90094729 | Inverse variance weighted | 16 | -0.063045956 | 0.0826642118152172 | 0.445657041934418 | -0.225067811 | 0.0989758994577539 | 0.938900324875947 | 0.79846207252541 | 1.10403969128303 | 0.998735809224318 |
| ebi-fl187-GCST90094729 | prot-a-1522 | Interleukin-31 receptor subunit alpha \|\| id:prot-a-1522 | F-cheese liking (derived food-liking factor) \|\| id:ebi-fl187-GCST90094729 | Inverse variance weighted | 16 | -0.122672878 | 0.0826642118152172 | 0.137811527829408 | -0.284694733 | 0.039348977222059 | 0.88455297203262 | 0.752243854427247 | 1.04013340318676 | 0.998735809224318 |
| ebi-fl187-GCST90094729 | prot-a-1540 | Interleukin-6 receptor subunit alpha \|\| id:prot-a-1540 | F-cheese liking (derived food-liking factor) \|\| id:ebi-fl187-GCST90094729 | Inverse variance weighted | 16 | 0.0271283591262384 | 0.0826642118152172 | 0.742779061835064 | -0.134893496 | 0.189150214284064 | 1.02749968326083 | 0.873808970855392 | 1.2082224311197 | 0.998735809224318 |
| ebi-fl187-GCST90094729 | prot-a-1541 | Interleukin-6 receptor subunit alpha \|\| id:prot-a-1541 | F-cheese liking (derived food-liking factor) \|\| id:ebi-fl187-GCST90094729 | Inverse variance weighted | 16 | -0.055567204 | 0.0865063965128551 | 0.520647303476181 | -0.225119741 | 0.113985332869207 | 0.945948449734631 | 0.798420608984904 | 1.1207356867867 | 0.998735809224318 |
| ebi-fl187-GCST90094729 | prot-a-1545 | Interleukin-9 \|\| id:prot-a-1545 | F-cheese liking (derived food-liking factor) \|\| id:ebi-fl187-GCST90094729 | Inverse variance weighted (multiplicative random effects) | 16 | -0.088628657 | 0.131092970285595 | 0.498993140254563 | -0.345570879 | 0.168313564978016 | 0.915185358358428 | 0.707816161048706 | 1.18330759630114 | 0.998735809224318 |
| ebi-fl187-GCST90094729 | prot-a-1548 | Inositol monophosphatase 3 \|\| id:prot-a-1548 | F-cheese liking (derived food-liking factor) \|\| id:ebi-fl187-GCST90094729 | Inverse variance weighted | 16 | -0.029069232 | 0.0826476971547762 | 0.725044772609921 | -0.191058719 | 0.132920254099641 | 0.971349213375471 | 0.826084080104874 | 1.14215891220826 | 0.998735809224318 |
| ebi-fl187-GCST90094729 | prot-a-1555 | Inhibin beta A chain:Inhibin beta B chain heterodimer \|\| id:prot-a-1555 | F-cheese liking (derived food-liking factor) \|\| id:ebi-fl187-GCST90094729 | Inverse variance weighted | 16 | -0.052948992 | 0.0826576086395749 | 0.521794175096288 | -0.214957905 | 0.109059921121515 | 0.948428388862007 | 0.806575392459031 | 1.11522917412221 | 0.998735809224318 |
| ebi-fl187-GCST90094729 | prot-a-1603 | Junctophilin-3 \|\| id:prot-a-1603 | F-cheese liking (derived food-liking factor) \|\| id:ebi-fl187-GCST90094729 | Inverse variance weighted | 16 | -0.076682173 | 0.0826576086395749 | 0.353558754076061 | -0.238691086 | 0.0853267397936495 | 0.926184173040283 | 0.787658163370308 | 1.08907285200956 | 0.998735809224318 |
| ebi-fl187-GCST90094729 | prot-a-1627 | UPF0258 protein KIAA1024 \|\| id:prot-a-1627 | F-cheese liking (derived food-liking factor) \|\| id:ebi-fl187-GCST90094729 | Inverse variance weighted | 16 | 0.0278099938948779 | 0.0890981010336932 | 0.754943402957577 | -0.146822284 | 0.202442271920917 | 1.02820030152546 | 0.863447412089064 | 1.22438940143352 | 0.998735809224318 |
| ebi-fl187-GCST90094729 | prot-a-1666 | Kallikrein-7 \|\| id:prot-a-1666 | F-cheese liking (derived food-liking factor) \|\| id:ebi-fl187-GCST90094729 | Inverse variance weighted | 16 | -0.083504 | 0.0871018512035971 | 0.337713048317375 | -0.254223629 | 0.0872156280580053 | 0.919887406837321 | 0.775518354522779 | 1.0911319330135 | 0.998735809224318 |
| ebi-fl187-GCST90094729 | prot-a-1678 | Importin subunit alpha-3 \|\| id:prot-a-1678 | F-cheese liking (derived food-liking factor) \|\| id:ebi-fl187-GCST90094729 | Inverse variance weighted | 16 | 0.0121551699419935 | 0.101645518238907 | 0.904812755009266 | -0.187070046 | 0.211380385690251 | 1.01222934424926 | 0.829385639364888 | 1.23538218740306 | 0.998735809224318 |
| ebi-fl187-GCST90094729 | prot-a-1718 | Protein LDOC1 \|\| id:prot-a-1718 | F-cheese liking (derived food-liking factor) \|\| id:ebi-fl187-GCST90094729 | Inverse variance weighted | 16 | 0.0123990658210243 | 0.0826461340187161 | 0.880744131788672 | -0.149587357 | 0.174385488497708 | 1.01247625292373 | 0.861063214959141 | 1.19051440698593 | 0.998735809224318 |
| ebi-fl187-GCST90094729 | prot-a-1723 | Leptin \|\| id:prot-a-1723 | F-cheese liking (derived food-liking factor) \|\| id:ebi-fl187-GCST90094729 | Inverse variance weighted | 16 | 0.0129056311763973 | 0.0848590528692472 | 0.879121362233833 | -0.153418112 | 0.179229374800122 | 1.01298926824353 | 0.857771002091099 | 1.19629511265242 | 0.998735809224318 |
| ebi-fl187-GCST90094729 | prot-a-1730 | Galectin-7 \|\| id:prot-a-1730 | F-cheese liking (derived food-liking factor) \|\| id:ebi-fl187-GCST90094729 | Inverse variance weighted (multiplicative random effects) | 16 | -0.096224634 | 0.108177697828409 | 0.373731585607919 | -0.308252921 | 0.115803654100208 | 0.908259967405768 | 0.734729465711398 | 1.12277539814357 | 0.998735809224318 |
| ebi-fl187-GCST90094729 | prot-a-1753 | Lipase member N \|\| id:prot-a-1753 | F-cheese liking (derived food-liking factor) \|\| id:ebi-fl187-GCST90094729 | Inverse variance weighted | 16 | -0.09940959 | 0.0826642118152172 | 0.229142347486299 | -0.261431445 | 0.0626122652977786 | 0.905371800959602 | 0.769948657431535 | 1.0646139714137 | 0.998735809224318 |
| ebi-fl187-GCST90094729 | prot-a-1756 | VIP36-like protein \|\| id:prot-a-1756 | F-cheese liking (derived food-liking factor) \|\| id:ebi-fl187-GCST90094729 | Inverse variance weighted | 16 | 0.0277195606921927 | 0.0826642118152172 | 0.737378259383833 | -0.134302294 | 0.189741415850018 | 1.02810732228346 | 0.874325720823951 | 1.20893694530314 | 0.998735809224318 |
| ebi-fl187-GCST90094729 | prot-a-1760 | Ig Kappa chain V-I region HK102- like \|\| id:prot-a-1760 | F-cheese liking (derived food-liking factor) \|\| id:ebi-fl187-GCST90094729 | Inverse variance weighted | 16 | -0.092757577 | 0.0826576086395749 | 0.261781485266735 | -0.254766489 | 0.0692513364257339 | 0.911414421783719 | 0.775097470274039 | 1.0717055339397 | 0.998735809224318 |
| ebi-fl187-GCST90094729 | prot-a-179 | Neutral ceramidase \|\| id:prot-a-179 | F-cheese liking (derived food-liking factor) \|\| id:ebi-fl187-GCST90094729 | Inverse variance weighted | 16 | -0.046630159 | 0.105644029436648 | 0.658931088670401 | -0.253692456 | 0.160432139120684 | 0.954440323892291 | 0.775930397883296 | 1.17401810053669 | 0.998735809224318 |
| ebi-fl187-GCST90094729 | prot-a-1809 | Lactotransferrin \|\| id:prot-a-1809 | F-cheese liking (derived food-liking factor) \|\| id:ebi-fl187-GCST90094729 | Inverse variance weighted | 16 | -0.032775186 | 0.0826576086395749 | 0.691723319906574 | -0.194784099 | 0.129233726778064 | 0.967756100104456 | 0.823012327987098 | 1.1379560638903 | 0.998735809224318 |
| ebi-fl187-GCST90094729 | prot-a-1818 | Lysophospholipase-like protein 1 \|\| id:prot-a-1818 | F-cheese liking (derived food-liking factor) \|\| id:ebi-fl187-GCST90094729 | Inverse variance weighted | 16 | -0.055186879 | 0.0826642118152172 | 0.50438702060165 | -0.217208734 | 0.106834976558272 | 0.946308286660846 | 0.804761970782172 | 1.11275060939152 | 0.998735809224318 |
| ebi-fl187-GCST90094729 | prot-a-1852 | MAP kinase-activated protein kinase 3 \|\| id:prot-a-1852 | F-cheese liking (derived food-liking factor) \|\| id:ebi-fl187-GCST90094729 | Inverse variance weighted | 16 | 0.111352257371852 | 0.0865732653412525 | 0.198366325141872 | -0.058331343 | 0.281035857440707 | 1.11778858678193 | 0.94333732771318 | 1.3245010963033 | 0.998735809224318 |
| ebi-fl187-GCST90094729 | prot-a-1856 | Megakaryocyte-associated tyrosine-protein kinase \|\| id:prot-a-1856 | F-cheese liking (derived food-liking factor) \|\| id:ebi-fl187-GCST90094729 | Inverse variance weighted | 16 | -0.206607762 | 0.0826576086395749 | 0.012434717400782 | -0.368616675 | -0.044598849 | 0.813338609654981 | 0.691690501875094 | 0.956381057947439 | 0.998735809224318 |
| ebi-fl187-GCST90094729 | prot-a-1890 | Beta-1,3-N-acetylglucosaminyltransferase manic fringe \|\| id:prot-a-1890 | F-cheese liking (derived food-liking factor) \|\| id:ebi-fl187-GCST90094729 | Inverse variance weighted | 16 | 0.0347113161043298 | 0.0826642118152172 | 0.674553085327368 | -0.127310539 | 0.196733171262156 | 1.03532078521694 | 0.880460212858248 | 1.21741915494687 | 0.998735809224318 |
| ebi-fl187-GCST90094729 | prot-a-1919 | Matrilysin \|\| id:prot-a-1919 | F-cheese liking (derived food-liking factor) \|\| id:ebi-fl187-GCST90094729 | Inverse variance weighted | 16 | -0.028480393 | 0.0933540547285665 | 0.760305731016714 | -0.21145434 | 0.154493554492563 | 0.971921350639502 | 0.80940623766267 | 1.1670667556958 | 0.998735809224318 |
| ebi-fl187-GCST90094729 | prot-a-1923 | Multimerin-2 \|\| id:prot-a-1923 | F-cheese liking (derived food-liking factor) \|\| id:ebi-fl187-GCST90094729 | Inverse variance weighted | 16 | 0.0185839795962655 | 0.0988997081266755 | 0.850949300514545 | -0.175259448 | 0.212427407524549 | 1.01875773644064 | 0.839239253296293 | 1.23667633691014 | 0.998735809224318 |
| ebi-fl187-GCST90094729 | prot-a-1948 | Mesothelin \|\| id:prot-a-1948 | F-cheese liking (derived food-liking factor) \|\| id:ebi-fl187-GCST90094729 | Inverse variance weighted | 16 | -0.049910167 | 0.0954510100051958 | 0.601052350474701 | -0.236994146 | 0.137173813102255 | 0.951314880600031 | 0.788995906692939 | 1.14702749960306 | 0.998735809224318 |
| ebi-fl187-GCST90094729 | prot-a-195 | ATPase family AAA domain-containing protein 1 \|\| id:prot-a-195 | F-cheese liking (derived food-liking factor) \|\| id:ebi-fl187-GCST90094729 | Inverse variance weighted | 16 | 0.0181133075112786 | 0.0826642118152172 | 0.826557274371889 | -0.143908548 | 0.180135162669104 | 1.01827834843886 | 0.865966939152642 | 1.19737919315255 | 0.998735809224318 |
| ebi-fl187-GCST90094729 | prot-a-1966 | Mucin-1 \|\| id:prot-a-1966 | F-cheese liking (derived food-liking factor) \|\| id:ebi-fl187-GCST90094729 | Inverse variance weighted | 16 | -0.089913111 | 0.0921405648875791 | 0.329150954535264 | -0.270508618 | 0.090682395995229 | 0.91401059911947 | 0.762991324230861 | 1.0949211986714 | 0.998735809224318 |
| ebi-fl187-GCST90094729 | prot-a-1986 | Myocardial zonula adherens protein \|\| id:prot-a-1986 | F-cheese liking (derived food-liking factor) \|\| id:ebi-fl187-GCST90094729 | Inverse variance weighted | 16 | -0.062214121 | 0.0826576086395749 | 0.451646651444712 | -0.224223034 | 0.0997947919384938 | 0.939681659676361 | 0.799136879853867 | 1.10494415136189 | 0.998735809224318 |
| ebi-fl187-GCST90094729 | prot-a-2071 | Epididymal secretory protein E1 \|\| id:prot-a-2071 | F-cheese liking (derived food-liking factor) \|\| id:ebi-fl187-GCST90094729 | Inverse variance weighted | 16 | -0.142899132 | 0.104261672660619 | 0.170505417423698 | -0.34725201 | 0.0614537467303 | 0.866841501327867 | 0.706627228619168 | 1.06338131052875 | 0.998735809224318 |
| ebi-fl187-GCST90094729 | prot-a-2098 | Neuritin \|\| id:prot-a-2098 | F-cheese liking (derived food-liking factor) \|\| id:ebi-fl187-GCST90094729 | Inverse variance weighted | 16 | 0.0295386186905858 | 0.0826642118152172 | 0.720842818884707 | -0.132483236 | 0.191560473848412 | 1.02997921115231 | 0.875917617456806 | 1.21113807310566 | 0.998735809224318 |
| ebi-fl187-GCST90094729 | prot-a-2115 | Neurotrophin-4 \|\| id:prot-a-2115 | F-cheese liking (derived food-liking factor) \|\| id:ebi-fl187-GCST90094729 | Inverse variance weighted | 16 | -0.086842632 | 0.0926086911993764 | 0.348379383263911 | -0.268355667 | 0.0946704022788027 | 0.916821362192827 | 0.764635776851063 | 1.09929646979733 | 0.998735809224318 |
| ebi-fl187-GCST90094729 | prot-a-2118 | Netrin-1 \|\| id:prot-a-2118 | F-cheese liking (derived food-liking factor) \|\| id:ebi-fl187-GCST90094729 | Inverse variance weighted | 16 | 0.0806537932698088 | 0.0971254004912323 | 0.406307656955455 | -0.109711992 | 0.271019578232624 | 1.08399554504271 | 0.896092180126926 | 1.31130074308426 | 0.998735809224318 |
| ebi-fl187-GCST90094729 | prot-a-2120 | Netrin-G1 \|\| id:prot-a-2120 | F-cheese liking (derived food-liking factor) \|\| id:ebi-fl187-GCST90094729 | Inverse variance weighted | 16 | -0.02447214 | 0.0826642118152172 | 0.767197439133448 | -0.186493995 | 0.137549715115525 | 0.975824874979443 | 0.829863545100895 | 1.14745875059842 | 0.998735809224318 |
| ebi-fl187-GCST90094729 | prot-a-2145 | Olfactomedin-like protein 3 \|\| id:prot-a-2145 | F-cheese liking (derived food-liking factor) \|\| id:ebi-fl187-GCST90094729 | Inverse variance weighted | 16 | -0.134780028 | 0.0901969837536374 | 0.135101062973213 | -0.311566116 | 0.0420060603080107 | 0.87390812599651 | 0.732299192206807 | 1.04290079903168 | 0.998735809224318 |
| ebi-fl187-GCST90094729 | prot-a-2162 | Prolyl 4-hydroxylase subunit alpha-2 \|\| id:prot-a-2162 | F-cheese liking (derived food-liking factor) \|\| id:ebi-fl187-GCST90094729 | Inverse variance weighted | 16 | -0.151626322 | 0.0826642118152172 | 0.0666178046110513 | -0.313648177 | 0.0103955328230664 | 0.859309325456224 | 0.730776086412414 | 1.01044975409787 | 0.998735809224318 |
| ebi-fl187-GCST90094729 | prot-a-2168 | Polyadenylate-binding protein-interacting protein 1 \|\| id:prot-a-2168 | F-cheese liking (derived food-liking factor) \|\| id:ebi-fl187-GCST90094729 | Inverse variance weighted (multiplicative random effects) | 16 | 0.0480472211171958 | 0.111167585830286 | 0.665592198941275 | -0.169841247 | 0.265935689344557 | 1.0492201995047 | 0.843798761455856 | 1.30465115301817 | 0.998735809224318 |
| ebi-fl187-GCST90094729 | prot-a-219 | Beta-1,4-galactosyltransferase 5 \|\| id:prot-a-219 | F-cheese liking (derived food-liking factor) \|\| id:ebi-fl187-GCST90094729 | Inverse variance weighted | 16 | -0.107212271 | 0.0927322915218685 | 0.247620546919506 | -0.288967563 | 0.0745430202371989 | 0.898334962158211 | 0.749036501858772 | 1.07739169217144 | 0.998735809224318 |
| ebi-fl187-GCST90094729 | prot-a-2190 | Pyruvate carboxylase, mitochondrial \|\| id:prot-a-2190 | F-cheese liking (derived food-liking factor) \|\| id:ebi-fl187-GCST90094729 | Inverse variance weighted | 16 | -0.089443291 | 0.0941593320060023 | 0.342155828740812 | -0.273995581 | 0.0951090000856095 | 0.914440120962325 | 0.760335434888401 | 1.09977872456822 | 0.998735809224318 |
| ebi-fl187-GCST90094729 | prot-a-2197 | Protocadherin-9 \|\| id:prot-a-2197 | F-cheese liking (derived food-liking factor) \|\| id:ebi-fl187-GCST90094729 | Inverse variance weighted | 16 | -0.010897767 | 0.0826642118152172 | 0.895117383590991 | -0.172919622 | 0.15112408857452 | 0.989161398955827 | 0.8412052267388 | 1.16314098163356 | 0.998735809224318 |
| ebi-fl187-GCST90094729 | prot-a-2198 | Protocadherin alpha-4 \|\| id:prot-a-2198 | F-cheese liking (derived food-liking factor) \|\| id:ebi-fl187-GCST90094729 | Inverse variance weighted (multiplicative random effects) | 16 | -0.028563079 | 0.118031713795723 | 0.808784075386494 | -0.259905238 | 0.202779080226129 | 0.971840989636123 | 0.771124655769282 | 1.2248018554078 | 0.998735809224318 |
| ebi-fl187-GCST90094729 | prot-a-2254 | Placenta growth factor \|\| id:prot-a-2254 | F-cheese liking (derived food-liking factor) \|\| id:ebi-fl187-GCST90094729 | Inverse variance weighted | 16 | -0.003203288 | 0.10474527953281 | 0.975603139625408 | -0.208504036 | 0.202097459565513 | 0.996801836735429 | 0.811797758115301 | 1.22396728961895 | 0.998955228396052 |
| ebi-fl187-GCST90094729 | prot-a-2261 | Membrane-associated progesterone receptor component 2 \|\| id:prot-a-2261 | F-cheese liking (derived food-liking factor) \|\| id:ebi-fl187-GCST90094729 | Inverse variance weighted | 16 | -0.005804254 | 0.0927610773224579 | 0.950107261662792 | -0.187615965 | 0.176007457872021 | 0.994212558457364 | 0.828932985200514 | 1.19244695173427 | 0.998735809224318 |
| ebi-fl187-GCST90094729 | prot-a-2268 | Polymeric immunoglobulin receptor \|\| id:prot-a-2268 | F-cheese liking (derived food-liking factor) \|\| id:ebi-fl187-GCST90094729 | Inverse variance weighted | 16 | 0.0426771319536345 | 0.0871430175207931 | 0.624320243674318 | -0.128123182 | 0.213477446294389 | 1.04360089506813 | 0.879745003380215 | 1.23797557701651 | 0.998735809224318 |
| ebi-fl187-GCST90094729 | prot-a-2283 | Group 10 secretory phospholipase A2 \|\| id:prot-a-2283 | F-cheese liking (derived food-liking factor) \|\| id:ebi-fl187-GCST90094729 | Inverse variance weighted | 16 | 0.10120805406356 | 0.0826642118152172 | 0.220828769512751 | -0.060813801 | 0.263229909221386 | 1.10650683105924 | 0.940998436343943 | 1.30112582539218 | 0.998735809224318 |
| ebi-fl187-GCST90094729 | prot-a-2298 | Inactive phospholipase D5 \|\| id:prot-a-2298 | F-cheese liking (derived food-liking factor) \|\| id:ebi-fl187-GCST90094729 | Inverse variance weighted (multiplicative random effects) | 16 | 0.038820535753249 | 0.108322608404018 | 0.720059790115301 | -0.173491777 | 0.251132848225124 | 1.03958389876667 | 0.840724064643997 | 1.28548084683713 | 0.998735809224318 |
| ebi-fl187-GCST90094729 | prot-a-2304 | Procollagen-lysine,2-oxoglutarate 5-dioxygenase 2 \|\| id:prot-a-2304 | F-cheese liking (derived food-liking factor) \|\| id:ebi-fl187-GCST90094729 | Inverse variance weighted | 16 | 0.00122347835457798 | 0.0826576086395749 | 0.988190332859456 | -0.160785435 | 0.163232391288145 | 1.00122422710955 | 0.851474748546222 | 1.17731025454679 | 0.998955228396052 |
| ebi-fl187-GCST90094729 | prot-a-2331 | Periostin \|\| id:prot-a-2331 | F-cheese liking (derived food-liking factor) \|\| id:ebi-fl187-GCST90094729 | Inverse variance weighted (multiplicative random effects) | 16 | -0.175198936 | 0.107913865227067 | 0.104481028669674 | -0.386710111 | 0.0363122402818139 | 0.839290039523771 | 0.679287984224389 | 1.0369795827437 | 0.998735809224318 |
| ebi-fl187-GCST90094729 | prot-a-2333 | Periostin \|\| id:prot-a-2333 | F-cheese liking (derived food-liking factor) \|\| id:ebi-fl187-GCST90094729 | Inverse variance weighted | 16 | -0.175497153 | 0.0909290744747426 | 0.053601334279576 | -0.353718139 | 0.00272383326966916 | 0.839039786167353 | 0.702072826649391 | 1.00272754627395 | 0.998735809224318 |
| ebi-fl187-GCST90094729 | prot-a-2367 | cAMP-dependent protein kinase catalytic subunit alpha \|\| id:prot-a-2367 | F-cheese liking (derived food-liking factor) \|\| id:ebi-fl187-GCST90094729 | Inverse variance weighted | 16 | 0.0992023969995441 | 0.0826576086395749 | 0.23007701231901 | -0.062806516 | 0.261211309933111 | 1.10428978188052 | 0.939125161866462 | 1.29850202282104 | 0.998735809224318 |
| ebi-fl187-GCST90094729 | prot-a-2385 | Phosphoribosyl pyrophosphate synthase-associated protein 1 \|\| id:prot-a-2385 | F-cheese liking (derived food-liking factor) \|\| id:ebi-fl187-GCST90094729 | Inverse variance weighted | 16 | 0.162419928015695 | 0.0826642118152172 | 0.0494355908694424 | 0.000398072857869553 | 0.324441783173521 | 1.17635412166871 | 1.00039815209938 | 1.38325827238183 | 0.998735809224318 |
| ebi-fl187-GCST90094729 | prot-a-2393 | Trypsin-3 \|\| id:prot-a-2393 | F-cheese liking (derived food-liking factor) \|\| id:ebi-fl187-GCST90094729 | Inverse variance weighted | 16 | 0.00548253256069214 | 0.0826395351739165 | 0.947104962674416 | -0.156490956 | 0.167456021501568 | 1.00549758914583 | 0.855139251222218 | 1.18229329355781 | 0.998735809224318 |
| ebi-fl187-GCST90094729 | prot-a-2405 | Pregnancy-specific beta-1-glycoprotein 6 \|\| id:prot-a-2405 | F-cheese liking (derived food-liking factor) \|\| id:ebi-fl187-GCST90094729 | Inverse variance weighted | 16 | -0.006470402 | 0.0826642118152172 | 0.937610638611605 | -0.168492258 | 0.155551452720938 | 0.99355048554147 | 0.844937805244542 | 1.16830204683999 | 0.998735809224318 |
| ebi-fl187-GCST90094729 | prot-a-244 | Beclin-1 \|\| id:prot-a-244 | F-cheese liking (derived food-liking factor) \|\| id:ebi-fl187-GCST90094729 | Inverse variance weighted | 16 | 0.0350196143442608 | 0.0826476971547762 | 0.671768861664973 | -0.126969872 | 0.197009100767622 | 1.03564002200033 | 0.8807602076713 | 1.21775512316188 | 0.998735809224318 |
| ebi-fl187-GCST90094729 | prot-a-2454 | tRNA pseudouridine synthase A, mitochondrial \|\| id:prot-a-2454 | F-cheese liking (derived food-liking factor) \|\| id:ebi-fl187-GCST90094729 | Inverse variance weighted | 16 | 0.0653462624496436 | 0.0826576086395749 | 0.429197524058293 | -0.09666265 | 0.22735517538321 | 1.06752860546447 | 0.90786222136063 | 1.25527563177697 | 0.998735809224318 |
| ebi-fl187-GCST90094729 | prot-a-2466 | Glutaminyl-peptide cyclotransferase-like protein \|\| id:prot-a-2466 | F-cheese liking (derived food-liking factor) \|\| id:ebi-fl187-GCST90094729 | Inverse variance weighted | 16 | -0.117495061 | 0.0826642118152172 | 0.155214037905161 | -0.279516916 | 0.0445267941748088 | 0.889144903237381 | 0.75614893658507 | 1.04553299052887 | 0.998735809224318 |
| ebi-fl187-GCST90094729 | prot-a-2474 | Ras-related protein Rab-27A \|\| id:prot-a-2474 | F-cheese liking (derived food-liking factor) \|\| id:ebi-fl187-GCST90094729 | Inverse variance weighted | 16 | 0.0188647155424388 | 0.100115706206951 | 0.850540265273206 | -0.177362069 | 0.215091499708063 | 1.01904377850697 | 0.837476505657073 | 1.23997534915801 | 0.998735809224318 |
| ebi-fl187-GCST90094729 | prot-a-2490 | Rap1 GTPase-GDP dissociation stimulator 1 \|\| id:prot-a-2490 | F-cheese liking (derived food-liking factor) \|\| id:ebi-fl187-GCST90094729 | Inverse variance weighted | 16 | 0.101634752720029 | 0.0887577732622369 | 0.252176011504081 | -0.072330483 | 0.275599988314014 | 1.10697907678357 | 0.930223422096651 | 1.31732081490127 | 0.998735809224318 |
| ebi-fl187-GCST90094729 | prot-a-2545 | Receptor-interacting serine/threonine-protein kinase 2 \|\| id:prot-a-2545 | F-cheese liking (derived food-liking factor) \|\| id:ebi-fl187-GCST90094729 | Inverse variance weighted (multiplicative random effects) | 16 | 0.130782915493891 | 0.107264087865117 | 0.22274515850657 | -0.079454697 | 0.341020527709519 | 1.13972033882814 | 0.923619862028426 | 1.40638211036929 | 0.998735809224318 |
| ebi-fl187-GCST90094729 | prot-a-2610 | RUN and FYVE domain-containing protein 1 \|\| id:prot-a-2610 | F-cheese liking (derived food-liking factor) \|\| id:ebi-fl187-GCST90094729 | Inverse variance weighted | 16 | -0.132655919 | 0.0905060386454419 | 0.142726366122142 | -0.310047755 | 0.0447359168386487 | 0.875766374924945 | 0.733411931556815 | 1.04575165803635 | 0.998735809224318 |
| ebi-fl187-GCST90094729 | prot-a-2627 | Serine--tRNA ligase, mitochondrial \|\| id:prot-a-2627 | F-cheese liking (derived food-liking factor) \|\| id:ebi-fl187-GCST90094729 | Inverse variance weighted | 16 | -0.074483241 | 0.0826642118152172 | 0.367570402540081 | -0.236505096 | 0.0875386146192093 | 0.928223030446659 | 0.789381859841386 | 1.09148441088411 | 0.998735809224318 |
| ebi-fl187-GCST90094729 | prot-a-264 | BolA-like protein 2 \|\| id:prot-a-264 | F-cheese liking (derived food-liking factor) \|\| id:ebi-fl187-GCST90094729 | Inverse variance weighted | 16 | -0.098755336 | 0.0826576086395749 | 0.232183971424799 | -0.260764249 | 0.0632535765156586 | 0.905964337390159 | 0.770462535248585 | 1.06529693927035 | 0.998735809224318 |
| ebi-fl187-GCST90094729 | prot-a-2646 | Secretoglobin family 1C member 1 \|\| id:prot-a-2646 | F-cheese liking (derived food-liking factor) \|\| id:ebi-fl187-GCST90094729 | Inverse variance weighted | 16 | 0.0261274219803602 | 0.0989097165733593 | 0.791661062627865 | -0.167735623 | 0.219990466464144 | 1.026471735 | 0.845577356760995 | 1.24606485112682 | 0.998735809224318 |
| ebi-fl187-GCST90094729 | prot-a-2672 | Semaphorin-3E \|\| id:prot-a-2672 | F-cheese liking (derived food-liking factor) \|\| id:ebi-fl187-GCST90094729 | Inverse variance weighted | 16 | -0.147210237 | 0.0826642118152172 | 0.0749415541217578 | -0.309232092 | 0.0148116182138903 | 0.863112500206065 | 0.734010392240709 | 1.01492185381438 | 0.998735809224318 |
| ebi-fl187-GCST90094729 | prot-a-2701 | Plasma protease C1 inhibitor \|\| id:prot-a-2701 | F-cheese liking (derived food-liking factor) \|\| id:ebi-fl187-GCST90094729 | Inverse variance weighted | 16 | -0.032005834 | 0.0826642118152172 | 0.698623806703287 | -0.19402769 | 0.130016020689878 | 0.968500931375072 | 0.823635097805195 | 1.13884662828712 | 0.998735809224318 |
| ebi-fl187-GCST90094729 | prot-a-2738 | Signaling threshold-regulating transmembrane adapter 1 \|\| id:prot-a-2738 | F-cheese liking (derived food-liking factor) \|\| id:ebi-fl187-GCST90094729 | Inverse variance weighted (multiplicative random effects) | 16 | -0.058301319 | 0.113388020764695 | 0.6071294367849 | -0.28054184 | 0.163939201375985 | 0.94336565030792 | 0.755374338502383 | 1.17814268346114 | 0.998735809224318 |
| ebi-fl187-GCST90094729 | prot-a-274 | Serine/threonine-protein kinase BRSK2 \|\| id:prot-a-274 | F-cheese liking (derived food-liking factor) \|\| id:ebi-fl187-GCST90094729 | Inverse variance weighted | 16 | -0.085971442 | 0.0826576086395749 | 0.29829758340208 | -0.247980355 | 0.0760374707742883 | 0.917620436104229 | 0.780375273527247 | 1.07900300447784 | 0.998735809224318 |
| ebi-fl187-GCST90094729 | prot-a-2749 | Mitochondrial glutamate carrier 2 \|\| id:prot-a-2749 | F-cheese liking (derived food-liking factor) \|\| id:ebi-fl187-GCST90094729 | Inverse variance weighted | 16 | 0.00202820855570736 | 0.0945682447958795 | 0.982889052836531 | -0.183325551 | 0.187381968355631 | 1.00203026676194 | 0.832497091152947 | 1.2060878844831 | 0.998955228396052 |
| ebi-fl187-GCST90094729 | prot-a-2760 | Sodium-coupled monocarboxylate transporter 1 \|\| id:prot-a-2760 | F-cheese liking (derived food-liking factor) \|\| id:ebi-fl187-GCST90094729 | Inverse variance weighted | 16 | -0.063287675 | 0.0870540480507028 | 0.467230304000626 | -0.233913609 | 0.107338259165331 | 0.938673401960457 | 0.791430185234351 | 1.11331077836905 | 0.998735809224318 |
| ebi-fl187-GCST90094729 | prot-a-2777 | Small integral membrane protein 10 \|\| id:prot-a-2777 | F-cheese liking (derived food-liking factor) \|\| id:ebi-fl187-GCST90094729 | Inverse variance weighted (multiplicative random effects) | 16 | 0.0285588349029848 | 0.109718955596473 | 0.794639467397307 | -0.186490318 | 0.243607987872073 | 1.02897054843604 | 0.829866596625982 | 1.27584408608983 | 0.998735809224318 |
| ebi-fl187-GCST90094729 | prot-a-2799 | Superoxide dismutase [Mn], mitochondrial \|\| id:prot-a-2799 | F-cheese liking (derived food-liking factor) \|\| id:ebi-fl187-GCST90094729 | Inverse variance weighted | 16 | 0.121303009475809 | 0.082928777984555 | 0.143539299731564 | -0.041237395 | 0.283843414325537 | 1.12896694822786 | 0.959601297996793 | 1.32822493347148 | 0.998735809224318 |
| ebi-fl187-GCST90094729 | prot-a-2805 | Protein SPACA7 \|\| id:prot-a-2805 | F-cheese liking (derived food-liking factor) \|\| id:ebi-fl187-GCST90094729 | Inverse variance weighted | 16 | -0.014497228 | 0.0909972802831271 | 0.873420733236795 | -0.192851898 | 0.16385744109275 | 0.985607350573766 | 0.8246040909268 | 1.17804636211934 | 0.998735809224318 |
| ebi-fl187-GCST90094729 | prot-a-2811 | SPARC-like protein 1 \|\| id:prot-a-2811 | F-cheese liking (derived food-liking factor) \|\| id:ebi-fl187-GCST90094729 | Inverse variance weighted | 16 | -0.131630778 | 0.0826642118152172 | 0.111304941808196 | -0.293652633 | 0.0303910769696593 | 0.876664619030195 | 0.745535420613508 | 1.03085759979977 | 0.998735809224318 |
| ebi-fl187-GCST90094729 | prot-a-2831 | Sepiapterin reductase \|\| id:prot-a-2831 | F-cheese liking (derived food-liking factor) \|\| id:ebi-fl187-GCST90094729 | Inverse variance weighted | 16 | -0.013778794 | 0.0828190003129474 | 0.867863909599328 | -0.176104035 | 0.148546446529468 | 0.986315699001166 | 0.838530742507155 | 1.16014668131009 | 0.998735809224318 |
| ebi-fl187-GCST90094729 | prot-a-2834 | SPRY domain-containing SOCS box protein 1 \|\| id:prot-a-2834 | F-cheese liking (derived food-liking factor) \|\| id:ebi-fl187-GCST90094729 | Inverse variance weighted | 16 | -0.140640318 | 0.0826642118152172 | 0.0888782800236626 | -0.302662173 | 0.0213815370063767 | 0.868801747724162 | 0.738848657010731 | 1.02161175998131 | 0.998735809224318 |
| ebi-fl187-GCST90094729 | prot-a-2835 | Spectrin alpha chain, non-erythrocytic 1 \|\| id:prot-a-2835 | F-cheese liking (derived food-liking factor) \|\| id:ebi-fl187-GCST90094729 | Inverse variance weighted | 16 | -0.029219949 | 0.0826642118152172 | 0.723730401166035 | -0.191241804 | 0.132801906002073 | 0.971202825731052 | 0.825932849877552 | 1.14202374787226 | 0.998735809224318 |
| ebi-fl187-GCST90094729 | prot-a-2859 | Sia-alpha-2,3-Gal-beta-1,4-GlcNAc-R:alpha 2,8-sialyltransferase \|\| id:prot-a-2859 | F-cheese liking (derived food-liking factor) \|\| id:ebi-fl187-GCST90094729 | Inverse variance weighted | 16 | -0.045842939 | 0.0826576086395749 | 0.57915976697298 | -0.207851852 | 0.116165973556604 | 0.955191973456446 | 0.812327372221293 | 1.12318227522077 | 0.998735809224318 |
| ebi-fl187-GCST90094729 | prot-a-2861 | Alpha-2,8-sialyltransferase 8F \|\| id:prot-a-2861 | F-cheese liking (derived food-liking factor) \|\| id:ebi-fl187-GCST90094729 | Inverse variance weighted | 16 | -0.084032348 | 0.0949161275940129 | 0.37597723679503 | -0.270067958 | 0.102003261802972 | 0.919401514555269 | 0.76332761807685 | 1.10738708380052 | 0.998735809224318 |
| ebi-fl187-GCST90094729 | prot-a-289 | Uncharacterized protein C17orf89 \|\| id:prot-a-289 | F-cheese liking (derived food-liking factor) \|\| id:ebi-fl187-GCST90094729 | Inverse variance weighted | 16 | 0.0884913193580322 | 0.0896192050929298 | 0.323439387160862 | -0.087162323 | 0.264144961340175 | 1.09252476875296 | 0.916528310277469 | 1.30231696823129 | 0.998735809224318 |
| ebi-fl187-GCST90094729 | prot-a-2921 | Tachykinin-3 \|\| id:prot-a-2921 | F-cheese liking (derived food-liking factor) \|\| id:ebi-fl187-GCST90094729 | Inverse variance weighted | 16 | 0.0814895941204606 | 0.0951401582139207 | 0.391709367713583 | -0.104985116 | 0.267964304219745 | 1.08490192816649 | 0.900337923135252 | 1.3073004740716 | 0.998735809224318 |
| ebi-fl187-GCST90094729 | prot-a-2928 | Tax1-binding protein 1 \|\| id:prot-a-2928 | F-cheese liking (derived food-liking factor) \|\| id:ebi-fl187-GCST90094729 | Inverse variance weighted (multiplicative random effects) | 16 | 5.42124618508093e-05 | 0.111521268087402 | 0.99961213421124 | -0.218527473 | 0.218635897913158 | 1.00005421393137 | 0.803701399061288 | 1.24437811352576 | 0.999882738470636 |
| ebi-fl187-GCST90094729 | prot-a-296 | Uncharacterized protein C1orf185 \|\| id:prot-a-296 | F-cheese liking (derived food-liking factor) \|\| id:ebi-fl187-GCST90094729 | Inverse variance weighted | 16 | -0.039815178 | 0.0826642118152172 | 0.630054941986878 | -0.201837033 | 0.122206677243607 | 0.960967030671376 | 0.817228098243374 | 1.12998762037469 | 0.998735809224318 |
| ebi-fl187-GCST90094729 | prot-a-2964 | Transforming growth factor beta-2 \|\| id:prot-a-2964 | F-cheese liking (derived food-liking factor) \|\| id:ebi-fl187-GCST90094729 | Inverse variance weighted | 16 | -0.03546641 | 0.0826642118152172 | 0.667892733876402 | -0.197488266 | 0.126555444795618 | 0.965155152897531 | 0.820789772112352 | 1.13491237441632 | 0.998735809224318 |
| ebi-fl187-GCST90094729 | prot-a-2977 | Thymocyte nuclear protein 1 \|\| id:prot-a-2977 | F-cheese liking (derived food-liking factor) \|\| id:ebi-fl187-GCST90094729 | Inverse variance weighted | 16 | 0.0675350267280603 | 0.0831063298292882 | 0.41642794923011 | -0.09535338 | 0.230423433193465 | 1.06986773290776 | 0.909051637272804 | 1.25913305579767 | 0.998735809224318 |
| ebi-fl187-GCST90094729 | prot-a-3015 | Transmembrane protein 70, mitochondrial \|\| id:prot-a-3015 | F-cheese liking (derived food-liking factor) \|\| id:ebi-fl187-GCST90094729 | Inverse variance weighted | 16 | -0.012667952 | 0.0869873895988951 | 0.88421367914589 | -0.183163236 | 0.157827331581924 | 0.987411948723579 | 0.832632229383748 | 1.17096398875132 | 0.998735809224318 |
| ebi-fl187-GCST90094729 | prot-a-3030 | Tumor necrosis factor alpha-induced protein 3 \|\| id:prot-a-3030 | F-cheese liking (derived food-liking factor) \|\| id:ebi-fl187-GCST90094729 | Inverse variance weighted | 16 | -0.023877792 | 0.0920002993741896 | 0.795218334046442 | -0.204198379 | 0.156442794554677 | 0.976405026758964 | 0.815300616628847 | 1.16934386756901 | 0.998735809224318 |
| ebi-fl187-GCST90094729 | prot-a-3032 | Tumor necrosis factor receptor superfamily member 10A \|\| id:prot-a-3032 | F-cheese liking (derived food-liking factor) \|\| id:ebi-fl187-GCST90094729 | Inverse variance weighted | 16 | 0.00766977607743222 | 0.0826576086395749 | 0.92607062075754 | -0.154339137 | 0.169678689010999 | 1.00769926415074 | 0.856981337766886 | 1.18492406102565 | 0.998735809224318 |
| ebi-fl187-GCST90094729 | prot-a-3081 | Tumor protein p53-inducible protein 11 \|\| id:prot-a-3081 | F-cheese liking (derived food-liking factor) \|\| id:ebi-fl187-GCST90094729 | Inverse variance weighted | 16 | 0.153215733604148 | 0.0826576086395749 | 0.0637936532046339 | -0.008793179 | 0.315224646537715 | 1.16557640582009 | 0.991245367605828 | 1.37056716954536 | 0.998735809224318 |
| ebi-fl187-GCST90094729 | prot-a-315 | Complement C4 \|\| id:prot-a-315 | F-cheese liking (derived food-liking factor) \|\| id:ebi-fl187-GCST90094729 | Inverse variance weighted | 16 | -0.024574732 | 0.0834559990897911 | 0.768403872081194 | -0.188148491 | 0.13899902591627 | 0.975724768037859 | 0.828491674942269 | 1.14912298066106 | 0.998735809224318 |
| ebi-fl187-GCST90094729 | prot-a-3157 | UDP-glucuronosyltransferase 1-8 \|\| id:prot-a-3157 | F-cheese liking (derived food-liking factor) \|\| id:ebi-fl187-GCST90094729 | Inverse variance weighted | 16 | 0.0232181802574714 | 0.106492884253845 | 0.827409126700188 | -0.185507873 | 0.231944233395007 | 1.02348982046114 | 0.830682295693093 | 1.26104940242351 | 0.998735809224318 |
| ebi-fl187-GCST90094729 | prot-a-317 | Glutaredoxin-like protein C5orf63 \|\| id:prot-a-317 | F-cheese liking (derived food-liking factor) \|\| id:ebi-fl187-GCST90094729 | Inverse variance weighted | 16 | -0.179113915 | 0.0826576086395749 | 0.0302395805243715 | -0.341122828 | -0.017105002 | 0.836010660074296 | 0.710971575891396 | 0.983040458237141 | 0.998735809224318 |
| ebi-fl187-GCST90094729 | prot-a-3192 | Guanine nucleotide exchange factor VAV3 \|\| id:prot-a-3192 | F-cheese liking (derived food-liking factor) \|\| id:ebi-fl187-GCST90094729 | Inverse variance weighted | 16 | 0.0121147553493752 | 0.0988495527898599 | 0.902457500452651 | -0.181630368 | 0.205859878817501 | 1.01218843623932 | 0.833909523000951 | 1.22858104170544 | 0.998735809224318 |
| ebi-fl187-GCST90094729 | prot-a-3233 | Serine/threonine-protein kinase WNK3 \|\| id:prot-a-3233 | F-cheese liking (derived food-liking factor) \|\| id:ebi-fl187-GCST90094729 | Inverse variance weighted | 16 | -0.006376654 | 0.0826642118152172 | 0.938512777069797 | -0.16839851 | 0.155645200692771 | 0.99364363325054 | 0.84501702016317 | 1.16841157792145 | 0.998735809224318 |
| ebi-fl187-GCST90094729 | prot-a-3250 | 14-3-3 protein theta \|\| id:prot-a-3250 | F-cheese liking (derived food-liking factor) \|\| id:ebi-fl187-GCST90094729 | Inverse variance weighted | 16 | 0.0979431791534199 | 0.0826576086395749 | 0.236046564932802 | -0.064065734 | 0.259952092086987 | 1.1029001156098 | 0.937943342942796 | 1.29686795494031 | 0.998735809224318 |
| ebi-fl187-GCST90094729 | prot-a-3278 | E3 ubiquitin-protein ligase ZNRF3 \|\| id:prot-a-3278 | F-cheese liking (derived food-liking factor) \|\| id:ebi-fl187-GCST90094729 | Inverse variance weighted | 16 | -0.007168922 | 0.0826472287513788 | 0.930877209373207 | -0.16915749 | 0.154819646726364 | 0.992856713796279 | 0.844375912169207 | 1.16744738915859 | 0.998735809224318 |
| ebi-fl187-GCST90094729 | prot-a-355 | Calpain-3 \|\| id:prot-a-355 | F-cheese liking (derived food-liking factor) \|\| id:ebi-fl187-GCST90094729 | Inverse variance weighted | 16 | 0.103004088615716 | 0.0826576086395749 | 0.212707956504719 | -0.059004824 | 0.265013001549282 | 1.10849594128067 | 0.942702221250975 | 1.3034479225106 | 0.998735809224318 |
| ebi-fl187-GCST90094729 | prot-a-385 | Coiled-coil domain-containing protein 90B, mitochondrial \|\| id:prot-a-385 | F-cheese liking (derived food-liking factor) \|\| id:ebi-fl187-GCST90094729 | Inverse variance weighted (multiplicative random effects) | 16 | 0.116041185718691 | 0.113545920824559 | 0.306792344123898 | -0.106508819 | 0.338591190534826 | 1.1230421244778 | 0.898967120047641 | 1.40296968067616 | 0.998735809224318 |
| ebi-fl187-GCST90094729 | prot-a-390 | C-C motif chemokine 14 \|\| id:prot-a-390 | F-cheese liking (derived food-liking factor) \|\| id:ebi-fl187-GCST90094729 | Inverse variance weighted | 16 | -0.100447141 | 0.0826642118152172 | 0.224319386810402 | -0.262468996 | 0.0615747143799867 | 0.904432918769839 | 0.769150210781519 | 1.06350995304724 | 0.998735809224318 |
| ebi-fl187-GCST90094729 | prot-a-40 | Pituitary adenylate cyclase-activating polypeptide 27 \|\| id:prot-a-40 | F-cheese liking (derived food-liking factor) \|\| id:ebi-fl187-GCST90094729 | Inverse variance weighted | 16 | 0.072987234329861 | 0.0838986885843153 | 0.384330528977157 | -0.091454195 | 0.237428663955119 | 1.07571680458116 | 0.912603116710854 | 1.26798454056227 | 0.998735809224318 |
| ebi-fl187-GCST90094729 | prot-a-401 | C-C motif chemokine 24 \|\| id:prot-a-401 | F-cheese liking (derived food-liking factor) \|\| id:ebi-fl187-GCST90094729 | Inverse variance weighted | 16 | 0.161342666986803 | 0.0860372540014356 | 0.0607562505102518 | -0.007290351 | 0.329975684829616 | 1.1750875635466 | 0.992736159289916 | 1.39093430724792 | 0.998735809224318 |
| ebi-fl187-GCST90094729 | prot-a-412 | Cerebral cavernous malformations 2 protein \|\| id:prot-a-412 | F-cheese liking (derived food-liking factor) \|\| id:ebi-fl187-GCST90094729 | Inverse variance weighted (multiplicative random effects) | 16 | 0.127234244014158 | 0.132359147466835 | 0.336411238930094 | -0.132189685 | 0.386658173049155 | 1.13568301357422 | 0.876174782084016 | 1.4720532177989 | 0.998735809224318 |
| ebi-fl187-GCST90094729 | prot-a-44 | Adhesion G protein-coupled receptor F5 \|\| id:prot-a-44 | F-cheese liking (derived food-liking factor) \|\| id:ebi-fl187-GCST90094729 | Inverse variance weighted | 16 | 0.11059567484474 | 0.0826576086395749 | 0.180897247293034 | -0.051413238 | 0.272604587778307 | 1.11694320730816 | 0.949886060317098 | 1.31338081531101 | 0.998735809224318 |
| ebi-fl187-GCST90094729 | prot-a-48 | Adrenomedullin \|\| id:prot-a-48 | F-cheese liking (derived food-liking factor) \|\| id:ebi-fl187-GCST90094729 | Inverse variance weighted | 16 | -0.023796329 | 0.0914842467535553 | 0.794775879740812 | -0.203105452 | 0.155512795098051 | 0.976484571545398 | 0.816192167647337 | 1.168256884 | 0.998735809224318 |
| ebi-fl187-GCST90094729 | prot-a-502 | Carcinoembryonic antigen-related cell adhesion molecule 1 \|\| id:prot-a-502 | F-cheese liking (derived food-liking factor) \|\| id:ebi-fl187-GCST90094729 | Inverse variance weighted | 16 | 0.00889515713468282 | 0.0826642118152172 | 0.914308314492659 | -0.153126698 | 0.170917012292509 | 1.00893483660937 | 0.858021005359265 | 1.18639228896003 | 0.998735809224318 |
| ebi-fl187-GCST90094729 | prot-a-518 | Complement factor B \|\| id:prot-a-518 | F-cheese liking (derived food-liking factor) \|\| id:ebi-fl187-GCST90094729 | Inverse variance weighted | 16 | 0.0036367548149451 | 0.098100901016248 | 0.970427937076639 | -0.188641011 | 0.195914520806791 | 1.00364337583164 | 0.828083726144588 | 1.2164229220402 | 0.998810926879716 |
| ebi-fl187-GCST90094729 | prot-a-525 | Properdin \|\| id:prot-a-525 | F-cheese liking (derived food-liking factor) \|\| id:ebi-fl187-GCST90094729 | Inverse variance weighted (multiplicative random effects) | 16 | -0.153817378 | 0.135452170185894 | 0.256130521548496 | -0.419303631 | 0.111668875942436 | 0.857428592362881 | 0.657504526077225 | 1.11814255544006 | 0.998735809224318 |
| ebi-fl187-GCST90094729 | prot-a-533 | Chondroadherin \|\| id:prot-a-533 | F-cheese liking (derived food-liking factor) \|\| id:ebi-fl187-GCST90094729 | Inverse variance weighted | 16 | -0.01163294 | 0.0849020713683936 | 0.891018166989643 | -0.178041 | 0.154775119687968 | 0.988434460844115 | 0.836908109489128 | 1.16739540734118 | 0.998735809224318 |
| ebi-fl187-GCST90094729 | prot-a-535 | Coiled-coil-helix-coiled-coil-helix domain-containing protein 2 \|\| id:prot-a-535 | F-cheese liking (derived food-liking factor) \|\| id:ebi-fl187-GCST90094729 | Inverse variance weighted (multiplicative random effects) | 16 | -0.094078165 | 0.108107922139884 | 0.384177715982378 | -0.305969693 | 0.117813362241316 | 0.91021161263026 | 0.736408937797142 | 1.12503411792539 | 0.998735809224318 |
| ebi-fl187-GCST90094729 | prot-a-591 | Caseinolytic peptidase B protein homolog \|\| id:prot-a-591 | F-cheese liking (derived food-liking factor) \|\| id:ebi-fl187-GCST90094729 | Inverse variance weighted (multiplicative random effects) | 15 | -0.006108841 | 0.131044632534976 | 0.962818890512404 | -0.26295632 | 0.250738639182454 | 0.993909780443625 | 0.768775476417956 | 1.28497419853236 | 0.998735809224318 |
| ebi-fl187-GCST90094729 | prot-a-605 | Ciliary neurotrophic factor receptor subunit alpha \|\| id:prot-a-605 | F-cheese liking (derived food-liking factor) \|\| id:ebi-fl187-GCST90094729 | Inverse variance weighted | 16 | -0.105026146 | 0.0898758150520291 | 0.242577080411417 | -0.281182743 | 0.0711304518365926 | 0.900300983310287 | 0.754890371817077 | 1.07372128564633 | 0.998735809224318 |
| ebi-fl187-GCST90094729 | prot-a-606 | Ciliary neurotrophic factor receptor subunit alpha \|\| id:prot-a-606 | F-cheese liking (derived food-liking factor) \|\| id:ebi-fl187-GCST90094729 | Inverse variance weighted | 16 | -0.101475709 | 0.0901316358536489 | 0.260224286300532 | -0.278133716 | 0.0751822970404186 | 0.903503125859826 | 0.757195565956612 | 1.07808066388658 | 0.998735809224318 |
| ebi-fl187-GCST90094729 | prot-a-707 | Cancer/testis antigen 55 \|\| id:prot-a-707 | F-cheese liking (derived food-liking factor) \|\| id:ebi-fl187-GCST90094729 | Inverse variance weighted | 16 | 0.0190792741617923 | 0.103216007886693 | 0.853348246309191 | -0.183224101 | 0.22138264961971 | 1.01926244659087 | 0.832581552223844 | 1.24780080972908 | 0.998735809224318 |
| ebi-fl187-GCST90094729 | prot-a-719 | Dipeptidyl peptidase 1 \|\| id:prot-a-719 | F-cheese liking (derived food-liking factor) \|\| id:ebi-fl187-GCST90094729 | Inverse variance weighted | 16 | -0.055037214 | 0.100301660650099 | 0.583199764814153 | -0.251628468 | 0.141554041259402 | 0.946449926475145 | 0.777533562631299 | 1.15206276150111 | 0.998735809224318 |
| ebi-fl187-GCST90094729 | prot-a-752 | Cytochrome b5 \|\| id:prot-a-752 | F-cheese liking (derived food-liking factor) \|\| id:ebi-fl187-GCST90094729 | Inverse variance weighted | 16 | 0.0572931502231543 | 0.0853505044127421 | 0.502049160870428 | -0.109993838 | 0.224580138872129 | 1.05896620107589 | 0.895839655062011 | 1.25179702493017 | 0.998735809224318 |
| ebi-fl187-GCST90094729 | prot-a-755 | Cytoglobin \|\| id:prot-a-755 | F-cheese liking (derived food-liking factor) \|\| id:ebi-fl187-GCST90094729 | Inverse variance weighted | 16 | 0.038124847661495 | 0.0826461340187161 | 0.644581770177237 | -0.123861575 | 0.200111270338178 | 1.0388609241395 | 0.88350213118763 | 1.22153867161955 | 0.998735809224318 |
| ebi-fl187-GCST90094729 | prot-a-807 | Desmin \|\| id:prot-a-807 | F-cheese liking (derived food-liking factor) \|\| id:ebi-fl187-GCST90094729 | Inverse variance weighted | 16 | 0.00846973348526928 | 0.098258447378986 | 0.931308601182605 | -0.184116823 | 0.201056290348082 | 1.00850570315739 | 0.831838619952954 | 1.22269359573435 | 0.998735809224318 |
| ebi-fl187-GCST90094729 | prot-a-82 | Ameloblastin \|\| id:prot-a-82 | F-cheese liking (derived food-liking factor) \|\| id:ebi-fl187-GCST90094729 | Inverse variance weighted | 16 | 0.0102749844038929 | 0.0980549081482684 | 0.916543978106105 | -0.181912636 | 0.202462604374499 | 1.01032795331896 | 0.83367417070577 | 1.22441429652728 | 0.998735809224318 |
| ebi-fl187-GCST90094729 | prot-a-84 | Muellerian-inhibiting factor \|\| id:prot-a-84 | F-cheese liking (derived food-liking factor) \|\| id:ebi-fl187-GCST90094729 | Inverse variance weighted | 16 | -0.145778932 | 0.0972331383650197 | 0.133803056013589 | -0.336355883 | 0.0447980192009506 | 0.864348761922243 | 0.714368828714721 | 1.04581660370131 | 0.998735809224318 |
| ebi-fl187-GCST90094729 | prot-a-858 | Inactive dipeptidyl peptidase 10 \|\| id:prot-a-858 | F-cheese liking (derived food-liking factor) \|\| id:ebi-fl187-GCST90094729 | Inverse variance weighted | 16 | 0.129343704076106 | 0.098717078499322 | 0.190112468326239 | -0.06414177 | 0.322829177934777 | 1.13808122010554 | 0.937872028191783 | 1.3810294204574 | 0.998735809224318 |
| ebi-fl187-GCST90094729 | prot-a-863 | Dihydropyrimidinase-related protein 3 \|\| id:prot-a-863 | F-cheese liking (derived food-liking factor) \|\| id:ebi-fl187-GCST90094729 | Inverse variance weighted | 16 | 0.0168985863413241 | 0.0826642118152172 | 0.838021822353686 | -0.145123269 | 0.17892044149915 | 1.01704217512705 | 0.864915669408246 | 1.19592559433553 | 0.998735809224318 |
| ebi-fl187-GCST90094729 | prot-a-864 | Dihydropyrimidinase-related protein 5 \|\| id:prot-a-864 | F-cheese liking (derived food-liking factor) \|\| id:ebi-fl187-GCST90094729 | Inverse variance weighted | 16 | -0.144360577 | 0.103009089280268 | 0.161083933765074 | -0.346258392 | 0.0575372380420686 | 0.865575585181093 | 0.707329695201795 | 1.05922471337478 | 0.998735809224318 |
| ebi-fl187-GCST90094729 | prot-a-884 | Endothelin-converting enzyme 1 \|\| id:prot-a-884 | F-cheese liking (derived food-liking factor) \|\| id:ebi-fl187-GCST90094729 | Inverse variance weighted | 16 | 0.00655716740670445 | 0.0838181995904292 | 0.937644437572795 | -0.157726504 | 0.170840838603946 | 1.00657871269518 | 0.85408333858427 | 1.18630192052519 | 0.998735809224318 |
| ebi-fl187-GCST90094729 | prot-a-893 | Early endosome antigen 1 \|\| id:prot-a-893 | F-cheese liking (derived food-liking factor) \|\| id:ebi-fl187-GCST90094729 | Inverse variance weighted | 15 | 0.0128591513005621 | 0.0850891910078503 | 0.879876673251522 | -0.153915663 | 0.179633965675949 | 1.01294218572232 | 0.857344323746601 | 1.19677922066604 | 0.998735809224318 |
| ebi-fl187-GCST90094729 | prot-a-897 | Ephrin-A2 \|\| id:prot-a-897 | F-cheese liking (derived food-liking factor) \|\| id:ebi-fl187-GCST90094729 | Inverse variance weighted | 16 | -0.006512153 | 0.0900908496489033 | 0.942375664585679 | -0.183090219 | 0.17006591196412 | 0.993509004769666 | 0.832693027899356 | 1.18538297966596 | 0.998735809224318 |
| ebi-fl187-GCST90094729 | prot-a-90 | Anaphase-promoting complex subunit 10 \|\| id:prot-a-90 | F-cheese liking (derived food-liking factor) \|\| id:ebi-fl187-GCST90094729 | Inverse variance weighted | 16 | 0.104895935807316 | 0.0826395351739165 | 0.20432729669973 | -0.057077553 | 0.266869424748193 | 1.11059503116667 | 0.944520815977053 | 1.30586992090395 | 0.998735809224318 |
| ebi-fl187-GCST90094729 | prot-a-914 | Histone-lysine N-methyltransferase EHMT2 \|\| id:prot-a-914 | F-cheese liking (derived food-liking factor) \|\| id:ebi-fl187-GCST90094729 | Inverse variance weighted | 16 | 0.0508286103437897 | 0.0826461340187161 | 0.538544828556124 | -0.111157812 | 0.212815033020473 | 1.0521425514791 | 0.894797527700784 | 1.23715579710803 | 0.998735809224318 |
| ebi-fl187-GCST90094729 | prot-a-932 | RNA polymerase II elongation factor ELL \|\| id:prot-a-932 | F-cheese liking (derived food-liking factor) \|\| id:ebi-fl187-GCST90094729 | Inverse variance weighted | 16 | 0.0276450848548902 | 0.0826461340187161 | 0.738002719723333 | -0.134341338 | 0.189631507531574 | 1.028030756 | 0.874291584879828 | 1.20880408037799 | 0.998735809224318 |
| ebi-fl187-GCST90094729 | prot-a-991 | Estrogen receptor \|\| id:prot-a-991 | F-cheese liking (derived food-liking factor) \|\| id:ebi-fl187-GCST90094729 | Inverse variance weighted | 16 | -0.111671524 | 0.082646134018716 | 0.176631111948294 | -0.273657947 | 0.0503148986085087 | 0.89433797775831 | 0.760592193806875 | 1.05160219230953 | 0.998735809224318 |
| ebi-fl187-GCST90094729 | prot-a-992 | Persulfide dioxygenase ETHE1, mitochondrial \|\| id:prot-a-992 | F-cheese liking (derived food-liking factor) \|\| id:ebi-fl187-GCST90094729 | Inverse variance weighted | 16 | -0.034529919 | 0.0826642118152172 | 0.676156939742519 | -0.196551774 | 0.12749193623955 | 0.966059435800798 | 0.821558794746557 | 1.13597570796824 | 0.998735809224318 |
| ebi-fl187-GCST90094729 | prot-a-993 | Ethanolamine kinase 1 \|\| id:prot-a-993 | F-cheese liking (derived food-liking factor) \|\| id:ebi-fl187-GCST90094729 | Inverse variance weighted | 16 | 0.0155059150891185 | 0.0845690256363512 | 0.854521695686238 | -0.150249375 | 0.181261205336367 | 1.01562675556331 | 0.860493363997949 | 1.19872825261965 | 0.998735809224318 |
| ebi-fl187-GCST90094729 | prot-b-10 | coagulation factor III, tissue factor \|\| id:prot-b-10 | F-cheese liking (derived food-liking factor) \|\| id:ebi-fl187-GCST90094729 | Inverse variance weighted | 12 | -0.015702009 | 0.126126988373912 | 0.900924431864887 | -0.262910906 | 0.231506888708743 | 0.984420625326588 | 0.768810390870466 | 1.26049800975136 | 0.998735809224318 |
| ebi-fl187-GCST90094729 | prot-b-28 | prolactin \|\| id:prot-b-28 | F-cheese liking (derived food-liking factor) \|\| id:ebi-fl187-GCST90094729 | Inverse variance weighted | 12 | -0.028298955 | 0.148653999558679 | 0.849020819374952 | -0.319660795 | 0.263062883644266 | 0.972097709408831 | 0.726395392509749 | 1.30090852224839 | 0.998735809224318 |
| ebi-fl187-GCST90094729 | prot-b-54 | matrix metallopeptidase 7 \|\| id:prot-b-54 | F-cheese liking (derived food-liking factor) \|\| id:ebi-fl187-GCST90094729 | Inverse variance weighted | 12 | 0.0643665133483443 | 0.121230901917875 | 0.595459338768608 | -0.173246054 | 0.301979081107379 | 1.06648320747015 | 0.840930674684807 | 1.35253293292236 | 0.998735809224318 |
| ebi-fl187-GCST90094729 | prot-c-2421_7_3 | BDNF \|\| id:prot-c-2421_7_3 | F-cheese liking (derived food-liking factor) \|\| id:ebi-fl187-GCST90094729 | Inverse variance weighted | 2 | 1.23656705114449 | 0.658533948985515 | 0.0604143489018621 | -0.054159489 | 2.5272935911561 | 3.44377085963316 | 0.947281013677593 | 12.5195771502024 | 0.998735809224318 |
| ebi-fl187-GCST90094729 | prot-c-2443_10_4 | FGF-8B \|\| id:prot-c-2443_10_4 | F-cheese liking (derived food-liking factor) \|\| id:ebi-fl187-GCST90094729 | Inverse variance weighted | 2 | 0.714663402504133 | 0.680685210964677 | 0.293755985827199 | -0.619479611 | 2.0488064159949 | 2.04349872992823 | 0.538224450821385 | 7.75863499483437 | 0.998735809224318 |
| ebi-fl187-GCST90094729 | prot-c-2605_49_2 | CD30 \|\| id:prot-c-2605_49_2 | F-cheese liking (derived food-liking factor) \|\| id:ebi-fl187-GCST90094729 | Inverse variance weighted | 2 | 0.181907371543731 | 0.448718975500538 | 0.685188917772605 | -0.69758182 | 1.06139656352479 | 1.19950308061518 | 0.497787589309425 | 2.89040480579546 | 0.998735809224318 |
| ebi-fl187-GCST90094729 | prot-c-2624_31_2 | HPV E7 Type18 \|\| id:prot-c-2624_31_2 | F-cheese liking (derived food-liking factor) \|\| id:ebi-fl187-GCST90094729 | Inverse variance weighted | 2 | 0.36387055059357 | 0.454617243355733 | 0.423485567127104 | -0.527179246 | 1.25492034757081 | 1.43888793891208 | 0.590267623140621 | 3.50755897762231 | 0.998735809224318 |
| ebi-fl187-GCST90094729 | prot-c-2774_10_3 | IL-16 \|\| id:prot-c-2774_10_3 | F-cheese liking (derived food-liking factor) \|\| id:ebi-fl187-GCST90094729 | Inverse variance weighted | 2 | -1.00326266 | 0.434930286233346 | 0.0210703998539531 | -1.855726021 | -0.150799299 | 0.36668113137687 | 0.156339395706475 | 0.860020287914243 | 0.998735809224318 |
| ebi-fl187-GCST90094729 | prot-c-2851_63_3 | C5a \|\| id:prot-c-2851_63_3 | F-cheese liking (derived food-liking factor) \|\| id:ebi-fl187-GCST90094729 | Inverse variance weighted | 2 | -0.15664196 | 0.466396069792953 | 0.736979391829803 | -1.070778256 | 0.757494337135527 | 0.855010132140678 | 0.342741672676934 | 2.13292512799369 | 0.998735809224318 |
| ebi-fl187-GCST90094729 | prot-c-2891_1_3 | CD22 \|\| id:prot-c-2891_1_3 | F-cheese liking (derived food-liking factor) \|\| id:ebi-fl187-GCST90094729 | Inverse variance weighted | 2 | 0.0835623537064945 | 0.457035967740652 | 0.854927172047437 | -0.812228143 | 0.979352850478173 | 1.08715300119866 | 0.443867962263955 | 2.66273249816666 | 0.998735809224318 |
| ebi-fl187-GCST90094729 | prot-c-2961_1_2 | Protein C \|\| id:prot-c-2961_1_2 | F-cheese liking (derived food-liking factor) \|\| id:ebi-fl187-GCST90094729 | Inverse variance weighted | 2 | 0.724647411386651 | 0.443363270176341 | 0.102168034033152 | -0.144344598 | 1.59363942093228 | 2.0640032276183 | 0.865589416140609 | 4.92162825027743 | 0.998735809224318 |
| ebi-fl187-GCST90094729 | prot-c-2982_82_2 | Galectin-4 \|\| id:prot-c-2982_82_2 | F-cheese liking (derived food-liking factor) \|\| id:ebi-fl187-GCST90094729 | Inverse variance weighted | 2 | -0.184925843 | 0.418861415872683 | 0.658853604930966 | -1.005894218 | 0.636042531888212 | 0.831165918153066 | 0.365717457286854 | 1.88899044804789 | 0.998735809224318 |
| ebi-fl187-GCST90094729 | prot-c-2994_71_2 | IL-1Rrp2 \|\| id:prot-c-2994_71_2 | F-cheese liking (derived food-liking factor) \|\| id:ebi-fl187-GCST90094729 | Inverse variance weighted | 2 | 0.216021899051454 | 0.45198647486263 | 0.632693724190971 | -0.669871592 | 1.10191538978221 | 1.24112955826313 | 0.511774289644588 | 3.00992568709185 | 0.998735809224318 |
| ebi-fl187-GCST90094729 | prot-c-3336_50_1 | TFPI \|\| id:prot-c-3336_50_1 | F-cheese liking (derived food-liking factor) \|\| id:ebi-fl187-GCST90094729 | Inverse variance weighted | 2 | 0.673752337626296 | 0.439801260090701 | 0.125535448585869 | -0.188258132 | 1.53576280740407 | 1.96158405395662 | 0.828400842739394 | 4.64486731811229 | 0.998735809224318 |
| ebi-fl187-GCST90094729 | prot-c-3472_40_2 | Survivin \|\| id:prot-c-3472_40_2 | F-cheese liking (derived food-liking factor) \|\| id:ebi-fl187-GCST90094729 | Inverse variance weighted | 2 | -0.009879278 | 0.454427167528432 | 0.98265530136055 | -0.900556526 | 0.880797970653385 | 0.990169362054616 | 0.406343456080488 | 2.41282430141422 | 0.998955228396052 |
| ebi-fl187-GCST90094729 | prot-c-3473_78_2 | Thrombopoietin Receptor \|\| id:prot-c-3473_78_2 | F-cheese liking (derived food-liking factor) \|\| id:ebi-fl187-GCST90094729 | Inverse variance weighted | 2 | 0.396870836673504 | 0.453059563093493 | 0.381041241818494 | -0.491125907 | 1.28486758033675 | 1.48716383061807 | 0.611937022002407 | 3.61418933579394 | 0.998735809224318 |
| ebi-fl187-GCST90094729 | prot-c-3497_13_2 | IFN-aA \|\| id:prot-c-3497_13_2 | F-cheese liking (derived food-liking factor) \|\| id:ebi-fl187-GCST90094729 | Inverse variance weighted | 2 | -0.12015687 | 0.987898019034549 | 0.903192987765897 | -2.056436988 | 1.81612324683162 | 0.886781315998237 | 0.127908899925906 | 6.14797799730195 | 0.998735809224318 |
| ebi-fl187-GCST90094729 | prot-c-3665_64_3 | COLEC12 \|\| id:prot-c-3665_64_3 | F-cheese liking (derived food-liking factor) \|\| id:ebi-fl187-GCST90094729 | Inverse variance weighted | 2 | -0.205741497 | 0.449418639367719 | 0.647099902113437 | -1.08660203 | 0.675119035843848 | 0.814043481510737 | 0.337360890316556 | 1.96426678020775 | 0.998735809224318 |
| ebi-fl187-GCST90094729 | prot-c-3741_4_3 | IL-5 \|\| id:prot-c-3741_4_3 | F-cheese liking (derived food-liking factor) \|\| id:ebi-fl187-GCST90094729 | Inverse variance weighted | 2 | -0.38914822 | 0.443104836533406 | 0.379818597366679 | -1.257633699 | 0.4793372598917 | 0.677633823878686 | 0.284326031985725 | 1.61500371969987 | 0.998735809224318 |
| ebi-fl187-GCST90094729 | prot-c-3848_14_2 | GAPDH, liver \|\| id:prot-c-3848_14_2 | F-cheese liking (derived food-liking factor) \|\| id:ebi-fl187-GCST90094729 | Inverse variance weighted | 2 | -0.00344191 | 0.448633282098112 | 0.993878697444797 | -0.882763143 | 0.875879322727348 | 0.996564006397847 | 0.413638389110331 | 2.40098560722038 | 0.999107451094609 |
| ebi-fl187-GCST90094729 | prot-c-3859_50_2 | PSA1 \|\| id:prot-c-3859_50_2 | F-cheese liking (derived food-liking factor) \|\| id:ebi-fl187-GCST90094729 | Inverse variance weighted | 2 | 0.304291484786362 | 0.447565244625878 | 0.496579277257805 | -0.572936395 | 1.18151936425308 | 1.35566415398066 | 0.563867268522228 | 3.25932254093174 | 0.998735809224318 |
| ebi-fl187-GCST90094729 | prot-c-3868_8_1 | SGTA \|\| id:prot-c-3868_8_1 | F-cheese liking (derived food-liking factor) \|\| id:ebi-fl187-GCST90094729 | Inverse variance weighted | 2 | -0.254425221 | 0.437967061251268 | 0.561292224008367 | -1.112840661 | 0.603990218873233 | 0.775362031581335 | 0.328624124117071 | 1.82940397827813 | 0.998735809224318 |
| ebi-fl187-GCST90094729 | prot-c-3882_10_1 | EF-1-beta \|\| id:prot-c-3882_10_1 | F-cheese liking (derived food-liking factor) \|\| id:ebi-fl187-GCST90094729 | Inverse variance weighted | 2 | 0.0452540706156334 | 0.451601730210196 | 0.920179257426343 | -0.839885321 | 0.930393461827617 | 1.04629365861544 | 0.431760034573356 | 2.53550660644321 | 0.998735809224318 |
| ebi-fl187-GCST90094729 | prot-c-4134_4_2 | HB-EGF \|\| id:prot-c-4134_4_2 | F-cheese liking (derived food-liking factor) \|\| id:ebi-fl187-GCST90094729 | Inverse variance weighted | 2 | 0.324868557682791 | 0.456318780423568 | 0.476506049756069 | -0.569516252 | 1.21925336731298 | 1.38384873774104 | 0.565799076709793 | 3.38465969241889 | 0.998735809224318 |
| ebi-fl187-GCST90094729 | prot-c-4220_39_2 | FER \|\| id:prot-c-4220_39_2 | F-cheese liking (derived food-liking factor) \|\| id:ebi-fl187-GCST90094729 | Inverse variance weighted | 2 | 0.0537778502077635 | 0.447079707749591 | 0.904255848962034 | -0.822498377 | 0.930054077396963 | 1.05525015251244 | 0.439332663616904 | 2.53464624098274 | 0.998735809224318 |
| ebi-fl187-GCST90094729 | prot-c-4228_37_2 | HSP70 protein 8 \|\| id:prot-c-4228_37_2 | F-cheese liking (derived food-liking factor) \|\| id:ebi-fl187-GCST90094729 | Inverse variance weighted | 2 | -0.050629674 | 0.455446475802588 | 0.911485563806037 | -0.943304767 | 0.842045418566422 | 0.95063064859434 | 0.389339032312705 | 2.3211097656427 | 0.998735809224318 |
| ebi-fl187-GCST90094729 | prot-c-4246_40_2 | NCAM-L1 \|\| id:prot-c-4246_40_2 | F-cheese liking (derived food-liking factor) \|\| id:ebi-fl187-GCST90094729 | Inverse variance weighted | 2 | -0.334438879 | 0.450528309636539 | 0.457889840570355 | -1.217474366 | 0.54859660739388 | 0.715739589857113 | 0.295976752562869 | 1.73082228942969 | 0.998735809224318 |
| ebi-fl187-GCST90094729 | prot-c-4267_81_3 | PESC \|\| id:prot-c-4267_81_3 | F-cheese liking (derived food-liking factor) \|\| id:ebi-fl187-GCST90094729 | Inverse variance weighted | 2 | 0.365256044465634 | 0.456168211557412 | 0.423302603880927 | -0.52883365 | 1.25934573911816 | 1.44088289101186 | 0.589291889491259 | 3.52311569637088 | 0.998735809224318 |
| ebi-fl187-GCST90094729 | prot-c-4280_47_2 | PSA2 \|\| id:prot-c-4280_47_2 | F-cheese liking (derived food-liking factor) \|\| id:ebi-fl187-GCST90094729 | Inverse variance weighted | 2 | 0.29901240957554 | 0.45248479165886 | 0.508725757147719 | -0.587857782 | 1.18588260122691 | 1.34852635800878 | 0.555516047419478 | 3.27357480795014 | 0.998735809224318 |
| ebi-fl187-GCST90094729 | prot-c-4324_33_2 | CYTT \|\| id:prot-c-4324_33_2 | F-cheese liking (derived food-liking factor) \|\| id:ebi-fl187-GCST90094729 | Inverse variance weighted | 2 | -0.496290801 | 0.4392116354394 | 0.258493826895754 | -1.357145606 | 0.36456400467028 | 0.608784580291974 | 0.25739443444616 | 1.43988608766441 | 0.998735809224318 |
| ebi-fl187-GCST90094729 | prot-c-4462_8_2 | RASA1 \|\| id:prot-c-4462_8_2 | F-cheese liking (derived food-liking factor) \|\| id:ebi-fl187-GCST90094729 | Inverse variance weighted | 2 | 0.2208957768583 | 0.453813605022337 | 0.626432376636355 | -0.668578889 | 1.11037044270208 | 1.24719343737168 | 0.512436289439711 | 3.03548265858326 | 0.998735809224318 |
| ebi-fl187-GCST90094729 | prot-c-4499_21_1 | PDGF-AA \|\| id:prot-c-4499_21_1 | F-cheese liking (derived food-liking factor) \|\| id:ebi-fl187-GCST90094729 | Inverse variance weighted | 2 | 0.881524976376504 | 0.453735602544593 | 0.0520383439307352 | -0.007796805 | 1.7708467573639 | 2.41457907627863 | 0.992233511629073 | 5.87582665498809 | 0.998735809224318 |
| ebi-fl187-GCST90094729 | prot-c-4535_50_2 | BST1 \|\| id:prot-c-4535_50_2 | F-cheese liking (derived food-liking factor) \|\| id:ebi-fl187-GCST90094729 | Inverse variance weighted | 2 | 0.0662418307479688 | 0.451577887569607 | 0.883376938562089 | -0.818850829 | 0.951334490384398 | 1.0684850784706 | 0.44093807676715 | 2.58916256741695 | 0.998735809224318 |
| ebi-fl187-GCST90094729 | prot-c-4542_24_2 | Clusterin \|\| id:prot-c-4542_24_2 | F-cheese liking (derived food-liking factor) \|\| id:ebi-fl187-GCST90094729 | Inverse variance weighted | 2 | -0.110929594 | 0.591356128078885 | 0.851201913467496 | -1.269987605 | 1.04812841721297 | 0.895001760364379 | 0.280835102748273 | 2.85230778922014 | 0.998735809224318 |
| ebi-fl187-GCST90094729 | prot-c-4696_2_2 | FABP \|\| id:prot-c-4696_2_2 | F-cheese liking (derived food-liking factor) \|\| id:ebi-fl187-GCST90094729 | Inverse variance weighted | 2 | 0.388420948633642 | 0.432003485326738 | 0.368591242797509 | -0.458305883 | 1.23514777987405 | 1.47465040572749 | 0.632354020525311 | 3.43888668139688 | 0.998735809224318 |
| ebi-fl187-GCST90094729 | prot-c-4906_35_1 | Coagulation Factor V \|\| id:prot-c-4906_35_1 | F-cheese liking (derived food-liking factor) \|\| id:ebi-fl187-GCST90094729 | Inverse variance weighted | 2 | 0.14269949308959 | 0.689525525894845 | 0.836046431928244 | -1.208770538 | 1.49416952384349 | 1.15338314997609 | 0.298564127220114 | 4.45563471752256 | 0.998735809224318 |
| ebi-fl187-GCST90094729 | prot-c-4916_2_1 | IgD \|\| id:prot-c-4916_2_1 | F-cheese liking (derived food-liking factor) \|\| id:ebi-fl187-GCST90094729 | Inverse variance weighted | 2 | 0.128626531751623 | 0.453153997021558 | 0.776527415945337 | -0.759555302 | 1.01680836591388 | 1.13726531235966 | 0.467874443391393 | 2.76435785062654 | 0.998735809224318 |
| ebi-fl187-GCST90094729 | prot-c-4922_13_1 | MIP-3b \|\| id:prot-c-4922_13_1 | F-cheese liking (derived food-liking factor) \|\| id:ebi-fl187-GCST90094729 | Inverse variance weighted | 2 | 0.74949204873309 | 0.45560087473399 | 0.0999568619069864 | -0.143485666 | 1.64246976321171 | 2.11592495683429 | 0.866333218339049 | 5.1679172957697 | 0.998735809224318 |
| ebi-fl187-GCST90094729 | prot-c-4973_18_1 | cIAP-2 \|\| id:prot-c-4973_18_1 | F-cheese liking (derived food-liking factor) \|\| id:ebi-fl187-GCST90094729 | Inverse variance weighted | 2 | 0.946263278505608 | 0.454934268882399 | 0.0375255330250758 | 0.0545921114961074 | 1.83793444551511 | 2.57606561223436 | 1.05610975178286 | 6.28354584107732 | 0.998735809224318 |
| ebi-fl187-GCST90094729 | prot-c-4997_19_1 | IF4A3 \|\| id:prot-c-4997_19_1 | F-cheese liking (derived food-liking factor) \|\| id:ebi-fl187-GCST90094729 | Inverse variance weighted | 2 | -0.248982205 | 0.455425217306321 | 0.584582985501153 | -1.141615631 | 0.643651221049158 | 0.779593846234147 | 0.319302729538068 | 1.90341800699731 | 0.998735809224318 |
| ebi-fl187-GCST90094729 | prot-c-5000_52_1 | LG3BP \|\| id:prot-c-5000_52_1 | F-cheese liking (derived food-liking factor) \|\| id:ebi-fl187-GCST90094729 | Inverse variance weighted | 2 | -0.585061316 | 0.420246952341057 | 0.163866518990552 | -1.408745342 | 0.238622710851534 | 0.557071703502937 | 0.244449791638749 | 1.26949947784071 | 0.998735809224318 |
| ebi-fl187-GCST90094729 | prot-c-5006_71_1 | MK13 \|\| id:prot-c-5006_71_1 | F-cheese liking (derived food-liking factor) \|\| id:ebi-fl187-GCST90094729 | Inverse variance weighted | 2 | 0.32458374476876 | 0.454616502951361 | 0.475244097908952 | -0.566464601 | 1.21563209055343 | 1.38345465587193 | 0.567528335193919 | 3.37242506878488 | 0.998735809224318 |
| ebi-fl187-GCST90094729 | prot-c-5109_24_3 | Nr-CAM \|\| id:prot-c-5109_24_3 | F-cheese liking (derived food-liking factor) \|\| id:ebi-fl187-GCST90094729 | Inverse variance weighted | 2 | -0.424269647 | 0.446167613555684 | 0.341645057047707 | -1.298758169 | 0.450218875955322 | 0.654247442514311 | 0.272870441641843 | 1.56865548888707 | 0.998735809224318 |
| ebi-fl187-GCST90094729 | prot-c-5255_22_3 | PDE4D \|\| id:prot-c-5255_22_3 | F-cheese liking (derived food-liking factor) \|\| id:ebi-fl187-GCST90094729 | Inverse variance weighted | 2 | 0.211102184943485 | 0.447479813083916 | 0.637099527481563 | -0.665958249 | 1.08816261858796 | 1.23503855095816 | 0.513780961804763 | 2.96881421412505 | 0.998735809224318 |
| ebi-fl187-GCST90094729 | prot-c-5440_26_3 | Troponin I, skeletal, fast twitch \|\| id:prot-c-5440_26_3 | F-cheese liking (derived food-liking factor) \|\| id:ebi-fl187-GCST90094729 | Inverse variance weighted | 2 | 0.106970555160205 | 0.599093149281971 | 0.858287815693049 | -1.067252017 | 1.28119312775287 | 1.11290148479095 | 0.343952395130874 | 3.6009335372665 | 0.998735809224318 |
| ebi-fl187-GCST90094729 | ukb-rsfMRI191-edge-pheno716 | Net100_Pair10_33 (OccipitalPrecuneus)&(Temporal\|Frontal\|Supp_Motor_Area) (Default_mode\|Central_executive)&(Default_mode\|Salience) \|\| id:ukb-rsfMRI191-edge-pheno716 | F-cheese liking (derived food-liking factor) \|\| id:ebi-fl187-GCST90094729 | Inverse variance weighted | 15 | -0.006919936 | 0.0290455736213042 | 0.811691797 | -0.06384926 | 0.050009388575123 | 0.993103951900538 | 0.938146405042698 | 1.05128096636002 | 0.998735809224318 |
| ebi-fl187-GCST90094729 | ukb-rsfMRI191-edge-pheno903 | Net100_Pair27_38 (Precentral\|Frontal\|Supp_Motor_Area)&(Subcortical) (Attention\|Salience\|Motor)&(Subcortical-cerebellum) \|\| id:ukb-rsfMRI191-edge-pheno903 | F-cheese liking (derived food-liking factor) \|\| id:ebi-fl187-GCST90094729 | Inverse variance weighted | 15 | 0.0592639451513288 | 0.0305532235785588 | 0.0524167272360015 | -0.000620373 | 0.119148263365304 | 1.06105526417539 | 0.999379819328935 | 1.12653693006357 | 0.998735809224318 |
| ebi-fl187-GCST90094729 | ukb-rsfMRI191-node-pheno23 | Net100_Node2 RolandicOper\|SupraMarginal\|Insula Salience\|Motor \|\| id:ukb-rsfMRI191-node-pheno23 | F-cheese liking (derived food-liking factor) \|\| id:ebi-fl187-GCST90094729 | Inverse variance weighted (multiplicative random effects) | 15 | 0.00637144652313278 | 0.0353849598360454 | 0.857104784558363 | -0.062983075 | 0.0757259678017817 | 1.00639178736578 | 0.938959365671469 | 1.07866694417921 | 0.998735809224318 |
| ebi-fl187-GCST90094729 | ukb-rsfMRI191-node-pheno55 | Net100_Node34 Parietal\|Postcentral\|Precuneus Attention \|\| id:ukb-rsfMRI191-node-pheno55 | F-cheese liking (derived food-liking factor) \|\| id:ebi-fl187-GCST90094729 | Inverse variance weighted | 14 | 0.00603430262127887 | 0.0260867307518384 | 0.817068590608977 | -0.04509569 | 0.0571642948948822 | 1.00605254570164 | 0.955906007143101 | 1.05882975642942 | 0.998735809224318 |
| ebi-fl187-GCST90094729 | ebi-a-GCST90001414 | IgD- CD24- B cell Absolute Count \|\| id:ebi-a-GCST90001414 | F-cheese liking (derived food-liking factor) \|\| id:ebi-fl187-GCST90094729 | MR Egger | 14 | 0.197264465863128 | 0.473222667009738 | 0.684145651551043 | -0.730251961 | 1.12478089320221 | 1.2180661350243 | 0.481787582885393 | 3.07954202639958 | 0.999950574162623 |
| ebi-fl187-GCST90094729 | ebi-a-GCST90001422 | CD20- CD38- B cell %B cell \|\| id:ebi-a-GCST90001422 | F-cheese liking (derived food-liking factor) \|\| id:ebi-fl187-GCST90094729 | MR Egger | 14 | -0.083854412 | 0.450485128221077 | 0.85544262484503 | -0.966805264 | 0.799096439088388 | 0.919565123790549 | 0.380296045094399 | 2.22353092491937 | 0.999950574162623 |
| ebi-fl187-GCST90094729 | ebi-a-GCST90001444 | CD20- CD38- B cell %lymphocyte \|\| id:ebi-a-GCST90001444 | F-cheese liking (derived food-liking factor) \|\| id:ebi-fl187-GCST90094729 | MR Egger | 14 | -0.040829707 | 0.451053074692313 | 0.92936674114295 | -0.924893733 | 0.843234319486457 | 0.959992596133938 | 0.396573559396423 | 2.32387097625624 | 0.999950574162623 |
| ebi-fl187-GCST90094729 | ebi-a-GCST90001459 | Myeloid Dendritic Cell %Dendritic Cell \|\| id:ebi-a-GCST90001459 | F-cheese liking (derived food-liking factor) \|\| id:ebi-fl187-GCST90094729 | MR Egger | 14 | 0.212394406016142 | 0.542548563841139 | 0.702308439987034 | -0.851000779 | 1.27578959114478 | 1.23663552539904 | 0.426987397981681 | 3.58152823691664 | 0.999950574162623 |
| ebi-fl187-GCST90094729 | ebi-a-GCST90001476 | HLA DR++ monocyte %leukocyte \|\| id:ebi-a-GCST90001476 | F-cheese liking (derived food-liking factor) \|\| id:ebi-fl187-GCST90094729 | MR Egger | 14 | 0.310079105308019 | 0.480110502222698 | 0.530533717703859 | -0.630937479 | 1.25109568966451 | 1.36353297256182 | 0.532092741317619 | 3.49416938607224 | 0.999950574162623 |
| ebi-fl187-GCST90094729 | ebi-a-GCST90001478 | CD4 regulatory T cell %CD4+ T cell \|\| id:ebi-a-GCST90001478 | F-cheese liking (derived food-liking factor) \|\| id:ebi-fl187-GCST90094729 | MR Egger | 14 | -0.396976464 | 0.490213723309183 | 0.433818462563275 | -1.357795362 | 0.563842433570298 | 0.672349849794712 | 0.257227245305877 | 1.75741228337387 | 0.999950574162623 |
| ebi-fl187-GCST90094729 | ebi-a-GCST90001485 | CD39+ resting CD4 regulatory T cell %CD4 regulatory T cell \|\| id:ebi-a-GCST90001485 | F-cheese liking (derived food-liking factor) \|\| id:ebi-fl187-GCST90094729 | MR Egger | 13 | 0.334035599920808 | 0.516677653505488 | 0.531208857546672 | -0.678652601 | 1.34672380079156 | 1.39659286128479 | 0.507300067704032 | 3.84480851543977 | 0.999950574162623 |
| ebi-fl187-GCST90094729 | ebi-a-GCST90001515 | Immature Myeloid-Derived Suppressor Cells Absolute Count \|\| id:ebi-a-GCST90001515 | F-cheese liking (derived food-liking factor) \|\| id:ebi-fl187-GCST90094729 | MR Egger | 14 | 0.281713232894382 | 0.734819431754214 | 0.708145343866372 | -1.158532853 | 1.72195931913264 | 1.32539858476831 | 0.313946448642219 | 5.59548106405818 | 0.999950574162623 |
| ebi-fl187-GCST90094729 | ebi-a-GCST90001517 | CD33+ HLA DR+ Absolute Count \|\| id:ebi-a-GCST90001517 | F-cheese liking (derived food-liking factor) \|\| id:ebi-fl187-GCST90094729 | MR Egger | 14 | -1.118792992 | 0.650443960747763 | 0.1110839619964 | -2.393663155 | 0.156077170955436 | 0.326673854678396 | 0.0912946441526536 | 1.16891640600518 | 0.999950574162623 |
| ebi-fl187-GCST90094729 | ebi-a-GCST90001518 | CD33+ HLA DR+ CD14- Absolute Count \|\| id:ebi-a-GCST90001518 | F-cheese liking (derived food-liking factor) \|\| id:ebi-fl187-GCST90094729 | MR Egger | 14 | -1.360749497 | 0.619718361382258 | 0.0485048008864285 | -2.575397485 | -0.146101509 | 0.25646848258544 | 0.0761235588007728 | 0.864069988265061 | 0.999950574162623 |
| ebi-fl187-GCST90094729 | ebi-a-GCST90001520 | CD33+ HLA DR+ CD14dim Absolute Count \|\| id:ebi-a-GCST90001520 | F-cheese liking (derived food-liking factor) \|\| id:ebi-fl187-GCST90094729 | MR Egger | 14 | -0.391797713 | 0.725974744801809 | 0.599286071947417 | -1.814708213 | 1.03111278638085 | 0.675840813653492 | 0.16288542926817 | 2.80418455752611 | 0.999950574162623 |
| ebi-fl187-GCST90094729 | ebi-a-GCST90001531 | CD33dim HLA DR- Absolute Count \|\| id:ebi-a-GCST90001531 | F-cheese liking (derived food-liking factor) \|\| id:ebi-fl187-GCST90094729 | MR Egger | 14 | 0.218664187263192 | 0.624257879544267 | 0.732201122824197 | -1.004881257 | 1.44220963116996 | 1.24441331667172 | 0.366088102753269 | 4.23003230933617 | 0.999950574162623 |
| ebi-fl187-GCST90094729 | ebi-a-GCST90001532 | Basophil Absolute Count \|\| id:ebi-a-GCST90001532 | F-cheese liking (derived food-liking factor) \|\| id:ebi-fl187-GCST90094729 | MR Egger | 14 | 0.217988006334345 | 0.622101586096545 | 0.732108094605791 | -1.001331102 | 1.43730711508357 | 1.243572153 | 0.367390081724939 | 4.20934525862702 | 0.999950574162623 |
| ebi-fl187-GCST90094729 | ebi-a-GCST90001588 | T/B cell \|\| id:ebi-a-GCST90001588 | F-cheese liking (derived food-liking factor) \|\| id:ebi-fl187-GCST90094729 | MR Egger | 14 | -0.402786325 | 0.467433825159058 | 0.405744437413287 | -1.318956623 | 0.51338397211365 | 0.668454916036611 | 0.26741417038396 | 1.67093603952229 | 0.999950574162623 |
| ebi-fl187-GCST90094729 | ebi-a-GCST90001624 | HLA DR+ CD4+ T cell Absolute Count \|\| id:ebi-a-GCST90001624 | F-cheese liking (derived food-liking factor) \|\| id:ebi-fl187-GCST90094729 | MR Egger | 14 | -0.573193595 | 0.454332304786141 | 0.231059144960196 | -1.463684912 | 0.317297722690995 | 0.563722260504249 | 0.231382079261917 | 1.37341140680261 | 0.999950574162623 |
| ebi-fl187-GCST90094729 | ebi-a-GCST90001631 | CD8+ Natural Killer T %T cell \|\| id:ebi-a-GCST90001631 | F-cheese liking (derived food-liking factor) \|\| id:ebi-fl187-GCST90094729 | MR Egger | 14 | 0.16848351130169 | 0.513336510645282 | 0.748407052830748 | -0.83765605 | 1.17462307216644 | 1.18350871216587 | 0.432723618357415 | 3.23692262763341 | 0.999950574162623 |
| ebi-fl187-GCST90094729 | ebi-a-GCST90001632 | CD8+ Natural Killer T %lymphocyte \|\| id:ebi-a-GCST90001632 | F-cheese liking (derived food-liking factor) \|\| id:ebi-fl187-GCST90094729 | MR Egger | 14 | 0.0910624272894818 | 0.52787540320527 | 0.865913930569167 | -0.943573363 | 1.12569821757181 | 1.09533738206798 | 0.389234471288123 | 3.08236826143655 | 0.999950574162623 |
| ebi-fl187-GCST90094729 | ebi-a-GCST90001646 | Natural Killer %CD3- lymphocyte \|\| id:ebi-a-GCST90001646 | F-cheese liking (derived food-liking factor) \|\| id:ebi-fl187-GCST90094729 | MR Egger | 14 | 0.0820780838925643 | 0.451093073154695 | 0.858656463397695 | -0.802064339 | 0.966220507275766 | 1.08554056975354 | 0.448402353340979 | 2.62799318469398 | 0.999950574162623 |
| ebi-fl187-GCST90094729 | ebi-a-GCST90001684 | CD127- CD8+ T cell Absolute Count \|\| id:ebi-a-GCST90001684 | F-cheese liking (derived food-liking factor) \|\| id:ebi-fl187-GCST90094729 | MR Egger | 14 | -0.904367087 | 0.488677694242217 | 0.0889822443046749 | -1.862175368 | 0.0534411933962307 | 0.404798005834773 | 0.155334353176337 | 1.05489495515388 | 0.999950574162623 |
| ebi-fl187-GCST90094729 | ebi-a-GCST90001687 | CD28- CD8+ T cell Absolute Count \|\| id:ebi-a-GCST90001687 | F-cheese liking (derived food-liking factor) \|\| id:ebi-fl187-GCST90094729 | MR Egger | 14 | -0.682472978 | 0.475793171525296 | 0.177004168653631 | -1.615027594 | 0.250081638485247 | 0.50536568770081 | 0.198885182348478 | 1.28413024685681 | 0.999950574162623 |
| ebi-fl187-GCST90094729 | ebi-a-GCST90001701 | BAFF-R on CD20- CD38- B cell \|\| id:ebi-a-GCST90001701 | F-cheese liking (derived food-liking factor) \|\| id:ebi-fl187-GCST90094729 | MR Egger | 14 | -0.072691665 | 0.557933214783716 | 0.898497632999703 | -1.166240766 | 1.02085743639586 | 0.929887503081404 | 0.311535879140433 | 2.77557362180164 | 0.999950574162623 |
| ebi-fl187-GCST90094729 | ebi-a-GCST90001732 | CD19 on IgD- CD27- B cell \|\| id:ebi-a-GCST90001732 | F-cheese liking (derived food-liking factor) \|\| id:ebi-fl187-GCST90094729 | MR Egger | 14 | 0.550948240891229 | 0.575714505116006 | 0.357459947274633 | -0.577452189 | 1.6793486709186 | 1.73489733873672 | 0.561326700496096 | 5.36206236634681 | 0.999950574162623 |
| ebi-fl187-GCST90094729 | ebi-a-GCST90001798 | CD27 on CD24+ CD27+ B cell \|\| id:ebi-a-GCST90001798 | F-cheese liking (derived food-liking factor) \|\| id:ebi-fl187-GCST90094729 | MR Egger | 14 | 0.0689177886276952 | 0.459024289100661 | 0.883148425327413 | -0.830769818 | 0.968605395264991 | 1.07134812852792 | 0.435713736900416 | 2.63426813362693 | 0.999950574162623 |
| ebi-fl187-GCST90094729 | ebi-a-GCST90001836 | CD66b on CD66b++ myeloid cell \|\| id:ebi-a-GCST90001836 | F-cheese liking (derived food-liking factor) \|\| id:ebi-fl187-GCST90094729 | MR Egger | 14 | -0.855748642 | 0.696680873639023 | 0.242872726401831 | -2.221243154 | 0.509745870484351 | 0.424964925436478 | 0.108474174846516 | 1.66486804907031 | 0.999950574162623 |
| ebi-fl187-GCST90094729 | ebi-a-GCST90001943 | CD25 on activated & secreting CD4 regulatory T cell \|\| id:ebi-a-GCST90001943 | F-cheese liking (derived food-liking factor) \|\| id:ebi-fl187-GCST90094729 | MR Egger | 14 | 0.211463990324826 | 0.471339144539931 | 0.661681637452663 | -0.712360733 | 1.13528871362309 | 1.23548547539701 | 0.490484925696749 | 3.11207191077005 | 0.999950574162623 |
| ebi-fl187-GCST90094729 | ebi-a-GCST90001960 | CD25 on CD4+ T cell \|\| id:ebi-a-GCST90001960 | F-cheese liking (derived food-liking factor) \|\| id:ebi-fl187-GCST90094729 | MR Egger | 14 | 0.369981955948121 | 0.631312853757879 | 0.568700260049926 | -0.867391237 | 1.60735514931356 | 1.44770849190051 | 0.420045921230954 | 4.98959702162775 | 0.999950574162623 |
| ebi-fl187-GCST90094729 | ebi-a-GCST90001988 | HLA DR on CD14+ CD16- monocyte \|\| id:ebi-a-GCST90001988 | F-cheese liking (derived food-liking factor) \|\| id:ebi-fl187-GCST90094729 | MR Egger | 14 | -0.579327859 | 0.467456909472512 | 0.238919218479034 | -1.495543401 | 0.336887683703173 | 0.560274823792159 | 0.224126780820898 | 1.40058174674887 | 0.999950574162623 |
| ebi-fl187-GCST90094729 | ebi-a-GCST90001991 | HLA DR on CD14+ monocyte \|\| id:ebi-a-GCST90001991 | F-cheese liking (derived food-liking factor) \|\| id:ebi-fl187-GCST90094729 | MR Egger | 14 | -0.524467947 | 0.468355257287013 | 0.284708455365338 | -1.442444252 | 0.393508356859966 | 0.591870186666696 | 0.236349354774307 | 1.48217166998143 | 0.999950574162623 |
| ebi-fl187-GCST90094729 | ebi-a-GCST90002019 | CD14 on Monocytic Myeloid-Derived Suppressor Cells \|\| id:ebi-a-GCST90002019 | F-cheese liking (derived food-liking factor) \|\| id:ebi-fl187-GCST90094729 | MR Egger | 14 | -0.278489718 | 0.671284998786767 | 0.685564586150815 | -1.594208315 | 1.03722887984683 | 0.756926050593682 | 0.203069231665034 | 2.82138776696815 | 0.999950574162623 |
| ebi-fl187-GCST90094729 | ebi-a-GCST90002037 | CD80 on plasmacytoid Dendritic Cell \|\| id:ebi-a-GCST90002037 | F-cheese liking (derived food-liking factor) \|\| id:ebi-fl187-GCST90094729 | MR Egger | 14 | -0.217220791 | 0.52326684642921 | 0.685376756996568 | -1.24282381 | 0.808382227542023 | 0.804752267260771 | 0.288568204433308 | 2.24427432306052 | 0.999950574162623 |
| ebi-fl187-GCST90094729 | ebi-a-GCST90002042 | CD45 on CD33+ HLA DR+ CD14- \|\| id:ebi-a-GCST90002042 | F-cheese liking (derived food-liking factor) \|\| id:ebi-fl187-GCST90094729 | MR Egger | 14 | 0.0213587711399836 | 0.785539156797046 | 0.978755177674289 | -1.518297976 | 1.56101551846219 | 1.02158850236921 | 0.219084456764444 | 4.76365637063465 | 0.999950574162623 |
| ebi-fl187-GCST90094729 | ebi-a-GCST90002056 | CD8 on Effector Memory CD8+ T cell \|\| id:ebi-a-GCST90002056 | F-cheese liking (derived food-liking factor) \|\| id:ebi-fl187-GCST90094729 | MR Egger | 14 | -0.15426027 | 0.513162091425676 | 0.768858441689049 | -1.160057969 | 0.851537429103033 | 0.857048927777794 | 0.313468008839364 | 2.34324665960244 | 0.999950574162623 |
| ebi-fl187-GCST90094729 | ebi-a-GCST90002058 | CD8 on CD8+ T cell \|\| id:ebi-a-GCST90002058 | F-cheese liking (derived food-liking factor) \|\| id:ebi-fl187-GCST90094729 | MR Egger | 14 | -0.096169167 | 0.494202731695367 | 0.848965925594185 | -1.064806522 | 0.872468186735674 | 0.908310346583001 | 0.34479455864464 | 2.39280947168323 | 0.999950574162623 |
| ebi-fl187-GCST90094729 | ebi-a-GCST90002063 | CD4 on CD4 regulatory T cell \|\| id:ebi-a-GCST90002063 | F-cheese liking (derived food-liking factor) \|\| id:ebi-fl187-GCST90094729 | MR Egger | 14 | 0.226415566904349 | 0.566414225842293 | 0.696375348204949 | -0.883756316 | 1.33658744955524 | 1.25409671809543 | 0.413227778712828 | 3.80603303881637 | 0.999950574162623 |
| ebi-fl187-GCST90094729 | ebi-a-GCST90002066 | CD4 on activated CD4 regulatory T cell \|\| id:ebi-a-GCST90002066 | F-cheese liking (derived food-liking factor) \|\| id:ebi-fl187-GCST90094729 | MR Egger | 14 | -0.009144661 | 0.570709335452247 | 0.987479110824404 | -1.127734959 | 1.10944563598996 | 0.99089702375788 | 0.323765769270603 | 3.03267672152078 | 0.999950574162623 |
| ebi-fl187-GCST90094729 | ebi-a-GCST90002070 | CD4 on activated & secreting CD4 regulatory T cell \|\| id:ebi-a-GCST90002070 | F-cheese liking (derived food-liking factor) \|\| id:ebi-fl187-GCST90094729 | MR Egger | 14 | 0.125883493355261 | 0.582466743659253 | 0.832523354388199 | -1.015751324 | 1.2675183109274 | 1.13415002457137 | 0.362130250371017 | 3.55202658965215 | 0.999950574162623 |
| ebi-fl187-GCST90094729 | ebi-a-GCST90002081 | SSC-A on CD4+ T cell \|\| id:ebi-a-GCST90002081 | F-cheese liking (derived food-liking factor) \|\| id:ebi-fl187-GCST90094729 | MR Egger | 14 | 0.278352182090033 | 0.48317639174181 | 0.575201326498207 | -0.668673546 | 1.22537790990398 | 1.32095133069892 | 0.512387786187513 | 3.40545279398344 | 0.999950574162623 |
| ebi-fl187-GCST90094729 | ebi-a-GCST90002094 | CD11b on Monocytic Myeloid-Derived Suppressor Cells \|\| id:ebi-a-GCST90002094 | F-cheese liking (derived food-liking factor) \|\| id:ebi-fl187-GCST90094729 | MR Egger | 14 | -0.145684295 | 0.684946097609886 | 0.835135501851937 | -1.488178646 | 1.19681005663762 | 0.864430565440566 | 0.225783512973483 | 3.30954281217007 | 0.999950574162623 |
| ebi-fl187-GCST90094729 | ebi-a-GCST90002104 | HLA DR on myeloid Dendritic Cell \|\| id:ebi-a-GCST90002104 | F-cheese liking (derived food-liking factor) \|\| id:ebi-fl187-GCST90094729 | MR Egger | 14 | 0.0013806318512482 | 0.518709310958732 | 0.997920035439181 | -1.015289618 | 1.01805088133036 | 1.00138158536217 | 0.362297486897824 | 2.76779474262611 | 0.999950574162623 |
| ebi-fl187-GCST90094729 | ebi-a-GCST90002105 | HLA DR on plasmacytoid Dendritic Cell \|\| id:ebi-a-GCST90002105 | F-cheese liking (derived food-liking factor) \|\| id:ebi-fl187-GCST90094729 | MR Egger | 14 | 0.207896705736765 | 0.655688774423318 | 0.756641898116567 | -1.077253292 | 1.49304670360647 | 1.23108599885975 | 0.340529577543457 | 4.45063464830773 | 0.999950574162623 |
| ebi-fl187-GCST90094729 | ebi-a-GCST90002106 | HLA DR on Dendritic Cell \|\| id:ebi-a-GCST90002106 | F-cheese liking (derived food-liking factor) \|\| id:ebi-fl187-GCST90094729 | MR Egger | 14 | 0.245418357131819 | 0.640937010873758 | 0.708486100111922 | -1.010818184 | 1.50165489844438 | 1.27815592701498 | 0.363921103239579 | 4.48911195097151 | 0.999950574162623 |
| ebi-fl187-GCST90094729 | ebi-a-GCST90002111 | HLA DR on CD33dim HLA DR+ CD11b- \|\| id:ebi-a-GCST90002111 | F-cheese liking (derived food-liking factor) \|\| id:ebi-fl187-GCST90094729 | MR Egger | 14 | -0.814496901 | 0.664863979253513 | 0.244056338583669 | -2.117630301 | 0.48863649811732 | 0.442862074694377 | 0.120316404678448 | 1.63009207037699 | 0.999950574162623 |
| ebi-fl187-GCST90094729 | ebi-a-GCST90002112 | HLA DR on CD33- HLA DR+ \|\| id:ebi-a-GCST90002112 | F-cheese liking (derived food-liking factor) \|\| id:ebi-fl187-GCST90094729 | MR Egger | 14 | 0.451766956997777 | 0.671911409140709 | 0.514092200114534 | -0.865179405 | 1.76871331891357 | 1.57108577536641 | 0.420976020683646 | 5.86330430305806 | 0.999950574162623 |
| ebi-fl187-GCST90094729 | ebi-a-GCST90002115 | HLA DR on HLA DR+ CD8+ T cell \|\| id:ebi-a-GCST90002115 | F-cheese liking (derived food-liking factor) \|\| id:ebi-fl187-GCST90094729 | MR Egger | 14 | -0.00139438 | 0.505125227659229 | 0.997842830919881 | -0.991439826 | 0.988651066044532 | 0.99860659152878 | 0.371042070073284 | 2.68760662220262 | 0.999950574162623 |
| ebi-fl187-GCST90094729 | ebi-a-GCST90002116 | HLA DR on B cell \|\| id:ebi-a-GCST90002116 | F-cheese liking (derived food-liking factor) \|\| id:ebi-fl187-GCST90094729 | MR Egger | 14 | 0.415723518548657 | 0.501890038722033 | 0.423660200953384 | -0.567980957 | 1.39942799444384 | 1.5154668124788 | 0.566668412149689 | 4.05288103321699 | 0.999950574162623 |
| ebi-fl187-GCST90094729 | ebi-cfmet338-GCST90026023 | Sphingomyelin (d18:1/24:1, d18:2/24:0) levels \|\| id:ebi-cfmet338-GCST90026023 | F-cheese liking (derived food-liking factor) \|\| id:ebi-fl187-GCST90094729 | MR Egger | 15 | 0.613245397015896 | 0.275582381239714 | 0.0443859936701176 | 0.0731039297860565 | 1.15338686424573 | 1.84641403211032 | 1.07584234316916 | 3.16890741438112 | 0.999950574162623 |
| ebi-fl187-GCST90094729 | ebi-cfmet338-GCST90026046 | 1-arachidonoyl-gpc (20:4n6) levels \|\| id:ebi-cfmet338-GCST90026046 | F-cheese liking (derived food-liking factor) \|\| id:ebi-fl187-GCST90094729 | MR Egger | 15 | 0.316875287416727 | 0.417090305625313 | 0.460972920187292 | -0.500621712 | 1.13437228644234 | 1.37283135190474 | 0.606153689755787 | 3.10922122990279 | 0.999950574162623 |
| ebi-fl187-GCST90094729 | ebi-cfmet338-GCST90026047 | 1-linoleoyl-gpc (18:2) levels \|\| id:ebi-cfmet338-GCST90026047 | F-cheese liking (derived food-liking factor) \|\| id:ebi-fl187-GCST90094729 | MR Egger | 15 | 0.14087063430799 | 0.497603935414955 | 0.781559588471864 | -0.834433079 | 1.1161743477213 | 1.15127570277277 | 0.434120523678838 | 3.05315153626666 | 0.999950574162623 |
| ebi-fl187-GCST90094729 | ebi-cfmet338-GCST90026050 | 1-myristoyl-2-palmitoyl-gpc (14:0/16:0) levels \|\| id:ebi-cfmet338-GCST90026050 | F-cheese liking (derived food-liking factor) \|\| id:ebi-fl187-GCST90094729 | MR Egger | 15 | 0.0791007983287704 | 0.206118324713153 | 0.707356425892733 | -0.324891118 | 0.483092714766549 | 1.08231341195531 | 0.722606028069662 | 1.62108019611681 | 0.999950574162623 |
| ebi-fl187-GCST90094729 | ebi-cfmet338-GCST90026068 | 2,3-dihydroxyisovalerate levels \|\| id:ebi-cfmet338-GCST90026068 | F-cheese liking (derived food-liking factor) \|\| id:ebi-fl187-GCST90094729 | MR Egger | 15 | -0.1031282 | 0.671825692106656 | 0.88035775328699 | -1.419906556 | 1.21365015690175 | 0.902011328552721 | 0.241736604639168 | 3.36574776522535 | 0.999950574162623 |
| ebi-fl187-GCST90094729 | ebi-cfmet338-GCST90026082 | 3-hydroxyhexanoate levels \|\| id:ebi-cfmet338-GCST90026082 | F-cheese liking (derived food-liking factor) \|\| id:ebi-fl187-GCST90094729 | MR Egger | 15 | -0.002452923 | 0.193128597232591 | 0.990059205522644 | -0.380984973 | 0.376079128048005 | 0.997550083428296 | 0.6831881557403 | 1.45656238414951 | 0.999950574162623 |
| ebi-fl187-GCST90094729 | ebi-cfmet338-GCST90026094 | 3-ureidopropionate levels \|\| id:ebi-cfmet338-GCST90026094 | F-cheese liking (derived food-liking factor) \|\| id:ebi-fl187-GCST90094729 | MR Egger | 15 | 0.459828402469833 | 0.327648994644829 | 0.183925339776931 | -0.182363627 | 1.1020204319737 | 1.58380218513182 | 0.833298275537387 | 3.01024187288837 | 0.999950574162623 |
| ebi-fl187-GCST90094729 | ebi-cfmet338-GCST90026173 | Glycerol levels \|\| id:ebi-cfmet338-GCST90026173 | F-cheese liking (derived food-liking factor) \|\| id:ebi-fl187-GCST90094729 | MR Egger | 15 | -0.017490922 | 0.185453908485902 | 0.926297820733151 | -0.380980582 | 0.345998739094332 | 0.982661156676513 | 0.683191155580541 | 1.41340083365143 | 0.999950574162623 |
| ebi-fl187-GCST90094729 | ebi-cfmet338-GCST90026179 | Histidine levels \|\| id:ebi-cfmet338-GCST90026179 | F-cheese liking (derived food-liking factor) \|\| id:ebi-fl187-GCST90094729 | MR Egger | 15 | -0.124288436 | 0.128447678280967 | 0.350892477789919 | -0.376045886 | 0.127469013248192 | 0.883125078916796 | 0.686570828596031 | 1.13594966830536 | 0.999950574162623 |
| ebi-fl187-GCST90094729 | ebi-cfmet338-GCST90026188 | Isocitrate levels \|\| id:ebi-cfmet338-GCST90026188 | F-cheese liking (derived food-liking factor) \|\| id:ebi-fl187-GCST90094729 | MR Egger | 15 | -0.210214509 | 0.251282057448909 | 0.417948324395486 | -0.702727341 | 0.282298324040679 | 0.810410387359234 | 0.495232791473035 | 1.32617429065277 | 0.999950574162623 |
| ebi-fl187-GCST90094729 | ebi-cfmet338-GCST90026207 | N-acetyl-isoputreanine levels \|\| id:ebi-cfmet338-GCST90026207 | F-cheese liking (derived food-liking factor) \|\| id:ebi-fl187-GCST90094729 | MR Egger | 15 | 0.0416602510865067 | 0.159835762954375 | 0.7984500462378 | -0.271617844 | 0.354938346477082 | 1.04254021666821 | 0.762145463674002 | 1.42609272793034 | 0.999950574162623 |
| ebi-fl187-GCST90094729 | ebi-cfmet338-GCST90026213 | N-acetylglutamate levels \|\| id:ebi-cfmet338-GCST90026213 | F-cheese liking (derived food-liking factor) \|\| id:ebi-fl187-GCST90094729 | MR Egger | 15 | 0.272683205746095 | 0.275076677252092 | 0.339632869479362 | -0.266467082 | 0.811833493160194 | 1.31348407470061 | 0.766081221427112 | 2.25203329129802 | 0.999950574162623 |
| ebi-fl187-GCST90094729 | ebi-cfmet338-GCST90026224 | N-acetyltaurine levels \|\| id:ebi-cfmet338-GCST90026224 | F-cheese liking (derived food-liking factor) \|\| id:ebi-fl187-GCST90094729 | MR Egger | 15 | 0.11558013659151 | 0.218565720202406 | 0.605847511471699 | -0.312808675 | 0.543968948188226 | 1.12252446622863 | 0.731389832339246 | 1.72283113815207 | 0.999950574162623 |
| ebi-fl187-GCST90094729 | ebi-cfmet338-GCST90026250 | Phenyllactate (pla) levels \|\| id:ebi-cfmet338-GCST90026250 | F-cheese liking (derived food-liking factor) \|\| id:ebi-fl187-GCST90094729 | MR Egger | 15 | -0.163840611 | 0.341985099430531 | 0.639835050177911 | -0.834131406 | 0.506450183510529 | 0.848877312487093 | 0.434251505809548 | 1.65939019672932 | 0.999950574162623 |
| ebi-fl187-GCST90094729 | ebi-cfmet338-GCST90026251 | Pipecolate levels \|\| id:ebi-cfmet338-GCST90026251 | F-cheese liking (derived food-liking factor) \|\| id:ebi-fl187-GCST90094729 | MR Egger | 15 | -0.551493793 | 0.708726865990045 | 0.450413439380044 | -1.940598451 | 0.837610864039692 | 0.576088610006661 | 0.143617975784965 | 2.31083946675532 | 0.999950574162623 |
| ebi-fl187-GCST90094729 | ebi-cfmet338-GCST90026278 | Trigonelline (n'-methylnicotinate) levels \|\| id:ebi-cfmet338-GCST90026278 | F-cheese liking (derived food-liking factor) \|\| id:ebi-fl187-GCST90094729 | MR Egger | 15 | -0.327944834 | 0.579865834873565 | 0.581328122035159 | -1.464481871 | 0.808592202037245 | 0.720402760120918 | 0.231197750785622 | 2.24474561290633 | 0.999950574162623 |
| ebi-fl187-GCST90094729 | ebi-cfmet338-GCST90026299 | 1-carboxyethylvaline levels \|\| id:ebi-cfmet338-GCST90026299 | F-cheese liking (derived food-liking factor) \|\| id:ebi-fl187-GCST90094729 | MR Egger | 15 | 0.0204232282883794 | 0.349716572636244 | 0.95431865951059 | -0.665021254 | 0.705867710655417 | 1.02063320947694 | 0.514262597412779 | 2.02560356037533 | 0.999950574162623 |
| ebi-fl187-GCST90094729 | ebi-gm430-GCST90011306 | C_Erysipelotrichia abundance \|\| id:ebi-gm430-GCST90011306 | F-cheese liking (derived food-liking factor) \|\| id:ebi-fl187-GCST90094729 | MR Egger | 14 | -0.04248278 | 0.29093560278224 | 0.886328864412773 | -0.612716561 | 0.527751001406745 | 0.958406969102344 | 0.541876826093483 | 1.69511570562251 | 0.999950574162623 |
| ebi-fl187-GCST90094729 | ebi-gm430-GCST90011312 | F_Erysipelotrichaceae abundance \|\| id:ebi-gm430-GCST90011312 | F-cheese liking (derived food-liking factor) \|\| id:ebi-fl187-GCST90094729 | MR Egger | 14 | -0.04248278 | 0.29093560278224 | 0.886328864412773 | -0.612716561 | 0.527751001406745 | 0.958406969102344 | 0.541876826093483 | 1.69511570562251 | 0.999950574162623 |
| ebi-fl187-GCST90094729 | ebi-gm430-GCST90011352 | O_Erysipelotrichales abundance \|\| id:ebi-gm430-GCST90011352 | F-cheese liking (derived food-liking factor) \|\| id:ebi-fl187-GCST90094729 | MR Egger | 14 | -0.04248278 | 0.29093560278224 | 0.886328864412773 | -0.612716561 | 0.527751001406745 | 0.958406969102344 | 0.541876826093483 | 1.69511570562251 | 0.999950574162623 |
| ebi-fl187-GCST90094729 | ebi-gm430-GCST90011362 | OTU97_11 (Parabacteroides) abundance \|\| id:ebi-gm430-GCST90011362 | F-cheese liking (derived food-liking factor) \|\| id:ebi-fl187-GCST90094729 | MR Egger | 14 | 0.156497964054127 | 0.387991682428257 | 0.693782313554921 | -0.603965734 | 0.916961661613511 | 1.16940838146445 | 0.546639505293405 | 2.50167788715765 | 0.999950574162623 |
| ebi-fl187-GCST90094729 | ebi-gm430-GCST90011384 | OTU97_235 (Phascolarctobacterium) abundance \|\| id:ebi-gm430-GCST90011384 | F-cheese liking (derived food-liking factor) \|\| id:ebi-fl187-GCST90094729 | MR Egger | 14 | -0.372700763 | 1.46690097701615 | 0.803739992767687 | -3.247826678 | 2.50242515234292 | 0.688871338067341 | 0.0388585683257525 | 12.2120742182927 | 0.999950574162623 |
| ebi-fl187-GCST90094729 | ebi-gm430-GCST90011390 | OTU97_3 (Bacteroides) abundance \|\| id:ebi-gm430-GCST90011390 | F-cheese liking (derived food-liking factor) \|\| id:ebi-fl187-GCST90094729 | MR Egger | 14 | -0.078178052 | 0.316285597717734 | 0.808950634930895 | -0.698097823 | 0.541741719693048 | 0.924799749592228 | 0.497530795717056 | 1.7189982694704 | 0.999950574162623 |
| ebi-fl187-GCST90094729 | ebi-gm430-GCST90011398 | OTU97_4 (Alistipes) abundance \|\| id:ebi-gm430-GCST90011398 | F-cheese liking (derived food-liking factor) \|\| id:ebi-fl187-GCST90094729 | MR Egger | 14 | -0.232678396 | 0.354970129405895 | 0.524521954574109 | -0.92841985 | 0.46306305748819 | 0.792408374135671 | 0.395177657337835 | 1.58893353341467 | 0.999950574162623 |
| ebi-fl187-GCST90094729 | ebi-gm430-GCST90011421 | OTU99_108 (Cytophagales) abundance \|\| id:ebi-gm430-GCST90011421 | F-cheese liking (derived food-liking factor) \|\| id:ebi-fl187-GCST90094729 | MR Egger | 14 | 0.352976495479404 | 1.06894124670607 | 0.746933817549524 | -1.742148348 | 2.44810133902331 | 1.42329768911059 | 0.175143726462522 | 11.5663652518038 | 0.999950574162623 |
| ebi-fl187-GCST90094729 | ebi-gm430-GCST90011428 | OTU99_123 (Phascolarctobacterium) abundance \|\| id:ebi-gm430-GCST90011428 | F-cheese liking (derived food-liking factor) \|\| id:ebi-fl187-GCST90094729 | MR Egger | 14 | -0.335085276 | 0.661932401111625 | 0.621871645626277 | -1.632472783 | 0.962302229827129 | 0.71527708753127 | 0.19544568142609 | 2.61771612559622 | 0.999950574162623 |
| ebi-fl187-GCST90094729 | ebi-gm430-GCST90011450 | OTU99_298 (Phascolarctobacterium) abundance \|\| id:ebi-gm430-GCST90011450 | F-cheese liking (derived food-liking factor) \|\| id:ebi-fl187-GCST90094729 | MR Egger | 14 | -0.36348976 | 1.4697111713932 | 0.808840978202565 | -3.244123656 | 2.51714413572783 | 0.695245846356153 | 0.0390027291875287 | 12.393152913772 | 0.999950574162623 |
| ebi-fl187-GCST90094729 | ebi-gm430-GCST90011451 | OTU99_3 (Bacteroides) abundance \|\| id:ebi-gm430-GCST90011451 | F-cheese liking (derived food-liking factor) \|\| id:ebi-fl187-GCST90094729 | MR Egger | 14 | -0.080585774 | 0.315279641170349 | 0.802587566243977 | -0.698533871 | 0.537362322624628 | 0.922575767112134 | 0.49731389599855 | 1.71148655388673 | 0.999950574162623 |
| ebi-fl187-GCST90094729 | ebi-gm430-GCST90011472 | OTU99_72 (Parabacteroides) abundance \|\| id:ebi-gm430-GCST90011472 | F-cheese liking (derived food-liking factor) \|\| id:ebi-fl187-GCST90094729 | MR Egger | 14 | -0.965813511 | 0.878289750450613 | 0.293055204395121 | -2.687261422 | 0.755634399712335 | 0.38067339168368 | 0.0680670913948446 | 2.12896170772668 | 0.999950574162623 |
| ebi-fl187-GCST90094729 | ebi-gm430-GCST90011507 | TestASV_3 (Bacteroides) abundance \|\| id:ebi-gm430-GCST90011507 | F-cheese liking (derived food-liking factor) \|\| id:ebi-fl187-GCST90094729 | MR Egger | 14 | 0.350163424308514 | 0.382649159930041 | 0.378159404569795 | -0.399828929 | 1.10015577777139 | 1.41929947767703 | 0.670434728061856 | 3.00463404268705 | 0.999950574162623 |
| ebi-fl187-GCST90094729 | ebi-gm430-GCST90011512 | TestASV_34 (Phascolarctobacterium) abundance \|\| id:ebi-gm430-GCST90011512 | F-cheese liking (derived food-liking factor) \|\| id:ebi-fl187-GCST90094729 | MR Egger | 14 | 0.381647563431078 | 0.946559594797274 | 0.69389649643965 | -1.473609242 | 2.23690436923373 | 1.46469578180036 | 0.229097124160369 | 9.36429796352239 | 0.999950574162623 |
| ebi-fl187-GCST90094729 | ebi-gm430-GCST90011519 | TestASV_41 (Barnesiella) abundance \|\| id:ebi-gm430-GCST90011519 | F-cheese liking (derived food-liking factor) \|\| id:ebi-fl187-GCST90094729 | MR Egger | 14 | -0.855040044 | 1.95628872375418 | 0.669820551794478 | -4.689365943 | 2.97928585450631 | 0.42526616136108 | 0.00919251288953754 | 19.6737616984605 | 0.999950574162623 |
| ebi-fl187-GCST90094729 | ebi-gm430-GCST90011524 | TestASV_46 (Sutterella) abundance \|\| id:ebi-gm430-GCST90011524 | F-cheese liking (derived food-liking factor) \|\| id:ebi-fl187-GCST90094729 | MR Egger | 14 | 0.14024849530626 | 1.38636005203864 | 0.921091619694677 | -2.577017207 | 2.857514197 | 1.15055967201475 | 0.0760003596333314 | 17.4181749304003 | 0.999950574162623 |
| ebi-fl187-GCST90094729 | ebi-gm430-GCST90011581 | OTU97_19 (Bacteria) prevalence \|\| id:ebi-gm430-GCST90011581 | F-cheese liking (derived food-liking factor) \|\| id:ebi-fl187-GCST90094729 | MR Egger | 14 | 1.01951357054335 | 1.40850278821009 | 0.483044098969126 | -1.741151894 | 3.78017903543513 | 2.7718461283767 | 0.175318336060344 | 43.8238870619463 | 0.999950574162623 |
| ebi-fl187-GCST90094729 | ebi-gm430-GCST90011614 | OTU97_80 (Ruminococcus) prevalence \|\| id:ebi-gm430-GCST90011614 | F-cheese liking (derived food-liking factor) \|\| id:ebi-fl187-GCST90094729 | MR Egger | 14 | 0.649686751630568 | 0.772858346896262 | 0.416991869213339 | -0.865115608 | 2.16448911154724 | 1.9149408829436 | 0.421002878392586 | 8.71015086445398 | 0.999950574162623 |
| ebi-fl187-GCST90094729 | ebi-gm430-GCST90011623 | OTU99_11 (Parabacteroides) prevalence \|\| id:ebi-gm430-GCST90011623 | F-cheese liking (derived food-liking factor) \|\| id:ebi-fl187-GCST90094729 | MR Egger | 14 | 0.215718378791285 | 0.848711894965626 | 0.803666083020151 | -1.447756935 | 1.87919369292391 | 1.24075290746017 | 0.235097034959778 | 6.54822285459381 | 0.999950574162623 |
| ebi-fl187-GCST90094729 | ebi-gm430-GCST90011642 | OTU99_19 (Bacteria) prevalence \|\| id:ebi-gm430-GCST90011642 | F-cheese liking (derived food-liking factor) \|\| id:ebi-fl187-GCST90094729 | MR Egger | 14 | 1.01951357054335 | 1.40850278821009 | 0.483044098969126 | -1.741151894 | 3.78017903543513 | 2.7718461283767 | 0.175318336060344 | 43.8238870619463 | 0.999950574162623 |
| ebi-fl187-GCST90094729 | ebi-gm430-GCST90011653 | OTU99_30 (Parasutterella) prevalence \|\| id:ebi-gm430-GCST90011653 | F-cheese liking (derived food-liking factor) \|\| id:ebi-fl187-GCST90094729 | MR Egger | 14 | 0.933984891616545 | 0.868967825966391 | 0.303591559688201 | -0.769192047 | 2.63716183051067 | 2.54462907194137 | 0.46338731214559 | 13.9734881470704 | 0.999950574162623 |
| ebi-fl187-GCST90094729 | ebi-gm430-GCST90011675 | OTU99_8 (Ruminococcaceae) prevalence \|\| id:ebi-gm430-GCST90011675 | F-cheese liking (derived food-liking factor) \|\| id:ebi-fl187-GCST90094729 | MR Egger | 14 | -0.661029642 | 0.660405002214812 | 0.336609958902055 | -1.955423446 | 0.633364162799819 | 0.516319436766064 | 0.141504544489324 | 1.88393780386706 | 0.999950574162623 |
| ebi-fl187-GCST90094729 | ebi-gm430-GCST90011679 | OTU99_92 (Ruminococcus) prevalence \|\| id:ebi-gm430-GCST90011679 | F-cheese liking (derived food-liking factor) \|\| id:ebi-fl187-GCST90094729 | MR Egger | 14 | 1.11319375257547 | 0.732403310238332 | 0.154432576043915 | -0.322316735 | 2.5487042406426 | 3.04406487617276 | 0.7244686890398 | 12.7905196049675 | 0.999950574162623 |
| ebi-fl187-GCST90094729 | ebi-gm430-GCST90011684 | TestASV_11 (Lachnospiraceae) prevalence \|\| id:ebi-gm430-GCST90011684 | F-cheese liking (derived food-liking factor) \|\| id:ebi-fl187-GCST90094729 | MR Egger | 14 | 0.508418968882326 | 0.691199572673268 | 0.476130034903472 | -0.846332194 | 1.86317013132193 | 1.66266039798063 | 0.428985485672682 | 6.44413317312649 | 0.999950574162623 |
| ebi-fl187-GCST90094729 | ebi-gm430-GCST90011688 | TestASV_15 (Bacteroides) prevalence \|\| id:ebi-gm430-GCST90011688 | F-cheese liking (derived food-liking factor) \|\| id:ebi-fl187-GCST90094729 | MR Egger | 14 | -1.135336844 | 0.664348641769945 | 0.113171520280157 | -2.437460182 | 0.166786493423341 | 0.32131387031098 | 0.0873825054952431 | 1.18150197993397 | 0.999950574162623 |
| ebi-fl187-GCST90094729 | ebi-gm430-GCST90011692 | TestASV_19 (Prevotella) prevalence \|\| id:ebi-gm430-GCST90011692 | F-cheese liking (derived food-liking factor) \|\| id:ebi-fl187-GCST90094729 | MR Egger | 13 | 2.03104065733971 | 1.3906014892994 | 0.172105405263529 | -0.694538262 | 4.75661957636653 | 7.62201413796973 | 0.499304942988845 | 116.351941504229 | 0.999950574162623 |
| ebi-fl187-GCST90094729 | ebi-gm430-GCST90011693 | TestASV_2 (Bacteroides) prevalence \|\| id:ebi-gm430-GCST90011693 | F-cheese liking (derived food-liking factor) \|\| id:ebi-fl187-GCST90094729 | MR Egger | 14 | -0.38959986 | 0.65072223669993 | 0.560492527937331 | -1.665015444 | 0.885815723845898 | 0.677327846187402 | 0.18918773614065 | 2.4249616839846 | 0.999950574162623 |
| ebi-fl187-GCST90094729 | ebi-gm430-GCST90011716 | TestASV_40 (Barnesiella) prevalence \|\| id:ebi-gm430-GCST90011716 | F-cheese liking (derived food-liking factor) \|\| id:ebi-fl187-GCST90094729 | MR Egger | 14 | 0.151468182721729 | 1.46101868424234 | 0.919141372126396 | -2.712128438 | 3.01506480383672 | 1.16354128050402 | 0.0663953378283522 | 20.3904122746826 | 0.999950574162623 |
| ebi-fl187-GCST90094729 | ebi-gm430-GCST90011718 | TestASV_42 (Alphaproteobacteria) prevalence \|\| id:ebi-gm430-GCST90011718 | F-cheese liking (derived food-liking factor) \|\| id:ebi-fl187-GCST90094729 | MR Egger | 14 | 0.792690305221798 | 1.13676258823875 | 0.498888707575884 | -1.435364368 | 3.02074497816975 | 2.20933221613029 | 0.238028618265573 | 20.5065629368364 | 0.999950574162623 |
| ebi-fl187-GCST90094729 | ebi-hbsm44-GCST90104929 | Sulcal depth of the right inferior frontal sulcus \|\| id:ebi-hbsm44-GCST90104929 | F-cheese liking (derived food-liking factor) \|\| id:ebi-fl187-GCST90094729 | MR Egger | 13 | 0.633922897251599 | 0.598792637958971 | 0.31244488538269 | -0.539710673 | 1.80755646765118 | 1.88499071894566 | 0.582916881484656 | 6.09553458369831 | 0.999950574162623 |
| ebi-fl187-GCST90094729 | ebi-hbsm44-GCST90104935 | Sulcal opening distance of the left calloso-marginal posterior fissure \|\| id:ebi-hbsm44-GCST90104935 | F-cheese liking (derived food-liking factor) \|\| id:ebi-fl187-GCST90094729 | MR Egger | 13 | 0.0150324612739644 | 0.195361107961962 | 0.940047412560353 | -0.36787531 | 0.397940232879409 | 1.01514601701385 | 0.692203486931266 | 1.4887550486457 | 0.999950574162623 |
| ebi-fl187-GCST90094729 | ebi-hbsm44-GCST90104936 | Sulcal opening distance of the left anterior inferior temporal sulcus \|\| id:ebi-hbsm44-GCST90104936 | F-cheese liking (derived food-liking factor) \|\| id:ebi-fl187-GCST90094729 | MR Egger | 13 | 0.00477622815681591 | 0.0694585444369389 | 0.946411773002434 | -0.131362519 | 0.140914975253216 | 1.00478765251573 | 0.876899823968694 | 1.15132675255743 | 0.999950574162623 |
| ebi-fl187-GCST90094729 | ebi-hbsm44-GCST90104941 | Sulcal opening distance of the right posterior inferior temporal sulcus \|\| id:ebi-hbsm44-GCST90104941 | F-cheese liking (derived food-liking factor) \|\| id:ebi-fl187-GCST90094729 | MR Egger | 12 | -0.020375788 | 0.0761842067090781 | 0.79455458528547 | -0.169696834 | 0.128945256662955 | 0.979830395132196 | 0.843920626165203 | 1.13762784491651 | 0.999950574162623 |
| ebi-fl187-GCST90094729 | ebi-IPs91-GCST90274819 | Leukemia inhibitory factor levels \|\| id:ebi-IPs91-GCST90274819 | F-cheese liking (derived food-liking factor) \|\| id:ebi-fl187-GCST90094729 | MR Egger | 16 | -0.090611472 | 0.245555122500148 | 0.717643633275144 | -0.571899512 | 0.390676568165007 | 0.913372512824322 | 0.564452235925786 | 1.47798041018426 | 0.999950574162623 |
| ebi-fl187-GCST90094729 | ebi-IPs91-GCST90274837 | STAM binding protein levels \|\| id:ebi-IPs91-GCST90274837 | F-cheese liking (derived food-liking factor) \|\| id:ebi-fl187-GCST90094729 | MR Egger | 16 | 0.190430885349378 | 0.204642319906653 | 0.367853139304282 | -0.210668062 | 0.591529832366418 | 1.20977075786471 | 0.810042906551235 | 1.80675032735687 | 0.999950574162623 |
| ebi-fl187-GCST90094729 | ebi-IPs91-GCST90274840 | TNF-beta levels \|\| id:ebi-IPs91-GCST90274840 | F-cheese liking (derived food-liking factor) \|\| id:ebi-fl187-GCST90094729 | MR Egger | 16 | 0.245540539782404 | 0.233537103542321 | 0.310885977653349 | -0.212192183 | 0.703273262725353 | 1.27831210503493 | 0.808809243112496 | 2.02035504884996 | 0.999950574162623 |
| ebi-fl187-GCST90094729 | ebi-IPs91-GCST90274842 | Tumor necrosis factor ligand superfamily member 14 levels \|\| id:ebi-IPs91-GCST90274842 | F-cheese liking (derived food-liking factor) \|\| id:ebi-fl187-GCST90094729 | MR Egger | 16 | 0.0989799819469976 | 0.233131539653579 | 0.677604561531609 | -0.357957836 | 0.555917799668013 | 1.10404419852239 | 0.699102551503061 | 1.74354047152352 | 0.999950574162623 |
| ebi-fl187-GCST90094729 | ebi-met1400-GCST90199628 | 3-methylhistidine levels \|\| id:ebi-met1400-GCST90199628 | F-cheese liking (derived food-liking factor) \|\| id:ebi-fl187-GCST90094729 | MR Egger | 15 | 0.194094402827902 | 0.348211288929029 | 0.586721339768772 | -0.488399723 | 0.876588529128799 | 1.21421090248881 | 0.613607550674318 | 2.40268900554192 | 0.999950574162623 |
| ebi-fl187-GCST90094729 | ebi-met1400-GCST90199651 | Docosahexaenoate DHA; 22:6n3 levels \|\| id:ebi-met1400-GCST90199651 | F-cheese liking (derived food-liking factor) \|\| id:ebi-fl187-GCST90094729 | MR Egger | 15 | -0.096853372 | 0.299968316512253 | 0.751921524541862 | -0.684791272 | 0.491084528400466 | 0.907689089045026 | 0.504195458136817 | 1.63408747356827 | 0.999950574162623 |
| ebi-fl187-GCST90094729 | ebi-met1400-GCST90199652 | 1-stearoyl-2-oleoyl-GPS (18:0/18:1) levels \|\| id:ebi-met1400-GCST90199652 | F-cheese liking (derived food-liking factor) \|\| id:ebi-fl187-GCST90094729 | MR Egger | 15 | -0.653456152 | 0.276903498960538 | 0.0345836884942851 | -1.19618701 | -0.110725294 | 0.520244621420284 | 0.302344854677575 | 0.895184627518679 | 0.999950574162623 |
| ebi-fl187-GCST90094729 | ebi-met1400-GCST90199653 | 1-myristoyl-2-palmitoyl-gpc (14:0/16:0) levels \|\| id:ebi-met1400-GCST90199653 | F-cheese liking (derived food-liking factor) \|\| id:ebi-fl187-GCST90094729 | MR Egger | 15 | 0.219935349134549 | 0.271588948880154 | 0.432623294280998 | -0.312378991 | 0.75224968893965 | 1.24599617325241 | 0.731704166620148 | 2.12176796932961 | 0.999950574162623 |
| ebi-fl187-GCST90094729 | ebi-met1400-GCST90199667 | Erythritol levels in elite athletes \|\| id:ebi-met1400-GCST90199667 | F-cheese liking (derived food-liking factor) \|\| id:ebi-fl187-GCST90094729 | MR Egger | 15 | 0.494392032648518 | 0.297186006793009 | 0.120102733411028 | -0.088092541 | 1.07687660596282 | 1.63950117281011 | 0.915676135523163 | 2.93549650511529 | 0.999950574162623 |
| ebi-fl187-GCST90094729 | ebi-met1400-GCST90199674 | 3-hydroxymyristate levels \|\| id:ebi-met1400-GCST90199674 | F-cheese liking (derived food-liking factor) \|\| id:ebi-fl187-GCST90094729 | MR Egger | 15 | -0.17607709 | 0.266501325169246 | 0.520342323470714 | -0.698419687 | 0.346265507190628 | 0.838553336650259 | 0.497370684177589 | 1.41377793419809 | 0.999950574162623 |
| ebi-fl187-GCST90094729 | ebi-met1400-GCST90199685 | 2-linoleoylglycerol (18:2) levels \|\| id:ebi-met1400-GCST90199685 | F-cheese liking (derived food-liking factor) \|\| id:ebi-fl187-GCST90094729 | MR Egger | 15 | -0.134424953 | 0.31398744853566 | 0.675569804279226 | -0.749840352 | 0.480990446606079 | 0.874218484305662 | 0.472441971300067 | 1.61767583053343 | 0.999950574162623 |
| ebi-fl187-GCST90094729 | ebi-met1400-GCST90199703 | Gamma-glutamylvaline levels \|\| id:ebi-met1400-GCST90199703 | F-cheese liking (derived food-liking factor) \|\| id:ebi-fl187-GCST90094729 | MR Egger | 15 | 1.00645981198202 | 0.267119442353994 | 0.0023465290973467 | 0.482905704968192 | 1.53001391899585 | 2.73589825613044 | 1.62077706658119 | 4.61824110313116 | 0.999950574162623 |
| ebi-fl187-GCST90094729 | ebi-met1400-GCST90199779 | Gamma-glutamyl-2-aminobutyrate levels \|\| id:ebi-met1400-GCST90199779 | F-cheese liking (derived food-liking factor) \|\| id:ebi-fl187-GCST90094729 | MR Egger | 15 | 0.300602361170949 | 0.340286983646457 | 0.393074989208948 | -0.366360127 | 0.967564849118005 | 1.35067215504783 | 0.693253097246975 | 2.63152848168468 | 0.999950574162623 |
| ebi-fl187-GCST90094729 | ebi-met1400-GCST90199824 | 4-vinylphenol sulfate levels \|\| id:ebi-met1400-GCST90199824 | F-cheese liking (derived food-liking factor) \|\| id:ebi-fl187-GCST90094729 | MR Egger | 15 | -0.405025515 | 0.321220848513749 | 0.229506833138887 | -1.034618378 | 0.224567348111758 | 0.666959793178797 | 0.355361968974861 | 1.25178101359679 | 0.999950574162623 |
| ebi-fl187-GCST90094729 | ebi-met1400-GCST90199830 | 4-ethylphenylsulfate levels \|\| id:ebi-met1400-GCST90199830 | F-cheese liking (derived food-liking factor) \|\| id:ebi-fl187-GCST90094729 | MR Egger | 15 | -0.252652103 | 0.280226663803236 | 0.383669958141626 | -0.801896364 | 0.296592157696958 | 0.776738059387132 | 0.448477680087996 | 1.34526653095001 | 0.999950574162623 |
| ebi-fl187-GCST90094729 | ebi-met1400-GCST90199833 | Hexanoylglutamine levels \|\| id:ebi-met1400-GCST90199833 | F-cheese liking (derived food-liking factor) \|\| id:ebi-fl187-GCST90094729 | MR Egger | 15 | 0.132722964626515 | 0.270032174503011 | 0.631261357209477 | -0.396540097 | 0.661986026652417 | 1.14193359850499 | 0.672643304913248 | 1.93863870177484 | 0.999950574162623 |
| ebi-fl187-GCST90094729 | ebi-met1400-GCST90199837 | Carotenoid levels (cryptoxanthin) \|\| id:ebi-met1400-GCST90199837 | F-cheese liking (derived food-liking factor) \|\| id:ebi-fl187-GCST90094729 | MR Egger | 15 | -0.057493818 | 0.394603874821087 | 0.886393079349895 | -0.830917413 | 0.715929776301732 | 0.944127726637812 | 0.435649432482562 | 2.04608820244926 | 0.999950574162623 |
| ebi-fl187-GCST90094729 | ebi-met1400-GCST90199869 | 2-hydroxyhippurate levels \|\| id:ebi-met1400-GCST90199869 | F-cheese liking (derived food-liking factor) \|\| id:ebi-fl187-GCST90094729 | MR Egger | 15 | 0.281711530601981 | 0.316461682000388 | 0.389540214314719 | -0.338553366 | 0.901976427322742 | 1.3253963285543 | 0.71280073896496 | 2.46446914504611 | 0.999950574162623 |
| ebi-fl187-GCST90094729 | ebi-met1400-GCST90199902 | Hydantoin-5-propionate levels \|\| id:ebi-met1400-GCST90199902 | F-cheese liking (derived food-liking factor) \|\| id:ebi-fl187-GCST90094729 | MR Egger | 15 | 0.327647402977318 | 0.273058260759709 | 0.25158273054694 | -0.207546788 | 0.862841594066348 | 1.38769958727378 | 0.812575222023405 | 2.36988538701019 | 0.999950574162623 |
| ebi-fl187-GCST90094729 | ebi-met1400-GCST90199904 | Pantoate levels \|\| id:ebi-met1400-GCST90199904 | F-cheese liking (derived food-liking factor) \|\| id:ebi-fl187-GCST90094729 | MR Egger | 15 | 0.0289362212064146 | 0.281122138693616 | 0.919588502255736 | -0.522063171 | 0.579935613045902 | 1.02935894111008 | 0.593295215104909 | 1.78592343687771 | 0.999950574162623 |
| ebi-fl187-GCST90094729 | ebi-met1400-GCST90199936 | 3-methyl catechol sulfate (2) levels \|\| id:ebi-met1400-GCST90199936 | F-cheese liking (derived food-liking factor) \|\| id:ebi-fl187-GCST90094729 | MR Egger | 15 | 0.0923758328803251 | 0.383543078001034 | 0.813429206691525 | -0.6593686 | 0.844120265762352 | 1.09677694947045 | 0.517177777470621 | 2.32593071336681 | 0.999950574162623 |
| ebi-fl187-GCST90094729 | ebi-met1400-GCST90199977 | 4-hydroxychlorothalonil levels \|\| id:ebi-met1400-GCST90199977 | F-cheese liking (derived food-liking factor) \|\| id:ebi-fl187-GCST90094729 | MR Egger | 15 | 0.144229023453274 | 0.37293988618354 | 0.705208448086661 | -0.586733153 | 0.875191200373012 | 1.15514863436173 | 0.556141148096533 | 2.39933400366945 | 0.999950574162623 |
| ebi-fl187-GCST90094729 | ebi-met1400-GCST90200040 | Sphingomyelin (d18:2/24:1, d18:1/24:2) levels \|\| id:ebi-met1400-GCST90200040 | F-cheese liking (derived food-liking factor) \|\| id:ebi-fl187-GCST90094729 | MR Egger | 15 | 0.173675079741042 | 0.249258557568226 | 0.498219394764382 | -0.314871693 | 0.662221852574766 | 1.18966895546991 | 0.729882517229901 | 1.93909593694662 | 0.999950574162623 |
| ebi-fl187-GCST90094729 | ebi-met1400-GCST90200077 | 1-stearoyl-2-oleoyl-GPI (18:0/18:1) levels \|\| id:ebi-met1400-GCST90200077 | F-cheese liking (derived food-liking factor) \|\| id:ebi-fl187-GCST90094729 | MR Egger | 15 | 0.103841601433414 | 0.270236035186679 | 0.706995819015623 | -0.425821028 | 0.633504230399304 | 1.1094247097133 | 0.65323324242466 | 1.88420170099411 | 0.999950574162623 |
| ebi-fl187-GCST90094729 | ebi-met1400-GCST90200080 | 1-palmitoyl-2-oleoyl-GPI (16:0/18:1) levels \|\| id:ebi-met1400-GCST90200080 | F-cheese liking (derived food-liking factor) \|\| id:ebi-fl187-GCST90094729 | MR Egger | 15 | 0.304792559330783 | 0.272677814913393 | 0.283894526337698 | -0.229655958 | 0.839241076561032 | 1.3563436129946 | 0.794807002540748 | 2.31460969849335 | 0.999950574162623 |
| ebi-fl187-GCST90094729 | ebi-met1400-GCST90200082 | 1-oleoyl-2-linoleoyl-GPE (18:1/18:2) levels \|\| id:ebi-met1400-GCST90200082 | F-cheese liking (derived food-liking factor) \|\| id:ebi-fl187-GCST90094729 | MR Egger | 15 | -0.044946664 | 0.301617640193826 | 0.883825775757202 | -0.636117239 | 0.54622391073044 | 0.956048472227308 | 0.529343754428556 | 1.72672044130358 | 0.999950574162623 |
| ebi-fl187-GCST90094729 | ebi-met1400-GCST90200100 | Perfluorooctanesulfonate (PFOS) levels \|\| id:ebi-met1400-GCST90200100 | F-cheese liking (derived food-liking factor) \|\| id:ebi-fl187-GCST90094729 | MR Egger | 15 | -0.294055195 | 0.251964404213725 | 0.26415169208023 | -0.787905427 | 0.199795037448481 | 0.745235357183842 | 0.454796402562798 | 1.22115244198802 | 0.999950574162623 |
| ebi-fl187-GCST90094729 | ebi-met1400-GCST90200102 | 1-palmitoleoyl-2-linolenoyl-GPC (16:1/18:3) levels \|\| id:ebi-met1400-GCST90200102 | F-cheese liking (derived food-liking factor) \|\| id:ebi-fl187-GCST90094729 | MR Egger | 15 | 0.0638842363858507 | 0.267357347640106 | 0.81487231289908 | -0.460136165 | 0.587904637760459 | 1.0659689911956 | 0.631197692628452 | 1.80021236367134 | 0.999950574162623 |
| ebi-fl187-GCST90094729 | ebi-met1400-GCST90200114 | Glycosyl-N-tricosanoyl-sphingadienine (d18:2/23:0) levels \|\| id:ebi-met1400-GCST90200114 | F-cheese liking (derived food-liking factor) \|\| id:ebi-fl187-GCST90094729 | MR Egger | 15 | 0.0102328663970492 | 0.301255320641505 | 0.973419137223663 | -0.580227562 | 0.600693294854399 | 1.01028540121542 | 0.559770969435474 | 1.82338250398791 | 0.999950574162623 |
| ebi-fl187-GCST90094729 | ebi-met1400-GCST90200135 | Arachidoylcarnitine (C20) levels \|\| id:ebi-met1400-GCST90200135 | F-cheese liking (derived food-liking factor) \|\| id:ebi-fl187-GCST90094729 | MR Egger | 15 | -0.240304626 | 0.280077616159458 | 0.406437394209022 | -0.789256754 | 0.308647501723487 | 0.786388270302461 | 0.454182239169249 | 1.36158233047693 | 0.999950574162623 |
| ebi-fl187-GCST90094729 | ebi-met1400-GCST90200146 | Docosahexaenoylcarnitine (C22:6) levels \|\| id:ebi-met1400-GCST90200146 | F-cheese liking (derived food-liking factor) \|\| id:ebi-fl187-GCST90094729 | MR Egger | 15 | -0.110161021 | 0.416065265607995 | 0.795339259774937 | -0.925648942 | 0.705326899296534 | 0.895689898536742 | 0.396274176704423 | 2.02450838712904 | 0.999950574162623 |
| ebi-fl187-GCST90094729 | ebi-met1400-GCST90200162 | 3-hydroxyphenylacetoylglutamine levels \|\| id:ebi-met1400-GCST90200162 | F-cheese liking (derived food-liking factor) \|\| id:ebi-fl187-GCST90094729 | MR Egger | 15 | -0.102444089 | 0.336137218272987 | 0.765366672065301 | -0.761273037 | 0.556384858772165 | 0.902628615171916 | 0.467071449205792 | 1.74435499817544 | 0.999950574162623 |
| ebi-fl187-GCST90094729 | ebi-met1400-GCST90200172 | 3-carboxy-4-methyl-5-pentyl-2-furanpropionate (3-CMPFP) levels \|\| id:ebi-met1400-GCST90200172 | F-cheese liking (derived food-liking factor) \|\| id:ebi-fl187-GCST90094729 | MR Egger | 15 | 0.263071819783487 | 0.271576935100043 | 0.350381260114822 | -0.269218973 | 0.795362612579571 | 1.300920147 | 0.763975947216138 | 2.21524412656991 | 0.999950574162623 |
| ebi-fl187-GCST90094729 | ebi-met1400-GCST90200222 | Dihydrocaffeate sulfate (2) levels \|\| id:ebi-met1400-GCST90200222 | F-cheese liking (derived food-liking factor) \|\| id:ebi-fl187-GCST90094729 | MR Egger | 15 | -0.097719212 | 0.346387334034783 | 0.782300635571192 | -0.776638386 | 0.581199963063067 | 0.906903515952468 | 0.459949588209566 | 1.78818289727918 | 0.999950574162623 |
| ebi-fl187-GCST90094729 | ebi-met1400-GCST90200223 | 4-methylhexanoylglutamine levels \|\| id:ebi-met1400-GCST90200223 | F-cheese liking (derived food-liking factor) \|\| id:ebi-fl187-GCST90094729 | MR Egger | 15 | -0.284837754 | 0.347869424138209 | 0.427652252633204 | -0.966661825 | 0.396986317537432 | 0.752136275692938 | 0.380350598083531 | 1.48733557949869 | 0.999950574162623 |
| ebi-fl187-GCST90094729 | ebi-met1400-GCST90200224 | 2-ketocaprylate levels \|\| id:ebi-met1400-GCST90200224 | F-cheese liking (derived food-liking factor) \|\| id:ebi-fl187-GCST90094729 | MR Egger | 15 | -0.422271259 | 0.276894123381724 | 0.151205694715322 | -0.964983741 | 0.120441222643664 | 0.655556189635438 | 0.380989394190703 | 1.12799443848619 | 0.999950574162623 |
| ebi-fl187-GCST90094729 | ebi-met1400-GCST90200243 | Hydroxypalmitoyl sphingomyelin (d18:1/16:0(OH)) levels \|\| id:ebi-met1400-GCST90200243 | F-cheese liking (derived food-liking factor) \|\| id:ebi-fl187-GCST90094729 | MR Egger | 15 | -0.208614295 | 0.369470166878698 | 0.581937027767451 | -0.932775822 | 0.515547231855259 | 0.811708255021251 | 0.393460018030219 | 1.67455462074177 | 0.999950574162623 |
| ebi-fl187-GCST90094729 | ebi-met1400-GCST90200273 | 2-methoxyhydroquinone sulfate (2) levels \|\| id:ebi-met1400-GCST90200273 | F-cheese liking (derived food-liking factor) \|\| id:ebi-fl187-GCST90094729 | MR Egger | 15 | 0.213153995862827 | 0.324813767346252 | 0.523119520642043 | -0.423480988 | 0.84978897986148 | 1.23757521803349 | 0.654763623825184 | 2.3391531914112 | 0.999950574162623 |
| ebi-fl187-GCST90094729 | ebi-met1400-GCST90200276 | 2,4-di-tert-butylphenol levels \|\| id:ebi-met1400-GCST90200276 | F-cheese liking (derived food-liking factor) \|\| id:ebi-fl187-GCST90094729 | MR Egger | 15 | -0.349383005 | 0.271971819632331 | 0.221341293978787 | -0.882447772 | 0.183681761389937 | 0.705123012842488 | 0.413768859453564 | 1.20163335611259 | 0.999950574162623 |
| ebi-fl187-GCST90094729 | ebi-met1400-GCST90200289 | N-acetylmethionine levels \|\| id:ebi-met1400-GCST90200289 | F-cheese liking (derived food-liking factor) \|\| id:ebi-fl187-GCST90094729 | MR Egger | 15 | -0.11331893 | 0.291519123691256 | 0.703776105090649 | -0.684696413 | 0.458058552017303 | 0.892865852622343 | 0.504243288121873 | 1.58100157118272 | 0.999950574162623 |
| ebi-fl187-GCST90094729 | ebi-met1400-GCST90200308 | 3-hydroxyisobutyrate levels \|\| id:ebi-met1400-GCST90200308 | F-cheese liking (derived food-liking factor) \|\| id:ebi-fl187-GCST90094729 | MR Egger | 15 | -0.0114323 | 0.276322929322014 | 0.967627242268261 | -0.553025241 | 0.530160641656163 | 0.98863280060587 | 0.575207035480557 | 1.69920524984059 | 0.999950574162623 |
| ebi-fl187-GCST90094729 | ebi-met1400-GCST90200383 | Cystathionine levels \|\| id:ebi-met1400-GCST90200383 | F-cheese liking (derived food-liking factor) \|\| id:ebi-fl187-GCST90094729 | MR Egger | 15 | 0.144068498254454 | 0.339868473966344 | 0.678572438904132 | -0.522073711 | 0.810210707228489 | 1.15496321877987 | 0.593288961754973 | 2.24838168704253 | 0.999950574162623 |
| ebi-fl187-GCST90094729 | ebi-met1400-GCST90200384 | Guanidinoacetate levels \|\| id:ebi-met1400-GCST90200384 | F-cheese liking (derived food-liking factor) \|\| id:ebi-fl187-GCST90094729 | MR Egger | 15 | 0.110673224885276 | 0.272978204946735 | 0.691755605817861 | -0.424364057 | 0.645710506580877 | 1.1170298296579 | 0.654185677800355 | 1.90734172680305 | 0.999950574162623 |
| ebi-fl187-GCST90094729 | ebi-met1400-GCST90200403 | Citrulline levels \|\| id:ebi-met1400-GCST90200403 | F-cheese liking (derived food-liking factor) \|\| id:ebi-fl187-GCST90094729 | MR Egger | 15 | 0.126587380875327 | 0.274063417876826 | 0.651794031434341 | -0.410576918 | 0.663751679913907 | 1.13494861964713 | 0.663267488674605 | 1.94206468918434 | 0.999950574162623 |
| ebi-fl187-GCST90094729 | ebi-met1400-GCST90200434 | 12,13-DiHOME levels \|\| id:ebi-met1400-GCST90200434 | F-cheese liking (derived food-liking factor) \|\| id:ebi-fl187-GCST90094729 | MR Egger | 15 | -0.059353925 | 0.276275621940755 | 0.833228728450884 | -0.600854144 | 0.482146293983932 | 0.942373180681157 | 0.548343072053925 | 1.61954669791066 | 0.999950574162623 |
| ebi-fl187-GCST90094729 | ebi-met1400-GCST90200437 | Fructose levels \|\| id:ebi-met1400-GCST90200437 | F-cheese liking (derived food-liking factor) \|\| id:ebi-fl187-GCST90094729 | MR Egger | 15 | -0.140958024 | 0.280685266099663 | 0.623930383783924 | -0.691101145 | 0.409185097623143 | 0.868525768229047 | 0.501024064815218 | 1.50559037589554 | 0.999950574162623 |
| ebi-fl187-GCST90094729 | ebi-met1400-GCST90200448 | N-stearoyl-sphinganine (d18:0/18:0) levels \|\| id:ebi-met1400-GCST90200448 | F-cheese liking (derived food-liking factor) \|\| id:ebi-fl187-GCST90094729 | MR Egger | 15 | 0.0931074290838562 | 0.327766751616567 | 0.780833787896108 | -0.549315404 | 0.735530262252328 | 1.097579641 | 0.577344923094899 | 2.08658813813128 | 0.999950574162623 |
| ebi-fl187-GCST90094729 | ebi-met1400-GCST90200468 | X-11847 levels \|\| id:ebi-met1400-GCST90200468 | F-cheese liking (derived food-liking factor) \|\| id:ebi-fl187-GCST90094729 | MR Egger | 15 | -0.105682737 | 0.328213732527937 | 0.752576667042901 | -0.748981653 | 0.537616178340644 | 0.899710047136412 | 0.4728478307355 | 1.71192107968242 | 0.999950574162623 |
| ebi-fl187-GCST90094729 | ebi-met1400-GCST90200476 | X-11843 levels \|\| id:ebi-met1400-GCST90200476 | F-cheese liking (derived food-liking factor) \|\| id:ebi-fl187-GCST90094729 | MR Egger | 15 | -0.382945862 | 0.449116083582296 | 0.409279644602553 | -1.263213386 | 0.497321661394335 | 0.681849811980794 | 0.282743999468991 | 1.64431134514398 | 0.999950574162623 |
| ebi-fl187-GCST90094729 | ebi-met1400-GCST90200511 | X-12906 levels \|\| id:ebi-met1400-GCST90200511 | F-cheese liking (derived food-liking factor) \|\| id:ebi-fl187-GCST90094729 | MR Egger | 15 | -0.209236976 | 0.281604319731411 | 0.470685850854866 | -0.761181442 | 0.342707491132956 | 0.811202977599923 | 0.467114232408123 | 1.4087566278478 | 0.999950574162623 |
| ebi-fl187-GCST90094729 | ebi-met1400-GCST90200515 | X-12849 levels \|\| id:ebi-met1400-GCST90200515 | F-cheese liking (derived food-liking factor) \|\| id:ebi-fl187-GCST90094729 | MR Egger | 15 | 0.00194511597371237 | 0.380584384970296 | 0.995999733527854 | -0.744000279 | 0.747890510515493 | 1.00194700893893 | 0.475209139308034 | 2.11253893429634 | 0.999950574162623 |
| ebi-fl187-GCST90094729 | ebi-met1400-GCST90200551 | X-18886 levels \|\| id:ebi-met1400-GCST90200551 | F-cheese liking (derived food-liking factor) \|\| id:ebi-fl187-GCST90094729 | MR Egger | 15 | -0.33091766 | 0.274057985842273 | 0.248760262392641 | -0.868071313 | 0.206235991851194 | 0.718264308201865 | 0.419760355517212 | 1.22904321395723 | 0.999950574162623 |
| ebi-fl187-GCST90094729 | ebi-met1400-GCST90200564 | X-21319 levels \|\| id:ebi-met1400-GCST90200564 | F-cheese liking (derived food-liking factor) \|\| id:ebi-fl187-GCST90094729 | MR Egger | 15 | -0.443097466 | 0.276116423875683 | 0.132556784785429 | -0.984285657 | 0.0980907248149613 | 0.642044626528494 | 0.373706086020499 | 1.10306285574247 | 0.999950574162623 |
| ebi-fl187-GCST90094729 | ebi-met1400-GCST90200579 | X-21442 levels \|\| id:ebi-met1400-GCST90200579 | F-cheese liking (derived food-liking factor) \|\| id:ebi-fl187-GCST90094729 | MR Egger | 15 | -0.2231807 | 0.298018106499938 | 0.467262303082584 | -0.807296189 | 0.360934788373253 | 0.799970281310081 | 0.4460625058632 | 1.43466990066992 | 0.999950574162623 |
| ebi-fl187-GCST90094729 | ebi-met1400-GCST90200581 | X-21441 levels \|\| id:ebi-met1400-GCST90200581 | F-cheese liking (derived food-liking factor) \|\| id:ebi-fl187-GCST90094729 | MR Egger | 15 | -0.224620261 | 0.274119788257689 | 0.427312593146846 | -0.761895046 | 0.3126545240391 | 0.798819504133922 | 0.466781016861742 | 1.36704916681256 | 0.999950574162623 |
| ebi-fl187-GCST90094729 | ebi-met1400-GCST90200648 | X-24585 levels \|\| id:ebi-met1400-GCST90200648 | F-cheese liking (derived food-liking factor) \|\| id:ebi-fl187-GCST90094729 | MR Egger | 15 | 0.564593982304458 | 0.294373018575431 | 0.0773494249033399 | -0.012377134 | 1.1415650987123 | 1.75873356079083 | 0.98769914758037 | 3.13166589788952 | 0.999950574162623 |
| ebi-fl187-GCST90094729 | ebi-met1400-GCST90200720 | Adenosine 5'-diphosphate (ADP) to pantothenate ratio \|\| id:ebi-met1400-GCST90200720 | F-cheese liking (derived food-liking factor) \|\| id:ebi-fl187-GCST90094729 | MR Egger | 15 | -0.403981992 | 0.459410127887765 | 0.395175983880757 | -1.304425842 | 0.496461858999482 | 0.667656144539164 | 0.271328275544574 | 1.64289816992432 | 0.999950574162623 |
| ebi-fl187-GCST90094729 | ebi-met1400-GCST90200724 | Cholate to taurocholate ratio \|\| id:ebi-met1400-GCST90200724 | F-cheese liking (derived food-liking factor) \|\| id:ebi-fl187-GCST90094729 | MR Egger | 15 | 0.267994390409878 | 0.2933126699668 | 0.37751871720156 | -0.306898443 | 0.842887223544805 | 1.3073398063539 | 0.735725315371432 | 2.32306451004014 | 0.999950574162623 |
| ebi-fl187-GCST90094729 | ebi-met1400-GCST90200726 | N-acetylputrescine to (N(1) + N(8))-acetylspermidine ratio \|\| id:ebi-met1400-GCST90200726 | F-cheese liking (derived food-liking factor) \|\| id:ebi-fl187-GCST90094729 | MR Egger | 15 | -0.082905564 | 0.271989453512863 | 0.765334631837896 | -0.616004893 | 0.450193765023628 | 0.920438065732515 | 0.540097882075724 | 1.5686160989808 | 0.999950574162623 |
| ebi-fl187-GCST90094729 | ebi-met1400-GCST90200744 | Arginine to glutamate ratio \|\| id:ebi-met1400-GCST90200744 | F-cheese liking (derived food-liking factor) \|\| id:ebi-fl187-GCST90094729 | MR Egger | 15 | -0.074537149 | 0.287145342650882 | 0.799253313930149 | -0.63734202 | 0.488267722932311 | 0.928172993032413 | 0.528695820897631 | 1.62949104370083 | 0.999950574162623 |
| ebi-fl187-GCST90094729 | ebi-met1400-GCST90200759 | Histidine to trans-urocanate ratio \|\| id:ebi-met1400-GCST90200759 | F-cheese liking (derived food-liking factor) \|\| id:ebi-fl187-GCST90094729 | MR Egger | 15 | -0.116443939 | 0.275727988469172 | 0.679696428066539 | -0.656870797 | 0.423982918036385 | 0.890079994029309 | 0.518471200483912 | 1.52803549171445 | 0.999950574162623 |
| ebi-fl187-GCST90094729 | ebi-met1400-GCST90200795 | Oleoyl-linoleoyl-glycerol (18:1 to 18:2) [2] to linoleoyl-arachidonoyl-glycerol (18:2 to 20:4) [2] ratio \|\| id:ebi-met1400-GCST90200795 | F-cheese liking (derived food-liking factor) \|\| id:ebi-fl187-GCST90094729 | MR Egger | 15 | 0.258105992825546 | 0.36235597845614 | 0.488866877421175 | -0.452111725 | 0.968323710599581 | 1.29447601652371 | 0.63628307705974 | 2.63352620518894 | 0.999950574162623 |
| ebi-fl187-GCST90094729 | ebi-met1400-GCST90200811 | Choline to taurocholate ratio \|\| id:ebi-met1400-GCST90200811 | F-cheese liking (derived food-liking factor) \|\| id:ebi-fl187-GCST90094729 | MR Egger | 15 | -0.099730441 | 0.285990365293995 | 0.73288281333259 | -0.660271557 | 0.460810674879039 | 0.905081357894188 | 0.516710998911332 | 1.58535867464311 | 0.999950574162623 |
| ebi-fl187-GCST90094729 | ebi-met1400-GCST90200831 | Cholate to phosphate ratio \|\| id:ebi-met1400-GCST90200831 | F-cheese liking (derived food-liking factor) \|\| id:ebi-fl187-GCST90094729 | MR Egger | 15 | 0.216972095162157 | 0.361538011397715 | 0.558732223751591 | -0.491642407 | 0.925586597501678 | 1.24230943521074 | 0.611621038025647 | 2.52334801594727 | 0.999950574162623 |
| ebi-fl187-GCST90094729 | ebi-met1400-GCST90200841 | Adenosine 5'-diphosphate (ADP) to oxalate (ethanedioate) ratio \|\| id:ebi-met1400-GCST90200841 | F-cheese liking (derived food-liking factor) \|\| id:ebi-fl187-GCST90094729 | MR Egger | 15 | -0.381274119 | 0.424171815164571 | 0.385070250882045 | -1.212650877 | 0.450102638872526 | 0.682990643347468 | 0.297407842060641 | 1.56847316354582 | 0.999950574162623 |
| ebi-fl187-GCST90094729 | ebi-met1400-GCST90200844 | Aspartate to phosphate ratio \|\| id:ebi-met1400-GCST90200844 | F-cheese liking (derived food-liking factor) \|\| id:ebi-fl187-GCST90094729 | MR Egger | 15 | 0.0216280322443064 | 0.257118782080804 | 0.934245050186831 | -0.482324781 | 0.525580845122682 | 1.02186361345422 | 0.617346527005059 | 1.69144102837588 | 0.999950574162623 |
| ebi-fl187-GCST90094729 | ebi-met1400-GCST90200870 | Adenosine 5'-monophosphate (AMP) to acetoacetate ratio \|\| id:ebi-met1400-GCST90200870 | F-cheese liking (derived food-liking factor) \|\| id:ebi-fl187-GCST90094729 | MR Egger | 15 | 0.00886561087222794 | 0.301207886597377 | 0.976965901231321 | -0.581501847 | 0.599233068603086 | 1.00890502679627 | 0.559058116084132 | 1.82072189600697 | 0.999950574162623 |
| ebi-fl187-GCST90094729 | ebi-met1400-GCST90200887 | Cholesterol to cortisol ratio \|\| id:ebi-met1400-GCST90200887 | F-cheese liking (derived food-liking factor) \|\| id:ebi-fl187-GCST90094729 | MR Egger | 14 | -0.420575331 | 0.328000189302679 | 0.223975528505553 | -1.063455702 | 0.22230503966131 | 0.656668908889214 | 0.345260628438868 | 1.2489522997497 | 0.999950574162623 |
| ebi-fl187-GCST90094729 | ebi-met1400-GCST90200890 | Cortisol to taurocholate ratio \|\| id:ebi-met1400-GCST90200890 | F-cheese liking (derived food-liking factor) \|\| id:ebi-fl187-GCST90094729 | MR Egger | 15 | 0.0707516241057785 | 0.285272323384165 | 0.807997791197581 | -0.48838213 | 0.629885377938742 | 1.07331460728355 | 0.61361834642456 | 1.87739537600326 | 0.999950574162623 |
| ebi-fl187-GCST90094729 | ebi-met1400-GCST90200948 | Adenosine 5'-diphosphate (ADP) to mannitol to sorbitol ratio \|\| id:ebi-met1400-GCST90200948 | F-cheese liking (derived food-liking factor) \|\| id:ebi-fl187-GCST90094729 | MR Egger | 15 | -0.559873181 | 0.405133177881147 | 0.190273660003621 | -1.35393421 | 0.234187847574463 | 0.571281508563205 | 0.258222358725477 | 1.26388188705695 | 0.999950574162623 |
| ebi-fl187-GCST90094729 | ebi-met1400-GCST90200972 | Leucine to N-palmitoyl-sphingosine (d18:1 to 16:0) ratio \|\| id:ebi-met1400-GCST90200972 | F-cheese liking (derived food-liking factor) \|\| id:ebi-fl187-GCST90094729 | MR Egger | 15 | 0.19555786787389 | 0.263423802712098 | 0.471064948749973 | -0.320752785 | 0.711868521189602 | 1.21598915859351 | 0.725602608347588 | 2.03779536733505 | 0.999950574162623 |
| ebi-fl187-GCST90094729 | ebi-met1400-GCST90200973 | Proline to glutamate ratio \|\| id:ebi-met1400-GCST90200973 | F-cheese liking (derived food-liking factor) \|\| id:ebi-fl187-GCST90094729 | MR Egger | 15 | 0.0120417674035994 | 0.268217095741221 | 0.964872833893047 | -0.51366374 | 0.537747275056392 | 1.01211456138064 | 0.598299544297175 | 1.71214552162506 | 0.999950574162623 |
| ebi-fl187-GCST90094729 | ebi-met1400-GCST90200983 | Cholesterol to linoleoyl-arachidonoyl-glycerol (18:2 to 20:4) [1] ratio \|\| id:ebi-met1400-GCST90200983 | F-cheese liking (derived food-liking factor) \|\| id:ebi-fl187-GCST90094729 | MR Egger | 15 | -0.198830918 | 0.3180399796255 | 0.542674498740874 | -0.822189278 | 0.424527442173485 | 0.819688476271713 | 0.43946848190355 | 1.52886777049941 | 0.999950574162623 |
| ebi-fl187-GCST90094729 | ebi-met1400-GCST90201001 | Serine to alpha-ketobutyrate ratio \|\| id:ebi-met1400-GCST90201001 | F-cheese liking (derived food-liking factor) \|\| id:ebi-fl187-GCST90094729 | MR Egger | 15 | -0.095341842 | 0.278148291387031 | 0.737248026197137 | -0.640512493 | 0.449828808762497 | 0.909062125408632 | 0.527022259351221 | 1.56804372716586 | 0.999950574162623 |
| ebi-fl187-GCST90094729 | ebi-met1400-GCST90201005 | Fructose to maltose ratio \|\| id:ebi-met1400-GCST90201005 | F-cheese liking (derived food-liking factor) \|\| id:ebi-fl187-GCST90094729 | MR Egger | 15 | 0.00507069969819091 | 0.311895136967297 | 0.987275664587074 | -0.606243769 | 0.616385168154094 | 1.00508357745311 | 0.545395658531256 | 1.8522204602552 | 0.999950574162623 |
| ebi-fl187-GCST90094729 | ebi-met1400-GCST90201020 | Paraxanthine to 5-acetylamino-6-formylamino-3-methyluracil ratio \|\| id:ebi-met1400-GCST90201020 | F-cheese liking (derived food-liking factor) \|\| id:ebi-fl187-GCST90094729 | MR Egger | 15 | 0.190043634556075 | 0.288939303544456 | 0.522189110417913 | -0.3762774 | 0.756364669503208 | 1.20930236387784 | 0.686411895701531 | 2.13051699196719 | 0.999950574162623 |
| ebi-fl187-GCST90094729 | ebi-pw11-GCST90275197 | Placental weight (fetal genotype, adjusted for sex) \|\| id:ebi-pw11-GCST90275197 | F-cheese liking (derived food-liking factor) \|\| id:ebi-fl187-GCST90094729 | MR Egger | 15 | -0.059279598 | 0.117646105633663 | 0.622775825027248 | -0.289865965 | 0.171306768832962 | 0.942443226877507 | 0.748363867619729 | 1.18685478323843 | 0.999950574162623 |
| ebi-fl187-GCST90094729 | prot-a-1017 | FAS-associated factor 2 \|\| id:prot-a-1017 | F-cheese liking (derived food-liking factor) \|\| id:ebi-fl187-GCST90094729 | MR Egger | 16 | 0.46160351572543 | 0.429457267508552 | 0.300616630720789 | -0.380132729 | 1.30333976004219 | 1.5866161101638 | 0.68377064727433 | 3.68157172447525 | 0.999950574162623 |
| ebi-fl187-GCST90094729 | prot-a-1019 | Fas apoptotic inhibitory molecule 3 \|\| id:prot-a-1019 | F-cheese liking (derived food-liking factor) \|\| id:ebi-fl187-GCST90094729 | MR Egger | 16 | -0.114013816 | 0.429457267508552 | 0.794506338983157 | -0.95575006 | 0.727722428782013 | 0.892245628946766 | 0.384523620631607 | 2.07035984178802 | 0.999950574162623 |
| ebi-fl187-GCST90094729 | prot-a-1052 | MIP18 family protein FAM96A \|\| id:prot-a-1052 | F-cheese liking (derived food-liking factor) \|\| id:ebi-fl187-GCST90094729 | MR Egger | 16 | -0.566290712 | 0.446422927740407 | 0.225304466116202 | -1.441279651 | 0.308698225947552 | 0.567627030477938 | 0.236624767790018 | 1.36165139743581 | 0.999950574162623 |
| ebi-fl187-GCST90094729 | prot-a-1066 | F-box/LRR-repeat protein 4 \|\| id:prot-a-1066 | F-cheese liking (derived food-liking factor) \|\| id:ebi-fl187-GCST90094729 | MR Egger | 16 | 0.109659981383606 | 0.55855652627144 | 0.847175199637454 | -0.98511081 | 1.20443077287563 | 1.11589857965447 | 0.37339784838806 | 3.33486024477766 | 0.999950574162623 |
| ebi-fl187-GCST90094729 | prot-a-1076 | Low affinity immunoglobulin gamma Fc region receptor III-B \|\| id:prot-a-1076 | F-cheese liking (derived food-liking factor) \|\| id:ebi-fl187-GCST90094729 | MR Egger | 16 | 0.501853657757539 | 0.429457267508552 | 0.26208852942591 | -0.339882587 | 1.3435899020743 | 1.65178026996654 | 0.711853899071644 | 3.83277813580695 | 0.999950574162623 |
| ebi-fl187-GCST90094729 | prot-a-1081 | Fc receptor-like protein 3 \|\| id:prot-a-1081 | F-cheese liking (derived food-liking factor) \|\| id:ebi-fl187-GCST90094729 | MR Egger | 16 | -0.051497167 | 0.514052913762547 | 0.921622881166135 | -1.059040878 | 0.956046543984701 | 0.949806340769353 | 0.346788263137999 | 2.60139162958545 | 0.999950574162623 |
| ebi-fl187-GCST90094729 | prot-a-1105 | Fibroblast growth factor receptor 4 \|\| id:prot-a-1105 | F-cheese liking (derived food-liking factor) \|\| id:ebi-fl187-GCST90094729 | MR Egger | 16 | 0.32317544029361 | 0.529329427745087 | 0.551289218697775 | -0.714310238 | 1.36066111867398 | 1.38150770176278 | 0.489529654281899 | 3.89876999960213 | 0.999950574162623 |
| ebi-fl187-GCST90094729 | prot-a-1143 | Follistatin-related protein 1 \|\| id:prot-a-1143 | F-cheese liking (derived food-liking factor) \|\| id:ebi-fl187-GCST90094729 | MR Egger | 16 | -0.538915113 | 0.429457267508552 | 0.230068679847568 | -1.380651357 | 0.302821131191377 | 0.583380811369629 | 0.25141473885353 | 1.35367231303237 | 0.999950574162623 |
| ebi-fl187-GCST90094729 | prot-a-1151 | Alpha-(1,3)-fucosyltransferase 10 \|\| id:prot-a-1151 | F-cheese liking (derived food-liking factor) \|\| id:ebi-fl187-GCST90094729 | MR Egger | 16 | -0.324944031 | 0.48685577803691 | 0.51534668440232 | -1.279181356 | 0.629293293574258 | 0.722567793634027 | 0.27826500712171 | 1.87628412856376 | 0.999950574162623 |
| ebi-fl187-GCST90094729 | prot-a-1165 | Growth arrest and DNA damage-inducible proteins-interacting protein 1 \|\| id:prot-a-1165 | F-cheese liking (derived food-liking factor) \|\| id:ebi-fl187-GCST90094729 | MR Egger | 16 | 0.713288282770668 | 0.454245424225189 | 0.138672367504678 | -0.177032749 | 1.60360931425204 | 2.04069060569543 | 0.837752348764443 | 4.97094177571146 | 0.999950574162623 |
| ebi-fl187-GCST90094729 | prot-a-1194 | Growth/differentiation factor 11/8 \|\| id:prot-a-1194 | F-cheese liking (derived food-liking factor) \|\| id:ebi-fl187-GCST90094729 | MR Egger | 16 | 0.744039352142472 | 0.449422629117611 | 0.120048593526533 | -0.136829001 | 1.62490770521299 | 2.10441885817679 | 0.872119344980778 | 5.07795034720585 | 0.999950574162623 |
| ebi-fl187-GCST90094729 | prot-a-1216 | D-glucuronyl C5-epimerase \|\| id:prot-a-1216 | F-cheese liking (derived food-liking factor) \|\| id:ebi-fl187-GCST90094729 | MR Egger | 16 | -0.125537708 | 0.550510111546243 | 0.822912961410945 | -1.204537526 | 0.953462110771213 | 0.882022504619345 | 0.299830631178427 | 2.59467718690831 | 0.999950574162623 |
| ebi-fl187-GCST90094729 | prot-a-1246 | Glypican-3 \|\| id:prot-a-1246 | F-cheese liking (derived food-liking factor) \|\| id:ebi-fl187-GCST90094729 | MR Egger | 16 | -0.127030963 | 0.429457267508552 | 0.771729216181828 | -0.968767208 | 0.714705280902295 | 0.880706402497607 | 0.379550656921204 | 2.04358431017366 | 0.999950574162623 |
| ebi-fl187-GCST90094729 | prot-a-1275 | Glutamate receptor 4 \|\| id:prot-a-1275 | F-cheese liking (derived food-liking factor) \|\| id:ebi-fl187-GCST90094729 | MR Egger | 16 | 0.0346217649941993 | 0.513164463371937 | 0.947163853998116 | -0.971180583 | 1.0404241132032 | 1.03522807524249 | 0.378635763106227 | 2.8304171771265 | 0.999950574162623 |
| ebi-fl187-GCST90094729 | prot-a-1289 | Glutathione S-transferase P \|\| id:prot-a-1289 | F-cheese liking (derived food-liking factor) \|\| id:ebi-fl187-GCST90094729 | MR Egger | 16 | 0.487174980944326 | 0.429279194836985 | 0.275488156396405 | -0.354212241 | 1.32856220282482 | 1.62771140293951 | 0.701726016562539 | 3.77561092039573 | 0.999950574162623 |
| ebi-fl187-GCST90094729 | prot-a-130 | Apolipoprotein D \|\| id:prot-a-130 | F-cheese liking (derived food-liking factor) \|\| id:ebi-fl187-GCST90094729 | MR Egger | 16 | 0.15135185620357 | 0.429329292748824 | 0.729690931919241 | -0.690133558 | 0.992837269991265 | 1.16340593767026 | 0.501509084251281 | 2.69888107376382 | 0.999950574162623 |
| ebi-fl187-GCST90094729 | prot-a-1313 | Heparin-binding EGF-like growth factor \|\| id:prot-a-1313 | F-cheese liking (derived food-liking factor) \|\| id:ebi-fl187-GCST90094729 | MR Egger | 16 | 0.46578604142864 | 0.429279194836985 | 0.296234458695702 | -0.37560118 | 1.30717326330913 | 1.59326606994528 | 0.686876218086248 | 3.69571212803313 | 0.999950574162623 |
| ebi-fl187-GCST90094729 | prot-a-1347 | HLA class II histocompatibility antigen, DQ alpha 2 chain \|\| id:prot-a-1347 | F-cheese liking (derived food-liking factor) \|\| id:ebi-fl187-GCST90094729 | MR Egger | 16 | -0.129195002 | 0.429457267508552 | 0.767963071184909 | -0.970931246 | 0.712541242289909 | 0.878802580551505 | 0.378730182733376 | 2.03916669648607 | 0.999950574162623 |
| ebi-fl187-GCST90094729 | prot-a-1357 | Non-histone chromosomal protein HMG-14 \|\| id:prot-a-1357 | F-cheese liking (derived food-liking factor) \|\| id:ebi-fl187-GCST90094729 | MR Egger | 16 | 0.0323008643585307 | 0.429279194836985 | 0.941084874047232 | -0.809086358 | 0.873688086239021 | 1.03282819975812 | 0.445264693176484 | 2.39573023992899 | 0.999950574162623 |
| ebi-fl187-GCST90094729 | prot-a-137 | Apolipoprotein M \|\| id:prot-a-137 | F-cheese liking (derived food-liking factor) \|\| id:ebi-fl187-GCST90094729 | MR Egger | 16 | 0.140258699672536 | 0.429457267508552 | 0.748805380500705 | -0.701477545 | 0.981994943989298 | 1.15057141280697 | 0.495852118625686 | 2.669776988 | 0.999950574162623 |
| ebi-fl187-GCST90094729 | prot-a-1381 | 17-beta-hydroxysteroid dehydrogenase 14 \|\| id:prot-a-1381 | F-cheese liking (derived food-liking factor) \|\| id:ebi-fl187-GCST90094729 | MR Egger | 16 | 0.285697021844528 | 0.429457267508552 | 0.516700162925033 | -0.556039222 | 1.12743326616129 | 1.33068922440359 | 0.5734759822888 | 3.08772096239612 | 0.999950574162623 |
| ebi-fl187-GCST90094729 | prot-a-1392 | Serine protease HTRA2, mitochondrial \|\| id:prot-a-1392 | F-cheese liking (derived food-liking factor) \|\| id:ebi-fl187-GCST90094729 | MR Egger | 16 | 0.404600194234359 | 0.439744700357025 | 0.373113256647746 | -0.457299418 | 1.26649980693413 | 1.49870319008458 | 0.632990782556935 | 3.54841067810914 | 0.999950574162623 |
| ebi-fl187-GCST90094729 | prot-a-1416 | Interferon-induced protein with tetratricopeptide repeats 2 \|\| id:prot-a-1416 | F-cheese liking (derived food-liking factor) \|\| id:ebi-fl187-GCST90094729 | MR Egger | 16 | -0.022905348 | 0.506981049037673 | 0.964602038822312 | -1.016588204 | 0.970777508415018 | 0.977354988294035 | 0.361827317730218 | 2.63999628092056 | 0.999950574162623 |
| ebi-fl187-GCST90094729 | prot-a-1428 | Interferon gamma \|\| id:prot-a-1428 | F-cheese liking (derived food-liking factor) \|\| id:ebi-fl187-GCST90094729 | MR Egger | 16 | 0.00453331298538314 | 0.429457267508552 | 0.991726705877399 | -0.837202931 | 0.846269557302145 | 1.00454360399361 | 0.432919737747453 | 2.33093519268723 | 0.999950574162623 |
| ebi-fl187-GCST90094729 | prot-a-1483 | Interleukin-17C \|\| id:prot-a-1483 | F-cheese liking (derived food-liking factor) \|\| id:ebi-fl187-GCST90094729 | MR Egger | 16 | -0.398687792 | 0.46619415460701 | 0.406840056018608 | -1.312428335 | 0.515052751034673 | 0.671200222727068 | 0.269165637786756 | 1.6737267902888 | 0.999950574162623 |
| ebi-fl187-GCST90094729 | prot-a-1503 | Interleukin-1 receptor-like 2 \|\| id:prot-a-1503 | F-cheese liking (derived food-liking factor) \|\| id:ebi-fl187-GCST90094729 | MR Egger | 16 | 0.563420868624603 | 0.429457267508552 | 0.210647123896899 | -0.278315376 | 1.40515711294136 | 1.75667157609915 | 0.757058026162185 | 4.07616710956521 | 0.999950574162623 |
| ebi-fl187-GCST90094729 | prot-a-1522 | Interleukin-31 receptor subunit alpha \|\| id:prot-a-1522 | F-cheese liking (derived food-liking factor) \|\| id:ebi-fl187-GCST90094729 | MR Egger | 16 | 0.617610981748745 | 0.429457267508552 | 0.172377326603854 | -0.224125263 | 1.45934722606551 | 1.85449232943526 | 0.799215016373629 | 4.30314962741694 | 0.999950574162623 |
| ebi-fl187-GCST90094729 | prot-a-1540 | Interleukin-6 receptor subunit alpha \|\| id:prot-a-1540 | F-cheese liking (derived food-liking factor) \|\| id:ebi-fl187-GCST90094729 | MR Egger | 16 | 0.0589491233788016 | 0.429457267508552 | 0.892776470174291 | -0.782787121 | 0.900685367695563 | 1.06072127345291 | 0.457130157118894 | 2.46128942147854 | 0.999950574162623 |
| ebi-fl187-GCST90094729 | prot-a-1541 | Interleukin-6 receptor subunit alpha \|\| id:prot-a-1541 | F-cheese liking (derived food-liking factor) \|\| id:ebi-fl187-GCST90094729 | MR Egger | 16 | 0.191144142107862 | 0.460313734699503 | 0.684254526477056 | -0.711070778 | 1.09335906211889 | 1.21063394283363 | 0.491118037468625 | 2.98428164254615 | 0.999950574162623 |
| ebi-fl187-GCST90094729 | prot-a-1545 | Interleukin-9 \|\| id:prot-a-1545 | F-cheese liking (derived food-liking factor) \|\| id:ebi-fl187-GCST90094729 | MR Egger | 16 | -0.80476346 | 0.676817175309449 | 0.254200705291645 | -2.131325123 | 0.52179820386068 | 0.447193693359696 | 0.118679924069409 | 1.68505499939254 | 0.999950574162623 |
| ebi-fl187-GCST90094729 | prot-a-1548 | Inositol monophosphatase 3 \|\| id:prot-a-1548 | F-cheese liking (derived food-liking factor) \|\| id:ebi-fl187-GCST90094729 | MR Egger | 16 | 0.435539680183469 | 0.429376079755664 | 0.327624038620338 | -0.406037436 | 1.27711679650457 | 1.5457970699278 | 0.666285223812653 | 3.58628481616945 | 0.999950574162623 |
| ebi-fl187-GCST90094729 | prot-a-1555 | Inhibin beta A chain:Inhibin beta B chain heterodimer \|\| id:prot-a-1555 | F-cheese liking (derived food-liking factor) \|\| id:ebi-fl187-GCST90094729 | MR Egger | 16 | -0.03999214 | 0.429279194836985 | 0.927095619855638 | -0.881379362 | 0.801395082113261 | 0.960796991210639 | 0.414211170450693 | 2.2286479075757 | 0.999950574162623 |
| ebi-fl187-GCST90094729 | prot-a-1603 | Junctophilin-3 \|\| id:prot-a-1603 | F-cheese liking (derived food-liking factor) \|\| id:ebi-fl187-GCST90094729 | MR Egger | 16 | -0.34509288 | 0.429279194836985 | 0.434899133136604 | -1.186480102 | 0.496294342127244 | 0.708154577190815 | 0.305293978812967 | 1.64262297981162 | 0.999950574162623 |
| ebi-fl187-GCST90094729 | prot-a-1627 | UPF0258 protein KIAA1024 \|\| id:prot-a-1627 | F-cheese liking (derived food-liking factor) \|\| id:ebi-fl187-GCST90094729 | MR Egger | 16 | -0.624427894 | 0.444981720454634 | 0.182326400995254 | -1.496592066 | 0.24773627810616 | 0.535567742415542 | 0.223891870182189 | 1.28112202771219 | 0.999950574162623 |
| ebi-fl187-GCST90094729 | prot-a-1666 | Kallikrein-7 \|\| id:prot-a-1666 | F-cheese liking (derived food-liking factor) \|\| id:ebi-fl187-GCST90094729 | MR Egger | 16 | 0.506438955789151 | 0.439973938129137 | 0.268985619836654 | -0.355909963 | 1.36878787452226 | 1.65937156566312 | 0.700535691567152 | 3.93058344646433 | 0.999950574162623 |
| ebi-fl187-GCST90094729 | prot-a-1678 | Importin subunit alpha-3 \|\| id:prot-a-1678 | F-cheese liking (derived food-liking factor) \|\| id:ebi-fl187-GCST90094729 | MR Egger | 16 | -0.405757067 | 0.534621514166951 | 0.460463773758666 | -1.453615235 | 0.642101100738479 | 0.666472055796852 | 0.233723792488333 | 1.90046976573963 | 0.999950574162623 |
| ebi-fl187-GCST90094729 | prot-a-1718 | Protein LDOC1 \|\| id:prot-a-1718 | F-cheese liking (derived food-liking factor) \|\| id:ebi-fl187-GCST90094729 | MR Egger | 16 | -0.004152315 | 0.44383478817737 | 0.99266747576341 | -0.8740685 | 0.865763869974371 | 0.995856294086249 | 0.417250507693116 | 2.37682097489647 | 0.999950574162623 |
| ebi-fl187-GCST90094729 | prot-a-1723 | Leptin \|\| id:prot-a-1723 | F-cheese liking (derived food-liking factor) \|\| id:ebi-fl187-GCST90094729 | MR Egger | 16 | 0.455961658316894 | 0.439933753043388 | 0.31757152694402 | -0.406308498 | 1.31823181428193 | 1.57768985232244 | 0.666104644008675 | 3.73680816146476 | 0.999950574162623 |
| ebi-fl187-GCST90094729 | prot-a-1730 | Galectin-7 \|\| id:prot-a-1730 | F-cheese liking (derived food-liking factor) \|\| id:ebi-fl187-GCST90094729 | MR Egger | 16 | 0.162997392483067 | 0.577240404671746 | 0.781788747812568 | -0.968393801 | 1.29438858563969 | 1.17703362054937 | 0.379692410279448 | 3.64876438505557 | 0.999950574162623 |
| ebi-fl187-GCST90094729 | prot-a-1753 | Lipase member N \|\| id:prot-a-1753 | F-cheese liking (derived food-liking factor) \|\| id:ebi-fl187-GCST90094729 | MR Egger | 16 | 0.208674519545165 | 0.429457267508552 | 0.634553012894359 | -0.633061725 | 1.05041076386193 | 1.23204392704558 | 0.530963645250893 | 2.85882517898685 | 0.999950574162623 |
| ebi-fl187-GCST90094729 | prot-a-1756 | VIP36-like protein \|\| id:prot-a-1756 | F-cheese liking (derived food-liking factor) \|\| id:ebi-fl187-GCST90094729 | MR Egger | 16 | -0.274930743 | 0.429457267508552 | 0.532392519363625 | -1.116666987 | 0.566805501612888 | 0.75962473095815 | 0.327369103745719 | 1.76262733801368 | 0.999950574162623 |
| ebi-fl187-GCST90094729 | prot-a-1760 | Ig Kappa chain V-I region HK102- like \|\| id:prot-a-1760 | F-cheese liking (derived food-liking factor) \|\| id:ebi-fl187-GCST90094729 | MR Egger | 16 | 0.471815953602006 | 0.429279194836985 | 0.290271227307534 | -0.369571268 | 1.3132031754825 | 1.60290234817377 | 0.691030533847358 | 3.7180642705848 | 0.999950574162623 |
| ebi-fl187-GCST90094729 | prot-a-179 | Neutral ceramidase \|\| id:prot-a-179 | F-cheese liking (derived food-liking factor) \|\| id:ebi-fl187-GCST90094729 | MR Egger | 16 | 0.948301865296214 | 0.499201707783116 | 0.0782792545553421 | -0.030133482 | 1.92673721255112 | 2.5813225020557 | 0.970316005222924 | 6.86706786629606 | 0.999950574162623 |
| ebi-fl187-GCST90094729 | prot-a-1809 | Lactotransferrin \|\| id:prot-a-1809 | F-cheese liking (derived food-liking factor) \|\| id:ebi-fl187-GCST90094729 | MR Egger | 16 | 0.0192694385182246 | 0.429279194836985 | 0.964830649828572 | -0.822117783 | 0.860656660398715 | 1.01945629240876 | 0.439499902648405 | 2.36471299736154 | 0.999950574162623 |
| ebi-fl187-GCST90094729 | prot-a-1818 | Lysophospholipase-like protein 1 \|\| id:prot-a-1818 | F-cheese liking (derived food-liking factor) \|\| id:ebi-fl187-GCST90094729 | MR Egger | 16 | -0.402273426 | 0.429457267508552 | 0.364790285342644 | -1.244009671 | 0.439462818128877 | 0.668797853840135 | 0.288226205751018 | 1.55187335633027 | 0.999950574162623 |
| ebi-fl187-GCST90094729 | prot-a-1852 | MAP kinase-activated protein kinase 3 \|\| id:prot-a-1852 | F-cheese liking (derived food-liking factor) \|\| id:ebi-fl187-GCST90094729 | MR Egger | 16 | 0.0358537707235891 | 0.4649412702097 | 0.939623605846499 | -0.875431119 | 0.947138660334601 | 1.03650426813947 | 0.416682341322525 | 2.57832164056064 | 0.999950574162623 |
| ebi-fl187-GCST90094729 | prot-a-1856 | Megakaryocyte-associated tyrosine-protein kinase \|\| id:prot-a-1856 | F-cheese liking (derived food-liking factor) \|\| id:ebi-fl187-GCST90094729 | MR Egger | 16 | -0.387654245 | 0.429279194836985 | 0.381782300706075 | -1.229041467 | 0.453732976831579 | 0.678646948246247 | 0.292572884102883 | 1.57417759945925 | 0.999950574162623 |
| ebi-fl187-GCST90094729 | prot-a-1890 | Beta-1,3-N-acetylglucosaminyltransferase manic fringe \|\| id:prot-a-1890 | F-cheese liking (derived food-liking factor) \|\| id:ebi-fl187-GCST90094729 | MR Egger | 16 | 0.426013373746912 | 0.429457267508552 | 0.338043221378011 | -0.415722871 | 1.26774961806367 | 1.53114125209191 | 0.65986311263613 | 3.55284829377993 | 0.999950574162623 |
| ebi-fl187-GCST90094729 | prot-a-1919 | Matrilysin \|\| id:prot-a-1919 | F-cheese liking (derived food-liking factor) \|\| id:ebi-fl187-GCST90094729 | MR Egger | 16 | -0.431499871 | 0.489868881283724 | 0.393271109622776 | -1.391642878 | 0.528643136726045 | 0.649534146594363 | 0.248666440265799 | 1.69662865299034 | 0.999950574162623 |
| ebi-fl187-GCST90094729 | prot-a-1923 | Multimerin-2 \|\| id:prot-a-1923 | F-cheese liking (derived food-liking factor) \|\| id:ebi-fl187-GCST90094729 | MR Egger | 16 | -0.457974859 | 0.515572756048012 | 0.389395051198303 | -1.468497461 | 0.552547742742475 | 0.632563379212759 | 0.230271216875824 | 1.7376745307115 | 0.999950574162623 |
| ebi-fl187-GCST90094729 | prot-a-1948 | Mesothelin \|\| id:prot-a-1948 | F-cheese liking (derived food-liking factor) \|\| id:ebi-fl187-GCST90094729 | MR Egger | 16 | 0.838255708107805 | 0.452722806917554 | 0.085290713342751 | -0.049080993 | 1.72559240966621 | 2.31233007843221 | 0.952104012386255 | 5.61584692645238 | 0.999950574162623 |
| ebi-fl187-GCST90094729 | prot-a-195 | ATPase family AAA domain-containing protein 1 \|\| id:prot-a-195 | F-cheese liking (derived food-liking factor) \|\| id:ebi-fl187-GCST90094729 | MR Egger | 16 | -0.199066834 | 0.429457267508552 | 0.650108006746469 | -1.040803078 | 0.642669410513675 | 0.819495121527059 | 0.35317094418439 | 1.90155012824618 | 0.999950574162623 |
| ebi-fl187-GCST90094729 | prot-a-1966 | Mucin-1 \|\| id:prot-a-1966 | F-cheese liking (derived food-liking factor) \|\| id:ebi-fl187-GCST90094729 | MR Egger | 16 | 0.520059431195461 | 0.466810057387328 | 0.284010212095027 | -0.394888281 | 1.43500714367462 | 1.68212761758348 | 0.67375530612233 | 4.19967500979256 | 0.999950574162623 |
| ebi-fl187-GCST90094729 | prot-a-1986 | Myocardial zonula adherens protein \|\| id:prot-a-1986 | F-cheese liking (derived food-liking factor) \|\| id:ebi-fl187-GCST90094729 | MR Egger | 16 | -0.560162503 | 0.429279194836985 | 0.212971874569791 | -1.401549724 | 0.28122471934601 | 0.571116248469792 | 0.246215102572296 | 1.32475126772711 | 0.999950574162623 |
| ebi-fl187-GCST90094729 | prot-a-2071 | Epididymal secretory protein E1 \|\| id:prot-a-2071 | F-cheese liking (derived food-liking factor) \|\| id:ebi-fl187-GCST90094729 | MR Egger | 16 | 0.334599516571951 | 0.545187405071243 | 0.54923471259638 | -0.733967797 | 1.40316683051159 | 1.39738064535554 | 0.480000661302463 | 4.06806245373862 | 0.999950574162623 |
| ebi-fl187-GCST90094729 | prot-a-2098 | Neuritin \|\| id:prot-a-2098 | F-cheese liking (derived food-liking factor) \|\| id:ebi-fl187-GCST90094729 | MR Egger | 16 | -0.246082704 | 0.429457267508552 | 0.575727341453117 | -1.087818948 | 0.595653540794746 | 0.781857559877584 | 0.336950599687678 | 1.81421622191606 | 0.999950574162623 |
| ebi-fl187-GCST90094729 | prot-a-2115 | Neurotrophin-4 \|\| id:prot-a-2115 | F-cheese liking (derived food-liking factor) \|\| id:ebi-fl187-GCST90094729 | MR Egger | 16 | 0.0498129591860826 | 0.49645003455836 | 0.9214984735154 | -0.923229109 | 1.02285502692047 | 1.05107448416234 | 0.397234255315029 | 2.78112362283809 | 0.999950574162623 |
| ebi-fl187-GCST90094729 | prot-a-2118 | Netrin-1 \|\| id:prot-a-2118 | F-cheese liking (derived food-liking factor) \|\| id:ebi-fl187-GCST90094729 | MR Egger | 16 | -0.049212879 | 0.521097203819959 | 0.926097307457557 | -1.070563398 | 0.972137640543111 | 0.951978451958787 | 0.34281532138641 | 2.64358946773075 | 0.999950574162623 |
| ebi-fl187-GCST90094729 | prot-a-2120 | Netrin-G1 \|\| id:prot-a-2120 | F-cheese liking (derived food-liking factor) \|\| id:ebi-fl187-GCST90094729 | MR Egger | 16 | -0.337898281 | 0.429457267508552 | 0.444512761882067 | -1.179634525 | 0.503837963549743 | 0.713267837265704 | 0.307391061796783 | 1.65506116119288 | 0.999950574162623 |
| ebi-fl187-GCST90094729 | prot-a-2145 | Olfactomedin-like protein 3 \|\| id:prot-a-2145 | F-cheese liking (derived food-liking factor) \|\| id:ebi-fl187-GCST90094729 | MR Egger | 16 | 0.0590715411549721 | 0.481992894861914 | 0.904199937724886 | -0.885634533 | 1.00377761508432 | 1.06085113254071 | 0.412452375678016 | 2.72856987079512 | 0.999950574162623 |
| ebi-fl187-GCST90094729 | prot-a-2162 | Prolyl 4-hydroxylase subunit alpha-2 \|\| id:prot-a-2162 | F-cheese liking (derived food-liking factor) \|\| id:ebi-fl187-GCST90094729 | MR Egger | 16 | 0.0162406599041163 | 0.429457267508552 | 0.970367780666032 | -0.825495584 | 0.857976904220878 | 1.01637325626611 | 0.438017863840833 | 2.35838462613105 | 0.999950574162623 |
| ebi-fl187-GCST90094729 | prot-a-2168 | Polyadenylate-binding protein-interacting protein 1 \|\| id:prot-a-2168 | F-cheese liking (derived food-liking factor) \|\| id:ebi-fl187-GCST90094729 | MR Egger | 16 | -0.93218593 | 0.534888380810865 | 0.103285932007326 | -1.980567156 | 0.116195296684124 | 0.3936921856551 | 0.137990952703388 | 1.12321521092074 | 0.999950574162623 |
| ebi-fl187-GCST90094729 | prot-a-219 | Beta-1,4-galactosyltransferase 5 \|\| id:prot-a-219 | F-cheese liking (derived food-liking factor) \|\| id:ebi-fl187-GCST90094729 | MR Egger | 16 | -0.160835737 | 0.498458146492539 | 0.751716228059184 | -1.137813704 | 0.816142229960524 | 0.851431918241819 | 0.320519005678641 | 2.26175764481117 | 0.999950574162623 |
| ebi-fl187-GCST90094729 | prot-a-2190 | Pyruvate carboxylase, mitochondrial \|\| id:prot-a-2190 | F-cheese liking (derived food-liking factor) \|\| id:ebi-fl187-GCST90094729 | MR Egger | 16 | -0.471349299 | 0.495374119440484 | 0.357488878480527 | -1.442282573 | 0.499583975473237 | 0.624159522264173 | 0.236387570584203 | 1.64803550487131 | 0.999950574162623 |
| ebi-fl187-GCST90094729 | prot-a-2197 | Protocadherin-9 \|\| id:prot-a-2197 | F-cheese liking (derived food-liking factor) \|\| id:ebi-fl187-GCST90094729 | MR Egger | 16 | -0.049895705 | 0.429457267508552 | 0.909157509281659 | -0.89163195 | 0.791840538893975 | 0.951328637744987 | 0.409986129747756 | 2.20745559746265 | 0.999950574162623 |
| ebi-fl187-GCST90094729 | prot-a-2198 | Protocadherin alpha-4 \|\| id:prot-a-2198 | F-cheese liking (derived food-liking factor) \|\| id:ebi-fl187-GCST90094729 | MR Egger | 16 | -0.390515215 | 0.627018875817431 | 0.543420254170555 | -1.619472212 | 0.838441781156019 | 0.676708134184417 | 0.198003175200839 | 2.31276038077098 | 0.999950574162623 |
| ebi-fl187-GCST90094729 | prot-a-2254 | Placenta growth factor \|\| id:prot-a-2254 | F-cheese liking (derived food-liking factor) \|\| id:ebi-fl187-GCST90094729 | MR Egger | 16 | -0.688597613 | 0.531243943144834 | 0.215868043023525 | -1.729835742 | 0.352640515360601 | 0.502279966175512 | 0.177313532785537 | 1.42281956970772 | 0.999950574162623 |
| ebi-fl187-GCST90094729 | prot-a-2261 | Membrane-associated progesterone receptor component 2 \|\| id:prot-a-2261 | F-cheese liking (derived food-liking factor) \|\| id:ebi-fl187-GCST90094729 | MR Egger | 16 | 0.161320265389627 | 0.496748429795295 | 0.750170233397003 | -0.812306657 | 1.13494718778841 | 1.1750612400032 | 0.443833113807699 | 3.11100923928833 | 0.999950574162623 |
| ebi-fl187-GCST90094729 | prot-a-2268 | Polymeric immunoglobulin receptor \|\| id:prot-a-2268 | F-cheese liking (derived food-liking factor) \|\| id:ebi-fl187-GCST90094729 | MR Egger | 16 | 0.326766945503404 | 0.462183812442207 | 0.491163807717958 | -0.579113327 | 1.23264721789013 | 1.38647831451888 | 0.560395033553201 | 3.43029827449142 | 0.999950574162623 |
| ebi-fl187-GCST90094729 | prot-a-2283 | Group 10 secretory phospholipase A2 \|\| id:prot-a-2283 | F-cheese liking (derived food-liking factor) \|\| id:ebi-fl187-GCST90094729 | MR Egger | 16 | 0.0108611982733206 | 0.429457267508552 | 0.98018018745573 | -0.830875046 | 0.852597442590082 | 1.01092039520972 | 0.435667890012797 | 2.34573184959985 | 0.999950574162623 |
| ebi-fl187-GCST90094729 | prot-a-2298 | Inactive phospholipase D5 \|\| id:prot-a-2298 | F-cheese liking (derived food-liking factor) \|\| id:ebi-fl187-GCST90094729 | MR Egger | 16 | -0.062297261 | 0.581663048079288 | 0.91622774879323 | -1.202356835 | 1.07776231367967 | 0.939603538203555 | 0.30048518277515 | 2.93809764877915 | 0.999950574162623 |
| ebi-fl187-GCST90094729 | prot-a-2304 | Procollagen-lysine,2-oxoglutarate 5-dioxygenase 2 \|\| id:prot-a-2304 | F-cheese liking (derived food-liking factor) \|\| id:ebi-fl187-GCST90094729 | MR Egger | 16 | 0.359724907372342 | 0.429279194836985 | 0.416122681592044 | -0.481662315 | 1.20111212925283 | 1.4329351704347 | 0.617755633661843 | 3.32381137586306 | 0.999950574162623 |
| ebi-fl187-GCST90094729 | prot-a-2331 | Periostin \|\| id:prot-a-2331 | F-cheese liking (derived food-liking factor) \|\| id:ebi-fl187-GCST90094729 | MR Egger | 16 | -0.215760276 | 0.580012167270685 | 0.715468411139847 | -1.352584124 | 0.92106357173529 | 0.805928479023786 | 0.258571216494277 | 2.51196062000957 | 0.999950574162623 |
| ebi-fl187-GCST90094729 | prot-a-2333 | Periostin \|\| id:prot-a-2333 | F-cheese liking (derived food-liking factor) \|\| id:ebi-fl187-GCST90094729 | MR Egger | 16 | -0.334494932 | 0.486889504980666 | 0.50330229224575 | -1.288798361 | 0.619808498028329 | 0.71569947217417 | 0.275601757877971 | 1.8585720875452 | 0.999950574162623 |
| ebi-fl187-GCST90094729 | prot-a-2367 | cAMP-dependent protein kinase catalytic subunit alpha \|\| id:prot-a-2367 | F-cheese liking (derived food-liking factor) \|\| id:ebi-fl187-GCST90094729 | MR Egger | 16 | 0.262004758950809 | 0.429279194836985 | 0.551418559052914 | -0.579382463 | 1.1033919808313 | 1.29953272682699 | 0.560244231343536 | 3.01437339933776 | 0.999950574162623 |
| ebi-fl187-GCST90094729 | prot-a-2385 | Phosphoribosyl pyrophosphate synthase-associated protein 1 \|\| id:prot-a-2385 | F-cheese liking (derived food-liking factor) \|\| id:ebi-fl187-GCST90094729 | MR Egger | 16 | 0.981334969248003 | 0.429457267508552 | 0.0384226941897905 | 0.139598724931241 | 1.82307121356477 | 2.66801558435278 | 1.14981231525662 | 6.19084268266911 | 0.999950574162623 |
| ebi-fl187-GCST90094729 | prot-a-2393 | Trypsin-3 \|\| id:prot-a-2393 | F-cheese liking (derived food-liking factor) \|\| id:ebi-fl187-GCST90094729 | MR Egger | 16 | 0.100920221803406 | 0.429187569369918 | 0.817502760012954 | -0.740287414 | 0.942127857768444 | 1.1061883885284 | 0.476976805929565 | 2.56543449430485 | 0.999950574162623 |
| ebi-fl187-GCST90094729 | prot-a-2405 | Pregnancy-specific beta-1-glycoprotein 6 \|\| id:prot-a-2405 | F-cheese liking (derived food-liking factor) \|\| id:ebi-fl187-GCST90094729 | MR Egger | 16 | -0.090441286 | 0.429457267508552 | 0.836238130925541 | -0.93217753 | 0.75129495849234 | 0.913527969367945 | 0.393695492511693 | 2.11974321954609 | 0.999950574162623 |
| ebi-fl187-GCST90094729 | prot-a-244 | Beclin-1 \|\| id:prot-a-244 | F-cheese liking (derived food-liking factor) \|\| id:ebi-fl187-GCST90094729 | MR Egger | 16 | -0.019249794 | 0.429376079755664 | 0.964874399883746 | -0.86082691 | 0.822327322324237 | 0.980934300137068 | 0.422812309860867 | 2.2757901762653 | 0.999950574162623 |
| ebi-fl187-GCST90094729 | prot-a-2454 | tRNA pseudouridine synthase A, mitochondrial \|\| id:prot-a-2454 | F-cheese liking (derived food-liking factor) \|\| id:ebi-fl187-GCST90094729 | MR Egger | 16 | -0.336458693 | 0.429279194836985 | 0.446229942102413 | -1.177845915 | 0.504928529189356 | 0.714295388586998 | 0.307941356666144 | 1.65686709859438 | 0.999950574162623 |
| ebi-fl187-GCST90094729 | prot-a-2466 | Glutaminyl-peptide cyclotransferase-like protein \|\| id:prot-a-2466 | F-cheese liking (derived food-liking factor) \|\| id:ebi-fl187-GCST90094729 | MR Egger | 16 | -0.285897208 | 0.429457267508552 | 0.516410918883088 | -1.127633452 | 0.555839036377373 | 0.751339843748374 | 0.323798635342094 | 1.74340314994665 | 0.999950574162623 |
| ebi-fl187-GCST90094729 | prot-a-2474 | Ras-related protein Rab-27A \|\| id:prot-a-2474 | F-cheese liking (derived food-liking factor) \|\| id:ebi-fl187-GCST90094729 | MR Egger | 16 | -0.239084201 | 0.533591361009362 | 0.660961260054751 | -1.284923269 | 0.806754866269759 | 0.787348583800442 | 0.276671812153618 | 2.24062504809258 | 0.999950574162623 |
| ebi-fl187-GCST90094729 | prot-a-2490 | Rap1 GTPase-GDP dissociation stimulator 1 \|\| id:prot-a-2490 | F-cheese liking (derived food-liking factor) \|\| id:ebi-fl187-GCST90094729 | MR Egger | 16 | 0.0679973332775479 | 0.47705363572803 | 0.88868763827512 | -0.867027793 | 1.00302245930449 | 1.07036245411538 | 0.420198612427031 | 2.72651015328817 | 0.999950574162623 |
| ebi-fl187-GCST90094729 | prot-a-2545 | Receptor-interacting serine/threonine-protein kinase 2 \|\| id:prot-a-2545 | F-cheese liking (derived food-liking factor) \|\| id:ebi-fl187-GCST90094729 | MR Egger | 16 | 0.900178994787676 | 0.53720012692443 | 0.115980148737122 | -0.152733254 | 1.95309124355956 | 2.46004340669773 | 0.858358655027876 | 7.05044858275521 | 0.999950574162623 |
| ebi-fl187-GCST90094729 | prot-a-2610 | RUN and FYVE domain-containing protein 1 \|\| id:prot-a-2610 | F-cheese liking (derived food-liking factor) \|\| id:ebi-fl187-GCST90094729 | MR Egger | 16 | -0.147181641 | 0.486523788444532 | 0.766706063772006 | -1.100768267 | 0.806404983962623 | 0.863137181740163 | 0.332615448126513 | 2.23984123016133 | 0.999950574162623 |
| ebi-fl187-GCST90094729 | prot-a-2627 | Serine--tRNA ligase, mitochondrial \|\| id:prot-a-2627 | F-cheese liking (derived food-liking factor) \|\| id:ebi-fl187-GCST90094729 | MR Egger | 16 | 0.209724520405264 | 0.429457267508552 | 0.632863677018968 | -0.632011724 | 1.05146076472203 | 1.23333825363176 | 0.531521450331746 | 2.86182852436534 | 0.999950574162623 |
| ebi-fl187-GCST90094729 | prot-a-264 | BolA-like protein 2 \|\| id:prot-a-264 | F-cheese liking (derived food-liking factor) \|\| id:ebi-fl187-GCST90094729 | MR Egger | 16 | -0.034446794 | 0.429279194836985 | 0.937179517621494 | -0.875834016 | 0.806940427548708 | 0.966139742429607 | 0.416514494936594 | 2.24104085992024 | 0.999950574162623 |
| ebi-fl187-GCST90094729 | prot-a-2646 | Secretoglobin family 1C member 1 \|\| id:prot-a-2646 | F-cheese liking (derived food-liking factor) \|\| id:ebi-fl187-GCST90094729 | MR Egger | 16 | -0.000782102 | 0.531840939308658 | 0.998847415839055 | -1.043190343 | 1.04162613935934 | 0.999218204076174 | 0.352328837253762 | 2.83382145821376 | 0.999974376584515 |
| ebi-fl187-GCST90094729 | prot-a-2672 | Semaphorin-3E \|\| id:prot-a-2672 | F-cheese liking (derived food-liking factor) \|\| id:ebi-fl187-GCST90094729 | MR Egger | 16 | 0.0282780928313789 | 0.429457267508552 | 0.948431392212366 | -0.813458151 | 0.870014337148141 | 1.02868171365858 | 0.443322336563807 | 2.3869450752641 | 0.999950574162623 |
| ebi-fl187-GCST90094729 | prot-a-2701 | Plasma protease C1 inhibitor \|\| id:prot-a-2701 | F-cheese liking (derived food-liking factor) \|\| id:ebi-fl187-GCST90094729 | MR Egger | 16 | 0.73888553688402 | 0.429457267508552 | 0.107355460939337 | -0.102850707 | 1.58062178120078 | 2.09360097279193 | 0.902261664387306 | 4.85797547017778 | 0.999950574162623 |
| ebi-fl187-GCST90094729 | prot-a-2738 | Signaling threshold-regulating transmembrane adapter 1 \|\| id:prot-a-2738 | F-cheese liking (derived food-liking factor) \|\| id:ebi-fl187-GCST90094729 | MR Egger | 16 | 0.60601401549356 | 0.582126554769608 | 0.315504539743085 | -0.534954032 | 1.74698206284199 | 1.83311006878837 | 0.58569621289451 | 5.73726182671857 | 0.999950574162623 |
| ebi-fl187-GCST90094729 | prot-a-274 | Serine/threonine-protein kinase BRSK2 \|\| id:prot-a-274 | F-cheese liking (derived food-liking factor) \|\| id:ebi-fl187-GCST90094729 | MR Egger | 16 | -0.507304507 | 0.429279194836985 | 0.256986297005183 | -1.348691729 | 0.334082714583129 | 0.602116393728951 | 0.259579639065857 | 1.39665866283595 | 0.999950574162623 |
| ebi-fl187-GCST90094729 | prot-a-2749 | Mitochondrial glutamate carrier 2 \|\| id:prot-a-2749 | F-cheese liking (derived food-liking factor) \|\| id:ebi-fl187-GCST90094729 | MR Egger | 16 | -0.101024315 | 0.507599338325656 | 0.845105643624854 | -1.095919018 | 0.893870388216567 | 0.903911054110217 | 0.334232300253242 | 2.44457281095686 | 0.999950574162623 |
| ebi-fl187-GCST90094729 | prot-a-2760 | Sodium-coupled monocarboxylate transporter 1 \|\| id:prot-a-2760 | F-cheese liking (derived food-liking factor) \|\| id:ebi-fl187-GCST90094729 | MR Egger | 16 | 0.115548495212562 | 0.465441132960584 | 0.807540893163994 | -0.796716125 | 1.02781311581531 | 1.12248894856853 | 0.450806929492249 | 2.79494692124105 | 0.999950574162623 |
| ebi-fl187-GCST90094729 | prot-a-2777 | Small integral membrane protein 10 \|\| id:prot-a-2777 | F-cheese liking (derived food-liking factor) \|\| id:ebi-fl187-GCST90094729 | MR Egger | 16 | 0.637487493854708 | 0.56602459017984 | 0.278993988960819 | -0.471920703 | 1.74689569060719 | 1.89172194037639 | 0.623802976724969 | 5.73676630799282 | 0.999950574162623 |
| ebi-fl187-GCST90094729 | prot-a-2799 | Superoxide dismutase [Mn], mitochondrial \|\| id:prot-a-2799 | F-cheese liking (derived food-liking factor) \|\| id:ebi-fl187-GCST90094729 | MR Egger | 16 | 0.656484243125569 | 0.429457267508552 | 0.148629879823945 | -0.185252001 | 1.49822048744233 | 1.92800201849601 | 0.830894870969846 | 4.47372094015439 | 0.999950574162623 |
| ebi-fl187-GCST90094729 | prot-a-2805 | Protein SPACA7 \|\| id:prot-a-2805 | F-cheese liking (derived food-liking factor) \|\| id:ebi-fl187-GCST90094729 | MR Egger | 16 | -0.913440223 | 0.429376079755664 | 0.0516458572949791 | -1.755017339 | -0.071863107 | 0.401141830151511 | 0.172904244213375 | 0.930658287940719 | 0.999950574162623 |
| ebi-fl187-GCST90094729 | prot-a-2811 | SPARC-like protein 1 \|\| id:prot-a-2811 | F-cheese liking (derived food-liking factor) \|\| id:ebi-fl187-GCST90094729 | MR Egger | 16 | 0.073263049997712 | 0.433812508608588 | 0.868305316298923 | -0.777009467 | 0.923535566870544 | 1.07601354505099 | 0.45977894154014 | 2.51817785576446 | 0.999950574162623 |
| ebi-fl187-GCST90094729 | prot-a-2831 | Sepiapterin reductase \|\| id:prot-a-2831 | F-cheese liking (derived food-liking factor) \|\| id:ebi-fl187-GCST90094729 | MR Egger | 16 | 0.808585877075784 | 0.429457267508552 | 0.0806734503800445 | -0.033150367 | 1.65032212139255 | 2.24473141502174 | 0.967393084422911 | 5.20865737694556 | 0.999950574162623 |
| ebi-fl187-GCST90094729 | prot-a-2834 | SPRY domain-containing SOCS box protein 1 \|\| id:prot-a-2834 | F-cheese liking (derived food-liking factor) \|\| id:ebi-fl187-GCST90094729 | MR Egger | 16 | -1.03516681 | 0.429457267508552 | 0.030259444347319 | -1.876903054 | -0.193430565 | 0.355167130631405 | 0.153063401566829 | 0.824127057086665 | 0.999950574162623 |
| ebi-fl187-GCST90094729 | prot-a-2835 | Spectrin alpha chain, non-erythrocytic 1 \|\| id:prot-a-2835 | F-cheese liking (derived food-liking factor) \|\| id:ebi-fl187-GCST90094729 | MR Egger | 16 | 0.0190966965648848 | 0.429457267508552 | 0.965160145654489 | -0.822639548 | 0.860832940881647 | 1.01928020474677 | 0.439270647063866 | 2.36512988685437 | 0.999950574162623 |
| ebi-fl187-GCST90094729 | prot-a-2859 | Sia-alpha-2,3-Gal-beta-1,4-GlcNAc-R:alpha 2,8-sialyltransferase \|\| id:prot-a-2859 | F-cheese liking (derived food-liking factor) \|\| id:ebi-fl187-GCST90094729 | MR Egger | 16 | -0.265885265 | 0.429279194836985 | 0.545616931774265 | -1.107272487 | 0.57550195639409 | 0.766527069566415 | 0.330459064268277 | 1.77802279286572 | 0.999950574162623 |
| ebi-fl187-GCST90094729 | prot-a-2861 | Alpha-2,8-sialyltransferase 8F \|\| id:prot-a-2861 | F-cheese liking (derived food-liking factor) \|\| id:ebi-fl187-GCST90094729 | MR Egger | 16 | -0.2031868 | 0.509217275913118 | 0.695900028889806 | -1.201252661 | 0.794879060358151 | 0.816125774538723 | 0.300817153815202 | 2.21417319929702 | 0.999950574162623 |
| ebi-fl187-GCST90094729 | prot-a-289 | Uncharacterized protein C17orf89 \|\| id:prot-a-289 | F-cheese liking (derived food-liking factor) \|\| id:ebi-fl187-GCST90094729 | MR Egger | 16 | -0.363680985 | 0.465933468222994 | 0.448068633010801 | -1.276910583 | 0.549548612439351 | 0.695112910627854 | 0.278897601847916 | 1.73247082556489 | 0.999950574162623 |
| ebi-fl187-GCST90094729 | prot-a-2921 | Tachykinin-3 \|\| id:prot-a-2921 | F-cheese liking (derived food-liking factor) \|\| id:ebi-fl187-GCST90094729 | MR Egger | 16 | -0.029682175 | 0.510726394405519 | 0.954476366655755 | -1.030705908 | 0.971341557753304 | 0.970754014145799 | 0.356755035315222 | 2.64148578911445 | 0.999950574162623 |
| ebi-fl187-GCST90094729 | prot-a-2928 | Tax1-binding protein 1 \|\| id:prot-a-2928 | F-cheese liking (derived food-liking factor) \|\| id:ebi-fl187-GCST90094729 | MR Egger | 16 | -0.694265403 | 0.569116561554048 | 0.242654941802425 | -1.809733864 | 0.421203057697355 | 0.499441201294523 | 0.163697696922626 | 1.52379366502889 | 0.999950574162623 |
| ebi-fl187-GCST90094729 | prot-a-296 | Uncharacterized protein C1orf185 \|\| id:prot-a-296 | F-cheese liking (derived food-liking factor) \|\| id:ebi-fl187-GCST90094729 | MR Egger | 16 | -0.228235619 | 0.429457267508552 | 0.603436840635326 | -1.069971863 | 0.613500625410437 | 0.795936700005242 | 0.343018168708618 | 1.84688534954364 | 0.999950574162623 |
| ebi-fl187-GCST90094729 | prot-a-2964 | Transforming growth factor beta-2 \|\| id:prot-a-2964 | F-cheese liking (derived food-liking factor) \|\| id:ebi-fl187-GCST90094729 | MR Egger | 16 | 0.214419838330423 | 0.429457267508552 | 0.625335514003833 | -0.627316406 | 1.05615608264718 | 1.23914278523857 | 0.534022980669484 | 2.87529731451602 | 0.999950574162623 |
| ebi-fl187-GCST90094729 | prot-a-2977 | Thymocyte nuclear protein 1 \|\| id:prot-a-2977 | F-cheese liking (derived food-liking factor) \|\| id:ebi-fl187-GCST90094729 | MR Egger | 16 | 0.0936649973237507 | 0.446701705141796 | 0.836937068027787 | -0.781870345 | 0.96920033940167 | 1.09819178709931 | 0.457549435322603 | 2.63583584231077 | 0.999950574162623 |
| ebi-fl187-GCST90094729 | prot-a-3015 | Transmembrane protein 70, mitochondrial \|\| id:prot-a-3015 | F-cheese liking (derived food-liking factor) \|\| id:ebi-fl187-GCST90094729 | MR Egger | 16 | -0.022479927 | 0.467614341451933 | 0.962336624097108 | -0.939004036 | 0.894044182030691 | 0.977770863580586 | 0.391017080244582 | 2.44499769951001 | 0.999950574162623 |
| ebi-fl187-GCST90094729 | prot-a-3030 | Tumor necrosis factor alpha-induced protein 3 \|\| id:prot-a-3030 | F-cheese liking (derived food-liking factor) \|\| id:ebi-fl187-GCST90094729 | MR Egger | 16 | -0.509687407 | 0.476714684701719 | 0.303083136181897 | -1.444048189 | 0.424673375362297 | 0.600683319076712 | 0.235970569163255 | 1.52909089932899 | 0.999950574162623 |
| ebi-fl187-GCST90094729 | prot-a-3032 | Tumor necrosis factor receptor superfamily member 10A \|\| id:prot-a-3032 | F-cheese liking (derived food-liking factor) \|\| id:ebi-fl187-GCST90094729 | MR Egger | 16 | 0.00143617569410694 | 0.441699878461797 | 0.997451581022545 | -0.864295586 | 0.867167937479229 | 1.00143720748831 | 0.421348251684595 | 2.38016053592811 | 0.999950574162623 |
| ebi-fl187-GCST90094729 | prot-a-3081 | Tumor protein p53-inducible protein 11 \|\| id:prot-a-3081 | F-cheese liking (derived food-liking factor) \|\| id:ebi-fl187-GCST90094729 | MR Egger | 16 | 0.352801608350561 | 0.429279194836985 | 0.424940016384836 | -0.488585614 | 1.19418883023105 | 1.42304879442913 | 0.61349349773277 | 3.300879111 | 0.999950574162623 |
| ebi-fl187-GCST90094729 | prot-a-315 | Complement C4 \|\| id:prot-a-315 | F-cheese liking (derived food-liking factor) \|\| id:ebi-fl187-GCST90094729 | MR Egger | 16 | -0.042514411 | 0.448761778441514 | 0.925866211374939 | -0.922087496 | 0.837058675125431 | 0.958376654619709 | 0.39768800173234 | 2.30956379905634 | 0.999950574162623 |
| ebi-fl187-GCST90094729 | prot-a-3157 | UDP-glucuronosyltransferase 1-8 \|\| id:prot-a-3157 | F-cheese liking (derived food-liking factor) \|\| id:ebi-fl187-GCST90094729 | MR Egger | 16 | -0.062938359 | 0.571997416661357 | 0.913945256359051 | -1.184053295 | 1.05817657803236 | 0.939001353240798 | 0.306035767905643 | 2.88111271248498 | 0.999950574162623 |
| ebi-fl187-GCST90094729 | prot-a-317 | Glutaredoxin-like protein C5orf63 \|\| id:prot-a-317 | F-cheese liking (derived food-liking factor) \|\| id:ebi-fl187-GCST90094729 | MR Egger | 16 | -0.619609751 | 0.429279194836985 | 0.170917680318336 | -1.460996973 | 0.221777470541312 | 0.538154410659231 | 0.232004856761144 | 1.24829356486337 | 0.999950574162623 |
| ebi-fl187-GCST90094729 | prot-a-3192 | Guanine nucleotide exchange factor VAV3 \|\| id:prot-a-3192 | F-cheese liking (derived food-liking factor) \|\| id:ebi-fl187-GCST90094729 | MR Egger | 16 | -0.319497068 | 0.523839164191633 | 0.551690258652289 | -1.34622183 | 0.707227694000718 | 0.726514332647378 | 0.260221567121464 | 2.02836022156339 | 0.999950574162623 |
| ebi-fl187-GCST90094729 | prot-a-3233 | Serine/threonine-protein kinase WNK3 \|\| id:prot-a-3233 | F-cheese liking (derived food-liking factor) \|\| id:ebi-fl187-GCST90094729 | MR Egger | 16 | 0.405786908317458 | 0.429457267508552 | 0.360741419959192 | -0.435949336 | 1.24752315263422 | 1.5004827779888 | 0.646650487006253 | 3.48170860809887 | 0.999950574162623 |
| ebi-fl187-GCST90094729 | prot-a-3250 | 14-3-3 protein theta \|\| id:prot-a-3250 | F-cheese liking (derived food-liking factor) \|\| id:ebi-fl187-GCST90094729 | MR Egger | 16 | 0.234291223928078 | 0.429279194836985 | 0.593808851459736 | -0.607095998 | 1.07567844580857 | 1.26401254931141 | 0.544931054431101 | 2.93198141641006 | 0.999950574162623 |
| ebi-fl187-GCST90094729 | prot-a-3278 | E3 ubiquitin-protein ligase ZNRF3 \|\| id:prot-a-3278 | F-cheese liking (derived food-liking factor) \|\| id:ebi-fl187-GCST90094729 | MR Egger | 16 | -0.345804025 | 0.429416578275629 | 0.434117977131123 | -1.187460519 | 0.495852468298555 | 0.707651155367377 | 0.304994810113164 | 1.64189730804593 | 0.999950574162623 |
| ebi-fl187-GCST90094729 | prot-a-355 | Calpain-3 \|\| id:prot-a-355 | F-cheese liking (derived food-liking factor) \|\| id:ebi-fl187-GCST90094729 | MR Egger | 16 | -0.316085883 | 0.429279194836985 | 0.473695466728272 | -1.157473105 | 0.525301338674704 | 0.728996838893501 | 0.314279329197788 | 1.69096832576687 | 0.999950574162623 |
| ebi-fl187-GCST90094729 | prot-a-385 | Coiled-coil domain-containing protein 90B, mitochondrial \|\| id:prot-a-385 | F-cheese liking (derived food-liking factor) \|\| id:ebi-fl187-GCST90094729 | MR Egger | 16 | -0.975255483 | 0.533388296535719 | 0.0888692173227477 | -2.020696545 | 0.0701855779008321 | 0.377095999521421 | 0.132563096816019 | 1.0727072335404 | 0.999950574162623 |
| ebi-fl187-GCST90094729 | prot-a-390 | C-C motif chemokine 14 \|\| id:prot-a-390 | F-cheese liking (derived food-liking factor) \|\| id:ebi-fl187-GCST90094729 | MR Egger | 16 | -0.731754591 | 0.429457267508552 | 0.110482560550251 | -1.573490836 | 0.109981652872589 | 0.481064178066031 | 0.207320196933299 | 1.11625759015076 | 0.999950574162623 |
| ebi-fl187-GCST90094729 | prot-a-40 | Pituitary adenylate cyclase-activating polypeptide 27 \|\| id:prot-a-40 | F-cheese liking (derived food-liking factor) \|\| id:ebi-fl187-GCST90094729 | MR Egger | 16 | -0.090308825 | 0.448971556043014 | 0.84347752147022 | -0.970293075 | 0.789675424924101 | 0.913648984123479 | 0.37897195470994 | 2.20268137474388 | 0.999950574162623 |
| ebi-fl187-GCST90094729 | prot-a-401 | C-C motif chemokine 24 \|\| id:prot-a-401 | F-cheese liking (derived food-liking factor) \|\| id:ebi-fl187-GCST90094729 | MR Egger | 16 | 0.255663890598935 | 0.461800323416443 | 0.588569333460753 | -0.649464743 | 1.16079252449516 | 1.29131863066017 | 0.522325280058481 | 3.19246237843083 | 0.999950574162623 |
| ebi-fl187-GCST90094729 | prot-a-412 | Cerebral cavernous malformations 2 protein \|\| id:prot-a-412 | F-cheese liking (derived food-liking factor) \|\| id:ebi-fl187-GCST90094729 | MR Egger | 16 | -0.489732343 | 0.691474741786617 | 0.490419020516212 | -1.845022837 | 0.865558150611143 | 0.612790389695429 | 0.158021712076199 | 2.37633206708963 | 0.999950574162623 |
| ebi-fl187-GCST90094729 | prot-a-44 | Adhesion G protein-coupled receptor F5 \|\| id:prot-a-44 | F-cheese liking (derived food-liking factor) \|\| id:ebi-fl187-GCST90094729 | MR Egger | 16 | 0.818591512767495 | 0.429279194836985 | 0.0772661361680793 | -0.022795709 | 1.65997873464799 | 2.26730411859282 | 0.977462149987223 | 5.2591990045396 | 0.999950574162623 |
| ebi-fl187-GCST90094729 | prot-a-48 | Adrenomedullin \|\| id:prot-a-48 | F-cheese liking (derived food-liking factor) \|\| id:ebi-fl187-GCST90094729 | MR Egger | 16 | 0.745906418208228 | 0.445064353929488 | 0.115927363492419 | -0.126419715 | 1.61823255191002 | 2.10835161743326 | 0.881244897015117 | 5.04416712969308 | 0.999950574162623 |
| ebi-fl187-GCST90094729 | prot-a-502 | Carcinoembryonic antigen-related cell adhesion molecule 1 \|\| id:prot-a-502 | F-cheese liking (derived food-liking factor) \|\| id:ebi-fl187-GCST90094729 | MR Egger | 16 | -0.131603186 | 0.429457267508552 | 0.763779175201021 | -0.97333943 | 0.710133058267619 | 0.876688808415957 | 0.377819228072034 | 2.03426191600618 | 0.999950574162623 |
| ebi-fl187-GCST90094729 | prot-a-518 | Complement factor B \|\| id:prot-a-518 | F-cheese liking (derived food-liking factor) \|\| id:ebi-fl187-GCST90094729 | MR Egger | 16 | 0.371679419968039 | 0.517759939478721 | 0.484650756925454 | -0.643130061 | 1.38648890134633 | 1.45016801185959 | 0.525644546698668 | 4.00077823660247 | 0.999950574162623 |
| ebi-fl187-GCST90094729 | prot-a-525 | Properdin \|\| id:prot-a-525 | F-cheese liking (derived food-liking factor) \|\| id:ebi-fl187-GCST90094729 | MR Egger | 16 | 1.69266242042409 | 0.526952628831838 | 0.00626697674924775 | 0.659835267913684 | 2.72548957293449 | 5.4339288662535 | 1.93447363827466 | 15.2638848828348 | 0.999950574162623 |
| ebi-fl187-GCST90094729 | prot-a-533 | Chondroadherin \|\| id:prot-a-533 | F-cheese liking (derived food-liking factor) \|\| id:ebi-fl187-GCST90094729 | MR Egger | 16 | -0.004511578 | 0.456412630322978 | 0.992252624563926 | -0.899080333 | 0.89005717736917 | 0.995498583816663 | 0.406943740226118 | 2.43526888957753 | 0.999950574162623 |
| ebi-fl187-GCST90094729 | prot-a-535 | Coiled-coil-helix-coiled-coil-helix domain-containing protein 2 \|\| id:prot-a-535 | F-cheese liking (derived food-liking factor) \|\| id:ebi-fl187-GCST90094729 | MR Egger | 16 | -1.217091165 | 0.494393668581132 | 0.0274147987733891 | -2.186102756 | -0.248079575 | 0.296090192924776 | 0.112353766561749 | 0.780297848742331 | 0.999950574162623 |
| ebi-fl187-GCST90094729 | prot-a-591 | Caseinolytic peptidase B protein homolog \|\| id:prot-a-591 | F-cheese liking (derived food-liking factor) \|\| id:ebi-fl187-GCST90094729 | MR Egger | 15 | 1.11366341776826 | 0.615497441668034 | 0.0935652296938875 | -0.092711568 | 2.3200384034376 | 3.04549490328038 | 0.911456355656083 | 10.1760650944504 | 0.999950574162623 |
| ebi-fl187-GCST90094729 | prot-a-605 | Ciliary neurotrophic factor receptor subunit alpha \|\| id:prot-a-605 | F-cheese liking (derived food-liking factor) \|\| id:ebi-fl187-GCST90094729 | MR Egger | 16 | -0.150590638 | 0.483151879717152 | 0.759874048016769 | -1.097568322 | 0.796387046453373 | 0.860199759866822 | 0.333681503917697 | 2.21751466049927 | 0.999950574162623 |
| ebi-fl187-GCST90094729 | prot-a-606 | Ciliary neurotrophic factor receptor subunit alpha \|\| id:prot-a-606 | F-cheese liking (derived food-liking factor) \|\| id:ebi-fl187-GCST90094729 | MR Egger | 16 | 0.511141724986894 | 0.455063528321277 | 0.280232157411523 | -0.380782791 | 1.4030662404966 | 1.66719358531026 | 0.683326298449451 | 4.06765326785577 | 0.999950574162623 |
| ebi-fl187-GCST90094729 | prot-a-707 | Cancer/testis antigen 55 \|\| id:prot-a-707 | F-cheese liking (derived food-liking factor) \|\| id:ebi-fl187-GCST90094729 | MR Egger | 16 | 0.696247901923023 | 0.523317129796992 | 0.204635694341967 | -0.329453672 | 1.72194947632513 | 2.0062110671441 | 0.719316608562417 | 5.59542598908617 | 0.999950574162623 |
| ebi-fl187-GCST90094729 | prot-a-719 | Dipeptidyl peptidase 1 \|\| id:prot-a-719 | F-cheese liking (derived food-liking factor) \|\| id:ebi-fl187-GCST90094729 | MR Egger | 16 | 0.00235213315858732 | 0.53896949798921 | 0.99657951573251 | -1.054028083 | 1.05873234921744 | 1.00235490159394 | 0.348531005988849 | 2.88271439695536 | 0.999950574162623 |
| ebi-fl187-GCST90094729 | prot-a-752 | Cytochrome b5 \|\| id:prot-a-752 | F-cheese liking (derived food-liking factor) \|\| id:ebi-fl187-GCST90094729 | MR Egger | 16 | 0.184411709357722 | 0.457514396585704 | 0.692982671622086 | -0.712316508 | 1.0811399266657 | 1.20251080614639 | 0.490506617883623 | 2.94803818374151 | 0.999950574162623 |
| ebi-fl187-GCST90094729 | prot-a-755 | Cytoglobin \|\| id:prot-a-755 | F-cheese liking (derived food-liking factor) \|\| id:ebi-fl187-GCST90094729 | MR Egger | 16 | -0.66449489 | 0.429365574861893 | 0.144016738493217 | -1.506051416 | 0.17706163705028 | 0.514533358189283 | 0.221783983880728 | 1.19370466729427 | 0.999950574162623 |
| ebi-fl187-GCST90094729 | prot-a-807 | Desmin \|\| id:prot-a-807 | F-cheese liking (derived food-liking factor) \|\| id:ebi-fl187-GCST90094729 | MR Egger | 16 | 0.356413440281575 | 0.519822029661506 | 0.504133124743343 | -0.662437738 | 1.37526461841813 | 1.42819790075992 | 0.515592920892632 | 3.95612344755176 | 0.999950574162623 |
| ebi-fl187-GCST90094729 | prot-a-82 | Ameloblastin \|\| id:prot-a-82 | F-cheese liking (derived food-liking factor) \|\| id:ebi-fl187-GCST90094729 | MR Egger | 16 | -0.341675805 | 0.518508843947082 | 0.520614059097158 | -1.357953139 | 0.674601528949433 | 0.710578533246317 | 0.257186663830175 | 1.96325052158963 | 0.999950574162623 |
| ebi-fl187-GCST90094729 | prot-a-84 | Muellerian-inhibiting factor \|\| id:prot-a-84 | F-cheese liking (derived food-liking factor) \|\| id:ebi-fl187-GCST90094729 | MR Egger | 16 | 0.335367164508536 | 0.506200233432589 | 0.518397617861707 | -0.656785293 | 1.32751962203641 | 1.39845375355652 | 0.518515533607712 | 3.77167659226946 | 0.999950574162623 |
| ebi-fl187-GCST90094729 | prot-a-858 | Inactive dipeptidyl peptidase 10 \|\| id:prot-a-858 | F-cheese liking (derived food-liking factor) \|\| id:ebi-fl187-GCST90094729 | MR Egger | 16 | -0.517684685 | 0.50056396619158 | 0.318576463834346 | -1.498790058 | 0.463420689230957 | 0.595898645356767 | 0.223400298039703 | 1.58950188810815 | 0.999950574162623 |
| ebi-fl187-GCST90094729 | prot-a-863 | Dihydropyrimidinase-related protein 3 \|\| id:prot-a-863 | F-cheese liking (derived food-liking factor) \|\| id:ebi-fl187-GCST90094729 | MR Egger | 16 | 0.570296218138802 | 0.429457267508552 | 0.205440812608899 | -0.271440026 | 1.41203246245556 | 1.76879092179474 | 0.762280999002136 | 4.1042887453826 | 0.999950574162623 |
| ebi-fl187-GCST90094729 | prot-a-864 | Dihydropyrimidinase-related protein 5 \|\| id:prot-a-864 | F-cheese liking (derived food-liking factor) \|\| id:ebi-fl187-GCST90094729 | MR Egger | 16 | -0.338487648 | 0.551223557238575 | 0.549020874412924 | -1.418885821 | 0.741910523814975 | 0.712847584162241 | 0.241983479772087 | 2.09994367683508 | 0.999950574162623 |
| ebi-fl187-GCST90094729 | prot-a-884 | Endothelin-converting enzyme 1 \|\| id:prot-a-884 | F-cheese liking (derived food-liking factor) \|\| id:ebi-fl187-GCST90094729 | MR Egger | 16 | -0.391082612 | 0.437532171947037 | 0.386517019466101 | -1.248645669 | 0.466480444871136 | 0.67632428113173 | 0.286893082015959 | 1.59437282361137 | 0.999950574162623 |
| ebi-fl187-GCST90094729 | prot-a-893 | Early endosome antigen 1 \|\| id:prot-a-893 | F-cheese liking (derived food-liking factor) \|\| id:ebi-fl187-GCST90094729 | MR Egger | 15 | -0.518732871 | 0.433251893578696 | 0.252566026226602 | -1.367906582 | 0.330440840341996 | 0.595274359642098 | 0.254639468699748 | 1.39158145850962 | 0.999950574162623 |
| ebi-fl187-GCST90094729 | prot-a-897 | Ephrin-A2 \|\| id:prot-a-897 | F-cheese liking (derived food-liking factor) \|\| id:ebi-fl187-GCST90094729 | MR Egger | 16 | 0.147056716713219 | 0.482658777521177 | 0.76509168790303 | -0.798954487 | 1.09306792065473 | 1.15841966292251 | 0.449798988953576 | 2.98341292088585 | 0.999950574162623 |
| ebi-fl187-GCST90094729 | prot-a-90 | Anaphase-promoting complex subunit 10 \|\| id:prot-a-90 | F-cheese liking (derived food-liking factor) \|\| id:ebi-fl187-GCST90094729 | MR Egger | 16 | 0.256337366388518 | 0.429187569369918 | 0.559869086325359 | -0.58487027 | 1.09754500235356 | 1.29218859541177 | 0.557178140079794 | 2.99679984909875 | 0.999950574162623 |
| ebi-fl187-GCST90094729 | prot-a-914 | Histone-lysine N-methyltransferase EHMT2 \|\| id:prot-a-914 | F-cheese liking (derived food-liking factor) \|\| id:ebi-fl187-GCST90094729 | MR Egger | 16 | -0.425358113 | 0.429365574861893 | 0.338662529469241 | -1.266914639 | 0.416198414030222 | 0.653535703783765 | 0.28169942664852 | 1.51618667173594 | 0.999950574162623 |
| ebi-fl187-GCST90094729 | prot-a-932 | RNA polymerase II elongation factor ELL \|\| id:prot-a-932 | F-cheese liking (derived food-liking factor) \|\| id:ebi-fl187-GCST90094729 | MR Egger | 16 | -0.246864303 | 0.429365574861893 | 0.574448427318628 | -1.08842083 | 0.59469222355749 | 0.781246699037994 | 0.336747856185786 | 1.81247302260784 | 0.999950574162623 |
| ebi-fl187-GCST90094729 | prot-a-991 | Estrogen receptor \|\| id:prot-a-991 | F-cheese liking (derived food-liking factor) \|\| id:ebi-fl187-GCST90094729 | MR Egger | 16 | 0.376793692668251 | 0.429365574861893 | 0.394993525935367 | -0.464762834 | 1.21835021939756 | 1.45760356404726 | 0.628284095108613 | 3.3816042240509 | 0.999950574162623 |
| ebi-fl187-GCST90094729 | prot-a-992 | Persulfide dioxygenase ETHE1, mitochondrial \|\| id:prot-a-992 | F-cheese liking (derived food-liking factor) \|\| id:ebi-fl187-GCST90094729 | MR Egger | 16 | 0.0107959113216707 | 0.433069596088153 | 0.980463589169666 | -0.838020497 | 0.85961231965445 | 1.01085439745318 | 0.4325659420731 | 2.36224472031538 | 0.999950574162623 |
| ebi-fl187-GCST90094729 | prot-a-993 | Ethanolamine kinase 1 \|\| id:prot-a-993 | F-cheese liking (derived food-liking factor) \|\| id:ebi-fl187-GCST90094729 | MR Egger | 16 | 0.0962153862819456 | 0.454242173657967 | 0.835303857768048 | -0.794099274 | 0.98653004665156 | 1.10099617793072 | 0.451988169082969 | 2.6819121975636 | 0.999950574162623 |
| ebi-fl187-GCST90094729 | prot-b-10 | coagulation factor III, tissue factor \|\| id:prot-b-10 | F-cheese liking (derived food-liking factor) \|\| id:ebi-fl187-GCST90094729 | MR Egger | 12 | -1.094137749 | 0.621169604071311 | 0.108654206579124 | -2.311630173 | 0.123354675153398 | 0.334828188514342 | 0.0990995703865806 | 1.13128558869087 | 0.999950574162623 |
| ebi-fl187-GCST90094729 | prot-b-28 | prolactin \|\| id:prot-b-28 | F-cheese liking (derived food-liking factor) \|\| id:ebi-fl187-GCST90094729 | MR Egger | 12 | -0.378125734 | 0.814807199801456 | 0.652537041431561 | -1.975147846 | 1.21889637720205 | 0.685144349030202 | 0.138740798451737 | 3.38345161802796 | 0.999950574162623 |
| ebi-fl187-GCST90094729 | prot-b-54 | matrix metallopeptidase 7 \|\| id:prot-b-54 | F-cheese liking (derived food-liking factor) \|\| id:ebi-fl187-GCST90094729 | MR Egger | 12 | -0.46819077 | 0.648511821650221 | 0.486865734691295 | -1.739273941 | 0.802892400189223 | 0.626134064514271 | 0.175647885114831 | 2.23198740189135 | 0.999950574162623 |
| ebi-fl187-GCST90094729 | ukb-rsfMRI191-edge-pheno716 | Net100_Pair10_33 (OccipitalPrecuneus)&(Temporal\|Frontal\|Supp_Motor_Area) (Default_mode\|Central_executive)&(Default_mode\|Salience) \|\| id:ukb-rsfMRI191-edge-pheno716 | F-cheese liking (derived food-liking factor) \|\| id:ebi-fl187-GCST90094729 | MR Egger | 15 | -0.080749638 | 0.155319785282421 | 0.611877089870228 | -0.385176417 | 0.223677141274632 | 0.922424602717763 | 0.680330603710813 | 1.25066716543109 | 0.999950574162623 |
| ebi-fl187-GCST90094729 | ukb-rsfMRI191-edge-pheno903 | Net100_Pair27_38 (Precentral\|Frontal\|Supp_Motor_Area)&(Subcortical) (Attention\|Salience\|Motor)&(Subcortical-cerebellum) \|\| id:ukb-rsfMRI191-edge-pheno903 | F-cheese liking (derived food-liking factor) \|\| id:ebi-fl187-GCST90094729 | MR Egger | 15 | -0.222666398 | 0.14431081315191 | 0.146824866211868 | -0.505515592 | 0.0601827956246293 | 0.800381813613418 | 0.603194493024802 | 1.06203066336145 | 0.999950574162623 |
| ebi-fl187-GCST90094729 | ukb-rsfMRI191-node-pheno23 | Net100_Node2 RolandicOper\|SupraMarginal\|Insula Salience\|Motor \|\| id:ukb-rsfMRI191-node-pheno23 | F-cheese liking (derived food-liking factor) \|\| id:ebi-fl187-GCST90094729 | MR Egger | 15 | 0.00457284246170903 | 0.190918734594058 | 0.981254829304428 | -0.369627877 | 0.378773562266063 | 1.00458331386107 | 0.690991416362743 | 1.46049228773386 | 0.999950574162623 |
| ebi-fl187-GCST90094729 | ukb-rsfMRI191-node-pheno55 | Net100_Node34 Parietal\|Postcentral\|Precuneus Attention \|\| id:ukb-rsfMRI191-node-pheno55 | F-cheese liking (derived food-liking factor) \|\| id:ebi-fl187-GCST90094729 | MR Egger | 14 | 0.129250340100575 | 0.132668713012577 | 0.34917082424746 | -0.130780337 | 0.389281017605226 | 1.13797496927843 | 0.877410487489711 | 1.47591925235499 | 0.999950574162623 |
| ebi-fl187-GCST90094729 | ebi-a-GCST90001414 | IgD- CD24- B cell Absolute Count \|\| id:ebi-a-GCST90001414 | F-cheese liking (derived food-liking factor) \|\| id:ebi-fl187-GCST90094729 | Weighted median | 14 | 0.0935291075518092 | 0.121795655437308 | 0.442535749565786 | -0.145190377 | 0.332248592208934 | 1.09804256420697 | 0.864857628345244 | 1.39409936768103 | 0.999332001504091 |
| ebi-fl187-GCST90094729 | ebi-a-GCST90001422 | CD20- CD38- B cell %B cell \|\| id:ebi-a-GCST90001422 | F-cheese liking (derived food-liking factor) \|\| id:ebi-fl187-GCST90094729 | Weighted median | 14 | -0.046292179 | 0.108459644406716 | 0.669514241178058 | -0.258873082 | 0.166288723885789 | 0.95476295960203 | 0.771920987349202 | 1.18091401058856 | 0.999332001504091 |
| ebi-fl187-GCST90094729 | ebi-a-GCST90001444 | CD20- CD38- B cell %lymphocyte \|\| id:ebi-a-GCST90001444 | F-cheese liking (derived food-liking factor) \|\| id:ebi-fl187-GCST90094729 | Weighted median | 14 | -0.016605481 | 0.111840586810604 | 0.881968268026723 | -0.235813031 | 0.202602069143426 | 0.983531630013839 | 0.789928352119069 | 1.22458507109246 | 0.999332001504091 |
| ebi-fl187-GCST90094729 | ebi-a-GCST90001459 | Myeloid Dendritic Cell %Dendritic Cell \|\| id:ebi-a-GCST90001459 | F-cheese liking (derived food-liking factor) \|\| id:ebi-fl187-GCST90094729 | Weighted median | 14 | 0.0860651267574184 | 0.124715774455885 | 0.490137500836465 | -0.158377791 | 0.330508044690953 | 1.08987730616876 | 0.853527265980809 | 1.39167498197811 | 0.999332001504091 |
| ebi-fl187-GCST90094729 | ebi-a-GCST90001476 | HLA DR++ monocyte %leukocyte \|\| id:ebi-a-GCST90001476 | F-cheese liking (derived food-liking factor) \|\| id:ebi-fl187-GCST90094729 | Weighted median | 14 | -0.020165726 | 0.122765651429856 | 0.869524926927927 | -0.260786403 | 0.220454950359346 | 0.980036241926986 | 0.770445466692153 | 1.24664376261967 | 0.999332001504091 |
| ebi-fl187-GCST90094729 | ebi-a-GCST90001478 | CD4 regulatory T cell %CD4+ T cell \|\| id:ebi-a-GCST90001478 | F-cheese liking (derived food-liking factor) \|\| id:ebi-fl187-GCST90094729 | Weighted median | 14 | -0.103085681 | 0.128668389079235 | 0.423031310119753 | -0.355275724 | 0.149104361166745 | 0.902049681264991 | 0.700980139495854 | 1.16079412471726 | 0.999332001504091 |
| ebi-fl187-GCST90094729 | ebi-a-GCST90001485 | CD39+ resting CD4 regulatory T cell %CD4 regulatory T cell \|\| id:ebi-a-GCST90001485 | F-cheese liking (derived food-liking factor) \|\| id:ebi-fl187-GCST90094729 | Weighted median | 13 | 0.0202246546786401 | 0.125586703218736 | 0.872060817696814 | -0.225925284 | 0.266374592987362 | 1.02043055877752 | 0.797777706493149 | 1.30522389484186 | 0.999332001504091 |
| ebi-fl187-GCST90094729 | ebi-a-GCST90001515 | Immature Myeloid-Derived Suppressor Cells Absolute Count \|\| id:ebi-a-GCST90001515 | F-cheese liking (derived food-liking factor) \|\| id:ebi-fl187-GCST90094729 | Weighted median | 14 | 0.00654706930086416 | 0.176773282000483 | 0.970455877454133 | -0.339928563 | 0.353022702021811 | 1.00656854820813 | 0.711821171016346 | 1.42336345629505 | 0.999332001504091 |
| ebi-fl187-GCST90094729 | ebi-a-GCST90001517 | CD33+ HLA DR+ Absolute Count \|\| id:ebi-a-GCST90001517 | F-cheese liking (derived food-liking factor) \|\| id:ebi-fl187-GCST90094729 | Weighted median | 14 | -0.080532887 | 0.160878137399311 | 0.616664512843285 | -0.395854037 | 0.234788262072228 | 0.922624560518294 | 0.673104937497655 | 1.26464096792417 | 0.999332001504091 |
| ebi-fl187-GCST90094729 | ebi-a-GCST90001518 | CD33+ HLA DR+ CD14- Absolute Count \|\| id:ebi-a-GCST90001518 | F-cheese liking (derived food-liking factor) \|\| id:ebi-fl187-GCST90094729 | Weighted median | 14 | -0.099763215 | 0.16269049183508 | 0.539738391675017 | -0.418636579 | 0.219110148531254 | 0.905051694910509 | 0.65794326191837 | 1.24496840057618 | 0.999332001504091 |
| ebi-fl187-GCST90094729 | ebi-a-GCST90001520 | CD33+ HLA DR+ CD14dim Absolute Count \|\| id:ebi-a-GCST90001520 | F-cheese liking (derived food-liking factor) \|\| id:ebi-fl187-GCST90094729 | Weighted median | 14 | 0.0414638275948024 | 0.164640005221972 | 0.801160565674284 | -0.281230583 | 0.364158237829867 | 1.04233545738903 | 0.754854259123405 | 1.4393019481563 | 0.999332001504091 |
| ebi-fl187-GCST90094729 | ebi-a-GCST90001531 | CD33dim HLA DR- Absolute Count \|\| id:ebi-a-GCST90001531 | F-cheese liking (derived food-liking factor) \|\| id:ebi-fl187-GCST90094729 | Weighted median | 14 | -0.016414764 | 0.158942124269549 | 0.917744637737418 | -0.327941327 | 0.295111799887278 | 0.983719224422882 | 0.720405286625076 | 1.3432765284561 | 0.999332001504091 |
| ebi-fl187-GCST90094729 | ebi-a-GCST90001532 | Basophil Absolute Count \|\| id:ebi-a-GCST90001532 | F-cheese liking (derived food-liking factor) \|\| id:ebi-fl187-GCST90094729 | Weighted median | 14 | -0.020028449 | 0.15609007240508 | 0.897900956437613 | -0.325964991 | 0.285908093098148 | 0.980170788211893 | 0.721830457746924 | 1.33097012429027 | 0.999332001504091 |
| ebi-fl187-GCST90094729 | ebi-a-GCST90001588 | T/B cell \|\| id:ebi-a-GCST90001588 | F-cheese liking (derived food-liking factor) \|\| id:ebi-fl187-GCST90094729 | Weighted median | 14 | -0.07832703 | 0.124321471936647 | 0.528670885957715 | -0.321997115 | 0.165343054556084 | 0.924661984476957 | 0.72470028077154 | 1.1797977843014 | 0.999332001504091 |
| ebi-fl187-GCST90094729 | ebi-a-GCST90001624 | HLA DR+ CD4+ T cell Absolute Count \|\| id:ebi-a-GCST90001624 | F-cheese liking (derived food-liking factor) \|\| id:ebi-fl187-GCST90094729 | Weighted median | 14 | 0.0559330704874319 | 0.116264424808782 | 0.63045609042856 | -0.171945202 | 0.283811343112646 | 1.05752690160788 | 0.842025313091395 | 1.32818233636994 | 0.999332001504091 |
| ebi-fl187-GCST90094729 | ebi-a-GCST90001631 | CD8+ Natural Killer T %T cell \|\| id:ebi-a-GCST90001631 | F-cheese liking (derived food-liking factor) \|\| id:ebi-fl187-GCST90094729 | Weighted median | 14 | -0.073582657 | 0.12270117373538 | 0.548712905568112 | -0.314076958 | 0.166911643142849 | 0.929059349007396 | 0.730462811112424 | 1.18164985382836 | 0.999332001504091 |
| ebi-fl187-GCST90094729 | ebi-a-GCST90001632 | CD8+ Natural Killer T %lymphocyte \|\| id:ebi-a-GCST90001632 | F-cheese liking (derived food-liking factor) \|\| id:ebi-fl187-GCST90094729 | Weighted median | 14 | -0.07625901 | 0.123304737439946 | 0.536272359587365 | -0.317936295 | 0.16541827531149 | 0.926576182913712 | 0.727649141496992 | 1.17988653291979 | 0.999332001504091 |
| ebi-fl187-GCST90094729 | ebi-a-GCST90001646 | Natural Killer %CD3- lymphocyte \|\| id:ebi-a-GCST90001646 | F-cheese liking (derived food-liking factor) \|\| id:ebi-fl187-GCST90094729 | Weighted median | 14 | -0.011603664 | 0.114213783394738 | 0.919077348641481 | -0.23546268 | 0.212255351064427 | 0.988463398482055 | 0.790205153038936 | 1.23646357706117 | 0.999332001504091 |
| ebi-fl187-GCST90094729 | ebi-a-GCST90001684 | CD127- CD8+ T cell Absolute Count \|\| id:ebi-a-GCST90001684 | F-cheese liking (derived food-liking factor) \|\| id:ebi-fl187-GCST90094729 | Weighted median | 14 | -0.088482364 | 0.122503784349336 | 0.470120685314461 | -0.328589782 | 0.151625052874381 | 0.915319252751818 | 0.719938287986709 | 1.16372381971941 | 0.999332001504091 |
| ebi-fl187-GCST90094729 | ebi-a-GCST90001687 | CD28- CD8+ T cell Absolute Count \|\| id:ebi-a-GCST90001687 | F-cheese liking (derived food-liking factor) \|\| id:ebi-fl187-GCST90094729 | Weighted median | 14 | -0.168876814 | 0.121876622543539 | 0.165857633539618 | -0.407754994 | 0.0700013664272796 | 0.844612941672042 | 0.665141822635526 | 1.07250964675965 | 0.999332001504091 |
| ebi-fl187-GCST90094729 | ebi-a-GCST90001701 | BAFF-R on CD20- CD38- B cell \|\| id:ebi-a-GCST90001701 | F-cheese liking (derived food-liking factor) \|\| id:ebi-fl187-GCST90094729 | Weighted median | 14 | 0.0651712438698735 | 0.13488908894047 | 0.628991442061108 | -0.19921137 | 0.329553858193194 | 1.06734178447312 | 0.819376683006767 | 1.39034769784005 | 0.999332001504091 |
| ebi-fl187-GCST90094729 | ebi-a-GCST90001732 | CD19 on IgD- CD27- B cell \|\| id:ebi-a-GCST90001732 | F-cheese liking (derived food-liking factor) \|\| id:ebi-fl187-GCST90094729 | Weighted median | 14 | -0.055302408 | 0.135672295678268 | 0.683554484194626 | -0.321220107 | 0.21061529196562 | 0.946198966959562 | 0.725263597758083 | 1.23443736572861 | 0.999332001504091 |
| ebi-fl187-GCST90094729 | ebi-a-GCST90001798 | CD27 on CD24+ CD27+ B cell \|\| id:ebi-a-GCST90001798 | F-cheese liking (derived food-liking factor) \|\| id:ebi-fl187-GCST90094729 | Weighted median | 14 | -0.007776102 | 0.113693631704392 | 0.94547102122598 | -0.23061562 | 0.215063416390127 | 0.992254053913607 | 0.794044622341895 | 1.23994052702501 | 0.999332001504091 |
| ebi-fl187-GCST90094729 | ebi-a-GCST90001836 | CD66b on CD66b++ myeloid cell \|\| id:ebi-a-GCST90001836 | F-cheese liking (derived food-liking factor) \|\| id:ebi-fl187-GCST90094729 | Weighted median | 14 | -0.236229874 | 0.181958040666731 | 0.19419606652452 | -0.592867633 | 0.120407886191433 | 0.789599145138053 | 0.552739954425182 | 1.12795683578024 | 0.999332001504091 |
| ebi-fl187-GCST90094729 | ebi-a-GCST90001943 | CD25 on activated & secreting CD4 regulatory T cell \|\| id:ebi-a-GCST90001943 | F-cheese liking (derived food-liking factor) \|\| id:ebi-fl187-GCST90094729 | Weighted median | 14 | 0.166388316433601 | 0.123275266714843 | 0.177102629888845 | -0.075231206 | 0.408007839194694 | 1.18103162668037 | 0.927529010959211 | 1.50381894985344 | 0.999332001504091 |
| ebi-fl187-GCST90094729 | ebi-a-GCST90001960 | CD25 on CD4+ T cell \|\| id:ebi-a-GCST90001960 | F-cheese liking (derived food-liking factor) \|\| id:ebi-fl187-GCST90094729 | Weighted median | 14 | -0.012640137 | 0.142049785298754 | 0.929094743558366 | -0.291057716 | 0.265777442134502 | 0.987439414 | 0.747472535471371 | 1.3044447119479 | 0.999332001504091 |
| ebi-fl187-GCST90094729 | ebi-a-GCST90001988 | HLA DR on CD14+ CD16- monocyte \|\| id:ebi-a-GCST90001988 | F-cheese liking (derived food-liking factor) \|\| id:ebi-fl187-GCST90094729 | Weighted median | 14 | 0.0300312263361922 | 0.122014895135359 | 0.805583502616116 | -0.209117968 | 0.269180420801496 | 1.03048671177562 | 0.811299522511153 | 1.30889127095663 | 0.999332001504091 |
| ebi-fl187-GCST90094729 | ebi-a-GCST90001991 | HLA DR on CD14+ monocyte \|\| id:ebi-a-GCST90001991 | F-cheese liking (derived food-liking factor) \|\| id:ebi-fl187-GCST90094729 | Weighted median | 14 | 0.0280203259582279 | 0.118739545376728 | 0.813447091483073 | -0.204709183 | 0.260749834896614 | 1.02841658776159 | 0.814884264168746 | 1.29790293479538 | 0.999332001504091 |
| ebi-fl187-GCST90094729 | ebi-a-GCST90002019 | CD14 on Monocytic Myeloid-Derived Suppressor Cells \|\| id:ebi-a-GCST90002019 | F-cheese liking (derived food-liking factor) \|\| id:ebi-fl187-GCST90094729 | Weighted median | 14 | -0.037794221 | 0.167899586084665 | 0.821901370691453 | -0.366877409 | 0.291288968094623 | 0.962911067736905 | 0.692894582230216 | 1.33815121109168 | 0.999332001504091 |
| ebi-fl187-GCST90094729 | ebi-a-GCST90002037 | CD80 on plasmacytoid Dendritic Cell \|\| id:ebi-a-GCST90002037 | F-cheese liking (derived food-liking factor) \|\| id:ebi-fl187-GCST90094729 | Weighted median | 14 | 0.00601496553181236 | 0.133719401251821 | 0.964121659778783 | -0.256075061 | 0.268104991985381 | 1.00603309176165 | 0.774083863163259 | 1.30748440819265 | 0.999332001504091 |
| ebi-fl187-GCST90094729 | ebi-a-GCST90002042 | CD45 on CD33+ HLA DR+ CD14- \|\| id:ebi-a-GCST90002042 | F-cheese liking (derived food-liking factor) \|\| id:ebi-fl187-GCST90094729 | Weighted median | 14 | -0.122511298 | 0.180829457908757 | 0.498091083076262 | -0.476937036 | 0.231914439 | 0.884695909334158 | 0.620681608788214 | 1.26101183104276 | 0.999332001504091 |
| ebi-fl187-GCST90094729 | ebi-a-GCST90002056 | CD8 on Effector Memory CD8+ T cell \|\| id:ebi-a-GCST90002056 | F-cheese liking (derived food-liking factor) \|\| id:ebi-fl187-GCST90094729 | Weighted median | 14 | -0.078113854 | 0.131135774625362 | 0.55139464988617 | -0.335139972 | 0.178912264646246 | 0.924859121990468 | 0.715237966139247 | 1.19591581546784 | 0.999332001504091 |
| ebi-fl187-GCST90094729 | ebi-a-GCST90002058 | CD8 on CD8+ T cell \|\| id:ebi-a-GCST90002058 | F-cheese liking (derived food-liking factor) \|\| id:ebi-fl187-GCST90094729 | Weighted median | 14 | -0.144078107 | 0.127706275923746 | 0.259235839331153 | -0.394382408 | 0.106226193852923 | 0.865820118842756 | 0.674096227311883 | 1.11207339222512 | 0.999332001504091 |
| ebi-fl187-GCST90094729 | ebi-a-GCST90002063 | CD4 on CD4 regulatory T cell \|\| id:ebi-a-GCST90002063 | F-cheese liking (derived food-liking factor) \|\| id:ebi-fl187-GCST90094729 | Weighted median | 14 | -0.192045303 | 0.132027080299897 | 0.145782733528975 | -0.45081838 | 0.0667277744553334 | 0.825269480517255 | 0.637106542757167 | 1.06900442824805 | 0.999332001504091 |
| ebi-fl187-GCST90094729 | ebi-a-GCST90002066 | CD4 on activated CD4 regulatory T cell \|\| id:ebi-a-GCST90002066 | F-cheese liking (derived food-liking factor) \|\| id:ebi-fl187-GCST90094729 | Weighted median | 14 | -0.266489975 | 0.129604371589222 | 0.0397651261157764 | -0.520514543 | -0.012465407 | 0.766063683556293 | 0.594214720146458 | 0.987611964777972 | 0.999332001504091 |
| ebi-fl187-GCST90094729 | ebi-a-GCST90002070 | CD4 on activated & secreting CD4 regulatory T cell \|\| id:ebi-a-GCST90002070 | F-cheese liking (derived food-liking factor) \|\| id:ebi-fl187-GCST90094729 | Weighted median | 14 | -0.178122547 | 0.139695495959386 | 0.202281904291881 | -0.45192572 | 0.0956806246271219 | 0.836839864610525 | 0.636401440165177 | 1.10040756479055 | 0.999332001504091 |
| ebi-fl187-GCST90094729 | ebi-a-GCST90002081 | SSC-A on CD4+ T cell \|\| id:ebi-a-GCST90002081 | F-cheese liking (derived food-liking factor) \|\| id:ebi-fl187-GCST90094729 | Weighted median | 14 | -0.042113937 | 0.121768524035705 | 0.729453640374245 | -0.280780244 | 0.196552370431427 | 0.9587605363581 | 0.755194275879311 | 1.21719906444918 | 0.999332001504091 |
| ebi-fl187-GCST90094729 | ebi-a-GCST90002094 | CD11b on Monocytic Myeloid-Derived Suppressor Cells \|\| id:ebi-a-GCST90002094 | F-cheese liking (derived food-liking factor) \|\| id:ebi-fl187-GCST90094729 | Weighted median | 14 | -0.078218327 | 0.179884519751336 | 0.663689294332239 | -0.430791985 | 0.274355332090869 | 0.924762504228364 | 0.649994105019488 | 1.31568222330428 | 0.999332001504091 |
| ebi-fl187-GCST90094729 | ebi-a-GCST90002104 | HLA DR on myeloid Dendritic Cell \|\| id:ebi-a-GCST90002104 | F-cheese liking (derived food-liking factor) \|\| id:ebi-fl187-GCST90094729 | Weighted median | 14 | -0.123706816 | 0.134951829855489 | 0.359313564427748 | -0.388212402 | 0.140798770866358 | 0.883638871996929 | 0.678268262314263 | 1.15119297111123 | 0.999332001504091 |
| ebi-fl187-GCST90094729 | ebi-a-GCST90002105 | HLA DR on plasmacytoid Dendritic Cell \|\| id:ebi-a-GCST90002105 | F-cheese liking (derived food-liking factor) \|\| id:ebi-fl187-GCST90094729 | Weighted median | 14 | -0.065402212 | 0.151635743665533 | 0.666242021848875 | -0.36260827 | 0.231803845104021 | 0.936690638935462 | 0.695858968904223 | 1.26087237827941 | 0.999332001504091 |
| ebi-fl187-GCST90094729 | ebi-a-GCST90002106 | HLA DR on Dendritic Cell \|\| id:ebi-a-GCST90002106 | F-cheese liking (derived food-liking factor) \|\| id:ebi-fl187-GCST90094729 | Weighted median | 14 | 0.0177011256545182 | 0.148710906672319 | 0.905251234250378 | -0.273772251 | 0.309174502732264 | 1.01785871906625 | 0.760505259529384 | 1.36230007484801 | 0.999332001504091 |
| ebi-fl187-GCST90094729 | ebi-a-GCST90002111 | HLA DR on CD33dim HLA DR+ CD11b- \|\| id:ebi-a-GCST90002111 | F-cheese liking (derived food-liking factor) \|\| id:ebi-fl187-GCST90094729 | Weighted median | 14 | -0.128242996 | 0.171846269273451 | 0.455506859376322 | -0.465061683 | 0.208575692101937 | 0.879639604553935 | 0.628096360844246 | 1.23192217331073 | 0.999332001504091 |
| ebi-fl187-GCST90094729 | ebi-a-GCST90002112 | HLA DR on CD33- HLA DR+ \|\| id:ebi-a-GCST90002112 | F-cheese liking (derived food-liking factor) \|\| id:ebi-fl187-GCST90094729 | Weighted median | 14 | -0.080328399 | 0.174188598675363 | 0.644685529026595 | -0.421738052 | 0.261081254852292 | 0.922813246087265 | 0.655905830142159 | 1.29833315701668 | 0.999332001504091 |
| ebi-fl187-GCST90094729 | ebi-a-GCST90002115 | HLA DR on HLA DR+ CD8+ T cell \|\| id:ebi-a-GCST90002115 | F-cheese liking (derived food-liking factor) \|\| id:ebi-fl187-GCST90094729 | Weighted median | 14 | -0.119150093 | 0.118911222613874 | 0.316339337206158 | -0.352216089 | 0.113915903493086 | 0.887674557176246 | 0.703128167188732 | 1.12065787750833 | 0.999332001504091 |
| ebi-fl187-GCST90094729 | ebi-a-GCST90002116 | HLA DR on B cell \|\| id:ebi-a-GCST90002116 | F-cheese liking (derived food-liking factor) \|\| id:ebi-fl187-GCST90094729 | Weighted median | 14 | 0.0194411518820098 | 0.127250157579304 | 0.878572558516699 | -0.229969157 | 0.268851460737446 | 1.0196313617084 | 0.79455810870227 | 1.30846076881319 | 0.999332001504091 |
| ebi-fl187-GCST90094729 | ebi-cfmet338-GCST90026023 | Sphingomyelin (d18:1/24:1, d18:2/24:0) levels \|\| id:ebi-cfmet338-GCST90026023 | F-cheese liking (derived food-liking factor) \|\| id:ebi-fl187-GCST90094729 | Weighted median | 15 | 0.00290956434350655 | 0.076897979652625 | 0.969817897906909 | -0.147810476 | 0.153629604462652 | 1.00291380123401 | 0.862594582020167 | 1.16605890376685 | 0.999332001504091 |
| ebi-fl187-GCST90094729 | ebi-cfmet338-GCST90026046 | 1-arachidonoyl-gpc (20:4n6) levels \|\| id:ebi-cfmet338-GCST90026046 | F-cheese liking (derived food-liking factor) \|\| id:ebi-fl187-GCST90094729 | Weighted median | 15 | 0.0259711146681836 | 0.0919524879372826 | 0.777605697331364 | -0.154255762 | 0.206197991025257 | 1.02631130270275 | 0.857052791707949 | 1.22899651018739 | 0.999332001504091 |
| ebi-fl187-GCST90094729 | ebi-cfmet338-GCST90026047 | 1-linoleoyl-gpc (18:2) levels \|\| id:ebi-cfmet338-GCST90026047 | F-cheese liking (derived food-liking factor) \|\| id:ebi-fl187-GCST90094729 | Weighted median | 15 | -0.018987249 | 0.108426488564586 | 0.860988227115938 | -0.231503166 | 0.193528668843155 | 0.98119187359236 | 0.793340183522308 | 1.21352417638708 | 0.999332001504091 |
| ebi-fl187-GCST90094729 | ebi-cfmet338-GCST90026050 | 1-myristoyl-2-palmitoyl-gpc (14:0/16:0) levels \|\| id:ebi-cfmet338-GCST90026050 | F-cheese liking (derived food-liking factor) \|\| id:ebi-fl187-GCST90094729 | Weighted median | 15 | -0.060480025 | 0.0544117695477757 | 0.266342571418361 | -0.167127093 | 0.0461670433932106 | 0.941312571624132 | 0.846092071925825 | 1.04724933242882 | 0.999332001504091 |
| ebi-fl187-GCST90094729 | ebi-cfmet338-GCST90026068 | 2,3-dihydroxyisovalerate levels \|\| id:ebi-cfmet338-GCST90026068 | F-cheese liking (derived food-liking factor) \|\| id:ebi-fl187-GCST90094729 | Weighted median | 15 | -0.12351966 | 0.17106584292909 | 0.47025810071874 | -0.458808712 | 0.211769391869985 | 0.883804265241877 | 0.632036134004407 | 1.23586285219302 | 0.999332001504091 |
| ebi-fl187-GCST90094729 | ebi-cfmet338-GCST90026082 | 3-hydroxyhexanoate levels \|\| id:ebi-cfmet338-GCST90026082 | F-cheese liking (derived food-liking factor) \|\| id:ebi-fl187-GCST90094729 | Weighted median | 15 | 0.0151599026355274 | 0.0388817706831932 | 0.696612391105087 | -0.061048368 | 0.091368173174586 | 1.01527539684844 | 0.940777735228797 | 1.09567232816694 | 0.999332001504091 |
| ebi-fl187-GCST90094729 | ebi-cfmet338-GCST90026094 | 3-ureidopropionate levels \|\| id:ebi-cfmet338-GCST90026094 | F-cheese liking (derived food-liking factor) \|\| id:ebi-fl187-GCST90094729 | Weighted median | 15 | -0.095377632 | 0.0922255716700755 | 0.301053085856331 | -0.276139752 | 0.0853844886381379 | 0.909029591130865 | 0.758706892312135 | 1.08913574652435 | 0.999332001504091 |
| ebi-fl187-GCST90094729 | ebi-cfmet338-GCST90026173 | Glycerol levels \|\| id:ebi-cfmet338-GCST90026173 | F-cheese liking (derived food-liking factor) \|\| id:ebi-fl187-GCST90094729 | Weighted median | 15 | -0.004341102 | 0.0481441070509249 | 0.928152976782972 | -0.098703552 | 0.0900213473702041 | 0.995668306515612 | 0.906011253196667 | 1.09419764169803 | 0.999332001504091 |
| ebi-fl187-GCST90094729 | ebi-cfmet338-GCST90026179 | Histidine levels \|\| id:ebi-cfmet338-GCST90026179 | F-cheese liking (derived food-liking factor) \|\| id:ebi-fl187-GCST90094729 | Weighted median | 15 | 0.0317032194702081 | 0.0335784956715024 | 0.345091742049185 | -0.034110632 | 0.0975170709863527 | 1.03221111967977 | 0.966464576769338 | 1.10243026097463 | 0.999332001504091 |
| ebi-fl187-GCST90094729 | ebi-cfmet338-GCST90026188 | Isocitrate levels \|\| id:ebi-cfmet338-GCST90026188 | F-cheese liking (derived food-liking factor) \|\| id:ebi-fl187-GCST90094729 | Weighted median | 15 | -0.086224114 | 0.0644097260579179 | 0.180674355520219 | -0.212467177 | 0.0400189492650407 | 0.917388608724636 | 0.808586856227686 | 1.04083049697847 | 0.999332001504091 |
| ebi-fl187-GCST90094729 | ebi-cfmet338-GCST90026207 | N-acetyl-isoputreanine levels \|\| id:ebi-cfmet338-GCST90026207 | F-cheese liking (derived food-liking factor) \|\| id:ebi-fl187-GCST90094729 | Weighted median | 15 | -0.040360616 | 0.0411062185757402 | 0.326168049222885 | -0.120928805 | 0.0402075719870815 | 0.960443025168126 | 0.88609704317701 | 1.04102683977677 | 0.999332001504091 |
| ebi-fl187-GCST90094729 | ebi-cfmet338-GCST90026213 | N-acetylglutamate levels \|\| id:ebi-cfmet338-GCST90026213 | F-cheese liking (derived food-liking factor) \|\| id:ebi-fl187-GCST90094729 | Weighted median | 15 | -0.056609477 | 0.0678898741861324 | 0.404369623287582 | -0.18967363 | 0.0764546766191638 | 0.944963027317376 | 0.827229072485892 | 1.07945326475702 | 0.999332001504091 |
| ebi-fl187-GCST90094729 | ebi-cfmet338-GCST90026224 | N-acetyltaurine levels \|\| id:ebi-cfmet338-GCST90026224 | F-cheese liking (derived food-liking factor) \|\| id:ebi-fl187-GCST90094729 | Weighted median | 15 | -0.044807045 | 0.0556608597639454 | 0.420819132392813 | -0.15390233 | 0.0642882402281818 | 0.956181964211893 | 0.857355754818972 | 1.06639973376882 | 0.999332001504091 |
| ebi-fl187-GCST90094729 | ebi-cfmet338-GCST90026250 | Phenyllactate (pla) levels \|\| id:ebi-cfmet338-GCST90026250 | F-cheese liking (derived food-liking factor) \|\| id:ebi-fl187-GCST90094729 | Weighted median | 15 | -0.002537823 | 0.091844272985313 | 0.977955817961605 | -0.182552598 | 0.177476952394189 | 0.997465394892463 | 0.833140821478011 | 1.19420053412211 | 0.999332001504091 |
| ebi-fl187-GCST90094729 | ebi-cfmet338-GCST90026251 | Pipecolate levels \|\| id:ebi-cfmet338-GCST90026251 | F-cheese liking (derived food-liking factor) \|\| id:ebi-fl187-GCST90094729 | Weighted median | 15 | -0.015951318 | 0.155962039730454 | 0.918536868714616 | -0.321636916 | 0.289734279694417 | 0.98417523033319 | 0.724961364386403 | 1.33607241928154 | 0.999332001504091 |
| ebi-fl187-GCST90094729 | ebi-cfmet338-GCST90026278 | Trigonelline (n'-methylnicotinate) levels \|\| id:ebi-cfmet338-GCST90026278 | F-cheese liking (derived food-liking factor) \|\| id:ebi-fl187-GCST90094729 | Weighted median | 15 | -0.019319009 | 0.142403323072627 | 0.892086881067789 | -0.298429523 | 0.259791503881192 | 0.980866406781412 | 0.74198257303708 | 1.29665970996356 | 0.999332001504091 |
| ebi-fl187-GCST90094729 | ebi-cfmet338-GCST90026299 | 1-carboxyethylvaline levels \|\| id:ebi-cfmet338-GCST90026299 | F-cheese liking (derived food-liking factor) \|\| id:ebi-fl187-GCST90094729 | Weighted median | 15 | -0.072433946 | 0.0912318125838589 | 0.42722167793341 | -0.251248299 | 0.106380406567449 | 0.930127183162009 | 0.777829213549292 | 1.11224490130579 | 0.999332001504091 |
| ebi-fl187-GCST90094729 | ebi-gm430-GCST90011306 | C_Erysipelotrichia abundance \|\| id:ebi-gm430-GCST90011306 | F-cheese liking (derived food-liking factor) \|\| id:ebi-fl187-GCST90094729 | Weighted median | 14 | -0.0169103 | 0.0801642782820392 | 0.832929734256354 | -0.174032286 | 0.140211685016084 | 0.983231876169046 | 0.840269768397198 | 1.15051732035886 | 0.999332001504091 |
| ebi-fl187-GCST90094729 | ebi-gm430-GCST90011312 | F_Erysipelotrichaceae abundance \|\| id:ebi-gm430-GCST90011312 | F-cheese liking (derived food-liking factor) \|\| id:ebi-fl187-GCST90094729 | Weighted median | 14 | -0.0169103 | 0.081740776080165 | 0.83610578663919 | -0.177122222 | 0.143301620700411 | 0.983231876169046 | 0.837677396049878 | 1.15407784294246 | 0.999332001504091 |
| ebi-fl187-GCST90094729 | ebi-gm430-GCST90011352 | O_Erysipelotrichales abundance \|\| id:ebi-gm430-GCST90011352 | F-cheese liking (derived food-liking factor) \|\| id:ebi-fl187-GCST90094729 | Weighted median | 14 | -0.0169103 | 0.0794760796287133 | 0.831504647368912 | -0.172683416 | 0.138862815655565 | 0.983231876169046 | 0.84140394729998 | 1.14896646897977 | 0.999332001504091 |
| ebi-fl187-GCST90094729 | ebi-gm430-GCST90011362 | OTU97_11 (Parabacteroides) abundance \|\| id:ebi-gm430-GCST90011362 | F-cheese liking (derived food-liking factor) \|\| id:ebi-fl187-GCST90094729 | Weighted median | 14 | -0.233398973 | 0.108647685691248 | 0.0316963983981445 | -0.446348437 | -0.020449509 | 0.791837588729233 | 0.639960747278275 | 0.97975816421735 | 0.999332001504091 |
| ebi-fl187-GCST90094729 | ebi-gm430-GCST90011384 | OTU97_235 (Phascolarctobacterium) abundance \|\| id:ebi-gm430-GCST90011384 | F-cheese liking (derived food-liking factor) \|\| id:ebi-fl187-GCST90094729 | Weighted median | 14 | 0.413542022840914 | 0.325231777619197 | 0.203540095472455 | -0.223912261 | 1.05099630697454 | 1.51216443150676 | 0.799385268322657 | 2.8604996345657 | 0.999332001504091 |
| ebi-fl187-GCST90094729 | ebi-gm430-GCST90011390 | OTU97_3 (Bacteroides) abundance \|\| id:ebi-gm430-GCST90011390 | F-cheese liking (derived food-liking factor) \|\| id:ebi-fl187-GCST90094729 | Weighted median | 14 | -0.026538207 | 0.077973193816648 | 0.733592668725008 | -0.179365667 | 0.126289252415752 | 0.973810836281411 | 0.835800218664376 | 1.13461030959589 | 0.999332001504091 |
| ebi-fl187-GCST90094729 | ebi-gm430-GCST90011398 | OTU97_4 (Alistipes) abundance \|\| id:ebi-gm430-GCST90011398 | F-cheese liking (derived food-liking factor) \|\| id:ebi-fl187-GCST90094729 | Weighted median | 14 | -0.085988762 | 0.0893930318505493 | 0.336090820941372 | -0.261199104 | 0.0892215804558116 | 0.917604543228432 | 0.770127568562966 | 1.09332288847236 | 0.999332001504091 |
| ebi-fl187-GCST90094729 | ebi-gm430-GCST90011421 | OTU99_108 (Cytophagales) abundance \|\| id:ebi-gm430-GCST90011421 | F-cheese liking (derived food-liking factor) \|\| id:ebi-fl187-GCST90094729 | Weighted median | 14 | 0.156538887721293 | 0.284038235184455 | 0.581552054258922 | -0.400176053 | 0.713253828682825 | 1.16945623892308 | 0.670202044407128 | 2.04062029677326 | 0.999332001504091 |
| ebi-fl187-GCST90094729 | ebi-gm430-GCST90011428 | OTU99_123 (Phascolarctobacterium) abundance \|\| id:ebi-gm430-GCST90011428 | F-cheese liking (derived food-liking factor) \|\| id:ebi-fl187-GCST90094729 | Weighted median | 14 | -0.095391669 | 0.178489460038682 | 0.593038071958525 | -0.445231011 | 0.254447672369045 | 0.909016830743393 | 0.640676255684467 | 1.28974906006462 | 0.999332001504091 |
| ebi-fl187-GCST90094729 | ebi-gm430-GCST90011450 | OTU99_298 (Phascolarctobacterium) abundance \|\| id:ebi-gm430-GCST90011450 | F-cheese liking (derived food-liking factor) \|\| id:ebi-fl187-GCST90094729 | Weighted median | 14 | 0.428490780985831 | 0.327888334831272 | 0.191274112860263 | -0.214170355 | 1.07115191725512 | 1.53493921512169 | 0.80721086067403 | 2.91873971089917 | 0.999332001504091 |
| ebi-fl187-GCST90094729 | ebi-gm430-GCST90011451 | OTU99_3 (Bacteroides) abundance \|\| id:ebi-gm430-GCST90011451 | F-cheese liking (derived food-liking factor) \|\| id:ebi-fl187-GCST90094729 | Weighted median | 14 | -0.026253096 | 0.0781509136378187 | 0.73692500713777 | -0.179428887 | 0.126922694581578 | 0.974088520354356 | 0.835747381434925 | 1.13532924728645 | 0.999332001504091 |
| ebi-fl187-GCST90094729 | ebi-gm430-GCST90011472 | OTU99_72 (Parabacteroides) abundance \|\| id:ebi-gm430-GCST90011472 | F-cheese liking (derived food-liking factor) \|\| id:ebi-fl187-GCST90094729 | Weighted median | 14 | -0.364212274 | 0.218375864161716 | 0.0953508822202058 | -0.792228968 | 0.0638044197566657 | 0.694743703064109 | 0.452834316447605 | 1.0658839125393 | 0.999332001504091 |
| ebi-fl187-GCST90094729 | ebi-gm430-GCST90011507 | TestASV_3 (Bacteroides) abundance \|\| id:ebi-gm430-GCST90011507 | F-cheese liking (derived food-liking factor) \|\| id:ebi-fl187-GCST90094729 | Weighted median | 14 | -0.188024234 | 0.0976726281274144 | 0.0542236905334523 | -0.379462585 | 0.00341411685883911 | 0.82859462660272 | 0.684229025089838 | 1.00341995159407 | 0.999332001504091 |
| ebi-fl187-GCST90094729 | ebi-gm430-GCST90011512 | TestASV_34 (Phascolarctobacterium) abundance \|\| id:ebi-gm430-GCST90011512 | F-cheese liking (derived food-liking factor) \|\| id:ebi-fl187-GCST90094729 | Weighted median | 14 | -0.523871847 | 0.262682179427426 | 0.0461172932099467 | -1.038728919 | -0.009014776 | 0.592223105802787 | 0.353904237187986 | 0.991025735756874 | 0.999332001504091 |
| ebi-fl187-GCST90094729 | ebi-gm430-GCST90011519 | TestASV_41 (Barnesiella) abundance \|\| id:ebi-gm430-GCST90011519 | F-cheese liking (derived food-liking factor) \|\| id:ebi-fl187-GCST90094729 | Weighted median | 14 | 0.135097885562632 | 0.363388744941294 | 0.710062356282042 | -0.577144055 | 0.847339825647569 | 1.14464882346182 | 0.561499691333054 | 2.33343125432881 | 0.999332001504091 |
| ebi-fl187-GCST90094729 | ebi-gm430-GCST90011524 | TestASV_46 (Sutterella) abundance \|\| id:ebi-gm430-GCST90011524 | F-cheese liking (derived food-liking factor) \|\| id:ebi-fl187-GCST90094729 | Weighted median | 14 | 0.112924350919448 | 0.345197937794528 | 0.743570111048327 | -0.563663607 | 0.789512308996723 | 1.11954723702593 | 0.569120206945823 | 2.20232211163028 | 0.999332001504091 |
| ebi-fl187-GCST90094729 | ebi-gm430-GCST90011581 | OTU97_19 (Bacteria) prevalence \|\| id:ebi-gm430-GCST90011581 | F-cheese liking (derived food-liking factor) \|\| id:ebi-fl187-GCST90094729 | Weighted median | 14 | -0.201546445 | 0.368280570397539 | 0.584197844824168 | -0.923376363 | 0.520283473043944 | 0.817465609541104 | 0.397175765143139 | 1.68250452678445 | 0.999332001504091 |
| ebi-fl187-GCST90094729 | ebi-gm430-GCST90011614 | OTU97_80 (Ruminococcus) prevalence \|\| id:ebi-gm430-GCST90011614 | F-cheese liking (derived food-liking factor) \|\| id:ebi-fl187-GCST90094729 | Weighted median | 14 | 0.26609642571253 | 0.186286473718761 | 0.153169305935109 | -0.099025063 | 0.631217914201302 | 1.30486087476048 | 0.905720007881191 | 1.87989874096282 | 0.999332001504091 |
| ebi-fl187-GCST90094729 | ebi-gm430-GCST90011623 | OTU99_11 (Parabacteroides) prevalence \|\| id:ebi-gm430-GCST90011623 | F-cheese liking (derived food-liking factor) \|\| id:ebi-fl187-GCST90094729 | Weighted median | 14 | 0.358451844024252 | 0.154518889906657 | 0.0203520762407154 | 0.0555948198072033 | 0.6613088682413 | 1.4311121138685 | 1.05716925290492 | 1.9373263806466 | 0.999332001504091 |
| ebi-fl187-GCST90094729 | ebi-gm430-GCST90011642 | OTU99_19 (Bacteria) prevalence \|\| id:ebi-gm430-GCST90011642 | F-cheese liking (derived food-liking factor) \|\| id:ebi-fl187-GCST90094729 | Weighted median | 14 | -0.201546445 | 0.360384716102155 | 0.575988666553992 | -0.907900488 | 0.50480759862499 | 0.817465609541104 | 0.403370216047077 | 1.65666674483576 | 0.999332001504091 |
| ebi-fl187-GCST90094729 | ebi-gm430-GCST90011653 | OTU99_30 (Parasutterella) prevalence \|\| id:ebi-gm430-GCST90011653 | F-cheese liking (derived food-liking factor) \|\| id:ebi-fl187-GCST90094729 | Weighted median | 14 | -0.333178166 | 0.183275137764933 | 0.069077505589652 | -0.692397436 | 0.0260411038699118 | 0.716642501345839 | 0.50037501275995 | 1.0263831359253 | 0.999332001504091 |
| ebi-fl187-GCST90094729 | ebi-gm430-GCST90011675 | OTU99_8 (Ruminococcaceae) prevalence \|\| id:ebi-gm430-GCST90011675 | F-cheese liking (derived food-liking factor) \|\| id:ebi-fl187-GCST90094729 | Weighted median | 14 | 0.192542728527727 | 0.173295664440915 | 0.26654041874253 | -0.147116774 | 0.53220223083192 | 1.2123283036043 | 0.863193173204135 | 1.70267787251431 | 0.999332001504091 |
| ebi-fl187-GCST90094729 | ebi-gm430-GCST90011679 | OTU99_92 (Ruminococcus) prevalence \|\| id:ebi-gm430-GCST90011679 | F-cheese liking (derived food-liking factor) \|\| id:ebi-fl187-GCST90094729 | Weighted median | 14 | 0.301494946719188 | 0.185624348439358 | 0.104328671373443 | -0.062328776 | 0.66531866966033 | 1.35187828370025 | 0.939573926438682 | 1.94511027021312 | 0.999332001504091 |
| ebi-fl187-GCST90094729 | ebi-gm430-GCST90011684 | TestASV_11 (Lachnospiraceae) prevalence \|\| id:ebi-gm430-GCST90011684 | F-cheese liking (derived food-liking factor) \|\| id:ebi-fl187-GCST90094729 | Weighted median | 14 | 0.166025336224171 | 0.183197738216263 | 0.364796651745843 | -0.193042231 | 0.525092903128046 | 1.18060301336695 | 0.824447156440129 | 1.69061590458933 | 0.999332001504091 |
| ebi-fl187-GCST90094729 | ebi-gm430-GCST90011688 | TestASV_15 (Bacteroides) prevalence \|\| id:ebi-gm430-GCST90011688 | F-cheese liking (derived food-liking factor) \|\| id:ebi-fl187-GCST90094729 | Weighted median | 14 | -0.099672966 | 0.181173062498945 | 0.582214202538573 | -0.454772168 | 0.255426236662545 | 0.905133379177142 | 0.634592531753669 | 1.29101178016801 | 0.999332001504091 |
| ebi-fl187-GCST90094729 | ebi-gm430-GCST90011692 | TestASV_19 (Prevotella) prevalence \|\| id:ebi-gm430-GCST90011692 | F-cheese liking (derived food-liking factor) \|\| id:ebi-fl187-GCST90094729 | Weighted median | 13 | 0.240643853460826 | 0.356022962886282 | 0.499089990029766 | -0.457161154 | 0.938448860717938 | 1.27206791203941 | 0.633078308868803 | 2.5560136086982 | 0.999332001504091 |
| ebi-fl187-GCST90094729 | ebi-gm430-GCST90011693 | TestASV_2 (Bacteroides) prevalence \|\| id:ebi-gm430-GCST90011693 | F-cheese liking (derived food-liking factor) \|\| id:ebi-fl187-GCST90094729 | Weighted median | 14 | -0.113143564 | 0.175076637459298 | 0.518116401703914 | -0.456293773 | 0.230006645455328 | 0.893022445069749 | 0.633627666809364 | 1.25860837392741 | 0.999332001504091 |
| ebi-fl187-GCST90094729 | ebi-gm430-GCST90011716 | TestASV_40 (Barnesiella) prevalence \|\| id:ebi-gm430-GCST90011716 | F-cheese liking (derived food-liking factor) \|\| id:ebi-fl187-GCST90094729 | Weighted median | 14 | 0.587176880028706 | 0.326152306984736 | 0.0718109188155904 | -0.052081642 | 1.22643540171879 | 1.79890272181368 | 0.949251365220732 | 3.40905593725027 | 0.999332001504091 |
| ebi-fl187-GCST90094729 | ebi-gm430-GCST90011718 | TestASV_42 (Alphaproteobacteria) prevalence \|\| id:ebi-gm430-GCST90011718 | F-cheese liking (derived food-liking factor) \|\| id:ebi-fl187-GCST90094729 | Weighted median | 14 | 0.031806821515065 | 0.280368318541007 | 0.909676527712584 | -0.517715083 | 0.581328725855439 | 1.03231806440224 | 0.595880531313919 | 1.78841316352685 | 0.999332001504091 |
| ebi-fl187-GCST90094729 | ebi-hbsm44-GCST90104929 | Sulcal depth of the right inferior frontal sulcus \|\| id:ebi-hbsm44-GCST90104929 | F-cheese liking (derived food-liking factor) \|\| id:ebi-fl187-GCST90094729 | Weighted median | 13 | 0.045885035302762 | 0.156192988853143 | 0.768932579515608 | -0.260253223 | 0.352023293454922 | 1.04695404128356 | 0.770856362642502 | 1.42194164526643 | 0.999332001504091 |
| ebi-fl187-GCST90094729 | ebi-hbsm44-GCST90104935 | Sulcal opening distance of the left calloso-marginal posterior fissure \|\| id:ebi-hbsm44-GCST90104935 | F-cheese liking (derived food-liking factor) \|\| id:ebi-fl187-GCST90094729 | Weighted median | 13 | 0.0135511523569344 | 0.0364854308138053 | 0.710330168026174 | -0.057960292 | 0.0850625967519927 | 1.01364338537235 | 0.943687418596883 | 1.08878521898365 | 0.999332001504091 |
| ebi-fl187-GCST90094729 | ebi-hbsm44-GCST90104936 | Sulcal opening distance of the left anterior inferior temporal sulcus \|\| id:ebi-hbsm44-GCST90104936 | F-cheese liking (derived food-liking factor) \|\| id:ebi-fl187-GCST90094729 | Weighted median | 13 | 0.00825216563535437 | 0.0194492139548882 | 0.671352132437456 | -0.029868294 | 0.0463726249869352 | 1.00828630860739 | 0.970573355740645 | 1.04746464974749 | 0.999332001504091 |
| ebi-fl187-GCST90094729 | ebi-hbsm44-GCST90104941 | Sulcal opening distance of the right posterior inferior temporal sulcus \|\| id:ebi-hbsm44-GCST90104941 | F-cheese liking (derived food-liking factor) \|\| id:ebi-fl187-GCST90094729 | Weighted median | 12 | -0.010508903 | 0.017487984309794 | 0.547892384513725 | -0.044785352 | 0.023767546427602 | 0.989546122778072 | 0.956202706741492 | 1.0240522456185 | 0.999332001504091 |
| ebi-fl187-GCST90094729 | ebi-IPs91-GCST90274819 | Leukemia inhibitory factor levels \|\| id:ebi-IPs91-GCST90274819 | F-cheese liking (derived food-liking factor) \|\| id:ebi-fl187-GCST90094729 | Weighted median | 16 | -0.055008114 | 0.0625742422470277 | 0.379354878925432 | -0.177653628 | 0.0676374011362332 | 0.946477468518436 | 0.837232366725886 | 1.06997726559031 | 0.999332001504091 |
| ebi-fl187-GCST90094729 | ebi-IPs91-GCST90274837 | STAM binding protein levels \|\| id:ebi-IPs91-GCST90274837 | F-cheese liking (derived food-liking factor) \|\| id:ebi-fl187-GCST90094729 | Weighted median | 16 | -0.002047071 | 0.0543078165531585 | 0.96993177067647 | -0.108490392 | 0.104396249152014 | 0.997955022529283 | 0.897187515189731 | 1.11004022027749 | 0.999332001504091 |
| ebi-fl187-GCST90094729 | ebi-IPs91-GCST90274840 | TNF-beta levels \|\| id:ebi-IPs91-GCST90274840 | F-cheese liking (derived food-liking factor) \|\| id:ebi-fl187-GCST90094729 | Weighted median | 16 | -0.032180833 | 0.0621975541661788 | 0.604879001115016 | -0.154088039 | 0.0897263735672054 | 0.968331460351627 | 0.857196551164316 | 1.09387492965656 | 0.999332001504091 |
| ebi-fl187-GCST90094729 | ebi-IPs91-GCST90274842 | Tumor necrosis factor ligand superfamily member 14 levels \|\| id:ebi-IPs91-GCST90274842 | F-cheese liking (derived food-liking factor) \|\| id:ebi-fl187-GCST90094729 | Weighted median | 16 | 0.0475352628067107 | 0.0630990771092117 | 0.451243685042508 | -0.076138928 | 0.171209453940766 | 1.04868317998157 | 0.926687454477912 | 1.18673929021283 | 0.999332001504091 |
| ebi-fl187-GCST90094729 | ebi-met1400-GCST90199628 | 3-methylhistidine levels \|\| id:ebi-met1400-GCST90199628 | F-cheese liking (derived food-liking factor) \|\| id:ebi-fl187-GCST90094729 | Weighted median | 15 | 0.0768420476737998 | 0.0796190396918651 | 0.33448395521305 | -0.07921127 | 0.232895365469855 | 1.07987149470648 | 0.923844723038119 | 1.26224939754459 | 0.999332001504091 |
| ebi-fl187-GCST90094729 | ebi-met1400-GCST90199651 | Docosahexaenoate DHA; 22:6n3 levels \|\| id:ebi-met1400-GCST90199651 | F-cheese liking (derived food-liking factor) \|\| id:ebi-fl187-GCST90094729 | Weighted median | 15 | 0.0368146078182896 | 0.0746228022876636 | 0.621770527846741 | -0.109446085 | 0.18307530030211 | 1.03750065849687 | 0.896330489017488 | 1.20090483317301 | 0.999332001504091 |
| ebi-fl187-GCST90094729 | ebi-met1400-GCST90199652 | 1-stearoyl-2-oleoyl-GPS (18:0/18:1) levels \|\| id:ebi-met1400-GCST90199652 | F-cheese liking (derived food-liking factor) \|\| id:ebi-fl187-GCST90094729 | Weighted median | 15 | -0.000295546 | 0.075050295589296 | 0.996857957142612 | -0.147394126 | 0.146803033023801 | 0.999704497338296 | 0.862953798126146 | 1.15812582802066 | 0.999364451047625 |
| ebi-fl187-GCST90094729 | ebi-met1400-GCST90199653 | 1-myristoyl-2-palmitoyl-gpc (14:0/16:0) levels \|\| id:ebi-met1400-GCST90199653 | F-cheese liking (derived food-liking factor) \|\| id:ebi-fl187-GCST90094729 | Weighted median | 15 | 0.00925915702221321 | 0.0731985105236737 | 0.899341064385125 | -0.134209924 | 0.152728237648614 | 1.0093021556244 | 0.87440648677412 | 1.16500833051484 | 0.999332001504091 |
| ebi-fl187-GCST90094729 | ebi-met1400-GCST90199667 | Erythritol levels in elite athletes \|\| id:ebi-met1400-GCST90199667 | F-cheese liking (derived food-liking factor) \|\| id:ebi-fl187-GCST90094729 | Weighted median | 15 | 0.0558585299672288 | 0.0756179912360302 | 0.460093042419607 | -0.092352733 | 0.204069792789848 | 1.05744807594039 | 0.911783476826879 | 1.22638374321226 | 0.999332001504091 |
| ebi-fl187-GCST90094729 | ebi-met1400-GCST90199674 | 3-hydroxymyristate levels \|\| id:ebi-met1400-GCST90199674 | F-cheese liking (derived food-liking factor) \|\| id:ebi-fl187-GCST90094729 | Weighted median | 15 | 0.0258519009748988 | 0.0724022469798464 | 0.721047357372247 | -0.116056503 | 0.167760305055398 | 1.02618895963453 | 0.890424910103336 | 1.18265310070176 | 0.999332001504091 |
| ebi-fl187-GCST90094729 | ebi-met1400-GCST90199685 | 2-linoleoylglycerol (18:2) levels \|\| id:ebi-met1400-GCST90199685 | F-cheese liking (derived food-liking factor) \|\| id:ebi-fl187-GCST90094729 | Weighted median | 15 | 0.00611868936901124 | 0.0824098060144173 | 0.940813766346267 | -0.15540453 | 0.167641909157269 | 1.00613744678623 | 0.85606880155734 | 1.18251308771437 | 0.999332001504091 |
| ebi-fl187-GCST90094729 | ebi-met1400-GCST90199703 | Gamma-glutamylvaline levels \|\| id:ebi-met1400-GCST90199703 | F-cheese liking (derived food-liking factor) \|\| id:ebi-fl187-GCST90094729 | Weighted median | 15 | -0.031053238 | 0.0750979098365655 | 0.679237865339033 | -0.178245141 | 0.116138665657374 | 0.969423961875484 | 0.836737279813763 | 1.12315160389117 | 0.999332001504091 |
| ebi-fl187-GCST90094729 | ebi-met1400-GCST90199779 | Gamma-glutamyl-2-aminobutyrate levels \|\| id:ebi-met1400-GCST90199779 | F-cheese liking (derived food-liking factor) \|\| id:ebi-fl187-GCST90094729 | Weighted median | 15 | -0.028886831 | 0.0823709144261175 | 0.725819332154074 | -0.190333824 | 0.132560161037909 | 0.971526404686755 | 0.826683121612865 | 1.14174770274986 | 0.999332001504091 |
| ebi-fl187-GCST90094729 | ebi-met1400-GCST90199824 | 4-vinylphenol sulfate levels \|\| id:ebi-met1400-GCST90199824 | F-cheese liking (derived food-liking factor) \|\| id:ebi-fl187-GCST90094729 | Weighted median | 15 | 0.0146216948162296 | 0.0791497582168585 | 0.853437247066072 | -0.140511831 | 0.169755220921272 | 1.01472911471096 | 0.868913384506756 | 1.18501474899779 | 0.999332001504091 |
| ebi-fl187-GCST90094729 | ebi-met1400-GCST90199830 | 4-ethylphenylsulfate levels \|\| id:ebi-met1400-GCST90199830 | F-cheese liking (derived food-liking factor) \|\| id:ebi-fl187-GCST90094729 | Weighted median | 15 | 0.117101736061865 | 0.0728036301657935 | 0.107734442658633 | -0.025593379 | 0.259796851186821 | 1.12423379899202 | 0.974731355215237 | 1.29666664361787 | 0.999332001504091 |
| ebi-fl187-GCST90094729 | ebi-met1400-GCST90199833 | Hexanoylglutamine levels \|\| id:ebi-met1400-GCST90199833 | F-cheese liking (derived food-liking factor) \|\| id:ebi-fl187-GCST90094729 | Weighted median | 15 | 0.143002990861657 | 0.0712700869223597 | 0.0448035846134794 | 0.00331362049383249 | 0.282692361229482 | 1.1537332523174 | 1.00331911660322 | 1.32669695561008 | 0.999332001504091 |
| ebi-fl187-GCST90094729 | ebi-met1400-GCST90199837 | Carotenoid levels (cryptoxanthin) \|\| id:ebi-met1400-GCST90199837 | F-cheese liking (derived food-liking factor) \|\| id:ebi-fl187-GCST90094729 | Weighted median | 15 | 0.0430396123885065 | 0.0840995136900168 | 0.608811953195713 | -0.121795434 | 0.207874659220939 | 1.04397924854313 | 0.885329457892532 | 1.23105885800197 | 0.999332001504091 |
| ebi-fl187-GCST90094729 | ebi-met1400-GCST90199869 | 2-hydroxyhippurate levels \|\| id:ebi-met1400-GCST90199869 | F-cheese liking (derived food-liking factor) \|\| id:ebi-fl187-GCST90094729 | Weighted median | 15 | -0.05704399 | 0.0769747440164041 | 0.45864850887612 | -0.207914488 | 0.0938265086623348 | 0.944552517955898 | 0.812276493225572 | 1.0983691718493 | 0.999332001504091 |
| ebi-fl187-GCST90094729 | ebi-met1400-GCST90199902 | Hydantoin-5-propionate levels \|\| id:ebi-met1400-GCST90199902 | F-cheese liking (derived food-liking factor) \|\| id:ebi-fl187-GCST90094729 | Weighted median | 15 | -0.022695597 | 0.0734287445528202 | 0.757258178205284 | -0.166615936 | 0.121224742175974 | 0.977560010542628 | 0.846524668163329 | 1.12887859049103 | 0.999332001504091 |
| ebi-fl187-GCST90094729 | ebi-met1400-GCST90199904 | Pantoate levels \|\| id:ebi-met1400-GCST90199904 | F-cheese liking (derived food-liking factor) \|\| id:ebi-fl187-GCST90094729 | Weighted median | 15 | 0.0975766521277427 | 0.0771053448245053 | 0.205692899902161 | -0.053549824 | 0.248703127983773 | 1.10249594698469 | 0.947858713972279 | 1.28236127937651 | 0.999332001504091 |
| ebi-fl187-GCST90094729 | ebi-met1400-GCST90199936 | 3-methyl catechol sulfate (2) levels \|\| id:ebi-met1400-GCST90199936 | F-cheese liking (derived food-liking factor) \|\| id:ebi-fl187-GCST90094729 | Weighted median | 15 | -0.0554033 | 0.0870582829472732 | 0.524520166636844 | -0.226037535 | 0.115230934599977 | 0.946103507478358 | 0.797688160234964 | 1.12213254688297 | 0.999332001504091 |
| ebi-fl187-GCST90094729 | ebi-met1400-GCST90199977 | 4-hydroxychlorothalonil levels \|\| id:ebi-met1400-GCST90199977 | F-cheese liking (derived food-liking factor) \|\| id:ebi-fl187-GCST90094729 | Weighted median | 15 | 0.0891498026849997 | 0.08249792439998 | 0.279861351162646 | -0.072546129 | 0.250845734508961 | 1.09324441500901 | 0.930022844517735 | 1.28511182063291 | 0.999332001504091 |
| ebi-fl187-GCST90094729 | ebi-met1400-GCST90200040 | Sphingomyelin (d18:2/24:1, d18:1/24:2) levels \|\| id:ebi-met1400-GCST90200040 | F-cheese liking (derived food-liking factor) \|\| id:ebi-fl187-GCST90094729 | Weighted median | 15 | 0.00930864685052227 | 0.0638185108021865 | 0.884030933609706 | -0.115775634 | 0.134392928022808 | 1.00935210705083 | 0.89067503778982 | 1.14384217900172 | 0.999332001504091 |
| ebi-fl187-GCST90094729 | ebi-met1400-GCST90200077 | 1-stearoyl-2-oleoyl-GPI (18:0/18:1) levels \|\| id:ebi-met1400-GCST90200077 | F-cheese liking (derived food-liking factor) \|\| id:ebi-fl187-GCST90094729 | Weighted median | 15 | -0.051443382 | 0.0718514945203224 | 0.474011015521177 | -0.192272311 | 0.0893855476366566 | 0.949857427825556 | 0.825082159046374 | 1.09350217224206 | 0.999332001504091 |
| ebi-fl187-GCST90094729 | ebi-met1400-GCST90200080 | 1-palmitoyl-2-oleoyl-GPI (16:0/18:1) levels \|\| id:ebi-met1400-GCST90200080 | F-cheese liking (derived food-liking factor) \|\| id:ebi-fl187-GCST90094729 | Weighted median | 15 | -0.030383663 | 0.0750750897254227 | 0.685690466053622 | -0.177530838 | 0.116763513356386 | 0.970073281397874 | 0.837335176887881 | 1.12385362189082 | 0.999332001504091 |
| ebi-fl187-GCST90094729 | ebi-met1400-GCST90200082 | 1-oleoyl-2-linoleoyl-GPE (18:1/18:2) levels \|\| id:ebi-met1400-GCST90200082 | F-cheese liking (derived food-liking factor) \|\| id:ebi-fl187-GCST90094729 | Weighted median | 15 | -0.063091336 | 0.0774195055835151 | 0.415113427496832 | -0.214833567 | 0.0886508949589993 | 0.938857718278732 | 0.80667568651781 | 1.09269912296047 | 0.999332001504091 |
| ebi-fl187-GCST90094729 | ebi-met1400-GCST90200100 | Perfluorooctanesulfonate (PFOS) levels \|\| id:ebi-met1400-GCST90200100 | F-cheese liking (derived food-liking factor) \|\| id:ebi-fl187-GCST90094729 | Weighted median | 15 | -0.061090671 | 0.0693549010576164 | 0.378403643530535 | -0.197026277 | 0.0748449347393879 | 0.940737937945507 | 0.821169054836113 | 1.07771702145618 | 0.999332001504091 |
| ebi-fl187-GCST90094729 | ebi-met1400-GCST90200102 | 1-palmitoleoyl-2-linolenoyl-GPC (16:1/18:3) levels \|\| id:ebi-met1400-GCST90200102 | F-cheese liking (derived food-liking factor) \|\| id:ebi-fl187-GCST90094729 | Weighted median | 15 | -0.003579206 | 0.0716495282824094 | 0.960158760610861 | -0.144012282 | 0.136853869309378 | 0.996427191598896 | 0.865877113674198 | 1.14666057397522 | 0.999332001504091 |
| ebi-fl187-GCST90094729 | ebi-met1400-GCST90200114 | Glycosyl-N-tricosanoyl-sphingadienine (d18:2/23:0) levels \|\| id:ebi-met1400-GCST90200114 | F-cheese liking (derived food-liking factor) \|\| id:ebi-fl187-GCST90094729 | Weighted median | 15 | -0.058956894 | 0.0728074153439608 | 0.418075271991302 | -0.201659428 | 0.0837456399228213 | 0.942747406208261 | 0.817373254804223 | 1.08735227962075 | 0.999332001504091 |
| ebi-fl187-GCST90094729 | ebi-met1400-GCST90200135 | Arachidoylcarnitine (C20) levels \|\| id:ebi-met1400-GCST90200135 | F-cheese liking (derived food-liking factor) \|\| id:ebi-fl187-GCST90094729 | Weighted median | 15 | -0.06045403 | 0.0726592147565916 | 0.405396800136713 | -0.202866091 | 0.081958030939156 | 0.941337041302857 | 0.816387555823746 | 1.08541025522457 | 0.999332001504091 |
| ebi-fl187-GCST90094729 | ebi-met1400-GCST90200146 | Docosahexaenoylcarnitine (C22:6) levels \|\| id:ebi-met1400-GCST90200146 | F-cheese liking (derived food-liking factor) \|\| id:ebi-fl187-GCST90094729 | Weighted median | 15 | 0.0651846490912519 | 0.0853403886446646 | 0.444974841785959 | -0.102082513 | 0.232451810834795 | 1.06735609252193 | 0.902955043379452 | 1.26168964512327 | 0.999332001504091 |
| ebi-fl187-GCST90094729 | ebi-met1400-GCST90200162 | 3-hydroxyphenylacetoylglutamine levels \|\| id:ebi-met1400-GCST90200162 | F-cheese liking (derived food-liking factor) \|\| id:ebi-fl187-GCST90094729 | Weighted median | 15 | 0.0640544592483189 | 0.0844209975578809 | 0.448001926873085 | -0.101410696 | 0.229519614461766 | 1.06615045893312 | 0.903561867459801 | 1.25799554188676 | 0.999332001504091 |
| ebi-fl187-GCST90094729 | ebi-met1400-GCST90200172 | 3-carboxy-4-methyl-5-pentyl-2-furanpropionate (3-CMPFP) levels \|\| id:ebi-met1400-GCST90200172 | F-cheese liking (derived food-liking factor) \|\| id:ebi-fl187-GCST90094729 | Weighted median | 15 | 0.0226744672472888 | 0.0724872960408385 | 0.754427992481024 | -0.119400633 | 0.164749567487332 | 1.02293348698654 | 0.887452186905818 | 1.17909779730983 | 0.999332001504091 |
| ebi-fl187-GCST90094729 | ebi-met1400-GCST90200222 | Dihydrocaffeate sulfate (2) levels \|\| id:ebi-met1400-GCST90200222 | F-cheese liking (derived food-liking factor) \|\| id:ebi-fl187-GCST90094729 | Weighted median | 15 | -0.063514279 | 0.0880548502933629 | 0.470722754996544 | -0.236101785 | 0.10907322784515 | 0.93846071917792 | 0.789700289957179 | 1.11524401426736 | 0.999332001504091 |
| ebi-fl187-GCST90094729 | ebi-met1400-GCST90200223 | 4-methylhexanoylglutamine levels \|\| id:ebi-met1400-GCST90200223 | F-cheese liking (derived food-liking factor) \|\| id:ebi-fl187-GCST90094729 | Weighted median | 15 | 0.074073398730065 | 0.0878340989695551 | 0.39904220278601 | -0.098081435 | 0.246228232710393 | 1.07688584464892 | 0.906575073579773 | 1.27919149356931 | 0.999332001504091 |
| ebi-fl187-GCST90094729 | ebi-met1400-GCST90200224 | 2-ketocaprylate levels \|\| id:ebi-met1400-GCST90200224 | F-cheese liking (derived food-liking factor) \|\| id:ebi-fl187-GCST90094729 | Weighted median | 15 | 0.0116929188468913 | 0.0725710126679805 | 0.871995879004975 | -0.130546266 | 0.153932103676133 | 1.01176154825432 | 0.877615888248242 | 1.16641168902405 | 0.999332001504091 |
| ebi-fl187-GCST90094729 | ebi-met1400-GCST90200243 | Hydroxypalmitoyl sphingomyelin (d18:1/16:0(OH)) levels \|\| id:ebi-met1400-GCST90200243 | F-cheese liking (derived food-liking factor) \|\| id:ebi-fl187-GCST90094729 | Weighted median | 15 | -0.100170625 | 0.0822806823156382 | 0.223442253762885 | -0.261440762 | 0.0610995122822489 | 0.904683043270949 | 0.769941483562905 | 1.0630046909469 | 0.999332001504091 |
| ebi-fl187-GCST90094729 | ebi-met1400-GCST90200273 | 2-methoxyhydroquinone sulfate (2) levels \|\| id:ebi-met1400-GCST90200273 | F-cheese liking (derived food-liking factor) \|\| id:ebi-fl187-GCST90094729 | Weighted median | 15 | 0.117399598894598 | 0.081775848438068 | 0.151108567011563 | -0.042881064 | 0.277680261833212 | 1.12456871633328 | 0.958025326949419 | 1.32006405486424 | 0.999332001504091 |
| ebi-fl187-GCST90094729 | ebi-met1400-GCST90200276 | 2,4-di-tert-butylphenol levels \|\| id:ebi-met1400-GCST90200276 | F-cheese liking (derived food-liking factor) \|\| id:ebi-fl187-GCST90094729 | Weighted median | 15 | -0.000502446 | 0.0722756558074296 | 0.994453312298394 | -0.142162731 | 0.141157839615473 | 0.999497680437648 | 0.867480078968686 | 1.1516064027521 | 0.999332001504091 |
| ebi-fl187-GCST90094729 | ebi-met1400-GCST90200289 | N-acetylmethionine levels \|\| id:ebi-met1400-GCST90200289 | F-cheese liking (derived food-liking factor) \|\| id:ebi-fl187-GCST90094729 | Weighted median | 15 | 0.0118289099191115 | 0.0733859292166275 | 0.871945560090287 | -0.132007511 | 0.155665331183701 | 1.01189914814806 | 0.876334412604124 | 1.16843509885687 | 0.999332001504091 |
| ebi-fl187-GCST90094729 | ebi-met1400-GCST90200308 | 3-hydroxyisobutyrate levels \|\| id:ebi-met1400-GCST90200308 | F-cheese liking (derived food-liking factor) \|\| id:ebi-fl187-GCST90094729 | Weighted median | 15 | -0.104207076 | 0.0701502261585943 | 0.137415516916298 | -0.241701519 | 0.0332873671418703 | 0.901038694496453 | 0.78529053656495 | 1.03384759038507 | 0.999332001504091 |
| ebi-fl187-GCST90094729 | ebi-met1400-GCST90200383 | Cystathionine levels \|\| id:ebi-met1400-GCST90200383 | F-cheese liking (derived food-liking factor) \|\| id:ebi-fl187-GCST90094729 | Weighted median | 15 | -0.14875407 | 0.0798724712715827 | 0.0625474709160595 | -0.305304114 | 0.00779597382698039 | 0.861781026764109 | 0.736899239262827 | 1.00782644155467 | 0.999332001504091 |
| ebi-fl187-GCST90094729 | ebi-met1400-GCST90200384 | Guanidinoacetate levels \|\| id:ebi-met1400-GCST90200384 | F-cheese liking (derived food-liking factor) \|\| id:ebi-fl187-GCST90094729 | Weighted median | 15 | -0.041254317 | 0.0712257559073236 | 0.562450824689477 | -0.180856799 | 0.0983481641671768 | 0.959585059725267 | 0.834554859238421 | 1.1033468640854 | 0.999332001504091 |
| ebi-fl187-GCST90094729 | ebi-met1400-GCST90200403 | Citrulline levels \|\| id:ebi-met1400-GCST90200403 | F-cheese liking (derived food-liking factor) \|\| id:ebi-fl187-GCST90094729 | Weighted median | 15 | 0.0257500555151942 | 0.0728247058907621 | 0.723646501509338 | -0.116986368 | 0.168486479061088 | 1.02608445227008 | 0.889597320044111 | 1.18351222454019 | 0.999332001504091 |
| ebi-fl187-GCST90094729 | ebi-met1400-GCST90200434 | 12,13-DiHOME levels \|\| id:ebi-met1400-GCST90200434 | F-cheese liking (derived food-liking factor) \|\| id:ebi-fl187-GCST90094729 | Weighted median | 15 | 0.00618475019227316 | 0.0687508830745915 | 0.928319917280629 | -0.128566981 | 0.140936481018472 | 1.00620391524974 | 0.879354660713181 | 1.15135151298655 | 0.999332001504091 |
| ebi-fl187-GCST90094729 | ebi-met1400-GCST90200437 | Fructose levels \|\| id:ebi-met1400-GCST90200437 | F-cheese liking (derived food-liking factor) \|\| id:ebi-fl187-GCST90094729 | Weighted median | 15 | -0.036792249 | 0.0751906311389769 | 0.624615452586183 | -0.184165886 | 0.110581388079469 | 0.963876360873024 | 0.831797808782169 | 1.11692724991672 | 0.999332001504091 |
| ebi-fl187-GCST90094729 | ebi-met1400-GCST90200448 | N-stearoyl-sphinganine (d18:0/18:0) levels \|\| id:ebi-met1400-GCST90200448 | F-cheese liking (derived food-liking factor) \|\| id:ebi-fl187-GCST90094729 | Weighted median | 15 | 0.0332629550334375 | 0.0796253652454613 | 0.676134882063319 | -0.122802761 | 0.189328670914542 | 1.03382235229365 | 0.884438091177574 | 1.20843806566376 | 0.999332001504091 |
| ebi-fl187-GCST90094729 | ebi-met1400-GCST90200468 | X-11847 levels \|\| id:ebi-met1400-GCST90200468 | F-cheese liking (derived food-liking factor) \|\| id:ebi-fl187-GCST90094729 | Weighted median | 15 | 0.0773330412421402 | 0.0815052346949989 | 0.342716883519218 | -0.082417219 | 0.237083301244338 | 1.08040183485116 | 0.920887666924508 | 1.26754670159509 | 0.999332001504091 |
| ebi-fl187-GCST90094729 | ebi-met1400-GCST90200476 | X-11843 levels \|\| id:ebi-met1400-GCST90200476 | F-cheese liking (derived food-liking factor) \|\| id:ebi-fl187-GCST90094729 | Weighted median | 15 | -0.046930416 | 0.0904783581382933 | 0.603975425405555 | -0.224267998 | 0.130407166370132 | 0.954153789517577 | 0.799100948587994 | 1.1392921703565 | 0.999332001504091 |
| ebi-fl187-GCST90094729 | ebi-met1400-GCST90200511 | X-12906 levels \|\| id:ebi-met1400-GCST90200511 | F-cheese liking (derived food-liking factor) \|\| id:ebi-fl187-GCST90094729 | Weighted median | 15 | -0.141226235 | 0.0715080386275049 | 0.0482715459367339 | -0.281381991 | -0.00107048 | 0.868292851030928 | 0.754739976565387 | 0.998930093225425 | 0.999332001504091 |
| ebi-fl187-GCST90094729 | ebi-met1400-GCST90200515 | X-12849 levels \|\| id:ebi-met1400-GCST90200515 | F-cheese liking (derived food-liking factor) \|\| id:ebi-fl187-GCST90094729 | Weighted median | 15 | 0.0290126202940043 | 0.0831654061161228 | 0.727198598478558 | -0.133991576 | 0.192016816281605 | 1.02943758619815 | 0.87459743244905 | 1.21169089292885 | 0.999332001504091 |
| ebi-fl187-GCST90094729 | ebi-met1400-GCST90200551 | X-18886 levels \|\| id:ebi-met1400-GCST90200551 | F-cheese liking (derived food-liking factor) \|\| id:ebi-fl187-GCST90094729 | Weighted median | 15 | 0.0738614034738724 | 0.0731854715193477 | 0.312861524702868 | -0.069582121 | 0.217304927651794 | 1.07665757415538 | 0.932783529399053 | 1.24272298497054 | 0.999332001504091 |
| ebi-fl187-GCST90094729 | ebi-met1400-GCST90200564 | X-21319 levels \|\| id:ebi-met1400-GCST90200564 | F-cheese liking (derived food-liking factor) \|\| id:ebi-fl187-GCST90094729 | Weighted median | 15 | 0.025335259146066 | 0.07221987974959 | 0.725732939718145 | -0.116215705 | 0.166886223455262 | 1.02565892442467 | 0.890283163909013 | 1.181619817 | 0.999332001504091 |
| ebi-fl187-GCST90094729 | ebi-met1400-GCST90200579 | X-21442 levels \|\| id:ebi-met1400-GCST90200579 | F-cheese liking (derived food-liking factor) \|\| id:ebi-fl187-GCST90094729 | Weighted median | 15 | 0.0468847394099456 | 0.0759042853211782 | 0.536784730753606 | -0.10188766 | 0.195657138639455 | 1.04800120888033 | 0.903131003870123 | 1.21610987675999 | 0.999332001504091 |
| ebi-fl187-GCST90094729 | ebi-met1400-GCST90200581 | X-21441 levels \|\| id:ebi-met1400-GCST90200581 | F-cheese liking (derived food-liking factor) \|\| id:ebi-fl187-GCST90094729 | Weighted median | 15 | 0.0401719446331746 | 0.0763756452176382 | 0.598903169621181 | -0.10952432 | 0.189868209259745 | 1.04098975140581 | 0.896260367050847 | 1.20909024025877 | 0.999332001504091 |
| ebi-fl187-GCST90094729 | ebi-met1400-GCST90200648 | X-24585 levels \|\| id:ebi-met1400-GCST90200648 | F-cheese liking (derived food-liking factor) \|\| id:ebi-fl187-GCST90094729 | Weighted median | 15 | 0.151378828361906 | 0.0761240129002214 | 0.046747379343665 | 0.0021757630774718 | 0.30058189364634 | 1.16343731766261 | 1.00217813176755 | 1.35064451041516 | 0.999332001504091 |
| ebi-fl187-GCST90094729 | ebi-met1400-GCST90200720 | Adenosine 5'-diphosphate (ADP) to pantothenate ratio \|\| id:ebi-met1400-GCST90200720 | F-cheese liking (derived food-liking factor) \|\| id:ebi-fl187-GCST90094729 | Weighted median | 15 | -0.040957915 | 0.107099939632167 | 0.702144621614961 | -0.250873797 | 0.168957966583866 | 0.959869525115383 | 0.778120566688112 | 1.18407036735546 | 0.999332001504091 |
| ebi-fl187-GCST90094729 | ebi-met1400-GCST90200724 | Cholate to taurocholate ratio \|\| id:ebi-met1400-GCST90200724 | F-cheese liking (derived food-liking factor) \|\| id:ebi-fl187-GCST90094729 | Weighted median | 15 | -0.015578624 | 0.0786643043543168 | 0.843014315826137 | -0.16976066 | 0.138603412781947 | 0.984542095314543 | 0.843866763257442 | 1.14866846242958 | 0.999332001504091 |
| ebi-fl187-GCST90094729 | ebi-met1400-GCST90200726 | N-acetylputrescine to (N(1) + N(8))-acetylspermidine ratio \|\| id:ebi-met1400-GCST90200726 | F-cheese liking (derived food-liking factor) \|\| id:ebi-fl187-GCST90094729 | Weighted median | 15 | 0.0617383906560801 | 0.0719029889652942 | 0.390542169617332 | -0.079191468 | 0.202668249028057 | 1.0636840386424 | 0.923863017567875 | 1.22466611667295 | 0.999332001504091 |
| ebi-fl187-GCST90094729 | ebi-met1400-GCST90200744 | Arginine to glutamate ratio \|\| id:ebi-met1400-GCST90200744 | F-cheese liking (derived food-liking factor) \|\| id:ebi-fl187-GCST90094729 | Weighted median | 15 | -0.036606261 | 0.0749773174603168 | 0.625386131225247 | -0.183561803 | 0.110349281186512 | 0.964055646901838 | 0.832300435269841 | 1.11666803348721 | 0.999332001504091 |
| ebi-fl187-GCST90094729 | ebi-met1400-GCST90200759 | Histidine to trans-urocanate ratio \|\| id:ebi-met1400-GCST90200759 | F-cheese liking (derived food-liking factor) \|\| id:ebi-fl187-GCST90094729 | Weighted median | 15 | -0.068385222 | 0.0724378433581335 | 0.345142154168925 | -0.210363395 | 0.073592951372496 | 0.933900645565153 | 0.810289737553962 | 1.07636858195915 | 0.999332001504091 |
| ebi-fl187-GCST90094729 | ebi-met1400-GCST90200795 | Oleoyl-linoleoyl-glycerol (18:1 to 18:2) [2] to linoleoyl-arachidonoyl-glycerol (18:2 to 20:4) [2] ratio \|\| id:ebi-met1400-GCST90200795 | F-cheese liking (derived food-liking factor) \|\| id:ebi-fl187-GCST90094729 | Weighted median | 15 | -0.024722349 | 0.0778865824111154 | 0.750928922941923 | -0.177380051 | 0.127935352095514 | 0.97558074497779 | 0.837461446011179 | 1.13647952930207 | 0.999332001504091 |
| ebi-fl187-GCST90094729 | ebi-met1400-GCST90200811 | Choline to taurocholate ratio \|\| id:ebi-met1400-GCST90200811 | F-cheese liking (derived food-liking factor) \|\| id:ebi-fl187-GCST90094729 | Weighted median | 15 | -0.029486423 | 0.07536002818393 | 0.695594683596367 | -0.177192078 | 0.118219232366513 | 0.970944060181557 | 0.837618880815158 | 1.12549082833997 | 0.999332001504091 |
| ebi-fl187-GCST90094729 | ebi-met1400-GCST90200831 | Cholate to phosphate ratio \|\| id:ebi-met1400-GCST90200831 | F-cheese liking (derived food-liking factor) \|\| id:ebi-fl187-GCST90094729 | Weighted median | 15 | -0.080608805 | 0.0829288680150786 | 0.331038879893269 | -0.243149386 | 0.0819317764669563 | 0.922554519723424 | 0.784154363218425 | 1.08538175872528 | 0.999332001504091 |
| ebi-fl187-GCST90094729 | ebi-met1400-GCST90200841 | Adenosine 5'-diphosphate (ADP) to oxalate (ethanedioate) ratio \|\| id:ebi-met1400-GCST90200841 | F-cheese liking (derived food-liking factor) \|\| id:ebi-fl187-GCST90094729 | Weighted median | 15 | 0.0871621434904884 | 0.103706762077266 | 0.400646422267906 | -0.11610311 | 0.29042739716193 | 1.09107357585458 | 0.890383410969539 | 1.33699879541986 | 0.999332001504091 |
| ebi-fl187-GCST90094729 | ebi-met1400-GCST90200844 | Aspartate to phosphate ratio \|\| id:ebi-met1400-GCST90200844 | F-cheese liking (derived food-liking factor) \|\| id:ebi-fl187-GCST90094729 | Weighted median | 15 | -0.013853457 | 0.0656425878690727 | 0.832853147099142 | -0.142512929 | 0.114806015544942 | 0.986242060861092 | 0.867176342581981 | 1.12165583267117 | 0.999332001504091 |
| ebi-fl187-GCST90094729 | ebi-met1400-GCST90200870 | Adenosine 5'-monophosphate (AMP) to acetoacetate ratio \|\| id:ebi-met1400-GCST90200870 | F-cheese liking (derived food-liking factor) \|\| id:ebi-fl187-GCST90094729 | Weighted median | 15 | 0.0335650527680517 | 0.0757230946211733 | 0.657577361386871 | -0.114852213 | 0.181982318225551 | 1.03413471486391 | 0.89149788624664 | 1.19959298275979 | 0.999332001504091 |
| ebi-fl187-GCST90094729 | ebi-met1400-GCST90200887 | Cholesterol to cortisol ratio \|\| id:ebi-met1400-GCST90200887 | F-cheese liking (derived food-liking factor) \|\| id:ebi-fl187-GCST90094729 | Weighted median | 14 | -0.006976518 | 0.0741575386260345 | 0.925048002935871 | -0.152325293 | 0.138372258090031 | 0.993047761787326 | 0.858708903030299 | 1.14840297301078 | 0.999332001504091 |
| ebi-fl187-GCST90094729 | ebi-met1400-GCST90200890 | Cortisol to taurocholate ratio \|\| id:ebi-met1400-GCST90200890 | F-cheese liking (derived food-liking factor) \|\| id:ebi-fl187-GCST90094729 | Weighted median | 15 | -0.011209194 | 0.07691904720358 | 0.884136783469478 | -0.161970526 | 0.139552138590603 | 0.988853395010382 | 0.850466270433171 | 1.14975875095613 | 0.999332001504091 |
| ebi-fl187-GCST90094729 | ebi-met1400-GCST90200948 | Adenosine 5'-diphosphate (ADP) to mannitol to sorbitol ratio \|\| id:ebi-met1400-GCST90200948 | F-cheese liking (derived food-liking factor) \|\| id:ebi-fl187-GCST90094729 | Weighted median | 15 | -0.165467704 | 0.0985960831388201 | 0.0933006261202808 | -0.358716026 | 0.0277806194456809 | 0.847497233951918 | 0.698572699350883 | 1.02817009915153 | 0.999332001504091 |
| ebi-fl187-GCST90094729 | ebi-met1400-GCST90200972 | Leucine to N-palmitoyl-sphingosine (d18:1 to 16:0) ratio \|\| id:ebi-met1400-GCST90200972 | F-cheese liking (derived food-liking factor) \|\| id:ebi-fl187-GCST90094729 | Weighted median | 15 | -0.006860148 | 0.0705040718925427 | 0.922487009869139 | -0.145048129 | 0.131327832957105 | 0.993163329146551 | 0.864980661574309 | 1.14034156158614 | 0.999332001504091 |
| ebi-fl187-GCST90094729 | ebi-met1400-GCST90200973 | Proline to glutamate ratio \|\| id:ebi-met1400-GCST90200973 | F-cheese liking (derived food-liking factor) \|\| id:ebi-fl187-GCST90094729 | Weighted median | 15 | -0.058906913 | 0.0698907638744439 | 0.399316480951446 | -0.19589281 | 0.078078984253558 | 0.942794527042849 | 0.822100350781749 | 1.08120805370867 | 0.999332001504091 |
| ebi-fl187-GCST90094729 | ebi-met1400-GCST90200983 | Cholesterol to linoleoyl-arachidonoyl-glycerol (18:2 to 20:4) [1] ratio \|\| id:ebi-met1400-GCST90200983 | F-cheese liking (derived food-liking factor) \|\| id:ebi-fl187-GCST90094729 | Weighted median | 15 | -0.091535445 | 0.076004409532359 | 0.228456695413362 | -0.240504088 | 0.0574331976373615 | 0.912528970947368 | 0.786231431540143 | 1.05911451693946 | 0.999332001504091 |
| ebi-fl187-GCST90094729 | ebi-met1400-GCST90201001 | Serine to alpha-ketobutyrate ratio \|\| id:ebi-met1400-GCST90201001 | F-cheese liking (derived food-liking factor) \|\| id:ebi-fl187-GCST90094729 | Weighted median | 15 | -0.050599567 | 0.075395475793584 | 0.502142774387644 | -0.1983747 | 0.097175565482746 | 0.950659269599353 | 0.820062518441647 | 1.10205383925187 | 0.999332001504091 |
| ebi-fl187-GCST90094729 | ebi-met1400-GCST90201005 | Fructose to maltose ratio \|\| id:ebi-met1400-GCST90201005 | F-cheese liking (derived food-liking factor) \|\| id:ebi-fl187-GCST90094729 | Weighted median | 15 | -0.103393753 | 0.0879757336206837 | 0.239893473559355 | -0.275826191 | 0.0690386845917932 | 0.901771827928863 | 0.758944830587528 | 1.07147765802244 | 0.999332001504091 |
| ebi-fl187-GCST90094729 | ebi-met1400-GCST90201020 | Paraxanthine to 5-acetylamino-6-formylamino-3-methyluracil ratio \|\| id:ebi-met1400-GCST90201020 | F-cheese liking (derived food-liking factor) \|\| id:ebi-fl187-GCST90094729 | Weighted median | 15 | -0.030049263 | 0.0763581174239718 | 0.693927557434282 | -0.179711173 | 0.119612647110685 | 0.970397727628622 | 0.835511494683711 | 1.12706019699139 | 0.999332001504091 |
| ebi-fl187-GCST90094729 | ebi-pw11-GCST90275197 | Placental weight (fetal genotype, adjusted for sex) \|\| id:ebi-pw11-GCST90275197 | F-cheese liking (derived food-liking factor) \|\| id:ebi-fl187-GCST90094729 | Weighted median | 15 | 0.0493158985818366 | 0.0271448400384971 | 0.0692525213667702 | -0.003887988 | 0.102519785057291 | 1.05055216626671 | 0.996119560545397 | 1.10795922272965 | 0.999332001504091 |
| ebi-fl187-GCST90094729 | prot-a-1017 | FAS-associated factor 2 \|\| id:prot-a-1017 | F-cheese liking (derived food-liking factor) \|\| id:ebi-fl187-GCST90094729 | Weighted median | 16 | 0.00780754551189355 | 0.113062644603191 | 0.94494580796769 | -0.213795238 | 0.229410328934149 | 1.00783810387218 | 0.807513716291275 | 1.25785806869228 | 0.999332001504091 |
| ebi-fl187-GCST90094729 | prot-a-1019 | Fas apoptotic inhibitory molecule 3 \|\| id:prot-a-1019 | F-cheese liking (derived food-liking factor) \|\| id:ebi-fl187-GCST90094729 | Weighted median | 16 | -0.06391253 | 0.115536404437498 | 0.580139555889205 | -0.290363883 | 0.162538822447823 | 0.938087050182266 | 0.747991336759415 | 1.17649399193872 | 0.999332001504091 |
| ebi-fl187-GCST90094729 | prot-a-1052 | MIP18 family protein FAM96A \|\| id:prot-a-1052 | F-cheese liking (derived food-liking factor) \|\| id:ebi-fl187-GCST90094729 | Weighted median | 16 | -0.047381892 | 0.115724879398909 | 0.682219974335961 | -0.274202656 | 0.179438871213583 | 0.953723108420459 | 0.760178004993084 | 1.19654575844175 | 0.999332001504091 |
| ebi-fl187-GCST90094729 | prot-a-1066 | F-box/LRR-repeat protein 4 \|\| id:prot-a-1066 | F-cheese liking (derived food-liking factor) \|\| id:ebi-fl187-GCST90094729 | Weighted median | 16 | 0.0298400765581034 | 0.117846700488068 | 0.800105194993947 | -0.201139456 | 0.260819609514717 | 1.03028975329423 | 0.817798376384905 | 1.29799349863648 | 0.999332001504091 |
| ebi-fl187-GCST90094729 | prot-a-1076 | Low affinity immunoglobulin gamma Fc region receptor III-B \|\| id:prot-a-1076 | F-cheese liking (derived food-liking factor) \|\| id:ebi-fl187-GCST90094729 | Weighted median | 16 | -0.144719061 | 0.118872059929972 | 0.223438577770971 | -0.377708299 | 0.0882701763391365 | 0.865265345641675 | 0.685430412527456 | 1.09228319123995 | 0.999332001504091 |
| ebi-fl187-GCST90094729 | prot-a-1081 | Fc receptor-like protein 3 \|\| id:prot-a-1081 | F-cheese liking (derived food-liking factor) \|\| id:ebi-fl187-GCST90094729 | Weighted median | 16 | -0.1442135 | 0.11658942945773 | 0.216111359998637 | -0.372728782 | 0.0843017813627113 | 0.865702900433992 | 0.688852036505331 | 1.08795716946977 | 0.999332001504091 |
| ebi-fl187-GCST90094729 | prot-a-1105 | Fibroblast growth factor receptor 4 \|\| id:prot-a-1105 | F-cheese liking (derived food-liking factor) \|\| id:ebi-fl187-GCST90094729 | Weighted median | 16 | 0.00630493229426306 | 0.126513287816079 | 0.960252977159274 | -0.241661112 | 0.254270976413778 | 1.00632485021826 | 0.785322268891972 | 1.28952118675514 | 0.999332001504091 |
| ebi-fl187-GCST90094729 | prot-a-1143 | Follistatin-related protein 1 \|\| id:prot-a-1143 | F-cheese liking (derived food-liking factor) \|\| id:ebi-fl187-GCST90094729 | Weighted median | 16 | -0.197273997 | 0.111299579630261 | 0.0763183127925011 | -0.415421173 | 0.0208731790227595 | 0.820965660321923 | 0.660062221683109 | 1.02109254747106 | 0.999332001504091 |
| ebi-fl187-GCST90094729 | prot-a-1151 | Alpha-(1,3)-fucosyltransferase 10 \|\| id:prot-a-1151 | F-cheese liking (derived food-liking factor) \|\| id:ebi-fl187-GCST90094729 | Weighted median | 16 | 0.0113990915889836 | 0.111038753996949 | 0.918233871222842 | -0.206236866 | 0.229035049423004 | 1.0114643088036 | 0.813640329526556 | 1.25738610889513 | 0.999332001504091 |
| ebi-fl187-GCST90094729 | prot-a-1165 | Growth arrest and DNA damage-inducible proteins-interacting protein 1 \|\| id:prot-a-1165 | F-cheese liking (derived food-liking factor) \|\| id:ebi-fl187-GCST90094729 | Weighted median | 16 | 0.0192268076531589 | 0.122440009210879 | 0.875220863628056 | -0.22075561 | 0.259209225706482 | 1.01941283303148 | 0.801912635452599 | 1.29590491308663 | 0.999332001504091 |
| ebi-fl187-GCST90094729 | prot-a-1194 | Growth/differentiation factor 11/8 \|\| id:prot-a-1194 | F-cheese liking (derived food-liking factor) \|\| id:ebi-fl187-GCST90094729 | Weighted median | 16 | -0.228991306 | 0.124295473025234 | 0.0654292147211179 | -0.472610433 | 0.0146278214086764 | 0.795335448343494 | 0.623372869473465 | 1.01473533156173 | 0.999332001504091 |
| ebi-fl187-GCST90094729 | prot-a-1216 | D-glucuronyl C5-epimerase \|\| id:prot-a-1216 | F-cheese liking (derived food-liking factor) \|\| id:ebi-fl187-GCST90094729 | Weighted median | 16 | -0.086183276 | 0.116976867988597 | 0.461271438947822 | -0.315457937 | 0.143091385356919 | 0.917426073720998 | 0.729454753334792 | 1.15383524049343 | 0.999332001504091 |
| ebi-fl187-GCST90094729 | prot-a-1246 | Glypican-3 \|\| id:prot-a-1246 | F-cheese liking (derived food-liking factor) \|\| id:ebi-fl187-GCST90094729 | Weighted median | 16 | -0.02250152 | 0.120901565785861 | 0.852355063095227 | -0.259468589 | 0.214465548639688 | 0.977749750718674 | 0.771461439802861 | 1.23919942813308 | 0.999332001504091 |
| ebi-fl187-GCST90094729 | prot-a-1275 | Glutamate receptor 4 \|\| id:prot-a-1275 | F-cheese liking (derived food-liking factor) \|\| id:ebi-fl187-GCST90094729 | Weighted median | 16 | -0.028923667 | 0.116500025423903 | 0.803924140790407 | -0.257263717 | 0.199416382491218 | 0.971490618099799 | 0.773164290178632 | 1.2206901340954 | 0.999332001504091 |
| ebi-fl187-GCST90094729 | prot-a-1289 | Glutathione S-transferase P \|\| id:prot-a-1289 | F-cheese liking (derived food-liking factor) \|\| id:ebi-fl187-GCST90094729 | Weighted median | 16 | 0.117580847855485 | 0.116128571297634 | 0.311296291917114 | -0.110031152 | 0.345192847598847 | 1.12477256171739 | 0.895806228806661 | 1.41226224479104 | 0.999332001504091 |
| ebi-fl187-GCST90094729 | prot-a-130 | Apolipoprotein D \|\| id:prot-a-130 | F-cheese liking (derived food-liking factor) \|\| id:ebi-fl187-GCST90094729 | Weighted median | 16 | 0.150366491358947 | 0.11229990673907 | 0.180579612587386 | -0.069741326 | 0.370474308567524 | 1.16226012297472 | 0.932635037282127 | 1.44842145046774 | 0.999332001504091 |
| ebi-fl187-GCST90094729 | prot-a-1313 | Heparin-binding EGF-like growth factor \|\| id:prot-a-1313 | F-cheese liking (derived food-liking factor) \|\| id:ebi-fl187-GCST90094729 | Weighted median | 16 | 0.0466068941673375 | 0.114904453367189 | 0.685025989733145 | -0.178605834 | 0.271819622767029 | 1.04771006717823 | 0.83643552851344 | 1.31235026185162 | 0.999332001504091 |
| ebi-fl187-GCST90094729 | prot-a-1347 | HLA class II histocompatibility antigen, DQ alpha 2 chain \|\| id:prot-a-1347 | F-cheese liking (derived food-liking factor) \|\| id:ebi-fl187-GCST90094729 | Weighted median | 16 | -0.184608788 | 0.108574481995206 | 0.0890752102219297 | -0.397414772 | 0.028197197098083 | 0.831429485750614 | 0.672055217911791 | 1.02859850106284 | 0.999332001504091 |
| ebi-fl187-GCST90094729 | prot-a-1357 | Non-histone chromosomal protein HMG-14 \|\| id:prot-a-1357 | F-cheese liking (derived food-liking factor) \|\| id:ebi-fl187-GCST90094729 | Weighted median | 16 | 0.107953121339393 | 0.113411339011291 | 0.341161707929759 | -0.114333103 | 0.330239345801524 | 1.11399552154441 | 0.891960791466961 | 1.39130109069035 | 0.999332001504091 |
| ebi-fl187-GCST90094729 | prot-a-137 | Apolipoprotein M \|\| id:prot-a-137 | F-cheese liking (derived food-liking factor) \|\| id:ebi-fl187-GCST90094729 | Weighted median | 16 | -0.0469463 | 0.110130540978334 | 0.669904800624778 | -0.262802161 | 0.168909560091055 | 0.954138633243199 | 0.768893999835361 | 1.18401305204896 | 0.999332001504091 |
| ebi-fl187-GCST90094729 | prot-a-1381 | 17-beta-hydroxysteroid dehydrogenase 14 \|\| id:prot-a-1381 | F-cheese liking (derived food-liking factor) \|\| id:ebi-fl187-GCST90094729 | Weighted median | 16 | -0.039556752 | 0.11086334893466 | 0.721236747536678 | -0.256848916 | 0.177735411903434 | 0.961215401537993 | 0.773485066217241 | 1.19450922649662 | 0.999332001504091 |
| ebi-fl187-GCST90094729 | prot-a-1392 | Serine protease HTRA2, mitochondrial \|\| id:prot-a-1392 | F-cheese liking (derived food-liking factor) \|\| id:ebi-fl187-GCST90094729 | Weighted median | 16 | 0.099964825038987 | 0.123899406835918 | 0.419768773394552 | -0.142878012 | 0.342807662437386 | 1.10513204441539 | 0.866859808628663 | 1.40889775190501 | 0.999332001504091 |
| ebi-fl187-GCST90094729 | prot-a-1416 | Interferon-induced protein with tetratricopeptide repeats 2 \|\| id:prot-a-1416 | F-cheese liking (derived food-liking factor) \|\| id:ebi-fl187-GCST90094729 | Weighted median | 16 | -0.022515682 | 0.119413424890853 | 0.850443681281604 | -0.256565995 | 0.211534630793958 | 0.977735904226371 | 0.773703932455171 | 1.23557275375326 | 0.999332001504091 |
| ebi-fl187-GCST90094729 | prot-a-1428 | Interferon gamma \|\| id:prot-a-1428 | F-cheese liking (derived food-liking factor) \|\| id:ebi-fl187-GCST90094729 | Weighted median | 16 | 0.0984022148053769 | 0.10953489335853 | 0.368991474500613 | -0.116286176 | 0.313090605788095 | 1.10340650229929 | 0.890220426962112 | 1.3676454420071 | 0.999332001504091 |
| ebi-fl187-GCST90094729 | prot-a-1483 | Interleukin-17C \|\| id:prot-a-1483 | F-cheese liking (derived food-liking factor) \|\| id:ebi-fl187-GCST90094729 | Weighted median | 16 | -0.100318664 | 0.117448466403044 | 0.393021616004319 | -0.330517658 | 0.129880329975475 | 0.904549124703911 | 0.718551672885094 | 1.13869210785827 | 0.999332001504091 |
| ebi-fl187-GCST90094729 | prot-a-1503 | Interleukin-1 receptor-like 2 \|\| id:prot-a-1503 | F-cheese liking (derived food-liking factor) \|\| id:ebi-fl187-GCST90094729 | Weighted median | 16 | -0.00742611 | 0.113820810159281 | 0.947979833632239 | -0.230514898 | 0.215662677960476 | 0.992601395474573 | 0.794124604153828 | 1.24068379841713 | 0.999332001504091 |
| ebi-fl187-GCST90094729 | prot-a-1522 | Interleukin-31 receptor subunit alpha \|\| id:prot-a-1522 | F-cheese liking (derived food-liking factor) \|\| id:ebi-fl187-GCST90094729 | Weighted median | 16 | -0.109417139 | 0.115675596695554 | 0.344201390539388 | -0.336141309 | 0.11730703012734 | 0.896356433920619 | 0.714522130331853 | 1.12446462121157 | 0.999332001504091 |
| ebi-fl187-GCST90094729 | prot-a-1540 | Interleukin-6 receptor subunit alpha \|\| id:prot-a-1540 | F-cheese liking (derived food-liking factor) \|\| id:ebi-fl187-GCST90094729 | Weighted median | 16 | 0.011535517180364 | 0.118010777057953 | 0.922131055478805 | -0.219765606 | 0.242836640213953 | 1.01160230783318 | 0.802706925718559 | 1.27486034619341 | 0.999332001504091 |
| ebi-fl187-GCST90094729 | prot-a-1541 | Interleukin-6 receptor subunit alpha \|\| id:prot-a-1541 | F-cheese liking (derived food-liking factor) \|\| id:ebi-fl187-GCST90094729 | Weighted median | 16 | -0.091197299 | 0.120232145008825 | 0.448145656857025 | -0.326852303 | 0.144457705455163 | 0.912837591404359 | 0.721190252812427 | 1.15541282627073 | 0.999332001504091 |
| ebi-fl187-GCST90094729 | prot-a-1545 | Interleukin-9 \|\| id:prot-a-1545 | F-cheese liking (derived food-liking factor) \|\| id:ebi-fl187-GCST90094729 | Weighted median | 16 | -0.021971238 | 0.126062427321767 | 0.861638696546395 | -0.269053595 | 0.225111119761585 | 0.978268371807819 | 0.764102302228268 | 1.25246188172539 | 0.999332001504091 |
| ebi-fl187-GCST90094729 | prot-a-1548 | Inositol monophosphatase 3 \|\| id:prot-a-1548 | F-cheese liking (derived food-liking factor) \|\| id:ebi-fl187-GCST90094729 | Weighted median | 16 | -0.067997816 | 0.114171861023942 | 0.55145971188697 | -0.291774663 | 0.155779032030516 | 0.934262514400061 | 0.746936829378677 | 1.16856795847004 | 0.999332001504091 |
| ebi-fl187-GCST90094729 | prot-a-1555 | Inhibin beta A chain:Inhibin beta B chain heterodimer \|\| id:prot-a-1555 | F-cheese liking (derived food-liking factor) \|\| id:ebi-fl187-GCST90094729 | Weighted median | 16 | -0.14715256 | 0.11088312699977 | 0.18447691197397 | -0.364483489 | 0.0701783691207765 | 0.863162283506697 | 0.694555303896052 | 1.07269950065776 | 0.999332001504091 |
| ebi-fl187-GCST90094729 | prot-a-1603 | Junctophilin-3 \|\| id:prot-a-1603 | F-cheese liking (derived food-liking factor) \|\| id:ebi-fl187-GCST90094729 | Weighted median | 16 | -0.128399331 | 0.111886306077502 | 0.251138874441899 | -0.347696491 | 0.0908978287580356 | 0.87950209642325 | 0.706313216057147 | 1.09515710598045 | 0.999332001504091 |
| ebi-fl187-GCST90094729 | prot-a-1627 | UPF0258 protein KIAA1024 \|\| id:prot-a-1627 | F-cheese liking (derived food-liking factor) \|\| id:ebi-fl187-GCST90094729 | Weighted median | 16 | -0.048403977 | 0.121411680261533 | 0.690131626812802 | -0.28637087 | 0.189562916351155 | 0.952748820750346 | 0.750984046634356 | 1.20872116992272 | 0.999332001504091 |
| ebi-fl187-GCST90094729 | prot-a-1666 | Kallikrein-7 \|\| id:prot-a-1666 | F-cheese liking (derived food-liking factor) \|\| id:ebi-fl187-GCST90094729 | Weighted median | 16 | -0.14854513 | 0.119398425058364 | 0.213457985159078 | -0.382566043 | 0.0854757829173341 | 0.86196110581813 | 0.682108840762221 | 1.0892351829262 | 0.999332001504091 |
| ebi-fl187-GCST90094729 | prot-a-1678 | Importin subunit alpha-3 \|\| id:prot-a-1678 | F-cheese liking (derived food-liking factor) \|\| id:ebi-fl187-GCST90094729 | Weighted median | 16 | 0.0729351088525402 | 0.117733275157604 | 0.535590686436339 | -0.15782211 | 0.303692328161443 | 1.07566073379063 | 0.854001686427238 | 1.35485214210708 | 0.999332001504091 |
| ebi-fl187-GCST90094729 | prot-a-1718 | Protein LDOC1 \|\| id:prot-a-1718 | F-cheese liking (derived food-liking factor) \|\| id:ebi-fl187-GCST90094729 | Weighted median | 16 | 0.0724752786352165 | 0.113703931031363 | 0.523862077619109 | -0.150384426 | 0.295334983456688 | 1.07516622618513 | 0.860377161331268 | 1.34357635916383 | 0.999332001504091 |
| ebi-fl187-GCST90094729 | prot-a-1723 | Leptin \|\| id:prot-a-1723 | F-cheese liking (derived food-liking factor) \|\| id:ebi-fl187-GCST90094729 | Weighted median | 16 | 0.068515676562694 | 0.114480678448916 | 0.54951224096179 | -0.155866453 | 0.292897806322569 | 1.07091741312336 | 0.855673455195196 | 1.34030581265279 | 0.999332001504091 |
| ebi-fl187-GCST90094729 | prot-a-1730 | Galectin-7 \|\| id:prot-a-1730 | F-cheese liking (derived food-liking factor) \|\| id:ebi-fl187-GCST90094729 | Weighted median | 16 | -0.061974761 | 0.123311507620439 | 0.61525467176613 | -0.303665315 | 0.17971579437522 | 0.939906609207396 | 0.738107858377914 | 1.19687715555987 | 0.999332001504091 |
| ebi-fl187-GCST90094729 | prot-a-1753 | Lipase member N \|\| id:prot-a-1753 | F-cheese liking (derived food-liking factor) \|\| id:ebi-fl187-GCST90094729 | Weighted median | 16 | -0.150665817 | 0.115387683756285 | 0.191642457558284 | -0.376825677 | 0.0754940436581718 | 0.86013509358769 | 0.686035655495174 | 1.0784168042798 | 0.999332001504091 |
| ebi-fl187-GCST90094729 | prot-a-1756 | VIP36-like protein \|\| id:prot-a-1756 | F-cheese liking (derived food-liking factor) \|\| id:ebi-fl187-GCST90094729 | Weighted median | 16 | 0.0181259602094212 | 0.109119608447114 | 0.868069648657109 | -0.195748472 | 0.232000392765764 | 1.01829123248894 | 0.822219019491336 | 1.26112022415308 | 0.999332001504091 |
| ebi-fl187-GCST90094729 | prot-a-1760 | Ig Kappa chain V-I region HK102- like \|\| id:prot-a-1760 | F-cheese liking (derived food-liking factor) \|\| id:ebi-fl187-GCST90094729 | Weighted median | 16 | -0.096221258 | 0.111868029704519 | 0.389716352097925 | -0.315482596 | 0.123040080034859 | 0.908263033203838 | 0.729436765750558 | 1.13092974774285 | 0.999332001504091 |
| ebi-fl187-GCST90094729 | prot-a-179 | Neutral ceramidase \|\| id:prot-a-179 | F-cheese liking (derived food-liking factor) \|\| id:ebi-fl187-GCST90094729 | Weighted median | 16 | 0.037879165915261 | 0.123081083285862 | 0.758266619250052 | -0.203359757 | 0.27911808915555 | 1.03860572632357 | 0.815984632166204 | 1.32196344419927 | 0.999332001504091 |
| ebi-fl187-GCST90094729 | prot-a-1809 | Lactotransferrin \|\| id:prot-a-1809 | F-cheese liking (derived food-liking factor) \|\| id:ebi-fl187-GCST90094729 | Weighted median | 16 | -0.140599441 | 0.115392698350917 | 0.223055622920068 | -0.36676913 | 0.0855702479969485 | 0.868837262789745 | 0.692969612791976 | 1.0893380824746 | 0.999332001504091 |
| ebi-fl187-GCST90094729 | prot-a-1818 | Lysophospholipase-like protein 1 \|\| id:prot-a-1818 | F-cheese liking (derived food-liking factor) \|\| id:ebi-fl187-GCST90094729 | Weighted median | 16 | -0.045052804 | 0.116495642098603 | 0.698953277483677 | -0.273384263 | 0.183278654085445 | 0.955947002265862 | 0.760800384059453 | 1.20114906654631 | 0.999332001504091 |
| ebi-fl187-GCST90094729 | prot-a-1852 | MAP kinase-activated protein kinase 3 \|\| id:prot-a-1852 | F-cheese liking (derived food-liking factor) \|\| id:ebi-fl187-GCST90094729 | Weighted median | 16 | 0.175041107138255 | 0.117771309654991 | 0.137205153098268 | -0.05579066 | 0.405872874062038 | 1.19129518634178 | 0.945737095975675 | 1.50061177365257 | 0.999332001504091 |
| ebi-fl187-GCST90094729 | prot-a-1856 | Megakaryocyte-associated tyrosine-protein kinase \|\| id:prot-a-1856 | F-cheese liking (derived food-liking factor) \|\| id:ebi-fl187-GCST90094729 | Weighted median | 16 | -0.121833688 | 0.111756946670573 | 0.275639771645593 | -0.340877304 | 0.0972099270462779 | 0.885295591161282 | 0.71114615771211 | 1.1020917081955 | 0.999332001504091 |
| ebi-fl187-GCST90094729 | prot-a-1890 | Beta-1,3-N-acetylglucosaminyltransferase manic fringe \|\| id:prot-a-1890 | F-cheese liking (derived food-liking factor) \|\| id:ebi-fl187-GCST90094729 | Weighted median | 16 | -0.016863456 | 0.109541389847483 | 0.87765233579168 | -0.23156458 | 0.197837668377804 | 0.983277936443707 | 0.793291463225521 | 1.21876453373877 | 0.999332001504091 |
| ebi-fl187-GCST90094729 | prot-a-1919 | Matrilysin \|\| id:prot-a-1919 | F-cheese liking (derived food-liking factor) \|\| id:ebi-fl187-GCST90094729 | Weighted median | 16 | 0.0614301406078892 | 0.125153691190615 | 0.623541295990328 | -0.183871094 | 0.306731375341494 | 1.06335620851565 | 0.832043052151134 | 1.35897586460873 | 0.999332001504091 |
| ebi-fl187-GCST90094729 | prot-a-1923 | Multimerin-2 \|\| id:prot-a-1923 | F-cheese liking (derived food-liking factor) \|\| id:ebi-fl187-GCST90094729 | Weighted median | 16 | 0.0996000835367128 | 0.123970186150593 | 0.421732221381445 | -0.143381481 | 0.342581648391876 | 1.10472903039573 | 0.866423481470987 | 1.40857935720658 | 0.999332001504091 |
| ebi-fl187-GCST90094729 | prot-a-1948 | Mesothelin \|\| id:prot-a-1948 | F-cheese liking (derived food-liking factor) \|\| id:ebi-fl187-GCST90094729 | Weighted median | 16 | 0.108029713170988 | 0.12559288378086 | 0.38970199938047 | -0.138132339 | 0.354191765381473 | 1.1140808477694 | 0.870983419009336 | 1.42502843140035 | 0.999332001504091 |
| ebi-fl187-GCST90094729 | prot-a-195 | ATPase family AAA domain-containing protein 1 \|\| id:prot-a-195 | F-cheese liking (derived food-liking factor) \|\| id:ebi-fl187-GCST90094729 | Weighted median | 16 | 0.032061052905754 | 0.117405922494217 | 0.784792744372335 | -0.198054555 | 0.262176660994419 | 1.03258054542343 | 0.820325098931236 | 1.29975613836037 | 0.999332001504091 |
| ebi-fl187-GCST90094729 | prot-a-1966 | Mucin-1 \|\| id:prot-a-1966 | F-cheese liking (derived food-liking factor) \|\| id:ebi-fl187-GCST90094729 | Weighted median | 16 | -0.057874078 | 0.119283512916648 | 0.627548053665078 | -0.291669763 | 0.175921607241158 | 0.943768781135987 | 0.74701518700592 | 1.19234458380543 | 0.999332001504091 |
| ebi-fl187-GCST90094729 | prot-a-1986 | Myocardial zonula adherens protein \|\| id:prot-a-1986 | F-cheese liking (derived food-liking factor) \|\| id:ebi-fl187-GCST90094729 | Weighted median | 16 | -0.074077167 | 0.116456113923438 | 0.524714418208055 | -0.30233115 | 0.154176816128979 | 0.928600033648152 | 0.739093273289639 | 1.16669715941716 | 0.999332001504091 |
| ebi-fl187-GCST90094729 | prot-a-2071 | Epididymal secretory protein E1 \|\| id:prot-a-2071 | F-cheese liking (derived food-liking factor) \|\| id:ebi-fl187-GCST90094729 | Weighted median | 16 | -0.25449264 | 0.129031716745869 | 0.048572376669879 | -0.507394805 | -0.001590475 | 0.775309759353497 | 0.602062026566785 | 0.998410788962286 | 0.999332001504091 |
| ebi-fl187-GCST90094729 | prot-a-2098 | Neuritin \|\| id:prot-a-2098 | F-cheese liking (derived food-liking factor) \|\| id:ebi-fl187-GCST90094729 | Weighted median | 16 | 0.0669834303730812 | 0.114687799020908 | 0.559186547669186 | -0.157804656 | 0.291771516454062 | 1.06927776049425 | 0.854016592941956 | 1.33879708958454 | 0.999332001504091 |
| ebi-fl187-GCST90094729 | prot-a-2115 | Neurotrophin-4 \|\| id:prot-a-2115 | F-cheese liking (derived food-liking factor) \|\| id:ebi-fl187-GCST90094729 | Weighted median | 16 | -0.060464039 | 0.116270002974299 | 0.603041769435505 | -0.288353245 | 0.167425166665967 | 0.941327619338221 | 0.749496789483303 | 1.18225681465538 | 0.999332001504091 |
| ebi-fl187-GCST90094729 | prot-a-2118 | Netrin-1 \|\| id:prot-a-2118 | F-cheese liking (derived food-liking factor) \|\| id:ebi-fl187-GCST90094729 | Weighted median | 16 | 0.0564600137717574 | 0.120909236575256 | 0.640527096143821 | -0.18052209 | 0.293442117459259 | 1.0580843051538 | 0.834834239075256 | 1.34103555461851 | 0.999332001504091 |
| ebi-fl187-GCST90094729 | prot-a-2120 | Netrin-G1 \|\| id:prot-a-2120 | F-cheese liking (derived food-liking factor) \|\| id:ebi-fl187-GCST90094729 | Weighted median | 16 | -0.080696082 | 0.110757681126994 | 0.466257229322287 | -0.297781137 | 0.136388972657647 | 0.922474004976951 | 0.742463819558273 | 1.14612761920775 | 0.999332001504091 |
| ebi-fl187-GCST90094729 | prot-a-2145 | Olfactomedin-like protein 3 \|\| id:prot-a-2145 | F-cheese liking (derived food-liking factor) \|\| id:ebi-fl187-GCST90094729 | Weighted median | 16 | -0.195150449 | 0.121888718163594 | 0.10936490682193 | -0.434052336 | 0.0437514389091583 | 0.822710872970291 | 0.647878347047603 | 1.0447226452126 | 0.999332001504091 |
| ebi-fl187-GCST90094729 | prot-a-2162 | Prolyl 4-hydroxylase subunit alpha-2 \|\| id:prot-a-2162 | F-cheese liking (derived food-liking factor) \|\| id:ebi-fl187-GCST90094729 | Weighted median | 16 | -0.121906405 | 0.113285681270617 | 0.281883783660856 | -0.343946341 | 0.100133529828669 | 0.885231217432511 | 0.708966969663202 | 1.10531850121214 | 0.999332001504091 |
| ebi-fl187-GCST90094729 | prot-a-2168 | Polyadenylate-binding protein-interacting protein 1 \|\| id:prot-a-2168 | F-cheese liking (derived food-liking factor) \|\| id:ebi-fl187-GCST90094729 | Weighted median | 16 | 0.0616673509355952 | 0.124729237786001 | 0.621016831501693 | -0.182801955 | 0.306136656996157 | 1.06360847750956 | 0.832933097534825 | 1.35816789701157 | 0.999332001504091 |
| ebi-fl187-GCST90094729 | prot-a-219 | Beta-1,4-galactosyltransferase 5 \|\| id:prot-a-219 | F-cheese liking (derived food-liking factor) \|\| id:ebi-fl187-GCST90094729 | Weighted median | 16 | -0.141602609 | 0.121710014252429 | 0.244649996735356 | -0.380154237 | 0.0969490193113653 | 0.86796611020781 | 0.683755940916086 | 1.10180420145224 | 0.999332001504091 |
| ebi-fl187-GCST90094729 | prot-a-2190 | Pyruvate carboxylase, mitochondrial \|\| id:prot-a-2190 | F-cheese liking (derived food-liking factor) \|\| id:ebi-fl187-GCST90094729 | Weighted median | 16 | -0.176186637 | 0.11899029230508 | 0.138691661034004 | -0.40940761 | 0.057034335447674 | 0.838461480403241 | 0.664043506119222 | 1.05869216044073 | 0.999332001504091 |
| ebi-fl187-GCST90094729 | prot-a-2197 | Protocadherin-9 \|\| id:prot-a-2197 | F-cheese liking (derived food-liking factor) \|\| id:ebi-fl187-GCST90094729 | Weighted median | 16 | -0.06433762 | 0.114108980241663 | 0.572872493929116 | -0.287991221 | 0.159315981076664 | 0.937688363552364 | 0.749768174072071 | 1.17270844181897 | 0.999332001504091 |
| ebi-fl187-GCST90094729 | prot-a-2198 | Protocadherin alpha-4 \|\| id:prot-a-2198 | F-cheese liking (derived food-liking factor) \|\| id:ebi-fl187-GCST90094729 | Weighted median | 16 | 0.00398350405012543 | 0.127636917710655 | 0.975102340820202 | -0.246184855 | 0.254151862763009 | 1.00399144874813 | 0.781777696315091 | 1.28936759632638 | 0.999332001504091 |
| ebi-fl187-GCST90094729 | prot-a-2254 | Placenta growth factor \|\| id:prot-a-2254 | F-cheese liking (derived food-liking factor) \|\| id:ebi-fl187-GCST90094729 | Weighted median | 16 | -0.112487665 | 0.12125359209966 | 0.353560180297356 | -0.350144706 | 0.125169375471705 | 0.893608369660788 | 0.70458612481242 | 1.1333403968754 | 0.999332001504091 |
| ebi-fl187-GCST90094729 | prot-a-2261 | Membrane-associated progesterone receptor component 2 \|\| id:prot-a-2261 | F-cheese liking (derived food-liking factor) \|\| id:ebi-fl187-GCST90094729 | Weighted median | 16 | -0.053835365 | 0.119431397373768 | 0.652159705159413 | -0.287920904 | 0.18025017356684 | 0.947588099556512 | 0.749820897623601 | 1.19751691272796 | 0.999332001504091 |
| ebi-fl187-GCST90094729 | prot-a-2268 | Polymeric immunoglobulin receptor \|\| id:prot-a-2268 | F-cheese liking (derived food-liking factor) \|\| id:ebi-fl187-GCST90094729 | Weighted median | 16 | 0.135656078255542 | 0.115160426136032 | 0.238806658591747 | -0.090058357 | 0.361370513482166 | 1.14528793642852 | 0.913877852571662 | 1.43529515857903 | 0.999332001504091 |
| ebi-fl187-GCST90094729 | prot-a-2283 | Group 10 secretory phospholipase A2 \|\| id:prot-a-2283 | F-cheese liking (derived food-liking factor) \|\| id:ebi-fl187-GCST90094729 | Weighted median | 16 | 0.221297232575031 | 0.115369481127758 | 0.0550906070635852 | -0.00482695 | 0.447421415585437 | 1.24769423082346 | 0.99518468056826 | 1.56427336958324 | 0.999332001504091 |
| ebi-fl187-GCST90094729 | prot-a-2298 | Inactive phospholipase D5 \|\| id:prot-a-2298 | F-cheese liking (derived food-liking factor) \|\| id:ebi-fl187-GCST90094729 | Weighted median | 16 | 0.088471674132185 | 0.127509657500389 | 0.487780717954137 | -0.161447255 | 0.338390602832947 | 1.09250330606795 | 0.850911411971269 | 1.40268829043476 | 0.999332001504091 |
| ebi-fl187-GCST90094729 | prot-a-2304 | Procollagen-lysine,2-oxoglutarate 5-dioxygenase 2 \|\| id:prot-a-2304 | F-cheese liking (derived food-liking factor) \|\| id:ebi-fl187-GCST90094729 | Weighted median | 16 | 0.100505804684319 | 0.111976086866972 | 0.369417561369963 | -0.118967326 | 0.319978934943583 | 1.10573006009939 | 0.887836809845324 | 1.37709875536745 | 0.999332001504091 |
| ebi-fl187-GCST90094729 | prot-a-2331 | Periostin \|\| id:prot-a-2331 | F-cheese liking (derived food-liking factor) \|\| id:ebi-fl187-GCST90094729 | Weighted median | 16 | -0.235779785 | 0.124371499698717 | 0.0579904352350534 | -0.479547925 | 0.00798835395330774 | 0.789954614275026 | 0.619063191631623 | 1.0080203459839 | 0.999332001504091 |
| ebi-fl187-GCST90094729 | prot-a-2333 | Periostin \|\| id:prot-a-2333 | F-cheese liking (derived food-liking factor) \|\| id:ebi-fl187-GCST90094729 | Weighted median | 16 | -0.195230193 | 0.118478322928805 | 0.0993909249861073 | -0.427447706 | 0.0369873197402861 | 0.822645268911752 | 0.652171505669429 | 1.03767986270457 | 0.999332001504091 |
| ebi-fl187-GCST90094729 | prot-a-2367 | cAMP-dependent protein kinase catalytic subunit alpha \|\| id:prot-a-2367 | F-cheese liking (derived food-liking factor) \|\| id:ebi-fl187-GCST90094729 | Weighted median | 16 | 0.141071080795239 | 0.11118578026945 | 0.204517016614422 | -0.076853049 | 0.35899521012336 | 1.15150649507322 | 0.926025924476534 | 1.43188994297904 | 0.999332001504091 |
| ebi-fl187-GCST90094729 | prot-a-2385 | Phosphoribosyl pyrophosphate synthase-associated protein 1 \|\| id:prot-a-2385 | F-cheese liking (derived food-liking factor) \|\| id:ebi-fl187-GCST90094729 | Weighted median | 16 | 0.0981000522259848 | 0.116755644545348 | 0.400786849231822 | -0.130741011 | 0.326941115534868 | 1.1030731445111 | 0.877444993494841 | 1.38671981852131 | 0.999332001504091 |
| ebi-fl187-GCST90094729 | prot-a-2393 | Trypsin-3 \|\| id:prot-a-2393 | F-cheese liking (derived food-liking factor) \|\| id:ebi-fl187-GCST90094729 | Weighted median | 16 | 0.0464921993898702 | 0.11407242987681 | 0.683591329571433 | -0.177089763 | 0.270074161948419 | 1.04758990719622 | 0.837704586129954 | 1.31006160385176 | 0.999332001504091 |
| ebi-fl187-GCST90094729 | prot-a-2405 | Pregnancy-specific beta-1-glycoprotein 6 \|\| id:prot-a-2405 | F-cheese liking (derived food-liking factor) \|\| id:ebi-fl187-GCST90094729 | Weighted median | 16 | -0.021667983 | 0.111757098288662 | 0.846266221744805 | -0.240711896 | 0.197375929411779 | 0.978565081134613 | 0.786068063215877 | 1.21820191256519 | 0.999332001504091 |
| ebi-fl187-GCST90094729 | prot-a-244 | Beclin-1 \|\| id:prot-a-244 | F-cheese liking (derived food-liking factor) \|\| id:ebi-fl187-GCST90094729 | Weighted median | 16 | 0.0289177313557642 | 0.111802790899497 | 0.795905718783011 | -0.190215739 | 0.248051201518778 | 1.02933990859294 | 0.826780746009453 | 1.28152554656857 | 0.999332001504091 |
| ebi-fl187-GCST90094729 | prot-a-2454 | tRNA pseudouridine synthase A, mitochondrial \|\| id:prot-a-2454 | F-cheese liking (derived food-liking factor) \|\| id:ebi-fl187-GCST90094729 | Weighted median | 16 | 0.105072701645107 | 0.120105331128229 | 0.381662085086143 | -0.130333747 | 0.340479150656436 | 1.11079136377973 | 0.877802417782102 | 1.40562093342733 | 0.999332001504091 |
| ebi-fl187-GCST90094729 | prot-a-2466 | Glutaminyl-peptide cyclotransferase-like protein \|\| id:prot-a-2466 | F-cheese liking (derived food-liking factor) \|\| id:ebi-fl187-GCST90094729 | Weighted median | 16 | -0.140639891 | 0.112098299129959 | 0.209619581205479 | -0.360352557 | 0.0790727753044175 | 0.868802118842591 | 0.697430398554003 | 1.08228308268517 | 0.999332001504091 |
| ebi-fl187-GCST90094729 | prot-a-2474 | Ras-related protein Rab-27A \|\| id:prot-a-2474 | F-cheese liking (derived food-liking factor) \|\| id:ebi-fl187-GCST90094729 | Weighted median | 16 | 0.00307077877992942 | 0.114238341800212 | 0.978555081320989 | -0.220836371 | 0.226977928708344 | 1.00307549845087 | 0.801847875003159 | 1.25480217252992 | 0.999332001504091 |
| ebi-fl187-GCST90094729 | prot-a-2490 | Rap1 GTPase-GDP dissociation stimulator 1 \|\| id:prot-a-2490 | F-cheese liking (derived food-liking factor) \|\| id:ebi-fl187-GCST90094729 | Weighted median | 16 | 0.0988116995428635 | 0.116343212169315 | 0.395707641553227 | -0.129220996 | 0.326844395394721 | 1.10385842294221 | 0.878779737006182 | 1.38658570127214 | 0.999332001504091 |
| ebi-fl187-GCST90094729 | prot-a-2545 | Receptor-interacting serine/threonine-protein kinase 2 \|\| id:prot-a-2545 | F-cheese liking (derived food-liking factor) \|\| id:ebi-fl187-GCST90094729 | Weighted median | 16 | 0.160599173364891 | 0.123914618196847 | 0.194959281220606 | -0.082273478 | 0.403471825030712 | 1.17421421814152 | 0.921020045254391 | 1.49701305328796 | 0.999332001504091 |
| ebi-fl187-GCST90094729 | prot-a-2610 | RUN and FYVE domain-containing protein 1 \|\| id:prot-a-2610 | F-cheese liking (derived food-liking factor) \|\| id:ebi-fl187-GCST90094729 | Weighted median | 16 | -0.247389855 | 0.119755902076946 | 0.0388488573400699 | -0.482111423 | -0.012668286 | 0.78083622163264 | 0.617478256886728 | 0.987411618487781 | 0.999332001504091 |
| ebi-fl187-GCST90094729 | prot-a-2627 | Serine--tRNA ligase, mitochondrial \|\| id:prot-a-2627 | F-cheese liking (derived food-liking factor) \|\| id:ebi-fl187-GCST90094729 | Weighted median | 16 | 0.00984847259624544 | 0.117604584721439 | 0.933261364045205 | -0.220656513 | 0.240353458650265 | 1.00989712839976 | 0.80199210648044 | 1.27169856375008 | 0.999332001504091 |
| ebi-fl187-GCST90094729 | prot-a-264 | BolA-like protein 2 \|\| id:prot-a-264 | F-cheese liking (derived food-liking factor) \|\| id:ebi-fl187-GCST90094729 | Weighted median | 16 | -0.089290373 | 0.11498741139284 | 0.437440230283844 | -0.314665699 | 0.136084953616063 | 0.914579965946921 | 0.730032884171597 | 1.14577922754896 | 0.999332001504091 |
| ebi-fl187-GCST90094729 | prot-a-2646 | Secretoglobin family 1C member 1 \|\| id:prot-a-2646 | F-cheese liking (derived food-liking factor) \|\| id:ebi-fl187-GCST90094729 | Weighted median | 16 | -0.056858778 | 0.117122386713856 | 0.627346784700751 | -0.286418656 | 0.172701099865377 | 0.944727476161361 | 0.75094816113417 | 1.1885108059473 | 0.999332001504091 |
| ebi-fl187-GCST90094729 | prot-a-2672 | Semaphorin-3E \|\| id:prot-a-2672 | F-cheese liking (derived food-liking factor) \|\| id:ebi-fl187-GCST90094729 | Weighted median | 16 | -0.059233823 | 0.112355265810062 | 0.59805392592594 | -0.279450144 | 0.160982498195241 | 0.942486368596186 | 0.756199428120263 | 1.1746644098868 | 0.999332001504091 |
| ebi-fl187-GCST90094729 | prot-a-2701 | Plasma protease C1 inhibitor \|\| id:prot-a-2701 | F-cheese liking (derived food-liking factor) \|\| id:ebi-fl187-GCST90094729 | Weighted median | 16 | 0.0015827165295907 | 0.113535432293682 | 0.988877620337271 | -0.220946731 | 0.224112163825208 | 1.00158396968644 | 0.801759388261119 | 1.25121135220949 | 0.999332001504091 |
| ebi-fl187-GCST90094729 | prot-a-2738 | Signaling threshold-regulating transmembrane adapter 1 \|\| id:prot-a-2738 | F-cheese liking (derived food-liking factor) \|\| id:ebi-fl187-GCST90094729 | Weighted median | 16 | -0.074300022 | 0.123683600573842 | 0.548022135257217 | -0.316719879 | 0.168119834991387 | 0.928393113570738 | 0.728534804255058 | 1.18307837634043 | 0.999332001504091 |
| ebi-fl187-GCST90094729 | prot-a-274 | Serine/threonine-protein kinase BRSK2 \|\| id:prot-a-274 | F-cheese liking (derived food-liking factor) \|\| id:ebi-fl187-GCST90094729 | Weighted median | 16 | -0.09865684 | 0.11055957868151 | 0.372210207517545 | -0.315353614 | 0.118039933943787 | 0.906053575780493 | 0.729530855972321 | 1.12528904769967 | 0.999332001504091 |
| ebi-fl187-GCST90094729 | prot-a-2749 | Mitochondrial glutamate carrier 2 \|\| id:prot-a-2749 | F-cheese liking (derived food-liking factor) \|\| id:ebi-fl187-GCST90094729 | Weighted median | 16 | -0.019045072 | 0.11641329656437 | 0.870047016143539 | -0.247215133 | 0.20912498959294 | 0.981135139843813 | 0.780972662513053 | 1.23259905095622 | 0.999332001504091 |
| ebi-fl187-GCST90094729 | prot-a-2760 | Sodium-coupled monocarboxylate transporter 1 \|\| id:prot-a-2760 | F-cheese liking (derived food-liking factor) \|\| id:ebi-fl187-GCST90094729 | Weighted median | 16 | -0.114682728 | 0.116633848258449 | 0.325473877160115 | -0.343285071 | 0.113919614134068 | 0.891648993890459 | 0.709435943089701 | 1.12066203587509 | 0.999332001504091 |
| ebi-fl187-GCST90094729 | prot-a-2777 | Small integral membrane protein 10 \|\| id:prot-a-2777 | F-cheese liking (derived food-liking factor) \|\| id:ebi-fl187-GCST90094729 | Weighted median | 16 | 0.104060884098643 | 0.123440061277436 | 0.399224309777155 | -0.137881636 | 0.346003404202417 | 1.10966801399575 | 0.87120180456918 | 1.41340742733447 | 0.999332001504091 |
| ebi-fl187-GCST90094729 | prot-a-2799 | Superoxide dismutase [Mn], mitochondrial \|\| id:prot-a-2799 | F-cheese liking (derived food-liking factor) \|\| id:ebi-fl187-GCST90094729 | Weighted median | 16 | 0.0152768900469322 | 0.120037351145479 | 0.898728441952206 | -0.219996318 | 0.250550098292071 | 1.01539417823681 | 0.802521752683067 | 1.28473195119033 | 0.999332001504091 |
| ebi-fl187-GCST90094729 | prot-a-2805 | Protein SPACA7 \|\| id:prot-a-2805 | F-cheese liking (derived food-liking factor) \|\| id:ebi-fl187-GCST90094729 | Weighted median | 16 | -0.035626454 | 0.117284802178601 | 0.761310596495403 | -0.265504666 | 0.19425175821324 | 0.965000698261064 | 0.766818864649804 | 1.21440198014641 | 0.999332001504091 |
| ebi-fl187-GCST90094729 | prot-a-2811 | SPARC-like protein 1 \|\| id:prot-a-2811 | F-cheese liking (derived food-liking factor) \|\| id:ebi-fl187-GCST90094729 | Weighted median | 16 | -0.123681159 | 0.113774256482986 | 0.277003629816345 | -0.346678702 | 0.0993163836868006 | 0.883661543483842 | 0.707032460075993 | 1.1044156633888 | 0.999332001504091 |
| ebi-fl187-GCST90094729 | prot-a-2831 | Sepiapterin reductase \|\| id:prot-a-2831 | F-cheese liking (derived food-liking factor) \|\| id:ebi-fl187-GCST90094729 | Weighted median | 16 | 0.0159031749657243 | 0.114438797853287 | 0.889476512417096 | -0.208396869 | 0.240203218758167 | 1.0160303034743 | 0.811884761013046 | 1.27150751824677 | 0.999332001504091 |
| ebi-fl187-GCST90094729 | prot-a-2834 | SPRY domain-containing SOCS box protein 1 \|\| id:prot-a-2834 | F-cheese liking (derived food-liking factor) \|\| id:ebi-fl187-GCST90094729 | Weighted median | 16 | -0.269190549 | 0.119893098885678 | 0.0247517834006976 | -0.504181023 | -0.034200075 | 0.763997662578653 | 0.604000035007281 | 0.966378136747309 | 0.999332001504091 |
| ebi-fl187-GCST90094729 | prot-a-2835 | Spectrin alpha chain, non-erythrocytic 1 \|\| id:prot-a-2835 | F-cheese liking (derived food-liking factor) \|\| id:ebi-fl187-GCST90094729 | Weighted median | 16 | 0.0226077457738133 | 0.113325553791074 | 0.841876489932785 | -0.19951034 | 0.244725831204318 | 1.0228652376339 | 0.819131751228045 | 1.27727107732218 | 0.999332001504091 |
| ebi-fl187-GCST90094729 | prot-a-2859 | Sia-alpha-2,3-Gal-beta-1,4-GlcNAc-R:alpha 2,8-sialyltransferase \|\| id:prot-a-2859 | F-cheese liking (derived food-liking factor) \|\| id:ebi-fl187-GCST90094729 | Weighted median | 16 | -0.112819769 | 0.111967881315988 | 0.313642530982035 | -0.332276817 | 0.106637278082746 | 0.893311647794764 | 0.717288737885795 | 1.11253064203673 | 0.999332001504091 |
| ebi-fl187-GCST90094729 | prot-a-2861 | Alpha-2,8-sialyltransferase 8F \|\| id:prot-a-2861 | F-cheese liking (derived food-liking factor) \|\| id:ebi-fl187-GCST90094729 | Weighted median | 16 | -0.087011627 | 0.121176860679552 | 0.472723493275788 | -0.324518274 | 0.150495019459292 | 0.916666437057279 | 0.722875497409883 | 1.16240951566081 | 0.999332001504091 |
| ebi-fl187-GCST90094729 | prot-a-289 | Uncharacterized protein C17orf89 \|\| id:prot-a-289 | F-cheese liking (derived food-liking factor) \|\| id:ebi-fl187-GCST90094729 | Weighted median | 16 | 0.0646202623967161 | 0.114432402273188 | 0.572276140930621 | -0.159667246 | 0.288907770852165 | 1.06675386090672 | 0.852427390352639 | 1.33496859983422 | 0.999332001504091 |
| ebi-fl187-GCST90094729 | prot-a-2921 | Tachykinin-3 \|\| id:prot-a-2921 | F-cheese liking (derived food-liking factor) \|\| id:ebi-fl187-GCST90094729 | Weighted median | 16 | 0.115220179779747 | 0.110242595289505 | 0.295953097737383 | -0.100855307 | 0.331295666547177 | 1.12212047861405 | 0.904063835142182 | 1.39277152738556 | 0.999332001504091 |
| ebi-fl187-GCST90094729 | prot-a-2928 | Tax1-binding protein 1 \|\| id:prot-a-2928 | F-cheese liking (derived food-liking factor) \|\| id:ebi-fl187-GCST90094729 | Weighted median | 16 | -0.002961104 | 0.123948308284066 | 0.98094048718133 | -0.245899788 | 0.239977580336159 | 0.997043275843518 | 0.782000586734386 | 1.27122064966228 | 0.999332001504091 |
| ebi-fl187-GCST90094729 | prot-a-296 | Uncharacterized protein C1orf185 \|\| id:prot-a-296 | F-cheese liking (derived food-liking factor) \|\| id:ebi-fl187-GCST90094729 | Weighted median | 16 | -0.146146739 | 0.110174659779587 | 0.184673977061034 | -0.362089072 | 0.069795594356509 | 0.864030907013532 | 0.696220351355002 | 1.07228897693334 | 0.999332001504091 |
| ebi-fl187-GCST90094729 | prot-a-2964 | Transforming growth factor beta-2 \|\| id:prot-a-2964 | F-cheese liking (derived food-liking factor) \|\| id:ebi-fl187-GCST90094729 | Weighted median | 16 | -0.104748903 | 0.109972429038163 | 0.340842710294946 | -0.320294864 | 0.110797058322061 | 0.900550620124646 | 0.725934953785849 | 1.11716816386572 | 0.999332001504091 |
| ebi-fl187-GCST90094729 | prot-a-2977 | Thymocyte nuclear protein 1 \|\| id:prot-a-2977 | F-cheese liking (derived food-liking factor) \|\| id:ebi-fl187-GCST90094729 | Weighted median | 16 | -0.038393525 | 0.118786779956171 | 0.746533337259209 | -0.271215614 | 0.194428563243208 | 0.962334163361998 | 0.762452083196287 | 1.21461671150713 | 0.999332001504091 |
| ebi-fl187-GCST90094729 | prot-a-3015 | Transmembrane protein 70, mitochondrial \|\| id:prot-a-3015 | F-cheese liking (derived food-liking factor) \|\| id:ebi-fl187-GCST90094729 | Weighted median | 16 | -0.048763448 | 0.111561311988267 | 0.662038838154193 | -0.267423619 | 0.169896723695724 | 0.952406396880989 | 0.76534878626771 | 1.18518244373689 | 0.999332001504091 |
| ebi-fl187-GCST90094729 | prot-a-3030 | Tumor necrosis factor alpha-induced protein 3 \|\| id:prot-a-3030 | F-cheese liking (derived food-liking factor) \|\| id:ebi-fl187-GCST90094729 | Weighted median | 16 | -0.043484103 | 0.118067376076152 | 0.712650258964437 | -0.27489616 | 0.187927954372151 | 0.957447774768965 | 0.759651001406232 | 1.20674657140329 | 0.999332001504091 |
| ebi-fl187-GCST90094729 | prot-a-3032 | Tumor necrosis factor receptor superfamily member 10A \|\| id:prot-a-3032 | F-cheese liking (derived food-liking factor) \|\| id:ebi-fl187-GCST90094729 | Weighted median | 16 | 0.0391438562206522 | 0.117890064748082 | 0.739861366598154 | -0.191920671 | 0.270208383126893 | 1.03992007186162 | 0.825372342116694 | 1.31023745366522 | 0.999332001504091 |
| ebi-fl187-GCST90094729 | prot-a-3081 | Tumor protein p53-inducible protein 11 \|\| id:prot-a-3081 | F-cheese liking (derived food-liking factor) \|\| id:ebi-fl187-GCST90094729 | Weighted median | 16 | 0.147668868412921 | 0.11329217632026 | 0.192426653655697 | -0.074383797 | 0.369721534000631 | 1.15912900857956 | 0.928315340656931 | 1.44733152592289 | 0.999332001504091 |
| ebi-fl187-GCST90094729 | prot-a-315 | Complement C4 \|\| id:prot-a-315 | F-cheese liking (derived food-liking factor) \|\| id:ebi-fl187-GCST90094729 | Weighted median | 16 | -0.080038284 | 0.118311479528407 | 0.498720200025384 | -0.311928784 | 0.151852216171892 | 0.923081006750341 | 0.73203365918892 | 1.16398820508788 | 0.999332001504091 |
| ebi-fl187-GCST90094729 | prot-a-3157 | UDP-glucuronosyltransferase 1-8 \|\| id:prot-a-3157 | F-cheese liking (derived food-liking factor) \|\| id:ebi-fl187-GCST90094729 | Weighted median | 16 | 0.135245445887172 | 0.117806279724168 | 0.250955119187239 | -0.095654862 | 0.366145754146541 | 1.14481774067613 | 0.90877761529849 | 1.44216542892765 | 0.999332001504091 |
| ebi-fl187-GCST90094729 | prot-a-317 | Glutaredoxin-like protein C5orf63 \|\| id:prot-a-317 | F-cheese liking (derived food-liking factor) \|\| id:ebi-fl187-GCST90094729 | Weighted median | 16 | -0.140805251 | 0.113470997168131 | 0.214645655007047 | -0.363208405 | 0.0815979038362297 | 0.868658465929278 | 0.695441484869248 | 1.08501943994968 | 0.999332001504091 |
| ebi-fl187-GCST90094729 | prot-a-3192 | Guanine nucleotide exchange factor VAV3 \|\| id:prot-a-3192 | F-cheese liking (derived food-liking factor) \|\| id:ebi-fl187-GCST90094729 | Weighted median | 16 | 0.0492593627653562 | 0.121477227158525 | 0.685107918232961 | -0.188836002 | 0.287354727996065 | 1.05049277412114 | 0.827922272773393 | 1.33289694548751 | 0.999332001504091 |
| ebi-fl187-GCST90094729 | prot-a-3233 | Serine/threonine-protein kinase WNK3 \|\| id:prot-a-3233 | F-cheese liking (derived food-liking factor) \|\| id:ebi-fl187-GCST90094729 | Weighted median | 16 | 0.0965064455651405 | 0.113860107677475 | 0.396667534889995 | -0.126659365 | 0.319672256612992 | 1.10131667972932 | 0.881033731988974 | 1.37667649377264 | 0.999332001504091 |
| ebi-fl187-GCST90094729 | prot-a-3250 | 14-3-3 protein theta \|\| id:prot-a-3250 | F-cheese liking (derived food-liking factor) \|\| id:ebi-fl187-GCST90094729 | Weighted median | 16 | 0.135259210961583 | 0.114851323174209 | 0.23892036718969 | -0.089849382 | 0.360367804383032 | 1.14483349928597 | 0.914068849705285 | 1.43385669636368 | 0.999332001504091 |
| ebi-fl187-GCST90094729 | prot-a-3278 | E3 ubiquitin-protein ligase ZNRF3 \|\| id:prot-a-3278 | F-cheese liking (derived food-liking factor) \|\| id:ebi-fl187-GCST90094729 | Weighted median | 16 | 0.0612385957504904 | 0.114660427892247 | 0.593281634341228 | -0.163495843 | 0.285973034419295 | 1.06315254760805 | 0.849170029063422 | 1.33105656205521 | 0.999332001504091 |
| ebi-fl187-GCST90094729 | prot-a-355 | Calpain-3 \|\| id:prot-a-355 | F-cheese liking (derived food-liking factor) \|\| id:ebi-fl187-GCST90094729 | Weighted median | 16 | 0.127699386323932 | 0.109063637787939 | 0.241650870508957 | -0.086065344 | 0.341464116388293 | 1.13621139067007 | 0.917534274139819 | 1.40700610393949 | 0.999332001504091 |
| ebi-fl187-GCST90094729 | prot-a-385 | Coiled-coil domain-containing protein 90B, mitochondrial \|\| id:prot-a-385 | F-cheese liking (derived food-liking factor) \|\| id:ebi-fl187-GCST90094729 | Weighted median | 16 | 0.0259752609850679 | 0.121226491423292 | 0.830336121035874 | -0.211628662 | 0.263579184174721 | 1.02631555812346 | 0.809265152515549 | 1.30158035542749 | 0.999332001504091 |
| ebi-fl187-GCST90094729 | prot-a-390 | C-C motif chemokine 14 \|\| id:prot-a-390 | F-cheese liking (derived food-liking factor) \|\| id:ebi-fl187-GCST90094729 | Weighted median | 16 | -0.161541038 | 0.115556392713611 | 0.162130737092836 | -0.388031567 | 0.0649494919975805 | 0.850831614558424 | 0.678390927861332 | 1.06710512567478 | 0.999332001504091 |
| ebi-fl187-GCST90094729 | prot-a-40 | Pituitary adenylate cyclase-activating polypeptide 27 \|\| id:prot-a-40 | F-cheese liking (derived food-liking factor) \|\| id:ebi-fl187-GCST90094729 | Weighted median | 16 | 0.107387379714541 | 0.118713237018817 | 0.365680078601665 | -0.125290565 | 0.340065324271422 | 1.11336546614906 | 0.882240517261314 | 1.40503937073904 | 0.999332001504091 |
| ebi-fl187-GCST90094729 | prot-a-401 | C-C motif chemokine 24 \|\| id:prot-a-401 | F-cheese liking (derived food-liking factor) \|\| id:ebi-fl187-GCST90094729 | Weighted median | 16 | 0.0501347375516597 | 0.12545164091824 | 0.689426130890119 | -0.195750479 | 0.296019953751411 | 1.05141275161261 | 0.82221736987401 | 1.344496984 | 0.999332001504091 |
| ebi-fl187-GCST90094729 | prot-a-412 | Cerebral cavernous malformations 2 protein \|\| id:prot-a-412 | F-cheese liking (derived food-liking factor) \|\| id:ebi-fl187-GCST90094729 | Weighted median | 16 | 0.294457977583548 | 0.122532266622724 | 0.0162564917170866 | 0.0542947350030098 | 0.534621220164086 | 1.3423985513535 | 1.05579573626128 | 1.70680161776107 | 0.999332001504091 |
| ebi-fl187-GCST90094729 | prot-a-44 | Adhesion G protein-coupled receptor F5 \|\| id:prot-a-44 | F-cheese liking (derived food-liking factor) \|\| id:ebi-fl187-GCST90094729 | Weighted median | 16 | 0.135862501837132 | 0.113257212944096 | 0.230297645514794 | -0.086121636 | 0.357846639207561 | 1.14552437526875 | 0.917482625944208 | 1.43024625995987 | 0.999332001504091 |
| ebi-fl187-GCST90094729 | prot-a-48 | Adrenomedullin \|\| id:prot-a-48 | F-cheese liking (derived food-liking factor) \|\| id:ebi-fl187-GCST90094729 | Weighted median | 16 | -0.062030042 | 0.118424380927732 | 0.600421465038225 | -0.294141828 | 0.170081744806797 | 0.939854651430641 | 0.745170797544034 | 1.18540174779677 | 0.999332001504091 |
| ebi-fl187-GCST90094729 | prot-a-502 | Carcinoembryonic antigen-related cell adhesion molecule 1 \|\| id:prot-a-502 | F-cheese liking (derived food-liking factor) \|\| id:ebi-fl187-GCST90094729 | Weighted median | 16 | -0.044064733 | 0.108238585003518 | 0.683929081654718 | -0.256212359 | 0.168082893714152 | 0.956892013080099 | 0.77397758984544 | 1.18303467272138 | 0.999332001504091 |
| ebi-fl187-GCST90094729 | prot-a-518 | Complement factor B \|\| id:prot-a-518 | F-cheese liking (derived food-liking factor) \|\| id:ebi-fl187-GCST90094729 | Weighted median | 16 | -0.030634089 | 0.116876893154666 | 0.793240021573981 | -0.2597128 | 0.19844462117557 | 0.969830379367043 | 0.771273063628858 | 1.21950449081913 | 0.999332001504091 |
| ebi-fl187-GCST90094729 | prot-a-525 | Properdin \|\| id:prot-a-525 | F-cheese liking (derived food-liking factor) \|\| id:ebi-fl187-GCST90094729 | Weighted median | 16 | -0.147874931 | 0.13694920146648 | 0.28024041305023 | -0.416295366 | 0.120545504117202 | 0.862538985294137 | 0.659485452377479 | 1.12811207354185 | 0.999332001504091 |
| ebi-fl187-GCST90094729 | prot-a-533 | Chondroadherin \|\| id:prot-a-533 | F-cheese liking (derived food-liking factor) \|\| id:ebi-fl187-GCST90094729 | Weighted median | 16 | -0.008009776 | 0.111892349522078 | 0.942932446849366 | -0.227318781 | 0.211299229062229 | 0.99202221677943 | 0.796666779522772 | 1.23528193201866 | 0.999332001504091 |
| ebi-fl187-GCST90094729 | prot-a-535 | Coiled-coil-helix-coiled-coil-helix domain-containing protein 2 \|\| id:prot-a-535 | F-cheese liking (derived food-liking factor) \|\| id:ebi-fl187-GCST90094729 | Weighted median | 16 | -0.137911057 | 0.129445681366993 | 0.286696256601139 | -0.391624592 | 0.115802478520703 | 0.871176173358473 | 0.675957826014475 | 1.1227740782326 | 0.999332001504091 |
| ebi-fl187-GCST90094729 | prot-a-591 | Caseinolytic peptidase B protein homolog \|\| id:prot-a-591 | F-cheese liking (derived food-liking factor) \|\| id:ebi-fl187-GCST90094729 | Weighted median | 15 | 0.00366494737614527 | 0.120144770274088 | 0.97566476277764 | -0.231818802 | 0.239148697113358 | 1.0036716715078 | 0.793089816290049 | 1.27016739276709 | 0.999332001504091 |
| ebi-fl187-GCST90094729 | prot-a-605 | Ciliary neurotrophic factor receptor subunit alpha \|\| id:prot-a-605 | F-cheese liking (derived food-liking factor) \|\| id:ebi-fl187-GCST90094729 | Weighted median | 16 | -0.129626064 | 0.123307108253804 | 0.293145770698489 | -0.371307996 | 0.112055868006268 | 0.878423843662117 | 0.689831443270412 | 1.11857535147416 | 0.999332001504091 |
| ebi-fl187-GCST90094729 | prot-a-606 | Ciliary neurotrophic factor receptor subunit alpha \|\| id:prot-a-606 | F-cheese liking (derived food-liking factor) \|\| id:ebi-fl187-GCST90094729 | Weighted median | 16 | -0.146689483 | 0.122700163645371 | 0.231887163676494 | -0.387181803 | 0.093802838230808 | 0.863562086915952 | 0.678967645174512 | 1.09834317328473 | 0.999332001504091 |
| ebi-fl187-GCST90094729 | prot-a-707 | Cancer/testis antigen 55 \|\| id:prot-a-707 | F-cheese liking (derived food-liking factor) \|\| id:ebi-fl187-GCST90094729 | Weighted median | 16 | -0.122482305 | 0.117354324107079 | 0.29662580006319 | -0.35249678 | 0.107532170410226 | 0.884721560094844 | 0.702930833181427 | 1.11352668278051 | 0.999332001504091 |
| ebi-fl187-GCST90094729 | prot-a-719 | Dipeptidyl peptidase 1 \|\| id:prot-a-719 | F-cheese liking (derived food-liking factor) \|\| id:ebi-fl187-GCST90094729 | Weighted median | 16 | 0.000485662732061937 | 0.120744881812733 | 0.996790739739852 | -0.236174306 | 0.237145631085019 | 1.0004857806853 | 0.789643022719101 | 1.26762571004132 | 0.999364451047625 |
| ebi-fl187-GCST90094729 | prot-a-752 | Cytochrome b5 \|\| id:prot-a-752 | F-cheese liking (derived food-liking factor) \|\| id:ebi-fl187-GCST90094729 | Weighted median | 16 | 0.104351001851695 | 0.117352947291829 | 0.373892117818002 | -0.125660775 | 0.33436277854368 | 1.10998999509056 | 0.8819139634517 | 1.39704987137175 | 0.999332001504091 |
| ebi-fl187-GCST90094729 | prot-a-755 | Cytoglobin \|\| id:prot-a-755 | F-cheese liking (derived food-liking factor) \|\| id:ebi-fl187-GCST90094729 | Weighted median | 16 | 0.0404905338754538 | 0.115610313398049 | 0.726163927270291 | -0.18610568 | 0.267086748135629 | 1.0413214523773 | 0.830185855985218 | 1.30615374781871 | 0.999332001504091 |
| ebi-fl187-GCST90094729 | prot-a-807 | Desmin \|\| id:prot-a-807 | F-cheese liking (derived food-liking factor) \|\| id:ebi-fl187-GCST90094729 | Weighted median | 16 | 0.087153475566195 | 0.11659016264755 | 0.454749749406686 | -0.141363243 | 0.315670194355394 | 1.09106411855241 | 0.868173896130694 | 1.37117795881455 | 0.999332001504091 |
| ebi-fl187-GCST90094729 | prot-a-82 | Ameloblastin \|\| id:prot-a-82 | F-cheese liking (derived food-liking factor) \|\| id:ebi-fl187-GCST90094729 | Weighted median | 16 | -0.081560621 | 0.120236601729963 | 0.497559701517887 | -0.317224361 | 0.154103118068914 | 0.921676834892282 | 0.728167364648404 | 1.16661117926815 | 0.999332001504091 |
| ebi-fl187-GCST90094729 | prot-a-84 | Muellerian-inhibiting factor \|\| id:prot-a-84 | F-cheese liking (derived food-liking factor) \|\| id:ebi-fl187-GCST90094729 | Weighted median | 16 | -0.221989303 | 0.113939968559844 | 0.0513790113852633 | -0.445311642 | 0.00133303495899981 | 0.800923931437112 | 0.640624599519669 | 1.00133392384503 | 0.999332001504091 |
| ebi-fl187-GCST90094729 | prot-a-858 | Inactive dipeptidyl peptidase 10 \|\| id:prot-a-858 | F-cheese liking (derived food-liking factor) \|\| id:ebi-fl187-GCST90094729 | Weighted median | 16 | 0.0168799880546458 | 0.114080279924165 | 0.882369702209365 | -0.206717361 | 0.24047733670601 | 1.01702326006101 | 0.81324947385341 | 1.27185610905365 | 0.999332001504091 |
| ebi-fl187-GCST90094729 | prot-a-863 | Dihydropyrimidinase-related protein 3 \|\| id:prot-a-863 | F-cheese liking (derived food-liking factor) \|\| id:ebi-fl187-GCST90094729 | Weighted median | 16 | -0.005906007 | 0.10907858475445 | 0.956820028938915 | -0.219700033 | 0.207888019397957 | 0.994111399453077 | 0.802759563356614 | 1.23107530527611 | 0.999332001504091 |
| ebi-fl187-GCST90094729 | prot-a-864 | Dihydropyrimidinase-related protein 5 \|\| id:prot-a-864 | F-cheese liking (derived food-liking factor) \|\| id:ebi-fl187-GCST90094729 | Weighted median | 16 | -0.042278072 | 0.133029466409581 | 0.750629196810069 | -0.303015826 | 0.218459682595775 | 0.958603183218337 | 0.738587407593257 | 1.24415885436052 | 0.999332001504091 |
| ebi-fl187-GCST90094729 | prot-a-884 | Endothelin-converting enzyme 1 \|\| id:prot-a-884 | F-cheese liking (derived food-liking factor) \|\| id:ebi-fl187-GCST90094729 | Weighted median | 16 | 0.026584580874399 | 0.112290008974598 | 0.812851287130214 | -0.193503837 | 0.24667299846461 | 1.02694110316492 | 0.824066674479405 | 1.27976056068014 | 0.999332001504091 |
| ebi-fl187-GCST90094729 | prot-a-893 | Early endosome antigen 1 \|\| id:prot-a-893 | F-cheese liking (derived food-liking factor) \|\| id:ebi-fl187-GCST90094729 | Weighted median | 15 | -0.039662462 | 0.119395000992115 | 0.739741766427939 | -0.273676664 | 0.19435173964833 | 0.961113796551781 | 0.760577957558837 | 1.21452340386911 | 0.999332001504091 |
| ebi-fl187-GCST90094729 | prot-a-897 | Ephrin-A2 \|\| id:prot-a-897 | F-cheese liking (derived food-liking factor) \|\| id:ebi-fl187-GCST90094729 | Weighted median | 16 | -0.000704114 | 0.120385493730611 | 0.995333337499764 | -0.236659682 | 0.235251453733811 | 0.99929613385189 | 0.789259841893517 | 1.26522687475852 | 0.999364451047625 |
| ebi-fl187-GCST90094729 | prot-a-90 | Anaphase-promoting complex subunit 10 \|\| id:prot-a-90 | F-cheese liking (derived food-liking factor) \|\| id:ebi-fl187-GCST90094729 | Weighted median | 16 | 0.0889774226421563 | 0.117867568508698 | 0.450313083129014 | -0.142043012 | 0.319997856919205 | 1.09305597773179 | 0.867583939479744 | 1.37712481304306 | 0.999332001504091 |
| ebi-fl187-GCST90094729 | prot-a-914 | Histone-lysine N-methyltransferase EHMT2 \|\| id:prot-a-914 | F-cheese liking (derived food-liking factor) \|\| id:ebi-fl187-GCST90094729 | Weighted median | 16 | 0.098313092155986 | 0.111244120611691 | 0.376825841042349 | -0.119725384 | 0.3163515685549 | 1.10330816817041 | 0.887164032490438 | 1.37211256246986 | 0.999332001504091 |
| ebi-fl187-GCST90094729 | prot-a-932 | RNA polymerase II elongation factor ELL \|\| id:prot-a-932 | F-cheese liking (derived food-liking factor) \|\| id:ebi-fl187-GCST90094729 | Weighted median | 16 | 0.0786895100387571 | 0.111353895305951 | 0.479776864031452 | -0.139564125 | 0.29694314483842 | 1.08186836065132 | 0.869737249722997 | 1.34573878507693 | 0.999332001504091 |
| ebi-fl187-GCST90094729 | prot-a-991 | Estrogen receptor \|\| id:prot-a-991 | F-cheese liking (derived food-liking factor) \|\| id:ebi-fl187-GCST90094729 | Weighted median | 16 | -0.079982957 | 0.109680228545784 | 0.465856276281807 | -0.294956205 | 0.134990290798485 | 0.923132079052969 | 0.744564194865608 | 1.14452567186698 | 0.999332001504091 |
| ebi-fl187-GCST90094729 | prot-a-992 | Persulfide dioxygenase ETHE1, mitochondrial \|\| id:prot-a-992 | F-cheese liking (derived food-liking factor) \|\| id:ebi-fl187-GCST90094729 | Weighted median | 16 | -0.030350415 | 0.118316738836027 | 0.797550611306119 | -0.262251223 | 0.201550393221151 | 0.970105534550214 | 0.76931772910834 | 1.22329788143024 | 0.999332001504091 |
| ebi-fl187-GCST90094729 | prot-a-993 | Ethanolamine kinase 1 \|\| id:prot-a-993 | F-cheese liking (derived food-liking factor) \|\| id:ebi-fl187-GCST90094729 | Weighted median | 16 | -0.0884008 | 0.117691515930264 | 0.452578658549974 | -0.319076171 | 0.14227457131431 | 0.91539391339161 | 0.726820184481316 | 1.15289315647225 | 0.999332001504091 |
| ebi-fl187-GCST90094729 | prot-b-10 | coagulation factor III, tissue factor \|\| id:prot-b-10 | F-cheese liking (derived food-liking factor) \|\| id:ebi-fl187-GCST90094729 | Weighted median | 12 | -0.020621094 | 0.166135079927593 | 0.901218317813777 | -0.346245851 | 0.305003662401435 | 0.979590066561033 | 0.707338565894585 | 1.35662997152068 | 0.999332001504091 |
| ebi-fl187-GCST90094729 | prot-b-28 | prolactin \|\| id:prot-b-28 | F-cheese liking (derived food-liking factor) \|\| id:ebi-fl187-GCST90094729 | Weighted median | 12 | 0.117702277318447 | 0.179311893212291 | 0.511559792985875 | -0.233749033 | 0.469153588014537 | 1.1249091505383 | 0.79156044622133 | 1.5986405119224 | 0.999332001504091 |
| ebi-fl187-GCST90094729 | prot-b-54 | matrix metallopeptidase 7 \|\| id:prot-b-54 | F-cheese liking (derived food-liking factor) \|\| id:ebi-fl187-GCST90094729 | Weighted median | 12 | 0.16792154657696 | 0.155950178657129 | 0.281585688035413 | -0.137740804 | 0.473583896744933 | 1.18284380886182 | 0.871324506662517 | 1.6057386949001 | 0.999332001504091 |
| ebi-fl187-GCST90094729 | ukb-rsfMRI191-edge-pheno716 | Net100_Pair10_33 (OccipitalPrecuneus)&(Temporal\|Frontal\|Supp_Motor_Area) (Default_mode\|Central_executive)&(Default_mode\|Salience) \|\| id:ukb-rsfMRI191-edge-pheno716 | F-cheese liking (derived food-liking factor) \|\| id:ebi-fl187-GCST90094729 | Weighted median | 15 | 0.00400392896819521 | 0.0373860838841715 | 0.914712206600374 | -0.069272795 | 0.0772806533811714 | 1.00401195540063 | 0.933072107536078 | 1.0803452363926 | 0.999332001504091 |
| ebi-fl187-GCST90094729 | ukb-rsfMRI191-edge-pheno903 | Net100_Pair27_38 (Precentral\|Frontal\|Supp_Motor_Area)&(Subcortical) (Attention\|Salience\|Motor)&(Subcortical-cerebellum) \|\| id:ukb-rsfMRI191-edge-pheno903 | F-cheese liking (derived food-liking factor) \|\| id:ebi-fl187-GCST90094729 | Weighted median | 15 | 0.0242418878877454 | 0.0381763569649024 | 0.525430102629616 | -0.050583772 | 0.0990675475389541 | 1.02453811127996 | 0.950674285675066 | 1.10414087903906 | 0.999332001504091 |
| ebi-fl187-GCST90094729 | ukb-rsfMRI191-node-pheno23 | Net100_Node2 RolandicOper\|SupraMarginal\|Insula Salience\|Motor \|\| id:ukb-rsfMRI191-node-pheno23 | F-cheese liking (derived food-liking factor) \|\| id:ebi-fl187-GCST90094729 | Weighted median | 15 | -0.027732406 | 0.0367410787664451 | 0.450365077299073 | -0.09974492 | 0.0442801083370583 | 0.972648606867727 | 0.905068253017211 | 1.04527510415697 | 0.999332001504091 |
| ebi-fl187-GCST90094729 | ukb-rsfMRI191-node-pheno55 | Net100_Node34 Parietal\|Postcentral\|Precuneus Attention \|\| id:ukb-rsfMRI191-node-pheno55 | F-cheese liking (derived food-liking factor) \|\| id:ebi-fl187-GCST90094729 | Weighted median | 14 | -0.000706579 | 0.0340145866114663 | 0.983426888160409 | -0.067375168 | 0.0659620112402457 | 0.99929367104959 | 0.934844411571421 | 1.06818613732867 | 0.999332001504091 |
| ebi-fl187-GCST90094729 | ebi-a-GCST90001414 | IgD- CD24- B cell Absolute Count \|\| id:ebi-a-GCST90001414 | F-cheese liking (derived food-liking factor) \|\| id:ebi-fl187-GCST90094729 | Weighted mode | 14 | 0.190759299171859 | 0.179641398452589 | 0.307615389417979 | -0.161337842 | 0.542856440138933 | 1.21016812855107 | 0.851004517642121 | 1.72091554039984 | 0.999714303421225 |
| ebi-fl187-GCST90094729 | ebi-a-GCST90001422 | CD20- CD38- B cell %B cell \|\| id:ebi-a-GCST90001422 | F-cheese liking (derived food-liking factor) \|\| id:ebi-fl187-GCST90094729 | Weighted mode | 14 | 0.00799300643486189 | 0.162900531324487 | 0.961611914009232 | -0.311292035 | 0.327278047830856 | 1.00802503579087 | 0.732499929042569 | 1.38718712793505 | 0.999714303421225 |
| ebi-fl187-GCST90094729 | ebi-a-GCST90001444 | CD20- CD38- B cell %lymphocyte \|\| id:ebi-a-GCST90001444 | F-cheese liking (derived food-liking factor) \|\| id:ebi-fl187-GCST90094729 | Weighted mode | 14 | 0.053204277410241 | 0.162670675710492 | 0.748823512358455 | -0.265630247 | 0.372038801802805 | 1.05464506327833 | 0.766722573080479 | 1.45068926956008 | 0.999714303421225 |
| ebi-fl187-GCST90094729 | ebi-a-GCST90001459 | Myeloid Dendritic Cell %Dendritic Cell \|\| id:ebi-a-GCST90001459 | F-cheese liking (derived food-liking factor) \|\| id:ebi-fl187-GCST90094729 | Weighted mode | 14 | 0.149338022470347 | 0.195981079061693 | 0.459660919173581 | -0.234784892 | 0.533460937431266 | 1.16106538907641 | 0.790740925646885 | 1.70482239376739 | 0.999714303421225 |
| ebi-fl187-GCST90094729 | ebi-a-GCST90001476 | HLA DR++ monocyte %leukocyte \|\| id:ebi-a-GCST90001476 | F-cheese liking (derived food-liking factor) \|\| id:ebi-fl187-GCST90094729 | Weighted mode | 14 | -0.050843222 | 0.167687431080592 | 0.766533329494898 | -0.379510586 | 0.277824143417792 | 0.950427665476111 | 0.68419618218868 | 1.32025400143678 | 0.999714303421225 |
| ebi-fl187-GCST90094729 | ebi-a-GCST90001478 | CD4 regulatory T cell %CD4+ T cell \|\| id:ebi-a-GCST90001478 | F-cheese liking (derived food-liking factor) \|\| id:ebi-fl187-GCST90094729 | Weighted mode | 14 | -0.129589864 | 0.220667258638149 | 0.567086961939834 | -0.562097691 | 0.302917962574635 | 0.878455643018357 | 0.570012099442397 | 1.35380339734135 | 0.999714303421225 |
| ebi-fl187-GCST90094729 | ebi-a-GCST90001485 | CD39+ resting CD4 regulatory T cell %CD4 regulatory T cell \|\| id:ebi-a-GCST90001485 | F-cheese liking (derived food-liking factor) \|\| id:ebi-fl187-GCST90094729 | Weighted mode | 13 | 0.09339733788164 | 0.184930853896373 | 0.622678106729496 | -0.269067136 | 0.455861811518531 | 1.09789788503283 | 0.764091956035649 | 1.57753233290591 | 0.999714303421225 |
| ebi-fl187-GCST90094729 | ebi-a-GCST90001515 | Immature Myeloid-Derived Suppressor Cells Absolute Count \|\| id:ebi-a-GCST90001515 | F-cheese liking (derived food-liking factor) \|\| id:ebi-fl187-GCST90094729 | Weighted mode | 14 | 0.13326991775911 | 0.273133031221025 | 0.633725170360664 | -0.402070823 | 0.66861065895232 | 1.14255835350418 | 0.668933367854341 | 1.95152410373771 | 0.999714303421225 |
| ebi-fl187-GCST90094729 | ebi-a-GCST90001517 | CD33+ HLA DR+ Absolute Count \|\| id:ebi-a-GCST90001517 | F-cheese liking (derived food-liking factor) \|\| id:ebi-fl187-GCST90094729 | Weighted mode | 14 | -0.119464639 | 0.203570233343885 | 0.567358321952395 | -0.518462297 | 0.279533017954368 | 0.887395386097874 | 0.595435447388451 | 1.32251207871752 | 0.999714303421225 |
| ebi-fl187-GCST90094729 | ebi-a-GCST90001518 | CD33+ HLA DR+ CD14- Absolute Count \|\| id:ebi-a-GCST90001518 | F-cheese liking (derived food-liking factor) \|\| id:ebi-fl187-GCST90094729 | Weighted mode | 14 | -0.16732002 | 0.202590445991293 | 0.423757695079314 | -0.564397294 | 0.22975725444413 | 0.845928854118572 | 0.568702804168595 | 1.25829452744921 | 0.999714303421225 |
| ebi-fl187-GCST90094729 | ebi-a-GCST90001520 | CD33+ HLA DR+ CD14dim Absolute Count \|\| id:ebi-a-GCST90001520 | F-cheese liking (derived food-liking factor) \|\| id:ebi-fl187-GCST90094729 | Weighted mode | 14 | 0.00644897741599326 | 0.245112940743822 | 0.979409490963239 | -0.473972386 | 0.486870341273885 | 1.00646981684443 | 0.62252444244521 | 1.62721561299662 | 0.999714303421225 |
| ebi-fl187-GCST90094729 | ebi-a-GCST90001531 | CD33dim HLA DR- Absolute Count \|\| id:ebi-a-GCST90001531 | F-cheese liking (derived food-liking factor) \|\| id:ebi-fl187-GCST90094729 | Weighted mode | 14 | 0.0882555585869813 | 0.228025757129412 | 0.704986846575166 | -0.358674925 | 0.535186042560628 | 1.09226722463168 | 0.698601412027633 | 1.70776592984813 | 0.999714303421225 |
| ebi-fl187-GCST90094729 | ebi-a-GCST90001532 | Basophil Absolute Count \|\| id:ebi-a-GCST90001532 | F-cheese liking (derived food-liking factor) \|\| id:ebi-fl187-GCST90094729 | Weighted mode | 14 | 0.108045617848235 | 0.215761844257815 | 0.624908573316609 | -0.314847597 | 0.530938832593553 | 1.11409856700662 | 0.729900104833695 | 1.70052807060359 | 0.999714303421225 |
| ebi-fl187-GCST90094729 | ebi-a-GCST90001588 | T/B cell \|\| id:ebi-a-GCST90001588 | F-cheese liking (derived food-liking factor) \|\| id:ebi-fl187-GCST90094729 | Weighted mode | 14 | -0.133929082 | 0.195454550152314 | 0.505241304497287 | -0.51702 | 0.249161836125101 | 0.874652090829207 | 0.596294861335995 | 1.28294964386896 | 0.999714303421225 |
| ebi-fl187-GCST90094729 | ebi-a-GCST90001624 | HLA DR+ CD4+ T cell Absolute Count \|\| id:ebi-a-GCST90001624 | F-cheese liking (derived food-liking factor) \|\| id:ebi-fl187-GCST90094729 | Weighted mode | 14 | -0.060911011 | 0.192339810153476 | 0.756511054239659 | -0.437897039 | 0.316075016422817 | 0.940906965970865 | 0.6453922294214 | 1.37173315428074 | 0.999714303421225 |
| ebi-fl187-GCST90094729 | ebi-a-GCST90001631 | CD8+ Natural Killer T %T cell \|\| id:ebi-a-GCST90001631 | F-cheese liking (derived food-liking factor) \|\| id:ebi-fl187-GCST90094729 | Weighted mode | 14 | -0.036356389 | 0.160317794239429 | 0.824124192915297 | -0.350579266 | 0.277866487230638 | 0.964296567085725 | 0.704280005941285 | 1.32030990720875 | 0.999714303421225 |
| ebi-fl187-GCST90094729 | ebi-a-GCST90001632 | CD8+ Natural Killer T %lymphocyte \|\| id:ebi-a-GCST90001632 | F-cheese liking (derived food-liking factor) \|\| id:ebi-fl187-GCST90094729 | Weighted mode | 14 | -0.050212882 | 0.160863113442266 | 0.759879294950261 | -0.365504585 | 0.265078820012496 | 0.951026946113129 | 0.693846458240566 | 1.30353371627312 | 0.999714303421225 |
| ebi-fl187-GCST90094729 | ebi-a-GCST90001646 | Natural Killer %CD3- lymphocyte \|\| id:ebi-a-GCST90001646 | F-cheese liking (derived food-liking factor) \|\| id:ebi-fl187-GCST90094729 | Weighted mode | 14 | -0.06618685 | 0.171022746220235 | 0.705012805640088 | -0.401391433 | 0.269017732467159 | 0.935955964463613 | 0.66938798939072 | 1.30867834693653 | 0.999714303421225 |
| ebi-fl187-GCST90094729 | ebi-a-GCST90001684 | CD127- CD8+ T cell Absolute Count \|\| id:ebi-a-GCST90001684 | F-cheese liking (derived food-liking factor) \|\| id:ebi-fl187-GCST90094729 | Weighted mode | 14 | -0.142161772 | 0.170115553787425 | 0.418431229514015 | -0.475588257 | 0.191264713593236 | 0.867480911159669 | 0.621519335649237 | 1.21077991956651 | 0.999714303421225 |
| ebi-fl187-GCST90094729 | ebi-a-GCST90001687 | CD28- CD8+ T cell Absolute Count \|\| id:ebi-a-GCST90001687 | F-cheese liking (derived food-liking factor) \|\| id:ebi-fl187-GCST90094729 | Weighted mode | 14 | -0.170474432 | 0.169117987332494 | 0.331839321258915 | -0.501945687 | 0.160996823570511 | 0.843264650279868 | 0.605351688357115 | 1.17468123751582 | 0.999714303421225 |
| ebi-fl187-GCST90094729 | ebi-a-GCST90001701 | BAFF-R on CD20- CD38- B cell \|\| id:ebi-a-GCST90001701 | F-cheese liking (derived food-liking factor) \|\| id:ebi-fl187-GCST90094729 | Weighted mode | 14 | 0.28264665759835 | 0.284559861554512 | 0.338704313024798 | -0.275090671 | 0.840383986245193 | 1.32663632212739 | 0.759503255146368 | 2.31725660062972 | 0.999714303421225 |
| ebi-fl187-GCST90094729 | ebi-a-GCST90001732 | CD19 on IgD- CD27- B cell \|\| id:ebi-a-GCST90001732 | F-cheese liking (derived food-liking factor) \|\| id:ebi-fl187-GCST90094729 | Weighted mode | 14 | -0.106216244 | 0.23483306510925 | 0.658504282254821 | -0.566489051 | 0.354056563985631 | 0.899230174254082 | 0.567514459167088 | 1.42483577859107 | 0.999714303421225 |
| ebi-fl187-GCST90094729 | ebi-a-GCST90001798 | CD27 on CD24+ CD27+ B cell \|\| id:ebi-a-GCST90001798 | F-cheese liking (derived food-liking factor) \|\| id:ebi-fl187-GCST90094729 | Weighted mode | 14 | 0.0467983075128363 | 0.178797953243682 | 0.797624287460716 | -0.303645681 | 0.397242295870452 | 1.04791063206209 | 0.738122351011221 | 1.48771635391389 | 0.999714303421225 |
| ebi-fl187-GCST90094729 | ebi-a-GCST90001836 | CD66b on CD66b++ myeloid cell \|\| id:ebi-a-GCST90001836 | F-cheese liking (derived food-liking factor) \|\| id:ebi-fl187-GCST90094729 | Weighted mode | 14 | -0.351631212 | 0.289989956391029 | 0.246872743809883 | -0.920011527 | 0.216749102078559 | 0.70353953076142 | 0.398514447392255 | 1.24203243968419 | 0.999714303421225 |
| ebi-fl187-GCST90094729 | ebi-a-GCST90001943 | CD25 on activated & secreting CD4 regulatory T cell \|\| id:ebi-a-GCST90001943 | F-cheese liking (derived food-liking factor) \|\| id:ebi-fl187-GCST90094729 | Weighted mode | 14 | 0.175302354382321 | 0.169070411683896 | 0.318703635079181 | -0.156075653 | 0.506680361282757 | 1.19160644958264 | 0.855494467612121 | 1.65977219543018 | 0.999714303421225 |
| ebi-fl187-GCST90094729 | ebi-a-GCST90001960 | CD25 on CD4+ T cell \|\| id:ebi-a-GCST90001960 | F-cheese liking (derived food-liking factor) \|\| id:ebi-fl187-GCST90094729 | Weighted mode | 14 | -0.014325107 | 0.211720943826126 | 0.947085611839964 | -0.429298157 | 0.40064794299053 | 0.985777009245491 | 0.65096581035549 | 1.49279162822132 | 0.999714303421225 |
| ebi-fl187-GCST90094729 | ebi-a-GCST90001988 | HLA DR on CD14+ CD16- monocyte \|\| id:ebi-a-GCST90001988 | F-cheese liking (derived food-liking factor) \|\| id:ebi-fl187-GCST90094729 | Weighted mode | 14 | 0.0534580923780806 | 0.207228274518604 | 0.800470978278348 | -0.352709326 | 0.459625510434545 | 1.05491278195521 | 0.702781444210108 | 1.58348087687952 | 0.999714303421225 |
| ebi-fl187-GCST90094729 | ebi-a-GCST90001991 | HLA DR on CD14+ monocyte \|\| id:ebi-a-GCST90001991 | F-cheese liking (derived food-liking factor) \|\| id:ebi-fl187-GCST90094729 | Weighted mode | 14 | 0.0860849214789283 | 0.213948760254288 | 0.693955103045602 | -0.333254649 | 0.505424491577333 | 1.08989888020004 | 0.716587692853114 | 1.65768904616785 | 0.999714303421225 |
| ebi-fl187-GCST90094729 | ebi-a-GCST90002019 | CD14 on Monocytic Myeloid-Derived Suppressor Cells \|\| id:ebi-a-GCST90002019 | F-cheese liking (derived food-liking factor) \|\| id:ebi-fl187-GCST90094729 | Weighted mode | 14 | -0.056170678 | 0.292668978701715 | 0.850765900733481 | -0.629801877 | 0.517460520001773 | 0.94537776669348 | 0.532697330407398 | 1.67776159320122 | 0.999714303421225 |
| ebi-fl187-GCST90094729 | ebi-a-GCST90002037 | CD80 on plasmacytoid Dendritic Cell \|\| id:ebi-a-GCST90002037 | F-cheese liking (derived food-liking factor) \|\| id:ebi-fl187-GCST90094729 | Weighted mode | 14 | 0.0837057550894533 | 0.213486856820695 | 0.701345158191829 | -0.334728484 | 0.502139994458015 | 1.08730891162113 | 0.715532338258787 | 1.65225330299908 | 0.999714303421225 |
| ebi-fl187-GCST90094729 | ebi-a-GCST90002042 | CD45 on CD33+ HLA DR+ CD14- \|\| id:ebi-a-GCST90002042 | F-cheese liking (derived food-liking factor) \|\| id:ebi-fl187-GCST90094729 | Weighted mode | 14 | -0.079601201 | 0.269793007379811 | 0.772618424986174 | -0.608395495 | 0.449193093509811 | 0.923484557720634 | 0.544223377817946 | 1.56704721463429 | 0.999714303421225 |
| ebi-fl187-GCST90094729 | ebi-a-GCST90002056 | CD8 on Effector Memory CD8+ T cell \|\| id:ebi-a-GCST90002056 | F-cheese liking (derived food-liking factor) \|\| id:ebi-fl187-GCST90094729 | Weighted mode | 14 | -0.067556931 | 0.168498232836537 | 0.694979483790508 | -0.397813467 | 0.262699605471598 | 0.934674507251598 | 0.671787326314482 | 1.30043601640834 | 0.999714303421225 |
| ebi-fl187-GCST90094729 | ebi-a-GCST90002058 | CD8 on CD8+ T cell \|\| id:ebi-a-GCST90002058 | F-cheese liking (derived food-liking factor) \|\| id:ebi-fl187-GCST90094729 | Weighted mode | 14 | -0.265900813 | 0.227279599429087 | 0.263032197306521 | -0.711368828 | 0.179567201805604 | 0.766515152011221 | 0.490971681523186 | 1.19669932172053 | 0.999714303421225 |
| ebi-fl187-GCST90094729 | ebi-a-GCST90002063 | CD4 on CD4 regulatory T cell \|\| id:ebi-a-GCST90002063 | F-cheese liking (derived food-liking factor) \|\| id:ebi-fl187-GCST90094729 | Weighted mode | 14 | -0.202335452 | 0.187363758458619 | 0.299811914871816 | -0.569568418 | 0.164897514941726 | 0.816820878078746 | 0.565769561852999 | 1.17927225473237 | 0.999714303421225 |
| ebi-fl187-GCST90094729 | ebi-a-GCST90002066 | CD4 on activated CD4 regulatory T cell \|\| id:ebi-a-GCST90002066 | F-cheese liking (derived food-liking factor) \|\| id:ebi-fl187-GCST90094729 | Weighted mode | 14 | -0.244941416 | 0.198306678804578 | 0.238627268773772 | -0.633622507 | 0.143739674046103 | 0.782750393226108 | 0.530665973817084 | 1.15458350134734 | 0.999714303421225 |
| ebi-fl187-GCST90094729 | ebi-a-GCST90002070 | CD4 on activated & secreting CD4 regulatory T cell \|\| id:ebi-a-GCST90002070 | F-cheese liking (derived food-liking factor) \|\| id:ebi-fl187-GCST90094729 | Weighted mode | 14 | -0.173942027 | 0.180149048012384 | 0.351891179276711 | -0.527034161 | 0.179150106791976 | 0.840345613340134 | 0.590353268312067 | 1.196200288 | 0.999714303421225 |
| ebi-fl187-GCST90094729 | ebi-a-GCST90002081 | SSC-A on CD4+ T cell \|\| id:ebi-a-GCST90002081 | F-cheese liking (derived food-liking factor) \|\| id:ebi-fl187-GCST90094729 | Weighted mode | 14 | 0.0225075206663907 | 0.168217278790618 | 0.895609653756377 | -0.307198346 | 0.352213387096003 | 1.02276272599278 | 0.735504702197026 | 1.42221197302417 | 0.999714303421225 |
| ebi-fl187-GCST90094729 | ebi-a-GCST90002094 | CD11b on Monocytic Myeloid-Derived Suppressor Cells \|\| id:ebi-a-GCST90002094 | F-cheese liking (derived food-liking factor) \|\| id:ebi-fl187-GCST90094729 | Weighted mode | 14 | -0.200427375 | 0.267192854605509 | 0.466540812042333 | -0.72412537 | 0.323270620238416 | 0.818380922955256 | 0.484748359145451 | 1.38163919984747 | 0.999714303421225 |
| ebi-fl187-GCST90094729 | ebi-a-GCST90002104 | HLA DR on myeloid Dendritic Cell \|\| id:ebi-a-GCST90002104 | F-cheese liking (derived food-liking factor) \|\| id:ebi-fl187-GCST90094729 | Weighted mode | 14 | -0.125466265 | 0.20696791104363 | 0.554812800797555 | -0.53112337 | 0.280190841035135 | 0.882085521423842 | 0.587944119622093 | 1.32338234389637 | 0.999714303421225 |
| ebi-fl187-GCST90094729 | ebi-a-GCST90002105 | HLA DR on plasmacytoid Dendritic Cell \|\| id:ebi-a-GCST90002105 | F-cheese liking (derived food-liking factor) \|\| id:ebi-fl187-GCST90094729 | Weighted mode | 14 | 0.0164992233901846 | 0.249302492092161 | 0.948240267138892 | -0.472133661 | 0.50513210789082 | 1.01663608725618 | 0.623670146902089 | 1.65720443578298 | 0.999714303421225 |
| ebi-fl187-GCST90094729 | ebi-a-GCST90002106 | HLA DR on Dendritic Cell \|\| id:ebi-a-GCST90002106 | F-cheese liking (derived food-liking factor) \|\| id:ebi-fl187-GCST90094729 | Weighted mode | 14 | 0.0588108272023113 | 0.261214008972855 | 0.825367579638648 | -0.45316863 | 0.570790284789106 | 1.06057458989959 | 0.635610941271316 | 1.76966503831869 | 0.999714303421225 |
| ebi-fl187-GCST90094729 | ebi-a-GCST90002111 | HLA DR on CD33dim HLA DR+ CD11b- \|\| id:ebi-a-GCST90002111 | F-cheese liking (derived food-liking factor) \|\| id:ebi-fl187-GCST90094729 | Weighted mode | 14 | -0.138730295 | 0.275625129825204 | 0.623152563297707 | -0.678955549 | 0.40149495986229 | 0.870462765314489 | 0.507146405388119 | 1.49405658356008 | 0.999714303421225 |
| ebi-fl187-GCST90094729 | ebi-a-GCST90002112 | HLA DR on CD33- HLA DR+ \|\| id:ebi-a-GCST90002112 | F-cheese liking (derived food-liking factor) \|\| id:ebi-fl187-GCST90094729 | Weighted mode | 14 | -0.022315994 | 0.301969140950277 | 0.942213738096508 | -0.614175511 | 0.569543521949519 | 0.977931165534838 | 0.541086831819621 | 1.76746006053819 | 0.999714303421225 |
| ebi-fl187-GCST90094729 | ebi-a-GCST90002115 | HLA DR on HLA DR+ CD8+ T cell \|\| id:ebi-a-GCST90002115 | F-cheese liking (derived food-liking factor) \|\| id:ebi-fl187-GCST90094729 | Weighted mode | 14 | -0.16801704 | 0.158195318629725 | 0.307529423314798 | -0.478079865 | 0.142045784284272 | 0.845339429783626 | 0.619972681045119 | 1.15262941964196 | 0.999714303421225 |
| ebi-fl187-GCST90094729 | ebi-a-GCST90002116 | HLA DR on B cell \|\| id:ebi-a-GCST90002116 | F-cheese liking (derived food-liking factor) \|\| id:ebi-fl187-GCST90094729 | Weighted mode | 14 | -0.030511286 | 0.180571225649506 | 0.86842031811944 | -0.384430888 | 0.323408316283086 | 0.9699494851653 | 0.680837998863979 | 1.38182945919913 | 0.999714303421225 |
| ebi-fl187-GCST90094729 | ebi-cfmet338-GCST90026023 | Sphingomyelin (d18:1/24:1, d18:2/24:0) levels \|\| id:ebi-cfmet338-GCST90026023 | F-cheese liking (derived food-liking factor) \|\| id:ebi-fl187-GCST90094729 | Weighted mode | 15 | 0.0040316242130537 | 0.11176770686812 | 0.971734663082055 | -0.215033081 | 0.223096329674568 | 1.00403976214263 | 0.806514759220872 | 1.2499409743441 | 0.999714303421225 |
| ebi-fl187-GCST90094729 | ebi-cfmet338-GCST90026046 | 1-arachidonoyl-gpc (20:4n6) levels \|\| id:ebi-cfmet338-GCST90026046 | F-cheese liking (derived food-liking factor) \|\| id:ebi-fl187-GCST90094729 | Weighted mode | 15 | 0.0612383672674168 | 0.137184163331401 | 0.662138615651592 | -0.207642593 | 0.330119327396962 | 1.06315230469571 | 0.812497377186078 | 1.39113411897322 | 0.999714303421225 |
| ebi-fl187-GCST90094729 | ebi-cfmet338-GCST90026047 | 1-linoleoyl-gpc (18:2) levels \|\| id:ebi-cfmet338-GCST90026047 | F-cheese liking (derived food-liking factor) \|\| id:ebi-fl187-GCST90094729 | Weighted mode | 15 | -0.048082621 | 0.158111735587629 | 0.765520734421536 | -0.357981622 | 0.261816381100665 | 0.953055041796481 | 0.699085922407935 | 1.29928794670202 | 0.999714303421225 |
| ebi-fl187-GCST90094729 | ebi-cfmet338-GCST90026050 | 1-myristoyl-2-palmitoyl-gpc (14:0/16:0) levels \|\| id:ebi-cfmet338-GCST90026050 | F-cheese liking (derived food-liking factor) \|\| id:ebi-fl187-GCST90094729 | Weighted mode | 15 | -0.102095004 | 0.0894576055072582 | 0.272905112464357 | -0.27743191 | 0.0732419032504325 | 0.902943764736414 | 0.757727156232467 | 1.07599079110507 | 0.999714303421225 |
| ebi-fl187-GCST90094729 | ebi-cfmet338-GCST90026068 | 2,3-dihydroxyisovalerate levels \|\| id:ebi-cfmet338-GCST90026068 | F-cheese liking (derived food-liking factor) \|\| id:ebi-fl187-GCST90094729 | Weighted mode | 15 | -0.453217176 | 0.357923112721022 | 0.226090755643424 | -1.154746477 | 0.248312125132567 | 0.635580086022607 | 0.315137421445065 | 1.28185997047299 | 0.999714303421225 |
| ebi-fl187-GCST90094729 | ebi-cfmet338-GCST90026082 | 3-hydroxyhexanoate levels \|\| id:ebi-cfmet338-GCST90026082 | F-cheese liking (derived food-liking factor) \|\| id:ebi-fl187-GCST90094729 | Weighted mode | 15 | 0.029450687493374 | 0.0610759190873129 | 0.637116905533055 | -0.090258114 | 0.149159488904507 | 1.0298886478289 | 0.913695317354141 | 1.16085811843526 | 0.999714303421225 |
| ebi-fl187-GCST90094729 | ebi-cfmet338-GCST90026094 | 3-ureidopropionate levels \|\| id:ebi-cfmet338-GCST90026094 | F-cheese liking (derived food-liking factor) \|\| id:ebi-fl187-GCST90094729 | Weighted mode | 15 | -0.109470993 | 0.163483875973076 | 0.513997100055328 | -0.42989939 | 0.210957403647628 | 0.89630816296324 | 0.650574545627259 | 1.2348597534199 | 0.999714303421225 |
| ebi-fl187-GCST90094729 | ebi-cfmet338-GCST90026173 | Glycerol levels \|\| id:ebi-cfmet338-GCST90026173 | F-cheese liking (derived food-liking factor) \|\| id:ebi-fl187-GCST90094729 | Weighted mode | 15 | 0.00181633172955575 | 0.0843835069423808 | 0.983130856707941 | -0.163575342 | 0.167208005336622 | 1.00181798225918 | 0.849102523613273 | 1.18200010206891 | 0.999714303421225 |
| ebi-fl187-GCST90094729 | ebi-cfmet338-GCST90026179 | Histidine levels \|\| id:ebi-cfmet338-GCST90026179 | F-cheese liking (derived food-liking factor) \|\| id:ebi-fl187-GCST90094729 | Weighted mode | 15 | 0.0358767091622642 | 0.0546736336606639 | 0.522336537952599 | -0.071283613 | 0.143037031137166 | 1.03652804420175 | 0.931197755058601 | 1.15377252638362 | 0.999714303421225 |
| ebi-fl187-GCST90094729 | ebi-cfmet338-GCST90026188 | Isocitrate levels \|\| id:ebi-cfmet338-GCST90026188 | F-cheese liking (derived food-liking factor) \|\| id:ebi-fl187-GCST90094729 | Weighted mode | 15 | -0.095287411 | 0.0923688600259248 | 0.31975400802533 | -0.276330377 | 0.0857555545737519 | 0.909111608169521 | 0.758562278035302 | 1.08953996268993 | 0.999714303421225 |
| ebi-fl187-GCST90094729 | ebi-cfmet338-GCST90026207 | N-acetyl-isoputreanine levels \|\| id:ebi-cfmet338-GCST90026207 | F-cheese liking (derived food-liking factor) \|\| id:ebi-fl187-GCST90094729 | Weighted mode | 15 | -0.02193019 | 0.067614675214958 | 0.750475316969916 | -0.154454953 | 0.110594573424574 | 0.978308528388956 | 0.856882090882039 | 1.11694197708506 | 0.999714303421225 |
| ebi-fl187-GCST90094729 | ebi-cfmet338-GCST90026213 | N-acetylglutamate levels \|\| id:ebi-cfmet338-GCST90026213 | F-cheese liking (derived food-liking factor) \|\| id:ebi-fl187-GCST90094729 | Weighted mode | 15 | -0.048610939 | 0.102714584566693 | 0.643320598181854 | -0.249931525 | 0.152709646690083 | 0.952551658257692 | 0.778854113427827 | 1.16498667209461 | 0.999714303421225 |
| ebi-fl187-GCST90094729 | ebi-cfmet338-GCST90026224 | N-acetyltaurine levels \|\| id:ebi-cfmet338-GCST90026224 | F-cheese liking (derived food-liking factor) \|\| id:ebi-fl187-GCST90094729 | Weighted mode | 15 | -0.064821774 | 0.0849906888150986 | 0.458305604291545 | -0.231403524 | 0.101759976207511 | 0.937234488168522 | 0.793419237766715 | 1.10711770524372 | 0.999714303421225 |
| ebi-fl187-GCST90094729 | ebi-cfmet338-GCST90026250 | Phenyllactate (pla) levels \|\| id:ebi-cfmet338-GCST90026250 | F-cheese liking (derived food-liking factor) \|\| id:ebi-fl187-GCST90094729 | Weighted mode | 15 | 0.0415982250340525 | 0.156569947252763 | 0.794354541050991 | -0.265278872 | 0.348475321649468 | 1.04247555401945 | 0.766992027869139 | 1.4169055755995 | 0.999714303421225 |
| ebi-fl187-GCST90094729 | ebi-cfmet338-GCST90026251 | Pipecolate levels \|\| id:ebi-cfmet338-GCST90026251 | F-cheese liking (derived food-liking factor) \|\| id:ebi-fl187-GCST90094729 | Weighted mode | 15 | 0.0299224272581067 | 0.257023118134451 | 0.908973843103302 | -0.473842884 | 0.533687738801631 | 1.03037460187024 | 0.622605065923313 | 1.7052090936729 | 0.999714303421225 |
| ebi-fl187-GCST90094729 | ebi-cfmet338-GCST90026278 | Trigonelline (n'-methylnicotinate) levels \|\| id:ebi-cfmet338-GCST90026278 | F-cheese liking (derived food-liking factor) \|\| id:ebi-fl187-GCST90094729 | Weighted mode | 15 | 0.053529359387787 | 0.228341710030007 | 0.818047577568565 | -0.394020392 | 0.5010791110466 | 1.05498796511369 | 0.674340304769974 | 1.65050138433351 | 0.999714303421225 |
| ebi-fl187-GCST90094729 | ebi-cfmet338-GCST90026299 | 1-carboxyethylvaline levels \|\| id:ebi-cfmet338-GCST90026299 | F-cheese liking (derived food-liking factor) \|\| id:ebi-fl187-GCST90094729 | Weighted mode | 15 | -0.029016728 | 0.161745508884146 | 0.860196158922514 | -0.346037926 | 0.28800446892897 | 0.971400214277785 | 0.707485654569699 | 1.33376326460336 | 0.999714303421225 |
| ebi-fl187-GCST90094729 | ebi-gm430-GCST90011306 | C_Erysipelotrichia abundance \|\| id:ebi-gm430-GCST90011306 | F-cheese liking (derived food-liking factor) \|\| id:ebi-fl187-GCST90094729 | Weighted mode | 14 | -0.093884287 | 0.144495347926661 | 0.527175397378555 | -0.377095169 | 0.189326594801255 | 0.910388099761481 | 0.685850799006432 | 1.20843555681204 | 0.999714303421225 |
| ebi-fl187-GCST90094729 | ebi-gm430-GCST90011312 | F_Erysipelotrichaceae abundance \|\| id:ebi-gm430-GCST90011312 | F-cheese liking (derived food-liking factor) \|\| id:ebi-fl187-GCST90094729 | Weighted mode | 14 | -0.093884287 | 0.14330409765644 | 0.523801517835375 | -0.374760319 | 0.186991744271621 | 0.910388099761481 | 0.68745402903064 | 1.20561733175962 | 0.999714303421225 |
| ebi-fl187-GCST90094729 | ebi-gm430-GCST90011352 | O_Erysipelotrichales abundance \|\| id:ebi-gm430-GCST90011352 | F-cheese liking (derived food-liking factor) \|\| id:ebi-fl187-GCST90094729 | Weighted mode | 14 | -0.093884287 | 0.141077246152581 | 0.517376487491336 | -0.37039569 | 0.182627115324058 | 0.910388099761481 | 0.690461068317556 | 1.20036672626144 | 0.999714303421225 |
| ebi-fl187-GCST90094729 | ebi-gm430-GCST90011362 | OTU97_11 (Parabacteroides) abundance \|\| id:ebi-gm430-GCST90011362 | F-cheese liking (derived food-liking factor) \|\| id:ebi-fl187-GCST90094729 | Weighted mode | 14 | -0.248359266 | 0.180861332636395 | 0.192910984974486 | -0.602847478 | 0.106128945947975 | 0.780079636822853 | 0.547251129849396 | 1.11196525067594 | 0.999714303421225 |
| ebi-fl187-GCST90094729 | ebi-gm430-GCST90011384 | OTU97_235 (Phascolarctobacterium) abundance \|\| id:ebi-gm430-GCST90011384 | F-cheese liking (derived food-liking factor) \|\| id:ebi-fl187-GCST90094729 | Weighted mode | 14 | 0.397885145283545 | 0.470728756920038 | 0.413256149740308 | -0.524743218 | 1.32051350884682 | 1.48867303896812 | 0.591707284475231 | 3.74534415089384 | 0.999714303421225 |
| ebi-fl187-GCST90094729 | ebi-gm430-GCST90011390 | OTU97_3 (Bacteroides) abundance \|\| id:ebi-gm430-GCST90011390 | F-cheese liking (derived food-liking factor) \|\| id:ebi-fl187-GCST90094729 | Weighted mode | 14 | -0.018121483 | 0.110694348177296 | 0.872479225210464 | -0.235082406 | 0.198839439120772 | 0.982041723435518 | 0.790505704741605 | 1.21998606813779 | 0.999714303421225 |
| ebi-fl187-GCST90094729 | ebi-gm430-GCST90011398 | OTU97_4 (Alistipes) abundance \|\| id:ebi-gm430-GCST90011398 | F-cheese liking (derived food-liking factor) \|\| id:ebi-fl187-GCST90094729 | Weighted mode | 14 | -0.091758609 | 0.131651784353132 | 0.498090522599328 | -0.349796107 | 0.166278887848364 | 0.912325349654031 | 0.704831785465908 | 1.18090239513128 | 0.999714303421225 |
| ebi-fl187-GCST90094729 | ebi-gm430-GCST90011421 | OTU99_108 (Cytophagales) abundance \|\| id:ebi-gm430-GCST90011421 | F-cheese liking (derived food-liking factor) \|\| id:ebi-fl187-GCST90094729 | Weighted mode | 14 | 0.185217462831896 | 0.429334431705768 | 0.673241133166118 | -0.656278023 | 1.0267129489752 | 1.20348012386929 | 0.518778627555155 | 2.79187370415419 | 0.999714303421225 |
| ebi-fl187-GCST90094729 | ebi-gm430-GCST90011428 | OTU99_123 (Phascolarctobacterium) abundance \|\| id:ebi-gm430-GCST90011428 | F-cheese liking (derived food-liking factor) \|\| id:ebi-fl187-GCST90094729 | Weighted mode | 14 | -0.119781793 | 0.301863355037947 | 0.697946423995585 | -0.711433969 | 0.471870382588478 | 0.88711398982785 | 0.490939700078458 | 1.60298959489835 | 0.999714303421225 |
| ebi-fl187-GCST90094729 | ebi-gm430-GCST90011450 | OTU99_298 (Phascolarctobacterium) abundance \|\| id:ebi-gm430-GCST90011450 | F-cheese liking (derived food-liking factor) \|\| id:ebi-fl187-GCST90094729 | Weighted mode | 14 | 0.426131747244691 | 0.434205123440444 | 0.344304793024127 | -0.424910295 | 1.27717378918796 | 1.53132250936533 | 0.653828434376017 | 3.58648921398906 | 0.999714303421225 |
| ebi-fl187-GCST90094729 | ebi-gm430-GCST90011451 | OTU99_3 (Bacteroides) abundance \|\| id:ebi-gm430-GCST90011451 | F-cheese liking (derived food-liking factor) \|\| id:ebi-fl187-GCST90094729 | Weighted mode | 14 | -0.020713678 | 0.118185172396651 | 0.863572110839126 | -0.252356616 | 0.21092925985436 | 0.979499376601798 | 0.77696760962282 | 1.23482500027133 | 0.999714303421225 |
| ebi-fl187-GCST90094729 | ebi-gm430-GCST90011472 | OTU99_72 (Parabacteroides) abundance \|\| id:ebi-gm430-GCST90011472 | F-cheese liking (derived food-liking factor) \|\| id:ebi-fl187-GCST90094729 | Weighted mode | 14 | -0.394412487 | 0.325112187547373 | 0.246652959240232 | -1.031632375 | 0.24280740019945 | 0.674075951054941 | 0.356424666727048 | 1.27482306980338 | 0.999714303421225 |
| ebi-fl187-GCST90094729 | ebi-gm430-GCST90011507 | TestASV_3 (Bacteroides) abundance \|\| id:ebi-gm430-GCST90011507 | F-cheese liking (derived food-liking factor) \|\| id:ebi-fl187-GCST90094729 | Weighted mode | 14 | -0.205627654 | 0.145016223505227 | 0.179724770461501 | -0.489859452 | 0.0786041442908361 | 0.814136160375673 | 0.612712503742099 | 1.08177601009141 | 0.999714303421225 |
| ebi-fl187-GCST90094729 | ebi-gm430-GCST90011512 | TestASV_34 (Phascolarctobacterium) abundance \|\| id:ebi-gm430-GCST90011512 | F-cheese liking (derived food-liking factor) \|\| id:ebi-fl187-GCST90094729 | Weighted mode | 14 | -0.507864158 | 0.401303398170736 | 0.227891109219267 | -1.294418818 | 0.278690502399177 | 0.601779513133345 | 0.274057094988708 | 1.32139831096848 | 0.999714303421225 |
| ebi-fl187-GCST90094729 | ebi-gm430-GCST90011519 | TestASV_41 (Barnesiella) abundance \|\| id:ebi-gm430-GCST90011519 | F-cheese liking (derived food-liking factor) \|\| id:ebi-fl187-GCST90094729 | Weighted mode | 14 | 0.190916254061591 | 0.526003263874554 | 0.722470695069873 | -0.840050143 | 1.22188265125572 | 1.21035808526317 | 0.43168887665381 | 3.39357063336321 | 0.999714303421225 |
| ebi-fl187-GCST90094729 | ebi-gm430-GCST90011524 | TestASV_46 (Sutterella) abundance \|\| id:ebi-gm430-GCST90011524 | F-cheese liking (derived food-liking factor) \|\| id:ebi-fl187-GCST90094729 | Weighted mode | 14 | 0.444829123560094 | 0.701446397459696 | 0.536976376227058 | -0.930005815 | 1.8196640625811 | 1.56022356760438 | 0.394551415866593 | 6.16978543990637 | 0.999714303421225 |
| ebi-fl187-GCST90094729 | ebi-gm430-GCST90011581 | OTU97_19 (Bacteria) prevalence \|\| id:ebi-gm430-GCST90011581 | F-cheese liking (derived food-liking factor) \|\| id:ebi-fl187-GCST90094729 | Weighted mode | 14 | -0.191519813 | 0.477443508074068 | 0.694835475116685 | -1.127309089 | 0.744269462449749 | 0.82570326497563 | 0.323903680692331 | 2.10490316236643 | 0.999714303421225 |
| ebi-fl187-GCST90094729 | ebi-gm430-GCST90011614 | OTU97_80 (Ruminococcus) prevalence \|\| id:ebi-gm430-GCST90011614 | F-cheese liking (derived food-liking factor) \|\| id:ebi-fl187-GCST90094729 | Weighted mode | 14 | 0.292660856710235 | 0.267828933422684 | 0.29435495646183 | -0.232283853 | 0.817605566218697 | 1.33998826533292 | 0.792721075272509 | 2.26506977957243 | 0.999714303421225 |
| ebi-fl187-GCST90094729 | ebi-gm430-GCST90011623 | OTU99_11 (Parabacteroides) prevalence \|\| id:ebi-gm430-GCST90011623 | F-cheese liking (derived food-liking factor) \|\| id:ebi-fl187-GCST90094729 | Weighted mode | 14 | 0.41424763177909 | 0.197110659562143 | 0.0556508102311244 | 0.0279107390372889 | 0.800584524520891 | 1.5132318047754 | 1.02830389292935 | 2.22684219201059 | 0.999714303421225 |
| ebi-fl187-GCST90094729 | ebi-gm430-GCST90011642 | OTU99_19 (Bacteria) prevalence \|\| id:ebi-gm430-GCST90011642 | F-cheese liking (derived food-liking factor) \|\| id:ebi-fl187-GCST90094729 | Weighted mode | 14 | -0.191519813 | 0.486038083514614 | 0.699936975269226 | -1.144154457 | 0.761114830313219 | 0.82570326497563 | 0.318493103564178 | 2.14066136491408 | 0.999714303421225 |
| ebi-fl187-GCST90094729 | ebi-gm430-GCST90011653 | OTU99_30 (Parasutterella) prevalence \|\| id:ebi-gm430-GCST90011653 | F-cheese liking (derived food-liking factor) \|\| id:ebi-fl187-GCST90094729 | Weighted mode | 14 | -0.516453044 | 0.381292438270137 | 0.19865777594141 | -1.263786223 | 0.230880135236883 | 0.596633030557033 | 0.282582079757576 | 1.25970823577012 | 0.999714303421225 |
| ebi-fl187-GCST90094729 | ebi-gm430-GCST90011675 | OTU99_8 (Ruminococcaceae) prevalence \|\| id:ebi-gm430-GCST90011675 | F-cheese liking (derived food-liking factor) \|\| id:ebi-fl187-GCST90094729 | Weighted mode | 14 | 0.126498844856129 | 0.24072175270167 | 0.60808417261495 | -0.34531579 | 0.598313480151402 | 1.13484814026244 | 0.707996739560793 | 1.81904835078204 | 0.999714303421225 |
| ebi-fl187-GCST90094729 | ebi-gm430-GCST90011679 | OTU99_92 (Ruminococcus) prevalence \|\| id:ebi-gm430-GCST90011679 | F-cheese liking (derived food-liking factor) \|\| id:ebi-fl187-GCST90094729 | Weighted mode | 14 | 0.310866321385278 | 0.276602142779222 | 0.281391383048486 | -0.231273878 | 0.853006521232553 | 1.36460679024752 | 0.793522107657307 | 2.3466916347009 | 0.999714303421225 |
| ebi-fl187-GCST90094729 | ebi-gm430-GCST90011684 | TestASV_11 (Lachnospiraceae) prevalence \|\| id:ebi-gm430-GCST90011684 | F-cheese liking (derived food-liking factor) \|\| id:ebi-fl187-GCST90094729 | Weighted mode | 14 | 0.240281256787938 | 0.267547986682522 | 0.3854709966665 | -0.284112797 | 0.76467531068568 | 1.27160674806013 | 0.752681739592932 | 2.14829673240986 | 0.999714303421225 |
| ebi-fl187-GCST90094729 | ebi-gm430-GCST90011688 | TestASV_15 (Bacteroides) prevalence \|\| id:ebi-gm430-GCST90011688 | F-cheese liking (derived food-liking factor) \|\| id:ebi-fl187-GCST90094729 | Weighted mode | 14 | -0.04656154 | 0.289165933040034 | 0.874552925756057 | -0.613326769 | 0.52020368887221 | 0.954505818582994 | 0.541546269846593 | 1.6823702949092 | 0.999714303421225 |
| ebi-fl187-GCST90094729 | ebi-gm430-GCST90011692 | TestASV_19 (Prevotella) prevalence \|\| id:ebi-gm430-GCST90011692 | F-cheese liking (derived food-liking factor) \|\| id:ebi-fl187-GCST90094729 | Weighted mode | 13 | 0.314831204097466 | 0.492010004070246 | 0.534272100478724 | -0.649508404 | 1.27917081207515 | 1.37002803631955 | 0.522302475530112 | 3.59365867143664 | 0.999714303421225 |
| ebi-fl187-GCST90094729 | ebi-gm430-GCST90011693 | TestASV_2 (Bacteroides) prevalence \|\| id:ebi-gm430-GCST90011693 | F-cheese liking (derived food-liking factor) \|\| id:ebi-fl187-GCST90094729 | Weighted mode | 14 | -0.242303637 | 0.339047581174976 | 0.487455101974913 | -0.906836896 | 0.422229622536654 | 0.784817841975255 | 0.403799465948262 | 1.5253587412164 | 0.999714303421225 |
| ebi-fl187-GCST90094729 | ebi-gm430-GCST90011716 | TestASV_40 (Barnesiella) prevalence \|\| id:ebi-gm430-GCST90011716 | F-cheese liking (derived food-liking factor) \|\| id:ebi-fl187-GCST90094729 | Weighted mode | 14 | 0.639634787174446 | 0.465651125019473 | 0.192779846496277 | -0.273041418 | 1.55231099221261 | 1.89578838662571 | 0.761061265444373 | 4.72237094442906 | 0.999714303421225 |
| ebi-fl187-GCST90094729 | ebi-gm430-GCST90011718 | TestASV_42 (Alphaproteobacteria) prevalence \|\| id:ebi-gm430-GCST90011718 | F-cheese liking (derived food-liking factor) \|\| id:ebi-fl187-GCST90094729 | Weighted mode | 14 | -0.022355073 | 0.393759954138032 | 0.955589078264689 | -0.794124583 | 0.749414437606358 | 0.977892950500503 | 0.451976730072915 | 2.11576074388677 | 0.999714303421225 |
| ebi-fl187-GCST90094729 | ebi-hbsm44-GCST90104929 | Sulcal depth of the right inferior frontal sulcus \|\| id:ebi-hbsm44-GCST90104929 | F-cheese liking (derived food-liking factor) \|\| id:ebi-fl187-GCST90094729 | Weighted mode | 13 | 0.202918915275577 | 0.25787342232222 | 0.44660712011413 | -0.302512992 | 0.708350823027128 | 1.22497313761117 | 0.738958887290986 | 2.03063961158919 | 0.999714303421225 |
| ebi-fl187-GCST90094729 | ebi-hbsm44-GCST90104935 | Sulcal opening distance of the left calloso-marginal posterior fissure \|\| id:ebi-hbsm44-GCST90104935 | F-cheese liking (derived food-liking factor) \|\| id:ebi-fl187-GCST90094729 | Weighted mode | 13 | -0.012998049 | 0.052416880203967 | 0.808346599986915 | -0.115735135 | 0.0897390358649171 | 0.98708606049278 | 0.890711110669719 | 1.09388878071427 | 0.999714303421225 |
| ebi-fl187-GCST90094729 | ebi-hbsm44-GCST90104936 | Sulcal opening distance of the left anterior inferior temporal sulcus \|\| id:ebi-hbsm44-GCST90104936 | F-cheese liking (derived food-liking factor) \|\| id:ebi-fl187-GCST90094729 | Weighted mode | 13 | -0.015564812 | 0.0310742998343232 | 0.625509241386254 | -0.076470439 | 0.0453408160434545 | 0.984555694022706 | 0.926380298327589 | 1.04638442374316 | 0.999714303421225 |
| ebi-fl187-GCST90094729 | ebi-hbsm44-GCST90104941 | Sulcal opening distance of the right posterior inferior temporal sulcus \|\| id:ebi-hbsm44-GCST90104941 | F-cheese liking (derived food-liking factor) \|\| id:ebi-fl187-GCST90094729 | Weighted mode | 12 | -0.008375562 | 0.0283823553128219 | 0.77341463611915 | -0.064004978 | 0.0472538543564522 | 0.991659415243569 | 0.938000329712782 | 1.04838811319217 | 0.999714303421225 |
| ebi-fl187-GCST90094729 | ebi-IPs91-GCST90274819 | Leukemia inhibitory factor levels \|\| id:ebi-IPs91-GCST90274819 | F-cheese liking (derived food-liking factor) \|\| id:ebi-fl187-GCST90094729 | Weighted mode | 16 | -0.120800881 | 0.109147720912319 | 0.285829504940085 | -0.334730414 | 0.0931286524717712 | 0.88621040378354 | 0.71553095783695 | 1.09760293551568 | 0.999714303421225 |
| ebi-fl187-GCST90094729 | ebi-IPs91-GCST90274837 | STAM binding protein levels \|\| id:ebi-IPs91-GCST90274837 | F-cheese liking (derived food-liking factor) \|\| id:ebi-fl187-GCST90094729 | Weighted mode | 16 | -0.048944815 | 0.110008588014151 | 0.662731932043261 | -0.264561648 | 0.166672017187157 | 0.952233676958786 | 0.767542330091247 | 1.18136673377565 | 0.999714303421225 |
| ebi-fl187-GCST90094729 | ebi-IPs91-GCST90274840 | TNF-beta levels \|\| id:ebi-IPs91-GCST90274840 | F-cheese liking (derived food-liking factor) \|\| id:ebi-fl187-GCST90094729 | Weighted mode | 16 | -0.041713702 | 0.0922955924104108 | 0.657766212989594 | -0.222613063 | 0.13918565913537 | 0.959144342384812 | 0.800424503147616 | 1.14933746519643 | 0.999714303421225 |
| ebi-fl187-GCST90094729 | ebi-IPs91-GCST90274842 | Tumor necrosis factor ligand superfamily member 14 levels \|\| id:ebi-IPs91-GCST90274842 | F-cheese liking (derived food-liking factor) \|\| id:ebi-fl187-GCST90094729 | Weighted mode | 16 | 0.128478814196346 | 0.10999917014061 | 0.261037758674107 | -0.087119559 | 0.344077187671942 | 1.13709733071524 | 0.916567504931717 | 1.41068751899082 | 0.999714303421225 |
| ebi-fl187-GCST90094729 | ebi-met1400-GCST90199628 | 3-methylhistidine levels \|\| id:ebi-met1400-GCST90199628 | F-cheese liking (derived food-liking factor) \|\| id:ebi-fl187-GCST90094729 | Weighted mode | 15 | 0.102471796232121 | 0.13564244586833 | 0.462497436135791 | -0.163387398 | 0.368330990134048 | 1.10790605434397 | 0.849262122511708 | 1.44532034658722 | 0.999714303421225 |
| ebi-fl187-GCST90094729 | ebi-met1400-GCST90199651 | Docosahexaenoate DHA; 22:6n3 levels \|\| id:ebi-met1400-GCST90199651 | F-cheese liking (derived food-liking factor) \|\| id:ebi-fl187-GCST90094729 | Weighted mode | 15 | 0.0435932541622195 | 0.101866320371302 | 0.675200599305243 | -0.156064734 | 0.243251242089972 | 1.0445573990954 | 0.855503808595544 | 1.27538901527063 | 0.999714303421225 |
| ebi-fl187-GCST90094729 | ebi-met1400-GCST90199652 | 1-stearoyl-2-oleoyl-GPS (18:0/18:1) levels \|\| id:ebi-met1400-GCST90199652 | F-cheese liking (derived food-liking factor) \|\| id:ebi-fl187-GCST90094729 | Weighted mode | 15 | 0.00877210950749907 | 0.107782920468738 | 0.936286409906091 | -0.202482415 | 0.220026633626226 | 1.00881069720942 | 0.816700844473692 | 1.24610991857123 | 0.999714303421225 |
| ebi-fl187-GCST90094729 | ebi-met1400-GCST90199653 | 1-myristoyl-2-palmitoyl-gpc (14:0/16:0) levels \|\| id:ebi-met1400-GCST90199653 | F-cheese liking (derived food-liking factor) \|\| id:ebi-fl187-GCST90094729 | Weighted mode | 15 | 0.0646046210673181 | 0.126155072053917 | 0.616560488833122 | -0.18265932 | 0.311868562292995 | 1.06673717558869 | 0.833051911392683 | 1.36597514058945 | 0.999714303421225 |
| ebi-fl187-GCST90094729 | ebi-met1400-GCST90199667 | Erythritol levels in elite athletes \|\| id:ebi-met1400-GCST90199667 | F-cheese liking (derived food-liking factor) \|\| id:ebi-fl187-GCST90094729 | Weighted mode | 15 | 0.0884333528743321 | 0.115163419501946 | 0.455307424329336 | -0.137286949 | 0.314153655098146 | 1.09246144076923 | 0.871720050738503 | 1.36910008959469 | 0.999714303421225 |
| ebi-fl187-GCST90094729 | ebi-met1400-GCST90199674 | 3-hydroxymyristate levels \|\| id:ebi-met1400-GCST90199674 | F-cheese liking (derived food-liking factor) \|\| id:ebi-fl187-GCST90094729 | Weighted mode | 15 | -0.008538995 | 0.101757470232218 | 0.934311997835346 | -0.207983636 | 0.190905646902725 | 0.991497358915087 | 0.812220327445598 | 1.21034524687076 | 0.999714303421225 |
| ebi-fl187-GCST90094729 | ebi-met1400-GCST90199685 | 2-linoleoylglycerol (18:2) levels \|\| id:ebi-met1400-GCST90199685 | F-cheese liking (derived food-liking factor) \|\| id:ebi-fl187-GCST90094729 | Weighted mode | 15 | 0.0710181658556293 | 0.130888868215976 | 0.595948857010648 | -0.185524016 | 0.327560347558942 | 1.07360072856705 | 0.830668886123927 | 1.38757878576392 | 0.999714303421225 |
| ebi-fl187-GCST90094729 | ebi-met1400-GCST90199703 | Gamma-glutamylvaline levels \|\| id:ebi-met1400-GCST90199703 | F-cheese liking (derived food-liking factor) \|\| id:ebi-fl187-GCST90094729 | Weighted mode | 15 | -0.075549104 | 0.129886120580211 | 0.570045860841941 | -0.3301259 | 0.179027692691936 | 0.927234198836933 | 0.718833226643995 | 1.19605386566051 | 0.999714303421225 |
| ebi-fl187-GCST90094729 | ebi-met1400-GCST90199779 | Gamma-glutamyl-2-aminobutyrate levels \|\| id:ebi-met1400-GCST90199779 | F-cheese liking (derived food-liking factor) \|\| id:ebi-fl187-GCST90094729 | Weighted mode | 15 | -0.076492296 | 0.149576319786412 | 0.617045939831207 | -0.369661882 | 0.216677291158223 | 0.926360051288268 | 0.690967919556387 | 1.24194325139399 | 0.999714303421225 |
| ebi-fl187-GCST90094729 | ebi-met1400-GCST90199824 | 4-vinylphenol sulfate levels \|\| id:ebi-met1400-GCST90199824 | F-cheese liking (derived food-liking factor) \|\| id:ebi-fl187-GCST90094729 | Weighted mode | 15 | 0.139477633634731 | 0.147967921240422 | 0.361857901447143 | -0.150539492 | 0.429494759265957 | 1.14967309142219 | 0.860243756593158 | 1.53648103460211 | 0.999714303421225 |
| ebi-fl187-GCST90094729 | ebi-met1400-GCST90199830 | 4-ethylphenylsulfate levels \|\| id:ebi-met1400-GCST90199830 | F-cheese liking (derived food-liking factor) \|\| id:ebi-fl187-GCST90094729 | Weighted mode | 15 | 0.165878143579767 | 0.107692585856439 | 0.145782711040799 | -0.045199325 | 0.376955611858387 | 1.18042925007608 | 0.955806946912718 | 1.45783959714452 | 0.999714303421225 |
| ebi-fl187-GCST90094729 | ebi-met1400-GCST90199833 | Hexanoylglutamine levels \|\| id:ebi-met1400-GCST90199833 | F-cheese liking (derived food-liking factor) \|\| id:ebi-fl187-GCST90094729 | Weighted mode | 15 | 0.14924412820277 | 0.0986730608036154 | 0.152646299498351 | -0.044155071 | 0.342643327377857 | 1.16095637680999 | 0.956805573197744 | 1.40866623963239 | 0.999714303421225 |
| ebi-fl187-GCST90094729 | ebi-met1400-GCST90199837 | Carotenoid levels (cryptoxanthin) \|\| id:ebi-met1400-GCST90199837 | F-cheese liking (derived food-liking factor) \|\| id:ebi-fl187-GCST90094729 | Weighted mode | 15 | 0.0547567166410173 | 0.173113788946901 | 0.756437983699602 | -0.28454631 | 0.394059742976943 | 1.05628360718994 | 0.752355513302951 | 1.4829891442145 | 0.999714303421225 |
| ebi-fl187-GCST90094729 | ebi-met1400-GCST90199869 | 2-hydroxyhippurate levels \|\| id:ebi-met1400-GCST90199869 | F-cheese liking (derived food-liking factor) \|\| id:ebi-fl187-GCST90094729 | Weighted mode | 15 | -0.105215987 | 0.162080069216578 | 0.526741598151676 | -0.422892922 | 0.212460948855172 | 0.900130085363972 | 0.655148781066865 | 1.23671781737568 | 0.999714303421225 |
| ebi-fl187-GCST90094729 | ebi-met1400-GCST90199902 | Hydantoin-5-propionate levels \|\| id:ebi-met1400-GCST90199902 | F-cheese liking (derived food-liking factor) \|\| id:ebi-fl187-GCST90094729 | Weighted mode | 15 | -0.041233242 | 0.130651562691464 | 0.756963924842925 | -0.297310304 | 0.214843821273606 | 0.959605283970318 | 0.742813478248078 | 1.23966827203453 | 0.999714303421225 |
| ebi-fl187-GCST90094729 | ebi-met1400-GCST90199904 | Pantoate levels \|\| id:ebi-met1400-GCST90199904 | F-cheese liking (derived food-liking factor) \|\| id:ebi-fl187-GCST90094729 | Weighted mode | 15 | 0.143767257962708 | 0.116045903296068 | 0.235758495158245 | -0.083682712 | 0.371217228423002 | 1.15461534972161 | 0.919723026427105 | 1.44949791133496 | 0.999714303421225 |
| ebi-fl187-GCST90094729 | ebi-met1400-GCST90199936 | 3-methyl catechol sulfate (2) levels \|\| id:ebi-met1400-GCST90199936 | F-cheese liking (derived food-liking factor) \|\| id:ebi-fl187-GCST90094729 | Weighted mode | 15 | -0.086878732 | 0.164881462767676 | 0.606500616847891 | -0.410046399 | 0.236288934957359 | 0.916788265910064 | 0.66361945808172 | 1.26654020504456 | 0.999714303421225 |
| ebi-fl187-GCST90094729 | ebi-met1400-GCST90199977 | 4-hydroxychlorothalonil levels \|\| id:ebi-met1400-GCST90199977 | F-cheese liking (derived food-liking factor) \|\| id:ebi-fl187-GCST90094729 | Weighted mode | 15 | 0.266667632099779 | 0.215826326002371 | 0.236952315148842 | -0.156351967 | 0.689687231064426 | 1.30560643254002 | 0.855258114872481 | 1.99309205846477 | 0.999714303421225 |
| ebi-fl187-GCST90094729 | ebi-met1400-GCST90200040 | Sphingomyelin (d18:2/24:1, d18:1/24:2) levels \|\| id:ebi-met1400-GCST90200040 | F-cheese liking (derived food-liking factor) \|\| id:ebi-fl187-GCST90094729 | Weighted mode | 15 | -0.043732115 | 0.119192964318685 | 0.719178462361887 | -0.277350325 | 0.189886095099752 | 0.957210345457281 | 0.757788978158134 | 1.20911186604676 | 0.999714303421225 |
| ebi-fl187-GCST90094729 | ebi-met1400-GCST90200077 | 1-stearoyl-2-oleoyl-GPI (18:0/18:1) levels \|\| id:ebi-met1400-GCST90200077 | F-cheese liking (derived food-liking factor) \|\| id:ebi-fl187-GCST90094729 | Weighted mode | 15 | -0.057306168 | 0.126909982220669 | 0.658509451960587 | -0.306049733 | 0.191437397327599 | 0.94430490932289 | 0.736349997667642 | 1.21098901961808 | 0.999714303421225 |
| ebi-fl187-GCST90094729 | ebi-met1400-GCST90200080 | 1-palmitoyl-2-oleoyl-GPI (16:0/18:1) levels \|\| id:ebi-met1400-GCST90200080 | F-cheese liking (derived food-liking factor) \|\| id:ebi-fl187-GCST90094729 | Weighted mode | 15 | -0.041914428 | 0.158874977197053 | 0.795762093879287 | -0.353309384 | 0.269480526825438 | 0.958951836027092 | 0.702359861005777 | 1.30928413605937 | 0.999714303421225 |
| ebi-fl187-GCST90094729 | ebi-met1400-GCST90200082 | 1-oleoyl-2-linoleoyl-GPE (18:1/18:2) levels \|\| id:ebi-met1400-GCST90200082 | F-cheese liking (derived food-liking factor) \|\| id:ebi-fl187-GCST90094729 | Weighted mode | 15 | -0.216412912 | 0.161106156150922 | 0.200547650649951 | -0.532180978 | 0.0993551540210792 | 0.805402672748542 | 0.587322634016802 | 1.10445848278331 | 0.999714303421225 |
| ebi-fl187-GCST90094729 | ebi-met1400-GCST90200100 | Perfluorooctanesulfonate (PFOS) levels \|\| id:ebi-met1400-GCST90200100 | F-cheese liking (derived food-liking factor) \|\| id:ebi-fl187-GCST90094729 | Weighted mode | 15 | -0.072661454 | 0.103194518787277 | 0.492903848975623 | -0.274922711 | 0.129599802905488 | 0.929915596023409 | 0.759630832260565 | 1.13837271869838 | 0.999714303421225 |
| ebi-fl187-GCST90094729 | ebi-met1400-GCST90200102 | 1-palmitoleoyl-2-linolenoyl-GPC (16:1/18:3) levels \|\| id:ebi-met1400-GCST90200102 | F-cheese liking (derived food-liking factor) \|\| id:ebi-fl187-GCST90094729 | Weighted mode | 15 | 0.00313052834621275 | 0.115152320242942 | 0.978695124937024 | -0.222568019 | 0.228829076022379 | 1.00313543356739 | 0.800460558107653 | 1.25712714747288 | 0.999714303421225 |
| ebi-fl187-GCST90094729 | ebi-met1400-GCST90200114 | Glycosyl-N-tricosanoyl-sphingadienine (d18:2/23:0) levels \|\| id:ebi-met1400-GCST90200114 | F-cheese liking (derived food-liking factor) \|\| id:ebi-fl187-GCST90094729 | Weighted mode | 15 | -0.056229957 | 0.127526727206029 | 0.665998552776503 | -0.306182343 | 0.193722427947618 | 0.945321727189939 | 0.736252356972917 | 1.21375933052564 | 0.999714303421225 |
| ebi-fl187-GCST90094729 | ebi-met1400-GCST90200135 | Arachidoylcarnitine (C20) levels \|\| id:ebi-met1400-GCST90200135 | F-cheese liking (derived food-liking factor) \|\| id:ebi-fl187-GCST90094729 | Weighted mode | 15 | -0.098312695 | 0.122832321132543 | 0.436861900758317 | -0.339064045 | 0.142438654074131 | 0.906365443182285 | 0.712436819779036 | 1.15308234188375 | 0.999714303421225 |
| ebi-fl187-GCST90094729 | ebi-met1400-GCST90200146 | Docosahexaenoylcarnitine (C22:6) levels \|\| id:ebi-met1400-GCST90200146 | F-cheese liking (derived food-liking factor) \|\| id:ebi-fl187-GCST90094729 | Weighted mode | 15 | 0.0370944227951879 | 0.1612858311817 | 0.821424620518523 | -0.279025806 | 0.353214651911321 | 1.03779100733974 | 0.75652037995541 | 1.42363669697664 | 0.999714303421225 |
| ebi-fl187-GCST90094729 | ebi-met1400-GCST90200162 | 3-hydroxyphenylacetoylglutamine levels \|\| id:ebi-met1400-GCST90200162 | F-cheese liking (derived food-liking factor) \|\| id:ebi-fl187-GCST90094729 | Weighted mode | 15 | 0.0134856401336383 | 0.127419850805923 | 0.917213781828264 | -0.236257267 | 0.263228547713248 | 1.0135769815157 | 0.789577515210126 | 1.30112405389999 | 0.999714303421225 |
| ebi-fl187-GCST90094729 | ebi-met1400-GCST90200172 | 3-carboxy-4-methyl-5-pentyl-2-furanpropionate (3-CMPFP) levels \|\| id:ebi-met1400-GCST90200172 | F-cheese liking (derived food-liking factor) \|\| id:ebi-fl187-GCST90094729 | Weighted mode | 15 | 0.0132684073631153 | 0.132757244327444 | 0.921805182201988 | -0.246935792 | 0.273472606244906 | 1.01335682329352 | 0.781190850999259 | 1.31452134904293 | 0.999714303421225 |
| ebi-fl187-GCST90094729 | ebi-met1400-GCST90200222 | Dihydrocaffeate sulfate (2) levels \|\| id:ebi-met1400-GCST90200222 | F-cheese liking (derived food-liking factor) \|\| id:ebi-fl187-GCST90094729 | Weighted mode | 15 | -0.136440647 | 0.161169775890428 | 0.411475358939965 | -0.452333408 | 0.17945211391256 | 0.872458101874607 | 0.636142039787491 | 1.19656160404196 | 0.999714303421225 |
| ebi-fl187-GCST90094729 | ebi-met1400-GCST90200223 | 4-methylhexanoylglutamine levels \|\| id:ebi-met1400-GCST90200223 | F-cheese liking (derived food-liking factor) \|\| id:ebi-fl187-GCST90094729 | Weighted mode | 15 | 0.0724788416698516 | 0.135146059076363 | 0.600169629974962 | -0.192407434 | 0.337365117459523 | 1.07517005704646 | 0.824970678806363 | 1.4012505914051 | 0.999714303421225 |
| ebi-fl187-GCST90094729 | ebi-met1400-GCST90200224 | 2-ketocaprylate levels \|\| id:ebi-met1400-GCST90200224 | F-cheese liking (derived food-liking factor) \|\| id:ebi-fl187-GCST90094729 | Weighted mode | 15 | 0.0273158214599176 | 0.113660876316913 | 0.81355990762738 | -0.195459496 | 0.250091139041067 | 1.0276923188047 | 0.822456655574215 | 1.28414244686585 | 0.999714303421225 |
| ebi-fl187-GCST90094729 | ebi-met1400-GCST90200243 | Hydroxypalmitoyl sphingomyelin (d18:1/16:0(OH)) levels \|\| id:ebi-met1400-GCST90200243 | F-cheese liking (derived food-liking factor) \|\| id:ebi-fl187-GCST90094729 | Weighted mode | 15 | -0.242849161 | 0.156156989727565 | 0.142218508543202 | -0.548916861 | 0.0632185388672504 | 0.784389821425862 | 0.577575065857363 | 1.06525961442463 | 0.999714303421225 |
| ebi-fl187-GCST90094729 | ebi-met1400-GCST90200273 | 2-methoxyhydroquinone sulfate (2) levels \|\| id:ebi-met1400-GCST90200273 | F-cheese liking (derived food-liking factor) \|\| id:ebi-fl187-GCST90094729 | Weighted mode | 15 | 0.171341380156029 | 0.135030861373383 | 0.225166521893836 | -0.093319108 | 0.436001868447861 | 1.18689586256367 | 0.91090277742539 | 1.54651168432313 | 0.999714303421225 |
| ebi-fl187-GCST90094729 | ebi-met1400-GCST90200276 | 2,4-di-tert-butylphenol levels \|\| id:ebi-met1400-GCST90200276 | F-cheese liking (derived food-liking factor) \|\| id:ebi-fl187-GCST90094729 | Weighted mode | 15 | 0.0026769438193302 | 0.114041576039132 | 0.981603946047572 | -0.220844545 | 0.226198432856029 | 1.00268053003275 | 0.801841320670182 | 1.25382444055948 | 0.999714303421225 |
| ebi-fl187-GCST90094729 | ebi-met1400-GCST90200289 | N-acetylmethionine levels \|\| id:ebi-met1400-GCST90200289 | F-cheese liking (derived food-liking factor) \|\| id:ebi-fl187-GCST90094729 | Weighted mode | 15 | 0.0177819820065395 | 0.123585825229091 | 0.887642928700984 | -0.224446235 | 0.260010199455558 | 1.01794102273649 | 0.798958531197104 | 1.29694331471401 | 0.999714303421225 |
| ebi-fl187-GCST90094729 | ebi-met1400-GCST90200308 | 3-hydroxyisobutyrate levels \|\| id:ebi-met1400-GCST90200308 | F-cheese liking (derived food-liking factor) \|\| id:ebi-fl187-GCST90094729 | Weighted mode | 15 | -0.113994531 | 0.114221781187787 | 0.335212309412515 | -0.337869222 | 0.109880159859071 | 0.892262835414502 | 0.713288563967544 | 1.11614430355307 | 0.999714303421225 |
| ebi-fl187-GCST90094729 | ebi-met1400-GCST90200383 | Cystathionine levels \|\| id:ebi-met1400-GCST90200383 | F-cheese liking (derived food-liking factor) \|\| id:ebi-fl187-GCST90094729 | Weighted mode | 15 | -0.198229324 | 0.169592586871536 | 0.26197951025677 | -0.530630795 | 0.13417214579794 | 0.820181743825693 | 0.588233797839368 | 1.14358966685667 | 0.999714303421225 |
| ebi-fl187-GCST90094729 | ebi-met1400-GCST90200384 | Guanidinoacetate levels \|\| id:ebi-met1400-GCST90200384 | F-cheese liking (derived food-liking factor) \|\| id:ebi-fl187-GCST90094729 | Weighted mode | 15 | -0.07210374 | 0.126418959495525 | 0.577476349255677 | -0.3198849 | 0.175677420833413 | 0.93043436774981 | 0.726232621355521 | 1.19205346500979 | 0.999714303421225 |
| ebi-fl187-GCST90094729 | ebi-met1400-GCST90200403 | Citrulline levels \|\| id:ebi-met1400-GCST90200403 | F-cheese liking (derived food-liking factor) \|\| id:ebi-fl187-GCST90094729 | Weighted mode | 15 | 0.0784353018886104 | 0.127070795783703 | 0.546974125172381 | -0.170623458 | 0.327494061624668 | 1.08159337584983 | 0.843138991077917 | 1.38748681185605 | 0.999714303421225 |
| ebi-fl187-GCST90094729 | ebi-met1400-GCST90200434 | 12,13-DiHOME levels \|\| id:ebi-met1400-GCST90200434 | F-cheese liking (derived food-liking factor) \|\| id:ebi-fl187-GCST90094729 | Weighted mode | 15 | 0.0300947972367936 | 0.109843307944279 | 0.788101419505546 | -0.185198086 | 0.24538768080758 | 1.03055222282622 | 0.830939669755811 | 1.27811671849073 | 0.999714303421225 |
| ebi-fl187-GCST90094729 | ebi-met1400-GCST90200437 | Fructose levels \|\| id:ebi-met1400-GCST90200437 | F-cheese liking (derived food-liking factor) \|\| id:ebi-fl187-GCST90094729 | Weighted mode | 15 | -0.053346143 | 0.0999181896156493 | 0.60178694568635 | -0.249185794 | 0.142493509021307 | 0.94805179454332 | 0.779435145345577 | 1.15314559588958 | 0.999714303421225 |
| ebi-fl187-GCST90094729 | ebi-met1400-GCST90200448 | N-stearoyl-sphinganine (d18:0/18:0) levels \|\| id:ebi-met1400-GCST90200448 | F-cheese liking (derived food-liking factor) \|\| id:ebi-fl187-GCST90094729 | Weighted mode | 15 | 0.0612977127212079 | 0.118043448043853 | 0.611676354089589 | -0.170067445 | 0.29266287088716 | 1.06321539982387 | 0.843607917166438 | 1.33999096430908 | 0.999714303421225 |
| ebi-fl187-GCST90094729 | ebi-met1400-GCST90200468 | X-11847 levels \|\| id:ebi-met1400-GCST90200468 | F-cheese liking (derived food-liking factor) \|\| id:ebi-fl187-GCST90094729 | Weighted mode | 15 | 0.124940224840705 | 0.131885123849591 | 0.359530990075628 | -0.133554618 | 0.383435067585903 | 1.13308072096191 | 0.874979678116017 | 1.46731627296757 | 0.999714303421225 |
| ebi-fl187-GCST90094729 | ebi-met1400-GCST90200476 | X-11843 levels \|\| id:ebi-met1400-GCST90200476 | F-cheese liking (derived food-liking factor) \|\| id:ebi-fl187-GCST90094729 | Weighted mode | 15 | -0.157529293 | 0.183659628590888 | 0.40548955277337 | -0.517502165 | 0.202443579303631 | 0.854251789871091 | 0.596007418544077 | 1.22439100218011 | 0.999714303421225 |
| ebi-fl187-GCST90094729 | ebi-met1400-GCST90200511 | X-12906 levels \|\| id:ebi-met1400-GCST90200511 | F-cheese liking (derived food-liking factor) \|\| id:ebi-fl187-GCST90094729 | Weighted mode | 15 | -0.183579028 | 0.118939988921926 | 0.145017367354481 | -0.416701407 | 0.0495433500091961 | 0.832286099001794 | 0.659217728645936 | 1.05079114303322 | 0.999714303421225 |
| ebi-fl187-GCST90094729 | ebi-met1400-GCST90200515 | X-12849 levels \|\| id:ebi-met1400-GCST90200515 | F-cheese liking (derived food-liking factor) \|\| id:ebi-fl187-GCST90094729 | Weighted mode | 15 | -0.029409644 | 0.108172944620607 | 0.789685526698229 | -0.241428616 | 0.182609327209432 | 0.971018610795333 | 0.785504874500659 | 1.20034537419043 | 0.999714303421225 |
| ebi-fl187-GCST90094729 | ebi-met1400-GCST90200551 | X-18886 levels \|\| id:ebi-met1400-GCST90200551 | F-cheese liking (derived food-liking factor) \|\| id:ebi-fl187-GCST90094729 | Weighted mode | 15 | 0.0760103507079583 | 0.108531285476455 | 0.495181176296537 | -0.136710969 | 0.28873167024181 | 1.07897374224159 | 0.872222289135632 | 1.33473353174741 | 0.999714303421225 |
| ebi-fl187-GCST90094729 | ebi-met1400-GCST90200564 | X-21319 levels \|\| id:ebi-met1400-GCST90200564 | F-cheese liking (derived food-liking factor) \|\| id:ebi-fl187-GCST90094729 | Weighted mode | 15 | 0.00638937526310629 | 0.108917929867865 | 0.954050204631165 | -0.207089767 | 0.219868517804123 | 1.0064098308642 | 0.812946670702329 | 1.24591290445296 | 0.999714303421225 |
| ebi-fl187-GCST90094729 | ebi-met1400-GCST90200579 | X-21442 levels \|\| id:ebi-met1400-GCST90200579 | F-cheese liking (derived food-liking factor) \|\| id:ebi-fl187-GCST90094729 | Weighted mode | 15 | -0.027286123 | 0.124126726498902 | 0.82918064460604 | -0.270574507 | 0.216002260487461 | 0.973082779886542 | 0.762941053133245 | 1.24110518450021 | 0.999714303421225 |
| ebi-fl187-GCST90094729 | ebi-met1400-GCST90200581 | X-21441 levels \|\| id:ebi-met1400-GCST90200581 | F-cheese liking (derived food-liking factor) \|\| id:ebi-fl187-GCST90094729 | Weighted mode | 15 | 0.0484352601465112 | 0.111020687241884 | 0.66929168043218 | -0.169165287 | 0.266035807140603 | 1.04962741689547 | 0.844369328706887 | 1.30478177835507 | 0.999714303421225 |
| ebi-fl187-GCST90094729 | ebi-met1400-GCST90200648 | X-24585 levels \|\| id:ebi-met1400-GCST90200648 | F-cheese liking (derived food-liking factor) \|\| id:ebi-fl187-GCST90094729 | Weighted mode | 15 | 0.218149577332679 | 0.119552054436689 | 0.0894514676477555 | -0.016172449 | 0.452471604028589 | 1.24377309396791 | 0.983957622558136 | 1.5721932264284 | 0.999714303421225 |
| ebi-fl187-GCST90094729 | ebi-met1400-GCST90200720 | Adenosine 5'-diphosphate (ADP) to pantothenate ratio \|\| id:ebi-met1400-GCST90200720 | F-cheese liking (derived food-liking factor) \|\| id:ebi-fl187-GCST90094729 | Weighted mode | 15 | 0.0526608165408367 | 0.193244289528064 | 0.789208736978731 | -0.326097991 | 0.431419624015842 | 1.05407206067167 | 0.721734460532466 | 1.53944140102293 | 0.999714303421225 |
| ebi-fl187-GCST90094729 | ebi-met1400-GCST90200724 | Cholate to taurocholate ratio \|\| id:ebi-met1400-GCST90200724 | F-cheese liking (derived food-liking factor) \|\| id:ebi-fl187-GCST90094729 | Weighted mode | 15 | 0.0731286632467724 | 0.137543239106626 | 0.603284131532862 | -0.196456085 | 0.342713411895759 | 1.07586895280256 | 0.821637412379495 | 1.40876496878633 | 0.999714303421225 |
| ebi-fl187-GCST90094729 | ebi-met1400-GCST90200726 | N-acetylputrescine to (N(1) + N(8))-acetylspermidine ratio \|\| id:ebi-met1400-GCST90200726 | F-cheese liking (derived food-liking factor) \|\| id:ebi-fl187-GCST90094729 | Weighted mode | 15 | 0.0942125580755778 | 0.119698047046966 | 0.444352927299557 | -0.140395614 | 0.328820730287631 | 1.09879327848141 | 0.869014373014116 | 1.38932876869266 | 0.999714303421225 |
| ebi-fl187-GCST90094729 | ebi-met1400-GCST90200744 | Arginine to glutamate ratio \|\| id:ebi-met1400-GCST90200744 | F-cheese liking (derived food-liking factor) \|\| id:ebi-fl187-GCST90094729 | Weighted mode | 15 | -0.031715157 | 0.168938882730725 | 0.853780823776675 | -0.362835367 | 0.299405052849966 | 0.968782493400617 | 0.695700959091334 | 1.34905595177755 | 0.999714303421225 |
| ebi-fl187-GCST90094729 | ebi-met1400-GCST90200759 | Histidine to trans-urocanate ratio \|\| id:ebi-met1400-GCST90200759 | F-cheese liking (derived food-liking factor) \|\| id:ebi-fl187-GCST90094729 | Weighted mode | 15 | 0.0176442516390428 | 0.126110541299234 | 0.890723225558691 | -0.229532409 | 0.264820912585541 | 1.01780083099989 | 0.794905205893151 | 1.30319756859576 | 0.999714303421225 |
| ebi-fl187-GCST90094729 | ebi-met1400-GCST90200795 | Oleoyl-linoleoyl-glycerol (18:1 to 18:2) [2] to linoleoyl-arachidonoyl-glycerol (18:2 to 20:4) [2] ratio \|\| id:ebi-met1400-GCST90200795 | F-cheese liking (derived food-liking factor) \|\| id:ebi-fl187-GCST90094729 | Weighted mode | 15 | -0.153102538 | 0.139492404599285 | 0.290912076802946 | -0.426507651 | 0.12030257461605 | 0.858041735075058 | 0.652784870829701 | 1.1278380551235 | 0.999714303421225 |
| ebi-fl187-GCST90094729 | ebi-met1400-GCST90200811 | Choline to taurocholate ratio \|\| id:ebi-met1400-GCST90200811 | F-cheese liking (derived food-liking factor) \|\| id:ebi-fl187-GCST90094729 | Weighted mode | 15 | -0.049085952 | 0.10729544251138 | 0.654342213122669 | -0.259385019 | 0.161213115297854 | 0.952099291319901 | 0.771525913447702 | 1.17493533882878 | 0.999714303421225 |
| ebi-fl187-GCST90094729 | ebi-met1400-GCST90200831 | Cholate to phosphate ratio \|\| id:ebi-met1400-GCST90200831 | F-cheese liking (derived food-liking factor) \|\| id:ebi-fl187-GCST90094729 | Weighted mode | 15 | -0.110171556 | 0.134559526785665 | 0.426642847688255 | -0.373908228 | 0.153565116990443 | 0.895680463197081 | 0.688040051736884 | 1.16598371000025 | 0.999714303421225 |
| ebi-fl187-GCST90094729 | ebi-met1400-GCST90200841 | Adenosine 5'-diphosphate (ADP) to oxalate (ethanedioate) ratio \|\| id:ebi-met1400-GCST90200841 | F-cheese liking (derived food-liking factor) \|\| id:ebi-fl187-GCST90094729 | Weighted mode | 15 | 0.17481312451961 | 0.202405776265716 | 0.402320439634195 | -0.221902197 | 0.571528446000412 | 1.19102362270269 | 0.800993700121795 | 1.77097181865492 | 0.999714303421225 |
| ebi-fl187-GCST90094729 | ebi-met1400-GCST90200844 | Aspartate to phosphate ratio \|\| id:ebi-met1400-GCST90200844 | F-cheese liking (derived food-liking factor) \|\| id:ebi-fl187-GCST90094729 | Weighted mode | 15 | -0.065341785 | 0.119748926571719 | 0.593890277482595 | -0.300049681 | 0.169366111271903 | 0.936747242680127 | 0.740781417087977 | 1.18455373802203 | 0.999714303421225 |
| ebi-fl187-GCST90094729 | ebi-met1400-GCST90200870 | Adenosine 5'-monophosphate (AMP) to acetoacetate ratio \|\| id:ebi-met1400-GCST90200870 | F-cheese liking (derived food-liking factor) \|\| id:ebi-fl187-GCST90094729 | Weighted mode | 15 | -0.00192694 | 0.118249576815564 | 0.987228577975168 | -0.23369611 | 0.2298422310256 | 0.998074915823166 | 0.791602339309946 | 1.25840145755985 | 0.999714303421225 |
| ebi-fl187-GCST90094729 | ebi-met1400-GCST90200887 | Cholesterol to cortisol ratio \|\| id:ebi-met1400-GCST90200887 | F-cheese liking (derived food-liking factor) \|\| id:ebi-fl187-GCST90094729 | Weighted mode | 14 | 0.0179751770151438 | 0.124260462477334 | 0.887200103708728 | -0.225575329 | 0.261525683470718 | 1.01813770285933 | 0.798056941000741 | 1.29891030166821 | 0.999714303421225 |
| ebi-fl187-GCST90094729 | ebi-met1400-GCST90200890 | Cortisol to taurocholate ratio \|\| id:ebi-met1400-GCST90200890 | F-cheese liking (derived food-liking factor) \|\| id:ebi-fl187-GCST90094729 | Weighted mode | 15 | -0.007936359 | 0.126866432260275 | 0.951003783124701 | -0.256594566 | 0.240721848357185 | 0.99209505087519 | 0.773681827024528 | 1.2721671307136 | 0.999714303421225 |
| ebi-fl187-GCST90094729 | ebi-met1400-GCST90200948 | Adenosine 5'-diphosphate (ADP) to mannitol to sorbitol ratio \|\| id:ebi-met1400-GCST90200948 | F-cheese liking (derived food-liking factor) \|\| id:ebi-fl187-GCST90094729 | Weighted mode | 15 | -0.280079047 | 0.150828989071551 | 0.0844839500179542 | -0.575703866 | 0.0155457712989895 | 0.755724001166935 | 0.562308939416645 | 1.01566723540312 | 0.999714303421225 |
| ebi-fl187-GCST90094729 | ebi-met1400-GCST90200972 | Leucine to N-palmitoyl-sphingosine (d18:1 to 16:0) ratio \|\| id:ebi-met1400-GCST90200972 | F-cheese liking (derived food-liking factor) \|\| id:ebi-fl187-GCST90094729 | Weighted mode | 15 | -0.014941874 | 0.117518156973833 | 0.900633585299631 | -0.245277461 | 0.215393713907747 | 0.985169202119352 | 0.782487398047081 | 1.24035014394709 | 0.999714303421225 |
| ebi-fl187-GCST90094729 | ebi-met1400-GCST90200973 | Proline to glutamate ratio \|\| id:ebi-met1400-GCST90200973 | F-cheese liking (derived food-liking factor) \|\| id:ebi-fl187-GCST90094729 | Weighted mode | 15 | -0.045100139 | 0.118848118005784 | 0.710027548329094 | -0.278042451 | 0.187842171989308 | 0.955901753705681 | 0.757264674497243 | 1.20664305824677 | 0.999714303421225 |
| ebi-fl187-GCST90094729 | ebi-met1400-GCST90200983 | Cholesterol to linoleoyl-arachidonoyl-glycerol (18:2 to 20:4) [1] ratio \|\| id:ebi-met1400-GCST90200983 | F-cheese liking (derived food-liking factor) \|\| id:ebi-fl187-GCST90094729 | Weighted mode | 15 | -0.111366127 | 0.146616305154729 | 0.460108222021699 | -0.398734085 | 0.176001830772189 | 0.894611147369007 | 0.671169151279517 | 1.1924402417351 | 0.999714303421225 |
| ebi-fl187-GCST90094729 | ebi-met1400-GCST90201001 | Serine to alpha-ketobutyrate ratio \|\| id:ebi-met1400-GCST90201001 | F-cheese liking (derived food-liking factor) \|\| id:ebi-fl187-GCST90094729 | Weighted mode | 15 | 0.0223930411983573 | 0.122773156963101 | 0.857888577617262 | -0.218242346 | 0.263028428846034 | 1.02264564736166 | 0.803930588332925 | 1.3008637004799 | 0.999714303421225 |
| ebi-fl187-GCST90094729 | ebi-met1400-GCST90201005 | Fructose to maltose ratio \|\| id:ebi-met1400-GCST90201005 | F-cheese liking (derived food-liking factor) \|\| id:ebi-fl187-GCST90094729 | Weighted mode | 15 | -0.136874354 | 0.124911031351991 | 0.291670008221701 | -0.381699976 | 0.107951267051307 | 0.872079792238685 | 0.682699848924927 | 1.11399345587771 | 0.999714303421225 |
| ebi-fl187-GCST90094729 | ebi-met1400-GCST90201020 | Paraxanthine to 5-acetylamino-6-formylamino-3-methyluracil ratio \|\| id:ebi-met1400-GCST90201020 | F-cheese liking (derived food-liking factor) \|\| id:ebi-fl187-GCST90094729 | Weighted mode | 15 | -0.055888797 | 0.127407701979316 | 0.667600759405033 | -0.305607893 | 0.19383029897215 | 0.945644288613113 | 0.736675418577725 | 1.21389026705016 | 0.999714303421225 |
| ebi-fl187-GCST90094729 | ebi-pw11-GCST90275197 | Placental weight (fetal genotype, adjusted for sex) \|\| id:ebi-pw11-GCST90275197 | F-cheese liking (derived food-liking factor) \|\| id:ebi-fl187-GCST90094729 | Weighted mode | 15 | 0.058924949152216 | 0.0472176904154837 | 0.232521786310015 | -0.033621724 | 0.151471622366564 | 1.06069563164644 | 0.966937204543316 | 1.16354528267966 | 0.999714303421225 |
| ebi-fl187-GCST90094729 | prot-a-1017 | FAS-associated factor 2 \|\| id:prot-a-1017 | F-cheese liking (derived food-liking factor) \|\| id:ebi-fl187-GCST90094729 | Weighted mode | 16 | -0.035684182 | 0.200040994314436 | 0.860808033698326 | -0.427764531 | 0.356396166787188 | 0.96494499229681 | 0.651964914300872 | 1.42817323100457 | 0.999714303421225 |
| ebi-fl187-GCST90094729 | prot-a-1019 | Fas apoptotic inhibitory molecule 3 \|\| id:prot-a-1019 | F-cheese liking (derived food-liking factor) \|\| id:ebi-fl187-GCST90094729 | Weighted mode | 16 | -0.019920325 | 0.203706318860749 | 0.923394247286388 | -0.41918471 | 0.379344060396019 | 0.980276774167828 | 0.657582722248651 | 1.46132573356987 | 0.999714303421225 |
| ebi-fl187-GCST90094729 | prot-a-1052 | MIP18 family protein FAM96A \|\| id:prot-a-1052 | F-cheese liking (derived food-liking factor) \|\| id:ebi-fl187-GCST90094729 | Weighted mode | 16 | -0.025821432 | 0.183358090693965 | 0.889881826312853 | -0.38520329 | 0.33356042549406 | 0.974509089952709 | 0.680312321436352 | 1.39592939371642 | 0.999714303421225 |
| ebi-fl187-GCST90094729 | prot-a-1066 | F-box/LRR-repeat protein 4 \|\| id:prot-a-1066 | F-cheese liking (derived food-liking factor) \|\| id:ebi-fl187-GCST90094729 | Weighted mode | 16 | 0.0342329236580647 | 0.196647059186392 | 0.864127878911997 | -0.351195312 | 0.419661159663394 | 1.03482561402636 | 0.703846270562749 | 1.52144594101327 | 0.999714303421225 |
| ebi-fl187-GCST90094729 | prot-a-1076 | Low affinity immunoglobulin gamma Fc region receptor III-B \|\| id:prot-a-1076 | F-cheese liking (derived food-liking factor) \|\| id:ebi-fl187-GCST90094729 | Weighted mode | 16 | -0.230103767 | 0.201242761950538 | 0.270787764382115 | -0.62453958 | 0.164332046788907 | 0.794451160703113 | 0.535507930298104 | 1.17860560233193 | 0.999714303421225 |
| ebi-fl187-GCST90094729 | prot-a-1081 | Fc receptor-like protein 3 \|\| id:prot-a-1081 | F-cheese liking (derived food-liking factor) \|\| id:ebi-fl187-GCST90094729 | Weighted mode | 16 | -0.153044381 | 0.170108686379752 | 0.382504001420256 | -0.486457406 | 0.180368644662813 | 0.85809163830894 | 0.61480052957166 | 1.1976587922732 | 0.999714303421225 |
| ebi-fl187-GCST90094729 | prot-a-1105 | Fibroblast growth factor receptor 4 \|\| id:prot-a-1105 | F-cheese liking (derived food-liking factor) \|\| id:ebi-fl187-GCST90094729 | Weighted mode | 16 | 0.0880430631453444 | 0.25371013907187 | 0.733395289939865 | -0.409228809 | 0.585314935726209 | 1.09203514748394 | 0.664162248346058 | 1.7955563814565 | 0.999714303421225 |
| ebi-fl187-GCST90094729 | prot-a-1143 | Follistatin-related protein 1 \|\| id:prot-a-1143 | F-cheese liking (derived food-liking factor) \|\| id:ebi-fl187-GCST90094729 | Weighted mode | 16 | -0.198964748 | 0.173858393062368 | 0.27038866022691 | -0.539727198 | 0.141797702419722 | 0.819578784629327 | 0.582907248724759 | 1.15234350865253 | 0.999714303421225 |
| ebi-fl187-GCST90094729 | prot-a-1151 | Alpha-(1,3)-fucosyltransferase 10 \|\| id:prot-a-1151 | F-cheese liking (derived food-liking factor) \|\| id:ebi-fl187-GCST90094729 | Weighted mode | 16 | -0.000136016 | 0.166736305151097 | 0.999359871834159 | -0.326939174 | 0.326667142093309 | 0.999863993246917 | 0.721127604928937 | 1.386339946 | 0.999714303421225 |
| ebi-fl187-GCST90094729 | prot-a-1165 | Growth arrest and DNA damage-inducible proteins-interacting protein 1 \|\| id:prot-a-1165 | F-cheese liking (derived food-liking factor) \|\| id:ebi-fl187-GCST90094729 | Weighted mode | 16 | 0.021442421910314 | 0.235026745031828 | 0.928513910379356 | -0.439209998 | 0.482094842172697 | 1.02167396260876 | 0.644545411942462 | 1.61946337144334 | 0.999714303421225 |
| ebi-fl187-GCST90094729 | prot-a-1194 | Growth/differentiation factor 11/8 \|\| id:prot-a-1194 | F-cheese liking (derived food-liking factor) \|\| id:ebi-fl187-GCST90094729 | Weighted mode | 16 | -0.134345384 | 0.247669693381901 | 0.595484882435159 | -0.619777983 | 0.351087215065871 | 0.874288047380078 | 0.53806388366866 | 1.4206112192105 | 0.999714303421225 |
| ebi-fl187-GCST90094729 | prot-a-1216 | D-glucuronyl C5-epimerase \|\| id:prot-a-1216 | F-cheese liking (derived food-liking factor) \|\| id:ebi-fl187-GCST90094729 | Weighted mode | 16 | 0.00272358402467865 | 0.197537563551063 | 0.989181088657725 | -0.384450041 | 0.389897208584762 | 1.00272729634916 | 0.680824959394014 | 1.47682898073924 | 0.999714303421225 |
| ebi-fl187-GCST90094729 | prot-a-1246 | Glypican-3 \|\| id:prot-a-1246 | F-cheese liking (derived food-liking factor) \|\| id:ebi-fl187-GCST90094729 | Weighted mode | 16 | 0.108450628724269 | 0.219488855513931 | 0.62838895337517 | -0.321747528 | 0.538648785531573 | 1.11454988043049 | 0.724881179370061 | 1.71368973470539 | 0.999714303421225 |
| ebi-fl187-GCST90094729 | prot-a-1275 | Glutamate receptor 4 \|\| id:prot-a-1275 | F-cheese liking (derived food-liking factor) \|\| id:ebi-fl187-GCST90094729 | Weighted mode | 16 | 0.0210618244934457 | 0.188285966286264 | 0.912416922522252 | -0.347978669 | 0.390102318414524 | 1.0212851901253 | 0.706113937868199 | 1.47713192394732 | 0.999714303421225 |
| ebi-fl187-GCST90094729 | prot-a-1289 | Glutathione S-transferase P \|\| id:prot-a-1289 | F-cheese liking (derived food-liking factor) \|\| id:ebi-fl187-GCST90094729 | Weighted mode | 16 | 0.182206804648608 | 0.177134641601878 | 0.319953753715601 | -0.164977093 | 0.529390702188289 | 1.19986230532611 | 0.847913127103815 | 1.69789746817569 | 0.999714303421225 |
| ebi-fl187-GCST90094729 | prot-a-130 | Apolipoprotein D \|\| id:prot-a-130 | F-cheese liking (derived food-liking factor) \|\| id:ebi-fl187-GCST90094729 | Weighted mode | 16 | 0.179273751712775 | 0.166069530348016 | 0.297413229131993 | -0.146222528 | 0.504770031194886 | 1.1963482017141 | 0.863965425492955 | 1.65660450929264 | 0.999714303421225 |
| ebi-fl187-GCST90094729 | prot-a-1313 | Heparin-binding EGF-like growth factor \|\| id:prot-a-1313 | F-cheese liking (derived food-liking factor) \|\| id:ebi-fl187-GCST90094729 | Weighted mode | 16 | 0.195380850550379 | 0.203869630527086 | 0.353073739971844 | -0.204203625 | 0.594965326383467 | 1.2157739264977 | 0.815296339336135 | 1.81296808171026 | 0.999714303421225 |
[truncated: 245,218 more chars]
