# Supplementary material for: Mendelian Randomization Mediation Analysis Reveals the Impact of Dietary Preferences on Preeclampsia and Fetal Growth Restriction via Immune Modulation
Source: Food Sci Nutr. 2025 Nov 26;13(12):e71280. doi: 10.1002/fsn3.71280 (PMC12648301; doi:10.1002/fsn3.71280)
Supplement: Supplementary file 1 — Data S1: fsn371280‐sup‐0001‐Supinfo1.zip. [file FSN3-13-e71280-s001.zip › fsn371280-sup-0009-Supinfo9@Supplementary Material 3_PE - MR (Dietary Preferences to Immune Cells) MR Analysis - 2.docx]

| id.exposure | id.outcome | outcome | exposure | method | nsnp | b | se | pval | lo_ci | up_ci | or | or_lci95 | or_uci95 | p_fdr |
| --- | --- | --- | --- | --- | --- | --- | --- | --- | --- | --- | --- | --- | --- | --- |
| ebi-fl187-GCST90094733 | ebi-met1400-GCST90200726 | N-acetylputrescine to (N(1) + N(8))-acetylspermidine ratio \|\| id:ebi-met1400-GCST90200726 | Chilli pepper liking \|\| id:ebi-fl187-GCST90094733 | MR Egger | 13 | -0.122695585 | 0.515665148541225 | 0.816304231847912 | -1.133399276 | 0.888008106179449 | 0.884532886693694 | 0.321937041417801 | 2.43028395923942 | 0.999950574162623 |
| ebi-fl187-GCST90094733 | ebi-met1400-GCST90200744 | Arginine to glutamate ratio \|\| id:ebi-met1400-GCST90200744 | Chilli pepper liking \|\| id:ebi-fl187-GCST90094733 | MR Egger | 13 | -0.362306539 | 0.580489991844568 | 0.545262665677561 | -1.500066923 | 0.775453844695439 | 0.696068962628479 | 0.223115228033572 | 2.17157746248401 | 0.999950574162623 |
| ebi-fl187-GCST90094733 | ebi-met1400-GCST90200759 | Histidine to trans-urocanate ratio \|\| id:ebi-met1400-GCST90200759 | Chilli pepper liking \|\| id:ebi-fl187-GCST90094733 | MR Egger | 13 | -0.848153236 | 0.677960714066211 | 0.236875234917801 | -2.176956235 | 0.480649764064911 | 0.428204995976293 | 0.113386126655112 | 1.61712481048749 | 0.999950574162623 |
| ebi-fl187-GCST90094733 | ebi-met1400-GCST90200795 | Oleoyl-linoleoyl-glycerol (18:1 to 18:2) [2] to linoleoyl-arachidonoyl-glycerol (18:2 to 20:4) [2] ratio \|\| id:ebi-met1400-GCST90200795 | Chilli pepper liking \|\| id:ebi-fl187-GCST90094733 | MR Egger | 13 | -0.682724853 | 0.607285129285451 | 0.284852469305106 | -1.873003707 | 0.507554000181145 | 0.505238414487635 | 0.153661414127936 | 1.66122287057342 | 0.999950574162623 |
| ebi-fl187-GCST90094733 | ebi-met1400-GCST90200811 | Choline to taurocholate ratio \|\| id:ebi-met1400-GCST90200811 | Chilli pepper liking \|\| id:ebi-fl187-GCST90094733 | MR Egger | 13 | 0.40524668896527 | 0.542045504440594 | 0.470367494685171 | -0.6571625 | 1.46765587766883 | 1.49967240706324 | 0.518319982948382 | 4.33905194184042 | 0.999950574162623 |
| ebi-fl187-GCST90094733 | ebi-met1400-GCST90200831 | Cholate to phosphate ratio \|\| id:ebi-met1400-GCST90200831 | Chilli pepper liking \|\| id:ebi-fl187-GCST90094733 | MR Egger | 13 | -0.920740183 | 0.540239514537299 | 0.11636813591361 | -1.979609632 | 0.13812926527976 | 0.398224173121726 | 0.138123145684829 | 1.14812395324631 | 0.999950574162623 |
| ebi-fl187-GCST90094733 | ebi-met1400-GCST90200841 | Adenosine 5'-diphosphate (ADP) to oxalate (ethanedioate) ratio \|\| id:ebi-met1400-GCST90200841 | Chilli pepper liking \|\| id:ebi-fl187-GCST90094733 | MR Egger | 13 | -0.940996841 | 0.696163556213196 | 0.203615382818509 | -2.305477411 | 0.423483729131199 | 0.390238635515684 | 0.0997111860492188 | 1.52727290370383 | 0.999950574162623 |
| ebi-fl187-GCST90094733 | ebi-met1400-GCST90200844 | Aspartate to phosphate ratio \|\| id:ebi-met1400-GCST90200844 | Chilli pepper liking \|\| id:ebi-fl187-GCST90094733 | MR Egger | 13 | 0.020075749845762 | 0.635676365658322 | 0.97537137550832 | -1.225849927 | 1.26600142653607 | 1.02027862304796 | 0.293508133839885 | 3.54664266039221 | 0.999950574162623 |
| ebi-fl187-GCST90094733 | ebi-met1400-GCST90200870 | Adenosine 5'-monophosphate (AMP) to acetoacetate ratio \|\| id:ebi-met1400-GCST90200870 | Chilli pepper liking \|\| id:ebi-fl187-GCST90094733 | MR Egger | 13 | -0.32118652 | 0.764044682768396 | 0.682305048093224 | -1.818714099 | 1.17634105782462 | 0.72528795737206 | 0.162234234085046 | 3.24248839386868 | 0.999950574162623 |
| ebi-fl187-GCST90094733 | ebi-met1400-GCST90200887 | Cholesterol to cortisol ratio \|\| id:ebi-met1400-GCST90200887 | Chilli pepper liking \|\| id:ebi-fl187-GCST90094733 | MR Egger | 13 | -0.440095609 | 0.503699569391307 | 0.400939787334742 | -1.427346765 | 0.547155547222835 | 0.643974848487475 | 0.23994470817389 | 1.72832986666215 | 0.999950574162623 |
| ebi-fl187-GCST90094733 | ebi-met1400-GCST90200890 | Cortisol to taurocholate ratio \|\| id:ebi-met1400-GCST90200890 | Chilli pepper liking \|\| id:ebi-fl187-GCST90094733 | MR Egger | 13 | 0.164098687894351 | 0.540084092718748 | 0.766920569612674 | -0.894466134 | 1.2226635096231 | 1.17833059632025 | 0.408825798652391 | 3.39622156625443 | 0.999950574162623 |
| ebi-fl187-GCST90094733 | ebi-met1400-GCST90200948 | Adenosine 5'-diphosphate (ADP) to mannitol to sorbitol ratio \|\| id:ebi-met1400-GCST90200948 | Chilli pepper liking \|\| id:ebi-fl187-GCST90094733 | MR Egger | 13 | 0.431659020411639 | 0.694300485413187 | 0.546796550824764 | -0.929169931 | 1.79248797182148 | 1.53980998186258 | 0.394881353140363 | 6.00437260809536 | 0.999950574162623 |
| ebi-fl187-GCST90094733 | ebi-met1400-GCST90200972 | Leucine to N-palmitoyl-sphingosine (d18:1 to 16:0) ratio \|\| id:ebi-met1400-GCST90200972 | Chilli pepper liking \|\| id:ebi-fl187-GCST90094733 | MR Egger | 13 | -0.086957691 | 0.620206681105604 | 0.891030354214667 | -1.302562786 | 1.1286474041619 | 0.916715880323537 | 0.271834246645153 | 3.09147215852592 | 0.999950574162623 |
| ebi-fl187-GCST90094733 | ebi-met1400-GCST90200973 | Proline to glutamate ratio \|\| id:ebi-met1400-GCST90200973 | Chilli pepper liking \|\| id:ebi-fl187-GCST90094733 | MR Egger | 13 | -0.598062373 | 0.507755533061383 | 0.263708957270134 | -1.593263218 | 0.397138472078219 | 0.549876059385335 | 0.203261242684647 | 1.48756190157832 | 0.999950574162623 |
| ebi-fl187-GCST90094733 | ebi-met1400-GCST90200983 | Cholesterol to linoleoyl-arachidonoyl-glycerol (18:2 to 20:4) [1] ratio \|\| id:ebi-met1400-GCST90200983 | Chilli pepper liking \|\| id:ebi-fl187-GCST90094733 | MR Egger | 13 | -0.629152404 | 0.533792473211124 | 0.263406533857148 | -1.675385652 | 0.417080843144922 | 0.533043414867187 | 0.187235957664524 | 1.51752518948506 | 0.999950574162623 |
| ebi-fl187-GCST90094733 | ebi-met1400-GCST90201001 | Serine to alpha-ketobutyrate ratio \|\| id:ebi-met1400-GCST90201001 | Chilli pepper liking \|\| id:ebi-fl187-GCST90094733 | MR Egger | 13 | 0.614341710072016 | 0.527102224970331 | 0.268464444587646 | -0.418778651 | 1.64746207101387 | 1.84843938993098 | 0.657849793632787 | 5.19378163726482 | 0.999950574162623 |
| ebi-fl187-GCST90094733 | ebi-met1400-GCST90201005 | Fructose to maltose ratio \|\| id:ebi-met1400-GCST90201005 | Chilli pepper liking \|\| id:ebi-fl187-GCST90094733 | MR Egger | 13 | -0.095812948 | 0.589495312279965 | 0.873832771191375 | -1.25122376 | 1.05959786407251 | 0.908633961977139 | 0.286154398177062 | 2.88521050914413 | 0.999950574162623 |
| ebi-fl187-GCST90094733 | ebi-met1400-GCST90201020 | Paraxanthine to 5-acetylamino-6-formylamino-3-methyluracil ratio \|\| id:ebi-met1400-GCST90201020 | Chilli pepper liking \|\| id:ebi-fl187-GCST90094733 | MR Egger | 13 | 0.998811929476004 | 0.547997128382927 | 0.0956247379423403 | -0.075262442 | 2.07288630110654 | 2.71505423562634 | 0.927500039275929 | 7.94772958516234 | 0.999950574162623 |
| ebi-fl187-GCST90094733 | ebi-pw11-GCST90275197 | Placental weight (fetal genotype, adjusted for sex) \|\| id:ebi-pw11-GCST90275197 | Chilli pepper liking \|\| id:ebi-fl187-GCST90094733 | MR Egger | 12 | -0.051574291 | 0.199201856601071 | 0.800960126878728 | -0.44200993 | 0.338861348350381 | 0.949733091111787 | 0.642743253288117 | 1.40334875510303 | 0.999950574162623 |
| ebi-fl187-GCST90094733 | prot-a-1017 | FAS-associated factor 2 \|\| id:prot-a-1017 | Chilli pepper liking \|\| id:ebi-fl187-GCST90094733 | MR Egger | 14 | 0.305533098402382 | 0.914382455099105 | 0.744039982853503 | -1.486656514 | 2.09772271039663 | 1.3573484104345 | 0.226127447064458 | 8.14759433773592 | 0.999950574162623 |
| ebi-fl187-GCST90094733 | prot-a-1019 | Fas apoptotic inhibitory molecule 3 \|\| id:prot-a-1019 | Chilli pepper liking \|\| id:ebi-fl187-GCST90094733 | MR Egger | 14 | 0.679141576204699 | 0.825116887985818 | 0.426514267852144 | -0.938087524 | 2.2963706766569 | 1.97218403567481 | 0.391375616450576 | 9.93804853211068 | 0.999950574162623 |
| ebi-fl187-GCST90094733 | prot-a-1052 | MIP18 family protein FAM96A \|\| id:prot-a-1052 | Chilli pepper liking \|\| id:ebi-fl187-GCST90094733 | MR Egger | 14 | 0.056492224924746 | 0.825403690641896 | 0.946561067902777 | -1.561299009 | 1.67428345858286 | 1.05811838781815 | 0.209863279826627 | 5.33497105145703 | 0.999950574162623 |
| ebi-fl187-GCST90094733 | prot-a-1066 | F-box/LRR-repeat protein 4 \|\| id:prot-a-1066 | Chilli pepper liking \|\| id:ebi-fl187-GCST90094733 | MR Egger | 14 | -0.424239756 | 0.870906726530496 | 0.634947096490477 | -2.13121694 | 1.28273742801505 | 0.654266998674227 | 0.118692763957813 | 3.60649875595053 | 0.999950574162623 |
| ebi-fl187-GCST90094733 | prot-a-1076 | Low affinity immunoglobulin gamma Fc region receptor III-B \|\| id:prot-a-1076 | Chilli pepper liking \|\| id:ebi-fl187-GCST90094733 | MR Egger | 14 | -1.096757148 | 0.989752255383979 | 0.289529292448475 | -3.036671568 | 0.843157272666885 | 0.333952287540214 | 0.0479943699127369 | 2.32369193628575 | 0.999950574162623 |
| ebi-fl187-GCST90094733 | prot-a-1081 | Fc receptor-like protein 3 \|\| id:prot-a-1081 | Chilli pepper liking \|\| id:ebi-fl187-GCST90094733 | MR Egger | 14 | -0.892920652 | 0.825116887985818 | 0.300442020237171 | -2.510149753 | 0.724308448211199 | 0.409458119893673 | 0.0812560700144031 | 2.06330372508962 | 0.999950574162623 |
| ebi-fl187-GCST90094733 | prot-a-1105 | Fibroblast growth factor receptor 4 \|\| id:prot-a-1105 | Chilli pepper liking \|\| id:ebi-fl187-GCST90094733 | MR Egger | 14 | 0.0322408198940931 | 0.909147360341253 | 0.972293871783302 | -1.749688006 | 1.81416964616295 | 1.03276618600382 | 0.173828168271493 | 6.13597902778914 | 0.999950574162623 |
| ebi-fl187-GCST90094733 | prot-a-1143 | Follistatin-related protein 1 \|\| id:prot-a-1143 | Chilli pepper liking \|\| id:ebi-fl187-GCST90094733 | MR Egger | 14 | -1.044500666 | 0.925250785024763 | 0.281009720553254 | -2.857992205 | 0.768990872675427 | 0.351867474922207 | 0.0573838597270698 | 2.15758787395969 | 0.999950574162623 |
| ebi-fl187-GCST90094733 | prot-a-1151 | Alpha-(1,3)-fucosyltransferase 10 \|\| id:prot-a-1151 | Chilli pepper liking \|\| id:ebi-fl187-GCST90094733 | MR Egger | 14 | -0.071790418 | 0.826491174853842 | 0.932214046140825 | -1.691713121 | 1.5481322843289 | 0.930725938417462 | 0.184203690311329 | 4.70267870843949 | 0.999950574162623 |
| ebi-fl187-GCST90094733 | prot-a-1165 | Growth arrest and DNA damage-inducible proteins-interacting protein 1 \|\| id:prot-a-1165 | Chilli pepper liking \|\| id:ebi-fl187-GCST90094733 | MR Egger | 14 | -0.071169999 | 0.859640706832432 | 0.935383188883813 | -1.756065785 | 1.61372578618581 | 0.931303557804474 | 0.17272305858363 | 5.02148539917919 | 0.999950574162623 |
| ebi-fl187-GCST90094733 | prot-a-1194 | Growth/differentiation factor 11/8 \|\| id:prot-a-1194 | Chilli pepper liking \|\| id:ebi-fl187-GCST90094733 | MR Egger | 14 | 1.11835899067008 | 0.903656341478537 | 0.239536006250912 | -0.652807439 | 2.88952541996802 | 3.05982887338492 | 0.520582220665067 | 17.984772362066 | 0.999950574162623 |
| ebi-fl187-GCST90094733 | prot-a-1216 | D-glucuronyl C5-epimerase \|\| id:prot-a-1216 | Chilli pepper liking \|\| id:ebi-fl187-GCST90094733 | MR Egger | 14 | -0.281873204 | 0.950517666619609 | 0.771882302021695 | -2.14488783 | 1.58114142299279 | 0.754369329798355 | 0.117081169284076 | 4.86050053326395 | 0.999950574162623 |
| ebi-fl187-GCST90094733 | prot-a-1246 | Glypican-3 \|\| id:prot-a-1246 | Chilli pepper liking \|\| id:ebi-fl187-GCST90094733 | MR Egger | 14 | 1.96215976498824 | 1.16787556090845 | 0.118759617282264 | -0.326876334 | 4.2511958643688 | 7.11467651282532 | 0.721172921799942 | 70.1892990599419 | 0.999950574162623 |
| ebi-fl187-GCST90094733 | prot-a-1275 | Glutamate receptor 4 \|\| id:prot-a-1275 | Chilli pepper liking \|\| id:ebi-fl187-GCST90094733 | MR Egger | 14 | -0.544299352 | 1.21427772552903 | 0.661959131803635 | -2.924283694 | 1.83568499046343 | 0.580248190930782 | 0.0537031460452723 | 6.269427173 | 0.999950574162623 |
| ebi-fl187-GCST90094733 | prot-a-1289 | Glutathione S-transferase P \|\| id:prot-a-1289 | Chilli pepper liking \|\| id:ebi-fl187-GCST90094733 | MR Egger | 14 | -0.160161492 | 0.825403690641896 | 0.849390386498204 | -1.777952726 | 1.4576297417744 | 0.852006185771736 | 0.168983749490762 | 4.29576537851047 | 0.999950574162623 |
| ebi-fl187-GCST90094733 | prot-a-130 | Apolipoprotein D \|\| id:prot-a-130 | Chilli pepper liking \|\| id:ebi-fl187-GCST90094733 | MR Egger | 14 | 1.9801841397951 | 0.826383043630783 | 0.0337513136152833 | 0.360473374278768 | 3.59989490531144 | 7.24407678516934 | 1.43400807645606 | 36.5943883657318 | 0.999950574162623 |
| ebi-fl187-GCST90094733 | prot-a-1313 | Heparin-binding EGF-like growth factor \|\| id:prot-a-1313 | Chilli pepper liking \|\| id:ebi-fl187-GCST90094733 | MR Egger | 13 | -1.679905023 | 0.827205142097293 | 0.0671560806794852 | -3.301227101 | -0.058582944 | 0.186391678158801 | 0.0368379357758193 | 0.943100012396966 | 0.999950574162623 |
| ebi-fl187-GCST90094733 | prot-a-1347 | HLA class II histocompatibility antigen, DQ alpha 2 chain \|\| id:prot-a-1347 | Chilli pepper liking \|\| id:ebi-fl187-GCST90094733 | MR Egger | 13 | 0.747685948146163 | 0.85044704323877 | 0.398108875115487 | -0.919190257 | 2.41456215289415 | 2.11210683252403 | 0.398841869933705 | 11.1848720214259 | 0.999950574162623 |
| ebi-fl187-GCST90094733 | prot-a-1357 | Non-histone chromosomal protein HMG-14 \|\| id:prot-a-1357 | Chilli pepper liking \|\| id:ebi-fl187-GCST90094733 | MR Egger | 14 | -0.127241045 | 0.826491174853842 | 0.880205361887112 | -1.747163747 | 1.49268165807985 | 0.880521402056039 | 0.174267509867204 | 4.44901026054452 | 0.999950574162623 |
| ebi-fl187-GCST90094733 | prot-a-137 | Apolipoprotein M \|\| id:prot-a-137 | Chilli pepper liking \|\| id:ebi-fl187-GCST90094733 | MR Egger | 14 | 1.18157132639721 | 0.95942010276116 | 0.241707814410471 | -0.698892075 | 3.06203472780908 | 3.25949190671963 | 0.497135787948095 | 21.3709971149371 | 0.999950574162623 |
| ebi-fl187-GCST90094733 | prot-a-1381 | 17-beta-hydroxysteroid dehydrogenase 14 \|\| id:prot-a-1381 | Chilli pepper liking \|\| id:ebi-fl187-GCST90094733 | MR Egger | 14 | -0.19767421 | 1.11678185655302 | 0.862458095355776 | -2.386566648 | 1.99121822919728 | 0.820637165263668 | 0.0919448214704543 | 7.32445118976489 | 0.999950574162623 |
| ebi-fl187-GCST90094733 | prot-a-1392 | Serine protease HTRA2, mitochondrial \|\| id:prot-a-1392 | Chilli pepper liking \|\| id:ebi-fl187-GCST90094733 | MR Egger | 14 | 0.396380142537125 | 0.826491174853842 | 0.640138453294853 | -1.22354256 | 2.01630284525066 | 1.48643426705747 | 0.294186146635991 | 7.5105060369024 | 0.999950574162623 |
| ebi-fl187-GCST90094733 | prot-a-1416 | Interferon-induced protein with tetratricopeptide repeats 2 \|\| id:prot-a-1416 | Chilli pepper liking \|\| id:ebi-fl187-GCST90094733 | MR Egger | 14 | -0.354795213 | 0.825116887985818 | 0.674820669559416 | -1.972024314 | 1.26243388708025 | 0.701317048857439 | 0.139174837316824 | 3.53401241560963 | 0.999950574162623 |
| ebi-fl187-GCST90094733 | prot-a-1428 | Interferon gamma \|\| id:prot-a-1428 | Chilli pepper liking \|\| id:ebi-fl187-GCST90094733 | MR Egger | 14 | 0.65467510110052 | 0.93651859137143 | 0.497845538722932 | -1.180901338 | 2.49025154018852 | 1.92451714228847 | 0.307001901381977 | 12.0643103977192 | 0.999950574162623 |
| ebi-fl187-GCST90094733 | prot-a-1483 | Interleukin-17C \|\| id:prot-a-1483 | Chilli pepper liking \|\| id:ebi-fl187-GCST90094733 | MR Egger | 14 | -0.085188601 | 1.15495096374681 | 0.942417011514465 | -2.34889249 | 2.17851528831505 | 0.918339068741963 | 0.0954748430676925 | 8.83318178988695 | 0.999950574162623 |
| ebi-fl187-GCST90094733 | prot-a-1503 | Interleukin-1 receptor-like 2 \|\| id:prot-a-1503 | Chilli pepper liking \|\| id:ebi-fl187-GCST90094733 | MR Egger | 14 | -1.104340108 | 0.9567219364646 | 0.270849492130085 | -2.979515103 | 0.770834887598687 | 0.331429517825921 | 0.0508174691125543 | 2.16157016877461 | 0.999950574162623 |
| ebi-fl187-GCST90094733 | prot-a-1522 | Interleukin-31 receptor subunit alpha \|\| id:prot-a-1522 | Chilli pepper liking \|\| id:ebi-fl187-GCST90094733 | MR Egger | 14 | 0.397840829017335 | 0.826202521321446 | 0.638802204844533 | -1.221516113 | 2.01719777080737 | 1.48860706799923 | 0.29478290383337 | 7.51723038914722 | 0.999950574162623 |
| ebi-fl187-GCST90094733 | prot-a-1540 | Interleukin-6 receptor subunit alpha \|\| id:prot-a-1540 | Chilli pepper liking \|\| id:ebi-fl187-GCST90094733 | MR Egger | 14 | 0.371259897064696 | 0.984010045735944 | 0.712543958546837 | -1.557399793 | 2.29991958670715 | 1.44955976076148 | 0.21068317956214 | 9.97338043020715 | 0.999950574162623 |
| ebi-fl187-GCST90094733 | prot-a-1541 | Interleukin-6 receptor subunit alpha \|\| id:prot-a-1541 | Chilli pepper liking \|\| id:ebi-fl187-GCST90094733 | MR Egger | 14 | 0.698939967286332 | 1.13048132789568 | 0.547954602460236 | -1.516803435 | 2.91468336996187 | 2.01161919471976 | 0.219412132223373 | 18.4429718792637 | 0.999950574162623 |
| ebi-fl187-GCST90094733 | prot-a-1545 | Interleukin-9 \|\| id:prot-a-1545 | Chilli pepper liking \|\| id:ebi-fl187-GCST90094733 | MR Egger | 14 | -0.900293945 | 0.970897730923449 | 0.372056268561996 | -2.803253497 | 1.00266560773367 | 0.40645016823508 | 0.060612538735304 | 2.72553736743751 | 0.999950574162623 |
| ebi-fl187-GCST90094733 | prot-a-1548 | Inositol monophosphatase 3 \|\| id:prot-a-1548 | Chilli pepper liking \|\| id:ebi-fl187-GCST90094733 | MR Egger | 14 | 1.18646224026373 | 0.826491174853842 | 0.176682770709524 | -0.433460462 | 2.80638494297726 | 3.2754728497352 | 0.648261922797259 | 16.5499808211129 | 0.999950574162623 |
| ebi-fl187-GCST90094733 | prot-a-1555 | Inhibin beta A chain:Inhibin beta B chain heterodimer \|\| id:prot-a-1555 | Chilli pepper liking \|\| id:ebi-fl187-GCST90094733 | MR Egger | 14 | 0.268776618619183 | 0.826491174853842 | 0.750628066401848 | -1.351146084 | 1.88869932133271 | 1.30836284450206 | 0.258943319698702 | 6.61076460619005 | 0.999950574162623 |
| ebi-fl187-GCST90094733 | prot-a-1603 | Junctophilin-3 \|\| id:prot-a-1603 | Chilli pepper liking \|\| id:ebi-fl187-GCST90094733 | MR Egger | 14 | 0.324507225222984 | 1.29711215624907 | 0.806682413746765 | -2.217832601 | 2.86684705147117 | 1.38334879860019 | 0.108844763382551 | 17.5814971627322 | 0.999950574162623 |
| ebi-fl187-GCST90094733 | prot-a-1627 | UPF0258 protein KIAA1024 \|\| id:prot-a-1627 | Chilli pepper liking \|\| id:ebi-fl187-GCST90094733 | MR Egger | 14 | -1.52958864 | 1.07285827389388 | 0.179442100154629 | -3.632390857 | 0.573213576722108 | 0.216624759727582 | 0.0264528637308312 | 1.77395865357063 | 0.999950574162623 |
| ebi-fl187-GCST90094733 | prot-a-1666 | Kallikrein-7 \|\| id:prot-a-1666 | Chilli pepper liking \|\| id:ebi-fl187-GCST90094733 | MR Egger | 14 | -0.198400433 | 1.20832479184042 | 0.872311123647114 | -2.566717025 | 2.16991615862281 | 0.820041415424172 | 0.0767872225749774 | 8.75754976492685 | 0.999950574162623 |
| ebi-fl187-GCST90094733 | prot-a-1678 | Importin subunit alpha-3 \|\| id:prot-a-1678 | Chilli pepper liking \|\| id:ebi-fl187-GCST90094733 | MR Egger | 14 | -0.7310047 | 0.954264975847835 | 0.458456850171083 | -2.601364052 | 1.13935465308871 | 0.481425059476758 | 0.0741723342407161 | 3.12475116584605 | 0.999950574162623 |
| ebi-fl187-GCST90094733 | prot-a-1718 | Protein LDOC1 \|\| id:prot-a-1718 | Chilli pepper liking \|\| id:ebi-fl187-GCST90094733 | MR Egger | 14 | -0.005841376 | 0.825403690641896 | 0.994469715185559 | -1.62363261 | 1.61194985733733 | 0.994175651346739 | 0.197181114436183 | 5.01257551240081 | 0.999950574162623 |
| ebi-fl187-GCST90094733 | prot-a-1723 | Leptin \|\| id:prot-a-1723 | Chilli pepper liking \|\| id:ebi-fl187-GCST90094733 | MR Egger | 14 | -0.88837961 | 0.834327400770667 | 0.307929616375439 | -2.523661315 | 0.746902095708653 | 0.411321714722326 | 0.0801655573915055 | 2.11045190113082 | 0.999950574162623 |
| ebi-fl187-GCST90094733 | prot-a-1730 | Galectin-7 \|\| id:prot-a-1730 | Chilli pepper liking \|\| id:ebi-fl187-GCST90094733 | MR Egger | 14 | 1.53020078053695 | 1.0446991692848 | 0.168697509174001 | -0.517409591 | 3.57781115233517 | 4.619104155 | 0.59606259559755 | 35.7951050043218 | 0.999950574162623 |
| ebi-fl187-GCST90094733 | prot-a-1753 | Lipase member N \|\| id:prot-a-1753 | Chilli pepper liking \|\| id:ebi-fl187-GCST90094733 | MR Egger | 14 | -0.537118876 | 0.825003520267324 | 0.527284624576529 | -2.154125775 | 1.07988802409608 | 0.584429643552181 | 0.116004560342415 | 2.94434983637142 | 0.999950574162623 |
| ebi-fl187-GCST90094733 | prot-a-1756 | VIP36-like protein \|\| id:prot-a-1756 | Chilli pepper liking \|\| id:ebi-fl187-GCST90094733 | MR Egger | 14 | 1.02343775196616 | 0.826491174853842 | 0.239285856240157 | -0.596484951 | 2.64336045467969 | 2.78274472551099 | 0.550744130441967 | 14.0603735552206 | 0.999950574162623 |
| ebi-fl187-GCST90094733 | prot-a-1760 | Ig Kappa chain V-I region HK102- like \|\| id:prot-a-1760 | Chilli pepper liking \|\| id:ebi-fl187-GCST90094733 | MR Egger | 14 | 0.935027452686379 | 1.13370492517779 | 0.425602482450521 | -1.287034201 | 3.15708910603486 | 2.54728338655107 | 0.276088392829891 | 23.502084187208 | 0.999950574162623 |
| ebi-fl187-GCST90094733 | prot-a-179 | Neutral ceramidase \|\| id:prot-a-179 | Chilli pepper liking \|\| id:ebi-fl187-GCST90094733 | MR Egger | 14 | 0.773844109952926 | 1.08689142984719 | 0.490088776577191 | -1.356463093 | 2.90415131245341 | 2.16808461083316 | 0.257570169639175 | 18.2497487434843 | 0.999950574162623 |
| ebi-fl187-GCST90094733 | prot-a-1809 | Lactotransferrin \|\| id:prot-a-1809 | Chilli pepper liking \|\| id:ebi-fl187-GCST90094733 | MR Egger | 14 | 1.41861039860837 | 0.999429102430386 | 0.181227861801747 | -0.540270642 | 3.37749143937192 | 4.13137548445401 | 0.582590557471442 | 29.2971850893486 | 0.999950574162623 |
| ebi-fl187-GCST90094733 | prot-a-1818 | Lysophospholipase-like protein 1 \|\| id:prot-a-1818 | Chilli pepper liking \|\| id:ebi-fl187-GCST90094733 | MR Egger | 14 | -1.087990332 | 1.01157401472577 | 0.303281684540013 | -3.070675401 | 0.894694737084302 | 0.336892856714758 | 0.0463898125651905 | 2.44658882261997 | 0.999950574162623 |
| ebi-fl187-GCST90094733 | prot-a-1852 | MAP kinase-activated protein kinase 3 \|\| id:prot-a-1852 | Chilli pepper liking \|\| id:ebi-fl187-GCST90094733 | MR Egger | 14 | -0.648779499 | 0.959285802512871 | 0.511665762029337 | -2.528979672 | 1.23142067347863 | 0.522683322904024 | 0.0797403400644829 | 3.42609344054801 | 0.999950574162623 |
| ebi-fl187-GCST90094733 | prot-a-1856 | Megakaryocyte-associated tyrosine-protein kinase \|\| id:prot-a-1856 | Chilli pepper liking \|\| id:ebi-fl187-GCST90094733 | MR Egger | 14 | -0.284054514 | 0.825403690641896 | 0.736696302666034 | -1.901845747 | 1.33373671989643 | 0.752725609683222 | 0.1492928079469 | 3.79519851803245 | 0.999950574162623 |
| ebi-fl187-GCST90094733 | prot-a-1890 | Beta-1,3-N-acetylglucosaminyltransferase manic fringe \|\| id:prot-a-1890 | Chilli pepper liking \|\| id:ebi-fl187-GCST90094733 | MR Egger | 14 | 1.40277567051292 | 0.911831325496882 | 0.149888584224949 | -0.384413727 | 3.18996506848681 | 4.06647150161334 | 0.680849682690328 | 24.2875790263893 | 0.999950574162623 |
| ebi-fl187-GCST90094733 | prot-a-1919 | Matrilysin \|\| id:prot-a-1919 | Chilli pepper liking \|\| id:ebi-fl187-GCST90094733 | MR Egger | 14 | -0.041227508 | 0.826202521321446 | 0.961023035955172 | -1.66058445 | 1.57812943391895 | 0.959610786104256 | 0.19002788590668 | 4.84588278406595 | 0.999950574162623 |
| ebi-fl187-GCST90094733 | prot-a-1923 | Multimerin-2 \|\| id:prot-a-1923 | Chilli pepper liking \|\| id:ebi-fl187-GCST90094733 | MR Egger | 14 | -0.125277656 | 0.825403690641896 | 0.881884330645555 | -1.74306889 | 1.49251357731221 | 0.882251905733394 | 0.174982573502279 | 4.44826253032602 | 0.999950574162623 |
| ebi-fl187-GCST90094733 | prot-a-1948 | Mesothelin \|\| id:prot-a-1948 | Chilli pepper liking \|\| id:ebi-fl187-GCST90094733 | MR Egger | 14 | 0.156739639972725 | 0.825403690641896 | 0.852566100398425 | -1.461051594 | 1.77453087363084 | 1.16969103346297 | 0.231992184893893 | 5.89751380801661 | 0.999950574162623 |
| ebi-fl187-GCST90094733 | prot-a-195 | ATPase family AAA domain-containing protein 1 \|\| id:prot-a-195 | Chilli pepper liking \|\| id:ebi-fl187-GCST90094733 | MR Egger | 14 | -0.987963537 | 0.826202521321446 | 0.254874069338299 | -2.607320478 | 0.631393405246929 | 0.372334164396388 | 0.0737318453852693 | 1.88022867530796 | 0.999950574162623 |
| ebi-fl187-GCST90094733 | prot-a-1966 | Mucin-1 \|\| id:prot-a-1966 | Chilli pepper liking \|\| id:ebi-fl187-GCST90094733 | MR Egger | 14 | -0.170323838 | 0.826094698726507 | 0.84010732158261 | -1.789469447 | 1.44882177197564 | 0.843391650500553 | 0.167048774383219 | 4.2580945520873 | 0.999950574162623 |
| ebi-fl187-GCST90094733 | prot-a-1986 | Myocardial zonula adherens protein \|\| id:prot-a-1986 | Chilli pepper liking \|\| id:ebi-fl187-GCST90094733 | MR Egger | 14 | 1.33652153368526 | 0.825403690641896 | 0.131362298370817 | -0.2812697 | 2.95431276734338 | 3.80578216910567 | 0.75482473181581 | 19.1885312022567 | 0.999950574162623 |
| ebi-fl187-GCST90094733 | prot-a-2071 | Epididymal secretory protein E1 \|\| id:prot-a-2071 | Chilli pepper liking \|\| id:ebi-fl187-GCST90094733 | MR Egger | 13 | -0.41897137 | 0.97920900450875 | 0.677004404446003 | -2.338221019 | 1.50027827879967 | 0.657723025583831 | 0.0964991557987804 | 4.48293640293811 | 0.999950574162623 |
| ebi-fl187-GCST90094733 | prot-a-2098 | Neuritin \|\| id:prot-a-2098 | Chilli pepper liking \|\| id:ebi-fl187-GCST90094733 | MR Egger | 14 | 0.69911046756552 | 0.825403690641895 | 0.413572901633442 | -0.918680766 | 2.31690170122364 | 2.01196220559497 | 0.399045127855714 | 10.1441958168858 | 0.999950574162623 |
| ebi-fl187-GCST90094733 | prot-a-2115 | Neurotrophin-4 \|\| id:prot-a-2115 | Chilli pepper liking \|\| id:ebi-fl187-GCST90094733 | MR Egger | 14 | -0.154008047 | 1.01505995009891 | 0.881926262536602 | -2.143525549 | 1.83550945494699 | 0.857265122359619 | 0.1172407753926 | 6.2683267621978 | 0.999950574162623 |
| ebi-fl187-GCST90094733 | prot-a-2118 | Netrin-1 \|\| id:prot-a-2118 | Chilli pepper liking \|\| id:ebi-fl187-GCST90094733 | MR Egger | 14 | -0.571262338 | 0.856542384133086 | 0.517429614049463 | -2.250085411 | 1.10756073460209 | 0.564812004671629 | 0.105390222672093 | 3.02696580890381 | 0.999950574162623 |
| ebi-fl187-GCST90094733 | prot-a-2120 | Netrin-G1 \|\| id:prot-a-2120 | Chilli pepper liking \|\| id:ebi-fl187-GCST90094733 | MR Egger | 14 | 0.415797529438115 | 0.826491174853842 | 0.62400898241361 | -1.204125173 | 2.03572023215165 | 1.51557897767621 | 0.299954292797424 | 7.65776550871151 | 0.999950574162623 |
| ebi-fl187-GCST90094733 | prot-a-2145 | Olfactomedin-like protein 3 \|\| id:prot-a-2145 | Chilli pepper liking \|\| id:ebi-fl187-GCST90094733 | MR Egger | 14 | -1.179269687 | 1.09970340670278 | 0.304654349761187 | -3.334688364 | 0.976148990633615 | 0.307503230375725 | 0.0356256867409105 | 2.65421512795488 | 0.999950574162623 |
| ebi-fl187-GCST90094733 | prot-a-2162 | Prolyl 4-hydroxylase subunit alpha-2 \|\| id:prot-a-2162 | Chilli pepper liking \|\| id:ebi-fl187-GCST90094733 | MR Egger | 14 | -1.027729395 | 0.932379015402348 | 0.291962674308435 | -2.855192265 | 0.799733475007218 | 0.357818503291835 | 0.0575447561933827 | 2.22494784525183 | 0.999950574162623 |
| ebi-fl187-GCST90094733 | prot-a-2168 | Polyadenylate-binding protein-interacting protein 1 \|\| id:prot-a-2168 | Chilli pepper liking \|\| id:ebi-fl187-GCST90094733 | MR Egger | 14 | -1.809595575 | 1.07719994393376 | 0.11879989386775 | -3.920907465 | 0.301716314975439 | 0.163720335990327 | 0.0198230978075013 | 1.35217757976468 | 0.999950574162623 |
| ebi-fl187-GCST90094733 | prot-a-219 | Beta-1,4-galactosyltransferase 5 \|\| id:prot-a-219 | Chilli pepper liking \|\| id:ebi-fl187-GCST90094733 | MR Egger | 14 | -1.319425385 | 0.826484966983556 | 0.136376317096109 | -2.93933592 | 0.300485150102333 | 0.26728884597796 | 0.0529008474459913 | 1.35051385059888 | 0.999950574162623 |
| ebi-fl187-GCST90094733 | prot-a-2190 | Pyruvate carboxylase, mitochondrial \|\| id:prot-a-2190 | Chilli pepper liking \|\| id:ebi-fl187-GCST90094733 | MR Egger | 14 | -0.447826592 | 0.826491174853842 | 0.597845535028179 | -2.067749295 | 1.17209611032429 | 0.639015484573227 | 0.126470108509723 | 3.22875337371094 | 0.999950574162623 |
| ebi-fl187-GCST90094733 | prot-a-2197 | Protocadherin-9 \|\| id:prot-a-2197 | Chilli pepper liking \|\| id:ebi-fl187-GCST90094733 | MR Egger | 14 | 0.389273832543325 | 0.825116887985818 | 0.645546288956404 | -1.227955268 | 2.00650293299553 | 1.47590864782189 | 0.29289084913842 | 7.43726320956508 | 0.999950574162623 |
| ebi-fl187-GCST90094733 | prot-a-2198 | Protocadherin alpha-4 \|\| id:prot-a-2198 | Chilli pepper liking \|\| id:ebi-fl187-GCST90094733 | MR Egger | 14 | -0.849352559 | 1.08302526120602 | 0.448103463205105 | -2.972082071 | 1.27337695303221 | 0.427691747530145 | 0.0511966043259233 | 3.57289772073356 | 0.999950574162623 |
| ebi-fl187-GCST90094733 | prot-a-2254 | Placenta growth factor \|\| id:prot-a-2254 | Chilli pepper liking \|\| id:ebi-fl187-GCST90094733 | MR Egger | 14 | -1.081807301 | 0.826094698726507 | 0.214868515317545 | -2.700952911 | 0.537338308154962 | 0.338982328476274 | 0.0671415023802091 | 1.7114454539383 | 0.999950574162623 |
| ebi-fl187-GCST90094733 | prot-a-2261 | Membrane-associated progesterone receptor component 2 \|\| id:prot-a-2261 | Chilli pepper liking \|\| id:ebi-fl187-GCST90094733 | MR Egger | 14 | 0.0938904913902395 | 0.826704045967789 | 0.911455003230934 | -1.526449439 | 1.71423042148711 | 1.09843945075346 | 0.217305856968684 | 5.55240085933551 | 0.999950574162623 |
| ebi-fl187-GCST90094733 | prot-a-2268 | Polymeric immunoglobulin receptor \|\| id:prot-a-2268 | Chilli pepper liking \|\| id:ebi-fl187-GCST90094733 | MR Egger | 14 | 1.32901412087414 | 0.956199221422023 | 0.189806925460193 | -0.545136353 | 3.20316459486131 | 3.77731757272233 | 0.579762725497641 | 24.610288688273 | 0.999950574162623 |
| ebi-fl187-GCST90094733 | prot-a-2283 | Group 10 secretory phospholipase A2 \|\| id:prot-a-2283 | Chilli pepper liking \|\| id:ebi-fl187-GCST90094733 | MR Egger | 14 | -0.958423962 | 1.21266404597057 | 0.444664540860832 | -3.335245492 | 1.41839756791134 | 0.383496815423031 | 0.0356058441780234 | 4.13049629449258 | 0.999950574162623 |
| ebi-fl187-GCST90094733 | prot-a-2298 | Inactive phospholipase D5 \|\| id:prot-a-2298 | Chilli pepper liking \|\| id:ebi-fl187-GCST90094733 | MR Egger | 14 | -0.895978913 | 0.982853752946244 | 0.379923157473299 | -2.822372269 | 1.03041444296175 | 0.408207803141491 | 0.0594647089918111 | 2.80222695731261 | 0.999950574162623 |
| ebi-fl187-GCST90094733 | prot-a-2304 | Procollagen-lysine,2-oxoglutarate 5-dioxygenase 2 \|\| id:prot-a-2304 | Chilli pepper liking \|\| id:ebi-fl187-GCST90094733 | MR Egger | 14 | -0.141799755 | 0.972496502150987 | 0.886492022622632 | -2.047892899 | 1.76429338959377 | 0.86779501102843 | 0.129006447037022 | 5.83744610026908 | 0.999950574162623 |
| ebi-fl187-GCST90094733 | prot-a-2331 | Periostin \|\| id:prot-a-2331 | Chilli pepper liking \|\| id:ebi-fl187-GCST90094733 | MR Egger | 14 | 1.53392429479229 | 0.825403690641896 | 0.0878048910161654 | -0.083866939 | 3.1517155284504 | 4.63633551628372 | 0.919553604800601 | 23.3761326227578 | 0.999950574162623 |
| ebi-fl187-GCST90094733 | prot-a-2333 | Periostin \|\| id:prot-a-2333 | Chilli pepper liking \|\| id:ebi-fl187-GCST90094733 | MR Egger | 14 | 2.000547172 | 0.90106506211346 | 0.0464215538516216 | 0.234459649787615 | 3.76663469327238 | 7.39310028639276 | 1.26422545964079 | 43.2343229823823 | 0.999950574162623 |
| ebi-fl187-GCST90094733 | prot-a-2367 | cAMP-dependent protein kinase catalytic subunit alpha \|\| id:prot-a-2367 | Chilli pepper liking \|\| id:ebi-fl187-GCST90094733 | MR Egger | 14 | 0.0474086581581457 | 0.825403690641895 | 0.955142476087609 | -1.570382575 | 1.66519989181626 | 1.04855042022029 | 0.207965604590587 | 5.28672991819299 | 0.999950574162623 |
| ebi-fl187-GCST90094733 | prot-a-2385 | Phosphoribosyl pyrophosphate synthase-associated protein 1 \|\| id:prot-a-2385 | Chilli pepper liking \|\| id:ebi-fl187-GCST90094733 | MR Egger | 14 | 1.40866722877644 | 0.825403690641896 | 0.113609832595685 | -0.209124005 | 3.02645846243455 | 4.09050006874013 | 0.811294624911463 | 20.6240622069806 | 0.999950574162623 |
| ebi-fl187-GCST90094733 | prot-a-2393 | Trypsin-3 \|\| id:prot-a-2393 | Chilli pepper liking \|\| id:ebi-fl187-GCST90094733 | MR Egger | 14 | -0.213545586 | 1.28823619242565 | 0.871101222534037 | -2.738488523 | 2.31139735115312 | 0.807715338798932 | 0.0646680172720066 | 10.0885120041168 | 0.999950574162623 |
| ebi-fl187-GCST90094733 | prot-a-2405 | Pregnancy-specific beta-1-glycoprotein 6 \|\| id:prot-a-2405 | Chilli pepper liking \|\| id:ebi-fl187-GCST90094733 | MR Egger | 14 | 1.11358963644351 | 0.826196275605451 | 0.202601188497909 | -0.505755064 | 2.73293433663019 | 3.04527021092107 | 0.60305006224062 | 15.3779449471693 | 0.999950574162623 |
| ebi-fl187-GCST90094733 | prot-a-244 | Beclin-1 \|\| id:prot-a-244 | Chilli pepper liking \|\| id:ebi-fl187-GCST90094733 | MR Egger | 14 | 1.07226559005629 | 0.843307531502178 | 0.227642633930984 | -0.580617172 | 2.72514835180056 | 2.92199204260941 | 0.559552919756372 | 15.2586774112271 | 0.999950574162623 |
| ebi-fl187-GCST90094733 | prot-a-2454 | tRNA pseudouridine synthase A, mitochondrial \|\| id:prot-a-2454 | Chilli pepper liking \|\| id:ebi-fl187-GCST90094733 | MR Egger | 14 | -1.491467567 | 0.825403690641896 | 0.0958893383864921 | -3.109258801 | 0.126323666185987 | 0.225042148541041 | 0.0446340257723322 | 1.13464935648626 | 0.999950574162623 |
| ebi-fl187-GCST90094733 | prot-a-2466 | Glutaminyl-peptide cyclotransferase-like protein \|\| id:prot-a-2466 | Chilli pepper liking \|\| id:ebi-fl187-GCST90094733 | MR Egger | 14 | 0.828816616677492 | 0.825403690641895 | 0.335132549158101 | -0.788974617 | 2.44660785033561 | 2.29060647025783 | 0.454310398698995 | 11.5491039091609 | 0.999950574162623 |
| ebi-fl187-GCST90094733 | prot-a-2474 | Ras-related protein Rab-27A \|\| id:prot-a-2474 | Chilli pepper liking \|\| id:ebi-fl187-GCST90094733 | MR Egger | 14 | 0.653228381643216 | 1.11921752950293 | 0.570265391796845 | -1.540437976 | 2.84689473946896 | 1.9217349189264 | 0.214287228169195 | 17.2341820376953 | 0.999950574162623 |
| ebi-fl187-GCST90094733 | prot-a-2490 | Rap1 GTPase-GDP dissociation stimulator 1 \|\| id:prot-a-2490 | Chilli pepper liking \|\| id:ebi-fl187-GCST90094733 | MR Egger | 14 | -0.694282434 | 1.00191067535748 | 0.501527261184113 | -2.658027358 | 1.26946248935548 | 0.499432695185778 | 0.0700863407263474 | 3.55893908050419 | 0.999950574162623 |
| ebi-fl187-GCST90094733 | prot-a-2545 | Receptor-interacting serine/threonine-protein kinase 2 \|\| id:prot-a-2545 | Chilli pepper liking \|\| id:ebi-fl187-GCST90094733 | MR Egger | 14 | 0.169043700005215 | 0.906263707509977 | 0.855146831560794 | -1.607233167 | 1.94532056672477 | 1.18417188611088 | 0.200441435591753 | 6.99587414007276 | 0.999950574162623 |
| ebi-fl187-GCST90094733 | prot-a-2610 | RUN and FYVE domain-containing protein 1 \|\| id:prot-a-2610 | Chilli pepper liking \|\| id:ebi-fl187-GCST90094733 | MR Egger | 14 | 1.63901044850233 | 0.825403690641896 | 0.0703966224071092 | 0.0212192148442176 | 3.25680168216045 | 5.15007072969314 | 1.02144594320977 | 25.9663555346805 | 0.999950574162623 |
| ebi-fl187-GCST90094733 | prot-a-2627 | Serine--tRNA ligase, mitochondrial \|\| id:prot-a-2627 | Chilli pepper liking \|\| id:ebi-fl187-GCST90094733 | MR Egger | 14 | 0.284108484418405 | 0.826202521321446 | 0.736892361397693 | -1.335248457 | 1.90346542620844 | 1.32857705284404 | 0.263092799989277 | 6.70910410857192 | 0.999950574162623 |
| ebi-fl187-GCST90094733 | prot-a-264 | BolA-like protein 2 \|\| id:prot-a-264 | Chilli pepper liking \|\| id:ebi-fl187-GCST90094733 | MR Egger | 14 | -0.224005099 | 0.826383043630783 | 0.790946049663291 | -1.843715865 | 1.39570566612342 | 0.799311058357826 | 0.158228377097327 | 4.03782292237073 | 0.999950574162623 |
| ebi-fl187-GCST90094733 | prot-a-2646 | Secretoglobin family 1C member 1 \|\| id:prot-a-2646 | Chilli pepper liking \|\| id:ebi-fl187-GCST90094733 | MR Egger | 14 | -1.084632165 | 1.29039360260263 | 0.417037575246907 | -3.613803626 | 1.44453929645053 | 0.338026100978519 | 0.0269491472043335 | 4.23989835657458 | 0.999950574162623 |
| ebi-fl187-GCST90094733 | prot-a-2672 | Semaphorin-3E \|\| id:prot-a-2672 | Chilli pepper liking \|\| id:ebi-fl187-GCST90094733 | MR Egger | 14 | -1.058830408 | 1.26733936238184 | 0.419774380955311 | -3.542815558 | 1.42515474253409 | 0.346861259425064 | 0.0289317532687356 | 4.15850128999802 | 0.999950574162623 |
| ebi-fl187-GCST90094733 | prot-a-2701 | Plasma protease C1 inhibitor \|\| id:prot-a-2701 | Chilli pepper liking \|\| id:ebi-fl187-GCST90094733 | MR Egger | 14 | 0.930061678064469 | 0.873041405351208 | 0.307700731477939 | -0.781099476 | 2.64122283255284 | 2.53466550605928 | 0.457902281673391 | 14.0303498906545 | 0.999950574162623 |
| ebi-fl187-GCST90094733 | prot-a-2738 | Signaling threshold-regulating transmembrane adapter 1 \|\| id:prot-a-2738 | Chilli pepper liking \|\| id:ebi-fl187-GCST90094733 | MR Egger | 14 | 0.390455650836803 | 0.825403690641896 | 0.644667344018647 | -1.227335583 | 2.00824688449492 | 1.47765393476427 | 0.293072405477984 | 7.45024475219099 | 0.999950574162623 |
| ebi-fl187-GCST90094733 | prot-a-274 | Serine/threonine-protein kinase BRSK2 \|\| id:prot-a-274 | Chilli pepper liking \|\| id:ebi-fl187-GCST90094733 | MR Egger | 14 | 0.224393824333162 | 0.930473842114489 | 0.813501831237877 | -1.599334906 | 2.04812255487756 | 1.25156381867012 | 0.202030842779085 | 7.7533309798493 | 0.999950574162623 |
| ebi-fl187-GCST90094733 | prot-a-2749 | Mitochondrial glutamate carrier 2 \|\| id:prot-a-2749 | Chilli pepper liking \|\| id:ebi-fl187-GCST90094733 | MR Egger | 14 | -0.028849832 | 1.4207678416872 | 0.984133158290325 | -2.813554802 | 2.75585513745147 | 0.971562350839531 | 0.0599913555106465 | 15.7344903033791 | 0.999950574162623 |
| ebi-fl187-GCST90094733 | prot-a-2760 | Sodium-coupled monocarboxylate transporter 1 \|\| id:prot-a-2760 | Chilli pepper liking \|\| id:ebi-fl187-GCST90094733 | MR Egger | 14 | 0.979531810247662 | 0.826491174853842 | 0.258890810811323 | -0.640390892 | 2.59945451296119 | 2.66320906280242 | 0.527086349686261 | 13.4563957431543 | 0.999950574162623 |
| ebi-fl187-GCST90094733 | prot-a-2777 | Small integral membrane protein 10 \|\| id:prot-a-2777 | Chilli pepper liking \|\| id:ebi-fl187-GCST90094733 | MR Egger | 14 | 0.707303151948448 | 1.02870392322507 | 0.504798156875381 | -1.308956538 | 2.72356284146959 | 2.02851328323775 | 0.270101750418668 | 15.2345037893824 | 0.999950574162623 |
| ebi-fl187-GCST90094733 | prot-a-2799 | Superoxide dismutase [Mn], mitochondrial \|\| id:prot-a-2799 | Chilli pepper liking \|\| id:ebi-fl187-GCST90094733 | MR Egger | 14 | 1.17051147508014 | 0.826202521321446 | 0.181994028705576 | -0.448845467 | 2.78986841687018 | 3.2236410289909 | 0.638364739675427 | 16.2788776351858 | 0.999950574162623 |
| ebi-fl187-GCST90094733 | prot-a-2805 | Protein SPACA7 \|\| id:prot-a-2805 | Chilli pepper liking \|\| id:ebi-fl187-GCST90094733 | MR Egger | 14 | -1.344172646 | 0.844438412864496 | 0.137415641919834 | -2.999271935 | 0.310926643710336 | 0.260755355754967 | 0.049823329802555 | 1.3646891089847 | 0.999950574162623 |
| ebi-fl187-GCST90094733 | prot-a-2811 | SPARC-like protein 1 \|\| id:prot-a-2811 | Chilli pepper liking \|\| id:ebi-fl187-GCST90094733 | MR Egger | 14 | 0.255411830372583 | 0.825403690641896 | 0.762295059433955 | -1.362379403 | 1.8732030640307 | 1.29099318161193 | 0.25605080343189 | 6.50911214739399 | 0.999950574162623 |
| ebi-fl187-GCST90094733 | prot-a-2831 | Sepiapterin reductase \|\| id:prot-a-2831 | Chilli pepper liking \|\| id:ebi-fl187-GCST90094733 | MR Egger | 14 | 0.844103682506558 | 0.97155710961633 | 0.401990944433011 | -1.060148252 | 2.74835561735457 | 2.32589214218265 | 0.346404451252113 | 15.616930548995 | 0.999950574162623 |
| ebi-fl187-GCST90094733 | prot-a-2834 | SPRY domain-containing SOCS box protein 1 \|\| id:prot-a-2834 | Chilli pepper liking \|\| id:ebi-fl187-GCST90094733 | MR Egger | 14 | 0.5888868619811 | 1.02236976058105 | 0.575257800605147 | -1.414957869 | 2.59273159271995 | 1.80198144453204 | 0.242935848422216 | 13.3662328862818 | 0.999950574162623 |
| ebi-fl187-GCST90094733 | prot-a-2835 | Spectrin alpha chain, non-erythrocytic 1 \|\| id:prot-a-2835 | Chilli pepper liking \|\| id:ebi-fl187-GCST90094733 | MR Egger | 14 | 0.472230945079777 | 0.825403690641895 | 0.577800694601316 | -1.145560289 | 2.09002217873789 | 1.60356767703132 | 0.31804567050355 | 8.08509447950785 | 0.999950574162623 |
| ebi-fl187-GCST90094733 | prot-a-2859 | Sia-alpha-2,3-Gal-beta-1,4-GlcNAc-R:alpha 2,8-sialyltransferase \|\| id:prot-a-2859 | Chilli pepper liking \|\| id:ebi-fl187-GCST90094733 | MR Egger | 14 | -0.460115978 | 0.826202521321446 | 0.587829051906927 | -2.079472919 | 1.15924096419129 | 0.631210434991065 | 0.124996077847916 | 3.18751292121644 | 0.999950574162623 |
| ebi-fl187-GCST90094733 | prot-a-2861 | Alpha-2,8-sialyltransferase 8F \|\| id:prot-a-2861 | Chilli pepper liking \|\| id:ebi-fl187-GCST90094733 | MR Egger | 14 | -1.222386207 | 1.12081142021312 | 0.296852787336901 | -3.419176591 | 0.974404176416183 | 0.294526526423485 | 0.0327393817508777 | 2.649588054 | 0.999950574162623 |
| ebi-fl187-GCST90094733 | prot-a-289 | Uncharacterized protein C17orf89 \|\| id:prot-a-289 | Chilli pepper liking \|\| id:ebi-fl187-GCST90094733 | MR Egger | 14 | -1.792511888 | 1.00962933062305 | 0.101173007433544 | -3.771385376 | 0.186361600265487 | 0.166541310744699 | 0.0230201496284494 | 1.2048578585382 | 0.999950574162623 |
| ebi-fl187-GCST90094733 | prot-a-2921 | Tachykinin-3 \|\| id:prot-a-2921 | Chilli pepper liking \|\| id:ebi-fl187-GCST90094733 | MR Egger | 14 | 0.999863096952677 | 0.82529633793052 | 0.249013841886432 | -0.617717725 | 2.6174439192965 | 2.71790971286571 | 0.539173576608428 | 13.7006588003747 | 0.999950574162623 |
| ebi-fl187-GCST90094733 | prot-a-2928 | Tax1-binding protein 1 \|\| id:prot-a-2928 | Chilli pepper liking \|\| id:ebi-fl187-GCST90094733 | MR Egger | 14 | -0.729663258 | 1.29939303611214 | 0.584763448974152 | -3.276473608 | 1.81714709321306 | 0.482071296622354 | 0.0377611830151015 | 6.15427580577174 | 0.999950574162623 |
| ebi-fl187-GCST90094733 | prot-a-296 | Uncharacterized protein C1orf185 \|\| id:prot-a-296 | Chilli pepper liking \|\| id:ebi-fl187-GCST90094733 | MR Egger | 14 | -0.392048446 | 0.825116887985818 | 0.643216360868762 | -2.009277546 | 1.22518065485385 | 0.675671379863431 | 0.134085510291427 | 3.4047811174698 | 0.999950574162623 |
| ebi-fl187-GCST90094733 | prot-a-2964 | Transforming growth factor beta-2 \|\| id:prot-a-2964 | Chilli pepper liking \|\| id:ebi-fl187-GCST90094733 | MR Egger | 14 | 0.408106957574925 | 0.825403690641896 | 0.629927642750525 | -1.209684276 | 2.02589819123304 | 1.50396801333925 | 0.298291442306885 | 7.58291879798727 | 0.999950574162623 |
| ebi-fl187-GCST90094733 | prot-a-2977 | Thymocyte nuclear protein 1 \|\| id:prot-a-2977 | Chilli pepper liking \|\| id:ebi-fl187-GCST90094733 | MR Egger | 14 | -0.714573847 | 0.898440391292154 | 0.441858890359533 | -2.475517014 | 1.04636931965093 | 0.489400626771004 | 0.0841194880416292 | 2.84729471208053 | 0.999950574162623 |
| ebi-fl187-GCST90094733 | prot-a-3015 | Transmembrane protein 70, mitochondrial \|\| id:prot-a-3015 | Chilli pepper liking \|\| id:ebi-fl187-GCST90094733 | MR Egger | 14 | 1.04646943156911 | 0.825116887985818 | 0.228755467308889 | -0.570759669 | 2.66369853202131 | 2.84757977448461 | 0.56509598976131 | 14.3492622828183 | 0.999950574162623 |
| ebi-fl187-GCST90094733 | prot-a-3030 | Tumor necrosis factor alpha-induced protein 3 \|\| id:prot-a-3030 | Chilli pepper liking \|\| id:ebi-fl187-GCST90094733 | MR Egger | 14 | 0.322218832776605 | 0.825403690641896 | 0.703098806680763 | -1.295572401 | 1.94001006643472 | 1.38018677301639 | 0.273741129814226 | 6.95882102080223 | 0.999950574162623 |
| ebi-fl187-GCST90094733 | prot-a-3032 | Tumor necrosis factor receptor superfamily member 10A \|\| id:prot-a-3032 | Chilli pepper liking \|\| id:ebi-fl187-GCST90094733 | MR Egger | 14 | -0.310461162 | 0.9470290637246 | 0.748691812012325 | -2.166638127 | 1.54571580287428 | 0.733108796319285 | 0.114562113519024 | 4.69132849187059 | 0.999950574162623 |
| ebi-fl187-GCST90094733 | prot-a-3081 | Tumor protein p53-inducible protein 11 \|\| id:prot-a-3081 | Chilli pepper liking \|\| id:ebi-fl187-GCST90094733 | MR Egger | 14 | -0.009429203 | 0.826383043630783 | 0.991083672006677 | -1.629139969 | 1.61028156229467 | 0.990615112318912 | 0.196098152166611 | 5.00422002916607 | 0.999950574162623 |
| ebi-fl187-GCST90094733 | prot-a-315 | Complement C4 \|\| id:prot-a-315 | Chilli pepper liking \|\| id:ebi-fl187-GCST90094733 | MR Egger | 13 | -0.852835192 | 0.897480261916375 | 0.362394343376764 | -2.611896505 | 0.906226121603338 | 0.426204844883682 | 0.0733952172835834 | 2.4749646710693 | 0.999950574162623 |
| ebi-fl187-GCST90094733 | prot-a-3157 | UDP-glucuronosyltransferase 1-8 \|\| id:prot-a-3157 | Chilli pepper liking \|\| id:ebi-fl187-GCST90094733 | MR Egger | 14 | -0.041376088 | 0.825504652618227 | 0.960849654261294 | -1.659365207 | 1.57661303106906 | 0.959468217541509 | 0.190259717275646 | 4.83854004228627 | 0.999950574162623 |
| ebi-fl187-GCST90094733 | prot-a-317 | Glutaredoxin-like protein C5orf63 \|\| id:prot-a-317 | Chilli pepper liking \|\| id:ebi-fl187-GCST90094733 | MR Egger | 14 | 0.562866731456914 | 0.890654330706261 | 0.539260334345857 | -1.182815757 | 2.30854921964119 | 1.75569840874641 | 0.306414733410809 | 10.0598194746139 | 0.999950574162623 |
| ebi-fl187-GCST90094733 | prot-a-3192 | Guanine nucleotide exchange factor VAV3 \|\| id:prot-a-3192 | Chilli pepper liking \|\| id:ebi-fl187-GCST90094733 | MR Egger | 14 | -0.710847454 | 0.91481267156826 | 0.452180567829186 | -2.50388029 | 1.08218538206341 | 0.491227728010139 | 0.08176710214905 | 2.95112183780389 | 0.999950574162623 |
| ebi-fl187-GCST90094733 | prot-a-3233 | Serine/threonine-protein kinase WNK3 \|\| id:prot-a-3233 | Chilli pepper liking \|\| id:ebi-fl187-GCST90094733 | MR Egger | 14 | 0.9531250299942 | 0.825403690641895 | 0.270675223098516 | -0.664666204 | 2.57091626365232 | 2.59380271849757 | 0.514445218979291 | 13.0778016672679 | 0.999950574162623 |
| ebi-fl187-GCST90094733 | prot-a-3250 | 14-3-3 protein theta \|\| id:prot-a-3250 | Chilli pepper liking \|\| id:ebi-fl187-GCST90094733 | MR Egger | 14 | 0.245793675373012 | 1.09772297777698 | 0.826592355660626 | -1.905743361 | 2.3973307118159 | 1.27863573228368 | 0.148712054771743 | 10.993791581873 | 0.999950574162623 |
| ebi-fl187-GCST90094733 | prot-a-3278 | E3 ubiquitin-protein ligase ZNRF3 \|\| id:prot-a-3278 | Chilli pepper liking \|\| id:ebi-fl187-GCST90094733 | MR Egger | 14 | 0.484885798347824 | 0.825009841069701 | 0.567606912929882 | -1.13213349 | 2.10190508684444 | 1.62398953596565 | 0.322344802817577 | 8.1817420038209 | 0.999950574162623 |
| ebi-fl187-GCST90094733 | prot-a-355 | Calpain-3 \|\| id:prot-a-355 | Chilli pepper liking \|\| id:ebi-fl187-GCST90094733 | MR Egger | 14 | 0.609255459192462 | 0.82529633793052 | 0.474567507992258 | -1.008325363 | 2.22683628153628 | 1.8390616324885 | 0.364829425090307 | 9.27049041412727 | 0.999950574162623 |
| ebi-fl187-GCST90094733 | prot-a-385 | Coiled-coil domain-containing protein 90B, mitochondrial \|\| id:prot-a-385 | Chilli pepper liking \|\| id:ebi-fl187-GCST90094733 | MR Egger | 14 | -1.610210139 | 0.825403690641896 | 0.0748213708642057 | -3.228001372 | 0.0075810951397195 | 0.199845614401106 | 0.0396366385652411 | 1.00760990439737 | 0.999950574162623 |
| ebi-fl187-GCST90094733 | prot-a-390 | C-C motif chemokine 14 \|\| id:prot-a-390 | Chilli pepper liking \|\| id:ebi-fl187-GCST90094733 | MR Egger | 14 | -1.184585504 | 1.16224727554345 | 0.32820788621934 | -3.462590164 | 1.09341915622705 | 0.305872936384935 | 0.0313484591226671 | 2.98446098567865 | 0.999950574162623 |
| ebi-fl187-GCST90094733 | prot-a-40 | Pituitary adenylate cyclase-activating polypeptide 27 \|\| id:prot-a-40 | Chilli pepper liking \|\| id:ebi-fl187-GCST90094733 | MR Egger | 14 | -0.87636284 | 0.9655087190645 | 0.381919219433641 | -2.768759929 | 1.01603424958148 | 0.416294290483098 | 0.0627397582675272 | 2.76221874413123 | 0.999950574162623 |
| ebi-fl187-GCST90094733 | prot-a-401 | C-C motif chemokine 24 \|\| id:prot-a-401 | Chilli pepper liking \|\| id:ebi-fl187-GCST90094733 | MR Egger | 14 | 0.00329674137290407 | 0.999558091664308 | 0.997422618942012 | -1.955837118 | 1.96243060103495 | 1.00330218160144 | 0.141446020069622 | 7.11660368464764 | 0.999950574162623 |
| ebi-fl187-GCST90094733 | prot-a-412 | Cerebral cavernous malformations 2 protein \|\| id:prot-a-412 | Chilli pepper liking \|\| id:ebi-fl187-GCST90094733 | MR Egger | 14 | -0.775078775 | 1.02243833800582 | 0.46303808411484 | -2.779057918 | 1.22890036735388 | 0.460667490424679 | 0.062096980300317 | 3.41746950830535 | 0.999950574162623 |
| ebi-fl187-GCST90094733 | prot-a-44 | Adhesion G protein-coupled receptor F5 \|\| id:prot-a-44 | Chilli pepper liking \|\| id:ebi-fl187-GCST90094733 | MR Egger | 14 | -0.12447383 | 0.825403690641896 | 0.882635986746627 | -1.742265064 | 1.49331740371487 | 0.882961368213154 | 0.175123285661363 | 4.45183959867223 | 0.999950574162623 |
| ebi-fl187-GCST90094733 | prot-a-48 | Adrenomedullin \|\| id:prot-a-48 | Chilli pepper liking \|\| id:ebi-fl187-GCST90094733 | MR Egger | 14 | 0.645063592677361 | 0.825116887985818 | 0.449491855416236 | -0.972165508 | 2.26229269312956 | 1.90610823994414 | 0.378263019036359 | 9.60508545519151 | 0.999950574162623 |
| ebi-fl187-GCST90094733 | prot-a-502 | Carcinoembryonic antigen-related cell adhesion molecule 1 \|\| id:prot-a-502 | Chilli pepper liking \|\| id:ebi-fl187-GCST90094733 | MR Egger | 14 | 0.00114866897949416 | 0.921393254315544 | 0.999025790435874 | -1.804782109 | 1.80707944743796 | 1.00114932895238 | 0.164510297906795 | 6.09262758389545 | 0.999974376584515 |
| ebi-fl187-GCST90094733 | prot-a-518 | Complement factor B \|\| id:prot-a-518 | Chilli pepper liking \|\| id:ebi-fl187-GCST90094733 | MR Egger | 14 | -0.465654298 | 0.825116887985818 | 0.582911202532716 | -2.082883398 | 1.15157480263731 | 0.627724252187628 | 0.124570507476423 | 3.16317035843413 | 0.999950574162623 |
| ebi-fl187-GCST90094733 | prot-a-525 | Properdin \|\| id:prot-a-525 | Chilli pepper liking \|\| id:ebi-fl187-GCST90094733 | MR Egger | 14 | 0.623404831940845 | 0.905743909262591 | 0.50436539979037 | -1.15185323 | 2.39866289409552 | 1.86526816682394 | 0.316050511954006 | 11.0084470759316 | 0.999950574162623 |
| ebi-fl187-GCST90094733 | prot-a-533 | Chondroadherin \|\| id:prot-a-533 | Chilli pepper liking \|\| id:ebi-fl187-GCST90094733 | MR Egger | 14 | 0.558980852785405 | 1.17294950484142 | 0.642235660133064 | -1.740000177 | 2.85796188227458 | 1.74888921616264 | 0.175520369601883 | 17.4259745313182 | 0.999950574162623 |
| ebi-fl187-GCST90094733 | prot-a-535 | Coiled-coil-helix-coiled-coil-helix domain-containing protein 2 \|\| id:prot-a-535 | Chilli pepper liking \|\| id:ebi-fl187-GCST90094733 | MR Egger | 14 | -1.529174968 | 1.04590252863449 | 0.169414015418369 | -3.579143924 | 0.520793988160876 | 0.216714389894543 | 0.0278995721865907 | 1.68336369006894 | 0.999950574162623 |
| ebi-fl187-GCST90094733 | prot-a-591 | Caseinolytic peptidase B protein homolog \|\| id:prot-a-591 | Chilli pepper liking \|\| id:ebi-fl187-GCST90094733 | MR Egger | 14 | 1.64733082464176 | 0.965364823943773 | 0.113649212525488 | -0.24478423 | 3.53944587957156 | 5.1931000169985 | 0.782873440395967 | 34.4478256573752 | 0.999950574162623 |
| ebi-fl187-GCST90094733 | prot-a-605 | Ciliary neurotrophic factor receptor subunit alpha \|\| id:prot-a-605 | Chilli pepper liking \|\| id:ebi-fl187-GCST90094733 | MR Egger | 14 | 0.917567816536806 | 0.825116887985818 | 0.287901174337184 | -0.699661284 | 2.53479691698901 | 2.50319475120631 | 0.496753533710663 | 12.6138689254226 | 0.999950574162623 |
| ebi-fl187-GCST90094733 | prot-a-606 | Ciliary neurotrophic factor receptor subunit alpha \|\| id:prot-a-606 | Chilli pepper liking \|\| id:ebi-fl187-GCST90094733 | MR Egger | 14 | 1.2540002777694 | 0.914148277024782 | 0.195237840849489 | -0.537730345 | 3.04573090073797 | 3.50433326269935 | 0.584072391843993 | 21.0253930635043 | 0.999950574162623 |
| ebi-fl187-GCST90094733 | prot-a-707 | Cancer/testis antigen 55 \|\| id:prot-a-707 | Chilli pepper liking \|\| id:ebi-fl187-GCST90094733 | MR Egger | 14 | 0.953799044341803 | 0.942967440811678 | 0.331744550737187 | -0.89441714 | 2.80201522833269 | 2.59555156805333 | 0.408845829230017 | 16.4778199036829 | 0.999950574162623 |
| ebi-fl187-GCST90094733 | prot-a-719 | Dipeptidyl peptidase 1 \|\| id:prot-a-719 | Chilli pepper liking \|\| id:ebi-fl187-GCST90094733 | MR Egger | 14 | -0.986058044 | 0.826491174853842 | 0.255899394654385 | -2.605980747 | 0.633864658921701 | 0.373044320831791 | 0.0738306924847706 | 1.88488094343092 | 0.999950574162623 |
| ebi-fl187-GCST90094733 | prot-a-752 | Cytochrome b5 \|\| id:prot-a-752 | Chilli pepper liking \|\| id:ebi-fl187-GCST90094733 | MR Egger | 14 | 1.95658773597395 | 0.865488082584011 | 0.0431582654314873 | 0.260231094109286 | 3.65294437783861 | 7.07514357050257 | 1.29722983420257 | 38.5881169422805 | 0.999950574162623 |
| ebi-fl187-GCST90094733 | prot-a-755 | Cytoglobin \|\| id:prot-a-755 | Chilli pepper liking \|\| id:ebi-fl187-GCST90094733 | MR Egger | 14 | -0.070696505 | 0.862852555219088 | 0.936050383621545 | -1.761887513 | 1.62049450296036 | 0.931744628806307 | 0.171720433100248 | 5.05558970261034 | 0.999950574162623 |
| ebi-fl187-GCST90094733 | prot-a-807 | Desmin \|\| id:prot-a-807 | Chilli pepper liking \|\| id:ebi-fl187-GCST90094733 | MR Egger | 14 | 1.05755912268886 | 0.953738013167804 | 0.289220669197208 | -0.811767383 | 2.92688562849776 | 2.87933430314612 | 0.444072525965661 | 18.6693964262834 | 0.999950574162623 |
| ebi-fl187-GCST90094733 | prot-a-82 | Ameloblastin \|\| id:prot-a-82 | Chilli pepper liking \|\| id:ebi-fl187-GCST90094733 | MR Egger | 14 | -1.191371392 | 1.19850560301848 | 0.339822032661327 | -3.540442374 | 1.15769959018076 | 0.303804343485473 | 0.0290004951892031 | 3.18260355619726 | 0.999950574162623 |
| ebi-fl187-GCST90094733 | prot-a-84 | Muellerian-inhibiting factor \|\| id:prot-a-84 | Chilli pepper liking \|\| id:ebi-fl187-GCST90094733 | MR Egger | 14 | 0.74547609348045 | 0.825116887985818 | 0.384049924481108 | -0.871753007 | 2.36270519393265 | 2.10744453678104 | 0.418217767611055 | 10.6196408176964 | 0.999950574162623 |
| ebi-fl187-GCST90094733 | prot-a-858 | Inactive dipeptidyl peptidase 10 \|\| id:prot-a-858 | Chilli pepper liking \|\| id:ebi-fl187-GCST90094733 | MR Egger | 14 | -1.284503744 | 1.04659191872563 | 0.243235467466482 | -3.335823905 | 0.766816416576586 | 0.276787907180235 | 0.0355852552664622 | 2.15290139097071 | 0.999950574162623 |
| ebi-fl187-GCST90094733 | prot-a-863 | Dihydropyrimidinase-related protein 3 \|\| id:prot-a-863 | Chilli pepper liking \|\| id:ebi-fl187-GCST90094733 | MR Egger | 14 | 0.612264347029802 | 0.826491174853842 | 0.473062313557728 | -1.007658356 | 2.23218704974333 | 1.84460349590827 | 0.365072850215416 | 9.32022760692635 | 0.999950574162623 |
| ebi-fl187-GCST90094733 | prot-a-864 | Dihydropyrimidinase-related protein 5 \|\| id:prot-a-864 | Chilli pepper liking \|\| id:ebi-fl187-GCST90094733 | MR Egger | 14 | -1.282772609 | 0.825043846615584 | 0.145961678232553 | -2.899858549 | 0.334313330146811 | 0.277267479370202 | 0.0550310037191639 | 1.39698079120323 | 0.999950574162623 |
| ebi-fl187-GCST90094733 | prot-a-884 | Endothelin-converting enzyme 1 \|\| id:prot-a-884 | Chilli pepper liking \|\| id:ebi-fl187-GCST90094733 | MR Egger | 14 | 0.62201695811034 | 0.826491174853842 | 0.466198297640751 | -0.997905745 | 2.24193966082387 | 1.86268120555151 | 0.368650681982224 | 9.41156884576747 | 0.999950574162623 |
| ebi-fl187-GCST90094733 | prot-a-893 | Early endosome antigen 1 \|\| id:prot-a-893 | Chilli pepper liking \|\| id:ebi-fl187-GCST90094733 | MR Egger | 14 | 0.31047761658197 | 1.00758734792354 | 0.763259286558855 | -1.664393585 | 2.28534881851211 | 1.36407646411035 | 0.18930542076237 | 9.82911420310303 | 0.999950574162623 |
| ebi-fl187-GCST90094733 | prot-a-897 | Ephrin-A2 \|\| id:prot-a-897 | Chilli pepper liking \|\| id:ebi-fl187-GCST90094733 | MR Egger | 14 | 0.707047075415327 | 0.863698835086776 | 0.428956946256906 | -0.985802641 | 2.39989679218541 | 2.02799389509319 | 0.373139609428421 | 11.0220387614041 | 0.999950574162623 |
| ebi-fl187-GCST90094733 | prot-a-90 | Anaphase-promoting complex subunit 10 \|\| id:prot-a-90 | Chilli pepper liking \|\| id:ebi-fl187-GCST90094733 | MR Egger | 14 | 0.337687037846207 | 0.844136628523317 | 0.696157797370204 | -1.316820754 | 1.99219482975191 | 1.40170175515283 | 0.267985942271065 | 7.33160774683917 | 0.999950574162623 |
| ebi-fl187-GCST90094733 | prot-a-914 | Histone-lysine N-methyltransferase EHMT2 \|\| id:prot-a-914 | Chilli pepper liking \|\| id:ebi-fl187-GCST90094733 | MR Egger | 14 | -0.076740572 | 0.850188878313367 | 0.929567372417535 | -1.743110773 | 1.58962962967918 | 0.926130086690974 | 0.174975244807223 | 4.90193306155513 | 0.999950574162623 |
| ebi-fl187-GCST90094733 | prot-a-932 | RNA polymerase II elongation factor ELL \|\| id:prot-a-932 | Chilli pepper liking \|\| id:ebi-fl187-GCST90094733 | MR Egger | 14 | 0.839643141386883 | 0.825403690641896 | 0.329105465650274 | -0.778148092 | 2.457434375 | 2.31554050874918 | 0.459255723491911 | 11.6748198735357 | 0.999950574162623 |
| ebi-fl187-GCST90094733 | prot-a-991 | Estrogen receptor \|\| id:prot-a-991 | Chilli pepper liking \|\| id:ebi-fl187-GCST90094733 | MR Egger | 14 | -0.631060085 | 0.844458856409058 | 0.469276092753168 | -2.286199443 | 1.02407927364932 | 0.532027507626457 | 0.10165206305176 | 2.78453048933294 | 0.999950574162623 |
| ebi-fl187-GCST90094733 | prot-a-992 | Persulfide dioxygenase ETHE1, mitochondrial \|\| id:prot-a-992 | Chilli pepper liking \|\| id:ebi-fl187-GCST90094733 | MR Egger | 14 | 1.00547021818853 | 0.825116887985818 | 0.246419019121661 | -0.611758882 | 2.62269931864073 | 2.7331921673817 | 0.542396018848661 | 13.7728507662978 | 0.999950574162623 |
| ebi-fl187-GCST90094733 | prot-a-993 | Ethanolamine kinase 1 \|\| id:prot-a-993 | Chilli pepper liking \|\| id:ebi-fl187-GCST90094733 | MR Egger | 14 | -0.649828245 | 0.98420061446624 | 0.52155953770857 | -2.578861449 | 1.2792049592361 | 0.522135448372491 | 0.0758603256866307 | 3.59378138677288 | 0.999950574162623 |
| ebi-fl187-GCST90094733 | prot-b-10 | coagulation factor III, tissue factor \|\| id:prot-b-10 | Chilli pepper liking \|\| id:ebi-fl187-GCST90094733 | MR Egger | 10 | 1.706137624 | 1.12857885549819 | 0.169045246388741 | -0.505876932 | 3.91815218109945 | 5.50764773862374 | 0.602976573785367 | 50.307399875148 | 0.999950574162623 |
| ebi-fl187-GCST90094733 | prot-b-28 | prolactin \|\| id:prot-b-28 | Chilli pepper liking \|\| id:ebi-fl187-GCST90094733 | MR Egger | 10 | -0.02468334 | 1.21676139050377 | 0.984311982799804 | -2.409535665 | 2.36016898579839 | 0.975618802970116 | 0.0898570086430767 | 10.5927413240478 | 0.999950574162623 |
| ebi-fl187-GCST90094733 | prot-b-54 | matrix metallopeptidase 7 \|\| id:prot-b-54 | Chilli pepper liking \|\| id:ebi-fl187-GCST90094733 | MR Egger | 10 | 1.56436266562992 | 1.12856470092313 | 0.20310948991792 | -0.647624148 | 3.77634947943926 | 4.77962774392095 | 0.523287554726262 | 43.6563819722586 | 0.999950574162623 |
| ebi-fl187-GCST90094733 | ukb-rsfMRI191-edge-pheno716 | Net100_Pair10_33 (OccipitalPrecuneus)&(Temporal\|Frontal\|Supp_Motor_Area) (Default_mode\|Central_executive)&(Default_mode\|Salience) \|\| id:ukb-rsfMRI191-edge-pheno716 | Chilli pepper liking \|\| id:ebi-fl187-GCST90094733 | MR Egger | 13 | -0.038161477 | 0.296268674265789 | 0.899835294559375 | -0.618848078 | 0.542525124676118 | 0.962557497555111 | 0.538564464429892 | 1.72034546891307 | 0.999950574162623 |
| ebi-fl187-GCST90094733 | ukb-rsfMRI191-edge-pheno903 | Net100_Pair27_38 (Precentral\|Frontal\|Supp_Motor_Area)&(Subcortical) (Attention\|Salience\|Motor)&(Subcortical-cerebellum) \|\| id:ukb-rsfMRI191-edge-pheno903 | Chilli pepper liking \|\| id:ebi-fl187-GCST90094733 | MR Egger | 13 | -0.312529833 | 0.341688130623191 | 0.379981308367813 | -0.982238569 | 0.357178903264211 | 0.731593803160751 | 0.37447187881955 | 1.42929155190616 | 0.999950574162623 |
| ebi-fl187-GCST90094733 | ukb-rsfMRI191-node-pheno23 | Net100_Node2 RolandicOper\|SupraMarginal\|Insula Salience\|Motor \|\| id:ukb-rsfMRI191-node-pheno23 | Chilli pepper liking \|\| id:ebi-fl187-GCST90094733 | MR Egger | 13 | -0.519088803 | 0.335276488831608 | 0.14983711594155 | -1.176230721 | 0.138053115395376 | 0.595062520364022 | 0.308439143522791 | 1.1480365270688 | 0.999950574162623 |
| ebi-fl187-GCST90094733 | ukb-rsfMRI191-node-pheno55 | Net100_Node34 Parietal\|Postcentral\|Precuneus Attention \|\| id:ukb-rsfMRI191-node-pheno55 | Chilli pepper liking \|\| id:ebi-fl187-GCST90094733 | MR Egger | 13 | -0.966672998 | 0.340773341263288 | 0.0161769846670982 | -1.634588746 | -0.298757249 | 0.380346348640572 | 0.195032562640475 | 0.741739445791366 | 0.999950574162623 |
| ebi-fl187-GCST90094733 | ebi-a-GCST90001414 | IgD- CD24- B cell Absolute Count \|\| id:ebi-a-GCST90001414 | Chilli pepper liking \|\| id:ebi-fl187-GCST90094733 | Weighted median | 12 | -0.089653139 | 0.172614172958553 | 0.60349258680006 | -0.427976918 | 0.248670640405425 | 0.914248247712926 | 0.651826460349324 | 1.2823196192407 | 0.999332001504091 |
| ebi-fl187-GCST90094733 | ebi-a-GCST90001422 | CD20- CD38- B cell %B cell \|\| id:ebi-a-GCST90001422 | Chilli pepper liking \|\| id:ebi-fl187-GCST90094733 | Weighted median | 12 | 0.266316367665429 | 0.144611858318534 | 0.0655345218392868 | -0.017122875 | 0.549755609969755 | 1.30514789997282 | 0.983022888631211 | 1.73282947986628 | 0.999332001504091 |
| ebi-fl187-GCST90094733 | ebi-a-GCST90001444 | CD20- CD38- B cell %lymphocyte \|\| id:ebi-a-GCST90001444 | Chilli pepper liking \|\| id:ebi-fl187-GCST90094733 | Weighted median | 12 | 0.237116360951269 | 0.150075692816977 | 0.114111801057752 | -0.057031997 | 0.531264718872544 | 1.26758860701025 | 0.944563845701954 | 1.70108233967828 | 0.999332001504091 |
| ebi-fl187-GCST90094733 | ebi-a-GCST90001459 | Myeloid Dendritic Cell %Dendritic Cell \|\| id:ebi-a-GCST90001459 | Chilli pepper liking \|\| id:ebi-fl187-GCST90094733 | Weighted median | 12 | 0.256772489574436 | 0.163932904511064 | 0.117272147338195 | -0.064536003 | 0.578080982416122 | 1.2927509789744 | 0.937502360506399 | 1.78261427815133 | 0.999332001504091 |
| ebi-fl187-GCST90094733 | ebi-a-GCST90001476 | HLA DR++ monocyte %leukocyte \|\| id:ebi-a-GCST90001476 | Chilli pepper liking \|\| id:ebi-fl187-GCST90094733 | Weighted median | 12 | -0.015998348 | 0.166455272010221 | 0.923431625997333 | -0.342250681 | 0.310253985123991 | 0.984128945819174 | 0.710170156117652 | 1.36377144780878 | 0.999332001504091 |
| ebi-fl187-GCST90094733 | ebi-a-GCST90001478 | CD4 regulatory T cell %CD4+ T cell \|\| id:ebi-a-GCST90001478 | Chilli pepper liking \|\| id:ebi-fl187-GCST90094733 | Weighted median | 12 | -0.14176824 | 0.1702154552544 | 0.404915090368737 | -0.475390532 | 0.191854052716511 | 0.867822360053909 | 0.621642237941591 | 1.2114936898485 | 0.999332001504091 |
| ebi-fl187-GCST90094733 | ebi-a-GCST90001485 | CD39+ resting CD4 regulatory T cell %CD4 regulatory T cell \|\| id:ebi-a-GCST90001485 | Chilli pepper liking \|\| id:ebi-fl187-GCST90094733 | Weighted median | 12 | -0.002062293 | 0.17798770092171 | 0.990755346955767 | -0.350918186 | 0.346793601290556 | 0.99793983254813 | 0.704041351711505 | 1.41452473915804 | 0.999332001504091 |
| ebi-fl187-GCST90094733 | ebi-a-GCST90001515 | Immature Myeloid-Derived Suppressor Cells Absolute Count \|\| id:ebi-a-GCST90001515 | Chilli pepper liking \|\| id:ebi-fl187-GCST90094733 | Weighted median | 12 | 0.0133326862242703 | 0.225383349244606 | 0.952828177382741 | -0.428418678 | 0.455084050743698 | 1.01342196280959 | 0.651538572627353 | 1.57630586714665 | 0.999332001504091 |
| ebi-fl187-GCST90094733 | ebi-a-GCST90001517 | CD33+ HLA DR+ Absolute Count \|\| id:ebi-a-GCST90001517 | Chilli pepper liking \|\| id:ebi-fl187-GCST90094733 | Weighted median | 12 | 0.271646875733179 | 0.194834685385185 | 0.163244268820337 | -0.110229108 | 0.653522859088142 | 1.31212357681666 | 0.895628916377785 | 1.92230090984684 | 0.999332001504091 |
| ebi-fl187-GCST90094733 | ebi-a-GCST90001518 | CD33+ HLA DR+ CD14- Absolute Count \|\| id:ebi-a-GCST90001518 | Chilli pepper liking \|\| id:ebi-fl187-GCST90094733 | Weighted median | 12 | 0.200054293138541 | 0.215197413959752 | 0.352561915989698 | -0.221732638 | 0.621841224499654 | 1.22146907374956 | 0.801129527118232 | 1.86235389861796 | 0.999332001504091 |
| ebi-fl187-GCST90094733 | ebi-a-GCST90001520 | CD33+ HLA DR+ CD14dim Absolute Count \|\| id:ebi-a-GCST90001520 | Chilli pepper liking \|\| id:ebi-fl187-GCST90094733 | Weighted median | 12 | 0.251770544139348 | 0.206604034581199 | 0.222990778515483 | -0.153173364 | 0.656714451918498 | 1.28630085415084 | 0.857980966214178 | 1.92844591260565 | 0.999332001504091 |
| ebi-fl187-GCST90094733 | ebi-a-GCST90001531 | CD33dim HLA DR- Absolute Count \|\| id:ebi-a-GCST90001531 | Chilli pepper liking \|\| id:ebi-fl187-GCST90094733 | Weighted median | 12 | -0.067845769 | 0.205328069294325 | 0.741077964642295 | -0.470288785 | 0.334597246791702 | 0.934404576593082 | 0.624821803160422 | 1.39737747361215 | 0.999332001504091 |
| ebi-fl187-GCST90094733 | ebi-a-GCST90001532 | Basophil Absolute Count \|\| id:ebi-a-GCST90001532 | Chilli pepper liking \|\| id:ebi-fl187-GCST90094733 | Weighted median | 12 | -0.096382122 | 0.205483190790318 | 0.639033093194679 | -0.499129176 | 0.306364932192132 | 0.908116938519978 | 0.607059071389688 | 1.35847796844389 | 0.999332001504091 |
| ebi-fl187-GCST90094733 | ebi-a-GCST90001588 | T/B cell \|\| id:ebi-a-GCST90001588 | Chilli pepper liking \|\| id:ebi-fl187-GCST90094733 | Weighted median | 12 | 0.0192755739736174 | 0.15685685556927 | 0.90219705685304 | -0.288163863 | 0.326715010889387 | 1.01946254725655 | 0.749638744164022 | 1.38640631017255 | 0.999332001504091 |
| ebi-fl187-GCST90094733 | ebi-a-GCST90001624 | HLA DR+ CD4+ T cell Absolute Count \|\| id:ebi-a-GCST90001624 | Chilli pepper liking \|\| id:ebi-fl187-GCST90094733 | Weighted median | 12 | 0.235057313940936 | 0.157877772092147 | 0.136525215914496 | -0.074383119 | 0.544497747241543 | 1.26498126771693 | 0.928315969883316 | 1.7237424105457 | 0.999332001504091 |
| ebi-fl187-GCST90094733 | ebi-a-GCST90001631 | CD8+ Natural Killer T %T cell \|\| id:ebi-a-GCST90001631 | Chilli pepper liking \|\| id:ebi-fl187-GCST90094733 | Weighted median | 12 | 0.0901125146951183 | 0.162040015677055 | 0.578133796531424 | -0.227485916 | 0.407710945422146 | 1.09429740131729 | 0.796533639772858 | 1.50337254164337 | 0.999332001504091 |
| ebi-fl187-GCST90094733 | ebi-a-GCST90001632 | CD8+ Natural Killer T %lymphocyte \|\| id:ebi-a-GCST90001632 | Chilli pepper liking \|\| id:ebi-fl187-GCST90094733 | Weighted median | 12 | 0.149920314904991 | 0.161854139496838 | 0.35430670604743 | -0.167313799 | 0.467154428318793 | 1.16174166554483 | 0.845934116819064 | 1.59544776671011 | 0.999332001504091 |
| ebi-fl187-GCST90094733 | ebi-a-GCST90001646 | Natural Killer %CD3- lymphocyte \|\| id:ebi-a-GCST90001646 | Chilli pepper liking \|\| id:ebi-fl187-GCST90094733 | Weighted median | 12 | -0.091871208 | 0.158440745166449 | 0.562018578849019 | -0.402415068 | 0.218672652869978 | 0.912222629270118 | 0.668703130686667 | 1.24442385143013 | 0.999332001504091 |
| ebi-fl187-GCST90094733 | ebi-a-GCST90001684 | CD127- CD8+ T cell Absolute Count \|\| id:ebi-a-GCST90001684 | Chilli pepper liking \|\| id:ebi-fl187-GCST90094733 | Weighted median | 12 | 0.0222743876325079 | 0.160119802092247 | 0.889362624307177 | -0.291560424 | 0.336109199733311 | 1.02252431400746 | 0.747096869307981 | 1.39949184060282 | 0.999332001504091 |
| ebi-fl187-GCST90094733 | ebi-a-GCST90001687 | CD28- CD8+ T cell Absolute Count \|\| id:ebi-a-GCST90001687 | Chilli pepper liking \|\| id:ebi-fl187-GCST90094733 | Weighted median | 12 | 0.0386305584998892 | 0.157905172766899 | 0.806732065430216 | -0.27086358 | 0.348124697123011 | 1.03938642023176 | 0.762720539550141 | 1.4164088608383 | 0.999332001504091 |
| ebi-fl187-GCST90094733 | ebi-a-GCST90001701 | BAFF-R on CD20- CD38- B cell \|\| id:ebi-a-GCST90001701 | Chilli pepper liking \|\| id:ebi-fl187-GCST90094733 | Weighted median | 12 | 0.0850070388239236 | 0.167678980490234 | 0.612180780665814 | -0.243643763 | 0.413657840584782 | 1.0887247300131 | 0.783766791316733 | 1.51233957712186 | 0.999332001504091 |
| ebi-fl187-GCST90094733 | ebi-a-GCST90001732 | CD19 on IgD- CD27- B cell \|\| id:ebi-a-GCST90001732 | Chilli pepper liking \|\| id:ebi-fl187-GCST90094733 | Weighted median | 12 | 0.0928327852030546 | 0.155524128549445 | 0.550572288911518 | -0.211994507 | 0.397660077159966 | 1.09727823876896 | 0.80896914142091 | 1.48833802382265 | 0.999332001504091 |
| ebi-fl187-GCST90094733 | ebi-a-GCST90001798 | CD27 on CD24+ CD27+ B cell \|\| id:ebi-a-GCST90001798 | Chilli pepper liking \|\| id:ebi-fl187-GCST90094733 | Weighted median | 12 | 0.189489390615335 | 0.15069059623176 | 0.208582327086679 | -0.105864178 | 0.484842959229584 | 1.20863230107641 | 0.899546818027948 | 1.62391996717604 | 0.999332001504091 |
| ebi-fl187-GCST90094733 | ebi-a-GCST90001836 | CD66b on CD66b++ myeloid cell \|\| id:ebi-a-GCST90001836 | Chilli pepper liking \|\| id:ebi-fl187-GCST90094733 | Weighted median | 12 | 0.00808832547659398 | 0.257058966205235 | 0.974898812526895 | -0.495747248 | 0.511923899238854 | 1.00812112435079 | 0.60911557662456 | 1.66849813133037 | 0.999332001504091 |
| ebi-fl187-GCST90094733 | ebi-a-GCST90001943 | CD25 on activated & secreting CD4 regulatory T cell \|\| id:ebi-a-GCST90001943 | Chilli pepper liking \|\| id:ebi-fl187-GCST90094733 | Weighted median | 12 | -0.026668524 | 0.160688451821495 | 0.868185154093129 | -0.34161789 | 0.288280841427627 | 0.973683940757073 | 0.710619687930772 | 1.3341319310317 | 0.999332001504091 |
| ebi-fl187-GCST90094733 | ebi-a-GCST90001960 | CD25 on CD4+ T cell \|\| id:ebi-a-GCST90001960 | Chilli pepper liking \|\| id:ebi-fl187-GCST90094733 | Weighted median | 12 | 0.11802314485646 | 0.175622464599529 | 0.501566120476107 | -0.226196886 | 0.462243175471536 | 1.12527015528217 | 0.797561057792425 | 1.58763132928478 | 0.999332001504091 |
| ebi-fl187-GCST90094733 | ebi-a-GCST90001988 | HLA DR on CD14+ CD16- monocyte \|\| id:ebi-a-GCST90001988 | Chilli pepper liking \|\| id:ebi-fl187-GCST90094733 | Weighted median | 12 | -0.025155266 | 0.160049480526245 | 0.875109446906239 | -0.338852248 | 0.288541715773773 | 0.975158491258845 | 0.712587727652392 | 1.33448001722828 | 0.999332001504091 |
| ebi-fl187-GCST90094733 | ebi-a-GCST90001991 | HLA DR on CD14+ monocyte \|\| id:ebi-a-GCST90001991 | Chilli pepper liking \|\| id:ebi-fl187-GCST90094733 | Weighted median | 12 | -0.003637003 | 0.163051002049578 | 0.982203925494791 | -0.323216967 | 0.315942961187471 | 0.996369603054124 | 0.723816793082676 | 1.37155202169624 | 0.999332001504091 |
| ebi-fl187-GCST90094733 | ebi-a-GCST90002019 | CD14 on Monocytic Myeloid-Derived Suppressor Cells \|\| id:ebi-a-GCST90002019 | Chilli pepper liking \|\| id:ebi-fl187-GCST90094733 | Weighted median | 12 | 0.134945956436528 | 0.224634010918521 | 0.54801518701861 | -0.305336705 | 0.575228617836828 | 1.14447493117636 | 0.736875223071792 | 1.77753685709626 | 0.999332001504091 |
| ebi-fl187-GCST90094733 | ebi-a-GCST90002037 | CD80 on plasmacytoid Dendritic Cell \|\| id:ebi-a-GCST90002037 | Chilli pepper liking \|\| id:ebi-fl187-GCST90094733 | Weighted median | 12 | 0.076811150824448 | 0.172207673564256 | 0.65556986188313 | -0.260715889 | 0.414338191010389 | 1.07983813059502 | 0.770499795710022 | 1.51336884808959 | 0.999332001504091 |
| ebi-fl187-GCST90094733 | ebi-a-GCST90002042 | CD45 on CD33+ HLA DR+ CD14- \|\| id:ebi-a-GCST90002042 | Chilli pepper liking \|\| id:ebi-fl187-GCST90094733 | Weighted median | 12 | -0.079913352 | 0.238845942912856 | 0.737941060822293 | -0.5480514 | 0.388224696126878 | 0.923196336053562 | 0.578075150791224 | 1.47436103028503 | 0.999332001504091 |
| ebi-fl187-GCST90094733 | ebi-a-GCST90002056 | CD8 on Effector Memory CD8+ T cell \|\| id:ebi-a-GCST90002056 | Chilli pepper liking \|\| id:ebi-fl187-GCST90094733 | Weighted median | 12 | -0.160986454 | 0.181549490739146 | 0.375221095619304 | -0.516823456 | 0.194850547486608 | 0.851303602479377 | 0.596412071187014 | 1.2151293687802 | 0.999332001504091 |
| ebi-fl187-GCST90094733 | ebi-a-GCST90002058 | CD8 on CD8+ T cell \|\| id:ebi-a-GCST90002058 | Chilli pepper liking \|\| id:ebi-fl187-GCST90094733 | Weighted median | 12 | -0.419933846 | 0.195741636411907 | 0.0319250668708273 | -0.803587454 | -0.036280239 | 0.657090287433544 | 0.447719905284741 | 0.964370001742276 | 0.999332001504091 |
| ebi-fl187-GCST90094733 | ebi-a-GCST90002063 | CD4 on CD4 regulatory T cell \|\| id:ebi-a-GCST90002063 | Chilli pepper liking \|\| id:ebi-fl187-GCST90094733 | Weighted median | 12 | -0.333569341 | 0.181223668636217 | 0.065672869227316 | -0.688767732 | 0.0216290494428384 | 0.716362223584497 | 0.502194526375013 | 1.02186465289292 | 0.999332001504091 |
| ebi-fl187-GCST90094733 | ebi-a-GCST90002066 | CD4 on activated CD4 regulatory T cell \|\| id:ebi-a-GCST90002066 | Chilli pepper liking \|\| id:ebi-fl187-GCST90094733 | Weighted median | 12 | -0.419856764 | 0.184150978730313 | 0.0226100937630174 | -0.780792682 | -0.058920845 | 0.657140939576978 | 0.458042785132611 | 0.942781391793107 | 0.999332001504091 |
| ebi-fl187-GCST90094733 | ebi-a-GCST90002070 | CD4 on activated & secreting CD4 regulatory T cell \|\| id:ebi-a-GCST90002070 | Chilli pepper liking \|\| id:ebi-fl187-GCST90094733 | Weighted median | 12 | -0.226690863 | 0.177474376329465 | 0.20149070203411 | -0.574540641 | 0.121158914575056 | 0.79716717804861 | 0.562963411934917 | 1.1288042815675 | 0.999332001504091 |
| ebi-fl187-GCST90094733 | ebi-a-GCST90002081 | SSC-A on CD4+ T cell \|\| id:ebi-a-GCST90002081 | Chilli pepper liking \|\| id:ebi-fl187-GCST90094733 | Weighted median | 12 | 0.122054997177142 | 0.168260310585673 | 0.46821043515181 | -0.207735212 | 0.451845205925062 | 1.12981623677539 | 0.812422128213007 | 1.57120871595293 | 0.999332001504091 |
| ebi-fl187-GCST90094733 | ebi-a-GCST90002094 | CD11b on Monocytic Myeloid-Derived Suppressor Cells \|\| id:ebi-a-GCST90002094 | Chilli pepper liking \|\| id:ebi-fl187-GCST90094733 | Weighted median | 12 | 0.193979615036506 | 0.241390009066142 | 0.42163136474157 | -0.279144803 | 0.667104032806144 | 1.21407153390008 | 0.756430362100456 | 1.94858610029029 | 0.999332001504091 |
| ebi-fl187-GCST90094733 | ebi-a-GCST90002104 | HLA DR on myeloid Dendritic Cell \|\| id:ebi-a-GCST90002104 | Chilli pepper liking \|\| id:ebi-fl187-GCST90094733 | Weighted median | 12 | 0.00351502976596695 | 0.176390674171737 | 0.984101186838095 | -0.342210692 | 0.349240751142571 | 1.00352121472775 | 0.710198556067224 | 1.41799053209189 | 0.999332001504091 |
| ebi-fl187-GCST90094733 | ebi-a-GCST90002105 | HLA DR on plasmacytoid Dendritic Cell \|\| id:ebi-a-GCST90002105 | Chilli pepper liking \|\| id:ebi-fl187-GCST90094733 | Weighted median | 12 | 0.0446067359530071 | 0.177340752135139 | 0.801403426881744 | -0.302981138 | 0.392194610137879 | 1.04561657563622 | 0.738613027786768 | 1.48022575031109 | 0.999332001504091 |
| ebi-fl187-GCST90094733 | ebi-a-GCST90002106 | HLA DR on Dendritic Cell \|\| id:ebi-a-GCST90002106 | Chilli pepper liking \|\| id:ebi-fl187-GCST90094733 | Weighted median | 12 | -0.018927387 | 0.17125858261587 | 0.911997506078491 | -0.354594209 | 0.316739434972557 | 0.981250611251206 | 0.701458030901921 | 1.37264486207799 | 0.999332001504091 |
| ebi-fl187-GCST90094733 | ebi-a-GCST90002111 | HLA DR on CD33dim HLA DR+ CD11b- \|\| id:ebi-a-GCST90002111 | Chilli pepper liking \|\| id:ebi-fl187-GCST90094733 | Weighted median | 12 | 0.138070095089397 | 0.269843701572542 | 0.608884143009255 | -0.39082356 | 0.666963750171579 | 1.14805602054325 | 0.676499507087741 | 1.94831276667089 | 0.999332001504091 |
| ebi-fl187-GCST90094733 | ebi-a-GCST90002112 | HLA DR on CD33- HLA DR+ \|\| id:ebi-a-GCST90002112 | Chilli pepper liking \|\| id:ebi-fl187-GCST90094733 | Weighted median | 12 | 0.0596220157833371 | 0.248866208180488 | 0.81066007788045 | -0.428155752 | 0.547399783817094 | 1.06143526493393 | 0.651709901609687 | 1.72875203961548 | 0.999332001504091 |
| ebi-fl187-GCST90094733 | ebi-a-GCST90002115 | HLA DR on HLA DR+ CD8+ T cell \|\| id:ebi-a-GCST90002115 | Chilli pepper liking \|\| id:ebi-fl187-GCST90094733 | Weighted median | 12 | -0.439130264 | 0.163223800336105 | 0.00713756569135865 | -0.759048913 | -0.119211615 | 0.644596806444242 | 0.468111430244012 | 0.887619946946234 | 0.999332001504091 |
| ebi-fl187-GCST90094733 | ebi-a-GCST90002116 | HLA DR on B cell \|\| id:ebi-a-GCST90002116 | Chilli pepper liking \|\| id:ebi-fl187-GCST90094733 | Weighted median | 12 | 0.0891338571748022 | 0.170321148885213 | 0.600745892603023 | -0.244695595 | 0.42296330898982 | 1.09322698280802 | 0.782942833965845 | 1.52647828690858 | 0.999332001504091 |
| ebi-fl187-GCST90094733 | ebi-cfmet338-GCST90026023 | Sphingomyelin (d18:1/24:1, d18:2/24:0) levels \|\| id:ebi-cfmet338-GCST90026023 | Chilli pepper liking \|\| id:ebi-fl187-GCST90094733 | Weighted median | 12 | -0.115150786 | 0.103283391593425 | 0.264892724369257 | -0.317586234 | 0.0872846611237277 | 0.891231748148239 | 0.727903908058588 | 1.09120725979594 | 0.999332001504091 |
| ebi-fl187-GCST90094733 | ebi-cfmet338-GCST90026046 | 1-arachidonoyl-gpc (20:4n6) levels \|\| id:ebi-cfmet338-GCST90026046 | Chilli pepper liking \|\| id:ebi-fl187-GCST90094733 | Weighted median | 12 | -0.072795386 | 0.113855409027134 | 0.522584243354618 | -0.295951987 | 0.150361215956483 | 0.929791059075916 | 0.743823140023524 | 1.16225399160098 | 0.999332001504091 |
| ebi-fl187-GCST90094733 | ebi-cfmet338-GCST90026047 | 1-linoleoyl-gpc (18:2) levels \|\| id:ebi-cfmet338-GCST90026047 | Chilli pepper liking \|\| id:ebi-fl187-GCST90094733 | Weighted median | 12 | -0.101395798 | 0.143494823054358 | 0.479804882473746 | -0.382645651 | 0.179854055123255 | 0.903575328736081 | 0.682054541645008 | 1.197042648 | 0.999332001504091 |
| ebi-fl187-GCST90094733 | ebi-cfmet338-GCST90026050 | 1-myristoyl-2-palmitoyl-gpc (14:0/16:0) levels \|\| id:ebi-cfmet338-GCST90026050 | Chilli pepper liking \|\| id:ebi-fl187-GCST90094733 | Weighted median | 12 | -0.049391385 | 0.0726909927413297 | 0.496839794149373 | -0.191865731 | 0.0930829607045939 | 0.951808533141038 | 0.825417689190221 | 1.09755278524364 | 0.999332001504091 |
| ebi-fl187-GCST90094733 | ebi-cfmet338-GCST90026068 | 2,3-dihydroxyisovalerate levels \|\| id:ebi-cfmet338-GCST90026068 | Chilli pepper liking \|\| id:ebi-fl187-GCST90094733 | Weighted median | 12 | 0.172757930609271 | 0.235241293584586 | 0.46271347032015 | -0.288315005 | 0.63383086603506 | 1.18857835181985 | 0.749525450921024 | 1.88481724893908 | 0.999332001504091 |
| ebi-fl187-GCST90094733 | ebi-cfmet338-GCST90026082 | 3-hydroxyhexanoate levels \|\| id:ebi-cfmet338-GCST90026082 | Chilli pepper liking \|\| id:ebi-fl187-GCST90094733 | Weighted median | 12 | -0.0884999 | 0.0584082340727242 | 0.129722875708518 | -0.202980038 | 0.0259802392070859 | 0.915303202654902 | 0.816294535842274 | 1.02632066736289 | 0.999332001504091 |
| ebi-fl187-GCST90094733 | ebi-cfmet338-GCST90026094 | 3-ureidopropionate levels \|\| id:ebi-cfmet338-GCST90026094 | Chilli pepper liking \|\| id:ebi-fl187-GCST90094733 | Weighted median | 12 | -0.079016045 | 0.126352495650724 | 0.531733521587918 | -0.326666936 | 0.168634846933431 | 0.924025098766657 | 0.721323950049776 | 1.18368783275782 | 0.999332001504091 |
| ebi-fl187-GCST90094733 | ebi-cfmet338-GCST90026173 | Glycerol levels \|\| id:ebi-cfmet338-GCST90026173 | Chilli pepper liking \|\| id:ebi-fl187-GCST90094733 | Weighted median | 12 | 0.0140372716722039 | 0.0632958921704484 | 0.824491192069335 | -0.110022677 | 0.138097220326283 | 1.01413625678823 | 0.895813820712417 | 1.14808716225713 | 0.999332001504091 |
| ebi-fl187-GCST90094733 | ebi-cfmet338-GCST90026179 | Histidine levels \|\| id:ebi-cfmet338-GCST90026179 | Chilli pepper liking \|\| id:ebi-fl187-GCST90094733 | Weighted median | 12 | -0.012257059 | 0.0473529675247683 | 0.795755155188447 | -0.105068876 | 0.0805547570724616 | 0.987817752505534 | 0.900262514307857 | 1.08388819556181 | 0.999332001504091 |
| ebi-fl187-GCST90094733 | ebi-cfmet338-GCST90026188 | Isocitrate levels \|\| id:ebi-cfmet338-GCST90026188 | Chilli pepper liking \|\| id:ebi-fl187-GCST90094733 | Weighted median | 12 | -0.066805657 | 0.0901268718854803 | 0.458547838286925 | -0.243454326 | 0.109843012120271 | 0.935376967849929 | 0.78391528001916 | 1.11610284208613 | 0.999332001504091 |
| ebi-fl187-GCST90094733 | ebi-cfmet338-GCST90026207 | N-acetyl-isoputreanine levels \|\| id:ebi-cfmet338-GCST90026207 | Chilli pepper liking \|\| id:ebi-fl187-GCST90094733 | Weighted median | 12 | 0.0102165808301249 | 0.0591949173944127 | 0.86297198593382 | -0.105805457 | 0.126238618923174 | 1.01026894827887 | 0.899599641630065 | 1.13455286176761 | 0.999332001504091 |
| ebi-fl187-GCST90094733 | ebi-cfmet338-GCST90026213 | N-acetylglutamate levels \|\| id:ebi-cfmet338-GCST90026213 | Chilli pepper liking \|\| id:ebi-fl187-GCST90094733 | Weighted median | 12 | -0.168505937 | 0.0949508782122442 | 0.0759533269631416 | -0.354609659 | 0.017597783825243 | 0.844926246679163 | 0.701447193539659 | 1.01775353711922 | 0.999332001504091 |
| ebi-fl187-GCST90094733 | ebi-cfmet338-GCST90026224 | N-acetyltaurine levels \|\| id:ebi-cfmet338-GCST90026224 | Chilli pepper liking \|\| id:ebi-fl187-GCST90094733 | Weighted median | 11 | -0.02555554 | 0.0887560987039259 | 0.773400350837697 | -0.199517494 | 0.148406413279279 | 0.974768238658435 | 0.81912589119404 | 1.15998423357391 | 0.999332001504091 |
| ebi-fl187-GCST90094733 | ebi-cfmet338-GCST90026250 | Phenyllactate (pla) levels \|\| id:ebi-cfmet338-GCST90026250 | Chilli pepper liking \|\| id:ebi-fl187-GCST90094733 | Weighted median | 12 | 0.155180774575057 | 0.115824191363634 | 0.180311714970399 | -0.07183464 | 0.382196189647779 | 1.16786906305721 | 0.930684780659855 | 1.46549957277599 | 0.999332001504091 |
| ebi-fl187-GCST90094733 | ebi-cfmet338-GCST90026251 | Pipecolate levels \|\| id:ebi-cfmet338-GCST90026251 | Chilli pepper liking \|\| id:ebi-fl187-GCST90094733 | Weighted median | 12 | -0.245059406 | 0.186517184149634 | 0.188889824485054 | -0.610633087 | 0.120514275353386 | 0.782658042605951 | 0.543006989831506 | 1.12807684454642 | 0.999332001504091 |
| ebi-fl187-GCST90094733 | ebi-cfmet338-GCST90026278 | Trigonelline (n'-methylnicotinate) levels \|\| id:ebi-cfmet338-GCST90026278 | Chilli pepper liking \|\| id:ebi-fl187-GCST90094733 | Weighted median | 12 | 0.209929653750322 | 0.199287410646649 | 0.292157025180986 | -0.180673671 | 0.600532978617754 | 1.23359127838433 | 0.834707703488806 | 1.8230902095973 | 0.999332001504091 |
| ebi-fl187-GCST90094733 | ebi-cfmet338-GCST90026299 | 1-carboxyethylvaline levels \|\| id:ebi-cfmet338-GCST90026299 | Chilli pepper liking \|\| id:ebi-fl187-GCST90094733 | Weighted median | 12 | -0.057416182 | 0.133935019625281 | 0.668151127657739 | -0.319928821 | 0.205096456057417 | 0.944201027726207 | 0.726200725567275 | 1.22764347290181 | 0.999332001504091 |
| ebi-fl187-GCST90094733 | ebi-gm430-GCST90011306 | C_Erysipelotrichia abundance \|\| id:ebi-gm430-GCST90011306 | Chilli pepper liking \|\| id:ebi-fl187-GCST90094733 | Weighted median | 12 | -0.230551431 | 0.099374298862194 | 0.0203392130392683 | -0.425325057 | -0.035777805 | 0.794095593002378 | 0.653557307519615 | 0.964854655545066 | 0.999332001504091 |
| ebi-fl187-GCST90094733 | ebi-gm430-GCST90011312 | F_Erysipelotrichaceae abundance \|\| id:ebi-gm430-GCST90011312 | Chilli pepper liking \|\| id:ebi-fl187-GCST90094733 | Weighted median | 12 | -0.230551431 | 0.0927765033858553 | 0.0129544296317491 | -0.412393377 | -0.048709484 | 0.794095593002378 | 0.662063783884026 | 0.952457793607783 | 0.999332001504091 |
| ebi-fl187-GCST90094733 | ebi-gm430-GCST90011352 | O_Erysipelotrichales abundance \|\| id:ebi-gm430-GCST90011352 | Chilli pepper liking \|\| id:ebi-fl187-GCST90094733 | Weighted median | 12 | -0.230551431 | 0.0967688965713061 | 0.0171957565535461 | -0.420218468 | -0.040884393 | 0.794095593002378 | 0.656903291754966 | 0.959940098855551 | 0.999332001504091 |
| ebi-fl187-GCST90094733 | ebi-gm430-GCST90011362 | OTU97_11 (Parabacteroides) abundance \|\| id:ebi-gm430-GCST90011362 | Chilli pepper liking \|\| id:ebi-fl187-GCST90094733 | Weighted median | 12 | -0.148990883 | 0.137233735221222 | 0.277623943357704 | -0.417969004 | 0.119987238518943 | 0.861576970278551 | 0.658382635634151 | 1.12748246314147 | 0.999332001504091 |
| ebi-fl187-GCST90094733 | ebi-gm430-GCST90011384 | OTU97_235 (Phascolarctobacterium) abundance \|\| id:ebi-gm430-GCST90011384 | Chilli pepper liking \|\| id:ebi-fl187-GCST90094733 | Weighted median | 12 | -0.036997145 | 0.389753911521313 | 0.924374897937656 | -0.800914812 | 0.726920521329625 | 0.963678886405358 | 0.44891810062357 | 2.06870027030207 | 0.999332001504091 |
| ebi-fl187-GCST90094733 | ebi-gm430-GCST90011390 | OTU97_3 (Bacteroides) abundance \|\| id:ebi-gm430-GCST90011390 | Chilli pepper liking \|\| id:ebi-fl187-GCST90094733 | Weighted median | 12 | -0.148181787 | 0.108653879086488 | 0.172631600552306 | -0.36114339 | 0.0647798155406758 | 0.862274350021991 | 0.696879065479768 | 1.06692407841807 | 0.999332001504091 |
| ebi-fl187-GCST90094733 | ebi-gm430-GCST90011398 | OTU97_4 (Alistipes) abundance \|\| id:ebi-gm430-GCST90011398 | Chilli pepper liking \|\| id:ebi-fl187-GCST90094733 | Weighted median | 12 | -0.169123065 | 0.120540104423419 | 0.160603731907581 | -0.40538167 | 0.0671355397728307 | 0.844404980379533 | 0.666722294681525 | 1.06944042006327 | 0.999332001504091 |
| ebi-fl187-GCST90094733 | ebi-gm430-GCST90011421 | OTU99_108 (Cytophagales) abundance \|\| id:ebi-gm430-GCST90011421 | Chilli pepper liking \|\| id:ebi-fl187-GCST90094733 | Weighted median | 12 | -0.308874471 | 0.371618496490872 | 0.405883185211529 | -1.037246724 | 0.419497782117947 | 0.734272936785767 | 0.354429181123992 | 1.52119739121418 | 0.999332001504091 |
| ebi-fl187-GCST90094733 | ebi-gm430-GCST90011428 | OTU99_123 (Phascolarctobacterium) abundance \|\| id:ebi-gm430-GCST90011428 | Chilli pepper liking \|\| id:ebi-fl187-GCST90094733 | Weighted median | 12 | 0.0704288666651998 | 0.230220886458361 | 0.759666572812297 | -0.380804071 | 0.521661804123587 | 1.07296824290681 | 0.683311757235784 | 1.68482517400806 | 0.999332001504091 |
| ebi-fl187-GCST90094733 | ebi-gm430-GCST90011450 | OTU99_298 (Phascolarctobacterium) abundance \|\| id:ebi-gm430-GCST90011450 | Chilli pepper liking \|\| id:ebi-fl187-GCST90094733 | Weighted median | 12 | -0.058192344 | 0.385938990763445 | 0.88014831817112 | -0.814632765 | 0.698248078351787 | 0.943468459915617 | 0.442801909670662 | 2.0102278590388 | 0.999332001504091 |
| ebi-fl187-GCST90094733 | ebi-gm430-GCST90011451 | OTU99_3 (Bacteroides) abundance \|\| id:ebi-gm430-GCST90011451 | Chilli pepper liking \|\| id:ebi-fl187-GCST90094733 | Weighted median | 12 | -0.14574787 | 0.104799595010579 | 0.164307520337622 | -0.351155077 | 0.0596593357560199 | 0.86437561033402 | 0.703874590853001 | 1.0614748784082 | 0.999332001504091 |
| ebi-fl187-GCST90094733 | ebi-gm430-GCST90011472 | OTU99_72 (Parabacteroides) abundance \|\| id:ebi-gm430-GCST90011472 | Chilli pepper liking \|\| id:ebi-fl187-GCST90094733 | Weighted median | 12 | 0.159811936423072 | 0.33264035967491 | 0.630918400077636 | -0.492163169 | 0.811787041385896 | 1.17329019709086 | 0.611302612339713 | 2.25192868278551 | 0.999332001504091 |
| ebi-fl187-GCST90094733 | ebi-gm430-GCST90011507 | TestASV_3 (Bacteroides) abundance \|\| id:ebi-gm430-GCST90011507 | Chilli pepper liking \|\| id:ebi-fl187-GCST90094733 | Weighted median | 12 | -0.012568499 | 0.135854728021513 | 0.926289466284012 | -0.278843766 | 0.253706768029862 | 0.987510154825337 | 0.756658109844365 | 1.28879383329909 | 0.999332001504091 |
| ebi-fl187-GCST90094733 | ebi-gm430-GCST90011512 | TestASV_34 (Phascolarctobacterium) abundance \|\| id:ebi-gm430-GCST90011512 | Chilli pepper liking \|\| id:ebi-fl187-GCST90094733 | Weighted median | 12 | -0.127877157 | 0.330122072686662 | 0.698487311246087 | -0.774916419 | 0.519162105630777 | 0.879961469757364 | 0.460742288552264 | 1.68061887848548 | 0.999332001504091 |
| ebi-fl187-GCST90094733 | ebi-gm430-GCST90011519 | TestASV_41 (Barnesiella) abundance \|\| id:ebi-gm430-GCST90011519 | Chilli pepper liking \|\| id:ebi-fl187-GCST90094733 | Weighted median | 12 | -0.109051398 | 0.419094043410115 | 0.794703747826851 | -0.930475723 | 0.712372927273859 | 0.896684328703088 | 0.394366056777842 | 2.03882350299394 | 0.999332001504091 |
| ebi-fl187-GCST90094733 | ebi-gm430-GCST90011524 | TestASV_46 (Sutterella) abundance \|\| id:ebi-gm430-GCST90011524 | Chilli pepper liking \|\| id:ebi-fl187-GCST90094733 | Weighted median | 12 | -0.301551095 | 0.389001690958998 | 0.438226068643577 | -1.063994409 | 0.460892219 | 0.739670031959437 | 0.345074684259012 | 1.5854879570598 | 0.999332001504091 |
| ebi-fl187-GCST90094733 | ebi-gm430-GCST90011581 | OTU97_19 (Bacteria) prevalence \|\| id:ebi-gm430-GCST90011581 | Chilli pepper liking \|\| id:ebi-fl187-GCST90094733 | Weighted median | 12 | 0.0527680383648053 | 0.474700720072912 | 0.911488958672753 | -0.877645373 | 0.983181449707712 | 1.05418508625991 | 0.415760721470554 | 2.67294657408256 | 0.999332001504091 |
| ebi-fl187-GCST90094733 | ebi-gm430-GCST90011614 | OTU97_80 (Ruminococcus) prevalence \|\| id:ebi-gm430-GCST90011614 | Chilli pepper liking \|\| id:ebi-fl187-GCST90094733 | Weighted median | 12 | 0.40638252267944 | 0.238947476100744 | 0.0889957576556823 | -0.06195453 | 0.874719575836899 | 1.50137675328711 | 0.939925623788277 | 2.39820268568262 | 0.999332001504091 |
| ebi-fl187-GCST90094733 | ebi-gm430-GCST90011623 | OTU99_11 (Parabacteroides) prevalence \|\| id:ebi-gm430-GCST90011623 | Chilli pepper liking \|\| id:ebi-fl187-GCST90094733 | Weighted median | 12 | 0.0373926602605189 | 0.210146889831774 | 0.858773375307606 | -0.374495244 | 0.449280564330795 | 1.03810056165736 | 0.687636279877035 | 1.5671842915357 | 0.999332001504091 |
| ebi-fl187-GCST90094733 | ebi-gm430-GCST90011642 | OTU99_19 (Bacteria) prevalence \|\| id:ebi-gm430-GCST90011642 | Chilli pepper liking \|\| id:ebi-fl187-GCST90094733 | Weighted median | 12 | 0.0527680383648053 | 0.49453387290334 | 0.91502493859248 | -0.916518353 | 1.02205442925535 | 1.05418508625991 | 0.399908962100812 | 2.77889795281124 | 0.999332001504091 |
| ebi-fl187-GCST90094733 | ebi-gm430-GCST90011653 | OTU99_30 (Parasutterella) prevalence \|\| id:ebi-gm430-GCST90011653 | Chilli pepper liking \|\| id:ebi-fl187-GCST90094733 | Weighted median | 12 | -0.126589627 | 0.240735607337548 | 0.598995873629981 | -0.598431417 | 0.345252163876332 | 0.881095176523055 | 0.549673168274469 | 1.41234601741475 | 0.999332001504091 |
| ebi-fl187-GCST90094733 | ebi-gm430-GCST90011675 | OTU99_8 (Ruminococcaceae) prevalence \|\| id:ebi-gm430-GCST90011675 | Chilli pepper liking \|\| id:ebi-fl187-GCST90094733 | Weighted median | 12 | -0.149005551 | 0.2439578431096 | 0.541342576717054 | -0.627162924 | 0.329151821398807 | 0.861564332259358 | 0.534104950086087 | 1.38978883925691 | 0.999332001504091 |
| ebi-fl187-GCST90094733 | ebi-gm430-GCST90011679 | OTU99_92 (Ruminococcus) prevalence \|\| id:ebi-gm430-GCST90011679 | Chilli pepper liking \|\| id:ebi-fl187-GCST90094733 | Weighted median | 12 | 0.230814254344032 | 0.238126295206843 | 0.332398804518272 | -0.235913284 | 0.697541792949444 | 1.25962524780051 | 0.789849163316801 | 2.00880856571865 | 0.999332001504091 |
| ebi-fl187-GCST90094733 | ebi-gm430-GCST90011684 | TestASV_11 (Lachnospiraceae) prevalence \|\| id:ebi-gm430-GCST90011684 | Chilli pepper liking \|\| id:ebi-fl187-GCST90094733 | Weighted median | 12 | 0.0796189219793581 | 0.251825681396158 | 0.751875899513897 | -0.413959414 | 0.573197257515828 | 1.08287432943115 | 0.661027779487697 | 1.77392970420965 | 0.999332001504091 |
| ebi-fl187-GCST90094733 | ebi-gm430-GCST90011688 | TestASV_15 (Bacteroides) prevalence \|\| id:ebi-gm430-GCST90011688 | Chilli pepper liking \|\| id:ebi-fl187-GCST90094733 | Weighted median | 12 | -0.220920153 | 0.225037486582156 | 0.326245799698152 | -0.661993626 | 0.220153321133954 | 0.801780697864838 | 0.515821952536951 | 1.24626779513145 | 0.999332001504091 |
| ebi-fl187-GCST90094733 | ebi-gm430-GCST90011692 | TestASV_19 (Prevotella) prevalence \|\| id:ebi-gm430-GCST90011692 | Chilli pepper liking \|\| id:ebi-fl187-GCST90094733 | Weighted median | 12 | 0.194644086194729 | 0.466090474276144 | 0.67623221636878 | -0.718893243 | 1.10818141577597 | 1.21487851749735 | 0.487291270462297 | 3.02884517277794 | 0.999332001504091 |
| ebi-fl187-GCST90094733 | ebi-gm430-GCST90011693 | TestASV_2 (Bacteroides) prevalence \|\| id:ebi-gm430-GCST90011693 | Chilli pepper liking \|\| id:ebi-fl187-GCST90094733 | Weighted median | 12 | -0.457120033 | 0.224696954573727 | 0.0419128295431098 | -0.897526063 | -0.016714002 | 0.633104342395334 | 0.40757673247336 | 0.983424902416447 | 0.999332001504091 |
| ebi-fl187-GCST90094733 | ebi-gm430-GCST90011716 | TestASV_40 (Barnesiella) prevalence \|\| id:ebi-gm430-GCST90011716 | Chilli pepper liking \|\| id:ebi-fl187-GCST90094733 | Weighted median | 12 | -0.241982813 | 0.464370998703473 | 0.602298483222724 | -1.15214997 | 0.668184344843574 | 0.785069670730372 | 0.315956741082735 | 1.95069231879216 | 0.999332001504091 |
| ebi-fl187-GCST90094733 | ebi-gm430-GCST90011718 | TestASV_42 (Alphaproteobacteria) prevalence \|\| id:ebi-gm430-GCST90011718 | Chilli pepper liking \|\| id:ebi-fl187-GCST90094733 | Weighted median | 12 | 0.317509457976818 | 0.388643182873857 | 0.413946106464802 | -0.44423118 | 1.07925009640958 | 1.37370223724782 | 0.64131714369891 | 2.94247215306882 | 0.999332001504091 |
| ebi-fl187-GCST90094733 | ebi-hbsm44-GCST90104929 | Sulcal depth of the right inferior frontal sulcus \|\| id:ebi-hbsm44-GCST90104929 | Chilli pepper liking \|\| id:ebi-fl187-GCST90094733 | Weighted median | 13 | 0.285466125684238 | 0.179048037225778 | 0.110856554996547 | -0.065468027 | 0.636400278646763 | 1.33038200883995 | 0.936628992859033 | 1.88966634915112 | 0.999332001504091 |
| ebi-fl187-GCST90094733 | ebi-hbsm44-GCST90104935 | Sulcal opening distance of the left calloso-marginal posterior fissure \|\| id:ebi-hbsm44-GCST90104935 | Chilli pepper liking \|\| id:ebi-fl187-GCST90094733 | Weighted median | 12 | -0.052804614 | 0.0451807880934602 | 0.242508226431546 | -0.141358958 | 0.0357497308423471 | 0.948565330933076 | 0.868177616037204 | 1.03639643596803 | 0.999332001504091 |
| ebi-fl187-GCST90094733 | ebi-hbsm44-GCST90104936 | Sulcal opening distance of the left anterior inferior temporal sulcus \|\| id:ebi-hbsm44-GCST90104936 | Chilli pepper liking \|\| id:ebi-fl187-GCST90094733 | Weighted median | 13 | -0.018137214 | 0.0265659196900021 | 0.49478070185114 | -0.070206417 | 0.0339319881209115 | 0.982026274896873 | 0.932201377773601 | 1.03451424507768 | 0.999332001504091 |
| ebi-fl187-GCST90094733 | ebi-hbsm44-GCST90104941 | Sulcal opening distance of the right posterior inferior temporal sulcus \|\| id:ebi-hbsm44-GCST90104941 | Chilli pepper liking \|\| id:ebi-fl187-GCST90094733 | Weighted median | 12 | -0.010093783 | 0.0180565101142562 | 0.576154391177207 | -0.045484543 | 0.0252969767626196 | 0.989956988198635 | 0.955534372262231 | 1.025619661 | 0.999332001504091 |
| ebi-fl187-GCST90094733 | ebi-IPs91-GCST90274819 | Leukemia inhibitory factor levels \|\| id:ebi-IPs91-GCST90274819 | Chilli pepper liking \|\| id:ebi-fl187-GCST90094733 | Weighted median | 14 | -0.006225919 | 0.0782251338193082 | 0.936563544375139 | -0.159547181 | 0.147095343117427 | 0.99379342170727 | 0.852529742854436 | 1.15846440937284 | 0.999332001504091 |
| ebi-fl187-GCST90094733 | ebi-IPs91-GCST90274837 | STAM binding protein levels \|\| id:ebi-IPs91-GCST90274837 | Chilli pepper liking \|\| id:ebi-fl187-GCST90094733 | Weighted median | 14 | -0.02559871 | 0.0722310043682024 | 0.723038698304389 | -0.167171479 | 0.115974058560248 | 0.974726158996335 | 0.846054518684231 | 1.12296674038136 | 0.999332001504091 |
| ebi-fl187-GCST90094733 | ebi-IPs91-GCST90274840 | TNF-beta levels \|\| id:ebi-IPs91-GCST90274840 | Chilli pepper liking \|\| id:ebi-fl187-GCST90094733 | Weighted median | 14 | -0.004330198 | 0.085594165416412 | 0.959652332376505 | -0.172094762 | 0.163434366226846 | 0.995679163800316 | 0.841899389145389 | 1.17754806572843 | 0.999332001504091 |
| ebi-fl187-GCST90094733 | ebi-IPs91-GCST90274842 | Tumor necrosis factor ligand superfamily member 14 levels \|\| id:ebi-IPs91-GCST90274842 | Chilli pepper liking \|\| id:ebi-fl187-GCST90094733 | Weighted median | 14 | -0.022921136 | 0.0800139916302771 | 0.774522684429051 | -0.17974856 | 0.133906287344107 | 0.977339557395392 | 0.83548025828738 | 1.14328567428731 | 0.999332001504091 |
| ebi-fl187-GCST90094733 | ebi-met1400-GCST90199628 | 3-methylhistidine levels \|\| id:ebi-met1400-GCST90199628 | Chilli pepper liking \|\| id:ebi-fl187-GCST90094733 | Weighted median | 13 | 0.12447521630162 | 0.103611004814465 | 0.229607470506599 | -0.078602353 | 0.327552785737971 | 1.13255395123689 | 0.924407439090107 | 1.38756829318123 | 0.999332001504091 |
| ebi-fl187-GCST90094733 | ebi-met1400-GCST90199651 | Docosahexaenoate DHA; 22:6n3 levels \|\| id:ebi-met1400-GCST90199651 | Chilli pepper liking \|\| id:ebi-fl187-GCST90094733 | Weighted median | 13 | 0.136503747040191 | 0.0904658695358508 | 0.131324914797399 | -0.040809357 | 0.313816851330458 | 1.14625917284691 | 0.960012131856028 | 1.36863904917061 | 0.999332001504091 |
| ebi-fl187-GCST90094733 | ebi-met1400-GCST90199652 | 1-stearoyl-2-oleoyl-GPS (18:0/18:1) levels \|\| id:ebi-met1400-GCST90199652 | Chilli pepper liking \|\| id:ebi-fl187-GCST90094733 | Weighted median | 13 | 0.00803895330653609 | 0.0978068674449367 | 0.93449394347092 | -0.183662507 | 0.199740413498612 | 1.00807135245188 | 0.832216623816877 | 1.22108573964004 | 0.999332001504091 |
| ebi-fl187-GCST90094733 | ebi-met1400-GCST90199653 | 1-myristoyl-2-palmitoyl-gpc (14:0/16:0) levels \|\| id:ebi-met1400-GCST90199653 | Chilli pepper liking \|\| id:ebi-fl187-GCST90094733 | Weighted median | 13 | 0.0425717127935683 | 0.096280037122592 | 0.658369434011921 | -0.14613716 | 0.231280585553849 | 1.043490885 | 0.864039183471106 | 1.2602127873496 | 0.999332001504091 |
| ebi-fl187-GCST90094733 | ebi-met1400-GCST90199667 | Erythritol levels in elite athletes \|\| id:ebi-met1400-GCST90199667 | Chilli pepper liking \|\| id:ebi-fl187-GCST90094733 | Weighted median | 13 | 0.0595389923909703 | 0.0922556177074908 | 0.518687549379703 | -0.121282018 | 0.240360003097652 | 1.06134714463553 | 0.885784117019675 | 1.27170688634166 | 0.999332001504091 |
| ebi-fl187-GCST90094733 | ebi-met1400-GCST90199674 | 3-hydroxymyristate levels \|\| id:ebi-met1400-GCST90199674 | Chilli pepper liking \|\| id:ebi-fl187-GCST90094733 | Weighted median | 13 | 0.0815918551199674 | 0.0938038256143775 | 0.384402208834887 | -0.102263643 | 0.265447353324147 | 1.0850128769948 | 0.902791505553725 | 1.30401420040219 | 0.999332001504091 |
| ebi-fl187-GCST90094733 | ebi-met1400-GCST90199685 | 2-linoleoylglycerol (18:2) levels \|\| id:ebi-met1400-GCST90199685 | Chilli pepper liking \|\| id:ebi-fl187-GCST90094733 | Weighted median | 13 | -0.120918885 | 0.114704068191735 | 0.29179991369609 | -0.345738859 | 0.103901088399012 | 0.886105832924939 | 0.707697271813131 | 1.10949070798584 | 0.999332001504091 |
| ebi-fl187-GCST90094733 | ebi-met1400-GCST90199703 | Gamma-glutamylvaline levels \|\| id:ebi-met1400-GCST90199703 | Chilli pepper liking \|\| id:ebi-fl187-GCST90094733 | Weighted median | 13 | 0.0629422224626764 | 0.0934062390295127 | 0.500403767524019 | -0.120134006 | 0.246018450960521 | 1.06496530638098 | 0.886801591989056 | 1.27892317068497 | 0.999332001504091 |
| ebi-fl187-GCST90094733 | ebi-met1400-GCST90199779 | Gamma-glutamyl-2-aminobutyrate levels \|\| id:ebi-met1400-GCST90199779 | Chilli pepper liking \|\| id:ebi-fl187-GCST90094733 | Weighted median | 13 | -0.024039802 | 0.0989111450492864 | 0.807970987268958 | -0.217905646 | 0.169826042487077 | 0.976246852593346 | 0.804201317612684 | 1.18509867656972 | 0.999332001504091 |
| ebi-fl187-GCST90094733 | ebi-met1400-GCST90199824 | 4-vinylphenol sulfate levels \|\| id:ebi-met1400-GCST90199824 | Chilli pepper liking \|\| id:ebi-fl187-GCST90094733 | Weighted median | 13 | -0.045959804 | 0.0911554009547818 | 0.614126614218457 | -0.22462439 | 0.132704781749681 | 0.955080351712751 | 0.79881620577738 | 1.14191283505578 | 0.999332001504091 |
| ebi-fl187-GCST90094733 | ebi-met1400-GCST90199830 | 4-ethylphenylsulfate levels \|\| id:ebi-met1400-GCST90199830 | Chilli pepper liking \|\| id:ebi-fl187-GCST90094733 | Weighted median | 13 | 0.00376574737380279 | 0.0959809517631228 | 0.968703571302406 | -0.184356918 | 0.191888412829524 | 1.00377284670908 | 0.831638923879353 | 1.21153531762376 | 0.999332001504091 |
| ebi-fl187-GCST90094733 | ebi-met1400-GCST90199833 | Hexanoylglutamine levels \|\| id:ebi-met1400-GCST90199833 | Chilli pepper liking \|\| id:ebi-fl187-GCST90094733 | Weighted median | 12 | -0.027072595 | 0.0973942464320013 | 0.781036050370576 | -0.217965318 | 0.163820128354134 | 0.973290583267902 | 0.804153331102717 | 1.1780024068034 | 0.999332001504091 |
| ebi-fl187-GCST90094733 | ebi-met1400-GCST90199837 | Carotenoid levels (cryptoxanthin) \|\| id:ebi-met1400-GCST90199837 | Chilli pepper liking \|\| id:ebi-fl187-GCST90094733 | Weighted median | 13 | 0.134601383727212 | 0.101154501234343 | 0.183303724945074 | -0.063661439 | 0.332864206146524 | 1.14408064428273 | 0.938322625495085 | 1.39495785890463 | 0.999332001504091 |
| ebi-fl187-GCST90094733 | ebi-met1400-GCST90199869 | 2-hydroxyhippurate levels \|\| id:ebi-met1400-GCST90199869 | Chilli pepper liking \|\| id:ebi-fl187-GCST90094733 | Weighted median | 13 | 0.128860573090668 | 0.101196393933663 | 0.202886353807369 | -0.069484359 | 0.327205505200648 | 1.1375315106057 | 0.932874724345995 | 1.3870865013821 | 0.999332001504091 |
| ebi-fl187-GCST90094733 | ebi-met1400-GCST90199902 | Hydantoin-5-propionate levels \|\| id:ebi-met1400-GCST90199902 | Chilli pepper liking \|\| id:ebi-fl187-GCST90094733 | Weighted median | 13 | -0.059180903 | 0.0923935694332991 | 0.521827739858615 | -0.240272299 | 0.121910493021621 | 0.942536246035213 | 0.786413692123517 | 1.12965298542973 | 0.999332001504091 |
| ebi-fl187-GCST90094733 | ebi-met1400-GCST90199904 | Pantoate levels \|\| id:ebi-met1400-GCST90199904 | Chilli pepper liking \|\| id:ebi-fl187-GCST90094733 | Weighted median | 13 | 0.0753535625953995 | 0.0965062116499604 | 0.434910914605937 | -0.113798612 | 0.264505737429322 | 1.07826531778177 | 0.892437663809685 | 1.30278689781851 | 0.999332001504091 |
| ebi-fl187-GCST90094733 | ebi-met1400-GCST90199936 | 3-methyl catechol sulfate (2) levels \|\| id:ebi-met1400-GCST90199936 | Chilli pepper liking \|\| id:ebi-fl187-GCST90094733 | Weighted median | 13 | -0.060656766 | 0.10945979152906 | 0.579478550598278 | -0.275197958 | 0.153884424907927 | 0.941146217264976 | 0.759421774815015 | 1.16635607727726 | 0.999332001504091 |
| ebi-fl187-GCST90094733 | ebi-met1400-GCST90199977 | 4-hydroxychlorothalonil levels \|\| id:ebi-met1400-GCST90199977 | Chilli pepper liking \|\| id:ebi-fl187-GCST90094733 | Weighted median | 13 | 0.0888288345021899 | 0.0884160007229531 | 0.315056153992944 | -0.084466527 | 0.262124195919178 | 1.09289357464307 | 0.919002416708186 | 1.29968794834559 | 0.999332001504091 |
| ebi-fl187-GCST90094733 | ebi-met1400-GCST90200040 | Sphingomyelin (d18:2/24:1, d18:1/24:2) levels \|\| id:ebi-met1400-GCST90200040 | Chilli pepper liking \|\| id:ebi-fl187-GCST90094733 | Weighted median | 13 | 0.0401627808260494 | 0.0889142939594966 | 0.651483544213285 | -0.134109235 | 0.214434796986663 | 1.04098021202021 | 0.874494533682792 | 1.23916132128817 | 0.999332001504091 |
| ebi-fl187-GCST90094733 | ebi-met1400-GCST90200077 | 1-stearoyl-2-oleoyl-GPI (18:0/18:1) levels \|\| id:ebi-met1400-GCST90200077 | Chilli pepper liking \|\| id:ebi-fl187-GCST90094733 | Weighted median | 12 | 0.0675025242683527 | 0.0968299523622649 | 0.485724933639167 | -0.122284182 | 0.257289230898392 | 1.06983296013998 | 0.884896860687558 | 1.29341916945277 | 0.999332001504091 |
| ebi-fl187-GCST90094733 | ebi-met1400-GCST90200080 | 1-palmitoyl-2-oleoyl-GPI (16:0/18:1) levels \|\| id:ebi-met1400-GCST90200080 | Chilli pepper liking \|\| id:ebi-fl187-GCST90094733 | Weighted median | 13 | 0.0227839581819491 | 0.0959662030438439 | 0.812333705525872 | -0.1653098 | 0.210877716147883 | 1.02304549506195 | 0.847631067486029 | 1.23476135445425 | 0.999332001504091 |
| ebi-fl187-GCST90094733 | ebi-met1400-GCST90200082 | 1-oleoyl-2-linoleoyl-GPE (18:1/18:2) levels \|\| id:ebi-met1400-GCST90200082 | Chilli pepper liking \|\| id:ebi-fl187-GCST90094733 | Weighted median | 13 | -0.102629814 | 0.0960090271809131 | 0.285087674974304 | -0.290807507 | 0.0855478795567785 | 0.902460990332306 | 0.747659583409127 | 1.0893137159534 | 0.999332001504091 |
| ebi-fl187-GCST90094733 | ebi-met1400-GCST90200100 | Perfluorooctanesulfonate (PFOS) levels \|\| id:ebi-met1400-GCST90200100 | Chilli pepper liking \|\| id:ebi-fl187-GCST90094733 | Weighted median | 13 | 0.136503615619124 | 0.0945347472233341 | 0.148752542444558 | -0.048784489 | 0.321791720176859 | 1.14625902220432 | 0.952386357378024 | 1.37959740372811 | 0.999332001504091 |
| ebi-fl187-GCST90094733 | ebi-met1400-GCST90200102 | 1-palmitoleoyl-2-linolenoyl-GPC (16:1/18:3) levels \|\| id:ebi-met1400-GCST90200102 | Chilli pepper liking \|\| id:ebi-fl187-GCST90094733 | Weighted median | 13 | -0.112390926 | 0.10014094968599 | 0.261723283120105 | -0.308667188 | 0.0838853350804046 | 0.893694820389568 | 0.734425155090188 | 1.08750418807899 | 0.999332001504091 |
| ebi-fl187-GCST90094733 | ebi-met1400-GCST90200114 | Glycosyl-N-tricosanoyl-sphingadienine (d18:2/23:0) levels \|\| id:ebi-met1400-GCST90200114 | Chilli pepper liking \|\| id:ebi-fl187-GCST90094733 | Weighted median | 13 | 0.0541861743438868 | 0.111032545402727 | 0.625535218445228 | -0.163437615 | 0.271809963333231 | 1.05568112460152 | 0.849219476207174 | 1.31233758535237 | 0.999332001504091 |
| ebi-fl187-GCST90094733 | ebi-met1400-GCST90200135 | Arachidoylcarnitine (C20) levels \|\| id:ebi-met1400-GCST90200135 | Chilli pepper liking \|\| id:ebi-fl187-GCST90094733 | Weighted median | 13 | -0.077085337 | 0.111384548394235 | 0.488896567632022 | -0.295399052 | 0.141228377547201 | 0.92581084403248 | 0.744234539801782 | 1.15168763755095 | 0.999332001504091 |
| ebi-fl187-GCST90094733 | ebi-met1400-GCST90200146 | Docosahexaenoylcarnitine (C22:6) levels \|\| id:ebi-met1400-GCST90200146 | Chilli pepper liking \|\| id:ebi-fl187-GCST90094733 | Weighted median | 13 | 0.243935449760144 | 0.109044306929433 | 0.0252843411109135 | 0.0302086081784544 | 0.457662291341833 | 1.27626194481582 | 1.03066951761972 | 1.58037520654224 | 0.999332001504091 |
| ebi-fl187-GCST90094733 | ebi-met1400-GCST90200162 | 3-hydroxyphenylacetoylglutamine levels \|\| id:ebi-met1400-GCST90200162 | Chilli pepper liking \|\| id:ebi-fl187-GCST90094733 | Weighted median | 13 | -0.040218728 | 0.11340252878515 | 0.722848368846088 | -0.262487685 | 0.182050228264776 | 0.960579310433172 | 0.769135836546482 | 1.19967444993249 | 0.999332001504091 |
| ebi-fl187-GCST90094733 | ebi-met1400-GCST90200172 | 3-carboxy-4-methyl-5-pentyl-2-furanpropionate (3-CMPFP) levels \|\| id:ebi-met1400-GCST90200172 | Chilli pepper liking \|\| id:ebi-fl187-GCST90094733 | Weighted median | 13 | 0.224592279076059 | 0.0939674361323513 | 0.0168434393532153 | 0.0404161042566503 | 0.408768453895468 | 1.25181222209358 | 1.04124395010282 | 1.50496321177005 | 0.999332001504091 |
| ebi-fl187-GCST90094733 | ebi-met1400-GCST90200222 | Dihydrocaffeate sulfate (2) levels \|\| id:ebi-met1400-GCST90200222 | Chilli pepper liking \|\| id:ebi-fl187-GCST90094733 | Weighted median | 13 | 0.132581694904981 | 0.106844912730396 | 0.214650152656203 | -0.076834334 | 0.341997723856557 | 1.14177228925784 | 0.926043254738162 | 1.40775709325338 | 0.999332001504091 |
| ebi-fl187-GCST90094733 | ebi-met1400-GCST90200223 | 4-methylhexanoylglutamine levels \|\| id:ebi-met1400-GCST90200223 | Chilli pepper liking \|\| id:ebi-fl187-GCST90094733 | Weighted median | 13 | -0.021339371 | 0.12080904765319 | 0.859793376038735 | -0.258125105 | 0.215446362089481 | 0.97888670212928 | 0.772498582849441 | 1.24041544784593 | 0.999332001504091 |
| ebi-fl187-GCST90094733 | ebi-met1400-GCST90200224 | 2-ketocaprylate levels \|\| id:ebi-met1400-GCST90200224 | Chilli pepper liking \|\| id:ebi-fl187-GCST90094733 | Weighted median | 13 | 0.00270748494981585 | 0.0936280540876327 | 0.976930425141455 | -0.180803501 | 0.186218470961576 | 1.00271115349729 | 0.834599340468244 | 1.20468542041236 | 0.999332001504091 |
| ebi-fl187-GCST90094733 | ebi-met1400-GCST90200243 | Hydroxypalmitoyl sphingomyelin (d18:1/16:0(OH)) levels \|\| id:ebi-met1400-GCST90200243 | Chilli pepper liking \|\| id:ebi-fl187-GCST90094733 | Weighted median | 13 | 0.0323750958998882 | 0.0944357195104627 | 0.731728759955357 | -0.152718914 | 0.217469106140395 | 1.03290487103303 | 0.858370963673309 | 1.24292703010141 | 0.999332001504091 |
| ebi-fl187-GCST90094733 | ebi-met1400-GCST90200273 | 2-methoxyhydroquinone sulfate (2) levels \|\| id:ebi-met1400-GCST90200273 | Chilli pepper liking \|\| id:ebi-fl187-GCST90094733 | Weighted median | 13 | -0.011716621 | 0.125345673194388 | 0.925526629980697 | -0.25739414 | 0.233960898952441 | 0.988351751798199 | 0.773063458503276 | 1.26359508335037 | 0.999332001504091 |
| ebi-fl187-GCST90094733 | ebi-met1400-GCST90200276 | 2,4-di-tert-butylphenol levels \|\| id:ebi-met1400-GCST90200276 | Chilli pepper liking \|\| id:ebi-fl187-GCST90094733 | Weighted median | 13 | 0.177039028485663 | 0.10010424171671 | 0.0769695486909287 | -0.019165285 | 0.373243342250415 | 1.19367767965024 | 0.981017201139871 | 1.45243773630022 | 0.999332001504091 |
| ebi-fl187-GCST90094733 | ebi-met1400-GCST90200289 | N-acetylmethionine levels \|\| id:ebi-met1400-GCST90200289 | Chilli pepper liking \|\| id:ebi-fl187-GCST90094733 | Weighted median | 13 | -0.022152687 | 0.104300467168195 | 0.831800464906589 | -0.226581602 | 0.182276229000881 | 0.978090882230398 | 0.797254281876638 | 1.19994560788106 | 0.999332001504091 |
| ebi-fl187-GCST90094733 | ebi-met1400-GCST90200308 | 3-hydroxyisobutyrate levels \|\| id:ebi-met1400-GCST90200308 | Chilli pepper liking \|\| id:ebi-fl187-GCST90094733 | Weighted median | 13 | 0.0480051350394632 | 0.0997044251841149 | 0.630179321904334 | -0.147415538 | 0.243425808400328 | 1.04917604287102 | 0.862935320209135 | 1.27561167465914 | 0.999332001504091 |
| ebi-fl187-GCST90094733 | ebi-met1400-GCST90200383 | Cystathionine levels \|\| id:ebi-met1400-GCST90200383 | Chilli pepper liking \|\| id:ebi-fl187-GCST90094733 | Weighted median | 13 | -0.057609796 | 0.0995166623260511 | 0.562659358822782 | -0.252662455 | 0.137442861799285 | 0.944018234930274 | 0.776730019287686 | 1.14733614737606 | 0.999332001504091 |
| ebi-fl187-GCST90094733 | ebi-met1400-GCST90200384 | Guanidinoacetate levels \|\| id:ebi-met1400-GCST90200384 | Chilli pepper liking \|\| id:ebi-fl187-GCST90094733 | Weighted median | 13 | 0.118327607273793 | 0.092404631242461 | 0.200356120085866 | -0.06278547 | 0.299440684509017 | 1.12561280991388 | 0.939144926876866 | 1.34910402173567 | 0.999332001504091 |
| ebi-fl187-GCST90094733 | ebi-met1400-GCST90200403 | Citrulline levels \|\| id:ebi-met1400-GCST90200403 | Chilli pepper liking \|\| id:ebi-fl187-GCST90094733 | Weighted median | 13 | 0.0198343505072299 | 0.0912311357430509 | 0.8278906385177 | -0.158978676 | 0.19864737656361 | 1.02003235818852 | 0.853014548842008 | 1.21975177699384 | 0.999332001504091 |
| ebi-fl187-GCST90094733 | ebi-met1400-GCST90200434 | 12,13-DiHOME levels \|\| id:ebi-met1400-GCST90200434 | Chilli pepper liking \|\| id:ebi-fl187-GCST90094733 | Weighted median | 13 | 0.0679411291632482 | 0.09781375888029 | 0.487307984582965 | -0.123773838 | 0.259656096568617 | 1.07030229703222 | 0.883579650214204 | 1.29648414464359 | 0.999332001504091 |
| ebi-fl187-GCST90094733 | ebi-met1400-GCST90200437 | Fructose levels \|\| id:ebi-met1400-GCST90200437 | Chilli pepper liking \|\| id:ebi-fl187-GCST90094733 | Weighted median | 13 | -0.095606634 | 0.100159342079234 | 0.339807667104942 | -0.291918944 | 0.10070567661625 | 0.908821445348579 | 0.746829068247337 | 1.10595108659046 | 0.999332001504091 |
| ebi-fl187-GCST90094733 | ebi-met1400-GCST90200448 | N-stearoyl-sphinganine (d18:0/18:0) levels \|\| id:ebi-met1400-GCST90200448 | Chilli pepper liking \|\| id:ebi-fl187-GCST90094733 | Weighted median | 13 | 0.0276965184783821 | 0.106352851731104 | 0.794539028013216 | -0.180755071 | 0.236148107871346 | 1.02808363268765 | 0.834639761216075 | 1.26636185443676 | 0.999332001504091 |
| ebi-fl187-GCST90094733 | ebi-met1400-GCST90200468 | X-11847 levels \|\| id:ebi-met1400-GCST90200468 | Chilli pepper liking \|\| id:ebi-fl187-GCST90094733 | Weighted median | 13 | 0.0979731111581918 | 0.102006457921433 | 0.336823809564018 | -0.101959546 | 0.2979057686842 | 1.10293312811538 | 0.903066083233123 | 1.34703484903259 | 0.999332001504091 |
| ebi-fl187-GCST90094733 | ebi-met1400-GCST90200476 | X-11843 levels \|\| id:ebi-met1400-GCST90200476 | Chilli pepper liking \|\| id:ebi-fl187-GCST90094733 | Weighted median | 13 | 0.0792598111523731 | 0.102925094368865 | 0.441256735393777 | -0.122473374 | 0.280992996115348 | 1.08248552735089 | 0.884729461604081 | 1.32444432764747 | 0.999332001504091 |
| ebi-fl187-GCST90094733 | ebi-met1400-GCST90200511 | X-12906 levels \|\| id:ebi-met1400-GCST90200511 | Chilli pepper liking \|\| id:ebi-fl187-GCST90094733 | Weighted median | 13 | 0.0767947425421023 | 0.0994915909611086 | 0.440190425620744 | -0.118208776 | 0.271798260825875 | 1.07982041245144 | 0.8885105338037 | 1.31232222780199 | 0.999332001504091 |
| ebi-fl187-GCST90094733 | ebi-met1400-GCST90200515 | X-12849 levels \|\| id:ebi-met1400-GCST90200515 | Chilli pepper liking \|\| id:ebi-fl187-GCST90094733 | Weighted median | 13 | -0.045152344 | 0.108013895734476 | 0.675929952025467 | -0.25685958 | 0.166554891326772 | 0.9558518521469 | 0.773476817791772 | 1.18122837328348 | 0.999332001504091 |
| ebi-fl187-GCST90094733 | ebi-met1400-GCST90200551 | X-18886 levels \|\| id:ebi-met1400-GCST90200551 | Chilli pepper liking \|\| id:ebi-fl187-GCST90094733 | Weighted median | 13 | -0.086155421 | 0.0933454053868435 | 0.356020535709248 | -0.269112416 | 0.0968015731290884 | 0.917451628495425 | 0.764057358557841 | 1.10164175660535 | 0.999332001504091 |
| ebi-fl187-GCST90094733 | ebi-met1400-GCST90200564 | X-21319 levels \|\| id:ebi-met1400-GCST90200564 | Chilli pepper liking \|\| id:ebi-fl187-GCST90094733 | Weighted median | 13 | -0.020525264 | 0.0968003450914983 | 0.832078098141413 | -0.21025394 | 0.169203412849744 | 0.979683945887517 | 0.81037843241383 | 1.18436102867507 | 0.999332001504091 |
| ebi-fl187-GCST90094733 | ebi-met1400-GCST90200579 | X-21442 levels \|\| id:ebi-met1400-GCST90200579 | Chilli pepper liking \|\| id:ebi-fl187-GCST90094733 | Weighted median | 13 | 0.0617877202521012 | 0.10408896423582 | 0.55277639622128 | -0.14222665 | 0.265802090154309 | 1.06373651104053 | 0.867424632714903 | 1.30447686432324 | 0.999332001504091 |
| ebi-fl187-GCST90094733 | ebi-met1400-GCST90200581 | X-21441 levels \|\| id:ebi-met1400-GCST90200581 | Chilli pepper liking \|\| id:ebi-fl187-GCST90094733 | Weighted median | 13 | -0.057393454 | 0.089670015080594 | 0.522138924461569 | -0.233146684 | 0.118359775133145 | 0.944222487755281 | 0.792037385804821 | 1.12564901905082 | 0.999332001504091 |
| ebi-fl187-GCST90094733 | ebi-met1400-GCST90200648 | X-24585 levels \|\| id:ebi-met1400-GCST90200648 | Chilli pepper liking \|\| id:ebi-fl187-GCST90094733 | Weighted median | 13 | 0.039713126250065 | 0.103429893839399 | 0.7010067746294 | -0.163009466 | 0.242435718175288 | 1.0405122357261 | 0.849583146498445 | 1.27434932903027 | 0.999332001504091 |
| ebi-fl187-GCST90094733 | ebi-met1400-GCST90200720 | Adenosine 5'-diphosphate (ADP) to pantothenate ratio \|\| id:ebi-met1400-GCST90200720 | Chilli pepper liking \|\| id:ebi-fl187-GCST90094733 | Weighted median | 13 | -0.0818246 | 0.137362995284762 | 0.551387996290229 | -0.351056071 | 0.187406870946595 | 0.921433564143927 | 0.703944282192112 | 1.20611791957033 | 0.999332001504091 |
| ebi-fl187-GCST90094733 | ebi-met1400-GCST90200724 | Cholate to taurocholate ratio \|\| id:ebi-met1400-GCST90200724 | Chilli pepper liking \|\| id:ebi-fl187-GCST90094733 | Weighted median | 13 | -0.006027647 | 0.0993607791017376 | 0.951626603243852 | -0.200774774 | 0.188719479853083 | 0.993990482633968 | 0.818096667261078 | 1.20770212018429 | 0.999332001504091 |
| ebi-fl187-GCST90094733 | ebi-met1400-GCST90200726 | N-acetylputrescine to (N(1) + N(8))-acetylspermidine ratio \|\| id:ebi-met1400-GCST90200726 | Chilli pepper liking \|\| id:ebi-fl187-GCST90094733 | Weighted median | 13 | -0.061041865 | 0.0937731645534807 | 0.515077221429525 | -0.244837268 | 0.122753537033433 | 0.940783852573255 | 0.782831919668332 | 1.13060573416266 | 0.999332001504091 |
| ebi-fl187-GCST90094733 | ebi-met1400-GCST90200744 | Arginine to glutamate ratio \|\| id:ebi-met1400-GCST90200744 | Chilli pepper liking \|\| id:ebi-fl187-GCST90094733 | Weighted median | 13 | 0.179831331796538 | 0.096380529549618 | 0.0620627601510225 | -0.009074506 | 0.368737169713789 | 1.19701544764886 | 0.990966542949436 | 1.44590752544015 | 0.999332001504091 |
| ebi-fl187-GCST90094733 | ebi-met1400-GCST90200759 | Histidine to trans-urocanate ratio \|\| id:ebi-met1400-GCST90200759 | Chilli pepper liking \|\| id:ebi-fl187-GCST90094733 | Weighted median | 13 | 0.0120520323775742 | 0.0997286493358959 | 0.903811235092794 | -0.18341612 | 0.20752018507593 | 1.01212495076359 | 0.832421696074441 | 1.23062255679914 | 0.999332001504091 |
| ebi-fl187-GCST90094733 | ebi-met1400-GCST90200795 | Oleoyl-linoleoyl-glycerol (18:1 to 18:2) [2] to linoleoyl-arachidonoyl-glycerol (18:2 to 20:4) [2] ratio \|\| id:ebi-met1400-GCST90200795 | Chilli pepper liking \|\| id:ebi-fl187-GCST90094733 | Weighted median | 13 | -0.058264014 | 0.103651080224682 | 0.574036442638834 | -0.261420131 | 0.144892103689028 | 0.94340084394774 | 0.769957368854047 | 1.15591484459189 | 0.999332001504091 |
| ebi-fl187-GCST90094733 | ebi-met1400-GCST90200811 | Choline to taurocholate ratio \|\| id:ebi-met1400-GCST90200811 | Chilli pepper liking \|\| id:ebi-fl187-GCST90094733 | Weighted median | 13 | 0.124530850437698 | 0.0974892737149857 | 0.201468115021323 | -0.066548126 | 0.31560982691907 | 1.13261696165028 | 0.935617887185513 | 1.37109518681482 | 0.999332001504091 |
| ebi-fl187-GCST90094733 | ebi-met1400-GCST90200831 | Cholate to phosphate ratio \|\| id:ebi-met1400-GCST90200831 | Chilli pepper liking \|\| id:ebi-fl187-GCST90094733 | Weighted median | 13 | -0.106579841 | 0.103738094377151 | 0.304235224808142 | -0.309906506 | 0.0967468241664949 | 0.898903276127976 | 0.733515532472308 | 1.10158144451306 | 0.999332001504091 |
| ebi-fl187-GCST90094733 | ebi-met1400-GCST90200841 | Adenosine 5'-diphosphate (ADP) to oxalate (ethanedioate) ratio \|\| id:ebi-met1400-GCST90200841 | Chilli pepper liking \|\| id:ebi-fl187-GCST90094733 | Weighted median | 13 | -0.061580248 | 0.124125910403042 | 0.619815994610921 | -0.304867032 | 0.1817065365313 | 0.940277487456869 | 0.737221394545821 | 1.19926220258825 | 0.999332001504091 |
| ebi-fl187-GCST90094733 | ebi-met1400-GCST90200844 | Aspartate to phosphate ratio \|\| id:ebi-met1400-GCST90200844 | Chilli pepper liking \|\| id:ebi-fl187-GCST90094733 | Weighted median | 13 | -0.068864705 | 0.094853759567759 | 0.467833114867428 | -0.254778073 | 0.117048664213448 | 0.933452963483904 | 0.775088491712617 | 1.1241741354095 | 0.999332001504091 |
| ebi-fl187-GCST90094733 | ebi-met1400-GCST90200870 | Adenosine 5'-monophosphate (AMP) to acetoacetate ratio \|\| id:ebi-met1400-GCST90200870 | Chilli pepper liking \|\| id:ebi-fl187-GCST90094733 | Weighted median | 13 | 0.0397681923283647 | 0.116454841438549 | 0.732734547404495 | -0.188483297 | 0.26801968154792 | 1.04056953423193 | 0.828214337077299 | 1.30737287088353 | 0.999332001504091 |
| ebi-fl187-GCST90094733 | ebi-met1400-GCST90200887 | Cholesterol to cortisol ratio \|\| id:ebi-met1400-GCST90200887 | Chilli pepper liking \|\| id:ebi-fl187-GCST90094733 | Weighted median | 13 | 0.0515690687201844 | 0.0909323345953573 | 0.570636218392276 | -0.126658307 | 0.229796444527085 | 1.05292190774934 | 0.881034664472041 | 1.25834384108242 | 0.999332001504091 |
| ebi-fl187-GCST90094733 | ebi-met1400-GCST90200890 | Cortisol to taurocholate ratio \|\| id:ebi-met1400-GCST90200890 | Chilli pepper liking \|\| id:ebi-fl187-GCST90094733 | Weighted median | 13 | 0.0434514650887281 | 0.0951109553982008 | 0.647778674671904 | -0.142966007 | 0.229868937669202 | 1.04440930276904 | 0.866783532541099 | 1.25843506568786 | 0.999332001504091 |
| ebi-fl187-GCST90094733 | ebi-met1400-GCST90200948 | Adenosine 5'-diphosphate (ADP) to mannitol to sorbitol ratio \|\| id:ebi-met1400-GCST90200948 | Chilli pepper liking \|\| id:ebi-fl187-GCST90094733 | Weighted median | 13 | 0.107243472792172 | 0.126160526647185 | 0.395294128272206 | -0.140031159 | 0.354518105020654 | 1.11320525667926 | 0.869331147108269 | 1.42549355055376 | 0.999332001504091 |
| ebi-fl187-GCST90094733 | ebi-met1400-GCST90200972 | Leucine to N-palmitoyl-sphingosine (d18:1 to 16:0) ratio \|\| id:ebi-met1400-GCST90200972 | Chilli pepper liking \|\| id:ebi-fl187-GCST90094733 | Weighted median | 13 | -0.05450391 | 0.107574144742956 | 0.612390831792661 | -0.265349234 | 0.156341413650488 | 0.946954806214013 | 0.766938062551526 | 1.16922532443951 | 0.999332001504091 |
| ebi-fl187-GCST90094733 | ebi-met1400-GCST90200973 | Proline to glutamate ratio \|\| id:ebi-met1400-GCST90200973 | Chilli pepper liking \|\| id:ebi-fl187-GCST90094733 | Weighted median | 13 | 0.03356283687514 | 0.0922305328271221 | 0.715931502698712 | -0.147209007 | 0.214334681216299 | 1.03413242333466 | 0.863113561384476 | 1.23903726790784 | 0.999332001504091 |
| ebi-fl187-GCST90094733 | ebi-met1400-GCST90200983 | Cholesterol to linoleoyl-arachidonoyl-glycerol (18:2 to 20:4) [1] ratio \|\| id:ebi-met1400-GCST90200983 | Chilli pepper liking \|\| id:ebi-fl187-GCST90094733 | Weighted median | 13 | -0.02842674 | 0.0977332551961817 | 0.771158042973856 | -0.21998392 | 0.163130440638891 | 0.971973498758023 | 0.802531702785073 | 1.1771902331202 | 0.999332001504091 |
| ebi-fl187-GCST90094733 | ebi-met1400-GCST90201001 | Serine to alpha-ketobutyrate ratio \|\| id:ebi-met1400-GCST90201001 | Chilli pepper liking \|\| id:ebi-fl187-GCST90094733 | Weighted median | 13 | -0.043386795 | 0.0914547129720038 | 0.635209398210314 | -0.222638032 | 0.135864442402839 | 0.957540946357069 | 0.800404517330392 | 1.14552659823623 | 0.999332001504091 |
| ebi-fl187-GCST90094733 | ebi-met1400-GCST90201005 | Fructose to maltose ratio \|\| id:ebi-met1400-GCST90201005 | Chilli pepper liking \|\| id:ebi-fl187-GCST90094733 | Weighted median | 13 | 0.0946574899289865 | 0.112497648619462 | 0.40011408669866 | -0.125837901 | 0.315152881223132 | 1.0992822753884 | 0.881757766929802 | 1.37046881389059 | 0.999332001504091 |
| ebi-fl187-GCST90094733 | ebi-met1400-GCST90201020 | Paraxanthine to 5-acetylamino-6-formylamino-3-methyluracil ratio \|\| id:ebi-met1400-GCST90201020 | Chilli pepper liking \|\| id:ebi-fl187-GCST90094733 | Weighted median | 13 | -0.073189633 | 0.107804687438742 | 0.497195165935085 | -0.284486821 | 0.138107554120702 | 0.929424563504136 | 0.752400271553319 | 1.14809902641514 | 0.999332001504091 |
| ebi-fl187-GCST90094733 | ebi-pw11-GCST90275197 | Placental weight (fetal genotype, adjusted for sex) \|\| id:ebi-pw11-GCST90275197 | Chilli pepper liking \|\| id:ebi-fl187-GCST90094733 | Weighted median | 12 | -0.023845903 | 0.0369242417404118 | 0.518404896786368 | -0.096217417 | 0.0485256109636195 | 0.976436164197727 | 0.90826652232769 | 1.04972225587457 | 0.999332001504091 |
| ebi-fl187-GCST90094733 | prot-a-1017 | FAS-associated factor 2 \|\| id:prot-a-1017 | Chilli pepper liking \|\| id:ebi-fl187-GCST90094733 | Weighted median | 14 | 0.00130604750103407 | 0.152794703797006 | 0.993179983197331 | -0.298171572 | 0.300783666943165 | 1.00130690075249 | 0.742173992590947 | 1.35091706190675 | 0.999332001504091 |
| ebi-fl187-GCST90094733 | prot-a-1019 | Fas apoptotic inhibitory molecule 3 \|\| id:prot-a-1019 | Chilli pepper liking \|\| id:ebi-fl187-GCST90094733 | Weighted median | 14 | 0.134937315188719 | 0.151453114282663 | 0.372955417205137 | -0.161910789 | 0.431785419182739 | 1.1444650415276 | 0.850517076800388 | 1.54000462425303 | 0.999332001504091 |
| ebi-fl187-GCST90094733 | prot-a-1052 | MIP18 family protein FAM96A \|\| id:prot-a-1052 | Chilli pepper liking \|\| id:ebi-fl187-GCST90094733 | Weighted median | 14 | 0.00879678251189978 | 0.150844224712203 | 0.953496127260368 | -0.286857898 | 0.304451462947818 | 1.00883558790726 | 0.750618385689685 | 1.35588104798826 | 0.999332001504091 |
| ebi-fl187-GCST90094733 | prot-a-1066 | F-box/LRR-repeat protein 4 \|\| id:prot-a-1066 | Chilli pepper liking \|\| id:ebi-fl187-GCST90094733 | Weighted median | 14 | -0.021574826 | 0.153260118817345 | 0.888049585229052 | -0.321964659 | 0.278815006649037 | 0.97865624556913 | 0.724723802257911 | 1.32156284091608 | 0.999332001504091 |
| ebi-fl187-GCST90094733 | prot-a-1076 | Low affinity immunoglobulin gamma Fc region receptor III-B \|\| id:prot-a-1076 | Chilli pepper liking \|\| id:ebi-fl187-GCST90094733 | Weighted median | 14 | 0.0436475095062036 | 0.160031273022135 | 0.785050297718946 | -0.270013786 | 0.357308804629588 | 1.04461407345383 | 0.763368970751918 | 1.42947723088999 | 0.999332001504091 |
| ebi-fl187-GCST90094733 | prot-a-1081 | Fc receptor-like protein 3 \|\| id:prot-a-1081 | Chilli pepper liking \|\| id:ebi-fl187-GCST90094733 | Weighted median | 14 | 0.073320003336643 | 0.148195780990679 | 0.620775932293598 | -0.217143727 | 0.363783734078373 | 1.07607482936027 | 0.804814287122785 | 1.43876302509784 | 0.999332001504091 |
| ebi-fl187-GCST90094733 | prot-a-1105 | Fibroblast growth factor receptor 4 \|\| id:prot-a-1105 | Chilli pepper liking \|\| id:ebi-fl187-GCST90094733 | Weighted median | 14 | 0.11502647251501 | 0.159003102353566 | 0.469420156138463 | -0.196619608 | 0.426672553127999 | 1.12190313677646 | 0.821503066999441 | 1.53215088156173 | 0.999332001504091 |
| ebi-fl187-GCST90094733 | prot-a-1143 | Follistatin-related protein 1 \|\| id:prot-a-1143 | Chilli pepper liking \|\| id:ebi-fl187-GCST90094733 | Weighted median | 14 | 0.0201611442226709 | 0.148880533323235 | 0.892281180008057 | -0.271644701 | 0.311966989536212 | 1.02036575282539 | 0.762124995170632 | 1.36610959637379 | 0.999332001504091 |
| ebi-fl187-GCST90094733 | prot-a-1151 | Alpha-(1,3)-fucosyltransferase 10 \|\| id:prot-a-1151 | Chilli pepper liking \|\| id:ebi-fl187-GCST90094733 | Weighted median | 14 | 0.133933385066798 | 0.14575461537234 | 0.358149785652161 | -0.151745661 | 0.419612431196585 | 1.14331665514522 | 0.859206782692952 | 1.52137180509151 | 0.999332001504091 |
| ebi-fl187-GCST90094733 | prot-a-1165 | Growth arrest and DNA damage-inducible proteins-interacting protein 1 \|\| id:prot-a-1165 | Chilli pepper liking \|\| id:ebi-fl187-GCST90094733 | Weighted median | 14 | 0.0882907104132769 | 0.162654236719066 | 0.587259085600805 | -0.230511594 | 0.407093014382645 | 1.09230562049427 | 0.794127228190298 | 1.50244384804981 | 0.999332001504091 |
| ebi-fl187-GCST90094733 | prot-a-1194 | Growth/differentiation factor 11/8 \|\| id:prot-a-1194 | Chilli pepper liking \|\| id:ebi-fl187-GCST90094733 | Weighted median | 14 | -0.023338017 | 0.153731095160902 | 0.879336285169086 | -0.324650963 | 0.277974929648793 | 0.97693220839316 | 0.722779586162674 | 1.32045309257134 | 0.999332001504091 |
| ebi-fl187-GCST90094733 | prot-a-1216 | D-glucuronyl C5-epimerase \|\| id:prot-a-1216 | Chilli pepper liking \|\| id:ebi-fl187-GCST90094733 | Weighted median | 14 | 0.230590702365492 | 0.153725045415033 | 0.133609121246021 | -0.070710387 | 0.531891791378955 | 1.25934368755699 | 0.931731694995828 | 1.70214937616429 | 0.999332001504091 |
| ebi-fl187-GCST90094733 | prot-a-1246 | Glypican-3 \|\| id:prot-a-1246 | Chilli pepper liking \|\| id:ebi-fl187-GCST90094733 | Weighted median | 14 | -0.095250231 | 0.158120526003176 | 0.546914533410826 | -0.405166462 | 0.214665999821866 | 0.909145409506293 | 0.666865793731157 | 1.23944785202101 | 0.999332001504091 |
| ebi-fl187-GCST90094733 | prot-a-1275 | Glutamate receptor 4 \|\| id:prot-a-1275 | Chilli pepper liking \|\| id:ebi-fl187-GCST90094733 | Weighted median | 14 | -0.197364064 | 0.158068128612968 | 0.211810952517636 | -0.507177596 | 0.112449468065814 | 0.82089172176789 | 0.60219281389241 | 1.11901570945588 | 0.999332001504091 |
| ebi-fl187-GCST90094733 | prot-a-1289 | Glutathione S-transferase P \|\| id:prot-a-1289 | Chilli pepper liking \|\| id:ebi-fl187-GCST90094733 | Weighted median | 14 | 0.036861244460103 | 0.140643249657262 | 0.793251753880663 | -0.238799525 | 0.312522013788337 | 1.03754904517175 | 0.787572755299336 | 1.36686803078612 | 0.999332001504091 |
| ebi-fl187-GCST90094733 | prot-a-130 | Apolipoprotein D \|\| id:prot-a-130 | Chilli pepper liking \|\| id:ebi-fl187-GCST90094733 | Weighted median | 14 | -0.016749337 | 0.145582893480975 | 0.908405354440115 | -0.302091808 | 0.268593134271008 | 0.983390153316819 | 0.739270190727268 | 1.30812280242102 | 0.999332001504091 |
| ebi-fl187-GCST90094733 | prot-a-1313 | Heparin-binding EGF-like growth factor \|\| id:prot-a-1313 | Chilli pepper liking \|\| id:ebi-fl187-GCST90094733 | Weighted median | 13 | 0.149998776281993 | 0.160178516033839 | 0.349042813987715 | -0.163951115 | 0.463948667708318 | 1.16183282097167 | 0.848783513525593 | 1.59034133247957 | 0.999332001504091 |
| ebi-fl187-GCST90094733 | prot-a-1347 | HLA class II histocompatibility antigen, DQ alpha 2 chain \|\| id:prot-a-1347 | Chilli pepper liking \|\| id:ebi-fl187-GCST90094733 | Weighted median | 13 | -0.061021146 | 0.153318667169959 | 0.69062866269547 | -0.361525734 | 0.239483441332856 | 0.940803345036822 | 0.696612669232945 | 1.27059264513103 | 0.999332001504091 |
| ebi-fl187-GCST90094733 | prot-a-1357 | Non-histone chromosomal protein HMG-14 \|\| id:prot-a-1357 | Chilli pepper liking \|\| id:ebi-fl187-GCST90094733 | Weighted median | 14 | -0.045755542 | 0.1493893136604 | 0.759388496787417 | -0.338558596 | 0.247047513224248 | 0.955275458807294 | 0.712797010880192 | 1.28023993966056 | 0.999332001504091 |
| ebi-fl187-GCST90094733 | prot-a-137 | Apolipoprotein M \|\| id:prot-a-137 | Chilli pepper liking \|\| id:ebi-fl187-GCST90094733 | Weighted median | 14 | -0.118022921 | 0.150962691734689 | 0.434330996305989 | -0.413909797 | 0.177863954527762 | 0.888675682803919 | 0.661060578176204 | 1.19466278171636 | 0.999332001504091 |
| ebi-fl187-GCST90094733 | prot-a-1381 | 17-beta-hydroxysteroid dehydrogenase 14 \|\| id:prot-a-1381 | Chilli pepper liking \|\| id:ebi-fl187-GCST90094733 | Weighted median | 14 | -0.105562949 | 0.1450507258997 | 0.466756998079576 | -0.389862372 | 0.178736473436265 | 0.899817828137093 | 0.677150062832886 | 1.19570560245695 | 0.999332001504091 |
| ebi-fl187-GCST90094733 | prot-a-1392 | Serine protease HTRA2, mitochondrial \|\| id:prot-a-1392 | Chilli pepper liking \|\| id:ebi-fl187-GCST90094733 | Weighted median | 14 | -0.04209983 | 0.135258637511503 | 0.755607319345745 | -0.30720676 | 0.223007099325845 | 0.958774061191602 | 0.73549851371884 | 1.24982944665098 | 0.999332001504091 |
| ebi-fl187-GCST90094733 | prot-a-1416 | Interferon-induced protein with tetratricopeptide repeats 2 \|\| id:prot-a-1416 | Chilli pepper liking \|\| id:ebi-fl187-GCST90094733 | Weighted median | 14 | -0.15966993 | 0.150728395481539 | 0.289453083436959 | -0.455097586 | 0.135757724771495 | 0.852425102173283 | 0.634386058036618 | 1.14540435687379 | 0.999332001504091 |
| ebi-fl187-GCST90094733 | prot-a-1428 | Interferon gamma \|\| id:prot-a-1428 | Chilli pepper liking \|\| id:ebi-fl187-GCST90094733 | Weighted median | 14 | -0.053018473 | 0.162653422672052 | 0.744454788572794 | -0.371819182 | 0.265782235224598 | 0.9483624930185 | 0.689478901691021 | 1.30445096428391 | 0.999332001504091 |
| ebi-fl187-GCST90094733 | prot-a-1483 | Interleukin-17C \|\| id:prot-a-1483 | Chilli pepper liking \|\| id:ebi-fl187-GCST90094733 | Weighted median | 14 | 0.181947385790968 | 0.161855571711309 | 0.260956092877371 | -0.135289535 | 0.499184306345133 | 1.1995510787883 | 0.873462977176876 | 1.64737696756528 | 0.999332001504091 |
| ebi-fl187-GCST90094733 | prot-a-1503 | Interleukin-1 receptor-like 2 \|\| id:prot-a-1503 | Chilli pepper liking \|\| id:ebi-fl187-GCST90094733 | Weighted median | 14 | -0.02533577 | 0.159614063207692 | 0.873880467719204 | -0.338179334 | 0.287507794003164 | 0.97498248730515 | 0.713067399365562 | 1.33310098231599 | 0.999332001504091 |
| ebi-fl187-GCST90094733 | prot-a-1522 | Interleukin-31 receptor subunit alpha \|\| id:prot-a-1522 | Chilli pepper liking \|\| id:ebi-fl187-GCST90094733 | Weighted median | 14 | 0.0377442089113731 | 0.143180296512314 | 0.792078025003683 | -0.242889172 | 0.318377590075509 | 1.03846556866437 | 0.784358437633349 | 1.37489531005149 | 0.999332001504091 |
| ebi-fl187-GCST90094733 | prot-a-1540 | Interleukin-6 receptor subunit alpha \|\| id:prot-a-1540 | Chilli pepper liking \|\| id:ebi-fl187-GCST90094733 | Weighted median | 14 | 0.246881100826061 | 0.143162171991736 | 0.0846202204077875 | -0.033716756 | 0.527478957929863 | 1.28002690958786 | 0.966845318724539 | 1.69465462317232 | 0.999332001504091 |
| ebi-fl187-GCST90094733 | prot-a-1541 | Interleukin-6 receptor subunit alpha \|\| id:prot-a-1541 | Chilli pepper liking \|\| id:ebi-fl187-GCST90094733 | Weighted median | 14 | 0.31988743876576 | 0.151837724517152 | 0.0351373635535452 | 0.0222854987121411 | 0.617489378819378 | 1.3769727618589 | 1.02253567541966 | 1.8542668314464 | 0.999332001504091 |
| ebi-fl187-GCST90094733 | prot-a-1545 | Interleukin-9 \|\| id:prot-a-1545 | Chilli pepper liking \|\| id:ebi-fl187-GCST90094733 | Weighted median | 14 | 0.0991119866943109 | 0.155745563158228 | 0.524534525404098 | -0.206149317 | 0.404373290484438 | 1.10418994721739 | 0.813711566163486 | 1.49836316728841 | 0.999332001504091 |
| ebi-fl187-GCST90094733 | prot-a-1548 | Inositol monophosphatase 3 \|\| id:prot-a-1548 | Chilli pepper liking \|\| id:ebi-fl187-GCST90094733 | Weighted median | 14 | -0.004956446 | 0.145875838565286 | 0.972895369770223 | -0.290873089 | 0.280960197961614 | 0.995055817281766 | 0.747610551840234 | 1.32440088903116 | 0.999332001504091 |
| ebi-fl187-GCST90094733 | prot-a-1555 | Inhibin beta A chain:Inhibin beta B chain heterodimer \|\| id:prot-a-1555 | Chilli pepper liking \|\| id:ebi-fl187-GCST90094733 | Weighted median | 14 | -0.187669568 | 0.145912565526096 | 0.198380859244348 | -0.473658197 | 0.098319059962242 | 0.828888552900209 | 0.622720063844059 | 1.10331475251945 | 0.999332001504091 |
| ebi-fl187-GCST90094733 | prot-a-1603 | Junctophilin-3 \|\| id:prot-a-1603 | Chilli pepper liking \|\| id:ebi-fl187-GCST90094733 | Weighted median | 14 | 0.0155138478195649 | 0.174066190819777 | 0.928981662761267 | -0.325655886 | 0.356683581826327 | 1.01563481228856 | 0.7220536133078 | 1.42858376846414 | 0.999332001504091 |
| ebi-fl187-GCST90094733 | prot-a-1627 | UPF0258 protein KIAA1024 \|\| id:prot-a-1627 | Chilli pepper liking \|\| id:ebi-fl187-GCST90094733 | Weighted median | 14 | 0.119649525564668 | 0.165256781360765 | 0.469052302304767 | -0.204253766 | 0.443552817031767 | 1.12710176199542 | 0.815255460897268 | 1.55823351430855 | 0.999332001504091 |
| ebi-fl187-GCST90094733 | prot-a-1666 | Kallikrein-7 \|\| id:prot-a-1666 | Chilli pepper liking \|\| id:ebi-fl187-GCST90094733 | Weighted median | 14 | -0.023978201 | 0.165777205286159 | 0.884994225965724 | -0.348901524 | 0.30094512096841 | 0.976306991658899 | 0.705462598158261 | 1.35113519051256 | 0.999332001504091 |
| ebi-fl187-GCST90094733 | prot-a-1678 | Importin subunit alpha-3 \|\| id:prot-a-1678 | Chilli pepper liking \|\| id:ebi-fl187-GCST90094733 | Weighted median | 14 | 0.0950311407127422 | 0.167046675493521 | 0.569430964834205 | -0.232380343 | 0.422442624680043 | 1.09969309981982 | 0.792644588944371 | 1.52568368050287 | 0.999332001504091 |
| ebi-fl187-GCST90094733 | prot-a-1718 | Protein LDOC1 \|\| id:prot-a-1718 | Chilli pepper liking \|\| id:ebi-fl187-GCST90094733 | Weighted median | 14 | 0.0727362794227487 | 0.144180492750918 | 0.613923604164459 | -0.209857486 | 0.355330045214547 | 1.075446882 | 0.8106997735062 | 1.42665143606687 | 0.999332001504091 |
| ebi-fl187-GCST90094733 | prot-a-1723 | Leptin \|\| id:prot-a-1723 | Chilli pepper liking \|\| id:ebi-fl187-GCST90094733 | Weighted median | 14 | -0.034759958 | 0.154726660403155 | 0.822248480351801 | -0.338024212 | 0.268504296759052 | 0.965837230290559 | 0.713178020207814 | 1.30800659720769 | 0.999332001504091 |
| ebi-fl187-GCST90094733 | prot-a-1730 | Galectin-7 \|\| id:prot-a-1730 | Chilli pepper liking \|\| id:ebi-fl187-GCST90094733 | Weighted median | 14 | -0.030731351 | 0.154251707977283 | 0.842083931653133 | -0.333064699 | 0.271601996474466 | 0.969736056550889 | 0.716723821486927 | 1.31206469100458 | 0.999332001504091 |
| ebi-fl187-GCST90094733 | prot-a-1753 | Lipase member N \|\| id:prot-a-1753 | Chilli pepper liking \|\| id:ebi-fl187-GCST90094733 | Weighted median | 14 | 0.124155863921401 | 0.148499690067419 | 0.403116554491673 | -0.166903529 | 0.415215256453543 | 1.13219232518298 | 0.846281249327087 | 1.514696754 | 0.999332001504091 |
| ebi-fl187-GCST90094733 | prot-a-1756 | VIP36-like protein \|\| id:prot-a-1756 | Chilli pepper liking \|\| id:ebi-fl187-GCST90094733 | Weighted median | 14 | 0.150640205860335 | 0.138680388629851 | 0.277372759186439 | -0.121173356 | 0.422453767574842 | 1.16257829396678 | 0.885880373731873 | 1.52570068113034 | 0.999332001504091 |
| ebi-fl187-GCST90094733 | prot-a-1760 | Ig Kappa chain V-I region HK102- like \|\| id:prot-a-1760 | Chilli pepper liking \|\| id:ebi-fl187-GCST90094733 | Weighted median | 14 | 0.0615578402152454 | 0.158177052213753 | 0.697150034989366 | -0.248469182 | 0.3715848625542 | 1.0634920073565 | 0.779993898220203 | 1.45003089420561 | 0.999332001504091 |
| ebi-fl187-GCST90094733 | prot-a-179 | Neutral ceramidase \|\| id:prot-a-179 | Chilli pepper liking \|\| id:ebi-fl187-GCST90094733 | Weighted median | 14 | 0.263287991573212 | 0.160325826720191 | 0.100547375267766 | -0.050950629 | 0.577526611944786 | 1.3012014000349 | 0.950325588090459 | 1.78162632330554 | 0.999332001504091 |
| ebi-fl187-GCST90094733 | prot-a-1809 | Lactotransferrin \|\| id:prot-a-1809 | Chilli pepper liking \|\| id:ebi-fl187-GCST90094733 | Weighted median | 14 | 0.0131309149838045 | 0.154551271504475 | 0.932291961869817 | -0.289789577 | 0.316051407132575 | 1.01321750403069 | 0.748421035886691 | 1.37170076901687 | 0.999332001504091 |
| ebi-fl187-GCST90094733 | prot-a-1818 | Lysophospholipase-like protein 1 \|\| id:prot-a-1818 | Chilli pepper liking \|\| id:ebi-fl187-GCST90094733 | Weighted median | 14 | -0.187764928 | 0.162294438808156 | 0.24729687366227 | -0.505862029 | 0.130332171571432 | 0.828809513836846 | 0.602985560553028 | 1.13920673257326 | 0.999332001504091 |
| ebi-fl187-GCST90094733 | prot-a-1852 | MAP kinase-activated protein kinase 3 \|\| id:prot-a-1852 | Chilli pepper liking \|\| id:ebi-fl187-GCST90094733 | Weighted median | 14 | -0.101319036 | 0.161661532605263 | 0.530832571592386 | -0.41817564 | 0.215537568164519 | 0.903644691938164 | 0.658246604069106 | 1.24052858642973 | 0.999332001504091 |
| ebi-fl187-GCST90094733 | prot-a-1856 | Megakaryocyte-associated tyrosine-protein kinase \|\| id:prot-a-1856 | Chilli pepper liking \|\| id:ebi-fl187-GCST90094733 | Weighted median | 14 | -0.056685118 | 0.147788175560745 | 0.701307173084339 | -0.346349942 | 0.232979706101592 | 0.94489155187215 | 0.707264942019192 | 1.26235586094572 | 0.999332001504091 |
| ebi-fl187-GCST90094733 | prot-a-1890 | Beta-1,3-N-acetylglucosaminyltransferase manic fringe \|\| id:prot-a-1890 | Chilli pepper liking \|\| id:ebi-fl187-GCST90094733 | Weighted median | 14 | 0.0635291694176004 | 0.152769523215821 | 0.677519945145389 | -0.235899096 | 0.362957434920609 | 1.06559056800441 | 0.789860369915222 | 1.43757466745905 | 0.999332001504091 |
| ebi-fl187-GCST90094733 | prot-a-1919 | Matrilysin \|\| id:prot-a-1919 | Chilli pepper liking \|\| id:ebi-fl187-GCST90094733 | Weighted median | 14 | -0.097415957 | 0.149911222856519 | 0.51580571333641 | -0.391241954 | 0.196410039663157 | 0.907178580238486 | 0.676216522996015 | 1.21702583189965 | 0.999332001504091 |
| ebi-fl187-GCST90094733 | prot-a-1923 | Multimerin-2 \|\| id:prot-a-1923 | Chilli pepper liking \|\| id:ebi-fl187-GCST90094733 | Weighted median | 14 | -0.07355022 | 0.149809599226203 | 0.623456157510374 | -0.367177035 | 0.220076594111091 | 0.929089485400052 | 0.692687004445057 | 1.24617217638216 | 0.999332001504091 |
| ebi-fl187-GCST90094733 | prot-a-1948 | Mesothelin \|\| id:prot-a-1948 | Chilli pepper liking \|\| id:ebi-fl187-GCST90094733 | Weighted median | 14 | 0.100258364538799 | 0.146401619497069 | 0.493459205245099 | -0.18668881 | 0.387205538753053 | 1.10545649193968 | 0.829701891416512 | 1.47285918980523 | 0.999332001504091 |
| ebi-fl187-GCST90094733 | prot-a-195 | ATPase family AAA domain-containing protein 1 \|\| id:prot-a-195 | Chilli pepper liking \|\| id:ebi-fl187-GCST90094733 | Weighted median | 14 | -0.171734378 | 0.146365448671421 | 0.240665123837347 | -0.458610657 | 0.115141901773742 | 0.842202851384153 | 0.632161324566759 | 1.12203264469826 | 0.999332001504091 |
| ebi-fl187-GCST90094733 | prot-a-1966 | Mucin-1 \|\| id:prot-a-1966 | Chilli pepper liking \|\| id:ebi-fl187-GCST90094733 | Weighted median | 14 | 0.0238123413787664 | 0.156119302931518 | 0.878771637834852 | -0.282181492 | 0.329806175124542 | 1.02409811901628 | 0.75413680203865 | 1.39069855036583 | 0.999332001504091 |
| ebi-fl187-GCST90094733 | prot-a-1986 | Myocardial zonula adherens protein \|\| id:prot-a-1986 | Chilli pepper liking \|\| id:ebi-fl187-GCST90094733 | Weighted median | 14 | -0.15002687 | 0.150296627807076 | 0.318179883944867 | -0.44460826 | 0.14455452081996 | 0.860684849786219 | 0.641075361593336 | 1.15552469340014 | 0.999332001504091 |
| ebi-fl187-GCST90094733 | prot-a-2071 | Epididymal secretory protein E1 \|\| id:prot-a-2071 | Chilli pepper liking \|\| id:ebi-fl187-GCST90094733 | Weighted median | 13 | -0.171124408 | 0.151038219331207 | 0.257219522020678 | -0.467159317 | 0.124910502334771 | 0.842716726622878 | 0.62678022543428 | 1.13304704346395 | 0.999332001504091 |
| ebi-fl187-GCST90094733 | prot-a-2098 | Neuritin \|\| id:prot-a-2098 | Chilli pepper liking \|\| id:ebi-fl187-GCST90094733 | Weighted median | 14 | 0.227431501885788 | 0.158642132971911 | 0.151682591751206 | -0.083507079 | 0.538370082510734 | 1.25537144623226 | 0.919884575025227 | 1.71321219074911 | 0.999332001504091 |
| ebi-fl187-GCST90094733 | prot-a-2115 | Neurotrophin-4 \|\| id:prot-a-2115 | Chilli pepper liking \|\| id:ebi-fl187-GCST90094733 | Weighted median | 14 | 0.00840193155793229 | 0.155025482734287 | 0.956778131321776 | -0.295448015 | 0.312251877717134 | 1.00843732684503 | 0.744198101152565 | 1.36649884029451 | 0.999332001504091 |
| ebi-fl187-GCST90094733 | prot-a-2118 | Netrin-1 \|\| id:prot-a-2118 | Chilli pepper liking \|\| id:ebi-fl187-GCST90094733 | Weighted median | 14 | 0.199109746644829 | 0.143995937736685 | 0.166742890247214 | -0.083122291 | 0.481341784608731 | 1.22031588412511 | 0.920238603146043 | 1.61824428138202 | 0.999332001504091 |
| ebi-fl187-GCST90094733 | prot-a-2120 | Netrin-G1 \|\| id:prot-a-2120 | Chilli pepper liking \|\| id:ebi-fl187-GCST90094733 | Weighted median | 14 | 0.178513513949892 | 0.145345628389087 | 0.219371864726806 | -0.106363918 | 0.463390945592502 | 1.19543903826758 | 0.899097391084139 | 1.58945461124176 | 0.999332001504091 |
| ebi-fl187-GCST90094733 | prot-a-2145 | Olfactomedin-like protein 3 \|\| id:prot-a-2145 | Chilli pepper liking \|\| id:ebi-fl187-GCST90094733 | Weighted median | 14 | -0.360081351 | 0.158552362795541 | 0.0231435667784533 | -0.670843982 | -0.04931872 | 0.697619571872466 | 0.511276887229346 | 0.951877699179494 | 0.999332001504091 |
| ebi-fl187-GCST90094733 | prot-a-2162 | Prolyl 4-hydroxylase subunit alpha-2 \|\| id:prot-a-2162 | Chilli pepper liking \|\| id:ebi-fl187-GCST90094733 | Weighted median | 14 | 0.239584616040221 | 0.155849115852781 | 0.124223363492963 | -0.065879651 | 0.545048883111672 | 1.2707212034734 | 0.936243533455443 | 1.72469268866114 | 0.999332001504091 |
| ebi-fl187-GCST90094733 | prot-a-2168 | Polyadenylate-binding protein-interacting protein 1 \|\| id:prot-a-2168 | Chilli pepper liking \|\| id:ebi-fl187-GCST90094733 | Weighted median | 14 | -0.081816085 | 0.16581312636556 | 0.621713417138463 | -0.406809812 | 0.243177642987713 | 0.921441410297241 | 0.665770799635022 | 1.27529515123827 | 0.999332001504091 |
| ebi-fl187-GCST90094733 | prot-a-219 | Beta-1,4-galactosyltransferase 5 \|\| id:prot-a-219 | Chilli pepper liking \|\| id:ebi-fl187-GCST90094733 | Weighted median | 14 | 0.0390248260741863 | 0.149779993115442 | 0.794441360315499 | -0.25454396 | 0.332593612580452 | 1.03979629738975 | 0.775269971138737 | 1.39458044334849 | 0.999332001504091 |
| ebi-fl187-GCST90094733 | prot-a-2190 | Pyruvate carboxylase, mitochondrial \|\| id:prot-a-2190 | Chilli pepper liking \|\| id:ebi-fl187-GCST90094733 | Weighted median | 14 | -0.028298411 | 0.150084990032781 | 0.850446116643945 | -0.322464992 | 0.265868169075248 | 0.972098238329032 | 0.724361289909377 | 1.30456306559484 | 0.999332001504091 |
| ebi-fl187-GCST90094733 | prot-a-2197 | Protocadherin-9 \|\| id:prot-a-2197 | Chilli pepper liking \|\| id:ebi-fl187-GCST90094733 | Weighted median | 14 | -0.046761419 | 0.147192025631413 | 0.7507201761895 | -0.33525779 | 0.241734950901986 | 0.954315051551437 | 0.715153703419639 | 1.27345661955305 | 0.999332001504091 |
| ebi-fl187-GCST90094733 | prot-a-2198 | Protocadherin alpha-4 \|\| id:prot-a-2198 | Chilli pepper liking \|\| id:ebi-fl187-GCST90094733 | Weighted median | 14 | -0.039408037 | 0.160718280624149 | 0.806301990247322 | -0.354415867 | 0.275599793522257 | 0.96135835980397 | 0.701583141736609 | 1.31732055829806 | 0.999332001504091 |
| ebi-fl187-GCST90094733 | prot-a-2254 | Placenta growth factor \|\| id:prot-a-2254 | Chilli pepper liking \|\| id:ebi-fl187-GCST90094733 | Weighted median | 14 | -0.138270375 | 0.139974135515959 | 0.323236874133891 | -0.412619681 | 0.136078930141355 | 0.870863199864714 | 0.661913973370164 | 1.14577232599754 | 0.999332001504091 |
| ebi-fl187-GCST90094733 | prot-a-2261 | Membrane-associated progesterone receptor component 2 \|\| id:prot-a-2261 | Chilli pepper liking \|\| id:ebi-fl187-GCST90094733 | Weighted median | 14 | 0.0194257525085011 | 0.155079808718138 | 0.900315391998969 | -0.284530673 | 0.323382177596051 | 1.01961566014512 | 0.752367278065263 | 1.38179334046341 | 0.999332001504091 |
| ebi-fl187-GCST90094733 | prot-a-2268 | Polymeric immunoglobulin receptor \|\| id:prot-a-2268 | Chilli pepper liking \|\| id:ebi-fl187-GCST90094733 | Weighted median | 14 | 0.164120388656277 | 0.157047901607731 | 0.296007279720157 | -0.143693498 | 0.47193427580743 | 1.17835616726945 | 0.866153184633531 | 1.60309201833556 | 0.999332001504091 |
| ebi-fl187-GCST90094733 | prot-a-2283 | Group 10 secretory phospholipase A2 \|\| id:prot-a-2283 | Chilli pepper liking \|\| id:ebi-fl187-GCST90094733 | Weighted median | 14 | 0.10103772717882 | 0.168386566549323 | 0.548483330657071 | -0.228999943 | 0.431075397615494 | 1.10631837924743 | 0.795328578633744 | 1.53891157584605 | 0.999332001504091 |
| ebi-fl187-GCST90094733 | prot-a-2298 | Inactive phospholipase D5 \|\| id:prot-a-2298 | Chilli pepper liking \|\| id:ebi-fl187-GCST90094733 | Weighted median | 14 | -0.024613123 | 0.153094988833107 | 0.872274337628642 | -0.324679302 | 0.275453054631187 | 0.975687309529767 | 0.722759104171274 | 1.31712727032193 | 0.999332001504091 |
| ebi-fl187-GCST90094733 | prot-a-2304 | Procollagen-lysine,2-oxoglutarate 5-dioxygenase 2 \|\| id:prot-a-2304 | Chilli pepper liking \|\| id:ebi-fl187-GCST90094733 | Weighted median | 14 | 0.0744749078801705 | 0.155694606274545 | 0.632408459791821 | -0.23068652 | 0.379636336178278 | 1.07731831098295 | 0.793988326155568 | 1.46175290611484 | 0.999332001504091 |
| ebi-fl187-GCST90094733 | prot-a-2331 | Periostin \|\| id:prot-a-2331 | Chilli pepper liking \|\| id:ebi-fl187-GCST90094733 | Weighted median | 14 | -0.012628974 | 0.147741347978401 | 0.931879562822364 | -0.302202016 | 0.276944068471697 | 0.987450437276701 | 0.739188722149353 | 1.3190925901071 | 0.999332001504091 |
| ebi-fl187-GCST90094733 | prot-a-2333 | Periostin \|\| id:prot-a-2333 | Chilli pepper liking \|\| id:ebi-fl187-GCST90094733 | Weighted median | 14 | -0.116030164 | 0.157773804005948 | 0.462083155179467 | -0.42526682 | 0.193206492030245 | 0.890448363566962 | 0.653595369759275 | 1.21313327000941 | 0.999332001504091 |
| ebi-fl187-GCST90094733 | prot-a-2367 | cAMP-dependent protein kinase catalytic subunit alpha \|\| id:prot-a-2367 | Chilli pepper liking \|\| id:ebi-fl187-GCST90094733 | Weighted median | 14 | 0.0108585281602295 | 0.144708156988515 | 0.940184931214872 | -0.27276946 | 0.294486515857718 | 1.01091769594155 | 0.761268270539562 | 1.34243686163806 | 0.999332001504091 |
| ebi-fl187-GCST90094733 | prot-a-2385 | Phosphoribosyl pyrophosphate synthase-associated protein 1 \|\| id:prot-a-2385 | Chilli pepper liking \|\| id:ebi-fl187-GCST90094733 | Weighted median | 14 | 0.0562040461206312 | 0.153238356186101 | 0.713786623294133 | -0.244143132 | 0.35655122424539 | 1.05781350445911 | 0.783375500132744 | 1.42839469708518 | 0.999332001504091 |
| ebi-fl187-GCST90094733 | prot-a-2393 | Trypsin-3 \|\| id:prot-a-2393 | Chilli pepper liking \|\| id:ebi-fl187-GCST90094733 | Weighted median | 14 | 0.197049350687959 | 0.158244788607516 | 0.213051415328718 | -0.113110435 | 0.507209136358689 | 1.217804139 | 0.893052030484434 | 1.66065007367846 | 0.999332001504091 |
| ebi-fl187-GCST90094733 | prot-a-2405 | Pregnancy-specific beta-1-glycoprotein 6 \|\| id:prot-a-2405 | Chilli pepper liking \|\| id:ebi-fl187-GCST90094733 | Weighted median | 14 | 0.170334603188461 | 0.144311209747178 | 0.237869671314613 | -0.112515368 | 0.45318457429293 | 1.18570152446326 | 0.893583614485061 | 1.57331455313745 | 0.999332001504091 |
| ebi-fl187-GCST90094733 | prot-a-244 | Beclin-1 \|\| id:prot-a-244 | Chilli pepper liking \|\| id:ebi-fl187-GCST90094733 | Weighted median | 14 | -0.050407994 | 0.148552966994391 | 0.734363600193199 | -0.341571809 | 0.240755821193005 | 0.950841407652228 | 0.710652434244857 | 1.27221035057281 | 0.999332001504091 |
| ebi-fl187-GCST90094733 | prot-a-2454 | tRNA pseudouridine synthase A, mitochondrial \|\| id:prot-a-2454 | Chilli pepper liking \|\| id:ebi-fl187-GCST90094733 | Weighted median | 14 | -0.202165849 | 0.152751874656961 | 0.18567189166889 | -0.501559523 | 0.0972278254868119 | 0.816959424932407 | 0.605585498288552 | 1.10211143409493 | 0.999332001504091 |
| ebi-fl187-GCST90094733 | prot-a-2466 | Glutaminyl-peptide cyclotransferase-like protein \|\| id:prot-a-2466 | Chilli pepper liking \|\| id:ebi-fl187-GCST90094733 | Weighted median | 14 | 0.0286181004651629 | 0.145767327044999 | 0.844354023216057 | -0.257085861 | 0.314322061473362 | 1.02903153276117 | 0.773301814801215 | 1.3693306741935 | 0.999332001504091 |
| ebi-fl187-GCST90094733 | prot-a-2474 | Ras-related protein Rab-27A \|\| id:prot-a-2474 | Chilli pepper liking \|\| id:ebi-fl187-GCST90094733 | Weighted median | 14 | 0.165865862694581 | 0.152691073469248 | 0.277353384866791 | -0.133408641 | 0.465140366694307 | 1.180414753 | 0.875107413996924 | 1.5922376703404 | 0.999332001504091 |
| ebi-fl187-GCST90094733 | prot-a-2490 | Rap1 GTPase-GDP dissociation stimulator 1 \|\| id:prot-a-2490 | Chilli pepper liking \|\| id:ebi-fl187-GCST90094733 | Weighted median | 14 | -0.07868301 | 0.163318902273286 | 0.629965589458107 | -0.398788059 | 0.241422038043119 | 0.924332881909329 | 0.671132926953337 | 1.27305820093999 | 0.999332001504091 |
| ebi-fl187-GCST90094733 | prot-a-2545 | Receptor-interacting serine/threonine-protein kinase 2 \|\| id:prot-a-2545 | Chilli pepper liking \|\| id:ebi-fl187-GCST90094733 | Weighted median | 14 | 0.181786738874643 | 0.155527357842747 | 0.242467733328795 | -0.123046882 | 0.486620360246427 | 1.19935839008436 | 0.884222207043966 | 1.62680889080433 | 0.999332001504091 |
| ebi-fl187-GCST90094733 | prot-a-2610 | RUN and FYVE domain-containing protein 1 \|\| id:prot-a-2610 | Chilli pepper liking \|\| id:ebi-fl187-GCST90094733 | Weighted median | 14 | 0.120392135486027 | 0.147992457110112 | 0.415930394401332 | -0.16967308 | 0.410457351421846 | 1.12793906980433 | 0.84394067220759 | 1.50750708798415 | 0.999332001504091 |
| ebi-fl187-GCST90094733 | prot-a-2627 | Serine--tRNA ligase, mitochondrial \|\| id:prot-a-2627 | Chilli pepper liking \|\| id:ebi-fl187-GCST90094733 | Weighted median | 14 | 0.000393911450434607 | 0.149484967793485 | 0.997897477548906 | -0.292596625 | 0.293384448325664 | 1.00039398904374 | 0.746323127762502 | 1.34095822048987 | 0.999381480690152 |
| ebi-fl187-GCST90094733 | prot-a-264 | BolA-like protein 2 \|\| id:prot-a-264 | Chilli pepper liking \|\| id:ebi-fl187-GCST90094733 | Weighted median | 14 | -0.091878262 | 0.143588318805444 | 0.522255287960344 | -0.373311367 | 0.189554842909475 | 0.912216194207168 | 0.688450838728055 | 1.20871141142224 | 0.999332001504091 |
| ebi-fl187-GCST90094733 | prot-a-2646 | Secretoglobin family 1C member 1 \|\| id:prot-a-2646 | Chilli pepper liking \|\| id:ebi-fl187-GCST90094733 | Weighted median | 14 | -0.177139284 | 0.165290141760931 | 0.283860675154744 | -0.501107962 | 0.146829393973217 | 0.837663103431608 | 0.60585901909858 | 1.15815635771942 | 0.999332001504091 |
| ebi-fl187-GCST90094733 | prot-a-2672 | Semaphorin-3E \|\| id:prot-a-2672 | Chilli pepper liking \|\| id:ebi-fl187-GCST90094733 | Weighted median | 14 | 0.133684129750943 | 0.162955071763487 | 0.412002842762331 | -0.185707811 | 0.453076070407379 | 1.14303171290438 | 0.830516227317379 | 1.57314385165629 | 0.999332001504091 |
| ebi-fl187-GCST90094733 | prot-a-2701 | Plasma protease C1 inhibitor \|\| id:prot-a-2701 | Chilli pepper liking \|\| id:ebi-fl187-GCST90094733 | Weighted median | 14 | 0.173805673622229 | 0.164459309721216 | 0.290588787791275 | -0.148534573 | 0.496145920675811 | 1.18982432910134 | 0.861970205387602 | 1.64237919741652 | 0.999332001504091 |
| ebi-fl187-GCST90094733 | prot-a-2738 | Signaling threshold-regulating transmembrane adapter 1 \|\| id:prot-a-2738 | Chilli pepper liking \|\| id:ebi-fl187-GCST90094733 | Weighted median | 14 | 0.0420154070038992 | 0.146638282810874 | 0.774476711629358 | -0.245395627 | 0.329426441313212 | 1.04291054675384 | 0.782394940201226 | 1.39017055536001 | 0.999332001504091 |
| ebi-fl187-GCST90094733 | prot-a-274 | Serine/threonine-protein kinase BRSK2 \|\| id:prot-a-274 | Chilli pepper liking \|\| id:ebi-fl187-GCST90094733 | Weighted median | 14 | -0.056683256 | 0.14887903308313 | 0.703400903051762 | -0.348486161 | 0.235119648378976 | 0.944893310821074 | 0.705755681692636 | 1.26506012207103 | 0.999332001504091 |
| ebi-fl187-GCST90094733 | prot-a-2749 | Mitochondrial glutamate carrier 2 \|\| id:prot-a-2749 | Chilli pepper liking \|\| id:ebi-fl187-GCST90094733 | Weighted median | 14 | 0.206643379620261 | 0.165788988220127 | 0.212608761984734 | -0.118303037 | 0.531589796531709 | 1.22954401313306 | 0.888426785371212 | 1.7016354134344 | 0.999332001504091 |
| ebi-fl187-GCST90094733 | prot-a-2760 | Sodium-coupled monocarboxylate transporter 1 \|\| id:prot-a-2760 | Chilli pepper liking \|\| id:ebi-fl187-GCST90094733 | Weighted median | 14 | -0.113797097 | 0.151651382491405 | 0.453022169912834 | -0.411033807 | 0.183439612612612 | 0.892439016003683 | 0.662964518543687 | 1.20134241729128 | 0.999332001504091 |
| ebi-fl187-GCST90094733 | prot-a-2777 | Small integral membrane protein 10 \|\| id:prot-a-2777 | Chilli pepper liking \|\| id:ebi-fl187-GCST90094733 | Weighted median | 14 | 0.138051332622104 | 0.165886090915103 | 0.405292907580389 | -0.187085406 | 0.463188070815705 | 1.14803448038179 | 0.829372900294014 | 1.58913218369959 | 0.999332001504091 |
| ebi-fl187-GCST90094733 | prot-a-2799 | Superoxide dismutase [Mn], mitochondrial \|\| id:prot-a-2799 | Chilli pepper liking \|\| id:ebi-fl187-GCST90094733 | Weighted median | 14 | 0.214298339412784 | 0.147706240920016 | 0.146824009615397 | -0.075205893 | 0.503802571616016 | 1.2389922398771 | 0.927552490296361 | 1.65500258641449 | 0.999332001504091 |
| ebi-fl187-GCST90094733 | prot-a-2805 | Protein SPACA7 \|\| id:prot-a-2805 | Chilli pepper liking \|\| id:ebi-fl187-GCST90094733 | Weighted median | 14 | -0.307826 | 0.152376471003663 | 0.0433660125873813 | -0.606483883 | -0.009168117 | 0.735043204576588 | 0.545264717026747 | 0.990873782445228 | 0.999332001504091 |
| ebi-fl187-GCST90094733 | prot-a-2811 | SPARC-like protein 1 \|\| id:prot-a-2811 | Chilli pepper liking \|\| id:ebi-fl187-GCST90094733 | Weighted median | 14 | 0.162289285710975 | 0.140628012484089 | 0.248486877235909 | -0.113341619 | 0.43792019017979 | 1.17620045009331 | 0.892845595207857 | 1.54948123866551 | 0.999332001504091 |
| ebi-fl187-GCST90094733 | prot-a-2831 | Sepiapterin reductase \|\| id:prot-a-2831 | Chilli pepper liking \|\| id:ebi-fl187-GCST90094733 | Weighted median | 14 | 0.0559075304034268 | 0.154085925369797 | 0.716729290565501 | -0.246100883 | 0.357915944128228 | 1.05749989262688 | 0.781843345993221 | 1.43034538649839 | 0.999332001504091 |
| ebi-fl187-GCST90094733 | prot-a-2834 | SPRY domain-containing SOCS box protein 1 \|\| id:prot-a-2834 | Chilli pepper liking \|\| id:ebi-fl187-GCST90094733 | Weighted median | 14 | 0.0158042632931366 | 0.14917820745717 | 0.915628234014111 | -0.276585023 | 0.30819354990919 | 1.01592981118759 | 0.758369137326428 | 1.36096437797863 | 0.999332001504091 |
| ebi-fl187-GCST90094733 | prot-a-2835 | Spectrin alpha chain, non-erythrocytic 1 \|\| id:prot-a-2835 | Chilli pepper liking \|\| id:ebi-fl187-GCST90094733 | Weighted median | 14 | -0.046962087 | 0.144802544516524 | 0.74569721145344 | -0.330775075 | 0.236850899923411 | 0.954123570277702 | 0.718366729807765 | 1.26725215629499 | 0.999332001504091 |
| ebi-fl187-GCST90094733 | prot-a-2859 | Sia-alpha-2,3-Gal-beta-1,4-GlcNAc-R:alpha 2,8-sialyltransferase \|\| id:prot-a-2859 | Chilli pepper liking \|\| id:ebi-fl187-GCST90094733 | Weighted median | 14 | -0.027480132 | 0.147989203059099 | 0.852687783318511 | -0.31753897 | 0.26257870637562 | 0.972894012189156 | 0.727938312745017 | 1.30027880437317 | 0.999332001504091 |
| ebi-fl187-GCST90094733 | prot-a-2861 | Alpha-2,8-sialyltransferase 8F \|\| id:prot-a-2861 | Chilli pepper liking \|\| id:ebi-fl187-GCST90094733 | Weighted median | 14 | -0.111973031 | 0.170148450864656 | 0.510480162331511 | -0.445463995 | 0.221517932889003 | 0.89406836947877 | 0.640527006063636 | 1.24796962772091 | 0.999332001504091 |
| ebi-fl187-GCST90094733 | prot-a-289 | Uncharacterized protein C17orf89 \|\| id:prot-a-289 | Chilli pepper liking \|\| id:ebi-fl187-GCST90094733 | Weighted median | 14 | -0.006227705 | 0.158473325600741 | 0.968652700541198 | -0.316835423 | 0.304380013428946 | 0.993791647211107 | 0.728450631534498 | 1.35578417440056 | 0.999332001504091 |
| ebi-fl187-GCST90094733 | prot-a-2921 | Tachykinin-3 \|\| id:prot-a-2921 | Chilli pepper liking \|\| id:ebi-fl187-GCST90094733 | Weighted median | 14 | 0.0517074683332675 | 0.14814400522842 | 0.727062904657902 | -0.238654782 | 0.34206971858097 | 1.05306764181851 | 0.787686759156617 | 1.40785844798581 | 0.999332001504091 |
| ebi-fl187-GCST90094733 | prot-a-2928 | Tax1-binding protein 1 \|\| id:prot-a-2928 | Chilli pepper liking \|\| id:ebi-fl187-GCST90094733 | Weighted median | 14 | -0.058770978 | 0.169605794547997 | 0.728955595727488 | -0.391198336 | 0.273656378877793 | 0.942922694060287 | 0.676246018976044 | 1.31476294429085 | 0.999332001504091 |
| ebi-fl187-GCST90094733 | prot-a-296 | Uncharacterized protein C1orf185 \|\| id:prot-a-296 | Chilli pepper liking \|\| id:ebi-fl187-GCST90094733 | Weighted median | 14 | 0.033242721421287 | 0.144930846133343 | 0.818581709141042 | -0.250821737 | 0.317307179842638 | 1.03380143454476 | 0.778161076523543 | 1.37342439542397 | 0.999332001504091 |
| ebi-fl187-GCST90094733 | prot-a-2964 | Transforming growth factor beta-2 \|\| id:prot-a-2964 | Chilli pepper liking \|\| id:ebi-fl187-GCST90094733 | Weighted median | 14 | 0.124552519272294 | 0.150884417083292 | 0.409097437744252 | -0.171180938 | 0.420285976755546 | 1.13264150440579 | 0.842669088639542 | 1.52239686348738 | 0.999332001504091 |
| ebi-fl187-GCST90094733 | prot-a-2977 | Thymocyte nuclear protein 1 \|\| id:prot-a-2977 | Chilli pepper liking \|\| id:ebi-fl187-GCST90094733 | Weighted median | 14 | -0.161275777 | 0.154134598956037 | 0.295408332710482 | -0.463379591 | 0.140828036662614 | 0.851057336454477 | 0.629153765913867 | 1.15122666218317 | 0.999332001504091 |
| ebi-fl187-GCST90094733 | prot-a-3015 | Transmembrane protein 70, mitochondrial \|\| id:prot-a-3015 | Chilli pepper liking \|\| id:ebi-fl187-GCST90094733 | Weighted median | 14 | -0.050418843 | 0.156919496581967 | 0.747980118598122 | -0.357981056 | 0.257143370663697 | 0.950831092485243 | 0.699086318415913 | 1.29323052478737 | 0.999332001504091 |
| ebi-fl187-GCST90094733 | prot-a-3030 | Tumor necrosis factor alpha-induced protein 3 \|\| id:prot-a-3030 | Chilli pepper liking \|\| id:ebi-fl187-GCST90094733 | Weighted median | 14 | -0.119756113 | 0.142803803622742 | 0.401690494804066 | -0.399651568 | 0.160139341839949 | 0.88713677123004 | 0.670553647442497 | 1.17367440154883 | 0.999332001504091 |
| ebi-fl187-GCST90094733 | prot-a-3032 | Tumor necrosis factor receptor superfamily member 10A \|\| id:prot-a-3032 | Chilli pepper liking \|\| id:ebi-fl187-GCST90094733 | Weighted median | 14 | 0.125650595953586 | 0.159968903262043 | 0.432178706931188 | -0.187888454 | 0.439189646347189 | 1.13388591473398 | 0.828707140679383 | 1.55144948621788 | 0.999332001504091 |
| ebi-fl187-GCST90094733 | prot-a-3081 | Tumor protein p53-inducible protein 11 \|\| id:prot-a-3081 | Chilli pepper liking \|\| id:ebi-fl187-GCST90094733 | Weighted median | 14 | -0.020100171 | 0.147675634628779 | 0.891734105599906 | -0.309544415 | 0.269344072772591 | 0.980100490645226 | 0.733781179804062 | 1.30910549112137 | 0.999332001504091 |
| ebi-fl187-GCST90094733 | prot-a-315 | Complement C4 \|\| id:prot-a-315 | Chilli pepper liking \|\| id:ebi-fl187-GCST90094733 | Weighted median | 13 | -0.002732162 | 0.153122467244764 | 0.985764110136838 | -0.302852198 | 0.297387873337333 | 0.997271566496642 | 0.738708270668382 | 1.3463374065689 | 0.999332001504091 |
| ebi-fl187-GCST90094733 | prot-a-3157 | UDP-glucuronosyltransferase 1-8 \|\| id:prot-a-3157 | Chilli pepper liking \|\| id:ebi-fl187-GCST90094733 | Weighted median | 14 | 0.25761228145284 | 0.148866548162922 | 0.0835425163055156 | -0.034166153 | 0.549390715852168 | 1.29383707673155 | 0.966410919275255 | 1.73219729592941 | 0.999332001504091 |
| ebi-fl187-GCST90094733 | prot-a-317 | Glutaredoxin-like protein C5orf63 \|\| id:prot-a-317 | Chilli pepper liking \|\| id:ebi-fl187-GCST90094733 | Weighted median | 14 | -0.012902536 | 0.145393006674356 | 0.929286571788292 | -0.297872829 | 0.272067757140803 | 0.987180344935155 | 0.742395744937296 | 1.3126759414665 | 0.999332001504091 |
| ebi-fl187-GCST90094733 | prot-a-3192 | Guanine nucleotide exchange factor VAV3 \|\| id:prot-a-3192 | Chilli pepper liking \|\| id:ebi-fl187-GCST90094733 | Weighted median | 14 | -0.025004571 | 0.156955211571252 | 0.873424459477787 | -0.332636786 | 0.282627643457923 | 0.975305453680659 | 0.717030582481177 | 1.32661109751788 | 0.999332001504091 |
| ebi-fl187-GCST90094733 | prot-a-3233 | Serine/threonine-protein kinase WNK3 \|\| id:prot-a-3233 | Chilli pepper liking \|\| id:ebi-fl187-GCST90094733 | Weighted median | 14 | 0.184460499089748 | 0.153029466159233 | 0.228051996229948 | -0.115477255 | 0.484398252761845 | 1.20256947775765 | 0.89094083682805 | 1.62319796001608 | 0.999332001504091 |
| ebi-fl187-GCST90094733 | prot-a-3250 | 14-3-3 protein theta \|\| id:prot-a-3250 | Chilli pepper liking \|\| id:ebi-fl187-GCST90094733 | Weighted median | 14 | 0.162125601750359 | 0.151585317903481 | 0.28482926436666 | -0.134981621 | 0.459232824841182 | 1.17600794070094 | 0.873731969562868 | 1.58285918882377 | 0.999332001504091 |
| ebi-fl187-GCST90094733 | prot-a-3278 | E3 ubiquitin-protein ligase ZNRF3 \|\| id:prot-a-3278 | Chilli pepper liking \|\| id:ebi-fl187-GCST90094733 | Weighted median | 14 | 0.174080487620394 | 0.148499587552682 | 0.241091709753408 | -0.116978704 | 0.465139679223651 | 1.19015135441584 | 0.889604137986653 | 1.59223657572409 | 0.999332001504091 |
| ebi-fl187-GCST90094733 | prot-a-355 | Calpain-3 \|\| id:prot-a-355 | Chilli pepper liking \|\| id:ebi-fl187-GCST90094733 | Weighted median | 14 | 0.078418373807656 | 0.153347044392889 | 0.60908608209774 | -0.222141833 | 0.378978580817718 | 1.08157506670457 | 0.800801775999168 | 1.46079174644364 | 0.999332001504091 |
| ebi-fl187-GCST90094733 | prot-a-385 | Coiled-coil domain-containing protein 90B, mitochondrial \|\| id:prot-a-385 | Chilli pepper liking \|\| id:ebi-fl187-GCST90094733 | Weighted median | 14 | -0.106839832 | 0.161349381476082 | 0.507865805975814 | -0.423084619 | 0.209404956006755 | 0.898669599858059 | 0.655023203109616 | 1.23294418560298 | 0.999332001504091 |
| ebi-fl187-GCST90094733 | prot-a-390 | C-C motif chemokine 14 \|\| id:prot-a-390 | Chilli pepper liking \|\| id:ebi-fl187-GCST90094733 | Weighted median | 14 | 0.06527862625518 | 0.167853105651678 | 0.697347682896173 | -0.263713461 | 0.394270713332469 | 1.06745640433385 | 0.768193625693532 | 1.48330204396662 | 0.999332001504091 |
| ebi-fl187-GCST90094733 | prot-a-40 | Pituitary adenylate cyclase-activating polypeptide 27 \|\| id:prot-a-40 | Chilli pepper liking \|\| id:ebi-fl187-GCST90094733 | Weighted median | 14 | 0.0456235837689909 | 0.153861695936175 | 0.766830446283295 | -0.25594534 | 0.347192507803895 | 1.04668034932383 | 0.774184284342876 | 1.41508911484885 | 0.999332001504091 |
| ebi-fl187-GCST90094733 | prot-a-401 | C-C motif chemokine 24 \|\| id:prot-a-401 | Chilli pepper liking \|\| id:ebi-fl187-GCST90094733 | Weighted median | 14 | -0.097961187 | 0.155910218324437 | 0.52979568337363 | -0.403545215 | 0.207622841131946 | 0.90668409439663 | 0.66794782505579 | 1.2307488941418 | 0.999332001504091 |
| ebi-fl187-GCST90094733 | prot-a-412 | Cerebral cavernous malformations 2 protein \|\| id:prot-a-412 | Chilli pepper liking \|\| id:ebi-fl187-GCST90094733 | Weighted median | 14 | 0.0298619872929178 | 0.155576750327716 | 0.847786254804377 | -0.275068443 | 0.334792417935242 | 1.0303123279471 | 0.759520137343804 | 1.39765022798765 | 0.999332001504091 |
| ebi-fl187-GCST90094733 | prot-a-44 | Adhesion G protein-coupled receptor F5 \|\| id:prot-a-44 | Chilli pepper liking \|\| id:ebi-fl187-GCST90094733 | Weighted median | 14 | 0.0454382866339982 | 0.139609218049265 | 0.744827148889059 | -0.228195781 | 0.319072354010557 | 1.04648642042164 | 0.795968409293458 | 1.37585086963311 | 0.999332001504091 |
| ebi-fl187-GCST90094733 | prot-a-48 | Adrenomedullin \|\| id:prot-a-48 | Chilli pepper liking \|\| id:ebi-fl187-GCST90094733 | Weighted median | 14 | 0.11415190393594 | 0.150359513990468 | 0.44773705941592 | -0.180552743 | 0.408856551357257 | 1.12092238447436 | 0.834808648817995 | 1.5050958010494 | 0.999332001504091 |
| ebi-fl187-GCST90094733 | prot-a-502 | Carcinoembryonic antigen-related cell adhesion molecule 1 \|\| id:prot-a-502 | Chilli pepper liking \|\| id:ebi-fl187-GCST90094733 | Weighted median | 14 | 0.106991548745308 | 0.154490133112433 | 0.488594406939374 | -0.195809112 | 0.409792209645677 | 1.11292484882823 | 0.822169161799442 | 1.50650471543894 | 0.999332001504091 |
| ebi-fl187-GCST90094733 | prot-a-518 | Complement factor B \|\| id:prot-a-518 | Chilli pepper liking \|\| id:ebi-fl187-GCST90094733 | Weighted median | 14 | 0.128957359973386 | 0.149268958980284 | 0.387628449767463 | -0.1636098 | 0.421524519574742 | 1.1376416140628 | 0.84907326595448 | 1.52428358534233 | 0.999332001504091 |
| ebi-fl187-GCST90094733 | prot-a-525 | Properdin \|\| id:prot-a-525 | Chilli pepper liking \|\| id:ebi-fl187-GCST90094733 | Weighted median | 14 | 0.0520873980194471 | 0.147838861742701 | 0.724594180947203 | -0.237676771 | 0.341851567035141 | 1.05346780949017 | 0.788457502243327 | 1.4075513549867 | 0.999332001504091 |
| ebi-fl187-GCST90094733 | prot-a-533 | Chondroadherin \|\| id:prot-a-533 | Chilli pepper liking \|\| id:ebi-fl187-GCST90094733 | Weighted median | 14 | 0.0999245694022335 | 0.162064722859814 | 0.537517071460119 | -0.217722287 | 0.417571426207469 | 1.10508755751667 | 0.804348788442948 | 1.51826984428259 | 0.999332001504091 |
| ebi-fl187-GCST90094733 | prot-a-535 | Coiled-coil-helix-coiled-coil-helix domain-containing protein 2 \|\| id:prot-a-535 | Chilli pepper liking \|\| id:ebi-fl187-GCST90094733 | Weighted median | 14 | -0.047123953 | 0.162857929717064 | 0.772308804811745 | -0.366325495 | 0.272077589605239 | 0.953969143267572 | 0.693277106328035 | 1.31268884836947 | 0.999332001504091 |
| ebi-fl187-GCST90094733 | prot-a-591 | Caseinolytic peptidase B protein homolog \|\| id:prot-a-591 | Chilli pepper liking \|\| id:ebi-fl187-GCST90094733 | Weighted median | 14 | 0.153017560814394 | 0.166243108059469 | 0.357340503287771 | -0.172818931 | 0.478854052610952 | 1.16534544317809 | 0.841289932596004 | 1.61422352665673 | 0.999332001504091 |
| ebi-fl187-GCST90094733 | prot-a-605 | Ciliary neurotrophic factor receptor subunit alpha \|\| id:prot-a-605 | Chilli pepper liking \|\| id:ebi-fl187-GCST90094733 | Weighted median | 14 | 0.114354130698777 | 0.149438591858952 | 0.444137638124337 | -0.178545509 | 0.407253770742323 | 1.12114908790154 | 0.83648598808193 | 1.50268539486802 | 0.999332001504091 |
| ebi-fl187-GCST90094733 | prot-a-606 | Ciliary neurotrophic factor receptor subunit alpha \|\| id:prot-a-606 | Chilli pepper liking \|\| id:ebi-fl187-GCST90094733 | Weighted median | 14 | 0.239675166031852 | 0.151777504783988 | 0.114308123064316 | -0.057808743 | 0.537159075408469 | 1.27083627247741 | 0.943830444029496 | 1.71113873435703 | 0.999332001504091 |
| ebi-fl187-GCST90094733 | prot-a-707 | Cancer/testis antigen 55 \|\| id:prot-a-707 | Chilli pepper liking \|\| id:ebi-fl187-GCST90094733 | Weighted median | 14 | -0.028036067 | 0.149946220556722 | 0.851680899823322 | -0.32193066 | 0.265858524966034 | 0.972353295986318 | 0.724748442922724 | 1.30455048430683 | 0.999332001504091 |
| ebi-fl187-GCST90094733 | prot-a-719 | Dipeptidyl peptidase 1 \|\| id:prot-a-719 | Chilli pepper liking \|\| id:ebi-fl187-GCST90094733 | Weighted median | 14 | 0.00566754424693856 | 0.143324361905645 | 0.968457087421518 | -0.275248205 | 0.286583293582003 | 1.00568363516013 | 0.759383616954261 | 1.33186909942225 | 0.999332001504091 |
| ebi-fl187-GCST90094733 | prot-a-752 | Cytochrome b5 \|\| id:prot-a-752 | Chilli pepper liking \|\| id:ebi-fl187-GCST90094733 | Weighted median | 14 | -0.027596816 | 0.152571378194657 | 0.85646328591068 | -0.326636717 | 0.271443085495276 | 0.972780497504967 | 0.721345748060095 | 1.31185620608548 | 0.999332001504091 |
| ebi-fl187-GCST90094733 | prot-a-755 | Cytoglobin \|\| id:prot-a-755 | Chilli pepper liking \|\| id:ebi-fl187-GCST90094733 | Weighted median | 14 | 0.0720256706952487 | 0.153354512672891 | 0.638592404049822 | -0.228549174 | 0.372600515534115 | 1.07468293156774 | 0.795687169007321 | 1.45150437054793 | 0.999332001504091 |
| ebi-fl187-GCST90094733 | prot-a-807 | Desmin \|\| id:prot-a-807 | Chilli pepper liking \|\| id:ebi-fl187-GCST90094733 | Weighted median | 14 | 0.0321447288146282 | 0.159437008264462 | 0.840218520011062 | -0.280351807 | 0.344641265012974 | 1.03266695115404 | 0.755517897920589 | 1.41148348032633 | 0.999332001504091 |
| ebi-fl187-GCST90094733 | prot-a-82 | Ameloblastin \|\| id:prot-a-82 | Chilli pepper liking \|\| id:ebi-fl187-GCST90094733 | Weighted median | 14 | -0.034529667 | 0.15561633108697 | 0.824399720826027 | -0.339537676 | 0.270478342300337 | 0.966059679526178 | 0.712099467658074 | 1.31059121203324 | 0.999332001504091 |
| ebi-fl187-GCST90094733 | prot-a-84 | Muellerian-inhibiting factor \|\| id:prot-a-84 | Chilli pepper liking \|\| id:ebi-fl187-GCST90094733 | Weighted median | 14 | -0.013309397 | 0.144060488477558 | 0.926390124780307 | -0.295667954 | 0.269049160745352 | 0.986778781715334 | 0.744034440603527 | 1.308719477 | 0.999332001504091 |
| ebi-fl187-GCST90094733 | prot-a-858 | Inactive dipeptidyl peptidase 10 \|\| id:prot-a-858 | Chilli pepper liking \|\| id:ebi-fl187-GCST90094733 | Weighted median | 14 | 0.0688616267763376 | 0.151971730337867 | 0.650461404332051 | -0.229002965 | 0.366726218238556 | 1.07128796132314 | 0.795326175609315 | 1.44300279718148 | 0.999332001504091 |
| ebi-fl187-GCST90094733 | prot-a-863 | Dihydropyrimidinase-related protein 3 \|\| id:prot-a-863 | Chilli pepper liking \|\| id:ebi-fl187-GCST90094733 | Weighted median | 14 | -0.105969538 | 0.1481662186086 | 0.474481054813883 | -0.396375327 | 0.184436250306092 | 0.899452046616866 | 0.672754145993086 | 1.20254031726411 | 0.999332001504091 |
| ebi-fl187-GCST90094733 | prot-a-864 | Dihydropyrimidinase-related protein 5 \|\| id:prot-a-864 | Chilli pepper liking \|\| id:ebi-fl187-GCST90094733 | Weighted median | 14 | -0.042769672 | 0.14773549780399 | 0.772197509656237 | -0.332331248 | 0.246791903820884 | 0.958132049412533 | 0.717249696280485 | 1.27991274011285 | 0.999332001504091 |
| ebi-fl187-GCST90094733 | prot-a-884 | Endothelin-converting enzyme 1 \|\| id:prot-a-884 | Chilli pepper liking \|\| id:ebi-fl187-GCST90094733 | Weighted median | 14 | -0.055837993 | 0.145111407905051 | 0.700390076883654 | -0.340256352 | 0.228580366682166 | 0.945692332436333 | 0.711587882184817 | 1.25681452708689 | 0.999332001504091 |
| ebi-fl187-GCST90094733 | prot-a-893 | Early endosome antigen 1 \|\| id:prot-a-893 | Chilli pepper liking \|\| id:ebi-fl187-GCST90094733 | Weighted median | 14 | -0.025649247 | 0.161374773099982 | 0.873714483084974 | -0.341943802 | 0.290645308275353 | 0.974676900505934 | 0.710388125782847 | 1.33729017406219 | 0.999332001504091 |
| ebi-fl187-GCST90094733 | prot-a-897 | Ephrin-A2 \|\| id:prot-a-897 | Chilli pepper liking \|\| id:ebi-fl187-GCST90094733 | Weighted median | 14 | 0.239558643913406 | 0.149020013160643 | 0.107931532264218 | -0.052520582 | 0.531637869708266 | 1.27068820056973 | 0.94883479204962 | 1.70171721842036 | 0.999332001504091 |
| ebi-fl187-GCST90094733 | prot-a-90 | Anaphase-promoting complex subunit 10 \|\| id:prot-a-90 | Chilli pepper liking \|\| id:ebi-fl187-GCST90094733 | Weighted median | 14 | 0.177118782669509 | 0.149813850404501 | 0.237102922823658 | -0.116516364 | 0.47075392946233 | 1.19377288423579 | 0.890015532533586 | 1.60120093081954 | 0.999332001504091 |
| ebi-fl187-GCST90094733 | prot-a-914 | Histone-lysine N-methyltransferase EHMT2 \|\| id:prot-a-914 | Chilli pepper liking \|\| id:ebi-fl187-GCST90094733 | Weighted median | 14 | -0.100630578 | 0.151296204746252 | 0.50597185720097 | -0.397171139 | 0.195909983371483 | 0.904267027385627 | 0.672218972747949 | 1.2164174026124 | 0.999332001504091 |
| ebi-fl187-GCST90094733 | prot-a-932 | RNA polymerase II elongation factor ELL \|\| id:prot-a-932 | Chilli pepper liking \|\| id:ebi-fl187-GCST90094733 | Weighted median | 14 | -0.029547655 | 0.146110373726844 | 0.839738238270581 | -0.315923987 | 0.25682867782747 | 0.97088460934615 | 0.729114870137385 | 1.29282361843424 | 0.999332001504091 |
| ebi-fl187-GCST90094733 | prot-a-991 | Estrogen receptor \|\| id:prot-a-991 | Chilli pepper liking \|\| id:ebi-fl187-GCST90094733 | Weighted median | 14 | 0.117186991071698 | 0.151082479776826 | 0.437956194824411 | -0.178934669 | 0.413308651434278 | 1.12432964964143 | 0.836160524572672 | 1.51181157674102 | 0.999332001504091 |
| ebi-fl187-GCST90094733 | prot-a-992 | Persulfide dioxygenase ETHE1, mitochondrial \|\| id:prot-a-992 | Chilli pepper liking \|\| id:ebi-fl187-GCST90094733 | Weighted median | 14 | 0.0169868426741574 | 0.149359145448083 | 0.909450625089365 | -0.275757082 | 0.3097307677524 | 1.01713193950085 | 0.758997282164325 | 1.36305808553445 | 0.999332001504091 |
| ebi-fl187-GCST90094733 | prot-a-993 | Ethanolamine kinase 1 \|\| id:prot-a-993 | Chilli pepper liking \|\| id:ebi-fl187-GCST90094733 | Weighted median | 14 | -0.118768567 | 0.155303538299818 | 0.444419714422624 | -0.423163502 | 0.185626368493118 | 0.888013292940405 | 0.654971535435104 | 1.20397233433207 | 0.999332001504091 |
| ebi-fl187-GCST90094733 | prot-b-10 | coagulation factor III, tissue factor \|\| id:prot-b-10 | Chilli pepper liking \|\| id:ebi-fl187-GCST90094733 | Weighted median | 10 | 0.224893176273716 | 0.195085851816079 | 0.24899625915992 | -0.157475093 | 0.607261445833232 | 1.25218894555788 | 0.854298091101899 | 1.83539817273257 | 0.999332001504091 |
| ebi-fl187-GCST90094733 | prot-b-28 | prolactin \|\| id:prot-b-28 | Chilli pepper liking \|\| id:ebi-fl187-GCST90094733 | Weighted median | 10 | -0.180350093 | 0.191540857822886 | 0.34641024688951 | -0.555770175 | 0.195069987924337 | 0.834977839997563 | 0.573630295458409 | 1.21539604655965 | 0.999332001504091 |
| ebi-fl187-GCST90094733 | prot-b-54 | matrix metallopeptidase 7 \|\| id:prot-b-54 | Chilli pepper liking \|\| id:ebi-fl187-GCST90094733 | Weighted median | 10 | -0.00963007 | 0.188407282329368 | 0.959235440687999 | -0.378908343 | 0.359648203350576 | 0.990416150620949 | 0.684608358678495 | 1.43282526275943 | 0.999332001504091 |
| ebi-fl187-GCST90094733 | ukb-rsfMRI191-edge-pheno716 | Net100_Pair10_33 (OccipitalPrecuneus)&(Temporal\|Frontal\|Supp_Motor_Area) (Default_mode\|Central_executive)&(Default_mode\|Salience) \|\| id:ukb-rsfMRI191-edge-pheno716 | Chilli pepper liking \|\| id:ebi-fl187-GCST90094733 | Weighted median | 13 | 0.01836589430229 | 0.049070620988877 | 0.70819991730036 | -0.077812523 | 0.114544311440489 | 1.01853558458511 | 0.925137852507309 | 1.12136232914319 | 0.999332001504091 |
| ebi-fl187-GCST90094733 | ukb-rsfMRI191-edge-pheno903 | Net100_Pair27_38 (Precentral\|Frontal\|Supp_Motor_Area)&(Subcortical) (Attention\|Salience\|Motor)&(Subcortical-cerebellum) \|\| id:ukb-rsfMRI191-edge-pheno903 | Chilli pepper liking \|\| id:ebi-fl187-GCST90094733 | Weighted median | 13 | -0.00181842 | 0.0491637829580934 | 0.970495376997675 | -0.098179435 | 0.0945425941184062 | 0.998183231845371 | 0.906486233732564 | 1.09915597971587 | 0.999332001504091 |
| ebi-fl187-GCST90094733 | ukb-rsfMRI191-node-pheno23 | Net100_Node2 RolandicOper\|SupraMarginal\|Insula Salience\|Motor \|\| id:ukb-rsfMRI191-node-pheno23 | Chilli pepper liking \|\| id:ebi-fl187-GCST90094733 | Weighted median | 13 | -0.068254358 | 0.0518568674769281 | 0.188104667839325 | -0.169893818 | 0.0333851026504203 | 0.93402286754104 | 0.84375440348896 | 1.03394863894302 | 0.999332001504091 |
| ebi-fl187-GCST90094733 | ukb-rsfMRI191-node-pheno55 | Net100_Node34 Parietal\|Postcentral\|Precuneus Attention \|\| id:ukb-rsfMRI191-node-pheno55 | Chilli pepper liking \|\| id:ebi-fl187-GCST90094733 | Weighted median | 13 | 0.00105594768299352 | 0.0523694066632975 | 0.983912988128994 | -0.101588089 | 0.103699984743057 | 1.00105650539203 | 0.903401595753224 | 1.1092676077821 | 0.999332001504091 |
| ebi-fl187-GCST90094733 | ebi-a-GCST90001414 | IgD- CD24- B cell Absolute Count \|\| id:ebi-a-GCST90001414 | Chilli pepper liking \|\| id:ebi-fl187-GCST90094733 | Weighted mode | 12 | -0.098326088 | 0.319348833357122 | 0.763913405210164 | -0.724249801 | 0.527597625604727 | 0.906353304828196 | 0.484688045010116 | 1.6948557358287 | 0.999714303421225 |
| ebi-fl187-GCST90094733 | ebi-a-GCST90001422 | CD20- CD38- B cell %B cell \|\| id:ebi-a-GCST90001422 | Chilli pepper liking \|\| id:ebi-fl187-GCST90094733 | Weighted mode | 12 | 0.247487605221913 | 0.227307065209959 | 0.299529279979323 | -0.198034243 | 0.693009453033433 | 1.28080348701034 | 0.820341762030583 | 1.99972456391498 | 0.999714303421225 |
| ebi-fl187-GCST90094733 | ebi-a-GCST90001444 | CD20- CD38- B cell %lymphocyte \|\| id:ebi-a-GCST90001444 | Chilli pepper liking \|\| id:ebi-fl187-GCST90094733 | Weighted mode | 12 | 0.238249412002121 | 0.225595358437626 | 0.313569235158212 | -0.203917491 | 0.680416314539869 | 1.26902566358897 | 0.815529657326589 | 1.97469965730811 | 0.999714303421225 |
| ebi-fl187-GCST90094733 | ebi-a-GCST90001459 | Myeloid Dendritic Cell %Dendritic Cell \|\| id:ebi-a-GCST90001459 | Chilli pepper liking \|\| id:ebi-fl187-GCST90094733 | Weighted mode | 12 | 0.344569055798905 | 0.257714316935717 | 0.20820869155424 | -0.160551005 | 0.84968911699291 | 1.41138156189332 | 0.851674382475721 | 2.3389196085268 | 0.999714303421225 |
| ebi-fl187-GCST90094733 | ebi-a-GCST90001476 | HLA DR++ monocyte %leukocyte \|\| id:ebi-a-GCST90001476 | Chilli pepper liking \|\| id:ebi-fl187-GCST90094733 | Weighted mode | 12 | 0.0540762713637704 | 0.236286666211701 | 0.82317791664743 | -0.409045594 | 0.517198137138704 | 1.05556510847525 | 0.664283943996461 | 1.67732143505835 | 0.999714303421225 |
| ebi-fl187-GCST90094733 | ebi-a-GCST90001478 | CD4 regulatory T cell %CD4+ T cell \|\| id:ebi-a-GCST90001478 | Chilli pepper liking \|\| id:ebi-fl187-GCST90094733 | Weighted mode | 12 | 0.0679535804190419 | 0.28622003715045 | 0.816696997168557 | -0.493037692 | 0.628944853233924 | 1.07031562372286 | 0.61076824731347 | 1.87563046937067 | 0.999714303421225 |
| ebi-fl187-GCST90094733 | ebi-a-GCST90001485 | CD39+ resting CD4 regulatory T cell %CD4 regulatory T cell \|\| id:ebi-a-GCST90001485 | Chilli pepper liking \|\| id:ebi-fl187-GCST90094733 | Weighted mode | 12 | -0.024367772 | 0.294058302723125 | 0.935445917410165 | -0.600722045 | 0.551986501201148 | 0.975926725093236 | 0.548415512163334 | 1.73669954920521 | 0.999714303421225 |
| ebi-fl187-GCST90094733 | ebi-a-GCST90001515 | Immature Myeloid-Derived Suppressor Cells Absolute Count \|\| id:ebi-a-GCST90001515 | Chilli pepper liking \|\| id:ebi-fl187-GCST90094733 | Weighted mode | 12 | 0.032212430779782 | 0.344745166205901 | 0.92723547327665 | -0.643488095 | 0.707912956543347 | 1.03273686710268 | 0.525456381989859 | 2.02975065719851 | 0.999714303421225 |
| ebi-fl187-GCST90094733 | ebi-a-GCST90001517 | CD33+ HLA DR+ Absolute Count \|\| id:ebi-a-GCST90001517 | Chilli pepper liking \|\| id:ebi-fl187-GCST90094733 | Weighted mode | 12 | 0.400254542022732 | 0.313987613038706 | 0.228667618336899 | -0.21516118 | 1.0156702635786 | 1.49220447805036 | 0.806411452680491 | 2.76121351812667 | 0.999714303421225 |
| ebi-fl187-GCST90094733 | ebi-a-GCST90001518 | CD33+ HLA DR+ CD14- Absolute Count \|\| id:ebi-a-GCST90001518 | Chilli pepper liking \|\| id:ebi-fl187-GCST90094733 | Weighted mode | 12 | 0.199982897834481 | 0.342657039904056 | 0.571246137941861 | -0.4716249 | 0.87159069604643 | 1.22138186970665 | 0.623987526511462 | 2.39071072460087 | 0.999714303421225 |
| ebi-fl187-GCST90094733 | ebi-a-GCST90001520 | CD33+ HLA DR+ CD14dim Absolute Count \|\| id:ebi-a-GCST90001520 | Chilli pepper liking \|\| id:ebi-fl187-GCST90094733 | Weighted mode | 12 | 0.276871226100188 | 0.305062374195011 | 0.383547706624125 | -0.321051027 | 0.87479347952241 | 1.31899650777407 | 0.72538623552871 | 2.39837992824905 | 0.999714303421225 |
| ebi-fl187-GCST90094733 | ebi-a-GCST90001531 | CD33dim HLA DR- Absolute Count \|\| id:ebi-a-GCST90001531 | Chilli pepper liking \|\| id:ebi-fl187-GCST90094733 | Weighted mode | 12 | -0.084490487 | 0.329383249263543 | 0.802290338890437 | -0.730081655 | 0.561100682055568 | 0.918980398054364 | 0.481869641390429 | 1.75260049496227 | 0.999714303421225 |
| ebi-fl187-GCST90094733 | ebi-a-GCST90001532 | Basophil Absolute Count \|\| id:ebi-a-GCST90001532 | Chilli pepper liking \|\| id:ebi-fl187-GCST90094733 | Weighted mode | 12 | -0.124785383 | 0.323773559800292 | 0.707282815076857 | -0.759381561 | 0.509810793805578 | 0.882686321392179 | 0.467955739801012 | 1.66497614134227 | 0.999714303421225 |
| ebi-fl187-GCST90094733 | ebi-a-GCST90001588 | T/B cell \|\| id:ebi-a-GCST90001588 | Chilli pepper liking \|\| id:ebi-fl187-GCST90094733 | Weighted mode | 12 | -0.029153608 | 0.219108666895493 | 0.896552385244286 | -0.458606595 | 0.400299379358509 | 0.971267258822584 | 0.632163892503828 | 1.49227138602356 | 0.999714303421225 |
| ebi-fl187-GCST90094733 | ebi-a-GCST90001624 | HLA DR+ CD4+ T cell Absolute Count \|\| id:ebi-a-GCST90001624 | Chilli pepper liking \|\| id:ebi-fl187-GCST90094733 | Weighted mode | 12 | 0.322187766151615 | 0.238252037318801 | 0.20342661053084 | -0.144786227 | 0.789161759296464 | 1.38014389593752 | 0.86520723129395 | 2.20155022357464 | 0.999714303421225 |
| ebi-fl187-GCST90094733 | ebi-a-GCST90001631 | CD8+ Natural Killer T %T cell \|\| id:ebi-a-GCST90001631 | Chilli pepper liking \|\| id:ebi-fl187-GCST90094733 | Weighted mode | 12 | 0.122092809291387 | 0.252365473104383 | 0.638019720026359 | -0.372543518 | 0.616729136575978 | 1.1298589583237 | 0.688979667892966 | 1.85285767518852 | 0.999714303421225 |
| ebi-fl187-GCST90094733 | ebi-a-GCST90001632 | CD8+ Natural Killer T %lymphocyte \|\| id:ebi-a-GCST90001632 | Chilli pepper liking \|\| id:ebi-fl187-GCST90094733 | Weighted mode | 12 | 0.168556808309997 | 0.245894519060409 | 0.507227445805721 | -0.313396449 | 0.650510065668398 | 1.18359546299303 | 0.730960066695255 | 1.91651812984981 | 0.999714303421225 |
| ebi-fl187-GCST90094733 | ebi-a-GCST90001646 | Natural Killer %CD3- lymphocyte \|\| id:ebi-a-GCST90001646 | Chilli pepper liking \|\| id:ebi-fl187-GCST90094733 | Weighted mode | 12 | -0.163590024 | 0.205909751522889 | 0.443722419897421 | -0.567173137 | 0.239993088946482 | 0.849090057044944 | 0.567126363355827 | 1.27124036468085 | 0.999714303421225 |
| ebi-fl187-GCST90094733 | ebi-a-GCST90001684 | CD127- CD8+ T cell Absolute Count \|\| id:ebi-a-GCST90001684 | Chilli pepper liking \|\| id:ebi-fl187-GCST90094733 | Weighted mode | 12 | 0.198086588154351 | 0.238570082875337 | 0.424019291242598 | -0.269510774 | 0.665683950590012 | 1.21906794609527 | 0.763753050587815 | 1.94582091168494 | 0.999714303421225 |
| ebi-fl187-GCST90094733 | ebi-a-GCST90001687 | CD28- CD8+ T cell Absolute Count \|\| id:ebi-a-GCST90001687 | Chilli pepper liking \|\| id:ebi-fl187-GCST90094733 | Weighted mode | 12 | 0.0562084036304359 | 0.227532577709371 | 0.809431409470302 | -0.389755449 | 0.502172255940804 | 1.05781811390187 | 0.677222469898046 | 1.65230660800042 | 0.999714303421225 |
| ebi-fl187-GCST90094733 | ebi-a-GCST90001701 | BAFF-R on CD20- CD38- B cell \|\| id:ebi-a-GCST90001701 | Chilli pepper liking \|\| id:ebi-fl187-GCST90094733 | Weighted mode | 12 | 0.211580091152126 | 0.272131341777208 | 0.453265442929966 | -0.321797339 | 0.744957521035454 | 1.23562892460995 | 0.72484507346791 | 2.10635195743016 | 0.999714303421225 |
| ebi-fl187-GCST90094733 | ebi-a-GCST90001732 | CD19 on IgD- CD27- B cell \|\| id:ebi-a-GCST90001732 | Chilli pepper liking \|\| id:ebi-fl187-GCST90094733 | Weighted mode | 12 | -0.032919222 | 0.278029049677179 | 0.907883557625675 | -0.57785616 | 0.512017715003697 | 0.967616718223719 | 0.561099986810988 | 1.66865467010149 | 0.999714303421225 |
| ebi-fl187-GCST90094733 | ebi-a-GCST90001798 | CD27 on CD24+ CD27+ B cell \|\| id:ebi-a-GCST90001798 | Chilli pepper liking \|\| id:ebi-fl187-GCST90094733 | Weighted mode | 12 | 0.244086197498892 | 0.251538452360892 | 0.352714018339454 | -0.248929169 | 0.737101564126241 | 1.27645435292023 | 0.779635193669281 | 2.08986937521469 | 0.999714303421225 |
| ebi-fl187-GCST90094733 | ebi-a-GCST90001836 | CD66b on CD66b++ myeloid cell \|\| id:ebi-a-GCST90001836 | Chilli pepper liking \|\| id:ebi-fl187-GCST90094733 | Weighted mode | 12 | 0.0586396001145007 | 0.442391854251737 | 0.896941896204823 | -0.808448434 | 0.925727634447906 | 1.06039300634761 | 0.445548828518932 | 2.52370392634338 | 0.999714303421225 |
| ebi-fl187-GCST90094733 | ebi-a-GCST90001943 | CD25 on activated & secreting CD4 regulatory T cell \|\| id:ebi-a-GCST90001943 | Chilli pepper liking \|\| id:ebi-fl187-GCST90094733 | Weighted mode | 12 | -0.083369542 | 0.256792926761913 | 0.751531348000166 | -0.586683679 | 0.41994459432286 | 0.920011101530857 | 0.55616866379522 | 1.52187723264407 | 0.999714303421225 |
| ebi-fl187-GCST90094733 | ebi-a-GCST90001960 | CD25 on CD4+ T cell \|\| id:ebi-a-GCST90001960 | Chilli pepper liking \|\| id:ebi-fl187-GCST90094733 | Weighted mode | 12 | 0.169679127923034 | 0.277219164733985 | 0.552928927902759 | -0.373670435 | 0.713028690801645 | 1.18492458110319 | 0.688203682336252 | 2.040160928 | 0.999714303421225 |
| ebi-fl187-GCST90094733 | ebi-a-GCST90001988 | HLA DR on CD14+ CD16- monocyte \|\| id:ebi-a-GCST90001988 | Chilli pepper liking \|\| id:ebi-fl187-GCST90094733 | Weighted mode | 12 | -0.010657173 | 0.212200133098312 | 0.960845612743915 | -0.426569434 | 0.405255088187648 | 0.989399413784122 | 0.652744541626253 | 1.49968500319819 | 0.999714303421225 |
| ebi-fl187-GCST90094733 | ebi-a-GCST90001991 | HLA DR on CD14+ monocyte \|\| id:ebi-a-GCST90001991 | Chilli pepper liking \|\| id:ebi-fl187-GCST90094733 | Weighted mode | 12 | -0.013934409 | 0.223006015750812 | 0.951297976115272 | -0.4510262 | 0.423157381485078 | 0.986162225126953 | 0.636974153072137 | 1.52677456310728 | 0.999714303421225 |
| ebi-fl187-GCST90094733 | ebi-a-GCST90002019 | CD14 on Monocytic Myeloid-Derived Suppressor Cells \|\| id:ebi-a-GCST90002019 | Chilli pepper liking \|\| id:ebi-fl187-GCST90094733 | Weighted mode | 12 | 0.0988044413016306 | 0.345318281079725 | 0.780098543495945 | -0.57801939 | 0.775628272217892 | 1.10385041090057 | 0.561008405999916 | 2.17195627839761 | 0.999714303421225 |
| ebi-fl187-GCST90094733 | ebi-a-GCST90002037 | CD80 on plasmacytoid Dendritic Cell \|\| id:ebi-a-GCST90002037 | Chilli pepper liking \|\| id:ebi-fl187-GCST90094733 | Weighted mode | 12 | 0.120777148504633 | 0.284695635436022 | 0.679574175316779 | -0.437226297 | 0.678780593959235 | 1.12837342464116 | 0.64582526658488 | 1.97147224073354 | 0.999714303421225 |
| ebi-fl187-GCST90094733 | ebi-a-GCST90002042 | CD45 on CD33+ HLA DR+ CD14- \|\| id:ebi-a-GCST90002042 | Chilli pepper liking \|\| id:ebi-fl187-GCST90094733 | Weighted mode | 12 | -0.121734791 | 0.387330492711835 | 0.759179925902883 | -0.880902557 | 0.637432974869247 | 0.88538314908425 | 0.414408715535187 | 1.89161880842678 | 0.999714303421225 |
| ebi-fl187-GCST90094733 | ebi-a-GCST90002056 | CD8 on Effector Memory CD8+ T cell \|\| id:ebi-a-GCST90002056 | Chilli pepper liking \|\| id:ebi-fl187-GCST90094733 | Weighted mode | 12 | -0.265993133 | 0.283911694179064 | 0.368930932774918 | -0.822460054 | 0.290473787300952 | 0.7664443904343 | 0.439349500529326 | 1.33706082041852 | 0.999714303421225 |
| ebi-fl187-GCST90094733 | ebi-a-GCST90002058 | CD8 on CD8+ T cell \|\| id:ebi-a-GCST90002058 | Chilli pepper liking \|\| id:ebi-fl187-GCST90094733 | Weighted mode | 12 | -0.686536446 | 0.392224790800284 | 0.107857442505274 | -1.455297036 | 0.0822241442336871 | 0.503316316981203 | 0.233331045955342 | 1.08569913575928 | 0.999714303421225 |
| ebi-fl187-GCST90094733 | ebi-a-GCST90002063 | CD4 on CD4 regulatory T cell \|\| id:ebi-a-GCST90002063 | Chilli pepper liking \|\| id:ebi-fl187-GCST90094733 | Weighted mode | 12 | -0.557361593 | 0.322118564083935 | 0.111494760470269 | -1.188713978 | 0.0739907930437027 | 0.572718135990377 | 0.304612750931239 | 1.07679689142866 | 0.999714303421225 |
| ebi-fl187-GCST90094733 | ebi-a-GCST90002066 | CD4 on activated CD4 regulatory T cell \|\| id:ebi-a-GCST90002066 | Chilli pepper liking \|\| id:ebi-fl187-GCST90094733 | Weighted mode | 12 | -0.549594097 | 0.305312292659958 | 0.0992985413159488 | -1.148006191 | 0.0488179966873785 | 0.577184043616763 | 0.317268712543118 | 1.05002922455054 | 0.999714303421225 |
| ebi-fl187-GCST90094733 | ebi-a-GCST90002070 | CD4 on activated & secreting CD4 regulatory T cell \|\| id:ebi-a-GCST90002070 | Chilli pepper liking \|\| id:ebi-fl187-GCST90094733 | Weighted mode | 12 | -0.553061073 | 0.347932301209658 | 0.1402401424449 | -1.235008383 | 0.128886237597827 | 0.575186425326407 | 0.290832324268369 | 1.13756070516593 | 0.999714303421225 |
| ebi-fl187-GCST90094733 | ebi-a-GCST90002081 | SSC-A on CD4+ T cell \|\| id:ebi-a-GCST90002081 | Chilli pepper liking \|\| id:ebi-fl187-GCST90094733 | Weighted mode | 12 | 0.319742145790392 | 0.323266982868931 | 0.343874015636858 | -0.313861141 | 0.953345432213497 | 1.3767727119226 | 0.730620474612699 | 2.59437446137753 | 0.999714303421225 |
| ebi-fl187-GCST90094733 | ebi-a-GCST90002094 | CD11b on Monocytic Myeloid-Derived Suppressor Cells \|\| id:ebi-a-GCST90002094 | Chilli pepper liking \|\| id:ebi-fl187-GCST90094733 | Weighted mode | 12 | 0.265500738686919 | 0.395592493789548 | 0.515972591472421 | -0.509860549 | 1.04086202651443 | 1.30408381753159 | 0.600579324275853 | 2.83165692591616 | 0.999714303421225 |
| ebi-fl187-GCST90094733 | ebi-a-GCST90002104 | HLA DR on myeloid Dendritic Cell \|\| id:ebi-a-GCST90002104 | Chilli pepper liking \|\| id:ebi-fl187-GCST90094733 | Weighted mode | 12 | 0.019421749891803 | 0.279561607684145 | 0.945860641676223 | -0.528519001 | 0.567362500952727 | 1.01961157902262 | 0.589477338779657 | 1.76360939375414 | 0.999714303421225 |
| ebi-fl187-GCST90094733 | ebi-a-GCST90002105 | HLA DR on plasmacytoid Dendritic Cell \|\| id:ebi-a-GCST90002105 | Chilli pepper liking \|\| id:ebi-fl187-GCST90094733 | Weighted mode | 12 | 0.449317712158198 | 0.322737548087471 | 0.191364372655157 | -0.183247882 | 1.08188330640964 | 1.56724251010861 | 0.832561753006287 | 2.95023051037631 | 0.999714303421225 |
| ebi-fl187-GCST90094733 | ebi-a-GCST90002106 | HLA DR on Dendritic Cell \|\| id:ebi-a-GCST90002106 | Chilli pepper liking \|\| id:ebi-fl187-GCST90094733 | Weighted mode | 12 | -0.044532898 | 0.278388266982726 | 0.875806810911822 | -0.590173901 | 0.50110810524243 | 0.9564441344351 | 0.554230894863922 | 1.65054923998763 | 0.999714303421225 |
| ebi-fl187-GCST90094733 | ebi-a-GCST90002111 | HLA DR on CD33dim HLA DR+ CD11b- \|\| id:ebi-a-GCST90002111 | Chilli pepper liking \|\| id:ebi-fl187-GCST90094733 | Weighted mode | 12 | -0.649756581 | 0.52311433669974 | 0.240032071194559 | -1.675060681 | 0.375547519313712 | 0.52217286828913 | 0.187296813863583 | 1.45578826864557 | 0.999714303421225 |
| ebi-fl187-GCST90094733 | ebi-a-GCST90002112 | HLA DR on CD33- HLA DR+ \|\| id:ebi-a-GCST90002112 | Chilli pepper liking \|\| id:ebi-fl187-GCST90094733 | Weighted mode | 12 | 0.0224175697241207 | 0.376952742910115 | 0.953644032897065 | -0.716409806 | 0.761244945827945 | 1.02267073165941 | 0.488502931555719 | 2.14093991629097 | 0.999714303421225 |
| ebi-fl187-GCST90094733 | ebi-a-GCST90002115 | HLA DR on HLA DR+ CD8+ T cell \|\| id:ebi-a-GCST90002115 | Chilli pepper liking \|\| id:ebi-fl187-GCST90094733 | Weighted mode | 12 | -0.505157389 | 0.274888464321809 | 0.0932470386628703 | -1.043938779 | 0.0336240008531495 | 0.603410597631146 | 0.352065240126783 | 1.03419567692186 | 0.999714303421225 |
| ebi-fl187-GCST90094733 | ebi-a-GCST90002116 | HLA DR on B cell \|\| id:ebi-a-GCST90002116 | Chilli pepper liking \|\| id:ebi-fl187-GCST90094733 | Weighted mode | 12 | -0.101588254 | 0.276438059745078 | 0.720226070372274 | -0.643406851 | 0.440230343507007 | 0.903401447399991 | 0.525499074054804 | 1.55306491573244 | 0.999714303421225 |
| ebi-fl187-GCST90094733 | ebi-cfmet338-GCST90026023 | Sphingomyelin (d18:1/24:1, d18:2/24:0) levels \|\| id:ebi-cfmet338-GCST90026023 | Chilli pepper liking \|\| id:ebi-fl187-GCST90094733 | Weighted mode | 12 | -0.110385701 | 0.193208289399286 | 0.579262035005205 | -0.489073948 | 0.268302546056826 | 0.895488677652194 | 0.613193980610674 | 1.30774273257652 | 0.999714303421225 |
| ebi-fl187-GCST90094733 | ebi-cfmet338-GCST90026046 | 1-arachidonoyl-gpc (20:4n6) levels \|\| id:ebi-cfmet338-GCST90026046 | Chilli pepper liking \|\| id:ebi-fl187-GCST90094733 | Weighted mode | 12 | -0.06498765 | 0.162325681373218 | 0.69656177661767 | -0.383145985 | 0.253170685551266 | 0.937079036288037 | 0.681713371800475 | 1.28810311866308 | 0.999714303421225 |
| ebi-fl187-GCST90094733 | ebi-cfmet338-GCST90026047 | 1-linoleoyl-gpc (18:2) levels \|\| id:ebi-cfmet338-GCST90026047 | Chilli pepper liking \|\| id:ebi-fl187-GCST90094733 | Weighted mode | 12 | -0.306279686 | 0.231248337 | 0.212208256646809 | -0.759526427 | 0.146967055025294 | 0.736180691386894 | 0.467887953844518 | 1.15831580171646 | 0.999714303421225 |
| ebi-fl187-GCST90094733 | ebi-cfmet338-GCST90026050 | 1-myristoyl-2-palmitoyl-gpc (14:0/16:0) levels \|\| id:ebi-cfmet338-GCST90026050 | Chilli pepper liking \|\| id:ebi-fl187-GCST90094733 | Weighted mode | 12 | -0.066383456 | 0.104314481384501 | 0.537546742086015 | -0.270839839 | 0.138072927843989 | 0.935771968418915 | 0.76273864746764 | 1.14805927270882 | 0.999714303421225 |
| ebi-fl187-GCST90094733 | ebi-cfmet338-GCST90026068 | 2,3-dihydroxyisovalerate levels \|\| id:ebi-cfmet338-GCST90026068 | Chilli pepper liking \|\| id:ebi-fl187-GCST90094733 | Weighted mode | 12 | 0.215552192838704 | 0.368033882571241 | 0.569907823027759 | -0.505794217 | 0.936898602678337 | 1.24054672888879 | 0.603026451328319 | 2.55205419789917 | 0.999714303421225 |
| ebi-fl187-GCST90094733 | ebi-cfmet338-GCST90026082 | 3-hydroxyhexanoate levels \|\| id:ebi-cfmet338-GCST90026082 | Chilli pepper liking \|\| id:ebi-fl187-GCST90094733 | Weighted mode | 12 | -0.124522474 | 0.0909179030201661 | 0.198117315717573 | -0.302721564 | 0.0536766158823138 | 0.882918418402023 | 0.73880477761364 | 1.05514333038219 | 0.999714303421225 |
| ebi-fl187-GCST90094733 | ebi-cfmet338-GCST90026094 | 3-ureidopropionate levels \|\| id:ebi-cfmet338-GCST90026094 | Chilli pepper liking \|\| id:ebi-fl187-GCST90094733 | Weighted mode | 12 | -0.132376702 | 0.188251001001722 | 0.496549548631683 | -0.501348664 | 0.236595259768471 | 0.87601093767362 | 0.605713204910883 | 1.26692823716254 | 0.999714303421225 |
| ebi-fl187-GCST90094733 | ebi-cfmet338-GCST90026173 | Glycerol levels \|\| id:ebi-cfmet338-GCST90026173 | Chilli pepper liking \|\| id:ebi-fl187-GCST90094733 | Weighted mode | 12 | 0.0283289347314328 | 0.096025790387041 | 0.77347689241231 | -0.159881614 | 0.216539483890033 | 1.028734015 | 0.852244676468498 | 1.2417721143795 | 0.999714303421225 |
| ebi-fl187-GCST90094733 | ebi-cfmet338-GCST90026179 | Histidine levels \|\| id:ebi-cfmet338-GCST90026179 | Chilli pepper liking \|\| id:ebi-fl187-GCST90094733 | Weighted mode | 12 | -0.031496682 | 0.0729429867706072 | 0.674229581036406 | -0.174464936 | 0.111471572436842 | 0.968994171926157 | 0.839906304436205 | 1.11792196375659 | 0.999714303421225 |
| ebi-fl187-GCST90094733 | ebi-cfmet338-GCST90026188 | Isocitrate levels \|\| id:ebi-cfmet338-GCST90026188 | Chilli pepper liking \|\| id:ebi-fl187-GCST90094733 | Weighted mode | 12 | -0.178989965 | 0.141082843898379 | 0.230742877294881 | -0.455512339 | 0.0975324087090215 | 0.83611428948441 | 0.634122998528789 | 1.10244716987391 | 0.999714303421225 |
| ebi-fl187-GCST90094733 | ebi-cfmet338-GCST90026207 | N-acetyl-isoputreanine levels \|\| id:ebi-cfmet338-GCST90026207 | Chilli pepper liking \|\| id:ebi-fl187-GCST90094733 | Weighted mode | 12 | -0.006672124 | 0.0924500915269865 | 0.943762122196444 | -0.187874304 | 0.174530054968661 | 0.993350084776299 | 0.828718867484589 | 1.19068652789353 | 0.999714303421225 |
| ebi-fl187-GCST90094733 | ebi-cfmet338-GCST90026213 | N-acetylglutamate levels \|\| id:ebi-cfmet338-GCST90026213 | Chilli pepper liking \|\| id:ebi-fl187-GCST90094733 | Weighted mode | 12 | -0.247371533 | 0.163526403239615 | 0.158534984416582 | -0.567883284 | 0.0731402170944081 | 0.780850527693177 | 0.566723763476916 | 1.07588138330033 | 0.999714303421225 |
| ebi-fl187-GCST90094733 | ebi-cfmet338-GCST90026224 | N-acetyltaurine levels \|\| id:ebi-cfmet338-GCST90026224 | Chilli pepper liking \|\| id:ebi-fl187-GCST90094733 | Weighted mode | 11 | -0.094032063 | 0.133321966090701 | 0.496723004149844 | -0.355343117 | 0.167278990428513 | 0.910253576213003 | 0.700932900197265 | 1.18208400943283 | 0.999714303421225 |
| ebi-fl187-GCST90094733 | ebi-cfmet338-GCST90026250 | Phenyllactate (pla) levels \|\| id:ebi-cfmet338-GCST90026250 | Chilli pepper liking \|\| id:ebi-fl187-GCST90094733 | Weighted mode | 12 | 0.184223395160058 | 0.180284750041144 | 0.32880125325022 | -0.169134715 | 0.5375815052407 | 1.20228437760927 | 0.844395143098959 | 1.71186172310077 | 0.999714303421225 |
| ebi-fl187-GCST90094733 | ebi-cfmet338-GCST90026251 | Pipecolate levels \|\| id:ebi-cfmet338-GCST90026251 | Chilli pepper liking \|\| id:ebi-fl187-GCST90094733 | Weighted mode | 12 | -0.515838911 | 0.335171897403249 | 0.152053075751952 | -1.17277583 | 0.141098007542212 | 0.596999554770329 | 0.309506609931771 | 1.15153750181471 | 0.999714303421225 |
| ebi-fl187-GCST90094733 | ebi-cfmet338-GCST90026278 | Trigonelline (n'-methylnicotinate) levels \|\| id:ebi-cfmet338-GCST90026278 | Chilli pepper liking \|\| id:ebi-fl187-GCST90094733 | Weighted mode | 12 | 0.359085222150356 | 0.368782273782061 | 0.351128954586458 | -0.363728034 | 1.0818984787632 | 1.43201883609613 | 0.695080206901436 | 2.95027527265626 | 0.999714303421225 |
| ebi-fl187-GCST90094733 | ebi-cfmet338-GCST90026299 | 1-carboxyethylvaline levels \|\| id:ebi-cfmet338-GCST90026299 | Chilli pepper liking \|\| id:ebi-fl187-GCST90094733 | Weighted mode | 12 | -0.065825692 | 0.192614368425767 | 0.738979702751666 | -0.443349855 | 0.311698469633177 | 0.936294053161839 | 0.641882602222195 | 1.36574281800329 | 0.999714303421225 |
| ebi-fl187-GCST90094733 | ebi-gm430-GCST90011306 | C_Erysipelotrichia abundance \|\| id:ebi-gm430-GCST90011306 | Chilli pepper liking \|\| id:ebi-fl187-GCST90094733 | Weighted mode | 12 | -0.254465851 | 0.148081502620856 | 0.113700311919973 | -0.544705596 | 0.0357738939025843 | 0.775330529219291 | 0.580012515995932 | 1.03642147878009 | 0.999714303421225 |
| ebi-fl187-GCST90094733 | ebi-gm430-GCST90011312 | F_Erysipelotrichaceae abundance \|\| id:ebi-gm430-GCST90011312 | Chilli pepper liking \|\| id:ebi-fl187-GCST90094733 | Weighted mode | 12 | -0.254465851 | 0.137209413879886 | 0.0906369958336015 | -0.523396302 | 0.0144645999702838 | 0.775330529219291 | 0.592504801363337 | 1.0145697185175 | 0.999714303421225 |
| ebi-fl187-GCST90094733 | ebi-gm430-GCST90011352 | O_Erysipelotrichales abundance \|\| id:ebi-gm430-GCST90011352 | Chilli pepper liking \|\| id:ebi-fl187-GCST90094733 | Weighted mode | 12 | -0.254465851 | 0.145500295623315 | 0.108118827064356 | -0.539646431 | 0.0307147281874033 | 0.775330529219291 | 0.58295433072065 | 1.03119129211432 | 0.999714303421225 |
| ebi-fl187-GCST90094733 | ebi-gm430-GCST90011362 | OTU97_11 (Parabacteroides) abundance \|\| id:ebi-gm430-GCST90011362 | Chilli pepper liking \|\| id:ebi-fl187-GCST90094733 | Weighted mode | 12 | -0.271108254 | 0.238177998031553 | 0.27919648113598 | -0.73793713 | 0.195720622231736 | 0.762533944655772 | 0.478099155340581 | 1.21618708223419 | 0.999714303421225 |
| ebi-fl187-GCST90094733 | ebi-gm430-GCST90011384 | OTU97_235 (Phascolarctobacterium) abundance \|\| id:ebi-gm430-GCST90011384 | Chilli pepper liking \|\| id:ebi-fl187-GCST90094733 | Weighted mode | 12 | -0.05741042 | 0.558401805393812 | 0.91996253490159 | -1.151877958 | 1.03705711894393 | 0.944206468964865 | 0.316042696758005 | 2.82090320447349 | 0.999714303421225 |
| ebi-fl187-GCST90094733 | ebi-gm430-GCST90011390 | OTU97_3 (Bacteroides) abundance \|\| id:ebi-gm430-GCST90011390 | Chilli pepper liking \|\| id:ebi-fl187-GCST90094733 | Weighted mode | 12 | -0.248060562 | 0.189381478655005 | 0.216943116735129 | -0.61924826 | 0.123127136574839 | 0.780312684870937 | 0.538348984117083 | 1.13102820685934 | 0.999714303421225 |
| ebi-fl187-GCST90094733 | ebi-gm430-GCST90011398 | OTU97_4 (Alistipes) abundance \|\| id:ebi-gm430-GCST90011398 | Chilli pepper liking \|\| id:ebi-fl187-GCST90094733 | Weighted mode | 12 | -0.1892654 | 0.188403285333769 | 0.33668801614766 | -0.558535839 | 0.180005039523932 | 0.827566841529975 | 0.572046018460472 | 1.19722339654257 | 0.999714303421225 |
| ebi-fl187-GCST90094733 | ebi-gm430-GCST90011421 | OTU99_108 (Cytophagales) abundance \|\| id:ebi-gm430-GCST90011421 | Chilli pepper liking \|\| id:ebi-fl187-GCST90094733 | Weighted mode | 12 | -0.450296899 | 0.587556609487634 | 0.459576135509132 | -1.601907854 | 0.701314055634856 | 0.63743886858647 | 0.201511696213248 | 2.01640062994063 | 0.999714303421225 |
| ebi-fl187-GCST90094733 | ebi-gm430-GCST90011428 | OTU99_123 (Phascolarctobacterium) abundance \|\| id:ebi-gm430-GCST90011428 | Chilli pepper liking \|\| id:ebi-fl187-GCST90094733 | Weighted mode | 12 | 0.123502469742913 | 0.34452155983231 | 0.726772619973948 | -0.551759788 | 0.798764727014241 | 1.13145279893616 | 0.575935394139974 | 2.22279347518159 | 0.999714303421225 |
| ebi-fl187-GCST90094733 | ebi-gm430-GCST90011450 | OTU99_298 (Phascolarctobacterium) abundance \|\| id:ebi-gm430-GCST90011450 | Chilli pepper liking \|\| id:ebi-fl187-GCST90094733 | Weighted mode | 12 | -0.059358351 | 0.587748488568941 | 0.921373500547197 | -1.211345389 | 1.09262868661218 | 0.942369009781561 | 0.29779635797418 | 2.98210279211567 | 0.999714303421225 |
| ebi-fl187-GCST90094733 | ebi-gm430-GCST90011451 | OTU99_3 (Bacteroides) abundance \|\| id:ebi-gm430-GCST90011451 | Chilli pepper liking \|\| id:ebi-fl187-GCST90094733 | Weighted mode | 12 | -0.225999259 | 0.188977760532887 | 0.256877039759357 | -0.59639567 | 0.144397151378962 | 0.797718692563215 | 0.550793303534207 | 1.1553428634327 | 0.999714303421225 |
| ebi-fl187-GCST90094733 | ebi-gm430-GCST90011472 | OTU99_72 (Parabacteroides) abundance \|\| id:ebi-gm430-GCST90011472 | Chilli pepper liking \|\| id:ebi-fl187-GCST90094733 | Weighted mode | 12 | 0.167663718314084 | 0.487589994197601 | 0.737433050282305 | -0.78801267 | 1.12334010694138 | 1.18253887760897 | 0.454747631336516 | 3.07510825938058 | 0.999714303421225 |
| ebi-fl187-GCST90094733 | ebi-gm430-GCST90011507 | TestASV_3 (Bacteroides) abundance \|\| id:ebi-gm430-GCST90011507 | Chilli pepper liking \|\| id:ebi-fl187-GCST90094733 | Weighted mode | 12 | 0.0114222064151746 | 0.221321806199976 | 0.959765488369108 | -0.422368534 | 0.445212946567127 | 1.01148768889551 | 0.655492423802055 | 1.5608225322466 | 0.999714303421225 |
| ebi-fl187-GCST90094733 | ebi-gm430-GCST90011512 | TestASV_34 (Phascolarctobacterium) abundance \|\| id:ebi-gm430-GCST90011512 | Chilli pepper liking \|\| id:ebi-fl187-GCST90094733 | Weighted mode | 12 | -0.316095927 | 0.53166725884685 | 0.564181426746888 | -1.358163754 | 0.725971900560519 | 0.728989517196922 | 0.257132502217257 | 2.06673878875876 | 0.999714303421225 |
| ebi-fl187-GCST90094733 | ebi-gm430-GCST90011519 | TestASV_41 (Barnesiella) abundance \|\| id:ebi-gm430-GCST90011519 | Chilli pepper liking \|\| id:ebi-fl187-GCST90094733 | Weighted mode | 12 | -0.615801263 | 0.747668624418647 | 0.427647050534017 | -2.081231766 | 0.849629241248747 | 0.540207873478763 | 0.124776422088008 | 2.33877956816725 | 0.999714303421225 |
| ebi-fl187-GCST90094733 | ebi-gm430-GCST90011524 | TestASV_46 (Sutterella) abundance \|\| id:ebi-gm430-GCST90011524 | Chilli pepper liking \|\| id:ebi-fl187-GCST90094733 | Weighted mode | 12 | -0.286431934 | 0.527591332202634 | 0.598023593015269 | -1.320510945 | 0.747647076910803 | 0.750938189995476 | 0.266998845296481 | 2.11202473391789 | 0.999714303421225 |
| ebi-fl187-GCST90094733 | ebi-gm430-GCST90011581 | OTU97_19 (Bacteria) prevalence \|\| id:ebi-gm430-GCST90011581 | Chilli pepper liking \|\| id:ebi-fl187-GCST90094733 | Weighted mode | 12 | 0.139046912513578 | 0.88910821067808 | 0.878559540148118 | -1.60360518 | 1.88169900544261 | 1.14917800956806 | 0.201169955104131 | 6.56464876671675 | 0.999714303421225 |
| ebi-fl187-GCST90094733 | ebi-gm430-GCST90011614 | OTU97_80 (Ruminococcus) prevalence \|\| id:ebi-gm430-GCST90011614 | Chilli pepper liking \|\| id:ebi-fl187-GCST90094733 | Weighted mode | 12 | 0.403676145601609 | 0.386463827346037 | 0.318648177179394 | -0.353792956 | 1.16114524719984 | 1.4973189550996 | 0.702020301403257 | 3.19358863101129 | 0.999714303421225 |
| ebi-fl187-GCST90094733 | ebi-gm430-GCST90011623 | OTU99_11 (Parabacteroides) prevalence \|\| id:ebi-gm430-GCST90011623 | Chilli pepper liking \|\| id:ebi-fl187-GCST90094733 | Weighted mode | 12 | 0.0715484424195259 | 0.315489895116714 | 0.824750194697441 | -0.546811752 | 0.689908636848285 | 1.07417018484371 | 0.578792204898866 | 1.99353338942909 | 0.999714303421225 |
| ebi-fl187-GCST90094733 | ebi-gm430-GCST90011642 | OTU99_19 (Bacteria) prevalence \|\| id:ebi-gm430-GCST90011642 | Chilli pepper liking \|\| id:ebi-fl187-GCST90094733 | Weighted mode | 12 | 0.139046912513578 | 0.824266326573109 | 0.869100998523805 | -1.476515088 | 1.75460891259687 | 1.14917800956806 | 0.22843236968678 | 5.7811863506279 | 0.999714303421225 |
| ebi-fl187-GCST90094733 | ebi-gm430-GCST90011653 | OTU99_30 (Parasutterella) prevalence \|\| id:ebi-gm430-GCST90011653 | Chilli pepper liking \|\| id:ebi-fl187-GCST90094733 | Weighted mode | 12 | 0.240647079567836 | 0.449152335981542 | 0.602774945626976 | -0.639691499 | 1.12098565809166 | 1.27207201587324 | 0.52745511940092 | 3.06787659091395 | 0.999714303421225 |
| ebi-fl187-GCST90094733 | ebi-gm430-GCST90011675 | OTU99_8 (Ruminococcaceae) prevalence \|\| id:ebi-gm430-GCST90011675 | Chilli pepper liking \|\| id:ebi-fl187-GCST90094733 | Weighted mode | 12 | -0.211083713 | 0.365687615992822 | 0.575411488693049 | -0.92783144 | 0.505664014576119 | 0.809706281288516 | 0.395410252115462 | 1.6580861483749 | 0.999714303421225 |
| ebi-fl187-GCST90094733 | ebi-gm430-GCST90011679 | OTU99_92 (Ruminococcus) prevalence \|\| id:ebi-gm430-GCST90011679 | Chilli pepper liking \|\| id:ebi-fl187-GCST90094733 | Weighted mode | 12 | 0.293670940261752 | 0.342140139796388 | 0.409021076778439 | -0.376923734 | 0.964265614262673 | 1.34134244924327 | 0.6859683881451 | 2.62286075748632 | 0.999714303421225 |
| ebi-fl187-GCST90094733 | ebi-gm430-GCST90011684 | TestASV_11 (Lachnospiraceae) prevalence \|\| id:ebi-gm430-GCST90011684 | Chilli pepper liking \|\| id:ebi-fl187-GCST90094733 | Weighted mode | 12 | -0.014159834 | 0.396327691967796 | 0.972139676855069 | -0.79096211 | 0.762642442611769 | 0.985939945293071 | 0.453408356695456 | 2.14393396453746 | 0.999714303421225 |
| ebi-fl187-GCST90094733 | ebi-gm430-GCST90011688 | TestASV_15 (Bacteroides) prevalence \|\| id:ebi-gm430-GCST90011688 | Chilli pepper liking \|\| id:ebi-fl187-GCST90094733 | Weighted mode | 12 | -0.306410145 | 0.368790856239609 | 0.423725287183257 | -1.029240223 | 0.416419933237261 | 0.736084656100292 | 0.357278309236524 | 1.51652257340815 | 0.999714303421225 |
| ebi-fl187-GCST90094733 | ebi-gm430-GCST90011692 | TestASV_19 (Prevotella) prevalence \|\| id:ebi-gm430-GCST90011692 | Chilli pepper liking \|\| id:ebi-fl187-GCST90094733 | Weighted mode | 12 | 0.348081171815949 | 0.731106313329046 | 0.643317547900951 | -1.084887202 | 1.78104954594088 | 1.41634721254935 | 0.33793990258565 | 5.93608334247501 | 0.999714303421225 |
| ebi-fl187-GCST90094733 | ebi-gm430-GCST90011693 | TestASV_2 (Bacteroides) prevalence \|\| id:ebi-gm430-GCST90011693 | Chilli pepper liking \|\| id:ebi-fl187-GCST90094733 | Weighted mode | 12 | -0.517851427 | 0.371621331093028 | 0.190986323006551 | -1.246229236 | 0.210526382123197 | 0.595799292120799 | 0.287587178322511 | 1.23432761697589 | 0.999714303421225 |
| ebi-fl187-GCST90094733 | ebi-gm430-GCST90011716 | TestASV_40 (Barnesiella) prevalence \|\| id:ebi-gm430-GCST90011716 | Chilli pepper liking \|\| id:ebi-fl187-GCST90094733 | Weighted mode | 12 | -1.182843015 | 0.878255155703185 | 0.205133673264769 | -2.90422312 | 0.538537090469865 | 0.306406381277653 | 0.0547913403740361 | 1.71349833471414 | 0.999714303421225 |
| ebi-fl187-GCST90094733 | ebi-gm430-GCST90011718 | TestASV_42 (Alphaproteobacteria) prevalence \|\| id:ebi-gm430-GCST90011718 | Chilli pepper liking \|\| id:ebi-fl187-GCST90094733 | Weighted mode | 12 | 0.233120735984666 | 0.636590881542482 | 0.721160213411469 | -1.014597392 | 1.48083886380793 | 1.26253390339954 | 0.362548365383903 | 4.39663231013441 | 0.999714303421225 |
| ebi-fl187-GCST90094733 | ebi-hbsm44-GCST90104929 | Sulcal depth of the right inferior frontal sulcus \|\| id:ebi-hbsm44-GCST90104929 | Chilli pepper liking \|\| id:ebi-fl187-GCST90094733 | Weighted mode | 13 | 0.350471167359092 | 0.281835544576342 | 0.237418262749674 | -0.2019265 | 0.902868834728723 | 1.4197363244428 | 0.817154986617958 | 2.46666943719544 | 0.999714303421225 |
| ebi-fl187-GCST90094733 | ebi-hbsm44-GCST90104935 | Sulcal opening distance of the left calloso-marginal posterior fissure \|\| id:ebi-hbsm44-GCST90104935 | Chilli pepper liking \|\| id:ebi-fl187-GCST90094733 | Weighted mode | 12 | -0.031844214 | 0.0651191455504387 | 0.634435584972838 | -0.159477739 | 0.0957893111732041 | 0.968657473496337 | 0.852588946339882 | 1.10052717078771 | 0.999714303421225 |
| ebi-fl187-GCST90094733 | ebi-hbsm44-GCST90104936 | Sulcal opening distance of the left anterior inferior temporal sulcus \|\| id:ebi-hbsm44-GCST90104936 | Chilli pepper liking \|\| id:ebi-fl187-GCST90094733 | Weighted mode | 13 | -0.045448904 | 0.0516311922313369 | 0.396005277022095 | -0.146646041 | 0.055748232924232 | 0.955568427193035 | 0.863599602501313 | 1.05733144897642 | 0.999714303421225 |
| ebi-fl187-GCST90094733 | ebi-hbsm44-GCST90104941 | Sulcal opening distance of the right posterior inferior temporal sulcus \|\| id:ebi-hbsm44-GCST90104941 | Chilli pepper liking \|\| id:ebi-fl187-GCST90094733 | Weighted mode | 12 | -0.015778813 | 0.0290589175030149 | 0.597964429741999 | -0.072734291 | 0.0411766655672299 | 0.9843450205574 | 0.929847866109685 | 1.04203618119811 | 0.999714303421225 |
| ebi-fl187-GCST90094733 | ebi-IPs91-GCST90274819 | Leukemia inhibitory factor levels \|\| id:ebi-IPs91-GCST90274819 | Chilli pepper liking \|\| id:ebi-fl187-GCST90094733 | Weighted mode | 14 | -0.03761722 | 0.113928840513833 | 0.746523098135381 | -0.260917747 | 0.185683307833437 | 0.963081519098902 | 0.77034428015212 | 1.20404088967428 | 0.999714303421225 |
| ebi-fl187-GCST90094733 | ebi-IPs91-GCST90274837 | STAM binding protein levels \|\| id:ebi-IPs91-GCST90274837 | Chilli pepper liking \|\| id:ebi-fl187-GCST90094733 | Weighted mode | 14 | -0.003722632 | 0.0969556495168912 | 0.969955945944741 | -0.193755705 | 0.18631044085949 | 0.99628428821157 | 0.823859144153007 | 1.20479622030255 | 0.999714303421225 |
| ebi-fl187-GCST90094733 | ebi-IPs91-GCST90274840 | TNF-beta levels \|\| id:ebi-IPs91-GCST90274840 | Chilli pepper liking \|\| id:ebi-fl187-GCST90094733 | Weighted mode | 14 | -0.041480538 | 0.121224642621878 | 0.737684864238869 | -0.279080838 | 0.196119761386902 | 0.95936800623412 | 0.756478748748054 | 1.21667260700825 | 0.999714303421225 |
| ebi-fl187-GCST90094733 | ebi-IPs91-GCST90274842 | Tumor necrosis factor ligand superfamily member 14 levels \|\| id:ebi-IPs91-GCST90274842 | Chilli pepper liking \|\| id:ebi-fl187-GCST90094733 | Weighted mode | 14 | -0.012002083 | 0.110598958422804 | 0.91524115079046 | -0.228776041 | 0.204771875910455 | 0.988069655107662 | 0.795506674350447 | 1.22724506886348 | 0.999714303421225 |
| ebi-fl187-GCST90094733 | ebi-met1400-GCST90199628 | 3-methylhistidine levels \|\| id:ebi-met1400-GCST90199628 | Chilli pepper liking \|\| id:ebi-fl187-GCST90094733 | Weighted mode | 13 | 0.175522626269703 | 0.166983537704555 | 0.313903230714593 | -0.151765108 | 0.502810360170631 | 1.19186895589462 | 0.859190074232085 | 1.65336128829815 | 0.999714303421225 |
| ebi-fl187-GCST90094733 | ebi-met1400-GCST90199651 | Docosahexaenoate DHA; 22:6n3 levels \|\| id:ebi-met1400-GCST90199651 | Chilli pepper liking \|\| id:ebi-fl187-GCST90094733 | Weighted mode | 13 | 0.159378362945478 | 0.149506563386947 | 0.307389449029358 | -0.133654501 | 0.452411227183894 | 1.17278159984499 | 0.874892286545503 | 1.57209830522769 | 0.999714303421225 |
| ebi-fl187-GCST90094733 | ebi-met1400-GCST90199652 | 1-stearoyl-2-oleoyl-GPS (18:0/18:1) levels \|\| id:ebi-met1400-GCST90199652 | Chilli pepper liking \|\| id:ebi-fl187-GCST90094733 | Weighted mode | 13 | -0.010450824 | 0.162119640079434 | 0.949662480752309 | -0.328205318 | 0.307303670960782 | 0.989603596518672 | 0.720215131284943 | 1.35975382313266 | 0.999714303421225 |
| ebi-fl187-GCST90094733 | ebi-met1400-GCST90199653 | 1-myristoyl-2-palmitoyl-gpc (14:0/16:0) levels \|\| id:ebi-met1400-GCST90199653 | Chilli pepper liking \|\| id:ebi-fl187-GCST90094733 | Weighted mode | 13 | 0.0436236435897817 | 0.155698446413056 | 0.784110581548904 | -0.261545311 | 0.348792598559372 | 1.04458914307915 | 0.76986099117025 | 1.41735519834584 | 0.999714303421225 |
| ebi-fl187-GCST90094733 | ebi-met1400-GCST90199667 | Erythritol levels in elite athletes \|\| id:ebi-met1400-GCST90199667 | Chilli pepper liking \|\| id:ebi-fl187-GCST90094733 | Weighted mode | 13 | 0.146886735655632 | 0.145255679660111 | 0.331862956853122 | -0.137814396 | 0.431587867789449 | 1.15822277025756 | 0.871260385735858 | 1.5397004242424 | 0.999714303421225 |
| ebi-fl187-GCST90094733 | ebi-met1400-GCST90199674 | 3-hydroxymyristate levels \|\| id:ebi-met1400-GCST90199674 | Chilli pepper liking \|\| id:ebi-fl187-GCST90094733 | Weighted mode | 13 | -0.022522911 | 0.168389203120897 | 0.895813249077632 | -0.352565749 | 0.307519927235074 | 0.977728836306843 | 0.70288235448032 | 1.36004791022635 | 0.999714303421225 |
| ebi-fl187-GCST90094733 | ebi-met1400-GCST90199685 | 2-linoleoylglycerol (18:2) levels \|\| id:ebi-met1400-GCST90199685 | Chilli pepper liking \|\| id:ebi-fl187-GCST90094733 | Weighted mode | 13 | -0.147338096 | 0.192862047258248 | 0.459651691775127 | -0.525347708 | 0.230671516808869 | 0.863002150668939 | 0.59134971132757 | 1.25944546482861 | 0.999714303421225 |
| ebi-fl187-GCST90094733 | ebi-met1400-GCST90199703 | Gamma-glutamylvaline levels \|\| id:ebi-met1400-GCST90199703 | Chilli pepper liking \|\| id:ebi-fl187-GCST90094733 | Weighted mode | 13 | 0.0846213321547216 | 0.125716497299142 | 0.513630539589061 | -0.161783003 | 0.33102566686104 | 1.08830488259799 | 0.850625768135826 | 1.39239553027214 | 0.999714303421225 |
| ebi-fl187-GCST90094733 | ebi-met1400-GCST90199779 | Gamma-glutamyl-2-aminobutyrate levels \|\| id:ebi-met1400-GCST90199779 | Chilli pepper liking \|\| id:ebi-fl187-GCST90094733 | Weighted mode | 13 | -0.045842713 | 0.144994961286998 | 0.757307077255919 | -0.330032838 | 0.2383474107155 | 0.955192189301149 | 0.718900126140202 | 1.26915003256514 | 0.999714303421225 |
| ebi-fl187-GCST90094733 | ebi-met1400-GCST90199824 | 4-vinylphenol sulfate levels \|\| id:ebi-met1400-GCST90199824 | Chilli pepper liking \|\| id:ebi-fl187-GCST90094733 | Weighted mode | 13 | -0.037802662 | 0.139068021217966 | 0.790374065769341 | -0.310375983 | 0.234770659880213 | 0.962902939766014 | 0.733171244256352 | 1.26461870766694 | 0.999714303421225 |
| ebi-fl187-GCST90094733 | ebi-met1400-GCST90199830 | 4-ethylphenylsulfate levels \|\| id:ebi-met1400-GCST90199830 | Chilli pepper liking \|\| id:ebi-fl187-GCST90094733 | Weighted mode | 13 | -0.014793621 | 0.128192217943996 | 0.910035215067472 | -0.266050369 | 0.236463125760954 | 0.985315266597308 | 0.766400524023095 | 1.26676084391674 | 0.999714303421225 |
| ebi-fl187-GCST90094733 | ebi-met1400-GCST90199833 | Hexanoylglutamine levels \|\| id:ebi-met1400-GCST90199833 | Chilli pepper liking \|\| id:ebi-fl187-GCST90094733 | Weighted mode | 12 | -0.059171105 | 0.146191746644432 | 0.693420668035335 | -0.345706929 | 0.227364718008923 | 0.942545480723984 | 0.7077198690011 | 1.25528761045965 | 0.999714303421225 |
| ebi-fl187-GCST90094733 | ebi-met1400-GCST90199837 | Carotenoid levels (cryptoxanthin) \|\| id:ebi-met1400-GCST90199837 | Chilli pepper liking \|\| id:ebi-fl187-GCST90094733 | Weighted mode | 13 | 0.112500374520376 | 0.136324828441057 | 0.425338345150722 | -0.154696289 | 0.379697038264848 | 1.11907267602822 | 0.85667531950363 | 1.46184164025944 | 0.999714303421225 |
| ebi-fl187-GCST90094733 | ebi-met1400-GCST90199869 | 2-hydroxyhippurate levels \|\| id:ebi-met1400-GCST90199869 | Chilli pepper liking \|\| id:ebi-fl187-GCST90094733 | Weighted mode | 13 | 0.137400615294064 | 0.160447699466839 | 0.408575352150617 | -0.177076876 | 0.451878106249069 | 1.14728767745737 | 0.837715382123856 | 1.57126041007914 | 0.999714303421225 |
| ebi-fl187-GCST90094733 | ebi-met1400-GCST90199902 | Hydantoin-5-propionate levels \|\| id:ebi-met1400-GCST90199902 | Chilli pepper liking \|\| id:ebi-fl187-GCST90094733 | Weighted mode | 13 | -0.075978315 | 0.133072783494846 | 0.578567302440633 | -0.336800971 | 0.18484434050341 | 0.926836304650966 | 0.714050942751419 | 1.20303116232718 | 0.999714303421225 |
| ebi-fl187-GCST90094733 | ebi-met1400-GCST90199904 | Pantoate levels \|\| id:ebi-met1400-GCST90199904 | Chilli pepper liking \|\| id:ebi-fl187-GCST90094733 | Weighted mode | 13 | 0.115234315902338 | 0.153806760765471 | 0.46816078086685 | -0.186226935 | 0.416695567002661 | 1.12213634115882 | 0.830085198057029 | 1.51694063584878 | 0.999714303421225 |
| ebi-fl187-GCST90094733 | ebi-met1400-GCST90199936 | 3-methyl catechol sulfate (2) levels \|\| id:ebi-met1400-GCST90199936 | Chilli pepper liking \|\| id:ebi-fl187-GCST90094733 | Weighted mode | 13 | -0.16691476 | 0.194097317142534 | 0.406667128357506 | -0.547345502 | 0.213515981493337 | 0.846271744376599 | 0.578483357166385 | 1.23802328357084 | 0.999714303421225 |
| ebi-fl187-GCST90094733 | ebi-met1400-GCST90199977 | 4-hydroxychlorothalonil levels \|\| id:ebi-met1400-GCST90199977 | Chilli pepper liking \|\| id:ebi-fl187-GCST90094733 | Weighted mode | 13 | 0.0684695914067601 | 0.15124609030729 | 0.658838166496447 | -0.227972746 | 0.364911928409048 | 1.07086806086459 | 0.796145958023726 | 1.44038714537532 | 0.999714303421225 |
| ebi-fl187-GCST90094733 | ebi-met1400-GCST90200040 | Sphingomyelin (d18:2/24:1, d18:1/24:2) levels \|\| id:ebi-met1400-GCST90200040 | Chilli pepper liking \|\| id:ebi-fl187-GCST90094733 | Weighted mode | 13 | 0.106095749432721 | 0.143367403228841 | 0.473513923385889 | -0.174904361 | 0.387095859761248 | 1.11192833791722 | 0.839537309525993 | 1.47269765695276 | 0.999714303421225 |
| ebi-fl187-GCST90094733 | ebi-met1400-GCST90200077 | 1-stearoyl-2-oleoyl-GPI (18:0/18:1) levels \|\| id:ebi-met1400-GCST90200077 | Chilli pepper liking \|\| id:ebi-fl187-GCST90094733 | Weighted mode | 12 | -0.004871678 | 0.142822733723827 | 0.973400552705922 | -0.284804237 | 0.275060879604412 | 0.995140168884705 | 0.752161485602707 | 1.3166108271741 | 0.999714303421225 |
| ebi-fl187-GCST90094733 | ebi-met1400-GCST90200080 | 1-palmitoyl-2-oleoyl-GPI (16:0/18:1) levels \|\| id:ebi-met1400-GCST90200080 | Chilli pepper liking \|\| id:ebi-fl187-GCST90094733 | Weighted mode | 13 | -0.012638664 | 0.141496301784575 | 0.930299871588455 | -0.289971415 | 0.264694087991041 | 0.98744086898682 | 0.748284956994988 | 1.30303230157275 | 0.999714303421225 |
| ebi-fl187-GCST90094733 | ebi-met1400-GCST90200082 | 1-oleoyl-2-linoleoyl-GPE (18:1/18:2) levels \|\| id:ebi-met1400-GCST90200082 | Chilli pepper liking \|\| id:ebi-fl187-GCST90094733 | Weighted mode | 13 | -0.18251015 | 0.146216131298597 | 0.235755702636181 | -0.469093767 | 0.10407346741815 | 0.833176187207851 | 0.625568922514597 | 1.10968197739077 | 0.999714303421225 |
| ebi-fl187-GCST90094733 | ebi-met1400-GCST90200100 | Perfluorooctanesulfonate (PFOS) levels \|\| id:ebi-met1400-GCST90200100 | Chilli pepper liking \|\| id:ebi-fl187-GCST90094733 | Weighted mode | 13 | 0.0902853319929808 | 0.150154152384046 | 0.558838391525182 | -0.204016807 | 0.384587470665711 | 1.09448653117923 | 0.815448666087541 | 1.46900818745608 | 0.999714303421225 |
| ebi-fl187-GCST90094733 | ebi-met1400-GCST90200102 | 1-palmitoleoyl-2-linolenoyl-GPC (16:1/18:3) levels \|\| id:ebi-met1400-GCST90200102 | Chilli pepper liking \|\| id:ebi-fl187-GCST90094733 | Weighted mode | 13 | -0.146917556 | 0.130107202724735 | 0.280879978033646 | -0.401927673 | 0.108092561463748 | 0.86336515386541 | 0.669029132665246 | 1.11415086804898 | 0.999714303421225 |
| ebi-fl187-GCST90094733 | ebi-met1400-GCST90200114 | Glycosyl-N-tricosanoyl-sphingadienine (d18:2/23:0) levels \|\| id:ebi-met1400-GCST90200114 | Chilli pepper liking \|\| id:ebi-fl187-GCST90094733 | Weighted mode | 13 | 0.0573400775174351 | 0.19519667846442 | 0.773963647261151 | -0.325245412 | 0.439925567307698 | 1.05901589666047 | 0.722350058318149 | 1.55259165063377 | 0.999714303421225 |
| ebi-fl187-GCST90094733 | ebi-met1400-GCST90200135 | Arachidoylcarnitine (C20) levels \|\| id:ebi-met1400-GCST90200135 | Chilli pepper liking \|\| id:ebi-fl187-GCST90094733 | Weighted mode | 13 | -0.241243729 | 0.192054254735683 | 0.232976443213324 | -0.617670068 | 0.135182610368652 | 0.785650117401817 | 0.539199272721499 | 1.14474580771978 | 0.999714303421225 |
| ebi-fl187-GCST90094733 | ebi-met1400-GCST90200146 | Docosahexaenoylcarnitine (C22:6) levels \|\| id:ebi-met1400-GCST90200146 | Chilli pepper liking \|\| id:ebi-fl187-GCST90094733 | Weighted mode | 13 | 0.349928936168761 | 0.188265654061679 | 0.0877588770833754 | -0.019071746 | 0.718929618129651 | 1.41896670779958 | 0.98110896927747 | 2.05223535906146 | 0.999714303421225 |
| ebi-fl187-GCST90094733 | ebi-met1400-GCST90200162 | 3-hydroxyphenylacetoylglutamine levels \|\| id:ebi-met1400-GCST90200162 | Chilli pepper liking \|\| id:ebi-fl187-GCST90094733 | Weighted mode | 13 | -0.031563165 | 0.204605346090094 | 0.879965944063946 | -0.432589644 | 0.369463313050965 | 0.968929751796238 | 0.64882668735296 | 1.44695784285026 | 0.999714303421225 |
| ebi-fl187-GCST90094733 | ebi-met1400-GCST90200172 | 3-carboxy-4-methyl-5-pentyl-2-furanpropionate (3-CMPFP) levels \|\| id:ebi-met1400-GCST90200172 | Chilli pepper liking \|\| id:ebi-fl187-GCST90094733 | Weighted mode | 13 | 0.290147861193309 | 0.153047452422451 | 0.0823196905751487 | -0.009825146 | 0.590120867941313 | 1.33662510839845 | 0.990222963499484 | 1.8042064729416 | 0.999714303421225 |
| ebi-fl187-GCST90094733 | ebi-met1400-GCST90200222 | Dihydrocaffeate sulfate (2) levels \|\| id:ebi-met1400-GCST90200222 | Chilli pepper liking \|\| id:ebi-fl187-GCST90094733 | Weighted mode | 13 | 0.200147114136032 | 0.195897622803485 | 0.327082935510004 | -0.183812227 | 0.584106454830864 | 1.22158245698948 | 0.832092033942888 | 1.79338779648367 | 0.999714303421225 |
| ebi-fl187-GCST90094733 | ebi-met1400-GCST90200223 | 4-methylhexanoylglutamine levels \|\| id:ebi-met1400-GCST90200223 | Chilli pepper liking \|\| id:ebi-fl187-GCST90094733 | Weighted mode | 13 | -0.053934388 | 0.19371663814058 | 0.785430237555293 | -0.433618998 | 0.325750223230504 | 0.947494271906559 | 0.648159158200922 | 1.38506936751089 | 0.999714303421225 |
| ebi-fl187-GCST90094733 | ebi-met1400-GCST90200224 | 2-ketocaprylate levels \|\| id:ebi-met1400-GCST90200224 | Chilli pepper liking \|\| id:ebi-fl187-GCST90094733 | Weighted mode | 13 | 0.00729675508588723 | 0.152043993560906 | 0.962512835784602 | -0.290709472 | 0.305302982465264 | 1.00732344127131 | 0.747732883584196 | 1.35703609886835 | 0.999714303421225 |
| ebi-fl187-GCST90094733 | ebi-met1400-GCST90200243 | Hydroxypalmitoyl sphingomyelin (d18:1/16:0(OH)) levels \|\| id:ebi-met1400-GCST90200243 | Chilli pepper liking \|\| id:ebi-fl187-GCST90094733 | Weighted mode | 13 | 0.047804067592128 | 0.148654656317508 | 0.753304768121763 | -0.243559059 | 0.339167193974444 | 1.04896510892896 | 0.783833182425801 | 1.40377802882121 | 0.999714303421225 |
| ebi-fl187-GCST90094733 | ebi-met1400-GCST90200273 | 2-methoxyhydroquinone sulfate (2) levels \|\| id:ebi-met1400-GCST90200273 | Chilli pepper liking \|\| id:ebi-fl187-GCST90094733 | Weighted mode | 13 | -0.068833858 | 0.229077613131987 | 0.768951183412663 | -0.51782598 | 0.380158263897342 | 0.933481757869676 | 0.595814453760746 | 1.4625160346066 | 0.999714303421225 |
| ebi-fl187-GCST90094733 | ebi-met1400-GCST90200276 | 2,4-di-tert-butylphenol levels \|\| id:ebi-met1400-GCST90200276 | Chilli pepper liking \|\| id:ebi-fl187-GCST90094733 | Weighted mode | 13 | 0.281953411346996 | 0.217691592175515 | 0.219615969077852 | -0.144722109 | 0.708628932011006 | 1.32571695518083 | 0.865262708149575 | 2.03120442924498 | 0.999714303421225 |
| ebi-fl187-GCST90094733 | ebi-met1400-GCST90200289 | N-acetylmethionine levels \|\| id:ebi-met1400-GCST90200289 | Chilli pepper liking \|\| id:ebi-fl187-GCST90094733 | Weighted mode | 13 | 0.0172494256320588 | 0.166839521770427 | 0.919361763607184 | -0.309756037 | 0.344254888302096 | 1.01739905608269 | 0.733625911944611 | 1.41093822132622 | 0.999714303421225 |
| ebi-fl187-GCST90094733 | ebi-met1400-GCST90200308 | 3-hydroxyisobutyrate levels \|\| id:ebi-met1400-GCST90200308 | Chilli pepper liking \|\| id:ebi-fl187-GCST90094733 | Weighted mode | 13 | 0.00551687181449301 | 0.140225483703003 | 0.969264007853742 | -0.269325076 | 0.280358819872379 | 1.00553211777558 | 0.763894891200117 | 1.32360466279564 | 0.999714303421225 |
| ebi-fl187-GCST90094733 | ebi-met1400-GCST90200383 | Cystathionine levels \|\| id:ebi-met1400-GCST90200383 | Chilli pepper liking \|\| id:ebi-fl187-GCST90094733 | Weighted mode | 13 | 0.247529769875723 | 0.230973262496401 | 0.304943720027654 | -0.205177825 | 0.700237364368667 | 1.28085749278453 | 0.814502464943663 | 2.0142307573443 | 0.999714303421225 |
| ebi-fl187-GCST90094733 | ebi-met1400-GCST90200384 | Guanidinoacetate levels \|\| id:ebi-met1400-GCST90200384 | Chilli pepper liking \|\| id:ebi-fl187-GCST90094733 | Weighted mode | 13 | 0.145494920406669 | 0.148964354937692 | 0.347992628358512 | -0.146475215 | 0.437465056084546 | 1.15661185944938 | 0.863747139808077 | 1.54877617738474 | 0.999714303421225 |
| ebi-fl187-GCST90094733 | ebi-met1400-GCST90200403 | Citrulline levels \|\| id:ebi-met1400-GCST90200403 | Chilli pepper liking \|\| id:ebi-fl187-GCST90094733 | Weighted mode | 13 | 0.0105731033612959 | 0.148233372788416 | 0.944312157749278 | -0.279964307 | 0.301110514026591 | 1.0106291961359 | 0.75581071789648 | 1.35135867737482 | 0.999714303421225 |
| ebi-fl187-GCST90094733 | ebi-met1400-GCST90200434 | 12,13-DiHOME levels \|\| id:ebi-met1400-GCST90200434 | Chilli pepper liking \|\| id:ebi-fl187-GCST90094733 | Weighted mode | 13 | 0.0848442596534477 | 0.164107413851902 | 0.614548919687766 | -0.236806271 | 0.406494790803176 | 1.08854752272789 | 0.789144152925951 | 1.50154531950036 | 0.999714303421225 |
| ebi-fl187-GCST90094733 | ebi-met1400-GCST90200437 | Fructose levels \|\| id:ebi-met1400-GCST90200437 | Chilli pepper liking \|\| id:ebi-fl187-GCST90094733 | Weighted mode | 13 | -0.121736623 | 0.160752550493916 | 0.463486188398747 | -0.436811622 | 0.19333837588572 | 0.885381526854498 | 0.646093129646627 | 1.21329327325296 | 0.999714303421225 |
| ebi-fl187-GCST90094733 | ebi-met1400-GCST90200448 | N-stearoyl-sphinganine (d18:0/18:0) levels \|\| id:ebi-met1400-GCST90200448 | Chilli pepper liking \|\| id:ebi-fl187-GCST90094733 | Weighted mode | 13 | 0.0695910754578257 | 0.161230315012335 | 0.673666872990817 | -0.246420342 | 0.385602492882003 | 1.07206969599702 | 0.781593619268131 | 1.47050002039597 | 0.999714303421225 |
| ebi-fl187-GCST90094733 | ebi-met1400-GCST90200468 | X-11847 levels \|\| id:ebi-met1400-GCST90200468 | Chilli pepper liking \|\| id:ebi-fl187-GCST90094733 | Weighted mode | 13 | 0.123861018763738 | 0.157320228741555 | 0.44636806088162 | -0.18448663 | 0.432208667097185 | 1.13185855296634 | 0.831531057753121 | 1.54065656595524 | 0.999714303421225 |
| ebi-fl187-GCST90094733 | ebi-met1400-GCST90200476 | X-11843 levels \|\| id:ebi-met1400-GCST90200476 | Chilli pepper liking \|\| id:ebi-fl187-GCST90094733 | Weighted mode | 13 | 0.079464373294605 | 0.156128420038892 | 0.620003072986757 | -0.22654733 | 0.385476076570834 | 1.08270698555951 | 0.797281606096202 | 1.47031413695741 | 0.999714303421225 |
| ebi-fl187-GCST90094733 | ebi-met1400-GCST90200511 | X-12906 levels \|\| id:ebi-met1400-GCST90200511 | Chilli pepper liking \|\| id:ebi-fl187-GCST90094733 | Weighted mode | 13 | 0.0580312162716004 | 0.164219499831364 | 0.729936917522496 | -0.263839003 | 0.379901435941073 | 1.05974807657774 | 0.76809719074055 | 1.46214046783251 | 0.999714303421225 |
| ebi-fl187-GCST90094733 | ebi-met1400-GCST90200515 | X-12849 levels \|\| id:ebi-met1400-GCST90200515 | Chilli pepper liking \|\| id:ebi-fl187-GCST90094733 | Weighted mode | 13 | -0.092233502 | 0.199880139747534 | 0.65273252370372 | -0.483998576 | 0.299531572305117 | 0.911892196396675 | 0.616314079847855 | 1.34922664439928 | 0.999714303421225 |
| ebi-fl187-GCST90094733 | ebi-met1400-GCST90200551 | X-18886 levels \|\| id:ebi-met1400-GCST90200551 | Chilli pepper liking \|\| id:ebi-fl187-GCST90094733 | Weighted mode | 13 | -0.076434417 | 0.141965860897683 | 0.600145655138602 | -0.354687504 | 0.201818670372568 | 0.92641366929637 | 0.70139259110163 | 1.22362610832712 | 0.999714303421225 |
| ebi-fl187-GCST90094733 | ebi-met1400-GCST90200564 | X-21319 levels \|\| id:ebi-met1400-GCST90200564 | Chilli pepper liking \|\| id:ebi-fl187-GCST90094733 | Weighted mode | 13 | -0.067948863 | 0.133629038687557 | 0.620329098693147 | -0.329861779 | 0.193964053107267 | 0.934308250337897 | 0.719023110982165 | 1.21405264075179 | 0.999714303421225 |
| ebi-fl187-GCST90094733 | ebi-met1400-GCST90200579 | X-21442 levels \|\| id:ebi-met1400-GCST90200579 | Chilli pepper liking \|\| id:ebi-fl187-GCST90094733 | Weighted mode | 13 | 0.234633911650323 | 0.198354622065611 | 0.259755033118802 | -0.154141148 | 0.62340897089892 | 1.26444578512088 | 0.857151027663785 | 1.86527588710666 | 0.999714303421225 |
| ebi-fl187-GCST90094733 | ebi-met1400-GCST90200581 | X-21441 levels \|\| id:ebi-met1400-GCST90200581 | Chilli pepper liking \|\| id:ebi-fl187-GCST90094733 | Weighted mode | 13 | -0.067572298 | 0.132841866639019 | 0.620207712300628 | -0.327942356 | 0.192797761106185 | 0.934660144575584 | 0.720404545422492 | 1.21263752624676 | 0.999714303421225 |
| ebi-fl187-GCST90094733 | ebi-met1400-GCST90200648 | X-24585 levels \|\| id:ebi-met1400-GCST90200648 | Chilli pepper liking \|\| id:ebi-fl187-GCST90094733 | Weighted mode | 13 | -0.00605002 | 0.217814263233066 | 0.978297355134812 | -0.432965976 | 0.420865935721475 | 0.993968244304833 | 0.648582558703872 | 1.52328004727848 | 0.999714303421225 |
| ebi-fl187-GCST90094733 | ebi-met1400-GCST90200720 | Adenosine 5'-diphosphate (ADP) to pantothenate ratio \|\| id:ebi-met1400-GCST90200720 | Chilli pepper liking \|\| id:ebi-fl187-GCST90094733 | Weighted mode | 13 | -0.168745762 | 0.228131508249064 | 0.473713128203457 | -0.615883518 | 0.278391994622348 | 0.84472363731983 | 0.540163440452378 | 1.32100392216336 | 0.999714303421225 |
| ebi-fl187-GCST90094733 | ebi-met1400-GCST90200724 | Cholate to taurocholate ratio \|\| id:ebi-met1400-GCST90200724 | Chilli pepper liking \|\| id:ebi-fl187-GCST90094733 | Weighted mode | 13 | -0.053057266 | 0.140543736345097 | 0.712383660693794 | -0.328522989 | 0.222408457011442 | 0.948325703894207 | 0.719986375936605 | 1.24908146976607 | 0.999714303421225 |
| ebi-fl187-GCST90094733 | ebi-met1400-GCST90200726 | N-acetylputrescine to (N(1) + N(8))-acetylspermidine ratio \|\| id:ebi-met1400-GCST90200726 | Chilli pepper liking \|\| id:ebi-fl187-GCST90094733 | Weighted mode | 13 | -0.107761913 | 0.14747917463928 | 0.478991293516519 | -0.396821095 | 0.181297269784791 | 0.897841335776765 | 0.672454320445545 | 1.19877148487246 | 0.999714303421225 |
| ebi-fl187-GCST90094733 | ebi-met1400-GCST90200744 | Arginine to glutamate ratio \|\| id:ebi-met1400-GCST90200744 | Chilli pepper liking \|\| id:ebi-fl187-GCST90094733 | Weighted mode | 13 | 0.237168039191084 | 0.161788655744922 | 0.168380118389463 | -0.079937726 | 0.55427380445113 | 1.26765411545094 | 0.92317383426031 | 1.74067645418835 | 0.999714303421225 |
| ebi-fl187-GCST90094733 | ebi-met1400-GCST90200759 | Histidine to trans-urocanate ratio \|\| id:ebi-met1400-GCST90200759 | Chilli pepper liking \|\| id:ebi-fl187-GCST90094733 | Weighted mode | 13 | 0.0151562206057819 | 0.14663697963191 | 0.919385488979869 | -0.272252259 | 0.302564700684325 | 1.01527165858111 | 0.761662100373813 | 1.3533252346574 | 0.999714303421225 |
| ebi-fl187-GCST90094733 | ebi-met1400-GCST90200795 | Oleoyl-linoleoyl-glycerol (18:1 to 18:2) [2] to linoleoyl-arachidonoyl-glycerol (18:2 to 20:4) [2] ratio \|\| id:ebi-met1400-GCST90200795 | Chilli pepper liking \|\| id:ebi-fl187-GCST90094733 | Weighted mode | 13 | -0.072104899 | 0.184011211418673 | 0.702038480859162 | -0.432766873 | 0.288557075761699 | 0.930433289524864 | 0.648711706392491 | 1.33450051498266 | 0.999714303421225 |
| ebi-fl187-GCST90094733 | ebi-met1400-GCST90200811 | Choline to taurocholate ratio \|\| id:ebi-met1400-GCST90200811 | Chilli pepper liking \|\| id:ebi-fl187-GCST90094733 | Weighted mode | 13 | 0.172994356425563 | 0.153549902730494 | 0.281923652397389 | -0.127963453 | 0.473952165777332 | 1.1888593956486 | 0.879885535798631 | 1.60633014763574 | 0.999714303421225 |
| ebi-fl187-GCST90094733 | ebi-met1400-GCST90200831 | Cholate to phosphate ratio \|\| id:ebi-met1400-GCST90200831 | Chilli pepper liking \|\| id:ebi-fl187-GCST90094733 | Weighted mode | 13 | -0.131865806 | 0.145321448188739 | 0.382052101309583 | -0.416695844 | 0.152964232617331 | 0.876458602820528 | 0.65922139541119 | 1.16528329906368 | 0.999714303421225 |
| ebi-fl187-GCST90094733 | ebi-met1400-GCST90200841 | Adenosine 5'-diphosphate (ADP) to oxalate (ethanedioate) ratio \|\| id:ebi-met1400-GCST90200841 | Chilli pepper liking \|\| id:ebi-fl187-GCST90094733 | Weighted mode | 13 | -0.095504355 | 0.195458884349805 | 0.633920994366275 | -0.478603769 | 0.28759505783324 | 0.908914402875297 | 0.6196479599007 | 1.3332173188895 | 0.999714303421225 |
| ebi-fl187-GCST90094733 | ebi-met1400-GCST90200844 | Aspartate to phosphate ratio \|\| id:ebi-met1400-GCST90200844 | Chilli pepper liking \|\| id:ebi-fl187-GCST90094733 | Weighted mode | 13 | -0.019672561 | 0.136266446279621 | 0.887605876335156 | -0.286754796 | 0.24740967322422 | 0.980519680658105 | 0.750695779735075 | 1.28070367532526 | 0.999714303421225 |
| ebi-fl187-GCST90094733 | ebi-met1400-GCST90200870 | Adenosine 5'-monophosphate (AMP) to acetoacetate ratio \|\| id:ebi-met1400-GCST90200870 | Chilli pepper liking \|\| id:ebi-fl187-GCST90094733 | Weighted mode | 13 | 0.0634382421961557 | 0.186825570441982 | 0.740057904210016 | -0.302739876 | 0.429616360262441 | 1.06549368121975 | 0.738791248808408 | 1.53666788358728 | 0.999714303421225 |
| ebi-fl187-GCST90094733 | ebi-met1400-GCST90200887 | Cholesterol to cortisol ratio \|\| id:ebi-met1400-GCST90200887 | Chilli pepper liking \|\| id:ebi-fl187-GCST90094733 | Weighted mode | 13 | 0.0672158398978702 | 0.12879037949178 | 0.611237379327512 | -0.185213304 | 0.319644983701758 | 1.06952629971075 | 0.83092702496775 | 1.37663894830882 | 0.999714303421225 |
| ebi-fl187-GCST90094733 | ebi-met1400-GCST90200890 | Cortisol to taurocholate ratio \|\| id:ebi-met1400-GCST90200890 | Chilli pepper liking \|\| id:ebi-fl187-GCST90094733 | Weighted mode | 13 | 0.0626805842680082 | 0.150484710544724 | 0.684379667475728 | -0.232269448 | 0.357630616935667 | 1.06468670722851 | 0.792732494025076 | 1.42993732828268 | 0.999714303421225 |
| ebi-fl187-GCST90094733 | ebi-met1400-GCST90200948 | Adenosine 5'-diphosphate (ADP) to mannitol to sorbitol ratio \|\| id:ebi-met1400-GCST90200948 | Chilli pepper liking \|\| id:ebi-fl187-GCST90094733 | Weighted mode | 13 | 0.135182474686936 | 0.224908044732162 | 0.558985076985657 | -0.305637293 | 0.576002242361974 | 1.14474565239871 | 0.736653760490479 | 1.77891253526383 | 0.999714303421225 |
| ebi-fl187-GCST90094733 | ebi-met1400-GCST90200972 | Leucine to N-palmitoyl-sphingosine (d18:1 to 16:0) ratio \|\| id:ebi-met1400-GCST90200972 | Chilli pepper liking \|\| id:ebi-fl187-GCST90094733 | Weighted mode | 13 | 0.235495989549016 | 0.218355138173624 | 0.302012852469495 | -0.192480081 | 0.663472060369319 | 1.26553630587571 | 0.824910749213387 | 1.94152172585551 | 0.999714303421225 |
| ebi-fl187-GCST90094733 | ebi-met1400-GCST90200973 | Proline to glutamate ratio \|\| id:ebi-met1400-GCST90200973 | Chilli pepper liking \|\| id:ebi-fl187-GCST90094733 | Weighted mode | 13 | -0.064499309 | 0.150608436813521 | 0.676049135849791 | -0.359691845 | 0.230693226878585 | 0.937536761841048 | 0.69789135134781 | 1.25947280777426 | 0.999714303421225 |
| ebi-fl187-GCST90094733 | ebi-met1400-GCST90200983 | Cholesterol to linoleoyl-arachidonoyl-glycerol (18:2 to 20:4) [1] ratio \|\| id:ebi-met1400-GCST90200983 | Chilli pepper liking \|\| id:ebi-fl187-GCST90094733 | Weighted mode | 13 | -0.027669213 | 0.134772576298676 | 0.840776816470208 | -0.291823462 | 0.236485036823552 | 0.972710073707746 | 0.74690038 | 1.26678860029697 | 0.999714303421225 |
| ebi-fl187-GCST90094733 | ebi-met1400-GCST90201001 | Serine to alpha-ketobutyrate ratio \|\| id:ebi-met1400-GCST90201001 | Chilli pepper liking \|\| id:ebi-fl187-GCST90094733 | Weighted mode | 13 | -0.034953128 | 0.13235423314243 | 0.79619308434188 | -0.294367425 | 0.224461168904573 | 0.965650677122649 | 0.745002708518599 | 1.25164810753725 | 0.999714303421225 |
| ebi-fl187-GCST90094733 | ebi-met1400-GCST90201005 | Fructose to maltose ratio \|\| id:ebi-met1400-GCST90201005 | Chilli pepper liking \|\| id:ebi-fl187-GCST90094733 | Weighted mode | 13 | -0.111748776 | 0.184673824000416 | 0.556373572943609 | -0.473709471 | 0.250211918638601 | 0.894268890730708 | 0.622688134975959 | 1.28429755444049 | 0.999714303421225 |
| ebi-fl187-GCST90094733 | ebi-met1400-GCST90201020 | Paraxanthine to 5-acetylamino-6-formylamino-3-methyluracil ratio \|\| id:ebi-met1400-GCST90201020 | Chilli pepper liking \|\| id:ebi-fl187-GCST90094733 | Weighted mode | 13 | -0.001554764 | 0.190398387603927 | 0.993618856551906 | -0.374735603 | 0.371626076132629 | 0.99844644444767 | 0.687471019850385 | 1.45009065639909 | 0.999714303421225 |
| ebi-fl187-GCST90094733 | ebi-pw11-GCST90275197 | Placental weight (fetal genotype, adjusted for sex) \|\| id:ebi-pw11-GCST90275197 | Chilli pepper liking \|\| id:ebi-fl187-GCST90094733 | Weighted mode | 12 | -0.021435268 | 0.0605629560320733 | 0.730079450294312 | -0.140138662 | 0.0972681257550616 | 0.978792834569915 | 0.869237696899461 | 1.10215585037635 | 0.999714303421225 |
| ebi-fl187-GCST90094733 | prot-a-1017 | FAS-associated factor 2 \|\| id:prot-a-1017 | Chilli pepper liking \|\| id:ebi-fl187-GCST90094733 | Weighted mode | 14 | -0.059623472 | 0.215463722170321 | 0.786344746344126 | -0.481932367 | 0.362685423860057 | 0.942119201450194 | 0.617588829708141 | 1.4371836844274 | 0.999714303421225 |
| ebi-fl187-GCST90094733 | prot-a-1019 | Fas apoptotic inhibitory molecule 3 \|\| id:prot-a-1019 | Chilli pepper liking \|\| id:ebi-fl187-GCST90094733 | Weighted mode | 14 | 0.294990472923969 | 0.248391539448229 | 0.256236507100485 | -0.191856944 | 0.781837890242497 | 1.34311356268029 | 0.825424941710745 | 2.18548525868014 | 0.999714303421225 |
| ebi-fl187-GCST90094733 | prot-a-1052 | MIP18 family protein FAM96A \|\| id:prot-a-1052 | Chilli pepper liking \|\| id:ebi-fl187-GCST90094733 | Weighted mode | 14 | -0.022567683 | 0.26971430445765 | 0.934591523684726 | -0.551207719 | 0.506072354190441 | 0.97768506273916 | 0.576253437564933 | 1.65876334888773 | 0.999714303421225 |
| ebi-fl187-GCST90094733 | prot-a-1066 | F-box/LRR-repeat protein 4 \|\| id:prot-a-1066 | Chilli pepper liking \|\| id:ebi-fl187-GCST90094733 | Weighted mode | 14 | -0.005124499 | 0.22126833530102 | 0.981874633474436 | -0.438810437 | 0.428561437744506 | 0.994888608401846 | 0.64480299907122 | 1.53504767278298 | 0.999714303421225 |
| ebi-fl187-GCST90094733 | prot-a-1076 | Low affinity immunoglobulin gamma Fc region receptor III-B \|\| id:prot-a-1076 | Chilli pepper liking \|\| id:ebi-fl187-GCST90094733 | Weighted mode | 14 | 0.00940172381774484 | 0.229552375333029 | 0.967952704538503 | -0.440520932 | 0.459324379470481 | 1.00944605885612 | 0.643701009379408 | 1.5830041135442 | 0.999714303421225 |
| ebi-fl187-GCST90094733 | prot-a-1081 | Fc receptor-like protein 3 \|\| id:prot-a-1081 | Chilli pepper liking \|\| id:ebi-fl187-GCST90094733 | Weighted mode | 14 | 0.13629112667803 | 0.265027691 | 0.615705483139335 | -0.383163147 | 0.655745400156031 | 1.14601548071432 | 0.681701672766665 | 1.92657805386729 | 0.999714303421225 |
| ebi-fl187-GCST90094733 | prot-a-1105 | Fibroblast growth factor receptor 4 \|\| id:prot-a-1105 | Chilli pepper liking \|\| id:ebi-fl187-GCST90094733 | Weighted mode | 14 | 0.104305382175054 | 0.29859008229397 | 0.732437915626458 | -0.480931179 | 0.689541943471236 | 1.10993935886092 | 0.618207461819795 | 1.99280250795114 | 0.999714303421225 |
| ebi-fl187-GCST90094733 | prot-a-1143 | Follistatin-related protein 1 \|\| id:prot-a-1143 | Chilli pepper liking \|\| id:ebi-fl187-GCST90094733 | Weighted mode | 14 | -0.100180302 | 0.216149709053916 | 0.650687043845608 | -0.523833732 | 0.323473127762737 | 0.904674288761958 | 0.592245679086875 | 1.38191902051328 | 0.999714303421225 |
| ebi-fl187-GCST90094733 | prot-a-1151 | Alpha-(1,3)-fucosyltransferase 10 \|\| id:prot-a-1151 | Chilli pepper liking \|\| id:ebi-fl187-GCST90094733 | Weighted mode | 14 | 0.165554784235579 | 0.219675067995621 | 0.464500088432163 | -0.265008349 | 0.596117917506996 | 1.18004760895485 | 0.767199544572623 | 1.81505889732478 | 0.999714303421225 |
| ebi-fl187-GCST90094733 | prot-a-1165 | Growth arrest and DNA damage-inducible proteins-interacting protein 1 \|\| id:prot-a-1165 | Chilli pepper liking \|\| id:ebi-fl187-GCST90094733 | Weighted mode | 14 | 0.11583497156412 | 0.274549471156918 | 0.679984792413987 | -0.422281992 | 0.653951935031679 | 1.12281056117219 | 0.655549153772873 | 1.92312589990253 | 0.999714303421225 |
| ebi-fl187-GCST90094733 | prot-a-1194 | Growth/differentiation factor 11/8 \|\| id:prot-a-1194 | Chilli pepper liking \|\| id:ebi-fl187-GCST90094733 | Weighted mode | 14 | -0.040827505 | 0.245904485566927 | 0.870687757019653 | -0.522800297 | 0.441145286911945 | 0.959994710146756 | 0.592858042994188 | 1.55448653248479 | 0.999714303421225 |
| ebi-fl187-GCST90094733 | prot-a-1216 | D-glucuronyl C5-epimerase \|\| id:prot-a-1216 | Chilli pepper liking \|\| id:ebi-fl187-GCST90094733 | Weighted mode | 14 | 0.304878762214275 | 0.261647945324575 | 0.264863733023143 | -0.207951211 | 0.817708735050442 | 1.35646053876464 | 0.81224666475486 | 2.26530347623031 | 0.999714303421225 |
| ebi-fl187-GCST90094733 | prot-a-1246 | Glypican-3 \|\| id:prot-a-1246 | Chilli pepper liking \|\| id:ebi-fl187-GCST90094733 | Weighted mode | 14 | 0.168766696615323 | 0.282831213758037 | 0.560952943475162 | -0.385582482 | 0.723115875581076 | 1.1838439119113 | 0.680054401129832 | 2.06084455220205 | 0.999714303421225 |
| ebi-fl187-GCST90094733 | prot-a-1275 | Glutamate receptor 4 \|\| id:prot-a-1275 | Chilli pepper liking \|\| id:ebi-fl187-GCST90094733 | Weighted mode | 14 | -0.307957694 | 0.250692601742829 | 0.241062131428758 | -0.799315194 | 0.183399805017056 | 0.734946409701035 | 0.449636772753624 | 1.20129459569005 | 0.999714303421225 |
| ebi-fl187-GCST90094733 | prot-a-1289 | Glutathione S-transferase P \|\| id:prot-a-1289 | Chilli pepper liking \|\| id:ebi-fl187-GCST90094733 | Weighted mode | 14 | -0.022943787 | 0.206966194071211 | 0.913422455704656 | -0.428597527 | 0.382709953432589 | 0.977317420225146 | 0.651422056004472 | 1.46625268682794 | 0.999714303421225 |
| ebi-fl187-GCST90094733 | prot-a-130 | Apolipoprotein D \|\| id:prot-a-130 | Chilli pepper liking \|\| id:ebi-fl187-GCST90094733 | Weighted mode | 14 | 0.141070844041193 | 0.232736326423204 | 0.554858517003233 | -0.315092356 | 0.597234043830672 | 1.15150622244943 | 0.729721477183785 | 1.81708586330373 | 0.999714303421225 |
| ebi-fl187-GCST90094733 | prot-a-1313 | Heparin-binding EGF-like growth factor \|\| id:prot-a-1313 | Chilli pepper liking \|\| id:ebi-fl187-GCST90094733 | Weighted mode | 13 | 0.192851186807648 | 0.251660037220859 | 0.458298295899441 | -0.300402486 | 0.686104859760532 | 1.21270231398787 | 0.740520111608165 | 1.98596483646848 | 0.999714303421225 |
| ebi-fl187-GCST90094733 | prot-a-1347 | HLA class II histocompatibility antigen, DQ alpha 2 chain \|\| id:prot-a-1347 | Chilli pepper liking \|\| id:ebi-fl187-GCST90094733 | Weighted mode | 13 | -0.130473489 | 0.236685804449182 | 0.591577479328687 | -0.594377666 | 0.333430687727822 | 0.877679760814965 | 0.551905928996252 | 1.39574830070261 | 0.999714303421225 |
| ebi-fl187-GCST90094733 | prot-a-1357 | Non-histone chromosomal protein HMG-14 \|\| id:prot-a-1357 | Chilli pepper liking \|\| id:ebi-fl187-GCST90094733 | Weighted mode | 14 | 0.00316373517237545 | 0.218462865121713 | 0.988665494602612 | -0.42502348 | 0.431350950810933 | 1.00316874506443 | 0.653754434490737 | 1.53933568627808 | 0.999714303421225 |
| ebi-fl187-GCST90094733 | prot-a-137 | Apolipoprotein M \|\| id:prot-a-137 | Chilli pepper liking \|\| id:ebi-fl187-GCST90094733 | Weighted mode | 14 | 0.0463267259274924 | 0.242610643868032 | 0.851513920833124 | -0.429190136 | 0.521843587908836 | 1.04741657320861 | 0.651036131971202 | 1.68513147574517 | 0.999714303421225 |
| ebi-fl187-GCST90094733 | prot-a-1381 | 17-beta-hydroxysteroid dehydrogenase 14 \|\| id:prot-a-1381 | Chilli pepper liking \|\| id:ebi-fl187-GCST90094733 | Weighted mode | 14 | -0.129200731 | 0.194141440543207 | 0.517366258056869 | -0.509717954 | 0.251316492645571 | 0.878797546088503 | 0.60066496990476 | 1.28571693990017 | 0.999714303421225 |
| ebi-fl187-GCST90094733 | prot-a-1392 | Serine protease HTRA2, mitochondrial \|\| id:prot-a-1392 | Chilli pepper liking \|\| id:ebi-fl187-GCST90094733 | Weighted mode | 14 | -0.065170649 | 0.219209433744299 | 0.77093600719898 | -0.49482114 | 0.364479840776877 | 0.936907567056167 | 0.609679945204099 | 1.43976490634484 | 0.999714303421225 |
| ebi-fl187-GCST90094733 | prot-a-1416 | Interferon-induced protein with tetratricopeptide repeats 2 \|\| id:prot-a-1416 | Chilli pepper liking \|\| id:ebi-fl187-GCST90094733 | Weighted mode | 14 | -0.249680588 | 0.239563100712522 | 0.316298943499079 | -0.719224266 | 0.219863089038557 | 0.779049580840663 | 0.487129992846756 | 1.24590614070224 | 0.999714303421225 |
| ebi-fl187-GCST90094733 | prot-a-1428 | Interferon gamma \|\| id:prot-a-1428 | Chilli pepper liking \|\| id:ebi-fl187-GCST90094733 | Weighted mode | 14 | -0.293922054 | 0.352975384259395 | 0.420049971747144 | -0.985753807 | 0.397909699445044 | 0.745334585249831 | 0.373157831960726 | 1.48870959253511 | 0.999714303421225 |
| ebi-fl187-GCST90094733 | prot-a-1483 | Interleukin-17C \|\| id:prot-a-1483 | Chilli pepper liking \|\| id:ebi-fl187-GCST90094733 | Weighted mode | 14 | 0.308629099821401 | 0.251500080020601 | 0.241524916757969 | -0.184311057 | 0.801569256661779 | 1.36155727500274 | 0.831677064598966 | 2.22903611512576 | 0.999714303421225 |
| ebi-fl187-GCST90094733 | prot-a-1503 | Interleukin-1 receptor-like 2 \|\| id:prot-a-1503 | Chilli pepper liking \|\| id:ebi-fl187-GCST90094733 | Weighted mode | 14 | -0.070195235 | 0.245619491580298 | 0.779543135760286 | -0.551609439 | 0.411218968235814 | 0.932211801523414 | 0.576021991827536 | 1.50865566806296 | 0.999714303421225 |
| ebi-fl187-GCST90094733 | prot-a-1522 | Interleukin-31 receptor subunit alpha \|\| id:prot-a-1522 | Chilli pepper liking \|\| id:ebi-fl187-GCST90094733 | Weighted mode | 14 | 0.01690745450706 | 0.204638082866008 | 0.935411317289155 | -0.384183188 | 0.417998096924435 | 1.01705119446562 | 0.681006663564694 | 1.51891778378417 | 0.999714303421225 |
| ebi-fl187-GCST90094733 | prot-a-1540 | Interleukin-6 receptor subunit alpha \|\| id:prot-a-1540 | Chilli pepper liking \|\| id:ebi-fl187-GCST90094733 | Weighted mode | 14 | 0.331279489077234 | 0.242365778203545 | 0.194843123376783 | -0.143757436 | 0.806316414356182 | 1.39274899604829 | 0.866097806555528 | 2.23964285709013 | 0.999714303421225 |
| ebi-fl187-GCST90094733 | prot-a-1541 | Interleukin-6 receptor subunit alpha \|\| id:prot-a-1541 | Chilli pepper liking \|\| id:ebi-fl187-GCST90094733 | Weighted mode | 14 | 0.440438708391837 | 0.227010426370684 | 0.07436415300903 | -0.004501727 | 0.885379144078378 | 1.55338855364096 | 0.995508390291715 | 2.42390322584388 | 0.999714303421225 |
| ebi-fl187-GCST90094733 | prot-a-1545 | Interleukin-9 \|\| id:prot-a-1545 | Chilli pepper liking \|\| id:ebi-fl187-GCST90094733 | Weighted mode | 14 | 0.195896285166374 | 0.27983825603213 | 0.496243353022343 | -0.352586697 | 0.74437926698935 | 1.21640073999145 | 0.702867630895524 | 2.105134303 | 0.999714303421225 |
| ebi-fl187-GCST90094733 | prot-a-1548 | Inositol monophosphatase 3 \|\| id:prot-a-1548 | Chilli pepper liking \|\| id:ebi-fl187-GCST90094733 | Weighted mode | 14 | 0.105237868848926 | 0.233239384432861 | 0.659279266866492 | -0.351911325 | 0.562387062337333 | 1.11097484523546 | 0.703342488359843 | 1.75485645638185 | 0.999714303421225 |
| ebi-fl187-GCST90094733 | prot-a-1555 | Inhibin beta A chain:Inhibin beta B chain heterodimer \|\| id:prot-a-1555 | Chilli pepper liking \|\| id:ebi-fl187-GCST90094733 | Weighted mode | 14 | -0.249356618 | 0.234060420857058 | 0.306104390592964 | -0.708115043 | 0.209401807144504 | 0.779302010906204 | 0.492571799792462 | 1.23294030323769 | 0.999714303421225 |
| ebi-fl187-GCST90094733 | prot-a-1603 | Junctophilin-3 \|\| id:prot-a-1603 | Chilli pepper liking \|\| id:ebi-fl187-GCST90094733 | Weighted mode | 14 | 0.160808287859785 | 0.285182909960716 | 0.582434736782295 | -0.398150216 | 0.719766791382787 | 1.17445978902996 | 0.671561141082628 | 2.0539541549778 | 0.999714303421225 |
| ebi-fl187-GCST90094733 | prot-a-1627 | UPF0258 protein KIAA1024 \|\| id:prot-a-1627 | Chilli pepper liking \|\| id:ebi-fl187-GCST90094733 | Weighted mode | 14 | 0.0185212597125668 | 0.281999690500092 | 0.948633236621417 | -0.534198134 | 0.571240653092748 | 1.01869384207764 | 0.586139106970447 | 1.77046221885896 | 0.999714303421225 |
| ebi-fl187-GCST90094733 | prot-a-1666 | Kallikrein-7 \|\| id:prot-a-1666 | Chilli pepper liking \|\| id:ebi-fl187-GCST90094733 | Weighted mode | 14 | -0.037158038 | 0.252290527643621 | 0.885168560013868 | -0.531647472 | 0.457331396359601 | 0.963523850105342 | 0.587636057816485 | 1.57985235482563 | 0.999714303421225 |
| ebi-fl187-GCST90094733 | prot-a-1678 | Importin subunit alpha-3 \|\| id:prot-a-1678 | Chilli pepper liking \|\| id:ebi-fl187-GCST90094733 | Weighted mode | 14 | 0.481147371823017 | 0.321692657868008 | 0.158615209327253 | -0.149370238 | 1.11166498124431 | 1.61792970458317 | 0.861250188661992 | 3.03941475245348 | 0.999714303421225 |
| ebi-fl187-GCST90094733 | prot-a-1718 | Protein LDOC1 \|\| id:prot-a-1718 | Chilli pepper liking \|\| id:ebi-fl187-GCST90094733 | Weighted mode | 14 | 0.189983368705049 | 0.247603985788397 | 0.456620772090277 | -0.295320443 | 0.675287180850306 | 1.20922948643776 | 0.744293045424336 | 1.96459708962736 | 0.999714303421225 |
| ebi-fl187-GCST90094733 | prot-a-1723 | Leptin \|\| id:prot-a-1723 | Chilli pepper liking \|\| id:ebi-fl187-GCST90094733 | Weighted mode | 14 | 0.239437956245163 | 0.311461398879562 | 0.455777847642728 | -0.371026386 | 0.849902298049105 | 1.27053485342748 | 0.690025734604011 | 2.33941827503054 | 0.999714303421225 |
| ebi-fl187-GCST90094733 | prot-a-1730 | Galectin-7 \|\| id:prot-a-1730 | Chilli pepper liking \|\| id:ebi-fl187-GCST90094733 | Weighted mode | 14 | 0.0837450132400956 | 0.284474183285546 | 0.773111609697939 | -0.473824386 | 0.641314412479765 | 1.08735159819607 | 0.622616583156315 | 1.89897527641456 | 0.999714303421225 |
| ebi-fl187-GCST90094733 | prot-a-1753 | Lipase member N \|\| id:prot-a-1753 | Chilli pepper liking \|\| id:ebi-fl187-GCST90094733 | Weighted mode | 14 | 0.0294818792018194 | 0.243676289074554 | 0.905550675831857 | -0.448123647 | 0.507087405787945 | 1.02992077231634 | 0.638825690022717 | 1.66044793410072 | 0.999714303421225 |
| ebi-fl187-GCST90094733 | prot-a-1756 | VIP36-like protein \|\| id:prot-a-1756 | Chilli pepper liking \|\| id:ebi-fl187-GCST90094733 | Weighted mode | 14 | 0.146703073718213 | 0.213578314452126 | 0.504226327445115 | -0.271910423 | 0.565316570044379 | 1.15801006835283 | 0.761922509064358 | 1.76000485935671 | 0.999714303421225 |
| ebi-fl187-GCST90094733 | prot-a-1760 | Ig Kappa chain V-I region HK102- like \|\| id:prot-a-1760 | Chilli pepper liking \|\| id:ebi-fl187-GCST90094733 | Weighted mode | 14 | -0.009402194 | 0.223016799451779 | 0.967012490768544 | -0.446515121 | 0.427710732989979 | 0.99064186848728 | 0.639854084967428 | 1.53374235572775 | 0.999714303421225 |
| ebi-fl187-GCST90094733 | prot-a-179 | Neutral ceramidase \|\| id:prot-a-179 | Chilli pepper liking \|\| id:ebi-fl187-GCST90094733 | Weighted mode | 14 | 0.423076430938253 | 0.266755454506976 | 0.136752452813652 | -0.09976426 | 0.945917121771927 | 1.52665097487386 | 0.905050749647935 | 2.57517404409633 | 0.999714303421225 |
| ebi-fl187-GCST90094733 | prot-a-1809 | Lactotransferrin \|\| id:prot-a-1809 | Chilli pepper liking \|\| id:ebi-fl187-GCST90094733 | Weighted mode | 14 | 0.0140114629655896 | 0.217829689562717 | 0.949691606950084 | -0.412934729 | 0.440957654508516 | 1.01411008358086 | 0.661705471875861 | 1.55419488780245 | 0.999714303421225 |
| ebi-fl187-GCST90094733 | prot-a-1818 | Lysophospholipase-like protein 1 \|\| id:prot-a-1818 | Chilli pepper liking \|\| id:ebi-fl187-GCST90094733 | Weighted mode | 14 | -0.175786112 | 0.29916209010942 | 0.566871161665867 | -0.762143808 | 0.410571584961137 | 0.83879737313503 | 0.466664913766875 | 1.50767930569065 | 0.999714303421225 |
| ebi-fl187-GCST90094733 | prot-a-1852 | MAP kinase-activated protein kinase 3 \|\| id:prot-a-1852 | Chilli pepper liking \|\| id:ebi-fl187-GCST90094733 | Weighted mode | 14 | -0.121768021 | 0.27170844654027 | 0.66141794017935 | -0.654316576 | 0.410780534414546 | 0.885353728327836 | 0.519797183082229 | 1.50799436737232 | 0.999714303421225 |
| ebi-fl187-GCST90094733 | prot-a-1856 | Megakaryocyte-associated tyrosine-protein kinase \|\| id:prot-a-1856 | Chilli pepper liking \|\| id:ebi-fl187-GCST90094733 | Weighted mode | 14 | -0.046073738 | 0.218322656739714 | 0.836132021243227 | -0.473986145 | 0.38183866938114 | 0.95497154206645 | 0.622515877441428 | 1.46497572062743 | 0.999714303421225 |
| ebi-fl187-GCST90094733 | prot-a-1890 | Beta-1,3-N-acetylglucosaminyltransferase manic fringe \|\| id:prot-a-1890 | Chilli pepper liking \|\| id:ebi-fl187-GCST90094733 | Weighted mode | 14 | 0.0796074157070281 | 0.27090121446056 | 0.773503037302179 | -0.451358965 | 0.610573796049726 | 1.0828618696559 | 0.636762226027406 | 1.84148773407958 | 0.999714303421225 |
| ebi-fl187-GCST90094733 | prot-a-1919 | Matrilysin \|\| id:prot-a-1919 | Chilli pepper liking \|\| id:ebi-fl187-GCST90094733 | Weighted mode | 14 | -0.037173351 | 0.231664653569086 | 0.874983829987029 | -0.491236072 | 0.416889369695532 | 0.963509095317053 | 0.611869611486624 | 1.51723465151853 | 0.999714303421225 |
| ebi-fl187-GCST90094733 | prot-a-1923 | Multimerin-2 \|\| id:prot-a-1923 | Chilli pepper liking \|\| id:ebi-fl187-GCST90094733 | Weighted mode | 14 | -0.077295979 | 0.23308014321284 | 0.745455392632637 | -0.534133059 | 0.379541101941683 | 0.925615850431471 | 0.586177250753769 | 1.46161370382125 | 0.999714303421225 |
| ebi-fl187-GCST90094733 | prot-a-1948 | Mesothelin \|\| id:prot-a-1948 | Chilli pepper liking \|\| id:ebi-fl187-GCST90094733 | Weighted mode | 14 | 0.205188528423048 | 0.236895536911284 | 0.402107766484173 | -0.259126724 | 0.669503780769164 | 1.22775651014424 | 0.771725220799382 | 1.9532678310554 | 0.999714303421225 |
| ebi-fl187-GCST90094733 | prot-a-195 | ATPase family AAA domain-containing protein 1 \|\| id:prot-a-195 | Chilli pepper liking \|\| id:ebi-fl187-GCST90094733 | Weighted mode | 14 | -0.079691449 | 0.246333882179242 | 0.751454381434717 | -0.562505858 | 0.403122960517651 | 0.923401219217151 | 0.569779487166591 | 1.49649088964556 | 0.999714303421225 |
| ebi-fl187-GCST90094733 | prot-a-1966 | Mucin-1 \|\| id:prot-a-1966 | Chilli pepper liking \|\| id:ebi-fl187-GCST90094733 | Weighted mode | 14 | 0.00968470306212343 | 0.254668084957201 | 0.970242564864514 | -0.489464743 | 0.508834149578237 | 1.00973175155974 | 0.612954394246421 | 1.66335084580211 | 0.999714303421225 |
| ebi-fl187-GCST90094733 | prot-a-1986 | Myocardial zonula adherens protein \|\| id:prot-a-1986 | Chilli pepper liking \|\| id:ebi-fl187-GCST90094733 | Weighted mode | 14 | -0.076600776 | 0.277569495631804 | 0.786909623836447 | -0.620636987 | 0.467435435637979 | 0.926259565036225 | 0.537601882965848 | 1.59589616220819 | 0.999714303421225 |
| ebi-fl187-GCST90094733 | prot-a-2071 | Epididymal secretory protein E1 \|\| id:prot-a-2071 | Chilli pepper liking \|\| id:ebi-fl187-GCST90094733 | Weighted mode | 13 | -0.224831236 | 0.244737303227649 | 0.376367704421642 | -0.70451635 | 0.254853878241768 | 0.798650990855119 | 0.494347607464689 | 1.29027307012794 | 0.999714303421225 |
| ebi-fl187-GCST90094733 | prot-a-2098 | Neuritin \|\| id:prot-a-2098 | Chilli pepper liking \|\| id:ebi-fl187-GCST90094733 | Weighted mode | 14 | 0.298540660573125 | 0.258227423790455 | 0.268439368753581 | -0.20758509 | 0.804666411202416 | 1.3478903420764 | 0.81254409940842 | 2.235950486 | 0.999714303421225 |
| ebi-fl187-GCST90094733 | prot-a-2115 | Neurotrophin-4 \|\| id:prot-a-2115 | Chilli pepper liking \|\| id:ebi-fl187-GCST90094733 | Weighted mode | 14 | -0.076623802 | 0.212243720793966 | 0.723885033384157 | -0.492621495 | 0.339373890528527 | 0.926238236833255 | 0.61102250038341 | 1.40406821489166 | 0.999714303421225 |
| ebi-fl187-GCST90094733 | prot-a-2118 | Netrin-1 \|\| id:prot-a-2118 | Chilli pepper liking \|\| id:ebi-fl187-GCST90094733 | Weighted mode | 14 | 0.249239122430093 | 0.221334840067106 | 0.280493781717176 | -0.184577164 | 0.683055408961621 | 1.28304880213816 | 0.831455778885911 | 1.97991795892498 | 0.999714303421225 |
| ebi-fl187-GCST90094733 | prot-a-2120 | Netrin-G1 \|\| id:prot-a-2120 | Chilli pepper liking \|\| id:ebi-fl187-GCST90094733 | Weighted mode | 14 | 0.185005949988709 | 0.239072389084283 | 0.452863714467845 | -0.283575933 | 0.653587832593903 | 1.20322559928513 | 0.75308593618338 | 1.92242581253372 | 0.999714303421225 |
| ebi-fl187-GCST90094733 | prot-a-2145 | Olfactomedin-like protein 3 \|\| id:prot-a-2145 | Chilli pepper liking \|\| id:ebi-fl187-GCST90094733 | Weighted mode | 14 | -0.438324134 | 0.227300659860181 | 0.0759338422025399 | -0.883833427 | 0.00718515936615916 | 0.645116644752151 | 0.413195915311303 | 1.00721103455905 | 0.999714303421225 |
| ebi-fl187-GCST90094733 | prot-a-2162 | Prolyl 4-hydroxylase subunit alpha-2 \|\| id:prot-a-2162 | Chilli pepper liking \|\| id:ebi-fl187-GCST90094733 | Weighted mode | 14 | 0.213231220072865 | 0.235141448940197 | 0.381003049838271 | -0.24764602 | 0.674108459995651 | 1.23767079249235 | 0.780636224103951 | 1.9622827423195 | 0.999714303421225 |
| ebi-fl187-GCST90094733 | prot-a-2168 | Polyadenylate-binding protein-interacting protein 1 \|\| id:prot-a-2168 | Chilli pepper liking \|\| id:ebi-fl187-GCST90094733 | Weighted mode | 14 | -0.081425444 | 0.266063331470564 | 0.764421781156801 | -0.602909573 | 0.440058686176925 | 0.921801433575578 | 0.547217149235522 | 1.55279834363574 | 0.999714303421225 |
| ebi-fl187-GCST90094733 | prot-a-219 | Beta-1,4-galactosyltransferase 5 \|\| id:prot-a-219 | Chilli pepper liking \|\| id:ebi-fl187-GCST90094733 | Weighted mode | 14 | 0.0627833092861299 | 0.254034481041264 | 0.808656304077686 | -0.435124274 | 0.560690892127008 | 1.06479608280751 | 0.647184234194938 | 1.75188244406571 | 0.999714303421225 |
| ebi-fl187-GCST90094733 | prot-a-2190 | Pyruvate carboxylase, mitochondrial \|\| id:prot-a-2190 | Chilli pepper liking \|\| id:ebi-fl187-GCST90094733 | Weighted mode | 14 | -0.048279018 | 0.219235728672335 | 0.829124159436771 | -0.477981046 | 0.381423010269975 | 0.952867882761077 | 0.620033948916226 | 1.46436691665774 | 0.999714303421225 |
| ebi-fl187-GCST90094733 | prot-a-2197 | Protocadherin-9 \|\| id:prot-a-2197 | Chilli pepper liking \|\| id:ebi-fl187-GCST90094733 | Weighted mode | 14 | -0.04751074 | 0.218305587000207 | 0.831092937178056 | -0.475389691 | 0.380368210261522 | 0.95360023116427 | 0.621642760805995 | 1.46282311676488 | 0.999714303421225 |
| ebi-fl187-GCST90094733 | prot-a-2198 | Protocadherin alpha-4 \|\| id:prot-a-2198 | Chilli pepper liking \|\| id:ebi-fl187-GCST90094733 | Weighted mode | 14 | -0.160481813 | 0.216696063367718 | 0.472110392838446 | -0.585206097 | 0.264242471324456 | 0.851733314010254 | 0.556991055753479 | 1.30244396332994 | 0.999714303421225 |
| ebi-fl187-GCST90094733 | prot-a-2254 | Placenta growth factor \|\| id:prot-a-2254 | Chilli pepper liking \|\| id:ebi-fl187-GCST90094733 | Weighted mode | 14 | -0.174705471 | 0.223208520645493 | 0.447826688458558 | -0.612194171 | 0.262783229755137 | 0.839704301863471 | 0.542159971254181 | 1.30054476898562 | 0.999714303421225 |
| ebi-fl187-GCST90094733 | prot-a-2261 | Membrane-associated progesterone receptor component 2 \|\| id:prot-a-2261 | Chilli pepper liking \|\| id:ebi-fl187-GCST90094733 | Weighted mode | 14 | -0.145416331 | 0.269337491102478 | 0.598390708058882 | -0.673317813 | 0.382485151753332 | 0.864662232638258 | 0.510013637528978 | 1.46592310780807 | 0.999714303421225 |
| ebi-fl187-GCST90094733 | prot-a-2268 | Polymeric immunoglobulin receptor \|\| id:prot-a-2268 | Chilli pepper liking \|\| id:ebi-fl187-GCST90094733 | Weighted mode | 14 | 0.198992223264321 | 0.293241503552033 | 0.509294942508074 | -0.375761124 | 0.773745570226306 | 1.22017247690414 | 0.686766365659766 | 2.16787097889411 | 0.999714303421225 |
| ebi-fl187-GCST90094733 | prot-a-2283 | Group 10 secretory phospholipase A2 \|\| id:prot-a-2283 | Chilli pepper liking \|\| id:ebi-fl187-GCST90094733 | Weighted mode | 14 | 0.0814100924336518 | 0.255880015476891 | 0.755418847455197 | -0.420114738 | 0.582934922768357 | 1.08481568006165 | 0.656971435966832 | 1.79128801540017 | 0.999714303421225 |
| ebi-fl187-GCST90094733 | prot-a-2298 | Inactive phospholipase D5 \|\| id:prot-a-2298 | Chilli pepper liking \|\| id:ebi-fl187-GCST90094733 | Weighted mode | 14 | 0.0461951721291511 | 0.23717981817692 | 0.848585013777699 | -0.418677271 | 0.511067615755915 | 1.04727879064306 | 0.657916489412613 | 1.66707003545391 | 0.999714303421225 |
| ebi-fl187-GCST90094733 | prot-a-2304 | Procollagen-lysine,2-oxoglutarate 5-dioxygenase 2 \|\| id:prot-a-2304 | Chilli pepper liking \|\| id:ebi-fl187-GCST90094733 | Weighted mode | 14 | 0.191080884688714 | 0.265065083474584 | 0.48374438439649 | -0.328446679 | 0.710608448298898 | 1.210557364 | 0.72004132058205 | 2.03522921374569 | 0.999714303421225 |
| ebi-fl187-GCST90094733 | prot-a-2331 | Periostin \|\| id:prot-a-2331 | Chilli pepper liking \|\| id:ebi-fl187-GCST90094733 | Weighted mode | 14 | -0.035291547 | 0.272616393487541 | 0.898979179767018 | -0.569619678 | 0.49903658408509 | 0.965323937784076 | 0.565740561152367 | 1.64713363129002 | 0.999714303421225 |
| ebi-fl187-GCST90094733 | prot-a-2333 | Periostin \|\| id:prot-a-2333 | Chilli pepper liking \|\| id:ebi-fl187-GCST90094733 | Weighted mode | 14 | -0.238419945 | 0.286035553094831 | 0.419596351736562 | -0.799049629 | 0.322209738953614 | 0.78787175871776 | 0.449756196236456 | 1.38017422189925 | 0.999714303421225 |
| ebi-fl187-GCST90094733 | prot-a-2367 | cAMP-dependent protein kinase catalytic subunit alpha \|\| id:prot-a-2367 | Chilli pepper liking \|\| id:ebi-fl187-GCST90094733 | Weighted mode | 14 | 0.0597522252348774 | 0.252115172665828 | 0.816347763856899 | -0.434393513 | 0.5538979636599 | 1.06157348283606 | 0.64765734362564 | 1.74002235989789 | 0.999714303421225 |
| ebi-fl187-GCST90094733 | prot-a-2385 | Phosphoribosyl pyrophosphate synthase-associated protein 1 \|\| id:prot-a-2385 | Chilli pepper liking \|\| id:ebi-fl187-GCST90094733 | Weighted mode | 14 | 0.0545797049604069 | 0.242899760189468 | 0.82570555022938 | -0.421503825 | 0.530663234931763 | 1.0560966492008 | 0.656059478954066 | 1.70005947361861 | 0.999714303421225 |
| ebi-fl187-GCST90094733 | prot-a-2393 | Trypsin-3 \|\| id:prot-a-2393 | Chilli pepper liking \|\| id:ebi-fl187-GCST90094733 | Weighted mode | 14 | 0.37762707862034 | 0.256350464247602 | 0.164523309014395 | -0.124819831 | 0.880073988545639 | 1.45881881669816 | 0.882655915224031 | 2.41107809197933 | 0.999714303421225 |
| ebi-fl187-GCST90094733 | prot-a-2405 | Pregnancy-specific beta-1-glycoprotein 6 \|\| id:prot-a-2405 | Chilli pepper liking \|\| id:ebi-fl187-GCST90094733 | Weighted mode | 14 | 0.203565709520875 | 0.223498514153467 | 0.378972133731867 | -0.234491378 | 0.64162279726167 | 1.22576569947181 | 0.790973053457697 | 1.8995609817977 | 0.999714303421225 |
| ebi-fl187-GCST90094733 | prot-a-244 | Beclin-1 \|\| id:prot-a-244 | Chilli pepper liking \|\| id:ebi-fl187-GCST90094733 | Weighted mode | 14 | -0.10778965 | 0.265741616241942 | 0.691619808664836 | -0.628643218 | 0.413063917899038 | 0.897816432313663 | 0.533314902533404 | 1.51144163102014 | 0.999714303421225 |
| ebi-fl187-GCST90094733 | prot-a-2454 | tRNA pseudouridine synthase A, mitochondrial \|\| id:prot-a-2454 | Chilli pepper liking \|\| id:ebi-fl187-GCST90094733 | Weighted mode | 14 | -0.204215949 | 0.293930172675285 | 0.499425069649598 | -0.780319087 | 0.371887189520256 | 0.815286291978984 | 0.458259763072346 | 1.45046934392079 | 0.999714303421225 |
| ebi-fl187-GCST90094733 | prot-a-2466 | Glutaminyl-peptide cyclotransferase-like protein \|\| id:prot-a-2466 | Chilli pepper liking \|\| id:ebi-fl187-GCST90094733 | Weighted mode | 14 | 0.0334853023693616 | 0.262122320664323 | 0.90030427866525 | -0.480274446 | 0.547245050871434 | 1.03405224549662 | 0.618613592398735 | 1.72848456541415 | 0.999714303421225 |
| ebi-fl187-GCST90094733 | prot-a-2474 | Ras-related protein Rab-27A \|\| id:prot-a-2474 | Chilli pepper liking \|\| id:ebi-fl187-GCST90094733 | Weighted mode | 14 | 0.224512536911665 | 0.257594751728828 | 0.399252089543031 | -0.280373176 | 0.729398250300167 | 1.25171240385749 | 0.755501753360772 | 2.07383230416213 | 0.999714303421225 |
| ebi-fl187-GCST90094733 | prot-a-2490 | Rap1 GTPase-GDP dissociation stimulator 1 \|\| id:prot-a-2490 | Chilli pepper liking \|\| id:ebi-fl187-GCST90094733 | Weighted mode | 14 | 0.254780366725626 | 0.355407703455134 | 0.486136454230106 | -0.441818732 | 0.951379465497688 | 1.29017822368451 | 0.642866155926999 | 2.58927901791541 | 0.999714303421225 |
| ebi-fl187-GCST90094733 | prot-a-2545 | Receptor-interacting serine/threonine-protein kinase 2 \|\| id:prot-a-2545 | Chilli pepper liking \|\| id:ebi-fl187-GCST90094733 | Weighted mode | 14 | 0.29461841068015 | 0.258579189274121 | 0.275106787253414 | -0.2121968 | 0.801433621657427 | 1.34261393378674 | 0.808805508738371 | 2.22873380030528 | 0.999714303421225 |
| ebi-fl187-GCST90094733 | prot-a-2610 | RUN and FYVE domain-containing protein 1 \|\| id:prot-a-2610 | Chilli pepper liking \|\| id:ebi-fl187-GCST90094733 | Weighted mode | 14 | 0.241288505643472 | 0.226673359830424 | 0.306485674008879 | -0.20299128 | 0.685568290911103 | 1.27288821777291 | 0.816285359709703 | 1.98489951543577 | 0.999714303421225 |
| ebi-fl187-GCST90094733 | prot-a-2627 | Serine--tRNA ligase, mitochondrial \|\| id:prot-a-2627 | Chilli pepper liking \|\| id:ebi-fl187-GCST90094733 | Weighted mode | 14 | 0.00130418409077415 | 0.210038309981057 | 0.995140027619741 | -0.410370903 | 0.412979271653645 | 1.00130503490868 | 0.663404145597621 | 1.51131369857552 | 0.999714303421225 |
| ebi-fl187-GCST90094733 | prot-a-264 | BolA-like protein 2 \|\| id:prot-a-264 | Chilli pepper liking \|\| id:ebi-fl187-GCST90094733 | Weighted mode | 14 | -0.09879286 | 0.209250634824817 | 0.644663036947299 | -0.508924104 | 0.311338384517888 | 0.905930343237444 | 0.601141997283464 | 1.36525112287457 | 0.999714303421225 |
| ebi-fl187-GCST90094733 | prot-a-2646 | Secretoglobin family 1C member 1 \|\| id:prot-a-2646 | Chilli pepper liking \|\| id:ebi-fl187-GCST90094733 | Weighted mode | 14 | 0.0173981820065627 | 0.255089074193225 | 0.946660863083844 | -0.482576403 | 0.517372767425283 | 1.01755041193499 | 0.617191208098597 | 1.67761437175831 | 0.999714303421225 |
| ebi-fl187-GCST90094733 | prot-a-2672 | Semaphorin-3E \|\| id:prot-a-2672 | Chilli pepper liking \|\| id:ebi-fl187-GCST90094733 | Weighted mode | 14 | 0.140491449448141 | 0.222195841475861 | 0.538161706227639 | -0.2950124 | 0.575995298740829 | 1.15083923921218 | 0.744522355447202 | 1.77890018321202 | 0.999714303421225 |
| ebi-fl187-GCST90094733 | prot-a-2701 | Plasma protease C1 inhibitor \|\| id:prot-a-2701 | Chilli pepper liking \|\| id:ebi-fl187-GCST90094733 | Weighted mode | 14 | 0.270371660768224 | 0.26430716664116 | 0.324993492083887 | -0.247670386 | 0.788413707384897 | 1.31045140361239 | 0.780617203354594 | 2.19990396554149 | 0.999714303421225 |
| ebi-fl187-GCST90094733 | prot-a-2738 | Signaling threshold-regulating transmembrane adapter 1 \|\| id:prot-a-2738 | Chilli pepper liking \|\| id:ebi-fl187-GCST90094733 | Weighted mode | 14 | 0.214796415301609 | 0.25477433630469 | 0.414424377031592 | -0.284561284 | 0.714154114458801 | 1.23960950574809 | 0.752344247494958 | 2.04245826542499 | 0.999714303421225 |
| ebi-fl187-GCST90094733 | prot-a-274 | Serine/threonine-protein kinase BRSK2 \|\| id:prot-a-274 | Chilli pepper liking \|\| id:ebi-fl187-GCST90094733 | Weighted mode | 14 | -0.05161263 | 0.253598750940216 | 0.841879133713477 | -0.548666181 | 0.445440922280033 | 0.94969668001647 | 0.577719870211495 | 1.56117840243945 | 0.999714303421225 |
| ebi-fl187-GCST90094733 | prot-a-2749 | Mitochondrial glutamate carrier 2 \|\| id:prot-a-2749 | Chilli pepper liking \|\| id:ebi-fl187-GCST90094733 | Weighted mode | 14 | 0.240588476475323 | 0.195783112341926 | 0.24090813778098 | -0.143146424 | 0.624323376665499 | 1.27199747070352 | 0.866627164835967 | 1.86698228618577 | 0.999714303421225 |
| ebi-fl187-GCST90094733 | prot-a-2760 | Sodium-coupled monocarboxylate transporter 1 \|\| id:prot-a-2760 | Chilli pepper liking \|\| id:ebi-fl187-GCST90094733 | Weighted mode | 14 | -0.114015286 | 0.235145064944351 | 0.63583501018999 | -0.574899613 | 0.34686904108418 | 0.892244316747068 | 0.562761359615987 | 1.41463145463768 | 0.999714303421225 |
| ebi-fl187-GCST90094733 | prot-a-2777 | Small integral membrane protein 10 \|\| id:prot-a-2777 | Chilli pepper liking \|\| id:ebi-fl187-GCST90094733 | Weighted mode | 14 | 0.0919200809966141 | 0.256808497379506 | 0.726138576441712 | -0.411424574 | 0.595264735860445 | 1.09627720519806 | 0.662705504422804 | 1.8135109828062 | 0.999714303421225 |
| ebi-fl187-GCST90094733 | prot-a-2799 | Superoxide dismutase [Mn], mitochondrial \|\| id:prot-a-2799 | Chilli pepper liking \|\| id:ebi-fl187-GCST90094733 | Weighted mode | 14 | 0.33219603127821 | 0.229461897592248 | 0.171380226576994 | -0.117549288 | 0.781941350559016 | 1.39402609444642 | 0.889096688866576 | 2.18571138137391 | 0.999714303421225 |
| ebi-fl187-GCST90094733 | prot-a-2805 | Protein SPACA7 \|\| id:prot-a-2805 | Chilli pepper liking \|\| id:ebi-fl187-GCST90094733 | Weighted mode | 14 | -0.357012311 | 0.264739582443868 | 0.200509612425849 | -0.875901893 | 0.161877270575231 | 0.699763882878098 | 0.416486224394733 | 1.17571593752532 | 0.999714303421225 |
| ebi-fl187-GCST90094733 | prot-a-2811 | SPARC-like protein 1 \|\| id:prot-a-2811 | Chilli pepper liking \|\| id:ebi-fl187-GCST90094733 | Weighted mode | 14 | 0.276281941252414 | 0.247447467589229 | 0.284408628035221 | -0.208715095 | 0.761278977727303 | 1.31821947208794 | 0.811626438956408 | 2.14101277778253 | 0.999714303421225 |
| ebi-fl187-GCST90094733 | prot-a-2831 | Sepiapterin reductase \|\| id:prot-a-2831 | Chilli pepper liking \|\| id:ebi-fl187-GCST90094733 | Weighted mode | 14 | 0.154279090206868 | 0.328365324109714 | 0.646253249996524 | -0.489316945 | 0.797875125461908 | 1.16681648839534 | 0.61304499462386 | 2.22081695394408 | 0.999714303421225 |
| ebi-fl187-GCST90094733 | prot-a-2834 | SPRY domain-containing SOCS box protein 1 \|\| id:prot-a-2834 | Chilli pepper liking \|\| id:ebi-fl187-GCST90094733 | Weighted mode | 14 | -0.016646602 | 0.215111785385374 | 0.939495000904647 | -0.438265701 | 0.404972497771001 | 0.98349118745529 | 0.64515434196741 | 1.49926126646308 | 0.999714303421225 |
| ebi-fl187-GCST90094733 | prot-a-2835 | Spectrin alpha chain, non-erythrocytic 1 \|\| id:prot-a-2835 | Chilli pepper liking \|\| id:ebi-fl187-GCST90094733 | Weighted mode | 14 | -0.119066829 | 0.231576250860064 | 0.615769521111973 | -0.572956281 | 0.334822622812966 | 0.887748471549872 | 0.563856055637936 | 1.39769244447946 | 0.999714303421225 |
| ebi-fl187-GCST90094733 | prot-a-2859 | Sia-alpha-2,3-Gal-beta-1,4-GlcNAc-R:alpha 2,8-sialyltransferase \|\| id:prot-a-2859 | Chilli pepper liking \|\| id:ebi-fl187-GCST90094733 | Weighted mode | 14 | -0.023914905 | 0.241839056443378 | 0.922736016718354 | -0.497919456 | 0.450089645518641 | 0.97636879021743 | 0.607793887240294 | 1.56845278395135 | 0.999714303421225 |
| ebi-fl187-GCST90094733 | prot-a-2861 | Alpha-2,8-sialyltransferase 8F \|\| id:prot-a-2861 | Chilli pepper liking \|\| id:ebi-fl187-GCST90094733 | Weighted mode | 14 | -0.103043889 | 0.312883585935056 | 0.747147401713089 | -0.716295718 | 0.510207939143414 | 0.902087380638653 | 0.488558667378814 | 1.6656375101755 | 0.999714303421225 |
| ebi-fl187-GCST90094733 | prot-a-289 | Uncharacterized protein C17orf89 \|\| id:prot-a-289 | Chilli pepper liking \|\| id:ebi-fl187-GCST90094733 | Weighted mode | 14 | -0.044013225 | 0.248059608836309 | 0.861905341470504 | -0.530210058 | 0.442183608799597 | 0.956941302300389 | 0.588481341575537 | 1.55610142812119 | 0.999714303421225 |
| ebi-fl187-GCST90094733 | prot-a-2921 | Tachykinin-3 \|\| id:prot-a-2921 | Chilli pepper liking \|\| id:ebi-fl187-GCST90094733 | Weighted mode | 14 | 0.105669870094835 | 0.2227451763973 | 0.643085792229512 | -0.330910676 | 0.542250415833542 | 1.11145489143559 | 0.71826932512017 | 1.71987293970742 | 0.999714303421225 |
| ebi-fl187-GCST90094733 | prot-a-2928 | Tax1-binding protein 1 \|\| id:prot-a-2928 | Chilli pepper liking \|\| id:ebi-fl187-GCST90094733 | Weighted mode | 14 | -0.093124397 | 0.265247200263005 | 0.731148750143871 | -0.613008909 | 0.426760115747149 | 0.911080157819236 | 0.541718432758168 | 1.53228504657969 | 0.999714303421225 |
| ebi-fl187-GCST90094733 | prot-a-296 | Uncharacterized protein C1orf185 \|\| id:prot-a-296 | Chilli pepper liking \|\| id:ebi-fl187-GCST90094733 | Weighted mode | 14 | -0.0261023 | 0.275502848469246 | 0.925962800572752 | -0.566087883 | 0.513883283104174 | 0.974235420329018 | 0.567742173677648 | 1.67177056457771 | 0.999714303421225 |
| ebi-fl187-GCST90094733 | prot-a-2964 | Transforming growth factor beta-2 \|\| id:prot-a-2964 | Chilli pepper liking \|\| id:ebi-fl187-GCST90094733 | Weighted mode | 14 | 0.1523658039939 | 0.234477895142302 | 0.527131810404329 | -0.30721087 | 0.611942478472813 | 1.16458616879527 | 0.735495490262943 | 1.84400987158245 | 0.999714303421225 |
| ebi-fl187-GCST90094733 | prot-a-2977 | Thymocyte nuclear protein 1 \|\| id:prot-a-2977 | Chilli pepper liking \|\| id:ebi-fl187-GCST90094733 | Weighted mode | 14 | -0.236585744 | 0.253554134362807 | 0.367786951813356 | -0.733551848 | 0.260380359063809 | 0.789318199873824 | 0.480200358976808 | 1.29742347960665 | 0.999714303421225 |
| ebi-fl187-GCST90094733 | prot-a-3015 | Transmembrane protein 70, mitochondrial \|\| id:prot-a-3015 | Chilli pepper liking \|\| id:ebi-fl187-GCST90094733 | Weighted mode | 14 | 0.205990786426987 | 0.304154023459745 | 0.510115429795967 | -0.3901511 | 0.802132672408087 | 1.22874188284011 | 0.676954579234897 | 2.23029234302817 | 0.999714303421225 |
| ebi-fl187-GCST90094733 | prot-a-3030 | Tumor necrosis factor alpha-induced protein 3 \|\| id:prot-a-3030 | Chilli pepper liking \|\| id:ebi-fl187-GCST90094733 | Weighted mode | 14 | -0.090530119 | 0.235406223439006 | 0.7067736816168 | -0.551926317 | 0.3708660792864 | 0.913446821697821 | 0.575839492142116 | 1.44898901075011 | 0.999714303421225 |
| ebi-fl187-GCST90094733 | prot-a-3032 | Tumor necrosis factor receptor superfamily member 10A \|\| id:prot-a-3032 | Chilli pepper liking \|\| id:ebi-fl187-GCST90094733 | Weighted mode | 14 | 0.289060063009907 | 0.26788479099124 | 0.300181385596582 | -0.235994127 | 0.814114253352738 | 1.33517192056449 | 0.789785312065383 | 2.25717550102563 | 0.999714303421225 |
| ebi-fl187-GCST90094733 | prot-a-3081 | Tumor protein p53-inducible protein 11 \|\| id:prot-a-3081 | Chilli pepper liking \|\| id:ebi-fl187-GCST90094733 | Weighted mode | 14 | 0.0807855487138274 | 0.209542929001739 | 0.706077869105419 | -0.329918592 | 0.491489689557237 | 1.08413837676628 | 0.718982261864363 | 1.63474967647972 | 0.999714303421225 |
| ebi-fl187-GCST90094733 | prot-a-315 | Complement C4 \|\| id:prot-a-315 | Chilli pepper liking \|\| id:ebi-fl187-GCST90094733 | Weighted mode | 13 | 0.0410676547252615 | 0.225346532182916 | 0.858435049822255 | -0.400611548 | 0.482746857803776 | 1.04192259414786 | 0.669910238236748 | 1.62051963118699 | 0.999714303421225 |
| ebi-fl187-GCST90094733 | prot-a-3157 | UDP-glucuronosyltransferase 1-8 \|\| id:prot-a-3157 | Chilli pepper liking \|\| id:ebi-fl187-GCST90094733 | Weighted mode | 14 | 0.377760114665563 | 0.238661962547837 | 0.137476486308077 | -0.090017332 | 0.845537561259323 | 1.45901290509432 | 0.913915345218818 | 2.32922958167652 | 0.999714303421225 |
| ebi-fl187-GCST90094733 | prot-a-317 | Glutaredoxin-like protein C5orf63 \|\| id:prot-a-317 | Chilli pepper liking \|\| id:ebi-fl187-GCST90094733 | Weighted mode | 14 | 0.068100343493912 | 0.234242505562567 | 0.775847286457359 | -0.391014967 | 0.527215654396543 | 1.07047271806243 | 0.676370032456807 | 1.69420847336127 | 0.999714303421225 |
| ebi-fl187-GCST90094733 | prot-a-3192 | Guanine nucleotide exchange factor VAV3 \|\| id:prot-a-3192 | Chilli pepper liking \|\| id:ebi-fl187-GCST90094733 | Weighted mode | 14 | 0.336644578628958 | 0.31194034892365 | 0.300117340368124 | -0.274758505 | 0.948047662519312 | 1.40024129960328 | 0.759755578047113 | 2.58066640610184 | 0.999714303421225 |
| ebi-fl187-GCST90094733 | prot-a-3233 | Serine/threonine-protein kinase WNK3 \|\| id:prot-a-3233 | Chilli pepper liking \|\| id:ebi-fl187-GCST90094733 | Weighted mode | 14 | 0.211542529644837 | 0.261976726198375 | 0.433913252981645 | -0.301931854 | 0.725016912993651 | 1.23558251339673 | 0.739388449756914 | 2.06476602104578 | 0.999714303421225 |
| ebi-fl187-GCST90094733 | prot-a-3250 | 14-3-3 protein theta \|\| id:prot-a-3250 | Chilli pepper liking \|\| id:ebi-fl187-GCST90094733 | Weighted mode | 14 | 0.162611737687776 | 0.201002379444256 | 0.433070229937318 | -0.231352926 | 0.556576401398518 | 1.17657977940802 | 0.793459384149224 | 1.74468914851408 | 0.999714303421225 |
| ebi-fl187-GCST90094733 | prot-a-3278 | E3 ubiquitin-protein ligase ZNRF3 \|\| id:prot-a-3278 | Chilli pepper liking \|\| id:ebi-fl187-GCST90094733 | Weighted mode | 14 | 0.261594672970722 | 0.207054816463407 | 0.228630890434633 | -0.144232767 | 0.667422113 | 1.29899991593166 | 0.865686221163447 | 1.94920600598523 | 0.999714303421225 |
| ebi-fl187-GCST90094733 | prot-a-355 | Calpain-3 \|\| id:prot-a-355 | Chilli pepper liking \|\| id:ebi-fl187-GCST90094733 | Weighted mode | 14 | 0.128171335559634 | 0.268472170702425 | 0.640996684688935 | -0.398034119 | 0.654376790136387 | 1.13674775132504 | 0.671639111604729 | 1.92394312334659 | 0.999714303421225 |
| ebi-fl187-GCST90094733 | prot-a-385 | Coiled-coil domain-containing protein 90B, mitochondrial \|\| id:prot-a-385 | Chilli pepper liking \|\| id:ebi-fl187-GCST90094733 | Weighted mode | 14 | 0.198994149499206 | 0.265250085426433 | 0.466487974903072 | -0.320896018 | 0.718884316935015 | 1.22017482724519 | 0.725498685918486 | 2.05214239245378 | 0.999714303421225 |
| ebi-fl187-GCST90094733 | prot-a-390 | C-C motif chemokine 14 \|\| id:prot-a-390 | Chilli pepper liking \|\| id:ebi-fl187-GCST90094733 | Weighted mode | 14 | 0.385038963115794 | 0.307967999797142 | 0.233243530952382 | -0.218578316 | 0.988656242718193 | 1.46967158330967 | 0.803660537110287 | 2.68762053510104 | 0.999714303421225 |
| ebi-fl187-GCST90094733 | prot-a-40 | Pituitary adenylate cyclase-activating polypeptide 27 \|\| id:prot-a-40 | Chilli pepper liking \|\| id:ebi-fl187-GCST90094733 | Weighted mode | 14 | -0.018760911 | 0.247097198607227 | 0.940634597196821 | -0.50307142 | 0.465549598113277 | 0.981413979327456 | 0.60467060701893 | 1.59288939736606 | 0.999714303421225 |
| ebi-fl187-GCST90094733 | prot-a-401 | C-C motif chemokine 24 \|\| id:prot-a-401 | Chilli pepper liking \|\| id:ebi-fl187-GCST90094733 | Weighted mode | 14 | -0.138667193 | 0.226385672864482 | 0.550754331670518 | -0.582383112 | 0.305048725433384 | 0.870517694304843 | 0.558565654571697 | 1.35669110675789 | 0.999714303421225 |
| ebi-fl187-GCST90094733 | prot-a-412 | Cerebral cavernous malformations 2 protein \|\| id:prot-a-412 | Chilli pepper liking \|\| id:ebi-fl187-GCST90094733 | Weighted mode | 14 | 0.054118809493318 | 0.262064776957802 | 0.839592329677882 | -0.459528153 | 0.567765772330609 | 1.05561001119561 | 0.631581584869381 | 1.76432075036965 | 0.999714303421225 |
| ebi-fl187-GCST90094733 | prot-a-44 | Adhesion G protein-coupled receptor F5 \|\| id:prot-a-44 | Chilli pepper liking \|\| id:ebi-fl187-GCST90094733 | Weighted mode | 14 | 0.0452791976837064 | 0.213834255936298 | 0.835586464004145 | -0.373835944 | 0.464394339318851 | 1.04631994923773 | 0.68808978786138 | 1.59105026042531 | 0.999714303421225 |
| ebi-fl187-GCST90094733 | prot-a-48 | Adrenomedullin \|\| id:prot-a-48 | Chilli pepper liking \|\| id:ebi-fl187-GCST90094733 | Weighted mode | 14 | 0.114910566053526 | 0.223767576504591 | 0.616199084322603 | -0.323673884 | 0.553495016002524 | 1.12177310848945 | 0.723486144395119 | 1.73932136320616 | 0.999714303421225 |
| ebi-fl187-GCST90094733 | prot-a-502 | Carcinoembryonic antigen-related cell adhesion molecule 1 \|\| id:prot-a-502 | Chilli pepper liking \|\| id:ebi-fl187-GCST90094733 | Weighted mode | 14 | 0.240151447426926 | 0.247983968708333 | 0.35050970910013 | -0.245897131 | 0.72620002609526 | 1.27144169231383 | 0.782002664431357 | 2.06721031843205 | 0.999714303421225 |
| ebi-fl187-GCST90094733 | prot-a-518 | Complement factor B \|\| id:prot-a-518 | Chilli pepper liking \|\| id:ebi-fl187-GCST90094733 | Weighted mode | 14 | 0.302515682305621 | 0.254764302213003 | 0.256300903288886 | -0.19682235 | 0.801853714643107 | 1.3532588984744 | 0.821336530761455 | 2.22967027243065 | 0.999714303421225 |
| ebi-fl187-GCST90094733 | prot-a-525 | Properdin \|\| id:prot-a-525 | Chilli pepper liking \|\| id:ebi-fl187-GCST90094733 | Weighted mode | 14 | -0.026798657 | 0.229254070190849 | 0.908729878392798 | -0.476136634 | 0.422539320666964 | 0.973557240818104 | 0.621178602032813 | 1.52583121512498 | 0.999714303421225 |
| ebi-fl187-GCST90094733 | prot-a-533 | Chondroadherin \|\| id:prot-a-533 | Chilli pepper liking \|\| id:ebi-fl187-GCST90094733 | Weighted mode | 14 | 0.129376987608794 | 0.308275413559395 | 0.68157208947773 | -0.474842823 | 0.733596798185207 | 1.13811910009941 | 0.621982810194796 | 2.08255769255975 | 0.999714303421225 |
| ebi-fl187-GCST90094733 | prot-a-535 | Coiled-coil-helix-coiled-coil-helix domain-containing protein 2 \|\| id:prot-a-535 | Chilli pepper liking \|\| id:ebi-fl187-GCST90094733 | Weighted mode | 14 | 0.106723829681123 | 0.28356389070138 | 0.712715792835945 | -0.449061396 | 0.662509055455828 | 1.11262693750924 | 0.638226912851584 | 1.93965293086766 | 0.999714303421225 |
| ebi-fl187-GCST90094733 | prot-a-591 | Caseinolytic peptidase B protein homolog \|\| id:prot-a-591 | Chilli pepper liking \|\| id:ebi-fl187-GCST90094733 | Weighted mode | 14 | 0.117264904448885 | 0.295864095060771 | 0.698277976271834 | -0.462628722 | 0.697158530767996 | 1.12441725337421 | 0.629626355613108 | 2.00803881288364 | 0.999714303421225 |
| ebi-fl187-GCST90094733 | prot-a-605 | Ciliary neurotrophic factor receptor subunit alpha \|\| id:prot-a-605 | Chilli pepper liking \|\| id:ebi-fl187-GCST90094733 | Weighted mode | 14 | 0.200587243748414 | 0.225317014087258 | 0.389512825297364 | -0.241034104 | 0.64220859135944 | 1.22212022993898 | 0.785814826610475 | 1.9006740593945 | 0.999714303421225 |
| ebi-fl187-GCST90094733 | prot-a-606 | Ciliary neurotrophic factor receptor subunit alpha \|\| id:prot-a-606 | Chilli pepper liking \|\| id:ebi-fl187-GCST90094733 | Weighted mode | 14 | 0.269230774469152 | 0.226883522872979 | 0.256600255390476 | -0.17546093 | 0.713922479300191 | 1.30895718009205 | 0.839070178701312 | 2.04198521507038 | 0.999714303421225 |
| ebi-fl187-GCST90094733 | prot-a-707 | Cancer/testis antigen 55 \|\| id:prot-a-707 | Chilli pepper liking \|\| id:ebi-fl187-GCST90094733 | Weighted mode | 14 | 0.0530013029792829 | 0.262108636171027 | 0.842881753833722 | -0.460731624 | 0.566734229874495 | 1.05443101902023 | 0.630821952207662 | 1.76250171697583 | 0.999714303421225 |
| ebi-fl187-GCST90094733 | prot-a-719 | Dipeptidyl peptidase 1 \|\| id:prot-a-719 | Chilli pepper liking \|\| id:ebi-fl187-GCST90094733 | Weighted mode | 14 | 0.145182898189987 | 0.260127798801479 | 0.586245550441061 | -0.364667587 | 0.655033383840886 | 1.15625102714993 | 0.694427448907278 | 1.92520678710063 | 0.999714303421225 |
| ebi-fl187-GCST90094733 | prot-a-752 | Cytochrome b5 \|\| id:prot-a-752 | Chilli pepper liking \|\| id:ebi-fl187-GCST90094733 | Weighted mode | 14 | -0.0282387 | 0.219828262319298 | 0.899752556 | -0.459102094 | 0.402624694377947 | 0.972156285623768 | 0.631850733492183 | 1.49574542464228 | 0.999714303421225 |
| ebi-fl187-GCST90094733 | prot-a-755 | Cytoglobin \|\| id:prot-a-755 | Chilli pepper liking \|\| id:ebi-fl187-GCST90094733 | Weighted mode | 14 | 0.266040546157395 | 0.247288710288972 | 0.301564786683563 | -0.218645326 | 0.75072641832378 | 1.30478796175248 | 0.803606686005824 | 2.11853840290453 | 0.999714303421225 |
| ebi-fl187-GCST90094733 | prot-a-807 | Desmin \|\| id:prot-a-807 | Chilli pepper liking \|\| id:ebi-fl187-GCST90094733 | Weighted mode | 14 | 0.119916619656227 | 0.249491127378649 | 0.638756746840208 | -0.36908599 | 0.60891922931838 | 1.12740284442352 | 0.69136595733139 | 1.83844338896917 | 0.999714303421225 |
| ebi-fl187-GCST90094733 | prot-a-82 | Ameloblastin \|\| id:prot-a-82 | Chilli pepper liking \|\| id:ebi-fl187-GCST90094733 | Weighted mode | 14 | -0.039103958 | 0.231277688112481 | 0.868337820092468 | -0.492408227 | 0.414200310616707 | 0.96165073258237 | 0.61115282594864 | 1.5131601985817 | 0.999714303421225 |
| ebi-fl187-GCST90094733 | prot-a-84 | Muellerian-inhibiting factor \|\| id:prot-a-84 | Chilli pepper liking \|\| id:ebi-fl187-GCST90094733 | Weighted mode | 14 | -0.00661113 | 0.211782438891637 | 0.975570969006412 | -0.421704711 | 0.408482449939952 | 0.993410675154849 | 0.655927699351526 | 1.5045328478844 | 0.999714303421225 |
| ebi-fl187-GCST90094733 | prot-a-858 | Inactive dipeptidyl peptidase 10 \|\| id:prot-a-858 | Chilli pepper liking \|\| id:ebi-fl187-GCST90094733 | Weighted mode | 14 | 0.0793864311062003 | 0.235309552464472 | 0.741222904561755 | -0.381820292 | 0.540593153936565 | 1.08262260029628 | 0.682617714235939 | 1.71702502034274 | 0.999714303421225 |
| ebi-fl187-GCST90094733 | prot-a-863 | Dihydropyrimidinase-related protein 3 \|\| id:prot-a-863 | Chilli pepper liking \|\| id:ebi-fl187-GCST90094733 | Weighted mode | 14 | -0.166721378 | 0.233178040963983 | 0.487254097740919 | -0.623750338 | 0.29030758233723 | 0.846435414054159 | 0.535930742377829 | 1.33683861273989 | 0.999714303421225 |
| ebi-fl187-GCST90094733 | prot-a-864 | Dihydropyrimidinase-related protein 5 \|\| id:prot-a-864 | Chilli pepper liking \|\| id:ebi-fl187-GCST90094733 | Weighted mode | 14 | -0.008842729 | 0.25910878369459 | 0.973293938288646 | -0.516695945 | 0.49901048658824 | 0.991196252492049 | 0.59648812496615 | 1.64709064578618 | 0.999714303421225 |
| ebi-fl187-GCST90094733 | prot-a-884 | Endothelin-converting enzyme 1 \|\| id:prot-a-884 | Chilli pepper liking \|\| id:ebi-fl187-GCST90094733 | Weighted mode | 14 | -0.136989393 | 0.258187209127647 | 0.604654595350925 | -0.643036323 | 0.369057536750305 | 0.871979475047375 | 0.525693822076488 | 1.44637082075746 | 0.999714303421225 |
| ebi-fl187-GCST90094733 | prot-a-893 | Early endosome antigen 1 \|\| id:prot-a-893 | Chilli pepper liking \|\| id:ebi-fl187-GCST90094733 | Weighted mode | 14 | -0.042957278 | 0.243939130791518 | 0.862930284843685 | -0.521077974 | 0.435163418477015 | 0.957952314951982 | 0.593880015443616 | 1.54521555509218 | 0.999714303421225 |
| ebi-fl187-GCST90094733 | prot-a-897 | Ephrin-A2 \|\| id:prot-a-897 | Chilli pepper liking \|\| id:ebi-fl187-GCST90094733 | Weighted mode | 14 | 0.3073054051439 | 0.20565747092217 | 0.158980200267955 | -0.095783238 | 0.710394048151354 | 1.35975618119683 | 0.908660958013721 | 2.03479290707576 | 0.999714303421225 |
| ebi-fl187-GCST90094733 | prot-a-90 | Anaphase-promoting complex subunit 10 \|\| id:prot-a-90 | Chilli pepper liking \|\| id:ebi-fl187-GCST90094733 | Weighted mode | 14 | 0.221764173880204 | 0.243925818304684 | 0.379819811550115 | -0.25633043 | 0.699858777757384 | 1.24827696683722 | 0.773886211321139 | 2.01346834087697 | 0.999714303421225 |
| ebi-fl187-GCST90094733 | prot-a-914 | Histone-lysine N-methyltransferase EHMT2 \|\| id:prot-a-914 | Chilli pepper liking \|\| id:ebi-fl187-GCST90094733 | Weighted mode | 14 | -0.249609474 | 0.247725208504193 | 0.332030899808881 | -0.735150882 | 0.235931935121403 | 0.779104984774494 | 0.479433115586925 | 1.26608813109909 | 0.999714303421225 |
| ebi-fl187-GCST90094733 | prot-a-932 | RNA polymerase II elongation factor ELL \|\| id:prot-a-932 | Chilli pepper liking \|\| id:ebi-fl187-GCST90094733 | Weighted mode | 14 | -0.020504127 | 0.229003134043448 | 0.930020336086033 | -0.46935027 | 0.428342015409981 | 0.97970465291629 | 0.625408482931886 | 1.53471088598964 | 0.999714303421225 |
| ebi-fl187-GCST90094733 | prot-a-991 | Estrogen receptor \|\| id:prot-a-991 | Chilli pepper liking \|\| id:ebi-fl187-GCST90094733 | Weighted mode | 14 | 0.118038738976911 | 0.243388079196175 | 0.635759429150922 | -0.359001896 | 0.595079374201414 | 1.12528770301733 | 0.698373027062147 | 1.81317485855499 | 0.999714303421225 |
| ebi-fl187-GCST90094733 | prot-a-992 | Persulfide dioxygenase ETHE1, mitochondrial \|\| id:prot-a-992 | Chilli pepper liking \|\| id:ebi-fl187-GCST90094733 | Weighted mode | 14 | -0.118434261 | 0.241006608599521 | 0.631325327757578 | -0.590807214 | 0.35393869173455 | 0.888310210255247 | 0.553880004552923 | 1.42466783988828 | 0.999714303421225 |
| ebi-fl187-GCST90094733 | prot-a-993 | Ethanolamine kinase 1 \|\| id:prot-a-993 | Chilli pepper liking \|\| id:ebi-fl187-GCST90094733 | Weighted mode | 14 | -0.185355477 | 0.30356003115349 | 0.55198694065798 | -0.780333138 | 0.409622183733797 | 0.830808897627471 | 0.458253324100018 | 1.50624859237534 | 0.999714303421225 |
| ebi-fl187-GCST90094733 | prot-b-10 | coagulation factor III, tissue factor \|\| id:prot-b-10 | Chilli pepper liking \|\| id:ebi-fl187-GCST90094733 | Weighted mode | 10 | 0.252622717002451 | 0.283663534198147 | 0.396341441619201 | -0.30335781 | 0.80860324403082 | 1.28739747202016 | 0.738334865483717 | 2.24477039950982 | 0.999714303421225 |
| ebi-fl187-GCST90094733 | prot-b-28 | prolactin \|\| id:prot-b-28 | Chilli pepper liking \|\| id:ebi-fl187-GCST90094733 | Weighted mode | 10 | -0.272861714 | 0.255618433304691 | 0.313560625687953 | -0.773873843 | 0.228150415658042 | 0.761198043673595 | 0.46122289808345 | 1.25627427454322 | 0.999714303421225 |
| ebi-fl187-GCST90094733 | prot-b-54 | matrix metallopeptidase 7 \|\| id:prot-b-54 | Chilli pepper liking \|\| id:ebi-fl187-GCST90094733 | Weighted mode | 10 | -0.190384064 | 0.323493112078944 | 0.570651932828056 | -0.824430564 | 0.443662435485992 | 0.826641589537086 | 0.438484610369934 | 1.55840433482008 | 0.999714303421225 |
| ebi-fl187-GCST90094733 | ukb-rsfMRI191-edge-pheno716 | Net100_Pair10_33 (OccipitalPrecuneus)&(Temporal\|Frontal\|Supp_Motor_Area) (Default_mode\|Central_executive)&(Default_mode\|Salience) \|\| id:ukb-rsfMRI191-edge-pheno716 | Chilli pepper liking \|\| id:ebi-fl187-GCST90094733 | Weighted mode | 13 | -0.004142497 | 0.0805688248880324 | 0.959840286468785 | -0.162057394 | 0.153772399523279 | 0.99586607104902 | 0.850392395686268 | 1.16622542310749 | 0.999714303421225 |
| ebi-fl187-GCST90094733 | ukb-rsfMRI191-edge-pheno903 | Net100_Pair27_38 (Precentral\|Frontal\|Supp_Motor_Area)&(Subcortical) (Attention\|Salience\|Motor)&(Subcortical-cerebellum) \|\| id:ukb-rsfMRI191-edge-pheno903 | Chilli pepper liking \|\| id:ebi-fl187-GCST90094733 | Weighted mode | 13 | 0.0415534373322586 | 0.0756449116220387 | 0.592859831180631 | -0.106710589 | 0.189817464111454 | 1.04242886498076 | 0.898785753435464 | 1.20902888635195 | 0.999714303421225 |
| ebi-fl187-GCST90094733 | ukb-rsfMRI191-node-pheno23 | Net100_Node2 RolandicOper\|SupraMarginal\|Insula Salience\|Motor \|\| id:ukb-rsfMRI191-node-pheno23 | Chilli pepper liking \|\| id:ebi-fl187-GCST90094733 | Weighted mode | 13 | -0.116041996 | 0.0922488558881133 | 0.232346749620856 | -0.296849753 | 0.0647657619905376 | 0.890437828085784 | 0.743155660815355 | 1.0669090844524 | 0.999714303421225 |
| ebi-fl187-GCST90094733 | ukb-rsfMRI191-node-pheno55 | Net100_Node34 Parietal\|Postcentral\|Precuneus Attention \|\| id:ukb-rsfMRI191-node-pheno55 | Chilli pepper liking \|\| id:ebi-fl187-GCST90094733 | Weighted mode | 13 | 0.0349726968971408 | 0.0902290961163339 | 0.70510014958275 | -0.141876331 | 0.211821725285155 | 1.03559143355419 | 0.86772856054812 | 1.23592753080917 | 0.999714303421225 |
| ebi-fl187-GCST90094734 | ebi-a-GCST90001414 | IgD- CD24- B cell Absolute Count \|\| id:ebi-a-GCST90001414 | Chips liking \|\| id:ebi-fl187-GCST90094734 | Inverse variance weighted | 4 | 0.350078033808695 | 0.331672089316156 | 0.291199268077743 | -0.299999261 | 1.00015532886836 | 1.41917828815953 | 0.740818767960662 | 2.71870408889311 | 0.998735809224318 |
| ebi-fl187-GCST90094734 | ebi-a-GCST90001422 | CD20- CD38- B cell %B cell \|\| id:ebi-a-GCST90001422 | Chips liking \|\| id:ebi-fl187-GCST90094734 | Inverse variance weighted | 4 | -0.646465685 | 0.308338385832708 | 0.0360280150628518 | -1.250808921 | -0.042122449 | 0.523894115299111 | 0.286273130725043 | 0.958752375222576 | 0.998735809224318 |
| ebi-fl187-GCST90094734 | ebi-a-GCST90001444 | CD20- CD38- B cell %lymphocyte \|\| id:ebi-a-GCST90001444 | Chips liking \|\| id:ebi-fl187-GCST90094734 | Inverse variance weighted | 4 | -0.678791634 | 0.30685117876991 | 0.0269583735866783 | -1.280219944 | -0.077363323 | 0.507229541190593 | 0.277976154466176 | 0.925553517173091 | 0.998735809224318 |
| ebi-fl187-GCST90094734 | ebi-a-GCST90001459 | Myeloid Dendritic Cell %Dendritic Cell \|\| id:ebi-a-GCST90001459 | Chips liking \|\| id:ebi-fl187-GCST90094734 | Inverse variance weighted | 4 | 0.18022307164406 | 0.343907860942784 | 0.600247490775226 | -0.493836336 | 0.854282479091917 | 1.19748445815686 | 0.61028065601067 | 2.34968782543575 | 0.998735809224318 |
| ebi-fl187-GCST90094734 | ebi-a-GCST90001476 | HLA DR++ monocyte %leukocyte \|\| id:ebi-a-GCST90001476 | Chips liking \|\| id:ebi-fl187-GCST90094734 | Inverse variance weighted | 4 | 0.00859005703832313 | 0.333379937212193 | 0.979443527344112 | -0.64483462 | 0.662014733974222 | 1.00862705744761 | 0.524749318026852 | 1.93869435569875 | 0.998955228396052 |
| ebi-fl187-GCST90094734 | ebi-a-GCST90001478 | CD4 regulatory T cell %CD4+ T cell \|\| id:ebi-a-GCST90001478 | Chips liking \|\| id:ebi-fl187-GCST90094734 | Inverse variance weighted | 4 | 0.096144850576038 | 0.482231699049791 | 0.841969568162054 | -0.84902928 | 1.04131898071363 | 1.10091852112693 | 0.427830033800197 | 2.83295115911926 | 0.998735809224318 |
| ebi-fl187-GCST90094734 | ebi-a-GCST90001485 | CD39+ resting CD4 regulatory T cell %CD4 regulatory T cell \|\| id:ebi-a-GCST90001485 | Chips liking \|\| id:ebi-fl187-GCST90094734 | Inverse variance weighted | 4 | -0.305834547 | 0.331769314571571 | 0.356617823147942 | -0.956102403 | 0.344433309687697 | 0.736508467005029 | 0.384388160067731 | 1.41118998533804 | 0.998735809224318 |
| ebi-fl187-GCST90094734 | ebi-a-GCST90001515 | Immature Myeloid-Derived Suppressor Cells Absolute Count \|\| id:ebi-a-GCST90001515 | Chips liking \|\| id:ebi-fl187-GCST90094734 | Inverse variance weighted | 4 | -0.311085834 | 0.429745792061959 | 0.46913733765101 | -1.153387587 | 0.531215918320322 | 0.732650986716823 | 0.315565949685134 | 1.70099932794625 | 0.998735809224318 |
| ebi-fl187-GCST90094734 | ebi-a-GCST90001517 | CD33+ HLA DR+ Absolute Count \|\| id:ebi-a-GCST90001517 | Chips liking \|\| id:ebi-fl187-GCST90094734 | Inverse variance weighted | 4 | 0.313989863506283 | 0.418472753934712 | 0.453060004532943 | -0.506216734 | 1.13419646121832 | 1.36887586087549 | 0.602771716096468 | 3.10867459844071 | 0.998735809224318 |
| ebi-fl187-GCST90094734 | ebi-a-GCST90001518 | CD33+ HLA DR+ CD14- Absolute Count \|\| id:ebi-a-GCST90001518 | Chips liking \|\| id:ebi-fl187-GCST90094734 | Inverse variance weighted | 4 | 0.421403909457333 | 0.425882941400452 | 0.322426907483268 | -0.413326656 | 1.25613447460222 | 1.52409975240642 | 0.661446182377065 | 3.51182018609206 | 0.998735809224318 |
| ebi-fl187-GCST90094734 | ebi-a-GCST90001520 | CD33+ HLA DR+ CD14dim Absolute Count \|\| id:ebi-a-GCST90001520 | Chips liking \|\| id:ebi-fl187-GCST90094734 | Inverse variance weighted | 4 | 0.0424672406849076 | 0.417967861034007 | 0.919070939520746 | -0.776749767 | 0.861684248311561 | 1.04338187533819 | 0.459898361606645 | 2.36714419677661 | 0.998735809224318 |
| ebi-fl187-GCST90094734 | ebi-a-GCST90001531 | CD33dim HLA DR- Absolute Count \|\| id:ebi-a-GCST90001531 | Chips liking \|\| id:ebi-fl187-GCST90094734 | Inverse variance weighted | 4 | -0.266364819 | 0.430949934341677 | 0.536517582475856 | -1.111026691 | 0.578297052022487 | 0.766159566722525 | 0.329220779622299 | 1.78299948853134 | 0.998735809224318 |
| ebi-fl187-GCST90094734 | ebi-a-GCST90001532 | Basophil Absolute Count \|\| id:ebi-a-GCST90001532 | Chips liking \|\| id:ebi-fl187-GCST90094734 | Inverse variance weighted | 4 | -0.287882917 | 0.429567346085614 | 0.50274976901966 | -1.129834915 | 0.554069081352784 | 0.749849381733504 | 0.323086588671616 | 1.74032013398616 | 0.998735809224318 |
| ebi-fl187-GCST90094734 | ebi-a-GCST90001588 | T/B cell \|\| id:ebi-a-GCST90001588 | Chips liking \|\| id:ebi-fl187-GCST90094734 | Inverse variance weighted | 4 | -0.002141443 | 0.328594938953695 | 0.99480024808929 | -0.646187523 | 0.641904637413763 | 0.997860848317621 | 0.524039862917413 | 1.90009642980554 | 0.999107451094609 |
| ebi-fl187-GCST90094734 | ebi-a-GCST90001624 | HLA DR+ CD4+ T cell Absolute Count \|\| id:ebi-a-GCST90001624 | Chips liking \|\| id:ebi-fl187-GCST90094734 | Inverse variance weighted | 4 | 0.398735387212967 | 0.458979223185241 | 0.384987239579798 | -0.50086389 | 1.29833466465604 | 1.4899393094469 | 0.606006910065123 | 3.66319114347481 | 0.998735809224318 |
| ebi-fl187-GCST90094734 | ebi-a-GCST90001631 | CD8+ Natural Killer T %T cell \|\| id:ebi-a-GCST90001631 | Chips liking \|\| id:ebi-fl187-GCST90094734 | Inverse variance weighted | 4 | 0.503355784096048 | 0.319871673435594 | 0.115575817479889 | -0.123592696 | 1.13030426402981 | 1.65426331707382 | 0.883739718453661 | 3.09659854035356 | 0.998735809224318 |
| ebi-fl187-GCST90094734 | ebi-a-GCST90001632 | CD8+ Natural Killer T %lymphocyte \|\| id:ebi-a-GCST90001632 | Chips liking \|\| id:ebi-fl187-GCST90094734 | Inverse variance weighted | 4 | 0.495371302857364 | 0.321832719013623 | 0.123750638232453 | -0.135420826 | 1.12616343212407 | 1.64110747383901 | 0.873348306312587 | 3.08380255760903 | 0.998735809224318 |
| ebi-fl187-GCST90094734 | ebi-a-GCST90001646 | Natural Killer %CD3- lymphocyte \|\| id:ebi-a-GCST90001646 | Chips liking \|\| id:ebi-fl187-GCST90094734 | Inverse variance weighted | 4 | 0.0263729573747356 | 0.317014258123531 | 0.933699106491826 | -0.594974989 | 0.647720903296856 | 1.02672380128862 | 0.551576361421333 | 1.91118009737787 | 0.998735809224318 |
| ebi-fl187-GCST90094734 | ebi-a-GCST90001684 | CD127- CD8+ T cell Absolute Count \|\| id:ebi-a-GCST90001684 | Chips liking \|\| id:ebi-fl187-GCST90094734 | Inverse variance weighted | 4 | 0.663949137316315 | 0.340767112833778 | 0.0513676479902771 | -0.003954404 | 1.33185267847052 | 1.94244820209566 | 0.996053404521107 | 3.78805493831804 | 0.998735809224318 |
| ebi-fl187-GCST90094734 | ebi-a-GCST90001687 | CD28- CD8+ T cell Absolute Count \|\| id:ebi-a-GCST90001687 | Chips liking \|\| id:ebi-fl187-GCST90094734 | Inverse variance weighted | 4 | 0.488982948918245 | 0.329302646917156 | 0.137569150658264 | -0.156450239 | 1.13441613687587 | 1.63065691492013 | 0.855174070927423 | 3.10935757359101 | 0.998735809224318 |
| ebi-fl187-GCST90094734 | ebi-a-GCST90001701 | BAFF-R on CD20- CD38- B cell \|\| id:ebi-a-GCST90001701 | Chips liking \|\| id:ebi-fl187-GCST90094734 | Inverse variance weighted | 4 | -0.137948924 | 0.420028740270376 | 0.742588569946259 | -0.961205255 | 0.685307406755112 | 0.871143184966543 | 0.382431680360755 | 1.98438175414175 | 0.998735809224318 |
| ebi-fl187-GCST90094734 | ebi-a-GCST90001732 | CD19 on IgD- CD27- B cell \|\| id:ebi-a-GCST90001732 | Chips liking \|\| id:ebi-fl187-GCST90094734 | Inverse variance weighted | 4 | -0.142127042 | 0.320468086127872 | 0.657405370635394 | -0.77024449 | 0.485990407184349 | 0.867511039471717 | 0.46289987988117 | 1.6257844002865 | 0.998735809224318 |
| ebi-fl187-GCST90094734 | ebi-a-GCST90001798 | CD27 on CD24+ CD27+ B cell \|\| id:ebi-a-GCST90001798 | Chips liking \|\| id:ebi-fl187-GCST90094734 | Inverse variance weighted | 4 | 0.0223197360428748 | 0.311764339408006 | 0.942926853263081 | -0.588738369 | 0.633377841282566 | 1.02257068491108 | 0.555027082462335 | 1.88396357345405 | 0.998735809224318 |
| ebi-fl187-GCST90094734 | ebi-a-GCST90001836 | CD66b on CD66b++ myeloid cell \|\| id:ebi-a-GCST90001836 | Chips liking \|\| id:ebi-fl187-GCST90094734 | Inverse variance weighted | 4 | 0.133924178369035 | 0.523177044178197 | 0.797964334471252 | -0.891502828 | 1.1593511849583 | 1.14330612902278 | 0.410039071197595 | 3.18786427069812 | 0.998735809224318 |
| ebi-fl187-GCST90094734 | ebi-a-GCST90001943 | CD25 on activated & secreting CD4 regulatory T cell \|\| id:ebi-a-GCST90001943 | Chips liking \|\| id:ebi-fl187-GCST90094734 | Inverse variance weighted | 4 | -0.246733211 | 0.415810543839215 | 0.552927593625042 | -1.061721877 | 0.568255454780173 | 0.781349120964663 | 0.345859769316579 | 1.76518491884331 | 0.998735809224318 |
| ebi-fl187-GCST90094734 | ebi-a-GCST90001960 | CD25 on CD4+ T cell \|\| id:ebi-a-GCST90001960 | Chips liking \|\| id:ebi-fl187-GCST90094734 | Inverse variance weighted | 4 | 0.0672029808439344 | 0.528557682011515 | 0.898826362791398 | -0.968770076 | 1.1031760375865 | 1.06951254670281 | 0.379549568307942 | 3.01372253604217 | 0.998735809224318 |
| ebi-fl187-GCST90094734 | ebi-a-GCST90001988 | HLA DR on CD14+ CD16- monocyte \|\| id:ebi-a-GCST90001988 | Chips liking \|\| id:ebi-fl187-GCST90094734 | Inverse variance weighted | 4 | 0.0635317368563192 | 0.327507487110163 | 0.846187156156925 | -0.578382938 | 0.705446411592239 | 1.0655933038464 | 0.560804489436283 | 2.02475035523285 | 0.998735809224318 |
| ebi-fl187-GCST90094734 | ebi-a-GCST90001991 | HLA DR on CD14+ monocyte \|\| id:ebi-a-GCST90001991 | Chips liking \|\| id:ebi-fl187-GCST90094734 | Inverse variance weighted | 4 | 0.0252975887354221 | 0.330175367424488 | 0.938926927263112 | -0.621846131 | 0.672441308887419 | 1.02562028815953 | 0.536952237615637 | 1.95901404593346 | 0.998735809224318 |
| ebi-fl187-GCST90094734 | ebi-a-GCST90002019 | CD14 on Monocytic Myeloid-Derived Suppressor Cells \|\| id:ebi-a-GCST90002019 | Chips liking \|\| id:ebi-fl187-GCST90094734 | Inverse variance weighted | 4 | -0.552025386 | 0.516872847431453 | 0.285515986601201 | -1.565096167 | 0.461045394695769 | 0.575782446736343 | 0.209067906723881 | 1.58573083341549 | 0.998735809224318 |
| ebi-fl187-GCST90094734 | ebi-a-GCST90002037 | CD80 on plasmacytoid Dendritic Cell \|\| id:ebi-a-GCST90002037 | Chips liking \|\| id:ebi-fl187-GCST90094734 | Inverse variance weighted | 4 | -0.650415792 | 0.347588501446185 | 0.0613141009756848 | -1.331689255 | 0.0308576709579796 | 0.521828759487992 | 0.264030868981519 | 1.03133870399009 | 0.998735809224318 |
| ebi-fl187-GCST90094734 | ebi-a-GCST90002042 | CD45 on CD33+ HLA DR+ CD14- \|\| id:ebi-a-GCST90002042 | Chips liking \|\| id:ebi-fl187-GCST90094734 | Inverse variance weighted | 4 | -0.188973582 | 0.632254353327673 | 0.765025090288603 | -1.428192114 | 1.05024495101877 | 0.827808375858559 | 0.239741956808413 | 2.85835118835377 | 0.998735809224318 |
| ebi-fl187-GCST90094734 | ebi-a-GCST90002056 | CD8 on Effector Memory CD8+ T cell \|\| id:ebi-a-GCST90002056 | Chips liking \|\| id:ebi-fl187-GCST90094734 | Inverse variance weighted | 4 | -0.064447554 | 0.339259968493614 | 0.849336477362687 | -0.729397093 | 0.600501983837203 | 0.937585285185833 | 0.482199624162522 | 1.82303370419202 | 0.998735809224318 |
| ebi-fl187-GCST90094734 | ebi-a-GCST90002058 | CD8 on CD8+ T cell \|\| id:ebi-a-GCST90002058 | Chips liking \|\| id:ebi-fl187-GCST90094734 | Inverse variance weighted | 4 | -0.007747389 | 0.38697455026604 | 0.984027092146056 | -0.766217507 | 0.750722729920394 | 0.992282545061568 | 0.464767728346826 | 2.11853058889472 | 0.998955228396052 |
| ebi-fl187-GCST90094734 | ebi-a-GCST90002063 | CD4 on CD4 regulatory T cell \|\| id:ebi-a-GCST90002063 | Chips liking \|\| id:ebi-fl187-GCST90094734 | Inverse variance weighted | 4 | -0.232607203 | 0.338824099416403 | 0.492389568759541 | -0.896702438 | 0.431488031790488 | 0.792464790137984 | 0.407912561366556 | 1.53954671438546 | 0.998735809224318 |
| ebi-fl187-GCST90094734 | ebi-a-GCST90002066 | CD4 on activated CD4 regulatory T cell \|\| id:ebi-a-GCST90002066 | Chips liking \|\| id:ebi-fl187-GCST90094734 | Inverse variance weighted | 4 | -0.117673347 | 0.338942037938383 | 0.728457312750959 | -0.781999742 | 0.546653046990336 | 0.888986394936417 | 0.457490233640578 | 1.72746159867302 | 0.998735809224318 |
| ebi-fl187-GCST90094734 | ebi-a-GCST90002070 | CD4 on activated & secreting CD4 regulatory T cell \|\| id:ebi-a-GCST90002070 | Chips liking \|\| id:ebi-fl187-GCST90094734 | Inverse variance weighted | 4 | -0.22301246 | 0.340451882294911 | 0.512436593788058 | -0.890298149 | 0.444273229120029 | 0.800104879783225 | 0.410533334205257 | 1.5593564890223 | 0.998735809224318 |
| ebi-fl187-GCST90094734 | ebi-a-GCST90002081 | SSC-A on CD4+ T cell \|\| id:ebi-a-GCST90002081 | Chips liking \|\| id:ebi-fl187-GCST90094734 | Inverse variance weighted | 4 | -0.128810505 | 0.324979196515735 | 0.691835496648172 | -0.76576973 | 0.508148720153576 | 0.879140542484257 | 0.464975887216315 | 1.66221112683195 | 0.998735809224318 |
| ebi-fl187-GCST90094734 | ebi-a-GCST90002094 | CD11b on Monocytic Myeloid-Derived Suppressor Cells \|\| id:ebi-a-GCST90002094 | Chips liking \|\| id:ebi-fl187-GCST90094734 | Inverse variance weighted (multiplicative random effects) | 4 | -0.160038581 | 0.919153752909318 | 0.86177497767255 | -1.961579937 | 1.64150277452908 | 0.852110912893316 | 0.140636049220497 | 5.16292239362806 | 0.998735809224318 |
| ebi-fl187-GCST90094734 | ebi-a-GCST90002104 | HLA DR on myeloid Dendritic Cell \|\| id:ebi-a-GCST90002104 | Chips liking \|\| id:ebi-fl187-GCST90094734 | Inverse variance weighted | 4 | -0.050330354 | 0.394935767651653 | 0.898592680897918 | -0.824404458 | 0.723743750854054 | 0.95091523419953 | 0.438496057389448 | 2.06213891184351 | 0.998735809224318 |
| ebi-fl187-GCST90094734 | ebi-a-GCST90002105 | HLA DR on plasmacytoid Dendritic Cell \|\| id:ebi-a-GCST90002105 | Chips liking \|\| id:ebi-fl187-GCST90094734 | Inverse variance weighted | 4 | 0.196444711320567 | 0.452464506786174 | 0.664167890903148 | -0.690385722 | 1.08327514462147 | 1.21706802893387 | 0.501382637459216 | 2.95433961287394 | 0.998735809224318 |
| ebi-fl187-GCST90094734 | ebi-a-GCST90002106 | HLA DR on Dendritic Cell \|\| id:ebi-a-GCST90002106 | Chips liking \|\| id:ebi-fl187-GCST90094734 | Inverse variance weighted | 4 | 0.10710375026286 | 0.427364496020094 | 0.802112150554168 | -0.730530662 | 0.944738162462244 | 1.11304972769086 | 0.481653327173581 | 2.5721398076551 | 0.998735809224318 |
| ebi-fl187-GCST90094734 | ebi-a-GCST90002111 | HLA DR on CD33dim HLA DR+ CD11b- \|\| id:ebi-a-GCST90002111 | Chips liking \|\| id:ebi-fl187-GCST90094734 | Inverse variance weighted | 4 | 0.553645852132199 | 0.605170151398782 | 0.360265229062806 | -0.632487645 | 1.73977934887381 | 1.73958373549596 | 0.531268548457538 | 5.69608643611235 | 0.998735809224318 |
| ebi-fl187-GCST90094734 | ebi-a-GCST90002112 | HLA DR on CD33- HLA DR+ \|\| id:ebi-a-GCST90002112 | Chips liking \|\| id:ebi-fl187-GCST90094734 | Inverse variance weighted | 4 | 0.183262212677756 | 0.451003333861426 | 0.684490326025111 | -0.700704322 | 1.06722874704615 | 1.20112931812716 | 0.496235671132084 | 2.90731143042035 | 0.998735809224318 |
| ebi-fl187-GCST90094734 | ebi-a-GCST90002115 | HLA DR on HLA DR+ CD8+ T cell \|\| id:ebi-a-GCST90002115 | Chips liking \|\| id:ebi-fl187-GCST90094734 | Inverse variance weighted | 4 | 0.213003436268392 | 0.431019038763238 | 0.621175052875078 | -0.63179388 | 1.05780075224434 | 1.23738890323667 | 0.531637251811855 | 2.88003011947537 | 0.998735809224318 |
| ebi-fl187-GCST90094734 | ebi-a-GCST90002116 | HLA DR on B cell \|\| id:ebi-a-GCST90002116 | Chips liking \|\| id:ebi-fl187-GCST90094734 | Inverse variance weighted | 4 | -0.217497519 | 0.340741019386936 | 0.523273585922788 | -0.885349917 | 0.450354878635557 | 0.804529600663378 | 0.412569782688453 | 1.56886884474608 | 0.998735809224318 |
| ebi-fl187-GCST90094734 | ebi-cfmet338-GCST90026023 | Sphingomyelin (d18:1/24:1, d18:2/24:0) levels \|\| id:ebi-cfmet338-GCST90026023 | Chips liking \|\| id:ebi-fl187-GCST90094734 | Inverse variance weighted | 4 | -0.106352488 | 0.161703379550734 | 0.510730226792361 | -0.423291112 | 0.210586136182135 | 0.899107667785963 | 0.654887959840576 | 1.23440137526472 | 0.998735809224318 |
| ebi-fl187-GCST90094734 | ebi-cfmet338-GCST90026046 | 1-arachidonoyl-gpc (20:4n6) levels \|\| id:ebi-cfmet338-GCST90026046 | Chips liking \|\| id:ebi-fl187-GCST90094734 | Inverse variance weighted | 4 | 0.0289874154532711 | 0.210984482656881 | 0.890721570737647 | -0.384542171 | 0.442517001460759 | 1.02941163971473 | 0.68076223786701 | 1.55662030740781 | 0.998735809224318 |
| ebi-fl187-GCST90094734 | ebi-cfmet338-GCST90026047 | 1-linoleoyl-gpc (18:2) levels \|\| id:ebi-cfmet338-GCST90026047 | Chips liking \|\| id:ebi-fl187-GCST90094734 | Inverse variance weighted | 4 | 0.0963584534187068 | 0.229180778514652 | 0.674158676176547 | -0.352835872 | 0.545552779307424 | 1.10115370556973 | 0.702692515100094 | 1.72556197374213 | 0.998735809224318 |
| ebi-fl187-GCST90094734 | ebi-cfmet338-GCST90026050 | 1-myristoyl-2-palmitoyl-gpc (14:0/16:0) levels \|\| id:ebi-cfmet338-GCST90026050 | Chips liking \|\| id:ebi-fl187-GCST90094734 | Inverse variance weighted | 4 | 0.220325859693124 | 0.117959165995298 | 0.0617879091320705 | -0.010874106 | 0.451525825043908 | 1.24648284293265 | 0.989184803707026 | 1.57070698205512 | 0.998735809224318 |
| ebi-fl187-GCST90094734 | ebi-cfmet338-GCST90026068 | 2,3-dihydroxyisovalerate levels \|\| id:ebi-cfmet338-GCST90026068 | Chips liking \|\| id:ebi-fl187-GCST90094734 | Inverse variance weighted | 4 | 0.209678039808349 | 0.405665322769643 | 0.605243805706443 | -0.585425993 | 1.00478207243685 | 1.23328092866579 | 0.556868589256769 | 2.73131197979894 | 0.998735809224318 |
| ebi-fl187-GCST90094734 | ebi-cfmet338-GCST90026082 | 3-hydroxyhexanoate levels \|\| id:ebi-cfmet338-GCST90026082 | Chips liking \|\| id:ebi-fl187-GCST90094734 | Inverse variance weighted | 4 | -0.013837298 | 0.126736526235008 | 0.913058443208838 | -0.26224089 | 0.234566292963052 | 0.986257996906898 | 0.769325678615592 | 1.2643602878474 | 0.998735809224318 |
| ebi-fl187-GCST90094734 | ebi-cfmet338-GCST90026094 | 3-ureidopropionate levels \|\| id:ebi-cfmet338-GCST90026094 | Chips liking \|\| id:ebi-fl187-GCST90094734 | Inverse variance weighted | 4 | 0.440603060347646 | 0.188069151013305 | 0.0191410981872772 | 0.071987524361568 | 0.809218596333723 | 1.55364387706875 | 1.07464193713593 | 2.24615214923248 | 0.998735809224318 |
| ebi-fl187-GCST90094734 | ebi-cfmet338-GCST90026173 | Glycerol levels \|\| id:ebi-cfmet338-GCST90026173 | Chips liking \|\| id:ebi-fl187-GCST90094734 | Inverse variance weighted (multiplicative random effects) | 4 | -0.116740307 | 0.185940237451172 | 0.530110210825198 | -0.481183172 | 0.24770255860204 | 0.889816242386546 | 0.618051697440655 | 1.28107882964101 | 0.998735809224318 |
| ebi-fl187-GCST90094734 | ebi-cfmet338-GCST90026179 | Histidine levels \|\| id:ebi-cfmet338-GCST90026179 | Chips liking \|\| id:ebi-fl187-GCST90094734 | Inverse variance weighted | 4 | -0.08525139 | 0.073402659568483 | 0.245470735021848 | -0.229120603 | 0.0586178228444495 | 0.918281408702279 | 0.79523262054899 | 1.06036991413419 | 0.998735809224318 |
| ebi-fl187-GCST90094734 | ebi-cfmet338-GCST90026188 | Isocitrate levels \|\| id:ebi-cfmet338-GCST90026188 | Chips liking \|\| id:ebi-fl187-GCST90094734 | Inverse variance weighted | 4 | -0.034183096 | 0.172538965078248 | 0.842952643453506 | -0.372359468 | 0.303993275138231 | 0.966394545060862 | 0.689106486287967 | 1.35525994212325 | 0.998735809224318 |
| ebi-fl187-GCST90094734 | ebi-cfmet338-GCST90026207 | N-acetyl-isoputreanine levels \|\| id:ebi-cfmet338-GCST90026207 | Chips liking \|\| id:ebi-fl187-GCST90094734 | Inverse variance weighted | 4 | 0.0220120454664094 | 0.0948035602100287 | 0.816393665446962 | -0.163802933 | 0.207827023478066 | 1.02225609794777 | 0.848909297791978 | 1.23100021699547 | 0.998735809224318 |
| ebi-fl187-GCST90094734 | ebi-cfmet338-GCST90026213 | N-acetylglutamate levels \|\| id:ebi-cfmet338-GCST90026213 | Chips liking \|\| id:ebi-fl187-GCST90094734 | Inverse variance weighted | 4 | 0.200755205936331 | 0.157244459561737 | 0.201705476281728 | -0.107443935 | 0.508954346677335 | 1.22232551716647 | 0.898126874697944 | 1.66355078776454 | 0.998735809224318 |
| ebi-fl187-GCST90094734 | ebi-cfmet338-GCST90026224 | N-acetyltaurine levels \|\| id:ebi-cfmet338-GCST90026224 | Chips liking \|\| id:ebi-fl187-GCST90094734 | Inverse variance weighted | 4 | 0.201573139716245 | 0.164657720080213 | 0.220878715965858 | -0.121155992 | 0.524302271073462 | 1.22332570748597 | 0.885895756481107 | 1.68927977772514 | 0.998735809224318 |
| ebi-fl187-GCST90094734 | ebi-cfmet338-GCST90026250 | Phenyllactate (pla) levels \|\| id:ebi-cfmet338-GCST90026250 | Chips liking \|\| id:ebi-fl187-GCST90094734 | Inverse variance weighted | 4 | -0.077999735 | 0.206249632219183 | 0.705295985286499 | -0.482249014 | 0.326249544310374 | 0.924964671807857 | 0.617393303052374 | 1.38576113453572 | 0.998735809224318 |
| ebi-fl187-GCST90094734 | ebi-cfmet338-GCST90026251 | Pipecolate levels \|\| id:ebi-cfmet338-GCST90026251 | Chips liking \|\| id:ebi-fl187-GCST90094734 | Inverse variance weighted | 4 | -0.234775653 | 0.29828188530107 | 0.431227291159066 | -0.819408148 | 0.349856842112477 | 0.790748231662586 | 0.440692401897958 | 1.41886441242137 | 0.998735809224318 |
| ebi-fl187-GCST90094734 | ebi-cfmet338-GCST90026278 | Trigonelline (n'-methylnicotinate) levels \|\| id:ebi-cfmet338-GCST90026278 | Chips liking \|\| id:ebi-fl187-GCST90094734 | Inverse variance weighted | 4 | 0.270650376512861 | 0.333988248647894 | 0.417733886356278 | -0.383966591 | 0.925267343862733 | 1.31081669795543 | 0.681154183590548 | 2.52254255649063 | 0.998735809224318 |
| ebi-fl187-GCST90094734 | ebi-cfmet338-GCST90026299 | 1-carboxyethylvaline levels \|\| id:ebi-cfmet338-GCST90026299 | Chips liking \|\| id:ebi-fl187-GCST90094734 | Inverse variance weighted | 4 | 0.361523474177225 | 0.23991966881082 | 0.131848553047287 | -0.108719077 | 0.831766025046433 | 1.43551471911574 | 0.896982365360875 | 2.29737237695735 | 0.998735809224318 |
| ebi-fl187-GCST90094734 | ebi-gm430-GCST90011306 | C_Erysipelotrichia abundance \|\| id:ebi-gm430-GCST90011306 | Chips liking \|\| id:ebi-fl187-GCST90094734 | Inverse variance weighted | 4 | 0.0849659011677506 | 0.224971021289842 | 0.705672138516093 | -0.355977301 | 0.525909102895842 | 1.08867994335071 | 0.700488520751694 | 1.69199634818032 | 0.998735809224318 |
| ebi-fl187-GCST90094734 | ebi-gm430-GCST90011312 | F_Erysipelotrichaceae abundance \|\| id:ebi-gm430-GCST90011312 | Chips liking \|\| id:ebi-fl187-GCST90094734 | Inverse variance weighted | 4 | 0.0849659011677506 | 0.224971021289842 | 0.705672138516093 | -0.355977301 | 0.525909102895842 | 1.08867994335071 | 0.700488520751694 | 1.69199634818032 | 0.998735809224318 |
| ebi-fl187-GCST90094734 | ebi-gm430-GCST90011352 | O_Erysipelotrichales abundance \|\| id:ebi-gm430-GCST90011352 | Chips liking \|\| id:ebi-fl187-GCST90094734 | Inverse variance weighted | 4 | 0.0849659011677506 | 0.224971021289842 | 0.705672138516093 | -0.355977301 | 0.525909102895842 | 1.08867994335071 | 0.700488520751694 | 1.69199634818032 | 0.998735809224318 |
| ebi-fl187-GCST90094734 | ebi-gm430-GCST90011362 | OTU97_11 (Parabacteroides) abundance \|\| id:ebi-gm430-GCST90011362 | Chips liking \|\| id:ebi-fl187-GCST90094734 | Inverse variance weighted | 4 | 0.000159560066577668 | 0.275018791035389 | 0.999537084335141 | -0.53887727 | 0.53919639049594 | 1.00015957279696 | 0.583402888528907 | 1.7146284167015 | 0.999882738470636 |
| ebi-fl187-GCST90094734 | ebi-gm430-GCST90011384 | OTU97_235 (Phascolarctobacterium) abundance \|\| id:ebi-gm430-GCST90011384 | Chips liking \|\| id:ebi-fl187-GCST90094734 | Inverse variance weighted | 4 | -0.423566502 | 0.920934182772837 | 0.645565496529181 | -2.228597501 | 1.38146449579505 | 0.654707634563661 | 0.107679344490439 | 3.98072711887659 | 0.998735809224318 |
| ebi-fl187-GCST90094734 | ebi-gm430-GCST90011390 | OTU97_3 (Bacteroides) abundance \|\| id:ebi-gm430-GCST90011390 | Chips liking \|\| id:ebi-fl187-GCST90094734 | Inverse variance weighted | 4 | 0.026329802934882 | 0.178761928622161 | 0.882903308611574 | -0.324043577 | 0.376703183034317 | 1.02667949455412 | 0.723218725871723 | 1.45747164285242 | 0.998735809224318 |
| ebi-fl187-GCST90094734 | ebi-gm430-GCST90011398 | OTU97_4 (Alistipes) abundance \|\| id:ebi-gm430-GCST90011398 | Chips liking \|\| id:ebi-fl187-GCST90094734 | Inverse variance weighted | 4 | 0.340518341739257 | 0.208892287003662 | 0.103077303994761 | -0.068910541 | 0.749947224266435 | 1.40567602231323 | 0.933410178482409 | 2.11688829333201 | 0.998735809224318 |
| ebi-fl187-GCST90094734 | ebi-gm430-GCST90011421 | OTU99_108 (Cytophagales) abundance \|\| id:ebi-gm430-GCST90011421 | Chips liking \|\| id:ebi-fl187-GCST90094734 | Inverse variance weighted | 4 | -0.63191311 | 0.649303263756824 | 0.330445359997202 | -1.904547507 | 0.640721287309891 | 0.531573868509736 | 0.148889999121224 | 1.89784928034247 | 0.998735809224318 |
| ebi-fl187-GCST90094734 | ebi-gm430-GCST90011428 | OTU99_123 (Phascolarctobacterium) abundance \|\| id:ebi-gm430-GCST90011428 | Chips liking \|\| id:ebi-fl187-GCST90094734 | Inverse variance weighted (multiplicative random effects) | 4 | 0.163156966953168 | 0.687427704235234 | 0.812389787939178 | -1.184201333 | 1.51051526725423 | 1.17722146005246 | 0.305990466315169 | 4.52906387148877 | 0.998735809224318 |
| ebi-fl187-GCST90094734 | ebi-gm430-GCST90011450 | OTU99_298 (Phascolarctobacterium) abundance \|\| id:ebi-gm430-GCST90011450 | Chips liking \|\| id:ebi-fl187-GCST90094734 | Inverse variance weighted | 4 | -0.419356806 | 0.91070047164856 | 0.64517373078255 | -2.204329731 | 1.36561611812551 | 0.657469564125104 | 0.110324447614002 | 3.91813634329943 | 0.998735809224318 |
| ebi-fl187-GCST90094734 | ebi-gm430-GCST90011451 | OTU99_3 (Bacteroides) abundance \|\| id:ebi-gm430-GCST90011451 | Chips liking \|\| id:ebi-fl187-GCST90094734 | Inverse variance weighted | 4 | 0.0234466196940169 | 0.178762459948502 | 0.895648138541566 | -0.326927802 | 0.37382104119308 | 1.02372365260626 | 0.721135805850662 | 1.45327705045688 | 0.998735809224318 |
| ebi-fl187-GCST90094734 | ebi-gm430-GCST90011472 | OTU99_72 (Parabacteroides) abundance \|\| id:ebi-gm430-GCST90011472 | Chips liking \|\| id:ebi-fl187-GCST90094734 | Inverse variance weighted | 4 | 0.117419637137593 | 0.544641393391655 | 0.829306780059177 | -0.950077494 | 1.18491676818524 | 1.12459125094026 | 0.386711054541636 | 3.27041460759486 | 0.998735809224318 |
| ebi-fl187-GCST90094734 | ebi-gm430-GCST90011507 | TestASV_3 (Bacteroides) abundance \|\| id:ebi-gm430-GCST90011507 | Chips liking \|\| id:ebi-fl187-GCST90094734 | Inverse variance weighted | 4 | 0.251406282254889 | 0.229723725560078 | 0.273786001723608 | -0.19885222 | 0.701664784352642 | 1.28583238910489 | 0.819671015494449 | 2.01710796358197 | 0.998735809224318 |
| ebi-fl187-GCST90094734 | ebi-gm430-GCST90011512 | TestASV_34 (Phascolarctobacterium) abundance \|\| id:ebi-gm430-GCST90011512 | Chips liking \|\| id:ebi-fl187-GCST90094734 | Inverse variance weighted | 4 | 0.00393078711449522 | 0.734070097553399 | 0.995727520610527 | -1.434846604 | 1.44270817831916 | 1.00393852279061 | 0.238151892739202 | 4.23214170565809 | 0.999107451094609 |
| ebi-fl187-GCST90094734 | ebi-gm430-GCST90011519 | TestASV_41 (Barnesiella) abundance \|\| id:ebi-gm430-GCST90011519 | Chips liking \|\| id:ebi-fl187-GCST90094734 | Inverse variance weighted | 4 | -0.361468639 | 1.00173230712475 | 0.718216407003773 | -2.324863961 | 1.60192668302389 | 0.696652443491566 | 0.0977967473274354 | 4.96258454688522 | 0.998735809224318 |
| ebi-fl187-GCST90094734 | ebi-gm430-GCST90011524 | TestASV_46 (Sutterella) abundance \|\| id:ebi-gm430-GCST90011524 | Chips liking \|\| id:ebi-fl187-GCST90094734 | Inverse variance weighted | 4 | -1.445063228 | 0.741579564387372 | 0.0513397813039397 | -2.898559174 | 0.00843271841447946 | 0.235731171324892 | 0.0551025560843291 | 1.00846837393827 | 0.998735809224318 |
| ebi-fl187-GCST90094734 | ebi-gm430-GCST90011581 | OTU97_19 (Bacteria) prevalence \|\| id:ebi-gm430-GCST90011581 | Chips liking \|\| id:ebi-fl187-GCST90094734 | Inverse variance weighted | 4 | -2.080054632 | 1.51360694859214 | 0.169368125726842 | -5.046724251 | 0.886614987030434 | 0.12492338717141 | 0.00643036323634177 | 2.42690064134792 | 0.998735809224318 |
| ebi-fl187-GCST90094734 | ebi-gm430-GCST90011614 | OTU97_80 (Ruminococcus) prevalence \|\| id:ebi-gm430-GCST90011614 | Chips liking \|\| id:ebi-fl187-GCST90094734 | Inverse variance weighted | 4 | -0.027648453 | 0.453152965837986 | 0.951348449151221 | -0.915828266 | 0.86053135973024 | 0.972730266804226 | 0.400185028989263 | 2.36441671580475 | 0.998735809224318 |
| ebi-fl187-GCST90094734 | ebi-gm430-GCST90011623 | OTU99_11 (Parabacteroides) prevalence \|\| id:ebi-gm430-GCST90011623 | Chips liking \|\| id:ebi-fl187-GCST90094734 | Inverse variance weighted | 4 | -0.38258165 | 0.394503591372542 | 0.332156218517658 | -1.155808689 | 0.390645389391919 | 0.682098195590871 | 0.314802856398118 | 1.4779343292868 | 0.998735809224318 |
| ebi-fl187-GCST90094734 | ebi-gm430-GCST90011642 | OTU99_19 (Bacteria) prevalence \|\| id:ebi-gm430-GCST90011642 | Chips liking \|\| id:ebi-fl187-GCST90094734 | Inverse variance weighted | 4 | -2.080054632 | 1.51360694859214 | 0.169368125726842 | -5.046724251 | 0.886614987030434 | 0.12492338717141 | 0.00643036323634177 | 2.42690064134792 | 0.998735809224318 |
| ebi-fl187-GCST90094734 | ebi-gm430-GCST90011653 | OTU99_30 (Parasutterella) prevalence \|\| id:ebi-gm430-GCST90011653 | Chips liking \|\| id:ebi-fl187-GCST90094734 | Inverse variance weighted | 4 | -0.403274782 | 0.531710477767782 | 0.448182793702159 | -1.445427319 | 0.638877754146572 | 0.668128484230548 | 0.235645359368862 | 1.89435375530334 | 0.998735809224318 |
| ebi-fl187-GCST90094734 | ebi-gm430-GCST90011675 | OTU99_8 (Ruminococcaceae) prevalence \|\| id:ebi-gm430-GCST90011675 | Chips liking \|\| id:ebi-fl187-GCST90094734 | Inverse variance weighted | 4 | -0.541626759 | 0.45785727928318 | 0.236825077169838 | -1.439027026 | 0.355773508806521 | 0.581801032307579 | 0.237158395418678 | 1.42728424434055 | 0.998735809224318 |
| ebi-fl187-GCST90094734 | ebi-gm430-GCST90011679 | OTU99_92 (Ruminococcus) prevalence \|\| id:ebi-gm430-GCST90011679 | Chips liking \|\| id:ebi-fl187-GCST90094734 | Inverse variance weighted | 4 | -0.057924589 | 0.475406427774347 | 0.903023951576344 | -0.989721188 | 0.873872009033561 | 0.943721111324822 | 0.371680305565003 | 2.39617090985306 | 0.998735809224318 |
| ebi-fl187-GCST90094734 | ebi-gm430-GCST90011684 | TestASV_11 (Lachnospiraceae) prevalence \|\| id:ebi-gm430-GCST90011684 | Chips liking \|\| id:ebi-fl187-GCST90094734 | Inverse variance weighted | 4 | 0.000285761996929651 | 0.482413733643238 | 0.999527366125424 | -0.945245156 | 0.945816679937676 | 1.00028580283078 | 0.388584295473852 | 2.5749154018813 | 0.999882738470636 |
| ebi-fl187-GCST90094734 | ebi-gm430-GCST90011688 | TestASV_15 (Bacteroides) prevalence \|\| id:ebi-gm430-GCST90011688 | Chips liking \|\| id:ebi-fl187-GCST90094734 | Inverse variance weighted | 4 | -0.336144616 | 0.449549193081982 | 0.454618740479461 | -1.217261034 | 0.544971802474258 | 0.714519767377353 | 0.296039900603332 | 1.72455975337279 | 0.998735809224318 |
| ebi-fl187-GCST90094734 | ebi-gm430-GCST90011692 | TestASV_19 (Prevotella) prevalence \|\| id:ebi-gm430-GCST90011692 | Chips liking \|\| id:ebi-fl187-GCST90094734 | Inverse variance weighted | 4 | 0.39293507866123 | 1.1696993609485 | 0.736924960859373 | -1.899675669 | 2.68554582612028 | 1.48132221678601 | 0.149617136860183 | 14.6662043933805 | 0.998735809224318 |
| ebi-fl187-GCST90094734 | ebi-gm430-GCST90011693 | TestASV_2 (Bacteroides) prevalence \|\| id:ebi-gm430-GCST90011693 | Chips liking \|\| id:ebi-fl187-GCST90094734 | Inverse variance weighted | 4 | 0.0718503307349093 | 0.4674060137159 | 0.877829370194261 | -0.844265456 | 0.987966117618073 | 1.07449451322426 | 0.429873002840404 | 2.68576638058305 | 0.998735809224318 |
| ebi-fl187-GCST90094734 | ebi-gm430-GCST90011716 | TestASV_40 (Barnesiella) prevalence \|\| id:ebi-gm430-GCST90011716 | Chips liking \|\| id:ebi-fl187-GCST90094734 | Inverse variance weighted | 4 | -0.173320296 | 0.890062967338123 | 0.845605790053874 | -1.917843712 | 1.57120312009805 | 0.840868245069463 | 0.146923430058348 | 4.81243464902367 | 0.998735809224318 |
| ebi-fl187-GCST90094734 | ebi-gm430-GCST90011718 | TestASV_42 (Alphaproteobacteria) prevalence \|\| id:ebi-gm430-GCST90011718 | Chips liking \|\| id:ebi-fl187-GCST90094734 | Inverse variance weighted | 4 | -0.656542671 | 0.697983399944555 | 0.346895570985037 | -2.024590135 | 0.711504793148794 | 0.518641352289625 | 0.132047953981009 | 2.03705429880039 | 0.998735809224318 |
| ebi-fl187-GCST90094734 | ebi-hbsm44-GCST90104929 | Sulcal depth of the right inferior frontal sulcus \|\| id:ebi-hbsm44-GCST90104929 | Chips liking \|\| id:ebi-fl187-GCST90094734 | Inverse variance weighted | 3 | 0.357334192620069 | 0.404439651398146 | 0.376950478487768 | -0.435367524 | 1.15003590936043 | 1.42951352290501 | 0.64702682540962 | 3.15830632041352 | 0.998735809224318 |
| ebi-fl187-GCST90094734 | ebi-hbsm44-GCST90104935 | Sulcal opening distance of the left calloso-marginal posterior fissure \|\| id:ebi-hbsm44-GCST90104935 | Chips liking \|\| id:ebi-fl187-GCST90094734 | Inverse variance weighted | 4 | -0.079574546 | 0.073067736785858 | 0.27613107121558 | -0.22278731 | 0.0636382184282953 | 0.9235091737906 | 0.800285044002797 | 1.06570677593772 | 0.998735809224318 |
| ebi-fl187-GCST90094734 | ebi-hbsm44-GCST90104936 | Sulcal opening distance of the left anterior inferior temporal sulcus \|\| id:ebi-hbsm44-GCST90104936 | Chips liking \|\| id:ebi-fl187-GCST90094734 | Inverse variance weighted | 4 | -0.025152422 | 0.0416365906560326 | 0.545781038047994 | -0.106760139 | 0.0564552961793353 | 0.975161265151026 | 0.898741219933556 | 1.05807931355508 | 0.998735809224318 |
| ebi-fl187-GCST90094734 | ebi-hbsm44-GCST90104941 | Sulcal opening distance of the right posterior inferior temporal sulcus \|\| id:ebi-hbsm44-GCST90104941 | Chips liking \|\| id:ebi-fl187-GCST90094734 | Inverse variance weighted | 4 | 0.0178565253007777 | 0.0339777988722538 | 0.599211272422267 | -0.04873996 | 0.0844530110903952 | 1.01801690624193 | 0.952428766610303 | 1.08812171337789 | 0.998735809224318 |
| ebi-fl187-GCST90094734 | ebi-IPs91-GCST90274819 | Leukemia inhibitory factor levels \|\| id:ebi-IPs91-GCST90274819 | Chips liking \|\| id:ebi-fl187-GCST90094734 | Inverse variance weighted | 4 | -0.023062409 | 0.139702651141493 | 0.868879350774955 | -0.296879605 | 0.250754787248317 | 0.977201495712789 | 0.743133476363021 | 1.28499494854789 | 0.998735809224318 |
| ebi-fl187-GCST90094734 | ebi-IPs91-GCST90274837 | STAM binding protein levels \|\| id:ebi-IPs91-GCST90274837 | Chips liking \|\| id:ebi-fl187-GCST90094734 | Inverse variance weighted | 4 | -0.055929486 | 0.141462152494164 | 0.692571957603387 | -0.333195305 | 0.221336332499163 | 0.945605811619579 | 0.716630218823101 | 1.24774301652697 | 0.998735809224318 |
| ebi-fl187-GCST90094734 | ebi-IPs91-GCST90274840 | TNF-beta levels \|\| id:ebi-IPs91-GCST90274840 | Chips liking \|\| id:ebi-fl187-GCST90094734 | Inverse variance weighted | 4 | 0.170089701374892 | 0.168861720679245 | 0.313804029465865 | -0.160879271 | 0.501058673906213 | 1.18541117956402 | 0.851394852818671 | 1.65046765314969 | 0.998735809224318 |
| ebi-fl187-GCST90094734 | ebi-IPs91-GCST90274842 | Tumor necrosis factor ligand superfamily member 14 levels \|\| id:ebi-IPs91-GCST90274842 | Chips liking \|\| id:ebi-fl187-GCST90094734 | Inverse variance weighted | 4 | -0.006050357 | 0.18627866599419 | 0.974089155371349 | -0.371156542 | 0.359055828598579 | 0.993967909800085 | 0.689935929086489 | 1.43197674459493 | 0.998955228396052 |
| ebi-fl187-GCST90094734 | ebi-met1400-GCST90199628 | 3-methylhistidine levels \|\| id:ebi-met1400-GCST90199628 | Chips liking \|\| id:ebi-fl187-GCST90094734 | Inverse variance weighted | 4 | 0.0879465633930014 | 0.168137447553999 | 0.600930058021866 | -0.241602834 | 0.41749596059884 | 1.09192977144712 | 0.78536803724654 | 1.51815527144793 | 0.998735809224318 |
| ebi-fl187-GCST90094734 | ebi-met1400-GCST90199651 | Docosahexaenoate DHA; 22:6n3 levels \|\| id:ebi-met1400-GCST90199651 | Chips liking \|\| id:ebi-fl187-GCST90094734 | Inverse variance weighted | 4 | -0.415837216 | 0.161257760810408 | 0.00991696170199233 | -0.731902428 | -0.099772005 | 0.659787664294886 | 0.480993064606122 | 0.905043739689215 | 0.998735809224318 |
| ebi-fl187-GCST90094734 | ebi-met1400-GCST90199652 | 1-stearoyl-2-oleoyl-GPS (18:0/18:1) levels \|\| id:ebi-met1400-GCST90199652 | Chips liking \|\| id:ebi-fl187-GCST90094734 | Inverse variance weighted | 4 | -0.031876598 | 0.25522884319894 | 0.900607321673979 | -0.532125131 | 0.468371934197475 | 0.968626104645352 | 0.58735543510941 | 1.59739141670777 | 0.998735809224318 |
| ebi-fl187-GCST90094734 | ebi-met1400-GCST90199653 | 1-myristoyl-2-palmitoyl-gpc (14:0/16:0) levels \|\| id:ebi-met1400-GCST90199653 | Chips liking \|\| id:ebi-fl187-GCST90094734 | Inverse variance weighted | 4 | 0.182332375601249 | 0.265787223015612 | 0.492707555175764 | -0.338610582 | 0.703275332711847 | 1.20001298263898 | 0.712759956958972 | 2.02035923096195 | 0.998735809224318 |
| ebi-fl187-GCST90094734 | ebi-met1400-GCST90199667 | Erythritol levels in elite athletes \|\| id:ebi-met1400-GCST90199667 | Chips liking \|\| id:ebi-fl187-GCST90094734 | Inverse variance weighted | 4 | -0.07226491 | 0.158245160303622 | 0.64791237882669 | -0.382425424 | 0.23789560416594 | 0.930284421492665 | 0.682204765029011 | 1.26857675178381 | 0.998735809224318 |
| ebi-fl187-GCST90094734 | ebi-met1400-GCST90199674 | 3-hydroxymyristate levels \|\| id:ebi-met1400-GCST90199674 | Chips liking \|\| id:ebi-fl187-GCST90094734 | Inverse variance weighted | 4 | -0.119181356 | 0.17247482102261 | 0.489560970467357 | -0.457232005 | 0.218869293461297 | 0.887646806317661 | 0.63303345613087 | 1.2446685797331 | 0.998735809224318 |
| ebi-fl187-GCST90094734 | ebi-met1400-GCST90199685 | 2-linoleoylglycerol (18:2) levels \|\| id:ebi-met1400-GCST90199685 | Chips liking \|\| id:ebi-fl187-GCST90094734 | Inverse variance weighted | 4 | 0.00445701952096565 | 0.295508798983522 | 0.987966340591738 | -0.574740226 | 0.583654265528669 | 1.00446696680539 | 0.562851063615603 | 1.79257702903141 | 0.998955228396052 |
| ebi-fl187-GCST90094734 | ebi-met1400-GCST90199703 | Gamma-glutamylvaline levels \|\| id:ebi-met1400-GCST90199703 | Chips liking \|\| id:ebi-fl187-GCST90094734 | Inverse variance weighted (multiplicative random effects) | 4 | 0.0705124786509531 | 0.297423422664722 | 0.81259646596304 | -0.51243743 | 0.653462387073808 | 1.0730579596629 | 0.599033695353468 | 1.92218466795339 | 0.998735809224318 |
| ebi-fl187-GCST90094734 | ebi-met1400-GCST90199779 | Gamma-glutamyl-2-aminobutyrate levels \|\| id:ebi-met1400-GCST90199779 | Chips liking \|\| id:ebi-fl187-GCST90094734 | Inverse variance weighted | 4 | 0.133518226915763 | 0.174890378924106 | 0.445201318593834 | -0.209266916 | 0.476303369607011 | 1.14284209643189 | 0.811178690355827 | 1.61011140073703 | 0.998735809224318 |
| ebi-fl187-GCST90094734 | ebi-met1400-GCST90199824 | 4-vinylphenol sulfate levels \|\| id:ebi-met1400-GCST90199824 | Chips liking \|\| id:ebi-fl187-GCST90094734 | Inverse variance weighted (multiplicative random effects) | 4 | 0.103893924043637 | 0.292739859788229 | 0.722663106523366 | -0.469876201 | 0.677664049228565 | 1.1094827592286 | 0.625079647639843 | 1.969272232 | 0.998735809224318 |
| ebi-fl187-GCST90094734 | ebi-met1400-GCST90199830 | 4-ethylphenylsulfate levels \|\| id:ebi-met1400-GCST90199830 | Chips liking \|\| id:ebi-fl187-GCST90094734 | Inverse variance weighted | 4 | 0.0510868124538309 | 0.191990723040717 | 0.790169892458509 | -0.325215005 | 0.427388629613637 | 1.05241425198127 | 0.722372023559791 | 1.53324841169133 | 0.998735809224318 |
| ebi-fl187-GCST90094734 | ebi-met1400-GCST90199833 | Hexanoylglutamine levels \|\| id:ebi-met1400-GCST90199833 | Chips liking \|\| id:ebi-fl187-GCST90094734 | Inverse variance weighted | 4 | -0.208312962 | 0.166432946642521 | 0.210703638553827 | -0.534521537 | 0.11789561370993 | 0.811952886781007 | 0.585949578203325 | 1.12512665743953 | 0.998735809224318 |
| ebi-fl187-GCST90094734 | ebi-met1400-GCST90199837 | Carotenoid levels (cryptoxanthin) \|\| id:ebi-met1400-GCST90199837 | Chips liking \|\| id:ebi-fl187-GCST90094734 | Inverse variance weighted | 4 | 0.0803861875039749 | 0.204884996317993 | 0.694801213923845 | -0.321188405 | 0.481960780287241 | 1.08370550039526 | 0.725286590294136 | 1.61924627768266 | 0.998735809224318 |
| ebi-fl187-GCST90094734 | ebi-met1400-GCST90199869 | 2-hydroxyhippurate levels \|\| id:ebi-met1400-GCST90199869 | Chips liking \|\| id:ebi-fl187-GCST90094734 | Inverse variance weighted | 4 | -0.341323066 | 0.247152588423724 | 0.167272315356976 | -0.82574214 | 0.143096006978348 | 0.710829226116266 | 0.437909881558013 | 1.15384057309543 | 0.998735809224318 |
| ebi-fl187-GCST90094734 | ebi-met1400-GCST90199902 | Hydantoin-5-propionate levels \|\| id:ebi-met1400-GCST90199902 | Chips liking \|\| id:ebi-fl187-GCST90094734 | Inverse variance weighted | 4 | 0.204231067684898 | 0.195468262698561 | 0.296101634911988 | -0.178886727 | 0.587348862574077 | 1.22658154407147 | 0.836200612813902 | 1.79921212828814 | 0.998735809224318 |
| ebi-fl187-GCST90094734 | ebi-met1400-GCST90199904 | Pantoate levels \|\| id:ebi-met1400-GCST90199904 | Chips liking \|\| id:ebi-fl187-GCST90094734 | Inverse variance weighted | 4 | -0.05062778 | 0.170664731833415 | 0.76673334110296 | -0.385130655 | 0.283875093949148 | 0.950632448674408 | 0.680361737844685 | 1.32826701180395 | 0.998735809224318 |
| ebi-fl187-GCST90094734 | ebi-met1400-GCST90199936 | 3-methyl catechol sulfate (2) levels \|\| id:ebi-met1400-GCST90199936 | Chips liking \|\| id:ebi-fl187-GCST90094734 | Inverse variance weighted | 4 | -0.027165806 | 0.191927531038742 | 0.887441787176378 | -0.403343766 | 0.349012155300891 | 0.973199866221724 | 0.668082395582968 | 1.41766642239918 | 0.998735809224318 |
| ebi-fl187-GCST90094734 | ebi-met1400-GCST90199977 | 4-hydroxychlorothalonil levels \|\| id:ebi-met1400-GCST90199977 | Chips liking \|\| id:ebi-fl187-GCST90094734 | Inverse variance weighted | 4 | 0.218390592920114 | 0.236703851023712 | 0.356198913538031 | -0.245548955 | 0.682330140926591 | 1.24407289879815 | 0.782274986517571 | 1.97848250832353 | 0.998735809224318 |
| ebi-fl187-GCST90094734 | ebi-met1400-GCST90200040 | Sphingomyelin (d18:2/24:1, d18:1/24:2) levels \|\| id:ebi-met1400-GCST90200040 | Chips liking \|\| id:ebi-fl187-GCST90094734 | Inverse variance weighted | 4 | 0.181476163556775 | 0.15378253926272 | 0.237967196777258 | -0.119937613 | 0.482889940511707 | 1.19898595680841 | 0.886975770395332 | 1.62075151611304 | 0.998735809224318 |
| ebi-fl187-GCST90094734 | ebi-met1400-GCST90200077 | 1-stearoyl-2-oleoyl-GPI (18:0/18:1) levels \|\| id:ebi-met1400-GCST90200077 | Chips liking \|\| id:ebi-fl187-GCST90094734 | Inverse variance weighted | 4 | -0.361599577 | 0.169049901737527 | 0.0324347883129297 | -0.692937384 | -0.030261769 | 0.696561231268533 | 0.500104909170793 | 0.97019153383402 | 0.998735809224318 |
| ebi-fl187-GCST90094734 | ebi-met1400-GCST90200080 | 1-palmitoyl-2-oleoyl-GPI (16:0/18:1) levels \|\| id:ebi-met1400-GCST90200080 | Chips liking \|\| id:ebi-fl187-GCST90094734 | Inverse variance weighted | 4 | -0.140978949 | 0.189871281301557 | 0.457785973108334 | -0.513126661 | 0.231168761910524 | 0.868507594076002 | 0.598620964998419 | 1.26007187364324 | 0.998735809224318 |
| ebi-fl187-GCST90094734 | ebi-met1400-GCST90200082 | 1-oleoyl-2-linoleoyl-GPE (18:1/18:2) levels \|\| id:ebi-met1400-GCST90200082 | Chips liking \|\| id:ebi-fl187-GCST90094734 | Inverse variance weighted (multiplicative random effects) | 4 | -0.331158104 | 0.3501655819791 | 0.344292132721888 | -1.017482645 | 0.355166436534781 | 0.718091626802904 | 0.36150382923073 | 1.42641804260205 | 0.998735809224318 |
| ebi-fl187-GCST90094734 | ebi-met1400-GCST90200100 | Perfluorooctanesulfonate (PFOS) levels \|\| id:ebi-met1400-GCST90200100 | Chips liking \|\| id:ebi-fl187-GCST90094734 | Inverse variance weighted | 4 | 0.0326764248027278 | 0.203667050758491 | 0.872534160575869 | -0.366510995 | 0.431863844289371 | 1.03321616202268 | 0.693148515491826 | 1.54012540401592 | 0.998735809224318 |
| ebi-fl187-GCST90094734 | ebi-met1400-GCST90200102 | 1-palmitoleoyl-2-linolenoyl-GPC (16:1/18:3) levels \|\| id:ebi-met1400-GCST90200102 | Chips liking \|\| id:ebi-fl187-GCST90094734 | Inverse variance weighted | 4 | 0.0936581533816463 | 0.165146456743232 | 0.570631442774827 | -0.230028902 | 0.417345208598381 | 1.09818427116402 | 0.794510639356023 | 1.51792642375381 | 0.998735809224318 |
| ebi-fl187-GCST90094734 | ebi-met1400-GCST90200114 | Glycosyl-N-tricosanoyl-sphingadienine (d18:2/23:0) levels \|\| id:ebi-met1400-GCST90200114 | Chips liking \|\| id:ebi-fl187-GCST90094734 | Inverse variance weighted | 4 | -0.043079153 | 0.221682905327224 | 0.845919214809956 | -0.477577647 | 0.391419341887865 | 0.957835571935124 | 0.620284120528351 | 1.47907862300717 | 0.998735809224318 |
| ebi-fl187-GCST90094734 | ebi-met1400-GCST90200135 | Arachidoylcarnitine (C20) levels \|\| id:ebi-met1400-GCST90200135 | Chips liking \|\| id:ebi-fl187-GCST90094734 | Inverse variance weighted (multiplicative random effects) | 4 | -0.569813126 | 0.279951359472651 | 0.0418105950750652 | -1.11851779 | -0.021108461 | 0.565631130629828 | 0.32676376823045 | 0.979112762930131 | 0.998735809224318 |
| ebi-fl187-GCST90094734 | ebi-met1400-GCST90200146 | Docosahexaenoylcarnitine (C22:6) levels \|\| id:ebi-met1400-GCST90200146 | Chips liking \|\| id:ebi-fl187-GCST90094734 | Inverse variance weighted | 4 | 0.0837489330852887 | 0.189963819215373 | 0.659308698347695 | -0.288580153 | 0.456078018747419 | 1.08735586045436 | 0.749326742271148 | 1.57787344367407 | 0.998735809224318 |
| ebi-fl187-GCST90094734 | ebi-met1400-GCST90200162 | 3-hydroxyphenylacetoylglutamine levels \|\| id:ebi-met1400-GCST90200162 | Chips liking \|\| id:ebi-fl187-GCST90094734 | Inverse variance weighted | 4 | -0.162168671 | 0.205966570333673 | 0.431074474825386 | -0.565863148 | 0.241525807295017 | 0.850297772243625 | 0.567869779259274 | 1.27319031209154 | 0.998735809224318 |
| ebi-fl187-GCST90094734 | ebi-met1400-GCST90200172 | 3-carboxy-4-methyl-5-pentyl-2-furanpropionate (3-CMPFP) levels \|\| id:ebi-met1400-GCST90200172 | Chips liking \|\| id:ebi-fl187-GCST90094734 | Inverse variance weighted (multiplicative random effects) | 4 | -0.134274809 | 0.275711439983084 | 0.626249752850735 | -0.674669231 | 0.406119613700691 | 0.874349752695659 | 0.509324861606828 | 1.50098207974207 | 0.998735809224318 |
| ebi-fl187-GCST90094734 | ebi-met1400-GCST90200222 | Dihydrocaffeate sulfate (2) levels \|\| id:ebi-met1400-GCST90200222 | Chips liking \|\| id:ebi-fl187-GCST90094734 | Inverse variance weighted | 4 | -0.159218643 | 0.201602120401456 | 0.429664026317218 | -0.554358799 | 0.235921513426412 | 0.852809878048235 | 0.574440475201334 | 1.26607493638351 | 0.998735809224318 |
| ebi-fl187-GCST90094734 | ebi-met1400-GCST90200223 | 4-methylhexanoylglutamine levels \|\| id:ebi-met1400-GCST90200223 | Chips liking \|\| id:ebi-fl187-GCST90094734 | Inverse variance weighted | 4 | 0.0917295999627874 | 0.202923079274124 | 0.651239274743744 | -0.305999635 | 0.489458835340071 | 1.09606840506952 | 0.736386887932035 | 1.6314331070796 | 0.998735809224318 |
| ebi-fl187-GCST90094734 | ebi-met1400-GCST90200224 | 2-ketocaprylate levels \|\| id:ebi-met1400-GCST90200224 | Chips liking \|\| id:ebi-fl187-GCST90094734 | Inverse variance weighted | 4 | -0.016926611 | 0.170856075977943 | 0.921083157001267 | -0.35180452 | 0.317951297659831 | 0.983215838961801 | 0.703417612490295 | 1.37430932751729 | 0.998735809224318 |
| ebi-fl187-GCST90094734 | ebi-met1400-GCST90200243 | Hydroxypalmitoyl sphingomyelin (d18:1/16:0(OH)) levels \|\| id:ebi-met1400-GCST90200243 | Chips liking \|\| id:ebi-fl187-GCST90094734 | Inverse variance weighted | 4 | 0.028445293965376 | 0.164757323074429 | 0.862926823749768 | -0.294479059 | 0.351369647191258 | 1.02885372478745 | 0.744919545344419 | 1.42101250212141 | 0.998735809224318 |
| ebi-fl187-GCST90094734 | ebi-met1400-GCST90200273 | 2-methoxyhydroquinone sulfate (2) levels \|\| id:ebi-met1400-GCST90200273 | Chips liking \|\| id:ebi-fl187-GCST90094734 | Inverse variance weighted | 4 | -0.138495163 | 0.26508448322992 | 0.601352391680952 | -0.65806075 | 0.381070424605038 | 0.870667463090488 | 0.517854611107624 | 1.46385069288662 | 0.998735809224318 |
| ebi-fl187-GCST90094734 | ebi-met1400-GCST90200276 | 2,4-di-tert-butylphenol levels \|\| id:ebi-met1400-GCST90200276 | Chips liking \|\| id:ebi-fl187-GCST90094734 | Inverse variance weighted | 4 | -0.168327132 | 0.167813587009072 | 0.315831811620551 | -0.497241762 | 0.160587498779388 | 0.845077337826171 | 0.608205924774306 | 1.17420050975725 | 0.998735809224318 |
| ebi-fl187-GCST90094734 | ebi-met1400-GCST90200289 | N-acetylmethionine levels \|\| id:ebi-met1400-GCST90200289 | Chips liking \|\| id:ebi-fl187-GCST90094734 | Inverse variance weighted | 4 | -0.168767794 | 0.238633377669134 | 0.479425874064129 | -0.636489214 | 0.298953625979501 | 0.844705025977151 | 0.529146888056251 | 1.34844708910998 | 0.998735809224318 |
| ebi-fl187-GCST90094734 | ebi-met1400-GCST90200308 | 3-hydroxyisobutyrate levels \|\| id:ebi-met1400-GCST90200308 | Chips liking \|\| id:ebi-fl187-GCST90094734 | Inverse variance weighted | 4 | 0.102172935030231 | 0.185912664492967 | 0.582610974710359 | -0.262215887 | 0.466561757436446 | 1.10757499368204 | 0.769344913923024 | 1.59450247142664 | 0.998735809224318 |
| ebi-fl187-GCST90094734 | ebi-met1400-GCST90200383 | Cystathionine levels \|\| id:ebi-met1400-GCST90200383 | Chips liking \|\| id:ebi-fl187-GCST90094734 | Inverse variance weighted | 4 | -0.17148756 | 0.19706426339104 | 0.384184910969517 | -0.557733516 | 0.214758396324933 | 0.842410747610666 | 0.572505168201698 | 1.23956237795908 | 0.998735809224318 |
| ebi-fl187-GCST90094734 | ebi-met1400-GCST90200384 | Guanidinoacetate levels \|\| id:ebi-met1400-GCST90200384 | Chips liking \|\| id:ebi-fl187-GCST90094734 | Inverse variance weighted | 4 | 0.100622203485817 | 0.161273632252703 | 0.532678663565559 | -0.215474116 | 0.416718522701115 | 1.10585877324405 | 0.806159136829238 | 1.51697545868028 | 0.998735809224318 |
| ebi-fl187-GCST90094734 | ebi-met1400-GCST90200403 | Citrulline levels \|\| id:ebi-met1400-GCST90200403 | Chips liking \|\| id:ebi-fl187-GCST90094734 | Inverse variance weighted | 4 | 0.182913855061811 | 0.168842537437237 | 0.278657587981863 | -0.148017518 | 0.513845228438796 | 1.20071096845333 | 0.862416006734294 | 1.67170694711877 | 0.998735809224318 |
| ebi-fl187-GCST90094734 | ebi-met1400-GCST90200434 | 12,13-DiHOME levels \|\| id:ebi-met1400-GCST90200434 | Chips liking \|\| id:ebi-fl187-GCST90094734 | Inverse variance weighted | 4 | -0.003888974 | 0.234743796921484 | 0.98678213996915 | -0.463986816 | 0.456208867623532 | 0.996118577924779 | 0.628771843946354 | 1.57807992014913 | 0.998955228396052 |
| ebi-fl187-GCST90094734 | ebi-met1400-GCST90200437 | Fructose levels \|\| id:ebi-met1400-GCST90200437 | Chips liking \|\| id:ebi-fl187-GCST90094734 | Inverse variance weighted | 4 | 0.0141122397271935 | 0.169653351739462 | 0.93370619410325 | -0.31840833 | 0.346632809136538 | 1.01421228746079 | 0.72730574724876 | 1.41429731296298 | 0.998735809224318 |
| ebi-fl187-GCST90094734 | ebi-met1400-GCST90200448 | N-stearoyl-sphinganine (d18:0/18:0) levels \|\| id:ebi-met1400-GCST90200448 | Chips liking \|\| id:ebi-fl187-GCST90094734 | Inverse variance weighted (multiplicative random effects) | 4 | -0.072278177 | 0.316037303176886 | 0.819100997891004 | -0.691711291 | 0.547154937003009 | 0.93027207931015 | 0.500718460245959 | 1.72832881200132 | 0.998735809224318 |
| ebi-fl187-GCST90094734 | ebi-met1400-GCST90200468 | X-11847 levels \|\| id:ebi-met1400-GCST90200468 | Chips liking \|\| id:ebi-fl187-GCST90094734 | Inverse variance weighted (multiplicative random effects) | 4 | -0.247229028 | 0.310734992233538 | 0.426249095960385 | -0.856269613 | 0.361811556726458 | 0.780961810885917 | 0.424743588702447 | 1.435928325 | 0.998735809224318 |
| ebi-fl187-GCST90094734 | ebi-met1400-GCST90200476 | X-11843 levels \|\| id:ebi-met1400-GCST90200476 | Chips liking \|\| id:ebi-fl187-GCST90094734 | Inverse variance weighted | 4 | 0.219062137765579 | 0.23180923946856 | 0.344653576105583 | -0.235283972 | 0.673408247123957 | 1.24490863012485 | 0.790346381838093 | 1.96090920762488 | 0.998735809224318 |
| ebi-fl187-GCST90094734 | ebi-met1400-GCST90200511 | X-12906 levels \|\| id:ebi-met1400-GCST90200511 | Chips liking \|\| id:ebi-fl187-GCST90094734 | Inverse variance weighted | 4 | 0.344713814987708 | 0.168971797141905 | 0.0413436366463201 | 0.013529092589575 | 0.675898537385841 | 1.41158588713193 | 1.01362102488172 | 1.9657985261134 | 0.998735809224318 |
| ebi-fl187-GCST90094734 | ebi-met1400-GCST90200515 | X-12849 levels \|\| id:ebi-met1400-GCST90200515 | Chips liking \|\| id:ebi-fl187-GCST90094734 | Inverse variance weighted | 4 | -0.031751379 | 0.264494588752136 | 0.904447123603719 | -0.550160773 | 0.486658015092587 | 0.968747403223556 | 0.576857059990975 | 1.62687014919617 | 0.998735809224318 |
| ebi-fl187-GCST90094734 | ebi-met1400-GCST90200551 | X-18886 levels \|\| id:ebi-met1400-GCST90200551 | Chips liking \|\| id:ebi-fl187-GCST90094734 | Inverse variance weighted | 4 | -0.30391541 | 0.208509435757921 | 0.144961336767507 | -0.712593904 | 0.104763083944983 | 0.737923284637519 | 0.49037057204474 | 1.11044749634883 | 0.998735809224318 |
| ebi-fl187-GCST90094734 | ebi-met1400-GCST90200564 | X-21319 levels \|\| id:ebi-met1400-GCST90200564 | Chips liking \|\| id:ebi-fl187-GCST90094734 | Inverse variance weighted | 4 | -0.150198448 | 0.174716353078941 | 0.389970847998876 | -0.4926425 | 0.192245604110813 | 0.86053718766094 | 0.611009666005971 | 1.21196814477263 | 0.998735809224318 |
| ebi-fl187-GCST90094734 | ebi-met1400-GCST90200579 | X-21442 levels \|\| id:ebi-met1400-GCST90200579 | Chips liking \|\| id:ebi-fl187-GCST90094734 | Inverse variance weighted | 4 | -0.163321369 | 0.184172462965203 | 0.375194688982311 | -0.524299397 | 0.197656658066437 | 0.849318199717993 | 0.591969955188289 | 1.21854394475607 | 0.998735809224318 |
| ebi-fl187-GCST90094734 | ebi-met1400-GCST90200581 | X-21441 levels \|\| id:ebi-met1400-GCST90200581 | Chips liking \|\| id:ebi-fl187-GCST90094734 | Inverse variance weighted | 4 | 0.340705493316243 | 0.168881723176121 | 0.0436516232354089 | 0.00969731589104556 | 0.67171367074144 | 1.40593912141648 | 1.0097444872139 | 1.95758911106648 | 0.998735809224318 |
| ebi-fl187-GCST90094734 | ebi-met1400-GCST90200648 | X-24585 levels \|\| id:ebi-met1400-GCST90200648 | Chips liking \|\| id:ebi-fl187-GCST90094734 | Inverse variance weighted | 4 | 0.057847894434199 | 0.21327740089316 | 0.786211742881814 | -0.360175811 | 0.475871600184793 | 1.05955381941949 | 0.69755367745955 | 1.6094163539286 | 0.998735809224318 |
| ebi-fl187-GCST90094734 | ebi-met1400-GCST90200720 | Adenosine 5'-diphosphate (ADP) to pantothenate ratio \|\| id:ebi-met1400-GCST90200720 | Chips liking \|\| id:ebi-fl187-GCST90094734 | Inverse variance weighted (multiplicative random effects) | 4 | -0.205191801 | 0.372858550724065 | 0.582099387418863 | -0.935994561 | 0.525610958289845 | 0.814491081119659 | 0.392195609216701 | 1.69149196378923 | 0.998735809224318 |
| ebi-fl187-GCST90094734 | ebi-met1400-GCST90200724 | Cholate to taurocholate ratio \|\| id:ebi-met1400-GCST90200724 | Chips liking \|\| id:ebi-fl187-GCST90094734 | Inverse variance weighted | 4 | 0.179927639447804 | 0.19198101638787 | 0.348647570494389 | -0.196355153 | 0.55621043156803 | 1.19713073494658 | 0.821720346671748 | 1.74405076174442 | 0.998735809224318 |
| ebi-fl187-GCST90094734 | ebi-met1400-GCST90200726 | N-acetylputrescine to (N(1) + N(8))-acetylspermidine ratio \|\| id:ebi-met1400-GCST90200726 | Chips liking \|\| id:ebi-fl187-GCST90094734 | Inverse variance weighted | 4 | -0.063482261 | 0.167798617217827 | 0.705189844998301 | -0.39236755 | 0.265403029052673 | 0.938490767327649 | 0.67545580425138 | 1.30395640220371 | 0.998735809224318 |
| ebi-fl187-GCST90094734 | ebi-met1400-GCST90200744 | Arginine to glutamate ratio \|\| id:ebi-met1400-GCST90200744 | Chips liking \|\| id:ebi-fl187-GCST90094734 | Inverse variance weighted (multiplicative random effects) | 4 | -0.086781027 | 0.286445406718062 | 0.761921603830645 | -0.648214025 | 0.474651969815343 | 0.916877844822578 | 0.522978970800815 | 1.60745465738177 | 0.998735809224318 |
| ebi-fl187-GCST90094734 | ebi-met1400-GCST90200759 | Histidine to trans-urocanate ratio \|\| id:ebi-met1400-GCST90200759 | Chips liking \|\| id:ebi-fl187-GCST90094734 | Inverse variance weighted | 4 | 0.0370030959516982 | 0.25039697897172 | 0.882518189014397 | -0.453774983 | 0.52778117473627 | 1.0376962334906 | 0.635225653842645 | 1.69516685367893 | 0.998735809224318 |
| ebi-fl187-GCST90094734 | ebi-met1400-GCST90200795 | Oleoyl-linoleoyl-glycerol (18:1 to 18:2) [2] to linoleoyl-arachidonoyl-glycerol (18:2 to 20:4) [2] ratio \|\| id:ebi-met1400-GCST90200795 | Chips liking \|\| id:ebi-fl187-GCST90094734 | Inverse variance weighted (multiplicative random effects) | 4 | 0.0461745149880232 | 0.367046455405549 | 0.899890088870984 | -0.673236538 | 0.7655855675829 | 1.04725715708073 | 0.510055090960315 | 2.15025312460247 | 0.998735809224318 |
| ebi-fl187-GCST90094734 | ebi-met1400-GCST90200811 | Choline to taurocholate ratio \|\| id:ebi-met1400-GCST90200811 | Chips liking \|\| id:ebi-fl187-GCST90094734 | Inverse variance weighted | 4 | 0.118831085501404 | 0.241407946105348 | 0.622548407518131 | -0.354328489 | 0.591990659867887 | 1.12617967414632 | 0.701644447107681 | 1.80758311946802 | 0.998735809224318 |
| ebi-fl187-GCST90094734 | ebi-met1400-GCST90200831 | Cholate to phosphate ratio \|\| id:ebi-met1400-GCST90200831 | Chips liking \|\| id:ebi-fl187-GCST90094734 | Inverse variance weighted | 4 | 0.124546487069663 | 0.176017759025686 | 0.479206677114836 | -0.220448321 | 0.469541294760007 | 1.13263467210333 | 0.802159092874497 | 1.59926043579902 | 0.998735809224318 |
| ebi-fl187-GCST90094734 | ebi-met1400-GCST90200841 | Adenosine 5'-diphosphate (ADP) to oxalate (ethanedioate) ratio \|\| id:ebi-met1400-GCST90200841 | Chips liking \|\| id:ebi-fl187-GCST90094734 | Inverse variance weighted (multiplicative random effects) | 4 | 0.0409609385827774 | 0.384428901225637 | 0.915145906081163 | -0.712519708 | 0.794441584985026 | 1.04181141012052 | 0.49040695712889 | 2.21320476489904 | 0.998735809224318 |
| ebi-fl187-GCST90094734 | ebi-met1400-GCST90200844 | Aspartate to phosphate ratio \|\| id:ebi-met1400-GCST90200844 | Chips liking \|\| id:ebi-fl187-GCST90094734 | Inverse variance weighted | 4 | 0.0149077162978119 | 0.241013726689776 | 0.950678924054032 | -0.457479188 | 0.487294620609772 | 1.01501939054634 | 0.632877000317197 | 1.62790615343691 | 0.998735809224318 |
| ebi-fl187-GCST90094734 | ebi-met1400-GCST90200870 | Adenosine 5'-monophosphate (AMP) to acetoacetate ratio \|\| id:ebi-met1400-GCST90200870 | Chips liking \|\| id:ebi-fl187-GCST90094734 | Inverse variance weighted | 4 | 0.0038980629256678 | 0.245237045677426 | 0.987318093584094 | -0.476766547 | 0.484562672453422 | 1.00390567025436 | 0.620787437314767 | 1.62346486766587 | 0.998955228396052 |
| ebi-fl187-GCST90094734 | ebi-met1400-GCST90200887 | Cholesterol to cortisol ratio \|\| id:ebi-met1400-GCST90200887 | Chips liking \|\| id:ebi-fl187-GCST90094734 | Inverse variance weighted | 4 | -0.080807363 | 0.176878827954155 | 0.647777765940685 | -0.427489866 | 0.265875139921824 | 0.922371357304152 | 0.652144011012788 | 1.30457215953551 | 0.998735809224318 |
| ebi-fl187-GCST90094734 | ebi-met1400-GCST90200890 | Cortisol to taurocholate ratio \|\| id:ebi-met1400-GCST90200890 | Chips liking \|\| id:ebi-fl187-GCST90094734 | Inverse variance weighted | 4 | 0.227657385956119 | 0.212656622762016 | 0.284376388189682 | -0.189149595 | 0.64446436656967 | 1.25565504667352 | 0.827662683517711 | 1.90496639226932 | 0.998735809224318 |
| ebi-fl187-GCST90094734 | ebi-met1400-GCST90200948 | Adenosine 5'-diphosphate (ADP) to mannitol to sorbitol ratio \|\| id:ebi-met1400-GCST90200948 | Chips liking \|\| id:ebi-fl187-GCST90094734 | Inverse variance weighted (multiplicative random effects) | 4 | -0.170653716 | 0.373327618238674 | 0.647588270358501 | -0.902375848 | 0.561068415760556 | 0.843113479646423 | 0.405604858699382 | 1.75254394594998 | 0.998735809224318 |
| ebi-fl187-GCST90094734 | ebi-met1400-GCST90200972 | Leucine to N-palmitoyl-sphingosine (d18:1 to 16:0) ratio \|\| id:ebi-met1400-GCST90200972 | Chips liking \|\| id:ebi-fl187-GCST90094734 | Inverse variance weighted | 4 | 0.420992776563483 | 0.226753846260504 | 0.0633670883679637 | -0.023444762 | 0.865430315234071 | 1.52347327365653 | 0.976827931095246 | 2.37602830719983 | 0.998735809224318 |
| ebi-fl187-GCST90094734 | ebi-met1400-GCST90200973 | Proline to glutamate ratio \|\| id:ebi-met1400-GCST90200973 | Chips liking \|\| id:ebi-fl187-GCST90094734 | Inverse variance weighted | 4 | -0.132482212 | 0.256640747596263 | 0.605703142085384 | -0.635498078 | 0.370533652795314 | 0.875918514374026 | 0.529671604947603 | 1.44850740847076 | 0.998735809224318 |
| ebi-fl187-GCST90094734 | ebi-met1400-GCST90200983 | Cholesterol to linoleoyl-arachidonoyl-glycerol (18:2 to 20:4) [1] ratio \|\| id:ebi-met1400-GCST90200983 | Chips liking \|\| id:ebi-fl187-GCST90094734 | Inverse variance weighted (multiplicative random effects) | 4 | 0.270811866615924 | 0.52791453105168 | 0.607962302447431 | -0.763900614 | 1.30552434747722 | 1.31102839897243 | 0.465845793784959 | 3.68962323121381 | 0.998735809224318 |
| ebi-fl187-GCST90094734 | ebi-met1400-GCST90201001 | Serine to alpha-ketobutyrate ratio \|\| id:ebi-met1400-GCST90201001 | Chips liking \|\| id:ebi-fl187-GCST90094734 | Inverse variance weighted | 4 | -0.05419131 | 0.239197981617969 | 0.820770401078318 | -0.523019354 | 0.414636734065739 | 0.947250870691711 | 0.59272818729615 | 1.51382072129782 | 0.998735809224318 |
| ebi-fl187-GCST90094734 | ebi-met1400-GCST90201005 | Fructose to maltose ratio \|\| id:ebi-met1400-GCST90201005 | Chips liking \|\| id:ebi-fl187-GCST90094734 | Inverse variance weighted | 4 | -0.1460122 | 0.192201536147531 | 0.447444217333821 | -0.52272721 | 0.230702811296065 | 0.864147160911165 | 0.592901374264675 | 1.2594848791453 | 0.998735809224318 |
| ebi-fl187-GCST90094734 | ebi-met1400-GCST90201020 | Paraxanthine to 5-acetylamino-6-formylamino-3-methyluracil ratio \|\| id:ebi-met1400-GCST90201020 | Chips liking \|\| id:ebi-fl187-GCST90094734 | Inverse variance weighted | 4 | -0.088382148 | 0.202913070500375 | 0.663151236363913 | -0.486091767 | 0.309327469836758 | 0.915410987079938 | 0.615025365983409 | 1.36250847788491 | 0.998735809224318 |
| ebi-fl187-GCST90094734 | ebi-pw11-GCST90275197 | Placental weight (fetal genotype, adjusted for sex) \|\| id:ebi-pw11-GCST90275197 | Chips liking \|\| id:ebi-fl187-GCST90094734 | Inverse variance weighted | 4 | 0.077296337840373 | 0.0710832077860233 | 0.276857226835989 | -0.062026749 | 0.216619425100979 | 1.08036218115627 | 0.939857745805036 | 1.24187138711398 | 0.998735809224318 |
| ebi-fl187-GCST90094734 | prot-a-1017 | FAS-associated factor 2 \|\| id:prot-a-1017 | Chips liking \|\| id:ebi-fl187-GCST90094734 | Inverse variance weighted | 4 | 0.158520844886324 | 0.266309222229548 | 0.551675669023156 | -0.363445231 | 0.680486920456238 | 1.17177634951543 | 0.695276806008757 | 1.97483908770921 | 0.998735809224318 |
| ebi-fl187-GCST90094734 | prot-a-1019 | Fas apoptotic inhibitory molecule 3 \|\| id:prot-a-1019 | Chips liking \|\| id:ebi-fl187-GCST90094734 | Inverse variance weighted | 4 | 0.0641536329760119 | 0.266601294027962 | 0.809837932115407 | -0.458384903 | 0.586692169270817 | 1.06625619829161 | 0.632304053434599 | 1.79803098559905 | 0.998735809224318 |
| ebi-fl187-GCST90094734 | prot-a-1052 | MIP18 family protein FAM96A \|\| id:prot-a-1052 | Chips liking \|\| id:ebi-fl187-GCST90094734 | Inverse variance weighted | 4 | 0.0110165430792644 | 0.26615717318277 | 0.966984093187986 | -0.510651516 | 0.532684602517493 | 1.01107744864074 | 0.60010447353876 | 1.70349939423314 | 0.998735809224318 |
| ebi-fl187-GCST90094734 | prot-a-1066 | F-box/LRR-repeat protein 4 \|\| id:prot-a-1066 | Chips liking \|\| id:ebi-fl187-GCST90094734 | Inverse variance weighted | 4 | -0.415775531 | 0.266309222229548 | 0.118464547160779 | -0.937741607 | 0.10619054414456 | 0.659828364595042 | 0.391511023446869 | 1.11203374783967 | 0.998735809224318 |
| ebi-fl187-GCST90094734 | prot-a-1076 | Low affinity immunoglobulin gamma Fc region receptor III-B \|\| id:prot-a-1076 | Chips liking \|\| id:ebi-fl187-GCST90094734 | Inverse variance weighted | 4 | -0.321270863 | 0.281877447584772 | 0.254388929163955 | -0.87375066 | 0.23120893425655 | 0.725226787273719 | 0.417383147424234 | 1.26012249470335 | 0.998735809224318 |
| ebi-fl187-GCST90094734 | prot-a-1081 | Fc receptor-like protein 3 \|\| id:prot-a-1081 | Chips liking \|\| id:ebi-fl187-GCST90094734 | Inverse variance weighted | 4 | 0.299604170605108 | 0.26615717318277 | 0.260306776964759 | -0.222063889 | 0.821272230043337 | 1.34932459951561 | 0.80086419642115 | 2.27339027389923 | 0.998735809224318 |
| ebi-fl187-GCST90094734 | prot-a-1105 | Fibroblast growth factor receptor 4 \|\| id:prot-a-1105 | Chips liking \|\| id:ebi-fl187-GCST90094734 | Inverse variance weighted | 4 | 0.017219050241671 | 0.266448744444477 | 0.948473176450705 | -0.505020489 | 0.539458589352846 | 1.01736815265854 | 0.603493210406734 | 1.71507804925637 | 0.998735809224318 |
| ebi-fl187-GCST90094734 | prot-a-1143 | Follistatin-related protein 1 \|\| id:prot-a-1143 | Chips liking \|\| id:ebi-fl187-GCST90094734 | Inverse variance weighted | 4 | 0.0981929930677848 | 0.26615717318277 | 0.712180945636002 | -0.423475066 | 0.619861052506014 | 1.10317566982203 | 0.654767501193226 | 1.85866976639721 | 0.998735809224318 |
| ebi-fl187-GCST90094734 | prot-a-1151 | Alpha-(1,3)-fucosyltransferase 10 \|\| id:prot-a-1151 | Chips liking \|\| id:ebi-fl187-GCST90094734 | Inverse variance weighted | 4 | -0.143422928 | 0.273555408893563 | 0.600075421208697 | -0.679591529 | 0.392745673622064 | 0.866387572002241 | 0.506823972862998 | 1.48104167346252 | 0.998735809224318 |
| ebi-fl187-GCST90094734 | prot-a-1165 | Growth arrest and DNA damage-inducible proteins-interacting protein 1 \|\| id:prot-a-1165 | Chips liking \|\| id:ebi-fl187-GCST90094734 | Inverse variance weighted | 4 | 0.201906398220019 | 0.266309222229548 | 0.448352061156599 | -0.320059677 | 0.723872473789933 | 1.22373345912045 | 0.726105703716553 | 2.06240437350357 | 0.998735809224318 |
| ebi-fl187-GCST90094734 | prot-a-1194 | Growth/differentiation factor 11/8 \|\| id:prot-a-1194 | Chips liking \|\| id:ebi-fl187-GCST90094734 | Inverse variance weighted | 4 | -0.042379584 | 0.26615717318277 | 0.873489511850635 | -0.564047643 | 0.479288475398072 | 0.958505877977369 | 0.568901686078252 | 1.61492493448296 | 0.998735809224318 |
| ebi-fl187-GCST90094734 | prot-a-1216 | D-glucuronyl C5-epimerase \|\| id:prot-a-1216 | Chips liking \|\| id:ebi-fl187-GCST90094734 | Inverse variance weighted | 4 | 0.361992510942978 | 0.394041827586536 | 0.358270721916988 | -0.410329471 | 1.13431449301259 | 1.43618818622486 | 0.663431632556785 | 3.10904154253648 | 0.998735809224318 |
| ebi-fl187-GCST90094734 | prot-a-1246 | Glypican-3 \|\| id:prot-a-1246 | Chips liking \|\| id:ebi-fl187-GCST90094734 | Inverse variance weighted | 4 | -0.05954792 | 0.26615717318277 | 0.822965718836731 | -0.581215979 | 0.462120139737424 | 0.942190383028182 | 0.559217955598289 | 1.58743600591481 | 0.998735809224318 |
| ebi-fl187-GCST90094734 | prot-a-1275 | Glutamate receptor 4 \|\| id:prot-a-1275 | Chips liking \|\| id:ebi-fl187-GCST90094734 | Inverse variance weighted | 4 | 0.102454319665319 | 0.26615717318277 | 0.700282489010212 | -0.41921374 | 0.624122379103548 | 1.10788669211899 | 0.657563632744915 | 1.86660706500858 | 0.998735809224318 |
| ebi-fl187-GCST90094734 | prot-a-1289 | Glutathione S-transferase P \|\| id:prot-a-1289 | Chips liking \|\| id:ebi-fl187-GCST90094734 | Inverse variance weighted | 4 | 0.151517193876674 | 0.413819684273621 | 0.714258348756995 | -0.659569387 | 0.96260377505297 | 1.16359830840349 | 0.51707394516659 | 2.6185056044223 | 0.998735809224318 |
| ebi-fl187-GCST90094734 | prot-a-130 | Apolipoprotein D \|\| id:prot-a-130 | Chips liking \|\| id:ebi-fl187-GCST90094734 | Inverse variance weighted | 4 | -0.078337605 | 0.266448744444477 | 0.768753103273858 | -0.600577144 | 0.443901934483504 | 0.924652206979057 | 0.548494984280044 | 1.55877761579441 | 0.998735809224318 |
| ebi-fl187-GCST90094734 | prot-a-1313 | Heparin-binding EGF-like growth factor \|\| id:prot-a-1313 | Chips liking \|\| id:ebi-fl187-GCST90094734 | Inverse variance weighted | 4 | 0.277966171339659 | 0.26615717318277 | 0.296314925335124 | -0.243701888 | 0.799634230777888 | 1.3204415276856 | 0.783721236069269 | 2.22472704297449 | 0.998735809224318 |
| ebi-fl187-GCST90094734 | prot-a-1347 | HLA class II histocompatibility antigen, DQ alpha 2 chain \|\| id:prot-a-1347 | Chips liking \|\| id:ebi-fl187-GCST90094734 | Inverse variance weighted | 4 | 0.0608828978243955 | 0.376743461690261 | 0.871618444155476 | -0.677534287 | 0.799300082737307 | 1.06277445369934 | 0.507867705741235 | 2.22398377897927 | 0.998735809224318 |
| ebi-fl187-GCST90094734 | prot-a-1357 | Non-histone chromosomal protein HMG-14 \|\| id:prot-a-1357 | Chips liking \|\| id:ebi-fl187-GCST90094734 | Inverse variance weighted | 4 | 0.25021744845448 | 0.26615717318277 | 0.347160314426712 | -0.271450611 | 0.771885507892709 | 1.28430465638913 | 0.762272930448507 | 2.16384235165259 | 0.998735809224318 |
| ebi-fl187-GCST90094734 | prot-a-137 | Apolipoprotein M \|\| id:prot-a-137 | Chips liking \|\| id:ebi-fl187-GCST90094734 | Inverse variance weighted | 4 | -0.044700188 | 0.266448744444477 | 0.86676988686709 | -0.566939727 | 0.477539350978548 | 0.956284144193573 | 0.567258751644973 | 1.61210269878458 | 0.998735809224318 |
| ebi-fl187-GCST90094734 | prot-a-1381 | 17-beta-hydroxysteroid dehydrogenase 14 \|\| id:prot-a-1381 | Chips liking \|\| id:ebi-fl187-GCST90094734 | Inverse variance weighted | 4 | -0.151393413 | 0.27326624441879 | 0.579569045558511 | -0.686995252 | 0.384208426493848 | 0.859509490300935 | 0.503085445455617 | 1.46845147398037 | 0.998735809224318 |
| ebi-fl187-GCST90094734 | prot-a-1392 | Serine protease HTRA2, mitochondrial \|\| id:prot-a-1392 | Chips liking \|\| id:ebi-fl187-GCST90094734 | Inverse variance weighted (multiplicative random effects) | 4 | 0.0760466655135006 | 0.459483198676675 | 0.868546688159128 | -0.824540404 | 0.976633734919783 | 1.07901292567469 | 0.438436449852502 | 2.6555020554626 | 0.998735809224318 |
| ebi-fl187-GCST90094734 | prot-a-1416 | Interferon-induced protein with tetratricopeptide repeats 2 \|\| id:prot-a-1416 | Chips liking \|\| id:ebi-fl187-GCST90094734 | Inverse variance weighted | 4 | 0.484560813945697 | 0.266309222229548 | 0.0688287817137678 | -0.037405262 | 1.00652688951561 | 1.62346185044667 | 0.963285673518165 | 2.7360817795927 | 0.998735809224318 |
| ebi-fl187-GCST90094734 | prot-a-1428 | Interferon gamma \|\| id:prot-a-1428 | Chips liking \|\| id:ebi-fl187-GCST90094734 | Inverse variance weighted | 4 | 0.0805972977077177 | 0.26615717318277 | 0.762028236240233 | -0.441070762 | 0.602265357145947 | 1.08393430583497 | 0.643347180602408 | 1.82625122918204 | 0.998735809224318 |
| ebi-fl187-GCST90094734 | prot-a-1483 | Interleukin-17C \|\| id:prot-a-1483 | Chips liking \|\| id:ebi-fl187-GCST90094734 | Inverse variance weighted | 4 | 0.0327252296436209 | 0.266601294027962 | 0.902305304188074 | -0.489813307 | 0.555263765938426 | 1.03326658920362 | 0.612740778134548 | 1.7424005100735 | 0.998735809224318 |
| ebi-fl187-GCST90094734 | prot-a-1503 | Interleukin-1 receptor-like 2 \|\| id:prot-a-1503 | Chips liking \|\| id:ebi-fl187-GCST90094734 | Inverse variance weighted | 4 | 0.188602582836667 | 0.294173864970177 | 0.521440669578464 | -0.387978193 | 0.765183358178214 | 1.20756095166098 | 0.678427137899286 | 2.14938844647579 | 0.998735809224318 |
| ebi-fl187-GCST90094734 | prot-a-1522 | Interleukin-31 receptor subunit alpha \|\| id:prot-a-1522 | Chips liking \|\| id:ebi-fl187-GCST90094734 | Inverse variance weighted | 4 | 0.238090234881507 | 0.371934607038225 | 0.522081558882649 | -0.490901595 | 0.967082064676429 | 1.26882367981387 | 0.612074302262629 | 2.63025832730622 | 0.998735809224318 |
| ebi-fl187-GCST90094734 | prot-a-1540 | Interleukin-6 receptor subunit alpha \|\| id:prot-a-1540 | Chips liking \|\| id:ebi-fl187-GCST90094734 | Inverse variance weighted | 4 | -0.051062119 | 0.266309222229548 | 0.847945905467173 | -0.573028194 | 0.470903956745923 | 0.950219642172355 | 0.563815508044335 | 1.60144117266664 | 0.998735809224318 |
| ebi-fl187-GCST90094734 | prot-a-1541 | Interleukin-6 receptor subunit alpha \|\| id:prot-a-1541 | Chips liking \|\| id:ebi-fl187-GCST90094734 | Inverse variance weighted | 4 | 0.0129359980435912 | 0.266309222229548 | 0.961257911033595 | -0.509030078 | 0.534902073613505 | 1.01302003002117 | 0.601078295518832 | 1.70728104620431 | 0.998735809224318 |
| ebi-fl187-GCST90094734 | prot-a-1545 | Interleukin-9 \|\| id:prot-a-1545 | Chips liking \|\| id:ebi-fl187-GCST90094734 | Inverse variance weighted | 4 | 0.428575717607028 | 0.26615717318277 | 0.107346463356195 | -0.093092342 | 0.950243777045257 | 1.53506959320922 | 0.911109362904505 | 2.58634007281353 | 0.998735809224318 |
| ebi-fl187-GCST90094734 | prot-a-1548 | Inositol monophosphatase 3 \|\| id:prot-a-1548 | Chips liking \|\| id:ebi-fl187-GCST90094734 | Inverse variance weighted (multiplicative random effects) | 4 | 0.497600556131215 | 0.450444568015463 | 0.269294951943847 | -0.385270797 | 1.38047190944152 | 1.64476999887902 | 0.680266397038641 | 3.97677786377388 | 0.998735809224318 |
| ebi-fl187-GCST90094734 | prot-a-1555 | Inhibin beta A chain:Inhibin beta B chain heterodimer \|\| id:prot-a-1555 | Chips liking \|\| id:ebi-fl187-GCST90094734 | Inverse variance weighted | 4 | 0.377152782261582 | 0.26615717318277 | 0.156474065234414 | -0.144515277 | 0.898820841699811 | 1.45812706830516 | 0.865441690796525 | 2.45670455899503 | 0.998735809224318 |
| ebi-fl187-GCST90094734 | prot-a-1603 | Junctophilin-3 \|\| id:prot-a-1603 | Chips liking \|\| id:ebi-fl187-GCST90094734 | Inverse variance weighted | 4 | 0.474339441226921 | 0.266309222229548 | 0.0748862505606554 | -0.047626634 | 0.996305516796835 | 1.60695236034193 | 0.953489720942739 | 2.70825771027224 | 0.998735809224318 |
| ebi-fl187-GCST90094734 | prot-a-1627 | UPF0258 protein KIAA1024 \|\| id:prot-a-1627 | Chips liking \|\| id:ebi-fl187-GCST90094734 | Inverse variance weighted | 4 | 0.61416641755952 | 0.26615717318277 | 0.0210251336912722 | 0.0924983581212915 | 1.13583447699775 | 1.8481154007434 | 1.09691134056345 | 3.1137708291998 | 0.998735809224318 |
| ebi-fl187-GCST90094734 | prot-a-1666 | Kallikrein-7 \|\| id:prot-a-1666 | Chips liking \|\| id:ebi-fl187-GCST90094734 | Inverse variance weighted | 4 | 0.35724146186328 | 0.266309222229548 | 0.179773228658381 | -0.164724614 | 0.879207537433194 | 1.42938096918019 | 0.848127234546321 | 2.40898991546641 | 0.998735809224318 |
| ebi-fl187-GCST90094734 | prot-a-1678 | Importin subunit alpha-3 \|\| id:prot-a-1678 | Chips liking \|\| id:ebi-fl187-GCST90094734 | Inverse variance weighted | 4 | 0.228018239082521 | 0.26615717318277 | 0.39160781882227 | -0.29364982 | 0.74968629852075 | 1.25610823548518 | 0.745537517800353 | 2.11633601473052 | 0.998735809224318 |
| ebi-fl187-GCST90094734 | prot-a-1718 | Protein LDOC1 \|\| id:prot-a-1718 | Chips liking \|\| id:ebi-fl187-GCST90094734 | Inverse variance weighted | 4 | -0.144515154 | 0.314467294675943 | 0.645835355032606 | -0.760871051 | 0.471840743949849 | 0.865441797731933 | 0.467259242981696 | 1.60294208517309 | 0.998735809224318 |
| ebi-fl187-GCST90094734 | prot-a-1723 | Leptin \|\| id:prot-a-1723 | Chips liking \|\| id:ebi-fl187-GCST90094734 | Inverse variance weighted | 4 | 0.102473359551527 | 0.386840424323112 | 0.791087830322325 | -0.655733872 | 0.860680591224826 | 1.10790778635636 | 0.519060998381741 | 2.3647695875742 | 0.998735809224318 |
| ebi-fl187-GCST90094734 | prot-a-1730 | Galectin-7 \|\| id:prot-a-1730 | Chips liking \|\| id:ebi-fl187-GCST90094734 | Inverse variance weighted | 4 | -0.244562045 | 0.386532332515589 | 0.526924435713588 | -1.002165417 | 0.513041326567882 | 0.783047402554652 | 0.367083690691566 | 1.67036359880881 | 0.998735809224318 |
| ebi-fl187-GCST90094734 | prot-a-1753 | Lipase member N \|\| id:prot-a-1753 | Chips liking \|\| id:ebi-fl187-GCST90094734 | Inverse variance weighted | 4 | -0.069856589 | 0.361960956040948 | 0.846963026427705 | -0.779300063 | 0.639586885002234 | 0.9325275451757 | 0.458726979096601 | 1.89569757641896 | 0.998735809224318 |
| ebi-fl187-GCST90094734 | prot-a-1756 | VIP36-like protein \|\| id:prot-a-1756 | Chips liking \|\| id:ebi-fl187-GCST90094734 | Inverse variance weighted | 4 | 0.307696963140676 | 0.26615717318277 | 0.247651542448235 | -0.213971096 | 0.829365022578905 | 1.36028870885417 | 0.807371720717416 | 2.29186299687574 | 0.998735809224318 |
| ebi-fl187-GCST90094734 | prot-a-1760 | Ig Kappa chain V-I region HK102- like \|\| id:prot-a-1760 | Chips liking \|\| id:ebi-fl187-GCST90094734 | Inverse variance weighted | 4 | 0.104344245987376 | 0.26615717318277 | 0.695028692772596 | -0.417323813 | 0.626012305425605 | 1.10998249617409 | 0.658807554652989 | 1.87013815052838 | 0.998735809224318 |
| ebi-fl187-GCST90094734 | prot-a-179 | Neutral ceramidase \|\| id:prot-a-179 | Chips liking \|\| id:ebi-fl187-GCST90094734 | Inverse variance weighted | 4 | -0.093008297 | 0.419962013935508 | 0.824727773229042 | -0.916133844 | 0.730117250568503 | 0.9111859403873 | 0.400062760049202 | 2.07532392631941 | 0.998735809224318 |
| ebi-fl187-GCST90094734 | prot-a-1809 | Lactotransferrin \|\| id:prot-a-1809 | Chips liking \|\| id:ebi-fl187-GCST90094734 | Inverse variance weighted | 4 | 0.283362620432571 | 0.281356776952341 | 0.313872699634312 | -0.268096662 | 0.834821903259161 | 1.32758648452845 | 0.764833846858928 | 2.30440360496717 | 0.998735809224318 |
| ebi-fl187-GCST90094734 | prot-a-1818 | Lysophospholipase-like protein 1 \|\| id:prot-a-1818 | Chips liking \|\| id:ebi-fl187-GCST90094734 | Inverse variance weighted | 4 | 0.153074122530983 | 0.266448744444477 | 0.565631229976616 | -0.369165417 | 0.675313661642158 | 1.16541135898091 | 0.691311046682546 | 1.96464911440279 | 0.998735809224318 |
| ebi-fl187-GCST90094734 | prot-a-1852 | MAP kinase-activated protein kinase 3 \|\| id:prot-a-1852 | Chips liking \|\| id:ebi-fl187-GCST90094734 | Inverse variance weighted | 4 | 0.213680909225399 | 0.266448744444477 | 0.422576804324018 | -0.30855863 | 0.735920448336574 | 1.238227485 | 0.734504886999102 | 2.08740245464349 | 0.998735809224318 |
| ebi-fl187-GCST90094734 | prot-a-1856 | Megakaryocyte-associated tyrosine-protein kinase \|\| id:prot-a-1856 | Chips liking \|\| id:ebi-fl187-GCST90094734 | Inverse variance weighted | 4 | 0.226513973329196 | 0.286928271012978 | 0.429852198925916 | -0.335865438 | 0.788893384514632 | 1.25422013534229 | 0.714719273503276 | 2.20095946228996 | 0.998735809224318 |
| ebi-fl187-GCST90094734 | prot-a-1890 | Beta-1,3-N-acetylglucosaminyltransferase manic fringe \|\| id:prot-a-1890 | Chips liking \|\| id:ebi-fl187-GCST90094734 | Inverse variance weighted (multiplicative random effects) | 4 | -0.193618162 | 0.522278854239324 | 0.710847596937726 | -1.217284716 | 0.830048392417194 | 0.823972468296925 | 0.296032889930448 | 2.29342972218674 | 0.998735809224318 |
| ebi-fl187-GCST90094734 | prot-a-1919 | Matrilysin \|\| id:prot-a-1919 | Chips liking \|\| id:ebi-fl187-GCST90094734 | Inverse variance weighted | 4 | 0.919907114951433 | 0.26615717318277 | 0.000547736468852078 | 0.398239055513204 | 1.44157517438966 | 2.50905732520084 | 1.4891999888262 | 4.22734938784552 | 0.998735809224318 |
| ebi-fl187-GCST90094734 | prot-a-1923 | Multimerin-2 \|\| id:prot-a-1923 | Chips liking \|\| id:ebi-fl187-GCST90094734 | Inverse variance weighted | 4 | -0.249623487 | 0.266601294027962 | 0.34910968323728 | -0.772162023 | 0.272915049495537 | 0.779094067056153 | 0.462013104726378 | 1.31378863307693 | 0.998735809224318 |
| ebi-fl187-GCST90094734 | prot-a-1948 | Mesothelin \|\| id:prot-a-1948 | Chips liking \|\| id:ebi-fl187-GCST90094734 | Inverse variance weighted | 4 | -0.171356119 | 0.266309222229548 | 0.519933489173232 | -0.693322194 | 0.350609956888271 | 0.842521482401205 | 0.499912500811196 | 1.41993338265332 | 0.998735809224318 |
| ebi-fl187-GCST90094734 | prot-a-195 | ATPase family AAA domain-containing protein 1 \|\| id:prot-a-195 | Chips liking \|\| id:ebi-fl187-GCST90094734 | Inverse variance weighted | 4 | 0.572370040439929 | 0.26615717318277 | 0.0315159711380612 | 0.0507019810017001 | 1.09403809987816 | 1.77246288603904 | 1.05200932779522 | 2.98630877063611 | 0.998735809224318 |
| ebi-fl187-GCST90094734 | prot-a-1966 | Mucin-1 \|\| id:prot-a-1966 | Chips liking \|\| id:ebi-fl187-GCST90094734 | Inverse variance weighted | 4 | 0.0392864385835447 | 0.299834842103712 | 0.895754004742206 | -0.548389852 | 0.62696272910682 | 1.04006835669384 | 0.577879533293451 | 1.87191641903417 | 0.998735809224318 |
| ebi-fl187-GCST90094734 | prot-a-1986 | Myocardial zonula adherens protein \|\| id:prot-a-1986 | Chips liking \|\| id:ebi-fl187-GCST90094734 | Inverse variance weighted | 4 | 0.293973563535268 | 0.266309222229548 | 0.269644968762364 | -0.227992512 | 0.815939639105181 | 1.341748432 | 0.796130221208648 | 2.26129947980692 | 0.998735809224318 |
| ebi-fl187-GCST90094734 | prot-a-2071 | Epididymal secretory protein E1 \|\| id:prot-a-2071 | Chips liking \|\| id:ebi-fl187-GCST90094734 | Inverse variance weighted | 4 | 0.296286889536473 | 0.345620478345513 | 0.391300764679316 | -0.381129248 | 0.973703027093678 | 1.34485592658615 | 0.683089595935873 | 2.64773094779165 | 0.998735809224318 |
| ebi-fl187-GCST90094734 | prot-a-2098 | Neuritin \|\| id:prot-a-2098 | Chips liking \|\| id:ebi-fl187-GCST90094734 | Inverse variance weighted | 4 | 0.358418975256425 | 0.26615717318277 | 0.178094897928207 | -0.163249084 | 0.880087034694654 | 1.43106507574974 | 0.849379595041932 | 2.41110954746859 | 0.998735809224318 |
| ebi-fl187-GCST90094734 | prot-a-2115 | Neurotrophin-4 \|\| id:prot-a-2115 | Chips liking \|\| id:ebi-fl187-GCST90094734 | Inverse variance weighted | 4 | -0.197892431 | 0.266601294027962 | 0.457918564864502 | -0.720430967 | 0.324646105401799 | 0.820458104336804 | 0.486542526905476 | 1.38354093167013 | 0.998735809224318 |
| ebi-fl187-GCST90094734 | prot-a-2118 | Netrin-1 \|\| id:prot-a-2118 | Chips liking \|\| id:ebi-fl187-GCST90094734 | Inverse variance weighted | 4 | 0.278686240071573 | 0.26615717318277 | 0.295065477288426 | -0.242981819 | 0.800354299509802 | 1.32139267874798 | 0.784285772454006 | 2.22632957625298 | 0.998735809224318 |
| ebi-fl187-GCST90094734 | prot-a-2120 | Netrin-G1 \|\| id:prot-a-2120 | Chips liking \|\| id:ebi-fl187-GCST90094734 | Inverse variance weighted | 4 | 0.120140936262818 | 0.266309222229548 | 0.651893538162249 | -0.401825139 | 0.642107011832732 | 1.12765576797025 | 0.66909773435514 | 1.90048099962875 | 0.998735809224318 |
| ebi-fl187-GCST90094734 | prot-a-2145 | Olfactomedin-like protein 3 \|\| id:prot-a-2145 | Chips liking \|\| id:ebi-fl187-GCST90094734 | Inverse variance weighted | 4 | 0.26219021957823 | 0.320955599324239 | 0.413982745649311 | -0.366882755 | 0.891263194253739 | 1.29977376133237 | 0.692890878205823 | 2.43820763670996 | 0.998735809224318 |
| ebi-fl187-GCST90094734 | prot-a-2162 | Prolyl 4-hydroxylase subunit alpha-2 \|\| id:prot-a-2162 | Chips liking \|\| id:ebi-fl187-GCST90094734 | Inverse variance weighted | 4 | 0.424577063409545 | 0.369492721110093 | 0.250522584462613 | -0.29962867 | 1.14878279678533 | 1.52894363669023 | 0.741093359817167 | 3.1543510857421 | 0.998735809224318 |
| ebi-fl187-GCST90094734 | prot-a-2168 | Polyadenylate-binding protein-interacting protein 1 \|\| id:prot-a-2168 | Chips liking \|\| id:ebi-fl187-GCST90094734 | Inverse variance weighted | 4 | 0.35418779774073 | 0.314464941392929 | 0.260030572937818 | -0.262163487 | 0.970539082870871 | 1.4250227774107 | 0.76938522864242 | 2.63936691340236 | 0.998735809224318 |
| ebi-fl187-GCST90094734 | prot-a-219 | Beta-1,4-galactosyltransferase 5 \|\| id:prot-a-219 | Chips liking \|\| id:ebi-fl187-GCST90094734 | Inverse variance weighted | 4 | -0.129979391 | 0.266309222229548 | 0.625495341042438 | -0.651945467 | 0.391986684112873 | 0.878113527374442 | 0.521031141205795 | 1.47991800484997 | 0.998735809224318 |
| ebi-fl187-GCST90094734 | prot-a-2190 | Pyruvate carboxylase, mitochondrial \|\| id:prot-a-2190 | Chips liking \|\| id:ebi-fl187-GCST90094734 | Inverse variance weighted | 4 | 0.594371787866321 | 0.26615717318277 | 0.0255383368667189 | 0.0727037284280924 | 1.11603984730455 | 1.81189233460366 | 1.07541187574504 | 3.0527409137277 | 0.998735809224318 |
| ebi-fl187-GCST90094734 | prot-a-2197 | Protocadherin-9 \|\| id:prot-a-2197 | Chips liking \|\| id:ebi-fl187-GCST90094734 | Inverse variance weighted | 4 | -0.10507652 | 0.305156856043253 | 0.730593543703798 | -0.703183957 | 0.493030918281441 | 0.900255632782681 | 0.495006711753144 | 1.63727114221658 | 0.998735809224318 |
| ebi-fl187-GCST90094734 | prot-a-2198 | Protocadherin alpha-4 \|\| id:prot-a-2198 | Chips liking \|\| id:ebi-fl187-GCST90094734 | Inverse variance weighted | 4 | -0.856725557 | 0.360042324885304 | 0.0173355100008314 | -1.562408514 | -0.151042601 | 0.424549973291369 | 0.209630564489056 | 0.85981106934963 | 0.998735809224318 |
| ebi-fl187-GCST90094734 | prot-a-2254 | Placenta growth factor \|\| id:prot-a-2254 | Chips liking \|\| id:ebi-fl187-GCST90094734 | Inverse variance weighted | 4 | 0.0631558715658497 | 0.266309222229548 | 0.812538561201938 | -0.458810204 | 0.585121947135763 | 1.06519286 | 0.632035191265059 | 1.79520989299661 | 0.998735809224318 |
| ebi-fl187-GCST90094734 | prot-a-2261 | Membrane-associated progesterone receptor component 2 \|\| id:prot-a-2261 | Chips liking \|\| id:ebi-fl187-GCST90094734 | Inverse variance weighted | 4 | -0.269146498 | 0.266309222229548 | 0.312182030137554 | -0.791112573 | 0.252819577892546 | 0.764031318635526 | 0.453340140489473 | 1.28765093518008 | 0.998735809224318 |
| ebi-fl187-GCST90094734 | prot-a-2268 | Polymeric immunoglobulin receptor \|\| id:prot-a-2268 | Chips liking \|\| id:ebi-fl187-GCST90094734 | Inverse variance weighted | 4 | 0.0362538457997289 | 0.26615717318277 | 0.891653555521475 | -0.485414214 | 0.557921905237958 | 1.036919031 | 0.615442219399396 | 1.74703821444626 | 0.998735809224318 |
| ebi-fl187-GCST90094734 | prot-a-2283 | Group 10 secretory phospholipase A2 \|\| id:prot-a-2283 | Chips liking \|\| id:ebi-fl187-GCST90094734 | Inverse variance weighted (multiplicative random effects) | 4 | -0.114414672 | 0.473833636821966 | 0.809194032787161 | -1.0431286 | 0.814299256666832 | 0.89188803863606 | 0.352350591784242 | 2.2575931246035 | 0.998735809224318 |
| ebi-fl187-GCST90094734 | prot-a-2298 | Inactive phospholipase D5 \|\| id:prot-a-2298 | Chips liking \|\| id:ebi-fl187-GCST90094734 | Inverse variance weighted | 4 | 0.496472669124255 | 0.26615717318277 | 0.0621342629373839 | -0.02519539 | 1.01814072856248 | 1.64291592995452 | 0.975119364534573 | 2.76804343249469 | 0.998735809224318 |
| ebi-fl187-GCST90094734 | prot-a-2304 | Procollagen-lysine,2-oxoglutarate 5-dioxygenase 2 \|\| id:prot-a-2304 | Chips liking \|\| id:ebi-fl187-GCST90094734 | Inverse variance weighted | 4 | -0.204877771 | 0.346978132724061 | 0.554880243015062 | -0.884954911 | 0.475199369486169 | 0.814746896306611 | 0.412732782653729 | 1.60833481841004 | 0.998735809224318 |
| ebi-fl187-GCST90094734 | prot-a-2331 | Periostin \|\| id:prot-a-2331 | Chips liking \|\| id:ebi-fl187-GCST90094734 | Inverse variance weighted | 4 | 0.165500620779248 | 0.26642109337285 | 0.534468440520324 | -0.356684722 | 0.687685963790034 | 1.17998369522862 | 0.699993155228501 | 1.98910733712944 | 0.998735809224318 |
| ebi-fl187-GCST90094734 | prot-a-2333 | Periostin \|\| id:prot-a-2333 | Chips liking \|\| id:ebi-fl187-GCST90094734 | Inverse variance weighted | 4 | 0.0859451370117328 | 0.266309222229548 | 0.746902114252308 | -0.436020939 | 0.607911212581646 | 1.08974653991343 | 0.64660418683431 | 1.83659114096888 | 0.998735809224318 |
| ebi-fl187-GCST90094734 | prot-a-2367 | cAMP-dependent protein kinase catalytic subunit alpha \|\| id:prot-a-2367 | Chips liking \|\| id:ebi-fl187-GCST90094734 | Inverse variance weighted | 4 | 0.119078954413251 | 0.266448744444477 | 0.654939105707403 | -0.403160585 | 0.641318493524426 | 1.12645885367523 | 0.668204787243541 | 1.89898302623329 | 0.998735809224318 |
| ebi-fl187-GCST90094734 | prot-a-2385 | Phosphoribosyl pyrophosphate synthase-associated protein 1 \|\| id:prot-a-2385 | Chips liking \|\| id:ebi-fl187-GCST90094734 | Inverse variance weighted | 4 | -0.602570844 | 0.266448744444477 | 0.0237290072702953 | -1.124810383 | -0.080331304 | 0.547402539293485 | 0.324714032928747 | 0.922810564490473 | 0.998735809224318 |
| ebi-fl187-GCST90094734 | prot-a-2393 | Trypsin-3 \|\| id:prot-a-2393 | Chips liking \|\| id:ebi-fl187-GCST90094734 | Inverse variance weighted | 4 | -0.051547744 | 0.266309222229548 | 0.846517684871119 | -0.573513819 | 0.470418331958216 | 0.949758303988045 | 0.563541771729885 | 1.60066366194166 | 0.998735809224318 |
| ebi-fl187-GCST90094734 | prot-a-2405 | Pregnancy-specific beta-1-glycoprotein 6 \|\| id:prot-a-2405 | Chips liking \|\| id:ebi-fl187-GCST90094734 | Inverse variance weighted | 4 | 0.410034194728301 | 0.276101583239291 | 0.137521428979014 | -0.131124908 | 0.951193297877311 | 1.50686931121857 | 0.877108209347167 | 2.58879702286948 | 0.998735809224318 |
| ebi-fl187-GCST90094734 | prot-a-244 | Beclin-1 \|\| id:prot-a-244 | Chips liking \|\| id:ebi-fl187-GCST90094734 | Inverse variance weighted | 4 | -0.115268958 | 0.266309222229548 | 0.665131899357931 | -0.637235034 | 0.406697117210972 | 0.891126435768749 | 0.528752387149204 | 1.50184915250668 | 0.998735809224318 |
| ebi-fl187-GCST90094734 | prot-a-2454 | tRNA pseudouridine synthase A, mitochondrial \|\| id:prot-a-2454 | Chips liking \|\| id:ebi-fl187-GCST90094734 | Inverse variance weighted | 4 | -0.048911203 | 0.26615717318277 | 0.854195367522413 | -0.570579263 | 0.472756856368586 | 0.952265684214001 | 0.565197945876965 | 1.60441123317345 | 0.998735809224318 |
| ebi-fl187-GCST90094734 | prot-a-2466 | Glutaminyl-peptide cyclotransferase-like protein \|\| id:prot-a-2466 | Chips liking \|\| id:ebi-fl187-GCST90094734 | Inverse variance weighted | 4 | 0.384345992851704 | 0.266448744444477 | 0.149168059323484 | -0.137893546 | 0.906585531962879 | 1.46865349739683 | 0.871191428395898 | 2.47585435888351 | 0.998735809224318 |
| ebi-fl187-GCST90094734 | prot-a-2474 | Ras-related protein Rab-27A \|\| id:prot-a-2474 | Chips liking \|\| id:ebi-fl187-GCST90094734 | Inverse variance weighted (multiplicative random effects) | 4 | 0.388366967555186 | 0.522223255644842 | 0.457070118736988 | -0.635190614 | 1.41192454861908 | 1.47457080465674 | 0.52983448508135 | 4.1038458597352 | 0.998735809224318 |
| ebi-fl187-GCST90094734 | prot-a-2490 | Rap1 GTPase-GDP dissociation stimulator 1 \|\| id:prot-a-2490 | Chips liking \|\| id:ebi-fl187-GCST90094734 | Inverse variance weighted | 4 | 0.358434336092424 | 0.266309222229548 | 0.178324166670771 | -0.163531739 | 0.880400411662338 | 1.43108705827451 | 0.849139547328323 | 2.41186525207136 | 0.998735809224318 |
| ebi-fl187-GCST90094734 | prot-a-2545 | Receptor-interacting serine/threonine-protein kinase 2 \|\| id:prot-a-2545 | Chips liking \|\| id:ebi-fl187-GCST90094734 | Inverse variance weighted | 4 | 0.454667014949189 | 0.28420167040718 | 0.109642035403265 | -0.102368259 | 1.01170228894726 | 1.57564862823256 | 0.902697064089603 | 2.75027880162099 | 0.998735809224318 |
| ebi-fl187-GCST90094734 | prot-a-2610 | RUN and FYVE domain-containing protein 1 \|\| id:prot-a-2610 | Chips liking \|\| id:ebi-fl187-GCST90094734 | Inverse variance weighted | 4 | 0.464334347480293 | 0.26615717318277 | 0.0810567832579588 | -0.057333712 | 0.986002406918521 | 1.590954813 | 0.944278899621061 | 2.68049748738911 | 0.998735809224318 |
| ebi-fl187-GCST90094734 | prot-a-2627 | Serine--tRNA ligase, mitochondrial \|\| id:prot-a-2627 | Chips liking \|\| id:ebi-fl187-GCST90094734 | Inverse variance weighted | 4 | 0.381671020409819 | 0.324649598494125 | 0.23973880555033 | -0.254642193 | 1.0179842334583 | 1.46473013954114 | 0.775193818399159 | 2.76761028114326 | 0.998735809224318 |
| ebi-fl187-GCST90094734 | prot-a-264 | BolA-like protein 2 \|\| id:prot-a-264 | Chips liking \|\| id:ebi-fl187-GCST90094734 | Inverse variance weighted | 4 | 0.178488788498271 | 0.266448744444477 | 0.50293404236997 | -0.343750751 | 0.700728327609446 | 1.19540948086289 | 0.709105650173347 | 2.01521991340436 | 0.998735809224318 |
| ebi-fl187-GCST90094734 | prot-a-2646 | Secretoglobin family 1C member 1 \|\| id:prot-a-2646 | Chips liking \|\| id:ebi-fl187-GCST90094734 | Inverse variance weighted | 4 | -0.029679116 | 0.266601294027962 | 0.911359443130123 | -0.552217652 | 0.492859420121174 | 0.970756983791596 | 0.575671753875702 | 1.63699037730385 | 0.998735809224318 |
| ebi-fl187-GCST90094734 | prot-a-2672 | Semaphorin-3E \|\| id:prot-a-2672 | Chips liking \|\| id:ebi-fl187-GCST90094734 | Inverse variance weighted | 4 | 0.00294478518658392 | 0.374223389476662 | 0.993721465699663 | -0.730533058 | 0.736422628560842 | 1.0029491253257 | 0.481652173012625 | 2.08845097012613 | 0.999107451094609 |
| ebi-fl187-GCST90094734 | prot-a-2701 | Plasma protease C1 inhibitor \|\| id:prot-a-2701 | Chips liking \|\| id:ebi-fl187-GCST90094734 | Inverse variance weighted | 4 | -0.012209118 | 0.266448744444477 | 0.963452411372791 | -0.534448657 | 0.510030420890164 | 0.987865111 | 0.585992283645546 | 1.66534185535698 | 0.998735809224318 |
| ebi-fl187-GCST90094734 | prot-a-2738 | Signaling threshold-regulating transmembrane adapter 1 \|\| id:prot-a-2738 | Chips liking \|\| id:ebi-fl187-GCST90094734 | Inverse variance weighted | 4 | 0.389978228188257 | 0.26615717318277 | 0.142861504255237 | -0.131689831 | 0.911646287626486 | 1.4769486376849 | 0.876612850828815 | 2.4884158112636 | 0.998735809224318 |
| ebi-fl187-GCST90094734 | prot-a-274 | Serine/threonine-protein kinase BRSK2 \|\| id:prot-a-274 | Chips liking \|\| id:ebi-fl187-GCST90094734 | Inverse variance weighted | 4 | 0.108285301035139 | 0.301304973684112 | 0.719305076063846 | -0.482272447 | 0.698843049455999 | 1.11436562970563 | 0.617378835599573 | 2.01142424239928 | 0.998735809224318 |
| ebi-fl187-GCST90094734 | prot-a-2749 | Mitochondrial glutamate carrier 2 \|\| id:prot-a-2749 | Chips liking \|\| id:ebi-fl187-GCST90094734 | Inverse variance weighted | 4 | 0.471633665884579 | 0.26615717318277 | 0.0763925895254146 | -0.050034394 | 0.993301725322809 | 1.60261018539312 | 0.951196708903075 | 2.70013487461244 | 0.998735809224318 |
| ebi-fl187-GCST90094734 | prot-a-2760 | Sodium-coupled monocarboxylate transporter 1 \|\| id:prot-a-2760 | Chips liking \|\| id:ebi-fl187-GCST90094734 | Inverse variance weighted | 4 | 0.0146419718225836 | 0.416796228815807 | 0.97197623209941 | -0.802278637 | 0.831562580301566 | 1.01474969058827 | 0.448306272282854 | 2.29690503616088 | 0.998955228396052 |
| ebi-fl187-GCST90094734 | prot-a-2777 | Small integral membrane protein 10 \|\| id:prot-a-2777 | Chips liking \|\| id:ebi-fl187-GCST90094734 | Inverse variance weighted (multiplicative random effects) | 4 | -0.272655463 | 0.461167626782287 | 0.554367301815664 | -1.176544012 | 0.631233085345288 | 0.761355057320261 | 0.308342527506846 | 1.87992726139364 | 0.998735809224318 |
| ebi-fl187-GCST90094734 | prot-a-2799 | Superoxide dismutase [Mn], mitochondrial \|\| id:prot-a-2799 | Chips liking \|\| id:ebi-fl187-GCST90094734 | Inverse variance weighted | 4 | 0.380612186298879 | 0.266601294027962 | 0.153393867797726 | -0.14192635 | 0.903150722593684 | 1.46318005409257 | 0.867685159148175 | 2.46736485938759 | 0.998735809224318 |
| ebi-fl187-GCST90094734 | prot-a-2805 | Protein SPACA7 \|\| id:prot-a-2805 | Chips liking \|\| id:ebi-fl187-GCST90094734 | Inverse variance weighted | 4 | 0.0662586566381877 | 0.33698279687697 | 0.844122365794425 | -0.594227625 | 0.726744938517048 | 1.06850305683448 | 0.551988743435107 | 2.06833707397668 | 0.998735809224318 |
| ebi-fl187-GCST90094734 | prot-a-2811 | SPARC-like protein 1 \|\| id:prot-a-2811 | Chips liking \|\| id:ebi-fl187-GCST90094734 | Inverse variance weighted | 4 | -0.348809175 | 0.26615717318277 | 0.190013789575006 | -0.870477234 | 0.172858884798009 | 0.705527750011846 | 0.418751658992134 | 1.18869834984016 | 0.998735809224318 |
| ebi-fl187-GCST90094734 | prot-a-2831 | Sepiapterin reductase \|\| id:prot-a-2831 | Chips liking \|\| id:ebi-fl187-GCST90094734 | Inverse variance weighted | 4 | -0.190267632 | 0.273295082296477 | 0.486304514874881 | -0.725925993 | 0.345390729687024 | 0.826737843149103 | 0.483876295416962 | 1.41254173384512 | 0.998735809224318 |
| ebi-fl187-GCST90094734 | prot-a-2834 | SPRY domain-containing SOCS box protein 1 \|\| id:prot-a-2834 | Chips liking \|\| id:ebi-fl187-GCST90094734 | Inverse variance weighted | 4 | 0.355214464011781 | 0.319044947691166 | 0.265550373937179 | -0.270113633 | 0.980542561486466 | 1.42648655150692 | 0.763292753809808 | 2.66590226551154 | 0.998735809224318 |
| ebi-fl187-GCST90094734 | prot-a-2835 | Spectrin alpha chain, non-erythrocytic 1 \|\| id:prot-a-2835 | Chips liking \|\| id:ebi-fl187-GCST90094734 | Inverse variance weighted | 4 | -0.119075577 | 0.27954929401649 | 0.670140227214111 | -0.666992193 | 0.428841039671044 | 0.887740705801217 | 0.513250015505024 | 1.5354769350781 | 0.998735809224318 |
| ebi-fl187-GCST90094734 | prot-a-2859 | Sia-alpha-2,3-Gal-beta-1,4-GlcNAc-R:alpha 2,8-sialyltransferase \|\| id:prot-a-2859 | Chips liking \|\| id:ebi-fl187-GCST90094734 | Inverse variance weighted | 4 | 0.245524905605649 | 0.281414341881002 | 0.382953316480961 | -0.306047204 | 0.797097015692413 | 1.27829211983376 | 0.736351859528248 | 2.21908958670377 | 0.998735809224318 |
| ebi-fl187-GCST90094734 | prot-a-2861 | Alpha-2,8-sialyltransferase 8F \|\| id:prot-a-2861 | Chips liking \|\| id:ebi-fl187-GCST90094734 | Inverse variance weighted | 4 | 0.135590975623471 | 0.366645908319541 | 0.711520705769087 | -0.583035005 | 0.854216955929771 | 1.1452133775964 | 0.558201648477047 | 2.34953387150321 | 0.998735809224318 |
| ebi-fl187-GCST90094734 | prot-a-289 | Uncharacterized protein C17orf89 \|\| id:prot-a-289 | Chips liking \|\| id:ebi-fl187-GCST90094734 | Inverse variance weighted | 4 | 0.327120514453289 | 0.266448744444477 | 0.219557646133018 | -0.195119025 | 0.849360053564464 | 1.38696861687332 | 0.822736726270611 | 2.33815008223998 | 0.998735809224318 |
| ebi-fl187-GCST90094734 | prot-a-2921 | Tachykinin-3 \|\| id:prot-a-2921 | Chips liking \|\| id:ebi-fl187-GCST90094734 | Inverse variance weighted | 4 | -0.007948878 | 0.283064688599563 | 0.977597157089336 | -0.562755668 | 0.546857911431953 | 0.992082630567526 | 0.569637168185656 | 1.72781553038162 | 0.998955228396052 |
| ebi-fl187-GCST90094734 | prot-a-2928 | Tax1-binding protein 1 \|\| id:prot-a-2928 | Chips liking \|\| id:ebi-fl187-GCST90094734 | Inverse variance weighted | 4 | 0.276718652621506 | 0.266309222229548 | 0.298763923951251 | -0.245247423 | 0.79868472819142 | 1.31879527923997 | 0.782510903133157 | 2.22261566143274 | 0.998735809224318 |
| ebi-fl187-GCST90094734 | prot-a-296 | Uncharacterized protein C1orf185 \|\| id:prot-a-296 | Chips liking \|\| id:ebi-fl187-GCST90094734 | Inverse variance weighted | 4 | 0.158904185125014 | 0.26615717318277 | 0.550486428231147 | -0.362763874 | 0.680572244563243 | 1.17222562464806 | 0.695750698716279 | 1.97500759627967 | 0.998735809224318 |
| ebi-fl187-GCST90094734 | prot-a-2964 | Transforming growth factor beta-2 \|\| id:prot-a-2964 | Chips liking \|\| id:ebi-fl187-GCST90094734 | Inverse variance weighted | 4 | -0.276856178 | 0.26615717318277 | 0.298247887929453 | -0.798524237 | 0.244811881533909 | 0.7581635299374 | 0.449992556555412 | 1.27738099164837 | 0.998735809224318 |
| ebi-fl187-GCST90094734 | prot-a-2977 | Thymocyte nuclear protein 1 \|\| id:prot-a-2977 | Chips liking \|\| id:ebi-fl187-GCST90094734 | Inverse variance weighted | 4 | 0.437841335429871 | 0.26615717318277 | 0.0999599050837432 | -0.083826724 | 0.9595093948681 | 1.54935905952719 | 0.919590585261333 | 2.61041547598794 | 0.998735809224318 |
| ebi-fl187-GCST90094734 | prot-a-3015 | Transmembrane protein 70, mitochondrial \|\| id:prot-a-3015 | Chips liking \|\| id:ebi-fl187-GCST90094734 | Inverse variance weighted | 4 | 0.205627374669407 | 0.281191980616751 | 0.464614001165359 | -0.345508907 | 0.756763656678238 | 1.22829542472183 | 0.707860026626254 | 2.13136721052504 | 0.998735809224318 |
| ebi-fl187-GCST90094734 | prot-a-3030 | Tumor necrosis factor alpha-induced protein 3 \|\| id:prot-a-3030 | Chips liking \|\| id:ebi-fl187-GCST90094734 | Inverse variance weighted | 4 | -0.417179576 | 0.266601294027962 | 0.11762816559697 | -0.939718112 | 0.105358960264483 | 0.658902586208687 | 0.390737964052002 | 1.11110938289763 | 0.998735809224318 |
| ebi-fl187-GCST90094734 | prot-a-3032 | Tumor necrosis factor receptor superfamily member 10A \|\| id:prot-a-3032 | Chips liking \|\| id:ebi-fl187-GCST90094734 | Inverse variance weighted | 4 | 0.168899678761837 | 0.26615717318277 | 0.525698307253372 | -0.352768381 | 0.690567738200066 | 1.184001352 | 0.702739942678768 | 1.99484776308602 | 0.998735809224318 |
| ebi-fl187-GCST90094734 | prot-a-3081 | Tumor protein p53-inducible protein 11 \|\| id:prot-a-3081 | Chips liking \|\| id:ebi-fl187-GCST90094734 | Inverse variance weighted | 4 | 0.158829771557023 | 0.266309222229548 | 0.550900638530895 | -0.363136304 | 0.680795847126937 | 1.17213839840229 | 0.695491628738179 | 1.97544926241851 | 0.998735809224318 |
| ebi-fl187-GCST90094734 | prot-a-315 | Complement C4 \|\| id:prot-a-315 | Chips liking \|\| id:ebi-fl187-GCST90094734 | Inverse variance weighted | 4 | 0.00581090773544356 | 0.26615717318277 | 0.982581475445285 | -0.515857152 | 0.527478967173673 | 1.00582782380984 | 0.59698866539799 | 1.69465463883738 | 0.998955228396052 |
| ebi-fl187-GCST90094734 | prot-a-3157 | UDP-glucuronosyltransferase 1-8 \|\| id:prot-a-3157 | Chips liking \|\| id:ebi-fl187-GCST90094734 | Inverse variance weighted | 4 | 0.0709236299845019 | 0.26615717318277 | 0.78987515452975 | -0.450744429 | 0.592591689422731 | 1.07349923958419 | 0.637153659080205 | 1.8086698568936 | 0.998735809224318 |
| ebi-fl187-GCST90094734 | prot-a-317 | Glutaredoxin-like protein C5orf63 \|\| id:prot-a-317 | Chips liking \|\| id:ebi-fl187-GCST90094734 | Inverse variance weighted | 4 | -0.22267321 | 0.266309222229548 | 0.403073155961024 | -0.744639285 | 0.299292866032952 | 0.800376361924229 | 0.474905574560974 | 1.34890461397356 | 0.998735809224318 |
| ebi-fl187-GCST90094734 | prot-a-3192 | Guanine nucleotide exchange factor VAV3 \|\| id:prot-a-3192 | Chips liking \|\| id:ebi-fl187-GCST90094734 | Inverse variance weighted | 3 | 0.137944360538568 | 0.465484733398956 | 0.766966152219345 | -0.774405717 | 1.05029443800052 | 1.14791167930971 | 0.460977650829388 | 2.85849264302694 | 0.998735809224318 |
| ebi-fl187-GCST90094734 | prot-a-3233 | Serine/threonine-protein kinase WNK3 \|\| id:prot-a-3233 | Chips liking \|\| id:ebi-fl187-GCST90094734 | Inverse variance weighted | 4 | -0.623565108 | 0.26615717318277 | 0.0191373113093375 | -1.145233167 | -0.101897049 | 0.536030022157501 | 0.318149726987032 | 0.903122524652937 | 0.998735809224318 |
| ebi-fl187-GCST90094734 | prot-a-3250 | 14-3-3 protein theta \|\| id:prot-a-3250 | Chips liking \|\| id:ebi-fl187-GCST90094734 | Inverse variance weighted | 4 | 0.157474370434564 | 0.329066138387666 | 0.632259293429604 | -0.487495261 | 0.80244400167439 | 1.17055075689002 | 0.614162786853889 | 2.23098680640474 | 0.998735809224318 |
| ebi-fl187-GCST90094734 | prot-a-3278 | E3 ubiquitin-protein ligase ZNRF3 \|\| id:prot-a-3278 | Chips liking \|\| id:ebi-fl187-GCST90094734 | Inverse variance weighted | 4 | 0.213051270943389 | 0.26615717318277 | 0.42343758406173 | -0.308616788 | 0.734719330381618 | 1.23744809474839 | 0.734462170458702 | 2.08489674320474 | 0.998735809224318 |
| ebi-fl187-GCST90094734 | prot-a-355 | Calpain-3 \|\| id:prot-a-355 | Chips liking \|\| id:ebi-fl187-GCST90094734 | Inverse variance weighted | 4 | 0.219572167191515 | 0.266448744444477 | 0.409900317342919 | -0.302667372 | 0.74181170630269 | 1.24554373210539 | 0.738844816034414 | 2.09973617587753 | 0.998735809224318 |
| ebi-fl187-GCST90094734 | prot-a-385 | Coiled-coil domain-containing protein 90B, mitochondrial \|\| id:prot-a-385 | Chips liking \|\| id:ebi-fl187-GCST90094734 | Inverse variance weighted | 4 | 0.620863070717077 | 0.26615717318277 | 0.01966424492292 | 0.0991950112788478 | 1.14253113015531 | 1.86053312075308 | 1.10428162593475 | 3.13469264734805 | 0.998735809224318 |
| ebi-fl187-GCST90094734 | prot-a-390 | C-C motif chemokine 14 \|\| id:prot-a-390 | Chips liking \|\| id:ebi-fl187-GCST90094734 | Inverse variance weighted | 4 | -0.109692302 | 0.26615717318277 | 0.680241536557681 | -0.631360361 | 0.411975757896013 | 0.896109824490784 | 0.531867776481315 | 1.50979783520898 | 0.998735809224318 |
| ebi-fl187-GCST90094734 | prot-a-40 | Pituitary adenylate cyclase-activating polypeptide 27 \|\| id:prot-a-40 | Chips liking \|\| id:ebi-fl187-GCST90094734 | Inverse variance weighted | 4 | -0.145165281 | 0.26615717318277 | 0.585470280702773 | -0.66683334 | 0.376502778772598 | 0.864879333464909 | 0.513331553167626 | 1.4571795885893 | 0.998735809224318 |
| ebi-fl187-GCST90094734 | prot-a-401 | C-C motif chemokine 24 \|\| id:prot-a-401 | Chips liking \|\| id:ebi-fl187-GCST90094734 | Inverse variance weighted | 4 | -0.295850882 | 0.317215037499218 | 0.351000257061783 | -0.917592356 | 0.32589059148725 | 0.743898348375432 | 0.399479689243637 | 1.3852638009293 | 0.998735809224318 |
| ebi-fl187-GCST90094734 | prot-a-412 | Cerebral cavernous malformations 2 protein \|\| id:prot-a-412 | Chips liking \|\| id:ebi-fl187-GCST90094734 | Inverse variance weighted | 3 | -0.48342472 | 0.335675110054076 | 0.14982310320828 | -1.141347936 | 0.174498495733951 | 0.616667856591415 | 0.319388216760267 | 1.19064895133087 | 0.998735809224318 |
| ebi-fl187-GCST90094734 | prot-a-44 | Adhesion G protein-coupled receptor F5 \|\| id:prot-a-44 | Chips liking \|\| id:ebi-fl187-GCST90094734 | Inverse variance weighted | 4 | 0.175764236714825 | 0.266309222229548 | 0.509253467782062 | -0.346201839 | 0.697730312284738 | 1.19215695867442 | 0.707369698006822 | 2.0091873006725 | 0.998735809224318 |
| ebi-fl187-GCST90094734 | prot-a-48 | Adrenomedullin \|\| id:prot-a-48 | Chips liking \|\| id:ebi-fl187-GCST90094734 | Inverse variance weighted | 4 | 0.414808350039638 | 0.26615717318277 | 0.119112636005266 | -0.106859709 | 0.936476409477867 | 1.51408053940904 | 0.89865173653991 | 2.55097696538571 | 0.998735809224318 |
| ebi-fl187-GCST90094734 | prot-a-502 | Carcinoembryonic antigen-related cell adhesion molecule 1 \|\| id:prot-a-502 | Chips liking \|\| id:ebi-fl187-GCST90094734 | Inverse variance weighted | 4 | 0.0600711890346187 | 0.266601294027962 | 0.821728633665175 | -0.462467347 | 0.582609725329424 | 1.06191214035472 | 0.629727969519447 | 1.79070558783228 | 0.998735809224318 |
| ebi-fl187-GCST90094734 | prot-a-518 | Complement factor B \|\| id:prot-a-518 | Chips liking \|\| id:ebi-fl187-GCST90094734 | Inverse variance weighted | 4 | 0.0984807070557394 | 0.266448744444477 | 0.711677051989453 | -0.423758832 | 0.620720246166914 | 1.10349311455787 | 0.654581727004156 | 1.86026740992251 | 0.998735809224318 |
| ebi-fl187-GCST90094734 | prot-a-525 | Properdin \|\| id:prot-a-525 | Chips liking \|\| id:ebi-fl187-GCST90094734 | Inverse variance weighted | 4 | 0.0675570281750897 | 0.334826590668838 | 0.8400987142749 | -0.58870309 | 0.723817145886011 | 1.06989127180495 | 0.555046663975054 | 2.06229026814918 | 0.998735809224318 |
| ebi-fl187-GCST90094734 | prot-a-533 | Chondroadherin \|\| id:prot-a-533 | Chips liking \|\| id:ebi-fl187-GCST90094734 | Inverse variance weighted | 4 | 0.314421676447595 | 0.290427320099151 | 0.278978271206994 | -0.254815871 | 0.883659223841931 | 1.36946708682758 | 0.775059195739205 | 2.4197378886852 | 0.998735809224318 |
| ebi-fl187-GCST90094734 | prot-a-535 | Coiled-coil-helix-coiled-coil-helix domain-containing protein 2 \|\| id:prot-a-535 | Chips liking \|\| id:ebi-fl187-GCST90094734 | Inverse variance weighted | 4 | 0.284975778759187 | 0.306518541291669 | 0.352517156893111 | -0.315800562 | 0.885752119690858 | 1.32972982002523 | 0.729204866700792 | 2.42480745125066 | 0.998735809224318 |
| ebi-fl187-GCST90094734 | prot-a-591 | Caseinolytic peptidase B protein homolog \|\| id:prot-a-591 | Chips liking \|\| id:ebi-fl187-GCST90094734 | Inverse variance weighted | 3 | 0.0983088972609413 | 0.335675110054076 | 0.769622140331292 | -0.559614318 | 0.75623211296693 | 1.10330353991815 | 0.571429411137974 | 2.13023459673133 | 0.998735809224318 |
| ebi-fl187-GCST90094734 | prot-a-605 | Ciliary neurotrophic factor receptor subunit alpha \|\| id:prot-a-605 | Chips liking \|\| id:ebi-fl187-GCST90094734 | Inverse variance weighted | 4 | 0.0210341315313954 | 0.26615717318277 | 0.937009567611898 | -0.500633928 | 0.542702190969624 | 1.02125690810489 | 0.606146284846941 | 1.72065011107893 | 0.998735809224318 |
| ebi-fl187-GCST90094734 | prot-a-606 | Ciliary neurotrophic factor receptor subunit alpha \|\| id:prot-a-606 | Chips liking \|\| id:ebi-fl187-GCST90094734 | Inverse variance weighted | 4 | 0.177146397696725 | 0.26615717318277 | 0.505685583674214 | -0.344521662 | 0.698814457134954 | 1.19380585076166 | 0.70855920339429 | 2.01136673193376 | 0.998735809224318 |
| ebi-fl187-GCST90094734 | prot-a-707 | Cancer/testis antigen 55 \|\| id:prot-a-707 | Chips liking \|\| id:ebi-fl187-GCST90094734 | Inverse variance weighted | 4 | 0.100185757218668 | 0.266309222229548 | 0.706767579490213 | -0.421780318 | 0.622151832788582 | 1.1053762306201 | 0.65587810795239 | 1.86293245102272 | 0.998735809224318 |
| ebi-fl187-GCST90094734 | prot-a-719 | Dipeptidyl peptidase 1 \|\| id:prot-a-719 | Chips liking \|\| id:ebi-fl187-GCST90094734 | Inverse variance weighted | 4 | -0.611577659 | 0.26615717318277 | 0.0215728096946821 | -1.133245718 | -0.0899096 | 0.542494322513412 | 0.321986481102254 | 0.914013808753122 | 0.998735809224318 |
| ebi-fl187-GCST90094734 | prot-a-752 | Cytochrome b5 \|\| id:prot-a-752 | Chips liking \|\| id:ebi-fl187-GCST90094734 | Inverse variance weighted | 4 | -0.031026937 | 0.26615717318277 | 0.907197849649627 | -0.552694996 | 0.490641122799609 | 0.969449459014577 | 0.575397026218408 | 1.63336307064423 | 0.998735809224318 |
| ebi-fl187-GCST90094734 | prot-a-755 | Cytoglobin \|\| id:prot-a-755 | Chips liking \|\| id:ebi-fl187-GCST90094734 | Inverse variance weighted | 4 | -0.079662247 | 0.276267722246685 | 0.773077593407197 | -0.621146982 | 0.461822488961191 | 0.923428184691417 | 0.537327778591587 | 1.58696357466868 | 0.998735809224318 |
| ebi-fl187-GCST90094734 | prot-a-807 | Desmin \|\| id:prot-a-807 | Chips liking \|\| id:ebi-fl187-GCST90094734 | Inverse variance weighted | 4 | -0.652900587 | 0.26615717318277 | 0.0141645123978472 | -1.174568647 | -0.131232528 | 0.520533731409206 | 0.308952218513461 | 0.877013820578813 | 0.998735809224318 |
| ebi-fl187-GCST90094734 | prot-a-82 | Ameloblastin \|\| id:prot-a-82 | Chips liking \|\| id:ebi-fl187-GCST90094734 | Inverse variance weighted | 4 | 0.0361760108985644 | 0.266309222229548 | 0.891946088041603 | -0.485790065 | 0.558142086468478 | 1.03683832527662 | 0.615210948270044 | 1.7474229218212 | 0.998735809224318 |
| ebi-fl187-GCST90094734 | prot-a-84 | Muellerian-inhibiting factor \|\| id:prot-a-84 | Chips liking \|\| id:ebi-fl187-GCST90094734 | Inverse variance weighted | 4 | -0.004587881 | 0.371813118409161 | 0.990154983869978 | -0.733341593 | 0.724165831557326 | 0.995422627722888 | 0.480301334133456 | 2.06300948459872 | 0.999107451094609 |
| ebi-fl187-GCST90094734 | prot-a-858 | Inactive dipeptidyl peptidase 10 \|\| id:prot-a-858 | Chips liking \|\| id:ebi-fl187-GCST90094734 | Inverse variance weighted (multiplicative random effects) | 4 | 0.672994388468921 | 0.476808259888783 | 0.158109886051654 | -0.261549801 | 1.60753857785094 | 1.96009783628567 | 0.769857534861463 | 4.9905123400567 | 0.998735809224318 |
| ebi-fl187-GCST90094734 | prot-a-863 | Dihydropyrimidinase-related protein 3 \|\| id:prot-a-863 | Chips liking \|\| id:ebi-fl187-GCST90094734 | Inverse variance weighted | 4 | 0.0701617653814454 | 0.26615717318277 | 0.792080240888335 | -0.451506294 | 0.591829824819674 | 1.07268168998274 | 0.636668419127687 | 1.8072924201278 | 0.998735809224318 |
| ebi-fl187-GCST90094734 | prot-a-864 | Dihydropyrimidinase-related protein 5 \|\| id:prot-a-864 | Chips liking \|\| id:ebi-fl187-GCST90094734 | Inverse variance weighted | 4 | -0.593280928 | 0.270679217861567 | 0.0283922915233448 | -1.123812195 | -0.062749661 | 0.552511556841745 | 0.325038320147443 | 0.939178556870506 | 0.998735809224318 |
| ebi-fl187-GCST90094734 | prot-a-884 | Endothelin-converting enzyme 1 \|\| id:prot-a-884 | Chips liking \|\| id:ebi-fl187-GCST90094734 | Inverse variance weighted | 4 | -0.673796784 | 0.26615717318277 | 0.0113551605343907 | -1.195464844 | -0.152128725 | 0.509769414395657 | 0.302563276891725 | 0.85887771484669 | 0.998735809224318 |
| ebi-fl187-GCST90094734 | prot-a-893 | Early endosome antigen 1 \|\| id:prot-a-893 | Chips liking \|\| id:ebi-fl187-GCST90094734 | Inverse variance weighted | 3 | 0.0264012176023047 | 0.335980283330465 | 0.937366963698113 | -0.632120138 | 0.684922572930017 | 1.02675281714689 | 0.531463829187661 | 1.98361824384257 | 0.998735809224318 |
| ebi-fl187-GCST90094734 | prot-a-897 | Ephrin-A2 \|\| id:prot-a-897 | Chips liking \|\| id:ebi-fl187-GCST90094734 | Inverse variance weighted | 4 | 0.239684857668489 | 0.266309222229548 | 0.36810714654523 | -0.282281218 | 0.761650933238403 | 1.27084858902047 | 0.754061599092934 | 2.14180928740845 | 0.998735809224318 |
| ebi-fl187-GCST90094734 | prot-a-90 | Anaphase-promoting complex subunit 10 \|\| id:prot-a-90 | Chips liking \|\| id:ebi-fl187-GCST90094734 | Inverse variance weighted | 4 | 0.0984073364610675 | 0.266601294027962 | 0.712039772758259 | -0.4241312 | 0.620945872755873 | 1.10341215358195 | 0.654338027236541 | 1.86068718306698 | 0.998735809224318 |
| ebi-fl187-GCST90094734 | prot-a-914 | Histone-lysine N-methyltransferase EHMT2 \|\| id:prot-a-914 | Chips liking \|\| id:ebi-fl187-GCST90094734 | Inverse variance weighted (multiplicative random effects) | 4 | 0.264921203320126 | 0.53631519542711 | 0.621330545205285 | -0.78625658 | 1.31609898635726 | 1.30332827379137 | 0.455546910973874 | 3.72884668591556 | 0.998735809224318 |
| ebi-fl187-GCST90094734 | prot-a-932 | RNA polymerase II elongation factor ELL \|\| id:prot-a-932 | Chips liking \|\| id:ebi-fl187-GCST90094734 | Inverse variance weighted | 4 | -0.303613996 | 0.26615717318277 | 0.253981479352611 | -0.825282055 | 0.218054063653012 | 0.738145738832398 | 0.438111403426514 | 1.24365430229624 | 0.998735809224318 |
| ebi-fl187-GCST90094734 | prot-a-991 | Estrogen receptor \|\| id:prot-a-991 | Chips liking \|\| id:ebi-fl187-GCST90094734 | Inverse variance weighted | 4 | 0.3544463886008 | 0.26615717318277 | 0.182952760655135 | -0.167221671 | 0.876114448039029 | 1.42539132292553 | 0.846012054349437 | 2.40155020608276 | 0.998735809224318 |
| ebi-fl187-GCST90094734 | prot-a-992 | Persulfide dioxygenase ETHE1, mitochondrial \|\| id:prot-a-992 | Chips liking \|\| id:ebi-fl187-GCST90094734 | Inverse variance weighted | 4 | 0.33521620379898 | 0.289505189614633 | 0.24690746302811 | -0.232213968 | 0.902646375443662 | 1.39824265791958 | 0.792776476483053 | 2.46612076470686 | 0.998735809224318 |
| ebi-fl187-GCST90094734 | prot-a-993 | Ethanolamine kinase 1 \|\| id:prot-a-993 | Chips liking \|\| id:ebi-fl187-GCST90094734 | Inverse variance weighted | 4 | 0.444066614728316 | 0.266309222229548 | 0.0954180185763523 | -0.077899461 | 0.96603269029823 | 1.55903433681165 | 0.92505742636352 | 2.62749964930566 | 0.998735809224318 |
| ebi-fl187-GCST90094734 | prot-b-10 | coagulation factor III, tissue factor \|\| id:prot-b-10 | Chips liking \|\| id:ebi-fl187-GCST90094734 | Inverse variance weighted | 4 | -0.238161397 | 0.353786925646166 | 0.500834376208246 | -0.931583771 | 0.455260977523689 | 0.788075488012122 | 0.393929322217953 | 1.57658478254108 | 0.998735809224318 |
| ebi-fl187-GCST90094734 | prot-b-28 | prolactin \|\| id:prot-b-28 | Chips liking \|\| id:ebi-fl187-GCST90094734 | Inverse variance weighted | 4 | 0.484471891868285 | 0.303642571208216 | 0.110592986732886 | -0.110667548 | 1.07961133143639 | 1.62331749526461 | 0.895236322836458 | 2.94353526908174 | 0.998735809224318 |
| ebi-fl187-GCST90094734 | prot-b-54 | matrix metallopeptidase 7 \|\| id:prot-b-54 | Chips liking \|\| id:ebi-fl187-GCST90094734 | Inverse variance weighted | 4 | -0.133411982 | 0.270167371948035 | 0.621438718570752 | -0.662940031 | 0.396116067329853 | 0.875104490807747 | 0.51533400728487 | 1.48604178844451 | 0.998735809224318 |
| ebi-fl187-GCST90094734 | ukb-rsfMRI191-edge-pheno716 | Net100_Pair10_33 (OccipitalPrecuneus)&(Temporal\|Frontal\|Supp_Motor_Area) (Default_mode\|Central_executive)&(Default_mode\|Salience) \|\| id:ukb-rsfMRI191-edge-pheno716 | Chips liking \|\| id:ebi-fl187-GCST90094734 | Inverse variance weighted | 4 | -0.023651031 | 0.0831331371987653 | 0.776030429521741 | -0.18659198 | 0.139289918070985 | 0.976626462816032 | 0.829782235280105 | 1.14945730014404 | 0.998735809224318 |
| ebi-fl187-GCST90094734 | ukb-rsfMRI191-edge-pheno903 | Net100_Pair27_38 (Precentral\|Frontal\|Supp_Motor_Area)&(Subcortical) (Attention\|Salience\|Motor)&(Subcortical-cerebellum) \|\| id:ukb-rsfMRI191-edge-pheno903 | Chips liking \|\| id:ebi-fl187-GCST90094734 | Inverse variance weighted | 4 | 0.065648840966781 | 0.0825439083020143 | 0.426427242857032 | -0.096137219 | 0.227434901238729 | 1.06785166555997 | 0.908339365820058 | 1.25537571369013 | 0.998735809224318 |
| ebi-fl187-GCST90094734 | ukb-rsfMRI191-node-pheno23 | Net100_Node2 RolandicOper\|SupraMarginal\|Insula Salience\|Motor \|\| id:ukb-rsfMRI191-node-pheno23 | Chips liking \|\| id:ebi-fl187-GCST90094734 | Inverse variance weighted (multiplicative random effects) | 4 | 0.0884060361374823 | 0.208824745096517 | 0.672039638685099 | -0.320890464 | 0.497702536526655 | 1.09243159869513 | 0.725502715120782 | 1.644937742 | 0.998735809224318 |
| ebi-fl187-GCST90094734 | ukb-rsfMRI191-node-pheno55 | Net100_Node34 Parietal\|Postcentral\|Precuneus Attention \|\| id:ukb-rsfMRI191-node-pheno55 | Chips liking \|\| id:ebi-fl187-GCST90094734 | Inverse variance weighted | 3 | -0.048563764 | 0.0991833206770574 | 0.624391213049319 | -0.242963073 | 0.145835544302532 | 0.952596595786032 | 0.784300475295343 | 1.1570058961923 | 0.998735809224318 |
| ebi-fl187-GCST90094734 | ebi-a-GCST90001414 | IgD- CD24- B cell Absolute Count \|\| id:ebi-a-GCST90001414 | Chips liking \|\| id:ebi-fl187-GCST90094734 | MR Egger | 4 | 1.33991354695152 | 1.27627826643228 | 0.403931624595512 | -1.161591855 | 3.84141894915878 | 3.81871335168463 | 0.312987553234781 | 46.5915385823527 | 0.999950574162623 |
| ebi-fl187-GCST90094734 | ebi-a-GCST90001422 | CD20- CD38- B cell %B cell \|\| id:ebi-a-GCST90001422 | Chips liking \|\| id:ebi-fl187-GCST90094734 | MR Egger | 4 | -1.699878357 | 1.21424302950427 | 0.296486464476701 | -4.079794695 | 0.680037980526665 | 0.182705747521169 | 0.016910937194092 | 1.97395270256999 | 0.999950574162623 |
| ebi-fl187-GCST90094734 | ebi-a-GCST90001444 | CD20- CD38- B cell %lymphocyte \|\| id:ebi-a-GCST90001444 | Chips liking \|\| id:ebi-fl187-GCST90094734 | MR Egger | 4 | -1.841113543 | 1.21515793783275 | 0.26896853803894 | -4.222823101 | 0.540596015399304 | 0.158640674541524 | 0.0146572072665701 | 1.71702993355289 | 0.999950574162623 |
| ebi-fl187-GCST90094734 | ebi-a-GCST90001459 | Myeloid Dendritic Cell %Dendritic Cell \|\| id:ebi-a-GCST90001459 | Chips liking \|\| id:ebi-fl187-GCST90094734 | MR Egger | 4 | 0.245592665646708 | 1.3220968100874 | 0.869766543807216 | -2.345717082 | 2.836902413 | 1.27837873989494 | 0.0957784964519102 | 17.0628300000087 | 0.999950574162623 |
| ebi-fl187-GCST90094734 | ebi-a-GCST90001476 | HLA DR++ monocyte %leukocyte \|\| id:ebi-a-GCST90001476 | Chips liking \|\| id:ebi-fl187-GCST90094734 | MR Egger | 4 | -0.341584369 | 1.29106641002429 | 0.816107413745043 | -2.872074532 | 2.18890579493311 | 0.710643509011258 | 0.0565814247445815 | 8.92544150628157 | 0.999950574162623 |
| ebi-fl187-GCST90094734 | ebi-a-GCST90001478 | CD4 regulatory T cell %CD4+ T cell \|\| id:ebi-a-GCST90001478 | Chips liking \|\| id:ebi-fl187-GCST90094734 | MR Egger | 4 | 1.64799975761068 | 1.98922897420755 | 0.494534888506794 | -2.250889032 | 5.54688854705749 | 5.19657501495155 | 0.105305562935979 | 256.438417241416 | 0.999950574162623 |
| ebi-fl187-GCST90094734 | ebi-a-GCST90001485 | CD39+ resting CD4 regulatory T cell %CD4 regulatory T cell \|\| id:ebi-a-GCST90001485 | Chips liking \|\| id:ebi-fl187-GCST90094734 | MR Egger | 4 | -0.816956685 | 1.27237857826525 | 0.586599380644849 | -3.310818698 | 1.67690532850956 | 0.441774068473195 | 0.0364862902591747 | 5.34897700448688 | 0.999950574162623 |
| ebi-fl187-GCST90094734 | ebi-a-GCST90001515 | Immature Myeloid-Derived Suppressor Cells Absolute Count \|\| id:ebi-a-GCST90001515 | Chips liking \|\| id:ebi-fl187-GCST90094734 | MR Egger | 4 | 0.774255213507109 | 1.71521393798325 | 0.695923327761051 | -2.587564105 | 4.13607453195428 | 2.16897610135735 | 0.0752030037800783 | 62.5567742216376 | 0.999950574162623 |
| ebi-fl187-GCST90094734 | ebi-a-GCST90001517 | CD33+ HLA DR+ Absolute Count \|\| id:ebi-a-GCST90001517 | Chips liking \|\| id:ebi-fl187-GCST90094734 | MR Egger | 4 | 1.20381650603173 | 1.67692108983997 | 0.547363922250934 | -2.08294883 | 4.49058184211807 | 3.33281237973246 | 0.124562356872105 | 89.1733155780184 | 0.999950574162623 |
| ebi-fl187-GCST90094734 | ebi-a-GCST90001518 | CD33+ HLA DR+ CD14- Absolute Count \|\| id:ebi-a-GCST90001518 | Chips liking \|\| id:ebi-fl187-GCST90094734 | MR Egger | 4 | 1.53581207180451 | 1.69877328810463 | 0.46137978066283 | -1.793783573 | 4.86540771648958 | 4.64509615035241 | 0.166329657243976 | 129.723818370943 | 0.999950574162623 |
| ebi-fl187-GCST90094734 | ebi-a-GCST90001520 | CD33+ HLA DR+ CD14dim Absolute Count \|\| id:ebi-a-GCST90001520 | Chips liking \|\| id:ebi-fl187-GCST90094734 | MR Egger | 4 | 0.408691482211703 | 1.68244590101094 | 0.830712267166195 | -2.888902484 | 3.70628544819315 | 1.5048473766757 | 0.055637241891027 | 40.7023344457514 | 0.999950574162623 |
| ebi-fl187-GCST90094734 | ebi-a-GCST90001531 | CD33dim HLA DR- Absolute Count \|\| id:ebi-a-GCST90001531 | Chips liking \|\| id:ebi-fl187-GCST90094734 | MR Egger | 4 | -0.976349753 | 1.71140065932756 | 0.625890616788446 | -4.330695046 | 2.37799553894227 | 0.376683580360619 | 0.0131583986236558 | 10.7832665487279 | 0.999950574162623 |
| ebi-fl187-GCST90094734 | ebi-a-GCST90001532 | Basophil Absolute Count \|\| id:ebi-a-GCST90001532 | Chips liking \|\| id:ebi-fl187-GCST90094734 | MR Egger | 4 | -1.276885471 | 1.70653093802157 | 0.53234013861783 | -4.621686109 | 2.06791516764674 | 0.278904605645689 | 0.00983619716686203 | 7.90831840098154 | 0.999950574162623 |
| ebi-fl187-GCST90094734 | ebi-a-GCST90001588 | T/B cell \|\| id:ebi-a-GCST90001588 | Chips liking \|\| id:ebi-fl187-GCST90094734 | MR Egger | 4 | 1.13759374656091 | 1.27204596101969 | 0.465530791682804 | -1.355616337 | 3.63080383015951 | 3.11925361287939 | 0.257788360963812 | 37.7431435037788 | 0.999950574162623 |
| ebi-fl187-GCST90094734 | ebi-a-GCST90001624 | HLA DR+ CD4+ T cell Absolute Count \|\| id:ebi-a-GCST90001624 | Chips liking \|\| id:ebi-fl187-GCST90094734 | MR Egger | 4 | 3.01534372341406 | 1.23666246947864 | 0.134969492631209 | 0.591485283235916 | 5.4392021635922 | 20.3961003530774 | 1.80666983999358 | 230.258401620454 | 0.999950574162623 |
| ebi-fl187-GCST90094734 | ebi-a-GCST90001631 | CD8+ Natural Killer T %T cell \|\| id:ebi-a-GCST90001631 | Chips liking \|\| id:ebi-fl187-GCST90094734 | MR Egger | 4 | -0.48731532 | 1.25268184229245 | 0.734774491974897 | -2.942571731 | 1.96794109061304 | 0.614273309571708 | 0.0527299469676933 | 7.15592790342392 | 0.999950574162623 |
| ebi-fl187-GCST90094734 | ebi-a-GCST90001632 | CD8+ Natural Killer T %lymphocyte \|\| id:ebi-a-GCST90001632 | Chips liking \|\| id:ebi-fl187-GCST90094734 | MR Egger | 4 | -0.476931382 | 1.26034586911292 | 0.741515107547507 | -2.947209286 | 1.99334652108281 | 0.620685117768737 | 0.0524859751097421 | 7.34005636008624 | 0.999950574162623 |
| ebi-fl187-GCST90094734 | ebi-a-GCST90001646 | Natural Killer %CD3- lymphocyte \|\| id:ebi-a-GCST90001646 | Chips liking \|\| id:ebi-fl187-GCST90094734 | MR Egger | 4 | 0.624168977261949 | 1.22620728574789 | 0.661335219890899 | -1.779197303 | 3.02753525732782 | 1.86669404748686 | 0.168773566979801 | 20.6462820528047 | 0.999950574162623 |
| ebi-fl187-GCST90094734 | ebi-a-GCST90001684 | CD127- CD8+ T cell Absolute Count \|\| id:ebi-a-GCST90001684 | Chips liking \|\| id:ebi-fl187-GCST90094734 | MR Egger | 4 | 0.912258520939411 | 1.33274097840108 | 0.564335359752596 | -1.699913797 | 3.52443083860553 | 2.48993976878085 | 0.182699272649266 | 33.9344539376376 | 0.999950574162623 |
| ebi-fl187-GCST90094734 | ebi-a-GCST90001687 | CD28- CD8+ T cell Absolute Count \|\| id:ebi-a-GCST90001687 | Chips liking \|\| id:ebi-fl187-GCST90094734 | MR Egger | 4 | 0.0378877845164098 | 1.29392266662325 | 0.979299405346055 | -2.498200642 | 2.57397621109798 | 1.03861467769065 | 0.0822328318800889 | 13.1178803411201 | 0.999950574162623 |
| ebi-fl187-GCST90094734 | ebi-a-GCST90001701 | BAFF-R on CD20- CD38- B cell \|\| id:ebi-a-GCST90001701 | Chips liking \|\| id:ebi-fl187-GCST90094734 | MR Egger | 4 | -0.430890037 | 2.09080575362146 | 0.855796871926239 | -4.528869315 | 3.66708923967808 | 0.649930374866334 | 0.0107928725205378 | 39.1378190903383 | 0.999950574162623 |
| ebi-fl187-GCST90094734 | ebi-a-GCST90001732 | CD19 on IgD- CD27- B cell \|\| id:ebi-a-GCST90001732 | Chips liking \|\| id:ebi-fl187-GCST90094734 | MR Egger | 4 | 1.42544471727881 | 1.23855576284959 | 0.368798348381481 | -1.002124578 | 3.853014012 | 4.15970732519942 | 0.367098682323699 | 47.1349145733524 | 0.999950574162623 |
| ebi-fl187-GCST90094734 | ebi-a-GCST90001798 | CD27 on CD24+ CD27+ B cell \|\| id:ebi-a-GCST90001798 | Chips liking \|\| id:ebi-fl187-GCST90094734 | MR Egger | 4 | 0.185934755764402 | 1.24443123519899 | 0.8949334998352 | -2.253150465 | 2.62501997675442 | 1.20434368133136 | 0.10506769048758 | 13.80484996 | 0.999950574162623 |
| ebi-fl187-GCST90094734 | ebi-a-GCST90001836 | CD66b on CD66b++ myeloid cell \|\| id:ebi-a-GCST90001836 | Chips liking \|\| id:ebi-fl187-GCST90094734 | MR Egger | 4 | -2.025861844 | 2.11823327546408 | 0.439803818620406 | -6.177599064 | 2.12587537589924 | 0.131880134091466 | 0.00207540479184088 | 8.38023012973581 | 0.999950574162623 |
| ebi-fl187-GCST90094734 | ebi-a-GCST90001943 | CD25 on activated & secreting CD4 regulatory T cell \|\| id:ebi-a-GCST90001943 | Chips liking \|\| id:ebi-fl187-GCST90094734 | MR Egger | 4 | 2.44470937598425 | 1.28218047817682 | 0.196817145471384 | -0.068364361 | 4.95778311321082 | 11.527199031112 | 0.933920127278686 | 142.278031730671 | 0.999950574162623 |
| ebi-fl187-GCST90094734 | ebi-a-GCST90001960 | CD25 on CD4+ T cell \|\| id:ebi-a-GCST90001960 | Chips liking \|\| id:ebi-fl187-GCST90094734 | MR Egger | 4 | 1.29187803282744 | 2.46345432928836 | 0.652315755935833 | -3.536492453 | 6.12024851823263 | 3.63961545860433 | 0.0291152713855369 | 454.97775071717 | 0.999950574162623 |
| ebi-fl187-GCST90094734 | ebi-a-GCST90001988 | HLA DR on CD14+ CD16- monocyte \|\| id:ebi-a-GCST90001988 | Chips liking \|\| id:ebi-fl187-GCST90094734 | MR Egger | 4 | 0.828551872086152 | 1.5093500685451 | 0.638141185006525 | -2.129774262 | 3.78687800643455 | 2.29000012485081 | 0.11886412294234 | 44.118447534927 | 0.999950574162623 |
| ebi-fl187-GCST90094734 | ebi-a-GCST90001991 | HLA DR on CD14+ monocyte \|\| id:ebi-a-GCST90001991 | Chips liking \|\| id:ebi-fl187-GCST90094734 | MR Egger | 4 | 0.891349111338972 | 1.49462315916329 | 0.611438261474101 | -2.038112281 | 3.82081050329902 | 2.43841712940267 | 0.130274400419262 | 45.6411856652475 | 0.999950574162623 |
| ebi-fl187-GCST90094734 | ebi-a-GCST90002019 | CD14 on Monocytic Myeloid-Derived Suppressor Cells \|\| id:ebi-a-GCST90002019 | Chips liking \|\| id:ebi-fl187-GCST90094734 | MR Egger | 4 | 1.96682225520874 | 1.81857532971106 | 0.392528366325263 | -1.597585391 | 5.53122990144243 | 7.1479260751341 | 0.202384608175359 | 252.454213965282 | 0.999950574162623 |
| ebi-fl187-GCST90094734 | ebi-a-GCST90002037 | CD80 on plasmacytoid Dendritic Cell \|\| id:ebi-a-GCST90002037 | Chips liking \|\| id:ebi-fl187-GCST90094734 | MR Egger | 4 | -0.893804473 | 1.4073606167646 | 0.590334610858479 | -3.652231282 | 1.86462233580137 | 0.409096392158731 | 0.025933199895008 | 6.45349817048633 | 0.999950574162623 |
| ebi-fl187-GCST90094734 | ebi-a-GCST90002042 | CD45 on CD33+ HLA DR+ CD14- \|\| id:ebi-a-GCST90002042 | Chips liking \|\| id:ebi-fl187-GCST90094734 | MR Egger | 4 | -0.919401556 | 3.1292506812374 | 0.796589185235036 | -7.052732892 | 5.21392977876359 | 0.398757603605517 | 0.000865041658836887 | 183.814992964631 | 0.999950574162623 |
| ebi-fl187-GCST90094734 | ebi-a-GCST90002056 | CD8 on Effector Memory CD8+ T cell \|\| id:ebi-a-GCST90002056 | Chips liking \|\| id:ebi-fl187-GCST90094734 | MR Egger | 4 | -1.123118185 | 1.37844364353681 | 0.500792391324168 | -3.824867726 | 1.57863135647281 | 0.325263978481867 | 0.0218213217294864 | 4.84831565243331 | 0.999950574162623 |
| ebi-fl187-GCST90094734 | ebi-a-GCST90002058 | CD8 on CD8+ T cell \|\| id:ebi-a-GCST90002058 | Chips liking \|\| id:ebi-fl187-GCST90094734 | MR Egger | 4 | -1.24664497 | 1.70155819631001 | 0.540002955997752 | -4.581699035 | 2.08840909441056 | 0.287467643232392 | 0.0102374876605261 | 8.07206305353815 | 0.999950574162623 |
| ebi-fl187-GCST90094734 | ebi-a-GCST90002063 | CD4 on CD4 regulatory T cell \|\| id:ebi-a-GCST90002063 | Chips liking \|\| id:ebi-fl187-GCST90094734 | MR Egger | 4 | 0.15104490955445 | 1.37235101951254 | 0.922408417371016 | -2.538763089 | 2.84085290779903 | 1.16304888891638 | 0.0789640108882348 | 17.1303699342757 | 0.999950574162623 |
| ebi-fl187-GCST90094734 | ebi-a-GCST90002066 | CD4 on activated CD4 regulatory T cell \|\| id:ebi-a-GCST90002066 | Chips liking \|\| id:ebi-fl187-GCST90094734 | MR Egger | 4 | 0.167058949322037 | 1.44766434873639 | 0.918670932105793 | -2.670363174 | 3.00448107284535 | 1.18182393097447 | 0.0692270792542081 | 20.1757436377621 | 0.999950574162623 |
| ebi-fl187-GCST90094734 | ebi-a-GCST90002070 | CD4 on activated & secreting CD4 regulatory T cell \|\| id:ebi-a-GCST90002070 | Chips liking \|\| id:ebi-fl187-GCST90094734 | MR Egger | 4 | -0.141977782 | 1.37896615571937 | 0.927388757401027 | -2.844751448 | 2.56079588285481 | 0.867640533200999 | 0.0581487179492377 | 12.9461168088089 | 0.999950574162623 |
| ebi-fl187-GCST90094734 | ebi-a-GCST90002081 | SSC-A on CD4+ T cell \|\| id:ebi-a-GCST90002081 | Chips liking \|\| id:ebi-fl187-GCST90094734 | MR Egger | 4 | 1.60026691679092 | 1.30626513294466 | 0.34524687712049 | -0.960012744 | 4.16054657736245 | 4.95435464836918 | 0.382888006503264 | 64.1065522160932 | 0.999950574162623 |
| ebi-fl187-GCST90094734 | ebi-a-GCST90002094 | CD11b on Monocytic Myeloid-Derived Suppressor Cells \|\| id:ebi-a-GCST90002094 | Chips liking \|\| id:ebi-fl187-GCST90094734 | MR Egger | 4 | 3.19597136866833 | 3.92220843013513 | 0.500761523578936 | -4.491557154 | 10.8834998917332 | 24.4338964901343 | 0.0112031851312057 | 53289.7823876578 | 0.999950574162623 |
| ebi-fl187-GCST90094734 | ebi-a-GCST90002104 | HLA DR on myeloid Dendritic Cell \|\| id:ebi-a-GCST90002104 | Chips liking \|\| id:ebi-fl187-GCST90094734 | MR Egger | 4 | -1.611647653 | 1.56621908468724 | 0.411646460107993 | -4.681437059 | 1.45814175306077 | 0.19955853983741 | 0.00926568897408815 | 4.29796542204335 | 0.999950574162623 |
| ebi-fl187-GCST90094734 | ebi-a-GCST90002105 | HLA DR on plasmacytoid Dendritic Cell \|\| id:ebi-a-GCST90002105 | Chips liking \|\| id:ebi-fl187-GCST90094734 | MR Egger | 4 | -0.806140594 | 2.07050363260085 | 0.734566905980882 | -4.864327713 | 3.25204652630108 | 0.446578271643032 | 0.00771701449263543 | 25.8431745714618 | 0.999950574162623 |
| ebi-fl187-GCST90094734 | ebi-a-GCST90002106 | HLA DR on Dendritic Cell \|\| id:ebi-a-GCST90002106 | Chips liking \|\| id:ebi-fl187-GCST90094734 | MR Egger | 4 | -0.667869679 | 2.01186383622064 | 0.771476318814682 | -4.611122798 | 3.27538343971557 | 0.512799843133879 | 0.00994065069413731 | 26.4533668075892 | 0.999950574162623 |
| ebi-fl187-GCST90094734 | ebi-a-GCST90002111 | HLA DR on CD33dim HLA DR+ CD11b- \|\| id:ebi-a-GCST90002111 | Chips liking \|\| id:ebi-fl187-GCST90094734 | MR Egger | 4 | -1.229222536 | 2.69284234735471 | 0.692826689465531 | -6.507193537 | 4.04874846492728 | 0.292519913031308 | 0.00149266295406669 | 57.3256670480891 | 0.999950574162623 |
| ebi-fl187-GCST90094734 | ebi-a-GCST90002112 | HLA DR on CD33- HLA DR+ \|\| id:ebi-a-GCST90002112 | Chips liking \|\| id:ebi-fl187-GCST90094734 | MR Egger | 4 | -0.155216 | 1.81827825417748 | 0.939748009220641 | -3.719041378 | 3.40860937788057 | 0.856230211519181 | 0.0242572101869401 | 30.2231859916394 | 0.999950574162623 |
| ebi-fl187-GCST90094734 | ebi-a-GCST90002115 | HLA DR on HLA DR+ CD8+ T cell \|\| id:ebi-a-GCST90002115 | Chips liking \|\| id:ebi-fl187-GCST90094734 | MR Egger | 4 | 1.04210701907406 | 2.10783794867553 | 0.669993644581626 | -3.08925536 | 5.17346939847809 | 2.83518451317463 | 0.0455358495832161 | 176.526216098273 | 0.999950574162623 |
| ebi-fl187-GCST90094734 | ebi-a-GCST90002116 | HLA DR on B cell \|\| id:ebi-a-GCST90002116 | Chips liking \|\| id:ebi-fl187-GCST90094734 | MR Egger | 4 | 0.151355679771455 | 1.47705533012255 | 0.927731360015425 | -2.743672767 | 3.04638412681165 | 1.16341038604034 | 0.06433363000568 | 21.0391318852524 | 0.999950574162623 |
| ebi-fl187-GCST90094734 | ebi-cfmet338-GCST90026023 | Sphingomyelin (d18:1/24:1, d18:2/24:0) levels \|\| id:ebi-cfmet338-GCST90026023 | Chips liking \|\| id:ebi-fl187-GCST90094734 | MR Egger | 4 | 0.464305830209214 | 0.549895294690242 | 0.487369244214509 | -0.613488947 | 1.54210060780209 | 1.59090944421522 | 0.541458449676948 | 4.67439904429096 | 0.999950574162623 |
| ebi-fl187-GCST90094734 | ebi-cfmet338-GCST90026046 | 1-arachidonoyl-gpc (20:4n6) levels \|\| id:ebi-cfmet338-GCST90026046 | Chips liking \|\| id:ebi-fl187-GCST90094734 | MR Egger | 4 | -0.385126459 | 0.846163493684267 | 0.693639670769903 | -2.043606906 | 1.27335398879036 | 0.680364592653315 | 0.129560554288282 | 3.57281567278829 | 0.999950574162623 |
| ebi-fl187-GCST90094734 | ebi-cfmet338-GCST90026047 | 1-linoleoyl-gpc (18:2) levels \|\| id:ebi-cfmet338-GCST90026047 | Chips liking \|\| id:ebi-fl187-GCST90094734 | MR Egger | 4 | -0.302516964 | 0.795354451183157 | 0.740277967158023 | -1.861411688 | 1.25637776071677 | 0.738955952797778 | 0.155453024240173 | 3.51267466711758 | 0.999950574162623 |
| ebi-fl187-GCST90094734 | ebi-cfmet338-GCST90026050 | 1-myristoyl-2-palmitoyl-gpc (14:0/16:0) levels \|\| id:ebi-cfmet338-GCST90026050 | Chips liking \|\| id:ebi-fl187-GCST90094734 | MR Egger | 4 | 0.0613054414506657 | 0.413241736509542 | 0.895671404257471 | -0.748648362 | 0.871259245009368 | 1.0632236171598 | 0.473005452956197 | 2.38991845235887 | 0.999950574162623 |
| ebi-fl187-GCST90094734 | ebi-cfmet338-GCST90026068 | 2,3-dihydroxyisovalerate levels \|\| id:ebi-cfmet338-GCST90026068 | Chips liking \|\| id:ebi-fl187-GCST90094734 | MR Egger | 4 | 0.804439990217879 | 1.67671019928359 | 0.678733459805336 | -2.481912 | 4.09079198081371 | 2.23544427756182 | 0.0835832614876595 | 59.7872232925688 | 0.999950574162623 |
| ebi-fl187-GCST90094734 | ebi-cfmet338-GCST90026082 | 3-hydroxyhexanoate levels \|\| id:ebi-cfmet338-GCST90026082 | Chips liking \|\| id:ebi-fl187-GCST90094734 | MR Egger | 4 | -0.600247808 | 0.327375001947195 | 0.208172997801026 | -1.241902812 | 0.0414071957603644 | 0.548675652998849 | 0.288834097766983 | 1.04227642969142 | 0.999950574162623 |
| ebi-fl187-GCST90094734 | ebi-cfmet338-GCST90026094 | 3-ureidopropionate levels \|\| id:ebi-cfmet338-GCST90026094 | Chips liking \|\| id:ebi-fl187-GCST90094734 | MR Egger | 4 | 0.562600596078024 | 0.654667516193555 | 0.48069571637326 | -0.720547736 | 1.84574892781739 | 1.755231217 | 0.486485717394146 | 6.33284085550249 | 0.999950574162623 |
| ebi-fl187-GCST90094734 | ebi-cfmet338-GCST90026173 | Glycerol levels \|\| id:ebi-cfmet338-GCST90026173 | Chips liking \|\| id:ebi-fl187-GCST90094734 | MR Egger | 4 | -0.836942811 | 0.585410760291482 | 0.289061194906182 | -1.984347902 | 0.310462278702909 | 0.43303236341777 | 0.137470229005842 | 1.36405554223097 | 0.999950574162623 |
| ebi-fl187-GCST90094734 | ebi-cfmet338-GCST90026179 | Histidine levels \|\| id:ebi-cfmet338-GCST90026179 | Chips liking \|\| id:ebi-fl187-GCST90094734 | MR Egger | 4 | -0.247870063 | 0.253848244843377 | 0.43182150200147 | -0.745412623 | 0.249672496445542 | 0.780461347146684 | 0.474538453895327 | 1.28360496265362 | 0.999950574162623 |
| ebi-fl187-GCST90094734 | ebi-cfmet338-GCST90026188 | Isocitrate levels \|\| id:ebi-cfmet338-GCST90026188 | Chips liking \|\| id:ebi-fl187-GCST90094734 | MR Egger | 4 | -0.471247416 | 0.661932113051416 | 0.550352477234472 | -1.768634358 | 0.826139525134105 | 0.624223116238609 | 0.170565761610226 | 2.28448250790842 | 0.999950574162623 |
| ebi-fl187-GCST90094734 | ebi-cfmet338-GCST90026207 | N-acetyl-isoputreanine levels \|\| id:ebi-cfmet338-GCST90026207 | Chips liking \|\| id:ebi-fl187-GCST90094734 | MR Egger | 4 | -0.128889425 | 0.38885486111642 | 0.771806947913806 | -0.891044953 | 0.633266102446992 | 0.879071163165623 | 0.410226860863398 | 1.88375307331883 | 0.999950574162623 |
| ebi-fl187-GCST90094734 | ebi-cfmet338-GCST90026213 | N-acetylglutamate levels \|\| id:ebi-cfmet338-GCST90026213 | Chips liking \|\| id:ebi-fl187-GCST90094734 | MR Egger | 4 | -0.421883542 | 0.54869429703107 | 0.52234665030901 | -1.497324364 | 0.653557280218199 | 0.655810409339522 | 0.223727974615833 | 1.92236707875527 | 0.999950574162623 |
| ebi-fl187-GCST90094734 | ebi-cfmet338-GCST90026224 | N-acetyltaurine levels \|\| id:ebi-cfmet338-GCST90026224 | Chips liking \|\| id:ebi-fl187-GCST90094734 | MR Egger | 4 | -0.625508379 | 0.439717751062971 | 0.290825119830626 | -1.487355171 | 0.236338413497318 | 0.534989382227877 | 0.225969516699121 | 1.26660287315506 | 0.999950574162623 |
| ebi-fl187-GCST90094734 | ebi-cfmet338-GCST90026250 | Phenyllactate (pla) levels \|\| id:ebi-cfmet338-GCST90026250 | Chips liking \|\| id:ebi-fl187-GCST90094734 | MR Egger | 4 | -0.891321343 | 0.689087414278353 | 0.325092817455956 | -2.241932675 | 0.459289988755243 | 0.410113493887387 | 0.106252953361977 | 1.58294967383662 | 0.999950574162623 |
| ebi-fl187-GCST90094734 | ebi-cfmet338-GCST90026251 | Pipecolate levels \|\| id:ebi-cfmet338-GCST90026251 | Chips liking \|\| id:ebi-fl187-GCST90094734 | MR Egger | 4 | -0.88366748 | 1.03857669114815 | 0.484472550506431 | -2.919277794 | 1.15194283505487 | 0.413264489908811 | 0.0539726525884502 | 3.16433472191688 | 0.999950574162623 |
| ebi-fl187-GCST90094734 | ebi-cfmet338-GCST90026278 | Trigonelline (n'-methylnicotinate) levels \|\| id:ebi-cfmet338-GCST90026278 | Chips liking \|\| id:ebi-fl187-GCST90094734 | MR Egger | 4 | 0.22068299632507 | 1.16639348366332 | 0.867396024670495 | -2.065448232 | 2.50681422430518 | 1.24692808711872 | 0.126761459333455 | 12.2657916895345 | 0.999950574162623 |
| ebi-fl187-GCST90094734 | ebi-cfmet338-GCST90026299 | 1-carboxyethylvaline levels \|\| id:ebi-cfmet338-GCST90026299 | Chips liking \|\| id:ebi-fl187-GCST90094734 | MR Egger | 4 | -0.597788391 | 0.744881897768615 | 0.506456813585472 | -2.057756911 | 0.86218012837455 | 0.550026735876831 | 0.127740181387132 | 2.368318307 | 0.999950574162623 |
| ebi-fl187-GCST90094734 | ebi-gm430-GCST90011306 | C_Erysipelotrichia abundance \|\| id:ebi-gm430-GCST90011306 | Chips liking \|\| id:ebi-fl187-GCST90094734 | MR Egger | 4 | -0.680938289 | 0.900060451328186 | 0.528295649378874 | -2.445056773 | 1.08318019593979 | 0.506141862324364 | 0.0867212113155101 | 2.9540591155391 | 0.999950574162623 |
| ebi-fl187-GCST90094734 | ebi-gm430-GCST90011312 | F_Erysipelotrichaceae abundance \|\| id:ebi-gm430-GCST90011312 | Chips liking \|\| id:ebi-fl187-GCST90094734 | MR Egger | 4 | -0.680938289 | 0.900060451328186 | 0.528295649378874 | -2.445056773 | 1.08318019593979 | 0.506141862324364 | 0.0867212113155101 | 2.9540591155391 | 0.999950574162623 |
| ebi-fl187-GCST90094734 | ebi-gm430-GCST90011352 | O_Erysipelotrichales abundance \|\| id:ebi-gm430-GCST90011352 | Chips liking \|\| id:ebi-fl187-GCST90094734 | MR Egger | 4 | -0.680938289 | 0.900060451328186 | 0.528295649378874 | -2.445056773 | 1.08318019593979 | 0.506141862324364 | 0.0867212113155101 | 2.9540591155391 | 0.999950574162623 |
| ebi-fl187-GCST90094734 | ebi-gm430-GCST90011362 | OTU97_11 (Parabacteroides) abundance \|\| id:ebi-gm430-GCST90011362 | Chips liking \|\| id:ebi-fl187-GCST90094734 | MR Egger | 4 | 1.4501912669688 | 0.866721602856605 | 0.236261823647286 | -0.248583075 | 3.14896560856775 | 4.26392998614363 | 0.779905067818785 | 23.3119384357678 | 0.999950574162623 |
| ebi-fl187-GCST90094734 | ebi-gm430-GCST90011384 | OTU97_235 (Phascolarctobacterium) abundance \|\| id:ebi-gm430-GCST90011384 | Chips liking \|\| id:ebi-fl187-GCST90094734 | MR Egger | 4 | -4.920500961 | 2.69086298077252 | 0.208967827999948 | -10.1945924 | 0.353590481752422 | 0.00729547518584702 | 3.73718652427054e-05 | 1.42417184268581 | 0.999950574162623 |
| ebi-fl187-GCST90094734 | ebi-gm430-GCST90011390 | OTU97_3 (Bacteroides) abundance \|\| id:ebi-gm430-GCST90011390 | Chips liking \|\| id:ebi-fl187-GCST90094734 | MR Egger | 4 | 0.0333370999623879 | 0.725650922144557 | 0.967531962688664 | -1.388938707 | 1.45561290736572 | 1.03389900782028 | 0.24933978672073 | 4.28711026198568 | 0.999950574162623 |
| ebi-fl187-GCST90094734 | ebi-gm430-GCST90011398 | OTU97_4 (Alistipes) abundance \|\| id:ebi-gm430-GCST90011398 | Chips liking \|\| id:ebi-fl187-GCST90094734 | MR Egger | 4 | 0.625198229526101 | 0.800646030161026 | 0.516632515557208 | -0.94406799 | 2.19446444864171 | 1.86861633565174 | 0.389041993172608 | 8.97519309262675 | 0.999950574162623 |
| ebi-fl187-GCST90094734 | ebi-gm430-GCST90011421 | OTU99_108 (Cytophagales) abundance \|\| id:ebi-gm430-GCST90011421 | Chips liking \|\| id:ebi-fl187-GCST90094734 | MR Egger | 4 | -0.537111362 | 2.77499679003787 | 0.864400846615233 | -5.97610507 | 4.90188234694343 | 0.58443403502976 | 0.00253869510028901 | 134.542797700395 | 0.999950574162623 |
| ebi-fl187-GCST90094734 | ebi-gm430-GCST90011428 | OTU99_123 (Phascolarctobacterium) abundance \|\| id:ebi-gm430-GCST90011428 | Chips liking \|\| id:ebi-fl187-GCST90094734 | MR Egger | 4 | 3.22123336722002 | 2.31428943107507 | 0.298541030130933 | -1.314773918 | 7.75724065212716 | 25.0590080884988 | 0.268535027396994 | 2338.44311658867 | 0.999950574162623 |
| ebi-fl187-GCST90094734 | ebi-gm430-GCST90011450 | OTU99_298 (Phascolarctobacterium) abundance \|\| id:ebi-gm430-GCST90011450 | Chips liking \|\| id:ebi-fl187-GCST90094734 | MR Egger | 4 | -4.841150077 | 2.69080275640933 | 0.213808615616428 | -10.11512348 | 0.432823325950951 | 0.00789796556089615 | 4.04629636842244e-05 | 1.54160383524802 | 0.999950574162623 |
| ebi-fl187-GCST90094734 | ebi-gm430-GCST90011451 | OTU99_3 (Bacteroides) abundance \|\| id:ebi-gm430-GCST90011451 | Chips liking \|\| id:ebi-fl187-GCST90094734 | MR Egger | 4 | 0.0186574738647872 | 0.734847224995596 | 0.982049737378482 | -1.421643087 | 1.45895803485615 | 1.01883261204671 | 0.241317185810862 | 4.30147520526575 | 0.999950574162623 |
| ebi-fl187-GCST90094734 | ebi-gm430-GCST90011472 | OTU99_72 (Parabacteroides) abundance \|\| id:ebi-gm430-GCST90011472 | Chips liking \|\| id:ebi-fl187-GCST90094734 | MR Egger | 4 | 1.30670382896021 | 2.42478977636126 | 0.64392064079858 | -3.445884133 | 6.05929179062828 | 3.69397764096228 | 0.0318765664651025 | 428.072164763036 | 0.999950574162623 |
| ebi-fl187-GCST90094734 | ebi-gm430-GCST90011507 | TestASV_3 (Bacteroides) abundance \|\| id:ebi-gm430-GCST90011507 | Chips liking \|\| id:ebi-fl187-GCST90094734 | MR Egger | 4 | 1.08134766444137 | 0.877914684136873 | 0.343222724883775 | -0.639365116 | 2.80206044534965 | 2.94865066625208 | 0.527627299612448 | 16.4785649983902 | 0.999950574162623 |
| ebi-fl187-GCST90094734 | ebi-gm430-GCST90011512 | TestASV_34 (Phascolarctobacterium) abundance \|\| id:ebi-gm430-GCST90011512 | Chips liking \|\| id:ebi-fl187-GCST90094734 | MR Egger | 4 | -2.854442445 | 2.66039798474634 | 0.395582603414791 | -8.068822495 | 2.35993760548146 | 0.0575879206263215 | 0.000313151801954411 | 10.5902906557322 | 0.999950574162623 |
| ebi-fl187-GCST90094734 | ebi-gm430-GCST90011519 | TestASV_41 (Barnesiella) abundance \|\| id:ebi-gm430-GCST90011519 | Chips liking \|\| id:ebi-fl187-GCST90094734 | MR Egger | 4 | -6.274144489 | 2.90613850563978 | 0.163493286126674 | -11.97017596 | -0.578113018 | 0.00188440249225226 | 6.33021751637246e-06 | 0.560955882419888 | 0.999950574162623 |
| ebi-fl187-GCST90094734 | ebi-gm430-GCST90011524 | TestASV_46 (Sutterella) abundance \|\| id:ebi-gm430-GCST90011524 | Chips liking \|\| id:ebi-fl187-GCST90094734 | MR Egger | 4 | -3.315926653 | 2.90389795170236 | 0.371783184753357 | -9.007566639 | 2.37571333208296 | 0.0363003951079636 | 0.000122479530660548 | 10.7586849646438 | 0.999950574162623 |
| ebi-fl187-GCST90094734 | ebi-gm430-GCST90011581 | OTU97_19 (Bacteria) prevalence \|\| id:ebi-gm430-GCST90011581 | Chips liking \|\| id:ebi-fl187-GCST90094734 | MR Egger | 4 | -1.357349315 | 6.35963783933897 | 0.850770783585057 | -13.82223948 | 11.1075408496464 | 0.257342006170573 | 9.93293665907717e-07 | 66672.0330682626 | 0.999950574162623 |
| ebi-fl187-GCST90094734 | ebi-gm430-GCST90011614 | OTU97_80 (Ruminococcus) prevalence \|\| id:ebi-gm430-GCST90011614 | Chips liking \|\| id:ebi-fl187-GCST90094734 | MR Egger | 4 | -0.767044971 | 1.57667327957441 | 0.674705014836643 | -3.857324599 | 2.32323465655254 | 0.464383308716786 | 0.0211244403292636 | 10.2086424091439 | 0.999950574162623 |
| ebi-fl187-GCST90094734 | ebi-gm430-GCST90011623 | OTU99_11 (Parabacteroides) prevalence \|\| id:ebi-gm430-GCST90011623 | Chips liking \|\| id:ebi-fl187-GCST90094734 | MR Egger | 4 | 0.381426281798895 | 1.36902836771591 | 0.806707641844014 | -2.301869319 | 3.06472188252208 | 1.4643717073843 | 0.100071603029715 | 21.4285015175669 | 0.999950574162623 |
| ebi-fl187-GCST90094734 | ebi-gm430-GCST90011642 | OTU99_19 (Bacteria) prevalence \|\| id:ebi-gm430-GCST90011642 | Chips liking \|\| id:ebi-fl187-GCST90094734 | MR Egger | 4 | -1.357349315 | 6.35963783933897 | 0.850770783585057 | -13.82223948 | 11.1075408496464 | 0.257342006170573 | 9.93293665907717e-07 | 66672.0330682626 | 0.999950574162623 |
| ebi-fl187-GCST90094734 | ebi-gm430-GCST90011653 | OTU99_30 (Parasutterella) prevalence \|\| id:ebi-gm430-GCST90011653 | Chips liking \|\| id:ebi-fl187-GCST90094734 | MR Egger | 4 | -1.320639434 | 2.13546041040952 | 0.599336323573207 | -5.506141839 | 2.86486297026401 | 0.266964541135106 | 0.0040617480715348 | 17.5466486272122 | 0.999950574162623 |
| ebi-fl187-GCST90094734 | ebi-gm430-GCST90011675 | OTU99_8 (Ruminococcaceae) prevalence \|\| id:ebi-gm430-GCST90011675 | Chips liking \|\| id:ebi-fl187-GCST90094734 | MR Egger | 4 | -1.495377963 | 1.59227962216369 | 0.446795807184922 | -4.616246023 | 1.62549009618837 | 0.224163863014165 | 0.00988985274546632 | 5.08090856099638 | 0.999950574162623 |
| ebi-fl187-GCST90094734 | ebi-gm430-GCST90011679 | OTU99_92 (Ruminococcus) prevalence \|\| id:ebi-gm430-GCST90011679 | Chips liking \|\| id:ebi-fl187-GCST90094734 | MR Egger | 4 | 0.412711872509702 | 1.66152064368309 | 0.827007001604818 | -2.843868589 | 3.66929233412856 | 1.51090962861268 | 0.0582000777049429 | 39.2241384523206 | 0.999950574162623 |
| ebi-fl187-GCST90094734 | ebi-gm430-GCST90011684 | TestASV_11 (Lachnospiraceae) prevalence \|\| id:ebi-gm430-GCST90011684 | Chips liking \|\| id:ebi-fl187-GCST90094734 | MR Egger | 4 | -0.675243114 | 1.67443507696672 | 0.725778579195062 | -3.957135865 | 2.60664963713768 | 0.509032652742992 | 0.0191177918972804 | 13.5535653359334 | 0.999950574162623 |
| ebi-fl187-GCST90094734 | ebi-gm430-GCST90011688 | TestASV_15 (Bacteroides) prevalence \|\| id:ebi-gm430-GCST90011688 | Chips liking \|\| id:ebi-fl187-GCST90094734 | MR Egger | 4 | -0.475901593 | 1.55370131968717 | 0.788319980625613 | -3.52115618 | 2.56935299339452 | 0.621324621811699 | 0.0295652326754009 | 13.0573735004173 | 0.999950574162623 |
| ebi-fl187-GCST90094734 | ebi-gm430-GCST90011692 | TestASV_19 (Prevotella) prevalence \|\| id:ebi-gm430-GCST90011692 | Chips liking \|\| id:ebi-fl187-GCST90094734 | MR Egger | 4 | -0.862881352 | 4.87509936014421 | 0.87581259145902 | -10.4180761 | 8.69231339410184 | 0.421944558397987 | 2.98873244866603e-05 | 5956.94708106562 | 0.999950574162623 |
| ebi-fl187-GCST90094734 | ebi-gm430-GCST90011693 | TestASV_2 (Bacteroides) prevalence \|\| id:ebi-gm430-GCST90011693 | Chips liking \|\| id:ebi-fl187-GCST90094734 | MR Egger | 4 | 1.9320027570988 | 1.53230344680217 | 0.334525367701503 | -1.071311999 | 4.93531751283105 | 6.90332208339033 | 0.342558785800738 | 139.117307050316 | 0.999950574162623 |
| ebi-fl187-GCST90094734 | ebi-gm430-GCST90011716 | TestASV_40 (Barnesiella) prevalence \|\| id:ebi-gm430-GCST90011716 | Chips liking \|\| id:ebi-fl187-GCST90094734 | MR Egger | 4 | 1.23770127702295 | 3.29377414267152 | 0.743201136397877 | -5.218096043 | 7.69349859665913 | 3.44767908965048 | 0.00541763425782647 | 2194.03720139313 | 0.999950574162623 |
| ebi-fl187-GCST90094734 | ebi-gm430-GCST90011718 | TestASV_42 (Alphaproteobacteria) prevalence \|\| id:ebi-gm430-GCST90011718 | Chips liking \|\| id:ebi-fl187-GCST90094734 | MR Egger | 4 | -2.469371533 | 2.38825507616494 | 0.409797009947525 | -7.150351482 | 2.21160841674159 | 0.0846380345407109 | 0.000784588264326455 | 9.13039006142198 | 0.999950574162623 |
| ebi-fl187-GCST90094734 | ebi-hbsm44-GCST90104929 | Sulcal depth of the right inferior frontal sulcus \|\| id:ebi-hbsm44-GCST90104929 | Chips liking \|\| id:ebi-fl187-GCST90094734 | MR Egger | 3 | 0.296441560573835 | 2.90690103070135 | 0.935302096232724 | -5.40108446 | 5.99396758074848 | 1.34506395293483 | 0.00451168554795524 | 401.0024675 | 0.999950574162623 |
| ebi-fl187-GCST90094734 | ebi-hbsm44-GCST90104935 | Sulcal opening distance of the left calloso-marginal posterior fissure \|\| id:ebi-hbsm44-GCST90104935 | Chips liking \|\| id:ebi-fl187-GCST90094734 | MR Egger | 4 | -0.144400597 | 0.267072596768635 | 0.642891068212552 | -0.667862887 | 0.379061692538144 | 0.865540945382548 | 0.512803326329407 | 1.46091316040427 | 0.999950574162623 |
| ebi-fl187-GCST90094734 | ebi-hbsm44-GCST90104936 | Sulcal opening distance of the left anterior inferior temporal sulcus \|\| id:ebi-hbsm44-GCST90104936 | Chips liking \|\| id:ebi-fl187-GCST90094734 | MR Egger | 4 | 0.0696776470767262 | 0.152208393710987 | 0.692034611504173 | -0.228650805 | 0.36800609875026 | 1.07216251082368 | 0.795606307068825 | 1.44485085073151 | 0.999950574162623 |
| ebi-fl187-GCST90094734 | ebi-hbsm44-GCST90104941 | Sulcal opening distance of the right posterior inferior temporal sulcus \|\| id:ebi-hbsm44-GCST90104941 | Chips liking \|\| id:ebi-fl187-GCST90094734 | MR Egger | 4 | -0.02821523 | 0.13410990439151 | 0.852852020683726 | -0.291070642 | 0.234640182939248 | 0.972179102496536 | 0.747462873674777 | 1.26445371485059 | 0.999950574162623 |
| ebi-fl187-GCST90094734 | ebi-IPs91-GCST90274819 | Leukemia inhibitory factor levels \|\| id:ebi-IPs91-GCST90274819 | Chips liking \|\| id:ebi-fl187-GCST90094734 | MR Egger | 4 | 0.0611874136982838 | 0.535799149424379 | 0.919511518026119 | -0.988978919 | 1.11135374657007 | 1.06309813467135 | 0.371956294626823 | 3.03846892838736 | 0.999950574162623 |
| ebi-fl187-GCST90094734 | ebi-IPs91-GCST90274837 | STAM binding protein levels \|\| id:ebi-IPs91-GCST90274837 | Chips liking \|\| id:ebi-fl187-GCST90094734 | MR Egger | 4 | -0.042388588 | 0.630346195053919 | 0.952503195405161 | -1.277867131 | 1.19308995408524 | 0.958497247456499 | 0.278630950588007 | 3.29725384578733 | 0.999950574162623 |
| ebi-fl187-GCST90094734 | ebi-IPs91-GCST90274840 | TNF-beta levels \|\| id:ebi-IPs91-GCST90274840 | Chips liking \|\| id:ebi-fl187-GCST90094734 | MR Egger | 4 | 0.945757511130574 | 0.513861755968558 | 0.20705379037674 | -0.061411531 | 1.95292655282895 | 2.57476305171581 | 0.94043614191071 | 7.04928753483653 | 0.999950574162623 |
| ebi-fl187-GCST90094734 | ebi-IPs91-GCST90274842 | Tumor necrosis factor ligand superfamily member 14 levels \|\| id:ebi-IPs91-GCST90274842 | Chips liking \|\| id:ebi-fl187-GCST90094734 | MR Egger | 4 | 0.506090641323581 | 0.736938256357388 | 0.563176349194983 | -0.938308341 | 1.95048962378406 | 1.6587936831913 | 0.391289203645421 | 7.03212983583574 | 0.999950574162623 |
| ebi-fl187-GCST90094734 | ebi-met1400-GCST90199628 | 3-methylhistidine levels \|\| id:ebi-met1400-GCST90199628 | Chips liking \|\| id:ebi-fl187-GCST90094734 | MR Egger | 4 | 0.627120026067171 | 0.621190999620833 | 0.41899350311843 | -0.590414333 | 1.844654385 | 1.87221088895592 | 0.554097656117171 | 6.32591308414399 | 0.999950574162623 |
| ebi-fl187-GCST90094734 | ebi-met1400-GCST90199651 | Docosahexaenoate DHA; 22:6n3 levels \|\| id:ebi-met1400-GCST90199651 | Chips liking \|\| id:ebi-fl187-GCST90094734 | MR Egger | 4 | -0.168127835 | 0.595840352867211 | 0.804332917838726 | -1.335974927 | 0.999719256494808 | 0.845245775678612 | 0.262901740535644 | 2.71751879560369 | 0.999950574162623 |
| ebi-fl187-GCST90094734 | ebi-met1400-GCST90199652 | 1-stearoyl-2-oleoyl-GPS (18:0/18:1) levels \|\| id:ebi-met1400-GCST90199652 | Chips liking \|\| id:ebi-fl187-GCST90094734 | MR Egger | 4 | -0.54857008 | 1.09033485861292 | 0.66481926511453 | -2.685626403 | 1.588486243 | 0.577775392392194 | 0.0681784734042825 | 4.89633145750349 | 0.999950574162623 |
| ebi-fl187-GCST90094734 | ebi-met1400-GCST90199653 | 1-myristoyl-2-palmitoyl-gpc (14:0/16:0) levels \|\| id:ebi-met1400-GCST90199653 | Chips liking \|\| id:ebi-fl187-GCST90094734 | MR Egger | 4 | 0.899405663442415 | 1.08143316819205 | 0.493075402930716 | -1.220203346 | 3.01901467309883 | 2.4581417134349 | 0.295170139090735 | 20.4711110071715 | 0.999950574162623 |
| ebi-fl187-GCST90094734 | ebi-met1400-GCST90199667 | Erythritol levels in elite athletes \|\| id:ebi-met1400-GCST90199667 | Chips liking \|\| id:ebi-fl187-GCST90094734 | MR Egger | 4 | 0.0919029877423349 | 0.584378366470801 | 0.889477256943213 | -1.053478611 | 1.2372845860251 | 1.09625846641319 | 0.34872256676704 | 3.44624277208143 | 0.999950574162623 |
| ebi-fl187-GCST90094734 | ebi-met1400-GCST90199674 | 3-hydroxymyristate levels \|\| id:ebi-met1400-GCST90199674 | Chips liking \|\| id:ebi-fl187-GCST90094734 | MR Egger | 4 | 0.705781488277549 | 0.607598808461914 | 0.365288025568781 | -0.485112176 | 1.8966751528629 | 2.02542891554899 | 0.615628133999703 | 6.6637017793341 | 0.999950574162623 |
| ebi-fl187-GCST90094734 | ebi-met1400-GCST90199685 | 2-linoleoylglycerol (18:2) levels \|\| id:ebi-met1400-GCST90199685 | Chips liking \|\| id:ebi-fl187-GCST90094734 | MR Egger | 4 | -0.091250118 | 1.33294319027302 | 0.951649767553548 | -2.703818771 | 2.52131853517622 | 0.912789377511883 | 0.0669493597033665 | 12.4449950139946 | 0.999950574162623 |
| ebi-fl187-GCST90094734 | ebi-met1400-GCST90199703 | Gamma-glutamylvaline levels \|\| id:ebi-met1400-GCST90199703 | Chips liking \|\| id:ebi-fl187-GCST90094734 | MR Egger | 4 | 1.65536010151572 | 0.674911069040786 | 0.133690517519967 | 0.332534406195774 | 2.97818579683566 | 5.23496470152549 | 1.39449787772652 | 19.6521313255037 | 0.999950574162623 |
| ebi-fl187-GCST90094734 | ebi-met1400-GCST90199779 | Gamma-glutamyl-2-aminobutyrate levels \|\| id:ebi-met1400-GCST90199779 | Chips liking \|\| id:ebi-fl187-GCST90094734 | MR Egger | 4 | -0.327274847 | 0.647237239037891 | 0.663325439322244 | -1.595859836 | 0.941310141505614 | 0.720885582550002 | 0.202734135527216 | 2.56333755426546 | 0.999950574162623 |
| ebi-fl187-GCST90094734 | ebi-met1400-GCST90199824 | 4-vinylphenol sulfate levels \|\| id:ebi-met1400-GCST90199824 | Chips liking \|\| id:ebi-fl187-GCST90094734 | MR Egger | 4 | -0.596803201 | 1.22051883755716 | 0.673223929417165 | -2.989020122 | 1.79541372108103 | 0.550568884129143 | 0.0503367364192161 | 6.02196562062952 | 0.999950574162623 |
| ebi-fl187-GCST90094734 | ebi-met1400-GCST90199830 | 4-ethylphenylsulfate levels \|\| id:ebi-met1400-GCST90199830 | Chips liking \|\| id:ebi-fl187-GCST90094734 | MR Egger | 4 | -0.100516041 | 0.862118982930679 | 0.917835872003386 | -1.790269248 | 1.5892371653395 | 0.90437060510247 | 0.166915222068515 | 4.9000096051017 | 0.999950574162623 |
| ebi-fl187-GCST90094734 | ebi-met1400-GCST90199833 | Hexanoylglutamine levels \|\| id:ebi-met1400-GCST90199833 | Chips liking \|\| id:ebi-fl187-GCST90094734 | MR Egger | 4 | 0.612969357749746 | 0.614532543595749 | 0.423630046963506 | -0.591514428 | 1.81745314319741 | 1.84590441967565 | 0.553488431492409 | 6.15615961004024 | 0.999950574162623 |
| ebi-fl187-GCST90094734 | ebi-met1400-GCST90199837 | Carotenoid levels (cryptoxanthin) \|\| id:ebi-met1400-GCST90199837 | Chips liking \|\| id:ebi-fl187-GCST90094734 | MR Egger | 4 | 0.0384937864078814 | 0.926714550797067 | 0.970640922844766 | -1.777866733 | 1.85485430597013 | 1.03924427089791 | 0.168998281431632 | 6.39076708617926 | 0.999950574162623 |
| ebi-fl187-GCST90094734 | ebi-met1400-GCST90199869 | 2-hydroxyhippurate levels \|\| id:ebi-met1400-GCST90199869 | Chips liking \|\| id:ebi-fl187-GCST90094734 | MR Egger | 4 | 1.0503688359604 | 0.627657233091018 | 0.236207873749809 | -0.179839341 | 2.2805770128188 | 2.85870531695907 | 0.835404415953675 | 9.78232330730611 | 0.999950574162623 |
| ebi-fl187-GCST90094734 | ebi-met1400-GCST90199902 | Hydantoin-5-propionate levels \|\| id:ebi-met1400-GCST90199902 | Chips liking \|\| id:ebi-fl187-GCST90094734 | MR Egger | 4 | 0.564089218943693 | 0.843676479654578 | 0.572583221010856 | -1.089516681 | 2.21769511906667 | 1.75784604054069 | 0.336379032741902 | 9.186133503 | 0.999950574162623 |
| ebi-fl187-GCST90094734 | ebi-met1400-GCST90199904 | Pantoate levels \|\| id:ebi-met1400-GCST90199904 | Chips liking \|\| id:ebi-fl187-GCST90094734 | MR Egger | 4 | 0.038961649034529 | 0.629415250902575 | 0.956271010818484 | -1.194692243 | 1.27261554080358 | 1.03973060821288 | 0.302797127860035 | 3.57017830814571 | 0.999950574162623 |
| ebi-fl187-GCST90094734 | ebi-met1400-GCST90199936 | 3-methyl catechol sulfate (2) levels \|\| id:ebi-met1400-GCST90199936 | Chips liking \|\| id:ebi-fl187-GCST90094734 | MR Egger | 4 | 0.0532536279924195 | 0.709922524146775 | 0.947032050419298 | -1.338194519 | 1.4447017753201 | 1.05469711191049 | 0.26231885290613 | 4.24058730643504 | 0.999950574162623 |
| ebi-fl187-GCST90094734 | ebi-met1400-GCST90199977 | 4-hydroxychlorothalonil levels \|\| id:ebi-met1400-GCST90199977 | Chips liking \|\| id:ebi-fl187-GCST90094734 | MR Egger | 4 | 1.08736956 | 0.860366858910744 | 0.333645926085829 | -0.598949483 | 2.77368860370306 | 2.96646070458162 | 0.54938847485928 | 16.0176077848755 | 0.999950574162623 |
| ebi-fl187-GCST90094734 | ebi-met1400-GCST90200040 | Sphingomyelin (d18:2/24:1, d18:1/24:2) levels \|\| id:ebi-met1400-GCST90200040 | Chips liking \|\| id:ebi-fl187-GCST90094734 | MR Egger | 4 | 0.451714587008554 | 0.59526607932034 | 0.527183007000824 | -0.715006928 | 1.61843610247642 | 1.57100349977569 | 0.48918872246043 | 5.04519397727349 | 0.999950574162623 |
| ebi-fl187-GCST90094734 | ebi-met1400-GCST90200077 | 1-stearoyl-2-oleoyl-GPI (18:0/18:1) levels \|\| id:ebi-met1400-GCST90200077 | Chips liking \|\| id:ebi-fl187-GCST90094734 | MR Egger | 4 | -0.206944157 | 0.756511606657502 | 0.810090659820416 | -1.689706906 | 1.27581859192801 | 0.813065052613454 | 0.184573613453354 | 3.58163210554681 | 0.999950574162623 |
| ebi-fl187-GCST90094734 | ebi-met1400-GCST90200080 | 1-palmitoyl-2-oleoyl-GPI (16:0/18:1) levels \|\| id:ebi-met1400-GCST90200080 | Chips liking \|\| id:ebi-fl187-GCST90094734 | MR Egger | 4 | 0.970313958923421 | 0.62248655772953 | 0.25938608577625 | -0.249759694 | 2.1903876120733 | 2.63877279558163 | 0.778987955884468 | 8.9386771824934 | 0.999950574162623 |
| ebi-fl187-GCST90094734 | ebi-met1400-GCST90200082 | 1-oleoyl-2-linoleoyl-GPE (18:1/18:2) levels \|\| id:ebi-met1400-GCST90200082 | Chips liking \|\| id:ebi-fl187-GCST90094734 | MR Egger | 4 | -2.34768813 | 0.62521979002892 | 0.064171243528011 | -3.573118919 | -1.122257342 | 0.0955898983582054 | 0.028068174665419 | 0.325544100286282 | 0.999950574162623 |
| ebi-fl187-GCST90094734 | ebi-met1400-GCST90200100 | Perfluorooctanesulfonate (PFOS) levels \|\| id:ebi-met1400-GCST90200100 | Chips liking \|\| id:ebi-fl187-GCST90094734 | MR Egger | 4 | -0.211392176 | 0.904292970322942 | 0.836916044437647 | -1.983806398 | 1.56102204548814 | 0.809456554912006 | 0.13754468976932 | 4.76368746324486 | 0.999950574162623 |
| ebi-fl187-GCST90094734 | ebi-met1400-GCST90200102 | 1-palmitoleoyl-2-linolenoyl-GPC (16:1/18:3) levels \|\| id:ebi-met1400-GCST90200102 | Chips liking \|\| id:ebi-fl187-GCST90094734 | MR Egger | 4 | 0.424378749301568 | 0.610623212797935 | 0.558947137411176 | -0.772442748 | 1.62120024638552 | 1.5286404556603 | 0.461883424444638 | 5.05915891112785 | 0.999950574162623 |
| ebi-fl187-GCST90094734 | ebi-met1400-GCST90200114 | Glycosyl-N-tricosanoyl-sphingadienine (d18:2/23:0) levels \|\| id:ebi-met1400-GCST90200114 | Chips liking \|\| id:ebi-fl187-GCST90094734 | MR Egger | 4 | -1.19114716 | 0.654401080399566 | 0.210331461293577 | -2.473773278 | 0.0914789572094294 | 0.303872473585284 | 0.0842662982047879 | 1.09579371789222 | 0.999950574162623 |
| ebi-fl187-GCST90094734 | ebi-met1400-GCST90200135 | Arachidoylcarnitine (C20) levels \|\| id:ebi-met1400-GCST90200135 | Chips liking \|\| id:ebi-fl187-GCST90094734 | MR Egger | 4 | -2.184805015 | 0.638951540870386 | 0.075916936674277 | -3.437150035 | -0.932459995 | 0.112499667260177 | 0.0321561988977304 | 0.393584303104429 | 0.999950574162623 |
| ebi-fl187-GCST90094734 | ebi-met1400-GCST90200146 | Docosahexaenoylcarnitine (C22:6) levels \|\| id:ebi-met1400-GCST90200146 | Chips liking \|\| id:ebi-fl187-GCST90094734 | MR Egger | 4 | -0.660873119 | 0.701590825667019 | 0.445644252266138 | -2.035991138 | 0.71424489906889 | 0.516400258598304 | 0.130551024337332 | 2.04264369761931 | 0.999950574162623 |
| ebi-fl187-GCST90094734 | ebi-met1400-GCST90200162 | 3-hydroxyphenylacetoylglutamine levels \|\| id:ebi-met1400-GCST90200162 | Chips liking \|\| id:ebi-fl187-GCST90094734 | MR Egger | 4 | -0.168689963 | 0.758179589300634 | 0.844585032443428 | -1.654721958 | 1.31734203173281 | 0.844770772735028 | 0.19114519464766 | 3.73348469357438 | 0.999950574162623 |
| ebi-fl187-GCST90094734 | ebi-met1400-GCST90200172 | 3-carboxy-4-methyl-5-pentyl-2-furanpropionate (3-CMPFP) levels \|\| id:ebi-met1400-GCST90200172 | Chips liking \|\| id:ebi-fl187-GCST90094734 | MR Egger | 4 | 1.47451817383886 | 0.619051142200845 | 0.140138947320152 | 0.261177935125204 | 2.68785841255251 | 4.36893020084367 | 1.29845868628864 | 14.7001605067633 | 0.999950574162623 |
| ebi-fl187-GCST90094734 | ebi-met1400-GCST90200222 | Dihydrocaffeate sulfate (2) levels \|\| id:ebi-met1400-GCST90200222 | Chips liking \|\| id:ebi-fl187-GCST90094734 | MR Egger | 4 | 0.99910067327046 | 0.709989490375146 | 0.294651919058751 | -0.392478728 | 2.39068007440575 | 2.71583830388046 | 0.6753807129896 | 10.9209184552741 | 0.999950574162623 |
| ebi-fl187-GCST90094734 | ebi-met1400-GCST90200223 | 4-methylhexanoylglutamine levels \|\| id:ebi-met1400-GCST90200223 | Chips liking \|\| id:ebi-fl187-GCST90094734 | MR Egger | 4 | 0.798772485827713 | 0.752238172830599 | 0.399565467832377 | -0.675614333 | 2.27315930457569 | 2.22281072148845 | 0.508843725116209 | 9.71002934630986 | 0.999950574162623 |
| ebi-fl187-GCST90094734 | ebi-met1400-GCST90200224 | 2-ketocaprylate levels \|\| id:ebi-met1400-GCST90200224 | Chips liking \|\| id:ebi-fl187-GCST90094734 | MR Egger | 4 | 0.243368689326755 | 0.631548514411284 | 0.737099950074432 | -0.994466399 | 1.48120377757287 | 1.2755388149829 | 0.369920782018668 | 4.39823699455167 | 0.999950574162623 |
| ebi-fl187-GCST90094734 | ebi-met1400-GCST90200243 | Hydroxypalmitoyl sphingomyelin (d18:1/16:0(OH)) levels \|\| id:ebi-met1400-GCST90200243 | Chips liking \|\| id:ebi-fl187-GCST90094734 | MR Egger | 4 | 0.130740738037845 | 0.665958398251706 | 0.862499568017785 | -1.174537723 | 1.43601919861119 | 1.13967226933738 | 0.308961772745403 | 4.20392746311341 | 0.999950574162623 |
| ebi-fl187-GCST90094734 | ebi-met1400-GCST90200273 | 2-methoxyhydroquinone sulfate (2) levels \|\| id:ebi-met1400-GCST90200273 | Chips liking \|\| id:ebi-fl187-GCST90094734 | MR Egger | 4 | -1.326930433 | 0.819893266000158 | 0.246984763341129 | -2.933921234 | 0.280060368473597 | 0.265290339263593 | 0.0531880658329908 | 1.3232096900756 | 0.999950574162623 |
| ebi-fl187-GCST90094734 | ebi-met1400-GCST90200276 | 2,4-di-tert-butylphenol levels \|\| id:ebi-met1400-GCST90200276 | Chips liking \|\| id:ebi-fl187-GCST90094734 | MR Egger | 4 | -0.567488765 | 0.620309868008231 | 0.456845871699828 | -1.783296106 | 0.648318576432151 | 0.566947390732194 | 0.168083213095941 | 1.91232269979616 | 0.999950574162623 |
| ebi-fl187-GCST90094734 | ebi-met1400-GCST90200289 | N-acetylmethionine levels \|\| id:ebi-met1400-GCST90200289 | Chips liking \|\| id:ebi-fl187-GCST90094734 | MR Egger | 4 | 0.946319163034974 | 0.705495067471003 | 0.311830037095913 | -0.436451169 | 2.32908949527814 | 2.57620957847141 | 0.64632605772883 | 10.2685876777575 | 0.999950574162623 |
| ebi-fl187-GCST90094734 | ebi-met1400-GCST90200308 | 3-hydroxyisobutyrate levels \|\| id:ebi-met1400-GCST90200308 | Chips liking \|\| id:ebi-fl187-GCST90094734 | MR Egger | 4 | 1.02432730284833 | 0.630041689940187 | 0.245501755850935 | -0.210554409 | 2.2592090151311 | 2.78522121985697 | 0.810134974968365 | 9.57551208530958 | 0.999950574162623 |
| ebi-fl187-GCST90094734 | ebi-met1400-GCST90200383 | Cystathionine levels \|\| id:ebi-met1400-GCST90200383 | Chips liking \|\| id:ebi-fl187-GCST90094734 | MR Egger | 4 | -0.189734136 | 0.892545456142739 | 0.851355640244578 | -1.93912323 | 1.55965495760255 | 0.82717902147373 | 0.143830000271745 | 4.75717953329276 | 0.999950574162623 |
| ebi-fl187-GCST90094734 | ebi-met1400-GCST90200384 | Guanidinoacetate levels \|\| id:ebi-met1400-GCST90200384 | Chips liking \|\| id:ebi-fl187-GCST90094734 | MR Egger | 4 | 0.378857570694534 | 0.596085476752659 | 0.590075283087718 | -0.789469964 | 1.54718510512975 | 1.46061498654955 | 0.454085413242449 | 4.69822653782109 | 0.999950574162623 |
| ebi-fl187-GCST90094734 | ebi-met1400-GCST90200403 | Citrulline levels \|\| id:ebi-met1400-GCST90200403 | Chips liking \|\| id:ebi-fl187-GCST90094734 | MR Egger | 4 | -0.4642389 | 0.623869020778314 | 0.534348701537925 | -1.687022181 | 0.758544380226715 | 0.628613360481745 | 0.185069808613328 | 2.13516596757152 | 0.999950574162623 |
| ebi-fl187-GCST90094734 | ebi-met1400-GCST90200434 | 12,13-DiHOME levels \|\| id:ebi-met1400-GCST90200434 | Chips liking \|\| id:ebi-fl187-GCST90094734 | MR Egger | 4 | -1.171029166 | 0.628811499219649 | 0.203605769610219 | -2.403499705 | 0.061441372128392 | 0.310047686367205 | 0.0904010221379109 | 1.06336815168978 | 0.999950574162623 |
| ebi-fl187-GCST90094734 | ebi-met1400-GCST90200437 | Fructose levels \|\| id:ebi-met1400-GCST90200437 | Chips liking \|\| id:ebi-fl187-GCST90094734 | MR Egger | 4 | -0.544102499 | 0.626785282248454 | 0.476864931701592 | -1.772601652 | 0.684396654677725 | 0.580362425796937 | 0.169890417666941 | 1.98257529707897 | 0.999950574162623 |
| ebi-fl187-GCST90094734 | ebi-met1400-GCST90200448 | N-stearoyl-sphinganine (d18:0/18:0) levels \|\| id:ebi-met1400-GCST90200448 | Chips liking \|\| id:ebi-fl187-GCST90094734 | MR Egger | 4 | 1.64181948799161 | 0.697469011469148 | 0.1428016904412 | 0.274780225512078 | 3.00885875047114 | 5.16455781962315 | 1.31624136680515 | 20.2642601462769 | 0.999950574162623 |
| ebi-fl187-GCST90094734 | ebi-met1400-GCST90200468 | X-11847 levels \|\| id:ebi-met1400-GCST90200468 | Chips liking \|\| id:ebi-fl187-GCST90094734 | MR Egger | 4 | -1.657152928 | 0.948472845050543 | 0.222718064593635 | -3.516159704 | 0.201853848367639 | 0.190681090828882 | 0.0297133242975501 | 1.22366915379745 | 0.999950574162623 |
| ebi-fl187-GCST90094734 | ebi-met1400-GCST90200476 | X-11843 levels \|\| id:ebi-met1400-GCST90200476 | Chips liking \|\| id:ebi-fl187-GCST90094734 | MR Egger | 4 | -0.268987374 | 0.986692060646055 | 0.810716622184594 | -2.202903813 | 1.66492906446843 | 0.764152903477847 | 0.110481873382929 | 5.28529832101714 | 0.999950574162623 |
| ebi-fl187-GCST90094734 | ebi-met1400-GCST90200511 | X-12906 levels \|\| id:ebi-met1400-GCST90200511 | Chips liking \|\| id:ebi-fl187-GCST90094734 | MR Egger | 4 | 0.866469865537537 | 0.623906862842521 | 0.299337943813569 | -0.356387586 | 2.08932731670888 | 2.37849959243773 | 0.700201179717397 | 8.07947840577722 | 0.999950574162623 |
| ebi-fl187-GCST90094734 | ebi-met1400-GCST90200515 | X-12849 levels \|\| id:ebi-met1400-GCST90200515 | Chips liking \|\| id:ebi-fl187-GCST90094734 | MR Egger | 4 | 0.669423585810076 | 1.07834656855565 | 0.598057272472354 | -1.444135689 | 2.78298286017914 | 1.95311119510239 | 0.235949922667577 | 16.1671735142233 | 0.999950574162623 |
| ebi-fl187-GCST90094734 | ebi-met1400-GCST90200551 | X-18886 levels \|\| id:ebi-met1400-GCST90200551 | Chips liking \|\| id:ebi-fl187-GCST90094734 | MR Egger | 4 | 0.90076412013626 | 0.614356428032847 | 0.280250423038211 | -0.303374479 | 2.10490271908064 | 2.46148326166034 | 0.738322558443224 | 8.20630465390278 | 0.999950574162623 |
| ebi-fl187-GCST90094734 | ebi-met1400-GCST90200564 | X-21319 levels \|\| id:ebi-met1400-GCST90200564 | Chips liking \|\| id:ebi-fl187-GCST90094734 | MR Egger | 4 | -0.868528373 | 0.630760316753297 | 0.302394429851128 | -2.104818594 | 0.367761847416051 | 0.419568543364191 | 0.121867779779141 | 1.44449798707895 | 0.999950574162623 |
| ebi-fl187-GCST90094734 | ebi-met1400-GCST90200579 | X-21442 levels \|\| id:ebi-met1400-GCST90200579 | Chips liking \|\| id:ebi-fl187-GCST90094734 | MR Egger | 4 | 0.159784859884655 | 0.681505482967596 | 0.836445145949875 | -1.175965887 | 1.49553560650114 | 1.17325842888385 | 0.308520839540986 | 4.46172564224511 | 0.999950574162623 |
| ebi-fl187-GCST90094734 | ebi-met1400-GCST90200581 | X-21441 levels \|\| id:ebi-met1400-GCST90200581 | Chips liking \|\| id:ebi-fl187-GCST90094734 | MR Egger | 4 | 1.04152349326469 | 0.623655849889466 | 0.236864317672925 | -0.180841973 | 2.26388895904805 | 2.83353059243653 | 0.834567232833461 | 9.6204299694521 | 0.999950574162623 |
| ebi-fl187-GCST90094734 | ebi-met1400-GCST90200648 | X-24585 levels \|\| id:ebi-met1400-GCST90200648 | Chips liking \|\| id:ebi-fl187-GCST90094734 | MR Egger | 4 | -0.08504589 | 0.95942992596306 | 0.937443328594247 | -1.965528544 | 1.79543676534484 | 0.91847013525982 | 0.140081827576034 | 6.02210439399292 | 0.999950574162623 |
| ebi-fl187-GCST90094734 | ebi-met1400-GCST90200720 | Adenosine 5'-diphosphate (ADP) to pantothenate ratio \|\| id:ebi-met1400-GCST90200720 | Chips liking \|\| id:ebi-fl187-GCST90094734 | MR Egger | 4 | -1.245020805 | 1.49303063669726 | 0.492076020806083 | -4.171360853 | 1.68131924249064 | 0.287934917456958 | 0.0154312461654611 | 5.37263911170769 | 0.999950574162623 |
| ebi-fl187-GCST90094734 | ebi-met1400-GCST90200724 | Cholate to taurocholate ratio \|\| id:ebi-met1400-GCST90200724 | Chips liking \|\| id:ebi-fl187-GCST90094734 | MR Egger | 4 | -0.450210647 | 0.734546559650076 | 0.60234682474358 | -1.889921904 | 0.989500610245872 | 0.637493851521465 | 0.151083607464147 | 2.68989083295561 | 0.999950574162623 |
| ebi-fl187-GCST90094734 | ebi-met1400-GCST90200726 | N-acetylputrescine to (N(1) + N(8))-acetylspermidine ratio \|\| id:ebi-met1400-GCST90200726 | Chips liking \|\| id:ebi-fl187-GCST90094734 | MR Egger | 4 | 0.155091633854429 | 0.619719573834804 | 0.825746129507501 | -1.059558731 | 1.36974199857064 | 1.16776496300718 | 0.346608724322543 | 3.93433550033256 | 0.999950574162623 |
| ebi-fl187-GCST90094734 | ebi-met1400-GCST90200744 | Arginine to glutamate ratio \|\| id:ebi-met1400-GCST90200744 | Chips liking \|\| id:ebi-fl187-GCST90094734 | MR Egger | 4 | -1.339295827 | 0.913093573931794 | 0.280114205973879 | -3.128959232 | 0.450367577848354 | 0.262030118149706 | 0.0437633210244577 | 1.5688887682719 | 0.999950574162623 |
| ebi-fl187-GCST90094734 | ebi-met1400-GCST90200759 | Histidine to trans-urocanate ratio \|\| id:ebi-met1400-GCST90200759 | Chips liking \|\| id:ebi-fl187-GCST90094734 | MR Egger | 4 | 0.501294717551988 | 1.08059290860085 | 0.688309487031492 | -1.616667383 | 2.61925681840966 | 1.65085728153462 | 0.198559319778319 | 13.7255192404897 | 0.999950574162623 |
| ebi-fl187-GCST90094734 | ebi-met1400-GCST90200795 | Oleoyl-linoleoyl-glycerol (18:1 to 18:2) [2] to linoleoyl-arachidonoyl-glycerol (18:2 to 20:4) [2] ratio \|\| id:ebi-met1400-GCST90200795 | Chips liking \|\| id:ebi-fl187-GCST90094734 | MR Egger | 4 | 1.56981518430777 | 1.22757297833648 | 0.329297448551605 | -0.836227853 | 3.97585822184727 | 4.80575993184685 | 0.433342074174674 | 53.295836935592 | 0.999950574162623 |
| ebi-fl187-GCST90094734 | ebi-met1400-GCST90200811 | Choline to taurocholate ratio \|\| id:ebi-met1400-GCST90200811 | Chips liking \|\| id:ebi-fl187-GCST90094734 | MR Egger | 4 | -1.071398329 | 0.655520522075066 | 0.243787446908291 | -2.356218552 | 0.213421894638595 | 0.342529213978929 | 0.0947779442959411 | 1.23790680733352 | 0.999950574162623 |
| ebi-fl187-GCST90094734 | ebi-met1400-GCST90200831 | Cholate to phosphate ratio \|\| id:ebi-met1400-GCST90200831 | Chips liking \|\| id:ebi-fl187-GCST90094734 | MR Egger | 4 | -0.122413236 | 0.648787824071785 | 0.867754941322521 | -1.394037371 | 1.14921089949671 | 0.884782669176347 | 0.248071722533426 | 3.15570176108782 | 0.999950574162623 |
| ebi-fl187-GCST90094734 | ebi-met1400-GCST90200841 | Adenosine 5'-diphosphate (ADP) to oxalate (ethanedioate) ratio \|\| id:ebi-met1400-GCST90200841 | Chips liking \|\| id:ebi-fl187-GCST90094734 | MR Egger | 4 | -0.349230168 | 1.70547460502069 | 0.85670006704923 | -3.691960394 | 2.99350005806294 | 0.705230790184254 | 0.0249230950429869 | 19.9554054809841 | 0.999950574162623 |
| ebi-fl187-GCST90094734 | ebi-met1400-GCST90200844 | Aspartate to phosphate ratio \|\| id:ebi-met1400-GCST90200844 | Chips liking \|\| id:ebi-fl187-GCST90094734 | MR Egger | 4 | 1.35307924813906 | 0.585875455736487 | 0.147187192453153 | 0.204763354895543 | 2.50139514138257 | 3.86932180747819 | 1.2272346115345 | 12.1995021238083 | 0.999950574162623 |
| ebi-fl187-GCST90094734 | ebi-met1400-GCST90200870 | Adenosine 5'-monophosphate (AMP) to acetoacetate ratio \|\| id:ebi-met1400-GCST90200870 | Chips liking \|\| id:ebi-fl187-GCST90094734 | MR Egger | 4 | -1.067322857 | 0.783139546598317 | 0.306083167484683 | -2.602276369 | 0.46763065387614 | 0.343928030397922 | 0.0741046964505348 | 1.59620774065718 | 0.999950574162623 |
| ebi-fl187-GCST90094734 | ebi-met1400-GCST90200887 | Cholesterol to cortisol ratio \|\| id:ebi-met1400-GCST90200887 | Chips liking \|\| id:ebi-fl187-GCST90094734 | MR Egger | 4 | -0.971086071 | 0.605712374718143 | 0.250072502379959 | -2.158282325 | 0.216110183627559 | 0.378671550570106 | 0.115523382309928 | 1.241239136 | 0.999950574162623 |
| ebi-fl187-GCST90094734 | ebi-met1400-GCST90200890 | Cortisol to taurocholate ratio \|\| id:ebi-met1400-GCST90200890 | Chips liking \|\| id:ebi-fl187-GCST90094734 | MR Egger | 4 | -0.786270398 | 0.651760971000478 | 0.351010581548266 | -2.063721901 | 0.49118110558175 | 0.455540616332933 | 0.126980480556682 | 1.63424529675138 | 0.999950574162623 |
| ebi-fl187-GCST90094734 | ebi-met1400-GCST90200948 | Adenosine 5'-diphosphate (ADP) to mannitol to sorbitol ratio \|\| id:ebi-met1400-GCST90200948 | Chips liking \|\| id:ebi-fl187-GCST90094734 | MR Egger | 4 | -1.376138806 | 1.42821066531493 | 0.436940865289747 | -4.17543171 | 1.42315409832195 | 0.252551824521934 | 0.0153685554680078 | 4.15018992527513 | 0.999950574162623 |
| ebi-fl187-GCST90094734 | ebi-met1400-GCST90200972 | Leucine to N-palmitoyl-sphingosine (d18:1 to 16:0) ratio \|\| id:ebi-met1400-GCST90200972 | Chips liking \|\| id:ebi-fl187-GCST90094734 | MR Egger | 4 | 1.75577489888801 | 0.601065659457518 | 0.099934572900976 | 0.577686206351277 | 2.93386359142475 | 5.78793106601797 | 1.78191068359175 | 18.800126366295 | 0.999950574162623 |
| ebi-fl187-GCST90094734 | ebi-met1400-GCST90200973 | Proline to glutamate ratio \|\| id:ebi-met1400-GCST90200973 | Chips liking \|\| id:ebi-fl187-GCST90094734 | MR Egger | 4 | -1.491028064 | 0.611389096123351 | 0.134928141323517 | -2.689350692 | -0.292705435 | 0.225141077186079 | 0.0679250293244301 | 0.746241924967079 | 0.999950574162623 |
| ebi-fl187-GCST90094734 | ebi-met1400-GCST90200983 | Cholesterol to linoleoyl-arachidonoyl-glycerol (18:2 to 20:4) [1] ratio \|\| id:ebi-met1400-GCST90200983 | Chips liking \|\| id:ebi-fl187-GCST90094734 | MR Egger | 4 | 2.17258815678394 | 1.93676443377695 | 0.378556304406265 | -1.623470133 | 5.96864644698676 | 8.7809812122459 | 0.197213154348154 | 390.976105547684 | 0.999950574162623 |
| ebi-fl187-GCST90094734 | ebi-met1400-GCST90201001 | Serine to alpha-ketobutyrate ratio \|\| id:ebi-met1400-GCST90201001 | Chips liking \|\| id:ebi-fl187-GCST90094734 | MR Egger | 4 | -1.487894369 | 0.634927121191492 | 0.143826404070281 | -2.732351526 | -0.243437211 | 0.225847707183494 | 0.0650661049654152 | 0.783928696318199 | 0.999950574162623 |
| ebi-fl187-GCST90094734 | ebi-met1400-GCST90201005 | Fructose to maltose ratio \|\| id:ebi-met1400-GCST90201005 | Chips liking \|\| id:ebi-fl187-GCST90094734 | MR Egger | 4 | -0.517426086 | 0.711112590203873 | 0.542493328816536 | -1.911206763 | 0.876354590307752 | 0.596052763488691 | 0.147901796400457 | 2.40212698904992 | 0.999950574162623 |
| ebi-fl187-GCST90094734 | ebi-met1400-GCST90201020 | Paraxanthine to 5-acetylamino-6-formylamino-3-methyluracil ratio \|\| id:ebi-met1400-GCST90201020 | Chips liking \|\| id:ebi-fl187-GCST90094734 | MR Egger | 4 | 0.400165526815557 | 0.847136924846355 | 0.683187053623597 | -1.260222846 | 2.06055389951441 | 1.49207165507131 | 0.283590822410331 | 7.85031689299883 | 0.999950574162623 |
| ebi-fl187-GCST90094734 | ebi-pw11-GCST90275197 | Placental weight (fetal genotype, adjusted for sex) \|\| id:ebi-pw11-GCST90275197 | Chips liking \|\| id:ebi-fl187-GCST90094734 | MR Egger | 4 | 0.263116332889444 | 0.276258253262591 | 0.441399896188752 | -0.278349844 | 0.804582509284122 | 1.30097805668521 | 0.75703193247735 | 2.23576289369679 | 0.999950574162623 |
| ebi-fl187-GCST90094734 | prot-a-1017 | FAS-associated factor 2 \|\| id:prot-a-1017 | Chips liking \|\| id:ebi-fl187-GCST90094734 | MR Egger | 4 | 1.2354600658141 | 0.967268920147747 | 0.329739119622199 | -0.660387018 | 3.13130714930369 | 3.43996076505881 | 0.516651342592352 | 22.9038987990799 | 0.999950574162623 |
| ebi-fl187-GCST90094734 | prot-a-1019 | Fas apoptotic inhibitory molecule 3 \|\| id:prot-a-1019 | Chips liking \|\| id:ebi-fl187-GCST90094734 | MR Egger | 4 | 0.562860667727268 | 0.96930320450575 | 0.620166138031714 | -1.336973613 | 2.46269494855854 | 1.7556877626982 | 0.262639315215628 | 11.7363979477235 | 0.999950574162623 |
| ebi-fl187-GCST90094734 | prot-a-1052 | MIP18 family protein FAM96A \|\| id:prot-a-1052 | Chips liking \|\| id:ebi-fl187-GCST90094734 | MR Egger | 4 | 0.991563571205321 | 0.966269467535104 | 0.412705270869208 | -0.902324585 | 2.88545172757413 | 2.69544570049658 | 0.40562565158042 | 17.9116569576346 | 0.999950574162623 |
| ebi-fl187-GCST90094734 | prot-a-1066 | F-box/LRR-repeat protein 4 \|\| id:prot-a-1066 | Chips liking \|\| id:ebi-fl187-GCST90094734 | MR Egger | 4 | 0.0733215829191721 | 1.12824627563692 | 0.954095541765747 | -2.138041117 | 2.28468428316754 | 1.07607652911062 | 0.117885540936215 | 9.82258457913244 | 0.999950574162623 |
| ebi-fl187-GCST90094734 | prot-a-1076 | Low affinity immunoglobulin gamma Fc region receptor III-B \|\| id:prot-a-1076 | Chips liking \|\| id:ebi-fl187-GCST90094734 | MR Egger | 4 | 1.33617731213031 | 0.966269467535105 | 0.300873582087244 | -0.557710844 | 3.23006546849912 | 3.80447236229442 | 0.572518148145619 | 25.28131205 | 0.999950574162623 |
| ebi-fl187-GCST90094734 | prot-a-1081 | Fc receptor-like protein 3 \|\| id:prot-a-1081 | Chips liking \|\| id:ebi-fl187-GCST90094734 | MR Egger | 4 | -0.017511477 | 0.966269467535104 | 0.987186320552322 | -1.911399633 | 1.87637667984133 | 0.982640958294401 | 0.147873273390128 | 6.5298023826813 | 0.999950574162623 |
| ebi-fl187-GCST90094734 | prot-a-1105 | Fibroblast growth factor receptor 4 \|\| id:prot-a-1105 | Chips liking \|\| id:ebi-fl187-GCST90094734 | MR Egger | 4 | 0.0564717399297601 | 0.968301588014475 | 0.958796268126503 | -1.841399373 | 1.95434285243813 | 1.05809671249029 | 0.158595336784917 | 7.05927851145519 | 0.999950574162623 |
| ebi-fl187-GCST90094734 | prot-a-1143 | Follistatin-related protein 1 \|\| id:prot-a-1143 | Chips liking \|\| id:ebi-fl187-GCST90094734 | MR Egger | 4 | 0.225637146781243 | 0.966269467535105 | 0.837086811948993 | -1.66825101 | 2.11952530315005 | 1.25312088382753 | 0.188576596040473 | 8.32718366147444 | 0.999950574162623 |
| ebi-fl187-GCST90094734 | prot-a-1151 | Alpha-(1,3)-fucosyltransferase 10 \|\| id:prot-a-1151 | Chips liking \|\| id:ebi-fl187-GCST90094734 | MR Egger | 4 | -0.512446564 | 1.1856544740149 | 0.707728991908383 | -2.836329334 | 1.81143620461311 | 0.599028223394827 | 0.058640521187507 | 6.1192295899991 | 0.999950574162623 |
| ebi-fl187-GCST90094734 | prot-a-1165 | Growth arrest and DNA damage-inducible proteins-interacting protein 1 \|\| id:prot-a-1165 | Chips liking \|\| id:ebi-fl187-GCST90094734 | MR Egger | 4 | 0.287757476058049 | 1.10431208196029 | 0.818795061995044 | -1.876694205 | 2.45220915670021 | 1.33343387526559 | 0.153095372122481 | 11.6139754915865 | 0.999950574162623 |
| ebi-fl187-GCST90094734 | prot-a-1194 | Growth/differentiation factor 11/8 \|\| id:prot-a-1194 | Chips liking \|\| id:ebi-fl187-GCST90094734 | MR Egger | 4 | -0.421609859 | 0.966269467535105 | 0.70518294182697 | -2.315498015 | 1.47227829767194 | 0.655989918237289 | 0.0987170092004324 | 4.35915528959401 | 0.999950574162623 |
| ebi-fl187-GCST90094734 | prot-a-1216 | D-glucuronyl C5-epimerase \|\| id:prot-a-1216 | Chips liking \|\| id:ebi-fl187-GCST90094734 | MR Egger | 4 | 0.220481380729219 | 1.7489614686142 | 0.911211232251936 | -3.207483098 | 3.64844585921304 | 1.24667671231085 | 0.0404583148553755 | 38.4149174421605 | 0.999950574162623 |
| ebi-fl187-GCST90094734 | prot-a-1246 | Glypican-3 \|\| id:prot-a-1246 | Chips liking \|\| id:ebi-fl187-GCST90094734 | MR Egger | 4 | -1.1670175 | 0.966269467535104 | 0.350582031161008 | -3.060905656 | 0.726870656605175 | 0.311293992519262 | 0.0468452503144225 | 2.06859711770498 | 0.999950574162623 |
| ebi-fl187-GCST90094734 | prot-a-1275 | Glutamate receptor 4 \|\| id:prot-a-1275 | Chips liking \|\| id:ebi-fl187-GCST90094734 | MR Egger | 4 | -1.160315009 | 0.966269467535105 | 0.35274665233171 | -3.054203166 | 0.733573147071709 | 0.313387445373234 | 0.0471602847362952 | 2.08250843833385 | 0.999950574162623 |
| ebi-fl187-GCST90094734 | prot-a-1289 | Glutathione S-transferase P \|\| id:prot-a-1289 | Chips liking \|\| id:ebi-fl187-GCST90094734 | MR Egger | 4 | -1.899852626 | 1.05298663350831 | 0.212959028958217 | -3.963706428 | 0.164001175625939 | 0.149590663365121 | 0.0189925890154023 | 1.17821570023285 | 0.999950574162623 |
| ebi-fl187-GCST90094734 | prot-a-130 | Apolipoprotein D \|\| id:prot-a-130 | Chips liking \|\| id:ebi-fl187-GCST90094734 | MR Egger | 4 | -0.532761189 | 0.968301588014475 | 0.637422218095312 | -2.430632302 | 1.3651099234059 | 0.58698196179743 | 0.0879811843464061 | 3.91615350526517 | 0.999950574162623 |
| ebi-fl187-GCST90094734 | prot-a-1313 | Heparin-binding EGF-like growth factor \|\| id:prot-a-1313 | Chips liking \|\| id:ebi-fl187-GCST90094734 | MR Egger | 4 | 1.15480274124552 | 0.966269467535105 | 0.35453869708172 | -0.739085415 | 3.04869089761432 | 3.17339737536974 | 0.477550476298151 | 21.0877203600907 | 0.999950574162623 |
| ebi-fl187-GCST90094734 | prot-a-1347 | HLA class II histocompatibility antigen, DQ alpha 2 chain \|\| id:prot-a-1347 | Chips liking \|\| id:ebi-fl187-GCST90094734 | MR Egger | 4 | -0.403158553 | 1.64251857766512 | 0.828996104177769 | -3.622494965 | 2.81617785899525 | 0.668206144682596 | 0.0267159379299007 | 16.7128495717851 | 0.999950574162623 |
| ebi-fl187-GCST90094734 | prot-a-1357 | Non-histone chromosomal protein HMG-14 \|\| id:prot-a-1357 | Chips liking \|\| id:ebi-fl187-GCST90094734 | MR Egger | 4 | 1.23838552602415 | 0.966269467535105 | 0.328485165491117 | -0.65550263 | 3.13227368239295 | 3.4500389679041 | 0.519181040848318 | 22.9260468768432 | 0.999950574162623 |
| ebi-fl187-GCST90094734 | prot-a-137 | Apolipoprotein M \|\| id:prot-a-137 | Chips liking \|\| id:ebi-fl187-GCST90094734 | MR Egger | 4 | -0.10042451 | 0.968301588014475 | 0.926860940355921 | -1.998295623 | 1.79744660219673 | 0.904453386740039 | 0.13556614228457 | 6.03422001245979 | 0.999950574162623 |
| ebi-fl187-GCST90094734 | prot-a-1381 | 17-beta-hydroxysteroid dehydrogenase 14 \|\| id:prot-a-1381 | Chips liking \|\| id:ebi-fl187-GCST90094734 | MR Egger | 4 | -0.805641917 | 1.11702707736412 | 0.545679579648742 | -2.995014988 | 1.38373115487896 | 0.446801025421647 | 0.0500358771238071 | 3.98976030386906 | 0.999950574162623 |
| ebi-fl187-GCST90094734 | prot-a-1392 | Serine protease HTRA2, mitochondrial \|\| id:prot-a-1392 | Chips liking \|\| id:ebi-fl187-GCST90094734 | MR Egger | 4 | 1.00354162550973 | 1.92677085151689 | 0.654402644235141 | -2.772929243 | 4.78001249448283 | 2.72792603272459 | 0.0624787210466992 | 119.105838201367 | 0.999950574162623 |
| ebi-fl187-GCST90094734 | prot-a-1416 | Interferon-induced protein with tetratricopeptide repeats 2 \|\| id:prot-a-1416 | Chips liking \|\| id:ebi-fl187-GCST90094734 | MR Egger | 4 | 1.71638979952728 | 0.967268920147747 | 0.217979689107432 | -0.179457284 | 3.61223688301686 | 5.56440354632678 | 0.835723648983603 | 37.0488341020747 | 0.999950574162623 |
| ebi-fl187-GCST90094734 | prot-a-1428 | Interferon gamma \|\| id:prot-a-1428 | Chips liking \|\| id:ebi-fl187-GCST90094734 | MR Egger | 4 | 0.197555994701606 | 0.966269467535104 | 0.856917909113048 | -1.696332162 | 2.09144415107041 | 1.21842128819602 | 0.18335480801298 | 8.09659943809132 | 0.999950574162623 |
| ebi-fl187-GCST90094734 | prot-a-1483 | Interleukin-17C \|\| id:prot-a-1483 | Chips liking \|\| id:ebi-fl187-GCST90094734 | MR Egger | 4 | -0.490277233 | 0.96930320450575 | 0.66323404978386 | -2.390111514 | 1.40955704814776 | 0.612456577665598 | 0.0916194664990958 | 4.09414149480513 | 0.999950574162623 |
| ebi-fl187-GCST90094734 | prot-a-1503 | Interleukin-1 receptor-like 2 \|\| id:prot-a-1503 | Chips liking \|\| id:ebi-fl187-GCST90094734 | MR Egger | 4 | 0.798603780564349 | 1.22863929954343 | 0.582384910892094 | -1.609529247 | 3.20673680766948 | 2.22243575325073 | 0.19998173401283 | 24.6983590861865 | 0.999950574162623 |
| ebi-fl187-GCST90094734 | prot-a-1522 | Interleukin-31 receptor subunit alpha \|\| id:prot-a-1522 | Chips liking \|\| id:ebi-fl187-GCST90094734 | MR Egger | 4 | -1.941280141 | 0.966269467535104 | 0.182277916381426 | -3.835168297 | -0.047391984 | 0.143520106228978 | 0.0215977033383738 | 0.953713483756374 | 0.999950574162623 |
| ebi-fl187-GCST90094734 | prot-a-1540 | Interleukin-6 receptor subunit alpha \|\| id:prot-a-1540 | Chips liking \|\| id:ebi-fl187-GCST90094734 | MR Egger | 4 | -0.436826021 | 0.967268920147747 | 0.695799129508267 | -2.332673104 | 1.45902106281456 | 0.646083826861174 | 0.0970360127259544 | 4.3017463270101 | 0.999950574162623 |
| ebi-fl187-GCST90094734 | prot-a-1541 | Interleukin-6 receptor subunit alpha \|\| id:prot-a-1541 | Chips liking \|\| id:ebi-fl187-GCST90094734 | MR Egger | 4 | -0.417873253 | 1.06974421228543 | 0.733753531304151 | -2.514571909 | 1.67882540320703 | 0.658445679234659 | 0.080897536306152 | 5.35925730620576 | 0.999950574162623 |
| ebi-fl187-GCST90094734 | prot-a-1545 | Interleukin-9 \|\| id:prot-a-1545 | Chips liking \|\| id:ebi-fl187-GCST90094734 | MR Egger | 4 | 1.20536142867815 | 0.966269467535105 | 0.338496833655735 | -0.688526728 | 3.09924958504695 | 3.33796529645812 | 0.502315571810338 | 22.1812998553939 | 0.999950574162623 |
| ebi-fl187-GCST90094734 | prot-a-1548 | Inositol monophosphatase 3 \|\| id:prot-a-1548 | Chips liking \|\| id:ebi-fl187-GCST90094734 | MR Egger | 4 | 2.51401660667412 | 1.3459226897012 | 0.202734495006394 | -0.123991865 | 5.15202507848848 | 12.3544535162568 | 0.883387027083102 | 172.781031423264 | 0.999950574162623 |
| ebi-fl187-GCST90094734 | prot-a-1555 | Inhibin beta A chain:Inhibin beta B chain heterodimer \|\| id:prot-a-1555 | Chips liking \|\| id:ebi-fl187-GCST90094734 | MR Egger | 4 | 0.167731917446049 | 0.966269467535105 | 0.87816971367216 | -1.726156239 | 2.06162007381485 | 1.18261952848406 | 0.177967160208311 | 7.85869116254268 | 0.999950574162623 |
| ebi-fl187-GCST90094734 | prot-a-1603 | Junctophilin-3 \|\| id:prot-a-1603 | Chips liking \|\| id:ebi-fl187-GCST90094734 | MR Egger | 4 | 1.86881402260213 | 0.967268920147747 | 0.193072558154904 | -0.027033061 | 3.76466110609171 | 6.48060598763507 | 0.973329061869824 | 43.149080421261 | 0.999950574162623 |
| ebi-fl187-GCST90094734 | prot-a-1627 | UPF0258 protein KIAA1024 \|\| id:prot-a-1627 | Chips liking \|\| id:ebi-fl187-GCST90094734 | MR Egger | 4 | 1.58925110512429 | 0.966269467535104 | 0.241757122264898 | -0.304637051 | 3.4831392614931 | 4.90007791065718 | 0.737390960960173 | 32.5617817436296 | 0.999950574162623 |
| ebi-fl187-GCST90094734 | prot-a-1666 | Kallikrein-7 \|\| id:prot-a-1666 | Chips liking \|\| id:ebi-fl187-GCST90094734 | MR Egger | 4 | 0.57214444668981 | 0.967268920147747 | 0.614134655187925 | -1.323702637 | 2.46799153017939 | 1.77206307458881 | 0.266148025856623 | 11.7987256535685 | 0.999950574162623 |
| ebi-fl187-GCST90094734 | prot-a-1678 | Importin subunit alpha-3 \|\| id:prot-a-1678 | Chips liking \|\| id:ebi-fl187-GCST90094734 | MR Egger | 4 | 1.00973468684263 | 0.966269467535104 | 0.405721404075814 | -0.88415347 | 2.90362284321144 | 2.74487266756711 | 0.413063696323794 | 18.2401068605433 | 0.999950574162623 |
| ebi-fl187-GCST90094734 | prot-a-1718 | Protein LDOC1 \|\| id:prot-a-1718 | Chips liking \|\| id:ebi-fl187-GCST90094734 | MR Egger | 4 | 0.637802018971219 | 1.27505755885849 | 0.666539685605713 | -1.861310796 | 3.13691483433386 | 1.8923170280202 | 0.155468708924812 | 23.032697443104 | 0.999950574162623 |
| ebi-fl187-GCST90094734 | prot-a-1723 | Leptin \|\| id:prot-a-1723 | Chips liking \|\| id:ebi-fl187-GCST90094734 | MR Egger | 4 | 0.5169939810979 | 1.69360651932172 | 0.789006457008667 | -2.802474797 | 3.83646275896846 | 1.67697903470775 | 0.060659756144199 | 46.3611933447954 | 0.999950574162623 |
| ebi-fl187-GCST90094734 | prot-a-1730 | Galectin-7 \|\| id:prot-a-1730 | Chips liking \|\| id:ebi-fl187-GCST90094734 | MR Egger | 4 | -1.938203985 | 1.1839983402355 | 0.243278023193467 | -4.258840732 | 0.382432762160215 | 0.143962276179623 | 0.0141386834427791 | 1.46584631070461 | 0.999950574162623 |
| ebi-fl187-GCST90094734 | prot-a-1753 | Lipase member N \|\| id:prot-a-1753 | Chips liking \|\| id:ebi-fl187-GCST90094734 | MR Egger | 4 | 1.54240878789426 | 1.08802717760924 | 0.292043214616526 | -0.59012448 | 3.67494205600837 | 4.67583982301755 | 0.554258286246798 | 39.4463711107812 | 0.999950574162623 |
| ebi-fl187-GCST90094734 | prot-a-1756 | VIP36-like protein \|\| id:prot-a-1756 | Chips liking \|\| id:ebi-fl187-GCST90094734 | MR Egger | 4 | 0.137970836824076 | 0.966269467535104 | 0.899544982426783 | -1.75591732 | 2.03185899319288 | 1.14794207214942 | 0.172748703825271 | 7.62825405823925 | 0.999950574162623 |
| ebi-fl187-GCST90094734 | prot-a-1760 | Ig Kappa chain V-I region HK102- like \|\| id:prot-a-1760 | Chips liking \|\| id:ebi-fl187-GCST90094734 | MR Egger | 4 | -0.754796425 | 0.966269467535104 | 0.516500819052831 | -2.648684582 | 1.13909173116559 | 0.470106306785832 | 0.0707442101196614 | 3.12392970825459 | 0.999950574162623 |
| ebi-fl187-GCST90094734 | prot-a-179 | Neutral ceramidase \|\| id:prot-a-179 | Chips liking \|\| id:ebi-fl187-GCST90094734 | MR Egger | 4 | 0.485297964197848 | 1.8210380771599 | 0.814818615495942 | -3.083936667 | 4.05453259543125 | 1.62465902695436 | 0.0457786860148439 | 57.6582069876013 | 0.999950574162623 |
| ebi-fl187-GCST90094734 | prot-a-1809 | Lactotransferrin \|\| id:prot-a-1809 | Chips liking \|\| id:ebi-fl187-GCST90094734 | MR Egger | 4 | 0.0080920548241194 | 1.23511021166364 | 0.995367307804794 | -2.41272396 | 2.42890806968485 | 1.00812488399182 | 0.0895709742096793 | 11.3464857415126 | 0.999950574162623 |
| ebi-fl187-GCST90094734 | prot-a-1818 | Lysophospholipase-like protein 1 \|\| id:prot-a-1818 | Chips liking \|\| id:ebi-fl187-GCST90094734 | MR Egger | 4 | -0.298391677 | 0.968301588014475 | 0.787093983336326 | -2.196262789 | 1.59947943562215 | 0.742010654401466 | 0.11121802784533 | 4.95045472314057 | 0.999950574162623 |
| ebi-fl187-GCST90094734 | prot-a-1852 | MAP kinase-activated protein kinase 3 \|\| id:prot-a-1852 | Chips liking \|\| id:ebi-fl187-GCST90094734 | MR Egger | 4 | 1.03112812146833 | 0.968301588014475 | 0.398474373763261 | -0.866742991 | 2.9289992339767 | 2.80422756027133 | 0.42031830275331 | 18.7088978954137 | 0.999950574162623 |
| ebi-fl187-GCST90094734 | prot-a-1856 | Megakaryocyte-associated tyrosine-protein kinase \|\| id:prot-a-1856 | Chips liking \|\| id:ebi-fl187-GCST90094734 | MR Egger | 4 | -1.138578189 | 0.96930320450575 | 0.361060563497518 | -3.03841247 | 0.761256091404746 | 0.320274067300704 | 0.0479108891138524 | 2.14096377843421 | 0.999950574162623 |
| ebi-fl187-GCST90094734 | prot-a-1890 | Beta-1,3-N-acetylglucosaminyltransferase manic fringe \|\| id:prot-a-1890 | Chips liking \|\| id:ebi-fl187-GCST90094734 | MR Egger | 4 | 0.516235094511969 | 2.2627845603545 | 0.840738609700714 | -3.918822644 | 4.9512928328068 | 1.67570688058483 | 0.0198644685375629 | 141.357597578286 | 0.999950574162623 |
| ebi-fl187-GCST90094734 | prot-a-1919 | Matrilysin \|\| id:prot-a-1919 | Chips liking \|\| id:ebi-fl187-GCST90094734 | MR Egger | 4 | 0.0979271597987939 | 0.966269467535105 | 0.92852114194033 | -1.795960997 | 1.9918153161676 | 1.10288244800324 | 0.165967881120924 | 7.32882583002542 | 0.999950574162623 |
| ebi-fl187-GCST90094734 | prot-a-1923 | Multimerin-2 \|\| id:prot-a-1923 | Chips liking \|\| id:ebi-fl187-GCST90094734 | MR Egger | 4 | -0.255305721 | 0.96930320450575 | 0.816902961863544 | -2.155140002 | 1.64452856002017 | 0.774679626070969 | 0.115886965111662 | 5.17856794740639 | 0.999950574162623 |
| ebi-fl187-GCST90094734 | prot-a-1948 | Mesothelin \|\| id:prot-a-1948 | Chips liking \|\| id:ebi-fl187-GCST90094734 | MR Egger | 4 | -0.325971277 | 0.967268920147747 | 0.768194483558799 | -2.22181836 | 1.56987580650079 | 0.721825920147952 | 0.108411797759566 | 4.8060512763839 | 0.999950574162623 |
| ebi-fl187-GCST90094734 | prot-a-195 | ATPase family AAA domain-containing protein 1 \|\| id:prot-a-195 | Chips liking \|\| id:ebi-fl187-GCST90094734 | MR Egger | 4 | 0.477073977168209 | 0.966269467535105 | 0.670391356986837 | -1.416814179 | 2.37096213353701 | 1.6113526429507 | 0.242485302375891 | 10.7076895568676 | 0.999950574162623 |
| ebi-fl187-GCST90094734 | prot-a-1966 | Mucin-1 \|\| id:prot-a-1966 | Chips liking \|\| id:ebi-fl187-GCST90094734 | MR Egger | 4 | 1.45387457173411 | 0.966269467535105 | 0.271339639190365 | -0.440013585 | 3.34776272810291 | 4.27966429912692 | 0.644027672143055 | 28.4390365592135 | 0.999950574162623 |
| ebi-fl187-GCST90094734 | prot-a-1986 | Myocardial zonula adherens protein \|\| id:prot-a-1986 | Chips liking \|\| id:ebi-fl187-GCST90094734 | MR Egger | 4 | 0.476849531121218 | 0.967268920147747 | 0.670833119157666 | -1.418997552 | 2.3726966146108 | 1.61099102180342 | 0.241956444030759 | 10.7262779577025 | 0.999950574162623 |
| ebi-fl187-GCST90094734 | prot-a-2071 | Epididymal secretory protein E1 \|\| id:prot-a-2071 | Chips liking \|\| id:ebi-fl187-GCST90094734 | MR Egger | 4 | -0.082826586 | 1.51124394747417 | 0.961274743693531 | -3.044864723 | 2.87921155089407 | 0.920510762690398 | 0.0476027510996061 | 17.8002330675353 | 0.999950574162623 |
| ebi-fl187-GCST90094734 | prot-a-2098 | Neuritin \|\| id:prot-a-2098 | Chips liking \|\| id:ebi-fl187-GCST90094734 | MR Egger | 4 | 1.36527356619569 | 0.966269467535104 | 0.293213693861495 | -0.52861459 | 3.2591617225645 | 3.91679440798816 | 0.589420993920986 | 26.0277095533924 | 0.999950574162623 |
| ebi-fl187-GCST90094734 | prot-a-2115 | Neurotrophin-4 \|\| id:prot-a-2115 | Chips liking \|\| id:ebi-fl187-GCST90094734 | MR Egger | 4 | 0.415282558509014 | 0.96930320450575 | 0.710064164396214 | -1.484551722 | 2.31511683934028 | 1.51479869948949 | 0.2266038993813 | 10.1261059771701 | 0.999950574162623 |
| ebi-fl187-GCST90094734 | prot-a-2118 | Netrin-1 \|\| id:prot-a-2118 | Chips liking \|\| id:ebi-fl187-GCST90094734 | MR Egger | 4 | 0.693516768572289 | 0.966269467535104 | 0.547437638571779 | -1.200371388 | 2.58740492494109 | 2.00073931263682 | 0.301082372826638 | 13.2952246906707 | 0.999950574162623 |
| ebi-fl187-GCST90094734 | prot-a-2120 | Netrin-G1 \|\| id:prot-a-2120 | Chips liking \|\| id:ebi-fl187-GCST90094734 | MR Egger | 4 | 0.604967931445982 | 0.967268920147747 | 0.595536338334737 | -1.290879152 | 2.50081501493557 | 1.831193484 | 0.275028884566626 | 12.1924269224401 | 0.999950574162623 |
| ebi-fl187-GCST90094734 | prot-a-2145 | Olfactomedin-like protein 3 \|\| id:prot-a-2145 | Chips liking \|\| id:ebi-fl187-GCST90094734 | MR Egger | 4 | 0.263222936926008 | 1.42708751333469 | 0.870671150245742 | -2.533868589 | 3.06031446306199 | 1.30111675359023 | 0.0793514475793636 | 21.3342649455767 | 0.999950574162623 |
| ebi-fl187-GCST90094734 | prot-a-2162 | Prolyl 4-hydroxylase subunit alpha-2 \|\| id:prot-a-2162 | Chips liking \|\| id:ebi-fl187-GCST90094734 | MR Egger | 4 | 1.45195227151147 | 1.45878292414151 | 0.424456223818344 | -1.40726226 | 4.31116680282882 | 4.27144540161703 | 0.244812599822367 | 74.5273970058478 | 0.999950574162623 |
| ebi-fl187-GCST90094734 | prot-a-2168 | Polyadenylate-binding protein-interacting protein 1 \|\| id:prot-a-2168 | Chips liking \|\| id:ebi-fl187-GCST90094734 | MR Egger | 4 | 1.19304319004217 | 1.2554499364381 | 0.442263279182623 | -1.267638685 | 3.65372506546085 | 3.29709965647173 | 0.28149553714166 | 38.6182539698144 | 0.999950574162623 |
| ebi-fl187-GCST90094734 | prot-a-219 | Beta-1,4-galactosyltransferase 5 \|\| id:prot-a-219 | Chips liking \|\| id:ebi-fl187-GCST90094734 | MR Egger | 4 | 0.156978041464679 | 0.967268920147747 | 0.885991895577293 | -1.738869042 | 2.05282512495426 | 1.16996992279297 | 0.175719019107279 | 7.78987742587215 | 0.999950574162623 |
| ebi-fl187-GCST90094734 | prot-a-2190 | Pyruvate carboxylase, mitochondrial \|\| id:prot-a-2190 | Chips liking \|\| id:ebi-fl187-GCST90094734 | MR Egger | 4 | -0.286451994 | 0.966269467535105 | 0.794836327400761 | -2.18034015 | 1.60743616270841 | 0.750923126736449 | 0.113003086099328 | 4.99000126219561 | 0.999950574162623 |
| ebi-fl187-GCST90094734 | prot-a-2197 | Protocadherin-9 \|\| id:prot-a-2197 | Chips liking \|\| id:ebi-fl187-GCST90094734 | MR Egger | 4 | -0.936921715 | 1.21259939183294 | 0.520542340214426 | -3.313616523 | 1.43977309290896 | 0.391832151795599 | 0.0363843506829743 | 4.21973821983344 | 0.999950574162623 |
| ebi-fl187-GCST90094734 | prot-a-2198 | Protocadherin alpha-4 \|\| id:prot-a-2198 | Chips liking \|\| id:ebi-fl187-GCST90094734 | MR Egger | 4 | -1.243021697 | 1.5754632526853 | 0.512794138793856 | -4.330929673 | 1.84488627780298 | 0.288511106186803 | 0.0131553116688802 | 6.32738018590922 | 0.999950574162623 |
| ebi-fl187-GCST90094734 | prot-a-2254 | Placenta growth factor \|\| id:prot-a-2254 | Chips liking \|\| id:ebi-fl187-GCST90094734 | MR Egger | 4 | 0.907186303315498 | 0.967268920147747 | 0.447310593813367 | -0.98866078 | 2.80303338680508 | 2.47734222814544 | 0.372074647255516 | 16.4946054793623 | 0.999950574162623 |
| ebi-fl187-GCST90094734 | prot-a-2261 | Membrane-associated progesterone receptor component 2 \|\| id:prot-a-2261 | Chips liking \|\| id:ebi-fl187-GCST90094734 | MR Egger | 4 | -1.238758484 | 0.967268920147747 | 0.328755347476077 | -3.134605568 | 0.657088599201353 | 0.289743716107496 | 0.0435169149180909 | 1.92916757039872 | 0.999950574162623 |
| ebi-fl187-GCST90094734 | prot-a-2268 | Polymeric immunoglobulin receptor \|\| id:prot-a-2268 | Chips liking \|\| id:ebi-fl187-GCST90094734 | MR Egger | 4 | 0.262581063165531 | 0.966269467535105 | 0.811297855845975 | -1.631307093 | 2.15646921953434 | 1.30028186886075 | 0.195673643211466 | 8.64057576042991 | 0.999950574162623 |
| ebi-fl187-GCST90094734 | prot-a-2283 | Group 10 secretory phospholipase A2 \|\| id:prot-a-2283 | Chips liking \|\| id:ebi-fl187-GCST90094734 | MR Egger | 4 | 1.38118100615002 | 1.79798554298868 | 0.522684241559409 | -2.142870658 | 4.90523267040783 | 3.97959878390187 | 0.117317580507029 | 134.994315535551 | 0.999950574162623 |
| ebi-fl187-GCST90094734 | prot-a-2298 | Inactive phospholipase D5 \|\| id:prot-a-2298 | Chips liking \|\| id:ebi-fl187-GCST90094734 | MR Egger | 4 | 0.867790819108023 | 0.966269467535105 | 0.463919422279953 | -1.026097337 | 2.76167897547683 | 2.38164355602456 | 0.358402960618644 | 15.8263927791288 | 0.999950574162623 |
| ebi-fl187-GCST90094734 | prot-a-2304 | Procollagen-lysine,2-oxoglutarate 5-dioxygenase 2 \|\| id:prot-a-2304 | Chips liking \|\| id:ebi-fl187-GCST90094734 | MR Egger | 4 | -0.461731944 | 1.53118146010145 | 0.7914582122009 | -3.462847606 | 2.53938371772033 | 0.630191243797632 | 0.031340389752208 | 12.6718591217018 | 0.999950574162623 |
| ebi-fl187-GCST90094734 | prot-a-2331 | Periostin \|\| id:prot-a-2331 | Chips liking \|\| id:ebi-fl187-GCST90094734 | MR Egger | 4 | 0.957877427959598 | 1.03130472348607 | 0.451044535065656 | -1.06347983 | 2.9792346859923 | 2.6061588386041 | 0.345252298205578 | 19.672755047064 | 0.999950574162623 |
| ebi-fl187-GCST90094734 | prot-a-2333 | Periostin \|\| id:prot-a-2333 | Chips liking \|\| id:ebi-fl187-GCST90094734 | MR Egger | 4 | -0.175207887 | 0.967268920147746 | 0.872954884874626 | -2.071054971 | 1.72063919647527 | 0.839282526693671 | 0.12605272962268 | 5.58809921627094 | 0.999950574162623 |
| ebi-fl187-GCST90094734 | prot-a-2367 | cAMP-dependent protein kinase catalytic subunit alpha \|\| id:prot-a-2367 | Chips liking \|\| id:ebi-fl187-GCST90094734 | MR Egger | 4 | -0.450134188 | 0.968301588014475 | 0.687725665661572 | -2.348005301 | 1.44773692408717 | 0.637542595047271 | 0.0955595848495556 | 4.25347767196269 | 0.999950574162623 |
| ebi-fl187-GCST90094734 | prot-a-2385 | Phosphoribosyl pyrophosphate synthase-associated protein 1 \|\| id:prot-a-2385 | Chips liking \|\| id:ebi-fl187-GCST90094734 | MR Egger | 4 | 0.406997920159965 | 0.968301588014475 | 0.715104730239125 | -1.490873192 | 2.30486903266834 | 1.50230098111298 | 0.225175947755446 | 10.0228654985129 | 0.999950574162623 |
| ebi-fl187-GCST90094734 | prot-a-2393 | Trypsin-3 \|\| id:prot-a-2393 | Chips liking \|\| id:ebi-fl187-GCST90094734 | MR Egger | 4 | -0.88275725 | 0.967268920147747 | 0.45777550295008 | -2.778604333 | 1.01308983363611 | 0.413640826789378 | 0.0621251529036231 | 2.75409758511873 | 0.999950574162623 |
| ebi-fl187-GCST90094734 | prot-a-2405 | Pregnancy-specific beta-1-glycoprotein 6 \|\| id:prot-a-2405 | Chips liking \|\| id:ebi-fl187-GCST90094734 | MR Egger | 4 | 1.23635470218106 | 1.06662671764373 | 0.366093470893811 | -0.854233664 | 3.32694306876277 | 3.44303965609873 | 0.425609225641394 | 27.8530665203595 | 0.999950574162623 |
| ebi-fl187-GCST90094734 | prot-a-244 | Beclin-1 \|\| id:prot-a-244 | Chips liking \|\| id:ebi-fl187-GCST90094734 | MR Egger | 4 | -0.692453815 | 0.967268920147747 | 0.548361217016551 | -2.588300899 | 1.20339326799082 | 0.500346802747152 | 0.0751476150620245 | 3.33140210521211 | 0.999950574162623 |
| ebi-fl187-GCST90094734 | prot-a-2454 | tRNA pseudouridine synthase A, mitochondrial \|\| id:prot-a-2454 | Chips liking \|\| id:ebi-fl187-GCST90094734 | MR Egger | 4 | 0.194307832043423 | 1.15033981220028 | 0.881403123374962 | -2.0603582 | 2.44897386395596 | 1.21447007822606 | 0.127408324075137 | 11.5764615978826 | 0.999950574162623 |
| ebi-fl187-GCST90094734 | prot-a-2466 | Glutaminyl-peptide cyclotransferase-like protein \|\| id:prot-a-2466 | Chips liking \|\| id:ebi-fl187-GCST90094734 | MR Egger | 4 | 0.203872630072762 | 0.968301588014475 | 0.852744069867894 | -1.693998482 | 2.10174374258113 | 1.22614196989639 | 0.183783198989595 | 8.18042203317245 | 0.999950574162623 |
| ebi-fl187-GCST90094734 | prot-a-2474 | Ras-related protein Rab-27A \|\| id:prot-a-2474 | Chips liking \|\| id:ebi-fl187-GCST90094734 | MR Egger | 4 | 3.15607585493915 | 1.11674324681599 | 0.105717424300236 | 0.967259091179806 | 5.34489261869849 | 23.478282735114 | 2.63072399395733 | 209.535383208617 | 0.999950574162623 |
| ebi-fl187-GCST90094734 | prot-a-2490 | Rap1 GTPase-GDP dissociation stimulator 1 \|\| id:prot-a-2490 | Chips liking \|\| id:ebi-fl187-GCST90094734 | MR Egger | 4 | 1.45665712593643 | 0.967268920147747 | 0.271039547202091 | -0.439189958 | 3.35250420942601 | 4.29158928026596 | 0.644558329276888 | 28.5741999039188 | 0.999950574162623 |
| ebi-fl187-GCST90094734 | prot-a-2545 | Receptor-interacting serine/threonine-protein kinase 2 \|\| id:prot-a-2545 | Chips liking \|\| id:ebi-fl187-GCST90094734 | MR Egger | 4 | 1.95295407410877 | 0.967268920147747 | 0.180936966304581 | 0.0571069906191866 | 3.84880115759835 | 7.04948154292098 | 1.05876908269559 | 46.9367597110613 | 0.999950574162623 |
| ebi-fl187-GCST90094734 | prot-a-2610 | RUN and FYVE domain-containing protein 1 \|\| id:prot-a-2610 | Chips liking \|\| id:ebi-fl187-GCST90094734 | MR Egger | 4 | 0.378891287467381 | 0.996856040883449 | 0.740449042612873 | -1.574946553 | 2.33272912759894 | 1.4606642346035 | 0.207018616979014 | 10.3060296575458 | 0.999950574162623 |
| ebi-fl187-GCST90094734 | prot-a-2627 | Serine--tRNA ligase, mitochondrial \|\| id:prot-a-2627 | Chips liking \|\| id:ebi-fl187-GCST90094734 | MR Egger | 4 | -0.409012845 | 1.32351055374562 | 0.786515991708705 | -3.003093531 | 2.18506783997902 | 0.664305699019968 | 0.0496332885271833 | 8.89125171524176 | 0.999950574162623 |
| ebi-fl187-GCST90094734 | prot-a-264 | BolA-like protein 2 \|\| id:prot-a-264 | Chips liking \|\| id:ebi-fl187-GCST90094734 | MR Egger | 4 | 0.480569784225081 | 0.968301588014475 | 0.668860913791175 | -1.417301328 | 2.37844089673345 | 1.61699547827621 | 0.242367204651207 | 10.7880700300543 | 0.999950574162623 |
| ebi-fl187-GCST90094734 | prot-a-2646 | Secretoglobin family 1C member 1 \|\| id:prot-a-2646 | Chips liking \|\| id:ebi-fl187-GCST90094734 | MR Egger | 4 | 0.652517993202207 | 0.96930320450575 | 0.570197831862543 | -1.247316288 | 2.55235227403348 | 1.92037022544194 | 0.287274725999929 | 12.8372650602227 | 0.999950574162623 |
| ebi-fl187-GCST90094734 | prot-a-2672 | Semaphorin-3E \|\| id:prot-a-2672 | Chips liking \|\| id:ebi-fl187-GCST90094734 | MR Egger | 4 | 1.42169253683481 | 1.2960063868363 | 0.387091867919541 | -1.118479981 | 3.96186505503396 | 4.1441285980962 | 0.32677612311002 | 52.5552530402494 | 0.999950574162623 |
| ebi-fl187-GCST90094734 | prot-a-2701 | Plasma protease C1 inhibitor \|\| id:prot-a-2701 | Chips liking \|\| id:ebi-fl187-GCST90094734 | MR Egger | 4 | -0.264345325 | 1.01568369787121 | 0.819005450242647 | -2.255085373 | 1.72639472272813 | 0.767708384902695 | 0.10486459075707 | 5.62035440175659 | 0.999950574162623 |
| ebi-fl187-GCST90094734 | prot-a-2738 | Signaling threshold-regulating transmembrane adapter 1 \|\| id:prot-a-2738 | Chips liking \|\| id:ebi-fl187-GCST90094734 | MR Egger | 4 | 0.52758730035112 | 0.966269467535104 | 0.639828240380975 | -1.366300856 | 2.42147545671992 | 1.69483823610374 | 0.25504867848621 | 11.2624643405653 | 0.999950574162623 |
| ebi-fl187-GCST90094734 | prot-a-274 | Serine/threonine-protein kinase BRSK2 \|\| id:prot-a-274 | Chips liking \|\| id:ebi-fl187-GCST90094734 | MR Egger | 4 | -1.499242544 | 0.966269467535105 | 0.260934936067289 | -3.393130701 | 0.394645612056549 | 0.223299235382757 | 0.0336033101437931 | 1.4838582362617 | 0.999950574162623 |
| ebi-fl187-GCST90094734 | prot-a-2749 | Mitochondrial glutamate carrier 2 \|\| id:prot-a-2749 | Chips liking \|\| id:ebi-fl187-GCST90094734 | MR Egger | 4 | -0.315741632 | 0.966269467535105 | 0.774874559483579 | -2.209629789 | 1.5781465240498 | 0.729247839902936 | 0.109741268455064 | 4.84596560154449 | 0.999950574162623 |
| ebi-fl187-GCST90094734 | prot-a-2760 | Sodium-coupled monocarboxylate transporter 1 \|\| id:prot-a-2760 | Chips liking \|\| id:ebi-fl187-GCST90094734 | MR Egger | 4 | -2.440887374 | 0.966269467535105 | 0.127435234879895 | -4.334775531 | -0.546999218 | 0.0870835414791059 | 0.0131048153735337 | 0.578683711321777 | 0.999950574162623 |
| ebi-fl187-GCST90094734 | prot-a-2777 | Small integral membrane protein 10 \|\| id:prot-a-2777 | Chips liking \|\| id:ebi-fl187-GCST90094734 | MR Egger | 4 | -3.031444707 | 0.966269467535104 | 0.0883444241173825 | -4.925332863 | -1.137556551 | 0.0482458865847188 | 0.00726030918686148 | 0.320601438924636 | 0.999950574162623 |
| ebi-fl187-GCST90094734 | prot-a-2799 | Superoxide dismutase [Mn], mitochondrial \|\| id:prot-a-2799 | Chips liking \|\| id:ebi-fl187-GCST90094734 | MR Egger | 4 | 0.0673307157870259 | 0.96930320450575 | 0.950941378992386 | -1.832503565 | 1.9671649966183 | 1.06964916955267 | 0.16001246427812 | 7.15037638527987 | 0.999950574162623 |
| ebi-fl187-GCST90094734 | prot-a-2805 | Protein SPACA7 \|\| id:prot-a-2805 | Chips liking \|\| id:ebi-fl187-GCST90094734 | MR Egger | 4 | 0.868470605023566 | 1.3772671194182 | 0.592763873511663 | -1.830972949 | 3.56791415908324 | 2.38326311418383 | 0.160257569450543 | 35.4425883963134 | 0.999950574162623 |
| ebi-fl187-GCST90094734 | prot-a-2811 | SPARC-like protein 1 \|\| id:prot-a-2811 | Chips liking \|\| id:ebi-fl187-GCST90094734 | MR Egger | 4 | 0.255988536074087 | 0.966269467535105 | 0.815872915057437 | -1.63789962 | 2.14987669244289 | 1.29173791946701 | 0.194387902215361 | 8.58379988452346 | 0.999950574162623 |
| ebi-fl187-GCST90094734 | prot-a-2831 | Sepiapterin reductase \|\| id:prot-a-2831 | Chips liking \|\| id:ebi-fl187-GCST90094734 | MR Egger | 4 | -0.973022043 | 1.07152509335882 | 0.459690946901835 | -3.073211226 | 1.12716714046657 | 0.377939162336045 | 0.0462723251519797 | 3.08689934984086 | 0.999950574162623 |
| ebi-fl187-GCST90094734 | prot-a-2834 | SPRY domain-containing SOCS box protein 1 \|\| id:prot-a-2834 | Chips liking \|\| id:ebi-fl187-GCST90094734 | MR Egger | 4 | -0.557737433 | 1.25033835379719 | 0.699190308457593 | -3.008400606 | 1.89292574042637 | 0.572502925790265 | 0.0493705786322696 | 6.63876359399571 | 0.999950574162623 |
| ebi-fl187-GCST90094734 | prot-a-2835 | Spectrin alpha chain, non-erythrocytic 1 \|\| id:prot-a-2835 | Chips liking \|\| id:ebi-fl187-GCST90094734 | MR Egger | 4 | 1.51172615472479 | 0.966269467535104 | 0.25816135836427 | -0.382162002 | 3.4056143110936 | 4.53455137989123 | 0.682384496840138 | 30.1328009532587 | 0.999950574162623 |
| ebi-fl187-GCST90094734 | prot-a-2859 | Sia-alpha-2,3-Gal-beta-1,4-GlcNAc-R:alpha 2,8-sialyltransferase \|\| id:prot-a-2859 | Chips liking \|\| id:ebi-fl187-GCST90094734 | MR Egger | 4 | 1.64762758628514 | 0.966269467535105 | 0.230284753521285 | -0.24626057 | 3.54151574265395 | 5.19464135858801 | 0.781718505928601 | 34.5192017838934 | 0.999950574162623 |
| ebi-fl187-GCST90094734 | prot-a-2861 | Alpha-2,8-sialyltransferase 8F \|\| id:prot-a-2861 | Chips liking \|\| id:ebi-fl187-GCST90094734 | MR Egger | 4 | 0.447813966899816 | 1.613986208 | 0.807477572245338 | -2.715599002 | 3.61122693546769 | 1.56488754769085 | 0.066165308004685 | 37.0114356113096 | 0.999950574162623 |
| ebi-fl187-GCST90094734 | prot-a-289 | Uncharacterized protein C17orf89 \|\| id:prot-a-289 | Chips liking \|\| id:ebi-fl187-GCST90094734 | MR Egger | 4 | -0.169630772 | 0.968301588014475 | 0.87706593379255 | -2.067501885 | 1.72824034048161 | 0.843976378761903 | 0.126501402422122 | 5.63073700583332 | 0.999950574162623 |
| ebi-fl187-GCST90094734 | prot-a-2921 | Tachykinin-3 \|\| id:prot-a-2921 | Chips liking \|\| id:ebi-fl187-GCST90094734 | MR Egger | 4 | 1.12525592600301 | 0.967268920147747 | 0.364720220998384 | -0.770591157 | 3.02110300949259 | 3.08100525738739 | 0.462739435557509 | 20.5139062431799 | 0.999950574162623 |
| ebi-fl187-GCST90094734 | prot-a-2928 | Tax1-binding protein 1 \|\| id:prot-a-2928 | Chips liking \|\| id:ebi-fl187-GCST90094734 | MR Egger | 4 | -0.140938849 | 0.967268920147747 | 0.897511407278935 | -2.036785932 | 1.75490823454797 | 0.868542422362146 | 0.130447304274456 | 5.78291704561061 | 0.999950574162623 |
| ebi-fl187-GCST90094734 | prot-a-296 | Uncharacterized protein C1orf185 \|\| id:prot-a-296 | Chips liking \|\| id:ebi-fl187-GCST90094734 | MR Egger | 4 | 1.06368272875876 | 0.966269467535105 | 0.385756999352392 | -0.830205428 | 2.95757088512756 | 2.89702030795874 | 0.435959718958903 | 19.2511516540282 | 0.999950574162623 |
| ebi-fl187-GCST90094734 | prot-a-2964 | Transforming growth factor beta-2 \|\| id:prot-a-2964 | Chips liking \|\| id:ebi-fl187-GCST90094734 | MR Egger | 4 | -0.506516293 | 0.966269467535104 | 0.652443713626437 | -2.40040445 | 1.3873718630081 | 0.602591177352933 | 0.0906812698565456 | 4.00431233040771 | 0.999950574162623 |
| ebi-fl187-GCST90094734 | prot-a-2977 | Thymocyte nuclear protein 1 \|\| id:prot-a-2977 | Chips liking \|\| id:ebi-fl187-GCST90094734 | MR Egger | 4 | 1.65869733501925 | 0.966269467535105 | 0.228189734537602 | -0.235190821 | 3.55258549138805 | 5.25246418480701 | 0.790420006224817 | 34.9034434799383 | 0.999950574162623 |
| ebi-fl187-GCST90094734 | prot-a-3015 | Transmembrane protein 70, mitochondrial \|\| id:prot-a-3015 | Chips liking \|\| id:ebi-fl187-GCST90094734 | MR Egger | 4 | -1.004240293 | 0.96930320450575 | 0.409024565033478 | -2.904074574 | 0.895593987823501 | 0.366322827131493 | 0.0547994800156313 | 2.44878990894315 | 0.999950574162623 |
| ebi-fl187-GCST90094734 | prot-a-3030 | Tumor necrosis factor alpha-induced protein 3 \|\| id:prot-a-3030 | Chips liking \|\| id:ebi-fl187-GCST90094734 | MR Egger | 4 | -0.477261003 | 0.96930320450575 | 0.671196436258707 | -2.377095283 | 1.42257327818586 | 0.620480561089425 | 0.0928198015225037 | 4.14778011129992 | 0.999950574162623 |
| ebi-fl187-GCST90094734 | prot-a-3032 | Tumor necrosis factor receptor superfamily member 10A \|\| id:prot-a-3032 | Chips liking \|\| id:ebi-fl187-GCST90094734 | MR Egger | 4 | 0.666240649915512 | 0.966269467535105 | 0.56176249051857 | -1.227647506 | 2.56012880628432 | 1.94690445044869 | 0.29298100352479 | 12.9374836374202 | 0.999950574162623 |
| ebi-fl187-GCST90094734 | prot-a-3081 | Tumor protein p53-inducible protein 11 \|\| id:prot-a-3081 | Chips liking \|\| id:ebi-fl187-GCST90094734 | MR Egger | 4 | 0.360764637921361 | 0.967268920147747 | 0.744988169772129 | -1.535082446 | 2.25661172141094 | 1.43442581170451 | 0.215437928535092 | 9.5506739378484 | 0.999950574162623 |
| ebi-fl187-GCST90094734 | prot-a-315 | Complement C4 \|\| id:prot-a-315 | Chips liking \|\| id:ebi-fl187-GCST90094734 | MR Egger | 4 | -0.800539622 | 0.966269467535105 | 0.49452431019513 | -2.694427778 | 1.09334853434884 | 0.449086561722693 | 0.0675810420448159 | 2.98425022488064 | 0.999950574162623 |
| ebi-fl187-GCST90094734 | prot-a-3157 | UDP-glucuronosyltransferase 1-8 \|\| id:prot-a-3157 | Chips liking \|\| id:ebi-fl187-GCST90094734 | MR Egger | 4 | 1.03166051119913 | 0.966269467535105 | 0.397469877105984 | -0.862227645 | 2.92554866756793 | 2.80572089971127 | 0.422220476520272 | 18.6444528506865 | 0.999950574162623 |
| ebi-fl187-GCST90094734 | prot-a-317 | Glutaredoxin-like protein C5orf63 \|\| id:prot-a-317 | Chips liking \|\| id:ebi-fl187-GCST90094734 | MR Egger | 4 | -1.536639065 | 0.967268920147747 | 0.253079261330052 | -3.432486149 | 0.359208018076914 | 0.215102834454998 | 0.0323065220235718 | 1.43219469297298 | 0.999950574162623 |
| ebi-fl187-GCST90094734 | prot-a-3192 | Guanine nucleotide exchange factor VAV3 \|\| id:prot-a-3192 | Chips liking \|\| id:ebi-fl187-GCST90094734 | MR Egger | 3 | 4.06649953599628 | 2.60051681361449 | 0.362209096023409 | -1.030513419 | 9.16351249068068 | 58.3523443904328 | 0.356823713569043 | 9542.51628010377 | 0.999950574162623 |
| ebi-fl187-GCST90094734 | prot-a-3233 | Serine/threonine-protein kinase WNK3 \|\| id:prot-a-3233 | Chips liking \|\| id:ebi-fl187-GCST90094734 | MR Egger | 4 | -0.301596128 | 1.00304185183791 | 0.792034623879575 | -2.267558157 | 1.66436590185728 | 0.739636723326855 | 0.103564760426192 | 5.28232267657843 | 0.999950574162623 |
| ebi-fl187-GCST90094734 | prot-a-3250 | 14-3-3 protein theta \|\| id:prot-a-3250 | Chips liking \|\| id:ebi-fl187-GCST90094734 | MR Egger | 4 | 0.869471781002095 | 1.36620942340403 | 0.589626912235643 | -1.808298689 | 3.547242251 | 2.38565037479885 | 0.163932800386662 | 34.7174433508973 | 0.999950574162623 |
| ebi-fl187-GCST90094734 | prot-a-3278 | E3 ubiquitin-protein ligase ZNRF3 \|\| id:prot-a-3278 | Chips liking \|\| id:ebi-fl187-GCST90094734 | MR Egger | 4 | 1.0574147276938 | 0.966269467535105 | 0.38801845641441 | -0.836473429 | 2.9513028840626 | 2.87891857169912 | 0.433235669068493 | 19.1308627941339 | 0.999950574162623 |
| ebi-fl187-GCST90094734 | prot-a-355 | Calpain-3 \|\| id:prot-a-355 | Chips liking \|\| id:ebi-fl187-GCST90094734 | MR Egger | 4 | 0.318763240195173 | 1.03544751133125 | 0.787297909398728 | -1.710713882 | 2.34824036240442 | 1.37542564084683 | 0.180736721856941 | 10.4671351458745 | 0.999950574162623 |
| ebi-fl187-GCST90094734 | prot-a-385 | Coiled-coil domain-containing protein 90B, mitochondrial \|\| id:prot-a-385 | Chips liking \|\| id:ebi-fl187-GCST90094734 | MR Egger | 4 | 0.997133931661963 | 0.966269467535105 | 0.410550237262683 | -0.896754225 | 2.89102208803077 | 2.71050220077431 | 0.407891437433405 | 18.0117097496117 | 0.999950574162623 |
| ebi-fl187-GCST90094734 | prot-a-390 | C-C motif chemokine 14 \|\| id:prot-a-390 | Chips liking \|\| id:ebi-fl187-GCST90094734 | MR Egger | 4 | 0.11258869845895 | 0.966269467535105 | 0.917886898326796 | -1.781299458 | 2.00647685482775 | 1.11917152129966 | 0.168419151412989 | 7.43706926189624 | 0.999950574162623 |
| ebi-fl187-GCST90094734 | prot-a-40 | Pituitary adenylate cyclase-activating polypeptide 27 \|\| id:prot-a-40 | Chips liking \|\| id:ebi-fl187-GCST90094734 | MR Egger | 4 | 0.525101718149593 | 0.984216656295598 | 0.647025568674215 | -1.403962928 | 2.45416636448896 | 1.69063080747064 | 0.245621651704291 | 11.6367287140057 | 0.999950574162623 |
| ebi-fl187-GCST90094734 | prot-a-401 | C-C motif chemokine 24 \|\| id:prot-a-401 | Chips liking \|\| id:ebi-fl187-GCST90094734 | MR Egger | 4 | 1.35811923473018 | 0.966269467535105 | 0.295074526488645 | -0.535768922 | 3.25200739109898 | 3.88887236339005 | 0.585219129445853 | 25.842163213392 | 0.999950574162623 |
| ebi-fl187-GCST90094734 | prot-a-412 | Cerebral cavernous malformations 2 protein \|\| id:prot-a-412 | Chips liking \|\| id:ebi-fl187-GCST90094734 | MR Egger | 3 | 1.19716994583991 | 2.57717629547932 | 0.723153590030764 | -3.854095593 | 6.24843548497937 | 3.31073409524476 | 0.0211927615209239 | 517.203019464654 | 0.999950574162623 |
| ebi-fl187-GCST90094734 | prot-a-44 | Adhesion G protein-coupled receptor F5 \|\| id:prot-a-44 | Chips liking \|\| id:ebi-fl187-GCST90094734 | MR Egger | 4 | 0.320556366326451 | 0.967268920147747 | 0.771843096450267 | -1.575290717 | 2.21640344981603 | 1.37789416503125 | 0.206947380779616 | 9.17427571624615 | 0.999950574162623 |
| ebi-fl187-GCST90094734 | prot-a-48 | Adrenomedullin \|\| id:prot-a-48 | Chips liking \|\| id:ebi-fl187-GCST90094734 | MR Egger | 4 | 0.67115382337658 | 0.966269467535104 | 0.559156935941263 | -1.222734333 | 2.56504197974538 | 1.9564934666866 | 0.294424011988585 | 13.0012041454545 | 0.999950574162623 |
| ebi-fl187-GCST90094734 | prot-a-502 | Carcinoembryonic antigen-related cell adhesion molecule 1 \|\| id:prot-a-502 | Chips liking \|\| id:ebi-fl187-GCST90094734 | MR Egger | 4 | -0.158729889 | 0.96930320450575 | 0.884975097511744 | -2.058564169 | 1.74110439217636 | 0.853226794083174 | 0.127637103636948 | 5.70363899992725 | 0.999950574162623 |
| ebi-fl187-GCST90094734 | prot-a-518 | Complement factor B \|\| id:prot-a-518 | Chips liking \|\| id:ebi-fl187-GCST90094734 | MR Egger | 4 | -0.752803064 | 0.968301588014475 | 0.518257550215813 | -2.650674177 | 1.14506804801269 | 0.471044332827667 | 0.0706035976896731 | 3.14265520100423 | 0.999950574162623 |
| ebi-fl187-GCST90094734 | prot-a-525 | Properdin \|\| id:prot-a-525 | Chips liking \|\| id:ebi-fl187-GCST90094734 | MR Egger | 4 | -1.800115412 | 0.966269467535105 | 0.20350192225086 | -3.694003569 | 0.0937727440283729 | 0.165279811790875 | 0.0248722247821248 | 1.09831012002029 | 0.999950574162623 |
| ebi-fl187-GCST90094734 | prot-a-533 | Chondroadherin \|\| id:prot-a-533 | Chips liking \|\| id:ebi-fl187-GCST90094734 | MR Egger | 4 | 1.75375120678037 | 0.967268920147747 | 0.211497450660001 | -0.142095877 | 3.64959829026995 | 5.77622991935248 | 0.867538075802604 | 38.4592135052462 | 0.999950574162623 |
| ebi-fl187-GCST90094734 | prot-a-535 | Coiled-coil-helix-coiled-coil-helix domain-containing protein 2 \|\| id:prot-a-535 | Chips liking \|\| id:ebi-fl187-GCST90094734 | MR Egger | 4 | 1.25319249283859 | 1.16201508814845 | 0.393612165964522 | -1.02435708 | 3.53074206560954 | 3.50150365810703 | 0.359027217023229 | 34.1492992352822 | 0.999950574162623 |
| ebi-fl187-GCST90094734 | prot-a-591 | Caseinolytic peptidase B protein homolog \|\| id:prot-a-591 | Chips liking \|\| id:ebi-fl187-GCST90094734 | MR Egger | 3 | -2.182842103 | 2.41870837984599 | 0.532603322777757 | -6.923510527 | 2.55782632153052 | 0.112720711087807 | 0.00098436821618783 | 12.9077295459084 | 0.999950574162623 |
| ebi-fl187-GCST90094734 | prot-a-605 | Ciliary neurotrophic factor receptor subunit alpha \|\| id:prot-a-605 | Chips liking \|\| id:ebi-fl187-GCST90094734 | MR Egger | 4 | 0.544818132361659 | 0.966269467535105 | 0.629656397421746 | -1.349070024 | 2.43870628873046 | 1.72429476044257 | 0.259481460002095 | 11.4582075377012 | 0.999950574162623 |
| ebi-fl187-GCST90094734 | prot-a-606 | Ciliary neurotrophic factor receptor subunit alpha \|\| id:prot-a-606 | Chips liking \|\| id:ebi-fl187-GCST90094734 | MR Egger | 4 | 0.53592054177045 | 0.966269467535105 | 0.634892558535912 | -1.357967615 | 2.42980869813925 | 1.70902074334751 | 0.257182941009376 | 11.3567093125574 | 0.999950574162623 |
| ebi-fl187-GCST90094734 | prot-a-707 | Cancer/testis antigen 55 \|\| id:prot-a-707 | Chips liking \|\| id:ebi-fl187-GCST90094734 | MR Egger | 4 | 0.0811886467515337 | 0.967268920147747 | 0.940752573933332 | -1.814658437 | 1.97703573024112 | 1.08457547891015 | 0.162893537337217 | 7.2213053303522 | 0.999950574162623 |
| ebi-fl187-GCST90094734 | prot-a-719 | Dipeptidyl peptidase 1 \|\| id:prot-a-719 | Chips liking \|\| id:ebi-fl187-GCST90094734 | MR Egger | 4 | -1.476539677 | 0.966269467535105 | 0.266077046608445 | -3.370427833 | 0.417348479813544 | 0.228426752835601 | 0.0343749274712787 | 1.51793138922587 | 0.999950574162623 |
| ebi-fl187-GCST90094734 | prot-a-752 | Cytochrome b5 \|\| id:prot-a-752 | Chips liking \|\| id:ebi-fl187-GCST90094734 | MR Egger | 4 | -0.694991847 | 1.0382667826899 | 0.572181822144485 | -2.729994741 | 1.34001104730077 | 0.499078517069772 | 0.065219632667472 | 3.81908569572165 | 0.999950574162623 |
| ebi-fl187-GCST90094734 | prot-a-755 | Cytoglobin \|\| id:prot-a-755 | Chips liking \|\| id:ebi-fl187-GCST90094734 | MR Egger | 4 | 0.296519215335968 | 1.19681816523988 | 0.827437944000744 | -2.049244389 | 2.64228281920614 | 1.3451684076118 | 0.128832213916774 | 14.0452297591175 | 0.999950574162623 |
| ebi-fl187-GCST90094734 | prot-a-807 | Desmin \|\| id:prot-a-807 | Chips liking \|\| id:ebi-fl187-GCST90094734 | MR Egger | 4 | -1.844996559 | 0.966269467535105 | 0.196410990117668 | -3.738884716 | 0.0488915970670252 | 0.158025864608024 | 0.0237806104891595 | 1.05010650994419 | 0.999950574162623 |
| ebi-fl187-GCST90094734 | prot-a-82 | Ameloblastin \|\| id:prot-a-82 | Chips liking \|\| id:ebi-fl187-GCST90094734 | MR Egger | 4 | 0.334161637057582 | 0.967268920147747 | 0.762694327938821 | -1.561685446 | 2.23000872054717 | 1.39676889494342 | 0.209782196411597 | 9.29994717975798 | 0.999950574162623 |
| ebi-fl187-GCST90094734 | prot-a-84 | Muellerian-inhibiting factor \|\| id:prot-a-84 | Chips liking \|\| id:ebi-fl187-GCST90094734 | MR Egger | 4 | -2.021959178 | 0.966269467535105 | 0.171472387093934 | -3.915847334 | -0.128071022 | 0.132395823840301 | 0.0199236594904712 | 0.879790892769238 | 0.999950574162623 |
| ebi-fl187-GCST90094734 | prot-a-858 | Inactive dipeptidyl peptidase 10 \|\| id:prot-a-858 | Chips liking \|\| id:ebi-fl187-GCST90094734 | MR Egger | 4 | 3.48771681225194 | 0.966269467535105 | 0.0689160826132443 | 1.59382865588314 | 5.38160496862075 | 32.7111766228128 | 4.92255968248449 | 217.37086903308 | 0.999950574162623 |
| ebi-fl187-GCST90094734 | prot-a-863 | Dihydropyrimidinase-related protein 3 \|\| id:prot-a-863 | Chips liking \|\| id:ebi-fl187-GCST90094734 | MR Egger | 4 | 0.526654175455207 | 0.966269467535105 | 0.640382817143625 | -1.367233981 | 2.42054233182401 | 1.69325747798817 | 0.254810797218372 | 11.2519599564132 | 0.999950574162623 |
| ebi-fl187-GCST90094734 | prot-a-864 | Dihydropyrimidinase-related protein 5 \|\| id:prot-a-864 | Chips liking \|\| id:ebi-fl187-GCST90094734 | MR Egger | 4 | -1.677637937 | 0.966269467535105 | 0.224662534789577 | -3.571526093 | 0.216250219606572 | 0.186814723488439 | 0.0281129179956598 | 1.24141296600552 | 0.999950574162623 |
| ebi-fl187-GCST90094734 | prot-a-884 | Endothelin-converting enzyme 1 \|\| id:prot-a-884 | Chips liking \|\| id:ebi-fl187-GCST90094734 | MR Egger | 4 | -1.918712678 | 0.966269467535105 | 0.185464623300837 | -3.812600834 | -0.024824521 | 0.146795814090703 | 0.0220906500653491 | 0.975481073250697 | 0.999950574162623 |
| ebi-fl187-GCST90094734 | prot-a-893 | Early endosome antigen 1 \|\| id:prot-a-893 | Chips liking \|\| id:ebi-fl187-GCST90094734 | MR Egger | 3 | -0.514398606 | 2.42409071515241 | 0.866882216709466 | -5.265616407 | 4.23681919592039 | 0.597860036095469 | 0.00516620755768073 | 69.1874298059639 | 0.999950574162623 |
| ebi-fl187-GCST90094734 | prot-a-897 | Ephrin-A2 \|\| id:prot-a-897 | Chips liking \|\| id:ebi-fl187-GCST90094734 | MR Egger | 4 | 1.16974398035649 | 0.967268920147747 | 0.350094020694393 | -0.726103103 | 3.06559106384607 | 3.22116785070723 | 0.483790603569504 | 21.447134867594 | 0.999950574162623 |
| ebi-fl187-GCST90094734 | prot-a-90 | Anaphase-promoting complex subunit 10 \|\| id:prot-a-90 | Chips liking \|\| id:ebi-fl187-GCST90094734 | MR Egger | 4 | -0.52857847 | 0.96930320450575 | 0.640222344459403 | -2.428412751 | 1.37125581041234 | 0.589442284046888 | 0.0881766798916982 | 3.94029585429111 | 0.999950574162623 |
| ebi-fl187-GCST90094734 | prot-a-914 | Histone-lysine N-methyltransferase EHMT2 \|\| id:prot-a-914 | Chips liking \|\| id:ebi-fl187-GCST90094734 | MR Egger | 4 | 3.12899184422283 | 1.11731973075529 | 0.107363809000378 | 0.939045171942454 | 5.3189385165032 | 22.8509306282184 | 2.55753824283613 | 204.167046978974 | 0.999950574162623 |
| ebi-fl187-GCST90094734 | prot-a-932 | RNA polymerase II elongation factor ELL \|\| id:prot-a-932 | Chips liking \|\| id:ebi-fl187-GCST90094734 | MR Egger | 4 | -1.439885341 | 0.966269467535104 | 0.274653604058446 | -3.333773498 | 0.454002814871963 | 0.236954926021742 | 0.0356582943759736 | 1.57460242977301 | 0.999950574162623 |
| ebi-fl187-GCST90094734 | prot-a-991 | Estrogen receptor \|\| id:prot-a-991 | Chips liking \|\| id:ebi-fl187-GCST90094734 | MR Egger | 4 | 0.233871606355938 | 0.966269467535104 | 0.831307708541425 | -1.66001655 | 2.12775976272474 | 1.26348225870172 | 0.190135833324993 | 8.39603661307416 | 0.999950574162623 |
| ebi-fl187-GCST90094734 | prot-a-992 | Persulfide dioxygenase ETHE1, mitochondrial \|\| id:prot-a-992 | Chips liking \|\| id:ebi-fl187-GCST90094734 | MR Egger | 4 | 1.3627468567422 | 1.04199555637748 | 0.321048525208679 | -0.679564434 | 3.40505814724207 | 3.90691029892457 | 0.506837705689402 | 30.1160468380717 | 0.999950574162623 |
| ebi-fl187-GCST90094734 | prot-a-993 | Ethanolamine kinase 1 \|\| id:prot-a-993 | Chips liking \|\| id:ebi-fl187-GCST90094734 | MR Egger | 4 | 0.97318244107453 | 0.967268920147747 | 0.420303778163881 | -0.922664642 | 2.86902952456411 | 2.64635293479604 | 0.397458543894819 | 17.6199102096969 | 0.999950574162623 |
| ebi-fl187-GCST90094734 | prot-b-10 | coagulation factor III, tissue factor \|\| id:prot-b-10 | Chips liking \|\| id:ebi-fl187-GCST90094734 | MR Egger | 4 | -1.290357931 | 1.40422163467604 | 0.455147340633427 | -4.042632335 | 1.46191647291353 | 0.275172272759916 | 0.0175512108874143 | 4.31421969581342 | 0.999950574162623 |
| ebi-fl187-GCST90094734 | prot-b-28 | prolactin \|\| id:prot-b-28 | Chips liking \|\| id:ebi-fl187-GCST90094734 | MR Egger | 4 | -1.02664885 | 0.999380867318454 | 0.412290171262821 | -2.98543535 | 0.932137649928826 | 0.358205351311626 | 0.0505175059669814 | 2.53993286588911 | 0.999950574162623 |
| ebi-fl187-GCST90094734 | prot-b-54 | matrix metallopeptidase 7 \|\| id:prot-b-54 | Chips liking \|\| id:ebi-fl187-GCST90094734 | MR Egger | 4 | 0.641795003395193 | 0.999380867318454 | 0.586534173932345 | -1.316991497 | 2.60058150333936 | 1.89988812601707 | 0.26794018959021 | 13.4715695204261 | 0.999950574162623 |
| ebi-fl187-GCST90094734 | ukb-rsfMRI191-edge-pheno716 | Net100_Pair10_33 (OccipitalPrecuneus)&(Temporal\|Frontal\|Supp_Motor_Area) (Default_mode\|Central_executive)&(Default_mode\|Salience) \|\| id:ukb-rsfMRI191-edge-pheno716 | Chips liking \|\| id:ebi-fl187-GCST90094734 | MR Egger | 4 | 0.396516850179494 | 0.303953701795786 | 0.32197179910213 | -0.199232405 | 0.992266105699235 | 1.48663748787225 | 0.819359447692265 | 2.69734000940841 | 0.999950574162623 |
| ebi-fl187-GCST90094734 | ukb-rsfMRI191-edge-pheno903 | Net100_Pair27_38 (Precentral\|Frontal\|Supp_Motor_Area)&(Subcortical) (Attention\|Salience\|Motor)&(Subcortical-cerebellum) \|\| id:ukb-rsfMRI191-edge-pheno903 | Chips liking \|\| id:ebi-fl187-GCST90094734 | MR Egger | 4 | 0.231223207301587 | 0.301795651357772 | 0.523656364635317 | -0.360296269 | 0.822742683962821 | 1.26014048061682 | 0.697469656569098 | 2.27673564854485 | 0.999950574162623 |
| ebi-fl187-GCST90094734 | ukb-rsfMRI191-node-pheno23 | Net100_Node2 RolandicOper\|SupraMarginal\|Insula Salience\|Motor \|\| id:ukb-rsfMRI191-node-pheno23 | Chips liking \|\| id:ebi-fl187-GCST90094734 | MR Egger | 4 | 0.837007064813196 | 0.756028177537735 | 0.383576033098697 | -0.644808163 | 2.31882229278716 | 2.30944460482339 | 0.524763201365105 | 10.1636973950791 | 0.999950574162623 |
| ebi-fl187-GCST90094734 | ukb-rsfMRI191-node-pheno55 | Net100_Node34 Parietal\|Postcentral\|Precuneus Attention \|\| id:ukb-rsfMRI191-node-pheno55 | Chips liking \|\| id:ebi-fl187-GCST90094734 | MR Egger | 3 | -0.353390059 | 0.713137665990736 | 0.707106969439248 | -1.751139884 | 1.0443597662869 | 0.702303200221401 | 0.173575974096263 | 2.84157866668621 | 0.999950574162623 |
| ebi-fl187-GCST90094734 | ebi-a-GCST90001414 | IgD- CD24- B cell Absolute Count \|\| id:ebi-a-GCST90001414 | Chips liking \|\| id:ebi-fl187-GCST90094734 | Weighted median | 4 | 0.206167739333191 | 0.386454420971311 | 0.593697708108578 | -0.551282926 | 0.96361840443696 | 1.22895933152577 | 0.576210101197863 | 2.62116376544679 | 0.999332001504091 |
| ebi-fl187-GCST90094734 | ebi-a-GCST90001422 | CD20- CD38- B cell %B cell \|\| id:ebi-a-GCST90001422 | Chips liking \|\| id:ebi-fl187-GCST90094734 | Weighted median | 4 | -0.636005401 | 0.369755576197658 | 0.0854197537975842 | -1.360726331 | 0.0887155278744886 | 0.529402958145229 | 0.256474424006022 | 1.09276974957292 | 0.999332001504091 |
| ebi-fl187-GCST90094734 | ebi-a-GCST90001444 | CD20- CD38- B cell %lymphocyte \|\| id:ebi-a-GCST90001444 | Chips liking \|\| id:ebi-fl187-GCST90094734 | Weighted median | 4 | -0.631164453 | 0.377010048044891 | 0.0941047294211423 | -1.370104147 | 0.107775241658466 | 0.531971984091383 | 0.254080496535273 | 1.11379738199951 | 0.999332001504091 |
| ebi-fl187-GCST90094734 | ebi-a-GCST90001459 | Myeloid Dendritic Cell %Dendritic Cell \|\| id:ebi-a-GCST90001459 | Chips liking \|\| id:ebi-fl187-GCST90094734 | Weighted median | 4 | 0.264634583426194 | 0.381050600574151 | 0.487377023421978 | -0.482224594 | 1.01149376055153 | 1.3029547675095 | 0.617408380159775 | 2.7497053501872 | 0.999332001504091 |
| ebi-fl187-GCST90094734 | ebi-a-GCST90001476 | HLA DR++ monocyte %leukocyte \|\| id:ebi-a-GCST90001476 | Chips liking \|\| id:ebi-fl187-GCST90094734 | Weighted median | 4 | 0.111783342575672 | 0.377310465901191 | 0.76702881086277 | -0.627745171 | 0.851311855742006 | 1.11827055277943 | 0.533794059597611 | 2.34271814518934 | 0.999332001504091 |
| ebi-fl187-GCST90094734 | ebi-a-GCST90001478 | CD4 regulatory T cell %CD4+ T cell \|\| id:ebi-a-GCST90001478 | Chips liking \|\| id:ebi-fl187-GCST90094734 | Weighted median | 4 | 0.250948346433229 | 0.417112426019222 | 0.547418862488408 | -0.566592009 | 1.0684887014309 | 1.28524369519557 | 0.567456032406064 | 2.91097681883116 | 0.999332001504091 |
| ebi-fl187-GCST90094734 | ebi-a-GCST90001485 | CD39+ resting CD4 regulatory T cell %CD4 regulatory T cell \|\| id:ebi-a-GCST90001485 | Chips liking \|\| id:ebi-fl187-GCST90094734 | Weighted median | 4 | -0.305730007 | 0.385625146671789 | 0.427884699385694 | -1.061555295 | 0.450095280321761 | 0.736585465416832 | 0.345917388279143 | 1.56846162189889 | 0.999332001504091 |
| ebi-fl187-GCST90094734 | ebi-a-GCST90001515 | Immature Myeloid-Derived Suppressor Cells Absolute Count \|\| id:ebi-a-GCST90001515 | Chips liking \|\| id:ebi-fl187-GCST90094734 | Weighted median | 4 | -0.469926478 | 0.502476433653356 | 0.349674454353741 | -1.454780288 | 0.514927332404266 | 0.625048221666057 | 0.23345165050861 | 1.67351688693026 | 0.999332001504091 |
| ebi-fl187-GCST90094734 | ebi-a-GCST90001517 | CD33+ HLA DR+ Absolute Count \|\| id:ebi-a-GCST90001517 | Chips liking \|\| id:ebi-fl187-GCST90094734 | Weighted median | 4 | 0.41541216292629 | 0.489849201128987 | 0.396415187025136 | -0.544692271 | 1.3755165971391 | 1.51499503681504 | 0.580020244763297 | 3.95712043208228 | 0.999332001504091 |
| ebi-fl187-GCST90094734 | ebi-a-GCST90001518 | CD33+ HLA DR+ CD14- Absolute Count \|\| id:ebi-a-GCST90001518 | Chips liking \|\| id:ebi-fl187-GCST90094734 | Weighted median | 4 | 0.583788703518822 | 0.476049768037513 | 0.220078789984515 | -0.349268842 | 1.51684624887235 | 1.79281803568425 | 0.705203516575814 | 4.55782824890266 | 0.999332001504091 |
| ebi-fl187-GCST90094734 | ebi-a-GCST90001520 | CD33+ HLA DR+ CD14dim Absolute Count \|\| id:ebi-a-GCST90001520 | Chips liking \|\| id:ebi-fl187-GCST90094734 | Weighted median | 4 | 0.0322648496347198 | 0.459001922145287 | 0.943960059392656 | -0.867378918 | 0.931908617039483 | 1.03279100340558 | 0.42005109608042 | 2.53935120433845 | 0.999332001504091 |
| ebi-fl187-GCST90094734 | ebi-a-GCST90001531 | CD33dim HLA DR- Absolute Count \|\| id:ebi-a-GCST90001531 | Chips liking \|\| id:ebi-fl187-GCST90094734 | Weighted median | 4 | -0.283802284 | 0.502020084391654 | 0.571855734375678 | -1.26776165 | 0.700157081037502 | 0.752915493151839 | 0.281460925339531 | 2.01406905468046 | 0.999332001504091 |
| ebi-fl187-GCST90094734 | ebi-a-GCST90001532 | Basophil Absolute Count \|\| id:ebi-a-GCST90001532 | Chips liking \|\| id:ebi-fl187-GCST90094734 | Weighted median | 4 | -0.250691957 | 0.491282092009814 | 0.609854554602713 | -1.213604858 | 0.712220942983885 | 0.778262072545056 | 0.297124255883219 | 2.03851365739789 | 0.999332001504091 |
| ebi-fl187-GCST90094734 | ebi-a-GCST90001588 | T/B cell \|\| id:ebi-a-GCST90001588 | Chips liking \|\| id:ebi-fl187-GCST90094734 | Weighted median | 4 | -0.204643863 | 0.391439184735544 | 0.601114404475623 | -0.971864665 | 0.562576938976446 | 0.814937494045437 | 0.378376833781237 | 1.75518969426396 | 0.999332001504091 |
| ebi-fl187-GCST90094734 | ebi-a-GCST90001624 | HLA DR+ CD4+ T cell Absolute Count \|\| id:ebi-a-GCST90001624 | Chips liking \|\| id:ebi-fl187-GCST90094734 | Weighted median | 4 | 0.552395229021927 | 0.400469313401823 | 0.167780802350909 | -0.232524625 | 1.3373150832895 | 1.73740953171223 | 0.792530232854815 | 3.80880344464725 | 0.999332001504091 |
| ebi-fl187-GCST90094734 | ebi-a-GCST90001631 | CD8+ Natural Killer T %T cell \|\| id:ebi-a-GCST90001631 | Chips liking \|\| id:ebi-fl187-GCST90094734 | Weighted median | 4 | 0.369932145995958 | 0.377228857918149 | 0.32676188155141 | -0.369436416 | 1.10930070751553 | 1.44763638340566 | 0.691123727502116 | 3.03223723215812 | 0.999332001504091 |
| ebi-fl187-GCST90094734 | ebi-a-GCST90001632 | CD8+ Natural Killer T %lymphocyte \|\| id:ebi-a-GCST90001632 | Chips liking \|\| id:ebi-fl187-GCST90094734 | Weighted median | 4 | 0.35213435248465 | 0.36986321117402 | 0.341063285349195 | -0.372797541 | 1.07706624638573 | 1.4220995734954 | 0.68880467314652 | 2.93605324670267 | 0.999332001504091 |
| ebi-fl187-GCST90094734 | ebi-a-GCST90001646 | Natural Killer %CD3- lymphocyte \|\| id:ebi-a-GCST90001646 | Chips liking \|\| id:ebi-fl187-GCST90094734 | Weighted median | 4 | -0.04871848 | 0.364205713607703 | 0.893587312508706 | -0.762561679 | 0.665124718518376 | 0.952449225320068 | 0.46646994897774 | 1.94473305043727 | 0.999332001504091 |
| ebi-fl187-GCST90094734 | ebi-a-GCST90001684 | CD127- CD8+ T cell Absolute Count \|\| id:ebi-a-GCST90001684 | Chips liking \|\| id:ebi-fl187-GCST90094734 | Weighted median | 4 | 0.657610810694494 | 0.395339260935655 | 0.0962306302281639 | -0.117254141 | 1.43247576212838 | 1.93017526696864 | 0.889359142050286 | 4.18905747415909 | 0.999332001504091 |
| ebi-fl187-GCST90094734 | ebi-a-GCST90001687 | CD28- CD8+ T cell Absolute Count \|\| id:ebi-a-GCST90001687 | Chips liking \|\| id:ebi-fl187-GCST90094734 | Weighted median | 4 | 0.46639296234036 | 0.371199133118086 | 0.208953047195286 | -0.261157339 | 1.19394326325181 | 1.5942333499426 | 0.770159734249666 | 3.30006862348544 | 0.999332001504091 |
| ebi-fl187-GCST90094734 | ebi-a-GCST90001701 | BAFF-R on CD20- CD38- B cell \|\| id:ebi-a-GCST90001701 | Chips liking \|\| id:ebi-fl187-GCST90094734 | Weighted median | 4 | -0.441710013 | 0.378084399812978 | 0.242692165122928 | -1.182755437 | 0.299335410593518 | 0.642936051496214 | 0.306433216921533 | 1.34896200374841 | 0.999332001504091 |
| ebi-fl187-GCST90094734 | ebi-a-GCST90001732 | CD19 on IgD- CD27- B cell \|\| id:ebi-a-GCST90001732 | Chips liking \|\| id:ebi-fl187-GCST90094734 | Weighted median | 4 | -0.042526257 | 0.373417400144529 | 0.90932977090794 | -0.774424361 | 0.689371847314897 | 0.958365301423166 | 0.46096905629086 | 1.99246356873114 | 0.999332001504091 |
| ebi-fl187-GCST90094734 | ebi-a-GCST90001798 | CD27 on CD24+ CD27+ B cell \|\| id:ebi-a-GCST90001798 | Chips liking \|\| id:ebi-fl187-GCST90094734 | Weighted median | 4 | -0.035556574 | 0.362379141669933 | 0.921837153384932 | -0.745819692 | 0.674706543347299 | 0.96506813460651 | 0.474345323474905 | 1.96345670198678 | 0.999332001504091 |
| ebi-fl187-GCST90094734 | ebi-a-GCST90001836 | CD66b on CD66b++ myeloid cell \|\| id:ebi-a-GCST90001836 | Chips liking \|\| id:ebi-fl187-GCST90094734 | Weighted median | 4 | 0.526805644024351 | 0.552170269933626 | 0.340051368766401 | -0.555448085 | 1.60905937309426 | 1.6935139727005 | 0.573815085623692 | 4.99810766148567 | 0.999332001504091 |
| ebi-fl187-GCST90094734 | ebi-a-GCST90001943 | CD25 on activated & secreting CD4 regulatory T cell \|\| id:ebi-a-GCST90001943 | Chips liking \|\| id:ebi-fl187-GCST90094734 | Weighted median | 4 | -0.284242748 | 0.374738870375035 | 0.448146419299961 | -1.018730934 | 0.450245437775557 | 0.752583934165873 | 0.361052849414366 | 1.56869715578555 | 0.999332001504091 |
| ebi-fl187-GCST90094734 | ebi-a-GCST90001960 | CD25 on CD4+ T cell \|\| id:ebi-a-GCST90001960 | Chips liking \|\| id:ebi-fl187-GCST90094734 | Weighted median | 4 | 0.470837652987008 | 0.407494555158629 | 0.247908135975943 | -0.327851675 | 1.26952698109792 | 1.60133499461725 | 0.720469875385424 | 3.55916861008803 | 0.999332001504091 |
| ebi-fl187-GCST90094734 | ebi-a-GCST90001988 | HLA DR on CD14+ CD16- monocyte \|\| id:ebi-a-GCST90001988 | Chips liking \|\| id:ebi-fl187-GCST90094734 | Weighted median | 4 | 0.0956871131442225 | 0.385754189517434 | 0.804094143609093 | -0.660391098 | 0.851765324598393 | 1.10041470482702 | 0.516649234331377 | 2.34378073581515 | 0.999332001504091 |
| ebi-fl187-GCST90094734 | ebi-a-GCST90001991 | HLA DR on CD14+ monocyte \|\| id:ebi-a-GCST90001991 | Chips liking \|\| id:ebi-fl187-GCST90094734 | Weighted median | 4 | 0.0897770552809588 | 0.371185836375803 | 0.808884368157557 | -0.637747184 | 0.817301294577532 | 1.09393037051754 | 0.528481655901685 | 2.26438068791409 | 0.999332001504091 |
| ebi-fl187-GCST90094734 | ebi-a-GCST90002019 | CD14 on Monocytic Myeloid-Derived Suppressor Cells \|\| id:ebi-a-GCST90002019 | Chips liking \|\| id:ebi-fl187-GCST90094734 | Weighted median | 4 | -0.317473143 | 0.541392255170173 | 0.557605845540131 | -1.378601963 | 0.743655677447999 | 0.727986232266923 | 0.251930515201611 | 2.10361160078635 | 0.999332001504091 |
| ebi-fl187-GCST90094734 | ebi-a-GCST90002037 | CD80 on plasmacytoid Dendritic Cell \|\| id:ebi-a-GCST90002037 | Chips liking \|\| id:ebi-fl187-GCST90094734 | Weighted median | 4 | -0.621838082 | 0.399009780481149 | 0.119125846518382 | -1.403897251 | 0.160221088219354 | 0.536956560041028 | 0.245637783908356 | 1.17377034910339 | 0.999332001504091 |
| ebi-fl187-GCST90094734 | ebi-a-GCST90002042 | CD45 on CD33+ HLA DR+ CD14- \|\| id:ebi-a-GCST90002042 | Chips liking \|\| id:ebi-fl187-GCST90094734 | Weighted median | 4 | -0.750602374 | 0.571318645939502 | 0.188911197795957 | -1.87038692 | 0.369182171605618 | 0.472082096888382 | 0.154064039749592 | 1.44655110020975 | 0.999332001504091 |
| ebi-fl187-GCST90094734 | ebi-a-GCST90002056 | CD8 on Effector Memory CD8+ T cell \|\| id:ebi-a-GCST90002056 | Chips liking \|\| id:ebi-fl187-GCST90094734 | Weighted median | 4 | -0.024490888 | 0.39946628962977 | 0.951113104657193 | -0.807444816 | 0.758463039560185 | 0.975806580316056 | 0.445996213999477 | 2.13499229881183 | 0.999332001504091 |
| ebi-fl187-GCST90094734 | ebi-a-GCST90002058 | CD8 on CD8+ T cell \|\| id:ebi-a-GCST90002058 | Chips liking \|\| id:ebi-fl187-GCST90094734 | Weighted median | 4 | -0.298942083 | 0.404913077753577 | 0.460340031948828 | -1.092571716 | 0.494687549217214 | 0.741602359441748 | 0.335352951381248 | 1.63998574416698 | 0.999332001504091 |
| ebi-fl187-GCST90094734 | ebi-a-GCST90002063 | CD4 on CD4 regulatory T cell \|\| id:ebi-a-GCST90002063 | Chips liking \|\| id:ebi-fl187-GCST90094734 | Weighted median | 4 | -0.278708912 | 0.39573384140276 | 0.481256087429964 | -1.054347241 | 0.496929417476693 | 0.756760155204833 | 0.348419787306378 | 1.6436665004966 | 0.999332001504091 |
| ebi-fl187-GCST90094734 | ebi-a-GCST90002066 | CD4 on activated CD4 regulatory T cell \|\| id:ebi-a-GCST90002066 | Chips liking \|\| id:ebi-fl187-GCST90094734 | Weighted median | 4 | -0.229355328 | 0.408887875726695 | 0.574848838752468 | -1.030775565 | 0.572064908066896 | 0.795045980926114 | 0.356730185885912 | 1.77192213273738 | 0.999332001504091 |
| ebi-fl187-GCST90094734 | ebi-a-GCST90002070 | CD4 on activated & secreting CD4 regulatory T cell \|\| id:ebi-a-GCST90002070 | Chips liking \|\| id:ebi-fl187-GCST90094734 | Weighted median | 4 | -0.247929614 | 0.376487866217467 | 0.510195818968231 | -0.985845832 | 0.489986603921208 | 0.780414871731408 | 0.373123493766074 | 1.63229435346515 | 0.999332001504091 |
| ebi-fl187-GCST90094734 | ebi-a-GCST90002081 | SSC-A on CD4+ T cell \|\| id:ebi-a-GCST90002081 | Chips liking \|\| id:ebi-fl187-GCST90094734 | Weighted median | 4 | 0.0114647394597344 | 0.384504969601238 | 0.976213094546967 | -0.742165001 | 0.76509447987816 | 1.01153071146139 | 0.476082080801142 | 2.14919742097365 | 0.999332001504091 |
| ebi-fl187-GCST90094734 | ebi-a-GCST90002094 | CD11b on Monocytic Myeloid-Derived Suppressor Cells \|\| id:ebi-a-GCST90002094 | Chips liking \|\| id:ebi-fl187-GCST90094734 | Weighted median | 4 | 0.708568313593549 | 0.577308814893789 | 0.219685643351065 | -0.422956964 | 1.84009359078538 | 2.0310813045788 | 0.655106825945675 | 6.29712758656494 | 0.999332001504091 |
| ebi-fl187-GCST90094734 | ebi-a-GCST90002104 | HLA DR on myeloid Dendritic Cell \|\| id:ebi-a-GCST90002104 | Chips liking \|\| id:ebi-fl187-GCST90094734 | Weighted median | 4 | -0.169521096 | 0.424252747176188 | 0.68946904466896 | -1.00105648 | 0.662014288742437 | 0.844068948047913 | 0.3674909890623 | 1.93869349253059 | 0.999332001504091 |
| ebi-fl187-GCST90094734 | ebi-a-GCST90002105 | HLA DR on plasmacytoid Dendritic Cell \|\| id:ebi-a-GCST90002105 | Chips liking \|\| id:ebi-fl187-GCST90094734 | Weighted median | 4 | -0.190983192 | 0.435586919906572 | 0.661059930619373 | -1.044733555 | 0.662767171433063 | 0.826146474248258 | 0.351785538530276 | 1.94015365089855 | 0.999332001504091 |
| ebi-fl187-GCST90094734 | ebi-a-GCST90002106 | HLA DR on Dendritic Cell \|\| id:ebi-a-GCST90002106 | Chips liking \|\| id:ebi-fl187-GCST90094734 | Weighted median | 4 | -0.273254183 | 0.442375554142119 | 0.536774895075664 | -1.14031027 | 0.593801902722946 | 0.760899355064517 | 0.31971980711623 | 1.81086005824819 | 0.999332001504091 |
| ebi-fl187-GCST90094734 | ebi-a-GCST90002111 | HLA DR on CD33dim HLA DR+ CD11b- \|\| id:ebi-a-GCST90002111 | Chips liking \|\| id:ebi-fl187-GCST90094734 | Weighted median | 4 | 0.0142480650142248 | 0.55766072492392 | 0.979616505410631 | -1.078766956 | 1.10726308586511 | 1.01435005249162 | 0.340014520192036 | 3.02606497042728 | 0.999332001504091 |
| ebi-fl187-GCST90094734 | ebi-a-GCST90002112 | HLA DR on CD33- HLA DR+ \|\| id:ebi-a-GCST90002112 | Chips liking \|\| id:ebi-fl187-GCST90094734 | Weighted median | 4 | 0.174563951052004 | 0.506037117054962 | 0.730122381542312 | -0.817268798 | 1.16639670047973 | 1.19072688818734 | 0.441636206344363 | 3.21040372570077 | 0.999332001504091 |
| ebi-fl187-GCST90094734 | ebi-a-GCST90002115 | HLA DR on HLA DR+ CD8+ T cell \|\| id:ebi-a-GCST90002115 | Chips liking \|\| id:ebi-fl187-GCST90094734 | Weighted median | 4 | 0.05077533939701 | 0.413021626526146 | 0.90215756184485 | -0.758747049 | 0.860297727388256 | 1.05208650434209 | 0.468252757562199 | 2.36386437611473 | 0.999332001504091 |
| ebi-fl187-GCST90094734 | ebi-a-GCST90002116 | HLA DR on B cell \|\| id:ebi-a-GCST90002116 | Chips liking \|\| id:ebi-fl187-GCST90094734 | Weighted median | 4 | -0.038621634 | 0.379236107659263 | 0.918883196294152 | -0.781924405 | 0.704681136961842 | 0.962114671717974 | 0.457524700727652 | 2.02320145789462 | 0.999332001504091 |
| ebi-fl187-GCST90094734 | ebi-cfmet338-GCST90026023 | Sphingomyelin (d18:1/24:1, d18:2/24:0) levels \|\| id:ebi-cfmet338-GCST90026023 | Chips liking \|\| id:ebi-fl187-GCST90094734 | Weighted median | 4 | -0.050041175 | 0.192543244250623 | 0.79494417172699 | -0.427425934 | 0.327343583380922 | 0.951190258102296 | 0.652185704940572 | 1.38727804098551 | 0.999332001504091 |
| ebi-fl187-GCST90094734 | ebi-cfmet338-GCST90026046 | 1-arachidonoyl-gpc (20:4n6) levels \|\| id:ebi-cfmet338-GCST90026046 | Chips liking \|\| id:ebi-fl187-GCST90094734 | Weighted median | 4 | -0.134371426 | 0.223148850536291 | 0.547067297267745 | -0.571743173 | 0.303000320725375 | 0.874265279149765 | 0.564540488529713 | 1.35391489867708 | 0.999332001504091 |
| ebi-fl187-GCST90094734 | ebi-cfmet338-GCST90026047 | 1-linoleoyl-gpc (18:2) levels \|\| id:ebi-cfmet338-GCST90026047 | Chips liking \|\| id:ebi-fl187-GCST90094734 | Weighted median | 4 | 0.0144846048903666 | 0.263760377096864 | 0.956205566120435 | -0.502485734 | 0.53145494400022 | 1.01459001510665 | 0.605024857983408 | 1.70140595906283 | 0.999332001504091 |
| ebi-fl187-GCST90094734 | ebi-cfmet338-GCST90026050 | 1-myristoyl-2-palmitoyl-gpc (14:0/16:0) levels \|\| id:ebi-cfmet338-GCST90026050 | Chips liking \|\| id:ebi-fl187-GCST90094734 | Weighted median | 4 | 0.174451022029796 | 0.142492989559155 | 0.220847400351637 | -0.104835238 | 0.453737281565741 | 1.1905924281565 | 0.900472874520965 | 1.57418437588992 | 0.999332001504091 |
| ebi-fl187-GCST90094734 | ebi-cfmet338-GCST90026068 | 2,3-dihydroxyisovalerate levels \|\| id:ebi-cfmet338-GCST90026068 | Chips liking \|\| id:ebi-fl187-GCST90094734 | Weighted median | 4 | 0.179170208254768 | 0.46561604843619 | 0.700383764619972 | -0.733437247 | 1.0917776631897 | 1.19622433409727 | 0.480255393551537 | 2.97956603236542 | 0.999332001504091 |
| ebi-fl187-GCST90094734 | ebi-cfmet338-GCST90026082 | 3-hydroxyhexanoate levels \|\| id:ebi-cfmet338-GCST90026082 | Chips liking \|\| id:ebi-fl187-GCST90094734 | Weighted median | 4 | -0.003359926 | 0.114013959864272 | 0.976490201923032 | -0.226827287 | 0.220107435246289 | 0.996645712147521 | 0.797058432419796 | 1.2462106103394 | 0.999332001504091 |
| ebi-fl187-GCST90094734 | ebi-cfmet338-GCST90026094 | 3-ureidopropionate levels \|\| id:ebi-cfmet338-GCST90026094 | Chips liking \|\| id:ebi-fl187-GCST90094734 | Weighted median | 4 | 0.478245553424566 | 0.222868285242923 | 0.0318836433037297 | 0.0414237143484381 | 0.915067392500694 | 1.6132415717438 | 1.04229364676862 | 2.49694352150278 | 0.999332001504091 |
| ebi-fl187-GCST90094734 | ebi-cfmet338-GCST90026173 | Glycerol levels \|\| id:ebi-cfmet338-GCST90026173 | Chips liking \|\| id:ebi-fl187-GCST90094734 | Weighted median | 4 | -0.151696691 | 0.153878801577967 | 0.32422179692178 | -0.453299142 | 0.149905759812055 | 0.859248858892213 | 0.635527991836149 | 1.16172475640997 | 0.999332001504091 |
| ebi-fl187-GCST90094734 | ebi-cfmet338-GCST90026179 | Histidine levels \|\| id:ebi-cfmet338-GCST90026179 | Chips liking \|\| id:ebi-fl187-GCST90094734 | Weighted median | 4 | -0.070085289 | 0.0827149404413467 | 0.396821729357075 | -0.232206572 | 0.0920359943259429 | 0.932314300417343 | 0.792782339595395 | 1.0964042857038 | 0.999332001504091 |
| ebi-fl187-GCST90094734 | ebi-cfmet338-GCST90026188 | Isocitrate levels \|\| id:ebi-cfmet338-GCST90026188 | Chips liking \|\| id:ebi-fl187-GCST90094734 | Weighted median | 4 | -0.030620976 | 0.182657743972645 | 0.866865504492847 | -0.388630154 | 0.327388202621911 | 0.969843097653867 | 0.677984973849731 | 1.38733994165971 | 0.999332001504091 |
| ebi-fl187-GCST90094734 | ebi-cfmet338-GCST90026207 | N-acetyl-isoputreanine levels \|\| id:ebi-cfmet338-GCST90026207 | Chips liking \|\| id:ebi-fl187-GCST90094734 | Weighted median | 4 | -0.027613022 | 0.109687550051777 | 0.801240077461969 | -0.24260062 | 0.18737457621054 | 0.972764732630673 | 0.78458479869037 | 1.20607896893942 | 0.999332001504091 |
| ebi-fl187-GCST90094734 | ebi-cfmet338-GCST90026213 | N-acetylglutamate levels \|\| id:ebi-cfmet338-GCST90026213 | Chips liking \|\| id:ebi-fl187-GCST90094734 | Weighted median | 4 | 0.215711654651594 | 0.193501382049782 | 0.26494430149255 | -0.163551054 | 0.594974363469167 | 1.24074456449234 | 0.849123146620869 | 1.81298446573222 | 0.999332001504091 |
| ebi-fl187-GCST90094734 | ebi-cfmet338-GCST90026224 | N-acetyltaurine levels \|\| id:ebi-cfmet338-GCST90026224 | Chips liking \|\| id:ebi-fl187-GCST90094734 | Weighted median | 4 | 0.191605446333306 | 0.176662886089684 | 0.278106680300966 | -0.15465381 | 0.537864703069087 | 1.21119254221915 | 0.85671171083464 | 1.71234658727625 | 0.999332001504091 |
| ebi-fl187-GCST90094734 | ebi-cfmet338-GCST90026250 | Phenyllactate (pla) levels \|\| id:ebi-cfmet338-GCST90026250 | Chips liking \|\| id:ebi-fl187-GCST90094734 | Weighted median | 4 | -0.012295898 | 0.249089761847298 | 0.960629757351375 | -0.500511831 | 0.475920035111609 | 0.987779387561829 | 0.606220297751772 | 1.60949430777975 | 0.999332001504091 |
| ebi-fl187-GCST90094734 | ebi-cfmet338-GCST90026251 | Pipecolate levels \|\| id:ebi-cfmet338-GCST90026251 | Chips liking \|\| id:ebi-fl187-GCST90094734 | Weighted median | 4 | -0.282292265 | 0.36380585238335 | 0.437783193385203 | -0.995351735 | 0.430767205906256 | 0.754053269123248 | 0.369593422574924 | 1.53843736913416 | 0.999332001504091 |
| ebi-fl187-GCST90094734 | ebi-cfmet338-GCST90026278 | Trigonelline (n'-methylnicotinate) levels \|\| id:ebi-cfmet338-GCST90026278 | Chips liking \|\| id:ebi-fl187-GCST90094734 | Weighted median | 4 | 0.198423586274846 | 0.401957587588488 | 0.621558262292159 | -0.589413285 | 0.986260457948282 | 1.21947883893303 | 0.554652612074239 | 2.68118928178129 | 0.999332001504091 |
| ebi-fl187-GCST90094734 | ebi-cfmet338-GCST90026299 | 1-carboxyethylvaline levels \|\| id:ebi-cfmet338-GCST90026299 | Chips liking \|\| id:ebi-fl187-GCST90094734 | Weighted median | 4 | 0.322739664527881 | 0.270257298512008 | 0.2324020603687 | -0.206964641 | 0.852443969611417 | 1.38090580534164 | 0.813048398418897 | 2.34537187076996 | 0.999332001504091 |
| ebi-fl187-GCST90094734 | ebi-gm430-GCST90011306 | C_Erysipelotrichia abundance \|\| id:ebi-gm430-GCST90011306 | Chips liking \|\| id:ebi-fl187-GCST90094734 | Weighted median | 4 | 0.0756567522260237 | 0.232375879966047 | 0.744742754282174 | -0.379799973 | 0.531113476959476 | 1.07859228620939 | 0.683998213977208 | 1.70082508418534 | 0.999332001504091 |
| ebi-fl187-GCST90094734 | ebi-gm430-GCST90011312 | F_Erysipelotrichaceae abundance \|\| id:ebi-gm430-GCST90011312 | Chips liking \|\| id:ebi-fl187-GCST90094734 | Weighted median | 4 | 0.0756567522260237 | 0.216907630896359 | 0.727241642099936 | -0.349482204 | 0.500795708782888 | 1.07859228620939 | 0.705053068643777 | 1.65003369478019 | 0.999332001504091 |
| ebi-fl187-GCST90094734 | ebi-gm430-GCST90011352 | O_Erysipelotrichales abundance \|\| id:ebi-gm430-GCST90011352 | Chips liking \|\| id:ebi-fl187-GCST90094734 | Weighted median | 4 | 0.0756567522260237 | 0.228101981785144 | 0.740131278675337 | -0.371423132 | 0.522736636524905 | 1.07859228620939 | 0.689752023599715 | 1.68663705225393 | 0.999332001504091 |
| ebi-fl187-GCST90094734 | ebi-gm430-GCST90011362 | OTU97_11 (Parabacteroides) abundance \|\| id:ebi-gm430-GCST90011362 | Chips liking \|\| id:ebi-fl187-GCST90094734 | Weighted median | 4 | 0.030428783632238 | 0.283145145460277 | 0.914418435820172 | -0.524535701 | 0.58539326873438 | 1.0308964707323 | 0.591830086424549 | 1.79569703829816 | 0.999332001504091 |
| ebi-fl187-GCST90094734 | ebi-gm430-GCST90011384 | OTU97_235 (Phascolarctobacterium) abundance \|\| id:ebi-gm430-GCST90011384 | Chips liking \|\| id:ebi-fl187-GCST90094734 | Weighted median | 4 | -1.237410747 | 0.891188260273606 | 0.164986262452545 | -2.984139738 | 0.509318242706737 | 0.290134477655994 | 0.0505829994917615 | 1.66415625744824 | 0.999332001504091 |
| ebi-fl187-GCST90094734 | ebi-gm430-GCST90011390 | OTU97_3 (Bacteroides) abundance \|\| id:ebi-gm430-GCST90011390 | Chips liking \|\| id:ebi-fl187-GCST90094734 | Weighted median | 4 | 0.0314644760285903 | 0.218438238489844 | 0.885466598300339 | -0.396674471 | 0.459603423468685 | 1.03196471545945 | 0.672552925206121 | 1.58344590297769 | 0.999332001504091 |
| ebi-fl187-GCST90094734 | ebi-gm430-GCST90011398 | OTU97_4 (Alistipes) abundance \|\| id:ebi-gm430-GCST90011398 | Chips liking \|\| id:ebi-fl187-GCST90094734 | Weighted median | 4 | 0.337293921133355 | 0.242383607851467 | 0.164052443706037 | -0.13777795 | 0.812365792522229 | 1.40115083106228 | 0.871292140464542 | 2.25323236628738 | 0.999332001504091 |
| ebi-fl187-GCST90094734 | ebi-gm430-GCST90011421 | OTU99_108 (Cytophagales) abundance \|\| id:ebi-gm430-GCST90011421 | Chips liking \|\| id:ebi-fl187-GCST90094734 | Weighted median | 4 | -0.659443741 | 0.798439623371052 | 0.408851970037965 | -2.224385403 | 0.905497920399461 | 0.517138917465235 | 0.108133856937171 | 2.47316305486543 | 0.999332001504091 |
| ebi-fl187-GCST90094734 | ebi-gm430-GCST90011428 | OTU99_123 (Phascolarctobacterium) abundance \|\| id:ebi-gm430-GCST90011428 | Chips liking \|\| id:ebi-fl187-GCST90094734 | Weighted median | 4 | 0.60782756926137 | 0.466198153491952 | 0.192302940867467 | -0.305920812 | 1.52157595010559 | 1.83643752881227 | 0.736444935055831 | 4.57943647473716 | 0.999332001504091 |
| ebi-fl187-GCST90094734 | ebi-gm430-GCST90011450 | OTU99_298 (Phascolarctobacterium) abundance \|\| id:ebi-gm430-GCST90011450 | Chips liking \|\| id:ebi-fl187-GCST90094734 | Weighted median | 4 | -1.217537996 | 0.858507128677603 | 0.156131298732802 | -2.900211968 | 0.465135976250589 | 0.295957920286702 | 0.0550115581214088 | 1.59223067972586 | 0.999332001504091 |
| ebi-fl187-GCST90094734 | ebi-gm430-GCST90011451 | OTU99_3 (Bacteroides) abundance \|\| id:ebi-gm430-GCST90011451 | Chips liking \|\| id:ebi-fl187-GCST90094734 | Weighted median | 4 | 0.0315120017714245 | 0.217822696669406 | 0.884972904522011 | -0.395420484 | 0.458444487243459 | 1.03201376151459 | 0.67339682732011 | 1.58161185313873 | 0.999332001504091 |
| ebi-fl187-GCST90094734 | ebi-gm430-GCST90011472 | OTU99_72 (Parabacteroides) abundance \|\| id:ebi-gm430-GCST90011472 | Chips liking \|\| id:ebi-fl187-GCST90094734 | Weighted median | 4 | 0.238696323430531 | 0.622321268175639 | 0.70130604762194 | -0.981053362 | 1.45844600905478 | 1.26959293241099 | 0.374915968473222 | 4.29927330274025 | 0.999332001504091 |
| ebi-fl187-GCST90094734 | ebi-gm430-GCST90011507 | TestASV_3 (Bacteroides) abundance \|\| id:ebi-gm430-GCST90011507 | Chips liking \|\| id:ebi-fl187-GCST90094734 | Weighted median | 4 | 0.344446079965793 | 0.272657124785586 | 0.206483581014021 | -0.189961885 | 0.878854044545541 | 1.41120800674167 | 0.826990654410699 | 2.40813850515748 | 0.999332001504091 |
| ebi-fl187-GCST90094734 | ebi-gm430-GCST90011512 | TestASV_34 (Phascolarctobacterium) abundance \|\| id:ebi-gm430-GCST90011512 | Chips liking \|\| id:ebi-fl187-GCST90094734 | Weighted median | 4 | -0.039024112 | 0.729269473980389 | 0.957324577317252 | -1.468392281 | 1.39034405704085 | 0.961727519730623 | 0.230295438076982 | 4.01623162807957 | 0.999332001504091 |
| ebi-fl187-GCST90094734 | ebi-gm430-GCST90011519 | TestASV_41 (Barnesiella) abundance \|\| id:ebi-gm430-GCST90011519 | Chips liking \|\| id:ebi-fl187-GCST90094734 | Weighted median | 4 | 0.588114771521083 | 0.885148649954324 | 0.506418423390617 | -1.146776582 | 2.32300612543156 | 1.80059068881329 | 0.317659068681246 | 10.2063096832108 | 0.999332001504091 |
| ebi-fl187-GCST90094734 | ebi-gm430-GCST90011524 | TestASV_46 (Sutterella) abundance \|\| id:ebi-gm430-GCST90011524 | Chips liking \|\| id:ebi-fl187-GCST90094734 | Weighted median | 4 | -1.481613355 | 0.910862721908737 | 0.103821133916869 | -3.26690429 | 0.30367757946445 | 0.227270723974179 | 0.0381242662394627 | 1.35483215995072 | 0.999332001504091 |
| ebi-fl187-GCST90094734 | ebi-gm430-GCST90011581 | OTU97_19 (Bacteria) prevalence \|\| id:ebi-gm430-GCST90011581 | Chips liking \|\| id:ebi-fl187-GCST90094734 | Weighted median | 4 | -1.171745176 | 1.15168052687977 | 0.308952691526791 | -3.429039008 | 1.08554865710049 | 0.309825768815378 | 0.0324180793147525 | 2.96106398192309 | 0.999332001504091 |
| ebi-fl187-GCST90094734 | ebi-gm430-GCST90011614 | OTU97_80 (Ruminococcus) prevalence \|\| id:ebi-gm430-GCST90011614 | Chips liking \|\| id:ebi-fl187-GCST90094734 | Weighted median | 4 | -0.202382324 | 0.544992609845816 | 0.710377792405862 | -1.27056784 | 0.865803190932244 | 0.81678259235287 | 0.280672199712179 | 2.37691443561136 | 0.999332001504091 |
| ebi-fl187-GCST90094734 | ebi-gm430-GCST90011623 | OTU99_11 (Parabacteroides) prevalence \|\| id:ebi-gm430-GCST90011623 | Chips liking \|\| id:ebi-fl187-GCST90094734 | Weighted median | 4 | -0.319277652 | 0.449458870358567 | 0.477480853582712 | -1.200217038 | 0.561661733655682 | 0.72667375869181 | 0.301128848371136 | 1.75358407016675 | 0.999332001504091 |
| ebi-fl187-GCST90094734 | ebi-gm430-GCST90011642 | OTU99_19 (Bacteria) prevalence \|\| id:ebi-gm430-GCST90011642 | Chips liking \|\| id:ebi-fl187-GCST90094734 | Weighted median | 4 | -1.171745176 | 1.23119706843789 | 0.341242999556269 | -3.58489143 | 1.2414010785544 | 0.309825768815378 | 0.0277396791713718 | 3.46045844398613 | 0.999332001504091 |
| ebi-fl187-GCST90094734 | ebi-gm430-GCST90011653 | OTU99_30 (Parasutterella) prevalence \|\| id:ebi-gm430-GCST90011653 | Chips liking \|\| id:ebi-fl187-GCST90094734 | Weighted median | 4 | -0.813615674 | 0.553389417928037 | 0.141496560992176 | -1.898258933 | 0.271027584786768 | 0.443252508658324 | 0.14982925496258 | 1.31131124212669 | 0.999332001504091 |
| ebi-fl187-GCST90094734 | ebi-gm430-GCST90011675 | OTU99_8 (Ruminococcaceae) prevalence \|\| id:ebi-gm430-GCST90011675 | Chips liking \|\| id:ebi-fl187-GCST90094734 | Weighted median | 4 | -0.778911606 | 0.551751025154673 | 0.158035812870438 | -1.860343615 | 0.302520403448609 | 0.458905209336996 | 0.155619148081751 | 1.35326528741824 | 0.999332001504091 |
| ebi-fl187-GCST90094734 | ebi-gm430-GCST90011679 | OTU99_92 (Ruminococcus) prevalence \|\| id:ebi-gm430-GCST90011679 | Chips liking \|\| id:ebi-fl187-GCST90094734 | Weighted median | 4 | -0.164002152 | 0.58778959136769 | 0.780233262722976 | -1.316069751 | 0.988065447443451 | 0.848740195697246 | 0.268187276200977 | 2.68603317053852 | 0.999332001504091 |
| ebi-fl187-GCST90094734 | ebi-gm430-GCST90011684 | TestASV_11 (Lachnospiraceae) prevalence \|\| id:ebi-gm430-GCST90011684 | Chips liking \|\| id:ebi-fl187-GCST90094734 | Weighted median | 4 | -0.110211069 | 0.55738570989726 | 0.843257449678182 | -1.202687061 | 0.982264922230471 | 0.895645072284172 | 0.300385971204606 | 2.6704978674271 | 0.999332001504091 |
| ebi-fl187-GCST90094734 | ebi-gm430-GCST90011688 | TestASV_15 (Bacteroides) prevalence \|\| id:ebi-gm430-GCST90011688 | Chips liking \|\| id:ebi-fl187-GCST90094734 | Weighted median | 4 | -0.335221515 | 0.511657278273034 | 0.512359544925965 | -1.338069781 | 0.667626750113688 | 0.715179645569893 | 0.262351576238378 | 1.94960492622593 | 0.999332001504091 |
| ebi-fl187-GCST90094734 | ebi-gm430-GCST90011692 | TestASV_19 (Prevotella) prevalence \|\| id:ebi-gm430-GCST90011692 | Chips liking \|\| id:ebi-fl187-GCST90094734 | Weighted median | 4 | 0.345607273532187 | 1.04719874913086 | 0.741377099117763 | -1.706902275 | 2.39811682182867 | 1.41284764418393 | 0.181426933829113 | 11.0024373093151 | 0.999332001504091 |
| ebi-fl187-GCST90094734 | ebi-gm430-GCST90011693 | TestASV_2 (Bacteroides) prevalence \|\| id:ebi-gm430-GCST90011693 | Chips liking \|\| id:ebi-fl187-GCST90094734 | Weighted median | 4 | 0.0615755218504467 | 0.553953361982758 | 0.911492238654579 | -1.024173068 | 1.14732411133665 | 1.06351081180046 | 0.359093288524778 | 3.14975323393834 | 0.999332001504091 |
| ebi-fl187-GCST90094734 | ebi-gm430-GCST90011716 | TestASV_40 (Barnesiella) prevalence \|\| id:ebi-gm430-GCST90011716 | Chips liking \|\| id:ebi-fl187-GCST90094734 | Weighted median | 4 | -0.012882295 | 1.03775857701788 | 0.990095653856646 | -2.046889106 | 2.02112451614474 | 0.987200326783701 | 0.129136007830132 | 7.54680666978496 | 0.999332001504091 |
| ebi-fl187-GCST90094734 | ebi-gm430-GCST90011718 | TestASV_42 (Alphaproteobacteria) prevalence \|\| id:ebi-gm430-GCST90011718 | Chips liking \|\| id:ebi-fl187-GCST90094734 | Weighted median | 4 | -0.731074691 | 0.817348108351948 | 0.371082691712526 | -2.333076983 | 0.870927601599535 | 0.481391365139634 | 0.0969968298336545 | 2.38912598306997 | 0.999332001504091 |
| ebi-fl187-GCST90094734 | ebi-hbsm44-GCST90104929 | Sulcal depth of the right inferior frontal sulcus \|\| id:ebi-hbsm44-GCST90104929 | Chips liking \|\| id:ebi-fl187-GCST90094734 | Weighted median | 3 | 0.124534645222734 | 0.473333846471961 | 0.792472673147215 | -0.803199694 | 1.05226898430778 | 1.13262125969633 | 0.447893546661444 | 2.8641424451819 | 0.999332001504091 |
| ebi-fl187-GCST90094734 | ebi-hbsm44-GCST90104935 | Sulcal opening distance of the left calloso-marginal posterior fissure \|\| id:ebi-hbsm44-GCST90104935 | Chips liking \|\| id:ebi-fl187-GCST90094734 | Weighted median | 4 | -0.110292137 | 0.0869928388605696 | 0.204858641753152 | -0.280798101 | 0.0602138272964285 | 0.895572467339236 | 0.755180790307892 | 1.06206362045979 | 0.999332001504091 |
| ebi-fl187-GCST90094734 | ebi-hbsm44-GCST90104936 | Sulcal opening distance of the left anterior inferior temporal sulcus \|\| id:ebi-hbsm44-GCST90104936 | Chips liking \|\| id:ebi-fl187-GCST90094734 | Weighted median | 4 | -0.014242385 | 0.0489484882722244 | 0.771076717863413 | -0.110181422 | 0.081696652441353 | 0.985858558395855 | 0.895671626388877 | 1.08512658939625 | 0.999332001504091 |
| ebi-fl187-GCST90094734 | ebi-hbsm44-GCST90104941 | Sulcal opening distance of the right posterior inferior temporal sulcus \|\| id:ebi-hbsm44-GCST90104941 | Chips liking \|\| id:ebi-fl187-GCST90094734 | Weighted median | 4 | 0.016186412924487 | 0.0426653045871113 | 0.704404835766584 | -0.067437584 | 0.0998104099152252 | 1.01631812258222 | 0.934786064340281 | 1.1049614084887 | 0.999332001504091 |
| ebi-fl187-GCST90094734 | ebi-IPs91-GCST90274819 | Leukemia inhibitory factor levels \|\| id:ebi-IPs91-GCST90274819 | Chips liking \|\| id:ebi-fl187-GCST90094734 | Weighted median | 4 | 0.0274263108141503 | 0.172218279702671 | 0.873469397165472 | -0.310121517 | 0.364974139031386 | 1.02780587413857 | 0.733357835069863 | 1.44047675554336 | 0.999332001504091 |
| ebi-fl187-GCST90094734 | ebi-IPs91-GCST90274837 | STAM binding protein levels \|\| id:ebi-IPs91-GCST90274837 | Chips liking \|\| id:ebi-fl187-GCST90094734 | Weighted median | 4 | -0.042766858 | 0.150770628771114 | 0.776674706273671 | -0.33827729 | 0.252743574489104 | 0.958134745573716 | 0.712997553183688 | 1.28755307304554 | 0.999332001504091 |
| ebi-fl187-GCST90094734 | ebi-IPs91-GCST90274840 | TNF-beta levels \|\| id:ebi-IPs91-GCST90274840 | Chips liking \|\| id:ebi-fl187-GCST90094734 | Weighted median | 4 | 0.16179803923049 | 0.190000343181679 | 0.394454412267324 | -0.210602633 | 0.534198711866581 | 1.1756227876608 | 0.810095907984683 | 1.70608063223728 | 0.999332001504091 |
| ebi-fl187-GCST90094734 | ebi-IPs91-GCST90274842 | Tumor necrosis factor ligand superfamily member 14 levels \|\| id:ebi-IPs91-GCST90274842 | Chips liking \|\| id:ebi-fl187-GCST90094734 | Weighted median | 4 | 0.144745429801825 | 0.182503914466779 | 0.427714534495018 | -0.212962243 | 0.502453102156712 | 1.15574531450148 | 0.808186651705027 | 1.6527707172274 | 0.999332001504091 |
| ebi-fl187-GCST90094734 | ebi-met1400-GCST90199628 | 3-methylhistidine levels \|\| id:ebi-met1400-GCST90199628 | Chips liking \|\| id:ebi-fl187-GCST90094734 | Weighted median | 4 | 0.136911850167455 | 0.198588976760639 | 0.490556980515578 | -0.252322544 | 0.526146244618308 | 1.14672706026667 | 0.776994082647791 | 1.69239763868822 | 0.999332001504091 |
| ebi-fl187-GCST90094734 | ebi-met1400-GCST90199651 | Docosahexaenoate DHA; 22:6n3 levels \|\| id:ebi-met1400-GCST90199651 | Chips liking \|\| id:ebi-fl187-GCST90094734 | Weighted median | 4 | -0.358001705 | 0.193334140885275 | 0.0640652903558122 | -0.736936621 | 0.0209332112704694 | 0.699071883182555 | 0.478577737246163 | 1.02115384779178 | 0.999332001504091 |
| ebi-fl187-GCST90094734 | ebi-met1400-GCST90199652 | 1-stearoyl-2-oleoyl-GPS (18:0/18:1) levels \|\| id:ebi-met1400-GCST90199652 | Chips liking \|\| id:ebi-fl187-GCST90094734 | Weighted median | 4 | 0.0628108101763751 | 0.230307377027013 | 0.785063845398147 | -0.388591649 | 0.51421326914932 | 1.06482536605037 | 0.678011080132763 | 1.67232231656493 | 0.999332001504091 |
| ebi-fl187-GCST90094734 | ebi-met1400-GCST90199653 | 1-myristoyl-2-palmitoyl-gpc (14:0/16:0) levels \|\| id:ebi-met1400-GCST90199653 | Chips liking \|\| id:ebi-fl187-GCST90094734 | Weighted median | 4 | 0.318581939040311 | 0.206079631356147 | 0.122124692195009 | -0.085334138 | 0.722498016498358 | 1.37517629719355 | 0.918205425429617 | 2.05957163395994 | 0.999332001504091 |
| ebi-fl187-GCST90094734 | ebi-met1400-GCST90199667 | Erythritol levels in elite athletes \|\| id:ebi-met1400-GCST90199667 | Chips liking \|\| id:ebi-fl187-GCST90094734 | Weighted median | 4 | -0.066364738 | 0.184504296091038 | 0.719077397826237 | -0.427993159 | 0.295263681965537 | 0.935789483704442 | 0.651815874044031 | 1.34348056358117 | 0.999332001504091 |
| ebi-fl187-GCST90094734 | ebi-met1400-GCST90199674 | 3-hydroxymyristate levels \|\| id:ebi-met1400-GCST90199674 | Chips liking \|\| id:ebi-fl187-GCST90094734 | Weighted median | 4 | 0.0160607693753676 | 0.201400760014019 | 0.936439808843761 | -0.37868472 | 0.410806259002845 | 1.01619043678788 | 0.684761470060403 | 1.50803316040554 | 0.999332001504091 |
| ebi-fl187-GCST90094734 | ebi-met1400-GCST90199685 | 2-linoleoylglycerol (18:2) levels \|\| id:ebi-met1400-GCST90199685 | Chips liking \|\| id:ebi-fl187-GCST90094734 | Weighted median | 4 | -0.304999177 | 0.260165926152577 | 0.241066354753859 | -0.814924392 | 0.204926038100861 | 0.737123980927809 | 0.442672795516135 | 1.22743427823555 | 0.999332001504091 |
| ebi-fl187-GCST90094734 | ebi-met1400-GCST90199703 | Gamma-glutamylvaline levels \|\| id:ebi-met1400-GCST90199703 | Chips liking \|\| id:ebi-fl187-GCST90094734 | Weighted median | 4 | 0.261789714821735 | 0.231588809025849 | 0.258304576779009 | -0.192124351 | 0.7157037805124 | 1.29925329998917 | 0.825204247246105 | 2.04562584737802 | 0.999332001504091 |
| ebi-fl187-GCST90094734 | ebi-met1400-GCST90199779 | Gamma-glutamyl-2-aminobutyrate levels \|\| id:ebi-met1400-GCST90199779 | Chips liking \|\| id:ebi-fl187-GCST90094734 | Weighted median | 4 | 0.05381277636347 | 0.203798145489867 | 0.791741859726405 | -0.345631589 | 0.453257141523609 | 1.0552870089872 | 0.707773190653353 | 1.57342872836019 | 0.999332001504091 |
| ebi-fl187-GCST90094734 | ebi-met1400-GCST90199824 | 4-vinylphenol sulfate levels \|\| id:ebi-met1400-GCST90199824 | Chips liking \|\| id:ebi-fl187-GCST90094734 | Weighted median | 4 | 0.0710682292406421 | 0.222007064084161 | 0.748879659874293 | -0.364065616 | 0.506202074845598 | 1.07365447799911 | 0.694845600005015 | 1.65897853871308 | 0.999332001504091 |
| ebi-fl187-GCST90094734 | ebi-met1400-GCST90199830 | 4-ethylphenylsulfate levels \|\| id:ebi-met1400-GCST90199830 | Chips liking \|\| id:ebi-fl187-GCST90094734 | Weighted median | 4 | 0.0496768843121358 | 0.20099584509929 | 0.804789208486045 | -0.344274972 | 0.443628740706743 | 1.050931469 | 0.708734019184317 | 1.55835182561469 | 0.999332001504091 |
| ebi-fl187-GCST90094734 | ebi-met1400-GCST90199833 | Hexanoylglutamine levels \|\| id:ebi-met1400-GCST90199833 | Chips liking \|\| id:ebi-fl187-GCST90094734 | Weighted median | 4 | -0.053456047 | 0.201373352920658 | 0.790657120246245 | -0.448147819 | 0.341235724358171 | 0.947947604882005 | 0.638810248702254 | 1.406684792 | 0.999332001504091 |
| ebi-fl187-GCST90094734 | ebi-met1400-GCST90199837 | Carotenoid levels (cryptoxanthin) \|\| id:ebi-met1400-GCST90199837 | Chips liking \|\| id:ebi-fl187-GCST90094734 | Weighted median | 4 | -0.01639297 | 0.208622216597241 | 0.937368845630887 | -0.425292515 | 0.392506574543421 | 0.983740663532128 | 0.653578575943517 | 1.48068760009396 | 0.999332001504091 |
| ebi-fl187-GCST90094734 | ebi-met1400-GCST90199869 | 2-hydroxyhippurate levels \|\| id:ebi-met1400-GCST90199869 | Chips liking \|\| id:ebi-fl187-GCST90094734 | Weighted median | 4 | -0.563233873 | 0.245764410175456 | 0.0219193015431145 | -1.044932116 | -0.081535629 | 0.569364830177353 | 0.351715694277518 | 0.92169987042744 | 0.999332001504091 |
| ebi-fl187-GCST90094734 | ebi-met1400-GCST90199902 | Hydantoin-5-propionate levels \|\| id:ebi-met1400-GCST90199902 | Chips liking \|\| id:ebi-fl187-GCST90094734 | Weighted median | 4 | 0.240339659373949 | 0.217384397805082 | 0.268900747239772 | -0.18573376 | 0.666413079071909 | 1.27168101535129 | 0.830494676183831 | 1.94724018248483 | 0.999332001504091 |
| ebi-fl187-GCST90094734 | ebi-met1400-GCST90199904 | Pantoate levels \|\| id:ebi-met1400-GCST90199904 | Chips liking \|\| id:ebi-fl187-GCST90094734 | Weighted median | 4 | -0.050253152 | 0.200781516360578 | 0.80236483760011 | -0.443784924 | 0.343278620291953 | 0.950988649561267 | 0.641603399583274 | 1.40956143963976 | 0.999332001504091 |
| ebi-fl187-GCST90094734 | ebi-met1400-GCST90199936 | 3-methyl catechol sulfate (2) levels \|\| id:ebi-met1400-GCST90199936 | Chips liking \|\| id:ebi-fl187-GCST90094734 | Weighted median | 4 | -0.040147122 | 0.22383207413089 | 0.857652928090569 | -0.478857987 | 0.398563743155647 | 0.960648096150681 | 0.619490453873122 | 1.48968359216547 | 0.999332001504091 |
| ebi-fl187-GCST90094734 | ebi-met1400-GCST90199977 | 4-hydroxychlorothalonil levels \|\| id:ebi-met1400-GCST90199977 | Chips liking \|\| id:ebi-fl187-GCST90094734 | Weighted median | 4 | 0.321359062705358 | 0.198530703471582 | 0.105514631979934 | -0.067761116 | 0.710479241509658 | 1.37900063971024 | 0.934483680022897 | 2.03496626530134 | 0.999332001504091 |
| ebi-fl187-GCST90094734 | ebi-met1400-GCST90200040 | Sphingomyelin (d18:2/24:1, d18:1/24:2) levels \|\| id:ebi-met1400-GCST90200040 | Chips liking \|\| id:ebi-fl187-GCST90094734 | Weighted median | 4 | 0.261336325214111 | 0.190386656618811 | 0.169857780391726 | -0.111821522 | 0.63449417218698 | 1.29866436556375 | 0.894203839187534 | 1.88606787454352 | 0.999332001504091 |
| ebi-fl187-GCST90094734 | ebi-met1400-GCST90200077 | 1-stearoyl-2-oleoyl-GPI (18:0/18:1) levels \|\| id:ebi-met1400-GCST90200077 | Chips liking \|\| id:ebi-fl187-GCST90094734 | Weighted median | 4 | -0.31624229 | 0.210699094317211 | 0.133376492914269 | -0.729212515 | 0.0967279343679811 | 0.728882827391341 | 0.48228863548279 | 1.10156063605806 | 0.999332001504091 |
| ebi-fl187-GCST90094734 | ebi-met1400-GCST90200080 | 1-palmitoyl-2-oleoyl-GPI (16:0/18:1) levels \|\| id:ebi-met1400-GCST90200080 | Chips liking \|\| id:ebi-fl187-GCST90094734 | Weighted median | 4 | -0.061280542 | 0.223356988128967 | 0.783806881861192 | -0.499060239 | 0.37649915445705 | 0.940559336103066 | 0.607100921479692 | 1.45717430732023 | 0.999332001504091 |
| ebi-fl187-GCST90094734 | ebi-met1400-GCST90200082 | 1-oleoyl-2-linoleoyl-GPE (18:1/18:2) levels \|\| id:ebi-met1400-GCST90200082 | Chips liking \|\| id:ebi-fl187-GCST90094734 | Weighted median | 4 | -0.115172696 | 0.252192185342211 | 0.647896595080778 | -0.60946938 | 0.37912398680481 | 0.891212221415253 | 0.543639258561623 | 1.46100416975291 | 0.999332001504091 |
| ebi-fl187-GCST90094734 | ebi-met1400-GCST90200100 | Perfluorooctanesulfonate (PFOS) levels \|\| id:ebi-met1400-GCST90200100 | Chips liking \|\| id:ebi-fl187-GCST90094734 | Weighted median | 4 | 0.162519189107127 | 0.200266943590158 | 0.417071242457262 | -0.23000402 | 0.555042398543836 | 1.1764708936581 | 0.794530408222809 | 1.74201484210099 | 0.999332001504091 |
| ebi-fl187-GCST90094734 | ebi-met1400-GCST90200102 | 1-palmitoleoyl-2-linolenoyl-GPC (16:1/18:3) levels \|\| id:ebi-met1400-GCST90200102 | Chips liking \|\| id:ebi-fl187-GCST90094734 | Weighted median | 4 | 0.138325162376081 | 0.199851756667927 | 0.488850144438788 | -0.253384281 | 0.530034605445218 | 1.14834888942642 | 0.77616955753178 | 1.69899110194476 | 0.999332001504091 |
| ebi-fl187-GCST90094734 | ebi-met1400-GCST90200114 | Glycosyl-N-tricosanoyl-sphingadienine (d18:2/23:0) levels \|\| id:ebi-met1400-GCST90200114 | Chips liking \|\| id:ebi-fl187-GCST90094734 | Weighted median | 4 | -0.239410919 | 0.223708518660075 | 0.284533192907884 | -0.677879615 | 0.199057777873526 | 0.78709138534273 | 0.507692354986483 | 1.22025246745587 | 0.999332001504091 |
| ebi-fl187-GCST90094734 | ebi-met1400-GCST90200135 | Arachidoylcarnitine (C20) levels \|\| id:ebi-met1400-GCST90200135 | Chips liking \|\| id:ebi-fl187-GCST90094734 | Weighted median | 4 | -0.512701323 | 0.241664445576859 | 0.0338761992511753 | -0.986363637 | -0.03903901 | 0.598875635057976 | 0.372930338552941 | 0.961713191953623 | 0.999332001504091 |
| ebi-fl187-GCST90094734 | ebi-met1400-GCST90200146 | Docosahexaenoylcarnitine (C22:6) levels \|\| id:ebi-met1400-GCST90200146 | Chips liking \|\| id:ebi-fl187-GCST90094734 | Weighted median | 4 | 0.173885744397186 | 0.232129621724519 | 0.453803569263573 | -0.281088314 | 0.628859802977242 | 1.18991960307171 | 0.754961658714011 | 1.87547095330135 | 0.999332001504091 |
| ebi-fl187-GCST90094734 | ebi-met1400-GCST90200162 | 3-hydroxyphenylacetoylglutamine levels \|\| id:ebi-met1400-GCST90200162 | Chips liking \|\| id:ebi-fl187-GCST90094734 | Weighted median | 4 | -0.141734275 | 0.240493418454843 | 0.555627963979056 | -0.613101375 | 0.329632825298031 | 0.867851835888096 | 0.541668344666721 | 1.39045749390755 | 0.999332001504091 |
| ebi-fl187-GCST90094734 | ebi-met1400-GCST90200172 | 3-carboxy-4-methyl-5-pentyl-2-furanpropionate (3-CMPFP) levels \|\| id:ebi-met1400-GCST90200172 | Chips liking \|\| id:ebi-fl187-GCST90094734 | Weighted median | 4 | -0.271895634 | 0.247772571578354 | 0.272484185126542 | -0.757529874 | 0.213738606721265 | 0.761933777246826 | 0.468823049987542 | 1.23829892946826 | 0.999332001504091 |
| ebi-fl187-GCST90094734 | ebi-met1400-GCST90200222 | Dihydrocaffeate sulfate (2) levels \|\| id:ebi-met1400-GCST90200222 | Chips liking \|\| id:ebi-fl187-GCST90094734 | Weighted median | 4 | -0.155779808 | 0.242060579828113 | 0.519862669061953 | -0.630218544 | 0.318658928864605 | 0.85574759874606 | 0.532475418949299 | 1.37528217585078 | 0.999332001504091 |
| ebi-fl187-GCST90094734 | ebi-met1400-GCST90200223 | 4-methylhexanoylglutamine levels \|\| id:ebi-met1400-GCST90200223 | Chips liking \|\| id:ebi-fl187-GCST90094734 | Weighted median | 4 | 0.167610248912602 | 0.258604097586754 | 0.516897933091159 | -0.339253782 | 0.674474280182639 | 1.18247564965335 | 0.712301656555579 | 1.96300071627589 | 0.999332001504091 |
| ebi-fl187-GCST90094734 | ebi-met1400-GCST90200224 | 2-ketocaprylate levels \|\| id:ebi-met1400-GCST90200224 | Chips liking \|\| id:ebi-fl187-GCST90094734 | Weighted median | 4 | 0.0317719714417884 | 0.198235038020376 | 0.872665044550025 | -0.356768703 | 0.420312645961726 | 1.03228208866893 | 0.699934371679084 | 1.52243746514462 | 0.999332001504091 |
| ebi-fl187-GCST90094734 | ebi-met1400-GCST90200243 | Hydroxypalmitoyl sphingomyelin (d18:1/16:0(OH)) levels \|\| id:ebi-met1400-GCST90200243 | Chips liking \|\| id:ebi-fl187-GCST90094734 | Weighted median | 4 | 0.184009435967829 | 0.214647508890196 | 0.391299375471939 | -0.236699681 | 0.604718553392613 | 1.20202716533245 | 0.789228272315311 | 1.83073688168626 | 0.999332001504091 |
| ebi-fl187-GCST90094734 | ebi-met1400-GCST90200273 | 2-methoxyhydroquinone sulfate (2) levels \|\| id:ebi-met1400-GCST90200273 | Chips liking \|\| id:ebi-fl187-GCST90094734 | Weighted median | 4 | -0.12904525 | 0.264270588134434 | 0.625332210229769 | -0.647015603 | 0.388925102356725 | 0.878934192533565 | 0.523606095690412 | 1.47539404365795 | 0.999332001504091 |
| ebi-fl187-GCST90094734 | ebi-met1400-GCST90200276 | 2,4-di-tert-butylphenol levels \|\| id:ebi-met1400-GCST90200276 | Chips liking \|\| id:ebi-fl187-GCST90094734 | Weighted median | 4 | -0.163154446 | 0.200519686557312 | 0.415840905559702 | -0.556173031 | 0.229864140064217 | 0.849459982936154 | 0.573399251307764 | 1.258429028 | 0.999332001504091 |
| ebi-fl187-GCST90094734 | ebi-met1400-GCST90200289 | N-acetylmethionine levels \|\| id:ebi-met1400-GCST90200289 | Chips liking \|\| id:ebi-fl187-GCST90094734 | Weighted median | 4 | 0.0101479749402694 | 0.212445151145003 | 0.961901533781023 | -0.406244521 | 0.426540471184476 | 1.01019964025618 | 0.666147260311863 | 1.53194852545961 | 0.999332001504091 |
| ebi-fl187-GCST90094734 | ebi-met1400-GCST90200308 | 3-hydroxyisobutyrate levels \|\| id:ebi-met1400-GCST90200308 | Chips liking \|\| id:ebi-fl187-GCST90094734 | Weighted median | 4 | 0.221937707250173 | 0.213016497410747 | 0.297467145946465 | -0.195574628 | 0.639450042175237 | 1.24849360334222 | 0.822361970312376 | 1.89543818155204 | 0.999332001504091 |
| ebi-fl187-GCST90094734 | ebi-met1400-GCST90200383 | Cystathionine levels \|\| id:ebi-met1400-GCST90200383 | Chips liking \|\| id:ebi-fl187-GCST90094734 | Weighted median | 4 | -0.250377132 | 0.219590712564462 | 0.254203363803565 | -0.680774929 | 0.180020664544089 | 0.778507127687336 | 0.50622455239008 | 1.19724210332842 | 0.999332001504091 |
| ebi-fl187-GCST90094734 | ebi-met1400-GCST90200384 | Guanidinoacetate levels \|\| id:ebi-met1400-GCST90200384 | Chips liking \|\| id:ebi-fl187-GCST90094734 | Weighted median | 4 | 0.13615271118919 | 0.188434634684069 | 0.469958790489151 | -0.233179173 | 0.505484595169965 | 1.145856865 | 0.792011653871631 | 1.65778868222921 | 0.999332001504091 |
| ebi-fl187-GCST90094734 | ebi-met1400-GCST90200403 | Citrulline levels \|\| id:ebi-met1400-GCST90200403 | Chips liking \|\| id:ebi-fl187-GCST90094734 | Weighted median | 4 | 0.0793138424748644 | 0.203266038835725 | 0.696390720056974 | -0.319087594 | 0.477715278592886 | 1.08254401705563 | 0.726811882417301 | 1.61238633711562 | 0.999332001504091 |
| ebi-fl187-GCST90094734 | ebi-met1400-GCST90200434 | 12,13-DiHOME levels \|\| id:ebi-met1400-GCST90200434 | Chips liking \|\| id:ebi-fl187-GCST90094734 | Weighted median | 4 | 0.102553040402499 | 0.242046140364618 | 0.671790368615559 | -0.371857395 | 0.576963475517151 | 1.10799606890874 | 0.689452555094186 | 1.78062330706645 | 0.999332001504091 |
| ebi-fl187-GCST90094734 | ebi-met1400-GCST90200437 | Fructose levels \|\| id:ebi-met1400-GCST90200437 | Chips liking \|\| id:ebi-fl187-GCST90094734 | Weighted median | 4 | 0.0502664911208745 | 0.206122351802655 | 0.807333634136236 | -0.353733318 | 0.454266300654078 | 1.05155128812149 | 0.702062169446601 | 1.57501736978874 | 0.999332001504091 |
| ebi-fl187-GCST90094734 | ebi-met1400-GCST90200448 | N-stearoyl-sphinganine (d18:0/18:0) levels \|\| id:ebi-met1400-GCST90200448 | Chips liking \|\| id:ebi-fl187-GCST90094734 | Weighted median | 4 | 6.20121236398763e-05 | 0.242196471677491 | 0.999795709181263 | -0.474643072 | 0.474767096611523 | 1.00006201404643 | 0.6221070640459 | 1.60763972913964 | 0.99994023009904 |
| ebi-fl187-GCST90094734 | ebi-met1400-GCST90200468 | X-11847 levels \|\| id:ebi-met1400-GCST90200468 | Chips liking \|\| id:ebi-fl187-GCST90094734 | Weighted median | 4 | -0.50293805 | 0.221322617253171 | 0.0230611254662391 | -0.93673038 | -0.06914572 | 0.604751257734344 | 0.391907130381769 | 0.933190685698951 | 0.999332001504091 |
| ebi-fl187-GCST90094734 | ebi-met1400-GCST90200476 | X-11843 levels \|\| id:ebi-met1400-GCST90200476 | Chips liking \|\| id:ebi-fl187-GCST90094734 | Weighted median | 4 | 0.0169909092662358 | 0.231978398334675 | 0.941612353222882 | -0.437686751 | 0.471668570002199 | 1.01713607576995 | 0.645527961874808 | 1.60266612406377 | 0.999332001504091 |
| ebi-fl187-GCST90094734 | ebi-met1400-GCST90200511 | X-12906 levels \|\| id:ebi-met1400-GCST90200511 | Chips liking \|\| id:ebi-fl187-GCST90094734 | Weighted median | 4 | 0.252445131359357 | 0.199237042843667 | 0.205134116968653 | -0.138059473 | 0.642949735332945 | 1.28716886901108 | 0.871046886769852 | 1.902083255 | 0.999332001504091 |
| ebi-fl187-GCST90094734 | ebi-met1400-GCST90200515 | X-12849 levels \|\| id:ebi-met1400-GCST90200515 | Chips liking \|\| id:ebi-fl187-GCST90094734 | Weighted median | 4 | 0.0663900731402232 | 0.236312014310066 | 0.778754551030879 | -0.396781475 | 0.529561621187952 | 1.06864348499571 | 0.672480963542021 | 1.69818769591449 | 0.999332001504091 |
| ebi-fl187-GCST90094734 | ebi-met1400-GCST90200551 | X-18886 levels \|\| id:ebi-met1400-GCST90200551 | Chips liking \|\| id:ebi-fl187-GCST90094734 | Weighted median | 4 | -0.327225768 | 0.222835162732231 | 0.141977797273478 | -0.763982687 | 0.109531150868661 | 0.720920963705608 | 0.465807562086924 | 1.11575482712588 | 0.999332001504091 |
| ebi-fl187-GCST90094734 | ebi-met1400-GCST90200564 | X-21319 levels \|\| id:ebi-met1400-GCST90200564 | Chips liking \|\| id:ebi-fl187-GCST90094734 | Weighted median | 4 | -0.192468927 | 0.202274635312797 | 0.341339050759609 | -0.588927212 | 0.203989358126603 | 0.824919950471649 | 0.554922279321962 | 1.22628510341595 | 0.999332001504091 |
| ebi-fl187-GCST90094734 | ebi-met1400-GCST90200579 | X-21442 levels \|\| id:ebi-met1400-GCST90200579 | Chips liking \|\| id:ebi-fl187-GCST90094734 | Weighted median | 4 | -0.16640858 | 0.213772338223124 | 0.436310655319392 | -0.585402363 | 0.252585203045137 | 0.846700218839566 | 0.556881748234068 | 1.28734917755222 | 0.999332001504091 |
| ebi-fl187-GCST90094734 | ebi-met1400-GCST90200581 | X-21441 levels \|\| id:ebi-met1400-GCST90200581 | Chips liking \|\| id:ebi-fl187-GCST90094734 | Weighted median | 4 | 0.356256210068155 | 0.210212243926385 | 0.0901237502696636 | -0.055759788 | 0.76827220816387 | 1.42797336255174 | 0.945766292993084 | 2.15603784916473 | 0.999332001504091 |
| ebi-fl187-GCST90094734 | ebi-met1400-GCST90200648 | X-24585 levels \|\| id:ebi-met1400-GCST90200648 | Chips liking \|\| id:ebi-fl187-GCST90094734 | Weighted median | 4 | 0.0654106446762083 | 0.204935527624063 | 0.749592527118718 | -0.336262989 | 0.467084278819372 | 1.06759733754555 | 0.714435192177122 | 1.59533585077339 | 0.999332001504091 |
| ebi-fl187-GCST90094734 | ebi-met1400-GCST90200720 | Adenosine 5'-diphosphate (ADP) to pantothenate ratio \|\| id:ebi-met1400-GCST90200720 | Chips liking \|\| id:ebi-fl187-GCST90094734 | Weighted median | 4 | -0.344423292 | 0.300148182307271 | 0.25117055718675 | -0.932713729 | 0.243867145657943 | 0.708628907846186 | 0.393484450028603 | 1.27617477386661 | 0.999332001504091 |
| ebi-fl187-GCST90094734 | ebi-met1400-GCST90200724 | Cholate to taurocholate ratio \|\| id:ebi-met1400-GCST90200724 | Chips liking \|\| id:ebi-fl187-GCST90094734 | Weighted median | 4 | 0.143240116504308 | 0.222130923031247 | 0.519027340855662 | -0.292136493 | 0.578616725645553 | 1.15400686449525 | 0.746666614522623 | 1.78356955755092 | 0.999332001504091 |
| ebi-fl187-GCST90094734 | ebi-met1400-GCST90200726 | N-acetylputrescine to (N(1) + N(8))-acetylspermidine ratio \|\| id:ebi-met1400-GCST90200726 | Chips liking \|\| id:ebi-fl187-GCST90094734 | Weighted median | 4 | -0.027858102 | 0.191552934553535 | 0.884369078151331 | -0.403301853 | 0.347585649990931 | 0.972526356814417 | 0.668110397448472 | 1.41564555545129 | 0.999332001504091 |
| ebi-fl187-GCST90094734 | ebi-met1400-GCST90200744 | Arginine to glutamate ratio \|\| id:ebi-met1400-GCST90200744 | Chips liking \|\| id:ebi-fl187-GCST90094734 | Weighted median | 4 | -0.391479609 | 0.224424720512283 | 0.0810947021393054 | -0.831352061 | 0.0483928434409116 | 0.676055835968814 | 0.435460119486202 | 1.04958289610255 | 0.999332001504091 |
| ebi-fl187-GCST90094734 | ebi-met1400-GCST90200759 | Histidine to trans-urocanate ratio \|\| id:ebi-met1400-GCST90200759 | Chips liking \|\| id:ebi-fl187-GCST90094734 | Weighted median | 4 | 0.199353982225798 | 0.229197164910776 | 0.384413793794082 | -0.249872461 | 0.648580425450918 | 1.22061396508355 | 0.778900116879337 | 1.9128235051835 | 0.999332001504091 |
| ebi-fl187-GCST90094734 | ebi-met1400-GCST90200795 | Oleoyl-linoleoyl-glycerol (18:1 to 18:2) [2] to linoleoyl-arachidonoyl-glycerol (18:2 to 20:4) [2] ratio \|\| id:ebi-met1400-GCST90200795 | Chips liking \|\| id:ebi-fl187-GCST90094734 | Weighted median | 4 | 0.271027240984889 | 0.218932601945369 | 0.215735233784031 | -0.158080659 | 0.700135140797812 | 1.3113107912955 | 0.853780914223183 | 2.01402486600741 | 0.999332001504091 |
| ebi-fl187-GCST90094734 | ebi-met1400-GCST90200811 | Choline to taurocholate ratio \|\| id:ebi-met1400-GCST90200811 | Chips liking \|\| id:ebi-fl187-GCST90094734 | Weighted median | 4 | 0.104672523907792 | 0.235982675834479 | 0.657360583001287 | -0.357853521 | 0.567198568543371 | 1.11034693873558 | 0.699175482221861 | 1.76332030471324 | 0.999332001504091 |
| ebi-fl187-GCST90094734 | ebi-met1400-GCST90200831 | Cholate to phosphate ratio \|\| id:ebi-met1400-GCST90200831 | Chips liking \|\| id:ebi-fl187-GCST90094734 | Weighted median | 4 | 0.118840710854552 | 0.200052764115216 | 0.552480789930793 | -0.273262707 | 0.510944128520375 | 1.12619051407556 | 0.760892869630675 | 1.66686418629392 | 0.999332001504091 |
| ebi-fl187-GCST90094734 | ebi-met1400-GCST90200841 | Adenosine 5'-diphosphate (ADP) to oxalate (ethanedioate) ratio \|\| id:ebi-met1400-GCST90200841 | Chips liking \|\| id:ebi-fl187-GCST90094734 | Weighted median | 4 | 0.0518015181988416 | 0.302367678803643 | 0.863972447845553 | -0.540839132 | 0.644442168653981 | 1.0531666873462 | 0.58225945462978 | 1.90492410645529 | 0.999332001504091 |
| ebi-fl187-GCST90094734 | ebi-met1400-GCST90200844 | Aspartate to phosphate ratio \|\| id:ebi-met1400-GCST90200844 | Chips liking \|\| id:ebi-fl187-GCST90094734 | Weighted median | 4 | -0.144317049 | 0.220448640571299 | 0.512692111247898 | -0.576396385 | 0.287762286234439 | 0.865613262482554 | 0.561919664629604 | 1.33344028933316 | 0.999332001504091 |
| ebi-fl187-GCST90094734 | ebi-met1400-GCST90200870 | Adenosine 5'-monophosphate (AMP) to acetoacetate ratio \|\| id:ebi-met1400-GCST90200870 | Chips liking \|\| id:ebi-fl187-GCST90094734 | Weighted median | 4 | -0.17149331 | 0.221268581133869 | 0.438312529422979 | -0.605179729 | 0.262193109341098 | 0.842405903965156 | 0.545976290210134 | 1.29977751737575 | 0.999332001504091 |
| ebi-fl187-GCST90094734 | ebi-met1400-GCST90200887 | Cholesterol to cortisol ratio \|\| id:ebi-met1400-GCST90200887 | Chips liking \|\| id:ebi-fl187-GCST90094734 | Weighted median | 4 | -0.139193076 | 0.200527271686858 | 0.48759730526576 | -0.532226529 | 0.253840376188877 | 0.870060024254588 | 0.587295881649573 | 1.28896603817424 | 0.999332001504091 |
| ebi-fl187-GCST90094734 | ebi-met1400-GCST90200890 | Cortisol to taurocholate ratio \|\| id:ebi-met1400-GCST90200890 | Chips liking \|\| id:ebi-fl187-GCST90094734 | Weighted median | 4 | 0.280006800653821 | 0.24971915670416 | 0.262165791305004 | -0.209442746 | 0.769456347793975 | 1.32313881051585 | 0.811036072768721 | 2.15859241120651 | 0.999332001504091 |
| ebi-fl187-GCST90094734 | ebi-met1400-GCST90200948 | Adenosine 5'-diphosphate (ADP) to mannitol to sorbitol ratio \|\| id:ebi-met1400-GCST90200948 | Chips liking \|\| id:ebi-fl187-GCST90094734 | Weighted median | 4 | -0.342186574 | 0.323219247480013 | 0.289744382355345 | -0.975696299 | 0.29132315131353 | 0.710215684745497 | 0.376929806393186 | 1.33819695418927 | 0.999332001504091 |
| ebi-fl187-GCST90094734 | ebi-met1400-GCST90200972 | Leucine to N-palmitoyl-sphingosine (d18:1 to 16:0) ratio \|\| id:ebi-met1400-GCST90200972 | Chips liking \|\| id:ebi-fl187-GCST90094734 | Weighted median | 4 | 0.347433821915379 | 0.229975347383355 | 0.130853747535023 | -0.103317859 | 0.798185502786754 | 1.41543063702668 | 0.901840269911612 | 2.22150635215047 | 0.999332001504091 |
| ebi-fl187-GCST90094734 | ebi-met1400-GCST90200973 | Proline to glutamate ratio \|\| id:ebi-met1400-GCST90200973 | Chips liking \|\| id:ebi-fl187-GCST90094734 | Weighted median | 4 | -0.371144885 | 0.215763841848972 | 0.0854062635871506 | -0.794042015 | 0.051752245179785 | 0.689943971892045 | 0.452014050313708 | 1.05311479592238 | 0.999332001504091 |
| ebi-fl187-GCST90094734 | ebi-met1400-GCST90200983 | Cholesterol to linoleoyl-arachidonoyl-glycerol (18:2 to 20:4) [1] ratio \|\| id:ebi-met1400-GCST90200983 | Chips liking \|\| id:ebi-fl187-GCST90094734 | Weighted median | 4 | 0.466483161342285 | 0.252163269624206 | 0.0643243459052232 | -0.027756847 | 0.960723169805729 | 1.59437715468503 | 0.972624834579732 | 2.6135858565445 | 0.999332001504091 |
| ebi-fl187-GCST90094734 | ebi-met1400-GCST90201001 | Serine to alpha-ketobutyrate ratio \|\| id:ebi-met1400-GCST90201001 | Chips liking \|\| id:ebi-fl187-GCST90094734 | Weighted median | 4 | 0.0965357796953314 | 0.239401826969395 | 0.686773559686488 | -0.372691801 | 0.565763360555346 | 1.10134898637002 | 0.68887751137696 | 1.76079138852092 | 0.999332001504091 |
| ebi-fl187-GCST90094734 | ebi-met1400-GCST90201005 | Fructose to maltose ratio \|\| id:ebi-met1400-GCST90201005 | Chips liking \|\| id:ebi-fl187-GCST90094734 | Weighted median | 4 | -0.247094134 | 0.229825916636159 | 0.282313810319545 | -0.697552931 | 0.203364662671896 | 0.781067165144937 | 0.497801970548584 | 1.22551928791131 | 0.999332001504091 |
| ebi-fl187-GCST90094734 | ebi-met1400-GCST90201020 | Paraxanthine to 5-acetylamino-6-formylamino-3-methyluracil ratio \|\| id:ebi-met1400-GCST90201020 | Chips liking \|\| id:ebi-fl187-GCST90094734 | Weighted median | 4 | -0.112549607 | 0.22710746443647 | 0.620191729697166 | -0.557680237 | 0.332581023094922 | 0.893553019345195 | 0.572535671329292 | 1.39456288640869 | 0.999332001504091 |
| ebi-fl187-GCST90094734 | ebi-pw11-GCST90275197 | Placental weight (fetal genotype, adjusted for sex) \|\| id:ebi-pw11-GCST90275197 | Chips liking \|\| id:ebi-fl187-GCST90094734 | Weighted median | 4 | 0.137164600687341 | 0.0732484661350638 | 0.0611245412656629 | -0.006402393 | 0.280731594312066 | 1.14701693275849 | 0.993618058710506 | 1.32409816075818 | 0.999332001504091 |
| ebi-fl187-GCST90094734 | prot-a-1017 | FAS-associated factor 2 \|\| id:prot-a-1017 | Chips liking \|\| id:ebi-fl187-GCST90094734 | Weighted median | 4 | -0.065974815 | 0.326782157068408 | 0.84000088820443 | -0.706467843 | 0.574518212505593 | 0.936154440718087 | 0.493383832377934 | 1.77627453387017 | 0.999332001504091 |
| ebi-fl187-GCST90094734 | prot-a-1019 | Fas apoptotic inhibitory molecule 3 \|\| id:prot-a-1019 | Chips liking \|\| id:ebi-fl187-GCST90094734 | Weighted median | 4 | 0.190680361102057 | 0.323389914435869 | 0.555438805152189 | -0.443163871 | 0.82452459339636 | 1.21007260398518 | 0.642001992835248 | 2.28079620196947 | 0.999332001504091 |
| ebi-fl187-GCST90094734 | prot-a-1052 | MIP18 family protein FAM96A \|\| id:prot-a-1052 | Chips liking \|\| id:ebi-fl187-GCST90094734 | Weighted median | 4 | -0.038468405 | 0.316242783511669 | 0.903182547906987 | -0.658304261 | 0.581367450467914 | 0.96226210672396 | 0.51772852304087 | 1.78848242047452 | 0.999332001504091 |
| ebi-fl187-GCST90094734 | prot-a-1066 | F-box/LRR-repeat protein 4 \|\| id:prot-a-1066 | Chips liking \|\| id:ebi-fl187-GCST90094734 | Weighted median | 4 | -0.252670277 | 0.329005503689298 | 0.442497928026022 | -0.897521065 | 0.392180509749252 | 0.7767239429813 | 0.407578769859419 | 1.48020487869991 | 0.999332001504091 |
| ebi-fl187-GCST90094734 | prot-a-1076 | Low affinity immunoglobulin gamma Fc region receptor III-B \|\| id:prot-a-1076 | Chips liking \|\| id:ebi-fl187-GCST90094734 | Weighted median | 4 | -0.308283852 | 0.351378526893022 | 0.380293623974625 | -0.996985765 | 0.38041806082058 | 0.73470674051121 | 0.368989989291824 | 1.4628960411327 | 0.999332001504091 |
| ebi-fl187-GCST90094734 | prot-a-1081 | Fc receptor-like protein 3 \|\| id:prot-a-1081 | Chips liking \|\| id:ebi-fl187-GCST90094734 | Weighted median | 4 | 0.254004866300533 | 0.308593737479712 | 0.410448621041838 | -0.350838859 | 0.858848591760769 | 1.28917807778051 | 0.704097203529715 | 2.36044129688082 | 0.999332001504091 |
| ebi-fl187-GCST90094734 | prot-a-1105 | Fibroblast growth factor receptor 4 \|\| id:prot-a-1105 | Chips liking \|\| id:ebi-fl187-GCST90094734 | Weighted median | 4 | -0.032007235 | 0.295070488519336 | 0.913620351342568 | -0.610345393 | 0.546330922213095 | 0.968499574683592 | 0.543163232011955 | 1.72690523010522 | 0.999332001504091 |
| ebi-fl187-GCST90094734 | prot-a-1143 | Follistatin-related protein 1 \|\| id:prot-a-1143 | Chips liking \|\| id:ebi-fl187-GCST90094734 | Weighted median | 4 | 0.0514038697281284 | 0.31410296720623 | 0.870004377210038 | -0.564237946 | 0.66704568545234 | 1.05274798047816 | 0.568793432955817 | 1.94847240876451 | 0.999332001504091 |
| ebi-fl187-GCST90094734 | prot-a-1151 | Alpha-(1,3)-fucosyltransferase 10 \|\| id:prot-a-1151 | Chips liking \|\| id:ebi-fl187-GCST90094734 | Weighted median | 4 | -0.115499715 | 0.31377374349519 | 0.712799622218038 | -0.730496252 | 0.499496822610775 | 0.890920826470391 | 0.481669901171956 | 1.64789188011837 | 0.999332001504091 |
| ebi-fl187-GCST90094734 | prot-a-1165 | Growth arrest and DNA damage-inducible proteins-interacting protein 1 \|\| id:prot-a-1165 | Chips liking \|\| id:ebi-fl187-GCST90094734 | Weighted median | 4 | 0.0864909467538662 | 0.328909634816168 | 0.792579372900598 | -0.558171937 | 0.731153830993556 | 1.09034149654316 | 0.572254224744861 | 2.07747628182914 | 0.999332001504091 |
| ebi-fl187-GCST90094734 | prot-a-1194 | Growth/differentiation factor 11/8 \|\| id:prot-a-1194 | Chips liking \|\| id:ebi-fl187-GCST90094734 | Weighted median | 4 | -0.110869425 | 0.310535542418106 | 0.72107195084004 | -0.719519088 | 0.497780238500725 | 0.895055613509109 | 0.486986397365046 | 1.64506556160262 | 0.999332001504091 |
| ebi-fl187-GCST90094734 | prot-a-1216 | D-glucuronyl C5-epimerase \|\| id:prot-a-1216 | Chips liking \|\| id:ebi-fl187-GCST90094734 | Weighted median | 4 | 0.685871080050165 | 0.361429832733731 | 0.0577408326245831 | -0.022531392 | 1.39427355220828 | 1.9855006124494 | 0.977720544002716 | 4.03204443868778 | 0.999332001504091 |
| ebi-fl187-GCST90094734 | prot-a-1246 | Glypican-3 \|\| id:prot-a-1246 | Chips liking \|\| id:ebi-fl187-GCST90094734 | Weighted median | 4 | -0.199443588 | 0.333571521446037 | 0.549904308853222 | -0.85324377 | 0.454356594099667 | 0.819186431508069 | 0.426030742438147 | 1.5751595899546 | 0.999332001504091 |
| ebi-fl187-GCST90094734 | prot-a-1275 | Glutamate receptor 4 \|\| id:prot-a-1275 | Chips liking \|\| id:ebi-fl187-GCST90094734 | Weighted median | 4 | 0.382884702431196 | 0.339397159067399 | 0.259264292801862 | -0.282333729 | 1.0481031342033 | 1.4665089354056 | 0.754022003272534 | 2.85223567520634 | 0.999332001504091 |
| ebi-fl187-GCST90094734 | prot-a-1289 | Glutathione S-transferase P \|\| id:prot-a-1289 | Chips liking \|\| id:ebi-fl187-GCST90094734 | Weighted median | 4 | -0.1355007 | 0.371639484050681 | 0.715408118980778 | -0.863914088 | 0.592912689110111 | 0.873278551957071 | 0.421509025748494 | 1.8092505325456 | 0.999332001504091 |
| ebi-fl187-GCST90094734 | prot-a-130 | Apolipoprotein D \|\| id:prot-a-130 | Chips liking \|\| id:ebi-fl187-GCST90094734 | Weighted median | 4 | -0.117100668 | 0.306313021578098 | 0.702245633703273 | -0.717474191 | 0.483272853950741 | 0.889495644604082 | 0.487983253344224 | 1.62137224248455 | 0.999332001504091 |
| ebi-fl187-GCST90094734 | prot-a-1313 | Heparin-binding EGF-like growth factor \|\| id:prot-a-1313 | Chips liking \|\| id:ebi-fl187-GCST90094734 | Weighted median | 4 | 0.351347875265309 | 0.320536659086382 | 0.273024580773763 | -0.276903977 | 0.979599727074618 | 1.42098156427923 | 0.758127291618092 | 2.66338994565391 | 0.999332001504091 |
| ebi-fl187-GCST90094734 | prot-a-1347 | HLA class II histocompatibility antigen, DQ alpha 2 chain \|\| id:prot-a-1347 | Chips liking \|\| id:ebi-fl187-GCST90094734 | Weighted median | 4 | 0.151806460725895 | 0.350752303859668 | 0.665158284453313 | -0.535668055 | 0.839280976290844 | 1.16393494750684 | 0.585278161603631 | 2.31470205263738 | 0.999332001504091 |
| ebi-fl187-GCST90094734 | prot-a-1357 | Non-histone chromosomal protein HMG-14 \|\| id:prot-a-1357 | Chips liking \|\| id:ebi-fl187-GCST90094734 | Weighted median | 4 | 0.35965817992388 | 0.329303716676811 | 0.274754996855242 | -0.285777105 | 1.00509346461043 | 1.43283955751701 | 0.751430087469574 | 2.73216262140775 | 0.999332001504091 |
| ebi-fl187-GCST90094734 | prot-a-137 | Apolipoprotein M \|\| id:prot-a-137 | Chips liking \|\| id:ebi-fl187-GCST90094734 | Weighted median | 4 | -0.017108137 | 0.295601030052029 | 0.953847583217109 | -0.596486156 | 0.562269881569259 | 0.983037375847253 | 0.550743466527297 | 1.75465083300222 | 0.999332001504091 |
| ebi-fl187-GCST90094734 | prot-a-1381 | 17-beta-hydroxysteroid dehydrogenase 14 \|\| id:prot-a-1381 | Chips liking \|\| id:ebi-fl187-GCST90094734 | Weighted median | 4 | -0.28916624 | 0.310528662602188 | 0.351746870869724 | -0.897802419 | 0.319469938493274 | 0.748887699807792 | 0.40746411199342 | 1.37639799534655 | 0.999332001504091 |
| ebi-fl187-GCST90094734 | prot-a-1392 | Serine protease HTRA2, mitochondrial \|\| id:prot-a-1392 | Chips liking \|\| id:ebi-fl187-GCST90094734 | Weighted median | 4 | 0.355113177758535 | 0.36914066004663 | 0.336049790644286 | -0.368402516 | 1.07862887144993 | 1.42634207534566 | 0.691838649556266 | 2.9406447835868 | 0.999332001504091 |
| ebi-fl187-GCST90094734 | prot-a-1416 | Interferon-induced protein with tetratricopeptide repeats 2 \|\| id:prot-a-1416 | Chips liking \|\| id:ebi-fl187-GCST90094734 | Weighted median | 4 | 0.239869080202034 | 0.339734202133076 | 0.480157507527258 | -0.426009956 | 0.905748116382862 | 1.27108272953361 | 0.653109839740592 | 2.47378190774207 | 0.999332001504091 |
| ebi-fl187-GCST90094734 | prot-a-1428 | Interferon gamma \|\| id:prot-a-1428 | Chips liking \|\| id:ebi-fl187-GCST90094734 | Weighted median | 4 | 0.0346318625846988 | 0.318451609928261 | 0.913400160032447 | -0.589533293 | 0.658797018044091 | 1.03523852860444 | 0.554586053607986 | 1.93246621355654 | 0.999332001504091 |
| ebi-fl187-GCST90094734 | prot-a-1483 | Interleukin-17C \|\| id:prot-a-1483 | Chips liking \|\| id:ebi-fl187-GCST90094734 | Weighted median | 4 | -0.128612718 | 0.312507262202701 | 0.680668454964766 | -0.741126952 | 0.483901515875635 | 0.879314442230274 | 0.476576533917794 | 1.62239185794261 | 0.999332001504091 |
| ebi-fl187-GCST90094734 | prot-a-1503 | Interleukin-1 receptor-like 2 \|\| id:prot-a-1503 | Chips liking \|\| id:ebi-fl187-GCST90094734 | Weighted median | 4 | 0.37633540003676 | 0.321614014654085 | 0.241942160398951 | -0.254028069 | 1.00669886875877 | 1.45693570812264 | 0.775670029702998 | 2.73655236933104 | 0.999332001504091 |
| ebi-fl187-GCST90094734 | prot-a-1522 | Interleukin-31 receptor subunit alpha \|\| id:prot-a-1522 | Chips liking \|\| id:ebi-fl187-GCST90094734 | Weighted median | 4 | 0.431214809507158 | 0.373008292753579 | 0.247662271019439 | -0.299881444 | 1.16231106330417 | 1.53912613337553 | 0.740906054118432 | 3.19731393915812 | 0.999332001504091 |
| ebi-fl187-GCST90094734 | prot-a-1540 | Interleukin-6 receptor subunit alpha \|\| id:prot-a-1540 | Chips liking \|\| id:ebi-fl187-GCST90094734 | Weighted median | 4 | -0.097884862 | 0.329632835625758 | 0.766503775620132 | -0.74396522 | 0.548195495792862 | 0.906753299474756 | 0.475225799818514 | 1.73012817574793 | 0.999332001504091 |
| ebi-fl187-GCST90094734 | prot-a-1541 | Interleukin-6 receptor subunit alpha \|\| id:prot-a-1541 | Chips liking \|\| id:ebi-fl187-GCST90094734 | Weighted median | 4 | -0.187881409 | 0.325259292549387 | 0.563510037081347 | -0.825389622 | 0.449626804400805 | 0.828712979309723 | 0.438064279557525 | 1.5677270074841 | 0.999332001504091 |
| ebi-fl187-GCST90094734 | prot-a-1545 | Interleukin-9 \|\| id:prot-a-1545 | Chips liking \|\| id:ebi-fl187-GCST90094734 | Weighted median | 4 | 0.447508922225477 | 0.324795316656311 | 0.168259402866459 | -0.189089898 | 1.08410774287185 | 1.56441025987924 | 0.827712093339817 | 2.95680041515432 | 0.999332001504091 |
| ebi-fl187-GCST90094734 | prot-a-1548 | Inositol monophosphatase 3 \|\| id:prot-a-1548 | Chips liking \|\| id:ebi-fl187-GCST90094734 | Weighted median | 4 | 0.137459786489123 | 0.38534275666065 | 0.721300812963691 | -0.617812017 | 0.892731589543998 | 1.14735556584882 | 0.539122739695345 | 2.4417905192205 | 0.999332001504091 |
| ebi-fl187-GCST90094734 | prot-a-1555 | Inhibin beta A chain:Inhibin beta B chain heterodimer \|\| id:prot-a-1555 | Chips liking \|\| id:ebi-fl187-GCST90094734 | Weighted median | 4 | 0.435359876718418 | 0.31402675710269 | 0.165631423835639 | -0.180132567 | 1.05085232063969 | 1.54551915524412 | 0.835159489314939 | 2.86008779135809 | 0.999332001504091 |
| ebi-fl187-GCST90094734 | prot-a-1603 | Junctophilin-3 \|\| id:prot-a-1603 | Chips liking \|\| id:ebi-fl187-GCST90094734 | Weighted median | 4 | 0.410460101298058 | 0.343444545727824 | 0.232037128127116 | -0.262691208 | 1.08361141092459 | 1.50751123344773 | 0.768979315061027 | 2.95533322478347 | 0.999332001504091 |
| ebi-fl187-GCST90094734 | prot-a-1627 | UPF0258 protein KIAA1024 \|\| id:prot-a-1627 | Chips liking \|\| id:ebi-fl187-GCST90094734 | Weighted median | 4 | 0.525067106157611 | 0.32243682037083 | 0.103432876754893 | -0.106909062 | 1.15704327408444 | 1.69057229238336 | 0.898607387040731 | 3.18051544755973 | 0.999332001504091 |
| ebi-fl187-GCST90094734 | prot-a-1666 | Kallikrein-7 \|\| id:prot-a-1666 | Chips liking \|\| id:ebi-fl187-GCST90094734 | Weighted median | 4 | 0.351898830081028 | 0.317377955983916 | 0.267529858883102 | -0.270161964 | 0.973959623809504 | 1.42176467662507 | 0.763255864621593 | 2.64841043403049 | 0.999332001504091 |
| ebi-fl187-GCST90094734 | prot-a-1678 | Importin subunit alpha-3 \|\| id:prot-a-1678 | Chips liking \|\| id:ebi-fl187-GCST90094734 | Weighted median | 4 | 0.409340493935034 | 0.324765534824649 | 0.207518425075054 | -0.227199954 | 1.04588094219135 | 1.50582435726663 | 0.79676145046608 | 2.84590449702484 | 0.999332001504091 |
| ebi-fl187-GCST90094734 | prot-a-1718 | Protein LDOC1 \|\| id:prot-a-1718 | Chips liking \|\| id:ebi-fl187-GCST90094734 | Weighted median | 4 | -0.007669181 | 0.346698623015576 | 0.982351754440841 | -0.687198483 | 0.671860119710516 | 0.992360151736711 | 0.502983213345161 | 1.9578758189672 | 0.999332001504091 |
| ebi-fl187-GCST90094734 | prot-a-1723 | Leptin \|\| id:prot-a-1723 | Chips liking \|\| id:ebi-fl187-GCST90094734 | Weighted median | 4 | -0.012059114 | 0.376917932771769 | 0.974476832419527 | -0.750818262 | 0.726700034314634 | 0.988013305798038 | 0.471980191163735 | 2.0682441990353 | 0.999332001504091 |
| ebi-fl187-GCST90094734 | prot-a-1730 | Galectin-7 \|\| id:prot-a-1730 | Chips liking \|\| id:ebi-fl187-GCST90094734 | Weighted median | 4 | -0.511925006 | 0.309663128008282 | 0.0982967874155634 | -1.118864737 | 0.0950147247567488 | 0.599340733035546 | 0.326650418327746 | 1.09967504745447 | 0.999332001504091 |
| ebi-fl187-GCST90094734 | prot-a-1753 | Lipase member N \|\| id:prot-a-1753 | Chips liking \|\| id:ebi-fl187-GCST90094734 | Weighted median | 4 | 0.145362089485109 | 0.339781301257826 | 0.668788917966557 | -0.520609261 | 0.811333439950449 | 1.15645823583341 | 0.594158440118665 | 2.25090743633941 | 0.999332001504091 |
| ebi-fl187-GCST90094734 | prot-a-1756 | VIP36-like protein \|\| id:prot-a-1756 | Chips liking \|\| id:ebi-fl187-GCST90094734 | Weighted median | 4 | 0.291503400866574 | 0.317942909498346 | 0.359225460467182 | -0.331664702 | 0.914671503483333 | 1.3384381853324 | 0.717727935434296 | 2.49595520463045 | 0.999332001504091 |
| ebi-fl187-GCST90094734 | prot-a-1760 | Ig Kappa chain V-I region HK102- like \|\| id:prot-a-1760 | Chips liking \|\| id:ebi-fl187-GCST90094734 | Weighted median | 4 | 0.123765111604169 | 0.308476715984181 | 0.688262730535613 | -0.480849252 | 0.728379474933163 | 1.13175000483284 | 0.618258112022308 | 2.07172061074861 | 0.999332001504091 |
| ebi-fl187-GCST90094734 | prot-a-179 | Neutral ceramidase \|\| id:prot-a-179 | Chips liking \|\| id:ebi-fl187-GCST90094734 | Weighted median | 4 | -0.022730549 | 0.363951591463155 | 0.950200602070987 | -0.736075668 | 0.690614570763485 | 0.977525844091046 | 0.478989947715269 | 1.99494118910807 | 0.999332001504091 |
| ebi-fl187-GCST90094734 | prot-a-1809 | Lactotransferrin \|\| id:prot-a-1809 | Chips liking \|\| id:ebi-fl187-GCST90094734 | Weighted median | 4 | 0.318678037607825 | 0.310591595811462 | 0.304874798968745 | -0.29008149 | 0.92743756539829 | 1.37530845601582 | 0.748202593928191 | 2.5280229773838 | 0.999332001504091 |
| ebi-fl187-GCST90094734 | prot-a-1818 | Lysophospholipase-like protein 1 \|\| id:prot-a-1818 | Chips liking \|\| id:ebi-fl187-GCST90094734 | Weighted median | 4 | 0.132826726845437 | 0.318801779249275 | 0.676939099971028 | -0.49202476 | 0.757678214174016 | 1.14205209421661 | 0.611387227401855 | 2.13331736000967 | 0.999332001504091 |
| ebi-fl187-GCST90094734 | prot-a-1852 | MAP kinase-activated protein kinase 3 \|\| id:prot-a-1852 | Chips liking \|\| id:ebi-fl187-GCST90094734 | Weighted median | 4 | 0.378404604798101 | 0.312447976344005 | 0.225858478800665 | -0.233993429 | 0.990802638432351 | 1.4599535275933 | 0.791367016080828 | 2.69339542768413 | 0.999332001504091 |
| ebi-fl187-GCST90094734 | prot-a-1856 | Megakaryocyte-associated tyrosine-protein kinase \|\| id:prot-a-1856 | Chips liking \|\| id:ebi-fl187-GCST90094734 | Weighted median | 4 | 0.270637947217846 | 0.34309132257541 | 0.430216110688981 | -0.401821045 | 0.943096939465649 | 1.31080040552924 | 0.669100473832305 | 2.56792181493245 | 0.999332001504091 |
| ebi-fl187-GCST90094734 | prot-a-1890 | Beta-1,3-N-acetylglucosaminyltransferase manic fringe \|\| id:prot-a-1890 | Chips liking \|\| id:ebi-fl187-GCST90094734 | Weighted median | 4 | -0.11283975 | 0.360336932879032 | 0.754166198119071 | -0.819100139 | 0.59342063822055 | 0.893293798779367 | 0.440828160295845 | 1.81016977318813 | 0.999332001504091 |
| ebi-fl187-GCST90094734 | prot-a-1919 | Matrilysin \|\| id:prot-a-1919 | Chips liking \|\| id:ebi-fl187-GCST90094734 | Weighted median | 4 | 1.02686693687882 | 0.32936300845438 | 0.0018224591788544 | 0.381315440308234 | 1.6724184334494 | 2.79230365203559 | 1.46420940323655 | 5.32503046895927 | 0.999332001504091 |
| ebi-fl187-GCST90094734 | prot-a-1923 | Multimerin-2 \|\| id:prot-a-1923 | Chips liking \|\| id:ebi-fl187-GCST90094734 | Weighted median | 4 | -0.199450632 | 0.319223988942734 | 0.532103392982668 | -0.82512965 | 0.426228386653675 | 0.81918066139255 | 0.438178178980382 | 1.53147050261848 | 0.999332001504091 |
| ebi-fl187-GCST90094734 | prot-a-1948 | Mesothelin \|\| id:prot-a-1948 | Chips liking \|\| id:ebi-fl187-GCST90094734 | Weighted median | 4 | -0.224558651 | 0.3062527875308 | 0.463408364006182 | -0.824814115 | 0.375696812099691 | 0.798868720508497 | 0.438316461338987 | 1.45600562355635 | 0.999332001504091 |
| ebi-fl187-GCST90094734 | prot-a-195 | ATPase family AAA domain-containing protein 1 \|\| id:prot-a-195 | Chips liking \|\| id:ebi-fl187-GCST90094734 | Weighted median | 4 | 0.478955196653988 | 0.318870164122099 | 0.133087219896976 | -0.146030325 | 1.1039407183333 | 1.61438680400771 | 0.864131497977747 | 3.01602795298332 | 0.999332001504091 |
| ebi-fl187-GCST90094734 | prot-a-1966 | Mucin-1 \|\| id:prot-a-1966 | Chips liking \|\| id:ebi-fl187-GCST90094734 | Weighted median | 4 | 0.175807936845263 | 0.34265167158977 | 0.607894917719903 | -0.495789339 | 0.847405213161213 | 1.19220905722737 | 0.609089938767693 | 2.33358383658523 | 0.999332001504091 |
| ebi-fl187-GCST90094734 | prot-a-1986 | Myocardial zonula adherens protein \|\| id:prot-a-1986 | Chips liking \|\| id:ebi-fl187-GCST90094734 | Weighted median | 4 | 0.36862690163239 | 0.299118033412377 | 0.21780739350278 | -0.217644444 | 0.954898247120649 | 1.44574809678154 | 0.804411404242859 | 2.59840617415763 | 0.999332001504091 |
| ebi-fl187-GCST90094734 | prot-a-2071 | Epididymal secretory protein E1 \|\| id:prot-a-2071 | Chips liking \|\| id:ebi-fl187-GCST90094734 | Weighted median | 4 | 0.430510257809605 | 0.344397486672121 | 0.211285335027161 | -0.244508816 | 1.10552933168696 | 1.5380421213616 | 0.783089084568508 | 3.02082306304392 | 0.999332001504091 |
| ebi-fl187-GCST90094734 | prot-a-2098 | Neuritin \|\| id:prot-a-2098 | Chips liking \|\| id:ebi-fl187-GCST90094734 | Weighted median | 4 | 0.144605860076449 | 0.331179396520078 | 0.662373131410536 | -0.504505757 | 0.793717477255803 | 1.1555840187016 | 0.603803927494175 | 2.21160274630944 | 0.999332001504091 |
| ebi-fl187-GCST90094734 | prot-a-2115 | Neurotrophin-4 \|\| id:prot-a-2115 | Chips liking \|\| id:ebi-fl187-GCST90094734 | Weighted median | 4 | -0.192178881 | 0.316334645048648 | 0.54350743138967 | -0.812194785 | 0.427837023346601 | 0.825159250019498 | 0.443882768978984 | 1.53393606482836 | 0.999332001504091 |
| ebi-fl187-GCST90094734 | prot-a-2118 | Netrin-1 \|\| id:prot-a-2118 | Chips liking \|\| id:ebi-fl187-GCST90094734 | Weighted median | 4 | 0.354252426469008 | 0.313927100044264 | 0.259128052164695 | -0.26104469 | 0.969549542555764 | 1.42511487779671 | 0.770246496824399 | 2.63675644522995 | 0.999332001504091 |
| ebi-fl187-GCST90094734 | prot-a-2120 | Netrin-G1 \|\| id:prot-a-2120 | Chips liking \|\| id:ebi-fl187-GCST90094734 | Weighted median | 4 | 0.21773862811933 | 0.320287216083486 | 0.496616517775573 | -0.410024315 | 0.845501571642963 | 1.24326207140271 | 0.663634113408358 | 2.32914575510591 | 0.999332001504091 |
| ebi-fl187-GCST90094734 | prot-a-2145 | Olfactomedin-like protein 3 \|\| id:prot-a-2145 | Chips liking \|\| id:ebi-fl187-GCST90094734 | Weighted median | 4 | 0.414591856799584 | 0.339925668315811 | 0.222595892695703 | -0.251662453 | 1.08084616669857 | 1.51375278668677 | 0.777507138905087 | 2.94717229532946 | 0.999332001504091 |
| ebi-fl187-GCST90094734 | prot-a-2162 | Prolyl 4-hydroxylase subunit alpha-2 \|\| id:prot-a-2162 | Chips liking \|\| id:ebi-fl187-GCST90094734 | Weighted median | 4 | 0.55328305754272 | 0.325651561657767 | 0.0893185736188258 | -0.084994003 | 1.19156011839194 | 1.73895273839686 | 0.918517792454615 | 3.29221344563924 | 0.999332001504091 |
| ebi-fl187-GCST90094734 | prot-a-2168 | Polyadenylate-binding protein-interacting protein 1 \|\| id:prot-a-2168 | Chips liking \|\| id:ebi-fl187-GCST90094734 | Weighted median | 4 | 0.369791563682299 | 0.330791609620745 | 0.263610191497325 | -0.278559991 | 1.01814311853896 | 1.44743288563797 | 0.756872860696153 | 2.76805004806128 | 0.999332001504091 |
| ebi-fl187-GCST90094734 | prot-a-219 | Beta-1,4-galactosyltransferase 5 \|\| id:prot-a-219 | Chips liking \|\| id:ebi-fl187-GCST90094734 | Weighted median | 4 | -0.020761828 | 0.313040206885525 | 0.947120513480806 | -0.634320634 | 0.59279697745175 | 0.979452214841457 | 0.530295631035147 | 1.80904119327782 | 0.999332001504091 |
| ebi-fl187-GCST90094734 | prot-a-2190 | Pyruvate carboxylase, mitochondrial \|\| id:prot-a-2190 | Chips liking \|\| id:ebi-fl187-GCST90094734 | Weighted median | 4 | 0.475855457632798 | 0.338819681843455 | 0.160184754213114 | -0.188231119 | 1.13994203404597 | 1.60939037405112 | 0.828423220941037 | 3.12658712432779 | 0.999332001504091 |
| ebi-fl187-GCST90094734 | prot-a-2197 | Protocadherin-9 \|\| id:prot-a-2197 | Chips liking \|\| id:ebi-fl187-GCST90094734 | Weighted median | 4 | -0.070576339 | 0.324408583904149 | 0.827776738112627 | -0.706417164 | 0.565264484968343 | 0.931856599358673 | 0.493408837342346 | 1.75991319175706 | 0.999332001504091 |
| ebi-fl187-GCST90094734 | prot-a-2198 | Protocadherin alpha-4 \|\| id:prot-a-2198 | Chips liking \|\| id:ebi-fl187-GCST90094734 | Weighted median | 4 | -1.083120213 | 0.349089943037064 | 0.00191766064183848 | -1.767336502 | -0.398903925 | 0.338537566492453 | 0.170787275154232 | 0.671055169790222 | 0.999332001504091 |
| ebi-fl187-GCST90094734 | prot-a-2254 | Placenta growth factor \|\| id:prot-a-2254 | Chips liking \|\| id:ebi-fl187-GCST90094734 | Weighted median | 4 | 0.16852264830281 | 0.332219712151603 | 0.611970548495273 | -0.482627988 | 0.819673284119953 | 1.18355503205407 | 0.617159371665391 | 2.26975815034693 | 0.999332001504091 |
| ebi-fl187-GCST90094734 | prot-a-2261 | Membrane-associated progesterone receptor component 2 \|\| id:prot-a-2261 | Chips liking \|\| id:ebi-fl187-GCST90094734 | Weighted median | 4 | -0.366830038 | 0.322518267595872 | 0.255374009098006 | -0.998965842 | 0.265305766668097 | 0.692927406489331 | 0.368260083313436 | 1.30382958246214 | 0.999332001504091 |
| ebi-fl187-GCST90094734 | prot-a-2268 | Polymeric immunoglobulin receptor \|\| id:prot-a-2268 | Chips liking \|\| id:ebi-fl187-GCST90094734 | Weighted median | 4 | 0.076277862747493 | 0.304339199009021 | 0.80209680794275 | -0.520226967 | 0.672782692805174 | 1.07926241931855 | 0.594385626552525 | 1.95968293599105 | 0.999332001504091 |
| ebi-fl187-GCST90094734 | prot-a-2283 | Group 10 secretory phospholipase A2 \|\| id:prot-a-2283 | Chips liking \|\| id:ebi-fl187-GCST90094734 | Weighted median | 4 | -0.028814195 | 0.400991427579243 | 0.942715404845917 | -0.814757393 | 0.757129002998022 | 0.971596975216505 | 0.442746727738308 | 2.13214603995408 | 0.999332001504091 |
| ebi-fl187-GCST90094734 | prot-a-2298 | Inactive phospholipase D5 \|\| id:prot-a-2298 | Chips liking \|\| id:ebi-fl187-GCST90094734 | Weighted median | 4 | 0.621771929755914 | 0.306391082126786 | 0.042423588358824 | 0.0212454087874144 | 1.22229845072441 | 1.86222485175299 | 1.02147269925721 | 3.39498197162607 | 0.999332001504091 |
| ebi-fl187-GCST90094734 | prot-a-2304 | Procollagen-lysine,2-oxoglutarate 5-dioxygenase 2 \|\| id:prot-a-2304 | Chips liking \|\| id:ebi-fl187-GCST90094734 | Weighted median | 4 | -0.294208192 | 0.333798553691066 | 0.378104361633779 | -0.948453357 | 0.360036973251322 | 0.745121347003002 | 0.387339636467983 | 1.43338241038872 | 0.999332001504091 |
| ebi-fl187-GCST90094734 | prot-a-2331 | Periostin \|\| id:prot-a-2331 | Chips liking \|\| id:ebi-fl187-GCST90094734 | Weighted median | 4 | 0.216123175421783 | 0.314740196725414 | 0.492289655526308 | -0.40076761 | 0.833013961003596 | 1.24125526172519 | 0.669805698992095 | 2.30024114019765 | 0.999332001504091 |
| ebi-fl187-GCST90094734 | prot-a-2333 | Periostin \|\| id:prot-a-2333 | Chips liking \|\| id:ebi-fl187-GCST90094734 | Weighted median | 4 | 0.000245574383975137 | 0.315891273839266 | 0.99937972332257 | -0.618901322 | 0.619392471108935 | 1.00024560453983 | 0.538535789923362 | 1.85779903234218 | 0.99994023009904 |
| ebi-fl187-GCST90094734 | prot-a-2367 | cAMP-dependent protein kinase catalytic subunit alpha \|\| id:prot-a-2367 | Chips liking \|\| id:ebi-fl187-GCST90094734 | Weighted median | 4 | 0.0798685483503165 | 0.315714536851286 | 0.80028590833732 | -0.538931944 | 0.698669040578837 | 1.08314467716177 | 0.583370992714021 | 2.01107426717564 | 0.999332001504091 |
| ebi-fl187-GCST90094734 | prot-a-2385 | Phosphoribosyl pyrophosphate synthase-associated protein 1 \|\| id:prot-a-2385 | Chips liking \|\| id:ebi-fl187-GCST90094734 | Weighted median | 4 | -0.650750418 | 0.336806105572186 | 0.0533444746106485 | -1.310890385 | 0.00938954907208511 | 0.521654171244193 | 0.269579919638518 | 1.00943376918191 | 0.999332001504091 |
| ebi-fl187-GCST90094734 | prot-a-2393 | Trypsin-3 \|\| id:prot-a-2393 | Chips liking \|\| id:ebi-fl187-GCST90094734 | Weighted median | 4 | -0.099033388 | 0.320400206886825 | 0.757250958329307 | -0.727017793 | 0.528951017845958 | 0.905712467905839 | 0.48334828745586 | 1.69715109334074 | 0.999332001504091 |
| ebi-fl187-GCST90094734 | prot-a-2405 | Pregnancy-specific beta-1-glycoprotein 6 \|\| id:prot-a-2405 | Chips liking \|\| id:ebi-fl187-GCST90094734 | Weighted median | 4 | 0.405127398201095 | 0.316299445957568 | 0.200251630569867 | -0.214819516 | 1.02507431227793 | 1.49949352066575 | 0.806687021239979 | 2.78730258367413 | 0.999332001504091 |
| ebi-fl187-GCST90094734 | prot-a-244 | Beclin-1 \|\| id:prot-a-244 | Chips liking \|\| id:ebi-fl187-GCST90094734 | Weighted median | 4 | -0.104751627 | 0.307318993977982 | 0.733211734217344 | -0.707096856 | 0.497593600698951 | 0.900548166212962 | 0.493073585368784 | 1.64475855883248 | 0.999332001504091 |
| ebi-fl187-GCST90094734 | prot-a-2454 | tRNA pseudouridine synthase A, mitochondrial \|\| id:prot-a-2454 | Chips liking \|\| id:ebi-fl187-GCST90094734 | Weighted median | 4 | -0.199943372 | 0.334948947979084 | 0.550549539607554 | -0.85644331 | 0.456556566267532 | 0.81877711766293 | 0.424669818430216 | 1.57862871179902 | 0.999332001504091 |
| ebi-fl187-GCST90094734 | prot-a-2466 | Glutaminyl-peptide cyclotransferase-like protein \|\| id:prot-a-2466 | Chips liking \|\| id:ebi-fl187-GCST90094734 | Weighted median | 4 | 0.276366601613952 | 0.310450842490894 | 0.373352864399595 | -0.33211705 | 0.884850252896104 | 1.31833107774925 | 0.717403346116187 | 2.42262158375552 | 0.999332001504091 |
| ebi-fl187-GCST90094734 | prot-a-2474 | Ras-related protein Rab-27A \|\| id:prot-a-2474 | Chips liking \|\| id:ebi-fl187-GCST90094734 | Weighted median | 4 | 0.159406841673546 | 0.410971481585742 | 0.698106237862911 | -0.646097262 | 0.9649109455816 | 1.17281499964884 | 0.524087165440551 | 2.62455391794428 | 0.999332001504091 |
| ebi-fl187-GCST90094734 | prot-a-2490 | Rap1 GTPase-GDP dissociation stimulator 1 \|\| id:prot-a-2490 | Chips liking \|\| id:ebi-fl187-GCST90094734 | Weighted median | 4 | 0.145460399724147 | 0.334148287934374 | 0.663332413324538 | -0.509470245 | 0.80039104407552 | 1.15657193310773 | 0.600813778848117 | 2.22641138326938 | 0.999332001504091 |
| ebi-fl187-GCST90094734 | prot-a-2545 | Receptor-interacting serine/threonine-protein kinase 2 \|\| id:prot-a-2545 | Chips liking \|\| id:ebi-fl187-GCST90094734 | Weighted median | 4 | 0.751713875031112 | 0.339864255634349 | 0.0269800570668548 | 0.0855799339877877 | 1.41784781607444 | 2.12063140106269 | 1.08934863384439 | 4.12822617062693 | 0.999332001504091 |
| ebi-fl187-GCST90094734 | prot-a-2610 | RUN and FYVE domain-containing protein 1 \|\| id:prot-a-2610 | Chips liking \|\| id:ebi-fl187-GCST90094734 | Weighted median | 4 | 0.642738263024061 | 0.320552441501967 | 0.0449528367902158 | 0.0144554776802062 | 1.27102104836792 | 1.90168105925354 | 1.01456046336043 | 3.56449022184981 | 0.999332001504091 |
| ebi-fl187-GCST90094734 | prot-a-2627 | Serine--tRNA ligase, mitochondrial \|\| id:prot-a-2627 | Chips liking \|\| id:ebi-fl187-GCST90094734 | Weighted median | 4 | 0.224235529523815 | 0.334431980375048 | 0.50254122787889 | -0.431251152 | 0.879722211058908 | 1.25136571829361 | 0.649695717896229 | 2.41023007815266 | 0.999332001504091 |
| ebi-fl187-GCST90094734 | prot-a-264 | BolA-like protein 2 \|\| id:prot-a-264 | Chips liking \|\| id:ebi-fl187-GCST90094734 | Weighted median | 4 | 0.196682118567897 | 0.311687655801256 | 0.528025404224745 | -0.414225687 | 0.807589923938359 | 1.21735700400546 | 0.66085178890735 | 2.24249688065674 | 0.999332001504091 |
| ebi-fl187-GCST90094734 | prot-a-2646 | Secretoglobin family 1C member 1 \|\| id:prot-a-2646 | Chips liking \|\| id:ebi-fl187-GCST90094734 | Weighted median | 4 | 0.165663385559251 | 0.302252646124528 | 0.583626170492337 | -0.426751801 | 0.758078571963325 | 1.18017577064632 | 0.652625513228807 | 2.13417162122547 | 0.999332001504091 |
| ebi-fl187-GCST90094734 | prot-a-2672 | Semaphorin-3E \|\| id:prot-a-2672 | Chips liking \|\| id:ebi-fl187-GCST90094734 | Weighted median | 4 | 0.0728290808981052 | 0.375114655566399 | 0.846057241196263 | -0.662395644 | 0.808053805808248 | 1.0755466897294 | 0.515614624636858 | 2.24353737561767 | 0.999332001504091 |
| ebi-fl187-GCST90094734 | prot-a-2701 | Plasma protease C1 inhibitor \|\| id:prot-a-2701 | Chips liking \|\| id:ebi-fl187-GCST90094734 | Weighted median | 4 | 0.104662101344451 | 0.324982078948191 | 0.747411033159033 | -0.532302773 | 0.741626976082906 | 1.11033536613459 | 0.587251105234381 | 2.09934832697708 | 0.999332001504091 |
| ebi-fl187-GCST90094734 | prot-a-2738 | Signaling threshold-regulating transmembrane adapter 1 \|\| id:prot-a-2738 | Chips liking \|\| id:ebi-fl187-GCST90094734 | Weighted median | 4 | 0.388136109718269 | 0.320159844377782 | 0.225389997956242 | -0.239377185 | 1.01564940469872 | 1.47423042772125 | 0.787117937089045 | 2.76115592290628 | 0.999332001504091 |
| ebi-fl187-GCST90094734 | prot-a-274 | Serine/threonine-protein kinase BRSK2 \|\| id:prot-a-274 | Chips liking \|\| id:ebi-fl187-GCST90094734 | Weighted median | 4 | 0.0924137264129293 | 0.347088275928018 | 0.790043409718633 | -0.587879294 | 0.772706747231845 | 1.09681851101099 | 0.555504097103396 | 2.16562011399973 | 0.999332001504091 |
| ebi-fl187-GCST90094734 | prot-a-2749 | Mitochondrial glutamate carrier 2 \|\| id:prot-a-2749 | Chips liking \|\| id:ebi-fl187-GCST90094734 | Weighted median | 4 | 0.522229667515186 | 0.339776584231194 | 0.12429831299046 | -0.143732438 | 1.18819177260833 | 1.68578219624885 | 0.86611945807936 | 3.28114278772987 | 0.999332001504091 |
| ebi-fl187-GCST90094734 | prot-a-2760 | Sodium-coupled monocarboxylate transporter 1 \|\| id:prot-a-2760 | Chips liking \|\| id:ebi-fl187-GCST90094734 | Weighted median | 4 | 0.277446624934343 | 0.373120742075412 | 0.457128167924424 | -0.45387003 | 1.00876327940215 | 1.31975567521798 | 0.6351652806093 | 2.74220757249082 | 0.999332001504091 |
| ebi-fl187-GCST90094734 | prot-a-2777 | Small integral membrane protein 10 \|\| id:prot-a-2777 | Chips liking \|\| id:ebi-fl187-GCST90094734 | Weighted median | 4 | 0.0086405288147498 | 0.396958895438908 | 0.982633969774648 | -0.769398906 | 0.786679963875009 | 1.00867796593166 | 0.463291466238083 | 2.1960932007178 | 0.999332001504091 |
| ebi-fl187-GCST90094734 | prot-a-2799 | Superoxide dismutase [Mn], mitochondrial \|\| id:prot-a-2799 | Chips liking \|\| id:ebi-fl187-GCST90094734 | Weighted median | 4 | 0.356638125172464 | 0.309056414271765 | 0.248517383855319 | -0.249112447 | 0.962388697145123 | 1.42851883130219 | 0.779492317039627 | 2.61794248227496 | 0.999332001504091 |
| ebi-fl187-GCST90094734 | prot-a-2805 | Protein SPACA7 \|\| id:prot-a-2805 | Chips liking \|\| id:ebi-fl187-GCST90094734 | Weighted median | 4 | 0.00892608657909833 | 0.343867861182303 | 0.979290920319368 | -0.665054921 | 0.682907094496412 | 1.00896604288591 | 0.514245283891883 | 1.97962433022697 | 0.999332001504091 |
| ebi-fl187-GCST90094734 | prot-a-2811 | SPARC-like protein 1 \|\| id:prot-a-2811 | Chips liking \|\| id:ebi-fl187-GCST90094734 | Weighted median | 4 | -0.108990825 | 0.326637772854452 | 0.738624893814736 | -0.74920086 | 0.531219209394591 | 0.896738644678744 | 0.472744190528524 | 1.70100492607059 | 0.999332001504091 |
| ebi-fl187-GCST90094734 | prot-a-2831 | Sepiapterin reductase \|\| id:prot-a-2831 | Chips liking \|\| id:ebi-fl187-GCST90094734 | Weighted median | 4 | -0.184006048 | 0.328322425063565 | 0.575177219431293 | -0.827518001 | 0.459505905314871 | 0.831930772452448 | 0.437132904456095 | 1.58329149578544 | 0.999332001504091 |
| ebi-fl187-GCST90094734 | prot-a-2834 | SPRY domain-containing SOCS box protein 1 \|\| id:prot-a-2834 | Chips liking \|\| id:ebi-fl187-GCST90094734 | Weighted median | 4 | 0.114959778498315 | 0.337542209784412 | 0.733420562622811 | -0.546622953 | 0.776542509675763 | 1.12182831504503 | 0.578901490795646 | 2.17394287015411 | 0.999332001504091 |
| ebi-fl187-GCST90094734 | prot-a-2835 | Spectrin alpha chain, non-erythrocytic 1 \|\| id:prot-a-2835 | Chips liking \|\| id:ebi-fl187-GCST90094734 | Weighted median | 4 | -0.22322879 | 0.349380962591318 | 0.522870684562417 | -0.908015476 | 0.461557897158858 | 0.799931812341406 | 0.403323836098826 | 1.58654373266205 | 0.999332001504091 |
| ebi-fl187-GCST90094734 | prot-a-2859 | Sia-alpha-2,3-Gal-beta-1,4-GlcNAc-R:alpha 2,8-sialyltransferase \|\| id:prot-a-2859 | Chips liking \|\| id:ebi-fl187-GCST90094734 | Weighted median | 4 | 0.367566235771813 | 0.334831134809867 | 0.272306681252629 | -0.288702788 | 1.02383525999915 | 1.44421545408687 | 0.74923485356223 | 2.78385110877668 | 0.999332001504091 |
| ebi-fl187-GCST90094734 | prot-a-2861 | Alpha-2,8-sialyltransferase 8F \|\| id:prot-a-2861 | Chips liking \|\| id:ebi-fl187-GCST90094734 | Weighted median | 4 | 0.130193670970794 | 0.33304994464187 | 0.695861282641836 | -0.522584221 | 0.782971562468858 | 1.13904896268241 | 0.592986159219611 | 2.18796428755661 | 0.999332001504091 |
| ebi-fl187-GCST90094734 | prot-a-289 | Uncharacterized protein C17orf89 \|\| id:prot-a-289 | Chips liking \|\| id:ebi-fl187-GCST90094734 | Weighted median | 4 | 0.257000063926868 | 0.319631462529801 | 0.42136740093486 | -0.369477603 | 0.883477730485278 | 1.29304520941968 | 0.691095262700755 | 2.41929876218403 | 0.999332001504091 |
| ebi-fl187-GCST90094734 | prot-a-2921 | Tachykinin-3 \|\| id:prot-a-2921 | Chips liking \|\| id:ebi-fl187-GCST90094734 | Weighted median | 4 | -0.081623967 | 0.336262851015391 | 0.808207918541372 | -0.740699155 | 0.577451221012256 | 0.92161845251762 | 0.476780455540344 | 1.78149200989867 | 0.999332001504091 |
| ebi-fl187-GCST90094734 | prot-a-2928 | Tax1-binding protein 1 \|\| id:prot-a-2928 | Chips liking \|\| id:ebi-fl187-GCST90094734 | Weighted median | 4 | 0.204506024090507 | 0.314211465011967 | 0.515140254634267 | -0.411348447 | 0.820360495513962 | 1.22691884689368 | 0.662755955816417 | 2.27131849008971 | 0.999332001504091 |
| ebi-fl187-GCST90094734 | prot-a-296 | Uncharacterized protein C1orf185 \|\| id:prot-a-296 | Chips liking \|\| id:ebi-fl187-GCST90094734 | Weighted median | 4 | 0.270212512383438 | 0.311079064992397 | 0.385049708641939 | -0.339502455 | 0.879927479768536 | 1.31024286398298 | 0.712124548641017 | 2.41072487375199 | 0.999332001504091 |
| ebi-fl187-GCST90094734 | prot-a-2964 | Transforming growth factor beta-2 \|\| id:prot-a-2964 | Chips liking \|\| id:ebi-fl187-GCST90094734 | Weighted median | 4 | -0.32689826 | 0.314580488364158 | 0.298732104385575 | -0.943476017 | 0.289679497238651 | 0.721157109851109 | 0.389272363490539 | 1.33599922795815 | 0.999332001504091 |
| ebi-fl187-GCST90094734 | prot-a-2977 | Thymocyte nuclear protein 1 \|\| id:prot-a-2977 | Chips liking \|\| id:ebi-fl187-GCST90094734 | Weighted median | 4 | 0.535513892484375 | 0.322550731064305 | 0.0968647675129094 | -0.09668554 | 1.16771332537041 | 1.70832591256814 | 0.907841440706897 | 3.21463340699599 | 0.999332001504091 |
| ebi-fl187-GCST90094734 | prot-a-3015 | Transmembrane protein 70, mitochondrial \|\| id:prot-a-3015 | Chips liking \|\| id:ebi-fl187-GCST90094734 | Weighted median | 4 | 0.241147603223534 | 0.330571274334826 | 0.465703528425491 | -0.406772094 | 0.889067300919793 | 1.27270887737776 | 0.665795911580091 | 2.43285946696792 | 0.999332001504091 |
| ebi-fl187-GCST90094734 | prot-a-3030 | Tumor necrosis factor alpha-induced protein 3 \|\| id:prot-a-3030 | Chips liking \|\| id:ebi-fl187-GCST90094734 | Weighted median | 4 | -0.268485461 | 0.320515006077415 | 0.402216669277993 | -0.896694873 | 0.359723950516678 | 0.764536538023744 | 0.407915647079718 | 1.43293379932322 | 0.999332001504091 |
| ebi-fl187-GCST90094734 | prot-a-3032 | Tumor necrosis factor receptor superfamily member 10A \|\| id:prot-a-3032 | Chips liking \|\| id:ebi-fl187-GCST90094734 | Weighted median | 4 | 0.284603722573192 | 0.315304978995309 | 0.366722316897566 | -0.333394036 | 0.902601481403998 | 1.32923517784303 | 0.716487816348026 | 2.4660100530686 | 0.999332001504091 |
| ebi-fl187-GCST90094734 | prot-a-3081 | Tumor protein p53-inducible protein 11 \|\| id:prot-a-3081 | Chips liking \|\| id:ebi-fl187-GCST90094734 | Weighted median | 4 | 0.204772020297964 | 0.317654194695239 | 0.519161690011705 | -0.417830201 | 0.827374241900632 | 1.22724524606235 | 0.658474026963601 | 2.28730493885661 | 0.999332001504091 |
| ebi-fl187-GCST90094734 | prot-a-315 | Complement C4 \|\| id:prot-a-315 | Chips liking \|\| id:ebi-fl187-GCST90094734 | Weighted median | 4 | 0.105852631211805 | 0.321544900972491 | 0.742004405851426 | -0.524375375 | 0.736080637117887 | 1.11165804073634 | 0.591924980240831 | 2.08773685988207 | 0.999332001504091 |
| ebi-fl187-GCST90094734 | prot-a-3157 | UDP-glucuronosyltransferase 1-8 \|\| id:prot-a-3157 | Chips liking \|\| id:ebi-fl187-GCST90094734 | Weighted median | 4 | 0.213670034971899 | 0.311870483743309 | 0.493265511500934 | -0.397596113 | 0.824936183108784 | 1.23821402005565 | 0.671933357902229 | 2.28173514743923 | 0.999332001504091 |
| ebi-fl187-GCST90094734 | prot-a-317 | Glutaredoxin-like protein C5orf63 \|\| id:prot-a-317 | Chips liking \|\| id:ebi-fl187-GCST90094734 | Weighted median | 4 | -0.178443385 | 0.340057732502882 | 0.599760898275391 | -0.844956541 | 0.488069770206337 | 0.836571417609826 | 0.429576026661578 | 1.62916851343071 | 0.999332001504091 |
| ebi-fl187-GCST90094734 | prot-a-3192 | Guanine nucleotide exchange factor VAV3 \|\| id:prot-a-3192 | Chips liking \|\| id:ebi-fl187-GCST90094734 | Weighted median | 3 | -0.037744353 | 0.394418124133966 | 0.923761783224157 | -0.810803877 | 0.735315169976337 | 0.962959086714167 | 0.444500598919422 | 2.08613937740381 | 0.999332001504091 |
| ebi-fl187-GCST90094734 | prot-a-3233 | Serine/threonine-protein kinase WNK3 \|\| id:prot-a-3233 | Chips liking \|\| id:ebi-fl187-GCST90094734 | Weighted median | 4 | -0.743413474 | 0.319063407976749 | 0.0198068578339397 | -1.368777754 | -0.118049195 | 0.47548807587108 | 0.254417730653963 | 0.888652334546164 | 0.999332001504091 |
| ebi-fl187-GCST90094734 | prot-a-3250 | 14-3-3 protein theta \|\| id:prot-a-3250 | Chips liking \|\| id:ebi-fl187-GCST90094734 | Weighted median | 4 | 0.374578706863667 | 0.320843018976032 | 0.243015449492718 | -0.25427361 | 1.00343102405669 | 1.45437856582536 | 0.775479593789549 | 2.72762433682589 | 0.999332001504091 |
| ebi-fl187-GCST90094734 | prot-a-3278 | E3 ubiquitin-protein ligase ZNRF3 \|\| id:prot-a-3278 | Chips liking \|\| id:ebi-fl187-GCST90094734 | Weighted median | 4 | 0.367075785736013 | 0.329282326505388 | 0.264946747766365 | -0.278317574 | 1.01246914568657 | 1.443507312 | 0.757056361754995 | 2.75238868033895 | 0.999332001504091 |
| ebi-fl187-GCST90094734 | prot-a-355 | Calpain-3 \|\| id:prot-a-355 | Chips liking \|\| id:ebi-fl187-GCST90094734 | Weighted median | 4 | 0.277784984613904 | 0.313327108179256 | 0.375312909016264 | -0.336336147 | 0.891906116645246 | 1.32020230288149 | 0.714382927474522 | 2.43977571901834 | 0.999332001504091 |
| ebi-fl187-GCST90094734 | prot-a-385 | Coiled-coil domain-containing protein 90B, mitochondrial \|\| id:prot-a-385 | Chips liking \|\| id:ebi-fl187-GCST90094734 | Weighted median | 4 | 0.601447221291374 | 0.311821209446517 | 0.0537531180305753 | -0.009722349 | 1.21261679180655 | 1.82475771860257 | 0.990324760018965 | 3.36227151539247 | 0.999332001504091 |
| ebi-fl187-GCST90094734 | prot-a-390 | C-C motif chemokine 14 \|\| id:prot-a-390 | Chips liking \|\| id:ebi-fl187-GCST90094734 | Weighted median | 4 | -0.135276994 | 0.308542392787998 | 0.661068136865045 | -0.740020084 | 0.469466095785217 | 0.87347393106869 | 0.477104333288225 | 1.59914017757554 | 0.999332001504091 |
| ebi-fl187-GCST90094734 | prot-a-40 | Pituitary adenylate cyclase-activating polypeptide 27 \|\| id:prot-a-40 | Chips liking \|\| id:ebi-fl187-GCST90094734 | Weighted median | 4 | -0.142873104 | 0.330500213486587 | 0.665528087946176 | -0.790653523 | 0.504907314016217 | 0.866864063136696 | 0.453548294233397 | 1.65683194824488 | 0.999332001504091 |
| ebi-fl187-GCST90094734 | prot-a-401 | C-C motif chemokine 24 \|\| id:prot-a-401 | Chips liking \|\| id:ebi-fl187-GCST90094734 | Weighted median | 4 | -0.047072858 | 0.351703119165193 | 0.893527044347141 | -0.736410972 | 0.642265255465269 | 0.954017887129002 | 0.478829367445529 | 1.90078176244197 | 0.999332001504091 |
| ebi-fl187-GCST90094734 | prot-a-412 | Cerebral cavernous malformations 2 protein \|\| id:prot-a-412 | Chips liking \|\| id:ebi-fl187-GCST90094734 | Weighted median | 3 | -0.521319375 | 0.388459314711974 | 0.179590174044666 | -1.282699632 | 0.240060881745601 | 0.593736669597299 | 0.277287714339009 | 1.27132654854482 | 0.999332001504091 |
| ebi-fl187-GCST90094734 | prot-a-44 | Adhesion G protein-coupled receptor F5 \|\| id:prot-a-44 | Chips liking \|\| id:ebi-fl187-GCST90094734 | Weighted median | 4 | 0.191638236391586 | 0.3193179281107 | 0.548407145291023 | -0.434224903 | 0.817501375488557 | 1.21123225794434 | 0.64776655465111 | 2.26483379259232 | 0.999332001504091 |
| ebi-fl187-GCST90094734 | prot-a-48 | Adrenomedullin \|\| id:prot-a-48 | Chips liking \|\| id:ebi-fl187-GCST90094734 | Weighted median | 4 | 0.550454428168766 | 0.318714329579324 | 0.0841480663940096 | -0.074225658 | 1.17513451414424 | 1.7340408358521 | 0.928462155466577 | 3.23857854916186 | 0.999332001504091 |
| ebi-fl187-GCST90094734 | prot-a-502 | Carcinoembryonic antigen-related cell adhesion molecule 1 \|\| id:prot-a-502 | Chips liking \|\| id:ebi-fl187-GCST90094734 | Weighted median | 4 | 0.16410156788551 | 0.318645720105507 | 0.606555164693896 | -0.460444044 | 0.788647179292304 | 1.17833398990684 | 0.631003390321401 | 2.20041764127853 | 0.999332001504091 |
| ebi-fl187-GCST90094734 | prot-a-518 | Complement factor B \|\| id:prot-a-518 | Chips liking \|\| id:ebi-fl187-GCST90094734 | Weighted median | 4 | 0.0759659445120821 | 0.31770257549766 | 0.811020088417437 | -0.546731103 | 0.698662992487496 | 1.07892583018605 | 0.578838885530908 | 2.01106210405156 | 0.999332001504091 |
| ebi-fl187-GCST90094734 | prot-a-525 | Properdin \|\| id:prot-a-525 | Chips liking \|\| id:ebi-fl187-GCST90094734 | Weighted median | 4 | 0.372734169440043 | 0.369530957686147 | 0.313133731006016 | -0.351546508 | 1.09701484650489 | 1.45169838274148 | 0.70359912647687 | 2.99521149920516 | 0.999332001504091 |
| ebi-fl187-GCST90094734 | prot-a-533 | Chondroadherin \|\| id:prot-a-533 | Chips liking \|\| id:ebi-fl187-GCST90094734 | Weighted median | 4 | 0.186581854442873 | 0.339919699627595 | 0.583074217 | -0.479660757 | 0.852824465712958 | 1.20512326274182 | 0.618993345457903 | 2.34626444542327 | 0.999332001504091 |
| ebi-fl187-GCST90094734 | prot-a-535 | Coiled-coil-helix-coiled-coil-helix domain-containing protein 2 \|\| id:prot-a-535 | Chips liking \|\| id:ebi-fl187-GCST90094734 | Weighted median | 4 | 0.343715490877551 | 0.3260961560942 | 0.291868677668102 | -0.295432975 | 0.982863956822184 | 1.41017737010249 | 0.744209293629515 | 2.67209806726646 | 0.999332001504091 |
| ebi-fl187-GCST90094734 | prot-a-591 | Caseinolytic peptidase B protein homolog \|\| id:prot-a-591 | Chips liking \|\| id:ebi-fl187-GCST90094734 | Weighted median | 3 | -0.045677653 | 0.377792664712319 | 0.903764959837227 | -0.786151275 | 0.694795970177364 | 0.955349867051469 | 0.45559488451282 | 2.00330029923674 | 0.999332001504091 |
| ebi-fl187-GCST90094734 | prot-a-605 | Ciliary neurotrophic factor receptor subunit alpha \|\| id:prot-a-605 | Chips liking \|\| id:ebi-fl187-GCST90094734 | Weighted median | 4 | 0.0948804143038544 | 0.303494051685085 | 0.754564114156021 | -0.499967927 | 0.689728755606621 | 1.09952735951904 | 0.606550113283108 | 1.9931748224184 | 0.999332001504091 |
| ebi-fl187-GCST90094734 | prot-a-606 | Ciliary neurotrophic factor receptor subunit alpha \|\| id:prot-a-606 | Chips liking \|\| id:ebi-fl187-GCST90094734 | Weighted median | 4 | 0.282073574764581 | 0.31460985571434 | 0.369941936773512 | -0.334561742 | 0.898708891964686 | 1.32587626743247 | 0.715651657387481 | 2.45642954696442 | 0.999332001504091 |
| ebi-fl187-GCST90094734 | prot-a-707 | Cancer/testis antigen 55 \|\| id:prot-a-707 | Chips liking \|\| id:ebi-fl187-GCST90094734 | Weighted median | 4 | 0.0542328261116826 | 0.296707194058993 | 0.854968829906284 | -0.527313274 | 0.635778926467309 | 1.05573037514101 | 0.590188516135491 | 1.88849256555089 | 0.999332001504091 |
| ebi-fl187-GCST90094734 | prot-a-719 | Dipeptidyl peptidase 1 \|\| id:prot-a-719 | Chips liking \|\| id:ebi-fl187-GCST90094734 | Weighted median | 4 | -0.757703965 | 0.309150918437992 | 0.0142491879750557 | -1.363639765 | -0.151768165 | 0.468741439163982 | 0.255728290056132 | 0.859187447510376 | 0.999332001504091 |
| ebi-fl187-GCST90094734 | prot-a-752 | Cytochrome b5 \|\| id:prot-a-752 | Chips liking \|\| id:ebi-fl187-GCST90094734 | Weighted median | 4 | -0.141492232 | 0.307831055624802 | 0.645772857616069 | -0.744841101 | 0.461856637096895 | 0.868061918726433 | 0.474809740761649 | 1.58701776744147 | 0.999332001504091 |
| ebi-fl187-GCST90094734 | prot-a-755 | Cytoglobin \|\| id:prot-a-755 | Chips liking \|\| id:ebi-fl187-GCST90094734 | Weighted median | 4 | 0.192937105335974 | 0.33304584062686 | 0.562379372210396 | -0.459832742 | 0.845706952964621 | 1.21280651206219 | 0.631389241392764 | 2.32962416726621 | 0.999332001504091 |
| ebi-fl187-GCST90094734 | prot-a-807 | Desmin \|\| id:prot-a-807 | Chips liking \|\| id:ebi-fl187-GCST90094734 | Weighted median | 4 | -0.39878827 | 0.329040933534014 | 0.225523248781184 | -1.043708499 | 0.246131960179483 | 0.671132785559725 | 0.352146323050911 | 1.27906834849455 | 0.999332001504091 |
| ebi-fl187-GCST90094734 | prot-a-82 | Ameloblastin \|\| id:prot-a-82 | Chips liking \|\| id:ebi-fl187-GCST90094734 | Weighted median | 4 | 0.167874538024965 | 0.319779046093976 | 0.599603758710375 | -0.458892392 | 0.794641468369158 | 1.18278820639404 | 0.631983247492179 | 2.21364719197266 | 0.999332001504091 |
| ebi-fl187-GCST90094734 | prot-a-84 | Muellerian-inhibiting factor \|\| id:prot-a-84 | Chips liking \|\| id:ebi-fl187-GCST90094734 | Weighted median | 4 | 0.0983954678962914 | 0.390817603577632 | 0.801220192291239 | -0.667607035 | 0.864397970908449 | 1.10339905774105 | 0.512934544706971 | 2.37357669353183 | 0.999332001504091 |
| ebi-fl187-GCST90094734 | prot-a-858 | Inactive dipeptidyl peptidase 10 \|\| id:prot-a-858 | Chips liking \|\| id:ebi-fl187-GCST90094734 | Weighted median | 4 | 0.768522828558035 | 0.393711316407888 | 0.050938705516957 | -0.003151352 | 1.5401970087175 | 2.15657826393698 | 0.996853608695118 | 4.66550932646306 | 0.999332001504091 |
| ebi-fl187-GCST90094734 | prot-a-863 | Dihydropyrimidinase-related protein 3 \|\| id:prot-a-863 | Chips liking \|\| id:ebi-fl187-GCST90094734 | Weighted median | 4 | 0.309281709523467 | 0.331419442093519 | 0.350715022934013 | -0.340300397 | 0.958863816026765 | 1.36244613049663 | 0.711556541218592 | 2.6087307908466 | 0.999332001504091 |
| ebi-fl187-GCST90094734 | prot-a-864 | Dihydropyrimidinase-related protein 5 \|\| id:prot-a-864 | Chips liking \|\| id:ebi-fl187-GCST90094734 | Weighted median | 4 | -0.469798471 | 0.334860278658752 | 0.160626189756671 | -1.126124617 | 0.186527675421554 | 0.625128237214112 | 0.324287562927503 | 1.20505797211158 | 0.999332001504091 |
| ebi-fl187-GCST90094734 | prot-a-884 | Endothelin-converting enzyme 1 \|\| id:prot-a-884 | Chips liking \|\| id:ebi-fl187-GCST90094734 | Weighted median | 4 | -0.597201029 | 0.339464662954071 | 0.0785361448012133 | -1.262551769 | 0.0681497099683909 | 0.550349895483674 | 0.282931129726656 | 1.07052556483096 | 0.999332001504091 |
| ebi-fl187-GCST90094734 | prot-a-893 | Early endosome antigen 1 \|\| id:prot-a-893 | Chips liking \|\| id:ebi-fl187-GCST90094734 | Weighted median | 3 | 0.0485751798762579 | 0.373982420086812 | 0.896656410842581 | -0.684430363 | 0.78158072324641 | 1.04977429075501 | 0.504377459572542 | 2.18492329626336 | 0.999332001504091 |
| ebi-fl187-GCST90094734 | prot-a-897 | Ephrin-A2 \|\| id:prot-a-897 | Chips liking \|\| id:ebi-fl187-GCST90094734 | Weighted median | 4 | 0.197837372571835 | 0.33542399563643 | 0.555316147229267 | -0.459593659 | 0.855268404019238 | 1.218764173 | 0.63154021413694 | 2.35200558361431 | 0.999332001504091 |
| ebi-fl187-GCST90094734 | prot-a-90 | Anaphase-promoting complex subunit 10 \|\| id:prot-a-90 | Chips liking \|\| id:ebi-fl187-GCST90094734 | Weighted median | 4 | 0.052821192753811 | 0.316758810447159 | 0.867562635540097 | -0.568026076 | 0.673668461230243 | 1.05424112231334 | 0.566642845567914 | 1.96141953025554 | 0.999332001504091 |
| ebi-fl187-GCST90094734 | prot-a-914 | Histone-lysine N-methyltransferase EHMT2 \|\| id:prot-a-914 | Chips liking \|\| id:ebi-fl187-GCST90094734 | Weighted median | 4 | -0.344455761 | 0.387900751476305 | 0.374540898138878 | -1.104741234 | 0.415829711463492 | 0.708605899205084 | 0.331296599339791 | 1.51562775286217 | 0.999332001504091 |
| ebi-fl187-GCST90094734 | prot-a-932 | RNA polymerase II elongation factor ELL \|\| id:prot-a-932 | Chips liking \|\| id:ebi-fl187-GCST90094734 | Weighted median | 4 | -0.180599283 | 0.308919070301739 | 0.558805398019561 | -0.786080661 | 0.424882094495702 | 0.834769797885883 | 0.455627057211708 | 1.52941008316512 | 0.999332001504091 |
| ebi-fl187-GCST90094734 | prot-a-991 | Estrogen receptor \|\| id:prot-a-991 | Chips liking \|\| id:ebi-fl187-GCST90094734 | Weighted median | 4 | 0.371406811590978 | 0.304901248486529 | 0.223177748128795 | -0.226199635 | 0.969013258624575 | 1.44977273779137 | 0.797558864754558 | 2.63534277421632 | 0.999332001504091 |
| ebi-fl187-GCST90094734 | prot-a-992 | Persulfide dioxygenase ETHE1, mitochondrial \|\| id:prot-a-992 | Chips liking \|\| id:ebi-fl187-GCST90094734 | Weighted median | 4 | 0.495121405800607 | 0.32513199728132 | 0.127800744821543 | -0.142137309 | 1.13238012047199 | 1.64069741714959 | 0.867502132569492 | 3.10303331090164 | 0.999332001504091 |
| ebi-fl187-GCST90094734 | prot-a-993 | Ethanolamine kinase 1 \|\| id:prot-a-993 | Chips liking \|\| id:ebi-fl187-GCST90094734 | Weighted median | 4 | 0.50731770996628 | 0.305311348219847 | 0.096584872077115 | -0.091092533 | 1.10572795247718 | 1.66083038623632 | 0.912933230955825 | 3.02142312089783 | 0.999332001504091 |
| ebi-fl187-GCST90094734 | prot-b-10 | coagulation factor III, tissue factor \|\| id:prot-b-10 | Chips liking \|\| id:ebi-fl187-GCST90094734 | Weighted median | 4 | -0.322138807 | 0.336304844811354 | 0.338124588928199 | -0.981296303 | 0.337018688866529 | 0.724597604155679 | 0.374824897225695 | 1.40076524220856 | 0.999332001504091 |
| ebi-fl187-GCST90094734 | prot-b-28 | prolactin \|\| id:prot-b-28 | Chips liking \|\| id:ebi-fl187-GCST90094734 | Weighted median | 4 | 0.255050884718066 | 0.357882496906409 | 0.476052119375194 | -0.446398809 | 0.956500578654627 | 1.29052728731934 | 0.639928511775279 | 2.60257301975109 | 0.999332001504091 |
| ebi-fl187-GCST90094734 | prot-b-54 | matrix metallopeptidase 7 \|\| id:prot-b-54 | Chips liking \|\| id:ebi-fl187-GCST90094734 | Weighted median | 4 | 0.00744641285833965 | 0.316288673705999 | 0.981217066778172 | -0.612479388 | 0.627372213322097 | 1.00747420633497 | 0.5420053603724 | 1.87268309622048 | 0.999332001504091 |
| ebi-fl187-GCST90094734 | ukb-rsfMRI191-edge-pheno716 | Net100_Pair10_33 (OccipitalPrecuneus)&(Temporal\|Frontal\|Supp_Motor_Area) (Default_mode\|Central_executive)&(Default_mode\|Salience) \|\| id:ukb-rsfMRI191-edge-pheno716 | Chips liking \|\| id:ebi-fl187-GCST90094734 | Weighted median | 4 | -0.005694416 | 0.104107835587307 | 0.95637963409246 | -0.209745773 | 0.198356942020696 | 0.994321766723718 | 0.810790344177656 | 1.21939757038343 | 0.999332001504091 |
| ebi-fl187-GCST90094734 | ukb-rsfMRI191-edge-pheno903 | Net100_Pair27_38 (Precentral\|Frontal\|Supp_Motor_Area)&(Subcortical) (Attention\|Salience\|Motor)&(Subcortical-cerebellum) \|\| id:ukb-rsfMRI191-edge-pheno903 | Chips liking \|\| id:ebi-fl187-GCST90094734 | Weighted median | 4 | 0.0840024915643068 | 0.0951533138188689 | 0.377337831486626 | -0.102498004 | 0.27050298664929 | 1.08763160370953 | 0.902579951733338 | 1.3106235110984 | 0.999332001504091 |
| ebi-fl187-GCST90094734 | ukb-rsfMRI191-node-pheno23 | Net100_Node2 RolandicOper\|SupraMarginal\|Insula Salience\|Motor \|\| id:ukb-rsfMRI191-node-pheno23 | Chips liking \|\| id:ebi-fl187-GCST90094734 | Weighted median | 4 | 0.0594921310728919 | 0.12479436496675 | 0.633560598060029 | -0.185104824 | 0.304089086407723 | 1.06129740967472 | 0.831017168524956 | 1.35538979751951 | 0.999332001504091 |
| ebi-fl187-GCST90094734 | ukb-rsfMRI191-node-pheno55 | Net100_Node34 Parietal\|Postcentral\|Precuneus Attention \|\| id:ukb-rsfMRI191-node-pheno55 | Chips liking \|\| id:ebi-fl187-GCST90094734 | Weighted median | 3 | -0.03705066 | 0.112345306082293 | 0.741556615757007 | -0.25724746 | 0.18314613989818 | 0.963627316730348 | 0.773176859787461 | 1.2009899077996 | 0.999332001504091 |
| ebi-fl187-GCST90094734 | ebi-a-GCST90001414 | IgD- CD24- B cell Absolute Count \|\| id:ebi-a-GCST90001414 | Chips liking \|\| id:ebi-fl187-GCST90094734 | Weighted mode | 4 | 0.159531385890431 | 0.551900454343557 | 0.791360846306438 | -0.922193505 | 1.2412562764038 | 1.17296107607082 | 0.397645845754674 | 3.4599573984385 | 0.999714303421225 |
| ebi-fl187-GCST90094734 | ebi-a-GCST90001422 | CD20- CD38- B cell %B cell \|\| id:ebi-a-GCST90001422 | Chips liking \|\| id:ebi-fl187-GCST90094734 | Weighted mode | 4 | -0.605704087 | 0.511992502861971 | 0.32200870921769 | -1.609209392 | 0.397801219078997 | 0.545690078314398 | 0.200045709281385 | 1.48854810553282 | 0.999714303421225 |
| ebi-fl187-GCST90094734 | ebi-a-GCST90001444 | CD20- CD38- B cell %lymphocyte \|\| id:ebi-a-GCST90001444 | Chips liking \|\| id:ebi-fl187-GCST90094734 | Weighted mode | 4 | -0.510145464 | 0.560203559265927 | 0.429624665246074 | -1.60814444 | 0.587853511938939 | 0.6004082345428 | 0.200258861789275 | 1.80012032868804 | 0.999714303421225 |
| ebi-fl187-GCST90094734 | ebi-a-GCST90001459 | Myeloid Dendritic Cell %Dendritic Cell \|\| id:ebi-a-GCST90001459 | Chips liking \|\| id:ebi-fl187-GCST90094734 | Weighted mode | 4 | 0.294498395748709 | 0.515982735692886 | 0.608118859737226 | -0.716827766 | 1.30582455770676 | 1.34245280973637 | 0.488298799616113 | 3.69073106013346 | 0.999714303421225 |
| ebi-fl187-GCST90094734 | ebi-a-GCST90001476 | HLA DR++ monocyte %leukocyte \|\| id:ebi-a-GCST90001476 | Chips liking \|\| id:ebi-fl187-GCST90094734 | Weighted mode | 4 | 0.204253578951357 | 0.521052056066284 | 0.72124473128859 | -0.817008451 | 1.22551560884127 | 1.22660915628623 | 0.441751200167367 | 3.40592175350104 | 0.999714303421225 |
| ebi-fl187-GCST90094734 | ebi-a-GCST90001478 | CD4 regulatory T cell %CD4+ T cell \|\| id:ebi-a-GCST90001478 | Chips liking \|\| id:ebi-fl187-GCST90094734 | Weighted mode | 4 | 0.422008905610492 | 0.587093849197631 | 0.524187061896901 | -0.728695039 | 1.57271285003785 | 1.52502210587567 | 0.482538273121737 | 4.81970561291152 | 0.999714303421225 |
| ebi-fl187-GCST90094734 | ebi-a-GCST90001485 | CD39+ resting CD4 regulatory T cell %CD4 regulatory T cell \|\| id:ebi-a-GCST90001485 | Chips liking \|\| id:ebi-fl187-GCST90094734 | Weighted mode | 4 | -0.325410501 | 0.551571962787949 | 0.596700453075313 | -1.406491548 | 0.75567054575028 | 0.722230816082622 | 0.245001352418171 | 2.12903866264815 | 0.999714303421225 |
| ebi-fl187-GCST90094734 | ebi-a-GCST90001515 | Immature Myeloid-Derived Suppressor Cells Absolute Count \|\| id:ebi-a-GCST90001515 | Chips liking \|\| id:ebi-fl187-GCST90094734 | Weighted mode | 4 | -0.668403586 | 0.701178243287872 | 0.410785091802303 | -2.042712943 | 0.705905771141693 | 0.512526129077911 | 0.129676428533332 | 2.025680657 | 0.999714303421225 |
| ebi-fl187-GCST90094734 | ebi-a-GCST90001517 | CD33+ HLA DR+ Absolute Count \|\| id:ebi-a-GCST90001517 | Chips liking \|\| id:ebi-fl187-GCST90094734 | Weighted mode | 4 | 0.455692576443231 | 0.591459742419861 | 0.497143861087564 | -0.703568519 | 1.61495367158616 | 1.57726538169215 | 0.494816387930661 | 5.02765499479193 | 0.999714303421225 |
| ebi-fl187-GCST90094734 | ebi-a-GCST90001518 | CD33+ HLA DR+ CD14- Absolute Count \|\| id:ebi-a-GCST90001518 | Chips liking \|\| id:ebi-fl187-GCST90094734 | Weighted mode | 4 | 0.623844030479198 | 0.6334604551988 | 0.397327187134608 | -0.617738462 | 1.86542652266885 | 1.86608756980357 | 0.539162396248902 | 6.45869007631576 | 0.999714303421225 |
| ebi-fl187-GCST90094734 | ebi-a-GCST90001520 | CD33+ HLA DR+ CD14dim Absolute Count \|\| id:ebi-a-GCST90001520 | Chips liking \|\| id:ebi-fl187-GCST90094734 | Weighted mode | 4 | 0.19244680062152 | 0.569218976980052 | 0.757572883520871 | -0.923222394 | 1.30811599550242 | 1.21221201306635 | 0.397236922469543 | 3.69919783762053 | 0.999714303421225 |
| ebi-fl187-GCST90094734 | ebi-a-GCST90001531 | CD33dim HLA DR- Absolute Count \|\| id:ebi-a-GCST90001531 | Chips liking \|\| id:ebi-fl187-GCST90094734 | Weighted mode | 4 | -0.322389424 | 0.659829795386228 | 0.658616696499194 | -1.615655823 | 0.970876975324864 | 0.724416030671803 | 0.198760276208789 | 2.64025888625268 | 0.999714303421225 |
| ebi-fl187-GCST90094734 | ebi-a-GCST90001532 | Basophil Absolute Count \|\| id:ebi-a-GCST90001532 | Chips liking \|\| id:ebi-fl187-GCST90094734 | Weighted mode | 4 | -0.204581502 | 0.594242546926366 | 0.753360591777911 | -1.369296894 | 0.96013388990972 | 0.814988315979136 | 0.25428568649475 | 2.61204617663851 | 0.999714303421225 |
| ebi-fl187-GCST90094734 | ebi-a-GCST90001588 | T/B cell \|\| id:ebi-a-GCST90001588 | Chips liking \|\| id:ebi-fl187-GCST90094734 | Weighted mode | 4 | -0.217425561 | 0.544265494646619 | 0.716276825475601 | -1.28418593 | 0.849334808727407 | 0.804587495556313 | 0.276875888187463 | 2.33809105676719 | 0.999714303421225 |
| ebi-fl187-GCST90094734 | ebi-a-GCST90001624 | HLA DR+ CD4+ T cell Absolute Count \|\| id:ebi-a-GCST90001624 | Chips liking \|\| id:ebi-fl187-GCST90094734 | Weighted mode | 4 | 0.684433098363869 | 0.502606286575317 | 0.266513517369875 | -0.300675223 | 1.66954142005149 | 1.98264755074744 | 0.740318171781792 | 5.30973230202356 | 0.999714303421225 |
| ebi-fl187-GCST90094734 | ebi-a-GCST90001631 | CD8+ Natural Killer T %T cell \|\| id:ebi-a-GCST90001631 | Chips liking \|\| id:ebi-fl187-GCST90094734 | Weighted mode | 4 | 0.279922503561545 | 0.472291349395476 | 0.595096012385238 | -0.645768541 | 1.20561354837668 | 1.32302727846242 | 0.524259472206535 | 3.33880696935907 | 0.999714303421225 |
| ebi-fl187-GCST90094734 | ebi-a-GCST90001632 | CD8+ Natural Killer T %lymphocyte \|\| id:ebi-a-GCST90001632 | Chips liking \|\| id:ebi-fl187-GCST90094734 | Weighted mode | 4 | 0.264388731796202 | 0.461290791229367 | 0.606686037463687 | -0.639741219 | 1.16851868260576 | 1.30263447333014 | 0.527428894954069 | 3.21722337805528 | 0.999714303421225 |
| ebi-fl187-GCST90094734 | ebi-a-GCST90001646 | Natural Killer %CD3- lymphocyte \|\| id:ebi-a-GCST90001646 | Chips liking \|\| id:ebi-fl187-GCST90094734 | Weighted mode | 4 | -0.140383366 | 0.491245887855432 | 0.793650207119301 | -1.103225306 | 0.822458574529391 | 0.869025017175102 | 0.331799202142035 | 2.27608889834793 | 0.999714303421225 |
| ebi-fl187-GCST90094734 | ebi-a-GCST90001684 | CD127- CD8+ T cell Absolute Count \|\| id:ebi-a-GCST90001684 | Chips liking \|\| id:ebi-fl187-GCST90094734 | Weighted mode | 4 | 0.491310116483968 | 0.500052606338164 | 0.398294815690273 | -0.488792992 | 1.47141322490677 | 1.63445614581218 | 0.613366285618315 | 4.35538593369246 | 0.999714303421225 |
| ebi-fl187-GCST90094734 | ebi-a-GCST90001687 | CD28- CD8+ T cell Absolute Count \|\| id:ebi-a-GCST90001687 | Chips liking \|\| id:ebi-fl187-GCST90094734 | Weighted mode | 4 | 0.432608051126679 | 0.498375430346535 | 0.449239591789886 | -0.544207792 | 1.40942389460589 | 1.54127200247219 | 0.580301320435298 | 4.09359638165686 | 0.999714303421225 |
| ebi-fl187-GCST90094734 | ebi-a-GCST90001701 | BAFF-R on CD20- CD38- B cell \|\| id:ebi-a-GCST90001701 | Chips liking \|\| id:ebi-fl187-GCST90094734 | Weighted mode | 4 | -0.486891999 | 0.570947827933589 | 0.456455287990282 | -1.605949742 | 0.632165743328894 | 0.614533399323741 | 0.200698852201803 | 1.88168140844505 | 0.999714303421225 |
| ebi-fl187-GCST90094734 | ebi-a-GCST90001732 | CD19 on IgD- CD27- B cell \|\| id:ebi-a-GCST90001732 | Chips liking \|\| id:ebi-fl187-GCST90094734 | Weighted mode | 4 | 0.225882835773197 | 0.582376352622528 | 0.724000358185497 | -0.915574815 | 1.36734048691335 | 1.25342879965849 | 0.400286469134634 | 3.92489848385282 | 0.999714303421225 |
| ebi-fl187-GCST90094734 | ebi-a-GCST90001798 | CD27 on CD24+ CD27+ B cell \|\| id:ebi-a-GCST90001798 | Chips liking \|\| id:ebi-fl187-GCST90094734 | Weighted mode | 4 | -0.139318084 | 0.455002032238585 | 0.779477844529379 | -1.031122067 | 0.752485899415912 | 0.869951267063695 | 0.3566065995124 | 2.12226921234926 | 0.999714303421225 |
| ebi-fl187-GCST90094734 | ebi-a-GCST90001836 | CD66b on CD66b++ myeloid cell \|\| id:ebi-a-GCST90001836 | Chips liking \|\| id:ebi-fl187-GCST90094734 | Weighted mode | 4 | 0.72507977228396 | 0.727398707096265 | 0.392322541718049 | -0.700621694 | 2.15078123819264 | 2.06489581485185 | 0.496276675819875 | 8.59156783693021 | 0.999714303421225 |
| ebi-fl187-GCST90094734 | ebi-a-GCST90001943 | CD25 on activated & secreting CD4 regulatory T cell \|\| id:ebi-a-GCST90001943 | Chips liking \|\| id:ebi-fl187-GCST90094734 | Weighted mode | 4 | -0.270372402 | 0.554537225869805 | 0.659267055757326 | -1.357265365 | 0.816520560710878 | 0.763095263218432 | 0.257363611134242 | 2.26261349916586 | 0.999714303421225 |
| ebi-fl187-GCST90094734 | ebi-a-GCST90001960 | CD25 on CD4+ T cell \|\| id:ebi-a-GCST90001960 | Chips liking \|\| id:ebi-fl187-GCST90094734 | Weighted mode | 4 | 0.640999593961496 | 0.498301939386664 | 0.288598874664399 | -0.335672207 | 1.61767139515936 | 1.89837753792632 | 0.714857392495511 | 5.04133735530453 | 0.999714303421225 |
| ebi-fl187-GCST90094734 | ebi-a-GCST90001988 | HLA DR on CD14+ CD16- monocyte \|\| id:ebi-a-GCST90001988 | Chips liking \|\| id:ebi-fl187-GCST90094734 | Weighted mode | 4 | 0.442392254800196 | 0.56294945005502 | 0.489309865674383 | -0.660988667 | 1.54577317690804 | 1.55642613633405 | 0.51634059299269 | 4.69159766003141 | 0.999714303421225 |
| ebi-fl187-GCST90094734 | ebi-a-GCST90001991 | HLA DR on CD14+ monocyte \|\| id:ebi-a-GCST90001991 | Chips liking \|\| id:ebi-fl187-GCST90094734 | Weighted mode | 4 | 0.432327297132449 | 0.587572454138038 | 0.515168832142661 | -0.719314713 | 1.583969307 | 1.54083934493931 | 0.487085935283855 | 4.87426491904229 | 0.999714303421225 |
| ebi-fl187-GCST90094734 | ebi-a-GCST90002019 | CD14 on Monocytic Myeloid-Derived Suppressor Cells \|\| id:ebi-a-GCST90002019 | Chips liking \|\| id:ebi-fl187-GCST90094734 | Weighted mode | 4 | -0.131385132 | 0.684710481165686 | 0.860086446689388 | -1.473417675 | 1.21064741074422 | 0.876879994505601 | 0.229141015800813 | 3.35565643748627 | 0.999714303421225 |
| ebi-fl187-GCST90094734 | ebi-a-GCST90002037 | CD80 on plasmacytoid Dendritic Cell \|\| id:ebi-a-GCST90002037 | Chips liking \|\| id:ebi-fl187-GCST90094734 | Weighted mode | 4 | -0.379415065 | 0.536096490884728 | 0.530137750095275 | -1.430164187 | 0.671334057535515 | 0.684261540974496 | 0.239269634119108 | 1.95684612542065 | 0.999714303421225 |
| ebi-fl187-GCST90094734 | ebi-a-GCST90002042 | CD45 on CD33+ HLA DR+ CD14- \|\| id:ebi-a-GCST90002042 | Chips liking \|\| id:ebi-fl187-GCST90094734 | Weighted mode | 4 | -0.793195142 | 0.741064680931833 | 0.362925142312621 | -2.245681917 | 0.65929163268485 | 0.452397010911975 | 0.10585533125186 | 1.9334222760603 | 0.999714303421225 |
| ebi-fl187-GCST90094734 | ebi-a-GCST90002056 | CD8 on Effector Memory CD8+ T cell \|\| id:ebi-a-GCST90002056 | Chips liking \|\| id:ebi-fl187-GCST90094734 | Weighted mode | 4 | 0.0346286443843791 | 0.534076064067267 | 0.952381417892181 | -1.012160441 | 1.08141772995622 | 1.03523519700484 | 0.363432955272167 | 2.948857272 | 0.999714303421225 |
| ebi-fl187-GCST90094734 | ebi-a-GCST90002058 | CD8 on CD8+ T cell \|\| id:ebi-a-GCST90002058 | Chips liking \|\| id:ebi-fl187-GCST90094734 | Weighted mode | 4 | -0.400164152 | 0.564991121 | 0.529851852163264 | -1.507546749 | 0.707218444335826 | 0.670210020589643 | 0.221452590975116 | 2.02834145999783 | 0.999714303421225 |
| ebi-fl187-GCST90094734 | ebi-a-GCST90002063 | CD4 on CD4 regulatory T cell \|\| id:ebi-a-GCST90002063 | Chips liking \|\| id:ebi-fl187-GCST90094734 | Weighted mode | 4 | -0.277924494 | 0.515889087499514 | 0.627482438741381 | -1.289067106 | 0.73321811746858 | 0.757354004103854 | 0.275527701501432 | 2.0817692174199 | 0.999714303421225 |
| ebi-fl187-GCST90094734 | ebi-a-GCST90002066 | CD4 on activated CD4 regulatory T cell \|\| id:ebi-a-GCST90002066 | Chips liking \|\| id:ebi-fl187-GCST90094734 | Weighted mode | 4 | -0.406625233 | 0.589444371117483 | 0.539865428380561 | -1.5619362 | 0.748685734813227 | 0.665893698810239 | 0.209729599378924 | 2.11421954472937 | 0.999714303421225 |
| ebi-fl187-GCST90094734 | ebi-a-GCST90002070 | CD4 on activated & secreting CD4 regulatory T cell \|\| id:ebi-a-GCST90002070 | Chips liking \|\| id:ebi-fl187-GCST90094734 | Weighted mode | 4 | -0.265675402 | 0.524902548039518 | 0.647607927326346 | -1.294484396 | 0.763133592374951 | 0.766687952657541 | 0.274039123596127 | 2.14498721582731 | 0.999714303421225 |
| ebi-fl187-GCST90094734 | ebi-a-GCST90002081 | SSC-A on CD4+ T cell \|\| id:ebi-a-GCST90002081 | Chips liking \|\| id:ebi-fl187-GCST90094734 | Weighted mode | 4 | 0.0530547993117807 | 0.4615780645065 | 0.915752481623721 | -0.851638207 | 0.95774780574452 | 1.05448742872147 | 0.426715310982095 | 2.60582104441588 | 0.999714303421225 |
| ebi-fl187-GCST90094734 | ebi-a-GCST90002094 | CD11b on Monocytic Myeloid-Derived Suppressor Cells \|\| id:ebi-a-GCST90002094 | Chips liking \|\| id:ebi-fl187-GCST90094734 | Weighted mode | 4 | 0.742000970638032 | 0.620632926253493 | 0.317757365516845 | -0.474439565 | 1.95844150609488 | 2.10013361865893 | 0.622233680410754 | 7.08827142450716 | 0.999714303421225 |
| ebi-fl187-GCST90094734 | ebi-a-GCST90002104 | HLA DR on myeloid Dendritic Cell \|\| id:ebi-a-GCST90002104 | Chips liking \|\| id:ebi-fl187-GCST90094734 | Weighted mode | 4 | -0.251355076 | 0.677871155859731 | 0.735421673938573 | -1.579982541 | 1.07727238973439 | 0.77774616372158 | 0.205978694306954 | 2.93665855693898 | 0.999714303421225 |
| ebi-fl187-GCST90094734 | ebi-a-GCST90002105 | HLA DR on plasmacytoid Dendritic Cell \|\| id:ebi-a-GCST90002105 | Chips liking \|\| id:ebi-fl187-GCST90094734 | Weighted mode | 4 | -0.35356555 | 0.582065427118386 | 0.586457090455455 | -1.494413787 | 0.787282687244595 | 0.702179963247876 | 0.224380100703077 | 2.1974172363852 | 0.999714303421225 |
| ebi-fl187-GCST90094734 | ebi-a-GCST90002106 | HLA DR on Dendritic Cell \|\| id:ebi-a-GCST90002106 | Chips liking \|\| id:ebi-fl187-GCST90094734 | Weighted mode | 4 | -0.408171796 | 0.625206772502947 | 0.560415168494751 | -1.633577071 | 0.817233477699988 | 0.664864647653628 | 0.195229972233583 | 2.26422712989324 | 0.999714303421225 |
| ebi-fl187-GCST90094734 | ebi-a-GCST90002111 | HLA DR on CD33dim HLA DR+ CD11b- \|\| id:ebi-a-GCST90002111 | Chips liking \|\| id:ebi-fl187-GCST90094734 | Weighted mode | 4 | -0.144100833 | 0.698170216815097 | 0.849694080011473 | -1.512514458 | 1.22431279202324 | 0.865800442458463 | 0.220355206888433 | 3.4018275163373 | 0.999714303421225 |
| ebi-fl187-GCST90094734 | ebi-a-GCST90002112 | HLA DR on CD33- HLA DR+ \|\| id:ebi-a-GCST90002112 | Chips liking \|\| id:ebi-fl187-GCST90094734 | Weighted mode | 4 | 0.406605483218928 | 0.698653683252934 | 0.60142646542445 | -0.962755736 | 1.77596670239468 | 1.50171153837855 | 0.381839186806785 | 5.90598771005761 | 0.999714303421225 |
| ebi-fl187-GCST90094734 | ebi-a-GCST90002115 | HLA DR on HLA DR+ CD8+ T cell \|\| id:ebi-a-GCST90002115 | Chips liking \|\| id:ebi-fl187-GCST90094734 | Weighted mode | 4 | -0.058623003 | 0.656598118705673 | 0.934483640713183 | -1.345555316 | 1.22830930916452 | 0.943062233311084 | 0.260395066114099 | 3.41545018179411 | 0.999714303421225 |
| ebi-fl187-GCST90094734 | ebi-a-GCST90002116 | HLA DR on B cell \|\| id:ebi-a-GCST90002116 | Chips liking \|\| id:ebi-fl187-GCST90094734 | Weighted mode | 4 | 0.12655376937878 | 0.502440145769161 | 0.817404664090042 | -0.858228916 | 1.11133645508634 | 1.13491047296661 | 0.423912201838163 | 3.03841638920556 | 0.999714303421225 |
| ebi-fl187-GCST90094734 | ebi-cfmet338-GCST90026023 | Sphingomyelin (d18:1/24:1, d18:2/24:0) levels \|\| id:ebi-cfmet338-GCST90026023 | Chips liking \|\| id:ebi-fl187-GCST90094734 | Weighted mode | 4 | 0.056956224406155 | 0.225581716879027 | 0.816976297298714 | -0.385183941 | 0.499096389489048 | 1.05860946812306 | 0.680325485164614 | 1.64723214172791 | 0.999714303421225 |
| ebi-fl187-GCST90094734 | ebi-cfmet338-GCST90026046 | 1-arachidonoyl-gpc (20:4n6) levels \|\| id:ebi-cfmet338-GCST90026046 | Chips liking \|\| id:ebi-fl187-GCST90094734 | Weighted mode | 4 | -0.138591514 | 0.251723280328941 | 0.620273650076556 | -0.631969143 | 0.354786115430919 | 0.870583577026032 | 0.531544083237721 | 1.42587564886595 | 0.999714303421225 |
| ebi-fl187-GCST90094734 | ebi-cfmet338-GCST90026047 | 1-linoleoyl-gpc (18:2) levels \|\| id:ebi-cfmet338-GCST90026047 | Chips liking \|\| id:ebi-fl187-GCST90094734 | Weighted mode | 4 | -0.014308959 | 0.292366945809672 | 0.964041780403373 | -0.587348172 | 0.558730255245359 | 0.985792928063024 | 0.555799215958603 | 1.74845100373703 | 0.999714303421225 |
| ebi-fl187-GCST90094734 | ebi-cfmet338-GCST90026050 | 1-myristoyl-2-palmitoyl-gpc (14:0/16:0) levels \|\| id:ebi-cfmet338-GCST90026050 | Chips liking \|\| id:ebi-fl187-GCST90094734 | Weighted mode | 4 | 0.172936979000237 | 0.157642146129044 | 0.352796354228195 | -0.136041627 | 0.481915585413163 | 1.18879118391433 | 0.872806299064482 | 1.61917309770474 | 0.999714303421225 |
| ebi-fl187-GCST90094734 | ebi-cfmet338-GCST90026068 | 2,3-dihydroxyisovalerate levels \|\| id:ebi-cfmet338-GCST90026068 | Chips liking \|\| id:ebi-fl187-GCST90094734 | Weighted mode | 4 | 0.197536007620589 | 0.511263398786084 | 0.724997690899274 | -0.804540254 | 1.19961226924131 | 1.21839693575438 | 0.4472935207021 | 3.31882986081604 | 0.999714303421225 |
| ebi-fl187-GCST90094734 | ebi-cfmet338-GCST90026082 | 3-hydroxyhexanoate levels \|\| id:ebi-cfmet338-GCST90026082 | Chips liking \|\| id:ebi-fl187-GCST90094734 | Weighted mode | 4 | -0.13412856 | 0.117556877643513 | 0.336715528205139 | -0.364540041 | 0.0962829198246367 | 0.874477634219907 | 0.694516026640405 | 1.10107053461386 | 0.999714303421225 |
| ebi-fl187-GCST90094734 | ebi-cfmet338-GCST90026094 | 3-ureidopropionate levels \|\| id:ebi-cfmet338-GCST90026094 | Chips liking \|\| id:ebi-fl187-GCST90094734 | Weighted mode | 4 | 0.545530845490314 | 0.273374777611875 | 0.139929423271521 | 0.00971628137103853 | 1.08134540960959 | 1.72552412599646 | 1.00976363768437 | 2.94864401754833 | 0.999714303421225 |
| ebi-fl187-GCST90094734 | ebi-cfmet338-GCST90026173 | Glycerol levels \|\| id:ebi-cfmet338-GCST90026173 | Chips liking \|\| id:ebi-fl187-GCST90094734 | Weighted mode | 4 | -0.295228205 | 0.152712254815684 | 0.14869436835842 | -0.594544225 | 0.0040878139407417 | 0.744361700649505 | 0.55181401162818 | 1.00409618044851 | 0.999714303421225 |
| ebi-fl187-GCST90094734 | ebi-cfmet338-GCST90026179 | Histidine levels \|\| id:ebi-cfmet338-GCST90026179 | Chips liking \|\| id:ebi-fl187-GCST90094734 | Weighted mode | 4 | -0.139473573 | 0.0979310580416844 | 0.249587158480982 | -0.331418447 | 0.0524713004416237 | 0.869816009249943 | 0.717904701052827 | 1.05387231597446 | 0.999714303421225 |
| ebi-fl187-GCST90094734 | ebi-cfmet338-GCST90026188 | Isocitrate levels \|\| id:ebi-cfmet338-GCST90026188 | Chips liking \|\| id:ebi-fl187-GCST90094734 | Weighted mode | 4 | -0.039614349 | 0.21327018895976 | 0.864492416449503 | -0.45762392 | 0.378395220990802 | 0.961160039661082 | 0.632785409570299 | 1.45993982773501 | 0.999714303421225 |
| ebi-fl187-GCST90094734 | ebi-cfmet338-GCST90026207 | N-acetyl-isoputreanine levels \|\| id:ebi-cfmet338-GCST90026207 | Chips liking \|\| id:ebi-fl187-GCST90094734 | Weighted mode | 4 | -0.045088094 | 0.128711263063161 | 0.749262940463819 | -0.29736217 | 0.20718598128055 | 0.955913267591365 | 0.742774952891758 | 1.23021134678778 | 0.999714303421225 |
| ebi-fl187-GCST90094734 | ebi-cfmet338-GCST90026213 | N-acetylglutamate levels \|\| id:ebi-cfmet338-GCST90026213 | Chips liking \|\| id:ebi-fl187-GCST90094734 | Weighted mode | 4 | -0.035587961 | 0.227200388367892 | 0.885478623811478 | -0.480900722 | 0.409724800081216 | 0.965037844687048 | 0.61822629072768 | 1.50640316603495 | 0.999714303421225 |
| ebi-fl187-GCST90094734 | ebi-cfmet338-GCST90026224 | N-acetyltaurine levels \|\| id:ebi-cfmet338-GCST90026224 | Chips liking \|\| id:ebi-fl187-GCST90094734 | Weighted mode | 4 | -0.070885671 | 0.172767355881404 | 0.70913176089562 | -0.409509688 | 0.267738346964449 | 0.931568391729115 | 0.663975725543316 | 1.30700511341533 | 0.999714303421225 |
| ebi-fl187-GCST90094734 | ebi-cfmet338-GCST90026250 | Phenyllactate (pla) levels \|\| id:ebi-cfmet338-GCST90026250 | Chips liking \|\| id:ebi-fl187-GCST90094734 | Weighted mode | 4 | -0.418998577 | 0.319602259728084 | 0.281179044660119 | -1.045419007 | 0.207421851625588 | 0.657705130891205 | 0.351544489085092 | 1.2305015513866 | 0.999714303421225 |
| ebi-fl187-GCST90094734 | ebi-cfmet338-GCST90026251 | Pipecolate levels \|\| id:ebi-cfmet338-GCST90026251 | Chips liking \|\| id:ebi-fl187-GCST90094734 | Weighted mode | 4 | -0.379893781 | 0.428011167486699 | 0.440145304029723 | -1.218795669 | 0.459008107643867 | 0.683934052398355 | 0.295585935983389 | 1.58250353310559 | 0.999714303421225 |
| ebi-fl187-GCST90094734 | ebi-cfmet338-GCST90026278 | Trigonelline (n'-methylnicotinate) levels \|\| id:ebi-cfmet338-GCST90026278 | Chips liking \|\| id:ebi-fl187-GCST90094734 | Weighted mode | 4 | 0.176407892859666 | 0.484876732718352 | 0.740120948289732 | -0.773950503 | 1.12676628898764 | 1.19292454483076 | 0.461187541919754 | 3.08566220964246 | 0.999714303421225 |
| ebi-fl187-GCST90094734 | ebi-cfmet338-GCST90026299 | 1-carboxyethylvaline levels \|\| id:ebi-cfmet338-GCST90026299 | Chips liking \|\| id:ebi-fl187-GCST90094734 | Weighted mode | 4 | -0.040244686 | 0.303477798253064 | 0.902895463997447 | -0.635061171 | 0.554571798473954 | 0.960554376089069 | 0.529903072795871 | 1.74119524266146 | 0.999714303421225 |
| ebi-fl187-GCST90094734 | ebi-gm430-GCST90011306 | C_Erysipelotrichia abundance \|\| id:ebi-gm430-GCST90011306 | Chips liking \|\| id:ebi-fl187-GCST90094734 | Weighted mode | 4 | 0.032292823625337 | 0.291897959053031 | 0.918895307715198 | -0.539827176 | 0.604412823369278 | 1.03281989509552 | 0.582848973892133 | 1.83017725600825 | 0.999714303421225 |
| ebi-fl187-GCST90094734 | ebi-gm430-GCST90011312 | F_Erysipelotrichaceae abundance \|\| id:ebi-gm430-GCST90011312 | Chips liking \|\| id:ebi-fl187-GCST90094734 | Weighted mode | 4 | 0.032292823625337 | 0.32068526028619 | 0.926141626994871 | -0.596250287 | 0.66083593378627 | 1.03281989509552 | 0.550873385544404 | 1.93641036887439 | 0.999714303421225 |
| ebi-fl187-GCST90094734 | ebi-gm430-GCST90011352 | O_Erysipelotrichales abundance \|\| id:ebi-gm430-GCST90011352 | Chips liking \|\| id:ebi-fl187-GCST90094734 | Weighted mode | 4 | 0.032292823625337 | 0.323609912838433 | 0.926806171930783 | -0.601982606 | 0.666568252788665 | 1.03281989509552 | 0.547724637006256 | 1.94754236642626 | 0.999714303421225 |
| ebi-fl187-GCST90094734 | ebi-gm430-GCST90011362 | OTU97_11 (Parabacteroides) abundance \|\| id:ebi-gm430-GCST90011362 | Chips liking \|\| id:ebi-fl187-GCST90094734 | Weighted mode | 4 | 0.148128272650582 | 0.395012533866444 | 0.732604582970955 | -0.626096294 | 0.922352839028811 | 1.15966163969522 | 0.534674946309376 | 2.51520129728028 | 0.999714303421225 |
| ebi-fl187-GCST90094734 | ebi-gm430-GCST90011384 | OTU97_235 (Phascolarctobacterium) abundance \|\| id:ebi-gm430-GCST90011384 | Chips liking \|\| id:ebi-fl187-GCST90094734 | Weighted mode | 4 | -1.271322192 | 0.959354725148786 | 0.276997469519443 | -3.151657454 | 0.609013068906546 | 0.28046055371232 | 0.0427811602799719 | 1.83861591583446 | 0.999714303421225 |
| ebi-fl187-GCST90094734 | ebi-gm430-GCST90011390 | OTU97_3 (Bacteroides) abundance \|\| id:ebi-gm430-GCST90011390 | Chips liking \|\| id:ebi-fl187-GCST90094734 | Weighted mode | 4 | 0.0285259854863266 | 0.304018232003647 | 0.931159779589599 | -0.567349749 | 0.624401720213475 | 1.02893674790893 | 0.567026210755495 | 1.86712855793173 | 0.999714303421225 |
| ebi-fl187-GCST90094734 | ebi-gm430-GCST90011398 | OTU97_4 (Alistipes) abundance \|\| id:ebi-gm430-GCST90011398 | Chips liking \|\| id:ebi-fl187-GCST90094734 | Weighted mode | 4 | 0.35871527327795 | 0.297667175889295 | 0.314559108102785 | -0.224712391 | 0.942142938020967 | 1.43148916032497 | 0.79874591186844 | 2.56547318199656 | 0.999714303421225 |
| ebi-fl187-GCST90094734 | ebi-gm430-GCST90011421 | OTU99_108 (Cytophagales) abundance \|\| id:ebi-gm430-GCST90011421 | Chips liking \|\| id:ebi-fl187-GCST90094734 | Weighted mode | 4 | -0.58705554 | 1.12828091116166 | 0.638806787686212 | -2.798486126 | 1.6243750457127 | 0.555961884486008 | 0.0609021911133365 | 5.07524624895715 | 0.999714303421225 |
| ebi-fl187-GCST90094734 | ebi-gm430-GCST90011428 | OTU99_123 (Phascolarctobacterium) abundance \|\| id:ebi-gm430-GCST90011428 | Chips liking \|\| id:ebi-fl187-GCST90094734 | Weighted mode | 4 | 0.780867759299265 | 0.523574840556931 | 0.23265451979808 | -0.245338928 | 1.80707444679085 | 2.18336607991137 | 0.782439302558066 | 6.0925971168911 | 0.999714303421225 |
| ebi-fl187-GCST90094734 | ebi-gm430-GCST90011450 | OTU99_298 (Phascolarctobacterium) abundance \|\| id:ebi-gm430-GCST90011450 | Chips liking \|\| id:ebi-fl187-GCST90094734 | Weighted mode | 4 | -1.246723727 | 0.955543346279038 | 0.283049286841087 | -3.119588686 | 0.626141231372295 | 0.287445004041175 | 0.0441753346151791 | 1.87037927540317 | 0.999714303421225 |
| ebi-fl187-GCST90094734 | ebi-gm430-GCST90011451 | OTU99_3 (Bacteroides) abundance \|\| id:ebi-gm430-GCST90011451 | Chips liking \|\| id:ebi-fl187-GCST90094734 | Weighted mode | 4 | 0.028920593387291 | 0.343181076371471 | 0.938148692643151 | -0.643714316 | 0.701555503075373 | 1.02934285460043 | 0.525337525999481 | 2.01688754349144 | 0.999714303421225 |
| ebi-fl187-GCST90094734 | ebi-gm430-GCST90011472 | OTU99_72 (Parabacteroides) abundance \|\| id:ebi-gm430-GCST90011472 | Chips liking \|\| id:ebi-fl187-GCST90094734 | Weighted mode | 4 | 0.851426752355466 | 1.05012867361111 | 0.476840040292495 | -1.206825448 | 2.90967895263324 | 2.34298733103449 | 0.299145426397558 | 18.3509061111052 | 0.999714303421225 |
| ebi-fl187-GCST90094734 | ebi-gm430-GCST90011507 | TestASV_3 (Bacteroides) abundance \|\| id:ebi-gm430-GCST90011507 | Chips liking \|\| id:ebi-fl187-GCST90094734 | Weighted mode | 4 | 0.519387550010661 | 0.391636721671326 | 0.27670148347375 | -0.248220424 | 1.28699552448646 | 1.68099780727844 | 0.780187951811182 | 3.62188831744329 | 0.999714303421225 |
| ebi-fl187-GCST90094734 | ebi-gm430-GCST90011512 | TestASV_34 (Phascolarctobacterium) abundance \|\| id:ebi-gm430-GCST90011512 | Chips liking \|\| id:ebi-fl187-GCST90094734 | Weighted mode | 4 | -0.232796656 | 1.13072708200253 | 0.850062575485098 | -2.449021736 | 1.98342842498292 | 0.79231466978335 | 0.0863780456731026 | 7.26761680079755 | 0.999714303421225 |
| ebi-fl187-GCST90094734 | ebi-gm430-GCST90011519 | TestASV_41 (Barnesiella) abundance \|\| id:ebi-gm430-GCST90011519 | Chips liking \|\| id:ebi-fl187-GCST90094734 | Weighted mode | 4 | 0.650355644314023 | 1.221382142 | 0.631312315625915 | -1.743553354 | 3.0442646429339 | 1.91622220137394 | 0.174897821223358 | 20.9945870071706 | 0.999714303421225 |
| ebi-fl187-GCST90094734 | ebi-gm430-GCST90011524 | TestASV_46 (Sutterella) abundance \|\| id:ebi-gm430-GCST90011524 | Chips liking \|\| id:ebi-fl187-GCST90094734 | Weighted mode | 4 | -1.630391625 | 1.16511544700693 | 0.256185121119808 | -3.914017901 | 0.653234651530279 | 0.195852858308047 | 0.0199601418634481 | 1.92174696802508 | 0.999714303421225 |
| ebi-fl187-GCST90094734 | ebi-gm430-GCST90011581 | OTU97_19 (Bacteria) prevalence \|\| id:ebi-gm430-GCST90011581 | Chips liking \|\| id:ebi-fl187-GCST90094734 | Weighted mode | 4 | -1.020618079 | 1.26365588102796 | 0.478381541590458 | -3.497383606 | 1.45614744784381 | 0.360372132886857 | 0.0302764951301666 | 4.28940250854946 | 0.999714303421225 |
| ebi-fl187-GCST90094734 | ebi-gm430-GCST90011614 | OTU97_80 (Ruminococcus) prevalence \|\| id:ebi-gm430-GCST90011614 | Chips liking \|\| id:ebi-fl187-GCST90094734 | Weighted mode | 4 | -0.249171275 | 0.649624803148884 | 0.726871132549429 | -1.52243589 | 1.02409333878088 | 0.77944646195393 | 0.21817977729283 | 2.78456965439603 | 0.999714303421225 |
| ebi-fl187-GCST90094734 | ebi-gm430-GCST90011623 | OTU99_11 (Parabacteroides) prevalence \|\| id:ebi-gm430-GCST90011623 | Chips liking \|\| id:ebi-fl187-GCST90094734 | Weighted mode | 4 | -0.276205159 | 0.543927900137516 | 0.646575738411026 | -1.342303843 | 0.789893525642954 | 0.758657269710688 | 0.261243111661645 | 2.20316183352741 | 0.999714303421225 |
| ebi-fl187-GCST90094734 | ebi-gm430-GCST90011642 | OTU99_19 (Bacteria) prevalence \|\| id:ebi-gm430-GCST90011642 | Chips liking \|\| id:ebi-fl187-GCST90094734 | Weighted mode | 4 | -1.020618079 | 1.26525485660697 | 0.478888002238021 | -3.500517598 | 1.45928143997868 | 0.360372132886857 | 0.0301817573638066 | 4.30286654935333 | 0.999714303421225 |
| ebi-fl187-GCST90094734 | ebi-gm430-GCST90011653 | OTU99_30 (Parasutterella) prevalence \|\| id:ebi-gm430-GCST90011653 | Chips liking \|\| id:ebi-fl187-GCST90094734 | Weighted mode | 4 | -0.864250969 | 0.555581286587677 | 0.217658370316203 | -1.953190291 | 0.22468835265767 | 0.421367051414453 | 0.141820899189907 | 1.25193249395464 | 0.999714303421225 |
| ebi-fl187-GCST90094734 | ebi-gm430-GCST90011675 | OTU99_8 (Ruminococcaceae) prevalence \|\| id:ebi-gm430-GCST90011675 | Chips liking \|\| id:ebi-fl187-GCST90094734 | Weighted mode | 4 | -0.964789885 | 0.737262574911827 | 0.281889852306864 | -2.409824532 | 0.48024476182884 | 0.381063258435455 | 0.0898310556807726 | 1.61647000393124 | 0.999714303421225 |
| ebi-fl187-GCST90094734 | ebi-gm430-GCST90011679 | OTU99_92 (Ruminococcus) prevalence \|\| id:ebi-gm430-GCST90011679 | Chips liking \|\| id:ebi-fl187-GCST90094734 | Weighted mode | 4 | 0.385521903597328 | 0.744463146240294 | 0.640327815766346 | -1.073625863 | 1.8446696702283 | 1.47038151862626 | 0.341767067538885 | 6.32600977585906 | 0.999714303421225 |
| ebi-fl187-GCST90094734 | ebi-gm430-GCST90011684 | TestASV_11 (Lachnospiraceae) prevalence \|\| id:ebi-gm430-GCST90011684 | Chips liking \|\| id:ebi-fl187-GCST90094734 | Weighted mode | 4 | -0.177012789 | 0.712010214556558 | 0.819710029996906 | -1.57255281 | 1.21852723130022 | 0.837769070032624 | 0.20751475990653 | 3.38220286123003 | 0.999714303421225 |
| ebi-fl187-GCST90094734 | ebi-gm430-GCST90011688 | TestASV_15 (Bacteroides) prevalence \|\| id:ebi-gm430-GCST90011688 | Chips liking \|\| id:ebi-fl187-GCST90094734 | Weighted mode | 4 | -0.451604862 | 0.618555854041273 | 0.518177562031208 | -1.663974336 | 0.76076461213467 | 0.636605667259912 | 0.189384803631531 | 2.13991179765367 | 0.999714303421225 |
| ebi-fl187-GCST90094734 | ebi-gm430-GCST90011692 | TestASV_19 (Prevotella) prevalence \|\| id:ebi-gm430-GCST90011692 | Chips liking \|\| id:ebi-fl187-GCST90094734 | Weighted mode | 4 | 0.374435325402756 | 1.33525267885905 | 0.797379090131925 | -2.242659925 | 2.99153057596649 | 1.45417004985089 | 0.106175708998865 | 19.916142343876 | 0.999714303421225 |
| ebi-fl187-GCST90094734 | ebi-gm430-GCST90011693 | TestASV_2 (Bacteroides) prevalence \|\| id:ebi-gm430-GCST90011693 | Chips liking \|\| id:ebi-fl187-GCST90094734 | Weighted mode | 4 | 0.624704286150701 | 0.792728030239395 | 0.48820052448552 | -0.929042653 | 2.17845122541992 | 1.86769357290662 | 0.394931616000329 | 8.8326159288138 | 0.999714303421225 |
| ebi-fl187-GCST90094734 | ebi-gm430-GCST90011716 | TestASV_40 (Barnesiella) prevalence \|\| id:ebi-gm430-GCST90011716 | Chips liking \|\| id:ebi-fl187-GCST90094734 | Weighted mode | 4 | -0.1335964 | 1.39021284430531 | 0.929502575419261 | -2.858413574 | 2.59122077526252 | 0.874943120766357 | 0.0573596849956047 | 13.3460541952947 | 0.999714303421225 |
| ebi-fl187-GCST90094734 | ebi-gm430-GCST90011718 | TestASV_42 (Alphaproteobacteria) prevalence \|\| id:ebi-gm430-GCST90011718 | Chips liking \|\| id:ebi-fl187-GCST90094734 | Weighted mode | 4 | -0.89820178 | 0.97875546405229 | 0.426452270746728 | -2.816562489 | 1.02015892959615 | 0.407301419191028 | 0.0598111913335721 | 2.77363554171356 | 0.999714303421225 |
| ebi-fl187-GCST90094734 | ebi-hbsm44-GCST90104929 | Sulcal depth of the right inferior frontal sulcus \|\| id:ebi-hbsm44-GCST90104929 | Chips liking \|\| id:ebi-fl187-GCST90094734 | Weighted mode | 3 | 0.111430012240474 | 0.575251459883227 | 0.864295806448424 | -1.016062849 | 1.2389228736116 | 1.1178755036657 | 0.362017455346137 | 3.45189333619567 | 0.999714303421225 |
| ebi-fl187-GCST90094734 | ebi-hbsm44-GCST90104935 | Sulcal opening distance of the left calloso-marginal posterior fissure \|\| id:ebi-hbsm44-GCST90104935 | Chips liking \|\| id:ebi-fl187-GCST90094734 | Weighted mode | 4 | -0.114952208 | 0.108290137150274 | 0.366337790043308 | -0.327200877 | 0.0972964608230756 | 0.891408745103182 | 0.720938908574878 | 1.10218708047979 | 0.999714303421225 |
| ebi-fl187-GCST90094734 | ebi-hbsm44-GCST90104936 | Sulcal opening distance of the left anterior inferior temporal sulcus \|\| id:ebi-hbsm44-GCST90104936 | Chips liking \|\| id:ebi-fl187-GCST90094734 | Weighted mode | 4 | 0.0225790313999807 | 0.0805624302866667 | 0.797487509366513 | -0.135323332 | 0.180481394761848 | 1.02283586712076 | 0.87343345707394 | 1.1977938360336 | 0.999714303421225 |
| ebi-fl187-GCST90094734 | ebi-hbsm44-GCST90104941 | Sulcal opening distance of the right posterior inferior temporal sulcus \|\| id:ebi-hbsm44-GCST90104941 | Chips liking \|\| id:ebi-fl187-GCST90094734 | Weighted mode | 4 | 0.00829651763802552 | 0.0529437975000518 | 0.885429910029813 | -0.095473325 | 0.112066360738127 | 1.00833102911616 | 0.908942606954344 | 1.11858708844696 | 0.999714303421225 |
| ebi-fl187-GCST90094734 | ebi-IPs91-GCST90274819 | Leukemia inhibitory factor levels \|\| id:ebi-IPs91-GCST90274819 | Chips liking \|\| id:ebi-fl187-GCST90094734 | Weighted mode | 4 | 0.0496510443913168 | 0.196929710546897 | 0.817230248620148 | -0.336331188 | 0.435633277063235 | 1.0509043134299 | 0.714386470205997 | 1.54594175848139 | 0.999714303421225 |
| ebi-fl187-GCST90094734 | ebi-IPs91-GCST90274837 | STAM binding protein levels \|\| id:ebi-IPs91-GCST90274837 | Chips liking \|\| id:ebi-fl187-GCST90094734 | Weighted mode | 4 | -0.032063982 | 0.196979121468977 | 0.881039641269627 | -0.41814306 | 0.354015095699382 | 0.968444616705579 | 0.658268049559032 | 1.42477669431821 | 0.999714303421225 |
| ebi-fl187-GCST90094734 | ebi-IPs91-GCST90274840 | TNF-beta levels \|\| id:ebi-IPs91-GCST90274840 | Chips liking \|\| id:ebi-fl187-GCST90094734 | Weighted mode | 4 | 0.409286889072636 | 0.2914603146427 | 0.254863706136901 | -0.161975328 | 0.980549105772328 | 1.5057436399226 | 0.850462187201643 | 2.66591971199514 | 0.999714303421225 |
| ebi-fl187-GCST90094734 | ebi-IPs91-GCST90274842 | Tumor necrosis factor ligand superfamily member 14 levels \|\| id:ebi-IPs91-GCST90274842 | Chips liking \|\| id:ebi-fl187-GCST90094734 | Weighted mode | 4 | 0.230082099777162 | 0.273803747557672 | 0.462423429492282 | -0.306573245 | 0.7667374449902 | 1.25870334495166 | 0.735964610156466 | 2.15273137964565 | 0.999714303421225 |
| ebi-fl187-GCST90094734 | ebi-met1400-GCST90199628 | 3-methylhistidine levels \|\| id:ebi-met1400-GCST90199628 | Chips liking \|\| id:ebi-fl187-GCST90094734 | Weighted mode | 4 | 0.198540253546059 | 0.259668313093638 | 0.500154736373773 | -0.31040964 | 0.707490147209589 | 1.21962112050111 | 0.733146568456624 | 2.02889264107683 | 0.999714303421225 |
| ebi-fl187-GCST90094734 | ebi-met1400-GCST90199651 | Docosahexaenoate DHA; 22:6n3 levels \|\| id:ebi-met1400-GCST90199651 | Chips liking \|\| id:ebi-fl187-GCST90094734 | Weighted mode | 4 | -0.332512349 | 0.215771436704601 | 0.220960650774373 | -0.755424365 | 0.0903996664767571 | 0.717119812763803 | 0.469811200800711 | 1.09461167588581 | 0.999714303421225 |
| ebi-fl187-GCST90094734 | ebi-met1400-GCST90199652 | 1-stearoyl-2-oleoyl-GPS (18:0/18:1) levels \|\| id:ebi-met1400-GCST90199652 | Chips liking \|\| id:ebi-fl187-GCST90094734 | Weighted mode | 4 | 0.208710112106165 | 0.318015040846946 | 0.558483932211707 | -0.414599368 | 0.832019592166179 | 1.23208777942461 | 0.660604887184177 | 2.29795498891646 | 0.999714303421225 |
| ebi-fl187-GCST90094734 | ebi-met1400-GCST90199653 | 1-myristoyl-2-palmitoyl-gpc (14:0/16:0) levels \|\| id:ebi-met1400-GCST90199653 | Chips liking \|\| id:ebi-fl187-GCST90094734 | Weighted mode | 4 | 0.504354354613145 | 0.453200486213543 | 0.346910457361958 | -0.383918598 | 1.39262730759169 | 1.65591604069297 | 0.681186874647825 | 4.02541216790464 | 0.999714303421225 |
| ebi-fl187-GCST90094734 | ebi-met1400-GCST90199667 | Erythritol levels in elite athletes \|\| id:ebi-met1400-GCST90199667 | Chips liking \|\| id:ebi-fl187-GCST90094734 | Weighted mode | 4 | -0.03905161 | 0.207036403352597 | 0.86242771688706 | -0.44484296 | 0.366739741030062 | 0.961701074914495 | 0.640924918907045 | 1.44302231073938 | 0.999714303421225 |
| ebi-fl187-GCST90094734 | ebi-met1400-GCST90199674 | 3-hydroxymyristate levels \|\| id:ebi-met1400-GCST90199674 | Chips liking \|\| id:ebi-fl187-GCST90094734 | Weighted mode | 4 | 0.0416984605897377 | 0.22422887663695 | 0.864336805793167 | -0.397790138 | 0.481187058798159 | 1.04258005237303 | 0.671802999046352 | 1.61799391659332 | 0.999714303421225 |
| ebi-fl187-GCST90094734 | ebi-met1400-GCST90199685 | 2-linoleoylglycerol (18:2) levels \|\| id:ebi-met1400-GCST90199685 | Chips liking \|\| id:ebi-fl187-GCST90094734 | Weighted mode | 4 | -0.31728707 | 0.285626532068731 | 0.347657582701996 | -0.877115073 | 0.242540933135649 | 0.728121703428085 | 0.415981258019396 | 1.2744834166983 | 0.999714303421225 |
| ebi-fl187-GCST90094734 | ebi-met1400-GCST90199703 | Gamma-glutamylvaline levels \|\| id:ebi-met1400-GCST90199703 | Chips liking \|\| id:ebi-fl187-GCST90094734 | Weighted mode | 4 | 0.40800335038896 | 0.268653803634739 | 0.226143907776713 | -0.118558105 | 0.934564805513048 | 1.50381219951746 | 0.88820020551974 | 2.5461051656639 | 0.999714303421225 |
| ebi-fl187-GCST90094734 | ebi-met1400-GCST90199779 | Gamma-glutamyl-2-aminobutyrate levels \|\| id:ebi-met1400-GCST90199779 | Chips liking \|\| id:ebi-fl187-GCST90094734 | Weighted mode | 4 | 0.0381698789609052 | 0.23483604073875 | 0.881213113633256 | -0.422108761 | 0.498448518808855 | 1.03890770645015 | 0.655662725055828 | 1.64616529394683 | 0.999714303421225 |
| ebi-fl187-GCST90094734 | ebi-met1400-GCST90199824 | 4-vinylphenol sulfate levels \|\| id:ebi-met1400-GCST90199824 | Chips liking \|\| id:ebi-fl187-GCST90094734 | Weighted mode | 4 | 0.0324466229609262 | 0.239831943958814 | 0.900950653387048 | -0.437623987 | 0.502517233120202 | 1.03297875432508 | 0.645568479238498 | 1.65287671440474 | 0.999714303421225 |
| ebi-fl187-GCST90094734 | ebi-met1400-GCST90199830 | 4-ethylphenylsulfate levels \|\| id:ebi-met1400-GCST90199830 | Chips liking \|\| id:ebi-fl187-GCST90094734 | Weighted mode | 4 | 0.0328284569698667 | 0.260986972578507 | 0.907857775642728 | -0.478706009 | 0.54436292322374 | 1.03337325605629 | 0.619584610043032 | 1.72351002433423 | 0.999714303421225 |
| ebi-fl187-GCST90094734 | ebi-met1400-GCST90199833 | Hexanoylglutamine levels \|\| id:ebi-met1400-GCST90199833 | Chips liking \|\| id:ebi-fl187-GCST90094734 | Weighted mode | 4 | -0.054613009 | 0.229632325911346 | 0.827332219172831 | -0.504692368 | 0.395466349987725 | 0.946851500261068 | 0.603691261864945 | 1.48507659490888 | 0.999714303421225 |
| ebi-fl187-GCST90094734 | ebi-met1400-GCST90199837 | Carotenoid levels (cryptoxanthin) \|\| id:ebi-met1400-GCST90199837 | Chips liking \|\| id:ebi-fl187-GCST90094734 | Weighted mode | 4 | -0.033827651 | 0.269932611638071 | 0.908197441975255 | -0.56289557 | 0.49524026799213 | 0.966738106801665 | 0.569557480522874 | 1.64089244563075 | 0.999714303421225 |
| ebi-fl187-GCST90094734 | ebi-met1400-GCST90199869 | 2-hydroxyhippurate levels \|\| id:ebi-met1400-GCST90199869 | Chips liking \|\| id:ebi-fl187-GCST90094734 | Weighted mode | 4 | -0.669437372 | 0.415264546341842 | 0.205338535474115 | -1.483355883 | 0.144481138753358 | 0.511996560326935 | 0.226875043345916 | 1.15543990172126 | 0.999714303421225 |
| ebi-fl187-GCST90094734 | ebi-met1400-GCST90199902 | Hydantoin-5-propionate levels \|\| id:ebi-met1400-GCST90199902 | Chips liking \|\| id:ebi-fl187-GCST90094734 | Weighted mode | 4 | 0.302865958872467 | 0.301266687687767 | 0.388812997035771 | -0.287616749 | 0.893348666740491 | 1.35373299638326 | 0.750048994192386 | 2.44329775746187 | 0.999714303421225 |
| ebi-fl187-GCST90094734 | ebi-met1400-GCST90199904 | Pantoate levels \|\| id:ebi-met1400-GCST90199904 | Chips liking \|\| id:ebi-fl187-GCST90094734 | Weighted mode | 4 | 0.0432692835629913 | 0.265806115027035 | 0.881034911273058 | -0.477710702 | 0.564249269015979 | 1.04421904801974 | 0.620201594180125 | 1.75812740644226 | 0.999714303421225 |
| ebi-fl187-GCST90094734 | ebi-met1400-GCST90199936 | 3-methyl catechol sulfate (2) levels \|\| id:ebi-met1400-GCST90199936 | Chips liking \|\| id:ebi-fl187-GCST90094734 | Weighted mode | 4 | -0.047935188 | 0.288832824806406 | 0.878741298075438 | -0.614047524 | 0.51817714903548 | 0.953195563981926 | 0.541156087990876 | 1.67896435678673 | 0.999714303421225 |
| ebi-fl187-GCST90094734 | ebi-met1400-GCST90199977 | 4-hydroxychlorothalonil levels \|\| id:ebi-met1400-GCST90199977 | Chips liking \|\| id:ebi-fl187-GCST90094734 | Weighted mode | 4 | 0.366905369581824 | 0.229206716721266 | 0.207741786660458 | -0.082339795 | 0.816150534355506 | 1.44326133622905 | 0.920958968093752 | 2.261776427 | 0.999714303421225 |
| ebi-fl187-GCST90094734 | ebi-met1400-GCST90200040 | Sphingomyelin (d18:2/24:1, d18:1/24:2) levels \|\| id:ebi-met1400-GCST90200040 | Chips liking \|\| id:ebi-fl187-GCST90094734 | Weighted mode | 4 | 0.325392158744483 | 0.286492429443314 | 0.33857544653449 | -0.236133003 | 0.886917320453379 | 1.3845735121391 | 0.78967563774715 | 2.42763448545318 | 0.999714303421225 |
| ebi-fl187-GCST90094734 | ebi-met1400-GCST90200077 | 1-stearoyl-2-oleoyl-GPI (18:0/18:1) levels \|\| id:ebi-met1400-GCST90200077 | Chips liking \|\| id:ebi-fl187-GCST90094734 | Weighted mode | 4 | -0.275792293 | 0.278476702349262 | 0.395007411698734 | -0.82160663 | 0.270022043705995 | 0.758970557965204 | 0.439724612145412 | 1.30999332752274 | 0.999714303421225 |
| ebi-fl187-GCST90094734 | ebi-met1400-GCST90200080 | 1-palmitoyl-2-oleoyl-GPI (16:0/18:1) levels \|\| id:ebi-met1400-GCST90200080 | Chips liking \|\| id:ebi-fl187-GCST90094734 | Weighted mode | 4 | 0.0560961715576447 | 0.288671263670101 | 0.858335679965204 | -0.509699505 | 0.621891848351042 | 1.05769939944422 | 0.600676051703874 | 1.86244818053139 | 0.999714303421225 |
| ebi-fl187-GCST90094734 | ebi-met1400-GCST90200082 | 1-oleoyl-2-linoleoyl-GPE (18:1/18:2) levels \|\| id:ebi-met1400-GCST90200082 | Chips liking \|\| id:ebi-fl187-GCST90094734 | Weighted mode | 4 | 0.0303120950067268 | 0.400630634996246 | 0.944451874578289 | -0.75492395 | 0.815548139599369 | 1.03077618385828 | 0.470046360591639 | 2.26041435545226 | 0.999714303421225 |
| ebi-fl187-GCST90094734 | ebi-met1400-GCST90200100 | Perfluorooctanesulfonate (PFOS) levels \|\| id:ebi-met1400-GCST90200100 | Chips liking \|\| id:ebi-fl187-GCST90094734 | Weighted mode | 4 | 0.189315424855426 | 0.281931450040815 | 0.549987143205101 | -0.363270217 | 0.741901066935424 | 1.20842205872772 | 0.695398499456236 | 2.09992381801456 | 0.999714303421225 |
| ebi-fl187-GCST90094734 | ebi-met1400-GCST90200102 | 1-palmitoleoyl-2-linolenoyl-GPC (16:1/18:3) levels \|\| id:ebi-met1400-GCST90200102 | Chips liking \|\| id:ebi-fl187-GCST90094734 | Weighted mode | 4 | 0.220335053845548 | 0.265163193561581 | 0.466958903382375 | -0.299384806 | 0.740054913226247 | 1.24649430333858 | 0.74127410816589 | 2.09605061223564 | 0.999714303421225 |
| ebi-fl187-GCST90094734 | ebi-met1400-GCST90200114 | Glycosyl-N-tricosanoyl-sphingadienine (d18:2/23:0) levels \|\| id:ebi-met1400-GCST90200114 | Chips liking \|\| id:ebi-fl187-GCST90094734 | Weighted mode | 4 | -0.280148688 | 0.248768536477236 | 0.342057609983551 | -0.76773502 | 0.207437643221859 | 0.755671373630148 | 0.464062972314753 | 1.23052098312374 | 0.999714303421225 |
| ebi-fl187-GCST90094734 | ebi-met1400-GCST90200135 | Arachidoylcarnitine (C20) levels \|\| id:ebi-met1400-GCST90200135 | Chips liking \|\| id:ebi-fl187-GCST90094734 | Weighted mode | 4 | -0.563788164 | 0.358936199783535 | 0.214277900331427 | -1.267303116 | 0.139726787248533 | 0.569049323358436 | 0.281590014301818 | 1.149959572 | 0.999714303421225 |
| ebi-fl187-GCST90094734 | ebi-met1400-GCST90200146 | Docosahexaenoylcarnitine (C22:6) levels \|\| id:ebi-met1400-GCST90200146 | Chips liking \|\| id:ebi-fl187-GCST90094734 | Weighted mode | 4 | 0.318968839141796 | 0.374956872652774 | 0.457454924727801 | -0.415946631 | 1.05388430954123 | 1.375708456 | 0.659715477730259 | 2.86877270542712 | 0.999714303421225 |
| ebi-fl187-GCST90094734 | ebi-met1400-GCST90200162 | 3-hydroxyphenylacetoylglutamine levels \|\| id:ebi-met1400-GCST90200162 | Chips liking \|\| id:ebi-fl187-GCST90094734 | Weighted mode | 4 | -0.077526608 | 0.318168536471065 | 0.823202404131164 | -0.70113694 | 0.546083723165507 | 0.925402400667888 | 0.496021037024358 | 1.72647839353603 | 0.999714303421225 |
| ebi-fl187-GCST90094734 | ebi-met1400-GCST90200172 | 3-carboxy-4-methyl-5-pentyl-2-furanpropionate (3-CMPFP) levels \|\| id:ebi-met1400-GCST90200172 | Chips liking \|\| id:ebi-fl187-GCST90094734 | Weighted mode | 4 | -0.541227505 | 0.426881475544055 | 0.294307270784065 | -1.377915197 | 0.295460186997167 | 0.582033364793973 | 0.252103591858842 | 1.34374459021224 | 0.999714303421225 |
| ebi-fl187-GCST90094734 | ebi-met1400-GCST90200222 | Dihydrocaffeate sulfate (2) levels \|\| id:ebi-met1400-GCST90200222 | Chips liking \|\| id:ebi-fl187-GCST90094734 | Weighted mode | 4 | 0.111702453948331 | 0.330702904020881 | 0.757788723422681 | -0.536475238 | 0.759880145829258 | 1.11818010106772 | 0.584805925582672 | 2.13801995453117 | 0.999714303421225 |
| ebi-fl187-GCST90094734 | ebi-met1400-GCST90200223 | 4-methylhexanoylglutamine levels \|\| id:ebi-met1400-GCST90200223 | Chips liking \|\| id:ebi-fl187-GCST90094734 | Weighted mode | 4 | 0.293044569791027 | 0.330224369894126 | 0.440223075482367 | -0.354195195 | 0.940284334783515 | 1.34050253501813 | 0.701737978099999 | 2.56070941358394 | 0.999714303421225 |
| ebi-fl187-GCST90094734 | ebi-met1400-GCST90200224 | 2-ketocaprylate levels \|\| id:ebi-met1400-GCST90200224 | Chips liking \|\| id:ebi-fl187-GCST90094734 | Weighted mode | 4 | 0.115729027318207 | 0.269201450183909 | 0.69627908647443 | -0.411905815 | 0.643363869678669 | 1.12269161215507 | 0.662386659973843 | 1.90287113580024 | 0.999714303421225 |
| ebi-fl187-GCST90094734 | ebi-met1400-GCST90200243 | Hydroxypalmitoyl sphingomyelin (d18:1/16:0(OH)) levels \|\| id:ebi-met1400-GCST90200243 | Chips liking \|\| id:ebi-fl187-GCST90094734 | Weighted mode | 4 | 0.191848773352815 | 0.293741013500392 | 0.560266789647756 | -0.383883613 | 0.767581159813583 | 1.21148729394958 | 0.681210706562882 | 2.15454843745294 | 0.999714303421225 |
| ebi-fl187-GCST90094734 | ebi-met1400-GCST90200273 | 2-methoxyhydroquinone sulfate (2) levels \|\| id:ebi-met1400-GCST90200273 | Chips liking \|\| id:ebi-fl187-GCST90094734 | Weighted mode | 4 | -0.485361309 | 0.465222195156071 | 0.373486818231904 | -1.397196812 | 0.426474193414743 | 0.615474779952723 | 0.247289191459919 | 1.53184699469265 | 0.999714303421225 |
| ebi-fl187-GCST90094734 | ebi-met1400-GCST90200276 | 2,4-di-tert-butylphenol levels \|\| id:ebi-met1400-GCST90200276 | Chips liking \|\| id:ebi-fl187-GCST90094734 | Weighted mode | 4 | 0.00328161804776206 | 0.296484526510455 | 0.991863761567721 | -0.577828054 | 0.584391290008253 | 1.00328700845107 | 0.561115757206782 | 1.79389868917146 | 0.999714303421225 |
| ebi-fl187-GCST90094734 | ebi-met1400-GCST90200289 | N-acetylmethionine levels \|\| id:ebi-met1400-GCST90200289 | Chips liking \|\| id:ebi-fl187-GCST90094734 | Weighted mode | 4 | 0.0382857591791805 | 0.228778892959135 | 0.877740616899538 | -0.410120871 | 0.486692389379086 | 1.03902810227755 | 0.663570038900886 | 1.62692607265793 | 0.999714303421225 |
| ebi-fl187-GCST90094734 | ebi-met1400-GCST90200308 | 3-hydroxyisobutyrate levels \|\| id:ebi-met1400-GCST90200308 | Chips liking \|\| id:ebi-fl187-GCST90094734 | Weighted mode | 4 | 0.264511888985572 | 0.231279571733069 | 0.335743934231312 | -0.188796072 | 0.717819849582388 | 1.30279491201004 | 0.827955333076939 | 2.04995911609343 | 0.999714303421225 |
| ebi-fl187-GCST90094734 | ebi-met1400-GCST90200383 | Cystathionine levels \|\| id:ebi-met1400-GCST90200383 | Chips liking \|\| id:ebi-fl187-GCST90094734 | Weighted mode | 4 | -0.318514111 | 0.307632414328162 | 0.376632969835428 | -0.921473643 | 0.284445420957115 | 0.727228815865664 | 0.397932198710191 | 1.32902477442028 | 0.999714303421225 |
| ebi-fl187-GCST90094734 | ebi-met1400-GCST90200384 | Guanidinoacetate levels \|\| id:ebi-met1400-GCST90200384 | Chips liking \|\| id:ebi-fl187-GCST90094734 | Weighted mode | 4 | 0.140961325404679 | 0.226637640435311 | 0.578029007001321 | -0.30324845 | 0.585171100657889 | 1.15138011796354 | 0.738415614330851 | 1.79529813605452 | 0.999714303421225 |
| ebi-fl187-GCST90094734 | ebi-met1400-GCST90200403 | Citrulline levels \|\| id:ebi-met1400-GCST90200403 | Chips liking \|\| id:ebi-fl187-GCST90094734 | Weighted mode | 4 | 0.0500070140334689 | 0.236677797418594 | 0.84620209553594 | -0.413881469 | 0.513895496973913 | 1.05127847005254 | 0.661079305074746 | 1.67179098349032 | 0.999714303421225 |
| ebi-fl187-GCST90094734 | ebi-met1400-GCST90200434 | 12,13-DiHOME levels \|\| id:ebi-met1400-GCST90200434 | Chips liking \|\| id:ebi-fl187-GCST90094734 | Weighted mode | 4 | 0.358402339715277 | 0.420645493 | 0.456812114295347 | -0.466062826 | 1.18286750557192 | 1.4310412694058 | 0.627467861424406 | 3.26371953153698 | 0.999714303421225 |
| ebi-fl187-GCST90094734 | ebi-met1400-GCST90200437 | Fructose levels \|\| id:ebi-met1400-GCST90200437 | Chips liking \|\| id:ebi-fl187-GCST90094734 | Weighted mode | 4 | 0.152714730527767 | 0.308698298817887 | 0.654769661691644 | -0.452333935 | 0.757763396210826 | 1.16499259471281 | 0.636141704173432 | 2.13349908806743 | 0.999714303421225 |
| ebi-fl187-GCST90094734 | ebi-met1400-GCST90200448 | N-stearoyl-sphinganine (d18:0/18:0) levels \|\| id:ebi-met1400-GCST90200448 | Chips liking \|\| id:ebi-fl187-GCST90094734 | Weighted mode | 4 | 0.0539597771794105 | 0.268545502011903 | 0.853602158231851 | -0.472389407 | 0.580308961122739 | 1.0554421484411 | 0.623510666366918 | 1.78659033244259 | 0.999714303421225 |
| ebi-fl187-GCST90094734 | ebi-met1400-GCST90200468 | X-11847 levels \|\| id:ebi-met1400-GCST90200468 | Chips liking \|\| id:ebi-fl187-GCST90094734 | Weighted mode | 4 | -0.582606649 | 0.314313093392224 | 0.160850541580166 | -1.198660312 | 0.0334470141025141 | 0.558440808574486 | 0.301597988592072 | 1.03401265418626 | 0.999714303421225 |
| ebi-fl187-GCST90094734 | ebi-met1400-GCST90200476 | X-11843 levels \|\| id:ebi-met1400-GCST90200476 | Chips liking \|\| id:ebi-fl187-GCST90094734 | Weighted mode | 4 | -0.002094031 | 0.254899841089515 | 0.993961119449337 | -0.501697719 | 0.497509657924669 | 0.997908160341745 | 0.60550181459101 | 1.64462049903067 | 0.999714303421225 |
| ebi-fl187-GCST90094734 | ebi-met1400-GCST90200511 | X-12906 levels \|\| id:ebi-met1400-GCST90200511 | Chips liking \|\| id:ebi-fl187-GCST90094734 | Weighted mode | 4 | 0.226296956316259 | 0.255239297103872 | 0.440593766802076 | -0.273972066 | 0.726565978639849 | 1.25394797776744 | 0.760353314668148 | 2.06796695774688 | 0.999714303421225 |
| ebi-fl187-GCST90094734 | ebi-met1400-GCST90200515 | X-12849 levels \|\| id:ebi-met1400-GCST90200515 | Chips liking \|\| id:ebi-fl187-GCST90094734 | Weighted mode | 4 | 0.319328391298007 | 0.32022314268392 | 0.392159205431564 | -0.308308968 | 0.94696575095849 | 1.37620318385857 | 0.734688287501133 | 2.57787586311501 | 0.999714303421225 |
| ebi-fl187-GCST90094734 | ebi-met1400-GCST90200551 | X-18886 levels \|\| id:ebi-met1400-GCST90200551 | Chips liking \|\| id:ebi-fl187-GCST90094734 | Weighted mode | 4 | -0.270118768 | 0.234663420759589 | 0.333115874533935 | -0.730059073 | 0.189821536532742 | 0.763288834545798 | 0.481880523196019 | 1.20903381003695 | 0.999714303421225 |
| ebi-fl187-GCST90094734 | ebi-met1400-GCST90200564 | X-21319 levels \|\| id:ebi-met1400-GCST90200564 | Chips liking \|\| id:ebi-fl187-GCST90094734 | Weighted mode | 4 | -0.387278006 | 0.319980035770524 | 0.312818197660512 | -1.014438876 | 0.239882864532965 | 0.678902330054838 | 0.362605839710559 | 1.27110025067935 | 0.999714303421225 |
| ebi-fl187-GCST90094734 | ebi-met1400-GCST90200579 | X-21442 levels \|\| id:ebi-met1400-GCST90200579 | Chips liking \|\| id:ebi-fl187-GCST90094734 | Weighted mode | 4 | -0.167952847 | 0.29710822937683 | 0.61139124641108 | -0.750284976 | 0.414379283010117 | 0.845393696958636 | 0.47223195871977 | 1.5134310367196 | 0.999714303421225 |
| ebi-fl187-GCST90094734 | ebi-met1400-GCST90200581 | X-21441 levels \|\| id:ebi-met1400-GCST90200581 | Chips liking \|\| id:ebi-fl187-GCST90094734 | Weighted mode | 4 | 0.476243241946635 | 0.268747742757709 | 0.174500629066691 | -0.050502334 | 1.00298881775175 | 1.61001459141604 | 0.950751709749802 | 2.7264184307959 | 0.999714303421225 |
| ebi-fl187-GCST90094734 | ebi-met1400-GCST90200648 | X-24585 levels \|\| id:ebi-met1400-GCST90200648 | Chips liking \|\| id:ebi-fl187-GCST90094734 | Weighted mode | 4 | 0.0510012037831376 | 0.248134418385327 | 0.850308220212912 | -0.435342256 | 0.537344663818378 | 1.05232416005252 | 0.647043174604716 | 1.71145633134412 | 0.999714303421225 |
| ebi-fl187-GCST90094734 | ebi-met1400-GCST90200720 | Adenosine 5'-diphosphate (ADP) to pantothenate ratio \|\| id:ebi-met1400-GCST90200720 | Chips liking \|\| id:ebi-fl187-GCST90094734 | Weighted mode | 4 | -0.507165564 | 0.370389538918567 | 0.264414433194751 | -1.23312906 | 0.218797932725961 | 0.602200059846683 | 0.291379406145282 | 1.24457976243707 | 0.999714303421225 |
| ebi-fl187-GCST90094734 | ebi-met1400-GCST90200724 | Cholate to taurocholate ratio \|\| id:ebi-met1400-GCST90200724 | Chips liking \|\| id:ebi-fl187-GCST90094734 | Weighted mode | 4 | -0.088381251 | 0.331952980172847 | 0.807299943816631 | -0.739009092 | 0.562246590422534 | 0.915411808778595 | 0.477586925902221 | 1.75460996564815 | 0.999714303421225 |
| ebi-fl187-GCST90094734 | ebi-met1400-GCST90200726 | N-acetylputrescine to (N(1) + N(8))-acetylspermidine ratio \|\| id:ebi-met1400-GCST90200726 | Chips liking \|\| id:ebi-fl187-GCST90094734 | Weighted mode | 4 | -0.004836429 | 0.241699939290004 | 0.985291814293091 | -0.47856831 | 0.468895452253733 | 0.995175247934798 | 0.619669932421297 | 1.59822789889499 | 0.999714303421225 |
| ebi-fl187-GCST90094734 | ebi-met1400-GCST90200744 | Arginine to glutamate ratio \|\| id:ebi-met1400-GCST90200744 | Chips liking \|\| id:ebi-fl187-GCST90094734 | Weighted mode | 4 | -0.407462294 | 0.234128018297116 | 0.180174071462732 | -0.86635321 | 0.0514286216395996 | 0.665336537956369 | 0.420482166756199 | 1.05277403832547 | 0.999714303421225 |
| ebi-fl187-GCST90094734 | ebi-met1400-GCST90200759 | Histidine to trans-urocanate ratio \|\| id:ebi-met1400-GCST90200759 | Chips liking \|\| id:ebi-fl187-GCST90094734 | Weighted mode | 4 | 0.367439279410812 | 0.377198179392226 | 0.401839211165174 | -0.371869152 | 1.10674771101957 | 1.4440321133867 | 0.689444448913215 | 3.02450581446996 | 0.999714303421225 |
| ebi-fl187-GCST90094734 | ebi-met1400-GCST90200795 | Oleoyl-linoleoyl-glycerol (18:1 to 18:2) [2] to linoleoyl-arachidonoyl-glycerol (18:2 to 20:4) [2] ratio \|\| id:ebi-met1400-GCST90200795 | Chips liking \|\| id:ebi-fl187-GCST90094734 | Weighted mode | 4 | 0.341846470136334 | 0.206667444312847 | 0.196682101283043 | -0.063221721 | 0.746914660989514 | 1.40754418085816 | 0.93873531354663 | 2.1104784197183 | 0.999714303421225 |
| ebi-fl187-GCST90094734 | ebi-met1400-GCST90200811 | Choline to taurocholate ratio \|\| id:ebi-met1400-GCST90200811 | Chips liking \|\| id:ebi-fl187-GCST90094734 | Weighted mode | 4 | 0.458876265783692 | 0.405096196740335 | 0.33966300199359 | -0.33511228 | 1.25286481139475 | 1.5822949066492 | 0.715257772824596 | 3.50035646829941 | 0.999714303421225 |
| ebi-fl187-GCST90094734 | ebi-met1400-GCST90200831 | Cholate to phosphate ratio \|\| id:ebi-met1400-GCST90200831 | Chips liking \|\| id:ebi-fl187-GCST90094734 | Weighted mode | 4 | 0.068606464255764 | 0.256605131951547 | 0.806517353470789 | -0.434339594 | 0.571552522880796 | 1.07101464365836 | 0.647692265487405 | 1.77101445864488 | 0.999714303421225 |
| ebi-fl187-GCST90094734 | ebi-met1400-GCST90200841 | Adenosine 5'-diphosphate (ADP) to oxalate (ethanedioate) ratio \|\| id:ebi-met1400-GCST90200841 | Chips liking \|\| id:ebi-fl187-GCST90094734 | Weighted mode | 4 | -0.013913721 | 0.439807842705026 | 0.976749450012252 | -0.875937092 | 0.8481096510239 | 0.986182627760875 | 0.41647156443133 | 2.33522828053176 | 0.999714303421225 |
| ebi-fl187-GCST90094734 | ebi-met1400-GCST90200844 | Aspartate to phosphate ratio \|\| id:ebi-met1400-GCST90200844 | Chips liking \|\| id:ebi-fl187-GCST90094734 | Weighted mode | 4 | -0.245777523 | 0.397053869286037 | 0.57974091912562 | -1.024003106 | 0.532448061235852 | 0.782096204329148 | 0.359154325663097 | 1.7030964939563 | 0.999714303421225 |
| ebi-fl187-GCST90094734 | ebi-met1400-GCST90200870 | Adenosine 5'-monophosphate (AMP) to acetoacetate ratio \|\| id:ebi-met1400-GCST90200870 | Chips liking \|\| id:ebi-fl187-GCST90094734 | Weighted mode | 4 | -0.181437712 | 0.24227788623955 | 0.508297305798149 | -0.656302369 | 0.293426944643154 | 0.834070195927632 | 0.518765997470238 | 1.34101520748701 | 0.999714303421225 |
| ebi-fl187-GCST90094734 | ebi-met1400-GCST90200887 | Cholesterol to cortisol ratio \|\| id:ebi-met1400-GCST90200887 | Chips liking \|\| id:ebi-fl187-GCST90094734 | Weighted mode | 4 | -0.255895757 | 0.291651337526398 | 0.444860175712983 | -0.827532378 | 0.315740865031142 | 0.774222672250957 | 0.437126619781167 | 1.37127486431161 | 0.999714303421225 |
| ebi-fl187-GCST90094734 | ebi-met1400-GCST90200890 | Cortisol to taurocholate ratio \|\| id:ebi-met1400-GCST90200890 | Chips liking \|\| id:ebi-fl187-GCST90094734 | Weighted mode | 4 | 0.547171162500011 | 0.397800136948634 | 0.262689840716743 | -0.232517106 | 1.32685943091933 | 1.72835685522279 | 0.79253619217067 | 3.76918738665289 | 0.999714303421225 |
| ebi-fl187-GCST90094734 | ebi-met1400-GCST90200948 | Adenosine 5'-diphosphate (ADP) to mannitol to sorbitol ratio \|\| id:ebi-met1400-GCST90200948 | Chips liking \|\| id:ebi-fl187-GCST90094734 | Weighted mode | 4 | -0.527139022 | 0.352198095050933 | 0.231374646599211 | -1.217447288 | 0.163169244402741 | 0.590291366830242 | 0.295984767184475 | 1.17723591341829 | 0.999714303421225 |
| ebi-fl187-GCST90094734 | ebi-met1400-GCST90200972 | Leucine to N-palmitoyl-sphingosine (d18:1 to 16:0) ratio \|\| id:ebi-met1400-GCST90200972 | Chips liking \|\| id:ebi-fl187-GCST90094734 | Weighted mode | 4 | 0.0570024026398525 | 0.370954354985926 | 0.887629211465266 | -0.670068133 | 0.784072938412267 | 1.0586583539672 | 0.511673714665859 | 2.19037538630732 | 0.999714303421225 |
| ebi-fl187-GCST90094734 | ebi-met1400-GCST90200973 | Proline to glutamate ratio \|\| id:ebi-met1400-GCST90200973 | Chips liking \|\| id:ebi-fl187-GCST90094734 | Weighted mode | 4 | -0.436686403 | 0.244324136108106 | 0.171849549049722 | -0.91556171 | 0.0421889037542559 | 0.646174037868937 | 0.400291715154329 | 1.04309150404191 | 0.999714303421225 |
| ebi-fl187-GCST90094734 | ebi-met1400-GCST90200983 | Cholesterol to linoleoyl-arachidonoyl-glycerol (18:2 to 20:4) [1] ratio \|\| id:ebi-met1400-GCST90200983 | Chips liking \|\| id:ebi-fl187-GCST90094734 | Weighted mode | 4 | 0.990039419318118 | 0.293102331655571 | 0.0431615668347336 | 0.415558849273199 | 1.56451998936304 | 2.69134056106802 | 1.51521728220236 | 4.78037975195332 | 0.999714303421225 |
| ebi-fl187-GCST90094734 | ebi-met1400-GCST90201001 | Serine to alpha-ketobutyrate ratio \|\| id:ebi-met1400-GCST90201001 | Chips liking \|\| id:ebi-fl187-GCST90094734 | Weighted mode | 4 | 0.131771667745912 | 0.375197228801252 | 0.748650944208953 | -0.603614901 | 0.867158236196366 | 1.14084779717647 | 0.546831318007158 | 2.38013744542949 | 0.999714303421225 |
| ebi-fl187-GCST90094734 | ebi-met1400-GCST90201005 | Fructose to maltose ratio \|\| id:ebi-met1400-GCST90201005 | Chips liking \|\| id:ebi-fl187-GCST90094734 | Weighted mode | 4 | -0.330214814 | 0.29815832466996 | 0.34888904184403 | -0.91460513 | 0.25417550232462 | 0.718769315114766 | 0.400674809166906 | 1.28939807677138 | 0.999714303421225 |
| ebi-fl187-GCST90094734 | ebi-met1400-GCST90201020 | Paraxanthine to 5-acetylamino-6-formylamino-3-methyluracil ratio \|\| id:ebi-met1400-GCST90201020 | Chips liking \|\| id:ebi-fl187-GCST90094734 | Weighted mode | 4 | -0.075584982 | 0.276847797571807 | 0.802553419580056 | -0.618206665 | 0.467036701113669 | 0.927200931678401 | 0.538910017529994 | 1.59525995015939 | 0.999714303421225 |
| ebi-fl187-GCST90094734 | ebi-pw11-GCST90275197 | Placental weight (fetal genotype, adjusted for sex) \|\| id:ebi-pw11-GCST90275197 | Chips liking \|\| id:ebi-fl187-GCST90094734 | Weighted mode | 4 | 0.151363385360038 | 0.0930911545842287 | 0.202429189019182 | -0.031095278 | 0.333822048345126 | 1.16341935083667 | 0.969383208146104 | 1.39629464852171 | 0.999714303421225 |
| ebi-fl187-GCST90094734 | prot-a-1017 | FAS-associated factor 2 \|\| id:prot-a-1017 | Chips liking \|\| id:ebi-fl187-GCST90094734 | Weighted mode | 4 | -0.150614495 | 0.4822545708774 | 0.775251977553432 | -1.095833454 | 0.794604463996318 | 0.860179238213139 | 0.334260899788441 | 2.21356527886221 | 0.999714303421225 |
| ebi-fl187-GCST90094734 | prot-a-1019 | Fas apoptotic inhibitory molecule 3 \|\| id:prot-a-1019 | Chips liking \|\| id:ebi-fl187-GCST90094734 | Weighted mode | 4 | 0.235638672880216 | 0.409593036288807 | 0.605403265156799 | -0.567163678 | 1.03844102400628 | 1.26571688969443 | 0.567131727703225 | 2.82480976923244 | 0.999714303421225 |
| ebi-fl187-GCST90094734 | prot-a-1052 | MIP18 family protein FAM96A \|\| id:prot-a-1052 | Chips liking \|\| id:ebi-fl187-GCST90094734 | Weighted mode | 4 | -0.079698436 | 0.388195398901525 | 0.850475255423304 | -0.840561418 | 0.681164545747969 | 0.923394766931793 | 0.431468221416459 | 1.97617774212396 | 0.999714303421225 |
| ebi-fl187-GCST90094734 | prot-a-1066 | F-box/LRR-repeat protein 4 \|\| id:prot-a-1066 | Chips liking \|\| id:ebi-fl187-GCST90094734 | Weighted mode | 4 | -0.084597177 | 0.443406119476174 | 0.860871978078815 | -0.953673171 | 0.784478817063318 | 0.91888235670617 | 0.385323063232746 | 2.19126459335728 | 0.999714303421225 |
| ebi-fl187-GCST90094734 | prot-a-1076 | Low affinity immunoglobulin gamma Fc region receptor III-B \|\| id:prot-a-1076 | Chips liking \|\| id:ebi-fl187-GCST90094734 | Weighted mode | 4 | 0.0743200746401609 | 0.472616788777194 | 0.885033571104404 | -0.852008831 | 1.00064898064346 | 1.07715151921113 | 0.426557189247019 | 2.72004651331041 | 0.999714303421225 |
| ebi-fl187-GCST90094734 | prot-a-1081 | Fc receptor-like protein 3 \|\| id:prot-a-1081 | Chips liking \|\| id:ebi-fl187-GCST90094734 | Weighted mode | 4 | 0.235385586596978 | 0.375161467881404 | 0.574887956336194 | -0.49993089 | 0.970702063644529 | 1.26539659464404 | 0.606572578221708 | 2.63979711452017 | 0.999714303421225 |
| ebi-fl187-GCST90094734 | prot-a-1105 | Fibroblast growth factor receptor 4 \|\| id:prot-a-1105 | Chips liking \|\| id:ebi-fl187-GCST90094734 | Weighted mode | 4 | -0.041812556 | 0.401216522079285 | 0.923575715136608 | -0.828196939 | 0.744571827294456 | 0.95904953182403 | 0.43683621890243 | 2.1055397073138 | 0.999714303421225 |
| ebi-fl187-GCST90094734 | prot-a-1143 | Follistatin-related protein 1 \|\| id:prot-a-1143 | Chips liking \|\| id:ebi-fl187-GCST90094734 | Weighted mode | 4 | -0.02683442 | 0.381812491180496 | 0.94839220548614 | -0.775186902 | 0.721518062988204 | 0.973522424289805 | 0.460617682384755 | 2.05755433809735 | 0.999714303421225 |
| ebi-fl187-GCST90094734 | prot-a-1151 | Alpha-(1,3)-fucosyltransferase 10 \|\| id:prot-a-1151 | Chips liking \|\| id:ebi-fl187-GCST90094734 | Weighted mode | 4 | -0.140824867 | 0.401049832217312 | 0.748695588501047 | -0.926882539 | 0.645232804 | 0.868641425829486 | 0.395785635606761 | 1.90643080189187 | 0.999714303421225 |
| ebi-fl187-GCST90094734 | prot-a-1165 | Growth arrest and DNA damage-inducible proteins-interacting protein 1 \|\| id:prot-a-1165 | Chips liking \|\| id:ebi-fl187-GCST90094734 | Weighted mode | 4 | 0.0120069842051207 | 0.418308071182887 | 0.978903632415792 | -0.807876835 | 0.83189080372358 | 1.01207935741122 | 0.445803576541599 | 2.29765905792893 | 0.999714303421225 |
| ebi-fl187-GCST90094734 | prot-a-1194 | Growth/differentiation factor 11/8 \|\| id:prot-a-1194 | Chips liking \|\| id:ebi-fl187-GCST90094734 | Weighted mode | 4 | -0.138391231 | 0.370937922201648 | 0.733887489530197 | -0.865429558 | 0.588647096564385 | 0.870757957633558 | 0.420870725208991 | 1.8015494434916 | 0.999714303421225 |
| ebi-fl187-GCST90094734 | prot-a-1216 | D-glucuronyl C5-epimerase \|\| id:prot-a-1216 | Chips liking \|\| id:ebi-fl187-GCST90094734 | Weighted mode | 4 | 0.704302525105605 | 0.464565276393646 | 0.226767292884506 | -0.206245417 | 1.61485046683715 | 2.02243559482667 | 0.813633372621563 | 5.02713614369451 | 0.999714303421225 |
| ebi-fl187-GCST90094734 | prot-a-1246 | Glypican-3 \|\| id:prot-a-1246 | Chips liking \|\| id:ebi-fl187-GCST90094734 | Weighted mode | 4 | -0.23547232 | 0.361281418379666 | 0.561029213571573 | -0.9435839 | 0.472639260382209 | 0.790197535656815 | 0.389230370073295 | 1.60422257194505 | 0.999714303421225 |
| ebi-fl187-GCST90094734 | prot-a-1275 | Glutamate receptor 4 \|\| id:prot-a-1275 | Chips liking \|\| id:ebi-fl187-GCST90094734 | Weighted mode | 4 | 0.424233948935405 | 0.499668196814764 | 0.458242851203296 | -0.555115717 | 1.40358361469234 | 1.52841912398743 | 0.574005835222416 | 4.06975831119439 | 0.999714303421225 |
| ebi-fl187-GCST90094734 | prot-a-1289 | Glutathione S-transferase P \|\| id:prot-a-1289 | Chips liking \|\| id:ebi-fl187-GCST90094734 | Weighted mode | 4 | -0.245542672 | 0.445476556443174 | 0.619897712364278 | -1.118676723 | 0.627591378398949 | 0.782279901454643 | 0.326711839011252 | 1.87309356793407 | 0.999714303421225 |
| ebi-fl187-GCST90094734 | prot-a-130 | Apolipoprotein D \|\| id:prot-a-130 | Chips liking \|\| id:ebi-fl187-GCST90094734 | Weighted mode | 4 | -0.140740997 | 0.385341740351377 | 0.739165961585319 | -0.896010808 | 0.614528814527085 | 0.868714282548453 | 0.408194783627602 | 1.84878527353294 | 0.999714303421225 |
| ebi-fl187-GCST90094734 | prot-a-1313 | Heparin-binding EGF-like growth factor \|\| id:prot-a-1313 | Chips liking \|\| id:ebi-fl187-GCST90094734 | Weighted mode | 4 | 0.420527130349051 | 0.354186620790881 | 0.320552329507942 | -0.273678646 | 1.11473290709918 | 1.52276403923284 | 0.760576449972889 | 3.04875376993776 | 0.999714303421225 |
| ebi-fl187-GCST90094734 | prot-a-1347 | HLA class II histocompatibility antigen, DQ alpha 2 chain \|\| id:prot-a-1347 | Chips liking \|\| id:ebi-fl187-GCST90094734 | Weighted mode | 4 | 0.219060012232288 | 0.502334604984437 | 0.692250691309073 | -0.765515814 | 1.20363583800178 | 1.24490598403292 | 0.465093967326964 | 3.33221030147541 | 0.999714303421225 |
| ebi-fl187-GCST90094734 | prot-a-1357 | Non-histone chromosomal protein HMG-14 \|\| id:prot-a-1357 | Chips liking \|\| id:ebi-fl187-GCST90094734 | Weighted mode | 4 | 0.436545196773291 | 0.373530114443166 | 0.326945157145734 | -0.295573828 | 1.1686642210819 | 1.54735217623768 | 0.744104477295618 | 3.21769164191749 | 0.999714303421225 |
| ebi-fl187-GCST90094734 | prot-a-137 | Apolipoprotein M \|\| id:prot-a-137 | Chips liking \|\| id:ebi-fl187-GCST90094734 | Weighted mode | 4 | -0.001076182 | 0.366224865137175 | 0.997839837034937 | -0.718876918 | 0.716724553762908 | 0.998924396970116 | 0.48729922595276 | 2.04771503363489 | 0.999714303421225 |
| ebi-fl187-GCST90094734 | prot-a-1381 | 17-beta-hydroxysteroid dehydrogenase 14 \|\| id:prot-a-1381 | Chips liking \|\| id:ebi-fl187-GCST90094734 | Weighted mode | 4 | -0.342665246 | 0.392771411120896 | 0.447180987497391 | -1.112497212 | 0.427166719425803 | 0.709875805292161 | 0.328737009148805 | 1.53290820599724 | 0.999714303421225 |
| ebi-fl187-GCST90094734 | prot-a-1392 | Serine protease HTRA2, mitochondrial \|\| id:prot-a-1392 | Chips liking \|\| id:ebi-fl187-GCST90094734 | Weighted mode | 4 | 0.608264007180443 | 0.446522454133229 | 0.266385241659873 | -0.266920003 | 1.48344801728157 | 1.83723919471183 | 0.765734325524842 | 4.4081187770599 | 0.999714303421225 |
| ebi-fl187-GCST90094734 | prot-a-1416 | Interferon-induced protein with tetratricopeptide repeats 2 \|\| id:prot-a-1416 | Chips liking \|\| id:ebi-fl187-GCST90094734 | Weighted mode | 4 | 0.148941153069945 | 0.500330731573633 | 0.785367815437444 | -0.831707081 | 1.12958938695427 | 1.16060468917652 | 0.435305549940399 | 3.09438564411353 | 0.999714303421225 |
| ebi-fl187-GCST90094734 | prot-a-1428 | Interferon gamma \|\| id:prot-a-1428 | Chips liking \|\| id:ebi-fl187-GCST90094734 | Weighted mode | 4 | -0.071467961 | 0.435086327926685 | 0.879968669996099 | -0.924237164 | 0.781301241538543 | 0.931026106078318 | 0.39683402294664 | 2.18431273549322 | 0.999714303421225 |
| ebi-fl187-GCST90094734 | prot-a-1483 | Interleukin-17C \|\| id:prot-a-1483 | Chips liking \|\| id:ebi-fl187-GCST90094734 | Weighted mode | 4 | -0.209447858 | 0.383195656865324 | 0.622695619325666 | -0.960511345 | 0.541615629923897 | 0.811031927536797 | 0.38269714566654 | 1.71878153503971 | 0.999714303421225 |
| ebi-fl187-GCST90094734 | prot-a-1503 | Interleukin-1 receptor-like 2 \|\| id:prot-a-1503 | Chips liking \|\| id:ebi-fl187-GCST90094734 | Weighted mode | 4 | 0.60548699211288 | 0.551146823915742 | 0.352208133870445 | -0.474760783 | 1.68573476698773 | 1.83214423145454 | 0.622033839885668 | 5.39641458327917 | 0.999714303421225 |
| ebi-fl187-GCST90094734 | prot-a-1522 | Interleukin-31 receptor subunit alpha \|\| id:prot-a-1522 | Chips liking \|\| id:ebi-fl187-GCST90094734 | Weighted mode | 4 | 0.461549957793463 | 0.549618223813415 | 0.462689819218355 | -0.615701761 | 1.53880167646776 | 1.58653113656164 | 0.540261627771537 | 4.65900393048825 | 0.999714303421225 |
| ebi-fl187-GCST90094734 | prot-a-1540 | Interleukin-6 receptor subunit alpha \|\| id:prot-a-1540 | Chips liking \|\| id:ebi-fl187-GCST90094734 | Weighted mode | 4 | -0.163048902 | 0.390313678990817 | 0.704236098359998 | -0.928063713 | 0.601965909126005 | 0.849549642980403 | 0.395318419891785 | 1.82570444373854 | 0.999714303421225 |
| ebi-fl187-GCST90094734 | prot-a-1541 | Interleukin-6 receptor subunit alpha \|\| id:prot-a-1541 | Chips liking \|\| id:ebi-fl187-GCST90094734 | Weighted mode | 4 | -0.204708716 | 0.393929237364086 | 0.639209563897312 | -0.976810022 | 0.567392588757165 | 0.814884644315371 | 0.376510244716479 | 1.76366245768697 | 0.999714303421225 |
| ebi-fl187-GCST90094734 | prot-a-1545 | Interleukin-9 \|\| id:prot-a-1545 | Chips liking \|\| id:ebi-fl187-GCST90094734 | Weighted mode | 4 | 0.1379425857163 | 0.468983186074746 | 0.78783443696055 | -0.781264459 | 1.0571496304228 | 1.14790964197231 | 0.457826742011381 | 2.87815547939366 | 0.999714303421225 |
| ebi-fl187-GCST90094734 | prot-a-1548 | Inositol monophosphatase 3 \|\| id:prot-a-1548 | Chips liking \|\| id:ebi-fl187-GCST90094734 | Weighted mode | 4 | -0.170096557 | 0.552931380851834 | 0.778486450997987 | -1.253842064 | 0.913648949091227 | 0.843583358466228 | 0.285406139046123 | 2.49340425913599 | 0.999714303421225 |
| ebi-fl187-GCST90094734 | prot-a-1555 | Inhibin beta A chain:Inhibin beta B chain heterodimer \|\| id:prot-a-1555 | Chips liking \|\| id:ebi-fl187-GCST90094734 | Weighted mode | 4 | 0.44857883592553 | 0.422465477431747 | 0.366224665521598 | -0.3794535 | 1.27661117169175 | 1.5660849395704 | 0.684235241721892 | 3.58447195993174 | 0.999714303421225 |
| ebi-fl187-GCST90094734 | prot-a-1603 | Junctophilin-3 \|\| id:prot-a-1603 | Chips liking \|\| id:ebi-fl187-GCST90094734 | Weighted mode | 4 | 0.233443325867202 | 0.428144017824955 | 0.623508953323745 | -0.605718949 | 1.07260560080411 | 1.26294124976251 | 0.545681968034464 | 2.92298572022992 | 0.999714303421225 |
| ebi-fl187-GCST90094734 | prot-a-1627 | UPF0258 protein KIAA1024 \|\| id:prot-a-1627 | Chips liking \|\| id:ebi-fl187-GCST90094734 | Weighted mode | 4 | 0.539524345797374 | 0.429691587542351 | 0.298152470128289 | -0.302671166 | 1.38171985738038 | 1.71519083039898 | 0.738842012961524 | 3.98174377346617 | 0.999714303421225 |
| ebi-fl187-GCST90094734 | prot-a-1666 | Kallikrein-7 \|\| id:prot-a-1666 | Chips liking \|\| id:ebi-fl187-GCST90094734 | Weighted mode | 4 | 0.461215465202839 | 0.430685917795169 | 0.362717127148575 | -0.382928934 | 1.30535986408137 | 1.58600054239652 | 0.681861354944372 | 3.68901639936359 | 0.999714303421225 |
| ebi-fl187-GCST90094734 | prot-a-1678 | Importin subunit alpha-3 \|\| id:prot-a-1678 | Chips liking \|\| id:ebi-fl187-GCST90094734 | Weighted mode | 4 | 0.481183433629187 | 0.381592149271248 | 0.296457925382042 | -0.266737179 | 1.22910404620083 | 1.61798805110261 | 0.765874332918462 | 3.41816564544609 | 0.999714303421225 |
| ebi-fl187-GCST90094734 | prot-a-1718 | Protein LDOC1 \|\| id:prot-a-1718 | Chips liking \|\| id:ebi-fl187-GCST90094734 | Weighted mode | 4 | 0.226408693747988 | 0.500373889465075 | 0.681640722441624 | -0.75432413 | 1.20714151709953 | 1.25408809852222 | 0.470328388365847 | 3.34391246150276 | 0.999714303421225 |
| ebi-fl187-GCST90094734 | prot-a-1723 | Leptin \|\| id:prot-a-1723 | Chips liking \|\| id:ebi-fl187-GCST90094734 | Weighted mode | 4 | -0.086626258 | 0.49399989077702 | 0.871967087677667 | -1.054866044 | 0.881613527844301 | 0.917019760322215 | 0.348239072894585 | 2.41479290026702 | 0.999714303421225 |
| ebi-fl187-GCST90094734 | prot-a-1730 | Galectin-7 \|\| id:prot-a-1730 | Chips liking \|\| id:ebi-fl187-GCST90094734 | Weighted mode | 4 | -0.543558014 | 0.331077858316288 | 0.199174365863218 | -1.192470616 | 0.105354588722567 | 0.580678510448283 | 0.303470577891931 | 1.11110452564701 | 0.999714303421225 |
| ebi-fl187-GCST90094734 | prot-a-1753 | Lipase member N \|\| id:prot-a-1753 | Chips liking \|\| id:ebi-fl187-GCST90094734 | Weighted mode | 4 | 0.250339300942093 | 0.387647794007088 | 0.564409477391364 | -0.509450375 | 1.01012897719598 | 1.28446116164145 | 0.6008257167252 | 2.74595515777481 | 0.999714303421225 |
| ebi-fl187-GCST90094734 | prot-a-1756 | VIP36-like protein \|\| id:prot-a-1756 | Chips liking \|\| id:ebi-fl187-GCST90094734 | Weighted mode | 4 | 0.277465634211457 | 0.369760042642192 | 0.507509341180324 | -0.447264049 | 1.00219531779015 | 1.31978076305779 | 0.639375059403699 | 2.72425587598283 | 0.999714303421225 |
| ebi-fl187-GCST90094734 | prot-a-1760 | Ig Kappa chain V-I region HK102- like \|\| id:prot-a-1760 | Chips liking \|\| id:ebi-fl187-GCST90094734 | Weighted mode | 4 | 0.165476086507983 | 0.404172247545541 | 0.709709097443474 | -0.626701519 | 0.957653691697243 | 1.17995474554369 | 0.534351445595017 | 2.60557581159102 | 0.999714303421225 |
| ebi-fl187-GCST90094734 | prot-a-179 | Neutral ceramidase \|\| id:prot-a-179 | Chips liking \|\| id:ebi-fl187-GCST90094734 | Weighted mode | 4 | 0.0594485667853708 | 0.472473699850521 | 0.907829938312183 | -0.866599885 | 0.985497018492391 | 1.0612511760163 | 0.420378457178239 | 2.67914313724798 | 0.999714303421225 |
| ebi-fl187-GCST90094734 | prot-a-1809 | Lactotransferrin \|\| id:prot-a-1809 | Chips liking \|\| id:ebi-fl187-GCST90094734 | Weighted mode | 4 | 0.108676492999526 | 0.479555535257143 | 0.835283422271019 | -0.831252356 | 1.04860534210353 | 1.1148016458628 | 0.435503539142194 | 2.8536684502411 | 0.999714303421225 |
| ebi-fl187-GCST90094734 | prot-a-1818 | Lysophospholipase-like protein 1 \|\| id:prot-a-1818 | Chips liking \|\| id:ebi-fl187-GCST90094734 | Weighted mode | 4 | 0.0778989331834863 | 0.404721071988118 | 0.859662221542213 | -0.715354368 | 0.871152234280198 | 1.08101339856609 | 0.489018788520425 | 2.38966271912597 | 0.999714303421225 |
| ebi-fl187-GCST90094734 | prot-a-1852 | MAP kinase-activated protein kinase 3 \|\| id:prot-a-1852 | Chips liking \|\| id:ebi-fl187-GCST90094734 | Weighted mode | 4 | 0.42221834901247 | 0.363543871651176 | 0.329490855191408 | -0.290327639 | 1.13476433744878 | 1.52534154514467 | 0.748018447092167 | 3.11044044219629 | 0.999714303421225 |
| ebi-fl187-GCST90094734 | prot-a-1856 | Megakaryocyte-associated tyrosine-protein kinase \|\| id:prot-a-1856 | Chips liking \|\| id:ebi-fl187-GCST90094734 | Weighted mode | 4 | 0.183083840758258 | 0.484669144453156 | 0.730758714801255 | -0.766867682 | 1.13303536388644 | 1.20091508949188 | 0.464465646087889 | 3.10506721932321 | 0.999714303421225 |
| ebi-fl187-GCST90094734 | prot-a-1890 | Beta-1,3-N-acetylglucosaminyltransferase manic fringe \|\| id:prot-a-1890 | Chips liking \|\| id:ebi-fl187-GCST90094734 | Weighted mode | 4 | 0.460199544577022 | 0.53637748634123 | 0.453985453050347 | -0.591100329 | 1.51149941780583 | 1.58439010990725 | 0.553717677986037 | 4.53352334623349 | 0.999714303421225 |
| ebi-fl187-GCST90094734 | prot-a-1919 | Matrilysin \|\| id:prot-a-1919 | Chips liking \|\| id:ebi-fl187-GCST90094734 | Weighted mode | 4 | 1.0969925499452 | 0.421877480976988 | 0.0803575353277744 | 0.270112687230299 | 1.92387241266009 | 2.99514471703768 | 1.31011207531654 | 6.84742324341317 | 0.999714303421225 |
| ebi-fl187-GCST90094734 | prot-a-1923 | Multimerin-2 \|\| id:prot-a-1923 | Chips liking \|\| id:ebi-fl187-GCST90094734 | Weighted mode | 4 | -0.17071223 | 0.382831461972835 | 0.685873679711975 | -0.921061896 | 0.579637435067689 | 0.843064146800416 | 0.398096079972407 | 1.78539099322349 | 0.999714303421225 |
| ebi-fl187-GCST90094734 | prot-a-1948 | Mesothelin \|\| id:prot-a-1948 | Chips liking \|\| id:ebi-fl187-GCST90094734 | Weighted mode | 4 | -0.297102715 | 0.360541229472023 | 0.470318629252175 | -1.003763525 | 0.409558094389869 | 0.742967694236938 | 0.366497519725129 | 1.50615206098461 | 0.999714303421225 |
| ebi-fl187-GCST90094734 | prot-a-195 | ATPase family AAA domain-containing protein 1 \|\| id:prot-a-195 | Chips liking \|\| id:ebi-fl187-GCST90094734 | Weighted mode | 4 | 0.37201070061879 | 0.417837525857905 | 0.438881289153789 | -0.44695085 | 1.19097225130028 | 1.45064850404675 | 0.639575342590282 | 3.29027863045863 | 0.999714303421225 |
| ebi-fl187-GCST90094734 | prot-a-1966 | Mucin-1 \|\| id:prot-a-1966 | Chips liking \|\| id:ebi-fl187-GCST90094734 | Weighted mode | 4 | 0.401013941582437 | 0.480505317123789 | 0.465200472501556 | -0.54077648 | 1.34280436314506 | 1.49333808785089 | 0.582295935652696 | 3.82976852161346 | 0.999714303421225 |
| ebi-fl187-GCST90094734 | prot-a-1986 | Myocardial zonula adherens protein \|\| id:prot-a-1986 | Chips liking \|\| id:ebi-fl187-GCST90094734 | Weighted mode | 4 | 0.421368975649858 | 0.382504393850465 | 0.351084375253235 | -0.328339636 | 1.17107758759677 | 1.52404651072907 | 0.720118399820002 | 3.2254664891857 | 0.999714303421225 |
| ebi-fl187-GCST90094734 | prot-a-2071 | Epididymal secretory protein E1 \|\| id:prot-a-2071 | Chips liking \|\| id:ebi-fl187-GCST90094734 | Weighted mode | 4 | 0.529601321076711 | 0.474611062503089 | 0.34581055337912 | -0.400636361 | 1.45983900358276 | 1.69825511511537 | 0.66989361590926 | 4.30526634009029 | 0.999714303421225 |
| ebi-fl187-GCST90094734 | prot-a-2098 | Neuritin \|\| id:prot-a-2098 | Chips liking \|\| id:ebi-fl187-GCST90094734 | Weighted mode | 4 | 0.13280076309967 | 0.438515411241141 | 0.781795330562925 | -0.726689443 | 0.992290969132306 | 1.14202244265132 | 0.483507021029817 | 2.69740707537495 | 0.999714303421225 |
| ebi-fl187-GCST90094734 | prot-a-2115 | Neurotrophin-4 \|\| id:prot-a-2115 | Chips liking \|\| id:ebi-fl187-GCST90094734 | Weighted mode | 4 | 0.0275603582730484 | 0.46621020438206 | 0.956577429660801 | -0.886211642 | 0.941332358861887 | 1.02794365813883 | 0.412214414148174 | 2.5633945054818 | 0.999714303421225 |
| ebi-fl187-GCST90094734 | prot-a-2118 | Netrin-1 \|\| id:prot-a-2118 | Chips liking \|\| id:ebi-fl187-GCST90094734 | Weighted mode | 4 | 0.435264080778885 | 0.398060605629996 | 0.354132072132982 | -0.344934706 | 1.21546286781368 | 1.54537110787586 | 0.708266597336021 | 3.37185442605936 | 0.999714303421225 |
| ebi-fl187-GCST90094734 | prot-a-2120 | Netrin-G1 \|\| id:prot-a-2120 | Chips liking \|\| id:ebi-fl187-GCST90094734 | Weighted mode | 4 | 0.399427713601592 | 0.427337887184771 | 0.418899413689078 | -0.438154545 | 1.23700997248374 | 1.49097119090644 | 0.645226058509292 | 3.44529651708256 | 0.999714303421225 |
| ebi-fl187-GCST90094734 | prot-a-2145 | Olfactomedin-like protein 3 \|\| id:prot-a-2145 | Chips liking \|\| id:ebi-fl187-GCST90094734 | Weighted mode | 4 | 0.550873426242583 | 0.469747244313918 | 0.325559634564166 | -0.369831173 | 1.47157802509786 | 1.73476754785707 | 0.690850955354164 | 4.35610376127405 | 0.999714303421225 |
| ebi-fl187-GCST90094734 | prot-a-2162 | Prolyl 4-hydroxylase subunit alpha-2 \|\| id:prot-a-2162 | Chips liking \|\| id:ebi-fl187-GCST90094734 | Weighted mode | 4 | 0.587050732723418 | 0.377193233626761 | 0.217481348138367 | -0.152248005 | 1.32634947063187 | 1.79867580939534 | 0.858775273608145 | 3.76726574079287 | 0.999714303421225 |
| ebi-fl187-GCST90094734 | prot-a-2168 | Polyadenylate-binding protein-interacting protein 1 \|\| id:prot-a-2168 | Chips liking \|\| id:ebi-fl187-GCST90094734 | Weighted mode | 4 | 0.59307346772049 | 0.45919840730849 | 0.287023820303937 | -0.306955411 | 1.49310234604513 | 1.80954144471765 | 0.73568340385443 | 4.45088229936305 | 0.999714303421225 |
| ebi-fl187-GCST90094734 | prot-a-219 | Beta-1,4-galactosyltransferase 5 \|\| id:prot-a-219 | Chips liking \|\| id:ebi-fl187-GCST90094734 | Weighted mode | 4 | -0.01134884 | 0.381708506125158 | 0.97814837112654 | -0.759497512 | 0.736799832335817 | 0.988715315486551 | 0.467901482981064 | 2.0892388903098 | 0.999714303421225 |
| ebi-fl187-GCST90094734 | prot-a-2190 | Pyruvate carboxylase, mitochondrial \|\| id:prot-a-2190 | Chips liking \|\| id:ebi-fl187-GCST90094734 | Weighted mode | 4 | 0.361657782085806 | 0.404906702406853 | 0.437565738891147 | -0.431959355 | 1.15527491880324 | 1.43570753304333 | 0.649235764575684 | 3.17489613620488 | 0.999714303421225 |
| ebi-fl187-GCST90094734 | prot-a-2197 | Protocadherin-9 \|\| id:prot-a-2197 | Chips liking \|\| id:ebi-fl187-GCST90094734 | Weighted mode | 4 | -0.081356108 | 0.417194831506781 | 0.857846750554717 | -0.899057978 | 0.736345761863549 | 0.92186534946127 | 0.406952837902601 | 2.08829044396724 | 0.999714303421225 |
| ebi-fl187-GCST90094734 | prot-a-2198 | Protocadherin alpha-4 \|\| id:prot-a-2198 | Chips liking \|\| id:ebi-fl187-GCST90094734 | Weighted mode | 4 | -1.30337974 | 0.541113863629246 | 0.0951281661274722 | -2.363962913 | -0.242796568 | 0.27161226110594 | 0.0940467845032256 | 0.784431076221976 | 0.999714303421225 |
| ebi-fl187-GCST90094734 | prot-a-2254 | Placenta growth factor \|\| id:prot-a-2254 | Chips liking \|\| id:ebi-fl187-GCST90094734 | Weighted mode | 4 | 0.272247975417253 | 0.376702510812967 | 0.522102162554371 | -0.466088946 | 1.01058489661067 | 1.31291253098045 | 0.627451472407055 | 2.74720737747703 | 0.999714303421225 |
| ebi-fl187-GCST90094734 | prot-a-2261 | Membrane-associated progesterone receptor component 2 \|\| id:prot-a-2261 | Chips liking \|\| id:ebi-fl187-GCST90094734 | Weighted mode | 4 | -0.559448168 | 0.416830540697005 | 0.272082870904503 | -1.376436027 | 0.257539692138432 | 0.571524362489597 | 0.252476771793887 | 1.29374316139386 | 0.999714303421225 |
| ebi-fl187-GCST90094734 | prot-a-2268 | Polymeric immunoglobulin receptor \|\| id:prot-a-2268 | Chips liking \|\| id:ebi-fl187-GCST90094734 | Weighted mode | 4 | 0.100979896136513 | 0.368884186248958 | 0.802046632781341 | -0.622033109 | 0.823992901184471 | 1.1062544015524 | 0.536851849016879 | 2.27958384272152 | 0.999714303421225 |
| ebi-fl187-GCST90094734 | prot-a-2283 | Group 10 secretory phospholipase A2 \|\| id:prot-a-2283 | Chips liking \|\| id:ebi-fl187-GCST90094734 | Weighted mode | 4 | 0.497661207823945 | 0.714655295304957 | 0.536304115769483 | -0.903063171 | 1.89838558662166 | 1.64486975998891 | 0.405326172838862 | 6.67510935298417 | 0.999714303421225 |
| ebi-fl187-GCST90094734 | prot-a-2298 | Inactive phospholipase D5 \|\| id:prot-a-2298 | Chips liking \|\| id:ebi-fl187-GCST90094734 | Weighted mode | 4 | 0.639900626912016 | 0.389806070027508 | 0.199214328300752 | -0.12411927 | 1.40392052416593 | 1.89629242950725 | 0.883274486150075 | 4.07112968232564 | 0.999714303421225 |
| ebi-fl187-GCST90094734 | prot-a-2304 | Procollagen-lysine,2-oxoglutarate 5-dioxygenase 2 \|\| id:prot-a-2304 | Chips liking \|\| id:ebi-fl187-GCST90094734 | Weighted mode | 4 | -0.367259169 | 0.464581351207931 | 0.486954168232659 | -1.277838617 | 0.543320279510773 | 0.692630113626206 | 0.278638895389659 | 1.72171395393659 | 0.999714303421225 |
| ebi-fl187-GCST90094734 | prot-a-2331 | Periostin \|\| id:prot-a-2331 | Chips liking \|\| id:ebi-fl187-GCST90094734 | Weighted mode | 4 | 0.298074535038255 | 0.403653212257344 | 0.513768936627436 | -0.493085761 | 1.08923483106265 | 1.3472622023771 | 0.610738889250428 | 2.97199912090374 | 0.999714303421225 |
| ebi-fl187-GCST90094734 | prot-a-2333 | Periostin \|\| id:prot-a-2333 | Chips liking \|\| id:ebi-fl187-GCST90094734 | Weighted mode | 4 | -0.015458419 | 0.40058488433227 | 0.971641952121214 | -0.800604792 | 0.769687954247402 | 0.984660449023475 | 0.449057295563598 | 2.15909241303886 | 0.999714303421225 |
| ebi-fl187-GCST90094734 | prot-a-2367 | cAMP-dependent protein kinase catalytic subunit alpha \|\| id:prot-a-2367 | Chips liking \|\| id:ebi-fl187-GCST90094734 | Weighted mode | 4 | 0.0605217840072646 | 0.373201136343663 | 0.881479716584832 | -0.670952443 | 0.791996011240844 | 1.06239074044586 | 0.511221436441845 | 2.2077988224453 | 0.999714303421225 |
| ebi-fl187-GCST90094734 | prot-a-2385 | Phosphoribosyl pyrophosphate synthase-associated protein 1 \|\| id:prot-a-2385 | Chips liking \|\| id:ebi-fl187-GCST90094734 | Weighted mode | 4 | -0.546914656 | 0.45548970620484 | 0.316021269407769 | -1.43967448 | 0.345845168095236 | 0.578732647897193 | 0.237004895906335 | 1.41318379293929 | 0.999714303421225 |
| ebi-fl187-GCST90094734 | prot-a-2393 | Trypsin-3 \|\| id:prot-a-2393 | Chips liking \|\| id:ebi-fl187-GCST90094734 | Weighted mode | 4 | -0.314703827 | 0.459292948401473 | 0.542418114867433 | -1.214918005 | 0.58551035229099 | 0.730005050350047 | 0.296734343897723 | 1.79590729720269 | 0.999714303421225 |
| ebi-fl187-GCST90094734 | prot-a-2405 | Pregnancy-specific beta-1-glycoprotein 6 \|\| id:prot-a-2405 | Chips liking \|\| id:ebi-fl187-GCST90094734 | Weighted mode | 4 | 0.456882942234843 | 0.39546552702307 | 0.331628824735486 | -0.318229491 | 1.23199537520006 | 1.57914402235977 | 0.727435829477757 | 3.42806298824308 | 0.999714303421225 |
| ebi-fl187-GCST90094734 | prot-a-244 | Beclin-1 \|\| id:prot-a-244 | Chips liking \|\| id:ebi-fl187-GCST90094734 | Weighted mode | 4 | -0.138952922 | 0.399009669442636 | 0.750661013676664 | -0.921011874 | 0.643106030335376 | 0.870268998216007 | 0.39811599402704 | 1.90238056400329 | 0.999714303421225 |
| ebi-fl187-GCST90094734 | prot-a-2454 | tRNA pseudouridine synthase A, mitochondrial \|\| id:prot-a-2454 | Chips liking \|\| id:ebi-fl187-GCST90094734 | Weighted mode | 4 | -0.275688633 | 0.413413402849111 | 0.552566820634892 | -1.085978902 | 0.534601636960671 | 0.759049237139821 | 0.337571174934766 | 1.70676819344508 | 0.999714303421225 |
| ebi-fl187-GCST90094734 | prot-a-2466 | Glutaminyl-peptide cyclotransferase-like protein \|\| id:prot-a-2466 | Chips liking \|\| id:ebi-fl187-GCST90094734 | Weighted mode | 4 | 0.262299184585161 | 0.372105120645069 | 0.531667343452789 | -0.467026852 | 0.991625221049496 | 1.29991539890592 | 0.626863257729928 | 2.6956118794264 | 0.999714303421225 |
| ebi-fl187-GCST90094734 | prot-a-2474 | Ras-related protein Rab-27A \|\| id:prot-a-2474 | Chips liking \|\| id:ebi-fl187-GCST90094734 | Weighted mode | 4 | -0.344933976 | 0.803399166900462 | 0.696640678612021 | -1.919596343 | 1.22972839152703 | 0.708267114836887 | 0.146666153043122 | 3.42030042754225 | 0.999714303421225 |
| ebi-fl187-GCST90094734 | prot-a-2490 | Rap1 GTPase-GDP dissociation stimulator 1 \|\| id:prot-a-2490 | Chips liking \|\| id:ebi-fl187-GCST90094734 | Weighted mode | 4 | 0.034530127903994 | 0.493184787437409 | 0.94858789944715 | -0.932112055 | 1.00117231128132 | 1.03513321430043 | 0.393721270437193 | 2.72147036952851 | 0.999714303421225 |
| ebi-fl187-GCST90094734 | prot-a-2545 | Receptor-interacting serine/threonine-protein kinase 2 \|\| id:prot-a-2545 | Chips liking \|\| id:ebi-fl187-GCST90094734 | Weighted mode | 4 | 0.741307435275789 | 0.36887886769569 | 0.138033802259481 | 0.0183048545922367 | 1.46431001595934 | 2.09867760668521 | 1.01847341536576 | 4.32455833441684 | 0.999714303421225 |
| ebi-fl187-GCST90094734 | prot-a-2610 | RUN and FYVE domain-containing protein 1 \|\| id:prot-a-2610 | Chips liking \|\| id:ebi-fl187-GCST90094734 | Weighted mode | 4 | 0.68925519576249 | 0.467733415401449 | 0.23701554194589 | -0.227502298 | 1.60601268994933 | 1.99223115831852 | 0.796520590753066 | 4.9829031844898 | 0.999714303421225 |
| ebi-fl187-GCST90094734 | prot-a-2627 | Serine--tRNA ligase, mitochondrial \|\| id:prot-a-2627 | Chips liking \|\| id:ebi-fl187-GCST90094734 | Weighted mode | 4 | -0.027298571 | 0.437039644281595 | 0.954123285843823 | -0.883896274 | 0.829299132067213 | 0.973070667733619 | 0.413169948321379 | 2.29171198982517 | 0.999714303421225 |
| ebi-fl187-GCST90094734 | prot-a-264 | BolA-like protein 2 \|\| id:prot-a-264 | Chips liking \|\| id:ebi-fl187-GCST90094734 | Weighted mode | 4 | 0.215169182486008 | 0.370230623840886 | 0.601906303132761 | -0.51048284 | 0.940821205214144 | 1.2400716776292 | 0.600205705368509 | 2.56208455185203 | 0.999714303421225 |
| ebi-fl187-GCST90094734 | prot-a-2646 | Secretoglobin family 1C member 1 \|\| id:prot-a-2646 | Chips liking \|\| id:ebi-fl187-GCST90094734 | Weighted mode | 4 | 0.187153051099238 | 0.387558202158537 | 0.66221280332876 | -0.572461025 | 0.94676712732997 | 1.20581182175258 | 0.564135377571675 | 2.57736388690418 | 0.999714303421225 |
| ebi-fl187-GCST90094734 | prot-a-2672 | Semaphorin-3E \|\| id:prot-a-2672 | Chips liking \|\| id:ebi-fl187-GCST90094734 | Weighted mode | 4 | 0.424800854046772 | 0.515639959462717 | 0.470423306287895 | -0.585853467 | 1.4354551745937 | 1.52928583825029 | 0.556630593463734 | 4.20155701561392 | 0.999714303421225 |
| ebi-fl187-GCST90094734 | prot-a-2701 | Plasma protease C1 inhibitor \|\| id:prot-a-2701 | Chips liking \|\| id:ebi-fl187-GCST90094734 | Weighted mode | 4 | 0.180235038015758 | 0.440503357342089 | 0.709882325210545 | -0.683151542 | 1.04362161840625 | 1.19749878778672 | 0.505022880721009 | 2.83948193535979 | 0.999714303421225 |
| ebi-fl187-GCST90094734 | prot-a-2738 | Signaling threshold-regulating transmembrane adapter 1 \|\| id:prot-a-2738 | Chips liking \|\| id:ebi-fl187-GCST90094734 | Weighted mode | 4 | 0.574407718299161 | 0.440546987237801 | 0.283311621852609 | -0.289064377 | 1.43787981328525 | 1.77607827666799 | 0.74896398803047 | 4.211756634 | 0.999714303421225 |
| ebi-fl187-GCST90094734 | prot-a-274 | Serine/threonine-protein kinase BRSK2 \|\| id:prot-a-274 | Chips liking \|\| id:ebi-fl187-GCST90094734 | Weighted mode | 4 | -0.091017837 | 0.454437890810436 | 0.854065136572834 | -0.981716103 | 0.799680428766598 | 0.913001425345043 | 0.374667578601311 | 2.2248298232634 | 0.999714303421225 |
| ebi-fl187-GCST90094734 | prot-a-2749 | Mitochondrial glutamate carrier 2 \|\| id:prot-a-2749 | Chips liking \|\| id:ebi-fl187-GCST90094734 | Weighted mode | 4 | 0.604133870866746 | 0.480394639968358 | 0.297530887486497 | -0.337439623 | 1.54570736520473 | 1.82966679468315 | 0.713595057798419 | 4.69128890815802 | 0.999714303421225 |
| ebi-fl187-GCST90094734 | prot-a-2760 | Sodium-coupled monocarboxylate transporter 1 \|\| id:prot-a-2760 | Chips liking \|\| id:ebi-fl187-GCST90094734 | Weighted mode | 4 | 0.326951759830178 | 0.587567705919814 | 0.616716010872754 | -0.824680944 | 1.47858446343301 | 1.38673457925513 | 0.438374836376183 | 4.38673170476347 | 0.999714303421225 |
| ebi-fl187-GCST90094734 | prot-a-2777 | Small integral membrane protein 10 \|\| id:prot-a-2777 | Chips liking \|\| id:ebi-fl187-GCST90094734 | Weighted mode | 4 | -0.004076401 | 0.616879769721169 | 0.995142401334324 | -1.213160749 | 1.20500794782145 | 0.995931896411704 | 0.297256240509924 | 3.33678559813817 | 0.999714303421225 |
| ebi-fl187-GCST90094734 | prot-a-2799 | Superoxide dismutase [Mn], mitochondrial \|\| id:prot-a-2799 | Chips liking \|\| id:ebi-fl187-GCST90094734 | Weighted mode | 4 | 0.193776757731449 | 0.43970345172826 | 0.689254456493348 | -0.668042008 | 1.05559552311884 | 1.21382527559906 | 0.512711480782026 | 2.87368599087315 | 0.999714303421225 |
| ebi-fl187-GCST90094734 | prot-a-2805 | Protein SPACA7 \|\| id:prot-a-2805 | Chips liking \|\| id:ebi-fl187-GCST90094734 | Weighted mode | 4 | 0.442601960380025 | 0.47393464565416 | 0.419252326359424 | -0.486309945 | 1.37151386586218 | 1.55675256180485 | 0.614891195261105 | 3.94131280031889 | 0.999714303421225 |
| ebi-fl187-GCST90094734 | prot-a-2811 | SPARC-like protein 1 \|\| id:prot-a-2811 | Chips liking \|\| id:ebi-fl187-GCST90094734 | Weighted mode | 4 | -0.048344342 | 0.4385678570822 | 0.919185680703579 | -0.907937341 | 0.81124865829787 | 0.952805639980808 | 0.403355350930964 | 2.25071660877661 | 0.999714303421225 |
| ebi-fl187-GCST90094734 | prot-a-2831 | Sepiapterin reductase \|\| id:prot-a-2831 | Chips liking \|\| id:ebi-fl187-GCST90094734 | Weighted mode | 4 | -0.232300499 | 0.390127509322862 | 0.593473331019694 | -0.996950417 | 0.532349419733646 | 0.792707879952677 | 0.369003032502296 | 1.70292850624515 | 0.999714303421225 |
| ebi-fl187-GCST90094734 | prot-a-2834 | SPRY domain-containing SOCS box protein 1 \|\| id:prot-a-2834 | Chips liking \|\| id:ebi-fl187-GCST90094734 | Weighted mode | 4 | -0.100541395 | 0.520849468019555 | 0.859262084165079 | -1.121406352 | 0.920323562624827 | 0.904347676443066 | 0.325821252888574 | 2.51010243388777 | 0.999714303421225 |
| ebi-fl187-GCST90094734 | prot-a-2835 | Spectrin alpha chain, non-erythrocytic 1 \|\| id:prot-a-2835 | Chips liking \|\| id:ebi-fl187-GCST90094734 | Weighted mode | 4 | -0.523259501 | 0.498660061548265 | 0.371102831564662 | -1.500633221 | 0.454114219969349 | 0.592585862615577 | 0.222988914103106 | 1.57477785828168 | 0.999714303421225 |
| ebi-fl187-GCST90094734 | prot-a-2859 | Sia-alpha-2,3-Gal-beta-1,4-GlcNAc-R:alpha 2,8-sialyltransferase \|\| id:prot-a-2859 | Chips liking \|\| id:ebi-fl187-GCST90094734 | Weighted mode | 4 | 0.522409769980608 | 0.429650812982699 | 0.310973730922868 | -0.319705823 | 1.3645253634267 | 1.68608583712098 | 0.72636268450445 | 3.91386494761836 | 0.999714303421225 |
| ebi-fl187-GCST90094734 | prot-a-2861 | Alpha-2,8-sialyltransferase 8F \|\| id:prot-a-2861 | Chips liking \|\| id:ebi-fl187-GCST90094734 | Weighted mode | 4 | 0.505047749729543 | 0.542293317191053 | 0.420387649825442 | -0.557847152 | 1.56794265142401 | 1.65706464296033 | 0.572440114816902 | 4.79676940849531 | 0.999714303421225 |
| ebi-fl187-GCST90094734 | prot-a-289 | Uncharacterized protein C17orf89 \|\| id:prot-a-289 | Chips liking \|\| id:ebi-fl187-GCST90094734 | Weighted mode | 4 | 0.247222947554946 | 0.393776420828474 | 0.574657671695017 | -0.524578837 | 1.01902473237876 | 1.28046455739977 | 0.591804557911524 | 2.7704914753328 | 0.999714303421225 |
| ebi-fl187-GCST90094734 | prot-a-2921 | Tachykinin-3 \|\| id:prot-a-2921 | Chips liking \|\| id:ebi-fl187-GCST90094734 | Weighted mode | 4 | -0.450255552 | 0.601222242773465 | 0.5082880813423 | -1.628651148 | 0.728140043398339 | 0.637465225012343 | 0.19619403238861 | 2.07122463488155 | 0.999714303421225 |
| ebi-fl187-GCST90094734 | prot-a-2928 | Tax1-binding protein 1 \|\| id:prot-a-2928 | Chips liking \|\| id:ebi-fl187-GCST90094734 | Weighted mode | 4 | 0.143239563909896 | 0.383080161822147 | 0.733329855319137 | -0.607597553 | 0.894076681081305 | 1.15400622679768 | 0.544657809897009 | 2.4450771609052 | 0.999714303421225 |
| ebi-fl187-GCST90094734 | prot-a-296 | Uncharacterized protein C1orf185 \|\| id:prot-a-296 | Chips liking \|\| id:ebi-fl187-GCST90094734 | Weighted mode | 4 | 0.337787334927529 | 0.386216280749973 | 0.446163641478612 | -0.419196575 | 1.09477124519747 | 1.40184234879821 | 0.657574919547049 | 2.98849897170306 | 0.999714303421225 |
| ebi-fl187-GCST90094734 | prot-a-2964 | Transforming growth factor beta-2 \|\| id:prot-a-2964 | Chips liking \|\| id:ebi-fl187-GCST90094734 | Weighted mode | 4 | -0.341760158 | 0.337148949340134 | 0.385385383380968 | -1.002572099 | 0.319051782903225 | 0.710518596615686 | 0.366934434854567 | 1.37582256714831 | 0.999714303421225 |
| ebi-fl187-GCST90094734 | prot-a-2977 | Thymocyte nuclear protein 1 \|\| id:prot-a-2977 | Chips liking \|\| id:ebi-fl187-GCST90094734 | Weighted mode | 4 | 0.757350238733753 | 0.461060640580957 | 0.199002504523817 | -0.146328617 | 1.66102909427243 | 2.13261779903498 | 0.863873773095927 | 5.26472595696658 | 0.999714303421225 |
| ebi-fl187-GCST90094734 | prot-a-3015 | Transmembrane protein 70, mitochondrial \|\| id:prot-a-3015 | Chips liking \|\| id:ebi-fl187-GCST90094734 | Weighted mode | 4 | 0.107070141809022 | 0.449435182073207 | 0.827045709498781 | -0.773822815 | 0.987963098672508 | 1.11301232043907 | 0.461246433893053 | 2.68575827241278 | 0.999714303421225 |
| ebi-fl187-GCST90094734 | prot-a-3030 | Tumor necrosis factor alpha-induced protein 3 \|\| id:prot-a-3030 | Chips liking \|\| id:ebi-fl187-GCST90094734 | Weighted mode | 4 | -0.237918161 | 0.411135357662362 | 0.603386496807066 | -1.043743462 | 0.567907140326912 | 0.788267199696745 | 0.35213401137296 | 1.76457018648969 | 0.999714303421225 |
| ebi-fl187-GCST90094734 | prot-a-3032 | Tumor necrosis factor receptor superfamily member 10A \|\| id:prot-a-3032 | Chips liking \|\| id:ebi-fl187-GCST90094734 | Weighted mode | 4 | 0.3279932645156 | 0.438111681144032 | 0.508417588642543 | -0.530705631 | 1.1866921595579 | 1.38817962219573 | 0.588189778546577 | 3.276226031 | 0.999714303421225 |
| ebi-fl187-GCST90094734 | prot-a-3081 | Tumor protein p53-inducible protein 11 \|\| id:prot-a-3081 | Chips liking \|\| id:ebi-fl187-GCST90094734 | Weighted mode | 4 | 0.235664693513674 | 0.392965387082966 | 0.590972035141929 | -0.534547465 | 1.00587685219629 | 1.26574982487817 | 0.585934385876069 | 2.73430380226566 | 0.999714303421225 |
| ebi-fl187-GCST90094734 | prot-a-315 | Complement C4 \|\| id:prot-a-315 | Chips liking \|\| id:ebi-fl187-GCST90094734 | Weighted mode | 4 | 0.253192533674784 | 0.430431691749699 | 0.597728016842433 | -0.590453582 | 1.09683864950419 | 1.28813126160656 | 0.554075908784461 | 2.99468379841356 | 0.999714303421225 |
| ebi-fl187-GCST90094734 | prot-a-3157 | UDP-glucuronosyltransferase 1-8 \|\| id:prot-a-3157 | Chips liking \|\| id:ebi-fl187-GCST90094734 | Weighted mode | 4 | 0.331058729824435 | 0.452406149905453 | 0.517288932791325 | -0.555657324 | 1.21777478363912 | 1.39244156775565 | 0.573695033720931 | 3.37965888782119 | 0.999714303421225 |
| ebi-fl187-GCST90094734 | prot-a-317 | Glutaredoxin-like protein C5orf63 \|\| id:prot-a-317 | Chips liking \|\| id:ebi-fl187-GCST90094734 | Weighted mode | 4 | -0.01884833 | 0.443471652209063 | 0.968769262672417 | -0.888052769 | 0.850356107921514 | 0.981328188602043 | 0.411456173521279 | 2.34048016706976 | 0.999714303421225 |
| ebi-fl187-GCST90094734 | prot-a-3192 | Guanine nucleotide exchange factor VAV3 \|\| id:prot-a-3192 | Chips liking \|\| id:ebi-fl187-GCST90094734 | Weighted mode | 3 | -0.437121542 | 0.723814162875183 | 0.607277314075767 | -1.855797302 | 0.981554216759868 | 0.645892923214892 | 0.156328252190666 | 2.668600604 | 0.999714303421225 |
| ebi-fl187-GCST90094734 | prot-a-3233 | Serine/threonine-protein kinase WNK3 \|\| id:prot-a-3233 | Chips liking \|\| id:ebi-fl187-GCST90094734 | Weighted mode | 4 | -0.777983978 | 0.423105151822113 | 0.163241073557917 | -1.607270076 | 0.0513021191815404 | 0.459331099916252 | 0.200434037585865 | 1.05264086824519 | 0.999714303421225 |
| ebi-fl187-GCST90094734 | prot-a-3250 | 14-3-3 protein theta \|\| id:prot-a-3250 | Chips liking \|\| id:ebi-fl187-GCST90094734 | Weighted mode | 4 | 0.496970486561477 | 0.38374365827527 | 0.28595788157941 | -0.255167084 | 1.24910805678101 | 1.64373400576165 | 0.774787032893967 | 3.48723115770961 | 0.999714303421225 |
| ebi-fl187-GCST90094734 | prot-a-3278 | E3 ubiquitin-protein ligase ZNRF3 \|\| id:prot-a-3278 | Chips liking \|\| id:ebi-fl187-GCST90094734 | Weighted mode | 4 | 0.431154733823506 | 0.370078726482124 | 0.328220222735339 | -0.29419957 | 1.15650903772847 | 1.5390336720982 | 0.745127771393714 | 3.17881675436915 | 0.999714303421225 |
| ebi-fl187-GCST90094734 | prot-a-355 | Calpain-3 \|\| id:prot-a-355 | Chips liking \|\| id:ebi-fl187-GCST90094734 | Weighted mode | 4 | 0.345843168063177 | 0.393240658792406 | 0.4438990783994 | -0.424908523 | 1.11659485929629 | 1.41318096652923 | 0.653829592652841 | 3.05443569181007 | 0.999714303421225 |
| ebi-fl187-GCST90094734 | prot-a-385 | Coiled-coil domain-containing protein 90B, mitochondrial \|\| id:prot-a-385 | Chips liking \|\| id:ebi-fl187-GCST90094734 | Weighted mode | 4 | 0.468250301154848 | 0.401639253522465 | 0.327937123698228 | -0.318962636 | 1.25546323805888 | 1.59719713294539 | 0.726902708974073 | 3.50946371501273 | 0.999714303421225 |
| ebi-fl187-GCST90094734 | prot-a-390 | C-C motif chemokine 14 \|\| id:prot-a-390 | Chips liking \|\| id:ebi-fl187-GCST90094734 | Weighted mode | 4 | -0.117984946 | 0.397372777935113 | 0.785903919183392 | -0.896835591 | 0.660865698327335 | 0.88870943076756 | 0.407858250096587 | 1.93646800609811 | 0.999714303421225 |
| ebi-fl187-GCST90094734 | prot-a-40 | Pituitary adenylate cyclase-activating polypeptide 27 \|\| id:prot-a-40 | Chips liking \|\| id:ebi-fl187-GCST90094734 | Weighted mode | 4 | -0.035759816 | 0.410580508757437 | 0.936083145254869 | -0.840497613 | 0.768977980953222 | 0.964872012269927 | 0.431495751939107 | 2.15756005911548 | 0.999714303421225 |
| ebi-fl187-GCST90094734 | prot-a-401 | C-C motif chemokine 24 \|\| id:prot-a-401 | Chips liking \|\| id:ebi-fl187-GCST90094734 | Weighted mode | 4 | 0.0714580653236316 | 0.427866659518215 | 0.877984642488813 | -0.767160587 | 0.910076717979334 | 1.0740731088487 | 0.464329621717485 | 2.4845131329007 | 0.999714303421225 |
| ebi-fl187-GCST90094734 | prot-a-412 | Cerebral cavernous malformations 2 protein \|\| id:prot-a-412 | Chips liking \|\| id:ebi-fl187-GCST90094734 | Weighted mode | 3 | -0.175702469 | 0.561372940407713 | 0.783913470836875 | -1.275993432 | 0.924588494428203 | 0.838867535499329 | 0.27915351040471 | 2.52083071101098 | 0.999714303421225 |
| ebi-fl187-GCST90094734 | prot-a-44 | Adhesion G protein-coupled receptor F5 \|\| id:prot-a-44 | Chips liking \|\| id:ebi-fl187-GCST90094734 | Weighted mode | 4 | 0.221162047362479 | 0.380003572719572 | 0.601417204281647 | -0.523644955 | 0.96596904989284 | 1.24752557241398 | 0.592357491742754 | 2.62733243948352 | 0.999714303421225 |
| ebi-fl187-GCST90094734 | prot-a-48 | Adrenomedullin \|\| id:prot-a-48 | Chips liking \|\| id:ebi-fl187-GCST90094734 | Weighted mode | 4 | 0.634008634654156 | 0.445830886416887 | 0.25014212698628 | -0.239819903 | 1.50783717203125 | 1.88515234008214 | 0.786769543360304 | 4.51695083434322 | 0.999714303421225 |
| ebi-fl187-GCST90094734 | prot-a-502 | Carcinoembryonic antigen-related cell adhesion molecule 1 \|\| id:prot-a-502 | Chips liking \|\| id:ebi-fl187-GCST90094734 | Weighted mode | 4 | 0.179318669232313 | 0.402858755692054 | 0.686394003739634 | -0.610284492 | 0.968921830388739 | 1.19640193991471 | 0.543196312126432 | 2.63510184048989 | 0.999714303421225 |
| ebi-fl187-GCST90094734 | prot-a-518 | Complement factor B \|\| id:prot-a-518 | Chips liking \|\| id:ebi-fl187-GCST90094734 | Weighted mode | 4 | 0.0370701082320759 | 0.383257002179156 | 0.929045173935192 | -0.714113616 | 0.788253832503222 | 1.03776577421156 | 0.489625916068213 | 2.19955228426855 | 0.999714303421225 |
| ebi-fl187-GCST90094734 | prot-a-525 | Properdin \|\| id:prot-a-525 | Chips liking \|\| id:ebi-fl187-GCST90094734 | Weighted mode | 4 | 0.481938161506862 | 0.63940869909628 | 0.505775937439283 | -0.771302889 | 1.73517921173557 | 1.61920965272094 | 0.462410206622586 | 5.66994383323501 | 0.999714303421225 |
| ebi-fl187-GCST90094734 | prot-a-533 | Chondroadherin \|\| id:prot-a-533 | Chips liking \|\| id:ebi-fl187-GCST90094734 | Weighted mode | 4 | -0.159109553 | 0.565072257537415 | 0.796575828049077 | -1.266651178 | 0.948432071918482 | 0.852902915901399 | 0.28177365346412 | 2.5816586292222 | 0.999714303421225 |
| ebi-fl187-GCST90094734 | prot-a-535 | Coiled-coil-helix-coiled-coil-helix domain-containing protein 2 \|\| id:prot-a-535 | Chips liking \|\| id:ebi-fl187-GCST90094734 | Weighted mode | 4 | 0.559263527524482 | 0.452346909679696 | 0.304297177242619 | -0.327336415 | 1.44586347049669 | 1.74938365284458 | 0.72084120011623 | 4.24551643877512 | 0.999714303421225 |
| ebi-fl187-GCST90094734 | prot-a-591 | Caseinolytic peptidase B protein homolog \|\| id:prot-a-591 | Chips liking \|\| id:ebi-fl187-GCST90094734 | Weighted mode | 3 | -0.079428723 | 0.452878370131652 | 0.876925903197019 | -0.967070328 | 0.808212882625464 | 0.923643852339912 | 0.380195255375868 | 2.24389429879104 | 0.999714303421225 |
| ebi-fl187-GCST90094734 | prot-a-605 | Ciliary neurotrophic factor receptor subunit alpha \|\| id:prot-a-605 | Chips liking \|\| id:ebi-fl187-GCST90094734 | Weighted mode | 4 | 0.13396213555079 | 0.379799509561713 | 0.747626117890233 | -0.610444903 | 0.878369174291748 | 1.14334952652494 | 0.543109184306617 | 2.40697115345924 | 0.999714303421225 |
| ebi-fl187-GCST90094734 | prot-a-606 | Ciliary neurotrophic factor receptor subunit alpha \|\| id:prot-a-606 | Chips liking \|\| id:ebi-fl187-GCST90094734 | Weighted mode | 4 | 0.327851036406401 | 0.392790509319472 | 0.465149947616979 | -0.442018362 | 1.09772043467257 | 1.3879821980728 | 0.642737833485215 | 2.99732563076408 | 0.999714303421225 |
| ebi-fl187-GCST90094734 | prot-a-707 | Cancer/testis antigen 55 \|\| id:prot-a-707 | Chips liking \|\| id:ebi-fl187-GCST90094734 | Weighted mode | 4 | 0.0141318230288394 | 0.403711326125446 | 0.974274814311878 | -0.777142376 | 0.805406022234713 | 1.01423214928043 | 0.459717836702776 | 2.23760483172001 | 0.999714303421225 |
| ebi-fl187-GCST90094734 | prot-a-719 | Dipeptidyl peptidase 1 \|\| id:prot-a-719 | Chips liking \|\| id:ebi-fl187-GCST90094734 | Weighted mode | 4 | -0.852567501 | 0.379668909902287 | 0.11039160046673 | -1.596718565 | -0.108416438 | 0.42631895118224 | 0.20256011656434 | 0.897253868233219 | 0.999714303421225 |
| ebi-fl187-GCST90094734 | prot-a-752 | Cytochrome b5 \|\| id:prot-a-752 | Chips liking \|\| id:ebi-fl187-GCST90094734 | Weighted mode | 4 | -0.172880338 | 0.372893218634752 | 0.674487341915865 | -0.903751046 | 0.557990370704713 | 0.841238273228181 | 0.405047454820475 | 1.74715783032778 | 0.999714303421225 |
| ebi-fl187-GCST90094734 | prot-a-755 | Cytoglobin \|\| id:prot-a-755 | Chips liking \|\| id:ebi-fl187-GCST90094734 | Weighted mode | 4 | 0.282032733952985 | 0.4605729136742 | 0.583594694931954 | -0.620690177 | 1.18475564475442 | 1.32582211867538 | 0.53757328889195 | 3.26988770962203 | 0.999714303421225 |
| ebi-fl187-GCST90094734 | prot-a-807 | Desmin \|\| id:prot-a-807 | Chips liking \|\| id:ebi-fl187-GCST90094734 | Weighted mode | 4 | -0.348698445 | 0.532679986930676 | 0.559428079362255 | -1.392751219 | 0.695354329605123 | 0.705605877327124 | 0.248390985667873 | 2.00441917318329 | 0.999714303421225 |
| ebi-fl187-GCST90094734 | prot-a-82 | Ameloblastin \|\| id:prot-a-82 | Chips liking \|\| id:ebi-fl187-GCST90094734 | Weighted mode | 4 | 0.243055043369723 | 0.416627982956057 | 0.600594783747449 | -0.573535803 | 1.0596458899636 | 1.27513881012378 | 0.563529382939781 | 2.8853490772772 | 0.999714303421225 |
| ebi-fl187-GCST90094734 | prot-a-84 | Muellerian-inhibiting factor \|\| id:prot-a-84 | Chips liking \|\| id:ebi-fl187-GCST90094734 | Weighted mode | 4 | 0.371331756965825 | 0.606720221713782 | 0.583780984540518 | -0.817839878 | 1.56050339152484 | 1.44966392972531 | 0.441384069087265 | 4.76121739847191 | 0.999714303421225 |
| ebi-fl187-GCST90094734 | prot-a-858 | Inactive dipeptidyl peptidase 10 \|\| id:prot-a-858 | Chips liking \|\| id:ebi-fl187-GCST90094734 | Weighted mode | 4 | 1.01128684393571 | 0.535653039036399 | 0.155468682364118 | -0.038593113 | 2.06116680044706 | 2.7491364493212 | 0.96214211303856 | 7.85512983432154 | 0.999714303421225 |
| ebi-fl187-GCST90094734 | prot-a-863 | Dihydropyrimidinase-related protein 3 \|\| id:prot-a-863 | Chips liking \|\| id:ebi-fl187-GCST90094734 | Weighted mode | 4 | 0.348493350404546 | 0.433823921250066 | 0.480550569239922 | -0.501801535 | 1.19878823605468 | 1.41693112087337 | 0.605438957017339 | 3.31609616135415 | 0.999714303421225 |
| ebi-fl187-GCST90094734 | prot-a-864 | Dihydropyrimidinase-related protein 5 \|\| id:prot-a-864 | Chips liking \|\| id:ebi-fl187-GCST90094734 | Weighted mode | 4 | -0.208818571 | 0.537241896774876 | 0.723452352595527 | -1.261812688 | 0.844175547003102 | 0.811542459887786 | 0.283140315888538 | 2.32605929725665 | 0.999714303421225 |
| ebi-fl187-GCST90094734 | prot-a-884 | Endothelin-converting enzyme 1 \|\| id:prot-a-884 | Chips liking \|\| id:ebi-fl187-GCST90094734 | Weighted mode | 4 | -0.519523249 | 0.466626034062691 | 0.346730582090774 | -1.434110276 | 0.395063777423457 | 0.59480405360955 | 0.238327315219507 | 1.48447886413901 | 0.999714303421225 |
| ebi-fl187-GCST90094734 | prot-a-893 | Early endosome antigen 1 \|\| id:prot-a-893 | Chips liking \|\| id:ebi-fl187-GCST90094734 | Weighted mode | 3 | 0.120061530737646 | 0.437712893800857 | 0.809593956538939 | -0.737855741 | 0.977978802587326 | 1.12756622942676 | 0.478138068907555 | 2.65907628867238 | 0.999714303421225 |
| ebi-fl187-GCST90094734 | prot-a-897 | Ephrin-A2 \|\| id:prot-a-897 | Chips liking \|\| id:ebi-fl187-GCST90094734 | Weighted mode | 4 | 0.33253537464007 | 0.517165880128195 | 0.565994858585589 | -0.68110975 | 1.34618049969133 | 1.39449922822069 | 0.506055085795686 | 3.84272019408817 | 0.999714303421225 |
| ebi-fl187-GCST90094734 | prot-a-90 | Anaphase-promoting complex subunit 10 \|\| id:prot-a-90 | Chips liking \|\| id:ebi-fl187-GCST90094734 | Weighted mode | 4 | 0.020682214759956 | 0.381419984848155 | 0.960165489705789 | -0.726900956 | 0.76826538506234 | 1.02089757390256 | 0.483404764012811 | 2.15602313834977 | 0.999714303421225 |
| ebi-fl187-GCST90094734 | prot-a-914 | Histone-lysine N-methyltransferase EHMT2 \|\| id:prot-a-914 | Chips liking \|\| id:ebi-fl187-GCST90094734 | Weighted mode | 4 | -0.494006754 | 0.550041926542379 | 0.435306061176823 | -1.572088931 | 0.584075421528604 | 0.610176661641881 | 0.207611044026843 | 1.79333214260168 | 0.999714303421225 |
| ebi-fl187-GCST90094734 | prot-a-932 | RNA polymerase II elongation factor ELL \|\| id:prot-a-932 | Chips liking \|\| id:ebi-fl187-GCST90094734 | Weighted mode | 4 | -0.127537884 | 0.42379876825303 | 0.783112270608572 | -0.958183469 | 0.703107702117327 | 0.880260067730655 | 0.383589054720247 | 2.0200205853274 | 0.999714303421225 |
| ebi-fl187-GCST90094734 | prot-a-991 | Estrogen receptor \|\| id:prot-a-991 | Chips liking \|\| id:ebi-fl187-GCST90094734 | Weighted mode | 4 | 0.387226550053108 | 0.385278423241902 | 0.388916690555803 | -0.36791916 | 1.14237225960724 | 1.47289013681672 | 0.692173135048669 | 3.13419467656663 | 0.999714303421225 |
| ebi-fl187-GCST90094734 | prot-a-992 | Persulfide dioxygenase ETHE1, mitochondrial \|\| id:prot-a-992 | Chips liking \|\| id:ebi-fl187-GCST90094734 | Weighted mode | 4 | 0.506837947262777 | 0.379337951254178 | 0.273820941605488 | -0.236664437 | 1.25034033172097 | 1.66003377286813 | 0.789256088573136 | 3.49153103404592 | 0.999714303421225 |
| ebi-fl187-GCST90094734 | prot-a-993 | Ethanolamine kinase 1 \|\| id:prot-a-993 | Chips liking \|\| id:ebi-fl187-GCST90094734 | Weighted mode | 4 | 0.5350378711857 | 0.369124800543712 | 0.243067475570604 | -0.188446738 | 1.25852248025138 | 1.70751290656812 | 0.828244616328023 | 3.52021645371249 | 0.999714303421225 |
| ebi-fl187-GCST90094734 | prot-b-10 | coagulation factor III, tissue factor \|\| id:prot-b-10 | Chips liking \|\| id:ebi-fl187-GCST90094734 | Weighted mode | 4 | -0.601121529 | 0.514130389013037 | 0.326771979425124 | -1.608817092 | 0.406574033016959 | 0.548196472708823 | 0.20012420265378 | 1.50166430999004 | 0.999714303421225 |
| ebi-fl187-GCST90094734 | prot-b-28 | prolactin \|\| id:prot-b-28 | Chips liking \|\| id:ebi-fl187-GCST90094734 | Weighted mode | 4 | 0.158656324400958 | 0.402384431764964 | 0.719723482491832 | -0.630017162 | 0.947329810660287 | 1.17193511196081 | 0.532582660820271 | 2.57881453468889 | 0.999714303421225 |
| ebi-fl187-GCST90094734 | prot-b-54 | matrix metallopeptidase 7 \|\| id:prot-b-54 | Chips liking \|\| id:ebi-fl187-GCST90094734 | Weighted mode | 4 | 0.0109679817978634 | 0.363569448816775 | 0.977828196850815 | -0.701628138 | 0.723564101478742 | 1.01102835061638 | 0.495777452271447 | 2.06176848315083 | 0.999714303421225 |
| ebi-fl187-GCST90094734 | ukb-rsfMRI191-edge-pheno716 | Net100_Pair10_33 (OccipitalPrecuneus)&(Temporal\|Frontal\|Supp_Motor_Area) (Default_mode\|Central_executive)&(Default_mode\|Salience) \|\| id:ukb-rsfMRI191-edge-pheno716 | Chips liking \|\| id:ebi-fl187-GCST90094734 | Weighted mode | 4 | 0.0243596477087797 | 0.133628635488303 | 0.866974831701507 | -0.237552478 | 0.286271773265853 | 1.02465876780869 | 0.788555508198916 | 1.33145425975818 | 0.999714303421225 |
| ebi-fl187-GCST90094734 | ukb-rsfMRI191-edge-pheno903 | Net100_Pair27_38 (Precentral\|Frontal\|Supp_Motor_Area)&(Subcortical) (Attention\|Salience\|Motor)&(Subcortical-cerebellum) \|\| id:ukb-rsfMRI191-edge-pheno903 | Chips liking \|\| id:ebi-fl187-GCST90094734 | Weighted mode | 4 | 0.0997953321984096 | 0.110206403254515 | 0.431936700706589 | -0.116209218 | 0.315799882577258 | 1.10494474831909 | 0.89028893917922 | 1.37135579597733 | 0.999714303421225 |
| ebi-fl187-GCST90094734 | ukb-rsfMRI191-node-pheno23 | Net100_Node2 RolandicOper\|SupraMarginal\|Insula Salience\|Motor \|\| id:ukb-rsfMRI191-node-pheno23 | Chips liking \|\| id:ebi-fl187-GCST90094734 | Weighted mode | 4 | 0.0945591661839422 | 0.298566501986407 | 0.772222562316515 | -0.490631178 | 0.6797495100773 | 1.09917419515174 | 0.612239840065212 | 1.97338335767039 | 0.999714303421225 |
| ebi-fl187-GCST90094734 | ukb-rsfMRI191-node-pheno55 | Net100_Node34 Parietal\|Postcentral\|Precuneus Attention \|\| id:ukb-rsfMRI191-node-pheno55 | Chips liking \|\| id:ebi-fl187-GCST90094734 | Weighted mode | 3 | -0.004119806 | 0.143222320418731 | 0.979664202544374 | -0.284835554 | 0.276595942361625 | 0.995888669098134 | 0.752137930465007 | 1.3186334594572 | 0.999714303421225 |
| ebi-fl187-GCST90094750 | ebi-a-GCST90001414 | IgD- CD24- B cell Absolute Count \|\| id:ebi-a-GCST90001414 | Curry liking \|\| id:ebi-fl187-GCST90094750 | Inverse variance weighted | 6 | -0.044365764 | 0.21338320058676 | 0.835294618520337 | -0.462596838 | 0.373865308710064 | 0.95660400174928 | 0.62964643111629 | 1.45334138484735 | 0.998735809224318 |
| ebi-fl187-GCST90094750 | ebi-a-GCST90001422 | CD20- CD38- B cell %B cell \|\| id:ebi-a-GCST90001422 | Curry liking \|\| id:ebi-fl187-GCST90094750 | Inverse variance weighted | 6 | 0.123494607538865 | 0.199928557455629 | 0.536777275029484 | -0.268365365 | 0.515354580151897 | 1.13144390325835 | 0.76462836156287 | 1.674232046 | 0.998735809224318 |
| ebi-fl187-GCST90094750 | ebi-a-GCST90001444 | CD20- CD38- B cell %lymphocyte \|\| id:ebi-a-GCST90001444 | Curry liking \|\| id:ebi-fl187-GCST90094750 | Inverse variance weighted | 6 | 0.158241166076939 | 0.19934102981526 | 0.427299439689429 | -0.232467252 | 0.548949584514849 | 1.17144867432517 | 0.792575703904855 | 1.73143333793505 | 0.998735809224318 |
| ebi-fl187-GCST90094750 | ebi-a-GCST90001459 | Myeloid Dendritic Cell %Dendritic Cell \|\| id:ebi-a-GCST90001459 | Curry liking \|\| id:ebi-fl187-GCST90094750 | Inverse variance weighted | 6 | 0.266114536496973 | 0.220431930988776 | 0.227339219767354 | -0.165932048 | 0.698161121234974 | 1.30488450702851 | 0.847103794426581 | 2.01005306302003 | 0.998735809224318 |
| ebi-fl187-GCST90094750 | ebi-a-GCST90001476 | HLA DR++ monocyte %leukocyte \|\| id:ebi-a-GCST90001476 | Curry liking \|\| id:ebi-fl187-GCST90094750 | Inverse variance weighted | 6 | -0.147567828 | 0.214357993777038 | 0.491189807334184 | -0.567709496 | 0.272573839804132 | 0.862803914073705 | 0.566822261713342 | 1.31334043213247 | 0.998735809224318 |
| ebi-fl187-GCST90094750 | ebi-a-GCST90001478 | CD4 regulatory T cell %CD4+ T cell \|\| id:ebi-a-GCST90001478 | Curry liking \|\| id:ebi-fl187-GCST90094750 | Inverse variance weighted | 6 | -0.112692567 | 0.216032295070082 | 0.60191623378686 | -0.536115866 | 0.31073073106881 | 0.893425286075393 | 0.585016126416727 | 1.36442177532437 | 0.998735809224318 |
| ebi-fl187-GCST90094750 | ebi-a-GCST90001485 | CD39+ resting CD4 regulatory T cell %CD4 regulatory T cell \|\| id:ebi-a-GCST90001485 | Curry liking \|\| id:ebi-fl187-GCST90094750 | Inverse variance weighted | 6 | 0.109481918033399 | 0.21300436725724 | 0.607259627804179 | -0.308006642 | 0.526970477857589 | 1.11569989670448 | 0.734910436871487 | 1.69379314410808 | 0.998735809224318 |
| ebi-fl187-GCST90094750 | ebi-a-GCST90001515 | Immature Myeloid-Derived Suppressor Cells Absolute Count \|\| id:ebi-a-GCST90001515 | Curry liking \|\| id:ebi-fl187-GCST90094750 | Inverse variance weighted (multiplicative random effects) | 6 | 0.407361525184424 | 0.436034371804694 | 0.350179191540873 | -0.447265844 | 1.26198889392162 | 1.50284732461856 | 0.639373912247243 | 3.53244015411094 | 0.998735809224318 |
| ebi-fl187-GCST90094750 | ebi-a-GCST90001517 | CD33+ HLA DR+ Absolute Count \|\| id:ebi-a-GCST90001517 | Curry liking \|\| id:ebi-fl187-GCST90094750 | Inverse variance weighted | 6 | 0.302298101143395 | 0.270883572026128 | 0.264434721423584 | -0.2286337 | 0.833229902314605 | 1.35296448686085 | 0.795619915688179 | 2.30073791091983 | 0.998735809224318 |
| ebi-fl187-GCST90094750 | ebi-a-GCST90001518 | CD33+ HLA DR+ CD14- Absolute Count \|\| id:ebi-a-GCST90001518 | Curry liking \|\| id:ebi-fl187-GCST90094750 | Inverse variance weighted | 6 | 0.158779397858381 | 0.275275317386624 | 0.564073109774411 | -0.380760224 | 0.698319019936164 | 1.17207935494297 | 0.683341718772016 | 2.01037047284664 | 0.998735809224318 |
| ebi-fl187-GCST90094750 | ebi-a-GCST90001520 | CD33+ HLA DR+ CD14dim Absolute Count \|\| id:ebi-a-GCST90001520 | Curry liking \|\| id:ebi-fl187-GCST90094750 | Inverse variance weighted | 6 | 0.346785852385264 | 0.273055465796275 | 0.204077571683352 | -0.188402861 | 0.881974565345962 | 1.41451377818227 | 0.828280958266575 | 2.41566488846351 | 0.998735809224318 |
| ebi-fl187-GCST90094750 | ebi-a-GCST90001531 | CD33dim HLA DR- Absolute Count \|\| id:ebi-a-GCST90001531 | Curry liking \|\| id:ebi-fl187-GCST90094750 | Inverse variance weighted | 6 | 0.311659309790134 | 0.371956872479866 | 0.40209193301647 | -0.41737616 | 1.04069477985067 | 1.36568933677606 | 0.658773069075421 | 2.83118338034294 | 0.998735809224318 |
| ebi-fl187-GCST90094750 | ebi-a-GCST90001532 | Basophil Absolute Count \|\| id:ebi-a-GCST90001532 | Curry liking \|\| id:ebi-fl187-GCST90094750 | Inverse variance weighted | 6 | 0.192409796398555 | 0.355898860770552 | 0.588762257363196 | -0.505151971 | 0.889971563508837 | 1.21216715693267 | 0.603413867223869 | 2.43506040573162 | 0.998735809224318 |
| ebi-fl187-GCST90094750 | ebi-a-GCST90001588 | T/B cell \|\| id:ebi-a-GCST90001588 | Curry liking \|\| id:ebi-fl187-GCST90094750 | Inverse variance weighted | 6 | 0.191573077247984 | 0.22544769422753 | 0.39546680898879 | -0.250304403 | 0.633450557933943 | 1.21115333765892 | 0.778563749514286 | 1.88410057395748 | 0.998735809224318 |
| ebi-fl187-GCST90094750 | ebi-a-GCST90001624 | HLA DR+ CD4+ T cell Absolute Count \|\| id:ebi-a-GCST90001624 | Curry liking \|\| id:ebi-fl187-GCST90094750 | Inverse variance weighted | 6 | 0.259279107782193 | 0.204269695962308 | 0.204334814715829 | -0.141089496 | 0.659647711868318 | 1.29599547677623 | 0.868411588592284 | 1.93411085007182 | 0.998735809224318 |
| ebi-fl187-GCST90094750 | ebi-a-GCST90001631 | CD8+ Natural Killer T %T cell \|\| id:ebi-a-GCST90001631 | Curry liking \|\| id:ebi-fl187-GCST90094750 | Inverse variance weighted | 6 | -0.169576027 | 0.206492242098849 | 0.411519674671125 | -0.574300822 | 0.235148767098223 | 0.844022583185364 | 0.563098437282712 | 1.26509695953789 | 0.998735809224318 |
| ebi-fl187-GCST90094750 | ebi-a-GCST90001632 | CD8+ Natural Killer T %lymphocyte \|\| id:ebi-a-GCST90001632 | Curry liking \|\| id:ebi-fl187-GCST90094750 | Inverse variance weighted | 6 | -0.116984917 | 0.207741969937467 | 0.573349046572119 | -0.524159178 | 0.290189343845725 | 0.889598610671941 | 0.592052966116373 | 1.33668055630329 | 0.998735809224318 |
| ebi-fl187-GCST90094750 | ebi-a-GCST90001646 | Natural Killer %CD3- lymphocyte \|\| id:ebi-a-GCST90001646 | Curry liking \|\| id:ebi-fl187-GCST90094750 | Inverse variance weighted | 6 | -0.256194284 | 0.203893771028396 | 0.208931616794771 | -0.655826075 | 0.143437507077064 | 0.773991579896256 | 0.519013141486153 | 1.15423467705448 | 0.998735809224318 |
| ebi-fl187-GCST90094750 | ebi-a-GCST90001684 | CD127- CD8+ T cell Absolute Count \|\| id:ebi-a-GCST90001684 | Curry liking \|\| id:ebi-fl187-GCST90094750 | Inverse variance weighted | 6 | 0.273004627500302 | 0.220822227267645 | 0.216343524175802 | -0.159806938 | 0.705816192944887 | 1.31390632491259 | 0.852308321479858 | 2.02549920860546 | 0.998735809224318 |
| ebi-fl187-GCST90094750 | ebi-a-GCST90001687 | CD28- CD8+ T cell Absolute Count \|\| id:ebi-a-GCST90001687 | Curry liking \|\| id:ebi-fl187-GCST90094750 | Inverse variance weighted | 6 | 0.233203191800911 | 0.243739325564797 | 0.338681871839608 | -0.244525886 | 0.710932269907914 | 1.26263801095516 | 0.783075717165285 | 2.03588837166343 | 0.998735809224318 |
| ebi-fl187-GCST90094750 | ebi-a-GCST90001701 | BAFF-R on CD20- CD38- B cell \|\| id:ebi-a-GCST90001701 | Curry liking \|\| id:ebi-fl187-GCST90094750 | Inverse variance weighted | 6 | -0.045034845 | 0.211282188549664 | 0.831209795601686 | -0.459147934 | 0.369078244842169 | 0.955964170953498 | 0.631821769891701 | 1.44640077264741 | 0.998735809224318 |
| ebi-fl187-GCST90094750 | ebi-a-GCST90001732 | CD19 on IgD- CD27- B cell \|\| id:ebi-a-GCST90001732 | Curry liking \|\| id:ebi-fl187-GCST90094750 | Inverse variance weighted | 6 | 0.143266628845562 | 0.226995667315537 | 0.527947308734888 | -0.301644879 | 0.588178136784015 | 1.15403746032463 | 0.739600665918593 | 1.80070478733062 | 0.998735809224318 |
| ebi-fl187-GCST90094750 | ebi-a-GCST90001798 | CD27 on CD24+ CD27+ B cell \|\| id:ebi-a-GCST90001798 | Curry liking \|\| id:ebi-fl187-GCST90094750 | Inverse variance weighted | 6 | 0.112150315480255 | 0.203516974115318 | 0.581591696771918 | -0.286742954 | 0.511043584746279 | 1.11868100307975 | 0.750704669832015 | 1.66702997455918 | 0.998735809224318 |
| ebi-fl187-GCST90094750 | ebi-a-GCST90001836 | CD66b on CD66b++ myeloid cell \|\| id:ebi-a-GCST90001836 | Curry liking \|\| id:ebi-fl187-GCST90094750 | Inverse variance weighted (multiplicative random effects) | 6 | -0.338987142 | 0.484204725928207 | 0.483870770802865 | -1.288028405 | 0.610054120594599 | 0.712491610087332 | 0.275814040943477 | 1.84053100671865 | 0.998735809224318 |
| ebi-fl187-GCST90094750 | ebi-a-GCST90001943 | CD25 on activated & secreting CD4 regulatory T cell \|\| id:ebi-a-GCST90001943 | Curry liking \|\| id:ebi-fl187-GCST90094750 | Inverse variance weighted | 6 | -0.066832169 | 0.205160041798123 | 0.744608769704701 | -0.468945851 | 0.335281512701756 | 0.935352169046108 | 0.625661461089632 | 1.39833397859537 | 0.998735809224318 |
| ebi-fl187-GCST90094750 | ebi-a-GCST90001960 | CD25 on CD4+ T cell \|\| id:ebi-a-GCST90001960 | Curry liking \|\| id:ebi-fl187-GCST90094750 | Inverse variance weighted | 6 | 0.296345636271288 | 0.219391764864206 | 0.17677230194286 | -0.133662223 | 0.726353495405133 | 1.34493493480135 | 0.874885531029885 | 2.06752759611855 | 0.998735809224318 |
| ebi-fl187-GCST90094750 | ebi-a-GCST90001988 | HLA DR on CD14+ CD16- monocyte \|\| id:ebi-a-GCST90001988 | Curry liking \|\| id:ebi-fl187-GCST90094750 | Inverse variance weighted (multiplicative random effects) | 6 | 0.00692758983386889 | 0.324045692276008 | 0.982943775852729 | -0.628201967 | 0.642057146694845 | 1.00695164109133 | 0.533550280056444 | 1.90038623424441 | 0.998955228396052 |
| ebi-fl187-GCST90094750 | ebi-a-GCST90001991 | HLA DR on CD14+ monocyte \|\| id:ebi-a-GCST90001991 | Curry liking \|\| id:ebi-fl187-GCST90094750 | Inverse variance weighted (multiplicative random effects) | 6 | -0.011145065 | 0.326012768810644 | 0.972728847906952 | -0.650130092 | 0.627839961512562 | 0.988916810799993 | 0.521977867081636 | 1.87355924524263 | 0.998955228396052 |
| ebi-fl187-GCST90094750 | ebi-a-GCST90002019 | CD14 on Monocytic Myeloid-Derived Suppressor Cells \|\| id:ebi-a-GCST90002019 | Curry liking \|\| id:ebi-fl187-GCST90094750 | Inverse variance weighted | 6 | 0.155339058598166 | 0.294544585615526 | 0.597924696707246 | -0.421968329 | 0.732646446404597 | 1.16805393270155 | 0.655754807338504 | 2.08057947030081 | 0.998735809224318 |
| ebi-fl187-GCST90094750 | ebi-a-GCST90002037 | CD80 on plasmacytoid Dendritic Cell \|\| id:ebi-a-GCST90002037 | Curry liking \|\| id:ebi-fl187-GCST90094750 | Inverse variance weighted | 6 | 0.109591371259325 | 0.228614991253419 | 0.631674800448106 | -0.338494012 | 0.557676754116026 | 1.11582202034064 | 0.712843048167287 | 1.74660997856133 | 0.998735809224318 |
| ebi-fl187-GCST90094750 | ebi-a-GCST90002042 | CD45 on CD33+ HLA DR+ CD14- \|\| id:ebi-a-GCST90002042 | Curry liking \|\| id:ebi-fl187-GCST90094750 | Inverse variance weighted | 6 | 0.0441807208240197 | 0.297405739654146 | 0.881905815402951 | -0.538734529 | 0.627095970546147 | 1.0451712220263 | 0.583486170255449 | 1.87216585248921 | 0.998735809224318 |
| ebi-fl187-GCST90094750 | ebi-a-GCST90002056 | CD8 on Effector Memory CD8+ T cell \|\| id:ebi-a-GCST90002056 | Curry liking \|\| id:ebi-fl187-GCST90094750 | Inverse variance weighted | 6 | -0.119196231 | 0.223294434118074 | 0.593474762714482 | -0.556853322 | 0.318460859637308 | 0.887633602233702 | 0.573009305688305 | 1.37500980174825 | 0.998735809224318 |
| ebi-fl187-GCST90094750 | ebi-a-GCST90002058 | CD8 on CD8+ T cell \|\| id:ebi-a-GCST90002058 | Curry liking \|\| id:ebi-fl187-GCST90094750 | Inverse variance weighted (multiplicative random effects) | 6 | -0.425115016 | 0.383395487686176 | 0.267509605624868 | -1.176570172 | 0.326340139372142 | 0.653694595146214 | 0.308334461150943 | 1.38588668333827 | 0.998735809224318 |
| ebi-fl187-GCST90094750 | ebi-a-GCST90002063 | CD4 on CD4 regulatory T cell \|\| id:ebi-a-GCST90002063 | Curry liking \|\| id:ebi-fl187-GCST90094750 | Inverse variance weighted | 6 | -0.412919674 | 0.306503558142765 | 0.177917844894212 | -1.013666648 | 0.187827300399405 | 0.661715433937927 | 0.362885962298951 | 1.20662511367948 | 0.998735809224318 |
| ebi-fl187-GCST90094750 | ebi-a-GCST90002066 | CD4 on activated CD4 regulatory T cell \|\| id:ebi-a-GCST90002066 | Curry liking \|\| id:ebi-fl187-GCST90094750 | Inverse variance weighted (multiplicative random effects) | 6 | -0.479076634 | 0.36809112204862 | 0.193081752638625 | -1.200535233 | 0.242381965019352 | 0.619355019100187 | 0.301033045841296 | 1.27428083057309 | 0.998735809224318 |
| ebi-fl187-GCST90094750 | ebi-a-GCST90002070 | CD4 on activated & secreting CD4 regulatory T cell \|\| id:ebi-a-GCST90002070 | Curry liking \|\| id:ebi-fl187-GCST90094750 | Inverse variance weighted | 6 | -0.371642622 | 0.306435116341891 | 0.225208650397185 | -0.97225545 | 0.228970205965905 | 0.689600646547508 | 0.378228998713015 | 1.25730457827632 | 0.998735809224318 |
| ebi-fl187-GCST90094750 | ebi-a-GCST90002081 | SSC-A on CD4+ T cell \|\| id:ebi-a-GCST90002081 | Curry liking \|\| id:ebi-fl187-GCST90094750 | Inverse variance weighted | 6 | 0.0768268254105569 | 0.213184302902204 | 0.718564852730522 | -0.341014408 | 0.494668059098877 | 1.07985505674343 | 0.711048663145962 | 1.63995378096223 | 0.998735809224318 |
| ebi-fl187-GCST90094750 | ebi-a-GCST90002094 | CD11b on Monocytic Myeloid-Derived Suppressor Cells \|\| id:ebi-a-GCST90002094 | Curry liking \|\| id:ebi-fl187-GCST90094750 | Inverse variance weighted | 6 | 0.223640089673429 | 0.381777218639036 | 0.558018734624785 | -0.524643259 | 0.97192343820594 | 1.2506208270685 | 0.591766434148815 | 2.64302326533135 | 0.998735809224318 |
| ebi-fl187-GCST90094750 | ebi-a-GCST90002104 | HLA DR on myeloid Dendritic Cell \|\| id:ebi-a-GCST90002104 | Curry liking \|\| id:ebi-fl187-GCST90094750 | Inverse variance weighted | 6 | 0.181460296896533 | 0.303083907319484 | 0.549364239343547 | -0.412584161 | 0.775504755242722 | 1.19896693305652 | 0.661937484728171 | 2.17168802149538 | 0.998735809224318 |
| ebi-fl187-GCST90094750 | ebi-a-GCST90002105 | HLA DR on plasmacytoid Dendritic Cell \|\| id:ebi-a-GCST90002105 | Curry liking \|\| id:ebi-fl187-GCST90094750 | Inverse variance weighted | 5 | -0.048925488 | 0.273180286147916 | 0.857862347848965 | -0.584358849 | 0.486507873048357 | 0.952252081451146 | 0.557463165517383 | 1.62662590592232 | 0.998735809224318 |
| ebi-fl187-GCST90094750 | ebi-a-GCST90002106 | HLA DR on Dendritic Cell \|\| id:ebi-a-GCST90002106 | Curry liking \|\| id:ebi-fl187-GCST90094750 | Inverse variance weighted (multiplicative random effects) | 6 | 0.369111301308864 | 0.429477333094234 | 0.390096137144498 | -0.472664272 | 1.21088687417356 | 1.44644858633665 | 0.62333930878851 | 3.35646009070341 | 0.998735809224318 |
| ebi-fl187-GCST90094750 | ebi-a-GCST90002111 | HLA DR on CD33dim HLA DR+ CD11b- \|\| id:ebi-a-GCST90002111 | Curry liking \|\| id:ebi-fl187-GCST90094750 | Inverse variance weighted (multiplicative random effects) | 6 | 0.191355374510094 | 0.55368205510043 | 0.729639468554963 | -0.893861453 | 1.27657220250694 | 1.21088969496022 | 0.409073082334704 | 3.58433227870311 | 0.998735809224318 |
| ebi-fl187-GCST90094750 | ebi-a-GCST90002112 | HLA DR on CD33- HLA DR+ \|\| id:ebi-a-GCST90002112 | Curry liking \|\| id:ebi-fl187-GCST90094750 | Inverse variance weighted (multiplicative random effects) | 6 | 0.461148192943201 | 0.452089166590821 | 0.307710372315615 | -0.424946574 | 1.34724295946121 | 1.58589385214494 | 0.653804714645436 | 3.84680509934077 | 0.998735809224318 |
| ebi-fl187-GCST90094750 | ebi-a-GCST90002115 | HLA DR on HLA DR+ CD8+ T cell \|\| id:ebi-a-GCST90002115 | Curry liking \|\| id:ebi-fl187-GCST90094750 | Inverse variance weighted | 6 | -0.457626575 | 0.216339719100726 | 0.0344031393453276 | -0.881652425 | -0.033600726 | 0.632783729052728 | 0.414098080034692 | 0.966957508521484 | 0.998735809224318 |
| ebi-fl187-GCST90094750 | ebi-a-GCST90002116 | HLA DR on B cell \|\| id:ebi-a-GCST90002116 | Curry liking \|\| id:ebi-fl187-GCST90094750 | Inverse variance weighted | 6 | -0.118933786 | 0.223105415359969 | 0.593975873946578 | -0.5562204 | 0.318352828593134 | 0.887866588446836 | 0.573372090958845 | 1.374861266 | 0.998735809224318 |
| ebi-fl187-GCST90094750 | ebi-cfmet338-GCST90026023 | Sphingomyelin (d18:1/24:1, d18:2/24:0) levels \|\| id:ebi-cfmet338-GCST90026023 | Curry liking \|\| id:ebi-fl187-GCST90094750 | Inverse variance weighted (multiplicative random effects) | 6 | 0.0525810026730178 | 0.200853378076267 | 0.793484896765895 | -0.341091618 | 0.446253623702501 | 1.05398793446082 | 0.710993765142086 | 1.5624476900539 | 0.998735809224318 |
| ebi-fl187-GCST90094750 | ebi-cfmet338-GCST90026046 | 1-arachidonoyl-gpc (20:4n6) levels \|\| id:ebi-cfmet338-GCST90026046 | Curry liking \|\| id:ebi-fl187-GCST90094750 | Inverse variance weighted | 6 | 0.0196778667924454 | 0.145656548405631 | 0.892534617173069 | -0.265808968 | 0.305164701667481 | 1.0198727522241 | 0.76658555582292 | 1.35684845980771 | 0.998735809224318 |
| ebi-fl187-GCST90094750 | ebi-cfmet338-GCST90026047 | 1-linoleoyl-gpc (18:2) levels \|\| id:ebi-cfmet338-GCST90026047 | Curry liking \|\| id:ebi-fl187-GCST90094750 | Inverse variance weighted | 6 | 0.0134266357089992 | 0.189865774056996 | 0.943623419222605 | -0.358710281 | 0.385563552860711 | 1.01351717775344 | 0.698576712673594 | 1.47044276020873 | 0.998735809224318 |
| ebi-fl187-GCST90094750 | ebi-cfmet338-GCST90026050 | 1-myristoyl-2-palmitoyl-gpc (14:0/16:0) levels \|\| id:ebi-cfmet338-GCST90026050 | Curry liking \|\| id:ebi-fl187-GCST90094750 | Inverse variance weighted | 6 | -0.011296355 | 0.0938147361237256 | 0.90415733347937 | -0.195173238 | 0.172580527643948 | 0.988767209088134 | 0.822692124204064 | 1.18836751319797 | 0.998735809224318 |
| ebi-fl187-GCST90094750 | ebi-cfmet338-GCST90026068 | 2,3-dihydroxyisovalerate levels \|\| id:ebi-cfmet338-GCST90026068 | Curry liking \|\| id:ebi-fl187-GCST90094750 | Inverse variance weighted | 6 | 0.00202737204250413 | 0.302508975245719 | 0.994652731406189 | -0.590890219 | 0.594944963524113 | 1.00202942855074 | 0.553834031394441 | 1.81293116487207 | 0.999107451094609 |
| ebi-fl187-GCST90094750 | ebi-cfmet338-GCST90026082 | 3-hydroxyhexanoate levels \|\| id:ebi-cfmet338-GCST90026082 | Curry liking \|\| id:ebi-fl187-GCST90094750 | Inverse variance weighted (multiplicative random effects) | 6 | 0.0895682122984759 | 0.101329133741324 | 0.376732130795374 | -0.10903689 | 0.28817331443147 | 1.09370193469077 | 0.896697337871681 | 1.33398848354505 | 0.998735809224318 |
| ebi-fl187-GCST90094750 | ebi-cfmet338-GCST90026094 | 3-ureidopropionate levels \|\| id:ebi-cfmet338-GCST90026094 | Curry liking \|\| id:ebi-fl187-GCST90094750 | Inverse variance weighted (multiplicative random effects) | 6 | 0.0820202432425333 | 0.288377866924791 | 0.776089037804053 | -0.483200376 | 0.647240862415124 | 1.08547778319717 | 0.616806217870606 | 1.910262873 | 0.998735809224318 |
| ebi-fl187-GCST90094750 | ebi-cfmet338-GCST90026173 | Glycerol levels \|\| id:ebi-cfmet338-GCST90026173 | Curry liking \|\| id:ebi-fl187-GCST90094750 | Inverse variance weighted | 6 | 0.044758407254437 | 0.0830664453535545 | 0.590006533060496 | -0.118051826 | 0.207568640147404 | 1.04577517769043 | 0.888649996567684 | 1.2306821881478 | 0.998735809224318 |
| ebi-fl187-GCST90094750 | ebi-cfmet338-GCST90026179 | Histidine levels \|\| id:ebi-cfmet338-GCST90026179 | Curry liking \|\| id:ebi-fl187-GCST90094750 | Inverse variance weighted (multiplicative random effects) | 6 | 0.0143564150912271 | 0.0936387976654161 | 0.878148332584135 | -0.169175628 | 0.197888458515443 | 1.01445996335224 | 0.844360596718946 | 1.2188264365292 | 0.998735809224318 |
| ebi-fl187-GCST90094750 | ebi-cfmet338-GCST90026188 | Isocitrate levels \|\| id:ebi-cfmet338-GCST90026188 | Curry liking \|\| id:ebi-fl187-GCST90094750 | Inverse variance weighted (multiplicative random effects) | 6 | -0.130224181 | 0.176991915549105 | 0.461874672572968 | -0.477128336 | 0.216679973362568 | 0.877898600572591 | 0.62056288387909 | 1.24194658254405 | 0.998735809224318 |
| ebi-fl187-GCST90094750 | ebi-cfmet338-GCST90026207 | N-acetyl-isoputreanine levels \|\| id:ebi-cfmet338-GCST90026207 | Curry liking \|\| id:ebi-fl187-GCST90094750 | Inverse variance weighted | 6 | 0.0818384457239383 | 0.0751088383558293 | 0.275890231215223 | -0.065374877 | 0.229051768901364 | 1.08528046396631 | 0.93671624374926 | 1.25740713191072 | 0.998735809224318 |
| ebi-fl187-GCST90094750 | ebi-cfmet338-GCST90026213 | N-acetylglutamate levels \|\| id:ebi-cfmet338-GCST90026213 | Curry liking \|\| id:ebi-fl187-GCST90094750 | Inverse variance weighted | 6 | -0.057641748 | 0.158554774913095 | 0.716198025968955 | -0.368409107 | 0.253125610344941 | 0.943988072023564 | 0.691834089398799 | 1.28804505845779 | 0.998735809224318 |
| ebi-fl187-GCST90094750 | ebi-cfmet338-GCST90026224 | N-acetyltaurine levels \|\| id:ebi-cfmet338-GCST90026224 | Curry liking \|\| id:ebi-fl187-GCST90094750 | Inverse variance weighted | 6 | 0.175927431223167 | 0.131428975889622 | 0.180709342343348 | -0.081673362 | 0.433528223966825 | 1.19235152801905 | 0.921572930719997 | 1.54269089181975 | 0.998735809224318 |
| ebi-fl187-GCST90094750 | ebi-cfmet338-GCST90026250 | Phenyllactate (pla) levels \|\| id:ebi-cfmet338-GCST90026250 | Curry liking \|\| id:ebi-fl187-GCST90094750 | Inverse variance weighted | 6 | 0.193863772696173 | 0.157191732305203 | 0.217465734047054 | -0.114232023 | 0.501959568014372 | 1.21393090115803 | 0.892050955867233 | 1.65195521970348 | 0.998735809224318 |
| ebi-fl187-GCST90094750 | ebi-cfmet338-GCST90026251 | Pipecolate levels \|\| id:ebi-cfmet338-GCST90026251 | Curry liking \|\| id:ebi-fl187-GCST90094750 | Inverse variance weighted | 6 | 0.160173226511128 | 0.287274858511116 | 0.577144267450167 | -0.402885496 | 0.723231949192916 | 1.1737141717938 | 0.668388627999457 | 2.06108377575616 | 0.998735809224318 |
| ebi-fl187-GCST90094750 | ebi-cfmet338-GCST90026278 | Trigonelline (n'-methylnicotinate) levels \|\| id:ebi-cfmet338-GCST90026278 | Curry liking \|\| id:ebi-fl187-GCST90094750 | Inverse variance weighted | 6 | 0.16382256597079 | 0.262696052453154 | 0.532877038408798 | -0.351061697 | 0.678706828778972 | 1.17800527832519 | 0.703940321624262 | 1.97132682009187 | 0.998735809224318 |
| ebi-fl187-GCST90094750 | ebi-cfmet338-GCST90026299 | 1-carboxyethylvaline levels \|\| id:ebi-cfmet338-GCST90026299 | Curry liking \|\| id:ebi-fl187-GCST90094750 | Inverse variance weighted | 6 | 0.0738306020316633 | 0.158207925267774 | 0.640737310285263 | -0.236256931 | 0.383918135556499 | 1.07662441206005 | 0.789577780470946 | 1.46802525769189 | 0.998735809224318 |
| ebi-fl187-GCST90094750 | ebi-gm430-GCST90011306 | C_Erysipelotrichia abundance \|\| id:ebi-gm430-GCST90011306 | Curry liking \|\| id:ebi-fl187-GCST90094750 | Inverse variance weighted | 5 | -0.180048606 | 0.139692865628478 | 0.197436658664829 | -0.453846623 | 0.0937494106683898 | 0.835229613284588 | 0.635180148057783 | 1.09828449305387 | 0.998735809224318 |
| ebi-fl187-GCST90094750 | ebi-gm430-GCST90011312 | F_Erysipelotrichaceae abundance \|\| id:ebi-gm430-GCST90011312 | Curry liking \|\| id:ebi-fl187-GCST90094750 | Inverse variance weighted | 5 | -0.180048606 | 0.139692865628478 | 0.197436658664829 | -0.453846623 | 0.0937494106683898 | 0.835229613284588 | 0.635180148057783 | 1.09828449305387 | 0.998735809224318 |
| ebi-fl187-GCST90094750 | ebi-gm430-GCST90011352 | O_Erysipelotrichales abundance \|\| id:ebi-gm430-GCST90011352 | Curry liking \|\| id:ebi-fl187-GCST90094750 | Inverse variance weighted | 5 | -0.180048606 | 0.139692865628478 | 0.197436658664829 | -0.453846623 | 0.0937494106683898 | 0.835229613284588 | 0.635180148057783 | 1.09828449305387 | 0.998735809224318 |
| ebi-fl187-GCST90094750 | ebi-gm430-GCST90011362 | OTU97_11 (Parabacteroides) abundance \|\| id:ebi-gm430-GCST90011362 | Curry liking \|\| id:ebi-fl187-GCST90094750 | Inverse variance weighted | 5 | -0.109593203 | 0.181445007880135 | 0.54584211023964 | -0.465225418 | 0.246039012601491 | 0.896198632208524 | 0.627993528006838 | 1.27894946771439 | 0.998735809224318 |
| ebi-fl187-GCST90094750 | ebi-gm430-GCST90011384 | OTU97_235 (Phascolarctobacterium) abundance \|\| id:ebi-gm430-GCST90011384 | Curry liking \|\| id:ebi-fl187-GCST90094750 | Inverse variance weighted | 5 | 0.491199098224288 | 0.558029070890005 | 0.378729707618467 | -0.602537881 | 1.5849360771687 | 1.63427470140736 | 0.547420583533171 | 4.87897949036156 | 0.998735809224318 |
| ebi-fl187-GCST90094750 | ebi-gm430-GCST90011390 | OTU97_3 (Bacteroides) abundance \|\| id:ebi-gm430-GCST90011390 | Curry liking \|\| id:ebi-fl187-GCST90094750 | Inverse variance weighted (multiplicative random effects) | 5 | -0.186043417 | 0.238930550445908 | 0.436185563121112 | -0.654347295 | 0.282260462261836 | 0.830237548097808 | 0.519781215437253 | 1.3261240802856 | 0.998735809224318 |
| ebi-fl187-GCST90094750 | ebi-gm430-GCST90011398 | OTU97_4 (Alistipes) abundance \|\| id:ebi-gm430-GCST90011398 | Curry liking \|\| id:ebi-fl187-GCST90094750 | Inverse variance weighted | 5 | -0.234509694 | 0.16873373179279 | 0.164583221162703 | -0.565227808 | 0.0962084202484632 | 0.790958566250139 | 0.568230684300539 | 1.10098850838119 | 0.998735809224318 |
| ebi-fl187-GCST90094750 | ebi-gm430-GCST90011421 | OTU99_108 (Cytophagales) abundance \|\| id:ebi-gm430-GCST90011421 | Curry liking \|\| id:ebi-fl187-GCST90094750 | Inverse variance weighted | 5 | 0.348586143464423 | 0.535663717340485 | 0.515204290146789 | -0.701314743 | 1.39848702945177 | 1.41706260834818 | 0.495932850973969 | 4.04906920772615 | 0.998735809224318 |
| ebi-fl187-GCST90094750 | ebi-gm430-GCST90011428 | OTU99_123 (Phascolarctobacterium) abundance \|\| id:ebi-gm430-GCST90011428 | Curry liking \|\| id:ebi-fl187-GCST90094750 | Inverse variance weighted | 5 | -0.324735442 | 0.321925363880381 | 0.31310463174448 | -0.955709156 | 0.306238270848468 | 0.722718529063043 | 0.384539349618558 | 1.3583059126957 | 0.998735809224318 |
| ebi-fl187-GCST90094750 | ebi-gm430-GCST90011450 | OTU99_298 (Phascolarctobacterium) abundance \|\| id:ebi-gm430-GCST90011450 | Curry liking \|\| id:ebi-fl187-GCST90094750 | Inverse variance weighted | 5 | 0.464046650214332 | 0.558045277629447 | 0.405658846391504 | -0.629722094 | 1.55781539436805 | 1.59049716574309 | 0.532739832064642 | 4.74843644492091 | 0.998735809224318 |
| ebi-fl187-GCST90094750 | ebi-gm430-GCST90011451 | OTU99_3 (Bacteroides) abundance \|\| id:ebi-gm430-GCST90011451 | Curry liking \|\| id:ebi-fl187-GCST90094750 | Inverse variance weighted (multiplicative random effects) | 5 | -0.187002473 | 0.237497231574835 | 0.431054761631646 | -0.652497047 | 0.278492100614709 | 0.829441684947999 | 0.520743830025167 | 1.32113616919115 | 0.998735809224318 |
| ebi-fl187-GCST90094750 | ebi-gm430-GCST90011472 | OTU99_72 (Parabacteroides) abundance \|\| id:ebi-gm430-GCST90011472 | Curry liking \|\| id:ebi-fl187-GCST90094750 | Inverse variance weighted | 5 | -0.637325579 | 0.616109176979264 | 0.300932331351651 | -1.844899566 | 0.570248407615624 | 0.528704513254653 | 0.158041192779138 | 1.76870635699693 | 0.998735809224318 |
| ebi-fl187-GCST90094750 | ebi-gm430-GCST90011507 | TestASV_3 (Bacteroides) abundance \|\| id:ebi-gm430-GCST90011507 | Curry liking \|\| id:ebi-fl187-GCST90094750 | Inverse variance weighted | 5 | -0.008925826 | 0.275709554115145 | 0.974173783136229 | -0.549316552 | 0.531464900302169 | 0.991113891162635 | 0.577344260450772 | 1.70142289885862 | 0.998955228396052 |
| ebi-fl187-GCST90094750 | ebi-gm430-GCST90011512 | TestASV_34 (Phascolarctobacterium) abundance \|\| id:ebi-gm430-GCST90011512 | Curry liking \|\| id:ebi-fl187-GCST90094750 | Inverse variance weighted | 5 | -0.064719788 | 0.469151662728765 | 0.890279401958665 | -0.984257047 | 0.854817471340611 | 0.937330078085229 | 0.373716777987371 | 2.35094522652914 | 0.998735809224318 |
| ebi-fl187-GCST90094750 | ebi-gm430-GCST90011519 | TestASV_41 (Barnesiella) abundance \|\| id:ebi-gm430-GCST90011519 | Curry liking \|\| id:ebi-fl187-GCST90094750 | Inverse variance weighted | 5 | -0.641644362 | 0.59153897724753 | 0.278053024697606 | -1.801060758 | 0.517772033092041 | 0.526426076742596 | 0.165123639114936 | 1.67828431931364 | 0.998735809224318 |
| ebi-fl187-GCST90094750 | ebi-gm430-GCST90011524 | TestASV_46 (Sutterella) abundance \|\| id:ebi-gm430-GCST90011524 | Curry liking \|\| id:ebi-fl187-GCST90094750 | Inverse variance weighted | 5 | -0.543738654 | 0.678446327083483 | 0.422873017074583 | -1.873493455 | 0.786016147053733 | 0.580573625892859 | 0.153586177106632 | 2.19463588086031 | 0.998735809224318 |
| ebi-fl187-GCST90094750 | ebi-gm430-GCST90011581 | OTU97_19 (Bacteria) prevalence \|\| id:ebi-gm430-GCST90011581 | Curry liking \|\| id:ebi-fl187-GCST90094750 | Inverse variance weighted | 5 | -0.149884463 | 0.70157698783277 | 0.830828297781756 | -1.52497536 | 1.22520643269694 | 0.860807425395543 | 0.217626419199551 | 3.40486888651446 | 0.998735809224318 |
| ebi-fl187-GCST90094750 | ebi-gm430-GCST90011614 | OTU97_80 (Ruminococcus) prevalence \|\| id:ebi-gm430-GCST90011614 | Curry liking \|\| id:ebi-fl187-GCST90094750 | Inverse variance weighted | 5 | 0.665625138724922 | 0.32924641255748 | 0.0432112263132582 | 0.0203021701122605 | 1.31094810733758 | 1.94570647769307 | 1.02050966096054 | 3.70968922898138 | 0.998735809224318 |
| ebi-fl187-GCST90094750 | ebi-gm430-GCST90011623 | OTU99_11 (Parabacteroides) prevalence \|\| id:ebi-gm430-GCST90011623 | Curry liking \|\| id:ebi-fl187-GCST90094750 | Inverse variance weighted | 5 | -0.062945655 | 0.313919821720241 | 0.841077772093099 | -0.678228506 | 0.552337195114108 | 0.938994501529114 | 0.507515256713079 | 1.7373087059733 | 0.998735809224318 |
| ebi-fl187-GCST90094750 | ebi-gm430-GCST90011642 | OTU99_19 (Bacteria) prevalence \|\| id:ebi-gm430-GCST90011642 | Curry liking \|\| id:ebi-fl187-GCST90094750 | Inverse variance weighted | 5 | -0.149884463 | 0.70157698783277 | 0.830828297781756 | -1.52497536 | 1.22520643269694 | 0.860807425395543 | 0.217626419199551 | 3.40486888651446 | 0.998735809224318 |
| ebi-fl187-GCST90094750 | ebi-gm430-GCST90011653 | OTU99_30 (Parasutterella) prevalence \|\| id:ebi-gm430-GCST90011653 | Curry liking \|\| id:ebi-fl187-GCST90094750 | Inverse variance weighted | 5 | -0.321246026 | 0.314544273992273 | 0.307109376107084 | -0.937752803 | 0.295260750659923 | 0.725244799697425 | 0.391506639959532 | 1.34347662543482 | 0.998735809224318 |
| ebi-fl187-GCST90094750 | ebi-gm430-GCST90011675 | OTU99_8 (Ruminococcaceae) prevalence \|\| id:ebi-gm430-GCST90011675 | Curry liking \|\| id:ebi-fl187-GCST90094750 | Inverse variance weighted | 5 | 0.416624125485842 | 0.332324455131441 | 0.209963151802497 | -0.234731807 | 1.06798005754347 | 1.51683226717989 | 0.790782903969665 | 2.90949654476388 | 0.998735809224318 |
| ebi-fl187-GCST90094750 | ebi-gm430-GCST90011679 | OTU99_92 (Ruminococcus) prevalence \|\| id:ebi-gm430-GCST90011679 | Curry liking \|\| id:ebi-fl187-GCST90094750 | Inverse variance weighted | 5 | 0.377029529567799 | 0.344366908703154 | 0.273583095693606 | -0.297929611 | 1.05198867062598 | 1.45794736129102 | 0.742353591071691 | 2.86333969938347 | 0.998735809224318 |
| ebi-fl187-GCST90094750 | ebi-gm430-GCST90011684 | TestASV_11 (Lachnospiraceae) prevalence \|\| id:ebi-gm430-GCST90011684 | Curry liking \|\| id:ebi-fl187-GCST90094750 | Inverse variance weighted | 5 | 0.233206767704244 | 0.349914070556647 | 0.505111578182906 | -0.452624811 | 0.919038345995272 | 1.26264252603471 | 0.635956693089634 | 2.50687848068076 | 0.998735809224318 |
| ebi-fl187-GCST90094750 | ebi-gm430-GCST90011688 | TestASV_15 (Bacteroides) prevalence \|\| id:ebi-gm430-GCST90011688 | Curry liking \|\| id:ebi-fl187-GCST90094750 | Inverse variance weighted | 5 | -0.192510476 | 0.329819088123508 | 0.559432217062495 | -0.838955889 | 0.453934936760982 | 0.824885676688084 | 0.432161512672703 | 1.57449555236189 | 0.998735809224318 |
| ebi-fl187-GCST90094750 | ebi-gm430-GCST90011692 | TestASV_19 (Prevotella) prevalence \|\| id:ebi-gm430-GCST90011692 | Curry liking \|\| id:ebi-fl187-GCST90094750 | Inverse variance weighted | 5 | 0.244681227396975 | 0.647516895133772 | 0.705522559350224 | -1.024451887 | 1.51381434185917 | 1.27721410743967 | 0.358993180295657 | 4.5440302651416 | 0.998735809224318 |
| ebi-fl187-GCST90094750 | ebi-gm430-GCST90011693 | TestASV_2 (Bacteroides) prevalence \|\| id:ebi-gm430-GCST90011693 | Curry liking \|\| id:ebi-fl187-GCST90094750 | Inverse variance weighted | 5 | -0.270933651 | 0.324244163064195 | 0.403388250065108 | -0.906452211 | 0.36458490835583 | 0.762667096734953 | 0.403954831351926 | 1.43991618690501 | 0.998735809224318 |
| ebi-fl187-GCST90094750 | ebi-gm430-GCST90011716 | TestASV_40 (Barnesiella) prevalence \|\| id:ebi-gm430-GCST90011716 | Curry liking \|\| id:ebi-fl187-GCST90094750 | Inverse variance weighted | 5 | 0.551367727060342 | 0.751521288586822 | 0.463150614186637 | -0.921613999 | 2.02434945269051 | 1.73562525684026 | 0.397876350712342 | 7.57118392884763 | 0.998735809224318 |
| ebi-fl187-GCST90094750 | ebi-gm430-GCST90011718 | TestASV_42 (Alphaproteobacteria) prevalence \|\| id:ebi-gm430-GCST90011718 | Curry liking \|\| id:ebi-fl187-GCST90094750 | Inverse variance weighted | 5 | 0.226651471880504 | 0.634076851550736 | 0.72075412328602 | -1.016139157 | 1.46944210091995 | 1.25439260065052 | 0.361989831561885 | 4.34680938350548 | 0.998735809224318 |
| ebi-fl187-GCST90094750 | ebi-hbsm44-GCST90104929 | Sulcal depth of the right inferior frontal sulcus \|\| id:ebi-hbsm44-GCST90104929 | Curry liking \|\| id:ebi-fl187-GCST90094750 | Inverse variance weighted | 5 | 0.250559273458089 | 0.269619459217304 | 0.352729964984084 | -0.277894867 | 0.779013413524005 | 1.28474373887335 | 0.757376442883421 | 2.1793211157854 | 0.998735809224318 |
| ebi-fl187-GCST90094750 | ebi-hbsm44-GCST90104935 | Sulcal opening distance of the left calloso-marginal posterior fissure \|\| id:ebi-hbsm44-GCST90104935 | Curry liking \|\| id:ebi-fl187-GCST90094750 | Inverse variance weighted | 5 | -0.094804245 | 0.0609309572617751 | 0.119724970081134 | -0.214228921 | 0.0246204311807088 | 0.909550966144116 | 0.80716358694549 | 1.02492601672524 | 0.998735809224318 |
| ebi-fl187-GCST90094750 | ebi-hbsm44-GCST90104936 | Sulcal opening distance of the left anterior inferior temporal sulcus \|\| id:ebi-hbsm44-GCST90104936 | Curry liking \|\| id:ebi-fl187-GCST90094750 | Inverse variance weighted | 5 | -0.014985227 | 0.0402014574422819 | 0.709332050627929 | -0.093780083 | 0.0638096299922978 | 0.985126493168638 | 0.910482970743624 | 1.0658894660601 | 0.998735809224318 |
| ebi-fl187-GCST90094750 | ebi-hbsm44-GCST90104941 | Sulcal opening distance of the right posterior inferior temporal sulcus \|\| id:ebi-hbsm44-GCST90104941 | Curry liking \|\| id:ebi-fl187-GCST90094750 | Inverse variance weighted | 5 | -0.012918865 | 0.0283754690314324 | 0.648905756731271 | -0.068534784 | 0.0426970546536638 | 0.98716422568814 | 0.933760979643818 | 1.04362168662283 | 0.998735809224318 |
| ebi-fl187-GCST90094750 | ebi-IPs91-GCST90274819 | Leukemia inhibitory factor levels \|\| id:ebi-IPs91-GCST90274819 | Curry liking \|\| id:ebi-fl187-GCST90094750 | Inverse variance weighted | 7 | 0.0192626749591117 | 0.113935314783529 | 0.865744127161374 | -0.204050542 | 0.242575891934829 | 1.01944939727918 | 0.815421157116117 | 1.27452797188691 | 0.998735809224318 |
| ebi-fl187-GCST90094750 | ebi-IPs91-GCST90274837 | STAM binding protein levels \|\| id:ebi-IPs91-GCST90274837 | Curry liking \|\| id:ebi-fl187-GCST90094750 | Inverse variance weighted | 7 | -0.0837164 | 0.0921802532030069 | 0.363782338987927 | -0.264389696 | 0.0969568960663864 | 0.919692043582777 | 0.767674321370294 | 1.1018128801282 | 0.998735809224318 |
| ebi-fl187-GCST90094750 | ebi-IPs91-GCST90274840 | TNF-beta levels \|\| id:ebi-IPs91-GCST90274840 | Curry liking \|\| id:ebi-fl187-GCST90094750 | Inverse variance weighted | 7 | 0.00529001849729701 | 0.131682914927943 | 0.967955682889719 | -0.252808495 | 0.263388531756064 | 1.00530403535072 | 0.776616593729757 | 1.30133222963829 | 0.998735809224318 |
| ebi-fl187-GCST90094750 | ebi-IPs91-GCST90274842 | Tumor necrosis factor ligand superfamily member 14 levels \|\| id:ebi-IPs91-GCST90274842 | Curry liking \|\| id:ebi-fl187-GCST90094750 | Inverse variance weighted | 7 | 0.00399775242995549 | 0.105026465305811 | 0.969636464133797 | -0.20185412 | 0.209849624429346 | 1.00400575410155 | 0.8172141347969 | 1.23349255886224 | 0.998735809224318 |
| ebi-fl187-GCST90094750 | ebi-met1400-GCST90199628 | 3-methylhistidine levels \|\| id:ebi-met1400-GCST90199628 | Curry liking \|\| id:ebi-fl187-GCST90094750 | Inverse variance weighted | 7 | 0.00938681812988416 | 0.172837375039666 | 0.956688088930346 | -0.329374437 | 0.348148073207629 | 1.00943101248039 | 0.719373606254104 | 1.41644197131868 | 0.998735809224318 |
| ebi-fl187-GCST90094750 | ebi-met1400-GCST90199651 | Docosahexaenoate DHA; 22:6n3 levels \|\| id:ebi-met1400-GCST90199651 | Curry liking \|\| id:ebi-fl187-GCST90094750 | Inverse variance weighted | 7 | 0.0463252857125961 | 0.16408126165574 | 0.777689441640561 | -0.275273987 | 0.367924558557846 | 1.04741506470474 | 0.759364038744419 | 1.44473304211827 | 0.998735809224318 |
| ebi-fl187-GCST90094750 | ebi-met1400-GCST90199652 | 1-stearoyl-2-oleoyl-GPS (18:0/18:1) levels \|\| id:ebi-met1400-GCST90199652 | Curry liking \|\| id:ebi-fl187-GCST90094750 | Inverse variance weighted | 7 | -0.097844511 | 0.130293409157985 | 0.452679628166173 | -0.353219593 | 0.15753057103677 | 0.906789888724832 | 0.702422929378455 | 1.1706165443961 | 0.998735809224318 |
| ebi-fl187-GCST90094750 | ebi-met1400-GCST90199653 | 1-myristoyl-2-palmitoyl-gpc (14:0/16:0) levels \|\| id:ebi-met1400-GCST90199653 | Curry liking \|\| id:ebi-fl187-GCST90094750 | Inverse variance weighted | 7 | -0.197617601 | 0.120073132831443 | 0.0998028167542676 | -0.432960942 | 0.0377257390866131 | 0.820683621522024 | 0.648585824026603 | 1.03844638856442 | 0.998735809224318 |
| ebi-fl187-GCST90094750 | ebi-met1400-GCST90199667 | Erythritol levels in elite athletes \|\| id:ebi-met1400-GCST90199667 | Curry liking \|\| id:ebi-fl187-GCST90094750 | Inverse variance weighted (multiplicative random effects) | 7 | 0.0351057821122529 | 0.188170771769788 | 0.852002952282044 | -0.333708931 | 0.403920494781037 | 1.03572926463433 | 0.716262233938585 | 1.49768486846112 | 0.998735809224318 |
| ebi-fl187-GCST90094750 | ebi-met1400-GCST90199674 | 3-hydroxymyristate levels \|\| id:ebi-met1400-GCST90199674 | Curry liking \|\| id:ebi-fl187-GCST90094750 | Inverse variance weighted | 7 | 0.0277803458055335 | 0.128995823054734 | 0.829487830017247 | -0.225051467 | 0.280612158992812 | 1.02816981780295 | 0.79847512227789 | 1.32394002611524 | 0.998735809224318 |
| ebi-fl187-GCST90094750 | ebi-met1400-GCST90199685 | 2-linoleoylglycerol (18:2) levels \|\| id:ebi-met1400-GCST90199685 | Curry liking \|\| id:ebi-fl187-GCST90094750 | Inverse variance weighted | 7 | -0.155022605 | 0.177752610624157 | 0.383139854128896 | -0.503417722 | 0.19337251214211 | 0.856395818710319 | 0.604461245189373 | 1.21333469125013 | 0.998735809224318 |
| ebi-fl187-GCST90094750 | ebi-met1400-GCST90199703 | Gamma-glutamylvaline levels \|\| id:ebi-met1400-GCST90199703 | Curry liking \|\| id:ebi-fl187-GCST90094750 | Inverse variance weighted | 7 | 0.0165055788806657 | 0.158196504369501 | 0.91690276812046 | -0.29355957 | 0.326570727444889 | 1.01664254849769 | 0.745604806098809 | 1.38620628912485 | 0.998735809224318 |
| ebi-fl187-GCST90094750 | ebi-met1400-GCST90199779 | Gamma-glutamyl-2-aminobutyrate levels \|\| id:ebi-met1400-GCST90199779 | Curry liking \|\| id:ebi-fl187-GCST90094750 | Inverse variance weighted | 7 | 0.111535526883977 | 0.12515518370873 | 0.372833768170265 | -0.133768633 | 0.356839686953087 | 1.11799346212401 | 0.874792439131367 | 1.42880679512176 | 0.998735809224318 |
| ebi-fl187-GCST90094750 | ebi-met1400-GCST90199824 | 4-vinylphenol sulfate levels \|\| id:ebi-met1400-GCST90199824 | Curry liking \|\| id:ebi-fl187-GCST90094750 | Inverse variance weighted | 7 | -0.083754901 | 0.12102332677966 | 0.48890225679945 | -0.320960622 | 0.15345081932758 | 0.9196566352479 | 0.725451817524217 | 1.16585044840315 | 0.998735809224318 |
| ebi-fl187-GCST90094750 | ebi-met1400-GCST90199830 | 4-ethylphenylsulfate levels \|\| id:ebi-met1400-GCST90199830 | Curry liking \|\| id:ebi-fl187-GCST90094750 | Inverse variance weighted | 7 | 0.0813229894971207 | 0.121177913192832 | 0.50215424088233 | -0.15618572 | 0.318831699355072 | 1.08472119354541 | 0.855400310363554 | 1.37551980455386 | 0.998735809224318 |
| ebi-fl187-GCST90094750 | ebi-met1400-GCST90199833 | Hexanoylglutamine levels \|\| id:ebi-met1400-GCST90199833 | Curry liking \|\| id:ebi-fl187-GCST90094750 | Inverse variance weighted | 6 | 0.086697801213674 | 0.127659127850069 | 0.497052231295645 | -0.163514089 | 0.33690969179981 | 1.09056706187322 | 0.849154534862781 | 1.40061257122649 | 0.998735809224318 |
| ebi-fl187-GCST90094750 | ebi-met1400-GCST90199837 | Carotenoid levels (cryptoxanthin) \|\| id:ebi-met1400-GCST90199837 | Curry liking \|\| id:ebi-fl187-GCST90094750 | Inverse variance weighted | 7 | 0.136246318871247 | 0.119859539758077 | 0.255656811192983 | -0.098678379 | 0.371171016797077 | 1.14596413142452 | 0.906034060699657 | 1.44943092922739 | 0.998735809224318 |
| ebi-fl187-GCST90094750 | ebi-met1400-GCST90199869 | 2-hydroxyhippurate levels \|\| id:ebi-met1400-GCST90199869 | Curry liking \|\| id:ebi-fl187-GCST90094750 | Inverse variance weighted | 7 | -0.006835949 | 0.132173919209292 | 0.958752318249549 | -0.265896831 | 0.252224932370039 | 0.993187362671088 | 0.766518204391794 | 1.28688546693062 | 0.998735809224318 |
| ebi-fl187-GCST90094750 | ebi-met1400-GCST90199902 | Hydantoin-5-propionate levels \|\| id:ebi-met1400-GCST90199902 | Curry liking \|\| id:ebi-fl187-GCST90094750 | Inverse variance weighted | 7 | 0.113422134717185 | 0.124575175877573 | 0.362573958314941 | -0.13074521 | 0.357589479437229 | 1.12010466822992 | 0.877441309181279 | 1.429878505 | 0.998735809224318 |
| ebi-fl187-GCST90094750 | ebi-met1400-GCST90199904 | Pantoate levels \|\| id:ebi-met1400-GCST90199904 | Curry liking \|\| id:ebi-fl187-GCST90094750 | Inverse variance weighted | 7 | -0.139581919 | 0.122757619394975 | 0.25551613120171 | -0.380186853 | 0.101023014914367 | 0.869721773461337 | 0.68373364 | 1.10630210291859 | 0.998735809224318 |
| ebi-fl187-GCST90094750 | ebi-met1400-GCST90199936 | 3-methyl catechol sulfate (2) levels \|\| id:ebi-met1400-GCST90199936 | Curry liking \|\| id:ebi-fl187-GCST90094750 | Inverse variance weighted | 7 | -0.086514914 | 0.137506070512145 | 0.52923672117565 | -0.356026812 | 0.182996984153251 | 0.917121870680765 | 0.70045383923697 | 1.20081078661406 | 0.998735809224318 |
| ebi-fl187-GCST90094750 | ebi-met1400-GCST90199977 | 4-hydroxychlorothalonil levels \|\| id:ebi-met1400-GCST90199977 | Curry liking \|\| id:ebi-fl187-GCST90094750 | Inverse variance weighted | 7 | 0.0923857831412796 | 0.120017418233642 | 0.441436461406647 | -0.142848357 | 0.327619922879218 | 1.0967878627416 | 0.866885516398678 | 1.38766145367695 | 0.998735809224318 |
| ebi-fl187-GCST90094750 | ebi-met1400-GCST90200040 | Sphingomyelin (d18:2/24:1, d18:1/24:2) levels \|\| id:ebi-met1400-GCST90200040 | Curry liking \|\| id:ebi-fl187-GCST90094750 | Inverse variance weighted | 7 | -0.008147351 | 0.130390063758962 | 0.950177048569908 | -0.263711876 | 0.247417174158287 | 0.991885748900677 | 0.768194843316164 | 1.28071328183512 | 0.998735809224318 |
| ebi-fl187-GCST90094750 | ebi-met1400-GCST90200077 | 1-stearoyl-2-oleoyl-GPI (18:0/18:1) levels \|\| id:ebi-met1400-GCST90200077 | Curry liking \|\| id:ebi-fl187-GCST90094750 | Inverse variance weighted | 7 | 0.0636714963905632 | 0.133765911479273 | 0.634080116447032 | -0.19850969 | 0.325852682889939 | 1.0657422410777 | 0.819951825279501 | 1.38521128851704 | 0.998735809224318 |
| ebi-fl187-GCST90094750 | ebi-met1400-GCST90200080 | 1-palmitoyl-2-oleoyl-GPI (16:0/18:1) levels \|\| id:ebi-met1400-GCST90200080 | Curry liking \|\| id:ebi-fl187-GCST90094750 | Inverse variance weighted | 7 | -0.019861421 | 0.126853340480799 | 0.875583789583741 | -0.268493968 | 0.22877112660873 | 0.980334517932199 | 0.764530034383004 | 1.25705429980256 | 0.998735809224318 |
| ebi-fl187-GCST90094750 | ebi-met1400-GCST90200082 | 1-oleoyl-2-linoleoyl-GPE (18:1/18:2) levels \|\| id:ebi-met1400-GCST90200082 | Curry liking \|\| id:ebi-fl187-GCST90094750 | Inverse variance weighted | 7 | -0.222765293 | 0.144906486656653 | 0.1242189306354 | -0.506782007 | 0.0612514208316473 | 0.800302663878007 | 0.602431082011776 | 1.06316618271319 | 0.998735809224318 |
| ebi-fl187-GCST90094750 | ebi-met1400-GCST90200100 | Perfluorooctanesulfonate (PFOS) levels \|\| id:ebi-met1400-GCST90200100 | Curry liking \|\| id:ebi-fl187-GCST90094750 | Inverse variance weighted | 7 | 0.164981719075508 | 0.142964093292557 | 0.248496855548877 | -0.115227904 | 0.44519134192892 | 1.17937155851191 | 0.891163021342218 | 1.56078881160475 | 0.998735809224318 |
| ebi-fl187-GCST90094750 | ebi-met1400-GCST90200102 | 1-palmitoleoyl-2-linolenoyl-GPC (16:1/18:3) levels \|\| id:ebi-met1400-GCST90200102 | Curry liking \|\| id:ebi-fl187-GCST90094750 | Inverse variance weighted | 7 | -0.240199216 | 0.118511691257173 | 0.0426831123093891 | -0.472482131 | -0.007916301 | 0.786471168047853 | 0.62345285476318 | 0.99211495054507 | 0.998735809224318 |
| ebi-fl187-GCST90094750 | ebi-met1400-GCST90200114 | Glycosyl-N-tricosanoyl-sphingadienine (d18:2/23:0) levels \|\| id:ebi-met1400-GCST90200114 | Curry liking \|\| id:ebi-fl187-GCST90094750 | Inverse variance weighted (multiplicative random effects) | 7 | -0.226118741 | 0.206933340402834 | 0.274519851899333 | -0.631708088 | 0.179470606243184 | 0.797623385486801 | 0.531682863763756 | 1.19658373145934 | 0.998735809224318 |
| ebi-fl187-GCST90094750 | ebi-met1400-GCST90200135 | Arachidoylcarnitine (C20) levels \|\| id:ebi-met1400-GCST90200135 | Curry liking \|\| id:ebi-fl187-GCST90094750 | Inverse variance weighted | 7 | -0.277542147 | 0.127148392289948 | 0.0290490143946195 | -0.526752995 | -0.028331298 | 0.757643631847245 | 0.590519278889735 | 0.972066270144362 | 0.998735809224318 |
| ebi-fl187-GCST90094750 | ebi-met1400-GCST90200146 | Docosahexaenoylcarnitine (C22:6) levels \|\| id:ebi-met1400-GCST90200146 | Curry liking \|\| id:ebi-fl187-GCST90094750 | Inverse variance weighted | 7 | 0.0393898881246741 | 0.136198323835708 | 0.772420843356597 | -0.227558827 | 0.306338602842662 | 1.04017595685358 | 0.79647556617512 | 1.35844220107358 | 0.998735809224318 |
| ebi-fl187-GCST90094750 | ebi-met1400-GCST90200162 | 3-hydroxyphenylacetoylglutamine levels \|\| id:ebi-met1400-GCST90200162 | Curry liking \|\| id:ebi-fl187-GCST90094750 | Inverse variance weighted | 7 | 0.0404839335534443 | 0.165802503865666 | 0.807099458588102 | -0.284488974 | 0.36545684113015 | 1.04131457934308 | 0.752398651348286 | 1.44117224453998 | 0.998735809224318 |
| ebi-fl187-GCST90094750 | ebi-met1400-GCST90200172 | 3-carboxy-4-methyl-5-pentyl-2-furanpropionate (3-CMPFP) levels \|\| id:ebi-met1400-GCST90200172 | Curry liking \|\| id:ebi-fl187-GCST90094750 | Inverse variance weighted | 7 | 0.108898190050716 | 0.120156101278239 | 0.364773934731077 | -0.126607768 | 0.344404148556063 | 1.1150488214984 | 0.88107919188397 | 1.41114883404113 | 0.998735809224318 |
| ebi-fl187-GCST90094750 | ebi-met1400-GCST90200222 | Dihydrocaffeate sulfate (2) levels \|\| id:ebi-met1400-GCST90200222 | Curry liking \|\| id:ebi-fl187-GCST90094750 | Inverse variance weighted | 7 | -0.032622597 | 0.137559611862751 | 0.812538533492558 | -0.302239436 | 0.236994242049036 | 0.967903780261954 | 0.739161061597112 | 1.26743381993249 | 0.998735809224318 |
| ebi-fl187-GCST90094750 | ebi-met1400-GCST90200223 | 4-methylhexanoylglutamine levels \|\| id:ebi-met1400-GCST90200223 | Curry liking \|\| id:ebi-fl187-GCST90094750 | Inverse variance weighted | 7 | -0.074029357 | 0.179588160618166 | 0.680180117601935 | -0.426022151 | 0.277963438224181 | 0.928644431609684 | 0.653101874840205 | 1.32043791877136 | 0.998735809224318 |
| ebi-fl187-GCST90094750 | ebi-met1400-GCST90200224 | 2-ketocaprylate levels \|\| id:ebi-met1400-GCST90200224 | Curry liking \|\| id:ebi-fl187-GCST90094750 | Inverse variance weighted | 7 | -0.25229552 | 0.122477023187268 | 0.0394041200822468 | -0.492350486 | -0.012240555 | 0.777015080397441 | 0.611188115558216 | 0.987834055990464 | 0.998735809224318 |
| ebi-fl187-GCST90094750 | ebi-met1400-GCST90200243 | Hydroxypalmitoyl sphingomyelin (d18:1/16:0(OH)) levels \|\| id:ebi-met1400-GCST90200243 | Curry liking \|\| id:ebi-fl187-GCST90094750 | Inverse variance weighted | 7 | -0.067349656 | 0.154714190439245 | 0.663332632390988 | -0.37058947 | 0.235890156859166 | 0.934868261509371 | 0.690327283687176 | 1.26603523724205 | 0.998735809224318 |
| ebi-fl187-GCST90094750 | ebi-met1400-GCST90200273 | 2-methoxyhydroquinone sulfate (2) levels \|\| id:ebi-met1400-GCST90200273 | Curry liking \|\| id:ebi-fl187-GCST90094750 | Inverse variance weighted | 7 | -0.12016953 | 0.161062666095584 | 0.455604479160094 | -0.435852355 | 0.195513295714594 | 0.886770089988346 | 0.646713202611944 | 1.2159349605389 | 0.998735809224318 |
| ebi-fl187-GCST90094750 | ebi-met1400-GCST90200276 | 2,4-di-tert-butylphenol levels \|\| id:ebi-met1400-GCST90200276 | Curry liking \|\| id:ebi-fl187-GCST90094750 | Inverse variance weighted | 7 | -0.104457221 | 0.133008050620298 | 0.432251260286178 | -0.365153 | 0.156238558496667 | 0.900813332729205 | 0.69409044696039 | 1.16910506977343 | 0.998735809224318 |
| ebi-fl187-GCST90094750 | ebi-met1400-GCST90200289 | N-acetylmethionine levels \|\| id:ebi-met1400-GCST90200289 | Curry liking \|\| id:ebi-fl187-GCST90094750 | Inverse variance weighted | 7 | 0.113532043229187 | 0.120343104274063 | 0.345474819352866 | -0.122340441 | 0.349404527606351 | 1.12022778403291 | 0.88484707886453 | 1.41822278458513 | 0.998735809224318 |
| ebi-fl187-GCST90094750 | ebi-met1400-GCST90200308 | 3-hydroxyisobutyrate levels \|\| id:ebi-met1400-GCST90200308 | Curry liking \|\| id:ebi-fl187-GCST90094750 | Inverse variance weighted | 7 | 0.0957433104960029 | 0.122149669942194 | 0.433145947099729 | -0.143670043 | 0.335156663582703 | 1.10047654695695 | 0.866173501277905 | 1.39815940872768 | 0.998735809224318 |
| ebi-fl187-GCST90094750 | ebi-met1400-GCST90200383 | Cystathionine levels \|\| id:ebi-met1400-GCST90200383 | Curry liking \|\| id:ebi-fl187-GCST90094750 | Inverse variance weighted | 7 | 0.093568750220681 | 0.126027638169988 | 0.457816992645356 | -0.153445421 | 0.340582921033858 | 1.09808609440757 | 0.857747578275884 | 1.40576680281043 | 0.998735809224318 |
| ebi-fl187-GCST90094750 | ebi-met1400-GCST90200384 | Guanidinoacetate levels \|\| id:ebi-met1400-GCST90200384 | Curry liking \|\| id:ebi-fl187-GCST90094750 | Inverse variance weighted | 7 | 0.0343189000681168 | 0.11564415950102 | 0.76664750571356 | -0.192343653 | 0.260981452690115 | 1.03491458844248 | 0.825023298406172 | 1.29820358702617 | 0.998735809224318 |
| ebi-fl187-GCST90094750 | ebi-met1400-GCST90200403 | Citrulline levels \|\| id:ebi-met1400-GCST90200403 | Curry liking \|\| id:ebi-fl187-GCST90094750 | Inverse variance weighted | 7 | -0.043245109 | 0.121128686482198 | 0.721078405068644 | -0.280657335 | 0.194167116178116 | 0.957676625823521 | 0.755287101724111 | 1.21429919504138 | 0.998735809224318 |
| ebi-fl187-GCST90094750 | ebi-met1400-GCST90200434 | 12,13-DiHOME levels \|\| id:ebi-met1400-GCST90200434 | Curry liking \|\| id:ebi-fl187-GCST90094750 | Inverse variance weighted | 7 | 0.0427406270837643 | 0.122153594322862 | 0.726419372264211 | -0.196680418 | 0.282161671956573 | 1.04366716074652 | 0.821453113170581 | 1.32599307855385 | 0.998735809224318 |
| ebi-fl187-GCST90094750 | ebi-met1400-GCST90200437 | Fructose levels \|\| id:ebi-met1400-GCST90200437 | Curry liking \|\| id:ebi-fl187-GCST90094750 | Inverse variance weighted | 7 | -0.075320129 | 0.130452748696615 | 0.563686294851751 | -0.331007517 | 0.180367257962519 | 0.927446535819902 | 0.718199770364164 | 1.19765713148056 | 0.998735809224318 |
| ebi-fl187-GCST90094750 | ebi-met1400-GCST90200448 | N-stearoyl-sphinganine (d18:0/18:0) levels \|\| id:ebi-met1400-GCST90200448 | Curry liking \|\| id:ebi-fl187-GCST90094750 | Inverse variance weighted | 7 | -0.040995619 | 0.134908023026927 | 0.761220619955524 | -0.305415344 | 0.22342410609642 | 0.959833334933532 | 0.736817278068204 | 1.25035074267673 | 0.998735809224318 |
| ebi-fl187-GCST90094750 | ebi-met1400-GCST90200468 | X-11847 levels \|\| id:ebi-met1400-GCST90200468 | Curry liking \|\| id:ebi-fl187-GCST90094750 | Inverse variance weighted | 7 | -0.038572427 | 0.128199042490318 | 0.763506603123262 | -0.28984255 | 0.212697696475582 | 0.96216201589505 | 0.748381390887974 | 1.23701064203747 | 0.998735809224318 |
| ebi-fl187-GCST90094750 | ebi-met1400-GCST90200476 | X-11843 levels \|\| id:ebi-met1400-GCST90200476 | Curry liking \|\| id:ebi-fl187-GCST90094750 | Inverse variance weighted | 7 | 0.114979668684725 | 0.127636992586328 | 0.367676894942123 | -0.135188837 | 0.365148174153928 | 1.12185062864125 | 0.873550937561805 | 1.44072747090804 | 0.998735809224318 |
| ebi-fl187-GCST90094750 | ebi-met1400-GCST90200511 | X-12906 levels \|\| id:ebi-met1400-GCST90200511 | Curry liking \|\| id:ebi-fl187-GCST90094750 | Inverse variance weighted | 7 | 0.0612681232849409 | 0.121130701911078 | 0.612995816675881 | -0.176148052 | 0.298684299030654 | 1.06318394034499 | 0.838493833071597 | 1.34808396487156 | 0.998735809224318 |
| ebi-fl187-GCST90094750 | ebi-met1400-GCST90200515 | X-12849 levels \|\| id:ebi-met1400-GCST90200515 | Curry liking \|\| id:ebi-fl187-GCST90094750 | Inverse variance weighted (multiplicative random effects) | 7 | -0.244232076 | 0.18963918010735 | 0.197788529640334 | -0.615924869 | 0.1274607166662 | 0.783305826414439 | 0.54014110426973 | 1.13594024384489 | 0.998735809224318 |
| ebi-fl187-GCST90094750 | ebi-met1400-GCST90200551 | X-18886 levels \|\| id:ebi-met1400-GCST90200551 | Curry liking \|\| id:ebi-fl187-GCST90094750 | Inverse variance weighted | 7 | -0.118666208 | 0.136356814031314 | 0.384157024710466 | -0.385925563 | 0.148593147967899 | 0.888104193781692 | 0.679821127618555 | 1.16020086309407 | 0.998735809224318 |
| ebi-fl187-GCST90094750 | ebi-met1400-GCST90200564 | X-21319 levels \|\| id:ebi-met1400-GCST90200564 | Curry liking \|\| id:ebi-fl187-GCST90094750 | Inverse variance weighted | 7 | 0.028750611581871 | 0.12228782024939 | 0.814126390073138 | -0.210933516 | 0.268434739270675 | 1.02916789991359 | 0.809827905603431 | 1.30791561871816 | 0.998735809224318 |
| ebi-fl187-GCST90094750 | ebi-met1400-GCST90200579 | X-21442 levels \|\| id:ebi-met1400-GCST90200579 | Curry liking \|\| id:ebi-fl187-GCST90094750 | Inverse variance weighted | 7 | 0.214088194565666 | 0.132209015787544 | 0.105379257382471 | -0.045041476 | 0.473217865509251 | 1.23873189939783 | 0.955957831342538 | 1.60515105193583 | 0.998735809224318 |
| ebi-fl187-GCST90094750 | ebi-met1400-GCST90200581 | X-21441 levels \|\| id:ebi-met1400-GCST90200581 | Curry liking \|\| id:ebi-fl187-GCST90094750 | Inverse variance weighted | 7 | -0.065341801 | 0.121240875185441 | 0.589926868583495 | -0.302973916 | 0.172290314367051 | 0.936747227516301 | 0.738618361974766 | 1.18802268320735 | 0.998735809224318 |
| ebi-fl187-GCST90094750 | ebi-met1400-GCST90200648 | X-24585 levels \|\| id:ebi-met1400-GCST90200648 | Curry liking \|\| id:ebi-fl187-GCST90094750 | Inverse variance weighted (multiplicative random effects) | 7 | 0.100251104575319 | 0.190094738217712 | 0.597933595291946 | -0.272334582 | 0.472836791482035 | 1.10544846639505 | 0.761599400753239 | 1.60453948709332 | 0.998735809224318 |
| ebi-fl187-GCST90094750 | ebi-met1400-GCST90200720 | Adenosine 5'-diphosphate (ADP) to pantothenate ratio \|\| id:ebi-met1400-GCST90200720 | Curry liking \|\| id:ebi-fl187-GCST90094750 | Inverse variance weighted | 7 | -0.169364745 | 0.160632545339042 | 0.29171751674337 | -0.484204534 | 0.145475043717313 | 0.84420092903124 | 0.616187157791215 | 1.15658887006322 | 0.998735809224318 |
| ebi-fl187-GCST90094750 | ebi-met1400-GCST90200724 | Cholate to taurocholate ratio \|\| id:ebi-met1400-GCST90200724 | Curry liking \|\| id:ebi-fl187-GCST90094750 | Inverse variance weighted | 7 | 0.0896710218940281 | 0.129701929391546 | 0.489337900898251 | -0.16454476 | 0.343886803501458 | 1.09381438352464 | 0.848279787334426 | 1.41041897198207 | 0.998735809224318 |
| ebi-fl187-GCST90094750 | ebi-met1400-GCST90200726 | N-acetylputrescine to (N(1) + N(8))-acetylspermidine ratio \|\| id:ebi-met1400-GCST90200726 | Curry liking \|\| id:ebi-fl187-GCST90094750 | Inverse variance weighted | 7 | -0.170953839 | 0.120356403777083 | 0.155492222101246 | -0.406852391 | 0.0649447122833184 | 0.842860479755301 | 0.665742452944492 | 1.06710002522938 | 0.998735809224318 |
| ebi-fl187-GCST90094750 | ebi-met1400-GCST90200744 | Arginine to glutamate ratio \|\| id:ebi-met1400-GCST90200744 | Curry liking \|\| id:ebi-fl187-GCST90094750 | Inverse variance weighted | 7 | 0.0152974599043123 | 0.135169555907343 | 0.909893895116354 | -0.24963487 | 0.280229789482706 | 1.01541506496506 | 0.779085198776394 | 1.32343388858799 | 0.998735809224318 |
| ebi-fl187-GCST90094750 | ebi-met1400-GCST90200759 | Histidine to trans-urocanate ratio \|\| id:ebi-met1400-GCST90200759 | Curry liking \|\| id:ebi-fl187-GCST90094750 | Inverse variance weighted | 7 | -0.328045597 | 0.135573796344384 | 0.0155340463272174 | -0.593770238 | -0.062320957 | 0.720330173705642 | 0.552241273609311 | 0.939581273524812 | 0.998735809224318 |
| ebi-fl187-GCST90094750 | ebi-met1400-GCST90200795 | Oleoyl-linoleoyl-glycerol (18:1 to 18:2) [2] to linoleoyl-arachidonoyl-glycerol (18:2 to 20:4) [2] ratio \|\| id:ebi-met1400-GCST90200795 | Curry liking \|\| id:ebi-fl187-GCST90094750 | Inverse variance weighted | 7 | 0.0586891471924235 | 0.126496866047793 | 0.642678322931619 | -0.18924471 | 0.306623004646098 | 1.06044554702413 | 0.827583963625615 | 1.35882859942902 | 0.998735809224318 |
| ebi-fl187-GCST90094750 | ebi-met1400-GCST90200811 | Choline to taurocholate ratio \|\| id:ebi-met1400-GCST90200811 | Curry liking \|\| id:ebi-fl187-GCST90094750 | Inverse variance weighted | 7 | 0.238054886858746 | 0.126743738077066 | 0.0603486335996166 | -0.01036284 | 0.486472613489795 | 1.26877883019824 | 0.989690669456407 | 1.62656855282207 | 0.998735809224318 |
| ebi-fl187-GCST90094750 | ebi-met1400-GCST90200831 | Cholate to phosphate ratio \|\| id:ebi-met1400-GCST90200831 | Curry liking \|\| id:ebi-fl187-GCST90094750 | Inverse variance weighted | 7 | 0.023242725058203 | 0.137384399518389 | 0.86565487551622 | -0.246030698 | 0.292516148114246 | 1.02351494212313 | 0.781898221847131 | 1.33979437154179 | 0.998735809224318 |
| ebi-fl187-GCST90094750 | ebi-met1400-GCST90200841 | Adenosine 5'-diphosphate (ADP) to oxalate (ethanedioate) ratio \|\| id:ebi-met1400-GCST90200841 | Curry liking \|\| id:ebi-fl187-GCST90094750 | Inverse variance weighted | 7 | -0.05502846 | 0.162854935551062 | 0.735439579418578 | -0.374224133 | 0.264167213941794 | 0.946458211617182 | 0.687822730491213 | 1.30234594849441 | 0.998735809224318 |
| ebi-fl187-GCST90094750 | ebi-met1400-GCST90200844 | Aspartate to phosphate ratio \|\| id:ebi-met1400-GCST90200844 | Curry liking \|\| id:ebi-fl187-GCST90094750 | Inverse variance weighted | 7 | -0.149769472 | 0.113693286716185 | 0.187734239173248 | -0.372608314 | 0.0730693703307825 | 0.860906416901579 | 0.688935026485913 | 1.07580516328628 | 0.998735809224318 |
| ebi-fl187-GCST90094750 | ebi-met1400-GCST90200870 | Adenosine 5'-monophosphate (AMP) to acetoacetate ratio \|\| id:ebi-met1400-GCST90200870 | Curry liking \|\| id:ebi-fl187-GCST90094750 | Inverse variance weighted | 7 | -0.056718858 | 0.13972462771742 | 0.684791719720548 | -0.330579129 | 0.21714141203301 | 0.944859671489649 | 0.718507504659449 | 1.24251979696533 | 0.998735809224318 |
| ebi-fl187-GCST90094750 | ebi-met1400-GCST90200887 | Cholesterol to cortisol ratio \|\| id:ebi-met1400-GCST90200887 | Curry liking \|\| id:ebi-fl187-GCST90094750 | Inverse variance weighted | 7 | -0.003364436 | 0.117485381324176 | 0.977154052440562 | -0.233635784 | 0.226906911164612 | 0.996641217142887 | 0.791650095321424 | 1.25471306272598 | 0.998955228396052 |
| ebi-fl187-GCST90094750 | ebi-met1400-GCST90200890 | Cortisol to taurocholate ratio \|\| id:ebi-met1400-GCST90200890 | Curry liking \|\| id:ebi-fl187-GCST90094750 | Inverse variance weighted | 7 | 0.120611611354934 | 0.126336694922356 | 0.33973756316359 | -0.127008311 | 0.368231533402752 | 1.12818665237995 | 0.880726353120507 | 1.44517660689793 | 0.998735809224318 |
| ebi-fl187-GCST90094750 | ebi-met1400-GCST90200948 | Adenosine 5'-diphosphate (ADP) to mannitol to sorbitol ratio \|\| id:ebi-met1400-GCST90200948 | Curry liking \|\| id:ebi-fl187-GCST90094750 | Inverse variance weighted | 7 | -0.181241152 | 0.16239641715052 | 0.264404341418844 | -0.49953813 | 0.137055825394711 | 0.834234157017428 | 0.606810862832064 | 1.14689217244152 | 0.998735809224318 |
| ebi-fl187-GCST90094750 | ebi-met1400-GCST90200972 | Leucine to N-palmitoyl-sphingosine (d18:1 to 16:0) ratio \|\| id:ebi-met1400-GCST90200972 | Curry liking \|\| id:ebi-fl187-GCST90094750 | Inverse variance weighted | 7 | 0.174752517300242 | 0.116573563915002 | 0.133854171966874 | -0.053731668 | 0.403236702573646 | 1.19095144026012 | 0.947686366990818 | 1.49666111327673 | 0.998735809224318 |
| ebi-fl187-GCST90094750 | ebi-met1400-GCST90200973 | Proline to glutamate ratio \|\| id:ebi-met1400-GCST90200973 | Curry liking \|\| id:ebi-fl187-GCST90094750 | Inverse variance weighted | 7 | 0.063753539572855 | 0.118672664300626 | 0.591114430629953 | -0.168844882 | 0.296351961602082 | 1.06582968154956 | 0.84463991169328 | 1.34494344198662 | 0.998735809224318 |
| ebi-fl187-GCST90094750 | ebi-met1400-GCST90200983 | Cholesterol to linoleoyl-arachidonoyl-glycerol (18:2 to 20:4) [1] ratio \|\| id:ebi-met1400-GCST90200983 | Curry liking \|\| id:ebi-fl187-GCST90094750 | Inverse variance weighted | 7 | 0.00508905293083575 | 0.135973640117376 | 0.970144735422913 | -0.261419282 | 0.271597387560894 | 1.00510202415512 | 0.769958022619355 | 1.31205864382576 | 0.998735809224318 |
| ebi-fl187-GCST90094750 | ebi-met1400-GCST90201001 | Serine to alpha-ketobutyrate ratio \|\| id:ebi-met1400-GCST90201001 | Curry liking \|\| id:ebi-fl187-GCST90094750 | Inverse variance weighted | 7 | -0.068745344 | 0.123050704443935 | 0.57638359087297 | -0.309924725 | 0.17243403682659 | 0.93356438769152 | 0.733502168820015 | 1.18819344101994 | 0.998735809224318 |
| ebi-fl187-GCST90094750 | ebi-met1400-GCST90201005 | Fructose to maltose ratio \|\| id:ebi-met1400-GCST90201005 | Curry liking \|\| id:ebi-fl187-GCST90094750 | Inverse variance weighted | 7 | -0.04096057 | 0.13780741621219 | 0.766290524287288 | -0.311063105 | 0.229141966101144 | 0.959866977068735 | 0.732667639088915 | 1.25752055162799 | 0.998735809224318 |
| ebi-fl187-GCST90094750 | ebi-met1400-GCST90201020 | Paraxanthine to 5-acetylamino-6-formylamino-3-methyluracil ratio \|\| id:ebi-met1400-GCST90201020 | Curry liking \|\| id:ebi-fl187-GCST90094750 | Inverse variance weighted | 7 | 0.119510676940773 | 0.139424407688325 | 0.391349919443706 | -0.153761162 | 0.392782516009891 | 1.12694527633094 | 0.857476794489243 | 1.48109623957941 | 0.998735809224318 |
| ebi-fl187-GCST90094750 | ebi-pw11-GCST90275197 | Placental weight (fetal genotype, adjusted for sex) \|\| id:ebi-pw11-GCST90275197 | Curry liking \|\| id:ebi-fl187-GCST90094750 | Inverse variance weighted | 6 | -0.030549225 | 0.0535083654245677 | 0.568050971986327 | -0.135425621 | 0.0743271712548498 | 0.969912686962093 | 0.873344118792065 | 1.07715916336755 | 0.998735809224318 |
| ebi-fl187-GCST90094750 | prot-a-1017 | FAS-associated factor 2 \|\| id:prot-a-1017 | Curry liking \|\| id:ebi-fl187-GCST90094750 | Inverse variance weighted | 7 | 0.0847904093776129 | 0.237234027602331 | 0.720783012438868 | -0.380188285 | 0.549769103478181 | 1.08848890572182 | 0.683732660677454 | 1.73285286197322 | 0.998735809224318 |
| ebi-fl187-GCST90094750 | prot-a-1019 | Fas apoptotic inhibitory molecule 3 \|\| id:prot-a-1019 | Curry liking \|\| id:ebi-fl187-GCST90094750 | Inverse variance weighted | 7 | 0.116332981859887 | 0.246143637719706 | 0.636482641789967 | -0.366108548 | 0.598774511790511 | 1.12336987165148 | 0.693427526904148 | 1.81988718297405 | 0.998735809224318 |
| ebi-fl187-GCST90094750 | prot-a-1052 | MIP18 family protein FAM96A \|\| id:prot-a-1052 | Curry liking \|\| id:ebi-fl187-GCST90094750 | Inverse variance weighted | 7 | -0.250035572 | 0.191353959577593 | 0.191326421765128 | -0.625089332 | 0.125018189224696 | 0.778773080415161 | 0.535213614509758 | 1.1331690643461 | 0.998735809224318 |
| ebi-fl187-GCST90094750 | prot-a-1066 | F-box/LRR-repeat protein 4 \|\| id:prot-a-1066 | Curry liking \|\| id:ebi-fl187-GCST90094750 | Inverse variance weighted | 7 | -0.19279441 | 0.208157768835779 | 0.354345336138214 | -0.600783637 | 0.21519481688416 | 0.824651496785647 | 0.548381735481247 | 1.24010346652777 | 0.998735809224318 |
| ebi-fl187-GCST90094750 | prot-a-1076 | Low affinity immunoglobulin gamma Fc region receptor III-B \|\| id:prot-a-1076 | Curry liking \|\| id:ebi-fl187-GCST90094750 | Inverse variance weighted (multiplicative random effects) | 7 | -0.179195927 | 0.319646993868912 | 0.575066363507535 | -0.805704035 | 0.447312180572762 | 0.835942099380406 | 0.446773271612216 | 1.56410250549423 | 0.998735809224318 |
| ebi-fl187-GCST90094750 | prot-a-1081 | Fc receptor-like protein 3 \|\| id:prot-a-1081 | Curry liking \|\| id:ebi-fl187-GCST90094750 | Inverse variance weighted | 7 | 0.0615545686566573 | 0.191160766747636 | 0.747449517107821 | -0.313120534 | 0.436229671482023 | 1.06348852808578 | 0.731161777280316 | 1.54686402450774 | 0.998735809224318 |
| ebi-fl187-GCST90094750 | prot-a-1105 | Fibroblast growth factor receptor 4 \|\| id:prot-a-1105 | Curry liking \|\| id:ebi-fl187-GCST90094750 | Inverse variance weighted | 7 | 0.379298589200346 | 0.191221045205977 | 0.0473048112798304 | 0.00450534059663071 | 0.75409183780406 | 1.46125928685221 | 1.0045155049024 | 2.12568018411952 | 0.998735809224318 |
| ebi-fl187-GCST90094750 | prot-a-1143 | Follistatin-related protein 1 \|\| id:prot-a-1143 | Curry liking \|\| id:ebi-fl187-GCST90094750 | Inverse variance weighted | 7 | -0.161243401 | 0.191353959577593 | 0.399427179761211 | -0.536297162 | 0.213810359601629 | 0.851084890835628 | 0.58491007474982 | 1.23838778417095 | 0.998735809224318 |
| ebi-fl187-GCST90094750 | prot-a-1151 | Alpha-(1,3)-fucosyltransferase 10 \|\| id:prot-a-1151 | Curry liking \|\| id:ebi-fl187-GCST90094750 | Inverse variance weighted | 7 | -0.139784743 | 0.221928329169234 | 0.528783338810488 | -0.574764268 | 0.295194782240763 | 0.86954539104709 | 0.562837531929187 | 1.34338800132889 | 0.998735809224318 |
| ebi-fl187-GCST90094750 | prot-a-1165 | Growth arrest and DNA damage-inducible proteins-interacting protein 1 \|\| id:prot-a-1165 | Curry liking \|\| id:ebi-fl187-GCST90094750 | Inverse variance weighted | 7 | 0.251724726135273 | 0.248447929980325 | 0.310969862078571 | -0.235233217 | 0.73868266889671 | 1.2862419197632 | 0.790386496860343 | 2.09317629125495 | 0.998735809224318 |
| ebi-fl187-GCST90094750 | prot-a-1194 | Growth/differentiation factor 11/8 \|\| id:prot-a-1194 | Curry liking \|\| id:ebi-fl187-GCST90094750 | Inverse variance weighted | 7 | -0.146308136 | 0.191221045205977 | 0.444196697779318 | -0.521101385 | 0.228485112589052 | 0.863891466094668 | 0.593866112642008 | 1.25669481606047 | 0.998735809224318 |
| ebi-fl187-GCST90094750 | prot-a-1216 | D-glucuronyl C5-epimerase \|\| id:prot-a-1216 | Curry liking \|\| id:ebi-fl187-GCST90094750 | Inverse variance weighted | 7 | -0.23543782 | 0.191221045205977 | 0.218235421035063 | -0.610231069 | 0.13935542822799 | 0.790224797362209 | 0.54322533204812 | 1.14953260373879 | 0.998735809224318 |
| ebi-fl187-GCST90094750 | prot-a-1246 | Glypican-3 \|\| id:prot-a-1246 | Curry liking \|\| id:ebi-fl187-GCST90094750 | Inverse variance weighted (multiplicative random effects) | 7 | 0.107267812995334 | 0.311186205094705 | 0.730315259620121 | -0.502657149 | 0.717192774980956 | 1.11323235265113 | 0.604921156674269 | 2.04867404175861 | 0.998735809224318 |
| ebi-fl187-GCST90094750 | prot-a-1275 | Glutamate receptor 4 \|\| id:prot-a-1275 | Curry liking \|\| id:ebi-fl187-GCST90094750 | Inverse variance weighted | 6 | -0.020304361 | 0.245068394288115 | 0.933969367710003 | -0.500638414 | 0.460029691355493 | 0.979900384014216 | 0.606143565470424 | 1.58412101899655 | 0.998735809224318 |
| ebi-fl187-GCST90094750 | prot-a-1289 | Glutathione S-transferase P \|\| id:prot-a-1289 | Curry liking \|\| id:ebi-fl187-GCST90094750 | Inverse variance weighted | 7 | 0.0414591288303549 | 0.207239234780985 | 0.841438005492237 | -0.364729771 | 0.447648029001086 | 1.04233055971175 | 0.694384268057088 | 1.56462789508313 | 0.998735809224318 |
| ebi-fl187-GCST90094750 | prot-a-130 | Apolipoprotein D \|\| id:prot-a-130 | Curry liking \|\| id:ebi-fl187-GCST90094750 | Inverse variance weighted | 7 | 0.097085886293661 | 0.191353959577593 | 0.611900261739334 | -0.277967874 | 0.472139647065744 | 1.10195501238866 | 0.757321150460504 | 1.60342128116997 | 0.998735809224318 |
| ebi-fl187-GCST90094750 | prot-a-1313 | Heparin-binding EGF-like growth factor \|\| id:prot-a-1313 | Curry liking \|\| id:ebi-fl187-GCST90094750 | Inverse variance weighted (multiplicative random effects) | 7 | -0.109717461 | 0.365689595415647 | 0.764155082213066 | -0.826469068 | 0.607034145918891 | 0.896087279051276 | 0.437591668072013 | 1.83498103429469 | 0.998735809224318 |
| ebi-fl187-GCST90094750 | prot-a-1347 | HLA class II histocompatibility antigen, DQ alpha 2 chain \|\| id:prot-a-1347 | Curry liking \|\| id:ebi-fl187-GCST90094750 | Inverse variance weighted | 6 | -0.157885288 | 0.207033359509123 | 0.445697236016235 | -0.563670673 | 0.24790009670883 | 0.853947734463309 | 0.569116185892935 | 1.28133191652399 | 0.998735809224318 |
| ebi-fl187-GCST90094750 | prot-a-1357 | Non-histone chromosomal protein HMG-14 \|\| id:prot-a-1357 | Curry liking \|\| id:ebi-fl187-GCST90094750 | Inverse variance weighted (multiplicative random effects) | 7 | -0.066977232 | 0.291724800771668 | 0.818410019069688 | -0.638757841 | 0.504803377701944 | 0.935216494280716 | 0.527947811866459 | 1.65665975218768 | 0.998735809224318 |
| ebi-fl187-GCST90094750 | prot-a-137 | Apolipoprotein M \|\| id:prot-a-137 | Curry liking \|\| id:ebi-fl187-GCST90094750 | Inverse variance weighted | 7 | -0.105452803 | 0.215714207973496 | 0.624944900436518 | -0.528252651 | 0.3173470442666 | 0.899916944899277 | 0.58963436708605 | 1.37347914728766 | 0.998735809224318 |
| ebi-fl187-GCST90094750 | prot-a-1381 | 17-beta-hydroxysteroid dehydrogenase 14 \|\| id:prot-a-1381 | Curry liking \|\| id:ebi-fl187-GCST90094750 | Inverse variance weighted (multiplicative random effects) | 7 | 0.0373497898834648 | 0.284397034723123 | 0.895514462917807 | -0.520068398 | 0.594767977940786 | 1.0380560588488 | 0.594479885241037 | 1.81261033058468 | 0.998735809224318 |
| ebi-fl187-GCST90094750 | prot-a-1392 | Serine protease HTRA2, mitochondrial \|\| id:prot-a-1392 | Curry liking \|\| id:ebi-fl187-GCST90094750 | Inverse variance weighted | 7 | 0.0113586619759371 | 0.191353959577593 | 0.952665826190355 | -0.363695099 | 0.38641242274802 | 1.01142341651962 | 0.69510310020821 | 1.4716915047247 | 0.998735809224318 |
| ebi-fl187-GCST90094750 | prot-a-1416 | Interferon-induced protein with tetratricopeptide repeats 2 \|\| id:prot-a-1416 | Curry liking \|\| id:ebi-fl187-GCST90094750 | Inverse variance weighted | 7 | -0.022985619 | 0.227691814475168 | 0.919589696348858 | -0.469261576 | 0.423290337138009 | 0.977276537658094 | 0.625463955644377 | 1.52697756991135 | 0.998735809224318 |
| ebi-fl187-GCST90094750 | prot-a-1428 | Interferon gamma \|\| id:prot-a-1428 | Curry liking \|\| id:ebi-fl187-GCST90094750 | Inverse variance weighted | 7 | 0.268339545784232 | 0.214941213882024 | 0.211872800534741 | -0.152945233 | 0.689624324992998 | 1.30779111959629 | 0.858176719924514 | 1.9929666848168 | 0.998735809224318 |
| ebi-fl187-GCST90094750 | prot-a-1483 | Interleukin-17C \|\| id:prot-a-1483 | Curry liking \|\| id:ebi-fl187-GCST90094750 | Inverse variance weighted | 7 | 0.0148011039940019 | 0.256527470319755 | 0.953989242669967 | -0.487992738 | 0.517594945820722 | 1.01491118275894 | 0.613857330961255 | 1.67798714283689 | 0.998735809224318 |
| ebi-fl187-GCST90094750 | prot-a-1503 | Interleukin-1 receptor-like 2 \|\| id:prot-a-1503 | Curry liking \|\| id:ebi-fl187-GCST90094750 | Inverse variance weighted | 7 | -0.01653674 | 0.270294597077458 | 0.951215515113549 | -0.54631415 | 0.513240670590272 | 0.983599241603925 | 0.579080284758566 | 1.67069660899815 | 0.998735809224318 |
| ebi-fl187-GCST90094750 | prot-a-1522 | Interleukin-31 receptor subunit alpha \|\| id:prot-a-1522 | Curry liking \|\| id:ebi-fl187-GCST90094750 | Inverse variance weighted | 7 | 0.0621394768900033 | 0.224406786404895 | 0.781852657317931 | -0.377697824 | 0.501976778243598 | 1.0641107532365 | 0.685437591847341 | 1.65198365047613 | 0.998735809224318 |
| ebi-fl187-GCST90094750 | prot-a-1540 | Interleukin-6 receptor subunit alpha \|\| id:prot-a-1540 | Curry liking \|\| id:ebi-fl187-GCST90094750 | Inverse variance weighted (multiplicative random effects) | 7 | 0.210035542676891 | 0.293582858583732 | 0.474348518411488 | -0.36538686 | 0.785457945501006 | 1.23372190895667 | 0.693928145799723 | 2.1934111735525 | 0.998735809224318 |
| ebi-fl187-GCST90094750 | prot-a-1541 | Interleukin-6 receptor subunit alpha \|\| id:prot-a-1541 | Curry liking \|\| id:ebi-fl187-GCST90094750 | Inverse variance weighted (multiplicative random effects) | 7 | 0.0958191496194981 | 0.316755963155902 | 0.762269800664927 | -0.525022538 | 0.716660837405067 | 1.10056000929852 | 0.591542031944018 | 2.04758456484759 | 0.998735809224318 |
| ebi-fl187-GCST90094750 | prot-a-1545 | Interleukin-9 \|\| id:prot-a-1545 | Curry liking \|\| id:ebi-fl187-GCST90094750 | Inverse variance weighted | 7 | -0.258072703 | 0.191221045205977 | 0.177143014059098 | -0.632865952 | 0.116720545399529 | 0.772539063999841 | 0.531067603753138 | 1.12380533323433 | 0.998735809224318 |
| ebi-fl187-GCST90094750 | prot-a-1548 | Inositol monophosphatase 3 \|\| id:prot-a-1548 | Curry liking \|\| id:ebi-fl187-GCST90094750 | Inverse variance weighted | 7 | -0.302963514 | 0.19392182339641 | 0.118218001209648 | -0.683050287 | 0.0771232602765174 | 0.738626045698635 | 0.505074019370122 | 1.08017521088252 | 0.998735809224318 |
| ebi-fl187-GCST90094750 | prot-a-1555 | Inhibin beta A chain:Inhibin beta B chain heterodimer \|\| id:prot-a-1555 | Curry liking \|\| id:ebi-fl187-GCST90094750 | Inverse variance weighted | 7 | -0.197285446 | 0.225094679745639 | 0.380782516918766 | -0.638471018 | 0.243900126635327 | 0.820956261457123 | 0.52809926134793 | 1.27621686405204 | 0.998735809224318 |
| ebi-fl187-GCST90094750 | prot-a-1603 | Junctophilin-3 \|\| id:prot-a-1603 | Curry liking \|\| id:ebi-fl187-GCST90094750 | Inverse variance weighted | 7 | 0.0451550798219522 | 0.206758104681367 | 0.827120820035215 | -0.360090805 | 0.450400964997432 | 1.04619009030199 | 0.697612976201891 | 1.56894114986952 | 0.998735809224318 |
| ebi-fl187-GCST90094750 | prot-a-1627 | UPF0258 protein KIAA1024 \|\| id:prot-a-1627 | Curry liking \|\| id:ebi-fl187-GCST90094750 | Inverse variance weighted | 7 | 0.0456845377557753 | 0.191353959577593 | 0.811304387713836 | -0.329369223 | 0.420738298527858 | 1.04674415060846 | 0.719377357028627 | 1.5230856324957 | 0.998735809224318 |
| ebi-fl187-GCST90094750 | prot-a-1666 | Kallikrein-7 \|\| id:prot-a-1666 | Curry liking \|\| id:ebi-fl187-GCST90094750 | Inverse variance weighted | 7 | 0.0424274408686048 | 0.191221045205977 | 0.824410087439325 | -0.332365808 | 0.41722068947232 | 1.04334034975758 | 0.717224908441437 | 1.51773742464935 | 0.998735809224318 |
| ebi-fl187-GCST90094750 | prot-a-1678 | Importin subunit alpha-3 \|\| id:prot-a-1678 | Curry liking \|\| id:ebi-fl187-GCST90094750 | Inverse variance weighted | 7 | 0.108026486957246 | 0.206996544665062 | 0.601756810075626 | -0.297686741 | 0.513739714500767 | 1.11407725351226 | 0.742533909055632 | 1.67153056804094 | 0.998735809224318 |
| ebi-fl187-GCST90094750 | prot-a-1718 | Protein LDOC1 \|\| id:prot-a-1718 | Curry liking \|\| id:ebi-fl187-GCST90094750 | Inverse variance weighted | 7 | -0.28929045 | 0.191353959577593 | 0.130582710007799 | -0.66434421 | 0.0857633111090261 | 0.748794686650638 | 0.514610893527988 | 1.08954841377786 | 0.998735809224318 |
| ebi-fl187-GCST90094750 | prot-a-1723 | Leptin \|\| id:prot-a-1723 | Curry liking \|\| id:ebi-fl187-GCST90094750 | Inverse variance weighted | 7 | 0.0698735788180523 | 0.220899776827376 | 0.751764574709707 | -0.363089984 | 0.502837141399709 | 1.07237260207251 | 0.695523844829921 | 1.65340556793849 | 0.998735809224318 |
| ebi-fl187-GCST90094750 | prot-a-1730 | Galectin-7 \|\| id:prot-a-1730 | Curry liking \|\| id:ebi-fl187-GCST90094750 | Inverse variance weighted | 7 | -0.035881616 | 0.191353959577593 | 0.851257363149196 | -0.410935377 | 0.339172144879779 | 0.964754498323401 | 0.66302977741212 | 1.40378497881055 | 0.998735809224318 |
| ebi-fl187-GCST90094750 | prot-a-1753 | Lipase member N \|\| id:prot-a-1753 | Curry liking \|\| id:ebi-fl187-GCST90094750 | Inverse variance weighted | 7 | 0.222086287785829 | 0.191114833028976 | 0.245212401093147 | -0.152498785 | 0.596671360522622 | 1.24867911897223 | 0.858559937148185 | 1.81606370702126 | 0.998735809224318 |
| ebi-fl187-GCST90094750 | prot-a-1756 | VIP36-like protein \|\| id:prot-a-1756 | Curry liking \|\| id:ebi-fl187-GCST90094750 | Inverse variance weighted | 7 | 0.270697851552012 | 0.191323812985303 | 0.157107338448161 | -0.104296822 | 0.645692525003206 | 1.31087893050672 | 0.900957833713341 | 1.90730743 | 0.998735809224318 |
| ebi-fl187-GCST90094750 | prot-a-1760 | Ig Kappa chain V-I region HK102- like \|\| id:prot-a-1760 | Curry liking \|\| id:ebi-fl187-GCST90094750 | Inverse variance weighted | 7 | 0.0210342620555788 | 0.191353959577593 | 0.912470189901737 | -0.354019499 | 0.396088022827662 | 1.02125704140363 | 0.70186128182781 | 1.4860001137267 | 0.998735809224318 |
| ebi-fl187-GCST90094750 | prot-a-179 | Neutral ceramidase \|\| id:prot-a-179 | Curry liking \|\| id:ebi-fl187-GCST90094750 | Inverse variance weighted | 7 | 0.179363724852479 | 0.243444323742033 | 0.461259105678534 | -0.29778715 | 0.656514599386863 | 1.19645584576045 | 0.742459355640332 | 1.9280605463174 | 0.998735809224318 |
| ebi-fl187-GCST90094750 | prot-a-1809 | Lactotransferrin \|\| id:prot-a-1809 | Curry liking \|\| id:ebi-fl187-GCST90094750 | Inverse variance weighted (multiplicative random effects) | 7 | 0.0213815322544085 | 0.285545983933139 | 0.940310610375235 | -0.538288596 | 0.581051660763362 | 1.02161175512664 | 0.583746423809298 | 1.78791772530652 | 0.998735809224318 |
| ebi-fl187-GCST90094750 | prot-a-1818 | Lysophospholipase-like protein 1 \|\| id:prot-a-1818 | Curry liking \|\| id:ebi-fl187-GCST90094750 | Inverse variance weighted | 7 | -0.20387193 | 0.191353959577593 | 0.286686869500012 | -0.578925691 | 0.171181831001243 | 0.815566814327928 | 0.560500193892117 | 1.18670650943794 | 0.998735809224318 |
| ebi-fl187-GCST90094750 | prot-a-1852 | MAP kinase-activated protein kinase 3 \|\| id:prot-a-1852 | Curry liking \|\| id:ebi-fl187-GCST90094750 | Inverse variance weighted | 7 | 0.361621618062741 | 0.191323812985303 | 0.058744252255154 | -0.013373055 | 0.736616291513935 | 1.43565561302181 | 0.98671596664273 | 2.08885546487495 | 0.998735809224318 |
| ebi-fl187-GCST90094750 | prot-a-1856 | Megakaryocyte-associated tyrosine-protein kinase \|\| id:prot-a-1856 | Curry liking \|\| id:ebi-fl187-GCST90094750 | Inverse variance weighted | 7 | 0.00238325643782854 | 0.191353959577593 | 0.990062842912193 | -0.372670504 | 0.377437017209911 | 1.00238609865091 | 0.688892182440729 | 1.45854157788334 | 0.999107451094609 |
| ebi-fl187-GCST90094750 | prot-a-1890 | Beta-1,3-N-acetylglucosaminyltransferase manic fringe \|\| id:prot-a-1890 | Curry liking \|\| id:ebi-fl187-GCST90094750 | Inverse variance weighted | 7 | -0.52948354 | 0.233701049052198 | 0.0234731466524892 | -0.987537596 | -0.071429484 | 0.588909038919529 | 0.372492790216742 | 0.931061929868022 | 0.998735809224318 |
| ebi-fl187-GCST90094750 | prot-a-1919 | Matrilysin \|\| id:prot-a-1919 | Curry liking \|\| id:ebi-fl187-GCST90094750 | Inverse variance weighted | 7 | -0.052700203 | 0.205051813791142 | 0.797171668452798 | -0.454601758 | 0.349201351732736 | 0.948664376305914 | 0.634700681884708 | 1.41793466520234 | 0.998735809224318 |
| ebi-fl187-GCST90094750 | prot-a-1923 | Multimerin-2 \|\| id:prot-a-1923 | Curry liking \|\| id:ebi-fl187-GCST90094750 | Inverse variance weighted | 7 | 0.120151518175511 | 0.191353959577593 | 0.530068194664526 | -0.254902243 | 0.495205278947594 | 1.12766770078828 | 0.774992255488665 | 1.6408350333763 | 0.998735809224318 |
| ebi-fl187-GCST90094750 | prot-a-1948 | Mesothelin \|\| id:prot-a-1948 | Curry liking \|\| id:ebi-fl187-GCST90094750 | Inverse variance weighted | 7 | 0.0039493136151993 | 0.191353959577593 | 0.983533799488668 | -0.371104447 | 0.379003074387282 | 1.00395712243065 | 0.689971872194754 | 1.4608275268861 | 0.998955228396052 |
| ebi-fl187-GCST90094750 | prot-a-195 | ATPase family AAA domain-containing protein 1 \|\| id:prot-a-195 | Curry liking \|\| id:ebi-fl187-GCST90094750 | Inverse variance weighted | 7 | -0.263909561 | 0.191221045205977 | 0.167547168694309 | -0.63870281 | 0.11088368715532 | 0.768042997212077 | 0.527976866304951 | 1.11726494703236 | 0.998735809224318 |
| ebi-fl187-GCST90094750 | prot-a-1966 | Mucin-1 \|\| id:prot-a-1966 | Curry liking \|\| id:ebi-fl187-GCST90094750 | Inverse variance weighted | 7 | -0.416361374 | 0.191221045205977 | 0.029452033932949 | -0.791154622 | -0.041568125 | 0.659441922416025 | 0.453321078339559 | 0.959283981747721 | 0.998735809224318 |
| ebi-fl187-GCST90094750 | prot-a-1986 | Myocardial zonula adherens protein \|\| id:prot-a-1986 | Curry liking \|\| id:ebi-fl187-GCST90094750 | Inverse variance weighted | 7 | -0.264217668 | 0.191353959577593 | 0.167346698683999 | -0.639271429 | 0.110836092696381 | 0.767806394525879 | 0.527676734073589 | 1.11721177267712 | 0.998735809224318 |
| ebi-fl187-GCST90094750 | prot-a-2071 | Epididymal secretory protein E1 \|\| id:prot-a-2071 | Curry liking \|\| id:ebi-fl187-GCST90094750 | Inverse variance weighted | 7 | -0.202480631 | 0.257969843966993 | 0.43251197377912 | -0.708101525 | 0.303140263213349 | 0.816702301182855 | 0.49257845816588 | 1.35410438215459 | 0.998735809224318 |
| ebi-fl187-GCST90094750 | prot-a-2098 | Neuritin \|\| id:prot-a-2098 | Curry liking \|\| id:ebi-fl187-GCST90094750 | Inverse variance weighted | 7 | -0.036670806 | 0.219804215109047 | 0.867500703685901 | -0.467487067 | 0.394145455869384 | 0.963993424219091 | 0.626574831929663 | 1.4831162609512 | 0.998735809224318 |
| ebi-fl187-GCST90094750 | prot-a-2115 | Neurotrophin-4 \|\| id:prot-a-2115 | Curry liking \|\| id:ebi-fl187-GCST90094750 | Inverse variance weighted (multiplicative random effects) | 7 | 0.186141567782173 | 0.302520870519394 | 0.538355578324378 | -0.406799338 | 0.779082474000186 | 1.20459277983558 | 0.66577777290792 | 2.17947162593649 | 0.998735809224318 |
| ebi-fl187-GCST90094750 | prot-a-2118 | Netrin-1 \|\| id:prot-a-2118 | Curry liking \|\| id:ebi-fl187-GCST90094750 | Inverse variance weighted | 7 | 0.201555041142961 | 0.191190961379617 | 0.291787697862991 | -0.173179243 | 0.57628932544701 | 1.22330356723636 | 0.840986860190902 | 1.77942330427553 | 0.998735809224318 |
| ebi-fl187-GCST90094750 | prot-a-2120 | Netrin-G1 \|\| id:prot-a-2120 | Curry liking \|\| id:ebi-fl187-GCST90094750 | Inverse variance weighted | 7 | 0.24465503854662 | 0.191925349801204 | 0.202401065907903 | -0.131518647 | 0.620828724156979 | 1.27718065910852 | 0.8767629259312 | 1.86046921893784 | 0.998735809224318 |
| ebi-fl187-GCST90094750 | prot-a-2145 | Olfactomedin-like protein 3 \|\| id:prot-a-2145 | Curry liking \|\| id:ebi-fl187-GCST90094750 | Inverse variance weighted | 7 | -0.13408114 | 0.259791210913809 | 0.605776781464582 | -0.643271914 | 0.375109632985519 | 0.874519102889775 | 0.525569988053628 | 1.45515093841527 | 0.998735809224318 |
| ebi-fl187-GCST90094750 | prot-a-2162 | Prolyl 4-hydroxylase subunit alpha-2 \|\| id:prot-a-2162 | Curry liking \|\| id:ebi-fl187-GCST90094750 | Inverse variance weighted | 7 | 0.364185531453024 | 0.219014741291994 | 0.0963453275892058 | -0.065083361 | 0.793454424385332 | 1.43934123245739 | 0.936989351303281 | 2.21102105436991 | 0.998735809224318 |
| ebi-fl187-GCST90094750 | prot-a-2168 | Polyadenylate-binding protein-interacting protein 1 \|\| id:prot-a-2168 | Curry liking \|\| id:ebi-fl187-GCST90094750 | Inverse variance weighted | 7 | 0.00433329268969681 | 0.238749773084675 | 0.985519242708746 | -0.463616263 | 0.472282847935659 | 1.00434269497852 | 0.629004880886233 | 1.60365090893333 | 0.998955228396052 |
| ebi-fl187-GCST90094750 | prot-a-219 | Beta-1,4-galactosyltransferase 5 \|\| id:prot-a-219 | Curry liking \|\| id:ebi-fl187-GCST90094750 | Inverse variance weighted | 7 | -0.18064663 | 0.191726879872452 | 0.346086077576368 | -0.556431315 | 0.195138054062439 | 0.834730274815762 | 0.5732511706961 | 1.21547877669034 | 0.998735809224318 |
| ebi-fl187-GCST90094750 | prot-a-2190 | Pyruvate carboxylase, mitochondrial \|\| id:prot-a-2190 | Curry liking \|\| id:ebi-fl187-GCST90094750 | Inverse variance weighted | 7 | -0.229331523 | 0.191353959577593 | 0.230735023955477 | -0.604385284 | 0.14572223752512 | 0.795064907308785 | 0.546410210511838 | 1.15687480700954 | 0.998735809224318 |
| ebi-fl187-GCST90094750 | prot-a-2197 | Protocadherin-9 \|\| id:prot-a-2197 | Curry liking \|\| id:ebi-fl187-GCST90094750 | Inverse variance weighted | 7 | -0.11774011 | 0.191190961379617 | 0.538010245818897 | -0.492474394 | 0.256994174308774 | 0.888927045851046 | 0.611112388822622 | 1.29303759389965 | 0.998735809224318 |
| ebi-fl187-GCST90094750 | prot-a-2198 | Protocadherin alpha-4 \|\| id:prot-a-2198 | Curry liking \|\| id:ebi-fl187-GCST90094750 | Inverse variance weighted | 7 | 0.156403891350356 | 0.207729483611372 | 0.451497385626304 | -0.250745897 | 0.563553679228644 | 1.16929837723047 | 0.778220094864942 | 1.75690489620562 | 0.998735809224318 |
| ebi-fl187-GCST90094750 | prot-a-2254 | Placenta growth factor \|\| id:prot-a-2254 | Curry liking \|\| id:ebi-fl187-GCST90094750 | Inverse variance weighted | 7 | -0.139943579 | 0.196193777660596 | 0.475664685617786 | -0.524483384 | 0.244596224925302 | 0.869407286591894 | 0.591861050580677 | 1.27710554569775 | 0.998735809224318 |
| ebi-fl187-GCST90094750 | prot-a-2261 | Membrane-associated progesterone receptor component 2 \|\| id:prot-a-2261 | Curry liking \|\| id:ebi-fl187-GCST90094750 | Inverse variance weighted | 7 | 0.120829592774664 | 0.191221045205977 | 0.52746242676977 | -0.253963656 | 0.495622841378379 | 1.12843260291351 | 0.775719994434245 | 1.64152032750794 | 0.998735809224318 |
| ebi-fl187-GCST90094750 | prot-a-2268 | Polymeric immunoglobulin receptor \|\| id:prot-a-2268 | Curry liking \|\| id:ebi-fl187-GCST90094750 | Inverse variance weighted | 7 | 0.0432559449649397 | 0.229569732419587 | 0.850546002406266 | -0.406700731 | 0.493212620507329 | 1.04420511969448 | 0.665843427065225 | 1.63756866505697 | 0.998735809224318 |
| ebi-fl187-GCST90094750 | prot-a-2283 | Group 10 secretory phospholipase A2 \|\| id:prot-a-2283 | Curry liking \|\| id:ebi-fl187-GCST90094750 | Inverse variance weighted | 7 | 0.0775368311042741 | 0.263862968963506 | 0.768870505790996 | -0.439634588 | 0.594708250272746 | 1.08062203222837 | 0.6442718026815 | 1.81250207082965 | 0.998735809224318 |
| ebi-fl187-GCST90094750 | prot-a-2298 | Inactive phospholipase D5 \|\| id:prot-a-2298 | Curry liking \|\| id:ebi-fl187-GCST90094750 | Inverse variance weighted | 7 | 0.0221292840538426 | 0.224677525301446 | 0.921540404079323 | -0.418238666 | 0.462497233644678 | 1.02237595283436 | 0.658205118799117 | 1.58803473124154 | 0.998735809224318 |
| ebi-fl187-GCST90094750 | prot-a-2304 | Procollagen-lysine,2-oxoglutarate 5-dioxygenase 2 \|\| id:prot-a-2304 | Curry liking \|\| id:ebi-fl187-GCST90094750 | Inverse variance weighted | 7 | -0.121898766 | 0.191353959577593 | 0.52410341882489 | -0.496952527 | 0.253154994476912 | 0.885237979887064 | 0.60838186479699 | 1.28808290709984 | 0.998735809224318 |
| ebi-fl187-GCST90094750 | prot-a-2331 | Periostin \|\| id:prot-a-2331 | Curry liking \|\| id:ebi-fl187-GCST90094750 | Inverse variance weighted | 7 | -0.030114276 | 0.191353959577593 | 0.874949533231247 | -0.405168037 | 0.344939484363527 | 0.970334640854565 | 0.666864743371642 | 1.41190447478438 | 0.998735809224318 |
| ebi-fl187-GCST90094750 | prot-a-2333 | Periostin \|\| id:prot-a-2333 | Curry liking \|\| id:ebi-fl187-GCST90094750 | Inverse variance weighted (multiplicative random effects) | 7 | -0.041789402 | 0.281799723091173 | 0.882110206495477 | -0.59411686 | 0.510538054931192 | 0.959071737581589 | 0.552049888215841 | 1.66618745418193 | 0.998735809224318 |
| ebi-fl187-GCST90094750 | prot-a-2367 | cAMP-dependent protein kinase catalytic subunit alpha \|\| id:prot-a-2367 | Curry liking \|\| id:ebi-fl187-GCST90094750 | Inverse variance weighted | 7 | 0.0865621944695809 | 0.191353959577593 | 0.65100469959303 | -0.288491566 | 0.461615955241664 | 1.09041918365162 | 0.749393125275777 | 1.58663584702342 | 0.998735809224318 |
| ebi-fl187-GCST90094750 | prot-a-2385 | Phosphoribosyl pyrophosphate synthase-associated protein 1 \|\| id:prot-a-2385 | Curry liking \|\| id:ebi-fl187-GCST90094750 | Inverse variance weighted (multiplicative random effects) | 7 | 0.261776750476873 | 0.282010048480906 | 0.35327620635989 | -0.290962945 | 0.81451644549945 | 1.29923645613051 | 0.747543378064349 | 2.25808350186906 | 0.998735809224318 |
| ebi-fl187-GCST90094750 | prot-a-2393 | Trypsin-3 \|\| id:prot-a-2393 | Curry liking \|\| id:ebi-fl187-GCST90094750 | Inverse variance weighted | 6 | 0.0268366008366017 | 0.229497777331399 | 0.906910601645004 | -0.422979043 | 0.476652244406143 | 1.02719994543826 | 0.655092361913525 | 1.61067322602618 | 0.998735809224318 |
| ebi-fl187-GCST90094750 | prot-a-2405 | Pregnancy-specific beta-1-glycoprotein 6 \|\| id:prot-a-2405 | Curry liking \|\| id:ebi-fl187-GCST90094750 | Inverse variance weighted | 7 | 0.324274198201046 | 0.191221045205977 | 0.0899222271693479 | -0.05051905 | 0.699067446804761 | 1.38302647850545 | 0.950735816599669 | 2.0118756513121 | 0.998735809224318 |
| ebi-fl187-GCST90094750 | prot-a-244 | Beclin-1 \|\| id:prot-a-244 | Curry liking \|\| id:ebi-fl187-GCST90094750 | Inverse variance weighted | 7 | 0.233464064964952 | 0.231624068897407 | 0.313481399839918 | -0.22051911 | 0.687447240003869 | 1.26296744229615 | 0.802102310480686 | 1.98863254656899 | 0.998735809224318 |
| ebi-fl187-GCST90094750 | prot-a-2454 | tRNA pseudouridine synthase A, mitochondrial \|\| id:prot-a-2454 | Curry liking \|\| id:ebi-fl187-GCST90094750 | Inverse variance weighted | 7 | -0.058916825 | 0.191353959577593 | 0.758162638631827 | -0.433970586 | 0.316136936013501 | 0.942785182281181 | 0.647931313760815 | 1.37181809406588 | 0.998735809224318 |
| ebi-fl187-GCST90094750 | prot-a-2466 | Glutaminyl-peptide cyclotransferase-like protein \|\| id:prot-a-2466 | Curry liking \|\| id:ebi-fl187-GCST90094750 | Inverse variance weighted | 7 | -0.067673873 | 0.191353959577593 | 0.723595145897973 | -0.442727634 | 0.307379887809017 | 0.934565210866019 | 0.642282119248428 | 1.35985746323294 | 0.998735809224318 |
| ebi-fl187-GCST90094750 | prot-a-2474 | Ras-related protein Rab-27A \|\| id:prot-a-2474 | Curry liking \|\| id:ebi-fl187-GCST90094750 | Inverse variance weighted | 7 | -0.122976551 | 0.193903833853364 | 0.525941087893357 | -0.503028065 | 0.257074963222748 | 0.884284397787734 | 0.604696823047899 | 1.29314206122243 | 0.998735809224318 |
| ebi-fl187-GCST90094750 | prot-a-2490 | Rap1 GTPase-GDP dissociation stimulator 1 \|\| id:prot-a-2490 | Curry liking \|\| id:ebi-fl187-GCST90094750 | Inverse variance weighted | 7 | -0.229412221 | 0.191221045205977 | 0.230247070305859 | -0.604205469 | 0.145381028003494 | 0.795000750263784 | 0.546508471996986 | 1.15648013764637 | 0.998735809224318 |
| ebi-fl187-GCST90094750 | prot-a-2545 | Receptor-interacting serine/threonine-protein kinase 2 \|\| id:prot-a-2545 | Curry liking \|\| id:ebi-fl187-GCST90094750 | Inverse variance weighted | 7 | 0.0749236427377105 | 0.191353959577593 | 0.695394600439548 | -0.300130118 | 0.449977403509793 | 1.07780184974399 | 0.74072183314203 | 1.56827674753962 | 0.998735809224318 |
| ebi-fl187-GCST90094750 | prot-a-2610 | RUN and FYVE domain-containing protein 1 \|\| id:prot-a-2610 | Curry liking \|\| id:ebi-fl187-GCST90094750 | Inverse variance weighted | 7 | 0.115529272434763 | 0.191353959577593 | 0.546012231568348 | -0.259524488 | 0.490583033206846 | 1.12246737142028 | 0.771418317010717 | 1.63326819200438 | 0.998735809224318 |
| ebi-fl187-GCST90094750 | prot-a-2627 | Serine--tRNA ligase, mitochondrial \|\| id:prot-a-2627 | Curry liking \|\| id:ebi-fl187-GCST90094750 | Inverse variance weighted | 7 | 0.0691585039630309 | 0.191221045205977 | 0.717600256197605 | -0.305634745 | 0.443951752566746 | 1.07160604949346 | 0.73665563774258 | 1.55885527304178 | 0.998735809224318 |
| ebi-fl187-GCST90094750 | prot-a-264 | BolA-like protein 2 \|\| id:prot-a-264 | Curry liking \|\| id:ebi-fl187-GCST90094750 | Inverse variance weighted | 7 | -0.250328925 | 0.191277636430961 | 0.19062911340559 | -0.625233093 | 0.124575242033008 | 0.778544657859721 | 0.535136677486386 | 1.13266724144007 | 0.998735809224318 |
| ebi-fl187-GCST90094750 | prot-a-2646 | Secretoglobin family 1C member 1 \|\| id:prot-a-2646 | Curry liking \|\| id:ebi-fl187-GCST90094750 | Inverse variance weighted | 7 | 0.0453436155810662 | 0.191353959577593 | 0.812686275967188 | -0.329710145 | 0.420397376353149 | 1.04638735313981 | 0.719132147136752 | 1.52256646733208 | 0.998735809224318 |
| ebi-fl187-GCST90094750 | prot-a-2672 | Semaphorin-3E \|\| id:prot-a-2672 | Curry liking \|\| id:ebi-fl187-GCST90094750 | Inverse variance weighted | 7 | 0.00252695508327513 | 0.242870589017157 | 0.991698533452947 | -0.473499399 | 0.478553309556902 | 1.00253015052528 | 0.622818958091311 | 1.61373813313643 | 0.999107451094609 |
| ebi-fl187-GCST90094750 | prot-a-2701 | Plasma protease C1 inhibitor \|\| id:prot-a-2701 | Curry liking \|\| id:ebi-fl187-GCST90094750 | Inverse variance weighted | 7 | -0.079703481 | 0.214149862807427 | 0.709754715951498 | -0.499437212 | 0.340030250073934 | 0.923390108481949 | 0.606872103881293 | 1.4049900909749 | 0.998735809224318 |
| ebi-fl187-GCST90094750 | prot-a-2738 | Signaling threshold-regulating transmembrane adapter 1 \|\| id:prot-a-2738 | Curry liking \|\| id:ebi-fl187-GCST90094750 | Inverse variance weighted | 7 | -0.038281214 | 0.191353959577593 | 0.841437983511329 | -0.413334975 | 0.336772546381016 | 0.962442250220595 | 0.661440679514583 | 1.40042049680021 | 0.998735809224318 |
| ebi-fl187-GCST90094750 | prot-a-274 | Serine/threonine-protein kinase BRSK2 \|\| id:prot-a-274 | Curry liking \|\| id:ebi-fl187-GCST90094750 | Inverse variance weighted | 7 | 0.109997920128679 | 0.191353959577593 | 0.565399476193606 | -0.265055841 | 0.485051680900762 | 1.11627574874654 | 0.767163109898106 | 1.62425894984074 | 0.998735809224318 |
| ebi-fl187-GCST90094750 | prot-a-2749 | Mitochondrial glutamate carrier 2 \|\| id:prot-a-2749 | Curry liking \|\| id:ebi-fl187-GCST90094750 | Inverse variance weighted | 7 | 0.120984379229006 | 0.237882246472655 | 0.611040089283819 | -0.345264824 | 0.58723358231541 | 1.12860728251375 | 0.708032824654055 | 1.79900472660349 | 0.998735809224318 |
| ebi-fl187-GCST90094750 | prot-a-2760 | Sodium-coupled monocarboxylate transporter 1 \|\| id:prot-a-2760 | Curry liking \|\| id:ebi-fl187-GCST90094750 | Inverse variance weighted | 7 | -0.116311186 | 0.191353959577593 | 0.543298466193112 | -0.491364947 | 0.258742574700595 | 0.890198162921878 | 0.611790762147779 | 1.29530031883363 | 0.998735809224318 |
| ebi-fl187-GCST90094750 | prot-a-2777 | Small integral membrane protein 10 \|\| id:prot-a-2777 | Curry liking \|\| id:ebi-fl187-GCST90094750 | Inverse variance weighted | 7 | 0.26474175419168 | 0.191643094180486 | 0.16714641086563 | -0.11087871 | 0.640362218785432 | 1.30309441365219 | 0.895047302273122 | 1.89716794473214 | 0.998735809224318 |
| ebi-fl187-GCST90094750 | prot-a-2799 | Superoxide dismutase [Mn], mitochondrial \|\| id:prot-a-2799 | Curry liking \|\| id:ebi-fl187-GCST90094750 | Inverse variance weighted | 7 | 0.406145969739399 | 0.191221045205977 | 0.0336734366414204 | 0.0313527211356842 | 0.780939218343114 | 1.50102164020519 | 1.03184939479713 | 2.18352210673851 | 0.998735809224318 |
| ebi-fl187-GCST90094750 | prot-a-2805 | Protein SPACA7 \|\| id:prot-a-2805 | Curry liking \|\| id:ebi-fl187-GCST90094750 | Inverse variance weighted | 7 | -0.238237724 | 0.222725002946376 | 0.284777031632967 | -0.67477873 | 0.198303281292621 | 0.788015338287159 | 0.509269093982941 | 1.21933213837757 | 0.998735809224318 |
| ebi-fl187-GCST90094750 | prot-a-2811 | SPARC-like protein 1 \|\| id:prot-a-2811 | Curry liking \|\| id:ebi-fl187-GCST90094750 | Inverse variance weighted | 7 | -0.108413088 | 0.201723919055391 | 0.590968564347199 | -0.503791969 | 0.286965793484768 | 0.897256873939892 | 0.604235069279469 | 1.33237863666604 | 0.998735809224318 |
| ebi-fl187-GCST90094750 | prot-a-2831 | Sepiapterin reductase \|\| id:prot-a-2831 | Curry liking \|\| id:ebi-fl187-GCST90094750 | Inverse variance weighted | 7 | 0.429613029308127 | 0.257789766005932 | 0.0956089397539793 | -0.075654912 | 0.934880970679753 | 1.53666276502529 | 0.927136094843238 | 2.54691028269633 | 0.998735809224318 |
| ebi-fl187-GCST90094750 | prot-a-2834 | SPRY domain-containing SOCS box protein 1 \|\| id:prot-a-2834 | Curry liking \|\| id:ebi-fl187-GCST90094750 | Inverse variance weighted | 7 | 0.0835140420844897 | 0.206276471656773 | 0.685577037528778 | -0.320787842 | 0.487815926531764 | 1.08710048034249 | 0.72557717140017 | 1.62875501179337 | 0.998735809224318 |
| ebi-fl187-GCST90094750 | prot-a-2835 | Spectrin alpha chain, non-erythrocytic 1 \|\| id:prot-a-2835 | Curry liking \|\| id:ebi-fl187-GCST90094750 | Inverse variance weighted | 7 | -0.141345672 | 0.191353959577593 | 0.460112984407223 | -0.516399433 | 0.233708088657472 | 0.868189151042349 | 0.596665017439768 | 1.26327567388132 | 0.998735809224318 |
| ebi-fl187-GCST90094750 | prot-a-2859 | Sia-alpha-2,3-Gal-beta-1,4-GlcNAc-R:alpha 2,8-sialyltransferase \|\| id:prot-a-2859 | Curry liking \|\| id:ebi-fl187-GCST90094750 | Inverse variance weighted | 7 | -0.041139183 | 0.191221045205977 | 0.829658629645867 | -0.415932431 | 0.333654066040828 | 0.95969554776593 | 0.659724845816616 | 1.39606011542843 | 0.998735809224318 |
| ebi-fl187-GCST90094750 | prot-a-2861 | Alpha-2,8-sialyltransferase 8F \|\| id:prot-a-2861 | Curry liking \|\| id:ebi-fl187-GCST90094750 | Inverse variance weighted | 7 | -0.336978618 | 0.191221045205977 | 0.0780276586640755 | -0.711771866 | 0.0378146308989065 | 0.713924105075477 | 0.490773840977076 | 1.03853870204877 | 0.998735809224318 |
| ebi-fl187-GCST90094750 | prot-a-289 | Uncharacterized protein C17orf89 \|\| id:prot-a-289 | Curry liking \|\| id:ebi-fl187-GCST90094750 | Inverse variance weighted | 7 | -0.326782305 | 0.231856853708244 | 0.158712774468301 | -0.781221738 | 0.127657128037081 | 0.721240736273104 | 0.457846301012359 | 1.13616337733769 | 0.998735809224318 |
| ebi-fl187-GCST90094750 | prot-a-2921 | Tachykinin-3 \|\| id:prot-a-2921 | Curry liking \|\| id:ebi-fl187-GCST90094750 | Inverse variance weighted | 7 | -0.148220564 | 0.2275267552303 | 0.514760980423599 | -0.594173004 | 0.29773187622598 | 0.86224091464014 | 0.552018894415753 | 1.34680063019642 | 0.998735809224318 |
| ebi-fl187-GCST90094750 | prot-a-2928 | Tax1-binding protein 1 \|\| id:prot-a-2928 | Curry liking \|\| id:ebi-fl187-GCST90094750 | Inverse variance weighted | 7 | -0.116628056 | 0.239544532626463 | 0.626347474318836 | -0.58613534 | 0.352879228183515 | 0.889916130789482 | 0.556473716321191 | 1.42315925552577 | 0.998735809224318 |
| ebi-fl187-GCST90094750 | prot-a-296 | Uncharacterized protein C1orf185 \|\| id:prot-a-296 | Curry liking \|\| id:ebi-fl187-GCST90094750 | Inverse variance weighted | 7 | -0.184877342 | 0.191221045205977 | 0.333631380868235 | -0.559670591 | 0.189915906618219 | 0.831206231705662 | 0.571397256484486 | 1.20914791204477 | 0.998735809224318 |
| ebi-fl187-GCST90094750 | prot-a-2964 | Transforming growth factor beta-2 \|\| id:prot-a-2964 | Curry liking \|\| id:ebi-fl187-GCST90094750 | Inverse variance weighted | 7 | 0.135419853309042 | 0.191353959577593 | 0.479135939661277 | -0.239633907 | 0.510473614081124 | 1.14501742279931 | 0.786915892375693 | 1.66608008710546 | 0.998735809224318 |
| ebi-fl187-GCST90094750 | prot-a-2977 | Thymocyte nuclear protein 1 \|\| id:prot-a-2977 | Curry liking \|\| id:ebi-fl187-GCST90094750 | Inverse variance weighted | 7 | 0.242201406107272 | 0.191353959577593 | 0.205611712532046 | -0.132852355 | 0.617255166879355 | 1.27405076858313 | 0.875594359988407 | 1.85383259086863 | 0.998735809224318 |
| ebi-fl187-GCST90094750 | prot-a-3015 | Transmembrane protein 70, mitochondrial \|\| id:prot-a-3015 | Curry liking \|\| id:ebi-fl187-GCST90094750 | Inverse variance weighted | 7 | -0.235689636 | 0.191221045205977 | 0.217743435181723 | -0.610482884 | 0.139103613062449 | 0.790025831826367 | 0.543088556892956 | 1.14924317043926 | 0.998735809224318 |
| ebi-fl187-GCST90094750 | prot-a-3030 | Tumor necrosis factor alpha-induced protein 3 \|\| id:prot-a-3030 | Curry liking \|\| id:ebi-fl187-GCST90094750 | Inverse variance weighted | 7 | -0.288338165 | 0.234785918804432 | 0.219413303055199 | -0.748518566 | 0.171842235972571 | 0.749508092061932 | 0.473066851330229 | 1.18749047515522 | 0.998735809224318 |
| ebi-fl187-GCST90094750 | prot-a-3032 | Tumor necrosis factor receptor superfamily member 10A \|\| id:prot-a-3032 | Curry liking \|\| id:ebi-fl187-GCST90094750 | Inverse variance weighted | 7 | 0.100920409240023 | 0.191353959577593 | 0.597914642702835 | -0.274133352 | 0.475974170012106 | 1.10618859586863 | 0.760230690573824 | 1.60958143995238 | 0.998735809224318 |
| ebi-fl187-GCST90094750 | prot-a-3081 | Tumor protein p53-inducible protein 11 \|\| id:prot-a-3081 | Curry liking \|\| id:ebi-fl187-GCST90094750 | Inverse variance weighted | 7 | -0.17618335 | 0.191353959577593 | 0.357196815456045 | -0.551237111 | 0.198870410487513 | 0.83846423658637 | 0.576236500703549 | 1.22002385335885 | 0.998735809224318 |
| ebi-fl187-GCST90094750 | prot-a-315 | Complement C4 \|\| id:prot-a-315 | Curry liking \|\| id:ebi-fl187-GCST90094750 | Inverse variance weighted | 6 | -0.143830438 | 0.207033359509123 | 0.487230118623187 | -0.549615822 | 0.261954946794709 | 0.86603458230175 | 0.577171504109342 | 1.29946799591215 | 0.998735809224318 |
| ebi-fl187-GCST90094750 | prot-a-3157 | UDP-glucuronosyltransferase 1-8 \|\| id:prot-a-3157 | Curry liking \|\| id:ebi-fl187-GCST90094750 | Inverse variance weighted | 7 | 0.304532620717425 | 0.191221045205977 | 0.111257015866506 | -0.070260628 | 0.67932586932114 | 1.3559910927354 | 0.932150843740034 | 1.97254752911057 | 0.998735809224318 |
| ebi-fl187-GCST90094750 | prot-a-317 | Glutaredoxin-like protein C5orf63 \|\| id:prot-a-317 | Curry liking \|\| id:ebi-fl187-GCST90094750 | Inverse variance weighted (multiplicative random effects) | 7 | 0.0155528880235151 | 0.299989213770983 | 0.958652339077563 | -0.572425971 | 0.603531747014642 | 1.01567446365276 | 0.56415515321226 | 1.82856544027382 | 0.998735809224318 |
| ebi-fl187-GCST90094750 | prot-a-3192 | Guanine nucleotide exchange factor VAV3 \|\| id:prot-a-3192 | Curry liking \|\| id:ebi-fl187-GCST90094750 | Inverse variance weighted | 7 | -0.018751872 | 0.256890394775312 | 0.941809607354511 | -0.522257046 | 0.484753301543536 | 0.981422850310419 | 0.593180200941374 | 1.62377437679621 | 0.998735809224318 |
| ebi-fl187-GCST90094750 | prot-a-3233 | Serine/threonine-protein kinase WNK3 \|\| id:prot-a-3233 | Curry liking \|\| id:ebi-fl187-GCST90094750 | Inverse variance weighted | 7 | 0.359914096612551 | 0.218935488876906 | 0.100191154672782 | -0.069199462 | 0.789027654811288 | 1.4332062919967 | 0.933140535823096 | 2.20125500561079 | 0.998735809224318 |
| ebi-fl187-GCST90094750 | prot-a-3250 | 14-3-3 protein theta \|\| id:prot-a-3250 | Curry liking \|\| id:ebi-fl187-GCST90094750 | Inverse variance weighted | 7 | 0.189132903985581 | 0.191221045205977 | 0.322624020999165 | -0.185660345 | 0.563926152589295 | 1.2082015166098 | 0.830555649774892 | 1.75755941836512 | 0.998735809224318 |
| ebi-fl187-GCST90094750 | prot-a-3278 | E3 ubiquitin-protein ligase ZNRF3 \|\| id:prot-a-3278 | Curry liking \|\| id:ebi-fl187-GCST90094750 | Inverse variance weighted | 7 | 0.23362845236292 | 0.191221045205977 | 0.221793991379128 | -0.141164796 | 0.608421700966635 | 1.26317507529336 | 0.868346199716412 | 1.8375289387614 | 0.998735809224318 |
| ebi-fl187-GCST90094750 | prot-a-355 | Calpain-3 \|\| id:prot-a-355 | Curry liking \|\| id:ebi-fl187-GCST90094750 | Inverse variance weighted | 7 | 0.281429299524072 | 0.210192269278873 | 0.180598783773544 | -0.130547548 | 0.693406147310662 | 1.32502231330168 | 0.877614762899514 | 2.000518001 | 0.998735809224318 |
| ebi-fl187-GCST90094750 | prot-a-385 | Coiled-coil domain-containing protein 90B, mitochondrial \|\| id:prot-a-385 | Curry liking \|\| id:ebi-fl187-GCST90094750 | Inverse variance weighted | 7 | -0.244030956 | 0.191353959577593 | 0.202208159324293 | -0.619084717 | 0.131022804384092 | 0.783463380691074 | 0.538437034305508 | 1.13999377787154 | 0.998735809224318 |
| ebi-fl187-GCST90094750 | prot-a-390 | C-C motif chemokine 14 \|\| id:prot-a-390 | Curry liking \|\| id:ebi-fl187-GCST90094750 | Inverse variance weighted | 7 | -0.044033677 | 0.243472455205696 | 0.856479887539656 | -0.521239689 | 0.433172334946473 | 0.956921730431642 | 0.593783983762982 | 1.54214196275426 | 0.998735809224318 |
| ebi-fl187-GCST90094750 | prot-a-40 | Pituitary adenylate cyclase-activating polypeptide 27 \|\| id:prot-a-40 | Curry liking \|\| id:ebi-fl187-GCST90094750 | Inverse variance weighted | 7 | 0.0888105781780989 | 0.191353959577593 | 0.642564100389992 | -0.286243183 | 0.463864338950182 | 1.0928736226059 | 0.751079944167376 | 1.59020722662456 | 0.998735809224318 |
| ebi-fl187-GCST90094750 | prot-a-401 | C-C motif chemokine 24 \|\| id:prot-a-401 | Curry liking \|\| id:ebi-fl187-GCST90094750 | Inverse variance weighted | 7 | -0.05597301 | 0.191353959577593 | 0.769896464215236 | -0.43102677 | 0.319080751240141 | 0.94556465677865 | 0.649841514086993 | 1.37586242301725 | 0.998735809224318 |
| ebi-fl187-GCST90094750 | prot-a-412 | Cerebral cavernous malformations 2 protein \|\| id:prot-a-412 | Curry liking \|\| id:ebi-fl187-GCST90094750 | Inverse variance weighted | 7 | 0.133258093165345 | 0.233089742583484 | 0.567523236377473 | -0.323597802 | 0.590113988628973 | 1.14254484329568 | 0.723541190470498 | 1.80419406128443 | 0.998735809224318 |
| ebi-fl187-GCST90094750 | prot-a-44 | Adhesion G protein-coupled receptor F5 \|\| id:prot-a-44 | Curry liking \|\| id:ebi-fl187-GCST90094750 | Inverse variance weighted | 7 | 0.0482302176451261 | 0.191353959577593 | 0.801004629643267 | -0.326823543 | 0.423283978417209 | 1.04941222072732 | 0.721210994435967 | 1.52696786031819 | 0.998735809224318 |
| ebi-fl187-GCST90094750 | prot-a-48 | Adrenomedullin \|\| id:prot-a-48 | Curry liking \|\| id:ebi-fl187-GCST90094750 | Inverse variance weighted | 7 | 0.192423135394507 | 0.191221045205977 | 0.314277825175265 | -0.182370113 | 0.567216383998222 | 1.21218332613331 | 0.833292870636325 | 1.76335171934635 | 0.998735809224318 |
| ebi-fl187-GCST90094750 | prot-a-502 | Carcinoembryonic antigen-related cell adhesion molecule 1 \|\| id:prot-a-502 | Curry liking \|\| id:ebi-fl187-GCST90094750 | Inverse variance weighted | 7 | -0.144179589 | 0.227479662393323 | 0.526202806950848 | -0.590039727 | 0.301680549691886 | 0.865732258454114 | 0.554305263472896 | 1.35212921961494 | 0.998735809224318 |
| ebi-fl187-GCST90094750 | prot-a-518 | Complement factor B \|\| id:prot-a-518 | Curry liking \|\| id:ebi-fl187-GCST90094750 | Inverse variance weighted | 7 | 0.142608156481912 | 0.191221045205977 | 0.455802454942692 | -0.232185092 | 0.517401405085626 | 1.15327780868265 | 0.792799368808215 | 1.6776624153968 | 0.998735809224318 |
| ebi-fl187-GCST90094750 | prot-a-525 | Properdin \|\| id:prot-a-525 | Curry liking \|\| id:ebi-fl187-GCST90094750 | Inverse variance weighted | 7 | 0.28482289920818 | 0.191353959577593 | 0.136629369311078 | -0.090230862 | 0.659876659980263 | 1.32952654706592 | 0.913720218041546 | 1.93455371179348 | 0.998735809224318 |
| ebi-fl187-GCST90094750 | prot-a-533 | Chondroadherin \|\| id:prot-a-533 | Curry liking \|\| id:ebi-fl187-GCST90094750 | Inverse variance weighted (multiplicative random effects) | 7 | 0.0946339426134562 | 0.328357363463467 | 0.773190500749601 | -0.54894649 | 0.738214375001851 | 1.09925639054657 | 0.577557953191157 | 2.09219629905698 | 0.998735809224318 |
| ebi-fl187-GCST90094750 | prot-a-535 | Coiled-coil-helix-coiled-coil-helix domain-containing protein 2 \|\| id:prot-a-535 | Curry liking \|\| id:ebi-fl187-GCST90094750 | Inverse variance weighted | 7 | -0.183194386 | 0.209216414509646 | 0.381235844546956 | -0.593258559 | 0.226869786069769 | 0.832606292691459 | 0.552523916464788 | 1.25466648224918 | 0.998735809224318 |
| ebi-fl187-GCST90094750 | prot-a-591 | Caseinolytic peptidase B protein homolog \|\| id:prot-a-591 | Curry liking \|\| id:ebi-fl187-GCST90094750 | Inverse variance weighted | 7 | 0.144224069488668 | 0.272922237949923 | 0.597191331435649 | -0.390703517 | 0.679151655870518 | 1.15514291181046 | 0.676580721059943 | 1.972203915 | 0.998735809224318 |
| ebi-fl187-GCST90094750 | prot-a-605 | Ciliary neurotrophic factor receptor subunit alpha \|\| id:prot-a-605 | Curry liking \|\| id:ebi-fl187-GCST90094750 | Inverse variance weighted | 7 | -0.013109822 | 0.191190961379617 | 0.945332490582474 | -0.387844106 | 0.361624462130496 | 0.986975737247448 | 0.678518111598018 | 1.43565969612946 | 0.998735809224318 |
| ebi-fl187-GCST90094750 | prot-a-606 | Ciliary neurotrophic factor receptor subunit alpha \|\| id:prot-a-606 | Curry liking \|\| id:ebi-fl187-GCST90094750 | Inverse variance weighted | 7 | 0.112126163804351 | 0.191221045205977 | 0.557627323200114 | -0.262667085 | 0.486919412408065 | 1.11865398538499 | 0.768997865779674 | 1.62729546427149 | 0.998735809224318 |
| ebi-fl187-GCST90094750 | prot-a-707 | Cancer/testis antigen 55 \|\| id:prot-a-707 | Curry liking \|\| id:ebi-fl187-GCST90094750 | Inverse variance weighted (multiplicative random effects) | 7 | 0.25709741401815 | 0.303879832034125 | 0.397525068303579 | -0.338507057 | 0.852701884805036 | 1.29317109361617 | 0.712833749068225 | 2.34597685582419 | 0.998735809224318 |
| ebi-fl187-GCST90094750 | prot-a-719 | Dipeptidyl peptidase 1 \|\| id:prot-a-719 | Curry liking \|\| id:ebi-fl187-GCST90094750 | Inverse variance weighted | 7 | -0.14324613 | 0.196454955551115 | 0.465906800273796 | -0.528297843 | 0.24180558244409 | 0.866540760590435 | 0.589607720739166 | 1.27354656893449 | 0.998735809224318 |
| ebi-fl187-GCST90094750 | prot-a-752 | Cytochrome b5 \|\| id:prot-a-752 | Curry liking \|\| id:ebi-fl187-GCST90094750 | Inverse variance weighted | 7 | -0.195483468 | 0.191221045205977 | 0.306643403514217 | -0.570276716 | 0.17930978071214 | 0.822436940068459 | 0.565368970131859 | 1.19639130571919 | 0.998735809224318 |
| ebi-fl187-GCST90094750 | prot-a-755 | Cytoglobin \|\| id:prot-a-755 | Curry liking \|\| id:ebi-fl187-GCST90094750 | Inverse variance weighted | 7 | -0.108517604 | 0.191353959577593 | 0.570643496393482 | -0.483571365 | 0.266536156699268 | 0.897163100953398 | 0.616577432042299 | 1.30543478869512 | 0.998735809224318 |
| ebi-fl187-GCST90094750 | prot-a-807 | Desmin \|\| id:prot-a-807 | Curry liking \|\| id:ebi-fl187-GCST90094750 | Inverse variance weighted | 7 | 0.0504542949118574 | 0.216754562369412 | 0.815938807890993 | -0.374384647 | 0.475293237155905 | 1.05174879198522 | 0.687712334233031 | 1.60848579613745 | 0.998735809224318 |
| ebi-fl187-GCST90094750 | prot-a-82 | Ameloblastin \|\| id:prot-a-82 | Curry liking \|\| id:ebi-fl187-GCST90094750 | Inverse variance weighted | 7 | 0.0774117362136341 | 0.212510002062711 | 0.715653877824132 | -0.339107868 | 0.493931340256547 | 1.08048686038826 | 0.712405599298894 | 1.63874604104825 | 0.998735809224318 |
| ebi-fl187-GCST90094750 | prot-a-84 | Muellerian-inhibiting factor \|\| id:prot-a-84 | Curry liking \|\| id:ebi-fl187-GCST90094750 | Inverse variance weighted | 7 | 0.145714851381295 | 0.191221045205977 | 0.446046224466555 | -0.229078397 | 0.520508099985009 | 1.15686626219739 | 0.795266184401186 | 1.6828825050801 | 0.998735809224318 |
| ebi-fl187-GCST90094750 | prot-a-858 | Inactive dipeptidyl peptidase 10 \|\| id:prot-a-858 | Curry liking \|\| id:ebi-fl187-GCST90094750 | Inverse variance weighted | 7 | 0.0707375366651318 | 0.207134725567482 | 0.732723469730151 | -0.335246525 | 0.476721598777396 | 1.07329948713423 | 0.715161759046448 | 1.61078493712885 | 0.998735809224318 |
| ebi-fl187-GCST90094750 | prot-a-863 | Dihydropyrimidinase-related protein 3 \|\| id:prot-a-863 | Curry liking \|\| id:ebi-fl187-GCST90094750 | Inverse variance weighted | 7 | 0.111665295800239 | 0.191353959577593 | 0.559520672026512 | -0.263388465 | 0.486719056572322 | 1.11813855233788 | 0.768443326007667 | 1.62696945878839 | 0.998735809224318 |
| ebi-fl187-GCST90094750 | prot-a-864 | Dihydropyrimidinase-related protein 5 \|\| id:prot-a-864 | Curry liking \|\| id:ebi-fl187-GCST90094750 | Inverse variance weighted | 7 | -0.047575811 | 0.191221045205977 | 0.803515430428944 | -0.42236906 | 0.327217437557446 | 0.953538181665206 | 0.655492079070023 | 1.3871030526919 | 0.998735809224318 |
| ebi-fl187-GCST90094750 | prot-a-884 | Endothelin-converting enzyme 1 \|\| id:prot-a-884 | Curry liking \|\| id:ebi-fl187-GCST90094750 | Inverse variance weighted | 7 | 0.209960556519355 | 0.191353959577593 | 0.272538003814896 | -0.165093204 | 0.585014317291438 | 1.23362940035972 | 0.84781468047156 | 1.79501668523294 | 0.998735809224318 |
| ebi-fl187-GCST90094750 | prot-a-893 | Early endosome antigen 1 \|\| id:prot-a-893 | Curry liking \|\| id:ebi-fl187-GCST90094750 | Inverse variance weighted | 7 | 0.180451988359678 | 0.191353959577593 | 0.345666991349743 | -0.194601772 | 0.555505749131761 | 1.19775861374422 | 0.823162398770252 | 1.742822193 | 0.998735809224318 |
| ebi-fl187-GCST90094750 | prot-a-897 | Ephrin-A2 \|\| id:prot-a-897 | Curry liking \|\| id:ebi-fl187-GCST90094750 | Inverse variance weighted | 7 | 0.227419204015066 | 0.191353959577593 | 0.234646720868554 | -0.147634557 | 0.602472964787148 | 1.25535600793144 | 0.862746342160859 | 1.82663041225126 | 0.998735809224318 |
| ebi-fl187-GCST90094750 | prot-a-90 | Anaphase-promoting complex subunit 10 \|\| id:prot-a-90 | Curry liking \|\| id:ebi-fl187-GCST90094750 | Inverse variance weighted | 7 | 0.253514489718696 | 0.191353959577593 | 0.185222300509173 | -0.121539271 | 0.628568250490778 | 1.28854605001982 | 0.885556275938209 | 1.87492423478406 | 0.998735809224318 |
| ebi-fl187-GCST90094750 | prot-a-914 | Histone-lysine N-methyltransferase EHMT2 \|\| id:prot-a-914 | Curry liking \|\| id:ebi-fl187-GCST90094750 | Inverse variance weighted | 7 | -0.276789975 | 0.191221045205977 | 0.147760577680431 | -0.651583224 | 0.0980032734933546 | 0.75821372414284 | 0.521219915441537 | 1.10296639565576 | 0.998735809224318 |
| ebi-fl187-GCST90094750 | prot-a-932 | RNA polymerase II elongation factor ELL \|\| id:prot-a-932 | Curry liking \|\| id:ebi-fl187-GCST90094750 | Inverse variance weighted | 7 | 0.0203092620539282 | 0.191353959577593 | 0.915475616993087 | -0.354744499 | 0.395363022826011 | 1.02051689838119 | 0.701352616810676 | 1.48492315408684 | 0.998735809224318 |
| ebi-fl187-GCST90094750 | prot-a-991 | Estrogen receptor \|\| id:prot-a-991 | Curry liking \|\| id:ebi-fl187-GCST90094750 | Inverse variance weighted | 7 | -0.134273986 | 0.191353959577593 | 0.482863296923377 | -0.509327747 | 0.240779774348329 | 0.874350471623394 | 0.600899399368553 | 1.27224082438991 | 0.998735809224318 |
| ebi-fl187-GCST90094750 | prot-a-992 | Persulfide dioxygenase ETHE1, mitochondrial \|\| id:prot-a-992 | Curry liking \|\| id:ebi-fl187-GCST90094750 | Inverse variance weighted | 7 | 0.186019326700088 | 0.202158295129829 | 0.35748566496347 | -0.210210932 | 0.582249585154554 | 1.20444553811038 | 0.810413286044187 | 1.79006079892291 | 0.998735809224318 |
| ebi-fl187-GCST90094750 | prot-a-993 | Ethanolamine kinase 1 \|\| id:prot-a-993 | Curry liking \|\| id:ebi-fl187-GCST90094750 | Inverse variance weighted | 7 | 0.203515536496039 | 0.224485844405858 | 0.364626209154894 | -0.236476719 | 0.64350779153152 | 1.22570420064173 | 0.789404260572188 | 1.90314502024835 | 0.998735809224318 |
| ebi-fl187-GCST90094750 | prot-b-10 | coagulation factor III, tissue factor \|\| id:prot-b-10 | Curry liking \|\| id:ebi-fl187-GCST90094750 | Inverse variance weighted | 5 | 0.292071208834129 | 0.258037271039267 | 0.257678398647724 | -0.213681842 | 0.797824260071093 | 1.33919837699913 | 0.80760528991089 | 2.22070399409469 | 0.998735809224318 |
| ebi-fl187-GCST90094750 | prot-b-28 | prolactin \|\| id:prot-b-28 | Curry liking \|\| id:ebi-fl187-GCST90094750 | Inverse variance weighted | 5 | -0.263048217 | 0.234402392114274 | 0.261774053808657 | -0.722476905 | 0.196380471971417 | 0.768704832105274 | 0.485548108701577 | 1.216989848 | 0.998735809224318 |
| ebi-fl187-GCST90094750 | prot-b-54 | matrix metallopeptidase 7 \|\| id:prot-b-54 | Curry liking \|\| id:ebi-fl187-GCST90094750 | Inverse variance weighted | 5 | 0.117562967483585 | 0.23432807822392 | 0.615876851260156 | -0.341720066 | 0.576846000802469 | 1.12475245054548 | 0.710547083275664 | 1.78041414113759 | 0.998735809224318 |
| ebi-fl187-GCST90094750 | ukb-rsfMRI191-edge-pheno716 | Net100_Pair10_33 (OccipitalPrecuneus)&(Temporal\|Frontal\|Supp_Motor_Area) (Default_mode\|Central_executive)&(Default_mode\|Salience) \|\| id:ukb-rsfMRI191-edge-pheno716 | Curry liking \|\| id:ebi-fl187-GCST90094750 | Inverse variance weighted | 7 | -0.003970939 | 0.0700478737841172 | 0.954792991648517 | -0.141264772 | 0.133322893535813 | 0.996036934672024 | 0.868259390747602 | 1.14261888302367 | 0.998735809224318 |
| ebi-fl187-GCST90094750 | ukb-rsfMRI191-edge-pheno903 | Net100_Pair27_38 (Precentral\|Frontal\|Supp_Motor_Area)&(Subcortical) (Attention\|Salience\|Motor)&(Subcortical-cerebellum) \|\| id:ukb-rsfMRI191-edge-pheno903 | Curry liking \|\| id:ebi-fl187-GCST90094750 | Inverse variance weighted (multiplicative random effects) | 7 | -0.059983211 | 0.0876136039099554 | 0.493575203561601 | -0.231705875 | 0.111739452806355 | 0.941780345136258 | 0.793179383267451 | 1.11822147321988 | 0.998735809224318 |
| ebi-fl187-GCST90094750 | ukb-rsfMRI191-node-pheno23 | Net100_Node2 RolandicOper\|SupraMarginal\|Insula Salience\|Motor \|\| id:ukb-rsfMRI191-node-pheno23 | Curry liking \|\| id:ebi-fl187-GCST90094750 | Inverse variance weighted (multiplicative random effects) | 7 | -0.032138579 | 0.0881224068376896 | 0.715332504695025 | -0.204858496 | 0.140581338752873 | 0.968372377044697 | 0.814762600380211 | 1.15094269200083 | 0.998735809224318 |
| ebi-fl187-GCST90094750 | ukb-rsfMRI191-node-pheno55 | Net100_Node34 Parietal\|Postcentral\|Precuneus Attention \|\| id:ukb-rsfMRI191-node-pheno55 | Curry liking \|\| id:ebi-fl187-GCST90094750 | Inverse variance weighted (multiplicative random effects) | 7 | 0.0556116000374681 | 0.0914187727744551 | 0.542977201417443 | -0.123569195 | 0.2347923946754 | 1.05718699259725 | 0.883760487674504 | 1.26464619419425 | 0.998735809224318 |
| ebi-fl187-GCST90094750 | ebi-a-GCST90001414 | IgD- CD24- B cell Absolute Count \|\| id:ebi-a-GCST90001414 | Curry liking \|\| id:ebi-fl187-GCST90094750 | MR Egger | 6 | 0.987649384749402 | 1.59406941635175 | 0.569084013627693 | -2.136726671 | 4.11202544079883 | 2.68491584479618 | 0.118040597001485 | 61.0702866366128 | 0.999950574162623 |
| ebi-fl187-GCST90094750 | ebi-a-GCST90001422 | CD20- CD38- B cell %B cell \|\| id:ebi-a-GCST90001422 | Curry liking \|\| id:ebi-fl187-GCST90094750 | MR Egger | 6 | -1.017649991 | 1.46958012345362 | 0.526739272709572 | -3.898027033 | 1.86272705074527 | 0.361443337927767 | 0.020281887487933 | 6.44127853534801 | 0.999950574162623 |
| ebi-fl187-GCST90094750 | ebi-a-GCST90001444 | CD20- CD38- B cell %lymphocyte \|\| id:ebi-a-GCST90001444 | Curry liking \|\| id:ebi-fl187-GCST90094750 | MR Egger | 6 | -1.370253531 | 1.45922128473643 | 0.400880641613514 | -4.230327249 | 1.48982018729678 | 0.254042543781495 | 0.0145476290741892 | 4.43629774459107 | 0.999950574162623 |
| ebi-fl187-GCST90094750 | ebi-a-GCST90001459 | Myeloid Dendritic Cell %Dendritic Cell \|\| id:ebi-a-GCST90001459 | Curry liking \|\| id:ebi-fl187-GCST90094750 | MR Egger | 6 | -0.958174213 | 1.65105759458434 | 0.592808556012673 | -4.194247098 | 2.27789867244004 | 0.383592605424656 | 0.0150820935218281 | 9.75615797127352 | 0.999950574162623 |
| ebi-fl187-GCST90094750 | ebi-a-GCST90001476 | HLA DR++ monocyte %leukocyte \|\| id:ebi-a-GCST90001476 | Curry liking \|\| id:ebi-fl187-GCST90094750 | MR Egger | 6 | 2.95310401204464 | 1.59842165124513 | 0.138387835305171 | -0.179802424 | 6.08601044848509 | 19.1653509759241 | 0.835435256731881 | 439.663845966028 | 0.999950574162623 |
| ebi-fl187-GCST90094750 | ebi-a-GCST90001478 | CD4 regulatory T cell %CD4+ T cell \|\| id:ebi-a-GCST90001478 | Curry liking \|\| id:ebi-fl187-GCST90094750 | MR Egger | 6 | -1.081596631 | 1.60689743681382 | 0.537773154528542 | -4.231115608 | 2.06792234469072 | 0.339053749367151 | 0.0145361648430819 | 7.9083751595342 | 0.999950574162623 |
| ebi-fl187-GCST90094750 | ebi-a-GCST90001485 | CD39+ resting CD4 regulatory T cell %CD4 regulatory T cell \|\| id:ebi-a-GCST90001485 | Curry liking \|\| id:ebi-fl187-GCST90094750 | MR Egger | 6 | 0.251205922408624 | 1.59493208331509 | 0.882479610068638 | -2.874860961 | 3.3772728057062 | 1.28557478573263 | 0.0564239840990031 | 29.2907804385387 | 0.999950574162623 |
| ebi-fl187-GCST90094750 | ebi-a-GCST90001515 | Immature Myeloid-Derived Suppressor Cells Absolute Count \|\| id:ebi-a-GCST90001515 | Curry liking \|\| id:ebi-fl187-GCST90094750 | MR Egger | 6 | 2.23380565207726 | 3.44177514302357 | 0.551702874949824 | -4.512073628 | 8.97968493240346 | 9.33532556458218 | 0.0109756770880825 | 7940.13004367343 | 0.999950574162623 |
| ebi-fl187-GCST90094750 | ebi-a-GCST90001517 | CD33+ HLA DR+ Absolute Count \|\| id:ebi-a-GCST90001517 | Curry liking \|\| id:ebi-fl187-GCST90094750 | MR Egger | 6 | 1.28312561056013 | 1.97409994489834 | 0.55114666899829 | -2.586110281 | 5.15236150256087 | 3.60789900757547 | 0.0753124151873697 | 172.839168900363 | 0.999950574162623 |
| ebi-fl187-GCST90094750 | ebi-a-GCST90001518 | CD33+ HLA DR+ CD14- Absolute Count \|\| id:ebi-a-GCST90001518 | Curry liking \|\| id:ebi-fl187-GCST90094750 | MR Egger | 6 | -0.242433604 | 2.01234765420035 | 0.909917401959843 | -4.186635006 | 3.70176779848175 | 0.784715848037958 | 0.015197337879372 | 40.5188702817323 | 0.999950574162623 |
| ebi-fl187-GCST90094750 | ebi-a-GCST90001520 | CD33+ HLA DR+ CD14dim Absolute Count \|\| id:ebi-a-GCST90001520 | Curry liking \|\| id:ebi-fl187-GCST90094750 | MR Egger | 6 | 2.96201631141833 | 1.96802535371475 | 0.206757731960757 | -0.895313382 | 6.81934600469924 | 19.336921730748 | 0.408479568492738 | 915.386156034196 | 0.999950574162623 |
| ebi-fl187-GCST90094750 | ebi-a-GCST90001531 | CD33dim HLA DR- Absolute Count \|\| id:ebi-a-GCST90001531 | Curry liking \|\| id:ebi-fl187-GCST90094750 | MR Egger | 6 | 5.85738574325556 | 2.03881838790858 | 0.0453345223600155 | 1.86130170295475 | 9.85346978355637 | 349.808458501001 | 6.43210401321525 | 19024.2504454898 | 0.999950574162623 |
| ebi-fl187-GCST90094750 | ebi-a-GCST90001532 | Basophil Absolute Count \|\| id:ebi-a-GCST90001532 | Curry liking \|\| id:ebi-fl187-GCST90094750 | MR Egger | 6 | 5.49429628976815 | 2.03167501060375 | 0.0538529502197247 | 1.5122132689848 | 9.4763793105515 | 243.300253026943 | 4.53676076259833 | 13047.8586420042 | 0.999950574162623 |
| ebi-fl187-GCST90094750 | ebi-a-GCST90001588 | T/B cell \|\| id:ebi-a-GCST90001588 | Curry liking \|\| id:ebi-fl187-GCST90094750 | MR Egger | 6 | 2.39550719427478 | 1.57547044578883 | 0.203022793065151 | -0.692414879 | 5.48342926802089 | 10.9737624773418 | 0.500366284643259 | 240.67061791538 | 0.999950574162623 |
| ebi-fl187-GCST90094750 | ebi-a-GCST90001624 | HLA DR+ CD4+ T cell Absolute Count \|\| id:ebi-a-GCST90001624 | Curry liking \|\| id:ebi-fl187-GCST90094750 | MR Egger | 6 | 0.617976083079888 | 1.51772429695544 | 0.704728537414922 | -2.356763539 | 3.59271570511255 | 1.85516953066934 | 0.0947263056154847 | 36.3326107268909 | 0.999950574162623 |
| ebi-fl187-GCST90094750 | ebi-a-GCST90001631 | CD8+ Natural Killer T %T cell \|\| id:ebi-a-GCST90001631 | Curry liking \|\| id:ebi-fl187-GCST90094750 | MR Egger | 6 | -0.439187875 | 1.52858917913735 | 0.788140498924956 | -3.435222666 | 2.55684691658131 | 0.644559671909574 | 0.0322182355392256 | 12.8950938404544 | 0.999950574162623 |
| ebi-fl187-GCST90094750 | ebi-a-GCST90001632 | CD8+ Natural Killer T %lymphocyte \|\| id:ebi-a-GCST90001632 | Curry liking \|\| id:ebi-fl187-GCST90094750 | MR Egger | 6 | -0.222405035 | 1.53796210725566 | 0.892012273799393 | -3.236810765 | 2.7920006950472 | 0.800591031128406 | 0.0392889973326537 | 16.3136257638865 | 0.999950574162623 |
| ebi-fl187-GCST90094750 | ebi-a-GCST90001646 | Natural Killer %CD3- lymphocyte \|\| id:ebi-a-GCST90001646 | Curry liking \|\| id:ebi-fl187-GCST90094750 | MR Egger | 6 | 1.33938711798332 | 1.520729167 | 0.428191038961439 | -1.64124205 | 4.3200162858874 | 3.81670359939735 | 0.193739258958148 | 75.1898528155287 | 0.999950574162623 |
| ebi-fl187-GCST90094750 | ebi-a-GCST90001684 | CD127- CD8+ T cell Absolute Count \|\| id:ebi-a-GCST90001684 | Curry liking \|\| id:ebi-fl187-GCST90094750 | MR Egger | 6 | -0.162062047 | 1.81633982297541 | 0.933192366645231 | -3.7220881 | 3.39796400646296 | 0.850388439218458 | 0.0241834177023447 | 29.9031553958682 | 0.999950574162623 |
| ebi-fl187-GCST90094750 | ebi-a-GCST90001687 | CD28- CD8+ T cell Absolute Count \|\| id:ebi-a-GCST90001687 | Curry liking \|\| id:ebi-fl187-GCST90094750 | MR Egger | 6 | -0.365818352 | 1.98977690133811 | 0.863075730205681 | -4.265781078 | 3.53414437484296 | 0.693628786201468 | 0.0140408958084046 | 34.2656835868929 | 0.999950574162623 |
| ebi-fl187-GCST90094750 | ebi-a-GCST90001701 | BAFF-R on CD20- CD38- B cell \|\| id:ebi-a-GCST90001701 | Curry liking \|\| id:ebi-fl187-GCST90094750 | MR Egger | 6 | -1.201466461 | 1.58863022922002 | 0.491572655037954 | -4.31518171 | 1.9122487881648 | 0.300752846017004 | 0.0133641208660319 | 6.76829215285187 | 0.999950574162623 |
| ebi-fl187-GCST90094750 | ebi-a-GCST90001732 | CD19 on IgD- CD27- B cell \|\| id:ebi-a-GCST90001732 | Curry liking \|\| id:ebi-fl187-GCST90094750 | MR Egger | 6 | 2.68110309227904 | 1.45013255165884 | 0.138170674807267 | -0.161156709 | 5.52336289353037 | 14.6011908766108 | 0.851158676453915 | 250.475946392782 | 0.999950574162623 |
| ebi-fl187-GCST90094750 | ebi-a-GCST90001798 | CD27 on CD24+ CD27+ B cell \|\| id:ebi-a-GCST90001798 | Curry liking \|\| id:ebi-fl187-GCST90094750 | MR Egger | 6 | 0.900187286037729 | 1.48835407056719 | 0.577931247964465 | -2.016986692 | 3.81736126434942 | 2.46006380361731 | 0.1330557996807 | 45.4840295003382 | 0.999950574162623 |
| ebi-fl187-GCST90094750 | ebi-a-GCST90001836 | CD66b on CD66b++ myeloid cell \|\| id:ebi-a-GCST90001836 | Curry liking \|\| id:ebi-fl187-GCST90094750 | MR Egger | 6 | -4.231771314 | 3.30360176060548 | 0.269434227085137 | -10.70683076 | 2.24328813712893 | 0.0145266365162522 | 2.23914635233637e-05 | 9.42426868414052 | 0.999950574162623 |
| ebi-fl187-GCST90094750 | ebi-a-GCST90001943 | CD25 on activated & secreting CD4 regulatory T cell \|\| id:ebi-a-GCST90001943 | Curry liking \|\| id:ebi-fl187-GCST90094750 | MR Egger | 6 | 0.599479654427163 | 1.52376437677673 | 0.714078268057819 | -2.387098524 | 3.58605783290954 | 1.82117091557476 | 0.0918959312695199 | 36.0915163263109 | 0.999950574162623 |
| ebi-fl187-GCST90094750 | ebi-a-GCST90001960 | CD25 on CD4+ T cell \|\| id:ebi-a-GCST90001960 | Curry liking \|\| id:ebi-fl187-GCST90094750 | MR Egger | 6 | -1.036299993 | 1.58351847098999 | 0.548555072664254 | -4.139996196 | 2.06739621055991 | 0.354764889298201 | 0.015922912079599 | 7.90421538783842 | 0.999950574162623 |
| ebi-fl187-GCST90094750 | ebi-a-GCST90001988 | HLA DR on CD14+ CD16- monocyte \|\| id:ebi-a-GCST90001988 | Curry liking \|\| id:ebi-fl187-GCST90094750 | MR Egger | 6 | 3.25273010234721 | 2.07261412483501 | 0.191641222103244 | -0.809593582 | 7.31505378702383 | 25.8608463858811 | 0.445038901146725 | 1502.75262245816 | 0.999950574162623 |
| ebi-fl187-GCST90094750 | ebi-a-GCST90001991 | HLA DR on CD14+ monocyte \|\| id:ebi-a-GCST90001991 | Curry liking \|\| id:ebi-fl187-GCST90094750 | MR Egger | 6 | 3.2616771234253 | 2.08131464700556 | 0.192153110455934 | -0.817699585 | 7.3410538315562 | 26.093262087294 | 0.44144599647674 | 1542.33662053857 | 0.999950574162623 |
| ebi-fl187-GCST90094750 | ebi-a-GCST90002019 | CD14 on Monocytic Myeloid-Derived Suppressor Cells \|\| id:ebi-a-GCST90002019 | Curry liking \|\| id:ebi-fl187-GCST90094750 | MR Egger | 6 | -0.645533391 | 2.12222458094654 | 0.77615996703698 | -4.80509357 | 3.51402678737352 | 0.524382766302427 | 0.00818793483398321 | 33.5832283927957 | 0.999950574162623 |
| ebi-fl187-GCST90094750 | ebi-a-GCST90002037 | CD80 on plasmacytoid Dendritic Cell \|\| id:ebi-a-GCST90002037 | Curry liking \|\| id:ebi-fl187-GCST90094750 | MR Egger | 6 | -0.417029371 | 1.65069050038425 | 0.812998535044632 | -3.652382752 | 2.81832400925453 | 0.659001563796356 | 0.0259292720819907 | 16.7487563751424 | 0.999950574162623 |
| ebi-fl187-GCST90094750 | ebi-a-GCST90002042 | CD45 on CD33+ HLA DR+ CD14- \|\| id:ebi-a-GCST90002042 | Curry liking \|\| id:ebi-fl187-GCST90094750 | MR Egger | 6 | 1.15714472363081 | 2.12906103456822 | 0.615657760150475 | -3.015814904 | 5.33010435138453 | 3.18083812577663 | 0.0490058841139477 | 206.459517368742 | 0.999950574162623 |
| ebi-fl187-GCST90094750 | ebi-a-GCST90002056 | CD8 on Effector Memory CD8+ T cell \|\| id:ebi-a-GCST90002056 | Curry liking \|\| id:ebi-fl187-GCST90094750 | MR Egger | 6 | -1.544044555 | 1.63859249285798 | 0.399393167424678 | -4.755685841 | 1.66759673075302 | 0.213515776325202 | 0.0086026426223944 | 5.29941655614943 | 0.999950574162623 |
| ebi-fl187-GCST90094750 | ebi-a-GCST90002058 | CD8 on CD8+ T cell \|\| id:ebi-a-GCST90002058 | Curry liking \|\| id:ebi-fl187-GCST90094750 | MR Egger | 6 | -0.804259403 | 3.0848132616778 | 0.80718365708194 | -6.850493396 | 5.24197458955312 | 0.447419161026997 | 0.00105893309040505 | 189.043016473808 | 0.999950574162623 |
| ebi-fl187-GCST90094750 | ebi-a-GCST90002063 | CD4 on CD4 regulatory T cell \|\| id:ebi-a-GCST90002063 | Curry liking \|\| id:ebi-fl187-GCST90094750 | MR Egger | 6 | 0.681184665522866 | 2.40964268350309 | 0.791438993975556 | -4.041714994 | 5.40408432518893 | 1.97621750277528 | 0.0175673187175939 | 222.312561242713 | 0.999950574162623 |
| ebi-fl187-GCST90094750 | ebi-a-GCST90002066 | CD4 on activated CD4 regulatory T cell \|\| id:ebi-a-GCST90002066 | Curry liking \|\| id:ebi-fl187-GCST90094750 | MR Egger | 6 | 0.073720488627956 | 2.95590661659002 | 0.981297378427194 | -5.71985648 | 5.86729745714439 | 1.0765058678083 | 0.00328018164059745 | 353.29289972327 | 0.999950574162623 |
| ebi-fl187-GCST90094750 | ebi-a-GCST90002070 | CD4 on activated & secreting CD4 regulatory T cell \|\| id:ebi-a-GCST90002070 | Curry liking \|\| id:ebi-fl187-GCST90094750 | MR Egger | 6 | 0.324591747396164 | 2.44653353359046 | 0.90085761652753 | -4.470613978 | 5.11979747323346 | 1.38346572718837 | 0.0114402896025677 | 167.301483161341 | 0.999950574162623 |
| ebi-fl187-GCST90094750 | ebi-a-GCST90002081 | SSC-A on CD4+ T cell \|\| id:ebi-a-GCST90002081 | Curry liking \|\| id:ebi-fl187-GCST90094750 | MR Egger | 6 | 1.17331875707026 | 1.54835585496163 | 0.490771541219918 | -1.861458719 | 4.20809623279506 | 3.232703413 | 0.155445713342366 | 67.2284306223155 | 0.999950574162623 |
| ebi-fl187-GCST90094750 | ebi-a-GCST90002094 | CD11b on Monocytic Myeloid-Derived Suppressor Cells \|\| id:ebi-a-GCST90002094 | Curry liking \|\| id:ebi-fl187-GCST90094750 | MR Egger | 6 | -1.728926509 | 2.88799211486924 | 0.581651504296622 | -7.389391055 | 3.93153803564449 | 0.177474825286148 | 0.00061777202840186 | 50.9853346578845 | 0.999950574162623 |
| ebi-fl187-GCST90094750 | ebi-a-GCST90002104 | HLA DR on myeloid Dendritic Cell \|\| id:ebi-a-GCST90002104 | Curry liking \|\| id:ebi-fl187-GCST90094750 | MR Egger | 6 | 1.47366109829658 | 2.37596212430168 | 0.568690323828488 | -3.183224665 | 6.13054686192787 | 4.36518730182533 | 0.0414517712819859 | 459.687477536044 | 0.999950574162623 |
| ebi-fl187-GCST90094750 | ebi-a-GCST90002105 | HLA DR on plasmacytoid Dendritic Cell \|\| id:ebi-a-GCST90002105 | Curry liking \|\| id:ebi-fl187-GCST90094750 | MR Egger | 5 | 2.15950290389745 | 2.44859302974995 | 0.442754272595608 | -2.639739434 | 6.95874524220736 | 8.666828341 | 0.0713798662702428 | 1052.31233140833 | 0.999950574162623 |
| ebi-fl187-GCST90094750 | ebi-a-GCST90002106 | HLA DR on Dendritic Cell \|\| id:ebi-a-GCST90002106 | Curry liking \|\| id:ebi-fl187-GCST90094750 | MR Egger | 6 | 2.98004161213032 | 3.23873747786157 | 0.40957639952696 | -3.367883844 | 9.327967069 | 19.6886359138001 | 0.0344624882191296 | 11248.2413249257 | 0.999950574162623 |
| ebi-fl187-GCST90094750 | ebi-a-GCST90002111 | HLA DR on CD33dim HLA DR+ CD11b- \|\| id:ebi-a-GCST90002111 | Curry liking \|\| id:ebi-fl187-GCST90094750 | MR Egger | 6 | 7.20009182047951 | 2.72685622875422 | 0.0575540713267987 | 1.85545361212124 | 12.5447300288378 | 1339.55375721602 | 6.39459826011267 | 280612.510040614 | 0.999950574162623 |
| ebi-fl187-GCST90094750 | ebi-a-GCST90002112 | HLA DR on CD33- HLA DR+ \|\| id:ebi-a-GCST90002112 | Curry liking \|\| id:ebi-fl187-GCST90094750 | MR Egger | 6 | 6.38767730623547 | 2.12753757887698 | 0.0398483365845445 | 2.21770365163658 | 10.5576509608344 | 594.474193377339 | 9.18621188426676 | 38470.6526524721 | 0.999950574162623 |
| ebi-fl187-GCST90094750 | ebi-a-GCST90002115 | HLA DR on HLA DR+ CD8+ T cell \|\| id:ebi-a-GCST90002115 | Curry liking \|\| id:ebi-fl187-GCST90094750 | MR Egger | 6 | -2.15346502 | 1.48765065098833 | 0.221300102448721 | -5.069260296 | 0.762330255512525 | 0.116081236256447 | 0.00628706898900936 | 2.14326476047597 | 0.999950574162623 |
| ebi-fl187-GCST90094750 | ebi-a-GCST90002116 | HLA DR on B cell \|\| id:ebi-a-GCST90002116 | Curry liking \|\| id:ebi-fl187-GCST90094750 | MR Egger | 6 | 0.952551498990497 | 1.63010035482308 | 0.590353607265175 | -2.242445196 | 4.14754819444373 | 2.59231551873937 | 0.106198510418614 | 63.2786629700138 | 0.999950574162623 |
| ebi-fl187-GCST90094750 | ebi-cfmet338-GCST90026023 | Sphingomyelin (d18:1/24:1, d18:2/24:0) levels \|\| id:ebi-cfmet338-GCST90026023 | Curry liking \|\| id:ebi-fl187-GCST90094750 | MR Egger | 6 | -0.892029615 | 1.57437840856425 | 0.601271388693665 | -3.977811296 | 2.19375206536116 | 0.409823124745438 | 0.0187265814560976 | 8.96880159199723 | 0.999950574162623 |
| ebi-fl187-GCST90094750 | ebi-cfmet338-GCST90026046 | 1-arachidonoyl-gpc (20:4n6) levels \|\| id:ebi-cfmet338-GCST90026046 | Curry liking \|\| id:ebi-fl187-GCST90094750 | MR Egger | 6 | -0.203638192 | 1.06451413452204 | 0.85761077726087 | -2.290085896 | 1.88280951164494 | 0.815757465362498 | 0.1012577638757 | 6.57194289922836 | 0.999950574162623 |
| ebi-fl187-GCST90094750 | ebi-cfmet338-GCST90026047 | 1-linoleoyl-gpc (18:2) levels \|\| id:ebi-cfmet338-GCST90026047 | Curry liking \|\| id:ebi-fl187-GCST90094750 | MR Egger | 6 | -1.605255392 | 1.3198300782681 | 0.290748465247295 | -4.192122345 | 0.981611561597101 | 0.20083825591447 | 0.0151141733157973 | 2.66875363911613 | 0.999950574162623 |
| ebi-fl187-GCST90094750 | ebi-cfmet338-GCST90026050 | 1-myristoyl-2-palmitoyl-gpc (14:0/16:0) levels \|\| id:ebi-cfmet338-GCST90026050 | Curry liking \|\| id:ebi-fl187-GCST90094750 | MR Egger | 6 | -0.075417721 | 0.68578470123949 | 0.917727494471944 | -1.419555736 | 1.26872029312243 | 0.927356029037129 | 0.241821425653932 | 3.55629862931266 | 0.999950574162623 |
| ebi-fl187-GCST90094750 | ebi-cfmet338-GCST90026068 | 2,3-dihydroxyisovalerate levels \|\| id:ebi-cfmet338-GCST90026068 | Curry liking \|\| id:ebi-fl187-GCST90094750 | MR Egger | 6 | -0.638841326 | 2.21591438581929 | 0.787441428087752 | -4.982033522 | 3.7043508702369 | 0.527903738170087 | 0.00686009820208051 | 40.623668723785 | 0.999950574162623 |
| ebi-fl187-GCST90094750 | ebi-cfmet338-GCST90026082 | 3-hydroxyhexanoate levels \|\| id:ebi-cfmet338-GCST90026082 | Curry liking \|\| id:ebi-fl187-GCST90094750 | MR Egger | 6 | 1.12137295663985 | 0.643492026083546 | 0.156355447324553 | -0.139871414 | 2.3826173277636 | 3.06906500518434 | 0.869470029463573 | 10.8332198774676 | 0.999950574162623 |
| ebi-fl187-GCST90094750 | ebi-cfmet338-GCST90026094 | 3-ureidopropionate levels \|\| id:ebi-cfmet338-GCST90026094 | Curry liking \|\| id:ebi-fl187-GCST90094750 | MR Egger | 6 | -0.469602979 | 2.34874168054856 | 0.851282289528069 | -5.073136673 | 4.13393071488587 | 0.625250456579663 | 0.0062627451172099 | 62.4228075925981 | 0.999950574162623 |
| ebi-fl187-GCST90094750 | ebi-cfmet338-GCST90026173 | Glycerol levels \|\| id:ebi-cfmet338-GCST90026173 | Curry liking \|\| id:ebi-fl187-GCST90094750 | MR Egger | 6 | 0.194670867027858 | 0.608267756647193 | 0.764956897349207 | -0.997533936 | 1.38687567005636 | 1.21491105339187 | 0.368787774961716 | 4.00232591171699 | 0.999950574162623 |
| ebi-fl187-GCST90094750 | ebi-cfmet338-GCST90026179 | Histidine levels \|\| id:ebi-cfmet338-GCST90026179 | Curry liking \|\| id:ebi-fl187-GCST90094750 | MR Egger | 6 | 1.3304891406522 | 0.420809622401265 | 0.0341271386750966 | 0.505702280745726 | 2.15527600055868 | 3.78289330199468 | 1.65814959819466 | 8.63027181012905 | 0.999950574162623 |
| ebi-fl187-GCST90094750 | ebi-cfmet338-GCST90026188 | Isocitrate levels \|\| id:ebi-cfmet338-GCST90026188 | Curry liking \|\| id:ebi-fl187-GCST90094750 | MR Egger | 6 | -1.018415752 | 1.37398102817851 | 0.499718211344441 | -3.711418567 | 1.67458706307191 | 0.361166664685976 | 0.0244428248722314 | 5.33659101851937 | 0.999950574162623 |
| ebi-fl187-GCST90094750 | ebi-cfmet338-GCST90026207 | N-acetyl-isoputreanine levels \|\| id:ebi-cfmet338-GCST90026207 | Curry liking \|\| id:ebi-fl187-GCST90094750 | MR Egger | 6 | 0.25190887658527 | 0.607570278777908 | 0.699693740726363 | -0.93892887 | 1.44274662298997 | 1.28647880360199 | 0.391046472789912 | 4.23230441208038 | 0.999950574162623 |
| ebi-fl187-GCST90094750 | ebi-cfmet338-GCST90026213 | N-acetylglutamate levels \|\| id:ebi-cfmet338-GCST90026213 | Curry liking \|\| id:ebi-fl187-GCST90094750 | MR Egger | 6 | -0.139672458 | 1.2980178697467 | 0.919490771495991 | -2.683787482 | 2.4044425669209 | 0.869643033562081 | 0.0683039635584846 | 11.0722565195723 | 0.999950574162623 |
| ebi-fl187-GCST90094750 | ebi-cfmet338-GCST90026224 | N-acetyltaurine levels \|\| id:ebi-cfmet338-GCST90026224 | Curry liking \|\| id:ebi-fl187-GCST90094750 | MR Egger | 6 | 1.81142860084032 | 0.718383019686233 | 0.0652499190800307 | 0.403397882255307 | 3.21945931942534 | 6.1191830609446 | 1.49690236408037 | 25.0145916205801 | 0.999950574162623 |
| ebi-fl187-GCST90094750 | ebi-cfmet338-GCST90026250 | Phenyllactate (pla) levels \|\| id:ebi-cfmet338-GCST90026250 | Curry liking \|\| id:ebi-fl187-GCST90094750 | MR Egger | 6 | 1.49069176798392 | 1.14627280215116 | 0.263306016419645 | -0.756002924 | 3.7373864602002 | 4.44016602154052 | 0.469539465998289 | 41.9881090440965 | 0.999950574162623 |
| ebi-fl187-GCST90094750 | ebi-cfmet338-GCST90026251 | Pipecolate levels \|\| id:ebi-cfmet338-GCST90026251 | Curry liking \|\| id:ebi-fl187-GCST90094750 | MR Egger | 6 | 1.42686380429656 | 2.26496764001345 | 0.562907718828006 | -3.01247277 | 5.86620037872291 | 4.16561450227008 | 0.0491699423446928 | 352.905522237118 | 0.999950574162623 |
| ebi-fl187-GCST90094750 | ebi-cfmet338-GCST90026278 | Trigonelline (n'-methylnicotinate) levels \|\| id:ebi-cfmet338-GCST90026278 | Curry liking \|\| id:ebi-fl187-GCST90094750 | MR Egger | 6 | 1.57083121288681 | 1.92152413872887 | 0.459543005520028 | -2.195356099 | 5.3370185247954 | 4.8106452026487 | 0.111318913889751 | 207.891960648187 | 0.999950574162623 |
| ebi-fl187-GCST90094750 | ebi-cfmet338-GCST90026299 | 1-carboxyethylvaline levels \|\| id:ebi-cfmet338-GCST90026299 | Curry liking \|\| id:ebi-fl187-GCST90094750 | MR Egger | 6 | -1.370133136 | 1.15836626662688 | 0.302383560762008 | -3.640531019 | 0.900264746303409 | 0.254073130948101 | 0.0262384071762334 | 2.46025436819362 | 0.999950574162623 |
| ebi-fl187-GCST90094750 | ebi-gm430-GCST90011306 | C_Erysipelotrichia abundance \|\| id:ebi-gm430-GCST90011306 | Curry liking \|\| id:ebi-fl187-GCST90094750 | MR Egger | 5 | -0.235845013 | 1.19770191555721 | 0.856480009967779 | -2.583340767 | 2.11165074196466 | 0.7899030895295 | 0.0755212831051609 | 8.26186824685464 | 0.999950574162623 |
| ebi-fl187-GCST90094750 | ebi-gm430-GCST90011312 | F_Erysipelotrichaceae abundance \|\| id:ebi-gm430-GCST90011312 | Curry liking \|\| id:ebi-fl187-GCST90094750 | MR Egger | 5 | -0.235845013 | 1.19770191555721 | 0.856480009967779 | -2.583340767 | 2.11165074196466 | 0.7899030895295 | 0.0755212831051609 | 8.26186824685464 | 0.999950574162623 |
| ebi-fl187-GCST90094750 | ebi-gm430-GCST90011352 | O_Erysipelotrichales abundance \|\| id:ebi-gm430-GCST90011352 | Curry liking \|\| id:ebi-fl187-GCST90094750 | MR Egger | 5 | -0.235845013 | 1.19770191555721 | 0.856480009967779 | -2.583340767 | 2.11165074196466 | 0.7899030895295 | 0.0755212831051609 | 8.26186824685464 | 0.999950574162623 |
| ebi-fl187-GCST90094750 | ebi-gm430-GCST90011362 | OTU97_11 (Parabacteroides) abundance \|\| id:ebi-gm430-GCST90011362 | Curry liking \|\| id:ebi-fl187-GCST90094750 | MR Egger | 5 | -1.695554271 | 1.55949105367662 | 0.356474813546138 | -4.752156736 | 1.36104819418917 | 0.183497493493458 | 0.00863305588595458 | 3.90027941011742 | 0.999950574162623 |
| ebi-fl187-GCST90094750 | ebi-gm430-GCST90011384 | OTU97_235 (Phascolarctobacterium) abundance \|\| id:ebi-gm430-GCST90011384 | Curry liking \|\| id:ebi-fl187-GCST90094750 | MR Egger | 5 | 3.95283273003922 | 4.89791457421065 | 0.478692530825214 | -5.647079835 | 13.5527452954921 | 52.0826942794843 | 0.00352780351500731 | 768922.37106482 | 0.999950574162623 |
| ebi-fl187-GCST90094750 | ebi-gm430-GCST90011390 | OTU97_3 (Bacteroides) abundance \|\| id:ebi-gm430-GCST90011390 | Curry liking \|\| id:ebi-fl187-GCST90094750 | MR Egger | 5 | -1.434398388 | 2.25122568148709 | 0.569313781831073 | -5.846800724 | 2.97800394760173 | 0.23825866014817 | 0.00288912750498732 | 19.648557925397 | 0.999950574162623 |
| ebi-fl187-GCST90094750 | ebi-gm430-GCST90011398 | OTU97_4 (Alistipes) abundance \|\| id:ebi-gm430-GCST90011398 | Curry liking \|\| id:ebi-fl187-GCST90094750 | MR Egger | 5 | -1.728840707 | 1.45038822215896 | 0.318963721798142 | -4.571601623 | 1.11392020826506 | 0.177490053693462 | 0.0103413834532633 | 3.04627705785025 | 0.999950574162623 |
| ebi-fl187-GCST90094750 | ebi-gm430-GCST90011421 | OTU99_108 (Cytophagales) abundance \|\| id:ebi-gm430-GCST90011421 | Curry liking \|\| id:ebi-fl187-GCST90094750 | MR Egger | 5 | -2.134143276 | 4.60136967103463 | 0.674367518359287 | -11.15282783 | 6.88454127953825 | 0.118345936798554 | 1.43346936315174e-05 | 977.053372520857 | 0.999950574162623 |
| ebi-fl187-GCST90094750 | ebi-gm430-GCST90011428 | OTU99_123 (Phascolarctobacterium) abundance \|\| id:ebi-gm430-GCST90011428 | Curry liking \|\| id:ebi-fl187-GCST90094750 | MR Egger | 5 | 2.41780468205295 | 2.80199173325059 | 0.451663101906443 | -3.074099115 | 7.90970847922411 | 11.2211981575429 | 0.0462312586688171 | 2723.59636567226 | 0.999950574162623 |
| ebi-fl187-GCST90094750 | ebi-gm430-GCST90011450 | OTU99_298 (Phascolarctobacterium) abundance \|\| id:ebi-gm430-GCST90011450 | Curry liking \|\| id:ebi-fl187-GCST90094750 | MR Egger | 5 | 3.86073625534557 | 4.898060106 | 0.488112851763854 | -5.739461553 | 13.460934063625 | 47.5003108555036 | 0.00321649972395802 | 701470.457019978 | 0.999950574162623 |
| ebi-fl187-GCST90094750 | ebi-gm430-GCST90011451 | OTU99_3 (Bacteroides) abundance \|\| id:ebi-gm430-GCST90011451 | Curry liking \|\| id:ebi-fl187-GCST90094750 | MR Egger | 5 | -1.375984409 | 2.2471812821822 | 0.583615758088921 | -5.780459722 | 3.028490904 | 0.252590820624853 | 0.00308729578300392 | 20.6660220297576 | 0.999950574162623 |
| ebi-fl187-GCST90094750 | ebi-gm430-GCST90011472 | OTU99_72 (Parabacteroides) abundance \|\| id:ebi-gm430-GCST90011472 | Curry liking \|\| id:ebi-fl187-GCST90094750 | MR Egger | 5 | 1.30136243754949 | 6.03935463807111 | 0.843211223802434 | -10.53577265 | 13.1384975281689 | 3.67429926226697 | 2.65688082326378e-05 | 508132.504494926 | 0.999950574162623 |
| ebi-fl187-GCST90094750 | ebi-gm430-GCST90011507 | TestASV_3 (Bacteroides) abundance \|\| id:ebi-gm430-GCST90011507 | Curry liking \|\| id:ebi-fl187-GCST90094750 | MR Egger | 5 | -2.527521556 | 2.29779219087559 | 0.3516912936731 | -7.031194251 | 1.97615113762271 | 0.0798566955297383 | 0.000883875581086365 | 7.21492024147934 | 0.999950574162623 |
| ebi-fl187-GCST90094750 | ebi-gm430-GCST90011512 | TestASV_34 (Phascolarctobacterium) abundance \|\| id:ebi-gm430-GCST90011512 | Curry liking \|\| id:ebi-fl187-GCST90094750 | MR Egger | 5 | -4.733957117 | 4.01765353661177 | 0.323634377813926 | -12.60855805 | 3.14064381471766 | 0.00879161267555992 | 3.3432804952363e-06 | 23.1187462575146 | 0.999950574162623 |
| ebi-fl187-GCST90094750 | ebi-gm430-GCST90011519 | TestASV_41 (Barnesiella) abundance \|\| id:ebi-gm430-GCST90011519 | Curry liking \|\| id:ebi-fl187-GCST90094750 | MR Egger | 5 | -5.095650268 | 5.10759900705133 | 0.391970686817504 | -15.10654432 | 4.91524378627997 | 0.00612332354141221 | 2.74986365265642e-07 | 136.352546631147 | 0.999950574162623 |
| ebi-fl187-GCST90094750 | ebi-gm430-GCST90011524 | TestASV_46 (Sutterella) abundance \|\| id:ebi-gm430-GCST90011524 | Curry liking \|\| id:ebi-fl187-GCST90094750 | MR Egger | 5 | 2.25975564559514 | 6.59812727988688 | 0.754577653629846 | -10.67257382 | 15.1920851141734 | 9.58074778278967 | 2.31718165189619e-05 | 3961309.11887357 | 0.999950574162623 |
| ebi-fl187-GCST90094750 | ebi-gm430-GCST90011581 | OTU97_19 (Bacteria) prevalence \|\| id:ebi-gm430-GCST90011581 | Curry liking \|\| id:ebi-fl187-GCST90094750 | MR Egger | 5 | 4.02222813792815 | 6.33536560924006 | 0.570614261209239 | -8.395088456 | 16.4395447320387 | 55.8253539428949 | 0.000225974486607403 | 13791247.8069434 | 0.999950574162623 |
| ebi-fl187-GCST90094750 | ebi-gm430-GCST90011614 | OTU97_80 (Ruminococcus) prevalence \|\| id:ebi-gm430-GCST90011614 | Curry liking \|\| id:ebi-fl187-GCST90094750 | MR Egger | 5 | 3.03367079404907 | 2.87292100493711 | 0.368506667690088 | -2.597254376 | 8.66459596372581 | 20.7733474831914 | 0.0744777857712375 | 5794.10304950376 | 0.999950574162623 |
| ebi-fl187-GCST90094750 | ebi-gm430-GCST90011623 | OTU99_11 (Parabacteroides) prevalence \|\| id:ebi-gm430-GCST90011623 | Curry liking \|\| id:ebi-fl187-GCST90094750 | MR Egger | 5 | 1.18659376168367 | 3.07973683179929 | 0.725716803708304 | -4.849690429 | 7.22287795201028 | 3.275903673 | 0.00783080136584004 | 1370.42741512672 | 0.999950574162623 |
| ebi-fl187-GCST90094750 | ebi-gm430-GCST90011642 | OTU99_19 (Bacteria) prevalence \|\| id:ebi-gm430-GCST90011642 | Curry liking \|\| id:ebi-fl187-GCST90094750 | MR Egger | 5 | 4.02222813792815 | 6.33536560924006 | 0.570614261209239 | -8.395088456 | 16.4395447320387 | 55.8253539428949 | 0.000225974486607403 | 13791247.8069434 | 0.999950574162623 |
| ebi-fl187-GCST90094750 | ebi-gm430-GCST90011653 | OTU99_30 (Parasutterella) prevalence \|\| id:ebi-gm430-GCST90011653 | Curry liking \|\| id:ebi-fl187-GCST90094750 | MR Egger | 5 | -4.741063339 | 2.74562324032387 | 0.182663330562703 | -10.12248489 | 0.640358212277349 | 0.00872935898289309 | 4.01661928598246e-05 | 1.89716034372866 | 0.999950574162623 |
| ebi-fl187-GCST90094750 | ebi-gm430-GCST90011675 | OTU99_8 (Ruminococcaceae) prevalence \|\| id:ebi-gm430-GCST90011675 | Curry liking \|\| id:ebi-fl187-GCST90094750 | MR Egger | 5 | 2.3948415702472 | 2.89928739612939 | 0.469359508318726 | -3.287761726 | 8.07744486666081 | 10.9664605078168 | 0.03733732710065 | 3220.99264752706 | 0.999950574162623 |
| ebi-fl187-GCST90094750 | ebi-gm430-GCST90011679 | OTU99_92 (Ruminococcus) prevalence \|\| id:ebi-gm430-GCST90011679 | Curry liking \|\| id:ebi-fl187-GCST90094750 | MR Egger | 5 | 2.12754159012021 | 3.00444551501606 | 0.529924737819126 | -3.761171619 | 8.0162547995517 | 8.3942050277067 | 0.0232564766694958 | 3029.80881620811 | 0.999950574162623 |
| ebi-fl187-GCST90094750 | ebi-gm430-GCST90011684 | TestASV_11 (Lachnospiraceae) prevalence \|\| id:ebi-gm430-GCST90011684 | Curry liking \|\| id:ebi-fl187-GCST90094750 | MR Egger | 5 | -1.505316462 | 3.04807668895791 | 0.655302456247483 | -7.479546773 | 4.46891384807376 | 0.221947044848274 | 0.00056451321043302 | 87.2618918503177 | 0.999950574162623 |
| ebi-fl187-GCST90094750 | ebi-gm430-GCST90011688 | TestASV_15 (Bacteroides) prevalence \|\| id:ebi-gm430-GCST90011688 | Curry liking \|\| id:ebi-fl187-GCST90094750 | MR Egger | 5 | 0.541784792435391 | 2.88424156008036 | 0.86298698427716 | -5.111328665 | 6.19489825019289 | 1.71907231303456 | 0.00602806831508288 | 490.241560475974 | 0.999950574162623 |
| ebi-fl187-GCST90094750 | ebi-gm430-GCST90011692 | TestASV_19 (Prevotella) prevalence \|\| id:ebi-gm430-GCST90011692 | Curry liking \|\| id:ebi-fl187-GCST90094750 | MR Egger | 5 | 3.63426790283971 | 5.66529704939587 | 0.566846571074089 | -7.469714314 | 14.7382501196556 | 37.8741152128454 | 0.000570091140686645 | 2516174.17072675 | 0.999950574162623 |
| ebi-fl187-GCST90094750 | ebi-gm430-GCST90011693 | TestASV_2 (Bacteroides) prevalence \|\| id:ebi-gm430-GCST90011693 | Curry liking \|\| id:ebi-fl187-GCST90094750 | MR Egger | 5 | 3.74564735296444 | 2.83152891794831 | 0.277685406868038 | -1.804149326 | 9.29544403214314 | 42.3364049471271 | 0.164614430213301 | 10888.2992914087 | 0.999950574162623 |
| ebi-fl187-GCST90094750 | ebi-gm430-GCST90011716 | TestASV_40 (Barnesiella) prevalence \|\| id:ebi-gm430-GCST90011716 | Curry liking \|\| id:ebi-fl187-GCST90094750 | MR Egger | 5 | 1.49624856176749 | 7.52865568882488 | 0.855172161075444 | -13.25991659 | 16.2524137118642 | 4.46490778726746 | 1.74297595216418e-06 | 11437565.460412 | 0.999950574162623 |
| ebi-fl187-GCST90094750 | ebi-gm430-GCST90011718 | TestASV_42 (Alphaproteobacteria) prevalence \|\| id:ebi-gm430-GCST90011718 | Curry liking \|\| id:ebi-fl187-GCST90094750 | MR Egger | 5 | 9.6213924908936 | 4.45799806799023 | 0.119774969444966 | 0.883716277632757 | 18.3590687041545 | 15084.0397089139 | 2.41987594784291 | 94024759.4687284 | 0.999950574162623 |
| ebi-fl187-GCST90094750 | ebi-hbsm44-GCST90104929 | Sulcal depth of the right inferior frontal sulcus \|\| id:ebi-hbsm44-GCST90104929 | Curry liking \|\| id:ebi-fl187-GCST90094750 | MR Egger | 5 | 2.92542807221111 | 2.31099805967039 | 0.294928600863587 | -1.604128125 | 7.45498426916507 | 18.6422045518121 | 0.201064781919525 | 1728.45680498488 | 0.999950574162623 |
| ebi-fl187-GCST90094750 | ebi-hbsm44-GCST90104935 | Sulcal opening distance of the left calloso-marginal posterior fissure \|\| id:ebi-hbsm44-GCST90104935 | Curry liking \|\| id:ebi-fl187-GCST90094750 | MR Egger | 5 | 0.0315649835983761 | 0.552815996494293 | 0.958056950229039 | -1.05195437 | 1.11508433672719 | 1.03206844093761 | 0.349254509305744 | 3.04982538062787 | 0.999950574162623 |
| ebi-fl187-GCST90094750 | ebi-hbsm44-GCST90104936 | Sulcal opening distance of the left anterior inferior temporal sulcus \|\| id:ebi-hbsm44-GCST90104936 | Curry liking \|\| id:ebi-fl187-GCST90094750 | MR Egger | 5 | 0.410409261315318 | 0.311470084186574 | 0.279209777264719 | -0.200072104 | 1.020890626 | 1.50743459355084 | 0.818671721697484 | 2.77566574441113 | 0.999950574162623 |
| ebi-fl187-GCST90094750 | ebi-hbsm44-GCST90104941 | Sulcal opening distance of the right posterior inferior temporal sulcus \|\| id:ebi-hbsm44-GCST90104941 | Curry liking \|\| id:ebi-fl187-GCST90094750 | MR Egger | 5 | -0.115742283 | 0.243175045623554 | 0.66661430106622 | -0.592365372 | 0.360880806667908 | 0.890704743693835 | 0.553017643903174 | 1.43459245683236 | 0.999950574162623 |
| ebi-fl187-GCST90094750 | ebi-IPs91-GCST90274819 | Leukemia inhibitory factor levels \|\| id:ebi-IPs91-GCST90274819 | Curry liking \|\| id:ebi-fl187-GCST90094750 | MR Egger | 7 | -0.498289042 | 0.518328434955075 | 0.380533496309428 | -1.514212775 | 0.517634690359374 | 0.607569296382982 | 0.21998129154787 | 1.67805383498707 | 0.999950574162623 |
| ebi-fl187-GCST90094750 | ebi-IPs91-GCST90274837 | STAM binding protein levels \|\| id:ebi-IPs91-GCST90274837 | Curry liking \|\| id:ebi-fl187-GCST90094750 | MR Egger | 7 | -0.624960479 | 0.418916631598982 | 0.195943786754128 | -1.446037077 | 0.196116119284979 | 0.535282583191798 | 0.235501716552246 | 1.21666817577067 | 0.999950574162623 |
| ebi-fl187-GCST90094750 | ebi-IPs91-GCST90274840 | TNF-beta levels \|\| id:ebi-IPs91-GCST90274840 | Curry liking \|\| id:ebi-fl187-GCST90094750 | MR Egger | 7 | -0.597248473 | 0.600670049504202 | 0.365727309753365 | -1.77456177 | 0.580064824526344 | 0.550323785808754 | 0.169557738587862 | 1.78615421359812 | 0.999950574162623 |
| ebi-fl187-GCST90094750 | ebi-IPs91-GCST90274842 | Tumor necrosis factor ligand superfamily member 14 levels \|\| id:ebi-IPs91-GCST90274842 | Curry liking \|\| id:ebi-fl187-GCST90094750 | MR Egger | 7 | 0.184644047186148 | 0.481869820312715 | 0.717334907888364 | -0.759820801 | 1.12910889499907 | 1.20279022735455 | 0.467750240049907 | 3.09289917385236 | 0.999950574162623 |
| ebi-fl187-GCST90094750 | ebi-met1400-GCST90199628 | 3-methylhistidine levels \|\| id:ebi-met1400-GCST90199628 | Curry liking \|\| id:ebi-fl187-GCST90094750 | MR Egger | 7 | 0.819601951145604 | 0.735136595635363 | 0.315593899057601 | -0.621265776 | 2.26046967859091 | 2.26959624752158 | 0.537263951037742 | 9.58759119575138 | 0.999950574162623 |
| ebi-fl187-GCST90094750 | ebi-met1400-GCST90199651 | Docosahexaenoate DHA; 22:6n3 levels \|\| id:ebi-met1400-GCST90199651 | Curry liking \|\| id:ebi-fl187-GCST90094750 | MR Egger | 7 | 0.460276639334906 | 0.758601568347967 | 0.570513761735495 | -1.026582435 | 1.94713571329692 | 1.58451226278778 | 0.358229142449141 | 7.0085842088665 | 0.999950574162623 |
| ebi-fl187-GCST90094750 | ebi-met1400-GCST90199652 | 1-stearoyl-2-oleoyl-GPS (18:0/18:1) levels \|\| id:ebi-met1400-GCST90199652 | Curry liking \|\| id:ebi-fl187-GCST90094750 | MR Egger | 7 | -0.275139323 | 0.613232432508059 | 0.672445462152668 | -1.477074891 | 0.926796244531323 | 0.75946630458959 | 0.228304528271787 | 2.5264022232635 | 0.999950574162623 |
| ebi-fl187-GCST90094750 | ebi-met1400-GCST90199653 | 1-myristoyl-2-palmitoyl-gpc (14:0/16:0) levels \|\| id:ebi-met1400-GCST90199653 | Curry liking \|\| id:ebi-fl187-GCST90094750 | MR Egger | 7 | -0.681399896 | 0.52248092043339 | 0.248984590429409 | -1.7054625 | 0.342662708461277 | 0.505908277652126 | 0.181688335950731 | 1.40869354137486 | 0.999950574162623 |
| ebi-fl187-GCST90094750 | ebi-met1400-GCST90199667 | Erythritol levels in elite athletes \|\| id:ebi-met1400-GCST90199667 | Curry liking \|\| id:ebi-fl187-GCST90094750 | MR Egger | 7 | 0.37285094963677 | 0.882218665849874 | 0.69012559339999 | -1.356297635 | 2.10199953470252 | 1.45186792226344 | 0.257612789983098 | 8.18251478832191 | 0.999950574162623 |
| ebi-fl187-GCST90094750 | ebi-met1400-GCST90199674 | 3-hydroxymyristate levels \|\| id:ebi-met1400-GCST90199674 | Curry liking \|\| id:ebi-fl187-GCST90094750 | MR Egger | 7 | -0.072876424 | 0.613748510895155 | 0.910104442075373 | -1.275823506 | 1.1300706569965 | 0.929715713143398 | 0.279200949946508 | 3.09587523764279 | 0.999950574162623 |
| ebi-fl187-GCST90094750 | ebi-met1400-GCST90199685 | 2-linoleoylglycerol (18:2) levels \|\| id:ebi-met1400-GCST90199685 | Curry liking \|\| id:ebi-fl187-GCST90094750 | MR Egger | 7 | -0.221939759 | 0.844390681399048 | 0.803160482430859 | -1.876945495 | 1.4330659761733 | 0.800963613434982 | 0.15305690556972 | 4.19153064449347 | 0.999950574162623 |
| ebi-fl187-GCST90094750 | ebi-met1400-GCST90199703 | Gamma-glutamylvaline levels \|\| id:ebi-met1400-GCST90199703 | Curry liking \|\| id:ebi-fl187-GCST90094750 | MR Egger | 7 | -0.428070288 | 0.726504883369329 | 0.581328576545436 | -1.852019859 | 0.995879283868461 | 0.651765602190871 | 0.156919889952274 | 2.70710360763334 | 0.999950574162623 |
| ebi-fl187-GCST90094750 | ebi-met1400-GCST90199779 | Gamma-glutamyl-2-aminobutyrate levels \|\| id:ebi-met1400-GCST90199779 | Curry liking \|\| id:ebi-fl187-GCST90094750 | MR Egger | 7 | 0.241843111489552 | 0.543280381601024 | 0.674819674886968 | -0.822986436 | 1.30667265942756 | 1.27359436481843 | 0.439118295467713 | 3.69386250319998 | 0.999950574162623 |
| ebi-fl187-GCST90094750 | ebi-met1400-GCST90199824 | 4-vinylphenol sulfate levels \|\| id:ebi-met1400-GCST90199824 | Curry liking \|\| id:ebi-fl187-GCST90094750 | MR Egger | 7 | 0.466552130048672 | 0.52683401566028 | 0.416397092189403 | -0.566042541 | 1.49914680074282 | 1.59448712060694 | 0.567767916968731 | 4.477866927 | 0.999950574162623 |
| ebi-fl187-GCST90094750 | ebi-met1400-GCST90199830 | 4-ethylphenylsulfate levels \|\| id:ebi-met1400-GCST90199830 | Curry liking \|\| id:ebi-fl187-GCST90094750 | MR Egger | 7 | -0.164994792 | 0.528838689413523 | 0.767635691028433 | -1.201518624 | 0.871529038811793 | 0.847898119557842 | 0.300737158380936 | 2.39056332453296 | 0.999950574162623 |
| ebi-fl187-GCST90094750 | ebi-met1400-GCST90199833 | Hexanoylglutamine levels \|\| id:ebi-met1400-GCST90199833 | Curry liking \|\| id:ebi-fl187-GCST90094750 | MR Egger | 6 | 0.624700036703467 | 0.545645485200834 | 0.31610611978748 | -0.444765114 | 1.6941651876971 | 1.86768563625819 | 0.640974814176209 | 5.44210093553881 | 0.999950574162623 |
| ebi-fl187-GCST90094750 | ebi-met1400-GCST90199837 | Carotenoid levels (cryptoxanthin) \|\| id:ebi-met1400-GCST90199837 | Curry liking \|\| id:ebi-fl187-GCST90094750 | MR Egger | 7 | 0.271246822328658 | 0.521325519247587 | 0.625061798701302 | -0.750551195 | 1.29304484005393 | 1.31159876229652 | 0.472106258214793 | 3.64386466674436 | 0.999950574162623 |
| ebi-fl187-GCST90094750 | ebi-met1400-GCST90199869 | 2-hydroxyhippurate levels \|\| id:ebi-met1400-GCST90199869 | Curry liking \|\| id:ebi-fl187-GCST90094750 | MR Egger | 7 | 0.651803482 | 0.552357718808608 | 0.291063138056029 | -0.430817646 | 1.73442461127987 | 1.91899859028285 | 0.649977425659689 | 5.66566690492364 | 0.999950574162623 |
| ebi-fl187-GCST90094750 | ebi-met1400-GCST90199902 | Hydantoin-5-propionate levels \|\| id:ebi-met1400-GCST90199902 | Curry liking \|\| id:ebi-fl187-GCST90094750 | MR Egger | 7 | -0.122060853 | 0.583192313691118 | 0.84247600034202 | -1.265117788 | 1.02099608216188 | 0.885094506497495 | 0.28220605383517 | 2.77595847001073 | 0.999950574162623 |
| ebi-fl187-GCST90094750 | ebi-met1400-GCST90199904 | Pantoate levels \|\| id:ebi-met1400-GCST90199904 | Curry liking \|\| id:ebi-fl187-GCST90094750 | MR Egger | 7 | 0.127708495132214 | 0.55124192780899 | 0.825973991096107 | -0.952725683 | 1.20814267363784 | 1.13622174024893 | 0.385688325189874 | 3.34726191771232 | 0.999950574162623 |
| ebi-fl187-GCST90094750 | ebi-met1400-GCST90199936 | 3-methyl catechol sulfate (2) levels \|\| id:ebi-met1400-GCST90199936 | Curry liking \|\| id:ebi-fl187-GCST90094750 | MR Egger | 7 | 0.610640228337581 | 0.597768428021093 | 0.353858307886597 | -0.560985891 | 1.78226634725892 | 1.84161007238637 | 0.570646191722164 | 5.94331077279178 | 0.999950574162623 |
| ebi-fl187-GCST90094750 | ebi-met1400-GCST90199977 | 4-hydroxychlorothalonil levels \|\| id:ebi-met1400-GCST90199977 | Curry liking \|\| id:ebi-fl187-GCST90094750 | MR Egger | 7 | 0.0605061165942217 | 0.542188484639507 | 0.915485152507308 | -1.002183313 | 1.12319554648766 | 1.06237409566171 | 0.367077121271592 | 3.07466375246519 | 0.999950574162623 |
| ebi-fl187-GCST90094750 | ebi-met1400-GCST90200040 | Sphingomyelin (d18:2/24:1, d18:1/24:2) levels \|\| id:ebi-met1400-GCST90200040 | Curry liking \|\| id:ebi-fl187-GCST90094750 | MR Egger | 7 | 0.412880560853267 | 0.59105890129162 | 0.515984560081317 | -0.745594886 | 1.57135600738484 | 1.51116452295345 | 0.474451971288838 | 4.81317046534708 | 0.999950574162623 |
| ebi-fl187-GCST90094750 | ebi-met1400-GCST90200077 | 1-stearoyl-2-oleoyl-GPI (18:0/18:1) levels \|\| id:ebi-met1400-GCST90200077 | Curry liking \|\| id:ebi-fl187-GCST90094750 | MR Egger | 7 | 0.343975634167931 | 0.622479373543793 | 0.604337679660878 | -0.876083938 | 1.56403520631376 | 1.41054426600425 | 0.416410411905459 | 4.77806286651926 | 0.999950574162623 |
| ebi-fl187-GCST90094750 | ebi-met1400-GCST90200080 | 1-palmitoyl-2-oleoyl-GPI (16:0/18:1) levels \|\| id:ebi-met1400-GCST90200080 | Curry liking \|\| id:ebi-fl187-GCST90094750 | MR Egger | 7 | 0.234154566166823 | 0.591249448180233 | 0.708415546144169 | -0.924694352 | 1.39300348460008 | 1.26383982398862 | 0.396652636528497 | 4.02692672026353 | 0.999950574162623 |
| ebi-fl187-GCST90094750 | ebi-met1400-GCST90200082 | 1-oleoyl-2-linoleoyl-GPE (18:1/18:2) levels \|\| id:ebi-met1400-GCST90200082 | Curry liking \|\| id:ebi-fl187-GCST90094750 | MR Egger | 7 | -0.543825642 | 0.675396612525139 | 0.457272369203246 | -1.867603002 | 0.779951718878586 | 0.58052312535933 | 0.154493539010665 | 2.18136694411343 | 0.999950574162623 |
| ebi-fl187-GCST90094750 | ebi-met1400-GCST90200100 | Perfluorooctanesulfonate (PFOS) levels \|\| id:ebi-met1400-GCST90200100 | Curry liking \|\| id:ebi-fl187-GCST90094750 | MR Egger | 7 | -0.164530102 | 0.664329688520124 | 0.814243855409175 | -1.466616292 | 1.13755608713709 | 0.848292220960179 | 0.230704803671715 | 3.11913614579746 | 0.999950574162623 |
| ebi-fl187-GCST90094750 | ebi-met1400-GCST90200102 | 1-palmitoleoyl-2-linolenoyl-GPC (16:1/18:3) levels \|\| id:ebi-met1400-GCST90200102 | Curry liking \|\| id:ebi-fl187-GCST90094750 | MR Egger | 7 | -0.438471027 | 0.514887667329288 | 0.433326223094788 | -1.447650855 | 0.570708800734149 | 0.645021888417434 | 0.235121975410081 | 1.76952084471027 | 0.999950574162623 |
| ebi-fl187-GCST90094750 | ebi-met1400-GCST90200114 | Glycosyl-N-tricosanoyl-sphingadienine (d18:2/23:0) levels \|\| id:ebi-met1400-GCST90200114 | Curry liking \|\| id:ebi-fl187-GCST90094750 | MR Egger | 7 | 0.451422889677384 | 0.932699973516896 | 0.648842679131626 | -1.376669058 | 2.2795148377705 | 1.5705453090773 | 0.252417943728397 | 9.77193828390745 | 0.999950574162623 |
| ebi-fl187-GCST90094750 | ebi-met1400-GCST90200135 | Arachidoylcarnitine (C20) levels \|\| id:ebi-met1400-GCST90200135 | Curry liking \|\| id:ebi-fl187-GCST90094750 | MR Egger | 7 | 0.0389990159443362 | 0.587911887095753 | 0.949682009357221 | -1.113308283 | 1.19130631465201 | 1.03976946045863 | 0.328470488325332 | 3.29137797558128 | 0.999950574162623 |
| ebi-fl187-GCST90094750 | ebi-met1400-GCST90200146 | Docosahexaenoylcarnitine (C22:6) levels \|\| id:ebi-met1400-GCST90200146 | Curry liking \|\| id:ebi-fl187-GCST90094750 | MR Egger | 7 | -0.510112732 | 0.600595063802224 | 0.434460286590232 | -1.687279057 | 0.667053593478327 | 0.600427887815995 | 0.185022274837211 | 1.94848781739588 | 0.999950574162623 |
| ebi-fl187-GCST90094750 | ebi-met1400-GCST90200162 | 3-hydroxyphenylacetoylglutamine levels \|\| id:ebi-met1400-GCST90200162 | Curry liking \|\| id:ebi-fl187-GCST90094750 | MR Egger | 7 | 1.5350817430894 | 0.645770036413538 | 0.0633896600077773 | 0.26937247171887 | 2.80079101445994 | 4.64170494174864 | 1.30914266886579 | 16.457659870578 | 0.999950574162623 |
| ebi-fl187-GCST90094750 | ebi-met1400-GCST90200172 | 3-carboxy-4-methyl-5-pentyl-2-furanpropionate (3-CMPFP) levels \|\| id:ebi-met1400-GCST90200172 | Curry liking \|\| id:ebi-fl187-GCST90094750 | MR Egger | 7 | 0.327417209825482 | 0.523769189035107 | 0.559312300073967 | -0.699170401 | 1.35400482033429 | 1.38738018509549 | 0.496997441551069 | 3.87290480206189 | 0.999950574162623 |
| ebi-fl187-GCST90094750 | ebi-met1400-GCST90200222 | Dihydrocaffeate sulfate (2) levels \|\| id:ebi-met1400-GCST90200222 | Curry liking \|\| id:ebi-fl187-GCST90094750 | MR Egger | 7 | 0.462391762434912 | 0.600412078679806 | 0.476018410567153 | -0.714415912 | 1.63919943664733 | 1.58786724812976 | 0.489477926609775 | 5.15104412398405 | 0.999950574162623 |
| ebi-fl187-GCST90094750 | ebi-met1400-GCST90200223 | 4-methylhexanoylglutamine levels \|\| id:ebi-met1400-GCST90200223 | Curry liking \|\| id:ebi-fl187-GCST90094750 | MR Egger | 7 | -0.778394995 | 0.795660083018167 | 0.372854842689254 | -2.337888758 | 0.78109876743832 | 0.459142345870446 | 0.0965312240205854 | 2.18387051350826 | 0.999950574162623 |
| ebi-fl187-GCST90094750 | ebi-met1400-GCST90200224 | 2-ketocaprylate levels \|\| id:ebi-met1400-GCST90200224 | Curry liking \|\| id:ebi-fl187-GCST90094750 | MR Egger | 7 | 0.14099705446422 | 0.533965169321187 | 0.802274303724654 | -0.905574677 | 1.18756878633375 | 1.15142125642724 | 0.404309470810167 | 3.27909931740126 | 0.999950574162623 |
| ebi-fl187-GCST90094750 | ebi-met1400-GCST90200243 | Hydroxypalmitoyl sphingomyelin (d18:1/16:0(OH)) levels \|\| id:ebi-met1400-GCST90200243 | Curry liking \|\| id:ebi-fl187-GCST90094750 | MR Egger | 7 | 0.321101848194883 | 0.715796818720119 | 0.67249737224721 | -1.081859916 | 1.72406361288632 | 1.3786459863487 | 0.338964493340244 | 5.6072679971455 | 0.999950574162623 |
| ebi-fl187-GCST90094750 | ebi-met1400-GCST90200273 | 2-methoxyhydroquinone sulfate (2) levels \|\| id:ebi-met1400-GCST90200273 | Curry liking \|\| id:ebi-fl187-GCST90094750 | MR Egger | 7 | 0.310105162966291 | 0.768627164336595 | 0.703289649092449 | -1.196404079 | 1.81661440506602 | 1.36356850350099 | 0.302279232117937 | 6.15099836899975 | 0.999950574162623 |
| ebi-fl187-GCST90094750 | ebi-met1400-GCST90200276 | 2,4-di-tert-butylphenol levels \|\| id:ebi-met1400-GCST90200276 | Curry liking \|\| id:ebi-fl187-GCST90094750 | MR Egger | 7 | -0.302653595 | 0.627161406983361 | 0.64978206784539 | -1.531889952 | 0.926582762895245 | 0.73885499526378 | 0.216126811675223 | 2.52586294034904 | 0.999950574162623 |
| ebi-fl187-GCST90094750 | ebi-met1400-GCST90200289 | N-acetylmethionine levels \|\| id:ebi-met1400-GCST90200289 | Curry liking \|\| id:ebi-fl187-GCST90094750 | MR Egger | 7 | -0.377673102 | 0.523253290502494 | 0.502762518433562 | -1.403249552 | 0.647903347044045 | 0.68545453752909 | 0.245796934923706 | 1.91152881204583 | 0.999950574162623 |
| ebi-fl187-GCST90094750 | ebi-met1400-GCST90200308 | 3-hydroxyisobutyrate levels \|\| id:ebi-met1400-GCST90200308 | Curry liking \|\| id:ebi-fl187-GCST90094750 | MR Egger | 7 | -0.080142149 | 0.531169051349039 | 0.885969492084211 | -1.121233489 | 0.960949192061362 | 0.922985136032183 | 0.325877580126048 | 2.61417665187902 | 0.999950574162623 |
| ebi-fl187-GCST90094750 | ebi-met1400-GCST90200383 | Cystathionine levels \|\| id:ebi-met1400-GCST90200383 | Curry liking \|\| id:ebi-fl187-GCST90094750 | MR Egger | 7 | -0.241772416 | 0.578293674238499 | 0.693238058698779 | -1.375228017 | 0.89168318577947 | 0.785234864322864 | 0.2527819505914 | 2.43923187832669 | 0.999950574162623 |
| ebi-fl187-GCST90094750 | ebi-met1400-GCST90200384 | Guanidinoacetate levels \|\| id:ebi-met1400-GCST90200384 | Curry liking \|\| id:ebi-fl187-GCST90094750 | MR Egger | 7 | -0.093738246 | 0.502607459425424 | 0.859380644505502 | -1.078848866 | 0.891372374519403 | 0.910521063623213 | 0.339986670542141 | 2.43847385540013 | 0.999950574162623 |
| ebi-fl187-GCST90094750 | ebi-met1400-GCST90200403 | Citrulline levels \|\| id:ebi-met1400-GCST90200403 | Curry liking \|\| id:ebi-fl187-GCST90094750 | MR Egger | 7 | -0.384846784 | 0.52725974893361 | 0.498197371972473 | -1.418275892 | 0.64858232356566 | 0.680554899882253 | 0.242131117365617 | 1.91282713594544 | 0.999950574162623 |
| ebi-fl187-GCST90094750 | ebi-met1400-GCST90200434 | 12,13-DiHOME levels \|\| id:ebi-met1400-GCST90200434 | Curry liking \|\| id:ebi-fl187-GCST90094750 | MR Egger | 7 | -0.142786309 | 0.531170158978399 | 0.798811577700265 | -1.183879821 | 0.898307202265697 | 0.866939305942504 | 0.306088861866809 | 2.4554430226707 | 0.999950574162623 |
| ebi-fl187-GCST90094750 | ebi-met1400-GCST90200437 | Fructose levels \|\| id:ebi-met1400-GCST90200437 | Curry liking \|\| id:ebi-fl187-GCST90094750 | MR Egger | 7 | -0.18074584 | 0.619693403019206 | 0.782252632192669 | -1.39534491 | 1.03385323031032 | 0.834647466067725 | 0.247747571132119 | 2.81187980745842 | 0.999950574162623 |
| ebi-fl187-GCST90094750 | ebi-met1400-GCST90200448 | N-stearoyl-sphinganine (d18:0/18:0) levels \|\| id:ebi-met1400-GCST90200448 | Curry liking \|\| id:ebi-fl187-GCST90094750 | MR Egger | 7 | -0.69883119 | 0.585319236246529 | 0.286054552819243 | -1.846056893 | 0.448394513378652 | 0.497166057156069 | 0.157858393308171 | 1.56579630140779 | 0.999950574162623 |
| ebi-fl187-GCST90094750 | ebi-met1400-GCST90200468 | X-11847 levels \|\| id:ebi-met1400-GCST90200468 | Curry liking \|\| id:ebi-fl187-GCST90094750 | MR Egger | 7 | -0.414692335 | 0.555114095516631 | 0.488658433156695 | -1.502715962 | 0.673331292312789 | 0.660543475619996 | 0.222524969297057 | 1.96075831203323 | 0.999950574162623 |
| ebi-fl187-GCST90094750 | ebi-met1400-GCST90200476 | X-11843 levels \|\| id:ebi-met1400-GCST90200476 | Curry liking \|\| id:ebi-fl187-GCST90094750 | MR Egger | 7 | -0.409891514 | 0.555143999622897 | 0.493477310305295 | -1.497973753 | 0.678190725549229 | 0.663722250994202 | 0.223582735331886 | 1.9703096744518 | 0.999950574162623 |
| ebi-fl187-GCST90094750 | ebi-met1400-GCST90200511 | X-12906 levels \|\| id:ebi-met1400-GCST90200511 | Curry liking \|\| id:ebi-fl187-GCST90094750 | MR Egger | 7 | -0.25272831 | 0.52736843130864 | 0.652003837967285 | -1.286370435 | 0.780913815384459 | 0.776678869057976 | 0.27627171156297 | 2.18346663952123 | 0.999950574162623 |
| ebi-fl187-GCST90094750 | ebi-met1400-GCST90200515 | X-12849 levels \|\| id:ebi-met1400-GCST90200515 | Curry liking \|\| id:ebi-fl187-GCST90094750 | MR Egger | 7 | 1.47108528455565 | 0.553628384944803 | 0.0450366678738258 | 0.385973650063836 | 2.55619691904746 | 4.35395786107422 | 1.47104590833827 | 12.8867147847372 | 0.999950574162623 |
| ebi-fl187-GCST90094750 | ebi-met1400-GCST90200551 | X-18886 levels \|\| id:ebi-met1400-GCST90200551 | Curry liking \|\| id:ebi-fl187-GCST90094750 | MR Egger | 7 | 0.278976475951721 | 0.623790330093592 | 0.673418741340503 | -0.943652571 | 1.50160552293516 | 1.32177624997549 | 0.389203642009971 | 4.48889030425497 | 0.999950574162623 |
| ebi-fl187-GCST90094750 | ebi-met1400-GCST90200564 | X-21319 levels \|\| id:ebi-met1400-GCST90200564 | Curry liking \|\| id:ebi-fl187-GCST90094750 | MR Egger | 7 | -0.037988022 | 0.5329096834377 | 0.94593504202901 | -1.082491002 | 1.00651495740785 | 0.962724472210674 | 0.338750645360115 | 2.73604913256484 | 0.999950574162623 |
| ebi-fl187-GCST90094750 | ebi-met1400-GCST90200579 | X-21442 levels \|\| id:ebi-met1400-GCST90200579 | Curry liking \|\| id:ebi-fl187-GCST90094750 | MR Egger | 7 | 0.30210097745391 | 0.579893230250173 | 0.624635771460021 | -0.834489754 | 1.43869170874425 | 1.3526978117943 | 0.43409592071208 | 4.21517752811764 | 0.999950574162623 |
| ebi-fl187-GCST90094750 | ebi-met1400-GCST90200581 | X-21441 levels \|\| id:ebi-met1400-GCST90200581 | Curry liking \|\| id:ebi-fl187-GCST90094750 | MR Egger | 7 | -0.111920028 | 0.527639816130583 | 0.840393512980097 | -1.146094067 | 0.922254012060906 | 0.894115759264582 | 0.317875949833812 | 2.51495273984469 | 0.999950574162623 |
| ebi-fl187-GCST90094750 | ebi-met1400-GCST90200648 | X-24585 levels \|\| id:ebi-met1400-GCST90200648 | Curry liking \|\| id:ebi-fl187-GCST90094750 | MR Egger | 7 | 0.62773292599104 | 0.873256986837534 | 0.504421122344567 | -1.083850768 | 2.33931662019261 | 1.87335871858365 | 0.338290336593426 | 10.3741446587971 | 0.999950574162623 |
| ebi-fl187-GCST90094750 | ebi-met1400-GCST90200720 | Adenosine 5'-diphosphate (ADP) to pantothenate ratio \|\| id:ebi-met1400-GCST90200720 | Curry liking \|\| id:ebi-fl187-GCST90094750 | MR Egger | 7 | -0.104273132 | 0.699341196108634 | 0.887300456876837 | -1.474981877 | 1.26643561206403 | 0.900979177288095 | 0.228782873312458 | 3.54818289565791 | 0.999950574162623 |
| ebi-fl187-GCST90094750 | ebi-met1400-GCST90200724 | Cholate to taurocholate ratio \|\| id:ebi-met1400-GCST90200724 | Curry liking \|\| id:ebi-fl187-GCST90094750 | MR Egger | 7 | -0.301612999 | 0.563209130156831 | 0.615239621906859 | -1.405502894 | 0.802276895781938 | 0.739624244591661 | 0.245243693748043 | 2.23061402651114 | 0.999950574162623 |
| ebi-fl187-GCST90094750 | ebi-met1400-GCST90200726 | N-acetylputrescine to (N(1) + N(8))-acetylspermidine ratio \|\| id:ebi-met1400-GCST90200726 | Curry liking \|\| id:ebi-fl187-GCST90094750 | MR Egger | 7 | -0.324781628 | 0.523599935582037 | 0.562244087477325 | -1.351037502 | 0.701474245469959 | 0.722685150418207 | 0.258971437830196 | 2.01672366269764 | 0.999950574162623 |
| ebi-fl187-GCST90094750 | ebi-met1400-GCST90200744 | Arginine to glutamate ratio \|\| id:ebi-met1400-GCST90200744 | Curry liking \|\| id:ebi-fl187-GCST90094750 | MR Egger | 7 | 0.015271792206335 | 0.644241873013045 | 0.982004823699166 | -1.247442279 | 1.2779858633119 | 1.01538900193234 | 0.287238534172288 | 3.58940289197667 | 0.999950574162623 |
| ebi-fl187-GCST90094750 | ebi-met1400-GCST90200759 | Histidine to trans-urocanate ratio \|\| id:ebi-met1400-GCST90200759 | Curry liking \|\| id:ebi-fl187-GCST90094750 | MR Egger | 7 | 0.304553519757569 | 0.57849243084859 | 0.621077577668752 | -0.829291645 | 1.43839868422081 | 1.35601943194381 | 0.436358273565547 | 4.21394255867823 | 0.999950574162623 |
| ebi-fl187-GCST90094750 | ebi-met1400-GCST90200795 | Oleoyl-linoleoyl-glycerol (18:1 to 18:2) [2] to linoleoyl-arachidonoyl-glycerol (18:2 to 20:4) [2] ratio \|\| id:ebi-met1400-GCST90200795 | Curry liking \|\| id:ebi-fl187-GCST90094750 | MR Egger | 7 | -0.969952085 | 0.546809970511934 | 0.136272287335671 | -2.041699627 | 0.10179545731276 | 0.379101202343797 | 0.129807898261126 | 1.10715698770044 | 0.999950574162623 |
| ebi-fl187-GCST90094750 | ebi-met1400-GCST90200811 | Choline to taurocholate ratio \|\| id:ebi-met1400-GCST90200811 | Curry liking \|\| id:ebi-fl187-GCST90094750 | MR Egger | 7 | 0.596614013758795 | 0.555061440818846 | 0.331546384478177 | -0.49130641 | 1.68453443776373 | 1.8159595646309 | 0.611826575345513 | 5.38994099514581 | 0.999950574162623 |
| ebi-fl187-GCST90094750 | ebi-met1400-GCST90200831 | Cholate to phosphate ratio \|\| id:ebi-met1400-GCST90200831 | Curry liking \|\| id:ebi-fl187-GCST90094750 | MR Egger | 7 | -0.85329997 | 0.544991826685468 | 0.178192269702971 | -1.921483951 | 0.214884009907302 | 0.426006800001123 | 0.146389565968799 | 1.23971809360974 | 0.999950574162623 |
| ebi-fl187-GCST90094750 | ebi-met1400-GCST90200841 | Adenosine 5'-diphosphate (ADP) to oxalate (ethanedioate) ratio \|\| id:ebi-met1400-GCST90200841 | Curry liking \|\| id:ebi-fl187-GCST90094750 | MR Egger | 7 | -0.519929068 | 0.707185055267763 | 0.49522848233566 | -1.906011777 | 0.866153639922677 | 0.594562719758261 | 0.148672143484492 | 2.37774756885249 | 0.999950574162623 |
| ebi-fl187-GCST90094750 | ebi-met1400-GCST90200844 | Aspartate to phosphate ratio \|\| id:ebi-met1400-GCST90200844 | Curry liking \|\| id:ebi-fl187-GCST90094750 | MR Egger | 7 | -0.053292468 | 0.494931318463556 | 0.918439604756408 | -1.023357852 | 0.91677291596442 | 0.948102682021379 | 0.359386146194872 | 2.50120575089925 | 0.999950574162623 |
| ebi-fl187-GCST90094750 | ebi-met1400-GCST90200870 | Adenosine 5'-monophosphate (AMP) to acetoacetate ratio \|\| id:ebi-met1400-GCST90200870 | Curry liking \|\| id:ebi-fl187-GCST90094750 | MR Egger | 7 | -0.603506088 | 0.624557234495114 | 0.37827686073347 | -1.827638267 | 0.620626092082935 | 0.546890823697626 | 0.160792869498079 | 1.86009226639394 | 0.999950574162623 |
| ebi-fl187-GCST90094750 | ebi-met1400-GCST90200887 | Cholesterol to cortisol ratio \|\| id:ebi-met1400-GCST90200887 | Curry liking \|\| id:ebi-fl187-GCST90094750 | MR Egger | 7 | 0.313718262071173 | 0.511011313527277 | 0.566124039504499 | -0.687863912 | 1.31530043658464 | 1.36850412271178 | 0.502648624594865 | 3.72587020483468 | 0.999950574162623 |
| ebi-fl187-GCST90094750 | ebi-met1400-GCST90200890 | Cortisol to taurocholate ratio \|\| id:ebi-met1400-GCST90200890 | Curry liking \|\| id:ebi-fl187-GCST90094750 | MR Egger | 7 | 0.280049159863569 | 0.5522159243877 | 0.63362769653734 | -0.802294052 | 1.36239237166346 | 1.32319485881732 | 0.448299361569441 | 3.90552560296116 | 0.999950574162623 |
| ebi-fl187-GCST90094750 | ebi-met1400-GCST90200948 | Adenosine 5'-diphosphate (ADP) to mannitol to sorbitol ratio \|\| id:ebi-met1400-GCST90200948 | Curry liking \|\| id:ebi-fl187-GCST90094750 | MR Egger | 7 | -0.367440473 | 0.707565369859893 | 0.625709933993671 | -1.754268598 | 1.01938765227268 | 0.692504548540481 | 0.173033753327091 | 2.77149712427911 | 0.999950574162623 |
| ebi-fl187-GCST90094750 | ebi-met1400-GCST90200972 | Leucine to N-palmitoyl-sphingosine (d18:1 to 16:0) ratio \|\| id:ebi-met1400-GCST90200972 | Curry liking \|\| id:ebi-fl187-GCST90094750 | MR Egger | 7 | -0.171725396 | 0.507543473540676 | 0.748847111491439 | -1.166510605 | 0.823059811648753 | 0.842210415352485 | 0.311451825928095 | 2.27745777895027 | 0.999950574162623 |
| ebi-fl187-GCST90094750 | ebi-met1400-GCST90200973 | Proline to glutamate ratio \|\| id:ebi-met1400-GCST90200973 | Curry liking \|\| id:ebi-fl187-GCST90094750 | MR Egger | 7 | 0.185939512430222 | 0.518414107369093 | 0.734489303166093 | -0.830152138 | 1.20203116287364 | 1.20434941000541 | 0.435982951695594 | 3.32686747438025 | 0.999950574162623 |
| ebi-fl187-GCST90094750 | ebi-met1400-GCST90200983 | Cholesterol to linoleoyl-arachidonoyl-glycerol (18:2 to 20:4) [1] ratio \|\| id:ebi-met1400-GCST90200983 | Curry liking \|\| id:ebi-fl187-GCST90094750 | MR Egger | 7 | 0.685817828377551 | 0.564996706495529 | 0.279010017564049 | -0.421575716 | 1.79321137310879 | 1.98539488403595 | 0.656012315652547 | 6.00871775042079 | 0.999950574162623 |
| ebi-fl187-GCST90094750 | ebi-met1400-GCST90201001 | Serine to alpha-ketobutyrate ratio \|\| id:ebi-met1400-GCST90201001 | Curry liking \|\| id:ebi-fl187-GCST90094750 | MR Egger | 7 | 0.0229170325961986 | 0.535315778924375 | 0.967509750729575 | -1.026301894 | 1.07213595928797 | 1.02318164530081 | 0.358329654341345 | 2.92161328708563 | 0.999950574162623 |
| ebi-fl187-GCST90094750 | ebi-met1400-GCST90201005 | Fructose to maltose ratio \|\| id:ebi-met1400-GCST90201005 | Curry liking \|\| id:ebi-fl187-GCST90094750 | MR Egger | 7 | -0.782873975 | 0.593642012089413 | 0.244417918334108 | -1.946412318 | 0.380664369041041 | 0.457090455390085 | 0.142785422395965 | 1.46325640883225 | 0.999950574162623 |
| ebi-fl187-GCST90094750 | ebi-met1400-GCST90201020 | Paraxanthine to 5-acetylamino-6-formylamino-3-methyluracil ratio \|\| id:ebi-met1400-GCST90201020 | Curry liking \|\| id:ebi-fl187-GCST90094750 | MR Egger | 7 | -0.330955188 | 0.631338653454115 | 0.622530781273139 | -1.568378949 | 0.906468572283249 | 0.718237353622086 | 0.208382707653386 | 2.47556480068453 | 0.999950574162623 |
| ebi-fl187-GCST90094750 | ebi-pw11-GCST90275197 | Placental weight (fetal genotype, adjusted for sex) \|\| id:ebi-pw11-GCST90275197 | Curry liking \|\| id:ebi-fl187-GCST90094750 | MR Egger | 6 | -0.085032512 | 0.453792888146244 | 0.86048231078933 | -0.974466573 | 0.804401548666639 | 0.918482422123662 | 0.377393611872594 | 2.23535834526777 | 0.999950574162623 |
| ebi-fl187-GCST90094750 | prot-a-1017 | FAS-associated factor 2 \|\| id:prot-a-1017 | Curry liking \|\| id:ebi-fl187-GCST90094750 | MR Egger | 7 | 0.168325204318488 | 1.11286076322002 | 0.885688121165218 | -2.012881892 | 2.34953230022973 | 1.18332136930129 | 0.133603089707648 | 10.4806667728203 | 0.999950574162623 |
| ebi-fl187-GCST90094750 | prot-a-1019 | Fas apoptotic inhibitory molecule 3 \|\| id:prot-a-1019 | Curry liking \|\| id:ebi-fl187-GCST90094750 | MR Egger | 7 | -1.678024231 | 0.8191408011544 | 0.0958235678427169 | -3.283540201 | -0.072508261 | 0.186742572004862 | 0.0374952807310478 | 0.930058063816941 | 0.999950574162623 |
| ebi-fl187-GCST90094750 | prot-a-1052 | MIP18 family protein FAM96A \|\| id:prot-a-1052 | Curry liking \|\| id:ebi-fl187-GCST90094750 | MR Egger | 7 | -0.645633437 | 0.819915926718942 | 0.466694262301024 | -2.252668654 | 0.961401779170811 | 0.524330306572142 | 0.105118325523049 | 2.61536006231143 | 0.999950574162623 |
| ebi-fl187-GCST90094750 | prot-a-1066 | F-box/LRR-repeat protein 4 \|\| id:prot-a-1066 | Curry liking \|\| id:ebi-fl187-GCST90094750 | MR Egger | 7 | 0.497142315891822 | 0.924788500986499 | 0.613924935928528 | -1.315443146 | 2.30972777782536 | 1.6440164717424 | 0.268355376262978 | 10.0716825464741 | 0.999950574162623 |
| ebi-fl187-GCST90094750 | prot-a-1076 | Low affinity immunoglobulin gamma Fc region receptor III-B \|\| id:prot-a-1076 | Curry liking \|\| id:ebi-fl187-GCST90094750 | MR Egger | 7 | -0.39569005 | 1.49704433262166 | 0.802086746469328 | -3.329896942 | 2.53851684220252 | 0.673215326871445 | 0.035796794035218 | 12.6608789571696 | 0.999950574162623 |
| ebi-fl187-GCST90094750 | prot-a-1081 | Fc receptor-like protein 3 \|\| id:prot-a-1081 | Curry liking \|\| id:ebi-fl187-GCST90094750 | MR Egger | 7 | -0.374248974 | 0.818379439505932 | 0.666635218707719 | -1.978272675 | 1.22977472764791 | 0.6878056449355 | 0.138307933820348 | 3.42045891466813 | 0.999950574162623 |
| ebi-fl187-GCST90094750 | prot-a-1105 | Fibroblast growth factor receptor 4 \|\| id:prot-a-1105 | Curry liking \|\| id:ebi-fl187-GCST90094750 | MR Egger | 7 | 0.410049173011768 | 0.819901221252002 | 0.638220034527872 | -1.196957221 | 2.01705556666569 | 1.50689188170329 | 0.302112075162416 | 7.51616148385512 | 0.999950574162623 |
| ebi-fl187-GCST90094750 | prot-a-1143 | Follistatin-related protein 1 \|\| id:prot-a-1143 | Curry liking \|\| id:ebi-fl187-GCST90094750 | MR Egger | 7 | -0.970293302 | 0.819915926718942 | 0.289842137033019 | -2.577328519 | 0.636741914213206 | 0.378971868534984 | 0.075976703504278 | 1.89031203667333 | 0.999950574162623 |
| ebi-fl187-GCST90094750 | prot-a-1151 | Alpha-(1,3)-fucosyltransferase 10 \|\| id:prot-a-1151 | Curry liking \|\| id:ebi-fl187-GCST90094750 | MR Egger | 7 | -0.532550677 | 1.02590032049958 | 0.62583759364407 | -2.543315305 | 1.4782139514097 | 0.587105541746725 | 0.0786053665666252 | 4.38510666899004 | 0.999950574162623 |
| ebi-fl187-GCST90094750 | prot-a-1165 | Growth arrest and DNA damage-inducible proteins-interacting protein 1 \|\| id:prot-a-1165 | Curry liking \|\| id:ebi-fl187-GCST90094750 | MR Egger | 7 | -1.123109994 | 0.980811008755552 | 0.304010104639468 | -3.045499571 | 0.799279582843656 | 0.325266642581089 | 0.0475725401659547 | 2.22393818801562 | 0.999950574162623 |
| ebi-fl187-GCST90094750 | prot-a-1194 | Growth/differentiation factor 11/8 \|\| id:prot-a-1194 | Curry liking \|\| id:ebi-fl187-GCST90094750 | MR Egger | 7 | -0.383686762 | 0.819901221252002 | 0.659496932052222 | -1.990693156 | 1.22331963168125 | 0.681344816863282 | 0.136600707073325 | 3.39845063332849 | 0.999950574162623 |
| ebi-fl187-GCST90094750 | prot-a-1216 | D-glucuronyl C5-epimerase \|\| id:prot-a-1216 | Curry liking \|\| id:ebi-fl187-GCST90094750 | MR Egger | 7 | -0.900256044 | 0.819901221252002 | 0.322242036678314 | -2.507262438 | 0.706750349172638 | 0.406465573148932 | 0.0814910208735755 | 2.02739222535452 | 0.999950574162623 |
| ebi-fl187-GCST90094750 | prot-a-1246 | Glypican-3 \|\| id:prot-a-1246 | Curry liking \|\| id:ebi-fl187-GCST90094750 | MR Egger | 7 | 1.64219321200542 | 1.27986411970358 | 0.255710264166022 | -0.866340463 | 4.15072688662444 | 5.16648829961196 | 0.420487526874801 | 63.4801263866619 | 0.999950574162623 |
| ebi-fl187-GCST90094750 | prot-a-1275 | Glutamate receptor 4 \|\| id:prot-a-1275 | Curry liking \|\| id:ebi-fl187-GCST90094750 | MR Egger | 6 | -1.073063417 | 1.00616039894542 | 0.346293812878245 | -3.045137799 | 0.899010965269584 | 0.341959347253923 | 0.0475897537344107 | 2.45717168083969 | 0.999950574162623 |
| ebi-fl187-GCST90094750 | prot-a-1289 | Glutathione S-transferase P \|\| id:prot-a-1289 | Curry liking \|\| id:ebi-fl187-GCST90094750 | MR Egger | 7 | -0.690524362 | 0.912622684859138 | 0.483374298273367 | -2.479264824 | 1.09821610044625 | 0.501313130640161 | 0.0838048142213951 | 2.998811671 | 0.999950574162623 |
| ebi-fl187-GCST90094750 | prot-a-130 | Apolipoprotein D \|\| id:prot-a-130 | Curry liking \|\| id:ebi-fl187-GCST90094750 | MR Egger | 7 | 0.486791476632798 | 0.819915926718942 | 0.57854548620233 | -1.12024374 | 2.09382669300192 | 1.62708728828154 | 0.326200276963129 | 8.11591292421442 | 0.999950574162623 |
| ebi-fl187-GCST90094750 | prot-a-1313 | Heparin-binding EGF-like growth factor \|\| id:prot-a-1313 | Curry liking \|\| id:ebi-fl187-GCST90094750 | MR Egger | 7 | -1.074561936 | 1.65811456316873 | 0.54552387581508 | -4.32446648 | 2.17534260776973 | 0.341447298299194 | 0.0132406123459098 | 8.80520133586068 | 0.999950574162623 |
| ebi-fl187-GCST90094750 | prot-a-1347 | HLA class II histocompatibility antigen, DQ alpha 2 chain \|\| id:prot-a-1347 | Curry liking \|\| id:ebi-fl187-GCST90094750 | MR Egger | 6 | -0.967147104 | 0.863622949967657 | 0.325466451659582 | -2.659848086 | 0.725553877704271 | 0.380166066647606 | 0.0699588486541628 | 2.06587502525619 | 0.999950574162623 |
| ebi-fl187-GCST90094750 | prot-a-1357 | Non-histone chromosomal protein HMG-14 \|\| id:prot-a-1357 | Curry liking \|\| id:ebi-fl187-GCST90094750 | MR Egger | 7 | 0.117509424917413 | 1.36665995740419 | 0.934816962507402 | -2.561144092 | 2.79616294142964 | 1.12469223002516 | 0.0772163473140796 | 16.3816686010001 | 0.999950574162623 |
| ebi-fl187-GCST90094750 | prot-a-137 | Apolipoprotein M \|\| id:prot-a-137 | Curry liking \|\| id:ebi-fl187-GCST90094750 | MR Egger | 7 | -0.578228616 | 0.988891826050768 | 0.584122819999669 | -2.516456595 | 1.35999936319927 | 0.560891040595766 | 0.0807452134401106 | 3.89619082069727 | 0.999950574162623 |
| ebi-fl187-GCST90094750 | prot-a-1381 | 17-beta-hydroxysteroid dehydrogenase 14 \|\| id:prot-a-1381 | Curry liking \|\| id:ebi-fl187-GCST90094750 | MR Egger | 7 | -0.165765014 | 1.33162379165887 | 0.90578238190554 | -2.775747646 | 2.44421761725409 | 0.847245301247283 | 0.0623028787713336 | 11.5215318239176 | 0.999950574162623 |
| ebi-fl187-GCST90094750 | prot-a-1392 | Serine protease HTRA2, mitochondrial \|\| id:prot-a-1392 | Curry liking \|\| id:ebi-fl187-GCST90094750 | MR Egger | 7 | 0.661339367549639 | 0.819915926718942 | 0.456535719713678 | -0.945695849 | 2.26837458391877 | 1.93738546866231 | 0.388409202759768 | 9.6636805397873 | 0.999950574162623 |
| ebi-fl187-GCST90094750 | prot-a-1416 | Interferon-induced protein with tetratricopeptide repeats 2 \|\| id:prot-a-1416 | Curry liking \|\| id:ebi-fl187-GCST90094750 | MR Egger | 7 | 0.184195652180029 | 1.06520541869467 | 0.869496104408241 | -1.903606968 | 2.27199827282159 | 1.20225102312047 | 0.149030101721938 | 9.69876223590771 | 0.999950574162623 |
| ebi-fl187-GCST90094750 | prot-a-1428 | Interferon gamma \|\| id:prot-a-1428 | Curry liking \|\| id:ebi-fl187-GCST90094750 | MR Egger | 7 | -0.79302197 | 0.883857596650249 | 0.410714165699788 | -2.525382859 | 0.939338919526361 | 0.452475360206022 | 0.0800276675804922 | 2.55828962386849 | 0.999950574162623 |
| ebi-fl187-GCST90094750 | prot-a-1483 | Interleukin-17C \|\| id:prot-a-1483 | Curry liking \|\| id:ebi-fl187-GCST90094750 | MR Egger | 7 | -0.495289677 | 1.18184081313371 | 0.692550441987828 | -2.811697671 | 1.82111831633678 | 0.609394353950536 | 0.0601028708228101 | 6.17876440081549 | 0.999950574162623 |
| ebi-fl187-GCST90094750 | prot-a-1503 | Interleukin-1 receptor-like 2 \|\| id:prot-a-1503 | Curry liking \|\| id:ebi-fl187-GCST90094750 | MR Egger | 7 | -1.009561785 | 1.18380324481529 | 0.432706387405772 | -3.329816145 | 1.31069257479175 | 0.364378620750845 | 0.0357996864181221 | 3.70874140375369 | 0.999950574162623 |
| ebi-fl187-GCST90094750 | prot-a-1522 | Interleukin-31 receptor subunit alpha \|\| id:prot-a-1522 | Curry liking \|\| id:ebi-fl187-GCST90094750 | MR Egger | 7 | -1.803040232 | 0.8191408011544 | 0.0789808399562683 | -3.408556202 | -0.197524261 | 0.164797104477906 | 0.0330889396548637 | 0.820760227664475 | 0.999950574162623 |
| ebi-fl187-GCST90094750 | prot-a-1540 | Interleukin-6 receptor subunit alpha \|\| id:prot-a-1540 | Curry liking \|\| id:ebi-fl187-GCST90094750 | MR Egger | 7 | 0.500870268304614 | 1.37244349856737 | 0.730078574702177 | -2.189118989 | 3.19085952549666 | 1.65015672508979 | 0.112015391963105 | 24.3093129402785 | 0.999950574162623 |
| ebi-fl187-GCST90094750 | prot-a-1541 | Interleukin-6 receptor subunit alpha \|\| id:prot-a-1541 | Curry liking \|\| id:ebi-fl187-GCST90094750 | MR Egger | 7 | 0.380305367562287 | 1.48202488927478 | 0.807702625505471 | -2.524463415 | 3.28507415054085 | 1.46273119190015 | 0.0801012823705379 | 26.7109648739482 | 0.999950574162623 |
| ebi-fl187-GCST90094750 | prot-a-1545 | Interleukin-9 \|\| id:prot-a-1545 | Curry liking \|\| id:ebi-fl187-GCST90094750 | MR Egger | 7 | 0.0639474164214509 | 0.819901221252002 | 0.940857809832342 | -1.543058977 | 1.67095381007537 | 1.06603634128198 | 0.213726316515296 | 5.31723701349868 | 0.999950574162623 |
| ebi-fl187-GCST90094750 | prot-a-1548 | Inositol monophosphatase 3 \|\| id:prot-a-1548 | Curry liking \|\| id:ebi-fl187-GCST90094750 | MR Egger | 7 | -0.214314019 | 0.909312301588237 | 0.823024296615214 | -1.99656613 | 1.56793809202714 | 0.807094902022221 | 0.135800805793254 | 4.79674753816974 | 0.999950574162623 |
| ebi-fl187-GCST90094750 | prot-a-1555 | Inhibin beta A chain:Inhibin beta B chain heterodimer \|\| id:prot-a-1555 | Curry liking \|\| id:ebi-fl187-GCST90094750 | MR Egger | 7 | -0.91133981 | 1.00347445545329 | 0.405425936359123 | -2.878149743 | 1.05547012238788 | 0.401985279054687 | 0.0562387227192227 | 2.87332565114324 | 0.999950574162623 |
| ebi-fl187-GCST90094750 | prot-a-1603 | Junctophilin-3 \|\| id:prot-a-1603 | Curry liking \|\| id:ebi-fl187-GCST90094750 | MR Egger | 7 | 0.203848907317585 | 0.968387386040103 | 0.841584395425934 | -1.694190369 | 2.10188818395619 | 1.22611288277564 | 0.183747936787235 | 8.1816037099189 | 0.999950574162623 |
| ebi-fl187-GCST90094750 | prot-a-1627 | UPF0258 protein KIAA1024 \|\| id:prot-a-1627 | Curry liking \|\| id:ebi-fl187-GCST90094750 | MR Egger | 7 | -1.110893087 | 0.819915926718942 | 0.233452700856376 | -2.717928303 | 0.496142129682217 | 0.329264767744129 | 0.0660113684163638 | 1.64237297117931 | 0.999950574162623 |
| ebi-fl187-GCST90094750 | prot-a-1666 | Kallikrein-7 \|\| id:prot-a-1666 | Curry liking \|\| id:ebi-fl187-GCST90094750 | MR Egger | 7 | 0.271639888380238 | 0.819901221252002 | 0.753846639659396 | -1.335366505 | 1.87864628203416 | 1.31211440857816 | 0.26306174426926 | 6.5446392670316 | 0.999950574162623 |
| ebi-fl187-GCST90094750 | prot-a-1678 | Importin subunit alpha-3 \|\| id:prot-a-1678 | Curry liking \|\| id:ebi-fl187-GCST90094750 | MR Egger | 7 | -0.625473385 | 0.911150516854488 | 0.522951519999791 | -2.411328398 | 1.16038162770113 | 0.535008103573938 | 0.089696063292327 | 3.19115087533912 | 0.999950574162623 |
| ebi-fl187-GCST90094750 | prot-a-1718 | Protein LDOC1 \|\| id:prot-a-1718 | Curry liking \|\| id:ebi-fl187-GCST90094750 | MR Egger | 7 | -0.568686012 | 0.826756255188028 | 0.522148474344345 | -2.189128272 | 1.0517562479527 | 0.56626902063943 | 0.112014352073384 | 2.86267427164928 | 0.999950574162623 |
| ebi-fl187-GCST90094750 | prot-a-1723 | Leptin \|\| id:prot-a-1723 | Curry liking \|\| id:ebi-fl187-GCST90094750 | MR Egger | 7 | -0.414312485 | 1.01265929839006 | 0.699377814097998 | -2.399124709 | 1.57049974024921 | 0.660794430920044 | 0.0907973926187391 | 4.8090508696483 | 0.999950574162623 |
| ebi-fl187-GCST90094750 | prot-a-1730 | Galectin-7 \|\| id:prot-a-1730 | Curry liking \|\| id:ebi-fl187-GCST90094750 | MR Egger | 7 | 0.311357664385194 | 0.819915926718942 | 0.719730553383991 | -1.295677552 | 1.91839288075432 | 1.36527744498881 | 0.273712347145939 | 6.81000517963962 | 0.999950574162623 |
| ebi-fl187-GCST90094750 | prot-a-1753 | Lipase member N \|\| id:prot-a-1753 | Curry liking \|\| id:ebi-fl187-GCST90094750 | MR Egger | 7 | -0.234563653 | 0.818906400548675 | 0.786034011557434 | -1.839620198 | 1.37049289241431 | 0.790915888388086 | 0.158877756780978 | 3.93729087808729 | 0.999950574162623 |
| ebi-fl187-GCST90094750 | prot-a-1756 | VIP36-like protein \|\| id:prot-a-1756 | Curry liking \|\| id:ebi-fl187-GCST90094750 | MR Egger | 7 | 0.599252488946213 | 0.819155532212577 | 0.497273811651611 | -1.006292354 | 2.20479733208286 | 1.82075725539427 | 0.365571881034795 | 9.06841350512771 | 0.999950574162623 |
| ebi-fl187-GCST90094750 | prot-a-1760 | Ig Kappa chain V-I region HK102- like \|\| id:prot-a-1760 | Curry liking \|\| id:ebi-fl187-GCST90094750 | MR Egger | 7 | 0.195205358607021 | 0.819915926718942 | 0.821268273513543 | -1.411829858 | 1.80224057497615 | 1.21556058668891 | 0.243696944164667 | 6.06321734963192 | 0.999950574162623 |
| ebi-fl187-GCST90094750 | prot-a-179 | Neutral ceramidase \|\| id:prot-a-179 | Curry liking \|\| id:ebi-fl187-GCST90094750 | MR Egger | 7 | -0.35442856 | 1.11678407409456 | 0.763791100222888 | -2.543325345 | 1.8344682254383 | 0.701574236414054 | 0.0786045773676989 | 6.26180339215494 | 0.999950574162623 |
| ebi-fl187-GCST90094750 | prot-a-1809 | Lactotransferrin \|\| id:prot-a-1809 | Curry liking \|\| id:ebi-fl187-GCST90094750 | MR Egger | 7 | -0.411454953 | 1.32542351019336 | 0.768755067280989 | -3.009285033 | 2.18637512695619 | 0.662685372294977 | 0.0493269332854712 | 8.90288273370283 | 0.999950574162623 |
| ebi-fl187-GCST90094750 | prot-a-1818 | Lysophospholipase-like protein 1 \|\| id:prot-a-1818 | Curry liking \|\| id:ebi-fl187-GCST90094750 | MR Egger | 7 | -0.897089929 | 0.819915926718942 | 0.323786509126796 | -2.504125145 | 0.709945287248063 | 0.407754529458927 | 0.0817470835156922 | 2.03387997643188 | 0.999950574162623 |
| ebi-fl187-GCST90094750 | prot-a-1852 | MAP kinase-activated protein kinase 3 \|\| id:prot-a-1852 | Curry liking \|\| id:ebi-fl187-GCST90094750 | MR Egger | 7 | 1.92805456229325 | 0.819155532212577 | 0.0652586990888452 | 0.322509719156595 | 3.5335994054299 | 6.87612015758655 | 1.38058830894834 | 34.2470149248073 | 0.999950574162623 |
| ebi-fl187-GCST90094750 | prot-a-1856 | Megakaryocyte-associated tyrosine-protein kinase \|\| id:prot-a-1856 | Curry liking \|\| id:ebi-fl187-GCST90094750 | MR Egger | 7 | -0.57170399 | 0.819915926718942 | 0.516715472506242 | -2.178739207 | 1.03533122603763 | 0.564562609378677 | 0.113184142527998 | 2.81603882654867 | 0.999950574162623 |
| ebi-fl187-GCST90094750 | prot-a-1890 | Beta-1,3-N-acetylglucosaminyltransferase manic fringe \|\| id:prot-a-1890 | Curry liking \|\| id:ebi-fl187-GCST90094750 | MR Egger | 7 | -0.391410362 | 1.09510110997883 | 0.735369245462824 | -2.537808537 | 1.75498781380105 | 0.676102652432078 | 0.0790394220795405 | 5.78337726414141 | 0.999950574162623 |
| ebi-fl187-GCST90094750 | prot-a-1919 | Matrilysin \|\| id:prot-a-1919 | Curry liking \|\| id:ebi-fl187-GCST90094750 | MR Egger | 7 | -0.78418002 | 0.902458088528022 | 0.424619024505854 | -2.552997873 | 0.984637833683906 | 0.456493864277733 | 0.0778479375749989 | 2.67684224675133 | 0.999950574162623 |
| ebi-fl187-GCST90094750 | prot-a-1923 | Multimerin-2 \|\| id:prot-a-1923 | Curry liking \|\| id:ebi-fl187-GCST90094750 | MR Egger | 7 | 0.392195187050972 | 0.825202480512644 | 0.654630157644346 | -1.225201675 | 2.00959204885575 | 1.48022660427296 | 0.293698462785199 | 7.46027329942111 | 0.999950574162623 |
| ebi-fl187-GCST90094750 | prot-a-1948 | Mesothelin \|\| id:prot-a-1948 | Curry liking \|\| id:ebi-fl187-GCST90094750 | MR Egger | 7 | -0.076096467 | 0.819915926718942 | 0.929658480454717 | -1.683131683 | 1.53093874971084 | 0.926726804009343 | 0.185791224794263 | 4.62251416997975 | 0.999950574162623 |
| ebi-fl187-GCST90094750 | prot-a-195 | ATPase family AAA domain-containing protein 1 \|\| id:prot-a-195 | Curry liking \|\| id:ebi-fl187-GCST90094750 | MR Egger | 7 | -0.274809682 | 0.819901221252002 | 0.751098664779037 | -1.881816076 | 1.33219671168538 | 0.759716697253297 | 0.152313242064385 | 3.78935837923717 | 0.999950574162623 |
| ebi-fl187-GCST90094750 | prot-a-1966 | Mucin-1 \|\| id:prot-a-1966 | Curry liking \|\| id:ebi-fl187-GCST90094750 | MR Egger | 7 | -0.618790025 | 0.819901221252002 | 0.484430530636344 | -2.225796419 | 0.988216368176182 | 0.538595730603069 | 0.107981385938709 | 2.68643857922427 | 0.999950574162623 |
| ebi-fl187-GCST90094750 | prot-a-1986 | Myocardial zonula adherens protein \|\| id:prot-a-1986 | Curry liking \|\| id:ebi-fl187-GCST90094750 | MR Egger | 7 | -1.128431672 | 0.819915926718942 | 0.227177591923574 | -2.735466889 | 0.478603544147009 | 0.323540275933947 | 0.0648637159041703 | 1.61381920064627 | 0.999950574162623 |
| ebi-fl187-GCST90094750 | prot-a-2071 | Epididymal secretory protein E1 \|\| id:prot-a-2071 | Curry liking \|\| id:ebi-fl187-GCST90094750 | MR Egger | 7 | -1.267050059 | 1.10745050515364 | 0.304375956114389 | -3.437653049 | 0.903552931483136 | 0.281661281723287 | 0.0321400279603219 | 2.46835745506958 | 0.999950574162623 |
| ebi-fl187-GCST90094750 | prot-a-2098 | Neuritin \|\| id:prot-a-2098 | Curry liking \|\| id:ebi-fl187-GCST90094750 | MR Egger | 7 | -1.514829266 | 0.819915926718942 | 0.123939748786974 | -3.121864483 | 0.0922059501953072 | 0.219845716744078 | 0.0440749148540252 | 1.09659064188307 | 0.999950574162623 |
| ebi-fl187-GCST90094750 | prot-a-2115 | Neurotrophin-4 \|\| id:prot-a-2115 | Curry liking \|\| id:ebi-fl187-GCST90094750 | MR Egger | 7 | 0.868586054097372 | 1.38484317944888 | 0.558046150216981 | -1.845706578 | 3.58287868581718 | 2.38353827558626 | 0.157913703172063 | 35.9769582820461 | 0.999950574162623 |
| ebi-fl187-GCST90094750 | prot-a-2118 | Netrin-1 \|\| id:prot-a-2118 | Curry liking \|\| id:ebi-fl187-GCST90094750 | MR Egger | 7 | -0.159356041 | 0.8191408011544 | 0.853410157485911 | -1.764872012 | 1.44615992876633 | 0.852692710928014 | 0.171208697782805 | 4.24677524381469 | 0.999950574162623 |
| ebi-fl187-GCST90094750 | prot-a-2120 | Netrin-G1 \|\| id:prot-a-2120 | Curry liking \|\| id:ebi-fl187-GCST90094750 | MR Egger | 7 | 0.47455746595209 | 0.89462815606774 | 0.618503093992813 | -1.27891372 | 2.22802865184486 | 1.60730275388449 | 0.278339490930453 | 9.28155086440888 | 0.999950574162623 |
| ebi-fl187-GCST90094750 | prot-a-2145 | Olfactomedin-like protein 3 \|\| id:prot-a-2145 | Curry liking \|\| id:ebi-fl187-GCST90094750 | MR Egger | 7 | -0.245327758 | 1.21832827778196 | 0.84834938852005 | -2.633251183 | 2.14259566636702 | 0.78244804253737 | 0.0718445025298931 | 8.52152799047952 | 0.999950574162623 |
| ebi-fl187-GCST90094750 | prot-a-2162 | Prolyl 4-hydroxylase subunit alpha-2 \|\| id:prot-a-2162 | Curry liking \|\| id:ebi-fl187-GCST90094750 | MR Egger | 7 | 0.508076538251407 | 1.02657187373476 | 0.641631770074046 | -1.504004334 | 2.52015741077154 | 1.66209114960115 | 0.222238458928151 | 12.4305532125544 | 0.999950574162623 |
| ebi-fl187-GCST90094750 | prot-a-2168 | Polyadenylate-binding protein-interacting protein 1 \|\| id:prot-a-2168 | Curry liking \|\| id:ebi-fl187-GCST90094750 | MR Egger | 7 | -0.88231571 | 1.0446329414868 | 0.436860909059874 | -2.929796276 | 1.16516485481557 | 0.413823505820229 | 0.0534079175215572 | 3.20645143859471 | 0.999950574162623 |
| ebi-fl187-GCST90094750 | prot-a-219 | Beta-1,4-galactosyltransferase 5 \|\| id:prot-a-219 | Curry liking \|\| id:ebi-fl187-GCST90094750 | MR Egger | 7 | -1.311316648 | 0.819915926718942 | 0.170642315712018 | -2.918351865 | 0.295718568063842 | 0.269465032037107 | 0.0540226506072815 | 1.34409183323138 | 0.999950574162623 |
| ebi-fl187-GCST90094750 | prot-a-2190 | Pyruvate carboxylase, mitochondrial \|\| id:prot-a-2190 | Curry liking \|\| id:ebi-fl187-GCST90094750 | MR Egger | 7 | -0.794561576 | 0.819915926718942 | 0.377014590813085 | -2.401596792 | 0.812473640504216 | 0.451779262441785 | 0.0905732111584221 | 2.25347538595538 | 0.999950574162623 |
| ebi-fl187-GCST90094750 | prot-a-2197 | Protocadherin-9 \|\| id:prot-a-2197 | Curry liking \|\| id:ebi-fl187-GCST90094750 | MR Egger | 7 | -0.195786318 | 0.8191408011544 | 0.82058245911417 | -1.801302288 | 1.40972965261087 | 0.822187902950715 | 0.165083761586008 | 4.0948482228902 | 0.999950574162623 |
| ebi-fl187-GCST90094750 | prot-a-2198 | Protocadherin alpha-4 \|\| id:prot-a-2198 | Curry liking \|\| id:ebi-fl187-GCST90094750 | MR Egger | 7 | -0.428694374 | 0.938272668707919 | 0.666908857125273 | -2.267708805 | 1.41032005622063 | 0.651358970709089 | 0.103549159801414 | 4.09726654988666 | 0.999950574162623 |
| ebi-fl187-GCST90094750 | prot-a-2254 | Placenta growth factor \|\| id:prot-a-2254 | Curry liking \|\| id:ebi-fl187-GCST90094750 | MR Egger | 7 | -0.783145236 | 0.872746201173462 | 0.410662625964244 | -2.49372779 | 0.927437318654802 | 0.456966481395606 | 0.0826014709960271 | 2.52802235361067 | 0.999950574162623 |
| ebi-fl187-GCST90094750 | prot-a-2261 | Membrane-associated progesterone receptor component 2 \|\| id:prot-a-2261 | Curry liking \|\| id:ebi-fl187-GCST90094750 | MR Egger | 7 | -0.893468491 | 0.819901221252002 | 0.325544042138975 | -2.500474884 | 0.713537902866407 | 0.409233864385927 | 0.0820460269894206 | 2.041200067 | 0.999950574162623 |
| ebi-fl187-GCST90094750 | prot-a-2268 | Polymeric immunoglobulin receptor \|\| id:prot-a-2268 | Curry liking \|\| id:ebi-fl187-GCST90094750 | MR Egger | 7 | 0.169711269601729 | 1.07670941919646 | 0.880923636653858 | -1.940639192 | 2.28006173122679 | 1.18496266718043 | 0.143612124709335 | 9.77728395463314 | 0.999950574162623 |
| ebi-fl187-GCST90094750 | prot-a-2283 | Group 10 secretory phospholipase A2 \|\| id:prot-a-2283 | Curry liking \|\| id:ebi-fl187-GCST90094750 | MR Egger | 7 | -0.04212426 | 1.23742995023778 | 0.974161049419778 | -2.467486962 | 2.38323844284852 | 0.958750639182686 | 0.0847976912751698 | 10.8399506438257 | 0.999950574162623 |
| ebi-fl187-GCST90094750 | prot-a-2298 | Inactive phospholipase D5 \|\| id:prot-a-2298 | Curry liking \|\| id:ebi-fl187-GCST90094750 | MR Egger | 7 | -1.045026602 | 0.933417147047554 | 0.313775736852292 | -2.87452421 | 0.784471006189503 | 0.351682463788391 | 0.0564429881120664 | 2.19124747773289 | 0.999950574162623 |
| ebi-fl187-GCST90094750 | prot-a-2304 | Procollagen-lysine,2-oxoglutarate 5-dioxygenase 2 \|\| id:prot-a-2304 | Curry liking \|\| id:ebi-fl187-GCST90094750 | MR Egger | 7 | 0.810309741754333 | 0.819915926718942 | 0.368395224659146 | -0.796725475 | 2.41734495812346 | 2.24860436548307 | 0.450802714816711 | 11.2160406898288 | 0.999950574162623 |
| ebi-fl187-GCST90094750 | prot-a-2331 | Periostin \|\| id:prot-a-2331 | Curry liking \|\| id:ebi-fl187-GCST90094750 | MR Egger | 7 | -0.417471346 | 0.819915926718942 | 0.63230483581448 | -2.024506563 | 1.18956386996247 | 0.658710365996589 | 0.13205898993502 | 3.28564792510423 | 0.999950574162623 |
| ebi-fl187-GCST90094750 | prot-a-2333 | Periostin \|\| id:prot-a-2333 | Curry liking \|\| id:ebi-fl187-GCST90094750 | MR Egger | 7 | -0.445712599 | 1.30959478807339 | 0.747430561868536 | -3.012518384 | 2.12109318563839 | 0.64036778796915 | 0.0491676995836867 | 8.34024994743824 | 0.999950574162623 |
| ebi-fl187-GCST90094750 | prot-a-2367 | cAMP-dependent protein kinase catalytic subunit alpha \|\| id:prot-a-2367 | Curry liking \|\| id:ebi-fl187-GCST90094750 | MR Egger | 7 | 0.380400393881133 | 0.819915926718942 | 0.662181269534778 | -1.226634822 | 1.98743561025026 | 1.46287019646522 | 0.293277850970151 | 7.29679791578939 | 0.999950574162623 |
| ebi-fl187-GCST90094750 | prot-a-2385 | Phosphoribosyl pyrophosphate synthase-associated protein 1 \|\| id:prot-a-2385 | Curry liking \|\| id:ebi-fl187-GCST90094750 | MR Egger | 7 | 1.80113279619048 | 1.11845332257429 | 0.168230754232974 | -0.391035716 | 3.99330130843608 | 6.05650436501655 | 0.676355998839753 | 54.2336361123269 | 0.999950574162623 |
| ebi-fl187-GCST90094750 | prot-a-2393 | Trypsin-3 \|\| id:prot-a-2393 | Curry liking \|\| id:ebi-fl187-GCST90094750 | MR Egger | 6 | -1.016943919 | 0.925541899325571 | 0.333566177899362 | -2.831006042 | 0.79711820364769 | 0.361698633135988 | 0.0589535141313459 | 2.2191366051728 | 0.999950574162623 |
| ebi-fl187-GCST90094750 | prot-a-2405 | Pregnancy-specific beta-1-glycoprotein 6 \|\| id:prot-a-2405 | Curry liking \|\| id:ebi-fl187-GCST90094750 | MR Egger | 7 | -0.327810552 | 0.819901221252002 | 0.705799439619526 | -1.934816946 | 1.27919584148349 | 0.72049950384379 | 0.144450708708907 | 3.59374861971265 | 0.999950574162623 |
| ebi-fl187-GCST90094750 | prot-a-244 | Beclin-1 \|\| id:prot-a-244 | Curry liking \|\| id:ebi-fl187-GCST90094750 | MR Egger | 7 | -0.989186738 | 0.93135092409203 | 0.336774576630465 | -2.814634549 | 0.836261073076139 | 0.371879003084115 | 0.0599266149583878 | 2.30772242067844 | 0.999950574162623 |
| ebi-fl187-GCST90094750 | prot-a-2454 | tRNA pseudouridine synthase A, mitochondrial \|\| id:prot-a-2454 | Curry liking \|\| id:ebi-fl187-GCST90094750 | MR Egger | 7 | 0.0589033685063435 | 0.819915926718942 | 0.945513777587782 | -1.548131848 | 1.66593858487547 | 1.06067274139662 | 0.212644855935321 | 5.29063663164288 | 0.999950574162623 |
| ebi-fl187-GCST90094750 | prot-a-2466 | Glutaminyl-peptide cyclotransferase-like protein \|\| id:prot-a-2466 | Curry liking \|\| id:ebi-fl187-GCST90094750 | MR Egger | 7 | -0.306842324 | 0.819915926718942 | 0.723574413858229 | -1.91387754 | 1.30019289280429 | 0.735766604816834 | 0.147507310763246 | 3.67000451681056 | 0.999950574162623 |
| ebi-fl187-GCST90094750 | prot-a-2474 | Ras-related protein Rab-27A \|\| id:prot-a-2474 | Curry liking \|\| id:ebi-fl187-GCST90094750 | MR Egger | 7 | -1.713628859 | 0.819915926718942 | 0.0908992157529803 | -3.320664075 | -0.106593643 | 0.180210645614708 | 0.0361288315228054 | 0.898890869813224 | 0.999950574162623 |
| ebi-fl187-GCST90094750 | prot-a-2490 | Rap1 GTPase-GDP dissociation stimulator 1 \|\| id:prot-a-2490 | Curry liking \|\| id:ebi-fl187-GCST90094750 | MR Egger | 7 | 0.343089651074554 | 0.819901221252002 | 0.692982314341577 | -1.263916743 | 1.95009604472848 | 1.40929510108332 | 0.282545199608649 | 7.02936268139894 | 0.999950574162623 |
| ebi-fl187-GCST90094750 | prot-a-2545 | Receptor-interacting serine/threonine-protein kinase 2 \|\| id:prot-a-2545 | Curry liking \|\| id:ebi-fl187-GCST90094750 | MR Egger | 7 | -0.516103219 | 0.819915926718942 | 0.556686334512544 | -2.123138435 | 1.09093199754345 | 0.596841784186757 | 0.119655507548406 | 2.97704738084975 | 0.999950574162623 |
| ebi-fl187-GCST90094750 | prot-a-2610 | RUN and FYVE domain-containing protein 1 \|\| id:prot-a-2610 | Curry liking \|\| id:ebi-fl187-GCST90094750 | MR Egger | 7 | -0.71086649 | 0.819915926718942 | 0.42558508261937 | -2.317901707 | 0.896168725919913 | 0.49121837697079 | 0.0984800088915231 | 2.45019772631831 | 0.999950574162623 |
| ebi-fl187-GCST90094750 | prot-a-2627 | Serine--tRNA ligase, mitochondrial \|\| id:prot-a-2627 | Curry liking \|\| id:ebi-fl187-GCST90094750 | MR Egger | 7 | -0.413046617 | 0.819901221252001 | 0.635825059641292 | -2.02005301 | 1.19395977697105 | 0.661631439045964 | 0.132648433155624 | 3.30012312034214 | 0.999950574162623 |
| ebi-fl187-GCST90094750 | prot-a-264 | BolA-like protein 2 \|\| id:prot-a-264 | Curry liking \|\| id:ebi-fl187-GCST90094750 | MR Egger | 7 | 0.313375161521001 | 0.8196809305852 | 0.717939797006691 | -1.293199462 | 1.91994978546799 | 1.36803466873393 | 0.274391471973815 | 6.82061596665275 | 0.999950574162623 |
| ebi-fl187-GCST90094750 | prot-a-2646 | Secretoglobin family 1C member 1 \|\| id:prot-a-2646 | Curry liking \|\| id:ebi-fl187-GCST90094750 | MR Egger | 7 | 0.416744167198176 | 0.856872108555293 | 0.647282355269683 | -1.262725166 | 2.09621349996655 | 1.51701436125179 | 0.282882074638965 | 8.13530717766865 | 0.999950574162623 |
| ebi-fl187-GCST90094750 | prot-a-2672 | Semaphorin-3E \|\| id:prot-a-2672 | Curry liking \|\| id:ebi-fl187-GCST90094750 | MR Egger | 7 | -0.756426127 | 1.08604207833126 | 0.517160147185885 | -2.885068601 | 1.37221634632516 | 0.469340797542431 | 0.0558509579883843 | 3.94408246826453 | 0.999950574162623 |
| ebi-fl187-GCST90094750 | prot-a-2701 | Plasma protease C1 inhibitor \|\| id:prot-a-2701 | Curry liking \|\| id:ebi-fl187-GCST90094750 | MR Egger | 7 | 0.0904730412160972 | 1.00212008999306 | 0.931568491582759 | -1.873682335 | 2.0546284176025 | 1.09469199567889 | 0.153557170480252 | 7.80393752799417 | 0.999950574162623 |
| ebi-fl187-GCST90094750 | prot-a-2738 | Signaling threshold-regulating transmembrane adapter 1 \|\| id:prot-a-2738 | Curry liking \|\| id:ebi-fl187-GCST90094750 | MR Egger | 7 | 0.19070108028536 | 0.819915926718942 | 0.825303494677198 | -1.416334136 | 1.79773629665449 | 1.210097676 | 0.242601733720026 | 6.03596834578327 | 0.999950574162623 |
| ebi-fl187-GCST90094750 | prot-a-274 | Serine/threonine-protein kinase BRSK2 \|\| id:prot-a-274 | Curry liking \|\| id:ebi-fl187-GCST90094750 | MR Egger | 7 | -0.093858995 | 0.819915926718942 | 0.913316928930494 | -1.700894211 | 1.51317622164546 | 0.910411125962963 | 0.182520239435394 | 4.5411315525396 | 0.999950574162623 |
| ebi-fl187-GCST90094750 | prot-a-2749 | Mitochondrial glutamate carrier 2 \|\| id:prot-a-2749 | Curry liking \|\| id:ebi-fl187-GCST90094750 | MR Egger | 7 | -1.027380988 | 0.983757348182755 | 0.344164271935426 | -2.95554539 | 0.900783414657643 | 0.357943191626442 | 0.0520502651278852 | 2.46153075525995 | 0.999950574162623 |
| ebi-fl187-GCST90094750 | prot-a-2760 | Sodium-coupled monocarboxylate transporter 1 \|\| id:prot-a-2760 | Curry liking \|\| id:ebi-fl187-GCST90094750 | MR Egger | 7 | -0.30292144 | 0.819915926718942 | 0.726919733905049 | -1.909956656 | 1.30411377686218 | 0.738657123358943 | 0.148086805149192 | 3.68442242601712 | 0.999950574162623 |
| ebi-fl187-GCST90094750 | prot-a-2777 | Small integral membrane protein 10 \|\| id:prot-a-2777 | Curry liking \|\| id:ebi-fl187-GCST90094750 | MR Egger | 7 | -0.003868392 | 0.891622179153982 | 0.996706089497382 | -1.751447863 | 1.74371107946745 | 0.996139080913977 | 0.173522524673176 | 5.71852599766571 | 0.999950574162623 |
| ebi-fl187-GCST90094750 | prot-a-2799 | Superoxide dismutase [Mn], mitochondrial \|\| id:prot-a-2799 | Curry liking \|\| id:ebi-fl187-GCST90094750 | MR Egger | 7 | 0.548441324401306 | 0.819901221252002 | 0.533190269152472 | -1.058565069 | 2.15544771805523 | 1.73055354303138 | 0.346953307276379 | 8.63175390604605 | 0.999950574162623 |
| ebi-fl187-GCST90094750 | prot-a-2805 | Protein SPACA7 \|\| id:prot-a-2805 | Curry liking \|\| id:ebi-fl187-GCST90094750 | MR Egger | 7 | -0.169156001 | 1.04493869036874 | 0.877737693458469 | -2.217235834 | 1.8789238317599 | 0.844377169121821 | 0.10890973768067 | 6.54645598196795 | 0.999950574162623 |
| ebi-fl187-GCST90094750 | prot-a-2811 | SPARC-like protein 1 \|\| id:prot-a-2811 | Curry liking \|\| id:ebi-fl187-GCST90094750 | MR Egger | 7 | -0.027675948 | 0.946118799446929 | 0.977795225600486 | -1.882068794 | 1.82671689936063 | 0.972703522689422 | 0.152274754500656 | 6.21345374126568 | 0.999950574162623 |
| ebi-fl187-GCST90094750 | prot-a-2831 | Sepiapterin reductase \|\| id:prot-a-2831 | Curry liking \|\| id:ebi-fl187-GCST90094750 | MR Egger | 7 | -0.262981843 | 1.16832178771105 | 0.830816808065361 | -2.552892547 | 2.02692886042811 | 0.768755855111231 | 0.0778561374145442 | 7.59073830777276 | 0.999950574162623 |
| ebi-fl187-GCST90094750 | prot-a-2834 | SPRY domain-containing SOCS box protein 1 \|\| id:prot-a-2834 | Curry liking \|\| id:ebi-fl187-GCST90094750 | MR Egger | 7 | -1.209174306 | 0.819915926718942 | 0.200290144894303 | -2.816209522 | 0.397860910817638 | 0.298443600947234 | 0.0598323064705893 | 1.48863696220927 | 0.999950574162623 |
| ebi-fl187-GCST90094750 | prot-a-2835 | Spectrin alpha chain, non-erythrocytic 1 \|\| id:prot-a-2835 | Curry liking \|\| id:ebi-fl187-GCST90094750 | MR Egger | 7 | 0.436761396908419 | 0.819915926718942 | 0.617062134384783 | -1.170273819 | 2.04379661327754 | 1.54768675015344 | 0.31028196839115 | 7.71986296535568 | 0.999950574162623 |
| ebi-fl187-GCST90094750 | prot-a-2859 | Sia-alpha-2,3-Gal-beta-1,4-GlcNAc-R:alpha 2,8-sialyltransferase \|\| id:prot-a-2859 | Curry liking \|\| id:ebi-fl187-GCST90094750 | MR Egger | 7 | -0.702856506 | 0.819901221252002 | 0.430471358600501 | -2.309862899 | 0.904149887890198 | 0.495168829056229 | 0.0992748612679676 | 2.469831397 | 0.999950574162623 |
| ebi-fl187-GCST90094750 | prot-a-2861 | Alpha-2,8-sialyltransferase 8F \|\| id:prot-a-2861 | Curry liking \|\| id:ebi-fl187-GCST90094750 | MR Egger | 7 | 0.295512503698375 | 0.819901221252002 | 0.733255345398599 | -1.31149389 | 1.9025188973523 | 1.34381489233544 | 0.269417275842459 | 6.70275675238609 | 0.999950574162623 |
| ebi-fl187-GCST90094750 | prot-a-289 | Uncharacterized protein C17orf89 \|\| id:prot-a-289 | Curry liking \|\| id:ebi-fl187-GCST90094750 | MR Egger | 7 | -1.451267122 | 0.958398295671337 | 0.190382984824105 | -3.329727782 | 0.427193537030585 | 0.234273247040829 | 0.035802849921395 | 1.5329493154749 | 0.999950574162623 |
| ebi-fl187-GCST90094750 | prot-a-2921 | Tachykinin-3 \|\| id:prot-a-2921 | Curry liking \|\| id:ebi-fl187-GCST90094750 | MR Egger | 7 | -1.725102454 | 0.819155532212577 | 0.0890780100290435 | -3.330647297 | -0.11955761 | 0.178154798253463 | 0.0357699438076878 | 0.887312887919905 | 0.999950574162623 |
| ebi-fl187-GCST90094750 | prot-a-2928 | Tax1-binding protein 1 \|\| id:prot-a-2928 | Curry liking \|\| id:ebi-fl187-GCST90094750 | MR Egger | 7 | -0.959593026 | 1.05623136664613 | 0.405271283080198 | -3.029806505 | 1.11062045218313 | 0.383048744967848 | 0.0483249878616328 | 3.03624165290139 | 0.999950574162623 |
| ebi-fl187-GCST90094750 | prot-a-296 | Uncharacterized protein C1orf185 \|\| id:prot-a-296 | Curry liking \|\| id:ebi-fl187-GCST90094750 | MR Egger | 7 | -1.02280896 | 0.819901221252002 | 0.267464210608669 | -2.629815354 | 0.584197433199503 | 0.359583464508706 | 0.0720917724594964 | 1.79355096340195 | 0.999950574162623 |
| ebi-fl187-GCST90094750 | prot-a-2964 | Transforming growth factor beta-2 \|\| id:prot-a-2964 | Curry liking \|\| id:ebi-fl187-GCST90094750 | MR Egger | 7 | -0.000527585 | 0.819915926718942 | 0.999511474864205 | -1.607562801 | 1.60650763139322 | 0.999472554172573 | 0.200375374041841 | 4.98537004021089 | 0.999974376584515 |
| ebi-fl187-GCST90094750 | prot-a-2977 | Thymocyte nuclear protein 1 \|\| id:prot-a-2977 | Curry liking \|\| id:ebi-fl187-GCST90094750 | MR Egger | 7 | 0.844463839826519 | 0.819915926718942 | 0.350259474557806 | -0.762571377 | 2.45149905619565 | 2.32672998013077 | 0.466465425305286 | 11.6057313291682 | 0.999950574162623 |
| ebi-fl187-GCST90094750 | prot-a-3015 | Transmembrane protein 70, mitochondrial \|\| id:prot-a-3015 | Curry liking \|\| id:ebi-fl187-GCST90094750 | MR Egger | 7 | -0.868651286 | 0.819901221252002 | 0.337864933782766 | -2.47565768 | 0.738355107450757 | 0.419516976196167 | 0.0841076561519636 | 2.09249075968534 | 0.999950574162623 |
| ebi-fl187-GCST90094750 | prot-a-3030 | Tumor necrosis factor alpha-induced protein 3 \|\| id:prot-a-3030 | Curry liking \|\| id:ebi-fl187-GCST90094750 | MR Egger | 7 | 0.117654050726402 | 1.08609919363379 | 0.917949043587712 | -2.011100369 | 2.24640847024863 | 1.12485490131174 | 0.133841318799974 | 9.45372146919778 | 0.999950574162623 |
| ebi-fl187-GCST90094750 | prot-a-3032 | Tumor necrosis factor receptor superfamily member 10A \|\| id:prot-a-3032 | Curry liking \|\| id:ebi-fl187-GCST90094750 | MR Egger | 7 | -0.331802627 | 0.819915926718942 | 0.702444703532653 | -1.938837843 | 1.27523258935384 | 0.717628949445023 | 0.14387105335508 | 3.5795338747576 | 0.999950574162623 |
| ebi-fl187-GCST90094750 | prot-a-3081 | Tumor protein p53-inducible protein 11 \|\| id:prot-a-3081 | Curry liking \|\| id:ebi-fl187-GCST90094750 | MR Egger | 7 | -1.015607888 | 0.819915926718942 | 0.270444316088238 | -2.622643104 | 0.591427328339061 | 0.362182196678659 | 0.0726106913369544 | 1.80656513766339 | 0.999950574162623 |
| ebi-fl187-GCST90094750 | prot-a-315 | Complement C4 \|\| id:prot-a-315 | Curry liking \|\| id:ebi-fl187-GCST90094750 | MR Egger | 6 | -0.820905511 | 0.863622949967658 | 0.395665170060132 | -2.513606493 | 0.871795471098164 | 0.440033019380702 | 0.0809756738078996 | 2.39120033264123 | 0.999950574162623 |
| ebi-fl187-GCST90094750 | prot-a-3157 | UDP-glucuronosyltransferase 1-8 \|\| id:prot-a-3157 | Curry liking \|\| id:ebi-fl187-GCST90094750 | MR Egger | 7 | -0.856651935 | 0.819901221252001 | 0.343962941058016 | -2.463658329 | 0.750354458342058 | 0.424581230717571 | 0.0851229728187987 | 2.11775053793511 | 0.999950574162623 |
| ebi-fl187-GCST90094750 | prot-a-317 | Glutaredoxin-like protein C5orf63 \|\| id:prot-a-317 | Curry liking \|\| id:ebi-fl187-GCST90094750 | MR Egger | 7 | -0.784667726 | 1.35913520487441 | 0.588737957424684 | -3.448572727 | 1.87923727568162 | 0.456271283743695 | 0.03179097840423 | 6.54850825 | 0.999950574162623 |
| ebi-fl187-GCST90094750 | prot-a-3192 | Guanine nucleotide exchange factor VAV3 \|\| id:prot-a-3192 | Curry liking \|\| id:ebi-fl187-GCST90094750 | MR Egger | 7 | -0.976676973 | 1.12330293682828 | 0.424354702227911 | -3.178350729 | 1.22499678329954 | 0.376560342295213 | 0.041654297722945 | 3.40415513262583 | 0.999950574162623 |
| ebi-fl187-GCST90094750 | prot-a-3233 | Serine/threonine-protein kinase WNK3 \|\| id:prot-a-3233 | Curry liking \|\| id:ebi-fl187-GCST90094750 | MR Egger | 7 | 1.17521544333168 | 0.955925213360911 | 0.273614941368878 | -0.698397975 | 3.04882886151906 | 3.23884065529817 | 0.497381483514055 | 21.0906299050352 | 0.999950574162623 |
| ebi-fl187-GCST90094750 | prot-a-3250 | 14-3-3 protein theta \|\| id:prot-a-3250 | Curry liking \|\| id:ebi-fl187-GCST90094750 | MR Egger | 7 | 0.237205983540541 | 0.832632276815508 | 0.787153845510021 | -1.394753279 | 1.86916524609894 | 1.26770221667426 | 0.247894189520942 | 6.4828825284954 | 0.999950574162623 |
| ebi-fl187-GCST90094750 | prot-a-3278 | E3 ubiquitin-protein ligase ZNRF3 \|\| id:prot-a-3278 | Curry liking \|\| id:ebi-fl187-GCST90094750 | MR Egger | 7 | 0.679331426915767 | 0.819901221252002 | 0.44509399804159 | -0.927674967 | 2.28633782056969 | 1.97255849176059 | 0.395472128133991 | 9.83884002591069 | 0.999950574162623 |
| ebi-fl187-GCST90094750 | prot-a-355 | Calpain-3 \|\| id:prot-a-355 | Curry liking \|\| id:ebi-fl187-GCST90094750 | MR Egger | 7 | -0.715586982 | 0.873561961458962 | 0.449961581845215 | -2.427768427 | 0.996594462399696 | 0.488905049061457 | 0.0882335126146275 | 2.70904036249555 | 0.999950574162623 |
| ebi-fl187-GCST90094750 | prot-a-385 | Coiled-coil domain-containing protein 90B, mitochondrial \|\| id:prot-a-385 | Curry liking \|\| id:ebi-fl187-GCST90094750 | MR Egger | 7 | -0.549985506 | 0.869739535076201 | 0.554937930486092 | -2.254674995 | 1.15470398261422 | 0.576958172673673 | 0.104907633713891 | 3.17308399046337 | 0.999950574162623 |
| ebi-fl187-GCST90094750 | prot-a-390 | C-C motif chemokine 14 \|\| id:prot-a-390 | Curry liking \|\| id:ebi-fl187-GCST90094750 | MR Egger | 7 | -1.075239243 | 1.03877129809951 | 0.348062602498495 | -3.111230988 | 0.960752500923088 | 0.34121611184872 | 0.0445460858948323 | 2.61366251706225 | 0.999950574162623 |
| ebi-fl187-GCST90094750 | prot-a-40 | Pituitary adenylate cyclase-activating polypeptide 27 \|\| id:prot-a-40 | Curry liking \|\| id:ebi-fl187-GCST90094750 | MR Egger | 7 | -0.335471348 | 0.819915926718942 | 0.699363918260095 | -1.942506564 | 1.27156386862654 | 0.715000992810751 | 0.143344197673691 | 3.56642562459428 | 0.999950574162623 |
| ebi-fl187-GCST90094750 | prot-a-401 | C-C motif chemokine 24 \|\| id:prot-a-401 | Curry liking \|\| id:ebi-fl187-GCST90094750 | MR Egger | 7 | -0.532152509 | 0.819915926718942 | 0.544945627540294 | -2.139187726 | 1.07488270709991 | 0.587339354637931 | 0.117750449858525 | 2.92964925332323 | 0.999950574162623 |
| ebi-fl187-GCST90094750 | prot-a-412 | Cerebral cavernous malformations 2 protein \|\| id:prot-a-412 | Curry liking \|\| id:ebi-fl187-GCST90094750 | MR Egger | 7 | -0.483328575 | 1.05680943862908 | 0.666607023571139 | -2.554675075 | 1.58801792463241 | 0.616727148905834 | 0.0777174803335216 | 4.89403895449583 | 0.999950574162623 |
| ebi-fl187-GCST90094750 | prot-a-44 | Adhesion G protein-coupled receptor F5 \|\| id:prot-a-44 | Curry liking \|\| id:ebi-fl187-GCST90094750 | MR Egger | 7 | -0.884760037 | 0.819915926718942 | 0.329830872902116 | -2.491795253 | 0.722275179329592 | 0.412813221277346 | 0.0827612557018582 | 2.05911273597982 | 0.999950574162623 |
| ebi-fl187-GCST90094750 | prot-a-48 | Adrenomedullin \|\| id:prot-a-48 | Curry liking \|\| id:ebi-fl187-GCST90094750 | MR Egger | 7 | -0.428837731 | 0.819901221252002 | 0.623291471226994 | -2.035844124 | 1.17816866283754 | 0.651265600944551 | 0.130570218455791 | 3.24841980039482 | 0.999950574162623 |
| ebi-fl187-GCST90094750 | prot-a-502 | Carcinoembryonic antigen-related cell adhesion molecule 1 \|\| id:prot-a-502 | Curry liking \|\| id:ebi-fl187-GCST90094750 | MR Egger | 7 | 0.0045225719820906 | 1.06554640780242 | 0.996777629990491 | -2.083948387 | 2.09299353127483 | 1.00453281424539 | 0.124437911869777 | 8.10915387226819 | 0.999950574162623 |
| ebi-fl187-GCST90094750 | prot-a-518 | Complement factor B \|\| id:prot-a-518 | Curry liking \|\| id:ebi-fl187-GCST90094750 | MR Egger | 7 | -0.659024731 | 0.819901221252001 | 0.458014840158609 | -2.266031125 | 0.947981662713379 | 0.517355649 | 0.103723028016548 | 2.58049608824039 | 0.999950574162623 |
| ebi-fl187-GCST90094750 | prot-a-525 | Properdin \|\| id:prot-a-525 | Curry liking \|\| id:ebi-fl187-GCST90094750 | MR Egger | 7 | -0.659867669 | 0.819915926718942 | 0.457480595822352 | -2.266902885 | 0.947167547768057 | 0.516919734677478 | 0.103632645792217 | 2.57839612273125 | 0.999950574162623 |
| ebi-fl187-GCST90094750 | prot-a-533 | Chondroadherin \|\| id:prot-a-533 | Curry liking \|\| id:ebi-fl187-GCST90094750 | MR Egger | 7 | -0.815132032 | 1.48353460145834 | 0.606333232006677 | -3.722859851 | 2.0925957863614 | 0.442580888443718 | 0.0241647613072786 | 8.10592913891647 | 0.999950574162623 |
| ebi-fl187-GCST90094750 | prot-a-535 | Coiled-coil-helix-coiled-coil-helix domain-containing protein 2 \|\| id:prot-a-535 | Curry liking \|\| id:ebi-fl187-GCST90094750 | MR Egger | 7 | -0.105521307 | 0.981365319381474 | 0.918553823594165 | -2.028997333 | 1.8179547190702 | 0.8998552994999 | 0.131467272994512 | 6.15924816567738 | 0.999950574162623 |
| ebi-fl187-GCST90094750 | prot-a-591 | Caseinolytic peptidase B protein homolog \|\| id:prot-a-591 | Curry liking \|\| id:ebi-fl187-GCST90094750 | MR Egger | 7 | 0.417219425048654 | 1.27574070562186 | 0.756884492961587 | -2.083232358 | 2.91767120806749 | 1.51773550558744 | 0.124527044972892 | 18.4981588973079 | 0.999950574162623 |
| ebi-fl187-GCST90094750 | prot-a-605 | Ciliary neurotrophic factor receptor subunit alpha \|\| id:prot-a-605 | Curry liking \|\| id:ebi-fl187-GCST90094750 | MR Egger | 7 | -1.001154457 | 0.8191408011544 | 0.27610018339162 | -2.606670427 | 0.604361513264087 | 0.367454985231172 | 0.0737797904320773 | 1.83008335182986 | 0.999950574162623 |
| ebi-fl187-GCST90094750 | prot-a-606 | Ciliary neurotrophic factor receptor subunit alpha \|\| id:prot-a-606 | Curry liking \|\| id:ebi-fl187-GCST90094750 | MR Egger | 7 | -0.859999621 | 0.819901221252002 | 0.34225238204438 | -2.467006015 | 0.747006772728729 | 0.423162242727863 | 0.0848384843220417 | 2.11067282850965 | 0.999950574162623 |
| ebi-fl187-GCST90094750 | prot-a-707 | Cancer/testis antigen 55 \|\| id:prot-a-707 | Curry liking \|\| id:ebi-fl187-GCST90094750 | MR Egger | 7 | -0.769835247 | 1.34690887162194 | 0.59235534040341 | -3.409776636 | 1.87010614112859 | 0.46308935727156 | 0.0330485814271131 | 6.48898511093883 | 0.999950574162623 |
| ebi-fl187-GCST90094750 | prot-a-719 | Dipeptidyl peptidase 1 \|\| id:prot-a-719 | Curry liking \|\| id:ebi-fl187-GCST90094750 | MR Egger | 7 | -0.052483813 | 0.921170531735044 | 0.956771778777234 | -1.857978055 | 1.75301042925563 | 0.948869680337137 | 0.155987710271669 | 5.77195260251618 | 0.999950574162623 |
| ebi-fl187-GCST90094750 | prot-a-752 | Cytochrome b5 \|\| id:prot-a-752 | Curry liking \|\| id:ebi-fl187-GCST90094750 | MR Egger | 7 | -0.418730978 | 0.865322851393725 | 0.648905136862705 | -2.114763767 | 1.27730181093585 | 0.657881156102237 | 0.120661790540354 | 3.58694839199878 | 0.999950574162623 |
| ebi-fl187-GCST90094750 | prot-a-755 | Cytoglobin \|\| id:prot-a-755 | Curry liking \|\| id:ebi-fl187-GCST90094750 | MR Egger | 7 | -0.521344749 | 0.819915926718942 | 0.552834034585552 | -2.128379965 | 1.08569046756355 | 0.593721604482968 | 0.119029970436242 | 2.96148392155274 | 0.999950574162623 |
| ebi-fl187-GCST90094750 | prot-a-807 | Desmin \|\| id:prot-a-807 | Curry liking \|\| id:ebi-fl187-GCST90094750 | MR Egger | 7 | 0.228367453119405 | 1.01479334291691 | 0.830857535145222 | -1.760627499 | 2.21736240523654 | 1.25654696271325 | 0.171936939708115 | 9.18307765733353 | 0.999950574162623 |
| ebi-fl187-GCST90094750 | prot-a-82 | Ameloblastin \|\| id:prot-a-82 | Curry liking \|\| id:ebi-fl187-GCST90094750 | MR Egger | 7 | 0.960445317304522 | 0.911055732718546 | 0.340040629129406 | -0.825223919 | 2.74611455343287 | 2.61285976605484 | 0.438136874386529 | 15.5819711970768 | 0.999950574162623 |
| ebi-fl187-GCST90094750 | prot-a-84 | Muellerian-inhibiting factor \|\| id:prot-a-84 | Curry liking \|\| id:ebi-fl187-GCST90094750 | MR Egger | 7 | 0.322014272110067 | 0.819901221252001 | 0.710691135699539 | -1.284992122 | 1.92902066576399 | 1.37990446996519 | 0.27665276322005 | 6.8827664111034 | 0.999950574162623 |
| ebi-fl187-GCST90094750 | prot-a-858 | Inactive dipeptidyl peptidase 10 \|\| id:prot-a-858 | Curry liking \|\| id:ebi-fl187-GCST90094750 | MR Egger | 7 | -1.723466775 | 0.819915926718942 | 0.0895252800274152 | -3.330501991 | -0.116431558 | 0.178446440743735 | 0.0357751417597078 | 0.890091014257583 | 0.999950574162623 |
| ebi-fl187-GCST90094750 | prot-a-863 | Dihydropyrimidinase-related protein 3 \|\| id:prot-a-863 | Curry liking \|\| id:ebi-fl187-GCST90094750 | MR Egger | 7 | 0.122245728393014 | 0.819915926718942 | 0.887305327683547 | -1.484789488 | 1.72928094476214 | 1.1300317485517 | 0.226550027161733 | 5.63659942456414 | 0.999950574162623 |
| ebi-fl187-GCST90094750 | prot-a-864 | Dihydropyrimidinase-related protein 5 \|\| id:prot-a-864 | Curry liking \|\| id:ebi-fl187-GCST90094750 | MR Egger | 7 | -0.765693245 | 0.819901221252002 | 0.393232518167571 | -2.372699639 | 0.841313148693865 | 0.465011452363173 | 0.0932287024394419 | 2.31941070905032 | 0.999950574162623 |
| ebi-fl187-GCST90094750 | prot-a-884 | Endothelin-converting enzyme 1 \|\| id:prot-a-884 | Curry liking \|\| id:ebi-fl187-GCST90094750 | MR Egger | 7 | 0.194061324834193 | 0.877543021167487 | 0.833729360197251 | -1.525922997 | 1.91404564632247 | 1.21417073949247 | 0.217420286027548 | 6.78046474675741 | 0.999950574162623 |
| ebi-fl187-GCST90094750 | prot-a-893 | Early endosome antigen 1 \|\| id:prot-a-893 | Curry liking \|\| id:ebi-fl187-GCST90094750 | MR Egger | 7 | 0.36664163673645 | 0.819915926718942 | 0.673457998598813 | -1.24039358 | 1.97367685310558 | 1.44288075099884 | 0.289270344615432 | 7.19709054300315 | 0.999950574162623 |
| ebi-fl187-GCST90094750 | prot-a-897 | Ephrin-A2 \|\| id:prot-a-897 | Curry liking \|\| id:ebi-fl187-GCST90094750 | MR Egger | 7 | 0.450514172730682 | 0.819915926718942 | 0.606326097906843 | -1.156521044 | 2.05754938909981 | 1.56911877619485 | 0.314578684910872 | 7.82676593140693 | 0.999950574162623 |
| ebi-fl187-GCST90094750 | prot-a-90 | Anaphase-promoting complex subunit 10 \|\| id:prot-a-90 | Curry liking \|\| id:ebi-fl187-GCST90094750 | MR Egger | 7 | 0.473248692967217 | 0.819915926718942 | 0.588823630373263 | -1.133786523 | 2.08028390933634 | 1.60520053542513 | 0.321812396303619 | 8.00674178038228 | 0.999950574162623 |
| ebi-fl187-GCST90094750 | prot-a-914 | Histone-lysine N-methyltransferase EHMT2 \|\| id:prot-a-914 | Curry liking \|\| id:ebi-fl187-GCST90094750 | MR Egger | 7 | 0.888279575915538 | 0.819901221252002 | 0.328087903660297 | -0.718726818 | 2.49528596956946 | 2.43094379734344 | 0.487372374976559 | 12.1252004612013 | 0.999950574162623 |
| ebi-fl187-GCST90094750 | prot-a-932 | RNA polymerase II elongation factor ELL \|\| id:prot-a-932 | Curry liking \|\| id:ebi-fl187-GCST90094750 | MR Egger | 7 | -0.412436253 | 0.819915926718942 | 0.636318243203853 | -2.019471469 | 1.19459896335134 | 0.66203539810446 | 0.132725596085969 | 3.30223318838547 | 0.999950574162623 |
| ebi-fl187-GCST90094750 | prot-a-991 | Estrogen receptor \|\| id:prot-a-991 | Curry liking \|\| id:ebi-fl187-GCST90094750 | MR Egger | 7 | -0.413485571 | 0.819915926718942 | 0.635480671636262 | -2.020520788 | 1.19354964506386 | 0.661341076600105 | 0.132586397735239 | 3.29876991206874 | 0.999950574162623 |
| ebi-fl187-GCST90094750 | prot-a-992 | Persulfide dioxygenase ETHE1, mitochondrial \|\| id:prot-a-992 | Curry liking \|\| id:ebi-fl187-GCST90094750 | MR Egger | 7 | -1.230069069 | 0.819901221252002 | 0.193837708185956 | -2.837075462 | 0.376937325109047 | 0.292272390155011 | 0.0585967841319857 | 1.45781293824098 | 0.999950574162623 |
| ebi-fl187-GCST90094750 | prot-a-993 | Ethanolamine kinase 1 \|\| id:prot-a-993 | Curry liking \|\| id:ebi-fl187-GCST90094750 | MR Egger | 7 | -0.390619715 | 1.01763904184517 | 0.716871002743831 | -2.385192237 | 1.60395280691546 | 0.676637422112666 | 0.0920712783607541 | 4.97264955102905 | 0.999950574162623 |
| ebi-fl187-GCST90094750 | prot-b-10 | coagulation factor III, tissue factor \|\| id:prot-b-10 | Curry liking \|\| id:ebi-fl187-GCST90094750 | MR Egger | 5 | 1.86084626347885 | 2.05715820393606 | 0.4323720877578 | -2.171183816 | 5.89287634319353 | 6.42917524612324 | 0.114042531568056 | 362.446306452756 | 0.999950574162623 |
| ebi-fl187-GCST90094750 | prot-b-28 | prolactin \|\| id:prot-b-28 | Curry liking \|\| id:ebi-fl187-GCST90094750 | MR Egger | 5 | 0.0873816596782538 | 1.77084521519615 | 0.963746136505693 | -3.383474962 | 3.55823828146271 | 1.09131311045642 | 0.0339293464493419 | 35.1013040240029 | 0.999950574162623 |
| ebi-fl187-GCST90094750 | prot-b-54 | matrix metallopeptidase 7 \|\| id:prot-b-54 | Curry liking \|\| id:ebi-fl187-GCST90094750 | MR Egger | 5 | 3.41974937410633 | 1.76968592744153 | 0.148814995459423 | -0.048835044 | 6.88833379189174 | 30.561754494095 | 0.952338210949939 | 980.765894949937 | 0.999950574162623 |
| ebi-fl187-GCST90094750 | ukb-rsfMRI191-edge-pheno716 | Net100_Pair10_33 (OccipitalPrecuneus)&(Temporal\|Frontal\|Supp_Motor_Area) (Default_mode\|Central_executive)&(Default_mode\|Salience) \|\| id:ukb-rsfMRI191-edge-pheno716 | Curry liking \|\| id:ebi-fl187-GCST90094750 | MR Egger | 7 | -0.239661929 | 0.306717575984624 | 0.469943434672449 | -0.840828377 | 0.361504520384607 | 0.786893842449709 | 0.431353052236682 | 1.4354875109253 | 0.999950574162623 |
| ebi-fl187-GCST90094750 | ukb-rsfMRI191-edge-pheno903 | Net100_Pair27_38 (Precentral\|Frontal\|Supp_Motor_Area)&(Subcortical) (Attention\|Salience\|Motor)&(Subcortical-cerebellum) \|\| id:ukb-rsfMRI191-edge-pheno903 | Curry liking \|\| id:ebi-fl187-GCST90094750 | MR Egger | 7 | -0.164746225 | 0.404023111079614 | 0.700319567269518 | -0.956631523 | 0.627139072794166 | 0.848108905684157 | 0.384184826708579 | 1.8722465487852 | 0.999950574162623 |
| ebi-fl187-GCST90094750 | ukb-rsfMRI191-node-pheno23 | Net100_Node2 RolandicOper\|SupraMarginal\|Insula Salience\|Motor \|\| id:ukb-rsfMRI191-node-pheno23 | Curry liking \|\| id:ebi-fl187-GCST90094750 | MR Egger | 7 | -0.230900749 | 0.398896493959427 | 0.587787477160066 | -1.012737877 | 0.550936379218648 | 0.793818249426762 | 0.363223156609627 | 1.73487676007457 | 0.999950574162623 |
| ebi-fl187-GCST90094750 | ukb-rsfMRI191-node-pheno55 | Net100_Node34 Parietal\|Postcentral\|Precuneus Attention \|\| id:ukb-rsfMRI191-node-pheno55 | Curry liking \|\| id:ebi-fl187-GCST90094750 | MR Egger | 7 | -0.330055371 | 0.385650881194149 | 0.431179017051546 | -1.085931098 | 0.425820356452383 | 0.718883927232139 | 0.337587312700933 | 1.53084574386991 | 0.999950574162623 |
| ebi-fl187-GCST90094750 | ebi-a-GCST90001414 | IgD- CD24- B cell Absolute Count \|\| id:ebi-a-GCST90001414 | Curry liking \|\| id:ebi-fl187-GCST90094750 | Weighted median | 6 | -0.09158994 | 0.265532655960729 | 0.730147674396986 | -0.612033945 | 0.428854066136779 | 0.912479244492111 | 0.542246846308046 | 1.53549693704606 | 0.999332001504091 |
| ebi-fl187-GCST90094750 | ebi-a-GCST90001422 | CD20- CD38- B cell %B cell \|\| id:ebi-a-GCST90001422 | Curry liking \|\| id:ebi-fl187-GCST90094750 | Weighted median | 6 | 0.0505802610169589 | 0.253662928755362 | 0.841950252020512 | -0.446599079 | 0.547759601377468 | 1.05188128502873 | 0.639800366044508 | 1.72937418687991 | 0.999332001504091 |
| ebi-fl187-GCST90094750 | ebi-a-GCST90001444 | CD20- CD38- B cell %lymphocyte \|\| id:ebi-a-GCST90001444 | Curry liking \|\| id:ebi-fl187-GCST90094750 | Weighted median | 6 | 0.0132040450795146 | 0.245861181104633 | 0.957169971159412 | -0.46868387 | 0.495091960044595 | 1.01329160343315 | 0.625825394141124 | 1.64064910628504 | 0.999332001504091 |
| ebi-fl187-GCST90094750 | ebi-a-GCST90001459 | Myeloid Dendritic Cell %Dendritic Cell \|\| id:ebi-a-GCST90001459 | Curry liking \|\| id:ebi-fl187-GCST90094750 | Weighted median | 6 | 0.365033660773105 | 0.270397097002908 | 0.177018853904133 | -0.164944649 | 0.895011970898804 | 1.44056249778049 | 0.847940636852235 | 2.44736508644674 | 0.999332001504091 |
| ebi-fl187-GCST90094750 | ebi-a-GCST90001476 | HLA DR++ monocyte %leukocyte \|\| id:ebi-a-GCST90001476 | Curry liking \|\| id:ebi-fl187-GCST90094750 | Weighted median | 6 | -0.086894428 | 0.27399298776644 | 0.75113656547545 | -0.623920684 | 0.450131827695377 | 0.916773875876417 | 0.535839456437308 | 1.5685189460993 | 0.999332001504091 |
| ebi-fl187-GCST90094750 | ebi-a-GCST90001478 | CD4 regulatory T cell %CD4+ T cell \|\| id:ebi-a-GCST90001478 | Curry liking \|\| id:ebi-fl187-GCST90094750 | Weighted median | 6 | -0.105471864 | 0.260224023664491 | 0.68524834342148 | -0.61551095 | 0.404567222328665 | 0.899899792022782 | 0.540364725168698 | 1.49865377579898 | 0.999332001504091 |
| ebi-fl187-GCST90094750 | ebi-a-GCST90001485 | CD39+ resting CD4 regulatory T cell %CD4 regulatory T cell \|\| id:ebi-a-GCST90001485 | Curry liking \|\| id:ebi-fl187-GCST90094750 | Weighted median | 6 | 0.0891563840304949 | 0.265107308422147 | 0.736642385356218 | -0.43045394 | 0.608766708537903 | 1.09325161005189 | 0.650213869327069 | 1.83816300953101 | 0.999332001504091 |
| ebi-fl187-GCST90094750 | ebi-a-GCST90001515 | Immature Myeloid-Derived Suppressor Cells Absolute Count \|\| id:ebi-a-GCST90001515 | Curry liking \|\| id:ebi-fl187-GCST90094750 | Weighted median | 6 | 0.163897312850275 | 0.385594191244102 | 0.670799277167705 | -0.591867302 | 0.919661927688716 | 1.17809333383466 | 0.553293154111198 | 2.50844221171535 | 0.999332001504091 |
| ebi-fl187-GCST90094750 | ebi-a-GCST90001517 | CD33+ HLA DR+ Absolute Count \|\| id:ebi-a-GCST90001517 | Curry liking \|\| id:ebi-fl187-GCST90094750 | Weighted median | 6 | 0.280366043101496 | 0.336867870749415 | 0.405254850874658 | -0.379894984 | 0.94062706977035 | 1.32361422352991 | 0.683933229669071 | 2.56158720870805 | 0.999332001504091 |
| ebi-fl187-GCST90094750 | ebi-a-GCST90001518 | CD33+ HLA DR+ CD14- Absolute Count \|\| id:ebi-a-GCST90001518 | Curry liking \|\| id:ebi-fl187-GCST90094750 | Weighted median | 6 | 0.151806304058698 | 0.354849289971056 | 0.66879308906441 | -0.543698304 | 0.847310912401967 | 1.16393476515643 | 0.580597052363428 | 2.3333637882332 | 0.999332001504091 |
| ebi-fl187-GCST90094750 | ebi-a-GCST90001520 | CD33+ HLA DR+ CD14dim Absolute Count \|\| id:ebi-a-GCST90001520 | Curry liking \|\| id:ebi-fl187-GCST90094750 | Weighted median | 6 | 0.15452326973772 | 0.348140253462417 | 0.6571485059723 | -0.527831627 | 0.836878166524057 | 1.16710143588582 | 0.589882669537935 | 2.30914694054959 | 0.999332001504091 |
| ebi-fl187-GCST90094750 | ebi-a-GCST90001531 | CD33dim HLA DR- Absolute Count \|\| id:ebi-a-GCST90001531 | Curry liking \|\| id:ebi-fl187-GCST90094750 | Weighted median | 6 | -0.072378241 | 0.382736172387473 | 0.850008644842796 | -0.822541139 | 0.677784656932873 | 0.930178997479733 | 0.439313877343492 | 1.96950975595039 | 0.999332001504091 |
| ebi-fl187-GCST90094750 | ebi-a-GCST90001532 | Basophil Absolute Count \|\| id:ebi-a-GCST90001532 | Curry liking \|\| id:ebi-fl187-GCST90094750 | Weighted median | 6 | -0.073656121 | 0.362767003372335 | 0.839103965268521 | -0.784679447 | 0.63736720588296 | 0.928991099703771 | 0.456265935607447 | 1.89149440266637 | 0.999332001504091 |
| ebi-fl187-GCST90094750 | ebi-a-GCST90001588 | T/B cell \|\| id:ebi-a-GCST90001588 | Curry liking \|\| id:ebi-fl187-GCST90094750 | Weighted median | 6 | 0.0236465194620576 | 0.275599945431646 | 0.931625279148995 | -0.516529374 | 0.563822412508083 | 1.02392831518227 | 0.596587491408365 | 1.75737709846543 | 0.999332001504091 |
| ebi-fl187-GCST90094750 | ebi-a-GCST90001624 | HLA DR+ CD4+ T cell Absolute Count \|\| id:ebi-a-GCST90001624 | Curry liking \|\| id:ebi-fl187-GCST90094750 | Weighted median | 6 | 0.216018571207869 | 0.247147890984336 | 0.382093322953128 | -0.268391295 | 0.700428437537168 | 1.24112542798496 | 0.764608534970369 | 2.01461565956827 | 0.999332001504091 |
| ebi-fl187-GCST90094750 | ebi-a-GCST90001631 | CD8+ Natural Killer T %T cell \|\| id:ebi-a-GCST90001631 | Curry liking \|\| id:ebi-fl187-GCST90094750 | Weighted median | 6 | -0.276285379 | 0.248412946650856 | 0.266051303868204 | -0.763174754 | 0.210603996844164 | 0.758596412692119 | 0.46618405546497 | 1.23442342268738 | 0.999332001504091 |
| ebi-fl187-GCST90094750 | ebi-a-GCST90001632 | CD8+ Natural Killer T %lymphocyte \|\| id:ebi-a-GCST90001632 | Curry liking \|\| id:ebi-fl187-GCST90094750 | Weighted median | 6 | -0.185409741 | 0.248670451997645 | 0.455906526945078 | -0.672803827 | 0.301984345048832 | 0.830763816219179 | 0.510275845077381 | 1.35254005259527 | 0.999332001504091 |
| ebi-fl187-GCST90094750 | ebi-a-GCST90001646 | Natural Killer %CD3- lymphocyte \|\| id:ebi-a-GCST90001646 | Curry liking \|\| id:ebi-fl187-GCST90094750 | Weighted median | 6 | -0.190567363 | 0.260675647461671 | 0.464747603198703 | -0.701491632 | 0.320356905946711 | 0.826490080937874 | 0.49584513337859 | 1.37761935714541 | 0.999332001504091 |
| ebi-fl187-GCST90094750 | ebi-a-GCST90001684 | CD127- CD8+ T cell Absolute Count \|\| id:ebi-a-GCST90001684 | Curry liking \|\| id:ebi-fl187-GCST90094750 | Weighted median | 6 | 0.168155038957087 | 0.276401479495121 | 0.542940494492886 | -0.373591861 | 0.709901938767524 | 1.18312002612413 | 0.688257759447244 | 2.0337918127362 | 0.999332001504091 |
| ebi-fl187-GCST90094750 | ebi-a-GCST90001687 | CD28- CD8+ T cell Absolute Count \|\| id:ebi-a-GCST90001687 | Curry liking \|\| id:ebi-fl187-GCST90094750 | Weighted median | 6 | 0.190464677735405 | 0.272478532930046 | 0.48454702351967 | -0.343593247 | 0.724522602278294 | 1.2098116395959 | 0.709217345807707 | 2.06374563729096 | 0.999332001504091 |
| ebi-fl187-GCST90094750 | ebi-a-GCST90001701 | BAFF-R on CD20- CD38- B cell \|\| id:ebi-a-GCST90001701 | Curry liking \|\| id:ebi-fl187-GCST90094750 | Weighted median | 6 | -0.288836355 | 0.250924580091545 | 0.249695899923646 | -0.780648532 | 0.202975821982525 | 0.749134787537045 | 0.458108816729258 | 1.22504284878202 | 0.999332001504091 |
| ebi-fl187-GCST90094750 | ebi-a-GCST90001732 | CD19 on IgD- CD27- B cell \|\| id:ebi-a-GCST90001732 | Curry liking \|\| id:ebi-fl187-GCST90094750 | Weighted median | 6 | 0.153939495421603 | 0.285382967752903 | 0.589601398901667 | -0.405411121 | 0.713290112217292 | 1.16642031087424 | 0.666702658794259 | 2.04069433903338 | 0.999332001504091 |
| ebi-fl187-GCST90094750 | ebi-a-GCST90001798 | CD27 on CD24+ CD27+ B cell \|\| id:ebi-a-GCST90001798 | Curry liking \|\| id:ebi-fl187-GCST90094750 | Weighted median | 6 | 0.176222433899188 | 0.248024745954501 | 0.477392001748183 | -0.309906068 | 0.662350935970009 | 1.19270332679882 | 0.733515853473669 | 1.93934625818967 | 0.999332001504091 |
| ebi-fl187-GCST90094750 | ebi-a-GCST90001836 | CD66b on CD66b++ myeloid cell \|\| id:ebi-a-GCST90001836 | Curry liking \|\| id:ebi-fl187-GCST90094750 | Weighted median | 6 | -0.398424906 | 0.454454178738064 | 0.380643716451262 | -1.289155096 | 0.492305284729328 | 0.671376695330922 | 0.275503458776897 | 1.63608351428533 | 0.999332001504091 |
| ebi-fl187-GCST90094750 | ebi-a-GCST90001943 | CD25 on activated & secreting CD4 regulatory T cell \|\| id:ebi-a-GCST90001943 | Curry liking \|\| id:ebi-fl187-GCST90094750 | Weighted median | 6 | -0.210398576 | 0.264390733768115 | 0.426156141515185 | -0.728604414 | 0.307807262345397 | 0.810261231050619 | 0.482582005033578 | 1.36043875589183 | 0.999332001504091 |
| ebi-fl187-GCST90094750 | ebi-a-GCST90001960 | CD25 on CD4+ T cell \|\| id:ebi-a-GCST90001960 | Curry liking \|\| id:ebi-fl187-GCST90094750 | Weighted median | 6 | 0.209202774488607 | 0.268463408514416 | 0.435826618880587 | -0.316985506 | 0.735391055176863 | 1.23269493227381 | 0.728341311482757 | 2.08629769051552 | 0.999332001504091 |
| ebi-fl187-GCST90094750 | ebi-a-GCST90001988 | HLA DR on CD14+ CD16- monocyte \|\| id:ebi-a-GCST90001988 | Curry liking \|\| id:ebi-fl187-GCST90094750 | Weighted median | 6 | -0.112281074 | 0.272034042408893 | 0.679792420297894 | -0.645467797 | 0.420905649467207 | 0.893793000526376 | 0.524417164059093 | 1.52334054363622 | 0.999332001504091 |
| ebi-fl187-GCST90094750 | ebi-a-GCST90001991 | HLA DR on CD14+ monocyte \|\| id:ebi-a-GCST90001991 | Curry liking \|\| id:ebi-fl187-GCST90094750 | Weighted median | 6 | -0.146241372 | 0.284532453683041 | 0.607272407790416 | -0.703924981 | 0.411442237096852 | 0.863949144777266 | 0.494640035807561 | 1.50899254150094 | 0.999332001504091 |
| ebi-fl187-GCST90094750 | ebi-a-GCST90002019 | CD14 on Monocytic Myeloid-Derived Suppressor Cells \|\| id:ebi-a-GCST90002019 | Curry liking \|\| id:ebi-fl187-GCST90094750 | Weighted median | 6 | 0.126645447146255 | 0.369007508984632 | 0.731443784692322 | -0.59660927 | 0.849900164756134 | 1.13501452379455 | 0.550675666343746 | 2.3394132843713 | 0.999332001504091 |
| ebi-fl187-GCST90094750 | ebi-a-GCST90002037 | CD80 on plasmacytoid Dendritic Cell \|\| id:ebi-a-GCST90002037 | Curry liking \|\| id:ebi-fl187-GCST90094750 | Weighted median | 6 | 0.0476239453862689 | 0.278389780370861 | 0.864169273554149 | -0.498020024 | 0.593267914913156 | 1.04877618403497 | 0.607732765454295 | 1.80989333918292 | 0.999332001504091 |
| ebi-fl187-GCST90094750 | ebi-a-GCST90002042 | CD45 on CD33+ HLA DR+ CD14- \|\| id:ebi-a-GCST90002042 | Curry liking \|\| id:ebi-fl187-GCST90094750 | Weighted median | 6 | 0.00941894484447359 | 0.397120754597233 | 0.981077478247296 | -0.768937734 | 0.78777562385505 | 1.00946344270336 | 0.463505172600855 | 2.19850069080469 | 0.999332001504091 |
| ebi-fl187-GCST90094750 | ebi-a-GCST90002056 | CD8 on Effector Memory CD8+ T cell \|\| id:ebi-a-GCST90002056 | Curry liking \|\| id:ebi-fl187-GCST90094750 | Weighted median | 6 | -0.027508194 | 0.297826706858031 | 0.926409565757872 | -0.61124854 | 0.556232151190061 | 0.972866710606114 | 0.54267289727243 | 1.74408864227914 | 0.999332001504091 |
| ebi-fl187-GCST90094750 | ebi-a-GCST90002058 | CD8 on CD8+ T cell \|\| id:ebi-a-GCST90002058 | Curry liking \|\| id:ebi-fl187-GCST90094750 | Weighted median | 6 | -0.06758085 | 0.338031563861638 | 0.841539492420644 | -0.730122716 | 0.59496101474216 | 0.934652150535991 | 0.481849855969939 | 1.81296026485904 | 0.999332001504091 |
| ebi-fl187-GCST90094750 | ebi-a-GCST90002063 | CD4 on CD4 regulatory T cell \|\| id:ebi-a-GCST90002063 | Curry liking \|\| id:ebi-fl187-GCST90094750 | Weighted median | 6 | -0.738180499 | 0.305384530321384 | 0.0156397155195064 | -1.336734178 | -0.139626819 | 0.477982815157348 | 0.262702207784765 | 0.869682723690427 | 0.999332001504091 |
| ebi-fl187-GCST90094750 | ebi-a-GCST90002066 | CD4 on activated CD4 regulatory T cell \|\| id:ebi-a-GCST90002066 | Curry liking \|\| id:ebi-fl187-GCST90094750 | Weighted median | 6 | -0.679351577 | 0.334967536561137 | 0.0425488501177308 | -1.335887948 | -0.022815205 | 0.506945601219363 | 0.262924608332324 | 0.977443093766382 | 0.999332001504091 |
| ebi-fl187-GCST90094750 | ebi-a-GCST90002070 | CD4 on activated & secreting CD4 regulatory T cell \|\| id:ebi-a-GCST90002070 | Curry liking \|\| id:ebi-fl187-GCST90094750 | Weighted median | 6 | -0.680641626 | 0.313522269762786 | 0.0299348425249838 | -1.295145275 | -0.066137977 | 0.506292037891745 | 0.273858076727685 | 0.936001708240518 | 0.999332001504091 |
| ebi-fl187-GCST90094750 | ebi-a-GCST90002081 | SSC-A on CD4+ T cell \|\| id:ebi-a-GCST90002081 | Curry liking \|\| id:ebi-fl187-GCST90094750 | Weighted median | 6 | 0.196005154386527 | 0.265266634763955 | 0.459968551163987 | -0.32391745 | 0.715927758523879 | 1.21653317580037 | 0.723309949331945 | 2.04608407390196 | 0.999332001504091 |
| ebi-fl187-GCST90094750 | ebi-a-GCST90002094 | CD11b on Monocytic Myeloid-Derived Suppressor Cells \|\| id:ebi-a-GCST90002094 | Curry liking \|\| id:ebi-fl187-GCST90094750 | Weighted median | 6 | 0.360231108730881 | 0.3926158683565 | 0.358872622545609 | -0.409295993 | 1.12975821070962 | 1.43366070778318 | 0.664117628893072 | 3.09490809401823 | 0.999332001504091 |
| ebi-fl187-GCST90094750 | ebi-a-GCST90002104 | HLA DR on myeloid Dendritic Cell \|\| id:ebi-a-GCST90002104 | Curry liking \|\| id:ebi-fl187-GCST90094750 | Weighted median | 6 | 0.0129115328223839 | 0.319087310941393 | 0.967723247654843 | -0.612499597 | 0.638322662267513 | 1.01299524656522 | 0.541994407087359 | 1.89330250671448 | 0.999332001504091 |
| ebi-fl187-GCST90094750 | ebi-a-GCST90002105 | HLA DR on plasmacytoid Dendritic Cell \|\| id:ebi-a-GCST90002105 | Curry liking \|\| id:ebi-fl187-GCST90094750 | Weighted median | 5 | -0.205048657 | 0.345980894786617 | 0.553409451665567 | -0.883171211 | 0.473073896904306 | 0.814607679181084 | 0.413469631136013 | 1.60491997721231 | 0.999332001504091 |
| ebi-fl187-GCST90094750 | ebi-a-GCST90002106 | HLA DR on Dendritic Cell \|\| id:ebi-a-GCST90002106 | Curry liking \|\| id:ebi-fl187-GCST90094750 | Weighted median | 6 | -0.014901834 | 0.346819423275346 | 0.965727739816355 | -0.694667903 | 0.664864235988485 | 0.985208649211779 | 0.499240216510955 | 1.94422654742279 | 0.999332001504091 |
| ebi-fl187-GCST90094750 | ebi-a-GCST90002111 | HLA DR on CD33dim HLA DR+ CD11b- \|\| id:ebi-a-GCST90002111 | Curry liking \|\| id:ebi-fl187-GCST90094750 | Weighted median | 6 | -0.109604063 | 0.478925878700807 | 0.818982459394048 | -1.048298785 | 0.829090659251359 | 0.896188899402046 | 0.350533575039721 | 2.29123427997002 | 0.999332001504091 |
| ebi-fl187-GCST90094750 | ebi-a-GCST90002112 | HLA DR on CD33- HLA DR+ \|\| id:ebi-a-GCST90002112 | Curry liking \|\| id:ebi-fl187-GCST90094750 | Weighted median | 6 | 0.0828213936753182 | 0.39643756334259 | 0.834515302878613 | -0.69419623 | 0.859839017826795 | 1.08634776263863 | 0.499475750072143 | 2.36278029758101 | 0.999332001504091 |
| ebi-fl187-GCST90094750 | ebi-a-GCST90002115 | HLA DR on HLA DR+ CD8+ T cell \|\| id:ebi-a-GCST90002115 | Curry liking \|\| id:ebi-fl187-GCST90094750 | Weighted median | 6 | -0.490824546 | 0.264751985083076 | 0.0637530136069872 | -1.009738436 | 0.0280893450414356 | 0.612121463909935 | 0.364314258428526 | 1.02848757058119 | 0.999332001504091 |
| ebi-fl187-GCST90094750 | ebi-a-GCST90002116 | HLA DR on B cell \|\| id:ebi-a-GCST90002116 | Curry liking \|\| id:ebi-fl187-GCST90094750 | Weighted median | 6 | -0.029407604 | 0.273246805907282 | 0.914294860216808 | -0.564971343 | 0.506156136000686 | 0.971020592325293 | 0.568376434399057 | 1.65890232890579 | 0.999332001504091 |
| ebi-fl187-GCST90094750 | ebi-cfmet338-GCST90026023 | Sphingomyelin (d18:1/24:1, d18:2/24:0) levels \|\| id:ebi-cfmet338-GCST90026023 | Curry liking \|\| id:ebi-fl187-GCST90094750 | Weighted median | 6 | 0.209105933590312 | 0.172011748704216 | 0.22411860809017 | -0.128037094 | 0.546248961050575 | 1.23257556276926 | 0.879820742583133 | 1.72676369674519 | 0.999332001504091 |
| ebi-fl187-GCST90094750 | ebi-cfmet338-GCST90026046 | 1-arachidonoyl-gpc (20:4n6) levels \|\| id:ebi-cfmet338-GCST90026046 | Curry liking \|\| id:ebi-fl187-GCST90094750 | Weighted median | 6 | 0.0569326394932364 | 0.182073900741386 | 0.754516396235966 | -0.299932206 | 0.413797484946352 | 1.05858450120536 | 0.740868445444343 | 1.512550782 | 0.999332001504091 |
| ebi-fl187-GCST90094750 | ebi-cfmet338-GCST90026047 | 1-linoleoyl-gpc (18:2) levels \|\| id:ebi-cfmet338-GCST90026047 | Curry liking \|\| id:ebi-fl187-GCST90094750 | Weighted median | 6 | 0.0186391295436026 | 0.238409860404053 | 0.937684048037749 | -0.448644197 | 0.485922455935547 | 1.01881392242547 | 0.638493236189003 | 1.62567392995956 | 0.999332001504091 |
| ebi-fl187-GCST90094750 | ebi-cfmet338-GCST90026050 | 1-myristoyl-2-palmitoyl-gpc (14:0/16:0) levels \|\| id:ebi-cfmet338-GCST90026050 | Curry liking \|\| id:ebi-fl187-GCST90094750 | Weighted median | 6 | -0.038670159 | 0.115848258217008 | 0.738530413875427 | -0.265732745 | 0.188392426835862 | 0.962067986025398 | 0.766643989276681 | 1.20730720214507 | 0.999332001504091 |
| ebi-fl187-GCST90094750 | ebi-cfmet338-GCST90026068 | 2,3-dihydroxyisovalerate levels \|\| id:ebi-cfmet338-GCST90026068 | Curry liking \|\| id:ebi-fl187-GCST90094750 | Weighted median | 6 | 0.25426855777306 | 0.37425060566054 | 0.496879886251303 | -0.479262629 | 0.987799744867718 | 1.28951806787046 | 0.619239832797961 | 2.6853195794123 | 0.999332001504091 |
| ebi-fl187-GCST90094750 | ebi-cfmet338-GCST90026082 | 3-hydroxyhexanoate levels \|\| id:ebi-cfmet338-GCST90026082 | Curry liking \|\| id:ebi-fl187-GCST90094750 | Weighted median | 6 | 0.0186599131310136 | 0.102773536516504 | 0.855925370799379 | -0.182776218 | 0.220096044703362 | 1.01883509725373 | 0.832954534746304 | 1.24619641540479 | 0.999332001504091 |
| ebi-fl187-GCST90094750 | ebi-cfmet338-GCST90026094 | 3-ureidopropionate levels \|\| id:ebi-cfmet338-GCST90026094 | Curry liking \|\| id:ebi-fl187-GCST90094750 | Weighted median | 6 | 0.116224984559625 | 0.210265344836001 | 0.580431955298132 | -0.295895091 | 0.528345060438186 | 1.12324855728907 | 0.743865461871387 | 1.69612300358438 | 0.999332001504091 |
| ebi-fl187-GCST90094750 | ebi-cfmet338-GCST90026173 | Glycerol levels \|\| id:ebi-cfmet338-GCST90026173 | Curry liking \|\| id:ebi-fl187-GCST90094750 | Weighted median | 6 | 0.0112119405068235 | 0.109597719438636 | 0.918517875937895 | -0.20359959 | 0.22602347060655 | 1.01127502987607 | 0.815788956186965 | 1.25360508780469 | 0.999332001504091 |
| ebi-fl187-GCST90094750 | ebi-cfmet338-GCST90026179 | Histidine levels \|\| id:ebi-cfmet338-GCST90026179 | Curry liking \|\| id:ebi-fl187-GCST90094750 | Weighted median | 6 | -0.013117094 | 0.085141734501511 | 0.877560962685224 | -0.179994893 | 0.153760705878105 | 0.986968560409094 | 0.835274476839906 | 1.16621178576094 | 0.999332001504091 |
| ebi-fl187-GCST90094750 | ebi-cfmet338-GCST90026188 | Isocitrate levels \|\| id:ebi-cfmet338-GCST90026188 | Curry liking \|\| id:ebi-fl187-GCST90094750 | Weighted median | 6 | -0.265617893 | 0.150099664513964 | 0.076792103384272 | -0.559813235 | 0.0285774495064892 | 0.76673204526143 | 0.57131575545065 | 1.02898970249308 | 0.999332001504091 |
| ebi-fl187-GCST90094750 | ebi-cfmet338-GCST90026207 | N-acetyl-isoputreanine levels \|\| id:ebi-cfmet338-GCST90026207 | Curry liking \|\| id:ebi-fl187-GCST90094750 | Weighted median | 6 | 0.0224711308792287 | 0.0899119781281326 | 0.802646383727844 | -0.153756346 | 0.198698608010369 | 1.02272550855204 | 0.857480924001473 | 1.21981426824281 | 0.999332001504091 |
| ebi-fl187-GCST90094750 | ebi-cfmet338-GCST90026213 | N-acetylglutamate levels \|\| id:ebi-cfmet338-GCST90026213 | Curry liking \|\| id:ebi-fl187-GCST90094750 | Weighted median | 6 | -0.147005809 | 0.167294161891624 | 0.379549599816403 | -0.474902367 | 0.180890748028613 | 0.863288962315338 | 0.621945776189942 | 1.1982842572242 | 0.999332001504091 |
| ebi-fl187-GCST90094750 | ebi-cfmet338-GCST90026224 | N-acetyltaurine levels \|\| id:ebi-cfmet338-GCST90026224 | Curry liking \|\| id:ebi-fl187-GCST90094750 | Weighted median | 6 | 0.23956323182613 | 0.131337616773093 | 0.0681481369673358 | -0.017858497 | 0.496984960701393 | 1.27069403038967 | 0.982300020876017 | 1.64375779756981 | 0.999332001504091 |
| ebi-fl187-GCST90094750 | ebi-cfmet338-GCST90026250 | Phenyllactate (pla) levels \|\| id:ebi-cfmet338-GCST90026250 | Curry liking \|\| id:ebi-fl187-GCST90094750 | Weighted median | 6 | 0.184260751543798 | 0.201596959271836 | 0.360713898395612 | -0.210869289 | 0.579390791716597 | 1.20232929144475 | 0.809879920477741 | 1.78495069270674 | 0.999332001504091 |
| ebi-fl187-GCST90094750 | ebi-cfmet338-GCST90026251 | Pipecolate levels \|\| id:ebi-cfmet338-GCST90026251 | Curry liking \|\| id:ebi-fl187-GCST90094750 | Weighted median | 6 | 0.417526873605309 | 0.311501677383165 | 0.180125740899572 | -0.193016414 | 1.02807016127631 | 1.51820220291705 | 0.824468441148887 | 2.7956654420032 | 0.999332001504091 |
| ebi-fl187-GCST90094750 | ebi-cfmet338-GCST90026278 | Trigonelline (n'-methylnicotinate) levels \|\| id:ebi-cfmet338-GCST90026278 | Curry liking \|\| id:ebi-fl187-GCST90094750 | Weighted median | 6 | 0.37536559906597 | 0.333645754764321 | 0.260571095025108 | -0.27858008 | 1.02931127840404 | 1.45552345537126 | 0.756857655956005 | 2.79913734433972 | 0.999332001504091 |
| ebi-fl187-GCST90094750 | ebi-cfmet338-GCST90026299 | 1-carboxyethylvaline levels \|\| id:ebi-cfmet338-GCST90026299 | Curry liking \|\| id:ebi-fl187-GCST90094750 | Weighted median | 6 | -0.063338434 | 0.19043514545289 | 0.739437414414246 | -0.436591319 | 0.309914451104159 | 0.938625757075128 | 0.646235481567668 | 1.36330847960815 | 0.999332001504091 |
| ebi-fl187-GCST90094750 | ebi-gm430-GCST90011306 | C_Erysipelotrichia abundance \|\| id:ebi-gm430-GCST90011306 | Curry liking \|\| id:ebi-fl187-GCST90094750 | Weighted median | 5 | -0.333262078 | 0.176699909880994 | 0.0592903427628439 | -0.679593901 | 0.01306974575109 | 0.716582369345654 | 0.506822770808869 | 1.01315552818892 | 0.999332001504091 |
| ebi-fl187-GCST90094750 | ebi-gm430-GCST90011312 | F_Erysipelotrichaceae abundance \|\| id:ebi-gm430-GCST90011312 | Curry liking \|\| id:ebi-fl187-GCST90094750 | Weighted median | 5 | -0.333262078 | 0.167559386510415 | 0.0467101243700047 | -0.661678475 | -0.00484568 | 0.716582369345654 | 0.515984540007048 | 0.995166041312047 | 0.999332001504091 |
| ebi-fl187-GCST90094750 | ebi-gm430-GCST90011352 | O_Erysipelotrichales abundance \|\| id:ebi-gm430-GCST90011352 | Curry liking \|\| id:ebi-fl187-GCST90094750 | Weighted median | 5 | -0.333262078 | 0.177460017499815 | 0.0603872053691173 | -0.681083712 | 0.0145595566839786 | 0.716582369345654 | 0.506068262880516 | 1.014666063 | 0.999332001504091 |
| ebi-fl187-GCST90094750 | ebi-gm430-GCST90011362 | OTU97_11 (Parabacteroides) abundance \|\| id:ebi-gm430-GCST90011362 | Curry liking \|\| id:ebi-fl187-GCST90094750 | Weighted median | 5 | -0.10926355 | 0.22183822855736 | 0.622339727049067 | -0.544066478 | 0.325539378224443 | 0.896494115562721 | 0.580383331297445 | 1.38477736333659 | 0.999332001504091 |
| ebi-fl187-GCST90094750 | ebi-gm430-GCST90011384 | OTU97_235 (Phascolarctobacterium) abundance \|\| id:ebi-gm430-GCST90011384 | Curry liking \|\| id:ebi-fl187-GCST90094750 | Weighted median | 5 | 0.770832361450635 | 0.682515869858222 | 0.258729788934816 | -0.566898743 | 2.10856346637275 | 2.16156470833523 | 0.567282000524909 | 8.2364009152362 | 0.999332001504091 |
| ebi-fl187-GCST90094750 | ebi-gm430-GCST90011390 | OTU97_3 (Bacteroides) abundance \|\| id:ebi-gm430-GCST90011390 | Curry liking \|\| id:ebi-fl187-GCST90094750 | Weighted median | 5 | -0.328023156 | 0.201414433048414 | 0.103398134618995 | -0.722795444 | 0.0667491332734295 | 0.720346339531464 | 0.485393467245967 | 1.06902726116302 | 0.999332001504091 |
| ebi-fl187-GCST90094750 | ebi-gm430-GCST90011398 | OTU97_4 (Alistipes) abundance \|\| id:ebi-gm430-GCST90011398 | Curry liking \|\| id:ebi-fl187-GCST90094750 | Weighted median | 5 | -0.242510986 | 0.219343384393917 | 0.268889936246087 | -0.67242402 | 0.18740204697562 | 0.784655126967675 | 0.510469688190438 | 1.20611210130653 | 0.999332001504091 |
| ebi-fl187-GCST90094750 | ebi-gm430-GCST90011421 | OTU99_108 (Cytophagales) abundance \|\| id:ebi-gm430-GCST90011421 | Curry liking \|\| id:ebi-fl187-GCST90094750 | Weighted median | 5 | 0.571021257702872 | 0.650795273943679 | 0.380258163099636 | -0.704537479 | 1.84657999463248 | 1.77007383021709 | 0.494337162595334 | 6.33810605694682 | 0.999332001504091 |
| ebi-fl187-GCST90094750 | ebi-gm430-GCST90011428 | OTU99_123 (Phascolarctobacterium) abundance \|\| id:ebi-gm430-GCST90011428 | Curry liking \|\| id:ebi-fl187-GCST90094750 | Weighted median | 5 | -0.327799938 | 0.3993332801284 | 0.411721421425967 | -1.110493167 | 0.454893290898985 | 0.720507151278898 | 0.32939647347376 | 1.57600519996274 | 0.999332001504091 |
| ebi-fl187-GCST90094750 | ebi-gm430-GCST90011450 | OTU99_298 (Phascolarctobacterium) abundance \|\| id:ebi-gm430-GCST90011450 | Curry liking \|\| id:ebi-fl187-GCST90094750 | Weighted median | 5 | 0.710753055328379 | 0.670041972512846 | 0.28879943755595 | -0.602529211 | 2.02403532145356 | 2.03552354347717 | 0.54742532964823 | 7.56880595699201 | 0.999332001504091 |
| ebi-fl187-GCST90094750 | ebi-gm430-GCST90011451 | OTU99_3 (Bacteroides) abundance \|\| id:ebi-gm430-GCST90011451 | Curry liking \|\| id:ebi-fl187-GCST90094750 | Weighted median | 5 | -0.313502997 | 0.207609429799519 | 0.131027744245607 | -0.72041748 | 0.0934114852284816 | 0.730882188415885 | 0.486549089241777 | 1.09791341748488 | 0.999332001504091 |
| ebi-fl187-GCST90094750 | ebi-gm430-GCST90011472 | OTU99_72 (Parabacteroides) abundance \|\| id:ebi-gm430-GCST90011472 | Curry liking \|\| id:ebi-fl187-GCST90094750 | Weighted median | 5 | -0.703295052 | 0.577007026139901 | 0.222894526109221 | -1.834228823 | 0.427638719588245 | 0.494951722414326 | 0.159736639523105 | 1.53363190970018 | 0.999332001504091 |
| ebi-fl187-GCST90094750 | ebi-gm430-GCST90011507 | TestASV_3 (Bacteroides) abundance \|\| id:ebi-gm430-GCST90011507 | Curry liking \|\| id:ebi-fl187-GCST90094750 | Weighted median | 5 | -0.175769772 | 0.233752623798015 | 0.452082449728205 | -0.633924914 | 0.282385370953661 | 0.838811079164948 | 0.530505520726601 | 1.32628973505532 | 0.999332001504091 |
| ebi-fl187-GCST90094750 | ebi-gm430-GCST90011512 | TestASV_34 (Phascolarctobacterium) abundance \|\| id:ebi-gm430-GCST90011512 | Curry liking \|\| id:ebi-fl187-GCST90094750 | Weighted median | 5 | -0.107628171 | 0.595766121146973 | 0.856638326888699 | -1.275329768 | 1.06007342677395 | 0.897961422753876 | 0.27933883598771 | 2.88658293395925 | 0.999332001504091 |
| ebi-fl187-GCST90094750 | ebi-gm430-GCST90011519 | TestASV_41 (Barnesiella) abundance \|\| id:ebi-gm430-GCST90011519 | Curry liking \|\| id:ebi-fl187-GCST90094750 | Weighted median | 5 | -0.993264678 | 0.739798742990223 | 0.179396763414162 | -2.443270214 | 0.456740858551016 | 0.370365590882862 | 0.0868762823823315 | 1.57891966769873 | 0.999332001504091 |
| ebi-fl187-GCST90094750 | ebi-gm430-GCST90011524 | TestASV_46 (Sutterella) abundance \|\| id:ebi-gm430-GCST90011524 | Curry liking \|\| id:ebi-fl187-GCST90094750 | Weighted median | 5 | -0.558553085 | 0.779268054848262 | 0.473518305541133 | -2.085918472 | 0.968812302869469 | 0.572036153240875 | 0.124192999955775 | 2.6348132401274 | 0.999332001504091 |
| ebi-fl187-GCST90094750 | ebi-gm430-GCST90011581 | OTU97_19 (Bacteria) prevalence \|\| id:ebi-gm430-GCST90011581 | Curry liking \|\| id:ebi-fl187-GCST90094750 | Weighted median | 5 | -0.012220275 | 0.894318823639775 | 0.989097774584942 | -1.765085169 | 1.74064461924566 | 0.987854089247547 | 0.171172207226101 | 5.70101722386565 | 0.999332001504091 |
| ebi-fl187-GCST90094750 | ebi-gm430-GCST90011614 | OTU97_80 (Ruminococcus) prevalence \|\| id:ebi-gm430-GCST90011614 | Curry liking \|\| id:ebi-fl187-GCST90094750 | Weighted median | 5 | 0.713193008718946 | 0.41217104670472 | 0.083570864001665 | -0.094662243 | 1.5210482602602 | 2.04049619009464 | 0.909680133580481 | 4.57702059008676 | 0.999332001504091 |
| ebi-fl187-GCST90094750 | ebi-gm430-GCST90011623 | OTU99_11 (Parabacteroides) prevalence \|\| id:ebi-gm430-GCST90011623 | Curry liking \|\| id:ebi-fl187-GCST90094750 | Weighted median | 5 | 0.0607133105471444 | 0.395453326297659 | 0.877981607620486 | -0.714375209 | 0.835801830090556 | 1.06259423595515 | 0.489497850128213 | 2.30666285866091 | 0.999332001504091 |
| ebi-fl187-GCST90094750 | ebi-gm430-GCST90011642 | OTU99_19 (Bacteria) prevalence \|\| id:ebi-gm430-GCST90011642 | Curry liking \|\| id:ebi-fl187-GCST90094750 | Weighted median | 5 | -0.012220275 | 0.921087160556752 | 0.989414592663469 | -1.81755111 | 1.79311055960294 | 0.987854089247547 | 0.162423020446982 | 6.00811202105207 | 0.999332001504091 |
| ebi-fl187-GCST90094750 | ebi-gm430-GCST90011653 | OTU99_30 (Parasutterella) prevalence \|\| id:ebi-gm430-GCST90011653 | Curry liking \|\| id:ebi-fl187-GCST90094750 | Weighted median | 5 | -0.424730568 | 0.392858075057067 | 0.279639383610832 | -1.194732395 | 0.34527125884366 | 0.653945955187096 | 0.302784969998382 | 1.4123729863733 | 0.999332001504091 |
| ebi-fl187-GCST90094750 | ebi-gm430-GCST90011675 | OTU99_8 (Ruminococcaceae) prevalence \|\| id:ebi-gm430-GCST90011675 | Curry liking \|\| id:ebi-fl187-GCST90094750 | Weighted median | 5 | 0.228504042106646 | 0.408820909077073 | 0.576206537021942 | -0.57278494 | 1.02979302389771 | 1.25671860491226 | 0.563952675504588 | 2.8004861410038 | 0.999332001504091 |
| ebi-fl187-GCST90094750 | ebi-gm430-GCST90011679 | OTU99_92 (Ruminococcus) prevalence \|\| id:ebi-gm430-GCST90011679 | Curry liking \|\| id:ebi-fl187-GCST90094750 | Weighted median | 5 | 0.390871000590068 | 0.426923456107207 | 0.359901454142562 | -0.445898973 | 1.22764097456019 | 1.47826780543818 | 0.640248450931369 | 3.41316828086986 | 0.999332001504091 |
| ebi-fl187-GCST90094750 | ebi-gm430-GCST90011684 | TestASV_11 (Lachnospiraceae) prevalence \|\| id:ebi-gm430-GCST90011684 | Curry liking \|\| id:ebi-fl187-GCST90094750 | Weighted median | 5 | 0.204265328995549 | 0.41567666276732 | 0.623140473362971 | -0.61046093 | 1.0189915880195 | 1.2266235690827 | 0.54310048005333 | 2.77039965068976 | 0.999332001504091 |
| ebi-fl187-GCST90094750 | ebi-gm430-GCST90011688 | TestASV_15 (Bacteroides) prevalence \|\| id:ebi-gm430-GCST90011688 | Curry liking \|\| id:ebi-fl187-GCST90094750 | Weighted median | 5 | -0.413931069 | 0.421107784046627 | 0.325628339324054 | -1.239302326 | 0.411440187639596 | 0.661046516232209 | 0.289586184280244 | 1.5089894488884 | 0.999332001504091 |
| ebi-fl187-GCST90094750 | ebi-gm430-GCST90011692 | TestASV_19 (Prevotella) prevalence \|\| id:ebi-gm430-GCST90011692 | Curry liking \|\| id:ebi-fl187-GCST90094750 | Weighted median | 5 | 0.51209447803809 | 0.759193670969194 | 0.499978126934395 | -0.975925117 | 2.00011407313771 | 1.66878276601377 | 0.376843567840084 | 7.38989903982212 | 0.999332001504091 |
| ebi-fl187-GCST90094750 | ebi-gm430-GCST90011693 | TestASV_2 (Bacteroides) prevalence \|\| id:ebi-gm430-GCST90011693 | Curry liking \|\| id:ebi-fl187-GCST90094750 | Weighted median | 5 | -0.330740867 | 0.398143444094132 | 0.406138714893905 | -1.111102018 | 0.449620283069633 | 0.718391303561497 | 0.329195981282563 | 1.56771678385043 | 0.999332001504091 |
| ebi-fl187-GCST90094750 | ebi-gm430-GCST90011716 | TestASV_40 (Barnesiella) prevalence \|\| id:ebi-gm430-GCST90011716 | Curry liking \|\| id:ebi-fl187-GCST90094750 | Weighted median | 5 | 0.557751861762594 | 0.812417938615677 | 0.492376955629952 | -1.034587298 | 2.15009102144932 | 1.74674116725287 | 0.355373013845536 | 8.58563983899487 | 0.999332001504091 |
| ebi-fl187-GCST90094750 | ebi-gm430-GCST90011718 | TestASV_42 (Alphaproteobacteria) prevalence \|\| id:ebi-gm430-GCST90011718 | Curry liking \|\| id:ebi-fl187-GCST90094750 | Weighted median | 5 | -0.052488537 | 0.687529664620511 | 0.939145684124084 | -1.40004668 | 1.29506960531126 | 0.948865197507922 | 0.246585453063714 | 3.65125011169703 | 0.999332001504091 |
| ebi-fl187-GCST90094750 | ebi-hbsm44-GCST90104929 | Sulcal depth of the right inferior frontal sulcus \|\| id:ebi-hbsm44-GCST90104929 | Curry liking \|\| id:ebi-fl187-GCST90094750 | Weighted median | 5 | 0.327486158313324 | 0.328345626759225 | 0.318578917542037 | -0.31607127 | 0.971043586761404 | 1.38747584615912 | 0.729007491853925 | 2.64069882022655 | 0.999332001504091 |
| ebi-fl187-GCST90094750 | ebi-hbsm44-GCST90104935 | Sulcal opening distance of the left calloso-marginal posterior fissure \|\| id:ebi-hbsm44-GCST90104935 | Curry liking \|\| id:ebi-fl187-GCST90094750 | Weighted median | 5 | -0.092990074 | 0.0756117560363569 | 0.218758453196174 | -0.241189116 | 0.05520896788791 | 0.911202544899333 | 0.78569302539229 | 1.05676142080613 | 0.999332001504091 |
| ebi-fl187-GCST90094750 | ebi-hbsm44-GCST90104936 | Sulcal opening distance of the left anterior inferior temporal sulcus \|\| id:ebi-hbsm44-GCST90104936 | Curry liking \|\| id:ebi-fl187-GCST90094750 | Weighted median | 5 | 0.0114292167076012 | 0.0458569738398455 | 0.803178099359088 | -0.078450452 | 0.101308885433698 | 1.01149477974485 | 0.924547868277365 | 1.10661840728418 | 0.999332001504091 |
| ebi-fl187-GCST90094750 | ebi-hbsm44-GCST90104941 | Sulcal opening distance of the right posterior inferior temporal sulcus \|\| id:ebi-hbsm44-GCST90104941 | Curry liking \|\| id:ebi-fl187-GCST90094750 | Weighted median | 5 | -0.01693695 | 0.0347383450938868 | 0.625863116694472 | -0.085024106 | 0.0511502064510871 | 0.983205673864356 | 0.918490142720086 | 1.05248097084224 | 0.999332001504091 |
| ebi-fl187-GCST90094750 | ebi-IPs91-GCST90274819 | Leukemia inhibitory factor levels \|\| id:ebi-IPs91-GCST90274819 | Curry liking \|\| id:ebi-fl187-GCST90094750 | Weighted median | 7 | -0.040170085 | 0.137891696368802 | 0.770809500515726 | -0.31043781 | 0.230097640215336 | 0.960626037496465 | 0.73312591642444 | 1.25872290590517 | 0.999332001504091 |
| ebi-fl187-GCST90094750 | ebi-IPs91-GCST90274837 | STAM binding protein levels \|\| id:ebi-IPs91-GCST90274837 | Curry liking \|\| id:ebi-fl187-GCST90094750 | Weighted median | 7 | -0.075477524 | 0.115559055441891 | 0.513658871117583 | -0.301973273 | 0.151018224393264 | 0.927300572054434 | 0.7393578254872 | 1.16301785318342 | 0.999332001504091 |
| ebi-fl187-GCST90094750 | ebi-IPs91-GCST90274840 | TNF-beta levels \|\| id:ebi-IPs91-GCST90274840 | Curry liking \|\| id:ebi-fl187-GCST90094750 | Weighted median | 7 | -0.015083648 | 0.139680836587319 | 0.914006381771059 | -0.288858088 | 0.258690791660853 | 0.985029540356311 | 0.749118506944014 | 1.29523324598237 | 0.999332001504091 |
| ebi-fl187-GCST90094750 | ebi-IPs91-GCST90274842 | Tumor necrosis factor ligand superfamily member 14 levels \|\| id:ebi-IPs91-GCST90274842 | Curry liking \|\| id:ebi-fl187-GCST90094750 | Weighted median | 7 | -0.013069031 | 0.125920555441198 | 0.917337665076556 | -0.259873319 | 0.233735258024693 | 0.987015998322447 | 0.771149269341668 | 1.26330999674827 | 0.999332001504091 |
| ebi-fl187-GCST90094750 | ebi-met1400-GCST90199628 | 3-methylhistidine levels \|\| id:ebi-met1400-GCST90199628 | Curry liking \|\| id:ebi-fl187-GCST90094750 | Weighted median | 7 | 0.00301724721030474 | 0.180504612069297 | 0.986663482820795 | -0.350771792 | 0.356806286866127 | 1.00302180368218 | 0.704144426599154 | 1.42875907364751 | 0.999332001504091 |
| ebi-fl187-GCST90094750 | ebi-met1400-GCST90199651 | Docosahexaenoate DHA; 22:6n3 levels \|\| id:ebi-met1400-GCST90199651 | Curry liking \|\| id:ebi-fl187-GCST90094750 | Weighted median | 7 | -0.001727856 | 0.15978720611922 | 0.991372260591755 | -0.31491078 | 0.311455068474323 | 0.998273636363623 | 0.729853989292566 | 1.36541043507153 | 0.999332001504091 |
| ebi-fl187-GCST90094750 | ebi-met1400-GCST90199652 | 1-stearoyl-2-oleoyl-GPS (18:0/18:1) levels \|\| id:ebi-met1400-GCST90199652 | Curry liking \|\| id:ebi-fl187-GCST90094750 | Weighted median | 7 | -0.092149459 | 0.167572333040829 | 0.582382098870044 | -0.420591232 | 0.236292313902722 | 0.911968837538472 | 0.656658467775433 | 1.26654448462194 | 0.999332001504091 |
| ebi-fl187-GCST90094750 | ebi-met1400-GCST90199653 | 1-myristoyl-2-palmitoyl-gpc (14:0/16:0) levels \|\| id:ebi-met1400-GCST90199653 | Curry liking \|\| id:ebi-fl187-GCST90094750 | Weighted median | 7 | -0.284236464 | 0.170451159657838 | 0.0954043531124196 | -0.618320737 | 0.0498478085973569 | 0.752588663288357 | 0.538848546549961 | 1.05111111412758 | 0.999332001504091 |
| ebi-fl187-GCST90094750 | ebi-met1400-GCST90199667 | Erythritol levels in elite athletes \|\| id:ebi-met1400-GCST90199667 | Curry liking \|\| id:ebi-fl187-GCST90094750 | Weighted median | 7 | 0.059781391461788 | 0.173179712698893 | 0.72994508382286 | -0.279650845 | 0.399213628351619 | 1.06160444538067 | 0.756047672878216 | 1.49065203013136 | 0.999332001504091 |
| ebi-fl187-GCST90094750 | ebi-met1400-GCST90199674 | 3-hydroxymyristate levels \|\| id:ebi-met1400-GCST90199674 | Curry liking \|\| id:ebi-fl187-GCST90094750 | Weighted median | 7 | -0.000511939 | 0.158567863199387 | 0.997424018448587 | -0.311304951 | 0.310281072399958 | 0.999488191547812 | 0.732490467855868 | 1.36380838916266 | 0.999364451047625 |
| ebi-fl187-GCST90094750 | ebi-met1400-GCST90199685 | 2-linoleoylglycerol (18:2) levels \|\| id:ebi-met1400-GCST90199685 | Curry liking \|\| id:ebi-fl187-GCST90094750 | Weighted median | 7 | -0.205718404 | 0.204254575897535 | 0.313854680075795 | -0.606057373 | 0.194620564781334 | 0.814062280709922 | 0.545497327586777 | 1.21484994217358 | 0.999332001504091 |
| ebi-fl187-GCST90094750 | ebi-met1400-GCST90199703 | Gamma-glutamylvaline levels \|\| id:ebi-met1400-GCST90199703 | Curry liking \|\| id:ebi-fl187-GCST90094750 | Weighted median | 7 | 0.0672138248652044 | 0.158970476606291 | 0.672436104982753 | -0.244368309 | 0.378795959013535 | 1.0695241445825 | 0.783199121628152 | 1.46052499837712 | 0.999332001504091 |
| ebi-fl187-GCST90094750 | ebi-met1400-GCST90199779 | Gamma-glutamyl-2-aminobutyrate levels \|\| id:ebi-met1400-GCST90199779 | Curry liking \|\| id:ebi-fl187-GCST90094750 | Weighted median | 7 | 0.0567817188923963 | 0.161636583411921 | 0.72536892370873 | -0.260025985 | 0.373589422379761 | 1.0584247510515 | 0.771031550600714 | 1.4529404831302 | 0.999332001504091 |
| ebi-fl187-GCST90094750 | ebi-met1400-GCST90199824 | 4-vinylphenol sulfate levels \|\| id:ebi-met1400-GCST90199824 | Curry liking \|\| id:ebi-fl187-GCST90094750 | Weighted median | 7 | -0.107193995 | 0.145159454771021 | 0.460236872260725 | -0.391706527 | 0.17731853599148 | 0.898351380085709 | 0.67590244417028 | 1.19401136815327 | 0.999332001504091 |
| ebi-fl187-GCST90094750 | ebi-met1400-GCST90199830 | 4-ethylphenylsulfate levels \|\| id:ebi-met1400-GCST90199830 | Curry liking \|\| id:ebi-fl187-GCST90094750 | Weighted median | 7 | 0.0575698389545422 | 0.154072817379158 | 0.708662166103884 | -0.244412883 | 0.359552561017693 | 1.05925924562984 | 0.783164212225215 | 1.43268823056183 | 0.999332001504091 |
| ebi-fl187-GCST90094750 | ebi-met1400-GCST90199833 | Hexanoylglutamine levels \|\| id:ebi-met1400-GCST90199833 | Curry liking \|\| id:ebi-fl187-GCST90094750 | Weighted median | 6 | 0.0619077527479045 | 0.157491704224036 | 0.694256110720606 | -0.246775988 | 0.370591493027016 | 1.06386420165218 | 0.781315698387513 | 1.44859119289792 | 0.999332001504091 |
| ebi-fl187-GCST90094750 | ebi-met1400-GCST90199837 | Carotenoid levels (cryptoxanthin) \|\| id:ebi-met1400-GCST90199837 | Curry liking \|\| id:ebi-fl187-GCST90094750 | Weighted median | 7 | 0.148424124490085 | 0.144447476373807 | 0.304170936943306 | -0.134692929 | 0.431541178182748 | 1.16000477848114 | 0.873984245526392 | 1.53962853791334 | 0.999332001504091 |
| ebi-fl187-GCST90094750 | ebi-met1400-GCST90199869 | 2-hydroxyhippurate levels \|\| id:ebi-met1400-GCST90199869 | Curry liking \|\| id:ebi-fl187-GCST90094750 | Weighted median | 7 | 0.0822369564645677 | 0.159840171307369 | 0.606906009781237 | -0.231049779 | 0.39552369222701 | 1.08571304607638 | 0.793699955225347 | 1.48516175496795 | 0.999332001504091 |
| ebi-fl187-GCST90094750 | ebi-met1400-GCST90199902 | Hydantoin-5-propionate levels \|\| id:ebi-met1400-GCST90199902 | Curry liking \|\| id:ebi-fl187-GCST90094750 | Weighted median | 7 | 0.0339581181743825 | 0.151885522 | 0.823086416868563 | -0.263737505 | 0.331653741431583 | 1.0345412773434 | 0.768175155267775 | 1.39327033318882 | 0.999332001504091 |
| ebi-fl187-GCST90094750 | ebi-met1400-GCST90199904 | Pantoate levels \|\| id:ebi-met1400-GCST90199904 | Curry liking \|\| id:ebi-fl187-GCST90094750 | Weighted median | 7 | -0.145215536 | 0.163766257082461 | 0.375227299197488 | -0.4661974 | 0.175766328089959 | 0.864835869937144 | 0.627383426539603 | 1.19215945192445 | 0.999332001504091 |
| ebi-fl187-GCST90094750 | ebi-met1400-GCST90199936 | 3-methyl catechol sulfate (2) levels \|\| id:ebi-met1400-GCST90199936 | Curry liking \|\| id:ebi-fl187-GCST90094750 | Weighted median | 7 | -0.168227064 | 0.170978868162525 | 0.325161936656543 | -0.503345645 | 0.1668915180419 | 0.845161907426995 | 0.604504814119398 | 1.18162607324506 | 0.999332001504091 |
| ebi-fl187-GCST90094750 | ebi-met1400-GCST90199977 | 4-hydroxychlorothalonil levels \|\| id:ebi-met1400-GCST90199977 | Curry liking \|\| id:ebi-fl187-GCST90094750 | Weighted median | 7 | 0.020377455593479 | 0.159416491606413 | 0.898287253964395 | -0.292078868 | 0.332833779142049 | 1.0205864934136 | 0.746709642188445 | 1.39491541516134 | 0.999332001504091 |
| ebi-fl187-GCST90094750 | ebi-met1400-GCST90200040 | Sphingomyelin (d18:2/24:1, d18:1/24:2) levels \|\| id:ebi-met1400-GCST90200040 | Curry liking \|\| id:ebi-fl187-GCST90094750 | Weighted median | 7 | -0.162264452 | 0.157514842897298 | 0.302937997113034 | -0.470993544 | 0.14646463978534 | 0.850216333148509 | 0.624381609173756 | 1.15773399237218 | 0.999332001504091 |
| ebi-fl187-GCST90094750 | ebi-met1400-GCST90200077 | 1-stearoyl-2-oleoyl-GPI (18:0/18:1) levels \|\| id:ebi-met1400-GCST90200077 | Curry liking \|\| id:ebi-fl187-GCST90094750 | Weighted median | 7 | -0.007865218 | 0.154261780933251 | 0.959336535836611 | -0.310218309 | 0.294487872250994 | 0.992165631518515 | 0.733286855623669 | 1.34243868251163 | 0.999332001504091 |
| ebi-fl187-GCST90094750 | ebi-met1400-GCST90200080 | 1-palmitoyl-2-oleoyl-GPI (16:0/18:1) levels \|\| id:ebi-met1400-GCST90200080 | Curry liking \|\| id:ebi-fl187-GCST90094750 | Weighted median | 7 | 0.0118861428568994 | 0.151710631000551 | 0.937551664457615 | -0.285466694 | 0.309238979617979 | 1.01195706376638 | 0.751663375733852 | 1.36238791454603 | 0.999332001504091 |
| ebi-fl187-GCST90094750 | ebi-met1400-GCST90200082 | 1-oleoyl-2-linoleoyl-GPE (18:1/18:2) levels \|\| id:ebi-met1400-GCST90200082 | Curry liking \|\| id:ebi-fl187-GCST90094750 | Weighted median | 7 | -0.339543206 | 0.157796575679796 | 0.0314148403366142 | -0.648824494 | -0.030261918 | 0.712095529364893 | 0.522659805297155 | 0.970191389891118 | 0.999332001504091 |
| ebi-fl187-GCST90094750 | ebi-met1400-GCST90200100 | Perfluorooctanesulfonate (PFOS) levels \|\| id:ebi-met1400-GCST90200100 | Curry liking \|\| id:ebi-fl187-GCST90094750 | Weighted median | 7 | 0.225334716061882 | 0.159893545935274 | 0.158752501855398 | -0.088056634 | 0.538726066095019 | 1.25274195887935 | 0.915709015016737 | 1.71382217473111 | 0.999332001504091 |
| ebi-fl187-GCST90094750 | ebi-met1400-GCST90200102 | 1-palmitoleoyl-2-linolenoyl-GPC (16:1/18:3) levels \|\| id:ebi-met1400-GCST90200102 | Curry liking \|\| id:ebi-fl187-GCST90094750 | Weighted median | 7 | -0.264450601 | 0.151357981238838 | 0.0806050159927871 | -0.561112244 | 0.0322110420815789 | 0.767627567852722 | 0.570574092967647 | 1.03273543294385 | 0.999332001504091 |
| ebi-fl187-GCST90094750 | ebi-met1400-GCST90200114 | Glycosyl-N-tricosanoyl-sphingadienine (d18:2/23:0) levels \|\| id:ebi-met1400-GCST90200114 | Curry liking \|\| id:ebi-fl187-GCST90094750 | Weighted median | 7 | -0.475306307 | 0.183345846953399 | 0.00953080786876311 | -0.834664167 | -0.115948447 | 0.621694597761327 | 0.434020215169249 | 0.890521130991325 | 0.999332001504091 |
| ebi-fl187-GCST90094750 | ebi-met1400-GCST90200135 | Arachidoylcarnitine (C20) levels \|\| id:ebi-met1400-GCST90200135 | Curry liking \|\| id:ebi-fl187-GCST90094750 | Weighted median | 7 | -0.391883904 | 0.154999605345632 | 0.011461973507671 | -0.69568313 | -0.088084677 | 0.67578256528985 | 0.498733631613705 | 0.915683335956486 | 0.999332001504091 |
| ebi-fl187-GCST90094750 | ebi-met1400-GCST90200146 | Docosahexaenoylcarnitine (C22:6) levels \|\| id:ebi-met1400-GCST90200146 | Curry liking \|\| id:ebi-fl187-GCST90094750 | Weighted median | 7 | 0.0819947889080165 | 0.174246826184117 | 0.637950049155539 | -0.25952899 | 0.423518568228885 | 1.08545015343422 | 0.771414844035004 | 1.5273261134406 | 0.999332001504091 |
| ebi-fl187-GCST90094750 | ebi-met1400-GCST90200162 | 3-hydroxyphenylacetoylglutamine levels \|\| id:ebi-met1400-GCST90200162 | Curry liking \|\| id:ebi-fl187-GCST90094750 | Weighted median | 7 | -0.089763176 | 0.199267534244193 | 0.652374730487965 | -0.480327543 | 0.300801191508776 | 0.914147652098126 | 0.618580746994891 | 1.35094073634888 | 0.999332001504091 |
| ebi-fl187-GCST90094750 | ebi-met1400-GCST90200172 | 3-carboxy-4-methyl-5-pentyl-2-furanpropionate (3-CMPFP) levels \|\| id:ebi-met1400-GCST90200172 | Curry liking \|\| id:ebi-fl187-GCST90094750 | Weighted median | 7 | 0.0760499767580385 | 0.156426168068147 | 0.626845431265469 | -0.230545313 | 0.382645266171607 | 1.07901649855626 | 0.794100451386771 | 1.46615784202538 | 0.999332001504091 |
| ebi-fl187-GCST90094750 | ebi-met1400-GCST90200222 | Dihydrocaffeate sulfate (2) levels \|\| id:ebi-met1400-GCST90200222 | Curry liking \|\| id:ebi-fl187-GCST90094750 | Weighted median | 7 | -0.058130794 | 0.172753666011663 | 0.736497407942016 | -0.396727979 | 0.280466391627328 | 0.943526531987427 | 0.67251693939065 | 1.32374705293051 | 0.999332001504091 |
| ebi-fl187-GCST90094750 | ebi-met1400-GCST90200223 | 4-methylhexanoylglutamine levels \|\| id:ebi-met1400-GCST90200223 | Curry liking \|\| id:ebi-fl187-GCST90094750 | Weighted median | 7 | -0.117842028 | 0.198860040074781 | 0.553457065615386 | -0.507607706 | 0.271923650783599 | 0.888836453007477 | 0.601933860306529 | 1.31248679014767 | 0.999332001504091 |
| ebi-fl187-GCST90094750 | ebi-met1400-GCST90200224 | 2-ketocaprylate levels \|\| id:ebi-met1400-GCST90200224 | Curry liking \|\| id:ebi-fl187-GCST90094750 | Weighted median | 7 | -0.208459724 | 0.159149592187279 | 0.190251620918363 | -0.520392925 | 0.103473476196971 | 0.811833731061584 | 0.594286991766618 | 1.109016378 | 0.999332001504091 |
| ebi-fl187-GCST90094750 | ebi-met1400-GCST90200243 | Hydroxypalmitoyl sphingomyelin (d18:1/16:0(OH)) levels \|\| id:ebi-met1400-GCST90200243 | Curry liking \|\| id:ebi-fl187-GCST90094750 | Weighted median | 7 | -0.105337256 | 0.164811938201352 | 0.522734409011545 | -0.428368655 | 0.217694142454577 | 0.900020933557426 | 0.651571165356841 | 1.24320676529317 | 0.999332001504091 |
| ebi-fl187-GCST90094750 | ebi-met1400-GCST90200273 | 2-methoxyhydroquinone sulfate (2) levels \|\| id:ebi-met1400-GCST90200273 | Curry liking \|\| id:ebi-fl187-GCST90094750 | Weighted median | 7 | -0.119528699 | 0.191492764561421 | 0.532499967178433 | -0.494854517 | 0.255797119816272 | 0.887338541969623 | 0.609659595790552 | 1.29149068349164 | 0.999332001504091 |
| ebi-fl187-GCST90094750 | ebi-met1400-GCST90200276 | 2,4-di-tert-butylphenol levels \|\| id:ebi-met1400-GCST90200276 | Curry liking \|\| id:ebi-fl187-GCST90094750 | Weighted median | 7 | -0.190637741 | 0.157973572205572 | 0.227520797281964 | -0.500265943 | 0.11899046036253 | 0.826431916197782 | 0.606369378768172 | 1.12635917317893 | 0.999332001504091 |
| ebi-fl187-GCST90094750 | ebi-met1400-GCST90200289 | N-acetylmethionine levels \|\| id:ebi-met1400-GCST90200289 | Curry liking \|\| id:ebi-fl187-GCST90094750 | Weighted median | 7 | 0.0813783420723572 | 0.159887041488018 | 0.610770470537017 | -0.232000259 | 0.394756943388872 | 1.08478123731866 | 0.79294591774006 | 1.48402344537243 | 0.999332001504091 |
| ebi-fl187-GCST90094750 | ebi-met1400-GCST90200308 | 3-hydroxyisobutyrate levels \|\| id:ebi-met1400-GCST90200308 | Curry liking \|\| id:ebi-fl187-GCST90094750 | Weighted median | 7 | 0.0280938494998944 | 0.159856174265978 | 0.86049464352221 | -0.285224252 | 0.341411951061211 | 1.02849220337116 | 0.751845632480236 | 1.40693270892023 | 0.999332001504091 |
| ebi-fl187-GCST90094750 | ebi-met1400-GCST90200383 | Cystathionine levels \|\| id:ebi-met1400-GCST90200383 | Curry liking \|\| id:ebi-fl187-GCST90094750 | Weighted median | 7 | -0.034085064 | 0.162036279702635 | 0.833390983185731 | -0.351676173 | 0.283506043743645 | 0.966489287238285 | 0.703507900164344 | 1.32777690503284 | 0.999332001504091 |
| ebi-fl187-GCST90094750 | ebi-met1400-GCST90200384 | Guanidinoacetate levels \|\| id:ebi-met1400-GCST90200384 | Curry liking \|\| id:ebi-fl187-GCST90094750 | Weighted median | 7 | 0.0395178458640715 | 0.144208097553836 | 0.784058897849615 | -0.243130025 | 0.322165717069591 | 1.04030906393345 | 0.784169545229582 | 1.38011346536704 | 0.999332001504091 |
| ebi-fl187-GCST90094750 | ebi-met1400-GCST90200403 | Citrulline levels \|\| id:ebi-met1400-GCST90200403 | Curry liking \|\| id:ebi-fl187-GCST90094750 | Weighted median | 7 | -0.114882358 | 0.16043283076871 | 0.473943428645114 | -0.429330706 | 0.199565990416004 | 0.891471012268535 | 0.650944622160639 | 1.22087277267492 | 0.999332001504091 |
| ebi-fl187-GCST90094750 | ebi-met1400-GCST90200434 | 12,13-DiHOME levels \|\| id:ebi-met1400-GCST90200434 | Curry liking \|\| id:ebi-fl187-GCST90094750 | Weighted median | 7 | 0.0754940572749256 | 0.158314287070373 | 0.633460439081473 | -0.234801945 | 0.385790059932856 | 1.07841681896434 | 0.79072744134191 | 1.47077586361681 | 0.999332001504091 |
| ebi-fl187-GCST90094750 | ebi-met1400-GCST90200437 | Fructose levels \|\| id:ebi-met1400-GCST90200437 | Curry liking \|\| id:ebi-fl187-GCST90094750 | Weighted median | 7 | -0.132695939 | 0.173106752963382 | 0.443345824538108 | -0.471985174 | 0.206593297180928 | 0.875731327700356 | 0.623762760484221 | 1.22948243611159 | 0.999332001504091 |
| ebi-fl187-GCST90094750 | ebi-met1400-GCST90200448 | N-stearoyl-sphinganine (d18:0/18:0) levels \|\| id:ebi-met1400-GCST90200448 | Curry liking \|\| id:ebi-fl187-GCST90094750 | Weighted median | 7 | -0.009708475 | 0.170301197479139 | 0.95453908080577 | -0.343498822 | 0.324081871631157 | 0.990338499677816 | 0.709284316335387 | 1.38276051134387 | 0.999332001504091 |
| ebi-fl187-GCST90094750 | ebi-met1400-GCST90200468 | X-11847 levels \|\| id:ebi-met1400-GCST90200468 | Curry liking \|\| id:ebi-fl187-GCST90094750 | Weighted median | 7 | -0.10727341 | 0.156070629732245 | 0.491869212398555 | -0.413171844 | 0.198625024649989 | 0.898280041003415 | 0.661548589969404 | 1.21972451351218 | 0.999332001504091 |
| ebi-fl187-GCST90094750 | ebi-met1400-GCST90200476 | X-11843 levels \|\| id:ebi-met1400-GCST90200476 | Curry liking \|\| id:ebi-fl187-GCST90094750 | Weighted median | 7 | 0.060439061348571 | 0.158291289795192 | 0.702593565637173 | -0.249811867 | 0.370689989347147 | 1.06230286029413 | 0.778947315255066 | 1.4487338808268 | 0.999332001504091 |
| ebi-fl187-GCST90094750 | ebi-met1400-GCST90200511 | X-12906 levels \|\| id:ebi-met1400-GCST90200511 | Curry liking \|\| id:ebi-fl187-GCST90094750 | Weighted median | 7 | 0.100056581684383 | 0.148762818190045 | 0.50120731924392 | -0.191518542 | 0.391631705336871 | 1.10523345227685 | 0.825704314781473 | 1.47939275859918 | 0.999332001504091 |
| ebi-fl187-GCST90094750 | ebi-met1400-GCST90200515 | X-12849 levels \|\| id:ebi-met1400-GCST90200515 | Curry liking \|\| id:ebi-fl187-GCST90094750 | Weighted median | 7 | -0.124009172 | 0.174768409217183 | 0.477975257334078 | -0.466555254 | 0.21853690966701 | 0.883371738207834 | 0.627158954541168 | 1.24425494081518 | 0.999332001504091 |
| ebi-fl187-GCST90094750 | ebi-met1400-GCST90200551 | X-18886 levels \|\| id:ebi-met1400-GCST90200551 | Curry liking \|\| id:ebi-fl187-GCST90094750 | Weighted median | 7 | -0.101463058 | 0.162269594674202 | 0.531790837639011 | -0.419511464 | 0.216585347205945 | 0.903514556039259 | 0.657367889315436 | 1.2418290674723 | 0.999332001504091 |
| ebi-fl187-GCST90094750 | ebi-met1400-GCST90200564 | X-21319 levels \|\| id:ebi-met1400-GCST90200564 | Curry liking \|\| id:ebi-fl187-GCST90094750 | Weighted median | 7 | 0.0219339620885993 | 0.154955939276552 | 0.887435723925985 | -0.281779679 | 0.325647603070641 | 1.02217627985493 | 0.754439885254004 | 1.38492723876373 | 0.999332001504091 |
| ebi-fl187-GCST90094750 | ebi-met1400-GCST90200579 | X-21442 levels \|\| id:ebi-met1400-GCST90200579 | Curry liking \|\| id:ebi-fl187-GCST90094750 | Weighted median | 7 | 0.310725029710161 | 0.170436432026559 | 0.0682861405126758 | -0.023330377 | 0.644780436482216 | 1.36441399628866 | 0.976939671992929 | 1.90556858999361 | 0.999332001504091 |
| ebi-fl187-GCST90094750 | ebi-met1400-GCST90200581 | X-21441 levels \|\| id:ebi-met1400-GCST90200581 | Curry liking \|\| id:ebi-fl187-GCST90094750 | Weighted median | 7 | -0.0454368 | 0.154636455465876 | 0.768887954465626 | -0.348524253 | 0.25765065228669 | 0.955579992911679 | 0.705728798677332 | 1.29388672329154 | 0.999332001504091 |
| ebi-fl187-GCST90094750 | ebi-met1400-GCST90200648 | X-24585 levels \|\| id:ebi-met1400-GCST90200648 | Curry liking \|\| id:ebi-fl187-GCST90094750 | Weighted median | 7 | 0.201876040047154 | 0.17915451586178 | 0.259815881784857 | -0.149266811 | 0.553018891136243 | 1.22369630937246 | 0.861339269409735 | 1.73849342617103 | 0.999332001504091 |
| ebi-fl187-GCST90094750 | ebi-met1400-GCST90200720 | Adenosine 5'-diphosphate (ADP) to pantothenate ratio \|\| id:ebi-met1400-GCST90200720 | Curry liking \|\| id:ebi-fl187-GCST90094750 | Weighted median | 7 | -0.218354286 | 0.209685539824576 | 0.297717012681727 | -0.629337944 | 0.192629371908464 | 0.803840601621981 | 0.53294452324374 | 1.21243334837774 | 0.999332001504091 |
| ebi-fl187-GCST90094750 | ebi-met1400-GCST90200724 | Cholate to taurocholate ratio \|\| id:ebi-met1400-GCST90200724 | Curry liking \|\| id:ebi-fl187-GCST90094750 | Weighted median | 7 | 0.0991036593060455 | 0.164906553316339 | 0.547860905170882 | -0.224113185 | 0.422320503806069 | 1.10418075223726 | 0.799224668850607 | 1.52549737405459 | 0.999332001504091 |
| ebi-fl187-GCST90094750 | ebi-met1400-GCST90200726 | N-acetylputrescine to (N(1) + N(8))-acetylspermidine ratio \|\| id:ebi-met1400-GCST90200726 | Curry liking \|\| id:ebi-fl187-GCST90094750 | Weighted median | 7 | -0.136841477 | 0.152111776354678 | 0.368327125776299 | -0.434980559 | 0.161297604276996 | 0.872108464095144 | 0.647277250651165 | 1.1750346121098 | 0.999332001504091 |
| ebi-fl187-GCST90094750 | ebi-met1400-GCST90200744 | Arginine to glutamate ratio \|\| id:ebi-met1400-GCST90200744 | Curry liking \|\| id:ebi-fl187-GCST90094750 | Weighted median | 7 | 0.0667709378866117 | 0.163611614951215 | 0.683195603269546 | -0.253907827 | 0.387449703190993 | 1.06905057114306 | 0.775763302858042 | 1.47321885354823 | 0.999332001504091 |
| ebi-fl187-GCST90094750 | ebi-met1400-GCST90200759 | Histidine to trans-urocanate ratio \|\| id:ebi-met1400-GCST90200759 | Curry liking \|\| id:ebi-fl187-GCST90094750 | Weighted median | 7 | -0.276505447 | 0.16740752102374 | 0.0985976183108883 | -0.604624188 | 0.0516132946480244 | 0.75842948828984 | 0.546279686657638 | 1.05296847522739 | 0.999332001504091 |
| ebi-fl187-GCST90094750 | ebi-met1400-GCST90200795 | Oleoyl-linoleoyl-glycerol (18:1 to 18:2) [2] to linoleoyl-arachidonoyl-glycerol (18:2 to 20:4) [2] ratio \|\| id:ebi-met1400-GCST90200795 | Curry liking \|\| id:ebi-fl187-GCST90094750 | Weighted median | 7 | 0.121208850836431 | 0.169691597018197 | 0.475048457409166 | -0.211386679 | 0.453804380992096 | 1.12886065124058 | 0.809461004527631 | 1.57429000630235 | 0.999332001504091 |
| ebi-fl187-GCST90094750 | ebi-met1400-GCST90200811 | Choline to taurocholate ratio \|\| id:ebi-met1400-GCST90200811 | Curry liking \|\| id:ebi-fl187-GCST90094750 | Weighted median | 7 | 0.288249713002649 | 0.16032740695719 | 0.0721957547006246 | -0.025992005 | 0.602491430638741 | 1.33409040225234 | 0.974342879806082 | 1.8266641428488 | 0.999332001504091 |
| ebi-fl187-GCST90094750 | ebi-met1400-GCST90200831 | Cholate to phosphate ratio \|\| id:ebi-met1400-GCST90200831 | Curry liking \|\| id:ebi-fl187-GCST90094750 | Weighted median | 7 | -0.205322872 | 0.176525899731652 | 0.244776066534592 | -0.551313636 | 0.140667891104526 | 0.814384331759571 | 0.576192406015319 | 1.1510423131086 | 0.999332001504091 |
| ebi-fl187-GCST90094750 | ebi-met1400-GCST90200841 | Adenosine 5'-diphosphate (ADP) to oxalate (ethanedioate) ratio \|\| id:ebi-met1400-GCST90200841 | Curry liking \|\| id:ebi-fl187-GCST90094750 | Weighted median | 7 | -0.111356495 | 0.208815512526482 | 0.593841897712907 | -0.520634899 | 0.297921909919618 | 0.894619764930233 | 0.594143207158665 | 1.34705659201468 | 0.999332001504091 |
| ebi-fl187-GCST90094750 | ebi-met1400-GCST90200844 | Aspartate to phosphate ratio \|\| id:ebi-met1400-GCST90200844 | Curry liking \|\| id:ebi-fl187-GCST90094750 | Weighted median | 7 | -0.156941089 | 0.143301570812212 | 0.273437558650065 | -0.437812167 | 0.123929990116108 | 0.85475441204874 | 0.645447007417809 | 1.13193662147364 | 0.999332001504091 |
| ebi-fl187-GCST90094750 | ebi-met1400-GCST90200870 | Adenosine 5'-monophosphate (AMP) to acetoacetate ratio \|\| id:ebi-met1400-GCST90200870 | Curry liking \|\| id:ebi-fl187-GCST90094750 | Weighted median | 7 | -0.052112185 | 0.189457762649384 | 0.78327041426913 | -0.4234494 | 0.319225029736921 | 0.949222372304581 | 0.654784307013227 | 1.37606094470028 | 0.999332001504091 |
| ebi-fl187-GCST90094750 | ebi-met1400-GCST90200887 | Cholesterol to cortisol ratio \|\| id:ebi-met1400-GCST90200887 | Curry liking \|\| id:ebi-fl187-GCST90094750 | Weighted median | 7 | -0.074353906 | 0.145294095171728 | 0.608827659554312 | -0.359130333 | 0.210422520348637 | 0.928343089333277 | 0.698283336250867 | 1.23419942417648 | 0.999332001504091 |
| ebi-fl187-GCST90094750 | ebi-met1400-GCST90200890 | Cortisol to taurocholate ratio \|\| id:ebi-met1400-GCST90200890 | Curry liking \|\| id:ebi-fl187-GCST90094750 | Weighted median | 7 | 0.224628643067946 | 0.161129910109078 | 0.163292214958049 | -0.091185981 | 0.540443266881738 | 1.25185774381074 | 0.912847922973554 | 1.71676767980587 | 0.999332001504091 |
| ebi-fl187-GCST90094750 | ebi-met1400-GCST90200948 | Adenosine 5'-diphosphate (ADP) to mannitol to sorbitol ratio \|\| id:ebi-met1400-GCST90200948 | Curry liking \|\| id:ebi-fl187-GCST90094750 | Weighted median | 7 | -0.208493623 | 0.214711706740225 | 0.331528435002638 | -0.629328569 | 0.212341321798011 | 0.81180621123911 | 0.532949519931284 | 1.23656988131141 | 0.999332001504091 |
| ebi-fl187-GCST90094750 | ebi-met1400-GCST90200972 | Leucine to N-palmitoyl-sphingosine (d18:1 to 16:0) ratio \|\| id:ebi-met1400-GCST90200972 | Curry liking \|\| id:ebi-fl187-GCST90094750 | Weighted median | 7 | 0.27404219432051 | 0.144120825662909 | 0.0572397810421551 | -0.008434624 | 0.556519012619812 | 1.31527029800442 | 0.991600847661998 | 1.74458902580761 | 0.999332001504091 |
| ebi-fl187-GCST90094750 | ebi-met1400-GCST90200973 | Proline to glutamate ratio \|\| id:ebi-met1400-GCST90200973 | Curry liking \|\| id:ebi-fl187-GCST90094750 | Weighted median | 7 | 0.00829758379783563 | 0.151926029652515 | 0.956444434825763 | -0.289477434 | 0.306072601916766 | 1.00833210415875 | 0.748654686621551 | 1.35808090224536 | 0.999332001504091 |
| ebi-fl187-GCST90094750 | ebi-met1400-GCST90200983 | Cholesterol to linoleoyl-arachidonoyl-glycerol (18:2 to 20:4) [1] ratio \|\| id:ebi-met1400-GCST90200983 | Curry liking \|\| id:ebi-fl187-GCST90094750 | Weighted median | 7 | -0.061251951 | 0.162912185211368 | 0.706930726160641 | -0.380559834 | 0.258055932040454 | 0.940586228303438 | 0.68347866749823 | 1.29441121566004 | 0.999332001504091 |
| ebi-fl187-GCST90094750 | ebi-met1400-GCST90201001 | Serine to alpha-ketobutyrate ratio \|\| id:ebi-met1400-GCST90201001 | Curry liking \|\| id:ebi-fl187-GCST90094750 | Weighted median | 7 | -0.038673113 | 0.156143546783852 | 0.804384587412719 | -0.344714465 | 0.267368238638261 | 0.962065144284131 | 0.708422604213823 | 1.30652146944634 | 0.999332001504091 |
| ebi-fl187-GCST90094750 | ebi-met1400-GCST90201005 | Fructose to maltose ratio \|\| id:ebi-met1400-GCST90201005 | Curry liking \|\| id:ebi-fl187-GCST90094750 | Weighted median | 7 | -0.082082823 | 0.172282226065104 | 0.6337581357638 | -0.419755986 | 0.255590340217987 | 0.921195659468736 | 0.657207168028668 | 1.29122365717567 | 0.999332001504091 |
| ebi-fl187-GCST90094750 | ebi-met1400-GCST90201020 | Paraxanthine to 5-acetylamino-6-formylamino-3-methyluracil ratio \|\| id:ebi-met1400-GCST90201020 | Curry liking \|\| id:ebi-fl187-GCST90094750 | Weighted median | 7 | 0.27496201178536 | 0.164914543728133 | 0.0954547750600325 | -0.048270494 | 0.5981945174925 | 1.3164806631677 | 0.952876005047265 | 1.81883196482474 | 0.999332001504091 |
| ebi-fl187-GCST90094750 | ebi-pw11-GCST90275197 | Placental weight (fetal genotype, adjusted for sex) \|\| id:ebi-pw11-GCST90275197 | Curry liking \|\| id:ebi-fl187-GCST90094750 | Weighted median | 6 | -0.037126408 | 0.0636941060150817 | 0.55996999001395 | -0.161966856 | 0.0877140398323743 | 0.963554326716348 | 0.850469392246022 | 1.09167590156521 | 0.999332001504091 |
| ebi-fl187-GCST90094750 | prot-a-1017 | FAS-associated factor 2 \|\| id:prot-a-1017 | Curry liking \|\| id:ebi-fl187-GCST90094750 | Weighted median | 7 | 0.443422488482593 | 0.269914727632417 | 0.100419279476194 | -0.085610378 | 0.972455354642131 | 1.55803044522849 | 0.917951816073138 | 2.64442950681574 | 0.999332001504091 |
| ebi-fl187-GCST90094750 | prot-a-1019 | Fas apoptotic inhibitory molecule 3 \|\| id:prot-a-1019 | Curry liking \|\| id:ebi-fl187-GCST90094750 | Weighted median | 7 | 0.286513702961558 | 0.260158930452546 | 0.270764985928141 | -0.223397801 | 0.796425206648549 | 1.33177641705022 | 0.799796626325936 | 2.21759928290612 | 0.999332001504091 |
| ebi-fl187-GCST90094750 | prot-a-1052 | MIP18 family protein FAM96A \|\| id:prot-a-1052 | Curry liking \|\| id:ebi-fl187-GCST90094750 | Weighted median | 7 | -0.240232897 | 0.233274010943185 | 0.303089165536979 | -0.697449959 | 0.2169841639549 | 0.786444678741383 | 0.497853232652931 | 1.24232442847631 | 0.999332001504091 |
| ebi-fl187-GCST90094750 | prot-a-1066 | F-box/LRR-repeat protein 4 \|\| id:prot-a-1066 | Curry liking \|\| id:ebi-fl187-GCST90094750 | Weighted median | 7 | -0.085024463 | 0.258385320991867 | 0.742109783890773 | -0.591459692 | 0.42141076617881 | 0.918489815142197 | 0.553518727836634 | 1.52411020276975 | 0.999332001504091 |
| ebi-fl187-GCST90094750 | prot-a-1076 | Low affinity immunoglobulin gamma Fc region receptor III-B \|\| id:prot-a-1076 | Curry liking \|\| id:ebi-fl187-GCST90094750 | Weighted median | 7 | -0.172409612 | 0.278486263463503 | 0.535853401061435 | -0.718242689 | 0.373423464172207 | 0.841634358838236 | 0.487608383266861 | 1.45269937573936 | 0.999332001504091 |
| ebi-fl187-GCST90094750 | prot-a-1081 | Fc receptor-like protein 3 \|\| id:prot-a-1081 | Curry liking \|\| id:ebi-fl187-GCST90094750 | Weighted median | 7 | 0.0636635297515589 | 0.250144831818088 | 0.799104146891757 | -0.426620341 | 0.553947400115011 | 1.06573375072781 | 0.652711313170309 | 1.74010838256149 | 0.999332001504091 |
| ebi-fl187-GCST90094750 | prot-a-1105 | Fibroblast growth factor receptor 4 \|\| id:prot-a-1105 | Curry liking \|\| id:ebi-fl187-GCST90094750 | Weighted median | 7 | 0.284580709895416 | 0.244062454196817 | 0.243608004211268 | -0.1937817 | 0.762943120121176 | 1.32920458893416 | 0.823837728144019 | 2.14457869418525 | 0.999332001504091 |
| ebi-fl187-GCST90094750 | prot-a-1143 | Follistatin-related protein 1 \|\| id:prot-a-1143 | Curry liking \|\| id:ebi-fl187-GCST90094750 | Weighted median | 7 | -0.271398785 | 0.243154764038213 | 0.264355125828237 | -0.747982122 | 0.205184552880323 | 0.762312437295306 | 0.473320693090702 | 1.22775162915548 | 0.999332001504091 |
| ebi-fl187-GCST90094750 | prot-a-1151 | Alpha-(1,3)-fucosyltransferase 10 \|\| id:prot-a-1151 | Curry liking \|\| id:ebi-fl187-GCST90094750 | Weighted median | 7 | -0.030446658 | 0.260983864514989 | 0.907128638088776 | -0.541975032 | 0.481081716538193 | 0.9700121731627 | 0.581598441548044 | 1.61782348243466 | 0.999332001504091 |
| ebi-fl187-GCST90094750 | prot-a-1165 | Growth arrest and DNA damage-inducible proteins-interacting protein 1 \|\| id:prot-a-1165 | Curry liking \|\| id:ebi-fl187-GCST90094750 | Weighted median | 7 | 0.413867970242202 | 0.275547321688981 | 0.133100958126197 | -0.12620478 | 0.953940720752604 | 1.51265739790972 | 0.881434327942622 | 2.59591932253399 | 0.999332001504091 |
| ebi-fl187-GCST90094750 | prot-a-1194 | Growth/differentiation factor 11/8 \|\| id:prot-a-1194 | Curry liking \|\| id:ebi-fl187-GCST90094750 | Weighted median | 7 | -0.169821923 | 0.231765752125683 | 0.46372258716399 | -0.624082797 | 0.284438951145535 | 0.843815067256072 | 0.535752597022975 | 1.32901617590822 | 0.999332001504091 |
| ebi-fl187-GCST90094750 | prot-a-1216 | D-glucuronyl C5-epimerase \|\| id:prot-a-1216 | Curry liking \|\| id:ebi-fl187-GCST90094750 | Weighted median | 7 | -0.474047095 | 0.253407100259106 | 0.0613875324710546 | -0.970725012 | 0.0226308211478305 | 0.622477936054855 | 0.378808298008817 | 1.02288884090415 | 0.999332001504091 |
| ebi-fl187-GCST90094750 | prot-a-1246 | Glypican-3 \|\| id:prot-a-1246 | Curry liking \|\| id:ebi-fl187-GCST90094750 | Weighted median | 7 | 0.177338056960924 | 0.274920354396356 | 0.518893175871482 | -0.361505838 | 0.716181951577781 | 1.19403467664021 | 0.696626529397633 | 2.0466042403698 | 0.999332001504091 |
| ebi-fl187-GCST90094750 | prot-a-1275 | Glutamate receptor 4 \|\| id:prot-a-1275 | Curry liking \|\| id:ebi-fl187-GCST90094750 | Weighted median | 6 | 0.110007202845183 | 0.293625303895129 | 0.70791955942533 | -0.465498393 | 0.685512798479635 | 1.11628611086595 | 0.627822125182495 | 1.98478937159154 | 0.999332001504091 |
| ebi-fl187-GCST90094750 | prot-a-1289 | Glutathione S-transferase P \|\| id:prot-a-1289 | Curry liking \|\| id:ebi-fl187-GCST90094750 | Weighted median | 7 | 0.167100233874888 | 0.247544988579724 | 0.499656858494465 | -0.318087944 | 0.652288411491147 | 1.18187272305418 | 0.727538803116745 | 1.91992939416505 | 0.999332001504091 |
| ebi-fl187-GCST90094750 | prot-a-130 | Apolipoprotein D \|\| id:prot-a-130 | Curry liking \|\| id:ebi-fl187-GCST90094750 | Weighted median | 7 | 0.197570519671812 | 0.234337322288146 | 0.399170792780314 | -0.261730632 | 0.656871671356579 | 1.21843898585745 | 0.769718333263066 | 1.92874912562327 | 0.999332001504091 |
| ebi-fl187-GCST90094750 | prot-a-1313 | Heparin-binding EGF-like growth factor \|\| id:prot-a-1313 | Curry liking \|\| id:ebi-fl187-GCST90094750 | Weighted median | 7 | 0.0855392583716364 | 0.29121207800517 | 0.768960200963538 | -0.485236415 | 0.65631493126177 | 1.08930432481866 | 0.615551654212836 | 1.92767561251383 | 0.999332001504091 |
| ebi-fl187-GCST90094750 | prot-a-1347 | HLA class II histocompatibility antigen, DQ alpha 2 chain \|\| id:prot-a-1347 | Curry liking \|\| id:ebi-fl187-GCST90094750 | Weighted median | 6 | -0.246697524 | 0.25308245275499 | 0.329673692064961 | -0.742739131 | 0.24934408352111 | 0.781377005676157 | 0.475808826093273 | 1.28318347940808 | 0.999332001504091 |
| ebi-fl187-GCST90094750 | prot-a-1357 | Non-histone chromosomal protein HMG-14 \|\| id:prot-a-1357 | Curry liking \|\| id:ebi-fl187-GCST90094750 | Weighted median | 7 | 0.0167478840093834 | 0.282981291962761 | 0.95280578080196 | -0.537895448 | 0.571391216256395 | 1.01688891604812 | 0.583975967677602 | 1.77072880532029 | 0.999332001504091 |
| ebi-fl187-GCST90094750 | prot-a-137 | Apolipoprotein M \|\| id:prot-a-137 | Curry liking \|\| id:ebi-fl187-GCST90094750 | Weighted median | 7 | -0.177856027 | 0.262422965827554 | 0.497932969148661 | -0.69220504 | 0.336492986310885 | 0.837062929516697 | 0.50047129239045 | 1.40002904986693 | 0.999332001504091 |
| ebi-fl187-GCST90094750 | prot-a-1381 | 17-beta-hydroxysteroid dehydrogenase 14 \|\| id:prot-a-1381 | Curry liking \|\| id:ebi-fl187-GCST90094750 | Weighted median | 7 | -0.190223813 | 0.263141934400775 | 0.469744806125522 | -0.705982004 | 0.325534378561142 | 0.826774070561427 | 0.493623595681043 | 1.38477043993334 | 0.999332001504091 |
| ebi-fl187-GCST90094750 | prot-a-1392 | Serine protease HTRA2, mitochondrial \|\| id:prot-a-1392 | Curry liking \|\| id:ebi-fl187-GCST90094750 | Weighted median | 7 | 0.112284476821324 | 0.249984075985247 | 0.653311539109372 | -0.377684312 | 0.602253265752407 | 1.11883109689152 | 0.685446853785187 | 1.82622914739322 | 0.999332001504091 |
| ebi-fl187-GCST90094750 | prot-a-1416 | Interferon-induced protein with tetratricopeptide repeats 2 \|\| id:prot-a-1416 | Curry liking \|\| id:ebi-fl187-GCST90094750 | Weighted median | 7 | -0.165141187 | 0.254008051089285 | 0.515600794546595 | -0.662996967 | 0.332714592953877 | 0.847774000816427 | 0.515304666748928 | 1.39474917041742 | 0.999332001504091 |
| ebi-fl187-GCST90094750 | prot-a-1428 | Interferon gamma \|\| id:prot-a-1428 | Curry liking \|\| id:ebi-fl187-GCST90094750 | Weighted median | 7 | 0.338414315368727 | 0.257872666783555 | 0.189408449792334 | -0.167016112 | 0.843844742264494 | 1.40272155212539 | 0.846185977879097 | 2.3252899530772 | 0.999332001504091 |
| ebi-fl187-GCST90094750 | prot-a-1483 | Interleukin-17C \|\| id:prot-a-1483 | Curry liking \|\| id:ebi-fl187-GCST90094750 | Weighted median | 7 | 0.190641491686999 | 0.293344085599865 | 0.515763049274153 | -0.384312916 | 0.765595899462734 | 1.21002557008498 | 0.680918323540956 | 2.15027534087413 | 0.999332001504091 |
| ebi-fl187-GCST90094750 | prot-a-1503 | Interleukin-1 receptor-like 2 \|\| id:prot-a-1503 | Curry liking \|\| id:ebi-fl187-GCST90094750 | Weighted median | 7 | -0.105506481 | 0.249904621429296 | 0.672888534594964 | -0.595319539 | 0.384306577314149 | 0.899868641060638 | 0.551386348444442 | 1.46859561077057 | 0.999332001504091 |
| ebi-fl187-GCST90094750 | prot-a-1522 | Interleukin-31 receptor subunit alpha \|\| id:prot-a-1522 | Curry liking \|\| id:ebi-fl187-GCST90094750 | Weighted median | 7 | 0.106714996138073 | 0.246503018869972 | 0.665076104585136 | -0.376430921 | 0.589860913123218 | 1.1126171091147 | 0.686306525522736 | 1.80373752173182 | 0.999332001504091 |
| ebi-fl187-GCST90094750 | prot-a-1540 | Interleukin-6 receptor subunit alpha \|\| id:prot-a-1540 | Curry liking \|\| id:ebi-fl187-GCST90094750 | Weighted median | 7 | 0.47109597726518 | 0.287802416321872 | 0.101657028420187 | -0.092996759 | 1.03518871325605 | 1.60174871175814 | 0.911196453729012 | 2.81563753361782 | 0.999332001504091 |
| ebi-fl187-GCST90094750 | prot-a-1541 | Interleukin-6 receptor subunit alpha \|\| id:prot-a-1541 | Curry liking \|\| id:ebi-fl187-GCST90094750 | Weighted median | 7 | 0.40348823528626 | 0.290853206218859 | 0.165363316750579 | -0.166584049 | 0.973560519475224 | 1.49703761985629 | 0.846551662206952 | 2.64735365284431 | 0.999332001504091 |
| ebi-fl187-GCST90094750 | prot-a-1545 | Interleukin-9 \|\| id:prot-a-1545 | Curry liking \|\| id:ebi-fl187-GCST90094750 | Weighted median | 7 | -0.229194656 | 0.233589781001494 | 0.326501802152503 | -0.687030626 | 0.228641315254825 | 0.795173733492122 | 0.503067649302243 | 1.25689113047282 | 0.999332001504091 |
| ebi-fl187-GCST90094750 | prot-a-1548 | Inositol monophosphatase 3 \|\| id:prot-a-1548 | Curry liking \|\| id:ebi-fl187-GCST90094750 | Weighted median | 7 | -0.226639823 | 0.260491557517269 | 0.384274877006704 | -0.737203276 | 0.283923629549412 | 0.797207866377175 | 0.478450139151741 | 1.32833148160521 | 0.999332001504091 |
| ebi-fl187-GCST90094750 | prot-a-1555 | Inhibin beta A chain:Inhibin beta B chain heterodimer \|\| id:prot-a-1555 | Curry liking \|\| id:ebi-fl187-GCST90094750 | Weighted median | 7 | -0.294945398 | 0.26008206269779 | 0.256774647363796 | -0.804706241 | 0.214815444476343 | 0.744572241183273 | 0.44721928182033 | 1.23963309471841 | 0.999332001504091 |
| ebi-fl187-GCST90094750 | prot-a-1603 | Junctophilin-3 \|\| id:prot-a-1603 | Curry liking \|\| id:ebi-fl187-GCST90094750 | Weighted median | 7 | 0.104514286512121 | 0.249232130248722 | 0.6749638996729 | -0.383980689 | 0.593009261799615 | 1.110171254 | 0.681144580788544 | 1.80942526517258 | 0.999332001504091 |
| ebi-fl187-GCST90094750 | prot-a-1627 | UPF0258 protein KIAA1024 \|\| id:prot-a-1627 | Curry liking \|\| id:ebi-fl187-GCST90094750 | Weighted median | 7 | -0.156148481 | 0.260237804341696 | 0.548491386140271 | -0.666214578 | 0.353917615427331 | 0.855432165447013 | 0.51364928177796 | 1.42463781346762 | 0.999332001504091 |
| ebi-fl187-GCST90094750 | prot-a-1666 | Kallikrein-7 \|\| id:prot-a-1666 | Curry liking \|\| id:ebi-fl187-GCST90094750 | Weighted median | 7 | 0.168045668162819 | 0.256547306230236 | 0.512449728546819 | -0.334787052 | 0.670878388374083 | 1.18299063442312 | 0.715490432353027 | 1.95595465411104 | 0.999332001504091 |
| ebi-fl187-GCST90094750 | prot-a-1678 | Importin subunit alpha-3 \|\| id:prot-a-1678 | Curry liking \|\| id:ebi-fl187-GCST90094750 | Weighted median | 7 | 0.0333235030949874 | 0.250549297774394 | 0.89419197115133 | -0.457753121 | 0.5244001267328 | 1.03388495012814 | 0.632703658463229 | 1.6894450913999 | 0.999332001504091 |
| ebi-fl187-GCST90094750 | prot-a-1718 | Protein LDOC1 \|\| id:prot-a-1718 | Curry liking \|\| id:ebi-fl187-GCST90094750 | Weighted median | 7 | -0.190574938 | 0.248146419621532 | 0.442490804525315 | -0.676941921 | 0.295792044042954 | 0.826483820020627 | 0.508168638534247 | 1.34419059532312 | 0.999332001504091 |
| ebi-fl187-GCST90094750 | prot-a-1723 | Leptin \|\| id:prot-a-1723 | Curry liking \|\| id:ebi-fl187-GCST90094750 | Weighted median | 7 | 0.218644365169349 | 0.247931892378402 | 0.37784565644965 | -0.267302144 | 0.704590874231017 | 1.24438865003865 | 0.765441762969302 | 2.02301884644765 | 0.999332001504091 |
| ebi-fl187-GCST90094750 | prot-a-1730 | Galectin-7 \|\| id:prot-a-1730 | Curry liking \|\| id:ebi-fl187-GCST90094750 | Weighted median | 7 | 0.160274387452734 | 0.24635961953091 | 0.515323551524044 | -0.322590467 | 0.643139241733317 | 1.17383291183042 | 0.724270406396851 | 1.90244374577041 | 0.999332001504091 |
| ebi-fl187-GCST90094750 | prot-a-1753 | Lipase member N \|\| id:prot-a-1753 | Curry liking \|\| id:ebi-fl187-GCST90094750 | Weighted median | 7 | 0.155026777655084 | 0.250315336219799 | 0.535701667670154 | -0.335591281 | 0.64564483664589 | 1.1676892286659 | 0.714915245314694 | 1.90721647590851 | 0.999332001504091 |
| ebi-fl187-GCST90094750 | prot-a-1756 | VIP36-like protein \|\| id:prot-a-1756 | Curry liking \|\| id:ebi-fl187-GCST90094750 | Weighted median | 7 | 0.264135440357092 | 0.237632381500666 | 0.266340430301384 | -0.201624027 | 0.729894908098398 | 1.3023045689525 | 0.817402191017272 | 2.0748625449656 | 0.999332001504091 |
| ebi-fl187-GCST90094750 | prot-a-1760 | Ig Kappa chain V-I region HK102- like \|\| id:prot-a-1760 | Curry liking \|\| id:ebi-fl187-GCST90094750 | Weighted median | 7 | 0.103630600001806 | 0.259421547659254 | 0.689548417191098 | -0.404835633 | 0.612096833413943 | 1.10919064420624 | 0.667086448572405 | 1.84429452558594 | 0.999332001504091 |
| ebi-fl187-GCST90094750 | prot-a-179 | Neutral ceramidase \|\| id:prot-a-179 | Curry liking \|\| id:ebi-fl187-GCST90094750 | Weighted median | 7 | 0.444876366019445 | 0.277017437804535 | 0.108284728322442 | -0.098077812 | 0.987830544116334 | 1.56029727814398 | 0.906578358263888 | 2.68540228651129 | 0.999332001504091 |
| ebi-fl187-GCST90094750 | prot-a-1809 | Lactotransferrin \|\| id:prot-a-1809 | Curry liking \|\| id:ebi-fl187-GCST90094750 | Weighted median | 7 | 0.0838177183913086 | 0.266743660397241 | 0.753349610544671 | -0.438999856 | 0.606635292769902 | 1.0874306571324 | 0.644680872472038 | 1.83424929226933 | 0.999332001504091 |
| ebi-fl187-GCST90094750 | prot-a-1818 | Lysophospholipase-like protein 1 \|\| id:prot-a-1818 | Curry liking \|\| id:ebi-fl187-GCST90094750 | Weighted median | 7 | -0.29518708 | 0.243458513760085 | 0.225331049192354 | -0.772365767 | 0.181991607063481 | 0.744392313594377 | 0.46191898201788 | 1.19960412563634 | 0.999332001504091 |
| ebi-fl187-GCST90094750 | prot-a-1852 | MAP kinase-activated protein kinase 3 \|\| id:prot-a-1852 | Curry liking \|\| id:ebi-fl187-GCST90094750 | Weighted median | 7 | 0.0926167035975698 | 0.256135848419624 | 0.717656856558157 | -0.409409559 | 0.594642966500032 | 1.09704116274027 | 0.664042211955035 | 1.81238374771875 | 0.999332001504091 |
| ebi-fl187-GCST90094750 | prot-a-1856 | Megakaryocyte-associated tyrosine-protein kinase \|\| id:prot-a-1856 | Curry liking \|\| id:ebi-fl187-GCST90094750 | Weighted median | 7 | 0.015305600156279 | 0.241057813092023 | 0.949373548591695 | -0.457167714 | 0.487778913816643 | 1.01542333073318 | 0.633074156073703 | 1.62869472826375 | 0.999332001504091 |
| ebi-fl187-GCST90094750 | prot-a-1890 | Beta-1,3-N-acetylglucosaminyltransferase manic fringe \|\| id:prot-a-1890 | Curry liking \|\| id:ebi-fl187-GCST90094750 | Weighted median | 7 | -0.332590169 | 0.250918023826134 | 0.185007577161571 | -0.824389496 | 0.159209157412717 | 0.717064008798801 | 0.438502618372076 | 1.17258317549729 | 0.999332001504091 |
| ebi-fl187-GCST90094750 | prot-a-1919 | Matrilysin \|\| id:prot-a-1919 | Curry liking \|\| id:ebi-fl187-GCST90094750 | Weighted median | 7 | -0.10445818 | 0.258715333832314 | 0.686391268989814 | -0.611540234 | 0.402623874262786 | 0.900812468552877 | 0.54251462556729 | 1.49574419795928 | 0.999332001504091 |
| ebi-fl187-GCST90094750 | prot-a-1923 | Multimerin-2 \|\| id:prot-a-1923 | Curry liking \|\| id:ebi-fl187-GCST90094750 | Weighted median | 7 | 0.123242157481747 | 0.255306408694431 | 0.629292758421237 | -0.377158404 | 0.623642718522832 | 1.13115830623136 | 0.68580743095345 | 1.86571194187461 | 0.999332001504091 |
| ebi-fl187-GCST90094750 | prot-a-1948 | Mesothelin \|\| id:prot-a-1948 | Curry liking \|\| id:ebi-fl187-GCST90094750 | Weighted median | 7 | 0.0077811104228708 | 0.237179049073705 | 0.973828572147925 | -0.457089826 | 0.472652046607333 | 1.00781146193433 | 0.633123466710874 | 1.60424308402713 | 0.999332001504091 |
| ebi-fl187-GCST90094750 | prot-a-195 | ATPase family AAA domain-containing protein 1 \|\| id:prot-a-195 | Curry liking \|\| id:ebi-fl187-GCST90094750 | Weighted median | 7 | -0.029031682 | 0.253330755654633 | 0.908762250284127 | -0.525559963 | 0.467496599040174 | 0.971385688496024 | 0.591224207902148 | 1.59599377563218 | 0.999332001504091 |
| ebi-fl187-GCST90094750 | prot-a-1966 | Mucin-1 \|\| id:prot-a-1966 | Curry liking \|\| id:ebi-fl187-GCST90094750 | Weighted median | 7 | -0.47553457 | 0.251188688907701 | 0.0583396689590448 | -0.967864401 | 0.0167952597837012 | 0.621552703915591 | 0.379893472634819 | 1.01693709308912 | 0.999332001504091 |
| ebi-fl187-GCST90094750 | prot-a-1986 | Myocardial zonula adherens protein \|\| id:prot-a-1986 | Curry liking \|\| id:ebi-fl187-GCST90094750 | Weighted median | 7 | -0.15194907 | 0.256428523628343 | 0.553476218943902 | -0.654548976 | 0.350650836266757 | 0.859032030089849 | 0.519676396079782 | 1.41999142983396 | 0.999332001504091 |
| ebi-fl187-GCST90094750 | prot-a-2071 | Epididymal secretory protein E1 \|\| id:prot-a-2071 | Curry liking \|\| id:ebi-fl187-GCST90094750 | Weighted median | 7 | -0.090576304 | 0.280261004954724 | 0.746554731247166 | -0.639887874 | 0.458735265494203 | 0.913404634616311 | 0.527351550585936 | 1.58207181833743 | 0.999332001504091 |
| ebi-fl187-GCST90094750 | prot-a-2098 | Neuritin \|\| id:prot-a-2098 | Curry liking \|\| id:ebi-fl187-GCST90094750 | Weighted median | 7 | -0.280264503 | 0.260054808081217 | 0.281161759504104 | -0.789971927 | 0.229442920737609 | 0.755583860747689 | 0.453857536273017 | 1.25789906522331 | 0.999332001504091 |
| ebi-fl187-GCST90094750 | prot-a-2115 | Neurotrophin-4 \|\| id:prot-a-2115 | Curry liking \|\| id:ebi-fl187-GCST90094750 | Weighted median | 7 | -0.044473616 | 0.270767172601277 | 0.869534028491211 | -0.575177274 | 0.486230042624555 | 0.956500836390634 | 0.562605124720821 | 1.62617404253112 | 0.999332001504091 |
| ebi-fl187-GCST90094750 | prot-a-2118 | Netrin-1 \|\| id:prot-a-2118 | Curry liking \|\| id:ebi-fl187-GCST90094750 | Weighted median | 7 | 0.262670553495366 | 0.243100321011512 | 0.279918384618607 | -0.213806076 | 0.73914718267793 | 1.30039823672089 | 0.80750496468525 | 2.09414882634925 | 0.999332001504091 |
| ebi-fl187-GCST90094750 | prot-a-2120 | Netrin-G1 \|\| id:prot-a-2120 | Curry liking \|\| id:ebi-fl187-GCST90094750 | Weighted median | 7 | 0.286963593541063 | 0.25030658902939 | 0.251609657739146 | -0.203637321 | 0.777564508038668 | 1.33237570551127 | 0.815758175937901 | 2.17616577191595 | 0.999332001504091 |
| ebi-fl187-GCST90094750 | prot-a-2145 | Olfactomedin-like protein 3 \|\| id:prot-a-2145 | Curry liking \|\| id:ebi-fl187-GCST90094750 | Weighted median | 7 | -0.149618658 | 0.272146765396872 | 0.582475799405901 | -0.683026318 | 0.383789002012995 | 0.861036262975015 | 0.505086125682194 | 1.46783569862789 | 0.999332001504091 |
| ebi-fl187-GCST90094750 | prot-a-2162 | Prolyl 4-hydroxylase subunit alpha-2 \|\| id:prot-a-2162 | Curry liking \|\| id:ebi-fl187-GCST90094750 | Weighted median | 7 | 0.414774053644344 | 0.259643262455628 | 0.110159494630949 | -0.094126741 | 0.923674848057375 | 1.51402861279481 | 0.910167399614486 | 2.51852861499137 | 0.999332001504091 |
| ebi-fl187-GCST90094750 | prot-a-2168 | Polyadenylate-binding protein-interacting protein 1 \|\| id:prot-a-2168 | Curry liking \|\| id:ebi-fl187-GCST90094750 | Weighted median | 7 | -0.179505216 | 0.254559388975354 | 0.4807103847781 | -0.678441618 | 0.319431186619289 | 0.835683592196371 | 0.507407110577282 | 1.37634465837834 | 0.999332001504091 |
| ebi-fl187-GCST90094750 | prot-a-219 | Beta-1,4-galactosyltransferase 5 \|\| id:prot-a-219 | Curry liking \|\| id:ebi-fl187-GCST90094750 | Weighted median | 7 | -0.077714166 | 0.261356322333991 | 0.766199711560024 | -0.589972558 | 0.434544225995341 | 0.925228850818547 | 0.55434249703987 | 1.54425906539408 | 0.999332001504091 |
| ebi-fl187-GCST90094750 | prot-a-2190 | Pyruvate carboxylase, mitochondrial \|\| id:prot-a-2190 | Curry liking \|\| id:ebi-fl187-GCST90094750 | Weighted median | 7 | -0.226979852 | 0.22761978161435 | 0.31867297287268 | -0.673114624 | 0.219154920366067 | 0.796936839132136 | 0.510117277630095 | 1.24502414134355 | 0.999332001504091 |
| ebi-fl187-GCST90094750 | prot-a-2197 | Protocadherin-9 \|\| id:prot-a-2197 | Curry liking \|\| id:ebi-fl187-GCST90094750 | Weighted median | 7 | -0.057407486 | 0.247314042911318 | 0.81644182790983 | -0.54214301 | 0.42732803847322 | 0.944209239265967 | 0.581500754371293 | 1.53315551323596 | 0.999332001504091 |
| ebi-fl187-GCST90094750 | prot-a-2198 | Protocadherin alpha-4 \|\| id:prot-a-2198 | Curry liking \|\| id:ebi-fl187-GCST90094750 | Weighted median | 7 | 0.0238810095980974 | 0.241432787061521 | 0.921206793433188 | -0.449327253 | 0.497089272238679 | 1.02416844442506 | 0.63805725834474 | 1.64392926941569 | 0.999332001504091 |
| ebi-fl187-GCST90094750 | prot-a-2254 | Placenta growth factor \|\| id:prot-a-2254 | Curry liking \|\| id:ebi-fl187-GCST90094750 | Weighted median | 7 | -0.279299128 | 0.23509624988512 | 0.234825394699555 | -0.740087777 | 0.181489522094782 | 0.756313635032156 | 0.477072037513838 | 1.19900197361486 | 0.999332001504091 |
| ebi-fl187-GCST90094750 | prot-a-2261 | Membrane-associated progesterone receptor component 2 \|\| id:prot-a-2261 | Curry liking \|\| id:ebi-fl187-GCST90094750 | Weighted median | 7 | 0.274678984391507 | 0.260499370204688 | 0.291684980446392 | -0.235899781 | 0.785257749992697 | 1.31610811579959 | 0.789859828762895 | 2.19297210643878 | 0.999332001504091 |
| ebi-fl187-GCST90094750 | prot-a-2268 | Polymeric immunoglobulin receptor \|\| id:prot-a-2268 | Curry liking \|\| id:ebi-fl187-GCST90094750 | Weighted median | 7 | 0.0821634281239101 | 0.277202564874919 | 0.76692300588503 | -0.461153599 | 0.625480455278752 | 1.08563321833251 | 0.630555817196958 | 1.86914378172942 | 0.999332001504091 |
| ebi-fl187-GCST90094750 | prot-a-2283 | Group 10 secretory phospholipase A2 \|\| id:prot-a-2283 | Curry liking \|\| id:ebi-fl187-GCST90094750 | Weighted median | 7 | 0.0539616655136233 | 0.256300863056231 | 0.833245986638308 | -0.448388026 | 0.556311357103837 | 1.0554441414705 | 0.638656820445934 | 1.74422678988474 | 0.999332001504091 |
| ebi-fl187-GCST90094750 | prot-a-2298 | Inactive phospholipase D5 \|\| id:prot-a-2298 | Curry liking \|\| id:ebi-fl187-GCST90094750 | Weighted median | 7 | 0.0225333086768484 | 0.259525492947277 | 0.93081059179246 | -0.486136657 | 0.53120327485351 | 1.02278910134874 | 0.614997757514709 | 1.70097782155366 | 0.999332001504091 |
| ebi-fl187-GCST90094750 | prot-a-2304 | Procollagen-lysine,2-oxoglutarate 5-dioxygenase 2 \|\| id:prot-a-2304 | Curry liking \|\| id:ebi-fl187-GCST90094750 | Weighted median | 7 | 0.056373029389217 | 0.242823935863405 | 0.81641686470936 | -0.419561885 | 0.532307943681491 | 1.05799227234661 | 0.657334745013799 | 1.70285787695834 | 0.999332001504091 |
| ebi-fl187-GCST90094750 | prot-a-2331 | Periostin \|\| id:prot-a-2331 | Curry liking \|\| id:ebi-fl187-GCST90094750 | Weighted median | 7 | -0.032230931 | 0.255118504070724 | 0.899465092986118 | -0.532263199 | 0.467801336990281 | 0.968282949719802 | 0.587274345820268 | 1.59648020961743 | 0.999332001504091 |
| ebi-fl187-GCST90094750 | prot-a-2333 | Periostin \|\| id:prot-a-2333 | Curry liking \|\| id:ebi-fl187-GCST90094750 | Weighted median | 7 | -0.267099738 | 0.264540516668333 | 0.312651401203897 | -0.785599151 | 0.251399674814815 | 0.765596708683697 | 0.455846499279713 | 1.2858238930725 | 0.999332001504091 |
| ebi-fl187-GCST90094750 | prot-a-2367 | cAMP-dependent protein kinase catalytic subunit alpha \|\| id:prot-a-2367 | Curry liking \|\| id:ebi-fl187-GCST90094750 | Weighted median | 7 | 0.0965163612279681 | 0.236370195534226 | 0.683033531228775 | -0.366769222 | 0.559801944475051 | 1.10132760006832 | 0.692969548705825 | 1.75032580426871 | 0.999332001504091 |
| ebi-fl187-GCST90094750 | prot-a-2385 | Phosphoribosyl pyrophosphate synthase-associated protein 1 \|\| id:prot-a-2385 | Curry liking \|\| id:ebi-fl187-GCST90094750 | Weighted median | 7 | 0.485908756990654 | 0.260821970900196 | 0.0624636551418554 | -0.025302306 | 0.997119819955038 | 1.62565166009451 | 0.975015114577448 | 2.71046395123152 | 0.999332001504091 |
| ebi-fl187-GCST90094750 | prot-a-2393 | Trypsin-3 \|\| id:prot-a-2393 | Curry liking \|\| id:ebi-fl187-GCST90094750 | Weighted median | 6 | 0.206173652249917 | 0.291515187544413 | 0.479412120769974 | -0.365196115 | 0.777543419836965 | 1.22896659828144 | 0.694060521616711 | 2.1761198809771 | 0.999332001504091 |
| ebi-fl187-GCST90094750 | prot-a-2405 | Pregnancy-specific beta-1-glycoprotein 6 \|\| id:prot-a-2405 | Curry liking \|\| id:ebi-fl187-GCST90094750 | Weighted median | 7 | 0.254520337646645 | 0.243354851329818 | 0.295615745377694 | -0.222455171 | 0.731495846253088 | 1.28984278344328 | 0.800550893874036 | 2.07818693193851 | 0.999332001504091 |
| ebi-fl187-GCST90094750 | prot-a-244 | Beclin-1 \|\| id:prot-a-244 | Curry liking \|\| id:ebi-fl187-GCST90094750 | Weighted median | 7 | 0.0588626862666497 | 0.274529586240476 | 0.830225078445618 | -0.479215303 | 0.596940675297982 | 1.06062959173164 | 0.619269139980652 | 1.81655286567642 | 0.999332001504091 |
| ebi-fl187-GCST90094750 | prot-a-2454 | tRNA pseudouridine synthase A, mitochondrial \|\| id:prot-a-2454 | Curry liking \|\| id:ebi-fl187-GCST90094750 | Weighted median | 7 | 0.0153975325231213 | 0.251487338208705 | 0.951179322513133 | -0.47751765 | 0.508312715412183 | 1.01551668529441 | 0.62032133660096 | 1.66248374392889 | 0.999332001504091 |
| ebi-fl187-GCST90094750 | prot-a-2466 | Glutaminyl-peptide cyclotransferase-like protein \|\| id:prot-a-2466 | Curry liking \|\| id:ebi-fl187-GCST90094750 | Weighted median | 7 | -0.064551862 | 0.243225347730642 | 0.790701796313375 | -0.541273544 | 0.412169819564481 | 0.937487493036547 | 0.582006569484917 | 1.51009085752721 | 0.999332001504091 |
| ebi-fl187-GCST90094750 | prot-a-2474 | Ras-related protein Rab-27A \|\| id:prot-a-2474 | Curry liking \|\| id:ebi-fl187-GCST90094750 | Weighted median | 7 | -0.074928846 | 0.244340420227152 | 0.75910400732047 | -0.55383607 | 0.403978377214803 | 0.927809500937824 | 0.574740830088184 | 1.49777156061527 | 0.999332001504091 |
| ebi-fl187-GCST90094750 | prot-a-2490 | Rap1 GTPase-GDP dissociation stimulator 1 \|\| id:prot-a-2490 | Curry liking \|\| id:ebi-fl187-GCST90094750 | Weighted median | 7 | -0.379935008 | 0.236961225696531 | 0.10885439880966 | -0.84437901 | 0.0845089945486283 | 0.683905856302844 | 0.429824191798429 | 1.08818263189959 | 0.999332001504091 |
| ebi-fl187-GCST90094750 | prot-a-2545 | Receptor-interacting serine/threonine-protein kinase 2 \|\| id:prot-a-2545 | Curry liking \|\| id:ebi-fl187-GCST90094750 | Weighted median | 7 | 0.0189605395825308 | 0.241520630055916 | 0.937426476512696 | -0.454419895 | 0.492340974492127 | 1.01914143207756 | 0.634816120952484 | 1.63614190675988 | 0.999332001504091 |
| ebi-fl187-GCST90094750 | prot-a-2610 | RUN and FYVE domain-containing protein 1 \|\| id:prot-a-2610 | Curry liking \|\| id:ebi-fl187-GCST90094750 | Weighted median | 7 | -0.047294938 | 0.253190080786756 | 0.851820447800403 | -0.543547496 | 0.448957620271269 | 0.953806042387196 | 0.580684617571837 | 1.56667826039284 | 0.999332001504091 |
| ebi-fl187-GCST90094750 | prot-a-2627 | Serine--tRNA ligase, mitochondrial \|\| id:prot-a-2627 | Curry liking \|\| id:ebi-fl187-GCST90094750 | Weighted median | 7 | 0.104629211318827 | 0.24331098118259 | 0.667179229572381 | -0.372260312 | 0.581518734436703 | 1.1102988477765 | 0.689174818835234 | 1.78875300966059 | 0.999332001504091 |
| ebi-fl187-GCST90094750 | prot-a-264 | BolA-like protein 2 \|\| id:prot-a-264 | Curry liking \|\| id:ebi-fl187-GCST90094750 | Weighted median | 7 | -0.115024516 | 0.241920786450844 | 0.634456608679296 | -0.589189257 | 0.359140225877094 | 0.89134429182866 | 0.554776883924722 | 1.43209760463513 | 0.999332001504091 |
| ebi-fl187-GCST90094750 | prot-a-2646 | Secretoglobin family 1C member 1 \|\| id:prot-a-2646 | Curry liking \|\| id:ebi-fl187-GCST90094750 | Weighted median | 7 | -0.144323702 | 0.245628638522418 | 0.556821818271063 | -0.625755834 | 0.337108429128187 | 0.865607503498385 | 0.534857012651683 | 1.40089095288849 | 0.999332001504091 |
| ebi-fl187-GCST90094750 | prot-a-2672 | Semaphorin-3E \|\| id:prot-a-2672 | Curry liking \|\| id:ebi-fl187-GCST90094750 | Weighted median | 7 | 0.0467827439375682 | 0.269417856304896 | 0.862145397017012 | -0.481276254 | 0.574841742295165 | 1.04789432295301 | 0.617994170498025 | 1.77684930456904 | 0.999332001504091 |
| ebi-fl187-GCST90094750 | prot-a-2701 | Plasma protease C1 inhibitor \|\| id:prot-a-2701 | Curry liking \|\| id:ebi-fl187-GCST90094750 | Weighted median | 7 | -0.164558045 | 0.269277795586277 | 0.541127519541787 | -0.692342524 | 0.363226434649427 | 0.84826851802395 | 0.500402490167033 | 1.43796142667148 | 0.999332001504091 |
| ebi-fl187-GCST90094750 | prot-a-2738 | Signaling threshold-regulating transmembrane adapter 1 \|\| id:prot-a-2738 | Curry liking \|\| id:ebi-fl187-GCST90094750 | Weighted median | 7 | 0.0590235238043947 | 0.236892199337463 | 0.80323877125217 | -0.405285187 | 0.523332234505822 | 1.06080019450293 | 0.666786624931949 | 1.68764190909244 | 0.999332001504091 |
| ebi-fl187-GCST90094750 | prot-a-274 | Serine/threonine-protein kinase BRSK2 \|\| id:prot-a-274 | Curry liking \|\| id:ebi-fl187-GCST90094750 | Weighted median | 7 | 0.176213374394906 | 0.241557986915356 | 0.465703910204502 | -0.29724028 | 0.649667028749003 | 1.19269252154687 | 0.742865495224877 | 1.91490311516381 | 0.999332001504091 |
| ebi-fl187-GCST90094750 | prot-a-2749 | Mitochondrial glutamate carrier 2 \|\| id:prot-a-2749 | Curry liking \|\| id:ebi-fl187-GCST90094750 | Weighted median | 7 | 0.111354466996936 | 0.256203308383657 | 0.66382868346144 | -0.390804017 | 0.613512951428904 | 1.11779105667835 | 0.67651272774761 | 1.84690811442686 | 0.999332001504091 |
| ebi-fl187-GCST90094750 | prot-a-2760 | Sodium-coupled monocarboxylate transporter 1 \|\| id:prot-a-2760 | Curry liking \|\| id:ebi-fl187-GCST90094750 | Weighted median | 7 | -0.146783224 | 0.254953521590242 | 0.564800789293825 | -0.646492126 | 0.35292567852453 | 0.863481139296224 | 0.523880263203516 | 1.42322536329386 | 0.999332001504091 |
| ebi-fl187-GCST90094750 | prot-a-2777 | Small integral membrane protein 10 \|\| id:prot-a-2777 | Curry liking \|\| id:ebi-fl187-GCST90094750 | Weighted median | 7 | 0.25339033133223 | 0.258505858764975 | 0.326981889896623 | -0.253281152 | 0.760061814511581 | 1.28838607615261 | 0.776249607130138 | 2.13840840108231 | 0.999332001504091 |
| ebi-fl187-GCST90094750 | prot-a-2799 | Superoxide dismutase [Mn], mitochondrial \|\| id:prot-a-2799 | Curry liking \|\| id:ebi-fl187-GCST90094750 | Weighted median | 7 | 0.517830319534023 | 0.249590491807908 | 0.0380125170624307 | 0.0286329555905235 | 1.00702768347752 | 1.67838214338613 | 1.02904681926713 | 2.73745233598176 | 0.999332001504091 |
| ebi-fl187-GCST90094750 | prot-a-2805 | Protein SPACA7 \|\| id:prot-a-2805 | Curry liking \|\| id:ebi-fl187-GCST90094750 | Weighted median | 7 | -0.051897031 | 0.264476230170492 | 0.844433520767816 | -0.570270442 | 0.466476380233087 | 0.949426623413757 | 0.565372517528004 | 1.59436634307609 | 0.999332001504091 |
| ebi-fl187-GCST90094750 | prot-a-2811 | SPARC-like protein 1 \|\| id:prot-a-2811 | Curry liking \|\| id:ebi-fl187-GCST90094750 | Weighted median | 7 | 0.13902907804465 | 0.277610809425581 | 0.61650786786427 | -0.405088108 | 0.683146264518788 | 1.14915751477131 | 0.66691804716799 | 1.98009785364638 | 0.999332001504091 |
| ebi-fl187-GCST90094750 | prot-a-2831 | Sepiapterin reductase \|\| id:prot-a-2831 | Curry liking \|\| id:ebi-fl187-GCST90094750 | Weighted median | 7 | 0.197493314475978 | 0.270205452848654 | 0.464839899196956 | -0.332109373 | 0.72709600205934 | 1.21834491966818 | 0.71740885332774 | 2.06906331918817 | 0.999332001504091 |
| ebi-fl187-GCST90094750 | prot-a-2834 | SPRY domain-containing SOCS box protein 1 \|\| id:prot-a-2834 | Curry liking \|\| id:ebi-fl187-GCST90094750 | Weighted median | 7 | -0.027307486 | 0.241162514195079 | 0.909846060730376 | -0.499986014 | 0.445371041917886 | 0.973061992672372 | 0.606539142875454 | 1.56106931053904 | 0.999332001504091 |
| ebi-fl187-GCST90094750 | prot-a-2835 | Spectrin alpha chain, non-erythrocytic 1 \|\| id:prot-a-2835 | Curry liking \|\| id:ebi-fl187-GCST90094750 | Weighted median | 7 | -0.059074147 | 0.233252038085191 | 0.800064866515871 | -0.516248142 | 0.398099847678569 | 0.942636872899402 | 0.596755294477698 | 1.48899269494922 | 0.999332001504091 |
| ebi-fl187-GCST90094750 | prot-a-2859 | Sia-alpha-2,3-Gal-beta-1,4-GlcNAc-R:alpha 2,8-sialyltransferase \|\| id:prot-a-2859 | Curry liking \|\| id:ebi-fl187-GCST90094750 | Weighted median | 7 | -0.10127027 | 0.237651584588999 | 0.670013733582451 | -0.567067376 | 0.364526835833691 | 0.903688759951854 | 0.567186346531059 | 1.43983256976832 | 0.999332001504091 |
| ebi-fl187-GCST90094750 | prot-a-2861 | Alpha-2,8-sialyltransferase 8F \|\| id:prot-a-2861 | Curry liking \|\| id:ebi-fl187-GCST90094750 | Weighted median | 7 | -0.353451403 | 0.24514599085561 | 0.149358903077209 | -0.833937545 | 0.127034739494032 | 0.702260119786723 | 0.434335698660664 | 1.13545646227934 | 0.999332001504091 |
| ebi-fl187-GCST90094750 | prot-a-289 | Uncharacterized protein C17orf89 \|\| id:prot-a-289 | Curry liking \|\| id:ebi-fl187-GCST90094750 | Weighted median | 7 | -0.541907716 | 0.266493338424578 | 0.0420044905955913 | -1.06423466 | -0.019580773 | 0.581637593828228 | 0.344991789932088 | 0.98060968529392 | 0.999332001504091 |
| ebi-fl187-GCST90094750 | prot-a-2921 | Tachykinin-3 \|\| id:prot-a-2921 | Curry liking \|\| id:ebi-fl187-GCST90094750 | Weighted median | 7 | -0.092293073 | 0.24957602735771 | 0.7115319381848 | -0.581462087 | 0.396875940362617 | 0.911837875084234 | 0.559080344665001 | 1.48717142065929 | 0.999332001504091 |
| ebi-fl187-GCST90094750 | prot-a-2928 | Tax1-binding protein 1 \|\| id:prot-a-2928 | Curry liking \|\| id:ebi-fl187-GCST90094750 | Weighted median | 7 | 0.0501314628867405 | 0.258133543793056 | 0.846013432726498 | -0.455810283 | 0.55607320872113 | 1.05140930859379 | 0.633934093798475 | 1.74381145455334 | 0.999332001504091 |
| ebi-fl187-GCST90094750 | prot-a-296 | Uncharacterized protein C1orf185 \|\| id:prot-a-296 | Curry liking \|\| id:ebi-fl187-GCST90094750 | Weighted median | 7 | -0.186883027 | 0.242980169843302 | 0.44181690013566 | -0.66312416 | 0.289358105856446 | 0.829540764552093 | 0.515239127969895 | 1.33556991831156 | 0.999332001504091 |
| ebi-fl187-GCST90094750 | prot-a-2964 | Transforming growth factor beta-2 \|\| id:prot-a-2964 | Curry liking \|\| id:ebi-fl187-GCST90094750 | Weighted median | 7 | 0.145021085912419 | 0.245553080285874 | 0.55479546094097 | -0.336262951 | 0.626305123272732 | 1.15606394667415 | 0.714435219339278 | 1.87068584053824 | 0.999332001504091 |
| ebi-fl187-GCST90094750 | prot-a-2977 | Thymocyte nuclear protein 1 \|\| id:prot-a-2977 | Curry liking \|\| id:ebi-fl187-GCST90094750 | Weighted median | 7 | 0.224556822993044 | 0.246644326056157 | 0.362586315636623 | -0.258866056 | 0.707979702063112 | 1.25176783852235 | 0.77192641097119 | 2.02988613848245 | 0.999332001504091 |
| ebi-fl187-GCST90094750 | prot-a-3015 | Transmembrane protein 70, mitochondrial \|\| id:prot-a-3015 | Curry liking \|\| id:ebi-fl187-GCST90094750 | Weighted median | 7 | -0.302889121 | 0.247431946212373 | 0.22090280584983 | -0.787855735 | 0.18207749391845 | 0.738680996292858 | 0.454819002792235 | 1.1997071602865 | 0.999332001504091 |
| ebi-fl187-GCST90094750 | prot-a-3030 | Tumor necrosis factor alpha-induced protein 3 \|\| id:prot-a-3030 | Curry liking \|\| id:ebi-fl187-GCST90094750 | Weighted median | 7 | -0.107029432 | 0.274318617932378 | 0.696414786473588 | -0.644693924 | 0.430635058756481 | 0.89849922762057 | 0.524823153539429 | 1.5382340824529 | 0.999332001504091 |
| ebi-fl187-GCST90094750 | prot-a-3032 | Tumor necrosis factor receptor superfamily member 10A \|\| id:prot-a-3032 | Curry liking \|\| id:ebi-fl187-GCST90094750 | Weighted median | 7 | 0.0493834946479114 | 0.252896993006648 | 0.845180656529331 | -0.446294612 | 0.545061600940942 | 1.05062318186052 | 0.639995194247356 | 1.72471462314778 | 0.999332001504091 |
| ebi-fl187-GCST90094750 | prot-a-3081 | Tumor protein p53-inducible protein 11 \|\| id:prot-a-3081 | Curry liking \|\| id:ebi-fl187-GCST90094750 | Weighted median | 7 | -0.102754437 | 0.246480869447294 | 0.676761755613071 | -0.585856941 | 0.380348067370734 | 0.902348529918627 | 0.556628659530632 | 1.46279365157536 | 0.999332001504091 |
| ebi-fl187-GCST90094750 | prot-a-315 | Complement C4 \|\| id:prot-a-315 | Curry liking \|\| id:ebi-fl187-GCST90094750 | Weighted median | 6 | -0.185280603 | 0.268124205258937 | 0.489549656276886 | -0.710804046 | 0.340242839053951 | 0.830871106002786 | 0.491249052005128 | 1.40528880813614 | 0.999332001504091 |
| ebi-fl187-GCST90094750 | prot-a-3157 | UDP-glucuronosyltransferase 1-8 \|\| id:prot-a-3157 | Curry liking \|\| id:ebi-fl187-GCST90094750 | Weighted median | 7 | 0.300119199014868 | 0.244131059468331 | 0.218945703220186 | -0.178377678 | 0.778616075572798 | 1.35001971900613 | 0.836626388813937 | 2.17845536080827 | 0.999332001504091 |
| ebi-fl187-GCST90094750 | prot-a-317 | Glutaredoxin-like protein C5orf63 \|\| id:prot-a-317 | Curry liking \|\| id:ebi-fl187-GCST90094750 | Weighted median | 7 | -0.013376775 | 0.2715762005828 | 0.960715231658034 | -0.545666128 | 0.518912578023456 | 0.986712296332199 | 0.579455662958112 | 1.68019956999461 | 0.999332001504091 |
| ebi-fl187-GCST90094750 | prot-a-3192 | Guanine nucleotide exchange factor VAV3 \|\| id:prot-a-3192 | Curry liking \|\| id:ebi-fl187-GCST90094750 | Weighted median | 7 | 0.121281833146862 | 0.260400204646548 | 0.641393254658485 | -0.389102568 | 0.631666234254096 | 1.12894304110553 | 0.677664759757046 | 1.88074172621509 | 0.999332001504091 |
| ebi-fl187-GCST90094750 | prot-a-3233 | Serine/threonine-protein kinase WNK3 \|\| id:prot-a-3233 | Curry liking \|\| id:ebi-fl187-GCST90094750 | Weighted median | 7 | 0.52274001065291 | 0.274401668939946 | 0.0567777824542798 | -0.01508726 | 1.0605672817752 | 1.68664274319284 | 0.985025982023221 | 2.888008839 | 0.999332001504091 |
| ebi-fl187-GCST90094750 | prot-a-3250 | 14-3-3 protein theta \|\| id:prot-a-3250 | Curry liking \|\| id:ebi-fl187-GCST90094750 | Weighted median | 7 | 0.27251747811462 | 0.244869718310831 | 0.265747926915084 | -0.20742717 | 0.752462126003849 | 1.31326641213278 | 0.812672426733853 | 2.12221875936848 | 0.999332001504091 |
| ebi-fl187-GCST90094750 | prot-a-3278 | E3 ubiquitin-protein ligase ZNRF3 \|\| id:prot-a-3278 | Curry liking \|\| id:ebi-fl187-GCST90094750 | Weighted median | 7 | 0.332879691167233 | 0.231066538073858 | 0.14969131455822 | -0.120010723 | 0.785770105791994 | 1.39497946002321 | 0.886910925914517 | 2.19409597630124 | 0.999332001504091 |
| ebi-fl187-GCST90094750 | prot-a-355 | Calpain-3 \|\| id:prot-a-355 | Curry liking \|\| id:ebi-fl187-GCST90094750 | Weighted median | 7 | 0.27250670697225 | 0.255243650126421 | 0.285685803517655 | -0.227770847 | 0.772784261220034 | 1.31325226682946 | 0.796306714782834 | 2.16578798635781 | 0.999332001504091 |
| ebi-fl187-GCST90094750 | prot-a-385 | Coiled-coil domain-containing protein 90B, mitochondrial \|\| id:prot-a-385 | Curry liking \|\| id:ebi-fl187-GCST90094750 | Weighted median | 7 | -0.371045122 | 0.250227618426602 | 0.138119474572707 | -0.861491254 | 0.119401010331271 | 0.690012806246953 | 0.422531510499932 | 1.1268216948399 | 0.999332001504091 |
| ebi-fl187-GCST90094750 | prot-a-390 | C-C motif chemokine 14 \|\| id:prot-a-390 | Curry liking \|\| id:ebi-fl187-GCST90094750 | Weighted median | 7 | 0.0830490481218697 | 0.259442671087914 | 0.748888291680848 | -0.425458587 | 0.59155668345418 | 1.08659510269026 | 0.653470043401929 | 1.80679884121979 | 0.999332001504091 |
| ebi-fl187-GCST90094750 | prot-a-40 | Pituitary adenylate cyclase-activating polypeptide 27 \|\| id:prot-a-40 | Curry liking \|\| id:ebi-fl187-GCST90094750 | Weighted median | 7 | -0.101383775 | 0.237824928151428 | 0.669892297781662 | -0.567520634 | 0.364753084592119 | 0.903586192920029 | 0.566929323031812 | 1.44015836695379 | 0.999332001504091 |
| ebi-fl187-GCST90094750 | prot-a-401 | C-C motif chemokine 24 \|\| id:prot-a-401 | Curry liking \|\| id:ebi-fl187-GCST90094750 | Weighted median | 7 | -0.111507675 | 0.246206110212234 | 0.6506180377805 | -0.594071651 | 0.371056301068739 | 0.894484526255378 | 0.552074846195261 | 1.44926466623934 | 0.999332001504091 |
| ebi-fl187-GCST90094750 | prot-a-412 | Cerebral cavernous malformations 2 protein \|\| id:prot-a-412 | Curry liking \|\| id:ebi-fl187-GCST90094750 | Weighted median | 7 | 0.0744293723200301 | 0.249502527466594 | 0.76546573655549 | -0.414595582 | 0.563454326154554 | 1.0772692558071 | 0.660607388529255 | 1.75673035097424 | 0.999332001504091 |
| ebi-fl187-GCST90094750 | prot-a-44 | Adhesion G protein-coupled receptor F5 \|\| id:prot-a-44 | Curry liking \|\| id:ebi-fl187-GCST90094750 | Weighted median | 7 | 0.0490568708898354 | 0.240537568792719 | 0.838395061249646 | -0.422396764 | 0.520510505723566 | 1.05028007940429 | 0.65547391937636 | 1.68288655366029 | 0.999332001504091 |
| ebi-fl187-GCST90094750 | prot-a-48 | Adrenomedullin \|\| id:prot-a-48 | Curry liking \|\| id:ebi-fl187-GCST90094750 | Weighted median | 7 | 0.128167101185756 | 0.250958587146916 | 0.609554022598123 | -0.36371173 | 0.620045931993712 | 1.13674293792024 | 0.695091540165587 | 1.85901342807844 | 0.999332001504091 |
| ebi-fl187-GCST90094750 | prot-a-502 | Carcinoembryonic antigen-related cell adhesion molecule 1 \|\| id:prot-a-502 | Curry liking \|\| id:ebi-fl187-GCST90094750 | Weighted median | 7 | 0.0723857984977243 | 0.264895189028807 | 0.784651796510103 | -0.446808772 | 0.591580368994185 | 1.07507002446752 | 0.639666218672314 | 1.80684163673284 | 0.999332001504091 |
| ebi-fl187-GCST90094750 | prot-a-518 | Complement factor B \|\| id:prot-a-518 | Curry liking \|\| id:ebi-fl187-GCST90094750 | Weighted median | 7 | 0.279548174795064 | 0.244733553100575 | 0.253347955234353 | -0.200129589 | 0.75922593887219 | 1.32253212397432 | 0.81862466122182 | 2.13662170442491 | 0.999332001504091 |
| ebi-fl187-GCST90094750 | prot-a-525 | Properdin \|\| id:prot-a-525 | Curry liking \|\| id:ebi-fl187-GCST90094750 | Weighted median | 7 | 0.26372862479064 | 0.238998821829313 | 0.269822032265682 | -0.204709066 | 0.732166315576094 | 1.30177487893197 | 0.814884359498268 | 2.07958075973102 | 0.999332001504091 |
| ebi-fl187-GCST90094750 | prot-a-533 | Chondroadherin \|\| id:prot-a-533 | Curry liking \|\| id:ebi-fl187-GCST90094750 | Weighted median | 7 | -0.169136645 | 0.283726882026528 | 0.551091824596997 | -0.725241334 | 0.386968043878915 | 0.844393513441129 | 0.484207699234917 | 1.47250943483147 | 0.999332001504091 |
| ebi-fl187-GCST90094750 | prot-a-535 | Coiled-coil-helix-coiled-coil-helix domain-containing protein 2 \|\| id:prot-a-535 | Curry liking \|\| id:ebi-fl187-GCST90094750 | Weighted median | 7 | -0.1870113 | 0.260709907057021 | 0.473179344611974 | -0.698002718 | 0.323980117914772 | 0.82943436379299 | 0.497578115937904 | 1.38261981748116 | 0.999332001504091 |
| ebi-fl187-GCST90094750 | prot-a-591 | Caseinolytic peptidase B protein homolog \|\| id:prot-a-591 | Curry liking \|\| id:ebi-fl187-GCST90094750 | Weighted median | 7 | 0.0908704901330226 | 0.283603831071697 | 0.748654909074417 | -0.464993019 | 0.646733999033548 | 1.09512716630026 | 0.628139490362157 | 1.90929487601134 | 0.999332001504091 |
| ebi-fl187-GCST90094750 | prot-a-605 | Ciliary neurotrophic factor receptor subunit alpha \|\| id:prot-a-605 | Curry liking \|\| id:ebi-fl187-GCST90094750 | Weighted median | 7 | -0.057528065 | 0.25219370518161 | 0.819560236810693 | -0.551827727 | 0.436771597321219 | 0.944095394133405 | 0.575896266728152 | 1.54770253727769 | 0.999332001504091 |
| ebi-fl187-GCST90094750 | prot-a-606 | Ciliary neurotrophic factor receptor subunit alpha \|\| id:prot-a-606 | Curry liking \|\| id:ebi-fl187-GCST90094750 | Weighted median | 7 | 0.298114270872392 | 0.242298699517621 | 0.218562876794805 | -0.17679118 | 0.77301972192693 | 1.34731573802815 | 0.837954747812266 | 2.16629800437031 | 0.999332001504091 |
| ebi-fl187-GCST90094750 | prot-a-707 | Cancer/testis antigen 55 \|\| id:prot-a-707 | Curry liking \|\| id:ebi-fl187-GCST90094750 | Weighted median | 7 | 0.192286114974919 | 0.29351182909939 | 0.51238908037335 | -0.38299707 | 0.767569300009724 | 1.21201724364395 | 0.681814896959881 | 2.15452288508258 | 0.999332001504091 |
| ebi-fl187-GCST90094750 | prot-a-719 | Dipeptidyl peptidase 1 \|\| id:prot-a-719 | Curry liking \|\| id:ebi-fl187-GCST90094750 | Weighted median | 7 | -0.247013923 | 0.251789357614902 | 0.326575971028835 | -0.740521064 | 0.246493218120293 | 0.781129817937598 | 0.476865373523253 | 1.27953050556621 | 0.999332001504091 |
| ebi-fl187-GCST90094750 | prot-a-752 | Cytochrome b5 \|\| id:prot-a-752 | Curry liking \|\| id:ebi-fl187-GCST90094750 | Weighted median | 7 | -0.310561407 | 0.243344025688125 | 0.201876461957136 | -0.787515697 | 0.166392883660415 | 0.733035309758895 | 0.454973684923338 | 1.181037021 | 0.999332001504091 |
| ebi-fl187-GCST90094750 | prot-a-755 | Cytoglobin \|\| id:prot-a-755 | Curry liking \|\| id:ebi-fl187-GCST90094750 | Weighted median | 7 | -0.206377061 | 0.258208716197114 | 0.424137057732023 | -0.712466145 | 0.299712022825364 | 0.813526269479716 | 0.490433225574853 | 1.34947013501754 | 0.999332001504091 |
| ebi-fl187-GCST90094750 | prot-a-807 | Desmin \|\| id:prot-a-807 | Curry liking \|\| id:ebi-fl187-GCST90094750 | Weighted median | 7 | 0.00508081903459706 | 0.262885150030301 | 0.98458013054028 | -0.510174075 | 0.520335713093986 | 1.00509374828341 | 0.600391056627112 | 1.68259242320096 | 0.999332001504091 |
| ebi-fl187-GCST90094750 | prot-a-82 | Ameloblastin \|\| id:prot-a-82 | Curry liking \|\| id:ebi-fl187-GCST90094750 | Weighted median | 7 | -0.011010599 | 0.242885702985981 | 0.963842340065849 | -0.487066577 | 0.465045379171103 | 0.989049796096122 | 0.614426125221119 | 1.5920864348106 | 0.999332001504091 |
| ebi-fl187-GCST90094750 | prot-a-84 | Muellerian-inhibiting factor \|\| id:prot-a-84 | Curry liking \|\| id:ebi-fl187-GCST90094750 | Weighted median | 7 | 0.155311202215548 | 0.249946177571235 | 0.534350558669038 | -0.334583306 | 0.645205710255168 | 1.16802139539747 | 0.715636225679108 | 1.90637915068038 | 0.999332001504091 |
| ebi-fl187-GCST90094750 | prot-a-858 | Inactive dipeptidyl peptidase 10 \|\| id:prot-a-858 | Curry liking \|\| id:ebi-fl187-GCST90094750 | Weighted median | 7 | 0.0855322219220533 | 0.256583454851384 | 0.73886972664859 | -0.41737135 | 0.588435793430767 | 1.08929666001066 | 0.658776238231937 | 1.8011688106647 | 0.999332001504091 |
| ebi-fl187-GCST90094750 | prot-a-863 | Dihydropyrimidinase-related protein 3 \|\| id:prot-a-863 | Curry liking \|\| id:ebi-fl187-GCST90094750 | Weighted median | 7 | -0.030637844 | 0.242096149441073 | 0.89929469003908 | -0.505146297 | 0.443870609306716 | 0.969826738446161 | 0.603417291130243 | 1.55872878757481 | 0.999332001504091 |
| ebi-fl187-GCST90094750 | prot-a-864 | Dihydropyrimidinase-related protein 5 \|\| id:prot-a-864 | Curry liking \|\| id:ebi-fl187-GCST90094750 | Weighted median | 7 | -0.064893417 | 0.2458456293897 | 0.791810727168107 | -0.54675085 | 0.416964016772661 | 0.937167344319794 | 0.578827455328712 | 1.51734791287785 | 0.999332001504091 |
| ebi-fl187-GCST90094750 | prot-a-884 | Endothelin-converting enzyme 1 \|\| id:prot-a-884 | Curry liking \|\| id:ebi-fl187-GCST90094750 | Weighted median | 7 | 0.171443521213826 | 0.244623835559963 | 0.483399421806204 | -0.308019196 | 0.650906238911354 | 1.1870170995541 | 0.734901210354554 | 1.91727755347422 | 0.999332001504091 |
| ebi-fl187-GCST90094750 | prot-a-893 | Early endosome antigen 1 \|\| id:prot-a-893 | Curry liking \|\| id:ebi-fl187-GCST90094750 | Weighted median | 7 | 0.172416626554445 | 0.243309790467764 | 0.478554089578202 | -0.304470563 | 0.649303815871263 | 1.18817275442885 | 0.737513738282228 | 1.91420772398791 | 0.999332001504091 |
| ebi-fl187-GCST90094750 | prot-a-897 | Ephrin-A2 \|\| id:prot-a-897 | Curry liking \|\| id:ebi-fl187-GCST90094750 | Weighted median | 7 | 0.269498471935588 | 0.235362201005428 | 0.252193754378203 | -0.191811442 | 0.730808385906227 | 1.30930763151822 | 0.82546250134771 | 2.07675875179429 | 0.999332001504091 |
| ebi-fl187-GCST90094750 | prot-a-90 | Anaphase-promoting complex subunit 10 \|\| id:prot-a-90 | Curry liking \|\| id:ebi-fl187-GCST90094750 | Weighted median | 7 | 0.258767159809133 | 0.235795034627867 | 0.272456031661225 | -0.203391108 | 0.720925427679752 | 1.29533216432402 | 0.815959050848045 | 2.05633531999979 | 0.999332001504091 |
| ebi-fl187-GCST90094750 | prot-a-914 | Histone-lysine N-methyltransferase EHMT2 \|\| id:prot-a-914 | Curry liking \|\| id:ebi-fl187-GCST90094750 | Weighted median | 7 | -0.241279677 | 0.245767862090677 | 0.326228884647447 | -0.722984686 | 0.2404253329494 | 0.785621875488619 | 0.485301619024122 | 1.27178996947788 | 0.999332001504091 |
| ebi-fl187-GCST90094750 | prot-a-932 | RNA polymerase II elongation factor ELL \|\| id:prot-a-932 | Curry liking \|\| id:ebi-fl187-GCST90094750 | Weighted median | 7 | -0.025637138 | 0.248961500806882 | 0.917981769564683 | -0.513601679 | 0.462327403861698 | 0.974688703213692 | 0.598336676486248 | 1.58776505854764 | 0.999332001504091 |
| ebi-fl187-GCST90094750 | prot-a-991 | Estrogen receptor \|\| id:prot-a-991 | Curry liking \|\| id:ebi-fl187-GCST90094750 | Weighted median | 7 | -0.243100503 | 0.231382982339338 | 0.293423393837335 | -0.696611148 | 0.210410142451214 | 0.784192696144225 | 0.498271012427933 | 1.23418414747716 | 0.999332001504091 |
| ebi-fl187-GCST90094750 | prot-a-992 | Persulfide dioxygenase ETHE1, mitochondrial \|\| id:prot-a-992 | Curry liking \|\| id:ebi-fl187-GCST90094750 | Weighted median | 7 | -0.039831371 | 0.259152049286909 | 0.877847195019005 | -0.547769387 | 0.468106645783727 | 0.960951469950108 | 0.578238198297935 | 1.59696770347828 | 0.999332001504091 |
| ebi-fl187-GCST90094750 | prot-a-993 | Ethanolamine kinase 1 \|\| id:prot-a-993 | Curry liking \|\| id:ebi-fl187-GCST90094750 | Weighted median | 7 | -0.010574214 | 0.259028236248622 | 0.967437298092846 | -0.518269557 | 0.497121129144008 | 0.989481496558853 | 0.595550222559839 | 1.64398164074899 | 0.999332001504091 |
| ebi-fl187-GCST90094750 | prot-b-10 | coagulation factor III, tissue factor \|\| id:prot-b-10 | Curry liking \|\| id:ebi-fl187-GCST90094750 | Weighted median | 5 | 0.30508857048744 | 0.291319570216201 | 0.294977720730298 | -0.265897787 | 0.876074928111194 | 1.35674516526535 | 0.766517471442893 | 2.4014552988673 | 0.999332001504091 |
| ebi-fl187-GCST90094750 | prot-b-28 | prolactin \|\| id:prot-b-28 | Curry liking \|\| id:ebi-fl187-GCST90094750 | Weighted median | 5 | -0.405619145 | 0.288473309731019 | 0.159697701323174 | -0.971026832 | 0.159788542358284 | 0.666563983504453 | 0.378693983370911 | 1.17326274938503 | 0.999332001504091 |
| ebi-fl187-GCST90094750 | prot-b-54 | matrix metallopeptidase 7 \|\| id:prot-b-54 | Curry liking \|\| id:ebi-fl187-GCST90094750 | Weighted median | 5 | -0.009598809 | 0.287997968692213 | 0.973411881733003 | -0.574074828 | 0.55487720930269 | 0.990447112188167 | 0.563225708508289 | 1.74172710375745 | 0.999332001504091 |
| ebi-fl187-GCST90094750 | ukb-rsfMRI191-edge-pheno716 | Net100_Pair10_33 (OccipitalPrecuneus)&(Temporal\|Frontal\|Supp_Motor_Area) (Default_mode\|Central_executive)&(Default_mode\|Salience) \|\| id:ukb-rsfMRI191-edge-pheno716 | Curry liking \|\| id:ebi-fl187-GCST90094750 | Weighted median | 7 | 0.0468940742461302 | 0.0835451930291615 | 0.574591757463377 | -0.116854504 | 0.210642652583287 | 1.04801099184559 | 0.889714633585998 | 1.23447114115947 | 0.999332001504091 |
| ebi-fl187-GCST90094750 | ukb-rsfMRI191-edge-pheno903 | Net100_Pair27_38 (Precentral\|Frontal\|Supp_Motor_Area)&(Subcortical) (Attention\|Salience\|Motor)&(Subcortical-cerebellum) \|\| id:ukb-rsfMRI191-edge-pheno903 | Curry liking \|\| id:ebi-fl187-GCST90094750 | Weighted median | 7 | 0.0431404973113144 | 0.0842909297732783 | 0.608787673358569 | -0.122069725 | 0.20835071966694 | 1.0440845756219 | 0.885086653644972 | 1.23164505595272 | 0.999332001504091 |
| ebi-fl187-GCST90094750 | ukb-rsfMRI191-node-pheno23 | Net100_Node2 RolandicOper\|SupraMarginal\|Insula Salience\|Motor \|\| id:ukb-rsfMRI191-node-pheno23 | Curry liking \|\| id:ebi-fl187-GCST90094750 | Weighted median | 7 | -0.05849643 | 0.0805186899663175 | 0.467535336196748 | -0.216313063 | 0.0993202020181687 | 0.943181607254126 | 0.805483095725063 | 1.10441988042182 | 0.999332001504091 |
| ebi-fl187-GCST90094750 | ukb-rsfMRI191-node-pheno55 | Net100_Node34 Parietal\|Postcentral\|Precuneus Attention \|\| id:ukb-rsfMRI191-node-pheno55 | Curry liking \|\| id:ebi-fl187-GCST90094750 | Weighted median | 7 | -0.00548638 | 0.0895456734326357 | 0.951144927956553 | -0.1809959 | 0.170023140343332 | 0.994528643109882 | 0.834438780294479 | 1.18533227999893 | 0.999332001504091 |
| ebi-fl187-GCST90094750 | ebi-a-GCST90001414 | IgD- CD24- B cell Absolute Count \|\| id:ebi-a-GCST90001414 | Curry liking \|\| id:ebi-fl187-GCST90094750 | Weighted mode | 6 | -0.214945187 | 0.379288217958904 | 0.595405695137657 | -0.958350094 | 0.528459720008062 | 0.80658565019055 | 0.383525144535435 | 1.69631749146816 | 0.999714303421225 |
| ebi-fl187-GCST90094750 | ebi-a-GCST90001422 | CD20- CD38- B cell %B cell \|\| id:ebi-a-GCST90001422 | Curry liking \|\| id:ebi-fl187-GCST90094750 | Weighted mode | 6 | -0.042952696 | 0.335148299616324 | 0.903017388957552 | -0.699843363 | 0.613937971103566 | 0.957956704040831 | 0.496663093320952 | 1.84769325355075 | 0.999714303421225 |
| ebi-fl187-GCST90094750 | ebi-a-GCST90001444 | CD20- CD38- B cell %lymphocyte \|\| id:ebi-a-GCST90001444 | Curry liking \|\| id:ebi-fl187-GCST90094750 | Weighted mode | 6 | -0.038171968 | 0.344873763854767 | 0.916172605794387 | -0.714124545 | 0.637780609243153 | 0.962547399391037 | 0.489620564961882 | 1.89227651446091 | 0.999714303421225 |
| ebi-fl187-GCST90094750 | ebi-a-GCST90001459 | Myeloid Dendritic Cell %Dendritic Cell \|\| id:ebi-a-GCST90001459 | Curry liking \|\| id:ebi-fl187-GCST90094750 | Weighted mode | 6 | 0.493665814172846 | 0.420035627367511 | 0.292791010866426 | -0.329604015 | 1.31693564381317 | 1.63831096899384 | 0.719208472482839 | 3.73196775874962 | 0.999714303421225 |
| ebi-fl187-GCST90094750 | ebi-a-GCST90001476 | HLA DR++ monocyte %leukocyte \|\| id:ebi-a-GCST90001476 | Curry liking \|\| id:ebi-fl187-GCST90094750 | Weighted mode | 6 | -0.096285223 | 0.356839968736915 | 0.798075138616988 | -0.795691562 | 0.603121115550516 | 0.908204938028014 | 0.451269046507831 | 1.82781472791344 | 0.999714303421225 |
| ebi-fl187-GCST90094750 | ebi-a-GCST90001478 | CD4 regulatory T cell %CD4+ T cell \|\| id:ebi-a-GCST90001478 | Curry liking \|\| id:ebi-fl187-GCST90094750 | Weighted mode | 6 | -0.206597158 | 0.358272911384367 | 0.58916421787257 | -0.908812064 | 0.49561774845603 | 0.813347234543408 | 0.40300268111342 | 1.64151196739366 | 0.999714303421225 |
| ebi-fl187-GCST90094750 | ebi-a-GCST90001485 | CD39+ resting CD4 regulatory T cell %CD4 regulatory T cell \|\| id:ebi-a-GCST90001485 | Curry liking \|\| id:ebi-fl187-GCST90094750 | Weighted mode | 6 | 0.0171580503643247 | 0.347121932371877 | 0.962490818336932 | -0.663200937 | 0.697517037813203 | 1.01730609521878 | 0.515199570893931 | 2.00875883800444 | 0.999714303421225 |
| ebi-fl187-GCST90094750 | ebi-a-GCST90001515 | Immature Myeloid-Derived Suppressor Cells Absolute Count \|\| id:ebi-a-GCST90001515 | Curry liking \|\| id:ebi-fl187-GCST90094750 | Weighted mode | 6 | -0.074250897 | 0.479165111634776 | 0.882914888775554 | -1.013414516 | 0.86491272210811 | 0.928438722408689 | 0.362977469005063 | 2.37479880949816 | 0.999714303421225 |
| ebi-fl187-GCST90094750 | ebi-a-GCST90001517 | CD33+ HLA DR+ Absolute Count \|\| id:ebi-a-GCST90001517 | Curry liking \|\| id:ebi-fl187-GCST90094750 | Weighted mode | 6 | 0.176247373841058 | 0.497979046218467 | 0.7378304374804 | -0.799791557 | 1.15228630442925 | 1.19273307312139 | 0.449422633470141 | 3.16542176065574 | 0.999714303421225 |
| ebi-fl187-GCST90094750 | ebi-a-GCST90001518 | CD33+ HLA DR+ CD14- Absolute Count \|\| id:ebi-a-GCST90001518 | Curry liking \|\| id:ebi-fl187-GCST90094750 | Weighted mode | 6 | 0.520189247087865 | 0.525393943409157 | 0.367591465674116 | -0.509582882 | 1.54996137616981 | 1.68234599865566 | 0.600746108577268 | 4.71128821108072 | 0.999714303421225 |
| ebi-fl187-GCST90094750 | ebi-a-GCST90001520 | CD33+ HLA DR+ CD14dim Absolute Count \|\| id:ebi-a-GCST90001520 | Curry liking \|\| id:ebi-fl187-GCST90094750 | Weighted mode | 6 | 0.00469982927435919 | 0.51402138138945 | 0.993058433484904 | -1.002782078 | 1.01218173679768 | 1.00471089079426 | 0.366857394146534 | 2.75159773303463 | 0.999714303421225 |
| ebi-fl187-GCST90094750 | ebi-a-GCST90001531 | CD33dim HLA DR- Absolute Count \|\| id:ebi-a-GCST90001531 | Curry liking \|\| id:ebi-fl187-GCST90094750 | Weighted mode | 6 | -0.075649295 | 0.498458541526829 | 0.885304733700231 | -1.052628036 | 0.901329446876594 | 0.927141303088928 | 0.349019307518076 | 2.46287519738121 | 0.999714303421225 |
| ebi-fl187-GCST90094750 | ebi-a-GCST90001532 | Basophil Absolute Count \|\| id:ebi-a-GCST90001532 | Curry liking \|\| id:ebi-fl187-GCST90094750 | Weighted mode | 6 | -0.022511103 | 0.513166095116909 | 0.966708330325081 | -1.02831665 | 0.983294443277981 | 0.97774038113382 | 0.357608434489713 | 2.67324861692323 | 0.999714303421225 |
| ebi-fl187-GCST90094750 | ebi-a-GCST90001588 | T/B cell \|\| id:ebi-a-GCST90001588 | Curry liking \|\| id:ebi-fl187-GCST90094750 | Weighted mode | 6 | 0.0227299438710307 | 0.328635017402525 | 0.947539413065382 | -0.62139469 | 0.66685457797998 | 1.02299023745686 | 0.537194694690105 | 1.94810007670636 | 0.999714303421225 |
| ebi-fl187-GCST90094750 | ebi-a-GCST90001624 | HLA DR+ CD4+ T cell Absolute Count \|\| id:ebi-a-GCST90001624 | Curry liking \|\| id:ebi-fl187-GCST90094750 | Weighted mode | 6 | 0.168585606307582 | 0.339448394392613 | 0.640500810889509 | -0.496733247 | 0.833904459317104 | 1.18362954866311 | 0.608515285622302 | 2.30229041335538 | 0.999714303421225 |
| ebi-fl187-GCST90094750 | ebi-a-GCST90001631 | CD8+ Natural Killer T %T cell \|\| id:ebi-a-GCST90001631 | Curry liking \|\| id:ebi-fl187-GCST90094750 | Weighted mode | 6 | -0.339366141 | 0.385236971442465 | 0.418681646056184 | -1.094430605 | 0.415698323480073 | 0.71222162812692 | 0.334730146511519 | 1.51542863066952 | 0.999714303421225 |
| ebi-fl187-GCST90094750 | ebi-a-GCST90001632 | CD8+ Natural Killer T %lymphocyte \|\| id:ebi-a-GCST90001632 | Curry liking \|\| id:ebi-fl187-GCST90094750 | Weighted mode | 6 | -0.266355132 | 0.364760159191732 | 0.498018021091402 | -0.981285045 | 0.448574779516084 | 0.766166988383376 | 0.374829117132521 | 1.56607858690155 | 0.999714303421225 |
| ebi-fl187-GCST90094750 | ebi-a-GCST90001646 | Natural Killer %CD3- lymphocyte \|\| id:ebi-a-GCST90001646 | Curry liking \|\| id:ebi-fl187-GCST90094750 | Weighted mode | 6 | -0.248694637 | 0.392432624279515 | 0.554113113007259 | -1.017862581 | 0.520473306201548 | 0.779818064313144 | 0.361366506945833 | 1.68282395224927 | 0.999714303421225 |
| ebi-fl187-GCST90094750 | ebi-a-GCST90001684 | CD127- CD8+ T cell Absolute Count \|\| id:ebi-a-GCST90001684 | Curry liking \|\| id:ebi-fl187-GCST90094750 | Weighted mode | 6 | 0.107749014988109 | 0.346913643455614 | 0.768640090426846 | -0.572201726 | 0.787699756161111 | 1.11376817118578 | 0.564281676247546 | 2.19833390195418 | 0.999714303421225 |
| ebi-fl187-GCST90094750 | ebi-a-GCST90001687 | CD28- CD8+ T cell Absolute Count \|\| id:ebi-a-GCST90001687 | Curry liking \|\| id:ebi-fl187-GCST90094750 | Weighted mode | 6 | 0.209522332845109 | 0.368451796059145 | 0.59417864812806 | -0.512643187 | 0.931687853121033 | 1.23308891318702 | 0.598910452262242 | 2.53879066909151 | 0.999714303421225 |
| ebi-fl187-GCST90094750 | ebi-a-GCST90001701 | BAFF-R on CD20- CD38- B cell \|\| id:ebi-a-GCST90001701 | Curry liking \|\| id:ebi-fl187-GCST90094750 | Weighted mode | 6 | -0.398287264 | 0.403812174938488 | 0.369269918285683 | -1.189759127 | 0.393184598852352 | 0.671469111033435 | 0.304294551609827 | 1.48169188270629 | 0.999714303421225 |
| ebi-fl187-GCST90094750 | ebi-a-GCST90001732 | CD19 on IgD- CD27- B cell \|\| id:ebi-a-GCST90001732 | Curry liking \|\| id:ebi-fl187-GCST90094750 | Weighted mode | 6 | 0.124459242171396 | 0.411697789696743 | 0.774589284636445 | -0.682468426 | 0.931386909977011 | 1.13253585981709 | 0.50536798816609 | 2.53802675239906 | 0.999714303421225 |
| ebi-fl187-GCST90094750 | ebi-a-GCST90001798 | CD27 on CD24+ CD27+ B cell \|\| id:ebi-a-GCST90001798 | Curry liking \|\| id:ebi-fl187-GCST90094750 | Weighted mode | 6 | 0.258701133709645 | 0.333374010257941 | 0.47283432928819 | -0.394711926 | 0.912114193815209 | 1.29524664141707 | 0.673874136641597 | 2.48958042886645 | 0.999714303421225 |
| ebi-fl187-GCST90094750 | ebi-a-GCST90001836 | CD66b on CD66b++ myeloid cell \|\| id:ebi-a-GCST90001836 | Curry liking \|\| id:ebi-fl187-GCST90094750 | Weighted mode | 6 | -0.747292256 | 0.6226742416646 | 0.28384360326115 | -1.967733769 | 0.473149257857668 | 0.473647333763281 | 0.139773255982556 | 1.60504093006936 | 0.999714303421225 |
| ebi-fl187-GCST90094750 | ebi-a-GCST90001943 | CD25 on activated & secreting CD4 regulatory T cell \|\| id:ebi-a-GCST90001943 | Curry liking \|\| id:ebi-fl187-GCST90094750 | Weighted mode | 6 | -0.328629179 | 0.407787714248464 | 0.456909898195081 | -1.127893099 | 0.470634740438171 | 0.719909924622812 | 0.32371457285989 | 1.60101009661601 | 0.999714303421225 |
| ebi-fl187-GCST90094750 | ebi-a-GCST90001960 | CD25 on CD4+ T cell \|\| id:ebi-a-GCST90001960 | Curry liking \|\| id:ebi-fl187-GCST90094750 | Weighted mode | 6 | 0.126188562473014 | 0.347626469390972 | 0.731445496242846 | -0.555159318 | 0.807536442479319 | 1.1344960715002 | 0.573980808705038 | 2.24237695185868 | 0.999714303421225 |
| ebi-fl187-GCST90094750 | ebi-a-GCST90001988 | HLA DR on CD14+ CD16- monocyte \|\| id:ebi-a-GCST90001988 | Curry liking \|\| id:ebi-fl187-GCST90094750 | Weighted mode | 6 | -0.144953193 | 0.340413937610997 | 0.687950341334701 | -0.812164511 | 0.522258124701245 | 0.865062783142708 | 0.443896207515838 | 1.68583016954905 | 0.999714303421225 |
| ebi-fl187-GCST90094750 | ebi-a-GCST90001991 | HLA DR on CD14+ monocyte \|\| id:ebi-a-GCST90001991 | Curry liking \|\| id:ebi-fl187-GCST90094750 | Weighted mode | 6 | -0.168098987 | 0.353234047350202 | 0.654221866219058 | -0.86043772 | 0.524239746095204 | 0.845270160030178 | 0.422976896547886 | 1.68917415884571 | 0.999714303421225 |
| ebi-fl187-GCST90094750 | ebi-a-GCST90002019 | CD14 on Monocytic Myeloid-Derived Suppressor Cells \|\| id:ebi-a-GCST90002019 | Curry liking \|\| id:ebi-fl187-GCST90094750 | Weighted mode | 6 | 0.113682629268242 | 0.50389994001286 | 0.830439780506048 | -0.873961253 | 1.10132651169345 | 1.12039648739961 | 0.41729525875934 | 3.0081537295906 | 0.999714303421225 |
| ebi-fl187-GCST90094750 | ebi-a-GCST90002037 | CD80 on plasmacytoid Dendritic Cell \|\| id:ebi-a-GCST90002037 | Curry liking \|\| id:ebi-fl187-GCST90094750 | Weighted mode | 6 | -0.039362854 | 0.367579463744638 | 0.918884336375549 | -0.759818603 | 0.681092895339444 | 0.961401797744913 | 0.467751268206869 | 1.97603615325393 | 0.999714303421225 |
| ebi-fl187-GCST90094750 | ebi-a-GCST90002042 | CD45 on CD33+ HLA DR+ CD14- \|\| id:ebi-a-GCST90002042 | Curry liking \|\| id:ebi-fl187-GCST90094750 | Weighted mode | 6 | 0.029121391397184 | 0.559952104843409 | 0.960536985886392 | -1.068384734 | 1.12662751689026 | 1.02954956534999 | 0.343563015091742 | 3.08523403553584 | 0.999714303421225 |
| ebi-fl187-GCST90094750 | ebi-a-GCST90002056 | CD8 on Effector Memory CD8+ T cell \|\| id:ebi-a-GCST90002056 | Curry liking \|\| id:ebi-fl187-GCST90094750 | Weighted mode | 6 | 0.186985136608406 | 0.49853376399836 | 0.722991814821686 | -0.790141041 | 1.16411131404519 | 1.20560936547264 | 0.453780789150318 | 3.20307509014858 | 0.999714303421225 |
| ebi-fl187-GCST90094750 | ebi-a-GCST90002058 | CD8 on CD8+ T cell \|\| id:ebi-a-GCST90002058 | Curry liking \|\| id:ebi-fl187-GCST90094750 | Weighted mode | 6 | 0.331664974617794 | 0.502146946627209 | 0.538147361039951 | -0.652543041 | 1.31587299000712 | 1.39328598414182 | 0.52071987968582 | 3.72800407539136 | 0.999714303421225 |
| ebi-fl187-GCST90094750 | ebi-a-GCST90002063 | CD4 on CD4 regulatory T cell \|\| id:ebi-a-GCST90002063 | Curry liking \|\| id:ebi-fl187-GCST90094750 | Weighted mode | 6 | -0.756565479 | 0.445937064376203 | 0.150540103720958 | -1.630602125 | 0.117471167546427 | 0.46927539878944 | 0.195811635580076 | 1.12464920308033 | 0.999714303421225 |
| ebi-fl187-GCST90094750 | ebi-a-GCST90002066 | CD4 on activated CD4 regulatory T cell \|\| id:ebi-a-GCST90002066 | Curry liking \|\| id:ebi-fl187-GCST90094750 | Weighted mode | 6 | -0.693580744 | 0.510547129223215 | 0.232379980923668 | -1.694253118 | 0.307091628822902 | 0.499783265040295 | 0.183736407257896 | 1.35946552859139 | 0.999714303421225 |
| ebi-fl187-GCST90094750 | ebi-a-GCST90002070 | CD4 on activated & secreting CD4 regulatory T cell \|\| id:ebi-a-GCST90002070 | Curry liking \|\| id:ebi-fl187-GCST90094750 | Weighted mode | 6 | -0.71024085 | 0.485442248705889 | 0.203311300574275 | -1.661707657 | 0.241225957640607 | 0.49152579930172 | 0.189814564960482 | 1.2728086036469 | 0.999714303421225 |
| ebi-fl187-GCST90094750 | ebi-a-GCST90002081 | SSC-A on CD4+ T cell \|\| id:ebi-a-GCST90002081 | Curry liking \|\| id:ebi-fl187-GCST90094750 | Weighted mode | 6 | 0.300130600765508 | 0.372068860145857 | 0.456504554765683 | -0.429124365 | 1.02938556665139 | 1.35003511168208 | 0.651078952633492 | 2.79934529507118 | 0.999714303421225 |
| ebi-fl187-GCST90094750 | ebi-a-GCST90002094 | CD11b on Monocytic Myeloid-Derived Suppressor Cells \|\| id:ebi-a-GCST90002094 | Curry liking \|\| id:ebi-fl187-GCST90094750 | Weighted mode | 6 | 0.310337485005038 | 0.618861249423616 | 0.637338230734949 | -0.902630564 | 1.52330553387533 | 1.36388532731685 | 0.405501557756148 | 4.58736384728963 | 0.999714303421225 |
| ebi-fl187-GCST90094750 | ebi-a-GCST90002104 | HLA DR on myeloid Dendritic Cell \|\| id:ebi-a-GCST90002104 | Curry liking \|\| id:ebi-fl187-GCST90094750 | Weighted mode | 6 | -0.049405636 | 0.474097212132224 | 0.921053820236938 | -0.978636172 | 0.879824899323095 | 0.951794968645318 | 0.375823307751408 | 2.4104775932039 | 0.999714303421225 |
| ebi-fl187-GCST90094750 | ebi-a-GCST90002105 | HLA DR on plasmacytoid Dendritic Cell \|\| id:ebi-a-GCST90002105 | Curry liking \|\| id:ebi-fl187-GCST90094750 | Weighted mode | 5 | -0.533811788 | 0.573821536806422 | 0.404888247313887 | -1.658502 | 0.590878424288106 | 0.586365603111611 | 0.190424021737914 | 1.80557377884634 | 0.999714303421225 |
| ebi-fl187-GCST90094750 | ebi-a-GCST90002106 | HLA DR on Dendritic Cell \|\| id:ebi-a-GCST90002106 | Curry liking \|\| id:ebi-fl187-GCST90094750 | Weighted mode | 6 | -0.078579187 | 0.368676666057452 | 0.839636760616991 | -0.801185453 | 0.644027078190802 | 0.924428854024933 | 0.448796621454953 | 1.90413355471221 | 0.999714303421225 |
| ebi-fl187-GCST90094750 | ebi-a-GCST90002111 | HLA DR on CD33dim HLA DR+ CD11b- \|\| id:ebi-a-GCST90002111 | Curry liking \|\| id:ebi-fl187-GCST90094750 | Weighted mode | 6 | -0.903838878 | 1.18537606098809 | 0.48017145465661 | -3.227175957 | 1.41949820172885 | 0.405011880471707 | 0.0396693687407335 | 4.13504496114639 | 0.999714303421225 |
| ebi-fl187-GCST90094750 | ebi-a-GCST90002112 | HLA DR on CD33- HLA DR+ \|\| id:ebi-a-GCST90002112 | Curry liking \|\| id:ebi-fl187-GCST90094750 | Weighted mode | 6 | 0.0173080282890665 | 0.450899724437128 | 0.970865771388624 | -0.866455432 | 0.901071488185837 | 1.01745868011766 | 0.420439186625669 | 2.46223995925592 | 0.999714303421225 |
| ebi-fl187-GCST90094750 | ebi-a-GCST90002115 | HLA DR on HLA DR+ CD8+ T cell \|\| id:ebi-a-GCST90002115 | Curry liking \|\| id:ebi-fl187-GCST90094750 | Weighted mode | 6 | -0.495259696 | 0.371684012811715 | 0.240200735365355 | -1.223760361 | 0.233240969557813 | 0.60941262499585 | 0.294122079726933 | 1.262685711 | 0.999714303421225 |
| ebi-fl187-GCST90094750 | ebi-a-GCST90002116 | HLA DR on B cell \|\| id:ebi-a-GCST90002116 | Curry liking \|\| id:ebi-fl187-GCST90094750 | Weighted mode | 6 | 0.0379607741550425 | 0.357220952088892 | 0.919502448617929 | -0.662192292 | 0.73811384024927 | 1.03869048856732 | 0.515719486601158 | 2.09198597119251 | 0.999714303421225 |
| ebi-fl187-GCST90094750 | ebi-cfmet338-GCST90026023 | Sphingomyelin (d18:1/24:1, d18:2/24:0) levels \|\| id:ebi-cfmet338-GCST90026023 | Curry liking \|\| id:ebi-fl187-GCST90094750 | Weighted mode | 6 | 0.318985567519079 | 0.266080619074885 | 0.284306864030673 | -0.202532446 | 0.840503580905853 | 1.37573146954457 | 0.81665998492642 | 2.31753374871892 | 0.999714303421225 |
| ebi-fl187-GCST90094750 | ebi-cfmet338-GCST90026046 | 1-arachidonoyl-gpc (20:4n6) levels \|\| id:ebi-cfmet338-GCST90026046 | Curry liking \|\| id:ebi-fl187-GCST90094750 | Weighted mode | 6 | 0.157949822082472 | 0.266657397124201 | 0.57939782074569 | -0.364698676 | 0.680598320445905 | 1.17110742950131 | 0.694405860312865 | 1.97505909701747 | 0.999714303421225 |
| ebi-fl187-GCST90094750 | ebi-cfmet338-GCST90026047 | 1-linoleoyl-gpc (18:2) levels \|\| id:ebi-cfmet338-GCST90026047 | Curry liking \|\| id:ebi-fl187-GCST90094750 | Weighted mode | 6 | 0.367826947736808 | 0.413800514662539 | 0.414770473188687 | -0.443222061 | 1.17887595647538 | 1.44459202742224 | 0.641964635948478 | 3.25071819977849 | 0.999714303421225 |
| ebi-fl187-GCST90094750 | ebi-cfmet338-GCST90026050 | 1-myristoyl-2-palmitoyl-gpc (14:0/16:0) levels \|\| id:ebi-cfmet338-GCST90026050 | Curry liking \|\| id:ebi-fl187-GCST90094750 | Weighted mode | 6 | -0.04713139 | 0.159878267180892 | 0.779997901390668 | -0.360492793 | 0.266230013984894 | 0.953962048578262 | 0.697332600506975 | 1.30503520051409 | 0.999714303421225 |
| ebi-fl187-GCST90094750 | ebi-cfmet338-GCST90026068 | 2,3-dihydroxyisovalerate levels \|\| id:ebi-cfmet338-GCST90026068 | Curry liking \|\| id:ebi-fl187-GCST90094750 | Weighted mode | 6 | 0.34052073706368 | 0.561567905048453 | 0.570739915676198 | -0.760152357 | 1.44119383095865 | 1.40567938936737 | 0.467595180262593 | 4.22573762326331 | 0.999714303421225 |
| ebi-fl187-GCST90094750 | ebi-cfmet338-GCST90026082 | 3-hydroxyhexanoate levels \|\| id:ebi-cfmet338-GCST90026082 | Curry liking \|\| id:ebi-fl187-GCST90094750 | Weighted mode | 6 | -0.043357755 | 0.197004325989111 | 0.8345084008675 | -0.429486234 | 0.342770724335688 | 0.957568754151439 | 0.650843390344637 | 1.40884571085772 | 0.999714303421225 |
| ebi-fl187-GCST90094750 | ebi-cfmet338-GCST90026094 | 3-ureidopropionate levels \|\| id:ebi-cfmet338-GCST90026094 | Curry liking \|\| id:ebi-fl187-GCST90094750 | Weighted mode | 6 | 0.272042259631015 | 0.304742572028788 | 0.412917867868385 | -0.325253182 | 0.869337700807439 | 1.31264247192553 | 0.722344446205448 | 2.38533052777527 | 0.999714303421225 |
| ebi-fl187-GCST90094750 | ebi-cfmet338-GCST90026173 | Glycerol levels \|\| id:ebi-cfmet338-GCST90026173 | Curry liking \|\| id:ebi-fl187-GCST90094750 | Weighted mode | 6 | -0.035944875 | 0.137851364498935 | 0.804683134765522 | -0.30613355 | 0.234243798996574 | 0.964693470338524 | 0.736288281708088 | 1.26395260502428 | 0.999714303421225 |
| ebi-fl187-GCST90094750 | ebi-cfmet338-GCST90026179 | Histidine levels \|\| id:ebi-cfmet338-GCST90026179 | Curry liking \|\| id:ebi-fl187-GCST90094750 | Weighted mode | 6 | 0.0484451890164191 | 0.116854985349991 | 0.695639581659442 | -0.180590582 | 0.277480960302402 | 1.04963783856128 | 0.834777061271316 | 1.31980099029279 | 0.999714303421225 |
| ebi-fl187-GCST90094750 | ebi-cfmet338-GCST90026188 | Isocitrate levels \|\| id:ebi-cfmet338-GCST90026188 | Curry liking \|\| id:ebi-fl187-GCST90094750 | Weighted mode | 6 | -0.329726572 | 0.187043073501513 | 0.138217084378748 | -0.696330996 | 0.0368778522448187 | 0.719120334318136 | 0.498410623822112 | 1.03756627670601 | 0.999714303421225 |
| ebi-fl187-GCST90094750 | ebi-cfmet338-GCST90026207 | N-acetyl-isoputreanine levels \|\| id:ebi-cfmet338-GCST90026207 | Curry liking \|\| id:ebi-fl187-GCST90094750 | Weighted mode | 6 | -0.01621423 | 0.114853468910028 | 0.893244613765739 | -0.241327029 | 0.208898568979458 | 0.983916512958066 | 0.78558467528846 | 1.23231999671593 | 0.999714303421225 |
| ebi-fl187-GCST90094750 | ebi-cfmet338-GCST90026213 | N-acetylglutamate levels \|\| id:ebi-cfmet338-GCST90026213 | Curry liking \|\| id:ebi-fl187-GCST90094750 | Weighted mode | 6 | -0.294661853 | 0.258696569054834 | 0.306304646717157 | -0.801707128 | 0.212383422211052 | 0.744783391057943 | 0.44856255620845 | 1.23662194251005 | 0.999714303421225 |
| ebi-fl187-GCST90094750 | ebi-cfmet338-GCST90026224 | N-acetyltaurine levels \|\| id:ebi-cfmet338-GCST90026224 | Curry liking \|\| id:ebi-fl187-GCST90094750 | Weighted mode | 6 | 0.342949398268717 | 0.241509963610415 | 0.214835863102906 | -0.13041013 | 0.816308926945131 | 1.40909745735151 | 0.877735371124264 | 2.26213470441695 | 0.999714303421225 |
| ebi-fl187-GCST90094750 | ebi-cfmet338-GCST90026250 | Phenyllactate (pla) levels \|\| id:ebi-cfmet338-GCST90026250 | Curry liking \|\| id:ebi-fl187-GCST90094750 | Weighted mode | 6 | 0.182816445319179 | 0.278809583920337 | 0.540981961812023 | -0.363650339 | 0.729283229803039 | 1.20059401320331 | 0.695134213463109 | 2.07359378465712 | 0.999714303421225 |
| ebi-fl187-GCST90094750 | ebi-cfmet338-GCST90026251 | Pipecolate levels \|\| id:ebi-cfmet338-GCST90026251 | Curry liking \|\| id:ebi-fl187-GCST90094750 | Weighted mode | 6 | 0.551762082820065 | 0.422616393862959 | 0.248535862582952 | -0.276566049 | 1.38009021479146 | 1.73630984563391 | 0.758383526889076 | 3.97526023859193 | 0.999714303421225 |
| ebi-fl187-GCST90094750 | ebi-cfmet338-GCST90026278 | Trigonelline (n'-methylnicotinate) levels \|\| id:ebi-cfmet338-GCST90026278 | Curry liking \|\| id:ebi-fl187-GCST90094750 | Weighted mode | 6 | 0.50853478383232 | 0.513705030351366 | 0.367661733 | -0.498327076 | 1.515396643 | 1.66285297006263 | 0.607546188833281 | 4.55122598226828 | 0.999714303421225 |
| ebi-fl187-GCST90094750 | ebi-cfmet338-GCST90026299 | 1-carboxyethylvaline levels \|\| id:ebi-cfmet338-GCST90026299 | Curry liking \|\| id:ebi-fl187-GCST90094750 | Weighted mode | 6 | -0.135590028 | 0.271300119722656 | 0.638444001762574 | -0.667338262 | 0.396158206977956 | 0.873200547171652 | 0.513072426079579 | 1.48610441104198 | 0.999714303421225 |
| ebi-fl187-GCST90094750 | ebi-gm430-GCST90011306 | C_Erysipelotrichia abundance \|\| id:ebi-gm430-GCST90011306 | Curry liking \|\| id:ebi-fl187-GCST90094750 | Weighted mode | 5 | -0.346422126 | 0.244011064674844 | 0.228711664415213 | -0.824683813 | 0.131839560305142 | 0.707213890393865 | 0.43837357848437 | 1.14092525488248 | 0.999714303421225 |
| ebi-fl187-GCST90094750 | ebi-gm430-GCST90011312 | F_Erysipelotrichaceae abundance \|\| id:ebi-gm430-GCST90011312 | Curry liking \|\| id:ebi-fl187-GCST90094750 | Weighted mode | 5 | -0.346422126 | 0.254230143842275 | 0.244671984423544 | -0.844713208 | 0.151868955473307 | 0.707213890393865 | 0.429680569324882 | 1.16400768960037 | 0.999714303421225 |
| ebi-fl187-GCST90094750 | ebi-gm430-GCST90011352 | O_Erysipelotrichales abundance \|\| id:ebi-gm430-GCST90011352 | Curry liking \|\| id:ebi-fl187-GCST90094750 | Weighted mode | 5 | -0.346422126 | 0.242932392151894 | 0.22701338449882 | -0.822569615 | 0.12972536216016 | 0.707213890393865 | 0.439301367510141 | 1.13851566090215 | 0.999714303421225 |
| ebi-fl187-GCST90094750 | ebi-gm430-GCST90011362 | OTU97_11 (Parabacteroides) abundance \|\| id:ebi-gm430-GCST90011362 | Curry liking \|\| id:ebi-fl187-GCST90094750 | Weighted mode | 5 | -0.073310933 | 0.300967421986889 | 0.819535394708748 | -0.663207081 | 0.516585213594404 | 0.929311830918285 | 0.515196405770121 | 1.67629368025918 | 0.999714303421225 |
| ebi-fl187-GCST90094750 | ebi-gm430-GCST90011384 | OTU97_235 (Phascolarctobacterium) abundance \|\| id:ebi-gm430-GCST90011384 | Curry liking \|\| id:ebi-fl187-GCST90094750 | Weighted mode | 5 | 1.01335608282874 | 0.942568405621129 | 0.34285598693927 | -0.834077992 | 2.86079015784616 | 2.75483095900296 | 0.434274701568703 | 17.4753297515779 | 0.999714303421225 |
| ebi-fl187-GCST90094750 | ebi-gm430-GCST90011390 | OTU97_3 (Bacteroides) abundance \|\| id:ebi-gm430-GCST90011390 | Curry liking \|\| id:ebi-fl187-GCST90094750 | Weighted mode | 5 | -0.422215207 | 0.251161412661098 | 0.168047493880917 | -0.914491575 | 0.070061162260633 | 0.655592936313448 | 0.400720310382566 | 1.0725737802852 | 0.999714303421225 |
| ebi-fl187-GCST90094750 | ebi-gm430-GCST90011398 | OTU97_4 (Alistipes) abundance \|\| id:ebi-gm430-GCST90011398 | Curry liking \|\| id:ebi-fl187-GCST90094750 | Weighted mode | 5 | -0.285691801 | 0.307485716853894 | 0.405418378679366 | -0.888363806 | 0.316980203999284 | 0.751494189991662 | 0.411328215192608 | 1.37297539223459 | 0.999714303421225 |
| ebi-fl187-GCST90094750 | ebi-gm430-GCST90011421 | OTU99_108 (Cytophagales) abundance \|\| id:ebi-gm430-GCST90011421 | Curry liking \|\| id:ebi-fl187-GCST90094750 | Weighted mode | 5 | 0.883776940474881 | 0.908053083514139 | 0.385532616548327 | -0.896007103 | 2.66356098416259 | 2.42002274884814 | 0.408196295762459 | 14.3472887082507 | 0.999714303421225 |
| ebi-fl187-GCST90094750 | ebi-gm430-GCST90011428 | OTU99_123 (Phascolarctobacterium) abundance \|\| id:ebi-gm430-GCST90011428 | Curry liking \|\| id:ebi-fl187-GCST90094750 | Weighted mode | 5 | 0.0921872727434359 | 0.610143231550909 | 0.887217326823622 | -1.103693461 | 1.28806800658322 | 1.0965701605554 | 0.331643904963861 | 3.62577481154535 | 0.999714303421225 |
| ebi-fl187-GCST90094750 | ebi-gm430-GCST90011450 | OTU99_298 (Phascolarctobacterium) abundance \|\| id:ebi-gm430-GCST90011450 | Curry liking \|\| id:ebi-fl187-GCST90094750 | Weighted mode | 5 | 0.960489049634519 | 0.928157620769614 | 0.359205083655502 | -0.858699887 | 2.77967798634296 | 2.61297403499897 | 0.423712598599917 | 16.1138312387677 | 0.999714303421225 |
| ebi-fl187-GCST90094750 | ebi-gm430-GCST90011451 | OTU99_3 (Bacteroides) abundance \|\| id:ebi-gm430-GCST90011451 | Curry liking \|\| id:ebi-fl187-GCST90094750 | Weighted mode | 5 | -0.398371946 | 0.26799583583015 | 0.211348358084937 | -0.923643784 | 0.126899892640526 | 0.67141225238944 | 0.397069566243398 | 1.13530335987103 | 0.999714303421225 |
| ebi-fl187-GCST90094750 | ebi-gm430-GCST90011472 | OTU99_72 (Parabacteroides) abundance \|\| id:ebi-gm430-GCST90011472 | Curry liking \|\| id:ebi-fl187-GCST90094750 | Weighted mode | 5 | -1.177132632 | 0.92341075665816 | 0.27140722593074 | -2.987017715 | 0.632752450898801 | 0.308161084176948 | 0.0504376320329781 | 1.8827857290965 | 0.999714303421225 |
| ebi-fl187-GCST90094750 | ebi-gm430-GCST90011507 | TestASV_3 (Bacteroides) abundance \|\| id:ebi-gm430-GCST90011507 | Curry liking \|\| id:ebi-fl187-GCST90094750 | Weighted mode | 5 | -0.248020032 | 0.271124553159231 | 0.412061837951329 | -0.779424156 | 0.283384092419615 | 0.780344311441767 | 0.458670057690111 | 1.32761499075429 | 0.999714303421225 |
| ebi-fl187-GCST90094750 | ebi-gm430-GCST90011512 | TestASV_34 (Phascolarctobacterium) abundance \|\| id:ebi-gm430-GCST90011512 | Curry liking \|\| id:ebi-fl187-GCST90094750 | Weighted mode | 5 | -0.593732569 | 0.765798218010938 | 0.481439225575541 | -2.094697076 | 0.907231938354991 | 0.552262076588953 | 0.123107530201154 | 2.47745528433551 | 0.999714303421225 |
| ebi-fl187-GCST90094750 | ebi-gm430-GCST90011519 | TestASV_41 (Barnesiella) abundance \|\| id:ebi-gm430-GCST90011519 | Curry liking \|\| id:ebi-fl187-GCST90094750 | Weighted mode | 5 | -1.19013227 | 0.963567614633901 | 0.284370737287293 | -3.078724794 | 0.698460255063979 | 0.304181027497273 | 0.046017901539251 | 2.01065442782907 | 0.999714303421225 |
| ebi-fl187-GCST90094750 | ebi-gm430-GCST90011524 | TestASV_46 (Sutterella) abundance \|\| id:ebi-gm430-GCST90011524 | Curry liking \|\| id:ebi-fl187-GCST90094750 | Weighted mode | 5 | -0.645853175 | 0.939435667878497 | 0.529562393133426 | -2.487147084 | 1.19544073384174 | 0.524215103935955 | 0.0831468394538355 | 3.30501408110839 | 0.999714303421225 |
| ebi-fl187-GCST90094750 | ebi-gm430-GCST90011581 | OTU97_19 (Bacteria) prevalence \|\| id:ebi-gm430-GCST90011581 | Curry liking \|\| id:ebi-fl187-GCST90094750 | Weighted mode | 5 | 1.00110774010482 | 1.46555977117121 | 0.532063569308692 | -1.871389411 | 3.8736048916004 | 2.72129464665832 | 0.153909669340037 | 48.1155250718537 | 0.999714303421225 |
| ebi-fl187-GCST90094750 | ebi-gm430-GCST90011614 | OTU97_80 (Ruminococcus) prevalence \|\| id:ebi-gm430-GCST90011614 | Curry liking \|\| id:ebi-fl187-GCST90094750 | Weighted mode | 5 | 0.640545369534278 | 0.499628599918369 | 0.269089490759992 | -0.338726686 | 1.61981742537428 | 1.89751544428333 | 0.712677206913236 | 5.05216783470405 | 0.999714303421225 |
| ebi-fl187-GCST90094750 | ebi-gm430-GCST90011623 | OTU99_11 (Parabacteroides) prevalence \|\| id:ebi-gm430-GCST90011623 | Curry liking \|\| id:ebi-fl187-GCST90094750 | Weighted mode | 5 | 0.277510975508504 | 0.489857902134853 | 0.601319873099802 | -0.682610513 | 1.23763246369282 | 1.31984060498605 | 0.505296187024865 | 3.44744185153374 | 0.999714303421225 |
| ebi-fl187-GCST90094750 | ebi-gm430-GCST90011642 | OTU99_19 (Bacteria) prevalence \|\| id:ebi-gm430-GCST90011642 | Curry liking \|\| id:ebi-fl187-GCST90094750 | Weighted mode | 5 | 1.00110774010482 | 1.38825990852409 | 0.510730179697562 | -1.719881681 | 3.72209716081203 | 2.72129464665832 | 0.179087336163713 | 41.3510229844592 | 0.999714303421225 |
| ebi-fl187-GCST90094750 | ebi-gm430-GCST90011653 | OTU99_30 (Parasutterella) prevalence \|\| id:ebi-gm430-GCST90011653 | Curry liking \|\| id:ebi-fl187-GCST90094750 | Weighted mode | 5 | -0.811526946 | 0.5762272807681 | 0.231802330093055 | -1.940932416 | 0.317878524183139 | 0.444179310267499 | 0.143570020302913 | 1.37420931788855 | 0.999714303421225 |
| ebi-fl187-GCST90094750 | ebi-gm430-GCST90011675 | OTU99_8 (Ruminococcaceae) prevalence \|\| id:ebi-gm430-GCST90011675 | Curry liking \|\| id:ebi-fl187-GCST90094750 | Weighted mode | 5 | 0.131449669157405 | 0.569899285058306 | 0.828900304650665 | -0.98555293 | 1.24845226787168 | 1.14048050493304 | 0.373232798425863 | 3.48494501988599 | 0.999714303421225 |
| ebi-fl187-GCST90094750 | ebi-gm430-GCST90011679 | OTU99_92 (Ruminococcus) prevalence \|\| id:ebi-gm430-GCST90011679 | Curry liking \|\| id:ebi-fl187-GCST90094750 | Weighted mode | 5 | 0.437811294496433 | 0.540129188769153 | 0.463083170893626 | -0.620841915 | 1.49646450448397 | 1.54931251603392 | 0.537491724439159 | 4.46587205569353 | 0.999714303421225 |
| ebi-fl187-GCST90094750 | ebi-gm430-GCST90011684 | TestASV_11 (Lachnospiraceae) prevalence \|\| id:ebi-gm430-GCST90011684 | Curry liking \|\| id:ebi-fl187-GCST90094750 | Weighted mode | 5 | 0.0517802280699742 | 0.553162957629939 | 0.929922204997785 | -1.032419169 | 1.13597962502466 | 1.05314426553039 | 0.356144344266471 | 3.11422281969387 | 0.999714303421225 |
| ebi-fl187-GCST90094750 | ebi-gm430-GCST90011688 | TestASV_15 (Bacteroides) prevalence \|\| id:ebi-gm430-GCST90011688 | Curry liking \|\| id:ebi-fl187-GCST90094750 | Weighted mode | 5 | -0.430953657 | 0.51781232378941 | 0.452065360004012 | -1.445865812 | 0.58395849756987 | 0.649889027846819 | 0.235542053183834 | 1.79312247136628 | 0.999714303421225 |
| ebi-fl187-GCST90094750 | ebi-gm430-GCST90011692 | TestASV_19 (Prevotella) prevalence \|\| id:ebi-gm430-GCST90011692 | Curry liking \|\| id:ebi-fl187-GCST90094750 | Weighted mode | 5 | 0.565909959680807 | 0.938686782763699 | 0.579105082178901 | -1.273916135 | 2.40573605389766 | 1.76104953792038 | 0.279733997989507 | 11.0865876057222 | 0.999714303421225 |
| ebi-fl187-GCST90094750 | ebi-gm430-GCST90011693 | TestASV_2 (Bacteroides) prevalence \|\| id:ebi-gm430-GCST90011693 | Curry liking \|\| id:ebi-fl187-GCST90094750 | Weighted mode | 5 | -0.738868562 | 0.569560079960542 | 0.264303847069132 | -1.855206319 | 0.377469194770592 | 0.47765404586264 | 0.156420666840832 | 1.45858851094856 | 0.999714303421225 |
| ebi-fl187-GCST90094750 | ebi-gm430-GCST90011716 | TestASV_40 (Barnesiella) prevalence \|\| id:ebi-gm430-GCST90011716 | Curry liking \|\| id:ebi-fl187-GCST90094750 | Weighted mode | 5 | 0.656268214916671 | 0.980059689816621 | 0.539769166321153 | -1.264648777 | 2.57718520695725 | 1.92758556065814 | 0.282338442448067 | 13.1600431788214 | 0.999714303421225 |
| ebi-fl187-GCST90094750 | ebi-gm430-GCST90011718 | TestASV_42 (Alphaproteobacteria) prevalence \|\| id:ebi-gm430-GCST90011718 | Curry liking \|\| id:ebi-fl187-GCST90094750 | Weighted mode | 5 | -0.151905686 | 1.10874558351016 | 0.897644762610199 | -2.32504703 | 2.02123565769175 | 0.859069299192528 | 0.0977788454365695 | 7.54764548016566 | 0.999714303421225 |
| ebi-fl187-GCST90094750 | ebi-hbsm44-GCST90104929 | Sulcal depth of the right inferior frontal sulcus \|\| id:ebi-hbsm44-GCST90104929 | Curry liking \|\| id:ebi-fl187-GCST90094750 | Weighted mode | 5 | 0.468661231509454 | 0.465553316584597 | 0.371044459991742 | -0.443823269 | 1.38114573201526 | 1.59785360460243 | 0.641578797673253 | 3.97945840947391 | 0.999714303421225 |
| ebi-fl187-GCST90094750 | ebi-hbsm44-GCST90104935 | Sulcal opening distance of the left calloso-marginal posterior fissure \|\| id:ebi-hbsm44-GCST90104935 | Curry liking \|\| id:ebi-fl187-GCST90094750 | Weighted mode | 5 | -0.001040176 | 0.117196016255548 | 0.993343469426948 | -0.230744367 | 0.228664016303468 | 0.998960365237666 | 0.793942397641019 | 1.25691966354338 | 0.999714303421225 |
| ebi-fl187-GCST90094750 | ebi-hbsm44-GCST90104936 | Sulcal opening distance of the left anterior inferior temporal sulcus \|\| id:ebi-hbsm44-GCST90104936 | Curry liking \|\| id:ebi-fl187-GCST90094750 | Weighted mode | 5 | 0.0329297216459951 | 0.0523849426217544 | 0.563713591771319 | -0.069744766 | 0.135604209184634 | 1.03347790556294 | 0.932631828983002 | 1.14522853294795 | 0.999714303421225 |
| ebi-fl187-GCST90094750 | ebi-hbsm44-GCST90104941 | Sulcal opening distance of the right posterior inferior temporal sulcus \|\| id:ebi-hbsm44-GCST90104941 | Curry liking \|\| id:ebi-fl187-GCST90094750 | Weighted mode | 5 | -0.005704778 | 0.0442181778601156 | 0.90357332790848 | -0.092372407 | 0.0809628501922691 | 0.994311462935707 | 0.911765538425691 | 1.08433061314471 | 0.999714303421225 |
| ebi-fl187-GCST90094750 | ebi-IPs91-GCST90274819 | Leukemia inhibitory factor levels \|\| id:ebi-IPs91-GCST90274819 | Curry liking \|\| id:ebi-fl187-GCST90094750 | Weighted mode | 7 | -0.061010718 | 0.184689893613076 | 0.75237186736024 | -0.423002909 | 0.300981473512372 | 0.940813156115487 | 0.655076727195468 | 1.35118430860675 | 0.999714303421225 |
| ebi-fl187-GCST90094750 | ebi-IPs91-GCST90274837 | STAM binding protein levels \|\| id:ebi-IPs91-GCST90274837 | Curry liking \|\| id:ebi-fl187-GCST90094750 | Weighted mode | 7 | -0.017047181 | 0.151304555287293 | 0.913968797875448 | -0.31360411 | 0.279509747130982 | 0.983097299798782 | 0.730808290887585 | 1.32248130313059 | 0.999714303421225 |
| ebi-fl187-GCST90094750 | ebi-IPs91-GCST90274840 | TNF-beta levels \|\| id:ebi-IPs91-GCST90274840 | Curry liking \|\| id:ebi-fl187-GCST90094750 | Weighted mode | 7 | -0.024140787 | 0.178377517284056 | 0.896772861943664 | -0.373760721 | 0.325479146462896 | 0.976148270692722 | 0.688141549752925 | 1.38469395826849 | 0.999714303421225 |
| ebi-fl187-GCST90094750 | ebi-IPs91-GCST90274842 | Tumor necrosis factor ligand superfamily member 14 levels \|\| id:ebi-IPs91-GCST90274842 | Curry liking \|\| id:ebi-fl187-GCST90094750 | Weighted mode | 7 | -0.037120605 | 0.166513638358496 | 0.830986801268686 | -0.363487336 | 0.289246126446385 | 0.963559918451199 | 0.695247531831524 | 1.33542037035331 | 0.999714303421225 |
| ebi-fl187-GCST90094750 | ebi-met1400-GCST90199628 | 3-methylhistidine levels \|\| id:ebi-met1400-GCST90199628 | Curry liking \|\| id:ebi-fl187-GCST90094750 | Weighted mode | 7 | -0.294408921 | 0.34563302127346 | 0.427011406161656 | -0.971849642 | 0.383031800993305 | 0.744971794759357 | 0.378382518098979 | 1.46672467262825 | 0.999714303421225 |
| ebi-fl187-GCST90094750 | ebi-met1400-GCST90199651 | Docosahexaenoate DHA; 22:6n3 levels \|\| id:ebi-met1400-GCST90199651 | Curry liking \|\| id:ebi-fl187-GCST90094750 | Weighted mode | 7 | 0.0326141524257806 | 0.239915148607451 | 0.896314696906929 | -0.437619539 | 0.502847843696385 | 1.03315182319966 | 0.645571350961705 | 1.65342326327009 | 0.999714303421225 |
| ebi-fl187-GCST90094750 | ebi-met1400-GCST90199652 | 1-stearoyl-2-oleoyl-GPS (18:0/18:1) levels \|\| id:ebi-met1400-GCST90199652 | Curry liking \|\| id:ebi-fl187-GCST90094750 | Weighted mode | 7 | -0.278049887 | 0.258304230030126 | 0.323095505521446 | -0.784326177 | 0.228226404358436 | 0.757259043568299 | 0.45642714913827 | 1.25636974081982 | 0.999714303421225 |
| ebi-fl187-GCST90094750 | ebi-met1400-GCST90199653 | 1-myristoyl-2-palmitoyl-gpc (14:0/16:0) levels \|\| id:ebi-met1400-GCST90199653 | Curry liking \|\| id:ebi-fl187-GCST90094750 | Weighted mode | 7 | -0.326821562 | 0.241463932579687 | 0.224659797861151 | -0.80009087 | 0.146447745448497 | 0.721212422953894 | 0.449288135330775 | 1.15771443338935 | 0.999714303421225 |
| ebi-fl187-GCST90094750 | ebi-met1400-GCST90199667 | Erythritol levels in elite athletes \|\| id:ebi-met1400-GCST90199667 | Curry liking \|\| id:ebi-fl187-GCST90094750 | Weighted mode | 7 | 0.168106560475244 | 0.24584955807525 | 0.519635063032154 | -0.313758573 | 0.649971694302734 | 1.18306267165167 | 0.730695416211046 | 1.91548660906246 | 0.999714303421225 |
| ebi-fl187-GCST90094750 | ebi-met1400-GCST90199674 | 3-hydroxymyristate levels \|\| id:ebi-met1400-GCST90199674 | Curry liking \|\| id:ebi-fl187-GCST90094750 | Weighted mode | 7 | -0.128791939 | 0.264882967867773 | 0.644057448471745 | -0.647962556 | 0.390378678106324 | 0.879156864849425 | 0.523110500119177 | 1.4775402000835 | 0.999714303421225 |
| ebi-fl187-GCST90094750 | ebi-met1400-GCST90199685 | 2-linoleoylglycerol (18:2) levels \|\| id:ebi-met1400-GCST90199685 | Curry liking \|\| id:ebi-fl187-GCST90094750 | Weighted mode | 7 | -0.360202075 | 0.299223605034027 | 0.274004019693574 | -0.94668034 | 0.226276191284803 | 0.697535357662692 | 0.388027005316949 | 1.25392193976857 | 0.999714303421225 |
| ebi-fl187-GCST90094750 | ebi-met1400-GCST90199703 | Gamma-glutamylvaline levels \|\| id:ebi-met1400-GCST90199703 | Curry liking \|\| id:ebi-fl187-GCST90094750 | Weighted mode | 7 | 0.0333471729324732 | 0.212725553231937 | 0.880574709282459 | -0.383594911 | 0.450289257267069 | 1.03390942230651 | 0.681407401647635 | 1.56876589680334 | 0.999714303421225 |
| ebi-fl187-GCST90094750 | ebi-met1400-GCST90199779 | Gamma-glutamyl-2-aminobutyrate levels \|\| id:ebi-met1400-GCST90199779 | Curry liking \|\| id:ebi-fl187-GCST90094750 | Weighted mode | 7 | 0.00947753021767794 | 0.249414938810141 | 0.970921201981835 | -0.47937575 | 0.498330810285554 | 1.00952258422829 | 0.619169788022571 | 1.64597153766458 | 0.999714303421225 |
| ebi-fl187-GCST90094750 | ebi-met1400-GCST90199824 | 4-vinylphenol sulfate levels \|\| id:ebi-met1400-GCST90199824 | Curry liking \|\| id:ebi-fl187-GCST90094750 | Weighted mode | 7 | -0.079261062 | 0.208280862712128 | 0.716646442709879 | -0.487491553 | 0.328969428772561 | 0.92379872408751 | 0.614165064017899 | 1.38953537513629 | 0.999714303421225 |
| ebi-fl187-GCST90094750 | ebi-met1400-GCST90199830 | 4-ethylphenylsulfate levels \|\| id:ebi-met1400-GCST90199830 | Curry liking \|\| id:ebi-fl187-GCST90094750 | Weighted mode | 7 | 0.044820589580195 | 0.218314656180898 | 0.8441237178928 | -0.383076137 | 0.472717315694755 | 1.04584020844506 | 0.681760990390861 | 1.60434779492639 | 0.999714303421225 |
| ebi-fl187-GCST90094750 | ebi-met1400-GCST90199833 | Hexanoylglutamine levels \|\| id:ebi-met1400-GCST90199833 | Curry liking \|\| id:ebi-fl187-GCST90094750 | Weighted mode | 6 | 0.0319208916573455 | 0.210085265002088 | 0.885172883169016 | -0.379846228 | 0.443688011061437 | 1.03243582778724 | 0.68396657620784 | 1.55844419241741 | 0.999714303421225 |
| ebi-fl187-GCST90094750 | ebi-met1400-GCST90199837 | Carotenoid levels (cryptoxanthin) \|\| id:ebi-met1400-GCST90199837 | Curry liking \|\| id:ebi-fl187-GCST90094750 | Weighted mode | 7 | 0.246593115678842 | 0.203288982691399 | 0.270708230742996 | -0.15185329 | 0.645039521753983 | 1.27965833392457 | 0.859114311816133 | 1.90606235871095 | 0.999714303421225 |
| ebi-fl187-GCST90094750 | ebi-met1400-GCST90199869 | 2-hydroxyhippurate levels \|\| id:ebi-met1400-GCST90199869 | Curry liking \|\| id:ebi-fl187-GCST90094750 | Weighted mode | 7 | 0.166069639249438 | 0.240630207663194 | 0.515896230277528 | -0.305565568 | 0.637704846269298 | 1.18065531881072 | 0.736706599036711 | 1.89213315539554 | 0.999714303421225 |
| ebi-fl187-GCST90094750 | ebi-met1400-GCST90199902 | Hydantoin-5-propionate levels \|\| id:ebi-met1400-GCST90199902 | Curry liking \|\| id:ebi-fl187-GCST90094750 | Weighted mode | 7 | 0.0113778136592838 | 0.191737923881605 | 0.9546080105407 | -0.364428517 | 0.38718414446723 | 1.01144278716611 | 0.694593485740771 | 1.47282767937181 | 0.999714303421225 |
| ebi-fl187-GCST90094750 | ebi-met1400-GCST90199904 | Pantoate levels \|\| id:ebi-met1400-GCST90199904 | Curry liking \|\| id:ebi-fl187-GCST90094750 | Weighted mode | 7 | 0.0403632684791651 | 0.233607232917128 | 0.868503490796732 | -0.417506908 | 0.498233444996736 | 1.04118893662251 | 0.658686941597629 | 1.64581128497208 | 0.999714303421225 |
| ebi-fl187-GCST90094750 | ebi-met1400-GCST90199936 | 3-methyl catechol sulfate (2) levels \|\| id:ebi-met1400-GCST90199936 | Curry liking \|\| id:ebi-fl187-GCST90094750 | Weighted mode | 7 | -0.222244144 | 0.230041566916855 | 0.371301971858394 | -0.673125615 | 0.228637327036287 | 0.800719849425119 | 0.510111670596872 | 1.25688611772631 | 0.999714303421225 |
| ebi-fl187-GCST90094750 | ebi-met1400-GCST90199977 | 4-hydroxychlorothalonil levels \|\| id:ebi-met1400-GCST90199977 | Curry liking \|\| id:ebi-fl187-GCST90094750 | Weighted mode | 7 | -0.0880745 | 0.230188796531899 | 0.715189765539428 | -0.539244541 | 0.363095540935973 | 0.915692654835221 | 0.583188660846853 | 1.43777321887842 | 0.999714303421225 |
| ebi-fl187-GCST90094750 | ebi-met1400-GCST90200040 | Sphingomyelin (d18:2/24:1, d18:1/24:2) levels \|\| id:ebi-met1400-GCST90200040 | Curry liking \|\| id:ebi-fl187-GCST90094750 | Weighted mode | 7 | -0.23025067 | 0.202449379496744 | 0.29877347850466 | -0.627051454 | 0.166550113645494 | 0.794334462 | 0.53416448987446 | 1.18122272976428 | 0.999714303421225 |
| ebi-fl187-GCST90094750 | ebi-met1400-GCST90200077 | 1-stearoyl-2-oleoyl-GPI (18:0/18:1) levels \|\| id:ebi-met1400-GCST90200077 | Curry liking \|\| id:ebi-fl187-GCST90094750 | Weighted mode | 7 | -0.010371175 | 0.203610407004595 | 0.961029624104699 | -0.409447572 | 0.388705223167532 | 0.989682420627709 | 0.664016970207763 | 1.47506967087461 | 0.999714303421225 |
| ebi-fl187-GCST90094750 | ebi-met1400-GCST90200080 | 1-palmitoyl-2-oleoyl-GPI (16:0/18:1) levels \|\| id:ebi-met1400-GCST90200080 | Curry liking \|\| id:ebi-fl187-GCST90094750 | Weighted mode | 7 | -0.016542502 | 0.189660805991951 | 0.93333353439969 | -0.388277681 | 0.355192678192815 | 0.983593574249426 | 0.678223986998369 | 1.42645547466767 | 0.999714303421225 |
| ebi-fl187-GCST90094750 | ebi-met1400-GCST90200082 | 1-oleoyl-2-linoleoyl-GPE (18:1/18:2) levels \|\| id:ebi-met1400-GCST90200082 | Curry liking \|\| id:ebi-fl187-GCST90094750 | Weighted mode | 7 | -0.369910353 | 0.219671172150525 | 0.143180554638323 | -0.80046585 | 0.0606451445171855 | 0.690796255744118 | 0.449119692827037 | 1.0625218055933 | 0.999714303421225 |
| ebi-fl187-GCST90094750 | ebi-met1400-GCST90200100 | Perfluorooctanesulfonate (PFOS) levels \|\| id:ebi-met1400-GCST90200100 | Curry liking \|\| id:ebi-fl187-GCST90094750 | Weighted mode | 7 | 0.240094151432129 | 0.224063348402282 | 0.325121449708577 | -0.199070011 | 0.679258314300601 | 1.27136884588417 | 0.819492517476246 | 1.97241427812267 | 0.999714303421225 |
| ebi-fl187-GCST90094750 | ebi-met1400-GCST90200102 | 1-palmitoleoyl-2-linolenoyl-GPC (16:1/18:3) levels \|\| id:ebi-met1400-GCST90200102 | Curry liking \|\| id:ebi-fl187-GCST90094750 | Weighted mode | 7 | -0.318827091 | 0.205261006413281 | 0.171348174939237 | -0.721138664 | 0.0834844810782222 | 0.727001243139663 | 0.486198324090097 | 1.08706834503336 | 0.999714303421225 |
| ebi-fl187-GCST90094750 | ebi-met1400-GCST90200114 | Glycosyl-N-tricosanoyl-sphingadienine (d18:2/23:0) levels \|\| id:ebi-met1400-GCST90200114 | Curry liking \|\| id:ebi-fl187-GCST90094750 | Weighted mode | 7 | -0.574103463 | 0.219038065924265 | 0.0395320993914406 | -1.003418072 | -0.144788854 | 0.563209580791746 | 0.366624149261954 | 0.865204958631817 | 0.999714303421225 |
| ebi-fl187-GCST90094750 | ebi-met1400-GCST90200135 | Arachidoylcarnitine (C20) levels \|\| id:ebi-met1400-GCST90200135 | Curry liking \|\| id:ebi-fl187-GCST90094750 | Weighted mode | 7 | -0.400463791 | 0.184543028086305 | 0.0730589882989286 | -0.762168126 | -0.038759456 | 0.670009229595033 | 0.466653565566899 | 0.961982080212295 | 0.999714303421225 |
| ebi-fl187-GCST90094750 | ebi-met1400-GCST90200146 | Docosahexaenoylcarnitine (C22:6) levels \|\| id:ebi-met1400-GCST90200146 | Curry liking \|\| id:ebi-fl187-GCST90094750 | Weighted mode | 7 | 0.177039910601371 | 0.262315435489338 | 0.524871155411086 | -0.337098343 | 0.691178164160473 | 1.19367873261253 | 0.713838635447967 | 1.99606584168325 | 0.999714303421225 |
| ebi-fl187-GCST90094750 | ebi-met1400-GCST90200162 | 3-hydroxyphenylacetoylglutamine levels \|\| id:ebi-met1400-GCST90200162 | Curry liking \|\| id:ebi-fl187-GCST90094750 | Weighted mode | 7 | -0.183686392 | 0.297778524248647 | 0.559992984351787 | -0.767332299 | 0.399959515614257 | 0.832196746537275 | 0.464249897544161 | 1.49176430325727 | 0.999714303421225 |
| ebi-fl187-GCST90094750 | ebi-met1400-GCST90200172 | 3-carboxy-4-methyl-5-pentyl-2-furanpropionate (3-CMPFP) levels \|\| id:ebi-met1400-GCST90200172 | Curry liking \|\| id:ebi-fl187-GCST90094750 | Weighted mode | 7 | -0.09743219 | 0.261201162744615 | 0.721959544242979 | -0.609386469 | 0.414522088801927 | 0.907163854090108 | 0.543684333876278 | 1.51364717886996 | 0.999714303421225 |
| ebi-fl187-GCST90094750 | ebi-met1400-GCST90200222 | Dihydrocaffeate sulfate (2) levels \|\| id:ebi-met1400-GCST90200222 | Curry liking \|\| id:ebi-fl187-GCST90094750 | Weighted mode | 7 | -0.187258343 | 0.249353139578175 | 0.48107179831345 | -0.675990496 | 0.301473810938803 | 0.829229483381977 | 0.508652352783685 | 1.3518497109997 | 0.999714303421225 |
| ebi-fl187-GCST90094750 | ebi-met1400-GCST90200223 | 4-methylhexanoylglutamine levels \|\| id:ebi-met1400-GCST90200223 | Curry liking \|\| id:ebi-fl187-GCST90094750 | Weighted mode | 7 | -0.157250041 | 0.298030509296426 | 0.616689090826386 | -0.741389839 | 0.426889757587745 | 0.854490374789535 | 0.476451264658967 | 1.53248370771056 | 0.999714303421225 |
| ebi-fl187-GCST90094750 | ebi-met1400-GCST90200224 | 2-ketocaprylate levels \|\| id:ebi-met1400-GCST90200224 | Curry liking \|\| id:ebi-fl187-GCST90094750 | Weighted mode | 7 | -0.1533363 | 0.251263582133242 | 0.564072815514249 | -0.64581292 | 0.339140321478553 | 0.857841181733443 | 0.524236206491011 | 1.40374030630875 | 0.999714303421225 |
| ebi-fl187-GCST90094750 | ebi-met1400-GCST90200243 | Hydroxypalmitoyl sphingomyelin (d18:1/16:0(OH)) levels \|\| id:ebi-met1400-GCST90200243 | Curry liking \|\| id:ebi-fl187-GCST90094750 | Weighted mode | 7 | -0.252105639 | 0.300604556667831 | 0.433792984355636 | -0.84129057 | 0.337079291926654 | 0.777162634938192 | 0.431153730055415 | 1.40085013544113 | 0.999714303421225 |
| ebi-fl187-GCST90094750 | ebi-met1400-GCST90200273 | 2-methoxyhydroquinone sulfate (2) levels \|\| id:ebi-met1400-GCST90200273 | Curry liking \|\| id:ebi-fl187-GCST90094750 | Weighted mode | 7 | -0.024344641 | 0.312137511467428 | 0.940369319308506 | -0.636134163 | 0.587444881896114 | 0.975949300058155 | 0.529334795770585 | 1.79938489571127 | 0.999714303421225 |
| ebi-fl187-GCST90094750 | ebi-met1400-GCST90200276 | 2,4-di-tert-butylphenol levels \|\| id:ebi-met1400-GCST90200276 | Curry liking \|\| id:ebi-fl187-GCST90094750 | Weighted mode | 7 | -0.277131746 | 0.269617381136618 | 0.343652367463796 | -0.805581813 | 0.251318320560944 | 0.757954632687389 | 0.446827880426922 | 1.28571929008407 | 0.999714303421225 |
| ebi-fl187-GCST90094750 | ebi-met1400-GCST90200289 | N-acetylmethionine levels \|\| id:ebi-met1400-GCST90200289 | Curry liking \|\| id:ebi-fl187-GCST90094750 | Weighted mode | 7 | 0.0514416318894851 | 0.233929481816752 | 0.833238066132617 | -0.407060152 | 0.50994341625032 | 1.05278773526788 | 0.665604151362668 | 1.66519696919161 | 0.999714303421225 |
| ebi-fl187-GCST90094750 | ebi-met1400-GCST90200308 | 3-hydroxyisobutyrate levels \|\| id:ebi-met1400-GCST90200308 | Curry liking \|\| id:ebi-fl187-GCST90094750 | Weighted mode | 7 | -0.071988096 | 0.231670918253542 | 0.766514292179516 | -0.526063096 | 0.382086903640022 | 0.93054197278951 | 0.590926818437635 | 1.46533942292954 | 0.999714303421225 |
| ebi-fl187-GCST90094750 | ebi-met1400-GCST90200383 | Cystathionine levels \|\| id:ebi-met1400-GCST90200383 | Curry liking \|\| id:ebi-fl187-GCST90094750 | Weighted mode | 7 | -0.14415387 | 0.266798432950511 | 0.608439136867834 | -0.667078798 | 0.378771059054938 | 0.865754524569836 | 0.513205567290304 | 1.4604886318179 | 0.999714303421225 |
| ebi-fl187-GCST90094750 | ebi-met1400-GCST90200384 | Guanidinoacetate levels \|\| id:ebi-met1400-GCST90200384 | Curry liking \|\| id:ebi-fl187-GCST90094750 | Weighted mode | 7 | 0.0575058700770457 | 0.196914470116372 | 0.780094639127938 | -0.328446491 | 0.443458231505135 | 1.05919148817213 | 0.72004145564052 | 1.55808613494107 | 0.999714303421225 |
| ebi-fl187-GCST90094750 | ebi-met1400-GCST90200403 | Citrulline levels \|\| id:ebi-met1400-GCST90200403 | Curry liking \|\| id:ebi-fl187-GCST90094750 | Weighted mode | 7 | -0.14363214 | 0.22632662144283 | 0.549092204432327 | -0.587232318 | 0.299968037936656 | 0.866206332040704 | 0.555863611367476 | 1.34981566399277 | 0.999714303421225 |
| ebi-fl187-GCST90094750 | ebi-met1400-GCST90200434 | 12,13-DiHOME levels \|\| id:ebi-met1400-GCST90200434 | Curry liking \|\| id:ebi-fl187-GCST90094750 | Weighted mode | 7 | 0.0591830788329352 | 0.235838100617033 | 0.810227330825027 | -0.403059598 | 0.521425756042321 | 1.06096946401178 | 0.668272270194366 | 1.6844275211929 | 0.999714303421225 |
| ebi-fl187-GCST90094750 | ebi-met1400-GCST90200437 | Fructose levels \|\| id:ebi-met1400-GCST90200437 | Curry liking \|\| id:ebi-fl187-GCST90094750 | Weighted mode | 7 | -0.265002753 | 0.255292888089453 | 0.33926515840858 | -0.765376813 | 0.235371307871229 | 0.767203838026423 | 0.465158619927155 | 1.26537852652209 | 0.999714303421225 |
| ebi-fl187-GCST90094750 | ebi-met1400-GCST90200448 | N-stearoyl-sphinganine (d18:0/18:0) levels \|\| id:ebi-met1400-GCST90200448 | Curry liking \|\| id:ebi-fl187-GCST90094750 | Weighted mode | 7 | 0.10995869550824 | 0.239032696757081 | 0.661703764911435 | -0.35854539 | 0.578462781152117 | 1.11623196411271 | 0.698691911398211 | 1.78329500797212 | 0.999714303421225 |
| ebi-fl187-GCST90094750 | ebi-met1400-GCST90200468 | X-11847 levels \|\| id:ebi-met1400-GCST90200468 | Curry liking \|\| id:ebi-fl187-GCST90094750 | Weighted mode | 7 | -0.167307646 | 0.214386296293451 | 0.464818348383773 | -0.587504787 | 0.252889494270899 | 0.845939321059442 | 0.55571217635219 | 1.28774096621734 | 0.999714303421225 |
| ebi-fl187-GCST90094750 | ebi-met1400-GCST90200476 | X-11843 levels \|\| id:ebi-met1400-GCST90200476 | Curry liking \|\| id:ebi-fl187-GCST90094750 | Weighted mode | 7 | 0.04898030767997 | 0.213654219476698 | 0.826289072894731 | -0.369781962 | 0.467742577854299 | 1.05019966966841 | 0.690884953047938 | 1.59638640457568 | 0.999714303421225 |
| ebi-fl187-GCST90094750 | ebi-met1400-GCST90200511 | X-12906 levels \|\| id:ebi-met1400-GCST90200511 | Curry liking \|\| id:ebi-fl187-GCST90094750 | Weighted mode | 7 | 0.132426588253698 | 0.216689019955148 | 0.563529951214926 | -0.292283891 | 0.557137067365788 | 1.14159520651524 | 0.746556565302351 | 1.74566761061269 | 0.999714303421225 |
| ebi-fl187-GCST90094750 | ebi-met1400-GCST90200515 | X-12849 levels \|\| id:ebi-met1400-GCST90200515 | Curry liking \|\| id:ebi-fl187-GCST90094750 | Weighted mode | 7 | -0.141763102 | 0.236987624574861 | 0.571588213858408 | -0.606258846 | 0.322732641995127 | 0.867826818425077 | 0.54538743534624 | 1.38089610791945 | 0.999714303421225 |
| ebi-fl187-GCST90094750 | ebi-met1400-GCST90200551 | X-18886 levels \|\| id:ebi-met1400-GCST90200551 | Curry liking \|\| id:ebi-fl187-GCST90094750 | Weighted mode | 7 | -0.022015264 | 0.222926103438353 | 0.924548816474876 | -0.458950426 | 0.414919899148905 | 0.978225303707033 | 0.631946572034164 | 1.51424944316493 | 0.999714303421225 |
| ebi-fl187-GCST90094750 | ebi-met1400-GCST90200564 | X-21319 levels \|\| id:ebi-met1400-GCST90200564 | Curry liking \|\| id:ebi-fl187-GCST90094750 | Weighted mode | 7 | 0.0571209769917476 | 0.225819691332857 | 0.808750596478486 | -0.385485618 | 0.499727572004147 | 1.05878389113799 | 0.680120277334085 | 1.64827217404466 | 0.999714303421225 |
| ebi-fl187-GCST90094750 | ebi-met1400-GCST90200579 | X-21442 levels \|\| id:ebi-met1400-GCST90200579 | Curry liking \|\| id:ebi-fl187-GCST90094750 | Weighted mode | 7 | 0.367808168488565 | 0.236159108655276 | 0.17036988077922 | -0.095063684 | 0.830680021452906 | 1.44456489932467 | 0.909315023373654 | 2.29487877657488 | 0.999714303421225 |
| ebi-fl187-GCST90094750 | ebi-met1400-GCST90200581 | X-21441 levels \|\| id:ebi-met1400-GCST90200581 | Curry liking \|\| id:ebi-fl187-GCST90094750 | Weighted mode | 7 | 0.000648431310390274 | 0.196248224366825 | 0.997470801205475 | -0.383998088 | 0.385294951069366 | 1.00064864158742 | 0.681132729198534 | 1.47004784968848 | 0.999714303421225 |
| ebi-fl187-GCST90094750 | ebi-met1400-GCST90200648 | X-24585 levels \|\| id:ebi-met1400-GCST90200648 | Curry liking \|\| id:ebi-fl187-GCST90094750 | Weighted mode | 7 | 0.37727733049686 | 0.362511319568775 | 0.338108070239025 | -0.333244856 | 1.08779951685166 | 1.45830868676822 | 0.71659471025987 | 2.96773642821396 | 0.999714303421225 |
| ebi-fl187-GCST90094750 | ebi-met1400-GCST90200720 | Adenosine 5'-diphosphate (ADP) to pantothenate ratio \|\| id:ebi-met1400-GCST90200720 | Curry liking \|\| id:ebi-fl187-GCST90094750 | Weighted mode | 7 | -0.392232531 | 0.329590438858023 | 0.278970902220172 | -1.038229792 | 0.253764728712742 | 0.675547009770464 | 0.354080924528409 | 1.28886853483461 | 0.999714303421225 |
| ebi-fl187-GCST90094750 | ebi-met1400-GCST90200724 | Cholate to taurocholate ratio \|\| id:ebi-met1400-GCST90200724 | Curry liking \|\| id:ebi-fl187-GCST90094750 | Weighted mode | 7 | 0.18070135816267 | 0.237334379149907 | 0.475281680059262 | -0.284474025 | 0.645876741296488 | 1.19805733581835 | 0.752409899079006 | 1.90765881956776 | 0.999714303421225 |
| ebi-fl187-GCST90094750 | ebi-met1400-GCST90200726 | N-acetylputrescine to (N(1) + N(8))-acetylspermidine ratio \|\| id:ebi-met1400-GCST90200726 | Curry liking \|\| id:ebi-fl187-GCST90094750 | Weighted mode | 7 | -0.322597114 | 0.211228665610521 | 0.177557710543018 | -0.736605299 | 0.0914110702179657 | 0.724265591788529 | 0.478736326861628 | 1.09571933027845 | 0.999714303421225 |
| ebi-fl187-GCST90094750 | ebi-met1400-GCST90200744 | Arginine to glutamate ratio \|\| id:ebi-met1400-GCST90200744 | Curry liking \|\| id:ebi-fl187-GCST90094750 | Weighted mode | 7 | 0.133866808367251 | 0.2532717628162 | 0.61608879202953 | -0.362545847 | 0.630279463487004 | 1.14324053942958 | 0.695902408081795 | 1.87813537619143 | 0.999714303421225 |
| ebi-fl187-GCST90094750 | ebi-met1400-GCST90200759 | Histidine to trans-urocanate ratio \|\| id:ebi-met1400-GCST90200759 | Curry liking \|\| id:ebi-fl187-GCST90094750 | Weighted mode | 7 | -0.303131239 | 0.271319534576896 | 0.306618884327344 | -0.834917527 | 0.228655048421063 | 0.738502169465871 | 0.433910265786472 | 1.25690839168618 | 0.999714303421225 |
| ebi-fl187-GCST90094750 | ebi-met1400-GCST90200795 | Oleoyl-linoleoyl-glycerol (18:1 to 18:2) [2] to linoleoyl-arachidonoyl-glycerol (18:2 to 20:4) [2] ratio \|\| id:ebi-met1400-GCST90200795 | Curry liking \|\| id:ebi-fl187-GCST90094750 | Weighted mode | 7 | 0.333745915503283 | 0.256506567065888 | 0.240945607114821 | -0.169006956 | 0.836498786952425 | 1.39618834868858 | 0.844503029048257 | 2.30827106352788 | 0.999714303421225 |
| ebi-fl187-GCST90094750 | ebi-met1400-GCST90200811 | Choline to taurocholate ratio \|\| id:ebi-met1400-GCST90200811 | Curry liking \|\| id:ebi-fl187-GCST90094750 | Weighted mode | 7 | 0.389665707142712 | 0.249113504938606 | 0.168802171090755 | -0.098596763 | 0.87792817682238 | 1.47648713227127 | 0.906108011062288 | 2.40590991928972 | 0.999714303421225 |
| ebi-fl187-GCST90094750 | ebi-met1400-GCST90200831 | Cholate to phosphate ratio \|\| id:ebi-met1400-GCST90200831 | Curry liking \|\| id:ebi-fl187-GCST90094750 | Weighted mode | 7 | -0.237958809 | 0.272643883019842 | 0.416338547646851 | -0.77234082 | 0.296423201364472 | 0.788235158340135 | 0.461930505563346 | 1.3450392588508 | 0.999714303421225 |
| ebi-fl187-GCST90094750 | ebi-met1400-GCST90200841 | Adenosine 5'-diphosphate (ADP) to oxalate (ethanedioate) ratio \|\| id:ebi-met1400-GCST90200841 | Curry liking \|\| id:ebi-fl187-GCST90094750 | Weighted mode | 7 | -0.137704986 | 0.30520058946598 | 0.667697652695363 | -0.735898142 | 0.460488168967074 | 0.871355715629694 | 0.479074988448549 | 1.58484746953572 | 0.999714303421225 |
| ebi-fl187-GCST90094750 | ebi-met1400-GCST90200844 | Aspartate to phosphate ratio \|\| id:ebi-met1400-GCST90200844 | Curry liking \|\| id:ebi-fl187-GCST90094750 | Weighted mode | 7 | -0.163325426 | 0.205571601443843 | 0.457173156670413 | -0.566245765 | 0.23959491248396 | 0.849314754040528 | 0.567652544324086 | 1.27073428745013 | 0.999714303421225 |
| ebi-fl187-GCST90094750 | ebi-met1400-GCST90200870 | Adenosine 5'-monophosphate (AMP) to acetoacetate ratio \|\| id:ebi-met1400-GCST90200870 | Curry liking \|\| id:ebi-fl187-GCST90094750 | Weighted mode | 7 | -0.337236574 | 0.310445823342723 | 0.319050697981257 | -0.945710388 | 0.271237239846896 | 0.713739967676933 | 0.388403555782696 | 1.31158619398552 | 0.999714303421225 |
| ebi-fl187-GCST90094750 | ebi-met1400-GCST90200887 | Cholesterol to cortisol ratio \|\| id:ebi-met1400-GCST90200887 | Curry liking \|\| id:ebi-fl187-GCST90094750 | Weighted mode | 7 | -0.154386867 | 0.216692261275965 | 0.502925113719155 | -0.579103699 | 0.270329965394711 | 0.856940435152288 | 0.560400429105289 | 1.31039676499074 | 0.999714303421225 |
| ebi-fl187-GCST90094750 | ebi-met1400-GCST90200890 | Cortisol to taurocholate ratio \|\| id:ebi-met1400-GCST90200890 | Curry liking \|\| id:ebi-fl187-GCST90094750 | Weighted mode | 7 | 0.240766757065973 | 0.212691451837066 | 0.300837514700496 | -0.176108489 | 0.657642002666622 | 1.27222426337967 | 0.838527007835898 | 1.93023547388075 | 0.999714303421225 |
| ebi-fl187-GCST90094750 | ebi-met1400-GCST90200948 | Adenosine 5'-diphosphate (ADP) to mannitol to sorbitol ratio \|\| id:ebi-met1400-GCST90200948 | Curry liking \|\| id:ebi-fl187-GCST90094750 | Weighted mode | 7 | -0.244248989 | 0.296356162460509 | 0.441364695679059 | -0.825107067 | 0.336609089372244 | 0.7832925787052 | 0.438188074263499 | 1.40019160696211 | 0.999714303421225 |
| ebi-fl187-GCST90094750 | ebi-met1400-GCST90200972 | Leucine to N-palmitoyl-sphingosine (d18:1 to 16:0) ratio \|\| id:ebi-met1400-GCST90200972 | Curry liking \|\| id:ebi-fl187-GCST90094750 | Weighted mode | 7 | 0.312347487575375 | 0.204880725802185 | 0.178215566837717 | -0.089218735 | 0.713913710147657 | 1.36662949730086 | 0.914645486714542 | 2.04196730866907 | 0.999714303421225 |
| ebi-fl187-GCST90094750 | ebi-met1400-GCST90200973 | Proline to glutamate ratio \|\| id:ebi-met1400-GCST90200973 | Curry liking \|\| id:ebi-fl187-GCST90094750 | Weighted mode | 7 | -0.02702773 | 0.215392168841745 | 0.904241281526379 | -0.449196381 | 0.395140920783686 | 0.973334250449104 | 0.638140767617292 | 1.48459338624403 | 0.999714303421225 |
| ebi-fl187-GCST90094750 | ebi-met1400-GCST90200983 | Cholesterol to linoleoyl-arachidonoyl-glycerol (18:2 to 20:4) [1] ratio \|\| id:ebi-met1400-GCST90200983 | Curry liking \|\| id:ebi-fl187-GCST90094750 | Weighted mode | 7 | -0.08092007 | 0.218342079709862 | 0.72365904900416 | -0.508870546 | 0.347030406598271 | 0.922267405670719 | 0.601174194187502 | 1.41485974578827 | 0.999714303421225 |
| ebi-fl187-GCST90094750 | ebi-met1400-GCST90201001 | Serine to alpha-ketobutyrate ratio \|\| id:ebi-met1400-GCST90201001 | Curry liking \|\| id:ebi-fl187-GCST90094750 | Weighted mode | 7 | 0.0158387564105196 | 0.213977771852181 | 0.943400094625227 | -0.403557676 | 0.435235189240795 | 1.01596485437819 | 0.667939501328945 | 1.54532646037261 | 0.999714303421225 |
| ebi-fl187-GCST90094750 | ebi-met1400-GCST90201005 | Fructose to maltose ratio \|\| id:ebi-met1400-GCST90201005 | Curry liking \|\| id:ebi-fl187-GCST90094750 | Weighted mode | 7 | -0.119289163 | 0.234718862035368 | 0.629437234807111 | -0.579338133 | 0.340759806459695 | 0.887551116593362 | 0.560269067638869 | 1.4060154844634 | 0.999714303421225 |
| ebi-fl187-GCST90094750 | ebi-met1400-GCST90201020 | Paraxanthine to 5-acetylamino-6-formylamino-3-methyluracil ratio \|\| id:ebi-met1400-GCST90201020 | Curry liking \|\| id:ebi-fl187-GCST90094750 | Weighted mode | 7 | 0.285539757519405 | 0.210346237316099 | 0.2234654207384 | -0.126738868 | 0.697818382658959 | 1.33047997091525 | 0.880963690708378 | 2.0093642583422 | 0.999714303421225 |
| ebi-fl187-GCST90094750 | ebi-pw11-GCST90275197 | Placental weight (fetal genotype, adjusted for sex) \|\| id:ebi-pw11-GCST90275197 | Curry liking \|\| id:ebi-fl187-GCST90094750 | Weighted mode | 6 | -0.080340436 | 0.0946511985859354 | 0.434734112829471 | -0.265856785 | 0.105175913018926 | 0.922802137643789 | 0.766548900605375 | 1.11090601599902 | 0.999714303421225 |
| ebi-fl187-GCST90094750 | prot-a-1017 | FAS-associated factor 2 \|\| id:prot-a-1017 | Curry liking \|\| id:ebi-fl187-GCST90094750 | Weighted mode | 7 | 0.53098906697303 | 0.385482717807844 | 0.217544693292879 | -0.22455706 | 1.2865351938764 | 1.70061349772167 | 0.798869991933308 | 3.62022143507249 | 0.999714303421225 |
| ebi-fl187-GCST90094750 | prot-a-1019 | Fas apoptotic inhibitory molecule 3 \|\| id:prot-a-1019 | Curry liking \|\| id:ebi-fl187-GCST90094750 | Weighted mode | 7 | 0.515595159924595 | 0.391190351790979 | 0.235580653058948 | -0.25113793 | 1.28232824943492 | 1.67463488083508 | 0.777915066655994 | 3.60502335578196 | 0.999714303421225 |
| ebi-fl187-GCST90094750 | prot-a-1052 | MIP18 family protein FAM96A \|\| id:prot-a-1052 | Curry liking \|\| id:ebi-fl187-GCST90094750 | Weighted mode | 7 | -0.155202736 | 0.340071338477277 | 0.664168910276462 | -0.82174256 | 0.511337087185255 | 0.856241568698027 | 0.439664844378409 | 1.66751932373164 | 0.999714303421225 |
| ebi-fl187-GCST90094750 | prot-a-1066 | F-box/LRR-repeat protein 4 \|\| id:prot-a-1066 | Curry liking \|\| id:ebi-fl187-GCST90094750 | Weighted mode | 7 | 0.0825168580124207 | 0.379780703430492 | 0.835194100373133 | -0.661853321 | 0.826887036736184 | 1.0860169813725 | 0.515894330300571 | 2.28619082350115 | 0.999714303421225 |
| ebi-fl187-GCST90094750 | prot-a-1076 | Low affinity immunoglobulin gamma Fc region receptor III-B \|\| id:prot-a-1076 | Curry liking \|\| id:ebi-fl187-GCST90094750 | Weighted mode | 7 | 0.0750896066893239 | 0.371972312041389 | 0.846689671976628 | -0.653976125 | 0.804155338290446 | 1.07798074084236 | 0.519974178738445 | 2.2348080445963 | 0.999714303421225 |
| ebi-fl187-GCST90094750 | prot-a-1081 | Fc receptor-like protein 3 \|\| id:prot-a-1081 | Curry liking \|\| id:ebi-fl187-GCST90094750 | Weighted mode | 7 | -0.106008132 | 0.354243985461645 | 0.77484296882696 | -0.800326344 | 0.588310079167447 | 0.899417333680983 | 0.449182352300893 | 1.80094239228686 | 0.999714303421225 |
| ebi-fl187-GCST90094750 | prot-a-1105 | Fibroblast growth factor receptor 4 \|\| id:prot-a-1105 | Curry liking \|\| id:ebi-fl187-GCST90094750 | Weighted mode | 7 | 0.244823328395869 | 0.348995630640518 | 0.509266710641601 | -0.439208108 | 0.928854764451284 | 1.27739561373594 | 0.644546630580804 | 2.53160822906087 | 0.999714303421225 |
| ebi-fl187-GCST90094750 | prot-a-1143 | Follistatin-related protein 1 \|\| id:prot-a-1143 | Curry liking \|\| id:ebi-fl187-GCST90094750 | Weighted mode | 7 | -0.341340911 | 0.328855834736752 | 0.339294797128224 | -0.985898347 | 0.303216525161099 | 0.710816541772781 | 0.373103899567578 | 1.35420765272999 | 0.999714303421225 |
| ebi-fl187-GCST90094750 | prot-a-1151 | Alpha-(1,3)-fucosyltransferase 10 \|\| id:prot-a-1151 | Curry liking \|\| id:ebi-fl187-GCST90094750 | Weighted mode | 7 | 0.0560185517430014 | 0.420513785918685 | 0.898379322919053 | -0.768188469 | 0.880225572143625 | 1.05761730419904 | 0.463852591177813 | 2.41144359957332 | 0.999714303421225 |
| ebi-fl187-GCST90094750 | prot-a-1165 | Growth arrest and DNA damage-inducible proteins-interacting protein 1 \|\| id:prot-a-1165 | Curry liking \|\| id:ebi-fl187-GCST90094750 | Weighted mode | 7 | 0.655951528420287 | 0.524488904774406 | 0.257622879773249 | -0.372046725 | 1.68394978177812 | 1.92697521698935 | 0.689322033242785 | 5.38679065490329 | 0.999714303421225 |
| ebi-fl187-GCST90094750 | prot-a-1194 | Growth/differentiation factor 11/8 \|\| id:prot-a-1194 | Curry liking \|\| id:ebi-fl187-GCST90094750 | Weighted mode | 7 | -0.288542846 | 0.327612886123129 | 0.412342422246468 | -0.930664103 | 0.353578410547886 | 0.749354697418373 | 0.394291773033589 | 1.42415465131996 | 0.999714303421225 |
| ebi-fl187-GCST90094750 | prot-a-1216 | D-glucuronyl C5-epimerase \|\| id:prot-a-1216 | Curry liking \|\| id:ebi-fl187-GCST90094750 | Weighted mode | 7 | -0.541844514 | 0.39090033537696 | 0.215018551755729 | -1.308009172 | 0.224320142972866 | 0.581674355564256 | 0.270357756844597 | 1.25147160514272 | 0.999714303421225 |
| ebi-fl187-GCST90094750 | prot-a-1246 | Glypican-3 \|\| id:prot-a-1246 | Curry liking \|\| id:ebi-fl187-GCST90094750 | Weighted mode | 7 | 0.229730475953518 | 0.393331233366535 | 0.580456574827087 | -0.541198741 | 1.00065969335193 | 1.25826083267217 | 0.582050106423776 | 2.7200756525318 | 0.999714303421225 |
| ebi-fl187-GCST90094750 | prot-a-1275 | Glutamate receptor 4 \|\| id:prot-a-1275 | Curry liking \|\| id:ebi-fl187-GCST90094750 | Weighted mode | 6 | 0.241926241283703 | 0.464723532874131 | 0.624881123496069 | -0.668931883 | 1.152784366 | 1.27370024285656 | 0.512255434342333 | 3.16699872737455 | 0.999714303421225 |
| ebi-fl187-GCST90094750 | prot-a-1289 | Glutathione S-transferase P \|\| id:prot-a-1289 | Curry liking \|\| id:ebi-fl187-GCST90094750 | Weighted mode | 7 | 0.140030341676623 | 0.323422994405811 | 0.680172140052891 | -0.493878727 | 0.773939410712012 | 1.15030870062235 | 0.610254785813038 | 2.16829124078817 | 0.999714303421225 |
| ebi-fl187-GCST90094750 | prot-a-130 | Apolipoprotein D \|\| id:prot-a-130 | Curry liking \|\| id:ebi-fl187-GCST90094750 | Weighted mode | 7 | 0.28380185267495 | 0.362236471 | 0.463145907589183 | -0.426181631 | 0.993785336599351 | 1.32816973139804 | 0.652997726555892 | 2.70144100609049 | 0.999714303421225 |
| ebi-fl187-GCST90094750 | prot-a-1313 | Heparin-binding EGF-like growth factor \|\| id:prot-a-1313 | Curry liking \|\| id:ebi-fl187-GCST90094750 | Weighted mode | 7 | 0.279907604864096 | 0.430175637226735 | 0.539354579717948 | -0.563236644 | 1.1230518538285 | 1.32300756722612 | 0.569363252131444 | 3.07422197759523 | 0.999714303421225 |
| ebi-fl187-GCST90094750 | prot-a-1347 | HLA class II histocompatibility antigen, DQ alpha 2 chain \|\| id:prot-a-1347 | Curry liking \|\| id:ebi-fl187-GCST90094750 | Weighted mode | 6 | -0.225544041 | 0.329923415773337 | 0.524598949894489 | -0.872193936 | 0.421105853487264 | 0.79808191100648 | 0.418033403761935 | 1.52364555306801 | 0.999714303421225 |
| ebi-fl187-GCST90094750 | prot-a-1357 | Non-histone chromosomal protein HMG-14 \|\| id:prot-a-1357 | Curry liking \|\| id:ebi-fl187-GCST90094750 | Weighted mode | 7 | 0.0948419913998864 | 0.429674293957443 | 0.83262213736746 | -0.747319625 | 0.937003607556474 | 1.09948511329652 | 0.473634370709648 | 2.55232219010922 | 0.999714303421225 |
| ebi-fl187-GCST90094750 | prot-a-137 | Apolipoprotein M \|\| id:prot-a-137 | Curry liking \|\| id:ebi-fl187-GCST90094750 | Weighted mode | 7 | -0.058822572 | 0.401686348259639 | 0.888371103233958 | -0.846127814 | 0.728482670928327 | 0.942874046893192 | 0.429073170389475 | 2.07193441505042 | 0.999714303421225 |
| ebi-fl187-GCST90094750 | prot-a-1381 | 17-beta-hydroxysteroid dehydrogenase 14 \|\| id:prot-a-1381 | Curry liking \|\| id:ebi-fl187-GCST90094750 | Weighted mode | 7 | -0.199769123 | 0.301551433780301 | 0.532276193197459 | -0.790809934 | 0.391271686764492 | 0.818919800636292 | 0.453477359928048 | 1.47886024559328 | 0.999714303421225 |
| ebi-fl187-GCST90094750 | prot-a-1392 | Serine protease HTRA2, mitochondrial \|\| id:prot-a-1392 | Curry liking \|\| id:ebi-fl187-GCST90094750 | Weighted mode | 7 | 0.220907772095587 | 0.344016905695004 | 0.544519293381859 | -0.453365363 | 0.895180907257795 | 1.24720839784263 | 0.6354859081255 | 2.44777857031881 | 0.999714303421225 |
| ebi-fl187-GCST90094750 | prot-a-1416 | Interferon-induced protein with tetratricopeptide repeats 2 \|\| id:prot-a-1416 | Curry liking \|\| id:ebi-fl187-GCST90094750 | Weighted mode | 7 | -0.422649369 | 0.420028475875256 | 0.35314164078867 | -1.245905182 | 0.40060644383756 | 0.655308364341076 | 0.287680387247873 | 1.4927296799186 | 0.999714303421225 |
| ebi-fl187-GCST90094750 | prot-a-1428 | Interferon gamma \|\| id:prot-a-1428 | Curry liking \|\| id:ebi-fl187-GCST90094750 | Weighted mode | 7 | 0.416058656927612 | 0.352560282445507 | 0.282620403427308 | -0.274959497 | 1.1070768105208 | 1.5159747886861 | 0.759602889051746 | 3.02550134162973 | 0.999714303421225 |
| ebi-fl187-GCST90094750 | prot-a-1483 | Interleukin-17C \|\| id:prot-a-1483 | Curry liking \|\| id:ebi-fl187-GCST90094750 | Weighted mode | 7 | 0.507029473089612 | 0.605439996372765 | 0.434419547467895 | -0.67963292 | 1.69369186598023 | 1.6603517426578 | 0.506802995568918 | 5.43952568049077 | 0.999714303421225 |
| ebi-fl187-GCST90094750 | prot-a-1503 | Interleukin-1 receptor-like 2 \|\| id:prot-a-1503 | Curry liking \|\| id:ebi-fl187-GCST90094750 | Weighted mode | 7 | -0.107010667 | 0.383021286840231 | 0.789329717919952 | -0.857732389 | 0.643711055367533 | 0.898516088612445 | 0.424122738075972 | 1.90353190012367 | 0.999714303421225 |
| ebi-fl187-GCST90094750 | prot-a-1522 | Interleukin-31 receptor subunit alpha \|\| id:prot-a-1522 | Curry liking \|\| id:ebi-fl187-GCST90094750 | Weighted mode | 7 | 0.145416296809946 | 0.326166167881016 | 0.67135329410412 | -0.493869392 | 0.784701985856738 | 1.15652092603978 | 0.610260482642455 | 2.19175366980391 | 0.999714303421225 |
| ebi-fl187-GCST90094750 | prot-a-1540 | Interleukin-6 receptor subunit alpha \|\| id:prot-a-1540 | Curry liking \|\| id:ebi-fl187-GCST90094750 | Weighted mode | 7 | 0.705365917329791 | 0.439410494774311 | 0.159560222952892 | -0.155878652 | 1.56661048708744 | 2.0245873810057 | 0.855663016701003 | 4.79038357779093 | 0.999714303421225 |
| ebi-fl187-GCST90094750 | prot-a-1541 | Interleukin-6 receptor subunit alpha \|\| id:prot-a-1541 | Curry liking \|\| id:ebi-fl187-GCST90094750 | Weighted mode | 7 | 0.507528204202078 | 0.421011214728019 | 0.273391053191088 | -0.317653777 | 1.33271018506899 | 1.66118001825573 | 0.727854745092797 | 3.79130461353288 | 0.999714303421225 |
| ebi-fl187-GCST90094750 | prot-a-1545 | Interleukin-9 \|\| id:prot-a-1545 | Curry liking \|\| id:ebi-fl187-GCST90094750 | Weighted mode | 7 | -0.109509857 | 0.365599533744808 | 0.774637115957257 | -0.826084943 | 0.607065229628339 | 0.8962733301902 | 0.437759790460403 | 1.83503807319849 | 0.999714303421225 |
| ebi-fl187-GCST90094750 | prot-a-1548 | Inositol monophosphatase 3 \|\| id:prot-a-1548 | Curry liking \|\| id:ebi-fl187-GCST90094750 | Weighted mode | 7 | -0.029156605 | 0.363858927028961 | 0.938738479521706 | -0.742320102 | 0.684006892204432 | 0.971264347923748 | 0.476008245820194 | 1.98180271419937 | 0.999714303421225 |
| ebi-fl187-GCST90094750 | prot-a-1555 | Inhibin beta A chain:Inhibin beta B chain heterodimer \|\| id:prot-a-1555 | Curry liking \|\| id:ebi-fl187-GCST90094750 | Weighted mode | 7 | -0.425136883 | 0.352625175494032 | 0.273342369190829 | -1.116282227 | 0.266008461398137 | 0.653680301565872 | 0.327495086674996 | 1.30474609861643 | 0.999714303421225 |
| ebi-fl187-GCST90094750 | prot-a-1603 | Junctophilin-3 \|\| id:prot-a-1603 | Curry liking \|\| id:ebi-fl187-GCST90094750 | Weighted mode | 7 | 0.146686123105418 | 0.37053357775113 | 0.705888461506457 | -0.579559689 | 0.872931935497631 | 1.15799043953891 | 0.560144950097208 | 2.39391939145539 | 0.999714303421225 |
| ebi-fl187-GCST90094750 | prot-a-1627 | UPF0258 protein KIAA1024 \|\| id:prot-a-1627 | Curry liking \|\| id:ebi-fl187-GCST90094750 | Weighted mode | 7 | -0.185726163 | 0.322433918670853 | 0.585547770875768 | -0.817696644 | 0.44624431736005 | 0.830500985549924 | 0.441447294716541 | 1.56243314944829 | 0.999714303421225 |
| ebi-fl187-GCST90094750 | prot-a-1666 | Kallikrein-7 \|\| id:prot-a-1666 | Curry liking \|\| id:ebi-fl187-GCST90094750 | Weighted mode | 7 | 0.235304701291209 | 0.428744817729481 | 0.602935651503818 | -0.605035141 | 1.07564454404099 | 1.26529424679284 | 0.546055237125043 | 2.93188201874243 | 0.999714303421225 |
| ebi-fl187-GCST90094750 | prot-a-1678 | Importin subunit alpha-3 \|\| id:prot-a-1678 | Curry liking \|\| id:ebi-fl187-GCST90094750 | Weighted mode | 7 | -0.216399095 | 0.364017467272203 | 0.573911832467624 | -0.929873331 | 0.497075140956587 | 0.805413801185134 | 0.394603691359462 | 1.64390603875159 | 0.999714303421225 |
| ebi-fl187-GCST90094750 | prot-a-1718 | Protein LDOC1 \|\| id:prot-a-1718 | Curry liking \|\| id:ebi-fl187-GCST90094750 | Weighted mode | 7 | -0.097193729 | 0.358107541012233 | 0.795172710925658 | -0.799084509 | 0.604697051460523 | 0.907380203314825 | 0.449740508955763 | 1.83069751772936 | 0.999714303421225 |
| ebi-fl187-GCST90094750 | prot-a-1723 | Leptin \|\| id:prot-a-1723 | Curry liking \|\| id:ebi-fl187-GCST90094750 | Weighted mode | 7 | 0.285627568821822 | 0.353605746591612 | 0.450059436475756 | -0.407439694 | 0.978694832141382 | 1.33059680722404 | 0.665351574549077 | 2.66098094769626 | 0.999714303421225 |
| ebi-fl187-GCST90094750 | prot-a-1730 | Galectin-7 \|\| id:prot-a-1730 | Curry liking \|\| id:ebi-fl187-GCST90094750 | Weighted mode | 7 | 0.254466310437764 | 0.394856768746458 | 0.543119207930285 | -0.519452956 | 1.02838557718082 | 1.28977309872025 | 0.594845865660718 | 2.79654737842837 | 0.999714303421225 |
| ebi-fl187-GCST90094750 | prot-a-1753 | Lipase member N \|\| id:prot-a-1753 | Curry liking \|\| id:ebi-fl187-GCST90094750 | Weighted mode | 7 | 0.154694220549656 | 0.360801732049293 | 0.683070232602181 | -0.552477174 | 0.86186561536627 | 1.16730096987866 | 0.575522373891298 | 2.36757355768244 | 0.999714303421225 |
| ebi-fl187-GCST90094750 | prot-a-1756 | VIP36-like protein \|\| id:prot-a-1756 | Curry liking \|\| id:ebi-fl187-GCST90094750 | Weighted mode | 7 | 0.254109406011036 | 0.370071809130738 | 0.517946963191836 | -0.47123134 | 0.979450151907282 | 1.28931285512821 | 0.624233151680569 | 2.66299159844927 | 0.999714303421225 |
| ebi-fl187-GCST90094750 | prot-a-1760 | Ig Kappa chain V-I region HK102- like \|\| id:prot-a-1760 | Curry liking \|\| id:ebi-fl187-GCST90094750 | Weighted mode | 7 | 0.269113489350378 | 0.420362192371554 | 0.545701662012181 | -0.554796408 | 1.09302338639862 | 1.30880366789624 | 0.574189149788089 | 2.98328005976924 | 0.999714303421225 |
| ebi-fl187-GCST90094750 | prot-a-179 | Neutral ceramidase \|\| id:prot-a-179 | Curry liking \|\| id:ebi-fl187-GCST90094750 | Weighted mode | 7 | 0.668705429971894 | 0.513772183005896 | 0.240804570164294 | -0.338288049 | 1.67569890866345 | 1.95170906043088 | 0.712989882493537 | 5.34252778348857 | 0.999714303421225 |
| ebi-fl187-GCST90094750 | prot-a-1809 | Lactotransferrin \|\| id:prot-a-1809 | Curry liking \|\| id:ebi-fl187-GCST90094750 | Weighted mode | 7 | 0.132638006734451 | 0.372627923470763 | 0.73405679912673 | -0.597712723 | 0.862988736737146 | 1.14183658635462 | 0.550068356865579 | 2.37023412393193 | 0.999714303421225 |
| ebi-fl187-GCST90094750 | prot-a-1818 | Lysophospholipase-like protein 1 \|\| id:prot-a-1818 | Curry liking \|\| id:ebi-fl187-GCST90094750 | Weighted mode | 7 | -0.293342455 | 0.31420295904894 | 0.386536442319848 | -0.909180255 | 0.322495344253004 | 0.745766705064923 | 0.402854326446957 | 1.38056846326713 | 0.999714303421225 |
| ebi-fl187-GCST90094750 | prot-a-1852 | MAP kinase-activated protein kinase 3 \|\| id:prot-a-1852 | Curry liking \|\| id:ebi-fl187-GCST90094750 | Weighted mode | 7 | 0.0407149228789215 | 0.389398120563955 | 0.920133713384975 | -0.722505393 | 0.803935239184273 | 1.04155513967774 | 0.485534276453618 | 2.23431621947034 | 0.999714303421225 |
| ebi-fl187-GCST90094750 | prot-a-1856 | Megakaryocyte-associated tyrosine-protein kinase \|\| id:prot-a-1856 | Curry liking \|\| id:ebi-fl187-GCST90094750 | Weighted mode | 7 | 0.00578498373645553 | 0.397490902665697 | 0.98886006364646 | -0.773297185 | 0.784867152961221 | 1.00580174906834 | 0.461488942385223 | 2.19211570530865 | 0.999714303421225 |
| ebi-fl187-GCST90094750 | prot-a-1890 | Beta-1,3-N-acetylglucosaminyltransferase manic fringe \|\| id:prot-a-1890 | Curry liking \|\| id:ebi-fl187-GCST90094750 | Weighted mode | 7 | -0.21857336 | 0.337342475060396 | 0.541017301097457 | -0.879764611 | 0.442617891568444 | 0.803664520814549 | 0.414880558646035 | 1.55677736292079 | 0.999714303421225 |
| ebi-fl187-GCST90094750 | prot-a-1919 | Matrilysin \|\| id:prot-a-1919 | Curry liking \|\| id:ebi-fl187-GCST90094750 | Weighted mode | 7 | -0.112505852 | 0.36142598313598 | 0.76611695572391 | -0.820900779 | 0.595889075113676 | 0.89359211794151 | 0.440035101647873 | 1.81464358242534 | 0.999714303421225 |
| ebi-fl187-GCST90094750 | prot-a-1923 | Multimerin-2 \|\| id:prot-a-1923 | Curry liking \|\| id:ebi-fl187-GCST90094750 | Weighted mode | 7 | 0.107495762349766 | 0.34820052114821 | 0.767974349031574 | -0.574977259 | 0.789968783800257 | 1.11348614217173 | 0.562717665367201 | 2.20332764566652 | 0.999714303421225 |
| ebi-fl187-GCST90094750 | prot-a-1948 | Mesothelin \|\| id:prot-a-1948 | Curry liking \|\| id:ebi-fl187-GCST90094750 | Weighted mode | 7 | -0.218216796 | 0.343125245501458 | 0.548272383456756 | -0.890742277 | 0.4543086848926 | 0.80395112914974 | 0.410351045340586 | 1.57508412711534 | 0.999714303421225 |
| ebi-fl187-GCST90094750 | prot-a-195 | ATPase family AAA domain-containing protein 1 \|\| id:prot-a-195 | Curry liking \|\| id:ebi-fl187-GCST90094750 | Weighted mode | 7 | 0.010807836454227 | 0.367564908101124 | 0.977496121934645 | -0.709619383 | 0.73123505633243 | 1.01086645209774 | 0.49183136100854 | 2.07764503239746 | 0.999714303421225 |
| ebi-fl187-GCST90094750 | prot-a-1966 | Mucin-1 \|\| id:prot-a-1966 | Curry liking \|\| id:ebi-fl187-GCST90094750 | Weighted mode | 7 | -0.532771578 | 0.41355687960788 | 0.245099586703088 | -1.343343062 | 0.27779990641798 | 0.58697586396054 | 0.26097176394125 | 1.32022200282857 | 0.999714303421225 |
| ebi-fl187-GCST90094750 | prot-a-1986 | Myocardial zonula adherens protein \|\| id:prot-a-1986 | Curry liking \|\| id:ebi-fl187-GCST90094750 | Weighted mode | 7 | 0.107395514311599 | 0.424162523281698 | 0.808570075768268 | -0.723963031 | 0.938754059943728 | 1.11337452296535 | 0.484827058852182 | 2.55679382112674 | 0.999714303421225 |
| ebi-fl187-GCST90094750 | prot-a-2071 | Epididymal secretory protein E1 \|\| id:prot-a-2071 | Curry liking \|\| id:ebi-fl187-GCST90094750 | Weighted mode | 7 | 0.0782499697970982 | 0.522928453333553 | 0.885953472388707 | -0.946689799 | 1.10318973833086 | 1.08139294046144 | 0.388023335263104 | 3.01376382656706 | 0.999714303421225 |
| ebi-fl187-GCST90094750 | prot-a-2098 | Neuritin \|\| id:prot-a-2098 | Curry liking \|\| id:ebi-fl187-GCST90094750 | Weighted mode | 7 | -0.469546917 | 0.43413487279165 | 0.320986297520868 | -1.320451268 | 0.38135743331397 | 0.625285510123015 | 0.26701477954058 | 1.46427089108144 | 0.999714303421225 |
| ebi-fl187-GCST90094750 | prot-a-2115 | Neurotrophin-4 \|\| id:prot-a-2115 | Curry liking \|\| id:ebi-fl187-GCST90094750 | Weighted mode | 7 | -0.073963683 | 0.360388935321751 | 0.844175824698719 | -0.780325996 | 0.632398630448619 | 0.928705421226071 | 0.458256597128903 | 1.88211967884029 | 0.999714303421225 |
| ebi-fl187-GCST90094750 | prot-a-2118 | Netrin-1 \|\| id:prot-a-2118 | Curry liking \|\| id:ebi-fl187-GCST90094750 | Weighted mode | 7 | 0.348813434029921 | 0.344309411490429 | 0.350117826861599 | -0.326033012 | 1.02365988055116 | 1.41738472991598 | 0.721781359237547 | 2.78336292131621 | 0.999714303421225 |
| ebi-fl187-GCST90094750 | prot-a-2120 | Netrin-G1 \|\| id:prot-a-2120 | Curry liking \|\| id:ebi-fl187-GCST90094750 | Weighted mode | 7 | 0.404211871108963 | 0.412890065222913 | 0.365396536247216 | -0.405052657 | 1.21347639894587 | 1.4981213219615 | 0.666941690966656 | 3.36516299057976 | 0.999714303421225 |
| ebi-fl187-GCST90094750 | prot-a-2145 | Olfactomedin-like protein 3 \|\| id:prot-a-2145 | Curry liking \|\| id:ebi-fl187-GCST90094750 | Weighted mode | 7 | -0.376355297 | 0.367709915032309 | 0.345547963040355 | -1.097066731 | 0.344356136116943 | 0.686358428387256 | 0.333848917616171 | 1.41108108297011 | 0.999714303421225 |
| ebi-fl187-GCST90094750 | prot-a-2162 | Prolyl 4-hydroxylase subunit alpha-2 \|\| id:prot-a-2162 | Curry liking \|\| id:ebi-fl187-GCST90094750 | Weighted mode | 7 | 0.415753497928486 | 0.464580809632413 | 0.405307176045175 | -0.494824889 | 1.32633188480801 | 1.51551224591502 | 0.60967765924375 | 3.76719949090367 | 0.999714303421225 |
| ebi-fl187-GCST90094750 | prot-a-2168 | Polyadenylate-binding protein-interacting protein 1 \|\| id:prot-a-2168 | Curry liking \|\| id:ebi-fl187-GCST90094750 | Weighted mode | 7 | -0.278447429 | 0.314003393115372 | 0.409338483143632 | -0.893894079 | 0.336999221802851 | 0.756958060970735 | 0.409059736247487 | 1.40073797368781 | 0.999714303421225 |
| ebi-fl187-GCST90094750 | prot-a-219 | Beta-1,4-galactosyltransferase 5 \|\| id:prot-a-219 | Curry liking \|\| id:ebi-fl187-GCST90094750 | Weighted mode | 7 | 0.0272585320865577 | 0.384962848937798 | 0.945851532904333 | -0.727268652 | 0.781785716004643 | 1.0276334446422 | 0.483227050549243 | 2.18537123562699 | 0.999714303421225 |
| ebi-fl187-GCST90094750 | prot-a-2190 | Pyruvate carboxylase, mitochondrial \|\| id:prot-a-2190 | Curry liking \|\| id:ebi-fl187-GCST90094750 | Weighted mode | 7 | -0.228254916 | 0.3405943725126 | 0.527688021304236 | -0.895819886 | 0.439310054369151 | 0.795921341082942 | 0.408272724338278 | 1.55163630442866 | 0.999714303421225 |
| ebi-fl187-GCST90094750 | prot-a-2197 | Protocadherin-9 \|\| id:prot-a-2197 | Curry liking \|\| id:ebi-fl187-GCST90094750 | Weighted mode | 7 | -0.107523594 | 0.319776869154568 | 0.748133406584626 | -0.734286258 | 0.519239069176319 | 0.898055333154042 | 0.47984782436931 | 1.68074822985068 | 0.999714303421225 |
| ebi-fl187-GCST90094750 | prot-a-2198 | Protocadherin alpha-4 \|\| id:prot-a-2198 | Curry liking \|\| id:ebi-fl187-GCST90094750 | Weighted mode | 7 | 0.0258599584859655 | 0.318823076389314 | 0.937991871720681 | -0.599033271 | 0.650753188209021 | 1.02619722819674 | 0.549342444620654 | 1.9169841352526 | 0.999714303421225 |
| ebi-fl187-GCST90094750 | prot-a-2254 | Placenta growth factor \|\| id:prot-a-2254 | Curry liking \|\| id:ebi-fl187-GCST90094750 | Weighted mode | 7 | -0.377884426 | 0.307104243406709 | 0.264567244039877 | -0.979808743 | 0.224039891338392 | 0.685309700251394 | 0.375382886660073 | 1.25112092732119 | 0.999714303421225 |
| ebi-fl187-GCST90094750 | prot-a-2261 | Membrane-associated progesterone receptor component 2 \|\| id:prot-a-2261 | Curry liking \|\| id:ebi-fl187-GCST90094750 | Weighted mode | 7 | 0.299461491311499 | 0.437832638253282 | 0.519526043339423 | -0.55869048 | 1.15761346228793 | 1.34913209256861 | 0.571957563714433 | 3.18232945706322 | 0.999714303421225 |
| ebi-fl187-GCST90094750 | prot-a-2268 | Polymeric immunoglobulin receptor \|\| id:prot-a-2268 | Curry liking \|\| id:ebi-fl187-GCST90094750 | Weighted mode | 7 | 0.296731867556035 | 0.530242218609251 | 0.595998277759871 | -0.742542881 | 1.33600661603017 | 1.34545449107711 | 0.475902212910151 | 3.80382300912168 | 0.999714303421225 |
| ebi-fl187-GCST90094750 | prot-a-2283 | Group 10 secretory phospholipase A2 \|\| id:prot-a-2283 | Curry liking \|\| id:ebi-fl187-GCST90094750 | Weighted mode | 7 | 0.0333001775916071 | 0.337215163052567 | 0.924552885321329 | -0.627641542 | 0.694241897174639 | 1.03386083452249 | 0.533849378794565 | 2.00219063207128 | 0.999714303421225 |
| ebi-fl187-GCST90094750 | prot-a-2298 | Inactive phospholipase D5 \|\| id:prot-a-2298 | Curry liking \|\| id:ebi-fl187-GCST90094750 | Weighted mode | 7 | 0.0654944394352603 | 0.388120289114752 | 0.871540043150499 | -0.695221327 | 0.826210206100175 | 1.06768680035542 | 0.498964001443037 | 2.28464398304559 | 0.999714303421225 |
| ebi-fl187-GCST90094750 | prot-a-2304 | Procollagen-lysine,2-oxoglutarate 5-dioxygenase 2 \|\| id:prot-a-2304 | Curry liking \|\| id:ebi-fl187-GCST90094750 | Weighted mode | 7 | 0.15148025719187 | 0.36621011012018 | 0.693513716814437 | -0.566291559 | 0.869252073027423 | 1.16355532973329 | 0.567626550140773 | 2.38512628596209 | 0.999714303421225 |
| ebi-fl187-GCST90094750 | prot-a-2331 | Periostin \|\| id:prot-a-2331 | Curry liking \|\| id:ebi-fl187-GCST90094750 | Weighted mode | 7 | -0.146192751 | 0.3504904211046 | 0.691111167428644 | -0.833153976 | 0.540768474633617 | 0.863991152207236 | 0.434676163831642 | 1.7173260767561 | 0.999714303421225 |
| ebi-fl187-GCST90094750 | prot-a-2333 | Periostin \|\| id:prot-a-2333 | Curry liking \|\| id:ebi-fl187-GCST90094750 | Weighted mode | 7 | -0.430990927 | 0.318707141697982 | 0.225018621373903 | -1.055656924 | 0.193675070991964 | 0.649864807142907 | 0.347963766297205 | 1.21370185193986 | 0.999714303421225 |
| ebi-fl187-GCST90094750 | prot-a-2367 | cAMP-dependent protein kinase catalytic subunit alpha \|\| id:prot-a-2367 | Curry liking \|\| id:ebi-fl187-GCST90094750 | Weighted mode | 7 | 0.102680351184947 | 0.333285912442439 | 0.768433009638342 | -0.550560037 | 0.755920739572128 | 1.10813713773482 | 0.576626787483714 | 2.12957140160924 | 0.999714303421225 |
| ebi-fl187-GCST90094750 | prot-a-2385 | Phosphoribosyl pyrophosphate synthase-associated protein 1 \|\| id:prot-a-2385 | Curry liking \|\| id:ebi-fl187-GCST90094750 | Weighted mode | 7 | 0.642148009467128 | 0.348807694900989 | 0.115221554161159 | -0.041515073 | 1.32581109147307 | 1.90055891645114 | 0.959334875620356 | 3.76523806930936 | 0.999714303421225 |
| ebi-fl187-GCST90094750 | prot-a-2393 | Trypsin-3 \|\| id:prot-a-2393 | Curry liking \|\| id:ebi-fl187-GCST90094750 | Weighted mode | 6 | 0.254607190683568 | 0.481686098488936 | 0.619713146954741 | -0.689497562 | 1.19871194372188 | 1.28995481507125 | 0.501828143085548 | 3.31584317829269 | 0.999714303421225 |
| ebi-fl187-GCST90094750 | prot-a-2405 | Pregnancy-specific beta-1-glycoprotein 6 \|\| id:prot-a-2405 | Curry liking \|\| id:ebi-fl187-GCST90094750 | Weighted mode | 7 | 0.239618935563931 | 0.312054853642873 | 0.471690715439308 | -0.372008578 | 0.851246448703963 | 1.27076481476822 | 0.689348329561107 | 2.34256491994555 | 0.999714303421225 |
| ebi-fl187-GCST90094750 | prot-a-244 | Beclin-1 \|\| id:prot-a-244 | Curry liking \|\| id:ebi-fl187-GCST90094750 | Weighted mode | 7 | -0.119019053 | 0.409970114395351 | 0.781350494365206 | -0.922560477 | 0.68452237114006 | 0.887790885454635 | 0.397499947370223 | 1.98282455509922 | 0.999714303421225 |
| ebi-fl187-GCST90094750 | prot-a-2454 | tRNA pseudouridine synthase A, mitochondrial \|\| id:prot-a-2454 | Curry liking \|\| id:ebi-fl187-GCST90094750 | Weighted mode | 7 | 0.172112973436851 | 0.349290243809473 | 0.639700094919308 | -0.512495904 | 0.856721851303417 | 1.18781201684005 | 0.598998668087227 | 2.35542658526272 | 0.999714303421225 |
| ebi-fl187-GCST90094750 | prot-a-2466 | Glutaminyl-peptide cyclotransferase-like protein \|\| id:prot-a-2466 | Curry liking \|\| id:ebi-fl187-GCST90094750 | Weighted mode | 7 | -0.019254872 | 0.396583428444586 | 0.962852232328972 | -0.796558392 | 0.758048647548838 | 0.980929318763574 | 0.450878042426617 | 2.13410775834482 | 0.999714303421225 |
| ebi-fl187-GCST90094750 | prot-a-2474 | Ras-related protein Rab-27A \|\| id:prot-a-2474 | Curry liking \|\| id:ebi-fl187-GCST90094750 | Weighted mode | 7 | 0.0609205786552924 | 0.350729338281025 | 0.867815795714005 | -0.626508924 | 0.748350081686101 | 1.0628145006783 | 0.534454368551583 | 2.1135100194116 | 0.999714303421225 |
| ebi-fl187-GCST90094750 | prot-a-2490 | Rap1 GTPase-GDP dissociation stimulator 1 \|\| id:prot-a-2490 | Curry liking \|\| id:ebi-fl187-GCST90094750 | Weighted mode | 7 | -0.387213568 | 0.334265043224057 | 0.290719617286323 | -1.042373053 | 0.267945916222161 | 0.678946077948254 | 0.352616909621319 | 1.30727643565465 | 0.999714303421225 |
| ebi-fl187-GCST90094750 | prot-a-2545 | Receptor-interacting serine/threonine-protein kinase 2 \|\| id:prot-a-2545 | Curry liking \|\| id:ebi-fl187-GCST90094750 | Weighted mode | 7 | 0.0200022225074386 | 0.362970332655766 | 0.957842346881236 | -0.691419629 | 0.731424074512741 | 1.02020360743434 | 0.500864522069041 | 2.07803778219807 | 0.999714303421225 |
| ebi-fl187-GCST90094750 | prot-a-2610 | RUN and FYVE domain-containing protein 1 \|\| id:prot-a-2610 | Curry liking \|\| id:ebi-fl187-GCST90094750 | Weighted mode | 7 | -0.140923163 | 0.345059040956132 | 0.697153262004944 | -0.817238884 | 0.535392556857307 | 0.868556046012786 | 0.441649417949965 | 1.70811864434707 | 0.999714303421225 |
| ebi-fl187-GCST90094750 | prot-a-2627 | Serine--tRNA ligase, mitochondrial \|\| id:prot-a-2627 | Curry liking \|\| id:ebi-fl187-GCST90094750 | Weighted mode | 7 | 0.139734583711131 | 0.349007140643468 | 0.702744763882475 | -0.544319412 | 0.823788579372328 | 1.14996853796675 | 0.580236551050294 | 2.27911812159995 | 0.999714303421225 |
| ebi-fl187-GCST90094750 | prot-a-264 | BolA-like protein 2 \|\| id:prot-a-264 | Curry liking \|\| id:ebi-fl187-GCST90094750 | Weighted mode | 7 | -0.087369543 | 0.314242578640066 | 0.790320186770044 | -0.703284997 | 0.528545910977003 | 0.916338406468786 | 0.494956698859127 | 1.69646370501744 | 0.999714303421225 |
| ebi-fl187-GCST90094750 | prot-a-2646 | Secretoglobin family 1C member 1 \|\| id:prot-a-2646 | Curry liking \|\| id:ebi-fl187-GCST90094750 | Weighted mode | 7 | -0.157293564 | 0.364179994278117 | 0.680895301872879 | -0.871086353 | 0.556499224500392 | 0.854453185057604 | 0.418496667072066 | 1.7445545040132 | 0.999714303421225 |
| ebi-fl187-GCST90094750 | prot-a-2672 | Semaphorin-3E \|\| id:prot-a-2672 | Curry liking \|\| id:ebi-fl187-GCST90094750 | Weighted mode | 7 | 0.133234613955425 | 0.510444420651725 | 0.802807422719812 | -0.867236451 | 1.13370567843281 | 1.14251801756038 | 0.420110943867208 | 3.10714928879051 | 0.999714303421225 |
| ebi-fl187-GCST90094750 | prot-a-2701 | Plasma protease C1 inhibitor \|\| id:prot-a-2701 | Curry liking \|\| id:ebi-fl187-GCST90094750 | Weighted mode | 7 | -0.508055133 | 0.423239755989912 | 0.275224572911057 | -1.337605055 | 0.321494788759967 | 0.601664599285193 | 0.262473526170436 | 1.37918781872862 | 0.999714303421225 |
| ebi-fl187-GCST90094750 | prot-a-2738 | Signaling threshold-regulating transmembrane adapter 1 \|\| id:prot-a-2738 | Curry liking \|\| id:ebi-fl187-GCST90094750 | Weighted mode | 7 | 0.18002677258892 | 0.360677522327863 | 0.635454116079864 | -0.526901171 | 0.886954716351532 | 1.19724941615919 | 0.590431784757447 | 2.42772527072264 | 0.999714303421225 |
| ebi-fl187-GCST90094750 | prot-a-274 | Serine/threonine-protein kinase BRSK2 \|\| id:prot-a-274 | Curry liking \|\| id:ebi-fl187-GCST90094750 | Weighted mode | 7 | 0.177587271371466 | 0.376336635816637 | 0.653681441475758 | -0.560032535 | 0.915207077572074 | 1.19433228437084 | 0.571190479961831 | 2.49729233159802 | 0.999714303421225 |
| ebi-fl187-GCST90094750 | prot-a-2749 | Mitochondrial glutamate carrier 2 \|\| id:prot-a-2749 | Curry liking \|\| id:ebi-fl187-GCST90094750 | Weighted mode | 7 | 0.0612943445514953 | 0.350684874457131 | 0.866997672293736 | -0.626048009 | 0.748636698487473 | 1.06321181873999 | 0.534700763361241 | 2.1141158737129 | 0.999714303421225 |
| ebi-fl187-GCST90094750 | prot-a-2760 | Sodium-coupled monocarboxylate transporter 1 \|\| id:prot-a-2760 | Curry liking \|\| id:ebi-fl187-GCST90094750 | Weighted mode | 7 | -0.334995718 | 0.365447488859311 | 0.394665354328323 | -1.051272796 | 0.38128136037312 | 0.715341149586218 | 0.349492633090479 | 1.46415950392538 | 0.999714303421225 |
| ebi-fl187-GCST90094750 | prot-a-2777 | Small integral membrane protein 10 \|\| id:prot-a-2777 | Curry liking \|\| id:ebi-fl187-GCST90094750 | Weighted mode | 7 | -0.173561806 | 0.414536936333337 | 0.690017782356876 | -0.986054201 | 0.638930589638152 | 0.840665191760489 | 0.373045754445102 | 1.8944538470594 | 0.999714303421225 |
| ebi-fl187-GCST90094750 | prot-a-2799 | Superoxide dismutase [Mn], mitochondrial \|\| id:prot-a-2799 | Curry liking \|\| id:ebi-fl187-GCST90094750 | Weighted mode | 7 | 0.543736357865871 | 0.426363152673792 | 0.249357856470165 | -0.291935421 | 1.3794081371065 | 1.72243047089975 | 0.746816762815976 | 3.97254972678617 | 0.999714303421225 |
| ebi-fl187-GCST90094750 | prot-a-2805 | Protein SPACA7 \|\| id:prot-a-2805 | Curry liking \|\| id:ebi-fl187-GCST90094750 | Weighted mode | 7 | 0.135608413870393 | 0.401056805279456 | 0.746784404106211 | -0.650462924 | 0.921679752218126 | 1.14523334828418 | 0.521804164920993 | 2.51350891808382 | 0.999714303421225 |
| ebi-fl187-GCST90094750 | prot-a-2811 | SPARC-like protein 1 \|\| id:prot-a-2811 | Curry liking \|\| id:ebi-fl187-GCST90094750 | Weighted mode | 7 | 0.272374820635107 | 0.443322709745947 | 0.561513746860896 | -0.59653769 | 1.14128733173716 | 1.3130790782191 | 0.550715085116893 | 3.13079614532577 | 0.999714303421225 |
| ebi-fl187-GCST90094750 | prot-a-2831 | Sepiapterin reductase \|\| id:prot-a-2831 | Curry liking \|\| id:ebi-fl187-GCST90094750 | Weighted mode | 7 | 0.0839504650712961 | 0.40192322287692 | 0.841458890160427 | -0.703819052 | 0.87171998191006 | 1.08757501952346 | 0.494692435590738 | 2.39101982968262 | 0.999714303421225 |
| ebi-fl187-GCST90094750 | prot-a-2834 | SPRY domain-containing SOCS box protein 1 \|\| id:prot-a-2834 | Curry liking \|\| id:ebi-fl187-GCST90094750 | Weighted mode | 7 | -0.104382743 | 0.369507311266669 | 0.787058486259657 | -0.828617073 | 0.619851586861913 | 0.900880425551137 | 0.436652727682022 | 1.85865217297396 | 0.999714303421225 |
| ebi-fl187-GCST90094750 | prot-a-2835 | Spectrin alpha chain, non-erythrocytic 1 \|\| id:prot-a-2835 | Curry liking \|\| id:ebi-fl187-GCST90094750 | Weighted mode | 7 | -0.050504619 | 0.315896151999173 | 0.878224216982459 | -0.669661077 | 0.56865183864512 | 0.950749536890271 | 0.511882036887751 | 1.76588474835519 | 0.999714303421225 |
| ebi-fl187-GCST90094750 | prot-a-2859 | Sia-alpha-2,3-Gal-beta-1,4-GlcNAc-R:alpha 2,8-sialyltransferase \|\| id:prot-a-2859 | Curry liking \|\| id:ebi-fl187-GCST90094750 | Weighted mode | 7 | -0.068868828 | 0.330773088077476 | 0.841956144023135 | -0.71718408 | 0.579446424968024 | 0.933449114749084 | 0.488124842868929 | 1.78504999807973 | 0.999714303421225 |
| ebi-fl187-GCST90094750 | prot-a-2861 | Alpha-2,8-sialyltransferase 8F \|\| id:prot-a-2861 | Curry liking \|\| id:ebi-fl187-GCST90094750 | Weighted mode | 7 | -0.363670608 | 0.374275031132776 | 0.368741964268679 | -1.097249669 | 0.369908453197255 | 0.695120124168046 | 0.333787849537696 | 1.44760208525454 | 0.999714303421225 |
| ebi-fl187-GCST90094750 | prot-a-289 | Uncharacterized protein C17orf89 \|\| id:prot-a-289 | Curry liking \|\| id:ebi-fl187-GCST90094750 | Weighted mode | 7 | -0.725739017 | 0.347610834585273 | 0.0818478757322068 | -1.407056253 | -0.044421781 | 0.483966776979114 | 0.244863038075854 | 0.956550417164201 | 0.999714303421225 |
| ebi-fl187-GCST90094750 | prot-a-2921 | Tachykinin-3 \|\| id:prot-a-2921 | Curry liking \|\| id:ebi-fl187-GCST90094750 | Weighted mode | 7 | -0.12797361 | 0.329975320841607 | 0.711529048835928 | -0.774725239 | 0.518778019086481 | 0.879876598990171 | 0.460830382000741 | 1.67997349933682 | 0.999714303421225 |
| ebi-fl187-GCST90094750 | prot-a-2928 | Tax1-binding protein 1 \|\| id:prot-a-2928 | Curry liking \|\| id:ebi-fl187-GCST90094750 | Weighted mode | 7 | 0.0989044661018135 | 0.353946567198277 | 0.789294868756373 | -0.594830806 | 0.792639737810436 | 1.10396082883952 | 0.551655895056634 | 2.20922049874393 | 0.999714303421225 |
| ebi-fl187-GCST90094750 | prot-a-296 | Uncharacterized protein C1orf185 \|\| id:prot-a-296 | Curry liking \|\| id:ebi-fl187-GCST90094750 | Weighted mode | 7 | -0.211442763 | 0.378124430105144 | 0.59627209432437 | -0.952566646 | 0.529681119815713 | 0.809415608093979 | 0.385749668850129 | 1.69839063913931 | 0.999714303421225 |
| ebi-fl187-GCST90094750 | prot-a-2964 | Transforming growth factor beta-2 \|\| id:prot-a-2964 | Curry liking \|\| id:ebi-fl187-GCST90094750 | Weighted mode | 7 | 0.198699831967409 | 0.357008943780102 | 0.597952343533129 | -0.501037698 | 0.898437361776409 | 1.21981576124398 | 0.605901590604468 | 2.45576264273345 | 0.999714303421225 |
| ebi-fl187-GCST90094750 | prot-a-2977 | Thymocyte nuclear protein 1 \|\| id:prot-a-2977 | Curry liking \|\| id:ebi-fl187-GCST90094750 | Weighted mode | 7 | -0.089363096 | 0.396140825569418 | 0.829012374333727 | -0.865799114 | 0.687072922118338 | 0.914513457106883 | 0.420715218791443 | 1.98788830513918 | 0.999714303421225 |
| ebi-fl187-GCST90094750 | prot-a-3015 | Transmembrane protein 70, mitochondrial \|\| id:prot-a-3015 | Curry liking \|\| id:ebi-fl187-GCST90094750 | Weighted mode | 7 | -0.497329156 | 0.391322921734409 | 0.250815492948216 | -1.264322082 | 0.26966377085639 | 0.608152773884684 | 0.282430696012366 | 1.30952407654528 | 0.999714303421225 |
| ebi-fl187-GCST90094750 | prot-a-3030 | Tumor necrosis factor alpha-induced protein 3 \|\| id:prot-a-3030 | Curry liking \|\| id:ebi-fl187-GCST90094750 | Weighted mode | 7 | -0.086890894 | 0.380045367881994 | 0.826747510608428 | -0.831779815 | 0.657998026809371 | 0.916777115841245 | 0.435273889371761 | 1.93092280665692 | 0.999714303421225 |
| ebi-fl187-GCST90094750 | prot-a-3032 | Tumor necrosis factor receptor superfamily member 10A \|\| id:prot-a-3032 | Curry liking \|\| id:ebi-fl187-GCST90094750 | Weighted mode | 7 | 0.0235956941095559 | 0.356655105886811 | 0.949401161109097 | -0.675448313 | 0.722639701647705 | 1.02387627498721 | 0.508928210105675 | 2.05986346534809 | 0.999714303421225 |
| ebi-fl187-GCST90094750 | prot-a-3081 | Tumor protein p53-inducible protein 11 \|\| id:prot-a-3081 | Curry liking \|\| id:ebi-fl187-GCST90094750 | Weighted mode | 7 | -0.125768461 | 0.3643917477597 | 0.741762246653711 | -0.839976286 | 0.588439364911643 | 0.881818998903938 | 0.43172076100155 | 1.80117524351615 | 0.999714303421225 |
| ebi-fl187-GCST90094750 | prot-a-315 | Complement C4 \|\| id:prot-a-315 | Curry liking \|\| id:ebi-fl187-GCST90094750 | Weighted mode | 6 | -0.327806747 | 0.370995844745909 | 0.417374356043772 | -1.054958603 | 0.399345108224187 | 0.720502245128162 | 0.348206841663867 | 1.49084803375531 | 0.999714303421225 |
| ebi-fl187-GCST90094750 | prot-a-3157 | UDP-glucuronosyltransferase 1-8 \|\| id:prot-a-3157 | Curry liking \|\| id:ebi-fl187-GCST90094750 | Weighted mode | 7 | 0.305282691277876 | 0.319981868331226 | 0.376891687783826 | -0.321881771 | 0.932447153207079 | 1.35700856327394 | 0.724783875990111 | 2.54071910510317 | 0.999714303421225 |
| ebi-fl187-GCST90094750 | prot-a-317 | Glutaredoxin-like protein C5orf63 \|\| id:prot-a-317 | Curry liking \|\| id:ebi-fl187-GCST90094750 | Weighted mode | 7 | 0.0162424687902765 | 0.436581219830136 | 0.97152945538695 | -0.839456722 | 0.871941659657344 | 1.01637509477129 | 0.43194512594709 | 2.39154992432509 | 0.999714303421225 |
| ebi-fl187-GCST90094750 | prot-a-3192 | Guanine nucleotide exchange factor VAV3 \|\| id:prot-a-3192 | Curry liking \|\| id:ebi-fl187-GCST90094750 | Weighted mode | 7 | 0.522441546521366 | 0.509913496069259 | 0.345087299204912 | -0.476988906 | 1.52187199881711 | 1.68613941594757 | 0.62064941487988 | 4.58079241170678 | 0.999714303421225 |
| ebi-fl187-GCST90094750 | prot-a-3233 | Serine/threonine-protein kinase WNK3 \|\| id:prot-a-3233 | Curry liking \|\| id:ebi-fl187-GCST90094750 | Weighted mode | 7 | 0.6478336772726 | 0.471695139837437 | 0.218732391323364 | -0.276688797 | 1.57235615135398 | 1.91139564090944 | 0.758290442800753 | 4.81798673684138 | 0.999714303421225 |
| ebi-fl187-GCST90094750 | prot-a-3250 | 14-3-3 protein theta \|\| id:prot-a-3250 | Curry liking \|\| id:ebi-fl187-GCST90094750 | Weighted mode | 7 | 0.412179754476537 | 0.363489233307173 | 0.300079054738973 | -0.300259143 | 1.1246186517586 | 1.5101058602216 | 0.740626267842302 | 3.07904243758363 | 0.999714303421225 |
| ebi-fl187-GCST90094750 | prot-a-3278 | E3 ubiquitin-protein ligase ZNRF3 \|\| id:prot-a-3278 | Curry liking \|\| id:ebi-fl187-GCST90094750 | Weighted mode | 7 | 0.383291697775288 | 0.332130340111285 | 0.292371347570477 | -0.267683769 | 1.03426716439341 | 1.46710591919095 | 0.765149707025851 | 2.81304398127726 | 0.999714303421225 |
| ebi-fl187-GCST90094750 | prot-a-355 | Calpain-3 \|\| id:prot-a-355 | Curry liking \|\| id:ebi-fl187-GCST90094750 | Weighted mode | 7 | 0.267804230172966 | 0.325298888338292 | 0.441847619144766 | -0.369781591 | 0.905390051316017 | 1.30709122594241 | 0.690885209728516 | 2.47289629142151 | 0.999714303421225 |
| ebi-fl187-GCST90094750 | prot-a-385 | Coiled-coil domain-containing protein 90B, mitochondrial \|\| id:prot-a-385 | Curry liking \|\| id:ebi-fl187-GCST90094750 | Weighted mode | 7 | -0.423209246 | 0.335246439024163 | 0.253657568241881 | -1.080292267 | 0.233873774098879 | 0.654941574613654 | 0.339496287624309 | 1.26348499760944 | 0.999714303421225 |
| ebi-fl187-GCST90094750 | prot-a-390 | C-C motif chemokine 14 \|\| id:prot-a-390 | Curry liking \|\| id:ebi-fl187-GCST90094750 | Weighted mode | 7 | 0.108975271201862 | 0.467555038257638 | 0.823451095443841 | -0.807432604 | 1.02538314618683 | 1.11513477405776 | 0.446001660540909 | 2.78816353016424 | 0.999714303421225 |
| ebi-fl187-GCST90094750 | prot-a-40 | Pituitary adenylate cyclase-activating polypeptide 27 \|\| id:prot-a-40 | Curry liking \|\| id:ebi-fl187-GCST90094750 | Weighted mode | 7 | -0.139273687 | 0.325923046281802 | 0.684055851432376 | -0.778082858 | 0.499535483325621 | 0.86998989061245 | 0.459285683636059 | 1.64795559002797 | 0.999714303421225 |
| ebi-fl187-GCST90094750 | prot-a-401 | C-C motif chemokine 24 \|\| id:prot-a-401 | Curry liking \|\| id:ebi-fl187-GCST90094750 | Weighted mode | 7 | -0.267349242 | 0.353201193436709 | 0.477750971808508 | -0.959623581 | 0.424925097283954 | 0.765405713072959 | 0.383037041266667 | 1.52947585347722 | 0.999714303421225 |
| ebi-fl187-GCST90094750 | prot-a-412 | Cerebral cavernous malformations 2 protein \|\| id:prot-a-412 | Curry liking \|\| id:ebi-fl187-GCST90094750 | Weighted mode | 7 | 0.0423776219263174 | 0.312777792352273 | 0.896657308320033 | -0.570666851 | 0.655422094936773 | 1.04328837293964 | 0.565148443161545 | 1.92595528180532 | 0.999714303421225 |
| ebi-fl187-GCST90094750 | prot-a-44 | Adhesion G protein-coupled receptor F5 \|\| id:prot-a-44 | Curry liking \|\| id:ebi-fl187-GCST90094750 | Weighted mode | 7 | 0.0564783932580129 | 0.297014005959347 | 0.855458805956628 | -0.525669058 | 0.638625844938333 | 1.05810375237846 | 0.591159711640178 | 1.89387661024986 | 0.999714303421225 |
| ebi-fl187-GCST90094750 | prot-a-48 | Adrenomedullin \|\| id:prot-a-48 | Curry liking \|\| id:ebi-fl187-GCST90094750 | Weighted mode | 7 | 0.0220067956523344 | 0.374483485938409 | 0.95504704058812 | -0.711980837 | 0.755994428091615 | 1.0222507313074 | 0.490671294447701 | 2.12972833235489 | 0.999714303421225 |
| ebi-fl187-GCST90094750 | prot-a-502 | Carcinoembryonic antigen-related cell adhesion molecule 1 \|\| id:prot-a-502 | Curry liking \|\| id:ebi-fl187-GCST90094750 | Weighted mode | 7 | 0.191986519464265 | 0.340004811194505 | 0.592773068413558 | -0.47442291 | 0.858395949405495 | 1.21165418310729 | 0.622244043389472 | 2.35937310294587 | 0.999714303421225 |
| ebi-fl187-GCST90094750 | prot-a-518 | Complement factor B \|\| id:prot-a-518 | Curry liking \|\| id:ebi-fl187-GCST90094750 | Weighted mode | 7 | 0.442049545566863 | 0.402040163377366 | 0.313691018734618 | -0.345949175 | 1.2300482657865 | 1.55589282611651 | 0.70754844758817 | 3.42139466860913 | 0.999714303421225 |
| ebi-fl187-GCST90094750 | prot-a-525 | Properdin \|\| id:prot-a-525 | Curry liking \|\| id:ebi-fl187-GCST90094750 | Weighted mode | 7 | 0.3224515156275 | 0.356195213577034 | 0.400213657863859 | -0.375691103 | 1.02059413423849 | 1.38050795617496 | 0.686814455214776 | 2.77484290348314 | 0.999714303421225 |
| ebi-fl187-GCST90094750 | prot-a-533 | Chondroadherin \|\| id:prot-a-533 | Curry liking \|\| id:ebi-fl187-GCST90094750 | Weighted mode | 7 | -0.347897021 | 0.320353758392708 | 0.31918139426126 | -0.975790387 | 0.279996345904259 | 0.706171593635652 | 0.376894343419413 | 1.32312497750326 | 0.999714303421225 |
| ebi-fl187-GCST90094750 | prot-a-535 | Coiled-coil-helix-coiled-coil-helix domain-containing protein 2 \|\| id:prot-a-535 | Curry liking \|\| id:ebi-fl187-GCST90094750 | Weighted mode | 7 | -0.155670005 | 0.415274125868751 | 0.720656720710758 | -0.969607292 | 0.658267281498107 | 0.855841567039812 | 0.379231936315083 | 1.93144278667658 | 0.999714303421225 |
| ebi-fl187-GCST90094750 | prot-a-591 | Caseinolytic peptidase B protein homolog \|\| id:prot-a-591 | Curry liking \|\| id:ebi-fl187-GCST90094750 | Weighted mode | 7 | 0.0812132255738662 | 0.456267225711404 | 0.864584468670381 | -0.813070537 | 0.975496987968217 | 1.08460213682576 | 0.44349420811055 | 2.65248513665767 | 0.999714303421225 |
| ebi-fl187-GCST90094750 | prot-a-605 | Ciliary neurotrophic factor receptor subunit alpha \|\| id:prot-a-605 | Curry liking \|\| id:ebi-fl187-GCST90094750 | Weighted mode | 7 | 0.00168057300419977 | 0.335515040172306 | 0.996165851600858 | -0.655928906 | 0.65929005174192 | 1.00168198595842 | 0.518959773911916 | 1.93341921943244 | 0.999714303421225 |
| ebi-fl187-GCST90094750 | prot-a-606 | Ciliary neurotrophic factor receptor subunit alpha \|\| id:prot-a-606 | Curry liking \|\| id:ebi-fl187-GCST90094750 | Weighted mode | 7 | 0.425779732741249 | 0.386285530431582 | 0.312595418021107 | -0.331339907 | 1.18289937238715 | 1.53078355649777 | 0.717961087629485 | 3.26382353754143 | 0.999714303421225 |
| ebi-fl187-GCST90094750 | prot-a-707 | Cancer/testis antigen 55 \|\| id:prot-a-707 | Curry liking \|\| id:ebi-fl187-GCST90094750 | Weighted mode | 7 | -0.165671036 | 0.541468983742883 | 0.769968903281391 | -1.226950244 | 0.895608172164643 | 0.847324927768571 | 0.293185359383136 | 2.44882464366095 | 0.999714303421225 |
| ebi-fl187-GCST90094750 | prot-a-719 | Dipeptidyl peptidase 1 \|\| id:prot-a-719 | Curry liking \|\| id:ebi-fl187-GCST90094750 | Weighted mode | 7 | -0.331582671 | 0.406795807428885 | 0.446150890269355 | -1.128902454 | 0.465737111091211 | 0.717786813273213 | 0.323387994906296 | 1.59318811280611 | 0.999714303421225 |
| ebi-fl187-GCST90094750 | prot-a-752 | Cytochrome b5 \|\| id:prot-a-752 | Curry liking \|\| id:ebi-fl187-GCST90094750 | Weighted mode | 7 | -0.377701391 | 0.373548998584435 | 0.350984302107794 | -1.109857428 | 0.354454646643053 | 0.685435147499784 | 0.329605950368289 | 1.4254030939159 | 0.999714303421225 |
| ebi-fl187-GCST90094750 | prot-a-755 | Cytoglobin \|\| id:prot-a-755 | Curry liking \|\| id:ebi-fl187-GCST90094750 | Weighted mode | 7 | -0.294172027 | 0.35679802857351 | 0.441204888793933 | -0.993496163 | 0.405152108511664 | 0.745148294424331 | 0.370279866435078 | 1.49953057407414 | 0.999714303421225 |
| ebi-fl187-GCST90094750 | prot-a-807 | Desmin \|\| id:prot-a-807 | Curry liking \|\| id:ebi-fl187-GCST90094750 | Weighted mode | 7 | 0.0834411056146491 | 0.417827532623165 | 0.848309664339077 | -0.735500858 | 0.902383069556052 | 1.08702119396257 | 0.479265354807148 | 2.46547150607052 | 0.999714303421225 |
| ebi-fl187-GCST90094750 | prot-a-82 | Ameloblastin \|\| id:prot-a-82 | Curry liking \|\| id:ebi-fl187-GCST90094750 | Weighted mode | 7 | -0.025646519 | 0.308225900029692 | 0.936393600680195 | -0.629769283 | 0.578476244828442 | 0.974679559204804 | 0.532714693012165 | 1.78331901784053 | 0.999714303421225 |
| ebi-fl187-GCST90094750 | prot-a-84 | Muellerian-inhibiting factor \|\| id:prot-a-84 | Curry liking \|\| id:ebi-fl187-GCST90094750 | Weighted mode | 7 | 0.153645035013805 | 0.424930265243096 | 0.730059964502831 | -0.679218285 | 0.986508354890273 | 1.16607689683723 | 0.507013177369086 | 2.68185402279537 | 0.999714303421225 |
| ebi-fl187-GCST90094750 | prot-a-858 | Inactive dipeptidyl peptidase 10 \|\| id:prot-a-858 | Curry liking \|\| id:ebi-fl187-GCST90094750 | Weighted mode | 7 | 0.131549281135759 | 0.373930472628377 | 0.737012983033775 | -0.601354445 | 0.864453007487379 | 1.14059411611084 | 0.548068803975377 | 2.37370733066775 | 0.999714303421225 |
| ebi-fl187-GCST90094750 | prot-a-863 | Dihydropyrimidinase-related protein 3 \|\| id:prot-a-863 | Curry liking \|\| id:ebi-fl187-GCST90094750 | Weighted mode | 7 | -0.154024141 | 0.34370214686299 | 0.669785065 | -0.827680349 | 0.519632066565816 | 0.857251325612531 | 0.437061942475103 | 1.68140888932764 | 0.999714303421225 |
| ebi-fl187-GCST90094750 | prot-a-864 | Dihydropyrimidinase-related protein 5 \|\| id:prot-a-864 | Curry liking \|\| id:ebi-fl187-GCST90094750 | Weighted mode | 7 | 0.0435223248366015 | 0.355484775010102 | 0.90655558359587 | -0.653227834 | 0.740272483856402 | 1.04448331197102 | 0.520363416208739 | 2.09650670090213 | 0.999714303421225 |
| ebi-fl187-GCST90094750 | prot-a-884 | Endothelin-converting enzyme 1 \|\| id:prot-a-884 | Curry liking \|\| id:ebi-fl187-GCST90094750 | Weighted mode | 7 | 0.177917236233432 | 0.375023880051724 | 0.651976739257503 | -0.557129569 | 0.912964041134812 | 1.19472643708289 | 0.57285103569916 | 2.49169709141346 | 0.999714303421225 |
| ebi-fl187-GCST90094750 | prot-a-893 | Early endosome antigen 1 \|\| id:prot-a-893 | Curry liking \|\| id:ebi-fl187-GCST90094750 | Weighted mode | 7 | -0.137990883 | 0.358518266014127 | 0.713590970137843 | -0.840686684 | 0.564704918797779 | 0.871106633946006 | 0.431414176489673 | 1.75892867934744 | 0.999714303421225 |
| ebi-fl187-GCST90094750 | prot-a-897 | Ephrin-A2 \|\| id:prot-a-897 | Curry liking \|\| id:ebi-fl187-GCST90094750 | Weighted mode | 7 | 0.34003799847362 | 0.31923661133892 | 0.327778820396934 | -0.28566576 | 0.965741756697903 | 1.40500097744185 | 0.751513760119864 | 2.62673533256093 | 0.999714303421225 |
| ebi-fl187-GCST90094750 | prot-a-90 | Anaphase-promoting complex subunit 10 \|\| id:prot-a-90 | Curry liking \|\| id:ebi-fl187-GCST90094750 | Weighted mode | 7 | 0.219308256160456 | 0.338089832862499 | 0.540569513407556 | -0.443347816 | 0.881964328570954 | 1.24521506274647 | 0.641883910602225 | 2.41564015997212 | 0.999714303421225 |
| ebi-fl187-GCST90094750 | prot-a-914 | Histone-lysine N-methyltransferase EHMT2 \|\| id:prot-a-914 | Curry liking \|\| id:ebi-fl187-GCST90094750 | Weighted mode | 7 | -0.473944923 | 0.361561259596691 | 0.23784967842807 | -1.182604992 | 0.234715146099231 | 0.622541539524194 | 0.306479321781947 | 1.26454850584958 | 0.999714303421225 |
| ebi-fl187-GCST90094750 | prot-a-932 | RNA polymerase II elongation factor ELL \|\| id:prot-a-932 | Curry liking \|\| id:ebi-fl187-GCST90094750 | Weighted mode | 7 | -0.057504694 | 0.31028478320535 | 0.859077387377707 | -0.665662869 | 0.550653480719373 | 0.944117458345849 | 0.513932744458315 | 1.73438603545862 | 0.999714303421225 |
| ebi-fl187-GCST90094750 | prot-a-991 | Estrogen receptor \|\| id:prot-a-991 | Curry liking \|\| id:ebi-fl187-GCST90094750 | Weighted mode | 7 | -0.292515961 | 0.336274468147075 | 0.417806425344638 | -0.951613919 | 0.366581996378895 | 0.746383331775104 | 0.386117358270242 | 1.44279469964104 | 0.999714303421225 |
| ebi-fl187-GCST90094750 | prot-a-992 | Persulfide dioxygenase ETHE1, mitochondrial \|\| id:prot-a-992 | Curry liking \|\| id:ebi-fl187-GCST90094750 | Weighted mode | 7 | -0.110801534 | 0.385516283557795 | 0.783466146214696 | -0.866413449 | 0.644810382142498 | 0.895116381799697 | 0.42045683795974 | 1.90562565435766 | 0.999714303421225 |
| ebi-fl187-GCST90094750 | prot-a-993 | Ethanolamine kinase 1 \|\| id:prot-a-993 | Curry liking \|\| id:ebi-fl187-GCST90094750 | Weighted mode | 7 | -0.238255135 | 0.390315043868245 | 0.563975626255735 | -1.003272621 | 0.526762350573736 | 0.788001618330054 | 0.366677478899879 | 1.69344065622403 | 0.999714303421225 |
| ebi-fl187-GCST90094750 | prot-b-10 | coagulation factor III, tissue factor \|\| id:prot-b-10 | Curry liking \|\| id:ebi-fl187-GCST90094750 | Weighted mode | 5 | 0.309672238606942 | 0.403876819369564 | 0.485981089540454 | -0.481926327 | 1.10127080457129 | 1.36297830924414 | 0.617592559764607 | 3.00798615867079 | 0.999714303421225 |
| ebi-fl187-GCST90094750 | prot-b-28 | prolactin \|\| id:prot-b-28 | Curry liking \|\| id:ebi-fl187-GCST90094750 | Weighted mode | 5 | -0.420612068 | 0.352127421027849 | 0.298273701652055 | -1.110781813 | 0.269557677248867 | 0.65664478555337 | 0.329301408227958 | 1.30938515178151 | 0.999714303421225 |
| ebi-fl187-GCST90094750 | prot-b-54 | matrix metallopeptidase 7 \|\| id:prot-b-54 | Curry liking \|\| id:ebi-fl187-GCST90094750 | Weighted mode | 5 | -0.292592015 | 0.436127718611136 | 0.539041836878624 | -1.147402343 | 0.562218313944458 | 0.746326568985347 | 0.317460352325826 | 1.75456035215937 | 0.999714303421225 |
| ebi-fl187-GCST90094750 | ukb-rsfMRI191-edge-pheno716 | Net100_Pair10_33 (OccipitalPrecuneus)&(Temporal\|Frontal\|Supp_Motor_Area) (Default_mode\|Central_executive)&(Default_mode\|Salience) \|\| id:ukb-rsfMRI191-edge-pheno716 | Curry liking \|\| id:ebi-fl187-GCST90094750 | Weighted mode | 7 | 0.0708508426616441 | 0.14591154593743 | 0.64449087372504 | -0.215135787 | 0.356837472699007 | 1.07342110529207 | 0.806431929467011 | 1.42880363138398 | 0.999714303421225 |
| ebi-fl187-GCST90094750 | ukb-rsfMRI191-edge-pheno903 | Net100_Pair27_38 (Precentral\|Frontal\|Supp_Motor_Area)&(Subcortical) (Attention\|Salience\|Motor)&(Subcortical-cerebellum) \|\| id:ukb-rsfMRI191-edge-pheno903 | Curry liking \|\| id:ebi-fl187-GCST90094750 | Weighted mode | 7 | 0.070330735969237 | 0.106310771075806 | 0.532822361954113 | -0.138038375 | 0.278699847277817 | 1.07286295695237 | 0.871065263679313 | 1.32141065933302 | 0.999714303421225 |
| ebi-fl187-GCST90094750 | ukb-rsfMRI191-node-pheno23 | Net100_Node2 RolandicOper\|SupraMarginal\|Insula Salience\|Motor \|\| id:ukb-rsfMRI191-node-pheno23 | Curry liking \|\| id:ebi-fl187-GCST90094750 | Weighted mode | 7 | -0.086706249 | 0.122066484934813 | 0.504165120235751 | -0.32595656 | 0.152544061069025 | 0.916946409630697 | 0.721836543420014 | 1.16479378302325 | 0.999714303421225 |
| ebi-fl187-GCST90094750 | ukb-rsfMRI191-node-pheno55 | Net100_Node34 Parietal\|Postcentral\|Precuneus Attention \|\| id:ukb-rsfMRI191-node-pheno55 | Curry liking \|\| id:ebi-fl187-GCST90094750 | Weighted mode | 7 | -0.019745344 | 0.13790872980004 | 0.890837869473089 | -0.290046455 | 0.250555766255829 | 0.980448318416314 | 0.748228808130897 | 1.28473923302511 | 0.999714303421225 |
| ebi-fl187-GCST90094764 | ebi-a-GCST90001414 | IgD- CD24- B cell Absolute Count \|\| id:ebi-a-GCST90001414 | Fried chicken liking \|\| id:ebi-fl187-GCST90094764 | Inverse variance weighted | 2 | 0.37479579511692 | 0.38503431361945 | 0.330350159335488 | -0.37987146 | 1.12946304981104 | 1.45469432860074 | 0.683949318696872 | 3.09399473296504 | 0.998735809224318 |
| ebi-fl187-GCST90094764 | ebi-a-GCST90001422 | CD20- CD38- B cell %B cell \|\| id:ebi-a-GCST90001422 | Fried chicken liking \|\| id:ebi-fl187-GCST90094764 | Inverse variance weighted | 2 | -0.734323373 | 0.364422568692131 | 0.0439012284497459 | -1.448591608 | -0.020055139 | 0.479830014958447 | 0.234900887777211 | 0.980144627947891 | 0.998735809224318 |
| ebi-fl187-GCST90094764 | ebi-a-GCST90001444 | CD20- CD38- B cell %lymphocyte \|\| id:ebi-a-GCST90001444 | Fried chicken liking \|\| id:ebi-fl187-GCST90094764 | Inverse variance weighted | 2 | -0.814277982 | 0.366519583780728 | 0.0263070143570934 | -1.532656366 | -0.095899598 | 0.442959036209938 | 0.215961232521194 | 0.908555232202526 | 0.998735809224318 |
| ebi-fl187-GCST90094764 | ebi-a-GCST90001459 | Myeloid Dendritic Cell %Dendritic Cell \|\| id:ebi-a-GCST90001459 | Fried chicken liking \|\| id:ebi-fl187-GCST90094764 | Inverse variance weighted | 2 | 0.136988230594166 | 0.398046220096042 | 0.730732303489937 | -0.643182361 | 0.917158821982408 | 1.14681465111392 | 0.525617056531632 | 2.50217116751881 | 0.998735809224318 |
| ebi-fl187-GCST90094764 | ebi-a-GCST90001476 | HLA DR++ monocyte %leukocyte \|\| id:ebi-a-GCST90001476 | Fried chicken liking \|\| id:ebi-fl187-GCST90094764 | Inverse variance weighted | 2 | -0.280389128 | 0.387780279987522 | 0.469641883694275 | -1.040438477 | 0.479660220559673 | 0.755489701890086 | 0.353299734186233 | 1.61552538661437 | 0.998735809224318 |
| ebi-fl187-GCST90094764 | ebi-a-GCST90001478 | CD4 regulatory T cell %CD4+ T cell \|\| id:ebi-a-GCST90001478 | Fried chicken liking \|\| id:ebi-fl187-GCST90094764 | Inverse variance weighted | 2 | 0.619839216677578 | 0.391149879550797 | 0.113043808943179 | -0.146814547 | 1.38649298059714 | 1.85862918124617 | 0.863454092511844 | 4.00079455681362 | 0.998735809224318 |
| ebi-fl187-GCST90094764 | ebi-a-GCST90001485 | CD39+ resting CD4 regulatory T cell %CD4 regulatory T cell \|\| id:ebi-a-GCST90001485 | Fried chicken liking \|\| id:ebi-fl187-GCST90094764 | Inverse variance weighted | 2 | 0.114037610291973 | 0.383177637619653 | 0.76600061513675 | -0.636990559 | 0.865065780026494 | 1.12079427749148 | 0.5288816694199 | 2.37516231907882 | 0.998735809224318 |
| ebi-fl187-GCST90094764 | ebi-a-GCST90001515 | Immature Myeloid-Derived Suppressor Cells Absolute Count \|\| id:ebi-a-GCST90001515 | Fried chicken liking \|\| id:ebi-fl187-GCST90094764 | Inverse variance weighted | 2 | 0.23938956217006 | 0.511181417207013 | 0.639565446245974 | -0.762526016 | 1.24130513989581 | 1.27047336855613 | 0.466486585117254 | 3.4601264681698 | 0.998735809224318 |
| ebi-fl187-GCST90094764 | ebi-a-GCST90001517 | CD33+ HLA DR+ Absolute Count \|\| id:ebi-a-GCST90001517 | Fried chicken liking \|\| id:ebi-fl187-GCST90094764 | Inverse variance weighted | 2 | 0.613695408938864 | 0.498767041051887 | 0.218538017012929 | -0.363887992 | 1.59127880940056 | 1.84724512742688 | 0.69496903280661 | 4.91002389994536 | 0.998735809224318 |
| ebi-fl187-GCST90094764 | ebi-a-GCST90001518 | CD33+ HLA DR+ CD14- Absolute Count \|\| id:ebi-a-GCST90001518 | Fried chicken liking \|\| id:ebi-fl187-GCST90094764 | Inverse variance weighted | 2 | 0.705714845426888 | 0.506250786030054 | 0.163316859207211 | -0.286536695 | 1.69796638604579 | 2.02529393968988 | 0.750859525091102 | 5.46282680724719 | 0.998735809224318 |
| ebi-fl187-GCST90094764 | ebi-a-GCST90001520 | CD33+ HLA DR+ CD14dim Absolute Count \|\| id:ebi-a-GCST90001520 | Fried chicken liking \|\| id:ebi-fl187-GCST90094764 | Inverse variance weighted | 2 | 0.415494917850834 | 0.499341874034391 | 0.405360928845215 | -0.563215155 | 1.39420499095824 | 1.51512041530277 | 0.569375487220854 | 4.03176800615725 | 0.998735809224318 |
| ebi-fl187-GCST90094764 | ebi-a-GCST90001531 | CD33dim HLA DR- Absolute Count \|\| id:ebi-a-GCST90001531 | Fried chicken liking \|\| id:ebi-fl187-GCST90094764 | Inverse variance weighted | 2 | -0.168225205 | 0.510993735478118 | 0.741995549260018 | -1.169772926 | 0.833322516740429 | 0.845163478381575 | 0.310437425426953 | 2.30095100230794 | 0.998735809224318 |
| ebi-fl187-GCST90094764 | ebi-a-GCST90001532 | Basophil Absolute Count \|\| id:ebi-a-GCST90001532 | Fried chicken liking \|\| id:ebi-fl187-GCST90094764 | Inverse variance weighted | 2 | -0.264125777 | 0.509547396629643 | 0.604211513695582 | -1.262838675 | 0.734587120035184 | 0.767876952047565 | 0.282849966748143 | 2.08462111650479 | 0.998735809224318 |
| ebi-fl187-GCST90094764 | ebi-a-GCST90001588 | T/B cell \|\| id:ebi-a-GCST90001588 | Fried chicken liking \|\| id:ebi-fl187-GCST90094764 | Inverse variance weighted | 2 | 0.0205745585729722 | 0.481844725643458 | 0.965941028997475 | -0.923841104 | 0.964990220834149 | 1.02078767387827 | 0.396991224255872 | 2.62476198836627 | 0.998735809224318 |
| ebi-fl187-GCST90094764 | ebi-a-GCST90001624 | HLA DR+ CD4+ T cell Absolute Count \|\| id:ebi-a-GCST90001624 | Fried chicken liking \|\| id:ebi-fl187-GCST90094764 | Inverse variance weighted | 2 | 0.460551041314088 | 0.613959015115711 | 0.453174485124738 | -0.742808628 | 1.66391071094088 | 1.58494711574825 | 0.47577576 | 5.27991875844094 | 0.998735809224318 |
| ebi-fl187-GCST90094764 | ebi-a-GCST90001631 | CD8+ Natural Killer T %T cell \|\| id:ebi-a-GCST90001631 | Fried chicken liking \|\| id:ebi-fl187-GCST90094764 | Inverse variance weighted | 2 | -0.026252728 | 0.375693742842344 | 0.94429076246991 | -0.762612464 | 0.710107007993117 | 0.974088878985245 | 0.466446259844581 | 2.03420892361509 | 0.998735809224318 |
| ebi-fl187-GCST90094764 | ebi-a-GCST90001632 | CD8+ Natural Killer T %lymphocyte \|\| id:ebi-a-GCST90001632 | Fried chicken liking \|\| id:ebi-fl187-GCST90094764 | Inverse variance weighted | 2 | -0.04382305 | 0.377945183813413 | 0.907691527938526 | -0.78459561 | 0.696949510678046 | 0.957123305844888 | 0.456304189390866 | 2.00761913629229 | 0.998735809224318 |
| ebi-fl187-GCST90094764 | ebi-a-GCST90001646 | Natural Killer %CD3- lymphocyte \|\| id:ebi-a-GCST90001646 | Fried chicken liking \|\| id:ebi-fl187-GCST90094764 | Inverse variance weighted | 2 | 0.25846642808084 | 0.368678060499241 | 0.483263901138445 | -0.46414257 | 0.981075426659351 | 1.2949426754123 | 0.628673917724149 | 2.66732321053558 | 0.998735809224318 |
| ebi-fl187-GCST90094764 | ebi-a-GCST90001684 | CD127- CD8+ T cell Absolute Count \|\| id:ebi-a-GCST90001684 | Fried chicken liking \|\| id:ebi-fl187-GCST90094764 | Inverse variance weighted | 2 | -0.0243883 | 0.836129178058796 | 0.976730522742369 | -1.663201489 | 1.61442488933512 | 0.975906691939637 | 0.189531225692306 | 5.02499716283545 | 0.998955228396052 |
| ebi-fl187-GCST90094764 | ebi-a-GCST90001687 | CD28- CD8+ T cell Absolute Count \|\| id:ebi-a-GCST90001687 | Fried chicken liking \|\| id:ebi-fl187-GCST90094764 | Inverse variance weighted | 2 | -0.32180726 | 0.713808021265963 | 0.652110875835986 | -1.720870982 | 1.0772564612835 | 0.724837881832444 | 0.178910252406645 | 2.93661178088993 | 0.998735809224318 |
| ebi-fl187-GCST90094764 | ebi-a-GCST90001701 | BAFF-R on CD20- CD38- B cell \|\| id:ebi-a-GCST90001701 | Fried chicken liking \|\| id:ebi-fl187-GCST90094764 | Inverse variance weighted | 2 | -0.459885169 | 0.362760072505158 | 0.204891096039116 | -1.170894911 | 0.251124572958904 | 0.631356140506022 | 0.310089314638733 | 1.28547020918492 | 0.998735809224318 |
| ebi-fl187-GCST90094764 | ebi-a-GCST90001732 | CD19 on IgD- CD27- B cell \|\| id:ebi-a-GCST90001732 | Fried chicken liking \|\| id:ebi-fl187-GCST90094764 | Inverse variance weighted | 2 | 0.0500629123354854 | 0.372760954108753 | 0.893162985500098 | -0.680548558 | 0.780674382388642 | 1.05133723637641 | 0.506339159915059 | 2.18294390814453 | 0.998735809224318 |
| ebi-fl187-GCST90094764 | ebi-a-GCST90001798 | CD27 on CD24+ CD27+ B cell \|\| id:ebi-a-GCST90001798 | Fried chicken liking \|\| id:ebi-fl187-GCST90094764 | Inverse variance weighted | 2 | -0.202039291 | 0.43292966096452 | 0.640729374583357 | -1.050581427 | 0.646502844175983 | 0.817062823839177 | 0.349734345061777 | 1.90885358423156 | 0.998735809224318 |
| ebi-fl187-GCST90094764 | ebi-a-GCST90001836 | CD66b on CD66b++ myeloid cell \|\| id:ebi-a-GCST90001836 | Fried chicken liking \|\| id:ebi-fl187-GCST90094764 | Inverse variance weighted | 2 | -0.60310821 | 0.545652888936118 | 0.269031148372388 | -1.672587873 | 0.466371452045373 | 0.547108462412898 | 0.18776053602837 | 1.59419905788178 | 0.998735809224318 |
| ebi-fl187-GCST90094764 | ebi-a-GCST90001943 | CD25 on activated & secreting CD4 regulatory T cell \|\| id:ebi-a-GCST90001943 | Fried chicken liking \|\| id:ebi-fl187-GCST90094764 | Inverse variance weighted | 2 | 0.662689716190352 | 0.379329971902536 | 0.0806372213756831 | -0.080797029 | 1.40617646111932 | 1.94000338164627 | 0.922380889258644 | 4.08032426151407 | 0.998735809224318 |
| ebi-fl187-GCST90094764 | ebi-a-GCST90001960 | CD25 on CD4+ T cell \|\| id:ebi-a-GCST90001960 | Fried chicken liking \|\| id:ebi-fl187-GCST90094764 | Inverse variance weighted | 2 | 0.264241407659015 | 0.434029668993341 | 0.542650682513601 | -0.586456744 | 1.11493955888596 | 1.30244257796604 | 0.556294892262072 | 3.04938386545472 | 0.998735809224318 |
| ebi-fl187-GCST90094764 | ebi-a-GCST90001988 | HLA DR on CD14+ CD16- monocyte \|\| id:ebi-a-GCST90001988 | Fried chicken liking \|\| id:ebi-fl187-GCST90094764 | Inverse variance weighted | 2 | -0.052666714 | 0.380423311437722 | 0.889890793413191 | -0.798296405 | 0.692962976163327 | 0.948696146700262 | 0.450095091240987 | 1.99963162513594 | 0.998735809224318 |
| ebi-fl187-GCST90094764 | ebi-a-GCST90001991 | HLA DR on CD14+ monocyte \|\| id:ebi-a-GCST90001991 | Fried chicken liking \|\| id:ebi-fl187-GCST90094764 | Inverse variance weighted | 2 | -0.047500795 | 0.381043050738567 | 0.900792791356832 | -0.794345175 | 0.699343584351452 | 0.953609714920921 | 0.451877038650128 | 2.01243128243047 | 0.998735809224318 |
| ebi-fl187-GCST90094764 | ebi-a-GCST90002019 | CD14 on Monocytic Myeloid-Derived Suppressor Cells \|\| id:ebi-a-GCST90002019 | Fried chicken liking \|\| id:ebi-fl187-GCST90094764 | Inverse variance weighted | 2 | -0.22329541 | 0.536562288034956 | 0.677293406783016 | -1.274957495 | 0.828366674249528 | 0.799878522036173 | 0.279442845729751 | 2.28957606105088 | 0.998735809224318 |
| ebi-fl187-GCST90094764 | ebi-a-GCST90002037 | CD80 on plasmacytoid Dendritic Cell \|\| id:ebi-a-GCST90002037 | Fried chicken liking \|\| id:ebi-fl187-GCST90094764 | Inverse variance weighted | 2 | -0.927699995 | 0.41782401708713 | 0.0263975446025732 | -1.746635069 | -0.108764922 | 0.395462230252851 | 0.174359665762292 | 0.896941244254096 | 0.998735809224318 |
| ebi-fl187-GCST90094764 | ebi-a-GCST90002042 | CD45 on CD33+ HLA DR+ CD14- \|\| id:ebi-a-GCST90002042 | Fried chicken liking \|\| id:ebi-fl187-GCST90094764 | Inverse variance weighted | 2 | -1.204707163 | 0.612677967855746 | 0.0492642543013197 | -2.40555598 | -0.003858346 | 0.29977977340075 | 0.090215323787583 | 0.996149088283538 | 0.998735809224318 |
| ebi-fl187-GCST90094764 | ebi-a-GCST90002056 | CD8 on Effector Memory CD8+ T cell \|\| id:ebi-a-GCST90002056 | Fried chicken liking \|\| id:ebi-fl187-GCST90094764 | Inverse variance weighted | 2 | -0.385338896 | 0.408082048530487 | 0.345032618576809 | -1.185179711 | 0.414501919101486 | 0.680220073263988 | 0.305691238441756 | 1.51361664936767 | 0.998735809224318 |
| ebi-fl187-GCST90094764 | ebi-a-GCST90002058 | CD8 on CD8+ T cell \|\| id:ebi-a-GCST90002058 | Fried chicken liking \|\| id:ebi-fl187-GCST90094764 | Inverse variance weighted | 2 | 0.0305213631031468 | 0.565971910690499 | 0.956993049334586 | -1.078783582 | 1.13982630805652 | 1.03099191500014 | 0.340008867153003 | 3.12622531787484 | 0.998735809224318 |
| ebi-fl187-GCST90094764 | ebi-a-GCST90002063 | CD4 on CD4 regulatory T cell \|\| id:ebi-a-GCST90002063 | Fried chicken liking \|\| id:ebi-fl187-GCST90094764 | Inverse variance weighted | 2 | -0.164397312 | 0.406970228719095 | 0.686246436315981 | -0.962058961 | 0.633264335867426 | 0.848404873112971 | 0.382105335612055 | 1.88374974552208 | 0.998735809224318 |
| ebi-fl187-GCST90094764 | ebi-a-GCST90002066 | CD4 on activated CD4 regulatory T cell \|\| id:ebi-a-GCST90002066 | Fried chicken liking \|\| id:ebi-fl187-GCST90094764 | Inverse variance weighted | 2 | 0.1166717087415 | 0.407034601274744 | 0.774389280366329 | -0.68111611 | 0.914459527239997 | 1.1237504516777 | 0.506051867626632 | 2.49542617749521 | 0.998735809224318 |
| ebi-fl187-GCST90094764 | ebi-a-GCST90002070 | CD4 on activated & secreting CD4 regulatory T cell \|\| id:ebi-a-GCST90002070 | Fried chicken liking \|\| id:ebi-fl187-GCST90094764 | Inverse variance weighted | 2 | -0.119721302 | 0.408932606127023 | 0.769701507320191 | -0.92122921 | 0.681786606326438 | 0.887167654398578 | 0.398029478566107 | 1.97740742682292 | 0.998735809224318 |
| ebi-fl187-GCST90094764 | ebi-a-GCST90002081 | SSC-A on CD4+ T cell \|\| id:ebi-a-GCST90002081 | Fried chicken liking \|\| id:ebi-fl187-GCST90094764 | Inverse variance weighted | 2 | 0.308242037960781 | 0.389083139213312 | 0.42822852301253 | -0.454360915 | 1.07084499081887 | 1.36103037008951 | 0.634853563784299 | 2.91784400998553 | 0.998735809224318 |
| ebi-fl187-GCST90094764 | ebi-a-GCST90002094 | CD11b on Monocytic Myeloid-Derived Suppressor Cells \|\| id:ebi-a-GCST90002094 | Fried chicken liking \|\| id:ebi-fl187-GCST90094764 | Inverse variance weighted | 2 | 0.0476746248561877 | 0.538875876985485 | 0.929502720262917 | -1.008522094 | 1.10387134374774 | 1.0488293368029 | 0.364757658934539 | 3.01581872455168 | 0.998735809224318 |
| ebi-fl187-GCST90094764 | ebi-a-GCST90002104 | HLA DR on myeloid Dendritic Cell \|\| id:ebi-a-GCST90002104 | Fried chicken liking \|\| id:ebi-fl187-GCST90094764 | Inverse variance weighted | 2 | -0.859369416 | 0.417240907332055 | 0.0394322003790009 | -1.677161594 | -0.041577237 | 0.423429005808844 | 0.18690373250356 | 0.959275240567233 | 0.998735809224318 |
| ebi-fl187-GCST90094764 | ebi-a-GCST90002105 | HLA DR on plasmacytoid Dendritic Cell \|\| id:ebi-a-GCST90002105 | Fried chicken liking \|\| id:ebi-fl187-GCST90094764 | Inverse variance weighted | 2 | -0.226596801 | 0.409768949993461 | 0.580272480961249 | -1.029743943 | 0.576550340745813 | 0.797242164546394 | 0.357098386323446 | 1.77988782160142 | 0.998735809224318 |
| ebi-fl187-GCST90094764 | ebi-a-GCST90002106 | HLA DR on Dendritic Cell \|\| id:ebi-a-GCST90002106 | Fried chicken liking \|\| id:ebi-fl187-GCST90094764 | Inverse variance weighted | 2 | -0.51178787 | 0.416686124978828 | 0.21935931509218 | -1.328492675 | 0.304916934702507 | 0.599422929792414 | 0.264876215031871 | 1.35651231922688 | 0.998735809224318 |
| ebi-fl187-GCST90094764 | ebi-a-GCST90002111 | HLA DR on CD33dim HLA DR+ CD11b- \|\| id:ebi-a-GCST90002111 | Fried chicken liking \|\| id:ebi-fl187-GCST90094764 | Inverse variance weighted | 2 | -0.962127053 | 0.753793355621761 | 0.201821086788102 | -2.43956203 | 0.515307923865817 | 0.382079318012689 | 0.087199033645861 | 1.67415393438791 | 0.998735809224318 |
| ebi-fl187-GCST90094764 | ebi-a-GCST90002112 | HLA DR on CD33- HLA DR+ \|\| id:ebi-a-GCST90002112 | Fried chicken liking \|\| id:ebi-fl187-GCST90094764 | Inverse variance weighted | 2 | -0.151506338 | 0.539426102552341 | 0.778813340911905 | -1.208781499 | 0.905768822855774 | 0.859412435173478 | 0.29856085453182 | 2.47383313157047 | 0.998735809224318 |
| ebi-fl187-GCST90094764 | ebi-a-GCST90002115 | HLA DR on HLA DR+ CD8+ T cell \|\| id:ebi-a-GCST90002115 | Fried chicken liking \|\| id:ebi-fl187-GCST90094764 | Inverse variance weighted | 2 | -0.290319158 | 0.375183306191767 | 0.439045421461899 | -1.025678438 | 0.445040121774639 | 0.748024791110354 | 0.358553126625733 | 1.56055280672465 | 0.998735809224318 |
| ebi-fl187-GCST90094764 | ebi-a-GCST90002116 | HLA DR on B cell \|\| id:ebi-a-GCST90002116 | Fried chicken liking \|\| id:ebi-fl187-GCST90094764 | Inverse variance weighted | 2 | -0.199905973 | 0.40528552747724 | 0.621837893819392 | -0.994265607 | 0.594453660963152 | 0.818807739582082 | 0.369995066673498 | 1.81204068591313 | 0.998735809224318 |
| ebi-fl187-GCST90094764 | ebi-cfmet338-GCST90026023 | Sphingomyelin (d18:1/24:1, d18:2/24:0) levels \|\| id:ebi-cfmet338-GCST90026023 | Fried chicken liking \|\| id:ebi-fl187-GCST90094764 | Inverse variance weighted | 2 | 0.0525105560840263 | 0.164143294471558 | 0.749038982859151 | -0.269210301 | 0.37423141324828 | 1.05391368722126 | 0.763982572392611 | 1.45387355713332 | 0.998735809224318 |
| ebi-fl187-GCST90094764 | ebi-cfmet338-GCST90026046 | 1-arachidonoyl-gpc (20:4n6) levels \|\| id:ebi-cfmet338-GCST90026046 | Fried chicken liking \|\| id:ebi-fl187-GCST90094764 | Inverse variance weighted | 2 | -0.054291338 | 0.194046014690762 | 0.779641773496477 | -0.434621527 | 0.326038850810169 | 0.947156123746256 | 0.647509685785903 | 1.38546919442786 | 0.998735809224318 |
| ebi-fl187-GCST90094764 | ebi-cfmet338-GCST90026047 | 1-linoleoyl-gpc (18:2) levels \|\| id:ebi-cfmet338-GCST90026047 | Fried chicken liking \|\| id:ebi-fl187-GCST90094764 | Inverse variance weighted | 2 | 0.094402162653253 | 0.237215359960914 | 0.69065986376626 | -0.370539943 | 0.559344268176644 | 1.09900163446893 | 0.690361474230005 | 1.74952490492391 | 0.998735809224318 |
| ebi-fl187-GCST90094764 | ebi-cfmet338-GCST90026050 | 1-myristoyl-2-palmitoyl-gpc (14:0/16:0) levels \|\| id:ebi-cfmet338-GCST90026050 | Fried chicken liking \|\| id:ebi-fl187-GCST90094764 | Inverse variance weighted | 2 | 0.0851913021134816 | 0.123488449838086 | 0.490274286901524 | -0.15684606 | 0.32722866379613 | 1.08892536049713 | 0.854835642456496 | 1.38711862472925 | 0.998735809224318 |
| ebi-fl187-GCST90094764 | ebi-cfmet338-GCST90026068 | 2,3-dihydroxyisovalerate levels \|\| id:ebi-cfmet338-GCST90026068 | Fried chicken liking \|\| id:ebi-fl187-GCST90094764 | Inverse variance weighted | 2 | 0.239432633168401 | 0.400638106420823 | 0.550088038871057 | -0.545818055 | 1.02468332175321 | 1.27052809029093 | 0.57936763459474 | 2.78621298779911 | 0.998735809224318 |
| ebi-fl187-GCST90094764 | ebi-cfmet338-GCST90026082 | 3-hydroxyhexanoate levels \|\| id:ebi-cfmet338-GCST90026082 | Fried chicken liking \|\| id:ebi-fl187-GCST90094764 | Inverse variance weighted | 2 | -0.134201029 | 0.086200730628806 | 0.11950769792859 | -0.303154461 | 0.0347524035280104 | 0.874414264741624 | 0.738485020767813 | 1.0353633247546 | 0.998735809224318 |
| ebi-fl187-GCST90094764 | ebi-cfmet338-GCST90026094 | 3-ureidopropionate levels \|\| id:ebi-cfmet338-GCST90026094 | Fried chicken liking \|\| id:ebi-fl187-GCST90094764 | Inverse variance weighted | 2 | 0.416445682891294 | 0.194994007475367 | 0.032705137161082 | 0.0342574282395748 | 0.798633937543013 | 1.51656162384251 | 1.03485097230566 | 2.22250277620892 | 0.998735809224318 |
| ebi-fl187-GCST90094764 | ebi-cfmet338-GCST90026173 | Glycerol levels \|\| id:ebi-cfmet338-GCST90026173 | Fried chicken liking \|\| id:ebi-fl187-GCST90094764 | Inverse variance weighted | 2 | -0.244652996 | 0.135723898935755 | 0.0714544203962175 | -0.510671838 | 0.0213658462018663 | 0.78297618720166 | 0.600092278779236 | 1.02159573019666 | 0.998735809224318 |
| ebi-fl187-GCST90094764 | ebi-cfmet338-GCST90026179 | Histidine levels \|\| id:ebi-cfmet338-GCST90026179 | Fried chicken liking \|\| id:ebi-fl187-GCST90094764 | Inverse variance weighted | 2 | -0.104859976 | 0.0756197658107596 | 0.165541141566655 | -0.253074717 | 0.0433547652999988 | 0.900450598733793 | 0.776409868890189 | 1.04430831349301 | 0.998735809224318 |
| ebi-fl187-GCST90094764 | ebi-cfmet338-GCST90026188 | Isocitrate levels \|\| id:ebi-cfmet338-GCST90026188 | Fried chicken liking \|\| id:ebi-fl187-GCST90094764 | Inverse variance weighted | 2 | -0.080851753 | 0.150592234174639 | 0.591342275632935 | -0.376012532 | 0.2143090259027 | 0.922330413953478 | 0.686593728553288 | 1.23900548042593 | 0.998735809224318 |
| ebi-fl187-GCST90094764 | ebi-cfmet338-GCST90026207 | N-acetyl-isoputreanine levels \|\| id:ebi-cfmet338-GCST90026207 | Fried chicken liking \|\| id:ebi-fl187-GCST90094764 | Inverse variance weighted | 2 | -0.056732559 | 0.0957675335079762 | 0.553583662013343 | -0.244436924 | 0.130971806949722 | 0.944846726591911 | 0.783145384171298 | 1.13993564259606 | 0.998735809224318 |
| ebi-fl187-GCST90094764 | ebi-cfmet338-GCST90026213 | N-acetylglutamate levels \|\| id:ebi-cfmet338-GCST90026213 | Fried chicken liking \|\| id:ebi-fl187-GCST90094764 | Inverse variance weighted | 2 | 0.0336736571281361 | 0.16381412175271 | 0.837134500328709 | -0.287402022 | 0.354749335763447 | 1.03424703250184 | 0.7502100676219 | 1.42582320659813 | 0.998735809224318 |
| ebi-fl187-GCST90094764 | ebi-cfmet338-GCST90026224 | N-acetyltaurine levels \|\| id:ebi-cfmet338-GCST90026224 | Fried chicken liking \|\| id:ebi-fl187-GCST90094764 | Inverse variance weighted | 2 | 0.0341108664480581 | 0.171614322914597 | 0.842446823809942 | -0.302253206 | 0.370474939360669 | 1.03469931380715 | 0.739150883410793 | 1.44842236412235 | 0.998735809224318 |
| ebi-fl187-GCST90094764 | ebi-cfmet338-GCST90026250 | Phenyllactate (pla) levels \|\| id:ebi-cfmet338-GCST90026250 | Fried chicken liking \|\| id:ebi-fl187-GCST90094764 | Inverse variance weighted | 2 | -0.273208809 | 0.205703166705511 | 0.184121898563773 | -0.676387015 | 0.12996939811901 | 0.760933881482437 | 0.508450702362332 | 1.1387935335672 | 0.998735809224318 |
| ebi-fl187-GCST90094764 | ebi-cfmet338-GCST90026251 | Pipecolate levels \|\| id:ebi-cfmet338-GCST90026251 | Fried chicken liking \|\| id:ebi-fl187-GCST90094764 | Inverse variance weighted | 2 | -0.208071882 | 0.310327772730802 | 0.502545040561795 | -0.816314316 | 0.400170552671527 | 0.812148655840793 | 0.442057941365688 | 1.49207915402739 | 0.998735809224318 |
| ebi-fl187-GCST90094764 | ebi-cfmet338-GCST90026278 | Trigonelline (n'-methylnicotinate) levels \|\| id:ebi-cfmet338-GCST90026278 | Fried chicken liking \|\| id:ebi-fl187-GCST90094764 | Inverse variance weighted | 2 | 0.502581159292582 | 0.378723444473018 | 0.184495451611447 | -0.239716792 | 1.2448791104597 | 1.65298237986387 | 0.786850672017973 | 3.47251498322163 | 0.998735809224318 |
| ebi-fl187-GCST90094764 | ebi-cfmet338-GCST90026299 | 1-carboxyethylvaline levels \|\| id:ebi-cfmet338-GCST90026299 | Fried chicken liking \|\| id:ebi-fl187-GCST90094764 | Inverse variance weighted | 2 | 0.0641721625351309 | 0.210109688883224 | 0.760044619090995 | -0.347642828 | 0.47598715274625 | 1.06627595573192 | 0.706351120235595 | 1.60960233685594 | 0.998735809224318 |
| ebi-fl187-GCST90094764 | ebi-gm430-GCST90011306 | C_Erysipelotrichia abundance \|\| id:ebi-gm430-GCST90011306 | Fried chicken liking \|\| id:ebi-fl187-GCST90094764 | Inverse variance weighted | 2 | 0.180718072121897 | 0.196042192297232 | 0.356615931238429 | -0.203524625 | 0.564960769024473 | 1.19807736026715 | 0.815850113945192 | 1.75937875922285 | 0.998735809224318 |
| ebi-fl187-GCST90094764 | ebi-gm430-GCST90011312 | F_Erysipelotrichaceae abundance \|\| id:ebi-gm430-GCST90011312 | Fried chicken liking \|\| id:ebi-fl187-GCST90094764 | Inverse variance weighted | 2 | 0.180718072121897 | 0.196042192297232 | 0.356615931238429 | -0.203524625 | 0.564960769024473 | 1.19807736026715 | 0.815850113945192 | 1.75937875922285 | 0.998735809224318 |
| ebi-fl187-GCST90094764 | ebi-gm430-GCST90011352 | O_Erysipelotrichales abundance \|\| id:ebi-gm430-GCST90011352 | Fried chicken liking \|\| id:ebi-fl187-GCST90094764 | Inverse variance weighted | 2 | 0.180718072121897 | 0.196042192297232 | 0.356615931238429 | -0.203524625 | 0.564960769024473 | 1.19807736026715 | 0.815850113945192 | 1.75937875922285 | 0.998735809224318 |
| ebi-fl187-GCST90094764 | ebi-gm430-GCST90011362 | OTU97_11 (Parabacteroides) abundance \|\| id:ebi-gm430-GCST90011362 | Fried chicken liking \|\| id:ebi-fl187-GCST90094764 | Inverse variance weighted | 2 | 0.302857074647766 | 0.255495186338763 | 0.235870034434016 | -0.197913491 | 0.803627639871742 | 1.35372096956855 | 0.820440825930984 | 2.23362905102894 | 0.998735809224318 |
| ebi-fl187-GCST90094764 | ebi-gm430-GCST90011384 | OTU97_235 (Phascolarctobacterium) abundance \|\| id:ebi-gm430-GCST90011384 | Fried chicken liking \|\| id:ebi-fl187-GCST90094764 | Inverse variance weighted | 2 | -0.929350101 | 0.789686932376624 | 0.239251825619449 | -2.477136488 | 0.618436286462449 | 0.394810213776727 | 0.0839833689481298 | 1.85602348244326 | 0.998735809224318 |
| ebi-fl187-GCST90094764 | ebi-gm430-GCST90011390 | OTU97_3 (Bacteroides) abundance \|\| id:ebi-gm430-GCST90011390 | Fried chicken liking \|\| id:ebi-fl187-GCST90094764 | Inverse variance weighted | 2 | -0.133742282 | 0.24993187081912 | 0.592570367651299 | -0.623608749 | 0.356124184410797 | 0.874815490907337 | 0.536006629670193 | 1.42778484587465 | 0.998735809224318 |
| ebi-fl187-GCST90094764 | ebi-gm430-GCST90011398 | OTU97_4 (Alistipes) abundance \|\| id:ebi-gm430-GCST90011398 | Fried chicken liking \|\| id:ebi-fl187-GCST90094764 | Inverse variance weighted | 2 | 0.432799684431329 | 0.236230205292182 | 0.0669350571304944 | -0.030211518 | 0.895810886804006 | 1.54156738982151 | 0.97024028861432 | 2.4493211065839 | 0.998735809224318 |
| ebi-fl187-GCST90094764 | ebi-gm430-GCST90011421 | OTU99_108 (Cytophagales) abundance \|\| id:ebi-gm430-GCST90011421 | Fried chicken liking \|\| id:ebi-fl187-GCST90094764 | Inverse variance weighted | 2 | -0.109936519 | 0.732896899794291 | 0.880762479487565 | -1.546414443 | 1.32654140461883 | 0.895891005568068 | 0.213010367110942 | 3.76798887652137 | 0.998735809224318 |
| ebi-fl187-GCST90094764 | ebi-gm430-GCST90011428 | OTU99_123 (Phascolarctobacterium) abundance \|\| id:ebi-gm430-GCST90011428 | Fried chicken liking \|\| id:ebi-fl187-GCST90094764 | Inverse variance weighted | 2 | 0.603656043401429 | 0.439537498814621 | 0.169631392841676 | -0.257837454 | 1.46514954107809 | 1.82879273847693 | 0.772720824365852 | 4.32819043417222 | 0.998735809224318 |
| ebi-fl187-GCST90094764 | ebi-gm430-GCST90011450 | OTU99_298 (Phascolarctobacterium) abundance \|\| id:ebi-gm430-GCST90011450 | Fried chicken liking \|\| id:ebi-fl187-GCST90094764 | Inverse variance weighted | 2 | -0.912168894 | 0.789682147376929 | 0.248045825006465 | -2.459945903 | 0.635608114765135 | 0.4016521376949 | 0.0854395728709426 | 1.88817001646959 | 0.998735809224318 |
| ebi-fl187-GCST90094764 | ebi-gm430-GCST90011451 | OTU99_3 (Bacteroides) abundance \|\| id:ebi-gm430-GCST90011451 | Fried chicken liking \|\| id:ebi-fl187-GCST90094764 | Inverse variance weighted | 2 | -0.141925537 | 0.254641682310525 | 0.577285650043054 | -0.641023234 | 0.357172160360493 | 0.867685864600609 | 0.526753156296021 | 1.42928191436334 | 0.998735809224318 |
| ebi-fl187-GCST90094764 | ebi-gm430-GCST90011472 | OTU99_72 (Parabacteroides) abundance \|\| id:ebi-gm430-GCST90011472 | Fried chicken liking \|\| id:ebi-fl187-GCST90094764 | Inverse variance weighted | 2 | 0.00512827435561191 | 0.811804848533327 | 0.994959695393509 | -1.586009229 | 1.59626577748093 | 1.00514144646164 | 0.204741058246632 | 4.93457118980982 | 0.999107451094609 |
| ebi-fl187-GCST90094764 | ebi-gm430-GCST90011507 | TestASV_3 (Bacteroides) abundance \|\| id:ebi-gm430-GCST90011507 | Fried chicken liking \|\| id:ebi-fl187-GCST90094764 | Inverse variance weighted | 2 | 0.041064104873417 | 0.523414746425907 | 0.937466731082819 | -0.984828798 | 1.0669570078682 | 1.04191889548358 | 0.37350316590695 | 2.90652150733357 | 0.998735809224318 |
| ebi-fl187-GCST90094764 | ebi-gm430-GCST90011512 | TestASV_34 (Phascolarctobacterium) abundance \|\| id:ebi-gm430-GCST90011512 | Fried chicken liking \|\| id:ebi-fl187-GCST90094764 | Inverse variance weighted | 2 | -0.763606315 | 0.649395224684911 | 0.239645627137431 | -2.036420956 | 0.509208325263006 | 0.465982911970788 | 0.130494923220392 | 1.66397334769912 | 0.998735809224318 |
| ebi-fl187-GCST90094764 | ebi-gm430-GCST90011519 | TestASV_41 (Barnesiella) abundance \|\| id:ebi-gm430-GCST90011519 | Fried chicken liking \|\| id:ebi-fl187-GCST90094764 | Inverse variance weighted | 2 | -1.294215465 | 1.39336701836711 | 0.35297184618173 | -4.025214821 | 1.43678389076627 | 0.274112831041951 | 0.017859587122668 | 4.20714340291022 | 0.998735809224318 |
| ebi-fl187-GCST90094764 | ebi-gm430-GCST90011524 | TestASV_46 (Sutterella) abundance \|\| id:ebi-gm430-GCST90011524 | Fried chicken liking \|\| id:ebi-fl187-GCST90094764 | Inverse variance weighted | 2 | -1.36005571 | 0.871477559050918 | 0.118610619024485 | -3.068151726 | 0.348040305344683 | 0.256646478678546 | 0.0465070332036452 | 1.41628933261937 | 0.998735809224318 |
| ebi-fl187-GCST90094764 | ebi-gm430-GCST90011581 | OTU97_19 (Bacteria) prevalence \|\| id:ebi-gm430-GCST90011581 | Fried chicken liking \|\| id:ebi-fl187-GCST90094764 | Inverse variance weighted | 2 | 0.107956941882524 | 1.85226598454678 | 0.953522639381909 | -3.522484388 | 3.73839827159421 | 1.11399977762048 | 0.0295259899623746 | 42.0306145914126 | 0.998735809224318 |
| ebi-fl187-GCST90094764 | ebi-gm430-GCST90011614 | OTU97_80 (Ruminococcus) prevalence \|\| id:ebi-gm430-GCST90011614 | Fried chicken liking \|\| id:ebi-fl187-GCST90094764 | Inverse variance weighted | 2 | -0.257622988 | 0.507104706969966 | 0.611434262593351 | -1.251548214 | 0.736302237320946 | 0.772886564432139 | 0.286061569316324 | 2.08819955405883 | 0.998735809224318 |
| ebi-fl187-GCST90094764 | ebi-gm430-GCST90011623 | OTU99_11 (Parabacteroides) prevalence \|\| id:ebi-gm430-GCST90011623 | Fried chicken liking \|\| id:ebi-fl187-GCST90094764 | Inverse variance weighted | 2 | -0.080985839 | 0.440374834319202 | 0.854090288213185 | -0.944120515 | 0.782148835774128 | 0.922206750268666 | 0.389021559213353 | 2.18616493122086 | 0.998735809224318 |
| ebi-fl187-GCST90094764 | ebi-gm430-GCST90011642 | OTU99_19 (Bacteria) prevalence \|\| id:ebi-gm430-GCST90011642 | Fried chicken liking \|\| id:ebi-fl187-GCST90094764 | Inverse variance weighted | 2 | 0.107956941882524 | 1.85226598454678 | 0.953522639381909 | -3.522484388 | 3.73839827159421 | 1.11399977762048 | 0.0295259899623746 | 42.0306145914126 | 0.998735809224318 |
| ebi-fl187-GCST90094764 | ebi-gm430-GCST90011653 | OTU99_30 (Parasutterella) prevalence \|\| id:ebi-gm430-GCST90011653 | Fried chicken liking \|\| id:ebi-fl187-GCST90094764 | Inverse variance weighted | 2 | 0.000767406935712274 | 1.10438184526238 | 0.999445570298706 | -2.16382101 | 2.16535582364998 | 1.00076770146775 | 0.114885303431666 | 8.71770333005878 | 0.999882738470636 |
| ebi-fl187-GCST90094764 | ebi-gm430-GCST90011675 | OTU99_8 (Ruminococcaceae) prevalence \|\| id:ebi-gm430-GCST90011675 | Fried chicken liking \|\| id:ebi-fl187-GCST90094764 | Inverse variance weighted | 2 | -0.436973947 | 0.696321382092197 | 0.530301158869467 | -1.801763856 | 0.927815962026751 | 0.645988261204983 | 0.165007581809826 | 2.52897975376418 | 0.998735809224318 |
| ebi-fl187-GCST90094764 | ebi-gm430-GCST90011679 | OTU99_92 (Ruminococcus) prevalence \|\| id:ebi-gm430-GCST90011679 | Fried chicken liking \|\| id:ebi-fl187-GCST90094764 | Inverse variance weighted | 2 | 0.0352526539379823 | 0.53426309674865 | 0.947390805605476 | -1.011903016 | 1.08240832356534 | 1.03588139525397 | 0.363526524224702 | 2.9517798386839 | 0.998735809224318 |
| ebi-fl187-GCST90094764 | ebi-gm430-GCST90011684 | TestASV_11 (Lachnospiraceae) prevalence \|\| id:ebi-gm430-GCST90011684 | Fried chicken liking \|\| id:ebi-fl187-GCST90094764 | Inverse variance weighted | 2 | 0.195515124076074 | 0.614674990402363 | 0.750425114051254 | -1.009247857 | 1.4002781052647 | 1.21593718370957 | 0.364493027335099 | 4.05632789613915 | 0.998735809224318 |
| ebi-fl187-GCST90094764 | ebi-gm430-GCST90011688 | TestASV_15 (Bacteroides) prevalence \|\| id:ebi-gm430-GCST90011688 | Fried chicken liking \|\| id:ebi-fl187-GCST90094764 | Inverse variance weighted | 2 | -0.183851102 | 0.500979167542036 | 0.71363130594219 | -1.16577027 | 0.798068066757622 | 0.832059686938927 | 0.31168248987211 | 2.22124548258383 | 0.998735809224318 |
| ebi-fl187-GCST90094764 | ebi-gm430-GCST90011692 | TestASV_19 (Prevotella) prevalence \|\| id:ebi-gm430-GCST90011692 | Fried chicken liking \|\| id:ebi-fl187-GCST90094764 | Inverse variance weighted | 2 | -0.956705826 | 2.67779541844708 | 0.720886931152797 | -6.205184846 | 4.29177319439472 | 0.384156281636412 | 0.00201893558227585 | 73.0959670116661 | 0.998735809224318 |
| ebi-fl187-GCST90094764 | ebi-gm430-GCST90011693 | TestASV_2 (Bacteroides) prevalence \|\| id:ebi-gm430-GCST90011693 | Fried chicken liking \|\| id:ebi-fl187-GCST90094764 | Inverse variance weighted | 2 | 0.434185126268925 | 0.493343188344785 | 0.37881196179269 | -0.532767523 | 1.4011377754247 | 1.54370462194238 | 0.586978243992021 | 4.05981649949989 | 0.998735809224318 |
| ebi-fl187-GCST90094764 | ebi-gm430-GCST90011716 | TestASV_40 (Barnesiella) prevalence \|\| id:ebi-gm430-GCST90011716 | Fried chicken liking \|\| id:ebi-fl187-GCST90094764 | Inverse variance weighted | 2 | 0.72722939997727 | 0.999177723145365 | 0.46671895803258 | -1.231158937 | 2.68561773734219 | 2.06933934633733 | 0.291954025102968 | 14.6672590959811 | 0.998735809224318 |
| ebi-fl187-GCST90094764 | ebi-gm430-GCST90011718 | TestASV_42 (Alphaproteobacteria) prevalence \|\| id:ebi-gm430-GCST90011718 | Fried chicken liking \|\| id:ebi-fl187-GCST90094764 | Inverse variance weighted | 2 | -0.551536511 | 0.772190261250386 | 0.475072911122164 | -2.065029423 | 0.961956401538598 | 0.576064001633348 | 0.126814559303754 | 2.61681100182637 | 0.998735809224318 |
| ebi-fl187-GCST90094764 | ebi-hbsm44-GCST90104935 | Sulcal opening distance of the left calloso-marginal posterior fissure \|\| id:ebi-hbsm44-GCST90104935 | Fried chicken liking \|\| id:ebi-fl187-GCST90094764 | Inverse variance weighted | 2 | -0.118373893 | 0.079816354638848 | 0.138053642190036 | -0.274813949 | 0.0380661616114997 | 0.888363837064757 | 0.759713455849728 | 1.03879995928428 | 0.998735809224318 |
| ebi-fl187-GCST90094764 | ebi-hbsm44-GCST90104936 | Sulcal opening distance of the left anterior inferior temporal sulcus \|\| id:ebi-hbsm44-GCST90104936 | Fried chicken liking \|\| id:ebi-fl187-GCST90094764 | Inverse variance weighted | 2 | 0.0088364183053354 | 0.0454780894152954 | 0.845940546068649 | -0.080300637 | 0.0979734735593143 | 1.00887557469868 | 0.922838865217651 | 1.10293352781966 | 0.998735809224318 |
| ebi-fl187-GCST90094764 | ebi-hbsm44-GCST90104941 | Sulcal opening distance of the right posterior inferior temporal sulcus \|\| id:ebi-hbsm44-GCST90104941 | Fried chicken liking \|\| id:ebi-fl187-GCST90094764 | Inverse variance weighted | 2 | 0.0248049092106925 | 0.0371207686818178 | 0.503991981562391 | -0.047951797 | 0.0975616158270554 | 1.02511511049897 | 0.953179731706101 | 1.10247936964875 | 0.998735809224318 |
| ebi-fl187-GCST90094764 | ebi-IPs91-GCST90274819 | Leukemia inhibitory factor levels \|\| id:ebi-IPs91-GCST90274819 | Fried chicken liking \|\| id:ebi-fl187-GCST90094764 | Inverse variance weighted | 3 | 0.04708127277246 | 0.134964933983515 | 0.727208846588196 | -0.217449998 | 0.31161254338015 | 1.04820719632286 | 0.804567834047455 | 1.36562546988205 | 0.998735809224318 |
| ebi-fl187-GCST90094764 | ebi-IPs91-GCST90274837 | STAM binding protein levels \|\| id:ebi-IPs91-GCST90274837 | Fried chicken liking \|\| id:ebi-fl187-GCST90094764 | Inverse variance weighted | 3 | 0.0290118922639376 | 0.119420242766148 | 0.808052277137993 | -0.205051784 | 0.263075568085588 | 1.02943683673691 | 0.814605132167324 | 1.30092502365086 | 0.998735809224318 |
| ebi-fl187-GCST90094764 | ebi-IPs91-GCST90274840 | TNF-beta levels \|\| id:ebi-IPs91-GCST90274840 | Fried chicken liking \|\| id:ebi-fl187-GCST90094764 | Inverse variance weighted | 3 | 0.272034619180279 | 0.136515291624536 | 0.046293827794164 | 0.00446464759618864 | 0.53960459076437 | 1.31263244278371 | 1.0044746289842 | 1.71532847135298 | 0.998735809224318 |
| ebi-fl187-GCST90094764 | ebi-IPs91-GCST90274842 | Tumor necrosis factor ligand superfamily member 14 levels \|\| id:ebi-IPs91-GCST90274842 | Fried chicken liking \|\| id:ebi-fl187-GCST90094764 | Inverse variance weighted | 3 | 0.0363170700600188 | 0.232817180460847 | 0.876041180926888 | -0.420004604 | 0.492638743763278 | 1.03698459113718 | 0.657043795012869 | 1.63662917208567 | 0.998735809224318 |
| ebi-fl187-GCST90094764 | ebi-met1400-GCST90199628 | 3-methylhistidine levels \|\| id:ebi-met1400-GCST90199628 | Fried chicken liking \|\| id:ebi-fl187-GCST90094764 | Inverse variance weighted | 2 | 0.105665209419015 | 0.185565316755956 | 0.569068928874478 | -0.258042811 | 0.46937323026069 | 1.11144971131672 | 0.772562156913905 | 1.59899167947944 | 0.998735809224318 |
| ebi-fl187-GCST90094764 | ebi-met1400-GCST90199651 | Docosahexaenoate DHA; 22:6n3 levels \|\| id:ebi-met1400-GCST90199651 | Fried chicken liking \|\| id:ebi-fl187-GCST90094764 | Inverse variance weighted | 2 | -0.401660027 | 0.17797805592811 | 0.0240208730300178 | -0.750497017 | -0.052823038 | 0.669208219467355 | 0.472131836850528 | 0.948547854747717 | 0.998735809224318 |
| ebi-fl187-GCST90094764 | ebi-met1400-GCST90199652 | 1-stearoyl-2-oleoyl-GPS (18:0/18:1) levels \|\| id:ebi-met1400-GCST90199652 | Fried chicken liking \|\| id:ebi-fl187-GCST90094764 | Inverse variance weighted | 2 | -0.275210106 | 0.347602835890819 | 0.428513910363905 | -0.956511664 | 0.406091452575168 | 0.759412549502778 | 0.384230877293451 | 1.50093981099249 | 0.998735809224318 |
| ebi-fl187-GCST90094764 | ebi-met1400-GCST90199653 | 1-myristoyl-2-palmitoyl-gpc (14:0/16:0) levels \|\| id:ebi-met1400-GCST90199653 | Fried chicken liking \|\| id:ebi-fl187-GCST90094764 | Inverse variance weighted | 2 | 0.381576941860369 | 0.194854632550665 | 0.0501989646800486 | -0.000338138 | 0.763492021659673 | 1.46459234633607 | 0.999661919223255 | 2.1457561798622 | 0.998735809224318 |
| ebi-fl187-GCST90094764 | ebi-met1400-GCST90199667 | Erythritol levels in elite athletes \|\| id:ebi-met1400-GCST90199667 | Fried chicken liking \|\| id:ebi-fl187-GCST90094764 | Inverse variance weighted | 2 | -0.000220374 | 0.174471921232502 | 0.998992198761041 | -0.34218534 | 0.341744591549363 | 0.999779650214239 | 0.710216561198534 | 1.40740078955029 | 0.999882738470636 |
| ebi-fl187-GCST90094764 | ebi-met1400-GCST90199674 | 3-hydroxymyristate levels \|\| id:ebi-met1400-GCST90199674 | Fried chicken liking \|\| id:ebi-fl187-GCST90094764 | Inverse variance weighted | 2 | 0.122362450555246 | 0.181537759718952 | 0.500290299558596 | -0.233451558 | 0.478176459604392 | 1.13016365599889 | 0.791795950599628 | 1.61313011057143 | 0.998735809224318 |
| ebi-fl187-GCST90094764 | ebi-met1400-GCST90199685 | 2-linoleoylglycerol (18:2) levels \|\| id:ebi-met1400-GCST90199685 | Fried chicken liking \|\| id:ebi-fl187-GCST90094764 | Inverse variance weighted | 2 | 0.161827548034391 | 0.520788224431851 | 0.756001475485312 | -0.858917372 | 1.18257246792082 | 1.17565747939495 | 0.423620457579426 | 3.26275675342743 | 0.998735809224318 |
| ebi-fl187-GCST90094764 | ebi-met1400-GCST90199703 | Gamma-glutamylvaline levels \|\| id:ebi-met1400-GCST90199703 | Fried chicken liking \|\| id:ebi-fl187-GCST90094764 | Inverse variance weighted | 2 | 0.425740991553274 | 0.181589403360687 | 0.0190513063607433 | 0.069825760966327 | 0.781656222140221 | 1.530724253 | 1.07232132474443 | 2.18508826178257 | 0.998735809224318 |
| ebi-fl187-GCST90094764 | ebi-met1400-GCST90199779 | Gamma-glutamyl-2-aminobutyrate levels \|\| id:ebi-met1400-GCST90199779 | Fried chicken liking \|\| id:ebi-fl187-GCST90094764 | Inverse variance weighted | 2 | -0.025880126 | 0.193414911956889 | 0.893555778467909 | -0.404973353 | 0.353213101809486 | 0.974451894418478 | 0.66699458398532 | 1.42363449019649 | 0.998735809224318 |
| ebi-fl187-GCST90094764 | ebi-met1400-GCST90199824 | 4-vinylphenol sulfate levels \|\| id:ebi-met1400-GCST90199824 | Fried chicken liking \|\| id:ebi-fl187-GCST90094764 | Inverse variance weighted | 2 | -0.116622955 | 0.186196426317339 | 0.53108954199692 | -0.48156795 | 0.248322040746278 | 0.889920670199743 | 0.617813930360852 | 1.2818726809643 | 0.998735809224318 |
| ebi-fl187-GCST90094764 | ebi-met1400-GCST90199830 | 4-ethylphenylsulfate levels \|\| id:ebi-met1400-GCST90199830 | Fried chicken liking \|\| id:ebi-fl187-GCST90094764 | Inverse variance weighted | 2 | -0.002225026 | 0.186446797949424 | 0.990478402317888 | -0.36766075 | 0.363210698426448 | 0.997777447980036 | 0.692352022596939 | 1.43793879876788 | 0.999107451094609 |
| ebi-fl187-GCST90094764 | ebi-met1400-GCST90199833 | Hexanoylglutamine levels \|\| id:ebi-met1400-GCST90199833 | Fried chicken liking \|\| id:ebi-fl187-GCST90094764 | Inverse variance weighted | 2 | 0.0525641129613751 | 0.183548691113515 | 0.774589432727712 | -0.307191322 | 0.412319547543865 | 1.05397013305886 | 0.735509868504719 | 1.5103169773078 | 0.998735809224318 |
| ebi-fl187-GCST90094764 | ebi-met1400-GCST90199837 | Carotenoid levels (cryptoxanthin) \|\| id:ebi-met1400-GCST90199837 | Fried chicken liking \|\| id:ebi-fl187-GCST90094764 | Inverse variance weighted | 2 | -0.010388615 | 0.184563306451696 | 0.955112743946711 | -0.372132695 | 0.351355465967547 | 0.989665160601699 | 0.689262774509102 | 1.42099235056811 | 0.998735809224318 |
| ebi-fl187-GCST90094764 | ebi-met1400-GCST90199869 | 2-hydroxyhippurate levels \|\| id:ebi-met1400-GCST90199869 | Fried chicken liking \|\| id:ebi-fl187-GCST90094764 | Inverse variance weighted | 2 | -0.019571573 | 0.273261264474689 | 0.942902572851426 | -0.555163651 | 0.516020505589233 | 0.980618707068796 | 0.573978321296715 | 1.67534733102956 | 0.998735809224318 |
| ebi-fl187-GCST90094764 | ebi-met1400-GCST90199902 | Hydantoin-5-propionate levels \|\| id:ebi-met1400-GCST90199902 | Fried chicken liking \|\| id:ebi-fl187-GCST90094764 | Inverse variance weighted | 2 | 0.352916963769978 | 0.185783370314685 | 0.0574836009556394 | -0.011218442 | 0.71705236958676 | 1.42321296028819 | 0.988844250019401 | 2.04838641706469 | 0.998735809224318 |
| ebi-fl187-GCST90094764 | ebi-met1400-GCST90199904 | Pantoate levels \|\| id:ebi-met1400-GCST90199904 | Fried chicken liking \|\| id:ebi-fl187-GCST90094764 | Inverse variance weighted | 2 | -0.038004844 | 0.188023593804124 | 0.839816614776941 | -0.406531087 | 0.33052140030421 | 0.962708277952423 | 0.66595639243621 | 1.39169356877507 | 0.998735809224318 |
| ebi-fl187-GCST90094764 | ebi-met1400-GCST90199936 | 3-methyl catechol sulfate (2) levels \|\| id:ebi-met1400-GCST90199936 | Fried chicken liking \|\| id:ebi-fl187-GCST90094764 | Inverse variance weighted | 2 | 0.0207836325974511 | 0.212033464357298 | 0.921915991123482 | -0.394801958 | 0.436369222737755 | 1.02100111637725 | 0.673813469711184 | 1.54707990638775 | 0.998735809224318 |
| ebi-fl187-GCST90094764 | ebi-met1400-GCST90199977 | 4-hydroxychlorothalonil levels \|\| id:ebi-met1400-GCST90199977 | Fried chicken liking \|\| id:ebi-fl187-GCST90094764 | Inverse variance weighted | 2 | 0.238301467214125 | 0.184822476069936 | 0.197275293090918 | -0.123950586 | 0.600553520311199 | 1.26909172470832 | 0.883423493396126 | 1.82312765934214 | 0.998735809224318 |
| ebi-fl187-GCST90094764 | ebi-met1400-GCST90200040 | Sphingomyelin (d18:2/24:1, d18:1/24:2) levels \|\| id:ebi-met1400-GCST90200040 | Fried chicken liking \|\| id:ebi-fl187-GCST90094764 | Inverse variance weighted | 2 | 0.204890604368427 | 0.169711686707784 | 0.227321882068548 | -0.127744302 | 0.537525510315684 | 1.2273907864283 | 0.880078385030151 | 1.71176587021561 | 0.998735809224318 |
| ebi-fl187-GCST90094764 | ebi-met1400-GCST90200077 | 1-stearoyl-2-oleoyl-GPI (18:0/18:1) levels \|\| id:ebi-met1400-GCST90200077 | Fried chicken liking \|\| id:ebi-fl187-GCST90094764 | Inverse variance weighted | 2 | -0.242625134 | 0.214278409865607 | 0.25751285899607 | -0.662610818 | 0.177360548885353 | 0.784565565254387 | 0.515503689826781 | 1.19406153307995 | 0.998735809224318 |
| ebi-fl187-GCST90094764 | ebi-met1400-GCST90200080 | 1-palmitoyl-2-oleoyl-GPI (16:0/18:1) levels \|\| id:ebi-met1400-GCST90200080 | Fried chicken liking \|\| id:ebi-fl187-GCST90094764 | Inverse variance weighted | 2 | 0.149090018629682 | 0.185899550142157 | 0.422557378631114 | -0.2152731 | 0.51345313690831 | 1.1607774761039 | 0.806321204067725 | 1.67105161346713 | 0.998735809224318 |
| ebi-fl187-GCST90094764 | ebi-met1400-GCST90200082 | 1-oleoyl-2-linoleoyl-GPE (18:1/18:2) levels \|\| id:ebi-met1400-GCST90200082 | Fried chicken liking \|\| id:ebi-fl187-GCST90094764 | Inverse variance weighted | 2 | -0.643985377 | 0.35246070130202 | 0.0676828461685811 | -1.334808351 | 0.046837597944541 | 0.5251951471461 | 0.263208614248369 | 1.04795180573207 | 0.998735809224318 |
| ebi-fl187-GCST90094764 | ebi-met1400-GCST90200100 | Perfluorooctanesulfonate (PFOS) levels \|\| id:ebi-met1400-GCST90200100 | Fried chicken liking \|\| id:ebi-fl187-GCST90094764 | Inverse variance weighted | 2 | -0.118329099 | 0.312951680202479 | 0.705351396764051 | -0.731714393 | 0.49505619372519 | 0.888403631333712 | 0.481083516645687 | 1.64059042735444 | 0.998735809224318 |
| ebi-fl187-GCST90094764 | ebi-met1400-GCST90200102 | 1-palmitoleoyl-2-linolenoyl-GPC (16:1/18:3) levels \|\| id:ebi-met1400-GCST90200102 | Fried chicken liking \|\| id:ebi-fl187-GCST90094764 | Inverse variance weighted | 2 | 0.0826513744351106 | 0.215059833496819 | 0.700742736236574 | -0.338865899 | 0.504168648088876 | 1.08616307831781 | 0.712577999948889 | 1.65560855483252 | 0.998735809224318 |
| ebi-fl187-GCST90094764 | ebi-met1400-GCST90200114 | Glycosyl-N-tricosanoyl-sphingadienine (d18:2/23:0) levels \|\| id:ebi-met1400-GCST90200114 | Fried chicken liking \|\| id:ebi-fl187-GCST90094764 | Inverse variance weighted | 2 | -0.138952504 | 0.195464821118994 | 0.47715710451815 | -0.522063553 | 0.244158545491518 | 0.870269361875806 | 0.593294988073499 | 1.27654670517105 | 0.998735809224318 |
| ebi-fl187-GCST90094764 | ebi-met1400-GCST90200135 | Arachidoylcarnitine (C20) levels \|\| id:ebi-met1400-GCST90200135 | Fried chicken liking \|\| id:ebi-fl187-GCST90094764 | Inverse variance weighted | 2 | -0.509045814 | 0.532742697631644 | 0.339315259355814 | -1.553221502 | 0.535129873078633 | 0.601068836572548 | 0.211565316812256 | 1.70767000821444 | 0.998735809224318 |
| ebi-fl187-GCST90094764 | ebi-met1400-GCST90200146 | Docosahexaenoylcarnitine (C22:6) levels \|\| id:ebi-met1400-GCST90200146 | Fried chicken liking \|\| id:ebi-fl187-GCST90094764 | Inverse variance weighted | 2 | -0.108983936 | 0.209542463185849 | 0.602990882883183 | -0.519687164 | 0.301719292116391 | 0.896744822935393 | 0.594706564749609 | 1.35218160539392 | 0.998735809224318 |
| ebi-fl187-GCST90094764 | ebi-met1400-GCST90200162 | 3-hydroxyphenylacetoylglutamine levels \|\| id:ebi-met1400-GCST90200162 | Fried chicken liking \|\| id:ebi-fl187-GCST90094764 | Inverse variance weighted | 2 | -0.109961596 | 0.226977167089336 | 0.628058701231033 | -0.554836844 | 0.334913651017812 | 0.895868539143701 | 0.574165932187422 | 1.39781967970452 | 0.998735809224318 |
| ebi-fl187-GCST90094764 | ebi-met1400-GCST90200172 | 3-carboxy-4-methyl-5-pentyl-2-furanpropionate (3-CMPFP) levels \|\| id:ebi-met1400-GCST90200172 | Fried chicken liking \|\| id:ebi-fl187-GCST90094764 | Inverse variance weighted | 2 | 0.154153592840582 | 0.316488947943414 | 0.6262057676949 | -0.466164745 | 0.774471930809673 | 1.16667006518716 | 0.627403913794297 | 2.16944620694553 | 0.998735809224318 |
| ebi-fl187-GCST90094764 | ebi-met1400-GCST90200222 | Dihydrocaffeate sulfate (2) levels \|\| id:ebi-met1400-GCST90200222 | Fried chicken liking \|\| id:ebi-fl187-GCST90094764 | Inverse variance weighted | 2 | 0.120167924580201 | 0.21197240964207 | 0.570779670315115 | -0.295297998 | 0.53563384747866 | 1.1276862019127 | 0.744309751359991 | 1.70853084708443 | 0.998735809224318 |
| ebi-fl187-GCST90094764 | ebi-met1400-GCST90200223 | 4-methylhexanoylglutamine levels \|\| id:ebi-met1400-GCST90200223 | Fried chicken liking \|\| id:ebi-fl187-GCST90094764 | Inverse variance weighted | 2 | 0.361488127253867 | 0.224642424314201 | 0.107578904445196 | -0.078811024 | 0.801787278909702 | 1.43546397898374 | 0.924214561943008 | 2.2295221475713 | 0.998735809224318 |
| ebi-fl187-GCST90094764 | ebi-met1400-GCST90200224 | 2-ketocaprylate levels \|\| id:ebi-met1400-GCST90200224 | Fried chicken liking \|\| id:ebi-fl187-GCST90094764 | Inverse variance weighted | 2 | -0.105265821 | 0.292816091364505 | 0.719225071964024 | -0.67918536 | 0.46865371789374 | 0.900085229064721 | 0.507029870853783 | 1.59784159898959 | 0.998735809224318 |
| ebi-fl187-GCST90094764 | ebi-met1400-GCST90200243 | Hydroxypalmitoyl sphingomyelin (d18:1/16:0(OH)) levels \|\| id:ebi-met1400-GCST90200243 | Fried chicken liking \|\| id:ebi-fl187-GCST90094764 | Inverse variance weighted | 2 | 0.0585975316369799 | 0.181888379735346 | 0.747329939635789 | -0.297903693 | 0.415098755918258 | 1.06034839816656 | 0.742372832269512 | 1.514520301 | 0.998735809224318 |
| ebi-fl187-GCST90094764 | ebi-met1400-GCST90200273 | 2-methoxyhydroquinone sulfate (2) levels \|\| id:ebi-met1400-GCST90200273 | Fried chicken liking \|\| id:ebi-fl187-GCST90094764 | Inverse variance weighted | 2 | -0.240941363 | 0.395828703014253 | 0.542722584652236 | -1.016765621 | 0.534882895073712 | 0.785887707265079 | 0.361763129132738 | 1.7072483033608 | 0.998735809224318 |
| ebi-fl187-GCST90094764 | ebi-met1400-GCST90200276 | 2,4-di-tert-butylphenol levels \|\| id:ebi-met1400-GCST90200276 | Fried chicken liking \|\| id:ebi-fl187-GCST90094764 | Inverse variance weighted | 2 | -0.237170941 | 0.185238934959207 | 0.200421557569749 | -0.600239253 | 0.125897371977988 | 0.788856428945927 | 0.548680346935866 | 1.13416576512091 | 0.998735809224318 |
| ebi-fl187-GCST90094764 | ebi-met1400-GCST90200289 | N-acetylmethionine levels \|\| id:ebi-met1400-GCST90200289 | Fried chicken liking \|\| id:ebi-fl187-GCST90094764 | Inverse variance weighted | 2 | -0.042502403 | 0.185512753953181 | 0.818785197564114 | -0.406107401 | 0.321102594665563 | 0.958388162432194 | 0.66623860900205 | 1.37864701546789 | 0.998735809224318 |
| ebi-fl187-GCST90094764 | ebi-met1400-GCST90200308 | 3-hydroxyisobutyrate levels \|\| id:ebi-met1400-GCST90200308 | Fried chicken liking \|\| id:ebi-fl187-GCST90094764 | Inverse variance weighted | 2 | 0.221944314161405 | 0.188184404953884 | 0.238239724115126 | -0.14689712 | 0.590785747871017 | 1.24850185205588 | 0.863382798059621 | 1.80540645249145 | 0.998735809224318 |
| ebi-fl187-GCST90094764 | ebi-met1400-GCST90200383 | Cystathionine levels \|\| id:ebi-met1400-GCST90200383 | Fried chicken liking \|\| id:ebi-fl187-GCST90094764 | Inverse variance weighted | 2 | -0.003734099 | 0.305805068929585 | 0.990257499248996 | -0.603112034 | 0.595643835838989 | 0.996272863816016 | 0.547106370221853 | 1.81419861511333 | 0.999107451094609 |
| ebi-fl187-GCST90094764 | ebi-met1400-GCST90200384 | Guanidinoacetate levels \|\| id:ebi-met1400-GCST90200384 | Fried chicken liking \|\| id:ebi-fl187-GCST90094764 | Inverse variance weighted | 2 | 0.0788650877410513 | 0.178006187189408 | 0.657731818569808 | -0.270027039 | 0.427757214632291 | 1.08205832928891 | 0.763358853483112 | 1.53381364824821 | 0.998735809224318 |
| ebi-fl187-GCST90094764 | ebi-met1400-GCST90200403 | Citrulline levels \|\| id:ebi-met1400-GCST90200403 | Fried chicken liking \|\| id:ebi-fl187-GCST90094764 | Inverse variance weighted | 2 | 0.0318723470210909 | 0.186204466607834 | 0.864091242442263 | -0.333088408 | 0.396833101572445 | 1.03238570978202 | 0.716706829074084 | 1.48710771339943 | 0.998735809224318 |
| ebi-fl187-GCST90094764 | ebi-met1400-GCST90200434 | 12,13-DiHOME levels \|\| id:ebi-met1400-GCST90200434 | Fried chicken liking \|\| id:ebi-fl187-GCST90094764 | Inverse variance weighted | 2 | -0.344123183 | 0.187800904581266 | 0.0668943804043644 | -0.712212956 | 0.023966589796692 | 0.708841605306381 | 0.490557413351057 | 1.02425609671453 | 0.998735809224318 |
| ebi-fl187-GCST90094764 | ebi-met1400-GCST90200437 | Fructose levels \|\| id:ebi-met1400-GCST90200437 | Fried chicken liking \|\| id:ebi-fl187-GCST90094764 | Inverse variance weighted | 2 | -0.156338577 | 0.187279972952764 | 0.403838577144907 | -0.523407324 | 0.210730169652112 | 0.855269566452961 | 0.592498270880225 | 1.23457918318298 | 0.998735809224318 |
| ebi-fl187-GCST90094764 | ebi-met1400-GCST90200448 | N-stearoyl-sphinganine (d18:0/18:0) levels \|\| id:ebi-met1400-GCST90200448 | Fried chicken liking \|\| id:ebi-fl187-GCST90094764 | Inverse variance weighted | 2 | 0.560654018511904 | 0.293135456316451 | 0.0557979052575578 | -0.013891476 | 1.13519951289215 | 1.75181784701773 | 0.986204565449672 | 3.11179432406151 | 0.998735809224318 |
| ebi-fl187-GCST90094764 | ebi-met1400-GCST90200468 | X-11847 levels \|\| id:ebi-met1400-GCST90200468 | Fried chicken liking \|\| id:ebi-fl187-GCST90094764 | Inverse variance weighted | 2 | -0.322864737 | 0.328678874456073 | 0.325946861809749 | -0.967075331 | 0.321345856766745 | 0.724071787745585 | 0.380193353335829 | 1.37898242883269 | 0.998735809224318 |
| ebi-fl187-GCST90094764 | ebi-met1400-GCST90200476 | X-11843 levels \|\| id:ebi-met1400-GCST90200476 | Fried chicken liking \|\| id:ebi-fl187-GCST90094764 | Inverse variance weighted | 2 | 0.015293772900617 | 0.196708339502233 | 0.938028135388162 | -0.370254573 | 0.400842118324993 | 1.01541132113286 | 0.690558511035927 | 1.49308151967899 | 0.998735809224318 |
| ebi-fl187-GCST90094764 | ebi-met1400-GCST90200511 | X-12906 levels \|\| id:ebi-met1400-GCST90200511 | Fried chicken liking \|\| id:ebi-fl187-GCST90094764 | Inverse variance weighted | 2 | 0.364702622806067 | 0.186307452846472 | 0.0502850655202699 | -0.000459985 | 0.729865230385153 | 1.44008569582397 | 0.999540121003758 | 2.07480096870369 | 0.998735809224318 |
| ebi-fl187-GCST90094764 | ebi-met1400-GCST90200515 | X-12849 levels \|\| id:ebi-met1400-GCST90200515 | Fried chicken liking \|\| id:ebi-fl187-GCST90094764 | Inverse variance weighted | 2 | 0.15980178069761 | 0.195667269600825 | 0.414098951780301 | -0.223706068 | 0.543309629115226 | 1.17327828153824 | 0.799550113421516 | 1.72169561709961 | 0.998735809224318 |
| ebi-fl187-GCST90094764 | ebi-met1400-GCST90200551 | X-18886 levels \|\| id:ebi-met1400-GCST90200551 | Fried chicken liking \|\| id:ebi-fl187-GCST90094764 | Inverse variance weighted | 2 | 0.064416106755246 | 0.18351087580838 | 0.725572857085301 | -0.29526521 | 0.424097423339671 | 1.06653609931734 | 0.744334156552246 | 1.52821046989963 | 0.998735809224318 |
| ebi-fl187-GCST90094764 | ebi-met1400-GCST90200564 | X-21319 levels \|\| id:ebi-met1400-GCST90200564 | Fried chicken liking \|\| id:ebi-fl187-GCST90094764 | Inverse variance weighted | 2 | -0.252415266 | 0.188402442090608 | 0.180321294839183 | -0.621684053 | 0.116853520446744 | 0.776922041723082 | 0.537039273279683 | 1.12395478123778 | 0.998735809224318 |
| ebi-fl187-GCST90094764 | ebi-met1400-GCST90200579 | X-21442 levels \|\| id:ebi-met1400-GCST90200579 | Fried chicken liking \|\| id:ebi-fl187-GCST90094764 | Inverse variance weighted | 2 | -0.084612193 | 0.203395583594352 | 0.677411332949617 | -0.483267537 | 0.314043150680498 | 0.918868558822264 | 0.616764793890347 | 1.36894880634548 | 0.998735809224318 |
| ebi-fl187-GCST90094764 | ebi-met1400-GCST90200581 | X-21441 levels \|\| id:ebi-met1400-GCST90200581 | Fried chicken liking \|\| id:ebi-fl187-GCST90094764 | Inverse variance weighted | 2 | 0.451792462692776 | 0.186327911953818 | 0.0153203226863315 | 0.0865897552632928 | 0.816995170122259 | 1.57112584751205 | 1.09044923688394 | 2.26368761169868 | 0.998735809224318 |
| ebi-fl187-GCST90094764 | ebi-met1400-GCST90200648 | X-24585 levels \|\| id:ebi-met1400-GCST90200648 | Fried chicken liking \|\| id:ebi-fl187-GCST90094764 | Inverse variance weighted | 2 | 0.203627386863592 | 0.182033443648969 | 0.263299623671469 | -0.153158163 | 0.56041293641557 | 1.22584130377446 | 0.857994008440287 | 1.75139556600302 | 0.998735809224318 |
| ebi-fl187-GCST90094764 | ebi-met1400-GCST90200720 | Adenosine 5'-diphosphate (ADP) to pantothenate ratio \|\| id:ebi-met1400-GCST90200720 | Fried chicken liking \|\| id:ebi-fl187-GCST90094764 | Inverse variance weighted | 2 | -0.385301476 | 0.245509293459481 | 0.116555531627326 | -0.866499691 | 0.0958967392622531 | 0.680245527643359 | 0.420420578612977 | 1.10064540466932 | 0.998735809224318 |
| ebi-fl187-GCST90094764 | ebi-met1400-GCST90200724 | Cholate to taurocholate ratio \|\| id:ebi-met1400-GCST90200724 | Fried chicken liking \|\| id:ebi-fl187-GCST90094764 | Inverse variance weighted | 2 | 0.0660383489615931 | 0.199963797082983 | 0.741209917263735 | -0.325890693 | 0.457967391244241 | 1.06826768333684 | 0.721884089871803 | 1.58085745242637 | 0.998735809224318 |
| ebi-fl187-GCST90094764 | ebi-met1400-GCST90200726 | N-acetylputrescine to (N(1) + N(8))-acetylspermidine ratio \|\| id:ebi-met1400-GCST90200726 | Fried chicken liking \|\| id:ebi-fl187-GCST90094764 | Inverse variance weighted | 2 | 0.0627926707298672 | 0.185094428502215 | 0.734423904959664 | -0.299992409 | 0.425577750594209 | 1.06480605088279 | 0.740823844154553 | 1.5304743967718 | 0.998735809224318 |
| ebi-fl187-GCST90094764 | ebi-met1400-GCST90200744 | Arginine to glutamate ratio \|\| id:ebi-met1400-GCST90200744 | Fried chicken liking \|\| id:ebi-fl187-GCST90094764 | Inverse variance weighted | 2 | -0.374427805 | 0.179577050924725 | 0.0370644211017832 | -0.726398825 | -0.022456785 | 0.687682654920365 | 0.483647557427231 | 0.977793491595323 | 0.998735809224318 |
| ebi-fl187-GCST90094764 | ebi-met1400-GCST90200759 | Histidine to trans-urocanate ratio \|\| id:ebi-met1400-GCST90200759 | Fried chicken liking \|\| id:ebi-fl187-GCST90094764 | Inverse variance weighted | 2 | 0.0558905681200552 | 0.400954275474578 | 0.889138916164172 | -0.729979812 | 0.841760948050228 | 1.05748195516617 | 0.481918719058605 | 2.32044957225675 | 0.998735809224318 |
| ebi-fl187-GCST90094764 | ebi-met1400-GCST90200795 | Oleoyl-linoleoyl-glycerol (18:1 to 18:2) [2] to linoleoyl-arachidonoyl-glycerol (18:2 to 20:4) [2] ratio \|\| id:ebi-met1400-GCST90200795 | Fried chicken liking \|\| id:ebi-fl187-GCST90094764 | Inverse variance weighted | 2 | 0.263594498589996 | 0.19340116047359 | 0.172900897940746 | -0.115471776 | 0.642660773118231 | 1.3016002885222 | 0.890945717989188 | 1.9015337038767 | 0.998735809224318 |
| ebi-fl187-GCST90094764 | ebi-met1400-GCST90200811 | Choline to taurocholate ratio \|\| id:ebi-met1400-GCST90200811 | Fried chicken liking \|\| id:ebi-fl187-GCST90094764 | Inverse variance weighted | 2 | -0.025395183 | 0.364725500497573 | 0.944489585242626 | -0.740257164 | 0.689466798219788 | 0.974924562516631 | 0.476991234901731 | 1.99265276393194 | 0.998735809224318 |
| ebi-fl187-GCST90094764 | ebi-met1400-GCST90200831 | Cholate to phosphate ratio \|\| id:ebi-met1400-GCST90200831 | Fried chicken liking \|\| id:ebi-fl187-GCST90094764 | Inverse variance weighted | 2 | -0.120017838 | 0.194145563293372 | 0.5364535240614 | -0.500543142 | 0.260507466542617 | 0.886904616403975 | 0.606201317147355 | 1.29758840231532 | 0.998735809224318 |
| ebi-fl187-GCST90094764 | ebi-met1400-GCST90200841 | Adenosine 5'-diphosphate (ADP) to oxalate (ethanedioate) ratio \|\| id:ebi-met1400-GCST90200841 | Fried chicken liking \|\| id:ebi-fl187-GCST90094764 | Inverse variance weighted | 2 | -0.04496047 | 0.249141389492329 | 0.856790148510562 | -0.533277593 | 0.443356653860483 | 0.956035273595988 | 0.586678920306881 | 1.55792787625923 | 0.998735809224318 |
| ebi-fl187-GCST90094764 | ebi-met1400-GCST90200844 | Aspartate to phosphate ratio \|\| id:ebi-met1400-GCST90200844 | Fried chicken liking \|\| id:ebi-fl187-GCST90094764 | Inverse variance weighted | 2 | 0.202118435349016 | 0.335178805390196 | 0.54649754348377 | -0.454832023 | 0.8590688939138 | 1.2239929635611 | 0.634554549429002 | 2.36096136446453 | 0.998735809224318 |
| ebi-fl187-GCST90094764 | ebi-met1400-GCST90200870 | Adenosine 5'-monophosphate (AMP) to acetoacetate ratio \|\| id:ebi-met1400-GCST90200870 | Fried chicken liking \|\| id:ebi-fl187-GCST90094764 | Inverse variance weighted | 2 | -0.129222159 | 0.204078664817473 | 0.526604489076911 | -0.529216342 | 0.270772023599498 | 0.878778714868401 | 0.589066415170212 | 1.31097616468699 | 0.998735809224318 |
| ebi-fl187-GCST90094764 | ebi-met1400-GCST90200887 | Cholesterol to cortisol ratio \|\| id:ebi-met1400-GCST90200887 | Fried chicken liking \|\| id:ebi-fl187-GCST90094764 | Inverse variance weighted | 2 | -0.087992443 | 0.319671251829264 | 0.78311713086535 | -0.714548097 | 0.538563210144717 | 0.915767796750895 | 0.489413229124536 | 1.71354309131801 | 0.998735809224318 |
| ebi-fl187-GCST90094764 | ebi-met1400-GCST90200890 | Cortisol to taurocholate ratio \|\| id:ebi-met1400-GCST90200890 | Fried chicken liking \|\| id:ebi-fl187-GCST90094764 | Inverse variance weighted | 2 | 0.0616546401207949 | 0.320718560611026 | 0.84755493755674 | -0.566953739 | 0.690263018918406 | 1.0635949582651 | 0.567250803592655 | 1.99423998711387 | 0.998735809224318 |
| ebi-fl187-GCST90094764 | ebi-met1400-GCST90200948 | Adenosine 5'-diphosphate (ADP) to mannitol to sorbitol ratio \|\| id:ebi-met1400-GCST90200948 | Fried chicken liking \|\| id:ebi-fl187-GCST90094764 | Inverse variance weighted | 2 | -0.410153195 | 0.24814610749238 | 0.098357374354655 | -0.896519566 | 0.0762131755200874 | 0.663548589913889 | 0.407987164002916 | 1.07919260708301 | 0.998735809224318 |
| ebi-fl187-GCST90094764 | ebi-met1400-GCST90200972 | Leucine to N-palmitoyl-sphingosine (d18:1 to 16:0) ratio \|\| id:ebi-met1400-GCST90200972 | Fried chicken liking \|\| id:ebi-fl187-GCST90094764 | Inverse variance weighted | 2 | 0.430896372157171 | 0.407612355069711 | 0.29045548185306 | -0.368023844 | 1.2298165880938 | 1.53863609615554 | 0.692100679196031 | 3.42060209960033 | 0.998735809224318 |
| ebi-fl187-GCST90094764 | ebi-met1400-GCST90200973 | Proline to glutamate ratio \|\| id:ebi-met1400-GCST90200973 | Fried chicken liking \|\| id:ebi-fl187-GCST90094764 | Inverse variance weighted | 2 | -0.438157302 | 0.182572821053103 | 0.0163993578024561 | -0.796000031 | -0.080314573 | 0.645224279945766 | 0.451129865397634 | 0.922826005023155 | 0.998735809224318 |
| ebi-fl187-GCST90094764 | ebi-met1400-GCST90200983 | Cholesterol to linoleoyl-arachidonoyl-glycerol (18:2 to 20:4) [1] ratio \|\| id:ebi-met1400-GCST90200983 | Fried chicken liking \|\| id:ebi-fl187-GCST90094764 | Inverse variance weighted | 2 | 0.456196103620845 | 0.587393200220293 | 0.437368132214996 | -0.695094569 | 1.60748677605262 | 1.57805977766134 | 0.499027253339631 | 4.99025382923869 | 0.998735809224318 |
| ebi-fl187-GCST90094764 | ebi-met1400-GCST90201001 | Serine to alpha-ketobutyrate ratio \|\| id:ebi-met1400-GCST90201001 | Fried chicken liking \|\| id:ebi-fl187-GCST90094764 | Inverse variance weighted | 2 | -0.25415969 | 0.282617465616456 | 0.368489489259915 | -0.808089923 | 0.299770542284878 | 0.775567941461699 | 0.445708591439682 | 1.34954910759116 | 0.998735809224318 |
| ebi-fl187-GCST90094764 | ebi-met1400-GCST90201005 | Fructose to maltose ratio \|\| id:ebi-met1400-GCST90201005 | Fried chicken liking \|\| id:ebi-fl187-GCST90094764 | Inverse variance weighted | 2 | -0.368767161 | 0.212040846251702 | 0.0820114308995317 | -0.78436722 | 0.0468328975544987 | 0.691586419927021 | 0.456408416660474 | 1.04794687996141 | 0.998735809224318 |
| ebi-fl187-GCST90094764 | ebi-met1400-GCST90201020 | Paraxanthine to 5-acetylamino-6-formylamino-3-methyluracil ratio \|\| id:ebi-met1400-GCST90201020 | Fried chicken liking \|\| id:ebi-fl187-GCST90094764 | Inverse variance weighted | 2 | 0.12924810926636 | 0.299795074873386 | 0.666380011714228 | -0.458350237 | 0.716846456018196 | 1.13797243064776 | 0.632325973161452 | 2.04796466993098 | 0.998735809224318 |
| ebi-fl187-GCST90094764 | ebi-pw11-GCST90275197 | Placental weight (fetal genotype, adjusted for sex) \|\| id:ebi-pw11-GCST90275197 | Fried chicken liking \|\| id:ebi-fl187-GCST90094764 | Inverse variance weighted | 2 | 0.145424259246657 | 0.0641407138745214 | 0.0233737912885647 | 0.0197084600525948 | 0.271140058440719 | 1.15653013480112 | 1.01990395393381 | 1.31145873838813 | 0.998735809224318 |
| ebi-fl187-GCST90094764 | prot-a-1017 | FAS-associated factor 2 \|\| id:prot-a-1017 | Fried chicken liking \|\| id:ebi-fl187-GCST90094764 | Inverse variance weighted | 3 | 0.247634238206299 | 0.239769524731267 | 0.301696970682371 | -0.22231403 | 0.717582506679582 | 1.28099130881815 | 0.80066389215597 | 2.04947263058041 | 0.998735809224318 |
| ebi-fl187-GCST90094764 | prot-a-1019 | Fas apoptotic inhibitory molecule 3 \|\| id:prot-a-1019 | Fried chicken liking \|\| id:ebi-fl187-GCST90094764 | Inverse variance weighted | 3 | 0.283574026266149 | 0.240075455324726 | 0.237528412324558 | -0.186973866 | 0.754121918702611 | 1.32786717372448 | 0.829465413209993 | 2.12574412745122 | 0.998735809224318 |
| ebi-fl187-GCST90094764 | prot-a-1052 | MIP18 family protein FAM96A \|\| id:prot-a-1052 | Fried chicken liking \|\| id:ebi-fl187-GCST90094764 | Inverse variance weighted | 3 | 0.155367743405628 | 0.239769524731267 | 0.516992796379945 | -0.314580525 | 0.625316011878912 | 1.16808743858427 | 0.730095066623236 | 1.86883643864212 | 0.998735809224318 |
| ebi-fl187-GCST90094764 | prot-a-1066 | F-box/LRR-repeat protein 4 \|\| id:prot-a-1066 | Fried chicken liking \|\| id:ebi-fl187-GCST90094764 | Inverse variance weighted (multiplicative random effects) | 3 | -0.182095626 | 0.498254802531527 | 0.714762929023092 | -1.158675039 | 0.794483787232102 | 0.833521630490507 | 0.313901813230614 | 2.21329816908428 | 0.998735809224318 |
| ebi-fl187-GCST90094764 | prot-a-1076 | Low affinity immunoglobulin gamma Fc region receptor III-B \|\| id:prot-a-1076 | Fried chicken liking \|\| id:ebi-fl187-GCST90094764 | Inverse variance weighted | 3 | -0.127446573 | 0.239769524731267 | 0.595046134468075 | -0.597394841 | 0.342501695910368 | 0.880340448911729 | 0.550243241617542 | 1.40846674229355 | 0.998735809224318 |
| ebi-fl187-GCST90094764 | prot-a-1081 | Fc receptor-like protein 3 \|\| id:prot-a-1081 | Fried chicken liking \|\| id:ebi-fl187-GCST90094764 | Inverse variance weighted | 3 | -0.00666093 | 0.239769524731267 | 0.977837175168387 | -0.476609199 | 0.463287338103145 | 0.99336120445312 | 0.620885124511866 | 1.58928994037076 | 0.998955228396052 |
| ebi-fl187-GCST90094764 | prot-a-1105 | Fibroblast growth factor receptor 4 \|\| id:prot-a-1105 | Fried chicken liking \|\| id:ebi-fl187-GCST90094764 | Inverse variance weighted | 3 | 0.225347626886453 | 0.308667856648852 | 0.46535039829358 | -0.379641372 | 0.830336625918203 | 1.25275813291542 | 0.684106704944768 | 2.29409086074145 | 0.998735809224318 |
| ebi-fl187-GCST90094764 | prot-a-1143 | Follistatin-related protein 1 \|\| id:prot-a-1143 | Fried chicken liking \|\| id:ebi-fl187-GCST90094764 | Inverse variance weighted | 3 | -0.143613015 | 0.239769524731267 | 0.549197720624186 | -0.613561284 | 0.326335253012677 | 0.866222898075309 | 0.541419283857184 | 1.3858799114143 | 0.998735809224318 |
| ebi-fl187-GCST90094764 | prot-a-1151 | Alpha-(1,3)-fucosyltransferase 10 \|\| id:prot-a-1151 | Fried chicken liking \|\| id:ebi-fl187-GCST90094764 | Inverse variance weighted | 3 | -0.07028454 | 0.239769524731267 | 0.769419919490102 | -0.540232809 | 0.399663728239397 | 0.932128554091547 | 0.582612599297971 | 1.49132312346103 | 0.998735809224318 |
| ebi-fl187-GCST90094764 | prot-a-1165 | Growth arrest and DNA damage-inducible proteins-interacting protein 1 \|\| id:prot-a-1165 | Fried chicken liking \|\| id:ebi-fl187-GCST90094764 | Inverse variance weighted | 3 | -0.184518203 | 0.239769524731267 | 0.441558051315153 | -0.654466471 | 0.28543006568334 | 0.831504804054363 | 0.519719273798026 | 1.33033403612847 | 0.998735809224318 |
| ebi-fl187-GCST90094764 | prot-a-1194 | Growth/differentiation factor 11/8 \|\| id:prot-a-1194 | Fried chicken liking \|\| id:ebi-fl187-GCST90094764 | Inverse variance weighted | 3 | -0.468608945 | 0.331353174899596 | 0.157294963157393 | -1.118061168 | 0.180843278054542 | 0.625872285951121 | 0.326913010127046 | 1.19822737605166 | 0.998735809224318 |
| ebi-fl187-GCST90094764 | prot-a-1216 | D-glucuronyl C5-epimerase \|\| id:prot-a-1216 | Fried chicken liking \|\| id:ebi-fl187-GCST90094764 | Inverse variance weighted | 3 | 0.15939726457413 | 0.30537191984835 | 0.601686011826091 | -0.439131698 | 0.757926227476896 | 1.17280376753678 | 0.644595881839192 | 2.13384651671045 | 0.998735809224318 |
| ebi-fl187-GCST90094764 | prot-a-1246 | Glypican-3 \|\| id:prot-a-1246 | Fried chicken liking \|\| id:ebi-fl187-GCST90094764 | Inverse variance weighted | 3 | -0.069352858 | 0.239769524731267 | 0.772391592606036 | -0.539301127 | 0.4005954099783 | 0.932997405927549 | 0.583155661758998 | 1.49271320944027 | 0.998735809224318 |
| ebi-fl187-GCST90094764 | prot-a-1275 | Glutamate receptor 4 \|\| id:prot-a-1275 | Fried chicken liking \|\| id:ebi-fl187-GCST90094764 | Inverse variance weighted | 3 | -0.084913561 | 0.239769524731267 | 0.723229024810093 | -0.554861829 | 0.385034707911663 | 0.918591683519024 | 0.574151586794922 | 1.46966532957038 | 0.998735809224318 |
| ebi-fl187-GCST90094764 | prot-a-1289 | Glutathione S-transferase P \|\| id:prot-a-1289 | Fried chicken liking \|\| id:ebi-fl187-GCST90094764 | Inverse variance weighted | 3 | -0.07238153 | 0.257557624489959 | 0.778687060938542 | -0.577194474 | 0.432431414288052 | 0.930175938343986 | 0.561471381686953 | 1.54099978110108 | 0.998735809224318 |
| ebi-fl187-GCST90094764 | prot-a-130 | Apolipoprotein D \|\| id:prot-a-130 | Fried chicken liking \|\| id:ebi-fl187-GCST90094764 | Inverse variance weighted | 3 | 0.0161403585527409 | 0.240075455324726 | 0.946398318449517 | -0.454407534 | 0.486688250989203 | 1.01627131776738 | 0.634823968244499 | 1.62691933981747 | 0.998735809224318 |
| ebi-fl187-GCST90094764 | prot-a-1313 | Heparin-binding EGF-like growth factor \|\| id:prot-a-1313 | Fried chicken liking \|\| id:ebi-fl187-GCST90094764 | Inverse variance weighted | 3 | 0.462828900186708 | 0.272413994162867 | 0.089320896108119 | -0.071102528 | 0.996760328745928 | 1.58856151658318 | 0.931366395751363 | 2.70948973838941 | 0.998735809224318 |
| ebi-fl187-GCST90094764 | prot-a-1347 | HLA class II histocompatibility antigen, DQ alpha 2 chain \|\| id:prot-a-1347 | Fried chicken liking \|\| id:ebi-fl187-GCST90094764 | Inverse variance weighted | 3 | -0.242782976 | 0.287108526349869 | 0.397768139246144 | -0.805515688 | 0.319949735781635 | 0.784441738089862 | 0.44685742833746 | 1.37705854582492 | 0.998735809224318 |
| ebi-fl187-GCST90094764 | prot-a-1357 | Non-histone chromosomal protein HMG-14 \|\| id:prot-a-1357 | Fried chicken liking \|\| id:ebi-fl187-GCST90094764 | Inverse variance weighted | 3 | 0.20643676738247 | 0.239769524731267 | 0.389249053860181 | -0.263511501 | 0.676385035855753 | 1.229290001 | 0.768348785539253 | 1.96675511675958 | 0.998735809224318 |
| ebi-fl187-GCST90094764 | prot-a-137 | Apolipoprotein M \|\| id:prot-a-137 | Fried chicken liking \|\| id:ebi-fl187-GCST90094764 | Inverse variance weighted | 3 | -0.196084814 | 0.240075455324726 | 0.414063726231328 | -0.666632706 | 0.274463078499945 | 0.821942519541148 | 0.513434555124804 | 1.31582399097667 | 0.998735809224318 |
| ebi-fl187-GCST90094764 | prot-a-1381 | 17-beta-hydroxysteroid dehydrogenase 14 \|\| id:prot-a-1381 | Fried chicken liking \|\| id:ebi-fl187-GCST90094764 | Inverse variance weighted | 3 | 0.032942676446085 | 0.240075455324726 | 0.890858474928208 | -0.437605216 | 0.503490568882547 | 1.03349129414933 | 0.645580597452418 | 1.65448630162895 | 0.998735809224318 |
| ebi-fl187-GCST90094764 | prot-a-1392 | Serine protease HTRA2, mitochondrial \|\| id:prot-a-1392 | Fried chicken liking \|\| id:ebi-fl187-GCST90094764 | Inverse variance weighted | 3 | 0.335846543929265 | 0.253853383520681 | 0.185837319902583 | -0.161706088 | 0.833399175629799 | 1.39912430421807 | 0.850691196346088 | 2.30112739741731 | 0.998735809224318 |
| ebi-fl187-GCST90094764 | prot-a-1416 | Interferon-induced protein with tetratricopeptide repeats 2 \|\| id:prot-a-1416 | Fried chicken liking \|\| id:ebi-fl187-GCST90094764 | Inverse variance weighted | 3 | 0.199340371452028 | 0.410862399964375 | 0.627551857662384 | -0.605949932 | 1.0046306753822 | 1.22059735169607 | 0.545555939109421 | 2.73089849851059 | 0.998735809224318 |
| ebi-fl187-GCST90094764 | prot-a-1428 | Interferon gamma \|\| id:prot-a-1428 | Fried chicken liking \|\| id:ebi-fl187-GCST90094764 | Inverse variance weighted | 3 | 0.0951918673726308 | 0.239769524731267 | 0.691357108192786 | -0.374756401 | 0.565140135845914 | 1.09986986402364 | 0.687456722096494 | 1.75969436170206 | 0.998735809224318 |
| ebi-fl187-GCST90094764 | prot-a-1483 | Interleukin-17C \|\| id:prot-a-1483 | Fried chicken liking \|\| id:ebi-fl187-GCST90094764 | Inverse variance weighted | 3 | -0.335823985 | 0.240075455324726 | 0.161864921980501 | -0.806371878 | 0.13472390701726 | 0.714748900973022 | 0.446474997061707 | 1.14422082939515 | 0.998735809224318 |
| ebi-fl187-GCST90094764 | prot-a-1503 | Interleukin-1 receptor-like 2 \|\| id:prot-a-1503 | Fried chicken liking \|\| id:ebi-fl187-GCST90094764 | Inverse variance weighted | 3 | 0.146024170385002 | 0.29317335764293 | 0.618426732466045 | -0.428595611 | 0.720643951365146 | 1.15722415826621 | 0.651423304606789 | 2.05575659176529 | 0.998735809224318 |
| ebi-fl187-GCST90094764 | prot-a-1522 | Interleukin-31 receptor subunit alpha \|\| id:prot-a-1522 | Fried chicken liking \|\| id:ebi-fl187-GCST90094764 | Inverse variance weighted | 3 | 0.0779556477076576 | 0.334964538129684 | 0.815972577332355 | -0.578574847 | 0.734486142441839 | 1.0810747094652 | 0.560696876251432 | 2.08441062710895 | 0.998735809224318 |
| ebi-fl187-GCST90094764 | prot-a-1540 | Interleukin-6 receptor subunit alpha \|\| id:prot-a-1540 | Fried chicken liking \|\| id:ebi-fl187-GCST90094764 | Inverse variance weighted | 3 | 0.0886114371285793 | 0.243563375791711 | 0.715997915917544 | -0.388772779 | 0.565995653680333 | 1.09265600827439 | 0.6778882826825 | 1.76120045576493 | 0.998735809224318 |
| ebi-fl187-GCST90094764 | prot-a-1541 | Interleukin-6 receptor subunit alpha \|\| id:prot-a-1541 | Fried chicken liking \|\| id:ebi-fl187-GCST90094764 | Inverse variance weighted | 3 | 0.178588205907574 | 0.262669454565076 | 0.496569604771507 | -0.336243925 | 0.693420336855123 | 1.19552833128433 | 0.714448812604507 | 2.00054638721151 | 0.998735809224318 |
| ebi-fl187-GCST90094764 | prot-a-1545 | Interleukin-9 \|\| id:prot-a-1545 | Fried chicken liking \|\| id:ebi-fl187-GCST90094764 | Inverse variance weighted | 3 | 0.650650795734486 | 0.239769524731267 | 0.0066546244879699 | 0.180702527261203 | 1.12059906420777 | 1.91678786055204 | 1.19805873646624 | 3.06669079781232 | 0.998735809224318 |
| ebi-fl187-GCST90094764 | prot-a-1548 | Inositol monophosphatase 3 \|\| id:prot-a-1548 | Fried chicken liking \|\| id:ebi-fl187-GCST90094764 | Inverse variance weighted | 3 | 0.633019221055579 | 0.371708617323688 | 0.088568347445246 | -0.095529669 | 1.36156811101001 | 1.88328806714205 | 0.908891395446768 | 3.90230775822917 | 0.998735809224318 |
| ebi-fl187-GCST90094764 | prot-a-1555 | Inhibin beta A chain:Inhibin beta B chain heterodimer \|\| id:prot-a-1555 | Fried chicken liking \|\| id:ebi-fl187-GCST90094764 | Inverse variance weighted | 3 | 0.240536043259379 | 0.239769524731267 | 0.315765869761047 | -0.229412225 | 0.710484311732662 | 1.27193077753394 | 0.795000746595902 | 2.03497658306027 | 0.998735809224318 |
| ebi-fl187-GCST90094764 | prot-a-1603 | Junctophilin-3 \|\| id:prot-a-1603 | Fried chicken liking \|\| id:ebi-fl187-GCST90094764 | Inverse variance weighted | 3 | 0.275478208049153 | 0.322840856794209 | 0.393496214435744 | -0.357289871 | 0.908246287365804 | 1.31716040099135 | 0.699569683190208 | 2.47996956361527 | 0.998735809224318 |
| ebi-fl187-GCST90094764 | prot-a-1627 | UPF0258 protein KIAA1024 \|\| id:prot-a-1627 | Fried chicken liking \|\| id:ebi-fl187-GCST90094764 | Inverse variance weighted | 3 | 0.390008023476984 | 0.324586297596449 | 0.229536302042082 | -0.24618112 | 1.02619716676602 | 1.47699264445159 | 0.781780616143522 | 2.79043407666635 | 0.998735809224318 |
| ebi-fl187-GCST90094764 | prot-a-1666 | Kallikrein-7 \|\| id:prot-a-1666 | Fried chicken liking \|\| id:ebi-fl187-GCST90094764 | Inverse variance weighted | 3 | 0.220688480344064 | 0.239769524731267 | 0.357353725515222 | -0.249259788 | 0.690636748817347 | 1.24693492531479 | 0.779377474066436 | 1.99498543351184 | 0.998735809224318 |
| ebi-fl187-GCST90094764 | prot-a-1678 | Importin subunit alpha-3 \|\| id:prot-a-1678 | Fried chicken liking \|\| id:ebi-fl187-GCST90094764 | Inverse variance weighted | 3 | 0.255378731671767 | 0.239769524731267 | 0.286830281319807 | -0.214569537 | 0.72532700014505 | 1.29095045212201 | 0.806888701321585 | 2.06540637773761 | 0.998735809224318 |
| ebi-fl187-GCST90094764 | prot-a-1718 | Protein LDOC1 \|\| id:prot-a-1718 | Fried chicken liking \|\| id:ebi-fl187-GCST90094764 | Inverse variance weighted | 3 | 0.0296528157953513 | 0.326442947076249 | 0.927622796699211 | -0.61017536 | 0.6694809920648 | 1.03009683851242 | 0.543255595162381 | 1.95322331911944 | 0.998735809224318 |
| ebi-fl187-GCST90094764 | prot-a-1723 | Leptin \|\| id:prot-a-1723 | Fried chicken liking \|\| id:ebi-fl187-GCST90094764 | Inverse variance weighted | 3 | 0.0539534349091084 | 0.239769524731267 | 0.821962104709027 | -0.415994834 | 0.523901703382391 | 1.05543545456294 | 0.659683678688976 | 1.68860324233316 | 0.998735809224318 |
| ebi-fl187-GCST90094764 | prot-a-1730 | Galectin-7 \|\| id:prot-a-1730 | Fried chicken liking \|\| id:ebi-fl187-GCST90094764 | Inverse variance weighted | 3 | -0.636433136 | 0.275984273915978 | 0.0211079193514095 | -1.177362313 | -0.095503959 | 0.529176562484795 | 0.308090313551311 | 0.908914762867372 | 0.998735809224318 |
| ebi-fl187-GCST90094764 | prot-a-1753 | Lipase member N \|\| id:prot-a-1753 | Fried chicken liking \|\| id:ebi-fl187-GCST90094764 | Inverse variance weighted | 3 | 0.11404415642297 | 0.239769524731267 | 0.634330339229467 | -0.355904112 | 0.583992424896253 | 1.12080161438166 | 0.700539790338969 | 1.79318330824962 | 0.998735809224318 |
| ebi-fl187-GCST90094764 | prot-a-1756 | VIP36-like protein \|\| id:prot-a-1756 | Fried chicken liking \|\| id:ebi-fl187-GCST90094764 | Inverse variance weighted | 3 | 0.247521077457436 | 0.239769524731267 | 0.301917934670201 | -0.222427191 | 0.717469345930719 | 1.28084635908383 | 0.800573293556545 | 2.04924072384438 | 0.998735809224318 |
| ebi-fl187-GCST90094764 | prot-a-1760 | Ig Kappa chain V-I region HK102- like \|\| id:prot-a-1760 | Fried chicken liking \|\| id:ebi-fl187-GCST90094764 | Inverse variance weighted | 3 | 0.0146543945374648 | 0.239769524731267 | 0.951264700172612 | -0.455293874 | 0.484602663010748 | 1.01476229661266 | 0.634261547620195 | 1.6235297922289 | 0.998735809224318 |
| ebi-fl187-GCST90094764 | prot-a-179 | Neutral ceramidase \|\| id:prot-a-179 | Fried chicken liking \|\| id:ebi-fl187-GCST90094764 | Inverse variance weighted | 3 | -0.118924284 | 0.240075455324726 | 0.620344534511919 | -0.589472177 | 0.351623607945163 | 0.887875024126107 | 0.554619948695562 | 1.4213734293565 | 0.998735809224318 |
| ebi-fl187-GCST90094764 | prot-a-1809 | Lactotransferrin \|\| id:prot-a-1809 | Fried chicken liking \|\| id:ebi-fl187-GCST90094764 | Inverse variance weighted | 3 | 0.371354288377504 | 0.267444778441417 | 0.164976637484286 | -0.152837477 | 0.895546054122682 | 1.44969659306808 | 0.858269198646425 | 2.44867253219346 | 0.998735809224318 |
| ebi-fl187-GCST90094764 | prot-a-1818 | Lysophospholipase-like protein 1 \|\| id:prot-a-1818 | Fried chicken liking \|\| id:ebi-fl187-GCST90094764 | Inverse variance weighted | 3 | 0.199315452683387 | 0.240075455324726 | 0.406414390218377 | -0.27123244 | 0.66986334511985 | 1.22056693629202 | 0.762439254614767 | 1.95397028281553 | 0.998735809224318 |
| ebi-fl187-GCST90094764 | prot-a-1852 | MAP kinase-activated protein kinase 3 \|\| id:prot-a-1852 | Fried chicken liking \|\| id:ebi-fl187-GCST90094764 | Inverse variance weighted | 3 | 0.438444736896201 | 0.240075455324726 | 0.0678082628406751 | -0.032103156 | 0.908992629332663 | 1.55029422716797 | 0.968406680412271 | 2.48182115985317 | 0.998735809224318 |
| ebi-fl187-GCST90094764 | prot-a-1856 | Megakaryocyte-associated tyrosine-protein kinase \|\| id:prot-a-1856 | Fried chicken liking \|\| id:ebi-fl187-GCST90094764 | Inverse variance weighted | 3 | 0.0942789610735929 | 0.286085359908117 | 0.74174124760942 | -0.466448344 | 0.655006266493503 | 1.09886624407184 | 0.627226007763293 | 1.92515458130724 | 0.998735809224318 |
| ebi-fl187-GCST90094764 | prot-a-1890 | Beta-1,3-N-acetylglucosaminyltransferase manic fringe \|\| id:prot-a-1890 | Fried chicken liking \|\| id:ebi-fl187-GCST90094764 | Inverse variance weighted | 3 | 0.050625438045377 | 0.363633204463579 | 0.889275384800388 | -0.662095643 | 0.763346518793993 | 1.05192880697288 | 0.515769332904266 | 2.14544398890187 | 0.998735809224318 |
| ebi-fl187-GCST90094764 | prot-a-1919 | Matrilysin \|\| id:prot-a-1919 | Fried chicken liking \|\| id:ebi-fl187-GCST90094764 | Inverse variance weighted (multiplicative random effects) | 3 | 0.468666154416514 | 0.596285482795102 | 0.431881473234384 | -0.700053392 | 1.63738570069491 | 1.59786147070659 | 0.496558790885247 | 5.14170995747942 | 0.998735809224318 |
| ebi-fl187-GCST90094764 | prot-a-1923 | Multimerin-2 \|\| id:prot-a-1923 | Fried chicken liking \|\| id:ebi-fl187-GCST90094764 | Inverse variance weighted | 3 | -0.227973228 | 0.240075455324726 | 0.34232067065275 | -0.698521121 | 0.242574663975133 | 0.796145573592162 | 0.497320236724182 | 1.27452640681889 | 0.998735809224318 |
| ebi-fl187-GCST90094764 | prot-a-1948 | Mesothelin \|\| id:prot-a-1948 | Fried chicken liking \|\| id:ebi-fl187-GCST90094764 | Inverse variance weighted | 3 | 0.0612850435039768 | 0.239769524731267 | 0.798259869832949 | -0.408663225 | 0.53123331197726 | 1.06320192980233 | 0.66453799444483 | 1.70102891480233 | 0.998735809224318 |
| ebi-fl187-GCST90094764 | prot-a-195 | ATPase family AAA domain-containing protein 1 \|\| id:prot-a-195 | Fried chicken liking \|\| id:ebi-fl187-GCST90094764 | Inverse variance weighted | 3 | 0.296782277597864 | 0.239769524731267 | 0.215797096663543 | -0.173165991 | 0.766730546071147 | 1.34552231720383 | 0.840998005262866 | 2.15271652817735 | 0.998735809224318 |
| ebi-fl187-GCST90094764 | prot-a-1966 | Mucin-1 \|\| id:prot-a-1966 | Fried chicken liking \|\| id:ebi-fl187-GCST90094764 | Inverse variance weighted | 3 | 0.280457879415243 | 0.239769524731267 | 0.242122614166905 | -0.189490389 | 0.750406147888526 | 1.32373578496281 | 0.827380668766831 | 2.11786000632911 | 0.998735809224318 |
| ebi-fl187-GCST90094764 | prot-a-1986 | Myocardial zonula adherens protein \|\| id:prot-a-1986 | Fried chicken liking \|\| id:ebi-fl187-GCST90094764 | Inverse variance weighted | 3 | -0.097811465 | 0.281715759023574 | 0.72844172774406 | -0.649974353 | 0.454351422501655 | 0.90681985475227 | 0.522059165908221 | 1.57515144388346 | 0.998735809224318 |
| ebi-fl187-GCST90094764 | prot-a-2071 | Epididymal secretory protein E1 \|\| id:prot-a-2071 | Fried chicken liking \|\| id:ebi-fl187-GCST90094764 | Inverse variance weighted | 3 | -0.171790838 | 0.311133693190327 | 0.58084917738276 | -0.781612877 | 0.438031200252321 | 0.842155301297898 | 0.457667254690228 | 1.54965325623786 | 0.998735809224318 |
| ebi-fl187-GCST90094764 | prot-a-2098 | Neuritin \|\| id:prot-a-2098 | Fried chicken liking \|\| id:ebi-fl187-GCST90094764 | Inverse variance weighted | 3 | 0.388257561588638 | 0.239769524731267 | 0.105383833746133 | -0.081690707 | 0.858205830061921 | 1.47440948663735 | 0.921556945840552 | 2.35892458311779 | 0.998735809224318 |
| ebi-fl187-GCST90094764 | prot-a-2115 | Neurotrophin-4 \|\| id:prot-a-2115 | Fried chicken liking \|\| id:ebi-fl187-GCST90094764 | Inverse variance weighted | 3 | -0.199608783 | 0.297032313015866 | 0.501577119273897 | -0.781792117 | 0.382574550451634 | 0.819051117080151 | 0.457585229983896 | 1.46605416528381 | 0.998735809224318 |
| ebi-fl187-GCST90094764 | prot-a-2118 | Netrin-1 \|\| id:prot-a-2118 | Fried chicken liking \|\| id:ebi-fl187-GCST90094764 | Inverse variance weighted | 3 | -0.10391263 | 0.327307150231955 | 0.750881368376602 | -0.745434645 | 0.537609384065823 | 0.901304040564866 | 0.47452800396043 | 1.71190944845964 | 0.998735809224318 |
| ebi-fl187-GCST90094764 | prot-a-2120 | Netrin-G1 \|\| id:prot-a-2120 | Fried chicken liking \|\| id:ebi-fl187-GCST90094764 | Inverse variance weighted | 3 | -0.019531698 | 0.262496867647569 | 0.9406862563507 | -0.534025558 | 0.494962162894494 | 0.980657810104096 | 0.586240268880474 | 1.64043616852638 | 0.998735809224318 |
| ebi-fl187-GCST90094764 | prot-a-2145 | Olfactomedin-like protein 3 \|\| id:prot-a-2145 | Fried chicken liking \|\| id:ebi-fl187-GCST90094764 | Inverse variance weighted | 3 | 0.134202481492274 | 0.280625028808111 | 0.632488607159034 | -0.415822575 | 0.684227537956172 | 1.14362435896945 | 0.659797324658962 | 1.98224003879418 | 0.998735809224318 |
| ebi-fl187-GCST90094764 | prot-a-2162 | Prolyl 4-hydroxylase subunit alpha-2 \|\| id:prot-a-2162 | Fried chicken liking \|\| id:ebi-fl187-GCST90094764 | Inverse variance weighted | 3 | 0.197987949785756 | 0.373930478302075 | 0.59647392420856 | -0.534915788 | 0.930891687257823 | 1.21894770515214 | 0.585718612787531 | 2.53677017505787 | 0.998735809224318 |
| ebi-fl187-GCST90094764 | prot-a-2168 | Polyadenylate-binding protein-interacting protein 1 \|\| id:prot-a-2168 | Fried chicken liking \|\| id:ebi-fl187-GCST90094764 | Inverse variance weighted | 3 | 0.327219192204293 | 0.239769524731267 | 0.172339856297952 | -0.142729076 | 0.797167460677576 | 1.38710548657003 | 0.866988924954306 | 2.21924591594303 | 0.998735809224318 |
| ebi-fl187-GCST90094764 | prot-a-219 | Beta-1,4-galactosyltransferase 5 \|\| id:prot-a-219 | Fried chicken liking \|\| id:ebi-fl187-GCST90094764 | Inverse variance weighted | 3 | -0.212139805 | 0.239769524731267 | 0.37628311627624 | -0.682088074 | 0.257808463215755 | 0.808851607954071 | 0.505560242402125 | 1.29409092886996 | 0.998735809224318 |
| ebi-fl187-GCST90094764 | prot-a-2190 | Pyruvate carboxylase, mitochondrial \|\| id:prot-a-2190 | Fried chicken liking \|\| id:ebi-fl187-GCST90094764 | Inverse variance weighted | 3 | 0.324522428617529 | 0.239769524731267 | 0.175903437307975 | -0.14542584 | 0.794470697090812 | 1.38336983035764 | 0.864654010562478 | 2.21326919688815 | 0.998735809224318 |
| ebi-fl187-GCST90094764 | prot-a-2197 | Protocadherin-9 \|\| id:prot-a-2197 | Fried chicken liking \|\| id:ebi-fl187-GCST90094764 | Inverse variance weighted | 3 | -0.085861536 | 0.240075455324726 | 0.720609776665913 | -0.556409429 | 0.384686356100119 | 0.917721293475705 | 0.573263717160297 | 1.4691534581513 | 0.998735809224318 |
| ebi-fl187-GCST90094764 | prot-a-2198 | Protocadherin alpha-4 \|\| id:prot-a-2198 | Fried chicken liking \|\| id:ebi-fl187-GCST90094764 | Inverse variance weighted | 3 | -0.743784579 | 0.397458651596571 | 0.0612964690156859 | -1.522803536 | 0.0352343783528608 | 0.475311652876053 | 0.218099579038804 | 1.03586246408834 | 0.998735809224318 |
| ebi-fl187-GCST90094764 | prot-a-2254 | Placenta growth factor \|\| id:prot-a-2254 | Fried chicken liking \|\| id:ebi-fl187-GCST90094764 | Inverse variance weighted | 3 | 0.175073585748401 | 0.239769524731267 | 0.465283545495784 | -0.294874683 | 0.645021854221684 | 1.19133387858203 | 0.744624895982152 | 1.90602868359015 | 0.998735809224318 |
| ebi-fl187-GCST90094764 | prot-a-2261 | Membrane-associated progesterone receptor component 2 \|\| id:prot-a-2261 | Fried chicken liking \|\| id:ebi-fl187-GCST90094764 | Inverse variance weighted | 3 | -0.261432694 | 0.239769524731267 | 0.275559011503413 | -0.731380962 | 0.208515574570365 | 0.769947695854771 | 0.481243951208661 | 1.23184811541669 | 0.998735809224318 |
| ebi-fl187-GCST90094764 | prot-a-2268 | Polymeric immunoglobulin receptor \|\| id:prot-a-2268 | Fried chicken liking \|\| id:ebi-fl187-GCST90094764 | Inverse variance weighted | 3 | 0.0946302425147042 | 0.239769524731267 | 0.693085201445949 | -0.375318026 | 0.564578510987987 | 1.09925232319689 | 0.687070737711974 | 1.75870635107781 | 0.998735809224318 |
| ebi-fl187-GCST90094764 | prot-a-2283 | Group 10 secretory phospholipase A2 \|\| id:prot-a-2283 | Fried chicken liking \|\| id:ebi-fl187-GCST90094764 | Inverse variance weighted (multiplicative random effects) | 3 | -0.124949807 | 0.499207490026389 | 0.802358188289008 | -1.103396487 | 0.853496873844734 | 0.882541199210148 | 0.331742409219254 | 2.34784262324601 | 0.998735809224318 |
| ebi-fl187-GCST90094764 | prot-a-2298 | Inactive phospholipase D5 \|\| id:prot-a-2298 | Fried chicken liking \|\| id:ebi-fl187-GCST90094764 | Inverse variance weighted | 3 | 0.705886878621487 | 0.314160270680105 | 0.0246463848595064 | 0.090132748088482 | 1.32164100915449 | 2.02564238744776 | 1.09431954289106 | 3.74956940911867 | 0.998735809224318 |
| ebi-fl187-GCST90094764 | prot-a-2304 | Procollagen-lysine,2-oxoglutarate 5-dioxygenase 2 \|\| id:prot-a-2304 | Fried chicken liking \|\| id:ebi-fl187-GCST90094764 | Inverse variance weighted | 3 | -0.15644957 | 0.272202332097382 | 0.565457206219408 | -0.689966141 | 0.37706700066538 | 0.855174642862809 | 0.501593052139265 | 1.45800199320242 | 0.998735809224318 |
| ebi-fl187-GCST90094764 | prot-a-2331 | Periostin \|\| id:prot-a-2331 | Fried chicken liking \|\| id:ebi-fl187-GCST90094764 | Inverse variance weighted | 3 | -0.215734711 | 0.331828806843368 | 0.51560270825487 | -0.866119172 | 0.434649750911442 | 0.805949083343326 | 0.420580587149865 | 1.54442203180075 | 0.998735809224318 |
| ebi-fl187-GCST90094764 | prot-a-2333 | Periostin \|\| id:prot-a-2333 | Fried chicken liking \|\| id:ebi-fl187-GCST90094764 | Inverse variance weighted | 3 | -0.320665241 | 0.239769524731267 | 0.1810955356586 | -0.790613509 | 0.149283027804005 | 0.725666133843516 | 0.45356644274579 | 1.16100153842848 | 0.998735809224318 |
| ebi-fl187-GCST90094764 | prot-a-2367 | cAMP-dependent protein kinase catalytic subunit alpha \|\| id:prot-a-2367 | Fried chicken liking \|\| id:ebi-fl187-GCST90094764 | Inverse variance weighted | 3 | 0.216102722735695 | 0.240075455324726 | 0.368043086567953 | -0.25444517 | 0.686650615172157 | 1.24122987498058 | 0.775346564409441 | 1.98704898333788 | 0.998735809224318 |
| ebi-fl187-GCST90094764 | prot-a-2385 | Phosphoribosyl pyrophosphate synthase-associated protein 1 \|\| id:prot-a-2385 | Fried chicken liking \|\| id:ebi-fl187-GCST90094764 | Inverse variance weighted | 3 | -0.493237079 | 0.274214942965824 | 0.0720623043676689 | -1.030698367 | 0.0442242091535758 | 0.610646480410157 | 0.356757725630739 | 1.0452166757652 | 0.998735809224318 |
| ebi-fl187-GCST90094764 | prot-a-2393 | Trypsin-3 \|\| id:prot-a-2393 | Fried chicken liking \|\| id:ebi-fl187-GCST90094764 | Inverse variance weighted | 3 | -0.299502365 | 0.239769524731267 | 0.211618965542014 | -0.769450633 | 0.170445904 | 0.741186969550707 | 0.463267502106059 | 1.18583350080531 | 0.998735809224318 |
| ebi-fl187-GCST90094764 | prot-a-2405 | Pregnancy-specific beta-1-glycoprotein 6 \|\| id:prot-a-2405 | Fried chicken liking \|\| id:ebi-fl187-GCST90094764 | Inverse variance weighted | 3 | 0.170742966825023 | 0.277044767819869 | 0.537695880483913 | -0.372264778 | 0.713750711751967 | 1.18618582072743 | 0.689171740778634 | 2.04163449839821 | 0.998735809224318 |
| ebi-fl187-GCST90094764 | prot-a-244 | Beclin-1 \|\| id:prot-a-244 | Fried chicken liking \|\| id:ebi-fl187-GCST90094764 | Inverse variance weighted | 3 | -0.217636305 | 0.239769524731267 | 0.364042237494194 | -0.687584574 | 0.252311963261809 | 0.804417951087867 | 0.502789053449833 | 1.28699747059423 | 0.998735809224318 |
| ebi-fl187-GCST90094764 | prot-a-2454 | tRNA pseudouridine synthase A, mitochondrial \|\| id:prot-a-2454 | Fried chicken liking \|\| id:ebi-fl187-GCST90094764 | Inverse variance weighted | 3 | 0.241079543889846 | 0.253215511201902 | 0.341060176 | -0.255222858 | 0.737381945845573 | 1.27262226060716 | 0.774743820811088 | 2.09045541853737 | 0.998735809224318 |
| ebi-fl187-GCST90094764 | prot-a-2466 | Glutaminyl-peptide cyclotransferase-like protein \|\| id:prot-a-2466 | Fried chicken liking \|\| id:ebi-fl187-GCST90094764 | Inverse variance weighted | 3 | 0.283509300116082 | 0.240075455324726 | 0.237635509190034 | -0.187038592 | 0.754057192552544 | 1.327781229 | 0.829411726844657 | 2.12560654067061 | 0.998735809224318 |
| ebi-fl187-GCST90094764 | prot-a-2474 | Ras-related protein Rab-27A \|\| id:prot-a-2474 | Fried chicken liking \|\| id:ebi-fl187-GCST90094764 | Inverse variance weighted (multiplicative random effects) | 3 | 0.395037106261765 | 0.548730469422442 | 0.471579788903214 | -0.680474614 | 1.47054882632975 | 1.48443927189118 | 0.506376601997353 | 4.35162277095958 | 0.998735809224318 |
| ebi-fl187-GCST90094764 | prot-a-2490 | Rap1 GTPase-GDP dissociation stimulator 1 \|\| id:prot-a-2490 | Fried chicken liking \|\| id:ebi-fl187-GCST90094764 | Inverse variance weighted | 3 | 0.188315038042163 | 0.346829199396994 | 0.587156041217924 | -0.491470193 | 0.86810026886027 | 1.20721377371228 | 0.611726377046793 | 2.38238066907675 | 0.998735809224318 |
| ebi-fl187-GCST90094764 | prot-a-2545 | Receptor-interacting serine/threonine-protein kinase 2 \|\| id:prot-a-2545 | Fried chicken liking \|\| id:ebi-fl187-GCST90094764 | Inverse variance weighted | 3 | 0.633567027049045 | 0.239769524731267 | 0.00823207351879077 | 0.163618758575762 | 1.10351529552233 | 1.88432002626355 | 1.17776521660209 | 3.01474513878214 | 0.998735809224318 |
| ebi-fl187-GCST90094764 | prot-a-2610 | RUN and FYVE domain-containing protein 1 \|\| id:prot-a-2610 | Fried chicken liking \|\| id:ebi-fl187-GCST90094764 | Inverse variance weighted | 3 | 0.0234552875198392 | 0.350695683225212 | 0.946675509309761 | -0.663908252 | 0.710818826641255 | 1.02373252610302 | 0.514835291603477 | 2.03565742693581 | 0.998735809224318 |
| ebi-fl187-GCST90094764 | prot-a-2627 | Serine--tRNA ligase, mitochondrial \|\| id:prot-a-2627 | Fried chicken liking \|\| id:ebi-fl187-GCST90094764 | Inverse variance weighted | 3 | 0.025187279687587 | 0.240075455324726 | 0.916444038753513 | -0.445360613 | 0.495735172124049 | 1.02550715920179 | 0.640593228290477 | 1.64170473106726 | 0.998735809224318 |
| ebi-fl187-GCST90094764 | prot-a-264 | BolA-like protein 2 \|\| id:prot-a-264 | Fried chicken liking \|\| id:ebi-fl187-GCST90094764 | Inverse variance weighted | 3 | 0.383105778557518 | 0.26171576551827 | 0.143242085083815 | -0.129857122 | 0.896068678973327 | 1.46683318136046 | 0.878220900527193 | 2.44995260377935 | 0.998735809224318 |
| ebi-fl187-GCST90094764 | prot-a-2646 | Secretoglobin family 1C member 1 \|\| id:prot-a-2646 | Fried chicken liking \|\| id:ebi-fl187-GCST90094764 | Inverse variance weighted | 3 | 0.492105743470814 | 0.404958542513372 | 0.224289704993163 | -0.301613 | 1.28582448679702 | 1.63575708069143 | 0.739624244199701 | 3.61764943214826 | 0.998735809224318 |
| ebi-fl187-GCST90094764 | prot-a-2672 | Semaphorin-3E \|\| id:prot-a-2672 | Fried chicken liking \|\| id:ebi-fl187-GCST90094764 | Inverse variance weighted | 3 | 0.221958394962535 | 0.277349969897378 | 0.423546998588205 | -0.321647546 | 0.765564335961396 | 1.24851943208594 | 0.724953658097221 | 2.15020747172663 | 0.998735809224318 |
| ebi-fl187-GCST90094764 | prot-a-2701 | Plasma protease C1 inhibitor \|\| id:prot-a-2701 | Fried chicken liking \|\| id:ebi-fl187-GCST90094764 | Inverse variance weighted | 3 | -0.081689239 | 0.240075455324726 | 0.733657085732552 | -0.552237131 | 0.388858653819282 | 0.921558298933626 | 0.575660540713602 | 1.47529600913876 | 0.998735809224318 |
| ebi-fl187-GCST90094764 | prot-a-2738 | Signaling threshold-regulating transmembrane adapter 1 \|\| id:prot-a-2738 | Fried chicken liking \|\| id:ebi-fl187-GCST90094764 | Inverse variance weighted | 3 | 0.342752012105729 | 0.280832694194567 | 0.222281178551689 | -0.207680069 | 0.893184092727079 | 1.40881934845945 | 0.812466928886444 | 2.44289568723003 | 0.998735809224318 |
| ebi-fl187-GCST90094764 | prot-a-274 | Serine/threonine-protein kinase BRSK2 \|\| id:prot-a-274 | Fried chicken liking \|\| id:ebi-fl187-GCST90094764 | Inverse variance weighted | 3 | -0.168874298 | 0.239769524731267 | 0.481233965239285 | -0.638822566 | 0.301073970620982 | 0.844615066641276 | 0.527913641548818 | 1.35130929502885 | 0.998735809224318 |
| ebi-fl187-GCST90094764 | prot-a-2749 | Mitochondrial glutamate carrier 2 \|\| id:prot-a-2749 | Fried chicken liking \|\| id:ebi-fl187-GCST90094764 | Inverse variance weighted | 3 | -0.175539015 | 0.357420356934571 | 0.623336177013292 | -0.876082914 | 0.525004885003208 | 0.839004663113192 | 0.416410838225655 | 1.69046710629616 | 0.998735809224318 |
| ebi-fl187-GCST90094764 | prot-a-2760 | Sodium-coupled monocarboxylate transporter 1 \|\| id:prot-a-2760 | Fried chicken liking \|\| id:ebi-fl187-GCST90094764 | Inverse variance weighted | 3 | -0.176674395 | 0.335152673536922 | 0.598092197775761 | -0.833573635 | 0.480224845507735 | 0.838052614539259 | 0.434493786485734 | 1.61643781011618 | 0.998735809224318 |
| ebi-fl187-GCST90094764 | prot-a-2777 | Small integral membrane protein 10 \|\| id:prot-a-2777 | Fried chicken liking \|\| id:ebi-fl187-GCST90094764 | Inverse variance weighted | 3 | -0.544729121 | 0.382746062806903 | 0.154674457156715 | -1.294911404 | 0.205453162310027 | 0.579998871698195 | 0.273922131690913 | 1.22808145911614 | 0.998735809224318 |
| ebi-fl187-GCST90094764 | prot-a-2799 | Superoxide dismutase [Mn], mitochondrial \|\| id:prot-a-2799 | Fried chicken liking \|\| id:ebi-fl187-GCST90094764 | Inverse variance weighted | 3 | 0.195844325629668 | 0.240075455324726 | 0.414636528140301 | -0.274703567 | 0.66639221806613 | 1.21633753801454 | 0.759797318991027 | 1.94719956151983 | 0.998735809224318 |
| ebi-fl187-GCST90094764 | prot-a-2805 | Protein SPACA7 \|\| id:prot-a-2805 | Fried chicken liking \|\| id:ebi-fl187-GCST90094764 | Inverse variance weighted | 3 | -0.063373805 | 0.253618626322407 | 0.802681423054883 | -0.560466313 | 0.433718702614524 | 0.938592557536365 | 0.570942763976951 | 1.54298476948246 | 0.998735809224318 |
| ebi-fl187-GCST90094764 | prot-a-2811 | SPARC-like protein 1 \|\| id:prot-a-2811 | Fried chicken liking \|\| id:ebi-fl187-GCST90094764 | Inverse variance weighted | 3 | -0.219503463 | 0.239769524731267 | 0.359941320909924 | -0.689451731 | 0.250444805855546 | 0.802917377492265 | 0.501851143030574 | 1.28459668575425 | 0.998735809224318 |
| ebi-fl187-GCST90094764 | prot-a-2831 | Sepiapterin reductase \|\| id:prot-a-2831 | Fried chicken liking \|\| id:ebi-fl187-GCST90094764 | Inverse variance weighted | 3 | -0.057091621 | 0.240075455324726 | 0.81203079928988 | -0.527639513 | 0.413456271758666 | 0.944507528982151 | 0.589996005104712 | 1.51203476732972 | 0.998735809224318 |
| ebi-fl187-GCST90094764 | prot-a-2834 | SPRY domain-containing SOCS box protein 1 \|\| id:prot-a-2834 | Fried chicken liking \|\| id:ebi-fl187-GCST90094764 | Inverse variance weighted | 3 | -0.161193465 | 0.239769524731267 | 0.501402251316809 | -0.631141733 | 0.308754803797383 | 0.851127392092818 | 0.531984070291874 | 1.3617284389235 | 0.998735809224318 |
| ebi-fl187-GCST90094764 | prot-a-2835 | Spectrin alpha chain, non-erythrocytic 1 \|\| id:prot-a-2835 | Fried chicken liking \|\| id:ebi-fl187-GCST90094764 | Inverse variance weighted | 3 | 0.0627926390699416 | 0.239769524731267 | 0.793408184456724 | -0.407155629 | 0.532740907543225 | 1.06480601717111 | 0.66554060455405 | 1.70359531251068 | 0.998735809224318 |
| ebi-fl187-GCST90094764 | prot-a-2859 | Sia-alpha-2,3-Gal-beta-1,4-GlcNAc-R:alpha 2,8-sialyltransferase \|\| id:prot-a-2859 | Fried chicken liking \|\| id:ebi-fl187-GCST90094764 | Inverse variance weighted | 3 | 0.327541590780744 | 0.239769524731267 | 0.171917469666223 | -0.142406678 | 0.797489859254027 | 1.38755275950049 | 0.867268486012132 | 2.21996151301466 | 0.998735809224318 |
| ebi-fl187-GCST90094764 | prot-a-2861 | Alpha-2,8-sialyltransferase 8F \|\| id:prot-a-2861 | Fried chicken liking \|\| id:ebi-fl187-GCST90094764 | Inverse variance weighted | 3 | 0.0938461422461359 | 0.316555838837594 | 0.766878814225624 | -0.526603302 | 0.714295586367821 | 1.09839073698418 | 0.590607682454736 | 2.04274723633504 | 0.998735809224318 |
| ebi-fl187-GCST90094764 | prot-a-289 | Uncharacterized protein C17orf89 \|\| id:prot-a-289 | Fried chicken liking \|\| id:ebi-fl187-GCST90094764 | Inverse variance weighted | 3 | 0.4400018379331 | 0.240075455324726 | 0.066837559638278 | -0.030546055 | 0.910549730369562 | 1.55271007228595 | 0.969915762049866 | 2.48568861638346 | 0.998735809224318 |
| ebi-fl187-GCST90094764 | prot-a-2921 | Tachykinin-3 \|\| id:prot-a-2921 | Fried chicken liking \|\| id:ebi-fl187-GCST90094764 | Inverse variance weighted | 3 | 0.0295405083001703 | 0.291043822030921 | 0.919154756611347 | -0.540905383 | 0.599986399480776 | 1.02998115741274 | 0.582220880855283 | 1.82209401879726 | 0.998735809224318 |
| ebi-fl187-GCST90094764 | prot-a-2928 | Tax1-binding protein 1 \|\| id:prot-a-2928 | Fried chicken liking \|\| id:ebi-fl187-GCST90094764 | Inverse variance weighted | 3 | -0.079039005 | 0.266945215580375 | 0.76716365038249 | -0.602251628 | 0.444173617554346 | 0.924003882986271 | 0.54757730685683 | 1.55920116681705 | 0.998735809224318 |
| ebi-fl187-GCST90094764 | prot-a-296 | Uncharacterized protein C1orf185 \|\| id:prot-a-296 | Fried chicken liking \|\| id:ebi-fl187-GCST90094764 | Inverse variance weighted | 3 | 0.0740151971172949 | 0.282263790734259 | 0.793151873436714 | -0.479221833 | 0.627252226956443 | 1.07682316997989 | 0.619265096192893 | 1.87245841326145 | 0.998735809224318 |
| ebi-fl187-GCST90094764 | prot-a-2964 | Transforming growth factor beta-2 \|\| id:prot-a-2964 | Fried chicken liking \|\| id:ebi-fl187-GCST90094764 | Inverse variance weighted | 3 | -0.32389097 | 0.239769524731267 | 0.176745749232048 | -0.793839238 | 0.146057298449681 | 0.723329102635687 | 0.452105717376251 | 1.15726249549799 | 0.998735809224318 |
| ebi-fl187-GCST90094764 | prot-a-2977 | Thymocyte nuclear protein 1 \|\| id:prot-a-2977 | Fried chicken liking \|\| id:ebi-fl187-GCST90094764 | Inverse variance weighted | 3 | 0.534059342890718 | 0.239769524731267 | 0.0259214704877818 | 0.0641110744174349 | 1.004007611 | 1.70584287409526 | 1.06621082093034 | 2.72919750388811 | 0.998735809224318 |
| ebi-fl187-GCST90094764 | prot-a-3015 | Transmembrane protein 70, mitochondrial \|\| id:prot-a-3015 | Fried chicken liking \|\| id:ebi-fl187-GCST90094764 | Inverse variance weighted | 3 | -0.068070249 | 0.240075455324726 | 0.77676530396748 | -0.538618141 | 0.402477643445438 | 0.934194845026787 | 0.583554084687065 | 1.49552549005398 | 0.998735809224318 |
| ebi-fl187-GCST90094764 | prot-a-3030 | Tumor necrosis factor alpha-induced protein 3 \|\| id:prot-a-3030 | Fried chicken liking \|\| id:ebi-fl187-GCST90094764 | Inverse variance weighted | 3 | -0.196535878 | 0.240075455324726 | 0.412990632725649 | -0.66708377 | 0.274012014551457 | 0.821571854505955 | 0.513203015530587 | 1.31523060404957 | 0.998735809224318 |
| ebi-fl187-GCST90094764 | prot-a-3032 | Tumor necrosis factor receptor superfamily member 10A \|\| id:prot-a-3032 | Fried chicken liking \|\| id:ebi-fl187-GCST90094764 | Inverse variance weighted | 3 | 0.0085642028704468 | 0.251796895108862 | 0.972867306498163 | -0.484957712 | 0.502086117283816 | 1.00860098057144 | 0.615723234199301 | 1.65216428665805 | 0.998955228396052 |
| ebi-fl187-GCST90094764 | prot-a-3081 | Tumor protein p53-inducible protein 11 \|\| id:prot-a-3081 | Fried chicken liking \|\| id:ebi-fl187-GCST90094764 | Inverse variance weighted | 3 | -0.042082282 | 0.239769524731267 | 0.860677824401485 | -0.51203055 | 0.427865986712303 | 0.958790886324212 | 0.599277479498491 | 1.53398049342297 | 0.998735809224318 |
| ebi-fl187-GCST90094764 | prot-a-315 | Complement C4 \|\| id:prot-a-315 | Fried chicken liking \|\| id:ebi-fl187-GCST90094764 | Inverse variance weighted | 3 | -0.117169751 | 0.239769524731267 | 0.625070687878566 | -0.58711802 | 0.352778517435018 | 0.889434197969404 | 0.555927149435407 | 1.42301593530898 | 0.998735809224318 |
| ebi-fl187-GCST90094764 | prot-a-3157 | UDP-glucuronosyltransferase 1-8 \|\| id:prot-a-3157 | Fried chicken liking \|\| id:ebi-fl187-GCST90094764 | Inverse variance weighted | 3 | 0.0846143068210714 | 0.239769524731267 | 0.724164531660863 | -0.385333962 | 0.554562575294354 | 1.08829723691994 | 0.680223429727109 | 1.74117918337908 | 0.998735809224318 |
| ebi-fl187-GCST90094764 | prot-a-317 | Glutaredoxin-like protein C5orf63 \|\| id:prot-a-317 | Fried chicken liking \|\| id:ebi-fl187-GCST90094764 | Inverse variance weighted | 3 | -0.444015847 | 0.239769524731267 | 0.0640481726427246 | -0.913964115 | 0.0259324214753644 | 0.641455255606676 | 0.400931730030068 | 1.02627159220989 | 0.998735809224318 |
| ebi-fl187-GCST90094764 | prot-a-3192 | Guanine nucleotide exchange factor VAV3 \|\| id:prot-a-3192 | Fried chicken liking \|\| id:ebi-fl187-GCST90094764 | Inverse variance weighted | 2 | 0.0546793131084925 | 0.318240197229555 | 0.863580814366312 | -0.569071473 | 0.67843009967842 | 1.05620185027158 | 0.566050787940215 | 1.9707813720682 | 0.998735809224318 |
| ebi-fl187-GCST90094764 | prot-a-3233 | Serine/threonine-protein kinase WNK3 \|\| id:prot-a-3233 | Fried chicken liking \|\| id:ebi-fl187-GCST90094764 | Inverse variance weighted | 3 | -0.354297134 | 0.239769524731267 | 0.139499589426136 | -0.824245403 | 0.115651134072843 | 0.701666447138584 | 0.438565808131475 | 1.12260416546766 | 0.998735809224318 |
| ebi-fl187-GCST90094764 | prot-a-3250 | 14-3-3 protein theta \|\| id:prot-a-3250 | Fried chicken liking \|\| id:ebi-fl187-GCST90094764 | Inverse variance weighted | 3 | 0.284315182961213 | 0.239769524731267 | 0.235707089698131 | -0.185633086 | 0.754263451434496 | 1.32885169616808 | 0.83057829028802 | 2.12604501111681 | 0.998735809224318 |
| ebi-fl187-GCST90094764 | prot-a-3278 | E3 ubiquitin-protein ligase ZNRF3 \|\| id:prot-a-3278 | Fried chicken liking \|\| id:ebi-fl187-GCST90094764 | Inverse variance weighted | 3 | 0.465735449793026 | 0.240419783386295 | 0.0527238250677707 | -0.005487326 | 0.936958225230165 | 1.59318546604779 | 0.994527702227077 | 2.55220636241902 | 0.998735809224318 |
| ebi-fl187-GCST90094764 | prot-a-355 | Calpain-3 \|\| id:prot-a-355 | Fried chicken liking \|\| id:ebi-fl187-GCST90094764 | Inverse variance weighted | 3 | 0.0498784114614531 | 0.240075455324726 | 0.835415277297484 | -0.420669481 | 0.520426303897915 | 1.05114328163036 | 0.656607086682408 | 1.68274485750573 | 0.998735809224318 |
| ebi-fl187-GCST90094764 | prot-a-385 | Coiled-coil domain-containing protein 90B, mitochondrial \|\| id:prot-a-385 | Fried chicken liking \|\| id:ebi-fl187-GCST90094764 | Inverse variance weighted | 3 | 0.54700188748408 | 0.239769524731267 | 0.0225268362005086 | 0.0770536190107973 | 1.01695015595736 | 1.72806431234938 | 1.08009998873294 | 2.7647498368356 | 0.998735809224318 |
| ebi-fl187-GCST90094764 | prot-a-390 | C-C motif chemokine 14 \|\| id:prot-a-390 | Fried chicken liking \|\| id:ebi-fl187-GCST90094764 | Inverse variance weighted | 3 | -0.178261211 | 0.239769524731267 | 0.457197777884304 | -0.648209479 | 0.291687057623161 | 0.836723833597094 | 0.522981347848173 | 1.33868402112242 | 0.998735809224318 |
| ebi-fl187-GCST90094764 | prot-a-40 | Pituitary adenylate cyclase-activating polypeptide 27 \|\| id:prot-a-40 | Fried chicken liking \|\| id:ebi-fl187-GCST90094764 | Inverse variance weighted | 3 | -0.251278531 | 0.242152359229767 | 0.299415460381397 | -0.725897155 | 0.223340092737901 | 0.777805698112151 | 0.483890249387463 | 1.25024570092403 | 0.998735809224318 |
| ebi-fl187-GCST90094764 | prot-a-401 | C-C motif chemokine 24 \|\| id:prot-a-401 | Fried chicken liking \|\| id:ebi-fl187-GCST90094764 | Inverse variance weighted | 3 | -0.109681079 | 0.239769524731267 | 0.647352098824702 | -0.579629348 | 0.360267189444652 | 0.896119881151893 | 0.560105932758731 | 1.43371243621801 | 0.998735809224318 |
| ebi-fl187-GCST90094764 | prot-a-412 | Cerebral cavernous malformations 2 protein \|\| id:prot-a-412 | Fried chicken liking \|\| id:ebi-fl187-GCST90094764 | Inverse variance weighted | 2 | -0.639102721 | 0.318240197229555 | 0.0446177850844935 | -1.262853508 | -0.015351935 | 0.527765764605955 | 0.282845771219068 | 0.984765305451139 | 0.998735809224318 |
| ebi-fl187-GCST90094764 | prot-a-44 | Adhesion G protein-coupled receptor F5 \|\| id:prot-a-44 | Fried chicken liking \|\| id:ebi-fl187-GCST90094764 | Inverse variance weighted | 3 | 0.262065344228332 | 0.239769524731267 | 0.274398828758271 | -0.207882924 | 0.732013612701615 | 1.29961146176296 | 0.812302132030588 | 2.07926322601607 | 0.998735809224318 |
| ebi-fl187-GCST90094764 | prot-a-48 | Adrenomedullin \|\| id:prot-a-48 | Fried chicken liking \|\| id:ebi-fl187-GCST90094764 | Inverse variance weighted | 3 | 0.300025428195669 | 0.239769524731267 | 0.210822274270541 | -0.16992284 | 0.769973696668952 | 1.3498931324863 | 0.843729916050915 | 2.15970944548528 | 0.998735809224318 |
| ebi-fl187-GCST90094764 | prot-a-502 | Carcinoembryonic antigen-related cell adhesion molecule 1 \|\| id:prot-a-502 | Fried chicken liking \|\| id:ebi-fl187-GCST90094764 | Inverse variance weighted | 3 | 0.0620493632267179 | 0.240075455324726 | 0.796053648123064 | -0.408498529 | 0.53259725566318 | 1.06401486663879 | 0.664647450047773 | 1.70335060541794 | 0.998735809224318 |
| ebi-fl187-GCST90094764 | prot-a-518 | Complement factor B \|\| id:prot-a-518 | Fried chicken liking \|\| id:ebi-fl187-GCST90094764 | Inverse variance weighted | 3 | -0.095879738 | 0.240075455324726 | 0.689618129785996 | -0.566427631 | 0.374668154313672 | 0.90857327622644 | 0.567549317363493 | 1.45450866209772 | 0.998735809224318 |
| ebi-fl187-GCST90094764 | prot-a-525 | Properdin \|\| id:prot-a-525 | Fried chicken liking \|\| id:ebi-fl187-GCST90094764 | Inverse variance weighted | 3 | -0.199246589 | 0.290082003283116 | 0.492169078423453 | -0.767807315 | 0.369314137639737 | 0.819347826426888 | 0.464029423880869 | 1.44674200841802 | 0.998735809224318 |
| ebi-fl187-GCST90094764 | prot-a-533 | Chondroadherin \|\| id:prot-a-533 | Fried chicken liking \|\| id:ebi-fl187-GCST90094764 | Inverse variance weighted | 3 | 0.360766472322454 | 0.259887461191624 | 0.165087047744399 | -0.148612952 | 0.870145896258037 | 1.4344284430192 | 0.861902648377692 | 2.38725912029086 | 0.998735809224318 |
| ebi-fl187-GCST90094764 | prot-a-535 | Coiled-coil-helix-coiled-coil-helix domain-containing protein 2 \|\| id:prot-a-535 | Fried chicken liking \|\| id:ebi-fl187-GCST90094764 | Inverse variance weighted | 3 | 0.317668683616806 | 0.239769524731267 | 0.185207032103415 | -0.152279585 | 0.787616952090089 | 1.37392098328023 | 0.858748154195374 | 2.19815187849388 | 0.998735809224318 |
| ebi-fl187-GCST90094764 | prot-a-591 | Caseinolytic peptidase B protein homolog \|\| id:prot-a-591 | Fried chicken liking \|\| id:ebi-fl187-GCST90094764 | Inverse variance weighted | 2 | 0.182428673324316 | 0.318240197229555 | 0.56648079464117 | -0.441322113 | 0.806179459894244 | 1.20012854672105 | 0.643185494634686 | 2.2393361490107 | 0.998735809224318 |
| ebi-fl187-GCST90094764 | prot-a-605 | Ciliary neurotrophic factor receptor subunit alpha \|\| id:prot-a-605 | Fried chicken liking \|\| id:ebi-fl187-GCST90094764 | Inverse variance weighted | 3 | -0.01564702 | 0.239769524731267 | 0.947968115097416 | -0.485595289 | 0.454301248170125 | 0.984474758334094 | 0.615330788203637 | 1.57507241369536 | 0.998735809224318 |
| ebi-fl187-GCST90094764 | prot-a-606 | Ciliary neurotrophic factor receptor subunit alpha \|\| id:prot-a-606 | Fried chicken liking \|\| id:ebi-fl187-GCST90094764 | Inverse variance weighted | 3 | 0.0343551413759979 | 0.277749729537843 | 0.901559939093472 | -0.510034329 | 0.578744611270169 | 1.03495209578036 | 0.600474965042698 | 1.78379766504336 | 0.998735809224318 |
| ebi-fl187-GCST90094764 | prot-a-707 | Cancer/testis antigen 55 \|\| id:prot-a-707 | Fried chicken liking \|\| id:ebi-fl187-GCST90094764 | Inverse variance weighted | 3 | -0.232329449 | 0.239769524731267 | 0.332560213961396 | -0.702277718 | 0.237618819198216 | 0.792684930808395 | 0.495455509795759 | 1.26822567739681 | 0.998735809224318 |
| ebi-fl187-GCST90094764 | prot-a-719 | Dipeptidyl peptidase 1 \|\| id:prot-a-719 | Fried chicken liking \|\| id:ebi-fl187-GCST90094764 | Inverse variance weighted | 3 | -0.393341358 | 0.312149780345864 | 0.207631650071154 | -1.005154927 | 0.218472211808718 | 0.674798360670638 | 0.365987928945797 | 1.24417444278939 | 0.998735809224318 |
| ebi-fl187-GCST90094764 | prot-a-752 | Cytochrome b5 \|\| id:prot-a-752 | Fried chicken liking \|\| id:ebi-fl187-GCST90094764 | Inverse variance weighted | 3 | -0.003318852 | 0.239769524731267 | 0.98895615967746 | -0.473267121 | 0.466629416307288 | 0.996686649136169 | 0.622963642503948 | 1.59461035731309 | 0.999095833221658 |
| ebi-fl187-GCST90094764 | prot-a-755 | Cytoglobin \|\| id:prot-a-755 | Fried chicken liking \|\| id:ebi-fl187-GCST90094764 | Inverse variance weighted | 3 | -0.178424159 | 0.27276401750348 | 0.513025617169139 | -0.713041633 | 0.356193315300915 | 0.836587502099294 | 0.490151068019229 | 1.42788355332377 | 0.998735809224318 |
| ebi-fl187-GCST90094764 | prot-a-807 | Desmin \|\| id:prot-a-807 | Fried chicken liking \|\| id:ebi-fl187-GCST90094764 | Inverse variance weighted | 3 | -0.639375925 | 0.239769524731267 | 0.00766166181478119 | -1.109324193 | -0.169427656 | 0.527621596917538 | 0.329781754540782 | 0.844147821099018 | 0.998735809224318 |
| ebi-fl187-GCST90094764 | prot-a-82 | Ameloblastin \|\| id:prot-a-82 | Fried chicken liking \|\| id:ebi-fl187-GCST90094764 | Inverse variance weighted | 3 | 0.437317721104035 | 0.375751962348222 | 0.244486100039229 | -0.299156125 | 1.17379156730655 | 1.5485480052861 | 0.741443642436486 | 3.23423222943204 | 0.998735809224318 |
| ebi-fl187-GCST90094764 | prot-a-84 | Muellerian-inhibiting factor \|\| id:prot-a-84 | Fried chicken liking \|\| id:ebi-fl187-GCST90094764 | Inverse variance weighted | 3 | -0.228291415 | 0.288907158926771 | 0.429417084636852 | -0.794549446 | 0.337966616884506 | 0.795892291394335 | 0.451784742447454 | 1.40209369636824 | 0.998735809224318 |
| ebi-fl187-GCST90094764 | prot-a-858 | Inactive dipeptidyl peptidase 10 \|\| id:prot-a-858 | Fried chicken liking \|\| id:ebi-fl187-GCST90094764 | Inverse variance weighted | 3 | 0.776549468449923 | 0.310458788178931 | 0.0123739546903543 | 0.168050243619219 | 1.38504869328063 | 2.17395799818421 | 1.18299604715758 | 3.99502043073149 | 0.998735809224318 |
| ebi-fl187-GCST90094764 | prot-a-863 | Dihydropyrimidinase-related protein 3 \|\| id:prot-a-863 | Fried chicken liking \|\| id:ebi-fl187-GCST90094764 | Inverse variance weighted | 3 | 0.103599841012807 | 0.239769524731267 | 0.66568258883712 | -0.366348427 | 0.57354810948609 | 1.10915652714812 | 0.693261207881216 | 1.77455220013705 | 0.998735809224318 |
| ebi-fl187-GCST90094764 | prot-a-864 | Dihydropyrimidinase-related protein 5 \|\| id:prot-a-864 | Fried chicken liking \|\| id:ebi-fl187-GCST90094764 | Inverse variance weighted (multiplicative random effects) | 3 | -0.315507267 | 0.41520126253262 | 0.447320368688254 | -1.129301742 | 0.498287207414163 | 0.729418770225618 | 0.323258895838426 | 1.64589977014395 | 0.998735809224318 |
| ebi-fl187-GCST90094764 | prot-a-884 | Endothelin-converting enzyme 1 \|\| id:prot-a-884 | Fried chicken liking \|\| id:ebi-fl187-GCST90094764 | Inverse variance weighted | 3 | -0.515917161 | 0.286514550216126 | 0.0717554559749971 | -1.077485679 | 0.0456513573649664 | 0.59695284156762 | 0.340450451971818 | 1.04670941980466 | 0.998735809224318 |
| ebi-fl187-GCST90094764 | prot-a-893 | Early endosome antigen 1 \|\| id:prot-a-893 | Fried chicken liking \|\| id:ebi-fl187-GCST90094764 | Inverse variance weighted | 2 | -0.309678659 | 0.443238843564977 | 0.484756910173327 | -1.178426792 | 0.559069474678157 | 0.733682680887942 | 0.307762532395588 | 1.74904421290314 | 0.998735809224318 |
| ebi-fl187-GCST90094764 | prot-a-897 | Ephrin-A2 \|\| id:prot-a-897 | Fried chicken liking \|\| id:ebi-fl187-GCST90094764 | Inverse variance weighted | 3 | 0.283638798279269 | 0.259957858856525 | 0.275230950249336 | -0.225878605 | 0.793156201638059 | 1.32795318514002 | 0.797814946469306 | 2.2103617759076 | 0.998735809224318 |
| ebi-fl187-GCST90094764 | prot-a-90 | Anaphase-promoting complex subunit 10 \|\| id:prot-a-90 | Fried chicken liking \|\| id:ebi-fl187-GCST90094764 | Inverse variance weighted | 3 | -0.092072157 | 0.240075455324726 | 0.70133912779954 | -0.562620049 | 0.378475735447309 | 0.912039337158148 | 0.569714426735741 | 1.46005737872899 | 0.998735809224318 |
| ebi-fl187-GCST90094764 | prot-a-914 | Histone-lysine N-methyltransferase EHMT2 \|\| id:prot-a-914 | Fried chicken liking \|\| id:ebi-fl187-GCST90094764 | Inverse variance weighted (multiplicative random effects) | 3 | 0.475077473811688 | 0.464136403911047 | 0.306037021381174 | -0.434629878 | 1.38478482547734 | 1.60813878130084 | 0.647504278405661 | 3.9939664125332 | 0.998735809224318 |
| ebi-fl187-GCST90094764 | prot-a-932 | RNA polymerase II elongation factor ELL \|\| id:prot-a-932 | Fried chicken liking \|\| id:ebi-fl187-GCST90094764 | Inverse variance weighted | 3 | -0.531138499 | 0.239769524731267 | 0.0267460399368342 | -1.001086768 | -0.061190231 | 0.587935224559085 | 0.367479858755151 | 0.940644283058945 | 0.998735809224318 |
| ebi-fl187-GCST90094764 | prot-a-991 | Estrogen receptor \|\| id:prot-a-991 | Fried chicken liking \|\| id:ebi-fl187-GCST90094764 | Inverse variance weighted | 3 | 0.109174496597783 | 0.239769524731267 | 0.648871129902718 | -0.360773772 | 0.579122765071066 | 1.11535695935637 | 0.697136692555258 | 1.7844723424683 | 0.998735809224318 |
| ebi-fl187-GCST90094764 | prot-a-992 | Persulfide dioxygenase ETHE1, mitochondrial \|\| id:prot-a-992 | Fried chicken liking \|\| id:ebi-fl187-GCST90094764 | Inverse variance weighted | 3 | 0.219923368084097 | 0.239769524731267 | 0.35902307579416 | -0.2500249 | 0.68987163655738 | 1.24598124499882 | 0.778781390870246 | 1.99345962947833 | 0.998735809224318 |
| ebi-fl187-GCST90094764 | prot-a-993 | Ethanolamine kinase 1 \|\| id:prot-a-993 | Fried chicken liking \|\| id:ebi-fl187-GCST90094764 | Inverse variance weighted | 3 | 0.311228024106089 | 0.239769524731267 | 0.194277043621676 | -0.158720244 | 0.781176292579372 | 1.36510046151212 | 0.853235022887547 | 2.18403982494072 | 0.998735809224318 |
| ebi-fl187-GCST90094764 | prot-b-10 | coagulation factor III, tissue factor \|\| id:prot-b-10 | Fried chicken liking \|\| id:ebi-fl187-GCST90094764 | Inverse variance weighted | 2 | -0.225930904 | 0.571426142854328 | 0.692561904353418 | -1.345926144 | 0.894064336001221 | 0.797773222705302 | 0.2602985222795 | 2.44504697641815 | 0.998735809224318 |
| ebi-fl187-GCST90094764 | prot-b-28 | prolactin \|\| id:prot-b-28 | Fried chicken liking \|\| id:ebi-fl187-GCST90094764 | Inverse variance weighted | 2 | -0.008590046 | 0.291709323211857 | 0.976507866116388 | -0.580340319 | 0.563160227729457 | 0.991446743262172 | 0.559707854786297 | 1.75621377531047 | 0.998955228396052 |
| ebi-fl187-GCST90094764 | prot-b-54 | matrix metallopeptidase 7 \|\| id:prot-b-54 | Fried chicken liking \|\| id:ebi-fl187-GCST90094764 | Inverse variance weighted | 2 | 0.0954213498444722 | 0.291709323211857 | 0.743583876756624 | -0.476328924 | 0.667171623339713 | 1.10012229384174 | 0.621059167598594 | 1.94871781071565 | 0.998735809224318 |
| ebi-fl187-GCST90094764 | ukb-rsfMRI191-edge-pheno716 | Net100_Pair10_33 (OccipitalPrecuneus)&(Temporal\|Frontal\|Supp_Motor_Area) (Default_mode\|Central_executive)&(Default_mode\|Salience) \|\| id:ukb-rsfMRI191-edge-pheno716 | Fried chicken liking \|\| id:ebi-fl187-GCST90094764 | Inverse variance weighted | 2 | -0.052155822 | 0.181692616058913 | 0.774070006299404 | -0.408273349 | 0.303961705821352 | 0.949180952372994 | 0.664797132265945 | 1.35521715816802 | 0.998735809224318 |
| ebi-fl187-GCST90094764 | ukb-rsfMRI191-edge-pheno903 | Net100_Pair27_38 (Precentral\|Frontal\|Supp_Motor_Area)&(Subcortical) (Attention\|Salience\|Motor)&(Subcortical-cerebellum) \|\| id:ukb-rsfMRI191-edge-pheno903 | Fried chicken liking \|\| id:ebi-fl187-GCST90094764 | Inverse variance weighted | 2 | 0.0985934124501998 | 0.0900834897137582 | 0.273750185110542 | -0.077970227 | 0.275157052289166 | 1.10361749119346 | 0.924991965559768 | 1.31673745526111 | 0.998735809224318 |
| ebi-fl187-GCST90094764 | ukb-rsfMRI191-node-pheno23 | Net100_Node2 RolandicOper\|SupraMarginal\|Insula Salience\|Motor \|\| id:ukb-rsfMRI191-node-pheno23 | Fried chicken liking \|\| id:ebi-fl187-GCST90094764 | Inverse variance weighted | 2 | 0.147261009544194 | 0.34842499480835 | 0.672552346560951 | -0.53565198 | 0.830173999368561 | 1.1586563439302 | 0.585287569767518 | 2.29371781099492 | 0.998735809224318 |
| ebi-fl187-GCST90094764 | ebi-IPs91-GCST90274819 | Leukemia inhibitory factor levels \|\| id:ebi-IPs91-GCST90274819 | Fried chicken liking \|\| id:ebi-fl187-GCST90094764 | MR Egger | 3 | -0.017931162 | 0.549496740874535 | 0.979233211156141 | -1.094944774 | 1.05908245045071 | 0.982228645017209 | 0.334558082817559 | 2.88372381550994 | 0.999950574162623 |
| ebi-fl187-GCST90094764 | ebi-IPs91-GCST90274837 | STAM binding protein levels \|\| id:ebi-IPs91-GCST90274837 | Fried chicken liking \|\| id:ebi-fl187-GCST90094764 | MR Egger | 3 | -0.190210033 | 0.618058072318585 | 0.80993362270117 | -1.401603855 | 1.02118378834807 | 0.826785463146786 | 0.246201775130804 | 2.77647958349476 | 0.999950574162623 |
| ebi-fl187-GCST90094764 | ebi-IPs91-GCST90274840 | TNF-beta levels \|\| id:ebi-IPs91-GCST90274840 | Fried chicken liking \|\| id:ebi-fl187-GCST90094764 | MR Egger | 3 | 0.957468605754439 | 0.557420751313564 | 0.335635370779533 | -0.135076067 | 2.05001327832903 | 2.60509360076198 | 0.873649453424588 | 7.7680042517383 | 0.999950574162623 |
| ebi-fl187-GCST90094764 | ebi-IPs91-GCST90274842 | Tumor necrosis factor ligand superfamily member 14 levels \|\| id:ebi-IPs91-GCST90274842 | Fried chicken liking \|\| id:ebi-fl187-GCST90094764 | MR Egger | 3 | 0.410295937097964 | 1.28705241291493 | 0.803537556601805 | -2.112326792 | 2.93291866641124 | 1.50726377438447 | 0.120956198811363 | 18.7823700471505 | 0.999950574162623 |
| ebi-fl187-GCST90094764 | prot-a-1017 | FAS-associated factor 2 \|\| id:prot-a-1017 | Fried chicken liking \|\| id:ebi-fl187-GCST90094764 | MR Egger | 3 | 1.17545216573843 | 1.04062328643286 | 0.461314920668752 | -0.864169476 | 3.21507380714684 | 3.23960745220861 | 0.421401391440688 | 24.9051300199201 | 0.999950574162623 |
| ebi-fl187-GCST90094764 | prot-a-1019 | Fas apoptotic inhibitory molecule 3 \|\| id:prot-a-1019 | Fried chicken liking \|\| id:ebi-fl187-GCST90094764 | MR Egger | 3 | 0.728299357390967 | 1.375128306 | 0.689925374001325 | -1.966952122 | 3.42355083712157 | 2.07155463623398 | 0.139882552056572 | 30.6781549793786 | 0.999950574162623 |
| ebi-fl187-GCST90094764 | prot-a-1052 | MIP18 family protein FAM96A \|\| id:prot-a-1052 | Fried chicken liking \|\| id:ebi-fl187-GCST90094764 | MR Egger | 3 | 0.993609000538459 | 1.04062328643286 | 0.514710658592655 | -1.046012641 | 3.03323064194686 | 2.70096468662212 | 0.351335862126987 | 20.7642060625823 | 0.999950574162623 |
| ebi-fl187-GCST90094764 | prot-a-1066 | F-box/LRR-repeat protein 4 \|\| id:prot-a-1066 | Fried chicken liking \|\| id:ebi-fl187-GCST90094764 | MR Egger | 3 | 1.5341190451535 | 2.4984131396572 | 0.649427998842456 | -3.362770709 | 6.4310087988816 | 4.6372385322287 | 0.0346391508701561 | 620.799894471827 | 0.999950574162623 |
| ebi-fl187-GCST90094764 | prot-a-1076 | Low affinity immunoglobulin gamma Fc region receptor III-B \|\| id:prot-a-1076 | Fried chicken liking \|\| id:ebi-fl187-GCST90094764 | MR Egger | 3 | 1.26364893571021 | 1.04062328643286 | 0.438574247888555 | -0.775972706 | 3.30327057711861 | 3.53830902231792 | 0.460255869684268 | 27.2014580628917 | 0.999950574162623 |
| ebi-fl187-GCST90094764 | prot-a-1081 | Fc receptor-like protein 3 \|\| id:prot-a-1081 | Fried chicken liking \|\| id:ebi-fl187-GCST90094764 | MR Egger | 3 | 0.41871742517226 | 1.04062328643286 | 0.756461535341258 | -1.620904216 | 2.45833906658066 | 1.5200107773156 | 0.197719836744094 | 11.6853867634229 | 0.999950574162623 |
| ebi-fl187-GCST90094764 | prot-a-1105 | Fibroblast growth factor receptor 4 \|\| id:prot-a-1105 | Fried chicken liking \|\| id:ebi-fl187-GCST90094764 | MR Egger | 3 | 0.132828026707471 | 1.8933367866093 | 0.955410666308911 | -3.578112075 | 3.84376812846169 | 1.14205357872774 | 0.0279283751909769 | 46.7011191222545 | 0.999950574162623 |
| ebi-fl187-GCST90094764 | prot-a-1143 | Follistatin-related protein 1 \|\| id:prot-a-1143 | Fried chicken liking \|\| id:ebi-fl187-GCST90094764 | MR Egger | 3 | 0.507614417603617 | 1.04062328643286 | 0.711077244930861 | -1.532007224 | 2.54723605901202 | 1.66132324040942 | 0.216101467683685 | 12.771754577643 | 0.999950574162623 |
| ebi-fl187-GCST90094764 | prot-a-1151 | Alpha-(1,3)-fucosyltransferase 10 \|\| id:prot-a-1151 | Fried chicken liking \|\| id:ebi-fl187-GCST90094764 | MR Egger | 3 | 0.143777149920225 | 1.04062328643286 | 0.912595141721614 | -1.895844491 | 2.18339879132863 | 1.15462677118409 | 0.150191446077804 | 8.87642416096296 | 0.999950574162623 |
| ebi-fl187-GCST90094764 | prot-a-1165 | Growth arrest and DNA damage-inducible proteins-interacting protein 1 \|\| id:prot-a-1165 | Fried chicken liking \|\| id:ebi-fl187-GCST90094764 | MR Egger | 3 | 0.194141034926805 | 1.04062328643286 | 0.882580635327406 | -1.845480606 | 2.23376267633521 | 1.21426752501191 | 0.157949391143797 | 9.3349243806594 | 0.999950574162623 |
| ebi-fl187-GCST90094764 | prot-a-1194 | Growth/differentiation factor 11/8 \|\| id:prot-a-1194 | Fried chicken liking \|\| id:ebi-fl187-GCST90094764 | MR Egger | 3 | 0.544460702973801 | 1.74712440714597 | 0.807680776307753 | -2.879903135 | 3.9688245409799 | 1.72367855695304 | 0.0561402005894822 | 52.9222862851036 | 0.999950574162623 |
| ebi-fl187-GCST90094764 | prot-a-1216 | D-glucuronyl C5-epimerase \|\| id:prot-a-1216 | Fried chicken liking \|\| id:ebi-fl187-GCST90094764 | MR Egger | 3 | 1.97307074893845 | 1.04062328643286 | 0.308974998899758 | -0.066550892 | 4.01269239034685 | 7.19272967813652 | 0.935615298871181 | 55.2955475238216 | 0.999950574162623 |
| ebi-fl187-GCST90094764 | prot-a-1246 | Glypican-3 \|\| id:prot-a-1246 | Fried chicken liking \|\| id:ebi-fl187-GCST90094764 | MR Egger | 3 | -0.495986993 | 1.0609419157098 | 0.721600664066721 | -2.575433147 | 1.583459162 | 0.608969562125842 | 0.0761208441109883 | 4.8717789710139 | 0.999950574162623 |
| ebi-fl187-GCST90094764 | prot-a-1275 | Glutamate receptor 4 \|\| id:prot-a-1275 | Fried chicken liking \|\| id:ebi-fl187-GCST90094764 | MR Egger | 3 | -1.314948222 | 1.04062328643286 | 0.426193060838388 | -3.354569864 | 0.72467341926478 | 0.268488224624075 | 0.034924389177118 | 2.06405690866077 | 0.999950574162623 |
| ebi-fl187-GCST90094764 | prot-a-1289 | Glutathione S-transferase P \|\| id:prot-a-1289 | Fried chicken liking \|\| id:ebi-fl187-GCST90094764 | MR Egger | 3 | -1.609727694 | 1.04062328643286 | 0.365344664588041 | -3.649349335 | 0.429893947503261 | 0.19994205210231 | 0.0260080457914673 | 1.53709450219434 | 0.999950574162623 |
| ebi-fl187-GCST90094764 | prot-a-130 | Apolipoprotein D \|\| id:prot-a-130 | Fried chicken liking \|\| id:ebi-fl187-GCST90094764 | MR Egger | 3 | -0.422781592 | 1.04259609642894 | 0.75474474330639 | -2.466269941 | 1.62070675670812 | 0.655221722959624 | 0.0849009546525088 | 5.05666288436125 | 0.999950574162623 |
| ebi-fl187-GCST90094764 | prot-a-1313 | Heparin-binding EGF-like growth factor \|\| id:prot-a-1313 | Fried chicken liking \|\| id:ebi-fl187-GCST90094764 | MR Egger | 3 | -0.694721402 | 1.17500539182974 | 0.660070190829739 | -2.99773197 | 1.60828916643053 | 0.499213508720051 | 0.0499001151035942 | 4.99425956776268 | 0.999950574162623 |
| ebi-fl187-GCST90094764 | prot-a-1347 | HLA class II histocompatibility antigen, DQ alpha 2 chain \|\| id:prot-a-1347 | Fried chicken liking \|\| id:ebi-fl187-GCST90094764 | MR Egger | 3 | 1.41229116547885 | 1.04259609642894 | 0.404842959071468 | -0.631197184 | 3.45577951447957 | 4.10535067464569 | 0.531954572394753 | 31.6829763976667 | 0.999950574162623 |
| ebi-fl187-GCST90094764 | prot-a-1357 | Non-histone chromosomal protein HMG-14 \|\| id:prot-a-1357 | Fried chicken liking \|\| id:ebi-fl187-GCST90094764 | MR Egger | 3 | 0.761336471869039 | 1.04062328643286 | 0.597891186046171 | -1.27828517 | 2.80095811327744 | 2.14113587701334 | 0.278514496323272 | 16.4604101558595 | 0.999950574162623 |
| ebi-fl187-GCST90094764 | prot-a-137 | Apolipoprotein M \|\| id:prot-a-137 | Fried chicken liking \|\| id:ebi-fl187-GCST90094764 | MR Egger | 3 | -0.176604582 | 1.38698351331559 | 0.919373082306641 | -2.895092268 | 2.54188310426185 | 0.838111123371018 | 0.0552939230032076 | 12.7035706089706 | 0.999950574162623 |
| ebi-fl187-GCST90094764 | prot-a-1381 | 17-beta-hydroxysteroid dehydrogenase 14 \|\| id:prot-a-1381 | Fried chicken liking \|\| id:ebi-fl187-GCST90094764 | MR Egger | 3 | -0.631777764 | 1.04259609642894 | 0.653172918474323 | -2.675266113 | 1.41171058485911 | 0.531645819516082 | 0.0688884938216741 | 4.10296787937607 | 0.999950574162623 |
| ebi-fl187-GCST90094764 | prot-a-1392 | Serine protease HTRA2, mitochondrial \|\| id:prot-a-1392 | Fried chicken liking \|\| id:ebi-fl187-GCST90094764 | MR Egger | 3 | 1.83017392791901 | 1.04062328643286 | 0.329135980569436 | -0.209447713 | 3.86979556932742 | 6.23497099975447 | 0.811032044360071 | 47.9325861883209 | 0.999950574162623 |
| ebi-fl187-GCST90094764 | prot-a-1416 | Interferon-induced protein with tetratricopeptide repeats 2 \|\| id:prot-a-1416 | Fried chicken liking \|\| id:ebi-fl187-GCST90094764 | MR Egger | 3 | 2.58921534567342 | 1.04062328643286 | 0.243283672657876 | 0.549593704265012 | 4.62883698708182 | 13.3193164426149 | 1.73254894759864 | 102.394908232981 | 0.999950574162623 |
| ebi-fl187-GCST90094764 | prot-a-1428 | Interferon gamma \|\| id:prot-a-1428 | Fried chicken liking \|\| id:ebi-fl187-GCST90094764 | MR Egger | 3 | 0.382265613336895 | 1.04062328643286 | 0.775883874006586 | -1.657356028 | 2.4218872547453 | 1.46560131669441 | 0.190642367405123 | 11.2671031562036 | 0.999950574162623 |
| ebi-fl187-GCST90094764 | prot-a-1483 | Interleukin-17C \|\| id:prot-a-1483 | Fried chicken liking \|\| id:ebi-fl187-GCST90094764 | MR Egger | 3 | -0.102551011 | 1.04259609642894 | 0.937582089838524 | -2.14603936 | 1.9409373377698 | 0.902532109304803 | 0.116946424392651 | 6.96527672869462 | 0.999950574162623 |
| ebi-fl187-GCST90094764 | prot-a-1503 | Interleukin-1 receptor-like 2 \|\| id:prot-a-1503 | Fried chicken liking \|\| id:ebi-fl187-GCST90094764 | MR Egger | 3 | 1.89705395492568 | 1.04062328643286 | 0.319409323379748 | -0.142567686 | 3.93667559633409 | 6.66622648146488 | 0.867128859403293 | 51.2479489297169 | 0.999950574162623 |
| ebi-fl187-GCST90094764 | prot-a-1522 | Interleukin-31 receptor subunit alpha \|\| id:prot-a-1522 | Fried chicken liking \|\| id:ebi-fl187-GCST90094764 | MR Egger | 3 | -1.903836721 | 1.04062328643286 | 0.318452165388555 | -3.943458362 | 0.135784920475173 | 0.148995865620774 | 0.0193810719408917 | 1.14543550737485 | 0.999950574162623 |
| ebi-fl187-GCST90094764 | prot-a-1540 | Interleukin-6 receptor subunit alpha \|\| id:prot-a-1540 | Fried chicken liking \|\| id:ebi-fl187-GCST90094764 | MR Egger | 3 | 0.0795348438264442 | 1.49492046235441 | 0.966161570432878 | -2.850509262 | 3.00957895004109 | 1.08278328718508 | 0.0578148704374821 | 20.27885971 | 0.999950574162623 |
| ebi-fl187-GCST90094764 | prot-a-1541 | Interleukin-6 receptor subunit alpha \|\| id:prot-a-1541 | Fried chicken liking \|\| id:ebi-fl187-GCST90094764 | MR Egger | 3 | -0.345121328 | 1.51973764432517 | 0.857839359969044 | -3.323807111 | 2.63356445511966 | 0.708134431892811 | 0.0360154555928401 | 13.9233105725822 | 0.999950574162623 |
| ebi-fl187-GCST90094764 | prot-a-1545 | Interleukin-9 \|\| id:prot-a-1545 | Fried chicken liking \|\| id:ebi-fl187-GCST90094764 | MR Egger | 3 | 0.887934175203086 | 1.04062328643286 | 0.550297990025977 | -1.151687466 | 2.92755581661149 | 2.43010429261502 | 0.316102906096172 | 18.681912627495 | 0.999950574162623 |
| ebi-fl187-GCST90094764 | prot-a-1548 | Inositol monophosphatase 3 \|\| id:prot-a-1548 | Fried chicken liking \|\| id:ebi-fl187-GCST90094764 | MR Egger | 3 | 2.61493882463711 | 1.04062328643286 | 0.241113789165954 | 0.575317183228703 | 4.65456046604551 | 13.6663803065459 | 1.77769429231613 | 105.063031079323 | 0.999950574162623 |
| ebi-fl187-GCST90094764 | prot-a-1555 | Inhibin beta A chain:Inhibin beta B chain heterodimer \|\| id:prot-a-1555 | Fried chicken liking \|\| id:ebi-fl187-GCST90094764 | MR Egger | 3 | 0.450796153093054 | 1.08291211353423 | 0.748877190720445 | -1.671711589 | 2.57330389562014 | 1.56956129926444 | 0.187925139531465 | 13.1090639511602 | 0.999950574162623 |
| ebi-fl187-GCST90094764 | prot-a-1603 | Junctophilin-3 \|\| id:prot-a-1603 | Fried chicken liking \|\| id:ebi-fl187-GCST90094764 | MR Egger | 3 | 1.8672598751827 | 1.11833801570455 | 0.343535628997781 | -0.324682636 | 4.05920238596362 | 6.47054199305437 | 0.722756694493821 | 57.928088391076 | 0.999950574162623 |
| ebi-fl187-GCST90094764 | prot-a-1627 | UPF0258 protein KIAA1024 \|\| id:prot-a-1627 | Fried chicken liking \|\| id:ebi-fl187-GCST90094764 | MR Egger | 3 | 1.49643056589279 | 1.63593172823678 | 0.528333425303636 | -1.709995621 | 4.70285675323689 | 4.46572049285948 | 0.180866584548536 | 110.261713462022 | 0.999950574162623 |
| ebi-fl187-GCST90094764 | prot-a-1666 | Kallikrein-7 \|\| id:prot-a-1666 | Fried chicken liking \|\| id:ebi-fl187-GCST90094764 | MR Egger | 3 | 0.904870694106279 | 1.04062328643286 | 0.544350139612347 | -1.134750947 | 2.94449233551468 | 2.47161230835542 | 0.321502182350159 | 19.0010137976627 | 0.999950574162623 |
| ebi-fl187-GCST90094764 | prot-a-1678 | Importin subunit alpha-3 \|\| id:prot-a-1678 | Fried chicken liking \|\| id:ebi-fl187-GCST90094764 | MR Egger | 3 | 0.709860344783894 | 1.04062328643286 | 0.61889086436391 | -1.329761297 | 2.7494819861923 | 2.0337072209921 | 0.264540400450301 | 15.6345308832797 | 0.999950574162623 |
| ebi-fl187-GCST90094764 | prot-a-1718 | Protein LDOC1 \|\| id:prot-a-1718 | Fried chicken liking \|\| id:ebi-fl187-GCST90094764 | MR Egger | 3 | 1.75405739479098 | 1.04062328643286 | 0.340880348842074 | -0.285564247 | 3.79367903619938 | 5.77799880249192 | 0.751590052508601 | 44.4195210542865 | 0.999950574162623 |
| ebi-fl187-GCST90094764 | prot-a-1723 | Leptin \|\| id:prot-a-1723 | Fried chicken liking \|\| id:ebi-fl187-GCST90094764 | MR Egger | 3 | -0.466858875 | 1.04062328643286 | 0.731526435692036 | -2.506480516 | 1.57276276673399 | 0.626968565312117 | 0.0815547654182531 | 4.81994620269676 | 0.999950574162623 |
| ebi-fl187-GCST90094764 | prot-a-1730 | Galectin-7 \|\| id:prot-a-1730 | Fried chicken liking \|\| id:ebi-fl187-GCST90094764 | MR Egger | 3 | -0.719943528 | 1.69176807251195 | 0.743860982534626 | -4.03580895 | 2.5959218944014 | 0.486779744744841 | 0.0176713790701782 | 13.408943294852 | 0.999950574162623 |
| ebi-fl187-GCST90094764 | prot-a-1753 | Lipase member N \|\| id:prot-a-1753 | Fried chicken liking \|\| id:ebi-fl187-GCST90094764 | MR Egger | 3 | 0.0400184025961303 | 1.04062328643286 | 0.975530089580228 | -1.999603239 | 2.07964004400453 | 1.04082992798895 | 0.135388989677942 | 8.00158817622071 | 0.999950574162623 |
| ebi-fl187-GCST90094764 | prot-a-1756 | VIP36-like protein \|\| id:prot-a-1756 | Fried chicken liking \|\| id:ebi-fl187-GCST90094764 | MR Egger | 3 | 0.263691826361992 | 1.04062328643286 | 0.842007406840813 | -1.775929815 | 2.3033134677704 | 1.30172697654334 | 0.169325934479274 | 10.0072864010567 | 0.999950574162623 |
| ebi-fl187-GCST90094764 | prot-a-1760 | Ig Kappa chain V-I region HK102- like \|\| id:prot-a-1760 | Fried chicken liking \|\| id:ebi-fl187-GCST90094764 | MR Egger | 3 | -1.07349426 | 1.04062328643286 | 0.490102430396869 | -3.113115901 | 0.966127381529518 | 0.341812048122895 | 0.0444621994532022 | 2.62774846226267 | 0.999950574162623 |
| ebi-fl187-GCST90094764 | prot-a-179 | Neutral ceramidase \|\| id:prot-a-179 | Fried chicken liking \|\| id:ebi-fl187-GCST90094764 | MR Egger | 3 | -0.138528644 | 1.04259609642894 | 0.915905565756183 | -2.182016993 | 1.90495970497832 | 0.870638312328623 | 0.112813756448524 | 6.71913687441397 | 0.999950574162623 |
| ebi-fl187-GCST90094764 | prot-a-1809 | Lactotransferrin \|\| id:prot-a-1809 | Fried chicken liking \|\| id:ebi-fl187-GCST90094764 | MR Egger | 3 | -1.14587511 | 1.04062328643286 | 0.469378569205804 | -3.185496752 | 0.893746531098251 | 0.317945558574298 | 0.0413576961907076 | 2.44427005196279 | 0.999950574162623 |
| ebi-fl187-GCST90094764 | prot-a-1818 | Lysophospholipase-like protein 1 \|\| id:prot-a-1818 | Fried chicken liking \|\| id:ebi-fl187-GCST90094764 | MR Egger | 3 | 0.00189777072026245 | 1.04259609642894 | 0.99884120319099 | -2.041590578 | 2.04538611972098 | 1.0018995726268 | 0.129822054430265 | 7.73214349468622 | 0.999974376584515 |
| ebi-fl187-GCST90094764 | prot-a-1852 | MAP kinase-activated protein kinase 3 \|\| id:prot-a-1852 | Fried chicken liking \|\| id:ebi-fl187-GCST90094764 | MR Egger | 3 | -0.048802749 | 1.04259609642894 | 0.970222281434847 | -2.092291098 | 1.99468560047098 | 0.952368967351308 | 0.123404080903057 | 7.34989186205532 | 0.999950574162623 |
| ebi-fl187-GCST90094764 | prot-a-1856 | Megakaryocyte-associated tyrosine-protein kinase \|\| id:prot-a-1856 | Fried chicken liking \|\| id:ebi-fl187-GCST90094764 | MR Egger | 3 | -1.592443167 | 1.04259609642894 | 0.369037356341392 | -3.635931516 | 0.451045181682215 | 0.203427995471639 | 0.0263593687653921 | 1.5699522135728 | 0.999950574162623 |
| ebi-fl187-GCST90094764 | prot-a-1890 | Beta-1,3-N-acetylglucosaminyltransferase manic fringe \|\| id:prot-a-1890 | Fried chicken liking \|\| id:ebi-fl187-GCST90094764 | MR Egger | 3 | 2.1403644397494 | 1.04062328643286 | 0.2880949624006 | 0.100742798340998 | 4.17998608115781 | 8.50253572630628 | 1.1059921421643 | 65.3649434033459 | 0.999950574162623 |
| ebi-fl187-GCST90094764 | prot-a-1919 | Matrilysin \|\| id:prot-a-1919 | Fried chicken liking \|\| id:ebi-fl187-GCST90094764 | MR Egger | 3 | -1.321144177 | 3.16414616202182 | 0.748196876509014 | -7.522870654 | 4.88058230102961 | 0.266829826814237 | 0.000540578522402704 | 131.707334877572 | 0.999950574162623 |
| ebi-fl187-GCST90094764 | prot-a-1923 | Multimerin-2 \|\| id:prot-a-1923 | Fried chicken liking \|\| id:ebi-fl187-GCST90094764 | MR Egger | 3 | -0.519868378 | 1.04259609642894 | 0.705531524647655 | -2.563356727 | 1.52361997105441 | 0.594598805135752 | 0.0770456845710794 | 4.58880651183902 | 0.999950574162623 |
| ebi-fl187-GCST90094764 | prot-a-1948 | Mesothelin \|\| id:prot-a-1948 | Fried chicken liking \|\| id:ebi-fl187-GCST90094764 | MR Egger | 3 | -0.864930002 | 1.04062328643286 | 0.55853128474802 | -2.904551643 | 1.1746916397934 | 0.42108102658756 | 0.0547733431074491 | 3.23714458334605 | 0.999950574162623 |
| ebi-fl187-GCST90094764 | prot-a-195 | ATPase family AAA domain-containing protein 1 \|\| id:prot-a-195 | Fried chicken liking \|\| id:ebi-fl187-GCST90094764 | MR Egger | 3 | 1.28101297166385 | 1.04062328643286 | 0.434316409434394 | -0.75860867 | 3.32063461307225 | 3.60028486559644 | 0.468317558323573 | 27.6779097496211 | 0.999950574162623 |
| ebi-fl187-GCST90094764 | prot-a-1966 | Mucin-1 \|\| id:prot-a-1966 | Fried chicken liking \|\| id:ebi-fl187-GCST90094764 | MR Egger | 3 | 1.30393229499974 | 1.04062328643286 | 0.428801748889357 | -0.735689346 | 3.34355393640815 | 3.68375383084404 | 0.479175027512772 | 28.3195941088487 | 0.999950574162623 |
| ebi-fl187-GCST90094764 | prot-a-1986 | Myocardial zonula adherens protein \|\| id:prot-a-1986 | Fried chicken liking \|\| id:ebi-fl187-GCST90094764 | MR Egger | 3 | 1.29848001252223 | 1.04062328643286 | 0.430102900023291 | -0.741141629 | 3.33810165393064 | 3.66372361918715 | 0.476569539290021 | 28.1656078518714 | 0.999950574162623 |
| ebi-fl187-GCST90094764 | prot-a-2071 | Epididymal secretory protein E1 \|\| id:prot-a-2071 | Fried chicken liking \|\| id:ebi-fl187-GCST90094764 | MR Egger | 3 | 1.52860842436047 | 1.04062328643286 | 0.380507422639052 | -0.511013217 | 3.56823006576887 | 4.61175474937128 | 0.599887454587543 | 35.4537867156627 | 0.999950574162623 |
| ebi-fl187-GCST90094764 | prot-a-2098 | Neuritin \|\| id:prot-a-2098 | Fried chicken liking \|\| id:ebi-fl187-GCST90094764 | MR Egger | 3 | 1.03633181964712 | 1.04062328643286 | 0.501315400950321 | -1.003289822 | 3.07595346105552 | 2.81885794716442 | 0.366671171965262 | 21.6705340747235 | 0.999950574162623 |
| ebi-fl187-GCST90094764 | prot-a-2115 | Neurotrophin-4 \|\| id:prot-a-2115 | Fried chicken liking \|\| id:ebi-fl187-GCST90094764 | MR Egger | 3 | 1.48665450189727 | 1.04259609642894 | 0.389356845032227 | -0.556833847 | 3.53014285089799 | 4.42227602747567 | 0.573020465154396 | 34.1288426023609 | 0.999950574162623 |
| ebi-fl187-GCST90094764 | prot-a-2118 | Netrin-1 \|\| id:prot-a-2118 | Fried chicken liking \|\| id:ebi-fl187-GCST90094764 | MR Egger | 3 | 1.44493954994457 | 1.22574772474085 | 0.447867429830623 | -0.957525991 | 3.84740509043664 | 4.24159573037343 | 0.38384133935185 | 46.8712785608286 | 0.999950574162623 |
| ebi-fl187-GCST90094764 | prot-a-2120 | Netrin-G1 \|\| id:prot-a-2120 | Fried chicken liking \|\| id:ebi-fl187-GCST90094764 | MR Egger | 3 | 1.5418935022507 | 1.04062328643286 | 0.37794885943719 | -0.497728139 | 3.5815151436591 | 4.67343105054138 | 0.607910179413131 | 35.9279356125428 | 0.999950574162623 |
| ebi-fl187-GCST90094764 | prot-a-2145 | Olfactomedin-like protein 3 \|\| id:prot-a-2145 | Fried chicken liking \|\| id:ebi-fl187-GCST90094764 | MR Egger | 3 | 1.78152725126156 | 1.04062328643286 | 0.336555524204641 | -0.25809439 | 3.82114889266997 | 5.93891971849375 | 0.77252231017113 | 45.6566327707748 | 0.999950574162623 |
| ebi-fl187-GCST90094764 | prot-a-2162 | Prolyl 4-hydroxylase subunit alpha-2 \|\| id:prot-a-2162 | Fried chicken liking \|\| id:ebi-fl187-GCST90094764 | MR Egger | 3 | -0.280896568 | 2.24173785103792 | 0.92064320549614 | -4.674702756 | 4.11290962028626 | 0.755106433800093 | 0.0093282975081668 | 61.1243076099475 | 0.999950574162623 |
| ebi-fl187-GCST90094764 | prot-a-2168 | Polyadenylate-binding protein-interacting protein 1 \|\| id:prot-a-2168 | Fried chicken liking \|\| id:ebi-fl187-GCST90094764 | MR Egger | 3 | 0.605957800323695 | 1.15677891642017 | 0.692811273616126 | -1.661328876 | 2.87324447650723 | 1.83300702309087 | 0.189886476810771 | 17.6943340206829 | 0.999950574162623 |
| ebi-fl187-GCST90094764 | prot-a-219 | Beta-1,4-galactosyltransferase 5 \|\| id:prot-a-219 | Fried chicken liking \|\| id:ebi-fl187-GCST90094764 | MR Egger | 3 | 0.405365651682386 | 1.04062328643286 | 0.763522626723078 | -1.63425599 | 2.44498729309079 | 1.49985082277977 | 0.195097471837819 | 11.5304030821228 | 0.999950574162623 |
| ebi-fl187-GCST90094764 | prot-a-2190 | Pyruvate carboxylase, mitochondrial \|\| id:prot-a-2190 | Fried chicken liking \|\| id:ebi-fl187-GCST90094764 | MR Egger | 3 | 0.725304428368391 | 1.04062328643286 | 0.612487486061284 | -1.314317213 | 2.7649260697768 | 2.06535975837229 | 0.268657696601582 | 15.8778660930368 | 0.999950574162623 |
| ebi-fl187-GCST90094764 | prot-a-2197 | Protocadherin-9 \|\| id:prot-a-2197 | Fried chicken liking \|\| id:ebi-fl187-GCST90094764 | MR Egger | 3 | 0.335273856896276 | 1.04259609642894 | 0.80192729598799 | -1.708214492 | 2.378762206 | 1.39832327326342 | 0.181189018393092 | 10.7915368927494 | 0.999950574162623 |
| ebi-fl187-GCST90094764 | prot-a-2198 | Protocadherin alpha-4 \|\| id:prot-a-2198 | Fried chicken liking \|\| id:ebi-fl187-GCST90094764 | MR Egger | 3 | -2.849347721 | 1.126659579 | 0.239714670610547 | -5.057600497 | -0.641094946 | 0.0578820637943157 | 0.00636080398567553 | 0.526715383249373 | 0.999950574162623 |
| ebi-fl187-GCST90094764 | prot-a-2254 | Placenta growth factor \|\| id:prot-a-2254 | Fried chicken liking \|\| id:ebi-fl187-GCST90094764 | MR Egger | 3 | 0.376842500017148 | 1.17578879755093 | 0.802547205728504 | -1.927703543 | 2.68138854321697 | 1.45767470754912 | 0.145481908081738 | 14.6053593951668 | 0.999950574162623 |
| ebi-fl187-GCST90094764 | prot-a-2261 | Membrane-associated progesterone receptor component 2 \|\| id:prot-a-2261 | Fried chicken liking \|\| id:ebi-fl187-GCST90094764 | MR Egger | 3 | -0.808679177 | 1.04062328643286 | 0.579431658629604 | -2.848300818 | 1.23094246447528 | 0.445446033233012 | 0.0579426924358238 | 3.42445544350211 | 0.999950574162623 |
| ebi-fl187-GCST90094764 | prot-a-2268 | Polymeric immunoglobulin receptor \|\| id:prot-a-2268 | Fried chicken liking \|\| id:ebi-fl187-GCST90094764 | MR Egger | 3 | -0.317639848 | 1.04062328643286 | 0.811396827490438 | -2.35726149 | 1.7219817931949 | 0.727864883052796 | 0.0946791483301942 | 5.59560681866109 | 0.999950574162623 |
| ebi-fl187-GCST90094764 | prot-a-2283 | Group 10 secretory phospholipase A2 \|\| id:prot-a-2283 | Fried chicken liking \|\| id:ebi-fl187-GCST90094764 | MR Egger | 3 | 2.74841128437824 | 1.04062328643286 | 0.230422545715144 | 0.708789642969836 | 4.78803292578665 | 15.6177999212351 | 2.03153089228878 | 120.064959536465 | 0.999950574162623 |
| ebi-fl187-GCST90094764 | prot-a-2298 | Inactive phospholipase D5 \|\| id:prot-a-2298 | Fried chicken liking \|\| id:ebi-fl187-GCST90094764 | MR Egger | 3 | 0.915860109313491 | 1.91614975889762 | 0.716151645089189 | -2.839793418 | 4.67151363675283 | 2.49892367535981 | 0.0584377368948492 | 106.85936634593 | 0.999950574162623 |
| ebi-fl187-GCST90094764 | prot-a-2304 | Procollagen-lysine,2-oxoglutarate 5-dioxygenase 2 \|\| id:prot-a-2304 | Fried chicken liking \|\| id:ebi-fl187-GCST90094764 | MR Egger | 3 | 1.19453606726229 | 1.04062328643286 | 0.456231537166726 | -0.845085574 | 3.2341577086707 | 3.30202549736434 | 0.429520600779369 | 25.3849812219949 | 0.999950574162623 |
| ebi-fl187-GCST90094764 | prot-a-2331 | Periostin \|\| id:prot-a-2331 | Fried chicken liking \|\| id:ebi-fl187-GCST90094764 | MR Egger | 3 | 1.32703294191362 | 1.27851593205845 | 0.488147096747138 | -1.178858285 | 3.83292416874818 | 3.76984143884498 | 0.307629763717672 | 46.1974299959987 | 0.999950574162623 |
| ebi-fl187-GCST90094764 | prot-a-2333 | Periostin \|\| id:prot-a-2333 | Fried chicken liking \|\| id:ebi-fl187-GCST90094764 | MR Egger | 3 | 0.664212819058524 | 1.04062328643286 | 0.638339732315624 | -1.375408822 | 2.70383446046693 | 1.94296045775501 | 0.252736250453431 | 14.936896996876 | 0.999950574162623 |
| ebi-fl187-GCST90094764 | prot-a-2367 | cAMP-dependent protein kinase catalytic subunit alpha \|\| id:prot-a-2367 | Fried chicken liking \|\| id:ebi-fl187-GCST90094764 | MR Egger | 3 | -0.498367169 | 1.04259609642894 | 0.716132460774957 | -2.541855518 | 1.54512117962029 | 0.607521830532333 | 0.0787201974187494 | 4.68853974806534 | 0.999950574162623 |
| ebi-fl187-GCST90094764 | prot-a-2385 | Phosphoribosyl pyrophosphate synthase-associated protein 1 \|\| id:prot-a-2385 | Fried chicken liking \|\| id:ebi-fl187-GCST90094764 | MR Egger | 3 | 1.1246168512969 | 1.04259609642894 | 0.475917860087773 | -0.918871498 | 3.16810520029762 | 3.07903689389066 | 0.398969024593406 | 23.7624166527752 | 0.999950574162623 |
| ebi-fl187-GCST90094764 | prot-a-2393 | Trypsin-3 \|\| id:prot-a-2393 | Fried chicken liking \|\| id:ebi-fl187-GCST90094764 | MR Egger | 3 | -0.541785456 | 1.04062328643286 | 0.694410145392024 | -2.581407097 | 1.49783618578937 | 0.581708709537265 | 0.0756674576251671 | 4.4720020121168 | 0.999950574162623 |
| ebi-fl187-GCST90094764 | prot-a-2405 | Pregnancy-specific beta-1-glycoprotein 6 \|\| id:prot-a-2405 | Fried chicken liking \|\| id:ebi-fl187-GCST90094764 | MR Egger | 3 | 0.122740861079981 | 1.69973684993084 | 0.954108271147452 | -3.208743365 | 3.45422508694442 | 1.13059140274783 | 0.0404073586910111 | 31.6337657638498 | 0.999950574162623 |
| ebi-fl187-GCST90094764 | prot-a-244 | Beclin-1 \|\| id:prot-a-244 | Fried chicken liking \|\| id:ebi-fl187-GCST90094764 | MR Egger | 3 | -0.18354875 | 1.04062328643286 | 0.888854008896387 | -2.223170392 | 1.8560728909329 | 0.832311299177236 | 0.108265320647407 | 6.39855952576159 | 0.999950574162623 |
| ebi-fl187-GCST90094764 | prot-a-2454 | tRNA pseudouridine synthase A, mitochondrial \|\| id:prot-a-2454 | Fried chicken liking \|\| id:ebi-fl187-GCST90094764 | MR Egger | 3 | -0.719454407 | 1.20048426830354 | 0.656284401203076 | -3.072403572 | 1.63349475930835 | 0.487017897254007 | 0.0463097122327752 | 5.12174273624289 | 0.999950574162623 |
| ebi-fl187-GCST90094764 | prot-a-2466 | Glutaminyl-peptide cyclotransferase-like protein \|\| id:prot-a-2466 | Fried chicken liking \|\| id:ebi-fl187-GCST90094764 | MR Egger | 3 | -0.093136638 | 1.04259609642894 | 0.943280378941594 | -2.136624987 | 1.95035171115975 | 0.911069005289113 | 0.118052600505926 | 7.03116008322769 | 0.999950574162623 |
| ebi-fl187-GCST90094764 | prot-a-2474 | Ras-related protein Rab-27A \|\| id:prot-a-2474 | Fried chicken liking \|\| id:ebi-fl187-GCST90094764 | MR Egger | 3 | 3.6233363528897 | 1.04062328643286 | 0.178045349531793 | 1.5837147114813 | 5.66295799429811 | 37.4623471625621 | 4.87302410981157 | 287.999284079593 | 0.999950574162623 |
| ebi-fl187-GCST90094764 | prot-a-2490 | Rap1 GTPase-GDP dissociation stimulator 1 \|\| id:prot-a-2490 | Fried chicken liking \|\| id:ebi-fl187-GCST90094764 | MR Egger | 3 | 1.56445261270417 | 1.59115192206709 | 0.505386258792251 | -1.554205155 | 4.68311037995567 | 4.78005767678784 | 0.211357312291635 | 108.105800294673 | 0.999950574162623 |
| ebi-fl187-GCST90094764 | prot-a-2545 | Receptor-interacting serine/threonine-protein kinase 2 \|\| id:prot-a-2545 | Fried chicken liking \|\| id:ebi-fl187-GCST90094764 | MR Egger | 3 | 0.210230268444835 | 1.17742285885115 | 0.887516126125113 | -2.097518535 | 2.51797907179309 | 1.23396216979455 | 0.122760676940671 | 12.4035047250509 | 0.999950574162623 |
| ebi-fl187-GCST90094764 | prot-a-2610 | RUN and FYVE domain-containing protein 1 \|\| id:prot-a-2610 | Fried chicken liking \|\| id:ebi-fl187-GCST90094764 | MR Egger | 3 | 1.19764270602537 | 1.78249298156861 | 0.623368487414047 | -2.296043538 | 4.69132894989985 | 3.31229964854613 | 0.100656299784751 | 108.997936395641 | 0.999950574162623 |
| ebi-fl187-GCST90094764 | prot-a-2627 | Serine--tRNA ligase, mitochondrial \|\| id:prot-a-2627 | Fried chicken liking \|\| id:ebi-fl187-GCST90094764 | MR Egger | 3 | 0.0586282610200477 | 1.04259609642894 | 0.964238650446108 | -1.984860088 | 2.10211661002077 | 1.06038098251932 | 0.137399836660793 | 8.18347281492433 | 0.999950574162623 |
| ebi-fl187-GCST90094764 | prot-a-264 | BolA-like protein 2 \|\| id:prot-a-264 | Fried chicken liking \|\| id:ebi-fl187-GCST90094764 | MR Egger | 3 | -0.904784084 | 1.04259609642894 | 0.544977263004514 | -2.948272433 | 1.13870426484655 | 0.404629241536794 | 0.0524302044376988 | 3.12271952517733 | 0.999950574162623 |
| ebi-fl187-GCST90094764 | prot-a-2646 | Secretoglobin family 1C member 1 \|\| id:prot-a-2646 | Fried chicken liking \|\| id:ebi-fl187-GCST90094764 | MR Egger | 3 | 0.496197702555535 | 2.4871001586651 | 0.874634956266469 | -4.378518608 | 5.37091401353913 | 1.64246424510053 | 0.0125439273321414 | 215.059345052274 | 0.999950574162623 |
| ebi-fl187-GCST90094764 | prot-a-2672 | Semaphorin-3E \|\| id:prot-a-2672 | Fried chicken liking \|\| id:ebi-fl187-GCST90094764 | MR Egger | 3 | 1.86843106414518 | 1.04062328643286 | 0.323507050648343 | -0.171190577 | 3.90805270555358 | 6.47812465991899 | 0.842660966147298 | 49.8018785672751 | 0.999950574162623 |
| ebi-fl187-GCST90094764 | prot-a-2701 | Plasma protease C1 inhibitor \|\| id:prot-a-2701 | Fried chicken liking \|\| id:ebi-fl187-GCST90094764 | MR Egger | 3 | 0.862922675394556 | 1.04259609642894 | 0.559850610408102 | -1.180565674 | 2.90641102439528 | 2.3700775482553 | 0.307104968282244 | 18.2910345481006 | 0.999950574162623 |
| ebi-fl187-GCST90094764 | prot-a-2738 | Signaling threshold-regulating transmembrane adapter 1 \|\| id:prot-a-2738 | Fried chicken liking \|\| id:ebi-fl187-GCST90094764 | MR Egger | 3 | 0.298799482941014 | 1.72311025462541 | 0.890692612049535 | -3.078496616 | 3.67609558200681 | 1.34823925139721 | 0.0460284030181519 | 39.4918997796047 | 0.999950574162623 |
| ebi-fl187-GCST90094764 | prot-a-274 | Serine/threonine-protein kinase BRSK2 \|\| id:prot-a-274 | Fried chicken liking \|\| id:ebi-fl187-GCST90094764 | MR Egger | 3 | -1.255215657 | 1.04062328643286 | 0.440667454255767 | -3.294837298 | 0.784405984455231 | 0.285014376264268 | 0.0370740765695178 | 2.19110500365368 | 0.999950574162623 |
| ebi-fl187-GCST90094764 | prot-a-2749 | Mitochondrial glutamate carrier 2 \|\| id:prot-a-2749 | Fried chicken liking \|\| id:ebi-fl187-GCST90094764 | MR Egger | 3 | -0.312172068 | 2.18928582829792 | 0.909831689111211 | -4.603172292 | 3.97882815515939 | 0.73185558824325 | 0.0100199989134008 | 53.4543572980373 | 0.999950574162623 |
| ebi-fl187-GCST90094764 | prot-a-2760 | Sodium-coupled monocarboxylate transporter 1 \|\| id:prot-a-2760 | Fried chicken liking \|\| id:ebi-fl187-GCST90094764 | MR Egger | 3 | -2.118259358 | 1.04062328643286 | 0.290702188757999 | -4.157881 | -0.078637717 | 0.120240742514879 | 0.0156406654050284 | 0.924374749163884 | 0.999950574162623 |
| ebi-fl187-GCST90094764 | prot-a-2777 | Small integral membrane protein 10 \|\| id:prot-a-2777 | Fried chicken liking \|\| id:ebi-fl187-GCST90094764 | MR Egger | 3 | -2.761268845 | 1.04062328643286 | 0.229440316893818 | -4.800890487 | -0.721647204 | 0.063211511829342 | 0.00822242183132222 | 0.485951135774861 | 0.999950574162623 |
| ebi-fl187-GCST90094764 | prot-a-2799 | Superoxide dismutase [Mn], mitochondrial \|\| id:prot-a-2799 | Fried chicken liking \|\| id:ebi-fl187-GCST90094764 | MR Egger | 3 | -0.686419467 | 1.04259609642894 | 0.629334245572889 | -2.729907816 | 1.35706888215515 | 0.503375197808772 | 0.0652253021302741 | 3.88478982071916 | 0.999950574162623 |
| ebi-fl187-GCST90094764 | prot-a-2805 | Protein SPACA7 \|\| id:prot-a-2805 | Fried chicken liking \|\| id:ebi-fl187-GCST90094764 | MR Egger | 3 | 1.36718351857226 | 1.04062328643286 | 0.414181696976503 | -0.672438123 | 3.40680515998066 | 3.92428244739923 | 0.510462489093514 | 30.1687059402018 | 0.999950574162623 |
| ebi-fl187-GCST90094764 | prot-a-2811 | SPARC-like protein 1 \|\| id:prot-a-2811 | Fried chicken liking \|\| id:ebi-fl187-GCST90094764 | MR Egger | 3 | 0.808023012494377 | 1.04062328643286 | 0.579682012615063 | -1.231598629 | 2.84764465390278 | 2.24346829073077 | 0.291825683609438 | 17.2471110467799 | 0.999950574162623 |
| ebi-fl187-GCST90094764 | prot-a-2831 | Sepiapterin reductase \|\| id:prot-a-2831 | Fried chicken liking \|\| id:ebi-fl187-GCST90094764 | MR Egger | 3 | -0.091767649 | 1.04259609642894 | 0.944109775563781 | -2.135255998 | 1.95172069997902 | 0.912317102691706 | 0.118214323869585 | 7.04079225443113 | 0.999950574162623 |
| ebi-fl187-GCST90094764 | prot-a-2834 | SPRY domain-containing SOCS box protein 1 \|\| id:prot-a-2834 | Fried chicken liking \|\| id:ebi-fl187-GCST90094764 | MR Egger | 3 | -0.824421261 | 1.19577523481428 | 0.615731917714251 | -3.168140721 | 1.51929819950935 | 0.438488689671234 | 0.0420817669590457 | 4.56901753095863 | 0.999950574162623 |
| ebi-fl187-GCST90094764 | prot-a-2835 | Spectrin alpha chain, non-erythrocytic 1 \|\| id:prot-a-2835 | Fried chicken liking \|\| id:ebi-fl187-GCST90094764 | MR Egger | 3 | 1.10243064829988 | 1.04062328643286 | 0.481644472995104 | -0.937190993 | 3.14205228970829 | 3.01147697656292 | 0.391726654212392 | 23.1513313757082 | 0.999950574162623 |
| ebi-fl187-GCST90094764 | prot-a-2859 | Sia-alpha-2,3-Gal-beta-1,4-GlcNAc-R:alpha 2,8-sialyltransferase \|\| id:prot-a-2859 | Fried chicken liking \|\| id:ebi-fl187-GCST90094764 | MR Egger | 3 | 1.58026853224469 | 1.04062328643286 | 0.370725778796317 | -0.459353109 | 3.61989017365309 | 4.85625969847932 | 0.631692149226709 | 37.3334673985486 | 0.999950574162623 |
| ebi-fl187-GCST90094764 | prot-a-2861 | Alpha-2,8-sialyltransferase 8F \|\| id:prot-a-2861 | Fried chicken liking \|\| id:ebi-fl187-GCST90094764 | MR Egger | 3 | 0.970118971584401 | 1.72168742402625 | 0.673333904344992 | -2.40438838 | 4.34462632267586 | 2.63825831845583 | 0.0903207207233892 | 77.063235314717 | 0.999950574162623 |
| ebi-fl187-GCST90094764 | prot-a-289 | Uncharacterized protein C17orf89 \|\| id:prot-a-289 | Fried chicken liking \|\| id:ebi-fl187-GCST90094764 | MR Egger | 3 | -0.601483373 | 1.04259609642894 | 0.666877259260281 | -2.644971722 | 1.44200497598501 | 0.547998147225224 | 0.0710073616562535 | 4.22916669987035 | 0.999950574162623 |
| ebi-fl187-GCST90094764 | prot-a-2921 | Tachykinin-3 \|\| id:prot-a-2921 | Fried chicken liking \|\| id:ebi-fl187-GCST90094764 | MR Egger | 3 | 1.32123024392213 | 1.19546456252012 | 0.468212923000388 | -1.021880299 | 3.66434078646157 | 3.74802953265458 | 0.359917551052151 | 39.0303983136833 | 0.999950574162623 |
| ebi-fl187-GCST90094764 | prot-a-2928 | Tax1-binding protein 1 \|\| id:prot-a-2928 | Fried chicken liking \|\| id:ebi-fl187-GCST90094764 | MR Egger | 3 | 0.034540216750338 | 1.63430053332394 | 0.986547326261071 | -3.168688829 | 3.23776926206527 | 1.03514365765306 | 0.0420587079426602 | 25.4768261887689 | 0.999950574162623 |
| ebi-fl187-GCST90094764 | prot-a-296 | Uncharacterized protein C1orf185 \|\| id:prot-a-296 | Fried chicken liking \|\| id:ebi-fl187-GCST90094764 | MR Egger | 3 | 1.5026414091454 | 1.04062328643286 | 0.385597089808058 | -0.536980232 | 3.54226305055381 | 4.49354269307713 | 0.584510676461706 | 34.5450078974387 | 0.999950574162623 |
| ebi-fl187-GCST90094764 | prot-a-2964 | Transforming growth factor beta-2 \|\| id:prot-a-2964 | Fried chicken liking \|\| id:ebi-fl187-GCST90094764 | MR Egger | 3 | -0.080725405 | 1.04062328643286 | 0.950713506693651 | -2.120347046 | 1.95889623683016 | 0.922446956381355 | 0.119989979244034 | 7.09149541235153 | 0.999950574162623 |
| ebi-fl187-GCST90094764 | prot-a-2977 | Thymocyte nuclear protein 1 \|\| id:prot-a-2977 | Fried chicken liking \|\| id:ebi-fl187-GCST90094764 | MR Egger | 3 | 1.19704548847054 | 1.04062328643286 | 0.455569949691479 | -0.842576153 | 3.23666712987894 | 3.31032207562856 | 0.430599802403737 | 25.448762826229 | 0.999950574162623 |
| ebi-fl187-GCST90094764 | prot-a-3015 | Transmembrane protein 70, mitochondrial \|\| id:prot-a-3015 | Fried chicken liking \|\| id:ebi-fl187-GCST90094764 | MR Egger | 3 | -1.193662157 | 1.04259609642894 | 0.457059632992404 | -3.237150506 | 0.849826191780975 | 0.303109195495941 | 0.0392756515234539 | 2.33924023741101 | 0.999950574162623 |
| ebi-fl187-GCST90094764 | prot-a-3030 | Tumor necrosis factor alpha-induced protein 3 \|\| id:prot-a-3030 | Fried chicken liking \|\| id:ebi-fl187-GCST90094764 | MR Egger | 3 | 0.215765529597568 | 1.12772513432938 | 0.879651183508847 | -1.994575734 | 2.42610679288316 | 1.24081141133939 | 0.136071372421371 | 11.3147455714811 | 0.999950574162623 |
| ebi-fl187-GCST90094764 | prot-a-3032 | Tumor necrosis factor receptor superfamily member 10A \|\| id:prot-a-3032 | Fried chicken liking \|\| id:ebi-fl187-GCST90094764 | MR Egger | 3 | 0.598187826152746 | 1.42175173504215 | 0.746461806072137 | -2.188445575 | 3.38482122683535 | 1.81881979444282 | 0.11209085014078 | 29.5127161628467 | 0.999950574162623 |
| ebi-fl187-GCST90094764 | prot-a-3081 | Tumor protein p53-inducible protein 11 \|\| id:prot-a-3081 | Fried chicken liking \|\| id:ebi-fl187-GCST90094764 | MR Egger | 3 | 0.782940809106355 | 1.04062328643286 | 0.589367183027205 | -1.256680832 | 2.82256245051476 | 2.18789700133238 | 0.284597086002444 | 16.8198956485384 | 0.999950574162623 |
| ebi-fl187-GCST90094764 | prot-a-315 | Complement C4 \|\| id:prot-a-315 | Fried chicken liking \|\| id:ebi-fl187-GCST90094764 | MR Egger | 3 | -0.821481665 | 1.04062328643286 | 0.574577499097808 | -2.861103307 | 1.21813997616154 | 0.439779565438051 | 0.05720561010453 | 3.38089333936787 | 0.999950574162623 |
| ebi-fl187-GCST90094764 | prot-a-3157 | UDP-glucuronosyltransferase 1-8 \|\| id:prot-a-3157 | Fried chicken liking \|\| id:ebi-fl187-GCST90094764 | MR Egger | 3 | 1.19385848238152 | 1.04062328643286 | 0.456410447750303 | -0.845763159 | 3.23348012378992 | 3.29978885265529 | 0.429229662693055 | 25.3677865685965 | 0.999950574162623 |
| ebi-fl187-GCST90094764 | prot-a-317 | Glutaredoxin-like protein C5orf63 \|\| id:prot-a-317 | Fried chicken liking \|\| id:ebi-fl187-GCST90094764 | MR Egger | 3 | -1.03625411 | 1.04062328643286 | 0.501339270339018 | -3.075875751 | 1.00336753186614 | 0.354781167362609 | 0.0461491954778747 | 2.7274511594795 | 0.999950574162623 |
| ebi-fl187-GCST90094764 | prot-a-3233 | Serine/threonine-protein kinase WNK3 \|\| id:prot-a-3233 | Fried chicken liking \|\| id:ebi-fl187-GCST90094764 | MR Egger | 3 | -1.12649974 | 1.04062328643286 | 0.474785898933658 | -3.166121382 | 0.913121901252184 | 0.324165937941281 | 0.0421668301858615 | 2.49209046205672 | 0.999950574162623 |
| ebi-fl187-GCST90094764 | prot-a-3250 | 14-3-3 protein theta \|\| id:prot-a-3250 | Fried chicken liking \|\| id:ebi-fl187-GCST90094764 | MR Egger | 3 | 0.61503562044588 | 1.04062328643286 | 0.660175559171627 | -1.424586021 | 2.65465726185428 | 1.84972248632458 | 0.240608049282297 | 14.2201114493908 | 0.999950574162623 |
| ebi-fl187-GCST90094764 | prot-a-3278 | E3 ubiquitin-protein ligase ZNRF3 \|\| id:prot-a-3278 | Fried chicken liking \|\| id:ebi-fl187-GCST90094764 | MR Egger | 3 | -0.472293002 | 1.11728605153462 | 0.74539461241766 | -2.662173664 | 1.71758765850848 | 0.623570778351625 | 0.0697963429741245 | 5.57107291077135 | 0.999950574162623 |
| ebi-fl187-GCST90094764 | prot-a-355 | Calpain-3 \|\| id:prot-a-355 | Fried chicken liking \|\| id:ebi-fl187-GCST90094764 | MR Egger | 3 | 0.319674906084329 | 1.04259609642894 | 0.810596776055932 | -1.723813443 | 2.36316325508505 | 1.37668014124238 | 0.178384589745709 | 10.6245063768842 | 0.999950574162623 |
| ebi-fl187-GCST90094764 | prot-a-385 | Coiled-coil domain-containing protein 90B, mitochondrial \|\| id:prot-a-385 | Fried chicken liking \|\| id:ebi-fl187-GCST90094764 | MR Egger | 3 | 1.38499649201838 | 1.04062328643286 | 0.410217025911426 | -0.654625149 | 3.42461813342678 | 3.99481189106534 | 0.51963681225979 | 30.7109151401281 | 0.999950574162623 |
| ebi-fl187-GCST90094764 | prot-a-390 | C-C motif chemokine 14 \|\| id:prot-a-390 | Fried chicken liking \|\| id:ebi-fl187-GCST90094764 | MR Egger | 3 | -0.295552921 | 1.04062328643286 | 0.823829367337519 | -2.335174563 | 1.74406872000871 | 0.744120033807346 | 0.0967935844917038 | 5.72057154016137 | 0.999950574162623 |
| ebi-fl187-GCST90094764 | prot-a-40 | Pituitary adenylate cyclase-activating polypeptide 27 \|\| id:prot-a-40 | Fried chicken liking \|\| id:ebi-fl187-GCST90094764 | MR Egger | 3 | -0.216098445 | 1.48584922824937 | 0.908056157968153 | -3.128362932 | 2.69616604246957 | 0.805655985247131 | 0.043789424861576 | 14.8227926860505 | 0.999950574162623 |
| ebi-fl187-GCST90094764 | prot-a-401 | C-C motif chemokine 24 \|\| id:prot-a-401 | Fried chicken liking \|\| id:ebi-fl187-GCST90094764 | MR Egger | 3 | 0.689497766466561 | 1.04062328643286 | 0.627469783771239 | -1.350123875 | 2.72911940787497 | 1.99271447384999 | 0.259208149262619 | 15.3193909434848 | 0.999950574162623 |
| ebi-fl187-GCST90094764 | prot-a-44 | Adhesion G protein-coupled receptor F5 \|\| id:prot-a-44 | Fried chicken liking \|\| id:ebi-fl187-GCST90094764 | MR Egger | 3 | 0.241339334286396 | 1.04062328643286 | 0.854921155547735 | -1.798282307 | 2.2809609756948 | 1.27295291859793 | 0.165583064938915 | 9.78608007748114 | 0.999950574162623 |
| ebi-fl187-GCST90094764 | prot-a-48 | Adrenomedullin \|\| id:prot-a-48 | Fried chicken liking \|\| id:ebi-fl187-GCST90094764 | MR Egger | 3 | 1.14876350762176 | 1.04062328643286 | 0.468581020273578 | -0.890858134 | 3.18838514903016 | 3.15429024153487 | 0.41030350633512 | 24.2492369044385 | 0.999950574162623 |
| ebi-fl187-GCST90094764 | prot-a-502 | Carcinoembryonic antigen-related cell adhesion molecule 1 \|\| id:prot-a-502 | Fried chicken liking \|\| id:ebi-fl187-GCST90094764 | MR Egger | 3 | 0.191417664951302 | 1.04259609642894 | 0.884405768754098 | -1.852070684 | 2.23490601395202 | 1.21096512416281 | 0.156911914684263 | 9.34560345458405 | 0.999950574162623 |
| ebi-fl187-GCST90094764 | prot-a-518 | Complement factor B \|\| id:prot-a-518 | Fried chicken liking \|\| id:ebi-fl187-GCST90094764 | MR Egger | 3 | -0.317448888 | 1.04259609642894 | 0.811839944393668 | -2.360937237 | 1.72603946123508 | 0.728003889728812 | 0.0943317705486461 | 5.61835806089284 | 0.999950574162623 |
| ebi-fl187-GCST90094764 | prot-a-525 | Properdin \|\| id:prot-a-525 | Fried chicken liking \|\| id:ebi-fl187-GCST90094764 | MR Egger | 3 | -1.89667437 | 1.04062328643286 | 0.319463044439878 | -3.936296012 | 0.142947271154465 | 0.150066857089934 | 0.0195203842810388 | 1.15366896842936 | 0.999950574162623 |
| ebi-fl187-GCST90094764 | prot-a-533 | Chondroadherin \|\| id:prot-a-533 | Fried chicken liking \|\| id:ebi-fl187-GCST90094764 | MR Egger | 3 | 1.85617011229184 | 1.04062328643286 | 0.325291843049923 | -0.183451529 | 3.89579175370024 | 6.39918163265449 | 0.83239222154643 | 49.1949882612141 | 0.999950574162623 |
| ebi-fl187-GCST90094764 | prot-a-535 | Coiled-coil-helix-coiled-coil-helix domain-containing protein 2 \|\| id:prot-a-535 | Fried chicken liking \|\| id:ebi-fl187-GCST90094764 | MR Egger | 3 | 0.814408039177627 | 1.04062328643286 | 0.57725234893801 | -1.225213602 | 2.85402968058603 | 2.2578387246102 | 0.29369495972444 | 17.3575866304701 | 0.999950574162623 |
| ebi-fl187-GCST90094764 | prot-a-605 | Ciliary neurotrophic factor receptor subunit alpha \|\| id:prot-a-605 | Fried chicken liking \|\| id:ebi-fl187-GCST90094764 | MR Egger | 3 | 0.233518161947266 | 1.04062328643286 | 0.859469125538635 | -1.806103479 | 2.27313980335567 | 1.2630357668716 | 0.16429306249317 | 9.70983999073755 | 0.999950574162623 |
| ebi-fl187-GCST90094764 | prot-a-606 | Ciliary neurotrophic factor receptor subunit alpha \|\| id:prot-a-606 | Fried chicken liking \|\| id:ebi-fl187-GCST90094764 | MR Egger | 3 | 0.229786353608365 | 1.69290823321678 | 0.914113508402995 | -3.088313783 | 3.54788649071325 | 1.25833114330106 | 0.0455787452759014 | 34.7398169172185 | 0.999950574162623 |
| ebi-fl187-GCST90094764 | prot-a-707 | Cancer/testis antigen 55 \|\| id:prot-a-707 | Fried chicken liking \|\| id:ebi-fl187-GCST90094764 | MR Egger | 3 | 0.316483548114102 | 1.04062328643286 | 0.812044124319795 | -1.723138093 | 2.35610518952251 | 1.3722936652317 | 0.178505102400488 | 10.5497819295384 | 0.999950574162623 |
| ebi-fl187-GCST90094764 | prot-a-719 | Dipeptidyl peptidase 1 \|\| id:prot-a-719 | Fried chicken liking \|\| id:ebi-fl187-GCST90094764 | MR Egger | 3 | -2.211300787 | 1.04062328643286 | 0.28001511327171 | -4.250922429 | -0.171679146 | 0.109558044080751 | 0.0142510822376562 | 0.842249369039773 | 0.999950574162623 |
| ebi-fl187-GCST90094764 | prot-a-752 | Cytochrome b5 \|\| id:prot-a-752 | Fried chicken liking \|\| id:ebi-fl187-GCST90094764 | MR Egger | 3 | -0.259727496 | 1.04062328643286 | 0.844288384886295 | -2.299349138 | 1.77989414513148 | 0.771261728862461 | 0.100324119666417 | 5.92922874764119 | 0.999950574162623 |
| ebi-fl187-GCST90094764 | prot-a-755 | Cytoglobin \|\| id:prot-a-755 | Fried chicken liking \|\| id:ebi-fl187-GCST90094764 | MR Egger | 3 | 1.34833867037352 | 1.04062328643286 | 0.418447860619475 | -0.691282971 | 3.38796031178192 | 3.85102239571254 | 0.500932974121957 | 29.6055046451562 | 0.999950574162623 |
| ebi-fl187-GCST90094764 | prot-a-807 | Desmin \|\| id:prot-a-807 | Fried chicken liking \|\| id:ebi-fl187-GCST90094764 | MR Egger | 3 | -1.878765532 | 1.04062328643286 | 0.322016516997467 | -3.918387173 | 0.160856109357985 | 0.152778589666601 | 0.0198731208078804 | 1.1745159547996 | 0.999950574162623 |
| ebi-fl187-GCST90094764 | prot-a-82 | Ameloblastin \|\| id:prot-a-82 | Fried chicken liking \|\| id:ebi-fl187-GCST90094764 | MR Egger | 3 | 0.803682363527047 | 2.27536189800038 | 0.7838469996965 | -3.656026957 | 5.2633916836078 | 2.23375128671977 | 0.0258349524814514 | 193.135435975918 | 0.999950574162623 |
| ebi-fl187-GCST90094764 | prot-a-84 | Muellerian-inhibiting factor \|\| id:prot-a-84 | Fried chicken liking \|\| id:ebi-fl187-GCST90094764 | MR Egger | 3 | -1.953448577 | 1.04062328643286 | 0.311607924034638 | -3.993070218 | 0.086173064664739 | 0.141784273570455 | 0.0184430030639248 | 1.08999495159351 | 0.999950574162623 |
| ebi-fl187-GCST90094764 | prot-a-858 | Inactive dipeptidyl peptidase 10 \|\| id:prot-a-858 | Fried chicken liking \|\| id:ebi-fl187-GCST90094764 | MR Egger | 3 | 2.51796223475217 | 1.04062328643286 | 0.249492513606332 | 0.478340593343769 | 4.55758387616058 | 12.4032958884924 | 1.61339490137854 | 95.3528170728978 | 0.999950574162623 |
| ebi-fl187-GCST90094764 | prot-a-863 | Dihydropyrimidinase-related protein 3 \|\| id:prot-a-863 | Fried chicken liking \|\| id:ebi-fl187-GCST90094764 | MR Egger | 3 | 0.902711688412017 | 1.04062328643286 | 0.545103034611545 | -1.136909953 | 2.94233332982042 | 2.46628183963441 | 0.320808806078742 | 18.9600347535895 | 0.999950574162623 |
| ebi-fl187-GCST90094764 | prot-a-864 | Dihydropyrimidinase-related protein 5 \|\| id:prot-a-864 | Fried chicken liking \|\| id:ebi-fl187-GCST90094764 | MR Egger | 3 | -2.71456415 | 1.04062328643286 | 0.233047068781383 | -4.754185791 | -0.674942509 | 0.0662338147290393 | 0.00861555669907276 | 0.509185693599213 | 0.999950574162623 |
| ebi-fl187-GCST90094764 | prot-a-884 | Endothelin-converting enzyme 1 \|\| id:prot-a-884 | Fried chicken liking \|\| id:ebi-fl187-GCST90094764 | MR Egger | 3 | -2.15282487 | 1.04062328643286 | 0.286644393970711 | -4.192446511 | -0.113203228 | 0.116155569518181 | 0.0151092746083099 | 0.892969164957298 | 0.999950574162623 |
| ebi-fl187-GCST90094764 | prot-a-897 | Ephrin-A2 \|\| id:prot-a-897 | Fried chicken liking \|\| id:ebi-fl187-GCST90094764 | MR Egger | 3 | 1.82394758723494 | 1.04062328643286 | 0.330068981270788 | -0.215674054 | 3.86356922864334 | 6.19627055226599 | 0.805997970738002 | 47.6350687604386 | 0.999950574162623 |
| ebi-fl187-GCST90094764 | prot-a-90 | Anaphase-promoting complex subunit 10 \|\| id:prot-a-90 | Fried chicken liking \|\| id:ebi-fl187-GCST90094764 | MR Egger | 3 | -0.377080373 | 1.04259609642894 | 0.779069300766828 | -2.420568722 | 1.66640797563397 | 0.685860946727262 | 0.088871060124492 | 5.29312059051242 | 0.999950574162623 |
| ebi-fl187-GCST90094764 | prot-a-914 | Histone-lysine N-methyltransferase EHMT2 \|\| id:prot-a-914 | Fried chicken liking \|\| id:ebi-fl187-GCST90094764 | MR Egger | 3 | 2.97643249725878 | 1.22801966747969 | 0.249112403173533 | 0.569513948998587 | 5.38335104551896 | 19.6177054402299 | 1.76740779230142 | 217.750746837259 | 0.999950574162623 |
| ebi-fl187-GCST90094764 | prot-a-932 | RNA polymerase II elongation factor ELL \|\| id:prot-a-932 | Fried chicken liking \|\| id:ebi-fl187-GCST90094764 | MR Egger | 3 | -1.09703283 | 1.04062328643286 | 0.483204455489537 | -3.136654471 | 0.942588811628373 | 0.333860235630079 | 0.043427844242461 | 2.56661731382906 | 0.999950574162623 |
| ebi-fl187-GCST90094764 | prot-a-991 | Estrogen receptor \|\| id:prot-a-991 | Fried chicken liking \|\| id:ebi-fl187-GCST90094764 | MR Egger | 3 | 0.780336131709093 | 1.04062328643286 | 0.59038589475066 | -1.25928551 | 2.8199577731175 | 2.18220565074926 | 0.283856766969881 | 16.776142252995 | 0.999950574162623 |
| ebi-fl187-GCST90094764 | prot-a-992 | Persulfide dioxygenase ETHE1, mitochondrial \|\| id:prot-a-992 | Fried chicken liking \|\| id:ebi-fl187-GCST90094764 | MR Egger | 3 | 0.773520322185917 | 1.04062328643286 | 0.593063222331017 | -1.266101319 | 2.81314196359432 | 2.16738272519545 | 0.281928631680114 | 16.6621880490864 | 0.999950574162623 |
| ebi-fl187-GCST90094764 | prot-a-993 | Ethanolamine kinase 1 \|\| id:prot-a-993 | Fried chicken liking \|\| id:ebi-fl187-GCST90094764 | MR Egger | 3 | 0.196197035716321 | 1.25775733413211 | 0.901487854549485 | -2.269007339 | 2.66140141061525 | 1.21676662820074 | 0.10341478495372 | 14.3163381151502 | 0.999950574162623 |
| ebi-fl187-GCST90094764 | ebi-IPs91-GCST90274819 | Leukemia inhibitory factor levels \|\| id:ebi-IPs91-GCST90274819 | Fried chicken liking \|\| id:ebi-fl187-GCST90094764 | Weighted median | 3 | 0.0605697293219737 | 0.145485436839957 | 0.677169662463861 | -0.224581727 | 0.345721185528289 | 1.06244167832536 | 0.798850286487005 | 1.41300859364613 | 0.999332001504091 |
| ebi-fl187-GCST90094764 | ebi-IPs91-GCST90274837 | STAM binding protein levels \|\| id:ebi-IPs91-GCST90274837 | Fried chicken liking \|\| id:ebi-fl187-GCST90094764 | Weighted median | 3 | 0.0537956719656349 | 0.146621267914618 | 0.713691938819566 | -0.233582013 | 0.341173357078286 | 1.05526895909273 | 0.791692663870817 | 1.4065970632846 | 0.999332001504091 |
| ebi-fl187-GCST90094764 | ebi-IPs91-GCST90274840 | TNF-beta levels \|\| id:ebi-IPs91-GCST90274840 | Fried chicken liking \|\| id:ebi-fl187-GCST90094764 | Weighted median | 3 | 0.317135583790544 | 0.157052978981451 | 0.0434570377914228 | 0.00931174498689979 | 0.624959422594189 | 1.37318874143909 | 1.00935523416615 | 1.86817015039589 | 0.999332001504091 |
| ebi-fl187-GCST90094764 | ebi-IPs91-GCST90274842 | Tumor necrosis factor ligand superfamily member 14 levels \|\| id:ebi-IPs91-GCST90274842 | Fried chicken liking \|\| id:ebi-fl187-GCST90094764 | Weighted median | 3 | 0.174415756251443 | 0.164366125921264 | 0.288625492945953 | -0.147741851 | 0.49657336305712 | 1.19055044172816 | 0.862653779795546 | 1.64308136995013 | 0.999332001504091 |
| ebi-fl187-GCST90094764 | prot-a-1017 | FAS-associated factor 2 \|\| id:prot-a-1017 | Fried chicken liking \|\| id:ebi-fl187-GCST90094764 | Weighted median | 3 | 0.116580519180243 | 0.276897890244661 | 0.673737921062067 | -0.426139346 | 0.659300384059778 | 1.1236479820392 | 0.653025339507822 | 1.93343919623757 | 0.999332001504091 |
| ebi-fl187-GCST90094764 | prot-a-1019 | Fas apoptotic inhibitory molecule 3 \|\| id:prot-a-1019 | Fried chicken liking \|\| id:ebi-fl187-GCST90094764 | Weighted median | 3 | 0.242294962571402 | 0.30068200925763 | 0.420347822178272 | -0.347041776 | 0.831631700716357 | 1.2741699698441 | 0.70677580167652 | 2.29706380467673 | 0.999332001504091 |
| ebi-fl187-GCST90094764 | prot-a-1052 | MIP18 family protein FAM96A \|\| id:prot-a-1052 | Fried chicken liking \|\| id:ebi-fl187-GCST90094764 | Weighted median | 3 | 0.276831986666893 | 0.279739754007246 | 0.322367002454895 | -0.271457931 | 0.825121904521095 | 1.31894475211403 | 0.762267350475912 | 2.28215895386707 | 0.999332001504091 |
| ebi-fl187-GCST90094764 | prot-a-1066 | F-box/LRR-repeat protein 4 \|\| id:prot-a-1066 | Fried chicken liking \|\| id:ebi-fl187-GCST90094764 | Weighted median | 3 | -0.005079197 | 0.306504494151511 | 0.986778570463996 | -0.605828006 | 0.595669611313786 | 0.994933680087681 | 0.545622461009882 | 1.81424537754667 | 0.999332001504091 |
| ebi-fl187-GCST90094764 | prot-a-1076 | Low affinity immunoglobulin gamma Fc region receptor III-B \|\| id:prot-a-1076 | Fried chicken liking \|\| id:ebi-fl187-GCST90094764 | Weighted median | 3 | -0.089057709 | 0.285651886833266 | 0.755215272103985 | -0.648935407 | 0.470819989565769 | 0.914792780615408 | 0.522601839071826 | 1.6013067098125 | 0.999332001504091 |
| ebi-fl187-GCST90094764 | prot-a-1081 | Fc receptor-like protein 3 \|\| id:prot-a-1081 | Fried chicken liking \|\| id:ebi-fl187-GCST90094764 | Weighted median | 3 | 0.0467088306256471 | 0.282714749710934 | 0.868774443924996 | -0.507412079 | 0.600829740059078 | 1.0478168724754 | 0.602051626642637 | 1.82363131276087 | 0.999332001504091 |
| ebi-fl187-GCST90094764 | prot-a-1105 | Fibroblast growth factor receptor 4 \|\| id:prot-a-1105 | Fried chicken liking \|\| id:ebi-fl187-GCST90094764 | Weighted median | 3 | -0.002594849 | 0.30728883871613 | 0.993262477395753 | -0.604880973 | 0.599691274698911 | 0.997408514526373 | 0.546139428071567 | 1.82155635304057 | 0.999332001504091 |
| ebi-fl187-GCST90094764 | prot-a-1143 | Follistatin-related protein 1 \|\| id:prot-a-1143 | Fried chicken liking \|\| id:ebi-fl187-GCST90094764 | Weighted median | 3 | -0.111292944 | 0.287086351937773 | 0.698264957857969 | -0.673982193 | 0.451396306208357 | 0.894676620755638 | 0.509674907193911 | 1.57050355908965 | 0.999332001504091 |
| ebi-fl187-GCST90094764 | prot-a-1151 | Alpha-(1,3)-fucosyltransferase 10 \|\| id:prot-a-1151 | Fried chicken liking \|\| id:ebi-fl187-GCST90094764 | Weighted median | 3 | -0.077948465 | 0.287771678763598 | 0.786491687469691 | -0.641980955 | 0.486084025756514 | 0.925012096164945 | 0.52624891539395 | 1.62593661102547 | 0.999332001504091 |
| ebi-fl187-GCST90094764 | prot-a-1165 | Growth arrest and DNA damage-inducible proteins-interacting protein 1 \|\| id:prot-a-1165 | Fried chicken liking \|\| id:ebi-fl187-GCST90094764 | Weighted median | 3 | -0.157983105 | 0.270196778761678 | 0.558751822883719 | -0.687568791 | 0.371602581549639 | 0.853864208033331 | 0.502796988774965 | 1.45005658752404 | 0.999332001504091 |
| ebi-fl187-GCST90094764 | prot-a-1194 | Growth/differentiation factor 11/8 \|\| id:prot-a-1194 | Fried chicken liking \|\| id:ebi-fl187-GCST90094764 | Weighted median | 3 | -0.35316657 | 0.300860223182828 | 0.240452719429278 | -0.942852608 | 0.23651946714851 | 0.702460174636704 | 0.389515115208138 | 1.266832217 | 0.999332001504091 |
| ebi-fl187-GCST90094764 | prot-a-1216 | D-glucuronyl C5-epimerase \|\| id:prot-a-1216 | Fried chicken liking \|\| id:ebi-fl187-GCST90094764 | Weighted median | 3 | 0.236230663706457 | 0.305134126973339 | 0.43882062302734 | -0.361832225 | 0.834293552574201 | 1.26646640431275 | 0.696399196303898 | 2.30318639332968 | 0.999332001504091 |
| ebi-fl187-GCST90094764 | prot-a-1246 | Glypican-3 \|\| id:prot-a-1246 | Fried chicken liking \|\| id:ebi-fl187-GCST90094764 | Weighted median | 3 | -0.166778069 | 0.281268186617455 | 0.553214382505903 | -0.718063715 | 0.38450757695644 | 0.846387430261386 | 0.487695660309691 | 1.46889082763125 | 0.999332001504091 |
| ebi-fl187-GCST90094764 | prot-a-1275 | Glutamate receptor 4 \|\| id:prot-a-1275 | Fried chicken liking \|\| id:ebi-fl187-GCST90094764 | Weighted median | 3 | -0.13294291 | 0.289465675922841 | 0.646040266753729 | -0.700295634 | 0.434409815155509 | 0.875515074141172 | 0.496438517760846 | 1.54405151418506 | 0.999332001504091 |
| ebi-fl187-GCST90094764 | prot-a-1289 | Glutathione S-transferase P \|\| id:prot-a-1289 | Fried chicken liking \|\| id:ebi-fl187-GCST90094764 | Weighted median | 3 | -0.113414862 | 0.28175588926861 | 0.687294892104166 | -0.665656405 | 0.438826680940727 | 0.892780202673537 | 0.513936066763318 | 1.55088646590913 | 0.999332001504091 |
| ebi-fl187-GCST90094764 | prot-a-130 | Apolipoprotein D \|\| id:prot-a-130 | Fried chicken liking \|\| id:ebi-fl187-GCST90094764 | Weighted median | 3 | -0.047195541 | 0.261884748972949 | 0.856983838624764 | -0.560489649 | 0.466098566531186 | 0.953900852190965 | 0.570929440113383 | 1.59376408340397 | 0.999332001504091 |
| ebi-fl187-GCST90094764 | prot-a-1313 | Heparin-binding EGF-like growth factor \|\| id:prot-a-1313 | Fried chicken liking \|\| id:ebi-fl187-GCST90094764 | Weighted median | 3 | 0.305680835946239 | 0.298857509533334 | 0.306387583049754 | -0.280079883 | 0.891441554631573 | 1.35754895656855 | 0.755723369791653 | 2.43864255513023 | 0.999332001504091 |
| ebi-fl187-GCST90094764 | prot-a-1347 | HLA class II histocompatibility antigen, DQ alpha 2 chain \|\| id:prot-a-1347 | Fried chicken liking \|\| id:ebi-fl187-GCST90094764 | Weighted median | 3 | -0.095130321 | 0.287967648006516 | 0.741134968847873 | -0.659546912 | 0.469286268672622 | 0.909254431417876 | 0.517085566940895 | 1.59885263466955 | 0.999332001504091 |
| ebi-fl187-GCST90094764 | prot-a-1357 | Non-histone chromosomal protein HMG-14 \|\| id:prot-a-1357 | Fried chicken liking \|\| id:ebi-fl187-GCST90094764 | Weighted median | 3 | 0.192618527637964 | 0.271143966500658 | 0.47746103018849 | -0.338823647 | 0.724060701979253 | 1.21242020049384 | 0.712608108797838 | 2.06279261268207 | 0.999332001504091 |
| ebi-fl187-GCST90094764 | prot-a-137 | Apolipoprotein M \|\| id:prot-a-137 | Fried chicken liking \|\| id:ebi-fl187-GCST90094764 | Weighted median | 3 | -0.049473647 | 0.300364080601042 | 0.869170465312642 | -0.638187245 | 0.539239950908146 | 0.951730238686445 | 0.528249142882336 | 1.7147031082489 | 0.999332001504091 |
| ebi-fl187-GCST90094764 | prot-a-1381 | 17-beta-hydroxysteroid dehydrogenase 14 \|\| id:prot-a-1381 | Fried chicken liking \|\| id:ebi-fl187-GCST90094764 | Weighted median | 3 | -0.036435987 | 0.282118406857094 | 0.89723785262426 | -0.589388064 | 0.516516090728877 | 0.964219814802463 | 0.554666601281545 | 1.67617781404108 | 0.999332001504091 |
| ebi-fl187-GCST90094764 | prot-a-1392 | Serine protease HTRA2, mitochondrial \|\| id:prot-a-1392 | Fried chicken liking \|\| id:ebi-fl187-GCST90094764 | Weighted median | 3 | 0.418818608000007 | 0.294156322769182 | 0.154505521884804 | -0.157727785 | 0.995365000627604 | 1.52016458408543 | 0.854082244643383 | 2.70571174755251 | 0.999332001504091 |
| ebi-fl187-GCST90094764 | prot-a-1416 | Interferon-induced protein with tetratricopeptide repeats 2 \|\| id:prot-a-1416 | Fried chicken liking \|\| id:ebi-fl187-GCST90094764 | Weighted median | 3 | 0.0835436841923714 | 0.320043138354301 | 0.794062568322958 | -0.543740867 | 0.710828235366802 | 1.08713270476981 | 0.580572341112618 | 2.03567657996795 | 0.999332001504091 |
| ebi-fl187-GCST90094764 | prot-a-1428 | Interferon gamma \|\| id:prot-a-1428 | Fried chicken liking \|\| id:ebi-fl187-GCST90094764 | Weighted median | 3 | 0.196204780583104 | 0.284490356342216 | 0.490401036033321 | -0.361396318 | 0.753805879013846 | 1.21677605193267 | 0.696702827979612 | 2.12507241408842 | 0.999332001504091 |
| ebi-fl187-GCST90094764 | prot-a-1483 | Interleukin-17C \|\| id:prot-a-1483 | Fried chicken liking \|\| id:ebi-fl187-GCST90094764 | Weighted median | 3 | -0.255166207 | 0.274147596068698 | 0.351976610271407 | -0.792495496 | 0.282163081058473 | 0.774787711934571 | 0.45271363960803 | 1.32599494701454 | 0.999332001504091 |
| ebi-fl187-GCST90094764 | prot-a-1503 | Interleukin-1 receptor-like 2 \|\| id:prot-a-1503 | Fried chicken liking \|\| id:ebi-fl187-GCST90094764 | Weighted median | 3 | 0.174417161034572 | 0.288002926739806 | 0.544774432429285 | -0.390068575 | 0.738902897444592 | 1.19055211419451 | 0.677010446660718 | 2.09363731919393 | 0.999332001504091 |
| ebi-fl187-GCST90094764 | prot-a-1522 | Interleukin-31 receptor subunit alpha \|\| id:prot-a-1522 | Fried chicken liking \|\| id:ebi-fl187-GCST90094764 | Weighted median | 3 | 0.0995129916087713 | 0.301032787653979 | 0.740967846818348 | -0.490511272 | 0.689537255410571 | 1.10463282160419 | 0.612313255400752 | 1.99279316559399 | 0.999332001504091 |
| ebi-fl187-GCST90094764 | prot-a-1540 | Interleukin-6 receptor subunit alpha \|\| id:prot-a-1540 | Fried chicken liking \|\| id:ebi-fl187-GCST90094764 | Weighted median | 3 | -0.062029209 | 0.292921098494912 | 0.832293645023283 | -0.636154562 | 0.512096144276746 | 0.939855434365865 | 0.529323998103496 | 1.66878554660644 | 0.999332001504091 |
| ebi-fl187-GCST90094764 | prot-a-1541 | Interleukin-6 receptor subunit alpha \|\| id:prot-a-1541 | Fried chicken liking \|\| id:ebi-fl187-GCST90094764 | Weighted median | 3 | 0.0132238837902186 | 0.296811345651859 | 0.964463477339855 | -0.568526354 | 0.594974121267861 | 1.01331170603153 | 0.566359437535671 | 1.81298402662507 | 0.999332001504091 |
| ebi-fl187-GCST90094764 | prot-a-1545 | Interleukin-9 \|\| id:prot-a-1545 | Fried chicken liking \|\| id:ebi-fl187-GCST90094764 | Weighted median | 3 | 0.658475243566151 | 0.284688097090381 | 0.0207242469669026 | 0.100486573269004 | 1.2164639138633 | 1.93184449528144 | 1.10570879554985 | 3.37523149763253 | 0.999332001504091 |
| ebi-fl187-GCST90094764 | prot-a-1548 | Inositol monophosphatase 3 \|\| id:prot-a-1548 | Fried chicken liking \|\| id:ebi-fl187-GCST90094764 | Weighted median | 3 | 0.347285589432389 | 0.335782520955985 | 0.301015801344265 | -0.310848152 | 1.00541933050612 | 1.41522083977862 | 0.732825145716607 | 2.73305308510554 | 0.999332001504091 |
| ebi-fl187-GCST90094764 | prot-a-1555 | Inhibin beta A chain:Inhibin beta B chain heterodimer \|\| id:prot-a-1555 | Fried chicken liking \|\| id:ebi-fl187-GCST90094764 | Weighted median | 3 | 0.393192968744523 | 0.280027074438499 | 0.160281680428756 | -0.155660097 | 0.942046034643981 | 1.48170428435948 | 0.855850046802612 | 2.56522459102651 | 0.999332001504091 |
| ebi-fl187-GCST90094764 | prot-a-1603 | Junctophilin-3 \|\| id:prot-a-1603 | Fried chicken liking \|\| id:ebi-fl187-GCST90094764 | Weighted median | 3 | 0.0749044077147919 | 0.307766286658473 | 0.807710409412074 | -0.528317514 | 0.6781263295654 | 1.07778111840009 | 0.589596122786165 | 1.97018279850705 | 0.999332001504091 |
| ebi-fl187-GCST90094764 | prot-a-1627 | UPF0258 protein KIAA1024 \|\| id:prot-a-1627 | Fried chicken liking \|\| id:ebi-fl187-GCST90094764 | Weighted median | 3 | 0.411773270454103 | 0.307902957026758 | 0.181109210993041 | -0.191716525 | 1.01526306622655 | 1.50949215105718 | 0.825540855256593 | 2.76008938818055 | 0.999332001504091 |
| ebi-fl187-GCST90094764 | prot-a-1666 | Kallikrein-7 \|\| id:prot-a-1666 | Fried chicken liking \|\| id:ebi-fl187-GCST90094764 | Weighted median | 3 | 0.170682589432058 | 0.270295939765114 | 0.527736130949995 | -0.359097453 | 0.70046263137168 | 1.18611420408204 | 0.698306296335935 | 2.01468454818051 | 0.999332001504091 |
| ebi-fl187-GCST90094764 | prot-a-1678 | Importin subunit alpha-3 \|\| id:prot-a-1678 | Fried chicken liking \|\| id:ebi-fl187-GCST90094764 | Weighted median | 3 | 0.218979545361518 | 0.273035644873087 | 0.422542453893777 | -0.316170319 | 0.754129409312769 | 1.24480581437421 | 0.728935288364072 | 2.12576005063142 | 0.999332001504091 |
| ebi-fl187-GCST90094764 | prot-a-1718 | Protein LDOC1 \|\| id:prot-a-1718 | Fried chicken liking \|\| id:ebi-fl187-GCST90094764 | Weighted median | 3 | 0.281128173522148 | 0.302848544813495 | 0.353262471901439 | -0.312454974 | 0.874711321356598 | 1.32462337469842 | 0.731648571185084 | 2.39818288984749 | 0.999332001504091 |
| ebi-fl187-GCST90094764 | prot-a-1723 | Leptin \|\| id:prot-a-1723 | Fried chicken liking \|\| id:ebi-fl187-GCST90094764 | Weighted median | 3 | 0.00430027901469934 | 0.283088813994697 | 0.987880148876733 | -0.550553796 | 0.559154354444305 | 1.00430953848251 | 0.576630386100092 | 1.74919267766765 | 0.999332001504091 |
| ebi-fl187-GCST90094764 | prot-a-1730 | Galectin-7 \|\| id:prot-a-1730 | Fried chicken liking \|\| id:ebi-fl187-GCST90094764 | Weighted median | 3 | -0.451267831 | 0.304633488449527 | 0.138514194508511 | -1.048349468 | 0.145813806763959 | 0.636820259385031 | 0.350515809501069 | 1.15698074600536 | 0.999332001504091 |
| ebi-fl187-GCST90094764 | prot-a-1753 | Lipase member N \|\| id:prot-a-1753 | Fried chicken liking \|\| id:ebi-fl187-GCST90094764 | Weighted median | 3 | 0.164635257576364 | 0.288092470568467 | 0.567683317198376 | -0.400025985 | 0.729296499890559 | 1.1789630224488 | 0.670302628171281 | 2.0736213016107 | 0.999332001504091 |
| ebi-fl187-GCST90094764 | prot-a-1756 | VIP36-like protein \|\| id:prot-a-1756 | Fried chicken liking \|\| id:ebi-fl187-GCST90094764 | Weighted median | 3 | 0.238809023819385 | 0.27307163555976 | 0.381830315161553 | -0.296411382 | 0.774029429516514 | 1.26973602409125 | 0.743481510280219 | 2.16848643655899 | 0.999332001504091 |
| ebi-fl187-GCST90094764 | prot-a-1760 | Ig Kappa chain V-I region HK102- like \|\| id:prot-a-1760 | Fried chicken liking \|\| id:ebi-fl187-GCST90094764 | Weighted median | 3 | -0.144783987 | 0.30054110506053 | 0.62998754867943 | -0.733844553 | 0.44427657909675 | 0.86520916950857 | 0.480059822450573 | 1.55936171283899 | 0.999332001504091 |
| ebi-fl187-GCST90094764 | prot-a-179 | Neutral ceramidase \|\| id:prot-a-179 | Fried chicken liking \|\| id:ebi-fl187-GCST90094764 | Weighted median | 3 | -0.037455383 | 0.271729146077116 | 0.890366180956429 | -0.570044509 | 0.495133743690889 | 0.963237393890657 | 0.565500268326736 | 1.64071766001919 | 0.999332001504091 |
| ebi-fl187-GCST90094764 | prot-a-1809 | Lactotransferrin \|\| id:prot-a-1809 | Fried chicken liking \|\| id:ebi-fl187-GCST90094764 | Weighted median | 3 | 0.442999506531245 | 0.297207682376163 | 0.136082689337255 | -0.139527551 | 1.02552656398853 | 1.55737156582738 | 0.869769059931257 | 2.78856343112427 | 0.999332001504091 |
| ebi-fl187-GCST90094764 | prot-a-1818 | Lysophospholipase-like protein 1 \|\| id:prot-a-1818 | Fried chicken liking \|\| id:ebi-fl187-GCST90094764 | Weighted median | 3 | 0.142933544142035 | 0.26677895393179 | 0.592113932402948 | -0.379953206 | 0.665820293848345 | 1.15365313210978 | 0.683893410869859 | 1.9460862293349 | 0.999332001504091 |
| ebi-fl187-GCST90094764 | prot-a-1852 | MAP kinase-activated protein kinase 3 \|\| id:prot-a-1852 | Fried chicken liking \|\| id:ebi-fl187-GCST90094764 | Weighted median | 3 | 0.433363830313398 | 0.283225837204785 | 0.125991998530582 | -0.121758811 | 0.988486471234777 | 1.54243730407261 | 0.885361882647111 | 2.68716429250553 | 0.999332001504091 |
| ebi-fl187-GCST90094764 | prot-a-1856 | Megakaryocyte-associated tyrosine-protein kinase \|\| id:prot-a-1856 | Fried chicken liking \|\| id:ebi-fl187-GCST90094764 | Weighted median | 3 | -0.010236108 | 0.288725518829454 | 0.971718737670276 | -0.576138125 | 0.555665908686687 | 0.989816102440185 | 0.562064804563624 | 1.74310134471154 | 0.999332001504091 |
| ebi-fl187-GCST90094764 | prot-a-1890 | Beta-1,3-N-acetylglucosaminyltransferase manic fringe \|\| id:prot-a-1890 | Fried chicken liking \|\| id:ebi-fl187-GCST90094764 | Weighted median | 3 | 0.235423105468742 | 0.301975192542615 | 0.435620028114368 | -0.356448272 | 0.827294482852267 | 1.26544407178724 | 0.700158688401195 | 2.28712251286652 | 0.999332001504091 |
| ebi-fl187-GCST90094764 | prot-a-1919 | Matrilysin \|\| id:prot-a-1919 | Fried chicken liking \|\| id:ebi-fl187-GCST90094764 | Weighted median | 3 | 0.286221998459922 | 0.339960505035942 | 0.39982870030128 | -0.380100591 | 0.952544588330368 | 1.33138798853012 | 0.683792622088355 | 2.59229760418978 | 0.999332001504091 |
| ebi-fl187-GCST90094764 | prot-a-1923 | Multimerin-2 \|\| id:prot-a-1923 | Fried chicken liking \|\| id:ebi-fl187-GCST90094764 | Weighted median | 3 | -0.213492154 | 0.272259289354608 | 0.432952029500706 | -0.747120361 | 0.320136052803409 | 0.807758497531003 | 0.473728758056289 | 1.3773151391751 | 0.999332001504091 |
| ebi-fl187-GCST90094764 | prot-a-1948 | Mesothelin \|\| id:prot-a-1948 | Fried chicken liking \|\| id:ebi-fl187-GCST90094764 | Weighted median | 3 | 0.160529884932888 | 0.271178425237087 | 0.553869708256768 | -0.370979829 | 0.692039598397578 | 1.17413286149808 | 0.690057860898598 | 1.99778606196073 | 0.999332001504091 |
| ebi-fl187-GCST90094764 | prot-a-195 | ATPase family AAA domain-containing protein 1 \|\| id:prot-a-195 | Fried chicken liking \|\| id:ebi-fl187-GCST90094764 | Weighted median | 3 | 0.184808995962978 | 0.277011916491326 | 0.504675181801925 | -0.35813436 | 0.727752352285977 | 1.20298864249505 | 0.698979153606284 | 2.07042179513588 | 0.999332001504091 |
| ebi-fl187-GCST90094764 | prot-a-1966 | Mucin-1 \|\| id:prot-a-1966 | Fried chicken liking \|\| id:ebi-fl187-GCST90094764 | Weighted median | 3 | 0.369217436082563 | 0.291329473531372 | 0.2050289086846 | -0.201788332 | 0.940223204204052 | 1.44660211297714 | 0.817267899065216 | 2.56055288071817 | 0.999332001504091 |
| ebi-fl187-GCST90094764 | prot-a-1986 | Myocardial zonula adherens protein \|\| id:prot-a-1986 | Fried chicken liking \|\| id:ebi-fl187-GCST90094764 | Weighted median | 3 | -0.243878806 | 0.282835117970383 | 0.38854174173535 | -0.798235637 | 0.310478025492916 | 0.783582594229693 | 0.450122443325219 | 1.36407702189627 | 0.999332001504091 |
| ebi-fl187-GCST90094764 | prot-a-2071 | Epididymal secretory protein E1 \|\| id:prot-a-2071 | Fried chicken liking \|\| id:ebi-fl187-GCST90094764 | Weighted median | 3 | -0.309810467 | 0.292132109436885 | 0.288910395489456 | -0.882389402 | 0.262768467118965 | 0.733585981523991 | 0.413793011720637 | 1.30052556965809 | 0.999332001504091 |
| ebi-fl187-GCST90094764 | prot-a-2098 | Neuritin \|\| id:prot-a-2098 | Fried chicken liking \|\| id:ebi-fl187-GCST90094764 | Weighted median | 3 | 0.39387116083138 | 0.286856293949154 | 0.169733415377402 | -0.168367175 | 0.956109496971722 | 1.48270950530811 | 0.845043498606585 | 2.60155540011381 | 0.999332001504091 |
| ebi-fl187-GCST90094764 | prot-a-2115 | Neurotrophin-4 \|\| id:prot-a-2115 | Fried chicken liking \|\| id:ebi-fl187-GCST90094764 | Weighted median | 3 | -0.028420229 | 0.296173079385346 | 0.923553787983917 | -0.608919465 | 0.552079006389323 | 0.971979826656248 | 0.543938296124462 | 1.7368602103547 | 0.999332001504091 |
| ebi-fl187-GCST90094764 | prot-a-2118 | Netrin-1 \|\| id:prot-a-2118 | Fried chicken liking \|\| id:ebi-fl187-GCST90094764 | Weighted median | 3 | -0.234429311 | 0.296682959521517 | 0.429429799261523 | -0.815927911 | 0.347069289862079 | 0.791022148637865 | 0.4422287877575 | 1.4149147612226 | 0.999332001504091 |
| ebi-fl187-GCST90094764 | prot-a-2120 | Netrin-G1 \|\| id:prot-a-2120 | Fried chicken liking \|\| id:ebi-fl187-GCST90094764 | Weighted median | 3 | 0.0463568244410554 | 0.285064880630294 | 0.870818816818393 | -0.512370342 | 0.605083990476432 | 1.04744809936498 | 0.599073884780501 | 1.83140602309064 | 0.999332001504091 |
| ebi-fl187-GCST90094764 | prot-a-2145 | Olfactomedin-like protein 3 \|\| id:prot-a-2145 | Fried chicken liking \|\| id:ebi-fl187-GCST90094764 | Weighted median | 3 | 0.238063514293518 | 0.300131235143353 | 0.42766243601976 | -0.350193707 | 0.826320735174489 | 1.26878977655206 | 0.704551600213521 | 2.28489651658607 | 0.999332001504091 |
| ebi-fl187-GCST90094764 | prot-a-2162 | Prolyl 4-hydroxylase subunit alpha-2 \|\| id:prot-a-2162 | Fried chicken liking \|\| id:ebi-fl187-GCST90094764 | Weighted median | 3 | 0.287261833747891 | 0.31780428112505 | 0.366050858658764 | -0.335634557 | 0.910158224752988 | 1.3327731327787 | 0.714884307368109 | 2.48471564580324 | 0.999332001504091 |
| ebi-fl187-GCST90094764 | prot-a-2168 | Polyadenylate-binding protein-interacting protein 1 \|\| id:prot-a-2168 | Fried chicken liking \|\| id:ebi-fl187-GCST90094764 | Weighted median | 3 | 0.476616585764285 | 0.287380088362852 | 0.0972188969162574 | -0.086648387 | 1.03988155895548 | 1.61061579263047 | 0.916999467497124 | 2.82888193877694 | 0.999332001504091 |
| ebi-fl187-GCST90094764 | prot-a-219 | Beta-1,4-galactosyltransferase 5 \|\| id:prot-a-219 | Fried chicken liking \|\| id:ebi-fl187-GCST90094764 | Weighted median | 3 | -0.263111015 | 0.265983921728373 | 0.32256581365892 | -0.784439502 | 0.258217471369441 | 0.768656559998672 | 0.456375427715104 | 1.29462033086896 | 0.999332001504091 |
| ebi-fl187-GCST90094764 | prot-a-2190 | Pyruvate carboxylase, mitochondrial \|\| id:prot-a-2190 | Fried chicken liking \|\| id:ebi-fl187-GCST90094764 | Weighted median | 3 | 0.356991789388188 | 0.283092247142583 | 0.207292982598629 | -0.197869015 | 0.91185259378765 | 1.42902413664325 | 0.820477316311673 | 2.48892923973691 | 0.999332001504091 |
| ebi-fl187-GCST90094764 | prot-a-2197 | Protocadherin-9 \|\| id:prot-a-2197 | Fried chicken liking \|\| id:ebi-fl187-GCST90094764 | Weighted median | 3 | -0.074769331 | 0.285314405116415 | 0.793275623126981 | -0.633985565 | 0.48444690327623 | 0.92795751290466 | 0.530473346306104 | 1.62327693135276 | 0.999332001504091 |
| ebi-fl187-GCST90094764 | prot-a-2198 | Protocadherin alpha-4 \|\| id:prot-a-2198 | Fried chicken liking \|\| id:ebi-fl187-GCST90094764 | Weighted median | 3 | -1.041353335 | 0.326496033429176 | 0.00142527843122286 | -1.681285561 | -0.40142111 | 0.352976662756908 | 0.186134534879323 | 0.669368124146233 | 0.999332001504091 |
| ebi-fl187-GCST90094764 | prot-a-2254 | Placenta growth factor \|\| id:prot-a-2254 | Fried chicken liking \|\| id:ebi-fl187-GCST90094764 | Weighted median | 3 | 0.347389439305569 | 0.291375802468459 | 0.23316775525342 | -0.223707134 | 0.918486012143748 | 1.41536781791505 | 0.799549261251382 | 2.50549422915391 | 0.999332001504091 |
| ebi-fl187-GCST90094764 | prot-a-2261 | Membrane-associated progesterone receptor component 2 \|\| id:prot-a-2261 | Fried chicken liking \|\| id:ebi-fl187-GCST90094764 | Weighted median | 3 | -0.308443131 | 0.279422709607003 | 0.269654526193186 | -0.856111642 | 0.239225379480855 | 0.734589726138204 | 0.424810691023374 | 1.27026479594439 | 0.999332001504091 |
| ebi-fl187-GCST90094764 | prot-a-2268 | Polymeric immunoglobulin receptor \|\| id:prot-a-2268 | Fried chicken liking \|\| id:ebi-fl187-GCST90094764 | Weighted median | 3 | 0.0907996561883314 | 0.288046218454124 | 0.752590068807594 | -0.473770932 | 0.655370244358415 | 1.09504959687044 | 0.622649865403774 | 1.92585542249897 | 0.999332001504091 |
| ebi-fl187-GCST90094764 | prot-a-2283 | Group 10 secretory phospholipase A2 \|\| id:prot-a-2283 | Fried chicken liking \|\| id:ebi-fl187-GCST90094764 | Weighted median | 3 | -0.244866356 | 0.313704814300053 | 0.435059588788295 | -0.859727792 | 0.369995080158713 | 0.782809149099563 | 0.423277286144225 | 1.44772749205632 | 0.999332001504091 |
| ebi-fl187-GCST90094764 | prot-a-2298 | Inactive phospholipase D5 \|\| id:prot-a-2298 | Fried chicken liking \|\| id:ebi-fl187-GCST90094764 | Weighted median | 3 | 0.517938410408384 | 0.320076608665494 | 0.105625936204188 | -0.109411743 | 1.14528856339275 | 1.67856357098467 | 0.896361271407968 | 3.14334828122492 | 0.999332001504091 |
| ebi-fl187-GCST90094764 | prot-a-2304 | Procollagen-lysine,2-oxoglutarate 5-dioxygenase 2 \|\| id:prot-a-2304 | Fried chicken liking \|\| id:ebi-fl187-GCST90094764 | Weighted median | 3 | 0.0577061504134024 | 0.297612567060787 | 0.846256620920367 | -0.525614481 | 0.641026781852544 | 1.05940364464432 | 0.591191976478653 | 1.89842915150962 | 0.999332001504091 |
| ebi-fl187-GCST90094764 | prot-a-2331 | Periostin \|\| id:prot-a-2331 | Fried chicken liking \|\| id:ebi-fl187-GCST90094764 | Weighted median | 3 | -0.323327611 | 0.303681669719797 | 0.287015082683822 | -0.918543684 | 0.271888461442544 | 0.723736711266229 | 0.399099833602629 | 1.31244060541499 | 0.999332001504091 |
| ebi-fl187-GCST90094764 | prot-a-2333 | Periostin \|\| id:prot-a-2333 | Fried chicken liking \|\| id:ebi-fl187-GCST90094764 | Weighted median | 3 | -0.406193028 | 0.276518674081665 | 0.141845446916528 | -0.94816963 | 0.135783572825593 | 0.666181563068919 | 0.387449551022287 | 1.14543396373021 | 0.999332001504091 |
| ebi-fl187-GCST90094764 | prot-a-2367 | cAMP-dependent protein kinase catalytic subunit alpha \|\| id:prot-a-2367 | Fried chicken liking \|\| id:ebi-fl187-GCST90094764 | Weighted median | 3 | 0.162525014247684 | 0.264054376778901 | 0.538225706077277 | -0.355021564 | 0.680071592734329 | 1.17647774678638 | 0.701158323099917 | 1.97401905259322 | 0.999332001504091 |
| ebi-fl187-GCST90094764 | prot-a-2385 | Phosphoribosyl pyrophosphate synthase-associated protein 1 \|\| id:prot-a-2385 | Fried chicken liking \|\| id:ebi-fl187-GCST90094764 | Weighted median | 3 | -0.384300122 | 0.291217415283742 | 0.18695756680885 | -0.955086256 | 0.18648601154592 | 0.680927035046814 | 0.384778953487154 | 1.205007766 | 0.999332001504091 |
| ebi-fl187-GCST90094764 | prot-a-2393 | Trypsin-3 \|\| id:prot-a-2393 | Fried chicken liking \|\| id:ebi-fl187-GCST90094764 | Weighted median | 3 | -0.266662098 | 0.276854797489079 | 0.335455217326521 | -0.809297501 | 0.275973305200152 | 0.765931837737015 | 0.445170688384245 | 1.3178126848119 | 0.999332001504091 |
| ebi-fl187-GCST90094764 | prot-a-2405 | Pregnancy-specific beta-1-glycoprotein 6 \|\| id:prot-a-2405 | Fried chicken liking \|\| id:ebi-fl187-GCST90094764 | Weighted median | 3 | 0.313596451704289 | 0.29853420143795 | 0.293509420795408 | -0.271530583 | 0.898723486522672 | 1.36833743487487 | 0.762211972295865 | 2.45646539772949 | 0.999332001504091 |
| ebi-fl187-GCST90094764 | prot-a-244 | Beclin-1 \|\| id:prot-a-244 | Fried chicken liking \|\| id:ebi-fl187-GCST90094764 | Weighted median | 3 | -0.234754404 | 0.270172791303828 | 0.384899266299786 | -0.764293075 | 0.294784266572545 | 0.79076503420883 | 0.465663003306939 | 1.34283663268633 | 0.999332001504091 |
| ebi-fl187-GCST90094764 | prot-a-2454 | tRNA pseudouridine synthase A, mitochondrial \|\| id:prot-a-2454 | Fried chicken liking \|\| id:ebi-fl187-GCST90094764 | Weighted median | 3 | 0.232281577106199 | 0.291986234231523 | 0.426309711542706 | -0.340011442 | 0.804574596199984 | 1.2614748812712 | 0.711762178742005 | 2.2357452019869 | 0.999332001504091 |
| ebi-fl187-GCST90094764 | prot-a-2466 | Glutaminyl-peptide cyclotransferase-like protein \|\| id:prot-a-2466 | Fried chicken liking \|\| id:ebi-fl187-GCST90094764 | Weighted median | 3 | 0.236971269688464 | 0.259745006600351 | 0.361598860074822 | -0.272128943 | 0.746071482625152 | 1.2674047043202 | 0.761756031459899 | 2.10869965998759 | 0.999332001504091 |
| ebi-fl187-GCST90094764 | prot-a-2474 | Ras-related protein Rab-27A \|\| id:prot-a-2474 | Fried chicken liking \|\| id:ebi-fl187-GCST90094764 | Weighted median | 3 | 0.241168089827749 | 0.352988911956751 | 0.494469616799354 | -0.450690178 | 0.933026357262981 | 1.27273495112789 | 0.637188226780224 | 2.54219112617289 | 0.999332001504091 |
| ebi-fl187-GCST90094764 | prot-a-2490 | Rap1 GTPase-GDP dissociation stimulator 1 \|\| id:prot-a-2490 | Fried chicken liking \|\| id:ebi-fl187-GCST90094764 | Weighted median | 3 | 0.144545884699721 | 0.310869100868224 | 0.641950455091847 | -0.464757553 | 0.753849322401439 | 1.15551471419304 | 0.628287413122789 | 2.12516473643835 | 0.999332001504091 |
| ebi-fl187-GCST90094764 | prot-a-2545 | Receptor-interacting serine/threonine-protein kinase 2 \|\| id:prot-a-2545 | Fried chicken liking \|\| id:ebi-fl187-GCST90094764 | Weighted median | 3 | 0.631784725365729 | 0.300743034294119 | 0.035663269261632 | 0.0423283781492561 | 1.2212410725822 | 1.88096459059639 | 1.04323699874453 | 3.39139408910462 | 0.999332001504091 |
| ebi-fl187-GCST90094764 | prot-a-2610 | RUN and FYVE domain-containing protein 1 \|\| id:prot-a-2610 | Fried chicken liking \|\| id:ebi-fl187-GCST90094764 | Weighted median | 3 | 0.0813191956738409 | 0.309184834891542 | 0.792541644390604 | -0.524683081 | 0.687321472061264 | 1.0847170783127 | 0.591742869381175 | 1.98838245607202 | 0.999332001504091 |
| ebi-fl187-GCST90094764 | prot-a-2627 | Serine--tRNA ligase, mitochondrial \|\| id:prot-a-2627 | Fried chicken liking \|\| id:ebi-fl187-GCST90094764 | Weighted median | 3 | -0.049843973 | 0.292318541108805 | 0.864607082993299 | -0.622788314 | 0.523100367250397 | 0.951377853246136 | 0.536446568878523 | 1.68725064555719 | 0.999332001504091 |
| ebi-fl187-GCST90094764 | prot-a-264 | BolA-like protein 2 \|\| id:prot-a-264 | Fried chicken liking \|\| id:ebi-fl187-GCST90094764 | Weighted median | 3 | 0.180209021779615 | 0.291238977636919 | 0.53606997038063 | -0.390619374 | 0.751037417947977 | 1.19746763378073 | 0.676637652651415 | 2.11919737001563 | 0.999332001504091 |
| ebi-fl187-GCST90094764 | prot-a-2646 | Secretoglobin family 1C member 1 \|\| id:prot-a-2646 | Fried chicken liking \|\| id:ebi-fl187-GCST90094764 | Weighted median | 3 | 0.184086726612909 | 0.313314756402543 | 0.556837291383874 | -0.430010196 | 0.798183649161894 | 1.20212007437792 | 0.650502462207983 | 2.22150223431489 | 0.999332001504091 |
| ebi-fl187-GCST90094764 | prot-a-2672 | Semaphorin-3E \|\| id:prot-a-2672 | Fried chicken liking \|\| id:ebi-fl187-GCST90094764 | Weighted median | 3 | 0.291251484632031 | 0.287599146781738 | 0.311203762479928 | -0.272442843 | 0.854945812324239 | 1.33810105349086 | 0.761516953910033 | 2.35124696851449 | 0.999332001504091 |
| ebi-fl187-GCST90094764 | prot-a-2701 | Plasma protease C1 inhibitor \|\| id:prot-a-2701 | Fried chicken liking \|\| id:ebi-fl187-GCST90094764 | Weighted median | 3 | 0.0634041054899564 | 0.288407707760562 | 0.825994267298384 | -0.501875002 | 0.628683212700658 | 1.06545730939581 | 0.605394479185076 | 1.87513979260772 | 0.999332001504091 |
| ebi-fl187-GCST90094764 | prot-a-2738 | Signaling threshold-regulating transmembrane adapter 1 \|\| id:prot-a-2738 | Fried chicken liking \|\| id:ebi-fl187-GCST90094764 | Weighted median | 3 | 0.477042791706763 | 0.29266275880503 | 0.103099695305764 | -0.096576216 | 1.05066179896462 | 1.61130239295852 | 0.907940695762166 | 2.8595429345464 | 0.999332001504091 |
| ebi-fl187-GCST90094764 | prot-a-274 | Serine/threonine-protein kinase BRSK2 \|\| id:prot-a-274 | Fried chicken liking \|\| id:ebi-fl187-GCST90094764 | Weighted median | 3 | -0.242020295 | 0.274228731073661 | 0.377479988448643 | -0.779508608 | 0.295468017665409 | 0.785040244810783 | 0.458631323640011 | 1.34375511267153 | 0.999332001504091 |
| ebi-fl187-GCST90094764 | prot-a-2749 | Mitochondrial glutamate carrier 2 \|\| id:prot-a-2749 | Fried chicken liking \|\| id:ebi-fl187-GCST90094764 | Weighted median | 3 | 0.0304416967215877 | 0.32488258545266 | 0.925346985000972 | -0.606328171 | 0.667211564208801 | 1.03090978287649 | 0.54534962798513 | 1.94879564575301 | 0.999332001504091 |
| ebi-fl187-GCST90094764 | prot-a-2760 | Sodium-coupled monocarboxylate transporter 1 \|\| id:prot-a-2760 | Fried chicken liking \|\| id:ebi-fl187-GCST90094764 | Weighted median | 3 | -0.079382507 | 0.300817934145691 | 0.791865547496106 | -0.668985657 | 0.510220644370562 | 0.923686540707018 | 0.5122278888897 | 1.66565867261285 | 0.999332001504091 |
| ebi-fl187-GCST90094764 | prot-a-2777 | Small integral membrane protein 10 \|\| id:prot-a-2777 | Fried chicken liking \|\| id:ebi-fl187-GCST90094764 | Weighted median | 3 | -0.703108395 | 0.305070857277769 | 0.0211812517886131 | -1.301047275 | -0.105169515 | 0.495044117137862 | 0.272246526670482 | 0.900171917379236 | 0.999332001504091 |
| ebi-fl187-GCST90094764 | prot-a-2799 | Superoxide dismutase [Mn], mitochondrial \|\| id:prot-a-2799 | Fried chicken liking \|\| id:ebi-fl187-GCST90094764 | Weighted median | 3 | 0.0737381620918858 | 0.279523201588001 | 0.79193419581178 | -0.474127313 | 0.621603637204367 | 1.07652489356405 | 0.622428004333793 | 1.86191147955097 | 0.999332001504091 |
| ebi-fl187-GCST90094764 | prot-a-2805 | Protein SPACA7 \|\| id:prot-a-2805 | Fried chicken liking \|\| id:ebi-fl187-GCST90094764 | Weighted median | 3 | -0.154243551 | 0.289381662480682 | 0.594026105301649 | -0.721431609 | 0.412944507483523 | 0.857063256995173 | 0.486055915397823 | 1.51126115992223 | 0.999332001504091 |
| ebi-fl187-GCST90094764 | prot-a-2811 | SPARC-like protein 1 \|\| id:prot-a-2811 | Fried chicken liking \|\| id:ebi-fl187-GCST90094764 | Weighted median | 3 | -0.13938725 | 0.278118798932972 | 0.616245282827203 | -0.684500096 | 0.405725595984955 | 0.86989109796302 | 0.504342289379113 | 1.50039078271005 | 0.999332001504091 |
| ebi-fl187-GCST90094764 | prot-a-2831 | Sepiapterin reductase \|\| id:prot-a-2831 | Fried chicken liking \|\| id:ebi-fl187-GCST90094764 | Weighted median | 3 | -0.140590141 | 0.285688659369301 | 0.622641844650789 | -0.700539913 | 0.419359631816049 | 0.868845343207675 | 0.496317263339938 | 1.52098725185108 | 0.999332001504091 |
| ebi-fl187-GCST90094764 | prot-a-2834 | SPRY domain-containing SOCS box protein 1 \|\| id:prot-a-2834 | Fried chicken liking \|\| id:ebi-fl187-GCST90094764 | Weighted median | 3 | -0.19403097 | 0.289816658234723 | 0.503179402806064 | -0.76207162 | 0.374009680037502 | 0.823632395893812 | 0.466698602601371 | 1.45355122081908 | 0.999332001504091 |
| ebi-fl187-GCST90094764 | prot-a-2835 | Spectrin alpha chain, non-erythrocytic 1 \|\| id:prot-a-2835 | Fried chicken liking \|\| id:ebi-fl187-GCST90094764 | Weighted median | 3 | -0.057582333 | 0.281292192514733 | 0.837801461264909 | -0.60891503 | 0.49375036416975 | 0.944044161048482 | 0.543940708122594 | 1.63844949403726 | 0.999332001504091 |
| ebi-fl187-GCST90094764 | prot-a-2859 | Sia-alpha-2,3-Gal-beta-1,4-GlcNAc-R:alpha 2,8-sialyltransferase \|\| id:prot-a-2859 | Fried chicken liking \|\| id:ebi-fl187-GCST90094764 | Weighted median | 3 | 0.361856414240552 | 0.277585367357982 | 0.192374757505399 | -0.182210906 | 0.905923734262196 | 1.43599273904881 | 0.833425547612426 | 2.47421638622462 | 0.999332001504091 |
| ebi-fl187-GCST90094764 | prot-a-2861 | Alpha-2,8-sialyltransferase 8F \|\| id:prot-a-2861 | Fried chicken liking \|\| id:ebi-fl187-GCST90094764 | Weighted median | 3 | 0.208009296748036 | 0.308239004170794 | 0.4997830161611 | -0.396139151 | 0.812157744922792 | 1.23122461588069 | 0.672913052610907 | 2.25276363546345 | 0.999332001504091 |
| ebi-fl187-GCST90094764 | prot-a-289 | Uncharacterized protein C17orf89 \|\| id:prot-a-289 | Fried chicken liking \|\| id:ebi-fl187-GCST90094764 | Weighted median | 3 | 0.337894564451778 | 0.283890522953754 | 0.233956631548237 | -0.218530861 | 0.894319989441135 | 1.40199267574594 | 0.803698676488734 | 2.44567214099776 | 0.999332001504091 |
| ebi-fl187-GCST90094764 | prot-a-2921 | Tachykinin-3 \|\| id:prot-a-2921 | Fried chicken liking \|\| id:ebi-fl187-GCST90094764 | Weighted median | 3 | -0.05599396 | 0.290216743939067 | 0.847007194834663 | -0.624818778 | 0.512830857846607 | 0.945544846704978 | 0.535358438244273 | 1.67001207651163 | 0.999332001504091 |
| ebi-fl187-GCST90094764 | prot-a-2928 | Tax1-binding protein 1 \|\| id:prot-a-2928 | Fried chicken liking \|\| id:ebi-fl187-GCST90094764 | Weighted median | 3 | 0.110974851056111 | 0.296618103269117 | 0.708304790705577 | -0.470396631 | 0.69234633346358 | 1.117366806 | 0.624754421943494 | 1.99839894699217 | 0.999332001504091 |
| ebi-fl187-GCST90094764 | prot-a-296 | Uncharacterized protein C1orf185 \|\| id:prot-a-296 | Fried chicken liking \|\| id:ebi-fl187-GCST90094764 | Weighted median | 3 | 0.305334140530044 | 0.294976187938809 | 0.300615423887219 | -0.272819188 | 0.88348746889011 | 1.35707838214575 | 0.761230414909389 | 2.41932232240951 | 0.999332001504091 |
| ebi-fl187-GCST90094764 | prot-a-2964 | Transforming growth factor beta-2 \|\| id:prot-a-2964 | Fried chicken liking \|\| id:ebi-fl187-GCST90094764 | Weighted median | 3 | -0.298661821 | 0.258222198647546 | 0.247432615180184 | -0.80477733 | 0.207453688345267 | 0.741810231660639 | 0.447187490554619 | 1.23054072714317 | 0.999332001504091 |
| ebi-fl187-GCST90094764 | prot-a-2977 | Thymocyte nuclear protein 1 \|\| id:prot-a-2977 | Fried chicken liking \|\| id:ebi-fl187-GCST90094764 | Weighted median | 3 | 0.472221934551439 | 0.276940780890954 | 0.0881689018097089 | -0.070581996 | 1.01502586509771 | 1.60355322810442 | 0.931851328316437 | 2.75943476950319 | 0.999332001504091 |
| ebi-fl187-GCST90094764 | prot-a-3015 | Transmembrane protein 70, mitochondrial \|\| id:prot-a-3015 | Fried chicken liking \|\| id:ebi-fl187-GCST90094764 | Weighted median | 3 | -0.226696031 | 0.279635498487032 | 0.417547150929281 | -0.774781608 | 0.321389546213709 | 0.797163058466541 | 0.460804406073161 | 1.37904267712848 | 0.999332001504091 |
| ebi-fl187-GCST90094764 | prot-a-3030 | Tumor necrosis factor alpha-induced protein 3 \|\| id:prot-a-3030 | Fried chicken liking \|\| id:ebi-fl187-GCST90094764 | Weighted median | 3 | -0.204692296 | 0.28216074365718 | 0.468178650526437 | -0.757727353 | 0.34834276166651 | 0.814898025299552 | 0.468730476138423 | 1.41671776306903 | 0.999332001504091 |
| ebi-fl187-GCST90094764 | prot-a-3032 | Tumor necrosis factor receptor superfamily member 10A \|\| id:prot-a-3032 | Fried chicken liking \|\| id:ebi-fl187-GCST90094764 | Weighted median | 3 | 0.136646320621513 | 0.284262367910066 | 0.630726229393452 | -0.42050792 | 0.693800561725241 | 1.14642261077299 | 0.656713177016677 | 2.00130718933053 | 0.999332001504091 |
| ebi-fl187-GCST90094764 | prot-a-3081 | Tumor protein p53-inducible protein 11 \|\| id:prot-a-3081 | Fried chicken liking \|\| id:ebi-fl187-GCST90094764 | Weighted median | 3 | -0.028690439 | 0.284205795206149 | 0.919590531014539 | -0.585733798 | 0.528352919435919 | 0.971717223504641 | 0.556697208724604 | 1.6961363334636 | 0.999332001504091 |
| ebi-fl187-GCST90094764 | prot-a-315 | Complement C4 \|\| id:prot-a-315 | Fried chicken liking \|\| id:ebi-fl187-GCST90094764 | Weighted median | 3 | -0.082437997 | 0.275085352180136 | 0.764420094170232 | -0.621605287 | 0.45672929313983 | 0.920868532575699 | 0.5370815749203 | 1.57890140694914 | 0.999332001504091 |
| ebi-fl187-GCST90094764 | prot-a-3157 | UDP-glucuronosyltransferase 1-8 \|\| id:prot-a-3157 | Fried chicken liking \|\| id:ebi-fl187-GCST90094764 | Weighted median | 3 | 0.0798969576885588 | 0.288919855268833 | 0.782135837516037 | -0.486385959 | 0.646179874015472 | 1.08317544902237 | 0.614844456983353 | 1.90823718102834 | 0.999332001504091 |
| ebi-fl187-GCST90094764 | prot-a-317 | Glutaredoxin-like protein C5orf63 \|\| id:prot-a-317 | Fried chicken liking \|\| id:ebi-fl187-GCST90094764 | Weighted median | 3 | -0.506317688 | 0.273281250739439 | 0.0639203771687386 | -1.041948939 | 0.0293135636075893 | 0.602710867171586 | 0.352766491080474 | 1.02974743517762 | 0.999332001504091 |
| ebi-fl187-GCST90094764 | prot-a-3233 | Serine/threonine-protein kinase WNK3 \|\| id:prot-a-3233 | Fried chicken liking \|\| id:ebi-fl187-GCST90094764 | Weighted median | 3 | -0.276191411 | 0.295725685817048 | 0.350332465335331 | -0.855813755 | 0.30343093362482 | 0.758667699840448 | 0.424937255625929 | 1.35449803744173 | 0.999332001504091 |
| ebi-fl187-GCST90094764 | prot-a-3250 | 14-3-3 protein theta \|\| id:prot-a-3250 | Fried chicken liking \|\| id:ebi-fl187-GCST90094764 | Weighted median | 3 | 0.335512708044223 | 0.290398709243272 | 0.247946339948572 | -0.233668762 | 0.904694178161036 | 1.39865730427269 | 0.791623988361648 | 2.47117606787537 | 0.999332001504091 |
| ebi-fl187-GCST90094764 | prot-a-3278 | E3 ubiquitin-protein ligase ZNRF3 \|\| id:prot-a-3278 | Fried chicken liking \|\| id:ebi-fl187-GCST90094764 | Weighted median | 3 | 0.351606548279554 | 0.302612408385537 | 0.24527448830443 | -0.241513772 | 0.944726868715206 | 1.42134918140792 | 0.785437986539941 | 2.5721107587228 | 0.999332001504091 |
| ebi-fl187-GCST90094764 | prot-a-355 | Calpain-3 \|\| id:prot-a-355 | Fried chicken liking \|\| id:ebi-fl187-GCST90094764 | Weighted median | 3 | 0.0497338630843898 | 0.279213322639611 | 0.858627649621735 | -0.497524249 | 0.596991975458027 | 1.05099135155583 | 0.608034138776246 | 1.81664605751952 | 0.999332001504091 |
| ebi-fl187-GCST90094764 | prot-a-385 | Coiled-coil domain-containing protein 90B, mitochondrial \|\| id:prot-a-385 | Fried chicken liking \|\| id:ebi-fl187-GCST90094764 | Weighted median | 3 | 0.68061010206983 | 0.2799773968135 | 0.0150594337071706 | 0.13185440431537 | 1.22936579982429 | 1.97508236655806 | 1.14094219091433 | 3.41906047979732 | 0.999332001504091 |
| ebi-fl187-GCST90094764 | prot-a-390 | C-C motif chemokine 14 \|\| id:prot-a-390 | Fried chicken liking \|\| id:ebi-fl187-GCST90094764 | Weighted median | 3 | -0.134017611 | 0.280608053697757 | 0.632936949175841 | -0.684009396 | 0.415974174366287 | 0.874574662437128 | 0.504589830720175 | 1.51584672066288 | 0.999332001504091 |
| ebi-fl187-GCST90094764 | prot-a-40 | Pituitary adenylate cyclase-activating polypeptide 27 \|\| id:prot-a-40 | Fried chicken liking \|\| id:ebi-fl187-GCST90094764 | Weighted median | 3 | -0.08721054 | 0.288314371802083 | 0.762282980615154 | -0.652306709 | 0.477885628274876 | 0.916484118333804 | 0.520842956781822 | 1.61266103001166 | 0.999332001504091 |
| ebi-fl187-GCST90094764 | prot-a-401 | C-C motif chemokine 24 \|\| id:prot-a-401 | Fried chicken liking \|\| id:ebi-fl187-GCST90094764 | Weighted median | 3 | -0.140129575 | 0.272693684504926 | 0.607341674549081 | -0.674609197 | 0.394350046564236 | 0.869245595546397 | 0.509355439505535 | 1.48341972377935 | 0.999332001504091 |
| ebi-fl187-GCST90094764 | prot-a-44 | Adhesion G protein-coupled receptor F5 \|\| id:prot-a-44 | Fried chicken liking \|\| id:ebi-fl187-GCST90094764 | Weighted median | 3 | 0.169984478689482 | 0.278502530995845 | 0.541628890773684 | -0.375880482 | 0.715849439441337 | 1.18528645397849 | 0.686684399241209 | 2.04592383274954 | 0.999332001504091 |
| ebi-fl187-GCST90094764 | prot-a-48 | Adrenomedullin \|\| id:prot-a-48 | Fried chicken liking \|\| id:ebi-fl187-GCST90094764 | Weighted median | 3 | 0.223001221978424 | 0.285655842039141 | 0.435000160508973 | -0.336884228 | 0.782886672375141 | 1.24982210099069 | 0.713991495042777 | 2.18777855894656 | 0.999332001504091 |
| ebi-fl187-GCST90094764 | prot-a-502 | Carcinoembryonic antigen-related cell adhesion molecule 1 \|\| id:prot-a-502 | Fried chicken liking \|\| id:ebi-fl187-GCST90094764 | Weighted median | 3 | 0.096138785178207 | 0.270226284628053 | 0.72201176467591 | -0.433504733 | 0.625782303049192 | 1.10091184363837 | 0.648233224719665 | 1.86970806377192 | 0.999332001504091 |
| ebi-fl187-GCST90094764 | prot-a-518 | Complement factor B \|\| id:prot-a-518 | Fried chicken liking \|\| id:ebi-fl187-GCST90094764 | Weighted median | 3 | -0.116241278 | 0.277018636836291 | 0.674766457730548 | -0.659197806 | 0.426715250171354 | 0.890260397109273 | 0.517266115759075 | 1.53221630127095 | 0.999332001504091 |
| ebi-fl187-GCST90094764 | prot-a-525 | Properdin \|\| id:prot-a-525 | Fried chicken liking \|\| id:ebi-fl187-GCST90094764 | Weighted median | 3 | -0.311434101 | 0.299004249234721 | 0.297610703175141 | -0.89748243 | 0.274614227431644 | 0.732395873020661 | 0.407594517028215 | 1.31602289139862 | 0.999332001504091 |
| ebi-fl187-GCST90094764 | prot-a-533 | Chondroadherin \|\| id:prot-a-533 | Fried chicken liking \|\| id:ebi-fl187-GCST90094764 | Weighted median | 3 | 0.268324265135736 | 0.303818302255693 | 0.377142551758115 | -0.327159607 | 0.863808137556894 | 1.30777113585257 | 0.720968661991951 | 2.37217709164203 | 0.999332001504091 |
| ebi-fl187-GCST90094764 | prot-a-535 | Coiled-coil-helix-coiled-coil-helix domain-containing protein 2 \|\| id:prot-a-535 | Fried chicken liking \|\| id:ebi-fl187-GCST90094764 | Weighted median | 3 | 0.299533565151425 | 0.269589248274156 | 0.266536560916834 | -0.228861361 | 0.827928491768771 | 1.3492293332033 | 0.795438804330807 | 2.28857302870422 | 0.999332001504091 |
| ebi-fl187-GCST90094764 | prot-a-605 | Ciliary neurotrophic factor receptor subunit alpha \|\| id:prot-a-605 | Fried chicken liking \|\| id:ebi-fl187-GCST90094764 | Weighted median | 3 | 0.044001385593159 | 0.278192119290927 | 0.874323584542627 | -0.501255168 | 0.589257939403375 | 1.04498380280985 | 0.605769839284588 | 1.80265024324184 | 0.999332001504091 |
| ebi-fl187-GCST90094764 | prot-a-606 | Ciliary neurotrophic factor receptor subunit alpha \|\| id:prot-a-606 | Fried chicken liking \|\| id:ebi-fl187-GCST90094764 | Weighted median | 3 | 0.242503765597833 | 0.294291517108663 | 0.409924970383384 | -0.334307608 | 0.819315139130813 | 1.274436048 | 0.715833552275781 | 2.26894539338992 | 0.999332001504091 |
| ebi-fl187-GCST90094764 | prot-a-707 | Cancer/testis antigen 55 \|\| id:prot-a-707 | Fried chicken liking \|\| id:ebi-fl187-GCST90094764 | Weighted median | 3 | -0.201744021 | 0.275210669150921 | 0.463526072348769 | -0.741156933 | 0.337668890359526 | 0.817304113713182 | 0.476562246008565 | 1.40167631801971 | 0.999332001504091 |
| ebi-fl187-GCST90094764 | prot-a-719 | Dipeptidyl peptidase 1 \|\| id:prot-a-719 | Fried chicken liking \|\| id:ebi-fl187-GCST90094764 | Weighted median | 3 | -0.517597104 | 0.323570031470032 | 0.109677413579235 | -1.151794366 | 0.116600157435509 | 0.595950836599766 | 0.31606911658956 | 1.12367004874178 | 0.999332001504091 |
| ebi-fl187-GCST90094764 | prot-a-752 | Cytochrome b5 \|\| id:prot-a-752 | Fried chicken liking \|\| id:ebi-fl187-GCST90094764 | Weighted median | 3 | -0.020243779 | 0.275996323875183 | 0.941529195321404 | -0.561196573 | 0.520709016176079 | 0.979959750950821 | 0.570525979030882 | 1.68322065739203 | 0.999332001504091 |
| ebi-fl187-GCST90094764 | prot-a-755 | Cytoglobin \|\| id:prot-a-755 | Fried chicken liking \|\| id:ebi-fl187-GCST90094764 | Weighted median | 3 | -0.277245821 | 0.287377892170493 | 0.334673532427458 | -0.84050649 | 0.286014847625061 | 0.757868174275885 | 0.431491921867195 | 1.33111221896071 | 0.999332001504091 |
| ebi-fl187-GCST90094764 | prot-a-807 | Desmin \|\| id:prot-a-807 | Fried chicken liking \|\| id:ebi-fl187-GCST90094764 | Weighted median | 3 | -0.539395717 | 0.30124846055617 | 0.0733680501766277 | -1.129842699 | 0.05105126604568 | 0.583100503862533 | 0.32308407376516 | 1.05237684309961 | 0.999332001504091 |
| ebi-fl187-GCST90094764 | prot-a-82 | Ameloblastin \|\| id:prot-a-82 | Fried chicken liking \|\| id:ebi-fl187-GCST90094764 | Weighted median | 3 | 0.248146164585741 | 0.329977204165311 | 0.452045031831405 | -0.398609156 | 0.894901484749752 | 1.28164724994343 | 0.671253005582836 | 2.44709470144019 | 0.999332001504091 |
| ebi-fl187-GCST90094764 | prot-a-84 | Muellerian-inhibiting factor \|\| id:prot-a-84 | Fried chicken liking \|\| id:ebi-fl187-GCST90094764 | Weighted median | 3 | -0.242466486 | 0.300147655270215 | 0.419191894279053 | -0.83075589 | 0.345822918228445 | 0.784690045160834 | 0.435719805380082 | 1.41315235013795 | 0.999332001504091 |
| ebi-fl187-GCST90094764 | prot-a-858 | Inactive dipeptidyl peptidase 10 \|\| id:prot-a-858 | Fried chicken liking \|\| id:ebi-fl187-GCST90094764 | Weighted median | 3 | 0.592719874544239 | 0.318881615122568 | 0.0630631730320068 | -0.032288091 | 1.21772784018447 | 1.80890171631912 | 0.968227604143949 | 3.37950023868125 | 0.999332001504091 |
| ebi-fl187-GCST90094764 | prot-a-863 | Dihydropyrimidinase-related protein 3 \|\| id:prot-a-863 | Fried chicken liking \|\| id:ebi-fl187-GCST90094764 | Weighted median | 3 | 0.0904237851793213 | 0.277875135122701 | 0.744869596778927 | -0.45421148 | 0.635059050019816 | 1.09463807681762 | 0.634948440365291 | 1.88713357344358 | 0.999332001504091 |
| ebi-fl187-GCST90094764 | prot-a-864 | Dihydropyrimidinase-related protein 5 \|\| id:prot-a-864 | Fried chicken liking \|\| id:ebi-fl187-GCST90094764 | Weighted median | 3 | -0.165128129 | 0.315932314076356 | 0.601204390072901 | -0.784355464 | 0.454099207019118 | 0.847785071639251 | 0.456413782043133 | 1.57475421639754 | 0.999332001504091 |
| ebi-fl187-GCST90094764 | prot-a-884 | Endothelin-converting enzyme 1 \|\| id:prot-a-884 | Fried chicken liking \|\| id:ebi-fl187-GCST90094764 | Weighted median | 3 | -0.39096148 | 0.297309188124142 | 0.188510018116843 | -0.973687489 | 0.19176452863996 | 0.676406210648326 | 0.377687747783357 | 1.21138523684932 | 0.999332001504091 |
| ebi-fl187-GCST90094764 | prot-a-897 | Ephrin-A2 \|\| id:prot-a-897 | Fried chicken liking \|\| id:ebi-fl187-GCST90094764 | Weighted median | 3 | 0.352976876768696 | 0.291671577786827 | 0.22620750693266 | -0.218699416 | 0.924653169230876 | 1.42329823179886 | 0.803563220349254 | 2.52099375051201 | 0.999332001504091 |
| ebi-fl187-GCST90094764 | prot-a-90 | Anaphase-promoting complex subunit 10 \|\| id:prot-a-90 | Fried chicken liking \|\| id:ebi-fl187-GCST90094764 | Weighted median | 3 | -0.071590447 | 0.281570734309523 | 0.799299440304776 | -0.623469086 | 0.480288192174561 | 0.930912075515386 | 0.536081495128375 | 1.61654020930687 | 0.999332001504091 |
| ebi-fl187-GCST90094764 | prot-a-914 | Histone-lysine N-methyltransferase EHMT2 \|\| id:prot-a-914 | Fried chicken liking \|\| id:ebi-fl187-GCST90094764 | Weighted median | 3 | 0.133700909384195 | 0.333895924924976 | 0.68884211625523 | -0.520735103 | 0.788136922237147 | 1.14305089271823 | 0.594083674446428 | 2.19929514905692 | 0.999332001504091 |
| ebi-fl187-GCST90094764 | prot-a-932 | RNA polymerase II elongation factor ELL \|\| id:prot-a-932 | Fried chicken liking \|\| id:ebi-fl187-GCST90094764 | Weighted median | 3 | -0.592967257 | 0.287555881161965 | 0.0391987876216134 | -1.156576784 | -0.02935773 | 0.552684891234946 | 0.314561150707792 | 0.971069022071126 | 0.999332001504091 |
| ebi-fl187-GCST90094764 | prot-a-991 | Estrogen receptor \|\| id:prot-a-991 | Fried chicken liking \|\| id:ebi-fl187-GCST90094764 | Weighted median | 3 | 0.0577772557056895 | 0.272797681840198 | 0.832266767318678 | -0.476906201 | 0.592460712112478 | 1.05947897652834 | 0.62070074785794 | 1.80843297769384 | 0.999332001504091 |
| ebi-fl187-GCST90094764 | prot-a-992 | Persulfide dioxygenase ETHE1, mitochondrial \|\| id:prot-a-992 | Fried chicken liking \|\| id:ebi-fl187-GCST90094764 | Weighted median | 3 | 0.152802493200544 | 0.268323564899499 | 0.56903653463947 | -0.373111694 | 0.678716680403562 | 1.16509484206337 | 0.688588317363314 | 1.97134624095931 | 0.999332001504091 |
| ebi-fl187-GCST90094764 | prot-a-993 | Ethanolamine kinase 1 \|\| id:prot-a-993 | Fried chicken liking \|\| id:ebi-fl187-GCST90094764 | Weighted median | 3 | 0.393264881574544 | 0.300083408326704 | 0.190020463581029 | -0.194898599 | 0.981428361894883 | 1.4818108417392 | 0.822918098752772 | 2.66826476902594 | 0.999332001504091 |
| ebi-fl187-GCST90094764 | ebi-IPs91-GCST90274819 | Leukemia inhibitory factor levels \|\| id:ebi-IPs91-GCST90274819 | Fried chicken liking \|\| id:ebi-fl187-GCST90094764 | Weighted mode | 3 | 0.0691407806001443 | 0.178710284323194 | 0.736125376555987 | -0.281131377 | 0.419412937873605 | 1.07158705719888 | 0.754929148884778 | 1.52106833184608 | 0.999714303421225 |
| ebi-fl187-GCST90094764 | ebi-IPs91-GCST90274837 | STAM binding protein levels \|\| id:ebi-IPs91-GCST90274837 | Fried chicken liking \|\| id:ebi-fl187-GCST90094764 | Weighted mode | 3 | 0.0737587471012805 | 0.158877971009335 | 0.68810284060619 | -0.237642076 | 0.385159570279578 | 1.07654705406719 | 0.788484858187237 | 1.46984884692044 | 0.999714303421225 |
| ebi-fl187-GCST90094764 | ebi-IPs91-GCST90274840 | TNF-beta levels \|\| id:ebi-IPs91-GCST90274840 | Fried chicken liking \|\| id:ebi-fl187-GCST90094764 | Weighted mode | 3 | 0.371605543164834 | 0.193735407745114 | 0.195118623756842 | -0.008115856 | 0.751326942345257 | 1.45006088204002 | 0.991916988629645 | 2.11981101818568 | 0.999714303421225 |
| ebi-fl187-GCST90094764 | ebi-IPs91-GCST90274842 | Tumor necrosis factor ligand superfamily member 14 levels \|\| id:ebi-IPs91-GCST90274842 | Fried chicken liking \|\| id:ebi-fl187-GCST90094764 | Weighted mode | 3 | 0.211357413266206 | 0.161162880554285 | 0.320035199816405 | -0.104521833 | 0.527236659152605 | 1.23535380800556 | 0.900755131347533 | 1.69424406017071 | 0.999714303421225 |
| ebi-fl187-GCST90094764 | prot-a-1017 | FAS-associated factor 2 \|\| id:prot-a-1017 | Fried chicken liking \|\| id:ebi-fl187-GCST90094764 | Weighted mode | 3 | 0.0284151133446855 | 0.363896858356663 | 0.944869099932005 | -0.684822729 | 0.741652955723744 | 1.02882267381201 | 0.50417959805757 | 2.09940286800108 | 0.999714303421225 |
| ebi-fl187-GCST90094764 | prot-a-1019 | Fas apoptotic inhibitory molecule 3 \|\| id:prot-a-1019 | Fried chicken liking \|\| id:ebi-fl187-GCST90094764 | Weighted mode | 3 | 0.192425340759014 | 0.366262288061267 | 0.651757277569596 | -0.525448744 | 0.910299425359097 | 1.21218599944234 | 0.591289967092554 | 2.48506651392925 | 0.999714303421225 |
| ebi-fl187-GCST90094764 | prot-a-1052 | MIP18 family protein FAM96A \|\| id:prot-a-1052 | Fried chicken liking \|\| id:ebi-fl187-GCST90094764 | Weighted mode | 3 | 0.288358676030849 | 0.344487595054497 | 0.490642176104007 | -0.38683701 | 0.963554362337663 | 1.33423577670254 | 0.679201788817531 | 2.62099590599175 | 0.999714303421225 |
| ebi-fl187-GCST90094764 | prot-a-1066 | F-box/LRR-repeat protein 4 \|\| id:prot-a-1066 | Fried chicken liking \|\| id:ebi-fl187-GCST90094764 | Weighted mode | 3 | 0.0942407989440075 | 0.384394439440413 | 0.829188544606589 | -0.659172302 | 0.847653900247217 | 1.09882430979599 | 0.517279308213888 | 2.33416424091603 | 0.999714303421225 |
| ebi-fl187-GCST90094764 | prot-a-1076 | Low affinity immunoglobulin gamma Fc region receptor III-B \|\| id:prot-a-1076 | Fried chicken liking \|\| id:ebi-fl187-GCST90094764 | Weighted mode | 3 | 0.0206985484775115 | 0.376281307028589 | 0.961132736508974 | -0.716812813 | 0.758209910253547 | 1.02091424909137 | 0.488306101159108 | 2.13445193808501 | 0.999714303421225 |
| ebi-fl187-GCST90094764 | prot-a-1081 | Fc receptor-like protein 3 \|\| id:prot-a-1081 | Fried chicken liking \|\| id:ebi-fl187-GCST90094764 | Weighted mode | 3 | 0.115963805462882 | 0.34356687166388 | 0.767851275767263 | -0.557427263 | 0.789354873924087 | 1.12295522655306 | 0.572680526574741 | 2.2019754161804 | 0.999714303421225 |
| ebi-fl187-GCST90094764 | prot-a-1105 | Fibroblast growth factor receptor 4 \|\| id:prot-a-1105 | Fried chicken liking \|\| id:ebi-fl187-GCST90094764 | Weighted mode | 3 | -0.064446398 | 0.382971853977669 | 0.881841828736338 | -0.815071232 | 0.686178436042456 | 0.93758636965058 | 0.442607798598904 | 1.98611096175279 | 0.999714303421225 |
| ebi-fl187-GCST90094764 | prot-a-1143 | Follistatin-related protein 1 \|\| id:prot-a-1143 | Fried chicken liking \|\| id:ebi-fl187-GCST90094764 | Weighted mode | 3 | 0.00476744536579021 | 0.350413956502744 | 0.990380129280733 | -0.682043909 | 0.691578800111169 | 1.0047788277145 | 0.505582570635287 | 1.99686569763421 | 0.999714303421225 |
| ebi-fl187-GCST90094764 | prot-a-1151 | Alpha-(1,3)-fucosyltransferase 10 \|\| id:prot-a-1151 | Fried chicken liking \|\| id:ebi-fl187-GCST90094764 | Weighted mode | 3 | -0.095327633 | 0.32764530910164 | 0.798489247843977 | -0.737512439 | 0.546857172872893 | 0.909075042718462 | 0.478302242988493 | 1.72781425428828 | 0.999714303421225 |
| ebi-fl187-GCST90094764 | prot-a-1165 | Growth arrest and DNA damage-inducible proteins-interacting protein 1 \|\| id:prot-a-1165 | Fried chicken liking \|\| id:ebi-fl187-GCST90094764 | Weighted mode | 3 | -0.091821567 | 0.344304213604507 | 0.814689722389723 | -0.766657825 | 0.583014692029513 | 0.912267914056743 | 0.464563127715634 | 1.79143091082093 | 0.999714303421225 |
| ebi-fl187-GCST90094764 | prot-a-1194 | Growth/differentiation factor 11/8 \|\| id:prot-a-1194 | Fried chicken liking \|\| id:ebi-fl187-GCST90094764 | Weighted mode | 3 | -0.195431353 | 0.405777303275786 | 0.677623563691983 | -0.990754867 | 0.599892161429017 | 0.822479802404262 | 0.371296305727086 | 1.82192231629739 | 0.999714303421225 |
| ebi-fl187-GCST90094764 | prot-a-1216 | D-glucuronyl C5-epimerase \|\| id:prot-a-1216 | Fried chicken liking \|\| id:ebi-fl187-GCST90094764 | Weighted mode | 3 | 0.358362375711435 | 0.394907353353784 | 0.459949171715109 | -0.415656037 | 1.13238078828485 | 1.43098408040977 | 0.659907215208386 | 3.10303538314787 | 0.999714303421225 |
| ebi-fl187-GCST90094764 | prot-a-1246 | Glypican-3 \|\| id:prot-a-1246 | Fried chicken liking \|\| id:ebi-fl187-GCST90094764 | Weighted mode | 3 | -0.244799715 | 0.381325650586893 | 0.586652881983162 | -0.992197991 | 0.502598559750349 | 0.78286131760698 | 0.3707608658829 | 1.6530111427642 | 0.999714303421225 |
| ebi-fl187-GCST90094764 | prot-a-1275 | Glutamate receptor 4 \|\| id:prot-a-1275 | Fried chicken liking \|\| id:ebi-fl187-GCST90094764 | Weighted mode | 3 | -0.218372597 | 0.373619855466409 | 0.618046507382218 | -0.950667514 | 0.513922319524312 | 0.803825882597607 | 0.386482954585955 | 1.67183582578962 | 0.999714303421225 |
| ebi-fl187-GCST90094764 | prot-a-1289 | Glutathione S-transferase P \|\| id:prot-a-1289 | Fried chicken liking \|\| id:ebi-fl187-GCST90094764 | Weighted mode | 3 | -0.22762565 | 0.388606731239833 | 0.617337951588058 | -0.989294843 | 0.53404354306436 | 0.796422344610708 | 0.371838803183892 | 1.70581592228698 | 0.999714303421225 |
| ebi-fl187-GCST90094764 | prot-a-130 | Apolipoprotein D \|\| id:prot-a-130 | Fried chicken liking \|\| id:ebi-fl187-GCST90094764 | Weighted mode | 3 | -0.053304614 | 0.318442469914612 | 0.882456747360845 | -0.677451855 | 0.570842627202498 | 0.948091166809694 | 0.507909572132145 | 1.76975766928193 | 0.999714303421225 |
| ebi-fl187-GCST90094764 | prot-a-1313 | Heparin-binding EGF-like growth factor \|\| id:prot-a-1313 | Fried chicken liking \|\| id:ebi-fl187-GCST90094764 | Weighted mode | 3 | 0.243198449224713 | 0.362703269119027 | 0.571587192442002 | -0.467699958 | 0.954096856698006 | 1.27532168560747 | 0.626441454053699 | 2.59632467049547 | 0.999714303421225 |
| ebi-fl187-GCST90094764 | prot-a-1347 | HLA class II histocompatibility antigen, DQ alpha 2 chain \|\| id:prot-a-1347 | Fried chicken liking \|\| id:ebi-fl187-GCST90094764 | Weighted mode | 3 | -0.010417399 | 0.367739692478065 | 0.979972964503205 | -0.731187196 | 0.710352398573662 | 0.989636674484179 | 0.481337209168699 | 2.03470816057533 | 0.999714303421225 |
| ebi-fl187-GCST90094764 | prot-a-1357 | Non-histone chromosomal protein HMG-14 \|\| id:prot-a-1357 | Fried chicken liking \|\| id:ebi-fl187-GCST90094764 | Weighted mode | 3 | 0.145543039574634 | 0.326523912086635 | 0.699395489270095 | -0.494443828 | 0.785529907264438 | 1.15666751598878 | 0.609910027792659 | 2.1935690209679 | 0.999714303421225 |
| ebi-fl187-GCST90094764 | prot-a-137 | Apolipoprotein M \|\| id:prot-a-137 | Fried chicken liking \|\| id:ebi-fl187-GCST90094764 | Weighted mode | 3 | 0.0122519438356418 | 0.366096326821477 | 0.976342270641003 | -0.705296857 | 0.729800744405737 | 1.01232730636418 | 0.493961916567561 | 2.07466717744501 | 0.999714303421225 |
| ebi-fl187-GCST90094764 | prot-a-1381 | 17-beta-hydroxysteroid dehydrogenase 14 \|\| id:prot-a-1381 | Fried chicken liking \|\| id:ebi-fl187-GCST90094764 | Weighted mode | 3 | -0.065179151 | 0.318341601928143 | 0.856716610802915 | -0.689128691 | 0.558770388614091 | 0.936899601686348 | 0.502013287285627 | 1.74852117637399 | 0.999714303421225 |
| ebi-fl187-GCST90094764 | prot-a-1392 | Serine protease HTRA2, mitochondrial \|\| id:prot-a-1392 | Fried chicken liking \|\| id:ebi-fl187-GCST90094764 | Weighted mode | 3 | 0.509899971733968 | 0.358942650031722 | 0.291311660214326 | -0.193627622 | 1.21342756579614 | 1.6651246270861 | 0.823964673194727 | 3.36499866308392 | 0.999714303421225 |
| ebi-fl187-GCST90094764 | prot-a-1416 | Interferon-induced protein with tetratricopeptide repeats 2 \|\| id:prot-a-1416 | Fried chicken liking \|\| id:ebi-fl187-GCST90094764 | Weighted mode | 3 | -0.24820782 | 0.54991327430774 | 0.695951657062436 | -1.326037837 | 0.829622198061186 | 0.780197786051132 | 0.265527241982914 | 2.29245248364481 | 0.999714303421225 |
| ebi-fl187-GCST90094764 | prot-a-1428 | Interferon gamma \|\| id:prot-a-1428 | Fried chicken liking \|\| id:ebi-fl187-GCST90094764 | Weighted mode | 3 | 0.241640036898893 | 0.337793485317571 | 0.548631007732223 | -0.420435194 | 0.903715268121332 | 1.27333575642342 | 0.656760938980139 | 2.468758192 | 0.999714303421225 |
| ebi-fl187-GCST90094764 | prot-a-1483 | Interleukin-17C \|\| id:prot-a-1483 | Fried chicken liking \|\| id:ebi-fl187-GCST90094764 | Weighted mode | 3 | -0.219275173 | 0.33252645501524 | 0.577401150583073 | -0.871027024 | 0.432476679225021 | 0.803100696434866 | 0.418521496644665 | 1.54106953593775 | 0.999714303421225 |
| ebi-fl187-GCST90094764 | prot-a-1503 | Interleukin-1 receptor-like 2 \|\| id:prot-a-1503 | Fried chicken liking \|\| id:ebi-fl187-GCST90094764 | Weighted mode | 3 | 0.306135567375378 | 0.391365918597835 | 0.515989564572911 | -0.460941633 | 1.07321276782714 | 1.35816641712441 | 0.630689487728949 | 2.92476099965903 | 0.999714303421225 |
| ebi-fl187-GCST90094764 | prot-a-1522 | Interleukin-31 receptor subunit alpha \|\| id:prot-a-1522 | Fried chicken liking \|\| id:ebi-fl187-GCST90094764 | Weighted mode | 3 | 0.151069284534161 | 0.518095898507488 | 0.798065425968897 | -0.864398677 | 1.16653724560884 | 1.16307723855496 | 0.421304816942825 | 3.21085496401581 | 0.999714303421225 |
| ebi-fl187-GCST90094764 | prot-a-1540 | Interleukin-6 receptor subunit alpha \|\| id:prot-a-1540 | Fried chicken liking \|\| id:ebi-fl187-GCST90094764 | Weighted mode | 3 | -0.136255734 | 0.369892414113978 | 0.747936529858476 | -0.861244866 | 0.588733397385609 | 0.872619445248007 | 0.422635630003135 | 1.80170492539706 | 0.999714303421225 |
| ebi-fl187-GCST90094764 | prot-a-1541 | Interleukin-6 receptor subunit alpha \|\| id:prot-a-1541 | Fried chicken liking \|\| id:ebi-fl187-GCST90094764 | Weighted mode | 3 | -0.071714703 | 0.404208781230716 | 0.875520906375834 | -0.863963915 | 0.720534507883542 | 0.930796411051865 | 0.421488024090368 | 2.05553161491791 | 0.999714303421225 |
| ebi-fl187-GCST90094764 | prot-a-1545 | Interleukin-9 \|\| id:prot-a-1545 | Fried chicken liking \|\| id:ebi-fl187-GCST90094764 | Weighted mode | 3 | 0.676089925010889 | 0.329957380497549 | 0.176992814382243 | 0.0293734592356928 | 1.32280639078609 | 1.96617479162977 | 1.02980911439418 | 3.75394163559583 | 0.999714303421225 |
| ebi-fl187-GCST90094764 | prot-a-1548 | Inositol monophosphatase 3 \|\| id:prot-a-1548 | Fried chicken liking \|\| id:ebi-fl187-GCST90094764 | Weighted mode | 3 | 0.17500917881777 | 0.49170414530648 | 0.755934884629376 | -0.788730946 | 1.13874930361847 | 1.19125715089448 | 0.454421114455669 | 3.12286017179705 | 0.999714303421225 |
| ebi-fl187-GCST90094764 | prot-a-1555 | Inhibin beta A chain:Inhibin beta B chain heterodimer \|\| id:prot-a-1555 | Fried chicken liking \|\| id:ebi-fl187-GCST90094764 | Weighted mode | 3 | 0.411308198209504 | 0.365321120307731 | 0.377158457980382 | -0.304721198 | 1.12733759401266 | 1.50879029137466 | 0.737328914813436 | 3.08742556762802 | 0.999714303421225 |
| ebi-fl187-GCST90094764 | prot-a-1603 | Junctophilin-3 \|\| id:prot-a-1603 | Fried chicken liking \|\| id:ebi-fl187-GCST90094764 | Weighted mode | 3 | -0.120819716 | 0.52495124624153 | 0.839369711905616 | -1.149724159 | 0.908084726697651 | 0.886193711796132 | 0.316724122965895 | 2.47956893043973 | 0.999714303421225 |
| ebi-fl187-GCST90094764 | prot-a-1627 | UPF0258 protein KIAA1024 \|\| id:prot-a-1627 | Fried chicken liking \|\| id:ebi-fl187-GCST90094764 | Weighted mode | 3 | 0.607873674053767 | 0.491018759898549 | 0.341330348470223 | -0.354523095 | 1.57027044345492 | 1.83652219933513 | 0.701507915835363 | 4.80794829611346 | 0.999714303421225 |
| ebi-fl187-GCST90094764 | prot-a-1666 | Kallikrein-7 \|\| id:prot-a-1666 | Fried chicken liking \|\| id:ebi-fl187-GCST90094764 | Weighted mode | 3 | 0.07189529777742 | 0.327789020881661 | 0.846739683836777 | -0.570571183 | 0.714361778705475 | 1.07454283115106 | 0.565202512331526 | 2.04288245502502 | 0.999714303421225 |
| ebi-fl187-GCST90094764 | prot-a-1678 | Importin subunit alpha-3 \|\| id:prot-a-1678 | Fried chicken liking \|\| id:ebi-fl187-GCST90094764 | Weighted mode | 3 | 0.154844872982046 | 0.329557995408795 | 0.684707464685386 | -0.491088798 | 0.800778543983285 | 1.16747683985639 | 0.611959730776681 | 2.22727428465135 | 0.999714303421225 |
| ebi-fl187-GCST90094764 | prot-a-1718 | Protein LDOC1 \|\| id:prot-a-1718 | Fried chicken liking \|\| id:ebi-fl187-GCST90094764 | Weighted mode | 3 | 0.304356507370769 | 0.335167142101474 | 0.45969022218663 | -0.352571091 | 0.961284105889658 | 1.35575230563344 | 0.702878599587885 | 2.61505232241824 | 0.999714303421225 |
| ebi-fl187-GCST90094764 | prot-a-1723 | Leptin \|\| id:prot-a-1723 | Fried chicken liking \|\| id:ebi-fl187-GCST90094764 | Weighted mode | 3 | -0.031604015 | 0.323979339118263 | 0.93118561753472 | -0.666603519 | 0.603395489943625 | 0.96889017236441 | 0.513449540944004 | 1.82831630227656 | 0.999714303421225 |
| ebi-fl187-GCST90094764 | prot-a-1730 | Galectin-7 \|\| id:prot-a-1730 | Fried chicken liking \|\| id:ebi-fl187-GCST90094764 | Weighted mode | 3 | -0.385160777 | 0.352315399888941 | 0.388403355979674 | -1.075698961 | 0.305377406666865 | 0.680341244108194 | 0.341059285 | 1.35713709895493 | 0.999714303421225 |
| ebi-fl187-GCST90094764 | prot-a-1753 | Lipase member N \|\| id:prot-a-1753 | Fried chicken liking \|\| id:ebi-fl187-GCST90094764 | Weighted mode | 3 | 0.220548477502268 | 0.328049292618191 | 0.57065588874377 | -0.422428136 | 0.863525091033922 | 1.2467603631016 | 0.655453356115083 | 2.37150575017928 | 0.999714303421225 |
| ebi-fl187-GCST90094764 | prot-a-1756 | VIP36-like protein \|\| id:prot-a-1756 | Fried chicken liking \|\| id:ebi-fl187-GCST90094764 | Weighted mode | 3 | 0.229076821197412 | 0.306432861764443 | 0.532669857613416 | -0.371531588 | 0.829685230255721 | 1.25743863324103 | 0.689677220056956 | 2.29259698650984 | 0.999714303421225 |
| ebi-fl187-GCST90094764 | prot-a-1760 | Ig Kappa chain V-I region HK102- like \|\| id:prot-a-1760 | Fried chicken liking \|\| id:ebi-fl187-GCST90094764 | Weighted mode | 3 | -0.158633827 | 0.334494579546805 | 0.682056275801791 | -0.814243203 | 0.496975548985541 | 0.853308760460855 | 0.442974442339155 | 1.64374232706129 | 0.999714303421225 |
| ebi-fl187-GCST90094764 | prot-a-179 | Neutral ceramidase \|\| id:prot-a-179 | Fried chicken liking \|\| id:ebi-fl187-GCST90094764 | Weighted mode | 3 | 0.0147563208295833 | 0.354429355454823 | 0.970573047577351 | -0.679925216 | 0.709437857521036 | 1.01486573284228 | 0.506654880697432 | 2.03284818707307 | 0.999714303421225 |
| ebi-fl187-GCST90094764 | prot-a-1809 | Lactotransferrin \|\| id:prot-a-1809 | Fried chicken liking \|\| id:ebi-fl187-GCST90094764 | Weighted mode | 3 | 0.692647223281876 | 0.424214253177037 | 0.244113967703577 | -0.138812713 | 1.52410715950887 | 1.99900033535949 | 0.870391026165983 | 4.59104267006232 | 0.999714303421225 |
| ebi-fl187-GCST90094764 | prot-a-1818 | Lysophospholipase-like protein 1 \|\| id:prot-a-1818 | Fried chicken liking \|\| id:ebi-fl187-GCST90094764 | Weighted mode | 3 | 0.108867209642962 | 0.312740611611625 | 0.760985526216128 | -0.504104389 | 0.721838808401746 | 1.11501427736634 | 0.60404632370293 | 2.05821439506387 | 0.999714303421225 |
| ebi-fl187-GCST90094764 | prot-a-1852 | MAP kinase-activated protein kinase 3 \|\| id:prot-a-1852 | Fried chicken liking \|\| id:ebi-fl187-GCST90094764 | Weighted mode | 3 | 0.338108355948854 | 0.337978452615283 | 0.422501821356308 | -0.324329411 | 1.00054612307481 | 1.40229244190157 | 0.723012034902418 | 2.71976675032754 | 0.999714303421225 |
| ebi-fl187-GCST90094764 | prot-a-1856 | Megakaryocyte-associated tyrosine-protein kinase \|\| id:prot-a-1856 | Fried chicken liking \|\| id:ebi-fl187-GCST90094764 | Weighted mode | 3 | -0.104103036 | 0.361906417762884 | 0.800680766390104 | -0.813439614 | 0.605233543179081 | 0.901132443883045 | 0.443330554521183 | 1.83167993529268 | 0.999714303421225 |
| ebi-fl187-GCST90094764 | prot-a-1890 | Beta-1,3-N-acetylglucosaminyltransferase manic fringe \|\| id:prot-a-1890 | Fried chicken liking \|\| id:ebi-fl187-GCST90094764 | Weighted mode | 3 | 0.350691905162087 | 0.356434847022961 | 0.42890144933039 | -0.347920395 | 1.04930420532709 | 1.42004974851089 | 0.706155087450684 | 2.85566347121542 | 0.999714303421225 |
| ebi-fl187-GCST90094764 | prot-a-1919 | Matrilysin \|\| id:prot-a-1919 | Fried chicken liking \|\| id:ebi-fl187-GCST90094764 | Weighted mode | 3 | 0.185138686926499 | 0.403209424373897 | 0.691191815943352 | -0.605151785 | 0.975429158699337 | 1.20338532236699 | 0.545991547106859 | 2.65230522663179 | 0.999714303421225 |
| ebi-fl187-GCST90094764 | prot-a-1923 | Multimerin-2 \|\| id:prot-a-1923 | Fried chicken liking \|\| id:ebi-fl187-GCST90094764 | Weighted mode | 3 | -0.187305562 | 0.336364280402975 | 0.633624211839637 | -0.846579551 | 0.471968427920035 | 0.829190328890093 | 0.42887938593144 | 1.6031467682496 | 0.999714303421225 |
| ebi-fl187-GCST90094764 | prot-a-1948 | Mesothelin \|\| id:prot-a-1948 | Fried chicken liking \|\| id:ebi-fl187-GCST90094764 | Weighted mode | 3 | 0.291325018445428 | 0.403934220250622 | 0.545688892353885 | -0.500386053 | 1.08303609013665 | 1.33819945278183 | 0.606296551774752 | 2.95363344914896 | 0.999714303421225 |
| ebi-fl187-GCST90094764 | prot-a-195 | ATPase family AAA domain-containing protein 1 \|\| id:prot-a-195 | Fried chicken liking \|\| id:ebi-fl187-GCST90094764 | Weighted mode | 3 | 0.0493859403486034 | 0.386409268885749 | 0.909993374757124 | -0.707976227 | 0.806748107364671 | 1.0506257513735 | 0.492640181359776 | 2.24060990397172 | 0.999714303421225 |
| ebi-fl187-GCST90094764 | prot-a-1966 | Mucin-1 \|\| id:prot-a-1966 | Fried chicken liking \|\| id:ebi-fl187-GCST90094764 | Weighted mode | 3 | 0.425561044020955 | 0.344346815059092 | 0.341974037843962 | -0.249358713 | 1.10048080153678 | 1.53044882800276 | 0.779300377678295 | 3.00561077887984 | 0.999714303421225 |
| ebi-fl187-GCST90094764 | prot-a-1986 | Myocardial zonula adherens protein \|\| id:prot-a-1986 | Fried chicken liking \|\| id:ebi-fl187-GCST90094764 | Weighted mode | 3 | -0.444779619 | 0.502787427963992 | 0.46968032876988 | -1.430242978 | 0.540683740052743 | 0.640965517245905 | 0.23925078260788 | 1.71718056601575 | 0.999714303421225 |
| ebi-fl187-GCST90094764 | prot-a-2071 | Epididymal secretory protein E1 \|\| id:prot-a-2071 | Fried chicken liking \|\| id:ebi-fl187-GCST90094764 | Weighted mode | 3 | -0.553479287 | 0.494018828164919 | 0.37903304305529 | -1.52175619 | 0.414797616040883 | 0.574945924380815 | 0.218328124322258 | 1.51406428735764 | 0.999714303421225 |
| ebi-fl187-GCST90094764 | prot-a-2098 | Neuritin \|\| id:prot-a-2098 | Fried chicken liking \|\| id:ebi-fl187-GCST90094764 | Weighted mode | 3 | 0.514839159202401 | 0.385166671480022 | 0.313099531713086 | -0.240087517 | 1.26976583530324 | 1.67336933409316 | 0.78655902084831 | 3.56001883401374 | 0.999714303421225 |
| ebi-fl187-GCST90094764 | prot-a-2115 | Neurotrophin-4 \|\| id:prot-a-2115 | Fried chicken liking \|\| id:ebi-fl187-GCST90094764 | Weighted mode | 3 | 0.0480880584566521 | 0.350908372573326 | 0.903550705449314 | -0.639692352 | 0.73586846870037 | 1.04926304774105 | 0.527454669570993 | 2.08729395504319 | 0.999714303421225 |
| ebi-fl187-GCST90094764 | prot-a-2118 | Netrin-1 \|\| id:prot-a-2118 | Fried chicken liking \|\| id:ebi-fl187-GCST90094764 | Weighted mode | 3 | -0.50198139 | 0.55775679946697 | 0.463104806461641 | -1.595184717 | 0.591221936966034 | 0.605330075740633 | 0.20287105132059 | 1.80619412287223 | 0.999714303421225 |
| ebi-fl187-GCST90094764 | prot-a-2120 | Netrin-G1 \|\| id:prot-a-2120 | Fried chicken liking \|\| id:ebi-fl187-GCST90094764 | Weighted mode | 3 | 0.154952458076199 | 0.36869887659805 | 0.715137813758649 | -0.56769734 | 0.877602256208378 | 1.16760244971888 | 0.56682915190259 | 2.40512591142069 | 0.999714303421225 |
| ebi-fl187-GCST90094764 | prot-a-2145 | Olfactomedin-like protein 3 \|\| id:prot-a-2145 | Fried chicken liking \|\| id:ebi-fl187-GCST90094764 | Weighted mode | 3 | 0.328827809118986 | 0.389550768848524 | 0.487473367855716 | -0.434691698 | 1.09234731606209 | 1.38933860355152 | 0.647464250947754 | 2.98126383424719 | 0.999714303421225 |
| ebi-fl187-GCST90094764 | prot-a-2162 | Prolyl 4-hydroxylase subunit alpha-2 \|\| id:prot-a-2162 | Fried chicken liking \|\| id:ebi-fl187-GCST90094764 | Weighted mode | 3 | 0.423786726393102 | 0.410032686185987 | 0.409953635506922 | -0.379877339 | 1.22745079131764 | 1.52773573332634 | 0.683945297801951 | 3.41251921518148 | 0.999714303421225 |
| ebi-fl187-GCST90094764 | prot-a-2168 | Polyadenylate-binding protein-interacting protein 1 \|\| id:prot-a-2168 | Fried chicken liking \|\| id:ebi-fl187-GCST90094764 | Weighted mode | 3 | 0.512312994529912 | 0.389825471811427 | 0.319267222499998 | -0.25174493 | 1.27637091928031 | 1.66914746241405 | 0.777443014999067 | 3.58361088534137 | 0.999714303421225 |
| ebi-fl187-GCST90094764 | prot-a-219 | Beta-1,4-galactosyltransferase 5 \|\| id:prot-a-219 | Fried chicken liking \|\| id:ebi-fl187-GCST90094764 | Weighted mode | 3 | -0.372763027 | 0.342272209827368 | 0.389857482886345 | -1.043616558 | 0.298090504225122 | 0.688828447222963 | 0.352178701215855 | 1.34728371723076 | 0.999714303421225 |
| ebi-fl187-GCST90094764 | prot-a-2190 | Pyruvate carboxylase, mitochondrial \|\| id:prot-a-2190 | Fried chicken liking \|\| id:ebi-fl187-GCST90094764 | Weighted mode | 3 | 0.384650807241611 | 0.330691323870048 | 0.364772766631954 | -0.263504188 | 1.0328058020269 | 1.46910123235118 | 0.768354404914857 | 2.80893610694524 | 0.999714303421225 |
| ebi-fl187-GCST90094764 | prot-a-2197 | Protocadherin-9 \|\| id:prot-a-2197 | Fried chicken liking \|\| id:ebi-fl187-GCST90094764 | Weighted mode | 3 | -0.064579974 | 0.323002059251919 | 0.860015424099101 | -0.69766401 | 0.568504061952001 | 0.937461138576541 | 0.497746677889367 | 1.76562381102735 | 0.999714303421225 |
| ebi-fl187-GCST90094764 | prot-a-2198 | Protocadherin alpha-4 \|\| id:prot-a-2198 | Fried chicken liking \|\| id:ebi-fl187-GCST90094764 | Weighted mode | 3 | -1.074932722 | 0.379658419367277 | 0.105390363243799 | -1.819063224 | -0.33080222 | 0.341320717801119 | 0.16217760384145 | 0.718347229461872 | 0.999714303421225 |
| ebi-fl187-GCST90094764 | prot-a-2254 | Placenta growth factor \|\| id:prot-a-2254 | Fried chicken liking \|\| id:ebi-fl187-GCST90094764 | Weighted mode | 3 | 0.359453395153094 | 0.369694920041015 | 0.43346107304889 | -0.365148648 | 1.08405543843348 | 1.43254616383895 | 0.694093467515048 | 2.95664576541372 | 0.999714303421225 |
| ebi-fl187-GCST90094764 | prot-a-2261 | Membrane-associated progesterone receptor component 2 \|\| id:prot-a-2261 | Fried chicken liking \|\| id:ebi-fl187-GCST90094764 | Weighted mode | 3 | -0.39879797 | 0.34698791109384 | 0.369319716910408 | -1.078894276 | 0.281298335605329 | 0.671126275206366 | 0.339971232283499 | 1.3248487945497 | 0.999714303421225 |
| ebi-fl187-GCST90094764 | prot-a-2268 | Polymeric immunoglobulin receptor \|\| id:prot-a-2268 | Fried chicken liking \|\| id:ebi-fl187-GCST90094764 | Weighted mode | 3 | 0.0933552990123189 | 0.334080565039659 | 0.806154394308193 | -0.561442608 | 0.748153206490051 | 1.09785173161724 | 0.570385626909142 | 2.11309396266915 | 0.999714303421225 |
| ebi-fl187-GCST90094764 | prot-a-2283 | Group 10 secretory phospholipase A2 \|\| id:prot-a-2283 | Fried chicken liking \|\| id:ebi-fl187-GCST90094764 | Weighted mode | 3 | -0.705862107 | 0.675577508052027 | 0.40577834594589 | -2.029994023 | 0.61826980858597 | 0.49368278326378 | 0.131336306146142 | 1.85571452131351 | 0.999714303421225 |
| ebi-fl187-GCST90094764 | prot-a-2298 | Inactive phospholipase D5 \|\| id:prot-a-2298 | Fried chicken liking \|\| id:ebi-fl187-GCST90094764 | Weighted mode | 3 | 0.427120836701786 | 0.36740298406357 | 0.36497847657451 | -0.292989012 | 1.14723068546638 | 1.53283787360662 | 0.74603033798668 | 3.14945897924704 | 0.999714303421225 |
| ebi-fl187-GCST90094764 | prot-a-2304 | Procollagen-lysine,2-oxoglutarate 5-dioxygenase 2 \|\| id:prot-a-2304 | Fried chicken liking \|\| id:ebi-fl187-GCST90094764 | Weighted mode | 3 | 0.0706276484334838 | 0.340819782225357 | 0.855015442278767 | -0.597379125 | 0.738634421595184 | 1.07318155063159 | 0.550251889477748 | 2.09307530358349 | 0.999714303421225 |
| ebi-fl187-GCST90094764 | prot-a-2331 | Periostin \|\| id:prot-a-2331 | Fried chicken liking \|\| id:ebi-fl187-GCST90094764 | Weighted mode | 3 | -0.617359058 | 0.554596071458319 | 0.381491244308718 | -1.704367358 | 0.46964924192859 | 0.53936699520297 | 0.181887419370694 | 1.599433081 | 0.999714303421225 |
| ebi-fl187-GCST90094764 | prot-a-2333 | Periostin \|\| id:prot-a-2333 | Fried chicken liking \|\| id:ebi-fl187-GCST90094764 | Weighted mode | 3 | -0.569689566 | 0.398268375759417 | 0.288877148370508 | -1.350295583 | 0.210916450005538 | 0.565701024002843 | 0.259163644963053 | 1.23480918245106 | 0.999714303421225 |
| ebi-fl187-GCST90094764 | prot-a-2367 | cAMP-dependent protein kinase catalytic subunit alpha \|\| id:prot-a-2367 | Fried chicken liking \|\| id:ebi-fl187-GCST90094764 | Weighted mode | 3 | 0.120490153677146 | 0.324140812186789 | 0.745788017276456 | -0.514825838 | 0.755806145563253 | 1.12804963377019 | 0.597604665454261 | 2.12932737946715 | 0.999714303421225 |
| ebi-fl187-GCST90094764 | prot-a-2385 | Phosphoribosyl pyrophosphate synthase-associated protein 1 \|\| id:prot-a-2385 | Fried chicken liking \|\| id:ebi-fl187-GCST90094764 | Weighted mode | 3 | -0.296099568 | 0.393343597377445 | 0.530126942144199 | -1.067053019 | 0.474853882836701 | 0.743713374263126 | 0.34402084796941 | 1.6077792561776 | 0.999714303421225 |
| ebi-fl187-GCST90094764 | prot-a-2393 | Trypsin-3 \|\| id:prot-a-2393 | Fried chicken liking \|\| id:ebi-fl187-GCST90094764 | Weighted mode | 3 | -0.24235021 | 0.318675756563752 | 0.526386947816778 | -0.866954693 | 0.382254273042947 | 0.784781291304365 | 0.420229330094551 | 1.46558469643891 | 0.999714303421225 |
| ebi-fl187-GCST90094764 | prot-a-2405 | Pregnancy-specific beta-1-glycoprotein 6 \|\| id:prot-a-2405 | Fried chicken liking \|\| id:ebi-fl187-GCST90094764 | Weighted mode | 3 | 0.42290702378953 | 0.36436474827296 | 0.365588964221958 | -0.291247883 | 1.13706193040453 | 1.52639237119038 | 0.747330404683728 | 3.11759518444081 | 0.999714303421225 |
| ebi-fl187-GCST90094764 | prot-a-244 | Beclin-1 \|\| id:prot-a-244 | Fried chicken liking \|\| id:ebi-fl187-GCST90094764 | Weighted mode | 3 | -0.256106157 | 0.333264315178802 | 0.52254267282362 | -0.909304215 | 0.397091900722583 | 0.774059792543563 | 0.402804391897113 | 1.48749262541712 | 0.999714303421225 |
| ebi-fl187-GCST90094764 | prot-a-2454 | tRNA pseudouridine synthase A, mitochondrial \|\| id:prot-a-2454 | Fried chicken liking \|\| id:ebi-fl187-GCST90094764 | Weighted mode | 3 | 0.0447567686332366 | 0.442309383390961 | 0.928631149103504 | -0.822169623 | 0.91168316007952 | 1.04577346406246 | 0.439477119805363 | 2.48850756695035 | 0.999714303421225 |
| ebi-fl187-GCST90094764 | prot-a-2466 | Glutaminyl-peptide cyclotransferase-like protein \|\| id:prot-a-2466 | Fried chicken liking \|\| id:ebi-fl187-GCST90094764 | Weighted mode | 3 | 0.226055417534013 | 0.306449797553588 | 0.537528447898714 | -0.374586186 | 0.826697020739045 | 1.25364513727522 | 0.687573747797307 | 2.28575645194225 | 0.999714303421225 |
| ebi-fl187-GCST90094764 | prot-a-2474 | Ras-related protein Rab-27A \|\| id:prot-a-2474 | Fried chicken liking \|\| id:ebi-fl187-GCST90094764 | Weighted mode | 3 | -0.03759403 | 0.59278697517619 | 0.95520093106976 | -1.199456501 | 1.12426844152771 | 0.963103852983346 | 0.301357955109189 | 3.07796431421659 | 0.999714303421225 |
| ebi-fl187-GCST90094764 | prot-a-2490 | Rap1 GTPase-GDP dissociation stimulator 1 \|\| id:prot-a-2490 | Fried chicken liking \|\| id:ebi-fl187-GCST90094764 | Weighted mode | 3 | -0.00653911 | 0.512536806418076 | 0.990978871240865 | -1.01111125 | 0.998033031073604 | 0.993482223944821 | 0.363814465998921 | 2.71294030759424 | 0.999714303421225 |
| ebi-fl187-GCST90094764 | prot-a-2545 | Receptor-interacting serine/threonine-protein kinase 2 \|\| id:prot-a-2545 | Fried chicken liking \|\| id:ebi-fl187-GCST90094764 | Weighted mode | 3 | 0.66457267731683 | 0.379981169938626 | 0.222404552195101 | -0.080190416 | 1.40933577039654 | 1.9436597739411 | 0.922940587217503 | 4.09323565260705 | 0.999714303421225 |
| ebi-fl187-GCST90094764 | prot-a-2610 | RUN and FYVE domain-containing protein 1 \|\| id:prot-a-2610 | Fried chicken liking \|\| id:ebi-fl187-GCST90094764 | Weighted mode | 3 | 0.284460196690199 | 0.489713900215012 | 0.620062980592401 | -0.675379048 | 1.24429944111162 | 1.32904441188068 | 0.508963462593938 | 3.47050265602368 | 0.999714303421225 |
| ebi-fl187-GCST90094764 | prot-a-2627 | Serine--tRNA ligase, mitochondrial \|\| id:prot-a-2627 | Fried chicken liking \|\| id:ebi-fl187-GCST90094764 | Weighted mode | 3 | -0.105471639 | 0.326371132865204 | 0.777230283061831 | -0.74515906 | 0.534215781197504 | 0.899899994352172 | 0.474658794880933 | 1.70610975414072 | 0.999714303421225 |
| ebi-fl187-GCST90094764 | prot-a-264 | BolA-like protein 2 \|\| id:prot-a-264 | Fried chicken liking \|\| id:ebi-fl187-GCST90094764 | Weighted mode | 3 | 0.166276293258606 | 0.336450165124611 | 0.670105701792448 | -0.49316603 | 0.825718616902842 | 1.180899331 | 0.610689867574038 | 2.28352115275201 | 0.999714303421225 |
| ebi-fl187-GCST90094764 | prot-a-2646 | Secretoglobin family 1C member 1 \|\| id:prot-a-2646 | Fried chicken liking \|\| id:ebi-fl187-GCST90094764 | Weighted mode | 3 | 0.116209118051254 | 0.34719293503011 | 0.769686619362842 | -0.564289035 | 0.796707270710269 | 1.12323073539782 | 0.568764374831271 | 2.21822487619167 | 0.999714303421225 |
| ebi-fl187-GCST90094764 | prot-a-2672 | Semaphorin-3E \|\| id:prot-a-2672 | Fried chicken liking \|\| id:ebi-fl187-GCST90094764 | Weighted mode | 3 | 0.401656028327318 | 0.401988628066652 | 0.422968323576866 | -0.386241683 | 1.18955373933796 | 1.49429724834189 | 0.679606256766909 | 3.28561463960741 | 0.999714303421225 |
| ebi-fl187-GCST90094764 | prot-a-2701 | Plasma protease C1 inhibitor \|\| id:prot-a-2701 | Fried chicken liking \|\| id:ebi-fl187-GCST90094764 | Weighted mode | 3 | 0.0797384206814538 | 0.354350785412104 | 0.842858885383675 | -0.614789119 | 0.774265960089178 | 1.08300373924005 | 0.540754918372192 | 2.16899941056227 | 0.999714303421225 |
| ebi-fl187-GCST90094764 | prot-a-2738 | Signaling threshold-regulating transmembrane adapter 1 \|\| id:prot-a-2738 | Fried chicken liking \|\| id:ebi-fl187-GCST90094764 | Weighted mode | 3 | 0.597872256467664 | 0.3680652675539 | 0.245790617335097 | -0.123535668 | 1.31928018087331 | 1.81824592060643 | 0.883790117710787 | 3.74072776052897 | 0.999714303421225 |
| ebi-fl187-GCST90094764 | prot-a-274 | Serine/threonine-protein kinase BRSK2 \|\| id:prot-a-274 | Fried chicken liking \|\| id:ebi-fl187-GCST90094764 | Weighted mode | 3 | -0.305964417 | 0.35150596596884 | 0.475835535295274 | -0.99491611 | 0.382987276684223 | 0.736412823051111 | 0.369754461976765 | 1.46665936917939 | 0.999714303421225 |
| ebi-fl187-GCST90094764 | prot-a-2749 | Mitochondrial glutamate carrier 2 \|\| id:prot-a-2749 | Fried chicken liking \|\| id:ebi-fl187-GCST90094764 | Weighted mode | 3 | 0.146614558260663 | 0.367054612556007 | 0.728190315962628 | -0.572812482 | 0.866041598870437 | 1.15790757109815 | 0.563937142959048 | 2.37748117843653 | 0.999714303421225 |
| ebi-fl187-GCST90094764 | prot-a-2760 | Sodium-coupled monocarboxylate transporter 1 \|\| id:prot-a-2760 | Fried chicken liking \|\| id:ebi-fl187-GCST90094764 | Weighted mode | 3 | 0.202674751275439 | 0.500568195640762 | 0.724758289252327 | -0.778438912 | 1.18378841473133 | 1.22467407978089 | 0.459122182203269 | 3.26672650510958 | 0.999714303421225 |
| ebi-fl187-GCST90094764 | prot-a-2777 | Small integral membrane protein 10 \|\| id:prot-a-2777 | Fried chicken liking \|\| id:ebi-fl187-GCST90094764 | Weighted mode | 3 | -0.846747431 | 0.407134258372379 | 0.173067384290578 | -1.644730577 | -0.048764284 | 0.42880739188116 | 0.193064571774104 | 0.952405600065596 | 0.999714303421225 |
| ebi-fl187-GCST90094764 | prot-a-2799 | Superoxide dismutase [Mn], mitochondrial \|\| id:prot-a-2799 | Fried chicken liking \|\| id:ebi-fl187-GCST90094764 | Weighted mode | 3 | 0.0359524163351403 | 0.353941194743969 | 0.928358512397257 | -0.657772325 | 0.72967715803332 | 1.03660651978013 | 0.518003994499682 | 2.07441079269773 | 0.999714303421225 |
| ebi-fl187-GCST90094764 | prot-a-2805 | Protein SPACA7 \|\| id:prot-a-2805 | Fried chicken liking \|\| id:ebi-fl187-GCST90094764 | Weighted mode | 3 | -0.371470145 | 0.419864676113393 | 0.469632838473223 | -1.19440491 | 0.451464620513539 | 0.689719597328818 | 0.302884143932607 | 1.57061085061381 | 0.999714303421225 |
| ebi-fl187-GCST90094764 | prot-a-2811 | SPARC-like protein 1 \|\| id:prot-a-2811 | Fried chicken liking \|\| id:ebi-fl187-GCST90094764 | Weighted mode | 3 | -0.080090113 | 0.341024194606674 | 0.8361783150042 | -0.748497534 | 0.588317308587079 | 0.923033165497054 | 0.473076800726014 | 1.80095541210221 | 0.999714303421225 |
| ebi-fl187-GCST90094764 | prot-a-2831 | Sepiapterin reductase \|\| id:prot-a-2831 | Fried chicken liking \|\| id:ebi-fl187-GCST90094764 | Weighted mode | 3 | -0.180682865 | 0.336543949178635 | 0.64508476607615 | -0.840309006 | 0.478943274926206 | 0.834700028931977 | 0.431577142958822 | 1.61436755784242 | 0.999714303421225 |
| ebi-fl187-GCST90094764 | prot-a-2834 | SPRY domain-containing SOCS box protein 1 \|\| id:prot-a-2834 | Fried chicken liking \|\| id:ebi-fl187-GCST90094764 | Weighted mode | 3 | -0.233423641 | 0.365911128702573 | 0.588816284488435 | -0.950609453 | 0.483762171683531 | 0.791818056204508 | 0.386505394852617 | 1.62216580280999 | 0.999714303421225 |
| ebi-fl187-GCST90094764 | prot-a-2835 | Spectrin alpha chain, non-erythrocytic 1 \|\| id:prot-a-2835 | Fried chicken liking \|\| id:ebi-fl187-GCST90094764 | Weighted mode | 3 | -0.192759712 | 0.408261318455286 | 0.683323551936311 | -0.992951896 | 0.607432472395711 | 0.824680111251913 | 0.370481452603295 | 1.83571210141714 | 0.999714303421225 |
| ebi-fl187-GCST90094764 | prot-a-2859 | Sia-alpha-2,3-Gal-beta-1,4-GlcNAc-R:alpha 2,8-sialyltransferase \|\| id:prot-a-2859 | Fried chicken liking \|\| id:ebi-fl187-GCST90094764 | Weighted mode | 3 | 0.45465521201299 | 0.367119083229043 | 0.341193002596605 | -0.264898191 | 1.17420861514191 | 1.57563003106208 | 0.76728406233367 | 3.23558134028471 | 0.999714303421225 |
| ebi-fl187-GCST90094764 | prot-a-2861 | Alpha-2,8-sialyltransferase 8F \|\| id:prot-a-2861 | Fried chicken liking \|\| id:ebi-fl187-GCST90094764 | Weighted mode | 3 | 0.348211895202816 | 0.410072488227165 | 0.485228334015101 | -0.455530182 | 1.15195397212806 | 1.41653237435618 | 0.634111684385368 | 3.16436996354053 | 0.999714303421225 |
| ebi-fl187-GCST90094764 | prot-a-289 | Uncharacterized protein C17orf89 \|\| id:prot-a-289 | Fried chicken liking \|\| id:ebi-fl187-GCST90094764 | Weighted mode | 3 | 0.28597798527784 | 0.338368739896826 | 0.48700526582518 | -0.377224745 | 0.949180715475619 | 1.33106315194427 | 0.685761935064516 | 2.58359209496972 | 0.999714303421225 |
| ebi-fl187-GCST90094764 | prot-a-2921 | Tachykinin-3 \|\| id:prot-a-2921 | Fried chicken liking \|\| id:ebi-fl187-GCST90094764 | Weighted mode | 3 | -0.299119285 | 0.473968622496902 | 0.592483887467702 | -1.228097785 | 0.629859214788225 | 0.741470957569768 | 0.292849110043883 | 1.877346258 | 0.999714303421225 |
| ebi-fl187-GCST90094764 | prot-a-2928 | Tax1-binding protein 1 \|\| id:prot-a-2928 | Fried chicken liking \|\| id:ebi-fl187-GCST90094764 | Weighted mode | 3 | 0.169259874650397 | 0.376132091571369 | 0.696781590666502 | -0.567959025 | 0.906478774130279 | 1.18442790171924 | 0.56668084075059 | 2.47559005614676 | 0.999714303421225 |
| ebi-fl187-GCST90094764 | prot-a-296 | Uncharacterized protein C1orf185 \|\| id:prot-a-296 | Fried chicken liking \|\| id:ebi-fl187-GCST90094764 | Weighted mode | 3 | 0.309748796946261 | 0.32116772055275 | 0.43657979482174 | -0.319739935 | 0.939237529229651 | 1.36308266059445 | 0.726337907336388 | 2.55803025127361 | 0.999714303421225 |
| ebi-fl187-GCST90094764 | prot-a-2964 | Transforming growth factor beta-2 \|\| id:prot-a-2964 | Fried chicken liking \|\| id:ebi-fl187-GCST90094764 | Weighted mode | 3 | -0.279658088 | 0.317745841786435 | 0.471621824475986 | -0.902439938 | 0.343123762089904 | 0.756042197310878 | 0.405578864325958 | 1.40934317439006 | 0.999714303421225 |
| ebi-fl187-GCST90094764 | prot-a-2977 | Thymocyte nuclear protein 1 \|\| id:prot-a-2977 | Fried chicken liking \|\| id:ebi-fl187-GCST90094764 | Weighted mode | 3 | 0.361152916149413 | 0.355579799131552 | 0.416664176239621 | -0.33578349 | 1.05808932244726 | 1.43498287615795 | 0.714777845509356 | 2.88086133027692 | 0.999714303421225 |
| ebi-fl187-GCST90094764 | prot-a-3015 | Transmembrane protein 70, mitochondrial \|\| id:prot-a-3015 | Fried chicken liking \|\| id:ebi-fl187-GCST90094764 | Weighted mode | 3 | -0.245208236 | 0.367992345933783 | 0.57376923146787 | -0.966473234 | 0.476056762502858 | 0.782541568318236 | 0.38042233574788 | 1.60971438478262 | 0.999714303421225 |
| ebi-fl187-GCST90094764 | prot-a-3030 | Tumor necrosis factor alpha-induced protein 3 \|\| id:prot-a-3030 | Fried chicken liking \|\| id:ebi-fl187-GCST90094764 | Weighted mode | 3 | -0.236553256 | 0.361503846915838 | 0.580071769663261 | -0.945100796 | 0.471994283629106 | 0.789343843629554 | 0.388640395420918 | 1.60318821928191 | 0.999714303421225 |
| ebi-fl187-GCST90094764 | prot-a-3032 | Tumor necrosis factor receptor superfamily member 10A \|\| id:prot-a-3032 | Fried chicken liking \|\| id:ebi-fl187-GCST90094764 | Weighted mode | 3 | 0.242585829600771 | 0.386801589601774 | 0.594607127259902 | -0.515545286 | 1.00071694522025 | 1.27454063778307 | 0.59717487471115 | 2.72023138640286 | 0.999714303421225 |
| ebi-fl187-GCST90094764 | prot-a-3081 | Tumor protein p53-inducible protein 11 \|\| id:prot-a-3081 | Fried chicken liking \|\| id:ebi-fl187-GCST90094764 | Weighted mode | 3 | 0.0410865600584457 | 0.34253243907268 | 0.915486414474287 | -0.630277021 | 0.712450140640898 | 1.04194229222785 | 0.53244428258086 | 2.0389809334992 | 0.999714303421225 |
| ebi-fl187-GCST90094764 | prot-a-315 | Complement C4 \|\| id:prot-a-315 | Fried chicken liking \|\| id:ebi-fl187-GCST90094764 | Weighted mode | 3 | 0.0265536297718063 | 0.330375396791963 | 0.943258482675548 | -0.620982148 | 0.674089407484055 | 1.02690931869736 | 0.537416355942822 | 1.96224535626095 | 0.999714303421225 |
| ebi-fl187-GCST90094764 | prot-a-3157 | UDP-glucuronosyltransferase 1-8 \|\| id:prot-a-3157 | Fried chicken liking \|\| id:ebi-fl187-GCST90094764 | Weighted mode | 3 | 0.161542011349406 | 0.349234266258114 | 0.689126627098139 | -0.522957151 | 0.846041173215309 | 1.17532183397743 | 0.592765058127818 | 2.33040290496711 | 0.999714303421225 |
| ebi-fl187-GCST90094764 | prot-a-317 | Glutaredoxin-like protein C5orf63 \|\| id:prot-a-317 | Fried chicken liking \|\| id:ebi-fl187-GCST90094764 | Weighted mode | 3 | -0.532482596 | 0.325861572414783 | 0.243856338680641 | -1.171171278 | 0.106206085638431 | 0.58714551353148 | 0.310003628036612 | 1.11205103063965 | 0.999714303421225 |
| ebi-fl187-GCST90094764 | prot-a-3233 | Serine/threonine-protein kinase WNK3 \|\| id:prot-a-3233 | Fried chicken liking \|\| id:ebi-fl187-GCST90094764 | Weighted mode | 3 | -0.154920254 | 0.347192123130421 | 0.699104561942762 | -0.835416816 | 0.525576306963112 | 0.856483475572532 | 0.433693673418197 | 1.69143335236401 | 0.999714303421225 |
| ebi-fl187-GCST90094764 | prot-a-3250 | 14-3-3 protein theta \|\| id:prot-a-3250 | Fried chicken liking \|\| id:ebi-fl187-GCST90094764 | Weighted mode | 3 | 0.390937134207064 | 0.333040367119969 | 0.36131581062118 | -0.261821985 | 1.0436962537622 | 1.47836557186783 | 0.769648020137937 | 2.83969386901353 | 0.999714303421225 |
| ebi-fl187-GCST90094764 | prot-a-3278 | E3 ubiquitin-protein ligase ZNRF3 \|\| id:prot-a-3278 | Fried chicken liking \|\| id:ebi-fl187-GCST90094764 | Weighted mode | 3 | 0.28727026467036 | 0.340703576413766 | 0.487900109467193 | -0.380508745 | 0.955049274441341 | 1.33278436933302 | 0.683513586554953 | 2.59879863411552 | 0.999714303421225 |
| ebi-fl187-GCST90094764 | prot-a-355 | Calpain-3 \|\| id:prot-a-355 | Fried chicken liking \|\| id:ebi-fl187-GCST90094764 | Weighted mode | 3 | 0.0462923932124592 | 0.315409971693612 | 0.896773090953664 | -0.571911151 | 0.664495937731938 | 1.04738061317118 | 0.564445666151228 | 1.9435106240198 | 0.999714303421225 |
| ebi-fl187-GCST90094764 | prot-a-385 | Coiled-coil domain-containing protein 90B, mitochondrial \|\| id:prot-a-385 | Fried chicken liking \|\| id:ebi-fl187-GCST90094764 | Weighted mode | 3 | 0.68295502657956 | 0.352538172603319 | 0.192317005351147 | -0.008019792 | 1.37392984488206 | 1.97971922001909 | 0.992012281010543 | 3.95084644125623 | 0.999714303421225 |
| ebi-fl187-GCST90094764 | prot-a-390 | C-C motif chemokine 14 \|\| id:prot-a-390 | Fried chicken liking \|\| id:ebi-fl187-GCST90094764 | Weighted mode | 3 | -0.081485265 | 0.337879492061927 | 0.831896195452071 | -0.74372907 | 0.580758538976623 | 0.921746291257046 | 0.475338037621226 | 1.78739372447012 | 0.999714303421225 |
| ebi-fl187-GCST90094764 | prot-a-40 | Pituitary adenylate cyclase-activating polypeptide 27 \|\| id:prot-a-40 | Fried chicken liking \|\| id:ebi-fl187-GCST90094764 | Weighted mode | 3 | -0.027969942 | 0.364454120850578 | 0.945812991507814 | -0.742300019 | 0.686360134529375 | 0.972417594961608 | 0.476017805372822 | 1.98647186789645 | 0.999714303421225 |
| ebi-fl187-GCST90094764 | prot-a-401 | C-C motif chemokine 24 \|\| id:prot-a-401 | Fried chicken liking \|\| id:ebi-fl187-GCST90094764 | Weighted mode | 3 | -0.25787025 | 0.417582107565006 | 0.599826664477326 | -1.076331181 | 0.560590680651263 | 0.772695482705811 | 0.340843728475569 | 1.75170689413686 | 0.999714303421225 |
| ebi-fl187-GCST90094764 | prot-a-44 | Adhesion G protein-coupled receptor F5 \|\| id:prot-a-44 | Fried chicken liking \|\| id:ebi-fl187-GCST90094764 | Weighted mode | 3 | 0.129435803295685 | 0.33788875833875 | 0.738548873665515 | -0.532826163 | 0.791697769639635 | 1.13818604132463 | 0.586943824502241 | 2.20714046316929 | 0.999714303421225 |
| ebi-fl187-GCST90094764 | prot-a-48 | Adrenomedullin \|\| id:prot-a-48 | Fried chicken liking \|\| id:ebi-fl187-GCST90094764 | Weighted mode | 3 | 0.111666431572267 | 0.352324974829267 | 0.78131306800907 | -0.578890519 | 0.80222338223763 | 1.11813982228909 | 0.560519907843247 | 2.23049466164244 | 0.999714303421225 |
| ebi-fl187-GCST90094764 | prot-a-502 | Carcinoembryonic antigen-related cell adhesion molecule 1 \|\| id:prot-a-502 | Fried chicken liking \|\| id:ebi-fl187-GCST90094764 | Weighted mode | 3 | 0.11881815760624 | 0.306485906703303 | 0.735623237649747 | -0.48189422 | 0.719530534744714 | 1.12616511510767 | 0.617612389636887 | 2.05346895199286 | 0.999714303421225 |
| ebi-fl187-GCST90094764 | prot-a-518 | Complement factor B \|\| id:prot-a-518 | Fried chicken liking \|\| id:ebi-fl187-GCST90094764 | Weighted mode | 3 | -0.131823048 | 0.321503298219142 | 0.721538889781143 | -0.761969512 | 0.498323416851295 | 0.876496079391508 | 0.466746258730223 | 1.64595936832721 | 0.999714303421225 |
| ebi-fl187-GCST90094764 | prot-a-525 | Properdin \|\| id:prot-a-525 | Fried chicken liking \|\| id:ebi-fl187-GCST90094764 | Weighted mode | 3 | -0.416282833 | 0.388164991834622 | 0.395761513834457 | -1.177086216 | 0.344520551492714 | 0.659493717845031 | 0.308175388006606 | 1.41131310547012 | 0.999714303421225 |
| ebi-fl187-GCST90094764 | prot-a-533 | Chondroadherin \|\| id:prot-a-533 | Fried chicken liking \|\| id:ebi-fl187-GCST90094764 | Weighted mode | 3 | 0.0511822431434152 | 0.425909563641984 | 0.915330962292345 | -0.783600502 | 0.885964987881704 | 1.0525146893914 | 0.456758487466346 | 2.42532367056735 | 0.999714303421225 |
| ebi-fl187-GCST90094764 | prot-a-535 | Coiled-coil-helix-coiled-coil-helix domain-containing protein 2 \|\| id:prot-a-535 | Fried chicken liking \|\| id:ebi-fl187-GCST90094764 | Weighted mode | 3 | 0.230886163251965 | 0.355013362927293 | 0.582189391223531 | -0.464940028 | 0.926712354589458 | 1.25971582933326 | 0.628172776784053 | 2.52619029241742 | 0.999714303421225 |
| ebi-fl187-GCST90094764 | prot-a-605 | Ciliary neurotrophic factor receptor subunit alpha \|\| id:prot-a-605 | Fried chicken liking \|\| id:ebi-fl187-GCST90094764 | Weighted mode | 3 | 0.0871850608074833 | 0.340220398512257 | 0.821700013053689 | -0.57964692 | 0.754017041891506 | 1.0910985806201 | 0.560096090229889 | 2.12552119787619 | 0.999714303421225 |
| ebi-fl187-GCST90094764 | prot-a-606 | Ciliary neurotrophic factor receptor subunit alpha \|\| id:prot-a-606 | Fried chicken liking \|\| id:ebi-fl187-GCST90094764 | Weighted mode | 3 | 0.296298462361269 | 0.389408210088051 | 0.526192438128924 | -0.466941629 | 1.05953855413385 | 1.34487149045823 | 0.626916682840242 | 2.88503939256031 | 0.999714303421225 |
| ebi-fl187-GCST90094764 | prot-a-707 | Cancer/testis antigen 55 \|\| id:prot-a-707 | Fried chicken liking \|\| id:ebi-fl187-GCST90094764 | Weighted mode | 3 | -0.103453659 | 0.345246866834213 | 0.792716517466877 | -0.780137518 | 0.573230199621018 | 0.901717807941331 | 0.458342976392493 | 1.77398814215109 | 0.999714303421225 |
| ebi-fl187-GCST90094764 | prot-a-719 | Dipeptidyl peptidase 1 \|\| id:prot-a-719 | Fried chicken liking \|\| id:ebi-fl187-GCST90094764 | Weighted mode | 3 | -0.629448481 | 0.383182432445104 | 0.242157806296218 | -1.380486049 | 0.121589086233283 | 0.532885616327997 | 0.251456303279911 | 1.12928996563339 | 0.999714303421225 |
| ebi-fl187-GCST90094764 | prot-a-752 | Cytochrome b5 \|\| id:prot-a-752 | Fried chicken liking \|\| id:ebi-fl187-GCST90094764 | Weighted mode | 3 | -0.038465833 | 0.310112087820517 | 0.91262697405428 | -0.646285525 | 0.569353859174861 | 0.962264581917013 | 0.523988508585679 | 1.76712487094652 | 0.999714303421225 |
| ebi-fl187-GCST90094764 | prot-a-755 | Cytoglobin \|\| id:prot-a-755 | Fried chicken liking \|\| id:ebi-fl187-GCST90094764 | Weighted mode | 3 | -0.508747704 | 0.466650282026362 | 0.389460884797618 | -1.423382256 | 0.405886849236983 | 0.60124804836219 | 0.240897859144452 | 1.50063274511117 | 0.999714303421225 |
| ebi-fl187-GCST90094764 | prot-a-807 | Desmin \|\| id:prot-a-807 | Fried chicken liking \|\| id:ebi-fl187-GCST90094764 | Weighted mode | 3 | -0.374812688 | 0.398491221910638 | 0.446208914059664 | -1.155855483 | 0.406230107011041 | 0.687418028423656 | 0.314788125829511 | 1.50114793738375 | 0.999714303421225 |
| ebi-fl187-GCST90094764 | prot-a-82 | Ameloblastin \|\| id:prot-a-82 | Fried chicken liking \|\| id:ebi-fl187-GCST90094764 | Weighted mode | 3 | 0.135477802007602 | 0.3743156735812 | 0.752064483266152 | -0.598180918 | 0.869136522226755 | 1.14508377699134 | 0.549810877922295 | 2.38485069863252 | 0.999714303421225 |
| ebi-fl187-GCST90094764 | prot-a-84 | Muellerian-inhibiting factor \|\| id:prot-a-84 | Fried chicken liking \|\| id:ebi-fl187-GCST90094764 | Weighted mode | 3 | -0.373083273 | 0.419518883528658 | 0.467666282242389 | -1.195340285 | 0.449173738424163 | 0.688607887810615 | 0.302600966088265 | 1.56701688459475 | 0.999714303421225 |
| ebi-fl187-GCST90094764 | prot-a-858 | Inactive dipeptidyl peptidase 10 \|\| id:prot-a-858 | Fried chicken liking \|\| id:ebi-fl187-GCST90094764 | Weighted mode | 3 | 0.397030988426882 | 0.47559869240244 | 0.49166300928595 | -0.535142449 | 1.32920442553567 | 1.48740202158594 | 0.585585868268192 | 3.77803648226823 | 0.999714303421225 |
| ebi-fl187-GCST90094764 | prot-a-863 | Dihydropyrimidinase-related protein 3 \|\| id:prot-a-863 | Fried chicken liking \|\| id:ebi-fl187-GCST90094764 | Weighted mode | 3 | 0.0515334880419025 | 0.339767206908589 | 0.893362578536736 | -0.614410237 | 0.717477213582737 | 1.05288444474017 | 0.540959839077206 | 2.04925684662072 | 0.999714303421225 |
| ebi-fl187-GCST90094764 | prot-a-864 | Dihydropyrimidinase-related protein 5 \|\| id:prot-a-864 | Fried chicken liking \|\| id:ebi-fl187-GCST90094764 | Weighted mode | 3 | 0.165709752349593 | 0.56894998345371 | 0.798284771769045 | -0.949432215 | 1.28085171991887 | 1.18023049287748 | 0.386960671472044 | 3.59970436018444 | 0.999714303421225 |
| ebi-fl187-GCST90094764 | prot-a-884 | Endothelin-converting enzyme 1 \|\| id:prot-a-884 | Fried chicken liking \|\| id:ebi-fl187-GCST90094764 | Weighted mode | 3 | -0.172009086 | 0.493876752326496 | 0.760871345599788 | -1.14000752 | 0.795989349001573 | 0.841971523352336 | 0.319816616748471 | 2.2166329359109 | 0.999714303421225 |
| ebi-fl187-GCST90094764 | prot-a-897 | Ephrin-A2 \|\| id:prot-a-897 | Fried chicken liking \|\| id:ebi-fl187-GCST90094764 | Weighted mode | 3 | 0.454274377547137 | 0.37051064519622 | 0.344939893362393 | -0.271926487 | 1.18047524213173 | 1.57503009108697 | 0.761910269312239 | 3.255921186 | 0.999714303421225 |
| ebi-fl187-GCST90094764 | prot-a-90 | Anaphase-promoting complex subunit 10 \|\| id:prot-a-90 | Fried chicken liking \|\| id:ebi-fl187-GCST90094764 | Weighted mode | 3 | -0.037172159 | 0.342047045161087 | 0.92338065807472 | -0.707584367 | 0.633240049791374 | 0.963510244375098 | 0.492833264887476 | 1.88370399718802 | 0.999714303421225 |
| ebi-fl187-GCST90094764 | prot-a-914 | Histone-lysine N-methyltransferase EHMT2 \|\| id:prot-a-914 | Fried chicken liking \|\| id:ebi-fl187-GCST90094764 | Weighted mode | 3 | -0.094993978 | 0.495583856129132 | 0.865689192785748 | -1.066338336 | 0.876350379992902 | 0.909378410710166 | 0.344266801648324 | 2.40211687536028 | 0.999714303421225 |
| ebi-fl187-GCST90094764 | prot-a-932 | RNA polymerase II elongation factor ELL \|\| id:prot-a-932 | Fried chicken liking \|\| id:ebi-fl187-GCST90094764 | Weighted mode | 3 | -0.637180816 | 0.314465206832123 | 0.179979472973082 | -1.253532622 | -0.020829011 | 0.528781055696843 | 0.285494469440956 | 0.979386414774997 | 0.999714303421225 |
| ebi-fl187-GCST90094764 | prot-a-991 | Estrogen receptor \|\| id:prot-a-991 | Fried chicken liking \|\| id:ebi-fl187-GCST90094764 | Weighted mode | 3 | -0.03788359 | 0.360791319287297 | 0.925956611788904 | -0.745034576 | 0.669267395620359 | 0.962825016651952 | 0.474717885817246 | 1.95280616211649 | 0.999714303421225 |
| ebi-fl187-GCST90094764 | prot-a-992 | Persulfide dioxygenase ETHE1, mitochondrial \|\| id:prot-a-992 | Fried chicken liking \|\| id:ebi-fl187-GCST90094764 | Weighted mode | 3 | 0.0843684718519945 | 0.330828636644585 | 0.822534773150768 | -0.564055656 | 0.73279259967538 | 1.08802972828519 | 0.568897127775733 | 2.080883576 | 0.999714303421225 |
| ebi-fl187-GCST90094764 | prot-a-993 | Ethanolamine kinase 1 \|\| id:prot-a-993 | Fried chicken liking \|\| id:ebi-fl187-GCST90094764 | Weighted mode | 3 | 0.491750809495073 | 0.351062180857967 | 0.29628292489257 | -0.196331065 | 1.17983268397669 | 1.63517659795005 | 0.821740140251729 | 3.25382973948967 | 0.999714303421225 |
| ebi-fl187-GCST90094766 | ebi-a-GCST90001414 | IgD- CD24- B cell Absolute Count \|\| id:ebi-a-GCST90001414 | Fruit liking \|\| id:ebi-fl187-GCST90094766 | Inverse variance weighted | 9 | -0.457147146 | 0.300170734347637 | 0.127769442801091 | -1.045481785 | 0.13118749315397 | 0.63308717685692 | 0.351522420173289 | 1.14018153750502 | 0.998735809224318 |
| ebi-fl187-GCST90094766 | ebi-a-GCST90001422 | CD20- CD38- B cell %B cell \|\| id:ebi-a-GCST90001422 | Fruit liking \|\| id:ebi-fl187-GCST90094766 | Inverse variance weighted | 9 | -0.177274707 | 0.299983747499955 | 0.554555462132757 | -0.765242852 | 0.410693438596693 | 0.83754967257599 | 0.46522093760405 | 1.50786303308899 | 0.998735809224318 |
| ebi-fl187-GCST90094766 | ebi-a-GCST90001444 | CD20- CD38- B cell %lymphocyte \|\| id:ebi-a-GCST90001444 | Fruit liking \|\| id:ebi-fl187-GCST90094766 | Inverse variance weighted | 9 | -0.218435992 | 0.302172504798294 | 0.469750496405709 | -0.810694102 | 0.373822116914482 | 0.80377492542961 | 0.444549396532745 | 1.45327861377895 | 0.998735809224318 |
| ebi-fl187-GCST90094766 | ebi-a-GCST90001459 | Myeloid Dendritic Cell %Dendritic Cell \|\| id:ebi-a-GCST90001459 | Fruit liking \|\| id:ebi-fl187-GCST90094766 | Inverse variance weighted | 9 | -0.206355235 | 0.306854452751742 | 0.50127453302214 | -0.807789963 | 0.395079492203569 | 0.813544025479113 | 0.445842306397632 | 1.48450219258124 | 0.998735809224318 |
| ebi-fl187-GCST90094766 | ebi-a-GCST90001476 | HLA DR++ monocyte %leukocyte \|\| id:ebi-a-GCST90001476 | Fruit liking \|\| id:ebi-fl187-GCST90094766 | Inverse variance weighted | 9 | 0.365635053766966 | 0.299826265280521 | 0.222658368355403 | -0.222024426 | 0.953294533716787 | 1.44142910253277 | 0.800895801268445 | 2.59424241497806 | 0.998735809224318 |
| ebi-fl187-GCST90094766 | ebi-a-GCST90001478 | CD4 regulatory T cell %CD4+ T cell \|\| id:ebi-a-GCST90001478 | Fruit liking \|\| id:ebi-fl187-GCST90094766 | Inverse variance weighted (multiplicative random effects) | 9 | -0.439079141 | 0.459381351412395 | 0.339170598953133 | -1.339466589 | 0.461308308171581 | 0.644629761227052 | 0.261985377102367 | 1.58614779823103 | 0.998735809224318 |
| ebi-fl187-GCST90094766 | ebi-a-GCST90001485 | CD39+ resting CD4 regulatory T cell %CD4 regulatory T cell \|\| id:ebi-a-GCST90001485 | Fruit liking \|\| id:ebi-fl187-GCST90094766 | Inverse variance weighted | 9 | 0.441640952110085 | 0.296181391700155 | 0.135930918175481 | -0.138874576 | 1.02215647984239 | 1.5552572283477 | 0.870337183112434 | 2.7791815554493 | 0.998735809224318 |
| ebi-fl187-GCST90094766 | ebi-a-GCST90001515 | Immature Myeloid-Derived Suppressor Cells Absolute Count \|\| id:ebi-a-GCST90001515 | Fruit liking \|\| id:ebi-fl187-GCST90094766 | Inverse variance weighted | 9 | -0.280986241 | 0.424946927234643 | 0.508466960353557 | -1.113882219 | 0.551909735923049 | 0.755038723641603 | 0.328282021352202 | 1.73656623609832 | 0.998735809224318 |
| ebi-fl187-GCST90094766 | ebi-a-GCST90001517 | CD33+ HLA DR+ Absolute Count \|\| id:ebi-a-GCST90001517 | Fruit liking \|\| id:ebi-fl187-GCST90094766 | Inverse variance weighted | 9 | -0.225022004 | 0.389473813323449 | 0.563426835053234 | -0.988390678 | 0.53834667057661 | 0.798498648771235 | 0.372175159130045 | 1.71317208160768 | 0.998735809224318 |
| ebi-fl187-GCST90094766 | ebi-a-GCST90001518 | CD33+ HLA DR+ CD14- Absolute Count \|\| id:ebi-a-GCST90001518 | Fruit liking \|\| id:ebi-fl187-GCST90094766 | Inverse variance weighted | 9 | -0.357878166 | 0.394400901917808 | 0.364196934052004 | -1.130903934 | 0.41514760146545 | 0.699158250858941 | 0.322741387596688 | 1.51459428052961 | 0.998735809224318 |
| ebi-fl187-GCST90094766 | ebi-a-GCST90001520 | CD33+ HLA DR+ CD14dim Absolute Count \|\| id:ebi-a-GCST90001520 | Fruit liking \|\| id:ebi-fl187-GCST90094766 | Inverse variance weighted | 9 | -0.108602947 | 0.390954802532654 | 0.781174328700593 | -0.87487436 | 0.657668465752172 | 0.897086537505282 | 0.416914397439661 | 1.9302865545431 | 0.998735809224318 |
| ebi-fl187-GCST90094766 | ebi-a-GCST90001531 | CD33dim HLA DR- Absolute Count \|\| id:ebi-a-GCST90001531 | Fruit liking \|\| id:ebi-fl187-GCST90094766 | Inverse variance weighted | 9 | -0.831076908 | 0.460568082900027 | 0.0711591479198031 | -1.73379035 | 0.0716365347751296 | 0.435579954242651 | 0.176613711865661 | 1.07426481519366 | 0.998735809224318 |
| ebi-fl187-GCST90094766 | ebi-a-GCST90001532 | Basophil Absolute Count \|\| id:ebi-a-GCST90001532 | Fruit liking \|\| id:ebi-fl187-GCST90094766 | Inverse variance weighted | 9 | -0.784401346 | 0.474105848207362 | 0.0980288665000496 | -1.713648809 | 0.144846116419137 | 0.45639284138879 | 0.180207050508728 | 1.15586168844623 | 0.998735809224318 |
| ebi-fl187-GCST90094766 | ebi-a-GCST90001588 | T/B cell \|\| id:ebi-a-GCST90001588 | Fruit liking \|\| id:ebi-fl187-GCST90094766 | Inverse variance weighted | 9 | 0.186072432351148 | 0.389530693876394 | 0.632875403717584 | -0.577407728 | 0.949552592348881 | 1.20450950267327 | 0.561351658472159 | 2.58455305178752 | 0.998735809224318 |
| ebi-fl187-GCST90094766 | ebi-a-GCST90001624 | HLA DR+ CD4+ T cell Absolute Count \|\| id:ebi-a-GCST90001624 | Fruit liking \|\| id:ebi-fl187-GCST90094766 | Inverse variance weighted | 9 | -0.285235734 | 0.286958510670149 | 0.320224607670025 | -0.847674415 | 0.277202947033699 | 0.7518369999748 | 0.428410078492127 | 1.31943411910533 | 0.998735809224318 |
| ebi-fl187-GCST90094766 | ebi-a-GCST90001631 | CD8+ Natural Killer T %T cell \|\| id:ebi-a-GCST90001631 | Fruit liking \|\| id:ebi-fl187-GCST90094766 | Inverse variance weighted | 9 | 0.208397205251374 | 0.355939321589398 | 0.558221514553154 | -0.489243865 | 0.906038275566593 | 1.23170231102372 | 0.613089797579539 | 2.47449980242798 | 0.998735809224318 |
| ebi-fl187-GCST90094766 | ebi-a-GCST90001632 | CD8+ Natural Killer T %lymphocyte \|\| id:ebi-a-GCST90001632 | Fruit liking \|\| id:ebi-fl187-GCST90094766 | Inverse variance weighted | 9 | 0.265899142635105 | 0.324572473271187 | 0.412655842584032 | -0.370262905 | 0.902061190246631 | 1.30460347318282 | 0.690552757013796 | 2.46467804951023 | 0.998735809224318 |
| ebi-fl187-GCST90094766 | ebi-a-GCST90001646 | Natural Killer %CD3- lymphocyte \|\| id:ebi-a-GCST90001646 | Fruit liking \|\| id:ebi-fl187-GCST90094766 | Inverse variance weighted | 9 | -0.366045809 | 0.285197308784986 | 0.199323019748884 | -0.925032535 | 0.192940915764925 | 0.693471032952978 | 0.396518518265111 | 1.21281113338404 | 0.998735809224318 |
| ebi-fl187-GCST90094766 | ebi-a-GCST90001684 | CD127- CD8+ T cell Absolute Count \|\| id:ebi-a-GCST90001684 | Fruit liking \|\| id:ebi-fl187-GCST90094766 | Inverse variance weighted | 9 | 0.441033690906587 | 0.308399798969073 | 0.152696566161634 | -0.163429915 | 1.04549729688597 | 1.55431306767655 | 0.849226014859408 | 2.84481288853363 | 0.998735809224318 |
| ebi-fl187-GCST90094766 | ebi-a-GCST90001687 | CD28- CD8+ T cell Absolute Count \|\| id:ebi-a-GCST90001687 | Fruit liking \|\| id:ebi-fl187-GCST90094766 | Inverse variance weighted | 9 | 0.292244507922662 | 0.300185248870771 | 0.330281414530033 | -0.29611858 | 0.880607595709373 | 1.33943047896818 | 0.743699235037142 | 2.41236500384369 | 0.998735809224318 |
| ebi-fl187-GCST90094766 | ebi-a-GCST90001701 | BAFF-R on CD20- CD38- B cell \|\| id:ebi-a-GCST90001701 | Fruit liking \|\| id:ebi-fl187-GCST90094766 | Inverse variance weighted | 9 | -0.236015737 | 0.334770392683127 | 0.480805384205249 | -0.892165707 | 0.420134232743428 | 0.789768245318867 | 0.409767355240185 | 1.52216586640593 | 0.998735809224318 |
| ebi-fl187-GCST90094766 | ebi-a-GCST90001732 | CD19 on IgD- CD27- B cell \|\| id:ebi-a-GCST90001732 | Fruit liking \|\| id:ebi-fl187-GCST90094766 | Inverse variance weighted | 9 | 0.166908821443116 | 0.365884824316484 | 0.648261563523357 | -0.550225434 | 0.884043077103425 | 1.18164651957198 | 0.576819760810986 | 2.42066689125466 | 0.998735809224318 |
| ebi-fl187-GCST90094766 | ebi-a-GCST90001798 | CD27 on CD24+ CD27+ B cell \|\| id:ebi-a-GCST90001798 | Fruit liking \|\| id:ebi-fl187-GCST90094766 | Inverse variance weighted | 9 | -0.250261125 | 0.292149609688442 | 0.391655068080845 | -0.82287436 | 0.322352109904796 | 0.778597445200543 | 0.439167513012282 | 1.38037073260439 | 0.998735809224318 |
| ebi-fl187-GCST90094766 | ebi-a-GCST90001836 | CD66b on CD66b++ myeloid cell \|\| id:ebi-a-GCST90001836 | Fruit liking \|\| id:ebi-fl187-GCST90094766 | Inverse variance weighted | 9 | 0.662121605517117 | 0.602153810976138 | 0.27151131022377 | -0.518099864 | 1.84234307503035 | 1.93890155802756 | 0.595651291811771 | 6.31130882011025 | 0.998735809224318 |
| ebi-fl187-GCST90094766 | ebi-a-GCST90001943 | CD25 on activated & secreting CD4 regulatory T cell \|\| id:ebi-a-GCST90001943 | Fruit liking \|\| id:ebi-fl187-GCST90094766 | Inverse variance weighted | 9 | -0.075313532 | 0.297450787588769 | 0.800116283360293 | -0.658317076 | 0.507690011592739 | 0.927452654577344 | 0.517721888466181 | 1.66144883120728 | 0.998735809224318 |
| ebi-fl187-GCST90094766 | ebi-a-GCST90001960 | CD25 on CD4+ T cell \|\| id:ebi-a-GCST90001960 | Fruit liking \|\| id:ebi-fl187-GCST90094766 | Inverse variance weighted | 9 | -0.467725137 | 0.320685513407593 | 0.14469820981413 | -1.096268743 | 0.160818469566609 | 0.62642568141886 | 0.334115431308863 | 1.17447174709609 | 0.998735809224318 |
| ebi-fl187-GCST90094766 | ebi-a-GCST90001988 | HLA DR on CD14+ CD16- monocyte \|\| id:ebi-a-GCST90001988 | Fruit liking \|\| id:ebi-fl187-GCST90094766 | Inverse variance weighted | 9 | -0.29572772 | 0.297842955731704 | 0.320759590642001 | -0.879499913 | 0.28804447300892 | 0.743989973866883 | 0.414990390907461 | 1.33381662164287 | 0.998735809224318 |
| ebi-fl187-GCST90094766 | ebi-a-GCST90001991 | HLA DR on CD14+ monocyte \|\| id:ebi-a-GCST90001991 | Fruit liking \|\| id:ebi-fl187-GCST90094766 | Inverse variance weighted | 9 | -0.297521522 | 0.298376742084814 | 0.318699589119342 | -0.882339937 | 0.287296892400487 | 0.742656599529766 | 0.413813480623026 | 1.33281985882802 | 0.998735809224318 |
| ebi-fl187-GCST90094766 | ebi-a-GCST90002019 | CD14 on Monocytic Myeloid-Derived Suppressor Cells \|\| id:ebi-a-GCST90002019 | Fruit liking \|\| id:ebi-fl187-GCST90094766 | Inverse variance weighted | 9 | -0.035642767 | 0.43096666300113 | 0.93408672032365 | -0.880337426 | 0.809051892909638 | 0.964984956800358 | 0.414642976730274 | 2.24577773918677 | 0.998735809224318 |
| ebi-fl187-GCST90094766 | ebi-a-GCST90002037 | CD80 on plasmacytoid Dendritic Cell \|\| id:ebi-a-GCST90002037 | Fruit liking \|\| id:ebi-fl187-GCST90094766 | Inverse variance weighted | 9 | 0.722147368063046 | 0.331109410841887 | 0.0291839206587206 | 0.0731729228129471 | 1.37112181331314 | 2.0588495749909 | 1.07591657134946 | 3.93976790144954 | 0.998735809224318 |
| ebi-fl187-GCST90094766 | ebi-a-GCST90002042 | CD45 on CD33+ HLA DR+ CD14- \|\| id:ebi-a-GCST90002042 | Fruit liking \|\| id:ebi-fl187-GCST90094766 | Inverse variance weighted | 9 | 0.39715743878471 | 0.512971336838055 | 0.438795204606937 | -0.608266381 | 1.4025812589873 | 1.48759011599586 | 0.5442936492122 | 4.06568100952768 | 0.998735809224318 |
| ebi-fl187-GCST90094766 | ebi-a-GCST90002056 | CD8 on Effector Memory CD8+ T cell \|\| id:ebi-a-GCST90002056 | Fruit liking \|\| id:ebi-fl187-GCST90094766 | Inverse variance weighted | 9 | -0.053087365 | 0.387800639008716 | 0.891115021180809 | -0.813176617 | 0.707001887495793 | 0.948297160918434 | 0.443447164475047 | 2.02790225633874 | 0.998735809224318 |
| ebi-fl187-GCST90094766 | ebi-a-GCST90002058 | CD8 on CD8+ T cell \|\| id:ebi-a-GCST90002058 | Fruit liking \|\| id:ebi-fl187-GCST90094766 | Inverse variance weighted | 9 | -0.155629238 | 0.313305570282431 | 0.619377297994858 | -0.769708156 | 0.458449679665209 | 0.855876457943697 | 0.463148215690357 | 1.58162006555583 | 0.998735809224318 |
| ebi-fl187-GCST90094766 | ebi-a-GCST90002063 | CD4 on CD4 regulatory T cell \|\| id:ebi-a-GCST90002063 | Fruit liking \|\| id:ebi-fl187-GCST90094766 | Inverse variance weighted | 9 | 0.0860060298677296 | 0.322923649279511 | 0.789980839183649 | -0.546924323 | 0.718936382455571 | 1.08981289971295 | 0.578727053516048 | 2.05224924109724 | 0.998735809224318 |
| ebi-fl187-GCST90094766 | ebi-a-GCST90002066 | CD4 on activated CD4 regulatory T cell \|\| id:ebi-a-GCST90002066 | Fruit liking \|\| id:ebi-fl187-GCST90094766 | Inverse variance weighted | 9 | 0.254147711127902 | 0.322966683004982 | 0.43133093038315 | -0.378866988 | 0.887162409817667 | 1.28936224335371 | 0.684636671803099 | 2.42822954576444 | 0.998735809224318 |
| ebi-fl187-GCST90094766 | ebi-a-GCST90002070 | CD4 on activated & secreting CD4 regulatory T cell \|\| id:ebi-a-GCST90002070 | Fruit liking \|\| id:ebi-fl187-GCST90094766 | Inverse variance weighted | 9 | 0.101319607218873 | 0.3244612422161 | 0.754835114657376 | -0.534624428 | 0.737263641962428 | 1.10663027227262 | 0.585889292720681 | 2.09020812417204 | 0.998735809224318 |
| ebi-fl187-GCST90094766 | ebi-a-GCST90002081 | SSC-A on CD4+ T cell \|\| id:ebi-a-GCST90002081 | Fruit liking \|\| id:ebi-fl187-GCST90094766 | Inverse variance weighted | 9 | -0.176345367 | 0.346885501397674 | 0.611195535461279 | -0.85624095 | 0.503550215636428 | 0.838328402282397 | 0.4247557632967 | 1.65458498930935 | 0.998735809224318 |
| ebi-fl187-GCST90094766 | ebi-a-GCST90002094 | CD11b on Monocytic Myeloid-Derived Suppressor Cells \|\| id:ebi-a-GCST90002094 | Fruit liking \|\| id:ebi-fl187-GCST90094766 | Inverse variance weighted | 9 | 0.398238748607879 | 0.433467564859383 | 0.358237909153465 | -0.451357679 | 1.24783517573227 | 1.48919953178287 | 0.636763044980031 | 3.48279515110972 | 0.998735809224318 |
| ebi-fl187-GCST90094766 | ebi-a-GCST90002104 | HLA DR on myeloid Dendritic Cell \|\| id:ebi-a-GCST90002104 | Fruit liking \|\| id:ebi-fl187-GCST90094766 | Inverse variance weighted | 9 | -0.48371574 | 0.391604322816426 | 0.216750302209775 | -1.251260213 | 0.283828732877911 | 0.616488420102864 | 0.286143967324688 | 1.32820543334983 | 0.998735809224318 |
| ebi-fl187-GCST90094766 | ebi-a-GCST90002105 | HLA DR on plasmacytoid Dendritic Cell \|\| id:ebi-a-GCST90002105 | Fruit liking \|\| id:ebi-fl187-GCST90094766 | Inverse variance weighted | 9 | -0.002262768 | 0.322622088869132 | 0.994403938748107 | -0.634602062 | 0.630076526032412 | 0.997739789978916 | 0.53014641157298 | 1.87775427085039 | 0.999107451094609 |
| ebi-fl187-GCST90094766 | ebi-a-GCST90002106 | HLA DR on Dendritic Cell \|\| id:ebi-a-GCST90002106 | Fruit liking \|\| id:ebi-fl187-GCST90094766 | Inverse variance weighted | 9 | -0.291102656 | 0.408253751179044 | 0.475818881335502 | -1.091280009 | 0.509074696068791 | 0.747438944806287 | 0.33578640903386 | 1.6637510071374 | 0.998735809224318 |
| ebi-fl187-GCST90094766 | ebi-a-GCST90002111 | HLA DR on CD33dim HLA DR+ CD11b- \|\| id:ebi-a-GCST90002111 | Fruit liking \|\| id:ebi-fl187-GCST90094766 | Inverse variance weighted | 9 | 0.262452637187263 | 0.423307134765746 | 0.535254344089627 | -0.567229347 | 1.09213462132813 | 1.30011488961218 | 0.567094486118358 | 2.98062980255907 | 0.998735809224318 |
| ebi-fl187-GCST90094766 | ebi-a-GCST90002112 | HLA DR on CD33- HLA DR+ \|\| id:ebi-a-GCST90002112 | Fruit liking \|\| id:ebi-fl187-GCST90094766 | Inverse variance weighted | 9 | -0.441688338 | 0.42765492710186 | 0.3016905758545 | -1.279891996 | 0.396515318697055 | 0.642949987040121 | 0.278067331342774 | 1.48663521111479 | 0.998735809224318 |
| ebi-fl187-GCST90094766 | ebi-a-GCST90002115 | HLA DR on HLA DR+ CD8+ T cell \|\| id:ebi-a-GCST90002115 | Fruit liking \|\| id:ebi-fl187-GCST90094766 | Inverse variance weighted | 9 | 0.0744886147488124 | 0.360701944125064 | 0.836392432381003 | -0.632487196 | 0.781464425233938 | 1.07733307774473 | 0.531268786929753 | 2.18466920880182 | 0.998735809224318 |
| ebi-fl187-GCST90094766 | ebi-a-GCST90002116 | HLA DR on B cell \|\| id:ebi-a-GCST90002116 | Fruit liking \|\| id:ebi-fl187-GCST90094766 | Inverse variance weighted | 9 | 0.565148147465115 | 0.319044510001153 | 0.0764980095999561 | -0.060179092 | 1.19047538706737 | 1.75970845975967 | 0.941595886063416 | 3.28864421476583 | 0.998735809224318 |
| ebi-fl187-GCST90094766 | ebi-cfmet338-GCST90026023 | Sphingomyelin (d18:1/24:1, d18:2/24:0) levels \|\| id:ebi-cfmet338-GCST90026023 | Fruit liking \|\| id:ebi-fl187-GCST90094766 | Inverse variance weighted | 8 | 0.221126053466062 | 0.200092653319451 | 0.26910833073633 | -0.171055547 | 0.613307653972186 | 1.24748066991586 | 0.84277475852814 | 1.84652898780637 | 0.998735809224318 |
| ebi-fl187-GCST90094766 | ebi-cfmet338-GCST90026046 | 1-arachidonoyl-gpc (20:4n6) levels \|\| id:ebi-cfmet338-GCST90026046 | Fruit liking \|\| id:ebi-fl187-GCST90094766 | Inverse variance weighted (multiplicative random effects) | 8 | -0.319862561 | 0.327493160033168 | 0.328717707191744 | -0.961749155 | 0.322024032209835 | 0.726248844799231 | 0.382223732320506 | 1.37991793803621 | 0.998735809224318 |
| ebi-fl187-GCST90094766 | ebi-cfmet338-GCST90026047 | 1-linoleoyl-gpc (18:2) levels \|\| id:ebi-cfmet338-GCST90026047 | Fruit liking \|\| id:ebi-fl187-GCST90094766 | Inverse variance weighted | 8 | 0.0499924372663398 | 0.323772690130865 | 0.877289644327303 | -0.584602035 | 0.684586909922835 | 1.05126314592278 | 0.557327614351017 | 1.98295252831203 | 0.998735809224318 |
| ebi-fl187-GCST90094766 | ebi-cfmet338-GCST90026050 | 1-myristoyl-2-palmitoyl-gpc (14:0/16:0) levels \|\| id:ebi-cfmet338-GCST90026050 | Fruit liking \|\| id:ebi-fl187-GCST90094766 | Inverse variance weighted | 8 | -0.00469021 | 0.143605364655564 | 0.973945394648269 | -0.286156725 | 0.276776304523536 | 0.995320771658712 | 0.751144883594828 | 1.31887131248792 | 0.998955228396052 |
| ebi-fl187-GCST90094766 | ebi-cfmet338-GCST90026068 | 2,3-dihydroxyisovalerate levels \|\| id:ebi-cfmet338-GCST90026068 | Fruit liking \|\| id:ebi-fl187-GCST90094766 | Inverse variance weighted | 8 | -0.040841929 | 0.461446067677338 | 0.929472477095532 | -0.945276222 | 0.863592363328416 | 0.959980862783794 | 0.388572223892727 | 2.37166529217882 | 0.998735809224318 |
| ebi-fl187-GCST90094766 | ebi-cfmet338-GCST90026082 | 3-hydroxyhexanoate levels \|\| id:ebi-cfmet338-GCST90026082 | Fruit liking \|\| id:ebi-fl187-GCST90094766 | Inverse variance weighted | 7 | -0.174412786 | 0.108369208198184 | 0.107522086157619 | -0.386816434 | 0.0379908625467071 | 0.839950106845158 | 0.679215764683117 | 1.03872174156374 | 0.998735809224318 |
| ebi-fl187-GCST90094766 | ebi-cfmet338-GCST90026094 | 3-ureidopropionate levels \|\| id:ebi-cfmet338-GCST90026094 | Fruit liking \|\| id:ebi-fl187-GCST90094766 | Inverse variance weighted | 8 | 0.297764972765858 | 0.224738962085154 | 0.185192037945375 | -0.142723393 | 0.73825333845276 | 1.34684520537482 | 0.866993852368033 | 2.09227781983293 | 0.998735809224318 |
| ebi-fl187-GCST90094766 | ebi-cfmet338-GCST90026173 | Glycerol levels \|\| id:ebi-cfmet338-GCST90026173 | Fruit liking \|\| id:ebi-fl187-GCST90094766 | Inverse variance weighted | 8 | -0.10026364 | 0.127138487191931 | 0.430335582786374 | -0.349455074 | 0.148927795319668 | 0.904598898525225 | 0.705072196892981 | 1.16058918621245 | 0.998735809224318 |
| ebi-fl187-GCST90094766 | ebi-cfmet338-GCST90026179 | Histidine levels \|\| id:ebi-cfmet338-GCST90026179 | Fruit liking \|\| id:ebi-fl187-GCST90094766 | Inverse variance weighted | 8 | -0.047115765 | 0.088470751507331 | 0.594339910768954 | -0.220518437 | 0.126286908436377 | 0.953976954515486 | 0.802102849976169 | 1.13460765009584 | 0.998735809224318 |
| ebi-fl187-GCST90094766 | ebi-cfmet338-GCST90026188 | Isocitrate levels \|\| id:ebi-cfmet338-GCST90026188 | Fruit liking \|\| id:ebi-fl187-GCST90094766 | Inverse variance weighted | 8 | -0.239211319 | 0.17350314363607 | 0.167982762336995 | -0.579277481 | 0.100854842075027 | 0.787248503871635 | 0.560303049963549 | 1.10611606859618 | 0.998735809224318 |
| ebi-fl187-GCST90094766 | ebi-cfmet338-GCST90026207 | N-acetyl-isoputreanine levels \|\| id:ebi-cfmet338-GCST90026207 | Fruit liking \|\| id:ebi-fl187-GCST90094766 | Inverse variance weighted | 8 | -0.040910678 | 0.110122952922256 | 0.710265151409943 | -0.256751665 | 0.174930310045785 | 0.959914867939828 | 0.773560291692963 | 1.19116320161075 | 0.998735809224318 |
| ebi-fl187-GCST90094766 | ebi-cfmet338-GCST90026213 | N-acetylglutamate levels \|\| id:ebi-cfmet338-GCST90026213 | Fruit liking \|\| id:ebi-fl187-GCST90094766 | Inverse variance weighted | 8 | 0.0238520137334828 | 0.194126519413864 | 0.90221131363577 | -0.356635964 | 0.404339991784657 | 1.02413874820604 | 0.70002728626653 | 1.49831327457383 | 0.998735809224318 |
| ebi-fl187-GCST90094766 | ebi-cfmet338-GCST90026224 | N-acetyltaurine levels \|\| id:ebi-cfmet338-GCST90026224 | Fruit liking \|\| id:ebi-fl187-GCST90094766 | Inverse variance weighted | 8 | -0.282094913 | 0.150466151435856 | 0.0608193536052784 | -0.57700857 | 0.0128187434914247 | 0.754202097308693 | 0.561575770927191 | 1.01290125577476 | 0.998735809224318 |
| ebi-fl187-GCST90094766 | ebi-cfmet338-GCST90026250 | Phenyllactate (pla) levels \|\| id:ebi-cfmet338-GCST90026250 | Fruit liking \|\| id:ebi-fl187-GCST90094766 | Inverse variance weighted | 8 | 0.12277779827315 | 0.241371762745569 | 0.610985808696848 | -0.350310857 | 0.595866453254465 | 1.13063316439214 | 0.704469066743005 | 1.81460253227802 | 0.998735809224318 |
| ebi-fl187-GCST90094766 | ebi-cfmet338-GCST90026251 | Pipecolate levels \|\| id:ebi-cfmet338-GCST90026251 | Fruit liking \|\| id:ebi-fl187-GCST90094766 | Inverse variance weighted | 8 | -0.277377969 | 0.422864682184205 | 0.511856477004615 | -1.106192746 | 0.551436807960979 | 0.757768030060535 | 0.330816067224285 | 1.73574515953762 | 0.998735809224318 |
| ebi-fl187-GCST90094766 | ebi-cfmet338-GCST90026278 | Trigonelline (n'-methylnicotinate) levels \|\| id:ebi-cfmet338-GCST90026278 | Fruit liking \|\| id:ebi-fl187-GCST90094766 | Inverse variance weighted | 8 | -0.326169267 | 0.39975457459455 | 0.414543725279524 | -1.109688233 | 0.457349699040411 | 0.721683019853795 | 0.329661722579767 | 1.57988127062362 | 0.998735809224318 |
| ebi-fl187-GCST90094766 | ebi-cfmet338-GCST90026299 | 1-carboxyethylvaline levels \|\| id:ebi-cfmet338-GCST90026299 | Fruit liking \|\| id:ebi-fl187-GCST90094766 | Inverse variance weighted | 8 | 0.320592327396753 | 0.323946412828294 | 0.32234709359783 | -0.314342642 | 0.955527296540209 | 1.37794371647115 | 0.730268764721416 | 2.60004121425986 | 0.998735809224318 |
| ebi-fl187-GCST90094766 | ebi-gm430-GCST90011306 | C_Erysipelotrichia abundance \|\| id:ebi-gm430-GCST90011306 | Fruit liking \|\| id:ebi-fl187-GCST90094766 | Inverse variance weighted | 8 | -0.008603564 | 0.198544509713212 | 0.965435944681111 | -0.397750803 | 0.380543674668681 | 0.99143334037742 | 0.671829424407359 | 1.46307981267568 | 0.998735809224318 |
| ebi-fl187-GCST90094766 | ebi-gm430-GCST90011312 | F_Erysipelotrichaceae abundance \|\| id:ebi-gm430-GCST90011312 | Fruit liking \|\| id:ebi-fl187-GCST90094766 | Inverse variance weighted | 8 | -0.008603564 | 0.198544509713212 | 0.965435944681111 | -0.397750803 | 0.380543674668681 | 0.99143334037742 | 0.671829424407359 | 1.46307981267568 | 0.998735809224318 |
| ebi-fl187-GCST90094766 | ebi-gm430-GCST90011352 | O_Erysipelotrichales abundance \|\| id:ebi-gm430-GCST90011352 | Fruit liking \|\| id:ebi-fl187-GCST90094766 | Inverse variance weighted | 8 | -0.008603564 | 0.198544509713212 | 0.965435944681111 | -0.397750803 | 0.380543674668681 | 0.99143334037742 | 0.671829424407359 | 1.46307981267568 | 0.998735809224318 |
| ebi-fl187-GCST90094766 | ebi-gm430-GCST90011362 | OTU97_11 (Parabacteroides) abundance \|\| id:ebi-gm430-GCST90011362 | Fruit liking \|\| id:ebi-fl187-GCST90094766 | Inverse variance weighted | 8 | -0.502653119 | 0.339598295032724 | 0.138835810835313 | -1.168265778 | 0.162959538810407 | 0.604923594231095 | 0.310905653519045 | 1.17698906634726 | 0.998735809224318 |
| ebi-fl187-GCST90094766 | ebi-gm430-GCST90011384 | OTU97_235 (Phascolarctobacterium) abundance \|\| id:ebi-gm430-GCST90011384 | Fruit liking \|\| id:ebi-fl187-GCST90094766 | Inverse variance weighted (multiplicative random effects) | 8 | 2.05641723344014 | 1.19817971466939 | 0.086109989796349 | -0.292015007 | 4.40484947419215 | 7.81790982825042 | 0.746757329069142 | 81.8468218568973 | 0.998735809224318 |
| ebi-fl187-GCST90094766 | ebi-gm430-GCST90011390 | OTU97_3 (Bacteroides) abundance \|\| id:ebi-gm430-GCST90011390 | Fruit liking \|\| id:ebi-fl187-GCST90094766 | Inverse variance weighted | 8 | -0.2654278 | 0.204987596954551 | 0.195372635253561 | -0.66720349 | 0.13634788958481 | 0.766877809122605 | 0.513141578463434 | 1.1460805337305 | 0.998735809224318 |
| ebi-fl187-GCST90094766 | ebi-gm430-GCST90011398 | OTU97_4 (Alistipes) abundance \|\| id:ebi-gm430-GCST90011398 | Fruit liking \|\| id:ebi-fl187-GCST90094766 | Inverse variance weighted | 8 | 0.0220285616639778 | 0.296963495225429 | 0.94086766988235 | -0.560019889 | 0.604077012305818 | 1.02227298187087 | 0.571197703197365 | 1.82956276541973 | 0.998735809224318 |
| ebi-fl187-GCST90094766 | ebi-gm430-GCST90011421 | OTU99_108 (Cytophagales) abundance \|\| id:ebi-gm430-GCST90011421 | Fruit liking \|\| id:ebi-fl187-GCST90094766 | Inverse variance weighted | 8 | 0.499450467934867 | 0.764010469891567 | 0.513290913707834 | -0.998010053 | 1.99691098892234 | 1.64781549439477 | 0.368612230606647 | 7.3662664396641 | 0.998735809224318 |
| ebi-fl187-GCST90094766 | ebi-gm430-GCST90011428 | OTU99_123 (Phascolarctobacterium) abundance \|\| id:ebi-gm430-GCST90011428 | Fruit liking \|\| id:ebi-fl187-GCST90094766 | Inverse variance weighted | 8 | 0.037919169022339 | 0.451602986828588 | 0.933083688587054 | -0.847222685 | 0.923061023206372 | 1.03864727461068 | 0.428603647736245 | 2.51698315390926 | 0.998735809224318 |
| ebi-fl187-GCST90094766 | ebi-gm430-GCST90011450 | OTU99_298 (Phascolarctobacterium) abundance \|\| id:ebi-gm430-GCST90011450 | Fruit liking \|\| id:ebi-fl187-GCST90094766 | Inverse variance weighted (multiplicative random effects) | 8 | 2.04058751680942 | 1.20780556571828 | 0.0911236497169811 | -0.326711392 | 4.40788642561726 | 7.69512888862489 | 0.721291883598734 | 82.0957645012011 | 0.998735809224318 |
| ebi-fl187-GCST90094766 | ebi-gm430-GCST90011451 | OTU99_3 (Bacteroides) abundance \|\| id:ebi-gm430-GCST90011451 | Fruit liking \|\| id:ebi-fl187-GCST90094766 | Inverse variance weighted | 8 | -0.264865392 | 0.20498856989371 | 0.196323089555747 | -0.666642989 | 0.136912205429104 | 0.76730922932095 | 0.513429275924784 | 1.14672746765489 | 0.998735809224318 |
| ebi-fl187-GCST90094766 | ebi-gm430-GCST90011472 | OTU99_72 (Parabacteroides) abundance \|\| id:ebi-gm430-GCST90011472 | Fruit liking \|\| id:ebi-fl187-GCST90094766 | Inverse variance weighted | 8 | 0.955759311198863 | 0.809530815487737 | 0.237748294936656 | -0.630921087 | 2.54243970955483 | 2.60064453192124 | 0.532101463395517 | 12.7106434518196 | 0.998735809224318 |
| ebi-fl187-GCST90094766 | ebi-gm430-GCST90011507 | TestASV_3 (Bacteroides) abundance \|\| id:ebi-gm430-GCST90011507 | Fruit liking \|\| id:ebi-fl187-GCST90094766 | Inverse variance weighted | 8 | 0.14886634934105 | 0.354401468209195 | 0.67444890180026 | -0.545760528 | 0.843493227031073 | 1.16051787486505 | 0.579400964874415 | 2.32447272187959 | 0.998735809224318 |
| ebi-fl187-GCST90094766 | ebi-gm430-GCST90011512 | TestASV_34 (Phascolarctobacterium) abundance \|\| id:ebi-gm430-GCST90011512 | Fruit liking \|\| id:ebi-fl187-GCST90094766 | Inverse variance weighted | 8 | 0.0768926168306 | 0.658099386184525 | 0.906986502759722 | -1.21298218 | 1.36676741375227 | 1.07992610427819 | 0.297309326116384 | 3.9226498742422 | 0.998735809224318 |
| ebi-fl187-GCST90094766 | ebi-gm430-GCST90011519 | TestASV_41 (Barnesiella) abundance \|\| id:ebi-gm430-GCST90011519 | Fruit liking \|\| id:ebi-fl187-GCST90094766 | Inverse variance weighted | 8 | -0.559720143 | 1.08986271909719 | 0.607553078040068 | -2.695851072 | 1.57641078652854 | 0.571368943130437 | 0.0674849227761797 | 4.83756157292699 | 0.998735809224318 |
| ebi-fl187-GCST90094766 | ebi-gm430-GCST90011524 | TestASV_46 (Sutterella) abundance \|\| id:ebi-gm430-GCST90011524 | Fruit liking \|\| id:ebi-fl187-GCST90094766 | Inverse variance weighted | 8 | -0.400480582 | 1.10483240872153 | 0.71699273701176 | -2.565952103 | 1.76499093873005 | 0.669997979439142 | 0.0768459812757661 | 5.84151942626173 | 0.998735809224318 |
| ebi-fl187-GCST90094766 | ebi-gm430-GCST90011581 | OTU97_19 (Bacteria) prevalence \|\| id:ebi-gm430-GCST90011581 | Fruit liking \|\| id:ebi-fl187-GCST90094766 | Inverse variance weighted | 8 | 0.562371709585254 | 1.10111349876331 | 0.609540123025557 | -1.595810748 | 2.72055416716133 | 1.75482951471256 | 0.202744087489857 | 15.1887370124191 | 0.998735809224318 |
| ebi-fl187-GCST90094766 | ebi-gm430-GCST90011614 | OTU97_80 (Ruminococcus) prevalence \|\| id:ebi-gm430-GCST90011614 | Fruit liking \|\| id:ebi-fl187-GCST90094766 | Inverse variance weighted | 8 | 0.666292512378511 | 0.58404270141565 | 0.25394133132797 | -0.478431182 | 1.81101620715318 | 1.94700542432706 | 0.619754911953866 | 6.1166600687486 | 0.998735809224318 |
| ebi-fl187-GCST90094766 | ebi-gm430-GCST90011623 | OTU99_11 (Parabacteroides) prevalence \|\| id:ebi-gm430-GCST90011623 | Fruit liking \|\| id:ebi-fl187-GCST90094766 | Inverse variance weighted | 8 | 0.708136276607862 | 0.524407878296103 | 0.176902458292768 | -0.319703165 | 1.73597571806822 | 2.0302039918637 | 0.726364615624281 | 5.67446177845109 | 0.998735809224318 |
| ebi-fl187-GCST90094766 | ebi-gm430-GCST90011642 | OTU99_19 (Bacteria) prevalence \|\| id:ebi-gm430-GCST90011642 | Fruit liking \|\| id:ebi-fl187-GCST90094766 | Inverse variance weighted | 8 | 0.562371709585254 | 1.10111349876331 | 0.609540123025557 | -1.595810748 | 2.72055416716133 | 1.75482951471256 | 0.202744087489857 | 15.1887370124191 | 0.998735809224318 |
| ebi-fl187-GCST90094766 | ebi-gm430-GCST90011653 | OTU99_30 (Parasutterella) prevalence \|\| id:ebi-gm430-GCST90011653 | Fruit liking \|\| id:ebi-fl187-GCST90094766 | Inverse variance weighted | 8 | -0.280987471 | 0.430213078152411 | 0.51366887205846 | -1.124205104 | 0.562230162089555 | 0.755037795222157 | 0.32491063481358 | 1.75458114056816 | 0.998735809224318 |
| ebi-fl187-GCST90094766 | ebi-gm430-GCST90011675 | OTU99_8 (Ruminococcaceae) prevalence \|\| id:ebi-gm430-GCST90011675 | Fruit liking \|\| id:ebi-fl187-GCST90094766 | Inverse variance weighted | 8 | 0.0618750693284467 | 0.596444629177282 | 0.917375810884804 | -1.107156404 | 1.23090654251592 | 1.06382943150044 | 0.330497427342281 | 3.42433243256224 | 0.998735809224318 |
| ebi-fl187-GCST90094766 | ebi-gm430-GCST90011679 | OTU99_92 (Ruminococcus) prevalence \|\| id:ebi-gm430-GCST90011679 | Fruit liking \|\| id:ebi-fl187-GCST90094766 | Inverse variance weighted | 8 | 0.736242217158531 | 0.557503849513651 | 0.186633196983279 | -0.356465328 | 1.82894976220529 | 2.08807422374365 | 0.700146746615042 | 6.22734303193143 | 0.998735809224318 |
| ebi-fl187-GCST90094766 | ebi-gm430-GCST90011684 | TestASV_11 (Lachnospiraceae) prevalence \|\| id:ebi-gm430-GCST90011684 | Fruit liking \|\| id:ebi-fl187-GCST90094766 | Inverse variance weighted | 8 | 0.0800568505774427 | 0.541049825457136 | 0.882369787453806 | -0.980400807 | 1.14051450847343 | 1.08334865492091 | 0.37516070155843 | 3.12837752793293 | 0.998735809224318 |
| ebi-fl187-GCST90094766 | ebi-gm430-GCST90011688 | TestASV_15 (Bacteroides) prevalence \|\| id:ebi-gm430-GCST90011688 | Fruit liking \|\| id:ebi-fl187-GCST90094766 | Inverse variance weighted | 8 | -0.147238586 | 0.451697262416761 | 0.744449013643348 | -1.03256522 | 0.738088048203485 | 0.863088032013124 | 0.356092332618603 | 2.09193201529038 | 0.998735809224318 |
| ebi-fl187-GCST90094766 | ebi-gm430-GCST90011692 | TestASV_19 (Prevotella) prevalence \|\| id:ebi-gm430-GCST90011692 | Fruit liking \|\| id:ebi-fl187-GCST90094766 | Inverse variance weighted | 8 | 0.27711263408708 | 0.884465920956638 | 0.75404462401624 | -1.456440571 | 2.01066583916209 | 1.31931496250294 | 0.233064376173534 | 7.46828837105563 | 0.998735809224318 |
| ebi-fl187-GCST90094766 | ebi-gm430-GCST90011693 | TestASV_2 (Bacteroides) prevalence \|\| id:ebi-gm430-GCST90011693 | Fruit liking \|\| id:ebi-fl187-GCST90094766 | Inverse variance weighted | 8 | -0.115318268 | 0.573977089127586 | 0.840768224658804 | -1.240313363 | 1.00967682654845 | 0.891082495601212 | 0.289293549887812 | 2.74471385302164 | 0.998735809224318 |
| ebi-fl187-GCST90094766 | ebi-gm430-GCST90011716 | TestASV_40 (Barnesiella) prevalence \|\| id:ebi-gm430-GCST90011716 | Fruit liking \|\| id:ebi-fl187-GCST90094766 | Inverse variance weighted | 8 | -1.407981439 | 0.917001657089635 | 0.124681027466919 | -3.205304687 | 0.389341808877021 | 0.244636598985206 | 0.0405465457537163 | 1.47600897809065 | 0.998735809224318 |
| ebi-fl187-GCST90094766 | ebi-gm430-GCST90011718 | TestASV_42 (Alphaproteobacteria) prevalence \|\| id:ebi-gm430-GCST90011718 | Fruit liking \|\| id:ebi-fl187-GCST90094766 | Inverse variance weighted | 8 | 0.369237030179935 | 0.768365118263273 | 0.630837124386389 | -1.136758602 | 1.87523266197595 | 1.4466304581175 | 0.320857364608965 | 6.52233644349632 | 0.998735809224318 |
| ebi-fl187-GCST90094766 | ebi-hbsm44-GCST90104929 | Sulcal depth of the right inferior frontal sulcus \|\| id:ebi-hbsm44-GCST90104929 | Fruit liking \|\| id:ebi-fl187-GCST90094766 | Inverse variance weighted | 8 | -0.088133948 | 0.384927072227286 | 0.818897894420648 | -0.84259101 | 0.666323113508759 | 0.915638220548428 | 0.430593405165919 | 1.94706500580527 | 0.998735809224318 |
| ebi-fl187-GCST90094766 | ebi-hbsm44-GCST90104935 | Sulcal opening distance of the left calloso-marginal posterior fissure \|\| id:ebi-hbsm44-GCST90104935 | Fruit liking \|\| id:ebi-fl187-GCST90094766 | Inverse variance weighted | 9 | -0.115427527 | 0.103000934917462 | 0.26243882069011 | -0.317309359 | 0.086454305833148 | 0.890985142615344 | 0.72810547426822 | 1.09030154615874 | 0.998735809224318 |
| ebi-fl187-GCST90094766 | ebi-hbsm44-GCST90104936 | Sulcal opening distance of the left anterior inferior temporal sulcus \|\| id:ebi-hbsm44-GCST90104936 | Fruit liking \|\| id:ebi-fl187-GCST90094766 | Inverse variance weighted (multiplicative random effects) | 9 | -0.071987859 | 0.0759092961021265 | 0.342956144928894 | -0.220770079 | 0.0767943613844533 | 0.930542193477993 | 0.801901032714165 | 1.07982000086971 | 0.998735809224318 |
| ebi-fl187-GCST90094766 | ebi-hbsm44-GCST90104941 | Sulcal opening distance of the right posterior inferior temporal sulcus \|\| id:ebi-hbsm44-GCST90104941 | Fruit liking \|\| id:ebi-fl187-GCST90094766 | Inverse variance weighted | 9 | 0.0159908092980254 | 0.0451400523556755 | 0.723152435911322 | -0.072483693 | 0.104465311915149 | 1.01611934651309 | 0.930080913069283 | 1.11011688536961 | 0.998735809224318 |
| ebi-fl187-GCST90094766 | ebi-IPs91-GCST90274819 | Leukemia inhibitory factor levels \|\| id:ebi-IPs91-GCST90274819 | Fruit liking \|\| id:ebi-fl187-GCST90094766 | Inverse variance weighted | 10 | 0.310427340720202 | 0.160960541710036 | 0.0537814344387384 | -0.005055321 | 0.625910002471873 | 1.36400788571454 | 0.994957435598556 | 1.86994683965769 | 0.998735809224318 |
| ebi-fl187-GCST90094766 | ebi-IPs91-GCST90274837 | STAM binding protein levels \|\| id:ebi-IPs91-GCST90274837 | Fruit liking \|\| id:ebi-fl187-GCST90094766 | Inverse variance weighted | 10 | -0.115177277 | 0.139158920305557 | 0.407858987176931 | -0.387928761 | 0.157574206837739 | 0.891208139231273 | 0.678460674565336 | 1.17066762630113 | 0.998735809224318 |
| ebi-fl187-GCST90094766 | ebi-IPs91-GCST90274840 | TNF-beta levels \|\| id:ebi-IPs91-GCST90274840 | Fruit liking \|\| id:ebi-fl187-GCST90094766 | Inverse variance weighted | 10 | -0.03810075 | 0.165268420851795 | 0.81767327219894 | -0.362026855 | 0.285825354402798 | 0.962615951999006 | 0.696263669195745 | 1.33086000611421 | 0.998735809224318 |
| ebi-fl187-GCST90094766 | ebi-IPs91-GCST90274842 | Tumor necrosis factor ligand superfamily member 14 levels \|\| id:ebi-IPs91-GCST90274842 | Fruit liking \|\| id:ebi-fl187-GCST90094766 | Inverse variance weighted | 10 | -0.160059632 | 0.15846771448053 | 0.312473399929985 | -0.470656353 | 0.150537088221651 | 0.852092975306365 | 0.624592181050763 | 1.1624584178191 | 0.998735809224318 |
| ebi-fl187-GCST90094766 | ebi-met1400-GCST90199628 | 3-methylhistidine levels \|\| id:ebi-met1400-GCST90199628 | Fruit liking \|\| id:ebi-fl187-GCST90094766 | Inverse variance weighted | 9 | -0.115974916 | 0.187816440300162 | 0.53691107399211 | -0.484095139 | 0.252145306516973 | 0.890497559838391 | 0.616254568996473 | 1.28678300165701 | 0.998735809224318 |
| ebi-fl187-GCST90094766 | ebi-met1400-GCST90199651 | Docosahexaenoate DHA; 22:6n3 levels \|\| id:ebi-met1400-GCST90199651 | Fruit liking \|\| id:ebi-fl187-GCST90094766 | Inverse variance weighted | 9 | 0.198785783348224 | 0.180180977450205 | 0.269915914803906 | -0.154368932 | 0.551940499150626 | 1.21992061059891 | 0.856955803875816 | 1.73661965930237 | 0.998735809224318 |
| ebi-fl187-GCST90094766 | ebi-met1400-GCST90199652 | 1-stearoyl-2-oleoyl-GPS (18:0/18:1) levels \|\| id:ebi-met1400-GCST90199652 | Fruit liking \|\| id:ebi-fl187-GCST90094766 | Inverse variance weighted (multiplicative random effects) | 9 | 0.245584737896953 | 0.285264475051735 | 0.389292057133726 | -0.313533633 | 0.804703108998354 | 1.27836860526837 | 0.730859797433237 | 2.23603254232231 | 0.998735809224318 |
| ebi-fl187-GCST90094766 | ebi-met1400-GCST90199653 | 1-myristoyl-2-palmitoyl-gpc (14:0/16:0) levels \|\| id:ebi-met1400-GCST90199653 | Fruit liking \|\| id:ebi-fl187-GCST90094766 | Inverse variance weighted | 9 | 0.149375132299654 | 0.187121890325766 | 0.424709665800955 | -0.217383773 | 0.516134037338155 | 1.1611084768143 | 0.804621118394172 | 1.67553754693967 | 0.998735809224318 |
| ebi-fl187-GCST90094766 | ebi-met1400-GCST90199667 | Erythritol levels in elite athletes \|\| id:ebi-met1400-GCST90199667 | Fruit liking \|\| id:ebi-fl187-GCST90094766 | Inverse variance weighted | 9 | 0.137812221824874 | 0.176856661995183 | 0.435843557991676 | -0.208826836 | 0.484451279335433 | 1.14776000575817 | 0.811535752508932 | 1.62328403492426 | 0.998735809224318 |
| ebi-fl187-GCST90094766 | ebi-met1400-GCST90199674 | 3-hydroxymyristate levels \|\| id:ebi-met1400-GCST90199674 | Fruit liking \|\| id:ebi-fl187-GCST90094766 | Inverse variance weighted | 9 | 0.22931161107959 | 0.187533313809338 | 0.221413555628375 | -0.138253684 | 0.596876906145892 | 1.25773390177125 | 0.870877735984511 | 1.81643702933389 | 0.998735809224318 |
| ebi-fl187-GCST90094766 | ebi-met1400-GCST90199685 | 2-linoleoylglycerol (18:2) levels \|\| id:ebi-met1400-GCST90199685 | Fruit liking \|\| id:ebi-fl187-GCST90094766 | Inverse variance weighted | 9 | -0.433312562 | 0.214951440656162 | 0.0438143493409585 | -0.854617386 | -0.012007738 | 0.648357808159667 | 0.425445941679491 | 0.988064066946231 | 0.998735809224318 |
| ebi-fl187-GCST90094766 | ebi-met1400-GCST90199703 | Gamma-glutamylvaline levels \|\| id:ebi-met1400-GCST90199703 | Fruit liking \|\| id:ebi-fl187-GCST90094766 | Inverse variance weighted | 9 | 0.126659001170963 | 0.183857292223845 | 0.490887183349732 | -0.233701292 | 0.487019293929698 | 1.13502990791371 | 0.791598237635931 | 1.62745800913609 | 0.998735809224318 |
| ebi-fl187-GCST90094766 | ebi-met1400-GCST90199779 | Gamma-glutamyl-2-aminobutyrate levels \|\| id:ebi-met1400-GCST90199779 | Fruit liking \|\| id:ebi-fl187-GCST90094766 | Inverse variance weighted (multiplicative random effects) | 9 | -0.092970539 | 0.287280080518006 | 0.746223215454638 | -0.656039497 | 0.470098418673307 | 0.911220345233914 | 0.518902384688871 | 1.60015167027237 | 0.998735809224318 |
| ebi-fl187-GCST90094766 | ebi-met1400-GCST90199824 | 4-vinylphenol sulfate levels \|\| id:ebi-met1400-GCST90199824 | Fruit liking \|\| id:ebi-fl187-GCST90094766 | Inverse variance weighted (multiplicative random effects) | 9 | -0.136519776 | 0.303735597582111 | 0.653093269427035 | -0.731841547 | 0.458801995305973 | 0.872389067762116 | 0.481022348577861 | 1.58217739321453 | 0.998735809224318 |
| ebi-fl187-GCST90094766 | ebi-met1400-GCST90199830 | 4-ethylphenylsulfate levels \|\| id:ebi-met1400-GCST90199830 | Fruit liking \|\| id:ebi-fl187-GCST90094766 | Inverse variance weighted | 9 | 0.209675982324963 | 0.188865261298935 | 0.26691794142914 | -0.16049993 | 0.579851894470875 | 1.23327839121338 | 0.851717883344701 | 1.78577392817088 | 0.998735809224318 |
| ebi-fl187-GCST90094766 | ebi-met1400-GCST90199833 | Hexanoylglutamine levels \|\| id:ebi-met1400-GCST90199833 | Fruit liking \|\| id:ebi-fl187-GCST90094766 | Inverse variance weighted | 9 | 0.226833361780662 | 0.185865836730487 | 0.222307784201164 | -0.137463678 | 0.591130401772417 | 1.25462078274679 | 0.871566006258747 | 1.80602880011002 | 0.998735809224318 |
| ebi-fl187-GCST90094766 | ebi-met1400-GCST90199837 | Carotenoid levels (cryptoxanthin) \|\| id:ebi-met1400-GCST90199837 | Fruit liking \|\| id:ebi-fl187-GCST90094766 | Inverse variance weighted | 9 | 0.187846069176488 | 0.186761018870697 | 0.314507056398535 | -0.178205528 | 0.553897666163054 | 1.20664776076979 | 0.836770426221024 | 1.7400218422468 | 0.998735809224318 |
| ebi-fl187-GCST90094766 | ebi-met1400-GCST90199869 | 2-hydroxyhippurate levels \|\| id:ebi-met1400-GCST90199869 | Fruit liking \|\| id:ebi-fl187-GCST90094766 | Inverse variance weighted | 9 | 0.0725982528692126 | 0.189758216162092 | 0.702029034649222 | -0.299327851 | 0.444524356546913 | 1.07529845205823 | 0.741316328432414 | 1.5597481353795 | 0.998735809224318 |
| ebi-fl187-GCST90094766 | ebi-met1400-GCST90199902 | Hydantoin-5-propionate levels \|\| id:ebi-met1400-GCST90199902 | Fruit liking \|\| id:ebi-fl187-GCST90094766 | Inverse variance weighted | 9 | -0.008284585 | 0.188260603875342 | 0.964899667526214 | -0.377275369 | 0.360706198404185 | 0.991749637412509 | 0.685727220022084 | 1.43434198700201 | 0.998735809224318 |
| ebi-fl187-GCST90094766 | ebi-met1400-GCST90199904 | Pantoate levels \|\| id:ebi-met1400-GCST90199904 | Fruit liking \|\| id:ebi-fl187-GCST90094766 | Inverse variance weighted | 9 | 0.194963190620566 | 0.212016812138561 | 0.357800245194409 | -0.220589761 | 0.610516142412146 | 1.21526625246982 | 0.802045643074336 | 1.84138156867359 | 0.998735809224318 |
| ebi-fl187-GCST90094766 | ebi-met1400-GCST90199936 | 3-methyl catechol sulfate (2) levels \|\| id:ebi-met1400-GCST90199936 | Fruit liking \|\| id:ebi-fl187-GCST90094766 | Inverse variance weighted | 9 | 0.209219392531097 | 0.214747110254216 | 0.329927753899128 | -0.211684944 | 0.630123728629361 | 1.23271541742078 | 0.809219607251762 | 1.87784290782034 | 0.998735809224318 |
| ebi-fl187-GCST90094766 | ebi-met1400-GCST90199977 | 4-hydroxychlorothalonil levels \|\| id:ebi-met1400-GCST90199977 | Fruit liking \|\| id:ebi-fl187-GCST90094766 | Inverse variance weighted | 9 | -0.138998865 | 0.186954294837258 | 0.457184371923449 | -0.505429283 | 0.227431552459431 | 0.87022901580074 | 0.603246556160771 | 1.25537150972097 | 0.998735809224318 |
| ebi-fl187-GCST90094766 | ebi-met1400-GCST90200040 | Sphingomyelin (d18:2/24:1, d18:1/24:2) levels \|\| id:ebi-met1400-GCST90200040 | Fruit liking \|\| id:ebi-fl187-GCST90094766 | Inverse variance weighted | 9 | 0.0516212051847268 | 0.171780478423736 | 0.763790595634226 | -0.285068533 | 0.38831094289525 | 1.0529768048061 | 0.751962718649054 | 1.47448819464298 | 0.998735809224318 |
| ebi-fl187-GCST90094766 | ebi-met1400-GCST90200077 | 1-stearoyl-2-oleoyl-GPI (18:0/18:1) levels \|\| id:ebi-met1400-GCST90200077 | Fruit liking \|\| id:ebi-fl187-GCST90094766 | Inverse variance weighted | 9 | 0.34534550966729 | 0.18651113889979 | 0.064082138988289 | -0.020216323 | 0.710907341910879 | 1.41247786012424 | 0.979986657137229 | 2.03583762167669 | 0.998735809224318 |
| ebi-fl187-GCST90094766 | ebi-met1400-GCST90200080 | 1-palmitoyl-2-oleoyl-GPI (16:0/18:1) levels \|\| id:ebi-met1400-GCST90200080 | Fruit liking \|\| id:ebi-fl187-GCST90094766 | Inverse variance weighted | 9 | 0.462655132426677 | 0.188057099645838 | 0.0138865667476609 | 0.0940632171208347 | 0.83124704773252 | 1.58828549978888 | 1.09862919589657 | 2.2961804021428 | 0.998735809224318 |
| ebi-fl187-GCST90094766 | ebi-met1400-GCST90200082 | 1-oleoyl-2-linoleoyl-GPE (18:1/18:2) levels \|\| id:ebi-met1400-GCST90200082 | Fruit liking \|\| id:ebi-fl187-GCST90094766 | Inverse variance weighted | 9 | 0.094497079965838 | 0.189103825266686 | 0.617279262429954 | -0.276146418 | 0.465140577488542 | 1.09910595370137 | 0.758701835359158 | 1.59223800597495 | 0.998735809224318 |
| ebi-fl187-GCST90094766 | ebi-met1400-GCST90200100 | Perfluorooctanesulfonate (PFOS) levels \|\| id:ebi-met1400-GCST90200100 | Fruit liking \|\| id:ebi-fl187-GCST90094766 | Inverse variance weighted | 9 | -0.056772943 | 0.173579991744162 | 0.743614352942727 | -0.396989727 | 0.283443840825985 | 0.94480857042021 | 0.672340932682565 | 1.32769431600402 | 0.998735809224318 |
| ebi-fl187-GCST90094766 | ebi-met1400-GCST90200102 | 1-palmitoleoyl-2-linolenoyl-GPC (16:1/18:3) levels \|\| id:ebi-met1400-GCST90200102 | Fruit liking \|\| id:ebi-fl187-GCST90094766 | Inverse variance weighted | 9 | 0.156276510053255 | 0.184445219281246 | 0.396839782671666 | -0.20523612 | 0.517789139844498 | 1.1691494399726 | 0.814454984808049 | 1.67831302955361 | 0.998735809224318 |
| ebi-fl187-GCST90094766 | ebi-met1400-GCST90200114 | Glycosyl-N-tricosanoyl-sphingadienine (d18:2/23:0) levels \|\| id:ebi-met1400-GCST90200114 | Fruit liking \|\| id:ebi-fl187-GCST90094766 | Inverse variance weighted | 9 | -0.043078503 | 0.236126334721917 | 0.855238716902657 | -0.505886119 | 0.419729112663163 | 0.957836193725494 | 0.602971034269066 | 1.52154933134175 | 0.998735809224318 |
| ebi-fl187-GCST90094766 | ebi-met1400-GCST90200135 | Arachidoylcarnitine (C20) levels \|\| id:ebi-met1400-GCST90200135 | Fruit liking \|\| id:ebi-fl187-GCST90094766 | Inverse variance weighted | 9 | -0.007493423 | 0.193134605043448 | 0.969050670146138 | -0.386037249 | 0.371050403133121 | 0.992534582943734 | 0.679745205626206 | 1.44925611859485 | 0.998735809224318 |
| ebi-fl187-GCST90094766 | ebi-met1400-GCST90200146 | Docosahexaenoylcarnitine (C22:6) levels \|\| id:ebi-met1400-GCST90200146 | Fruit liking \|\| id:ebi-fl187-GCST90094766 | Inverse variance weighted | 9 | 0.0860045134070684 | 0.230127581257175 | 0.708608654918852 | -0.365045546 | 0.537054572671131 | 1.08981124705581 | 0.69416503381661 | 1.71095992501852 | 0.998735809224318 |
| ebi-fl187-GCST90094766 | ebi-met1400-GCST90200162 | 3-hydroxyphenylacetoylglutamine levels \|\| id:ebi-met1400-GCST90200162 | Fruit liking \|\| id:ebi-fl187-GCST90094766 | Inverse variance weighted | 9 | -0.225413705 | 0.229702669626793 | 0.326430925834135 | -0.675630937 | 0.22480352757588 | 0.798185937017126 | 0.508835276120801 | 1.25207669348116 | 0.998735809224318 |
| ebi-fl187-GCST90094766 | ebi-met1400-GCST90200172 | 3-carboxy-4-methyl-5-pentyl-2-furanpropionate (3-CMPFP) levels \|\| id:ebi-met1400-GCST90200172 | Fruit liking \|\| id:ebi-fl187-GCST90094766 | Inverse variance weighted | 9 | -0.084366118 | 0.18715147687114 | 0.652140507900348 | -0.451183013 | 0.282450776193848 | 0.919094696941097 | 0.636874275150129 | 1.32637648419089 | 0.998735809224318 |
| ebi-fl187-GCST90094766 | ebi-met1400-GCST90200222 | Dihydrocaffeate sulfate (2) levels \|\| id:ebi-met1400-GCST90200222 | Fruit liking \|\| id:ebi-fl187-GCST90094766 | Inverse variance weighted | 9 | 0.253269391076125 | 0.213369060739282 | 0.235227036373804 | -0.164933968 | 0.671472750125118 | 1.28823026783254 | 0.847949694076535 | 1.9571175442987 | 0.998735809224318 |
| ebi-fl187-GCST90094766 | ebi-met1400-GCST90200223 | 4-methylhexanoylglutamine levels \|\| id:ebi-met1400-GCST90200223 | Fruit liking \|\| id:ebi-fl187-GCST90094766 | Inverse variance weighted | 9 | -0.048949394 | 0.22619096085401 | 0.828670212789228 | -0.492283677 | 0.39438488965876 | 0.95222931736254 | 0.611228949709602 | 1.48347141161348 | 0.998735809224318 |
| ebi-fl187-GCST90094766 | ebi-met1400-GCST90200224 | 2-ketocaprylate levels \|\| id:ebi-met1400-GCST90200224 | Fruit liking \|\| id:ebi-fl187-GCST90094766 | Inverse variance weighted | 9 | -0.399352062 | 0.1907024811355 | 0.0362501408264575 | -0.773128925 | -0.025575199 | 0.670754512643845 | 0.461566599288652 | 0.974749076136505 | 0.998735809224318 |
| ebi-fl187-GCST90094766 | ebi-met1400-GCST90200243 | Hydroxypalmitoyl sphingomyelin (d18:1/16:0(OH)) levels \|\| id:ebi-met1400-GCST90200243 | Fruit liking \|\| id:ebi-fl187-GCST90094766 | Inverse variance weighted | 9 | -0.24657655 | 0.184091663813632 | 0.180433109454478 | -0.607396211 | 0.114243110994901 | 0.781471537538694 | 0.544767483488621 | 1.12102462517084 | 0.998735809224318 |
| ebi-fl187-GCST90094766 | ebi-met1400-GCST90200273 | 2-methoxyhydroquinone sulfate (2) levels \|\| id:ebi-met1400-GCST90200273 | Fruit liking \|\| id:ebi-fl187-GCST90094766 | Inverse variance weighted (multiplicative random effects) | 9 | -0.065728909 | 0.37137447930122 | 0.85951764140839 | -0.793622888 | 0.66216507082998 | 0.936384675719311 | 0.452203541240662 | 1.93898583482193 | 0.998735809224318 |
| ebi-fl187-GCST90094766 | ebi-met1400-GCST90200276 | 2,4-di-tert-butylphenol levels \|\| id:ebi-met1400-GCST90200276 | Fruit liking \|\| id:ebi-fl187-GCST90094766 | Inverse variance weighted | 9 | 0.27483231874503 | 0.21407244210194 | 0.199201942198088 | -0.144749668 | 0.694414305264833 | 1.31630993585927 | 0.865238863172348 | 2.00253585569317 | 0.998735809224318 |
| ebi-fl187-GCST90094766 | ebi-met1400-GCST90200289 | N-acetylmethionine levels \|\| id:ebi-met1400-GCST90200289 | Fruit liking \|\| id:ebi-fl187-GCST90094766 | Inverse variance weighted | 9 | -0.02430936 | 0.187702977726816 | 0.896954336213242 | -0.392207196 | 0.343588476715242 | 0.975983733084735 | 0.675564125291726 | 1.40999826897955 | 0.998735809224318 |
| ebi-fl187-GCST90094766 | ebi-met1400-GCST90200308 | 3-hydroxyisobutyrate levels \|\| id:ebi-met1400-GCST90200308 | Fruit liking \|\| id:ebi-fl187-GCST90094766 | Inverse variance weighted | 9 | 0.0971327041290489 | 0.190283582340212 | 0.609727218763144 | -0.275823117 | 0.470088525515864 | 1.10200660474475 | 0.758947163544659 | 1.60013583979827 | 0.998735809224318 |
| ebi-fl187-GCST90094766 | ebi-met1400-GCST90200383 | Cystathionine levels \|\| id:ebi-met1400-GCST90200383 | Fruit liking \|\| id:ebi-fl187-GCST90094766 | Inverse variance weighted | 9 | -0.184784952 | 0.193683180871669 | 0.340054377746884 | -0.564403987 | 0.194834082069542 | 0.831283030020139 | 0.568698997793375 | 1.21510936133307 | 0.998735809224318 |
| ebi-fl187-GCST90094766 | ebi-met1400-GCST90200384 | Guanidinoacetate levels \|\| id:ebi-met1400-GCST90200384 | Fruit liking \|\| id:ebi-fl187-GCST90094766 | Inverse variance weighted | 9 | -0.046626528 | 0.182442538539929 | 0.798284203987157 | -0.404213904 | 0.310960847393771 | 0.954443788927993 | 0.667501325005492 | 1.36473578717725 | 0.998735809224318 |
| ebi-fl187-GCST90094766 | ebi-met1400-GCST90200403 | Citrulline levels \|\| id:ebi-met1400-GCST90200403 | Fruit liking \|\| id:ebi-fl187-GCST90094766 | Inverse variance weighted | 9 | -0.081521966 | 0.223520209199078 | 0.715321513693748 | -0.519621576 | 0.35657764448875 | 0.921712463718209 | 0.594745571643252 | 1.42843243611922 | 0.998735809224318 |
| ebi-fl187-GCST90094766 | ebi-met1400-GCST90200434 | 12,13-DiHOME levels \|\| id:ebi-met1400-GCST90200434 | Fruit liking \|\| id:ebi-fl187-GCST90094766 | Inverse variance weighted | 9 | -0.344010238 | 0.190144177984336 | 0.0704188015701629 | -0.716692827 | 0.0286723510620796 | 0.708921670223143 | 0.488364694893385 | 1.02908735985038 | 0.998735809224318 |
| ebi-fl187-GCST90094766 | ebi-met1400-GCST90200437 | Fructose levels \|\| id:ebi-met1400-GCST90200437 | Fruit liking \|\| id:ebi-fl187-GCST90094766 | Inverse variance weighted | 9 | 0.179479917687465 | 0.189502975478367 | 0.343583389442002 | -0.191945914 | 0.550905749625065 | 1.19659487343396 | 0.825351507039677 | 1.73482362231829 | 0.998735809224318 |
| ebi-fl187-GCST90094766 | ebi-met1400-GCST90200448 | N-stearoyl-sphinganine (d18:0/18:0) levels \|\| id:ebi-met1400-GCST90200448 | Fruit liking \|\| id:ebi-fl187-GCST90094766 | Inverse variance weighted | 9 | 0.414751624376948 | 0.209624717587838 | 0.0478674252831023 | 0.00388717790478527 | 0.82561607084911 | 1.51399465462304 | 1.00389474277965 | 2.28328699867518 | 0.998735809224318 |
| ebi-fl187-GCST90094766 | ebi-met1400-GCST90200468 | X-11847 levels \|\| id:ebi-met1400-GCST90200468 | Fruit liking \|\| id:ebi-fl187-GCST90094766 | Inverse variance weighted | 9 | 0.11836264063397 | 0.2001263359886 | 0.55422591199485 | -0.273884978 | 0.510610259171625 | 1.12565224460363 | 0.760419535279895 | 1.66630776432487 | 0.998735809224318 |
| ebi-fl187-GCST90094766 | ebi-met1400-GCST90200476 | X-11843 levels \|\| id:ebi-met1400-GCST90200476 | Fruit liking \|\| id:ebi-fl187-GCST90094766 | Inverse variance weighted | 9 | -0.124592385 | 0.198551478944114 | 0.530327141118851 | -0.513753283 | 0.264568514008627 | 0.882856695128509 | 0.59824597303807 | 1.30286868489064 | 0.998735809224318 |
| ebi-fl187-GCST90094766 | ebi-met1400-GCST90200511 | X-12906 levels \|\| id:ebi-met1400-GCST90200511 | Fruit liking \|\| id:ebi-fl187-GCST90094766 | Inverse variance weighted | 9 | -0.052418417 | 0.188950507483075 | 0.781458571519945 | -0.422761412 | 0.31792457750563 | 0.948931734442683 | 0.655234945771816 | 1.37427260625074 | 0.998735809224318 |
| ebi-fl187-GCST90094766 | ebi-met1400-GCST90200515 | X-12849 levels \|\| id:ebi-met1400-GCST90200515 | Fruit liking \|\| id:ebi-fl187-GCST90094766 | Inverse variance weighted (multiplicative random effects) | 9 | 0.146069990143699 | 0.309949651732267 | 0.637447895287715 | -0.461431327 | 0.753571307538943 | 1.15727718321268 | 0.630380718367919 | 2.12457399117816 | 0.998735809224318 |
| ebi-fl187-GCST90094766 | ebi-met1400-GCST90200551 | X-18886 levels \|\| id:ebi-met1400-GCST90200551 | Fruit liking \|\| id:ebi-fl187-GCST90094766 | Inverse variance weighted | 9 | -0.037991758 | 0.185845908271565 | 0.838020518699412 | -0.402249739 | 0.326266221877481 | 0.962720875281652 | 0.66881369627112 | 1.38578424585276 | 0.998735809224318 |
| ebi-fl187-GCST90094766 | ebi-met1400-GCST90200564 | X-21319 levels \|\| id:ebi-met1400-GCST90200564 | Fruit liking \|\| id:ebi-fl187-GCST90094766 | Inverse variance weighted | 9 | -0.534599848 | 0.190377650159174 | 0.00498344498689129 | -0.907740042 | -0.161459653 | 0.585903693952338 | 0.403434940571068 | 0.85090086173764 | 0.998735809224318 |
| ebi-fl187-GCST90094766 | ebi-met1400-GCST90200579 | X-21442 levels \|\| id:ebi-met1400-GCST90200579 | Fruit liking \|\| id:ebi-fl187-GCST90094766 | Inverse variance weighted | 9 | 0.166238778676322 | 0.205639093197181 | 0.418859972109263 | -0.236813844 | 0.569291401342796 | 1.18085503106382 | 0.789138177159316 | 1.76701450360478 | 0.998735809224318 |
| ebi-fl187-GCST90094766 | ebi-met1400-GCST90200581 | X-21441 levels \|\| id:ebi-met1400-GCST90200581 | Fruit liking \|\| id:ebi-fl187-GCST90094766 | Inverse variance weighted | 9 | 0.0908903610103901 | 0.188901685616036 | 0.630408709067362 | -0.279356943 | 0.461137664817821 | 1.09514892765409 | 0.756269909934866 | 1.58587715574351 | 0.998735809224318 |
| ebi-fl187-GCST90094766 | ebi-met1400-GCST90200648 | X-24585 levels \|\| id:ebi-met1400-GCST90200648 | Fruit liking \|\| id:ebi-fl187-GCST90094766 | Inverse variance weighted | 9 | -0.125860378 | 0.184152240066781 | 0.494317289399304 | -0.486798768 | 0.235078012983065 | 0.881737948603887 | 0.614590695768273 | 1.26500745188848 | 0.998735809224318 |
| ebi-fl187-GCST90094766 | ebi-met1400-GCST90200720 | Adenosine 5'-diphosphate (ADP) to pantothenate ratio \|\| id:ebi-met1400-GCST90200720 | Fruit liking \|\| id:ebi-fl187-GCST90094766 | Inverse variance weighted | 9 | 0.175263634202235 | 0.249867949647216 | 0.483037805710526 | -0.314477547 | 0.665004815510778 | 1.19156031125957 | 0.730170254196051 | 1.94449988507444 | 0.998735809224318 |
| ebi-fl187-GCST90094766 | ebi-met1400-GCST90200724 | Cholate to taurocholate ratio \|\| id:ebi-met1400-GCST90200724 | Fruit liking \|\| id:ebi-fl187-GCST90094766 | Inverse variance weighted | 9 | 0.168733044036037 | 0.2232142495792 | 0.449694667840322 | -0.268766885 | 0.606232973211269 | 1.18380407318053 | 0.76432140956123 | 1.83351148633047 | 0.998735809224318 |
| ebi-fl187-GCST90094766 | ebi-met1400-GCST90200726 | N-acetylputrescine to (N(1) + N(8))-acetylspermidine ratio \|\| id:ebi-met1400-GCST90200726 | Fruit liking \|\| id:ebi-fl187-GCST90094766 | Inverse variance weighted | 9 | 0.141682182866794 | 0.18753926592251 | 0.44996096978081 | -0.225894778 | 0.509259144074913 | 1.15221039813418 | 0.797802043303648 | 1.66405791099638 | 0.998735809224318 |
| ebi-fl187-GCST90094766 | ebi-met1400-GCST90200744 | Arginine to glutamate ratio \|\| id:ebi-met1400-GCST90200744 | Fruit liking \|\| id:ebi-fl187-GCST90094766 | Inverse variance weighted | 9 | 0.144491611814417 | 0.22980696233204 | 0.529511358816598 | -0.305930034 | 0.594913257985216 | 1.15545200277727 | 0.736438143022301 | 1.81287368582371 | 0.998735809224318 |
| ebi-fl187-GCST90094766 | ebi-met1400-GCST90200759 | Histidine to trans-urocanate ratio \|\| id:ebi-met1400-GCST90200759 | Fruit liking \|\| id:ebi-fl187-GCST90094766 | Inverse variance weighted | 9 | 0.162891853028198 | 0.212488781155419 | 0.443325103805857 | -0.253586158 | 0.57936986409282 | 1.1769094036177 | 0.776012882298623 | 1.78491333832105 | 0.998735809224318 |
| ebi-fl187-GCST90094766 | ebi-met1400-GCST90200795 | Oleoyl-linoleoyl-glycerol (18:1 to 18:2) [2] to linoleoyl-arachidonoyl-glycerol (18:2 to 20:4) [2] ratio \|\| id:ebi-met1400-GCST90200795 | Fruit liking \|\| id:ebi-fl187-GCST90094766 | Inverse variance weighted | 9 | -0.054902091 | 0.208546746987711 | 0.792349912813556 | -0.463653715 | 0.353849533050616 | 0.946577821861691 | 0.628981323468648 | 1.424540824 | 0.998735809224318 |
| ebi-fl187-GCST90094766 | ebi-met1400-GCST90200811 | Choline to taurocholate ratio \|\| id:ebi-met1400-GCST90200811 | Fruit liking \|\| id:ebi-fl187-GCST90094766 | Inverse variance weighted | 9 | -0.202332067 | 0.220411670076644 | 0.358632752012109 | -0.63433894 | 0.229674806321457 | 0.81682364270223 | 0.530285923087013 | 1.25819078770428 | 0.998735809224318 |
| ebi-fl187-GCST90094766 | ebi-met1400-GCST90200831 | Cholate to phosphate ratio \|\| id:ebi-met1400-GCST90200831 | Fruit liking \|\| id:ebi-fl187-GCST90094766 | Inverse variance weighted | 9 | 0.329039491535247 | 0.196571549882414 | 0.0941518799807686 | -0.056240746 | 0.714319729304779 | 1.38963273323407 | 0.945311528302996 | 2.04279655484813 | 0.998735809224318 |
| ebi-fl187-GCST90094766 | ebi-met1400-GCST90200841 | Adenosine 5'-diphosphate (ADP) to oxalate (ethanedioate) ratio \|\| id:ebi-met1400-GCST90200841 | Fruit liking \|\| id:ebi-fl187-GCST90094766 | Inverse variance weighted | 9 | -0.006983821 | 0.287792091151503 | 0.98063971700359 | -0.57105632 | 0.557088677365686 | 0.993040508916431 | 0.564928378296344 | 1.74558313980062 | 0.998955228396052 |
| ebi-fl187-GCST90094766 | ebi-met1400-GCST90200844 | Aspartate to phosphate ratio \|\| id:ebi-met1400-GCST90200844 | Fruit liking \|\| id:ebi-fl187-GCST90094766 | Inverse variance weighted | 9 | 0.0709251546384919 | 0.177153720714657 | 0.688891834436359 | -0.276296138 | 0.41814644723922 | 1.07350087630033 | 0.758588250715995 | 1.51914313243038 | 0.998735809224318 |
| ebi-fl187-GCST90094766 | ebi-met1400-GCST90200870 | Adenosine 5'-monophosphate (AMP) to acetoacetate ratio \|\| id:ebi-met1400-GCST90200870 | Fruit liking \|\| id:ebi-fl187-GCST90094766 | Inverse variance weighted | 9 | 0.0822807831350539 | 0.20750138438983 | 0.691713159701627 | -0.32442193 | 0.488983496539121 | 1.08576063030702 | 0.722945145579825 | 1.63065780790214 | 0.998735809224318 |
| ebi-fl187-GCST90094766 | ebi-met1400-GCST90200887 | Cholesterol to cortisol ratio \|\| id:ebi-met1400-GCST90200887 | Fruit liking \|\| id:ebi-fl187-GCST90094766 | Inverse variance weighted | 9 | 0.108366169361034 | 0.183316767017277 | 0.554425539819611 | -0.250934694 | 0.467667032714897 | 1.11445575023243 | 0.778073182752653 | 1.59626580989744 | 0.998735809224318 |
| ebi-fl187-GCST90094766 | ebi-met1400-GCST90200890 | Cortisol to taurocholate ratio \|\| id:ebi-met1400-GCST90200890 | Fruit liking \|\| id:ebi-fl187-GCST90094766 | Inverse variance weighted | 9 | -0.136514019 | 0.196747095762652 | 0.487772946644983 | -0.522138327 | 0.249110288831384 | 0.872394090200303 | 0.593250627129684 | 1.28288351299129 | 0.998735809224318 |
| ebi-fl187-GCST90094766 | ebi-met1400-GCST90200948 | Adenosine 5'-diphosphate (ADP) to mannitol to sorbitol ratio \|\| id:ebi-met1400-GCST90200948 | Fruit liking \|\| id:ebi-fl187-GCST90094766 | Inverse variance weighted | 9 | -0.029709154 | 0.278186357278764 | 0.914950924728801 | -0.574954414 | 0.51553610647408 | 0.970727825001432 | 0.56273052082277 | 1.67453599078695 | 0.998735809224318 |
| ebi-fl187-GCST90094766 | ebi-met1400-GCST90200972 | Leucine to N-palmitoyl-sphingosine (d18:1 to 16:0) ratio \|\| id:ebi-met1400-GCST90200972 | Fruit liking \|\| id:ebi-fl187-GCST90094766 | Inverse variance weighted | 9 | 0.173042138558509 | 0.214524037180652 | 0.419878033701198 | -0.247424974 | 0.593509251432586 | 1.18891620324349 | 0.780808799328032 | 1.81033018525328 | 0.998735809224318 |
| ebi-fl187-GCST90094766 | ebi-met1400-GCST90200973 | Proline to glutamate ratio \|\| id:ebi-met1400-GCST90200973 | Fruit liking \|\| id:ebi-fl187-GCST90094766 | Inverse variance weighted | 9 | -0.220455235 | 0.243699146532857 | 0.365666490317981 | -0.698105562 | 0.25719509223948 | 0.802153546489569 | 0.497526945436228 | 1.29329741443777 | 0.998735809224318 |
| ebi-fl187-GCST90094766 | ebi-met1400-GCST90200983 | Cholesterol to linoleoyl-arachidonoyl-glycerol (18:2 to 20:4) [1] ratio \|\| id:ebi-met1400-GCST90200983 | Fruit liking \|\| id:ebi-fl187-GCST90094766 | Inverse variance weighted | 9 | 0.25405840274118 | 0.194153586163276 | 0.190689048508244 | -0.126482626 | 0.634599431621202 | 1.28924709763367 | 0.881189459073839 | 1.88626641142966 | 0.998735809224318 |
| ebi-fl187-GCST90094766 | ebi-met1400-GCST90201001 | Serine to alpha-ketobutyrate ratio \|\| id:ebi-met1400-GCST90201001 | Fruit liking \|\| id:ebi-fl187-GCST90094766 | Inverse variance weighted | 9 | -0.122755852 | 0.191783969150062 | 0.522124763490361 | -0.498652432 | 0.253140727560237 | 0.884479580145455 | 0.607348552278447 | 1.28806453025943 | 0.998735809224318 |
| ebi-fl187-GCST90094766 | ebi-met1400-GCST90201005 | Fructose to maltose ratio \|\| id:ebi-met1400-GCST90201005 | Fruit liking \|\| id:ebi-fl187-GCST90094766 | Inverse variance weighted | 9 | -0.033437359 | 0.215073564247316 | 0.876451271305759 | -0.454981545 | 0.388106826692958 | 0.967115490191289 | 0.634459676694213 | 1.47418725842639 | 0.998735809224318 |
| ebi-fl187-GCST90094766 | ebi-met1400-GCST90201020 | Paraxanthine to 5-acetylamino-6-formylamino-3-methyluracil ratio \|\| id:ebi-met1400-GCST90201020 | Fruit liking \|\| id:ebi-fl187-GCST90094766 | Inverse variance weighted | 9 | 0.551109470522414 | 0.215815673538766 | 0.0106612017636791 | 0.128110750386432 | 0.974108190658396 | 1.73517707814532 | 1.13667888335185 | 2.64880392925269 | 0.998735809224318 |
| ebi-fl187-GCST90094766 | ebi-pw11-GCST90275197 | Placental weight (fetal genotype, adjusted for sex) \|\| id:ebi-pw11-GCST90275197 | Fruit liking \|\| id:ebi-fl187-GCST90094766 | Inverse variance weighted (multiplicative random effects) | 8 | 0.0471061809877661 | 0.107244970409485 | 0.660488318650909 | -0.163093961 | 0.257306322990358 | 1.04823330561856 | 0.849511363714604 | 1.2934412768811 | 0.998735809224318 |
| ebi-fl187-GCST90094766 | prot-a-1017 | FAS-associated factor 2 \|\| id:prot-a-1017 | Fruit liking \|\| id:ebi-fl187-GCST90094766 | Inverse variance weighted | 10 | -0.288624049 | 0.290692718478284 | 0.320766655504544 | -0.858381777 | 0.281133679093154 | 0.749293850136189 | 0.423847407142009 | 1.32463066752654 | 0.998735809224318 |
| ebi-fl187-GCST90094766 | prot-a-1019 | Fas apoptotic inhibitory molecule 3 \|\| id:prot-a-1019 | Fruit liking \|\| id:ebi-fl187-GCST90094766 | Inverse variance weighted | 10 | 0.308338084004607 | 0.290692718478284 | 0.288825763439738 | -0.261419644 | 0.878095812222043 | 1.36116109794993 | 0.769957743499146 | 2.40631326876747 | 0.998735809224318 |
| ebi-fl187-GCST90094766 | prot-a-1052 | MIP18 family protein FAM96A \|\| id:prot-a-1052 | Fruit liking \|\| id:ebi-fl187-GCST90094766 | Inverse variance weighted | 10 | -0.298235519 | 0.290692718478284 | 0.304916248324504 | -0.867993247 | 0.271522209303164 | 0.742126534327973 | 0.419793125606229 | 1.31196000925051 | 0.998735809224318 |
| ebi-fl187-GCST90094766 | prot-a-1066 | F-box/LRR-repeat protein 4 \|\| id:prot-a-1066 | Fruit liking \|\| id:ebi-fl187-GCST90094766 | Inverse variance weighted | 10 | -0.151040874 | 0.290692718478284 | 0.603349741405779 | -0.720798603 | 0.41871685373872 | 0.859812553551021 | 0.486363689471777 | 1.52000990873071 | 0.998735809224318 |
| ebi-fl187-GCST90094766 | prot-a-1076 | Low affinity immunoglobulin gamma Fc region receptor III-B \|\| id:prot-a-1076 | Fruit liking \|\| id:ebi-fl187-GCST90094766 | Inverse variance weighted (multiplicative random effects) | 10 | 0.000620860583272645 | 0.466592448034464 | 0.998938313447402 | -0.913900338 | 0.915142058730821 | 1.0006210533571 | 0.400957301432066 | 2.49712996582283 | 0.999882738470636 |
| ebi-fl187-GCST90094766 | prot-a-1081 | Fc receptor-like protein 3 \|\| id:prot-a-1081 | Fruit liking \|\| id:ebi-fl187-GCST90094766 | Inverse variance weighted | 10 | 0.117636820821162 | 0.305062267346758 | 0.699781252863132 | -0.480285223 | 0.715558864820807 | 1.12483552033535 | 0.618606925607659 | 2.04532942557221 | 0.998735809224318 |
| ebi-fl187-GCST90094766 | prot-a-1105 | Fibroblast growth factor receptor 4 \|\| id:prot-a-1105 | Fruit liking \|\| id:ebi-fl187-GCST90094766 | Inverse variance weighted | 10 | -0.085108964 | 0.290692718478284 | 0.769690447362907 | -0.654866693 | 0.484648763903608 | 0.918412204793297 | 0.519511312709243 | 1.62360464012717 | 0.998735809224318 |
| ebi-fl187-GCST90094766 | prot-a-1143 | Follistatin-related protein 1 \|\| id:prot-a-1143 | Fruit liking \|\| id:ebi-fl187-GCST90094766 | Inverse variance weighted | 10 | 0.716158266237403 | 0.298965580583454 | 0.0165998014894081 | 0.130185728293833 | 1.30213080418097 | 2.04655576642581 | 1.13903991562038 | 3.67712355612182 | 0.998735809224318 |
| ebi-fl187-GCST90094766 | prot-a-1151 | Alpha-(1,3)-fucosyltransferase 10 \|\| id:prot-a-1151 | Fruit liking \|\| id:ebi-fl187-GCST90094766 | Inverse variance weighted | 10 | 0.238578235687833 | 0.290692718478284 | 0.411803996816833 | -0.331179493 | 0.80833596390527 | 1.26944301789916 | 0.718076268146725 | 2.24417049717016 | 0.998735809224318 |
| ebi-fl187-GCST90094766 | prot-a-1165 | Growth arrest and DNA damage-inducible proteins-interacting protein 1 \|\| id:prot-a-1165 | Fruit liking \|\| id:ebi-fl187-GCST90094766 | Inverse variance weighted | 10 | -0.076038818 | 0.290620986518272 | 0.793597031977014 | -0.645655951 | 0.493578315973748 | 0.926780230474946 | 0.524318501943221 | 1.63816762600571 | 0.998735809224318 |
| ebi-fl187-GCST90094766 | prot-a-1194 | Growth/differentiation factor 11/8 \|\| id:prot-a-1194 | Fruit liking \|\| id:ebi-fl187-GCST90094766 | Inverse variance weighted | 10 | 0.167836106373145 | 0.290692718478284 | 0.563692204024066 | -0.401921622 | 0.737593834590581 | 1.18274275076299 | 0.669033181222272 | 2.09089840944323 | 0.998735809224318 |
| ebi-fl187-GCST90094766 | prot-a-1216 | D-glucuronyl C5-epimerase \|\| id:prot-a-1216 | Fruit liking \|\| id:ebi-fl187-GCST90094766 | Inverse variance weighted | 10 | 0.215767533109603 | 0.290692718478284 | 0.457934081638686 | -0.353990195 | 0.785525261327039 | 1.24081389732248 | 0.701881849197501 | 2.19355882980722 | 0.998735809224318 |
| ebi-fl187-GCST90094766 | prot-a-1246 | Glypican-3 \|\| id:prot-a-1246 | Fruit liking \|\| id:ebi-fl187-GCST90094766 | Inverse variance weighted | 10 | 0.0533125664259033 | 0.290692718478284 | 0.854485466371886 | -0.516445162 | 0.62307029464334 | 1.05475927593798 | 0.596637733225808 | 1.86464426941659 | 0.998735809224318 |
| ebi-fl187-GCST90094766 | prot-a-1275 | Glutamate receptor 4 \|\| id:prot-a-1275 | Fruit liking \|\| id:ebi-fl187-GCST90094766 | Inverse variance weighted | 10 | -0.140746998 | 0.290692718478284 | 0.628259601017962 | -0.710504726 | 0.429010730570998 | 0.868709069335999 | 0.491396114530885 | 1.53573751364936 | 0.998735809224318 |
| ebi-fl187-GCST90094766 | prot-a-1289 | Glutathione S-transferase P \|\| id:prot-a-1289 | Fruit liking \|\| id:ebi-fl187-GCST90094766 | Inverse variance weighted | 10 | -0.005211107 | 0.290692718478284 | 0.985697477067547 | -0.574968835 | 0.564546621046191 | 0.994802447093268 | 0.562722405558741 | 1.7586502669289 | 0.998955228396052 |
| ebi-fl187-GCST90094766 | prot-a-130 | Apolipoprotein D \|\| id:prot-a-130 | Fruit liking \|\| id:ebi-fl187-GCST90094766 | Inverse variance weighted | 10 | 0.371281590811168 | 0.342026486776064 | 0.27768494098166 | -0.299090323 | 1.04165350489225 | 1.44959120748452 | 0.74149243238915 | 2.83389900830925 | 0.998735809224318 |
| ebi-fl187-GCST90094766 | prot-a-1313 | Heparin-binding EGF-like growth factor \|\| id:prot-a-1313 | Fruit liking \|\| id:ebi-fl187-GCST90094766 | Inverse variance weighted | 10 | -0.337292758 | 0.290692718478284 | 0.245923889237907 | -0.907050486 | 0.232464970481189 | 0.713699868157403 | 0.40371322751565 | 1.26170624862211 | 0.998735809224318 |
| ebi-fl187-GCST90094766 | prot-a-1347 | HLA class II histocompatibility antigen, DQ alpha 2 chain \|\| id:prot-a-1347 | Fruit liking \|\| id:ebi-fl187-GCST90094766 | Inverse variance weighted | 10 | 0.0820744914530025 | 0.290692718478284 | 0.777682001251159 | -0.487683237 | 0.651832219670439 | 1.08553667002165 | 0.614047349864971 | 1.91905373782791 | 0.998735809224318 |
| ebi-fl187-GCST90094766 | prot-a-1357 | Non-histone chromosomal protein HMG-14 \|\| id:prot-a-1357 | Fruit liking \|\| id:ebi-fl187-GCST90094766 | Inverse variance weighted | 10 | -0.03444109 | 0.290692718478284 | 0.905687840953787 | -0.604198818 | 0.535316638304877 | 0.966145253711446 | 0.54651210687733 | 1.70798897137456 | 0.998735809224318 |
| ebi-fl187-GCST90094766 | prot-a-137 | Apolipoprotein M \|\| id:prot-a-137 | Fruit liking \|\| id:ebi-fl187-GCST90094766 | Inverse variance weighted | 10 | 0.345331664523678 | 0.290692718478284 | 0.234848704684373 | -0.224426064 | 0.915089392741114 | 1.41245830430079 | 0.798974647750383 | 2.49699845546484 | 0.998735809224318 |
| ebi-fl187-GCST90094766 | prot-a-1381 | 17-beta-hydroxysteroid dehydrogenase 14 \|\| id:prot-a-1381 | Fruit liking \|\| id:ebi-fl187-GCST90094766 | Inverse variance weighted | 10 | 0.144966402913021 | 0.290692718478284 | 0.617995727489612 | -0.424791325 | 0.714724131130458 | 1.15600073135847 | 0.653906224575977 | 2.04362283256725 | 0.998735809224318 |
| ebi-fl187-GCST90094766 | prot-a-1392 | Serine protease HTRA2, mitochondrial \|\| id:prot-a-1392 | Fruit liking \|\| id:ebi-fl187-GCST90094766 | Inverse variance weighted | 10 | -0.184147658 | 0.290692718478284 | 0.526421058837842 | -0.753905386 | 0.385610070707003 | 0.831812971325832 | 0.470525376738997 | 1.47051116363001 | 0.998735809224318 |
| ebi-fl187-GCST90094766 | prot-a-1416 | Interferon-induced protein with tetratricopeptide repeats 2 \|\| id:prot-a-1416 | Fruit liking \|\| id:ebi-fl187-GCST90094766 | Inverse variance weighted | 10 | 0.381811917174698 | 0.329524424564229 | 0.246588431803828 | -0.264055955 | 1.02767978932059 | 1.46493652981875 | 0.767930568921644 | 2.79457430560543 | 0.998735809224318 |
| ebi-fl187-GCST90094766 | prot-a-1428 | Interferon gamma \|\| id:prot-a-1428 | Fruit liking \|\| id:ebi-fl187-GCST90094766 | Inverse variance weighted | 10 | 0.172691607270628 | 0.290692718478284 | 0.55246579205176 | -0.397066121 | 0.742449335488064 | 1.18849952394941 | 0.672289571739994 | 2.10107545588149 | 0.998735809224318 |
| ebi-fl187-GCST90094766 | prot-a-1483 | Interleukin-17C \|\| id:prot-a-1483 | Fruit liking \|\| id:ebi-fl187-GCST90094766 | Inverse variance weighted | 10 | -0.424264127 | 0.290692718478284 | 0.144429334336597 | -0.994021855 | 0.145493601415315 | 0.654251053846965 | 0.370085264602881 | 1.15661033388934 | 0.998735809224318 |
| ebi-fl187-GCST90094766 | prot-a-1503 | Interleukin-1 receptor-like 2 \|\| id:prot-a-1503 | Fruit liking \|\| id:ebi-fl187-GCST90094766 | Inverse variance weighted | 10 | 0.598027406342704 | 0.301047108920212 | 0.0469788186966244 | 0.00797507285908816 | 1.18807973982632 | 1.81852804311887 | 1.00800695845961 | 3.28077521276581 | 0.998735809224318 |
| ebi-fl187-GCST90094766 | prot-a-1522 | Interleukin-31 receptor subunit alpha \|\| id:prot-a-1522 | Fruit liking \|\| id:ebi-fl187-GCST90094766 | Inverse variance weighted | 10 | 0.451486714067273 | 0.290692718478284 | 0.120389506652751 | -0.118271014 | 1.02124444228471 | 1.57064555137236 | 0.888455236042988 | 2.77664799301879 | 0.998735809224318 |
| ebi-fl187-GCST90094766 | prot-a-1540 | Interleukin-6 receptor subunit alpha \|\| id:prot-a-1540 | Fruit liking \|\| id:ebi-fl187-GCST90094766 | Inverse variance weighted | 10 | -0.159382143 | 0.290620986518272 | 0.583404062441459 | -0.728999277 | 0.410234990482001 | 0.852670454576528 | 0.482391489043417 | 1.50717191455736 | 0.998735809224318 |
| ebi-fl187-GCST90094766 | prot-a-1541 | Interleukin-6 receptor subunit alpha \|\| id:prot-a-1541 | Fruit liking \|\| id:ebi-fl187-GCST90094766 | Inverse variance weighted | 10 | -0.08700302 | 0.292298139961768 | 0.76596905809017 | -0.659907375 | 0.485901334057501 | 0.916674327027235 | 0.51689921027433 | 1.6256395930357 | 0.998735809224318 |
| ebi-fl187-GCST90094766 | prot-a-1545 | Interleukin-9 \|\| id:prot-a-1545 | Fruit liking \|\| id:ebi-fl187-GCST90094766 | Inverse variance weighted | 10 | 0.162740440679995 | 0.369705915755838 | 0.659800297861461 | -0.561883154 | 0.887364035561437 | 1.17673121849133 | 0.570134401295561 | 2.4287191887133 | 0.998735809224318 |
| ebi-fl187-GCST90094766 | prot-a-1548 | Inositol monophosphatase 3 \|\| id:prot-a-1548 | Fruit liking \|\| id:ebi-fl187-GCST90094766 | Inverse variance weighted | 10 | 0.345786503152031 | 0.290692718478284 | 0.234232804858009 | -0.223971225 | 0.915544231369467 | 1.41310089102408 | 0.799338134940995 | 2.49813444514373 | 0.998735809224318 |
| ebi-fl187-GCST90094766 | prot-a-1555 | Inhibin beta A chain:Inhibin beta B chain heterodimer \|\| id:prot-a-1555 | Fruit liking \|\| id:ebi-fl187-GCST90094766 | Inverse variance weighted | 10 | -0.239898609 | 0.290692718478284 | 0.409220997313364 | -0.809656337 | 0.329859119676875 | 0.786707622456916 | 0.445010973861073 | 1.3907721822272 | 0.998735809224318 |
| ebi-fl187-GCST90094766 | prot-a-1603 | Junctophilin-3 \|\| id:prot-a-1603 | Fruit liking \|\| id:ebi-fl187-GCST90094766 | Inverse variance weighted | 10 | -0.028168435 | 0.290692718478284 | 0.922804958967344 | -0.597926164 | 0.541589292717873 | 0.972224595873722 | 0.549950962557436 | 1.71873626773242 | 0.998735809224318 |
| ebi-fl187-GCST90094766 | prot-a-1627 | UPF0258 protein KIAA1024 \|\| id:prot-a-1627 | Fruit liking \|\| id:ebi-fl187-GCST90094766 | Inverse variance weighted | 10 | -0.038265847 | 0.290534807322622 | 0.895215015632154 | -0.60771407 | 0.531182375087598 | 0.962457040305876 | 0.54459435205098 | 1.70094227188686 | 0.998735809224318 |
| ebi-fl187-GCST90094766 | prot-a-1666 | Kallikrein-7 \|\| id:prot-a-1666 | Fruit liking \|\| id:ebi-fl187-GCST90094766 | Inverse variance weighted | 10 | 0.259490366574518 | 0.290692718478284 | 0.372038163366947 | -0.310267362 | 0.829248094791955 | 1.29626929613787 | 0.733250886852963 | 2.29159503007421 | 0.998735809224318 |
| ebi-fl187-GCST90094766 | prot-a-1678 | Importin subunit alpha-3 \|\| id:prot-a-1678 | Fruit liking \|\| id:ebi-fl187-GCST90094766 | Inverse variance weighted | 10 | -0.287340665 | 0.290692718478284 | 0.322923137532137 | -0.857098393 | 0.282417063574677 | 0.750256099571475 | 0.424391715530628 | 1.32633176931933 | 0.998735809224318 |
| ebi-fl187-GCST90094766 | prot-a-1718 | Protein LDOC1 \|\| id:prot-a-1718 | Fruit liking \|\| id:ebi-fl187-GCST90094766 | Inverse variance weighted | 10 | 0.000315756145214132 | 0.290692718478284 | 0.999133322275764 | -0.569441972 | 0.57007348436265 | 1.00031580600143 | 0.565841105755525 | 1.7683969961854 | 0.999882738470636 |
| ebi-fl187-GCST90094766 | prot-a-1723 | Leptin \|\| id:prot-a-1723 | Fruit liking \|\| id:ebi-fl187-GCST90094766 | Inverse variance weighted | 10 | -0.448936648 | 0.290692718478284 | 0.122499111041432 | -1.018694376 | 0.120821079968264 | 0.638306535249465 | 0.361066048891382 | 1.12842299682611 | 0.998735809224318 |
| ebi-fl187-GCST90094766 | prot-a-1730 | Galectin-7 \|\| id:prot-a-1730 | Fruit liking \|\| id:ebi-fl187-GCST90094766 | Inverse variance weighted | 10 | -0.154806223 | 0.333980212871542 | 0.642992294813732 | -0.80940744 | 0.499794994335429 | 0.856581147219322 | 0.445121749381141 | 1.64838330814364 | 0.998735809224318 |
| ebi-fl187-GCST90094766 | prot-a-1753 | Lipase member N \|\| id:prot-a-1753 | Fruit liking \|\| id:ebi-fl187-GCST90094766 | Inverse variance weighted | 10 | -0.116315426 | 0.290692718478284 | 0.689059378364869 | -0.686073155 | 0.453442301884664 | 0.890194388257075 | 0.503549553017885 | 1.57372009196529 | 0.998735809224318 |
| ebi-fl187-GCST90094766 | prot-a-1756 | VIP36-like protein \|\| id:prot-a-1756 | Fruit liking \|\| id:ebi-fl187-GCST90094766 | Inverse variance weighted | 10 | -0.546096681 | 0.381813788220924 | 0.152639636253041 | -1.294451706 | 0.202258344034762 | 0.579206230506666 | 0.274048082122253 | 1.22416422278804 | 0.998735809224318 |
| ebi-fl187-GCST90094766 | prot-a-1760 | Ig Kappa chain V-I region HK102- like \|\| id:prot-a-1760 | Fruit liking \|\| id:ebi-fl187-GCST90094766 | Inverse variance weighted | 10 | -0.18217329 | 0.290692718478284 | 0.53086455766105 | -0.751931018 | 0.387584438476393 | 0.833456898370845 | 0.471455284565456 | 1.47341736148363 | 0.998735809224318 |
| ebi-fl187-GCST90094766 | prot-a-179 | Neutral ceramidase \|\| id:prot-a-179 | Fruit liking \|\| id:ebi-fl187-GCST90094766 | Inverse variance weighted | 10 | -0.009550192 | 0.290692718478284 | 0.973791637071448 | -0.57930792 | 0.560207536166502 | 0.990495266206379 | 0.560285994996092 | 1.75103586586006 | 0.998955228396052 |
| ebi-fl187-GCST90094766 | prot-a-1809 | Lactotransferrin \|\| id:prot-a-1809 | Fruit liking \|\| id:ebi-fl187-GCST90094766 | Inverse variance weighted | 10 | -0.305495783 | 0.314046154098269 | 0.330665874994556 | -0.921026246 | 0.310034678557525 | 0.736758011381463 | 0.398110272493213 | 1.36347239656827 | 0.998735809224318 |
| ebi-fl187-GCST90094766 | prot-a-1818 | Lysophospholipase-like protein 1 \|\| id:prot-a-1818 | Fruit liking \|\| id:ebi-fl187-GCST90094766 | Inverse variance weighted | 10 | -0.084574981 | 0.290692718478284 | 0.771094994707035 | -0.654332709 | 0.485182747352195 | 0.918902752670204 | 0.519788797231061 | 1.62447184964921 | 0.998735809224318 |
| ebi-fl187-GCST90094766 | prot-a-1852 | MAP kinase-activated protein kinase 3 \|\| id:prot-a-1852 | Fruit liking \|\| id:ebi-fl187-GCST90094766 | Inverse variance weighted | 10 | -0.015685566 | 0.290692718478284 | 0.956967618543676 | -0.585443294 | 0.554072162710993 | 0.984436812285728 | 0.556858955010257 | 1.74032549654416 | 0.998735809224318 |
| ebi-fl187-GCST90094766 | prot-a-1856 | Megakaryocyte-associated tyrosine-protein kinase \|\| id:prot-a-1856 | Fruit liking \|\| id:ebi-fl187-GCST90094766 | Inverse variance weighted | 10 | -0.074488538 | 0.290692718478284 | 0.797761710869257 | -0.644246266 | 0.495269190493305 | 0.92821811349009 | 0.525058147205552 | 1.64093990503076 | 0.998735809224318 |
| ebi-fl187-GCST90094766 | prot-a-1890 | Beta-1,3-N-acetylglucosaminyltransferase manic fringe \|\| id:prot-a-1890 | Fruit liking \|\| id:ebi-fl187-GCST90094766 | Inverse variance weighted | 10 | 0.208634642575427 | 0.355083263887424 | 0.556824035928404 | -0.487328555 | 0.904597839794777 | 1.23199479784675 | 0.614265180109078 | 2.47093801027748 | 0.998735809224318 |
| ebi-fl187-GCST90094766 | prot-a-1919 | Matrilysin \|\| id:prot-a-1919 | Fruit liking \|\| id:ebi-fl187-GCST90094766 | Inverse variance weighted | 10 | -0.367514212 | 0.306659780767438 | 0.230744670840987 | -0.968567383 | 0.23353895783779 | 0.692453485266822 | 0.379626508194528 | 1.26306203309825 | 0.998735809224318 |
| ebi-fl187-GCST90094766 | prot-a-1923 | Multimerin-2 \|\| id:prot-a-1923 | Fruit liking \|\| id:ebi-fl187-GCST90094766 | Inverse variance weighted | 10 | 0.487114985439546 | 0.290692718478284 | 0.0937961628158147 | -0.082642743 | 1.05687271365698 | 1.62761375050164 | 0.920680008054802 | 2.87735858022924 | 0.998735809224318 |
| ebi-fl187-GCST90094766 | prot-a-1948 | Mesothelin \|\| id:prot-a-1948 | Fruit liking \|\| id:ebi-fl187-GCST90094766 | Inverse variance weighted | 10 | -0.053430423 | 0.290692718478284 | 0.854167382882277 | -0.623188152 | 0.516327304863977 | 0.947971895414831 | 0.536232120204967 | 1.67586140523043 | 0.998735809224318 |
| ebi-fl187-GCST90094766 | prot-a-195 | ATPase family AAA domain-containing protein 1 \|\| id:prot-a-195 | Fruit liking \|\| id:ebi-fl187-GCST90094766 | Inverse variance weighted | 10 | -0.560833847 | 0.290534807322622 | 0.0535634935479995 | -1.130282069 | 0.00861437544805222 | 0.57073296146506 | 0.322942151521299 | 1.0086515859519 | 0.998735809224318 |
| ebi-fl187-GCST90094766 | prot-a-1966 | Mucin-1 \|\| id:prot-a-1966 | Fruit liking \|\| id:ebi-fl187-GCST90094766 | Inverse variance weighted | 10 | -0.44172875 | 0.295759278292652 | 0.135295505126624 | -1.021416935 | 0.137959435632978 | 0.642924005057269 | 0.360084362295969 | 1.14792898431709 | 0.998735809224318 |
| ebi-fl187-GCST90094766 | prot-a-1986 | Myocardial zonula adherens protein \|\| id:prot-a-1986 | Fruit liking \|\| id:ebi-fl187-GCST90094766 | Inverse variance weighted | 10 | -0.133012121 | 0.290692718478284 | 0.647261762012648 | -0.702769849 | 0.436745607148158 | 0.875454480600051 | 0.495211740501731 | 1.54766231274362 | 0.998735809224318 |
| ebi-fl187-GCST90094766 | prot-a-2071 | Epididymal secretory protein E1 \|\| id:prot-a-2071 | Fruit liking \|\| id:ebi-fl187-GCST90094766 | Inverse variance weighted | 10 | 0.000837090727383442 | 0.290692718478284 | 0.997702381924836 | -0.568920637 | 0.57059481894482 | 1.00083744118561 | 0.566136175200238 | 1.76931916305237 | 0.999867182319574 |
| ebi-fl187-GCST90094766 | prot-a-2098 | Neuritin \|\| id:prot-a-2098 | Fruit liking \|\| id:ebi-fl187-GCST90094766 | Inverse variance weighted | 10 | -0.140907582 | 0.290692718478284 | 0.627867636411333 | -0.710665311 | 0.428850145748134 | 0.868569579044291 | 0.491317210108473 | 1.53549091731311 | 0.998735809224318 |
| ebi-fl187-GCST90094766 | prot-a-2115 | Neurotrophin-4 \|\| id:prot-a-2115 | Fruit liking \|\| id:ebi-fl187-GCST90094766 | Inverse variance weighted | 10 | 0.160724098577643 | 0.290692718478284 | 0.580331664757132 | -0.40903363 | 0.73048182679508 | 1.17436091626549 | 0.664291892049524 | 2.07608067802385 | 0.998735809224318 |
| ebi-fl187-GCST90094766 | prot-a-2118 | Netrin-1 \|\| id:prot-a-2118 | Fruit liking \|\| id:ebi-fl187-GCST90094766 | Inverse variance weighted | 10 | 0.0706843359586579 | 0.290692718478284 | 0.807882799350883 | -0.499073392 | 0.640442064176094 | 1.07324238836211 | 0.607092936181845 | 1.89731943089549 | 0.998735809224318 |
| ebi-fl187-GCST90094766 | prot-a-2120 | Netrin-G1 \|\| id:prot-a-2120 | Fruit liking \|\| id:ebi-fl187-GCST90094766 | Inverse variance weighted | 10 | 0.10074002103259 | 0.290692718478284 | 0.7289282271643 | -0.469017707 | 0.670497749250026 | 1.10598907048729 | 0.625616505151131 | 1.9552102829222 | 0.998735809224318 |
| ebi-fl187-GCST90094766 | prot-a-2145 | Olfactomedin-like protein 3 \|\| id:prot-a-2145 | Fruit liking \|\| id:ebi-fl187-GCST90094766 | Inverse variance weighted | 10 | 0.328628130951387 | 0.290692718478284 | 0.258265580028831 | -0.241129597 | 0.898385859168823 | 1.38906121066056 | 0.785739790061015 | 2.45563616781066 | 0.998735809224318 |
| ebi-fl187-GCST90094766 | prot-a-2162 | Prolyl 4-hydroxylase subunit alpha-2 \|\| id:prot-a-2162 | Fruit liking \|\| id:ebi-fl187-GCST90094766 | Inverse variance weighted | 10 | -0.406500542 | 0.290692718478284 | 0.161997273881693 | -0.97625827 | 0.163257186498087 | 0.665976734843484 | 0.376718042232706 | 1.17733944656363 | 0.998735809224318 |
| ebi-fl187-GCST90094766 | prot-a-2168 | Polyadenylate-binding protein-interacting protein 1 \|\| id:prot-a-2168 | Fruit liking \|\| id:ebi-fl187-GCST90094766 | Inverse variance weighted | 10 | 0.0735508749786106 | 0.290692718478284 | 0.800253280934046 | -0.496206853 | 0.643308603196047 | 1.07632329320353 | 0.608835688412427 | 1.90276597371168 | 0.998735809224318 |
| ebi-fl187-GCST90094766 | prot-a-219 | Beta-1,4-galactosyltransferase 5 \|\| id:prot-a-219 | Fruit liking \|\| id:ebi-fl187-GCST90094766 | Inverse variance weighted | 10 | -0.220326091 | 0.393941309656369 | 0.575966146735395 | -0.992451058 | 0.551798875714389 | 0.802257146298396 | 0.370667050196089 | 1.73637373067383 | 0.998735809224318 |
| ebi-fl187-GCST90094766 | prot-a-2190 | Pyruvate carboxylase, mitochondrial \|\| id:prot-a-2190 | Fruit liking \|\| id:ebi-fl187-GCST90094766 | Inverse variance weighted | 10 | 0.127165260174956 | 0.290692718478284 | 0.661780713354118 | -0.442592468 | 0.696922988392392 | 1.13560467250199 | 0.642368939623401 | 2.00756589035018 | 0.998735809224318 |
| ebi-fl187-GCST90094766 | prot-a-2197 | Protocadherin-9 \|\| id:prot-a-2197 | Fruit liking \|\| id:ebi-fl187-GCST90094766 | Inverse variance weighted | 10 | -0.001642129 | 0.290692718478284 | 0.995492757062951 | -0.571399858 | 0.568115598847977 | 0.998359218187252 | 0.564734337467309 | 1.76493806452341 | 0.999107451094609 |
| ebi-fl187-GCST90094766 | prot-a-2198 | Protocadherin alpha-4 \|\| id:prot-a-2198 | Fruit liking \|\| id:ebi-fl187-GCST90094766 | Inverse variance weighted | 10 | 0.171667660103082 | 0.290692718478284 | 0.554824106995156 | -0.398090068 | 0.741425388320519 | 1.18728318606737 | 0.671601535053964 | 2.09892516669877 | 0.998735809224318 |
| ebi-fl187-GCST90094766 | prot-a-2254 | Placenta growth factor \|\| id:prot-a-2254 | Fruit liking \|\| id:ebi-fl187-GCST90094766 | Inverse variance weighted | 10 | -0.414777709 | 0.32995780391574 | 0.208730865092712 | -1.061495005 | 0.231939586484191 | 0.660487084596394 | 0.34593824418628 | 1.26104354245302 | 0.998735809224318 |
| ebi-fl187-GCST90094766 | prot-a-2261 | Membrane-associated progesterone receptor component 2 \|\| id:prot-a-2261 | Fruit liking \|\| id:ebi-fl187-GCST90094766 | Inverse variance weighted | 10 | -0.062740245 | 0.290692718478284 | 0.829120166184865 | -0.632497974 | 0.507017482768026 | 0.939187400208311 | 0.53126306098253 | 1.66033183462581 | 0.998735809224318 |
| ebi-fl187-GCST90094766 | prot-a-2268 | Polymeric immunoglobulin receptor \|\| id:prot-a-2268 | Fruit liking \|\| id:ebi-fl187-GCST90094766 | Inverse variance weighted | 10 | -0.053450964 | 0.290692718478284 | 0.854111948829995 | -0.623208692 | 0.516306764430446 | 0.947952423861102 | 0.536221105877865 | 1.67582698266415 | 0.998735809224318 |
| ebi-fl187-GCST90094766 | prot-a-2283 | Group 10 secretory phospholipase A2 \|\| id:prot-a-2283 | Fruit liking \|\| id:ebi-fl187-GCST90094766 | Inverse variance weighted | 10 | 0.217823487633964 | 0.290692718478284 | 0.453660998328317 | -0.351934241 | 0.7875812158514 | 1.24336757849523 | 0.703326370787448 | 2.19807332621727 | 0.998735809224318 |
| ebi-fl187-GCST90094766 | prot-a-2298 | Inactive phospholipase D5 \|\| id:prot-a-2298 | Fruit liking \|\| id:ebi-fl187-GCST90094766 | Inverse variance weighted (multiplicative random effects) | 10 | -0.080946192 | 0.401011175793443 | 0.84002992365474 | -0.866928097 | 0.705035712114273 | 0.922243313771201 | 0.420240506532558 | 2.02391896205701 | 0.998735809224318 |
| ebi-fl187-GCST90094766 | prot-a-2304 | Procollagen-lysine,2-oxoglutarate 5-dioxygenase 2 \|\| id:prot-a-2304 | Fruit liking \|\| id:ebi-fl187-GCST90094766 | Inverse variance weighted | 10 | -0.371821825 | 0.290692718478284 | 0.20086575710923 | -0.941579553 | 0.197935902945398 | 0.689477078971704 | 0.390011304847741 | 1.21888426442649 | 0.998735809224318 |
| ebi-fl187-GCST90094766 | prot-a-2331 | Periostin \|\| id:prot-a-2331 | Fruit liking \|\| id:ebi-fl187-GCST90094766 | Inverse variance weighted | 10 | 0.066118425185584 | 0.319831562594718 | 0.836221590698331 | -0.560751438 | 0.692988287871232 | 1.0683532296042 | 0.57077999716645 | 1.99968223986812 | 0.998735809224318 |
| ebi-fl187-GCST90094766 | prot-a-2333 | Periostin \|\| id:prot-a-2333 | Fruit liking \|\| id:ebi-fl187-GCST90094766 | Inverse variance weighted | 10 | 0.200660004426674 | 0.334688271847739 | 0.548810856769683 | -0.455329008 | 0.856649017248242 | 1.22220915547093 | 0.634239263575277 | 2.35525503624023 | 0.998735809224318 |
| ebi-fl187-GCST90094766 | prot-a-2367 | cAMP-dependent protein kinase catalytic subunit alpha \|\| id:prot-a-2367 | Fruit liking \|\| id:ebi-fl187-GCST90094766 | Inverse variance weighted | 10 | -0.153311725 | 0.290692718478284 | 0.59791492392645 | -0.723069453 | 0.416446003466438 | 0.857862263226434 | 0.485260483437055 | 1.51656211001455 | 0.998735809224318 |
| ebi-fl187-GCST90094766 | prot-a-2385 | Phosphoribosyl pyrophosphate synthase-associated protein 1 \|\| id:prot-a-2385 | Fruit liking \|\| id:ebi-fl187-GCST90094766 | Inverse variance weighted | 10 | -0.30244339 | 0.396889321024041 | 0.446039361485206 | -1.080346459 | 0.475459679249939 | 0.739010322480814 | 0.339477890042067 | 1.60875353816274 | 0.998735809224318 |
| ebi-fl187-GCST90094766 | prot-a-2393 | Trypsin-3 \|\| id:prot-a-2393 | Fruit liking \|\| id:ebi-fl187-GCST90094766 | Inverse variance weighted | 10 | -0.175411899 | 0.290692718478284 | 0.546224518886011 | -0.745169627 | 0.394345829308852 | 0.83911132054021 | 0.474653778954455 | 1.48341346783272 | 0.998735809224318 |
| ebi-fl187-GCST90094766 | prot-a-2405 | Pregnancy-specific beta-1-glycoprotein 6 \|\| id:prot-a-2405 | Fruit liking \|\| id:ebi-fl187-GCST90094766 | Inverse variance weighted | 10 | 0.2640078032765 | 0.290692718478284 | 0.36377133505067 | -0.305749925 | 0.833765531493936 | 1.30213835720689 | 0.736570794411276 | 2.30197058337711 | 0.998735809224318 |
| ebi-fl187-GCST90094766 | prot-a-244 | Beclin-1 \|\| id:prot-a-244 | Fruit liking \|\| id:ebi-fl187-GCST90094766 | Inverse variance weighted | 10 | -0.103211283 | 0.290692718478284 | 0.722549986624879 | -0.672969011 | 0.466546445422586 | 0.901936389707495 | 0.510191562515912 | 1.59447805656962 | 0.998735809224318 |
| ebi-fl187-GCST90094766 | prot-a-2454 | tRNA pseudouridine synthase A, mitochondrial \|\| id:prot-a-2454 | Fruit liking \|\| id:ebi-fl187-GCST90094766 | Inverse variance weighted | 10 | 0.0854337651303653 | 0.290692718478284 | 0.76883648637979 | -0.484323963 | 0.655191493347802 | 1.08918941663583 | 0.61611357152298 | 1.92551120466146 | 0.998735809224318 |
| ebi-fl187-GCST90094766 | prot-a-2466 | Glutaminyl-peptide cyclotransferase-like protein \|\| id:prot-a-2466 | Fruit liking \|\| id:ebi-fl187-GCST90094766 | Inverse variance weighted | 9 | 0.537811732748114 | 0.306347260647144 | 0.0791631567914472 | -0.062628898 | 1.13825236361652 | 1.71225588613016 | 0.939291982039216 | 3.12130868318747 | 0.998735809224318 |
| ebi-fl187-GCST90094766 | prot-a-2474 | Ras-related protein Rab-27A \|\| id:prot-a-2474 | Fruit liking \|\| id:ebi-fl187-GCST90094766 | Inverse variance weighted | 10 | 0.398369710014205 | 0.389771738824597 | 0.306752981128895 | -0.365582898 | 1.16232231811042 | 1.48939457221896 | 0.693792122892395 | 3.1973499245095 | 0.998735809224318 |
| ebi-fl187-GCST90094766 | prot-a-2490 | Rap1 GTPase-GDP dissociation stimulator 1 \|\| id:prot-a-2490 | Fruit liking \|\| id:ebi-fl187-GCST90094766 | Inverse variance weighted | 10 | -0.007233473 | 0.361221636507544 | 0.984023408536044 | -0.71522788 | 0.700760934815297 | 0.9927926258587 | 0.489080647254706 | 2.01528562516625 | 0.998955228396052 |
| ebi-fl187-GCST90094766 | prot-a-2545 | Receptor-interacting serine/threonine-protein kinase 2 \|\| id:prot-a-2545 | Fruit liking \|\| id:ebi-fl187-GCST90094766 | Inverse variance weighted | 10 | 0.0656429929624443 | 0.339320991428313 | 0.84660345152126 | -0.59942615 | 0.730712136161937 | 1.06784542077706 | 0.549126661901402 | 2.07655887391473 | 0.998735809224318 |
| ebi-fl187-GCST90094766 | prot-a-2610 | RUN and FYVE domain-containing protein 1 \|\| id:prot-a-2610 | Fruit liking \|\| id:ebi-fl187-GCST90094766 | Inverse variance weighted | 10 | -0.038270656 | 0.290692718478284 | 0.895258523657354 | -0.608028384 | 0.531487072016482 | 0.962452411922489 | 0.544423204883872 | 1.70146062273926 | 0.998735809224318 |
| ebi-fl187-GCST90094766 | prot-a-2627 | Serine--tRNA ligase, mitochondrial \|\| id:prot-a-2627 | Fruit liking \|\| id:ebi-fl187-GCST90094766 | Inverse variance weighted | 10 | -0.551274642 | 0.290692718478284 | 0.0579049126465012 | -1.12103237 | 0.0184830864268807 | 0.576214874530601 | 0.325943126961556 | 1.01865495592879 | 0.998735809224318 |
| ebi-fl187-GCST90094766 | prot-a-264 | BolA-like protein 2 \|\| id:prot-a-264 | Fruit liking \|\| id:ebi-fl187-GCST90094766 | Inverse variance weighted | 10 | 0.196079799580365 | 0.31901643796466 | 0.538793452540229 | -0.429192419 | 0.821352017991097 | 1.21662398754437 | 0.651034645802904 | 2.27357167028017 | 0.998735809224318 |
| ebi-fl187-GCST90094766 | prot-a-2646 | Secretoglobin family 1C member 1 \|\| id:prot-a-2646 | Fruit liking \|\| id:ebi-fl187-GCST90094766 | Inverse variance weighted | 10 | -0.190657333 | 0.366023994352852 | 0.602445124525906 | -0.908064362 | 0.526749695510159 | 0.826415724686563 | 0.403304119629632 | 1.69341922576046 | 0.998735809224318 |
| ebi-fl187-GCST90094766 | prot-a-2672 | Semaphorin-3E \|\| id:prot-a-2672 | Fruit liking \|\| id:ebi-fl187-GCST90094766 | Inverse variance weighted | 10 | 0.379121371761982 | 0.290692718478284 | 0.192165483944193 | -0.190636356 | 0.948879099979418 | 1.46100034916943 | 0.826433060562929 | 2.58281296106335 | 0.998735809224318 |
| ebi-fl187-GCST90094766 | prot-a-2701 | Plasma protease C1 inhibitor \|\| id:prot-a-2701 | Fruit liking \|\| id:ebi-fl187-GCST90094766 | Inverse variance weighted | 10 | 0.400987827654433 | 0.290692718478284 | 0.167764789698602 | -0.168769901 | 0.97074555587187 | 1.49329909143676 | 0.844703246767536 | 2.63991192767313 | 0.998735809224318 |
| ebi-fl187-GCST90094766 | prot-a-2738 | Signaling threshold-regulating transmembrane adapter 1 \|\| id:prot-a-2738 | Fruit liking \|\| id:ebi-fl187-GCST90094766 | Inverse variance weighted | 10 | -0.310748658 | 0.380192686591794 | 0.41373119776682 | -1.055926323 | 0.434429008115788 | 0.732898061076189 | 0.347870037881004 | 1.54408114938883 | 0.998735809224318 |
| ebi-fl187-GCST90094766 | prot-a-274 | Serine/threonine-protein kinase BRSK2 \|\| id:prot-a-274 | Fruit liking \|\| id:ebi-fl187-GCST90094766 | Inverse variance weighted | 10 | -0.313310898 | 0.301918587790261 | 0.299394352649317 | -0.90507133 | 0.278449534451339 | 0.731022604049883 | 0.404513030286299 | 1.32107993468999 | 0.998735809224318 |
| ebi-fl187-GCST90094766 | prot-a-2749 | Mitochondrial glutamate carrier 2 \|\| id:prot-a-2749 | Fruit liking \|\| id:ebi-fl187-GCST90094766 | Inverse variance weighted | 10 | -0.57277445 | 0.290692718478284 | 0.0487950994416646 | -1.142532178 | -0.003016722 | 0.56395859128624 | 0.31901020755567 | 0.99698782406534 | 0.998735809224318 |
| ebi-fl187-GCST90094766 | prot-a-2760 | Sodium-coupled monocarboxylate transporter 1 \|\| id:prot-a-2760 | Fruit liking \|\| id:ebi-fl187-GCST90094766 | Inverse variance weighted | 10 | -0.234766689 | 0.351607496437005 | 0.504328081825403 | -0.923917382 | 0.454384004265848 | 0.790755320220032 | 0.39696094368279 | 1.57520276593245 | 0.998735809224318 |
| ebi-fl187-GCST90094766 | prot-a-2777 | Small integral membrane protein 10 \|\| id:prot-a-2777 | Fruit liking \|\| id:ebi-fl187-GCST90094766 | Inverse variance weighted | 10 | -0.024632989 | 0.290534807322622 | 0.932432330301369 | -0.594081212 | 0.544815232893599 | 0.975667926740611 | 0.552069567916882 | 1.72428976091224 | 0.998735809224318 |
| ebi-fl187-GCST90094766 | prot-a-2799 | Superoxide dismutase [Mn], mitochondrial \|\| id:prot-a-2799 | Fruit liking \|\| id:ebi-fl187-GCST90094766 | Inverse variance weighted | 10 | 0.642971924500496 | 0.358227034507595 | 0.0726739844954075 | -0.059153063 | 1.34509691213538 | 1.90212546077529 | 0.942562486546658 | 3.83855852547822 | 0.998735809224318 |
| ebi-fl187-GCST90094766 | prot-a-2805 | Protein SPACA7 \|\| id:prot-a-2805 | Fruit liking \|\| id:ebi-fl187-GCST90094766 | Inverse variance weighted | 10 | -0.155313156 | 0.290692718478284 | 0.59314342482679 | -0.725070884 | 0.41444457256198 | 0.856147028215787 | 0.484290239370899 | 1.5135298511795 | 0.998735809224318 |
| ebi-fl187-GCST90094766 | prot-a-2811 | SPARC-like protein 1 \|\| id:prot-a-2811 | Fruit liking \|\| id:ebi-fl187-GCST90094766 | Inverse variance weighted | 10 | 0.357222033312923 | 0.290692718478284 | 0.219122521667156 | -0.212535695 | 0.926979761530359 | 1.42935319864983 | 0.808531455353266 | 2.52686590356318 | 0.998735809224318 |
| ebi-fl187-GCST90094766 | prot-a-2831 | Sepiapterin reductase \|\| id:prot-a-2831 | Fruit liking \|\| id:ebi-fl187-GCST90094766 | Inverse variance weighted | 10 | 0.080951802289767 | 0.290692718478284 | 0.780644723590918 | -0.488805926 | 0.650709530507203 | 1.08431863363199 | 0.613358352396929 | 1.91690044595769 | 0.998735809224318 |
| ebi-fl187-GCST90094766 | prot-a-2834 | SPRY domain-containing SOCS box protein 1 \|\| id:prot-a-2834 | Fruit liking \|\| id:ebi-fl187-GCST90094766 | Inverse variance weighted | 10 | -0.022674207 | 0.290692718478284 | 0.937827573999868 | -0.592431935 | 0.547083521132878 | 0.97758092083647 | 0.552980834545386 | 1.72820538630267 | 0.998735809224318 |
| ebi-fl187-GCST90094766 | prot-a-2835 | Spectrin alpha chain, non-erythrocytic 1 \|\| id:prot-a-2835 | Fruit liking \|\| id:ebi-fl187-GCST90094766 | Inverse variance weighted | 10 | 0.15258770453353 | 0.290692718478284 | 0.599645303785259 | -0.417170024 | 0.722345432750967 | 1.16484461976872 | 0.658908880304507 | 2.05925740077606 | 0.998735809224318 |
| ebi-fl187-GCST90094766 | prot-a-2859 | Sia-alpha-2,3-Gal-beta-1,4-GlcNAc-R:alpha 2,8-sialyltransferase \|\| id:prot-a-2859 | Fruit liking \|\| id:ebi-fl187-GCST90094766 | Inverse variance weighted | 10 | 0.0539537417339032 | 0.290692718478284 | 0.852755288433276 | -0.515803986 | 0.62371146995134 | 1.05543577839675 | 0.597020405274998 | 1.86584021664529 | 0.998735809224318 |
| ebi-fl187-GCST90094766 | prot-a-2861 | Alpha-2,8-sialyltransferase 8F \|\| id:prot-a-2861 | Fruit liking \|\| id:ebi-fl187-GCST90094766 | Inverse variance weighted | 10 | 0.252405228769688 | 0.290692718478284 | 0.385236257447035 | -0.317352499 | 0.822162956987125 | 1.28711750866458 | 0.728074064181108 | 2.27541614598542 | 0.998735809224318 |
| ebi-fl187-GCST90094766 | prot-a-289 | Uncharacterized protein C17orf89 \|\| id:prot-a-289 | Fruit liking \|\| id:ebi-fl187-GCST90094766 | Inverse variance weighted | 10 | 0.0660124332365189 | 0.322770580001832 | 0.837948631047585 | -0.566617904 | 0.698642770040109 | 1.06823999876399 | 0.567441338320868 | 2.01102143586518 | 0.998735809224318 |
| ebi-fl187-GCST90094766 | prot-a-2921 | Tachykinin-3 \|\| id:prot-a-2921 | Fruit liking \|\| id:ebi-fl187-GCST90094766 | Inverse variance weighted | 10 | -0.445899426 | 0.290692718478284 | 0.12504929197962 | -1.015657154 | 0.123858302591145 | 0.640248161381484 | 0.362164353917569 | 1.13185547864733 | 0.998735809224318 |
| ebi-fl187-GCST90094766 | prot-a-2928 | Tax1-binding protein 1 \|\| id:prot-a-2928 | Fruit liking \|\| id:ebi-fl187-GCST90094766 | Inverse variance weighted | 10 | -0.187536421 | 0.295531285871332 | 0.525706397681445 | -0.766777742 | 0.391704898970513 | 0.828998924381111 | 0.464507422343418 | 1.47950104469364 | 0.998735809224318 |
| ebi-fl187-GCST90094766 | prot-a-296 | Uncharacterized protein C1orf185 \|\| id:prot-a-296 | Fruit liking \|\| id:ebi-fl187-GCST90094766 | Inverse variance weighted | 10 | -0.31199628 | 0.318594216410329 | 0.327436478154351 | -0.936440944 | 0.312448384402572 | 0.731984251379115 | 0.392020578684314 | 1.36676739283759 | 0.998735809224318 |
| ebi-fl187-GCST90094766 | prot-a-2964 | Transforming growth factor beta-2 \|\| id:prot-a-2964 | Fruit liking \|\| id:ebi-fl187-GCST90094766 | Inverse variance weighted | 10 | -0.456724075 | 0.290692718478284 | 0.116146045267953 | -1.026481803 | 0.113033653400964 | 0.633355074570015 | 0.35826519343238 | 1.11966961300499 | 0.998735809224318 |
| ebi-fl187-GCST90094766 | prot-a-2977 | Thymocyte nuclear protein 1 \|\| id:prot-a-2977 | Fruit liking \|\| id:ebi-fl187-GCST90094766 | Inverse variance weighted | 10 | -0.153326193 | 0.36730061771754 | 0.676356158013629 | -0.873235404 | 0.566583017729931 | 0.857849851554437 | 0.417598262240929 | 1.76223522546029 | 0.998735809224318 |
| ebi-fl187-GCST90094766 | prot-a-3015 | Transmembrane protein 70, mitochondrial \|\| id:prot-a-3015 | Fruit liking \|\| id:ebi-fl187-GCST90094766 | Inverse variance weighted | 10 | -0.296028051 | 0.290692718478284 | 0.30850982194247 | -0.86578578 | 0.273729676877102 | 0.743766564078532 | 0.420720828879496 | 1.31485931731616 | 0.998735809224318 |
| ebi-fl187-GCST90094766 | prot-a-3030 | Tumor necrosis factor alpha-induced protein 3 \|\| id:prot-a-3030 | Fruit liking \|\| id:ebi-fl187-GCST90094766 | Inverse variance weighted | 10 | 0.0819094959375677 | 0.290692718478284 | 0.778117215004937 | -0.487848232 | 0.651667224155004 | 1.08535757611451 | 0.613946043163784 | 1.91873712868756 | 0.998735809224318 |
| ebi-fl187-GCST90094766 | prot-a-3032 | Tumor necrosis factor receptor superfamily member 10A \|\| id:prot-a-3032 | Fruit liking \|\| id:ebi-fl187-GCST90094766 | Inverse variance weighted | 10 | -0.095318894 | 0.290692718478284 | 0.742985318384347 | -0.665076622 | 0.474438834091381 | 0.909082987014762 | 0.51423412437335 | 1.60711208787783 | 0.998735809224318 |
| ebi-fl187-GCST90094766 | prot-a-3081 | Tumor protein p53-inducible protein 11 \|\| id:prot-a-3081 | Fruit liking \|\| id:ebi-fl187-GCST90094766 | Inverse variance weighted | 10 | -0.051929232 | 0.338598831038995 | 0.878110392626332 | -0.715582941 | 0.611724476936727 | 0.949396051420588 | 0.48890702488903 | 1.84360791841269 | 0.998735809224318 |
| ebi-fl187-GCST90094766 | prot-a-315 | Complement C4 \|\| id:prot-a-315 | Fruit liking \|\| id:ebi-fl187-GCST90094766 | Inverse variance weighted | 10 | -0.050357732 | 0.290692718478284 | 0.862467851115469 | -0.620115461 | 0.519399995862998 | 0.950889199817393 | 0.537882329807842 | 1.68101872885912 | 0.998735809224318 |
| ebi-fl187-GCST90094766 | prot-a-3157 | UDP-glucuronosyltransferase 1-8 \|\| id:prot-a-3157 | Fruit liking \|\| id:ebi-fl187-GCST90094766 | Inverse variance weighted | 10 | 0.204463541099026 | 0.332285282705028 | 0.538339912901695 | -0.446815613 | 0.85574269520088 | 1.22686672481791 | 0.639661842728063 | 2.35312138370795 | 0.998735809224318 |
| ebi-fl187-GCST90094766 | prot-a-317 | Glutaredoxin-like protein C5orf63 \|\| id:prot-a-317 | Fruit liking \|\| id:ebi-fl187-GCST90094766 | Inverse variance weighted | 10 | -0.249435986 | 0.304009486901619 | 0.411938248284165 | -0.84529458 | 0.346422608314928 | 0.779240161502875 | 0.429430837694501 | 1.41400005774853 | 0.998735809224318 |
| ebi-fl187-GCST90094766 | prot-a-3192 | Guanine nucleotide exchange factor VAV3 \|\| id:prot-a-3192 | Fruit liking \|\| id:ebi-fl187-GCST90094766 | Inverse variance weighted | 10 | -0.150317737 | 0.290692718478284 | 0.605085074883231 | -0.720075465 | 0.419439990966314 | 0.860434540881239 | 0.486715524358905 | 1.52110948200529 | 0.998735809224318 |
| ebi-fl187-GCST90094766 | prot-a-3233 | Serine/threonine-protein kinase WNK3 \|\| id:prot-a-3233 | Fruit liking \|\| id:ebi-fl187-GCST90094766 | Inverse variance weighted | 10 | -0.094358716 | 0.291171936131944 | 0.745888610928604 | -0.66505571 | 0.476338279309145 | 0.909956288255095 | 0.514244878157729 | 1.61016761022755 | 0.998735809224318 |
| ebi-fl187-GCST90094766 | prot-a-3250 | 14-3-3 protein theta \|\| id:prot-a-3250 | Fruit liking \|\| id:ebi-fl187-GCST90094766 | Inverse variance weighted | 10 | -0.244818311 | 0.348906984207778 | 0.482883649337116 | -0.928676 | 0.439039377699153 | 0.782846759693885 | 0.395076445302211 | 1.55121636951659 | 0.998735809224318 |
| ebi-fl187-GCST90094766 | prot-a-3278 | E3 ubiquitin-protein ligase ZNRF3 \|\| id:prot-a-3278 | Fruit liking \|\| id:ebi-fl187-GCST90094766 | Inverse variance weighted | 10 | 0.315574068547362 | 0.290620986518272 | 0.277540381735438 | -0.254043065 | 0.885191202123174 | 1.37104615956005 | 0.775658397576239 | 2.42344771553848 | 0.998735809224318 |
| ebi-fl187-GCST90094766 | prot-a-355 | Calpain-3 \|\| id:prot-a-355 | Fruit liking \|\| id:ebi-fl187-GCST90094766 | Inverse variance weighted | 10 | -0.226207125 | 0.389158478711788 | 0.561056806862508 | -0.988957743 | 0.536543493686092 | 0.797552891741829 | 0.371964171371795 | 1.71008571277139 | 0.998735809224318 |
| ebi-fl187-GCST90094766 | prot-a-385 | Coiled-coil domain-containing protein 90B, mitochondrial \|\| id:prot-a-385 | Fruit liking \|\| id:ebi-fl187-GCST90094766 | Inverse variance weighted | 10 | 0.202245905413097 | 0.290692718478284 | 0.486593063630054 | -0.367511823 | 0.772003633630533 | 1.22414899596712 | 0.692455139998614 | 2.1640979722243 | 0.998735809224318 |
| ebi-fl187-GCST90094766 | prot-a-390 | C-C motif chemokine 14 \|\| id:prot-a-390 | Fruit liking \|\| id:ebi-fl187-GCST90094766 | Inverse variance weighted | 10 | 0.460798145912875 | 0.290692718478284 | 0.112927113502158 | -0.108959582 | 1.03055587413031 | 1.58533881186235 | 0.896766662007595 | 2.80262330757322 | 0.998735809224318 |
| ebi-fl187-GCST90094766 | prot-a-40 | Pituitary adenylate cyclase-activating polypeptide 27 \|\| id:prot-a-40 | Fruit liking \|\| id:ebi-fl187-GCST90094766 | Inverse variance weighted | 10 | 0.0635380766274481 | 0.290692718478284 | 0.826981354979763 | -0.506219652 | 0.633295804844885 | 1.06560005948548 | 0.60276995758233 | 1.8838090261331 | 0.998735809224318 |
| ebi-fl187-GCST90094766 | prot-a-401 | C-C motif chemokine 24 \|\| id:prot-a-401 | Fruit liking \|\| id:ebi-fl187-GCST90094766 | Inverse variance weighted | 10 | 1.00451846137393e-06 | 0.290692718478284 | 0.999997242828181 | -0.569756724 | 0.569758732735898 | 1.00000100451897 | 0.565663034372604 | 1.76784047794121 | 0.999997242828181 |
| ebi-fl187-GCST90094766 | prot-a-412 | Cerebral cavernous malformations 2 protein \|\| id:prot-a-412 | Fruit liking \|\| id:ebi-fl187-GCST90094766 | Inverse variance weighted | 10 | 0.0672567662462954 | 0.290692718478284 | 0.81702952870614 | -0.502500962 | 0.637014494463732 | 1.06957007241247 | 0.605015644885273 | 1.89082736863332 | 0.998735809224318 |
| ebi-fl187-GCST90094766 | prot-a-44 | Adhesion G protein-coupled receptor F5 \|\| id:prot-a-44 | Fruit liking \|\| id:ebi-fl187-GCST90094766 | Inverse variance weighted | 10 | 0.11127980745231 | 0.290692718478284 | 0.701861340457041 | -0.458477921 | 0.681037535669747 | 1.11770760602231 | 0.632245240861582 | 1.9759267635731 | 0.998735809224318 |
| ebi-fl187-GCST90094766 | prot-a-48 | Adrenomedullin \|\| id:prot-a-48 | Fruit liking \|\| id:ebi-fl187-GCST90094766 | Inverse variance weighted | 10 | 0.101976934696792 | 0.290692718478284 | 0.725733418369716 | -0.467780794 | 0.671734662914228 | 1.10735792988696 | 0.626390817534965 | 1.95763020554668 | 0.998735809224318 |
| ebi-fl187-GCST90094766 | prot-a-502 | Carcinoembryonic antigen-related cell adhesion molecule 1 \|\| id:prot-a-502 | Fruit liking \|\| id:ebi-fl187-GCST90094766 | Inverse variance weighted | 10 | -0.14005945 | 0.290692718478284 | 0.629938991789491 | -0.709817178 | 0.429698278555937 | 0.869306553882229 | 0.491734089112795 | 1.5367937699541 | 0.998735809224318 |
| ebi-fl187-GCST90094766 | prot-a-518 | Complement factor B \|\| id:prot-a-518 | Fruit liking \|\| id:ebi-fl187-GCST90094766 | Inverse variance weighted | 10 | 0.318126315374096 | 0.299637440015944 | 0.288370012020707 | -0.269163067 | 0.905415697805347 | 1.37454987704409 | 0.764018659215319 | 2.47295971334314 | 0.998735809224318 |
| ebi-fl187-GCST90094766 | prot-a-525 | Properdin \|\| id:prot-a-525 | Fruit liking \|\| id:ebi-fl187-GCST90094766 | Inverse variance weighted (multiplicative random effects) | 10 | -0.162545864 | 0.465316961310762 | 0.726846918582685 | -1.074567108 | 0.749475380196055 | 0.849977106004347 | 0.341445532303796 | 2.11588968775473 | 0.998735809224318 |
| ebi-fl187-GCST90094766 | prot-a-533 | Chondroadherin \|\| id:prot-a-533 | Fruit liking \|\| id:ebi-fl187-GCST90094766 | Inverse variance weighted | 10 | 0.353124071775046 | 0.304515559105217 | 0.246201228198778 | -0.243726424 | 0.949974567621272 | 1.42350774961074 | 0.783702006942391 | 2.58564389940473 | 0.998735809224318 |
| ebi-fl187-GCST90094766 | prot-a-535 | Coiled-coil-helix-coiled-coil-helix domain-containing protein 2 \|\| id:prot-a-535 | Fruit liking \|\| id:ebi-fl187-GCST90094766 | Inverse variance weighted | 10 | 0.0343089779991347 | 0.305897182247893 | 0.910697710661779 | -0.565249499 | 0.633867455205004 | 1.03490431999948 | 0.568218359040482 | 1.8848862140994 | 0.998735809224318 |
| ebi-fl187-GCST90094766 | prot-a-591 | Caseinolytic peptidase B protein homolog \|\| id:prot-a-591 | Fruit liking \|\| id:ebi-fl187-GCST90094766 | Inverse variance weighted | 10 | 0.152721069141825 | 0.296046587093034 | 0.605946349568743 | -0.427530242 | 0.732972379844172 | 1.16499997917465 | 0.652117680641632 | 2.08125771124857 | 0.998735809224318 |
| ebi-fl187-GCST90094766 | prot-a-605 | Ciliary neurotrophic factor receptor subunit alpha \|\| id:prot-a-605 | Fruit liking \|\| id:ebi-fl187-GCST90094766 | Inverse variance weighted | 10 | 0.451354066127933 | 0.290692718478284 | 0.120498537072981 | -0.118403662 | 1.02111179434537 | 1.57043722229406 | 0.888337392102782 | 2.77627970081138 | 0.998735809224318 |
| ebi-fl187-GCST90094766 | prot-a-606 | Ciliary neurotrophic factor receptor subunit alpha \|\| id:prot-a-606 | Fruit liking \|\| id:ebi-fl187-GCST90094766 | Inverse variance weighted | 10 | 0.314423493611621 | 0.290692718478284 | 0.279414631220906 | -0.255334235 | 0.884181221829058 | 1.36946957537617 | 0.774657537330059 | 2.42100131671722 | 0.998735809224318 |
| ebi-fl187-GCST90094766 | prot-a-707 | Cancer/testis antigen 55 \|\| id:prot-a-707 | Fruit liking \|\| id:ebi-fl187-GCST90094766 | Inverse variance weighted | 10 | -0.083445797 | 0.290692718478284 | 0.774067576313856 | -0.653203525 | 0.486311930964137 | 0.919940948646115 | 0.520376065727146 | 1.6263072126758 | 0.998735809224318 |
| ebi-fl187-GCST90094766 | prot-a-719 | Dipeptidyl peptidase 1 \|\| id:prot-a-719 | Fruit liking \|\| id:ebi-fl187-GCST90094766 | Inverse variance weighted | 10 | 0.357418336850109 | 0.290692718478284 | 0.218869394996901 | -0.212339391 | 0.927176065067545 | 1.42963381328053 | 0.808690188517309 | 2.52736198496772 | 0.998735809224318 |
| ebi-fl187-GCST90094766 | prot-a-752 | Cytochrome b5 \|\| id:prot-a-752 | Fruit liking \|\| id:ebi-fl187-GCST90094766 | Inverse variance weighted | 10 | -0.246736243 | 0.290692718478284 | 0.395999743714889 | -0.816493971 | 0.323021485217873 | 0.781346752031113 | 0.441978530675281 | 1.38129502801144 | 0.998735809224318 |
| ebi-fl187-GCST90094766 | prot-a-755 | Cytoglobin \|\| id:prot-a-755 | Fruit liking \|\| id:ebi-fl187-GCST90094766 | Inverse variance weighted | 10 | 0.510581159167306 | 0.368142904399009 | 0.165469138892782 | -0.210978933 | 1.23214125178936 | 1.66625927546725 | 0.809791126202987 | 3.42856309885605 | 0.998735809224318 |
| ebi-fl187-GCST90094766 | prot-a-807 | Desmin \|\| id:prot-a-807 | Fruit liking \|\| id:ebi-fl187-GCST90094766 | Inverse variance weighted | 10 | 0.128436084767932 | 0.290692718478284 | 0.658613918337749 | -0.441321643 | 0.698193812985369 | 1.13704874423429 | 0.643185796800799 | 2.01011877624718 | 0.998735809224318 |
| ebi-fl187-GCST90094766 | prot-a-82 | Ameloblastin \|\| id:prot-a-82 | Fruit liking \|\| id:ebi-fl187-GCST90094766 | Inverse variance weighted | 10 | -0.078625603 | 0.29790276644438 | 0.791833532439949 | -0.662515025 | 0.505263819081628 | 0.924385946853476 | 0.515553073531552 | 1.65742272252772 | 0.998735809224318 |
| ebi-fl187-GCST90094766 | prot-a-84 | Muellerian-inhibiting factor \|\| id:prot-a-84 | Fruit liking \|\| id:ebi-fl187-GCST90094766 | Inverse variance weighted | 10 | 0.561505531066131 | 0.290692718478284 | 0.0534074188137192 | -0.008252197 | 1.13126325928357 | 1.75331017718595 | 0.991781758759786 | 3.09956958803916 | 0.998735809224318 |
| ebi-fl187-GCST90094766 | prot-a-858 | Inactive dipeptidyl peptidase 10 \|\| id:prot-a-858 | Fruit liking \|\| id:ebi-fl187-GCST90094766 | Inverse variance weighted | 10 | -0.177786776 | 0.353597590718159 | 0.615109054458478 | -0.870838054 | 0.515264501586579 | 0.837120898541986 | 0.418600592294987 | 1.67408124038656 | 0.998735809224318 |
| ebi-fl187-GCST90094766 | prot-a-863 | Dihydropyrimidinase-related protein 3 \|\| id:prot-a-863 | Fruit liking \|\| id:ebi-fl187-GCST90094766 | Inverse variance weighted | 10 | 0.0709083770645793 | 0.290692718478284 | 0.807285827512379 | -0.498849351 | 0.640666105282016 | 1.07348286571112 | 0.60722896519214 | 1.89774455606005 | 0.998735809224318 |
| ebi-fl187-GCST90094766 | prot-a-864 | Dihydropyrimidinase-related protein 5 \|\| id:prot-a-864 | Fruit liking \|\| id:ebi-fl187-GCST90094766 | Inverse variance weighted | 10 | -0.249813043 | 0.290692718478284 | 0.390135578088875 | -0.819570771 | 0.319944685336353 | 0.778946399033564 | 0.440620741079048 | 1.37705159108364 | 0.998735809224318 |
| ebi-fl187-GCST90094766 | prot-a-884 | Endothelin-converting enzyme 1 \|\| id:prot-a-884 | Fruit liking \|\| id:ebi-fl187-GCST90094766 | Inverse variance weighted | 10 | 0.00557720010008574 | 0.290534807322622 | 0.984684490620857 | -0.563871022 | 0.575025422452424 | 1.00559278163421 | 0.569002175065579 | 1.7771757065046 | 0.998955228396052 |
| ebi-fl187-GCST90094766 | prot-a-893 | Early endosome antigen 1 \|\| id:prot-a-893 | Fruit liking \|\| id:ebi-fl187-GCST90094766 | Inverse variance weighted | 10 | 0.0853092683849795 | 0.290692718478284 | 0.76916377809707 | -0.48444846 | 0.655066996602416 | 1.08905382453891 | 0.616036872163047 | 1.92527149970483 | 0.998735809224318 |
| ebi-fl187-GCST90094766 | prot-a-897 | Ephrin-A2 \|\| id:prot-a-897 | Fruit liking \|\| id:ebi-fl187-GCST90094766 | Inverse variance weighted | 10 | 0.360814426888107 | 0.396170811386446 | 0.362424637839976 | -0.415680363 | 1.13730921720554 | 1.43449723206151 | 0.659891162126333 | 3.11836621991034 | 0.998735809224318 |
| ebi-fl187-GCST90094766 | prot-a-90 | Anaphase-promoting complex subunit 10 \|\| id:prot-a-90 | Fruit liking \|\| id:ebi-fl187-GCST90094766 | Inverse variance weighted | 10 | -0.371339716 | 0.290692718478284 | 0.201450337951869 | -0.941097444 | 0.198418012653845 | 0.689809562705606 | 0.390199378416616 | 1.2194720420391 | 0.998735809224318 |
| ebi-fl187-GCST90094766 | prot-a-914 | Histone-lysine N-methyltransferase EHMT2 \|\| id:prot-a-914 | Fruit liking \|\| id:ebi-fl187-GCST90094766 | Inverse variance weighted (multiplicative random effects) | 10 | 0.57117337702311 | 0.423926714970181 | 0.17787078774188 | -0.259722984 | 1.40206973836467 | 1.77034311312596 | 0.771265208771211 | 4.06360186165511 | 0.998735809224318 |
| ebi-fl187-GCST90094766 | prot-a-932 | RNA polymerase II elongation factor ELL \|\| id:prot-a-932 | Fruit liking \|\| id:ebi-fl187-GCST90094766 | Inverse variance weighted | 10 | -0.106882532 | 0.290534807322622 | 0.712961207333222 | -0.676330754 | 0.462565690489303 | 0.898631227326641 | 0.508479309188903 | 1.58814344680951 | 0.998735809224318 |
| ebi-fl187-GCST90094766 | prot-a-991 | Estrogen receptor \|\| id:prot-a-991 | Fruit liking \|\| id:ebi-fl187-GCST90094766 | Inverse variance weighted | 10 | -0.230884641 | 0.290692718478284 | 0.42704600232989 | -0.800642369 | 0.338873087083453 | 0.793831036201571 | 0.449040421647309 | 1.40336522873616 | 0.998735809224318 |
| ebi-fl187-GCST90094766 | prot-a-992 | Persulfide dioxygenase ETHE1, mitochondrial \|\| id:prot-a-992 | Fruit liking \|\| id:ebi-fl187-GCST90094766 | Inverse variance weighted (multiplicative random effects) | 10 | -0.296496029 | 0.429053767051692 | 0.489536428995741 | -1.137441413 | 0.544449354108773 | 0.743418579141071 | 0.320638354412996 | 1.72365899526875 | 0.998735809224318 |
| ebi-fl187-GCST90094766 | prot-a-993 | Ethanolamine kinase 1 \|\| id:prot-a-993 | Fruit liking \|\| id:ebi-fl187-GCST90094766 | Inverse variance weighted | 10 | 0.0797273183964643 | 0.290692718478284 | 0.783879710774069 | -0.49003041 | 0.649485046613901 | 1.08299171549064 | 0.612607764608709 | 1.91455466871287 | 0.998735809224318 |
| ebi-fl187-GCST90094766 | prot-b-10 | coagulation factor III, tissue factor \|\| id:prot-b-10 | Fruit liking \|\| id:ebi-fl187-GCST90094766 | Inverse variance weighted | 6 | -0.13520202 | 0.412167260076761 | 0.742891192590053 | -0.94304985 | 0.672645809697051 | 0.87353942138079 | 0.389438294014743 | 1.95941470685828 | 0.998735809224318 |
| ebi-fl187-GCST90094766 | prot-b-28 | prolactin \|\| id:prot-b-28 | Fruit liking \|\| id:ebi-fl187-GCST90094766 | Inverse variance weighted | 6 | 0.0905342065256771 | 0.412167260076761 | 0.82614059610985 | -0.717313623 | 0.898382036276128 | 1.09475895490147 | 0.488061613842547 | 2.45562678019504 | 0.998735809224318 |
| ebi-fl187-GCST90094766 | prot-b-54 | matrix metallopeptidase 7 \|\| id:prot-b-54 | Fruit liking \|\| id:ebi-fl187-GCST90094766 | Inverse variance weighted | 6 | 0.0690229098163374 | 0.504662728059364 | 0.891212302048577 | -0.920116037 | 1.05816185681269 | 1.07146075583629 | 0.398472800741656 | 2.88107029930404 | 0.998735809224318 |
| ebi-fl187-GCST90094766 | prot-c-2421_7_3 | BDNF \|\| id:prot-c-2421_7_3 | Fruit liking \|\| id:ebi-fl187-GCST90094766 | Inverse variance weighted | 2 | 0.766105502999696 | 1.46541357209961 | 0.601119477167614 | -2.106105098 | 3.63831610431492 | 2.15137140804942 | 0.121711097193836 | 38.027748020391 | 0.998735809224318 |
| ebi-fl187-GCST90094766 | prot-c-2443_10_4 | FGF-8B \|\| id:prot-c-2443_10_4 | Fruit liking \|\| id:ebi-fl187-GCST90094766 | Inverse variance weighted | 2 | 1.89389344252005 | 1.29786288260518 | 0.14449906091919 | -0.649917807 | 4.4377046924262 | 6.64519104885425 | 0.5220886868314 | 84.5805802530883 | 0.998735809224318 |
| ebi-fl187-GCST90094766 | prot-c-2605_49_2 | CD30 \|\| id:prot-c-2605_49_2 | Fruit liking \|\| id:ebi-fl187-GCST90094766 | Inverse variance weighted | 2 | 1.51849412126133 | 1.27668248378075 | 0.234279843886831 | -0.983803547 | 4.02079178947159 | 4.56534515998184 | 0.373886296834855 | 55.7452268409175 | 0.998735809224318 |
| ebi-fl187-GCST90094766 | prot-c-2624_31_2 | HPV E7 Type18 \|\| id:prot-c-2624_31_2 | Fruit liking \|\| id:ebi-fl187-GCST90094766 | Inverse variance weighted | 2 | 0.352411296250659 | 2.42718652027785 | 0.88455823109665 | -4.404874283 | 5.10969687599524 | 1.42249346964795 | 0.0122176422775994 | 165.620143822762 | 0.998735809224318 |
| ebi-fl187-GCST90094766 | prot-c-2774_10_3 | IL-16 \|\| id:prot-c-2774_10_3 | Fruit liking \|\| id:ebi-fl187-GCST90094766 | Inverse variance weighted | 2 | 1.44885909163798 | 1.50755337922112 | 0.336518718362849 | -1.505945532 | 4.40366371491137 | 4.25825346570349 | 0.221807468670818 | 81.7498287448848 | 0.998735809224318 |
| ebi-fl187-GCST90094766 | prot-c-2851_63_3 | C5a \|\| id:prot-c-2851_63_3 | Fruit liking \|\| id:ebi-fl187-GCST90094766 | Inverse variance weighted | 2 | -0.730597449 | 1.12403838999071 | 0.515708027418047 | -2.933712694 | 1.47251779508636 | 0.48162115989415 | 0.0531991588591796 | 4.36019942104331 | 0.998735809224318 |
| ebi-fl187-GCST90094766 | prot-c-2891_1_3 | CD22 \|\| id:prot-c-2891_1_3 | Fruit liking \|\| id:ebi-fl187-GCST90094766 | Inverse variance weighted | 2 | 0.636913160048085 | 2.07073952095151 | 0.758403742055429 | -3.421736301 | 4.69556262111304 | 1.89063577245464 | 0.0326556855862073 | 109.460376039231 | 0.998735809224318 |
| ebi-fl187-GCST90094766 | prot-c-2961_1_2 | Protein C \|\| id:prot-c-2961_1_2 | Fruit liking \|\| id:ebi-fl187-GCST90094766 | Inverse variance weighted | 2 | -0.358189476 | 1.26198927274278 | 0.776541233559419 | -2.83168845 | 2.11530949900463 | 0.698940630284227 | 0.0589132974794736 | 8.29215178173179 | 0.998735809224318 |
| ebi-fl187-GCST90094766 | prot-c-2982_82_2 | Galectin-4 \|\| id:prot-c-2982_82_2 | Fruit liking \|\| id:ebi-fl187-GCST90094766 | Inverse variance weighted | 2 | 1.16898458748771 | 1.19261550159222 | 0.326994492065773 | -1.168541796 | 3.50651097060845 | 3.21872264736475 | 0.310819849830969 | 33.3317691463169 | 0.998735809224318 |
| ebi-fl187-GCST90094766 | prot-c-2994_71_2 | IL-1Rrp2 \|\| id:prot-c-2994_71_2 | Fruit liking \|\| id:ebi-fl187-GCST90094766 | Inverse variance weighted | 2 | 2.46440326829287 | 1.28472565799404 | 0.0550814622554406 | -0.053659021 | 4.98246555796119 | 11.7564646031992 | 0.947755215681854 | 145.833499704708 | 0.998735809224318 |
| ebi-fl187-GCST90094766 | prot-c-3336_50_1 | TFPI \|\| id:prot-c-3336_50_1 | Fruit liking \|\| id:ebi-fl187-GCST90094766 | Inverse variance weighted | 2 | 1.23094712659125 | 1.25042390913502 | 0.324907153084085 | -1.219883735 | 3.68177798849589 | 3.42447140874772 | 0.295264493762308 | 39.7169476082383 | 0.998735809224318 |
| ebi-fl187-GCST90094766 | prot-c-3472_40_2 | Survivin \|\| id:prot-c-3472_40_2 | Fruit liking \|\| id:ebi-fl187-GCST90094766 | Inverse variance weighted | 2 | 1.26902044810328 | 2.78939488131586 | 0.649149015601778 | -4.198193519 | 6.73623441548235 | 3.55736623027448 | 0.0150226905237698 | 842.382692785555 | 0.998735809224318 |
| ebi-fl187-GCST90094766 | prot-c-3473_78_2 | Thrombopoietin Receptor \|\| id:prot-c-3473_78_2 | Fruit liking \|\| id:ebi-fl187-GCST90094766 | Inverse variance weighted | 2 | -1.706993129 | 1.28854800898444 | 0.185256876038092 | -4.232547227 | 0.818560968210545 | 0.1814104511001 | 0.0145153694767276 | 2.26723486585069 | 0.998735809224318 |
| ebi-fl187-GCST90094766 | prot-c-3497_13_2 | IFN-aA \|\| id:prot-c-3497_13_2 | Fruit liking \|\| id:ebi-fl187-GCST90094766 | Inverse variance weighted | 2 | 0.255515650130262 | 1.2938173579427 | 0.843444311746418 | -2.280366371 | 2.79139767169796 | 1.29112721916897 | 0.102246739567562 | 16.3037912321642 | 0.998735809224318 |
| ebi-fl187-GCST90094766 | prot-c-3665_64_3 | COLEC12 \|\| id:prot-c-3665_64_3 | Fruit liking \|\| id:ebi-fl187-GCST90094766 | Inverse variance weighted | 2 | 0.277359175616843 | 1.28013662763153 | 0.82847041645525 | -2.231708615 | 2.78642696577464 | 1.31964026853111 | 0.107344862364439 | 16.2229509635645 | 0.998735809224318 |
| ebi-fl187-GCST90094766 | prot-c-3741_4_3 | IL-5 \|\| id:prot-c-3741_4_3 | Fruit liking \|\| id:ebi-fl187-GCST90094766 | Inverse variance weighted | 2 | -0.782859498 | 1.26011655072582 | 0.534428833768569 | -3.252687938 | 1.68696894113335 | 0.457097072446231 | 0.0386701251228297 | 5.40307881019926 | 0.998735809224318 |
| ebi-fl187-GCST90094766 | prot-c-3848_14_2 | GAPDH, liver \|\| id:prot-c-3848_14_2 | Fruit liking \|\| id:ebi-fl187-GCST90094766 | Inverse variance weighted | 2 | -0.294850023 | 1.27728964973831 | 0.817438812913535 | -2.798337736 | 2.20863769082847 | 0.744643258707843 | 0.0609112290536516 | 9.10330642401959 | 0.998735809224318 |
| ebi-fl187-GCST90094766 | prot-c-3859_50_2 | PSA1 \|\| id:prot-c-3859_50_2 | Fruit liking \|\| id:ebi-fl187-GCST90094766 | Inverse variance weighted | 2 | -0.18925131 | 1.27321630031598 | 0.881837436276427 | -2.684755258 | 2.30625263906461 | 0.827578502174201 | 0.0682378926192493 | 10.0367427974716 | 0.998735809224318 |
| ebi-fl187-GCST90094766 | prot-c-3868_8_1 | SGTA \|\| id:prot-c-3868_8_1 | Fruit liking \|\| id:ebi-fl187-GCST90094766 | Inverse variance weighted | 2 | -0.713261847 | 1.24509125985594 | 0.566740105805928 | -3.153640716 | 1.72711702264154 | 0.490043142087784 | 0.042696398097514 | 5.62441544972503 | 0.998735809224318 |
| ebi-fl187-GCST90094766 | prot-c-3882_10_1 | EF-1-beta \|\| id:prot-c-3882_10_1 | Fruit liking \|\| id:ebi-fl187-GCST90094766 | Inverse variance weighted | 2 | -0.849590276 | 1.28460099834756 | 0.508378197433118 | -3.367408233 | 1.66822768033524 | 0.427590089802933 | 0.0344788828660781 | 5.30276127587532 | 0.998735809224318 |
| ebi-fl187-GCST90094766 | prot-c-4134_4_2 | HB-EGF \|\| id:prot-c-4134_4_2 | Fruit liking \|\| id:ebi-fl187-GCST90094766 | Inverse variance weighted | 2 | -0.113933379 | 1.29749637764164 | 0.930027553723525 | -2.657026279 | 2.42915952141414 | 0.892317401190871 | 0.070156537828142 | 11.3493391937114 | 0.998735809224318 |
| ebi-fl187-GCST90094766 | prot-c-4220_39_2 | FER \|\| id:prot-c-4220_39_2 | Fruit liking \|\| id:ebi-fl187-GCST90094766 | Inverse variance weighted | 2 | -0.566765077 | 2.15113798356646 | 0.792186640675001 | -4.782995525 | 3.64946537034713 | 0.567357831924801 | 0.00837088617250877 | 38.4541018492834 | 0.998735809224318 |
| ebi-fl187-GCST90094766 | prot-c-4228_37_2 | HSP70 protein 8 \|\| id:prot-c-4228_37_2 | Fruit liking \|\| id:ebi-fl187-GCST90094766 | Inverse variance weighted | 2 | 2.20730609461501 | 1.30143590259421 | 0.0898755774326674 | -0.343508274 | 4.75812046369965 | 9.09119256283118 | 0.709277612224075 | 116.52670377585 | 0.998735809224318 |
| ebi-fl187-GCST90094766 | prot-c-4246_40_2 | NCAM-L1 \|\| id:prot-c-4246_40_2 | Fruit liking \|\| id:ebi-fl187-GCST90094766 | Inverse variance weighted | 2 | 2.75820854583434 | 1.54350834922624 | 0.0739417687270625 | -0.267067819 | 5.78348491031776 | 15.7715635919423 | 0.765621146312786 | 324.889430409044 | 0.998735809224318 |
| ebi-fl187-GCST90094766 | prot-c-4267_81_3 | PESC \|\| id:prot-c-4267_81_3 | Fruit liking \|\| id:ebi-fl187-GCST90094766 | Inverse variance weighted | 2 | 1.26573630622197 | 2.5457455785025 | 0.619050346785141 | -3.723925028 | 6.25539764008687 | 3.54570249800965 | 0.0241390352803468 | 520.816431078652 | 0.998735809224318 |
| ebi-fl187-GCST90094766 | prot-c-4280_47_2 | PSA2 \|\| id:prot-c-4280_47_2 | Fruit liking \|\| id:ebi-fl187-GCST90094766 | Inverse variance weighted | 2 | 1.27262272133893 | 1.28739617879013 | 0.322895819859359 | -1.250673789 | 3.79591923176759 | 3.57020394402932 | 0.286311818074713 | 44.5191410109252 | 0.998735809224318 |
| ebi-fl187-GCST90094766 | prot-c-4324_33_2 | CYTT \|\| id:prot-c-4324_33_2 | Fruit liking \|\| id:ebi-fl187-GCST90094766 | Inverse variance weighted | 2 | 2.90870727311688 | 1.24729668890598 | 0.0197002105756603 | 0.464005762861164 | 5.35340878337259 | 18.3330835718307 | 1.59043213585322 | 211.327440935681 | 0.998735809224318 |
| ebi-fl187-GCST90094766 | prot-c-4462_8_2 | RASA1 \|\| id:prot-c-4462_8_2 | Fruit liking \|\| id:ebi-fl187-GCST90094766 | Inverse variance weighted | 2 | 0.958018997418525 | 1.29083189323887 | 0.45798326719619 | -1.572011513 | 3.48804950816672 | 2.60652781721819 | 0.207627117312206 | 32.7220613081875 | 0.998735809224318 |
| ebi-fl187-GCST90094766 | prot-c-4499_21_1 | PDGF-AA \|\| id:prot-c-4499_21_1 | Fruit liking \|\| id:ebi-fl187-GCST90094766 | Inverse variance weighted | 2 | 1.33380500129425 | 1.2917881630749 | 0.301825724676769 | -1.198099798 | 3.86570980092106 | 3.79545766833974 | 0.301767085771458 | 47.7371442791108 | 0.998735809224318 |
| ebi-fl187-GCST90094766 | prot-c-4535_50_2 | BST1 \|\| id:prot-c-4535_50_2 | Fruit liking \|\| id:ebi-fl187-GCST90094766 | Inverse variance weighted | 2 | 0.957370053476234 | 1.28514310261988 | 0.456300677213096 | -1.561510428 | 3.47625053461119 | 2.60483687550333 | 0.209818915447435 | 32.338243353859 | 0.998735809224318 |
| ebi-fl187-GCST90094766 | prot-c-4542_24_2 | Clusterin \|\| id:prot-c-4542_24_2 | Fruit liking \|\| id:ebi-fl187-GCST90094766 | Inverse variance weighted | 2 | -0.447014352 | 1.28458583648352 | 0.727852764381741 | -2.964802591 | 2.0707738878272 | 0.639534729810829 | 0.0515706487408118 | 7.9309584157031 | 0.998735809224318 |
| ebi-fl187-GCST90094766 | prot-c-4696_2_2 | FABP \|\| id:prot-c-4696_2_2 | Fruit liking \|\| id:ebi-fl187-GCST90094766 | Inverse variance weighted | 2 | -0.702996207 | 1.23010566592307 | 0.567665818087923 | -3.114003312 | 1.70801089796092 | 0.495099658067456 | 0.0444227607025372 | 5.51797473956884 | 0.998735809224318 |
| ebi-fl187-GCST90094766 | prot-c-4906_35_1 | Coagulation Factor V \|\| id:prot-c-4906_35_1 | Fruit liking \|\| id:ebi-fl187-GCST90094766 | Inverse variance weighted | 2 | 0.725628114641521 | 1.29564526428925 | 0.575444288952783 | -1.813836603 | 3.26509283264845 | 2.06602839518359 | 0.163027463707431 | 26.1825414726752 | 0.998735809224318 |
| ebi-fl187-GCST90094766 | prot-c-4916_2_1 | IgD \|\| id:prot-c-4916_2_1 | Fruit liking \|\| id:ebi-fl187-GCST90094766 | Inverse variance weighted | 2 | -0.228343746 | 1.28852354152131 | 0.859340704681617 | -2.753849887 | 2.29716239551918 | 0.795850642445148 | 0.063682219299513 | 9.94591978808752 | 0.998735809224318 |
| ebi-fl187-GCST90094766 | prot-c-4922_13_1 | MIP-3b \|\| id:prot-c-4922_13_1 | Fruit liking \|\| id:ebi-fl187-GCST90094766 | Inverse variance weighted | 2 | -0.341935692 | 1.29750412317915 | 0.792139472141057 | -2.885043774 | 2.20117238903363 | 0.710393886967997 | 0.0558523446120104 | 9.03560053113649 | 0.998735809224318 |
| ebi-fl187-GCST90094766 | prot-c-4973_18_1 | cIAP-2 \|\| id:prot-c-4973_18_1 | Fruit liking \|\| id:ebi-fl187-GCST90094766 | Inverse variance weighted | 2 | 0.730357392980683 | 1.93279480678854 | 0.705522472797257 | -3.057920428 | 4.51863521428622 | 2.0758223594582 | 0.0469853029988904 | 91.7103475554553 | 0.998735809224318 |
| ebi-fl187-GCST90094766 | prot-c-4997_19_1 | IF4A3 \|\| id:prot-c-4997_19_1 | Fruit liking \|\| id:ebi-fl187-GCST90094766 | Inverse variance weighted | 2 | -0.151075415 | 1.29533360178927 | 0.907152861507248 | -2.689929274 | 2.38777844463105 | 0.859782855796794 | 0.067885740462867 | 10.8892759227756 | 0.998735809224318 |
| ebi-fl187-GCST90094766 | prot-c-5000_52_1 | LG3BP \|\| id:prot-c-5000_52_1 | Fruit liking \|\| id:ebi-fl187-GCST90094766 | Inverse variance weighted | 2 | 1.54492211520964 | 1.19593830595688 | 0.196423805220652 | -0.799116964 | 3.88896119488513 | 4.68760651955297 | 0.449725912793159 | 48.8601040257642 | 0.998735809224318 |
| ebi-fl187-GCST90094766 | prot-c-5006_71_1 | MK13 \|\| id:prot-c-5006_71_1 | Fruit liking \|\| id:ebi-fl187-GCST90094766 | Inverse variance weighted | 2 | -0.523987048 | 1.82547050198949 | 0.774080016837335 | -4.101909232 | 3.05393513545641 | 0.592154884885574 | 0.0165410644997651 | 21.1985998663404 | 0.998735809224318 |
| ebi-fl187-GCST90094766 | prot-c-5109_24_3 | Nr-CAM \|\| id:prot-c-5109_24_3 | Fruit liking \|\| id:ebi-fl187-GCST90094766 | Inverse variance weighted | 2 | 1.58383900787007 | 1.2675488024683 | 0.211471647579249 | -0.900556645 | 4.06823466070793 | 4.87362984675549 | 0.406343407762303 | 58.4536808754634 | 0.998735809224318 |
| ebi-fl187-GCST90094766 | prot-c-5255_22_3 | PDE4D \|\| id:prot-c-5255_22_3 | Fruit liking \|\| id:ebi-fl187-GCST90094766 | Inverse variance weighted | 2 | -0.020624971 | 1.2726932968124 | 0.987070234856174 | -2.515103832 | 2.4738538910221 | 0.9795862692134 | 0.0808545164456695 | 11.8680971826259 | 0.998955228396052 |
| ebi-fl187-GCST90094766 | prot-c-5440_26_3 | Troponin I, skeletal, fast twitch \|\| id:prot-c-5440_26_3 | Fruit liking \|\| id:ebi-fl187-GCST90094766 | Inverse variance weighted | 2 | -0.774614849 | 1.23413249800105 | 0.530227094723137 | -3.193514545 | 1.64428484676377 | 0.460881255549315 | 0.0410274245098862 | 5.17730601552943 | 0.998735809224318 |
| ebi-fl187-GCST90094766 | ukb-rsfMRI191-edge-pheno716 | Net100_Pair10_33 (OccipitalPrecuneus)&(Temporal\|Frontal\|Supp_Motor_Area) (Default_mode\|Central_executive)&(Default_mode\|Salience) \|\| id:ukb-rsfMRI191-edge-pheno716 | Fruit liking \|\| id:ebi-fl187-GCST90094766 | Inverse variance weighted | 10 | -0.053809347 | 0.0906166588131619 | 0.552637211973726 | -0.231417999 | 0.123799303806352 | 0.947612754052235 | 0.793407753270154 | 1.1317887025194 | 0.998735809224318 |
| ebi-fl187-GCST90094766 | ukb-rsfMRI191-edge-pheno903 | Net100_Pair27_38 (Precentral\|Frontal\|Supp_Motor_Area)&(Subcortical) (Attention\|Salience\|Motor)&(Subcortical-cerebellum) \|\| id:ukb-rsfMRI191-edge-pheno903 | Fruit liking \|\| id:ebi-fl187-GCST90094766 | Inverse variance weighted | 10 | -0.054369682 | 0.104104845605985 | 0.601490084557292 | -0.258415179 | 0.149675815727833 | 0.947081922960231 | 0.772274533332572 | 1.16145765538507 | 0.998735809224318 |
| ebi-fl187-GCST90094766 | ukb-rsfMRI191-node-pheno23 | Net100_Node2 RolandicOper\|SupraMarginal\|Insula Salience\|Motor \|\| id:ukb-rsfMRI191-node-pheno23 | Fruit liking \|\| id:ebi-fl187-GCST90094766 | Inverse variance weighted | 10 | 0.00833002383377365 | 0.0965560500246835 | 0.931250687842657 | -0.180919834 | 0.197579881882153 | 1.00836481501901 | 0.834502254543023 | 1.21845039319293 | 0.998735809224318 |
| ebi-fl187-GCST90094766 | ukb-rsfMRI191-node-pheno55 | Net100_Node34 Parietal\|Postcentral\|Precuneus Attention \|\| id:ukb-rsfMRI191-node-pheno55 | Fruit liking \|\| id:ebi-fl187-GCST90094766 | Inverse variance weighted | 10 | -0.010563549 | 0.105071199765452 | 0.919917964204372 | -0.216503101 | 0.195376002284477 | 0.989492049086472 | 0.805330037754563 | 1.21576803211672 | 0.998735809224318 |
| ebi-fl187-GCST90094766 | ebi-a-GCST90001414 | IgD- CD24- B cell Absolute Count \|\| id:ebi-a-GCST90001414 | Fruit liking \|\| id:ebi-fl187-GCST90094766 | MR Egger | 9 | -1.400108285 | 1.36887300507516 | 0.3404349212699 | -4.083099375 | 1.28288280497695 | 0.246570262642377 | 0.0168551442015206 | 3.6070230958953 | 0.999950574162623 |
| ebi-fl187-GCST90094766 | ebi-a-GCST90001422 | CD20- CD38- B cell %B cell \|\| id:ebi-a-GCST90001422 | Fruit liking \|\| id:ebi-fl187-GCST90094766 | MR Egger | 9 | -0.04237861 | 1.46337915886797 | 0.97770530384026 | -2.910601762 | 2.82584454111381 | 0.95850681134473 | 0.0544429583254922 | 16.8751907620725 | 0.999950574162623 |
| ebi-fl187-GCST90094766 | ebi-a-GCST90001444 | CD20- CD38- B cell %lymphocyte \|\| id:ebi-a-GCST90001444 | Fruit liking \|\| id:ebi-fl187-GCST90094766 | MR Egger | 9 | -0.186762259 | 1.47517578571267 | 0.902814150647428 | -3.078106799 | 2.70458228098282 | 0.829640952599362 | 0.046046349175007 | 14.948071292557 | 0.999950574162623 |
| ebi-fl187-GCST90094766 | ebi-a-GCST90001459 | Myeloid Dendritic Cell %Dendritic Cell \|\| id:ebi-a-GCST90001459 | Fruit liking \|\| id:ebi-fl187-GCST90094766 | MR Egger | 9 | 1.01046923139619 | 1.40089987743062 | 0.494105490172014 | -1.735294528 | 3.7562329911602 | 2.74688963952248 | 0.176348253073607 | 42.7869432228881 | 0.999950574162623 |
| ebi-fl187-GCST90094766 | ebi-a-GCST90001476 | HLA DR++ monocyte %leukocyte \|\| id:ebi-a-GCST90001476 | Fruit liking \|\| id:ebi-fl187-GCST90094766 | MR Egger | 9 | -0.876136932 | 1.36782080901345 | 0.54221655823451 | -3.557065718 | 1.80479185325408 | 0.416388345055926 | 0.0285223947925316 | 6.07870605394646 | 0.999950574162623 |
| ebi-fl187-GCST90094766 | ebi-a-GCST90001478 | CD4 regulatory T cell %CD4+ T cell \|\| id:ebi-a-GCST90001478 | Fruit liking \|\| id:ebi-fl187-GCST90094766 | MR Egger | 9 | -1.138941935 | 2.22657916862564 | 0.624722543070638 | -5.503037105 | 3.22515323593472 | 0.320157590348901 | 0.00407437831331447 | 25.1574288825995 | 0.999950574162623 |
| ebi-fl187-GCST90094766 | ebi-a-GCST90001485 | CD39+ resting CD4 regulatory T cell %CD4 regulatory T cell \|\| id:ebi-a-GCST90001485 | Fruit liking \|\| id:ebi-fl187-GCST90094766 | MR Egger | 9 | -0.551154887 | 1.35215914611084 | 0.695737036707594 | -3.201386814 | 2.09907703907123 | 0.57628388297783 | 0.0407057135760134 | 8.15863633393478 | 0.999950574162623 |
| ebi-fl187-GCST90094766 | ebi-a-GCST90001515 | Immature Myeloid-Derived Suppressor Cells Absolute Count \|\| id:ebi-a-GCST90001515 | Fruit liking \|\| id:ebi-fl187-GCST90094766 | MR Egger | 9 | -0.048371696 | 2.07397150073931 | 0.982043379139743 | -4.113355837 | 4.01661244576595 | 0.952779577196651 | 0.0163528049932686 | 55.512734549009 | 0.999950574162623 |
| ebi-fl187-GCST90094766 | ebi-a-GCST90001517 | CD33+ HLA DR+ Absolute Count \|\| id:ebi-a-GCST90001517 | Fruit liking \|\| id:ebi-fl187-GCST90094766 | MR Egger | 9 | 2.29377351354146 | 1.78023171319254 | 0.238531863003299 | -1.195480644 | 5.78302767139885 | 9.91227128736778 | 0.302558496206734 | 324.740912273839 | 0.999950574162623 |
| ebi-fl187-GCST90094766 | ebi-a-GCST90001518 | CD33+ HLA DR+ CD14- Absolute Count \|\| id:ebi-a-GCST90001518 | Fruit liking \|\| id:ebi-fl187-GCST90094766 | MR Egger | 9 | 2.66291128214224 | 1.80224177937293 | 0.183049554 | -0.869482605 | 6.19530516971318 | 14.3379702732212 | 0.419168368590595 | 490.441089930053 | 0.999950574162623 |
| ebi-fl187-GCST90094766 | ebi-a-GCST90001520 | CD33+ HLA DR+ CD14dim Absolute Count \|\| id:ebi-a-GCST90001520 | Fruit liking \|\| id:ebi-fl187-GCST90094766 | MR Egger | 9 | 1.43762818363091 | 1.78745973205939 | 0.447681382863928 | -2.065792891 | 4.94104925846731 | 4.21069696397785 | 0.126717777314011 | 139.916981644311 | 0.999950574162623 |
| ebi-fl187-GCST90094766 | ebi-a-GCST90001531 | CD33dim HLA DR- Absolute Count \|\| id:ebi-a-GCST90001531 | Fruit liking \|\| id:ebi-fl187-GCST90094766 | MR Egger | 9 | -1.893952748 | 2.21212441890983 | 0.420243191357759 | -6.229716609 | 2.44181111305097 | 0.150475838680167 | 0.00197001010052453 | 11.4938385445186 | 0.999950574162623 |
| ebi-fl187-GCST90094766 | ebi-a-GCST90001532 | Basophil Absolute Count \|\| id:ebi-a-GCST90001532 | Fruit liking \|\| id:ebi-fl187-GCST90094766 | MR Egger | 9 | -2.134222723 | 2.25648387594896 | 0.375736963849079 | -6.55693112 | 2.28848567390779 | 0.118336534911324 | 0.00142023756820125 | 9.85999512219261 | 0.999950574162623 |
| ebi-fl187-GCST90094766 | ebi-a-GCST90001588 | T/B cell \|\| id:ebi-a-GCST90001588 | Fruit liking \|\| id:ebi-fl187-GCST90094766 | MR Egger | 9 | 2.420037902 | 1.69201429574011 | 0.195721111533552 | -0.896310118 | 5.73638592170961 | 11.2462855641829 | 0.408072625153411 | 309.942228895162 | 0.999950574162623 |
| ebi-fl187-GCST90094766 | ebi-a-GCST90001624 | HLA DR+ CD4+ T cell Absolute Count \|\| id:ebi-a-GCST90001624 | Fruit liking \|\| id:ebi-fl187-GCST90094766 | MR Egger | 9 | 1.05454852439551 | 1.30896551075095 | 0.446952187776613 | -1.511023877 | 3.62012092546737 | 2.87067881983628 | 0.220683909138417 | 37.3420831578972 | 0.999950574162623 |
| ebi-fl187-GCST90094766 | ebi-a-GCST90001631 | CD8+ Natural Killer T %T cell \|\| id:ebi-a-GCST90001631 | Fruit liking \|\| id:ebi-fl187-GCST90094766 | MR Egger | 9 | -1.61933268 | 1.58640837282216 | 0.341346381771188 | -4.72869309 | 1.49002773115351 | 0.1980308050006 | 0.00883801398141083 | 4.43721856648675 | 0.999950574162623 |
| ebi-fl187-GCST90094766 | ebi-a-GCST90001632 | CD8+ Natural Killer T %lymphocyte \|\| id:ebi-a-GCST90001632 | Fruit liking \|\| id:ebi-fl187-GCST90094766 | MR Egger | 9 | -1.237980057 | 1.47314335002874 | 0.428470140279693 | -4.125341023 | 1.6493809093476 | 0.289969348414912 | 0.0161579834083206 | 5.20375723228372 | 0.999950574162623 |
| ebi-fl187-GCST90094766 | ebi-a-GCST90001646 | Natural Killer %CD3- lymphocyte \|\| id:ebi-a-GCST90001646 | Fruit liking \|\| id:ebi-fl187-GCST90094766 | MR Egger | 9 | 0.722992442677838 | 1.30189933403069 | 0.595957188361793 | -1.828730252 | 3.27471513737799 | 2.06059019187439 | 0.160617381947382 | 26.4356938668066 | 0.999950574162623 |
| ebi-fl187-GCST90094766 | ebi-a-GCST90001684 | CD127- CD8+ T cell Absolute Count \|\| id:ebi-a-GCST90001684 | Fruit liking \|\| id:ebi-fl187-GCST90094766 | MR Egger | 9 | 0.187338112141105 | 1.41083767731522 | 0.898099885986911 | -2.577903735 | 2.95257995967893 | 1.20603499119396 | 0.0759330129895334 | 19.1553099596445 | 0.999950574162623 |
| ebi-fl187-GCST90094766 | ebi-a-GCST90001687 | CD28- CD8+ T cell Absolute Count \|\| id:ebi-a-GCST90001687 | Fruit liking \|\| id:ebi-fl187-GCST90094766 | MR Egger | 9 | -0.306816759 | 1.37140456689999 | 0.829360884171284 | -2.99476971 | 2.38113619248843 | 0.735785414878498 | 0.0500481513603839 | 10.8171862902504 | 0.999950574162623 |
| ebi-fl187-GCST90094766 | ebi-a-GCST90001701 | BAFF-R on CD20- CD38- B cell \|\| id:ebi-a-GCST90001701 | Fruit liking \|\| id:ebi-fl187-GCST90094766 | MR Egger | 9 | -2.387866699 | 1.40628268391449 | 0.133316079542738 | -5.14418076 | 0.368447361413541 | 0.0918253662188074 | 0.0058332512425166 | 1.44548855015207 | 0.999950574162623 |
| ebi-fl187-GCST90094766 | ebi-a-GCST90001732 | CD19 on IgD- CD27- B cell \|\| id:ebi-a-GCST90001732 | Fruit liking \|\| id:ebi-fl187-GCST90094766 | MR Egger | 9 | 2.19938695085105 | 1.60050735410348 | 0.211767331068557 | -0.937607463 | 5.33638136489387 | 9.0194824181746 | 0.391563545747358 | 207.759542417285 | 0.999950574162623 |
| ebi-fl187-GCST90094766 | ebi-a-GCST90001798 | CD27 on CD24+ CD27+ B cell \|\| id:ebi-a-GCST90001798 | Fruit liking \|\| id:ebi-fl187-GCST90094766 | MR Egger | 9 | 0.678158427964909 | 1.33235813592455 | 0.62640575579789 | -1.933263518 | 3.28958037437703 | 1.97024603923658 | 0.144675276774394 | 26.8316020655058 | 0.999950574162623 |
| ebi-fl187-GCST90094766 | ebi-a-GCST90001836 | CD66b on CD66b++ myeloid cell \|\| id:ebi-a-GCST90001836 | Fruit liking \|\| id:ebi-fl187-GCST90094766 | MR Egger | 9 | 2.31521257414831 | 2.8772183324034 | 0.447473354934435 | -3.324135357 | 7.95456050565896 | 10.127075444387 | 0.0360036355774554 | 2848.53613840388 | 0.999950574162623 |
| ebi-fl187-GCST90094766 | ebi-a-GCST90001943 | CD25 on activated & secreting CD4 regulatory T cell \|\| id:ebi-a-GCST90001943 | Fruit liking \|\| id:ebi-fl187-GCST90094766 | MR Egger | 9 | -0.641706663 | 1.35813625626452 | 0.650952913709014 | -3.303653726 | 2.02024039902709 | 0.526393280925712 | 0.0367486523211211 | 7.54013735748562 | 0.999950574162623 |
| ebi-fl187-GCST90094766 | ebi-a-GCST90001960 | CD25 on CD4+ T cell \|\| id:ebi-a-GCST90001960 | Fruit liking \|\| id:ebi-fl187-GCST90094766 | MR Egger | 9 | 0.680944319179944 | 1.49922194735553 | 0.663434314307337 | -2.257530698 | 3.61941933599678 | 1.97574258320055 | 0.104608476051529 | 37.3158935338003 | 0.999950574162623 |
| ebi-fl187-GCST90094766 | ebi-a-GCST90001988 | HLA DR on CD14+ CD16- monocyte \|\| id:ebi-a-GCST90001988 | Fruit liking \|\| id:ebi-fl187-GCST90094766 | MR Egger | 9 | -0.93589718 | 1.35623639035455 | 0.512372590824245 | -3.594120505 | 1.72232614524317 | 0.392233803358044 | 0.0274848453347229 | 5.59753400912735 | 0.999950574162623 |
| ebi-fl187-GCST90094766 | ebi-a-GCST90001991 | HLA DR on CD14+ monocyte \|\| id:ebi-a-GCST90001991 | Fruit liking \|\| id:ebi-fl187-GCST90094766 | MR Egger | 9 | -0.947187509 | 1.35877336047355 | 0.508228711085605 | -3.610383296 | 1.71600827746596 | 0.387830260094332 | 0.0270414799996493 | 5.56228100853903 | 0.999950574162623 |
| ebi-fl187-GCST90094766 | ebi-a-GCST90002019 | CD14 on Monocytic Myeloid-Derived Suppressor Cells \|\| id:ebi-a-GCST90002019 | Fruit liking \|\| id:ebi-fl187-GCST90094766 | MR Egger | 9 | 2.67786397987692 | 1.95536092770856 | 0.213158315754404 | -1.154643438 | 6.5103713981857 | 14.5539724922678 | 0.315169894342812 | 672.075979044156 | 0.999950574162623 |
| ebi-fl187-GCST90094766 | ebi-a-GCST90002037 | CD80 on plasmacytoid Dendritic Cell \|\| id:ebi-a-GCST90002037 | Fruit liking \|\| id:ebi-fl187-GCST90094766 | MR Egger | 9 | -0.010125954 | 1.5103590132645 | 0.994837820079502 | -2.97042962 | 2.95017771163464 | 0.989925140505243 | 0.0512812741190943 | 19.1093493802145 | 0.999950574162623 |
| ebi-fl187-GCST90094766 | ebi-a-GCST90002042 | CD45 on CD33+ HLA DR+ CD14- \|\| id:ebi-a-GCST90002042 | Fruit liking \|\| id:ebi-fl187-GCST90094766 | MR Egger | 9 | 0.658539190820136 | 2.50489024159014 | 0.800196503775476 | -4.251045683 | 5.56812406433681 | 1.93196803538202 | 0.0142493258423333 | 261.942251236123 | 0.999950574162623 |
| ebi-fl187-GCST90094766 | ebi-a-GCST90002056 | CD8 on Effector Memory CD8+ T cell \|\| id:ebi-a-GCST90002056 | Fruit liking \|\| id:ebi-fl187-GCST90094766 | MR Egger | 9 | 1.94163234132473 | 1.72643261970632 | 0.297817925273452 | -1.442175593 | 5.32544027594912 | 6.97011930339052 | 0.236412860545389 | 205.498816737043 | 0.999950574162623 |
| ebi-fl187-GCST90094766 | ebi-a-GCST90002058 | CD8 on CD8+ T cell \|\| id:ebi-a-GCST90002058 | Fruit liking \|\| id:ebi-fl187-GCST90094766 | MR Egger | 9 | -1.38354291 | 1.42838879989627 | 0.365010303017617 | -4.183184958 | 1.41609913774635 | 0.250688809946267 | 0.0152498599763885 | 4.12101353911309 | 0.999950574162623 |
| ebi-fl187-GCST90094766 | ebi-a-GCST90002063 | CD4 on CD4 regulatory T cell \|\| id:ebi-a-GCST90002063 | Fruit liking \|\| id:ebi-fl187-GCST90094766 | MR Egger | 9 | 0.0514431727079674 | 1.47305759978039 | 0.973116350537918 | -2.835749723 | 2.93863606827753 | 1.05278935742393 | 0.0586745197109152 | 18.8900639760823 | 0.999950574162623 |
| ebi-fl187-GCST90094766 | ebi-a-GCST90002066 | CD4 on activated CD4 regulatory T cell \|\| id:ebi-a-GCST90002066 | Fruit liking \|\| id:ebi-fl187-GCST90094766 | MR Egger | 9 | -0.250961743 | 1.47318151472662 | 0.86955123762006 | -3.138397512 | 2.63647402597448 | 0.778052137016003 | 0.0433522136809927 | 13.9638804230333 | 0.999950574162623 |
| ebi-fl187-GCST90094766 | ebi-a-GCST90002070 | CD4 on activated & secreting CD4 regulatory T cell \|\| id:ebi-a-GCST90002070 | Fruit liking \|\| id:ebi-fl187-GCST90094766 | MR Egger | 9 | 0.14249952907881 | 1.48007085005263 | 0.925997559581768 | -2.758439337 | 3.04343839518196 | 1.15315253791328 | 0.0633906225981942 | 20.9772474412282 | 0.999950574162623 |
| ebi-fl187-GCST90094766 | ebi-a-GCST90002081 | SSC-A on CD4+ T cell \|\| id:ebi-a-GCST90002081 | Fruit liking \|\| id:ebi-fl187-GCST90094766 | MR Egger | 9 | 0.46149384423324 | 1.67105762221637 | 0.790394288623119 | -2.813779095 | 3.73676678377732 | 1.5864421131489 | 0.0599779013575345 | 41.9620980629089 | 0.999950574162623 |
| ebi-fl187-GCST90094766 | ebi-a-GCST90002094 | CD11b on Monocytic Myeloid-Derived Suppressor Cells \|\| id:ebi-a-GCST90002094 | Fruit liking \|\| id:ebi-fl187-GCST90094766 | MR Egger | 9 | -0.940720955 | 1.98241323273503 | 0.649566499261496 | -4.826250892 | 2.94480898067479 | 0.390346311572939 | 0.00801651979828512 | 19.0070313293797 | 0.999950574162623 |
| ebi-fl187-GCST90094766 | ebi-a-GCST90002104 | HLA DR on myeloid Dendritic Cell \|\| id:ebi-a-GCST90002104 | Fruit liking \|\| id:ebi-fl187-GCST90094766 | MR Egger | 9 | 1.29227743838163 | 1.7797687219857 | 0.491340282602524 | -2.196069257 | 4.78062413347361 | 3.64106943157673 | 0.11123955425176 | 119.178710259464 | 0.999950574162623 |
| ebi-fl187-GCST90094766 | ebi-a-GCST90002105 | HLA DR on plasmacytoid Dendritic Cell \|\| id:ebi-a-GCST90002105 | Fruit liking \|\| id:ebi-fl187-GCST90094766 | MR Egger | 9 | 0.563607336235674 | 1.46915548788195 | 0.712640803983887 | -2.31593742 | 3.4431520924843 | 1.75699916899316 | 0.0986736419867906 | 31.2854174396028 | 0.999950574162623 |
| ebi-fl187-GCST90094766 | ebi-a-GCST90002106 | HLA DR on Dendritic Cell \|\| id:ebi-a-GCST90002106 | Fruit liking \|\| id:ebi-fl187-GCST90094766 | MR Egger | 9 | 1.37435244769781 | 1.88132697585497 | 0.4887934677359 | -2.313048425 | 5.06175332037356 | 3.95251643293362 | 0.0989591218249363 | 157.867065354996 | 0.999950574162623 |
| ebi-fl187-GCST90094766 | ebi-a-GCST90002111 | HLA DR on CD33dim HLA DR+ CD11b- \|\| id:ebi-a-GCST90002111 | Fruit liking \|\| id:ebi-fl187-GCST90094766 | MR Egger | 9 | 1.240238303 | 1.931105558 | 0.541170150181698 | -2.54472859 | 5.02520519657076 | 3.45643704620443 | 0.0784943532092885 | 152.201484131227 | 0.999950574162623 |
| ebi-fl187-GCST90094766 | ebi-a-GCST90002112 | HLA DR on CD33- HLA DR+ \|\| id:ebi-a-GCST90002112 | Fruit liking \|\| id:ebi-fl187-GCST90094766 | MR Egger | 9 | -1.589434137 | 1.95053006674244 | 0.441982602252305 | -5.412473068 | 2.23360479381612 | 0.204041038347584 | 0.00446059520501901 | 9.33345067562188 | 0.999950574162623 |
| ebi-fl187-GCST90094766 | ebi-a-GCST90002115 | HLA DR on HLA DR+ CD8+ T cell \|\| id:ebi-a-GCST90002115 | Fruit liking \|\| id:ebi-fl187-GCST90094766 | MR Egger | 9 | 0.366985666967158 | 1.75573202465288 | 0.840383974194964 | -3.074249101 | 3.8082204352868 | 1.44337723099366 | 0.0462243251364041 | 45.0701621884836 | 0.999950574162623 |
| ebi-fl187-GCST90094766 | ebi-a-GCST90002116 | HLA DR on B cell \|\| id:ebi-a-GCST90002116 | Fruit liking \|\| id:ebi-fl187-GCST90094766 | MR Egger | 9 | 0.105142255733437 | 1.45134410398453 | 0.944274483745697 | -2.739492188 | 2.94977669954311 | 1.11086862654731 | 0.064603144812125 | 19.1016878363403 | 0.999950574162623 |
| ebi-fl187-GCST90094766 | ebi-cfmet338-GCST90026023 | Sphingomyelin (d18:1/24:1, d18:2/24:0) levels \|\| id:ebi-cfmet338-GCST90026023 | Fruit liking \|\| id:ebi-fl187-GCST90094766 | MR Egger | 8 | -1.508167958 | 0.856392952293245 | 0.128704540913745 | -3.186698145 | 0.170362228276815 | 0.221315065224168 | 0.0413080391790156 | 1.18573428002507 | 0.999950574162623 |
| ebi-fl187-GCST90094766 | ebi-cfmet338-GCST90026046 | 1-arachidonoyl-gpc (20:4n6) levels \|\| id:ebi-cfmet338-GCST90026046 | Fruit liking \|\| id:ebi-fl187-GCST90094766 | MR Egger | 8 | -3.217069579 | 1.07365866835194 | 0.0241212168427054 | -5.321440569 | -1.112698589 | 0.0400723151191436 | 0.00488571044977077 | 0.328670815742526 | 0.999950574162623 |
| ebi-fl187-GCST90094766 | ebi-cfmet338-GCST90026047 | 1-linoleoyl-gpc (18:2) levels \|\| id:ebi-cfmet338-GCST90026047 | Fruit liking \|\| id:ebi-fl187-GCST90094766 | MR Egger | 8 | -3.135134672 | 1.2386649359579 | 0.0446158489446883 | -5.562917947 | -0.707351398 | 0.043493896013477 | 0.00383756224217824 | 0.492948093359756 | 0.999950574162623 |
| ebi-fl187-GCST90094766 | ebi-cfmet338-GCST90026050 | 1-myristoyl-2-palmitoyl-gpc (14:0/16:0) levels \|\| id:ebi-cfmet338-GCST90026050 | Fruit liking \|\| id:ebi-fl187-GCST90094766 | MR Egger | 8 | -0.74320978 | 0.659942675519586 | 0.303110062633426 | -2.036697424 | 0.55027786445895 | 0.475584940177087 | 0.130458850526748 | 1.7337346941967 | 0.999950574162623 |
| ebi-fl187-GCST90094766 | ebi-cfmet338-GCST90026068 | 2,3-dihydroxyisovalerate levels \|\| id:ebi-cfmet338-GCST90026068 | Fruit liking \|\| id:ebi-fl187-GCST90094766 | MR Egger | 8 | 3.53213815035308 | 2.11681920737176 | 0.146238009838319 | -0.616827496 | 7.68110379680172 | 34.1970078458298 | 0.539653778434882 | 2167.01039136495 | 0.999950574162623 |
| ebi-fl187-GCST90094766 | ebi-cfmet338-GCST90026082 | 3-hydroxyhexanoate levels \|\| id:ebi-cfmet338-GCST90026082 | Fruit liking \|\| id:ebi-fl187-GCST90094766 | MR Egger | 7 | -0.867198918 | 0.462206569432611 | 0.119455983421077 | -1.773123794 | 0.0387259577387138 | 0.42012671183963 | 0.169801733780239 | 1.03948558163498 | 0.999950574162623 |
| ebi-fl187-GCST90094766 | ebi-cfmet338-GCST90026094 | 3-ureidopropionate levels \|\| id:ebi-cfmet338-GCST90026094 | Fruit liking \|\| id:ebi-fl187-GCST90094766 | MR Egger | 8 | -0.064373515 | 1.03326715169935 | 0.952346737225725 | -2.089577133 | 1.96083010207772 | 0.937654705780111 | 0.123739450137235 | 7.10522267794547 | 0.999950574162623 |
| ebi-fl187-GCST90094766 | ebi-cfmet338-GCST90026173 | Glycerol levels \|\| id:ebi-cfmet338-GCST90026173 | Fruit liking \|\| id:ebi-fl187-GCST90094766 | MR Egger | 8 | 0.100211229911885 | 0.584428757482512 | 0.869492068442483 | -1.045269135 | 1.24569159457761 | 1.10540438788833 | 0.351597179622827 | 3.4753374929616 | 0.999950574162623 |
| ebi-fl187-GCST90094766 | ebi-cfmet338-GCST90026179 | Histidine levels \|\| id:ebi-cfmet338-GCST90026179 | Fruit liking \|\| id:ebi-fl187-GCST90094766 | MR Egger | 8 | -0.095092161 | 0.407738820737246 | 0.823344611609328 | -0.89426025 | 0.704075927590235 | 0.909289129561248 | 0.408909978063465 | 2.02197736786439 | 0.999950574162623 |
| ebi-fl187-GCST90094766 | ebi-cfmet338-GCST90026188 | Isocitrate levels \|\| id:ebi-cfmet338-GCST90026188 | Fruit liking \|\| id:ebi-fl187-GCST90094766 | MR Egger | 8 | -0.202667448 | 0.81233407000111 | 0.811305109617069 | -1.794842225 | 1.38950732913998 | 0.816549741478016 | 0.166153665129511 | 4.01287254053712 | 0.999950574162623 |
| ebi-fl187-GCST90094766 | ebi-cfmet338-GCST90026207 | N-acetyl-isoputreanine levels \|\| id:ebi-cfmet338-GCST90026207 | Fruit liking \|\| id:ebi-fl187-GCST90094766 | MR Egger | 8 | -0.84639292 | 0.5059265298612 | 0.145363633794465 | -1.838008919 | 0.145223078300033 | 0.428959435603359 | 0.159133959522584 | 1.1562974863768 | 0.999950574162623 |
| ebi-fl187-GCST90094766 | ebi-cfmet338-GCST90026213 | N-acetylglutamate levels \|\| id:ebi-cfmet338-GCST90026213 | Fruit liking \|\| id:ebi-fl187-GCST90094766 | MR Egger | 8 | -0.675581855 | 0.91926392078073 | 0.490106381764098 | -2.47733914 | 1.12617542939431 | 0.508860251399573 | 0.0839663513073959 | 3.08383955504362 | 0.999950574162623 |
| ebi-fl187-GCST90094766 | ebi-cfmet338-GCST90026224 | N-acetyltaurine levels \|\| id:ebi-cfmet338-GCST90026224 | Fruit liking \|\| id:ebi-fl187-GCST90094766 | MR Egger | 8 | -0.427757125 | 0.692566520419837 | 0.559508726430783 | -1.785187505 | 0.929673255208176 | 0.651969742843077 | 0.167765601189042 | 2.53368117522434 | 0.999950574162623 |
| ebi-fl187-GCST90094766 | ebi-cfmet338-GCST90026250 | Phenyllactate (pla) levels \|\| id:ebi-cfmet338-GCST90026250 | Fruit liking \|\| id:ebi-fl187-GCST90094766 | MR Egger | 8 | -1.074721293 | 1.11232243808708 | 0.371259732065985 | -3.254873272 | 1.10543068524621 | 0.341392890493224 | 0.0385857102413043 | 3.02052508429811 | 0.999950574162623 |
| ebi-fl187-GCST90094766 | ebi-cfmet338-GCST90026251 | Pipecolate levels \|\| id:ebi-cfmet338-GCST90026251 | Fruit liking \|\| id:ebi-fl187-GCST90094766 | MR Egger | 8 | -1.222170029 | 2.06239594728831 | 0.575088715545478 | -5.264466085 | 2.82012602804576 | 0.294590203627071 | 0.00517215378005943 | 16.7789651590836 | 0.999950574162623 |
| ebi-fl187-GCST90094766 | ebi-cfmet338-GCST90026278 | Trigonelline (n'-methylnicotinate) levels \|\| id:ebi-cfmet338-GCST90026278 | Fruit liking \|\| id:ebi-fl187-GCST90094766 | MR Egger | 8 | -2.614122678 | 1.8319882927101 | 0.203502223096128 | -6.204819732 | 0.976574375569371 | 0.0732320085629032 | 0.00201967285863724 | 2.65534443126385 | 0.999950574162623 |
| ebi-fl187-GCST90094766 | ebi-cfmet338-GCST90026299 | 1-carboxyethylvaline levels \|\| id:ebi-cfmet338-GCST90026299 | Fruit liking \|\| id:ebi-fl187-GCST90094766 | MR Egger | 8 | 0.142550407192699 | 1.60978468396294 | 0.932319307614849 | -3.012627573 | 3.29772838776006 | 1.15321120963197 | 0.0491623312671932 | 27.0511194189096 | 0.999950574162623 |
| ebi-fl187-GCST90094766 | ebi-gm430-GCST90011306 | C_Erysipelotrichia abundance \|\| id:ebi-gm430-GCST90011306 | Fruit liking \|\| id:ebi-fl187-GCST90094766 | MR Egger | 8 | -0.632048042 | 0.912873499490719 | 0.514592326957451 | -2.421280101 | 1.15718401703409 | 0.531502146856365 | 0.0888078616356687 | 3.18096311418745 | 0.999950574162623 |
| ebi-fl187-GCST90094766 | ebi-gm430-GCST90011312 | F_Erysipelotrichaceae abundance \|\| id:ebi-gm430-GCST90011312 | Fruit liking \|\| id:ebi-fl187-GCST90094766 | MR Egger | 8 | -0.632048042 | 0.912873499490719 | 0.514592326957451 | -2.421280101 | 1.15718401703409 | 0.531502146856365 | 0.0888078616356687 | 3.18096311418745 | 0.999950574162623 |
| ebi-fl187-GCST90094766 | ebi-gm430-GCST90011352 | O_Erysipelotrichales abundance \|\| id:ebi-gm430-GCST90011352 | Fruit liking \|\| id:ebi-fl187-GCST90094766 | MR Egger | 8 | -0.632048042 | 0.912873499490719 | 0.514592326957451 | -2.421280101 | 1.15718401703409 | 0.531502146856365 | 0.0888078616356687 | 3.18096311418745 | 0.999950574162623 |
| ebi-fl187-GCST90094766 | ebi-gm430-GCST90011362 | OTU97_11 (Parabacteroides) abundance \|\| id:ebi-gm430-GCST90011362 | Fruit liking \|\| id:ebi-fl187-GCST90094766 | MR Egger | 8 | -0.761572998 | 1.68028545253865 | 0.666305384774626 | -4.054932485 | 2.5317864893849 | 0.466931367122182 | 0.0173366506354352 | 12.575952886595 | 0.999950574162623 |
| ebi-fl187-GCST90094766 | ebi-gm430-GCST90011384 | OTU97_235 (Phascolarctobacterium) abundance \|\| id:ebi-gm430-GCST90011384 | Fruit liking \|\| id:ebi-fl187-GCST90094766 | MR Egger | 8 | 5.42761245431726 | 5.64122523659262 | 0.373137903378123 | -5.629189009 | 16.4844139180388 | 227.605178428924 | 0.00359148680860689 | 14424142.4257817 | 0.999950574162623 |
| ebi-fl187-GCST90094766 | ebi-gm430-GCST90011390 | OTU97_3 (Bacteroides) abundance \|\| id:ebi-gm430-GCST90011390 | Fruit liking \|\| id:ebi-fl187-GCST90094766 | MR Egger | 8 | 0.569748442531007 | 0.944784083674357 | 0.56855661569027 | -1.282028361 | 2.42152524653275 | 1.76782228659408 | 0.27747391187653 | 11.2630251105169 | 0.999950574162623 |
| ebi-fl187-GCST90094766 | ebi-gm430-GCST90011398 | OTU97_4 (Alistipes) abundance \|\| id:ebi-gm430-GCST90011398 | Fruit liking \|\| id:ebi-fl187-GCST90094766 | MR Egger | 8 | 1.7516059499371 | 1.2873005777472 | 0.222506036935177 | -0.771503182 | 4.27471508232161 | 5.7638517045619 | 0.462317598034334 | 71.8596623045136 | 0.999950574162623 |
| ebi-fl187-GCST90094766 | ebi-gm430-GCST90011421 | OTU99_108 (Cytophagales) abundance \|\| id:ebi-gm430-GCST90011421 | Fruit liking \|\| id:ebi-fl187-GCST90094766 | MR Egger | 8 | -2.830419448 | 3.5445547342649 | 0.455002147570177 | -9.777746728 | 4.11690783078508 | 0.058988106014468 | 5.66994114398875e-05 | 61.3691846671667 | 0.999950574162623 |
| ebi-fl187-GCST90094766 | ebi-gm430-GCST90011428 | OTU99_123 (Phascolarctobacterium) abundance \|\| id:ebi-gm430-GCST90011428 | Fruit liking \|\| id:ebi-fl187-GCST90094766 | MR Egger | 8 | -0.147496502 | 2.07252856251299 | 0.945577383780866 | -4.209652484 | 3.914659481 | 0.862865457017718 | 0.0148515285842594 | 50.1319977058458 | 0.999950574162623 |
| ebi-fl187-GCST90094766 | ebi-gm430-GCST90011450 | OTU99_298 (Phascolarctobacterium) abundance \|\| id:ebi-gm430-GCST90011450 | Fruit liking \|\| id:ebi-fl187-GCST90094766 | MR Egger | 8 | 5.39967447840389 | 5.69072600739948 | 0.37932915653762 | -5.754148496 | 16.5534974529069 | 221.334355363512 | 0.00316960439281795 | 15455839.5284868 | 0.999950574162623 |
| ebi-fl187-GCST90094766 | ebi-gm430-GCST90011451 | OTU99_3 (Bacteroides) abundance \|\| id:ebi-gm430-GCST90011451 | Fruit liking \|\| id:ebi-fl187-GCST90094766 | MR Egger | 8 | 0.536898024860807 | 0.952518512163095 | 0.593409773007499 | -1.330038259 | 2.40383430870047 | 1.71069209895305 | 0.264467142863437 | 11.0655237763563 | 0.999950574162623 |
| ebi-fl187-GCST90094766 | ebi-gm430-GCST90011472 | OTU99_72 (Parabacteroides) abundance \|\| id:ebi-gm430-GCST90011472 | Fruit liking \|\| id:ebi-fl187-GCST90094766 | MR Egger | 8 | -1.83766414 | 3.88139702922117 | 0.652623861275985 | -9.445202317 | 5.76987403761305 | 0.159188835044625 | 7.90679999454968e-05 | 320.497359492249 | 0.999950574162623 |
| ebi-fl187-GCST90094766 | ebi-gm430-GCST90011507 | TestASV_3 (Bacteroides) abundance \|\| id:ebi-gm430-GCST90011507 | Fruit liking \|\| id:ebi-fl187-GCST90094766 | MR Egger | 8 | 2.45971811955537 | 1.47159743221871 | 0.145663567285698 | -0.424612848 | 5.34404908670403 | 11.7015126474681 | 0.654022942677706 | 209.358707935008 | 0.999950574162623 |
| ebi-fl187-GCST90094766 | ebi-gm430-GCST90011512 | TestASV_34 (Phascolarctobacterium) abundance \|\| id:ebi-gm430-GCST90011512 | Fruit liking \|\| id:ebi-fl187-GCST90094766 | MR Egger | 8 | 0.439300188544785 | 3.01030996808376 | 0.888754532827806 | -5.460907349 | 6.33950772598896 | 1.55162099633292 | 0.00424969803610014 | 566.517360953603 | 0.999950574162623 |
| ebi-fl187-GCST90094766 | ebi-gm430-GCST90011519 | TestASV_41 (Barnesiella) abundance \|\| id:ebi-gm430-GCST90011519 | Fruit liking \|\| id:ebi-fl187-GCST90094766 | MR Egger | 8 | -2.808797509 | 5.2406115498642 | 0.611257636601482 | -13.08039615 | 7.46280112865746 | 0.060277431894476 | 2.08572060855652e-06 | 1742.02085403364 | 0.999950574162623 |
| ebi-fl187-GCST90094766 | ebi-gm430-GCST90011524 | TestASV_46 (Sutterella) abundance \|\| id:ebi-gm430-GCST90011524 | Fruit liking \|\| id:ebi-fl187-GCST90094766 | MR Egger | 8 | -4.570448476 | 5.19477409868881 | 0.412806109093905 | -14.75220571 | 5.61130875702577 | 0.0103533154585817 | 3.91920936604367e-07 | 273.501951474243 | 0.999950574162623 |
| ebi-fl187-GCST90094766 | ebi-gm430-GCST90011581 | OTU97_19 (Bacteria) prevalence \|\| id:ebi-gm430-GCST90011581 | Fruit liking \|\| id:ebi-fl187-GCST90094766 | MR Egger | 8 | 0.372123707610003 | 5.48448885830005 | 0.948109447404249 | -10.37747445 | 11.1217218698781 | 1.45081244673266 | 3.11257703072577e-05 | 67624.2462376459 | 0.999950574162623 |
| ebi-fl187-GCST90094766 | ebi-gm430-GCST90011614 | OTU97_80 (Ruminococcus) prevalence \|\| id:ebi-gm430-GCST90011614 | Fruit liking \|\| id:ebi-fl187-GCST90094766 | MR Egger | 8 | -4.338977027 | 2.09028448656443 | 0.0832188832799118 | -8.43593462 | -0.242019433 | 0.0130498710482994 | 0.00021693026396588 | 0.785040921740775 | 0.999950574162623 |
| ebi-fl187-GCST90094766 | ebi-gm430-GCST90011623 | OTU99_11 (Parabacteroides) prevalence \|\| id:ebi-gm430-GCST90011623 | Fruit liking \|\| id:ebi-fl187-GCST90094766 | MR Egger | 8 | -0.880528238 | 2.51875242343318 | 0.738590406975878 | -5.817282988 | 4.05622651199993 | 0.414563865474638 | 0.00297567912757926 | 57.7559579473496 | 0.999950574162623 |
| ebi-fl187-GCST90094766 | ebi-gm430-GCST90011642 | OTU99_19 (Bacteria) prevalence \|\| id:ebi-gm430-GCST90011642 | Fruit liking \|\| id:ebi-fl187-GCST90094766 | MR Egger | 8 | 0.372123707610003 | 5.48448885830005 | 0.948109447404249 | -10.37747445 | 11.1217218698781 | 1.45081244673266 | 3.11257703072577e-05 | 67624.2462376459 | 0.999950574162623 |
| ebi-fl187-GCST90094766 | ebi-gm430-GCST90011653 | OTU99_30 (Parasutterella) prevalence \|\| id:ebi-gm430-GCST90011653 | Fruit liking \|\| id:ebi-fl187-GCST90094766 | MR Egger | 8 | -3.000696249 | 1.98210498177702 | 0.180822579020428 | -6.885622013 | 0.884229515126476 | 0.0497524162281708 | 0.00102238002342094 | 2.42111823767709 | 0.999950574162623 |
| ebi-fl187-GCST90094766 | ebi-gm430-GCST90011675 | OTU99_8 (Ruminococcaceae) prevalence \|\| id:ebi-gm430-GCST90011675 | Fruit liking \|\| id:ebi-fl187-GCST90094766 | MR Egger | 8 | -1.502455354 | 2.89184209256622 | 0.621979426945781 | -7.170465856 | 4.16555514720965 | 0.222582968617495 | 0.000768964421352963 | 64.428439785819 | 0.999950574162623 |
| ebi-fl187-GCST90094766 | ebi-gm430-GCST90011679 | OTU99_92 (Ruminococcus) prevalence \|\| id:ebi-gm430-GCST90011679 | Fruit liking \|\| id:ebi-fl187-GCST90094766 | MR Egger | 8 | -3.819240557 | 2.18442374407147 | 0.130980590816077 | -8.100711096 | 0.46222998097543 | 0.0219444601253473 | 0.000303323369402471 | 1.58761038142759 | 0.999950574162623 |
| ebi-fl187-GCST90094766 | ebi-gm430-GCST90011684 | TestASV_11 (Lachnospiraceae) prevalence \|\| id:ebi-gm430-GCST90011684 | Fruit liking \|\| id:ebi-fl187-GCST90094766 | MR Egger | 8 | -2.584827091 | 2.44619917293749 | 0.331338833454466 | -7.37937747 | 2.20972328755695 | 0.0754091173587945 | 0.000623989216896953 | 9.11319431 | 0.999950574162623 |
| ebi-fl187-GCST90094766 | ebi-gm430-GCST90011688 | TestASV_15 (Bacteroides) prevalence \|\| id:ebi-gm430-GCST90011688 | Fruit liking \|\| id:ebi-fl187-GCST90094766 | MR Egger | 8 | 1.48535054744976 | 2.07973959193828 | 0.501929367350164 | -2.590939053 | 5.5616401476488 | 4.41651333893976 | 0.0749496253771716 | 260.249333800859 | 0.999950574162623 |
| ebi-fl187-GCST90094766 | ebi-gm430-GCST90011692 | TestASV_19 (Prevotella) prevalence \|\| id:ebi-gm430-GCST90011692 | Fruit liking \|\| id:ebi-fl187-GCST90094766 | MR Egger | 8 | -0.768188044 | 4.05292974345411 | 0.855919820075948 | -8.711930341 | 7.17555425311068 | 0.463852788128854 | 0.000164610193745403 | 1307.08435583091 | 0.999950574162623 |
| ebi-fl187-GCST90094766 | ebi-gm430-GCST90011693 | TestASV_2 (Bacteroides) prevalence \|\| id:ebi-gm430-GCST90011693 | Fruit liking \|\| id:ebi-fl187-GCST90094766 | MR Egger | 8 | 0.992046546693379 | 2.81363613654125 | 0.736454489012171 | -4.522680281 | 6.50677337431422 | 2.69674784912693 | 0.0108598771040363 | 669.662178687805 | 0.999950574162623 |
| ebi-fl187-GCST90094766 | ebi-gm430-GCST90011716 | TestASV_40 (Barnesiella) prevalence \|\| id:ebi-gm430-GCST90011716 | Fruit liking \|\| id:ebi-fl187-GCST90094766 | MR Egger | 8 | -2.506055015 | 4.55414964834584 | 0.601996366002708 | -11.43218833 | 6.42007829612119 | 0.0815894744727333 | 1.08408589583218e-05 | 614.051190069841 | 0.999950574162623 |
| ebi-fl187-GCST90094766 | ebi-gm430-GCST90011718 | TestASV_42 (Alphaproteobacteria) prevalence \|\| id:ebi-gm430-GCST90011718 | Fruit liking \|\| id:ebi-fl187-GCST90094766 | MR Egger | 8 | 6.54618945600094 | 3.2246457440921 | 0.0886577491751256 | 0.225883797580419 | 12.8664951144215 | 696.584743281966 | 1.25343000521594 | 387121.97933191 | 0.999950574162623 |
| ebi-fl187-GCST90094766 | ebi-hbsm44-GCST90104929 | Sulcal depth of the right inferior frontal sulcus \|\| id:ebi-hbsm44-GCST90104929 | Fruit liking \|\| id:ebi-fl187-GCST90094766 | MR Egger | 8 | -0.784164929 | 1.64625374072227 | 0.650687307153186 | -4.010822261 | 2.44249240305986 | 0.456500753312961 | 0.0181184909892988 | 11.501671849956 | 0.999950574162623 |
| ebi-fl187-GCST90094766 | ebi-hbsm44-GCST90104935 | Sulcal opening distance of the left calloso-marginal posterior fissure \|\| id:ebi-hbsm44-GCST90104935 | Fruit liking \|\| id:ebi-fl187-GCST90094766 | MR Egger | 9 | -0.452440699 | 0.474856403124582 | 0.372427412600289 | -1.383159249 | 0.478277851011531 | 0.636073790793069 | 0.250785007871536 | 1.61329367639518 | 0.999950574162623 |
| ebi-fl187-GCST90094766 | ebi-hbsm44-GCST90104936 | Sulcal opening distance of the left anterior inferior temporal sulcus \|\| id:ebi-hbsm44-GCST90104936 | Fruit liking \|\| id:ebi-fl187-GCST90094766 | MR Egger | 9 | -0.114837333 | 0.362625639159362 | 0.760719460711232 | -0.825583586 | 0.595908919613376 | 0.891511151433126 | 0.43797931931714 | 1.81467959347667 | 0.999950574162623 |
| ebi-fl187-GCST90094766 | ebi-hbsm44-GCST90104941 | Sulcal opening distance of the right posterior inferior temporal sulcus \|\| id:ebi-hbsm44-GCST90104941 | Fruit liking \|\| id:ebi-fl187-GCST90094766 | MR Egger | 9 | 0.10198555758497 | 0.213662251063358 | 0.647676448864866 | -0.316792454 | 0.520763569669153 | 1.10736747855173 | 0.728481932584571 | 1.68331248546326 | 0.999950574162623 |
| ebi-fl187-GCST90094766 | ebi-IPs91-GCST90274819 | Leukemia inhibitory factor levels \|\| id:ebi-IPs91-GCST90274819 | Fruit liking \|\| id:ebi-fl187-GCST90094766 | MR Egger | 10 | 0.923074743762249 | 0.750746871715554 | 0.253800826217494 | -0.548389125 | 2.39453861232473 | 2.51701768855418 | 0.577879953492636 | 10.9631386349438 | 0.999950574162623 |
| ebi-fl187-GCST90094766 | ebi-IPs91-GCST90274837 | STAM binding protein levels \|\| id:ebi-IPs91-GCST90274837 | Fruit liking \|\| id:ebi-fl187-GCST90094766 | MR Egger | 10 | -0.373695976 | 0.635295406177375 | 0.572609381650244 | -1.618874972 | 0.871483019832405 | 0.688186104930478 | 0.198121465871369 | 2.39045331577987 | 0.999950574162623 |
| ebi-fl187-GCST90094766 | ebi-IPs91-GCST90274840 | TNF-beta levels \|\| id:ebi-IPs91-GCST90274840 | Fruit liking \|\| id:ebi-fl187-GCST90094766 | MR Egger | 10 | -0.196360087 | 0.798499866937411 | 0.811942598969398 | -1.761419826 | 1.36869965210283 | 0.821716291966737 | 0.171800763333176 | 3.93023669617872 | 0.999950574162623 |
| ebi-fl187-GCST90094766 | ebi-IPs91-GCST90274842 | Tumor necrosis factor ligand superfamily member 14 levels \|\| id:ebi-IPs91-GCST90274842 | Fruit liking \|\| id:ebi-fl187-GCST90094766 | MR Egger | 10 | -0.298638662 | 0.723713305721941 | 0.690701893353595 | -1.717116741 | 1.11983941732069 | 0.741827411524033 | 0.179583186995059 | 3.0643620803076 | 0.999950574162623 |
| ebi-fl187-GCST90094766 | ebi-met1400-GCST90199628 | 3-methylhistidine levels \|\| id:ebi-met1400-GCST90199628 | Fruit liking \|\| id:ebi-fl187-GCST90094766 | MR Egger | 9 | 0.779830540726129 | 0.870567937496513 | 0.400128416672013 | -0.926482617 | 2.48614369821929 | 2.18110262611242 | 0.395943950547372 | 12.0148537666957 | 0.999950574162623 |
| ebi-fl187-GCST90094766 | ebi-met1400-GCST90199651 | Docosahexaenoate DHA; 22:6n3 levels \|\| id:ebi-met1400-GCST90199651 | Fruit liking \|\| id:ebi-fl187-GCST90094766 | MR Egger | 9 | -0.098567026 | 0.835198659535094 | 0.90936984913574 | -1.735556399 | 1.53842234681463 | 0.906134956091299 | 0.176302078768266 | 4.65723696729533 | 0.999950574162623 |
| ebi-fl187-GCST90094766 | ebi-met1400-GCST90199652 | 1-stearoyl-2-oleoyl-GPS (18:0/18:1) levels \|\| id:ebi-met1400-GCST90199652 | Fruit liking \|\| id:ebi-fl187-GCST90094766 | MR Egger | 9 | -1.991008555 | 1.11778352356573 | 0.118085666130957 | -4.181864261 | 0.19984715090227 | 0.136557630050361 | 0.0152700137166841 | 1.22121608211762 | 0.999950574162623 |
| ebi-fl187-GCST90094766 | ebi-met1400-GCST90199653 | 1-myristoyl-2-palmitoyl-gpc (14:0/16:0) levels \|\| id:ebi-met1400-GCST90199653 | Fruit liking \|\| id:ebi-fl187-GCST90094766 | MR Egger | 9 | -0.394978708 | 0.867450978727562 | 0.662657062669042 | -2.095182627 | 1.30522521007578 | 0.673694383241862 | 0.123047769813875 | 3.68851969197132 | 0.999950574162623 |
| ebi-fl187-GCST90094766 | ebi-met1400-GCST90199667 | Erythritol levels in elite athletes \|\| id:ebi-met1400-GCST90199667 | Fruit liking \|\| id:ebi-fl187-GCST90094766 | MR Egger | 9 | 0.575299457767504 | 0.819987696880284 | 0.505581046403642 | -1.031876428 | 2.18247534365286 | 1.7776627821442 | 0.356337690784761 | 8.86823103124791 | 0.999950574162623 |
| ebi-fl187-GCST90094766 | ebi-met1400-GCST90199674 | 3-hydroxymyristate levels \|\| id:ebi-met1400-GCST90199674 | Fruit liking \|\| id:ebi-fl187-GCST90094766 | MR Egger | 9 | -0.295374469 | 0.906801518336338 | 0.754147748737805 | -2.072705445 | 1.48195650681061 | 0.744252835566371 | 0.125844854392301 | 4.40154892246806 | 0.999950574162623 |
| ebi-fl187-GCST90094766 | ebi-met1400-GCST90199685 | 2-linoleoylglycerol (18:2) levels \|\| id:ebi-met1400-GCST90199685 | Fruit liking \|\| id:ebi-fl187-GCST90094766 | MR Egger | 9 | -0.537605324 | 0.995917137120466 | 0.606066373686866 | -2.489602913 | 1.41439226488009 | 0.58414541791205 | 0.0829428956129895 | 4.11398549261891 | 0.999950574162623 |
| ebi-fl187-GCST90094766 | ebi-met1400-GCST90199703 | Gamma-glutamylvaline levels \|\| id:ebi-met1400-GCST90199703 | Fruit liking \|\| id:ebi-fl187-GCST90094766 | MR Egger | 9 | 0.555210631322113 | 0.852105439214718 | 0.53547370407234 | -1.11491603 | 2.22533729218296 | 1.74230793075054 | 0.327942815252918 | 9.25660445774693 | 0.999950574162623 |
| ebi-fl187-GCST90094766 | ebi-met1400-GCST90199779 | Gamma-glutamyl-2-aminobutyrate levels \|\| id:ebi-met1400-GCST90199779 | Fruit liking \|\| id:ebi-fl187-GCST90094766 | MR Egger | 9 | 1.97601222083105 | 1.1781640322219 | 0.137405100411348 | -0.333189282 | 4.28521372398597 | 7.2139180375204 | 0.71663453506701 | 72.6180652837168 | 0.999950574162623 |
| ebi-fl187-GCST90094766 | ebi-met1400-GCST90199824 | 4-vinylphenol sulfate levels \|\| id:ebi-met1400-GCST90199824 | Fruit liking \|\| id:ebi-fl187-GCST90094766 | MR Egger | 9 | 0.629831762704315 | 1.47565303690852 | 0.682333713551274 | -2.26244819 | 3.52211171504502 | 1.87729472170839 | 0.104095327443622 | 33.8558469309097 | 0.999950574162623 |
| ebi-fl187-GCST90094766 | ebi-met1400-GCST90199830 | 4-ethylphenylsulfate levels \|\| id:ebi-met1400-GCST90199830 | Fruit liking \|\| id:ebi-fl187-GCST90094766 | MR Egger | 9 | 0.315068897081889 | 0.87568419247066 | 0.729608719679902 | -1.40127212 | 2.03140991432438 | 1.37035372107721 | 0.246283462420313 | 7.62482913962501 | 0.999950574162623 |
| ebi-fl187-GCST90094766 | ebi-met1400-GCST90199833 | Hexanoylglutamine levels \|\| id:ebi-met1400-GCST90199833 | Fruit liking \|\| id:ebi-fl187-GCST90094766 | MR Egger | 9 | 0.72384101693642 | 0.860974795982612 | 0.428283373454306 | -0.963669583 | 2.41135161706234 | 2.06233949777171 | 0.38149040351419 | 11.1490201716465 | 0.999950574162623 |
| ebi-fl187-GCST90094766 | ebi-met1400-GCST90199837 | Carotenoid levels (cryptoxanthin) \|\| id:ebi-met1400-GCST90199837 | Fruit liking \|\| id:ebi-fl187-GCST90094766 | MR Egger | 9 | 1.10874055533601 | 0.865894353359464 | 0.241177813083646 | -0.588412377 | 2.80589348792056 | 3.03053919348813 | 0.555208046317071 | 16.5418492476652 | 0.999950574162623 |
| ebi-fl187-GCST90094766 | ebi-met1400-GCST90199869 | 2-hydroxyhippurate levels \|\| id:ebi-met1400-GCST90199869 | Fruit liking \|\| id:ebi-fl187-GCST90094766 | MR Egger | 9 | -1.222517319 | 0.879381274629673 | 0.207068855173361 | -2.946104617 | 0.501069979355876 | 0.29448791307636 | 0.0525439867567977 | 1.65048631253414 | 0.999950574162623 |
| ebi-fl187-GCST90094766 | ebi-met1400-GCST90199902 | Hydantoin-5-propionate levels \|\| id:ebi-met1400-GCST90199902 | Fruit liking \|\| id:ebi-fl187-GCST90094766 | MR Egger | 9 | -0.23036763 | 0.873011242655654 | 0.79947433993449 | -1.941469666 | 1.48073440567942 | 0.794241561858843 | 0.143492908154369 | 4.39617307013766 | 0.999950574162623 |
| ebi-fl187-GCST90094766 | ebi-met1400-GCST90199904 | Pantoate levels \|\| id:ebi-met1400-GCST90199904 | Fruit liking \|\| id:ebi-fl187-GCST90094766 | MR Egger | 9 | -1.324052645 | 0.885367946341889 | 0.178442513821912 | -3.05937382 | 0.411268530152615 | 0.266054888251334 | 0.0469170645743682 | 1.50873044178261 | 0.999950574162623 |
| ebi-fl187-GCST90094766 | ebi-met1400-GCST90199936 | 3-methyl catechol sulfate (2) levels \|\| id:ebi-met1400-GCST90199936 | Fruit liking \|\| id:ebi-fl187-GCST90094766 | MR Egger | 9 | -0.96656848 | 0.99594153656236 | 0.364124964225596 | -2.918613892 | 0.98547693178879 | 0.380386103647442 | 0.0540084970760902 | 2.67908932263436 | 0.999950574162623 |
| ebi-fl187-GCST90094766 | ebi-met1400-GCST90199977 | 4-hydroxychlorothalonil levels \|\| id:ebi-met1400-GCST90199977 | Fruit liking \|\| id:ebi-fl187-GCST90094766 | MR Egger | 9 | -2.065418196 | 0.866435540555381 | 0.0486090354655398 | -3.763631856 | -0.367204537 | 0.126765266703199 | 0.0231993305601978 | 0.692667954389288 | 0.999950574162623 |
| ebi-fl187-GCST90094766 | ebi-met1400-GCST90200040 | Sphingomyelin (d18:2/24:1, d18:1/24:2) levels \|\| id:ebi-met1400-GCST90200040 | Fruit liking \|\| id:ebi-fl187-GCST90094766 | MR Egger | 9 | 0.045714922204185 | 0.79624797770509 | 0.955820768544234 | -1.514931114 | 1.60636095850616 | 1.04677595583529 | 0.219823327054424 | 4.9846388752164 | 0.999950574162623 |
| ebi-fl187-GCST90094766 | ebi-met1400-GCST90200077 | 1-stearoyl-2-oleoyl-GPI (18:0/18:1) levels \|\| id:ebi-met1400-GCST90200077 | Fruit liking \|\| id:ebi-fl187-GCST90094766 | MR Egger | 9 | 1.52610950211304 | 0.864667922490614 | 0.120922253195132 | -0.168639626 | 3.22085863019464 | 4.60024472015207 | 0.844813297308666 | 25.0496193096201 | 0.999950574162623 |
| ebi-fl187-GCST90094766 | ebi-met1400-GCST90200080 | 1-palmitoyl-2-oleoyl-GPI (16:0/18:1) levels \|\| id:ebi-met1400-GCST90200080 | Fruit liking \|\| id:ebi-fl187-GCST90094766 | MR Egger | 9 | 0.0800734530015084 | 0.871730442909826 | 0.92938610715182 | -1.628518215 | 1.78866512110477 | 1.083366641 | 0.196220114817213 | 5.98146260662576 | 0.999950574162623 |
| ebi-fl187-GCST90094766 | ebi-met1400-GCST90200082 | 1-oleoyl-2-linoleoyl-GPE (18:1/18:2) levels \|\| id:ebi-met1400-GCST90200082 | Fruit liking \|\| id:ebi-fl187-GCST90094766 | MR Egger | 9 | 0.732961887329089 | 0.876483731385672 | 0.430630426436793 | -0.984946226 | 2.45087000084501 | 2.08123587373521 | 0.373459308727814 | 11.5984329775518 | 0.999950574162623 |
| ebi-fl187-GCST90094766 | ebi-met1400-GCST90200100 | Perfluorooctanesulfonate (PFOS) levels \|\| id:ebi-met1400-GCST90200100 | Fruit liking \|\| id:ebi-fl187-GCST90094766 | MR Egger | 9 | -0.303695159 | 0.804555069942056 | 0.717008784225201 | -1.880623096 | 1.27323277854829 | 0.738085831323336 | 0.152495057144896 | 3.5723826371804 | 0.999950574162623 |
| ebi-fl187-GCST90094766 | ebi-met1400-GCST90200102 | 1-palmitoleoyl-2-linolenoyl-GPC (16:1/18:3) levels \|\| id:ebi-met1400-GCST90200102 | Fruit liking \|\| id:ebi-fl187-GCST90094766 | MR Egger | 9 | 0.366466490265167 | 0.854987416056384 | 0.681079146567072 | -1.309308845 | 2.04224182573568 | 1.44262805765664 | 0.27000660827104 | 7.70786954461884 | 0.999950574162623 |
| ebi-fl187-GCST90094766 | ebi-met1400-GCST90200114 | Glycosyl-N-tricosanoyl-sphingadienine (d18:2/23:0) levels \|\| id:ebi-met1400-GCST90200114 | Fruit liking \|\| id:ebi-fl187-GCST90094766 | MR Egger | 9 | 0.786507429346153 | 1.12626387288671 | 0.507497280630347 | -1.420969762 | 2.9939846202041 | 2.19571433149704 | 0.241479725568306 | 19.9650774581395 | 0.999950574162623 |
| ebi-fl187-GCST90094766 | ebi-met1400-GCST90200135 | Arachidoylcarnitine (C20) levels \|\| id:ebi-met1400-GCST90200135 | Fruit liking \|\| id:ebi-fl187-GCST90094766 | MR Egger | 9 | 1.25416931551582 | 0.895721255125581 | 0.204189943533157 | -0.501444345 | 3.00978297556195 | 3.50492567736557 | 0.605655252818569 | 20.2829975414025 | 0.999950574162623 |
| ebi-fl187-GCST90094766 | ebi-met1400-GCST90200146 | Docosahexaenoylcarnitine (C22:6) levels \|\| id:ebi-met1400-GCST90200146 | Fruit liking \|\| id:ebi-fl187-GCST90094766 | MR Egger | 9 | -1.047035519 | 1.0525857293194 | 0.353002876151676 | -3.110103548 | 1.01603251071161 | 0.350976672178646 | 0.0445963372298227 | 2.76221394099648 | 0.999950574162623 |
| ebi-fl187-GCST90094766 | ebi-met1400-GCST90200162 | 3-hydroxyphenylacetoylglutamine levels \|\| id:ebi-met1400-GCST90200162 | Fruit liking \|\| id:ebi-fl187-GCST90094766 | MR Egger | 9 | -1.603098268 | 1.06154114673756 | 0.174749372441292 | -3.683718916 | 0.477522379328159 | 0.201271956446848 | 0.0251293469185755 | 1.61207533897336 | 0.999950574162623 |
| ebi-fl187-GCST90094766 | ebi-met1400-GCST90200172 | 3-carboxy-4-methyl-5-pentyl-2-furanpropionate (3-CMPFP) levels \|\| id:ebi-met1400-GCST90200172 | Fruit liking \|\| id:ebi-fl187-GCST90094766 | MR Egger | 9 | 0.307524541171184 | 0.86743596555723 | 0.733388097352372 | -1.392649951 | 2.00769903366335 | 1.36005418541499 | 0.248416140960688 | 7.44616424726422 | 0.999950574162623 |
| ebi-fl187-GCST90094766 | ebi-met1400-GCST90200222 | Dihydrocaffeate sulfate (2) levels \|\| id:ebi-met1400-GCST90200222 | Fruit liking \|\| id:ebi-fl187-GCST90094766 | MR Egger | 9 | 0.314765971799725 | 0.995807809244385 | 0.761149975996235 | -1.637017334 | 2.26654927791872 | 1.36993866915762 | 0.194559483616084 | 9.64605745437024 | 0.999950574162623 |
| ebi-fl187-GCST90094766 | ebi-met1400-GCST90200223 | 4-methylhexanoylglutamine levels \|\| id:ebi-met1400-GCST90200223 | Fruit liking \|\| id:ebi-fl187-GCST90094766 | MR Egger | 9 | 1.4341686971962 | 1.04606601725064 | 0.212707960298735 | -0.616120697 | 3.48445809100745 | 4.1961552828276 | 0.540035340273101 | 32.604753512423 | 0.999950574162623 |
| ebi-fl187-GCST90094766 | ebi-met1400-GCST90200224 | 2-ketocaprylate levels \|\| id:ebi-met1400-GCST90200224 | Fruit liking \|\| id:ebi-fl187-GCST90094766 | MR Egger | 9 | -0.165345871 | 0.909611781642414 | 0.860910069829271 | -1.948184963 | 1.61749322098587 | 0.847600492925935 | 0.142532538775922 | 5.04043919920447 | 0.999950574162623 |
| ebi-fl187-GCST90094766 | ebi-met1400-GCST90200243 | Hydroxypalmitoyl sphingomyelin (d18:1/16:0(OH)) levels \|\| id:ebi-met1400-GCST90200243 | Fruit liking \|\| id:ebi-fl187-GCST90094766 | MR Egger | 9 | 0.923274613750838 | 0.853462245525315 | 0.315194887088442 | -0.749511387 | 2.59606061498046 | 2.51752081512915 | 0.472597413349389 | 13.4108035202532 | 0.999950574162623 |
| ebi-fl187-GCST90094766 | ebi-met1400-GCST90200273 | 2-methoxyhydroquinone sulfate (2) levels \|\| id:ebi-met1400-GCST90200273 | Fruit liking \|\| id:ebi-fl187-GCST90094766 | MR Egger | 9 | 1.24833042987077 | 1.76783924815782 | 0.502922932297449 | -2.216634497 | 4.7132953562601 | 3.48452044701657 | 0.108975248936141 | 111.418719977338 | 0.999950574162623 |
| ebi-fl187-GCST90094766 | ebi-met1400-GCST90200276 | 2,4-di-tert-butylphenol levels \|\| id:ebi-met1400-GCST90200276 | Fruit liking \|\| id:ebi-fl187-GCST90094766 | MR Egger | 9 | 0.320048610309156 | 1.06087326585511 | 0.771658263617113 | -1.759262991 | 2.39936021138517 | 1.37719470856941 | 0.172171709213573 | 11.0161261334684 | 0.999950574162623 |
| ebi-fl187-GCST90094766 | ebi-met1400-GCST90200289 | N-acetylmethionine levels \|\| id:ebi-met1400-GCST90200289 | Fruit liking \|\| id:ebi-fl187-GCST90094766 | MR Egger | 9 | -0.137460515 | 0.870725943017213 | 0.879017655509466 | -1.844083363 | 1.56916233351115 | 0.871568763382425 | 0.158170239135888 | 4.80262351156561 | 0.999950574162623 |
| ebi-fl187-GCST90094766 | ebi-met1400-GCST90200308 | 3-hydroxyisobutyrate levels \|\| id:ebi-met1400-GCST90200308 | Fruit liking \|\| id:ebi-fl187-GCST90094766 | MR Egger | 9 | 0.912905135433472 | 0.882060828132108 | 0.335108752432595 | -0.815934088 | 2.6417443585724 | 2.49155032057163 | 0.442226056453396 | 14.037668991571 | 0.999950574162623 |
| ebi-fl187-GCST90094766 | ebi-met1400-GCST90200383 | Cystathionine levels \|\| id:ebi-met1400-GCST90200383 | Fruit liking \|\| id:ebi-fl187-GCST90094766 | MR Egger | 9 | -0.400509645 | 0.956147334708037 | 0.687858595426186 | -2.274558421 | 1.47353913111034 | 0.669978507870127 | 0.102842311420719 | 4.36465492467966 | 0.999950574162623 |
| ebi-fl187-GCST90094766 | ebi-met1400-GCST90200384 | Guanidinoacetate levels \|\| id:ebi-met1400-GCST90200384 | Fruit liking \|\| id:ebi-fl187-GCST90094766 | MR Egger | 9 | -0.131281195 | 0.903520326957861 | 0.888569620143606 | -1.902181036 | 1.63961864572538 | 0.876971139718557 | 0.149242760171529 | 5.15320394111809 | 0.999950574162623 |
| ebi-fl187-GCST90094766 | ebi-met1400-GCST90200403 | Citrulline levels \|\| id:ebi-met1400-GCST90200403 | Fruit liking \|\| id:ebi-fl187-GCST90094766 | MR Egger | 9 | 0.507302915647997 | 1.08428288941034 | 0.654094968034021 | -1.617891548 | 2.63249737889227 | 1.66080581556473 | 0.198316399267126 | 13.9084612629453 | 0.999950574162623 |
| ebi-fl187-GCST90094766 | ebi-met1400-GCST90200434 | 12,13-DiHOME levels \|\| id:ebi-met1400-GCST90200434 | Fruit liking \|\| id:ebi-fl187-GCST90094766 | MR Egger | 9 | -0.264996613 | 0.881225681134143 | 0.77236797142731 | -1.992198948 | 1.46220572206376 | 0.767208548538143 | 0.136395169560105 | 4.31546776068651 | 0.999950574162623 |
| ebi-fl187-GCST90094766 | ebi-met1400-GCST90200437 | Fructose levels \|\| id:ebi-met1400-GCST90200437 | Fruit liking \|\| id:ebi-fl187-GCST90094766 | MR Egger | 9 | 0.740762270509505 | 0.878376845836336 | 0.426918114972454 | -0.980856347 | 2.46238088834872 | 2.09753379340994 | 0.374989839768398 | 11.7327125908638 | 0.999950574162623 |
| ebi-fl187-GCST90094766 | ebi-met1400-GCST90200448 | N-stearoyl-sphinganine (d18:0/18:0) levels \|\| id:ebi-met1400-GCST90200448 | Fruit liking \|\| id:ebi-fl187-GCST90094766 | MR Egger | 9 | -0.067018432 | 0.969615525971778 | 0.946828268015196 | -1.967464862 | 1.83342799934002 | 0.935177964384798 | 0.139810847043276 | 6.25529308752556 | 0.999950574162623 |
| ebi-fl187-GCST90094766 | ebi-met1400-GCST90200468 | X-11847 levels \|\| id:ebi-met1400-GCST90200468 | Fruit liking \|\| id:ebi-fl187-GCST90094766 | MR Egger | 9 | -0.607709259 | 0.927544845889969 | 0.53328292399914 | -2.425697157 | 1.21027863867936 | 0.544596971747868 | 0.0884164573799642 | 3.35441919327743 | 0.999950574162623 |
| ebi-fl187-GCST90094766 | ebi-met1400-GCST90200476 | X-11843 levels \|\| id:ebi-met1400-GCST90200476 | Fruit liking \|\| id:ebi-fl187-GCST90094766 | MR Egger | 9 | 0.578129124687985 | 0.919944427875689 | 0.549663392917393 | -1.224961954 | 2.38122020332434 | 1.78270009931833 | 0.293768876856778 | 10.8180950892867 | 0.999950574162623 |
| ebi-fl187-GCST90094766 | ebi-met1400-GCST90200511 | X-12906 levels \|\| id:ebi-met1400-GCST90200511 | Fruit liking \|\| id:ebi-fl187-GCST90094766 | MR Egger | 9 | -1.237500113 | 0.875770633336373 | 0.200530619797256 | -2.954010555 | 0.479010327916242 | 0.290108550658657 | 0.0521302150596196 | 1.61447580964346 | 0.999950574162623 |
| ebi-fl187-GCST90094766 | ebi-met1400-GCST90200515 | X-12849 levels \|\| id:ebi-met1400-GCST90200515 | Fruit liking \|\| id:ebi-fl187-GCST90094766 | MR Egger | 9 | -0.883333293 | 1.48275192914495 | 0.570101724471339 | -3.789527074 | 2.02286048782243 | 0.413402620316424 | 0.0226062904163511 | 7.55991909052322 | 0.999950574162623 |
| ebi-fl187-GCST90094766 | ebi-met1400-GCST90200551 | X-18886 levels \|\| id:ebi-met1400-GCST90200551 | Fruit liking \|\| id:ebi-fl187-GCST90094766 | MR Egger | 9 | 0.473983835114593 | 0.861094328837676 | 0.599132694529375 | -1.213761049 | 2.16172871963644 | 1.60638101985248 | 0.297077851160929 | 8.68614058859755 | 0.999950574162623 |
| ebi-fl187-GCST90094766 | ebi-met1400-GCST90200564 | X-21319 levels \|\| id:ebi-met1400-GCST90200564 | Fruit liking \|\| id:ebi-fl187-GCST90094766 | MR Egger | 9 | -0.655560976 | 0.882383821188315 | 0.481699711147419 | -2.385033266 | 1.07391131324953 | 0.519150749628798 | 0.0920859162158594 | 2.92680479182467 | 0.999950574162623 |
| ebi-fl187-GCST90094766 | ebi-met1400-GCST90200579 | X-21442 levels \|\| id:ebi-met1400-GCST90200579 | Fruit liking \|\| id:ebi-fl187-GCST90094766 | MR Egger | 9 | -0.706387222 | 0.952742078522113 | 0.482562821961655 | -2.573761696 | 1.16098725176412 | 0.493423611110617 | 0.0762481827898731 | 3.19308409844197 | 0.999950574162623 |
| ebi-fl187-GCST90094766 | ebi-met1400-GCST90200581 | X-21441 levels \|\| id:ebi-met1400-GCST90200581 | Fruit liking \|\| id:ebi-fl187-GCST90094766 | MR Egger | 9 | 0.444739918309821 | 0.875697407241406 | 0.627152823421605 | -1.271627 | 2.16110683650298 | 1.56008439367817 | 0.280375080259719 | 8.68074050355556 | 0.999950574162623 |
| ebi-fl187-GCST90094766 | ebi-met1400-GCST90200648 | X-24585 levels \|\| id:ebi-met1400-GCST90200648 | Fruit liking \|\| id:ebi-fl187-GCST90094766 | MR Egger | 9 | -0.826649534 | 0.853433410839394 | 0.365004501375156 | -2.499379019 | 0.846079951350622 | 0.437512704873791 | 0.0821359876669251 | 2.33049327539848 | 0.999950574162623 |
| ebi-fl187-GCST90094766 | ebi-met1400-GCST90200720 | Adenosine 5'-diphosphate (ADP) to pantothenate ratio \|\| id:ebi-met1400-GCST90200720 | Fruit liking \|\| id:ebi-fl187-GCST90094766 | MR Egger | 9 | -0.887580011 | 1.15878268993915 | 0.468736900498059 | -3.158794083 | 1.38363406150182 | 0.411650738689621 | 0.0424769338613039 | 3.98937294337253 | 0.999950574162623 |
| ebi-fl187-GCST90094766 | ebi-met1400-GCST90200724 | Cholate to taurocholate ratio \|\| id:ebi-met1400-GCST90200724 | Fruit liking \|\| id:ebi-fl187-GCST90094766 | MR Egger | 9 | 1.48620451387168 | 0.98133571466539 | 0.173678316632488 | -0.437213487 | 3.40962251461585 | 4.42028650388195 | 0.645833539709579 | 30.2538217280993 | 0.999950574162623 |
| ebi-fl187-GCST90094766 | ebi-met1400-GCST90200726 | N-acetylputrescine to (N(1) + N(8))-acetylspermidine ratio \|\| id:ebi-met1400-GCST90200726 | Fruit liking \|\| id:ebi-fl187-GCST90094766 | MR Egger | 9 | -1.057576764 | 0.869345371308165 | 0.263209149841124 | -2.761493691 | 0.646340164082619 | 0.347296372661709 | 0.0631973005571167 | 1.90854307700961 | 0.999950574162623 |
| ebi-fl187-GCST90094766 | ebi-met1400-GCST90200744 | Arginine to glutamate ratio \|\| id:ebi-met1400-GCST90200744 | Fruit liking \|\| id:ebi-fl187-GCST90094766 | MR Egger | 9 | 0.459513874376705 | 1.13175492292824 | 0.696853515442558 | -1.758725775 | 2.67775352331606 | 1.58330411318355 | 0.172264227494604 | 14.5523649993001 | 0.999950574162623 |
| ebi-fl187-GCST90094766 | ebi-met1400-GCST90200759 | Histidine to trans-urocanate ratio \|\| id:ebi-met1400-GCST90200759 | Fruit liking \|\| id:ebi-fl187-GCST90094766 | MR Egger | 9 | -0.655572001 | 1.00383299068426 | 0.534565370364849 | -2.623084663 | 1.31194066096593 | 0.519145026285161 | 0.0725786365743412 | 3.71337312241412 | 0.999950574162623 |
| ebi-fl187-GCST90094766 | ebi-met1400-GCST90200795 | Oleoyl-linoleoyl-glycerol (18:1 to 18:2) [2] to linoleoyl-arachidonoyl-glycerol (18:2 to 20:4) [2] ratio \|\| id:ebi-met1400-GCST90200795 | Fruit liking \|\| id:ebi-fl187-GCST90094766 | MR Egger | 9 | 0.69748160392813 | 0.990557780384509 | 0.504095180086182 | -1.244011646 | 2.63897485348177 | 2.00868766113567 | 0.288225636469988 | 13.998845381746 | 0.999950574162623 |
| ebi-fl187-GCST90094766 | ebi-met1400-GCST90200811 | Choline to taurocholate ratio \|\| id:ebi-met1400-GCST90200811 | Fruit liking \|\| id:ebi-fl187-GCST90094766 | MR Egger | 9 | 2.02224838240669 | 0.914523423090118 | 0.0626806371759072 | 0.229782473150059 | 3.81471429166332 | 7.55529303906538 | 1.25832626 | 45.3637937172162 | 0.999950574162623 |
| ebi-fl187-GCST90094766 | ebi-met1400-GCST90200831 | Cholate to phosphate ratio \|\| id:ebi-met1400-GCST90200831 | Fruit liking \|\| id:ebi-fl187-GCST90094766 | MR Egger | 9 | 0.40313943106714 | 0.910515597507476 | 0.671298521367253 | -1.38147114 | 2.18775000218179 | 1.4965155378758 | 0.251208717881627 | 8.91513150494644 | 0.999950574162623 |
| ebi-fl187-GCST90094766 | ebi-met1400-GCST90200841 | Adenosine 5'-diphosphate (ADP) to oxalate (ethanedioate) ratio \|\| id:ebi-met1400-GCST90200841 | Fruit liking \|\| id:ebi-fl187-GCST90094766 | MR Egger | 9 | -1.182654937 | 1.35241716453667 | 0.410856180829178 | -3.83339258 | 1.46808270514315 | 0.306464014800437 | 0.0216360888188261 | 4.34090436372584 | 0.999950574162623 |
| ebi-fl187-GCST90094766 | ebi-met1400-GCST90200844 | Aspartate to phosphate ratio \|\| id:ebi-met1400-GCST90200844 | Fruit liking \|\| id:ebi-fl187-GCST90094766 | MR Egger | 9 | 0.153359602530645 | 0.869090862348743 | 0.864929484787367 | -1.550058488 | 1.85677769273418 | 1.16574410810963 | 0.212235560299701 | 6.40307083164247 | 0.999950574162623 |
| ebi-fl187-GCST90094766 | ebi-met1400-GCST90200870 | Adenosine 5'-monophosphate (AMP) to acetoacetate ratio \|\| id:ebi-met1400-GCST90200870 | Fruit liking \|\| id:ebi-fl187-GCST90094766 | MR Egger | 9 | -0.967706743 | 0.960678572294322 | 0.347326730570403 | -2.850636745 | 0.915223258608535 | 0.379953370467053 | 0.0578075005289932 | 2.49733274070322 | 0.999950574162623 |
| ebi-fl187-GCST90094766 | ebi-met1400-GCST90200887 | Cholesterol to cortisol ratio \|\| id:ebi-met1400-GCST90200887 | Fruit liking \|\| id:ebi-fl187-GCST90094766 | MR Egger | 9 | 1.01746082723694 | 0.849852856774416 | 0.270186400621684 | -0.648250772 | 2.6831724265148 | 2.76616207573673 | 0.522959752972974 | 14.631436904552 | 0.999950574162623 |
| ebi-fl187-GCST90094766 | ebi-met1400-GCST90200890 | Cortisol to taurocholate ratio \|\| id:ebi-met1400-GCST90200890 | Fruit liking \|\| id:ebi-fl187-GCST90094766 | MR Egger | 9 | 1.65311721152254 | 0.911804823183988 | 0.11271576924704 | -0.134020242 | 3.44025466496316 | 5.22323640913329 | 0.87457236140207 | 31.1949014052515 | 0.999950574162623 |
| ebi-fl187-GCST90094766 | ebi-met1400-GCST90200948 | Adenosine 5'-diphosphate (ADP) to mannitol to sorbitol ratio \|\| id:ebi-met1400-GCST90200948 | Fruit liking \|\| id:ebi-fl187-GCST90094766 | MR Egger | 9 | -0.552304481 | 1.36484111626434 | 0.697803055060015 | -3.227393069 | 2.12278410707106 | 0.575621771423707 | 0.0396607570057881 | 8.35436458483662 | 0.999950574162623 |
| ebi-fl187-GCST90094766 | ebi-met1400-GCST90200972 | Leucine to N-palmitoyl-sphingosine (d18:1 to 16:0) ratio \|\| id:ebi-met1400-GCST90200972 | Fruit liking \|\| id:ebi-fl187-GCST90094766 | MR Egger | 9 | -0.350159817 | 1.04375408861764 | 0.747092360538036 | -2.395917831 | 1.69559819668149 | 0.704575477574788 | 0.0910890362297012 | 5.44990510546067 | 0.999950574162623 |
| ebi-fl187-GCST90094766 | ebi-met1400-GCST90200973 | Proline to glutamate ratio \|\| id:ebi-met1400-GCST90200973 | Fruit liking \|\| id:ebi-fl187-GCST90094766 | MR Egger | 9 | 0.681681631763567 | 1.15618129941759 | 0.573989545204363 | -1.584433715 | 2.94779697862204 | 1.97719986023747 | 0.20506388482576 | 19.0639092331873 | 0.999950574162623 |
| ebi-fl187-GCST90094766 | ebi-met1400-GCST90200983 | Cholesterol to linoleoyl-arachidonoyl-glycerol (18:2 to 20:4) [1] ratio \|\| id:ebi-met1400-GCST90200983 | Fruit liking \|\| id:ebi-fl187-GCST90094766 | MR Egger | 9 | 1.23605067557738 | 0.89899793251581 | 0.211548424121838 | -0.525985272 | 2.99808662330837 | 3.44199303955346 | 0.590972808374215 | 20.0471424682411 | 0.999950574162623 |
| ebi-fl187-GCST90094766 | ebi-met1400-GCST90201001 | Serine to alpha-ketobutyrate ratio \|\| id:ebi-met1400-GCST90201001 | Fruit liking \|\| id:ebi-fl187-GCST90094766 | MR Egger | 9 | 0.000277440169456053 | 0.889374809081358 | 0.999759804099942 | -1.742897186 | 1.74345206596892 | 1.00027747865954 | 0.175012621355125 | 5.71704501404627 | 0.999974376584515 |
| ebi-fl187-GCST90094766 | ebi-met1400-GCST90201005 | Fructose to maltose ratio \|\| id:ebi-met1400-GCST90201005 | Fruit liking \|\| id:ebi-fl187-GCST90094766 | MR Egger | 9 | 1.55296194510754 | 0.997257063204052 | 0.163372346176764 | -0.401661899 | 3.50758578898748 | 4.72544598621135 | 0.669206967142424 | 33.3676140043064 | 0.999950574162623 |
| ebi-fl187-GCST90094766 | ebi-met1400-GCST90201020 | Paraxanthine to 5-acetylamino-6-formylamino-3-methyluracil ratio \|\| id:ebi-met1400-GCST90201020 | Fruit liking \|\| id:ebi-fl187-GCST90094766 | MR Egger | 9 | 1.38194226164836 | 1.01937377356311 | 0.217311063540059 | -0.616030335 | 3.37991485783206 | 3.9826294287585 | 0.540084141194413 | 29.3682705286169 | 0.999950574162623 |
| ebi-fl187-GCST90094766 | ebi-pw11-GCST90275197 | Placental weight (fetal genotype, adjusted for sex) \|\| id:ebi-pw11-GCST90275197 | Fruit liking \|\| id:ebi-fl187-GCST90094766 | MR Egger | 8 | 0.301666956406062 | 0.511196741214369 | 0.576644448487758 | -0.700278656 | 1.30361256918622 | 1.35211083986091 | 0.496446946409202 | 3.68257622791873 | 0.999950574162623 |
| ebi-fl187-GCST90094766 | prot-a-1017 | FAS-associated factor 2 \|\| id:prot-a-1017 | Fruit liking \|\| id:ebi-fl187-GCST90094766 | MR Egger | 10 | -1.559694084 | 1.31430137447594 | 0.269384882353329 | -4.135724778 | 1.01633660971039 | 0.210200364965706 | 0.0159910709485389 | 2.7630540552228 | 0.999950574162623 |
| ebi-fl187-GCST90094766 | prot-a-1019 | Fas apoptotic inhibitory molecule 3 \|\| id:prot-a-1019 | Fruit liking \|\| id:ebi-fl187-GCST90094766 | MR Egger | 10 | 0.0741802564378233 | 1.31430137447594 | 0.956374830438288 | -2.501850438 | 2.65021095041067 | 1.07700092435026 | 0.0819332459091441 | 14.1570247605907 | 0.999950574162623 |
| ebi-fl187-GCST90094766 | prot-a-1052 | MIP18 family protein FAM96A \|\| id:prot-a-1052 | Fruit liking \|\| id:ebi-fl187-GCST90094766 | MR Egger | 10 | -2.032886784 | 1.31430137447594 | 0.160511337162818 | -4.608917478 | 0.543143910102975 | 0.130956930601958 | 0.0099625972048167 | 1.72141032304252 | 0.999950574162623 |
| ebi-fl187-GCST90094766 | prot-a-1066 | F-box/LRR-repeat protein 4 \|\| id:prot-a-1066 | Fruit liking \|\| id:ebi-fl187-GCST90094766 | MR Egger | 10 | -0.926598253 | 1.31430137447594 | 0.500810134989655 | -3.502628947 | 1.6494324407773 | 0.395898167650176 | 0.0301181003578342 | 5.20402539624308 | 0.999950574162623 |
| ebi-fl187-GCST90094766 | prot-a-1076 | Low affinity immunoglobulin gamma Fc region receptor III-B \|\| id:prot-a-1076 | Fruit liking \|\| id:ebi-fl187-GCST90094766 | MR Egger | 10 | -0.474142494 | 2.23093086088798 | 0.837008008303214 | -4.846766981 | 3.89848199332109 | 0.622418555326756 | 0.00785372779659524 | 49.327512749168 | 0.999950574162623 |
| ebi-fl187-GCST90094766 | prot-a-1081 | Fc receptor-like protein 3 \|\| id:prot-a-1081 | Fruit liking \|\| id:ebi-fl187-GCST90094766 | MR Egger | 10 | -2.371480452 | 1.31430137447594 | 0.108824294173761 | -4.947511146 | 0.204550242137535 | 0.0933424349573518 | 0.0071010604579869 | 1.22697310004842 | 0.999950574162623 |
| ebi-fl187-GCST90094766 | prot-a-1105 | Fibroblast growth factor receptor 4 \|\| id:prot-a-1105 | Fruit liking \|\| id:ebi-fl187-GCST90094766 | MR Egger | 10 | -1.51474406 | 1.31430137447594 | 0.28238500029176 | -4.090774754 | 1.06128663431345 | 0.219864449829392 | 0.0167262697990885 | 2.89008708333854 | 0.999950574162623 |
| ebi-fl187-GCST90094766 | prot-a-1143 | Follistatin-related protein 1 \|\| id:prot-a-1143 | Fruit liking \|\| id:ebi-fl187-GCST90094766 | MR Egger | 10 | 1.15097187275636 | 1.42507291189345 | 0.442637569271168 | -1.642171035 | 3.94411478006751 | 3.16126376333581 | 0.193559361737383 | 51.6306134287588 | 0.999950574162623 |
| ebi-fl187-GCST90094766 | prot-a-1151 | Alpha-(1,3)-fucosyltransferase 10 \|\| id:prot-a-1151 | Fruit liking \|\| id:ebi-fl187-GCST90094766 | MR Egger | 10 | -0.775785824 | 1.31430137447594 | 0.571305674571711 | -3.351816518 | 1.80024486981701 | 0.460341891048727 | 0.0350206805851443 | 6.05112902186748 | 0.999950574162623 |
| ebi-fl187-GCST90094766 | prot-a-1165 | Growth arrest and DNA damage-inducible proteins-interacting protein 1 \|\| id:prot-a-1165 | Fruit liking \|\| id:ebi-fl187-GCST90094766 | MR Egger | 10 | -1.391090935 | 1.31403638987749 | 0.320678716202236 | -3.96660226 | 1.18442038875954 | 0.248803727727862 | 0.0189376692291413 | 3.26879164390638 | 0.999950574162623 |
| ebi-fl187-GCST90094766 | prot-a-1194 | Growth/differentiation factor 11/8 \|\| id:prot-a-1194 | Fruit liking \|\| id:ebi-fl187-GCST90094766 | MR Egger | 10 | -0.142203691 | 1.34027580469476 | 0.918115021784473 | -2.769144269 | 2.48473688575675 | 0.867444547456156 | 0.0627156495337626 | 11.9979630045342 | 0.999950574162623 |
| ebi-fl187-GCST90094766 | prot-a-1216 | D-glucuronyl C5-epimerase \|\| id:prot-a-1216 | Fruit liking \|\| id:ebi-fl187-GCST90094766 | MR Egger | 10 | 1.63220771243721 | 1.31430137447594 | 0.249452301910092 | -0.943822982 | 4.20823840641006 | 5.11515505351705 | 0.389137323272076 | 67.2379894108157 | 0.999950574162623 |
| ebi-fl187-GCST90094766 | prot-a-1246 | Glypican-3 \|\| id:prot-a-1246 | Fruit liking \|\| id:ebi-fl187-GCST90094766 | MR Egger | 10 | -0.160294478 | 1.31430137447594 | 0.905937299170907 | -2.736325172 | 2.41573621575412 | 0.851892888125369 | 0.0648080683246721 | 11.1980114760233 | 0.999950574162623 |
| ebi-fl187-GCST90094766 | prot-a-1275 | Glutamate receptor 4 \|\| id:prot-a-1275 | Fruit liking \|\| id:ebi-fl187-GCST90094766 | MR Egger | 10 | -1.729032305 | 1.31430137447594 | 0.224773646246576 | -4.305062999 | 0.846998389324383 | 0.177456050303688 | 0.0135000350314174 | 2.33263467213971 | 0.999950574162623 |
| ebi-fl187-GCST90094766 | prot-a-1289 | Glutathione S-transferase P \|\| id:prot-a-1289 | Fruit liking \|\| id:ebi-fl187-GCST90094766 | MR Egger | 10 | 0.129560990887645 | 1.31430137447594 | 0.923898726297531 | -2.446469703 | 2.70559168486049 | 1.1383285370135 | 0.0865987668532319 | 14.9631675515128 | 0.999950574162623 |
| ebi-fl187-GCST90094766 | prot-a-130 | Apolipoprotein D \|\| id:prot-a-130 | Fruit liking \|\| id:ebi-fl187-GCST90094766 | MR Egger | 10 | -2.645633673 | 1.3142586672039 | 0.0789075039773384 | -5.22158066 | -0.069686685 | 0.0709603738351993 | 0.00539878872660115 | 0.932685998624232 | 0.999950574162623 |
| ebi-fl187-GCST90094766 | prot-a-1313 | Heparin-binding EGF-like growth factor \|\| id:prot-a-1313 | Fruit liking \|\| id:ebi-fl187-GCST90094766 | MR Egger | 10 | 0.507167451322595 | 1.31430137447594 | 0.709642054497434 | -2.068863243 | 3.08319814529544 | 1.66058085086299 | 0.126329305880451 | 21.8281003210955 | 0.999950574162623 |
| ebi-fl187-GCST90094766 | prot-a-1347 | HLA class II histocompatibility antigen, DQ alpha 2 chain \|\| id:prot-a-1347 | Fruit liking \|\| id:ebi-fl187-GCST90094766 | MR Egger | 10 | 0.470221976260403 | 1.31430137447594 | 0.729768648149317 | -2.105808718 | 3.04625267023325 | 1.60034939336658 | 0.121747175348394 | 21.0363663347412 | 0.999950574162623 |
| ebi-fl187-GCST90094766 | prot-a-1357 | Non-histone chromosomal protein HMG-14 \|\| id:prot-a-1357 | Fruit liking \|\| id:ebi-fl187-GCST90094766 | MR Egger | 10 | -0.276088028 | 1.3858253557359 | 0.847058775749584 | -2.992305726 | 2.44012966897843 | 0.758746136716231 | 0.0501716212687271 | 11.4745285367258 | 0.999950574162623 |
| ebi-fl187-GCST90094766 | prot-a-137 | Apolipoprotein M \|\| id:prot-a-137 | Fruit liking \|\| id:ebi-fl187-GCST90094766 | MR Egger | 10 | -1.295673355 | 1.31430137447594 | 0.353091188069426 | -3.871704049 | 1.28035733861754 | 0.273713495817419 | 0.0208228560017156 | 3.59792517349301 | 0.999950574162623 |
| ebi-fl187-GCST90094766 | prot-a-1381 | 17-beta-hydroxysteroid dehydrogenase 14 \|\| id:prot-a-1381 | Fruit liking \|\| id:ebi-fl187-GCST90094766 | MR Egger | 10 | -0.05445773 | 1.31430137447594 | 0.967964768469041 | -2.630488424 | 2.52157296371075 | 0.94699853739179 | 0.0720432659670468 | 12.4481617786789 | 0.999950574162623 |
| ebi-fl187-GCST90094766 | prot-a-1392 | Serine protease HTRA2, mitochondrial \|\| id:prot-a-1392 | Fruit liking \|\| id:ebi-fl187-GCST90094766 | MR Egger | 10 | -1.660355186 | 1.31430137447594 | 0.242053477738008 | -4.23638588 | 0.915675508306003 | 0.190071457453328 | 0.0144597568226118 | 2.49846241410766 | 0.999950574162623 |
| ebi-fl187-GCST90094766 | prot-a-1416 | Interferon-induced protein with tetratricopeptide repeats 2 \|\| id:prot-a-1416 | Fruit liking \|\| id:ebi-fl187-GCST90094766 | MR Egger | 10 | -1.071343321 | 1.48984662166068 | 0.492548889838801 | -3.9914427 | 1.84875605723539 | 0.342548056141714 | 0.0184730438315144 | 6.35191318965478 | 0.999950574162623 |
| ebi-fl187-GCST90094766 | prot-a-1428 | Interferon gamma \|\| id:prot-a-1428 | Fruit liking \|\| id:ebi-fl187-GCST90094766 | MR Egger | 10 | -1.341869038 | 1.31430137447594 | 0.33714527767785 | -3.917899732 | 1.23416165643123 | 0.261356726263747 | 0.0198828101618344 | 3.4354971861282 | 0.999950574162623 |
| ebi-fl187-GCST90094766 | prot-a-1483 | Interleukin-17C \|\| id:prot-a-1483 | Fruit liking \|\| id:ebi-fl187-GCST90094766 | MR Egger | 10 | -0.724233158 | 1.31430137447594 | 0.596655015559899 | -3.300263852 | 1.85179753578421 | 0.484696111724167 | 0.0368734369815515 | 6.37126180665139 | 0.999950574162623 |
| ebi-fl187-GCST90094766 | prot-a-1503 | Interleukin-1 receptor-like 2 \|\| id:prot-a-1503 | Fruit liking \|\| id:ebi-fl187-GCST90094766 | MR Egger | 10 | 0.197326089576942 | 1.43635479751481 | 0.894125284667961 | -2.617929314 | 3.01258149270598 | 1.21814119909605 | 0.0729537709166076 | 20.3398393570547 | 0.999950574162623 |
| ebi-fl187-GCST90094766 | prot-a-1522 | Interleukin-31 receptor subunit alpha \|\| id:prot-a-1522 | Fruit liking \|\| id:ebi-fl187-GCST90094766 | MR Egger | 10 | 0.370279713138857 | 1.31430137447594 | 0.785300072366658 | -2.205750981 | 2.9463104071117 | 1.44813962169688 | 0.11016776035438 | 19.0355904230289 | 0.999950574162623 |
| ebi-fl187-GCST90094766 | prot-a-1540 | Interleukin-6 receptor subunit alpha \|\| id:prot-a-1540 | Fruit liking \|\| id:ebi-fl187-GCST90094766 | MR Egger | 10 | -1.378426937 | 1.31403638987749 | 0.324831352004821 | -3.953938261 | 1.19708438717622 | 0.251974613409553 | 0.0191790208549858 | 3.31045084537716 | 0.999950574162623 |
| ebi-fl187-GCST90094766 | prot-a-1541 | Interleukin-6 receptor subunit alpha \|\| id:prot-a-1541 | Fruit liking \|\| id:ebi-fl187-GCST90094766 | MR Egger | 10 | -0.779438106 | 1.3791292486271 | 0.58745506443665 | -3.482531434 | 1.9236552210484 | 0.458663659151604 | 0.0307295227443704 | 6.84593620201532 | 0.999950574162623 |
| ebi-fl187-GCST90094766 | prot-a-1545 | Interleukin-9 \|\| id:prot-a-1545 | Fruit liking \|\| id:ebi-fl187-GCST90094766 | MR Egger | 10 | -2.76684045 | 1.41962053414463 | 0.0871266885253507 | -5.549296697 | 0.0156157973123441 | 0.0628603016031031 | 0.00389019226729016 | 1.0157383610208 | 0.999950574162623 |
| ebi-fl187-GCST90094766 | prot-a-1548 | Inositol monophosphatase 3 \|\| id:prot-a-1548 | Fruit liking \|\| id:ebi-fl187-GCST90094766 | MR Egger | 10 | -1.009190684 | 1.31430137447594 | 0.464632820662797 | -3.585221378 | 1.56684001 | 0.364513867259345 | 0.027730528032249 | 4.79148320831984 | 0.999950574162623 |
| ebi-fl187-GCST90094766 | prot-a-1555 | Inhibin beta A chain:Inhibin beta B chain heterodimer \|\| id:prot-a-1555 | Fruit liking \|\| id:ebi-fl187-GCST90094766 | MR Egger | 10 | -0.417567628 | 1.31430137447594 | 0.758838696900799 | -2.993598322 | 2.15846306591093 | 0.658646947325288 | 0.0501068115007108 | 8.6578209274954 | 0.999950574162623 |
| ebi-fl187-GCST90094766 | prot-a-1603 | Junctophilin-3 \|\| id:prot-a-1603 | Fruit liking \|\| id:ebi-fl187-GCST90094766 | MR Egger | 10 | 1.09521866827183 | 1.31430137447594 | 0.428846606771148 | -1.480812026 | 3.67124936224467 | 2.98983639427522 | 0.227452915760537 | 39.3009763565476 | 0.999950574162623 |
| ebi-fl187-GCST90094766 | prot-a-1627 | UPF0258 protein KIAA1024 \|\| id:prot-a-1627 | Fruit liking \|\| id:ebi-fl187-GCST90094766 | MR Egger | 10 | -1.507005334 | 1.3142586672039 | 0.284659212250986 | -4.082952321 | 1.0689416541693 | 0.221572521211648 | 0.0168576229942107 | 2.91229565241473 | 0.999950574162623 |
| ebi-fl187-GCST90094766 | prot-a-1666 | Kallikrein-7 \|\| id:prot-a-1666 | Fruit liking \|\| id:ebi-fl187-GCST90094766 | MR Egger | 10 | -1.040150105 | 1.31430137447594 | 0.451529446556715 | -3.616180799 | 1.53588058904543 | 0.35340163065113 | 0.0268851604990959 | 4.64541443042816 | 0.999950574162623 |
| ebi-fl187-GCST90094766 | prot-a-1678 | Importin subunit alpha-3 \|\| id:prot-a-1678 | Fruit liking \|\| id:ebi-fl187-GCST90094766 | MR Egger | 10 | 0.41448385904343 | 1.31430137447594 | 0.760554489984788 | -2.161546835 | 2.99051455301628 | 1.51358931360995 | 0.115146870010601 | 19.8959173624375 | 0.999950574162623 |
| ebi-fl187-GCST90094766 | prot-a-1718 | Protein LDOC1 \|\| id:prot-a-1718 | Fruit liking \|\| id:ebi-fl187-GCST90094766 | MR Egger | 10 | -0.824477236 | 1.31430137447594 | 0.54793659542154 | -3.40050793 | 1.75155345811752 | 0.438464145897333 | 0.0333563229853511 | 5.76354915743884 | 0.999950574162623 |
| ebi-fl187-GCST90094766 | prot-a-1723 | Leptin \|\| id:prot-a-1723 | Fruit liking \|\| id:ebi-fl187-GCST90094766 | MR Egger | 10 | 0.802449119202024 | 1.31430137447594 | 0.558438599787988 | -1.773581575 | 3.37847981317487 | 2.23099822357059 | 0.169724019675237 | 29.3261559742644 | 0.999950574162623 |
| ebi-fl187-GCST90094766 | prot-a-1730 | Galectin-7 \|\| id:prot-a-1730 | Fruit liking \|\| id:ebi-fl187-GCST90094766 | MR Egger | 10 | -0.971913238 | 1.57398098977379 | 0.554078678610208 | -4.056915978 | 2.11308950197654 | 0.378358455407889 | 0.0173022975846272 | 8.27376364777325 | 0.999950574162623 |
| ebi-fl187-GCST90094766 | prot-a-1753 | Lipase member N \|\| id:prot-a-1753 | Fruit liking \|\| id:ebi-fl187-GCST90094766 | MR Egger | 10 | -0.889036968 | 1.31430137447594 | 0.517840940158149 | -3.465067662 | 1.68699372637091 | 0.41105141803536 | 0.0312708895171203 | 5.40321272845072 | 0.999950574162623 |
| ebi-fl187-GCST90094766 | prot-a-1756 | VIP36-like protein \|\| id:prot-a-1756 | Fruit liking \|\| id:ebi-fl187-GCST90094766 | MR Egger | 10 | -3.237133067 | 1.54925059216343 | 0.0700743660408781 | -6.273664228 | -0.200601907 | 0.0392763364499552 | 0.00188530771371792 | 0.818238101772751 | 0.999950574162623 |
| ebi-fl187-GCST90094766 | prot-a-1760 | Ig Kappa chain V-I region HK102- like \|\| id:prot-a-1760 | Fruit liking \|\| id:ebi-fl187-GCST90094766 | MR Egger | 10 | 0.374621140460847 | 1.31430137447594 | 0.782857652661745 | -2.201409554 | 2.95065183443369 | 1.45444028164897 | 0.110647085403758 | 19.1184117065888 | 0.999950574162623 |
| ebi-fl187-GCST90094766 | prot-a-179 | Neutral ceramidase \|\| id:prot-a-179 | Fruit liking \|\| id:ebi-fl187-GCST90094766 | MR Egger | 10 | 2.36963359577283 | 1.31430137447594 | 0.109058341576905 | -0.206397098 | 4.94566428974568 | 10.6934734323442 | 0.813509968790163 | 140.564195197647 | 0.999950574162623 |
| ebi-fl187-GCST90094766 | prot-a-1809 | Lactotransferrin \|\| id:prot-a-1809 | Fruit liking \|\| id:ebi-fl187-GCST90094766 | MR Egger | 10 | -0.819566538 | 1.49444356565077 | 0.598377281976575 | -3.748675927 | 2.10954285078866 | 0.440622606323863 | 0.0235489057030211 | 8.24447146937814 | 0.999950574162623 |
| ebi-fl187-GCST90094766 | prot-a-1818 | Lysophospholipase-like protein 1 \|\| id:prot-a-1818 | Fruit liking \|\| id:ebi-fl187-GCST90094766 | MR Egger | 10 | 0.138906407669298 | 1.33784751056734 | 0.919861362666813 | -2.483274713 | 2.76108752838129 | 1.14901655583355 | 0.0834694390898948 | 15.8170350726537 | 0.999950574162623 |
| ebi-fl187-GCST90094766 | prot-a-1852 | MAP kinase-activated protein kinase 3 \|\| id:prot-a-1852 | Fruit liking \|\| id:ebi-fl187-GCST90094766 | MR Egger | 10 | -1.923651885 | 1.31430137447594 | 0.181447815087415 | -4.499682579 | 0.652378808782775 | 0.146072546742331 | 0.0111125233249308 | 1.92010295842715 | 0.999950574162623 |
| ebi-fl187-GCST90094766 | prot-a-1856 | Megakaryocyte-associated tyrosine-protein kinase \|\| id:prot-a-1856 | Fruit liking \|\| id:ebi-fl187-GCST90094766 | MR Egger | 10 | -2.539919046 | 1.31430137447594 | 0.0893718391570334 | -5.11594974 | 0.0361116475966385 | 0.0788727845699654 | 0.00600027642279743 | 1.03677159308602 | 0.999950574162623 |
| ebi-fl187-GCST90094766 | prot-a-1890 | Beta-1,3-N-acetylglucosaminyltransferase manic fringe \|\| id:prot-a-1890 | Fruit liking \|\| id:ebi-fl187-GCST90094766 | MR Egger | 10 | -0.372162233 | 1.68974610739194 | 0.831195019166112 | -3.684064604 | 2.93974013734977 | 0.689242415493232 | 0.0251206615126481 | 18.9109314289259 | 0.999950574162623 |
| ebi-fl187-GCST90094766 | prot-a-1919 | Matrilysin \|\| id:prot-a-1919 | Fruit liking \|\| id:ebi-fl187-GCST90094766 | MR Egger | 10 | -1.159410722 | 1.44230311902144 | 0.444704913790379 | -3.986324836 | 1.66750339089934 | 0.313670965711772 | 0.0185678286988335 | 5.29892193246789 | 0.999950574162623 |
| ebi-fl187-GCST90094766 | prot-a-1923 | Multimerin-2 \|\| id:prot-a-1923 | Fruit liking \|\| id:ebi-fl187-GCST90094766 | MR Egger | 10 | 1.76884339279816 | 1.31430137447594 | 0.215246662020353 | -0.807187301 | 4.34487408677101 | 5.86406701542873 | 0.446111079331411 | 77.0823311830221 | 0.999950574162623 |
| ebi-fl187-GCST90094766 | prot-a-1948 | Mesothelin \|\| id:prot-a-1948 | Fruit liking \|\| id:ebi-fl187-GCST90094766 | MR Egger | 10 | -0.991032628 | 1.31430137447594 | 0.47243485406041 | -3.567063322 | 1.58499806622088 | 0.371193188660581 | 0.0282386598922135 | 4.87928194305006 | 0.999950574162623 |
| ebi-fl187-GCST90094766 | prot-a-195 | ATPase family AAA domain-containing protein 1 \|\| id:prot-a-195 | Fruit liking \|\| id:ebi-fl187-GCST90094766 | MR Egger | 10 | -1.406038684 | 1.3142586672039 | 0.315907406320782 | -3.981985672 | 1.16990830376505 | 0.245112329941473 | 0.0186485726063431 | 3.22169720747966 | 0.999950574162623 |
| ebi-fl187-GCST90094766 | prot-a-1966 | Mucin-1 \|\| id:prot-a-1966 | Fruit liking \|\| id:ebi-fl187-GCST90094766 | MR Egger | 10 | -1.938129837 | 1.31430137447594 | 0.17853967715657 | -4.514160531 | 0.637900857057424 | 0.143972951059416 | 0.0109527958024109 | 1.89250407025704 | 0.999950574162623 |
| ebi-fl187-GCST90094766 | prot-a-1986 | Myocardial zonula adherens protein \|\| id:prot-a-1986 | Fruit liking \|\| id:ebi-fl187-GCST90094766 | MR Egger | 10 | -0.625580114 | 1.31430137447594 | 0.646816208021133 | -3.201610808 | 1.95045057969222 | 0.53495100576948 | 0.040696596738296 | 7.03185527807257 | 0.999950574162623 |
| ebi-fl187-GCST90094766 | prot-a-2071 | Epididymal secretory protein E1 \|\| id:prot-a-2071 | Fruit liking \|\| id:ebi-fl187-GCST90094766 | MR Egger | 10 | 0.503009487956336 | 1.31430137447594 | 0.711895457188418 | -2.073021206 | 3.07904018192918 | 1.65369055125207 | 0.125805123774712 | 21.7375283076513 | 0.999950574162623 |
| ebi-fl187-GCST90094766 | prot-a-2098 | Neuritin \|\| id:prot-a-2098 | Fruit liking \|\| id:ebi-fl187-GCST90094766 | MR Egger | 10 | -0.918218614 | 1.31430137447594 | 0.504578603820007 | -3.494249308 | 1.65781207974986 | 0.399229589929073 | 0.0303715395468198 | 5.24781647072036 | 0.999950574162623 |
| ebi-fl187-GCST90094766 | prot-a-2115 | Neurotrophin-4 \|\| id:prot-a-2115 | Fruit liking \|\| id:ebi-fl187-GCST90094766 | MR Egger | 10 | -0.869863317 | 1.31430137447594 | 0.526671376096665 | -3.445894011 | 1.7061673771654 | 0.419008816796582 | 0.0318762515876252 | 5.5078116092367 | 0.999950574162623 |
| ebi-fl187-GCST90094766 | prot-a-2118 | Netrin-1 \|\| id:prot-a-2118 | Fruit liking \|\| id:ebi-fl187-GCST90094766 | MR Egger | 10 | 0.157400424514921 | 1.31430137447594 | 0.907626376051046 | -2.418630269 | 2.73343111848777 | 1.17046420263802 | 0.0890434995684389 | 15.3855863291186 | 0.999950574162623 |
| ebi-fl187-GCST90094766 | prot-a-2120 | Netrin-G1 \|\| id:prot-a-2120 | Fruit liking \|\| id:ebi-fl187-GCST90094766 | MR Egger | 10 | -1.132502217 | 1.31430137447594 | 0.413946189505395 | -3.708532911 | 1.44352847685308 | 0.322225967505061 | 0.0245134603295084 | 4.23561474956625 | 0.999950574162623 |
| ebi-fl187-GCST90094766 | prot-a-2145 | Olfactomedin-like protein 3 \|\| id:prot-a-2145 | Fruit liking \|\| id:ebi-fl187-GCST90094766 | MR Egger | 10 | 0.472850320599363 | 1.31430137447594 | 0.728329200318805 | -2.103180373 | 3.04888101457221 | 1.60456119524314 | 0.122067589742728 | 21.0917298744607 | 0.999950574162623 |
| ebi-fl187-GCST90094766 | prot-a-2162 | Prolyl 4-hydroxylase subunit alpha-2 \|\| id:prot-a-2162 | Fruit liking \|\| id:ebi-fl187-GCST90094766 | MR Egger | 10 | -0.934121521 | 1.31430137447594 | 0.497442037983861 | -3.510152215 | 1.64190917257207 | 0.392930895341206 | 0.029892364013256 | 5.16502102159517 | 0.999950574162623 |
| ebi-fl187-GCST90094766 | prot-a-2168 | Polyadenylate-binding protein-interacting protein 1 \|\| id:prot-a-2168 | Fruit liking \|\| id:ebi-fl187-GCST90094766 | MR Egger | 10 | -0.89638964 | 1.31430137447594 | 0.514479087646535 | -3.472420334 | 1.67964105435135 | 0.408040175680159 | 0.0310418081349204 | 5.36363037376663 | 0.999950574162623 |
| ebi-fl187-GCST90094766 | prot-a-219 | Beta-1,4-galactosyltransferase 5 \|\| id:prot-a-219 | Fruit liking \|\| id:ebi-fl187-GCST90094766 | MR Egger | 10 | -2.130775209 | 1.75762053814617 | 0.259979488622494 | -5.575711464 | 1.31416104548151 | 0.118745205777191 | 0.00378877904292902 | 3.72162739903848 | 0.999950574162623 |
| ebi-fl187-GCST90094766 | prot-a-2190 | Pyruvate carboxylase, mitochondrial \|\| id:prot-a-2190 | Fruit liking \|\| id:ebi-fl187-GCST90094766 | MR Egger | 10 | -1.897694187 | 1.31430137447594 | 0.186767047937272 | -4.473724881 | 0.678336507234964 | 0.14991389444555 | 0.0114047552802379 | 1.97059693045538 | 0.999950574162623 |
| ebi-fl187-GCST90094766 | prot-a-2197 | Protocadherin-9 \|\| id:prot-a-2197 | Fruit liking \|\| id:ebi-fl187-GCST90094766 | MR Egger | 10 | -1.024023887 | 1.31430137447594 | 0.458323370150792 | -3.600054581 | 1.55200680703253 | 0.359146862307278 | 0.0273222311342694 | 4.72093468762802 | 0.999950574162623 |
| ebi-fl187-GCST90094766 | prot-a-2198 | Protocadherin alpha-4 \|\| id:prot-a-2198 | Fruit liking \|\| id:ebi-fl187-GCST90094766 | MR Egger | 10 | 0.598135705547714 | 1.31430137447594 | 0.661135321905778 | -1.977894988 | 3.17416639952056 | 1.81872499892511 | 0.138360180765803 | 23.906882770803 | 0.999950574162623 |
| ebi-fl187-GCST90094766 | prot-a-2254 | Placenta growth factor \|\| id:prot-a-2254 | Fruit liking \|\| id:ebi-fl187-GCST90094766 | MR Egger | 10 | -2.337787791 | 1.42046760251234 | 0.138425657514832 | -5.121904292 | 0.446328709949273 | 0.096540970982469 | 0.00596465363165186 | 1.56256501279133 | 0.999950574162623 |
| ebi-fl187-GCST90094766 | prot-a-2261 | Membrane-associated progesterone receptor component 2 \|\| id:prot-a-2261 | Fruit liking \|\| id:ebi-fl187-GCST90094766 | MR Egger | 10 | 1.01241842647312 | 1.31430137447594 | 0.463254962042183 | -1.563612267 | 3.58844912044597 | 2.7522490848901 | 0.209378372828749 | 36.1779247920408 | 0.999950574162623 |
| ebi-fl187-GCST90094766 | prot-a-2268 | Polymeric immunoglobulin receptor \|\| id:prot-a-2268 | Fruit liking \|\| id:ebi-fl187-GCST90094766 | MR Egger | 10 | -1.180248558 | 1.31430137447594 | 0.395405044030514 | -3.756279251 | 1.39578213646119 | 0.307202371653663 | 0.0233705346871049 | 4.03813170785979 | 0.999950574162623 |
| ebi-fl187-GCST90094766 | prot-a-2283 | Group 10 secretory phospholipase A2 \|\| id:prot-a-2283 | Fruit liking \|\| id:ebi-fl187-GCST90094766 | MR Egger | 10 | -1.740219932 | 1.31430137447594 | 0.222060602291345 | -4.316250626 | 0.835810761690249 | 0.175481802259315 | 0.0133498433771226 | 2.30668346094219 | 0.999950574162623 |
| ebi-fl187-GCST90094766 | prot-a-2298 | Inactive phospholipase D5 \|\| id:prot-a-2298 | Fruit liking \|\| id:ebi-fl187-GCST90094766 | MR Egger | 10 | -1.314120053 | 1.87046512386901 | 0.502255395997933 | -4.980231696 | 2.35199158981366 | 0.268710670393946 | 0.00687247005089234 | 10.5064734875328 | 0.999950574162623 |
| ebi-fl187-GCST90094766 | prot-a-2304 | Procollagen-lysine,2-oxoglutarate 5-dioxygenase 2 \|\| id:prot-a-2304 | Fruit liking \|\| id:ebi-fl187-GCST90094766 | MR Egger | 10 | -1.425784451 | 1.31430137447594 | 0.309606296365071 | -4.001815145 | 1.15024624274437 | 0.240319870029666 | 0.0182824234991933 | 3.15897068753625 | 0.999950574162623 |
| ebi-fl187-GCST90094766 | prot-a-2331 | Periostin \|\| id:prot-a-2331 | Fruit liking \|\| id:ebi-fl187-GCST90094766 | MR Egger | 10 | 2.70255992675245 | 1.31430137447594 | 0.0737861115066763 | 0.126529232779607 | 5.2785906207253 | 14.9178715449293 | 1.13488262646487 | 196.093310657356 | 0.999950574162623 |
| ebi-fl187-GCST90094766 | prot-a-2333 | Periostin \|\| id:prot-a-2333 | Fruit liking \|\| id:ebi-fl187-GCST90094766 | MR Egger | 10 | 3.50547118405844 | 1.3142586672039 | 0.0284829403094825 | 0.929524196338791 | 6.08141817177809 | 33.2971292332493 | 2.53330353571894 | 437.64941687537 | 0.999950574162623 |
| ebi-fl187-GCST90094766 | prot-a-2367 | cAMP-dependent protein kinase catalytic subunit alpha \|\| id:prot-a-2367 | Fruit liking \|\| id:ebi-fl187-GCST90094766 | MR Egger | 10 | -2.07907998 | 1.31430137447594 | 0.15233152915237 | -4.655110674 | 0.496950713690956 | 0.125045203346143 | 0.00951286035497075 | 1.6437015047433 | 0.999950574162623 |
| ebi-fl187-GCST90094766 | prot-a-2385 | Phosphoribosyl pyrophosphate synthase-associated protein 1 \|\| id:prot-a-2385 | Fruit liking \|\| id:ebi-fl187-GCST90094766 | MR Egger | 10 | 1.57856231071286 | 1.77694032636147 | 0.400268224889752 | -1.904240729 | 5.06136535038135 | 4.84798090835107 | 0.14893568225386 | 157.805829550475 | 0.999950574162623 |
| ebi-fl187-GCST90094766 | prot-a-2393 | Trypsin-3 \|\| id:prot-a-2393 | Fruit liking \|\| id:ebi-fl187-GCST90094766 | MR Egger | 10 | 0.552430867103042 | 1.31430137447594 | 0.685312207623478 | -2.023599827 | 3.12846156107589 | 1.73747145075738 | 0.132178786866783 | 22.8388163770899 | 0.999950574162623 |
| ebi-fl187-GCST90094766 | prot-a-2405 | Pregnancy-specific beta-1-glycoprotein 6 \|\| id:prot-a-2405 | Fruit liking \|\| id:ebi-fl187-GCST90094766 | MR Egger | 10 | -0.825437581 | 1.31430137447594 | 0.547481413786513 | -3.401468275 | 1.75059311339655 | 0.438043271294181 | 0.0333243047933725 | 5.75801682033196 | 0.999950574162623 |
| ebi-fl187-GCST90094766 | prot-a-244 | Beclin-1 \|\| id:prot-a-244 | Fruit liking \|\| id:ebi-fl187-GCST90094766 | MR Egger | 10 | -0.818240452 | 1.31430137447594 | 0.550898129669705 | -3.394271146 | 1.75779024227304 | 0.441207297465545 | 0.0335650092612147 | 5.7996074966614 | 0.999950574162623 |
| ebi-fl187-GCST90094766 | prot-a-2454 | tRNA pseudouridine synthase A, mitochondrial \|\| id:prot-a-2454 | Fruit liking \|\| id:ebi-fl187-GCST90094766 | MR Egger | 10 | 0.440427159230132 | 1.31430137447594 | 0.746164662334844 | -2.135603535 | 3.01645785320298 | 1.55337061340896 | 0.118173247189417 | 20.4188369194497 | 0.999950574162623 |
| ebi-fl187-GCST90094766 | prot-a-2466 | Glutaminyl-peptide cyclotransferase-like protein \|\| id:prot-a-2466 | Fruit liking \|\| id:ebi-fl187-GCST90094766 | MR Egger | 9 | -0.592383654 | 1.33434402953399 | 0.670477140042761 | -3.207697951 | 2.02293064434999 | 0.553007534080838 | 0.0404496231717556 | 7.5604494868004 | 0.999950574162623 |
| ebi-fl187-GCST90094766 | prot-a-2474 | Ras-related protein Rab-27A \|\| id:prot-a-2474 | Fruit liking \|\| id:ebi-fl187-GCST90094766 | MR Egger | 10 | -2.806578764 | 1.46416237095405 | 0.0915593015109807 | -5.676337011 | 0.0631794827906163 | 0.0604113206103915 | 0.00342608519790563 | 1.06521801037594 | 0.999950574162623 |
| ebi-fl187-GCST90094766 | prot-a-2490 | Rap1 GTPase-GDP dissociation stimulator 1 \|\| id:prot-a-2490 | Fruit liking \|\| id:ebi-fl187-GCST90094766 | MR Egger | 10 | -3.212212763 | 1.31430137447594 | 0.0403093299181214 | -5.788243457 | -0.636182069 | 0.0402674123888272 | 0.00306335837489562 | 0.529309438223052 | 0.999950574162623 |
| ebi-fl187-GCST90094766 | prot-a-2545 | Receptor-interacting serine/threonine-protein kinase 2 \|\| id:prot-a-2545 | Fruit liking \|\| id:ebi-fl187-GCST90094766 | MR Egger | 10 | -1.800018208 | 1.47999941211285 | 0.258562542111206 | -4.700817056 | 1.10078063994357 | 0.165295878520285 | 0.00908784879024778 | 3.00651211154754 | 0.999950574162623 |
| ebi-fl187-GCST90094766 | prot-a-2610 | RUN and FYVE domain-containing protein 1 \|\| id:prot-a-2610 | Fruit liking \|\| id:ebi-fl187-GCST90094766 | MR Egger | 10 | 0.628869320703706 | 1.31430137447594 | 0.645110363498354 | -1.947161373 | 3.20490001467655 | 1.87548880360582 | 0.142678508320116 | 24.6530349515496 | 0.999950574162623 |
| ebi-fl187-GCST90094766 | prot-a-2627 | Serine--tRNA ligase, mitochondrial \|\| id:prot-a-2627 | Fruit liking \|\| id:ebi-fl187-GCST90094766 | MR Egger | 10 | -1.718948706 | 1.32586323426126 | 0.230956861438982 | -4.317640645 | 0.879743233378697 | 0.179254498107365 | 0.0133312997365757 | 2.41028074731271 | 0.999950574162623 |
| ebi-fl187-GCST90094766 | prot-a-264 | BolA-like protein 2 \|\| id:prot-a-264 | Fruit liking \|\| id:ebi-fl187-GCST90094766 | MR Egger | 10 | 0.844732429678481 | 1.51167320272767 | 0.591587723072946 | -2.118147048 | 3.80761190702471 | 2.32735500012495 | 0.120254247585022 | 45.0427440642127 | 0.999950574162623 |
| ebi-fl187-GCST90094766 | prot-a-2646 | Secretoglobin family 1C member 1 \|\| id:prot-a-2646 | Fruit liking \|\| id:ebi-fl187-GCST90094766 | MR Egger | 10 | -4.267904048 | 1.31430137447594 | 0.011748833573656 | -6.843934742 | -1.691873354 | 0.014011119028381 | 0.00106590109150295 | 0.184174177128064 | 0.999950574162623 |
| ebi-fl187-GCST90094766 | prot-a-2672 | Semaphorin-3E \|\| id:prot-a-2672 | Fruit liking \|\| id:ebi-fl187-GCST90094766 | MR Egger | 10 | 0.100903571608031 | 1.31430137447594 | 0.940689033791018 | -2.475127122 | 2.67693426558088 | 1.10616997042894 | 0.084152291938971 | 14.5404477440276 | 0.999950574162623 |
| ebi-fl187-GCST90094766 | prot-a-2701 | Plasma protease C1 inhibitor \|\| id:prot-a-2701 | Fruit liking \|\| id:ebi-fl187-GCST90094766 | MR Egger | 10 | 0.422901450367774 | 1.31430137447594 | 0.755874344105089 | -2.153129244 | 2.99893214434062 | 1.52638386398564 | 0.116120220189349 | 20.064099916764 | 0.999950574162623 |
| ebi-fl187-GCST90094766 | prot-a-2738 | Signaling threshold-regulating transmembrane adapter 1 \|\| id:prot-a-2738 | Fruit liking \|\| id:ebi-fl187-GCST90094766 | MR Egger | 10 | -1.744292935 | 1.74758807925425 | 0.347452642135559 | -5.16956557 | 1.68097970077419 | 0.174768518068489 | 0.00568703890350684 | 5.37081518626875 | 0.999950574162623 |
| ebi-fl187-GCST90094766 | prot-a-274 | Serine/threonine-protein kinase BRSK2 \|\| id:prot-a-274 | Fruit liking \|\| id:ebi-fl187-GCST90094766 | MR Egger | 10 | -0.710767039 | 1.44067341418503 | 0.635017130824752 | -3.534486931 | 2.11295285285989 | 0.491267231807673 | 0.0291737212896426 | 8.27263312252375 | 0.999950574162623 |
| ebi-fl187-GCST90094766 | prot-a-2749 | Mitochondrial glutamate carrier 2 \|\| id:prot-a-2749 | Fruit liking \|\| id:ebi-fl187-GCST90094766 | MR Egger | 10 | -0.159610238 | 1.31430137447594 | 0.906336601637451 | -2.735640932 | 2.41642045628165 | 0.85247598723181 | 0.0648524278060775 | 11.205676231272 | 0.999950574162623 |
| ebi-fl187-GCST90094766 | prot-a-2760 | Sodium-coupled monocarboxylate transporter 1 \|\| id:prot-a-2760 | Fruit liking \|\| id:ebi-fl187-GCST90094766 | MR Egger | 10 | -0.137077404 | 1.68577410363826 | 0.937189602198322 | -3.441194647 | 3.16703983935354 | 0.871902734954858 | 0.0320264022226138 | 23.7371145824483 | 0.999950574162623 |
| ebi-fl187-GCST90094766 | prot-a-2777 | Small integral membrane protein 10 \|\| id:prot-a-2777 | Fruit liking \|\| id:ebi-fl187-GCST90094766 | MR Egger | 10 | -0.390988167 | 1.3142586672039 | 0.77366593535287 | -2.966935155 | 2.18495882079625 | 0.676388159744843 | 0.0514607882438406 | 8.89028245108109 | 0.999950574162623 |
| ebi-fl187-GCST90094766 | prot-a-2799 | Superoxide dismutase [Mn], mitochondrial \|\| id:prot-a-2799 | Fruit liking \|\| id:ebi-fl187-GCST90094766 | MR Egger | 10 | 0.560514426961703 | 1.71763018958788 | 0.752547858693077 | -2.806040745 | 3.92706959855394 | 1.75157332511579 | 0.0604438318329423 | 50.7580181504159 | 0.999950574162623 |
| ebi-fl187-GCST90094766 | prot-a-2805 | Protein SPACA7 \|\| id:prot-a-2805 | Fruit liking \|\| id:ebi-fl187-GCST90094766 | MR Egger | 10 | 0.094147576843699 | 1.31430137447594 | 0.944652290847989 | -2.481883117 | 2.67017827081655 | 1.0987218798604 | 0.0835856756786614 | 14.44254365 | 0.999950574162623 |
| ebi-fl187-GCST90094766 | prot-a-2811 | SPARC-like protein 1 \|\| id:prot-a-2811 | Fruit liking \|\| id:ebi-fl187-GCST90094766 | MR Egger | 10 | -0.733386455 | 1.31430137447594 | 0.592109590025137 | -3.309417149 | 1.8426442392099 | 0.480279787263228 | 0.036537463455539 | 6.31320984649915 | 0.999950574162623 |
| ebi-fl187-GCST90094766 | prot-a-2831 | Sepiapterin reductase \|\| id:prot-a-2831 | Fruit liking \|\| id:ebi-fl187-GCST90094766 | MR Egger | 10 | -1.289121801 | 1.31430137447594 | 0.355398237604567 | -3.865152495 | 1.28690889289909 | 0.275512631775972 | 0.020959725938949 | 3.62157456109987 | 0.999950574162623 |
| ebi-fl187-GCST90094766 | prot-a-2834 | SPRY domain-containing SOCS box protein 1 \|\| id:prot-a-2834 | Fruit liking \|\| id:ebi-fl187-GCST90094766 | MR Egger | 10 | 0.795390604711431 | 1.31430137447594 | 0.561828429224505 | -1.780640089 | 3.37142129868428 | 2.2153061368435 | 0.168530238340821 | 29.119885714585 | 0.999950574162623 |
| ebi-fl187-GCST90094766 | prot-a-2835 | Spectrin alpha chain, non-erythrocytic 1 \|\| id:prot-a-2835 | Fruit liking \|\| id:ebi-fl187-GCST90094766 | MR Egger | 10 | -0.216031182 | 1.36505642403676 | 0.878175351489601 | -2.891541773 | 2.4594794091852 | 0.805710177885949 | 0.0554905927254512 | 11.6987197084175 | 0.999950574162623 |
| ebi-fl187-GCST90094766 | prot-a-2859 | Sia-alpha-2,3-Gal-beta-1,4-GlcNAc-R:alpha 2,8-sialyltransferase \|\| id:prot-a-2859 | Fruit liking \|\| id:ebi-fl187-GCST90094766 | MR Egger | 10 | 0.545674290856436 | 1.31430137447594 | 0.688920152143285 | -2.030356403 | 3.12170498482928 | 1.725771662 | 0.131288721099797 | 22.6850243118305 | 0.999950574162623 |
| ebi-fl187-GCST90094766 | prot-a-2861 | Alpha-2,8-sialyltransferase 8F \|\| id:prot-a-2861 | Fruit liking \|\| id:ebi-fl187-GCST90094766 | MR Egger | 10 | -2.11137228 | 1.31430137447594 | 0.146840087758678 | -4.687402974 | 0.4646584139822 | 0.121071708100521 | 0.00921057522622325 | 1.59147047196837 | 0.999950574162623 |
| ebi-fl187-GCST90094766 | prot-a-289 | Uncharacterized protein C17orf89 \|\| id:prot-a-289 | Fruit liking \|\| id:ebi-fl187-GCST90094766 | MR Egger | 10 | -2.383097458 | 1.31430137447594 | 0.107362936156885 | -4.959128152 | 0.192933236124463 | 0.0922643495160801 | 0.00701904470704957 | 1.21280181946635 | 0.999950574162623 |
| ebi-fl187-GCST90094766 | prot-a-2921 | Tachykinin-3 \|\| id:prot-a-2921 | Fruit liking \|\| id:ebi-fl187-GCST90094766 | MR Egger | 10 | -1.210232056 | 1.31430137447594 | 0.384072426406654 | -3.78626275 | 1.36579863839205 | 0.298128089115327 | 0.0226802052678325 | 3.91885154785685 | 0.999950574162623 |
| ebi-fl187-GCST90094766 | prot-a-2928 | Tax1-binding protein 1 \|\| id:prot-a-2928 | Fruit liking \|\| id:ebi-fl187-GCST90094766 | MR Egger | 10 | 2.54185085278621 | 1.31430137447594 | 0.0891693097402379 | -0.034179841 | 5.11788154675906 | 12.7031609066793 | 0.966397690901043 | 166.981252687523 | 0.999950574162623 |
| ebi-fl187-GCST90094766 | prot-a-296 | Uncharacterized protein C1orf185 \|\| id:prot-a-296 | Fruit liking \|\| id:ebi-fl187-GCST90094766 | MR Egger | 10 | 0.226718037511299 | 1.51529573199419 | 0.884767914871963 | -2.743261597 | 3.19669767221991 | 1.2544761028644 | 0.0643600875078184 | 24.4516493621406 | 0.999950574162623 |
| ebi-fl187-GCST90094766 | prot-a-2964 | Transforming growth factor beta-2 \|\| id:prot-a-2964 | Fruit liking \|\| id:ebi-fl187-GCST90094766 | MR Egger | 10 | -2.664820833 | 1.31430137447594 | 0.0771490719248366 | -5.240851527 | -0.08879014 | 0.0696118245238599 | 0.005295745442687 | 0.915037583657335 | 0.999950574162623 |
| ebi-fl187-GCST90094766 | prot-a-2977 | Thymocyte nuclear protein 1 \|\| id:prot-a-2977 | Fruit liking \|\| id:ebi-fl187-GCST90094766 | MR Egger | 10 | -3.503168475 | 1.31430137447594 | 0.0285642630433248 | -6.079199169 | -0.927137781 | 0.0301018552001436 | 0.00229000983069974 | 0.395684627350938 | 0.999950574162623 |
| ebi-fl187-GCST90094766 | prot-a-3015 | Transmembrane protein 70, mitochondrial \|\| id:prot-a-3015 | Fruit liking \|\| id:ebi-fl187-GCST90094766 | MR Egger | 10 | -0.192461987 | 1.32884893424063 | 0.888424639139235 | -2.797005899 | 2.41208192362044 | 0.824925675102119 | 0.0609924069611817 | 11.1571653480701 | 0.999950574162623 |
| ebi-fl187-GCST90094766 | prot-a-3030 | Tumor necrosis factor alpha-induced protein 3 \|\| id:prot-a-3030 | Fruit liking \|\| id:ebi-fl187-GCST90094766 | MR Egger | 10 | 0.0250477671151111 | 1.31430137447594 | 0.985261694704513 | -2.550982927 | 2.60107846108796 | 1.02536409803956 | 0.0780049551413022 | 13.4782659850772 | 0.999950574162623 |
| ebi-fl187-GCST90094766 | prot-a-3032 | Tumor necrosis factor receptor superfamily member 10A \|\| id:prot-a-3032 | Fruit liking \|\| id:ebi-fl187-GCST90094766 | MR Egger | 10 | 0.735175055465269 | 1.31430137447594 | 0.591223615681258 | -1.840855639 | 3.31120574943812 | 2.08584709948159 | 0.15868159392138 | 27.4181649862388 | 0.999950574162623 |
| ebi-fl187-GCST90094766 | prot-a-3081 | Tumor protein p53-inducible protein 11 \|\| id:prot-a-3081 | Fruit liking \|\| id:ebi-fl187-GCST90094766 | MR Egger | 10 | 1.87752955253613 | 1.46539748853574 | 0.235997144998835 | -0.994649525 | 4.74970863006618 | 6.53733475464947 | 0.369853046080256 | 115.550611647725 | 0.999950574162623 |
| ebi-fl187-GCST90094766 | prot-a-315 | Complement C4 \|\| id:prot-a-315 | Fruit liking \|\| id:ebi-fl187-GCST90094766 | MR Egger | 10 | -0.87656143 | 1.31430137447594 | 0.52357612387788 | -3.452592124 | 1.69946926387223 | 0.416211626676923 | 0.0316634543088607 | 5.47104294090155 | 0.999950574162623 |
| ebi-fl187-GCST90094766 | prot-a-3157 | UDP-glucuronosyltransferase 1-8 \|\| id:prot-a-3157 | Fruit liking \|\| id:ebi-fl187-GCST90094766 | MR Egger | 10 | -1.504042201 | 1.46818097421172 | 0.335610130435285 | -4.381676911 | 1.37359250831482 | 0.222230043612332 | 0.0125043723154919 | 3.94951390104991 | 0.999950574162623 |
| ebi-fl187-GCST90094766 | prot-a-317 | Glutaredoxin-like protein C5orf63 \|\| id:prot-a-317 | Fruit liking \|\| id:ebi-fl187-GCST90094766 | MR Egger | 10 | 1.39585358700756 | 1.33028620125429 | 0.324707485890073 | -1.211507367 | 4.00321454146596 | 4.03842024488456 | 0.297748125162253 | 54.7739404417953 | 0.999950574162623 |
| ebi-fl187-GCST90094766 | prot-a-3192 | Guanine nucleotide exchange factor VAV3 \|\| id:prot-a-3192 | Fruit liking \|\| id:ebi-fl187-GCST90094766 | MR Egger | 10 | 0.713157100270579 | 1.31430137447594 | 0.602180583164703 | -1.862873594 | 3.28918779424343 | 2.04042292035806 | 0.155225932599089 | 26.8210705789436 | 0.999950574162623 |
| ebi-fl187-GCST90094766 | prot-a-3233 | Serine/threonine-protein kinase WNK3 \|\| id:prot-a-3233 | Fruit liking \|\| id:ebi-fl187-GCST90094766 | MR Egger | 10 | 1.84491138793054 | 1.31430137447594 | 0.198008631901469 | -0.731119306 | 4.42094208190339 | 6.3275390692276 | 0.481369888212032 | 83.1746057513385 | 0.999950574162623 |
| ebi-fl187-GCST90094766 | prot-a-3250 | 14-3-3 protein theta \|\| id:prot-a-3250 | Fruit liking \|\| id:ebi-fl187-GCST90094766 | MR Egger | 10 | -1.218331341 | 1.63555020807383 | 0.477638724430058 | -4.424009749 | 1.98734706705276 | 0.295723216718089 | 0.0119860746532293 | 7.29615186257265 | 0.999950574162623 |
| ebi-fl187-GCST90094766 | prot-a-3278 | E3 ubiquitin-protein ligase ZNRF3 \|\| id:prot-a-3278 | Fruit liking \|\| id:ebi-fl187-GCST90094766 | MR Egger | 10 | 0.0493300754848095 | 1.36403006004038 | 0.97203695769301 | -2.624168842 | 2.72282899316396 | 1.05056705994841 | 0.0724999909322203 | 15.2233280757302 | 0.999950574162623 |
| ebi-fl187-GCST90094766 | prot-a-355 | Calpain-3 \|\| id:prot-a-355 | Fruit liking \|\| id:ebi-fl187-GCST90094766 | MR Egger | 10 | 2.70880257615299 | 1.53317278502659 | 0.115247619744958 | -0.296216082 | 5.7138212348051 | 15.0112898723382 | 0.743626725936991 | 303.026795261333 | 0.999950574162623 |
| ebi-fl187-GCST90094766 | prot-a-385 | Coiled-coil domain-containing protein 90B, mitochondrial \|\| id:prot-a-385 | Fruit liking \|\| id:ebi-fl187-GCST90094766 | MR Egger | 10 | -0.638639661 | 1.31430137447594 | 0.64005647734583 | -3.214670355 | 1.93739103272841 | 0.528010208467287 | 0.0401685730019105 | 6.94061947962174 | 0.999950574162623 |
| ebi-fl187-GCST90094766 | prot-a-390 | C-C motif chemokine 14 \|\| id:prot-a-390 | Fruit liking \|\| id:ebi-fl187-GCST90094766 | MR Egger | 10 | -1.027962532 | 1.31430137447594 | 0.456657723179644 | -3.603993226 | 1.54806816190141 | 0.357735092322238 | 0.0272148302075513 | 4.70237717093273 | 0.999950574162623 |
| ebi-fl187-GCST90094766 | prot-a-40 | Pituitary adenylate cyclase-activating polypeptide 27 \|\| id:prot-a-40 | Fruit liking \|\| id:ebi-fl187-GCST90094766 | MR Egger | 10 | 0.785689673196919 | 1.31430137447594 | 0.566506631651919 | -1.790341021 | 3.36172036716977 | 2.19391950656508 | 0.166903242487659 | 28.8387614856714 | 0.999950574162623 |
| ebi-fl187-GCST90094766 | prot-a-401 | C-C motif chemokine 24 \|\| id:prot-a-401 | Fruit liking \|\| id:ebi-fl187-GCST90094766 | MR Egger | 10 | -0.540569314 | 1.31430137447594 | 0.691651794538367 | -3.116600008 | 2.03546138026921 | 0.58241658022985 | 0.044307557970132 | 7.6557835382238 | 0.999950574162623 |
| ebi-fl187-GCST90094766 | prot-a-412 | Cerebral cavernous malformations 2 protein \|\| id:prot-a-412 | Fruit liking \|\| id:ebi-fl187-GCST90094766 | MR Egger | 10 | 1.34874582560871 | 1.31430137447594 | 0.334819498332036 | -1.227284868 | 3.92477651958156 | 3.85259067888748 | 0.293087268862832 | 50.6417593525603 | 0.999950574162623 |
| ebi-fl187-GCST90094766 | prot-a-44 | Adhesion G protein-coupled receptor F5 \|\| id:prot-a-44 | Fruit liking \|\| id:ebi-fl187-GCST90094766 | MR Egger | 10 | 0.275045869609166 | 1.31430137447594 | 0.839466928949132 | -2.300984824 | 2.85107656358201 | 1.31659106500016 | 0.100160154974349 | 17.3064032586828 | 0.999950574162623 |
| ebi-fl187-GCST90094766 | prot-a-48 | Adrenomedullin \|\| id:prot-a-48 | Fruit liking \|\| id:ebi-fl187-GCST90094766 | MR Egger | 10 | 0.245810015207347 | 1.31430137447594 | 0.85629546158629 | -2.330220679 | 2.82184070918019 | 1.27865662515042 | 0.0972742783531108 | 16.8077604143826 | 0.999950574162623 |
| ebi-fl187-GCST90094766 | prot-a-502 | Carcinoembryonic antigen-related cell adhesion molecule 1 \|\| id:prot-a-502 | Fruit liking \|\| id:ebi-fl187-GCST90094766 | MR Egger | 10 | -0.603351342 | 1.31430137447594 | 0.658402369236107 | -3.179382036 | 1.97267935166125 | 0.546975458984492 | 0.041611361489109 | 7.18991501418672 | 0.999950574162623 |
| ebi-fl187-GCST90094766 | prot-a-518 | Complement factor B \|\| id:prot-a-518 | Fruit liking \|\| id:ebi-fl187-GCST90094766 | MR Egger | 10 | 1.14844279811974 | 1.4050386526963 | 0.437378167992947 | -1.605432961 | 3.9023185574045 | 3.15327879288156 | 0.200802596360695 | 49.5171244089693 | 0.999950574162623 |
| ebi-fl187-GCST90094766 | prot-a-525 | Properdin \|\| id:prot-a-525 | Fruit liking \|\| id:ebi-fl187-GCST90094766 | MR Egger | 10 | 2.03530572022114 | 2.08433800020844 | 0.357428219942352 | -2.04999676 | 6.1206082006297 | 7.65459193133545 | 0.128735320665442 | 455.14142763925 | 0.999950574162623 |
| ebi-fl187-GCST90094766 | prot-a-533 | Chondroadherin \|\| id:prot-a-533 | Fruit liking \|\| id:ebi-fl187-GCST90094766 | MR Egger | 10 | -0.21748884 | 1.44558870894002 | 0.884133793451986 | -3.050842709 | 2.61586503000381 | 0.804536583885277 | 0.04731903140883 | 13.6790440450352 | 0.999950574162623 |
| ebi-fl187-GCST90094766 | prot-a-535 | Coiled-coil-helix-coiled-coil-helix domain-containing protein 2 \|\| id:prot-a-535 | Fruit liking \|\| id:ebi-fl187-GCST90094766 | MR Egger | 10 | -2.564939023 | 1.31430137447594 | 0.0867826445197212 | -5.140969717 | 0.0110916705094546 | 0.0769238718501185 | 0.00585201216273933 | 1.01115341114458 | 0.999950574162623 |
| ebi-fl187-GCST90094766 | prot-a-591 | Caseinolytic peptidase B protein homolog \|\| id:prot-a-591 | Fruit liking \|\| id:ebi-fl187-GCST90094766 | MR Egger | 10 | 1.1312706097063 | 1.37466422206188 | 0.434383308600229 | -1.563071266 | 3.82561248494757 | 3.09959237126964 | 0.209491677586109 | 45.8608808652265 | 0.999950574162623 |
| ebi-fl187-GCST90094766 | prot-a-605 | Ciliary neurotrophic factor receptor subunit alpha \|\| id:prot-a-605 | Fruit liking \|\| id:ebi-fl187-GCST90094766 | MR Egger | 10 | 0.254822426016387 | 1.31430137447594 | 0.851099009098307 | -2.321208268 | 2.83085311998923 | 1.29023248880672 | 0.0981549164863929 | 16.9599235042206 | 0.999950574162623 |
| ebi-fl187-GCST90094766 | prot-a-606 | Ciliary neurotrophic factor receptor subunit alpha \|\| id:prot-a-606 | Fruit liking \|\| id:ebi-fl187-GCST90094766 | MR Egger | 10 | 2.15764175657975 | 1.31430137447594 | 0.139286590622562 | -0.418388937 | 4.7336724505526 | 8.65071309764302 | 0.65810621652552 | 113.712399637894 | 0.999950574162623 |
| ebi-fl187-GCST90094766 | prot-a-707 | Cancer/testis antigen 55 \|\| id:prot-a-707 | Fruit liking \|\| id:ebi-fl187-GCST90094766 | MR Egger | 10 | 0.0281886736399032 | 1.31430137447594 | 0.983413861348119 | -2.54784202 | 2.60421936761275 | 1.0285897338839 | 0.0782503465879314 | 13.5206665118404 | 0.999950574162623 |
| ebi-fl187-GCST90094766 | prot-a-719 | Dipeptidyl peptidase 1 \|\| id:prot-a-719 | Fruit liking \|\| id:ebi-fl187-GCST90094766 | MR Egger | 10 | -1.022586451 | 1.31430137447594 | 0.458932273837567 | -3.598617145 | 1.55344424276352 | 0.359663484056156 | 0.0273615333259721 | 4.72772560741775 | 0.999950574162623 |
| ebi-fl187-GCST90094766 | prot-a-752 | Cytochrome b5 \|\| id:prot-a-752 | Fruit liking \|\| id:ebi-fl187-GCST90094766 | MR Egger | 10 | 0.615836689176689 | 1.31430137447594 | 0.651882355274545 | -1.960194005 | 3.19186738314954 | 1.85120483482129 | 0.14083109636244 | 24.3338256179315 | 0.999950574162623 |
| ebi-fl187-GCST90094766 | prot-a-755 | Cytoglobin \|\| id:prot-a-755 | Fruit liking \|\| id:ebi-fl187-GCST90094766 | MR Egger | 10 | -2.645920141 | 1.3443545715903 | 0.0845825475326748 | -5.280855101 | -0.010985181 | 0.0709400488545767 | 0.00508807810987125 | 0.989074936119066 | 0.999950574162623 |
| ebi-fl187-GCST90094766 | prot-a-807 | Desmin \|\| id:prot-a-807 | Fruit liking \|\| id:ebi-fl187-GCST90094766 | MR Egger | 10 | 1.57609374297624 | 1.31430137447594 | 0.264765625661438 | -0.999936951 | 4.15212443694909 | 4.83602809832342 | 0.367902636334785 | 63.5689050797985 | 0.999950574162623 |
| ebi-fl187-GCST90094766 | prot-a-82 | Ameloblastin \|\| id:prot-a-82 | Fruit liking \|\| id:ebi-fl187-GCST90094766 | MR Egger | 10 | -3.205639628 | 1.31430137447594 | 0.0406250064619639 | -5.781670322 | -0.629608934 | 0.0405329673411597 | 0.00308356056666715 | 0.532800120496825 | 0.999950574162623 |
| ebi-fl187-GCST90094766 | prot-a-84 | Muellerian-inhibiting factor \|\| id:prot-a-84 | Fruit liking \|\| id:ebi-fl187-GCST90094766 | MR Egger | 10 | 1.04828222904981 | 1.31430137447594 | 0.448129369766605 | -1.527748465 | 3.62431292302266 | 2.85274654166223 | 0.217023754232598 | 37.4989496414464 | 0.999950574162623 |
| ebi-fl187-GCST90094766 | prot-a-858 | Inactive dipeptidyl peptidase 10 \|\| id:prot-a-858 | Fruit liking \|\| id:ebi-fl187-GCST90094766 | MR Egger | 10 | -3.320217056 | 1.31430137447594 | 0.035463461355523 | -5.89624775 | -0.744186362 | 0.0361449854011111 | 0.00274974320847335 | 0.475120718771364 | 0.999950574162623 |
| ebi-fl187-GCST90094766 | prot-a-863 | Dihydropyrimidinase-related protein 3 \|\| id:prot-a-863 | Fruit liking \|\| id:ebi-fl187-GCST90094766 | MR Egger | 10 | -0.532453359 | 1.31430137447594 | 0.696004485029178 | -3.108484053 | 2.04357733537847 | 0.587162680566917 | 0.0446686193185807 | 7.71817035560619 | 0.999950574162623 |
| ebi-fl187-GCST90094766 | prot-a-864 | Dihydropyrimidinase-related protein 5 \|\| id:prot-a-864 | Fruit liking \|\| id:ebi-fl187-GCST90094766 | MR Egger | 10 | -0.820419087 | 1.31430137447594 | 0.549862535443072 | -3.396449781 | 1.75561160742565 | 0.440247114196517 | 0.0334919629618844 | 5.78698602345093 | 0.999950574162623 |
| ebi-fl187-GCST90094766 | prot-a-884 | Endothelin-converting enzyme 1 \|\| id:prot-a-884 | Fruit liking \|\| id:ebi-fl187-GCST90094766 | MR Egger | 10 | 1.45934743779521 | 1.3142586672039 | 0.299085741083804 | -1.11659955 | 4.03529442551485 | 4.30315053852162 | 0.327391181311875 | 56.5595703677167 | 0.999950574162623 |
| ebi-fl187-GCST90094766 | prot-a-893 | Early endosome antigen 1 \|\| id:prot-a-893 | Fruit liking \|\| id:ebi-fl187-GCST90094766 | MR Egger | 10 | 1.23213943191433 | 1.31430137447594 | 0.375943763888504 | -1.343891262 | 3.80817012588718 | 3.42855685930534 | 0.260828738319252 | 45.0678947927191 | 0.999950574162623 |
| ebi-fl187-GCST90094766 | prot-a-897 | Ephrin-A2 \|\| id:prot-a-897 | Fruit liking \|\| id:ebi-fl187-GCST90094766 | MR Egger | 10 | -1.300254316 | 1.80291691148045 | 0.491325565623599 | -4.833971462 | 2.23346283084147 | 0.272462492743588 | 0.00795486599939626 | 9.33212576524659 | 0.999950574162623 |
| ebi-fl187-GCST90094766 | prot-a-90 | Anaphase-promoting complex subunit 10 \|\| id:prot-a-90 | Fruit liking \|\| id:ebi-fl187-GCST90094766 | MR Egger | 10 | -0.863966447 | 1.31430137447594 | 0.529405601997422 | -3.439997141 | 1.71206424667964 | 0.421486956566938 | 0.0320647769923961 | 5.54038640587422 | 0.999950574162623 |
| ebi-fl187-GCST90094766 | prot-a-914 | Histone-lysine N-methyltransferase EHMT2 \|\| id:prot-a-914 | Fruit liking \|\| id:ebi-fl187-GCST90094766 | MR Egger | 10 | -3.047658353 | 1.55288356841182 | 0.0853170494918855 | -6.091310147 | -0.004006559 | 0.0474699522276246 | 0.00226244283982202 | 0.996001456845747 | 0.999950574162623 |
| ebi-fl187-GCST90094766 | prot-a-932 | RNA polymerase II elongation factor ELL \|\| id:prot-a-932 | Fruit liking \|\| id:ebi-fl187-GCST90094766 | MR Egger | 10 | 0.962137480993383 | 1.3142586672039 | 0.48501338701575 | -1.613809507 | 3.53808446871303 | 2.61728489544076 | 0.199127589443446 | 34.4009599224715 | 0.999950574162623 |
| ebi-fl187-GCST90094766 | prot-a-991 | Estrogen receptor \|\| id:prot-a-991 | Fruit liking \|\| id:ebi-fl187-GCST90094766 | MR Egger | 10 | -0.042940983 | 1.31430137447594 | 0.97473652013234 | -2.618971677 | 2.53308971129344 | 0.957967925098893 | 0.0728777662169153 | 12.5923528279778 | 0.999950574162623 |
| ebi-fl187-GCST90094766 | prot-a-992 | Persulfide dioxygenase ETHE1, mitochondrial \|\| id:prot-a-992 | Fruit liking \|\| id:ebi-fl187-GCST90094766 | MR Egger | 10 | 0.936741144912277 | 2.00838071287388 | 0.653355025555583 | -2.999685052 | 4.87316734214509 | 2.55165238878112 | 0.0498027511590136 | 130.73434221302 | 0.999950574162623 |
| ebi-fl187-GCST90094766 | prot-a-993 | Ethanolamine kinase 1 \|\| id:prot-a-993 | Fruit liking \|\| id:ebi-fl187-GCST90094766 | MR Egger | 10 | -0.345766196 | 1.31430137447594 | 0.799137757416201 | -2.92179689 | 2.23026449748036 | 0.707677925346862 | 0.0538368613907153 | 9.30232619596224 | 0.999950574162623 |
| ebi-fl187-GCST90094766 | prot-b-10 | coagulation factor III, tissue factor \|\| id:prot-b-10 | Fruit liking \|\| id:ebi-fl187-GCST90094766 | MR Egger | 6 | -0.647132275 | 1.75622996837859 | 0.731189857681625 | -4.089343013 | 2.79507846281244 | 0.523545009042094 | 0.0167502346269236 | 16.363912661397 | 0.999950574162623 |
| ebi-fl187-GCST90094766 | prot-b-28 | prolactin \|\| id:prot-b-28 | Fruit liking \|\| id:ebi-fl187-GCST90094766 | MR Egger | 6 | 0.69564645260725 | 1.75622996837859 | 0.71225009301752 | -2.746564285 | 4.13785719062929 | 2.00500479566241 | 0.0641478768308768 | 62.6683910556847 | 0.999950574162623 |
| ebi-fl187-GCST90094766 | prot-b-54 | matrix metallopeptidase 7 \|\| id:prot-b-54 | Fruit liking \|\| id:ebi-fl187-GCST90094766 | MR Egger | 6 | 1.18032976083013 | 2.33522119445836 | 0.639825095031615 | -3.39670378 | 5.75736330196851 | 3.25544754499175 | 0.0334834570906762 | 316.512679365593 | 0.999950574162623 |
| ebi-fl187-GCST90094766 | ukb-rsfMRI191-edge-pheno716 | Net100_Pair10_33 (OccipitalPrecuneus)&(Temporal\|Frontal\|Supp_Motor_Area) (Default_mode\|Central_executive)&(Default_mode\|Salience) \|\| id:ukb-rsfMRI191-edge-pheno716 | Fruit liking \|\| id:ebi-fl187-GCST90094766 | MR Egger | 10 | 0.287139902048106 | 0.411590324772489 | 0.505171919259399 | -0.519577135 | 1.09385693860218 | 1.33261063539219 | 0.594772003357578 | 2.98576781613025 | 0.999950574162623 |
| ebi-fl187-GCST90094766 | ukb-rsfMRI191-edge-pheno903 | Net100_Pair27_38 (Precentral\|Frontal\|Supp_Motor_Area)&(Subcortical) (Attention\|Salience\|Motor)&(Subcortical-cerebellum) \|\| id:ukb-rsfMRI191-edge-pheno903 | Fruit liking \|\| id:ebi-fl187-GCST90094766 | MR Egger | 10 | -0.457335777 | 0.479806340248816 | 0.368413166517297 | -1.397756204 | 0.483084649941719 | 0.632967768392261 | 0.247150898489638 | 1.62106712244171 | 0.999950574162623 |
| ebi-fl187-GCST90094766 | ukb-rsfMRI191-node-pheno23 | Net100_Node2 RolandicOper\|SupraMarginal\|Insula Salience\|Motor \|\| id:ukb-rsfMRI191-node-pheno23 | Fruit liking \|\| id:ebi-fl187-GCST90094766 | MR Egger | 10 | -0.552400867 | 0.418425540024279 | 0.22329230216839 | -1.372514926 | 0.267713191188588 | 0.57556629195727 | 0.25346870234913 | 1.30697223510119 | 0.999950574162623 |
| ebi-fl187-GCST90094766 | ukb-rsfMRI191-node-pheno55 | Net100_Node34 Parietal\|Postcentral\|Precuneus Attention \|\| id:ukb-rsfMRI191-node-pheno55 | Fruit liking \|\| id:ebi-fl187-GCST90094766 | MR Egger | 10 | -0.791866433 | 0.419574354489113 | 0.095821951079006 | -1.614232168 | 0.0304993018171202 | 0.452998514396891 | 0.19904344375043 | 1.03096917024351 | 0.999950574162623 |
| ebi-fl187-GCST90094766 | ebi-a-GCST90001414 | IgD- CD24- B cell Absolute Count \|\| id:ebi-a-GCST90001414 | Fruit liking \|\| id:ebi-fl187-GCST90094766 | Weighted median | 9 | -0.511689153 | 0.390393238154558 | 0.18995850164116 | -1.2768599 | 0.253481593702969 | 0.5994821060521 | 0.278911737610072 | 1.28850366268588 | 0.999332001504091 |
| ebi-fl187-GCST90094766 | ebi-a-GCST90001422 | CD20- CD38- B cell %B cell \|\| id:ebi-a-GCST90001422 | Fruit liking \|\| id:ebi-fl187-GCST90094766 | Weighted median | 9 | -0.217311262 | 0.375185583490983 | 0.562447448682157 | -0.952675005 | 0.518052482119574 | 0.804679464565312 | 0.385707871678454 | 1.678755059 | 0.999332001504091 |
| ebi-fl187-GCST90094766 | ebi-a-GCST90001444 | CD20- CD38- B cell %lymphocyte \|\| id:ebi-a-GCST90001444 | Fruit liking \|\| id:ebi-fl187-GCST90094766 | Weighted median | 9 | -0.182311945 | 0.368260288889732 | 0.620555838414917 | -0.904102112 | 0.539478220869994 | 0.833341342905221 | 0.40490528169835 | 1.715111719 | 0.999332001504091 |
| ebi-fl187-GCST90094766 | ebi-a-GCST90001459 | Myeloid Dendritic Cell %Dendritic Cell \|\| id:ebi-a-GCST90001459 | Fruit liking \|\| id:ebi-fl187-GCST90094766 | Weighted median | 9 | -0.346411971 | 0.403786941890999 | 0.390943371308615 | -1.137834377 | 0.445010435078263 | 0.70722107249111 | 0.320512379707655 | 1.56050647975495 | 0.999332001504091 |
| ebi-fl187-GCST90094766 | ebi-a-GCST90001476 | HLA DR++ monocyte %leukocyte \|\| id:ebi-a-GCST90001476 | Fruit liking \|\| id:ebi-fl187-GCST90094766 | Weighted median | 9 | -0.002332343 | 0.390849700915249 | 0.995238759253667 | -0.768397757 | 0.763733070673635 | 0.997670374678606 | 0.463755522435752 | 2.14627347461775 | 0.999364451047625 |
| ebi-fl187-GCST90094766 | ebi-a-GCST90001478 | CD4 regulatory T cell %CD4+ T cell \|\| id:ebi-a-GCST90001478 | Fruit liking \|\| id:ebi-fl187-GCST90094766 | Weighted median | 9 | 0.0407016794879292 | 0.462389896706081 | 0.929857134737724 | -0.865582518 | 0.946985877031848 | 1.04154134604712 | 0.420806353918684 | 2.57792774615584 | 0.999332001504091 |
| ebi-fl187-GCST90094766 | ebi-a-GCST90001485 | CD39+ resting CD4 regulatory T cell %CD4 regulatory T cell \|\| id:ebi-a-GCST90001485 | Fruit liking \|\| id:ebi-fl187-GCST90094766 | Weighted median | 9 | 0.500707987618478 | 0.376262147075151 | 0.183273790896941 | -0.236765821 | 1.23818179588577 | 1.6498889582515 | 0.789176075121344 | 3.44933616258152 | 0.999332001504091 |
| ebi-fl187-GCST90094766 | ebi-a-GCST90001515 | Immature Myeloid-Derived Suppressor Cells Absolute Count \|\| id:ebi-a-GCST90001515 | Fruit liking \|\| id:ebi-fl187-GCST90094766 | Weighted median | 9 | 0.0053344992930248 | 0.542687863880656 | 0.99215710104137 | -1.058333714 | 1.06900271249911 | 1.00534875306869 | 0.347033586062699 | 2.912473478 | 0.999332001504091 |
| ebi-fl187-GCST90094766 | ebi-a-GCST90001517 | CD33+ HLA DR+ Absolute Count \|\| id:ebi-a-GCST90001517 | Fruit liking \|\| id:ebi-fl187-GCST90094766 | Weighted median | 9 | -0.357845634 | 0.538160418897774 | 0.506087390056832 | -1.412640055 | 0.696948786791911 | 0.699180996277104 | 0.243499581465774 | 2.00761768300516 | 0.999332001504091 |
| ebi-fl187-GCST90094766 | ebi-a-GCST90001518 | CD33+ HLA DR+ CD14- Absolute Count \|\| id:ebi-a-GCST90001518 | Fruit liking \|\| id:ebi-fl187-GCST90094766 | Weighted median | 9 | -0.422633347 | 0.527202095149117 | 0.422753484163778 | -1.455949454 | 0.610682759272232 | 0.655318863551619 | 0.233178866226726 | 1.84168839944966 | 0.999332001504091 |
| ebi-fl187-GCST90094766 | ebi-a-GCST90001520 | CD33+ HLA DR+ CD14dim Absolute Count \|\| id:ebi-a-GCST90001520 | Fruit liking \|\| id:ebi-fl187-GCST90094766 | Weighted median | 9 | -0.250970764 | 0.500429881437623 | 0.616011996459671 | -1.231813332 | 0.729871803252722 | 0.778045117869518 | 0.291763034465354 | 2.07481460614051 | 0.999332001504091 |
| ebi-fl187-GCST90094766 | ebi-a-GCST90001531 | CD33dim HLA DR- Absolute Count \|\| id:ebi-a-GCST90001531 | Fruit liking \|\| id:ebi-fl187-GCST90094766 | Weighted median | 9 | -0.825744886 | 0.525917772350511 | 0.116391233205661 | -1.85654372 | 0.205053948061867 | 0.43790867901425 | 0.15621160953163 | 1.22759128934765 | 0.999332001504091 |
| ebi-fl187-GCST90094766 | ebi-a-GCST90001532 | Basophil Absolute Count \|\| id:ebi-a-GCST90001532 | Fruit liking \|\| id:ebi-fl187-GCST90094766 | Weighted median | 9 | -0.807268789 | 0.560749447139925 | 0.14997359041009 | -1.906337705 | 0.291800127702943 | 0.446074728328513 | 0.148623694912607 | 1.33883539478925 | 0.999332001504091 |
| ebi-fl187-GCST90094766 | ebi-a-GCST90001588 | T/B cell \|\| id:ebi-a-GCST90001588 | Fruit liking \|\| id:ebi-fl187-GCST90094766 | Weighted median | 9 | 0.272789746467135 | 0.414924379852823 | 0.510895153935871 | -0.540462038 | 1.08604153097867 | 1.31362402169589 | 0.58247906270381 | 2.96252377272823 | 0.999332001504091 |
| ebi-fl187-GCST90094766 | ebi-a-GCST90001624 | HLA DR+ CD4+ T cell Absolute Count \|\| id:ebi-a-GCST90001624 | Fruit liking \|\| id:ebi-fl187-GCST90094766 | Weighted median | 9 | -0.264333884 | 0.358131721013033 | 0.46045901049515 | -0.966272057 | 0.437604289415341 | 0.767717168557326 | 0.380498875519391 | 1.54899183366349 | 0.999332001504091 |
| ebi-fl187-GCST90094766 | ebi-a-GCST90001631 | CD8+ Natural Killer T %T cell \|\| id:ebi-a-GCST90001631 | Fruit liking \|\| id:ebi-fl187-GCST90094766 | Weighted median | 9 | 0.557116977247891 | 0.409950826260079 | 0.174151377804093 | -0.246386642 | 1.36062059671765 | 1.74563254029687 | 0.781619959217216 | 3.89861201701536 | 0.999332001504091 |
| ebi-fl187-GCST90094766 | ebi-a-GCST90001632 | CD8+ Natural Killer T %lymphocyte \|\| id:ebi-a-GCST90001632 | Fruit liking \|\| id:ebi-fl187-GCST90094766 | Weighted median | 9 | 0.564763501033633 | 0.411512771293232 | 0.169936399371837 | -0.241801531 | 1.37132853276837 | 1.75903172434045 | 0.785212002563713 | 3.94058241230856 | 0.999332001504091 |
| ebi-fl187-GCST90094766 | ebi-a-GCST90001646 | Natural Killer %CD3- lymphocyte \|\| id:ebi-a-GCST90001646 | Fruit liking \|\| id:ebi-fl187-GCST90094766 | Weighted median | 9 | -0.400430083 | 0.371074526509356 | 0.280538872950935 | -1.127736155 | 0.326875988727604 | 0.670031814610809 | 0.323765381980207 | 1.38662950882778 | 0.999332001504091 |
| ebi-fl187-GCST90094766 | ebi-a-GCST90001684 | CD127- CD8+ T cell Absolute Count \|\| id:ebi-a-GCST90001684 | Fruit liking \|\| id:ebi-fl187-GCST90094766 | Weighted median | 9 | 0.357190351672835 | 0.392322956265116 | 0.362585351818273 | -0.411762643 | 1.12614334595246 | 1.42930791511356 | 0.66248150227441 | 3.08374061644375 | 0.999332001504091 |
| ebi-fl187-GCST90094766 | ebi-a-GCST90001687 | CD28- CD8+ T cell Absolute Count \|\| id:ebi-a-GCST90001687 | Fruit liking \|\| id:ebi-fl187-GCST90094766 | Weighted median | 9 | 0.0186056797262467 | 0.40768268533327 | 0.963599060019038 | -0.780452384 | 0.817663742979457 | 1.01877984385581 | 0.458198682876567 | 2.26520155782828 | 0.999332001504091 |
| ebi-fl187-GCST90094766 | ebi-a-GCST90001701 | BAFF-R on CD20- CD38- B cell \|\| id:ebi-a-GCST90001701 | Fruit liking \|\| id:ebi-fl187-GCST90094766 | Weighted median | 9 | -0.205324269 | 0.390661426847975 | 0.599179278841318 | -0.971020666 | 0.560372127166022 | 0.814383193995014 | 0.378696318295115 | 1.75132409432267 | 0.999332001504091 |
| ebi-fl187-GCST90094766 | ebi-a-GCST90001732 | CD19 on IgD- CD27- B cell \|\| id:ebi-a-GCST90001732 | Fruit liking \|\| id:ebi-fl187-GCST90094766 | Weighted median | 9 | 0.61728460026729 | 0.384252856420148 | 0.108174158782411 | -0.135850998 | 1.37042019885078 | 1.85388715624546 | 0.872972697200342 | 3.93700467278547 | 0.999332001504091 |
| ebi-fl187-GCST90094766 | ebi-a-GCST90001798 | CD27 on CD24+ CD27+ B cell \|\| id:ebi-a-GCST90001798 | Fruit liking \|\| id:ebi-fl187-GCST90094766 | Weighted median | 9 | 0.097778426972344 | 0.390202084660178 | 0.802135698198369 | -0.667017659 | 0.862574512906294 | 1.10271842537758 | 0.513236945201385 | 2.36925251978905 | 0.999332001504091 |
| ebi-fl187-GCST90094766 | ebi-a-GCST90001836 | CD66b on CD66b++ myeloid cell \|\| id:ebi-a-GCST90001836 | Fruit liking \|\| id:ebi-fl187-GCST90094766 | Weighted median | 9 | 0.010736169270003 | 0.60344986548738 | 0.985805329818714 | -1.172025567 | 1.19349790562527 | 1.01079400874143 | 0.309738908480904 | 3.29859923998072 | 0.999332001504091 |
| ebi-fl187-GCST90094766 | ebi-a-GCST90001943 | CD25 on activated & secreting CD4 regulatory T cell \|\| id:ebi-a-GCST90001943 | Fruit liking \|\| id:ebi-fl187-GCST90094766 | Weighted median | 9 | -0.13547739 | 0.385859500441588 | 0.725509207001475 | -0.891762011 | 0.620807230658508 | 0.873298907812794 | 0.409932809872725 | 1.86042923137527 | 0.999332001504091 |
| ebi-fl187-GCST90094766 | ebi-a-GCST90001960 | CD25 on CD4+ T cell \|\| id:ebi-a-GCST90001960 | Fruit liking \|\| id:ebi-fl187-GCST90094766 | Weighted median | 9 | -0.217199914 | 0.425566706263078 | 0.609786618539917 | -1.051310658 | 0.616910830627137 | 0.804769068901684 | 0.349479400861827 | 1.85319435899154 | 0.999332001504091 |
| ebi-fl187-GCST90094766 | ebi-a-GCST90001988 | HLA DR on CD14+ CD16- monocyte \|\| id:ebi-a-GCST90001988 | Fruit liking \|\| id:ebi-fl187-GCST90094766 | Weighted median | 9 | -0.42326306 | 0.39018186326197 | 0.278017313158235 | -1.188019512 | 0.341493391643339 | 0.654906330561201 | 0.304824367547376 | 1.40704729500497 | 0.999332001504091 |
| ebi-fl187-GCST90094766 | ebi-a-GCST90001991 | HLA DR on CD14+ monocyte \|\| id:ebi-a-GCST90001991 | Fruit liking \|\| id:ebi-fl187-GCST90094766 | Weighted median | 9 | -0.276607812 | 0.383886135725558 | 0.471188579572394 | -1.029024638 | 0.475809014048838 | 0.758351855314247 | 0.357355341465008 | 1.60931562992986 | 0.999332001504091 |
| ebi-fl187-GCST90094766 | ebi-a-GCST90002019 | CD14 on Monocytic Myeloid-Derived Suppressor Cells \|\| id:ebi-a-GCST90002019 | Fruit liking \|\| id:ebi-fl187-GCST90094766 | Weighted median | 9 | 0.337245163988011 | 0.550669921770078 | 0.540255239573959 | -0.742067883 | 1.41655821065736 | 1.40108251661298 | 0.476128319317973 | 4.12290581910881 | 0.999332001504091 |
| ebi-fl187-GCST90094766 | ebi-a-GCST90002037 | CD80 on plasmacytoid Dendritic Cell \|\| id:ebi-a-GCST90002037 | Fruit liking \|\| id:ebi-fl187-GCST90094766 | Weighted median | 9 | 0.618118767665426 | 0.433055449446438 | 0.153480983199353 | -0.230669913 | 1.46690744858044 | 1.85543425365042 | 0.794001512162855 | 4.3358056840995 | 0.999332001504091 |
| ebi-fl187-GCST90094766 | ebi-a-GCST90002042 | CD45 on CD33+ HLA DR+ CD14- \|\| id:ebi-a-GCST90002042 | Fruit liking \|\| id:ebi-fl187-GCST90094766 | Weighted median | 9 | 0.423330030187909 | 0.597281570793614 | 0.478472650876367 | -0.747341849 | 1.59400190894339 | 1.52703818151115 | 0.473623844865934 | 4.92341260489734 | 0.999332001504091 |
| ebi-fl187-GCST90094766 | ebi-a-GCST90002056 | CD8 on Effector Memory CD8+ T cell \|\| id:ebi-a-GCST90002056 | Fruit liking \|\| id:ebi-fl187-GCST90094766 | Weighted median | 9 | 0.498611525467129 | 0.430396563146302 | 0.246662793077405 | -0.344965738 | 1.34218878923388 | 1.64643365172182 | 0.708244618717033 | 3.82741174148629 | 0.999332001504091 |
| ebi-fl187-GCST90094766 | ebi-a-GCST90002058 | CD8 on CD8+ T cell \|\| id:ebi-a-GCST90002058 | Fruit liking \|\| id:ebi-fl187-GCST90094766 | Weighted median | 9 | -0.262459959 | 0.405058271013802 | 0.517013374854984 | -1.05637417 | 0.531454252645005 | 0.769157161926135 | 0.347714280415425 | 1.70140478278735 | 0.999332001504091 |
| ebi-fl187-GCST90094766 | ebi-a-GCST90002063 | CD4 on CD4 regulatory T cell \|\| id:ebi-a-GCST90002063 | Fruit liking \|\| id:ebi-fl187-GCST90094766 | Weighted median | 9 | -0.021392889 | 0.412032789626566 | 0.958592153802658 | -0.828977157 | 0.78619137823415 | 0.978834315352042 | 0.436495524414059 | 2.19502048319247 | 0.999332001504091 |
| ebi-fl187-GCST90094766 | ebi-a-GCST90002066 | CD4 on activated CD4 regulatory T cell \|\| id:ebi-a-GCST90002066 | Fruit liking \|\| id:ebi-fl187-GCST90094766 | Weighted median | 9 | 0.149535158689468 | 0.4347984384842 | 0.730907696354801 | -0.702669781 | 1.0017400981185 | 1.16129429967991 | 0.495261298100672 | 2.72301602334153 | 0.999332001504091 |
| ebi-fl187-GCST90094766 | ebi-a-GCST90002070 | CD4 on activated & secreting CD4 regulatory T cell \|\| id:ebi-a-GCST90002070 | Fruit liking \|\| id:ebi-fl187-GCST90094766 | Weighted median | 9 | -0.018592464 | 0.420761062851486 | 0.964754786517331 | -0.843284147 | 0.806099219604364 | 0.981579310054124 | 0.430295048292774 | 2.23915647123776 | 0.999332001504091 |
| ebi-fl187-GCST90094766 | ebi-a-GCST90002081 | SSC-A on CD4+ T cell \|\| id:ebi-a-GCST90002081 | Fruit liking \|\| id:ebi-fl187-GCST90094766 | Weighted median | 9 | -0.186578626 | 0.42310185024962 | 0.659228892771636 | -1.015858252 | 0.64270100052628 | 0.829793316087823 | 0.362091530492537 | 1.90161019918751 | 0.999332001504091 |
| ebi-fl187-GCST90094766 | ebi-a-GCST90002094 | CD11b on Monocytic Myeloid-Derived Suppressor Cells \|\| id:ebi-a-GCST90002094 | Fruit liking \|\| id:ebi-fl187-GCST90094766 | Weighted median | 9 | 0.388172543982952 | 0.574863443847589 | 0.499521360890206 | -0.738559806 | 1.51490489392423 | 1.47428414120136 | 0.477801547182025 | 4.54898846982971 | 0.999332001504091 |
| ebi-fl187-GCST90094766 | ebi-a-GCST90002104 | HLA DR on myeloid Dendritic Cell \|\| id:ebi-a-GCST90002104 | Fruit liking \|\| id:ebi-fl187-GCST90094766 | Weighted median | 9 | -0.151053094 | 0.469338637058975 | 0.747572057426929 | -1.070956823 | 0.768850634293711 | 0.859802046823466 | 0.342680475951606 | 2.15728531854328 | 0.999332001504091 |
| ebi-fl187-GCST90094766 | ebi-a-GCST90002105 | HLA DR on plasmacytoid Dendritic Cell \|\| id:ebi-a-GCST90002105 | Fruit liking \|\| id:ebi-fl187-GCST90094766 | Weighted median | 9 | -0.26472851 | 0.42528647840485 | 0.53363219150178 | -1.098290007 | 0.568832988155882 | 0.767414267366111 | 0.333440777805078 | 1.76620466648904 | 0.999332001504091 |
| ebi-fl187-GCST90094766 | ebi-a-GCST90002106 | HLA DR on Dendritic Cell \|\| id:ebi-a-GCST90002106 | Fruit liking \|\| id:ebi-fl187-GCST90094766 | Weighted median | 9 | -0.102689799 | 0.46465910064614 | 0.825092202887084 | -1.013421636 | 0.808042038714325 | 0.902406857982916 | 0.362974884499138 | 2.24351097585798 | 0.999332001504091 |
| ebi-fl187-GCST90094766 | ebi-a-GCST90002111 | HLA DR on CD33dim HLA DR+ CD11b- \|\| id:ebi-a-GCST90002111 | Fruit liking \|\| id:ebi-fl187-GCST90094766 | Weighted median | 9 | 0.326335907972047 | 0.543345408711135 | 0.548103181999101 | -0.738621093 | 1.39129290904587 | 1.38588081910963 | 0.47777226498738 | 4.02004424603156 | 0.999332001504091 |
| ebi-fl187-GCST90094766 | ebi-a-GCST90002112 | HLA DR on CD33- HLA DR+ \|\| id:ebi-a-GCST90002112 | Fruit liking \|\| id:ebi-fl187-GCST90094766 | Weighted median | 9 | -0.429990806 | 0.557595083770704 | 0.440616584734828 | -1.52287717 | 0.662895558566497 | 0.650515075775967 | 0.218083520105504 | 1.94040275765492 | 0.999332001504091 |
| ebi-fl187-GCST90094766 | ebi-a-GCST90002115 | HLA DR on HLA DR+ CD8+ T cell \|\| id:ebi-a-GCST90002115 | Fruit liking \|\| id:ebi-fl187-GCST90094766 | Weighted median | 9 | 0.0603727978055202 | 0.412025731994199 | 0.883505562139216 | -0.747197637 | 0.86794323251415 | 1.06223247067497 | 0.473692151874147 | 2.38200657809512 | 0.999332001504091 |
| ebi-fl187-GCST90094766 | ebi-a-GCST90002116 | HLA DR on B cell \|\| id:ebi-a-GCST90002116 | Fruit liking \|\| id:ebi-fl187-GCST90094766 | Weighted median | 9 | 0.572850012025893 | 0.405481399523352 | 0.157724772522413 | -0.221893531 | 1.36759355509166 | 1.77331382205774 | 0.801000641500251 | 3.92589187645492 | 0.999332001504091 |
| ebi-fl187-GCST90094766 | ebi-cfmet338-GCST90026023 | Sphingomyelin (d18:1/24:1, d18:2/24:0) levels \|\| id:ebi-cfmet338-GCST90026023 | Fruit liking \|\| id:ebi-fl187-GCST90094766 | Weighted median | 8 | 0.208054580462603 | 0.258890953268364 | 0.421606131589536 | -0.299371688 | 0.715480848868597 | 1.23128037156717 | 0.741283831960801 | 2.04516986347377 | 0.999332001504091 |
| ebi-fl187-GCST90094766 | ebi-cfmet338-GCST90026046 | 1-arachidonoyl-gpc (20:4n6) levels \|\| id:ebi-cfmet338-GCST90026046 | Fruit liking \|\| id:ebi-fl187-GCST90094766 | Weighted median | 8 | -0.662356454 | 0.342224419502139 | 0.0529357430481594 | -1.333116316 | 0.00840340802933692 | 0.51563483187569 | 0.263654349360935 | 1.00843881577501 | 0.999332001504091 |
| ebi-fl187-GCST90094766 | ebi-cfmet338-GCST90026047 | 1-linoleoyl-gpc (18:2) levels \|\| id:ebi-cfmet338-GCST90026047 | Fruit liking \|\| id:ebi-fl187-GCST90094766 | Weighted median | 8 | 0.187582 | 0.386164409 | 0.62713958398539 | -0.569300242 | 0.944464242036079 | 1.20632916442565 | 0.565921308183818 | 2.57143534251093 | 0.999332001504091 |
| ebi-fl187-GCST90094766 | ebi-cfmet338-GCST90026050 | 1-myristoyl-2-palmitoyl-gpc (14:0/16:0) levels \|\| id:ebi-cfmet338-GCST90026050 | Fruit liking \|\| id:ebi-fl187-GCST90094766 | Weighted median | 8 | -0.11082986 | 0.17197498011621 | 0.519281468348648 | -0.447900821 | 0.226241100880271 | 0.895091026629655 | 0.63896805298997 | 1.25387793991245 | 0.999332001504091 |
| ebi-fl187-GCST90094766 | ebi-cfmet338-GCST90026068 | 2,3-dihydroxyisovalerate levels \|\| id:ebi-cfmet338-GCST90026068 | Fruit liking \|\| id:ebi-fl187-GCST90094766 | Weighted median | 8 | 0.257863540639745 | 0.597993473624339 | 0.666312309703799 | -0.914203668 | 1.42993074894345 | 1.29416220602759 | 0.400835697457859 | 4.17840982260888 | 0.999332001504091 |
| ebi-fl187-GCST90094766 | ebi-cfmet338-GCST90026082 | 3-hydroxyhexanoate levels \|\| id:ebi-cfmet338-GCST90026082 | Fruit liking \|\| id:ebi-fl187-GCST90094766 | Weighted median | 7 | -0.328645385 | 0.141148173878497 | 0.0198923859309745 | -0.605295805 | -0.051994964 | 0.719898258516165 | 0.545912918777681 | 0.949333647892041 | 0.999332001504091 |
| ebi-fl187-GCST90094766 | ebi-cfmet338-GCST90026094 | 3-ureidopropionate levels \|\| id:ebi-cfmet338-GCST90026094 | Fruit liking \|\| id:ebi-fl187-GCST90094766 | Weighted median | 8 | 0.247988273791214 | 0.293266398230583 | 0.397771725085105 | -0.326813867 | 0.822790414323158 | 1.28144490561541 | 0.721217973185865 | 2.2768443205512 | 0.999332001504091 |
| ebi-fl187-GCST90094766 | ebi-cfmet338-GCST90026173 | Glycerol levels \|\| id:ebi-cfmet338-GCST90026173 | Fruit liking \|\| id:ebi-fl187-GCST90094766 | Weighted median | 8 | -0.126197108 | 0.165222900214629 | 0.444986952800244 | -0.450033993 | 0.197639776268056 | 0.881441090434607 | 0.637606477368483 | 1.21852337371652 | 0.999332001504091 |
| ebi-fl187-GCST90094766 | ebi-cfmet338-GCST90026179 | Histidine levels \|\| id:ebi-cfmet338-GCST90026179 | Fruit liking \|\| id:ebi-fl187-GCST90094766 | Weighted median | 8 | -0.04598684 | 0.109623998065754 | 0.674853603493576 | -0.260849876 | 0.168876196424144 | 0.955054530831229 | 0.770396565953206 | 1.18397354969085 | 0.999332001504091 |
| ebi-fl187-GCST90094766 | ebi-cfmet338-GCST90026188 | Isocitrate levels \|\| id:ebi-cfmet338-GCST90026188 | Fruit liking \|\| id:ebi-fl187-GCST90094766 | Weighted median | 8 | -0.183082003 | 0.228568008433246 | 0.423134106973113 | -0.6310753 | 0.264911293469215 | 0.832699868999993 | 0.532019413061526 | 1.30331535806649 | 0.999332001504091 |
| ebi-fl187-GCST90094766 | ebi-cfmet338-GCST90026207 | N-acetyl-isoputreanine levels \|\| id:ebi-cfmet338-GCST90026207 | Fruit liking \|\| id:ebi-fl187-GCST90094766 | Weighted median | 8 | -0.114415003 | 0.140686130882549 | 0.416066561950246 | -0.390159819 | 0.161329814016233 | 0.891887743412838 | 0.67694867656246 | 1.17507246027779 | 0.999332001504091 |
| ebi-fl187-GCST90094766 | ebi-cfmet338-GCST90026213 | N-acetylglutamate levels \|\| id:ebi-cfmet338-GCST90026213 | Fruit liking \|\| id:ebi-fl187-GCST90094766 | Weighted median | 8 | 0.165471372196191 | 0.262343901578967 | 0.528209095220433 | -0.348722675 | 0.679665419290966 | 1.17994918288223 | 0.705588780619467 | 1.97321742128906 | 0.999332001504091 |
| ebi-fl187-GCST90094766 | ebi-cfmet338-GCST90026224 | N-acetyltaurine levels \|\| id:ebi-cfmet338-GCST90026224 | Fruit liking \|\| id:ebi-fl187-GCST90094766 | Weighted median | 8 | -0.225623274 | 0.185444770812867 | 0.223733523796703 | -0.589095025 | 0.137848476866471 | 0.798018679487874 | 0.554829164284281 | 1.14780161859926 | 0.999332001504091 |
| ebi-fl187-GCST90094766 | ebi-cfmet338-GCST90026250 | Phenyllactate (pla) levels \|\| id:ebi-cfmet338-GCST90026250 | Fruit liking \|\| id:ebi-fl187-GCST90094766 | Weighted median | 8 | 0.127068745195554 | 0.318326618097265 | 0.689762602218114 | -0.496851426 | 0.750988916666193 | 1.13549507492941 | 0.608443375794815 | 2.11909458871937 | 0.999332001504091 |
| ebi-fl187-GCST90094766 | ebi-cfmet338-GCST90026251 | Pipecolate levels \|\| id:ebi-cfmet338-GCST90026251 | Fruit liking \|\| id:ebi-fl187-GCST90094766 | Weighted median | 8 | 0.121550892910409 | 0.479879977193393 | 0.800040752156726 | -0.819013862 | 1.06211564820946 | 1.12924683512077 | 0.440866194948863 | 2.89248399909225 | 0.999332001504091 |
| ebi-fl187-GCST90094766 | ebi-cfmet338-GCST90026278 | Trigonelline (n'-methylnicotinate) levels \|\| id:ebi-cfmet338-GCST90026278 | Fruit liking \|\| id:ebi-fl187-GCST90094766 | Weighted median | 8 | -0.650370214 | 0.5156275495159 | 0.207194365335437 | -1.661000211 | 0.360259783144425 | 0.521852543925453 | 0.189948896088002 | 1.43370181775259 | 0.999332001504091 |
| ebi-fl187-GCST90094766 | ebi-cfmet338-GCST90026299 | 1-carboxyethylvaline levels \|\| id:ebi-cfmet338-GCST90026299 | Fruit liking \|\| id:ebi-fl187-GCST90094766 | Weighted median | 8 | 0.314432286767922 | 0.327214536602263 | 0.336584257983417 | -0.326908205 | 0.955772778508358 | 1.36948161738914 | 0.721149937966754 | 2.60067955584187 | 0.999332001504091 |
| ebi-fl187-GCST90094766 | ebi-gm430-GCST90011306 | C_Erysipelotrichia abundance \|\| id:ebi-gm430-GCST90011306 | Fruit liking \|\| id:ebi-fl187-GCST90094766 | Weighted median | 8 | -0.05533627 | 0.268896729621566 | 0.836954918556944 | -0.58237386 | 0.471701320260246 | 0.946166927090981 | 0.558570822634447 | 1.6027186126524 | 0.999332001504091 |
| ebi-fl187-GCST90094766 | ebi-gm430-GCST90011312 | F_Erysipelotrichaceae abundance \|\| id:ebi-gm430-GCST90011312 | Fruit liking \|\| id:ebi-fl187-GCST90094766 | Weighted median | 8 | -0.05533627 | 0.2668029980292 | 0.835693595650088 | -0.578270146 | 0.467597606339209 | 0.946166927090981 | 0.560867747232462 | 1.59615499079455 | 0.999332001504091 |
| ebi-fl187-GCST90094766 | ebi-gm430-GCST90011352 | O_Erysipelotrichales abundance \|\| id:ebi-gm430-GCST90011352 | Fruit liking \|\| id:ebi-fl187-GCST90094766 | Weighted median | 8 | -0.05533627 | 0.273361339819159 | 0.839581338523822 | -0.591124496 | 0.480451956247528 | 0.946166927090981 | 0.553704296346416 | 1.61680496219358 | 0.999332001504091 |
| ebi-fl187-GCST90094766 | ebi-gm430-GCST90011362 | OTU97_11 (Parabacteroides) abundance \|\| id:ebi-gm430-GCST90011362 | Fruit liking \|\| id:ebi-fl187-GCST90094766 | Weighted median | 8 | -0.619558504 | 0.391381233793658 | 0.113420293400701 | -1.386665722 | 0.147548714375178 | 0.538181990422665 | 0.24990717699247 | 1.15898974291574 | 0.999332001504091 |
| ebi-fl187-GCST90094766 | ebi-gm430-GCST90011384 | OTU97_235 (Phascolarctobacterium) abundance \|\| id:ebi-gm430-GCST90011384 | Fruit liking \|\| id:ebi-fl187-GCST90094766 | Weighted median | 8 | 2.61907162769582 | 1.07716448371392 | 0.0150385221228836 | 0.507829239616547 | 4.7303140157751 | 13.7229776371317 | 1.66168016754858 | 113.331144528876 | 0.999332001504091 |
| ebi-fl187-GCST90094766 | ebi-gm430-GCST90011390 | OTU97_3 (Bacteroides) abundance \|\| id:ebi-gm430-GCST90011390 | Fruit liking \|\| id:ebi-fl187-GCST90094766 | Weighted median | 8 | -0.199727231 | 0.273286590953181 | 0.464880332067618 | -0.73536895 | 0.335914486887123 | 0.818954107595408 | 0.479328578235996 | 1.39921936809113 | 0.999332001504091 |
| ebi-fl187-GCST90094766 | ebi-gm430-GCST90011398 | OTU97_4 (Alistipes) abundance \|\| id:ebi-gm430-GCST90011398 | Fruit liking \|\| id:ebi-fl187-GCST90094766 | Weighted median | 8 | -0.014805018 | 0.318278231617947 | 0.962899013321477 | -0.638630352 | 0.609020316363199 | 0.985304037812732 | 0.528015124088472 | 1.8386292411719 | 0.999332001504091 |
| ebi-fl187-GCST90094766 | ebi-gm430-GCST90011421 | OTU99_108 (Cytophagales) abundance \|\| id:ebi-gm430-GCST90011421 | Fruit liking \|\| id:ebi-fl187-GCST90094766 | Weighted median | 8 | 0.117869951063864 | 0.960245602221558 | 0.902305231081805 | -1.764211429 | 1.99995133141812 | 1.12509778408283 | 0.171321832610347 | 7.3886964927997 | 0.999332001504091 |
| ebi-fl187-GCST90094766 | ebi-gm430-GCST90011428 | OTU99_123 (Phascolarctobacterium) abundance \|\| id:ebi-gm430-GCST90011428 | Fruit liking \|\| id:ebi-fl187-GCST90094766 | Weighted median | 8 | 0.0735450261397379 | 0.556130559084192 | 0.894791162523222 | -1.01647087 | 1.16356092194475 | 1.07631699798042 | 0.361869774920834 | 3.20131262798897 | 0.999332001504091 |
| ebi-fl187-GCST90094766 | ebi-gm430-GCST90011450 | OTU99_298 (Phascolarctobacterium) abundance \|\| id:ebi-gm430-GCST90011450 | Fruit liking \|\| id:ebi-fl187-GCST90094766 | Weighted median | 8 | 2.59325429671513 | 1.11771210004686 | 0.0203330271430658 | 0.402538580623286 | 4.78397001280698 | 13.3732212958877 | 1.4956166259335 | 119.578135685111 | 0.999332001504091 |
| ebi-fl187-GCST90094766 | ebi-gm430-GCST90011451 | OTU99_3 (Bacteroides) abundance \|\| id:ebi-gm430-GCST90011451 | Fruit liking \|\| id:ebi-fl187-GCST90094766 | Weighted median | 8 | -0.20314035 | 0.279911069401278 | 0.468003235423443 | -0.751766046 | 0.345485346506387 | 0.816163685205286 | 0.471533068096725 | 1.41267539037417 | 0.999332001504091 |
| ebi-fl187-GCST90094766 | ebi-gm430-GCST90011472 | OTU99_72 (Parabacteroides) abundance \|\| id:ebi-gm430-GCST90011472 | Fruit liking \|\| id:ebi-fl187-GCST90094766 | Weighted median | 8 | 0.667666465061027 | 0.925061424260699 | 0.470445947005076 | -1.145453926 | 2.480786857 | 1.94968235622046 | 0.318079500304342 | 11.9506641783588 | 0.999332001504091 |
| ebi-fl187-GCST90094766 | ebi-gm430-GCST90011507 | TestASV_3 (Bacteroides) abundance \|\| id:ebi-gm430-GCST90011507 | Fruit liking \|\| id:ebi-fl187-GCST90094766 | Weighted median | 8 | 0.0938074957643804 | 0.379857752099195 | 0.804943611461341 | -0.650713698 | 0.838328689878802 | 1.09834828886685 | 0.521673326475882 | 2.31249884253474 | 0.999332001504091 |
| ebi-fl187-GCST90094766 | ebi-gm430-GCST90011512 | TestASV_34 (Phascolarctobacterium) abundance \|\| id:ebi-gm430-GCST90011512 | Fruit liking \|\| id:ebi-fl187-GCST90094766 | Weighted median | 8 | 0.066816756259507 | 0.833718204714164 | 0.936123449080157 | -1.567270925 | 1.70090443749927 | 1.06909955442294 | 0.208613728717133 | 5.47890047455659 | 0.999332001504091 |
| ebi-fl187-GCST90094766 | ebi-gm430-GCST90011519 | TestASV_41 (Barnesiella) abundance \|\| id:ebi-gm430-GCST90011519 | Fruit liking \|\| id:ebi-fl187-GCST90094766 | Weighted median | 8 | -0.756118619 | 1.13439184302625 | 0.50506539692762 | -2.979526632 | 1.46728939310727 | 0.469485145775966 | 0.0508168832813193 | 4.33746203764737 | 0.999332001504091 |
| ebi-fl187-GCST90094766 | ebi-gm430-GCST90011524 | TestASV_46 (Sutterella) abundance \|\| id:ebi-gm430-GCST90011524 | Fruit liking \|\| id:ebi-fl187-GCST90094766 | Weighted median | 8 | -1.411456164 | 1.27062461985422 | 0.266638746525936 | -3.901880419 | 1.07896809131474 | 0.243788029308072 | 0.0202038839457799 | 2.94164247792205 | 0.999332001504091 |
| ebi-fl187-GCST90094766 | ebi-gm430-GCST90011581 | OTU97_19 (Bacteria) prevalence \|\| id:ebi-gm430-GCST90011581 | Fruit liking \|\| id:ebi-fl187-GCST90094766 | Weighted median | 8 | 0.530766872689008 | 1.4139823489762 | 0.707385145559765 | -2.240638531 | 3.30217227668236 | 1.70023567309994 | 0.106390548989724 | 27.1715990896976 | 0.999332001504091 |
| ebi-fl187-GCST90094766 | ebi-gm430-GCST90011614 | OTU97_80 (Ruminococcus) prevalence \|\| id:ebi-gm430-GCST90011614 | Fruit liking \|\| id:ebi-fl187-GCST90094766 | Weighted median | 8 | 0.307568688073392 | 0.612851768502098 | 0.61576267571849 | -0.893620778 | 1.5087581543375 | 1.36011422891947 | 0.409171547968539 | 4.52111278238592 | 0.999332001504091 |
| ebi-fl187-GCST90094766 | ebi-gm430-GCST90011623 | OTU99_11 (Parabacteroides) prevalence \|\| id:ebi-gm430-GCST90011623 | Fruit liking \|\| id:ebi-fl187-GCST90094766 | Weighted median | 8 | 0.74335859046448 | 0.519707946636257 | 0.152619721299194 | -0.275268985 | 1.76198616587154 | 2.10298673798528 | 0.759367837237174 | 5.82399333139079 | 0.999332001504091 |
| ebi-fl187-GCST90094766 | ebi-gm430-GCST90011642 | OTU99_19 (Bacteria) prevalence \|\| id:ebi-gm430-GCST90011642 | Fruit liking \|\| id:ebi-fl187-GCST90094766 | Weighted median | 8 | 0.530766872689008 | 1.37317372998648 | 0.699107407643929 | -2.160653638 | 3.22218738346251 | 1.70023567309994 | 0.115249764777397 | 25.0829261965535 | 0.999332001504091 |
| ebi-fl187-GCST90094766 | ebi-gm430-GCST90011653 | OTU99_30 (Parasutterella) prevalence \|\| id:ebi-gm430-GCST90011653 | Fruit liking \|\| id:ebi-fl187-GCST90094766 | Weighted median | 8 | -0.325794717 | 0.559690456412301 | 0.560500646600908 | -1.422788011 | 0.771198577968035 | 0.721953377264611 | 0.241041054068274 | 2.16235645400118 | 0.999332001504091 |
| ebi-fl187-GCST90094766 | ebi-gm430-GCST90011675 | OTU99_8 (Ruminococcaceae) prevalence \|\| id:ebi-gm430-GCST90011675 | Fruit liking \|\| id:ebi-fl187-GCST90094766 | Weighted median | 8 | -0.155897854 | 0.621197723919449 | 0.801842454764436 | -1.373445393 | 1.06164968492616 | 0.855646586821279 | 0.25323296774139 | 2.891136522 | 0.999332001504091 |
| ebi-fl187-GCST90094766 | ebi-gm430-GCST90011679 | OTU99_92 (Ruminococcus) prevalence \|\| id:ebi-gm430-GCST90011679 | Fruit liking \|\| id:ebi-fl187-GCST90094766 | Weighted median | 8 | 0.211260856008486 | 0.637707345904173 | 0.740431615371265 | -1.038645542 | 1.46116725398066 | 1.23523453138815 | 0.353933745855964 | 4.31098861128278 | 0.999332001504091 |
| ebi-fl187-GCST90094766 | ebi-gm430-GCST90011684 | TestASV_11 (Lachnospiraceae) prevalence \|\| id:ebi-gm430-GCST90011684 | Fruit liking \|\| id:ebi-fl187-GCST90094766 | Weighted median | 8 | 0.259581130344381 | 0.675771332649189 | 0.70088529054293 | -1.064930682 | 1.58409294233679 | 1.29638695576548 | 0.344751751562224 | 4.87486758649736 | 0.999332001504091 |
| ebi-fl187-GCST90094766 | ebi-gm430-GCST90011688 | TestASV_15 (Bacteroides) prevalence \|\| id:ebi-gm430-GCST90011688 | Fruit liking \|\| id:ebi-fl187-GCST90094766 | Weighted median | 8 | -0.341010161 | 0.609720977027193 | 0.575964585047043 | -1.536063276 | 0.854042954258722 | 0.711051683376501 | 0.215226724120627 | 2.34912508424924 | 0.999332001504091 |
| ebi-fl187-GCST90094766 | ebi-gm430-GCST90011692 | TestASV_19 (Prevotella) prevalence \|\| id:ebi-gm430-GCST90011692 | Fruit liking \|\| id:ebi-fl187-GCST90094766 | Weighted median | 8 | 0.31349140795056 | 1.16486415397569 | 0.787835248230131 | -1.969642334 | 2.59662514974292 | 1.36819370712333 | 0.139506744134127 | 13.4183765224433 | 0.999332001504091 |
| ebi-fl187-GCST90094766 | ebi-gm430-GCST90011693 | TestASV_2 (Bacteroides) prevalence \|\| id:ebi-gm430-GCST90011693 | Fruit liking \|\| id:ebi-fl187-GCST90094766 | Weighted median | 8 | 0.322186685834192 | 0.626308890681308 | 0.606957412355672 | -0.90537874 | 1.54975211156956 | 1.38014240494483 | 0.404388697960308 | 4.71030240838693 | 0.999332001504091 |
| ebi-fl187-GCST90094766 | ebi-gm430-GCST90011716 | TestASV_40 (Barnesiella) prevalence \|\| id:ebi-gm430-GCST90011716 | Fruit liking \|\| id:ebi-fl187-GCST90094766 | Weighted median | 8 | -1.658326155 | 1.19619063165138 | 0.165643303403525 | -4.002859793 | 0.686207482822293 | 0.190457509752128 | 0.0182633347733755 | 1.98616865271849 | 0.999332001504091 |
| ebi-fl187-GCST90094766 | ebi-gm430-GCST90011718 | TestASV_42 (Alphaproteobacteria) prevalence \|\| id:ebi-gm430-GCST90011718 | Fruit liking \|\| id:ebi-fl187-GCST90094766 | Weighted median | 8 | 0.979522368750103 | 0.951074552305648 | 0.303051675224887 | -0.884583754 | 2.84362849126917 | 2.66318391823925 | 0.412885999756752 | 17.1779827520107 | 0.999332001504091 |
| ebi-fl187-GCST90094766 | ebi-hbsm44-GCST90104929 | Sulcal depth of the right inferior frontal sulcus \|\| id:ebi-hbsm44-GCST90104929 | Fruit liking \|\| id:ebi-fl187-GCST90094766 | Weighted median | 8 | -0.262025515 | 0.484588149245232 | 0.588701998245147 | -1.211818287 | 0.687767257630398 | 0.769491389968732 | 0.297655563717413 | 1.98926904587664 | 0.999332001504091 |
| ebi-fl187-GCST90094766 | ebi-hbsm44-GCST90104935 | Sulcal opening distance of the left calloso-marginal posterior fissure \|\| id:ebi-hbsm44-GCST90104935 | Fruit liking \|\| id:ebi-fl187-GCST90094766 | Weighted median | 9 | -0.092962725 | 0.10834790295381 | 0.390891322875456 | -0.305324614 | 0.119399165259422 | 0.911227466095125 | 0.736884132421838 | 1.12681961577483 | 0.999332001504091 |
| ebi-fl187-GCST90094766 | ebi-hbsm44-GCST90104936 | Sulcal opening distance of the left anterior inferior temporal sulcus \|\| id:ebi-hbsm44-GCST90104936 | Fruit liking \|\| id:ebi-fl187-GCST90094766 | Weighted median | 9 | -0.067394027 | 0.0650368052598947 | 0.300088125459223 | -0.194866165 | 0.0600781113512307 | 0.934826781804686 | 0.822944789281852 | 1.06191949127222 | 0.999332001504091 |
| ebi-fl187-GCST90094766 | ebi-hbsm44-GCST90104941 | Sulcal opening distance of the right posterior inferior temporal sulcus \|\| id:ebi-hbsm44-GCST90104941 | Fruit liking \|\| id:ebi-fl187-GCST90094766 | Weighted median | 9 | -0.000732576 | 0.0543699475385523 | 0.9892496917719 | -0.107297674 | 0.105832520799703 | 0.999267691892701 | 0.898258245467208 | 1.11163568505981 | 0.999332001504091 |
| ebi-fl187-GCST90094766 | ebi-IPs91-GCST90274819 | Leukemia inhibitory factor levels \|\| id:ebi-IPs91-GCST90274819 | Fruit liking \|\| id:ebi-fl187-GCST90094766 | Weighted median | 10 | 0.392473727788258 | 0.221413949392724 | 0.0762984859623639 | -0.041497613 | 0.826445068597996 | 1.48063896510964 | 0.959351625290462 | 2.28518062325395 | 0.999332001504091 |
| ebi-fl187-GCST90094766 | ebi-IPs91-GCST90274837 | STAM binding protein levels \|\| id:ebi-IPs91-GCST90274837 | Fruit liking \|\| id:ebi-fl187-GCST90094766 | Weighted median | 10 | -0.204833367 | 0.185873273872657 | 0.270459288677937 | -0.569144983 | 0.159478250169366 | 0.814783075157062 | 0.566009179104437 | 1.17289875159411 | 0.999332001504091 |
| ebi-fl187-GCST90094766 | ebi-IPs91-GCST90274840 | TNF-beta levels \|\| id:ebi-IPs91-GCST90274840 | Fruit liking \|\| id:ebi-fl187-GCST90094766 | Weighted median | 10 | 0.0624947103848215 | 0.206519349246547 | 0.762187498256837 | -0.342283214 | 0.467272634908053 | 1.0644888281667 | 0.710147052540323 | 1.59563637029583 | 0.999332001504091 |
| ebi-fl187-GCST90094766 | ebi-IPs91-GCST90274842 | Tumor necrosis factor ligand superfamily member 14 levels \|\| id:ebi-IPs91-GCST90274842 | Fruit liking \|\| id:ebi-fl187-GCST90094766 | Weighted median | 10 | -0.290534627 | 0.196896561432325 | 0.140059407311736 | -0.676451888 | 0.0953826332460402 | 0.747863632469127 | 0.50841771911548 | 1.10007970167318 | 0.999332001504091 |
| ebi-fl187-GCST90094766 | ebi-met1400-GCST90199628 | 3-methylhistidine levels \|\| id:ebi-met1400-GCST90199628 | Fruit liking \|\| id:ebi-fl187-GCST90094766 | Weighted median | 9 | -0.062838784 | 0.239110020707316 | 0.792702575430327 | -0.531494424 | 0.405816857059499 | 0.939094859047036 | 0.58772600115799 | 1.50052771623338 | 0.999332001504091 |
| ebi-fl187-GCST90094766 | ebi-met1400-GCST90199651 | Docosahexaenoate DHA; 22:6n3 levels \|\| id:ebi-met1400-GCST90199651 | Fruit liking \|\| id:ebi-fl187-GCST90094766 | Weighted median | 9 | 0.1172067910456 | 0.233653168058743 | 0.615929762710569 | -0.340753418 | 0.575167000440736 | 1.12435191155954 | 0.711234263904432 | 1.77742733327799 | 0.999332001504091 |
| ebi-fl187-GCST90094766 | ebi-met1400-GCST90199652 | 1-stearoyl-2-oleoyl-GPS (18:0/18:1) levels \|\| id:ebi-met1400-GCST90199652 | Fruit liking \|\| id:ebi-fl187-GCST90094766 | Weighted median | 9 | 0.12650499691182 | 0.254253611954316 | 0.618798168767969 | -0.371832083 | 0.624842076342279 | 1.13485512193288 | 0.68947000687155 | 1.86795094049279 | 0.999332001504091 |
| ebi-fl187-GCST90094766 | ebi-met1400-GCST90199653 | 1-myristoyl-2-palmitoyl-gpc (14:0/16:0) levels \|\| id:ebi-met1400-GCST90199653 | Fruit liking \|\| id:ebi-fl187-GCST90094766 | Weighted median | 9 | 0.00393484907994353 | 0.238963091477562 | 0.986862349999192 | -0.46443281 | 0.472302508375966 | 1.00394260076248 | 0.628491478060198 | 1.60368243772623 | 0.999332001504091 |
| ebi-fl187-GCST90094766 | ebi-met1400-GCST90199667 | Erythritol levels in elite athletes \|\| id:ebi-met1400-GCST90199667 | Fruit liking \|\| id:ebi-fl187-GCST90094766 | Weighted median | 9 | 0.274045238636744 | 0.221306386033746 | 0.215602263195012 | -0.159715278 | 0.707805755262885 | 1.31527430210924 | 0.852386447602617 | 2.02953307699168 | 0.999332001504091 |
| ebi-fl187-GCST90094766 | ebi-met1400-GCST90199674 | 3-hydroxymyristate levels \|\| id:ebi-met1400-GCST90199674 | Fruit liking \|\| id:ebi-fl187-GCST90094766 | Weighted median | 9 | 0.255287711513875 | 0.243580303890195 | 0.294609189441292 | -0.222129684 | 0.732705107138658 | 1.29083295495541 | 0.800811505072317 | 2.0807015221996 | 0.999332001504091 |
| ebi-fl187-GCST90094766 | ebi-met1400-GCST90199685 | 2-linoleoylglycerol (18:2) levels \|\| id:ebi-met1400-GCST90199685 | Fruit liking \|\| id:ebi-fl187-GCST90094766 | Weighted median | 9 | -0.324252318 | 0.283000936103785 | 0.251892765834402 | -0.878934153 | 0.230429516891087 | 0.723067776438199 | 0.415225242641502 | 1.25914071600575 | 0.999332001504091 |
| ebi-fl187-GCST90094766 | ebi-met1400-GCST90199703 | Gamma-glutamylvaline levels \|\| id:ebi-met1400-GCST90199703 | Fruit liking \|\| id:ebi-fl187-GCST90094766 | Weighted median | 9 | 0.20459378680258 | 0.24685062834228 | 0.407208449563106 | -0.279233445 | 0.688421018353449 | 1.22702652934436 | 0.756363313560537 | 1.99056997704893 | 0.999332001504091 |
| ebi-fl187-GCST90094766 | ebi-met1400-GCST90199779 | Gamma-glutamyl-2-aminobutyrate levels \|\| id:ebi-met1400-GCST90199779 | Fruit liking \|\| id:ebi-fl187-GCST90094766 | Weighted median | 9 | -0.205444308 | 0.298534633125541 | 0.491342085369746 | -0.790572189 | 0.379683572808418 | 0.814285442393453 | 0.453585184543247 | 1.46182195602705 | 0.999332001504091 |
| ebi-fl187-GCST90094766 | ebi-met1400-GCST90199824 | 4-vinylphenol sulfate levels \|\| id:ebi-met1400-GCST90199824 | Fruit liking \|\| id:ebi-fl187-GCST90094766 | Weighted median | 9 | -0.130288907 | 0.286189127009308 | 0.648926107981997 | -0.691219596 | 0.430641781939132 | 0.87784177964726 | 0.500964721804045 | 1.53824442431636 | 0.999332001504091 |
| ebi-fl187-GCST90094766 | ebi-met1400-GCST90199830 | 4-ethylphenylsulfate levels \|\| id:ebi-met1400-GCST90199830 | Fruit liking \|\| id:ebi-fl187-GCST90094766 | Weighted median | 9 | 0.263444834942605 | 0.250122519441242 | 0.29222039878478 | -0.226795303 | 0.753684973047439 | 1.30140550085225 | 0.797083926151175 | 2.12481549568631 | 0.999332001504091 |
| ebi-fl187-GCST90094766 | ebi-met1400-GCST90199833 | Hexanoylglutamine levels \|\| id:ebi-met1400-GCST90199833 | Fruit liking \|\| id:ebi-fl187-GCST90094766 | Weighted median | 9 | 0.192028337762013 | 0.22339174919899 | 0.390007624599277 | -0.245819491 | 0.629876166192033 | 1.21170485348216 | 0.782063381923664 | 1.87737808199224 | 0.999332001504091 |
| ebi-fl187-GCST90094766 | ebi-met1400-GCST90199837 | Carotenoid levels (cryptoxanthin) \|\| id:ebi-met1400-GCST90199837 | Fruit liking \|\| id:ebi-fl187-GCST90094766 | Weighted median | 9 | 0.297959392 | 0.229025964862467 | 0.193263518639569 | -0.150931499 | 0.746850283206435 | 1.34710708354678 | 0.859906601057017 | 2.11034255616975 | 0.999332001504091 |
| ebi-fl187-GCST90094766 | ebi-met1400-GCST90199869 | 2-hydroxyhippurate levels \|\| id:ebi-met1400-GCST90199869 | Fruit liking \|\| id:ebi-fl187-GCST90094766 | Weighted median | 9 | -0.151080166 | 0.276554200324807 | 0.584863199671848 | -0.693126399 | 0.390966066639995 | 0.859778770874364 | 0.500010391071321 | 1.47840834519935 | 0.999332001504091 |
| ebi-fl187-GCST90094766 | ebi-met1400-GCST90199902 | Hydantoin-5-propionate levels \|\| id:ebi-met1400-GCST90199902 | Fruit liking \|\| id:ebi-fl187-GCST90094766 | Weighted median | 9 | -0.109993319 | 0.239949440188567 | 0.646663607582575 | -0.580294222 | 0.360307583628845 | 0.895840120258294 | 0.559733656430251 | 1.43377035103195 | 0.999332001504091 |
| ebi-fl187-GCST90094766 | ebi-met1400-GCST90199904 | Pantoate levels \|\| id:ebi-met1400-GCST90199904 | Fruit liking \|\| id:ebi-fl187-GCST90094766 | Weighted median | 9 | 0.0320447991916489 | 0.257330079495755 | 0.900897235041951 | -0.472322157 | 0.536411755003329 | 1.03256376229085 | 0.623552598958917 | 1.70986044317086 | 0.999332001504091 |
| ebi-fl187-GCST90094766 | ebi-met1400-GCST90199936 | 3-methyl catechol sulfate (2) levels \|\| id:ebi-met1400-GCST90199936 | Fruit liking \|\| id:ebi-fl187-GCST90094766 | Weighted median | 9 | 0.244372734834903 | 0.281797303070394 | 0.385836103463182 | -0.307949979 | 0.796695448852877 | 1.27682015715584 | 0.734952079993075 | 2.21819865280852 | 0.999332001504091 |
| ebi-fl187-GCST90094766 | ebi-met1400-GCST90199977 | 4-hydroxychlorothalonil levels \|\| id:ebi-met1400-GCST90199977 | Fruit liking \|\| id:ebi-fl187-GCST90094766 | Weighted median | 9 | -0.177948032 | 0.245882832257458 | 0.469243374928755 | -0.659878383 | 0.303982319456432 | 0.83698591903683 | 0.516914196226615 | 1.35524509440791 | 0.999332001504091 |
| ebi-fl187-GCST90094766 | ebi-met1400-GCST90200040 | Sphingomyelin (d18:2/24:1, d18:1/24:2) levels \|\| id:ebi-met1400-GCST90200040 | Fruit liking \|\| id:ebi-fl187-GCST90094766 | Weighted median | 9 | 0.0482403551826472 | 0.207233109718851 | 0.815929876132814 | -0.35793654 | 0.454417250231594 | 1.049422859 | 0.699117439685011 | 1.57525513594019 | 0.999332001504091 |
| ebi-fl187-GCST90094766 | ebi-met1400-GCST90200077 | 1-stearoyl-2-oleoyl-GPI (18:0/18:1) levels \|\| id:ebi-met1400-GCST90200077 | Fruit liking \|\| id:ebi-fl187-GCST90094766 | Weighted median | 9 | 0.338828311387402 | 0.242084914206564 | 0.161625379509242 | -0.13565812 | 0.813314743232268 | 1.40330239348799 | 0.873141090544066 | 2.2553715875885 | 0.999332001504091 |
| ebi-fl187-GCST90094766 | ebi-met1400-GCST90200080 | 1-palmitoyl-2-oleoyl-GPI (16:0/18:1) levels \|\| id:ebi-met1400-GCST90200080 | Fruit liking \|\| id:ebi-fl187-GCST90094766 | Weighted median | 9 | 0.462642706907852 | 0.26197672576436 | 0.0774010943565971 | -0.050831676 | 0.976117089405998 | 1.58826576464011 | 0.95043863909163 | 2.65413045658451 | 0.999332001504091 |
| ebi-fl187-GCST90094766 | ebi-met1400-GCST90200082 | 1-oleoyl-2-linoleoyl-GPE (18:1/18:2) levels \|\| id:ebi-met1400-GCST90200082 | Fruit liking \|\| id:ebi-fl187-GCST90094766 | Weighted median | 9 | 0.211121876993713 | 0.243807709183823 | 0.386525249494831 | -0.266741233 | 0.688984986994007 | 1.2350628716388 | 0.765871228021102 | 1.99169291271331 | 0.999332001504091 |
| ebi-fl187-GCST90094766 | ebi-met1400-GCST90200100 | Perfluorooctanesulfonate (PFOS) levels \|\| id:ebi-met1400-GCST90200100 | Fruit liking \|\| id:ebi-fl187-GCST90094766 | Weighted median | 9 | 0.0636182941131161 | 0.221938699584363 | 0.774381823615281 | -0.371381557 | 0.498618145298467 | 1.06568554267156 | 0.689780700636646 | 1.64644455087098 | 0.999332001504091 |
| ebi-fl187-GCST90094766 | ebi-met1400-GCST90200102 | 1-palmitoleoyl-2-linolenoyl-GPC (16:1/18:3) levels \|\| id:ebi-met1400-GCST90200102 | Fruit liking \|\| id:ebi-fl187-GCST90094766 | Weighted median | 9 | 0.17114000397823 | 0.227240613945636 | 0.451376345342092 | -0.274251599 | 0.616531607311677 | 1.18665687407552 | 0.760140800264338 | 1.85249171771993 | 0.999332001504091 |
| ebi-fl187-GCST90094766 | ebi-met1400-GCST90200114 | Glycosyl-N-tricosanoyl-sphingadienine (d18:2/23:0) levels \|\| id:ebi-met1400-GCST90200114 | Fruit liking \|\| id:ebi-fl187-GCST90094766 | Weighted median | 9 | -0.198017663 | 0.273055501180884 | 0.468334253827681 | -0.733206446 | 0.337171118996883 | 0.8203553628125 | 0.480366249792045 | 1.40097877731121 | 0.999332001504091 |
| ebi-fl187-GCST90094766 | ebi-met1400-GCST90200135 | Arachidoylcarnitine (C20) levels \|\| id:ebi-met1400-GCST90200135 | Fruit liking \|\| id:ebi-fl187-GCST90094766 | Weighted median | 9 | 0.193726065211231 | 0.250006495740472 | 0.438408132298046 | -0.296286666 | 0.683738796862557 | 1.21376374529631 | 0.743574239684433 | 1.98127147333797 | 0.999332001504091 |
| ebi-fl187-GCST90094766 | ebi-met1400-GCST90200146 | Docosahexaenoylcarnitine (C22:6) levels \|\| id:ebi-met1400-GCST90200146 | Fruit liking \|\| id:ebi-fl187-GCST90094766 | Weighted median | 9 | -0.008158129 | 0.295974791196591 | 0.978010218230694 | -0.58826872 | 0.571952461701935 | 0.991875058181486 | 0.555287811824004 | 1.77172289773639 | 0.999332001504091 |
| ebi-fl187-GCST90094766 | ebi-met1400-GCST90200162 | 3-hydroxyphenylacetoylglutamine levels \|\| id:ebi-met1400-GCST90200162 | Fruit liking \|\| id:ebi-fl187-GCST90094766 | Weighted median | 9 | -0.341222405 | 0.302920259710573 | 0.259978055962866 | -0.934946114 | 0.252501303938907 | 0.710900782667835 | 0.392607020933774 | 1.28724117463755 | 0.999332001504091 |
| ebi-fl187-GCST90094766 | ebi-met1400-GCST90200172 | 3-carboxy-4-methyl-5-pentyl-2-furanpropionate (3-CMPFP) levels \|\| id:ebi-met1400-GCST90200172 | Fruit liking \|\| id:ebi-fl187-GCST90094766 | Weighted median | 9 | -0.186628866 | 0.249359004493937 | 0.454198157618871 | -0.675372515 | 0.302114782762884 | 0.829751628250575 | 0.508966787600944 | 1.35271648633445 | 0.999332001504091 |
| ebi-fl187-GCST90094766 | ebi-met1400-GCST90200222 | Dihydrocaffeate sulfate (2) levels \|\| id:ebi-met1400-GCST90200222 | Fruit liking \|\| id:ebi-fl187-GCST90094766 | Weighted median | 9 | 0.153368080745404 | 0.28674932776498 | 0.592753096165125 | -0.408660602 | 0.715396763164765 | 1.16575399158043 | 0.664539737726547 | 2.04499790115623 | 0.999332001504091 |
| ebi-fl187-GCST90094766 | ebi-met1400-GCST90200223 | 4-methylhexanoylglutamine levels \|\| id:ebi-met1400-GCST90200223 | Fruit liking \|\| id:ebi-fl187-GCST90094766 | Weighted median | 9 | -0.080934202 | 0.313650652912554 | 0.796376559855131 | -0.695689482 | 0.533821077835537 | 0.922254372058487 | 0.498730463944324 | 1.70543647976544 | 0.999332001504091 |
| ebi-fl187-GCST90094766 | ebi-met1400-GCST90200224 | 2-ketocaprylate levels \|\| id:ebi-met1400-GCST90200224 | Fruit liking \|\| id:ebi-fl187-GCST90094766 | Weighted median | 9 | -0.384573173 | 0.254107791997802 | 0.13017165048969 | -0.882624446 | 0.113478098925394 | 0.680741132634101 | 0.413695763654583 | 1.1201673557549 | 0.999332001504091 |
| ebi-fl187-GCST90094766 | ebi-met1400-GCST90200243 | Hydroxypalmitoyl sphingomyelin (d18:1/16:0(OH)) levels \|\| id:ebi-met1400-GCST90200243 | Fruit liking \|\| id:ebi-fl187-GCST90094766 | Weighted median | 9 | -0.15298862 | 0.23131525469325 | 0.508364943669585 | -0.606366519 | 0.300389279325388 | 0.858139487491849 | 0.54532871515142 | 1.35038438199298 | 0.999332001504091 |
| ebi-fl187-GCST90094766 | ebi-met1400-GCST90200273 | 2-methoxyhydroquinone sulfate (2) levels \|\| id:ebi-met1400-GCST90200273 | Fruit liking \|\| id:ebi-fl187-GCST90094766 | Weighted median | 9 | 0.0430133750266664 | 0.328097085792264 | 0.895696569862028 | -0.600056913 | 0.686083663179504 | 1.04395185764117 | 0.548780402396949 | 1.98592274125004 | 0.999332001504091 |
| ebi-fl187-GCST90094766 | ebi-met1400-GCST90200276 | 2,4-di-tert-butylphenol levels \|\| id:ebi-met1400-GCST90200276 | Fruit liking \|\| id:ebi-fl187-GCST90094766 | Weighted median | 9 | 0.451397218492632 | 0.258157193620414 | 0.0803712094024553 | -0.054590881 | 0.957385317988644 | 1.57050499183601 | 0.946872452228896 | 2.60487663737161 | 0.999332001504091 |
| ebi-fl187-GCST90094766 | ebi-met1400-GCST90200289 | N-acetylmethionine levels \|\| id:ebi-met1400-GCST90200289 | Fruit liking \|\| id:ebi-fl187-GCST90094766 | Weighted median | 9 | 0.150416485214329 | 0.241511053035963 | 0.533406702199441 | -0.322945179 | 0.623778149164817 | 1.16231823029172 | 0.724013544617525 | 1.86596463355137 | 0.999332001504091 |
| ebi-fl187-GCST90094766 | ebi-met1400-GCST90200308 | 3-hydroxyisobutyrate levels \|\| id:ebi-met1400-GCST90200308 | Fruit liking \|\| id:ebi-fl187-GCST90094766 | Weighted median | 9 | 0.253595710964292 | 0.254256429132226 | 0.318569726214389 | -0.24474689 | 0.751938312063455 | 1.28865071158532 | 0.782902673555922 | 2.12110740269523 | 0.999332001504091 |
| ebi-fl187-GCST90094766 | ebi-met1400-GCST90200383 | Cystathionine levels \|\| id:ebi-met1400-GCST90200383 | Fruit liking \|\| id:ebi-fl187-GCST90094766 | Weighted median | 9 | -0.538453909 | 0.255189373285032 | 0.0348568939390469 | -1.038625081 | -0.038282737 | 0.583649931091383 | 0.353940987901717 | 0.962440784500402 | 0.999332001504091 |
| ebi-fl187-GCST90094766 | ebi-met1400-GCST90200384 | Guanidinoacetate levels \|\| id:ebi-met1400-GCST90200384 | Fruit liking \|\| id:ebi-fl187-GCST90094766 | Weighted median | 9 | 0.0169750908567157 | 0.252305793264075 | 0.946358933898287 | -0.477544264 | 0.511494445654302 | 1.01711998642222 | 0.620304827852267 | 1.6677817426659 | 0.999332001504091 |
| ebi-fl187-GCST90094766 | ebi-met1400-GCST90200403 | Citrulline levels \|\| id:ebi-met1400-GCST90200403 | Fruit liking \|\| id:ebi-fl187-GCST90094766 | Weighted median | 9 | 0.0980490843379419 | 0.250983030871413 | 0.696048411649546 | -0.393877656 | 0.589975824845912 | 1.10301692463528 | 0.674436564345518 | 1.8039448042271 | 0.999332001504091 |
| ebi-fl187-GCST90094766 | ebi-met1400-GCST90200434 | 12,13-DiHOME levels \|\| id:ebi-met1400-GCST90200434 | Fruit liking \|\| id:ebi-fl187-GCST90094766 | Weighted median | 9 | -0.287262644 | 0.248627588272631 | 0.247929413356289 | -0.774572717 | 0.2000474287948 | 0.750314637153408 | 0.460900673846322 | 1.22146068919474 | 0.999332001504091 |
| ebi-fl187-GCST90094766 | ebi-met1400-GCST90200437 | Fructose levels \|\| id:ebi-met1400-GCST90200437 | Fruit liking \|\| id:ebi-fl187-GCST90094766 | Weighted median | 9 | 0.244021768755819 | 0.25323901847029 | 0.335245218383966 | -0.252326707 | 0.740370244957587 | 1.27637211521994 | 0.776990847901856 | 2.09671166772458 | 0.999332001504091 |
| ebi-fl187-GCST90094766 | ebi-met1400-GCST90200448 | N-stearoyl-sphinganine (d18:0/18:0) levels \|\| id:ebi-met1400-GCST90200448 | Fruit liking \|\| id:ebi-fl187-GCST90094766 | Weighted median | 9 | 0.377424260658019 | 0.256902247608123 | 0.141795989246652 | -0.126104145 | 0.880952665969941 | 1.45852297204068 | 0.881523036091202 | 2.41319758290503 | 0.999332001504091 |
| ebi-fl187-GCST90094766 | ebi-met1400-GCST90200468 | X-11847 levels \|\| id:ebi-met1400-GCST90200468 | Fruit liking \|\| id:ebi-fl187-GCST90094766 | Weighted median | 9 | 0.129922388602194 | 0.254389300249623 | 0.609545247857855 | -0.36868064 | 0.628525417091455 | 1.13874000069173 | 0.691646259410772 | 1.87484392712555 | 0.999332001504091 |
| ebi-fl187-GCST90094766 | ebi-met1400-GCST90200476 | X-11843 levels \|\| id:ebi-met1400-GCST90200476 | Fruit liking \|\| id:ebi-fl187-GCST90094766 | Weighted median | 9 | -0.103076275 | 0.266940761965383 | 0.699393627635671 | -0.626280168 | 0.420127618525564 | 0.902058166437001 | 0.534576642178273 | 1.52215579850256 | 0.999332001504091 |
| ebi-fl187-GCST90094766 | ebi-met1400-GCST90200511 | X-12906 levels \|\| id:ebi-met1400-GCST90200511 | Fruit liking \|\| id:ebi-fl187-GCST90094766 | Weighted median | 9 | -0.088907087 | 0.263321144424662 | 0.73563639643865 | -0.60501653 | 0.427202356229713 | 0.91493057871419 | 0.546065400150478 | 1.53296283491979 | 0.999332001504091 |
| ebi-fl187-GCST90094766 | ebi-met1400-GCST90200515 | X-12849 levels \|\| id:ebi-met1400-GCST90200515 | Fruit liking \|\| id:ebi-fl187-GCST90094766 | Weighted median | 9 | 0.171991390661771 | 0.274390426744533 | 0.530782050100643 | -0.365813846 | 0.709796627081056 | 1.18766760813775 | 0.693631911715239 | 2.03357764196801 | 0.999332001504091 |
| ebi-fl187-GCST90094766 | ebi-met1400-GCST90200551 | X-18886 levels \|\| id:ebi-met1400-GCST90200551 | Fruit liking \|\| id:ebi-fl187-GCST90094766 | Weighted median | 9 | 0.0116731590322323 | 0.244638805636197 | 0.961942667882016 | -0.4678189 | 0.491165218079178 | 1.01174155623117 | 0.626366948431778 | 1.63421933288127 | 0.999332001504091 |
| ebi-fl187-GCST90094766 | ebi-met1400-GCST90200564 | X-21319 levels \|\| id:ebi-met1400-GCST90200564 | Fruit liking \|\| id:ebi-fl187-GCST90094766 | Weighted median | 9 | -0.573511339 | 0.242400893458026 | 0.017983282753274 | -1.048617091 | -0.098405588 | 0.563543169163312 | 0.350422016075191 | 0.906281252153073 | 0.999332001504091 |
| ebi-fl187-GCST90094766 | ebi-met1400-GCST90200579 | X-21442 levels \|\| id:ebi-met1400-GCST90200579 | Fruit liking \|\| id:ebi-fl187-GCST90094766 | Weighted median | 9 | 0.133287184674966 | 0.275292602033391 | 0.628268407977493 | -0.406286315 | 0.672860684660413 | 1.14257808213346 | 0.666119419930691 | 1.95983578125918 | 0.999332001504091 |
| ebi-fl187-GCST90094766 | ebi-met1400-GCST90200581 | X-21441 levels \|\| id:ebi-met1400-GCST90200581 | Fruit liking \|\| id:ebi-fl187-GCST90094766 | Weighted median | 9 | 0.155721103436971 | 0.236983265978913 | 0.511118248494874 | -0.308766098 | 0.620208304755641 | 1.1685002669324 | 0.734352516548744 | 1.85931530573091 | 0.999332001504091 |
| ebi-fl187-GCST90094766 | ebi-met1400-GCST90200648 | X-24585 levels \|\| id:ebi-met1400-GCST90200648 | Fruit liking \|\| id:ebi-fl187-GCST90094766 | Weighted median | 9 | -0.2054931 | 0.237333964329427 | 0.38657823307013 | -0.67066767 | 0.25968147012499 | 0.814245712875199 | 0.511367039326873 | 1.29651704147448 | 0.999332001504091 |
| ebi-fl187-GCST90094766 | ebi-met1400-GCST90200720 | Adenosine 5'-diphosphate (ADP) to pantothenate ratio \|\| id:ebi-met1400-GCST90200720 | Fruit liking \|\| id:ebi-fl187-GCST90094766 | Weighted median | 9 | 0.0963872469363937 | 0.315957263100504 | 0.760317263280647 | -0.522888989 | 0.715663482613381 | 1.10118541211489 | 0.592805463423907 | 2.04554341461513 | 0.999332001504091 |
| ebi-fl187-GCST90094766 | ebi-met1400-GCST90200724 | Cholate to taurocholate ratio \|\| id:ebi-met1400-GCST90200724 | Fruit liking \|\| id:ebi-fl187-GCST90094766 | Weighted median | 9 | 0.264169632774306 | 0.273319054845426 | 0.333781665462224 | -0.271535715 | 0.799874980271342 | 1.30234909865494 | 0.762208060932361 | 2.22526270936127 | 0.999332001504091 |
| ebi-fl187-GCST90094766 | ebi-met1400-GCST90200726 | N-acetylputrescine to (N(1) + N(8))-acetylspermidine ratio \|\| id:ebi-met1400-GCST90200726 | Fruit liking \|\| id:ebi-fl187-GCST90094766 | Weighted median | 9 | 0.0218562598068419 | 0.252761977193335 | 0.93109301824615 | -0.473557215 | 0.517269735105779 | 1.02209685751131 | 0.622782950167991 | 1.67744153216253 | 0.999332001504091 |
| ebi-fl187-GCST90094766 | ebi-met1400-GCST90200744 | Arginine to glutamate ratio \|\| id:ebi-met1400-GCST90200744 | Fruit liking \|\| id:ebi-fl187-GCST90094766 | Weighted median | 9 | 0.228711465930167 | 0.258322099519598 | 0.375955377582239 | -0.277599849 | 0.735022780988579 | 1.25697930532718 | 0.757599915135186 | 2.08552950238764 | 0.999332001504091 |
| ebi-fl187-GCST90094766 | ebi-met1400-GCST90200759 | Histidine to trans-urocanate ratio \|\| id:ebi-met1400-GCST90200759 | Fruit liking \|\| id:ebi-fl187-GCST90094766 | Weighted median | 9 | 0.22163112031592 | 0.261518977313649 | 0.396729723538967 | -0.290946075 | 0.734208315850672 | 1.24811089018636 | 0.747555988724311 | 2.08383160284772 | 0.999332001504091 |
| ebi-fl187-GCST90094766 | ebi-met1400-GCST90200795 | Oleoyl-linoleoyl-glycerol (18:1 to 18:2) [2] to linoleoyl-arachidonoyl-glycerol (18:2 to 20:4) [2] ratio \|\| id:ebi-met1400-GCST90200795 | Fruit liking \|\| id:ebi-fl187-GCST90094766 | Weighted median | 9 | 0.192677975017808 | 0.257523839152899 | 0.454342719331018 | -0.31206875 | 0.697424699757491 | 1.21249227784041 | 0.731931206431654 | 2.00857336168233 | 0.999332001504091 |
| ebi-fl187-GCST90094766 | ebi-met1400-GCST90200811 | Choline to taurocholate ratio \|\| id:ebi-met1400-GCST90200811 | Fruit liking \|\| id:ebi-fl187-GCST90094766 | Weighted median | 9 | -0.314408841 | 0.276510289626956 | 0.255513126633394 | -0.856369009 | 0.227551326528479 | 0.730220422972104 | 0.424701372995196 | 1.25552187967989 | 0.999332001504091 |
| ebi-fl187-GCST90094766 | ebi-met1400-GCST90200831 | Cholate to phosphate ratio \|\| id:ebi-met1400-GCST90200831 | Fruit liking \|\| id:ebi-fl187-GCST90094766 | Weighted median | 9 | 0.164964876616198 | 0.248816279472978 | 0.507331369200323 | -0.322715031 | 0.652644784383234 | 1.1793516951617 | 0.724180193762771 | 1.92061372688743 | 0.999332001504091 |
| ebi-fl187-GCST90094766 | ebi-met1400-GCST90200841 | Adenosine 5'-diphosphate (ADP) to oxalate (ethanedioate) ratio \|\| id:ebi-met1400-GCST90200841 | Fruit liking \|\| id:ebi-fl187-GCST90094766 | Weighted median | 9 | -0.063249453 | 0.342962181092532 | 0.853683139567917 | -0.735455328 | 0.608956421545973 | 0.93870928026293 | 0.479287176250722 | 1.83851176604565 | 0.999332001504091 |
| ebi-fl187-GCST90094766 | ebi-met1400-GCST90200844 | Aspartate to phosphate ratio \|\| id:ebi-met1400-GCST90200844 | Fruit liking \|\| id:ebi-fl187-GCST90094766 | Weighted median | 9 | -0.037888842 | 0.232544936217262 | 0.870572548393587 | -0.493676917 | 0.417899232752746 | 0.962819959859772 | 0.610377953844564 | 1.51876762465841 | 0.999332001504091 |
| ebi-fl187-GCST90094766 | ebi-met1400-GCST90200870 | Adenosine 5'-monophosphate (AMP) to acetoacetate ratio \|\| id:ebi-met1400-GCST90200870 | Fruit liking \|\| id:ebi-fl187-GCST90094766 | Weighted median | 9 | -0.035025652 | 0.267746525888835 | 0.895920502932435 | -0.559808843 | 0.489757538466168 | 0.965580646598672 | 0.571318264886453 | 1.63192049403708 | 0.999332001504091 |
| ebi-fl187-GCST90094766 | ebi-met1400-GCST90200887 | Cholesterol to cortisol ratio \|\| id:ebi-met1400-GCST90200887 | Fruit liking \|\| id:ebi-fl187-GCST90094766 | Weighted median | 9 | 0.175691378152953 | 0.232221819841646 | 0.449309285628208 | -0.279463389 | 0.63084614504258 | 1.19207010299702 | 0.756189412358083 | 1.87919998248588 | 0.999332001504091 |
| ebi-fl187-GCST90094766 | ebi-met1400-GCST90200890 | Cortisol to taurocholate ratio \|\| id:ebi-met1400-GCST90200890 | Fruit liking \|\| id:ebi-fl187-GCST90094766 | Weighted median | 9 | -0.050527101 | 0.267866498370488 | 0.850384446266535 | -0.575545438 | 0.474491235574523 | 0.950728162419031 | 0.562398031855339 | 1.60719630514135 | 0.999332001504091 |
| ebi-fl187-GCST90094766 | ebi-met1400-GCST90200948 | Adenosine 5'-diphosphate (ADP) to mannitol to sorbitol ratio \|\| id:ebi-met1400-GCST90200948 | Fruit liking \|\| id:ebi-fl187-GCST90094766 | Weighted median | 9 | -0.360502842 | 0.350361528339638 | 0.303505387016646 | -1.047211438 | 0.326205753098067 | 0.697325592988712 | 0.350914934060221 | 1.38570045170437 | 0.999332001504091 |
| ebi-fl187-GCST90094766 | ebi-met1400-GCST90200972 | Leucine to N-palmitoyl-sphingosine (d18:1 to 16:0) ratio \|\| id:ebi-met1400-GCST90200972 | Fruit liking \|\| id:ebi-fl187-GCST90094766 | Weighted median | 9 | 0.246710385164148 | 0.245730360206527 | 0.315384295139376 | -0.234921121 | 0.72834189116894 | 1.27980840759811 | 0.790633211652266 | 2.07164274915281 | 0.999332001504091 |
| ebi-fl187-GCST90094766 | ebi-met1400-GCST90200973 | Proline to glutamate ratio \|\| id:ebi-met1400-GCST90200973 | Fruit liking \|\| id:ebi-fl187-GCST90094766 | Weighted median | 9 | -0.007076809 | 0.251158926875893 | 0.977521286998772 | -0.499348305 | 0.485194687866607 | 0.992948172836475 | 0.606926061242108 | 1.62449124679455 | 0.999332001504091 |
| ebi-fl187-GCST90094766 | ebi-met1400-GCST90200983 | Cholesterol to linoleoyl-arachidonoyl-glycerol (18:2 to 20:4) [1] ratio \|\| id:ebi-met1400-GCST90200983 | Fruit liking \|\| id:ebi-fl187-GCST90094766 | Weighted median | 9 | 0.464843345238873 | 0.261770872461798 | 0.0757717898635062 | -0.048227565 | 0.977914255263998 | 1.59176481181819 | 0.952916912068473 | 2.65890465795463 | 0.999332001504091 |
| ebi-fl187-GCST90094766 | ebi-met1400-GCST90201001 | Serine to alpha-ketobutyrate ratio \|\| id:ebi-met1400-GCST90201001 | Fruit liking \|\| id:ebi-fl187-GCST90094766 | Weighted median | 9 | -0.091248637 | 0.252346645366448 | 0.717650698684266 | -0.585848062 | 0.40335078814803 | 0.912790729343636 | 0.556633601955413 | 1.49683187045978 | 0.999332001504091 |
| ebi-fl187-GCST90094766 | ebi-met1400-GCST90201005 | Fructose to maltose ratio \|\| id:ebi-met1400-GCST90201005 | Fruit liking \|\| id:ebi-fl187-GCST90094766 | Weighted median | 9 | 0.0773027937143247 | 0.274422353486462 | 0.778179096527227 | -0.460565019 | 0.61517060654779 | 1.08036915586084 | 0.630927058926218 | 1.84997219000551 | 0.999332001504091 |
| ebi-fl187-GCST90094766 | ebi-met1400-GCST90201020 | Paraxanthine to 5-acetylamino-6-formylamino-3-methyluracil ratio \|\| id:ebi-met1400-GCST90201020 | Fruit liking \|\| id:ebi-fl187-GCST90094766 | Weighted median | 9 | 0.735482565505201 | 0.28838461622102 | 0.0107613901793421 | 0.170248717712002 | 1.3007164132984 | 2.08648861703793 | 1.18559969429583 | 3.67192634240223 | 0.999332001504091 |
| ebi-fl187-GCST90094766 | ebi-pw11-GCST90275197 | Placental weight (fetal genotype, adjusted for sex) \|\| id:ebi-pw11-GCST90275197 | Fruit liking \|\| id:ebi-fl187-GCST90094766 | Weighted median | 8 | 0.0279054506556897 | 0.108125714283641 | 0.796342551977704 | -0.184020949 | 0.239831850651626 | 1.02829845488034 | 0.831918375503011 | 1.27103540857593 | 0.999332001504091 |
| ebi-fl187-GCST90094766 | prot-a-1017 | FAS-associated factor 2 \|\| id:prot-a-1017 | Fruit liking \|\| id:ebi-fl187-GCST90094766 | Weighted median | 10 | -0.186847264 | 0.386180948961085 | 0.628504067839204 | -0.943761924 | 0.570067395935832 | 0.829570431956026 | 0.389161083766689 | 1.76838622946248 | 0.999332001504091 |
| ebi-fl187-GCST90094766 | prot-a-1019 | Fas apoptotic inhibitory molecule 3 \|\| id:prot-a-1019 | Fruit liking \|\| id:ebi-fl187-GCST90094766 | Weighted median | 10 | 0.117634395872171 | 0.363975734269211 | 0.746549110205503 | -0.595758043 | 0.831026835039823 | 1.1248327926699 | 0.551144615994798 | 2.2956748097445 | 0.999332001504091 |
| ebi-fl187-GCST90094766 | prot-a-1052 | MIP18 family protein FAM96A \|\| id:prot-a-1052 | Fruit liking \|\| id:ebi-fl187-GCST90094766 | Weighted median | 10 | -0.125000239 | 0.379241564881061 | 0.74169780018332 | -0.868313706 | 0.618313228030796 | 0.882496691547768 | 0.419658620601932 | 1.85579509715705 | 0.999332001504091 |
| ebi-fl187-GCST90094766 | prot-a-1066 | F-box/LRR-repeat protein 4 \|\| id:prot-a-1066 | Fruit liking \|\| id:ebi-fl187-GCST90094766 | Weighted median | 10 | -0.080759092 | 0.41391355344996 | 0.845306042639398 | -0.892029657 | 0.730511472847574 | 0.922415882124105 | 0.409823107839617 | 2.07614222653294 | 0.999332001504091 |
| ebi-fl187-GCST90094766 | prot-a-1076 | Low affinity immunoglobulin gamma Fc region receptor III-B \|\| id:prot-a-1076 | Fruit liking \|\| id:ebi-fl187-GCST90094766 | Weighted median | 10 | -0.432631316 | 0.448876565728894 | 0.335141646525565 | -1.312429385 | 0.447166752718741 | 0.648799649694187 | 0.269165355186209 | 1.56387505796239 | 0.999332001504091 |
| ebi-fl187-GCST90094766 | prot-a-1081 | Fc receptor-like protein 3 \|\| id:prot-a-1081 | Fruit liking \|\| id:ebi-fl187-GCST90094766 | Weighted median | 10 | 0.145410322482332 | 0.408386731971995 | 0.721795370420663 | -0.655027672 | 0.945848317147442 | 1.15651401662551 | 0.519427688690229 | 2.57499686630864 | 0.999332001504091 |
| ebi-fl187-GCST90094766 | prot-a-1105 | Fibroblast growth factor receptor 4 \|\| id:prot-a-1105 | Fruit liking \|\| id:ebi-fl187-GCST90094766 | Weighted median | 10 | 0.202538088648473 | 0.396701150790522 | 0.609662105628065 | -0.574996167 | 0.980072344197896 | 1.22450672403987 | 0.562707025714587 | 2.6646490068517 | 0.999332001504091 |
| ebi-fl187-GCST90094766 | prot-a-1143 | Follistatin-related protein 1 \|\| id:prot-a-1143 | Fruit liking \|\| id:ebi-fl187-GCST90094766 | Weighted median | 10 | 0.315654924923418 | 0.418270261767599 | 0.450448580260436 | -0.504154788 | 1.13546463798791 | 1.37115702186582 | 0.604015881124151 | 3.1126194482054 | 0.999332001504091 |
| ebi-fl187-GCST90094766 | prot-a-1151 | Alpha-(1,3)-fucosyltransferase 10 \|\| id:prot-a-1151 | Fruit liking \|\| id:ebi-fl187-GCST90094766 | Weighted median | 10 | -0.086449971 | 0.385313648438645 | 0.822475172515066 | -0.841664722 | 0.66876478033083 | 0.91718143366554 | 0.430992443485356 | 1.95182489850163 | 0.999332001504091 |
| ebi-fl187-GCST90094766 | prot-a-1165 | Growth arrest and DNA damage-inducible proteins-interacting protein 1 \|\| id:prot-a-1165 | Fruit liking \|\| id:ebi-fl187-GCST90094766 | Weighted median | 10 | -0.107395986 | 0.400782374264531 | 0.788725612424542 | -0.89292944 | 0.678137467604134 | 0.898169939881487 | 0.409454521889681 | 1.97020474260159 | 0.999332001504091 |
| ebi-fl187-GCST90094766 | prot-a-1194 | Growth/differentiation factor 11/8 \|\| id:prot-a-1194 | Fruit liking \|\| id:ebi-fl187-GCST90094766 | Weighted median | 10 | 0.00200828280161324 | 0.39256417292616 | 0.995918193859156 | -0.767417496 | 0.771434061736887 | 1.00201030075216 | 0.464210346672887 | 2.16286571380742 | 0.999364451047625 |
| ebi-fl187-GCST90094766 | prot-a-1216 | D-glucuronyl C5-epimerase \|\| id:prot-a-1216 | Fruit liking \|\| id:ebi-fl187-GCST90094766 | Weighted median | 10 | 0.19165451484072 | 0.371976524198302 | 0.606390334832875 | -0.537419473 | 0.920728502269391 | 1.21125197508752 | 0.58425399217941 | 2.51111907970137 | 0.999332001504091 |
| ebi-fl187-GCST90094766 | prot-a-1246 | Glypican-3 \|\| id:prot-a-1246 | Fruit liking \|\| id:ebi-fl187-GCST90094766 | Weighted median | 10 | 0.0573417877129376 | 0.377965328746112 | 0.879414276251758 | -0.683470257 | 0.798153832055317 | 1.05901770778624 | 0.504861948377049 | 2.22143599653352 | 0.999332001504091 |
| ebi-fl187-GCST90094766 | prot-a-1275 | Glutamate receptor 4 \|\| id:prot-a-1275 | Fruit liking \|\| id:ebi-fl187-GCST90094766 | Weighted median | 10 | -0.36137312 | 0.371177472870802 | 0.330262235231028 | -1.088880967 | 0.366134726592886 | 0.696718990010177 | 0.33659294162747 | 1.44214952545869 | 0.999332001504091 |
| ebi-fl187-GCST90094766 | prot-a-1289 | Glutathione S-transferase P \|\| id:prot-a-1289 | Fruit liking \|\| id:ebi-fl187-GCST90094766 | Weighted median | 10 | 0.0554631724482213 | 0.362623748194101 | 0.878437987811118 | -0.655279374 | 0.766205718908659 | 1.05703008852529 | 0.519296964243206 | 2.15158702049431 | 0.999332001504091 |
| ebi-fl187-GCST90094766 | prot-a-130 | Apolipoprotein D \|\| id:prot-a-130 | Fruit liking \|\| id:ebi-fl187-GCST90094766 | Weighted median | 10 | 0.114124091384258 | 0.398770158845283 | 0.774732487912804 | -0.66746542 | 0.895713602721012 | 1.12089120919614 | 0.513007189159975 | 2.44908283821614 | 0.999332001504091 |
| ebi-fl187-GCST90094766 | prot-a-1313 | Heparin-binding EGF-like growth factor \|\| id:prot-a-1313 | Fruit liking \|\| id:ebi-fl187-GCST90094766 | Weighted median | 10 | -0.097587123 | 0.360229112677676 | 0.786465690437495 | -0.803636183 | 0.608461938277298 | 0.907023315910203 | 0.447698088466566 | 1.8376028774717 | 0.999332001504091 |
| ebi-fl187-GCST90094766 | prot-a-1347 | HLA class II histocompatibility antigen, DQ alpha 2 chain \|\| id:prot-a-1347 | Fruit liking \|\| id:ebi-fl187-GCST90094766 | Weighted median | 10 | 0.106800380377929 | 0.363278131098547 | 0.768765034412197 | -0.605224757 | 0.818825517331081 | 1.11271211313668 | 0.545951706613344 | 2.26783474018512 | 0.999332001504091 |
| ebi-fl187-GCST90094766 | prot-a-1357 | Non-histone chromosomal protein HMG-14 \|\| id:prot-a-1357 | Fruit liking \|\| id:ebi-fl187-GCST90094766 | Weighted median | 10 | 0.0230996210803905 | 0.38130307014695 | 0.951693114842168 | -0.724254396 | 0.770453638568412 | 1.02336848354322 | 0.484685817751313 | 2.16074622931693 | 0.999332001504091 |
| ebi-fl187-GCST90094766 | prot-a-137 | Apolipoprotein M \|\| id:prot-a-137 | Fruit liking \|\| id:ebi-fl187-GCST90094766 | Weighted median | 10 | 0.244226859863785 | 0.381882258004449 | 0.522475309898712 | -0.504262366 | 0.992716085552506 | 1.27663391463668 | 0.603950905989646 | 2.69855403119226 | 0.999332001504091 |
| ebi-fl187-GCST90094766 | prot-a-1381 | 17-beta-hydroxysteroid dehydrogenase 14 \|\| id:prot-a-1381 | Fruit liking \|\| id:ebi-fl187-GCST90094766 | Weighted median | 10 | 0.0876300740773399 | 0.367982235890342 | 0.811775073482498 | -0.633615108 | 0.80887525642241 | 1.09158424202207 | 0.530669900016942 | 2.2453810879284 | 0.999332001504091 |
| ebi-fl187-GCST90094766 | prot-a-1392 | Serine protease HTRA2, mitochondrial \|\| id:prot-a-1392 | Fruit liking \|\| id:ebi-fl187-GCST90094766 | Weighted median | 10 | -0.195931052 | 0.385750121060197 | 0.611507902714692 | -0.952001289 | 0.560139185759591 | 0.822068913127551 | 0.385967816939995 | 1.75091618593624 | 0.999332001504091 |
| ebi-fl187-GCST90094766 | prot-a-1416 | Interferon-induced protein with tetratricopeptide repeats 2 \|\| id:prot-a-1416 | Fruit liking \|\| id:ebi-fl187-GCST90094766 | Weighted median | 10 | 0.554494682482015 | 0.415847371208103 | 0.182397577064419 | -0.260566165 | 1.3695555300499 | 1.74106097384037 | 0.770615166870522 | 3.93360193900686 | 0.999332001504091 |
| ebi-fl187-GCST90094766 | prot-a-1428 | Interferon gamma \|\| id:prot-a-1428 | Fruit liking \|\| id:ebi-fl187-GCST90094766 | Weighted median | 10 | 0.0504234179342135 | 0.365629268557335 | 0.890312659184886 | -0.666209948 | 0.767056784306591 | 1.05171631766264 | 0.513651659545066 | 2.15341894118968 | 0.999332001504091 |
| ebi-fl187-GCST90094766 | prot-a-1483 | Interleukin-17C \|\| id:prot-a-1483 | Fruit liking \|\| id:ebi-fl187-GCST90094766 | Weighted median | 10 | -0.557016041 | 0.402627291016228 | 0.166525927744816 | -1.346165531 | 0.232133449835778 | 0.572916074087426 | 0.260236217665289 | 1.26128803627909 | 0.999332001504091 |
| ebi-fl187-GCST90094766 | prot-a-1503 | Interleukin-1 receptor-like 2 \|\| id:prot-a-1503 | Fruit liking \|\| id:ebi-fl187-GCST90094766 | Weighted median | 10 | 0.826414020667651 | 0.420666653246282 | 0.0494681759638007 | 0.00190738030493842 | 1.65092066103036 | 2.28510967422655 | 1.00190920051184 | 5.21177589803162 | 0.999332001504091 |
| ebi-fl187-GCST90094766 | prot-a-1522 | Interleukin-31 receptor subunit alpha \|\| id:prot-a-1522 | Fruit liking \|\| id:ebi-fl187-GCST90094766 | Weighted median | 10 | 0.500786398718213 | 0.404837083631863 | 0.216084399139915 | -0.292694285 | 1.29426708263667 | 1.65001833293129 | 0.746250245572323 | 3.64832107615769 | 0.999332001504091 |
| ebi-fl187-GCST90094766 | prot-a-1540 | Interleukin-6 receptor subunit alpha \|\| id:prot-a-1540 | Fruit liking \|\| id:ebi-fl187-GCST90094766 | Weighted median | 10 | -0.285832667 | 0.378848710870017 | 0.450562806948937 | -1.02837614 | 0.456710806670487 | 0.751388337767036 | 0.357587160868116 | 1.57887221890648 | 0.999332001504091 |
| ebi-fl187-GCST90094766 | prot-a-1541 | Interleukin-6 receptor subunit alpha \|\| id:prot-a-1541 | Fruit liking \|\| id:ebi-fl187-GCST90094766 | Weighted median | 10 | -0.269860057 | 0.386693921948186 | 0.485261781833486 | -1.027780144 | 0.488060030422035 | 0.763486331736943 | 0.357800345024082 | 1.62915264575807 | 0.999332001504091 |
| ebi-fl187-GCST90094766 | prot-a-1545 | Interleukin-9 \|\| id:prot-a-1545 | Fruit liking \|\| id:ebi-fl187-GCST90094766 | Weighted median | 10 | -0.250629977 | 0.414413813333325 | 0.545323915128149 | -1.062881052 | 0.561621096674094 | 0.778310310642353 | 0.345459089757095 | 1.75351281125106 | 0.999332001504091 |
| ebi-fl187-GCST90094766 | prot-a-1548 | Inositol monophosphatase 3 \|\| id:prot-a-1548 | Fruit liking \|\| id:ebi-fl187-GCST90094766 | Weighted median | 10 | 0.126903150394659 | 0.371959134698746 | 0.732971857477104 | -0.602136754 | 0.855943054404201 | 1.13530705841626 | 0.547640212813876 | 2.35359290046849 | 0.999332001504091 |
| ebi-fl187-GCST90094766 | prot-a-1555 | Inhibin beta A chain:Inhibin beta B chain heterodimer \|\| id:prot-a-1555 | Fruit liking \|\| id:ebi-fl187-GCST90094766 | Weighted median | 10 | -0.241411498 | 0.378327910019705 | 0.523407798228354 | -0.982934201 | 0.500111206048594 | 0.785518320977204 | 0.374211474612359 | 1.64890462867294 | 0.999332001504091 |
| ebi-fl187-GCST90094766 | prot-a-1603 | Junctophilin-3 \|\| id:prot-a-1603 | Fruit liking \|\| id:ebi-fl187-GCST90094766 | Weighted median | 10 | -0.053680357 | 0.372566053542689 | 0.885435093976488 | -0.783909822 | 0.676549108241616 | 0.947734995230498 | 0.456617224756597 | 1.96707783343772 | 0.999332001504091 |
| ebi-fl187-GCST90094766 | prot-a-1627 | UPF0258 protein KIAA1024 \|\| id:prot-a-1627 | Fruit liking \|\| id:ebi-fl187-GCST90094766 | Weighted median | 10 | -0.071874137 | 0.400812984383348 | 0.857685753470472 | -0.857467586 | 0.713719312706133 | 0.930648022885096 | 0.424235061907667 | 2.04157039402944 | 0.999332001504091 |
| ebi-fl187-GCST90094766 | prot-a-1666 | Kallikrein-7 \|\| id:prot-a-1666 | Fruit liking \|\| id:ebi-fl187-GCST90094766 | Weighted median | 10 | 0.277670300067105 | 0.370174724391225 | 0.45319083646072 | -0.44787216 | 1.00321275987391 | 1.32005090476037 | 0.638986366994107 | 2.72702905909533 | 0.999332001504091 |
| ebi-fl187-GCST90094766 | prot-a-1678 | Importin subunit alpha-3 \|\| id:prot-a-1678 | Fruit liking \|\| id:ebi-fl187-GCST90094766 | Weighted median | 10 | -0.149857306 | 0.357142177334689 | 0.674776770737988 | -0.849855974 | 0.55014136142405 | 0.860830802921355 | 0.427476495361271 | 1.7334980503009 | 0.999332001504091 |
| ebi-fl187-GCST90094766 | prot-a-1718 | Protein LDOC1 \|\| id:prot-a-1718 | Fruit liking \|\| id:ebi-fl187-GCST90094766 | Weighted median | 10 | -0.190712984 | 0.367416404597526 | 0.603715436598705 | -0.910849137 | 0.529423168630289 | 0.82636973513829 | 0.402182570739641 | 1.69795259376022 | 0.999332001504091 |
| ebi-fl187-GCST90094766 | prot-a-1723 | Leptin \|\| id:prot-a-1723 | Fruit liking \|\| id:ebi-fl187-GCST90094766 | Weighted median | 10 | -0.371869707 | 0.385869547081385 | 0.335186965464677 | -1.128174019 | 0.384434605608632 | 0.689444066635036 | 0.323623647884642 | 1.46878364459847 | 0.999332001504091 |
| ebi-fl187-GCST90094766 | prot-a-1730 | Galectin-7 \|\| id:prot-a-1730 | Fruit liking \|\| id:ebi-fl187-GCST90094766 | Weighted median | 10 | -0.226044937 | 0.387080535233467 | 0.559237859086685 | -0.984722786 | 0.532632911831644 | 0.79768225523252 | 0.373542763762881 | 1.70341134145688 | 0.999332001504091 |
| ebi-fl187-GCST90094766 | prot-a-1753 | Lipase member N \|\| id:prot-a-1753 | Fruit liking \|\| id:ebi-fl187-GCST90094766 | Weighted median | 10 | -0.292859673 | 0.375150152648579 | 0.435010403788297 | -1.028153973 | 0.442434625824287 | 0.746126834817938 | 0.357666613897033 | 1.55649208510051 | 0.999332001504091 |
| ebi-fl187-GCST90094766 | prot-a-1756 | VIP36-like protein \|\| id:prot-a-1756 | Fruit liking \|\| id:ebi-fl187-GCST90094766 | Weighted median | 10 | -0.326887488 | 0.426328607072648 | 0.443230129129036 | -1.162491558 | 0.50871658148408 | 0.721164877892132 | 0.312706084037205 | 1.66315530030844 | 0.999332001504091 |
| ebi-fl187-GCST90094766 | prot-a-1760 | Ig Kappa chain V-I region HK102- like \|\| id:prot-a-1760 | Fruit liking \|\| id:ebi-fl187-GCST90094766 | Weighted median | 10 | -0.07772598 | 0.379763706209019 | 0.837830491489799 | -0.822062844 | 0.666610884385253 | 0.92521792022471 | 0.439524049176236 | 1.94762539503657 | 0.999332001504091 |
| ebi-fl187-GCST90094766 | prot-a-179 | Neutral ceramidase \|\| id:prot-a-179 | Fruit liking \|\| id:ebi-fl187-GCST90094766 | Weighted median | 10 | 0.21688365923595 | 0.38686602220776 | 0.575058676726821 | -0.541373744 | 0.97514106276316 | 1.24219957528305 | 0.581948254910835 | 2.65154121833362 | 0.999332001504091 |
| ebi-fl187-GCST90094766 | prot-a-1809 | Lactotransferrin \|\| id:prot-a-1809 | Fruit liking \|\| id:ebi-fl187-GCST90094766 | Weighted median | 10 | -0.605367096 | 0.390374605992906 | 0.120965490903848 | -1.370501324 | 0.159767131522036 | 0.545874001569528 | 0.253979601569465 | 1.17323762911735 | 0.999332001504091 |
| ebi-fl187-GCST90094766 | prot-a-1818 | Lysophospholipase-like protein 1 \|\| id:prot-a-1818 | Fruit liking \|\| id:ebi-fl187-GCST90094766 | Weighted median | 10 | 0.0999959202279926 | 0.397848195215994 | 0.801549785179616 | -0.679786542 | 0.879778382851341 | 1.10516640923947 | 0.506725145157859 | 2.41036546889886 | 0.999332001504091 |
| ebi-fl187-GCST90094766 | prot-a-1852 | MAP kinase-activated protein kinase 3 \|\| id:prot-a-1852 | Fruit liking \|\| id:ebi-fl187-GCST90094766 | Weighted median | 10 | -0.171663432 | 0.400766087714162 | 0.668404898320413 | -0.957164964 | 0.613838100217588 | 0.842262604359923 | 0.38397994142854 | 1.84750873200282 | 0.999332001504091 |
| ebi-fl187-GCST90094766 | prot-a-1856 | Megakaryocyte-associated tyrosine-protein kinase \|\| id:prot-a-1856 | Fruit liking \|\| id:ebi-fl187-GCST90094766 | Weighted median | 10 | -0.300328842 | 0.414662854031111 | 0.46889854457121 | -1.113068036 | 0.512410351461704 | 0.74057464826164 | 0.328549411665388 | 1.66930997340036 | 0.999332001504091 |
| ebi-fl187-GCST90094766 | prot-a-1890 | Beta-1,3-N-acetylglucosaminyltransferase manic fringe \|\| id:prot-a-1890 | Fruit liking \|\| id:ebi-fl187-GCST90094766 | Weighted median | 10 | 0.127071408151431 | 0.412180260763954 | 0.757861018241508 | -0.680801903 | 0.93494471924878 | 1.13549809870671 | 0.506210897553034 | 2.54707264975757 | 0.999332001504091 |
| ebi-fl187-GCST90094766 | prot-a-1919 | Matrilysin \|\| id:prot-a-1919 | Fruit liking \|\| id:ebi-fl187-GCST90094766 | Weighted median | 10 | -0.609255175 | 0.389415165230708 | 0.117691064747593 | -1.372508899 | 0.153998549158031 | 0.543755721305115 | 0.253470230050219 | 1.16648919438573 | 0.999332001504091 |
| ebi-fl187-GCST90094766 | prot-a-1923 | Multimerin-2 \|\| id:prot-a-1923 | Fruit liking \|\| id:ebi-fl187-GCST90094766 | Weighted median | 10 | 0.626273441341528 | 0.390914891259314 | 0.109139892679461 | -0.139919746 | 1.39246662820978 | 1.87062657453697 | 0.869428008085821 | 4.02476541912657 | 0.999332001504091 |
| ebi-fl187-GCST90094766 | prot-a-1948 | Mesothelin \|\| id:prot-a-1948 | Fruit liking \|\| id:ebi-fl187-GCST90094766 | Weighted median | 10 | -0.081063592 | 0.404093240561126 | 0.841006544266535 | -0.873086343 | 0.710959159913215 | 0.92213504954921 | 0.417660514343222 | 2.03594311744859 | 0.999332001504091 |
| ebi-fl187-GCST90094766 | prot-a-195 | ATPase family AAA domain-containing protein 1 \|\| id:prot-a-195 | Fruit liking \|\| id:ebi-fl187-GCST90094766 | Weighted median | 10 | -0.642258498 | 0.355722329476436 | 0.0709956701555904 | -1.339474264 | 0.05495726747879 | 0.526102878800956 | 0.261983366449904 | 1.05649546699591 | 0.999332001504091 |
| ebi-fl187-GCST90094766 | prot-a-1966 | Mucin-1 \|\| id:prot-a-1966 | Fruit liking \|\| id:ebi-fl187-GCST90094766 | Weighted median | 10 | -0.792576922 | 0.409428315237782 | 0.0528905283474589 | -1.59505642 | 0.00990257599957545 | 0.452676778296016 | 0.202897080780579 | 1.00995176874952 | 0.999332001504091 |
| ebi-fl187-GCST90094766 | prot-a-1986 | Myocardial zonula adherens protein \|\| id:prot-a-1986 | Fruit liking \|\| id:ebi-fl187-GCST90094766 | Weighted median | 10 | -0.04539265 | 0.376680232541377 | 0.904081439692112 | -0.783685906 | 0.692900605742914 | 0.955622183070713 | 0.456719480027705 | 1.99950691116008 | 0.999332001504091 |
| ebi-fl187-GCST90094766 | prot-a-2071 | Epididymal secretory protein E1 \|\| id:prot-a-2071 | Fruit liking \|\| id:ebi-fl187-GCST90094766 | Weighted median | 10 | 0.304925134597566 | 0.416448862399651 | 0.46404463220308 | -0.511314636 | 1.12116490490088 | 1.35652344253115 | 0.599706664564255 | 3.06842654729145 | 0.999332001504091 |
| ebi-fl187-GCST90094766 | prot-a-2098 | Neuritin \|\| id:prot-a-2098 | Fruit liking \|\| id:ebi-fl187-GCST90094766 | Weighted median | 10 | -0.071454697 | 0.367626856010868 | 0.845888170010398 | -0.792003335 | 0.64909394088047 | 0.931038455566941 | 0.452936502374778 | 1.91380602181456 | 0.999332001504091 |
| ebi-fl187-GCST90094766 | prot-a-2115 | Neurotrophin-4 \|\| id:prot-a-2115 | Fruit liking \|\| id:ebi-fl187-GCST90094766 | Weighted median | 10 | 0.138288709276719 | 0.382752299665118 | 0.717874601195653 | -0.611905798 | 0.888483216620351 | 1.14830702931322 | 0.542316338155199 | 2.43143888686017 | 0.999332001504091 |
| ebi-fl187-GCST90094766 | prot-a-2118 | Netrin-1 \|\| id:prot-a-2118 | Fruit liking \|\| id:ebi-fl187-GCST90094766 | Weighted median | 10 | 0.277676112829545 | 0.371213003571512 | 0.454445881188947 | -0.449901374 | 1.00525359982971 | 1.32005857792499 | 0.637691041328287 | 2.73260017190093 | 0.999332001504091 |
| ebi-fl187-GCST90094766 | prot-a-2120 | Netrin-G1 \|\| id:prot-a-2120 | Fruit liking \|\| id:ebi-fl187-GCST90094766 | Weighted median | 10 | -0.019939235 | 0.36655619871387 | 0.956619570373599 | -0.738389384 | 0.698510914646214 | 0.980258237052542 | 0.477882981847154 | 2.01075628932251 | 0.999332001504091 |
| ebi-fl187-GCST90094766 | prot-a-2145 | Olfactomedin-like protein 3 \|\| id:prot-a-2145 | Fruit liking \|\| id:ebi-fl187-GCST90094766 | Weighted median | 10 | 0.188584270973754 | 0.385138202162852 | 0.62437910739035 | -0.566286605 | 0.943455147212944 | 1.20753883917283 | 0.567629361816743 | 2.56884182918928 | 0.999332001504091 |
| ebi-fl187-GCST90094766 | prot-a-2162 | Prolyl 4-hydroxylase subunit alpha-2 \|\| id:prot-a-2162 | Fruit liking \|\| id:ebi-fl187-GCST90094766 | Weighted median | 10 | -0.672042683 | 0.402552036732891 | 0.0950271566277922 | -1.461044675 | 0.116959309090877 | 0.510664386261222 | 0.231993790003108 | 1.1240736891796 | 0.999332001504091 |
| ebi-fl187-GCST90094766 | prot-a-2168 | Polyadenylate-binding protein-interacting protein 1 \|\| id:prot-a-2168 | Fruit liking \|\| id:ebi-fl187-GCST90094766 | Weighted median | 10 | -0.165139772 | 0.371518398641831 | 0.656681432329745 | -0.893315834 | 0.563036289120289 | 0.847775200386477 | 0.409296341663517 | 1.7559961261056 | 0.999332001504091 |
| ebi-fl187-GCST90094766 | prot-a-219 | Beta-1,4-galactosyltransferase 5 \|\| id:prot-a-219 | Fruit liking \|\| id:ebi-fl187-GCST90094766 | Weighted median | 10 | -0.308765475 | 0.416784310429141 | 0.458797752625219 | -1.125662724 | 0.508131773001702 | 0.734352973640943 | 0.32443738369377 | 1.66218295732623 | 0.999332001504091 |
| ebi-fl187-GCST90094766 | prot-a-2190 | Pyruvate carboxylase, mitochondrial \|\| id:prot-a-2190 | Fruit liking \|\| id:ebi-fl187-GCST90094766 | Weighted median | 10 | -0.184108851 | 0.398483837820631 | 0.64406455131205 | -0.965137173 | 0.596919470845745 | 0.831845251475768 | 0.380930942558477 | 1.81651434707633 | 0.999332001504091 |
| ebi-fl187-GCST90094766 | prot-a-2197 | Protocadherin-9 \|\| id:prot-a-2197 | Fruit liking \|\| id:ebi-fl187-GCST90094766 | Weighted median | 10 | -0.147247702 | 0.373534197191341 | 0.693432355280875 | -0.879374728 | 0.584879324594628 | 0.863080164339556 | 0.415042344757933 | 1.79477438744437 | 0.999332001504091 |
| ebi-fl187-GCST90094766 | prot-a-2198 | Protocadherin alpha-4 \|\| id:prot-a-2198 | Fruit liking \|\| id:ebi-fl187-GCST90094766 | Weighted median | 10 | 0.0229844344198913 | 0.391664058318475 | 0.953203761179552 | -0.74467712 | 0.790645988724103 | 1.02325061193389 | 0.474887606954155 | 2.20482025534128 | 0.999332001504091 |
| ebi-fl187-GCST90094766 | prot-a-2254 | Placenta growth factor \|\| id:prot-a-2254 | Fruit liking \|\| id:ebi-fl187-GCST90094766 | Weighted median | 10 | -0.850019291 | 0.418204089859623 | 0.0420983535589874 | -1.669699307 | -0.030339275 | 0.427406686667834 | 0.188303678657693 | 0.970116341383081 | 0.999332001504091 |
| ebi-fl187-GCST90094766 | prot-a-2261 | Membrane-associated progesterone receptor component 2 \|\| id:prot-a-2261 | Fruit liking \|\| id:ebi-fl187-GCST90094766 | Weighted median | 10 | -0.035057557 | 0.376734664842528 | 0.925858785289027 | -0.7734575 | 0.703342385866623 | 0.965549840289035 | 0.461414964794914 | 2.02049470696417 | 0.999332001504091 |
| ebi-fl187-GCST90094766 | prot-a-2268 | Polymeric immunoglobulin receptor \|\| id:prot-a-2268 | Fruit liking \|\| id:ebi-fl187-GCST90094766 | Weighted median | 10 | -0.063234137 | 0.381487943075239 | 0.868348516654632 | -0.810950506 | 0.684482231275303 | 0.938723657872688 | 0.444435427041121 | 1.98274496638709 | 0.999332001504091 |
| ebi-fl187-GCST90094766 | prot-a-2283 | Group 10 secretory phospholipase A2 \|\| id:prot-a-2283 | Fruit liking \|\| id:ebi-fl187-GCST90094766 | Weighted median | 10 | 0.292214232970127 | 0.392082381342375 | 0.456097727710419 | -0.476267234 | 1.06069570040118 | 1.33938992838784 | 0.621097481417249 | 2.88837973738557 | 0.999332001504091 |
| ebi-fl187-GCST90094766 | prot-a-2298 | Inactive phospholipase D5 \|\| id:prot-a-2298 | Fruit liking \|\| id:ebi-fl187-GCST90094766 | Weighted median | 10 | -0.761806659 | 0.424964145927186 | 0.0730309081836321 | -1.594736385 | 0.0711230673291099 | 0.466822276172217 | 0.202962025345033 | 1.07371335677274 | 0.999332001504091 |
| ebi-fl187-GCST90094766 | prot-a-2304 | Procollagen-lysine,2-oxoglutarate 5-dioxygenase 2 \|\| id:prot-a-2304 | Fruit liking \|\| id:ebi-fl187-GCST90094766 | Weighted median | 10 | -0.320144657 | 0.38645279888132 | 0.407433519498069 | -1.077592143 | 0.437302828589427 | 0.72604400197139 | 0.340414208339798 | 1.54852494368404 | 0.999332001504091 |
| ebi-fl187-GCST90094766 | prot-a-2331 | Periostin \|\| id:prot-a-2331 | Fruit liking \|\| id:ebi-fl187-GCST90094766 | Weighted median | 10 | -0.279053431 | 0.430659902565349 | 0.517006255273236 | -1.12314684 | 0.565039977910065 | 0.756499481522091 | 0.325254658082828 | 1.75951812317306 | 0.999332001504091 |
| ebi-fl187-GCST90094766 | prot-a-2333 | Periostin \|\| id:prot-a-2333 | Fruit liking \|\| id:ebi-fl187-GCST90094766 | Weighted median | 10 | 0.161292202844692 | 0.412167700139283 | 0.695555806951927 | -0.646556489 | 0.969140895117686 | 1.17502826525703 | 0.523846545616074 | 2.63567916159336 | 0.999332001504091 |
| ebi-fl187-GCST90094766 | prot-a-2367 | cAMP-dependent protein kinase catalytic subunit alpha \|\| id:prot-a-2367 | Fruit liking \|\| id:ebi-fl187-GCST90094766 | Weighted median | 10 | -0.410462439 | 0.417130561404793 | 0.325108480230348 | -1.228038339 | 0.407113461571234 | 0.663343423472608 | 0.292866519346866 | 1.50247456911659 | 0.999332001504091 |
| ebi-fl187-GCST90094766 | prot-a-2385 | Phosphoribosyl pyrophosphate synthase-associated protein 1 \|\| id:prot-a-2385 | Fruit liking \|\| id:ebi-fl187-GCST90094766 | Weighted median | 10 | -0.301061546 | 0.407438547217284 | 0.459959966146532 | -1.099641099 | 0.497518006097464 | 0.740032224989912 | 0.332990572905907 | 1.64463422866409 | 0.999332001504091 |
| ebi-fl187-GCST90094766 | prot-a-2393 | Trypsin-3 \|\| id:prot-a-2393 | Fruit liking \|\| id:ebi-fl187-GCST90094766 | Weighted median | 10 | -0.101047476 | 0.369465226072501 | 0.784471520369168 | -0.825199319 | 0.623104367071791 | 0.9038901187525 | 0.438147652551005 | 1.86470780345742 | 0.999332001504091 |
| ebi-fl187-GCST90094766 | prot-a-2405 | Pregnancy-specific beta-1-glycoprotein 6 \|\| id:prot-a-2405 | Fruit liking \|\| id:ebi-fl187-GCST90094766 | Weighted median | 10 | 0.0672298986784366 | 0.392217062996224 | 0.863901448593843 | -0.701515545 | 0.835975342151036 | 1.06954133605201 | 0.495833276528835 | 2.30706312721105 | 0.999332001504091 |
| ebi-fl187-GCST90094766 | prot-a-244 | Beclin-1 \|\| id:prot-a-244 | Fruit liking \|\| id:ebi-fl187-GCST90094766 | Weighted median | 10 | -0.286272806 | 0.367839603969445 | 0.436419026318952 | -1.00723843 | 0.434692817359091 | 0.751057694634579 | 0.365226185800732 | 1.54448854600358 | 0.999332001504091 |
| ebi-fl187-GCST90094766 | prot-a-2454 | tRNA pseudouridine synthase A, mitochondrial \|\| id:prot-a-2454 | Fruit liking \|\| id:ebi-fl187-GCST90094766 | Weighted median | 10 | 0.0512488229164101 | 0.362460556029164 | 0.887560709785187 | -0.659173867 | 0.761671512733571 | 1.05258476791337 | 0.517278498909552 | 2.14185336521588 | 0.999332001504091 |
| ebi-fl187-GCST90094766 | prot-a-2466 | Glutaminyl-peptide cyclotransferase-like protein \|\| id:prot-a-2466 | Fruit liking \|\| id:ebi-fl187-GCST90094766 | Weighted median | 9 | 0.310746987076921 | 0.394498389010825 | 0.430871306761972 | -0.462469855 | 1.08396382953814 | 1.3644439555561 | 0.629726390085526 | 2.95637492276721 | 0.999332001504091 |
| ebi-fl187-GCST90094766 | prot-a-2474 | Ras-related protein Rab-27A \|\| id:prot-a-2474 | Fruit liking \|\| id:ebi-fl187-GCST90094766 | Weighted median | 10 | 0.574871874354784 | 0.460076305126227 | 0.211477064647072 | -0.326877684 | 1.47662143240219 | 1.77690284550487 | 0.721171948721752 | 4.37812886089044 | 0.999332001504091 |
| ebi-fl187-GCST90094766 | prot-a-2490 | Rap1 GTPase-GDP dissociation stimulator 1 \|\| id:prot-a-2490 | Fruit liking \|\| id:ebi-fl187-GCST90094766 | Weighted median | 10 | -0.38891828 | 0.397170013315348 | 0.327469473695702 | -1.167371506 | 0.389534946472886 | 0.677789656975625 | 0.311183812094122 | 1.47629407844706 | 0.999332001504091 |
| ebi-fl187-GCST90094766 | prot-a-2545 | Receptor-interacting serine/threonine-protein kinase 2 \|\| id:prot-a-2545 | Fruit liking \|\| id:ebi-fl187-GCST90094766 | Weighted median | 10 | -0.451903257 | 0.390680897221833 | 0.247392163286015 | -1.217637816 | 0.313831301177548 | 0.636415735274426 | 0.295928379249056 | 1.36865882593848 | 0.999332001504091 |
| ebi-fl187-GCST90094766 | prot-a-2610 | RUN and FYVE domain-containing protein 1 \|\| id:prot-a-2610 | Fruit liking \|\| id:ebi-fl187-GCST90094766 | Weighted median | 10 | -0.084046842 | 0.37122017914354 | 0.820884894061102 | -0.811638393 | 0.643544708794161 | 0.919388188804104 | 0.44412981042939 | 1.90321528044981 | 0.999332001504091 |
| ebi-fl187-GCST90094766 | prot-a-2627 | Serine--tRNA ligase, mitochondrial \|\| id:prot-a-2627 | Fruit liking \|\| id:ebi-fl187-GCST90094766 | Weighted median | 10 | -0.65070438 | 0.402318172661855 | 0.105794664224475 | -1.439247998 | 0.137839238613183 | 0.521678187735416 | 0.237105995786987 | 1.14779101496616 | 0.999332001504091 |
| ebi-fl187-GCST90094766 | prot-a-264 | BolA-like protein 2 \|\| id:prot-a-264 | Fruit liking \|\| id:ebi-fl187-GCST90094766 | Weighted median | 10 | 0.308448064179246 | 0.369027760729334 | 0.403245071782097 | -0.414846347 | 1.03174247520874 | 1.36131080691755 | 0.660441751864504 | 2.80595087727087 | 0.999332001504091 |
| ebi-fl187-GCST90094766 | prot-a-2646 | Secretoglobin family 1C member 1 \|\| id:prot-a-2646 | Fruit liking \|\| id:ebi-fl187-GCST90094766 | Weighted median | 10 | -0.099787479 | 0.440421736759919 | 0.820756133432141 | -0.963014083 | 0.763439124843094 | 0.905029735237146 | 0.381740552425746 | 2.14564267919307 | 0.999332001504091 |
| ebi-fl187-GCST90094766 | prot-a-2672 | Semaphorin-3E \|\| id:prot-a-2672 | Fruit liking \|\| id:ebi-fl187-GCST90094766 | Weighted median | 10 | 0.122807437016161 | 0.387969749340003 | 0.751593661713892 | -0.637613272 | 0.883228145722568 | 1.13066667543455 | 0.528552430847855 | 2.41869501742243 | 0.999332001504091 |
| ebi-fl187-GCST90094766 | prot-a-2701 | Plasma protease C1 inhibitor \|\| id:prot-a-2701 | Fruit liking \|\| id:ebi-fl187-GCST90094766 | Weighted median | 10 | 0.437376542400096 | 0.394222692290232 | 0.267229347416517 | -0.335299934 | 1.21005301928895 | 1.54863909556578 | 0.71512356396228 | 3.35366245663429 | 0.999332001504091 |
| ebi-fl187-GCST90094766 | prot-a-2738 | Signaling threshold-regulating transmembrane adapter 1 \|\| id:prot-a-2738 | Fruit liking \|\| id:ebi-fl187-GCST90094766 | Weighted median | 10 | -0.421962388 | 0.410604625571207 | 0.304109287687184 | -1.226747454 | 0.382822678461243 | 0.655758703550017 | 0.293244820567663 | 1.46641797952021 | 0.999332001504091 |
| ebi-fl187-GCST90094766 | prot-a-274 | Serine/threonine-protein kinase BRSK2 \|\| id:prot-a-274 | Fruit liking \|\| id:ebi-fl187-GCST90094766 | Weighted median | 10 | -0.108144271 | 0.425469958187941 | 0.79935955637149 | -0.942065389 | 0.725776846835099 | 0.89749810394928 | 0.389821869425279 | 2.06633570297151 | 0.999332001504091 |
| ebi-fl187-GCST90094766 | prot-a-2749 | Mitochondrial glutamate carrier 2 \|\| id:prot-a-2749 | Fruit liking \|\| id:ebi-fl187-GCST90094766 | Weighted median | 10 | -0.321480688 | 0.388079948064363 | 0.407450923315643 | -1.082117386 | 0.439156010543487 | 0.72507463277813 | 0.338877231599019 | 1.55139730284512 | 0.999332001504091 |
| ebi-fl187-GCST90094766 | prot-a-2760 | Sodium-coupled monocarboxylate transporter 1 \|\| id:prot-a-2760 | Fruit liking \|\| id:ebi-fl187-GCST90094766 | Weighted median | 10 | 0.0876465206515608 | 0.41342394081387 | 0.832105816795441 | -0.722664403 | 0.897957444646746 | 1.09160219499096 | 0.485457077826361 | 2.45458436293576 | 0.999332001504091 |
| ebi-fl187-GCST90094766 | prot-a-2777 | Small integral membrane protein 10 \|\| id:prot-a-2777 | Fruit liking \|\| id:ebi-fl187-GCST90094766 | Weighted median | 10 | 0.0467045880354659 | 0.380531307374469 | 0.902316785021946 | -0.699136774 | 0.792545950489426 | 1.04781242702725 | 0.497014153999647 | 2.20901331158775 | 0.999332001504091 |
| ebi-fl187-GCST90094766 | prot-a-2799 | Superoxide dismutase [Mn], mitochondrial \|\| id:prot-a-2799 | Fruit liking \|\| id:ebi-fl187-GCST90094766 | Weighted median | 10 | 0.720723798234466 | 0.401825533207931 | 0.0728732516614016 | -0.066854247 | 1.50830184332201 | 2.05592074404666 | 0.935331518914473 | 4.51905021944192 | 0.999332001504091 |
| ebi-fl187-GCST90094766 | prot-a-2805 | Protein SPACA7 \|\| id:prot-a-2805 | Fruit liking \|\| id:ebi-fl187-GCST90094766 | Weighted median | 10 | -0.25132854 | 0.391476836114655 | 0.520872501305098 | -1.018623139 | 0.515966058985342 | 0.777766802229738 | 0.361091771388346 | 1.67525611654023 | 0.999332001504091 |
| ebi-fl187-GCST90094766 | prot-a-2811 | SPARC-like protein 1 \|\| id:prot-a-2811 | Fruit liking \|\| id:ebi-fl187-GCST90094766 | Weighted median | 10 | 0.400610545256046 | 0.371186257646385 | 0.280466958652628 | -0.32691452 | 1.12813561024296 | 1.49273580224026 | 0.721145384093538 | 3.08989036668486 | 0.999332001504091 |
| ebi-fl187-GCST90094766 | prot-a-2831 | Sepiapterin reductase \|\| id:prot-a-2831 | Fruit liking \|\| id:ebi-fl187-GCST90094766 | Weighted median | 10 | 0.111709509055774 | 0.397804694954735 | 0.778852329581207 | -0.667987693 | 0.891406711167054 | 1.11818798997631 | 0.512739329257525 | 2.43855758585521 | 0.999332001504091 |
| ebi-fl187-GCST90094766 | prot-a-2834 | SPRY domain-containing SOCS box protein 1 \|\| id:prot-a-2834 | Fruit liking \|\| id:ebi-fl187-GCST90094766 | Weighted median | 10 | -0.155961298 | 0.370333621010235 | 0.67365483266287 | -0.881815195 | 0.569892599410551 | 0.855592303060766 | 0.414030682762742 | 1.76807714870812 | 0.999332001504091 |
| ebi-fl187-GCST90094766 | prot-a-2835 | Spectrin alpha chain, non-erythrocytic 1 \|\| id:prot-a-2835 | Fruit liking \|\| id:ebi-fl187-GCST90094766 | Weighted median | 10 | 0.140957039502847 | 0.405952533950673 | 0.728421974331273 | -0.654709927 | 0.936624006046166 | 1.15137518327195 | 0.51959276053909 | 2.55135350861917 | 0.999332001504091 |
| ebi-fl187-GCST90094766 | prot-a-2859 | Sia-alpha-2,3-Gal-beta-1,4-GlcNAc-R:alpha 2,8-sialyltransferase \|\| id:prot-a-2859 | Fruit liking \|\| id:ebi-fl187-GCST90094766 | Weighted median | 10 | 0.0438958784746679 | 0.36776459872316 | 0.904991235819313 | -0.676922735 | 0.764714491972061 | 1.04487355539599 | 0.508178388275419 | 2.14838090708839 | 0.999332001504091 |
| ebi-fl187-GCST90094766 | prot-a-2861 | Alpha-2,8-sialyltransferase 8F \|\| id:prot-a-2861 | Fruit liking \|\| id:ebi-fl187-GCST90094766 | Weighted median | 10 | 0.28391448646668 | 0.384048026759528 | 0.459744166147325 | -0.468819646 | 1.03664861891535 | 1.3283193366161 | 0.625740427780393 | 2.81975110076707 | 0.999332001504091 |
| ebi-fl187-GCST90094766 | prot-a-289 | Uncharacterized protein C17orf89 \|\| id:prot-a-289 | Fruit liking \|\| id:ebi-fl187-GCST90094766 | Weighted median | 10 | 0.18285002201988 | 0.424420900029104 | 0.666597525869529 | -0.649014942 | 1.01471498607692 | 1.20063432586593 | 0.522560275474303 | 2.75857705245415 | 0.999332001504091 |
| ebi-fl187-GCST90094766 | prot-a-2921 | Tachykinin-3 \|\| id:prot-a-2921 | Fruit liking \|\| id:ebi-fl187-GCST90094766 | Weighted median | 10 | -0.389337399 | 0.365762577805325 | 0.28712313170002 | -1.106232052 | 0.327557253390441 | 0.677505641647484 | 0.330803064620213 | 1.38757449236799 | 0.999332001504091 |
| ebi-fl187-GCST90094766 | prot-a-2928 | Tax1-binding protein 1 \|\| id:prot-a-2928 | Fruit liking \|\| id:ebi-fl187-GCST90094766 | Weighted median | 10 | 0.125734178999675 | 0.378305660497864 | 0.739616421588173 | -0.615744916 | 0.867213273575488 | 1.1339806923335 | 0.540238313448669 | 2.38026844556136 | 0.999332001504091 |
| ebi-fl187-GCST90094766 | prot-a-296 | Uncharacterized protein C1orf185 \|\| id:prot-a-296 | Fruit liking \|\| id:ebi-fl187-GCST90094766 | Weighted median | 10 | -0.313785344 | 0.405156951551778 | 0.438647883343263 | -1.107892969 | 0.480322280854978 | 0.730675855146997 | 0.330254084012904 | 1.6165953159687 | 0.999332001504091 |
| ebi-fl187-GCST90094766 | prot-a-2964 | Transforming growth factor beta-2 \|\| id:prot-a-2964 | Fruit liking \|\| id:ebi-fl187-GCST90094766 | Weighted median | 10 | -0.641829523 | 0.398078759171008 | 0.10689277267106 | -1.422063891 | 0.138404845360896 | 0.526328612555216 | 0.241215660066112 | 1.14844039693929 | 0.999332001504091 |
| ebi-fl187-GCST90094766 | prot-a-2977 | Thymocyte nuclear protein 1 \|\| id:prot-a-2977 | Fruit liking \|\| id:ebi-fl187-GCST90094766 | Weighted median | 10 | -0.407750799 | 0.404587089140428 | 0.313541073530208 | -1.200741494 | 0.385239895358007 | 0.66514461263606 | 0.300970960969261 | 1.46996691738629 | 0.999332001504091 |
| ebi-fl187-GCST90094766 | prot-a-3015 | Transmembrane protein 70, mitochondrial \|\| id:prot-a-3015 | Fruit liking \|\| id:ebi-fl187-GCST90094766 | Weighted median | 10 | -0.147353466 | 0.389844921379785 | 0.705445675999137 | -0.911449511 | 0.616742580362445 | 0.862988886665454 | 0.401941183227726 | 1.8528825847789 | 0.999332001504091 |
| ebi-fl187-GCST90094766 | prot-a-3030 | Tumor necrosis factor alpha-induced protein 3 \|\| id:prot-a-3030 | Fruit liking \|\| id:ebi-fl187-GCST90094766 | Weighted median | 10 | -0.047813179 | 0.37724831134622 | 0.899144657668788 | -0.78721987 | 0.691593510973615 | 0.95331186886635 | 0.455108298576573 | 1.99689507346688 | 0.999332001504091 |
| ebi-fl187-GCST90094766 | prot-a-3032 | Tumor necrosis factor receptor superfamily member 10A \|\| id:prot-a-3032 | Fruit liking \|\| id:ebi-fl187-GCST90094766 | Weighted median | 10 | 0.187776478155556 | 0.392088726847097 | 0.632000359487804 | -0.580717426 | 0.956270382775866 | 1.20656379184199 | 0.559496824715254 | 2.60197398711784 | 0.999332001504091 |
| ebi-fl187-GCST90094766 | prot-a-3081 | Tumor protein p53-inducible protein 11 \|\| id:prot-a-3081 | Fruit liking \|\| id:ebi-fl187-GCST90094766 | Weighted median | 10 | 0.0145889057731801 | 0.415404528351137 | 0.971984247934079 | -0.79960397 | 0.828781781341409 | 1.01469584325981 | 0.449506947199984 | 2.29052667760141 | 0.999332001504091 |
| ebi-fl187-GCST90094766 | prot-a-315 | Complement C4 \|\| id:prot-a-315 | Fruit liking \|\| id:ebi-fl187-GCST90094766 | Weighted median | 10 | 0.0996590193124732 | 0.387713563930368 | 0.797145655012474 | -0.660259566 | 0.859577604615995 | 1.10479414037678 | 0.516717194872632 | 2.36216271632247 | 0.999332001504091 |
| ebi-fl187-GCST90094766 | prot-a-3157 | UDP-glucuronosyltransferase 1-8 \|\| id:prot-a-3157 | Fruit liking \|\| id:ebi-fl187-GCST90094766 | Weighted median | 10 | -0.229281658 | 0.405148950984605 | 0.571448561866045 | -1.023373602 | 0.564810286072264 | 0.795104554518491 | 0.359380486132376 | 1.75911402263278 | 0.999332001504091 |
| ebi-fl187-GCST90094766 | prot-a-317 | Glutaredoxin-like protein C5orf63 \|\| id:prot-a-317 | Fruit liking \|\| id:ebi-fl187-GCST90094766 | Weighted median | 10 | 0.152551029677273 | 0.406957432708259 | 0.707766477710747 | -0.645085538 | 0.950187597785461 | 1.1648019000431 | 0.524617665215358 | 2.58619477822397 | 0.999332001504091 |
| ebi-fl187-GCST90094766 | prot-a-3192 | Guanine nucleotide exchange factor VAV3 \|\| id:prot-a-3192 | Fruit liking \|\| id:ebi-fl187-GCST90094766 | Weighted median | 10 | -0.341304803 | 0.377918768682384 | 0.366463889089123 | -1.08202559 | 0.399415983712837 | 0.710842208412862 | 0.338908340718692 | 1.49095370208281 | 0.999332001504091 |
| ebi-fl187-GCST90094766 | prot-a-3233 | Serine/threonine-protein kinase WNK3 \|\| id:prot-a-3233 | Fruit liking \|\| id:ebi-fl187-GCST90094766 | Weighted median | 10 | -0.310797794 | 0.418291647784139 | 0.457471405114152 | -1.130649423 | 0.509053836094316 | 0.732862050312216 | 0.322823539229837 | 1.66371630169584 | 0.999332001504091 |
| ebi-fl187-GCST90094766 | prot-a-3250 | 14-3-3 protein theta \|\| id:prot-a-3250 | Fruit liking \|\| id:ebi-fl187-GCST90094766 | Weighted median | 10 | -0.060201923 | 0.420735459273792 | 0.886221192771963 | -0.884843423 | 0.764439576870982 | 0.941574388574484 | 0.412778799686472 | 2.14779036591222 | 0.999332001504091 |
| ebi-fl187-GCST90094766 | prot-a-3278 | E3 ubiquitin-protein ligase ZNRF3 \|\| id:prot-a-3278 | Fruit liking \|\| id:ebi-fl187-GCST90094766 | Weighted median | 10 | 0.272148368729348 | 0.376272788969342 | 0.469511492037992 | -0.465346298 | 1.00964303510926 | 1.31278176262454 | 0.62791762113776 | 2.7446211067574 | 0.999332001504091 |
| ebi-fl187-GCST90094766 | prot-a-355 | Calpain-3 \|\| id:prot-a-355 | Fruit liking \|\| id:ebi-fl187-GCST90094766 | Weighted median | 10 | -0.043182594 | 0.412565959875235 | 0.916638986131724 | -0.851811875 | 0.765446687808192 | 0.957736497595954 | 0.42664121071519 | 2.1499545186686 | 0.999332001504091 |
| ebi-fl187-GCST90094766 | prot-a-385 | Coiled-coil domain-containing protein 90B, mitochondrial \|\| id:prot-a-385 | Fruit liking \|\| id:ebi-fl187-GCST90094766 | Weighted median | 10 | -0.013932797 | 0.354960539587451 | 0.968689731033369 | -0.709655455 | 0.68178986023853 | 0.986163814854915 | 0.491813620223586 | 1.97741386114333 | 0.999332001504091 |
| ebi-fl187-GCST90094766 | prot-a-390 | C-C motif chemokine 14 \|\| id:prot-a-390 | Fruit liking \|\| id:ebi-fl187-GCST90094766 | Weighted median | 10 | 0.488459658462518 | 0.393133402491237 | 0.214060201227441 | -0.28208181 | 1.25900112734534 | 1.6298038309448 | 0.754211979610018 | 3.52190179839869 | 0.999332001504091 |
| ebi-fl187-GCST90094766 | prot-a-40 | Pituitary adenylate cyclase-activating polypeptide 27 \|\| id:prot-a-40 | Fruit liking \|\| id:ebi-fl187-GCST90094766 | Weighted median | 10 | 0.0930806946142293 | 0.368536460102045 | 0.800601687525961 | -0.629250767 | 0.815412156414238 | 1.09755029809266 | 0.53299098578329 | 2.26010699800669 | 0.999332001504091 |
| ebi-fl187-GCST90094766 | prot-a-401 | C-C motif chemokine 24 \|\| id:prot-a-401 | Fruit liking \|\| id:ebi-fl187-GCST90094766 | Weighted median | 10 | 0.0490933587361883 | 0.358816480344755 | 0.891172880967485 | -0.654186943 | 0.752373660211909 | 1.05031840256162 | 0.519864570465701 | 2.12203102390947 | 0.999332001504091 |
| ebi-fl187-GCST90094766 | prot-a-412 | Cerebral cavernous malformations 2 protein \|\| id:prot-a-412 | Fruit liking \|\| id:ebi-fl187-GCST90094766 | Weighted median | 10 | 0.418315102548323 | 0.388257585183428 | 0.28129426587788 | -0.342669764 | 1.17929996950784 | 1.51939936559189 | 0.709872598052091 | 3.25209683891987 | 0.999332001504091 |
| ebi-fl187-GCST90094766 | prot-a-44 | Adhesion G protein-coupled receptor F5 \|\| id:prot-a-44 | Fruit liking \|\| id:ebi-fl187-GCST90094766 | Weighted median | 10 | 0.241678176064322 | 0.410130093942572 | 0.555678832325307 | -0.562176808 | 1.04553316019176 | 1.27338432131259 | 0.569967003706626 | 2.84491491475766 | 0.999332001504091 |
| ebi-fl187-GCST90094766 | prot-a-48 | Adrenomedullin \|\| id:prot-a-48 | Fruit liking \|\| id:ebi-fl187-GCST90094766 | Weighted median | 10 | 0.490706407523148 | 0.384853548241222 | 0.202292565159408 | -0.263606547 | 1.24501936207594 | 1.63346970778158 | 0.768275760577997 | 3.47300204321515 | 0.999332001504091 |
| ebi-fl187-GCST90094766 | prot-a-502 | Carcinoembryonic antigen-related cell adhesion molecule 1 \|\| id:prot-a-502 | Fruit liking \|\| id:ebi-fl187-GCST90094766 | Weighted median | 10 | -0.242497077 | 0.381023604945704 | 0.524492389537019 | -0.989303342 | 0.504309189045307 | 0.784666041430196 | 0.371835642959387 | 1.65584125199365 | 0.999332001504091 |
| ebi-fl187-GCST90094766 | prot-a-518 | Complement factor B \|\| id:prot-a-518 | Fruit liking \|\| id:ebi-fl187-GCST90094766 | Weighted median | 10 | -0.009312752 | 0.39688864638644 | 0.981279838532951 | -0.787214499 | 0.768588994548892 | 0.990730477010895 | 0.455110742613738 | 2.15672096079546 | 0.999332001504091 |
| ebi-fl187-GCST90094766 | prot-a-525 | Properdin \|\| id:prot-a-525 | Fruit liking \|\| id:ebi-fl187-GCST90094766 | Weighted median | 10 | -0.464157473 | 0.462181368492643 | 0.315245789168253 | -1.370032955 | 0.441718009147429 | 0.628664548917731 | 0.254098585508589 | 1.55537707647954 | 0.999332001504091 |
| ebi-fl187-GCST90094766 | prot-a-533 | Chondroadherin \|\| id:prot-a-533 | Fruit liking \|\| id:ebi-fl187-GCST90094766 | Weighted median | 10 | 0.349295577773451 | 0.39302734595682 | 0.374147677994254 | -0.42103802 | 1.11962917584882 | 1.41806827786663 | 0.656365145733755 | 3.06371789203342 | 0.999332001504091 |
| ebi-fl187-GCST90094766 | prot-a-535 | Coiled-coil-helix-coiled-coil-helix domain-containing protein 2 \|\| id:prot-a-535 | Fruit liking \|\| id:ebi-fl187-GCST90094766 | Weighted median | 10 | 0.370111969357311 | 0.42421196116553 | 0.38295233640244 | -0.461343475 | 1.20156741324175 | 1.44789672565325 | 0.63043610146419 | 3.3253249985026 | 0.999332001504091 |
| ebi-fl187-GCST90094766 | prot-a-591 | Caseinolytic peptidase B protein homolog \|\| id:prot-a-591 | Fruit liking \|\| id:ebi-fl187-GCST90094766 | Weighted median | 10 | 0.237075878452896 | 0.380178993067104 | 0.532896775642854 | -0.508074948 | 0.982226704864421 | 1.2675372928952 | 0.60165267743229 | 2.67039580998276 | 0.999332001504091 |
| ebi-fl187-GCST90094766 | prot-a-605 | Ciliary neurotrophic factor receptor subunit alpha \|\| id:prot-a-605 | Fruit liking \|\| id:ebi-fl187-GCST90094766 | Weighted median | 10 | 0.397962375631017 | 0.360789110262133 | 0.270012737145923 | -0.30918428 | 1.1051090317448 | 1.48878801414392 | 0.734045487304893 | 3.01955367806514 | 0.999332001504091 |
| ebi-fl187-GCST90094766 | prot-a-606 | Ciliary neurotrophic factor receptor subunit alpha \|\| id:prot-a-606 | Fruit liking \|\| id:ebi-fl187-GCST90094766 | Weighted median | 10 | 0.198280759824425 | 0.388158436925056 | 0.609474095659724 | -0.562509777 | 0.959071296197535 | 1.21930467753682 | 0.569777254248627 | 2.60927210691082 | 0.999332001504091 |
| ebi-fl187-GCST90094766 | prot-a-707 | Cancer/testis antigen 55 \|\| id:prot-a-707 | Fruit liking \|\| id:ebi-fl187-GCST90094766 | Weighted median | 10 | 0.11704368100888 | 0.385226024268373 | 0.761256503876337 | -0.637999327 | 0.87208668857489 | 1.12416853343377 | 0.528348419991747 | 2.39189679337429 | 0.999332001504091 |
| ebi-fl187-GCST90094766 | prot-a-719 | Dipeptidyl peptidase 1 \|\| id:prot-a-719 | Fruit liking \|\| id:ebi-fl187-GCST90094766 | Weighted median | 10 | 0.170563029157513 | 0.381954829494129 | 0.655197923823196 | -0.578068437 | 0.919194494966006 | 1.18597240041937 | 0.560980890875007 | 2.50726995773885 | 0.999332001504091 |
| ebi-fl187-GCST90094766 | prot-a-752 | Cytochrome b5 \|\| id:prot-a-752 | Fruit liking \|\| id:ebi-fl187-GCST90094766 | Weighted median | 10 | -0.176679451 | 0.393812925298268 | 0.653692802401892 | -0.948552785 | 0.595193882580492 | 0.838048377037928 | 0.387301126220739 | 1.81338249415681 | 0.999332001504091 |
| ebi-fl187-GCST90094766 | prot-a-755 | Cytoglobin \|\| id:prot-a-755 | Fruit liking \|\| id:ebi-fl187-GCST90094766 | Weighted median | 10 | 0.196246670584986 | 0.396299306360031 | 0.620460279128504 | -0.58049997 | 0.972993311050646 | 1.21682702375138 | 0.559618504213084 | 2.64585247732953 | 0.999332001504091 |
| ebi-fl187-GCST90094766 | prot-a-807 | Desmin \|\| id:prot-a-807 | Fruit liking \|\| id:ebi-fl187-GCST90094766 | Weighted median | 10 | 0.181582308765758 | 0.38407309223259 | 0.636369712905766 | -0.571200952 | 0.934365569541634 | 1.19911323017797 | 0.564846677448584 | 2.54559794045833 | 0.999332001504091 |
| ebi-fl187-GCST90094766 | prot-a-82 | Ameloblastin \|\| id:prot-a-82 | Fruit liking \|\| id:ebi-fl187-GCST90094766 | Weighted median | 10 | -0.420236443 | 0.403817806083369 | 0.298034057458636 | -1.211719343 | 0.371246456434223 | 0.656891483737251 | 0.297685016406071 | 1.44954027789532 | 0.999332001504091 |
| ebi-fl187-GCST90094766 | prot-a-84 | Muellerian-inhibiting factor \|\| id:prot-a-84 | Fruit liking \|\| id:ebi-fl187-GCST90094766 | Weighted median | 10 | 0.55442641692543 | 0.385789415891135 | 0.150683360546848 | -0.201720838 | 1.31057367207205 | 1.74094212340068 | 0.817323061457372 | 3.70830045052994 | 0.999332001504091 |
| ebi-fl187-GCST90094766 | prot-a-858 | Inactive dipeptidyl peptidase 10 \|\| id:prot-a-858 | Fruit liking \|\| id:ebi-fl187-GCST90094766 | Weighted median | 10 | 0.00764470776425502 | 0.417629525239238 | 0.985395540040618 | -0.810909162 | 0.826198577233161 | 1.00767400314662 | 0.4444538021037 | 2.28461741537902 | 0.999332001504091 |
| ebi-fl187-GCST90094766 | prot-a-863 | Dihydropyrimidinase-related protein 3 \|\| id:prot-a-863 | Fruit liking \|\| id:ebi-fl187-GCST90094766 | Weighted median | 10 | 0.161542195061436 | 0.371229465718374 | 0.663450242446992 | -0.566067558 | 0.88915194786945 | 1.17532204989821 | 0.567753713239019 | 2.43306540981684 | 0.999332001504091 |
| ebi-fl187-GCST90094766 | prot-a-864 | Dihydropyrimidinase-related protein 5 \|\| id:prot-a-864 | Fruit liking \|\| id:ebi-fl187-GCST90094766 | Weighted median | 10 | -0.028411506 | 0.388918859446509 | 0.94176431785419 | -0.79069247 | 0.733869458654557 | 0.971988305608937 | 0.453530629994063 | 2.08312560113724 | 0.999332001504091 |
| ebi-fl187-GCST90094766 | prot-a-884 | Endothelin-converting enzyme 1 \|\| id:prot-a-884 | Fruit liking \|\| id:ebi-fl187-GCST90094766 | Weighted median | 10 | -0.184962593 | 0.392843843316718 | 0.637762273249565 | -0.954936526 | 0.585011339865516 | 0.831135373522257 | 0.384836570918876 | 1.79501134071169 | 0.999332001504091 |
| ebi-fl187-GCST90094766 | prot-a-893 | Early endosome antigen 1 \|\| id:prot-a-893 | Fruit liking \|\| id:ebi-fl187-GCST90094766 | Weighted median | 10 | 0.299620423703023 | 0.396460130742102 | 0.449805634333414 | -0.477441433 | 1.07668227995754 | 1.34934653039867 | 0.620368617939306 | 2.93492611722835 | 0.999332001504091 |
| ebi-fl187-GCST90094766 | prot-a-897 | Ephrin-A2 \|\| id:prot-a-897 | Fruit liking \|\| id:ebi-fl187-GCST90094766 | Weighted median | 10 | -0.004650939 | 0.416750050781433 | 0.991095776868298 | -0.821481039 | 0.812179160469633 | 0.99535985980699 | 0.439779841027443 | 2.2528118801452 | 0.999332001504091 |
| ebi-fl187-GCST90094766 | prot-a-90 | Anaphase-promoting complex subunit 10 \|\| id:prot-a-90 | Fruit liking \|\| id:ebi-fl187-GCST90094766 | Weighted median | 10 | -0.378044226 | 0.366479629925948 | 0.302280206335082 | -1.096344301 | 0.340255848470388 | 0.685200196205474 | 0.334090187219539 | 1.40530709024238 | 0.999332001504091 |
| ebi-fl187-GCST90094766 | prot-a-914 | Histone-lysine N-methyltransferase EHMT2 \|\| id:prot-a-914 | Fruit liking \|\| id:ebi-fl187-GCST90094766 | Weighted median | 10 | 0.376167107058549 | 0.457589399431375 | 0.411041300128775 | -0.520708116 | 1.27304232994404 | 1.45669053670415 | 0.594099707580188 | 3.57170234667557 | 0.999332001504091 |
| ebi-fl187-GCST90094766 | prot-a-932 | RNA polymerase II elongation factor ELL \|\| id:prot-a-932 | Fruit liking \|\| id:ebi-fl187-GCST90094766 | Weighted median | 10 | 0.0635115647793758 | 0.372477171058532 | 0.864608052343394 | -0.66654369 | 0.793566820054098 | 1.06557180883309 | 0.513480260986579 | 2.21126957752617 | 0.999332001504091 |
| ebi-fl187-GCST90094766 | prot-a-991 | Estrogen receptor \|\| id:prot-a-991 | Fruit liking \|\| id:ebi-fl187-GCST90094766 | Weighted median | 10 | 0.162437257205861 | 0.395148209493687 | 0.68101437338419 | -0.612053233 | 0.936927747813487 | 1.17637450710962 | 0.542236387458211 | 2.5521285789476 | 0.999332001504091 |
| ebi-fl187-GCST90094766 | prot-a-992 | Persulfide dioxygenase ETHE1, mitochondrial \|\| id:prot-a-992 | Fruit liking \|\| id:ebi-fl187-GCST90094766 | Weighted median | 10 | -0.286612947 | 0.458530891120043 | 0.531926486181488 | -1.185333494 | 0.612107599387817 | 0.750802272721826 | 0.305644232043001 | 1.8443143813195 | 0.999332001504091 |
| ebi-fl187-GCST90094766 | prot-a-993 | Ethanolamine kinase 1 \|\| id:prot-a-993 | Fruit liking \|\| id:ebi-fl187-GCST90094766 | Weighted median | 10 | 0.0104997645318133 | 0.374920113147939 | 0.977657893624357 | -0.724343657 | 0.745343186301773 | 1.01055508049143 | 0.48464255622349 | 2.1071644608859 | 0.999332001504091 |
| ebi-fl187-GCST90094766 | prot-b-10 | coagulation factor III, tissue factor \|\| id:prot-b-10 | Fruit liking \|\| id:ebi-fl187-GCST90094766 | Weighted median | 6 | -0.420268811 | 0.477942667027779 | 0.379222957214872 | -1.357036439 | 0.516498815980678 | 0.656870221880484 | 0.257422535082761 | 1.67614885874151 | 0.999332001504091 |
| ebi-fl187-GCST90094766 | prot-b-28 | prolactin \|\| id:prot-b-28 | Fruit liking \|\| id:ebi-fl187-GCST90094766 | Weighted median | 6 | 0.30280021181224 | 0.487298690761131 | 0.53434618400388 | -0.652305222 | 1.25790564570406 | 1.35364399534422 | 0.520843731333018 | 3.51804573214666 | 0.999332001504091 |
| ebi-fl187-GCST90094766 | prot-b-54 | matrix metallopeptidase 7 \|\| id:prot-b-54 | Fruit liking \|\| id:ebi-fl187-GCST90094766 | Weighted median | 6 | 0.0797892912419664 | 0.52505294059418 | 0.879215110079632 | -0.949314472 | 1.10889305480656 | 1.08305883364863 | 0.387006236024948 | 3.03100138435165 | 0.999332001504091 |
| ebi-fl187-GCST90094766 | ukb-rsfMRI191-edge-pheno716 | Net100_Pair10_33 (OccipitalPrecuneus)&(Temporal\|Frontal\|Supp_Motor_Area) (Default_mode\|Central_executive)&(Default_mode\|Salience) \|\| id:ukb-rsfMRI191-edge-pheno716 | Fruit liking \|\| id:ebi-fl187-GCST90094766 | Weighted median | 10 | 0.0183315205627896 | 0.119553845847603 | 0.878135871274594 | -0.215994017 | 0.252657058424092 | 1.01850057430997 | 0.805740122361689 | 1.28744168383871 | 0.999332001504091 |
| ebi-fl187-GCST90094766 | ukb-rsfMRI191-edge-pheno903 | Net100_Pair27_38 (Precentral\|Frontal\|Supp_Motor_Area)&(Subcortical) (Attention\|Salience\|Motor)&(Subcortical-cerebellum) \|\| id:ukb-rsfMRI191-edge-pheno903 | Fruit liking \|\| id:ebi-fl187-GCST90094766 | Weighted median | 10 | -0.104764636 | 0.121411319313372 | 0.388197846928693 | -0.342730822 | 0.133201549923316 | 0.900536451568674 | 0.709829256418623 | 1.14248024193248 | 0.999332001504091 |
| ebi-fl187-GCST90094766 | ukb-rsfMRI191-node-pheno23 | Net100_Node2 RolandicOper\|SupraMarginal\|Insula Salience\|Motor \|\| id:ukb-rsfMRI191-node-pheno23 | Fruit liking \|\| id:ebi-fl187-GCST90094766 | Weighted median | 10 | 0.00965283906876518 | 0.111945220255685 | 0.931284983810763 | -0.209759793 | 0.229065470769908 | 1.00969957798651 | 0.810778977665206 | 1.25742436085598 | 0.999332001504091 |
| ebi-fl187-GCST90094766 | ukb-rsfMRI191-node-pheno55 | Net100_Node34 Parietal\|Postcentral\|Precuneus Attention \|\| id:ukb-rsfMRI191-node-pheno55 | Fruit liking \|\| id:ebi-fl187-GCST90094766 | Weighted median | 10 | -0.014119798 | 0.126831560180458 | 0.911356915950747 | -0.262709656 | 0.234470060120127 | 0.985979418989087 | 0.768965129477645 | 1.2642386207167 | 0.999332001504091 |
| ebi-fl187-GCST90094766 | ebi-a-GCST90001414 | IgD- CD24- B cell Absolute Count \|\| id:ebi-a-GCST90001414 | Fruit liking \|\| id:ebi-fl187-GCST90094766 | Weighted mode | 9 | -0.494935195 | 0.486362688639368 | 0.338640613459399 | -1.448206065 | 0.45833567456503 | 0.609610411716494 | 0.234991469639949 | 1.58143976307971 | 0.999714303421225 |
| ebi-fl187-GCST90094766 | ebi-a-GCST90001422 | CD20- CD38- B cell %B cell \|\| id:ebi-a-GCST90001422 | Fruit liking \|\| id:ebi-fl187-GCST90094766 | Weighted mode | 9 | -0.237729535 | 0.506090677031847 | 0.651080675028212 | -1.229667262 | 0.754208191767479 | 0.788415900996785 | 0.292389850653185 | 2.12592752982345 | 0.999714303421225 |
| ebi-fl187-GCST90094766 | ebi-a-GCST90001444 | CD20- CD38- B cell %lymphocyte \|\| id:ebi-a-GCST90001444 | Fruit liking \|\| id:ebi-fl187-GCST90094766 | Weighted mode | 9 | -0.227195526 | 0.476241655011319 | 0.6460796924253 | -1.160629169 | 0.70623811816917 | 0.796764979066041 | 0.313289006981101 | 2.02635399812929 | 0.999714303421225 |
| ebi-fl187-GCST90094766 | ebi-a-GCST90001459 | Myeloid Dendritic Cell %Dendritic Cell \|\| id:ebi-a-GCST90001459 | Fruit liking \|\| id:ebi-fl187-GCST90094766 | Weighted mode | 9 | -0.501564977 | 0.558878784447471 | 0.395684190740775 | -1.596967395 | 0.593837440068328 | 0.605582195264543 | 0.20250971972089 | 1.81092441255101 | 0.999714303421225 |
| ebi-fl187-GCST90094766 | ebi-a-GCST90001476 | HLA DR++ monocyte %leukocyte \|\| id:ebi-a-GCST90001476 | Fruit liking \|\| id:ebi-fl187-GCST90094766 | Weighted mode | 9 | -0.033609058 | 0.556340848275878 | 0.953310266203064 | -1.12403712 | 1.05681900511392 | 0.966949452384491 | 0.324965219231799 | 2.87720404564199 | 0.999714303421225 |
| ebi-fl187-GCST90094766 | ebi-a-GCST90001478 | CD4 regulatory T cell %CD4+ T cell \|\| id:ebi-a-GCST90001478 | Fruit liking \|\| id:ebi-fl187-GCST90094766 | Weighted mode | 9 | 0.163023006018745 | 0.622876539444266 | 0.800145178862851 | -1.057815011 | 1.38386102332951 | 1.1770637689281 | 0.347213639986564 | 3.9902784815045 | 0.999714303421225 |
| ebi-fl187-GCST90094766 | ebi-a-GCST90001485 | CD39+ resting CD4 regulatory T cell %CD4 regulatory T cell \|\| id:ebi-a-GCST90001485 | Fruit liking \|\| id:ebi-fl187-GCST90094766 | Weighted mode | 9 | 0.536957349735447 | 0.535582935668335 | 0.345426831575059 | -0.512785204 | 1.58669990364538 | 1.71079358855777 | 0.598825402989413 | 4.88759275748712 | 0.999714303421225 |
| ebi-fl187-GCST90094766 | ebi-a-GCST90001515 | Immature Myeloid-Derived Suppressor Cells Absolute Count \|\| id:ebi-a-GCST90001515 | Fruit liking \|\| id:ebi-fl187-GCST90094766 | Weighted mode | 9 | 0.356161769738981 | 0.896402002683428 | 0.701518990158954 | -1.400786156 | 2.1131096949985 | 1.42783851064515 | 0.246403176560546 | 8.27393072175113 | 0.999714303421225 |
| ebi-fl187-GCST90094766 | ebi-a-GCST90001517 | CD33+ HLA DR+ Absolute Count \|\| id:ebi-a-GCST90001517 | Fruit liking \|\| id:ebi-fl187-GCST90094766 | Weighted mode | 9 | -0.468137533 | 0.854241464003179 | 0.598635203771416 | -2.142450803 | 1.20617573622072 | 0.626167398913104 | 0.117366847272686 | 3.34068453377335 | 0.999714303421225 |
| ebi-fl187-GCST90094766 | ebi-a-GCST90001518 | CD33+ HLA DR+ CD14- Absolute Count \|\| id:ebi-a-GCST90001518 | Fruit liking \|\| id:ebi-fl187-GCST90094766 | Weighted mode | 9 | -0.526310151 | 0.858098835914383 | 0.556680648788671 | -2.208183869 | 1.15556356755745 | 0.590780845091721 | 0.109900060263201 | 3.17581269829525 | 0.999714303421225 |
| ebi-fl187-GCST90094766 | ebi-a-GCST90001520 | CD33+ HLA DR+ CD14dim Absolute Count \|\| id:ebi-a-GCST90001520 | Fruit liking \|\| id:ebi-fl187-GCST90094766 | Weighted mode | 9 | -0.49819309 | 0.720238868393375 | 0.508695450398999 | -1.909861272 | 0.913475091947794 | 0.607627596699065 | 0.148100930893495 | 2.49297080067509 | 0.999714303421225 |
| ebi-fl187-GCST90094766 | ebi-a-GCST90001531 | CD33dim HLA DR- Absolute Count \|\| id:ebi-a-GCST90001531 | Fruit liking \|\| id:ebi-fl187-GCST90094766 | Weighted mode | 9 | -0.790067672 | 0.710888151216638 | 0.298686190030069 | -2.183408449 | 0.603273103940357 | 0.453814083534929 | 0.112656890253644 | 1.82809255564362 | 0.999714303421225 |
| ebi-fl187-GCST90094766 | ebi-a-GCST90001532 | Basophil Absolute Count \|\| id:ebi-a-GCST90001532 | Fruit liking \|\| id:ebi-fl187-GCST90094766 | Weighted mode | 9 | -0.709792742 | 0.718358149297016 | 0.352053692910478 | -2.117774714 | 0.698189230773673 | 0.491746105288786 | 0.12029903057007 | 2.01010956547851 | 0.999714303421225 |
| ebi-fl187-GCST90094766 | ebi-a-GCST90001588 | T/B cell \|\| id:ebi-a-GCST90001588 | Fruit liking \|\| id:ebi-fl187-GCST90094766 | Weighted mode | 9 | 0.382605332689739 | 0.527178274742608 | 0.488670461043204 | -0.650664086 | 1.41587475118525 | 1.46609929440679 | 0.521699208658921 | 4.12008894279415 | 0.999714303421225 |
| ebi-fl187-GCST90094766 | ebi-a-GCST90001624 | HLA DR+ CD4+ T cell Absolute Count \|\| id:ebi-a-GCST90001624 | Fruit liking \|\| id:ebi-fl187-GCST90094766 | Weighted mode | 9 | -0.177738551 | 0.513185484376294 | 0.738017377822908 | -1.183582101 | 0.82810499790882 | 0.837161269463391 | 0.306180004235067 | 2.28897701154741 | 0.999714303421225 |
| ebi-fl187-GCST90094766 | ebi-a-GCST90001631 | CD8+ Natural Killer T %T cell \|\| id:ebi-a-GCST90001631 | Fruit liking \|\| id:ebi-fl187-GCST90094766 | Weighted mode | 9 | 0.896902765098019 | 0.647136502412077 | 0.203167134067278 | -0.37148478 | 2.16529030982569 | 2.45199692770491 | 0.689709503383314 | 8.71713221868265 | 0.999714303421225 |
| ebi-fl187-GCST90094766 | ebi-a-GCST90001632 | CD8+ Natural Killer T %lymphocyte \|\| id:ebi-a-GCST90001632 | Fruit liking \|\| id:ebi-fl187-GCST90094766 | Weighted mode | 9 | 0.997712476539098 | 0.633123666032987 | 0.153708098156301 | -0.243209909 | 2.23863486196375 | 2.71207080164628 | 0.784106905489102 | 9.38051684234851 | 0.999714303421225 |
| ebi-fl187-GCST90094766 | ebi-a-GCST90001646 | Natural Killer %CD3- lymphocyte \|\| id:ebi-a-GCST90001646 | Fruit liking \|\| id:ebi-fl187-GCST90094766 | Weighted mode | 9 | -0.324758282 | 0.491197046191763 | 0.527089970825413 | -1.287504492 | 0.637987928671029 | 0.722702022716098 | 0.27595858126667 | 1.89266886081437 | 0.999714303421225 |
| ebi-fl187-GCST90094766 | ebi-a-GCST90001684 | CD127- CD8+ T cell Absolute Count \|\| id:ebi-a-GCST90001684 | Fruit liking \|\| id:ebi-fl187-GCST90094766 | Weighted mode | 9 | 0.325603972771722 | 0.497147338876666 | 0.530879868866615 | -0.648804811 | 1.30001275696999 | 1.38486681529248 | 0.522670092921675 | 3.66934347702528 | 0.999714303421225 |
| ebi-fl187-GCST90094766 | ebi-a-GCST90001687 | CD28- CD8+ T cell Absolute Count \|\| id:ebi-a-GCST90001687 | Fruit liking \|\| id:ebi-fl187-GCST90094766 | Weighted mode | 9 | 0.0601143657951542 | 0.524357738273104 | 0.911552617970011 | -0.967626801 | 1.08785553281044 | 1.06195799127076 | 0.379983745863436 | 2.96790267347154 | 0.999714303421225 |
| ebi-fl187-GCST90094766 | ebi-a-GCST90001701 | BAFF-R on CD20- CD38- B cell \|\| id:ebi-a-GCST90001701 | Fruit liking \|\| id:ebi-fl187-GCST90094766 | Weighted mode | 9 | -0.201368826 | 0.477216156883414 | 0.684161037097522 | -1.136712494 | 0.733974841470933 | 0.817610819791116 | 0.32087215907478 | 2.08334513834747 | 0.999714303421225 |
| ebi-fl187-GCST90094766 | ebi-a-GCST90001732 | CD19 on IgD- CD27- B cell \|\| id:ebi-a-GCST90001732 | Fruit liking \|\| id:ebi-fl187-GCST90094766 | Weighted mode | 9 | 0.727689664421067 | 0.443030565653421 | 0.139106064485536 | -0.140650244 | 1.59602957310177 | 2.07029200888213 | 0.86879312394682 | 4.93340576013072 | 0.999714303421225 |
| ebi-fl187-GCST90094766 | ebi-a-GCST90001798 | CD27 on CD24+ CD27+ B cell \|\| id:ebi-a-GCST90001798 | Fruit liking \|\| id:ebi-fl187-GCST90094766 | Weighted mode | 9 | 0.307663007240467 | 0.533992876964907 | 0.580353163679434 | -0.738963032 | 1.35428904609168 | 1.36024251981072 | 0.477608924179309 | 3.8740057378123 | 0.999714303421225 |
| ebi-fl187-GCST90094766 | ebi-a-GCST90001836 | CD66b on CD66b++ myeloid cell \|\| id:ebi-a-GCST90001836 | Fruit liking \|\| id:ebi-fl187-GCST90094766 | Weighted mode | 9 | -0.158132343 | 0.768643095826871 | 0.842141169841008 | -1.664672811 | 1.34840812490891 | 0.853736788479244 | 0.189252569262601 | 3.85128987597258 | 0.999714303421225 |
| ebi-fl187-GCST90094766 | ebi-a-GCST90001943 | CD25 on activated & secreting CD4 regulatory T cell \|\| id:ebi-a-GCST90001943 | Fruit liking \|\| id:ebi-fl187-GCST90094766 | Weighted mode | 9 | -0.24378223 | 0.498561704047967 | 0.637985078511663 | -1.22096317 | 0.733398710312715 | 0.783658273241065 | 0.294945947113329 | 2.08214520399292 | 0.999714303421225 |
| ebi-fl187-GCST90094766 | ebi-a-GCST90001960 | CD25 on CD4+ T cell \|\| id:ebi-a-GCST90001960 | Fruit liking \|\| id:ebi-fl187-GCST90094766 | Weighted mode | 9 | -0.089220924 | 0.577250730115266 | 0.880995004027747 | -1.220632355 | 1.0421905066747 | 0.914643484233695 | 0.29504353555536 | 2.83542122580817 | 0.999714303421225 |
| ebi-fl187-GCST90094766 | ebi-a-GCST90001988 | HLA DR on CD14+ CD16- monocyte \|\| id:ebi-a-GCST90001988 | Fruit liking \|\| id:ebi-fl187-GCST90094766 | Weighted mode | 9 | -0.506803515 | 0.528380308402987 | 0.365563143409601 | -1.542428919 | 0.528821889745641 | 0.602418125146668 | 0.213861018929748 | 1.6969319575927 | 0.999714303421225 |
| ebi-fl187-GCST90094766 | ebi-a-GCST90001991 | HLA DR on CD14+ monocyte \|\| id:ebi-a-GCST90001991 | Fruit liking \|\| id:ebi-fl187-GCST90094766 | Weighted mode | 9 | -0.390870048 | 0.507745958202001 | 0.46353245616096 | -1.386052126 | 0.604312029659538 | 0.676468058423097 | 0.250060565992191 | 1.82999279494955 | 0.999714303421225 |
| ebi-fl187-GCST90094766 | ebi-a-GCST90002019 | CD14 on Monocytic Myeloid-Derived Suppressor Cells \|\| id:ebi-a-GCST90002019 | Fruit liking \|\| id:ebi-fl187-GCST90094766 | Weighted mode | 9 | 0.323801969945158 | 0.668462848596643 | 0.641086762157427 | -0.986385213 | 1.63398915319458 | 1.38237352850652 | 0.372922292066364 | 5.12427552058349 | 0.999714303421225 |
| ebi-fl187-GCST90094766 | ebi-a-GCST90002037 | CD80 on plasmacytoid Dendritic Cell \|\| id:ebi-a-GCST90002037 | Fruit liking \|\| id:ebi-fl187-GCST90094766 | Weighted mode | 9 | 0.573894299047184 | 0.544039533202524 | 0.322295384761529 | -0.492423186 | 1.64021178412413 | 1.77516663793419 | 0.611143683631832 | 5.1562614109139 | 0.999714303421225 |
| ebi-fl187-GCST90094766 | ebi-a-GCST90002042 | CD45 on CD33+ HLA DR+ CD14- \|\| id:ebi-a-GCST90002042 | Fruit liking \|\| id:ebi-fl187-GCST90094766 | Weighted mode | 9 | 0.577126915892547 | 0.752670072712804 | 0.465239930110565 | -0.898106427 | 2.05236025840964 | 1.78091435659217 | 0.407340258586023 | 7.78625701403954 | 0.999714303421225 |
| ebi-fl187-GCST90094766 | ebi-a-GCST90002056 | CD8 on Effector Memory CD8+ T cell \|\| id:ebi-a-GCST90002056 | Fruit liking \|\| id:ebi-fl187-GCST90094766 | Weighted mode | 9 | 0.734616661062726 | 0.629495728269381 | 0.276818678152923 | -0.499194966 | 1.96842828847071 | 2.08468269926422 | 0.607019133899032 | 7.15941510557789 | 0.999714303421225 |
| ebi-fl187-GCST90094766 | ebi-a-GCST90002058 | CD8 on CD8+ T cell \|\| id:ebi-a-GCST90002058 | Fruit liking \|\| id:ebi-fl187-GCST90094766 | Weighted mode | 9 | -0.253944071 | 0.576955467006888 | 0.671481466592644 | -1.384776786 | 0.87688864442932 | 0.775735187000829 | 0.250379681745424 | 2.40341019748983 | 0.999714303421225 |
| ebi-fl187-GCST90094766 | ebi-a-GCST90002063 | CD4 on CD4 regulatory T cell \|\| id:ebi-a-GCST90002063 | Fruit liking \|\| id:ebi-fl187-GCST90094766 | Weighted mode | 9 | -0.151819728 | 0.551557343491906 | 0.790094852753415 | -1.232872122 | 0.929232664945348 | 0.859143145978304 | 0.291454282291053 | 2.53256510585213 | 0.999714303421225 |
| ebi-fl187-GCST90094766 | ebi-a-GCST90002066 | CD4 on activated CD4 regulatory T cell \|\| id:ebi-a-GCST90002066 | Fruit liking \|\| id:ebi-fl187-GCST90094766 | Weighted mode | 9 | -0.027880965 | 0.523612794422976 | 0.958840526717857 | -1.054162042 | 0.998400112560043 | 0.97250412241732 | 0.348484320363171 | 2.71393636056009 | 0.999714303421225 |
| ebi-fl187-GCST90094766 | ebi-a-GCST90002070 | CD4 on activated & secreting CD4 regulatory T cell \|\| id:ebi-a-GCST90002070 | Fruit liking \|\| id:ebi-fl187-GCST90094766 | Weighted mode | 9 | -0.231236227 | 0.590450436923715 | 0.705559081317946 | -1.388519083 | 0.926046629393372 | 0.793551985505421 | 0.249444437658162 | 2.52450910355664 | 0.999714303421225 |
| ebi-fl187-GCST90094766 | ebi-a-GCST90002081 | SSC-A on CD4+ T cell \|\| id:ebi-a-GCST90002081 | Fruit liking \|\| id:ebi-fl187-GCST90094766 | Weighted mode | 9 | -0.169736161 | 0.511929285932402 | 0.748739097771127 | -1.173117562 | 0.833645239070564 | 0.843887437343476 | 0.309400859841963 | 2.3016936904115 | 0.999714303421225 |
| ebi-fl187-GCST90094766 | ebi-a-GCST90002094 | CD11b on Monocytic Myeloid-Derived Suppressor Cells \|\| id:ebi-a-GCST90002094 | Fruit liking \|\| id:ebi-fl187-GCST90094766 | Weighted mode | 9 | 0.430601534095051 | 0.812436919004508 | 0.610493765047429 | -1.161774827 | 2.02297789534389 | 1.53818251454049 | 0.312930290546992 | 7.56080673399305 | 0.999714303421225 |
| ebi-fl187-GCST90094766 | ebi-a-GCST90002104 | HLA DR on myeloid Dendritic Cell \|\| id:ebi-a-GCST90002104 | Fruit liking \|\| id:ebi-fl187-GCST90094766 | Weighted mode | 9 | -0.018121221 | 0.619604069595366 | 0.977384478209458 | -1.232545197 | 1.19630275566369 | 0.982041981283863 | 0.291549581382264 | 3.30786430366867 | 0.999714303421225 |
| ebi-fl187-GCST90094766 | ebi-a-GCST90002105 | HLA DR on plasmacytoid Dendritic Cell \|\| id:ebi-a-GCST90002105 | Fruit liking \|\| id:ebi-fl187-GCST90094766 | Weighted mode | 9 | -0.315805377 | 0.615777795049752 | 0.621909483979102 | -1.522729855 | 0.891119101538878 | 0.729201355889591 | 0.218115649393105 | 2.43785633406287 | 0.999714303421225 |
| ebi-fl187-GCST90094766 | ebi-a-GCST90002106 | HLA DR on Dendritic Cell \|\| id:ebi-a-GCST90002106 | Fruit liking \|\| id:ebi-fl187-GCST90094766 | Weighted mode | 9 | 0.0950296562644084 | 0.667160190215132 | 0.890255254153488 | -1.212604317 | 1.40266362908607 | 1.09969146738345 | 0.2974216896967 | 4.06601591386687 | 0.999714303421225 |
| ebi-fl187-GCST90094766 | ebi-a-GCST90002111 | HLA DR on CD33dim HLA DR+ CD11b- \|\| id:ebi-a-GCST90002111 | Fruit liking \|\| id:ebi-fl187-GCST90094766 | Weighted mode | 9 | 0.372039722511935 | 0.742149969318917 | 0.629661033751783 | -1.082574217 | 1.82665366237701 | 1.45069060522355 | 0.338722457165911 | 6.21306083361649 | 0.999714303421225 |
| ebi-fl187-GCST90094766 | ebi-a-GCST90002112 | HLA DR on CD33- HLA DR+ \|\| id:ebi-a-GCST90002112 | Fruit liking \|\| id:ebi-fl187-GCST90094766 | Weighted mode | 9 | -0.035346899 | 0.82545344958171 | 0.966893557100084 | -1.653235661 | 1.58254186181429 | 0.965270506444355 | 0.191429504558315 | 4.86731213540546 | 0.999714303421225 |
| ebi-fl187-GCST90094766 | ebi-a-GCST90002115 | HLA DR on HLA DR+ CD8+ T cell \|\| id:ebi-a-GCST90002115 | Fruit liking \|\| id:ebi-fl187-GCST90094766 | Weighted mode | 9 | -0.068911909 | 0.545869241415726 | 0.902655083328525 | -1.138815622 | 1.0009918046614 | 0.933408901834373 | 0.320198032931396 | 2.72097917044457 | 0.999714303421225 |
| ebi-fl187-GCST90094766 | ebi-a-GCST90002116 | HLA DR on B cell \|\| id:ebi-a-GCST90002116 | Fruit liking \|\| id:ebi-fl187-GCST90094766 | Weighted mode | 9 | 0.668987202274633 | 0.499933267838893 | 0.217631893891343 | -0.310882003 | 1.64885640723886 | 1.95225907547297 | 0.732800339237102 | 5.20102856630012 | 0.999714303421225 |
| ebi-fl187-GCST90094766 | ebi-cfmet338-GCST90026023 | Sphingomyelin (d18:1/24:1, d18:2/24:0) levels \|\| id:ebi-cfmet338-GCST90026023 | Fruit liking \|\| id:ebi-fl187-GCST90094766 | Weighted mode | 8 | 0.444894510640399 | 0.430939666478434 | 0.336236858850066 | -0.399747236 | 1.28953625693813 | 1.56032558940351 | 0.670489500456568 | 3.63110226676118 | 0.999714303421225 |
| ebi-fl187-GCST90094766 | ebi-cfmet338-GCST90026046 | 1-arachidonoyl-gpc (20:4n6) levels \|\| id:ebi-cfmet338-GCST90026046 | Fruit liking \|\| id:ebi-fl187-GCST90094766 | Weighted mode | 8 | -0.884998015 | 0.399281020086206 | 0.0621996182872237 | -1.667588814 | -0.102407216 | 0.412714992517124 | 0.188701511915323 | 0.902661898781416 | 0.999714303421225 |
| ebi-fl187-GCST90094766 | ebi-cfmet338-GCST90026047 | 1-linoleoyl-gpc (18:2) levels \|\| id:ebi-cfmet338-GCST90026047 | Fruit liking \|\| id:ebi-fl187-GCST90094766 | Weighted mode | 8 | 0.32755157067138 | 0.529971766098203 | 0.556105746006533 | -0.711193091 | 1.36629623222386 | 1.38756660719438 | 0.491057971032472 | 3.92080202944839 | 0.999714303421225 |
| ebi-fl187-GCST90094766 | ebi-cfmet338-GCST90026050 | 1-myristoyl-2-palmitoyl-gpc (14:0/16:0) levels \|\| id:ebi-cfmet338-GCST90026050 | Fruit liking \|\| id:ebi-fl187-GCST90094766 | Weighted mode | 8 | -0.156656502 | 0.243443193508471 | 0.540398468999785 | -0.633805161 | 0.320492157672194 | 0.854997698720125 | 0.530569054398945 | 1.37780569514151 | 0.999714303421225 |
| ebi-fl187-GCST90094766 | ebi-cfmet338-GCST90026068 | 2,3-dihydroxyisovalerate levels \|\| id:ebi-cfmet338-GCST90026068 | Fruit liking \|\| id:ebi-fl187-GCST90094766 | Weighted mode | 8 | 0.779179712244061 | 1.0325412160826 | 0.47509152989903 | -1.244601071 | 2.80296049576595 | 2.17968356423406 | 0.288055798944411 | 16.4934032142466 | 0.999714303421225 |
| ebi-fl187-GCST90094766 | ebi-cfmet338-GCST90026082 | 3-hydroxyhexanoate levels \|\| id:ebi-cfmet338-GCST90026082 | Fruit liking \|\| id:ebi-fl187-GCST90094766 | Weighted mode | 7 | -0.347083443 | 0.178673409064546 | 0.100086325965834 | -0.697283324 | 0.00311643914055954 | 0.706746353025682 | 0.497936199114631 | 1.00312130028553 | 0.999714303421225 |
| ebi-fl187-GCST90094766 | ebi-cfmet338-GCST90026094 | 3-ureidopropionate levels \|\| id:ebi-cfmet338-GCST90026094 | Fruit liking \|\| id:ebi-fl187-GCST90094766 | Weighted mode | 8 | 0.166558607356874 | 0.36705750627231 | 0.663730088362375 | -0.552874105 | 0.885991319650602 | 1.18123276277183 | 0.575293976741805 | 2.42538753447056 | 0.999714303421225 |
| ebi-fl187-GCST90094766 | ebi-cfmet338-GCST90026173 | Glycerol levels \|\| id:ebi-cfmet338-GCST90026173 | Fruit liking \|\| id:ebi-fl187-GCST90094766 | Weighted mode | 8 | -0.109916954 | 0.205074011004911 | 0.608569443181069 | -0.511862016 | 0.292028107341232 | 0.895908533622715 | 0.599378486902024 | 1.33914065679372 | 0.999714303421225 |
| ebi-fl187-GCST90094766 | ebi-cfmet338-GCST90026179 | Histidine levels \|\| id:ebi-cfmet338-GCST90026179 | Fruit liking \|\| id:ebi-fl187-GCST90094766 | Weighted mode | 8 | -0.026984093 | 0.144504406251237 | 0.85716481957774 | -0.310212729 | 0.256244543121932 | 0.973376724777733 | 0.733290947100338 | 1.29206865581198 | 0.999714303421225 |
| ebi-fl187-GCST90094766 | ebi-cfmet338-GCST90026188 | Isocitrate levels \|\| id:ebi-cfmet338-GCST90026188 | Fruit liking \|\| id:ebi-fl187-GCST90094766 | Weighted mode | 8 | -0.086156438 | 0.325073945701902 | 0.798616439544797 | -0.723301371 | 0.550988495948173 | 0.917450696182993 | 0.485147955731412 | 1.73496717853357 | 0.999714303421225 |
| ebi-fl187-GCST90094766 | ebi-cfmet338-GCST90026207 | N-acetyl-isoputreanine levels \|\| id:ebi-cfmet338-GCST90026207 | Fruit liking \|\| id:ebi-fl187-GCST90094766 | Weighted mode | 8 | -0.161891651 | 0.187419690813432 | 0.416315576676682 | -0.529234245 | 0.205450942835646 | 0.850533353851401 | 0.589055869404143 | 1.22807873342383 | 0.999714303421225 |
| ebi-fl187-GCST90094766 | ebi-cfmet338-GCST90026213 | N-acetylglutamate levels \|\| id:ebi-cfmet338-GCST90026213 | Fruit liking \|\| id:ebi-fl187-GCST90094766 | Weighted mode | 8 | 0.144953059211457 | 0.333918945220955 | 0.677283832803632 | -0.509528073 | 0.799434191844529 | 1.15598530613262 | 0.600779035516225 | 2.22428205545869 | 0.999714303421225 |
| ebi-fl187-GCST90094766 | ebi-cfmet338-GCST90026224 | N-acetyltaurine levels \|\| id:ebi-cfmet338-GCST90026224 | Fruit liking \|\| id:ebi-fl187-GCST90094766 | Weighted mode | 8 | -0.208939602 | 0.230866044577055 | 0.39553225068133 | -0.661437049 | 0.243557845505432 | 0.811444243881897 | 0.516109127098033 | 1.27578011385177 | 0.999714303421225 |
| ebi-fl187-GCST90094766 | ebi-cfmet338-GCST90026250 | Phenyllactate (pla) levels \|\| id:ebi-cfmet338-GCST90026250 | Fruit liking \|\| id:ebi-fl187-GCST90094766 | Weighted mode | 8 | 0.114353328527094 | 0.404816140325551 | 0.785743900275973 | -0.679086307 | 0.907792963565175 | 1.12114818854785 | 0.507080096548358 | 2.47884558916868 | 0.999714303421225 |
| ebi-fl187-GCST90094766 | ebi-cfmet338-GCST90026251 | Pipecolate levels \|\| id:ebi-cfmet338-GCST90026251 | Fruit liking \|\| id:ebi-fl187-GCST90094766 | Weighted mode | 8 | 0.299680478728313 | 0.561662287803815 | 0.610161518084983 | -0.801177605 | 1.40053856282379 | 1.34942756787201 | 0.448800143349654 | 4.05738453500064 | 0.999714303421225 |
| ebi-fl187-GCST90094766 | ebi-cfmet338-GCST90026278 | Trigonelline (n'-methylnicotinate) levels \|\| id:ebi-cfmet338-GCST90026278 | Fruit liking \|\| id:ebi-fl187-GCST90094766 | Weighted mode | 8 | -0.897557085 | 0.678433468043177 | 0.22741843527767 | -2.227286683 | 0.432172511953145 | 0.407564088851771 | 0.107820585052585 | 1.54060086430213 | 0.999714303421225 |
| ebi-fl187-GCST90094766 | ebi-cfmet338-GCST90026299 | 1-carboxyethylvaline levels \|\| id:ebi-cfmet338-GCST90026299 | Fruit liking \|\| id:ebi-fl187-GCST90094766 | Weighted mode | 8 | 0.371899329036719 | 0.39933292973274 | 0.382694109985952 | -0.410793213 | 1.15459187131289 | 1.45048695202414 | 0.663124042696491 | 3.17272827182838 | 0.999714303421225 |
| ebi-fl187-GCST90094766 | ebi-gm430-GCST90011306 | C_Erysipelotrichia abundance \|\| id:ebi-gm430-GCST90011306 | Fruit liking \|\| id:ebi-fl187-GCST90094766 | Weighted mode | 8 | -0.300920707 | 0.394928266059258 | 0.470970432843581 | -1.074980108 | 0.473138694839244 | 0.740136458328921 | 0.341304544424883 | 1.60502397608199 | 0.999714303421225 |
| ebi-fl187-GCST90094766 | ebi-gm430-GCST90011312 | F_Erysipelotrichaceae abundance \|\| id:ebi-gm430-GCST90011312 | Fruit liking \|\| id:ebi-fl187-GCST90094766 | Weighted mode | 8 | -0.300920707 | 0.38018786090341 | 0.45462852717143 | -1.046088914 | 0.444247500733782 | 0.740136458328921 | 0.351309065660355 | 1.55931636981236 | 0.999714303421225 |
| ebi-fl187-GCST90094766 | ebi-gm430-GCST90011352 | O_Erysipelotrichales abundance \|\| id:ebi-gm430-GCST90011352 | Fruit liking \|\| id:ebi-fl187-GCST90094766 | Weighted mode | 8 | -0.300920707 | 0.396498398016736 | 0.47266171758527 | -1.078057567 | 0.4762161534759 | 0.740136458328921 | 0.340255808355543 | 1.6099709791736 | 0.999714303421225 |
| ebi-fl187-GCST90094766 | ebi-gm430-GCST90011362 | OTU97_11 (Parabacteroides) abundance \|\| id:ebi-gm430-GCST90011362 | Fruit liking \|\| id:ebi-fl187-GCST90094766 | Weighted mode | 8 | -1.002487343 | 0.580733062256003 | 0.127948095132428 | -2.140724145 | 0.135749458684651 | 0.366965535764031 | 0.117569674654116 | 1.14539488890104 | 0.999714303421225 |
| ebi-fl187-GCST90094766 | ebi-gm430-GCST90011384 | OTU97_235 (Phascolarctobacterium) abundance \|\| id:ebi-gm430-GCST90011384 | Fruit liking \|\| id:ebi-fl187-GCST90094766 | Weighted mode | 8 | 2.86683891414268 | 1.38997927719012 | 0.0780758770909448 | 0.142479530850058 | 5.59119829743531 | 17.5813540968967 | 1.15312947713563 | 268.056638919924 | 0.999714303421225 |
| ebi-fl187-GCST90094766 | ebi-gm430-GCST90011390 | OTU97_3 (Bacteroides) abundance \|\| id:ebi-gm430-GCST90011390 | Fruit liking \|\| id:ebi-fl187-GCST90094766 | Weighted mode | 8 | -0.121707289 | 0.358176195001251 | 0.743976985496605 | -0.823732632 | 0.580318052790912 | 0.885407498725677 | 0.438790749740254 | 1.78660657560289 | 0.999714303421225 |
| ebi-fl187-GCST90094766 | ebi-gm430-GCST90011398 | OTU97_4 (Alistipes) abundance \|\| id:ebi-gm430-GCST90011398 | Fruit liking \|\| id:ebi-fl187-GCST90094766 | Weighted mode | 8 | -0.03408286 | 0.398517706571364 | 0.93423952304369 | -0.815177565 | 0.747011844628525 | 0.966491417597748 | 0.442560737028762 | 2.11068353365788 | 0.999714303421225 |
| ebi-fl187-GCST90094766 | ebi-gm430-GCST90011421 | OTU99_108 (Cytophagales) abundance \|\| id:ebi-gm430-GCST90011421 | Fruit liking \|\| id:ebi-fl187-GCST90094766 | Weighted mode | 8 | -0.337015555 | 1.34274269040211 | 0.809029719436511 | -2.968791229 | 2.29476011781412 | 0.713897734870081 | 0.0513653618035841 | 9.92205560240151 | 0.999714303421225 |
| ebi-fl187-GCST90094766 | ebi-gm430-GCST90011428 | OTU99_123 (Phascolarctobacterium) abundance \|\| id:ebi-gm430-GCST90011428 | Fruit liking \|\| id:ebi-fl187-GCST90094766 | Weighted mode | 8 | 0.103401223791249 | 0.701189322978882 | 0.886922235241215 | -1.270929849 | 1.47773229682986 | 1.10893625143643 | 0.280570612074879 | 4.3829950708511 | 0.999714303421225 |
| ebi-fl187-GCST90094766 | ebi-gm430-GCST90011450 | OTU99_298 (Phascolarctobacterium) abundance \|\| id:ebi-gm430-GCST90011450 | Fruit liking \|\| id:ebi-fl187-GCST90094766 | Weighted mode | 8 | 2.8501823605307 | 1.40415103242426 | 0.0819338027636414 | 0.0980463369791544 | 5.60231838408224 | 17.2909347371119 | 1.1030138942562 | 271.054087024599 | 0.999714303421225 |
| ebi-fl187-GCST90094766 | ebi-gm430-GCST90011451 | OTU99_3 (Bacteroides) abundance \|\| id:ebi-gm430-GCST90011451 | Fruit liking \|\| id:ebi-fl187-GCST90094766 | Weighted mode | 8 | -0.143282549 | 0.376485519803283 | 0.714801401141926 | -0.881194168 | 0.594629069917415 | 0.866509203084247 | 0.41428788695129 | 1.81235856195326 | 0.999714303421225 |
| ebi-fl187-GCST90094766 | ebi-gm430-GCST90011472 | OTU99_72 (Parabacteroides) abundance \|\| id:ebi-gm430-GCST90011472 | Fruit liking \|\| id:ebi-fl187-GCST90094766 | Weighted mode | 8 | -0.589300309 | 1.34603476059165 | 0.674718758895323 | -3.22752844 | 2.04892782196317 | 0.554715278381555 | 0.0396553884579504 | 7.75957699660952 | 0.999714303421225 |
| ebi-fl187-GCST90094766 | ebi-gm430-GCST90011507 | TestASV_3 (Bacteroides) abundance \|\| id:ebi-gm430-GCST90011507 | Fruit liking \|\| id:ebi-fl187-GCST90094766 | Weighted mode | 8 | -0.100146307 | 0.807679102376153 | 0.904806410899003 | -1.683197348 | 1.48290473376791 | 0.904705043771832 | 0.185779025303357 | 4.4057245692278 | 0.999714303421225 |
| ebi-fl187-GCST90094766 | ebi-gm430-GCST90011512 | TestASV_34 (Phascolarctobacterium) abundance \|\| id:ebi-gm430-GCST90011512 | Fruit liking \|\| id:ebi-fl187-GCST90094766 | Weighted mode | 8 | 0.0891060881333683 | 1.09427663620019 | 0.937379972504577 | -2.055676119 | 2.23388829508574 | 1.09319662536414 | 0.128006258872187 | 9.33609709585237 | 0.999714303421225 |
| ebi-fl187-GCST90094766 | ebi-gm430-GCST90011519 | TestASV_41 (Barnesiella) abundance \|\| id:ebi-gm430-GCST90011519 | Fruit liking \|\| id:ebi-fl187-GCST90094766 | Weighted mode | 8 | -0.920171297 | 1.54382523915041 | 0.569915851522001 | -3.946068766 | 2.10572617131165 | 0.398450781646202 | 0.0193305454926412 | 8.21306493678139 | 0.999714303421225 |
| ebi-fl187-GCST90094766 | ebi-gm430-GCST90011524 | TestASV_46 (Sutterella) abundance \|\| id:ebi-gm430-GCST90011524 | Fruit liking \|\| id:ebi-fl187-GCST90094766 | Weighted mode | 8 | -1.8731512 | 1.92568350353717 | 0.363097712991262 | -5.647490867 | 1.90118846707424 | 0.153638751779294 | 0.00352635377503256 | 6.69384513131602 | 0.999714303421225 |
| ebi-fl187-GCST90094766 | ebi-gm430-GCST90011581 | OTU97_19 (Bacteria) prevalence \|\| id:ebi-gm430-GCST90011581 | Fruit liking \|\| id:ebi-fl187-GCST90094766 | Weighted mode | 8 | -1.269564405 | 1.97457300083837 | 0.540733140167056 | -5.139727487 | 2.60059867663013 | 0.280953977271782 | 0.00585928622947197 | 13.4718008735932 | 0.999714303421225 |
| ebi-fl187-GCST90094766 | ebi-gm430-GCST90011614 | OTU97_80 (Ruminococcus) prevalence \|\| id:ebi-gm430-GCST90011614 | Fruit liking \|\| id:ebi-fl187-GCST90094766 | Weighted mode | 8 | 0.0823348340876873 | 0.784549644561318 | 0.919362994869367 | -1.455382469 | 1.62005213742787 | 1.08581931828947 | 0.233311112507483 | 5.05335377856383 | 0.999714303421225 |
| ebi-fl187-GCST90094766 | ebi-gm430-GCST90011623 | OTU99_11 (Parabacteroides) prevalence \|\| id:ebi-gm430-GCST90011623 | Fruit liking \|\| id:ebi-fl187-GCST90094766 | Weighted mode | 8 | 0.778107478339112 | 0.632728451748309 | 0.258509833281264 | -0.462040287 | 2.0182552437658 | 2.17734768614207 | 0.629996958688002 | 7.52518386155585 | 0.999714303421225 |
| ebi-fl187-GCST90094766 | ebi-gm430-GCST90011642 | OTU99_19 (Bacteria) prevalence \|\| id:ebi-gm430-GCST90011642 | Fruit liking \|\| id:ebi-fl187-GCST90094766 | Weighted mode | 8 | -1.269564405 | 2.11384877540798 | 0.567039475784275 | -5.412708005 | 2.87357919478657 | 0.280953977271782 | 0.0044595473692626 | 17.700257629035 | 0.999714303421225 |
| ebi-fl187-GCST90094766 | ebi-gm430-GCST90011653 | OTU99_30 (Parasutterella) prevalence \|\| id:ebi-gm430-GCST90011653 | Fruit liking \|\| id:ebi-fl187-GCST90094766 | Weighted mode | 8 | 0.570264201085001 | 0.868901517702318 | 0.532600899539563 | -1.132782774 | 2.27331117578154 | 1.76873429122721 | 0.322135577598391 | 9.71150413216151 | 0.999714303421225 |
| ebi-fl187-GCST90094766 | ebi-gm430-GCST90011675 | OTU99_8 (Ruminococcaceae) prevalence \|\| id:ebi-gm430-GCST90011675 | Fruit liking \|\| id:ebi-fl187-GCST90094766 | Weighted mode | 8 | 0.291955515505438 | 0.836600913792104 | 0.737367283633843 | -1.347782276 | 1.93169330653796 | 1.33904344964332 | 0.259815822098752 | 6.90118617699574 | 0.999714303421225 |
| ebi-fl187-GCST90094766 | ebi-gm430-GCST90011679 | OTU99_92 (Ruminococcus) prevalence \|\| id:ebi-gm430-GCST90011679 | Fruit liking \|\| id:ebi-fl187-GCST90094766 | Weighted mode | 8 | 0.0178370214552444 | 0.761954165446855 | 0.981976910530801 | -1.475593143 | 1.51126718573108 | 1.01799705119107 | 0.228643068822099 | 4.53247063894179 | 0.999714303421225 |
| ebi-fl187-GCST90094766 | ebi-gm430-GCST90011684 | TestASV_11 (Lachnospiraceae) prevalence \|\| id:ebi-gm430-GCST90011684 | Fruit liking \|\| id:ebi-fl187-GCST90094766 | Weighted mode | 8 | 0.605828898783086 | 0.917392095848755 | 0.530132316812669 | -1.192259609 | 2.40391740664665 | 1.83277076088925 | 0.303534619002814 | 11.0664433368617 | 0.999714303421225 |
| ebi-fl187-GCST90094766 | ebi-gm430-GCST90011688 | TestASV_15 (Bacteroides) prevalence \|\| id:ebi-gm430-GCST90011688 | Fruit liking \|\| id:ebi-fl187-GCST90094766 | Weighted mode | 8 | -0.604859693 | 0.742599305452875 | 0.442173401157053 | -2.060354332 | 0.850634945212843 | 0.546151049820256 | 0.127408816854118 | 2.34113287121486 | 0.999714303421225 |
| ebi-fl187-GCST90094766 | ebi-gm430-GCST90011692 | TestASV_19 (Prevotella) prevalence \|\| id:ebi-gm430-GCST90011692 | Fruit liking \|\| id:ebi-fl187-GCST90094766 | Weighted mode | 8 | 0.437061860190363 | 1.42122283345755 | 0.767391479921326 | -2.348534893 | 3.22265861376716 | 1.54815184306198 | 0.0955089906125846 | 25.0947488168763 | 0.999714303421225 |
| ebi-fl187-GCST90094766 | ebi-gm430-GCST90011693 | TestASV_2 (Bacteroides) prevalence \|\| id:ebi-gm430-GCST90011693 | Fruit liking \|\| id:ebi-fl187-GCST90094766 | Weighted mode | 8 | 0.292605727142669 | 0.770549266 | 0.715399276939103 | -1.217670834 | 1.80288228836547 | 1.33991439439556 | 0.295918608403313 | 6.06710944605917 | 0.999714303421225 |
| ebi-fl187-GCST90094766 | ebi-gm430-GCST90011716 | TestASV_40 (Barnesiella) prevalence \|\| id:ebi-gm430-GCST90011716 | Fruit liking \|\| id:ebi-fl187-GCST90094766 | Weighted mode | 8 | -1.651938044 | 1.48356421516023 | 0.302265503201631 | -4.559723906 | 1.25584781729869 | 0.191678067806273 | 0.0104649478525614 | 3.5108136414606 | 0.999714303421225 |
| ebi-fl187-GCST90094766 | ebi-gm430-GCST90011718 | TestASV_42 (Alphaproteobacteria) prevalence \|\| id:ebi-gm430-GCST90011718 | Fruit liking \|\| id:ebi-fl187-GCST90094766 | Weighted mode | 8 | 1.05832264176909 | 1.21904489786785 | 0.414078495291656 | -1.331005358 | 3.44765064159007 | 2.88153356930892 | 0.264211500570363 | 31.4264734620926 | 0.999714303421225 |
| ebi-fl187-GCST90094766 | ebi-hbsm44-GCST90104929 | Sulcal depth of the right inferior frontal sulcus \|\| id:ebi-hbsm44-GCST90104929 | Fruit liking \|\| id:ebi-fl187-GCST90094766 | Weighted mode | 8 | -0.380442958 | 0.661369293098545 | 0.583141124051403 | -1.676726773 | 0.915840856417444 | 0.683558554373107 | 0.186985019954993 | 2.4988755643052 | 0.999714303421225 |
| ebi-fl187-GCST90094766 | ebi-hbsm44-GCST90104935 | Sulcal opening distance of the left calloso-marginal posterior fissure \|\| id:ebi-hbsm44-GCST90104935 | Fruit liking \|\| id:ebi-fl187-GCST90094766 | Weighted mode | 9 | -0.078913485 | 0.141952828523454 | 0.593472543048176 | -0.357141029 | 0.199314058600182 | 0.924119870934813 | 0.699673816329489 | 1.22056523472133 | 0.999714303421225 |
| ebi-fl187-GCST90094766 | ebi-hbsm44-GCST90104936 | Sulcal opening distance of the left anterior inferior temporal sulcus \|\| id:ebi-hbsm44-GCST90104936 | Fruit liking \|\| id:ebi-fl187-GCST90094766 | Weighted mode | 9 | -0.072467243 | 0.0664448116828388 | 0.307188633441052 | -0.202699074 | 0.0577645879916087 | 0.930096213409664 | 0.816523917894154 | 1.05946555543659 | 0.999714303421225 |
| ebi-fl187-GCST90094766 | ebi-hbsm44-GCST90104941 | Sulcal opening distance of the right posterior inferior temporal sulcus \|\| id:ebi-hbsm44-GCST90104941 | Fruit liking \|\| id:ebi-fl187-GCST90094766 | Weighted mode | 9 | -0.037077918 | 0.0853786448222858 | 0.675562786987194 | -0.204420062 | 0.13026422545755 | 0.963601050177422 | 0.815119898167307 | 1.13912933053248 | 0.999714303421225 |
| ebi-fl187-GCST90094766 | ebi-IPs91-GCST90274819 | Leukemia inhibitory factor levels \|\| id:ebi-IPs91-GCST90274819 | Fruit liking \|\| id:ebi-fl187-GCST90094766 | Weighted mode | 10 | 0.125592055299358 | 0.298896232244644 | 0.684203518126782 | -0.46024456 | 0.71142867049886 | 1.13381953825359 | 0.631129277718473 | 2.03689923873097 | 0.999714303421225 |
| ebi-fl187-GCST90094766 | ebi-IPs91-GCST90274837 | STAM binding protein levels \|\| id:ebi-IPs91-GCST90274837 | Fruit liking \|\| id:ebi-fl187-GCST90094766 | Weighted mode | 10 | -0.354878088 | 0.302744816735506 | 0.271209372102701 | -0.948257929 | 0.238501752668724 | 0.701258929783026 | 0.387415340985313 | 1.26934593077737 | 0.999714303421225 |
| ebi-fl187-GCST90094766 | ebi-IPs91-GCST90274840 | TNF-beta levels \|\| id:ebi-IPs91-GCST90274840 | Fruit liking \|\| id:ebi-fl187-GCST90094766 | Weighted mode | 10 | 0.145081504451091 | 0.256562837749602 | 0.585565582567087 | -0.357781658 | 0.647944666440312 | 1.15613379647851 | 0.699225729007569 | 1.91160779689408 | 0.999714303421225 |
| ebi-fl187-GCST90094766 | ebi-IPs91-GCST90274842 | Tumor necrosis factor ligand superfamily member 14 levels \|\| id:ebi-IPs91-GCST90274842 | Fruit liking \|\| id:ebi-fl187-GCST90094766 | Weighted mode | 10 | -0.326074912 | 0.293796582820691 | 0.295829306720976 | -0.901916214 | 0.249766390546016 | 0.721751117743733 | 0.405791331181706 | 1.28372549124532 | 0.999714303421225 |
| ebi-fl187-GCST90094766 | ebi-met1400-GCST90199628 | 3-methylhistidine levels \|\| id:ebi-met1400-GCST90199628 | Fruit liking \|\| id:ebi-fl187-GCST90094766 | Weighted mode | 9 | -0.240576343 | 0.325368252577262 | 0.480796296389977 | -0.878298118 | 0.397145431953187 | 0.78617462415047 | 0.4154894242207 | 1.48757225485919 | 0.999714303421225 |
| ebi-fl187-GCST90094766 | ebi-met1400-GCST90199651 | Docosahexaenoate DHA; 22:6n3 levels \|\| id:ebi-met1400-GCST90199651 | Fruit liking \|\| id:ebi-fl187-GCST90094766 | Weighted mode | 9 | 0.104177092149825 | 0.305517933022424 | 0.741897128428385 | -0.494638057 | 0.702992240873777 | 1.10979697384606 | 0.609791577411449 | 2.01978736470285 | 0.999714303421225 |
| ebi-fl187-GCST90094766 | ebi-met1400-GCST90199652 | 1-stearoyl-2-oleoyl-GPS (18:0/18:1) levels \|\| id:ebi-met1400-GCST90199652 | Fruit liking \|\| id:ebi-fl187-GCST90094766 | Weighted mode | 9 | 0.192629590046331 | 0.301253862174318 | 0.540419204543315 | -0.39782798 | 0.783087159907994 | 1.2124336128554 | 0.671777577026169 | 2.18821722524437 | 0.999714303421225 |
| ebi-fl187-GCST90094766 | ebi-met1400-GCST90199653 | 1-myristoyl-2-palmitoyl-gpc (14:0/16:0) levels \|\| id:ebi-met1400-GCST90199653 | Fruit liking \|\| id:ebi-fl187-GCST90094766 | Weighted mode | 9 | -0.024493595 | 0.314881824866472 | 0.939908143248601 | -0.641661971 | 0.592674781981439 | 0.975803939159892 | 0.526416806891613 | 1.8088201501439 | 0.999714303421225 |
| ebi-fl187-GCST90094766 | ebi-met1400-GCST90199667 | Erythritol levels in elite athletes \|\| id:ebi-met1400-GCST90199667 | Fruit liking \|\| id:ebi-fl187-GCST90094766 | Weighted mode | 9 | 0.367681314323239 | 0.314005022888275 | 0.275316731117578 | -0.247768531 | 0.983131159184259 | 1.44438166187257 | 0.780540593681162 | 2.67281215358026 | 0.999714303421225 |
| ebi-fl187-GCST90094766 | ebi-met1400-GCST90199674 | 3-hydroxymyristate levels \|\| id:ebi-met1400-GCST90199674 | Fruit liking \|\| id:ebi-fl187-GCST90094766 | Weighted mode | 9 | 0.16156188633787 | 0.365692412036544 | 0.670334557777726 | -0.555195241 | 0.878319013929497 | 1.17534519371746 | 0.573960189549274 | 2.40685042194225 | 0.999714303421225 |
| ebi-fl187-GCST90094766 | ebi-met1400-GCST90199685 | 2-linoleoylglycerol (18:2) levels \|\| id:ebi-met1400-GCST90199685 | Fruit liking \|\| id:ebi-fl187-GCST90094766 | Weighted mode | 9 | -0.288022875 | 0.413018892169939 | 0.505335078610482 | -1.097539904 | 0.521494153473809 | 0.749744441504372 | 0.333690986681991 | 1.68454273564902 | 0.999714303421225 |
| ebi-fl187-GCST90094766 | ebi-met1400-GCST90199703 | Gamma-glutamylvaline levels \|\| id:ebi-met1400-GCST90199703 | Fruit liking \|\| id:ebi-fl187-GCST90094766 | Weighted mode | 9 | 0.189800902678913 | 0.341769868811153 | 0.593841934906375 | -0.48006804 | 0.859669845548772 | 1.20900886326741 | 0.618741291098294 | 2.3623806144642 | 0.999714303421225 |
| ebi-fl187-GCST90094766 | ebi-met1400-GCST90199779 | Gamma-glutamyl-2-aminobutyrate levels \|\| id:ebi-met1400-GCST90199779 | Fruit liking \|\| id:ebi-fl187-GCST90094766 | Weighted mode | 9 | -0.869922483 | 0.532522985352979 | 0.14098754144346 | -1.913667534 | 0.173822568242297 | 0.418984026352874 | 0.14753829137639 | 1.18984443090113 | 0.999714303421225 |
| ebi-fl187-GCST90094766 | ebi-met1400-GCST90199824 | 4-vinylphenol sulfate levels \|\| id:ebi-met1400-GCST90199824 | Fruit liking \|\| id:ebi-fl187-GCST90094766 | Weighted mode | 9 | -0.102399601 | 0.435186375952461 | 0.81988683751193 | -0.955364898 | 0.750565695858903 | 0.902668772238561 | 0.384671753035097 | 2.11819793355175 | 0.999714303421225 |
| ebi-fl187-GCST90094766 | ebi-met1400-GCST90199830 | 4-ethylphenylsulfate levels \|\| id:ebi-met1400-GCST90199830 | Fruit liking \|\| id:ebi-fl187-GCST90094766 | Weighted mode | 9 | 0.318072796822035 | 0.333349110130344 | 0.367934005545949 | -0.335291459 | 0.97143705267751 | 1.37447631509342 | 0.715129624985779 | 2.64173804964428 | 0.999714303421225 |
| ebi-fl187-GCST90094766 | ebi-met1400-GCST90199833 | Hexanoylglutamine levels \|\| id:ebi-met1400-GCST90199833 | Fruit liking \|\| id:ebi-fl187-GCST90094766 | Weighted mode | 9 | 0.508818552336529 | 0.324699664890319 | 0.1557395226768 | -0.127592791 | 1.14522989552155 | 1.66332490231925 | 0.880211736450849 | 3.14316387308229 | 0.999714303421225 |
| ebi-fl187-GCST90094766 | ebi-met1400-GCST90199837 | Carotenoid levels (cryptoxanthin) \|\| id:ebi-met1400-GCST90199837 | Fruit liking \|\| id:ebi-fl187-GCST90094766 | Weighted mode | 9 | 0.321106787112204 | 0.283749877167465 | 0.29055883433827 | -0.235042972 | 0.877256546360435 | 1.37865279538406 | 0.790536877840572 | 2.40429457941568 | 0.999714303421225 |
| ebi-fl187-GCST90094766 | ebi-met1400-GCST90199869 | 2-hydroxyhippurate levels \|\| id:ebi-met1400-GCST90199869 | Fruit liking \|\| id:ebi-fl187-GCST90094766 | Weighted mode | 9 | -0.323980883 | 0.354004831570689 | 0.386846895075288 | -1.017830352 | 0.369868587270642 | 0.723264069170472 | 0.361378153429528 | 1.44754437640636 | 0.999714303421225 |
| ebi-fl187-GCST90094766 | ebi-met1400-GCST90199902 | Hydantoin-5-propionate levels \|\| id:ebi-met1400-GCST90199902 | Fruit liking \|\| id:ebi-fl187-GCST90094766 | Weighted mode | 9 | -0.14414608 | 0.374148804746795 | 0.710083176039309 | -0.877477737 | 0.589185577368699 | 0.865761268471525 | 0.415830423668623 | 1.8025198045219 | 0.999714303421225 |
| ebi-fl187-GCST90094766 | ebi-met1400-GCST90199904 | Pantoate levels \|\| id:ebi-met1400-GCST90199904 | Fruit liking \|\| id:ebi-fl187-GCST90094766 | Weighted mode | 9 | -0.129499436 | 0.336706815345473 | 0.710551814137838 | -0.789444794 | 0.530445921704909 | 0.878535083582887 | 0.454096842394336 | 1.69969006834833 | 0.999714303421225 |
| ebi-fl187-GCST90094766 | ebi-met1400-GCST90199936 | 3-methyl catechol sulfate (2) levels \|\| id:ebi-met1400-GCST90199936 | Fruit liking \|\| id:ebi-fl187-GCST90094766 | Weighted mode | 9 | -0.248386192 | 0.48589754780023 | 0.623023666054533 | -1.200745386 | 0.703973001383648 | 0.780058632458669 | 0.300969789616403 | 2.02176926411396 | 0.999714303421225 |
| ebi-fl187-GCST90094766 | ebi-met1400-GCST90199977 | 4-hydroxychlorothalonil levels \|\| id:ebi-met1400-GCST90199977 | Fruit liking \|\| id:ebi-fl187-GCST90094766 | Weighted mode | 9 | -0.154028073 | 0.329231355156413 | 0.652378409891714 | -0.799321529 | 0.491265383170949 | 0.857247955207003 | 0.449633924211463 | 1.6343830328091 | 0.999714303421225 |
| ebi-fl187-GCST90094766 | ebi-met1400-GCST90200040 | Sphingomyelin (d18:2/24:1, d18:1/24:2) levels \|\| id:ebi-met1400-GCST90200040 | Fruit liking \|\| id:ebi-fl187-GCST90094766 | Weighted mode | 9 | 0.0490758595820667 | 0.276517727051081 | 0.863544158636799 | -0.492898885 | 0.591050604602186 | 1.05030002303883 | 0.610853032080015 | 1.80588468987229 | 0.999714303421225 |
| ebi-fl187-GCST90094766 | ebi-met1400-GCST90200077 | 1-stearoyl-2-oleoyl-GPI (18:0/18:1) levels \|\| id:ebi-met1400-GCST90200077 | Fruit liking \|\| id:ebi-fl187-GCST90094766 | Weighted mode | 9 | 0.659751353540032 | 0.359127457873372 | 0.103505350404167 | -0.044138464 | 1.36364117097184 | 1.93431131494168 | 0.956821463076899 | 3.91040586723409 | 0.999714303421225 |
| ebi-fl187-GCST90094766 | ebi-met1400-GCST90200080 | 1-palmitoyl-2-oleoyl-GPI (16:0/18:1) levels \|\| id:ebi-met1400-GCST90200080 | Fruit liking \|\| id:ebi-fl187-GCST90094766 | Weighted mode | 9 | 0.472799706732286 | 0.352327407032956 | 0.216456684218479 | -0.217762011 | 1.16336142451688 | 1.60447998425131 | 0.804316837408366 | 3.20067403805451 | 0.999714303421225 |
| ebi-fl187-GCST90094766 | ebi-met1400-GCST90200082 | 1-oleoyl-2-linoleoyl-GPE (18:1/18:2) levels \|\| id:ebi-met1400-GCST90200082 | Fruit liking \|\| id:ebi-fl187-GCST90094766 | Weighted mode | 9 | 0.254162808903679 | 0.341229255784665 | 0.477673836279737 | -0.414646532 | 0.922972150241622 | 1.28938171000271 | 0.660573730832655 | 2.51675947209393 | 0.999714303421225 |
| ebi-fl187-GCST90094766 | ebi-met1400-GCST90200100 | Perfluorooctanesulfonate (PFOS) levels \|\| id:ebi-met1400-GCST90200100 | Fruit liking \|\| id:ebi-fl187-GCST90094766 | Weighted mode | 9 | 0.175823289577482 | 0.334673645141186 | 0.613580081148559 | -0.480137055 | 0.831783634054207 | 1.19222736103428 | 0.618698590322086 | 2.29741283176158 | 0.999714303421225 |
| ebi-fl187-GCST90094766 | ebi-met1400-GCST90200102 | 1-palmitoleoyl-2-linolenoyl-GPC (16:1/18:3) levels \|\| id:ebi-met1400-GCST90200102 | Fruit liking \|\| id:ebi-fl187-GCST90094766 | Weighted mode | 9 | 0.164115080271418 | 0.307992305499001 | 0.608613337315944 | -0.439549839 | 0.767779999049459 | 1.17834991211801 | 0.644326406745537 | 2.15497688881296 | 0.999714303421225 |
| ebi-fl187-GCST90094766 | ebi-met1400-GCST90200114 | Glycosyl-N-tricosanoyl-sphingadienine (d18:2/23:0) levels \|\| id:ebi-met1400-GCST90200114 | Fruit liking \|\| id:ebi-fl187-GCST90094766 | Weighted mode | 9 | -0.196860951 | 0.317721537968293 | 0.552753146277328 | -0.819595166 | 0.425873263023051 | 0.821304826663851 | 0.440609992393133 | 1.53092673781098 | 0.999714303421225 |
| ebi-fl187-GCST90094766 | ebi-met1400-GCST90200135 | Arachidoylcarnitine (C20) levels \|\| id:ebi-met1400-GCST90200135 | Fruit liking \|\| id:ebi-fl187-GCST90094766 | Weighted mode | 9 | 0.303137765205392 | 0.355938450025984 | 0.419166222467569 | -0.394501597 | 1.00077712725632 | 1.3541009995953 | 0.674015887192414 | 2.72039510039256 | 0.999714303421225 |
| ebi-fl187-GCST90094766 | ebi-met1400-GCST90200146 | Docosahexaenoylcarnitine (C22:6) levels \|\| id:ebi-met1400-GCST90200146 | Fruit liking \|\| id:ebi-fl187-GCST90094766 | Weighted mode | 9 | -0.339164883 | 0.448638780596062 | 0.471329022177649 | -1.218496893 | 0.540167127019258 | 0.712364982566237 | 0.295674263157426 | 1.71629367726337 | 0.999714303421225 |
| ebi-fl187-GCST90094766 | ebi-met1400-GCST90200162 | 3-hydroxyphenylacetoylglutamine levels \|\| id:ebi-met1400-GCST90200162 | Fruit liking \|\| id:ebi-fl187-GCST90094766 | Weighted mode | 9 | -0.4823287 | 0.406039003684368 | 0.268945986357064 | -1.278165147 | 0.313507747435499 | 0.617344107535051 | 0.278547926344497 | 1.36821606288644 | 0.999714303421225 |
| ebi-fl187-GCST90094766 | ebi-met1400-GCST90200172 | 3-carboxy-4-methyl-5-pentyl-2-furanpropionate (3-CMPFP) levels \|\| id:ebi-met1400-GCST90200172 | Fruit liking \|\| id:ebi-fl187-GCST90094766 | Weighted mode | 9 | -0.273857143 | 0.35402766913498 | 0.461442399710414 | -0.967751375 | 0.420037088353507 | 0.760440701664134 | 0.37993641293106 | 1.52201800371361 | 0.999714303421225 |
| ebi-fl187-GCST90094766 | ebi-met1400-GCST90200222 | Dihydrocaffeate sulfate (2) levels \|\| id:ebi-met1400-GCST90200222 | Fruit liking \|\| id:ebi-fl187-GCST90094766 | Weighted mode | 9 | 0.0790881131165353 | 0.353972486584199 | 0.828800537746457 | -0.614697961 | 0.772874186821566 | 1.08229968266706 | 0.540804214830396 | 2.16598275490248 | 0.999714303421225 |
| ebi-fl187-GCST90094766 | ebi-met1400-GCST90200223 | 4-methylhexanoylglutamine levels \|\| id:ebi-met1400-GCST90200223 | Fruit liking \|\| id:ebi-fl187-GCST90094766 | Weighted mode | 9 | -0.037776596 | 0.44767342960091 | 0.934824377282229 | -0.915216518 | 0.839663326477278 | 0.962928039281483 | 0.400429916596287 | 2.31558724861538 | 0.999714303421225 |
| ebi-fl187-GCST90094766 | ebi-met1400-GCST90200224 | 2-ketocaprylate levels \|\| id:ebi-met1400-GCST90200224 | Fruit liking \|\| id:ebi-fl187-GCST90094766 | Weighted mode | 9 | -0.406559233 | 0.352354594254966 | 0.28187142027662 | -1.097174238 | 0.284055771833681 | 0.665937649025608 | 0.333813028504507 | 1.32850702195935 | 0.999714303421225 |
| ebi-fl187-GCST90094766 | ebi-met1400-GCST90200243 | Hydroxypalmitoyl sphingomyelin (d18:1/16:0(OH)) levels \|\| id:ebi-met1400-GCST90200243 | Fruit liking \|\| id:ebi-fl187-GCST90094766 | Weighted mode | 9 | -0.122500247 | 0.326777357891302 | 0.717497239999943 | -0.762983868 | 0.517983374896894 | 0.884705686801524 | 0.466273051963826 | 1.67863904843396 | 0.999714303421225 |
| ebi-fl187-GCST90094766 | ebi-met1400-GCST90200273 | 2-methoxyhydroquinone sulfate (2) levels \|\| id:ebi-met1400-GCST90200273 | Fruit liking \|\| id:ebi-fl187-GCST90094766 | Weighted mode | 9 | 0.239785462207826 | 0.398800114177007 | 0.5643063902788 | -0.541862762 | 1.02143368599476 | 1.27097644858885 | 0.581663741725153 | 2.77717350590993 | 0.999714303421225 |
| ebi-fl187-GCST90094766 | ebi-met1400-GCST90200276 | 2,4-di-tert-butylphenol levels \|\| id:ebi-met1400-GCST90200276 | Fruit liking \|\| id:ebi-fl187-GCST90094766 | Weighted mode | 9 | 0.512021569244995 | 0.348371812781907 | 0.179825597757436 | -0.170787184 | 1.19483032229753 | 1.66866110151162 | 0.843000958637164 | 3.30299727796209 | 0.999714303421225 |
| ebi-fl187-GCST90094766 | ebi-met1400-GCST90200289 | N-acetylmethionine levels \|\| id:ebi-met1400-GCST90200289 | Fruit liking \|\| id:ebi-fl187-GCST90094766 | Weighted mode | 9 | 0.173163007959157 | 0.310989287061814 | 0.592885806378549 | -0.436375995 | 0.782702010600312 | 1.18905991551743 | 0.646374646810295 | 2.18737459717423 | 0.999714303421225 |
| ebi-fl187-GCST90094766 | ebi-met1400-GCST90200308 | 3-hydroxyisobutyrate levels \|\| id:ebi-met1400-GCST90200308 | Fruit liking \|\| id:ebi-fl187-GCST90094766 | Weighted mode | 9 | 0.461568759166801 | 0.345604238062778 | 0.218447093327891 | -0.215815547 | 1.13895306576985 | 1.58656096580627 | 0.805883935523127 | 3.12349655733754 | 0.999714303421225 |
| ebi-fl187-GCST90094766 | ebi-met1400-GCST90200383 | Cystathionine levels \|\| id:ebi-met1400-GCST90200383 | Fruit liking \|\| id:ebi-fl187-GCST90094766 | Weighted mode | 9 | -0.606152271 | 0.36659688958388 | 0.136837165811024 | -1.324682175 | 0.112377632192866 | 0.545445563080565 | 0.265887451346959 | 1.11893532687276 | 0.999714303421225 |
| ebi-fl187-GCST90094766 | ebi-met1400-GCST90200384 | Guanidinoacetate levels \|\| id:ebi-met1400-GCST90200384 | Fruit liking \|\| id:ebi-fl187-GCST90094766 | Weighted mode | 9 | 0.209945341202645 | 0.326959703105258 | 0.538759739089427 | -0.430895677 | 0.850786359288951 | 1.23361063044049 | 0.649926709617934 | 2.34148737852362 | 0.999714303421225 |
| ebi-fl187-GCST90094766 | ebi-met1400-GCST90200403 | Citrulline levels \|\| id:ebi-met1400-GCST90200403 | Fruit liking \|\| id:ebi-fl187-GCST90094766 | Weighted mode | 9 | 0.18689438623436 | 0.328297862272414 | 0.584789221011793 | -0.456569424 | 0.830358196288292 | 1.2054999609361 | 0.633453031138034 | 2.29414034566403 | 0.999714303421225 |
| ebi-fl187-GCST90094766 | ebi-met1400-GCST90200434 | 12,13-DiHOME levels \|\| id:ebi-met1400-GCST90200434 | Fruit liking \|\| id:ebi-fl187-GCST90094766 | Weighted mode | 9 | -0.244537978 | 0.35163169044386 | 0.50647581453793 | -0.933736092 | 0.444660134830071 | 0.783066248166185 | 0.393082371764631 | 1.55995992968169 | 0.999714303421225 |
| ebi-fl187-GCST90094766 | ebi-met1400-GCST90200437 | Fructose levels \|\| id:ebi-met1400-GCST90200437 | Fruit liking \|\| id:ebi-fl187-GCST90094766 | Weighted mode | 9 | 0.334330050175817 | 0.374556915678819 | 0.398124152671529 | -0.399801505 | 1.0684616049063 | 1.39700414895785 | 0.670453114718019 | 2.91089794254481 | 0.999714303421225 |
| ebi-fl187-GCST90094766 | ebi-met1400-GCST90200448 | N-stearoyl-sphinganine (d18:0/18:0) levels \|\| id:ebi-met1400-GCST90200448 | Fruit liking \|\| id:ebi-fl187-GCST90094766 | Weighted mode | 9 | 0.3984540893263 | 0.346214173119735 | 0.283013177415498 | -0.28012569 | 1.07703386864098 | 1.48952025161069 | 0.75568875297572 | 2.93595818545902 | 0.999714303421225 |
| ebi-fl187-GCST90094766 | ebi-met1400-GCST90200468 | X-11847 levels \|\| id:ebi-met1400-GCST90200468 | Fruit liking \|\| id:ebi-fl187-GCST90094766 | Weighted mode | 9 | 0.491221036145036 | 0.398177071748126 | 0.252337487226902 | -0.289206024 | 1.27164809677136 | 1.6343105543895 | 0.748857906446797 | 3.56672603065944 | 0.999714303421225 |
| ebi-fl187-GCST90094766 | ebi-met1400-GCST90200476 | X-11843 levels \|\| id:ebi-met1400-GCST90200476 | Fruit liking \|\| id:ebi-fl187-GCST90094766 | Weighted mode | 9 | -0.067723657 | 0.378242057874223 | 0.862351174119814 | -0.80907809 | 0.673630776861459 | 0.934518685995118 | 0.445268374424883 | 1.96134561679133 | 0.999714303421225 |
| ebi-fl187-GCST90094766 | ebi-met1400-GCST90200511 | X-12906 levels \|\| id:ebi-met1400-GCST90200511 | Fruit liking \|\| id:ebi-fl187-GCST90094766 | Weighted mode | 9 | -0.132010981 | 0.32753075734764 | 0.697468831774366 | -0.773971265 | 0.509949303516745 | 0.876331372132844 | 0.461177966835245 | 1.66520677267868 | 0.999714303421225 |
| ebi-fl187-GCST90094766 | ebi-met1400-GCST90200515 | X-12849 levels \|\| id:ebi-met1400-GCST90200515 | Fruit liking \|\| id:ebi-fl187-GCST90094766 | Weighted mode | 9 | 0.150417513099878 | 0.299552223049532 | 0.629095059603892 | -0.436704844 | 0.73753987027696 | 1.16231942502244 | 0.646162121844884 | 2.09078557859016 | 0.999714303421225 |
| ebi-fl187-GCST90094766 | ebi-met1400-GCST90200551 | X-18886 levels \|\| id:ebi-met1400-GCST90200551 | Fruit liking \|\| id:ebi-fl187-GCST90094766 | Weighted mode | 9 | 0.0397511281960443 | 0.323212854561556 | 0.905150665470862 | -0.593746067 | 0.673248323136694 | 1.0405517779672 | 0.552254622317221 | 1.96059563628019 | 0.999714303421225 |
| ebi-fl187-GCST90094766 | ebi-met1400-GCST90200564 | X-21319 levels \|\| id:ebi-met1400-GCST90200564 | Fruit liking \|\| id:ebi-fl187-GCST90094766 | Weighted mode | 9 | -0.647826356 | 0.32145007494899 | 0.0786264842774609 | -1.277868503 | -0.01778421 | 0.52318175233017 | 0.278630568065297 | 0.982372996157124 | 0.999714303421225 |
| ebi-fl187-GCST90094766 | ebi-met1400-GCST90200579 | X-21442 levels \|\| id:ebi-met1400-GCST90200579 | Fruit liking \|\| id:ebi-fl187-GCST90094766 | Weighted mode | 9 | -0.264249027 | 0.414280335536663 | 0.541393307365807 | -1.076238485 | 0.547740430382862 | 0.767782317114265 | 0.340875324818418 | 1.7293410333745 | 0.999714303421225 |
| ebi-fl187-GCST90094766 | ebi-met1400-GCST90200581 | X-21441 levels \|\| id:ebi-met1400-GCST90200581 | Fruit liking \|\| id:ebi-fl187-GCST90094766 | Weighted mode | 9 | 0.218119743717625 | 0.308553675131834 | 0.49969163118499 | -0.38664546 | 0.822884946976019 | 1.24373598827371 | 0.679331902880851 | 2.27705956685871 | 0.999714303421225 |
| ebi-fl187-GCST90094766 | ebi-met1400-GCST90200648 | X-24585 levels \|\| id:ebi-met1400-GCST90200648 | Fruit liking \|\| id:ebi-fl187-GCST90094766 | Weighted mode | 9 | -0.308449097 | 0.331839950500395 | 0.379816757660922 | -0.9588554 | 0.341957205654935 | 0.73458534360589 | 0.383331395966326 | 1.4077000546232 | 0.999714303421225 |
| ebi-fl187-GCST90094766 | ebi-met1400-GCST90200720 | Adenosine 5'-diphosphate (ADP) to pantothenate ratio \|\| id:ebi-met1400-GCST90200720 | Fruit liking \|\| id:ebi-fl187-GCST90094766 | Weighted mode | 9 | 0.0468164580084787 | 0.414283160464389 | 0.912810147949378 | -0.765178537 | 0.858811452518682 | 1.04792965233206 | 0.465250859298028 | 2.36035363350795 | 0.999714303421225 |
| ebi-fl187-GCST90094766 | ebi-met1400-GCST90200724 | Cholate to taurocholate ratio \|\| id:ebi-met1400-GCST90200724 | Fruit liking \|\| id:ebi-fl187-GCST90094766 | Weighted mode | 9 | 0.487465717834635 | 0.385779733171201 | 0.24195561441537 | -0.268662559 | 1.24359399485019 | 1.62818470749134 | 0.764401152284259 | 3.46805526625216 | 0.999714303421225 |
| ebi-fl187-GCST90094766 | ebi-met1400-GCST90200726 | N-acetylputrescine to (N(1) + N(8))-acetylspermidine ratio \|\| id:ebi-met1400-GCST90200726 | Fruit liking \|\| id:ebi-fl187-GCST90094766 | Weighted mode | 9 | -0.12922213 | 0.345050420709504 | 0.717762966228909 | -0.805520955 | 0.547076694109325 | 0.878778740319104 | 0.446855074494415 | 1.72819358784407 | 0.999714303421225 |
| ebi-fl187-GCST90094766 | ebi-met1400-GCST90200744 | Arginine to glutamate ratio \|\| id:ebi-met1400-GCST90200744 | Fruit liking \|\| id:ebi-fl187-GCST90094766 | Weighted mode | 9 | 0.259475822445952 | 0.312143821506027 | 0.429931603515776 | -0.352326068 | 0.871277712597765 | 1.29625044316767 | 0.70305084242275 | 2.38996258879669 | 0.999714303421225 |
| ebi-fl187-GCST90094766 | ebi-met1400-GCST90200759 | Histidine to trans-urocanate ratio \|\| id:ebi-met1400-GCST90200759 | Fruit liking \|\| id:ebi-fl187-GCST90094766 | Weighted mode | 9 | 0.107607852092098 | 0.379718650135522 | 0.784074433399821 | -0.636640702 | 0.851856406357723 | 1.11361095954174 | 0.529066734887761 | 2.34399422121021 | 0.999714303421225 |
| ebi-fl187-GCST90094766 | ebi-met1400-GCST90200795 | Oleoyl-linoleoyl-glycerol (18:1 to 18:2) [2] to linoleoyl-arachidonoyl-glycerol (18:2 to 20:4) [2] ratio \|\| id:ebi-met1400-GCST90200795 | Fruit liking \|\| id:ebi-fl187-GCST90094766 | Weighted mode | 9 | 0.344858272094153 | 0.366698285042715 | 0.374515926431732 | -0.373870367 | 1.06358691077787 | 1.41178981547378 | 0.688066102403273 | 2.89674273462073 | 0.999714303421225 |
| ebi-fl187-GCST90094766 | ebi-met1400-GCST90200811 | Choline to taurocholate ratio \|\| id:ebi-met1400-GCST90200811 | Fruit liking \|\| id:ebi-fl187-GCST90094766 | Weighted mode | 9 | -0.574990421 | 0.545668426073627 | 0.322786419614576 | -1.644500536 | 0.4945196939214 | 0.562710258879785 | 0.19310898964384 | 1.63971048697709 | 0.999714303421225 |
| ebi-fl187-GCST90094766 | ebi-met1400-GCST90200831 | Cholate to phosphate ratio \|\| id:ebi-met1400-GCST90200831 | Fruit liking \|\| id:ebi-fl187-GCST90094766 | Weighted mode | 9 | -0.053007263 | 0.370372540250171 | 0.889735535657564 | -0.778937442 | 0.672922915513201 | 0.948373124065618 | 0.45889335302568 | 1.95995774730616 | 0.999714303421225 |
| ebi-fl187-GCST90094766 | ebi-met1400-GCST90200841 | Adenosine 5'-diphosphate (ADP) to oxalate (ethanedioate) ratio \|\| id:ebi-met1400-GCST90200841 | Fruit liking \|\| id:ebi-fl187-GCST90094766 | Weighted mode | 9 | -0.126600527 | 0.476459901480706 | 0.797181126484738 | -1.060461933 | 0.807260880306792 | 0.881085572558561 | 0.346295807743959 | 2.24175912272328 | 0.999714303421225 |
| ebi-fl187-GCST90094766 | ebi-met1400-GCST90200844 | Aspartate to phosphate ratio \|\| id:ebi-met1400-GCST90200844 | Fruit liking \|\| id:ebi-fl187-GCST90094766 | Weighted mode | 9 | -0.065748188 | 0.331296962510251 | 0.847638090610431 | -0.715090235 | 0.583593858452595 | 0.936366622895801 | 0.489147971739382 | 1.79246874796496 | 0.999714303421225 |
| ebi-fl187-GCST90094766 | ebi-met1400-GCST90200870 | Adenosine 5'-monophosphate (AMP) to acetoacetate ratio \|\| id:ebi-met1400-GCST90200870 | Fruit liking \|\| id:ebi-fl187-GCST90094766 | Weighted mode | 9 | -0.099411341 | 0.342863705673795 | 0.77923208195913 | -0.771424204 | 0.572601522295999 | 0.905370215687024 | 0.46235411262765 | 1.77287322652907 | 0.999714303421225 |
| ebi-fl187-GCST90094766 | ebi-met1400-GCST90200887 | Cholesterol to cortisol ratio \|\| id:ebi-met1400-GCST90200887 | Fruit liking \|\| id:ebi-fl187-GCST90094766 | Weighted mode | 9 | 0.321658500628098 | 0.312957156584573 | 0.334112441045561 | -0.291737526 | 0.93505452753386 | 1.37941362662614 | 0.746964568816327 | 2.5473523547939 | 0.999714303421225 |
| ebi-fl187-GCST90094766 | ebi-met1400-GCST90200890 | Cortisol to taurocholate ratio \|\| id:ebi-met1400-GCST90200890 | Fruit liking \|\| id:ebi-fl187-GCST90094766 | Weighted mode | 9 | 0.299981275304933 | 0.388500818840768 | 0.462223435300242 | -0.46148033 | 1.06144288023284 | 1.34983353211809 | 0.630349828974651 | 2.89053868293135 | 0.999714303421225 |
| ebi-fl187-GCST90094766 | ebi-met1400-GCST90200948 | Adenosine 5'-diphosphate (ADP) to mannitol to sorbitol ratio \|\| id:ebi-met1400-GCST90200948 | Fruit liking \|\| id:ebi-fl187-GCST90094766 | Weighted mode | 9 | -0.55580284 | 0.510024674457555 | 0.307554092727107 | -1.555451202 | 0.443845521774266 | 0.573611557889251 | 0.21109411504225 | 1.55868968340628 | 0.999714303421225 |
| ebi-fl187-GCST90094766 | ebi-met1400-GCST90200972 | Leucine to N-palmitoyl-sphingosine (d18:1 to 16:0) ratio \|\| id:ebi-met1400-GCST90200972 | Fruit liking \|\| id:ebi-fl187-GCST90094766 | Weighted mode | 9 | 0.148835327698883 | 0.370972846184551 | 0.698773829267177 | -0.578271451 | 0.875942106220602 | 1.16048187425321 | 0.560867015363581 | 2.40113635421622 | 0.999714303421225 |
| ebi-fl187-GCST90094766 | ebi-met1400-GCST90200973 | Proline to glutamate ratio \|\| id:ebi-met1400-GCST90200973 | Fruit liking \|\| id:ebi-fl187-GCST90094766 | Weighted mode | 9 | -0.004118988 | 0.311666020259222 | 0.989779082316906 | -0.614984387 | 0.606746411957525 | 0.995889483644312 | 0.540649336153555 | 1.83445312388518 | 0.999714303421225 |
| ebi-fl187-GCST90094766 | ebi-met1400-GCST90200983 | Cholesterol to linoleoyl-arachidonoyl-glycerol (18:2 to 20:4) [1] ratio \|\| id:ebi-met1400-GCST90200983 | Fruit liking \|\| id:ebi-fl187-GCST90094766 | Weighted mode | 9 | 0.522040736313039 | 0.329044129534634 | 0.151278722232857 | -0.122885758 | 1.16696723020092 | 1.68546372947706 | 0.884364688756738 | 3.21223587904257 | 0.999714303421225 |
| ebi-fl187-GCST90094766 | ebi-met1400-GCST90201001 | Serine to alpha-ketobutyrate ratio \|\| id:ebi-met1400-GCST90201001 | Fruit liking \|\| id:ebi-fl187-GCST90094766 | Weighted mode | 9 | -0.086498645 | 0.340339782498999 | 0.805787275378291 | -0.753564619 | 0.580567328681803 | 0.917136791489327 | 0.470685743588765 | 1.78705198906181 | 0.999714303421225 |
| ebi-fl187-GCST90094766 | ebi-met1400-GCST90201005 | Fructose to maltose ratio \|\| id:ebi-met1400-GCST90201005 | Fruit liking \|\| id:ebi-fl187-GCST90094766 | Weighted mode | 9 | 0.174041795910222 | 0.434142194685461 | 0.698997380555493 | -0.676876906 | 1.02496049749373 | 1.19010530631542 | 0.508201678294034 | 2.78698536548446 | 0.999714303421225 |
| ebi-fl187-GCST90094766 | ebi-met1400-GCST90201020 | Paraxanthine to 5-acetylamino-6-formylamino-3-methyluracil ratio \|\| id:ebi-met1400-GCST90201020 | Fruit liking \|\| id:ebi-fl187-GCST90094766 | Weighted mode | 9 | -0.138513309 | 0.407739527159823 | 0.74282137813062 | -0.937682783 | 0.660656163965253 | 0.87065166345568 | 0.391534054562115 | 1.93606229201676 | 0.999714303421225 |
| ebi-fl187-GCST90094766 | ebi-pw11-GCST90275197 | Placental weight (fetal genotype, adjusted for sex) \|\| id:ebi-pw11-GCST90275197 | Fruit liking \|\| id:ebi-fl187-GCST90094766 | Weighted mode | 8 | 0.00928532382858738 | 0.145803318010119 | 0.951002333563959 | -0.276489179 | 0.29505982712842 | 1.00932856618402 | 0.758441825828855 | 1.34320671648319 | 0.999714303421225 |
| ebi-fl187-GCST90094766 | prot-a-1017 | FAS-associated factor 2 \|\| id:prot-a-1017 | Fruit liking \|\| id:ebi-fl187-GCST90094766 | Weighted mode | 10 | -0.093919824 | 0.537699528760039 | 0.865205336218713 | -1.147810901 | 0.959971251988826 | 0.910355747650616 | 0.317330677933494 | 2.61162139342292 | 0.999714303421225 |
| ebi-fl187-GCST90094766 | prot-a-1019 | Fas apoptotic inhibitory molecule 3 \|\| id:prot-a-1019 | Fruit liking \|\| id:ebi-fl187-GCST90094766 | Weighted mode | 10 | 0.0368601776790021 | 0.536970970629122 | 0.946773411343141 | -1.015602925 | 1.08932328011208 | 1.03754793833462 | 0.362183994293321 | 2.97226200302656 | 0.999714303421225 |
| ebi-fl187-GCST90094766 | prot-a-1052 | MIP18 family protein FAM96A \|\| id:prot-a-1052 | Fruit liking \|\| id:ebi-fl187-GCST90094766 | Weighted mode | 10 | -0.087893011 | 0.718705133225092 | 0.905353729330233 | -1.496555072 | 1.32076905018062 | 0.915858858359582 | 0.223900152994433 | 3.74630136343216 | 0.999714303421225 |
| ebi-fl187-GCST90094766 | prot-a-1066 | F-box/LRR-repeat protein 4 \|\| id:prot-a-1066 | Fruit liking \|\| id:ebi-fl187-GCST90094766 | Weighted mode | 10 | 0.0503299296181767 | 0.569424470002923 | 0.931504315270538 | -1.065742032 | 1.16640189082391 | 1.05161799907104 | 0.344472150691857 | 3.21042038884429 | 0.999714303421225 |
| ebi-fl187-GCST90094766 | prot-a-1076 | Low affinity immunoglobulin gamma Fc region receptor III-B \|\| id:prot-a-1076 | Fruit liking \|\| id:ebi-fl187-GCST90094766 | Weighted mode | 10 | -0.929325385 | 0.563899589717424 | 0.133748652007375 | -2.03456858 | 0.175917811245483 | 0.394819972182543 | 0.130736872782456 | 1.19234005767913 | 0.999714303421225 |
| ebi-fl187-GCST90094766 | prot-a-1081 | Fc receptor-like protein 3 \|\| id:prot-a-1081 | Fruit liking \|\| id:ebi-fl187-GCST90094766 | Weighted mode | 10 | 0.53512646527767 | 0.791143228666968 | 0.515793530530624 | -1.015514263 | 2.08576719346493 | 1.70766418882486 | 0.362216107617892 | 8.05076560778221 | 0.999714303421225 |
| ebi-fl187-GCST90094766 | prot-a-1105 | Fibroblast growth factor receptor 4 \|\| id:prot-a-1105 | Fruit liking \|\| id:ebi-fl187-GCST90094766 | Weighted mode | 10 | 0.291442459621011 | 0.549338158454697 | 0.608581490078174 | -0.785260331 | 1.36814525019222 | 1.3383566217276 | 0.456000975165048 | 3.92805836933526 | 0.999714303421225 |
| ebi-fl187-GCST90094766 | prot-a-1143 | Follistatin-related protein 1 \|\| id:prot-a-1143 | Fruit liking \|\| id:ebi-fl187-GCST90094766 | Weighted mode | 10 | 0.0701605506243666 | 0.652983074157356 | 0.916792106453874 | -1.209686275 | 1.35000737597278 | 1.07268038693585 | 0.298290846130016 | 3.85745398306764 | 0.999714303421225 |
| ebi-fl187-GCST90094766 | prot-a-1151 | Alpha-(1,3)-fucosyltransferase 10 \|\| id:prot-a-1151 | Fruit liking \|\| id:ebi-fl187-GCST90094766 | Weighted mode | 10 | -0.154782779 | 0.538416549506271 | 0.78025605176593 | -1.210079216 | 0.900513658443188 | 0.856601229403279 | 0.298173658482685 | 2.46086683159444 | 0.999714303421225 |
| ebi-fl187-GCST90094766 | prot-a-1165 | Growth arrest and DNA damage-inducible proteins-interacting protein 1 \|\| id:prot-a-1165 | Fruit liking \|\| id:ebi-fl187-GCST90094766 | Weighted mode | 10 | -0.180421273 | 0.555917607928934 | 0.752940433469031 | -1.270019784 | 0.909177238650539 | 0.834918408822885 | 0.28082606573953 | 2.48227936945816 | 0.999714303421225 |
| ebi-fl187-GCST90094766 | prot-a-1194 | Growth/differentiation factor 11/8 \|\| id:prot-a-1194 | Fruit liking \|\| id:ebi-fl187-GCST90094766 | Weighted mode | 10 | -0.197551502 | 0.558110883401033 | 0.73151628992598 | -1.291448833 | 0.896345829534026 | 0.820737869953256 | 0.274872250359191 | 2.45063170361928 | 0.999714303421225 |
| ebi-fl187-GCST90094766 | prot-a-1216 | D-glucuronyl C5-epimerase \|\| id:prot-a-1216 | Fruit liking \|\| id:ebi-fl187-GCST90094766 | Weighted mode | 10 | 0.217502193744715 | 0.552010080221649 | 0.702740167447022 | -0.864437563 | 1.29944195097915 | 1.24296815625956 | 0.421288434002352 | 3.66724959144421 | 0.999714303421225 |
| ebi-fl187-GCST90094766 | prot-a-1246 | Glypican-3 \|\| id:prot-a-1246 | Fruit liking \|\| id:ebi-fl187-GCST90094766 | Weighted mode | 10 | 0.0129017633942032 | 0.502248834154162 | 0.980066761510207 | -0.971505952 | 0.99730947833636 | 1.01298535022925 | 0.378512587058991 | 2.71097806218833 | 0.999714303421225 |
| ebi-fl187-GCST90094766 | prot-a-1275 | Glutamate receptor 4 \|\| id:prot-a-1275 | Fruit liking \|\| id:ebi-fl187-GCST90094766 | Weighted mode | 10 | -0.435446076 | 0.513231169868152 | 0.418186215210113 | -1.441379169 | 0.570487016722378 | 0.646976002090602 | 0.236601220451503 | 1.76912843679493 | 0.999714303421225 |
| ebi-fl187-GCST90094766 | prot-a-1289 | Glutathione S-transferase P \|\| id:prot-a-1289 | Fruit liking \|\| id:ebi-fl187-GCST90094766 | Weighted mode | 10 | 0.1273148436437 | 0.478088879343746 | 0.796009030817863 | -0.80973936 | 1.06436904715744 | 1.13577455289336 | 0.44497402919878 | 2.89900926874958 | 0.999714303421225 |
| ebi-fl187-GCST90094766 | prot-a-130 | Apolipoprotein D \|\| id:prot-a-130 | Fruit liking \|\| id:ebi-fl187-GCST90094766 | Weighted mode | 10 | 0.0481966184572795 | 0.493537673686301 | 0.924346180492476 | -0.919137222 | 1.01553045888243 | 1.04937696192132 | 0.398863022927198 | 2.7608275144929 | 0.999714303421225 |
| ebi-fl187-GCST90094766 | prot-a-1313 | Heparin-binding EGF-like growth factor \|\| id:prot-a-1313 | Fruit liking \|\| id:ebi-fl187-GCST90094766 | Weighted mode | 10 | -0.066461167 | 0.472674653175962 | 0.891277408618259 | -0.992903487 | 0.859981153065766 | 0.935699251010954 | 0.370499387512873 | 2.36311615579132 | 0.999714303421225 |
| ebi-fl187-GCST90094766 | prot-a-1347 | HLA class II histocompatibility antigen, DQ alpha 2 chain \|\| id:prot-a-1347 | Fruit liking \|\| id:ebi-fl187-GCST90094766 | Weighted mode | 10 | 0.0967403235087547 | 0.484036593531924 | 0.846031700745771 | -0.8519714 | 1.04545204683133 | 1.10157428353239 | 0.426573156242321 | 2.8446841635074 | 0.999714303421225 |
| ebi-fl187-GCST90094766 | prot-a-1357 | Non-histone chromosomal protein HMG-14 \|\| id:prot-a-1357 | Fruit liking \|\| id:ebi-fl187-GCST90094766 | Weighted mode | 10 | 0.155465498266179 | 0.515764737468943 | 0.769937134440109 | -0.855433387 | 1.16636438370531 | 1.16820163039025 | 0.425098918735908 | 3.21029997748417 | 0.999714303421225 |
| ebi-fl187-GCST90094766 | prot-a-137 | Apolipoprotein M \|\| id:prot-a-137 | Fruit liking \|\| id:ebi-fl187-GCST90094766 | Weighted mode | 10 | 0.149227719600329 | 0.500890270828025 | 0.772523436349466 | -0.832517211 | 1.13097265042326 | 1.16093732729464 | 0.434953038486928 | 3.09866895652582 | 0.999714303421225 |
| ebi-fl187-GCST90094766 | prot-a-1381 | 17-beta-hydroxysteroid dehydrogenase 14 \|\| id:prot-a-1381 | Fruit liking \|\| id:ebi-fl187-GCST90094766 | Weighted mode | 10 | 0.0812822277921812 | 0.519029650520834 | 0.879013477359452 | -0.936015887 | 1.09858034281302 | 1.08467697936131 | 0.392187245075573 | 2.99990416396551 | 0.999714303421225 |
| ebi-fl187-GCST90094766 | prot-a-1392 | Serine protease HTRA2, mitochondrial \|\| id:prot-a-1392 | Fruit liking \|\| id:ebi-fl187-GCST90094766 | Weighted mode | 10 | -0.259482768 | 0.565637397275829 | 0.657295139171987 | -1.368132066 | 0.849166531130902 | 0.771450501877282 | 0.254582058122015 | 2.33769764152616 | 0.999714303421225 |
| ebi-fl187-GCST90094766 | prot-a-1416 | Interferon-induced protein with tetratricopeptide repeats 2 \|\| id:prot-a-1416 | Fruit liking \|\| id:ebi-fl187-GCST90094766 | Weighted mode | 10 | 0.66490301351769 | 0.556118973389267 | 0.262398311128831 | -0.425090174 | 1.75489620136065 | 1.94430194118629 | 0.653710834538544 | 5.78284745910524 | 0.999714303421225 |
| ebi-fl187-GCST90094766 | prot-a-1428 | Interferon gamma \|\| id:prot-a-1428 | Fruit liking \|\| id:ebi-fl187-GCST90094766 | Weighted mode | 10 | -0.053451738 | 0.505078859443518 | 0.918039653334954 | -1.043406303 | 0.936502826081026 | 0.947951689538307 | 0.352252756460657 | 2.55104435442196 | 0.999714303421225 |
| ebi-fl187-GCST90094766 | prot-a-1483 | Interleukin-17C \|\| id:prot-a-1483 | Fruit liking \|\| id:ebi-fl187-GCST90094766 | Weighted mode | 10 | -0.797965263 | 0.548878198605786 | 0.179963459080774 | -1.873766533 | 0.277836005849436 | 0.450244160974414 | 0.153544241892382 | 1.32026966295251 | 0.999714303421225 |
| ebi-fl187-GCST90094766 | prot-a-1503 | Interleukin-1 receptor-like 2 \|\| id:prot-a-1503 | Fruit liking \|\| id:ebi-fl187-GCST90094766 | Weighted mode | 10 | 1.14376877774505 | 0.649805367849437 | 0.112231953191217 | -0.129849743 | 2.41738729872994 | 3.1385746939549 | 0.878227380608034 | 11.2165155938478 | 0.999714303421225 |
| ebi-fl187-GCST90094766 | prot-a-1522 | Interleukin-31 receptor subunit alpha \|\| id:prot-a-1522 | Fruit liking \|\| id:ebi-fl187-GCST90094766 | Weighted mode | 10 | 0.651027083089319 | 0.592170054204032 | 0.300133924234157 | -0.509626223 | 1.81168038932922 | 1.91750925930397 | 0.600720072110388 | 6.12072399478736 | 0.999714303421225 |
| ebi-fl187-GCST90094766 | prot-a-1540 | Interleukin-6 receptor subunit alpha \|\| id:prot-a-1540 | Fruit liking \|\| id:ebi-fl187-GCST90094766 | Weighted mode | 10 | -0.489350675 | 0.526958749014892 | 0.377313304335347 | -1.522189823 | 0.543488472863083 | 0.613024316868106 | 0.218233470586411 | 1.72200355913237 | 0.999714303421225 |
| ebi-fl187-GCST90094766 | prot-a-1541 | Interleukin-6 receptor subunit alpha \|\| id:prot-a-1541 | Fruit liking \|\| id:ebi-fl187-GCST90094766 | Weighted mode | 10 | -0.351407791 | 0.514129962508914 | 0.511502180013492 | -1.359102518 | 0.656286935129984 | 0.703696733869985 | 0.256891228803107 | 1.92762164580877 | 0.999714303421225 |
| ebi-fl187-GCST90094766 | prot-a-1545 | Interleukin-9 \|\| id:prot-a-1545 | Fruit liking \|\| id:ebi-fl187-GCST90094766 | Weighted mode | 10 | -0.519691776 | 0.558454186529789 | 0.376352545321152 | -1.614261982 | 0.574878429579668 | 0.594703821703672 | 0.199037509590122 | 1.7769144935408 | 0.999714303421225 |
| ebi-fl187-GCST90094766 | prot-a-1548 | Inositol monophosphatase 3 \|\| id:prot-a-1548 | Fruit liking \|\| id:ebi-fl187-GCST90094766 | Weighted mode | 10 | 0.045155771889634 | 0.474442328269368 | 0.926260055058965 | -0.884751192 | 0.975062735297594 | 1.04619081433659 | 0.4128168728416 | 2.65133353796379 | 0.999714303421225 |
| ebi-fl187-GCST90094766 | prot-a-1555 | Inhibin beta A chain:Inhibin beta B chain heterodimer \|\| id:prot-a-1555 | Fruit liking \|\| id:ebi-fl187-GCST90094766 | Weighted mode | 10 | -0.281533339 | 0.557913132148974 | 0.62594974668019 | -1.375043078 | 0.811976399583358 | 0.754625756464337 | 0.252828704103833 | 2.25235514431741 | 0.999714303421225 |
| ebi-fl187-GCST90094766 | prot-a-1603 | Junctophilin-3 \|\| id:prot-a-1603 | Fruit liking \|\| id:ebi-fl187-GCST90094766 | Weighted mode | 10 | 0.369381509395454 | 0.504836584462879 | 0.48297642012301 | -0.620098196 | 1.3588612149427 | 1.44683948125062 | 0.537891616114502 | 3.89175890047697 | 0.999714303421225 |
| ebi-fl187-GCST90094766 | prot-a-1627 | UPF0258 protein KIAA1024 \|\| id:prot-a-1627 | Fruit liking \|\| id:ebi-fl187-GCST90094766 | Weighted mode | 10 | 0.0901919709211993 | 0.521706932307859 | 0.866572270867786 | -0.932353616 | 1.1127375582446 | 1.09438435351341 | 0.393626174247612 | 3.04267650774045 | 0.999714303421225 |
| ebi-fl187-GCST90094766 | prot-a-1666 | Kallikrein-7 \|\| id:prot-a-1666 | Fruit liking \|\| id:ebi-fl187-GCST90094766 | Weighted mode | 10 | 0.350063875555049 | 0.558918373846997 | 0.546657599499197 | -0.745416137 | 1.44554388829516 | 1.4191581952156 | 0.474536786443887 | 4.24415986406512 | 0.999714303421225 |
| ebi-fl187-GCST90094766 | prot-a-1678 | Importin subunit alpha-3 \|\| id:prot-a-1678 | Fruit liking \|\| id:ebi-fl187-GCST90094766 | Weighted mode | 10 | -0.085861142 | 0.498833860605531 | 0.867148527896067 | -1.063575509 | 0.891853225009365 | 0.91772165557085 | 0.34521926656683 | 2.43964667870199 | 0.999714303421225 |
| ebi-fl187-GCST90094766 | prot-a-1718 | Protein LDOC1 \|\| id:prot-a-1718 | Fruit liking \|\| id:ebi-fl187-GCST90094766 | Weighted mode | 10 | -0.263172001 | 0.507864230720875 | 0.616820470107327 | -1.258585894 | 0.732241890783924 | 0.768609683977066 | 0.284055427206899 | 2.07973793041817 | 0.999714303421225 |
| ebi-fl187-GCST90094766 | prot-a-1723 | Leptin \|\| id:prot-a-1723 | Fruit liking \|\| id:ebi-fl187-GCST90094766 | Weighted mode | 10 | -0.250882593 | 0.5379103979636 | 0.652010640878347 | -1.305186973 | 0.80342178730027 | 0.77811372242094 | 0.271121837919572 | 2.23316930006716 | 0.999714303421225 |
| ebi-fl187-GCST90094766 | prot-a-1730 | Galectin-7 \|\| id:prot-a-1730 | Fruit liking \|\| id:ebi-fl187-GCST90094766 | Weighted mode | 10 | -0.231859271 | 0.526920544646722 | 0.670295213137343 | -1.264623538 | 0.800904996792001 | 0.793057721899615 | 0.282345568449921 | 2.22755594754858 | 0.999714303421225 |
| ebi-fl187-GCST90094766 | prot-a-1753 | Lipase member N \|\| id:prot-a-1753 | Fruit liking \|\| id:ebi-fl187-GCST90094766 | Weighted mode | 10 | -0.33696315 | 0.515057064971913 | 0.529327918329741 | -1.346474997 | 0.672548697302933 | 0.713935147898196 | 0.260155695749733 | 1.95922443264417 | 0.999714303421225 |
| ebi-fl187-GCST90094766 | prot-a-1756 | VIP36-like protein \|\| id:prot-a-1756 | Fruit liking \|\| id:ebi-fl187-GCST90094766 | Weighted mode | 10 | -0.300134173 | 0.768946970387438 | 0.70537843726245 | -1.807270235 | 1.20700188904869 | 0.740718829612702 | 0.164101484457944 | 3.34344559011849 | 0.999714303421225 |
| ebi-fl187-GCST90094766 | prot-a-1760 | Ig Kappa chain V-I region HK102- like \|\| id:prot-a-1760 | Fruit liking \|\| id:ebi-fl187-GCST90094766 | Weighted mode | 10 | 0.289623611158771 | 0.543130150408265 | 0.606776413992443 | -0.774911484 | 1.35415870595897 | 1.33592456628487 | 0.460744562624934 | 3.87350083229574 | 0.999714303421225 |
| ebi-fl187-GCST90094766 | prot-a-179 | Neutral ceramidase \|\| id:prot-a-179 | Fruit liking \|\| id:ebi-fl187-GCST90094766 | Weighted mode | 10 | 0.267973014990491 | 0.487644646727942 | 0.59601524031122 | -0.687810493 | 1.22375652257726 | 1.30731186171593 | 0.502675476724202 | 3.39993570985532 | 0.999714303421225 |
| ebi-fl187-GCST90094766 | prot-a-1809 | Lactotransferrin \|\| id:prot-a-1809 | Fruit liking \|\| id:ebi-fl187-GCST90094766 | Weighted mode | 10 | -0.661687591 | 0.519576904274719 | 0.23474223882667 | -1.680058323 | 0.356683141419015 | 0.515979836425202 | 0.186363106404013 | 1.42858313930555 | 0.999714303421225 |
| ebi-fl187-GCST90094766 | prot-a-1818 | Lysophospholipase-like protein 1 \|\| id:prot-a-1818 | Fruit liking \|\| id:ebi-fl187-GCST90094766 | Weighted mode | 10 | 0.0250023152774999 | 0.520591047308168 | 0.962743773456762 | -0.995356137 | 1.04536076800151 | 1.02531749441621 | 0.369591795624551 | 2.8444245159161 | 0.999714303421225 |
| ebi-fl187-GCST90094766 | prot-a-1852 | MAP kinase-activated protein kinase 3 \|\| id:prot-a-1852 | Fruit liking \|\| id:ebi-fl187-GCST90094766 | Weighted mode | 10 | -0.40912132 | 0.602791976029466 | 0.514393081901574 | -1.590593593 | 0.772350952708037 | 0.664233642402497 | 0.203804598771584 | 2.16484973527891 | 0.999714303421225 |
| ebi-fl187-GCST90094766 | prot-a-1856 | Megakaryocyte-associated tyrosine-protein kinase \|\| id:prot-a-1856 | Fruit liking \|\| id:ebi-fl187-GCST90094766 | Weighted mode | 10 | -0.410146652 | 0.54457216871808 | 0.470599072527523 | -1.477508103 | 0.657214798446718 | 0.663552931476258 | 0.228205645424285 | 1.92941104525314 | 0.999714303421225 |
| ebi-fl187-GCST90094766 | prot-a-1890 | Beta-1,3-N-acetylglucosaminyltransferase manic fringe \|\| id:prot-a-1890 | Fruit liking \|\| id:ebi-fl187-GCST90094766 | Weighted mode | 10 | 0.172826398463567 | 0.635915665136042 | 0.791926025092478 | -1.073568305 | 1.41922110213021 | 1.18865973401526 | 0.341786739475985 | 4.13389930058567 | 0.999714303421225 |
| ebi-fl187-GCST90094766 | prot-a-1919 | Matrilysin \|\| id:prot-a-1919 | Fruit liking \|\| id:ebi-fl187-GCST90094766 | Weighted mode | 10 | -1.016972802 | 0.513577089910604 | 0.0790372641453852 | -2.023583898 | -0.010361706 | 0.361688186285418 | 0.132180892286644 | 0.989691791569585 | 0.999714303421225 |
| ebi-fl187-GCST90094766 | prot-a-1923 | Multimerin-2 \|\| id:prot-a-1923 | Fruit liking \|\| id:ebi-fl187-GCST90094766 | Weighted mode | 10 | 0.816261500443015 | 0.581370792745721 | 0.193864079213091 | -0.323225253 | 1.95574825422463 | 2.26202742182465 | 0.723810795205661 | 7.06920660893542 | 0.999714303421225 |
| ebi-fl187-GCST90094766 | prot-a-1948 | Mesothelin \|\| id:prot-a-1948 | Fruit liking \|\| id:ebi-fl187-GCST90094766 | Weighted mode | 10 | -0.75677796 | 0.616409361488893 | 0.250693161382832 | -1.964940308 | 0.451384388614162 | 0.469175697147994 | 0.14016425299151 | 1.57048484257708 | 0.999714303421225 |
| ebi-fl187-GCST90094766 | prot-a-195 | ATPase family AAA domain-containing protein 1 \|\| id:prot-a-195 | Fruit liking \|\| id:ebi-fl187-GCST90094766 | Weighted mode | 10 | -0.849433439 | 0.555842986254027 | 0.160815521125066 | -1.938885692 | 0.24001881420882 | 0.42765715725574 | 0.143864169502431 | 1.27127306809338 | 0.999714303421225 |
| ebi-fl187-GCST90094766 | prot-a-1966 | Mucin-1 \|\| id:prot-a-1966 | Fruit liking \|\| id:ebi-fl187-GCST90094766 | Weighted mode | 10 | -0.911019298 | 0.599672504291734 | 0.163031481252831 | -2.086377406 | 0.264338810894073 | 0.402114141125005 | 0.124136016667919 | 1.30256944626521 | 0.999714303421225 |
| ebi-fl187-GCST90094766 | prot-a-1986 | Myocardial zonula adherens protein \|\| id:prot-a-1986 | Fruit liking \|\| id:ebi-fl187-GCST90094766 | Weighted mode | 10 | -0.057357601 | 0.495385394877283 | 0.910366169023713 | -1.028312975 | 0.913597772695415 | 0.94425634172281 | 0.357609748473098 | 2.49327665895779 | 0.999714303421225 |
| ebi-fl187-GCST90094766 | prot-a-2071 | Epididymal secretory protein E1 \|\| id:prot-a-2071 | Fruit liking \|\| id:ebi-fl187-GCST90094766 | Weighted mode | 10 | 0.553662859760285 | 0.595215601025287 | 0.376549195407292 | -0.612959718 | 1.72028543776985 | 1.73961332194076 | 0.541745081103736 | 5.58612272714714 | 0.999714303421225 |
| ebi-fl187-GCST90094766 | prot-a-2098 | Neuritin \|\| id:prot-a-2098 | Fruit liking \|\| id:ebi-fl187-GCST90094766 | Weighted mode | 10 | -0.167942444 | 0.489458639299067 | 0.739387616518913 | -1.127281377 | 0.791396488792343 | 0.845402491072516 | 0.323912656816263 | 2.20647559418163 | 0.999714303421225 |
| ebi-fl187-GCST90094766 | prot-a-2115 | Neurotrophin-4 \|\| id:prot-a-2115 | Fruit liking \|\| id:ebi-fl187-GCST90094766 | Weighted mode | 10 | -0.085813093 | 0.505845553753388 | 0.869042909015484 | -1.077270378 | 0.905644192529085 | 0.917765752192111 | 0.340523759287347 | 2.47352483615098 | 0.999714303421225 |
| ebi-fl187-GCST90094766 | prot-a-2118 | Netrin-1 \|\| id:prot-a-2118 | Fruit liking \|\| id:ebi-fl187-GCST90094766 | Weighted mode | 10 | 0.280110677561845 | 0.495045174495467 | 0.585339347769126 | -0.690177864 | 1.25039921957296 | 1.32327626122322 | 0.501486864448164 | 3.49173664886274 | 0.999714303421225 |
| ebi-fl187-GCST90094766 | prot-a-2120 | Netrin-G1 \|\| id:prot-a-2120 | Fruit liking \|\| id:ebi-fl187-GCST90094766 | Weighted mode | 10 | -0.228788202 | 0.508886457545224 | 0.663641108838193 | -1.226205659 | 0.768629254813893 | 0.795497000357762 | 0.293403742197061 | 2.1568077927008 | 0.999714303421225 |
| ebi-fl187-GCST90094766 | prot-a-2145 | Olfactomedin-like protein 3 \|\| id:prot-a-2145 | Fruit liking \|\| id:ebi-fl187-GCST90094766 | Weighted mode | 10 | 0.135213164223241 | 0.532671727958376 | 0.805323467602682 | -0.908823423 | 1.17924975102166 | 1.14478078465107 | 0.402998103671954 | 3.25193352764035 | 0.999714303421225 |
| ebi-fl187-GCST90094766 | prot-a-2162 | Prolyl 4-hydroxylase subunit alpha-2 \|\| id:prot-a-2162 | Fruit liking \|\| id:ebi-fl187-GCST90094766 | Weighted mode | 10 | -0.703833225 | 0.525545455407439 | 0.213328749678339 | -1.733902318 | 0.32623586761902 | 0.494685424259614 | 0.176593937997194 | 1.385742182 | 0.999714303421225 |
| ebi-fl187-GCST90094766 | prot-a-2168 | Polyadenylate-binding protein-interacting protein 1 \|\| id:prot-a-2168 | Fruit liking \|\| id:ebi-fl187-GCST90094766 | Weighted mode | 10 | -0.306561474 | 0.54922909370448 | 0.590343441591254 | -1.383050498 | 0.769927549195716 | 0.735973273225395 | 0.250812282502807 | 2.15960978265106 | 0.999714303421225 |
| ebi-fl187-GCST90094766 | prot-a-219 | Beta-1,4-galactosyltransferase 5 \|\| id:prot-a-219 | Fruit liking \|\| id:ebi-fl187-GCST90094766 | Weighted mode | 10 | -0.280570837 | 0.625383660186255 | 0.664299655110738 | -1.506322811 | 0.945181137355489 | 0.755352435541499 | 0.221723801168314 | 2.5732794534104 | 0.999714303421225 |
| ebi-fl187-GCST90094766 | prot-a-2190 | Pyruvate carboxylase, mitochondrial \|\| id:prot-a-2190 | Fruit liking \|\| id:ebi-fl187-GCST90094766 | Weighted mode | 10 | -0.425371201 | 0.533747611018395 | 0.445992956948552 | -1.471516519 | 0.620774116357671 | 0.653527150012004 | 0.229577063119011 | 1.86036762558204 | 0.999714303421225 |
| ebi-fl187-GCST90094766 | prot-a-2197 | Protocadherin-9 \|\| id:prot-a-2197 | Fruit liking \|\| id:ebi-fl187-GCST90094766 | Weighted mode | 10 | -0.293253688 | 0.49843434011965 | 0.570765141506308 | -1.270184995 | 0.683677618501716 | 0.745832907737415 | 0.280779674203018 | 1.98115026610451 | 0.999714303421225 |
| ebi-fl187-GCST90094766 | prot-a-2198 | Protocadherin alpha-4 \|\| id:prot-a-2198 | Fruit liking \|\| id:ebi-fl187-GCST90094766 | Weighted mode | 10 | -0.23344842 | 0.522310105821507 | 0.665470399412696 | -1.257176227 | 0.790279387381946 | 0.791798435627952 | 0.284456132906851 | 2.20401211341846 | 0.999714303421225 |
| ebi-fl187-GCST90094766 | prot-a-2254 | Placenta growth factor \|\| id:prot-a-2254 | Fruit liking \|\| id:ebi-fl187-GCST90094766 | Weighted mode | 10 | -1.015686791 | 0.526882195338687 | 0.0859847779396724 | -2.048375893 | 0.01700231231067 | 0.362153620716895 | 0.128944152667594 | 1.01714767428394 | 0.999714303421225 |
| ebi-fl187-GCST90094766 | prot-a-2261 | Membrane-associated progesterone receptor component 2 \|\| id:prot-a-2261 | Fruit liking \|\| id:ebi-fl187-GCST90094766 | Weighted mode | 10 | -0.427284714 | 0.556823248193565 | 0.462521883758844 | -1.51865828 | 0.664088852755992 | 0.652277813356071 | 0.219005533981544 | 1.94271961105984 | 0.999714303421225 |
| ebi-fl187-GCST90094766 | prot-a-2268 | Polymeric immunoglobulin receptor \|\| id:prot-a-2268 | Fruit liking \|\| id:ebi-fl187-GCST90094766 | Weighted mode | 10 | -0.399752972 | 0.53712138842788 | 0.475706981837649 | -1.452510893 | 0.653004949200582 | 0.67048565423091 | 0.233982045912744 | 1.92130558896428 | 0.999714303421225 |
| ebi-fl187-GCST90094766 | prot-a-2283 | Group 10 secretory phospholipase A2 \|\| id:prot-a-2283 | Fruit liking \|\| id:ebi-fl187-GCST90094766 | Weighted mode | 10 | 0.647076290214735 | 0.603149818422111 | 0.31127032355845 | -0.535097354 | 1.82924993432207 | 1.90994852267204 | 0.585612275734953 | 6.22921258725142 | 0.999714303421225 |
| ebi-fl187-GCST90094766 | prot-a-2298 | Inactive phospholipase D5 \|\| id:prot-a-2298 | Fruit liking \|\| id:ebi-fl187-GCST90094766 | Weighted mode | 10 | -0.897925001 | 0.550356030877482 | 0.137213651294602 | -1.976622821 | 0.180772819967825 | 0.407414167433606 | 0.138536310079988 | 1.19814295421743 | 0.999714303421225 |
| ebi-fl187-GCST90094766 | prot-a-2304 | Procollagen-lysine,2-oxoglutarate 5-dioxygenase 2 \|\| id:prot-a-2304 | Fruit liking \|\| id:ebi-fl187-GCST90094766 | Weighted mode | 10 | -0.129095343 | 0.582119304678153 | 0.829444874329949 | -1.27004918 | 1.01185849458379 | 0.878890165890119 | 0.280817810887756 | 2.75070844422724 | 0.999714303421225 |
| ebi-fl187-GCST90094766 | prot-a-2331 | Periostin \|\| id:prot-a-2331 | Fruit liking \|\| id:ebi-fl187-GCST90094766 | Weighted mode | 10 | -0.605881699 | 0.945888629289474 | 0.537785201169012 | -2.459823413 | 1.24806001414413 | 0.545593165415081 | 0.0854500390292908 | 3.48357830527861 | 0.999714303421225 |
| ebi-fl187-GCST90094766 | prot-a-2333 | Periostin \|\| id:prot-a-2333 | Fruit liking \|\| id:ebi-fl187-GCST90094766 | Weighted mode | 10 | -0.595903577 | 0.880947955277186 | 0.515770489709018 | -2.322561569 | 1.13075441555117 | 0.551064411828022 | 0.0980221731633004 | 3.09799279268641 | 0.999714303421225 |
| ebi-fl187-GCST90094766 | prot-a-2367 | cAMP-dependent protein kinase catalytic subunit alpha \|\| id:prot-a-2367 | Fruit liking \|\| id:ebi-fl187-GCST90094766 | Weighted mode | 10 | -0.587649309 | 0.480992739137283 | 0.25283936762853 | -1.530395078 | 0.355096459451625 | 0.555631869487993 | 0.216450135741816 | 1.42631822952042 | 0.999714303421225 |
| ebi-fl187-GCST90094766 | prot-a-2385 | Phosphoribosyl pyrophosphate synthase-associated protein 1 \|\| id:prot-a-2385 | Fruit liking \|\| id:ebi-fl187-GCST90094766 | Weighted mode | 10 | -0.290287065 | 0.524123921829457 | 0.59317179127923 | -1.317569952 | 0.736995821902169 | 0.748048798212508 | 0.267785243034736 | 2.08964839946237 | 0.999714303421225 |
| ebi-fl187-GCST90094766 | prot-a-2393 | Trypsin-3 \|\| id:prot-a-2393 | Fruit liking \|\| id:ebi-fl187-GCST90094766 | Weighted mode | 10 | 0.0459872803991743 | 0.4981633749592 | 0.928471027502729 | -0.930412935 | 1.02238749531921 | 1.0470610926731 | 0.394390819158274 | 2.77982366356709 | 0.999714303421225 |
| ebi-fl187-GCST90094766 | prot-a-2405 | Pregnancy-specific beta-1-glycoprotein 6 \|\| id:prot-a-2405 | Fruit liking \|\| id:ebi-fl187-GCST90094766 | Weighted mode | 10 | -0.072167712 | 0.555592079626765 | 0.899507606552579 | -1.161128189 | 1.01679276363688 | 0.930374847298014 | 0.31313270880747 | 2.7643147206861 | 0.999714303421225 |
| ebi-fl187-GCST90094766 | prot-a-244 | Beclin-1 \|\| id:prot-a-244 | Fruit liking \|\| id:ebi-fl187-GCST90094766 | Weighted mode | 10 | -0.366827717 | 0.481306970908633 | 0.465474557328506 | -1.31018938 | 0.57653394582618 | 0.692929014543627 | 0.269768962664026 | 1.77985864072283 | 0.999714303421225 |
| ebi-fl187-GCST90094766 | prot-a-2454 | tRNA pseudouridine synthase A, mitochondrial \|\| id:prot-a-2454 | Fruit liking \|\| id:ebi-fl187-GCST90094766 | Weighted mode | 10 | -0.154092846 | 0.492141804840972 | 0.761333941994879 | -1.118690783 | 0.810505091941607 | 0.857192430816852 | 0.326707245417355 | 2.24904367367505 | 0.999714303421225 |
| ebi-fl187-GCST90094766 | prot-a-2466 | Glutaminyl-peptide cyclotransferase-like protein \|\| id:prot-a-2466 | Fruit liking \|\| id:ebi-fl187-GCST90094766 | Weighted mode | 9 | 0.212629461816421 | 0.514325073877643 | 0.690162575363321 | -0.795447683 | 1.2207066066166 | 1.23692623791761 | 0.451379114934148 | 3.3895819886842 | 0.999714303421225 |
| ebi-fl187-GCST90094766 | prot-a-2474 | Ras-related protein Rab-27A \|\| id:prot-a-2474 | Fruit liking \|\| id:ebi-fl187-GCST90094766 | Weighted mode | 10 | 1.4373829904827 | 1.13499080944222 | 0.237153920651413 | -0.787198996 | 3.66196497698945 | 4.20966465649564 | 0.455117798369465 | 38.9377795894557 | 0.999714303421225 |
| ebi-fl187-GCST90094766 | prot-a-2490 | Rap1 GTPase-GDP dissociation stimulator 1 \|\| id:prot-a-2490 | Fruit liking \|\| id:ebi-fl187-GCST90094766 | Weighted mode | 10 | -0.679951285 | 0.586984581842887 | 0.276522168543097 | -1.830441066 | 0.470538494930988 | 0.506641672569792 | 0.160342830422696 | 1.60085601400225 | 0.999714303421225 |
| ebi-fl187-GCST90094766 | prot-a-2545 | Receptor-interacting serine/threonine-protein kinase 2 \|\| id:prot-a-2545 | Fruit liking \|\| id:ebi-fl187-GCST90094766 | Weighted mode | 10 | -0.45567877 | 0.500422945124113 | 0.386251109901303 | -1.436507742 | 0.525150202644654 | 0.634017469949845 | 0.237756617938192 | 1.69071277883883 | 0.999714303421225 |
| ebi-fl187-GCST90094766 | prot-a-2610 | RUN and FYVE domain-containing protein 1 \|\| id:prot-a-2610 | Fruit liking \|\| id:ebi-fl187-GCST90094766 | Weighted mode | 10 | -0.117862557 | 0.509312830969248 | 0.822168092157779 | -1.116115706 | 0.880390591304986 | 0.888818205709701 | 0.327549625843054 | 2.411841567 | 0.999714303421225 |
| ebi-fl187-GCST90094766 | prot-a-2627 | Serine--tRNA ligase, mitochondrial \|\| id:prot-a-2627 | Fruit liking \|\| id:ebi-fl187-GCST90094766 | Weighted mode | 10 | -0.547487925 | 0.532401642269412 | 0.330634578802171 | -1.590995144 | 0.496019293970368 | 0.578400973598348 | 0.203722777382587 | 1.64217124151633 | 0.999714303421225 |
| ebi-fl187-GCST90094766 | prot-a-264 | BolA-like protein 2 \|\| id:prot-a-264 | Fruit liking \|\| id:ebi-fl187-GCST90094766 | Weighted mode | 10 | 0.357698312375245 | 0.472162830916815 | 0.46807639856508 | -0.567740836 | 1.2831374609722 | 1.43003413179523 | 0.566804497544046 | 3.60794176291876 | 0.999714303421225 |
| ebi-fl187-GCST90094766 | prot-a-2646 | Secretoglobin family 1C member 1 \|\| id:prot-a-2646 | Fruit liking \|\| id:ebi-fl187-GCST90094766 | Weighted mode | 10 | 0.179033151411395 | 0.846867619923296 | 0.83727979056936 | -1.480827384 | 1.83889368646106 | 1.19606039460084 | 0.227449422579754 | 6.28957616733934 | 0.999714303421225 |
| ebi-fl187-GCST90094766 | prot-a-2672 | Semaphorin-3E \|\| id:prot-a-2672 | Fruit liking \|\| id:ebi-fl187-GCST90094766 | Weighted mode | 10 | 0.060299485039861 | 0.50556652446188 | 0.907680437038273 | -0.930610903 | 1.05120987298515 | 1.06215459832933 | 0.394312749972787 | 2.86111060529996 | 0.999714303421225 |
| ebi-fl187-GCST90094766 | prot-a-2701 | Plasma protease C1 inhibitor \|\| id:prot-a-2701 | Fruit liking \|\| id:ebi-fl187-GCST90094766 | Weighted mode | 10 | 0.309438521732399 | 0.56836919729887 | 0.599371507172021 | -0.804565105 | 1.42344214843818 | 1.3626597954361 | 0.447282405160899 | 4.15138556015861 | 0.999714303421225 |
| ebi-fl187-GCST90094766 | prot-a-2738 | Signaling threshold-regulating transmembrane adapter 1 \|\| id:prot-a-2738 | Fruit liking \|\| id:ebi-fl187-GCST90094766 | Weighted mode | 10 | -0.52070658 | 0.53294628329689 | 0.35408190742995 | -1.565281296 | 0.523868135016984 | 0.59410061986972 | 0.209029205926141 | 1.68854655963387 | 0.999714303421225 |
| ebi-fl187-GCST90094766 | prot-a-274 | Serine/threonine-protein kinase BRSK2 \|\| id:prot-a-274 | Fruit liking \|\| id:ebi-fl187-GCST90094766 | Weighted mode | 10 | -0.093810267 | 0.735093466943168 | 0.901258189202388 | -1.534593462 | 1.34697292795519 | 0.910455489074863 | 0.215543299802865 | 3.84576648100258 | 0.999714303421225 |
| ebi-fl187-GCST90094766 | prot-a-2749 | Mitochondrial glutamate carrier 2 \|\| id:prot-a-2749 | Fruit liking \|\| id:ebi-fl187-GCST90094766 | Weighted mode | 10 | -0.226448367 | 0.520601093422616 | 0.673824462908476 | -1.24682651 | 0.793929775760721 | 0.797360511088252 | 0.287415461064739 | 2.21207231610868 | 0.999714303421225 |
| ebi-fl187-GCST90094766 | prot-a-2760 | Sodium-coupled monocarboxylate transporter 1 \|\| id:prot-a-2760 | Fruit liking \|\| id:ebi-fl187-GCST90094766 | Weighted mode | 10 | 0.361168384616572 | 0.586933493960484 | 0.553564342134701 | -0.789221264 | 1.51155803277912 | 1.43500507331512 | 0.454198358417294 | 4.53378908637143 | 0.999714303421225 |
| ebi-fl187-GCST90094766 | prot-a-2777 | Small integral membrane protein 10 \|\| id:prot-a-2777 | Fruit liking \|\| id:ebi-fl187-GCST90094766 | Weighted mode | 10 | 0.179203685915027 | 0.515542695077948 | 0.736129956282763 | -0.831259996 | 1.18966736826781 | 1.19626438155946 | 0.435500211762723 | 3.28598800169477 | 0.999714303421225 |
| ebi-fl187-GCST90094766 | prot-a-2799 | Superoxide dismutase [Mn], mitochondrial \|\| id:prot-a-2799 | Fruit liking \|\| id:ebi-fl187-GCST90094766 | Weighted mode | 10 | 0.693736055708535 | 0.542688312595682 | 0.233113262525703 | -0.369933037 | 1.75740514839607 | 2.0011780971392 | 0.690780585843519 | 5.79737453330346 | 0.999714303421225 |
| ebi-fl187-GCST90094766 | prot-a-2805 | Protein SPACA7 \|\| id:prot-a-2805 | Fruit liking \|\| id:ebi-fl187-GCST90094766 | Weighted mode | 10 | -0.340342737 | 0.53122477306671 | 0.5377024537413 | -1.381543292 | 0.700857818112089 | 0.711526414467871 | 0.251190593258297 | 2.01548088214001 | 0.999714303421225 |
| ebi-fl187-GCST90094766 | prot-a-2811 | SPARC-like protein 1 \|\| id:prot-a-2811 | Fruit liking \|\| id:ebi-fl187-GCST90094766 | Weighted mode | 10 | 0.581315096687051 | 0.538124385770236 | 0.308122280254212 | -0.473408699 | 1.63603889279671 | 1.7883887891088 | 0.622875450312601 | 5.13478972305765 | 0.999714303421225 |
| ebi-fl187-GCST90094766 | prot-a-2831 | Sepiapterin reductase \|\| id:prot-a-2831 | Fruit liking \|\| id:ebi-fl187-GCST90094766 | Weighted mode | 10 | 0.083426042650077 | 0.533456381465318 | 0.879179198251987 | -0.962148465 | 1.1290005503221 | 1.08700482032415 | 0.382071137067927 | 3.09256409284292 | 0.999714303421225 |
| ebi-fl187-GCST90094766 | prot-a-2834 | SPRY domain-containing SOCS box protein 1 \|\| id:prot-a-2834 | Fruit liking \|\| id:ebi-fl187-GCST90094766 | Weighted mode | 10 | -0.371523657 | 0.601261605897077 | 0.551949690026294 | -1.549996404 | 0.806949090799915 | 0.6896826899794 | 0.212248737004626 | 2.24106027470427 | 0.999714303421225 |
| ebi-fl187-GCST90094766 | prot-a-2835 | Spectrin alpha chain, non-erythrocytic 1 \|\| id:prot-a-2835 | Fruit liking \|\| id:ebi-fl187-GCST90094766 | Weighted mode | 10 | 0.222730348639771 | 0.571148890979602 | 0.705627821950632 | -0.896721478 | 1.34218217495979 | 1.24948360335254 | 0.407904794883861 | 3.8273864260197 | 0.999714303421225 |
| ebi-fl187-GCST90094766 | prot-a-2859 | Sia-alpha-2,3-Gal-beta-1,4-GlcNAc-R:alpha 2,8-sialyltransferase \|\| id:prot-a-2859 | Fruit liking \|\| id:ebi-fl187-GCST90094766 | Weighted mode | 10 | 0.107688998169834 | 0.509283974596812 | 0.837245366823642 | -0.890507592 | 1.10588558837959 | 1.11370132836972 | 0.410447360054813 | 3.0218994431998 | 0.999714303421225 |
| ebi-fl187-GCST90094766 | prot-a-2861 | Alpha-2,8-sialyltransferase 8F \|\| id:prot-a-2861 | Fruit liking \|\| id:ebi-fl187-GCST90094766 | Weighted mode | 10 | -0.021701657 | 0.55749192308023 | 0.9697981697836 | -1.114385826 | 1.07098251205059 | 0.978532129535204 | 0.328116737658075 | 2.91824530308028 | 0.999714303421225 |
| ebi-fl187-GCST90094766 | prot-a-289 | Uncharacterized protein C17orf89 \|\| id:prot-a-289 | Fruit liking \|\| id:ebi-fl187-GCST90094766 | Weighted mode | 10 | -0.605263173 | 0.703750935161924 | 0.412083271226599 | -1.984615006 | 0.774088660183674 | 0.545930733648917 | 0.137433515059671 | 2.16861488126125 | 0.999714303421225 |
| ebi-fl187-GCST90094766 | prot-a-2921 | Tachykinin-3 \|\| id:prot-a-2921 | Fruit liking \|\| id:ebi-fl187-GCST90094766 | Weighted mode | 10 | -0.201231656 | 0.543305717770152 | 0.719663259873994 | -1.266110863 | 0.863647550896614 | 0.817722979231236 | 0.281925941095809 | 2.37179618223059 | 0.999714303421225 |
| ebi-fl187-GCST90094766 | prot-a-2928 | Tax1-binding protein 1 \|\| id:prot-a-2928 | Fruit liking \|\| id:ebi-fl187-GCST90094766 | Weighted mode | 10 | 0.214147003460152 | 0.496186742780743 | 0.676196843357085 | -0.758379012 | 1.18667301931041 | 1.23880474999351 | 0.468425123246127 | 3.27616332354604 | 0.999714303421225 |
| ebi-fl187-GCST90094766 | prot-a-296 | Uncharacterized protein C1orf185 \|\| id:prot-a-296 | Fruit liking \|\| id:ebi-fl187-GCST90094766 | Weighted mode | 10 | -0.349221767 | 0.520192930687672 | 0.518866868854676 | -1.368799911 | 0.670356376937582 | 0.705236714547901 | 0.254412093486051 | 1.95493388986081 | 0.999714303421225 |
| ebi-fl187-GCST90094766 | prot-a-2964 | Transforming growth factor beta-2 \|\| id:prot-a-2964 | Fruit liking \|\| id:ebi-fl187-GCST90094766 | Weighted mode | 10 | -0.685009374 | 0.553881523467553 | 0.24748132736223 | -1.77061716 | 0.400598412022228 | 0.504085504268179 | 0.170227898555127 | 1.49271769063762 | 0.999714303421225 |
| ebi-fl187-GCST90094766 | prot-a-2977 | Thymocyte nuclear protein 1 \|\| id:prot-a-2977 | Fruit liking \|\| id:ebi-fl187-GCST90094766 | Weighted mode | 10 | -0.757314107 | 0.510883903617076 | 0.172389472549389 | -1.758646558 | 0.244018344412226 | 0.468924217532933 | 0.17227787425528 | 1.27636774449075 | 0.999714303421225 |
| ebi-fl187-GCST90094766 | prot-a-3015 | Transmembrane protein 70, mitochondrial \|\| id:prot-a-3015 | Fruit liking \|\| id:ebi-fl187-GCST90094766 | Weighted mode | 10 | -0.13409867 | 0.459779936953405 | 0.777158600121722 | -1.035267346 | 0.767070006923455 | 0.874503773491612 | 0.355131425289967 | 2.15344741521153 | 0.999714303421225 |
| ebi-fl187-GCST90094766 | prot-a-3030 | Tumor necrosis factor alpha-induced protein 3 \|\| id:prot-a-3030 | Fruit liking \|\| id:ebi-fl187-GCST90094766 | Weighted mode | 10 | -0.084554229 | 0.475946176785467 | 0.86292806497706 | -1.017408735 | 0.848300277874726 | 0.918921822158947 | 0.36153054885734 | 2.33567348017699 | 0.999714303421225 |
| ebi-fl187-GCST90094766 | prot-a-3032 | Tumor necrosis factor receptor superfamily member 10A \|\| id:prot-a-3032 | Fruit liking \|\| id:ebi-fl187-GCST90094766 | Weighted mode | 10 | 0.373588110572074 | 0.596472753875896 | 0.546654411037169 | -0.795498487 | 1.54267470816883 | 1.45293857715295 | 0.451356183633627 | 4.6770833889645 | 0.999714303421225 |
| ebi-fl187-GCST90094766 | prot-a-3081 | Tumor protein p53-inducible protein 11 \|\| id:prot-a-3081 | Fruit liking \|\| id:ebi-fl187-GCST90094766 | Weighted mode | 10 | -0.045054415 | 0.754945689550444 | 0.953715443373077 | -1.524747966 | 1.43463913655611 | 0.955945462681051 | 0.217675911578189 | 4.19812978383803 | 0.999714303421225 |
| ebi-fl187-GCST90094766 | prot-a-315 | Complement C4 \|\| id:prot-a-315 | Fruit liking \|\| id:ebi-fl187-GCST90094766 | Weighted mode | 10 | 0.430640189017869 | 0.518421122655394 | 0.427642233433406 | -0.585465211 | 1.44674558942244 | 1.53824197401606 | 0.556846750097193 | 4.24926314145108 | 0.999714303421225 |
| ebi-fl187-GCST90094766 | prot-a-3157 | UDP-glucuronosyltransferase 1-8 \|\| id:prot-a-3157 | Fruit liking \|\| id:ebi-fl187-GCST90094766 | Weighted mode | 10 | -0.30304443 | 0.488657678690684 | 0.550532093951583 | -1.26081348 | 0.654724620597235 | 0.73856628141009 | 0.283423373487609 | 1.92461244576844 | 0.999714303421225 |
| ebi-fl187-GCST90094766 | prot-a-317 | Glutaredoxin-like protein C5orf63 \|\| id:prot-a-317 | Fruit liking \|\| id:ebi-fl187-GCST90094766 | Weighted mode | 10 | 0.345355664380952 | 0.631897997240832 | 0.597983219592113 | -0.89316441 | 1.58387573897298 | 1.41249220350529 | 0.409358323377129 | 4.87380886384267 | 0.999714303421225 |
| ebi-fl187-GCST90094766 | prot-a-3192 | Guanine nucleotide exchange factor VAV3 \|\| id:prot-a-3192 | Fruit liking \|\| id:ebi-fl187-GCST90094766 | Weighted mode | 10 | -0.412395773 | 0.536396546009716 | 0.4616943013144 | -1.463733003 | 0.63894145744375 | 0.662062198026827 | 0.231370952170074 | 1.8944744357274 | 0.999714303421225 |
| ebi-fl187-GCST90094766 | prot-a-3233 | Serine/threonine-protein kinase WNK3 \|\| id:prot-a-3233 | Fruit liking \|\| id:ebi-fl187-GCST90094766 | Weighted mode | 10 | -0.872836469 | 0.762716452734152 | 0.281988496135861 | -2.367760716 | 0.62208777843137 | 0.417764889952761 | 0.0936902906954621 | 1.86281312590373 | 0.999714303421225 |
| ebi-fl187-GCST90094766 | prot-a-3250 | 14-3-3 protein theta \|\| id:prot-a-3250 | Fruit liking \|\| id:ebi-fl187-GCST90094766 | Weighted mode | 10 | 0.350577726286428 | 0.662593912706305 | 0.609536531388162 | -0.948106343 | 1.64926179519078 | 1.41988761808334 | 0.38747407230114 | 5.20313742804319 | 0.999714303421225 |
| ebi-fl187-GCST90094766 | prot-a-3278 | E3 ubiquitin-protein ligase ZNRF3 \|\| id:prot-a-3278 | Fruit liking \|\| id:ebi-fl187-GCST90094766 | Weighted mode | 10 | 0.30137281080534 | 0.515629893840152 | 0.573258056548415 | -0.709261781 | 1.31200740273204 | 1.35171318089336 | 0.492007272487036 | 3.71362096776524 | 0.999714303421225 |
| ebi-fl187-GCST90094766 | prot-a-355 | Calpain-3 \|\| id:prot-a-355 | Fruit liking \|\| id:ebi-fl187-GCST90094766 | Weighted mode | 10 | -0.114462566 | 0.55114746298595 | 0.840101778505698 | -1.194711593 | 0.965786461611565 | 0.891845323272981 | 0.302791268623085 | 2.62685276316203 | 0.999714303421225 |
| ebi-fl187-GCST90094766 | prot-a-385 | Coiled-coil domain-containing protein 90B, mitochondrial \|\| id:prot-a-385 | Fruit liking \|\| id:ebi-fl187-GCST90094766 | Weighted mode | 10 | -0.080433963 | 0.506975374733511 | 0.877444033701309 | -1.074105698 | 0.913237771160462 | 0.922715834664762 | 0.3416031151578 | 2.49237923707982 | 0.999714303421225 |
| ebi-fl187-GCST90094766 | prot-a-390 | C-C motif chemokine 14 \|\| id:prot-a-390 | Fruit liking \|\| id:ebi-fl187-GCST90094766 | Weighted mode | 10 | 0.578027504740383 | 0.627924876760152 | 0.381304655622368 | -0.652705254 | 1.80876026319028 | 1.78251895063196 | 0.520635419034762 | 6.10287677940315 | 0.999714303421225 |
| ebi-fl187-GCST90094766 | prot-a-40 | Pituitary adenylate cyclase-activating polypeptide 27 \|\| id:prot-a-40 | Fruit liking \|\| id:ebi-fl187-GCST90094766 | Weighted mode | 10 | -0.061509595 | 0.576241890933376 | 0.917334634110812 | -1.190943701 | 1.06792451134412 | 0.940343923204062 | 0.30393430554352 | 2.90933493777718 | 0.999714303421225 |
| ebi-fl187-GCST90094766 | prot-a-401 | C-C motif chemokine 24 \|\| id:prot-a-401 | Fruit liking \|\| id:ebi-fl187-GCST90094766 | Weighted mode | 10 | 0.148265873664972 | 0.505039762018734 | 0.775741667227075 | -0.84161206 | 1.13814380722169 | 1.15982122129224 | 0.431015140859183 | 3.12096986356054 | 0.999714303421225 |
| ebi-fl187-GCST90094766 | prot-a-412 | Cerebral cavernous malformations 2 protein \|\| id:prot-a-412 | Fruit liking \|\| id:ebi-fl187-GCST90094766 | Weighted mode | 10 | 0.481572574581479 | 0.514752239821915 | 0.373930233562753 | -0.527341815 | 1.49048696463243 | 1.61861779903602 | 0.590171671672327 | 4.43925675377189 | 0.999714303421225 |
| ebi-fl187-GCST90094766 | prot-a-44 | Adhesion G protein-coupled receptor F5 \|\| id:prot-a-44 | Fruit liking \|\| id:ebi-fl187-GCST90094766 | Weighted mode | 10 | 0.399306371109461 | 0.566394743825302 | 0.498649729738783 | -0.710827327 | 1.50944006900705 | 1.49079028372253 | 0.491237615257542 | 4.52419684692983 | 0.999714303421225 |
| ebi-fl187-GCST90094766 | prot-a-48 | Adrenomedullin \|\| id:prot-a-48 | Fruit liking \|\| id:ebi-fl187-GCST90094766 | Weighted mode | 10 | 0.541062410807391 | 0.564307899452112 | 0.36270328526535 | -0.564981072 | 1.64710589373353 | 1.71783093520668 | 0.568370904712719 | 5.19193205965493 | 0.999714303421225 |
| ebi-fl187-GCST90094766 | prot-a-502 | Carcinoembryonic antigen-related cell adhesion molecule 1 \|\| id:prot-a-502 | Fruit liking \|\| id:ebi-fl187-GCST90094766 | Weighted mode | 10 | -0.33636516 | 0.510322545756448 | 0.526319948551393 | -1.33659735 | 0.663867029245539 | 0.714362201369266 | 0.262738155261915 | 1.94228871796876 | 0.999714303421225 |
| ebi-fl187-GCST90094766 | prot-a-518 | Complement factor B \|\| id:prot-a-518 | Fruit liking \|\| id:ebi-fl187-GCST90094766 | Weighted mode | 10 | -0.149036949 | 0.508039612460911 | 0.775901301957674 | -1.14479459 | 0.846720691073326 | 0.861537281068254 | 0.318289290951395 | 2.33198699350468 | 0.999714303421225 |
| ebi-fl187-GCST90094766 | prot-a-525 | Properdin \|\| id:prot-a-525 | Fruit liking \|\| id:ebi-fl187-GCST90094766 | Weighted mode | 10 | -0.907926333 | 0.838129625872734 | 0.306852371493124 | -2.5506604 | 0.734807733351184 | 0.40335979113345 | 0.0780301178865281 | 2.08508106241514 | 0.999714303421225 |
| ebi-fl187-GCST90094766 | prot-a-533 | Chondroadherin \|\| id:prot-a-533 | Fruit liking \|\| id:ebi-fl187-GCST90094766 | Weighted mode | 10 | 0.470694427392684 | 0.546748562119656 | 0.41164197131872 | -0.600932754 | 1.54232160914721 | 1.6011056588847 | 0.548299968313904 | 4.67543220692835 | 0.999714303421225 |
| ebi-fl187-GCST90094766 | prot-a-535 | Coiled-coil-helix-coiled-coil-helix domain-containing protein 2 \|\| id:prot-a-535 | Fruit liking \|\| id:ebi-fl187-GCST90094766 | Weighted mode | 10 | 0.431387453578759 | 0.800592578087664 | 0.603071557310113 | -1.137773999 | 2.00054890663058 | 1.5393918773168 | 0.320531732079808 | 7.3931131141765 | 0.999714303421225 |
| ebi-fl187-GCST90094766 | prot-a-591 | Caseinolytic peptidase B protein homolog \|\| id:prot-a-591 | Fruit liking \|\| id:ebi-fl187-GCST90094766 | Weighted mode | 10 | 0.278364674362634 | 0.503250975712832 | 0.593643834095444 | -0.708007238 | 1.26473658675978 | 1.32096783248611 | 0.492624904151172 | 3.54215956148165 | 0.999714303421225 |
| ebi-fl187-GCST90094766 | prot-a-605 | Ciliary neurotrophic factor receptor subunit alpha \|\| id:prot-a-605 | Fruit liking \|\| id:ebi-fl187-GCST90094766 | Weighted mode | 10 | 0.386381725851801 | 0.470757293106052 | 0.432980575462344 | -0.536302569 | 1.30906602033966 | 1.4716463290567 | 0.584906912328856 | 3.70271383732321 | 0.999714303421225 |
| ebi-fl187-GCST90094766 | prot-a-606 | Ciliary neurotrophic factor receptor subunit alpha \|\| id:prot-a-606 | Fruit liking \|\| id:ebi-fl187-GCST90094766 | Weighted mode | 10 | 0.0264782029799699 | 0.591504258303148 | 0.965272757330799 | -1.132870143 | 1.18582654925414 | 1.02683186514302 | 0.322107433944672 | 3.27339132276656 | 0.999714303421225 |
| ebi-fl187-GCST90094766 | prot-a-707 | Cancer/testis antigen 55 \|\| id:prot-a-707 | Fruit liking \|\| id:ebi-fl187-GCST90094766 | Weighted mode | 10 | 0.135091401702193 | 0.511188134049576 | 0.797524343055397 | -0.866837341 | 1.13702014443936 | 1.14464140174266 | 0.420278647594338 | 3.11746491543875 | 0.999714303421225 |
| ebi-fl187-GCST90094766 | prot-a-719 | Dipeptidyl peptidase 1 \|\| id:prot-a-719 | Fruit liking \|\| id:ebi-fl187-GCST90094766 | Weighted mode | 10 | 0.154688943650518 | 0.48091829930344 | 0.755060179929638 | -0.787910923 | 1.09728881028526 | 1.16729481016543 | 0.45479390304734 | 2.99603219108528 | 0.999714303421225 |
| ebi-fl187-GCST90094766 | prot-a-752 | Cytochrome b5 \|\| id:prot-a-752 | Fruit liking \|\| id:ebi-fl187-GCST90094766 | Weighted mode | 10 | -0.082643122 | 0.536158141315725 | 0.880900851778562 | -1.133513079 | 0.968226834823007 | 0.920679658769199 | 0.321900406090864 | 2.633271093 | 0.999714303421225 |
| ebi-fl187-GCST90094766 | prot-a-755 | Cytoglobin \|\| id:prot-a-755 | Fruit liking \|\| id:ebi-fl187-GCST90094766 | Weighted mode | 10 | -0.199239023 | 0.568297787328353 | 0.733962534121925 | -1.313102686 | 0.91462464023866 | 0.81935402552969 | 0.268984186840304 | 2.49583823881172 | 0.999714303421225 |
| ebi-fl187-GCST90094766 | prot-a-807 | Desmin \|\| id:prot-a-807 | Fruit liking \|\| id:ebi-fl187-GCST90094766 | Weighted mode | 10 | 0.0979724426036164 | 0.53493186629902 | 0.858740327261419 | -0.950494015 | 1.14643890054969 | 1.10293239074464 | 0.386550014639882 | 3.14696627210573 | 0.999714303421225 |
| ebi-fl187-GCST90094766 | prot-a-82 | Ameloblastin \|\| id:prot-a-82 | Fruit liking \|\| id:ebi-fl187-GCST90094766 | Weighted mode | 10 | -0.654036548 | 0.581029676873816 | 0.289434019952784 | -1.792854715 | 0.484781618306442 | 0.51994276105154 | 0.166484225625321 | 1.62382035748126 | 0.999714303421225 |
| ebi-fl187-GCST90094766 | prot-a-84 | Muellerian-inhibiting factor \|\| id:prot-a-84 | Fruit liking \|\| id:ebi-fl187-GCST90094766 | Weighted mode | 10 | 0.538503645834466 | 0.527185961229143 | 0.33370383277972 | -0.494780838 | 1.57178812984359 | 1.71344102834538 | 0.609704516609525 | 4.81525079384888 | 0.999714303421225 |
| ebi-fl187-GCST90094766 | prot-a-858 | Inactive dipeptidyl peptidase 10 \|\| id:prot-a-858 | Fruit liking \|\| id:ebi-fl187-GCST90094766 | Weighted mode | 10 | 0.347078868201872 | 0.958045270531874 | 0.725505102462624 | -1.530689862 | 2.22484759844435 | 1.41492831382187 | 0.216386339092548 | 9.25207266618867 | 0.999714303421225 |
| ebi-fl187-GCST90094766 | prot-a-863 | Dihydropyrimidinase-related protein 3 \|\| id:prot-a-863 | Fruit liking \|\| id:ebi-fl187-GCST90094766 | Weighted mode | 10 | 0.322687286812251 | 0.544061024865232 | 0.56771158142879 | -0.743672322 | 1.38904689554811 | 1.38083347854423 | 0.475365012861267 | 4.01102530451695 | 0.999714303421225 |
| ebi-fl187-GCST90094766 | prot-a-864 | Dihydropyrimidinase-related protein 5 \|\| id:prot-a-864 | Fruit liking \|\| id:ebi-fl187-GCST90094766 | Weighted mode | 10 | 0.0267564625485139 | 0.591667931348792 | 0.964917783699917 | -1.132912683 | 1.18642560799215 | 1.02711763069144 | 0.322093731914421 | 3.27535286392189 | 0.999714303421225 |
| ebi-fl187-GCST90094766 | prot-a-884 | Endothelin-converting enzyme 1 \|\| id:prot-a-884 | Fruit liking \|\| id:ebi-fl187-GCST90094766 | Weighted mode | 10 | -0.288396629 | 0.578986595047735 | 0.63035372943073 | -1.423210356 | 0.84641709706468 | 0.749464273843347 | 0.240939273234737 | 2.33127912368316 | 0.999714303421225 |
| ebi-fl187-GCST90094766 | prot-a-893 | Early endosome antigen 1 \|\| id:prot-a-893 | Fruit liking \|\| id:ebi-fl187-GCST90094766 | Weighted mode | 10 | 0.253064046713744 | 0.543269421313911 | 0.652413845053563 | -0.811744019 | 1.31787211248901 | 1.28796576416769 | 0.444082901423349 | 3.73546426658445 | 0.999714303421225 |
| ebi-fl187-GCST90094766 | prot-a-897 | Ephrin-A2 \|\| id:prot-a-897 | Fruit liking \|\| id:ebi-fl187-GCST90094766 | Weighted mode | 10 | -0.137288359 | 0.582915986381488 | 0.819076982754863 | -1.279803692 | 1.005226974 | 0.871718822051493 | 0.278091886714034 | 2.73252741637965 | 0.999714303421225 |
| ebi-fl187-GCST90094766 | prot-a-90 | Anaphase-promoting complex subunit 10 \|\| id:prot-a-90 | Fruit liking \|\| id:ebi-fl187-GCST90094766 | Weighted mode | 10 | -0.387940687 | 0.550602218630457 | 0.49889971162543 | -1.467121035 | 0.691239661559546 | 0.678452583158533 | 0.230588386279193 | 1.99618859831546 | 0.999714303421225 |
| ebi-fl187-GCST90094766 | prot-a-914 | Histone-lysine N-methyltransferase EHMT2 \|\| id:prot-a-914 | Fruit liking \|\| id:ebi-fl187-GCST90094766 | Weighted mode | 10 | 0.277400546699145 | 0.841592344099849 | 0.749233604772181 | -1.372120448 | 1.92692154113485 | 1.31969486460661 | 0.253568709892387 | 6.86833377985865 | 0.999714303421225 |
| ebi-fl187-GCST90094766 | prot-a-932 | RNA polymerase II elongation factor ELL \|\| id:prot-a-932 | Fruit liking \|\| id:ebi-fl187-GCST90094766 | Weighted mode | 10 | 0.105400949687641 | 0.502665362737364 | 0.83858388082333 | -0.879823161 | 1.09062506065287 | 1.1111560387192 | 0.414856267847674 | 2.97613375540343 | 0.999714303421225 |
| ebi-fl187-GCST90094766 | prot-a-991 | Estrogen receptor \|\| id:prot-a-991 | Fruit liking \|\| id:ebi-fl187-GCST90094766 | Weighted mode | 10 | 0.346739718480493 | 0.540131878046615 | 0.53690907541659 | -0.711918762 | 1.40539819945186 | 1.41444852264359 | 0.490701753468259 | 4.07714993693829 | 0.999714303421225 |
| ebi-fl187-GCST90094766 | prot-a-992 | Persulfide dioxygenase ETHE1, mitochondrial \|\| id:prot-a-992 | Fruit liking \|\| id:ebi-fl187-GCST90094766 | Weighted mode | 10 | -0.37801488 | 0.839504633292832 | 0.663156912689358 | -2.023443962 | 1.26741420091236 | 0.685220304277815 | 0.132199390555308 | 3.55165680735984 | 0.999714303421225 |
| ebi-fl187-GCST90094766 | prot-a-993 | Ethanolamine kinase 1 \|\| id:prot-a-993 | Fruit liking \|\| id:ebi-fl187-GCST90094766 | Weighted mode | 10 | -0.018986426 | 0.503325598942286 | 0.970732839869174 | -1.0055046 | 0.967531747443047 | 0.981192680387128 | 0.365859975124546 | 2.63144137512594 | 0.999714303421225 |
| ebi-fl187-GCST90094766 | prot-b-10 | coagulation factor III, tissue factor \|\| id:prot-b-10 | Fruit liking \|\| id:ebi-fl187-GCST90094766 | Weighted mode | 6 | -0.414921672 | 0.613702196123237 | 0.52898349189417 | -1.617777977 | 0.787934632011231 | 0.660392005606469 | 0.198338923499144 | 2.19885029814037 | 0.999714303421225 |
| ebi-fl187-GCST90094766 | prot-b-28 | prolactin \|\| id:prot-b-28 | Fruit liking \|\| id:ebi-fl187-GCST90094766 | Weighted mode | 6 | 0.31447222320599 | 0.562849524474212 | 0.600452606272848 | -0.788712845 | 1.41765729117545 | 1.36953631069906 | 0.454429340106438 | 4.12743971567477 | 0.999714303421225 |
| ebi-fl187-GCST90094766 | prot-b-54 | matrix metallopeptidase 7 \|\| id:prot-b-54 | Fruit liking \|\| id:ebi-fl187-GCST90094766 | Weighted mode | 6 | -0.02201018 | 0.590900606431385 | 0.971728260705349 | -1.180175369 | 1.13615500835258 | 0.978230276368882 | 0.307224856204304 | 3.11476905035437 | 0.999714303421225 |
| ebi-fl187-GCST90094766 | ukb-rsfMRI191-edge-pheno716 | Net100_Pair10_33 (OccipitalPrecuneus)&(Temporal\|Frontal\|Supp_Motor_Area) (Default_mode\|Central_executive)&(Default_mode\|Salience) \|\| id:ukb-rsfMRI191-edge-pheno716 | Fruit liking \|\| id:ebi-fl187-GCST90094766 | Weighted mode | 10 | 0.0187731444267575 | 0.154589252553379 | 0.906011583000778 | -0.284221791 | 0.32176807943138 | 1.01895046780359 | 0.752599706670396 | 1.37956478940254 | 0.999714303421225 |
| ebi-fl187-GCST90094766 | ukb-rsfMRI191-edge-pheno903 | Net100_Pair27_38 (Precentral\|Frontal\|Supp_Motor_Area)&(Subcortical) (Attention\|Salience\|Motor)&(Subcortical-cerebellum) \|\| id:ukb-rsfMRI191-edge-pheno903 | Fruit liking \|\| id:ebi-fl187-GCST90094766 | Weighted mode | 10 | -0.144025435 | 0.162377732268037 | 0.398172873755675 | -0.46228579 | 0.174234920723907 | 0.865865724898784 | 0.629842311730639 | 1.19033516737619 | 0.999714303421225 |
| ebi-fl187-GCST90094766 | ukb-rsfMRI191-node-pheno23 | Net100_Node2 RolandicOper\|SupraMarginal\|Insula Salience\|Motor \|\| id:ukb-rsfMRI191-node-pheno23 | Fruit liking \|\| id:ebi-fl187-GCST90094766 | Weighted mode | 10 | -0.018975365 | 0.138799170537533 | 0.894268612872271 | -0.291021739 | 0.25307100973387 | 0.981203534365404 | 0.747499428120884 | 1.28797473233045 | 0.999714303421225 |
| ebi-fl187-GCST90094766 | ukb-rsfMRI191-node-pheno55 | Net100_Node34 Parietal\|Postcentral\|Precuneus Attention \|\| id:ukb-rsfMRI191-node-pheno55 | Fruit liking \|\| id:ebi-fl187-GCST90094766 | Weighted mode | 10 | 0.0431744859181374 | 0.181484568244616 | 0.817289227687541 | -0.312535268 | 0.398884239677584 | 1.04412006320512 | 0.731589826897735 | 1.49016110709239 | 0.999714303421225 |
| ebi-fl187-GCST90094770 | ebi-a-GCST90001414 | IgD- CD24- B cell Absolute Count \|\| id:ebi-a-GCST90001414 | Gherkins liking \|\| id:ebi-fl187-GCST90094770 | Inverse variance weighted | 19 | -0.079694411 | 0.100159170555378 | 0.426219435698192 | -0.276006385 | 0.116617563420943 | 0.923398483816906 | 0.758808085637668 | 1.1236896074965 | 0.998735809224318 |
| ebi-fl187-GCST90094770 | ebi-a-GCST90001422 | CD20- CD38- B cell %B cell \|\| id:ebi-a-GCST90001422 | Gherkins liking \|\| id:ebi-fl187-GCST90094770 | Inverse variance weighted | 19 | -0.04214042 | 0.10463239885644 | 0.687134103968417 | -0.247219922 | 0.162939081407839 | 0.958735145194536 | 0.780968922311149 | 1.1769649884544 | 0.998735809224318 |
| ebi-fl187-GCST90094770 | ebi-a-GCST90001444 | CD20- CD38- B cell %lymphocyte \|\| id:ebi-a-GCST90001444 | Gherkins liking \|\| id:ebi-fl187-GCST90094770 | Inverse variance weighted | 19 | -0.068787918 | 0.105669628447122 | 0.515064873551693 | -0.27590039 | 0.138324553427852 | 0.933524642551926 | 0.758888519817524 | 1.14834819014161 | 0.998735809224318 |
| ebi-fl187-GCST90094770 | ebi-a-GCST90001459 | Myeloid Dendritic Cell %Dendritic Cell \|\| id:ebi-a-GCST90001459 | Gherkins liking \|\| id:ebi-fl187-GCST90094770 | Inverse variance weighted | 19 | -0.037404777 | 0.10497338910535 | 0.721596232052562 | -0.24315262 | 0.168343065609215 | 0.963286140313941 | 0.784151827634608 | 1.18334250513704 | 0.998735809224318 |
| ebi-fl187-GCST90094770 | ebi-a-GCST90001476 | HLA DR++ monocyte %leukocyte \|\| id:ebi-a-GCST90001476 | Gherkins liking \|\| id:ebi-fl187-GCST90094770 | Inverse variance weighted (multiplicative random effects) | 19 | -0.034968415 | 0.125770206751927 | 0.780986131321822 | -0.28147802 | 0.211541190636189 | 0.965635915774876 | 0.754667503211966 | 1.23558085894217 | 0.998735809224318 |
| ebi-fl187-GCST90094770 | ebi-a-GCST90001478 | CD4 regulatory T cell %CD4+ T cell \|\| id:ebi-a-GCST90001478 | Gherkins liking \|\| id:ebi-fl187-GCST90094770 | Inverse variance weighted | 19 | 0.158828405663571 | 0.094110176238397 | 0.0914715639827702 | -0.02562754 | 0.343284351090829 | 1.17213679738722 | 0.974698058277974 | 1.40956951757602 | 0.998735809224318 |
| ebi-fl187-GCST90094770 | ebi-a-GCST90001485 | CD39+ resting CD4 regulatory T cell %CD4 regulatory T cell \|\| id:ebi-a-GCST90001485 | Gherkins liking \|\| id:ebi-fl187-GCST90094770 | Inverse variance weighted | 18 | 0.0854488972105143 | 0.0951557653578602 | 0.369191040578502 | -0.101056403 | 0.27195419731192 | 1.08920589846208 | 0.903882049887445 | 1.31252688267492 | 0.998735809224318 |
| ebi-fl187-GCST90094770 | ebi-a-GCST90001515 | Immature Myeloid-Derived Suppressor Cells Absolute Count \|\| id:ebi-a-GCST90001515 | Gherkins liking \|\| id:ebi-fl187-GCST90094770 | Inverse variance weighted (multiplicative random effects) | 19 | -0.010141019 | 0.164483535241825 | 0.950838611276546 | -0.332528748 | 0.312246710423217 | 0.989910228101159 | 0.717108053342737 | 1.36649177921161 | 0.998735809224318 |
| ebi-fl187-GCST90094770 | ebi-a-GCST90001517 | CD33+ HLA DR+ Absolute Count \|\| id:ebi-a-GCST90001517 | Gherkins liking \|\| id:ebi-fl187-GCST90094770 | Inverse variance weighted | 19 | -0.053704592 | 0.121112974829117 | 0.657457929324511 | -0.291086023 | 0.183676838271966 | 0.947712026496288 | 0.747451377199151 | 1.20162744034439 | 0.998735809224318 |
| ebi-fl187-GCST90094770 | ebi-a-GCST90001518 | CD33+ HLA DR+ CD14- Absolute Count \|\| id:ebi-a-GCST90001518 | Gherkins liking \|\| id:ebi-fl187-GCST90094770 | Inverse variance weighted | 19 | -0.070755304 | 0.122657881930457 | 0.564040214101458 | -0.311164752 | 0.169654144907309 | 0.931689845316698 | 0.732593169545882 | 1.18489497848353 | 0.998735809224318 |
| ebi-fl187-GCST90094770 | ebi-a-GCST90001520 | CD33+ HLA DR+ CD14dim Absolute Count \|\| id:ebi-a-GCST90001520 | Gherkins liking \|\| id:ebi-fl187-GCST90094770 | Inverse variance weighted | 19 | -0.044893082 | 0.125129808585553 | 0.719765598217037 | -0.290147507 | 0.200361342675323 | 0.956099700490608 | 0.748153201619508 | 1.22184418284844 | 0.998735809224318 |
| ebi-fl187-GCST90094770 | ebi-a-GCST90001531 | CD33dim HLA DR- Absolute Count \|\| id:ebi-a-GCST90001531 | Gherkins liking \|\| id:ebi-fl187-GCST90094770 | Inverse variance weighted | 19 | -0.050542072 | 0.150317807519951 | 0.736693842933099 | -0.345164975 | 0.244080830881494 | 0.950713929529844 | 0.708103524737755 | 1.27644750269647 | 0.998735809224318 |
| ebi-fl187-GCST90094770 | ebi-a-GCST90001532 | Basophil Absolute Count \|\| id:ebi-a-GCST90001532 | Gherkins liking \|\| id:ebi-fl187-GCST90094770 | Inverse variance weighted | 19 | -0.074294743 | 0.151889841588956 | 0.624745632189133 | -0.371998833 | 0.223409346349778 | 0.928398014541926 | 0.689355047222426 | 1.25033228795275 | 0.998735809224318 |
| ebi-fl187-GCST90094770 | ebi-a-GCST90001588 | T/B cell \|\| id:ebi-a-GCST90001588 | Gherkins liking \|\| id:ebi-fl187-GCST90094770 | Inverse variance weighted | 19 | 0.00983930654081553 | 0.091829293500093 | 0.914671728506988 | -0.170146109 | 0.189824721800998 | 1.00988787166912 | 0.843541558815194 | 1.2090376611401 | 0.998735809224318 |
| ebi-fl187-GCST90094770 | ebi-a-GCST90001624 | HLA DR+ CD4+ T cell Absolute Count \|\| id:ebi-a-GCST90001624 | Gherkins liking \|\| id:ebi-fl187-GCST90094770 | Inverse variance weighted | 19 | -0.017808978 | 0.0892623939006498 | 0.841861736274401 | -0.19276327 | 0.157145313840938 | 0.982348664442671 | 0.824677176655258 | 1.17016564281093 | 0.998735809224318 |
| ebi-fl187-GCST90094770 | ebi-a-GCST90001631 | CD8+ Natural Killer T %T cell \|\| id:ebi-a-GCST90001631 | Gherkins liking \|\| id:ebi-fl187-GCST90094770 | Inverse variance weighted | 19 | -0.042142748 | 0.0905652062867289 | 0.641694836590503 | -0.219650552 | 0.135365056699921 | 0.958732913960359 | 0.80279928560136 | 1.14495468144617 | 0.998735809224318 |
| ebi-fl187-GCST90094770 | ebi-a-GCST90001632 | CD8+ Natural Killer T %lymphocyte \|\| id:ebi-a-GCST90001632 | Gherkins liking \|\| id:ebi-fl187-GCST90094770 | Inverse variance weighted | 19 | -0.039083436 | 0.0911189135349864 | 0.667975759 | -0.217676507 | 0.139509634292988 | 0.961670467635195 | 0.804385612887253 | 1.14970988230656 | 0.998735809224318 |
| ebi-fl187-GCST90094770 | ebi-a-GCST90001646 | Natural Killer %CD3- lymphocyte \|\| id:ebi-a-GCST90001646 | Gherkins liking \|\| id:ebi-fl187-GCST90094770 | Inverse variance weighted | 19 | 0.00824616127611123 | 0.0945249863510568 | 0.930482414592105 | -0.177022812 | 0.193515134524183 | 1.00828025451235 | 0.837760673332024 | 1.21350775227494 | 0.998735809224318 |
| ebi-fl187-GCST90094770 | ebi-a-GCST90001684 | CD127- CD8+ T cell Absolute Count \|\| id:ebi-a-GCST90001684 | Gherkins liking \|\| id:ebi-fl187-GCST90094770 | Inverse variance weighted | 19 | -0.040915435 | 0.0960425442080053 | 0.67009789682404 | -0.229158822 | 0.147327951462729 | 0.959910301152707 | 0.795202228000144 | 1.15873390920494 | 0.998735809224318 |
| ebi-fl187-GCST90094770 | ebi-a-GCST90001687 | CD28- CD8+ T cell Absolute Count \|\| id:ebi-a-GCST90001687 | Gherkins liking \|\| id:ebi-fl187-GCST90094770 | Inverse variance weighted | 19 | -0.062666059 | 0.0935028950203261 | 0.502727451955313 | -0.245931733 | 0.120599615628721 | 0.939257078136656 | 0.781975606348624 | 1.12817311904292 | 0.998735809224318 |
| ebi-fl187-GCST90094770 | ebi-a-GCST90001701 | BAFF-R on CD20- CD38- B cell \|\| id:ebi-a-GCST90001701 | Gherkins liking \|\| id:ebi-fl187-GCST90094770 | Inverse variance weighted | 19 | -0.042747777 | 0.100706161319846 | 0.671215599939424 | -0.240131854 | 0.154636298833926 | 0.958153027485385 | 0.786524148236113 | 1.16723335976178 | 0.998735809224318 |
| ebi-fl187-GCST90094770 | ebi-a-GCST90001732 | CD19 on IgD- CD27- B cell \|\| id:ebi-a-GCST90001732 | Gherkins liking \|\| id:ebi-fl187-GCST90094770 | Inverse variance weighted | 19 | -0.135738006 | 0.0905857357695541 | 0.134016918054611 | -0.313286048 | 0.0418100361488658 | 0.873071342015719 | 0.731040769858183 | 1.04269638531514 | 0.998735809224318 |
| ebi-fl187-GCST90094770 | ebi-a-GCST90001798 | CD27 on CD24+ CD27+ B cell \|\| id:ebi-a-GCST90001798 | Gherkins liking \|\| id:ebi-fl187-GCST90094770 | Inverse variance weighted | 19 | 0.0183281241887188 | 0.0909324652419406 | 0.840262640427831 | -0.159899508 | 0.196555756062922 | 1.0184971151069 | 0.852229427170783 | 1.21720318544364 | 0.998735809224318 |
| ebi-fl187-GCST90094770 | ebi-a-GCST90001836 | CD66b on CD66b++ myeloid cell \|\| id:ebi-a-GCST90001836 | Gherkins liking \|\| id:ebi-fl187-GCST90094770 | Inverse variance weighted | 19 | -0.055248215 | 0.138409225858954 | 0.689771116869705 | -0.326530298 | 0.216033867543974 | 0.946250245164799 | 0.721422517185668 | 1.24114441280186 | 0.998735809224318 |
| ebi-fl187-GCST90094770 | ebi-a-GCST90001943 | CD25 on activated & secreting CD4 regulatory T cell \|\| id:ebi-a-GCST90001943 | Gherkins liking \|\| id:ebi-fl187-GCST90094770 | Inverse variance weighted | 19 | 0.0785245286163989 | 0.0928802794124522 | 0.397866269654767 | -0.103520819 | 0.260569876264805 | 1.08168988719318 | 0.901657250915284 | 1.29766938697412 | 0.998735809224318 |
| ebi-fl187-GCST90094770 | ebi-a-GCST90001960 | CD25 on CD4+ T cell \|\| id:ebi-a-GCST90001960 | Gherkins liking \|\| id:ebi-fl187-GCST90094770 | Inverse variance weighted | 19 | 0.174592368582567 | 0.0993152174383661 | 0.0787539918581594 | -0.020065458 | 0.369250194761765 | 1.19076072618587 | 0.980134513957262 | 1.4466495025279 | 0.998735809224318 |
| ebi-fl187-GCST90094770 | ebi-a-GCST90001988 | HLA DR on CD14+ CD16- monocyte \|\| id:ebi-a-GCST90001988 | Gherkins liking \|\| id:ebi-fl187-GCST90094770 | Inverse variance weighted | 19 | 0.0122714739620418 | 0.0926158734518546 | 0.894589926954105 | -0.169255638 | 0.193798585927677 | 1.01234707743749 | 0.844293042408266 | 1.21385177150454 | 0.998735809224318 |
| ebi-fl187-GCST90094770 | ebi-a-GCST90001991 | HLA DR on CD14+ monocyte \|\| id:ebi-a-GCST90001991 | Gherkins liking \|\| id:ebi-fl187-GCST90094770 | Inverse variance weighted | 19 | 0.00358093678822563 | 0.0927853730950308 | 0.969214274228922 | -0.178278394 | 0.185440268054486 | 1.00358735600235 | 0.836709455769604 | 1.20374829540007 | 0.998735809224318 |
| ebi-fl187-GCST90094770 | ebi-a-GCST90002019 | CD14 on Monocytic Myeloid-Derived Suppressor Cells \|\| id:ebi-a-GCST90002019 | Gherkins liking \|\| id:ebi-fl187-GCST90094770 | Inverse variance weighted | 19 | 0.101987602827528 | 0.141958088070549 | 0.472489356611197 | -0.17625025 | 0.380225455445805 | 1.10736974338914 | 0.838408145619244 | 1.4626143066251 | 0.998735809224318 |
| ebi-fl187-GCST90094770 | ebi-a-GCST90002037 | CD80 on plasmacytoid Dendritic Cell \|\| id:ebi-a-GCST90002037 | Gherkins liking \|\| id:ebi-fl187-GCST90094770 | Inverse variance weighted | 19 | -0.129636882 | 0.113086010191358 | 0.251647678116788 | -0.351285462 | 0.0920116983063701 | 0.878414341365777 | 0.703782822176577 | 1.09637764776742 | 0.998735809224318 |
| ebi-fl187-GCST90094770 | ebi-a-GCST90002042 | CD45 on CD33+ HLA DR+ CD14- \|\| id:ebi-a-GCST90002042 | Gherkins liking \|\| id:ebi-fl187-GCST90094770 | Inverse variance weighted | 19 | 0.135936552281017 | 0.135981098876787 | 0.317469070158787 | -0.130586402 | 0.40245950607952 | 1.14560920499802 | 0.877580665371773 | 1.49549836540691 | 0.998735809224318 |
| ebi-fl187-GCST90094770 | ebi-a-GCST90002056 | CD8 on Effector Memory CD8+ T cell \|\| id:ebi-a-GCST90002056 | Gherkins liking \|\| id:ebi-fl187-GCST90094770 | Inverse variance weighted | 19 | -0.029036889 | 0.105797083588935 | 0.78373243719755 | -0.236399173 | 0.178325394999878 | 0.971380630706418 | 0.789465477986454 | 1.19521417468211 | 0.998735809224318 |
| ebi-fl187-GCST90094770 | ebi-a-GCST90002058 | CD8 on CD8+ T cell \|\| id:ebi-a-GCST90002058 | Gherkins liking \|\| id:ebi-fl187-GCST90094770 | Inverse variance weighted | 19 | -0.158331656 | 0.122044660317065 | 0.194519196189835 | -0.397539191 | 0.0808758779151184 | 0.853566644258433 | 0.671971607209605 | 1.08423631054302 | 0.998735809224318 |
| ebi-fl187-GCST90094770 | ebi-a-GCST90002063 | CD4 on CD4 regulatory T cell \|\| id:ebi-a-GCST90002063 | Gherkins liking \|\| id:ebi-fl187-GCST90094770 | Inverse variance weighted | 19 | -0.084043861 | 0.100975789371797 | 0.405230063166283 | -0.281956409 | 0.113868685690914 | 0.919390929365891 | 0.754306565060534 | 1.12060496375561 | 0.998735809224318 |
| ebi-fl187-GCST90094770 | ebi-a-GCST90002066 | CD4 on activated CD4 regulatory T cell \|\| id:ebi-a-GCST90002066 | Gherkins liking \|\| id:ebi-fl187-GCST90094770 | Inverse variance weighted | 19 | -0.09953506 | 0.100977122283265 | 0.324271059711129 | -0.29745022 | 0.0983800991845391 | 0.905258210515078 | 0.742709554258995 | 1.10338210004929 | 0.998735809224318 |
| ebi-fl187-GCST90094770 | ebi-a-GCST90002070 | CD4 on activated & secreting CD4 regulatory T cell \|\| id:ebi-a-GCST90002070 | Gherkins liking \|\| id:ebi-fl187-GCST90094770 | Inverse variance weighted | 19 | -0.059148215 | 0.10144812304651 | 0.559867025335827 | -0.257986537 | 0.139690105794824 | 0.942567055872619 | 0.772605633976163 | 1.14991739089983 | 0.998735809224318 |
| ebi-fl187-GCST90094770 | ebi-a-GCST90002081 | SSC-A on CD4+ T cell \|\| id:ebi-a-GCST90002081 | Gherkins liking \|\| id:ebi-fl187-GCST90094770 | Inverse variance weighted | 19 | 0.0250305668316026 | 0.095779422681601 | 0.793833764152448 | -0.162697102 | 0.21275823528754 | 1.02534646163805 | 0.849848567183378 | 1.23708553145895 | 0.998735809224318 |
| ebi-fl187-GCST90094770 | ebi-a-GCST90002094 | CD11b on Monocytic Myeloid-Derived Suppressor Cells \|\| id:ebi-a-GCST90002094 | Gherkins liking \|\| id:ebi-fl187-GCST90094770 | Inverse variance weighted | 19 | 0.117261079023538 | 0.142511258963451 | 0.410610501507136 | -0.162060989 | 0.396583146591903 | 1.12441295200817 | 0.850389338950328 | 1.48673604987137 | 0.998735809224318 |
| ebi-fl187-GCST90094770 | ebi-a-GCST90002104 | HLA DR on myeloid Dendritic Cell \|\| id:ebi-a-GCST90002104 | Gherkins liking \|\| id:ebi-fl187-GCST90094770 | Inverse variance weighted | 18 | -0.028755262 | 0.10432910760483 | 0.782839405165601 | -0.233240313 | 0.175729788718283 | 0.971654235902097 | 0.791963231520961 | 1.19211589196297 | 0.998735809224318 |
| ebi-fl187-GCST90094770 | ebi-a-GCST90002105 | HLA DR on plasmacytoid Dendritic Cell \|\| id:ebi-a-GCST90002105 | Gherkins liking \|\| id:ebi-fl187-GCST90094770 | Inverse variance weighted | 18 | 0.0947721213361448 | 0.119460355509582 | 0.427582474179271 | -0.139370175 | 0.328914418134925 | 1.09940829488525 | 0.869905951011481 | 1.38945893801173 | 0.998735809224318 |
| ebi-fl187-GCST90094770 | ebi-a-GCST90002106 | HLA DR on Dendritic Cell \|\| id:ebi-a-GCST90002106 | Gherkins liking \|\| id:ebi-fl187-GCST90094770 | Inverse variance weighted | 18 | 0.0416882548110347 | 0.104094737047275 | 0.688800220451562 | -0.16233743 | 0.245713939423694 | 1.04256941208603 | 0.850154288742984 | 1.2785337831143 | 0.998735809224318 |
| ebi-fl187-GCST90094770 | ebi-a-GCST90002111 | HLA DR on CD33dim HLA DR+ CD11b- \|\| id:ebi-a-GCST90002111 | Gherkins liking \|\| id:ebi-fl187-GCST90094770 | Inverse variance weighted | 19 | -0.421243141 | 0.132259509395024 | 0.00144766855520201 | -0.680471779 | -0.162014502 | 0.656230525715251 | 0.506378037463011 | 0.850428871358724 | 0.998735809224318 |
| ebi-fl187-GCST90094770 | ebi-a-GCST90002112 | HLA DR on CD33- HLA DR+ \|\| id:ebi-a-GCST90002112 | Gherkins liking \|\| id:ebi-fl187-GCST90094770 | Inverse variance weighted | 19 | -0.125028917 | 0.165870649928163 | 0.45098517737415 | -0.450135391 | 0.200077556524488 | 0.882471383495254 | 0.637541828228898 | 1.22149748958657 | 0.998735809224318 |
| ebi-fl187-GCST90094770 | ebi-a-GCST90002115 | HLA DR on HLA DR+ CD8+ T cell \|\| id:ebi-a-GCST90002115 | Gherkins liking \|\| id:ebi-fl187-GCST90094770 | Inverse variance weighted | 19 | -0.127989479 | 0.114353461974956 | 0.263035863421411 | -0.352122264 | 0.0961433068285694 | 0.87986263644543 | 0.703194141312138 | 1.10091682158806 | 0.998735809224318 |
| ebi-fl187-GCST90094770 | ebi-a-GCST90002116 | HLA DR on B cell \|\| id:ebi-a-GCST90002116 | Gherkins liking \|\| id:ebi-fl187-GCST90094770 | Inverse variance weighted (multiplicative random effects) | 19 | -0.014253156 | 0.135561676825221 | 0.916263538907808 | -0.279954043 | 0.251447730133533 | 0.985847938911152 | 0.755818475791144 | 1.2858856852342 | 0.998735809224318 |
| ebi-fl187-GCST90094770 | ebi-cfmet338-GCST90026023 | Sphingomyelin (d18:1/24:1, d18:2/24:0) levels \|\| id:ebi-cfmet338-GCST90026023 | Gherkins liking \|\| id:ebi-fl187-GCST90094770 | Inverse variance weighted (multiplicative random effects) | 18 | -0.024691787 | 0.0753033530610227 | 0.742989109856773 | -0.172286359 | 0.122902785392791 | 0.975610561935511 | 0.841738099704216 | 1.13077448780635 | 0.998735809224318 |
| ebi-fl187-GCST90094770 | ebi-cfmet338-GCST90026046 | 1-arachidonoyl-gpc (20:4n6) levels \|\| id:ebi-cfmet338-GCST90026046 | Gherkins liking \|\| id:ebi-fl187-GCST90094770 | Inverse variance weighted | 18 | 0.0575816478025869 | 0.0757916232517025 | 0.447412222960199 | -0.090969934 | 0.206133229375924 | 1.05927175433517 | 0.913045162311749 | 1.22891692092355 | 0.998735809224318 |
| ebi-fl187-GCST90094770 | ebi-cfmet338-GCST90026047 | 1-linoleoyl-gpc (18:2) levels \|\| id:ebi-cfmet338-GCST90026047 | Gherkins liking \|\| id:ebi-fl187-GCST90094770 | Inverse variance weighted | 18 | 0.0272883769835848 | 0.0969933458687465 | 0.778447505748373 | -0.162818581 | 0.217395334886328 | 1.0276641147142 | 0.849745334449177 | 1.24283534119773 | 0.998735809224318 |
| ebi-fl187-GCST90094770 | ebi-cfmet338-GCST90026050 | 1-myristoyl-2-palmitoyl-gpc (14:0/16:0) levels \|\| id:ebi-cfmet338-GCST90026050 | Gherkins liking \|\| id:ebi-fl187-GCST90094770 | Inverse variance weighted | 18 | -0.045198197 | 0.0414650248434848 | 0.275699125338717 | -0.126469645 | 0.0360732519887376 | 0.955808025058172 | 0.881200897640324 | 1.03673178637466 | 0.998735809224318 |
| ebi-fl187-GCST90094770 | ebi-cfmet338-GCST90026068 | 2,3-dihydroxyisovalerate levels \|\| id:ebi-cfmet338-GCST90026068 | Gherkins liking \|\| id:ebi-fl187-GCST90094770 | Inverse variance weighted | 18 | 0.306676752434354 | 0.134264859132811 | 0.022364609713333 | 0.0435176285340449 | 0.569835876334663 | 1.3589016354236 | 1.04447840677289 | 1.76797686077819 | 0.998735809224318 |
| ebi-fl187-GCST90094770 | ebi-cfmet338-GCST90026082 | 3-hydroxyhexanoate levels \|\| id:ebi-cfmet338-GCST90026082 | Gherkins liking \|\| id:ebi-fl187-GCST90094770 | Inverse variance weighted | 18 | -0.028507865 | 0.0357101804104275 | 0.424689469245033 | -0.098499818 | 0.0414840888742797 | 0.971894650426911 | 0.906195857238692 | 1.04235657665299 | 0.998735809224318 |
[truncated: 91,668 more chars]
