# Supplementary material for: Mendelian Randomization Mediation Analysis Reveals the Impact of Dietary Preferences on Preeclampsia and Fetal Growth Restriction via Immune Modulation
Source: Food Sci Nutr. 2025 Nov 26;13(12):e71280. doi: 10.1002/fsn3.71280 (PMC12648301; doi:10.1002/fsn3.71280)
Supplement: Supplementary file 1 — Data S1: fsn371280‐sup‐0001‐Supinfo1.zip. [file FSN3-13-e71280-s001.zip › fsn371280-sup-0010-Supinfo10@Supplementary Material 3_PE - MR (Dietary Preferences to Immune Cells) MR Analysis - 3.docx]

| id.exposure | id.outcome | outcome | exposure | method | nsnp | b | se | pval | lo_ci | up_ci | or | or_lci95 | or_uci95 | p_fdr |
| --- | --- | --- | --- | --- | --- | --- | --- | --- | --- | --- | --- | --- | --- | --- |
| ebi-fl187-GCST90094770 | prot-a-533 | Chondroadherin \|\| id:prot-a-533 | Gherkins liking \|\| id:ebi-fl187-GCST90094770 | Inverse variance weighted (multiplicative random effects) | 21 | 0.0220515886757854 | 0.107768148897635 | 0.837868520400852 | -0.189173983 | 0.233277160515151 | 1.02229652203393 | 0.827642498307411 | 1.26273141011965 | 0.998735809224318 |
| ebi-fl187-GCST90094770 | prot-a-535 | Coiled-coil-helix-coiled-coil-helix domain-containing protein 2 \|\| id:prot-a-535 | Gherkins liking \|\| id:ebi-fl187-GCST90094770 | Inverse variance weighted | 21 | -0.083007553 | 0.102040510785459 | 0.415944938425472 | -0.283006954 | 0.116991847838751 | 0.920344195557369 | 0.753514547569598 | 1.12411026572504 | 0.998735809224318 |
| ebi-fl187-GCST90094770 | prot-a-591 | Caseinolytic peptidase B protein homolog \|\| id:prot-a-591 | Gherkins liking \|\| id:ebi-fl187-GCST90094770 | Inverse variance weighted | 21 | -0.039978916 | 0.0925457554432304 | 0.665748090298633 | -0.221368597 | 0.1414107645355 | 0.960809696522402 | 0.801421224541127 | 1.15189770954724 | 0.998735809224318 |
| ebi-fl187-GCST90094770 | prot-a-605 | Ciliary neurotrophic factor receptor subunit alpha \|\| id:prot-a-605 | Gherkins liking \|\| id:ebi-fl187-GCST90094770 | Inverse variance weighted | 21 | -0.156183411 | 0.0861628797666366 | 0.0698853434654274 | -0.325062655 | 0.0126958338362585 | 0.855402286216076 | 0.722482085218021 | 1.01277676808122 | 0.998735809224318 |
| ebi-fl187-GCST90094770 | prot-a-606 | Ciliary neurotrophic factor receptor subunit alpha \|\| id:prot-a-606 | Gherkins liking \|\| id:ebi-fl187-GCST90094770 | Inverse variance weighted | 21 | -0.167016954 | 0.0836616105110475 | 0.0458970119854341 | -0.330993711 | -0.003040197 | 0.846185264916043 | 0.718209686069904 | 0.996964419234165 | 0.998735809224318 |
| ebi-fl187-GCST90094770 | prot-a-707 | Cancer/testis antigen 55 \|\| id:prot-a-707 | Gherkins liking \|\| id:ebi-fl187-GCST90094770 | Inverse variance weighted | 21 | 0.161951973216124 | 0.0942882263035363 | 0.0858647304652434 | -0.02285295 | 0.346756896771055 | 1.1758037698914 | 0.977406200456898 | 1.41447282065999 | 0.998735809224318 |
| ebi-fl187-GCST90094770 | prot-a-719 | Dipeptidyl peptidase 1 \|\| id:prot-a-719 | Gherkins liking \|\| id:ebi-fl187-GCST90094770 | Inverse variance weighted | 21 | -0.080249598 | 0.0821625506946115 | 0.328709046792959 | -0.241288198 | 0.0807890012090039 | 0.922885967004426 | 0.785615181417329 | 1.08414211975526 | 0.998735809224318 |
| ebi-fl187-GCST90094770 | prot-a-752 | Cytochrome b5 \|\| id:prot-a-752 | Gherkins liking \|\| id:ebi-fl187-GCST90094770 | Inverse variance weighted | 21 | -0.076202601 | 0.0916559660856668 | 0.405748738748662 | -0.255848295 | 0.103443092063334 | 0.926628451258935 | 0.774259418914106 | 1.1089826816531 | 0.998735809224318 |
| ebi-fl187-GCST90094770 | prot-a-755 | Cytoglobin \|\| id:prot-a-755 | Gherkins liking \|\| id:ebi-fl187-GCST90094770 | Inverse variance weighted | 21 | 0.0776676838284038 | 0.0821558175958591 | 0.344469773108408 | -0.083357719 | 0.238693086316288 | 1.08076344381689 | 0.92002197931975 | 1.26958882260027 | 0.998735809224318 |
| ebi-fl187-GCST90094770 | prot-a-807 | Desmin \|\| id:prot-a-807 | Gherkins liking \|\| id:ebi-fl187-GCST90094770 | Inverse variance weighted | 21 | 0.0489535977476029 | 0.0885578974482626 | 0.580409709868825 | -0.124619881 | 0.222527076746198 | 1.05017161928087 | 0.882832419967394 | 1.24922964426674 | 0.998735809224318 |
| ebi-fl187-GCST90094770 | prot-a-82 | Ameloblastin \|\| id:prot-a-82 | Gherkins liking \|\| id:ebi-fl187-GCST90094770 | Inverse variance weighted | 21 | 0.0463807231904148 | 0.101121046355058 | 0.6464744783863 | -0.151816528 | 0.244577974046328 | 1.04747313236371 | 0.859145895784858 | 1.2770822376117 | 0.998735809224318 |
| ebi-fl187-GCST90094770 | prot-a-84 | Muellerian-inhibiting factor \|\| id:prot-a-84 | Gherkins liking \|\| id:ebi-fl187-GCST90094770 | Inverse variance weighted | 21 | -0.037957168 | 0.0821381037511524 | 0.643999564914218 | -0.198947852 | 0.123033514975444 | 0.962754176332204 | 0.81959263255718 | 1.13092232314623 | 0.998735809224318 |
| ebi-fl187-GCST90094770 | prot-a-858 | Inactive dipeptidyl peptidase 10 \|\| id:prot-a-858 | Gherkins liking \|\| id:ebi-fl187-GCST90094770 | Inverse variance weighted | 21 | 0.0187797580686458 | 0.0821723147200182 | 0.819225543221772 | -0.142277979 | 0.179837494919881 | 1.01895720679937 | 0.867380109703684 | 1.19702282502544 | 0.998735809224318 |
| ebi-fl187-GCST90094770 | prot-a-863 | Dihydropyrimidinase-related protein 3 \|\| id:prot-a-863 | Gherkins liking \|\| id:ebi-fl187-GCST90094770 | Inverse variance weighted | 21 | -0.110203871 | 0.0821293558052917 | 0.17965018561083 | -0.271177408 | 0.0507696665856291 | 0.895651519496846 | 0.762481214007633 | 1.05208053607073 | 0.998735809224318 |
| ebi-fl187-GCST90094770 | prot-a-864 | Dihydropyrimidinase-related protein 5 \|\| id:prot-a-864 | Gherkins liking \|\| id:ebi-fl187-GCST90094770 | Inverse variance weighted | 21 | 0.0736832048068818 | 0.0821723147200181 | 0.369883631175854 | -0.087374532 | 0.234740941658117 | 1.07646573230435 | 0.916333834971584 | 1.26458112600575 | 0.998735809224318 |
| ebi-fl187-GCST90094770 | prot-a-884 | Endothelin-converting enzyme 1 \|\| id:prot-a-884 | Gherkins liking \|\| id:ebi-fl187-GCST90094770 | Inverse variance weighted | 21 | 0.0158782254080014 | 0.0821461156645631 | 0.846729925008285 | -0.145128161 | 0.176884612110545 | 1.01600495428382 | 0.864911437837678 | 1.19349337050043 | 0.998735809224318 |
| ebi-fl187-GCST90094770 | prot-a-893 | Early endosome antigen 1 \|\| id:prot-a-893 | Gherkins liking \|\| id:ebi-fl187-GCST90094770 | Inverse variance weighted | 21 | -0.06153106 | 0.0821558175958591 | 0.453883961917764 | -0.222556463 | 0.0994943421442515 | 0.940323738507394 | 0.800469808682294 | 1.10461222098567 | 0.998735809224318 |
| ebi-fl187-GCST90094770 | prot-a-897 | Ephrin-A2 \|\| id:prot-a-897 | Gherkins liking \|\| id:ebi-fl187-GCST90094770 | Inverse variance weighted | 21 | 0.052127758324346 | 0.0821548484058287 | 0.52575054055083 | -0.108895745 | 0.21315126119977 | 1.0535103286302 | 0.896823911404013 | 1.23757183368689 | 0.998735809224318 |
| ebi-fl187-GCST90094770 | prot-a-90 | Anaphase-promoting complex subunit 10 \|\| id:prot-a-90 | Gherkins liking \|\| id:ebi-fl187-GCST90094770 | Inverse variance weighted | 21 | 0.0682773986212443 | 0.0833427317966415 | 0.412651557416197 | -0.095074356 | 0.231629152942662 | 1.07066226752569 | 0.909305319920765 | 1.26065213299657 | 0.998735809224318 |
| ebi-fl187-GCST90094770 | prot-a-914 | Histone-lysine N-methyltransferase EHMT2 \|\| id:prot-a-914 | Gherkins liking \|\| id:ebi-fl187-GCST90094770 | Inverse variance weighted | 21 | -0.08053893 | 0.0821528463780298 | 0.326911057057667 | -0.241558509 | 0.0804806489408485 | 0.922618985364335 | 0.785402849418462 | 1.08380787360906 | 0.998735809224318 |
| ebi-fl187-GCST90094770 | prot-a-932 | RNA polymerase II elongation factor ELL \|\| id:prot-a-932 | Gherkins liking \|\| id:ebi-fl187-GCST90094770 | Inverse variance weighted | 21 | -0.079583252 | 0.0832675112906639 | 0.339196566351107 | -0.242787574 | 0.0836210705625319 | 0.92350113385153 | 0.78443813136623 | 1.08721683727903 | 0.998735809224318 |
| ebi-fl187-GCST90094770 | prot-a-991 | Estrogen receptor \|\| id:prot-a-991 | Gherkins liking \|\| id:ebi-fl187-GCST90094770 | Inverse variance weighted | 21 | 0.027844077061455 | 0.0995834946912975 | 0.779780308420313 | -0.167339573 | 0.223027726656398 | 1.02823534644483 | 0.845912313973237 | 1.24985522756201 | 0.998735809224318 |
| ebi-fl187-GCST90094770 | prot-a-992 | Persulfide dioxygenase ETHE1, mitochondrial \|\| id:prot-a-992 | Gherkins liking \|\| id:ebi-fl187-GCST90094770 | Inverse variance weighted | 21 | 0.0194962963920411 | 0.0991438203849194 | 0.844104091527544 | -0.174825592 | 0.213818184346483 | 1.01968759033059 | 0.839603441924803 | 1.2383974742773 | 0.998735809224318 |
| ebi-fl187-GCST90094770 | prot-a-993 | Ethanolamine kinase 1 \|\| id:prot-a-993 | Gherkins liking \|\| id:ebi-fl187-GCST90094770 | Inverse variance weighted | 21 | 0.0156973805205559 | 0.0821227391401409 | 0.848411710761512 | -0.145263188 | 0.176657949235232 | 1.01582123159537 | 0.864794659412109 | 1.19322288051758 | 0.998735809224318 |
| ebi-fl187-GCST90094770 | prot-b-10 | coagulation factor III, tissue factor \|\| id:prot-b-10 | Gherkins liking \|\| id:ebi-fl187-GCST90094770 | Inverse variance weighted | 15 | 0.0818262158075166 | 0.11770196588812 | 0.486930988508284 | -0.148869637 | 0.312522068948231 | 1.08526719115811 | 0.861681438667665 | 1.36686810618241 | 0.998735809224318 |
| ebi-fl187-GCST90094770 | prot-b-28 | prolactin \|\| id:prot-b-28 | Gherkins liking \|\| id:ebi-fl187-GCST90094770 | Inverse variance weighted | 15 | -0.01829865 | 0.11770196588812 | 0.876453978399042 | -0.248994504 | 0.212397202644478 | 0.981867753274755 | 0.779584258251036 | 1.23663898381382 | 0.998735809224318 |
| ebi-fl187-GCST90094770 | prot-b-54 | matrix metallopeptidase 7 \|\| id:prot-b-54 | Gherkins liking \|\| id:ebi-fl187-GCST90094770 | Inverse variance weighted | 15 | -0.020641077 | 0.133171595970789 | 0.876824398529355 | -0.281657405 | 0.240375251216357 | 0.97957049197101 | 0.754532139204348 | 1.27172627762706 | 0.998735809224318 |
| ebi-fl187-GCST90094770 | prot-c-2421_7_3 | BDNF \|\| id:prot-c-2421_7_3 | Gherkins liking \|\| id:ebi-fl187-GCST90094770 | Inverse variance weighted | 3 | 0.577546925521689 | 0.41023963041372 | 0.159181944232022 | -0.22652275 | 1.38161660113258 | 1.78166251487646 | 0.797301203433158 | 3.98133265477007 | 0.998735809224318 |
| ebi-fl187-GCST90094770 | prot-c-2443_10_4 | FGF-8B \|\| id:prot-c-2443_10_4 | Gherkins liking \|\| id:ebi-fl187-GCST90094770 | Inverse variance weighted (multiplicative random effects) | 3 | -0.002735148 | 0.779933780914205 | 0.997201906065959 | -1.531405359 | 1.5259350624743 | 0.99726858899211 | 0.216231570762472 | 4.59944232511177 | 0.999604360968858 |
| ebi-fl187-GCST90094770 | prot-c-2605_49_2 | CD30 \|\| id:prot-c-2605_49_2 | Gherkins liking \|\| id:ebi-fl187-GCST90094770 | Inverse variance weighted | 3 | 0.285073777754405 | 0.59743130144907 | 0.633244070879086 | -0.885891573 | 1.45603912859458 | 1.32986013859694 | 0.412346372415077 | 4.28893790885309 | 0.998735809224318 |
| ebi-fl187-GCST90094770 | prot-c-2624_31_2 | HPV E7 Type18 \|\| id:prot-c-2624_31_2 | Gherkins liking \|\| id:ebi-fl187-GCST90094770 | Inverse variance weighted | 3 | 0.681370235842255 | 0.48886816051512 | 0.163386786060559 | -0.276811359 | 1.63955183045189 | 1.97658426411741 | 0.758197510933963 | 5.15285963988984 | 0.998735809224318 |
| ebi-fl187-GCST90094770 | prot-c-2774_10_3 | IL-16 \|\| id:prot-c-2774_10_3 | Gherkins liking \|\| id:ebi-fl187-GCST90094770 | Inverse variance weighted | 3 | -0.302138549 | 0.398943064942396 | 0.448841675569128 | -1.084066957 | 0.47978985792263 | 0.739235637166328 | 0.338217210037783 | 1.61573483264101 | 0.998735809224318 |
| ebi-fl187-GCST90094770 | prot-c-2851_63_3 | C5a \|\| id:prot-c-2851_63_3 | Gherkins liking \|\| id:ebi-fl187-GCST90094770 | Inverse variance weighted | 3 | 0.612861112625804 | 0.44211206918704 | 0.165682138991383 | -0.253678543 | 1.4794007682324 | 1.8457046203368 | 0.775941193703186 | 4.39031407686254 | 0.998735809224318 |
| ebi-fl187-GCST90094770 | prot-c-2891_1_3 | CD22 \|\| id:prot-c-2891_1_3 | Gherkins liking \|\| id:ebi-fl187-GCST90094770 | Inverse variance weighted | 3 | 0.741595577163335 | 0.416601182999607 | 0.0750580996624592 | -0.074942742 | 1.55813389584256 | 2.09928241074266 | 0.927796609035066 | 4.74994906980411 | 0.998735809224318 |
| ebi-fl187-GCST90094770 | prot-c-2961_1_2 | Protein C \|\| id:prot-c-2961_1_2 | Gherkins liking \|\| id:ebi-fl187-GCST90094770 | Inverse variance weighted | 3 | 0.143427100316664 | 0.404326983837333 | 0.72279152303811 | -0.649053788 | 0.935907988637837 | 1.15422266527326 | 0.522539976509176 | 2.54952734895127 | 0.998735809224318 |
| ebi-fl187-GCST90094770 | prot-c-2982_82_2 | Galectin-4 \|\| id:prot-c-2982_82_2 | Gherkins liking \|\| id:ebi-fl187-GCST90094770 | Inverse variance weighted | 3 | 0.0600493446067154 | 0.436805314473219 | 0.890656195426516 | -0.796089072 | 0.916187760974225 | 1.06188894374489 | 0.451089698259866 | 2.49974258600348 | 0.998735809224318 |
| ebi-fl187-GCST90094770 | prot-c-2994_71_2 | IL-1Rrp2 \|\| id:prot-c-2994_71_2 | Gherkins liking \|\| id:ebi-fl187-GCST90094770 | Inverse variance weighted | 3 | -0.700734577 | 0.412882768948466 | 0.0896634594575337 | -1.509984804 | 0.108515650180293 | 0.496220657615952 | 0.220913334911328 | 1.11462235244262 | 0.998735809224318 |
| ebi-fl187-GCST90094770 | prot-c-3336_50_1 | TFPI \|\| id:prot-c-3336_50_1 | Gherkins liking \|\| id:ebi-fl187-GCST90094770 | Inverse variance weighted | 3 | 0.0194331264853167 | 0.401659559860441 | 0.961411737636203 | -0.767819611 | 0.80668586381178 | 1.01962317879508 | 0.464023718390626 | 2.24047044479091 | 0.998735809224318 |
| ebi-fl187-GCST90094770 | prot-c-3472_40_2 | Survivin \|\| id:prot-c-3472_40_2 | Gherkins liking \|\| id:ebi-fl187-GCST90094770 | Inverse variance weighted | 3 | 0.628354967835687 | 0.422306790125397 | 0.136774335486089 | -0.199366341 | 1.45607627648146 | 1.87452438860682 | 0.819249713748465 | 4.28909723679271 | 0.998735809224318 |
| ebi-fl187-GCST90094770 | prot-c-3473_78_2 | Thrombopoietin Receptor \|\| id:prot-c-3473_78_2 | Gherkins liking \|\| id:ebi-fl187-GCST90094770 | Inverse variance weighted | 3 | 0.244513683251247 | 0.413580266194907 | 0.554378274971088 | -0.566103638 | 1.05513100499326 | 1.27700013561849 | 0.567733228632079 | 2.87235142163297 | 0.998735809224318 |
| ebi-fl187-GCST90094770 | prot-c-3497_13_2 | IFN-aA \|\| id:prot-c-3497_13_2 | Gherkins liking \|\| id:ebi-fl187-GCST90094770 | Inverse variance weighted | 3 | 0.643573462637419 | 0.415150516634068 | 0.121089397976716 | -0.17012155 | 1.45726847524019 | 1.90327000599045 | 0.843562275399222 | 4.29421374253435 | 0.998735809224318 |
| ebi-fl187-GCST90094770 | prot-c-3665_64_3 | COLEC12 \|\| id:prot-c-3665_64_3 | Gherkins liking \|\| id:ebi-fl187-GCST90094770 | Inverse variance weighted | 3 | -0.401811645 | 0.564852987406165 | 0.476863565762249 | -1.508923501 | 0.705300209873647 | 0.669106763130327 | 0.221147915429863 | 2.02445435488961 | 0.998735809224318 |
| ebi-fl187-GCST90094770 | prot-c-3741_4_3 | IL-5 \|\| id:prot-c-3741_4_3 | Gherkins liking \|\| id:ebi-fl187-GCST90094770 | Inverse variance weighted | 3 | -0.345247187 | 0.647109146743706 | 0.593672050161573 | -1.613581114 | 0.923086740799811 | 0.708045312367105 | 0.199173073843696 | 2.51704788549107 | 0.998735809224318 |
| ebi-fl187-GCST90094770 | prot-c-3848_14_2 | GAPDH, liver \|\| id:prot-c-3848_14_2 | Gherkins liking \|\| id:ebi-fl187-GCST90094770 | Inverse variance weighted | 3 | -0.053362376 | 0.409703187561633 | 0.89637146543961 | -0.856380623 | 0.749655872077277 | 0.948036405021037 | 0.424696440391267 | 2.11627162313201 | 0.998735809224318 |
| ebi-fl187-GCST90094770 | prot-c-3859_50_2 | PSA1 \|\| id:prot-c-3859_50_2 | Gherkins liking \|\| id:ebi-fl187-GCST90094770 | Inverse variance weighted | 3 | 0.0820178308614776 | 0.408442762581812 | 0.840850130497991 | -0.718529984 | 0.882565645521829 | 1.08547516461429 | 0.487468315843035 | 2.41709316216117 | 0.998735809224318 |
| ebi-fl187-GCST90094770 | prot-c-3868_8_1 | SGTA \|\| id:prot-c-3868_8_1 | Gherkins liking \|\| id:ebi-fl187-GCST90094770 | Inverse variance weighted | 3 | 0.108844417866997 | 0.672849639221602 | 0.871489867256187 | -1.209940875 | 1.42762971074134 | 1.11498886450034 | 0.298214910863068 | 4.16880619537697 | 0.998735809224318 |
| ebi-fl187-GCST90094770 | prot-c-3882_10_1 | EF-1-beta \|\| id:prot-c-3882_10_1 | Gherkins liking \|\| id:ebi-fl187-GCST90094770 | Inverse variance weighted | 3 | 0.292795678930734 | 0.412427420770618 | 0.477745921590265 | -0.515562066 | 1.10115342364115 | 1.34016893770534 | 0.597164854343565 | 3.00763309917927 | 0.998735809224318 |
| ebi-fl187-GCST90094770 | prot-c-4134_4_2 | HB-EGF \|\| id:prot-c-4134_4_2 | Gherkins liking \|\| id:ebi-fl187-GCST90094770 | Inverse variance weighted | 3 | 0.675606132689174 | 0.415873432828242 | 0.104259018045693 | -0.139505796 | 1.49071806103253 | 1.96522380142186 | 0.869787982199437 | 4.44028276857637 | 0.998735809224318 |
| ebi-fl187-GCST90094770 | prot-c-4220_39_2 | FER \|\| id:prot-c-4220_39_2 | Gherkins liking \|\| id:ebi-fl187-GCST90094770 | Inverse variance weighted | 3 | 0.142283903716345 | 0.50906879714007 | 0.779862381668944 | -0.855490939 | 1.14005874611088 | 1.15290391578489 | 0.425074454357245 | 3.12695205606275 | 0.998735809224318 |
| ebi-fl187-GCST90094770 | prot-c-4228_37_2 | HSP70 protein 8 \|\| id:prot-c-4228_37_2 | Gherkins liking \|\| id:ebi-fl187-GCST90094770 | Inverse variance weighted | 3 | 0.429571662419492 | 0.415685816245165 | 0.301414563443724 | -0.385172537 | 1.24431586226002 | 1.53659919938258 | 0.680333243134318 | 3.47055964613071 | 0.998735809224318 |
| ebi-fl187-GCST90094770 | prot-c-4246_40_2 | NCAM-L1 \|\| id:prot-c-4246_40_2 | Gherkins liking \|\| id:ebi-fl187-GCST90094770 | Inverse variance weighted | 3 | 0.0648826321760316 | 0.411746524756846 | 0.874788501560645 | -0.742140556 | 0.87190582069945 | 1.06703378160155 | 0.476093718584797 | 2.39146421520393 | 0.998735809224318 |
| ebi-fl187-GCST90094770 | prot-c-4267_81_3 | PESC \|\| id:prot-c-4267_81_3 | Gherkins liking \|\| id:ebi-fl187-GCST90094770 | Inverse variance weighted | 3 | 0.655485795170932 | 0.575370873677368 | 0.254602862464573 | -0.472241117 | 1.78321270757857 | 1.92607796951533 | 0.623603133324615 | 5.94893794852243 | 0.998735809224318 |
| ebi-fl187-GCST90094770 | prot-c-4280_47_2 | PSA2 \|\| id:prot-c-4280_47_2 | Gherkins liking \|\| id:ebi-fl187-GCST90094770 | Inverse variance weighted | 3 | 0.564202796228853 | 0.412901728252969 | 0.171802907754196 | -0.245084591 | 1.37349018360467 | 1.75804570326007 | 0.782638331167549 | 3.94910978886046 | 0.998735809224318 |
| ebi-fl187-GCST90094770 | prot-c-4324_33_2 | CYTT \|\| id:prot-c-4324_33_2 | Gherkins liking \|\| id:ebi-fl187-GCST90094770 | Inverse variance weighted | 3 | -0.505727219 | 0.4003141343517 | 0.206472077348377 | -1.290342923 | 0.278888484102649 | 0.603066854111802 | 0.275176402712724 | 1.32165994955602 | 0.998735809224318 |
| ebi-fl187-GCST90094770 | prot-c-4462_8_2 | RASA1 \|\| id:prot-c-4462_8_2 | Gherkins liking \|\| id:ebi-fl187-GCST90094770 | Inverse variance weighted | 3 | 0.327275280244628 | 0.414355784541566 | 0.429620500229603 | -0.484862057 | 1.1394126179461 | 1.38718328878037 | 0.615782133459479 | 3.12493229685132 | 0.998735809224318 |
| ebi-fl187-GCST90094770 | prot-c-4499_21_1 | PDGF-AA \|\| id:prot-c-4499_21_1 | Gherkins liking \|\| id:ebi-fl187-GCST90094770 | Inverse variance weighted | 3 | 0.327528227154147 | 0.414254190529485 | 0.429150865657296 | -0.484409986 | 1.13946644059194 | 1.38753421688743 | 0.616060573743658 | 3.12510049350196 | 0.998735809224318 |
| ebi-fl187-GCST90094770 | prot-c-4535_50_2 | BST1 \|\| id:prot-c-4535_50_2 | Gherkins liking \|\| id:ebi-fl187-GCST90094770 | Inverse variance weighted | 3 | -0.326266475 | 0.423743096677729 | 0.44132188426833 | -1.156802945 | 0.504269994441779 | 0.721612869985819 | 0.314490017395898 | 1.65577635322412 | 0.998735809224318 |
| ebi-fl187-GCST90094770 | prot-c-4542_24_2 | Clusterin \|\| id:prot-c-4542_24_2 | Gherkins liking \|\| id:ebi-fl187-GCST90094770 | Inverse variance weighted | 3 | 0.235207497704186 | 0.412675022723664 | 0.568706074587289 | -0.573635547 | 1.04405054224257 | 1.2651712616308 | 0.563473177287885 | 2.84070011807978 | 0.998735809224318 |
| ebi-fl187-GCST90094770 | prot-c-4696_2_2 | FABP \|\| id:prot-c-4696_2_2 | Gherkins liking \|\| id:ebi-fl187-GCST90094770 | Inverse variance weighted | 3 | -0.45120637 | 0.394437890613166 | 0.252655786972682 | -1.224304636 | 0.321891895280252 | 0.636859399736431 | 0.293962039912545 | 1.37973561196307 | 0.998735809224318 |
| ebi-fl187-GCST90094770 | prot-c-4906_35_1 | Coagulation Factor V \|\| id:prot-c-4906_35_1 | Gherkins liking \|\| id:ebi-fl187-GCST90094770 | Inverse variance weighted | 3 | -0.542009759 | 0.416042144525517 | 0.192651215251429 | -1.357452362 | 0.273432844106182 | 0.581578244844069 | 0.25731548922135 | 1.31446908190183 | 0.998735809224318 |
| ebi-fl187-GCST90094770 | prot-c-4916_2_1 | IgD \|\| id:prot-c-4916_2_1 | Gherkins liking \|\| id:ebi-fl187-GCST90094770 | Inverse variance weighted | 3 | -0.407756528 | 0.413075713783739 | 0.32358235555152 | -1.217384927 | 0.401871871201954 | 0.6651408023947 | 0.296003225774598 | 1.49461981656631 | 0.998735809224318 |
| ebi-fl187-GCST90094770 | prot-c-4922_13_1 | MIP-3b \|\| id:prot-c-4922_13_1 | Gherkins liking \|\| id:ebi-fl187-GCST90094770 | Inverse variance weighted | 3 | 0.268184182720471 | 0.439656220468238 | 0.541871016192341 | -0.593542009 | 1.12991037483822 | 1.30758795294389 | 0.552367325429059 | 3.09537906384289 | 0.998735809224318 |
| ebi-fl187-GCST90094770 | prot-c-4973_18_1 | cIAP-2 \|\| id:prot-c-4973_18_1 | Gherkins liking \|\| id:ebi-fl187-GCST90094770 | Inverse variance weighted | 3 | -0.140532955 | 0.415475505074465 | 0.735177764159617 | -0.954864945 | 0.673799035020437 | 0.868895030089953 | 0.384864118916245 | 1.96167565695912 | 0.998735809224318 |
| ebi-fl187-GCST90094770 | prot-c-4997_19_1 | IF4A3 \|\| id:prot-c-4997_19_1 | Gherkins liking \|\| id:ebi-fl187-GCST90094770 | Inverse variance weighted | 3 | -0.073964665 | 0.415594407046864 | 0.85874402513312 | -0.888529703 | 0.740600372410358 | 0.928704508662478 | 0.411259982675844 | 2.09719423416363 | 0.998735809224318 |
| ebi-fl187-GCST90094770 | prot-c-5000_52_1 | LG3BP \|\| id:prot-c-5000_52_1 | Gherkins liking \|\| id:ebi-fl187-GCST90094770 | Inverse variance weighted | 3 | -0.106138203 | 0.383720684764962 | 0.782085140614123 | -0.858230745 | 0.645954339379701 | 0.899300353696596 | 0.423911426685662 | 1.90780685597918 | 0.998735809224318 |
| ebi-fl187-GCST90094770 | prot-c-5006_71_1 | MK13 \|\| id:prot-c-5006_71_1 | Gherkins liking \|\| id:ebi-fl187-GCST90094770 | Inverse variance weighted | 3 | -0.379969346 | 0.414326505250192 | 0.359102244717173 | -1.192049296 | 0.432110604109073 | 0.683882372497309 | 0.303598462873747 | 1.54050549197623 | 0.998735809224318 |
| ebi-fl187-GCST90094770 | prot-c-5109_24_3 | Nr-CAM \|\| id:prot-c-5109_24_3 | Gherkins liking \|\| id:ebi-fl187-GCST90094770 | Inverse variance weighted | 3 | -0.205764334 | 0.407193681316604 | 0.613331989203724 | -1.003863949 | 0.592335281719005 | 0.814024891945487 | 0.366460716462258 | 1.80820615945913 | 0.998735809224318 |
| ebi-fl187-GCST90094770 | prot-c-5255_22_3 | PDE4D \|\| id:prot-c-5255_22_3 | Gherkins liking \|\| id:ebi-fl187-GCST90094770 | Inverse variance weighted | 3 | 0.00929816451343328 | 0.407988781143685 | 0.981817588910204 | -0.790359847 | 0.808956175555056 | 1.00934152673726 | 0.453681510188986 | 2.24556278956997 | 0.998955228396052 |
| ebi-fl187-GCST90094770 | prot-c-5440_26_3 | Troponin I, skeletal, fast twitch \|\| id:prot-c-5440_26_3 | Gherkins liking \|\| id:ebi-fl187-GCST90094770 | Inverse variance weighted | 3 | -0.289040798 | 0.67262167636817 | 0.667397373763456 | -1.607379284 | 1.02929768744368 | 0.748981647647852 | 0.200412149789008 | 2.79909930163355 | 0.998735809224318 |
| ebi-fl187-GCST90094770 | ukb-rsfMRI191-edge-pheno716 | Net100_Pair10_33 (OccipitalPrecuneus)&(Temporal\|Frontal\|Supp_Motor_Area) (Default_mode\|Central_executive)&(Default_mode\|Salience) \|\| id:ukb-rsfMRI191-edge-pheno716 | Gherkins liking \|\| id:ebi-fl187-GCST90094770 | Inverse variance weighted | 21 | 0.0133847209512401 | 0.0293541723016164 | 0.648409125837544 | -0.044149457 | 0.0709188986624082 | 1.01347469731673 | 0.956810944922525 | 1.07349416052553 | 0.998735809224318 |
| ebi-fl187-GCST90094770 | ukb-rsfMRI191-edge-pheno903 | Net100_Pair27_38 (Precentral\|Frontal\|Supp_Motor_Area)&(Subcortical) (Attention\|Salience\|Motor)&(Subcortical-cerebellum) \|\| id:ukb-rsfMRI191-edge-pheno903 | Gherkins liking \|\| id:ebi-fl187-GCST90094770 | Inverse variance weighted (multiplicative random effects) | 21 | 0.0591243775933283 | 0.0325301633715317 | 0.0691367995653981 | -0.004634743 | 0.12288349780153 | 1.06090718561699 | 0.995375981230861 | 1.13075267810055 | 0.998735809224318 |
| ebi-fl187-GCST90094770 | ukb-rsfMRI191-node-pheno23 | Net100_Node2 RolandicOper\|SupraMarginal\|Insula Salience\|Motor \|\| id:ukb-rsfMRI191-node-pheno23 | Gherkins liking \|\| id:ebi-fl187-GCST90094770 | Inverse variance weighted | 21 | 0.00508916097981818 | 0.0268003030768805 | 0.849393824998231 | -0.047439433 | 0.057617755010504 | 1.00510213275537 | 0.953668232158771 | 1.05931000237115 | 0.998735809224318 |
| ebi-fl187-GCST90094770 | ukb-rsfMRI191-node-pheno55 | Net100_Node34 Parietal\|Postcentral\|Precuneus Attention \|\| id:ukb-rsfMRI191-node-pheno55 | Gherkins liking \|\| id:ebi-fl187-GCST90094770 | Inverse variance weighted | 21 | 0.00662050135947687 | 0.0297903167309991 | 0.824129634330871 | -0.051768519 | 0.0650095221522351 | 1.00664246532266 | 0.949548643463016 | 1.06716918608326 | 0.998735809224318 |
| ebi-fl187-GCST90094770 | ebi-a-GCST90001414 | IgD- CD24- B cell Absolute Count \|\| id:ebi-a-GCST90001414 | Gherkins liking \|\| id:ebi-fl187-GCST90094770 | MR Egger | 19 | 0.419585631229009 | 0.438955615872942 | 0.352533145082711 | -0.440767376 | 1.27993863833998 | 1.52133103292282 | 0.643542392643498 | 3.5964190365562 | 0.999950574162623 |
| ebi-fl187-GCST90094770 | ebi-a-GCST90001422 | CD20- CD38- B cell %B cell \|\| id:ebi-a-GCST90001422 | Gherkins liking \|\| id:ebi-fl187-GCST90094770 | MR Egger | 19 | -0.114884563 | 0.472731640488283 | 0.810895806103787 | -1.041438578 | 0.811669452679049 | 0.89146904676672 | 0.352946575380049 | 2.251663897 | 0.999950574162623 |
| ebi-fl187-GCST90094770 | ebi-a-GCST90001444 | CD20- CD38- B cell %lymphocyte \|\| id:ebi-a-GCST90001444 | Gherkins liking \|\| id:ebi-fl187-GCST90094770 | MR Egger | 19 | -0.060866884 | 0.476992906851197 | 0.899958536014151 | -0.995772981 | 0.874039213868728 | 0.940948487152775 | 0.369437765776974 | 2.39657159471236 | 0.999950574162623 |
| ebi-fl187-GCST90094770 | ebi-a-GCST90001459 | Myeloid Dendritic Cell %Dendritic Cell \|\| id:ebi-a-GCST90001459 | Gherkins liking \|\| id:ebi-fl187-GCST90094770 | MR Egger | 19 | -0.089097611 | 0.480029835502706 | 0.854948378994417 | -1.029956089 | 0.851760866442441 | 0.914756278810625 | 0.357022637543034 | 2.3437702868984 | 0.999950574162623 |
| ebi-fl187-GCST90094770 | ebi-a-GCST90001476 | HLA DR++ monocyte %leukocyte \|\| id:ebi-a-GCST90001476 | Gherkins liking \|\| id:ebi-fl187-GCST90094770 | MR Egger | 19 | -0.906665812 | 0.529963753038812 | 0.10530110147433 | -1.945394768 | 0.132063143675238 | 0.403868555238984 | 0.142930787664274 | 1.14118037531528 | 0.999950574162623 |
| ebi-fl187-GCST90094770 | ebi-a-GCST90001478 | CD4 regulatory T cell %CD4+ T cell \|\| id:ebi-a-GCST90001478 | Gherkins liking \|\| id:ebi-fl187-GCST90094770 | MR Egger | 19 | -0.405636209 | 0.417089444686568 | 0.344417689587502 | -1.22313152 | 0.411859102765872 | 0.666552609283504 | 0.294307093697639 | 1.50962171981861 | 0.999950574162623 |
| ebi-fl187-GCST90094770 | ebi-a-GCST90001485 | CD39+ resting CD4 regulatory T cell %CD4 regulatory T cell \|\| id:ebi-a-GCST90001485 | Gherkins liking \|\| id:ebi-fl187-GCST90094770 | MR Egger | 18 | -0.028281764 | 0.410148553301494 | 0.9458799228487 | -0.832172929 | 0.775609400041599 | 0.9721144209439 | 0.435102810909646 | 2.1719152892426 | 0.999950574162623 |
| ebi-fl187-GCST90094770 | ebi-a-GCST90001515 | Immature Myeloid-Derived Suppressor Cells Absolute Count \|\| id:ebi-a-GCST90001515 | Gherkins liking \|\| id:ebi-fl187-GCST90094770 | MR Egger | 19 | -0.570211109 | 0.733730461481347 | 0.447756235985736 | -2.008322814 | 0.867900595464049 | 0.565406063768428 | 0.134213587216791 | 2.38190501852643 | 0.999950574162623 |
| ebi-fl187-GCST90094770 | ebi-a-GCST90001517 | CD33+ HLA DR+ Absolute Count \|\| id:ebi-a-GCST90001517 | Gherkins liking \|\| id:ebi-fl187-GCST90094770 | MR Egger | 19 | -0.135868563 | 0.533683872430968 | 0.802096783613764 | -1.181888952 | 0.910151827459536 | 0.872957364277587 | 0.306698851530899 | 2.48469975039895 | 0.999950574162623 |
| ebi-fl187-GCST90094770 | ebi-a-GCST90001518 | CD33+ HLA DR+ CD14- Absolute Count \|\| id:ebi-a-GCST90001518 | Gherkins liking \|\| id:ebi-fl187-GCST90094770 | MR Egger | 19 | -0.109746357 | 0.541181476693423 | 0.84170620477671 | -1.170462051 | 0.950969337641957 | 0.896061386462478 | 0.310223569036258 | 2.58821730019879 | 0.999950574162623 |
| ebi-fl187-GCST90094770 | ebi-a-GCST90001520 | CD33+ HLA DR+ CD14dim Absolute Count \|\| id:ebi-a-GCST90001520 | Gherkins liking \|\| id:ebi-fl187-GCST90094770 | MR Egger | 19 | -0.181317309 | 0.565615801476037 | 0.752446025238743 | -1.28992428 | 0.927289661871428 | 0.834170626831655 | 0.275291627406102 | 2.52764910151892 | 0.999950574162623 |
| ebi-fl187-GCST90094770 | ebi-a-GCST90001531 | CD33dim HLA DR- Absolute Count \|\| id:ebi-a-GCST90001531 | Gherkins liking \|\| id:ebi-fl187-GCST90094770 | MR Egger | 19 | 0.0506545172858997 | 0.683004892195088 | 0.941745420630236 | -1.288035071 | 1.38934410598827 | 1.05195939670843 | 0.275812202270468 | 4.01221760028582 | 0.999950574162623 |
| ebi-fl187-GCST90094770 | ebi-a-GCST90001532 | Basophil Absolute Count \|\| id:ebi-a-GCST90001532 | Gherkins liking \|\| id:ebi-fl187-GCST90094770 | MR Egger | 19 | 0.0994644930205503 | 0.689185631920502 | 0.886943506255042 | -1.251339346 | 1.45026833158474 | 1.10457924977093 | 0.28612132479541 | 4.26425859693235 | 0.999950574162623 |
| ebi-fl187-GCST90094770 | ebi-a-GCST90001588 | T/B cell \|\| id:ebi-a-GCST90001588 | Gherkins liking \|\| id:ebi-fl187-GCST90094770 | MR Egger | 19 | 0.0277116924732134 | 0.406548542181992 | 0.946450953000438 | -0.76912345 | 0.824546835149917 | 1.02809923294174 | 0.463419100249647 | 2.28084693144066 | 0.999950574162623 |
| ebi-fl187-GCST90094770 | ebi-a-GCST90001624 | HLA DR+ CD4+ T cell Absolute Count \|\| id:ebi-a-GCST90001624 | Gherkins liking \|\| id:ebi-fl187-GCST90094770 | MR Egger | 19 | 0.393045891432499 | 0.394377759109805 | 0.332922868727648 | -0.379934516 | 1.16602629928772 | 1.48148637530126 | 0.683906192370061 | 3.20921480853569 | 0.999950574162623 |
| ebi-fl187-GCST90094770 | ebi-a-GCST90001631 | CD8+ Natural Killer T %T cell \|\| id:ebi-a-GCST90001631 | Gherkins liking \|\| id:ebi-fl187-GCST90094770 | MR Egger | 19 | 0.213386757182771 | 0.399491309859005 | 0.600156548647817 | -0.56961621 | 0.996389724506422 | 1.23786331120194 | 0.565742523282751 | 2.7084857760533 | 0.999950574162623 |
| ebi-fl187-GCST90094770 | ebi-a-GCST90001632 | CD8+ Natural Killer T %lymphocyte \|\| id:ebi-a-GCST90001632 | Gherkins liking \|\| id:ebi-fl187-GCST90094770 | MR Egger | 19 | 0.24746480413506 | 0.401991205102047 | 0.54631421912713 | -0.540437958 | 1.03536756613507 | 1.28077428363173 | 0.582493089073041 | 2.81614116353348 | 0.999950574162623 |
| ebi-fl187-GCST90094770 | ebi-a-GCST90001646 | Natural Killer %CD3- lymphocyte \|\| id:ebi-a-GCST90001646 | Gherkins liking \|\| id:ebi-fl187-GCST90094770 | MR Egger | 19 | -0.007770584 | 0.430700910233227 | 0.985815632043179 | -0.851944368 | 0.836403199721029 | 0.992259528605501 | 0.426584687276571 | 2.30805043283251 | 0.999950574162623 |
| ebi-fl187-GCST90094770 | ebi-a-GCST90001684 | CD127- CD8+ T cell Absolute Count \|\| id:ebi-a-GCST90001684 | Gherkins liking \|\| id:ebi-fl187-GCST90094770 | MR Egger | 19 | -0.134813657 | 0.42420052647093 | 0.75450227067604 | -0.966246689 | 0.696619375013374 | 0.87387873781635 | 0.38050852821457 | 2.00695645900705 | 0.999950574162623 |
| ebi-fl187-GCST90094770 | ebi-a-GCST90001687 | CD28- CD8+ T cell Absolute Count \|\| id:ebi-a-GCST90001687 | Gherkins liking \|\| id:ebi-fl187-GCST90094770 | MR Egger | 19 | -0.16798564 | 0.412908662681163 | 0.689205604992862 | -0.977286619 | 0.641315338548026 | 0.845365973793313 | 0.376330843647606 | 1.89897703499611 | 0.999950574162623 |
| ebi-fl187-GCST90094770 | ebi-a-GCST90001701 | BAFF-R on CD20- CD38- B cell \|\| id:ebi-a-GCST90001701 | Gherkins liking \|\| id:ebi-fl187-GCST90094770 | MR Egger | 19 | 0.262080587342774 | 0.44468927120553 | 0.56337309022863 | -0.609510384 | 1.13367155890561 | 1.29963127204018 | 0.543616967371688 | 3.10704327613442 | 0.999950574162623 |
| ebi-fl187-GCST90094770 | ebi-a-GCST90001732 | CD19 on IgD- CD27- B cell \|\| id:ebi-a-GCST90001732 | Gherkins liking \|\| id:ebi-fl187-GCST90094770 | MR Egger | 19 | 0.480086076089376 | 0.396434022333707 | 0.242458099506429 | -0.296924608 | 1.25709675986344 | 1.61621351354457 | 0.743100034282195 | 3.51520118537919 | 0.999950574162623 |
| ebi-fl187-GCST90094770 | ebi-a-GCST90001798 | CD27 on CD24+ CD27+ B cell \|\| id:ebi-a-GCST90001798 | Gherkins liking \|\| id:ebi-fl187-GCST90094770 | MR Egger | 19 | 0.0844043160239823 | 0.400821999707238 | 0.835719907334242 | -0.701206803 | 0.870015435450168 | 1.08806872850886 | 0.495986384418966 | 2.38694769685216 | 0.999950574162623 |
| ebi-fl187-GCST90094770 | ebi-a-GCST90001836 | CD66b on CD66b++ myeloid cell \|\| id:ebi-a-GCST90001836 | Gherkins liking \|\| id:ebi-fl187-GCST90094770 | MR Egger | 19 | -0.188793607 | 0.602085493204554 | 0.757665342995277 | -1.368881174 | 0.991293959293148 | 0.827957373346414 | 0.254391420109722 | 2.69471907418507 | 0.999950574162623 |
| ebi-fl187-GCST90094770 | ebi-a-GCST90001943 | CD25 on activated & secreting CD4 regulatory T cell \|\| id:ebi-a-GCST90001943 | Gherkins liking \|\| id:ebi-fl187-GCST90094770 | MR Egger | 19 | 0.228660233064256 | 0.406683720198433 | 0.581280659104104 | -0.568439859 | 1.02576032465318 | 1.25691490832461 | 0.566408427006043 | 2.7892153637605 | 0.999950574162623 |
| ebi-fl187-GCST90094770 | ebi-a-GCST90001960 | CD25 on CD4+ T cell \|\| id:ebi-a-GCST90001960 | Gherkins liking \|\| id:ebi-fl187-GCST90094770 | MR Egger | 19 | 0.177848644248224 | 0.43456540477799 | 0.687461631542097 | -0.673899549 | 1.02959683761308 | 1.19464449123524 | 0.509717030645708 | 2.79993677792316 | 0.999950574162623 |
| ebi-fl187-GCST90094770 | ebi-a-GCST90001988 | HLA DR on CD14+ CD16- monocyte \|\| id:ebi-a-GCST90001988 | Gherkins liking \|\| id:ebi-fl187-GCST90094770 | MR Egger | 19 | -0.260180258 | 0.407670068487848 | 0.531839148589497 | -1.059213592 | 0.538853076199614 | 0.770912610084738 | 0.346728373015105 | 1.71403986128869 | 0.999950574162623 |
| ebi-fl187-GCST90094770 | ebi-a-GCST90001991 | HLA DR on CD14+ monocyte \|\| id:ebi-a-GCST90001991 | Gherkins liking \|\| id:ebi-fl187-GCST90094770 | MR Egger | 19 | -0.259633392 | 0.408382737577102 | 0.53339881895769 | -1.060063558 | 0.540796773741499 | 0.771334311374596 | 0.346433791143599 | 1.71737467623968 | 0.999950574162623 |
| ebi-fl187-GCST90094770 | ebi-a-GCST90002019 | CD14 on Monocytic Myeloid-Derived Suppressor Cells \|\| id:ebi-a-GCST90002019 | Gherkins liking \|\| id:ebi-fl187-GCST90094770 | MR Egger | 19 | 0.428521826678714 | 0.635379607680694 | 0.509103636434935 | -0.816822204 | 1.67386585773287 | 1.53498686911288 | 0.441833482472309 | 5.33274362813059 | 0.999950574162623 |
| ebi-fl187-GCST90094770 | ebi-a-GCST90002037 | CD80 on plasmacytoid Dendritic Cell \|\| id:ebi-a-GCST90002037 | Gherkins liking \|\| id:ebi-fl187-GCST90094770 | MR Egger | 19 | -0.429210415 | 0.503892671556048 | 0.406170820531209 | -1.416840052 | 0.558419220880993 | 0.651022929538267 | 0.242479028775917 | 1.74790725995634 | 0.999950574162623 |
| ebi-fl187-GCST90094770 | ebi-a-GCST90002042 | CD45 on CD33+ HLA DR+ CD14- \|\| id:ebi-a-GCST90002042 | Gherkins liking \|\| id:ebi-fl187-GCST90094770 | MR Egger | 19 | -0.18946327 | 0.595367226838354 | 0.754187111178331 | -1.356383035 | 0.97745649461497 | 0.827403106865216 | 0.257590791005971 | 2.65768779457004 | 0.999950574162623 |
| ebi-fl187-GCST90094770 | ebi-a-GCST90002056 | CD8 on Effector Memory CD8+ T cell \|\| id:ebi-a-GCST90002056 | Gherkins liking \|\| id:ebi-fl187-GCST90094770 | MR Egger | 19 | 0.386710442208431 | 0.464492832488076 | 0.41664536809038 | -0.523695509 | 1.29711639388506 | 1.47213016279391 | 0.592327546281165 | 3.65873110209686 | 0.999950574162623 |
| ebi-fl187-GCST90094770 | ebi-a-GCST90002058 | CD8 on CD8+ T cell \|\| id:ebi-a-GCST90002058 | Gherkins liking \|\| id:ebi-fl187-GCST90094770 | MR Egger | 19 | 0.172503158373147 | 0.541423729101874 | 0.75390315577264 | -0.888687351 | 1.23369366741282 | 1.18827557362672 | 0.411195153697178 | 3.43388978732415 | 0.999950574162623 |
| ebi-fl187-GCST90094770 | ebi-a-GCST90002063 | CD4 on CD4 regulatory T cell \|\| id:ebi-a-GCST90002063 | Gherkins liking \|\| id:ebi-fl187-GCST90094770 | MR Egger | 19 | 0.139827563098404 | 0.441716770093128 | 0.755435465850604 | -0.725937306 | 1.00559243248093 | 1.15007546630778 | 0.483870821176865 | 2.73352622294122 | 0.999950574162623 |
| ebi-fl187-GCST90094770 | ebi-a-GCST90002066 | CD4 on activated CD4 regulatory T cell \|\| id:ebi-a-GCST90002066 | Gherkins liking \|\| id:ebi-fl187-GCST90094770 | MR Egger | 19 | 0.0715815135185393 | 0.441770912275575 | 0.873190061870799 | -0.794289475 | 0.937452501578666 | 1.07420570941967 | 0.451902208903135 | 2.55346816947547 | 0.999950574162623 |
| ebi-fl187-GCST90094770 | ebi-a-GCST90002070 | CD4 on activated & secreting CD4 regulatory T cell \|\| id:ebi-a-GCST90002070 | Gherkins liking \|\| id:ebi-fl187-GCST90094770 | MR Egger | 19 | 0.191438542477997 | 0.443779051261057 | 0.671610988250287 | -0.678368398 | 1.06124548294967 | 1.21099040638343 | 0.50744426437258 | 2.88996815476065 | 0.999950574162623 |
| ebi-fl187-GCST90094770 | ebi-a-GCST90002081 | SSC-A on CD4+ T cell \|\| id:ebi-a-GCST90002081 | Gherkins liking \|\| id:ebi-fl187-GCST90094770 | MR Egger | 19 | 0.478162934341137 | 0.419698961118002 | 0.270377864973777 | -0.344447029 | 1.30077289813242 | 1.61310829270956 | 0.708612086764581 | 3.67213375641003 | 0.999950574162623 |
| ebi-fl187-GCST90094770 | ebi-a-GCST90002094 | CD11b on Monocytic Myeloid-Derived Suppressor Cells \|\| id:ebi-a-GCST90002094 | Gherkins liking \|\| id:ebi-fl187-GCST90094770 | MR Egger | 19 | 0.424900919957149 | 0.635402634153389 | 0.512658630911395 | -0.820488243 | 1.67029008289779 | 1.52943887528669 | 0.440216669327752 | 5.31370898973532 | 0.999950574162623 |
| ebi-fl187-GCST90094770 | ebi-a-GCST90002104 | HLA DR on myeloid Dendritic Cell \|\| id:ebi-a-GCST90002104 | Gherkins liking \|\| id:ebi-fl187-GCST90094770 | MR Egger | 18 | 0.00551661595829206 | 0.479336437755903 | 0.990959744007398 | -0.933982802 | 0.945016033959862 | 1.00553186050398 | 0.392985406243323 | 2.57285463130549 | 0.999950574162623 |
| ebi-fl187-GCST90094770 | ebi-a-GCST90002105 | HLA DR on plasmacytoid Dendritic Cell \|\| id:ebi-a-GCST90002105 | Gherkins liking \|\| id:ebi-fl187-GCST90094770 | MR Egger | 18 | -0.724655788 | 0.526195680743643 | 0.187425596460217 | -1.755999322 | 0.306687746627908 | 0.484491308158472 | 0.172734538607702 | 1.35891657553333 | 0.999950574162623 |
| ebi-fl187-GCST90094770 | ebi-a-GCST90002106 | HLA DR on Dendritic Cell \|\| id:ebi-a-GCST90002106 | Gherkins liking \|\| id:ebi-fl187-GCST90094770 | MR Egger | 18 | -0.334299438 | 0.478400736551381 | 0.494719132938754 | -1.271964882 | 0.603366005585322 | 0.715839400573722 | 0.280280362621933 | 1.82826239633831 | 0.999950574162623 |
| ebi-fl187-GCST90094770 | ebi-a-GCST90002111 | HLA DR on CD33dim HLA DR+ CD11b- \|\| id:ebi-a-GCST90002111 | Gherkins liking \|\| id:ebi-fl187-GCST90094770 | MR Egger | 19 | -0.931746632 | 0.581428071837633 | 0.127458692346457 | -2.071345652 | 0.207852389231401 | 0.393865171891432 | 0.126016093704695 | 1.23103144263936 | 0.999950574162623 |
| ebi-fl187-GCST90094770 | ebi-a-GCST90002112 | HLA DR on CD33- HLA DR+ \|\| id:ebi-a-GCST90002112 | Gherkins liking \|\| id:ebi-fl187-GCST90094770 | MR Egger | 19 | -0.861637436 | 0.727984538836356 | 0.252861306840259 | -2.288487132 | 0.565212260457015 | 0.422469748613892 | 0.101419780611918 | 1.75982128355059 | 0.999950574162623 |
| ebi-fl187-GCST90094770 | ebi-a-GCST90002115 | HLA DR on HLA DR+ CD8+ T cell \|\| id:ebi-a-GCST90002115 | Gherkins liking \|\| id:ebi-fl187-GCST90094770 | MR Egger | 19 | -0.449625407 | 0.502590664529321 | 0.383484256154224 | -1.43470311 | 0.535452295388532 | 0.637867047348795 | 0.238186068683605 | 1.70822068789397 | 0.999950574162623 |
| ebi-fl187-GCST90094770 | ebi-a-GCST90002116 | HLA DR on B cell \|\| id:ebi-a-GCST90002116 | Gherkins liking \|\| id:ebi-fl187-GCST90094770 | MR Egger | 19 | 0.107873688501836 | 0.613018800686311 | 0.862395695750421 | -1.093643161 | 1.309390538 | 1.11390703723344 | 0.334993831471895 | 3.70391562777858 | 0.999950574162623 |
| ebi-fl187-GCST90094770 | ebi-cfmet338-GCST90026023 | Sphingomyelin (d18:1/24:1, d18:2/24:0) levels \|\| id:ebi-cfmet338-GCST90026023 | Gherkins liking \|\| id:ebi-fl187-GCST90094770 | MR Egger | 18 | 0.489141989874196 | 0.283960083095619 | 0.104235239717391 | -0.067419773 | 1.04570375274161 | 1.63091627677874 | 0.934802714031419 | 2.84540027744552 | 0.999950574162623 |
| ebi-fl187-GCST90094770 | ebi-cfmet338-GCST90026046 | 1-arachidonoyl-gpc (20:4n6) levels \|\| id:ebi-cfmet338-GCST90026046 | Gherkins liking \|\| id:ebi-fl187-GCST90094770 | MR Egger | 18 | 0.287283385577675 | 0.310376843686438 | 0.368407334670218 | -0.321055228 | 0.895621999203093 | 1.33280185678793 | 0.725383188386501 | 2.44885850388755 | 0.999950574162623 |
| ebi-fl187-GCST90094770 | ebi-cfmet338-GCST90026047 | 1-linoleoyl-gpc (18:2) levels \|\| id:ebi-cfmet338-GCST90026047 | Gherkins liking \|\| id:ebi-fl187-GCST90094770 | MR Egger | 18 | 0.549275405309615 | 0.380970525296771 | 0.168651938514599 | -0.197426824 | 1.29597763489129 | 1.73199756683506 | 0.820840204009561 | 3.65456706051843 | 0.999950574162623 |
| ebi-fl187-GCST90094770 | ebi-cfmet338-GCST90026050 | 1-myristoyl-2-palmitoyl-gpc (14:0/16:0) levels \|\| id:ebi-cfmet338-GCST90026050 | Gherkins liking \|\| id:ebi-fl187-GCST90094770 | MR Egger | 18 | 0.0868658536489417 | 0.167647022298373 | 0.61144633747594 | -0.24172231 | 0.415454017353752 | 1.09075034972435 | 0.785274210029226 | 1.51505844739191 | 0.999950574162623 |
| ebi-fl187-GCST90094770 | ebi-cfmet338-GCST90026068 | 2,3-dihydroxyisovalerate levels \|\| id:ebi-cfmet338-GCST90026068 | Gherkins liking \|\| id:ebi-fl187-GCST90094770 | MR Egger | 18 | 0.784326793680905 | 0.54169273682622 | 0.166951585053763 | -0.27739097 | 1.8460445578603 | 2.19093149522203 | 0.757758178095664 | 6.33471331027963 | 0.999950574162623 |
| ebi-fl187-GCST90094770 | ebi-cfmet338-GCST90026082 | 3-hydroxyhexanoate levels \|\| id:ebi-cfmet338-GCST90026082 | Gherkins liking \|\| id:ebi-fl187-GCST90094770 | MR Egger | 18 | -0.051246928 | 0.148649578316087 | 0.734772402033547 | -0.342600102 | 0.240106245353394 | 0.950044048950527 | 0.709922051462956 | 1.27138422181186 | 0.999950574162623 |
| ebi-fl187-GCST90094770 | ebi-cfmet338-GCST90026094 | 3-ureidopropionate levels \|\| id:ebi-cfmet338-GCST90026094 | Gherkins liking \|\| id:ebi-fl187-GCST90094770 | MR Egger | 18 | 0.219282065775333 | 0.264079240363532 | 0.418551078242704 | -0.298313245 | 0.736877376887855 | 1.24518245051148 | 0.742068853728791 | 2.08940090568528 | 0.999950574162623 |
| ebi-fl187-GCST90094770 | ebi-cfmet338-GCST90026173 | Glycerol levels \|\| id:ebi-cfmet338-GCST90026173 | Gherkins liking \|\| id:ebi-fl187-GCST90094770 | MR Egger | 18 | -0.062982734 | 0.149558037696437 | 0.679265935219529 | -0.356116488 | 0.230151019835088 | 0.938959685580226 | 0.700391028378562 | 1.25879009784858 | 0.999950574162623 |
| ebi-fl187-GCST90094770 | ebi-cfmet338-GCST90026179 | Histidine levels \|\| id:ebi-cfmet338-GCST90026179 | Gherkins liking \|\| id:ebi-fl187-GCST90094770 | MR Egger | 18 | 0.0879232100916205 | 0.118076344455064 | 0.467292346278657 | -0.143506425 | 0.319352845223547 | 1.09190427157983 | 0.866315234059067 | 1.37623683784024 | 0.999950574162623 |
| ebi-fl187-GCST90094770 | ebi-cfmet338-GCST90026188 | Isocitrate levels \|\| id:ebi-cfmet338-GCST90026188 | Gherkins liking \|\| id:ebi-fl187-GCST90094770 | MR Egger | 18 | -0.137009368 | 0.203526541619611 | 0.510442046479066 | -0.53592139 | 0.261902653137651 | 0.871962057172431 | 0.585129908839406 | 1.29940004375516 | 0.999950574162623 |
| ebi-fl187-GCST90094770 | ebi-cfmet338-GCST90026207 | N-acetyl-isoputreanine levels \|\| id:ebi-cfmet338-GCST90026207 | Gherkins liking \|\| id:ebi-fl187-GCST90094770 | MR Egger | 18 | 0.0360583246104418 | 0.131558824228113 | 0.787526182612361 | -0.221796971 | 0.293913620097544 | 1.03671631080259 | 0.801077989987262 | 1.34166800550996 | 0.999950574162623 |
| ebi-fl187-GCST90094770 | ebi-cfmet338-GCST90026213 | N-acetylglutamate levels \|\| id:ebi-cfmet338-GCST90026213 | Gherkins liking \|\| id:ebi-fl187-GCST90094770 | MR Egger | 18 | -0.148862222 | 0.220574545365696 | 0.509385120690393 | -0.58118833 | 0.283463887399888 | 0.861687828762636 | 0.559233417464543 | 1.32772093199302 | 0.999950574162623 |
| ebi-fl187-GCST90094770 | ebi-cfmet338-GCST90026224 | N-acetyltaurine levels \|\| id:ebi-cfmet338-GCST90026224 | Gherkins liking \|\| id:ebi-fl187-GCST90094770 | MR Egger | 18 | 0.138810003960162 | 0.178355424753041 | 0.447762693989411 | -0.210766629 | 0.488386636476122 | 1.14890579171483 | 0.809963067077508 | 1.62968482377676 | 0.999950574162623 |
| ebi-fl187-GCST90094770 | ebi-cfmet338-GCST90026250 | Phenyllactate (pla) levels \|\| id:ebi-cfmet338-GCST90026250 | Gherkins liking \|\| id:ebi-fl187-GCST90094770 | MR Egger | 18 | 0.425690125169022 | 0.281165549702728 | 0.149523797202739 | -0.125394352 | 0.97677460258637 | 1.53064639284521 | 0.882148956558079 | 2.6558761561895 | 0.999950574162623 |
| ebi-fl187-GCST90094770 | ebi-cfmet338-GCST90026251 | Pipecolate levels \|\| id:ebi-cfmet338-GCST90026251 | Gherkins liking \|\| id:ebi-fl187-GCST90094770 | MR Egger | 18 | -0.413233611 | 0.478566976068958 | 0.400632106717369 | -1.351224884 | 0.524757662350312 | 0.661507729462488 | 0.258922915834891 | 1.69004923618911 | 0.999950574162623 |
| ebi-fl187-GCST90094770 | ebi-cfmet338-GCST90026278 | Trigonelline (n'-methylnicotinate) levels \|\| id:ebi-cfmet338-GCST90026278 | Gherkins liking \|\| id:ebi-fl187-GCST90094770 | MR Egger | 17 | -0.374705583 | 0.48498570737906 | 0.451755266565532 | -1.325277569 | 0.575866403927072 | 0.687491658548152 | 0.26572919066607 | 1.77867090698079 | 0.999950574162623 |
| ebi-fl187-GCST90094770 | ebi-cfmet338-GCST90026299 | 1-carboxyethylvaline levels \|\| id:ebi-cfmet338-GCST90026299 | Gherkins liking \|\| id:ebi-fl187-GCST90094770 | MR Egger | 18 | 0.347637733145377 | 0.307830102411287 | 0.275411846263428 | -0.255709268 | 0.9509847338715 | 1.4157192886575 | 0.774367069680158 | 2.58825714929321 | 0.999950574162623 |
| ebi-fl187-GCST90094770 | ebi-gm430-GCST90011306 | C_Erysipelotrichia abundance \|\| id:ebi-gm430-GCST90011306 | Gherkins liking \|\| id:ebi-fl187-GCST90094770 | MR Egger | 15 | 0.427534666885046 | 0.240873403517432 | 0.0993121342549793 | -0.044577204 | 0.899646537779211 | 1.53347233945626 | 0.956401759193112 | 2.45873388800684 | 0.999950574162623 |
| ebi-fl187-GCST90094770 | ebi-gm430-GCST90011312 | F_Erysipelotrichaceae abundance \|\| id:ebi-gm430-GCST90011312 | Gherkins liking \|\| id:ebi-fl187-GCST90094770 | MR Egger | 15 | 0.427534666885046 | 0.240873403517432 | 0.0993121342549793 | -0.044577204 | 0.899646537779211 | 1.53347233945626 | 0.956401759193112 | 2.45873388800684 | 0.999950574162623 |
| ebi-fl187-GCST90094770 | ebi-gm430-GCST90011352 | O_Erysipelotrichales abundance \|\| id:ebi-gm430-GCST90011352 | Gherkins liking \|\| id:ebi-fl187-GCST90094770 | MR Egger | 15 | 0.427534666885046 | 0.240873403517432 | 0.0993121342549793 | -0.044577204 | 0.899646537779211 | 1.53347233945626 | 0.956401759193112 | 2.45873388800684 | 0.999950574162623 |
| ebi-fl187-GCST90094770 | ebi-gm430-GCST90011362 | OTU97_11 (Parabacteroides) abundance \|\| id:ebi-gm430-GCST90011362 | Gherkins liking \|\| id:ebi-fl187-GCST90094770 | MR Egger | 15 | -0.087110219 | 0.312721436448033 | 0.784967697026152 | -0.700044234 | 0.525823796898319 | 0.916576066389931 | 0.496563338333835 | 1.69185201690029 | 0.999950574162623 |
| ebi-fl187-GCST90094770 | ebi-gm430-GCST90011384 | OTU97_235 (Phascolarctobacterium) abundance \|\| id:ebi-gm430-GCST90011384 | Gherkins liking \|\| id:ebi-fl187-GCST90094770 | MR Egger | 15 | -1.392023648 | 0.994470631559234 | 0.184993343011055 | -3.341186086 | 0.557138789781615 | 0.24857177352776 | 0.0353949513611235 | 1.7456706173808 | 0.999950574162623 |
| ebi-fl187-GCST90094770 | ebi-gm430-GCST90011390 | OTU97_3 (Bacteroides) abundance \|\| id:ebi-gm430-GCST90011390 | Gherkins liking \|\| id:ebi-fl187-GCST90094770 | MR Egger | 15 | -0.07742442 | 0.248994501942615 | 0.760769648347283 | -0.565453644 | 0.410604803420052 | 0.925496970455777 | 0.568102371949739 | 1.50772938930556 | 0.999950574162623 |
| ebi-fl187-GCST90094770 | ebi-gm430-GCST90011398 | OTU97_4 (Alistipes) abundance \|\| id:ebi-gm430-GCST90011398 | Gherkins liking \|\| id:ebi-fl187-GCST90094770 | MR Egger | 15 | -0.1175909 | 0.292030108242682 | 0.693736410718988 | -0.689969912 | 0.454788112261433 | 0.88905969264126 | 0.50159116068884 | 1.57583944660004 | 0.999950574162623 |
| ebi-fl187-GCST90094770 | ebi-gm430-GCST90011421 | OTU99_108 (Cytophagales) abundance \|\| id:ebi-gm430-GCST90011421 | Gherkins liking \|\| id:ebi-fl187-GCST90094770 | MR Egger | 15 | 0.219191043665707 | 0.912309062224654 | 0.813876116862844 | -1.568934718 | 2.00731680562603 | 1.24506911653599 | 0.208266927173057 | 7.44331865838496 | 0.999950574162623 |
| ebi-fl187-GCST90094770 | ebi-gm430-GCST90011428 | OTU99_123 (Phascolarctobacterium) abundance \|\| id:ebi-gm430-GCST90011428 | Gherkins liking \|\| id:ebi-fl187-GCST90094770 | MR Egger | 15 | 1.00586226071623 | 0.685262514030283 | 0.165920145661282 | -0.337252267 | 2.34897678821558 | 2.73426390501733 | 0.713728767130376 | 10.4748462533456 | 0.999950574162623 |
| ebi-fl187-GCST90094770 | ebi-gm430-GCST90011450 | OTU99_298 (Phascolarctobacterium) abundance \|\| id:ebi-gm430-GCST90011450 | Gherkins liking \|\| id:ebi-fl187-GCST90094770 | MR Egger | 15 | -1.411082232 | 0.994401148356851 | 0.179420634292183 | -3.360108483 | 0.537944018704198 | 0.243879206383406 | 0.034731490969075 | 1.71248240851965 | 0.999950574162623 |
| ebi-fl187-GCST90094770 | ebi-gm430-GCST90011451 | OTU99_3 (Bacteroides) abundance \|\| id:ebi-gm430-GCST90011451 | Gherkins liking \|\| id:ebi-fl187-GCST90094770 | MR Egger | 15 | -0.093716874 | 0.248994782116206 | 0.712705057982921 | -0.581746647 | 0.394312898967966 | 0.910540523464231 | 0.558921275368769 | 1.48336461932586 | 0.999950574162623 |
| ebi-fl187-GCST90094770 | ebi-gm430-GCST90011472 | OTU99_72 (Parabacteroides) abundance \|\| id:ebi-gm430-GCST90011472 | Gherkins liking \|\| id:ebi-fl187-GCST90094770 | MR Egger | 15 | -1.979450102 | 0.746010738872611 | 0.0198834761641696 | -3.44163115 | -0.517269054 | 0.138145182221046 | 0.0320124256513654 | 0.596146370747515 | 0.999950574162623 |
| ebi-fl187-GCST90094770 | ebi-gm430-GCST90011507 | TestASV_3 (Bacteroides) abundance \|\| id:ebi-gm430-GCST90011507 | Gherkins liking \|\| id:ebi-fl187-GCST90094770 | MR Egger | 15 | -0.127113563 | 0.346186837295667 | 0.71939125031375 | -0.805639764 | 0.551412637910157 | 0.880633659351348 | 0.44680198714663 | 1.7357032069959 | 0.999950574162623 |
| ebi-fl187-GCST90094770 | ebi-gm430-GCST90011512 | TestASV_34 (Phascolarctobacterium) abundance \|\| id:ebi-gm430-GCST90011512 | Gherkins liking \|\| id:ebi-fl187-GCST90094770 | MR Egger | 15 | -1.51016316 | 0.842456116785843 | 0.0963305586456322 | -3.161377149 | 0.141050828429093 | 0.22087393712359 | 0.0423673547104547 | 1.15148317457821 | 0.999950574162623 |
| ebi-fl187-GCST90094770 | ebi-gm430-GCST90011519 | TestASV_41 (Barnesiella) abundance \|\| id:ebi-gm430-GCST90011519 | Gherkins liking \|\| id:ebi-fl187-GCST90094770 | MR Egger | 15 | -1.675393964 | 1.38651663608666 | 0.24843523143747 | -4.392966571 | 1.04217864274538 | 0.187234401339164 | 0.0123639960929512 | 2.8353875867707 | 0.999950574162623 |
| ebi-fl187-GCST90094770 | ebi-gm430-GCST90011524 | TestASV_46 (Sutterella) abundance \|\| id:ebi-gm430-GCST90011524 | Gherkins liking \|\| id:ebi-fl187-GCST90094770 | MR Egger | 15 | -1.5029075 | 1.45485448164025 | 0.320426174955858 | -4.354422284 | 1.34860728377483 | 0.222482351362692 | 0.0128498610072615 | 3.85205697088092 | 0.999950574162623 |
| ebi-fl187-GCST90094770 | ebi-gm430-GCST90011581 | OTU97_19 (Bacteria) prevalence \|\| id:ebi-gm430-GCST90011581 | Gherkins liking \|\| id:ebi-fl187-GCST90094770 | MR Egger | 15 | -0.979837253 | 1.29437081619086 | 0.462550742213272 | -3.516804053 | 1.55712954693732 | 0.375372184653718 | 0.0296941847344756 | 4.74518085853058 | 0.999950574162623 |
| ebi-fl187-GCST90094770 | ebi-gm430-GCST90011614 | OTU97_80 (Ruminococcus) prevalence \|\| id:ebi-gm430-GCST90011614 | Gherkins liking \|\| id:ebi-fl187-GCST90094770 | MR Egger | 15 | 1.69909447338751 | 0.602586336345471 | 0.0144742005265616 | 0.518025254150389 | 2.88016369262463 | 5.4689928302708 | 1.67870935005628 | 17.8171894834271 | 0.999950574162623 |
| ebi-fl187-GCST90094770 | ebi-gm430-GCST90011623 | OTU99_11 (Parabacteroides) prevalence \|\| id:ebi-gm430-GCST90011623 | Gherkins liking \|\| id:ebi-fl187-GCST90094770 | MR Egger | 15 | 0.0458233459397335 | 0.782725035251379 | 0.954206174794999 | -1.488317723 | 1.57996441503244 | 1.04688945734772 | 0.225752113827262 | 4.85478305086622 | 0.999950574162623 |
| ebi-fl187-GCST90094770 | ebi-gm430-GCST90011642 | OTU99_19 (Bacteria) prevalence \|\| id:ebi-gm430-GCST90011642 | Gherkins liking \|\| id:ebi-fl187-GCST90094770 | MR Egger | 15 | -0.979837253 | 1.29437081619086 | 0.462550742213272 | -3.516804053 | 1.55712954693732 | 0.375372184653718 | 0.0296941847344756 | 4.74518085853058 | 0.999950574162623 |
| ebi-fl187-GCST90094770 | ebi-gm430-GCST90011653 | OTU99_30 (Parasutterella) prevalence \|\| id:ebi-gm430-GCST90011653 | Gherkins liking \|\| id:ebi-fl187-GCST90094770 | MR Egger | 15 | -0.343943415 | 0.661441527420416 | 0.611810772463302 | -1.640368809 | 0.952481978644921 | 0.708969043857617 | 0.193908513926865 | 2.59213530633296 | 0.999950574162623 |
| ebi-fl187-GCST90094770 | ebi-gm430-GCST90011675 | OTU99_8 (Ruminococcaceae) prevalence \|\| id:ebi-gm430-GCST90011675 | Gherkins liking \|\| id:ebi-fl187-GCST90094770 | MR Egger | 15 | 0.58886952374461 | 0.607677866167456 | 0.350205251570929 | -0.602179094 | 1.77991814143282 | 1.80195020162245 | 0.547617026038116 | 5.92937102890808 | 0.999950574162623 |
| ebi-fl187-GCST90094770 | ebi-gm430-GCST90011679 | OTU99_92 (Ruminococcus) prevalence \|\| id:ebi-gm430-GCST90011679 | Gherkins liking \|\| id:ebi-fl187-GCST90094770 | MR Egger | 15 | 1.92426456967322 | 0.629181067013489 | 0.00915150321401519 | 0.691069678326785 | 3.15745946101966 | 6.85010903505155 | 1.99584930856192 | 23.5107899132451 | 0.999950574162623 |
| ebi-fl187-GCST90094770 | ebi-gm430-GCST90011684 | TestASV_11 (Lachnospiraceae) prevalence \|\| id:ebi-gm430-GCST90011684 | Gherkins liking \|\| id:ebi-fl187-GCST90094770 | MR Egger | 15 | 0.248857895616617 | 0.644701853238613 | 0.705736429637082 | -1.014757737 | 1.5124735279643 | 1.28255976275517 | 0.36249023726564 | 4.53794164898613 | 0.999950574162623 |
| ebi-fl187-GCST90094770 | ebi-gm430-GCST90011688 | TestASV_15 (Bacteroides) prevalence \|\| id:ebi-gm430-GCST90011688 | Gherkins liking \|\| id:ebi-fl187-GCST90094770 | MR Egger | 15 | -0.231634388 | 0.645173057652685 | 0.725339016055995 | -1.496173581 | 1.03290480460745 | 0.793236086617777 | 0.223985585108704 | 2.80921421263478 | 0.999950574162623 |
| ebi-fl187-GCST90094770 | ebi-gm430-GCST90011692 | TestASV_19 (Prevotella) prevalence \|\| id:ebi-gm430-GCST90011692 | Gherkins liking \|\| id:ebi-fl187-GCST90094770 | MR Egger | 15 | 0.491574952903732 | 1.23377961580886 | 0.696779803736485 | -1.926633094 | 2.90978299988909 | 1.63488906665017 | 0.145637722440254 | 18.3528155718638 | 0.999950574162623 |
| ebi-fl187-GCST90094770 | ebi-gm430-GCST90011693 | TestASV_2 (Bacteroides) prevalence \|\| id:ebi-gm430-GCST90011693 | Gherkins liking \|\| id:ebi-fl187-GCST90094770 | MR Egger | 15 | -0.124704304 | 0.591969137160824 | 0.836418548377501 | -1.284963813 | 1.03555520447714 | 0.88275789165747 | 0.276660594914308 | 2.81666962917111 | 0.999950574162623 |
| ebi-fl187-GCST90094770 | ebi-gm430-GCST90011716 | TestASV_40 (Barnesiella) prevalence \|\| id:ebi-gm430-GCST90011716 | Gherkins liking \|\| id:ebi-fl187-GCST90094770 | MR Egger | 15 | 0.265859759826196 | 1.49209855429439 | 0.861329454124529 | -2.658653407 | 3.19037292624319 | 1.30455209524524 | 0.0700424770065563 | 24.2974869242481 | 0.999950574162623 |
| ebi-fl187-GCST90094770 | ebi-gm430-GCST90011718 | TestASV_42 (Alphaproteobacteria) prevalence \|\| id:ebi-gm430-GCST90011718 | Gherkins liking \|\| id:ebi-fl187-GCST90094770 | MR Egger | 15 | 0.157199795558821 | 0.954810871761843 | 0.871759994735297 | -1.714229513 | 2.02862910421203 | 1.17022939718206 | 0.180102433834866 | 7.60365539137977 | 0.999950574162623 |
| ebi-fl187-GCST90094770 | ebi-hbsm44-GCST90104929 | Sulcal depth of the right inferior frontal sulcus \|\| id:ebi-hbsm44-GCST90104929 | Gherkins liking \|\| id:ebi-fl187-GCST90094770 | MR Egger | 17 | -0.142422937 | 0.565461379621817 | 0.804559597604552 | -1.250727241 | 0.965881366695418 | 0.867254384626693 | 0.28629651444926 | 2.62710207667416 | 0.999950574162623 |
| ebi-fl187-GCST90094770 | ebi-hbsm44-GCST90104935 | Sulcal opening distance of the left calloso-marginal posterior fissure \|\| id:ebi-hbsm44-GCST90104935 | Gherkins liking \|\| id:ebi-fl187-GCST90094770 | MR Egger | 17 | 0.036657474840382 | 0.102491001139975 | 0.725576588063444 | -0.164224887 | 0.237539837074733 | 1.03733764573649 | 0.848551171959429 | 1.26812551419536 | 0.999950574162623 |
| ebi-fl187-GCST90094770 | ebi-hbsm44-GCST90104936 | Sulcal opening distance of the left anterior inferior temporal sulcus \|\| id:ebi-hbsm44-GCST90104936 | Gherkins liking \|\| id:ebi-fl187-GCST90094770 | MR Egger | 17 | -0.108306488 | 0.0584275729363719 | 0.0835547123205846 | -0.222824531 | 0.00621155477926003 | 0.89735252634067 | 0.800255256860194 | 1.00623088649158 | 0.999950574162623 |
| ebi-fl187-GCST90094770 | ebi-hbsm44-GCST90104941 | Sulcal opening distance of the right posterior inferior temporal sulcus \|\| id:ebi-hbsm44-GCST90104941 | Gherkins liking \|\| id:ebi-fl187-GCST90094770 | MR Egger | 16 | 0.0160923598725264 | 0.0585494179710357 | 0.787445256706768 | -0.098664499 | 0.130849219095756 | 1.01622253925605 | 0.906046636271397 | 1.13979590889698 | 0.999950574162623 |
| ebi-fl187-GCST90094770 | ebi-IPs91-GCST90274819 | Leukemia inhibitory factor levels \|\| id:ebi-IPs91-GCST90274819 | Gherkins liking \|\| id:ebi-fl187-GCST90094770 | MR Egger | 22 | -0.048686875 | 0.194681939263185 | 0.805073115608372 | -0.430263476 | 0.33288972575889 | 0.952479327911399 | 0.650337723666765 | 1.3949934581427 | 0.999950574162623 |
| ebi-fl187-GCST90094770 | ebi-IPs91-GCST90274837 | STAM binding protein levels \|\| id:ebi-IPs91-GCST90274837 | Gherkins liking \|\| id:ebi-fl187-GCST90094770 | MR Egger | 22 | -0.055460388 | 0.161893256522389 | 0.735493247913653 | -0.372771171 | 0.261850394372378 | 0.946049497451621 | 0.6888228373175 | 1.29933214048757 | 0.999950574162623 |
| ebi-fl187-GCST90094770 | ebi-IPs91-GCST90274840 | TNF-beta levels \|\| id:ebi-IPs91-GCST90274840 | Gherkins liking \|\| id:ebi-fl187-GCST90094770 | MR Egger | 22 | 0.237780223222387 | 0.261277410029155 | 0.37362458343638 | -0.2743235 | 0.74988394687953 | 1.26843039064503 | 0.760086147285024 | 2.11675434641039 | 0.999950574162623 |
| ebi-fl187-GCST90094770 | ebi-IPs91-GCST90274842 | Tumor necrosis factor ligand superfamily member 14 levels \|\| id:ebi-IPs91-GCST90274842 | Gherkins liking \|\| id:ebi-fl187-GCST90094770 | MR Egger | 22 | 0.141601744014413 | 0.201552504351417 | 0.490430122863778 | -0.253441165 | 0.53664465254319 | 1.15211771937959 | 0.776125407297093 | 1.71025871183768 | 0.999950574162623 |
| ebi-fl187-GCST90094770 | ebi-met1400-GCST90199628 | 3-methylhistidine levels \|\| id:ebi-met1400-GCST90199628 | Gherkins liking \|\| id:ebi-fl187-GCST90094770 | MR Egger | 20 | 0.0486771102634747 | 0.254724618517885 | 0.850588548606567 | -0.450583142 | 0.547937362558529 | 1.04988130010861 | 0.637256432239176 | 1.72968162980277 | 0.999950574162623 |
| ebi-fl187-GCST90094770 | ebi-met1400-GCST90199651 | Docosahexaenoate DHA; 22:6n3 levels \|\| id:ebi-met1400-GCST90199651 | Gherkins liking \|\| id:ebi-fl187-GCST90094770 | MR Egger | 20 | -0.405136687 | 0.256439850458913 | 0.131553115073327 | -0.907758794 | 0.0974854200596321 | 0.666885650136331 | 0.403427375540982 | 1.10239536858742 | 0.999950574162623 |
| ebi-fl187-GCST90094770 | ebi-met1400-GCST90199652 | 1-stearoyl-2-oleoyl-GPS (18:0/18:1) levels \|\| id:ebi-met1400-GCST90199652 | Gherkins liking \|\| id:ebi-fl187-GCST90094770 | MR Egger | 20 | 0.140883558305997 | 0.238669942835996 | 0.562337090540192 | -0.32690953 | 0.60867664626455 | 1.15129058195381 | 0.721148982674458 | 1.83799746784622 | 0.999950574162623 |
| ebi-fl187-GCST90094770 | ebi-met1400-GCST90199653 | 1-myristoyl-2-palmitoyl-gpc (14:0/16:0) levels \|\| id:ebi-met1400-GCST90199653 | Gherkins liking \|\| id:ebi-fl187-GCST90094770 | MR Egger | 19 | -0.007820577 | 0.233904169312513 | 0.973717165199558 | -0.466272749 | 0.450631594970398 | 0.992209924265275 | 0.62733615556823 | 1.56930303645384 | 0.999950574162623 |
| ebi-fl187-GCST90094770 | ebi-met1400-GCST90199667 | Erythritol levels in elite athletes \|\| id:ebi-met1400-GCST90199667 | Gherkins liking \|\| id:ebi-fl187-GCST90094770 | MR Egger | 20 | 0.0849647939807494 | 0.289024637095184 | 0.772143156405214 | -0.481523495 | 0.651453082687309 | 1.0886787379791 | 0.617841396317085 | 1.91832629149291 | 0.999950574162623 |
| ebi-fl187-GCST90094770 | ebi-met1400-GCST90199674 | 3-hydroxymyristate levels \|\| id:ebi-met1400-GCST90199674 | Gherkins liking \|\| id:ebi-fl187-GCST90094770 | MR Egger | 20 | 0.0774431880458714 | 0.244369838440326 | 0.75495821322253 | -0.401521695 | 0.55640807138891 | 1.08052084421415 | 0.669300798862482 | 1.74439548968944 | 0.999950574162623 |
| ebi-fl187-GCST90094770 | ebi-met1400-GCST90199685 | 2-linoleoylglycerol (18:2) levels \|\| id:ebi-met1400-GCST90199685 | Gherkins liking \|\| id:ebi-fl187-GCST90094770 | MR Egger | 20 | -0.541543315 | 0.269533775122279 | 0.0597557608781846 | -1.069829515 | -0.013257116 | 0.581849581633123 | 0.343067000314921 | 0.986830372305896 | 0.999950574162623 |
| ebi-fl187-GCST90094770 | ebi-met1400-GCST90199703 | Gamma-glutamylvaline levels \|\| id:ebi-met1400-GCST90199703 | Gherkins liking \|\| id:ebi-fl187-GCST90094770 | MR Egger | 20 | -0.081044842 | 0.241654420290531 | 0.741219362385339 | -0.554687506 | 0.392597821732045 | 0.922152339327774 | 0.574251683477267 | 1.48082271483902 | 0.999950574162623 |
| ebi-fl187-GCST90094770 | ebi-met1400-GCST90199779 | Gamma-glutamyl-2-aminobutyrate levels \|\| id:ebi-met1400-GCST90199779 | Gherkins liking \|\| id:ebi-fl187-GCST90094770 | MR Egger | 20 | 0.118757736914066 | 0.24394198256738 | 0.632255472967994 | -0.359368549 | 0.59688402274613 | 1.12609707348749 | 0.698117013663701 | 1.81644995623608 | 0.999950574162623 |
| ebi-fl187-GCST90094770 | ebi-met1400-GCST90199824 | 4-vinylphenol sulfate levels \|\| id:ebi-met1400-GCST90199824 | Gherkins liking \|\| id:ebi-fl187-GCST90094770 | MR Egger | 20 | -0.504655249 | 0.256980910843158 | 0.0651913940018624 | -1.008337834 | -0.000972663 | 0.603713670722688 | 0.364824875461129 | 0.999027809594346 | 0.999950574162623 |
| ebi-fl187-GCST90094770 | ebi-met1400-GCST90199830 | 4-ethylphenylsulfate levels \|\| id:ebi-met1400-GCST90199830 | Gherkins liking \|\| id:ebi-fl187-GCST90094770 | MR Egger | 20 | -0.080492006 | 0.236234643729021 | 0.737252153475879 | -0.543511907 | 0.382527895989672 | 0.922662279575612 | 0.580705283915568 | 1.46598576891103 | 0.999950574162623 |
| ebi-fl187-GCST90094770 | ebi-met1400-GCST90199833 | Hexanoylglutamine levels \|\| id:ebi-met1400-GCST90199833 | Gherkins liking \|\| id:ebi-fl187-GCST90094770 | MR Egger | 20 | -0.245819477 | 0.301527156361996 | 0.425586534162782 | -0.836812704 | 0.345173749049488 | 0.782063392284426 | 0.433088707875484 | 1.41223527288842 | 0.999950574162623 |
| ebi-fl187-GCST90094770 | ebi-met1400-GCST90199837 | Carotenoid levels (cryptoxanthin) \|\| id:ebi-met1400-GCST90199837 | Gherkins liking \|\| id:ebi-fl187-GCST90094770 | MR Egger | 20 | 0.0243613024357854 | 0.23359003587484 | 0.918091824779097 | -0.433475168 | 0.482197772750473 | 1.02466046334063 | 0.648252389897608 | 1.61963007232301 | 0.999950574162623 |
| ebi-fl187-GCST90094770 | ebi-met1400-GCST90199869 | 2-hydroxyhippurate levels \|\| id:ebi-met1400-GCST90199869 | Gherkins liking \|\| id:ebi-fl187-GCST90094770 | MR Egger | 20 | -0.038790878 | 0.275549045217526 | 0.889609826795556 | -0.578867006 | 0.501285251021046 | 0.961951853788955 | 0.560533087425119 | 1.65084165371705 | 0.999950574162623 |
| ebi-fl187-GCST90094770 | ebi-met1400-GCST90199902 | Hydantoin-5-propionate levels \|\| id:ebi-met1400-GCST90199902 | Gherkins liking \|\| id:ebi-fl187-GCST90094770 | MR Egger | 20 | -0.083744776 | 0.23544849860995 | 0.72621303392748 | -0.545223833 | 0.37773428149456 | 0.919665947167588 | 0.579712010105539 | 1.45897521465128 | 0.999950574162623 |
| ebi-fl187-GCST90094770 | ebi-met1400-GCST90199904 | Pantoate levels \|\| id:ebi-met1400-GCST90199904 | Gherkins liking \|\| id:ebi-fl187-GCST90094770 | MR Egger | 20 | -0.201520922 | 0.281164768455685 | 0.482734133590997 | -0.752603868 | 0.349562024398902 | 0.817486474113727 | 0.471138172578831 | 1.41844616771543 | 0.999950574162623 |
| ebi-fl187-GCST90094770 | ebi-met1400-GCST90199936 | 3-methyl catechol sulfate (2) levels \|\| id:ebi-met1400-GCST90199936 | Gherkins liking \|\| id:ebi-fl187-GCST90094770 | MR Egger | 20 | 0.18270843850304 | 0.30674912540225 | 0.558839175223311 | -0.418519847 | 0.783936724291449 | 1.20046434786896 | 0.658020069550477 | 2.19007704656932 | 0.999950574162623 |
| ebi-fl187-GCST90094770 | ebi-met1400-GCST90199977 | 4-hydroxychlorothalonil levels \|\| id:ebi-met1400-GCST90199977 | Gherkins liking \|\| id:ebi-fl187-GCST90094770 | MR Egger | 20 | -0.207859818 | 0.263635563633001 | 0.440702537474686 | -0.724585523 | 0.308865886886621 | 0.812320901634266 | 0.484525352172562 | 1.361879712 | 0.999950574162623 |
| ebi-fl187-GCST90094770 | ebi-met1400-GCST90200040 | Sphingomyelin (d18:2/24:1, d18:1/24:2) levels \|\| id:ebi-met1400-GCST90200040 | Gherkins liking \|\| id:ebi-fl187-GCST90094770 | MR Egger | 20 | 0.0125766500419924 | 0.248404758237609 | 0.960178231408439 | -0.474296676 | 0.499449976187705 | 1.01265606869611 | 0.622322596934269 | 1.64781468408637 | 0.999950574162623 |
| ebi-fl187-GCST90094770 | ebi-met1400-GCST90200077 | 1-stearoyl-2-oleoyl-GPI (18:0/18:1) levels \|\| id:ebi-met1400-GCST90200077 | Gherkins liking \|\| id:ebi-fl187-GCST90094770 | MR Egger | 20 | 0.211295060031051 | 0.235096232570625 | 0.380646415662301 | -0.249493556 | 0.672083675869476 | 1.23527678210051 | 0.779195302097707 | 1.95831356309338 | 0.999950574162623 |
| ebi-fl187-GCST90094770 | ebi-met1400-GCST90200080 | 1-palmitoyl-2-oleoyl-GPI (16:0/18:1) levels \|\| id:ebi-met1400-GCST90200080 | Gherkins liking \|\| id:ebi-fl187-GCST90094770 | MR Egger | 20 | 0.202284553123245 | 0.235032664110928 | 0.40074143637412 | -0.258379469 | 0.662948574780664 | 1.22419630743692 | 0.772302112145109 | 1.9405056331901 | 0.999950574162623 |
| ebi-fl187-GCST90094770 | ebi-met1400-GCST90200082 | 1-oleoyl-2-linoleoyl-GPE (18:1/18:2) levels \|\| id:ebi-met1400-GCST90200082 | Gherkins liking \|\| id:ebi-fl187-GCST90094770 | MR Egger | 20 | 0.519509543628581 | 0.236358689687572 | 0.0412751115957566 | 0.0562465118409404 | 0.982772575416222 | 1.68120289079185 | 1.05785842622534 | 2.67185389834463 | 0.999950574162623 |
| ebi-fl187-GCST90094770 | ebi-met1400-GCST90200100 | Perfluorooctanesulfonate (PFOS) levels \|\| id:ebi-met1400-GCST90200100 | Gherkins liking \|\| id:ebi-fl187-GCST90094770 | MR Egger | 20 | -0.113085172 | 0.217151911608877 | 0.60887757346669 | -0.538702919 | 0.312532574343909 | 0.893074591561773 | 0.583504614390299 | 1.36688246574814 | 0.999950574162623 |
| ebi-fl187-GCST90094770 | ebi-met1400-GCST90200102 | 1-palmitoleoyl-2-linolenoyl-GPC (16:1/18:3) levels \|\| id:ebi-met1400-GCST90200102 | Gherkins liking \|\| id:ebi-fl187-GCST90094770 | MR Egger | 20 | 0.204195325216944 | 0.230609759553959 | 0.387585659755361 | -0.247799804 | 0.656190453942704 | 1.22653770380342 | 0.780516184239463 | 1.92743567555522 | 0.999950574162623 |
| ebi-fl187-GCST90094770 | ebi-met1400-GCST90200114 | Glycosyl-N-tricosanoyl-sphingadienine (d18:2/23:0) levels \|\| id:ebi-met1400-GCST90200114 | Gherkins liking \|\| id:ebi-fl187-GCST90094770 | MR Egger | 20 | -0.078531669 | 0.263888665983744 | 0.769420479232138 | -0.595753454 | 0.438690116708193 | 0.924472782690767 | 0.551147145394713 | 1.55067468475036 | 0.999950574162623 |
| ebi-fl187-GCST90094770 | ebi-met1400-GCST90200135 | Arachidoylcarnitine (C20) levels \|\| id:ebi-met1400-GCST90200135 | Gherkins liking \|\| id:ebi-fl187-GCST90094770 | MR Egger | 20 | 0.29401817980313 | 0.241509678219418 | 0.239165509348832 | -0.17934079 | 0.76737714911319 | 1.34180829725592 | 0.835821011825944 | 2.15410893135068 | 0.999950574162623 |
| ebi-fl187-GCST90094770 | ebi-met1400-GCST90200146 | Docosahexaenoylcarnitine (C22:6) levels \|\| id:ebi-met1400-GCST90200146 | Gherkins liking \|\| id:ebi-fl187-GCST90094770 | MR Egger | 20 | -0.714500554 | 0.269970665559624 | 0.0164068131189091 | -1.243643058 | -0.185358049 | 0.489436497979739 | 0.288331892379255 | 0.830806760840677 | 0.999950574162623 |
| ebi-fl187-GCST90094770 | ebi-met1400-GCST90200162 | 3-hydroxyphenylacetoylglutamine levels \|\| id:ebi-met1400-GCST90200162 | Gherkins liking \|\| id:ebi-fl187-GCST90094770 | MR Egger | 20 | -0.159706784 | 0.286664205579014 | 0.584306272104976 | -0.721568627 | 0.40215505870774 | 0.852393687601212 | 0.485989321686262 | 1.49504313416056 | 0.999950574162623 |
| ebi-fl187-GCST90094770 | ebi-met1400-GCST90200172 | 3-carboxy-4-methyl-5-pentyl-2-furanpropionate (3-CMPFP) levels \|\| id:ebi-met1400-GCST90200172 | Gherkins liking \|\| id:ebi-fl187-GCST90094770 | MR Egger | 20 | 0.207750670693644 | 0.290041704425447 | 0.483009683756904 | -0.36073107 | 0.77623241136752 | 1.23090623028939 | 0.697166462248898 | 2.17326883866127 | 0.999950574162623 |
| ebi-fl187-GCST90094770 | ebi-met1400-GCST90200222 | Dihydrocaffeate sulfate (2) levels \|\| id:ebi-met1400-GCST90200222 | Gherkins liking \|\| id:ebi-fl187-GCST90094770 | MR Egger | 20 | -0.078022869 | 0.308613524870204 | 0.803273971044807 | -0.682905378 | 0.526859639822712 | 0.924943273845266 | 0.505147214832798 | 1.6936054178083 | 0.999950574162623 |
| ebi-fl187-GCST90094770 | ebi-met1400-GCST90200223 | 4-methylhexanoylglutamine levels \|\| id:ebi-met1400-GCST90200223 | Gherkins liking \|\| id:ebi-fl187-GCST90094770 | MR Egger | 20 | 0.268970969015961 | 0.332038094738272 | 0.428485344598045 | -0.381823697 | 0.919765634702974 | 1.30861715005141 | 0.682615389962835 | 2.50870236825736 | 0.999950574162623 |
| ebi-fl187-GCST90094770 | ebi-met1400-GCST90200224 | 2-ketocaprylate levels \|\| id:ebi-met1400-GCST90200224 | Gherkins liking \|\| id:ebi-fl187-GCST90094770 | MR Egger | 20 | 0.0885694209309273 | 0.238491906274964 | 0.71469506321853 | -0.378874715 | 0.556013557229858 | 1.09261009998803 | 0.684631381084067 | 1.74370743670202 | 0.999950574162623 |
| ebi-fl187-GCST90094770 | ebi-met1400-GCST90200243 | Hydroxypalmitoyl sphingomyelin (d18:1/16:0(OH)) levels \|\| id:ebi-met1400-GCST90200243 | Gherkins liking \|\| id:ebi-fl187-GCST90094770 | MR Egger | 20 | -0.066074503 | 0.261590659735464 | 0.803448970187445 | -0.578792197 | 0.446643189612065 | 0.936061121892433 | 0.56057502229496 | 1.5630564849846 | 0.999950574162623 |
| ebi-fl187-GCST90094770 | ebi-met1400-GCST90200273 | 2-methoxyhydroquinone sulfate (2) levels \|\| id:ebi-met1400-GCST90200273 | Gherkins liking \|\| id:ebi-fl187-GCST90094770 | MR Egger | 20 | 0.414118621602612 | 0.280663153756505 | 0.157356593639011 | -0.13598116 | 0.964218402965362 | 1.5130365950655 | 0.872859077208188 | 2.6227369317503 | 0.999950574162623 |
| ebi-fl187-GCST90094770 | ebi-met1400-GCST90200276 | 2,4-di-tert-butylphenol levels \|\| id:ebi-met1400-GCST90200276 | Gherkins liking \|\| id:ebi-fl187-GCST90094770 | MR Egger | 20 | -0.359788565 | 0.234349918127952 | 0.142109994719489 | -0.819114404 | 0.0995372750134514 | 0.697823855197675 | 0.440821871758216 | 1.10465964617571 | 0.999950574162623 |
| ebi-fl187-GCST90094770 | ebi-met1400-GCST90200289 | N-acetylmethionine levels \|\| id:ebi-met1400-GCST90200289 | Gherkins liking \|\| id:ebi-fl187-GCST90094770 | MR Egger | 20 | 0.179250859196787 | 0.265699391568933 | 0.508479061812352 | -0.341519948 | 0.700021666671896 | 1.19632081460725 | 0.710689290450688 | 2.01379633926235 | 0.999950574162623 |
| ebi-fl187-GCST90094770 | ebi-met1400-GCST90200308 | 3-hydroxyisobutyrate levels \|\| id:ebi-met1400-GCST90200308 | Gherkins liking \|\| id:ebi-fl187-GCST90094770 | MR Egger | 20 | -0.248463151 | 0.2692069909601 | 0.368243050122776 | -0.776108853 | 0.279182551471775 | 0.779998602622265 | 0.460193211312695 | 1.32204866377155 | 0.999950574162623 |
| ebi-fl187-GCST90094770 | ebi-met1400-GCST90200383 | Cystathionine levels \|\| id:ebi-met1400-GCST90200383 | Gherkins liking \|\| id:ebi-fl187-GCST90094770 | MR Egger | 20 | -0.080271657 | 0.234716649350712 | 0.736316060285824 | -0.54031629 | 0.379772975813533 | 0.922865609507586 | 0.582563964284046 | 1.46195265314513 | 0.999950574162623 |
| ebi-fl187-GCST90094770 | ebi-met1400-GCST90200384 | Guanidinoacetate levels \|\| id:ebi-met1400-GCST90200384 | Gherkins liking \|\| id:ebi-fl187-GCST90094770 | MR Egger | 20 | 0.250956215146732 | 0.225262208223066 | 0.279907742787406 | -0.190557713 | 0.692470143263941 | 1.28525380844977 | 0.826498056694639 | 1.99864638368406 | 0.999950574162623 |
| ebi-fl187-GCST90094770 | ebi-met1400-GCST90200403 | Citrulline levels \|\| id:ebi-met1400-GCST90200403 | Gherkins liking \|\| id:ebi-fl187-GCST90094770 | MR Egger | 20 | 0.00965344484404611 | 0.263538727748347 | 0.971183140038893 | -0.506882462 | 0.526189351230805 | 1.00970018963774 | 0.602370568029548 | 1.69247059378983 | 0.999950574162623 |
| ebi-fl187-GCST90094770 | ebi-met1400-GCST90200434 | 12,13-DiHOME levels \|\| id:ebi-met1400-GCST90200434 | Gherkins liking \|\| id:ebi-fl187-GCST90094770 | MR Egger | 20 | 0.364750974827745 | 0.238181444861542 | 0.143056861508084 | -0.102084657 | 0.831586606756368 | 1.44015532856218 | 0.902953107040861 | 2.29696022330889 | 0.999950574162623 |
| ebi-fl187-GCST90094770 | ebi-met1400-GCST90200437 | Fructose levels \|\| id:ebi-met1400-GCST90200437 | Gherkins liking \|\| id:ebi-fl187-GCST90094770 | MR Egger | 20 | -0.056010385 | 0.237118864376681 | 0.815934953784909 | -0.52076336 | 0.408742588808761 | 0.945529316168052 | 0.594066888208305 | 1.5049242862695 | 0.999950574162623 |
| ebi-fl187-GCST90094770 | ebi-met1400-GCST90200448 | N-stearoyl-sphinganine (d18:0/18:0) levels \|\| id:ebi-met1400-GCST90200448 | Gherkins liking \|\| id:ebi-fl187-GCST90094770 | MR Egger | 20 | 0.060417061661091 | 0.262389550079674 | 0.820488540594021 | -0.453866456 | 0.574700579817251 | 1.06227949022026 | 0.63516755008329 | 1.776598498 | 0.999950574162623 |
| ebi-fl187-GCST90094770 | ebi-met1400-GCST90200468 | X-11847 levels \|\| id:ebi-met1400-GCST90200468 | Gherkins liking \|\| id:ebi-fl187-GCST90094770 | MR Egger | 20 | 0.165144862050586 | 0.256489287439294 | 0.527781576299607 | -0.337574141 | 0.667863865431602 | 1.1795639803924 | 0.713499072974894 | 1.95006726222912 | 0.999950574162623 |
| ebi-fl187-GCST90094770 | ebi-met1400-GCST90200476 | X-11843 levels \|\| id:ebi-met1400-GCST90200476 | Gherkins liking \|\| id:ebi-fl187-GCST90094770 | MR Egger | 19 | 0.166338901958505 | 0.322002517728891 | 0.612106603260073 | -0.464786033 | 0.797463836707132 | 1.18097326806436 | 0.628269519885181 | 2.21990374471373 | 0.999950574162623 |
| ebi-fl187-GCST90094770 | ebi-met1400-GCST90200511 | X-12906 levels \|\| id:ebi-met1400-GCST90200511 | Gherkins liking \|\| id:ebi-fl187-GCST90094770 | MR Egger | 20 | -0.004836657 | 0.283524784291448 | 0.986577193733183 | -0.560545234 | 0.55087192010473 | 0.995175020684732 | 0.570897705953815 | 1.73476493505997 | 0.999950574162623 |
| ebi-fl187-GCST90094770 | ebi-met1400-GCST90200515 | X-12849 levels \|\| id:ebi-met1400-GCST90200515 | Gherkins liking \|\| id:ebi-fl187-GCST90094770 | MR Egger | 20 | 0.0923229182027377 | 0.248070975576914 | 0.7141166553069 | -0.393896194 | 0.578542030333488 | 1.09671891540722 | 0.674424061919596 | 1.78343633824172 | 0.999950574162623 |
| ebi-fl187-GCST90094770 | ebi-met1400-GCST90200551 | X-18886 levels \|\| id:ebi-met1400-GCST90200551 | Gherkins liking \|\| id:ebi-fl187-GCST90094770 | MR Egger | 20 | -0.130743955 | 0.255355795335504 | 0.614870740929472 | -0.631241314 | 0.369753404184067 | 0.877442410659788 | 0.531931097752533 | 1.44737765337916 | 0.999950574162623 |
| ebi-fl187-GCST90094770 | ebi-met1400-GCST90200564 | X-21319 levels \|\| id:ebi-met1400-GCST90200564 | Gherkins liking \|\| id:ebi-fl187-GCST90094770 | MR Egger | 20 | 0.151872531371584 | 0.245492049428559 | 0.543900204777538 | -0.329291886 | 0.633036948251559 | 1.1640118519809 | 0.719432994032057 | 1.88332145285462 | 0.999950574162623 |
| ebi-fl187-GCST90094770 | ebi-met1400-GCST90200579 | X-21442 levels \|\| id:ebi-met1400-GCST90200579 | Gherkins liking \|\| id:ebi-fl187-GCST90094770 | MR Egger | 20 | 0.220577583764997 | 0.258427006898377 | 0.404576284995648 | -0.28593935 | 0.727094517285815 | 1.2467966521644 | 0.751308181589756 | 2.06906024710001 | 0.999950574162623 |
| ebi-fl187-GCST90094770 | ebi-met1400-GCST90200581 | X-21441 levels \|\| id:ebi-met1400-GCST90200581 | Gherkins liking \|\| id:ebi-fl187-GCST90094770 | MR Egger | 20 | -0.25127178 | 0.236058273713329 | 0.301197348593685 | -0.713945997 | 0.211402436350456 | 0.777810949270976 | 0.489707993765499 | 1.23540942869624 | 0.999950574162623 |
| ebi-fl187-GCST90094770 | ebi-met1400-GCST90200648 | X-24585 levels \|\| id:ebi-met1400-GCST90200648 | Gherkins liking \|\| id:ebi-fl187-GCST90094770 | MR Egger | 20 | 0.352521940038027 | 0.230388568870797 | 0.143371936293165 | -0.099039655 | 0.804083535024789 | 1.42265086842038 | 0.905706791555032 | 2.23464758384146 | 0.999950574162623 |
| ebi-fl187-GCST90094770 | ebi-met1400-GCST90200720 | Adenosine 5'-diphosphate (ADP) to pantothenate ratio \|\| id:ebi-met1400-GCST90200720 | Gherkins liking \|\| id:ebi-fl187-GCST90094770 | MR Egger | 20 | 0.0267077706990526 | 0.312256829246007 | 0.932783113212155 | -0.585315615 | 0.638731156021225 | 1.02706761965196 | 0.55693005880002 | 1.89407606694886 | 0.999950574162623 |
| ebi-fl187-GCST90094770 | ebi-met1400-GCST90200724 | Cholate to taurocholate ratio \|\| id:ebi-met1400-GCST90200724 | Gherkins liking \|\| id:ebi-fl187-GCST90094770 | MR Egger | 20 | -0.129427529 | 0.276010221050159 | 0.644754111936348 | -0.670407562 | 0.411552504597136 | 0.878598259301202 | 0.511500067349706 | 1.50915894351064 | 0.999950574162623 |
| ebi-fl187-GCST90094770 | ebi-met1400-GCST90200726 | N-acetylputrescine to (N(1) + N(8))-acetylspermidine ratio \|\| id:ebi-met1400-GCST90200726 | Gherkins liking \|\| id:ebi-fl187-GCST90094770 | MR Egger | 20 | -0.263913863 | 0.234572301529262 | 0.275332032420463 | -0.723675574 | 0.195847847648461 | 0.768039693174632 | 0.484966445804487 | 1.21634182198575 | 0.999950574162623 |
| ebi-fl187-GCST90094770 | ebi-met1400-GCST90200744 | Arginine to glutamate ratio \|\| id:ebi-met1400-GCST90200744 | Gherkins liking \|\| id:ebi-fl187-GCST90094770 | MR Egger | 20 | 0.0206395059949467 | 0.257210899684956 | 0.936929027762538 | -0.483493857 | 0.524772869377461 | 1.02085397355904 | 0.616625223243367 | 1.69007493700915 | 0.999950574162623 |
| ebi-fl187-GCST90094770 | ebi-met1400-GCST90200759 | Histidine to trans-urocanate ratio \|\| id:ebi-met1400-GCST90200759 | Gherkins liking \|\| id:ebi-fl187-GCST90094770 | MR Egger | 20 | -0.213682389 | 0.26730731608601 | 0.434487887400237 | -0.737604728 | 0.310239950940461 | 0.807604848808884 | 0.478258102840823 | 1.36375230852429 | 0.999950574162623 |
| ebi-fl187-GCST90094770 | ebi-met1400-GCST90200795 | Oleoyl-linoleoyl-glycerol (18:1 to 18:2) [2] to linoleoyl-arachidonoyl-glycerol (18:2 to 20:4) [2] ratio \|\| id:ebi-met1400-GCST90200795 | Gherkins liking \|\| id:ebi-fl187-GCST90094770 | MR Egger | 20 | 0.0275842505603145 | 0.304376641935926 | 0.928791128883959 | -0.568993968 | 0.62416246875473 | 1.0279682183574 | 0.566094661875068 | 1.86668189813472 | 0.999950574162623 |
| ebi-fl187-GCST90094770 | ebi-met1400-GCST90200811 | Choline to taurocholate ratio \|\| id:ebi-met1400-GCST90200811 | Gherkins liking \|\| id:ebi-fl187-GCST90094770 | MR Egger | 20 | 0.0231958050409468 | 0.246927654980394 | 0.926196439675303 | -0.460782399 | 0.50717400880252 | 1.02346692 | 0.630789923159381 | 1.66059174012428 | 0.999950574162623 |
| ebi-fl187-GCST90094770 | ebi-met1400-GCST90200831 | Cholate to phosphate ratio \|\| id:ebi-met1400-GCST90200831 | Gherkins liking \|\| id:ebi-fl187-GCST90094770 | MR Egger | 20 | -0.2650542 | 0.262896473977972 | 0.326716647988553 | -0.780331289 | 0.250222889032008 | 0.767164368567225 | 0.458254171606524 | 1.28431164376719 | 0.999950574162623 |
| ebi-fl187-GCST90094770 | ebi-met1400-GCST90200841 | Adenosine 5'-diphosphate (ADP) to oxalate (ethanedioate) ratio \|\| id:ebi-met1400-GCST90200841 | Gherkins liking \|\| id:ebi-fl187-GCST90094770 | MR Egger | 20 | 0.249246277178072 | 0.316510880083845 | 0.441247196462556 | -0.371115048 | 0.869607602142408 | 1.28305798206183 | 0.689964558097421 | 2.38597441855864 | 0.999950574162623 |
| ebi-fl187-GCST90094770 | ebi-met1400-GCST90200844 | Aspartate to phosphate ratio \|\| id:ebi-met1400-GCST90200844 | Gherkins liking \|\| id:ebi-fl187-GCST90094770 | MR Egger | 20 | -0.155335907 | 0.221489137733484 | 0.492068530797815 | -0.589454617 | 0.278782802683792 | 0.856127549706909 | 0.554629687738929 | 1.32152028203757 | 0.999950574162623 |
| ebi-fl187-GCST90094770 | ebi-met1400-GCST90200870 | Adenosine 5'-monophosphate (AMP) to acetoacetate ratio \|\| id:ebi-met1400-GCST90200870 | Gherkins liking \|\| id:ebi-fl187-GCST90094770 | MR Egger | 20 | 0.220095458465852 | 0.259220498672077 | 0.406995194805155 | -0.287976719 | 0.728167635863123 | 1.24619568483792 | 0.74977904769328 | 2.07128178586281 | 0.999950574162623 |
| ebi-fl187-GCST90094770 | ebi-met1400-GCST90200887 | Cholesterol to cortisol ratio \|\| id:ebi-met1400-GCST90200887 | Gherkins liking \|\| id:ebi-fl187-GCST90094770 | MR Egger | 20 | -0.07334783 | 0.294381307372832 | 0.80605853392403 | -0.650335192 | 0.50363953286405 | 0.929277543580843 | 0.521870820497072 | 1.65473277885344 | 0.999950574162623 |
| ebi-fl187-GCST90094770 | ebi-met1400-GCST90200890 | Cortisol to taurocholate ratio \|\| id:ebi-met1400-GCST90200890 | Gherkins liking \|\| id:ebi-fl187-GCST90094770 | MR Egger | 20 | 0.161245038084765 | 0.246180501096297 | 0.520759267468056 | -0.321268744 | 0.643758820233508 | 1.17497284663791 | 0.725228323991477 | 1.90362282424123 | 0.999950574162623 |
| ebi-fl187-GCST90094770 | ebi-met1400-GCST90200948 | Adenosine 5'-diphosphate (ADP) to mannitol to sorbitol ratio \|\| id:ebi-met1400-GCST90200948 | Gherkins liking \|\| id:ebi-fl187-GCST90094770 | MR Egger | 20 | -0.138843417 | 0.335336591400192 | 0.683735257220402 | -0.796103136 | 0.518416302304885 | 0.870364302182118 | 0.451083354078338 | 1.67936593461928 | 0.999950574162623 |
| ebi-fl187-GCST90094770 | ebi-met1400-GCST90200972 | Leucine to N-palmitoyl-sphingosine (d18:1 to 16:0) ratio \|\| id:ebi-met1400-GCST90200972 | Gherkins liking \|\| id:ebi-fl187-GCST90094770 | MR Egger | 20 | 0.0334219994354886 | 0.247425993188307 | 0.894048643993549 | -0.451532947 | 0.518376946084569 | 1.03398678902752 | 0.636651450130616 | 1.67929984242414 | 0.999950574162623 |
| ebi-fl187-GCST90094770 | ebi-met1400-GCST90200973 | Proline to glutamate ratio \|\| id:ebi-met1400-GCST90200973 | Gherkins liking \|\| id:ebi-fl187-GCST90094770 | MR Egger | 20 | -0.030830278 | 0.231896492823373 | 0.89570916201113 | -0.485347403 | 0.423686848405412 | 0.969640128830455 | 0.615483338576145 | 1.52758315377537 | 0.999950574162623 |
| ebi-fl187-GCST90094770 | ebi-met1400-GCST90200983 | Cholesterol to linoleoyl-arachidonoyl-glycerol (18:2 to 20:4) [1] ratio \|\| id:ebi-met1400-GCST90200983 | Gherkins liking \|\| id:ebi-fl187-GCST90094770 | MR Egger | 20 | -0.235020748 | 0.287573255762235 | 0.424468372643269 | -0.79866433 | 0.328622832880915 | 0.790554446708357 | 0.449929520449699 | 1.38905385133587 | 0.999950574162623 |
| ebi-fl187-GCST90094770 | ebi-met1400-GCST90201001 | Serine to alpha-ketobutyrate ratio \|\| id:ebi-met1400-GCST90201001 | Gherkins liking \|\| id:ebi-fl187-GCST90094770 | MR Egger | 20 | -0.009379898 | 0.239672930635187 | 0.969212571974332 | -0.479138842 | 0.460379045985823 | 0.990663955962129 | 0.619316491518408 | 1.58467453569072 | 0.999950574162623 |
| ebi-fl187-GCST90094770 | ebi-met1400-GCST90201005 | Fructose to maltose ratio \|\| id:ebi-met1400-GCST90201005 | Gherkins liking \|\| id:ebi-fl187-GCST90094770 | MR Egger | 20 | 0.231018687481401 | 0.269235495653232 | 0.402143879912174 | -0.296682884 | 0.758720258961736 | 1.25988278326532 | 0.743279680872909 | 2.13554153088679 | 0.999950574162623 |
| ebi-fl187-GCST90094770 | ebi-met1400-GCST90201020 | Paraxanthine to 5-acetylamino-6-formylamino-3-methyluracil ratio \|\| id:ebi-met1400-GCST90201020 | Gherkins liking \|\| id:ebi-fl187-GCST90094770 | MR Egger | 20 | 0.295353930468207 | 0.249451833425274 | 0.251808679154398 | -0.193571663 | 0.784279523981744 | 1.34360181616175 | 0.824010782957078 | 2.19082793299707 | 0.999950574162623 |
| ebi-fl187-GCST90094770 | ebi-pw11-GCST90275197 | Placental weight (fetal genotype, adjusted for sex) \|\| id:ebi-pw11-GCST90275197 | Gherkins liking \|\| id:ebi-fl187-GCST90094770 | MR Egger | 18 | -0.119067485 | 0.0902598600769384 | 0.205682757656155 | -0.295976811 | 0.0578418406826503 | 0.887747889013609 | 0.743804676041495 | 1.05954740516333 | 0.999950574162623 |
| ebi-fl187-GCST90094770 | prot-a-1017 | FAS-associated factor 2 \|\| id:prot-a-1017 | Gherkins liking \|\| id:ebi-fl187-GCST90094770 | MR Egger | 21 | -0.435511326 | 0.418540767635851 | 0.311146613837711 | -1.25585123 | 0.384828578964972 | 0.64693378868345 | 0.284833286315055 | 1.46936242022427 | 0.999950574162623 |
| ebi-fl187-GCST90094770 | prot-a-1019 | Fas apoptotic inhibitory molecule 3 \|\| id:prot-a-1019 | Gherkins liking \|\| id:ebi-fl187-GCST90094770 | MR Egger | 21 | 0.243166860282033 | 0.320187070147003 | 0.45690181510524 | -0.384399797 | 0.870733517770159 | 1.27528140018014 | 0.680859167166002 | 2.38866233734484 | 0.999950574162623 |
| ebi-fl187-GCST90094770 | prot-a-1052 | MIP18 family protein FAM96A \|\| id:prot-a-1052 | Gherkins liking \|\| id:ebi-fl187-GCST90094770 | MR Egger | 21 | 0.183901794639696 | 0.32018981064099 | 0.572467336755335 | -0.443670234 | 0.811473823496037 | 1.20189778449541 | 0.641676989056379 | 2.25122344888708 | 0.999950574162623 |
| ebi-fl187-GCST90094770 | prot-a-1066 | F-box/LRR-repeat protein 4 \|\| id:prot-a-1066 | Gherkins liking \|\| id:ebi-fl187-GCST90094770 | MR Egger | 21 | -0.03078343 | 0.366178876587478 | 0.933882887255565 | -0.748494029 | 0.68692716763724 | 0.969685554678133 | 0.473078459187358 | 1.98759858262548 | 0.999950574162623 |
| ebi-fl187-GCST90094770 | prot-a-1076 | Low affinity immunoglobulin gamma Fc region receptor III-B \|\| id:prot-a-1076 | Gherkins liking \|\| id:ebi-fl187-GCST90094770 | MR Egger | 21 | -0.895774269 | 0.320104266905722 | 0.0114639809874599 | -1.523178632 | -0.268369905 | 0.408291349049185 | 0.21801778613457 | 0.764624889849618 | 0.999950574162623 |
| ebi-fl187-GCST90094770 | prot-a-1081 | Fc receptor-like protein 3 \|\| id:prot-a-1081 | Gherkins liking \|\| id:ebi-fl187-GCST90094770 | MR Egger | 21 | -0.360425722 | 0.320179772553982 | 0.274312323187708 | -0.987978076 | 0.267126631980724 | 0.697379372967363 | 0.372328750738777 | 1.30620584329133 | 0.999950574162623 |
| ebi-fl187-GCST90094770 | prot-a-1105 | Fibroblast growth factor receptor 4 \|\| id:prot-a-1105 | Gherkins liking \|\| id:ebi-fl187-GCST90094770 | MR Egger | 21 | 0.0464145762099899 | 0.404627894595403 | 0.90987911624949 | -0.746656097 | 0.83948524961698 | 1.04750859309238 | 0.473948744454124 | 2.31517493282144 | 0.999950574162623 |
| ebi-fl187-GCST90094770 | prot-a-1143 | Follistatin-related protein 1 \|\| id:prot-a-1143 | Gherkins liking \|\| id:ebi-fl187-GCST90094770 | MR Egger | 21 | -0.67334116 | 0.320257102837477 | 0.049070593776528 | -1.301045082 | -0.045637238 | 0.510001730545298 | 0.272247123847536 | 0.95538847751009 | 0.999950574162623 |
| ebi-fl187-GCST90094770 | prot-a-1151 | Alpha-(1,3)-fucosyltransferase 10 \|\| id:prot-a-1151 | Gherkins liking \|\| id:ebi-fl187-GCST90094770 | MR Egger | 21 | 0.0557703058449856 | 0.368758246176853 | 0.881382092385849 | -0.666995857 | 0.778536468351618 | 1.05735478762728 | 0.513248135069196 | 2.17828194693312 | 0.999950574162623 |
| ebi-fl187-GCST90094770 | prot-a-1165 | Growth arrest and DNA damage-inducible proteins-interacting protein 1 \|\| id:prot-a-1165 | Gherkins liking \|\| id:ebi-fl187-GCST90094770 | MR Egger | 21 | 0.197671140511233 | 0.376427613424664 | 0.605571333264161 | -0.540126982 | 0.935469262823575 | 1.21856159237928 | 0.582674258649338 | 2.54840905082019 | 0.999950574162623 |
| ebi-fl187-GCST90094770 | prot-a-1194 | Growth/differentiation factor 11/8 \|\| id:prot-a-1194 | Gherkins liking \|\| id:ebi-fl187-GCST90094770 | MR Egger | 21 | -0.376880777 | 0.361609371451006 | 0.310385049395037 | -1.085635145 | 0.331873591033757 | 0.685997855736133 | 0.337687237388525 | 1.39357667679081 | 0.999950574162623 |
| ebi-fl187-GCST90094770 | prot-a-1216 | D-glucuronyl C5-epimerase \|\| id:prot-a-1216 | Gherkins liking \|\| id:ebi-fl187-GCST90094770 | MR Egger | 21 | -0.2053601 | 0.320274453913509 | 0.52905191748593 | -0.83309803 | 0.42237782974435 | 0.814354014785071 | 0.434700483121843 | 1.52558482712954 | 0.999950574162623 |
| ebi-fl187-GCST90094770 | prot-a-1246 | Glypican-3 \|\| id:prot-a-1246 | Gherkins liking \|\| id:ebi-fl187-GCST90094770 | MR Egger | 21 | 0.502953708108607 | 0.339258575818054 | 0.154602181759411 | -0.1619931 | 1.16790051671199 | 1.65359831121752 | 0.850447072184006 | 3.21523521486099 | 0.999950574162623 |
| ebi-fl187-GCST90094770 | prot-a-1275 | Glutamate receptor 4 \|\| id:prot-a-1275 | Gherkins liking \|\| id:ebi-fl187-GCST90094770 | MR Egger | 20 | -0.099806291 | 0.41743674274304 | 0.81373446110413 | -0.917982307 | 0.718369725050133 | 0.905012710412423 | 0.399323942111022 | 2.05108664829399 | 0.999950574162623 |
| ebi-fl187-GCST90094770 | prot-a-1289 | Glutathione S-transferase P \|\| id:prot-a-1289 | Gherkins liking \|\| id:ebi-fl187-GCST90094770 | MR Egger | 21 | -0.180353947 | 0.438289275876782 | 0.685314118214525 | -1.039400928 | 0.678693033445282 | 0.834974622112149 | 0.353666490202804 | 1.97129962516816 | 0.999950574162623 |
| ebi-fl187-GCST90094770 | prot-a-130 | Apolipoprotein D \|\| id:prot-a-130 | Gherkins liking \|\| id:ebi-fl187-GCST90094770 | MR Egger | 21 | -0.001769776 | 0.361463588911851 | 0.996144504247678 | -0.71023841 | 0.706698858183562 | 0.99823178904658 | 0.491526998366626 | 2.02728783561118 | 0.999950574162623 |
| ebi-fl187-GCST90094770 | prot-a-1313 | Heparin-binding EGF-like growth factor \|\| id:prot-a-1313 | Gherkins liking \|\| id:ebi-fl187-GCST90094770 | MR Egger | 21 | -0.506652454 | 0.409998526237096 | 0.231615653259429 | -1.310249565 | 0.296944657826474 | 0.602509133980739 | 0.269752727137987 | 1.34574082116518 | 0.999950574162623 |
| ebi-fl187-GCST90094770 | prot-a-1347 | HLA class II histocompatibility antigen, DQ alpha 2 chain \|\| id:prot-a-1347 | Gherkins liking \|\| id:ebi-fl187-GCST90094770 | MR Egger | 20 | -0.277950319 | 0.358964828389778 | 0.448798902077034 | -0.981521383 | 0.425620744740899 | 0.757334445785733 | 0.37474054122416 | 1.53054019962708 | 0.999950574162623 |
| ebi-fl187-GCST90094770 | prot-a-1357 | Non-histone chromosomal protein HMG-14 \|\| id:prot-a-1357 | Gherkins liking \|\| id:ebi-fl187-GCST90094770 | MR Egger | 21 | 0.126626234777167 | 0.336541473913945 | 0.710894126322767 | -0.532995054 | 0.786247523648499 | 1.13499271768608 | 0.586844703316412 | 2.19514372698676 | 0.999950574162623 |
| ebi-fl187-GCST90094770 | prot-a-137 | Apolipoprotein M \|\| id:prot-a-137 | Gherkins liking \|\| id:ebi-fl187-GCST90094770 | MR Egger | 21 | -0.123300244 | 0.326632322065451 | 0.709993627109054 | -0.763499595 | 0.516899107703735 | 0.883998207956968 | 0.466032644473026 | 1.67681994156176 | 0.999950574162623 |
| ebi-fl187-GCST90094770 | prot-a-1381 | 17-beta-hydroxysteroid dehydrogenase 14 \|\| id:prot-a-1381 | Gherkins liking \|\| id:ebi-fl187-GCST90094770 | MR Egger | 21 | 0.509497079717503 | 0.320019711310184 | 0.127867407951747 | -0.117741554 | 1.13673571388546 | 1.66445389679244 | 0.888925761836696 | 3.11657833925675 | 0.999950574162623 |
| ebi-fl187-GCST90094770 | prot-a-1392 | Serine protease HTRA2, mitochondrial \|\| id:prot-a-1392 | Gherkins liking \|\| id:ebi-fl187-GCST90094770 | MR Egger | 21 | 0.172109659326229 | 0.363669022068575 | 0.641420262819433 | -0.540681624 | 0.884900942580636 | 1.18780808030615 | 0.582351172565991 | 2.42274438879179 | 0.999950574162623 |
| ebi-fl187-GCST90094770 | prot-a-1416 | Interferon-induced protein with tetratricopeptide repeats 2 \|\| id:prot-a-1416 | Gherkins liking \|\| id:ebi-fl187-GCST90094770 | MR Egger | 21 | -0.080443459 | 0.338439943544024 | 0.814666601763909 | -0.743785749 | 0.582898830172483 | 0.922707072729129 | 0.47531109688358 | 1.79122336433246 | 0.999950574162623 |
| ebi-fl187-GCST90094770 | prot-a-1428 | Interferon gamma \|\| id:prot-a-1428 | Gherkins liking \|\| id:ebi-fl187-GCST90094770 | MR Egger | 21 | 0.0921161386321729 | 0.320257102837477 | 0.776742376344889 | -0.535587783 | 0.719820060193627 | 1.09649215978589 | 0.585325144885105 | 2.05406356958733 | 0.999950574162623 |
| ebi-fl187-GCST90094770 | prot-a-1483 | Interleukin-17C \|\| id:prot-a-1483 | Gherkins liking \|\| id:ebi-fl187-GCST90094770 | MR Egger | 21 | -0.376204018 | 0.352753667239382 | 0.299572446993579 | -1.067601206 | 0.315193169995385 | 0.686462268237436 | 0.343832311997171 | 1.37052402950879 | 0.999950574162623 |
| ebi-fl187-GCST90094770 | prot-a-1503 | Interleukin-1 receptor-like 2 \|\| id:prot-a-1503 | Gherkins liking \|\| id:ebi-fl187-GCST90094770 | MR Egger | 21 | -0.260405901 | 0.320104266905722 | 0.426007236983813 | -0.887810264 | 0.36699846181071 | 0.770738678452677 | 0.411555965503189 | 1.44339569889767 | 0.999950574162623 |
| ebi-fl187-GCST90094770 | prot-a-1522 | Interleukin-31 receptor subunit alpha \|\| id:prot-a-1522 | Gherkins liking \|\| id:ebi-fl187-GCST90094770 | MR Egger | 21 | 0.571533806026174 | 0.320267132167143 | 0.0903111681256865 | -0.056189773 | 1.19925738507377 | 1.77098131113493 | 0.945359715096853 | 3.31765226961027 | 0.999950574162623 |
| ebi-fl187-GCST90094770 | prot-a-1540 | Interleukin-6 receptor subunit alpha \|\| id:prot-a-1540 | Gherkins liking \|\| id:ebi-fl187-GCST90094770 | MR Egger | 21 | 0.550935192990447 | 0.320245908159522 | 0.101614885724723 | -0.076746787 | 1.17861717298311 | 1.73487470211605 | 0.926124330637007 | 3.24987707640946 | 0.999950574162623 |
| ebi-fl187-GCST90094770 | prot-a-1541 | Interleukin-6 receptor subunit alpha \|\| id:prot-a-1541 | Gherkins liking \|\| id:ebi-fl187-GCST90094770 | MR Egger | 21 | 0.735697386808735 | 0.320245908159522 | 0.0331361269806977 | 0.108015406816071 | 1.3633793668014 | 2.08693688738974 | 1.1140649094474 | 3.90938224067062 | 0.999950574162623 |
| ebi-fl187-GCST90094770 | prot-a-1545 | Interleukin-9 \|\| id:prot-a-1545 | Gherkins liking \|\| id:ebi-fl187-GCST90094770 | MR Egger | 21 | -0.103571089 | 0.351793514699244 | 0.77163798672685 | -0.793086378 | 0.585944199351891 | 0.901611925359893 | 0.452446217948184 | 1.79668661534545 | 0.999950574162623 |
| ebi-fl187-GCST90094770 | prot-a-1548 | Inositol monophosphatase 3 \|\| id:prot-a-1548 | Gherkins liking \|\| id:ebi-fl187-GCST90094770 | MR Egger | 21 | 0.169814939953374 | 0.320267165692591 | 0.602095586566821 | -0.457908705 | 0.797538584710851 | 1.18508551904476 | 0.63260522738928 | 2.22006968428884 | 0.999950574162623 |
| ebi-fl187-GCST90094770 | prot-a-1555 | Inhibin beta A chain:Inhibin beta B chain heterodimer \|\| id:prot-a-1555 | Gherkins liking \|\| id:ebi-fl187-GCST90094770 | MR Egger | 21 | 0.146407900873935 | 0.320253652346249 | 0.652738366064314 | -0.481289258 | 0.774105059472583 | 1.15766830566926 | 0.617986134583779 | 2.16865044529483 | 0.999950574162623 |
| ebi-fl187-GCST90094770 | prot-a-1603 | Junctophilin-3 \|\| id:prot-a-1603 | Gherkins liking \|\| id:ebi-fl187-GCST90094770 | MR Egger | 21 | -0.933900199 | 0.320197103820454 | 0.00884889707778719 | -1.561486523 | -0.306313876 | 0.393017869356362 | 0.209823931254625 | 0.736155521964606 | 0.999950574162623 |
| ebi-fl187-GCST90094770 | prot-a-1627 | UPF0258 protein KIAA1024 \|\| id:prot-a-1627 | Gherkins liking \|\| id:ebi-fl187-GCST90094770 | MR Egger | 21 | -0.255988424 | 0.320018724207081 | 0.433646793601657 | -0.883225123 | 0.371248275896385 | 0.774150930660308 | 0.413447340623148 | 1.4495429152814 | 0.999950574162623 |
| ebi-fl187-GCST90094770 | prot-a-1666 | Kallikrein-7 \|\| id:prot-a-1666 | Gherkins liking \|\| id:ebi-fl187-GCST90094770 | MR Egger | 21 | 0.820056586885086 | 0.320096971776192 | 0.019071136514732 | 0.19266652220375 | 1.44744665156642 | 2.27062832168102 | 1.21247839147133 | 4.25224318345463 | 0.999950574162623 |
| ebi-fl187-GCST90094770 | prot-a-1678 | Importin subunit alpha-3 \|\| id:prot-a-1678 | Gherkins liking \|\| id:ebi-fl187-GCST90094770 | MR Egger | 21 | -0.042932472 | 0.379417529907822 | 0.911095791061668 | -0.786590831 | 0.700725886683217 | 0.957976078152682 | 0.455394669481775 | 2.01521499440722 | 0.999950574162623 |
| ebi-fl187-GCST90094770 | prot-a-1718 | Protein LDOC1 \|\| id:prot-a-1718 | Gherkins liking \|\| id:ebi-fl187-GCST90094770 | MR Egger | 21 | -0.213961964 | 0.350401937738375 | 0.548687484673098 | -0.900749762 | 0.472825833787195 | 0.807379093764528 | 0.406264943446158 | 1.60452190513559 | 0.999950574162623 |
| ebi-fl187-GCST90094770 | prot-a-1723 | Leptin \|\| id:prot-a-1723 | Gherkins liking \|\| id:ebi-fl187-GCST90094770 | MR Egger | 21 | -0.059214726 | 0.356314822707663 | 0.869765861319279 | -0.757591778 | 0.639162326581493 | 0.942504367304833 | 0.468794028598077 | 1.89489291287514 | 0.999950574162623 |
| ebi-fl187-GCST90094770 | prot-a-1730 | Galectin-7 \|\| id:prot-a-1730 | Gherkins liking \|\| id:ebi-fl187-GCST90094770 | MR Egger | 21 | -0.689611851 | 0.393107738585341 | 0.0955018314881108 | -1.460103018 | 0.0808793169933272 | 0.501770793287891 | 0.232212351384838 | 1.0842400393229 | 0.999950574162623 |
| ebi-fl187-GCST90094770 | prot-a-1753 | Lipase member N \|\| id:prot-a-1753 | Gherkins liking \|\| id:ebi-fl187-GCST90094770 | MR Egger | 21 | -0.166342145 | 0.319961312915477 | 0.609150267283722 | -0.793466318 | 0.460782028432118 | 0.846756471228655 | 0.452274348217136 | 1.58531326040047 | 0.999950574162623 |
| ebi-fl187-GCST90094770 | prot-a-1756 | VIP36-like protein \|\| id:prot-a-1756 | Gherkins liking \|\| id:ebi-fl187-GCST90094770 | MR Egger | 21 | 0.4355834201776 | 0.343502132230638 | 0.220093421823003 | -0.237680759 | 1.10884759934965 | 1.54586468456129 | 0.788454357882494 | 3.03086361193012 | 0.999950574162623 |
| ebi-fl187-GCST90094770 | prot-a-1760 | Ig Kappa chain V-I region HK102- like \|\| id:prot-a-1760 | Gherkins liking \|\| id:ebi-fl187-GCST90094770 | MR Egger | 21 | 0.428557941552602 | 0.320267165692591 | 0.196650669242365 | -0.199165703 | 1.05628158631008 | 1.53504230597112 | 0.81941410253984 | 2.8756581975065 | 0.999950574162623 |
| ebi-fl187-GCST90094770 | prot-a-179 | Neutral ceramidase \|\| id:prot-a-179 | Gherkins liking \|\| id:ebi-fl187-GCST90094770 | MR Egger | 21 | -0.12439908 | 0.320274487800943 | 0.702027684529113 | -0.752137076 | 0.503338916013944 | 0.883027371925145 | 0.471358147343013 | 1.65423541305971 | 0.999950574162623 |
| ebi-fl187-GCST90094770 | prot-a-1809 | Lactotransferrin \|\| id:prot-a-1809 | Gherkins liking \|\| id:ebi-fl187-GCST90094770 | MR Egger | 21 | 1.0276310211125 | 0.320197103820454 | 0.00461617480596807 | 0.400044697624408 | 1.65521734460059 | 2.79443802254734 | 1.49189138015155 | 5.23421742745444 | 0.999950574162623 |
| ebi-fl187-GCST90094770 | prot-a-1818 | Lysophospholipase-like protein 1 \|\| id:prot-a-1818 | Gherkins liking \|\| id:ebi-fl187-GCST90094770 | MR Egger | 21 | 0.0208073046919206 | 0.320267165692591 | 0.948877719741009 | -0.60691634 | 0.648530949449398 | 1.0210252858982 | 0.545028964387707 | 1.91272886866599 | 0.999950574162623 |
| ebi-fl187-GCST90094770 | prot-a-1852 | MAP kinase-activated protein kinase 3 \|\| id:prot-a-1852 | Gherkins liking \|\| id:ebi-fl187-GCST90094770 | MR Egger | 21 | 0.362303721158649 | 0.32018981064099 | 0.271913010893772 | -0.265268308 | 0.98987575001499 | 1.43663521221596 | 0.767000130326496 | 2.69090010727927 | 0.999950574162623 |
| ebi-fl187-GCST90094770 | prot-a-1856 | Megakaryocyte-associated tyrosine-protein kinase \|\| id:prot-a-1856 | Gherkins liking \|\| id:ebi-fl187-GCST90094770 | MR Egger | 21 | -0.067135859 | 0.320274487800943 | 0.836195739820915 | -0.694873855 | 0.560602137006421 | 0.935068155204277 | 0.499137407615831 | 1.75172696242813 | 0.999950574162623 |
| ebi-fl187-GCST90094770 | prot-a-1890 | Beta-1,3-N-acetylglucosaminyltransferase manic fringe \|\| id:prot-a-1890 | Gherkins liking \|\| id:ebi-fl187-GCST90094770 | MR Egger | 21 | 0.0801377216559031 | 0.320274453913509 | 0.805106066675768 | -0.547600208 | 0.707875651326381 | 1.0834362700377 | 0.578336032568608 | 2.02967493832221 | 0.999950574162623 |
| ebi-fl187-GCST90094770 | prot-a-1919 | Matrilysin \|\| id:prot-a-1919 | Gherkins liking \|\| id:ebi-fl187-GCST90094770 | MR Egger | 21 | -0.51776274 | 0.350866425386669 | 0.156413280419123 | -1.205460934 | 0.169935453494197 | 0.595852133850906 | 0.299553893106102 | 1.18522834650299 | 0.999950574162623 |
| ebi-fl187-GCST90094770 | prot-a-1923 | Multimerin-2 \|\| id:prot-a-1923 | Gherkins liking \|\| id:ebi-fl187-GCST90094770 | MR Egger | 21 | -0.569596262 | 0.320274487800943 | 0.0913345357576661 | -1.197334258 | 0.0581417345507804 | 0.565753809167644 | 0.301998189206891 | 1.0598652045838 | 0.999950574162623 |
| ebi-fl187-GCST90094770 | prot-a-1948 | Mesothelin \|\| id:prot-a-1948 | Gherkins liking \|\| id:ebi-fl187-GCST90094770 | MR Egger | 21 | -0.000807534 | 0.320235805887805 | 0.9980142777103 | -0.628469713 | 0.626854645953181 | 0.999192792380582 | 0.533407443172717 | 1.8717141073377 | 0.999950574162623 |
| ebi-fl187-GCST90094770 | prot-a-195 | ATPase family AAA domain-containing protein 1 \|\| id:prot-a-195 | Gherkins liking \|\| id:ebi-fl187-GCST90094770 | MR Egger | 21 | -0.28179493 | 0.320257102837477 | 0.389906095716733 | -0.909498851 | 0.345908991839624 | 0.754428379508521 | 0.402725999087389 | 1.41327399049881 | 0.999950574162623 |
| ebi-fl187-GCST90094770 | prot-a-1966 | Mucin-1 \|\| id:prot-a-1966 | Gherkins liking \|\| id:ebi-fl187-GCST90094770 | MR Egger | 21 | -0.058834648 | 0.320197136562241 | 0.85616021009657 | -0.686421036 | 0.568751739690517 | 0.942862660521805 | 0.503374408120471 | 1.76606117089985 | 0.999950574162623 |
| ebi-fl187-GCST90094770 | prot-a-1986 | Myocardial zonula adherens protein \|\| id:prot-a-1986 | Gherkins liking \|\| id:ebi-fl187-GCST90094770 | MR Egger | 21 | 0.0594874840011393 | 0.349222799933454 | 0.866541581531284 | -0.624989204 | 0.74396417187071 | 1.06129247776097 | 0.535267207302991 | 2.10426065334214 | 0.999950574162623 |
| ebi-fl187-GCST90094770 | prot-a-2071 | Epididymal secretory protein E1 \|\| id:prot-a-2071 | Gherkins liking \|\| id:ebi-fl187-GCST90094770 | MR Egger | 21 | 0.670094605692349 | 0.32018981064099 | 0.0500217392816588 | 0.0425225768360084 | 1.29766663454869 | 1.95442221135639 | 1.0434396136728 | 3.66074483869559 | 0.999950574162623 |
| ebi-fl187-GCST90094770 | prot-a-2098 | Neuritin \|\| id:prot-a-2098 | Gherkins liking \|\| id:ebi-fl187-GCST90094770 | MR Egger | 21 | 0.133255755312803 | 0.320197103820454 | 0.681950375145042 | -0.494330568 | 0.760842078800893 | 1.14254217219743 | 0.609979110077794 | 2.14007757590771 | 0.999950574162623 |
| ebi-fl187-GCST90094770 | prot-a-2115 | Neurotrophin-4 \|\| id:prot-a-2115 | Gherkins liking \|\| id:ebi-fl187-GCST90094770 | MR Egger | 21 | 0.197048551063027 | 0.410504219163993 | 0.636695821141669 | -0.607539718 | 1.00163682062445 | 1.21780316490884 | 0.544689310963342 | 2.72273481159206 | 0.999950574162623 |
| ebi-fl187-GCST90094770 | prot-a-2118 | Netrin-1 \|\| id:prot-a-2118 | Gherkins liking \|\| id:ebi-fl187-GCST90094770 | MR Egger | 21 | -0.318352821 | 0.322625765184319 | 0.336162595597762 | -0.950699321 | 0.313993678961103 | 0.727346120298175 | 0.386470662050515 | 1.36888108376945 | 0.999950574162623 |
| ebi-fl187-GCST90094770 | prot-a-2120 | Netrin-G1 \|\| id:prot-a-2120 | Gherkins liking \|\| id:ebi-fl187-GCST90094770 | MR Egger | 21 | 0.408249195222511 | 0.329483310658554 | 0.230412471514829 | -0.237538094 | 1.05403648411328 | 1.50418194942604 | 0.788566851004895 | 2.86920929290377 | 0.999950574162623 |
| ebi-fl187-GCST90094770 | prot-a-2145 | Olfactomedin-like protein 3 \|\| id:prot-a-2145 | Gherkins liking \|\| id:ebi-fl187-GCST90094770 | MR Egger | 21 | 0.407931118595284 | 0.335671192929941 | 0.239149191237012 | -0.24998442 | 1.06584665673797 | 1.50370358038786 | 0.778812917234617 | 2.90329603892551 | 0.999950574162623 |
| ebi-fl187-GCST90094770 | prot-a-2162 | Prolyl 4-hydroxylase subunit alpha-2 \|\| id:prot-a-2162 | Gherkins liking \|\| id:ebi-fl187-GCST90094770 | MR Egger | 21 | -0.001843188 | 0.340830008558402 | 0.995741487263339 | -0.669870005 | 0.66618362843483 | 0.998158509288812 | 0.511775101608507 | 1.94679343823925 | 0.999950574162623 |
| ebi-fl187-GCST90094770 | prot-a-2168 | Polyadenylate-binding protein-interacting protein 1 \|\| id:prot-a-2168 | Gherkins liking \|\| id:ebi-fl187-GCST90094770 | MR Egger | 21 | -0.433636312 | 0.320274487800943 | 0.191638443189721 | -1.061374308 | 0.194101684202912 | 0.648147936325541 | 0.345980000375914 | 1.21421974364592 | 0.999950574162623 |
| ebi-fl187-GCST90094770 | prot-a-219 | Beta-1,4-galactosyltransferase 5 \|\| id:prot-a-219 | Gherkins liking \|\| id:ebi-fl187-GCST90094770 | MR Egger | 21 | -0.271387539 | 0.347505674217978 | 0.444449042604958 | -0.95249866 | 0.40972358245209 | 0.762321010019064 | 0.385775895208316 | 1.5064013317957 | 0.999950574162623 |
| ebi-fl187-GCST90094770 | prot-a-2190 | Pyruvate carboxylase, mitochondrial \|\| id:prot-a-2190 | Gherkins liking \|\| id:ebi-fl187-GCST90094770 | MR Egger | 21 | -0.130343572 | 0.320274487800943 | 0.688574866400087 | -0.758081568 | 0.497394423740605 | 0.877793793430617 | 0.468564474189058 | 1.64443099344834 | 0.999950574162623 |
| ebi-fl187-GCST90094770 | prot-a-2197 | Protocadherin-9 \|\| id:prot-a-2197 | Gherkins liking \|\| id:ebi-fl187-GCST90094770 | MR Egger | 21 | 0.000129336642220634 | 0.332213705642601 | 0.999693428121728 | -0.651009526 | 0.651268199701719 | 1.00012934500656 | 0.521519023688655 | 1.91797165838463 | 0.999974376584515 |
| ebi-fl187-GCST90094770 | prot-a-2198 | Protocadherin alpha-4 \|\| id:prot-a-2198 | Gherkins liking \|\| id:ebi-fl187-GCST90094770 | MR Egger | 21 | -0.615029525 | 0.362183866965797 | 0.105804231816769 | -1.324909904 | 0.0948508542019751 | 0.54062493309496 | 0.265826907870363 | 1.09949485785866 | 0.999950574162623 |
| ebi-fl187-GCST90094770 | prot-a-2254 | Placenta growth factor \|\| id:prot-a-2254 | Gherkins liking \|\| id:ebi-fl187-GCST90094770 | MR Egger | 21 | -0.460233793 | 0.320179739944204 | 0.166863517663704 | -1.087786083 | 0.167318497620464 | 0.631136073269131 | 0.336961673709988 | 1.18213071117521 | 0.999950574162623 |
| ebi-fl187-GCST90094770 | prot-a-2261 | Membrane-associated progesterone receptor component 2 \|\| id:prot-a-2261 | Gherkins liking \|\| id:ebi-fl187-GCST90094770 | MR Egger | 21 | 0.32433491302603 | 0.320267165692591 | 0.323926480685262 | -0.303388732 | 0.952058557783507 | 1.38311045126521 | 0.7383120352634 | 2.59103797450164 | 0.999950574162623 |
| ebi-fl187-GCST90094770 | prot-a-2268 | Polymeric immunoglobulin receptor \|\| id:prot-a-2268 | Gherkins liking \|\| id:ebi-fl187-GCST90094770 | MR Egger | 21 | 0.367376706124904 | 0.320245875263608 | 0.265552039887924 | -0.260305209 | 0.995058621641574 | 1.44394175837934 | 0.770816289527185 | 2.70488290130782 | 0.999950574162623 |
| ebi-fl187-GCST90094770 | prot-a-2283 | Group 10 secretory phospholipase A2 \|\| id:prot-a-2283 | Gherkins liking \|\| id:ebi-fl187-GCST90094770 | MR Egger | 21 | -0.91472167 | 0.320267165692591 | 0.0101059697201757 | -1.542445315 | -0.286998026 | 0.400628117272868 | 0.213857512519626 | 0.750513210682153 | 0.999950574162623 |
| ebi-fl187-GCST90094770 | prot-a-2298 | Inactive phospholipase D5 \|\| id:prot-a-2298 | Gherkins liking \|\| id:ebi-fl187-GCST90094770 | MR Egger | 21 | 0.284280257734633 | 0.352449978710436 | 0.429887734712965 | -0.406521701 | 0.975082216007086 | 1.32880528653194 | 0.665962643711721 | 2.6513851883253 | 0.999950574162623 |
| ebi-fl187-GCST90094770 | prot-a-2304 | Procollagen-lysine,2-oxoglutarate 5-dioxygenase 2 \|\| id:prot-a-2304 | Gherkins liking \|\| id:ebi-fl187-GCST90094770 | MR Egger | 21 | -0.141154287 | 0.406133925930438 | 0.731994166099192 | -0.937176782 | 0.654868207804316 | 0.868355325406875 | 0.391732221183444 | 1.92488881533534 | 0.999950574162623 |
| ebi-fl187-GCST90094770 | prot-a-2331 | Periostin \|\| id:prot-a-2331 | Gherkins liking \|\| id:ebi-fl187-GCST90094770 | MR Egger | 21 | -0.249617112 | 0.469110627902878 | 0.600819330235519 | -1.169073942 | 0.66983971899006 | 0.779099033874328 | 0.310654492057328 | 1.95392411860536 | 0.999950574162623 |
| ebi-fl187-GCST90094770 | prot-a-2333 | Periostin \|\| id:prot-a-2333 | Gherkins liking \|\| id:ebi-fl187-GCST90094770 | MR Egger | 21 | -0.247770731 | 0.459639799827289 | 0.596110118473035 | -1.148664738 | 0.653123277 | 0.780538876520513 | 0.317059844768122 | 1.92153294658131 | 0.999950574162623 |
| ebi-fl187-GCST90094770 | prot-a-2367 | cAMP-dependent protein kinase catalytic subunit alpha \|\| id:prot-a-2367 | Gherkins liking \|\| id:ebi-fl187-GCST90094770 | MR Egger | 21 | 0.27491805552326 | 0.434283490960583 | 0.534254571661163 | -0.576277587 | 1.126113698 | 1.31642279687042 | 0.561986423552866 | 3.08364919060562 | 0.999950574162623 |
| ebi-fl187-GCST90094770 | prot-a-2385 | Phosphoribosyl pyrophosphate synthase-associated protein 1 \|\| id:prot-a-2385 | Gherkins liking \|\| id:ebi-fl187-GCST90094770 | MR Egger | 21 | -0.197596741 | 0.345198845461901 | 0.573752961126643 | -0.874186478 | 0.478992996388879 | 0.820700741609496 | 0.417201284157656 | 1.61444782855426 | 0.999950574162623 |
| ebi-fl187-GCST90094770 | prot-a-2393 | Trypsin-3 \|\| id:prot-a-2393 | Gherkins liking \|\| id:ebi-fl187-GCST90094770 | MR Egger | 20 | -0.419284526 | 0.359036417666445 | 0.258121925302063 | -1.122995905 | 0.284426852497696 | 0.657517087859151 | 0.325303754227008 | 1.3290000967068 | 0.999950574162623 |
| ebi-fl187-GCST90094770 | prot-a-2405 | Pregnancy-specific beta-1-glycoprotein 6 \|\| id:prot-a-2405 | Gherkins liking \|\| id:ebi-fl187-GCST90094770 | MR Egger | 21 | -0.080222656 | 0.337011241466891 | 0.814397183816005 | -0.740764689 | 0.580319377375355 | 0.922910831966304 | 0.476749211135181 | 1.78660894211573 | 0.999950574162623 |
| ebi-fl187-GCST90094770 | prot-a-244 | Beclin-1 \|\| id:prot-a-244 | Gherkins liking \|\| id:ebi-fl187-GCST90094770 | MR Egger | 21 | 0.14231841655124 | 0.320094271376994 | 0.661621911146492 | -0.485066355 | 0.769703188450149 | 1.15294370645403 | 0.615656343318173 | 2.15912530534097 | 0.999950574162623 |
| ebi-fl187-GCST90094770 | prot-a-2454 | tRNA pseudouridine synthase A, mitochondrial \|\| id:prot-a-2454 | Gherkins liking \|\| id:ebi-fl187-GCST90094770 | MR Egger | 21 | 0.0536314685961773 | 0.374354445026968 | 0.887590262270081 | -0.680103244 | 0.787366180849034 | 1.05509569459966 | 0.506564690074748 | 2.19760071433023 | 0.999950574162623 |
| ebi-fl187-GCST90094770 | prot-a-2466 | Glutaminyl-peptide cyclotransferase-like protein \|\| id:prot-a-2466 | Gherkins liking \|\| id:ebi-fl187-GCST90094770 | MR Egger | 21 | -0.034199175 | 0.320257102837477 | 0.916077744001675 | -0.661903096 | 0.593504746813926 | 0.966379007173055 | 0.515868651991043 | 1.81032203042451 | 0.999950574162623 |
| ebi-fl187-GCST90094770 | prot-a-2474 | Ras-related protein Rab-27A \|\| id:prot-a-2474 | Gherkins liking \|\| id:ebi-fl187-GCST90094770 | MR Egger | 21 | -0.08481417 | 0.416871553327803 | 0.840943368937088 | -0.901882414 | 0.732254074859483 | 0.918682987709233 | 0.405805047130303 | 2.07976327025662 | 0.999950574162623 |
| ebi-fl187-GCST90094770 | prot-a-2490 | Rap1 GTPase-GDP dissociation stimulator 1 \|\| id:prot-a-2490 | Gherkins liking \|\| id:ebi-fl187-GCST90094770 | MR Egger | 21 | -0.143126789 | 0.320264394896774 | 0.659998296928275 | -0.770845003 | 0.484591425470528 | 0.866644181390078 | 0.462621986355368 | 1.62351154785007 | 0.999950574162623 |
| ebi-fl187-GCST90094770 | prot-a-2545 | Receptor-interacting serine/threonine-protein kinase 2 \|\| id:prot-a-2545 | Gherkins liking \|\| id:ebi-fl187-GCST90094770 | MR Egger | 21 | -0.003615941 | 0.377604570089674 | 0.992459408872336 | -0.743720898 | 0.736489016411063 | 0.996390588677194 | 0.475341921893087 | 2.08858962249869 | 0.999950574162623 |
| ebi-fl187-GCST90094770 | prot-a-2610 | RUN and FYVE domain-containing protein 1 \|\| id:prot-a-2610 | Gherkins liking \|\| id:ebi-fl187-GCST90094770 | MR Egger | 21 | -0.316086961 | 0.326764698025939 | 0.345535411056865 | -0.956545769 | 0.324371847327063 | 0.728996053328396 | 0.384217773392852 | 1.38316153642637 | 0.999950574162623 |
| ebi-fl187-GCST90094770 | prot-a-2627 | Serine--tRNA ligase, mitochondrial \|\| id:prot-a-2627 | Gherkins liking \|\| id:ebi-fl187-GCST90094770 | MR Egger | 21 | -0.12732006 | 0.320274453913509 | 0.695404310785309 | -0.75505799 | 0.500417869371376 | 0.880451829820223 | 0.469983359619556 | 1.64941036478672 | 0.999950574162623 |
| ebi-fl187-GCST90094770 | prot-a-264 | BolA-like protein 2 \|\| id:prot-a-264 | Gherkins liking \|\| id:ebi-fl187-GCST90094770 | MR Egger | 21 | 0.114967651954348 | 0.32018981064099 | 0.723508797645902 | -0.512604377 | 0.742539680810688 | 1.12183714774572 | 0.598933696744674 | 2.10126528679644 | 0.999950574162623 |
| ebi-fl187-GCST90094770 | prot-a-2646 | Secretoglobin family 1C member 1 \|\| id:prot-a-2646 | Gherkins liking \|\| id:ebi-fl187-GCST90094770 | MR Egger | 21 | 0.75060060928236 | 0.351277362360834 | 0.0458390272271464 | 0.062096979055126 | 1.43910423950959 | 2.11827188838417 | 1.06406553179433 | 4.2169167772516 | 0.999950574162623 |
| ebi-fl187-GCST90094770 | prot-a-2672 | Semaphorin-3E \|\| id:prot-a-2672 | Gherkins liking \|\| id:ebi-fl187-GCST90094770 | MR Egger | 21 | 0.516145260015095 | 0.320019711310184 | 0.123262477377816 | -0.111093374 | 1.14338389418306 | 1.67555635106178 | 0.894855188682728 | 3.13736694058423 | 0.999950574162623 |
| ebi-fl187-GCST90094770 | prot-a-2701 | Plasma protease C1 inhibitor \|\| id:prot-a-2701 | Gherkins liking \|\| id:ebi-fl187-GCST90094770 | MR Egger | 21 | 0.289806582266521 | 0.320187070147003 | 0.376738431013679 | -0.337760075 | 0.917373239754647 | 1.33616902424632 | 0.713366421648477 | 2.50270773500906 | 0.999950574162623 |
| ebi-fl187-GCST90094770 | prot-a-2738 | Signaling threshold-regulating transmembrane adapter 1 \|\| id:prot-a-2738 | Gherkins liking \|\| id:ebi-fl187-GCST90094770 | MR Egger | 21 | 0.0739626710735895 | 0.342870589014853 | 0.831507991279775 | -0.598063683 | 0.745989025542701 | 1.07676661020445 | 0.549875338677867 | 2.10852578993437 | 0.999950574162623 |
| ebi-fl187-GCST90094770 | prot-a-274 | Serine/threonine-protein kinase BRSK2 \|\| id:prot-a-274 | Gherkins liking \|\| id:ebi-fl187-GCST90094770 | MR Egger | 21 | -0.329268941 | 0.389819017017395 | 0.408804889503869 | -1.093314214 | 0.43477633230072 | 0.719449501219399 | 0.335104044624912 | 1.54461753926071 | 0.999950574162623 |
| ebi-fl187-GCST90094770 | prot-a-2749 | Mitochondrial glutamate carrier 2 \|\| id:prot-a-2749 | Gherkins liking \|\| id:ebi-fl187-GCST90094770 | MR Egger | 21 | -0.614723657 | 0.31996862215892 | 0.0698386329460342 | -1.241862156 | 0.0124148425547684 | 0.540790318347966 | 0.288845840718965 | 1.012492227 | 0.999950574162623 |
| ebi-fl187-GCST90094770 | prot-a-2760 | Sodium-coupled monocarboxylate transporter 1 \|\| id:prot-a-2760 | Gherkins liking \|\| id:ebi-fl187-GCST90094770 | MR Egger | 21 | 0.150823583854818 | 0.320187070147003 | 0.642969452821559 | -0.476743074 | 0.778390241342944 | 1.16279150479125 | 0.620802009209924 | 2.17796344656722 | 0.999950574162623 |
| ebi-fl187-GCST90094770 | prot-a-2777 | Small integral membrane protein 10 \|\| id:prot-a-2777 | Gherkins liking \|\| id:ebi-fl187-GCST90094770 | MR Egger | 21 | -0.210908667 | 0.359426359487689 | 0.564251010930391 | -0.915384332 | 0.493566997129794 | 0.809848028976232 | 0.400362724286663 | 1.63814908394691 | 0.999950574162623 |
| ebi-fl187-GCST90094770 | prot-a-2799 | Superoxide dismutase [Mn], mitochondrial \|\| id:prot-a-2799 | Gherkins liking \|\| id:ebi-fl187-GCST90094770 | MR Egger | 21 | 0.733114112899046 | 0.348915094268823 | 0.0492046651044573 | 0.0492405281321536 | 1.41698769766594 | 2.08155271516738 | 1.05047298866139 | 4.12467693390387 | 0.999950574162623 |
| ebi-fl187-GCST90094770 | prot-a-2805 | Protein SPACA7 \|\| id:prot-a-2805 | Gherkins liking \|\| id:ebi-fl187-GCST90094770 | MR Egger | 21 | -0.199440747 | 0.347133056069352 | 0.572345054 | -0.879821537 | 0.480940042819332 | 0.819188758703675 | 0.414856941701358 | 1.6175942956007 | 0.999950574162623 |
| ebi-fl187-GCST90094770 | prot-a-2811 | SPARC-like protein 1 \|\| id:prot-a-2811 | Gherkins liking \|\| id:ebi-fl187-GCST90094770 | MR Egger | 21 | 0.0637833507102903 | 0.320257069085294 | 0.84425111416488 | -0.563920505 | 0.691487206117466 | 1.06586145561829 | 0.568974020143549 | 1.99668280510613 | 0.999950574162623 |
| ebi-fl187-GCST90094770 | prot-a-2831 | Sepiapterin reductase \|\| id:prot-a-2831 | Gherkins liking \|\| id:ebi-fl187-GCST90094770 | MR Egger | 21 | 0.567682154230798 | 0.320264429012217 | 0.0923390024468483 | -0.060036127 | 1.19540043509474 | 1.76417322738893 | 0.94173051141699 | 3.30488089586573 | 0.999950574162623 |
| ebi-fl187-GCST90094770 | prot-a-2834 | SPRY domain-containing SOCS box protein 1 \|\| id:prot-a-2834 | Gherkins liking \|\| id:ebi-fl187-GCST90094770 | MR Egger | 21 | -0.565644824 | 0.367998942313208 | 0.140759813412815 | -1.286922751 | 0.155633102538971 | 0.567993772406067 | 0.276119164411906 | 1.16839744238399 | 0.999950574162623 |
| ebi-fl187-GCST90094770 | prot-a-2835 | Spectrin alpha chain, non-erythrocytic 1 \|\| id:prot-a-2835 | Gherkins liking \|\| id:ebi-fl187-GCST90094770 | MR Egger | 21 | -0.552381895 | 0.35839162007801 | 0.139739135091051 | -1.25482947 | 0.150065680409637 | 0.575577211886275 | 0.285124466269715 | 1.16191055498336 | 0.999950574162623 |
| ebi-fl187-GCST90094770 | prot-a-2859 | Sia-alpha-2,3-Gal-beta-1,4-GlcNAc-R:alpha 2,8-sialyltransferase \|\| id:prot-a-2859 | Gherkins liking \|\| id:ebi-fl187-GCST90094770 | MR Egger | 21 | 0.194178087280248 | 0.32018984302788 | 0.551396019313599 | -0.433394005 | 0.821750179614893 | 1.21431251731495 | 0.648305006027772 | 2.27447709950985 | 0.999950574162623 |
| ebi-fl187-GCST90094770 | prot-a-2861 | Alpha-2,8-sialyltransferase 8F \|\| id:prot-a-2861 | Gherkins liking \|\| id:ebi-fl187-GCST90094770 | MR Egger | 21 | -0.405052028 | 0.349149867641411 | 0.260374903054604 | -1.089385769 | 0.279281712348549 | 0.666942110139193 | 0.336423071802038 | 1.32217976577617 | 0.999950574162623 |
| ebi-fl187-GCST90094770 | prot-a-289 | Uncharacterized protein C17orf89 \|\| id:prot-a-289 | Gherkins liking \|\| id:ebi-fl187-GCST90094770 | MR Egger | 21 | -0.23177007 | 0.321587049280885 | 0.47986397435789 | -0.862080687 | 0.398540546333505 | 0.793128466167226 | 0.422282529892582 | 1.48964903664092 | 0.999950574162623 |
| ebi-fl187-GCST90094770 | prot-a-2921 | Tachykinin-3 \|\| id:prot-a-2921 | Gherkins liking \|\| id:ebi-fl187-GCST90094770 | MR Egger | 21 | -0.328194523 | 0.320179739944204 | 0.318222199119495 | -0.955746813 | 0.299357767707473 | 0.720222906457927 | 0.384524869173257 | 1.34899216298279 | 0.999950574162623 |
| ebi-fl187-GCST90094770 | prot-a-2928 | Tax1-binding protein 1 \|\| id:prot-a-2928 | Gherkins liking \|\| id:ebi-fl187-GCST90094770 | MR Egger | 21 | -0.598695397 | 0.320274453913509 | 0.077071706458617 | -1.226433326 | 0.0290425328746435 | 0.54952808475195 | 0.293336951244405 | 1.02946837979351 | 0.999950574162623 |
| ebi-fl187-GCST90094770 | prot-a-296 | Uncharacterized protein C1orf185 \|\| id:prot-a-296 | Gherkins liking \|\| id:ebi-fl187-GCST90094770 | MR Egger | 21 | -0.156108035 | 0.33560124585934 | 0.647104432752246 | -0.813886477 | 0.501670407053539 | 0.855466765171336 | 0.443132491082879 | 1.65147760780159 | 0.999950574162623 |
| ebi-fl187-GCST90094770 | prot-a-2964 | Transforming growth factor beta-2 \|\| id:prot-a-2964 | Gherkins liking \|\| id:ebi-fl187-GCST90094770 | MR Egger | 21 | -0.238160473 | 0.360806636534889 | 0.517126658074133 | -0.945341481 | 0.469020534672986 | 0.788076216042427 | 0.388546867049708 | 1.59842782161024 | 0.999950574162623 |
| ebi-fl187-GCST90094770 | prot-a-2977 | Thymocyte nuclear protein 1 \|\| id:prot-a-2977 | Gherkins liking \|\| id:ebi-fl187-GCST90094770 | MR Egger | 21 | 0.440291107975852 | 0.32018984302788 | 0.18510155472323 | -0.187280984 | 1.0678632003105 | 1.55315928976439 | 0.829210708409122 | 2.90915656891302 | 0.999950574162623 |
| ebi-fl187-GCST90094770 | prot-a-3015 | Transmembrane protein 70, mitochondrial \|\| id:prot-a-3015 | Gherkins liking \|\| id:ebi-fl187-GCST90094770 | MR Egger | 21 | -0.498620738 | 0.320019678045999 | 0.13571205984886 | -1.125859307 | 0.128617830635775 | 0.607367801386594 | 0.324373610950544 | 1.13725541692548 | 0.999950574162623 |
| ebi-fl187-GCST90094770 | prot-a-3030 | Tumor necrosis factor alpha-induced protein 3 \|\| id:prot-a-3030 | Gherkins liking \|\| id:ebi-fl187-GCST90094770 | MR Egger | 21 | -0.693904764 | 0.368741350327024 | 0.0752710736146115 | -1.41663781 | 0.0288282828011556 | 0.499621351806946 | 0.242528072969832 | 1.02924783974369 | 0.999950574162623 |
| ebi-fl187-GCST90094770 | prot-a-3032 | Tumor necrosis factor receptor superfamily member 10A \|\| id:prot-a-3032 | Gherkins liking \|\| id:ebi-fl187-GCST90094770 | MR Egger | 21 | -0.371457814 | 0.320274453913509 | 0.260493455286686 | -0.999195743 | 0.256280115993692 | 0.689728102308041 | 0.368175429668814 | 1.2921146192221 | 0.999950574162623 |
| ebi-fl187-GCST90094770 | prot-a-3081 | Tumor protein p53-inducible protein 11 \|\| id:prot-a-3081 | Gherkins liking \|\| id:ebi-fl187-GCST90094770 | MR Egger | 21 | -0.329978176 | 0.320019711310184 | 0.315433121813869 | -0.95721681 | 0.297260458324992 | 0.718939423507589 | 0.383960033971197 | 1.34616587389978 | 0.999950574162623 |
| ebi-fl187-GCST90094770 | prot-a-315 | Complement C4 \|\| id:prot-a-315 | Gherkins liking \|\| id:ebi-fl187-GCST90094770 | MR Egger | 20 | 0.0536610170262742 | 0.449131851778312 | 0.906220689454527 | -0.826637412 | 0.933959446511765 | 1.05512687148165 | 0.43751800818791 | 2.54456432441177 | 0.999950574162623 |
| ebi-fl187-GCST90094770 | prot-a-3157 | UDP-glucuronosyltransferase 1-8 \|\| id:prot-a-3157 | Gherkins liking \|\| id:ebi-fl187-GCST90094770 | MR Egger | 21 | -0.038344419 | 0.320274487800943 | 0.905958980439985 | -0.666082415 | 0.589393577526053 | 0.962381421776696 | 0.513717171662548 | 1.80289476791974 | 0.999950574162623 |
| ebi-fl187-GCST90094770 | prot-a-317 | Glutaredoxin-like protein C5orf63 \|\| id:prot-a-317 | Gherkins liking \|\| id:ebi-fl187-GCST90094770 | MR Egger | 21 | -0.195454664 | 0.390022857777458 | 0.622031485122636 | -0.959899466 | 0.568990136945634 | 0.82246062954884 | 0.382931381838924 | 1.76648224522485 | 0.999950574162623 |
| ebi-fl187-GCST90094770 | prot-a-3192 | Guanine nucleotide exchange factor VAV3 \|\| id:prot-a-3192 | Gherkins liking \|\| id:ebi-fl187-GCST90094770 | MR Egger | 21 | -0.175011611 | 0.383538734163045 | 0.653341847233912 | -0.92674753 | 0.57672430768763 | 0.839447273662048 | 0.395839073555974 | 1.78019749017778 | 0.999950574162623 |
| ebi-fl187-GCST90094770 | prot-a-3233 | Serine/threonine-protein kinase WNK3 \|\| id:prot-a-3233 | Gherkins liking \|\| id:ebi-fl187-GCST90094770 | MR Egger | 21 | -0.035689476 | 0.320264394896774 | 0.912438209056224 | -0.66340769 | 0.592028737888983 | 0.964939883853343 | 0.515093062836621 | 1.80765194996663 | 0.999950574162623 |
| ebi-fl187-GCST90094770 | prot-a-3250 | 14-3-3 protein theta \|\| id:prot-a-3250 | Gherkins liking \|\| id:ebi-fl187-GCST90094770 | MR Egger | 21 | -0.218307394 | 0.349498878821855 | 0.539643312966147 | -0.903325196 | 0.466710408665385 | 0.803878296458304 | 0.405219981022674 | 1.59473951379645 | 0.999950574162623 |
| ebi-fl187-GCST90094770 | prot-a-3278 | E3 ubiquitin-protein ligase ZNRF3 \|\| id:prot-a-3278 | Gherkins liking \|\| id:ebi-fl187-GCST90094770 | MR Egger | 21 | 0.232596827365544 | 0.329027266858412 | 0.488194719702841 | -0.412296616 | 0.87749027040803 | 1.26187262424567 | 0.662127849421674 | 2.40485658655113 | 0.999950574162623 |
| ebi-fl187-GCST90094770 | prot-a-355 | Calpain-3 \|\| id:prot-a-355 | Gherkins liking \|\| id:ebi-fl187-GCST90094770 | MR Egger | 21 | 0.245730407088498 | 0.331626652166719 | 0.467762334909024 | -0.404257831 | 0.895718645335267 | 1.27855483775344 | 0.667472004001381 | 2.44909518802731 | 0.999950574162623 |
| ebi-fl187-GCST90094770 | prot-a-385 | Coiled-coil domain-containing protein 90B, mitochondrial \|\| id:prot-a-385 | Gherkins liking \|\| id:ebi-fl187-GCST90094770 | MR Egger | 21 | -0.617314327 | 0.330713417955426 | 0.0774664317655592 | -1.265512626 | 0.0308839720166039 | 0.539391122142657 | 0.282094649912951 | 1.03136582964652 | 0.999950574162623 |
| ebi-fl187-GCST90094770 | prot-a-390 | C-C motif chemokine 14 \|\| id:prot-a-390 | Gherkins liking \|\| id:ebi-fl187-GCST90094770 | MR Egger | 21 | -0.370847449 | 0.387402759492298 | 0.35045611860475 | -1.130156858 | 0.388461959365304 | 0.69014921631645 | 0.322982590095756 | 1.47471088345969 | 0.999950574162623 |
| ebi-fl187-GCST90094770 | prot-a-40 | Pituitary adenylate cyclase-activating polypeptide 27 \|\| id:prot-a-40 | Gherkins liking \|\| id:ebi-fl187-GCST90094770 | MR Egger | 21 | -0.587925282 | 0.320038533467956 | 0.0818972029146638 | -1.215200808 | 0.0393502434101796 | 0.555478551290021 | 0.296650438595355 | 1.04013472019216 | 0.999950574162623 |
| ebi-fl187-GCST90094770 | prot-a-401 | C-C motif chemokine 24 \|\| id:prot-a-401 | Gherkins liking \|\| id:ebi-fl187-GCST90094770 | MR Egger | 21 | -0.73020671 | 0.319951284038043 | 0.0341720224535073 | -1.357311227 | -0.103102194 | 0.481809384853197 | 0.257351808124286 | 0.902034786639254 | 0.999950574162623 |
| ebi-fl187-GCST90094770 | prot-a-412 | Cerebral cavernous malformations 2 protein \|\| id:prot-a-412 | Gherkins liking \|\| id:ebi-fl187-GCST90094770 | MR Egger | 21 | -0.591981357 | 0.320264394896774 | 0.0801679340563434 | -1.219699571 | 0.0357368571928663 | 0.553230051960575 | 0.295318876010877 | 1.03638309384947 | 0.999950574162623 |
| ebi-fl187-GCST90094770 | prot-a-44 | Adhesion G protein-coupled receptor F5 \|\| id:prot-a-44 | Gherkins liking \|\| id:ebi-fl187-GCST90094770 | MR Egger | 21 | 0.282443992019574 | 0.320243279154194 | 0.388816565024982 | -0.345232835 | 0.910120819161794 | 1.32636748585219 | 0.70805547409054 | 2.48462270528382 | 0.999950574162623 |
| ebi-fl187-GCST90094770 | prot-a-48 | Adrenomedullin \|\| id:prot-a-48 | Gherkins liking \|\| id:ebi-fl187-GCST90094770 | MR Egger | 21 | 0.379935621614333 | 0.320274453913509 | 0.250134061294138 | -0.247802308 | 1.00767355128481 | 1.46219045294318 | 0.780514229402187 | 2.73922093940008 | 0.999950574162623 |
| ebi-fl187-GCST90094770 | prot-a-502 | Carcinoembryonic antigen-related cell adhesion molecule 1 \|\| id:prot-a-502 | Gherkins liking \|\| id:ebi-fl187-GCST90094770 | MR Egger | 21 | 0.200758085644554 | 0.320267132167143 | 0.538220302312368 | -0.426965493 | 0.828481664692154 | 1.22232903711238 | 0.652486066913174 | 2.28983935555345 | 0.999950574162623 |
| ebi-fl187-GCST90094770 | prot-a-518 | Complement factor B \|\| id:prot-a-518 | Gherkins liking \|\| id:ebi-fl187-GCST90094770 | MR Egger | 21 | 0.165400035371005 | 0.329152895676962 | 0.621088719548955 | -0.47973964 | 0.81053971089785 | 1.17986501205592 | 0.618944519128232 | 2.2491215345673 | 0.999950574162623 |
| ebi-fl187-GCST90094770 | prot-a-525 | Properdin \|\| id:prot-a-525 | Gherkins liking \|\| id:ebi-fl187-GCST90094770 | MR Egger | 21 | 0.623940861259425 | 0.346917448490796 | 0.0880006498787388 | -0.056017338 | 1.30389906030139 | 1.86626827326762 | 0.945522742480594 | 3.68363140443106 | 0.999950574162623 |
| ebi-fl187-GCST90094770 | prot-a-533 | Chondroadherin \|\| id:prot-a-533 | Gherkins liking \|\| id:ebi-fl187-GCST90094770 | MR Egger | 21 | 0.937695830894744 | 0.372051586547679 | 0.0208268544936464 | 0.208474721261294 | 1.66691694052819 | 2.55408957873925 | 1.23179779137286 | 5.29581528876912 | 0.999950574162623 |
| ebi-fl187-GCST90094770 | prot-a-535 | Coiled-coil-helix-coiled-coil-helix domain-containing protein 2 \|\| id:prot-a-535 | Gherkins liking \|\| id:ebi-fl187-GCST90094770 | MR Egger | 21 | -0.392671204 | 0.401396436245133 | 0.340236938997865 | -1.179408219 | 0.394065810944653 | 0.675250730765132 | 0.307460634094295 | 1.48299814297204 | 0.999950574162623 |
| ebi-fl187-GCST90094770 | prot-a-591 | Caseinolytic peptidase B protein homolog \|\| id:prot-a-591 | Gherkins liking \|\| id:ebi-fl187-GCST90094770 | MR Egger | 21 | 0.678723849076308 | 0.328412788509796 | 0.0526676726383782 | 0.0350347835971085 | 1.32241291455551 | 1.97136037294603 | 1.03565573200484 | 3.75246483935241 | 0.999950574162623 |
| ebi-fl187-GCST90094770 | prot-a-605 | Ciliary neurotrophic factor receptor subunit alpha \|\| id:prot-a-605 | Gherkins liking \|\| id:ebi-fl187-GCST90094770 | MR Egger | 21 | -0.396165782 | 0.339875612821573 | 0.258193002768058 | -1.062321984 | 0.269990418640565 | 0.672895132459629 | 0.345652278867891 | 1.30995189955313 | 0.999950574162623 |
| ebi-fl187-GCST90094770 | prot-a-606 | Ciliary neurotrophic factor receptor subunit alpha \|\| id:prot-a-606 | Gherkins liking \|\| id:ebi-fl187-GCST90094770 | MR Egger | 21 | 0.116166173913572 | 0.32770828789499 | 0.726882789054999 | -0.52614207 | 0.758474418187752 | 1.12318250025819 | 0.590880152161827 | 2.13501659223227 | 0.999950574162623 |
| ebi-fl187-GCST90094770 | prot-a-707 | Cancer/testis antigen 55 \|\| id:prot-a-707 | Gherkins liking \|\| id:ebi-fl187-GCST90094770 | MR Egger | 21 | 0.569142298118011 | 0.364193985801008 | 0.134615361806868 | -0.144677914 | 1.28296251028799 | 1.766751056 | 0.865300949509336 | 3.60731060625097 | 0.999950574162623 |
| ebi-fl187-GCST90094770 | prot-a-719 | Dipeptidyl peptidase 1 \|\| id:prot-a-719 | Gherkins liking \|\| id:ebi-fl187-GCST90094770 | MR Egger | 21 | -0.635235589 | 0.320274453913509 | 0.0619635813695524 | -1.262973519 | -0.00749766 | 0.529810655864658 | 0.282811828622533 | 0.992530377653994 | 0.999950574162623 |
| ebi-fl187-GCST90094770 | prot-a-752 | Cytochrome b5 \|\| id:prot-a-752 | Gherkins liking \|\| id:ebi-fl187-GCST90094770 | MR Egger | 21 | 0.0288428994902591 | 0.365657936683103 | 0.937953342412201 | -0.687846656 | 0.745532455389142 | 1.02926288404933 | 0.502657298391291 | 2.10756331972498 | 0.999950574162623 |
| ebi-fl187-GCST90094770 | prot-a-755 | Cytoglobin \|\| id:prot-a-755 | Gherkins liking \|\| id:ebi-fl187-GCST90094770 | MR Egger | 21 | -0.301776074 | 0.320267165692591 | 0.357885367278844 | -0.929499718 | 0.32594757104998 | 0.739503640659001 | 0.39475114769048 | 1.38534273490373 | 0.999950574162623 |
| ebi-fl187-GCST90094770 | prot-a-807 | Desmin \|\| id:prot-a-807 | Gherkins liking \|\| id:ebi-fl187-GCST90094770 | MR Egger | 21 | 0.119030320339939 | 0.353933541462245 | 0.740325197140914 | -0.574679421 | 0.812740061605939 | 1.12640407072493 | 0.562885289130644 | 2.25407583933359 | 0.999950574162623 |
| ebi-fl187-GCST90094770 | prot-a-82 | Ameloblastin \|\| id:prot-a-82 | Gherkins liking \|\| id:ebi-fl187-GCST90094770 | MR Egger | 21 | 0.725524441019498 | 0.370655066071185 | 0.0651529708216004 | -0.000959488 | 1.45200837051902 | 2.06581421363934 | 0.999040971681862 | 4.27168503218634 | 0.999950574162623 |
| ebi-fl187-GCST90094770 | prot-a-84 | Muellerian-inhibiting factor \|\| id:prot-a-84 | Gherkins liking \|\| id:ebi-fl187-GCST90094770 | MR Egger | 21 | 0.370298846568555 | 0.320257069085294 | 0.261909193768545 | -0.257405009 | 0.99800270197573 | 1.4481673298396 | 0.773055056223433 | 2.71285802780987 | 0.999950574162623 |
| ebi-fl187-GCST90094770 | prot-a-858 | Inactive dipeptidyl peptidase 10 \|\| id:prot-a-858 | Gherkins liking \|\| id:ebi-fl187-GCST90094770 | MR Egger | 21 | 0.0144583822166938 | 0.320274487800943 | 0.964463848125521 | -0.613279614 | 0.642196378306543 | 1.0145634101926 | 0.541571806939335 | 1.90065084650342 | 0.999950574162623 |
| ebi-fl187-GCST90094770 | prot-a-863 | Dihydropyrimidinase-related protein 3 \|\| id:prot-a-863 | Gherkins liking \|\| id:ebi-fl187-GCST90094770 | MR Egger | 21 | 0.0352985329698711 | 0.320019711310184 | 0.913327323507943 | -0.591940101 | 0.662537167137832 | 1.03592892157976 | 0.553252876272837 | 1.93970745854048 | 0.999950574162623 |
| ebi-fl187-GCST90094770 | prot-a-864 | Dihydropyrimidinase-related protein 5 \|\| id:prot-a-864 | Gherkins liking \|\| id:ebi-fl187-GCST90094770 | MR Egger | 21 | 0.475274878398154 | 0.320274487800943 | 0.154219740549701 | -0.152463118 | 1.103012874 | 1.60845626660744 | 0.858590560174178 | 3.01323084784895 | 0.999950574162623 |
| ebi-fl187-GCST90094770 | prot-a-884 | Endothelin-converting enzyme 1 \|\| id:prot-a-884 | Gherkins liking \|\| id:ebi-fl187-GCST90094770 | MR Egger | 21 | -0.03979592 | 0.32018984302788 | 0.902392349667279 | -0.667368012 | 0.587776172744603 | 0.960985537464181 | 0.513057162612391 | 1.79998111421555 | 0.999950574162623 |
| ebi-fl187-GCST90094770 | prot-a-893 | Early endosome antigen 1 \|\| id:prot-a-893 | Gherkins liking \|\| id:ebi-fl187-GCST90094770 | MR Egger | 21 | -0.569388868 | 0.320267165692591 | 0.0914370594958567 | -1.197112513 | 0.0583347769008774 | 0.56587115510148 | 0.302065163225141 | 1.06006982320308 | 0.999950574162623 |
| ebi-fl187-GCST90094770 | prot-a-897 | Ephrin-A2 \|\| id:prot-a-897 | Gherkins liking \|\| id:ebi-fl187-GCST90094770 | MR Egger | 21 | 0.326286211791419 | 0.320254503788483 | 0.321080439100156 | -0.301412616 | 0.953985039216846 | 1.38581194783745 | 0.739772468078361 | 2.59603437224106 | 0.999950574162623 |
| ebi-fl187-GCST90094770 | prot-a-90 | Anaphase-promoting complex subunit 10 \|\| id:prot-a-90 | Gherkins liking \|\| id:ebi-fl187-GCST90094770 | MR Egger | 21 | -0.224212263 | 0.325986052552271 | 0.499891835404107 | -0.863144926 | 0.414720399561466 | 0.799145486994117 | 0.421833359158659 | 1.51394738115736 | 0.999950574162623 |
| ebi-fl187-GCST90094770 | prot-a-914 | Histone-lysine N-methyltransferase EHMT2 \|\| id:prot-a-914 | Gherkins liking \|\| id:ebi-fl187-GCST90094770 | MR Egger | 21 | -0.336629156 | 0.320197103820454 | 0.306300587208551 | -0.964215479 | 0.29095716755322 | 0.714173637855382 | 0.381282206172053 | 1.33770728544682 | 0.999950574162623 |
| ebi-fl187-GCST90094770 | prot-a-932 | RNA polymerase II elongation factor ELL \|\| id:prot-a-932 | Gherkins liking \|\| id:ebi-fl187-GCST90094770 | MR Egger | 21 | -0.079975332 | 0.333033452287454 | 0.812792034940151 | -0.732720899 | 0.572770234414284 | 0.923139118037721 | 0.480599546855562 | 1.77317235695933 | 0.999950574162623 |
| ebi-fl187-GCST90094770 | prot-a-991 | Estrogen receptor \|\| id:prot-a-991 | Gherkins liking \|\| id:ebi-fl187-GCST90094770 | MR Egger | 21 | -0.166515513 | 0.395657971460554 | 0.678581494963162 | -0.942005137 | 0.608974111099858 | 0.846609683409023 | 0.389845357772206 | 1.83854428878626 | 0.999950574162623 |
| ebi-fl187-GCST90094770 | prot-a-992 | Persulfide dioxygenase ETHE1, mitochondrial \|\| id:prot-a-992 | Gherkins liking \|\| id:ebi-fl187-GCST90094770 | MR Egger | 21 | -0.360510858 | 0.386013850817766 | 0.362056910368397 | -1.117098006 | 0.396076289553932 | 0.697320003527207 | 0.327228031967232 | 1.48598267818289 | 0.999950574162623 |
| ebi-fl187-GCST90094770 | prot-a-993 | Ethanolamine kinase 1 \|\| id:prot-a-993 | Gherkins liking \|\| id:ebi-fl187-GCST90094770 | MR Egger | 21 | -0.166110011 | 0.322640680992513 | 0.61259877448845 | -0.798485746 | 0.466265723636661 | 0.846953054819563 | 0.450009877772112 | 1.59403051466228 | 0.999950574162623 |
| ebi-fl187-GCST90094770 | prot-b-10 | coagulation factor III, tissue factor \|\| id:prot-b-10 | Gherkins liking \|\| id:ebi-fl187-GCST90094770 | MR Egger | 15 | 0.431933554967436 | 0.533982485370121 | 0.4331330433264 | -0.614672116 | 1.47853922629287 | 1.54023277094441 | 0.540818191679786 | 4.38653326605501 | 0.999950574162623 |
| ebi-fl187-GCST90094770 | prot-b-28 | prolactin \|\| id:prot-b-28 | Gherkins liking \|\| id:ebi-fl187-GCST90094770 | MR Egger | 15 | 0.206864342426899 | 0.533982485370121 | 0.704728935476005 | -0.839741329 | 1.25347001375234 | 1.22981572664727 | 0.431822208909968 | 3.50247553345335 | 0.999950574162623 |
| ebi-fl187-GCST90094770 | prot-b-54 | matrix metallopeptidase 7 \|\| id:prot-b-54 | Gherkins liking \|\| id:ebi-fl187-GCST90094770 | MR Egger | 15 | 0.256489168235035 | 0.621999014773923 | 0.686792866625055 | -0.962628901 | 1.47560723719193 | 1.29238476691582 | 0.381887620541251 | 4.37369083446279 | 0.999950574162623 |
| ebi-fl187-GCST90094770 | prot-c-2421_7_3 | BDNF \|\| id:prot-c-2421_7_3 | Gherkins liking \|\| id:ebi-fl187-GCST90094770 | MR Egger | 3 | -2.320028207 | 3.69698282047787 | 0.643220244832815 | -9.566114535 | 4.92605812128717 | 0.0982708136552216 | 7.00630830727681e-05 | 137.835110773377 | 0.999950574162623 |
| ebi-fl187-GCST90094770 | prot-c-2443_10_4 | FGF-8B \|\| id:prot-c-2443_10_4 | Gherkins liking \|\| id:ebi-fl187-GCST90094770 | MR Egger | 3 | -8.724382148 | 4.64682581679958 | 0.311565442087553 | -17.83216075 | 0.383396452936747 | 0.000162573207818622 | 1.80132047835285e-08 | 1.46725961415842 | 0.999950574162623 |
| ebi-fl187-GCST90094770 | prot-c-2605_49_2 | CD30 \|\| id:prot-c-2605_49_2 | Gherkins liking \|\| id:ebi-fl187-GCST90094770 | MR Egger | 3 | 6.12468658783878 | 4.83412626767829 | 0.425372445044301 | -3.350200897 | 15.5995740724882 | 457.001461006004 | 0.0350773064739482 | 5954001.49999358 | 0.999950574162623 |
| ebi-fl187-GCST90094770 | prot-c-2624_31_2 | HPV E7 Type18 \|\| id:prot-c-2624_31_2 | Gherkins liking \|\| id:ebi-fl187-GCST90094770 | MR Egger | 3 | -3.025784744 | 4.99199401319525 | 0.653097723253488 | -12.81009301 | 6.75852352183249 | 0.048519730757255 | 2.73304821362116e-06 | 861.369463232908 | 0.999950574162623 |
| ebi-fl187-GCST90094770 | prot-c-2774_10_3 | IL-16 \|\| id:prot-c-2774_10_3 | Gherkins liking \|\| id:ebi-fl187-GCST90094770 | MR Egger | 3 | -1.771922719 | 3.88466049402793 | 0.72756328088442 | -9.385857287 | 5.84201184932289 | 0.170005801002178 | 8.3902320473227e-05 | 344.471669095431 | 0.999950574162623 |
| ebi-fl187-GCST90094770 | prot-c-2851_63_3 | C5a \|\| id:prot-c-2851_63_3 | Gherkins liking \|\| id:ebi-fl187-GCST90094770 | MR Egger | 3 | -0.385950628 | 5.54776290384102 | 0.955782435867239 | -11.25956592 | 10.4876646635489 | 0.679804088153162 | 1.28834641267703e-05 | 35870.290297894 | 0.999950574162623 |
| ebi-fl187-GCST90094770 | prot-c-2891_1_3 | CD22 \|\| id:prot-c-2891_1_3 | Gherkins liking \|\| id:ebi-fl187-GCST90094770 | MR Egger | 3 | -1.458011195 | 3.75356054164055 | 0.764134762010508 | -8.814989857 | 5.89896746648029 | 0.232698606954624 | 0.000148490458137738 | 364.660748964723 | 0.999950574162623 |
| ebi-fl187-GCST90094770 | prot-c-2961_1_2 | Protein C \|\| id:prot-c-2961_1_2 | Gherkins liking \|\| id:ebi-fl187-GCST90094770 | MR Egger | 3 | 2.59827440587361 | 3.64348422291167 | 0.605624771121959 | -4.542954671 | 9.73950348278048 | 13.4405251215228 | 0.0106419166923524 | 16975.110853114 | 0.999950574162623 |
| ebi-fl187-GCST90094770 | prot-c-2982_82_2 | Galectin-4 \|\| id:prot-c-2982_82_2 | Gherkins liking \|\| id:ebi-fl187-GCST90094770 | MR Egger | 3 | -5.410595613 | 3.44803875240363 | 0.361203935202472 | -12.16875157 | 1.34756034198956 | 0.00446897763856208 | 5.19013127416651e-06 | 3.84802620183731 | 0.999950574162623 |
| ebi-fl187-GCST90094770 | prot-c-2994_71_2 | IL-1Rrp2 \|\| id:prot-c-2994_71_2 | Gherkins liking \|\| id:ebi-fl187-GCST90094770 | MR Egger | 3 | 1.95967124387754 | 3.71855731454146 | 0.691231905326713 | -5.328701093 | 9.2480435803788 | 7.09699350152632 | 0.00485036609865045 | 10384.2299192056 | 0.999950574162623 |
| ebi-fl187-GCST90094770 | prot-c-3336_50_1 | TFPI \|\| id:prot-c-3336_50_1 | Gherkins liking \|\| id:ebi-fl187-GCST90094770 | MR Egger | 3 | 2.35381196966077 | 3.617546193 | 0.632770459622655 | -4.73657857 | 9.44420250886784 | 10.5256166787871 | 0.00876859606214984 | 12634.7029425825 | 0.999950574162623 |
| ebi-fl187-GCST90094770 | prot-c-3472_40_2 | Survivin \|\| id:prot-c-3472_40_2 | Gherkins liking \|\| id:ebi-fl187-GCST90094770 | MR Egger | 3 | 1.14370966538027 | 5.35659191509625 | 0.866083342910969 | -9.355210488 | 11.6426298189689 | 3.13838917086612 | 8.65134652762096e-05 | 113849.174303254 | 0.999950574162623 |
| ebi-fl187-GCST90094770 | prot-c-3473_78_2 | Thrombopoietin Receptor \|\| id:prot-c-3473_78_2 | Gherkins liking \|\| id:ebi-fl187-GCST90094770 | MR Egger | 3 | -0.937215968 | 3.89718575561374 | 0.849755302261714 | -8.575700049 | 6.70126811283158 | 0.39171687093676 | 0.000188634354466735 | 813.436700914125 | 0.999950574162623 |
| ebi-fl187-GCST90094770 | prot-c-3497_13_2 | IFN-aA \|\| id:prot-c-3497_13_2 | Gherkins liking \|\| id:ebi-fl187-GCST90094770 | MR Egger | 3 | -1.774466203 | 3.74264530078491 | 0.718148824246157 | -9.110050993 | 5.56111858612386 | 0.169573943336604 | 0.000110549077763449 | 260.113633152651 | 0.999950574162623 |
| ebi-fl187-GCST90094770 | prot-c-3665_64_3 | COLEC12 \|\| id:prot-c-3665_64_3 | Gherkins liking \|\| id:ebi-fl187-GCST90094770 | MR Egger | 3 | -0.84779587 | 7.17837582795665 | 0.925159261528851 | -14.91741249 | 13.221820753248 | 0.428358049211224 | 3.32238586415459e-07 | 552285.694156528 | 0.999950574162623 |
| ebi-fl187-GCST90094770 | prot-c-3741_4_3 | IL-5 \|\| id:prot-c-3741_4_3 | Gherkins liking \|\| id:ebi-fl187-GCST90094770 | MR Egger | 3 | -6.70501145 | 5.17171040788135 | 0.418263620017334 | -16.84156385 | 3.43154094991258 | 0.00122475868094943 | 4.8506702679114e-08 | 30.9242587871651 | 0.999950574162623 |
| ebi-fl187-GCST90094770 | prot-c-3848_14_2 | GAPDH, liver \|\| id:prot-c-3848_14_2 | Gherkins liking \|\| id:ebi-fl187-GCST90094770 | MR Egger | 3 | 2.32958466739102 | 3.68904490372845 | 0.641423480989991 | -4.900943344 | 9.56011267869879 | 10.2736736551274 | 0.00743956169439092 | 14187.4447323472 | 0.999950574162623 |
| ebi-fl187-GCST90094770 | prot-c-3859_50_2 | PSA1 \|\| id:prot-c-3859_50_2 | Gherkins liking \|\| id:ebi-fl187-GCST90094770 | MR Egger | 3 | -3.323175396 | 3.67662028502113 | 0.532117947204237 | -10.52935115 | 3.88300036280574 | 0.0360382142768851 | 2.6739968762708e-05 | 48.5697234649707 | 0.999950574162623 |
| ebi-fl187-GCST90094770 | prot-c-3868_8_1 | SGTA \|\| id:prot-c-3868_8_1 | Gherkins liking \|\| id:ebi-fl187-GCST90094770 | MR Egger | 3 | 4.5994323294876 | 7.28019260453619 | 0.641293225991649 | -9.669745175 | 18.8686098343785 | 99.4278573558661 | 6.31659495210367e-05 | 156506771.343413 | 0.999950574162623 |
| ebi-fl187-GCST90094770 | prot-c-3882_10_1 | EF-1-beta \|\| id:prot-c-3882_10_1 | Gherkins liking \|\| id:ebi-fl187-GCST90094770 | MR Egger | 3 | 2.10333538 | 3.71387828213518 | 0.671946466888441 | -5.175866053 | 9.38253681262095 | 8.19345266327089 | 0.00565132044895843 | 11879.1116433035 | 0.999950574162623 |
| ebi-fl187-GCST90094770 | prot-c-4134_4_2 | HB-EGF \|\| id:prot-c-4134_4_2 | Gherkins liking \|\| id:ebi-fl187-GCST90094770 | MR Egger | 3 | -3.665188031 | 3.74938017471323 | 0.50722848298258 | -11.01397317 | 3.68359711163428 | 0.0255993573634062 | 1.64699479636631e-05 | 39.7892633823251 | 0.999950574162623 |
| ebi-fl187-GCST90094770 | prot-c-4220_39_2 | FER \|\| id:prot-c-4220_39_2 | Gherkins liking \|\| id:ebi-fl187-GCST90094770 | MR Egger | 3 | 2.36415802998317 | 6.08637408246256 | 0.764134550255354 | -9.565135172 | 14.2934512316098 | 10.6350806277605 | 7.01317338995648e-05 | 1612749.802 | 0.999950574162623 |
| ebi-fl187-GCST90094770 | prot-c-4228_37_2 | HSP70 protein 8 \|\| id:prot-c-4228_37_2 | Gherkins liking \|\| id:ebi-fl187-GCST90094770 | MR Egger | 3 | 4.02611019107767 | 3.74400558716541 | 0.476896802342377 | -3.31214076 | 11.3643611419219 | 56.0424921322923 | 0.036438085012581 | 86194.456248555 | 0.999950574162623 |
| ebi-fl187-GCST90094770 | prot-c-4246_40_2 | NCAM-L1 \|\| id:prot-c-4246_40_2 | Gherkins liking \|\| id:ebi-fl187-GCST90094770 | MR Egger | 3 | 2.19813371803422 | 3.70767233369389 | 0.659310651298483 | -5.068904056 | 9.46517149207425 | 9.00818598682504 | 0.00628930909568823 | 12902.4370624213 | 0.999950574162623 |
| ebi-fl187-GCST90094770 | prot-c-4267_81_3 | PESC \|\| id:prot-c-4267_81_3 | Gherkins liking \|\| id:ebi-fl187-GCST90094770 | MR Egger | 3 | -3.119344813 | 6.27201229028829 | 0.706187124309855 | -15.4124889 | 9.173799276 | 0.0441861090995333 | 2.02507502435058e-07 | 9641.18471601797 | 0.999950574162623 |
| ebi-fl187-GCST90094770 | prot-c-4280_47_2 | PSA2 \|\| id:prot-c-4280_47_2 | Gherkins liking \|\| id:ebi-fl187-GCST90094770 | MR Egger | 3 | -1.597207542 | 3.72674291311007 | 0.742233263896697 | -8.901623652 | 5.70720856759458 | 0.202461093430774 | 0.000136167658036515 | 301.029590610953 | 0.999950574162623 |
| ebi-fl187-GCST90094770 | prot-c-4324_33_2 | CYTT \|\| id:prot-c-4324_33_2 | Gherkins liking \|\| id:ebi-fl187-GCST90094770 | MR Egger | 3 | -5.525186236 | 3.60615109627813 | 0.368127570971617 | -12.59324238 | 1.54286991292777 | 0.00398512644989208 | 3.39487918248919e-06 | 4.67799646701567 | 0.999950574162623 |
| ebi-fl187-GCST90094770 | prot-c-4462_8_2 | RASA1 \|\| id:prot-c-4462_8_2 | Gherkins liking \|\| id:ebi-fl187-GCST90094770 | MR Egger | 3 | 1.26910871484422 | 4.84448395113579 | 0.836890191587264 | -8.226079829 | 10.7642972590704 | 3.55768024125615 | 0.000267583248852163 | 47301.4987048659 | 0.999950574162623 |
| ebi-fl187-GCST90094770 | prot-c-4499_21_1 | PDGF-AA \|\| id:prot-c-4499_21_1 | Gherkins liking \|\| id:ebi-fl187-GCST90094770 | MR Egger | 3 | -1.426560015 | 3.73040714450417 | 0.767471608976656 | -8.738158019 | 5.88503798777088 | 0.240133558792501 | 0.000160348975870212 | 359.616428763633 | 0.999950574162623 |
| ebi-fl187-GCST90094770 | prot-c-4535_50_2 | BST1 \|\| id:prot-c-4535_50_2 | Gherkins liking \|\| id:ebi-fl187-GCST90094770 | MR Egger | 3 | -1.873287111 | 5.16796403073296 | 0.778615530963413 | -12.00249661 | 8.25592238933406 | 0.153617871995082 | 6.12889177704423e-06 | 3850.3617708972 | 0.999950574162623 |
| ebi-fl187-GCST90094770 | prot-c-4542_24_2 | Clusterin \|\| id:prot-c-4542_24_2 | Gherkins liking \|\| id:ebi-fl187-GCST90094770 | MR Egger | 3 | -0.189597642 | 3.71745199308177 | 0.967559200658609 | -7.475803549 | 7.09660826426185 | 0.827291934366964 | 0.000566630269684921 | 1207.86336573443 | 0.999950574162623 |
| ebi-fl187-GCST90094770 | prot-c-4696_2_2 | FABP \|\| id:prot-c-4696_2_2 | Gherkins liking \|\| id:ebi-fl187-GCST90094770 | MR Egger | 3 | -3.224997662 | 3.5511208823371 | 0.530615817647834 | -10.18519459 | 3.73519926759515 | 0.0397558745338726 | 3.77247344978202e-05 | 41.8963733209135 | 0.999950574162623 |
| ebi-fl187-GCST90094770 | prot-c-4906_35_1 | Coagulation Factor V \|\| id:prot-c-4906_35_1 | Gherkins liking \|\| id:ebi-fl187-GCST90094770 | MR Egger | 3 | 4.44686070003881 | 3.74747235991033 | 0.445795711309243 | -2.898185125 | 11.7919065254631 | 85.3585568345473 | 0.0551231709734549 | 132178.230972694 | 0.999950574162623 |
| ebi-fl187-GCST90094770 | prot-c-4916_2_1 | IgD \|\| id:prot-c-4916_2_1 | Gherkins liking \|\| id:ebi-fl187-GCST90094770 | MR Egger | 3 | 3.24376509855312 | 3.7220376977257 | 0.543641873560925 | -4.051428789 | 10.5389589860955 | 25.6300399361112 | 0.0173974995173242 | 37758.2391350275 | 0.999950574162623 |
| ebi-fl187-GCST90094770 | prot-c-4922_13_1 | MIP-3b \|\| id:prot-c-4922_13_1 | Gherkins liking \|\| id:ebi-fl187-GCST90094770 | MR Egger | 3 | 2.43139548669097 | 5.15830697038851 | 0.719587824276856 | -7.678886175 | 12.5416771486525 | 11.3747443139624 | 0.000462489744519045 | 279757.140004414 | 0.999950574162623 |
| ebi-fl187-GCST90094770 | prot-c-4973_18_1 | cIAP-2 \|\| id:prot-c-4973_18_1 | Gherkins liking \|\| id:ebi-fl187-GCST90094770 | MR Egger | 3 | -3.881945797 | 3.74233333976834 | 0.488343800489326 | -11.21691914 | 3.45302754858855 | 0.0206106819286292 | 1.34447865615136e-05 | 31.5959057899168 | 0.999950574162623 |
| ebi-fl187-GCST90094770 | prot-c-4997_19_1 | IF4A3 \|\| id:prot-c-4997_19_1 | Gherkins liking \|\| id:ebi-fl187-GCST90094770 | MR Egger | 3 | 3.60742974719574 | 3.74327303941663 | 0.511763593944421 | -3.72938541 | 10.9442449044523 | 36.8711627119278 | 0.0240075861244508 | 56627.2107775499 | 0.999950574162623 |
| ebi-fl187-GCST90094770 | prot-c-5000_52_1 | LG3BP \|\| id:prot-c-5000_52_1 | Gherkins liking \|\| id:ebi-fl187-GCST90094770 | MR Egger | 3 | -3.7825296 | 3.45770616786384 | 0.471458049812423 | -10.55963369 | 2.99457448913852 | 0.0227650321059751 | 2.59423526101604e-05 | 19.9768577111663 | 0.999950574162623 |
| ebi-fl187-GCST90094770 | prot-c-5006_71_1 | MK13 \|\| id:prot-c-5006_71_1 | Gherkins liking \|\| id:ebi-fl187-GCST90094770 | MR Egger | 3 | -0.598610742 | 3.72954388225431 | 0.898683429526997 | -7.908516751 | 6.71129526736119 | 0.54957460698735 | 0.000367599417871685 | 821.634186457631 | 0.999950574162623 |
| ebi-fl187-GCST90094770 | prot-c-5109_24_3 | Nr-CAM \|\| id:prot-c-5109_24_3 | Gherkins liking \|\| id:ebi-fl187-GCST90094770 | MR Egger | 3 | 1.25664879446489 | 3.66717837765193 | 0.789830179494508 | -5.931020826 | 8.44431841466268 | 3.51362684952949 | 0.00265576952666288 | 4648.58622474197 | 0.999950574162623 |
| ebi-fl187-GCST90094770 | prot-c-5255_22_3 | PDE4D \|\| id:prot-c-5255_22_3 | Gherkins liking \|\| id:ebi-fl187-GCST90094770 | MR Egger | 3 | 1.80967337403714 | 3.6734543636124 | 0.70859460253184 | -5.390297179 | 9.00964392671744 | 6.10845192736415 | 0.0045606178161148 | 8181.60750437668 | 0.999950574162623 |
| ebi-fl187-GCST90094770 | prot-c-5440_26_3 | Troponin I, skeletal, fast twitch \|\| id:prot-c-5440_26_3 | Gherkins liking \|\| id:ebi-fl187-GCST90094770 | MR Egger | 3 | -8.189923508 | 3.56473567845045 | 0.261350047379391 | -15.17680544 | -1.203041579 | 0.00027743510489077 | 2.56328647082503e-07 | 0.300279497831463 | 0.999950574162623 |
| ebi-fl187-GCST90094770 | ukb-rsfMRI191-edge-pheno716 | Net100_Pair10_33 (OccipitalPrecuneus)&(Temporal\|Frontal\|Supp_Motor_Area) (Default_mode\|Central_executive)&(Default_mode\|Salience) \|\| id:ukb-rsfMRI191-edge-pheno716 | Gherkins liking \|\| id:ebi-fl187-GCST90094770 | MR Egger | 21 | 0.0451391328740487 | 0.119408837960555 | 0.709604685103627 | -0.18890219 | 0.279180455276736 | 1.04617340689615 | 0.82786747684294 | 1.3220458925026 | 0.999950574162623 |
| ebi-fl187-GCST90094770 | ukb-rsfMRI191-edge-pheno903 | Net100_Pair27_38 (Precentral\|Frontal\|Supp_Motor_Area)&(Subcortical) (Attention\|Salience\|Motor)&(Subcortical-cerebellum) \|\| id:ukb-rsfMRI191-edge-pheno903 | Gherkins liking \|\| id:ebi-fl187-GCST90094770 | MR Egger | 21 | 0.104839154020693 | 0.132147468269715 | 0.43737007798034 | -0.154169884 | 0.363848191829335 | 1.11053197138692 | 0.857126396763175 | 1.43885576751556 | 0.999950574162623 |
| ebi-fl187-GCST90094770 | ukb-rsfMRI191-node-pheno23 | Net100_Node2 RolandicOper\|SupraMarginal\|Insula Salience\|Motor \|\| id:ukb-rsfMRI191-node-pheno23 | Gherkins liking \|\| id:ebi-fl187-GCST90094770 | MR Egger | 21 | -0.006853402 | 0.109195842652648 | 0.950611214834713 | -0.220877253 | 0.207170449923213 | 0.993170029323383 | 0.801815094426829 | 1.23019224008413 | 0.999950574162623 |
| ebi-fl187-GCST90094770 | ukb-rsfMRI191-node-pheno55 | Net100_Node34 Parietal\|Postcentral\|Precuneus Attention \|\| id:ukb-rsfMRI191-node-pheno55 | Gherkins liking \|\| id:ebi-fl187-GCST90094770 | MR Egger | 21 | 0.0073445128859704 | 0.121424485248779 | 0.952400066 | -0.230647478 | 0.245336503973578 | 1.00737154997161 | 0.794019325824681 | 1.2780513101973 | 0.999950574162623 |
| ebi-fl187-GCST90094770 | ebi-a-GCST90001414 | IgD- CD24- B cell Absolute Count \|\| id:ebi-a-GCST90001414 | Gherkins liking \|\| id:ebi-fl187-GCST90094770 | Weighted median | 19 | -0.038237838 | 0.131254900665457 | 0.770802747024923 | -0.295497443 | 0.219021767594242 | 0.962483998676525 | 0.744161317530495 | 1.24485837396458 | 0.999332001504091 |
| ebi-fl187-GCST90094770 | ebi-a-GCST90001422 | CD20- CD38- B cell %B cell \|\| id:ebi-a-GCST90001422 | Gherkins liking \|\| id:ebi-fl187-GCST90094770 | Weighted median | 19 | -0.052461147 | 0.128247543697356 | 0.682494444708651 | -0.303826333 | 0.198904038303498 | 0.948891187283148 | 0.737989019668634 | 1.22006488078632 | 0.999332001504091 |
| ebi-fl187-GCST90094770 | ebi-a-GCST90001444 | CD20- CD38- B cell %lymphocyte \|\| id:ebi-a-GCST90001444 | Gherkins liking \|\| id:ebi-fl187-GCST90094770 | Weighted median | 19 | -0.070069224 | 0.128722782548345 | 0.586206114009218 | -0.322365877 | 0.182227430161037 | 0.9323292784516 | 0.724433088120019 | 1.19988705335623 | 0.999332001504091 |
| ebi-fl187-GCST90094770 | ebi-a-GCST90001459 | Myeloid Dendritic Cell %Dendritic Cell \|\| id:ebi-a-GCST90001459 | Gherkins liking \|\| id:ebi-fl187-GCST90094770 | Weighted median | 19 | -0.054400558 | 0.145730046714153 | 0.708927567671097 | -0.34003145 | 0.2312303333834 | 0.947052680821115 | 0.711747938125807 | 1.26014946051298 | 0.999332001504091 |
| ebi-fl187-GCST90094770 | ebi-a-GCST90001476 | HLA DR++ monocyte %leukocyte \|\| id:ebi-a-GCST90001476 | Gherkins liking \|\| id:ebi-fl187-GCST90094770 | Weighted median | 19 | -0.036476409 | 0.14169435874801 | 0.796846683295683 | -0.314197352 | 0.24124453443203 | 0.964180839893902 | 0.730374873095432 | 1.27283224856647 | 0.999332001504091 |
| ebi-fl187-GCST90094770 | ebi-a-GCST90001478 | CD4 regulatory T cell %CD4+ T cell \|\| id:ebi-a-GCST90001478 | Gherkins liking \|\| id:ebi-fl187-GCST90094770 | Weighted median | 19 | 0.134616418003905 | 0.135256984916303 | 0.319607845321652 | -0.130487272 | 0.399720108439858 | 1.144097845 | 0.877667663452477 | 1.49140720692798 | 0.999332001504091 |
| ebi-fl187-GCST90094770 | ebi-a-GCST90001485 | CD39+ resting CD4 regulatory T cell %CD4 regulatory T cell \|\| id:ebi-a-GCST90001485 | Gherkins liking \|\| id:ebi-fl187-GCST90094770 | Weighted median | 18 | 0.0664961828351064 | 0.127050051376762 | 0.600705858334978 | -0.182521918 | 0.315514283533559 | 1.06875688444613 | 0.833166382501289 | 1.37096419399666 | 0.999332001504091 |
| ebi-fl187-GCST90094770 | ebi-a-GCST90001515 | Immature Myeloid-Derived Suppressor Cells Absolute Count \|\| id:ebi-a-GCST90001515 | Gherkins liking \|\| id:ebi-fl187-GCST90094770 | Weighted median | 19 | 0.0242376693006544 | 0.186473816861951 | 0.896583087722856 | -0.341251012 | 0.389726350350078 | 1.02453378918582 | 0.710880446465317 | 1.47657667390162 | 0.999332001504091 |
| ebi-fl187-GCST90094770 | ebi-a-GCST90001517 | CD33+ HLA DR+ Absolute Count \|\| id:ebi-a-GCST90001517 | Gherkins liking \|\| id:ebi-fl187-GCST90094770 | Weighted median | 19 | -0.090368725 | 0.172429071385085 | 0.600214438867362 | -0.428329705 | 0.247592254854546 | 0.913594258060472 | 0.651596544756327 | 1.28093752963837 | 0.999332001504091 |
| ebi-fl187-GCST90094770 | ebi-a-GCST90001518 | CD33+ HLA DR+ CD14- Absolute Count \|\| id:ebi-a-GCST90001518 | Gherkins liking \|\| id:ebi-fl187-GCST90094770 | Weighted median | 19 | -0.180438283 | 0.167343639694565 | 0.28092219528533 | -0.508431817 | 0.147555251063652 | 0.834904207108842 | 0.601438004802628 | 1.1589973188954 | 0.999332001504091 |
| ebi-fl187-GCST90094770 | ebi-a-GCST90001520 | CD33+ HLA DR+ CD14dim Absolute Count \|\| id:ebi-a-GCST90001520 | Gherkins liking \|\| id:ebi-fl187-GCST90094770 | Weighted median | 19 | 0.0213767782134104 | 0.183483578167693 | 0.907252220262442 | -0.338251035 | 0.381004591422089 | 1.02160689835402 | 0.713016273392953 | 1.46375432610825 | 0.999332001504091 |
| ebi-fl187-GCST90094770 | ebi-a-GCST90001531 | CD33dim HLA DR- Absolute Count \|\| id:ebi-a-GCST90001531 | Gherkins liking \|\| id:ebi-fl187-GCST90094770 | Weighted median | 19 | 0.170912651018409 | 0.177726072107847 | 0.336218734914673 | -0.17743045 | 0.51925575234979 | 1.18638711478936 | 0.837419239556459 | 1.68077627029886 | 0.999332001504091 |
| ebi-fl187-GCST90094770 | ebi-a-GCST90001532 | Basophil Absolute Count \|\| id:ebi-a-GCST90001532 | Gherkins liking \|\| id:ebi-fl187-GCST90094770 | Weighted median | 19 | 0.154412065689702 | 0.178486456941418 | 0.386973352610958 | -0.19542139 | 0.50424552129488 | 1.16697165669782 | 0.822487996873902 | 1.65573583166205 | 0.999332001504091 |
| ebi-fl187-GCST90094770 | ebi-a-GCST90001588 | T/B cell \|\| id:ebi-a-GCST90001588 | Gherkins liking \|\| id:ebi-fl187-GCST90094770 | Weighted median | 19 | -0.109896227 | 0.128826172871465 | 0.393627013970866 | -0.362395526 | 0.142603071934727 | 0.895927103611519 | 0.696007024712157 | 1.15327194480212 | 0.999332001504091 |
| ebi-fl187-GCST90094770 | ebi-a-GCST90001624 | HLA DR+ CD4+ T cell Absolute Count \|\| id:ebi-a-GCST90001624 | Gherkins liking \|\| id:ebi-fl187-GCST90094770 | Weighted median | 19 | 0.0778663599444757 | 0.123699310850381 | 0.529034235241095 | -0.164584289 | 0.320317009211222 | 1.08097818703176 | 0.848246255828969 | 1.37756439572671 | 0.999332001504091 |
| ebi-fl187-GCST90094770 | ebi-a-GCST90001631 | CD8+ Natural Killer T %T cell \|\| id:ebi-a-GCST90001631 | Gherkins liking \|\| id:ebi-fl187-GCST90094770 | Weighted median | 19 | 0.0515432161816393 | 0.128490095033217 | 0.688313044792978 | -0.20029737 | 0.303383802446745 | 1.05289468739699 | 0.818487323241772 | 1.35443419985791 | 0.999332001504091 |
| ebi-fl187-GCST90094770 | ebi-a-GCST90001632 | CD8+ Natural Killer T %lymphocyte \|\| id:ebi-a-GCST90001632 | Gherkins liking \|\| id:ebi-fl187-GCST90094770 | Weighted median | 19 | -0.031914863 | 0.122362694573764 | 0.794229903996308 | -0.271745744 | 0.207916018637988 | 0.968589041599017 | 0.762047991664919 | 1.23110977493163 | 0.999332001504091 |
| ebi-fl187-GCST90094770 | ebi-a-GCST90001646 | Natural Killer %CD3- lymphocyte \|\| id:ebi-a-GCST90001646 | Gherkins liking \|\| id:ebi-fl187-GCST90094770 | Weighted median | 19 | -0.02537346 | 0.129420708101047 | 0.844567826049264 | -0.279038048 | 0.228291128119611 | 0.974945741030021 | 0.756511119207403 | 1.2564510604265 | 0.999332001504091 |
| ebi-fl187-GCST90094770 | ebi-a-GCST90001684 | CD127- CD8+ T cell Absolute Count \|\| id:ebi-a-GCST90001684 | Gherkins liking \|\| id:ebi-fl187-GCST90094770 | Weighted median | 19 | -0.038798733 | 0.132790283811375 | 0.770148610278404 | -0.29906769 | 0.221470223029366 | 0.961944297075385 | 0.741509215339869 | 1.24791008868545 | 0.999332001504091 |
| ebi-fl187-GCST90094770 | ebi-a-GCST90001687 | CD28- CD8+ T cell Absolute Count \|\| id:ebi-a-GCST90001687 | Gherkins liking \|\| id:ebi-fl187-GCST90094770 | Weighted median | 19 | -0.020678256 | 0.133607088146095 | 0.877003312299532 | -0.282548148 | 0.241191637207564 | 0.979534073517603 | 0.753860343972542 | 1.27276492105405 | 0.999332001504091 |
| ebi-fl187-GCST90094770 | ebi-a-GCST90001701 | BAFF-R on CD20- CD38- B cell \|\| id:ebi-a-GCST90001701 | Gherkins liking \|\| id:ebi-fl187-GCST90094770 | Weighted median | 19 | -0.010066145 | 0.132493068551828 | 0.939439085299095 | -0.269752559 | 0.249620269714035 | 0.989984349417602 | 0.763568409087003 | 1.28353792591244 | 0.999332001504091 |
| ebi-fl187-GCST90094770 | ebi-a-GCST90001732 | CD19 on IgD- CD27- B cell \|\| id:ebi-a-GCST90001732 | Gherkins liking \|\| id:ebi-fl187-GCST90094770 | Weighted median | 19 | -0.199856663 | 0.130116454334226 | 0.124542546827686 | -0.454884914 | 0.0551715870362073 | 0.818848115523203 | 0.634520988258008 | 1.05672191890248 | 0.999332001504091 |
| ebi-fl187-GCST90094770 | ebi-a-GCST90001798 | CD27 on CD24+ CD27+ B cell \|\| id:ebi-a-GCST90001798 | Gherkins liking \|\| id:ebi-fl187-GCST90094770 | Weighted median | 19 | 0.0030467885683796 | 0.125506969176222 | 0.98063257502253 | -0.242946871 | 0.249040448153775 | 1.00305143474611 | 0.784313182426364 | 1.28279391866615 | 0.999332001504091 |
| ebi-fl187-GCST90094770 | ebi-a-GCST90001836 | CD66b on CD66b++ myeloid cell \|\| id:ebi-a-GCST90001836 | Gherkins liking \|\| id:ebi-fl187-GCST90094770 | Weighted median | 19 | -0.087992167 | 0.180714516896825 | 0.626320300918262 | -0.44219262 | 0.26620828588248 | 0.915768049690889 | 0.642625840716888 | 1.30500684488366 | 0.999332001504091 |
| ebi-fl187-GCST90094770 | ebi-a-GCST90001943 | CD25 on activated & secreting CD4 regulatory T cell \|\| id:ebi-a-GCST90001943 | Gherkins liking \|\| id:ebi-fl187-GCST90094770 | Weighted median | 19 | 0.136705141742645 | 0.136412747069574 | 0.316274312540964 | -0.130663843 | 0.40407412599901 | 1.14649004661956 | 0.877512707282175 | 1.49791497728709 | 0.999332001504091 |
| ebi-fl187-GCST90094770 | ebi-a-GCST90001960 | CD25 on CD4+ T cell \|\| id:ebi-a-GCST90001960 | Gherkins liking \|\| id:ebi-fl187-GCST90094770 | Weighted median | 19 | 0.162814471830461 | 0.132411501878204 | 0.218843282521473 | -0.096712072 | 0.42234101551174 | 1.17681833648191 | 0.907817354677393 | 1.52552866492864 | 0.999332001504091 |
| ebi-fl187-GCST90094770 | ebi-a-GCST90001988 | HLA DR on CD14+ CD16- monocyte \|\| id:ebi-a-GCST90001988 | Gherkins liking \|\| id:ebi-fl187-GCST90094770 | Weighted median | 19 | 0.0855627010120463 | 0.131578801482002 | 0.515513150430459 | -0.17233175 | 0.34345715191677 | 1.08932986128756 | 0.84169989299631 | 1.40981311339905 | 0.999332001504091 |
| ebi-fl187-GCST90094770 | ebi-a-GCST90001991 | HLA DR on CD14+ monocyte \|\| id:ebi-a-GCST90001991 | Gherkins liking \|\| id:ebi-fl187-GCST90094770 | Weighted median | 19 | 0.0567853250751193 | 0.130395203653462 | 0.66320947942964 | -0.198789274 | 0.312359924235905 | 1.05842856793143 | 0.819722611931043 | 1.36664649371368 | 0.999332001504091 |
| ebi-fl187-GCST90094770 | ebi-a-GCST90002019 | CD14 on Monocytic Myeloid-Derived Suppressor Cells \|\| id:ebi-a-GCST90002019 | Gherkins liking \|\| id:ebi-fl187-GCST90094770 | Weighted median | 19 | 0.0381692616091059 | 0.189869400436416 | 0.840675873870685 | -0.333974763 | 0.410313286464481 | 1.0389070650788 | 0.716071853328196 | 1.50728992468298 | 0.999332001504091 |
| ebi-fl187-GCST90094770 | ebi-a-GCST90002037 | CD80 on plasmacytoid Dendritic Cell \|\| id:ebi-a-GCST90002037 | Gherkins liking \|\| id:ebi-fl187-GCST90094770 | Weighted median | 19 | -0.157481571 | 0.143966125989482 | 0.274007899655062 | -0.439655178 | 0.124692035630781 | 0.854292556957308 | 0.644258537143241 | 1.13279953744779 | 0.999332001504091 |
| ebi-fl187-GCST90094770 | ebi-a-GCST90002042 | CD45 on CD33+ HLA DR+ CD14- \|\| id:ebi-a-GCST90002042 | Gherkins liking \|\| id:ebi-fl187-GCST90094770 | Weighted median | 19 | 0.0768665203368393 | 0.191994758794964 | 0.688893304189561 | -0.299443207 | 0.45317624757497 | 1.07989792236107 | 0.741230818009404 | 1.57330145264544 | 0.999332001504091 |
| ebi-fl187-GCST90094770 | ebi-a-GCST90002056 | CD8 on Effector Memory CD8+ T cell \|\| id:ebi-a-GCST90002056 | Gherkins liking \|\| id:ebi-fl187-GCST90094770 | Weighted median | 19 | -0.053226362 | 0.144297849118956 | 0.712228682157473 | -0.336050147 | 0.229597421973723 | 0.948165359297549 | 0.714587270815119 | 1.25809342719799 | 0.999332001504091 |
| ebi-fl187-GCST90094770 | ebi-a-GCST90002058 | CD8 on CD8+ T cell \|\| id:ebi-a-GCST90002058 | Gherkins liking \|\| id:ebi-fl187-GCST90094770 | Weighted median | 19 | -0.265032502 | 0.136790052774518 | 0.0526826685559542 | -0.533141006 | 0.00307600097274097 | 0.767181014296316 | 0.586759058520399 | 1.00308073671821 | 0.999332001504091 |
| ebi-fl187-GCST90094770 | ebi-a-GCST90002063 | CD4 on CD4 regulatory T cell \|\| id:ebi-a-GCST90002063 | Gherkins liking \|\| id:ebi-fl187-GCST90094770 | Weighted median | 19 | -0.140290368 | 0.142844137579873 | 0.326039754205713 | -0.420264878 | 0.139684141270708 | 0.869105837897175 | 0.656872805586955 | 1.14991053221027 | 0.999332001504091 |
| ebi-fl187-GCST90094770 | ebi-a-GCST90002066 | CD4 on activated CD4 regulatory T cell \|\| id:ebi-a-GCST90002066 | Gherkins liking \|\| id:ebi-fl187-GCST90094770 | Weighted median | 19 | -0.141284301 | 0.137777992449554 | 0.305151388535074 | -0.411329167 | 0.12876056375055 | 0.868242434022046 | 0.662768734325933 | 1.13741775251849 | 0.999332001504091 |
| ebi-fl187-GCST90094770 | ebi-a-GCST90002070 | CD4 on activated & secreting CD4 regulatory T cell \|\| id:ebi-a-GCST90002070 | Gherkins liking \|\| id:ebi-fl187-GCST90094770 | Weighted median | 19 | -0.090891381 | 0.145297911118127 | 0.531608910773849 | -0.375675287 | 0.193892524585765 | 0.913116887168247 | 0.68682531794862 | 1.21396580446704 | 0.999332001504091 |
| ebi-fl187-GCST90094770 | ebi-a-GCST90002081 | SSC-A on CD4+ T cell \|\| id:ebi-a-GCST90002081 | Gherkins liking \|\| id:ebi-fl187-GCST90094770 | Weighted median | 19 | 0.0847333605840808 | 0.124439803518545 | 0.495923080562599 | -0.159168654 | 0.328635375480428 | 1.08842681051422 | 0.852852509585594 | 1.38907127379129 | 0.999332001504091 |
| ebi-fl187-GCST90094770 | ebi-a-GCST90002094 | CD11b on Monocytic Myeloid-Derived Suppressor Cells \|\| id:ebi-a-GCST90002094 | Gherkins liking \|\| id:ebi-fl187-GCST90094770 | Weighted median | 19 | 0.236011063240664 | 0.198933398109071 | 0.235471356765902 | -0.153898397 | 0.625920523534443 | 1.26618831823548 | 0.857359126800102 | 1.86996651358889 | 0.999332001504091 |
| ebi-fl187-GCST90094770 | ebi-a-GCST90002104 | HLA DR on myeloid Dendritic Cell \|\| id:ebi-a-GCST90002104 | Gherkins liking \|\| id:ebi-fl187-GCST90094770 | Weighted median | 18 | -0.031272504 | 0.148843626504785 | 0.833587226120055 | -0.323006011 | 0.260461004436647 | 0.969211423569324 | 0.723969502239602 | 1.29752811502602 | 0.999332001504091 |
| ebi-fl187-GCST90094770 | ebi-a-GCST90002105 | HLA DR on plasmacytoid Dendritic Cell \|\| id:ebi-a-GCST90002105 | Gherkins liking \|\| id:ebi-fl187-GCST90094770 | Weighted median | 18 | 0.0991146792886982 | 0.143608628449637 | 0.490085919927451 | -0.182358232 | 0.380587591049988 | 1.10419292035704 | 0.833302770828257 | 1.46314406725752 | 0.999332001504091 |
| ebi-fl187-GCST90094770 | ebi-a-GCST90002106 | HLA DR on Dendritic Cell \|\| id:ebi-a-GCST90002106 | Gherkins liking \|\| id:ebi-fl187-GCST90094770 | Weighted median | 18 | 0.0578426675454786 | 0.13529542358518 | 0.668994334231251 | -0.207336363 | 0.323021697772432 | 1.05954828126406 | 0.812746226505315 | 1.38129532161203 | 0.999332001504091 |
| ebi-fl187-GCST90094770 | ebi-a-GCST90002111 | HLA DR on CD33dim HLA DR+ CD11b- \|\| id:ebi-a-GCST90002111 | Gherkins liking \|\| id:ebi-fl187-GCST90094770 | Weighted median | 19 | -0.311098931 | 0.174050899753994 | 0.0738725342236543 | -0.652238695 | 0.0300408324628783 | 0.732641391298164 | 0.520878382920525 | 1.03049661080907 | 0.999332001504091 |
| ebi-fl187-GCST90094770 | ebi-a-GCST90002112 | HLA DR on CD33- HLA DR+ \|\| id:ebi-a-GCST90002112 | Gherkins liking \|\| id:ebi-fl187-GCST90094770 | Weighted median | 19 | -0.145617571 | 0.194998319910045 | 0.455206507869037 | -0.527814278 | 0.236579136068643 | 0.864488245390203 | 0.589892903542367 | 1.26690780975662 | 0.999332001504091 |
| ebi-fl187-GCST90094770 | ebi-a-GCST90002115 | HLA DR on HLA DR+ CD8+ T cell \|\| id:ebi-a-GCST90002115 | Gherkins liking \|\| id:ebi-fl187-GCST90094770 | Weighted median | 19 | -0.191170522 | 0.140730321915947 | 0.174331267789859 | -0.467001953 | 0.0846609092528203 | 0.825991726626139 | 0.626878866331341 | 1.08834795539943 | 0.999332001504091 |
| ebi-fl187-GCST90094770 | ebi-a-GCST90002116 | HLA DR on B cell \|\| id:ebi-a-GCST90002116 | Gherkins liking \|\| id:ebi-fl187-GCST90094770 | Weighted median | 19 | 0.0679659866251525 | 0.152482980816073 | 0.65579348730253 | -0.230900656 | 0.366832629024655 | 1.07032890236146 | 0.79381832338481 | 1.4431563564135 | 0.999332001504091 |
| ebi-fl187-GCST90094770 | ebi-cfmet338-GCST90026023 | Sphingomyelin (d18:1/24:1, d18:2/24:0) levels \|\| id:ebi-cfmet338-GCST90026023 | Gherkins liking \|\| id:ebi-fl187-GCST90094770 | Weighted median | 18 | 0.0130268146575962 | 0.0871488913218832 | 0.88117671512803 | -0.157785012 | 0.183838641648487 | 1.01311203324786 | 0.854033368874577 | 1.20182188345191 | 0.999332001504091 |
| ebi-fl187-GCST90094770 | ebi-cfmet338-GCST90026046 | 1-arachidonoyl-gpc (20:4n6) levels \|\| id:ebi-cfmet338-GCST90026046 | Gherkins liking \|\| id:ebi-fl187-GCST90094770 | Weighted median | 18 | 0.0358520121072487 | 0.097687523973984 | 0.713613743144649 | -0.155615535 | 0.227319559096257 | 1.03650244532773 | 0.855888186275978 | 1.25523092431603 | 0.999332001504091 |
| ebi-fl187-GCST90094770 | ebi-cfmet338-GCST90026047 | 1-linoleoyl-gpc (18:2) levels \|\| id:ebi-cfmet338-GCST90026047 | Gherkins liking \|\| id:ebi-fl187-GCST90094770 | Weighted median | 18 | 0.0114957499153825 | 0.118052221235664 | 0.92242580672135 | -0.219886604 | 0.242878103537284 | 1.01156207997603 | 0.802609805779493 | 1.27491320723604 | 0.999332001504091 |
| ebi-fl187-GCST90094770 | ebi-cfmet338-GCST90026050 | 1-myristoyl-2-palmitoyl-gpc (14:0/16:0) levels \|\| id:ebi-cfmet338-GCST90026050 | Gherkins liking \|\| id:ebi-fl187-GCST90094770 | Weighted median | 18 | -0.05528132 | 0.0597156588616446 | 0.354579843154479 | -0.172324012 | 0.0617613712218215 | 0.946218920061917 | 0.841706406412479 | 1.06370848298425 | 0.999332001504091 |
| ebi-fl187-GCST90094770 | ebi-cfmet338-GCST90026068 | 2,3-dihydroxyisovalerate levels \|\| id:ebi-cfmet338-GCST90026068 | Gherkins liking \|\| id:ebi-fl187-GCST90094770 | Weighted median | 18 | 0.389600646639793 | 0.196310837392372 | 0.0471877679345556 | 0.00483140535074378 | 0.774369887928842 | 1.47639107440071 | 1.00484309540846 | 2.16922484169933 | 0.999332001504091 |
| ebi-fl187-GCST90094770 | ebi-cfmet338-GCST90026082 | 3-hydroxyhexanoate levels \|\| id:ebi-cfmet338-GCST90026082 | Gherkins liking \|\| id:ebi-fl187-GCST90094770 | Weighted median | 18 | -0.003485862 | 0.0421365291454832 | 0.934067993256461 | -0.086073459 | 0.0791017355019393 | 0.996520206938983 | 0.917526828372123 | 1.08231442627088 | 0.999332001504091 |
| ebi-fl187-GCST90094770 | ebi-cfmet338-GCST90026094 | 3-ureidopropionate levels \|\| id:ebi-cfmet338-GCST90026094 | Gherkins liking \|\| id:ebi-fl187-GCST90094770 | Weighted median | 18 | -0.064614711 | 0.0914617022061613 | 0.479897520599048 | -0.243879647 | 0.114650225593685 | 0.937428574977687 | 0.783581934982005 | 1.12148110357455 | 0.999332001504091 |
| ebi-fl187-GCST90094770 | ebi-cfmet338-GCST90026173 | Glycerol levels \|\| id:ebi-cfmet338-GCST90026173 | Gherkins liking \|\| id:ebi-fl187-GCST90094770 | Weighted median | 18 | -0.013682009 | 0.0509717539431507 | 0.788373473133333 | -0.113586646 | 0.086222629114257 | 0.986411164649027 | 0.892626850208211 | 1.09004897793214 | 0.999332001504091 |
| ebi-fl187-GCST90094770 | ebi-cfmet338-GCST90026179 | Histidine levels \|\| id:ebi-cfmet338-GCST90026179 | Gherkins liking \|\| id:ebi-fl187-GCST90094770 | Weighted median | 18 | -0.023292852 | 0.039069650026995 | 0.55104971183907 | -0.099869366 | 0.0532836616715934 | 0.976976332029887 | 0.904955627895242 | 1.05472878882086 | 0.999332001504091 |
| ebi-fl187-GCST90094770 | ebi-cfmet338-GCST90026188 | Isocitrate levels \|\| id:ebi-cfmet338-GCST90026188 | Gherkins liking \|\| id:ebi-fl187-GCST90094770 | Weighted median | 18 | -0.078567619 | 0.0690421063034343 | 0.255134381471782 | -0.213890147 | 0.0567549094807723 | 0.924439548256796 | 0.807437079351759 | 1.05839637568704 | 0.999332001504091 |
| ebi-fl187-GCST90094770 | ebi-cfmet338-GCST90026207 | N-acetyl-isoputreanine levels \|\| id:ebi-cfmet338-GCST90026207 | Gherkins liking \|\| id:ebi-fl187-GCST90094770 | Weighted median | 18 | 0.020358580691847 | 0.044974243639228 | 0.650784946619103 | -0.067790937 | 0.108508098224734 | 1.02056723012573 | 0.934455813441584 | 1.11461393489593 | 0.999332001504091 |
| ebi-fl187-GCST90094770 | ebi-cfmet338-GCST90026213 | N-acetylglutamate levels \|\| id:ebi-cfmet338-GCST90026213 | Gherkins liking \|\| id:ebi-fl187-GCST90094770 | Weighted median | 18 | -0.061368917 | 0.0736330681150927 | 0.404595299243023 | -0.205689731 | 0.0829518962707061 | 0.940476217882999 | 0.814085622845582 | 1.08648954309231 | 0.999332001504091 |
| ebi-fl187-GCST90094770 | ebi-cfmet338-GCST90026224 | N-acetyltaurine levels \|\| id:ebi-cfmet338-GCST90026224 | Gherkins liking \|\| id:ebi-fl187-GCST90094770 | Weighted median | 18 | 0.0102791371086776 | 0.0628364801964181 | 0.870057459278959 | -0.112880364 | 0.133438638293657 | 1.01033214892139 | 0.893257519412218 | 1.14275114282364 | 0.999332001504091 |
| ebi-fl187-GCST90094770 | ebi-cfmet338-GCST90026250 | Phenyllactate (pla) levels \|\| id:ebi-cfmet338-GCST90026250 | Gherkins liking \|\| id:ebi-fl187-GCST90094770 | Weighted median | 18 | 0.0930182926710582 | 0.0936716450969655 | 0.320697733430923 | -0.090578132 | 0.276614717061111 | 1.09748181095822 | 0.913402965369098 | 1.31865821663654 | 0.999332001504091 |
| ebi-fl187-GCST90094770 | ebi-cfmet338-GCST90026251 | Pipecolate levels \|\| id:ebi-cfmet338-GCST90026251 | Gherkins liking \|\| id:ebi-fl187-GCST90094770 | Weighted median | 18 | -0.180380501 | 0.157054138155742 | 0.250752168908448 | -0.488206612 | 0.127445609664694 | 0.834952450617863 | 0.613726056832149 | 1.13592308332354 | 0.999332001504091 |
| ebi-fl187-GCST90094770 | ebi-cfmet338-GCST90026278 | Trigonelline (n'-methylnicotinate) levels \|\| id:ebi-cfmet338-GCST90026278 | Gherkins liking \|\| id:ebi-fl187-GCST90094770 | Weighted median | 17 | 0.0325100598477094 | 0.162537846850796 | 0.841468597678219 | -0.28606412 | 0.351084239675269 | 1.03304428535989 | 0.751214446547416 | 1.42060699234351 | 0.999332001504091 |
| ebi-fl187-GCST90094770 | ebi-cfmet338-GCST90026299 | 1-carboxyethylvaline levels \|\| id:ebi-cfmet338-GCST90026299 | Gherkins liking \|\| id:ebi-fl187-GCST90094770 | Weighted median | 18 | 0.0707716075992261 | 0.106566281444665 | 0.506620664412471 | -0.138098304 | 0.279641519230769 | 1.07333605607328 | 0.871013063440726 | 1.32265558075105 | 0.999332001504091 |
| ebi-fl187-GCST90094770 | ebi-gm430-GCST90011306 | C_Erysipelotrichia abundance \|\| id:ebi-gm430-GCST90011306 | Gherkins liking \|\| id:ebi-fl187-GCST90094770 | Weighted median | 15 | -0.009683483 | 0.0916012456725332 | 0.915809711541456 | -0.189221925 | 0.16985495815406 | 0.990363250590121 | 0.827602820654697 | 1.18513294498384 | 0.999332001504091 |
| ebi-fl187-GCST90094770 | ebi-gm430-GCST90011312 | F_Erysipelotrichaceae abundance \|\| id:ebi-gm430-GCST90011312 | Gherkins liking \|\| id:ebi-fl187-GCST90094770 | Weighted median | 15 | -0.009683483 | 0.0924213989170352 | 0.916554079277118 | -0.190829425 | 0.171462458513284 | 0.990363250590121 | 0.826273517537068 | 1.18703957866522 | 0.999332001504091 |
| ebi-fl187-GCST90094770 | ebi-gm430-GCST90011352 | O_Erysipelotrichales abundance \|\| id:ebi-gm430-GCST90011352 | Gherkins liking \|\| id:ebi-fl187-GCST90094770 | Weighted median | 15 | -0.009683483 | 0.0892057882960185 | 0.913557686235804 | -0.184526828 | 0.165159861696092 | 0.990363250590121 | 0.831497631828888 | 1.17958167356665 | 0.999332001504091 |
| ebi-fl187-GCST90094770 | ebi-gm430-GCST90011362 | OTU97_11 (Parabacteroides) abundance \|\| id:ebi-gm430-GCST90011362 | Gherkins liking \|\| id:ebi-fl187-GCST90094770 | Weighted median | 15 | -0.137730284 | 0.117863900450395 | 0.24258353722085 | -0.368743529 | 0.0932829612370926 | 0.871333672996906 | 0.691602764084891 | 1.09777231833777 | 0.999332001504091 |
| ebi-fl187-GCST90094770 | ebi-gm430-GCST90011384 | OTU97_235 (Phascolarctobacterium) abundance \|\| id:ebi-gm430-GCST90011384 | Gherkins liking \|\| id:ebi-fl187-GCST90094770 | Weighted median | 15 | -0.141338095 | 0.351703925331996 | 0.687782132222778 | -0.830677789 | 0.548001598378645 | 0.868195729199767 | 0.435753837082903 | 1.72979274088942 | 0.999332001504091 |
| ebi-fl187-GCST90094770 | ebi-gm430-GCST90011390 | OTU97_3 (Bacteroides) abundance \|\| id:ebi-gm430-GCST90011390 | Gherkins liking \|\| id:ebi-fl187-GCST90094770 | Weighted median | 15 | -0.076102191 | 0.0867445490138543 | 0.380316209480137 | -0.246121507 | 0.0939171246716257 | 0.926721498757079 | 0.781827221311852 | 1.09846870619002 | 0.999332001504091 |
| ebi-fl187-GCST90094770 | ebi-gm430-GCST90011398 | OTU97_4 (Alistipes) abundance \|\| id:ebi-gm430-GCST90011398 | Gherkins liking \|\| id:ebi-fl187-GCST90094770 | Weighted median | 15 | -0.107630333 | 0.105613243812589 | 0.308156048469499 | -0.314632291 | 0.0993716244240646 | 0.897959480667911 | 0.730057273315061 | 1.10447667380941 | 0.999332001504091 |
| ebi-fl187-GCST90094770 | ebi-gm430-GCST90011421 | OTU99_108 (Cytophagales) abundance \|\| id:ebi-gm430-GCST90011421 | Gherkins liking \|\| id:ebi-fl187-GCST90094770 | Weighted median | 15 | 0.0653411292295376 | 0.322437524329165 | 0.839410630821502 | -0.566636418 | 0.697318676914702 | 1.06752312561923 | 0.567430832304986 | 2.00836041831321 | 0.999332001504091 |
| ebi-fl187-GCST90094770 | ebi-gm430-GCST90011428 | OTU99_123 (Phascolarctobacterium) abundance \|\| id:ebi-gm430-GCST90011428 | Gherkins liking \|\| id:ebi-fl187-GCST90094770 | Weighted median | 15 | -0.049755102 | 0.20142360433239 | 0.804894884599901 | -0.444545367 | 0.345035162396081 | 0.951462407120878 | 0.641115682396653 | 1.41203956948937 | 0.999332001504091 |
| ebi-fl187-GCST90094770 | ebi-gm430-GCST90011450 | OTU99_298 (Phascolarctobacterium) abundance \|\| id:ebi-gm430-GCST90011450 | Gherkins liking \|\| id:ebi-fl187-GCST90094770 | Weighted median | 15 | -0.140211075 | 0.333086966583618 | 0.673795722887727 | -0.793061529 | 0.512639379643427 | 0.869174755095296 | 0.452457460880862 | 1.66969233621256 | 0.999332001504091 |
| ebi-fl187-GCST90094770 | ebi-gm430-GCST90011451 | OTU99_3 (Bacteroides) abundance \|\| id:ebi-gm430-GCST90011451 | Gherkins liking \|\| id:ebi-fl187-GCST90094770 | Weighted median | 15 | -0.071020292 | 0.0869863623431667 | 0.414240991830156 | -0.241513562 | 0.0994729781071228 | 0.931442991015024 | 0.785438151386118 | 1.10458862226125 | 0.999332001504091 |
| ebi-fl187-GCST90094770 | ebi-gm430-GCST90011472 | OTU99_72 (Parabacteroides) abundance \|\| id:ebi-gm430-GCST90011472 | Gherkins liking \|\| id:ebi-fl187-GCST90094770 | Weighted median | 15 | 0.354819932353351 | 0.276940212468181 | 0.200118186711245 | -0.187982884 | 0.897622748790985 | 1.42592386840747 | 0.828628889853546 | 2.45376296118942 | 0.999332001504091 |
| ebi-fl187-GCST90094770 | ebi-gm430-GCST90011507 | TestASV_3 (Bacteroides) abundance \|\| id:ebi-gm430-GCST90011507 | Gherkins liking \|\| id:ebi-fl187-GCST90094770 | Weighted median | 15 | 0.0386541316037068 | 0.115006614827746 | 0.736792780915692 | -0.186758833 | 0.264067096666088 | 1.03941092208454 | 0.829643794585234 | 1.30221556769282 | 0.999332001504091 |
| ebi-fl187-GCST90094770 | ebi-gm430-GCST90011512 | TestASV_34 (Phascolarctobacterium) abundance \|\| id:ebi-gm430-GCST90011512 | Gherkins liking \|\| id:ebi-fl187-GCST90094770 | Weighted median | 15 | -0.448537911 | 0.299848793167385 | 0.134684843418233 | -1.036241546 | 0.139165723403249 | 0.638561102460034 | 0.354785624765182 | 1.149314553 | 0.999332001504091 |
| ebi-fl187-GCST90094770 | ebi-gm430-GCST90011519 | TestASV_41 (Barnesiella) abundance \|\| id:ebi-gm430-GCST90011519 | Gherkins liking \|\| id:ebi-fl187-GCST90094770 | Weighted median | 15 | -0.057756904 | 0.391620897368044 | 0.882751846874302 | -0.825333863 | 0.709820054957148 | 0.943879372958774 | 0.438088706556982 | 2.03362528493112 | 0.999332001504091 |
| ebi-fl187-GCST90094770 | ebi-gm430-GCST90011524 | TestASV_46 (Sutterella) abundance \|\| id:ebi-gm430-GCST90011524 | Gherkins liking \|\| id:ebi-fl187-GCST90094770 | Weighted median | 15 | -0.717376618 | 0.378388467235069 | 0.0579767911739595 | -1.459018014 | 0.0242647776165473 | 0.488030869399135 | 0.232464439522014 | 1.02456156294787 | 0.999332001504091 |
| ebi-fl187-GCST90094770 | ebi-gm430-GCST90011581 | OTU97_19 (Bacteria) prevalence \|\| id:ebi-gm430-GCST90011581 | Gherkins liking \|\| id:ebi-fl187-GCST90094770 | Weighted median | 15 | -0.30108457 | 0.456286904109799 | 0.509344950582791 | -1.195406902 | 0.593237762146507 | 0.740015187083505 | 0.302580808404533 | 1.80983876671417 | 0.999332001504091 |
| ebi-fl187-GCST90094770 | ebi-gm430-GCST90011614 | OTU97_80 (Ruminococcus) prevalence \|\| id:ebi-gm430-GCST90011614 | Gherkins liking \|\| id:ebi-fl187-GCST90094770 | Weighted median | 15 | 0.0452872258411136 | 0.220391823938778 | 0.837193174143846 | -0.386680749 | 0.477255200761118 | 1.0463283492927 | 0.679307929994765 | 1.61164468452771 | 0.999332001504091 |
| ebi-fl187-GCST90094770 | ebi-gm430-GCST90011623 | OTU99_11 (Parabacteroides) prevalence \|\| id:ebi-gm430-GCST90011623 | Gherkins liking \|\| id:ebi-fl187-GCST90094770 | Weighted median | 15 | 0.140604576669086 | 0.176347410873204 | 0.425267647407861 | -0.205036349 | 0.486245501980567 | 1.15096943782143 | 0.814617705625592 | 1.6261991823289 | 0.999332001504091 |
| ebi-fl187-GCST90094770 | ebi-gm430-GCST90011642 | OTU99_19 (Bacteria) prevalence \|\| id:ebi-gm430-GCST90011642 | Gherkins liking \|\| id:ebi-fl187-GCST90094770 | Weighted median | 15 | -0.30108457 | 0.43927962959021 | 0.493088224070526 | -1.162072644 | 0.559903504088112 | 0.740015187083505 | 0.312837108540614 | 1.75050357570717 | 0.999332001504091 |
| ebi-fl187-GCST90094770 | ebi-gm430-GCST90011653 | OTU99_30 (Parasutterella) prevalence \|\| id:ebi-gm430-GCST90011653 | Gherkins liking \|\| id:ebi-fl187-GCST90094770 | Weighted median | 15 | -0.193913407 | 0.198362611877521 | 0.328286869853171 | -0.582704126 | 0.194877312186644 | 0.823729230288756 | 0.558386375854363 | 1.21516189178849 | 0.999332001504091 |
| ebi-fl187-GCST90094770 | ebi-gm430-GCST90011675 | OTU99_8 (Ruminococcaceae) prevalence \|\| id:ebi-gm430-GCST90011675 | Gherkins liking \|\| id:ebi-fl187-GCST90094770 | Weighted median | 15 | 0.2839135648366 | 0.207595045639333 | 0.171427389187021 | -0.122972725 | 0.690799854289693 | 1.3283181123976 | 0.884287781520264 | 1.99531085309143 | 0.999332001504091 |
| ebi-fl187-GCST90094770 | ebi-gm430-GCST90011679 | OTU99_92 (Ruminococcus) prevalence \|\| id:ebi-gm430-GCST90011679 | Gherkins liking \|\| id:ebi-fl187-GCST90094770 | Weighted median | 15 | -0.006942718 | 0.223183070017243 | 0.975183627003783 | -0.444381535 | 0.430496099110269 | 0.993081326865903 | 0.64122072577165 | 1.53802034483981 | 0.999332001504091 |
| ebi-fl187-GCST90094770 | ebi-gm430-GCST90011684 | TestASV_11 (Lachnospiraceae) prevalence \|\| id:ebi-gm430-GCST90011684 | Gherkins liking \|\| id:ebi-fl187-GCST90094770 | Weighted median | 15 | 0.0910379892895436 | 0.210786352997351 | 0.66581628979653 | -0.322103263 | 0.504179241164352 | 1.09531061454018 | 0.724623359984886 | 1.65562609291179 | 0.999332001504091 |
| ebi-fl187-GCST90094770 | ebi-gm430-GCST90011688 | TestASV_15 (Bacteroides) prevalence \|\| id:ebi-gm430-GCST90011688 | Gherkins liking \|\| id:ebi-fl187-GCST90094770 | Weighted median | 15 | -0.108061569 | 0.20923844424089 | 0.60553890386931 | -0.518168919 | 0.302045782203597 | 0.897572332539367 | 0.59561016039823 | 1.35262315136045 | 0.999332001504091 |
| ebi-fl187-GCST90094770 | ebi-gm430-GCST90011692 | TestASV_19 (Prevotella) prevalence \|\| id:ebi-gm430-GCST90011692 | Gherkins liking \|\| id:ebi-fl187-GCST90094770 | Weighted median | 15 | 0.0304222778392924 | 0.379016196148998 | 0.936025355209943 | -0.712449467 | 0.773294022291329 | 1.03088976395513 | 0.490441405115167 | 2.16689230220664 | 0.999332001504091 |
| ebi-fl187-GCST90094770 | ebi-gm430-GCST90011693 | TestASV_2 (Bacteroides) prevalence \|\| id:ebi-gm430-GCST90011693 | Gherkins liking \|\| id:ebi-fl187-GCST90094770 | Weighted median | 15 | -0.247556575 | 0.191280128561568 | 0.195593037720328 | -0.622465627 | 0.127352476647446 | 0.780706050856493 | 0.536619700920569 | 1.13581729630564 | 0.999332001504091 |
| ebi-fl187-GCST90094770 | ebi-gm430-GCST90011716 | TestASV_40 (Barnesiella) prevalence \|\| id:ebi-gm430-GCST90011716 | Gherkins liking \|\| id:ebi-fl187-GCST90094770 | Weighted median | 15 | 0.614040928870541 | 0.429594918106696 | 0.152904041083211 | -0.227965111 | 1.45604696835967 | 1.84788349771558 | 0.796152036602964 | 4.28897153325056 | 0.999332001504091 |
| ebi-fl187-GCST90094770 | ebi-gm430-GCST90011718 | TestASV_42 (Alphaproteobacteria) prevalence \|\| id:ebi-gm430-GCST90011718 | Gherkins liking \|\| id:ebi-fl187-GCST90094770 | Weighted median | 15 | -0.287611287 | 0.322831083765256 | 0.372981034816887 | -0.920360211 | 0.345137637306666 | 0.750053091062927 | 0.398375515972247 | 1.41218427553224 | 0.999332001504091 |
| ebi-fl187-GCST90094770 | ebi-hbsm44-GCST90104929 | Sulcal depth of the right inferior frontal sulcus \|\| id:ebi-hbsm44-GCST90104929 | Gherkins liking \|\| id:ebi-fl187-GCST90094770 | Weighted median | 17 | 0.274227394037171 | 0.157936512273341 | 0.0825083104725006 | -0.03532817 | 0.583782958092919 | 1.31551390824853 | 0.965288585500196 | 1.79280773521066 | 0.999332001504091 |
| ebi-fl187-GCST90094770 | ebi-hbsm44-GCST90104935 | Sulcal opening distance of the left calloso-marginal posterior fissure \|\| id:ebi-hbsm44-GCST90104935 | Gherkins liking \|\| id:ebi-fl187-GCST90094770 | Weighted median | 17 | -0.007319307 | 0.0350329306390348 | 0.834505631553961 | -0.075983851 | 0.0613452371389604 | 0.992707413980746 | 0.926831173866615 | 1.06326592971738 | 0.999332001504091 |
| ebi-fl187-GCST90094770 | ebi-hbsm44-GCST90104936 | Sulcal opening distance of the left anterior inferior temporal sulcus \|\| id:ebi-hbsm44-GCST90104936 | Gherkins liking \|\| id:ebi-fl187-GCST90094770 | Weighted median | 17 | -0.02225931 | 0.0205083470731964 | 0.277754252381344 | -0.06245567 | 0.0179370506177736 | 0.977986600808782 | 0.939454708002951 | 1.01809888567668 | 0.999332001504091 |
| ebi-fl187-GCST90094770 | ebi-hbsm44-GCST90104941 | Sulcal opening distance of the right posterior inferior temporal sulcus \|\| id:ebi-hbsm44-GCST90104941 | Gherkins liking \|\| id:ebi-fl187-GCST90094770 | Weighted median | 16 | 0.014901785561368 | 0.016323233738252 | 0.361285294074911 | -0.017091753 | 0.046895323688342 | 1.0150133707517 | 0.983053482817672 | 1.04801230127558 | 0.999332001504091 |
| ebi-fl187-GCST90094770 | ebi-IPs91-GCST90274819 | Leukemia inhibitory factor levels \|\| id:ebi-IPs91-GCST90274819 | Gherkins liking \|\| id:ebi-fl187-GCST90094770 | Weighted median | 22 | -0.033090101 | 0.0649369331903683 | 0.610350717037628 | -0.16036649 | 0.0941862879995773 | 0.967451387272686 | 0.851831543918953 | 1.09876441347762 | 0.999332001504091 |
| ebi-fl187-GCST90094770 | ebi-IPs91-GCST90274837 | STAM binding protein levels \|\| id:ebi-IPs91-GCST90274837 | Gherkins liking \|\| id:ebi-fl187-GCST90094770 | Weighted median | 22 | -0.024862951 | 0.0576058818080974 | 0.666028971230035 | -0.13777048 | 0.0880445771812207 | 0.975443586277573 | 0.871298649693742 | 1.09203680086558 | 0.999332001504091 |
| ebi-fl187-GCST90094770 | ebi-IPs91-GCST90274840 | TNF-beta levels \|\| id:ebi-IPs91-GCST90274840 | Gherkins liking \|\| id:ebi-fl187-GCST90094770 | Weighted median | 22 | 0.0266937015735978 | 0.0746294589054036 | 0.720580428 | -0.119580038 | 0.172967441028189 | 1.02705316981042 | 0.887292987926374 | 1.18882739745617 | 0.999332001504091 |
| ebi-fl187-GCST90094770 | ebi-IPs91-GCST90274842 | Tumor necrosis factor ligand superfamily member 14 levels \|\| id:ebi-IPs91-GCST90274842 | Gherkins liking \|\| id:ebi-fl187-GCST90094770 | Weighted median | 22 | 0.0310375781921577 | 0.0656639111880517 | 0.636446271765279 | -0.097663688 | 0.159738844120739 | 1.0315242659753 | 0.906953872178448 | 1.17320444174312 | 0.999332001504091 |
| ebi-fl187-GCST90094770 | ebi-met1400-GCST90199628 | 3-methylhistidine levels \|\| id:ebi-met1400-GCST90199628 | Gherkins liking \|\| id:ebi-fl187-GCST90094770 | Weighted median | 20 | -0.074339351 | 0.0756330110811932 | 0.325658842103411 | -0.222580053 | 0.0739013507564304 | 0.928356601674381 | 0.800450925942034 | 1.07670058455878 | 0.999332001504091 |
| ebi-fl187-GCST90094770 | ebi-met1400-GCST90199651 | Docosahexaenoate DHA; 22:6n3 levels \|\| id:ebi-met1400-GCST90199651 | Gherkins liking \|\| id:ebi-fl187-GCST90094770 | Weighted median | 20 | 0.0588799272816815 | 0.0763896804813606 | 0.440835098212873 | -0.090843846 | 0.208603701025148 | 1.06064787822001 | 0.913160292977334 | 1.23195667860751 | 0.999332001504091 |
| ebi-fl187-GCST90094770 | ebi-met1400-GCST90199652 | 1-stearoyl-2-oleoyl-GPS (18:0/18:1) levels \|\| id:ebi-met1400-GCST90199652 | Gherkins liking \|\| id:ebi-fl187-GCST90094770 | Weighted median | 20 | 0.0722124065026877 | 0.0789867819435102 | 0.360593869624956 | -0.082601686 | 0.227026499111968 | 1.07488363209111 | 0.920717808887247 | 1.25486312025803 | 0.999332001504091 |
| ebi-fl187-GCST90094770 | ebi-met1400-GCST90199653 | 1-myristoyl-2-palmitoyl-gpc (14:0/16:0) levels \|\| id:ebi-met1400-GCST90199653 | Gherkins liking \|\| id:ebi-fl187-GCST90094770 | Weighted median | 19 | -0.049234623 | 0.0769952281501607 | 0.52253002336981 | -0.20014527 | 0.101676023976346 | 0.951957752122614 | 0.818611824395298 | 1.10702476414357 | 0.999332001504091 |
| ebi-fl187-GCST90094770 | ebi-met1400-GCST90199667 | Erythritol levels in elite athletes \|\| id:ebi-met1400-GCST90199667 | Gherkins liking \|\| id:ebi-fl187-GCST90094770 | Weighted median | 20 | 0.0387076802899944 | 0.0765945350107342 | 0.613307235854598 | -0.111417608 | 0.188832968911033 | 1.03946658266419 | 0.894565093078032 | 1.20783918893795 | 0.999332001504091 |
| ebi-fl187-GCST90094770 | ebi-met1400-GCST90199674 | 3-hydroxymyristate levels \|\| id:ebi-met1400-GCST90199674 | Gherkins liking \|\| id:ebi-fl187-GCST90094770 | Weighted median | 20 | 0.0267978048006286 | 0.0790102842987746 | 0.734482748192256 | -0.128062352 | 0.181657962026227 | 1.02716009492525 | 0.879798519863159 | 1.1992039504351 | 0.999332001504091 |
| ebi-fl187-GCST90094770 | ebi-met1400-GCST90199685 | 2-linoleoylglycerol (18:2) levels \|\| id:ebi-met1400-GCST90199685 | Gherkins liking \|\| id:ebi-fl187-GCST90094770 | Weighted median | 20 | -0.037772488 | 0.0888407996477853 | 0.670712379608357 | -0.211900455 | 0.136355479139178 | 0.962931994391369 | 0.809045229577096 | 1.14608923200403 | 0.999332001504091 |
| ebi-fl187-GCST90094770 | ebi-met1400-GCST90199703 | Gamma-glutamylvaline levels \|\| id:ebi-met1400-GCST90199703 | Gherkins liking \|\| id:ebi-fl187-GCST90094770 | Weighted median | 20 | -0.002262224 | 0.0777969719673955 | 0.976801936803627 | -0.154744289 | 0.150219841294581 | 0.997740333138202 | 0.856634200423379 | 1.16208968995019 | 0.999332001504091 |
| ebi-fl187-GCST90094770 | ebi-met1400-GCST90199779 | Gamma-glutamyl-2-aminobutyrate levels \|\| id:ebi-met1400-GCST90199779 | Gherkins liking \|\| id:ebi-fl187-GCST90094770 | Weighted median | 20 | 0.0849602112696719 | 0.0765221585469545 | 0.26688295378554 | -0.065023219 | 0.234943642021703 | 1.08867374889041 | 0.937045705408574 | 1.26483748304073 | 0.999332001504091 |
| ebi-fl187-GCST90094770 | ebi-met1400-GCST90199824 | 4-vinylphenol sulfate levels \|\| id:ebi-met1400-GCST90199824 | Gherkins liking \|\| id:ebi-fl187-GCST90094770 | Weighted median | 20 | -0.130197578 | 0.0800910931839582 | 0.104030797483787 | -0.28717612 | 0.0267809649295273 | 0.877921955973209 | 0.750379560086659 | 1.0271427978273 | 0.999332001504091 |
| ebi-fl187-GCST90094770 | ebi-met1400-GCST90199830 | 4-ethylphenylsulfate levels \|\| id:ebi-met1400-GCST90199830 | Gherkins liking \|\| id:ebi-fl187-GCST90094770 | Weighted median | 20 | 0.0958684788842284 | 0.0779033285893213 | 0.218469418066597 | -0.056822045 | 0.248559002919298 | 1.10061430045363 | 0.94476217941921 | 1.28217647229243 | 0.999332001504091 |
| ebi-fl187-GCST90094770 | ebi-met1400-GCST90199833 | Hexanoylglutamine levels \|\| id:ebi-met1400-GCST90199833 | Gherkins liking \|\| id:ebi-fl187-GCST90094770 | Weighted median | 20 | 0.0531665134520546 | 0.0763826107666706 | 0.48639426088098 | -0.096543404 | 0.202876430554729 | 1.05460523645827 | 0.907970487510672 | 1.22492109607486 | 0.999332001504091 |
| ebi-fl187-GCST90094770 | ebi-met1400-GCST90199837 | Carotenoid levels (cryptoxanthin) \|\| id:ebi-met1400-GCST90199837 | Gherkins liking \|\| id:ebi-fl187-GCST90094770 | Weighted median | 20 | -0.032458906 | 0.0751784535142483 | 0.665917237398782 | -0.179808675 | 0.114890862846949 | 0.968062230523559 | 0.83543003483248 | 1.12175100617991 | 0.999332001504091 |
| ebi-fl187-GCST90094770 | ebi-met1400-GCST90199869 | 2-hydroxyhippurate levels \|\| id:ebi-met1400-GCST90199869 | Gherkins liking \|\| id:ebi-fl187-GCST90094770 | Weighted median | 20 | 0.0948247541755015 | 0.0817969581916518 | 0.246346173814345 | -0.065497284 | 0.255146792231139 | 1.09946616138825 | 0.936601590678358 | 1.2906510645175 | 0.999332001504091 |
| ebi-fl187-GCST90094770 | ebi-met1400-GCST90199902 | Hydantoin-5-propionate levels \|\| id:ebi-met1400-GCST90199902 | Gherkins liking \|\| id:ebi-fl187-GCST90094770 | Weighted median | 20 | 0.0808739553035455 | 0.07815511838168 | 0.300768067729593 | -0.072310077 | 0.234057987331638 | 1.08423422597974 | 0.930242404568464 | 1.26371776970458 | 0.999332001504091 |
| ebi-fl187-GCST90094770 | ebi-met1400-GCST90199904 | Pantoate levels \|\| id:ebi-met1400-GCST90199904 | Gherkins liking \|\| id:ebi-fl187-GCST90094770 | Weighted median | 20 | -0.018983306 | 0.0809845081168039 | 0.814669311123225 | -0.177712942 | 0.139746330185362 | 0.98119574245903 | 0.837182709300915 | 1.14998204612188 | 0.999332001504091 |
| ebi-fl187-GCST90094770 | ebi-met1400-GCST90199936 | 3-methyl catechol sulfate (2) levels \|\| id:ebi-met1400-GCST90199936 | Gherkins liking \|\| id:ebi-fl187-GCST90094770 | Weighted median | 20 | -0.083819086 | 0.0893904649219896 | 0.348412132637946 | -0.259024398 | 0.0913862248380068 | 0.919597608752532 | 0.771804192600656 | 1.09569210705355 | 0.999332001504091 |
| ebi-fl187-GCST90094770 | ebi-met1400-GCST90199977 | 4-hydroxychlorothalonil levels \|\| id:ebi-met1400-GCST90199977 | Gherkins liking \|\| id:ebi-fl187-GCST90094770 | Weighted median | 20 | -0.009733636 | 0.0771857378247954 | 0.899647495960576 | -0.161017682 | 0.141550410570734 | 0.990313582938042 | 0.851277018947114 | 1.15205857872749 | 0.999332001504091 |
| ebi-fl187-GCST90094770 | ebi-met1400-GCST90200040 | Sphingomyelin (d18:2/24:1, d18:1/24:2) levels \|\| id:ebi-met1400-GCST90200040 | Gherkins liking \|\| id:ebi-fl187-GCST90094770 | Weighted median | 20 | 0.102075155820047 | 0.0718351551738864 | 0.155327280560942 | -0.038721748 | 0.242872059960864 | 1.10746670116841 | 0.962018355130932 | 1.27490550222392 | 0.999332001504091 |
| ebi-fl187-GCST90094770 | ebi-met1400-GCST90200077 | 1-stearoyl-2-oleoyl-GPI (18:0/18:1) levels \|\| id:ebi-met1400-GCST90200077 | Gherkins liking \|\| id:ebi-fl187-GCST90094770 | Weighted median | 20 | -0.002083035 | 0.0794003753822472 | 0.979070236490473 | -0.157707771 | 0.153541700445761 | 0.997919132708984 | 0.854099338053443 | 1.16595640701028 | 0.999332001504091 |
| ebi-fl187-GCST90094770 | ebi-met1400-GCST90200080 | 1-palmitoyl-2-oleoyl-GPI (16:0/18:1) levels \|\| id:ebi-met1400-GCST90200080 | Gherkins liking \|\| id:ebi-fl187-GCST90094770 | Weighted median | 20 | 0.00146693882154883 | 0.074585351872828 | 0.984308281020537 | -0.144720351 | 0.147654228492292 | 1.00146801530261 | 0.86526422968754 | 1.15911203914709 | 0.999332001504091 |
| ebi-fl187-GCST90094770 | ebi-met1400-GCST90200082 | 1-oleoyl-2-linoleoyl-GPE (18:1/18:2) levels \|\| id:ebi-met1400-GCST90200082 | Gherkins liking \|\| id:ebi-fl187-GCST90094770 | Weighted median | 20 | 0.0308242819035433 | 0.0839256255997939 | 0.713409476546844 | -0.133669944 | 0.195318508079139 | 1.03130426914084 | 0.874878775706518 | 1.21569813450921 | 0.999332001504091 |
| ebi-fl187-GCST90094770 | ebi-met1400-GCST90200100 | Perfluorooctanesulfonate (PFOS) levels \|\| id:ebi-met1400-GCST90200100 | Gherkins liking \|\| id:ebi-fl187-GCST90094770 | Weighted median | 20 | 0.00821449874900714 | 0.0726867822958078 | 0.910020822217507 | -0.134251595 | 0.15068059204879 | 1.00824833031687 | 0.874370050187292 | 1.16262524702098 | 0.999332001504091 |
| ebi-fl187-GCST90094770 | ebi-met1400-GCST90200102 | 1-palmitoleoyl-2-linolenoyl-GPC (16:1/18:3) levels \|\| id:ebi-met1400-GCST90200102 | Gherkins liking \|\| id:ebi-fl187-GCST90094770 | Weighted median | 20 | 0.0324425344123072 | 0.0758392565966986 | 0.668811133902775 | -0.116202409 | 0.181087477341837 | 1.03297453094985 | 0.890295001767704 | 1.19852001805294 | 0.999332001504091 |
| ebi-fl187-GCST90094770 | ebi-met1400-GCST90200114 | Glycosyl-N-tricosanoyl-sphingadienine (d18:2/23:0) levels \|\| id:ebi-met1400-GCST90200114 | Gherkins liking \|\| id:ebi-fl187-GCST90094770 | Weighted median | 20 | 0.027042212802706 | 0.0851317761864172 | 0.750749514742942 | -0.139816069 | 0.193900494128084 | 1.02741117175321 | 0.869518152449809 | 1.21397547925745 | 0.999332001504091 |
| ebi-fl187-GCST90094770 | ebi-met1400-GCST90200135 | Arachidoylcarnitine (C20) levels \|\| id:ebi-met1400-GCST90200135 | Gherkins liking \|\| id:ebi-fl187-GCST90094770 | Weighted median | 20 | -0.06077937 | 0.0797097644171538 | 0.445756591296474 | -0.217010509 | 0.0954517679357627 | 0.941030836204714 | 0.804921510678887 | 1.10015575797106 | 0.999332001504091 |
| ebi-fl187-GCST90094770 | ebi-met1400-GCST90200146 | Docosahexaenoylcarnitine (C22:6) levels \|\| id:ebi-met1400-GCST90200146 | Gherkins liking \|\| id:ebi-fl187-GCST90094770 | Weighted median | 20 | -0.020102155 | 0.0968064560501646 | 0.835499886065298 | -0.209842809 | 0.169638498853972 | 0.980098546221348 | 0.81071167264462 | 1.18487643969853 | 0.999332001504091 |
| ebi-fl187-GCST90094770 | ebi-met1400-GCST90200162 | 3-hydroxyphenylacetoylglutamine levels \|\| id:ebi-met1400-GCST90200162 | Gherkins liking \|\| id:ebi-fl187-GCST90094770 | Weighted median | 20 | 0.0435012455203773 | 0.0901537065153602 | 0.629434395989221 | -0.133200019 | 0.220202510290483 | 1.04446129520904 | 0.875289999749389 | 1.24632909950085 | 0.999332001504091 |
| ebi-fl187-GCST90094770 | ebi-met1400-GCST90200172 | 3-carboxy-4-methyl-5-pentyl-2-furanpropionate (3-CMPFP) levels \|\| id:ebi-met1400-GCST90200172 | Gherkins liking \|\| id:ebi-fl187-GCST90094770 | Weighted median | 20 | 0.0648539414415768 | 0.0776895548040297 | 0.403839741104533 | -0.087417586 | 0.217125468857475 | 1.06700316805784 | 0.916294384048091 | 1.24249998741202 | 0.999332001504091 |
| ebi-fl187-GCST90094770 | ebi-met1400-GCST90200222 | Dihydrocaffeate sulfate (2) levels \|\| id:ebi-met1400-GCST90200222 | Gherkins liking \|\| id:ebi-fl187-GCST90094770 | Weighted median | 20 | 0.041331762339337 | 0.0870532022028404 | 0.634938524514751 | -0.129292514 | 0.211956038656904 | 1.04219781017998 | 0.878716890974947 | 1.23609354355168 | 0.999332001504091 |
| ebi-fl187-GCST90094770 | ebi-met1400-GCST90200223 | 4-methylhexanoylglutamine levels \|\| id:ebi-met1400-GCST90200223 | Gherkins liking \|\| id:ebi-fl187-GCST90094770 | Weighted median | 20 | 0.0657759131255109 | 0.0936120013781329 | 0.482277621132777 | -0.11770361 | 0.249255435826651 | 1.06798736839816 | 0.888959492653412 | 1.2830697331928 | 0.999332001504091 |
| ebi-fl187-GCST90094770 | ebi-met1400-GCST90200224 | 2-ketocaprylate levels \|\| id:ebi-met1400-GCST90200224 | Gherkins liking \|\| id:ebi-fl187-GCST90094770 | Weighted median | 20 | 0.00022702247999664 | 0.0743160033453093 | 0.997562605066707 | -0.145432344 | 0.145886389036803 | 1.00022704825155 | 0.864648386679946 | 1.15706472534521 | 0.999364451047625 |
| ebi-fl187-GCST90094770 | ebi-met1400-GCST90200243 | Hydroxypalmitoyl sphingomyelin (d18:1/16:0(OH)) levels \|\| id:ebi-met1400-GCST90200243 | Gherkins liking \|\| id:ebi-fl187-GCST90094770 | Weighted median | 20 | 0.00649844545035865 | 0.0806694031741297 | 0.935794655050376 | -0.151613585 | 0.164610475671653 | 1.0065196061594 | 0.859320271033311 | 1.17893380586154 | 0.999332001504091 |
| ebi-fl187-GCST90094770 | ebi-met1400-GCST90200273 | 2-methoxyhydroquinone sulfate (2) levels \|\| id:ebi-met1400-GCST90200273 | Gherkins liking \|\| id:ebi-fl187-GCST90094770 | Weighted median | 20 | 0.0228142280739231 | 0.0906097541268126 | 0.801206815948684 | -0.15478089 | 0.200409346162476 | 1.02307646300726 | 0.856602847160022 | 1.22190283703776 | 0.999332001504091 |
| ebi-fl187-GCST90094770 | ebi-met1400-GCST90200276 | 2,4-di-tert-butylphenol levels \|\| id:ebi-met1400-GCST90200276 | Gherkins liking \|\| id:ebi-fl187-GCST90094770 | Weighted median | 20 | -0.091721591 | 0.077310772707485 | 0.235463909333724 | -0.243250706 | 0.0598075232032914 | 0.912359122903512 | 0.784074916991405 | 1.06163218731609 | 0.999332001504091 |
| ebi-fl187-GCST90094770 | ebi-met1400-GCST90200289 | N-acetylmethionine levels \|\| id:ebi-met1400-GCST90200289 | Gherkins liking \|\| id:ebi-fl187-GCST90094770 | Weighted median | 20 | 0.0622193587968925 | 0.0836465622124643 | 0.456975448035559 | -0.101727903 | 0.226166620733323 | 1.0641957598278 | 0.903275296606488 | 1.25378455437696 | 0.999332001504091 |
| ebi-fl187-GCST90094770 | ebi-met1400-GCST90200308 | 3-hydroxyisobutyrate levels \|\| id:ebi-met1400-GCST90200308 | Gherkins liking \|\| id:ebi-fl187-GCST90094770 | Weighted median | 20 | 0.0154313012927395 | 0.0841391036370993 | 0.854482407090347 | -0.149481342 | 0.180343944421454 | 1.01555097862241 | 0.86115450543182 | 1.19762921017731 | 0.999332001504091 |
| ebi-fl187-GCST90094770 | ebi-met1400-GCST90200383 | Cystathionine levels \|\| id:ebi-met1400-GCST90200383 | Gherkins liking \|\| id:ebi-fl187-GCST90094770 | Weighted median | 20 | -0.032740969 | 0.0761090933248431 | 0.667061276040287 | -0.181914792 | 0.116432853901142 | 0.96778921451691 | 0.833672373001464 | 1.12348207049631 | 0.999332001504091 |
| ebi-fl187-GCST90094770 | ebi-met1400-GCST90200384 | Guanidinoacetate levels \|\| id:ebi-met1400-GCST90200384 | Gherkins liking \|\| id:ebi-fl187-GCST90094770 | Weighted median | 20 | 0.127184041009447 | 0.0736832855542097 | 0.0843310794262099 | -0.017235199 | 0.271603280695698 | 1.13562600030567 | 0.98291247773202 | 1.312066376 | 0.999332001504091 |
| ebi-fl187-GCST90094770 | ebi-met1400-GCST90200403 | Citrulline levels \|\| id:ebi-met1400-GCST90200403 | Gherkins liking \|\| id:ebi-fl187-GCST90094770 | Weighted median | 20 | 0.0881898029156503 | 0.0774384466781893 | 0.254770959794131 | -0.063589553 | 0.239969158404901 | 1.09219540422837 | 0.938390080291034 | 1.27120994357444 | 0.999332001504091 |
| ebi-fl187-GCST90094770 | ebi-met1400-GCST90200434 | 12,13-DiHOME levels \|\| id:ebi-met1400-GCST90200434 | Gherkins liking \|\| id:ebi-fl187-GCST90094770 | Weighted median | 20 | 0.054402728760736 | 0.0791876032369472 | 0.492076001379703 | -0.100804974 | 0.209610431105152 | 1.05590976176714 | 0.904109340897662 | 1.23319755096008 | 0.999332001504091 |
| ebi-fl187-GCST90094770 | ebi-met1400-GCST90200437 | Fructose levels \|\| id:ebi-met1400-GCST90200437 | Gherkins liking \|\| id:ebi-fl187-GCST90094770 | Weighted median | 20 | -0.050768254 | 0.0741058302348783 | 0.493294871916492 | -0.196015681 | 0.0944791732448929 | 0.950498919318409 | 0.821999344578666 | 1.09908627249398 | 0.999332001504091 |
| ebi-fl187-GCST90094770 | ebi-met1400-GCST90200448 | N-stearoyl-sphinganine (d18:0/18:0) levels \|\| id:ebi-met1400-GCST90200448 | Gherkins liking \|\| id:ebi-fl187-GCST90094770 | Weighted median | 20 | -0.036748341 | 0.0821148243078518 | 0.654496878776483 | -0.197693397 | 0.12419671415217 | 0.963918683166553 | 0.820621419448988 | 1.13223857644542 | 0.999332001504091 |
| ebi-fl187-GCST90094770 | ebi-met1400-GCST90200468 | X-11847 levels \|\| id:ebi-met1400-GCST90200468 | Gherkins liking \|\| id:ebi-fl187-GCST90094770 | Weighted median | 20 | 0.0602081967739685 | 0.08079037213094 | 0.456126796407669 | -0.098140933 | 0.218557326150611 | 1.06205764050356 | 0.90652113636771 | 1.2442803443851 | 0.999332001504091 |
| ebi-fl187-GCST90094770 | ebi-met1400-GCST90200476 | X-11843 levels \|\| id:ebi-met1400-GCST90200476 | Gherkins liking \|\| id:ebi-fl187-GCST90094770 | Weighted median | 19 | -0.069849854 | 0.0897496647622509 | 0.436407344557579 | -0.245759197 | 0.106059488700657 | 0.932533825401207 | 0.782110536684231 | 1.11188801931268 | 0.999332001504091 |
| ebi-fl187-GCST90094770 | ebi-met1400-GCST90200511 | X-12906 levels \|\| id:ebi-met1400-GCST90200511 | Gherkins liking \|\| id:ebi-fl187-GCST90094770 | Weighted median | 20 | -0.062437873 | 0.0761761879039947 | 0.412415208149606 | -0.211743201 | 0.0868674553475362 | 0.939471427594158 | 0.809172465376972 | 1.09075209677905 | 0.999332001504091 |
| ebi-fl187-GCST90094770 | ebi-met1400-GCST90200515 | X-12849 levels \|\| id:ebi-met1400-GCST90200515 | Gherkins liking \|\| id:ebi-fl187-GCST90094770 | Weighted median | 20 | -0.001016381 | 0.0825488771968814 | 0.990176309784565 | -0.162812181 | 0.160779417831249 | 0.998984134866065 | 0.849750772954523 | 1.17442588283178 | 0.999332001504091 |
| ebi-fl187-GCST90094770 | ebi-met1400-GCST90200551 | X-18886 levels \|\| id:ebi-met1400-GCST90200551 | Gherkins liking \|\| id:ebi-fl187-GCST90094770 | Weighted median | 20 | 0.0552668672242516 | 0.0800413190679728 | 0.48989287592199 | -0.101614118 | 0.212147852597478 | 1.0568226083624 | 0.903378081625173 | 1.23633066626618 | 0.999332001504091 |
| ebi-fl187-GCST90094770 | ebi-met1400-GCST90200564 | X-21319 levels \|\| id:ebi-met1400-GCST90200564 | Gherkins liking \|\| id:ebi-fl187-GCST90094770 | Weighted median | 20 | -0.007897239 | 0.0812774155642294 | 0.922596159467085 | -0.167200973 | 0.151406495996368 | 0.992133862753302 | 0.846029565137579 | 1.16346950766634 | 0.999332001504091 |
| ebi-fl187-GCST90094770 | ebi-met1400-GCST90200579 | X-21442 levels \|\| id:ebi-met1400-GCST90200579 | Gherkins liking \|\| id:ebi-fl187-GCST90094770 | Weighted median | 20 | 0.0214787420378376 | 0.0860245880662693 | 0.802833721294799 | -0.147129451 | 0.190086934647725 | 1.02171107061124 | 0.863182230750086 | 1.2093547279147 | 0.999332001504091 |
| ebi-fl187-GCST90094770 | ebi-met1400-GCST90200581 | X-21441 levels \|\| id:ebi-met1400-GCST90200581 | Gherkins liking \|\| id:ebi-fl187-GCST90094770 | Weighted median | 20 | 0.0729398435079455 | 0.0794760655145355 | 0.358745255114515 | -0.082833245 | 0.228712931916435 | 1.0756658266856 | 0.920504633264148 | 1.25698114804293 | 0.999332001504091 |
| ebi-fl187-GCST90094770 | ebi-met1400-GCST90200648 | X-24585 levels \|\| id:ebi-met1400-GCST90200648 | Gherkins liking \|\| id:ebi-fl187-GCST90094770 | Weighted median | 20 | 0.0829582877305695 | 0.0730508677078363 | 0.256114136925847 | -0.060221413 | 0.226137988437929 | 1.0864964873688 | 0.941556037778123 | 1.25374865616116 | 0.999332001504091 |
| ebi-fl187-GCST90094770 | ebi-met1400-GCST90200720 | Adenosine 5'-diphosphate (ADP) to pantothenate ratio \|\| id:ebi-met1400-GCST90200720 | Gherkins liking \|\| id:ebi-fl187-GCST90094770 | Weighted median | 20 | 0.0251658310713973 | 0.0980873123725009 | 0.797514126206464 | -0.167085301 | 0.217416963321499 | 1.02548516372822 | 0.846127432591432 | 1.24286222207203 | 0.999332001504091 |
| ebi-fl187-GCST90094770 | ebi-met1400-GCST90200724 | Cholate to taurocholate ratio \|\| id:ebi-met1400-GCST90200724 | Gherkins liking \|\| id:ebi-fl187-GCST90094770 | Weighted median | 20 | -0.020222924 | 0.0851590781795222 | 0.812290386930622 | -0.187134717 | 0.14668886897129 | 0.97998018759608 | 0.829332003331488 | 1.1579936192297 | 0.999332001504091 |
| ebi-fl187-GCST90094770 | ebi-met1400-GCST90200726 | N-acetylputrescine to (N(1) + N(8))-acetylspermidine ratio \|\| id:ebi-met1400-GCST90200726 | Gherkins liking \|\| id:ebi-fl187-GCST90094770 | Weighted median | 20 | -0.066184161 | 0.072807055163999 | 0.363331653333748 | -0.208885989 | 0.0765176670324188 | 0.935958481285795 | 0.811487748728503 | 1.07952126210583 | 0.999332001504091 |
| ebi-fl187-GCST90094770 | ebi-met1400-GCST90200744 | Arginine to glutamate ratio \|\| id:ebi-met1400-GCST90200744 | Gherkins liking \|\| id:ebi-fl187-GCST90094770 | Weighted median | 20 | 0.0199289347639389 | 0.0799048426792115 | 0.803044928821037 | -0.136684557 | 0.176542426415193 | 1.02012884175378 | 0.872245326521389 | 1.19308504400727 | 0.999332001504091 |
| ebi-fl187-GCST90094770 | ebi-met1400-GCST90200759 | Histidine to trans-urocanate ratio \|\| id:ebi-met1400-GCST90200759 | Gherkins liking \|\| id:ebi-fl187-GCST90094770 | Weighted median | 20 | 0.0426564684507703 | 0.0833276733414397 | 0.608712326074032 | -0.120665771 | 0.205978708199992 | 1.04357933084085 | 0.886330147067138 | 1.22872704190635 | 0.999332001504091 |
| ebi-fl187-GCST90094770 | ebi-met1400-GCST90200795 | Oleoyl-linoleoyl-glycerol (18:1 to 18:2) [2] to linoleoyl-arachidonoyl-glycerol (18:2 to 20:4) [2] ratio \|\| id:ebi-met1400-GCST90200795 | Gherkins liking \|\| id:ebi-fl187-GCST90094770 | Weighted median | 20 | 0.0422631326504977 | 0.0864904192089476 | 0.625092870327064 | -0.127258089 | 0.211784354300035 | 1.04316893444669 | 0.880506394255457 | 1.23588134384283 | 0.999332001504091 |
| ebi-fl187-GCST90094770 | ebi-met1400-GCST90200811 | Choline to taurocholate ratio \|\| id:ebi-met1400-GCST90200811 | Gherkins liking \|\| id:ebi-fl187-GCST90094770 | Weighted median | 20 | -0.043543679 | 0.0798236614653228 | 0.585410787039726 | -0.199998055 | 0.112910697601778 | 0.957390735431962 | 0.818732345230604 | 1.1195319515962 | 0.999332001504091 |
| ebi-fl187-GCST90094770 | ebi-met1400-GCST90200831 | Cholate to phosphate ratio \|\| id:ebi-met1400-GCST90200831 | Gherkins liking \|\| id:ebi-fl187-GCST90094770 | Weighted median | 20 | -0.007111486 | 0.0819174970105642 | 0.930820329738133 | -0.16766978 | 0.153446808569911 | 0.992913741207339 | 0.845633033767896 | 1.1658457724689 | 0.999332001504091 |
| ebi-fl187-GCST90094770 | ebi-met1400-GCST90200841 | Adenosine 5'-diphosphate (ADP) to oxalate (ethanedioate) ratio \|\| id:ebi-met1400-GCST90200841 | Gherkins liking \|\| id:ebi-fl187-GCST90094770 | Weighted median | 20 | 0.0694558560007322 | 0.105338534591701 | 0.509665597898564 | -0.137007672 | 0.275919383800467 | 1.07192474111534 | 0.871963536577459 | 1.31774162842316 | 0.999332001504091 |
| ebi-fl187-GCST90094770 | ebi-met1400-GCST90200844 | Aspartate to phosphate ratio \|\| id:ebi-met1400-GCST90200844 | Gherkins liking \|\| id:ebi-fl187-GCST90094770 | Weighted median | 20 | -0.058704584 | 0.0719994619795857 | 0.414873407853898 | -0.199823529 | 0.0824143617962141 | 0.942985301257563 | 0.81887524792782 | 1.08590567444554 | 0.999332001504091 |
| ebi-fl187-GCST90094770 | ebi-met1400-GCST90200870 | Adenosine 5'-monophosphate (AMP) to acetoacetate ratio \|\| id:ebi-met1400-GCST90200870 | Gherkins liking \|\| id:ebi-fl187-GCST90094770 | Weighted median | 20 | -0.006478522 | 0.0868291264584132 | 0.940523173293203 | -0.17666361 | 0.163706565484865 | 0.993542418007136 | 0.838061652476338 | 1.17786863706586 | 0.999332001504091 |
| ebi-fl187-GCST90094770 | ebi-met1400-GCST90200887 | Cholesterol to cortisol ratio \|\| id:ebi-met1400-GCST90200887 | Gherkins liking \|\| id:ebi-fl187-GCST90094770 | Weighted median | 20 | -0.002306149 | 0.0766954793322873 | 0.976012101526985 | -0.152629288 | 0.148016990582353 | 0.997696508209502 | 0.858447899425813 | 1.15953259732969 | 0.999332001504091 |
| ebi-fl187-GCST90094770 | ebi-met1400-GCST90200890 | Cortisol to taurocholate ratio \|\| id:ebi-met1400-GCST90200890 | Gherkins liking \|\| id:ebi-fl187-GCST90094770 | Weighted median | 20 | -0.014846457 | 0.0779720927694502 | 0.848989959883694 | -0.167671759 | 0.137978844391202 | 0.985263207827948 | 0.845631359793685 | 1.14795126440942 | 0.999332001504091 |
| ebi-fl187-GCST90094770 | ebi-met1400-GCST90200948 | Adenosine 5'-diphosphate (ADP) to mannitol to sorbitol ratio \|\| id:ebi-met1400-GCST90200948 | Gherkins liking \|\| id:ebi-fl187-GCST90094770 | Weighted median | 20 | 0.00868698503302547 | 0.105249534141809 | 0.934219664962458 | -0.197602102 | 0.214976071950972 | 1.00872482638389 | 0.820696341706348 | 1.23983222984474 | 0.999332001504091 |
| ebi-fl187-GCST90094770 | ebi-met1400-GCST90200972 | Leucine to N-palmitoyl-sphingosine (d18:1 to 16:0) ratio \|\| id:ebi-met1400-GCST90200972 | Gherkins liking \|\| id:ebi-fl187-GCST90094770 | Weighted median | 20 | 0.0124849767923929 | 0.0736338662553149 | 0.865360257932752 | -0.131837401 | 0.15680735465281 | 1.01256323947861 | 0.87648349877438 | 1.16977024139884 | 0.999332001504091 |
| ebi-fl187-GCST90094770 | ebi-met1400-GCST90200973 | Proline to glutamate ratio \|\| id:ebi-met1400-GCST90200973 | Gherkins liking \|\| id:ebi-fl187-GCST90094770 | Weighted median | 20 | -0.002162321 | 0.0748251438200547 | 0.976945689457175 | -0.148819602 | 0.144494961307523 | 0.997840015551236 | 0.861724553861697 | 1.15545587296227 | 0.999332001504091 |
| ebi-fl187-GCST90094770 | ebi-met1400-GCST90200983 | Cholesterol to linoleoyl-arachidonoyl-glycerol (18:2 to 20:4) [1] ratio \|\| id:ebi-met1400-GCST90200983 | Gherkins liking \|\| id:ebi-fl187-GCST90094770 | Weighted median | 20 | -0.028503218 | 0.0793915450756371 | 0.719579508149942 | -0.184110647 | 0.127104210103053 | 0.97189916633128 | 0.831843758056383 | 1.1355353458713 | 0.999332001504091 |
| ebi-fl187-GCST90094770 | ebi-met1400-GCST90201001 | Serine to alpha-ketobutyrate ratio \|\| id:ebi-met1400-GCST90201001 | Gherkins liking \|\| id:ebi-fl187-GCST90094770 | Weighted median | 20 | -0.003527935 | 0.0751833818768154 | 0.962573482109466 | -0.150887363 | 0.143831493487054 | 0.996478280859294 | 0.859944554374901 | 1.15468951942616 | 0.999332001504091 |
| ebi-fl187-GCST90094770 | ebi-met1400-GCST90201005 | Fructose to maltose ratio \|\| id:ebi-met1400-GCST90201005 | Gherkins liking \|\| id:ebi-fl187-GCST90094770 | Weighted median | 20 | -0.091330915 | 0.0911607138069071 | 0.316407811273079 | -0.270005914 | 0.0873440841748376 | 0.912715629731243 | 0.763374979763387 | 1.09127210458733 | 0.999332001504091 |
| ebi-fl187-GCST90094770 | ebi-met1400-GCST90201020 | Paraxanthine to 5-acetylamino-6-formylamino-3-methyluracil ratio \|\| id:ebi-met1400-GCST90201020 | Gherkins liking \|\| id:ebi-fl187-GCST90094770 | Weighted median | 20 | 0.0469301155880671 | 0.0799453182742109 | 0.557185109323854 | -0.109762708 | 0.203622939405521 | 1.04804876424878 | 0.896046734587703 | 1.22583585190878 | 0.999332001504091 |
| ebi-fl187-GCST90094770 | ebi-pw11-GCST90275197 | Placental weight (fetal genotype, adjusted for sex) \|\| id:ebi-pw11-GCST90275197 | Gherkins liking \|\| id:ebi-fl187-GCST90094770 | Weighted median | 18 | 0.055178767606536 | 0.031955921970483 | 0.0842188727648274 | -0.007454839 | 0.117812374668683 | 1.05672950679578 | 0.992572878938549 | 1.12503300687303 | 0.999332001504091 |
| ebi-fl187-GCST90094770 | prot-a-1017 | FAS-associated factor 2 \|\| id:prot-a-1017 | Gherkins liking \|\| id:ebi-fl187-GCST90094770 | Weighted median | 21 | -0.202998794 | 0.129497393409241 | 0.116976892066618 | -0.456813685 | 0.0508160969341462 | 0.816279225736981 | 0.633298321902676 | 1.05212938567073 | 0.999332001504091 |
| ebi-fl187-GCST90094770 | prot-a-1019 | Fas apoptotic inhibitory molecule 3 \|\| id:prot-a-1019 | Gherkins liking \|\| id:ebi-fl187-GCST90094770 | Weighted median | 21 | 0.0716115695777411 | 0.110116900751074 | 0.515483507596448 | -0.144217556 | 0.287440695049847 | 1.07423799629527 | 0.86569938956575 | 1.33301153563633 | 0.999332001504091 |
| ebi-fl187-GCST90094770 | prot-a-1052 | MIP18 family protein FAM96A \|\| id:prot-a-1052 | Gherkins liking \|\| id:ebi-fl187-GCST90094770 | Weighted median | 21 | -0.043485569 | 0.121461179940554 | 0.720328119338317 | -0.281549481 | 0.194578344182284 | 0.957446371377422 | 0.754613575577772 | 1.21479865156404 | 0.999332001504091 |
| ebi-fl187-GCST90094770 | prot-a-1066 | F-box/LRR-repeat protein 4 \|\| id:prot-a-1066 | Gherkins liking \|\| id:ebi-fl187-GCST90094770 | Weighted median | 21 | 0.0692526231491311 | 0.122536429765628 | 0.571965304486387 | -0.170918779 | 0.309424025489761 | 1.07170691292918 | 0.842890030901286 | 1.36264004213225 | 0.999332001504091 |
| ebi-fl187-GCST90094770 | prot-a-1076 | Low affinity immunoglobulin gamma Fc region receptor III-B \|\| id:prot-a-1076 | Gherkins liking \|\| id:ebi-fl187-GCST90094770 | Weighted median | 21 | -0.14823535 | 0.12012282074361 | 0.217191150546857 | -0.383676079 | 0.0872053788072307 | 0.862228165791261 | 0.68135209602564 | 1.09112075 | 0.999332001504091 |
| ebi-fl187-GCST90094770 | prot-a-1081 | Fc receptor-like protein 3 \|\| id:prot-a-1081 | Gherkins liking \|\| id:ebi-fl187-GCST90094770 | Weighted median | 21 | -0.027969068 | 0.117398873782418 | 0.811695216728161 | -0.258070861 | 0.202132724604424 | 0.972418445174534 | 0.772540487467363 | 1.22401045363413 | 0.999332001504091 |
| ebi-fl187-GCST90094770 | prot-a-1105 | Fibroblast growth factor receptor 4 \|\| id:prot-a-1105 | Gherkins liking \|\| id:ebi-fl187-GCST90094770 | Weighted median | 21 | 0.283903203416766 | 0.116461669962453 | 0.014779423650471 | 0.0556383302903576 | 0.512168076543174 | 1.32830434920727 | 1.0572152518506 | 1.66890559045045 | 0.999332001504091 |
| ebi-fl187-GCST90094770 | prot-a-1143 | Follistatin-related protein 1 \|\| id:prot-a-1143 | Gherkins liking \|\| id:ebi-fl187-GCST90094770 | Weighted median | 21 | -0.155098054 | 0.119216693216071 | 0.193266676396843 | -0.388762773 | 0.0785666643077281 | 0.856331206327794 | 0.677895065886165 | 1.08173546590462 | 0.999332001504091 |
| ebi-fl187-GCST90094770 | prot-a-1151 | Alpha-(1,3)-fucosyltransferase 10 \|\| id:prot-a-1151 | Gherkins liking \|\| id:ebi-fl187-GCST90094770 | Weighted median | 21 | 0.033439195548874 | 0.119518654130873 | 0.779644586060992 | -0.200817367 | 0.267695757645385 | 1.03400456973446 | 0.81806182336652 | 1.30694945014288 | 0.999332001504091 |
| ebi-fl187-GCST90094770 | prot-a-1165 | Growth arrest and DNA damage-inducible proteins-interacting protein 1 \|\| id:prot-a-1165 | Gherkins liking \|\| id:ebi-fl187-GCST90094770 | Weighted median | 21 | -0.015315295 | 0.118038514124295 | 0.896765578215146 | -0.246670783 | 0.216040192718028 | 0.984801387728257 | 0.781397900937343 | 1.24115226328112 | 0.999332001504091 |
| ebi-fl187-GCST90094770 | prot-a-1194 | Growth/differentiation factor 11/8 \|\| id:prot-a-1194 | Gherkins liking \|\| id:ebi-fl187-GCST90094770 | Weighted median | 21 | -0.256887324 | 0.11781418379266 | 0.0292245066470663 | -0.487803124 | -0.025971524 | 0.773455358732169 | 0.613973737711098 | 0.974362835423106 | 0.999332001504091 |
| ebi-fl187-GCST90094770 | prot-a-1216 | D-glucuronyl C5-epimerase \|\| id:prot-a-1216 | Gherkins liking \|\| id:ebi-fl187-GCST90094770 | Weighted median | 21 | -0.074976649 | 0.112503022369174 | 0.505129135007604 | -0.295482573 | 0.145529274495043 | 0.927765149996278 | 0.744172383159269 | 1.15665159447794 | 0.999332001504091 |
| ebi-fl187-GCST90094770 | prot-a-1246 | Glypican-3 \|\| id:prot-a-1246 | Gherkins liking \|\| id:ebi-fl187-GCST90094770 | Weighted median | 21 | 0.145210617041784 | 0.12583089811074 | 0.248494432559622 | -0.101417943 | 0.391839177338834 | 1.15628307754499 | 0.903555319108518 | 1.47969972301866 | 0.999332001504091 |
| ebi-fl187-GCST90094770 | prot-a-1275 | Glutamate receptor 4 \|\| id:prot-a-1275 | Gherkins liking \|\| id:ebi-fl187-GCST90094770 | Weighted median | 20 | 0.0184637856188867 | 0.123063351947651 | 0.880737158145929 | -0.222740384 | 0.259667955436282 | 1.01863529525481 | 0.800322598718789 | 1.29649951956866 | 0.999332001504091 |
| ebi-fl187-GCST90094770 | prot-a-1289 | Glutathione S-transferase P \|\| id:prot-a-1289 | Gherkins liking \|\| id:ebi-fl187-GCST90094770 | Weighted median | 21 | -0.021880737 | 0.124584717826127 | 0.860585119573466 | -0.266066784 | 0.222305310247505 | 0.978356910175343 | 0.766387943622353 | 1.24895263769899 | 0.999332001504091 |
| ebi-fl187-GCST90094770 | prot-a-130 | Apolipoprotein D \|\| id:prot-a-130 | Gherkins liking \|\| id:ebi-fl187-GCST90094770 | Weighted median | 21 | 0.0140742730212952 | 0.114668909963699 | 0.902314293045928 | -0.210676791 | 0.238825336550144 | 1.01417378189213 | 0.810035835847258 | 1.26975673712209 | 0.999332001504091 |
| ebi-fl187-GCST90094770 | prot-a-1313 | Heparin-binding EGF-like growth factor \|\| id:prot-a-1313 | Gherkins liking \|\| id:ebi-fl187-GCST90094770 | Weighted median | 21 | -0.085473565 | 0.125902644120645 | 0.497209984377204 | -0.332242747 | 0.161295617644479 | 0.918077412264022 | 0.717313175875685 | 1.17503227775015 | 0.999332001504091 |
| ebi-fl187-GCST90094770 | prot-a-1347 | HLA class II histocompatibility antigen, DQ alpha 2 chain \|\| id:prot-a-1347 | Gherkins liking \|\| id:ebi-fl187-GCST90094770 | Weighted median | 20 | -0.054368352 | 0.114999889095855 | 0.636378184653658 | -0.279768134 | 0.171031430949319 | 0.947083182562354 | 0.755959002094804 | 1.18652804213839 | 0.999332001504091 |
| ebi-fl187-GCST90094770 | prot-a-1357 | Non-histone chromosomal protein HMG-14 \|\| id:prot-a-1357 | Gherkins liking \|\| id:ebi-fl187-GCST90094770 | Weighted median | 21 | -0.231553479 | 0.118992296146535 | 0.05166032181086 | -0.464778379 | 0.00167142160759978 | 0.793300269590811 | 0.628274328366428 | 1.00167281921125 | 0.999332001504091 |
| ebi-fl187-GCST90094770 | prot-a-137 | Apolipoprotein M \|\| id:prot-a-137 | Gherkins liking \|\| id:ebi-fl187-GCST90094770 | Weighted median | 21 | -0.076877674 | 0.116369610271295 | 0.508847319881557 | -0.30496211 | 0.151206762315184 | 0.926003121106227 | 0.737151304563532 | 1.16323714682454 | 0.999332001504091 |
| ebi-fl187-GCST90094770 | prot-a-1381 | 17-beta-hydroxysteroid dehydrogenase 14 \|\| id:prot-a-1381 | Gherkins liking \|\| id:ebi-fl187-GCST90094770 | Weighted median | 21 | -0.080233407 | 0.115947717496296 | 0.488950398726902 | -0.307490934 | 0.147024118854869 | 0.922900909308654 | 0.735289533850573 | 1.1583819015379 | 0.999332001504091 |
| ebi-fl187-GCST90094770 | prot-a-1392 | Serine protease HTRA2, mitochondrial \|\| id:prot-a-1392 | Gherkins liking \|\| id:ebi-fl187-GCST90094770 | Weighted median | 21 | -0.060702224 | 0.125256243874185 | 0.627942846975109 | -0.306204462 | 0.184800013852574 | 0.941103435940319 | 0.736236071667453 | 1.20297783716677 | 0.999332001504091 |
| ebi-fl187-GCST90094770 | prot-a-1416 | Interferon-induced protein with tetratricopeptide repeats 2 \|\| id:prot-a-1416 | Gherkins liking \|\| id:ebi-fl187-GCST90094770 | Weighted median | 21 | 0.0512458268475803 | 0.120454118486509 | 0.670517000632013 | -0.184844245 | 0.287335899081137 | 1.05258161430168 | 0.831233742260681 | 1.33287184872059 | 0.999332001504091 |
| ebi-fl187-GCST90094770 | prot-a-1428 | Interferon gamma \|\| id:prot-a-1428 | Gherkins liking \|\| id:ebi-fl187-GCST90094770 | Weighted median | 21 | 0.073357071037655 | 0.113220850268918 | 0.517042305916725 | -0.148555795 | 0.295269937564734 | 1.07611471771959 | 0.861951912799952 | 1.34348896788339 | 0.999332001504091 |
| ebi-fl187-GCST90094770 | prot-a-1483 | Interleukin-17C \|\| id:prot-a-1483 | Gherkins liking \|\| id:ebi-fl187-GCST90094770 | Weighted median | 21 | 0.111989185926552 | 0.123680877064267 | 0.365217036262778 | -0.130425333 | 0.354403704972515 | 1.11850076503022 | 0.877722027267887 | 1.42533048335058 | 0.999332001504091 |
| ebi-fl187-GCST90094770 | prot-a-1503 | Interleukin-1 receptor-like 2 \|\| id:prot-a-1503 | Gherkins liking \|\| id:ebi-fl187-GCST90094770 | Weighted median | 21 | -0.017746893 | 0.114613748253142 | 0.876946764594165 | -0.24238984 | 0.206896053274848 | 0.982409655357528 | 0.784750190994522 | 1.22985472576513 | 0.999332001504091 |
| ebi-fl187-GCST90094770 | prot-a-1522 | Interleukin-31 receptor subunit alpha \|\| id:prot-a-1522 | Gherkins liking \|\| id:ebi-fl187-GCST90094770 | Weighted median | 21 | -0.011606364 | 0.12086977973348 | 0.923501747647978 | -0.248511132 | 0.225298404503647 | 0.988460730242668 | 0.779961178218728 | 1.25269647069263 | 0.999332001504091 |
| ebi-fl187-GCST90094770 | prot-a-1540 | Interleukin-6 receptor subunit alpha \|\| id:prot-a-1540 | Gherkins liking \|\| id:ebi-fl187-GCST90094770 | Weighted median | 21 | -0.013986479 | 0.110067279119805 | 0.898883341212745 | -0.229718346 | 0.201745387862432 | 0.98611087716846 | 0.794757417360083 | 1.22353644121999 | 0.999332001504091 |
| ebi-fl187-GCST90094770 | prot-a-1541 | Interleukin-6 receptor subunit alpha \|\| id:prot-a-1541 | Gherkins liking \|\| id:ebi-fl187-GCST90094770 | Weighted median | 21 | -0.052988246 | 0.118382789289156 | 0.654441232435584 | -0.285018513 | 0.17904202052052 | 0.948391159345346 | 0.752000332037647 | 1.19607100263804 | 0.999332001504091 |
| ebi-fl187-GCST90094770 | prot-a-1545 | Interleukin-9 \|\| id:prot-a-1545 | Gherkins liking \|\| id:ebi-fl187-GCST90094770 | Weighted median | 21 | 0.00180924088700234 | 0.124519865682088 | 0.988407355151059 | -0.242249696 | 0.245868177623895 | 1.00181087855079 | 0.784860176753676 | 1.27873099707247 | 0.999332001504091 |
| ebi-fl187-GCST90094770 | prot-a-1548 | Inositol monophosphatase 3 \|\| id:prot-a-1548 | Gherkins liking \|\| id:ebi-fl187-GCST90094770 | Weighted median | 21 | -0.109767308 | 0.111990632649435 | 0.327013449539831 | -0.329268948 | 0.109734331879728 | 0.896042612886343 | 0.71944949614535 | 1.11598155035191 | 0.999332001504091 |
| ebi-fl187-GCST90094770 | prot-a-1555 | Inhibin beta A chain:Inhibin beta B chain heterodimer \|\| id:prot-a-1555 | Gherkins liking \|\| id:ebi-fl187-GCST90094770 | Weighted median | 21 | -0.121034872 | 0.113741461645163 | 0.287273117156642 | -0.343968136 | 0.101898393156841 | 0.886003062649803 | 0.708951517371827 | 1.1072709597053 | 0.999332001504091 |
| ebi-fl187-GCST90094770 | prot-a-1603 | Junctophilin-3 \|\| id:prot-a-1603 | Gherkins liking \|\| id:ebi-fl187-GCST90094770 | Weighted median | 21 | -0.204206164 | 0.122612159825521 | 0.0958201788225599 | -0.444525997 | 0.0361136696981467 | 0.815294269890682 | 0.641128100779589 | 1.03677368954554 | 0.999332001504091 |
| ebi-fl187-GCST90094770 | prot-a-1627 | UPF0258 protein KIAA1024 \|\| id:prot-a-1627 | Gherkins liking \|\| id:ebi-fl187-GCST90094770 | Weighted median | 21 | -0.038600577 | 0.12096868067013 | 0.749653996841222 | -0.275699192 | 0.198498036664474 | 0.962134930796347 | 0.759041222427657 | 1.21956963298751 | 0.999332001504091 |
| ebi-fl187-GCST90094770 | prot-a-1666 | Kallikrein-7 \|\| id:prot-a-1666 | Gherkins liking \|\| id:ebi-fl187-GCST90094770 | Weighted median | 21 | 0.198532915184159 | 0.113408696524483 | 0.0800153656711134 | -0.02374813 | 0.420813960372146 | 1.21961217051278 | 0.976531637805537 | 1.52320087632338 | 0.999332001504091 |
| ebi-fl187-GCST90094770 | prot-a-1678 | Importin subunit alpha-3 \|\| id:prot-a-1678 | Gherkins liking \|\| id:ebi-fl187-GCST90094770 | Weighted median | 21 | -0.079306967 | 0.120655193430852 | 0.51098639283744 | -0.315791146 | 0.157177211996635 | 0.923756318094615 | 0.729211732867936 | 1.17020296953212 | 0.999332001504091 |
| ebi-fl187-GCST90094770 | prot-a-1718 | Protein LDOC1 \|\| id:prot-a-1718 | Gherkins liking \|\| id:ebi-fl187-GCST90094770 | Weighted median | 21 | -0.071182135 | 0.120331139378685 | 0.554150577445362 | -0.307031168 | 0.164666898586721 | 0.931292256141404 | 0.735627672670154 | 1.17900032662017 | 0.999332001504091 |
| ebi-fl187-GCST90094770 | prot-a-1723 | Leptin \|\| id:prot-a-1723 | Gherkins liking \|\| id:ebi-fl187-GCST90094770 | Weighted median | 21 | -0.081304374 | 0.122769295793511 | 0.507808888476113 | -0.321932193 | 0.159323446183717 | 0.921913042770255 | 0.724747331369256 | 1.1727171962457 | 0.999332001504091 |
| ebi-fl187-GCST90094770 | prot-a-1730 | Galectin-7 \|\| id:prot-a-1730 | Gherkins liking \|\| id:ebi-fl187-GCST90094770 | Weighted median | 21 | 0.0705497567365785 | 0.122814305935785 | 0.565668401299156 | -0.170166283 | 0.311265796370717 | 1.073097962 | 0.843524541229132 | 1.36515202542183 | 0.999332001504091 |
| ebi-fl187-GCST90094770 | prot-a-1753 | Lipase member N \|\| id:prot-a-1753 | Gherkins liking \|\| id:ebi-fl187-GCST90094770 | Weighted median | 21 | -0.132408009 | 0.114064794221568 | 0.245717550446081 | -0.355975006 | 0.0911589872261475 | 0.875983512606674 | 0.700490127980978 | 1.09544315288275 | 0.999332001504091 |
| ebi-fl187-GCST90094770 | prot-a-1756 | VIP36-like protein \|\| id:prot-a-1756 | Gherkins liking \|\| id:ebi-fl187-GCST90094770 | Weighted median | 21 | 0.136996768834241 | 0.121389501834479 | 0.259078511499026 | -0.100926655 | 0.374920192429818 | 1.14682444293454 | 0.90399933450132 | 1.45487529992224 | 0.999332001504091 |
| ebi-fl187-GCST90094770 | prot-a-1760 | Ig Kappa chain V-I region HK102- like \|\| id:prot-a-1760 | Gherkins liking \|\| id:ebi-fl187-GCST90094770 | Weighted median | 21 | -0.118192622 | 0.119783748216952 | 0.323781550859886 | -0.352968769 | 0.116583524199825 | 0.888524886417906 | 0.702599136041264 | 1.12365135862846 | 0.999332001504091 |
| ebi-fl187-GCST90094770 | prot-a-179 | Neutral ceramidase \|\| id:prot-a-179 | Gherkins liking \|\| id:ebi-fl187-GCST90094770 | Weighted median | 21 | 0.21636433155293 | 0.121493721953189 | 0.0749339502306846 | -0.021763363 | 0.454492026581181 | 1.24155463413816 | 0.978471749812078 | 1.57537293217305 | 0.999332001504091 |
| ebi-fl187-GCST90094770 | prot-a-1809 | Lactotransferrin \|\| id:prot-a-1809 | Gherkins liking \|\| id:ebi-fl187-GCST90094770 | Weighted median | 21 | 0.0243489710784565 | 0.114984419098937 | 0.83229515490755 | -0.20102049 | 0.249718432512374 | 1.02464782796422 | 0.817895672409102 | 1.28366392777127 | 0.999332001504091 |
| ebi-fl187-GCST90094770 | prot-a-1818 | Lysophospholipase-like protein 1 \|\| id:prot-a-1818 | Gherkins liking \|\| id:ebi-fl187-GCST90094770 | Weighted median | 21 | -0.155006532 | 0.113687069041727 | 0.172741014521746 | -0.377833188 | 0.0678201229242924 | 0.856409583057528 | 0.685344815062765 | 1.07017279161235 | 0.999332001504091 |
| ebi-fl187-GCST90094770 | prot-a-1852 | MAP kinase-activated protein kinase 3 \|\| id:prot-a-1852 | Gherkins liking \|\| id:ebi-fl187-GCST90094770 | Weighted median | 21 | 0.0738374770042245 | 0.115177209047949 | 0.521472558741664 | -0.151909853 | 0.299584806738205 | 1.07663181384879 | 0.859065719680206 | 1.34929847162663 | 0.999332001504091 |
| ebi-fl187-GCST90094770 | prot-a-1856 | Megakaryocyte-associated tyrosine-protein kinase \|\| id:prot-a-1856 | Gherkins liking \|\| id:ebi-fl187-GCST90094770 | Weighted median | 21 | -0.073976248 | 0.111517717141222 | 0.507100850608002 | -0.292550973 | 0.144598477822676 | 0.928693752123093 | 0.746357199723864 | 1.15557548791861 | 0.999332001504091 |
| ebi-fl187-GCST90094770 | prot-a-1890 | Beta-1,3-N-acetylglucosaminyltransferase manic fringe \|\| id:prot-a-1890 | Gherkins liking \|\| id:ebi-fl187-GCST90094770 | Weighted median | 21 | 0.0139661091068643 | 0.109936424015222 | 0.898910281697544 | -0.201509282 | 0.229441500176699 | 1.01406409081838 | 0.817495989557379 | 1.25789727830234 | 0.999332001504091 |
| ebi-fl187-GCST90094770 | prot-a-1919 | Matrilysin \|\| id:prot-a-1919 | Gherkins liking \|\| id:ebi-fl187-GCST90094770 | Weighted median | 21 | -0.083453094 | 0.115189108870186 | 0.468766369635616 | -0.309223747 | 0.142317559683543 | 0.919934236368643 | 0.734016517593394 | 1.15294271853423 | 0.999332001504091 |
| ebi-fl187-GCST90094770 | prot-a-1923 | Multimerin-2 \|\| id:prot-a-1923 | Gherkins liking \|\| id:ebi-fl187-GCST90094770 | Weighted median | 21 | -0.066778833 | 0.112649106462877 | 0.55331237519439 | -0.287571082 | 0.154013415284782 | 0.935402058170269 | 0.750083247421312 | 1.16650653569085 | 0.999332001504091 |
| ebi-fl187-GCST90094770 | prot-a-1948 | Mesothelin \|\| id:prot-a-1948 | Gherkins liking \|\| id:ebi-fl187-GCST90094770 | Weighted median | 21 | 0.0999940552584299 | 0.120016303509875 | 0.404748522695763 | -0.1352379 | 0.335226010137784 | 1.10516434813968 | 0.873508079726401 | 1.39825636962804 | 0.999332001504091 |
| ebi-fl187-GCST90094770 | prot-a-195 | ATPase family AAA domain-containing protein 1 \|\| id:prot-a-195 | Gherkins liking \|\| id:ebi-fl187-GCST90094770 | Weighted median | 21 | -0.078473225 | 0.112957023493145 | 0.487232333821608 | -0.299868992 | 0.142922540583566 | 0.924526813377545 | 0.74091528051619 | 1.15364043788993 | 0.999332001504091 |
| ebi-fl187-GCST90094770 | prot-a-1966 | Mucin-1 \|\| id:prot-a-1966 | Gherkins liking \|\| id:ebi-fl187-GCST90094770 | Weighted median | 21 | -0.096562428 | 0.116095255710532 | 0.405549104893163 | -0.324109129 | 0.130984273445283 | 0.907953214356584 | 0.723171319154017 | 1.13994985368727 | 0.999332001504091 |
| ebi-fl187-GCST90094770 | prot-a-1986 | Myocardial zonula adherens protein \|\| id:prot-a-1986 | Gherkins liking \|\| id:ebi-fl187-GCST90094770 | Weighted median | 21 | -0.062835894 | 0.121510857283465 | 0.605071245913879 | -0.300997174 | 0.175325386071726 | 0.939097572399309 | 0.740079863854613 | 1.19163389460833 | 0.999332001504091 |
| ebi-fl187-GCST90094770 | prot-a-2071 | Epididymal secretory protein E1 \|\| id:prot-a-2071 | Gherkins liking \|\| id:ebi-fl187-GCST90094770 | Weighted median | 21 | 0.0448638088232006 | 0.11880954381718 | 0.705719478250136 | -0.188002897 | 0.277730514704874 | 1.04588540984395 | 0.8286123066909 | 1.32013039354061 | 0.999332001504091 |
| ebi-fl187-GCST90094770 | prot-a-2098 | Neuritin \|\| id:prot-a-2098 | Gherkins liking \|\| id:ebi-fl187-GCST90094770 | Weighted median | 21 | 0.0282790482582059 | 0.121511738167773 | 0.81597349150424 | -0.209883959 | 0.266442055067042 | 1.02868269648916 | 0.810678312798646 | 1.30531195093044 | 0.999332001504091 |
| ebi-fl187-GCST90094770 | prot-a-2115 | Neurotrophin-4 \|\| id:prot-a-2115 | Gherkins liking \|\| id:ebi-fl187-GCST90094770 | Weighted median | 21 | 0.0199829395678928 | 0.126974391029441 | 0.874947175170627 | -0.228886867 | 0.268852745985598 | 1.02018393509953 | 0.795418516617464 | 1.30846245051106 | 0.999332001504091 |
| ebi-fl187-GCST90094770 | prot-a-2118 | Netrin-1 \|\| id:prot-a-2118 | Gherkins liking \|\| id:ebi-fl187-GCST90094770 | Weighted median | 21 | 0.152783043918334 | 0.122637456410276 | 0.212833934817422 | -0.087586371 | 0.393152458482475 | 1.16507218202534 | 0.916139740652579 | 1.48164426134643 | 0.999332001504091 |
| ebi-fl187-GCST90094770 | prot-a-2120 | Netrin-G1 \|\| id:prot-a-2120 | Gherkins liking \|\| id:ebi-fl187-GCST90094770 | Weighted median | 21 | 0.0315769810504664 | 0.117837016678988 | 0.788721132696165 | -0.199383572 | 0.262537533741283 | 1.03208082320356 | 0.819235597517325 | 1.30022526957151 | 0.999332001504091 |
| ebi-fl187-GCST90094770 | prot-a-2145 | Olfactomedin-like protein 3 \|\| id:prot-a-2145 | Gherkins liking \|\| id:ebi-fl187-GCST90094770 | Weighted median | 21 | 0.104977284593427 | 0.123276628818722 | 0.394459075660814 | -0.136644908 | 0.346599477078122 | 1.11068538039916 | 0.872279910858505 | 1.41425017230798 | 0.999332001504091 |
| ebi-fl187-GCST90094770 | prot-a-2162 | Prolyl 4-hydroxylase subunit alpha-2 \|\| id:prot-a-2162 | Gherkins liking \|\| id:ebi-fl187-GCST90094770 | Weighted median | 21 | 0.010938546426007 | 0.119314309777534 | 0.926953550028576 | -0.222917501 | 0.244794593589973 | 1.01099859105892 | 0.800180860902086 | 1.27735890854829 | 0.999332001504091 |
| ebi-fl187-GCST90094770 | prot-a-2168 | Polyadenylate-binding protein-interacting protein 1 \|\| id:prot-a-2168 | Gherkins liking \|\| id:ebi-fl187-GCST90094770 | Weighted median | 21 | -0.057846547 | 0.118212152624977 | 0.624597342475307 | -0.289542366 | 0.173849272457073 | 0.943794764757777 | 0.748606076919144 | 1.18987620518661 | 0.999332001504091 |
| ebi-fl187-GCST90094770 | prot-a-219 | Beta-1,4-galactosyltransferase 5 \|\| id:prot-a-219 | Gherkins liking \|\| id:ebi-fl187-GCST90094770 | Weighted median | 21 | 0.0337555639899028 | 0.12537634944212 | 0.787749691801378 | -0.211982081 | 0.279493208896457 | 1.0343317478999 | 0.808979193602219 | 1.3224594318055 | 0.999332001504091 |
| ebi-fl187-GCST90094770 | prot-a-2190 | Pyruvate carboxylase, mitochondrial \|\| id:prot-a-2190 | Gherkins liking \|\| id:ebi-fl187-GCST90094770 | Weighted median | 21 | 0.0126556333770184 | 0.11576047000339 | 0.912943896167412 | -0.214234888 | 0.239546154583663 | 1.01273605480839 | 0.807158770982658 | 1.2706723306249 | 0.999332001504091 |
| ebi-fl187-GCST90094770 | prot-a-2197 | Protocadherin-9 \|\| id:prot-a-2197 | Gherkins liking \|\| id:ebi-fl187-GCST90094770 | Weighted median | 21 | -0.061396674 | 0.118582146326035 | 0.60462818935844 | -0.293817681 | 0.171024332678423 | 0.940450113554378 | 0.745412381955168 | 1.18651961987081 | 0.999332001504091 |
| ebi-fl187-GCST90094770 | prot-a-2198 | Protocadherin alpha-4 \|\| id:prot-a-2198 | Gherkins liking \|\| id:ebi-fl187-GCST90094770 | Weighted median | 21 | 0.101823522431801 | 0.124619306418473 | 0.413884639590631 | -0.142430318 | 0.346077363012008 | 1.10718806062912 | 0.867247983632106 | 1.41351196513096 | 0.999332001504091 |
| ebi-fl187-GCST90094770 | prot-a-2254 | Placenta growth factor \|\| id:prot-a-2254 | Gherkins liking \|\| id:ebi-fl187-GCST90094770 | Weighted median | 21 | -0.300327622 | 0.115129807456449 | 0.0090913429541512 | -0.525982045 | -0.074673199 | 0.740575552091226 | 0.590974715767173 | 0.928046723019702 | 0.999332001504091 |
| ebi-fl187-GCST90094770 | prot-a-2261 | Membrane-associated progesterone receptor component 2 \|\| id:prot-a-2261 | Gherkins liking \|\| id:ebi-fl187-GCST90094770 | Weighted median | 21 | 0.208075146315331 | 0.107740707237969 | 0.0534511046194813 | -0.00309664 | 0.419246932501751 | 1.23130569415835 | 0.996908149772947 | 1.52081584728951 | 0.999332001504091 |
| ebi-fl187-GCST90094770 | prot-a-2268 | Polymeric immunoglobulin receptor \|\| id:prot-a-2268 | Gherkins liking \|\| id:ebi-fl187-GCST90094770 | Weighted median | 21 | 0.00761336191675746 | 0.113871464538847 | 0.94669374580351 | -0.215574709 | 0.230801432412897 | 1.00764241724604 | 0.806078047062784 | 1.25960909707587 | 0.999332001504091 |
| ebi-fl187-GCST90094770 | prot-a-2283 | Group 10 secretory phospholipase A2 \|\| id:prot-a-2283 | Gherkins liking \|\| id:ebi-fl187-GCST90094770 | Weighted median | 21 | 0.185258836876169 | 0.128795225172446 | 0.150321066041068 | -0.067179804 | 0.437697478214163 | 1.20352991773929 | 0.935027064183257 | 1.5491361890779 | 0.999332001504091 |
| ebi-fl187-GCST90094770 | prot-a-2298 | Inactive phospholipase D5 \|\| id:prot-a-2298 | Gherkins liking \|\| id:ebi-fl187-GCST90094770 | Weighted median | 21 | -0.084710722 | 0.125279333912644 | 0.498929701413639 | -0.330258217 | 0.160836772083964 | 0.918778027879571 | 0.718738119172896 | 1.17449324308234 | 0.999332001504091 |
| ebi-fl187-GCST90094770 | prot-a-2304 | Procollagen-lysine,2-oxoglutarate 5-dioxygenase 2 \|\| id:prot-a-2304 | Gherkins liking \|\| id:ebi-fl187-GCST90094770 | Weighted median | 21 | -0.12711372 | 0.121807791705097 | 0.296689079097574 | -0.365856992 | 0.111629551472077 | 0.880633521020928 | 0.693601984741946 | 1.11809858594082 | 0.999332001504091 |
| ebi-fl187-GCST90094770 | prot-a-2331 | Periostin \|\| id:prot-a-2331 | Gherkins liking \|\| id:ebi-fl187-GCST90094770 | Weighted median | 21 | 0.130793889990901 | 0.124845686230433 | 0.294802450998842 | -0.113903655 | 0.37549143500255 | 1.13973284675423 | 0.892343924597917 | 1.45570662405284 | 0.999332001504091 |
| ebi-fl187-GCST90094770 | prot-a-2333 | Periostin \|\| id:prot-a-2333 | Gherkins liking \|\| id:ebi-fl187-GCST90094770 | Weighted median | 21 | -0.027811157 | 0.123194306468561 | 0.821395603162036 | -0.269271997 | 0.213649684147574 | 0.972572013333516 | 0.76393543907929 | 1.23818882163606 | 0.999332001504091 |
| ebi-fl187-GCST90094770 | prot-a-2367 | cAMP-dependent protein kinase catalytic subunit alpha \|\| id:prot-a-2367 | Gherkins liking \|\| id:ebi-fl187-GCST90094770 | Weighted median | 21 | -0.063316011 | 0.126370496480111 | 0.616346659170456 | -0.311002185 | 0.184370161613421 | 0.938646803643285 | 0.732712275192692 | 1.20246084557277 | 0.999332001504091 |
| ebi-fl187-GCST90094770 | prot-a-2385 | Phosphoribosyl pyrophosphate synthase-associated protein 1 \|\| id:prot-a-2385 | Gherkins liking \|\| id:ebi-fl187-GCST90094770 | Weighted median | 21 | 0.115991109567074 | 0.118550052537576 | 0.327869279538858 | -0.116366993 | 0.348349212540724 | 1.12298588825816 | 0.890148484720923 | 1.4167269021666 | 0.999332001504091 |
| ebi-fl187-GCST90094770 | prot-a-2393 | Trypsin-3 \|\| id:prot-a-2393 | Gherkins liking \|\| id:ebi-fl187-GCST90094770 | Weighted median | 20 | 0.0189148529475403 | 0.122649163241291 | 0.877436922447189 | -0.221477507 | 0.259307212900471 | 1.01909487199855 | 0.801333946345361 | 1.29603190139425 | 0.999332001504091 |
| ebi-fl187-GCST90094770 | prot-a-2405 | Pregnancy-specific beta-1-glycoprotein 6 \|\| id:prot-a-2405 | Gherkins liking \|\| id:ebi-fl187-GCST90094770 | Weighted median | 21 | 0.160544295694928 | 0.116669340498301 | 0.16880230719106 | -0.068127612 | 0.389216203071599 | 1.17414978176926 | 0.934141258633766 | 1.47582359443702 | 0.999332001504091 |
| ebi-fl187-GCST90094770 | prot-a-244 | Beclin-1 \|\| id:prot-a-244 | Gherkins liking \|\| id:ebi-fl187-GCST90094770 | Weighted median | 21 | 0.00362589403866439 | 0.111593105691936 | 0.974079618212633 | -0.215096593 | 0.222348381194859 | 1.00363247554466 | 0.80646353758769 | 1.24900643243079 | 0.999332001504091 |
| ebi-fl187-GCST90094770 | prot-a-2454 | tRNA pseudouridine synthase A, mitochondrial \|\| id:prot-a-2454 | Gherkins liking \|\| id:ebi-fl187-GCST90094770 | Weighted median | 21 | 0.0259830311291627 | 0.124054537836982 | 0.834098170841496 | -0.217163863 | 0.269129925289648 | 1.02632353277422 | 0.804798081846263 | 1.30882517949062 | 0.999332001504091 |
| ebi-fl187-GCST90094770 | prot-a-2466 | Glutaminyl-peptide cyclotransferase-like protein \|\| id:prot-a-2466 | Gherkins liking \|\| id:ebi-fl187-GCST90094770 | Weighted median | 21 | -0.055399798 | 0.106199596806766 | 0.601908939119878 | -0.263551008 | 0.152751411635977 | 0.946106820616968 | 0.768318431171072 | 1.165035329 | 0.999332001504091 |
| ebi-fl187-GCST90094770 | prot-a-2474 | Ras-related protein Rab-27A \|\| id:prot-a-2474 | Gherkins liking \|\| id:ebi-fl187-GCST90094770 | Weighted median | 21 | 0.0140958122019871 | 0.123311180683236 | 0.908991341173345 | -0.227594102 | 0.25578572634113 | 1.01419562659972 | 0.796447470721203 | 1.29147596900847 | 0.999332001504091 |
| ebi-fl187-GCST90094770 | prot-a-2490 | Rap1 GTPase-GDP dissociation stimulator 1 \|\| id:prot-a-2490 | Gherkins liking \|\| id:ebi-fl187-GCST90094770 | Weighted median | 21 | -0.099740043 | 0.114526822911447 | 0.383815277374902 | -0.324212615 | 0.124732530402771 | 0.905072667881897 | 0.72309648457917 | 1.13284541083558 | 0.999332001504091 |
| ebi-fl187-GCST90094770 | prot-a-2545 | Receptor-interacting serine/threonine-protein kinase 2 \|\| id:prot-a-2545 | Gherkins liking \|\| id:ebi-fl187-GCST90094770 | Weighted median | 21 | -0.062087299 | 0.127438803120429 | 0.626121531558746 | -0.311867353 | 0.187692755201673 | 0.939800839616806 | 0.732078629799802 | 1.20646277898589 | 0.999332001504091 |
| ebi-fl187-GCST90094770 | prot-a-2610 | RUN and FYVE domain-containing protein 1 \|\| id:prot-a-2610 | Gherkins liking \|\| id:ebi-fl187-GCST90094770 | Weighted median | 21 | -0.067839616 | 0.123473109468076 | 0.582711585234953 | -0.30984691 | 0.17416767896939 | 0.934410326410595 | 0.73355924810728 | 1.19025512984203 | 0.999332001504091 |
| ebi-fl187-GCST90094770 | prot-a-2627 | Serine--tRNA ligase, mitochondrial \|\| id:prot-a-2627 | Gherkins liking \|\| id:ebi-fl187-GCST90094770 | Weighted median | 21 | 0.0255659109794693 | 0.117536361234889 | 0.827807089016815 | -0.204805357 | 0.255937178999851 | 1.02589552182079 | 0.814805897208511 | 1.29167158129027 | 0.999332001504091 |
| ebi-fl187-GCST90094770 | prot-a-264 | BolA-like protein 2 \|\| id:prot-a-264 | Gherkins liking \|\| id:ebi-fl187-GCST90094770 | Weighted median | 21 | -0.029997608 | 0.118597481404882 | 0.800317439930492 | -0.262448671 | 0.202453455720406 | 0.970447855018908 | 0.76916584357148 | 1.22440309483566 | 0.999332001504091 |
| ebi-fl187-GCST90094770 | prot-a-2646 | Secretoglobin family 1C member 1 \|\| id:prot-a-2646 | Gherkins liking \|\| id:ebi-fl187-GCST90094770 | Weighted median | 21 | -0.024830239 | 0.120479792403814 | 0.836716987281077 | -0.260970632 | 0.211310153933869 | 0.975475495495481 | 0.770303541334623 | 1.23529542738892 | 0.999332001504091 |
| ebi-fl187-GCST90094770 | prot-a-2672 | Semaphorin-3E \|\| id:prot-a-2672 | Gherkins liking \|\| id:ebi-fl187-GCST90094770 | Weighted median | 21 | 0.101844157209961 | 0.117753328612578 | 0.387096925337626 | -0.128952367 | 0.332640681290615 | 1.10721090744486 | 0.879015834823316 | 1.39464608599602 | 0.999332001504091 |
| ebi-fl187-GCST90094770 | prot-a-2701 | Plasma protease C1 inhibitor \|\| id:prot-a-2701 | Gherkins liking \|\| id:ebi-fl187-GCST90094770 | Weighted median | 21 | 0.157225617008486 | 0.107877224294298 | 0.144992315061523 | -0.054213743 | 0.36866497662531 | 1.17025961459167 | 0.947229621532735 | 1.44580314467806 | 0.999332001504091 |
| ebi-fl187-GCST90094770 | prot-a-2738 | Signaling threshold-regulating transmembrane adapter 1 \|\| id:prot-a-2738 | Gherkins liking \|\| id:ebi-fl187-GCST90094770 | Weighted median | 21 | -0.102508461 | 0.120123367724802 | 0.393459770085503 | -0.337950262 | 0.132933339632155 | 0.902570512973619 | 0.713230761795352 | 1.14217385806363 | 0.999332001504091 |
| ebi-fl187-GCST90094770 | prot-a-274 | Serine/threonine-protein kinase BRSK2 \|\| id:prot-a-274 | Gherkins liking \|\| id:ebi-fl187-GCST90094770 | Weighted median | 21 | 0.0139277407699069 | 0.128165579921869 | 0.913464316652887 | -0.237276796 | 0.26513227741677 | 1.01402518361205 | 0.788772928704005 | 1.30360340166456 | 0.999332001504091 |
| ebi-fl187-GCST90094770 | prot-a-2749 | Mitochondrial glutamate carrier 2 \|\| id:prot-a-2749 | Gherkins liking \|\| id:ebi-fl187-GCST90094770 | Weighted median | 21 | 0.0259748282890485 | 0.115506588234093 | 0.822074603771433 | -0.200418085 | 0.252367741227871 | 1.0263151140409 | 0.81838852586278 | 1.28706925869754 | 0.999332001504091 |
| ebi-fl187-GCST90094770 | prot-a-2760 | Sodium-coupled monocarboxylate transporter 1 \|\| id:prot-a-2760 | Gherkins liking \|\| id:ebi-fl187-GCST90094770 | Weighted median | 21 | -0.055614782 | 0.113713411379189 | 0.624786141281543 | -0.278493068 | 0.167263504704006 | 0.945903445129019 | 0.756923514799388 | 1.18206570414725 | 0.999332001504091 |
| ebi-fl187-GCST90094770 | prot-a-2777 | Small integral membrane protein 10 \|\| id:prot-a-2777 | Gherkins liking \|\| id:ebi-fl187-GCST90094770 | Weighted median | 21 | -0.02934782 | 0.117429286697897 | 0.802649919753852 | -0.259509222 | 0.20081358143456 | 0.971078644666447 | 0.77143009350793 | 1.22239687311023 | 0.999332001504091 |
| ebi-fl187-GCST90094770 | prot-a-2799 | Superoxide dismutase [Mn], mitochondrial \|\| id:prot-a-2799 | Gherkins liking \|\| id:ebi-fl187-GCST90094770 | Weighted median | 21 | 0.147186750325613 | 0.128574816729026 | 0.252310383855434 | -0.10481989 | 0.399193391114503 | 1.15857030621008 | 0.90048669422282 | 1.49062186375801 | 0.999332001504091 |
| ebi-fl187-GCST90094770 | prot-a-2805 | Protein SPACA7 \|\| id:prot-a-2805 | Gherkins liking \|\| id:ebi-fl187-GCST90094770 | Weighted median | 21 | -0.102892883 | 0.116987067412698 | 0.379117458191288 | -0.332187535 | 0.126401768758145 | 0.902223611457751 | 0.717352781126941 | 1.13473797898028 | 0.999332001504091 |
| ebi-fl187-GCST90094770 | prot-a-2811 | SPARC-like protein 1 \|\| id:prot-a-2811 | Gherkins liking \|\| id:ebi-fl187-GCST90094770 | Weighted median | 21 | -0.041943801 | 0.12060374803428 | 0.728003983851503 | -0.278327147 | 0.194439545511062 | 0.95892366995845 | 0.757049114815596 | 1.21463005082645 | 0.999332001504091 |
| ebi-fl187-GCST90094770 | prot-a-2831 | Sepiapterin reductase \|\| id:prot-a-2831 | Gherkins liking \|\| id:ebi-fl187-GCST90094770 | Weighted median | 21 | -0.032716977 | 0.120668123646813 | 0.786289594682837 | -0.269226499 | 0.203792545839762 | 0.967812434485507 | 0.763970197674626 | 1.226043779 | 0.999332001504091 |
| ebi-fl187-GCST90094770 | prot-a-2834 | SPRY domain-containing SOCS box protein 1 \|\| id:prot-a-2834 | Gherkins liking \|\| id:ebi-fl187-GCST90094770 | Weighted median | 21 | 0.218038646203396 | 0.128299389698388 | 0.0892340633892665 | -0.033428158 | 0.469505450012237 | 1.2436351284665 | 0.967124389267596 | 1.59920311173948 | 0.999332001504091 |
| ebi-fl187-GCST90094770 | prot-a-2835 | Spectrin alpha chain, non-erythrocytic 1 \|\| id:prot-a-2835 | Gherkins liking \|\| id:ebi-fl187-GCST90094770 | Weighted median | 21 | -0.034105254 | 0.120968170473068 | 0.777992763404736 | -0.271202868 | 0.202992360199048 | 0.966469774543632 | 0.762461801571292 | 1.22506310897344 | 0.999332001504091 |
| ebi-fl187-GCST90094770 | prot-a-2859 | Sia-alpha-2,3-Gal-beta-1,4-GlcNAc-R:alpha 2,8-sialyltransferase \|\| id:prot-a-2859 | Gherkins liking \|\| id:ebi-fl187-GCST90094770 | Weighted median | 21 | -0.060979581 | 0.112364346063088 | 0.587339742315917 | -0.281213699 | 0.159254537248206 | 0.940842450608525 | 0.754867003677877 | 1.17263638833628 | 0.999332001504091 |
| ebi-fl187-GCST90094770 | prot-a-2861 | Alpha-2,8-sialyltransferase 8F \|\| id:prot-a-2861 | Gherkins liking \|\| id:ebi-fl187-GCST90094770 | Weighted median | 21 | -0.168979533 | 0.116740308232549 | 0.147761883548472 | -0.397790537 | 0.05983147147487 | 0.844526188412999 | 0.671802730877403 | 1.06165761187646 | 0.999332001504091 |
| ebi-fl187-GCST90094770 | prot-a-289 | Uncharacterized protein C17orf89 \|\| id:prot-a-289 | Gherkins liking \|\| id:ebi-fl187-GCST90094770 | Weighted median | 21 | -0.059734485 | 0.120194524220176 | 0.619201923534281 | -0.295315752 | 0.175846782915836 | 0.94201461981221 | 0.744296537218563 | 1.19225537076409 | 0.999332001504091 |
| ebi-fl187-GCST90094770 | prot-a-2921 | Tachykinin-3 \|\| id:prot-a-2921 | Gherkins liking \|\| id:ebi-fl187-GCST90094770 | Weighted median | 21 | -0.113984538 | 0.116768277839139 | 0.328985115741232 | -0.342850362 | 0.114881286873826 | 0.892271752357395 | 0.709744408166841 | 1.12174026437385 | 0.999332001504091 |
| ebi-fl187-GCST90094770 | prot-a-2928 | Tax1-binding protein 1 \|\| id:prot-a-2928 | Gherkins liking \|\| id:ebi-fl187-GCST90094770 | Weighted median | 21 | -0.127431094 | 0.115339300094799 | 0.269230346219098 | -0.353496122 | 0.0986339341795789 | 0.880354075416731 | 0.702228715690894 | 1.10366221258886 | 0.999332001504091 |
| ebi-fl187-GCST90094770 | prot-a-296 | Uncharacterized protein C1orf185 \|\| id:prot-a-296 | Gherkins liking \|\| id:ebi-fl187-GCST90094770 | Weighted median | 21 | 0.0350229694404113 | 0.116470638519004 | 0.763641642087799 | -0.193259482 | 0.263305420937658 | 1.03564349667801 | 0.824268063614806 | 1.30122407934596 | 0.999332001504091 |
| ebi-fl187-GCST90094770 | prot-a-2964 | Transforming growth factor beta-2 \|\| id:prot-a-2964 | Gherkins liking \|\| id:ebi-fl187-GCST90094770 | Weighted median | 21 | 0.0116060979470174 | 0.119203471163011 | 0.922437524687635 | -0.222032706 | 0.245244901426518 | 1.01167371001939 | 0.80088917039954 | 1.27793424280393 | 0.999332001504091 |
| ebi-fl187-GCST90094770 | prot-a-2977 | Thymocyte nuclear protein 1 \|\| id:prot-a-2977 | Gherkins liking \|\| id:ebi-fl187-GCST90094770 | Weighted median | 21 | -0.021016533 | 0.122027486543768 | 0.863258393268669 | -0.260190407 | 0.21815734033072 | 0.979202774987219 | 0.770904786221568 | 1.24378274941348 | 0.999332001504091 |
| ebi-fl187-GCST90094770 | prot-a-3015 | Transmembrane protein 70, mitochondrial \|\| id:prot-a-3015 | Gherkins liking \|\| id:ebi-fl187-GCST90094770 | Weighted median | 21 | -0.146369833 | 0.112188731559441 | 0.192003540104475 | -0.366259746 | 0.0735200812764348 | 0.863838168602479 | 0.69332268972106 | 1.0762901497349 | 0.999332001504091 |
| ebi-fl187-GCST90094770 | prot-a-3030 | Tumor necrosis factor alpha-induced protein 3 \|\| id:prot-a-3030 | Gherkins liking \|\| id:ebi-fl187-GCST90094770 | Weighted median | 21 | -0.096143246 | 0.122701264259484 | 0.43330099508202 | -0.336637724 | 0.14435123151204 | 0.908333891155859 | 0.714167518520299 | 1.15528981146024 | 0.999332001504091 |
| ebi-fl187-GCST90094770 | prot-a-3032 | Tumor necrosis factor receptor superfamily member 10A \|\| id:prot-a-3032 | Gherkins liking \|\| id:ebi-fl187-GCST90094770 | Weighted median | 21 | -0.091997087 | 0.108867758381727 | 0.398091189461419 | -0.305377894 | 0.121383719106926 | 0.912107806218238 | 0.736844872701215 | 1.12905807041097 | 0.999332001504091 |
| ebi-fl187-GCST90094770 | prot-a-3081 | Tumor protein p53-inducible protein 11 \|\| id:prot-a-3081 | Gherkins liking \|\| id:ebi-fl187-GCST90094770 | Weighted median | 21 | 0.11706671340378 | 0.117745308351979 | 0.320107617550098 | -0.113714091 | 0.34784751777366 | 1.12419442602555 | 0.892513096964397 | 1.41601631595702 | 0.999332001504091 |
| ebi-fl187-GCST90094770 | prot-a-315 | Complement C4 \|\| id:prot-a-315 | Gherkins liking \|\| id:ebi-fl187-GCST90094770 | Weighted median | 20 | -0.082251634 | 0.12684450830319 | 0.516697442774343 | -0.330866871 | 0.166363602002569 | 0.921040164262934 | 0.71830078966745 | 1.18100243851637 | 0.999332001504091 |
| ebi-fl187-GCST90094770 | prot-a-3157 | UDP-glucuronosyltransferase 1-8 \|\| id:prot-a-3157 | Gherkins liking \|\| id:ebi-fl187-GCST90094770 | Weighted median | 21 | 0.113902883704784 | 0.113455767962213 | 0.315407109863766 | -0.108470422 | 0.33627618891072 | 1.12064328687499 | 0.897205432414346 | 1.39972556010785 | 0.999332001504091 |
| ebi-fl187-GCST90094770 | prot-a-317 | Glutaredoxin-like protein C5orf63 \|\| id:prot-a-317 | Gherkins liking \|\| id:ebi-fl187-GCST90094770 | Weighted median | 21 | 0.0399122891554993 | 0.123983362290496 | 0.74751595828465 | -0.203095101 | 0.282919679244871 | 1.04071948780388 | 0.816200616293721 | 1.32699857200917 | 0.999332001504091 |
| ebi-fl187-GCST90094770 | prot-a-3192 | Guanine nucleotide exchange factor VAV3 \|\| id:prot-a-3192 | Gherkins liking \|\| id:ebi-fl187-GCST90094770 | Weighted median | 21 | -0.003122923 | 0.122644884075857 | 0.979685554602063 | -0.243506896 | 0.237261049801684 | 0.996881948264829 | 0.783874070593888 | 1.26777202621764 | 0.999332001504091 |
| ebi-fl187-GCST90094770 | prot-a-3233 | Serine/threonine-protein kinase WNK3 \|\| id:prot-a-3233 | Gherkins liking \|\| id:ebi-fl187-GCST90094770 | Weighted median | 21 | 0.159080746874283 | 0.116371187554586 | 0.171621713815754 | -0.069006781 | 0.38716827448127 | 1.17243261312747 | 0.933320351460914 | 1.47280430580269 | 0.999332001504091 |
| ebi-fl187-GCST90094770 | prot-a-3250 | 14-3-3 protein theta \|\| id:prot-a-3250 | Gherkins liking \|\| id:ebi-fl187-GCST90094770 | Weighted median | 21 | 0.132925186672954 | 0.116840450236961 | 0.255260765853427 | -0.096082096 | 0.361932469137399 | 1.14216454600473 | 0.908389438057579 | 1.43610195748169 | 0.999332001504091 |
| ebi-fl187-GCST90094770 | prot-a-3278 | E3 ubiquitin-protein ligase ZNRF3 \|\| id:prot-a-3278 | Gherkins liking \|\| id:ebi-fl187-GCST90094770 | Weighted median | 21 | 0.0827569891399988 | 0.119735947814553 | 0.489463468443316 | -0.151925469 | 0.317439446856522 | 1.08627779916879 | 0.859052304746307 | 1.3736060661818 | 0.999332001504091 |
| ebi-fl187-GCST90094770 | prot-a-355 | Calpain-3 \|\| id:prot-a-355 | Gherkins liking \|\| id:ebi-fl187-GCST90094770 | Weighted median | 21 | 0.0620352303648979 | 0.118696146129084 | 0.60122552307901 | -0.170609216 | 0.294679676777903 | 1.06399982916997 | 0.843150998979751 | 1.34269619302308 | 0.999332001504091 |
| ebi-fl187-GCST90094770 | prot-a-385 | Coiled-coil domain-containing protein 90B, mitochondrial \|\| id:prot-a-385 | Gherkins liking \|\| id:ebi-fl187-GCST90094770 | Weighted median | 21 | 0.0656221409178991 | 0.119156562927894 | 0.581824259455664 | -0.167924722 | 0.299169004256572 | 1.06782315424893 | 0.845417473270173 | 1.34873754659875 | 0.999332001504091 |
| ebi-fl187-GCST90094770 | prot-a-390 | C-C motif chemokine 14 \|\| id:prot-a-390 | Gherkins liking \|\| id:ebi-fl187-GCST90094770 | Weighted median | 21 | -0.19431809 | 0.122457927840703 | 0.112554526773529 | -0.434335628 | 0.0456994489596829 | 0.82339594891345 | 0.647694834365284 | 1.0467597589403 | 0.999332001504091 |
| ebi-fl187-GCST90094770 | prot-a-40 | Pituitary adenylate cyclase-activating polypeptide 27 \|\| id:prot-a-40 | Gherkins liking \|\| id:ebi-fl187-GCST90094770 | Weighted median | 21 | -0.016098419 | 0.121504033298541 | 0.894594459493303 | -0.254246324 | 0.222049486757188 | 0.984030468478883 | 0.775500754245814 | 1.24863316714176 | 0.999332001504091 |
| ebi-fl187-GCST90094770 | prot-a-401 | C-C motif chemokine 24 \|\| id:prot-a-401 | Gherkins liking \|\| id:ebi-fl187-GCST90094770 | Weighted median | 21 | 0.195119913763347 | 0.126266812707627 | 0.122274082947812 | -0.052363039 | 0.442602866670296 | 1.21545672774177 | 0.948984285856051 | 1.55675397267519 | 0.999332001504091 |
| ebi-fl187-GCST90094770 | prot-a-412 | Cerebral cavernous malformations 2 protein \|\| id:prot-a-412 | Gherkins liking \|\| id:ebi-fl187-GCST90094770 | Weighted median | 21 | -0.022956198 | 0.125301008966415 | 0.854634502845949 | -0.268546175 | 0.222633780070772 | 0.977305291247429 | 0.76449012160417 | 1.24936294833481 | 0.999332001504091 |
| ebi-fl187-GCST90094770 | prot-a-44 | Adhesion G protein-coupled receptor F5 \|\| id:prot-a-44 | Gherkins liking \|\| id:ebi-fl187-GCST90094770 | Weighted median | 21 | -0.009116228 | 0.118572922190261 | 0.938716716989261 | -0.241519155 | 0.223286699741566 | 0.990925199071835 | 0.785433758469396 | 1.25017894834198 | 0.999332001504091 |
| ebi-fl187-GCST90094770 | prot-a-48 | Adrenomedullin \|\| id:prot-a-48 | Gherkins liking \|\| id:ebi-fl187-GCST90094770 | Weighted median | 21 | 0.0595648629185805 | 0.1177435919587 | 0.61293651308945 | -0.171212577 | 0.290342303157632 | 1.06137460260131 | 0.842642427761728 | 1.33688502967908 | 0.999332001504091 |
| ebi-fl187-GCST90094770 | prot-a-502 | Carcinoembryonic antigen-related cell adhesion molecule 1 \|\| id:prot-a-502 | Gherkins liking \|\| id:ebi-fl187-GCST90094770 | Weighted median | 21 | 0.0602361106929402 | 0.11800322747992 | 0.609728143715044 | -0.171050215 | 0.291522436553583 | 1.06208728710825 | 0.842779252107561 | 1.33846366366528 | 0.999332001504091 |
| ebi-fl187-GCST90094770 | prot-a-518 | Complement factor B \|\| id:prot-a-518 | Gherkins liking \|\| id:ebi-fl187-GCST90094770 | Weighted median | 21 | -0.073291326 | 0.116116491649608 | 0.527917664439321 | -0.30087965 | 0.15429699765793 | 0.929330052601508 | 0.740166846756312 | 1.16683738329159 | 0.999332001504091 |
| ebi-fl187-GCST90094770 | prot-a-525 | Properdin \|\| id:prot-a-525 | Gherkins liking \|\| id:ebi-fl187-GCST90094770 | Weighted median | 21 | -0.037896175 | 0.127491459142625 | 0.766279613406704 | -0.287779434 | 0.211987085348786 | 0.96281290016459 | 0.749926982025754 | 1.23613192076281 | 0.999332001504091 |
| ebi-fl187-GCST90094770 | prot-a-533 | Chondroadherin \|\| id:prot-a-533 | Gherkins liking \|\| id:ebi-fl187-GCST90094770 | Weighted median | 21 | 0.0271423874769524 | 0.120536442209762 | 0.821839302987175 | -0.209109039 | 0.263393814208086 | 1.02751409748785 | 0.811306766535461 | 1.30133910388152 | 0.999332001504091 |
| ebi-fl187-GCST90094770 | prot-a-535 | Coiled-coil-helix-coiled-coil-helix domain-containing protein 2 \|\| id:prot-a-535 | Gherkins liking \|\| id:ebi-fl187-GCST90094770 | Weighted median | 21 | -0.013502778 | 0.128072865955159 | 0.916034265697475 | -0.264525595 | 0.23752003909273 | 0.986587975395554 | 0.767570002315381 | 1.26810040811778 | 0.999332001504091 |
| ebi-fl187-GCST90094770 | prot-a-591 | Caseinolytic peptidase B protein homolog \|\| id:prot-a-591 | Gherkins liking \|\| id:ebi-fl187-GCST90094770 | Weighted median | 21 | -0.112577632 | 0.119189846451476 | 0.344902109960742 | -0.346189731 | 0.121034467371045 | 0.893527978343356 | 0.707378262987427 | 1.12866381377138 | 0.999332001504091 |
| ebi-fl187-GCST90094770 | prot-a-605 | Ciliary neurotrophic factor receptor subunit alpha \|\| id:prot-a-605 | Gherkins liking \|\| id:ebi-fl187-GCST90094770 | Weighted median | 21 | -0.207017602 | 0.118921208 | 0.0817192878545031 | -0.44010317 | 0.0260679669062527 | 0.813005339731471 | 0.643969979306998 | 1.02641070806312 | 0.999332001504091 |
| ebi-fl187-GCST90094770 | prot-a-606 | Ciliary neurotrophic factor receptor subunit alpha \|\| id:prot-a-606 | Gherkins liking \|\| id:ebi-fl187-GCST90094770 | Weighted median | 21 | -0.157215271 | 0.119547356175626 | 0.188480393671255 | -0.391528089 | 0.0770975475435345 | 0.854520085998395 | 0.67602306164276 | 1.08014743698282 | 0.999332001504091 |
| ebi-fl187-GCST90094770 | prot-a-707 | Cancer/testis antigen 55 \|\| id:prot-a-707 | Gherkins liking \|\| id:ebi-fl187-GCST90094770 | Weighted median | 21 | 0.110240448764023 | 0.126379953091754 | 0.383048122393778 | -0.137464259 | 0.357945156823861 | 1.11654651041295 | 0.871565499805204 | 1.43038717135313 | 0.999332001504091 |
| ebi-fl187-GCST90094770 | prot-a-719 | Dipeptidyl peptidase 1 \|\| id:prot-a-719 | Gherkins liking \|\| id:ebi-fl187-GCST90094770 | Weighted median | 21 | -0.141804619 | 0.116611501879723 | 0.223968480857249 | -0.370363163 | 0.0867539243505849 | 0.86779078946632 | 0.690483527017309 | 1.09062826963532 | 0.999332001504091 |
| ebi-fl187-GCST90094770 | prot-a-752 | Cytochrome b5 \|\| id:prot-a-752 | Gherkins liking \|\| id:ebi-fl187-GCST90094770 | Weighted median | 21 | 0.00930638363970815 | 0.121562524865116 | 0.938976481772458 | -0.228956165 | 0.247568932375335 | 1.00934982267681 | 0.795363397419196 | 1.28090765534783 | 0.999332001504091 |
| ebi-fl187-GCST90094770 | prot-a-755 | Cytoglobin \|\| id:prot-a-755 | Gherkins liking \|\| id:ebi-fl187-GCST90094770 | Weighted median | 21 | 0.0728726279724882 | 0.111268840215706 | 0.512516721529724 | -0.145214299 | 0.290959554795271 | 1.07559352766092 | 0.86483693968907 | 1.33771047888171 | 0.999332001504091 |
| ebi-fl187-GCST90094770 | prot-a-807 | Desmin \|\| id:prot-a-807 | Gherkins liking \|\| id:ebi-fl187-GCST90094770 | Weighted median | 21 | 0.0188945871756649 | 0.126704051715067 | 0.881455961696312 | -0.229445354 | 0.267234528537196 | 1.01907421946362 | 0.794974409474722 | 1.30634678600734 | 0.999332001504091 |
| ebi-fl187-GCST90094770 | prot-a-82 | Ameloblastin \|\| id:prot-a-82 | Gherkins liking \|\| id:ebi-fl187-GCST90094770 | Weighted median | 21 | -0.113603504 | 0.124938200927769 | 0.36320362646454 | -0.358482378 | 0.131275369876896 | 0.89261180278987 | 0.698735939022545 | 1.14028173732463 | 0.999332001504091 |
| ebi-fl187-GCST90094770 | prot-a-84 | Muellerian-inhibiting factor \|\| id:prot-a-84 | Gherkins liking \|\| id:ebi-fl187-GCST90094770 | Weighted median | 21 | 0.0831344826520607 | 0.115924461759728 | 0.47328551249229 | -0.144077462 | 0.310346427701127 | 1.08668793939804 | 0.865820676916482 | 1.36389752418337 | 0.999332001504091 |
| ebi-fl187-GCST90094770 | prot-a-858 | Inactive dipeptidyl peptidase 10 \|\| id:prot-a-858 | Gherkins liking \|\| id:ebi-fl187-GCST90094770 | Weighted median | 21 | -0.114095653 | 0.112556962042642 | 0.310740086906487 | -0.334707298 | 0.106515992927794 | 0.892172613103136 | 0.715547497687307 | 1.1123957167678 | 0.999332001504091 |
| ebi-fl187-GCST90094770 | prot-a-863 | Dihydropyrimidinase-related protein 3 \|\| id:prot-a-863 | Gherkins liking \|\| id:ebi-fl187-GCST90094770 | Weighted median | 21 | -0.107236297 | 0.120058246887768 | 0.371748870859559 | -0.342550461 | 0.128077867079501 | 0.898313379313766 | 0.709957293525068 | 1.13664150620579 | 0.999332001504091 |
| ebi-fl187-GCST90094770 | prot-a-864 | Dihydropyrimidinase-related protein 5 \|\| id:prot-a-864 | Gherkins liking \|\| id:ebi-fl187-GCST90094770 | Weighted median | 21 | 0.104579756293898 | 0.112796448806738 | 0.353846225239893 | -0.116501283 | 0.325660795955104 | 1.11024393927706 | 0.890028954741881 | 1.38494551006931 | 0.999332001504091 |
| ebi-fl187-GCST90094770 | prot-a-884 | Endothelin-converting enzyme 1 \|\| id:prot-a-884 | Gherkins liking \|\| id:ebi-fl187-GCST90094770 | Weighted median | 21 | 0.0359789519694527 | 0.111585313514832 | 0.74712357 | -0.182728263 | 0.254686166458523 | 1.03663402715663 | 0.832994480806583 | 1.29005669427537 | 0.999332001504091 |
| ebi-fl187-GCST90094770 | prot-a-893 | Early endosome antigen 1 \|\| id:prot-a-893 | Gherkins liking \|\| id:ebi-fl187-GCST90094770 | Weighted median | 21 | -0.117856954 | 0.113788384888563 | 0.300316188667878 | -0.340882188 | 0.105168280569828 | 0.888823186290228 | 0.711142684275835 | 1.11089753709776 | 0.999332001504091 |
| ebi-fl187-GCST90094770 | prot-a-897 | Ephrin-A2 \|\| id:prot-a-897 | Gherkins liking \|\| id:ebi-fl187-GCST90094770 | Weighted median | 21 | 0.141266605161486 | 0.113411469754924 | 0.212907141822269 | -0.081019876 | 0.363553085881137 | 1.15173166466326 | 0.922175362512404 | 1.43843121526699 | 0.999332001504091 |
| ebi-fl187-GCST90094770 | prot-a-90 | Anaphase-promoting complex subunit 10 \|\| id:prot-a-90 | Gherkins liking \|\| id:ebi-fl187-GCST90094770 | Weighted median | 21 | 0.00229798619378244 | 0.12168882348541 | 0.9849335486812 | -0.236212108 | 0.240808080225186 | 1.00230062858773 | 0.789613173026616 | 1.2722768367917 | 0.999332001504091 |
| ebi-fl187-GCST90094770 | prot-a-914 | Histone-lysine N-methyltransferase EHMT2 \|\| id:prot-a-914 | Gherkins liking \|\| id:ebi-fl187-GCST90094770 | Weighted median | 21 | -0.07257605 | 0.116854070455058 | 0.534545166350133 | -0.301610028 | 0.156457928520241 | 0.929995018248085 | 0.739626442508089 | 1.16936156451275 | 0.999332001504091 |
| ebi-fl187-GCST90094770 | prot-a-932 | RNA polymerase II elongation factor ELL \|\| id:prot-a-932 | Gherkins liking \|\| id:ebi-fl187-GCST90094770 | Weighted median | 21 | 0.0306428964889251 | 0.120652202713027 | 0.799513250123158 | -0.205835421 | 0.267121213806458 | 1.03111722255265 | 0.813967027278737 | 1.30619876605962 | 0.999332001504091 |
| ebi-fl187-GCST90094770 | prot-a-991 | Estrogen receptor \|\| id:prot-a-991 | Gherkins liking \|\| id:ebi-fl187-GCST90094770 | Weighted median | 21 | -0.006746333 | 0.115357710351641 | 0.953364817043687 | -0.232847445 | 0.219354779642056 | 0.993276372766815 | 0.792274429781506 | 1.24527299583414 | 0.999332001504091 |
| ebi-fl187-GCST90094770 | prot-a-992 | Persulfide dioxygenase ETHE1, mitochondrial \|\| id:prot-a-992 | Gherkins liking \|\| id:ebi-fl187-GCST90094770 | Weighted median | 21 | 0.0222921380917786 | 0.125435031038148 | 0.858943977188862 | -0.223560523 | 0.268144798926548 | 1.02254246444474 | 0.799666492393379 | 1.30753645618349 | 0.999332001504091 |
| ebi-fl187-GCST90094770 | prot-a-993 | Ethanolamine kinase 1 \|\| id:prot-a-993 | Gherkins liking \|\| id:ebi-fl187-GCST90094770 | Weighted median | 21 | -0.08396603 | 0.119541872383737 | 0.482430689563204 | -0.3182681 | 0.150336039761657 | 0.919462489603823 | 0.727407744266522 | 1.16222473083639 | 0.999332001504091 |
| ebi-fl187-GCST90094770 | prot-b-10 | coagulation factor III, tissue factor \|\| id:prot-b-10 | Gherkins liking \|\| id:ebi-fl187-GCST90094770 | Weighted median | 15 | 0.141515390582165 | 0.164890313147536 | 0.390760193137863 | -0.181669623 | 0.464700404351336 | 1.15201823435566 | 0.833876788468209 | 1.59153729980401 | 0.999332001504091 |
| ebi-fl187-GCST90094770 | prot-b-28 | prolactin \|\| id:prot-b-28 | Gherkins liking \|\| id:ebi-fl187-GCST90094770 | Weighted median | 15 | 0.029414269082436 | 0.156896731565064 | 0.851287951790203 | -0.278103325 | 0.336931862949962 | 1.02985114160386 | 0.757218578025319 | 1.40064362476236 | 0.999332001504091 |
| ebi-fl187-GCST90094770 | prot-b-54 | matrix metallopeptidase 7 \|\| id:prot-b-54 | Gherkins liking \|\| id:ebi-fl187-GCST90094770 | Weighted median | 15 | 0.173867848874413 | 0.167464648423517 | 0.29916013470662 | -0.154362862 | 0.502098559784507 | 1.18989830902889 | 0.856961005971968 | 1.65218484384121 | 0.999332001504091 |
| ebi-fl187-GCST90094770 | prot-c-2421_7_3 | BDNF \|\| id:prot-c-2421_7_3 | Gherkins liking \|\| id:ebi-fl187-GCST90094770 | Weighted median | 3 | 0.767389342087007 | 0.496012494861129 | 0.121835045506848 | -0.204795148 | 1.73957383201482 | 2.1541351965046 | 0.814814215767426 | 5.69491591460453 | 0.999332001504091 |
| ebi-fl187-GCST90094770 | prot-c-2443_10_4 | FGF-8B \|\| id:prot-c-2443_10_4 | Gherkins liking \|\| id:ebi-fl187-GCST90094770 | Weighted median | 3 | 0.680838693271433 | 0.535720020693108 | 0.203769460693403 | -0.369172547 | 1.73084993382992 | 1.97553390461634 | 0.691306117163694 | 5.6454501289543 | 0.999332001504091 |
| ebi-fl187-GCST90094770 | prot-c-2605_49_2 | CD30 \|\| id:prot-c-2605_49_2 | Gherkins liking \|\| id:ebi-fl187-GCST90094770 | Weighted median | 3 | 0.764564218810525 | 0.526421572354592 | 0.146395944900748 | -0.267222063 | 1.79635050062553 | 2.14805808735347 | 0.765503062679708 | 6.02760951797165 | 0.999332001504091 |
| ebi-fl187-GCST90094770 | prot-c-2624_31_2 | HPV E7 Type18 \|\| id:prot-c-2624_31_2 | Gherkins liking \|\| id:ebi-fl187-GCST90094770 | Weighted median | 3 | 0.788660995096138 | 0.543485279245348 | 0.146747146669441 | -0.276570152 | 1.85389214241702 | 2.20044804202702 | 0.758380415192178 | 6.38462108021812 | 0.999332001504091 |
| ebi-fl187-GCST90094770 | prot-c-2774_10_3 | IL-16 \|\| id:prot-c-2774_10_3 | Gherkins liking \|\| id:ebi-fl187-GCST90094770 | Weighted median | 3 | -0.359777634 | 0.482402684562422 | 0.45578609632826 | -1.305286895 | 0.585731627995313 | 0.697831482991634 | 0.271094748027357 | 1.79630473182447 | 0.999332001504091 |
| ebi-fl187-GCST90094770 | prot-c-2851_63_3 | C5a \|\| id:prot-c-2851_63_3 | Gherkins liking \|\| id:ebi-fl187-GCST90094770 | Weighted median | 3 | 0.321222256956268 | 0.475569929769095 | 0.499391847247692 | -0.610894805 | 1.25333931930369 | 1.3788119973987 | 0.542864893246882 | 3.50201780925628 | 0.999332001504091 |
| ebi-fl187-GCST90094770 | prot-c-2891_1_3 | CD22 \|\| id:prot-c-2891_1_3 | Gherkins liking \|\| id:ebi-fl187-GCST90094770 | Weighted median | 3 | 0.904096111850188 | 0.497575722836556 | 0.0692167983968409 | -0.071152305 | 1.87934452860984 | 2.46969858258213 | 0.931320036711357 | 6.54921063477406 | 0.999332001504091 |
| ebi-fl187-GCST90094770 | prot-c-2961_1_2 | Protein C \|\| id:prot-c-2961_1_2 | Gherkins liking \|\| id:ebi-fl187-GCST90094770 | Weighted median | 3 | 0.332361746810699 | 0.458173686411687 | 0.46820325342777 | -0.565658679 | 1.2303821721776 | 1.39425712536514 | 0.567985903383242 | 3.42253728490829 | 0.999332001504091 |
| ebi-fl187-GCST90094770 | prot-c-2982_82_2 | Galectin-4 \|\| id:prot-c-2982_82_2 | Gherkins liking \|\| id:ebi-fl187-GCST90094770 | Weighted median | 3 | 0.199157309008308 | 0.508085117374671 | 0.695075747650601 | -0.796689521 | 1.19500413906266 | 1.22037392661306 | 0.450818923074479 | 3.30357144416259 | 0.999332001504091 |
| ebi-fl187-GCST90094770 | prot-c-2994_71_2 | IL-1Rrp2 \|\| id:prot-c-2994_71_2 | Gherkins liking \|\| id:ebi-fl187-GCST90094770 | Weighted median | 3 | -0.829086586 | 0.494352938641088 | 0.0935200526339183 | -1.798018346 | 0.139845173894553 | 0.436447761872156 | 0.165626778269379 | 1.15009572023072 | 0.999332001504091 |
| ebi-fl187-GCST90094770 | prot-c-3336_50_1 | TFPI \|\| id:prot-c-3336_50_1 | Gherkins liking \|\| id:ebi-fl187-GCST90094770 | Weighted median | 3 | -0.114731471 | 0.485818515273103 | 0.813307606481118 | -1.066935761 | 0.837472819252075 | 0.891605533988657 | 0.344061189622109 | 2.310520489 | 0.999332001504091 |
| ebi-fl187-GCST90094770 | prot-c-3472_40_2 | Survivin \|\| id:prot-c-3472_40_2 | Gherkins liking \|\| id:ebi-fl187-GCST90094770 | Weighted median | 3 | 0.378194453184948 | 0.537574241270662 | 0.481731488945172 | -0.67545106 | 1.43183996607545 | 1.45964674824057 | 0.508926812449778 | 4.18639493445733 | 0.999332001504091 |
| ebi-fl187-GCST90094770 | prot-c-3473_78_2 | Thrombopoietin Receptor \|\| id:prot-c-3473_78_2 | Gherkins liking \|\| id:ebi-fl187-GCST90094770 | Weighted median | 3 | 0.287274799646209 | 0.502290177718831 | 0.567369428082893 | -0.697213949 | 1.27176354797512 | 1.33279041349165 | 0.497970744990169 | 3.56713783724447 | 0.999332001504091 |
| ebi-fl187-GCST90094770 | prot-c-3497_13_2 | IFN-aA \|\| id:prot-c-3497_13_2 | Gherkins liking \|\| id:ebi-fl187-GCST90094770 | Weighted median | 3 | 0.81245477957846 | 0.518004964119615 | 0.11678070016613 | -0.20283495 | 1.8277445092529 | 2.25343288372425 | 0.816412979189929 | 6.21984202956734 | 0.999332001504091 |
| ebi-fl187-GCST90094770 | prot-c-3665_64_3 | COLEC12 \|\| id:prot-c-3665_64_3 | Gherkins liking \|\| id:ebi-fl187-GCST90094770 | Weighted median | 3 | -0.773922623 | 0.509970709107521 | 0.129119681366468 | -1.773465213 | 0.225619966423381 | 0.461200399934307 | 0.169743770164593 | 1.25309935494724 | 0.999332001504091 |
| ebi-fl187-GCST90094770 | prot-c-3741_4_3 | IL-5 \|\| id:prot-c-3741_4_3 | Gherkins liking \|\| id:ebi-fl187-GCST90094770 | Weighted median | 3 | 0.115432659362439 | 0.533952486103225 | 0.828843339309317 | -0.931114213 | 1.16197953212476 | 1.12235893163738 | 0.394114338163189 | 3.19625410559055 | 0.999332001504091 |
| ebi-fl187-GCST90094770 | prot-c-3848_14_2 | GAPDH, liver \|\| id:prot-c-3848_14_2 | Gherkins liking \|\| id:ebi-fl187-GCST90094770 | Weighted median | 3 | -0.125341484 | 0.500330771494345 | 0.802187374611334 | -1.105989796 | 0.855306828511256 | 0.882195595798321 | 0.330883213308928 | 2.35209595997041 | 0.999332001504091 |
| ebi-fl187-GCST90094770 | prot-c-3859_50_2 | PSA1 \|\| id:prot-c-3859_50_2 | Gherkins liking \|\| id:ebi-fl187-GCST90094770 | Weighted median | 3 | 0.310727733647643 | 0.493514822277899 | 0.52894200017871 | -0.656561318 | 1.27801678531233 | 1.36441768558379 | 0.51863168113213 | 3.58951388521047 | 0.999332001504091 |
| ebi-fl187-GCST90094770 | prot-c-3868_8_1 | SGTA \|\| id:prot-c-3868_8_1 | Gherkins liking \|\| id:ebi-fl187-GCST90094770 | Weighted median | 3 | 0.00730745232069996 | 0.531337282354224 | 0.989027083579422 | -1.034113621 | 1.04872852573498 | 1.00733421690433 | 0.35554138568217 | 2.85401999713573 | 0.999332001504091 |
| ebi-fl187-GCST90094770 | prot-c-3882_10_1 | EF-1-beta \|\| id:prot-c-3882_10_1 | Gherkins liking \|\| id:ebi-fl187-GCST90094770 | Weighted median | 3 | 0.161185483945499 | 0.489787582005807 | 0.742085673999509 | -0.798798177 | 1.12116914467688 | 1.17490287422496 | 0.449869302727779 | 3.06843955676025 | 0.999332001504091 |
| ebi-fl187-GCST90094770 | prot-c-4134_4_2 | HB-EGF \|\| id:prot-c-4134_4_2 | Gherkins liking \|\| id:ebi-fl187-GCST90094770 | Weighted median | 3 | 0.914630196112419 | 0.523698850588723 | 0.0807273713633037 | -0.111819551 | 1.94107994326632 | 2.49585210541242 | 0.894205601412229 | 6.96627008626835 | 0.999332001504091 |
| ebi-fl187-GCST90094770 | prot-c-4220_39_2 | FER \|\| id:prot-c-4220_39_2 | Gherkins liking \|\| id:ebi-fl187-GCST90094770 | Weighted median | 3 | 0.249832950707296 | 0.506409506655338 | 0.621771135372237 | -0.742729682 | 1.24239558375176 | 1.28381093906474 | 0.475813322004173 | 3.46390159972827 | 0.999332001504091 |
| ebi-fl187-GCST90094770 | prot-c-4228_37_2 | HSP70 protein 8 \|\| id:prot-c-4228_37_2 | Gherkins liking \|\| id:ebi-fl187-GCST90094770 | Weighted median | 3 | 0.458073995769504 | 0.49259616842878 | 0.352413592957632 | -0.507414494 | 1.42356248588991 | 1.58102598796776 | 0.602050172362728 | 4.15188515737758 | 0.999332001504091 |
| ebi-fl187-GCST90094770 | prot-c-4246_40_2 | NCAM-L1 \|\| id:prot-c-4246_40_2 | Gherkins liking \|\| id:ebi-fl187-GCST90094770 | Weighted median | 3 | -0.004356877 | 0.492765940505227 | 0.992945454249534 | -0.970178121 | 0.961464366048512 | 0.995652600079369 | 0.379015521568397 | 2.61552375465424 | 0.999332001504091 |
| ebi-fl187-GCST90094770 | prot-c-4267_81_3 | PESC \|\| id:prot-c-4267_81_3 | Gherkins liking \|\| id:ebi-fl187-GCST90094770 | Weighted median | 3 | 0.670672912063827 | 0.559013004275125 | 0.230238491079719 | -0.424992576 | 1.76633840044307 | 1.95555279305357 | 0.653774638528536 | 5.84939595550354 | 0.999332001504091 |
| ebi-fl187-GCST90094770 | prot-c-4280_47_2 | PSA2 \|\| id:prot-c-4280_47_2 | Gherkins liking \|\| id:ebi-fl187-GCST90094770 | Weighted median | 3 | 0.575592974955247 | 0.512946001332238 | 0.261806441948513 | -0.429781188 | 1.58096713756643 | 1.77818463330716 | 0.650651449717113 | 4.85965349267178 | 0.999332001504091 |
| ebi-fl187-GCST90094770 | prot-c-4324_33_2 | CYTT \|\| id:prot-c-4324_33_2 | Gherkins liking \|\| id:ebi-fl187-GCST90094770 | Weighted median | 3 | -0.426031001 | 0.508845987652489 | 0.402452391442247 | -1.423369137 | 0.571307134741851 | 0.653096095137549 | 0.240901019612711 | 1.77057992601958 | 0.999332001504091 |
| ebi-fl187-GCST90094770 | prot-c-4462_8_2 | RASA1 \|\| id:prot-c-4462_8_2 | Gherkins liking \|\| id:ebi-fl187-GCST90094770 | Weighted median | 3 | 0.1812923991905 | 0.511719070957595 | 0.723128224795326 | -0.82167698 | 1.18426177826739 | 1.19876564615712 | 0.439693678438524 | 3.26827322036978 | 0.999332001504091 |
| ebi-fl187-GCST90094770 | prot-c-4499_21_1 | PDGF-AA \|\| id:prot-c-4499_21_1 | Gherkins liking \|\| id:ebi-fl187-GCST90094770 | Weighted median | 3 | 0.382970937857329 | 0.498801146388693 | 0.442616238253943 | -0.594679309 | 1.36062118477917 | 1.46663540588161 | 0.551739475348177 | 3.89861430963974 | 0.999332001504091 |
| ebi-fl187-GCST90094770 | prot-c-4535_50_2 | BST1 \|\| id:prot-c-4535_50_2 | Gherkins liking \|\| id:ebi-fl187-GCST90094770 | Weighted median | 3 | -0.448807291 | 0.503672145940836 | 0.372891456576448 | -1.436004697 | 0.538390115374613 | 0.638389110378746 | 0.237876250429467 | 1.71324651163949 | 0.999332001504091 |
| ebi-fl187-GCST90094770 | prot-c-4542_24_2 | Clusterin \|\| id:prot-c-4542_24_2 | Gherkins liking \|\| id:ebi-fl187-GCST90094770 | Weighted median | 3 | 0.33590628469836 | 0.479220654009403 | 0.483338689763662 | -0.603366197 | 1.27517876655679 | 1.39920789147682 | 0.546967333807279 | 3.57934121941699 | 0.999332001504091 |
| ebi-fl187-GCST90094770 | prot-c-4696_2_2 | FABP \|\| id:prot-c-4696_2_2 | Gherkins liking \|\| id:ebi-fl187-GCST90094770 | Weighted median | 3 | -0.239668071 | 0.469243645420704 | 0.609523320787519 | -1.159385616 | 0.680049474505495 | 0.786889009383166 | 0.313678841097076 | 1.97397539127095 | 0.999332001504091 |
| ebi-fl187-GCST90094770 | prot-c-4906_35_1 | Coagulation Factor V \|\| id:prot-c-4906_35_1 | Gherkins liking \|\| id:ebi-fl187-GCST90094770 | Weighted median | 3 | -0.765374591 | 0.516122736906626 | 0.138092649183111 | -1.776975155 | 0.246225973566697 | 0.465159653822012 | 0.16914902377865 | 1.27918860369518 | 0.999332001504091 |
| ebi-fl187-GCST90094770 | prot-c-4916_2_1 | IgD \|\| id:prot-c-4916_2_1 | Gherkins liking \|\| id:ebi-fl187-GCST90094770 | Weighted median | 3 | -0.659675695 | 0.491568922814888 | 0.179602459133062 | -1.623150784 | 0.303799393237336 | 0.517018978898174 | 0.197276144272891 | 1.35499720722006 | 0.999332001504091 |
| ebi-fl187-GCST90094770 | prot-c-4922_13_1 | MIP-3b \|\| id:prot-c-4922_13_1 | Gherkins liking \|\| id:ebi-fl187-GCST90094770 | Weighted median | 3 | 0.318513792658517 | 0.527413555060264 | 0.54589910222438 | -0.715216775 | 1.35224436057663 | 1.37508258709761 | 0.48908607854241 | 3.8660927069816 | 0.999332001504091 |
| ebi-fl187-GCST90094770 | prot-c-4973_18_1 | cIAP-2 \|\| id:prot-c-4973_18_1 | Gherkins liking \|\| id:ebi-fl187-GCST90094770 | Weighted median | 3 | -0.11772193 | 0.503099919078044 | 0.814990304658491 | -1.103797771 | 0.868353911803711 | 0.888943207052571 | 0.331609313030155 | 2.38298501976346 | 0.999332001504091 |
| ebi-fl187-GCST90094770 | prot-c-4997_19_1 | IF4A3 \|\| id:prot-c-4997_19_1 | Gherkins liking \|\| id:ebi-fl187-GCST90094770 | Weighted median | 3 | -0.335698348 | 0.495047669826737 | 0.497699936679898 | -1.305991781 | 0.634595084836396 | 0.714838705804462 | 0.270903724631513 | 1.8862582122533 | 0.999332001504091 |
| ebi-fl187-GCST90094770 | prot-c-5000_52_1 | LG3BP \|\| id:prot-c-5000_52_1 | Gherkins liking \|\| id:ebi-fl187-GCST90094770 | Weighted median | 3 | -0.354217443 | 0.46954285979778 | 0.450616346731047 | -1.274521448 | 0.566086562362368 | 0.701722366259878 | 0.27956472245918 | 1.76136057145501 | 0.999332001504091 |
| ebi-fl187-GCST90094770 | prot-c-5006_71_1 | MK13 \|\| id:prot-c-5006_71_1 | Gherkins liking \|\| id:ebi-fl187-GCST90094770 | Weighted median | 3 | -0.53241051 | 0.483346619460218 | 0.270675317364618 | -1.479769884 | 0.414948864609205 | 0.587187840475797 | 0.2276900775597 | 1.51429330473229 | 0.999332001504091 |
| ebi-fl187-GCST90094770 | prot-c-5109_24_3 | Nr-CAM \|\| id:prot-c-5109_24_3 | Gherkins liking \|\| id:ebi-fl187-GCST90094770 | Weighted median | 3 | -0.305929908 | 0.464575928656497 | 0.510207690419689 | -1.216498728 | 0.604638912614929 | 0.736438236406033 | 0.2962656598376 | 1.83059108618298 | 0.999332001504091 |
| ebi-fl187-GCST90094770 | prot-c-5255_22_3 | PDE4D \|\| id:prot-c-5255_22_3 | Gherkins liking \|\| id:ebi-fl187-GCST90094770 | Weighted median | 3 | 0.124330247544011 | 0.477970581055469 | 0.794770339587498 | -0.812492091 | 1.06115258641273 | 1.13238977819792 | 0.443750819548345 | 2.8896996991967 | 0.999332001504091 |
| ebi-fl187-GCST90094770 | prot-c-5440_26_3 | Troponin I, skeletal, fast twitch \|\| id:prot-c-5440_26_3 | Gherkins liking \|\| id:ebi-fl187-GCST90094770 | Weighted median | 3 | -0.668949976 | 0.546513993267644 | 0.220940598347743 | -1.740117402 | 0.402217451236403 | 0.512246166486432 | 0.175499795315076 | 1.49513641659219 | 0.999332001504091 |
| ebi-fl187-GCST90094770 | ukb-rsfMRI191-edge-pheno716 | Net100_Pair10_33 (OccipitalPrecuneus)&(Temporal\|Frontal\|Supp_Motor_Area) (Default_mode\|Central_executive)&(Default_mode\|Salience) \|\| id:ukb-rsfMRI191-edge-pheno716 | Gherkins liking \|\| id:ebi-fl187-GCST90094770 | Weighted median | 21 | -0.026344198 | 0.0371681430005306 | 0.478458307608455 | -0.099193759 | 0.0465053620014109 | 0.973999782856438 | 0.905567229620875 | 1.04760369630597 | 0.999332001504091 |
| ebi-fl187-GCST90094770 | ukb-rsfMRI191-edge-pheno903 | Net100_Pair27_38 (Precentral\|Frontal\|Supp_Motor_Area)&(Subcortical) (Attention\|Salience\|Motor)&(Subcortical-cerebellum) \|\| id:ukb-rsfMRI191-edge-pheno903 | Gherkins liking \|\| id:ebi-fl187-GCST90094770 | Weighted median | 21 | 0.0922286065477321 | 0.0387859677746286 | 0.0174122120576025 | 0.01620810970946 | 0.168249103386004 | 1.09661548690857 | 1.0163401736572 | 1.18323132086808 | 0.999332001504091 |
| ebi-fl187-GCST90094770 | ukb-rsfMRI191-node-pheno23 | Net100_Node2 RolandicOper\|SupraMarginal\|Insula Salience\|Motor \|\| id:ukb-rsfMRI191-node-pheno23 | Gherkins liking \|\| id:ebi-fl187-GCST90094770 | Weighted median | 21 | 0.00680812748154735 | 0.0352633863619569 | 0.846908023052803 | -0.06230811 | 0.075924364750983 | 1.00683135546455 | 0.939593344281931 | 1.07888096964044 | 0.999332001504091 |
| ebi-fl187-GCST90094770 | ukb-rsfMRI191-node-pheno55 | Net100_Node34 Parietal\|Postcentral\|Precuneus Attention \|\| id:ukb-rsfMRI191-node-pheno55 | Gherkins liking \|\| id:ebi-fl187-GCST90094770 | Weighted median | 21 | -0.014036349 | 0.0368704107717836 | 0.703430215014051 | -0.086302355 | 0.0582296556622159 | 0.986061700810457 | 0.917316834355415 | 1.05995839320712 | 0.999332001504091 |
| ebi-fl187-GCST90094770 | ebi-a-GCST90001414 | IgD- CD24- B cell Absolute Count \|\| id:ebi-a-GCST90001414 | Gherkins liking \|\| id:ebi-fl187-GCST90094770 | Weighted mode | 19 | -0.009987868 | 0.179272075172411 | 0.956183835613453 | -0.361361136 | 0.341385398970888 | 0.990061844743222 | 0.696727339909098 | 1.40689535241181 | 0.999714303421225 |
| ebi-fl187-GCST90094770 | ebi-a-GCST90001422 | CD20- CD38- B cell %B cell \|\| id:ebi-a-GCST90001422 | Gherkins liking \|\| id:ebi-fl187-GCST90094770 | Weighted mode | 19 | -0.031597357 | 0.170493029042298 | 0.855043063317308 | -0.365763694 | 0.302568980341974 | 0.968896623399314 | 0.693666699791178 | 1.35333102643849 | 0.999714303421225 |
| ebi-fl187-GCST90094770 | ebi-a-GCST90001444 | CD20- CD38- B cell %lymphocyte \|\| id:ebi-a-GCST90001444 | Gherkins liking \|\| id:ebi-fl187-GCST90094770 | Weighted mode | 19 | -0.047247164 | 0.18936223194867 | 0.805793877705818 | -0.418397138 | 0.323902810907255 | 0.95385161094763 | 0.658100819459083 | 1.38251293541195 | 0.999714303421225 |
| ebi-fl187-GCST90094770 | ebi-a-GCST90001459 | Myeloid Dendritic Cell %Dendritic Cell \|\| id:ebi-a-GCST90001459 | Gherkins liking \|\| id:ebi-fl187-GCST90094770 | Weighted mode | 19 | -0.094535633 | 0.198618793037643 | 0.639824922361797 | -0.483828468 | 0.294757201083303 | 0.909795315065886 | 0.616418928647536 | 1.34280028864773 | 0.999714303421225 |
| ebi-fl187-GCST90094770 | ebi-a-GCST90001476 | HLA DR++ monocyte %leukocyte \|\| id:ebi-a-GCST90001476 | Gherkins liking \|\| id:ebi-fl187-GCST90094770 | Weighted mode | 19 | -0.11064928 | 0.163286535614459 | 0.50661832512877 | -0.43069089 | 0.209392329830773 | 0.895252676917911 | 0.650059819856915 | 1.232928618 | 0.999714303421225 |
| ebi-fl187-GCST90094770 | ebi-a-GCST90001478 | CD4 regulatory T cell %CD4+ T cell \|\| id:ebi-a-GCST90001478 | Gherkins liking \|\| id:ebi-fl187-GCST90094770 | Weighted mode | 19 | 0.0413653605018471 | 0.195523187845105 | 0.834825354339915 | -0.341860088 | 0.424590808678254 | 1.04223282669961 | 0.710447598131391 | 1.52896465257579 | 0.999714303421225 |
| ebi-fl187-GCST90094770 | ebi-a-GCST90001485 | CD39+ resting CD4 regulatory T cell %CD4 regulatory T cell \|\| id:ebi-a-GCST90001485 | Gherkins liking \|\| id:ebi-fl187-GCST90094770 | Weighted mode | 18 | -0.001915934 | 0.17988410049714 | 0.991625938260071 | -0.354488771 | 0.35065690279316 | 0.998085900049051 | 0.701531994940833 | 1.42000004427559 | 0.999714303421225 |
| ebi-fl187-GCST90094770 | ebi-a-GCST90001515 | Immature Myeloid-Derived Suppressor Cells Absolute Count \|\| id:ebi-a-GCST90001515 | Gherkins liking \|\| id:ebi-fl187-GCST90094770 | Weighted mode | 19 | 0.0213803199828004 | 0.273277987509032 | 0.93850307212756 | -0.514244536 | 0.557005175500504 | 1.02161051665647 | 0.59795215563302 | 1.74543738643804 | 0.999714303421225 |
| ebi-fl187-GCST90094770 | ebi-a-GCST90001517 | CD33+ HLA DR+ Absolute Count \|\| id:ebi-a-GCST90001517 | Gherkins liking \|\| id:ebi-fl187-GCST90094770 | Weighted mode | 19 | -0.149974573 | 0.25152048395015 | 0.558418547819066 | -0.642954721 | 0.343005575611822 | 0.860729861984861 | 0.525736721261333 | 1.40917661892636 | 0.999714303421225 |
| ebi-fl187-GCST90094770 | ebi-a-GCST90001518 | CD33+ HLA DR+ CD14- Absolute Count \|\| id:ebi-a-GCST90001518 | Gherkins liking \|\| id:ebi-fl187-GCST90094770 | Weighted mode | 19 | -0.234768228 | 0.239150204285029 | 0.339270619494331 | -0.703502629 | 0.233966171945765 | 0.790754102693255 | 0.494848992381462 | 1.26360174629637 | 0.999714303421225 |
| ebi-fl187-GCST90094770 | ebi-a-GCST90001520 | CD33+ HLA DR+ CD14dim Absolute Count \|\| id:ebi-a-GCST90001520 | Gherkins liking \|\| id:ebi-fl187-GCST90094770 | Weighted mode | 19 | 0.00577648975122802 | 0.257319925536721 | 0.982337044538888 | -0.498570564 | 0.5101235438032 | 1.00579320583942 | 0.607398276243609 | 1.6654969440628 | 0.999714303421225 |
| ebi-fl187-GCST90094770 | ebi-a-GCST90001531 | CD33dim HLA DR- Absolute Count \|\| id:ebi-a-GCST90001531 | Gherkins liking \|\| id:ebi-fl187-GCST90094770 | Weighted mode | 19 | 0.200331135633459 | 0.228503519462077 | 0.39219529554297 | -0.247535763 | 0.648198033779131 | 1.22180727510749 | 0.780722299720886 | 1.91209219723747 | 0.999714303421225 |
| ebi-fl187-GCST90094770 | ebi-a-GCST90001532 | Basophil Absolute Count \|\| id:ebi-a-GCST90001532 | Gherkins liking \|\| id:ebi-fl187-GCST90094770 | Weighted mode | 19 | 0.181701848200702 | 0.251315009524221 | 0.47896744166614 | -0.31087557 | 0.674279266868175 | 1.19925658006375 | 0.732805052787331 | 1.96261794232414 | 0.999714303421225 |
| ebi-fl187-GCST90094770 | ebi-a-GCST90001588 | T/B cell \|\| id:ebi-a-GCST90001588 | Gherkins liking \|\| id:ebi-fl187-GCST90094770 | Weighted mode | 19 | -0.142259934 | 0.187499755449473 | 0.457840344230328 | -0.509759455 | 0.225239586686454 | 0.867395761535162 | 0.60064004259049 | 1.25262279198746 | 0.999714303421225 |
| ebi-fl187-GCST90094770 | ebi-a-GCST90001624 | HLA DR+ CD4+ T cell Absolute Count \|\| id:ebi-a-GCST90001624 | Gherkins liking \|\| id:ebi-fl187-GCST90094770 | Weighted mode | 19 | 0.0903636391414686 | 0.182191464448004 | 0.625908935114143 | -0.266731631 | 0.447458909459556 | 1.09457224065433 | 0.765878581821711 | 1.56433202135158 | 0.999714303421225 |
| ebi-fl187-GCST90094770 | ebi-a-GCST90001631 | CD8+ Natural Killer T %T cell \|\| id:ebi-a-GCST90001631 | Gherkins liking \|\| id:ebi-fl187-GCST90094770 | Weighted mode | 19 | 0.0460869160191366 | 0.188435109542255 | 0.809550213049477 | -0.323245899 | 0.415419730721956 | 1.0471654224516 | 0.723795852036272 | 1.5150065020313 | 0.999714303421225 |
| ebi-fl187-GCST90094770 | ebi-a-GCST90001632 | CD8+ Natural Killer T %lymphocyte \|\| id:ebi-a-GCST90001632 | Gherkins liking \|\| id:ebi-fl187-GCST90094770 | Weighted mode | 19 | -0.041140251 | 0.175068754147942 | 0.816865328680629 | -0.384275009 | 0.301994507299147 | 0.959694522554499 | 0.6809441356882 | 1.35255379751568 | 0.999714303421225 |
| ebi-fl187-GCST90094770 | ebi-a-GCST90001646 | Natural Killer %CD3- lymphocyte \|\| id:ebi-a-GCST90001646 | Gherkins liking \|\| id:ebi-fl187-GCST90094770 | Weighted mode | 19 | -0.018574371 | 0.195083409042289 | 0.925198038806776 | -0.400937853 | 0.363789110535936 | 0.981597069337928 | 0.669691679133865 | 1.43877076056698 | 0.999714303421225 |
| ebi-fl187-GCST90094770 | ebi-a-GCST90001684 | CD127- CD8+ T cell Absolute Count \|\| id:ebi-a-GCST90001684 | Gherkins liking \|\| id:ebi-fl187-GCST90094770 | Weighted mode | 19 | 0.0136721084979223 | 0.198295045314526 | 0.945790942616409 | -0.37498618 | 0.402330397314394 | 1.01376599917988 | 0.687298776975501 | 1.49530529592346 | 0.999714303421225 |
| ebi-fl187-GCST90094770 | ebi-a-GCST90001687 | CD28- CD8+ T cell Absolute Count \|\| id:ebi-a-GCST90001687 | Gherkins liking \|\| id:ebi-fl187-GCST90094770 | Weighted mode | 19 | -0.022456418 | 0.183330641861921 | 0.903867148135074 | -0.381784476 | 0.336871640489802 | 0.977793850906992 | 0.68264216338849 | 1.40055927709731 | 0.999714303421225 |
| ebi-fl187-GCST90094770 | ebi-a-GCST90001701 | BAFF-R on CD20- CD38- B cell \|\| id:ebi-a-GCST90001701 | Gherkins liking \|\| id:ebi-fl187-GCST90094770 | Weighted mode | 19 | -0.000308229 | 0.219920730384644 | 0.99889714521448 | -0.43135286 | 0.430736402646949 | 0.999691818590697 | 0.649629641712376 | 1.53838998097881 | 0.999714303421225 |
| ebi-fl187-GCST90094770 | ebi-a-GCST90001732 | CD19 on IgD- CD27- B cell \|\| id:ebi-a-GCST90001732 | Gherkins liking \|\| id:ebi-fl187-GCST90094770 | Weighted mode | 19 | -0.246853072 | 0.302009514437775 | 0.424405310272721 | -0.83879172 | 0.345085576685151 | 0.781255473705611 | 0.432232465921643 | 1.41211075825486 | 0.999714303421225 |
| ebi-fl187-GCST90094770 | ebi-a-GCST90001798 | CD27 on CD24+ CD27+ B cell \|\| id:ebi-a-GCST90001798 | Gherkins liking \|\| id:ebi-fl187-GCST90094770 | Weighted mode | 19 | 0.0131315936659875 | 0.176039365261626 | 0.941359995466223 | -0.331905562 | 0.358168749578775 | 1.01321819168359 | 0.717555083944716 | 1.43070703131922 | 0.999714303421225 |
| ebi-fl187-GCST90094770 | ebi-a-GCST90001836 | CD66b on CD66b++ myeloid cell \|\| id:ebi-a-GCST90001836 | Gherkins liking \|\| id:ebi-fl187-GCST90094770 | Weighted mode | 19 | -0.144160406 | 0.266118177094802 | 0.594657087020574 | -0.665752033 | 0.37743122080733 | 0.865748865349767 | 0.513886922223192 | 1.45853312361367 | 0.999714303421225 |
| ebi-fl187-GCST90094770 | ebi-a-GCST90001943 | CD25 on activated & secreting CD4 regulatory T cell \|\| id:ebi-a-GCST90001943 | Gherkins liking \|\| id:ebi-fl187-GCST90094770 | Weighted mode | 19 | 0.167599867066498 | 0.198979388765253 | 0.410673754092681 | -0.222399735 | 0.557599469046394 | 1.18246337343686 | 0.800595274480664 | 1.74647499690362 | 0.999714303421225 |
| ebi-fl187-GCST90094770 | ebi-a-GCST90001960 | CD25 on CD4+ T cell \|\| id:ebi-a-GCST90001960 | Gherkins liking \|\| id:ebi-fl187-GCST90094770 | Weighted mode | 19 | 0.174149460150264 | 0.190491892835496 | 0.372691236791737 | -0.19921465 | 0.547513570107835 | 1.19023344499665 | 0.819373995985085 | 1.72894875908946 | 0.999714303421225 |
| ebi-fl187-GCST90094770 | ebi-a-GCST90001988 | HLA DR on CD14+ CD16- monocyte \|\| id:ebi-a-GCST90001988 | Gherkins liking \|\| id:ebi-fl187-GCST90094770 | Weighted mode | 19 | 0.165480545612256 | 0.203691687519897 | 0.427172660479774 | -0.233755162 | 0.564716253151254 | 1.17996000709667 | 0.791555595119135 | 1.75894861577981 | 0.999714303421225 |
| ebi-fl187-GCST90094770 | ebi-a-GCST90001991 | HLA DR on CD14+ monocyte \|\| id:ebi-a-GCST90001991 | Gherkins liking \|\| id:ebi-fl187-GCST90094770 | Weighted mode | 19 | 0.159451519058626 | 0.194785289184132 | 0.423719407708234 | -0.222327648 | 0.541230685859524 | 1.17286739912673 | 0.80065298920944 | 1.71812002761969 | 0.999714303421225 |
| ebi-fl187-GCST90094770 | ebi-a-GCST90002019 | CD14 on Monocytic Myeloid-Derived Suppressor Cells \|\| id:ebi-a-GCST90002019 | Gherkins liking \|\| id:ebi-fl187-GCST90094770 | Weighted mode | 19 | -0.003853902 | 0.314199571752414 | 0.990348498838432 | -0.619685063 | 0.611977258645518 | 0.996153514760191 | 0.538113883085273 | 1.84407400767958 | 0.999714303421225 |
| ebi-fl187-GCST90094770 | ebi-a-GCST90002037 | CD80 on plasmacytoid Dendritic Cell \|\| id:ebi-a-GCST90002037 | Gherkins liking \|\| id:ebi-fl187-GCST90094770 | Weighted mode | 19 | -0.138866123 | 0.19205869328592 | 0.478946061035795 | -0.515301161 | 0.237568916275988 | 0.870344540154046 | 0.597320677593175 | 1.26816239080857 | 0.999714303421225 |
| ebi-fl187-GCST90094770 | ebi-a-GCST90002042 | CD45 on CD33+ HLA DR+ CD14- \|\| id:ebi-a-GCST90002042 | Gherkins liking \|\| id:ebi-fl187-GCST90094770 | Weighted mode | 19 | 0.0243685191683121 | 0.29248105550954 | 0.934519402267733 | -0.54889435 | 0.59763138796701 | 1.0246678580678 | 0.577588067931411 | 1.81780801517864 | 0.999714303421225 |
| ebi-fl187-GCST90094770 | ebi-a-GCST90002056 | CD8 on Effector Memory CD8+ T cell \|\| id:ebi-a-GCST90002056 | Gherkins liking \|\| id:ebi-fl187-GCST90094770 | Weighted mode | 19 | -0.044298536 | 0.188862913339164 | 0.817202340210361 | -0.414469846 | 0.325872774505139 | 0.956668315250577 | 0.660690455703714 | 1.38523911992881 | 0.999714303421225 |
| ebi-fl187-GCST90094770 | ebi-a-GCST90002058 | CD8 on CD8+ T cell \|\| id:ebi-a-GCST90002058 | Gherkins liking \|\| id:ebi-fl187-GCST90094770 | Weighted mode | 19 | -0.360510148 | 0.201390909279182 | 0.0902738754162133 | -0.75523633 | 0.0342160340756319 | 0.697320498580881 | 0.469899550106023 | 1.03480813640144 | 0.999714303421225 |
| ebi-fl187-GCST90094770 | ebi-a-GCST90002063 | CD4 on CD4 regulatory T cell \|\| id:ebi-a-GCST90002063 | Gherkins liking \|\| id:ebi-fl187-GCST90094770 | Weighted mode | 19 | -0.142019335 | 0.203828300256173 | 0.494853936457886 | -0.541522804 | 0.257484133263947 | 0.867604480984473 | 0.581861516489367 | 1.29367128447666 | 0.999714303421225 |
| ebi-fl187-GCST90094770 | ebi-a-GCST90002066 | CD4 on activated CD4 regulatory T cell \|\| id:ebi-a-GCST90002066 | Gherkins liking \|\| id:ebi-fl187-GCST90094770 | Weighted mode | 19 | -0.125244108 | 0.202376418951526 | 0.543757378496376 | -0.521901889 | 0.271413673597006 | 0.882281504720792 | 0.593390910625022 | 1.31181762247162 | 0.999714303421225 |
| ebi-fl187-GCST90094770 | ebi-a-GCST90002070 | CD4 on activated & secreting CD4 regulatory T cell \|\| id:ebi-a-GCST90002070 | Gherkins liking \|\| id:ebi-fl187-GCST90094770 | Weighted mode | 19 | -0.052775784 | 0.202898143015417 | 0.79773117516213 | -0.450456144 | 0.344904576526749 | 0.948592678501225 | 0.637337367631185 | 1.41185518911366 | 0.999714303421225 |
| ebi-fl187-GCST90094770 | ebi-a-GCST90002081 | SSC-A on CD4+ T cell \|\| id:ebi-a-GCST90002081 | Gherkins liking \|\| id:ebi-fl187-GCST90094770 | Weighted mode | 19 | 0.096247593443584 | 0.213722213833838 | 0.65784253278414 | -0.322647946 | 0.515143132557906 | 1.10103163846362 | 0.724228777368358 | 1.67387807110198 | 0.999714303421225 |
| ebi-fl187-GCST90094770 | ebi-a-GCST90002094 | CD11b on Monocytic Myeloid-Derived Suppressor Cells \|\| id:ebi-a-GCST90002094 | Gherkins liking \|\| id:ebi-fl187-GCST90094770 | Weighted mode | 19 | 0.223266060565259 | 0.292291429197764 | 0.454854881758877 | -0.349625141 | 0.796157261792876 | 1.25015314594452 | 0.704952298146674 | 2.21700516818487 | 0.999714303421225 |
| ebi-fl187-GCST90094770 | ebi-a-GCST90002104 | HLA DR on myeloid Dendritic Cell \|\| id:ebi-a-GCST90002104 | Gherkins liking \|\| id:ebi-fl187-GCST90094770 | Weighted mode | 18 | 0.0181432792774274 | 0.199884566125155 | 0.928737247620034 | -0.37363047 | 0.40991702888273 | 1.01830886849676 | 0.688231186689796 | 1.50669276794418 | 0.999714303421225 |
| ebi-fl187-GCST90094770 | ebi-a-GCST90002105 | HLA DR on plasmacytoid Dendritic Cell \|\| id:ebi-a-GCST90002105 | Gherkins liking \|\| id:ebi-fl187-GCST90094770 | Weighted mode | 18 | -0.034251318 | 0.189887030200376 | 0.858989123166463 | -0.406429898 | 0.33792726084357 | 0.966328618004822 | 0.666023783883966 | 1.40203851659717 | 0.999714303421225 |
| ebi-fl187-GCST90094770 | ebi-a-GCST90002106 | HLA DR on Dendritic Cell \|\| id:ebi-a-GCST90002106 | Gherkins liking \|\| id:ebi-fl187-GCST90094770 | Weighted mode | 18 | -0.057486001 | 0.200731317250917 | 0.778045695399162 | -0.450919383 | 0.33594738056938 | 0.944135107012402 | 0.637042196504158 | 1.39926539432541 | 0.999714303421225 |
| ebi-fl187-GCST90094770 | ebi-a-GCST90002111 | HLA DR on CD33dim HLA DR+ CD11b- \|\| id:ebi-a-GCST90002111 | Gherkins liking \|\| id:ebi-fl187-GCST90094770 | Weighted mode | 19 | -0.231033499 | 0.25389226780703 | 0.374864643497384 | -0.728662344 | 0.266595345937319 | 0.793712877030434 | 0.482554049944662 | 1.30551205867234 | 0.999714303421225 |
| ebi-fl187-GCST90094770 | ebi-a-GCST90002112 | HLA DR on CD33- HLA DR+ \|\| id:ebi-a-GCST90002112 | Gherkins liking \|\| id:ebi-fl187-GCST90094770 | Weighted mode | 19 | -0.255561333 | 0.283058166417347 | 0.378525255552008 | -0.810355339 | 0.2992326734664 | 0.774481634045106 | 0.444700018933497 | 1.3488234223864 | 0.999714303421225 |
| ebi-fl187-GCST90094770 | ebi-a-GCST90002115 | HLA DR on HLA DR+ CD8+ T cell \|\| id:ebi-a-GCST90002115 | Gherkins liking \|\| id:ebi-fl187-GCST90094770 | Weighted mode | 19 | -0.217133377 | 0.200637968378905 | 0.293442643893052 | -0.610383795 | 0.176117040835894 | 0.804822617169479 | 0.543142373626255 | 1.19257763076546 | 0.999714303421225 |
| ebi-fl187-GCST90094770 | ebi-a-GCST90002116 | HLA DR on B cell \|\| id:ebi-a-GCST90002116 | Gherkins liking \|\| id:ebi-fl187-GCST90094770 | Weighted mode | 19 | 0.147664361578058 | 0.203879643855041 | 0.478207699010718 | -0.25193974 | 0.547268463533939 | 1.1591237845883 | 0.777291575954404 | 1.72852503431366 | 0.999714303421225 |
| ebi-fl187-GCST90094770 | ebi-cfmet338-GCST90026023 | Sphingomyelin (d18:1/24:1, d18:2/24:0) levels \|\| id:ebi-cfmet338-GCST90026023 | Gherkins liking \|\| id:ebi-fl187-GCST90094770 | Weighted mode | 18 | 0.0663202084855115 | 0.11822843615022 | 0.582150891423034 | -0.165407526 | 0.298047943339943 | 1.06856882719562 | 0.847548235444031 | 1.3472263768634 | 0.999714303421225 |
| ebi-fl187-GCST90094770 | ebi-cfmet338-GCST90026046 | 1-arachidonoyl-gpc (20:4n6) levels \|\| id:ebi-cfmet338-GCST90026046 | Gherkins liking \|\| id:ebi-fl187-GCST90094770 | Weighted mode | 18 | 0.0179862978330205 | 0.144953972504449 | 0.902705047235927 | -0.266123488 | 0.302096083941741 | 1.01814902544626 | 0.766344487098345 | 1.3526911923673 | 0.999714303421225 |
| ebi-fl187-GCST90094770 | ebi-cfmet338-GCST90026047 | 1-linoleoyl-gpc (18:2) levels \|\| id:ebi-cfmet338-GCST90026047 | Gherkins liking \|\| id:ebi-fl187-GCST90094770 | Weighted mode | 18 | 0.0273519693575193 | 0.163313890363445 | 0.868967784443101 | -0.292743256 | 0.347447194469871 | 1.02772946839284 | 0.746213702178715 | 1.41544956507656 | 0.999714303421225 |
| ebi-fl187-GCST90094770 | ebi-cfmet338-GCST90026050 | 1-myristoyl-2-palmitoyl-gpc (14:0/16:0) levels \|\| id:ebi-cfmet338-GCST90026050 | Gherkins liking \|\| id:ebi-fl187-GCST90094770 | Weighted mode | 18 | -0.064427511 | 0.0794572833344712 | 0.428661248453983 | -0.220163787 | 0.0913087639144116 | 0.937604077385837 | 0.802387366774995 | 1.09560723701806 | 0.999714303421225 |
| ebi-fl187-GCST90094770 | ebi-cfmet338-GCST90026068 | 2,3-dihydroxyisovalerate levels \|\| id:ebi-cfmet338-GCST90026068 | Gherkins liking \|\| id:ebi-fl187-GCST90094770 | Weighted mode | 18 | 0.384570852034178 | 0.270269839515813 | 0.172853078489054 | -0.145158033 | 0.914299737485172 | 1.46898377475315 | 0.864885601483346 | 2.49502746581399 | 0.999714303421225 |
| ebi-fl187-GCST90094770 | ebi-cfmet338-GCST90026082 | 3-hydroxyhexanoate levels \|\| id:ebi-cfmet338-GCST90026082 | Gherkins liking \|\| id:ebi-fl187-GCST90094770 | Weighted mode | 18 | 0.00845481274701576 | 0.0569650107600621 | 0.883756724944393 | -0.103196608 | 0.120106233836738 | 1.00849065562002 | 0.901949625226994 | 1.12761663625831 | 0.999714303421225 |
| ebi-fl187-GCST90094770 | ebi-cfmet338-GCST90026094 | 3-ureidopropionate levels \|\| id:ebi-cfmet338-GCST90026094 | Gherkins liking \|\| id:ebi-fl187-GCST90094770 | Weighted mode | 18 | -0.102805264 | 0.134338105510475 | 0.454603153679103 | -0.366107951 | 0.160497422808398 | 0.902302667193313 | 0.693427941073329 | 1.17409474726959 | 0.999714303421225 |
| ebi-fl187-GCST90094770 | ebi-cfmet338-GCST90026173 | Glycerol levels \|\| id:ebi-cfmet338-GCST90026173 | Gherkins liking \|\| id:ebi-fl187-GCST90094770 | Weighted mode | 18 | -0.00139813 | 0.0759340630806998 | 0.985524217271663 | -0.150228894 | 0.147432633394249 | 0.998602846684822 | 0.860510988180546 | 1.15885521405777 | 0.999714303421225 |
| ebi-fl187-GCST90094770 | ebi-cfmet338-GCST90026179 | Histidine levels \|\| id:ebi-cfmet338-GCST90026179 | Gherkins liking \|\| id:ebi-fl187-GCST90094770 | Weighted mode | 18 | -0.03226025 | 0.0498306023715153 | 0.526020874202321 | -0.12992823 | 0.0654077310576236 | 0.968254561433327 | 0.878158453881567 | 1.06759422697864 | 0.999714303421225 |
| ebi-fl187-GCST90094770 | ebi-cfmet338-GCST90026188 | Isocitrate levels \|\| id:ebi-cfmet338-GCST90026188 | Gherkins liking \|\| id:ebi-fl187-GCST90094770 | Weighted mode | 18 | -0.083107155 | 0.0997028911880648 | 0.416094303034749 | -0.278524821 | 0.112310512178692 | 0.920252532690773 | 0.756899480303929 | 1.11886022643816 | 0.999714303421225 |
| ebi-fl187-GCST90094770 | ebi-cfmet338-GCST90026207 | N-acetyl-isoputreanine levels \|\| id:ebi-cfmet338-GCST90026207 | Gherkins liking \|\| id:ebi-fl187-GCST90094770 | Weighted mode | 18 | 0.0345391778627324 | 0.0609515376619392 | 0.578346588831105 | -0.084925836 | 0.154004191680133 | 1.0351425822557 | 0.918580407514267 | 1.16649577634536 | 0.999714303421225 |
| ebi-fl187-GCST90094770 | ebi-cfmet338-GCST90026213 | N-acetylglutamate levels \|\| id:ebi-cfmet338-GCST90026213 | Gherkins liking \|\| id:ebi-fl187-GCST90094770 | Weighted mode | 18 | -0.084317222 | 0.103529966235681 | 0.426664486114587 | -0.287235956 | 0.118601511624067 | 0.919139638347534 | 0.750334661967534 | 1.12592116238686 | 0.999714303421225 |
| ebi-fl187-GCST90094770 | ebi-cfmet338-GCST90026224 | N-acetyltaurine levels \|\| id:ebi-cfmet338-GCST90026224 | Gherkins liking \|\| id:ebi-fl187-GCST90094770 | Weighted mode | 18 | -0.055828918 | 0.0999947629087949 | 0.583906281630912 | -0.251818654 | 0.140160816969575 | 0.945700914141494 | 0.777385701359746 | 1.15045879727879 | 0.999714303421225 |
| ebi-fl187-GCST90094770 | ebi-cfmet338-GCST90026250 | Phenyllactate (pla) levels \|\| id:ebi-cfmet338-GCST90026250 | Gherkins liking \|\| id:ebi-fl187-GCST90094770 | Weighted mode | 18 | 0.157200554202281 | 0.138462188808695 | 0.27199027759654 | -0.114185336 | 0.428586444267323 | 1.17023028496928 | 0.892092603807657 | 1.53508605946759 | 0.999714303421225 |
| ebi-fl187-GCST90094770 | ebi-cfmet338-GCST90026251 | Pipecolate levels \|\| id:ebi-cfmet338-GCST90026251 | Gherkins liking \|\| id:ebi-fl187-GCST90094770 | Weighted mode | 18 | -0.228890071 | 0.361878713288416 | 0.535472448996734 | -0.938172349 | 0.480392207000427 | 0.795415967945469 | 0.391342419483473 | 1.61670836220029 | 0.999714303421225 |
| ebi-fl187-GCST90094770 | ebi-cfmet338-GCST90026278 | Trigonelline (n'-methylnicotinate) levels \|\| id:ebi-cfmet338-GCST90026278 | Gherkins liking \|\| id:ebi-fl187-GCST90094770 | Weighted mode | 17 | -0.084685562 | 0.236452935963429 | 0.724912618215984 | -0.548133316 | 0.378762192825372 | 0.918801145288813 | 0.578027799092088 | 1.46047568284781 | 0.999714303421225 |
| ebi-fl187-GCST90094770 | ebi-cfmet338-GCST90026299 | 1-carboxyethylvaline levels \|\| id:ebi-cfmet338-GCST90026299 | Gherkins liking \|\| id:ebi-fl187-GCST90094770 | Weighted mode | 18 | 0.0324433070654511 | 0.150434511174877 | 0.83181600899359 | -0.262408335 | 0.32729494896821 | 1.03297532908118 | 0.769196869693283 | 1.38721057317336 | 0.999714303421225 |
| ebi-fl187-GCST90094770 | ebi-gm430-GCST90011306 | C_Erysipelotrichia abundance \|\| id:ebi-gm430-GCST90011306 | Gherkins liking \|\| id:ebi-fl187-GCST90094770 | Weighted mode | 15 | -0.064138496 | 0.179547849568446 | 0.726252812152489 | -0.416052281 | 0.287775289520039 | 0.937875098929056 | 0.659645782781371 | 1.3334576285508 | 0.999714303421225 |
| ebi-fl187-GCST90094770 | ebi-gm430-GCST90011312 | F_Erysipelotrichaceae abundance \|\| id:ebi-gm430-GCST90011312 | Gherkins liking \|\| id:ebi-fl187-GCST90094770 | Weighted mode | 15 | -0.064138496 | 0.169146595441457 | 0.710236825117366 | -0.395665823 | 0.26738883143114 | 0.937875098929056 | 0.673231637081358 | 1.30654837464938 | 0.999714303421225 |
| ebi-fl187-GCST90094770 | ebi-gm430-GCST90011352 | O_Erysipelotrichales abundance \|\| id:ebi-gm430-GCST90011352 | Gherkins liking \|\| id:ebi-fl187-GCST90094770 | Weighted mode | 15 | -0.064138496 | 0.15897573354081 | 0.692712912750506 | -0.375730933 | 0.247453942105872 | 0.937875098929056 | 0.686787099671537 | 1.28076037189963 | 0.999714303421225 |
| ebi-fl187-GCST90094770 | ebi-gm430-GCST90011362 | OTU97_11 (Parabacteroides) abundance \|\| id:ebi-gm430-GCST90011362 | Gherkins liking \|\| id:ebi-fl187-GCST90094770 | Weighted mode | 15 | -0.171213102 | 0.157768713627974 | 0.296160205495844 | -0.48043978 | 0.138013577085464 | 0.842641985960295 | 0.61851132286773 | 1.14799113654214 | 0.999714303421225 |
| ebi-fl187-GCST90094770 | ebi-gm430-GCST90011384 | OTU97_235 (Phascolarctobacterium) abundance \|\| id:ebi-gm430-GCST90011384 | Gherkins liking \|\| id:ebi-fl187-GCST90094770 | Weighted mode | 15 | -0.216614169 | 0.478277636057633 | 0.657556936588748 | -1.154038336 | 0.720809997469475 | 0.805240595997016 | 0.315360662162491 | 2.05609797048032 | 0.999714303421225 |
| ebi-fl187-GCST90094770 | ebi-gm430-GCST90011390 | OTU97_3 (Bacteroides) abundance \|\| id:ebi-gm430-GCST90011390 | Gherkins liking \|\| id:ebi-fl187-GCST90094770 | Weighted mode | 15 | -0.050480037 | 0.116136897386474 | 0.670434293824198 | -0.278108356 | 0.17714828172196 | 0.950772908614581 | 0.757214768280501 | 1.19380809992412 | 0.999714303421225 |
| ebi-fl187-GCST90094770 | ebi-gm430-GCST90011398 | OTU97_4 (Alistipes) abundance \|\| id:ebi-gm430-GCST90011398 | Gherkins liking \|\| id:ebi-fl187-GCST90094770 | Weighted mode | 15 | -0.114609021 | 0.139367500257768 | 0.424662305712902 | -0.387769322 | 0.15855127906932 | 0.891714717099741 | 0.678568856157912 | 1.17181201211396 | 0.999714303421225 |
| ebi-fl187-GCST90094770 | ebi-gm430-GCST90011421 | OTU99_108 (Cytophagales) abundance \|\| id:ebi-gm430-GCST90011421 | Gherkins liking \|\| id:ebi-fl187-GCST90094770 | Weighted mode | 15 | 0.19158651493417 | 0.448527201547585 | 0.67576941295138 | -0.6875268 | 1.07069982996744 | 1.21116961286675 | 0.502818102215399 | 2.91742048400512 | 0.999714303421225 |
| ebi-fl187-GCST90094770 | ebi-gm430-GCST90011428 | OTU99_123 (Phascolarctobacterium) abundance \|\| id:ebi-gm430-GCST90011428 | Gherkins liking \|\| id:ebi-fl187-GCST90094770 | Weighted mode | 15 | -0.015990644 | 0.239352583612692 | 0.94767934293888 | -0.485121708 | 0.453140420124584 | 0.984136527833406 | 0.615622266273154 | 1.57324508627544 | 0.999714303421225 |
| ebi-fl187-GCST90094770 | ebi-gm430-GCST90011450 | OTU99_298 (Phascolarctobacterium) abundance \|\| id:ebi-gm430-GCST90011450 | Gherkins liking \|\| id:ebi-fl187-GCST90094770 | Weighted mode | 15 | -0.236048317 | 0.474847149809593 | 0.626831218702875 | -1.166748731 | 0.694652096424222 | 0.789742514861865 | 0.311377669918309 | 2.00301209763684 | 0.999714303421225 |
| ebi-fl187-GCST90094770 | ebi-gm430-GCST90011451 | OTU99_3 (Bacteroides) abundance \|\| id:ebi-gm430-GCST90011451 | Gherkins liking \|\| id:ebi-fl187-GCST90094770 | Weighted mode | 15 | -0.050096895 | 0.116598759247409 | 0.673987889238351 | -0.278630463 | 0.178436672892074 | 0.951137259369389 | 0.756819524092494 | 1.1953471829965 | 0.999714303421225 |
| ebi-fl187-GCST90094770 | ebi-gm430-GCST90011472 | OTU99_72 (Parabacteroides) abundance \|\| id:ebi-gm430-GCST90011472 | Gherkins liking \|\| id:ebi-fl187-GCST90094770 | Weighted mode | 15 | 0.622667258248209 | 0.557499455091214 | 0.282842202975086 | -0.470031674 | 1.71536619022699 | 1.86389290133655 | 0.624982472442755 | 5.55871068523606 | 0.999714303421225 |
| ebi-fl187-GCST90094770 | ebi-gm430-GCST90011507 | TestASV_3 (Bacteroides) abundance \|\| id:ebi-gm430-GCST90011507 | Gherkins liking \|\| id:ebi-fl187-GCST90094770 | Weighted mode | 15 | -0.02301689 | 0.16200437912252 | 0.889044523641043 | -0.340545473 | 0.294511692774442 | 0.977245977650577 | 0.711382176865967 | 1.34247066048461 | 0.999714303421225 |
| ebi-fl187-GCST90094770 | ebi-gm430-GCST90011512 | TestASV_34 (Phascolarctobacterium) abundance \|\| id:ebi-gm430-GCST90011512 | Gherkins liking \|\| id:ebi-fl187-GCST90094770 | Weighted mode | 15 | -0.67009043 | 0.395814458064418 | 0.112591014098662 | -1.445886768 | 0.105705907813736 | 0.511662306075886 | 0.235537117189396 | 1.11149494645628 | 0.999714303421225 |
| ebi-fl187-GCST90094770 | ebi-gm430-GCST90011519 | TestASV_41 (Barnesiella) abundance \|\| id:ebi-gm430-GCST90011519 | Gherkins liking \|\| id:ebi-fl187-GCST90094770 | Weighted mode | 15 | 0.304043412783406 | 1.00795586991057 | 0.767357958859981 | -1.671550092 | 2.27963691780812 | 1.3553278933688 | 0.187955491364744 | 9.77313131532192 | 0.999714303421225 |
| ebi-fl187-GCST90094770 | ebi-gm430-GCST90011524 | TestASV_46 (Sutterella) abundance \|\| id:ebi-gm430-GCST90011524 | Gherkins liking \|\| id:ebi-fl187-GCST90094770 | Weighted mode | 15 | -0.738738863 | 0.496051725079841 | 0.158604367225732 | -1.711000244 | 0.233522518147285 | 0.477716001105123 | 0.180684973110188 | 1.26304126892001 | 0.999714303421225 |
| ebi-fl187-GCST90094770 | ebi-gm430-GCST90011581 | OTU97_19 (Bacteria) prevalence \|\| id:ebi-gm430-GCST90011581 | Gherkins liking \|\| id:ebi-fl187-GCST90094770 | Weighted mode | 15 | -0.383112824 | 0.609354167624219 | 0.539655829976493 | -1.577446992 | 0.811221344612731 | 0.681735978813982 | 0.206501626009464 | 2.25065513425137 | 0.999714303421225 |
| ebi-fl187-GCST90094770 | ebi-gm430-GCST90011614 | OTU97_80 (Ruminococcus) prevalence \|\| id:ebi-gm430-GCST90011614 | Gherkins liking \|\| id:ebi-fl187-GCST90094770 | Weighted mode | 15 | 0.465063019034523 | 0.352351636829493 | 0.208049868215121 | -0.225546189 | 1.15567222722033 | 1.59211451924556 | 0.798080196949598 | 3.17615779978137 | 0.999714303421225 |
| ebi-fl187-GCST90094770 | ebi-gm430-GCST90011623 | OTU99_11 (Parabacteroides) prevalence \|\| id:ebi-gm430-GCST90011623 | Gherkins liking \|\| id:ebi-fl187-GCST90094770 | Weighted mode | 15 | 0.185289876887513 | 0.220965093347458 | 0.415811120372716 | -0.247801706 | 0.618381459848532 | 1.20356727590139 | 0.780514699258344 | 1.85592172574988 | 0.999714303421225 |
| ebi-fl187-GCST90094770 | ebi-gm430-GCST90011642 | OTU99_19 (Bacteria) prevalence \|\| id:ebi-gm430-GCST90011642 | Gherkins liking \|\| id:ebi-fl187-GCST90094770 | Weighted mode | 15 | -0.383112824 | 0.659083622668558 | 0.570291889240103 | -1.674916724 | 0.908691076499635 | 0.681735978813982 | 0.187323778339766 | 2.48107287248111 | 0.999714303421225 |
| ebi-fl187-GCST90094770 | ebi-gm430-GCST90011653 | OTU99_30 (Parasutterella) prevalence \|\| id:ebi-gm430-GCST90011653 | Gherkins liking \|\| id:ebi-fl187-GCST90094770 | Weighted mode | 15 | -0.227955306 | 0.281931844583487 | 0.432301296942214 | -0.780541722 | 0.324631108950916 | 0.796159842263773 | 0.458157750018502 | 1.38352018362208 | 0.999714303421225 |
| ebi-fl187-GCST90094770 | ebi-gm430-GCST90011675 | OTU99_8 (Ruminococcaceae) prevalence \|\| id:ebi-gm430-GCST90011675 | Gherkins liking \|\| id:ebi-fl187-GCST90094770 | Weighted mode | 15 | 0.341814613634176 | 0.28231010431833 | 0.246024965191852 | -0.211513191 | 0.895142418098103 | 1.40749934213813 | 0.809358604870748 | 2.44768435919159 | 0.999714303421225 |
| ebi-fl187-GCST90094770 | ebi-gm430-GCST90011679 | OTU99_92 (Ruminococcus) prevalence \|\| id:ebi-gm430-GCST90011679 | Gherkins liking \|\| id:ebi-fl187-GCST90094770 | Weighted mode | 15 | -0.201630118 | 0.473055458849067 | 0.676421375054538 | -1.128818818 | 0.725558581110285 | 0.817397212358568 | 0.323415043061887 | 2.06588474192805 | 0.999714303421225 |
| ebi-fl187-GCST90094770 | ebi-gm430-GCST90011684 | TestASV_11 (Lachnospiraceae) prevalence \|\| id:ebi-gm430-GCST90011684 | Gherkins liking \|\| id:ebi-fl187-GCST90094770 | Weighted mode | 15 | 0.0609192289988989 | 0.269443697911419 | 0.824396914596562 | -0.467190419 | 0.58902887690528 | 1.06281306624489 | 0.626760731954853 | 1.80223737096252 | 0.999714303421225 |
| ebi-fl187-GCST90094770 | ebi-gm430-GCST90011688 | TestASV_15 (Bacteroides) prevalence \|\| id:ebi-gm430-GCST90011688 | Gherkins liking \|\| id:ebi-fl187-GCST90094770 | Weighted mode | 15 | -0.137338039 | 0.266747933938525 | 0.614682537024992 | -0.66016399 | 0.385487911112825 | 0.871675515648907 | 0.516766583028886 | 1.47033153755477 | 0.999714303421225 |
| ebi-fl187-GCST90094770 | ebi-gm430-GCST90011692 | TestASV_19 (Prevotella) prevalence \|\| id:ebi-gm430-GCST90011692 | Gherkins liking \|\| id:ebi-fl187-GCST90094770 | Weighted mode | 15 | 0.0903402488622893 | 0.577769756010005 | 0.877981386965554 | -1.042088473 | 1.2227689706419 | 1.09454663860346 | 0.35271727172669 | 3.39657975412798 | 0.999714303421225 |
| ebi-fl187-GCST90094770 | ebi-gm430-GCST90011693 | TestASV_2 (Bacteroides) prevalence \|\| id:ebi-gm430-GCST90011693 | Gherkins liking \|\| id:ebi-fl187-GCST90094770 | Weighted mode | 15 | -0.222417968 | 0.267289597713078 | 0.419307434342651 | -0.74630558 | 0.301469643459482 | 0.800580677244213 | 0.474114900959298 | 1.35184407720572 | 0.999714303421225 |
| ebi-fl187-GCST90094770 | ebi-gm430-GCST90011716 | TestASV_40 (Barnesiella) prevalence \|\| id:ebi-gm430-GCST90011716 | Gherkins liking \|\| id:ebi-fl187-GCST90094770 | Weighted mode | 15 | 0.683933443078486 | 0.555627682668122 | 0.238632294122172 | -0.405096815 | 1.772963701 | 1.98165715786778 | 0.666912240656922 | 5.88827862487642 | 0.999714303421225 |
| ebi-fl187-GCST90094770 | ebi-gm430-GCST90011718 | TestASV_42 (Alphaproteobacteria) prevalence \|\| id:ebi-gm430-GCST90011718 | Gherkins liking \|\| id:ebi-fl187-GCST90094770 | Weighted mode | 15 | -0.217313515 | 0.423623143951879 | 0.615958486407916 | -1.047614877 | 0.61298784711275 | 0.804677651213991 | 0.350773389779289 | 1.84593854958805 | 0.999714303421225 |
| ebi-fl187-GCST90094770 | ebi-hbsm44-GCST90104929 | Sulcal depth of the right inferior frontal sulcus \|\| id:ebi-hbsm44-GCST90104929 | Gherkins liking \|\| id:ebi-fl187-GCST90094770 | Weighted mode | 17 | 0.329470678792585 | 0.234610968962709 | 0.179335581244231 | -0.13036682 | 0.789308177959495 | 1.39023205436155 | 0.8777733866957 | 2.20187259521501 | 0.999714303421225 |
| ebi-fl187-GCST90094770 | ebi-hbsm44-GCST90104935 | Sulcal opening distance of the left calloso-marginal posterior fissure \|\| id:ebi-hbsm44-GCST90104935 | Gherkins liking \|\| id:ebi-fl187-GCST90094770 | Weighted mode | 17 | 0.00871464956674439 | 0.0528930883537297 | 0.871196698192882 | -0.094955804 | 0.112385102740055 | 1.00875273267187 | 0.909413126360383 | 1.11894368596315 | 0.999714303421225 |
| ebi-fl187-GCST90094770 | ebi-hbsm44-GCST90104936 | Sulcal opening distance of the left anterior inferior temporal sulcus \|\| id:ebi-hbsm44-GCST90104936 | Gherkins liking \|\| id:ebi-fl187-GCST90094770 | Weighted mode | 17 | -0.039612051 | 0.0304209292506335 | 0.211305613372782 | -0.099237073 | 0.0200129698927493 | 0.961162248343873 | 0.905528006542034 | 1.02021457201453 | 0.999714303421225 |
| ebi-fl187-GCST90094770 | ebi-hbsm44-GCST90104941 | Sulcal opening distance of the right posterior inferior temporal sulcus \|\| id:ebi-hbsm44-GCST90104941 | Gherkins liking \|\| id:ebi-fl187-GCST90094770 | Weighted mode | 16 | 0.0141936036212925 | 0.0208145268179673 | 0.50569050782624 | -0.026602869 | 0.0549900761845085 | 1.01429481107912 | 0.973747870270134 | 1.0565301298134 | 0.999714303421225 |
| ebi-fl187-GCST90094770 | ebi-IPs91-GCST90274819 | Leukemia inhibitory factor levels \|\| id:ebi-IPs91-GCST90274819 | Gherkins liking \|\| id:ebi-fl187-GCST90094770 | Weighted mode | 22 | -0.03804055 | 0.0908591303903678 | 0.679703827701878 | -0.216124445 | 0.140043345992295 | 0.962673904084161 | 0.805635038271478 | 1.15032365969708 | 0.999714303421225 |
| ebi-fl187-GCST90094770 | ebi-IPs91-GCST90274837 | STAM binding protein levels \|\| id:ebi-IPs91-GCST90274837 | Gherkins liking \|\| id:ebi-fl187-GCST90094770 | Weighted mode | 22 | -0.026885204 | 0.0830899361740071 | 0.749465702640477 | -0.189741479 | 0.135971070928006 | 0.973472985941438 | 0.827172947986274 | 1.14564875056028 | 0.999714303421225 |
| ebi-fl187-GCST90094770 | ebi-IPs91-GCST90274840 | TNF-beta levels \|\| id:ebi-IPs91-GCST90274840 | Gherkins liking \|\| id:ebi-fl187-GCST90094770 | Weighted mode | 22 | 0.0347040531001005 | 0.129735461056243 | 0.791694924274351 | -0.219577451 | 0.288985556770337 | 1.03531326570501 | 0.802857973477176 | 1.33507244563131 | 0.999714303421225 |
| ebi-fl187-GCST90094770 | ebi-IPs91-GCST90274842 | Tumor necrosis factor ligand superfamily member 14 levels \|\| id:ebi-IPs91-GCST90274842 | Gherkins liking \|\| id:ebi-fl187-GCST90094770 | Weighted mode | 22 | 0.0832541594744873 | 0.10690806832848 | 0.444816829462914 | -0.126285654 | 0.292793973398309 | 1.08681799853997 | 0.881363045545618 | 1.34016665200571 | 0.999714303421225 |
| ebi-fl187-GCST90094770 | ebi-met1400-GCST90199628 | 3-methylhistidine levels \|\| id:ebi-met1400-GCST90199628 | Gherkins liking \|\| id:ebi-fl187-GCST90094770 | Weighted mode | 20 | -0.20530195 | 0.145165952269395 | 0.17346460335674 | -0.489827216 | 0.0792233163961047 | 0.814401370745459 | 0.612732255062286 | 1.08244602302626 | 0.999714303421225 |
| ebi-fl187-GCST90094770 | ebi-met1400-GCST90199651 | Docosahexaenoate DHA; 22:6n3 levels \|\| id:ebi-met1400-GCST90199651 | Gherkins liking \|\| id:ebi-fl187-GCST90094770 | Weighted mode | 20 | 0.0422885343966408 | 0.119610683314173 | 0.727567749458352 | -0.192148405 | 0.276725473692419 | 1.0431954330957 | 0.825184397996942 | 1.31880427486677 | 0.999714303421225 |
| ebi-fl187-GCST90094770 | ebi-met1400-GCST90199652 | 1-stearoyl-2-oleoyl-GPS (18:0/18:1) levels \|\| id:ebi-met1400-GCST90199652 | Gherkins liking \|\| id:ebi-fl187-GCST90094770 | Weighted mode | 20 | 0.0423118340406136 | 0.114086107125694 | 0.714832529715854 | -0.181296936 | 0.265920604006975 | 1.04321973946105 | 0.834187621642915 | 1.30463147206354 | 0.999714303421225 |
| ebi-fl187-GCST90094770 | ebi-met1400-GCST90199653 | 1-myristoyl-2-palmitoyl-gpc (14:0/16:0) levels \|\| id:ebi-met1400-GCST90199653 | Gherkins liking \|\| id:ebi-fl187-GCST90094770 | Weighted mode | 19 | -0.005408699 | 0.111448365780761 | 0.961827507378489 | -0.223847496 | 0.213030097437967 | 0.994605901187352 | 0.79943704208226 | 1.23742189391183 | 0.999714303421225 |
| ebi-fl187-GCST90094770 | ebi-met1400-GCST90199667 | Erythritol levels in elite athletes \|\| id:ebi-met1400-GCST90199667 | Gherkins liking \|\| id:ebi-fl187-GCST90094770 | Weighted mode | 20 | 0.00712911439886277 | 0.127969780210599 | 0.956155002633112 | -0.243691655 | 0.257949883611637 | 1.00715458703138 | 0.783729256152807 | 1.29427395266277 | 0.999714303421225 |
| ebi-fl187-GCST90094770 | ebi-met1400-GCST90199674 | 3-hydroxymyristate levels \|\| id:ebi-met1400-GCST90199674 | Gherkins liking \|\| id:ebi-fl187-GCST90094770 | Weighted mode | 20 | 0.0148902561440989 | 0.124826800421516 | 0.906299960737629 | -0.229770273 | 0.259550784970271 | 1.01500166830647 | 0.794716149544058 | 1.29634761701519 | 0.999714303421225 |
| ebi-fl187-GCST90094770 | ebi-met1400-GCST90199685 | 2-linoleoylglycerol (18:2) levels \|\| id:ebi-met1400-GCST90199685 | Gherkins liking \|\| id:ebi-fl187-GCST90094770 | Weighted mode | 20 | -0.006750693 | 0.136526127119504 | 0.961079835802161 | -0.274341903 | 0.260840515674154 | 0.993272041263961 | 0.760072160156768 | 1.29802063497917 | 0.999714303421225 |
| ebi-fl187-GCST90094770 | ebi-met1400-GCST90199703 | Gamma-glutamylvaline levels \|\| id:ebi-met1400-GCST90199703 | Gherkins liking \|\| id:ebi-fl187-GCST90094770 | Weighted mode | 20 | 0.0270889199716894 | 0.108714302760478 | 0.805898987015547 | -0.185991113 | 0.240168953382226 | 1.02745916034112 | 0.830280973291792 | 1.27146395031008 | 0.999714303421225 |
| ebi-fl187-GCST90094770 | ebi-met1400-GCST90199779 | Gamma-glutamyl-2-aminobutyrate levels \|\| id:ebi-met1400-GCST90199779 | Gherkins liking \|\| id:ebi-fl187-GCST90094770 | Weighted mode | 20 | 0.0121317775027661 | 0.120247708412324 | 0.920694911296515 | -0.223553731 | 0.247817285990922 | 1.01220566601278 | 0.799671923552801 | 1.28122581290143 | 0.999714303421225 |
| ebi-fl187-GCST90094770 | ebi-met1400-GCST90199824 | 4-vinylphenol sulfate levels \|\| id:ebi-met1400-GCST90199824 | Gherkins liking \|\| id:ebi-fl187-GCST90094770 | Weighted mode | 20 | -0.172594997 | 0.12299186954961 | 0.176658133923034 | -0.413659062 | 0.0684690669265791 | 0.841478346767454 | 0.661226350222703 | 1.07086749921566 | 0.999714303421225 |
| ebi-fl187-GCST90094770 | ebi-met1400-GCST90199830 | 4-ethylphenylsulfate levels \|\| id:ebi-met1400-GCST90199830 | Gherkins liking \|\| id:ebi-fl187-GCST90094770 | Weighted mode | 20 | 0.128924125533907 | 0.126591240171826 | 0.321268121280374 | -0.119194705 | 0.377042956270686 | 1.1376038058097 | 0.887634956791348 | 1.45796693684849 | 0.999714303421225 |
| ebi-fl187-GCST90094770 | ebi-met1400-GCST90199833 | Hexanoylglutamine levels \|\| id:ebi-met1400-GCST90199833 | Gherkins liking \|\| id:ebi-fl187-GCST90094770 | Weighted mode | 20 | 0.0837480006256848 | 0.110356869397958 | 0.457234155644127 | -0.132551463 | 0.300047464645683 | 1.08735484653942 | 0.875857858328011 | 1.34992287966659 | 0.999714303421225 |
| ebi-fl187-GCST90094770 | ebi-met1400-GCST90199837 | Carotenoid levels (cryptoxanthin) \|\| id:ebi-met1400-GCST90199837 | Gherkins liking \|\| id:ebi-fl187-GCST90094770 | Weighted mode | 20 | -0.0329822 | 0.108240545443105 | 0.763898000111467 | -0.24513367 | 0.17916926862786 | 0.967555781502342 | 0.782599921502653 | 1.19622321009322 | 0.999714303421225 |
| ebi-fl187-GCST90094770 | ebi-met1400-GCST90199869 | 2-hydroxyhippurate levels \|\| id:ebi-met1400-GCST90199869 | Gherkins liking \|\| id:ebi-fl187-GCST90094770 | Weighted mode | 20 | 0.147742484983201 | 0.143116176043492 | 0.314881941198071 | -0.13276522 | 0.428250190028446 | 1.15921434282265 | 0.875670657879194 | 1.53456996704705 | 0.999714303421225 |
| ebi-fl187-GCST90094770 | ebi-met1400-GCST90199902 | Hydantoin-5-propionate levels \|\| id:ebi-met1400-GCST90199902 | Gherkins liking \|\| id:ebi-fl187-GCST90094770 | Weighted mode | 20 | 0.0840226732863344 | 0.133024125754925 | 0.535151916062089 | -0.176704613 | 0.344749959765987 | 1.08765355420973 | 0.838027290171398 | 1.41163690951293 | 0.999714303421225 |
| ebi-fl187-GCST90094770 | ebi-met1400-GCST90199904 | Pantoate levels \|\| id:ebi-met1400-GCST90199904 | Gherkins liking \|\| id:ebi-fl187-GCST90094770 | Weighted mode | 20 | 0.0281919804031061 | 0.117900169073722 | 0.813574921116314 | -0.202892351 | 0.259276311787601 | 1.02859313519221 | 0.816366117707026 | 1.29599185318495 | 0.999714303421225 |
| ebi-fl187-GCST90094770 | ebi-met1400-GCST90199936 | 3-methyl catechol sulfate (2) levels \|\| id:ebi-met1400-GCST90199936 | Gherkins liking \|\| id:ebi-fl187-GCST90094770 | Weighted mode | 20 | -0.082860452 | 0.129591791394959 | 0.530199750704603 | -0.336860363 | 0.171139459078393 | 0.920479589292435 | 0.714008534816244 | 1.18665622746656 | 0.999714303421225 |
| ebi-fl187-GCST90094770 | ebi-met1400-GCST90199977 | 4-hydroxychlorothalonil levels \|\| id:ebi-met1400-GCST90199977 | Gherkins liking \|\| id:ebi-fl187-GCST90094770 | Weighted mode | 20 | -0.090241069 | 0.120146054308539 | 0.46179787486156 | -0.325727335 | 0.14524519749025 | 0.913710891389997 | 0.722002024989083 | 1.15632306302372 | 0.999714303421225 |
| ebi-fl187-GCST90094770 | ebi-met1400-GCST90200040 | Sphingomyelin (d18:2/24:1, d18:1/24:2) levels \|\| id:ebi-met1400-GCST90200040 | Gherkins liking \|\| id:ebi-fl187-GCST90094770 | Weighted mode | 20 | 0.0905587576981581 | 0.113698323073327 | 0.43559132337953 | -0.132289956 | 0.313407470921878 | 1.09478583284734 | 0.876086932000851 | 1.36807886982851 | 0.999714303421225 |
| ebi-fl187-GCST90094770 | ebi-met1400-GCST90200077 | 1-stearoyl-2-oleoyl-GPI (18:0/18:1) levels \|\| id:ebi-met1400-GCST90200077 | Gherkins liking \|\| id:ebi-fl187-GCST90094770 | Weighted mode | 20 | -0.016194726 | 0.108499626502893 | 0.882920880496757 | -0.228853994 | 0.196464541463923 | 0.983935703061717 | 0.795444664380691 | 1.21709216380666 | 0.999714303421225 |
| ebi-fl187-GCST90094770 | ebi-met1400-GCST90200080 | 1-palmitoyl-2-oleoyl-GPI (16:0/18:1) levels \|\| id:ebi-met1400-GCST90200080 | Gherkins liking \|\| id:ebi-fl187-GCST90094770 | Weighted mode | 20 | 0.053206896038993 | 0.131245848272169 | 0.689713017838999 | -0.204034967 | 0.310448758652445 | 1.05464782500583 | 0.815433857760084 | 1.36403710025587 | 0.999714303421225 |
| ebi-fl187-GCST90094770 | ebi-met1400-GCST90200082 | 1-oleoyl-2-linoleoyl-GPE (18:1/18:2) levels \|\| id:ebi-met1400-GCST90200082 | Gherkins liking \|\| id:ebi-fl187-GCST90094770 | Weighted mode | 20 | 0.0758691753330291 | 0.133546540465892 | 0.576613083226805 | -0.185882044 | 0.337620394646178 | 1.07882142847072 | 0.830371536526892 | 1.40160834437502 | 0.999714303421225 |
| ebi-fl187-GCST90094770 | ebi-met1400-GCST90200100 | Perfluorooctanesulfonate (PFOS) levels \|\| id:ebi-met1400-GCST90200100 | Gherkins liking \|\| id:ebi-fl187-GCST90094770 | Weighted mode | 20 | -0.040384237 | 0.100575715996053 | 0.692509838440972 | -0.237512641 | 0.156744165940009 | 0.96042033882012 | 0.788586922576481 | 1.1696963274583 | 0.999714303421225 |
| ebi-fl187-GCST90094770 | ebi-met1400-GCST90200102 | 1-palmitoleoyl-2-linolenoyl-GPC (16:1/18:3) levels \|\| id:ebi-met1400-GCST90200102 | Gherkins liking \|\| id:ebi-fl187-GCST90094770 | Weighted mode | 20 | 0.0404885188474155 | 0.119186023811931 | 0.737802418408982 | -0.193116088 | 0.2740931255188 | 1.04131935408749 | 0.824386267376131 | 1.3153372880027 | 0.999714303421225 |
| ebi-fl187-GCST90094770 | ebi-met1400-GCST90200114 | Glycosyl-N-tricosanoyl-sphingadienine (d18:2/23:0) levels \|\| id:ebi-met1400-GCST90200114 | Gherkins liking \|\| id:ebi-fl187-GCST90094770 | Weighted mode | 20 | 0.0537930568782774 | 0.126079972938895 | 0.674423556338817 | -0.19332369 | 0.300909803838511 | 1.05526619947583 | 0.824215140689329 | 1.35108747313815 | 0.999714303421225 |
| ebi-fl187-GCST90094770 | ebi-met1400-GCST90200135 | Arachidoylcarnitine (C20) levels \|\| id:ebi-met1400-GCST90200135 | Gherkins liking \|\| id:ebi-fl187-GCST90094770 | Weighted mode | 20 | -0.099304235 | 0.125916807740827 | 0.440046949538671 | -0.346101178 | 0.147492708132316 | 0.905467191267862 | 0.707440905879575 | 1.15892483407239 | 0.999714303421225 |
| ebi-fl187-GCST90094770 | ebi-met1400-GCST90200146 | Docosahexaenoylcarnitine (C22:6) levels \|\| id:ebi-met1400-GCST90200146 | Gherkins liking \|\| id:ebi-fl187-GCST90094770 | Weighted mode | 20 | -0.093642109 | 0.166087667398781 | 0.57947722565908 | -0.419173937 | 0.231889718976816 | 0.910608602439369 | 0.657589805972864 | 1.2609806589228 | 0.999714303421225 |
| ebi-fl187-GCST90094770 | ebi-met1400-GCST90200162 | 3-hydroxyphenylacetoylglutamine levels \|\| id:ebi-met1400-GCST90200162 | Gherkins liking \|\| id:ebi-fl187-GCST90094770 | Weighted mode | 20 | 0.0257228685611505 | 0.127371867685295 | 0.842101644584567 | -0.223925992 | 0.275371729224329 | 1.02605655653843 | 0.799374292191323 | 1.31702015876629 | 0.999714303421225 |
| ebi-fl187-GCST90094770 | ebi-met1400-GCST90200172 | 3-carboxy-4-methyl-5-pentyl-2-furanpropionate (3-CMPFP) levels \|\| id:ebi-met1400-GCST90200172 | Gherkins liking \|\| id:ebi-fl187-GCST90094770 | Weighted mode | 20 | 0.137743708166282 | 0.119018641648551 | 0.261481059641519 | -0.095532829 | 0.371020245797442 | 1.14768137121479 | 0.908888522840039 | 1.44921241355061 | 0.999714303421225 |
| ebi-fl187-GCST90094770 | ebi-met1400-GCST90200222 | Dihydrocaffeate sulfate (2) levels \|\| id:ebi-met1400-GCST90200222 | Gherkins liking \|\| id:ebi-fl187-GCST90094770 | Weighted mode | 20 | -0.027775007 | 0.141080865265816 | 0.846017614449086 | -0.304293503 | 0.248743488850073 | 0.972607171921971 | 0.737644333856701 | 1.28241303763315 | 0.999714303421225 |
| ebi-fl187-GCST90094770 | ebi-met1400-GCST90200223 | 4-methylhexanoylglutamine levels \|\| id:ebi-met1400-GCST90200223 | Gherkins liking \|\| id:ebi-fl187-GCST90094770 | Weighted mode | 20 | 0.164544270664629 | 0.143678395410336 | 0.266336677861032 | -0.117065384 | 0.446153925668886 | 1.17885575712428 | 0.889527030124509 | 1.56229192485644 | 0.999714303421225 |
| ebi-fl187-GCST90094770 | ebi-met1400-GCST90200224 | 2-ketocaprylate levels \|\| id:ebi-met1400-GCST90200224 | Gherkins liking \|\| id:ebi-fl187-GCST90094770 | Weighted mode | 20 | -0.010173037 | 0.116697670587147 | 0.931445167301819 | -0.238900471 | 0.218554397802673 | 0.98987853376446 | 0.7874932569683 | 1.24427670070465 | 0.999714303421225 |
| ebi-fl187-GCST90094770 | ebi-met1400-GCST90200243 | Hydroxypalmitoyl sphingomyelin (d18:1/16:0(OH)) levels \|\| id:ebi-met1400-GCST90200243 | Gherkins liking \|\| id:ebi-fl187-GCST90094770 | Weighted mode | 20 | -0.222754989 | 0.178253922705485 | 0.226602506406711 | -0.572132677 | 0.126622699886019 | 0.800310910558194 | 0.564320640715136 | 1.13498870561746 | 0.999714303421225 |
| ebi-fl187-GCST90094770 | ebi-met1400-GCST90200273 | 2-methoxyhydroquinone sulfate (2) levels \|\| id:ebi-met1400-GCST90200273 | Gherkins liking \|\| id:ebi-fl187-GCST90094770 | Weighted mode | 20 | 0.106365788098251 | 0.153138075279259 | 0.495728554900149 | -0.193784839 | 0.4065164156456 | 1.1122286421068 | 0.823835142023618 | 1.50157779053237 | 0.999714303421225 |
| ebi-fl187-GCST90094770 | ebi-met1400-GCST90200276 | 2,4-di-tert-butylphenol levels \|\| id:ebi-met1400-GCST90200276 | Gherkins liking \|\| id:ebi-fl187-GCST90094770 | Weighted mode | 20 | -0.159530541 | 0.121672365280075 | 0.205433319804974 | -0.398008377 | 0.078947295153203 | 0.852543929628801 | 0.67165640134425 | 1.08214728616036 | 0.999714303421225 |
| ebi-fl187-GCST90094770 | ebi-met1400-GCST90200289 | N-acetylmethionine levels \|\| id:ebi-met1400-GCST90200289 | Gherkins liking \|\| id:ebi-fl187-GCST90094770 | Weighted mode | 20 | 0.290213636846094 | 0.164898860977329 | 0.0945072614347887 | -0.032988131 | 0.613415404361659 | 1.33671302867896 | 0.96755004369215 | 1.8467279627436 | 0.999714303421225 |
| ebi-fl187-GCST90094770 | ebi-met1400-GCST90200308 | 3-hydroxyisobutyrate levels \|\| id:ebi-met1400-GCST90200308 | Gherkins liking \|\| id:ebi-fl187-GCST90094770 | Weighted mode | 20 | -0.132639498 | 0.125028726573233 | 0.302047934743775 | -0.377695802 | 0.112416805859873 | 0.875780755724825 | 0.685438977910749 | 1.11897916053122 | 0.999714303421225 |
| ebi-fl187-GCST90094770 | ebi-met1400-GCST90200383 | Cystathionine levels \|\| id:ebi-met1400-GCST90200383 | Gherkins liking \|\| id:ebi-fl187-GCST90094770 | Weighted mode | 20 | -0.038685906 | 0.124067929260475 | 0.758578961338256 | -0.281859047 | 0.204487235763403 | 0.962052837116554 | 0.754380009211985 | 1.2268957953576 | 0.999714303421225 |
| ebi-fl187-GCST90094770 | ebi-met1400-GCST90200384 | Guanidinoacetate levels \|\| id:ebi-met1400-GCST90200384 | Gherkins liking \|\| id:ebi-fl187-GCST90094770 | Weighted mode | 20 | 0.153631397702815 | 0.0960677148758932 | 0.12627340617939 | -0.034661323 | 0.341924118859566 | 1.16606099479238 | 0.965932499549399 | 1.40765347911007 | 0.999714303421225 |
| ebi-fl187-GCST90094770 | ebi-met1400-GCST90200403 | Citrulline levels \|\| id:ebi-met1400-GCST90200403 | Gherkins liking \|\| id:ebi-fl187-GCST90094770 | Weighted mode | 20 | 0.109007152315229 | 0.119029941238524 | 0.371251528661982 | -0.124291533 | 0.342305837142735 | 1.11517032636263 | 0.883122344474554 | 1.40819090874625 | 0.999714303421225 |
| ebi-fl187-GCST90094770 | ebi-met1400-GCST90200434 | 12,13-DiHOME levels \|\| id:ebi-met1400-GCST90200434 | Gherkins liking \|\| id:ebi-fl187-GCST90094770 | Weighted mode | 20 | 0.0377303562642028 | 0.11644666711244 | 0.749467091970438 | -0.190505111 | 0.265965823804585 | 1.03845118326688 | 0.82654153303621 | 1.30469046856856 | 0.999714303421225 |
| ebi-fl187-GCST90094770 | ebi-met1400-GCST90200437 | Fructose levels \|\| id:ebi-met1400-GCST90200437 | Gherkins liking \|\| id:ebi-fl187-GCST90094770 | Weighted mode | 20 | -0.071324109 | 0.114642532663386 | 0.541242268096937 | -0.296023473 | 0.153375254831399 | 0.931160045687545 | 0.743769969147282 | 1.16576235482981 | 0.999714303421225 |
| ebi-fl187-GCST90094770 | ebi-met1400-GCST90200448 | N-stearoyl-sphinganine (d18:0/18:0) levels \|\| id:ebi-met1400-GCST90200448 | Gherkins liking \|\| id:ebi-fl187-GCST90094770 | Weighted mode | 20 | -0.072534653 | 0.123305389097274 | 0.563290975754478 | -0.314213215 | 0.169143909814336 | 0.930033517821207 | 0.73036328682213 | 1.18429055769549 | 0.999714303421225 |
| ebi-fl187-GCST90094770 | ebi-met1400-GCST90200468 | X-11847 levels \|\| id:ebi-met1400-GCST90200468 | Gherkins liking \|\| id:ebi-fl187-GCST90094770 | Weighted mode | 20 | 0.00410906191995664 | 0.124020852592692 | 0.973914892393342 | -0.238971809 | 0.247189933001632 | 1.00411751568994 | 0.787437080571189 | 1.28042228413219 | 0.999714303421225 |
| ebi-fl187-GCST90094770 | ebi-met1400-GCST90200476 | X-11843 levels \|\| id:ebi-met1400-GCST90200476 | Gherkins liking \|\| id:ebi-fl187-GCST90094770 | Weighted mode | 19 | -0.138201657 | 0.135778798225276 | 0.322238720414117 | -0.404328101 | 0.127924787868203 | 0.870923046609493 | 0.66742510238048 | 1.13646752333741 | 0.999714303421225 |
| ebi-fl187-GCST90094770 | ebi-met1400-GCST90200511 | X-12906 levels \|\| id:ebi-met1400-GCST90200511 | Gherkins liking \|\| id:ebi-fl187-GCST90094770 | Weighted mode | 20 | -0.02863798 | 0.119449091245581 | 0.813091162957656 | -0.262758199 | 0.205482238919115 | 0.971768200394517 | 0.768927802527582 | 1.2281171680798 | 0.999714303421225 |
| ebi-fl187-GCST90094770 | ebi-met1400-GCST90200515 | X-12849 levels \|\| id:ebi-met1400-GCST90200515 | Gherkins liking \|\| id:ebi-fl187-GCST90094770 | Weighted mode | 20 | -0.002749961 | 0.122472399080086 | 0.982320164499743 | -0.242795863 | 0.237295940991411 | 0.997253816474141 | 0.784431628631969 | 1.26781626106363 | 0.999714303421225 |
| ebi-fl187-GCST90094770 | ebi-met1400-GCST90200551 | X-18886 levels \|\| id:ebi-met1400-GCST90200551 | Gherkins liking \|\| id:ebi-fl187-GCST90094770 | Weighted mode | 20 | 0.0283144698670846 | 0.130568662386475 | 0.830632844226789 | -0.227600108 | 0.284229048144575 | 1.02871913473064 | 0.796442686895142 | 1.32873724070026 | 0.999714303421225 |
| ebi-fl187-GCST90094770 | ebi-met1400-GCST90200564 | X-21319 levels \|\| id:ebi-met1400-GCST90200564 | Gherkins liking \|\| id:ebi-fl187-GCST90094770 | Weighted mode | 20 | 0.00340139072706869 | 0.113726587532218 | 0.976451965929209 | -0.219502721 | 0.226305502290215 | 1.0034071820208 | 0.80291797308187 | 1.25395869401999 | 0.999714303421225 |
| ebi-fl187-GCST90094770 | ebi-met1400-GCST90200579 | X-21442 levels \|\| id:ebi-met1400-GCST90200579 | Gherkins liking \|\| id:ebi-fl187-GCST90094770 | Weighted mode | 20 | -0.039322275 | 0.130418016323885 | 0.766301102750169 | -0.294941587 | 0.216297037181939 | 0.9614408110554 | 0.744575079202941 | 1.24147108731116 | 0.999714303421225 |
| ebi-fl187-GCST90094770 | ebi-met1400-GCST90200581 | X-21441 levels \|\| id:ebi-met1400-GCST90200581 | Gherkins liking \|\| id:ebi-fl187-GCST90094770 | Weighted mode | 20 | 0.0433376844512482 | 0.118936995369579 | 0.719601629895488 | -0.189778826 | 0.276454195375623 | 1.044290476 | 0.82714205563957 | 1.31844656038519 | 0.999714303421225 |
| ebi-fl187-GCST90094770 | ebi-met1400-GCST90200648 | X-24585 levels \|\| id:ebi-met1400-GCST90200648 | Gherkins liking \|\| id:ebi-fl187-GCST90094770 | Weighted mode | 20 | 0.17875168595155 | 0.12166892303725 | 0.158151769340575 | -0.059719403 | 0.41722277510456 | 1.19572379228506 | 0.942028826775529 | 1.51774059009475 | 0.999714303421225 |
| ebi-fl187-GCST90094770 | ebi-met1400-GCST90200720 | Adenosine 5'-diphosphate (ADP) to pantothenate ratio \|\| id:ebi-met1400-GCST90200720 | Gherkins liking \|\| id:ebi-fl187-GCST90094770 | Weighted mode | 20 | 0.0119588689594121 | 0.134036252294811 | 0.929839929692187 | -0.250752186 | 0.274669923457241 | 1.01203066213577 | 0.778215200645996 | 1.31609619068449 | 0.999714303421225 |
| ebi-fl187-GCST90094770 | ebi-met1400-GCST90200724 | Cholate to taurocholate ratio \|\| id:ebi-met1400-GCST90200724 | Gherkins liking \|\| id:ebi-fl187-GCST90094770 | Weighted mode | 20 | -0.22206344 | 0.150046746950967 | 0.155274554208994 | -0.516155064 | 0.0720281843070862 | 0.800864556102405 | 0.59681084177728 | 1.07468563290688 | 0.999714303421225 |
| ebi-fl187-GCST90094770 | ebi-met1400-GCST90200726 | N-acetylputrescine to (N(1) + N(8))-acetylspermidine ratio \|\| id:ebi-met1400-GCST90200726 | Gherkins liking \|\| id:ebi-fl187-GCST90094770 | Weighted mode | 20 | -0.00318766 | 0.115120037083652 | 0.978198272511616 | -0.228822932 | 0.222447612979207 | 0.99681741548835 | 0.795469372897392 | 1.24913037971739 | 0.999714303421225 |
| ebi-fl187-GCST90094770 | ebi-met1400-GCST90200744 | Arginine to glutamate ratio \|\| id:ebi-met1400-GCST90200744 | Gherkins liking \|\| id:ebi-fl187-GCST90094770 | Weighted mode | 20 | -0.060695356 | 0.114827438236566 | 0.603218006011389 | -0.285757135 | 0.164366422596969 | 0.941109899267165 | 0.75144509328172 | 1.17864611854829 | 0.999714303421225 |
| ebi-fl187-GCST90094770 | ebi-met1400-GCST90200759 | Histidine to trans-urocanate ratio \|\| id:ebi-met1400-GCST90200759 | Gherkins liking \|\| id:ebi-fl187-GCST90094770 | Weighted mode | 20 | 0.140891939645893 | 0.211503257018603 | 0.513325786685153 | -0.273654444 | 0.555438323402355 | 1.15130023135193 | 0.76059485788784 | 1.74270468563498 | 0.999714303421225 |
| ebi-fl187-GCST90094770 | ebi-met1400-GCST90200795 | Oleoyl-linoleoyl-glycerol (18:1 to 18:2) [2] to linoleoyl-arachidonoyl-glycerol (18:2 to 20:4) [2] ratio \|\| id:ebi-met1400-GCST90200795 | Gherkins liking \|\| id:ebi-fl187-GCST90094770 | Weighted mode | 20 | 0.024354399657028 | 0.17822043086338 | 0.892742494473191 | -0.324957645 | 0.373666444149253 | 1.02465339036056 | 0.722557957055312 | 1.45305239548696 | 0.999714303421225 |
| ebi-fl187-GCST90094770 | ebi-met1400-GCST90200811 | Choline to taurocholate ratio \|\| id:ebi-met1400-GCST90200811 | Gherkins liking \|\| id:ebi-fl187-GCST90094770 | Weighted mode | 20 | -0.058608092 | 0.115472813061603 | 0.617610764584897 | -0.284934805 | 0.167718622062833 | 0.943076296322885 | 0.75206328338263 | 1.18260380520876 | 0.999714303421225 |
| ebi-fl187-GCST90094770 | ebi-met1400-GCST90200831 | Cholate to phosphate ratio \|\| id:ebi-met1400-GCST90200831 | Gherkins liking \|\| id:ebi-fl187-GCST90094770 | Weighted mode | 20 | 0.0111715650921369 | 0.132904770935087 | 0.933890522274482 | -0.249321786 | 0.271664916124908 | 1.01123420005165 | 0.779329155866605 | 1.31214724825351 | 0.999714303421225 |
| ebi-fl187-GCST90094770 | ebi-met1400-GCST90200841 | Adenosine 5'-diphosphate (ADP) to oxalate (ethanedioate) ratio \|\| id:ebi-met1400-GCST90200841 | Gherkins liking \|\| id:ebi-fl187-GCST90094770 | Weighted mode | 20 | 0.0918659725885036 | 0.160757452486467 | 0.574388468611898 | -0.223218634 | 0.40695057946198 | 1.09621788898841 | 0.799939935878314 | 1.50222986281935 | 0.999714303421225 |
| ebi-fl187-GCST90094770 | ebi-met1400-GCST90200844 | Aspartate to phosphate ratio \|\| id:ebi-met1400-GCST90200844 | Gherkins liking \|\| id:ebi-fl187-GCST90094770 | Weighted mode | 20 | -0.02931935 | 0.114284396162526 | 0.800285666264382 | -0.253316766 | 0.194678066952818 | 0.971106292608641 | 0.77622196214691 | 1.21491980069177 | 0.999714303421225 |
| ebi-fl187-GCST90094770 | ebi-met1400-GCST90200870 | Adenosine 5'-monophosphate (AMP) to acetoacetate ratio \|\| id:ebi-met1400-GCST90200870 | Gherkins liking \|\| id:ebi-fl187-GCST90094770 | Weighted mode | 20 | 0.0135804979187544 | 0.11429271414181 | 0.906663607686128 | -0.210433222 | 0.237594217636702 | 1.01367313174344 | 0.810233159259449 | 1.26819447744858 | 0.999714303421225 |
| ebi-fl187-GCST90094770 | ebi-met1400-GCST90200887 | Cholesterol to cortisol ratio \|\| id:ebi-met1400-GCST90200887 | Gherkins liking \|\| id:ebi-fl187-GCST90094770 | Weighted mode | 20 | -0.046646242 | 0.11949980666327 | 0.700622698817143 | -0.280865863 | 0.187573379008959 | 0.954424973297786 | 0.755129619424995 | 1.2063187646488 | 0.999714303421225 |
| ebi-fl187-GCST90094770 | ebi-met1400-GCST90200890 | Cortisol to taurocholate ratio \|\| id:ebi-met1400-GCST90200890 | Gherkins liking \|\| id:ebi-fl187-GCST90094770 | Weighted mode | 20 | -0.033742722 | 0.117794645007587 | 0.777630758209574 | -0.264620226 | 0.197134782182181 | 0.966820214181852 | 0.767497369991707 | 1.21790818196646 | 0.999714303421225 |
| ebi-fl187-GCST90094770 | ebi-met1400-GCST90200948 | Adenosine 5'-diphosphate (ADP) to mannitol to sorbitol ratio \|\| id:ebi-met1400-GCST90200948 | Gherkins liking \|\| id:ebi-fl187-GCST90094770 | Weighted mode | 20 | 0.00102489354173385 | 0.160189778516377 | 0.994961872817411 | -0.312947072 | 0.314996859433833 | 1.00102541892459 | 0.731288616932306 | 1.37025500757372 | 0.999714303421225 |
| ebi-fl187-GCST90094770 | ebi-met1400-GCST90200972 | Leucine to N-palmitoyl-sphingosine (d18:1 to 16:0) ratio \|\| id:ebi-met1400-GCST90200972 | Gherkins liking \|\| id:ebi-fl187-GCST90094770 | Weighted mode | 20 | 0.0513366314055977 | 0.11383837958826 | 0.657122288097316 | -0.171786593 | 0.274459855398588 | 1.05267719784959 | 0.842158876939692 | 1.31581974994941 | 0.999714303421225 |
| ebi-fl187-GCST90094770 | ebi-met1400-GCST90200973 | Proline to glutamate ratio \|\| id:ebi-met1400-GCST90200973 | Gherkins liking \|\| id:ebi-fl187-GCST90094770 | Weighted mode | 20 | -0.006400712 | 0.113756516831064 | 0.955716783803408 | -0.229363485 | 0.216562060710065 | 0.993619728644608 | 0.795039495833828 | 1.24180014996153 | 0.999714303421225 |
| ebi-fl187-GCST90094770 | ebi-met1400-GCST90200983 | Cholesterol to linoleoyl-arachidonoyl-glycerol (18:2 to 20:4) [1] ratio \|\| id:ebi-met1400-GCST90200983 | Gherkins liking \|\| id:ebi-fl187-GCST90094770 | Weighted mode | 20 | -0.042262066 | 0.118982655905147 | 0.72635592320843 | -0.275468072 | 0.190943939500691 | 0.958618526258245 | 0.75921667224462 | 1.21039159502209 | 0.999714303421225 |
| ebi-fl187-GCST90094770 | ebi-met1400-GCST90201001 | Serine to alpha-ketobutyrate ratio \|\| id:ebi-met1400-GCST90201001 | Gherkins liking \|\| id:ebi-fl187-GCST90094770 | Weighted mode | 20 | 0.00414421439475965 | 0.115704220506893 | 0.971801658258398 | -0.222636058 | 0.23092448658827 | 1.00415281352601 | 0.800406097849664 | 1.2597641067717 | 0.999714303421225 |
| ebi-fl187-GCST90094770 | ebi-met1400-GCST90201005 | Fructose to maltose ratio \|\| id:ebi-met1400-GCST90201005 | Gherkins liking \|\| id:ebi-fl187-GCST90094770 | Weighted mode | 20 | -0.066343043 | 0.122595968975138 | 0.594691656266506 | -0.306631142 | 0.173945056268147 | 0.935809786298422 | 0.735922001483494 | 1.18999018152271 | 0.999714303421225 |
| ebi-fl187-GCST90094770 | ebi-met1400-GCST90201020 | Paraxanthine to 5-acetylamino-6-formylamino-3-methyluracil ratio \|\| id:ebi-met1400-GCST90201020 | Gherkins liking \|\| id:ebi-fl187-GCST90094770 | Weighted mode | 20 | 0.122026183269281 | 0.12288861302478 | 0.333197259313256 | -0.118835498 | 0.362887864797849 | 1.12978368282345 | 0.887953858703657 | 1.43747465869181 | 0.999714303421225 |
| ebi-fl187-GCST90094770 | ebi-pw11-GCST90275197 | Placental weight (fetal genotype, adjusted for sex) \|\| id:ebi-pw11-GCST90275197 | Gherkins liking \|\| id:ebi-fl187-GCST90094770 | Weighted mode | 18 | 0.0502963558793675 | 0.0446102659864822 | 0.275209892480027 | -0.037139765 | 0.137732477212873 | 1.0515826929157 | 0.963541456128325 | 1.14766848173116 | 0.999714303421225 |
| ebi-fl187-GCST90094770 | prot-a-1017 | FAS-associated factor 2 \|\| id:prot-a-1017 | Gherkins liking \|\| id:ebi-fl187-GCST90094770 | Weighted mode | 21 | -0.259056638 | 0.216871572849065 | 0.246250601525617 | -0.684124921 | 0.166011644676607 | 0.77177930968624 | 0.504531541466658 | 1.1805868491953 | 0.999714303421225 |
| ebi-fl187-GCST90094770 | prot-a-1019 | Fas apoptotic inhibitory molecule 3 \|\| id:prot-a-1019 | Gherkins liking \|\| id:ebi-fl187-GCST90094770 | Weighted mode | 21 | 0.104830724885795 | 0.160368351885413 | 0.520759012782451 | -0.209491245 | 0.419152694581204 | 1.11052261060258 | 0.810996739832868 | 1.52067253551934 | 0.999714303421225 |
| ebi-fl187-GCST90094770 | prot-a-1052 | MIP18 family protein FAM96A \|\| id:prot-a-1052 | Gherkins liking \|\| id:ebi-fl187-GCST90094770 | Weighted mode | 21 | -0.137815217 | 0.20477537241573 | 0.508645526903028 | -0.539174946 | 0.263544513428404 | 0.871259671278028 | 0.583229249290285 | 1.30153522944745 | 0.999714303421225 |
| ebi-fl187-GCST90094770 | prot-a-1066 | F-box/LRR-repeat protein 4 \|\| id:prot-a-1066 | Gherkins liking \|\| id:ebi-fl187-GCST90094770 | Weighted mode | 21 | 0.180626829791486 | 0.262766506589673 | 0.499722583305112 | -0.334395523 | 0.695649182707245 | 1.19796804988373 | 0.715770622399936 | 2.00501026953347 | 0.999714303421225 |
| ebi-fl187-GCST90094770 | prot-a-1076 | Low affinity immunoglobulin gamma Fc region receptor III-B \|\| id:prot-a-1076 | Gherkins liking \|\| id:ebi-fl187-GCST90094770 | Weighted mode | 21 | -0.183352254 | 0.172716752372491 | 0.301079017555797 | -0.521877089 | 0.155172580263788 | 0.832474861161649 | 0.593405626698312 | 1.1678594932138 | 0.999714303421225 |
| ebi-fl187-GCST90094770 | prot-a-1081 | Fc receptor-like protein 3 \|\| id:prot-a-1081 | Gherkins liking \|\| id:ebi-fl187-GCST90094770 | Weighted mode | 21 | -0.04902976 | 0.154735452757185 | 0.754632182592963 | -0.352311248 | 0.254251726924741 | 0.952152792753328 | 0.703061261588606 | 1.2894963643701 | 0.999714303421225 |
| ebi-fl187-GCST90094770 | prot-a-1105 | Fibroblast growth factor receptor 4 \|\| id:prot-a-1105 | Gherkins liking \|\| id:ebi-fl187-GCST90094770 | Weighted mode | 21 | 0.320010442897088 | 0.164789224003422 | 0.0663630581863133 | -0.002976436 | 0.642997321943795 | 1.37714214561457 | 0.997027989044932 | 1.9021737705123 | 0.999714303421225 |
| ebi-fl187-GCST90094770 | prot-a-1143 | Follistatin-related protein 1 \|\| id:prot-a-1143 | Gherkins liking \|\| id:ebi-fl187-GCST90094770 | Weighted mode | 21 | -0.159403746 | 0.185708209747505 | 0.400866287288715 | -0.523391837 | 0.204584345376412 | 0.852652034846977 | 0.592507447261686 | 1.22701494451866 | 0.999714303421225 |
| ebi-fl187-GCST90094770 | prot-a-1151 | Alpha-(1,3)-fucosyltransferase 10 \|\| id:prot-a-1151 | Gherkins liking \|\| id:ebi-fl187-GCST90094770 | Weighted mode | 21 | 0.0611621506708298 | 0.18023624689561 | 0.737887742347281 | -0.292100893 | 0.414425194586224 | 1.06307127793323 | 0.746693195873541 | 1.51350052231891 | 0.999714303421225 |
| ebi-fl187-GCST90094770 | prot-a-1165 | Growth arrest and DNA damage-inducible proteins-interacting protein 1 \|\| id:prot-a-1165 | Gherkins liking \|\| id:ebi-fl187-GCST90094770 | Weighted mode | 21 | 0.0287639200734893 | 0.214464329798168 | 0.894648286970341 | -0.391586166 | 0.449114006477898 | 1.0291815966771 | 0.675983800941272 | 1.56692328642184 | 0.999714303421225 |
| ebi-fl187-GCST90094770 | prot-a-1194 | Growth/differentiation factor 11/8 \|\| id:prot-a-1194 | Gherkins liking \|\| id:ebi-fl187-GCST90094770 | Weighted mode | 21 | -0.398125553 | 0.21373037590086 | 0.0772531728251976 | -0.81703709 | 0.0207859834486401 | 0.6715777035602 | 0.441738548972218 | 1.02100351660176 | 0.999714303421225 |
| ebi-fl187-GCST90094770 | prot-a-1216 | D-glucuronyl C5-epimerase \|\| id:prot-a-1216 | Gherkins liking \|\| id:ebi-fl187-GCST90094770 | Weighted mode | 21 | -0.068830377 | 0.169706922105443 | 0.689354544045235 | -0.401455944 | 0.263795190522496 | 0.933485007360039 | 0.669344807617429 | 1.30186153541364 | 0.999714303421225 |
| ebi-fl187-GCST90094770 | prot-a-1246 | Glypican-3 \|\| id:prot-a-1246 | Gherkins liking \|\| id:ebi-fl187-GCST90094770 | Weighted mode | 21 | 0.116700672044809 | 0.192660459372461 | 0.551505441847135 | -0.260913828 | 0.494315172414833 | 1.12378299967422 | 0.770347298871943 | 1.63937516520939 | 0.999714303421225 |
| ebi-fl187-GCST90094770 | prot-a-1275 | Glutamate receptor 4 \|\| id:prot-a-1275 | Gherkins liking \|\| id:ebi-fl187-GCST90094770 | Weighted mode | 20 | 0.103483206795399 | 0.178151804389895 | 0.568154356785082 | -0.24569433 | 0.452660743399594 | 1.10902716908853 | 0.782161271774364 | 1.57249061818971 | 0.999714303421225 |
| ebi-fl187-GCST90094770 | prot-a-1289 | Glutathione S-transferase P \|\| id:prot-a-1289 | Gherkins liking \|\| id:ebi-fl187-GCST90094770 | Weighted mode | 21 | 0.00672221812024421 | 0.2035511843312 | 0.973982250958386 | -0.392238103 | 0.405682539409396 | 1.00674486294118 | 0.675543245822215 | 1.5003261824117 | 0.999714303421225 |
| ebi-fl187-GCST90094770 | prot-a-130 | Apolipoprotein D \|\| id:prot-a-130 | Gherkins liking \|\| id:ebi-fl187-GCST90094770 | Weighted mode | 21 | -0.008000352 | 0.155078213333734 | 0.959367859732815 | -0.31195365 | 0.295952946421264 | 0.992031565926745 | 0.732015456470442 | 1.344406896 | 0.999714303421225 |
| ebi-fl187-GCST90094770 | prot-a-1313 | Heparin-binding EGF-like growth factor \|\| id:prot-a-1313 | Gherkins liking \|\| id:ebi-fl187-GCST90094770 | Weighted mode | 21 | -0.139725298 | 0.196367659896089 | 0.484961251689791 | -0.524605911 | 0.245155315553437 | 0.869597082785809 | 0.591788535629353 | 1.27781976307701 | 0.999714303421225 |
| ebi-fl187-GCST90094770 | prot-a-1347 | HLA class II histocompatibility antigen, DQ alpha 2 chain \|\| id:prot-a-1347 | Gherkins liking \|\| id:ebi-fl187-GCST90094770 | Weighted mode | 20 | -0.053732692 | 0.16103877681649 | 0.74228811250915 | -0.369368694 | 0.261903310868888 | 0.947685396827466 | 0.691170532864797 | 1.29940089841144 | 0.999714303421225 |
| ebi-fl187-GCST90094770 | prot-a-1357 | Non-histone chromosomal protein HMG-14 \|\| id:prot-a-1357 | Gherkins liking \|\| id:ebi-fl187-GCST90094770 | Weighted mode | 21 | -0.368565395 | 0.186881714221497 | 0.0625765588325975 | -0.734853555 | -0.002277235 | 0.691725972799605 | 0.479575685423956 | 0.997725355952039 | 0.999714303421225 |
| ebi-fl187-GCST90094770 | prot-a-137 | Apolipoprotein M \|\| id:prot-a-137 | Gherkins liking \|\| id:ebi-fl187-GCST90094770 | Weighted mode | 21 | -0.165382004 | 0.187163674790264 | 0.387398834387551 | -0.532222806 | 0.201458799064164 | 0.847569867561623 | 0.587298067985855 | 1.22318583962331 | 0.999714303421225 |
| ebi-fl187-GCST90094770 | prot-a-1381 | 17-beta-hydroxysteroid dehydrogenase 14 \|\| id:prot-a-1381 | Gherkins liking \|\| id:ebi-fl187-GCST90094770 | Weighted mode | 21 | -0.063060106 | 0.183145554604706 | 0.734201983777996 | -0.422025393 | 0.295905181144581 | 0.938887039360807 | 0.655717388612112 | 1.34434268175425 | 0.999714303421225 |
| ebi-fl187-GCST90094770 | prot-a-1392 | Serine protease HTRA2, mitochondrial \|\| id:prot-a-1392 | Gherkins liking \|\| id:ebi-fl187-GCST90094770 | Weighted mode | 21 | -0.112419894 | 0.207954835951528 | 0.594754947081387 | -0.520011372 | 0.295171584762515 | 0.893668932750659 | 0.594513787021399 | 1.3433568384764 | 0.999714303421225 |
| ebi-fl187-GCST90094770 | prot-a-1416 | Interferon-induced protein with tetratricopeptide repeats 2 \|\| id:prot-a-1416 | Gherkins liking \|\| id:ebi-fl187-GCST90094770 | Weighted mode | 21 | 0.0612149509949675 | 0.169523152301842 | 0.721810408153969 | -0.271050428 | 0.393480329506578 | 1.06312740992316 | 0.762578040518626 | 1.4821301292144 | 0.999714303421225 |
| ebi-fl187-GCST90094770 | prot-a-1428 | Interferon gamma \|\| id:prot-a-1428 | Gherkins liking \|\| id:ebi-fl187-GCST90094770 | Weighted mode | 21 | 0.118171730168258 | 0.178970230556708 | 0.516602897666974 | -0.232609922 | 0.468953382059405 | 1.1254373663213 | 0.792462635700783 | 1.59832048660836 | 0.999714303421225 |
| ebi-fl187-GCST90094770 | prot-a-1483 | Interleukin-17C \|\| id:prot-a-1483 | Gherkins liking \|\| id:ebi-fl187-GCST90094770 | Weighted mode | 21 | 0.116767280595608 | 0.19246949660821 | 0.550889038637551 | -0.260472933 | 0.4940074939477 | 1.12385785572424 | 0.770687016466887 | 1.63887084236012 | 0.999714303421225 |
| ebi-fl187-GCST90094770 | prot-a-1503 | Interleukin-1 receptor-like 2 \|\| id:prot-a-1503 | Gherkins liking \|\| id:ebi-fl187-GCST90094770 | Weighted mode | 21 | -0.080805877 | 0.156245094894863 | 0.610707428011732 | -0.387046263 | 0.225434509013882 | 0.922372727845952 | 0.679059678879687 | 1.25286697993553 | 0.999714303421225 |
| ebi-fl187-GCST90094770 | prot-a-1522 | Interleukin-31 receptor subunit alpha \|\| id:prot-a-1522 | Gherkins liking \|\| id:ebi-fl187-GCST90094770 | Weighted mode | 21 | 0.0294518612712145 | 0.17313113009739 | 0.866630276340205 | -0.309885154 | 0.368788876262099 | 1.02988985669008 | 0.733531194716177 | 1.44598229026036 | 0.999714303421225 |
| ebi-fl187-GCST90094770 | prot-a-1540 | Interleukin-6 receptor subunit alpha \|\| id:prot-a-1540 | Gherkins liking \|\| id:ebi-fl187-GCST90094770 | Weighted mode | 21 | 0.0881125067744304 | 0.186554876528444 | 0.64181545507688 | -0.277535051 | 0.45376006477018 | 1.09211098500085 | 0.757649007614501 | 1.57422024126295 | 0.999714303421225 |
| ebi-fl187-GCST90094770 | prot-a-1541 | Interleukin-6 receptor subunit alpha \|\| id:prot-a-1541 | Gherkins liking \|\| id:ebi-fl187-GCST90094770 | Weighted mode | 21 | -0.279419627 | 0.213895911898948 | 0.206263260585907 | -0.698655614 | 0.139816360526505 | 0.756222505398816 | 0.497253355022051 | 1.15006258257685 | 0.999714303421225 |
| ebi-fl187-GCST90094770 | prot-a-1545 | Interleukin-9 \|\| id:prot-a-1545 | Gherkins liking \|\| id:ebi-fl187-GCST90094770 | Weighted mode | 21 | -0.083846421 | 0.240220225062455 | 0.730707335030425 | -0.554678062 | 0.386985220347087 | 0.919572472478253 | 0.574257106683575 | 1.47253472756013 | 0.999714303421225 |
| ebi-fl187-GCST90094770 | prot-a-1548 | Inositol monophosphatase 3 \|\| id:prot-a-1548 | Gherkins liking \|\| id:ebi-fl187-GCST90094770 | Weighted mode | 21 | -0.003003734 | 0.18074432088503 | 0.986905483341953 | -0.357262603 | 0.351255134821531 | 0.997000772582748 | 0.699588759470118 | 1.42084978792895 | 0.999714303421225 |
| ebi-fl187-GCST90094770 | prot-a-1555 | Inhibin beta A chain:Inhibin beta B chain heterodimer \|\| id:prot-a-1555 | Gherkins liking \|\| id:ebi-fl187-GCST90094770 | Weighted mode | 21 | -0.017584694 | 0.168876430193861 | 0.918105239260814 | -0.348582497 | 0.313413109212863 | 0.982569014473166 | 0.70568769540089 | 1.36808658347701 | 0.999714303421225 |
| ebi-fl187-GCST90094770 | prot-a-1603 | Junctophilin-3 \|\| id:prot-a-1603 | Gherkins liking \|\| id:ebi-fl187-GCST90094770 | Weighted mode | 21 | -0.265900037 | 0.163126778459712 | 0.11874615995497 | -0.585628522 | 0.0538284491403041 | 0.766515747160395 | 0.556755818303348 | 1.05530354839459 | 0.999714303421225 |
| ebi-fl187-GCST90094770 | prot-a-1627 | UPF0258 protein KIAA1024 \|\| id:prot-a-1627 | Gherkins liking \|\| id:ebi-fl187-GCST90094770 | Weighted mode | 21 | -0.091008926 | 0.179375488168937 | 0.617447527628102 | -0.442584883 | 0.260567030476635 | 0.913009561034164 | 0.642373811944045 | 1.29766569408718 | 0.999714303421225 |
| ebi-fl187-GCST90094770 | prot-a-1666 | Kallikrein-7 \|\| id:prot-a-1666 | Gherkins liking \|\| id:ebi-fl187-GCST90094770 | Weighted mode | 21 | 0.301965827868183 | 0.194620927182978 | 0.136448685686502 | -0.079491189 | 0.683422845146819 | 1.35251500759866 | 0.923586157271301 | 1.9806455860969 | 0.999714303421225 |
| ebi-fl187-GCST90094770 | prot-a-1678 | Importin subunit alpha-3 \|\| id:prot-a-1678 | Gherkins liking \|\| id:ebi-fl187-GCST90094770 | Weighted mode | 21 | -0.104377821 | 0.153648680635363 | 0.504717002799771 | -0.405529235 | 0.196773593040168 | 0.900884859889747 | 0.666623916742532 | 1.21746836618829 | 0.999714303421225 |
| ebi-fl187-GCST90094770 | prot-a-1718 | Protein LDOC1 \|\| id:prot-a-1718 | Gherkins liking \|\| id:ebi-fl187-GCST90094770 | Weighted mode | 21 | -0.083249713 | 0.184874160114302 | 0.657333972555094 | -0.445603067 | 0.279103640751038 | 0.920121352199524 | 0.640437932631802 | 1.32194434407467 | 0.999714303421225 |
| ebi-fl187-GCST90094770 | prot-a-1723 | Leptin \|\| id:prot-a-1723 | Gherkins liking \|\| id:ebi-fl187-GCST90094770 | Weighted mode | 21 | -0.179573003 | 0.224511537432131 | 0.433202226159651 | -0.619615616 | 0.260469610382704 | 0.835626945455636 | 0.538151254386372 | 1.29753928153103 | 0.999714303421225 |
| ebi-fl187-GCST90094770 | prot-a-1730 | Galectin-7 \|\| id:prot-a-1730 | Gherkins liking \|\| id:ebi-fl187-GCST90094770 | Weighted mode | 21 | 0.126602349657792 | 0.169021941152391 | 0.462558996897076 | -0.204680655 | 0.457885354316479 | 1.13496560857328 | 0.814907511501735 | 1.58072776905723 | 0.999714303421225 |
| ebi-fl187-GCST90094770 | prot-a-1753 | Lipase member N \|\| id:prot-a-1753 | Gherkins liking \|\| id:ebi-fl187-GCST90094770 | Weighted mode | 21 | -0.231167357 | 0.176024115590088 | 0.203959907838531 | -0.576174624 | 0.113839909535621 | 0.793606639277851 | 0.56204429044228 | 1.12057271751711 | 0.999714303421225 |
| ebi-fl187-GCST90094770 | prot-a-1756 | VIP36-like protein \|\| id:prot-a-1756 | Gherkins liking \|\| id:ebi-fl187-GCST90094770 | Weighted mode | 21 | 0.20222451789715 | 0.155126244856964 | 0.207172703533603 | -0.101822922 | 0.506271957816799 | 1.22412281474092 | 0.903189472474318 | 1.65909447711354 | 0.999714303421225 |
| ebi-fl187-GCST90094770 | prot-a-1760 | Ig Kappa chain V-I region HK102- like \|\| id:prot-a-1760 | Gherkins liking \|\| id:ebi-fl187-GCST90094770 | Weighted mode | 21 | 0.24763855031826 | 0.264872398986745 | 0.360973698056175 | -0.271511352 | 0.766788452332279 | 1.28099683260801 | 0.762226630854115 | 2.15284118755202 | 0.999714303421225 |
| ebi-fl187-GCST90094770 | prot-a-179 | Neutral ceramidase \|\| id:prot-a-179 | Gherkins liking \|\| id:ebi-fl187-GCST90094770 | Weighted mode | 21 | 0.308139010596888 | 0.196931930238958 | 0.133339486102092 | -0.077847573 | 0.694125593865245 | 1.36089015394147 | 0.925105427145962 | 2.00195778421548 | 0.999714303421225 |
| ebi-fl187-GCST90094770 | prot-a-1809 | Lactotransferrin \|\| id:prot-a-1809 | Gherkins liking \|\| id:ebi-fl187-GCST90094770 | Weighted mode | 21 | 0.0982729306347114 | 0.178804746007411 | 0.588671984374074 | -0.252184372 | 0.448730232809237 | 1.10326385852572 | 0.777101449469352 | 1.56632205789891 | 0.999714303421225 |
| ebi-fl187-GCST90094770 | prot-a-1818 | Lysophospholipase-like protein 1 \|\| id:prot-a-1818 | Gherkins liking \|\| id:ebi-fl187-GCST90094770 | Weighted mode | 21 | -0.170055803 | 0.186152383586407 | 0.371845806626216 | -0.534914475 | 0.194802868422151 | 0.843617738538648 | 0.585719381514282 | 1.21507143392985 | 0.999714303421225 |
| ebi-fl187-GCST90094770 | prot-a-1852 | MAP kinase-activated protein kinase 3 \|\| id:prot-a-1852 | Gherkins liking \|\| id:ebi-fl187-GCST90094770 | Weighted mode | 21 | 0.0505159532628605 | 0.184424714405663 | 0.786962004389533 | -0.310956487 | 0.411988393497961 | 1.05181364308069 | 0.732745759162218 | 1.50981691253398 | 0.999714303421225 |
| ebi-fl187-GCST90094770 | prot-a-1856 | Megakaryocyte-associated tyrosine-protein kinase \|\| id:prot-a-1856 | Gherkins liking \|\| id:ebi-fl187-GCST90094770 | Weighted mode | 21 | -0.050666099 | 0.155639816629624 | 0.748157888152182 | -0.35572014 | 0.254387941249889 | 0.950596022182748 | 0.70066868197966 | 1.28967202421058 | 0.999714303421225 |
| ebi-fl187-GCST90094770 | prot-a-1890 | Beta-1,3-N-acetylglucosaminyltransferase manic fringe \|\| id:prot-a-1890 | Gherkins liking \|\| id:ebi-fl187-GCST90094770 | Weighted mode | 21 | 0.0674432172936439 | 0.152382462973944 | 0.66281065584215 | -0.23122641 | 0.366112844722575 | 1.0697695134651 | 0.793559775717977 | 1.44211796887506 | 0.999714303421225 |
| ebi-fl187-GCST90094770 | prot-a-1919 | Matrilysin \|\| id:prot-a-1919 | Gherkins liking \|\| id:ebi-fl187-GCST90094770 | Weighted mode | 21 | 0.00118306261712675 | 0.217719558114497 | 0.99571824632697 | -0.425547271 | 0.42791339652154 | 1.00118376271176 | 0.653412093584034 | 1.53405322086952 | 0.999714303421225 |
| ebi-fl187-GCST90094770 | prot-a-1923 | Multimerin-2 \|\| id:prot-a-1923 | Gherkins liking \|\| id:ebi-fl187-GCST90094770 | Weighted mode | 21 | -0.025903427 | 0.1689033007412 | 0.879649270786502 | -0.356953896 | 0.305147042814714 | 0.974429188967703 | 0.699804760726033 | 1.35682449963209 | 0.999714303421225 |
| ebi-fl187-GCST90094770 | prot-a-1948 | Mesothelin \|\| id:prot-a-1948 | Gherkins liking \|\| id:ebi-fl187-GCST90094770 | Weighted mode | 21 | -0.094477884 | 0.175497836768495 | 0.596282077647899 | -0.438453644 | 0.249497876447025 | 0.909847856945164 | 0.645033101282421 | 1.28338083912573 | 0.999714303421225 |
| ebi-fl187-GCST90094770 | prot-a-195 | ATPase family AAA domain-containing protein 1 \|\| id:prot-a-195 | Gherkins liking \|\| id:ebi-fl187-GCST90094770 | Weighted mode | 21 | -0.154827613 | 0.184272032564794 | 0.410721796927491 | -0.516000797 | 0.206345571073117 | 0.856562825263527 | 0.59690291719272 | 1.22917789893553 | 0.999714303421225 |
| ebi-fl187-GCST90094770 | prot-a-1966 | Mucin-1 \|\| id:prot-a-1966 | Gherkins liking \|\| id:ebi-fl187-GCST90094770 | Weighted mode | 21 | -0.120188393 | 0.18158699486958 | 0.515603990781442 | -0.476098903 | 0.235722117101813 | 0.886753362993198 | 0.621202040596069 | 1.26582251086173 | 0.999714303421225 |
| ebi-fl187-GCST90094770 | prot-a-1986 | Myocardial zonula adherens protein \|\| id:prot-a-1986 | Gherkins liking \|\| id:ebi-fl187-GCST90094770 | Weighted mode | 21 | 0.023362823763708 | 0.183510696150603 | 0.899966042031219 | -0.336318141 | 0.38304378821889 | 1.02363787232448 | 0.714395791287947 | 1.46674225469315 | 0.999714303421225 |
| ebi-fl187-GCST90094770 | prot-a-2071 | Epididymal secretory protein E1 \|\| id:prot-a-2071 | Gherkins liking \|\| id:ebi-fl187-GCST90094770 | Weighted mode | 21 | 0.197262433530825 | 0.197424287373797 | 0.329643769214314 | -0.18968917 | 0.584214036783467 | 1.21806365951166 | 0.827216217833671 | 1.79358074302319 | 0.999714303421225 |
| ebi-fl187-GCST90094770 | prot-a-2098 | Neuritin \|\| id:prot-a-2098 | Gherkins liking \|\| id:ebi-fl187-GCST90094770 | Weighted mode | 21 | 0.0169339806847009 | 0.178136444887426 | 0.925211704572533 | -0.332213451 | 0.366081412664056 | 1.01707817330409 | 0.717334190600202 | 1.44207264085106 | 0.999714303421225 |
| ebi-fl187-GCST90094770 | prot-a-2115 | Neurotrophin-4 \|\| id:prot-a-2115 | Gherkins liking \|\| id:ebi-fl187-GCST90094770 | Weighted mode | 21 | -0.069868321 | 0.178130303791111 | 0.699035707900653 | -0.419003717 | 0.279267074185685 | 0.932516604447304 | 0.657701750799311 | 1.32216041163508 | 0.999714303421225 |
| ebi-fl187-GCST90094770 | prot-a-2118 | Netrin-1 \|\| id:prot-a-2118 | Gherkins liking \|\| id:ebi-fl187-GCST90094770 | Weighted mode | 21 | 0.213160155116199 | 0.195081389182225 | 0.287524952575209 | -0.169199368 | 0.59551967791336 | 1.2375828405963 | 0.844340552386846 | 1.81397338195913 | 0.999714303421225 |
| ebi-fl187-GCST90094770 | prot-a-2120 | Netrin-G1 \|\| id:prot-a-2120 | Gherkins liking \|\| id:ebi-fl187-GCST90094770 | Weighted mode | 21 | 0.107548550339779 | 0.179489498740287 | 0.555772653234363 | -0.244250867 | 0.459347967870741 | 1.11354492241851 | 0.783291107572809 | 1.58304145451925 | 0.999714303421225 |
| ebi-fl187-GCST90094770 | prot-a-2145 | Olfactomedin-like protein 3 \|\| id:prot-a-2145 | Gherkins liking \|\| id:ebi-fl187-GCST90094770 | Weighted mode | 21 | 0.148648161897254 | 0.186607271192493 | 0.43504791276212 | -0.21710209 | 0.51439841343454 | 1.16026469205811 | 0.804847798488651 | 1.67263196614893 | 0.999714303421225 |
| ebi-fl187-GCST90094770 | prot-a-2162 | Prolyl 4-hydroxylase subunit alpha-2 \|\| id:prot-a-2162 | Gherkins liking \|\| id:ebi-fl187-GCST90094770 | Weighted mode | 21 | 0.0310904682041513 | 0.17145252116544 | 0.857929188727559 | -0.304956473 | 0.367137409688414 | 1.03157882474889 | 0.737155459652547 | 1.44359626960083 | 0.999714303421225 |
| ebi-fl187-GCST90094770 | prot-a-2168 | Polyadenylate-binding protein-interacting protein 1 \|\| id:prot-a-2168 | Gherkins liking \|\| id:ebi-fl187-GCST90094770 | Weighted mode | 21 | -0.086619403 | 0.1987781915128 | 0.667678489967974 | -0.476224658 | 0.302985852269107 | 0.917026046498332 | 0.621123925826474 | 1.35389530976029 | 0.999714303421225 |
| ebi-fl187-GCST90094770 | prot-a-219 | Beta-1,4-galactosyltransferase 5 \|\| id:prot-a-219 | Gherkins liking \|\| id:ebi-fl187-GCST90094770 | Weighted mode | 21 | 0.0272303163213303 | 0.211840994380829 | 0.899004432400738 | -0.387978033 | 0.442438665307755 | 1.02760444958724 | 0.678427246338942 | 1.55649837253726 | 0.999714303421225 |
| ebi-fl187-GCST90094770 | prot-a-2190 | Pyruvate carboxylase, mitochondrial \|\| id:prot-a-2190 | Gherkins liking \|\| id:ebi-fl187-GCST90094770 | Weighted mode | 21 | -0.104208405 | 0.183534675823967 | 0.576499525021953 | -0.46393637 | 0.255519559594532 | 0.901037497114616 | 0.628803564194209 | 1.29113226679457 | 0.999714303421225 |
| ebi-fl187-GCST90094770 | prot-a-2197 | Protocadherin-9 \|\| id:prot-a-2197 | Gherkins liking \|\| id:ebi-fl187-GCST90094770 | Weighted mode | 21 | 0.0322649759173539 | 0.169420103255515 | 0.850881325703519 | -0.299798426 | 0.364328378298163 | 1.03279113382915 | 0.740967565081832 | 1.43954685249726 | 0.999714303421225 |
| ebi-fl187-GCST90094770 | prot-a-2198 | Protocadherin alpha-4 \|\| id:prot-a-2198 | Gherkins liking \|\| id:ebi-fl187-GCST90094770 | Weighted mode | 21 | 0.141342612537954 | 0.214093007413121 | 0.516662279484485 | -0.278279682 | 0.560964907067671 | 1.15181920809241 | 0.757085048846819 | 1.75236255180501 | 0.999714303421225 |
| ebi-fl187-GCST90094770 | prot-a-2254 | Placenta growth factor \|\| id:prot-a-2254 | Gherkins liking \|\| id:ebi-fl187-GCST90094770 | Weighted mode | 21 | -0.311378144 | 0.168030877681432 | 0.078681449161961 | -0.640718664 | 0.0179623763978407 | 0.732436856997467 | 0.526913614035519 | 1.01812467015165 | 0.999714303421225 |
| ebi-fl187-GCST90094770 | prot-a-2261 | Membrane-associated progesterone receptor component 2 \|\| id:prot-a-2261 | Gherkins liking \|\| id:ebi-fl187-GCST90094770 | Weighted mode | 21 | 0.230184129081863 | 0.17037885905972 | 0.191778333921424 | -0.103758435 | 0.564126692838914 | 1.25883177613053 | 0.901443028499985 | 1.75791191511331 | 0.999714303421225 |
| ebi-fl187-GCST90094770 | prot-a-2268 | Polymeric immunoglobulin receptor \|\| id:prot-a-2268 | Gherkins liking \|\| id:ebi-fl187-GCST90094770 | Weighted mode | 21 | -0.101247637 | 0.218638132416778 | 0.648304145178287 | -0.529778377 | 0.327283102443162 | 0.903709213250846 | 0.588735432751275 | 1.3871941396459 | 0.999714303421225 |
| ebi-fl187-GCST90094770 | prot-a-2283 | Group 10 secretory phospholipase A2 \|\| id:prot-a-2283 | Gherkins liking \|\| id:ebi-fl187-GCST90094770 | Weighted mode | 21 | 0.354574944429073 | 0.257946209823811 | 0.184460247856956 | -0.150999627 | 0.860149515683743 | 1.4255745770666 | 0.859848019532417 | 2.36351404970818 | 0.999714303421225 |
| ebi-fl187-GCST90094770 | prot-a-2298 | Inactive phospholipase D5 \|\| id:prot-a-2298 | Gherkins liking \|\| id:ebi-fl187-GCST90094770 | Weighted mode | 21 | -0.250767733 | 0.255786551351554 | 0.338610462723036 | -0.752109373 | 0.250573907872209 | 0.778203101642783 | 0.471371205436028 | 1.28476254048284 | 0.999714303421225 |
| ebi-fl187-GCST90094770 | prot-a-2304 | Procollagen-lysine,2-oxoglutarate 5-dioxygenase 2 \|\| id:prot-a-2304 | Gherkins liking \|\| id:ebi-fl187-GCST90094770 | Weighted mode | 21 | -0.104945986 | 0.188549693412518 | 0.583978528752596 | -0.474503385 | 0.26461141302295 | 0.900373153969354 | 0.622193970515793 | 1.30292457787189 | 0.999714303421225 |
| ebi-fl187-GCST90094770 | prot-a-2331 | Periostin \|\| id:prot-a-2331 | Gherkins liking \|\| id:ebi-fl187-GCST90094770 | Weighted mode | 21 | 0.158095434334392 | 0.18167258222672 | 0.394504251718661 | -0.197982827 | 0.514173695498764 | 1.17127796950742 | 0.82038394160977 | 1.67225613797543 | 0.999714303421225 |
| ebi-fl187-GCST90094770 | prot-a-2333 | Periostin \|\| id:prot-a-2333 | Gherkins liking \|\| id:ebi-fl187-GCST90094770 | Weighted mode | 21 | -0.013399903 | 0.183658451787878 | 0.942561913135394 | -0.373370468 | 0.346570662774567 | 0.986689476298085 | 0.68841015150407 | 1.41420942226131 | 0.999714303421225 |
| ebi-fl187-GCST90094770 | prot-a-2367 | cAMP-dependent protein kinase catalytic subunit alpha \|\| id:prot-a-2367 | Gherkins liking \|\| id:ebi-fl187-GCST90094770 | Weighted mode | 21 | 0.0621753330454089 | 0.200179200522319 | 0.759319685160369 | -0.3301759 | 0.454526566069155 | 1.06414890884108 | 0.718797285884176 | 1.5754273456872 | 0.999714303421225 |
| ebi-fl187-GCST90094770 | prot-a-2385 | Phosphoribosyl pyrophosphate synthase-associated protein 1 \|\| id:prot-a-2385 | Gherkins liking \|\| id:ebi-fl187-GCST90094770 | Weighted mode | 21 | 0.257831802962471 | 0.210986216642019 | 0.235903662647621 | -0.155701182 | 0.671364787580828 | 1.29412113297694 | 0.85581488535285 | 1.95690626031472 | 0.999714303421225 |
| ebi-fl187-GCST90094770 | prot-a-2393 | Trypsin-3 \|\| id:prot-a-2393 | Gherkins liking \|\| id:ebi-fl187-GCST90094770 | Weighted mode | 20 | -0.020008321 | 0.189802948924878 | 0.91715046209647 | -0.392022101 | 0.352005459240867 | 0.980190517448738 | 0.675689180696699 | 1.42191628628089 | 0.999714303421225 |
| ebi-fl187-GCST90094770 | prot-a-2405 | Pregnancy-specific beta-1-glycoprotein 6 \|\| id:prot-a-2405 | Gherkins liking \|\| id:ebi-fl187-GCST90094770 | Weighted mode | 21 | 0.186302436688709 | 0.187856566695046 | 0.333178329429087 | -0.181896434 | 0.554501307410998 | 1.20478657694644 | 0.833687677614885 | 1.74107250828388 | 0.999714303421225 |
| ebi-fl187-GCST90094770 | prot-a-244 | Beclin-1 \|\| id:prot-a-244 | Gherkins liking \|\| id:ebi-fl187-GCST90094770 | Weighted mode | 21 | 0.0264138681667714 | 0.156313502049904 | 0.867509653012902 | -0.279960596 | 0.332788332184583 | 1.02676580623176 | 0.755813523057611 | 1.39485202194032 | 0.999714303421225 |
| ebi-fl187-GCST90094770 | prot-a-2454 | tRNA pseudouridine synthase A, mitochondrial \|\| id:prot-a-2454 | Gherkins liking \|\| id:ebi-fl187-GCST90094770 | Weighted mode | 21 | -0.096432372 | 0.218707055134772 | 0.664000065088405 | -0.5250982 | 0.332233455800608 | 0.908071306330246 | 0.591497276288363 | 1.39407826618346 | 0.999714303421225 |
| ebi-fl187-GCST90094770 | prot-a-2466 | Glutaminyl-peptide cyclotransferase-like protein \|\| id:prot-a-2466 | Gherkins liking \|\| id:ebi-fl187-GCST90094770 | Weighted mode | 21 | -0.166100474 | 0.18416347481927 | 0.377834035333734 | -0.527060885 | 0.194859936270516 | 0.846961132023573 | 0.590337492155634 | 1.21514077742082 | 0.999714303421225 |
| ebi-fl187-GCST90094770 | prot-a-2474 | Ras-related protein Rab-27A \|\| id:prot-a-2474 | Gherkins liking \|\| id:ebi-fl187-GCST90094770 | Weighted mode | 21 | 0.0703790042260577 | 0.178347810788514 | 0.697302473742911 | -0.279182705 | 0.419940713371545 | 1.07291474342692 | 0.756401692279279 | 1.52187132632408 | 0.999714303421225 |
| ebi-fl187-GCST90094770 | prot-a-2490 | Rap1 GTPase-GDP dissociation stimulator 1 \|\| id:prot-a-2490 | Gherkins liking \|\| id:ebi-fl187-GCST90094770 | Weighted mode | 21 | -0.117146306 | 0.154291187351977 | 0.456556414604684 | -0.419557034 | 0.185264420809925 | 0.889455050676922 | 0.657337933945133 | 1.20353663818939 | 0.999714303421225 |
| ebi-fl187-GCST90094770 | prot-a-2545 | Receptor-interacting serine/threonine-protein kinase 2 \|\| id:prot-a-2545 | Gherkins liking \|\| id:ebi-fl187-GCST90094770 | Weighted mode | 21 | -0.091758173 | 0.226674018130062 | 0.689919655657914 | -0.536039248 | 0.352522902673646 | 0.912325747995892 | 0.585060950437095 | 1.42265223791544 | 0.999714303421225 |
| ebi-fl187-GCST90094770 | prot-a-2610 | RUN and FYVE domain-containing protein 1 \|\| id:prot-a-2610 | Gherkins liking \|\| id:ebi-fl187-GCST90094770 | Weighted mode | 21 | -0.209404481 | 0.191488574294314 | 0.287144365156494 | -0.584722087 | 0.16591312412213 | 0.81106710765101 | 0.557260710227575 | 1.18047054285369 | 0.999714303421225 |
| ebi-fl187-GCST90094770 | prot-a-2627 | Serine--tRNA ligase, mitochondrial \|\| id:prot-a-2627 | Gherkins liking \|\| id:ebi-fl187-GCST90094770 | Weighted mode | 21 | -0.049637635 | 0.192922929475393 | 0.799581507626209 | -0.427766576 | 0.328491307106768 | 0.951574179529646 | 0.651963580700589 | 1.38887116696686 | 0.999714303421225 |
| ebi-fl187-GCST90094770 | prot-a-264 | BolA-like protein 2 \|\| id:prot-a-264 | Gherkins liking \|\| id:ebi-fl187-GCST90094770 | Weighted mode | 21 | -0.031030283 | 0.164332449434447 | 0.852131940313541 | -0.353121884 | 0.291061317932324 | 0.969446214931337 | 0.70249156578191 | 1.33784661542323 | 0.999714303421225 |
| ebi-fl187-GCST90094770 | prot-a-2646 | Secretoglobin family 1C member 1 \|\| id:prot-a-2646 | Gherkins liking \|\| id:ebi-fl187-GCST90094770 | Weighted mode | 21 | -0.039091457 | 0.197801635418344 | 0.845329666950715 | -0.426782663 | 0.348599748147887 | 0.961662754072226 | 0.652605372310735 | 1.41708188716755 | 0.999714303421225 |
| ebi-fl187-GCST90094770 | prot-a-2672 | Semaphorin-3E \|\| id:prot-a-2672 | Gherkins liking \|\| id:ebi-fl187-GCST90094770 | Weighted mode | 21 | 0.0958194060681661 | 0.166756542392396 | 0.571964814976976 | -0.231023417 | 0.422662229157263 | 1.1005602915357 | 0.793720879239178 | 1.52601876426153 | 0.999714303421225 |
| ebi-fl187-GCST90094770 | prot-a-2701 | Plasma protease C1 inhibitor \|\| id:prot-a-2701 | Gherkins liking \|\| id:ebi-fl187-GCST90094770 | Weighted mode | 21 | 0.171434233098707 | 0.147650174245119 | 0.259278909933291 | -0.117960108 | 0.460828574619139 | 1.18700607445383 | 0.888731504809879 | 1.58538705240533 | 0.999714303421225 |
| ebi-fl187-GCST90094770 | prot-a-2738 | Signaling threshold-regulating transmembrane adapter 1 \|\| id:prot-a-2738 | Gherkins liking \|\| id:ebi-fl187-GCST90094770 | Weighted mode | 21 | -0.096356806 | 0.166072359846535 | 0.568253235366811 | -0.421858631 | 0.229145019403582 | 0.908139928573411 | 0.655826746283873 | 1.25752439122442 | 0.999714303421225 |
| ebi-fl187-GCST90094770 | prot-a-274 | Serine/threonine-protein kinase BRSK2 \|\| id:prot-a-274 | Gherkins liking \|\| id:ebi-fl187-GCST90094770 | Weighted mode | 21 | -0.159361496 | 0.183661827609939 | 0.395856251573023 | -0.519338678 | 0.200615685923832 | 0.852688059761725 | 0.594913847339792 | 1.22215499019127 | 0.999714303421225 |
| ebi-fl187-GCST90094770 | prot-a-2749 | Mitochondrial glutamate carrier 2 \|\| id:prot-a-2749 | Gherkins liking \|\| id:ebi-fl187-GCST90094770 | Weighted mode | 21 | 0.0135993213622361 | 0.183729327600113 | 0.941731184347366 | -0.346510161 | 0.373708803458457 | 1.01369221274193 | 0.707151634071127 | 1.4531139470863 | 0.999714303421225 |
| ebi-fl187-GCST90094770 | prot-a-2760 | Sodium-coupled monocarboxylate transporter 1 \|\| id:prot-a-2760 | Gherkins liking \|\| id:ebi-fl187-GCST90094770 | Weighted mode | 21 | -0.017326598 | 0.166693210594532 | 0.918249832060446 | -0.344045291 | 0.309392094417818 | 0.982822643960718 | 0.708896820596597 | 1.3625965322697 | 0.999714303421225 |
| ebi-fl187-GCST90094770 | prot-a-2777 | Small integral membrane protein 10 \|\| id:prot-a-2777 | Gherkins liking \|\| id:ebi-fl187-GCST90094770 | Weighted mode | 21 | 0.139536740664884 | 0.286014789339911 | 0.630950538350691 | -0.421052246 | 0.70012572777111 | 1.14974104719259 | 0.656355808258096 | 2.01400590802679 | 0.999714303421225 |
| ebi-fl187-GCST90094770 | prot-a-2799 | Superoxide dismutase [Mn], mitochondrial \|\| id:prot-a-2799 | Gherkins liking \|\| id:ebi-fl187-GCST90094770 | Weighted mode | 21 | 0.216644670893909 | 0.23786595671664 | 0.373255914947572 | -0.249572604 | 0.682861946058523 | 1.24190273953763 | 0.77913371034084 | 1.97953495529844 | 0.999714303421225 |
| ebi-fl187-GCST90094770 | prot-a-2805 | Protein SPACA7 \|\| id:prot-a-2805 | Gherkins liking \|\| id:ebi-fl187-GCST90094770 | Weighted mode | 21 | -0.188479807 | 0.179779504598847 | 0.306961812707729 | -0.540847636 | 0.163888022215959 | 0.828217227627743 | 0.582254503375406 | 1.17808238865115 | 0.999714303421225 |
| ebi-fl187-GCST90094770 | prot-a-2811 | SPARC-like protein 1 \|\| id:prot-a-2811 | Gherkins liking \|\| id:ebi-fl187-GCST90094770 | Weighted mode | 21 | -0.089733536 | 0.202802013563398 | 0.662898196575955 | -0.487225482 | 0.307758410951461 | 0.914174747815106 | 0.614328497174996 | 1.36037229818553 | 0.999714303421225 |
| ebi-fl187-GCST90094770 | prot-a-2831 | Sepiapterin reductase \|\| id:prot-a-2831 | Gherkins liking \|\| id:ebi-fl187-GCST90094770 | Weighted mode | 21 | -0.034866929 | 0.237049394640097 | 0.884535831696919 | -0.499483742 | 0.429749884838514 | 0.965733919217849 | 0.606843866767415 | 1.53687308021409 | 0.999714303421225 |
| ebi-fl187-GCST90094770 | prot-a-2834 | SPRY domain-containing SOCS box protein 1 \|\| id:prot-a-2834 | Gherkins liking \|\| id:ebi-fl187-GCST90094770 | Weighted mode | 21 | 0.278251053164129 | 0.258597659618157 | 0.294736525497525 | -0.22860036 | 0.785102466015716 | 1.32081775106417 | 0.795646442369164 | 2.19263159944701 | 0.999714303421225 |
| ebi-fl187-GCST90094770 | prot-a-2835 | Spectrin alpha chain, non-erythrocytic 1 \|\| id:prot-a-2835 | Gherkins liking \|\| id:ebi-fl187-GCST90094770 | Weighted mode | 21 | -0.024042875 | 0.174801233121206 | 0.891976056766172 | -0.366653292 | 0.318567541888445 | 0.976243852 | 0.69304988937266 | 1.37515649871408 | 0.999714303421225 |
| ebi-fl187-GCST90094770 | prot-a-2859 | Sia-alpha-2,3-Gal-beta-1,4-GlcNAc-R:alpha 2,8-sialyltransferase \|\| id:prot-a-2859 | Gherkins liking \|\| id:ebi-fl187-GCST90094770 | Weighted mode | 21 | -0.045248487 | 0.170299243182009 | 0.79318997472457 | -0.379035004 | 0.288538029668979 | 0.955759958429613 | 0.68452165152176 | 1.33447509820416 | 0.999714303421225 |
| ebi-fl187-GCST90094770 | prot-a-2861 | Alpha-2,8-sialyltransferase 8F \|\| id:prot-a-2861 | Gherkins liking \|\| id:ebi-fl187-GCST90094770 | Weighted mode | 21 | -0.261580452 | 0.179010491974972 | 0.159478855015767 | -0.612441016 | 0.0892801122707407 | 0.769833938252733 | 0.542026158240278 | 1.09338688451821 | 0.999714303421225 |
| ebi-fl187-GCST90094770 | prot-a-289 | Uncharacterized protein C17orf89 \|\| id:prot-a-289 | Gherkins liking \|\| id:ebi-fl187-GCST90094770 | Weighted mode | 21 | -0.167920434 | 0.202961298613504 | 0.417800390852011 | -0.56572458 | 0.229883710817805 | 0.845421098390966 | 0.567948473669127 | 1.25845365687342 | 0.999714303421225 |
| ebi-fl187-GCST90094770 | prot-a-2921 | Tachykinin-3 \|\| id:prot-a-2921 | Gherkins liking \|\| id:ebi-fl187-GCST90094770 | Weighted mode | 21 | -0.14587308 | 0.15442483470937 | 0.35612488660671 | -0.448545757 | 0.156799595533461 | 0.86426738861139 | 0.638556092761936 | 1.16976116504714 | 0.999714303421225 |
| ebi-fl187-GCST90094770 | prot-a-2928 | Tax1-binding protein 1 \|\| id:prot-a-2928 | Gherkins liking \|\| id:ebi-fl187-GCST90094770 | Weighted mode | 21 | -0.387398006 | 0.202071972384384 | 0.0696189130082772 | -0.783459072 | 0.00866305974335968 | 0.678820866287899 | 0.45682309120095 | 1.00870069263893 | 0.999714303421225 |
| ebi-fl187-GCST90094770 | prot-a-296 | Uncharacterized protein C1orf185 \|\| id:prot-a-296 | Gherkins liking \|\| id:ebi-fl187-GCST90094770 | Weighted mode | 21 | -0.01265892 | 0.188172905100298 | 0.947032510874105 | -0.381477814 | 0.356159973775797 | 0.987420866882076 | 0.682851535485778 | 1.42783594630206 | 0.999714303421225 |
| ebi-fl187-GCST90094770 | prot-a-2964 | Transforming growth factor beta-2 \|\| id:prot-a-2964 | Gherkins liking \|\| id:ebi-fl187-GCST90094770 | Weighted mode | 21 | -0.058272014 | 0.17603248574659 | 0.744065084451983 | -0.403295686 | 0.28675165845861 | 0.94339329672084 | 0.668114518226412 | 1.33209335827696 | 0.999714303421225 |
| ebi-fl187-GCST90094770 | prot-a-2977 | Thymocyte nuclear protein 1 \|\| id:prot-a-2977 | Gherkins liking \|\| id:ebi-fl187-GCST90094770 | Weighted mode | 21 | -0.0515786 | 0.210082683686185 | 0.808558940240253 | -0.46334066 | 0.360183459935252 | 0.949728998243997 | 0.629178260057992 | 1.43359239720458 | 0.999714303421225 |
| ebi-fl187-GCST90094770 | prot-a-3015 | Transmembrane protein 70, mitochondrial \|\| id:prot-a-3015 | Gherkins liking \|\| id:ebi-fl187-GCST90094770 | Weighted mode | 21 | -0.167448199 | 0.166852260425245 | 0.327573440830987 | -0.49447863 | 0.159582231328098 | 0.845820430409036 | 0.609888802424706 | 1.17302071730633 | 0.999714303421225 |
| ebi-fl187-GCST90094770 | prot-a-3030 | Tumor necrosis factor alpha-induced protein 3 \|\| id:prot-a-3030 | Gherkins liking \|\| id:ebi-fl187-GCST90094770 | Weighted mode | 21 | -0.068353247 | 0.185626815246019 | 0.716570857251451 | -0.432181805 | 0.295475311152891 | 0.93393050740377 | 0.649091358155117 | 1.34376491336836 | 0.999714303421225 |
| ebi-fl187-GCST90094770 | prot-a-3032 | Tumor necrosis factor receptor superfamily member 10A \|\| id:prot-a-3032 | Gherkins liking \|\| id:ebi-fl187-GCST90094770 | Weighted mode | 21 | -0.168575348 | 0.18293044584567 | 0.367758672320867 | -0.527119021 | 0.18996832625473 | 0.844867602272109 | 0.590303173033477 | 1.2092112968001 | 0.999714303421225 |
| ebi-fl187-GCST90094770 | prot-a-3081 | Tumor protein p53-inducible protein 11 \|\| id:prot-a-3081 | Gherkins liking \|\| id:ebi-fl187-GCST90094770 | Weighted mode | 21 | 0.124486900272189 | 0.185156231665994 | 0.509064445794143 | -0.238419314 | 0.487393114337537 | 1.13256718404123 | 0.787872256116403 | 1.62806649987885 | 0.999714303421225 |
| ebi-fl187-GCST90094770 | prot-a-315 | Complement C4 \|\| id:prot-a-315 | Gherkins liking \|\| id:ebi-fl187-GCST90094770 | Weighted mode | 20 | -0.086032852 | 0.216874321211048 | 0.696013102456272 | -0.511106522 | 0.339040817212375 | 0.917564086578123 | 0.599831484767552 | 1.40360063510872 | 0.999714303421225 |
| ebi-fl187-GCST90094770 | prot-a-3157 | UDP-glucuronosyltransferase 1-8 \|\| id:prot-a-3157 | Gherkins liking \|\| id:ebi-fl187-GCST90094770 | Weighted mode | 21 | 0.143809151034151 | 0.168663190398613 | 0.403953986011268 | -0.186770702 | 0.474389004215432 | 1.15466372111815 | 0.829633947859941 | 1.60703200767707 | 0.999714303421225 |
| ebi-fl187-GCST90094770 | prot-a-317 | Glutaredoxin-like protein C5orf63 \|\| id:prot-a-317 | Gherkins liking \|\| id:ebi-fl187-GCST90094770 | Weighted mode | 21 | -0.108710865 | 0.187888783358192 | 0.569323869154751 | -0.47697288 | 0.259551150540051 | 0.896989731275736 | 0.620659361208008 | 1.29634809092079 | 0.999714303421225 |
| ebi-fl187-GCST90094770 | prot-a-3192 | Guanine nucleotide exchange factor VAV3 \|\| id:prot-a-3192 | Gherkins liking \|\| id:ebi-fl187-GCST90094770 | Weighted mode | 21 | -0.019423121 | 0.193559882134263 | 0.921068083016297 | -0.39880049 | 0.359954248435797 | 0.98076429291237 | 0.671124584378382 | 1.43326383899771 | 0.999714303421225 |
| ebi-fl187-GCST90094770 | prot-a-3233 | Serine/threonine-protein kinase WNK3 \|\| id:prot-a-3233 | Gherkins liking \|\| id:ebi-fl187-GCST90094770 | Weighted mode | 21 | 0.141181910886808 | 0.160833106139579 | 0.390467053262541 | -0.174050977 | 0.456414798920383 | 1.15163412371593 | 0.84025406281196 | 1.57840492965729 | 0.999714303421225 |
| ebi-fl187-GCST90094770 | prot-a-3250 | 14-3-3 protein theta \|\| id:prot-a-3250 | Gherkins liking \|\| id:ebi-fl187-GCST90094770 | Weighted mode | 21 | 0.105162302845459 | 0.180018950821749 | 0.565635143787469 | -0.247674841 | 0.457999446456087 | 1.11089089647833 | 0.78061372577771 | 1.58090812795911 | 0.999714303421225 |
| ebi-fl187-GCST90094770 | prot-a-3278 | E3 ubiquitin-protein ligase ZNRF3 \|\| id:prot-a-3278 | Gherkins liking \|\| id:ebi-fl187-GCST90094770 | Weighted mode | 21 | 0.132768215158009 | 0.159301163391952 | 0.4144389201065 | -0.179462065 | 0.444998495406234 | 1.14198527277639 | 0.835719653291493 | 1.56048784793061 | 0.999714303421225 |
| ebi-fl187-GCST90094770 | prot-a-355 | Calpain-3 \|\| id:prot-a-355 | Gherkins liking \|\| id:ebi-fl187-GCST90094770 | Weighted mode | 21 | -0.018546325 | 0.187955005836984 | 0.922379180754894 | -0.386938137 | 0.34984548631519 | 0.981624599655908 | 0.679133107133408 | 1.41884830017621 | 0.999714303421225 |
| ebi-fl187-GCST90094770 | prot-a-385 | Coiled-coil domain-containing protein 90B, mitochondrial \|\| id:prot-a-385 | Gherkins liking \|\| id:ebi-fl187-GCST90094770 | Weighted mode | 21 | 0.0322301863148382 | 0.22875952888413 | 0.889365355021942 | -0.41613849 | 0.480598862927734 | 1.03275520406112 | 0.659588917493005 | 1.61704249909056 | 0.999714303421225 |
| ebi-fl187-GCST90094770 | prot-a-390 | C-C motif chemokine 14 \|\| id:prot-a-390 | Gherkins liking \|\| id:ebi-fl187-GCST90094770 | Weighted mode | 21 | -0.232835054 | 0.168580526494989 | 0.18247096578669 | -0.563252885 | 0.0975827784130323 | 0.792284247246936 | 0.569354004980378 | 1.10250270121008 | 0.999714303421225 |
| ebi-fl187-GCST90094770 | prot-a-40 | Pituitary adenylate cyclase-activating polypeptide 27 \|\| id:prot-a-40 | Gherkins liking \|\| id:ebi-fl187-GCST90094770 | Weighted mode | 21 | -0.041403322 | 0.194250845182538 | 0.833372591348354 | -0.422134979 | 0.339328334071576 | 0.959442087333523 | 0.655645535012955 | 1.40400425197545 | 0.999714303421225 |
| ebi-fl187-GCST90094770 | prot-a-401 | C-C motif chemokine 24 \|\| id:prot-a-401 | Gherkins liking \|\| id:ebi-fl187-GCST90094770 | Weighted mode | 21 | -0.115343273 | 0.206048305913306 | 0.581840404105861 | -0.519197952 | 0.288511406869922 | 0.891060214737529 | 0.59499757307508 | 1.33443957121469 | 0.999714303421225 |
| ebi-fl187-GCST90094770 | prot-a-412 | Cerebral cavernous malformations 2 protein \|\| id:prot-a-412 | Gherkins liking \|\| id:ebi-fl187-GCST90094770 | Weighted mode | 21 | -0.00284788 | 0.200059388346817 | 0.988783407461275 | -0.394964281 | 0.389268521423794 | 0.997156171626683 | 0.673704102926198 | 1.47590080911546 | 0.999714303421225 |
| ebi-fl187-GCST90094770 | prot-a-44 | Adhesion G protein-coupled receptor F5 \|\| id:prot-a-44 | Gherkins liking \|\| id:ebi-fl187-GCST90094770 | Weighted mode | 21 | -0.081874906 | 0.197403265112773 | 0.682733003480848 | -0.468785305 | 0.305035493833205 | 0.921387211694807 | 0.625761916434277 | 1.35667315568236 | 0.999714303421225 |
| ebi-fl187-GCST90094770 | prot-a-48 | Adrenomedullin \|\| id:prot-a-48 | Gherkins liking \|\| id:ebi-fl187-GCST90094770 | Weighted mode | 21 | 0.0507466392575617 | 0.238966699569736 | 0.833976618514035 | -0.417628092 | 0.519121370414243 | 1.05205631 | 0.658607124207432 | 1.68055041950591 | 0.999714303421225 |
| ebi-fl187-GCST90094770 | prot-a-502 | Carcinoembryonic antigen-related cell adhesion molecule 1 \|\| id:prot-a-502 | Gherkins liking \|\| id:ebi-fl187-GCST90094770 | Weighted mode | 21 | 0.14127965032322 | 0.178107510312683 | 0.436955603304011 | -0.20781107 | 0.490370370536079 | 1.1517466892871 | 0.812360501573631 | 1.63292089375858 | 0.999714303421225 |
| ebi-fl187-GCST90094770 | prot-a-518 | Complement factor B \|\| id:prot-a-518 | Gherkins liking \|\| id:ebi-fl187-GCST90094770 | Weighted mode | 21 | -0.195705057 | 0.206604220370949 | 0.354818900109717 | -0.600649329 | 0.209239214646004 | 0.822254716959121 | 0.54845539234121 | 1.23273985268961 | 0.999714303421225 |
| ebi-fl187-GCST90094770 | prot-a-525 | Properdin \|\| id:prot-a-525 | Gherkins liking \|\| id:ebi-fl187-GCST90094770 | Weighted mode | 21 | -0.082651158 | 0.240237841508299 | 0.734407870505974 | -0.553517327 | 0.388215011411999 | 0.920672260411953 | 0.574924053774391 | 1.47434675158796 | 0.999714303421225 |
| ebi-fl187-GCST90094770 | prot-a-533 | Chondroadherin \|\| id:prot-a-533 | Gherkins liking \|\| id:ebi-fl187-GCST90094770 | Weighted mode | 21 | 0.0309784502254522 | 0.174364301748754 | 0.860773087874726 | -0.310775581 | 0.372732481653011 | 1.03146327584597 | 0.732878329089062 | 1.45169593258584 | 0.999714303421225 |
| ebi-fl187-GCST90094770 | prot-a-535 | Coiled-coil-helix-coiled-coil-helix domain-containing protein 2 \|\| id:prot-a-535 | Gherkins liking \|\| id:ebi-fl187-GCST90094770 | Weighted mode | 21 | 0.129263406255491 | 0.260784282757221 | 0.625528674866999 | -0.381873788 | 0.640400600459645 | 1.13798983833281 | 0.682581197742208 | 1.89724076261155 | 0.999714303421225 |
| ebi-fl187-GCST90094770 | prot-a-591 | Caseinolytic peptidase B protein homolog \|\| id:prot-a-591 | Gherkins liking \|\| id:ebi-fl187-GCST90094770 | Weighted mode | 21 | -0.149489042 | 0.211074172052244 | 0.486975496193387 | -0.563194419 | 0.264216335524198 | 0.8611478746863 | 0.569387294104666 | 1.3024099233595 | 0.999714303421225 |
| ebi-fl187-GCST90094770 | prot-a-605 | Ciliary neurotrophic factor receptor subunit alpha \|\| id:prot-a-605 | Gherkins liking \|\| id:ebi-fl187-GCST90094770 | Weighted mode | 21 | -0.30649588 | 0.206657105455541 | 0.153632169114897 | -0.711543806 | 0.0985520469469343 | 0.736021550768906 | 0.490885779559184 | 1.10357184044471 | 0.999714303421225 |
| ebi-fl187-GCST90094770 | prot-a-606 | Ciliary neurotrophic factor receptor subunit alpha \|\| id:prot-a-606 | Gherkins liking \|\| id:ebi-fl187-GCST90094770 | Weighted mode | 21 | -0.256052477 | 0.245996564750003 | 0.310352124550446 | -0.738205744 | 0.226100789419378 | 0.774101344830268 | 0.477970748294124 | 1.25370201880908 | 0.999714303421225 |
| ebi-fl187-GCST90094770 | prot-a-707 | Cancer/testis antigen 55 \|\| id:prot-a-707 | Gherkins liking \|\| id:ebi-fl187-GCST90094770 | Weighted mode | 21 | 0.0385932401718152 | 0.404895375840439 | 0.925011942976612 | -0.755001696 | 0.832188176819076 | 1.03934763279208 | 0.470009817369747 | 2.29834242151732 | 0.999714303421225 |
| ebi-fl187-GCST90094770 | prot-a-719 | Dipeptidyl peptidase 1 \|\| id:prot-a-719 | Gherkins liking \|\| id:ebi-fl187-GCST90094770 | Weighted mode | 21 | -0.174042722 | 0.172660134990669 | 0.325491408267785 | -0.512456586 | 0.164371142836486 | 0.840260999475277 | 0.599022220041428 | 1.1786516820534 | 0.999714303421225 |
| ebi-fl187-GCST90094770 | prot-a-752 | Cytochrome b5 \|\| id:prot-a-752 | Gherkins liking \|\| id:ebi-fl187-GCST90094770 | Weighted mode | 21 | 0.0710345101494903 | 0.178039784141007 | 0.694135158348096 | -0.277923467 | 0.419992487065863 | 1.07361827595625 | 0.757354782106443 | 1.52195012126466 | 0.999714303421225 |
| ebi-fl187-GCST90094770 | prot-a-755 | Cytoglobin \|\| id:prot-a-755 | Gherkins liking \|\| id:ebi-fl187-GCST90094770 | Weighted mode | 21 | -0.035251207 | 0.164602141712553 | 0.83259056989433 | -0.357871405 | 0.287368990768848 | 0.965362879894277 | 0.699162978267759 | 1.33291595642936 | 0.999714303421225 |
| ebi-fl187-GCST90094770 | prot-a-807 | Desmin \|\| id:prot-a-807 | Gherkins liking \|\| id:ebi-fl187-GCST90094770 | Weighted mode | 21 | -0.011112394 | 0.288728965222343 | 0.9696807580649 | -0.577021166 | 0.554796378075398 | 0.988949120818227 | 0.561568697667107 | 1.74158632350784 | 0.999714303421225 |
| ebi-fl187-GCST90094770 | prot-a-82 | Ameloblastin \|\| id:prot-a-82 | Gherkins liking \|\| id:ebi-fl187-GCST90094770 | Weighted mode | 21 | -0.209827676 | 0.229544546383491 | 0.371551284684817 | -0.659734987 | 0.24007963509564 | 0.81072394127503 | 0.516988325106539 | 1.27135039040015 | 0.999714303421225 |
| ebi-fl187-GCST90094770 | prot-a-84 | Muellerian-inhibiting factor \|\| id:prot-a-84 | Gherkins liking \|\| id:ebi-fl187-GCST90094770 | Weighted mode | 21 | 0.114924780532043 | 0.163214289318575 | 0.489467343123258 | -0.204975227 | 0.434824787596451 | 1.12178905402253 | 0.814667498300328 | 1.54469238597371 | 0.999714303421225 |
| ebi-fl187-GCST90094770 | prot-a-858 | Inactive dipeptidyl peptidase 10 \|\| id:prot-a-858 | Gherkins liking \|\| id:ebi-fl187-GCST90094770 | Weighted mode | 21 | -0.128966362 | 0.166683884133828 | 0.448147028873008 | -0.455666775 | 0.197734050706632 | 0.879003532797132 | 0.634025074845203 | 1.21863825473855 | 0.999714303421225 |
| ebi-fl187-GCST90094770 | prot-a-863 | Dihydropyrimidinase-related protein 3 \|\| id:prot-a-863 | Gherkins liking \|\| id:ebi-fl187-GCST90094770 | Weighted mode | 21 | -0.108788538 | 0.173396448293956 | 0.537496285384891 | -0.448645577 | 0.231068500610382 | 0.896920061915319 | 0.638492355162431 | 1.25994554353204 | 0.999714303421225 |
| ebi-fl187-GCST90094770 | prot-a-864 | Dihydropyrimidinase-related protein 5 \|\| id:prot-a-864 | Gherkins liking \|\| id:ebi-fl187-GCST90094770 | Weighted mode | 21 | 0.225028469996721 | 0.177293374432217 | 0.218926329956309 | -0.122466544 | 0.572523483883866 | 1.25235837032302 | 0.884735504256319 | 1.77273487971582 | 0.999714303421225 |
| ebi-fl187-GCST90094770 | prot-a-884 | Endothelin-converting enzyme 1 \|\| id:prot-a-884 | Gherkins liking \|\| id:ebi-fl187-GCST90094770 | Weighted mode | 21 | 0.0727371367100065 | 0.174455607904766 | 0.681165008691565 | -0.269195855 | 0.414670128203348 | 1.0754478040083 | 0.763993609191392 | 1.51387127487939 | 0.999714303421225 |
| ebi-fl187-GCST90094770 | prot-a-893 | Early endosome antigen 1 \|\| id:prot-a-893 | Gherkins liking \|\| id:ebi-fl187-GCST90094770 | Weighted mode | 21 | -0.146376307 | 0.180730939985755 | 0.427522101211804 | -0.500608949 | 0.207856335404605 | 0.863832575797624 | 0.606161425701794 | 1.23103630051223 | 0.999714303421225 |
| ebi-fl187-GCST90094770 | prot-a-897 | Ephrin-A2 \|\| id:prot-a-897 | Gherkins liking \|\| id:ebi-fl187-GCST90094770 | Weighted mode | 21 | 0.23618401670064 | 0.164672283242527 | 0.16694782378339 | -0.086573658 | 0.558941691855993 | 1.26640732882488 | 0.91706799648535 | 1.74882072937651 | 0.999714303421225 |
| ebi-fl187-GCST90094770 | prot-a-90 | Anaphase-promoting complex subunit 10 \|\| id:prot-a-90 | Gherkins liking \|\| id:ebi-fl187-GCST90094770 | Weighted mode | 21 | -0.024006624 | 0.162658795335272 | 0.884145014269525 | -0.342817863 | 0.294804615205862 | 0.976279243203244 | 0.709767475055511 | 1.34286395785458 | 0.999714303421225 |
| ebi-fl187-GCST90094770 | prot-a-914 | Histone-lysine N-methyltransferase EHMT2 \|\| id:prot-a-914 | Gherkins liking \|\| id:ebi-fl187-GCST90094770 | Weighted mode | 21 | -0.120150943 | 0.181096825226597 | 0.514609920781109 | -0.47510072 | 0.234798834844358 | 0.88678657274379 | 0.621822423329089 | 1.26465433875564 | 0.999714303421225 |
| ebi-fl187-GCST90094770 | prot-a-932 | RNA polymerase II elongation factor ELL \|\| id:prot-a-932 | Gherkins liking \|\| id:ebi-fl187-GCST90094770 | Weighted mode | 21 | 0.14299497723472 | 0.213751206264557 | 0.51115804996961 | -0.275957387 | 0.561947341513251 | 1.15372400676657 | 0.758845266712376 | 1.75408497908446 | 0.999714303421225 |
| ebi-fl187-GCST90094770 | prot-a-991 | Estrogen receptor \|\| id:prot-a-991 | Gherkins liking \|\| id:ebi-fl187-GCST90094770 | Weighted mode | 21 | 0.00095096721764909 | 0.155141300062083 | 0.995169980328337 | -0.303125981 | 0.305027915339333 | 1.00095141953034 | 0.738506052849579 | 1.35666287418212 | 0.999714303421225 |
| ebi-fl187-GCST90094770 | prot-a-992 | Persulfide dioxygenase ETHE1, mitochondrial \|\| id:prot-a-992 | Gherkins liking \|\| id:ebi-fl187-GCST90094770 | Weighted mode | 21 | -0.006816765 | 0.168395851798745 | 0.968111357159797 | -0.336872634 | 0.323239104650858 | 0.993206416562952 | 0.713999773121061 | 1.38159565736243 | 0.999714303421225 |
| ebi-fl187-GCST90094770 | prot-a-993 | Ethanolamine kinase 1 \|\| id:prot-a-993 | Gherkins liking \|\| id:ebi-fl187-GCST90094770 | Weighted mode | 21 | -0.110352344 | 0.168482588715453 | 0.519944830193134 | -0.440578218 | 0.219873529978162 | 0.895518549200533 | 0.643664135410845 | 1.24591914920092 | 0.999714303421225 |
| ebi-fl187-GCST90094770 | prot-b-10 | coagulation factor III, tissue factor \|\| id:prot-b-10 | Gherkins liking \|\| id:ebi-fl187-GCST90094770 | Weighted mode | 15 | 0.345008881361691 | 0.250446571119599 | 0.189958923973047 | -0.145866398 | 0.835884160756106 | 1.41200246011653 | 0.864273164066554 | 2.30685277556714 | 0.999714303421225 |
| ebi-fl187-GCST90094770 | prot-b-28 | prolactin \|\| id:prot-b-28 | Gherkins liking \|\| id:ebi-fl187-GCST90094770 | Weighted mode | 15 | 0.00381175668265432 | 0.203516341055167 | 0.985321217414191 | -0.395080272 | 0.402703785150783 | 1.00381903066644 | 0.67362596391967 | 1.49586372898221 | 0.999714303421225 |
| ebi-fl187-GCST90094770 | prot-b-54 | matrix metallopeptidase 7 \|\| id:prot-b-54 | Gherkins liking \|\| id:ebi-fl187-GCST90094770 | Weighted mode | 15 | 0.237041109999948 | 0.231097323053849 | 0.32242094188748 | -0.215909643 | 0.689991863185491 | 1.26749322335059 | 0.805808108837864 | 1.9936993108156 | 0.999714303421225 |
| ebi-fl187-GCST90094770 | prot-c-2421_7_3 | BDNF \|\| id:prot-c-2421_7_3 | Gherkins liking \|\| id:ebi-fl187-GCST90094770 | Weighted mode | 3 | 0.851576407167133 | 0.59667904829724 | 0.28967049098848 | -0.317914527 | 2.02106734182972 | 2.34333799660099 | 0.727664981105078 | 7.54637519861768 | 0.999714303421225 |
| ebi-fl187-GCST90094770 | prot-c-2443_10_4 | FGF-8B \|\| id:prot-c-2443_10_4 | Gherkins liking \|\| id:ebi-fl187-GCST90094770 | Weighted mode | 3 | 0.715404213094388 | 0.594826104527244 | 0.352154590203966 | -0.450454952 | 1.88126337796779 | 2.04501313630342 | 0.637338127538335 | 6.56178964815188 | 0.999714303421225 |
| ebi-fl187-GCST90094770 | prot-c-2605_49_2 | CD30 \|\| id:prot-c-2605_49_2 | Gherkins liking \|\| id:ebi-fl187-GCST90094770 | Weighted mode | 3 | 0.830555535613503 | 0.641842280832449 | 0.324940208331512 | -0.427455335 | 2.0885664060451 | 2.29459311444486 | 0.652166530482335 | 8.07333298285594 | 0.999714303421225 |
| ebi-fl187-GCST90094770 | prot-c-2624_31_2 | HPV E7 Type18 \|\| id:prot-c-2624_31_2 | Gherkins liking \|\| id:ebi-fl187-GCST90094770 | Weighted mode | 3 | 1.0019940179314 | 0.751745223280413 | 0.314126324319761 | -0.47142662 | 2.47541465556101 | 2.72370753885173 | 0.624111263448984 | 11.8866349506351 | 0.999714303421225 |
| ebi-fl187-GCST90094770 | prot-c-2774_10_3 | IL-16 \|\| id:prot-c-2774_10_3 | Gherkins liking \|\| id:ebi-fl187-GCST90094770 | Weighted mode | 3 | -0.536734621 | 0.60290321963245 | 0.467264172509259 | -1.718424932 | 0.644955688986299 | 0.584654256210492 | 0.179348411509634 | 1.9059025749257 | 0.999714303421225 |
| ebi-fl187-GCST90094770 | prot-c-2851_63_3 | C5a \|\| id:prot-c-2851_63_3 | Gherkins liking \|\| id:ebi-fl187-GCST90094770 | Weighted mode | 3 | 0.100368777478371 | 0.63529087430704 | 0.888975775817448 | -1.144801336 | 1.34553889112017 | 1.10557855537909 | 0.318287143654955 | 3.84025546265602 | 0.999714303421225 |
| ebi-fl187-GCST90094770 | prot-c-2891_1_3 | CD22 \|\| id:prot-c-2891_1_3 | Gherkins liking \|\| id:ebi-fl187-GCST90094770 | Weighted mode | 3 | 0.940820111716114 | 0.622639855087475 | 0.269892640346951 | -0.279554004 | 2.16119422768756 | 2.56208175021915 | 0.756120892966783 | 8.68149915690059 | 0.999714303421225 |
| ebi-fl187-GCST90094770 | prot-c-2961_1_2 | Protein C \|\| id:prot-c-2961_1_2 | Gherkins liking \|\| id:ebi-fl187-GCST90094770 | Weighted mode | 3 | 0.384776411732332 | 0.598222715907212 | 0.585997066466099 | -0.787740111 | 1.55729293491047 | 1.4692857696524 | 0.454871593728628 | 4.74595622735452 | 0.999714303421225 |
| ebi-fl187-GCST90094770 | prot-c-2982_82_2 | Galectin-4 \|\| id:prot-c-2982_82_2 | Gherkins liking \|\| id:ebi-fl187-GCST90094770 | Weighted mode | 3 | 0.317317360263874 | 0.612080923113645 | 0.655816224757056 | -0.882361249 | 1.51699596956662 | 1.37343837753402 | 0.413804661330903 | 4.5585107011998 | 0.999714303421225 |
| ebi-fl187-GCST90094770 | prot-c-2994_71_2 | IL-1Rrp2 \|\| id:prot-c-2994_71_2 | Gherkins liking \|\| id:ebi-fl187-GCST90094770 | Weighted mode | 3 | -0.899778136 | 0.586686930098238 | 0.264844893942314 | -2.049684519 | 0.250128247452581 | 0.406659873105823 | 0.128775523471178 | 1.28419010023638 | 0.999714303421225 |
| ebi-fl187-GCST90094770 | prot-c-3336_50_1 | TFPI \|\| id:prot-c-3336_50_1 | Gherkins liking \|\| id:ebi-fl187-GCST90094770 | Weighted mode | 3 | -0.159094434 | 0.552962184208647 | 0.800640021200186 | -1.242900315 | 0.92471144754453 | 0.852915811336955 | 0.288546128629047 | 2.5211406740577 | 0.999714303421225 |
| ebi-fl187-GCST90094770 | prot-c-3472_40_2 | Survivin \|\| id:prot-c-3472_40_2 | Gherkins liking \|\| id:ebi-fl187-GCST90094770 | Weighted mode | 3 | 0.140119582812581 | 0.706448089350594 | 0.861109118319749 | -1.244518672 | 1.52475783793975 | 1.15041136005815 | 0.288079535421518 | 4.59403093459717 | 0.999714303421225 |
| ebi-fl187-GCST90094770 | prot-c-3473_78_2 | Thrombopoietin Receptor \|\| id:prot-c-3473_78_2 | Gherkins liking \|\| id:ebi-fl187-GCST90094770 | Weighted mode | 3 | 0.445582764804127 | 0.640621016866006 | 0.558663337661318 | -0.810034428 | 1.7011999578615 | 1.56139985963026 | 0.444842750800422 | 5.48051984047547 | 0.999714303421225 |
| ebi-fl187-GCST90094770 | prot-c-3497_13_2 | IFN-aA \|\| id:prot-c-3497_13_2 | Gherkins liking \|\| id:ebi-fl187-GCST90094770 | Weighted mode | 3 | 0.841010979174142 | 0.60953971688737 | 0.301669125685503 | -0.353686866 | 2.03570882427339 | 2.3187099597082 | 0.702094782738038 | 7.65767815035314 | 0.999714303421225 |
| ebi-fl187-GCST90094770 | prot-c-3665_64_3 | COLEC12 \|\| id:prot-c-3665_64_3 | Gherkins liking \|\| id:ebi-fl187-GCST90094770 | Weighted mode | 3 | -1.062521223 | 0.870204740380192 | 0.346491983847721 | -2.768122514 | 0.643080067886472 | 0.345583418092858 | 0.0627797622629142 | 1.90233117418625 | 0.999714303421225 |
| ebi-fl187-GCST90094770 | prot-c-3741_4_3 | IL-5 \|\| id:prot-c-3741_4_3 | Gherkins liking \|\| id:ebi-fl187-GCST90094770 | Weighted mode | 3 | 0.25517954997032 | 0.616383247538517 | 0.719052001703443 | -0.952931615 | 1.46329071514581 | 1.29069334402094 | 0.385608907864135 | 4.32015255437746 | 0.999714303421225 |
| ebi-fl187-GCST90094770 | prot-c-3848_14_2 | GAPDH, liver \|\| id:prot-c-3848_14_2 | Gherkins liking \|\| id:ebi-fl187-GCST90094770 | Weighted mode | 3 | -0.278358842 | 0.600875504117249 | 0.688704871057326 | -1.45607483 | 0.899357146117124 | 0.757025120395937 | 0.233149632953483 | 2.45802245386688 | 0.999714303421225 |
| ebi-fl187-GCST90094770 | prot-c-3859_50_2 | PSA1 \|\| id:prot-c-3859_50_2 | Gherkins liking \|\| id:ebi-fl187-GCST90094770 | Weighted mode | 3 | 0.40876899980122 | 0.625340543106458 | 0.580433763408505 | -0.816898465 | 1.63443646428988 | 1.50496403333835 | 0.441799789397826 | 5.12656817860668 | 0.999714303421225 |
| ebi-fl187-GCST90094770 | prot-c-3868_8_1 | SGTA \|\| id:prot-c-3868_8_1 | Gherkins liking \|\| id:ebi-fl187-GCST90094770 | Weighted mode | 3 | -0.300006618 | 0.850155165794115 | 0.757896281937519 | -1.966310743 | 1.36629750648522 | 0.740813317613854 | 0.139972298553287 | 3.92080702557812 | 0.999714303421225 |
| ebi-fl187-GCST90094770 | prot-c-3882_10_1 | EF-1-beta \|\| id:prot-c-3882_10_1 | Gherkins liking \|\| id:ebi-fl187-GCST90094770 | Weighted mode | 3 | 0.126348684386549 | 0.534902682498166 | 0.835257333353139 | -0.922060573 | 1.17475794208295 | 1.13467774372653 | 0.397698708852595 | 3.23735922055892 | 0.999714303421225 |
| ebi-fl187-GCST90094770 | prot-c-4134_4_2 | HB-EGF \|\| id:prot-c-4134_4_2 | Gherkins liking \|\| id:ebi-fl187-GCST90094770 | Weighted mode | 3 | 1.01230102599527 | 0.685437101592657 | 0.277738345037243 | -0.331155693 | 2.35575774511688 | 2.75192598849856 | 0.718093358136769 | 10.5461171035248 | 0.999714303421225 |
| ebi-fl187-GCST90094770 | prot-c-4220_39_2 | FER \|\| id:prot-c-4220_39_2 | Gherkins liking \|\| id:ebi-fl187-GCST90094770 | Weighted mode | 3 | 0.566665688450954 | 0.76223413283826 | 0.534692578668515 | -0.927313212 | 2.06064458881394 | 1.76238091673911 | 0.395615217965098 | 7.85102886502258 | 0.999714303421225 |
| ebi-fl187-GCST90094770 | prot-c-4228_37_2 | HSP70 protein 8 \|\| id:prot-c-4228_37_2 | Gherkins liking \|\| id:ebi-fl187-GCST90094770 | Weighted mode | 3 | 0.50958652088674 | 0.596417064581727 | 0.482887751002287 | -0.659390926 | 1.67856396746692 | 1.6646027741529 | 0.517166231247874 | 5.35785638793855 | 0.999714303421225 |
| ebi-fl187-GCST90094770 | prot-c-4246_40_2 | NCAM-L1 \|\| id:prot-c-4246_40_2 | Gherkins liking \|\| id:ebi-fl187-GCST90094770 | Weighted mode | 3 | -0.139104572 | 0.553717428029891 | 0.825099161003132 | -1.224390731 | 0.946181587089088 | 0.870137031861755 | 0.293936732379925 | 2.57585517838088 | 0.999714303421225 |
| ebi-fl187-GCST90094770 | prot-c-4267_81_3 | PESC \|\| id:prot-c-4267_81_3 | Gherkins liking \|\| id:ebi-fl187-GCST90094770 | Weighted mode | 3 | 0.924047760748114 | 0.830976932699855 | 0.381891893441437 | -0.704667027 | 2.55276254883983 | 2.51946798141384 | 0.494273126294806 | 12.8425329472261 | 0.999714303421225 |
| ebi-fl187-GCST90094770 | prot-c-4280_47_2 | PSA2 \|\| id:prot-c-4280_47_2 | Gherkins liking \|\| id:ebi-fl187-GCST90094770 | Weighted mode | 3 | 0.726851031695032 | 0.628649088401622 | 0.367048447887897 | -0.505301182 | 1.95900324496221 | 2.06855652207076 | 0.603323838038384 | 7.09225430063193 | 0.999714303421225 |
| ebi-fl187-GCST90094770 | prot-c-4324_33_2 | CYTT \|\| id:prot-c-4324_33_2 | Gherkins liking \|\| id:ebi-fl187-GCST90094770 | Weighted mode | 3 | -0.341443014 | 0.616757960180001 | 0.635474173986118 | -1.550288616 | 0.867402587924974 | 0.710743968901878 | 0.212186724508831 | 2.38071910719075 | 0.999714303421225 |
| ebi-fl187-GCST90094770 | prot-c-4462_8_2 | RASA1 \|\| id:prot-c-4462_8_2 | Gherkins liking \|\| id:ebi-fl187-GCST90094770 | Weighted mode | 3 | -0.095713298 | 0.697304005578741 | 0.903395116387656 | -1.462429149 | 1.27100255263945 | 0.908724511591626 | 0.231672822793489 | 3.56442429461625 | 0.999714303421225 |
| ebi-fl187-GCST90094770 | prot-c-4499_21_1 | PDGF-AA \|\| id:prot-c-4499_21_1 | Gherkins liking \|\| id:ebi-fl187-GCST90094770 | Weighted mode | 3 | 0.435322999897197 | 0.536222115016615 | 0.50214631134183 | -0.615672346 | 1.48631834532976 | 1.54546216246141 | 0.540277519987694 | 4.42078970017906 | 0.999714303421225 |
| ebi-fl187-GCST90094770 | prot-c-4535_50_2 | BST1 \|\| id:prot-c-4535_50_2 | Gherkins liking \|\| id:ebi-fl187-GCST90094770 | Weighted mode | 3 | -0.780996745 | 0.736219554217681 | 0.399941984796059 | -2.223987071 | 0.661993581649331 | 0.457949325218416 | 0.108176938728339 | 1.93865334823957 | 0.999714303421225 |
| ebi-fl187-GCST90094770 | prot-c-4542_24_2 | Clusterin \|\| id:prot-c-4542_24_2 | Gherkins liking \|\| id:ebi-fl187-GCST90094770 | Weighted mode | 3 | 0.52064276133729 | 0.619019658404906 | 0.488838151105022 | -0.692635769 | 1.7339212918109 | 1.68310913957307 | 0.500255771108369 | 5.66281598198839 | 0.999714303421225 |
| ebi-fl187-GCST90094770 | prot-c-4696_2_2 | FABP \|\| id:prot-c-4696_2_2 | Gherkins liking \|\| id:ebi-fl187-GCST90094770 | Weighted mode | 3 | -0.200732986 | 0.567005609929189 | 0.757161258071175 | -1.312063981 | 0.910598009696036 | 0.818130854975708 | 0.269263727177823 | 2.48580862665267 | 0.999714303421225 |
| ebi-fl187-GCST90094770 | prot-c-4906_35_1 | Coagulation Factor V \|\| id:prot-c-4906_35_1 | Gherkins liking \|\| id:ebi-fl187-GCST90094770 | Weighted mode | 3 | -0.91157525 | 0.669586366176119 | 0.306473228688158 | -2.223964528 | 0.400814027712275 | 0.401890646904775 | 0.108179377408663 | 1.49303957869334 | 0.999714303421225 |
| ebi-fl187-GCST90094770 | prot-c-4916_2_1 | IgD \|\| id:prot-c-4916_2_1 | Gherkins liking \|\| id:ebi-fl187-GCST90094770 | Weighted mode | 3 | -0.702612098 | 0.630446978824399 | 0.381046314814639 | -1.938288177 | 0.533063980478384 | 0.495289866944467 | 0.143950156244954 | 1.70414578696553 | 0.999714303421225 |
| ebi-fl187-GCST90094770 | prot-c-4922_13_1 | MIP-3b \|\| id:prot-c-4922_13_1 | Gherkins liking \|\| id:ebi-fl187-GCST90094770 | Weighted mode | 3 | 0.526741504217938 | 0.670684606484299 | 0.51449659381571 | -0.787800324 | 1.84128333292716 | 1.69340535452555 | 0.454844205349242 | 6.304624003 | 0.999714303421225 |
| ebi-fl187-GCST90094770 | prot-c-4973_18_1 | cIAP-2 \|\| id:prot-c-4973_18_1 | Gherkins liking \|\| id:ebi-fl187-GCST90094770 | Weighted mode | 3 | -0.08149716 | 0.606171288549235 | 0.905359202518445 | -1.269592886 | 1.10659856537977 | 0.921735327415603 | 0.280945975614126 | 3.02405475625269 | 0.999714303421225 |
| ebi-fl187-GCST90094770 | prot-c-4997_19_1 | IF4A3 \|\| id:prot-c-4997_19_1 | Gherkins liking \|\| id:ebi-fl187-GCST90094770 | Weighted mode | 3 | -0.371059379 | 0.623805017133079 | 0.612289627312231 | -1.593717212 | 0.851598454931758 | 0.690002968898197 | 0.203168984100265 | 2.34338966253514 | 0.999714303421225 |
| ebi-fl187-GCST90094770 | prot-c-5000_52_1 | LG3BP \|\| id:prot-c-5000_52_1 | Gherkins liking \|\| id:ebi-fl187-GCST90094770 | Weighted mode | 3 | -0.403924205 | 0.586847064070782 | 0.562380187862374 | -1.55414445 | 0.746296040903858 | 0.667694727490001 | 0.211370142977434 | 2.10917323912461 | 0.999714303421225 |
| ebi-fl187-GCST90094770 | prot-c-5006_71_1 | MK13 \|\| id:prot-c-5006_71_1 | Gherkins liking \|\| id:ebi-fl187-GCST90094770 | Weighted mode | 3 | -0.648728054 | 0.562540999820654 | 0.368034601916129 | -1.751308414 | 0.453852305417234 | 0.522710213151811 | 0.173546723891927 | 1.57436545505381 | 0.999714303421225 |
| ebi-fl187-GCST90094770 | prot-c-5109_24_3 | Nr-CAM \|\| id:prot-c-5109_24_3 | Gherkins liking \|\| id:ebi-fl187-GCST90094770 | Weighted mode | 3 | -0.343331266 | 0.574187775855281 | 0.610569180752135 | -1.468739307 | 0.782076774563293 | 0.709403171400758 | 0.230215533477381 | 2.18600739920488 | 0.999714303421225 |
| ebi-fl187-GCST90094770 | prot-c-5255_22_3 | PDE4D \|\| id:prot-c-5255_22_3 | Gherkins liking \|\| id:ebi-fl187-GCST90094770 | Weighted mode | 3 | 0.20703735250285 | 0.588969145171525 | 0.758774679444657 | -0.947342172 | 1.36141687703904 | 1.23002851556632 | 0.387770281752344 | 3.90171764135493 | 0.999714303421225 |
| ebi-fl187-GCST90094770 | prot-c-5440_26_3 | Troponin I, skeletal, fast twitch \|\| id:prot-c-5440_26_3 | Gherkins liking \|\| id:ebi-fl187-GCST90094770 | Weighted mode | 3 | -0.862919897 | 0.604231040275118 | 0.289439896152126 | -2.047212736 | 0.321372942092859 | 0.421928294830766 | 0.129094222303193 | 1.37901977952731 | 0.999714303421225 |
| ebi-fl187-GCST90094770 | ukb-rsfMRI191-edge-pheno716 | Net100_Pair10_33 (OccipitalPrecuneus)&(Temporal\|Frontal\|Supp_Motor_Area) (Default_mode\|Central_executive)&(Default_mode\|Salience) \|\| id:ukb-rsfMRI191-edge-pheno716 | Gherkins liking \|\| id:ebi-fl187-GCST90094770 | Weighted mode | 21 | -0.043530941 | 0.0587386665482063 | 0.467248830401417 | -0.158658727 | 0.0715968459191415 | 0.957402931092615 | 0.853287513317084 | 1.0742221796982 | 0.999714303421225 |
| ebi-fl187-GCST90094770 | ukb-rsfMRI191-edge-pheno903 | Net100_Pair27_38 (Precentral\|Frontal\|Supp_Motor_Area)&(Subcortical) (Attention\|Salience\|Motor)&(Subcortical-cerebellum) \|\| id:ukb-rsfMRI191-edge-pheno903 | Gherkins liking \|\| id:ebi-fl187-GCST90094770 | Weighted mode | 21 | 0.126779052675529 | 0.0617181483779326 | 0.0532639903161248 | 0.00581148185478093 | 0.247746623496277 | 1.13516617814144 | 1.00582840127521 | 1.28113528148791 | 0.999714303421225 |
| ebi-fl187-GCST90094770 | ukb-rsfMRI191-node-pheno23 | Net100_Node2 RolandicOper\|SupraMarginal\|Insula Salience\|Motor \|\| id:ukb-rsfMRI191-node-pheno23 | Gherkins liking \|\| id:ebi-fl187-GCST90094770 | Weighted mode | 21 | -0.084912931 | 0.0760579644792959 | 0.277474603336564 | -0.233986542 | 0.0641606790373875 | 0.918592261515087 | 0.791372466335002 | 1.0662637112247 | 0.999714303421225 |
| ebi-fl187-GCST90094770 | ukb-rsfMRI191-node-pheno55 | Net100_Node34 Parietal\|Postcentral\|Precuneus Attention \|\| id:ukb-rsfMRI191-node-pheno55 | Gherkins liking \|\| id:ebi-fl187-GCST90094770 | Weighted mode | 21 | -0.019060252 | 0.0555552700247577 | 0.735113502488038 | -0.127948582 | 0.0898280767982613 | 0.981120245563063 | 0.879898620873831 | 1.09398618592874 | 0.999714303421225 |
| ebi-fl187-GCST90094775 | ebi-a-GCST90001414 | IgD- CD24- B cell Absolute Count \|\| id:ebi-a-GCST90001414 | Green olives liking \|\| id:ebi-fl187-GCST90094775 | Inverse variance weighted | 25 | -0.021088739 | 0.0774959511752951 | 0.785524386465188 | -0.172980803 | 0.130803325292175 | 0.979132073501967 | 0.841153762053459 | 1.13974360052774 | 0.998735809224318 |
| ebi-fl187-GCST90094775 | ebi-a-GCST90001422 | CD20- CD38- B cell %B cell \|\| id:ebi-a-GCST90001422 | Green olives liking \|\| id:ebi-fl187-GCST90094775 | Inverse variance weighted | 25 | 0.0253833860605669 | 0.0737626622797459 | 0.730754220549975 | -0.119191432 | 0.169958204128869 | 1.02570828741186 | 0.887637862198526 | 1.18525531150684 | 0.998735809224318 |
| ebi-fl187-GCST90094775 | ebi-a-GCST90001444 | CD20- CD38- B cell %lymphocyte \|\| id:ebi-a-GCST90001444 | Green olives liking \|\| id:ebi-fl187-GCST90094775 | Inverse variance weighted | 25 | 0.00592255129026444 | 0.0738369074136147 | 0.93606923578083 | -0.138797787 | 0.150642889820949 | 1.00594012427232 | 0.870404017462361 | 1.16258141428532 | 0.998735809224318 |
| ebi-fl187-GCST90094775 | ebi-a-GCST90001459 | Myeloid Dendritic Cell %Dendritic Cell \|\| id:ebi-a-GCST90001459 | Green olives liking \|\| id:ebi-fl187-GCST90094775 | Inverse variance weighted | 25 | -0.037846829 | 0.0791981690667106 | 0.632739138781459 | -0.193075241 | 0.117381582184673 | 0.962860411709752 | 0.824419941989972 | 1.12454845548745 | 0.998735809224318 |
| ebi-fl187-GCST90094775 | ebi-a-GCST90001476 | HLA DR++ monocyte %leukocyte \|\| id:ebi-a-GCST90001476 | Green olives liking \|\| id:ebi-fl187-GCST90094775 | Inverse variance weighted | 25 | 0.0943144213567605 | 0.077450040272637 | 0.223320782470226 | -0.057487658 | 0.246116500291129 | 1.0989052108709 | 0.944133543209495 | 1.27904857439353 | 0.998735809224318 |
| ebi-fl187-GCST90094775 | ebi-a-GCST90001478 | CD4 regulatory T cell %CD4+ T cell \|\| id:ebi-a-GCST90001478 | Green olives liking \|\| id:ebi-fl187-GCST90094775 | Inverse variance weighted | 25 | 0.0263814758656947 | 0.0781058993358069 | 0.73553993244588 | -0.126706087 | 0.179469038563876 | 1.02673254746329 | 0.8809925698654 | 1.19658185560126 | 0.998735809224318 |
| ebi-fl187-GCST90094775 | ebi-a-GCST90001485 | CD39+ resting CD4 regulatory T cell %CD4 regulatory T cell \|\| id:ebi-a-GCST90001485 | Green olives liking \|\| id:ebi-fl187-GCST90094775 | Inverse variance weighted | 24 | 0.0520251294824008 | 0.0848936945366182 | 0.539990881373225 | -0.114366512 | 0.218416770774173 | 1.05340221363315 | 0.891930992726161 | 1.24410546638321 | 0.998735809224318 |
| ebi-fl187-GCST90094775 | ebi-a-GCST90001515 | Immature Myeloid-Derived Suppressor Cells Absolute Count \|\| id:ebi-a-GCST90001515 | Green olives liking \|\| id:ebi-fl187-GCST90094775 | Inverse variance weighted (multiplicative random effects) | 25 | -0.10695674 | 0.12886426645975 | 0.406541422853902 | -0.359530702 | 0.145617222628105 | 0.898564544381463 | 0.698003821089763 | 1.15675332429969 | 0.998735809224318 |
| ebi-fl187-GCST90094775 | ebi-a-GCST90001517 | CD33+ HLA DR+ Absolute Count \|\| id:ebi-a-GCST90001517 | Green olives liking \|\| id:ebi-fl187-GCST90094775 | Inverse variance weighted | 25 | -0.104086941 | 0.100133537338454 | 0.298580943039687 | -0.300348674 | 0.0921747919939964 | 0.901146947227932 | 0.740559961380121 | 1.09655647462339 | 0.998735809224318 |
| ebi-fl187-GCST90094775 | ebi-a-GCST90001518 | CD33+ HLA DR+ CD14- Absolute Count \|\| id:ebi-a-GCST90001518 | Green olives liking \|\| id:ebi-fl187-GCST90094775 | Inverse variance weighted | 25 | -0.139434453 | 0.101427396721681 | 0.169217710109416 | -0.338232151 | 0.0593632442117781 | 0.869850037080715 | 0.713029738160788 | 1.06116063119754 | 0.998735809224318 |
| ebi-fl187-GCST90094775 | ebi-a-GCST90001520 | CD33+ HLA DR+ CD14dim Absolute Count \|\| id:ebi-a-GCST90001520 | Green olives liking \|\| id:ebi-fl187-GCST90094775 | Inverse variance weighted | 25 | -0.112696925 | 0.10048619239453 | 0.26206806293907 | -0.309649862 | 0.0842560121339542 | 0.893421392812749 | 0.73370380880036 | 1.08790737564872 | 0.998735809224318 |
| ebi-fl187-GCST90094775 | ebi-a-GCST90001531 | CD33dim HLA DR- Absolute Count \|\| id:ebi-a-GCST90001531 | Green olives liking \|\| id:ebi-fl187-GCST90094775 | Inverse variance weighted | 25 | -0.097047068 | 0.10773771771493 | 0.367709810271532 | -0.308212995 | 0.114118858408105 | 0.907513290008264 | 0.734758801364839 | 1.12088534361452 | 0.998735809224318 |
| ebi-fl187-GCST90094775 | ebi-a-GCST90001532 | Basophil Absolute Count \|\| id:ebi-a-GCST90001532 | Green olives liking \|\| id:ebi-fl187-GCST90094775 | Inverse variance weighted | 25 | -0.08055015 | 0.106079751344105 | 0.447651724181072 | -0.288466462 | 0.127366162800526 | 0.922608633753798 | 0.749411938152633 | 1.1358328413814 | 0.998735809224318 |
| ebi-fl187-GCST90094775 | ebi-a-GCST90001588 | T/B cell \|\| id:ebi-a-GCST90001588 | Green olives liking \|\| id:ebi-fl187-GCST90094775 | Inverse variance weighted | 25 | 0.0619644682114041 | 0.0796508322318396 | 0.436597725757687 | -0.094151163 | 0.21808009938581 | 1.06392454091455 | 0.910145171600824 | 1.24368668216886 | 0.998735809224318 |
| ebi-fl187-GCST90094775 | ebi-a-GCST90001624 | HLA DR+ CD4+ T cell Absolute Count \|\| id:ebi-a-GCST90001624 | Green olives liking \|\| id:ebi-fl187-GCST90094775 | Inverse variance weighted | 25 | -0.046431452 | 0.0741203001996055 | 0.53103053004949 | -0.19170724 | 0.0988443366322271 | 0.954629996534253 | 0.825548520577808 | 1.10389445025612 | 0.998735809224318 |
| ebi-fl187-GCST90094775 | ebi-a-GCST90001631 | CD8+ Natural Killer T %T cell \|\| id:ebi-a-GCST90001631 | Green olives liking \|\| id:ebi-fl187-GCST90094775 | Inverse variance weighted (multiplicative random effects) | 25 | 0.0630053029925605 | 0.100819588704792 | 0.532016237195106 | -0.134601091 | 0.260611696853952 | 1.06503248707569 | 0.874064514469048 | 1.2977236574072 | 0.998735809224318 |
| ebi-fl187-GCST90094775 | ebi-a-GCST90001632 | CD8+ Natural Killer T %lymphocyte \|\| id:ebi-a-GCST90001632 | Green olives liking \|\| id:ebi-fl187-GCST90094775 | Inverse variance weighted (multiplicative random effects) | 25 | 0.0738310509141824 | 0.100701111573847 | 0.463454647726459 | -0.123543128 | 0.271205229598923 | 1.07662489533804 | 0.883783524809065 | 1.31154421045817 | 0.998735809224318 |
| ebi-fl187-GCST90094775 | ebi-a-GCST90001646 | Natural Killer %CD3- lymphocyte \|\| id:ebi-a-GCST90001646 | Green olives liking \|\| id:ebi-fl187-GCST90094775 | Inverse variance weighted | 25 | -0.013139386 | 0.0753587286337582 | 0.861584236390165 | -0.160842494 | 0.134563722093404 | 0.986946558870972 | 0.851426165147639 | 1.14403755714783 | 0.998735809224318 |
| ebi-fl187-GCST90094775 | ebi-a-GCST90001684 | CD127- CD8+ T cell Absolute Count \|\| id:ebi-a-GCST90001684 | Green olives liking \|\| id:ebi-fl187-GCST90094775 | Inverse variance weighted | 25 | -0.002997327 | 0.086112511322998 | 0.972233559419873 | -0.171777849 | 0.165783195436653 | 0.997007160742799 | 0.842166240504153 | 1.18031717583141 | 0.998955228396052 |
| ebi-fl187-GCST90094775 | ebi-a-GCST90001687 | CD28- CD8+ T cell Absolute Count \|\| id:ebi-a-GCST90001687 | Green olives liking \|\| id:ebi-fl187-GCST90094775 | Inverse variance weighted | 25 | -0.021162841 | 0.0917226101002534 | 0.817527253309796 | -0.200939156 | 0.158613475268451 | 0.979059521018485 | 0.817962197866365 | 1.17188489663376 | 0.998735809224318 |
| ebi-fl187-GCST90094775 | ebi-a-GCST90001701 | BAFF-R on CD20- CD38- B cell \|\| id:ebi-a-GCST90001701 | Green olives liking \|\| id:ebi-fl187-GCST90094775 | Inverse variance weighted | 25 | -0.062244947 | 0.0744514375557526 | 0.403128116668842 | -0.208169764 | 0.0836798707888824 | 0.939652693660117 | 0.812069164550805 | 1.0872807677547 | 0.998735809224318 |
| ebi-fl187-GCST90094775 | ebi-a-GCST90001732 | CD19 on IgD- CD27- B cell \|\| id:ebi-a-GCST90001732 | Green olives liking \|\| id:ebi-fl187-GCST90094775 | Inverse variance weighted | 25 | -0.131370022 | 0.0749321978807935 | 0.0795702187462709 | -0.27823713 | 0.0154970858352712 | 0.8768932445513 | 0.757117265116944 | 1.01561778837646 | 0.998735809224318 |
| ebi-fl187-GCST90094775 | ebi-a-GCST90001798 | CD27 on CD24+ CD27+ B cell \|\| id:ebi-a-GCST90001798 | Green olives liking \|\| id:ebi-fl187-GCST90094775 | Inverse variance weighted | 25 | -0.001693578 | 0.0780670910540686 | 0.982692146206349 | -0.154705077 | 0.151317920412414 | 0.998307855240505 | 0.856667791677578 | 1.16336645723923 | 0.998955228396052 |
| ebi-fl187-GCST90094775 | ebi-a-GCST90001836 | CD66b on CD66b++ myeloid cell \|\| id:ebi-a-GCST90001836 | Green olives liking \|\| id:ebi-fl187-GCST90094775 | Inverse variance weighted | 25 | -0.067625914 | 0.113226391038659 | 0.550331923142915 | -0.28954964 | 0.154297812778765 | 0.934610033039811 | 0.748600631383752 | 1.16683833440544 | 0.998735809224318 |
| ebi-fl187-GCST90094775 | ebi-a-GCST90001943 | CD25 on activated & secreting CD4 regulatory T cell \|\| id:ebi-a-GCST90001943 | Green olives liking \|\| id:ebi-fl187-GCST90094775 | Inverse variance weighted | 25 | 0.0421808900053672 | 0.0818138441365596 | 0.60615368856449 | -0.118174245 | 0.202536024513024 | 1.04308314500203 | 0.888541215703375 | 1.22450419649474 | 0.998735809224318 |
| ebi-fl187-GCST90094775 | ebi-a-GCST90001960 | CD25 on CD4+ T cell \|\| id:ebi-a-GCST90001960 | Green olives liking \|\| id:ebi-fl187-GCST90094775 | Inverse variance weighted | 25 | 0.0613391575085936 | 0.0817672412140462 | 0.453153604797306 | -0.098924635 | 0.221602950288124 | 1.0632594654732 | 0.905810971649642 | 1.24807573136311 | 0.998735809224318 |
| ebi-fl187-GCST90094775 | ebi-a-GCST90001988 | HLA DR on CD14+ CD16- monocyte \|\| id:ebi-a-GCST90001988 | Green olives liking \|\| id:ebi-fl187-GCST90094775 | Inverse variance weighted | 24 | -0.022906142 | 0.0782951669355261 | 0.769857433635677 | -0.17636467 | 0.130552384763965 | 0.977354211560189 | 0.838312220587582 | 1.13945762854908 | 0.998735809224318 |
| ebi-fl187-GCST90094775 | ebi-a-GCST90001991 | HLA DR on CD14+ monocyte \|\| id:ebi-a-GCST90001991 | Green olives liking \|\| id:ebi-fl187-GCST90094775 | Inverse variance weighted | 24 | -0.02214308 | 0.0784405664969877 | 0.777720245399263 | -0.175886591 | 0.131600429989232 | 0.978100278113802 | 0.838713095826396 | 1.14065245768419 | 0.998735809224318 |
| ebi-fl187-GCST90094775 | ebi-a-GCST90002019 | CD14 on Monocytic Myeloid-Derived Suppressor Cells \|\| id:ebi-a-GCST90002019 | Green olives liking \|\| id:ebi-fl187-GCST90094775 | Inverse variance weighted | 25 | 0.00616732053507201 | 0.109886844849382 | 0.955242798250737 | -0.209210895 | 0.221545536439861 | 1.00618637761326 | 0.811224134188091 | 1.24800407658945 | 0.998735809224318 |
| ebi-fl187-GCST90094775 | ebi-a-GCST90002037 | CD80 on plasmacytoid Dendritic Cell \|\| id:ebi-a-GCST90002037 | Green olives liking \|\| id:ebi-fl187-GCST90094775 | Inverse variance weighted | 25 | 0.0433662236840332 | 0.0853156582704141 | 0.611240713299981 | -0.123852467 | 0.210584913894045 | 1.04432027964728 | 0.883510178593908 | 1.23439986647154 | 0.998735809224318 |
| ebi-fl187-GCST90094775 | ebi-a-GCST90002042 | CD45 on CD33+ HLA DR+ CD14- \|\| id:ebi-a-GCST90002042 | Green olives liking \|\| id:ebi-fl187-GCST90094775 | Inverse variance weighted (multiplicative random effects) | 25 | 0.086229604264872 | 0.144785384934334 | 0.55146357267261 | -0.19754975 | 0.370008958736167 | 1.09005658121446 | 0.820739307662033 | 1.44774758459387 | 0.998735809224318 |
| ebi-fl187-GCST90094775 | ebi-a-GCST90002056 | CD8 on Effector Memory CD8+ T cell \|\| id:ebi-a-GCST90002056 | Green olives liking \|\| id:ebi-fl187-GCST90094775 | Inverse variance weighted | 25 | -0.023300282 | 0.087779850632716 | 0.790670539365742 | -0.195348789 | 0.148748225280471 | 0.976969073534661 | 0.822547712258794 | 1.1603807978775 | 0.998735809224318 |
| ebi-fl187-GCST90094775 | ebi-a-GCST90002058 | CD8 on CD8+ T cell \|\| id:ebi-a-GCST90002058 | Green olives liking \|\| id:ebi-fl187-GCST90094775 | Inverse variance weighted | 25 | -0.091796122 | 0.0806802763922408 | 0.255213821994758 | -0.249929464 | 0.0663372195943777 | 0.912291126553823 | 0.778855718607422 | 1.06858700489089 | 0.998735809224318 |
| ebi-fl187-GCST90094775 | ebi-a-GCST90002063 | CD4 on CD4 regulatory T cell \|\| id:ebi-a-GCST90002063 | Green olives liking \|\| id:ebi-fl187-GCST90094775 | Inverse variance weighted | 25 | 0.0201549983160663 | 0.0984630521829303 | 0.837809603032987 | -0.172832584 | 0.21314258059461 | 1.02035948177204 | 0.841278446559527 | 1.23756109086107 | 0.998735809224318 |
| ebi-fl187-GCST90094775 | ebi-a-GCST90002066 | CD4 on activated CD4 regulatory T cell \|\| id:ebi-a-GCST90002066 | Green olives liking \|\| id:ebi-fl187-GCST90094775 | Inverse variance weighted | 25 | 0.0483341166086586 | 0.0965223881105469 | 0.616543208065359 | -0.140849764 | 0.237517997305331 | 1.04952125923376 | 0.868619799783214 | 1.26809781892899 | 0.998735809224318 |
| ebi-fl187-GCST90094775 | ebi-a-GCST90002070 | CD4 on activated & secreting CD4 regulatory T cell \|\| id:ebi-a-GCST90002070 | Green olives liking \|\| id:ebi-fl187-GCST90094775 | Inverse variance weighted | 25 | 0.0132415581545897 | 0.0991888114294072 | 0.893799145600999 | -0.181168512 | 0.207651628556228 | 1.01332961583012 | 0.834294757965284 | 1.23078432474238 | 0.998735809224318 |
| ebi-fl187-GCST90094775 | ebi-a-GCST90002081 | SSC-A on CD4+ T cell \|\| id:ebi-a-GCST90002081 | Green olives liking \|\| id:ebi-fl187-GCST90094775 | Inverse variance weighted | 25 | 0.0109366479382391 | 0.078991568072986 | 0.889882128591228 | -0.143886825 | 0.165760121361292 | 1.01099667169228 | 0.865985750030856 | 1.18028994141815 | 0.998735809224318 |
| ebi-fl187-GCST90094775 | ebi-a-GCST90002094 | CD11b on Monocytic Myeloid-Derived Suppressor Cells \|\| id:ebi-a-GCST90002094 | Green olives liking \|\| id:ebi-fl187-GCST90094775 | Inverse variance weighted | 25 | -0.159735277 | 0.111304602758841 | 0.151253378414672 | -0.377892298 | 0.0584217447429514 | 0.852369401173548 | 0.685304305286498 | 1.06016201919703 | 0.998735809224318 |
| ebi-fl187-GCST90094775 | ebi-a-GCST90002104 | HLA DR on myeloid Dendritic Cell \|\| id:ebi-a-GCST90002104 | Green olives liking \|\| id:ebi-fl187-GCST90094775 | Inverse variance weighted | 24 | -0.027566537 | 0.0865558961431467 | 0.750118980325255 | -0.197216093 | 0.142083019755283 | 0.972809952850353 | 0.821013198833661 | 1.15267233914036 | 0.998735809224318 |
| ebi-fl187-GCST90094775 | ebi-a-GCST90002105 | HLA DR on plasmacytoid Dendritic Cell \|\| id:ebi-a-GCST90002105 | Green olives liking \|\| id:ebi-fl187-GCST90094775 | Inverse variance weighted | 24 | 0.0602667793595179 | 0.0846746415701604 | 0.476622501868118 | -0.105695518 | 0.226229076837032 | 1.06211986040862 | 0.8996985482822 | 1.25386286332052 | 0.998735809224318 |
| ebi-fl187-GCST90094775 | ebi-a-GCST90002106 | HLA DR on Dendritic Cell \|\| id:ebi-a-GCST90002106 | Green olives liking \|\| id:ebi-fl187-GCST90094775 | Inverse variance weighted | 24 | 0.0775903665823197 | 0.0863643843910965 | 0.368968611525744 | -0.091683827 | 0.246864559988869 | 1.08067988539404 | 0.912393578321177 | 1.28000573704626 | 0.998735809224318 |
| ebi-fl187-GCST90094775 | ebi-a-GCST90002111 | HLA DR on CD33dim HLA DR+ CD11b- \|\| id:ebi-a-GCST90002111 | Green olives liking \|\| id:ebi-fl187-GCST90094775 | Inverse variance weighted | 24 | 0.136376113392073 | 0.111080678164746 | 0.219551611836512 | -0.081342016 | 0.354094242594974 | 1.14611288094307 | 0.921878340552057 | 1.42488946543316 | 0.998735809224318 |
| ebi-fl187-GCST90094775 | ebi-a-GCST90002112 | HLA DR on CD33- HLA DR+ \|\| id:ebi-a-GCST90002112 | Green olives liking \|\| id:ebi-fl187-GCST90094775 | Inverse variance weighted | 24 | 0.00937722247109178 | 0.113895833703311 | 0.934383052734587 | -0.213858612 | 0.232613056529582 | 1.0094213263713 | 0.80746254279936 | 1.26189310354966 | 0.998735809224318 |
| ebi-fl187-GCST90094775 | ebi-a-GCST90002115 | HLA DR on HLA DR+ CD8+ T cell \|\| id:ebi-a-GCST90002115 | Green olives liking \|\| id:ebi-fl187-GCST90094775 | Inverse variance weighted | 25 | -0.023828686 | 0.0790226686406897 | 0.763000913731532 | -0.178713117 | 0.131055744407433 | 0.976452975369765 | 0.836345798656549 | 1.14003132991168 | 0.998735809224318 |
| ebi-fl187-GCST90094775 | ebi-a-GCST90002116 | HLA DR on B cell \|\| id:ebi-a-GCST90002116 | Green olives liking \|\| id:ebi-fl187-GCST90094775 | Inverse variance weighted | 25 | 0.187246483516736 | 0.101004370797632 | 0.0637612877758139 | -0.010722083 | 0.385215050280095 | 1.20592448892944 | 0.98933519339686 | 1.46993039639739 | 0.998735809224318 |
| ebi-fl187-GCST90094775 | ebi-cfmet338-GCST90026023 | Sphingomyelin (d18:1/24:1, d18:2/24:0) levels \|\| id:ebi-cfmet338-GCST90026023 | Green olives liking \|\| id:ebi-fl187-GCST90094775 | Inverse variance weighted | 23 | -0.027447596 | 0.0477247926922322 | 0.565208542585057 | -0.120988189 | 0.0660929979880473 | 0.972925666717031 | 0.886044424277922 | 1.06832606472096 | 0.998735809224318 |
| ebi-fl187-GCST90094775 | ebi-cfmet338-GCST90026046 | 1-arachidonoyl-gpc (20:4n6) levels \|\| id:ebi-cfmet338-GCST90026046 | Green olives liking \|\| id:ebi-fl187-GCST90094775 | Inverse variance weighted | 23 | 0.0032935238214017 | 0.0605789213441416 | 0.956642380791824 | -0.115441162 | 0.122028209655919 | 1.0032989534302 | 0.890972993752195 | 1.12978597220433 | 0.998735809224318 |
| ebi-fl187-GCST90094775 | ebi-cfmet338-GCST90026047 | 1-linoleoyl-gpc (18:2) levels \|\| id:ebi-cfmet338-GCST90026047 | Green olives liking \|\| id:ebi-fl187-GCST90094775 | Inverse variance weighted | 23 | -0.021282216 | 0.0687201238679792 | 0.756793596581849 | -0.155973659 | 0.113409226474875 | 0.978942652001947 | 0.855581726877517 | 1.12009020974077 | 0.998735809224318 |
| ebi-fl187-GCST90094775 | ebi-cfmet338-GCST90026050 | 1-myristoyl-2-palmitoyl-gpc (14:0/16:0) levels \|\| id:ebi-cfmet338-GCST90026050 | Green olives liking \|\| id:ebi-fl187-GCST90094775 | Inverse variance weighted | 23 | -0.009564292 | 0.0391680270572919 | 0.807086604295515 | -0.086333625 | 0.067205040831165 | 0.990481300172822 | 0.917288149691676 | 1.06951474988727 | 0.998735809224318 |
| ebi-fl187-GCST90094775 | ebi-cfmet338-GCST90026068 | 2,3-dihydroxyisovalerate levels \|\| id:ebi-cfmet338-GCST90026068 | Green olives liking \|\| id:ebi-fl187-GCST90094775 | Inverse variance weighted | 23 | 0.111041599867268 | 0.122512374636223 | 0.364739791871446 | -0.129082654 | 0.351165854154265 | 1.11744139130114 | 0.878901317464887 | 1.42072293917446 | 0.998735809224318 |
| ebi-fl187-GCST90094775 | ebi-cfmet338-GCST90026082 | 3-hydroxyhexanoate levels \|\| id:ebi-cfmet338-GCST90026082 | Green olives liking \|\| id:ebi-fl187-GCST90094775 | Inverse variance weighted | 23 | -0.051694684 | 0.0278850790295438 | 0.0637609794221713 | -0.106349439 | 0.00296007061433441 | 0.94961875611762 | 0.899110408770061 | 1.00296445594925 | 0.998735809224318 |
| ebi-fl187-GCST90094775 | ebi-cfmet338-GCST90026094 | 3-ureidopropionate levels \|\| id:ebi-cfmet338-GCST90026094 | Green olives liking \|\| id:ebi-fl187-GCST90094775 | Inverse variance weighted | 23 | -0.068876571 | 0.057022871945219 | 0.227094880831118 | -0.1806414 | 0.0428882584442221 | 0.933441887169641 | 0.834734641223229 | 1.04382125012326 | 0.998735809224318 |
| ebi-fl187-GCST90094775 | ebi-cfmet338-GCST90026173 | Glycerol levels \|\| id:ebi-cfmet338-GCST90026173 | Green olives liking \|\| id:ebi-fl187-GCST90094775 | Inverse variance weighted | 23 | 0.0310809115865382 | 0.0393132466781452 | 0.429179543965937 | -0.045973052 | 0.108134875075703 | 1.03156896639163 | 0.955067699101287 | 1.1141980127939 | 0.998735809224318 |
| ebi-fl187-GCST90094775 | ebi-cfmet338-GCST90026179 | Histidine levels \|\| id:ebi-cfmet338-GCST90026179 | Green olives liking \|\| id:ebi-fl187-GCST90094775 | Inverse variance weighted | 23 | 0.0098565431825941 | 0.0223898383712319 | 0.659774912028775 | -0.03402754 | 0.0537406263902087 | 1.00990527889463 | 0.966544885600631 | 1.05521087280436 | 0.998735809224318 |
| ebi-fl187-GCST90094775 | ebi-cfmet338-GCST90026188 | Isocitrate levels \|\| id:ebi-cfmet338-GCST90026188 | Green olives liking \|\| id:ebi-fl187-GCST90094775 | Inverse variance weighted | 23 | -0.024095915 | 0.043876826927749 | 0.582887575077665 | -0.110094496 | 0.0619026660329831 | 0.97619207405321 | 0.895749486980188 | 1.06385879009204 | 0.998735809224318 |
| ebi-fl187-GCST90094775 | ebi-cfmet338-GCST90026207 | N-acetyl-isoputreanine levels \|\| id:ebi-cfmet338-GCST90026207 | Green olives liking \|\| id:ebi-fl187-GCST90094775 | Inverse variance weighted | 23 | 0.00433848301632625 | 0.0279147016786662 | 0.876490780519141 | -0.050374332 | 0.059051298306512 | 1.00434790785868 | 0.950873415264309 | 1.06082965810935 | 0.998735809224318 |
| ebi-fl187-GCST90094775 | ebi-cfmet338-GCST90026213 | N-acetylglutamate levels \|\| id:ebi-cfmet338-GCST90026213 | Green olives liking \|\| id:ebi-fl187-GCST90094775 | Inverse variance weighted | 23 | 0.03572712293494 | 0.0538896456676161 | 0.507350902374224 | -0.069896583 | 0.141350828443468 | 1.03637300547822 | 0.93249025066143 | 1.15182867136907 | 0.998735809224318 |
| ebi-fl187-GCST90094775 | ebi-cfmet338-GCST90026224 | N-acetyltaurine levels \|\| id:ebi-cfmet338-GCST90026224 | Green olives liking \|\| id:ebi-fl187-GCST90094775 | Inverse variance weighted | 23 | 0.0262237382482402 | 0.0381429946040397 | 0.491760608115031 | -0.048536531 | 0.100984007672158 | 1.02657060588997 | 0.952622538248878 | 1.10625894996615 | 0.998735809224318 |
| ebi-fl187-GCST90094775 | ebi-cfmet338-GCST90026250 | Phenyllactate (pla) levels \|\| id:ebi-cfmet338-GCST90026250 | Green olives liking \|\| id:ebi-fl187-GCST90094775 | Inverse variance weighted | 23 | -0.011824184 | 0.0602952227046458 | 0.844528126788569 | -0.13000282 | 0.106354452943924 | 0.988245447387981 | 0.878092954643756 | 1.11221603489495 | 0.998735809224318 |
| ebi-fl187-GCST90094775 | ebi-cfmet338-GCST90026251 | Pipecolate levels \|\| id:ebi-cfmet338-GCST90026251 | Green olives liking \|\| id:ebi-fl187-GCST90094775 | Inverse variance weighted | 23 | 0.0242757154248481 | 0.0900469646897181 | 0.787476085656028 | -0.152216335 | 0.200767766216696 | 1.02457276946713 | 0.858802471295463 | 1.22234087001408 | 0.998735809224318 |
| ebi-fl187-GCST90094775 | ebi-cfmet338-GCST90026278 | Trigonelline (n'-methylnicotinate) levels \|\| id:ebi-cfmet338-GCST90026278 | Green olives liking \|\| id:ebi-fl187-GCST90094775 | Inverse variance weighted | 22 | 0.014434117612204 | 0.102970445996103 | 0.888519918192334 | -0.187387957 | 0.216256191764565 | 1.01453879251139 | 0.829122010675013 | 1.24142037994203 | 0.998735809224318 |
| ebi-fl187-GCST90094775 | ebi-cfmet338-GCST90026299 | 1-carboxyethylvaline levels \|\| id:ebi-cfmet338-GCST90026299 | Green olives liking \|\| id:ebi-fl187-GCST90094775 | Inverse variance weighted | 23 | 0.0568498857975542 | 0.0690949989347678 | 0.410633914028429 | -0.078576312 | 0.192276083709699 | 1.05849690305028 | 0.924431511916285 | 1.21200508563851 | 0.998735809224318 |
| ebi-fl187-GCST90094775 | ebi-gm430-GCST90011306 | C_Erysipelotrichia abundance \|\| id:ebi-gm430-GCST90011306 | Green olives liking \|\| id:ebi-fl187-GCST90094775 | Inverse variance weighted | 20 | 0.0683698280477216 | 0.0633672932791924 | 0.28061221998001 | -0.055830067 | 0.192569722874939 | 1.0707612327986 | 0.945699828053953 | 1.21236103005718 | 0.998735809224318 |
| ebi-fl187-GCST90094775 | ebi-gm430-GCST90011312 | F_Erysipelotrichaceae abundance \|\| id:ebi-gm430-GCST90011312 | Green olives liking \|\| id:ebi-fl187-GCST90094775 | Inverse variance weighted | 20 | 0.0683698280477216 | 0.0633672932791924 | 0.28061221998001 | -0.055830067 | 0.192569722874939 | 1.0707612327986 | 0.945699828053953 | 1.21236103005718 | 0.998735809224318 |
| ebi-fl187-GCST90094775 | ebi-gm430-GCST90011352 | O_Erysipelotrichales abundance \|\| id:ebi-gm430-GCST90011352 | Green olives liking \|\| id:ebi-fl187-GCST90094775 | Inverse variance weighted | 20 | 0.0683698280477216 | 0.0633672932791924 | 0.28061221998001 | -0.055830067 | 0.192569722874939 | 1.0707612327986 | 0.945699828053953 | 1.21236103005718 | 0.998735809224318 |
| ebi-fl187-GCST90094775 | ebi-gm430-GCST90011362 | OTU97_11 (Parabacteroides) abundance \|\| id:ebi-gm430-GCST90011362 | Green olives liking \|\| id:ebi-fl187-GCST90094775 | Inverse variance weighted | 20 | 0.104663813561864 | 0.0690351664024179 | 0.129495541350086 | -0.030645113 | 0.239972739710604 | 1.11033726727177 | 0.969819688811803 | 1.27121449617402 | 0.998735809224318 |
| ebi-fl187-GCST90094775 | ebi-gm430-GCST90011384 | OTU97_235 (Phascolarctobacterium) abundance \|\| id:ebi-gm430-GCST90011384 | Green olives liking \|\| id:ebi-fl187-GCST90094775 | Inverse variance weighted | 20 | 0.0866847916824643 | 0.250136052958765 | 0.728928986591132 | -0.403581872 | 0.576951455481644 | 1.09055287419928 | 0.667923340262698 | 1.78060190403971 | 0.998735809224318 |
| ebi-fl187-GCST90094775 | ebi-gm430-GCST90011390 | OTU97_3 (Bacteroides) abundance \|\| id:ebi-gm430-GCST90011390 | Green olives liking \|\| id:ebi-fl187-GCST90094775 | Inverse variance weighted | 20 | -0.000986963 | 0.0549899809436896 | 0.985680299172589 | -0.108767325 | 0.106793399884351 | 0.999013524122276 | 0.8969390881513 | 1.11270434588403 | 0.998955228396052 |
| ebi-fl187-GCST90094775 | ebi-gm430-GCST90011398 | OTU97_4 (Alistipes) abundance \|\| id:ebi-gm430-GCST90011398 | Green olives liking \|\| id:ebi-fl187-GCST90094775 | Inverse variance weighted | 20 | 0.0753389159951761 | 0.0763191344269002 | 0.32356601069333 | -0.074246587 | 0.2249244194719 | 1.07824952497638 | 0.928442723258918 | 1.25222806855643 | 0.998735809224318 |
| ebi-fl187-GCST90094775 | ebi-gm430-GCST90011421 | OTU99_108 (Cytophagales) abundance \|\| id:ebi-gm430-GCST90011421 | Green olives liking \|\| id:ebi-fl187-GCST90094775 | Inverse variance weighted | 20 | 0.0916633175303701 | 0.240803238864241 | 0.703458152663777 | -0.380311031 | 0.563637665704283 | 1.09599575739719 | 0.683648740433123 | 1.75705245865242 | 0.998735809224318 |
| ebi-fl187-GCST90094775 | ebi-gm430-GCST90011428 | OTU99_123 (Phascolarctobacterium) abundance \|\| id:ebi-gm430-GCST90011428 | Green olives liking \|\| id:ebi-fl187-GCST90094775 | Inverse variance weighted | 20 | 0.0500045408281683 | 0.121798704073511 | 0.681402034225498 | -0.188720919 | 0.288730000812251 | 1.05127587002827 | 0.828017558291283 | 1.33473130350566 | 0.998735809224318 |
| ebi-fl187-GCST90094775 | ebi-gm430-GCST90011450 | OTU99_298 (Phascolarctobacterium) abundance \|\| id:ebi-gm430-GCST90011450 | Green olives liking \|\| id:ebi-fl187-GCST90094775 | Inverse variance weighted | 20 | 0.0862696282563861 | 0.251049551256394 | 0.731120153415668 | -0.405787492 | 0.578326748718918 | 1.09010021050292 | 0.666451778574792 | 1.7830524385121 | 0.998735809224318 |
| ebi-fl187-GCST90094775 | ebi-gm430-GCST90011451 | OTU99_3 (Bacteroides) abundance \|\| id:ebi-gm430-GCST90011451 | Green olives liking \|\| id:ebi-fl187-GCST90094775 | Inverse variance weighted | 20 | -0.003415481 | 0.0549886130306292 | 0.950473237337671 | -0.111193162 | 0.104362200945696 | 0.996590345524621 | 0.894765897344885 | 1.11000242604246 | 0.998735809224318 |
| ebi-fl187-GCST90094775 | ebi-gm430-GCST90011472 | OTU99_72 (Parabacteroides) abundance \|\| id:ebi-gm430-GCST90011472 | Green olives liking \|\| id:ebi-fl187-GCST90094775 | Inverse variance weighted | 20 | 0.0594357901550358 | 0.172256383125473 | 0.730062345525986 | -0.278186721 | 0.397058301080964 | 1.06123761688895 | 0.75715543166865 | 1.48744264703763 | 0.998735809224318 |
| ebi-fl187-GCST90094775 | ebi-gm430-GCST90011507 | TestASV_3 (Bacteroides) abundance \|\| id:ebi-gm430-GCST90011507 | Green olives liking \|\| id:ebi-fl187-GCST90094775 | Inverse variance weighted | 20 | -0.021327097 | 0.0707773748970947 | 0.763165548873967 | -0.160050752 | 0.117396557397231 | 0.978898717 | 0.852100541892185 | 1.12456529596569 | 0.998735809224318 |
| ebi-fl187-GCST90094775 | ebi-gm430-GCST90011512 | TestASV_34 (Phascolarctobacterium) abundance \|\| id:ebi-gm430-GCST90011512 | Green olives liking \|\| id:ebi-fl187-GCST90094775 | Inverse variance weighted | 20 | 0.0627857288126299 | 0.214367919312919 | 0.769607983749808 | -0.357375393 | 0.482946850665952 | 1.06479865911297 | 0.699509857308672 | 1.62084375595649 | 0.998735809224318 |
| ebi-fl187-GCST90094775 | ebi-gm430-GCST90011519 | TestASV_41 (Barnesiella) abundance \|\| id:ebi-gm430-GCST90011519 | Green olives liking \|\| id:ebi-fl187-GCST90094775 | Inverse variance weighted (multiplicative random effects) | 20 | 0.0402029959386647 | 0.289707490264292 | 0.889631116847131 | -0.527623685 | 0.608029676856678 | 1.04102207599845 | 0.590005343714983 | 1.8368087242946 | 0.998735809224318 |
| ebi-fl187-GCST90094775 | ebi-gm430-GCST90011524 | TestASV_46 (Sutterella) abundance \|\| id:ebi-gm430-GCST90011524 | Green olives liking \|\| id:ebi-fl187-GCST90094775 | Inverse variance weighted | 20 | -0.366132304 | 0.242060841920688 | 0.130390666338737 | -0.840571554 | 0.108306946232191 | 0.693411054131438 | 0.431463848011624 | 1.11438975063032 | 0.998735809224318 |
| ebi-fl187-GCST90094775 | ebi-gm430-GCST90011581 | OTU97_19 (Bacteria) prevalence \|\| id:ebi-gm430-GCST90011581 | Green olives liking \|\| id:ebi-fl187-GCST90094775 | Inverse variance weighted | 20 | -0.166227421 | 0.28487712408737 | 0.559552932562393 | -0.724586584 | 0.392131742393109 | 0.846853620144873 | 0.484524837861461 | 1.48013269478155 | 0.998735809224318 |
| ebi-fl187-GCST90094775 | ebi-gm430-GCST90011614 | OTU97_80 (Ruminococcus) prevalence \|\| id:ebi-gm430-GCST90011614 | Green olives liking \|\| id:ebi-fl187-GCST90094775 | Inverse variance weighted (multiplicative random effects) | 20 | -0.021153562 | 0.173532673379112 | 0.902978539688215 | -0.361277602 | 0.318970477943838 | 0.979068605410104 | 0.696785542763503 | 1.37571071049767 | 0.998735809224318 |
| ebi-fl187-GCST90094775 | ebi-gm430-GCST90011623 | OTU99_11 (Parabacteroides) prevalence \|\| id:ebi-gm430-GCST90011623 | Green olives liking \|\| id:ebi-fl187-GCST90094775 | Inverse variance weighted | 20 | 0.146364321370333 | 0.125155602002304 | 0.242218827350643 | -0.098940659 | 0.39166930129485 | 1.15761785615846 | 0.905796457700156 | 1.47944837883271 | 0.998735809224318 |
| ebi-fl187-GCST90094775 | ebi-gm430-GCST90011642 | OTU99_19 (Bacteria) prevalence \|\| id:ebi-gm430-GCST90011642 | Green olives liking \|\| id:ebi-fl187-GCST90094775 | Inverse variance weighted | 20 | -0.166227421 | 0.28487712408737 | 0.559552932562393 | -0.724586584 | 0.392131742393109 | 0.846853620144873 | 0.484524837861461 | 1.48013269478155 | 0.998735809224318 |
| ebi-fl187-GCST90094775 | ebi-gm430-GCST90011653 | OTU99_30 (Parasutterella) prevalence \|\| id:ebi-gm430-GCST90011653 | Green olives liking \|\| id:ebi-fl187-GCST90094775 | Inverse variance weighted (multiplicative random effects) | 20 | 0.0734207259853587 | 0.148243851050117 | 0.620409573627459 | -0.217137222 | 0.363978674043589 | 1.0761832199259 | 0.804819522724125 | 1.43904352485132 | 0.998735809224318 |
| ebi-fl187-GCST90094775 | ebi-gm430-GCST90011675 | OTU99_8 (Ruminococcaceae) prevalence \|\| id:ebi-gm430-GCST90011675 | Green olives liking \|\| id:ebi-fl187-GCST90094775 | Inverse variance weighted | 20 | 0.149863694097774 | 0.122860105643243 | 0.222543981402988 | -0.090942113 | 0.390669501158529 | 1.16167588865613 | 0.913070564319044 | 1.47796996532404 | 0.998735809224318 |
| ebi-fl187-GCST90094775 | ebi-gm430-GCST90011679 | OTU99_92 (Ruminococcus) prevalence \|\| id:ebi-gm430-GCST90011679 | Green olives liking \|\| id:ebi-fl187-GCST90094775 | Inverse variance weighted (multiplicative random effects) | 20 | -0.049776275 | 0.173313409966434 | 0.773956254883223 | -0.389470558 | 0.289918008626509 | 0.951442262199186 | 0.677415431453814 | 1.33631791699216 | 0.998735809224318 |
| ebi-fl187-GCST90094775 | ebi-gm430-GCST90011684 | TestASV_11 (Lachnospiraceae) prevalence \|\| id:ebi-gm430-GCST90011684 | Green olives liking \|\| id:ebi-fl187-GCST90094775 | Inverse variance weighted | 20 | -0.103607145 | 0.129417513365507 | 0.423383457553484 | -0.357265472 | 0.150051180720944 | 0.901579417411202 | 0.699586752615851 | 1.16189370776416 | 0.998735809224318 |
| ebi-fl187-GCST90094775 | ebi-gm430-GCST90011688 | TestASV_15 (Bacteroides) prevalence \|\| id:ebi-gm430-GCST90011688 | Green olives liking \|\| id:ebi-fl187-GCST90094775 | Inverse variance weighted | 20 | -0.090009909 | 0.122034720772799 | 0.460772007615763 | -0.329197961 | 0.149178144108884 | 0.913922129532248 | 0.719500569365201 | 1.16087977468271 | 0.998735809224318 |
| ebi-fl187-GCST90094775 | ebi-gm430-GCST90011692 | TestASV_19 (Prevotella) prevalence \|\| id:ebi-gm430-GCST90011692 | Green olives liking \|\| id:ebi-fl187-GCST90094775 | Inverse variance weighted | 20 | -0.234660226 | 0.29346368159898 | 0.423929422075477 | -0.809849042 | 0.340528589779358 | 0.790839510565736 | 0.4449252261365 | 1.40569042781129 | 0.998735809224318 |
| ebi-fl187-GCST90094775 | ebi-gm430-GCST90011693 | TestASV_2 (Bacteroides) prevalence \|\| id:ebi-gm430-GCST90011693 | Green olives liking \|\| id:ebi-fl187-GCST90094775 | Inverse variance weighted | 20 | 0.0047911887339924 | 0.123260902183846 | 0.968993792677447 | -0.23680018 | 0.24638255701433 | 1.00480268483139 | 0.789148960367267 | 1.27938891913955 | 0.998735809224318 |
| ebi-fl187-GCST90094775 | ebi-gm430-GCST90011716 | TestASV_40 (Barnesiella) prevalence \|\| id:ebi-gm430-GCST90011716 | Green olives liking \|\| id:ebi-fl187-GCST90094775 | Inverse variance weighted | 20 | -0.192384313 | 0.279509139386703 | 0.491267999280982 | -0.740222226 | 0.355453600531843 | 0.824989753548253 | 0.477007900249019 | 1.42682771732774 | 0.998735809224318 |
| ebi-fl187-GCST90094775 | ebi-gm430-GCST90011718 | TestASV_42 (Alphaproteobacteria) prevalence \|\| id:ebi-gm430-GCST90011718 | Green olives liking \|\| id:ebi-fl187-GCST90094775 | Inverse variance weighted | 20 | -0.155821333 | 0.192455382637637 | 0.418141864384478 | -0.533033883 | 0.221391217434808 | 0.855712064619173 | 0.586821917511704 | 1.2478115007015 | 0.998735809224318 |
| ebi-fl187-GCST90094775 | ebi-hbsm44-GCST90104929 | Sulcal depth of the right inferior frontal sulcus \|\| id:ebi-hbsm44-GCST90104929 | Green olives liking \|\| id:ebi-fl187-GCST90094775 | Inverse variance weighted | 19 | 0.2127443951215 | 0.123277418633338 | 0.0843947492716003 | -0.028879345 | 0.454368135642843 | 1.23706841010827 | 0.971533677402709 | 1.57517776983193 | 0.998735809224318 |
| ebi-fl187-GCST90094775 | ebi-hbsm44-GCST90104935 | Sulcal opening distance of the left calloso-marginal posterior fissure \|\| id:ebi-hbsm44-GCST90104935 | Green olives liking \|\| id:ebi-fl187-GCST90094775 | Inverse variance weighted | 20 | -0.012162836 | 0.0229093552560626 | 0.595480219088667 | -0.057065173 | 0.0327394998030253 | 0.987910831822596 | 0.944532509531495 | 1.03328133418776 | 0.998735809224318 |
| ebi-fl187-GCST90094775 | ebi-hbsm44-GCST90104936 | Sulcal opening distance of the left anterior inferior temporal sulcus \|\| id:ebi-hbsm44-GCST90104936 | Green olives liking \|\| id:ebi-fl187-GCST90094775 | Inverse variance weighted | 18 | -0.016993377 | 0.0158358235308973 | 0.283227526344793 | -0.048031591 | 0.0140448369067841 | 0.983150195806923 | 0.953103676784987 | 1.01414392899593 | 0.998735809224318 |
| ebi-fl187-GCST90094775 | ebi-hbsm44-GCST90104941 | Sulcal opening distance of the right posterior inferior temporal sulcus \|\| id:ebi-hbsm44-GCST90104941 | Green olives liking \|\| id:ebi-fl187-GCST90094775 | Inverse variance weighted | 19 | -0.006900495 | 0.0110762232586793 | 0.533284048286736 | -0.028609893 | 0.0148089025150374 | 0.993123258675202 | 0.971795495087082 | 1.01491909759601 | 0.998735809224318 |
| ebi-fl187-GCST90094775 | ebi-IPs91-GCST90274819 | Leukemia inhibitory factor levels \|\| id:ebi-IPs91-GCST90274819 | Green olives liking \|\| id:ebi-fl187-GCST90094775 | Inverse variance weighted | 27 | 0.0441595175809822 | 0.0386996005539905 | 0.253834726043998 | -0.0316917 | 0.120010734666804 | 1.04514906124181 | 0.968805219170635 | 1.12750895494736 | 0.998735809224318 |
| ebi-fl187-GCST90094775 | ebi-IPs91-GCST90274837 | STAM binding protein levels \|\| id:ebi-IPs91-GCST90274837 | Green olives liking \|\| id:ebi-fl187-GCST90094775 | Inverse variance weighted | 27 | -0.00833197 | 0.0343354486113134 | 0.808265859069637 | -0.075629449 | 0.0589655091241087 | 0.991702644506439 | 0.927159702468304 | 1.06073865430393 | 0.998735809224318 |
| ebi-fl187-GCST90094775 | ebi-IPs91-GCST90274840 | TNF-beta levels \|\| id:ebi-IPs91-GCST90274840 | Green olives liking \|\| id:ebi-fl187-GCST90094775 | Inverse variance weighted | 27 | 0.00959335534498211 | 0.0455604501582761 | 0.833228110625004 | -0.079705127 | 0.0988918376552033 | 1.00963951908199 | 0.92338858864161 | 1.10394688761716 | 0.998735809224318 |
| ebi-fl187-GCST90094775 | ebi-IPs91-GCST90274842 | Tumor necrosis factor ligand superfamily member 14 levels \|\| id:ebi-IPs91-GCST90274842 | Green olives liking \|\| id:ebi-fl187-GCST90094775 | Inverse variance weighted | 27 | 0.0153006037099029 | 0.04227225453338 | 0.717386545464601 | -0.067553015 | 0.0981542225953276 | 1.01541825723763 | 0.934678167175408 | 1.10313290000922 | 0.998735809224318 |
| ebi-fl187-GCST90094775 | ebi-met1400-GCST90199628 | 3-methylhistidine levels \|\| id:ebi-met1400-GCST90199628 | Green olives liking \|\| id:ebi-fl187-GCST90094775 | Inverse variance weighted | 24 | -0.031388566 | 0.0477249852700708 | 0.510733031370964 | -0.124929537 | 0.0621524049251092 | 0.969098940810526 | 0.882559087860314 | 1.06412451018659 | 0.998735809224318 |
| ebi-fl187-GCST90094775 | ebi-met1400-GCST90199651 | Docosahexaenoate DHA; 22:6n3 levels \|\| id:ebi-met1400-GCST90199651 | Green olives liking \|\| id:ebi-fl187-GCST90094775 | Inverse variance weighted | 24 | 0.0460618703595862 | 0.0457708549675036 | 0.314243344392873 | -0.043649005 | 0.135772746095893 | 1.04713919583137 | 0.957289902120779 | 1.14542156249343 | 0.998735809224318 |
| ebi-fl187-GCST90094775 | ebi-met1400-GCST90199652 | 1-stearoyl-2-oleoyl-GPS (18:0/18:1) levels \|\| id:ebi-met1400-GCST90199652 | Green olives liking \|\| id:ebi-fl187-GCST90094775 | Inverse variance weighted | 24 | 0.0217076992448264 | 0.0549831699986731 | 0.692985880895548 | -0.086059314 | 0.129474712442226 | 1.02194502550613 | 0.917539806693522 | 1.13823032803367 | 0.998735809224318 |
| ebi-fl187-GCST90094775 | ebi-met1400-GCST90199653 | 1-myristoyl-2-palmitoyl-gpc (14:0/16:0) levels \|\| id:ebi-met1400-GCST90199653 | Green olives liking \|\| id:ebi-fl187-GCST90094775 | Inverse variance weighted | 23 | 0.0081263264914772 | 0.0516823604683552 | 0.875058874633182 | -0.0931711 | 0.109423753009453 | 1.00815943470455 | 0.911037608401028 | 1.11563500388054 | 0.998735809224318 |
| ebi-fl187-GCST90094775 | ebi-met1400-GCST90199667 | Erythritol levels in elite athletes \|\| id:ebi-met1400-GCST90199667 | Green olives liking \|\| id:ebi-fl187-GCST90094775 | Inverse variance weighted | 24 | -0.022528425 | 0.0549202810285181 | 0.681657562053354 | -0.130172176 | 0.0851153258363755 | 0.977723445029441 | 0.877944257155897 | 1.08884263114497 | 0.998735809224318 |
| ebi-fl187-GCST90094775 | ebi-met1400-GCST90199674 | 3-hydroxymyristate levels \|\| id:ebi-met1400-GCST90199674 | Green olives liking \|\| id:ebi-fl187-GCST90094775 | Inverse variance weighted | 24 | 0.0388117464975653 | 0.0466707557163772 | 0.405630575565313 | -0.052662935 | 0.130286427701665 | 1.03957476163814 | 0.948699732349731 | 1.13915462204072 | 0.998735809224318 |
| ebi-fl187-GCST90094775 | ebi-met1400-GCST90199685 | 2-linoleoylglycerol (18:2) levels \|\| id:ebi-met1400-GCST90199685 | Green olives liking \|\| id:ebi-fl187-GCST90094775 | Inverse variance weighted | 24 | -0.036317287 | 0.0547415405956945 | 0.507053950783168 | -0.143610706 | 0.0709761326964727 | 0.964334274333174 | 0.866224898205309 | 1.07355560268516 | 0.998735809224318 |
| ebi-fl187-GCST90094775 | ebi-met1400-GCST90199703 | Gamma-glutamylvaline levels \|\| id:ebi-met1400-GCST90199703 | Green olives liking \|\| id:ebi-fl187-GCST90094775 | Inverse variance weighted | 24 | 0.00023558654188129 | 0.0538403414726859 | 0.996508746795894 | -0.105291483 | 0.105762655828346 | 1.00023561429457 | 0.900062131766463 | 1.11155802337746 | 0.999391239686106 |
| ebi-fl187-GCST90094775 | ebi-met1400-GCST90199779 | Gamma-glutamyl-2-aminobutyrate levels \|\| id:ebi-met1400-GCST90199779 | Green olives liking \|\| id:ebi-fl187-GCST90094775 | Inverse variance weighted | 23 | 0.00321956444916648 | 0.0505313199029998 | 0.949197768973025 | -0.095821823 | 0.102260951459046 | 1.00322475281338 | 0.908625898282224 | 1.10767248276789 | 0.998735809224318 |
| ebi-fl187-GCST90094775 | ebi-met1400-GCST90199824 | 4-vinylphenol sulfate levels \|\| id:ebi-met1400-GCST90199824 | Green olives liking \|\| id:ebi-fl187-GCST90094775 | Inverse variance weighted | 24 | -0.048517791 | 0.0518958405795066 | 0.349836168017188 | -0.150233638 | 0.0531980567358611 | 0.952640390920433 | 0.860506905535681 | 1.05463850269521 | 0.998735809224318 |
| ebi-fl187-GCST90094775 | ebi-met1400-GCST90199830 | 4-ethylphenylsulfate levels \|\| id:ebi-met1400-GCST90199830 | Green olives liking \|\| id:ebi-fl187-GCST90094775 | Inverse variance weighted | 24 | -0.035555666 | 0.0479783028223167 | 0.458646131251073 | -0.12959314 | 0.0584818075083064 | 0.965069011180548 | 0.878452765905991 | 1.06022569737195 | 0.998735809224318 |
| ebi-fl187-GCST90094775 | ebi-met1400-GCST90199833 | Hexanoylglutamine levels \|\| id:ebi-met1400-GCST90199833 | Green olives liking \|\| id:ebi-fl187-GCST90094775 | Inverse variance weighted | 24 | 0.0339463981696406 | 0.0472227741862215 | 0.472229341176993 | -0.058610239 | 0.126503035574635 | 1.03452915258577 | 0.943074270882508 | 1.13485289610151 | 0.998735809224318 |
| ebi-fl187-GCST90094775 | ebi-met1400-GCST90199837 | Carotenoid levels (cryptoxanthin) \|\| id:ebi-met1400-GCST90199837 | Green olives liking \|\| id:ebi-fl187-GCST90094775 | Inverse variance weighted | 24 | 0.102560449041814 | 0.0474588166227061 | 0.0306921716152656 | 0.00954116846130962 | 0.195579729622318 | 1.10800427768239 | 1.00958683051669 | 1.21601574253317 | 0.998735809224318 |
| ebi-fl187-GCST90094775 | ebi-met1400-GCST90199869 | 2-hydroxyhippurate levels \|\| id:ebi-met1400-GCST90199869 | Green olives liking \|\| id:ebi-fl187-GCST90094775 | Inverse variance weighted | 24 | 0.0389120979697199 | 0.0538373870610443 | 0.46982063382317 | -0.066609181 | 0.144433376609367 | 1.03967908973053 | 0.935560765128896 | 1.15538471675219 | 0.998735809224318 |
| ebi-fl187-GCST90094775 | ebi-met1400-GCST90199902 | Hydantoin-5-propionate levels \|\| id:ebi-met1400-GCST90199902 | Green olives liking \|\| id:ebi-fl187-GCST90094775 | Inverse variance weighted | 24 | 0.0377235226427823 | 0.047810060560451 | 0.430095077071242 | -0.055984196 | 0.131431241341266 | 1.03844408690888 | 0.945554079256313 | 1.14045948856163 | 0.998735809224318 |
| ebi-fl187-GCST90094775 | ebi-met1400-GCST90199904 | Pantoate levels \|\| id:ebi-met1400-GCST90199904 | Green olives liking \|\| id:ebi-fl187-GCST90094775 | Inverse variance weighted | 24 | 0.0287618837377894 | 0.0558886984269203 | 0.60681306206516 | -0.080779965 | 0.138303732654553 | 1.02917950092001 | 0.922396628494246 | 1.14832428089318 | 0.998735809224318 |
| ebi-fl187-GCST90094775 | ebi-met1400-GCST90199936 | 3-methyl catechol sulfate (2) levels \|\| id:ebi-met1400-GCST90199936 | Green olives liking \|\| id:ebi-fl187-GCST90094775 | Inverse variance weighted | 24 | 0.0497358964896452 | 0.0623129829313833 | 0.42477610044977 | -0.07239755 | 0.171869343035157 | 1.05099348864934 | 0.930161036725212 | 1.18752266497013 | 0.998735809224318 |
| ebi-fl187-GCST90094775 | ebi-met1400-GCST90199977 | 4-hydroxychlorothalonil levels \|\| id:ebi-met1400-GCST90199977 | Green olives liking \|\| id:ebi-fl187-GCST90094775 | Inverse variance weighted | 24 | 0.103255979672503 | 0.0474929117692188 | 0.0296950330023077 | 0.0101698726048337 | 0.196342086740172 | 1.10877519666425 | 1.01022176151125 | 1.21694313424683 | 0.998735809224318 |
| ebi-fl187-GCST90094775 | ebi-met1400-GCST90200040 | Sphingomyelin (d18:2/24:1, d18:1/24:2) levels \|\| id:ebi-met1400-GCST90200040 | Green olives liking \|\| id:ebi-fl187-GCST90094775 | Inverse variance weighted | 24 | 0.0758245988557139 | 0.0436454004073636 | 0.082336476045745 | -0.009720386 | 0.161369583654147 | 1.07877333948361 | 0.990326704306739 | 1.17511919341333 | 0.998735809224318 |
| ebi-fl187-GCST90094775 | ebi-met1400-GCST90200077 | 1-stearoyl-2-oleoyl-GPI (18:0/18:1) levels \|\| id:ebi-met1400-GCST90200077 | Green olives liking \|\| id:ebi-fl187-GCST90094775 | Inverse variance weighted | 24 | 0.0453632300253837 | 0.0473751123163358 | 0.33829833444609 | -0.04749199 | 0.138218450165402 | 1.04640787764757 | 0.953618111473791 | 1.14822635311598 | 0.998735809224318 |
| ebi-fl187-GCST90094775 | ebi-met1400-GCST90200080 | 1-palmitoyl-2-oleoyl-GPI (16:0/18:1) levels \|\| id:ebi-met1400-GCST90200080 | Green olives liking \|\| id:ebi-fl187-GCST90094775 | Inverse variance weighted | 24 | -0.002515646 | 0.0477702769357401 | 0.958001754187613 | -0.096145389 | 0.0911140967008973 | 0.99748751549278 | 0.908331945097404 | 1.09539397896797 | 0.998735809224318 |
| ebi-fl187-GCST90094775 | ebi-met1400-GCST90200082 | 1-oleoyl-2-linoleoyl-GPE (18:1/18:2) levels \|\| id:ebi-met1400-GCST90200082 | Green olives liking \|\| id:ebi-fl187-GCST90094775 | Inverse variance weighted | 24 | 0.0118436473300152 | 0.0507641269605081 | 0.815522699695114 | -0.087654042 | 0.111341336172611 | 1.01191406103149 | 0.916077746779857 | 1.11777637925672 | 0.998735809224318 |
| ebi-fl187-GCST90094775 | ebi-met1400-GCST90200100 | Perfluorooctanesulfonate (PFOS) levels \|\| id:ebi-met1400-GCST90200100 | Green olives liking \|\| id:ebi-fl187-GCST90094775 | Inverse variance weighted | 24 | -0.01407606 | 0.0441135530347196 | 0.749660563130072 | -0.100538624 | 0.0723865039201889 | 0.986022544508449 | 0.904350182138551 | 1.07507078284633 | 0.998735809224318 |
| ebi-fl187-GCST90094775 | ebi-met1400-GCST90200102 | 1-palmitoleoyl-2-linolenoyl-GPC (16:1/18:3) levels \|\| id:ebi-met1400-GCST90200102 | Green olives liking \|\| id:ebi-fl187-GCST90094775 | Inverse variance weighted | 24 | 0.00694851624836668 | 0.0547649700006212 | 0.899036288985326 | -0.100390825 | 0.114287857449584 | 1.00697271319923 | 0.904483854090025 | 1.12107478817073 | 0.998735809224318 |
| ebi-fl187-GCST90094775 | ebi-met1400-GCST90200114 | Glycosyl-N-tricosanoyl-sphingadienine (d18:2/23:0) levels \|\| id:ebi-met1400-GCST90200114 | Green olives liking \|\| id:ebi-fl187-GCST90094775 | Inverse variance weighted | 24 | 0.114875052064017 | 0.0500571305111008 | 0.0217400659565682 | 0.0167630762622596 | 0.212987027865775 | 1.12173327055845 | 1.01690436499903 | 1.23736859982793 | 0.998735809224318 |
| ebi-fl187-GCST90094775 | ebi-met1400-GCST90200135 | Arachidoylcarnitine (C20) levels \|\| id:ebi-met1400-GCST90200135 | Green olives liking \|\| id:ebi-fl187-GCST90094775 | Inverse variance weighted | 24 | 0.0274503410793223 | 0.059166077974468 | 0.642680672186098 | -0.088515172 | 0.143415853909279 | 1.02783057288302 | 0.915289224090789 | 1.15420968448794 | 0.998735809224318 |
| ebi-fl187-GCST90094775 | ebi-met1400-GCST90200146 | Docosahexaenoylcarnitine (C22:6) levels \|\| id:ebi-met1400-GCST90200146 | Green olives liking \|\| id:ebi-fl187-GCST90094775 | Inverse variance weighted | 24 | -0.020530047 | 0.0536956830808303 | 0.702208639864335 | -0.125773586 | 0.0847134916388739 | 0.979679259414063 | 0.881814479292792 | 1.0884051848364 | 0.998735809224318 |
| ebi-fl187-GCST90094775 | ebi-met1400-GCST90200162 | 3-hydroxyphenylacetoylglutamine levels \|\| id:ebi-met1400-GCST90200162 | Green olives liking \|\| id:ebi-fl187-GCST90094775 | Inverse variance weighted | 24 | 0.0529205352603142 | 0.0583211562288888 | 0.364196051797425 | -0.061388931 | 0.167230001468936 | 1.05434585847118 | 0.940457395649837 | 1.1820261017855 | 0.998735809224318 |
| ebi-fl187-GCST90094775 | ebi-met1400-GCST90200172 | 3-carboxy-4-methyl-5-pentyl-2-furanpropionate (3-CMPFP) levels \|\| id:ebi-met1400-GCST90200172 | Green olives liking \|\| id:ebi-fl187-GCST90094775 | Inverse variance weighted | 24 | 0.0786759779490141 | 0.0475591319646972 | 0.0980712448245738 | -0.014539921 | 0.171891876599821 | 1.08185372081063 | 0.985565273489663 | 1.18754942439039 | 0.998735809224318 |
| ebi-fl187-GCST90094775 | ebi-met1400-GCST90200222 | Dihydrocaffeate sulfate (2) levels \|\| id:ebi-met1400-GCST90200222 | Green olives liking \|\| id:ebi-fl187-GCST90094775 | Inverse variance weighted (multiplicative random effects) | 24 | 0.0546410637264499 | 0.0693844915594594 | 0.430982678726613 | -0.08135254 | 0.19063466718299 | 1.0561614519761 | 0.921868638829882 | 1.2100173122888 | 0.998735809224318 |
| ebi-fl187-GCST90094775 | ebi-met1400-GCST90200223 | 4-methylhexanoylglutamine levels \|\| id:ebi-met1400-GCST90200223 | Green olives liking \|\| id:ebi-fl187-GCST90094775 | Inverse variance weighted | 24 | 0.0301849697696114 | 0.0573649487343058 | 0.598754900688002 | -0.08225033 | 0.142620269288851 | 1.03064515452023 | 0.921041365780926 | 1.15329177819869 | 0.998735809224318 |
| ebi-fl187-GCST90094775 | ebi-met1400-GCST90200224 | 2-ketocaprylate levels \|\| id:ebi-met1400-GCST90200224 | Green olives liking \|\| id:ebi-fl187-GCST90094775 | Inverse variance weighted | 24 | -0.046668826 | 0.0509257925721029 | 0.359452712662945 | -0.146483379 | 0.0531457275502557 | 0.954403418960273 | 0.863740088152369 | 1.05458331576521 | 0.998735809224318 |
| ebi-fl187-GCST90094775 | ebi-met1400-GCST90200243 | Hydroxypalmitoyl sphingomyelin (d18:1/16:0(OH)) levels \|\| id:ebi-met1400-GCST90200243 | Green olives liking \|\| id:ebi-fl187-GCST90094775 | Inverse variance weighted | 24 | 0.0651191504985317 | 0.0467729833325486 | 0.163850172688222 | -0.026555897 | 0.156794197830327 | 1.06728618448941 | 0.973793610335149 | 1.16975485104067 | 0.998735809224318 |
| ebi-fl187-GCST90094775 | ebi-met1400-GCST90200273 | 2-methoxyhydroquinone sulfate (2) levels \|\| id:ebi-met1400-GCST90200273 | Green olives liking \|\| id:ebi-fl187-GCST90094775 | Inverse variance weighted | 24 | -0.009719478 | 0.063026657725916 | 0.877442444417006 | -0.133251727 | 0.113812771304194 | 0.990327603626905 | 0.875244741659057 | 1.12054230756801 | 0.998735809224318 |
| ebi-fl187-GCST90094775 | ebi-met1400-GCST90200276 | 2,4-di-tert-butylphenol levels \|\| id:ebi-met1400-GCST90200276 | Green olives liking \|\| id:ebi-fl187-GCST90094775 | Inverse variance weighted | 24 | -0.047914924 | 0.0476356656579567 | 0.314481774849853 | -0.141280829 | 0.045450980636001 | 0.953214879285941 | 0.868245449179195 | 1.04649970460667 | 0.998735809224318 |
| ebi-fl187-GCST90094775 | ebi-met1400-GCST90200289 | N-acetylmethionine levels \|\| id:ebi-met1400-GCST90200289 | Green olives liking \|\| id:ebi-fl187-GCST90094775 | Inverse variance weighted | 24 | 0.0731897277308804 | 0.0476515802514185 | 0.124554334229685 | -0.02020737 | 0.166586825023661 | 1.07593465219106 | 0.979995431011158 | 1.1812660948746 | 0.998735809224318 |
| ebi-fl187-GCST90094775 | ebi-met1400-GCST90200308 | 3-hydroxyisobutyrate levels \|\| id:ebi-met1400-GCST90200308 | Green olives liking \|\| id:ebi-fl187-GCST90094775 | Inverse variance weighted | 24 | 0.0996489604024502 | 0.0483513320183067 | 0.0393092259669858 | 0.00488034964656918 | 0.194417571158331 | 1.10478302740782 | 1.00489227794977 | 1.21460336041052 | 0.998735809224318 |
| ebi-fl187-GCST90094775 | ebi-met1400-GCST90200383 | Cystathionine levels \|\| id:ebi-met1400-GCST90200383 | Green olives liking \|\| id:ebi-fl187-GCST90094775 | Inverse variance weighted | 24 | -0.056594259 | 0.0527285186302223 | 0.283130239010201 | -0.159942156 | 0.0467536372934101 | 0.944977407461977 | 0.852193082021248 | 1.04786382270971 | 0.998735809224318 |
| ebi-fl187-GCST90094775 | ebi-met1400-GCST90200384 | Guanidinoacetate levels \|\| id:ebi-met1400-GCST90200384 | Green olives liking \|\| id:ebi-fl187-GCST90094775 | Inverse variance weighted | 24 | 0.00649465882763861 | 0.0471128796315333 | 0.890356478268696 | -0.085846585 | 0.0988359029054439 | 1.00651579485661 | 0.917735014508422 | 1.10388514035118 | 0.998735809224318 |
| ebi-fl187-GCST90094775 | ebi-met1400-GCST90200403 | Citrulline levels \|\| id:ebi-met1400-GCST90200403 | Green olives liking \|\| id:ebi-fl187-GCST90094775 | Inverse variance weighted | 24 | 0.0891180794389901 | 0.0479518660632107 | 0.0630994151756636 | -0.004867578 | 0.183103736922883 | 1.09320973429758 | 0.995144249414966 | 1.20093898333389 | 0.998735809224318 |
| ebi-fl187-GCST90094775 | ebi-met1400-GCST90200434 | 12,13-DiHOME levels \|\| id:ebi-met1400-GCST90200434 | Green olives liking \|\| id:ebi-fl187-GCST90094775 | Inverse variance weighted | 24 | -0.011849244 | 0.0483022020905363 | 0.806212705092881 | -0.10652156 | 0.0828230718024469 | 0.988220681538246 | 0.898955666115329 | 1.08634958566981 | 0.998735809224318 |
| ebi-fl187-GCST90094775 | ebi-met1400-GCST90200437 | Fructose levels \|\| id:ebi-met1400-GCST90200437 | Green olives liking \|\| id:ebi-fl187-GCST90094775 | Inverse variance weighted | 24 | -0.01811025 | 0.0481774651883187 | 0.706985988549659 | -0.112538081 | 0.0763175822676889 | 0.982052755563014 | 0.893563318434136 | 1.07930528795537 | 0.998735809224318 |
| ebi-fl187-GCST90094775 | ebi-met1400-GCST90200448 | N-stearoyl-sphinganine (d18:0/18:0) levels \|\| id:ebi-met1400-GCST90200448 | Green olives liking \|\| id:ebi-fl187-GCST90094775 | Inverse variance weighted | 24 | -0.007439237 | 0.0547565812807224 | 0.89193184464829 | -0.114762136 | 0.0998836623552728 | 0.992588365678371 | 0.891578192805304 | 1.10504235257267 | 0.998735809224318 |
| ebi-fl187-GCST90094775 | ebi-met1400-GCST90200468 | X-11847 levels \|\| id:ebi-met1400-GCST90200468 | Green olives liking \|\| id:ebi-fl187-GCST90094775 | Inverse variance weighted | 24 | 0.154370564048214 | 0.050800673605804 | 0.00237561638864559 | 0.054801243780838 | 0.25393988431559 | 1.16692322646347 | 1.05633064152496 | 1.28909430715189 | 0.998735809224318 |
| ebi-fl187-GCST90094775 | ebi-met1400-GCST90200476 | X-11843 levels \|\| id:ebi-met1400-GCST90200476 | Green olives liking \|\| id:ebi-fl187-GCST90094775 | Inverse variance weighted | 24 | 0.131016631510212 | 0.0597228637212331 | 0.0282538698393346 | 0.0139598186165948 | 0.248073444403829 | 1.13998674085545 | 1.01405771187815 | 1.28155405171101 | 0.998735809224318 |
| ebi-fl187-GCST90094775 | ebi-met1400-GCST90200511 | X-12906 levels \|\| id:ebi-met1400-GCST90200511 | Green olives liking \|\| id:ebi-fl187-GCST90094775 | Inverse variance weighted | 24 | 0.00457286663197606 | 0.0518695244515974 | 0.92974875257894 | -0.097091401 | 0.106237134557107 | 1.00458333814212 | 0.907473058131537 | 1.1120855591577 | 0.998735809224318 |
| ebi-fl187-GCST90094775 | ebi-met1400-GCST90200515 | X-12849 levels \|\| id:ebi-met1400-GCST90200515 | Green olives liking \|\| id:ebi-fl187-GCST90094775 | Inverse variance weighted (multiplicative random effects) | 24 | 0.0292663079278966 | 0.0646147207211569 | 0.650595108201815 | -0.097378545 | 0.155911160541364 | 1.0296987749124 | 0.907212520646697 | 1.16872236981506 | 0.998735809224318 |
| ebi-fl187-GCST90094775 | ebi-met1400-GCST90200551 | X-18886 levels \|\| id:ebi-met1400-GCST90200551 | Green olives liking \|\| id:ebi-fl187-GCST90094775 | Inverse variance weighted (multiplicative random effects) | 24 | -0.017737028 | 0.0603007233223235 | 0.768648727621514 | -0.135926446 | 0.100452389423665 | 0.982419346889571 | 0.872906835917027 | 1.10567099881742 | 0.998735809224318 |
| ebi-fl187-GCST90094775 | ebi-met1400-GCST90200564 | X-21319 levels \|\| id:ebi-met1400-GCST90200564 | Green olives liking \|\| id:ebi-fl187-GCST90094775 | Inverse variance weighted | 24 | -0.032683272 | 0.0483853218261284 | 0.499371445386847 | -0.127518503 | 0.0621519586560384 | 0.967845054557947 | 0.880277128001764 | 1.06412403530084 | 0.998735809224318 |
| ebi-fl187-GCST90094775 | ebi-met1400-GCST90200579 | X-21442 levels \|\| id:ebi-met1400-GCST90200579 | Green olives liking \|\| id:ebi-fl187-GCST90094775 | Inverse variance weighted | 24 | 0.0543715298490826 | 0.0528903035654893 | 0.303947179275567 | -0.049293465 | 0.158036524837442 | 1.05587681904566 | 0.951901738728422 | 1.17120897214376 | 0.998735809224318 |
| ebi-fl187-GCST90094775 | ebi-met1400-GCST90200581 | X-21441 levels \|\| id:ebi-met1400-GCST90200581 | Green olives liking \|\| id:ebi-fl187-GCST90094775 | Inverse variance weighted (multiplicative random effects) | 24 | 0.0122124922871872 | 0.0600426108224649 | 0.838824674509939 | -0.105471025 | 0.129896009499218 | 1.01228736927219 | 0.899900547155016 | 1.13870996214818 | 0.998735809224318 |
| ebi-fl187-GCST90094775 | ebi-met1400-GCST90200648 | X-24585 levels \|\| id:ebi-met1400-GCST90200648 | Green olives liking \|\| id:ebi-fl187-GCST90094775 | Inverse variance weighted | 24 | -0.010865662 | 0.0467946008377544 | 0.81638341208495 | -0.10258308 | 0.0808517555714411 | 0.98919315601034 | 0.902503166934503 | 1.08421015653755 | 0.998735809224318 |
| ebi-fl187-GCST90094775 | ebi-met1400-GCST90200720 | Adenosine 5'-diphosphate (ADP) to pantothenate ratio \|\| id:ebi-met1400-GCST90200720 | Green olives liking \|\| id:ebi-fl187-GCST90094775 | Inverse variance weighted | 24 | -0.015390992 | 0.0729682264659581 | 0.832943994043525 | -0.158408716 | 0.127626732226997 | 0.984726844353445 | 0.853500871618614 | 1.13612884325629 | 0.998735809224318 |
| ebi-fl187-GCST90094775 | ebi-met1400-GCST90200724 | Cholate to taurocholate ratio \|\| id:ebi-met1400-GCST90200724 | Green olives liking \|\| id:ebi-fl187-GCST90094775 | Inverse variance weighted | 24 | 0.0646568633447478 | 0.0513524890769284 | 0.208001638728527 | -0.035994015 | 0.165307741935527 | 1.06679290582388 | 0.964646066635223 | 1.17975612328549 | 0.998735809224318 |
| ebi-fl187-GCST90094775 | ebi-met1400-GCST90200726 | N-acetylputrescine to (N(1) + N(8))-acetylspermidine ratio \|\| id:ebi-met1400-GCST90200726 | Green olives liking \|\| id:ebi-fl187-GCST90094775 | Inverse variance weighted | 24 | -0.058629557 | 0.047655882172837 | 0.218596370396215 | -0.152035086 | 0.0347759722032893 | 0.943056053107963 | 0.85895814288069 | 1.03538772718417 | 0.998735809224318 |
| ebi-fl187-GCST90094775 | ebi-met1400-GCST90200744 | Arginine to glutamate ratio \|\| id:ebi-met1400-GCST90200744 | Green olives liking \|\| id:ebi-fl187-GCST90094775 | Inverse variance weighted | 24 | 0.117481220365183 | 0.0568193139571929 | 0.0386749907348344 | 0.0061153650090848 | 0.228847075721281 | 1.12466050903176 | 1.00613410202878 | 1.25714977558667 | 0.998735809224318 |
| ebi-fl187-GCST90094775 | ebi-met1400-GCST90200759 | Histidine to trans-urocanate ratio \|\| id:ebi-met1400-GCST90200759 | Green olives liking \|\| id:ebi-fl187-GCST90094775 | Inverse variance weighted | 24 | 0.0346478997238425 | 0.0482962555515394 | 0.47312519953004 | -0.060012761 | 0.12930856060486 | 1.0352551310019 | 0.941752515655696 | 1.1380412246837 | 0.998735809224318 |
| ebi-fl187-GCST90094775 | ebi-met1400-GCST90200795 | Oleoyl-linoleoyl-glycerol (18:1 to 18:2) [2] to linoleoyl-arachidonoyl-glycerol (18:2 to 20:4) [2] ratio \|\| id:ebi-met1400-GCST90200795 | Green olives liking \|\| id:ebi-fl187-GCST90094775 | Inverse variance weighted | 24 | 0.0617888849802316 | 0.0497800525964764 | 0.214517899786448 | -0.035780018 | 0.159357788069325 | 1.06373775000509 | 0.964852520221315 | 1.17275747025705 | 0.998735809224318 |
| ebi-fl187-GCST90094775 | ebi-met1400-GCST90200811 | Choline to taurocholate ratio \|\| id:ebi-met1400-GCST90200811 | Green olives liking \|\| id:ebi-fl187-GCST90094775 | Inverse variance weighted | 24 | 0.0268863185735716 | 0.05014431028974 | 0.591834949969638 | -0.07139653 | 0.125169166741462 | 1.02725101676453 | 0.931092613141245 | 1.13334016031301 | 0.998735809224318 |
| ebi-fl187-GCST90094775 | ebi-met1400-GCST90200831 | Cholate to phosphate ratio \|\| id:ebi-met1400-GCST90200831 | Green olives liking \|\| id:ebi-fl187-GCST90094775 | Inverse variance weighted | 24 | 0.0452806477901041 | 0.0514546126476775 | 0.378853112012917 | -0.055570393 | 0.146131688579552 | 1.04632146651408 | 0.945945433390446 | 1.15734858760748 | 0.998735809224318 |
| ebi-fl187-GCST90094775 | ebi-met1400-GCST90200841 | Adenosine 5'-diphosphate (ADP) to oxalate (ethanedioate) ratio \|\| id:ebi-met1400-GCST90200841 | Green olives liking \|\| id:ebi-fl187-GCST90094775 | Inverse variance weighted | 24 | -0.053296079 | 0.06950664725536 | 0.443214279330714 | -0.189529108 | 0.0829369495466576 | 0.948099258571276 | 0.827348634335792 | 1.08647330375429 | 0.998735809224318 |
| ebi-fl187-GCST90094775 | ebi-met1400-GCST90200844 | Aspartate to phosphate ratio \|\| id:ebi-met1400-GCST90200844 | Green olives liking \|\| id:ebi-fl187-GCST90094775 | Inverse variance weighted | 24 | -0.083685203 | 0.0505055106711982 | 0.0975291938531118 | -0.182676004 | 0.0153055974744127 | 0.919720735451818 | 0.833038012705323 | 1.01542332800995 | 0.998735809224318 |
| ebi-fl187-GCST90094775 | ebi-met1400-GCST90200870 | Adenosine 5'-monophosphate (AMP) to acetoacetate ratio \|\| id:ebi-met1400-GCST90200870 | Green olives liking \|\| id:ebi-fl187-GCST90094775 | Inverse variance weighted (multiplicative random effects) | 24 | 0.0119002064398025 | 0.0686118496605673 | 0.862303670318422 | -0.122579019 | 0.146379431774515 | 1.01197129560852 | 0.884635999222528 | 1.15763534836431 | 0.998735809224318 |
| ebi-fl187-GCST90094775 | ebi-met1400-GCST90200887 | Cholesterol to cortisol ratio \|\| id:ebi-met1400-GCST90200887 | Green olives liking \|\| id:ebi-fl187-GCST90094775 | Inverse variance weighted (multiplicative random effects) | 24 | -0.03475977 | 0.058818734419416 | 0.554544362969092 | -0.15004449 | 0.0805249491509784 | 0.965837411211258 | 0.860669684574258 | 1.0838558875891 | 0.998735809224318 |
| ebi-fl187-GCST90094775 | ebi-met1400-GCST90200890 | Cortisol to taurocholate ratio \|\| id:ebi-met1400-GCST90200890 | Green olives liking \|\| id:ebi-fl187-GCST90094775 | Inverse variance weighted | 24 | 0.0853148444978804 | 0.0499898218471876 | 0.0878881859526618 | -0.012665206 | 0.183294895318368 | 1.08905989724292 | 0.987414659873475 | 1.2011685748465 | 0.998735809224318 |
| ebi-fl187-GCST90094775 | ebi-met1400-GCST90200948 | Adenosine 5'-diphosphate (ADP) to mannitol to sorbitol ratio \|\| id:ebi-met1400-GCST90200948 | Green olives liking \|\| id:ebi-fl187-GCST90094775 | Inverse variance weighted | 24 | -0.149028931 | 0.0664748172662125 | 0.0249687717895585 | -0.279319572 | -0.018738289 | 0.86154419 | 0.756298172526684 | 0.981436181501986 | 0.998735809224318 |
| ebi-fl187-GCST90094775 | ebi-met1400-GCST90200972 | Leucine to N-palmitoyl-sphingosine (d18:1 to 16:0) ratio \|\| id:ebi-met1400-GCST90200972 | Green olives liking \|\| id:ebi-fl187-GCST90094775 | Inverse variance weighted | 24 | -0.020193316 | 0.0504113942768872 | 0.68873639275266 | -0.118999649 | 0.078613017030949 | 0.980009203777673 | 0.887808112795863 | 1.08178560845139 | 0.998735809224318 |
| ebi-fl187-GCST90094775 | ebi-met1400-GCST90200973 | Proline to glutamate ratio \|\| id:ebi-met1400-GCST90200973 | Green olives liking \|\| id:ebi-fl187-GCST90094775 | Inverse variance weighted | 24 | 0.0701333119004992 | 0.0469388761088602 | 0.135138699445084 | -0.021866885 | 0.162133509073865 | 1.07265116888893 | 0.978370461900533 | 1.17601723981294 | 0.998735809224318 |
| ebi-fl187-GCST90094775 | ebi-met1400-GCST90200983 | Cholesterol to linoleoyl-arachidonoyl-glycerol (18:2 to 20:4) [1] ratio \|\| id:ebi-met1400-GCST90200983 | Green olives liking \|\| id:ebi-fl187-GCST90094775 | Inverse variance weighted | 24 | 0.0964033398900499 | 0.0493754767528167 | 0.0508843445238849 | -0.000372595 | 0.193179274325571 | 1.10120313358329 | 0.999627474859257 | 1.21310025175568 | 0.998735809224318 |
| ebi-fl187-GCST90094775 | ebi-met1400-GCST90201001 | Serine to alpha-ketobutyrate ratio \|\| id:ebi-met1400-GCST90201001 | Green olives liking \|\| id:ebi-fl187-GCST90094775 | Inverse variance weighted | 24 | -0.023941893 | 0.0487396165931912 | 0.623270581800128 | -0.119471542 | 0.0715877552411123 | 0.976342440164973 | 0.887389260957061 | 1.07421241433462 | 0.998735809224318 |
| ebi-fl187-GCST90094775 | ebi-met1400-GCST90201005 | Fructose to maltose ratio \|\| id:ebi-met1400-GCST90201005 | Green olives liking \|\| id:ebi-fl187-GCST90094775 | Inverse variance weighted | 24 | -0.075199055 | 0.0548588942995601 | 0.170445936424971 | -0.182722488 | 0.0323243779948719 | 0.927558832883186 | 0.832999291247158 | 1.03285248559034 | 0.998735809224318 |
| ebi-fl187-GCST90094775 | ebi-met1400-GCST90201020 | Paraxanthine to 5-acetylamino-6-formylamino-3-methyluracil ratio \|\| id:ebi-met1400-GCST90201020 | Green olives liking \|\| id:ebi-fl187-GCST90094775 | Inverse variance weighted | 24 | -0.008694124 | 0.0506373347405074 | 0.863678145123796 | -0.1079433 | 0.0905550522487399 | 0.991343560761424 | 0.897678493417269 | 1.09478177618133 | 0.998735809224318 |
| ebi-fl187-GCST90094775 | ebi-pw11-GCST90275197 | Placental weight (fetal genotype, adjusted for sex) \|\| id:ebi-pw11-GCST90275197 | Green olives liking \|\| id:ebi-fl187-GCST90094775 | Inverse variance weighted | 23 | 0.0160351622071491 | 0.018112145842358 | 0.375980575626158 | -0.019464644 | 0.0515349680581708 | 1.01616441536159 | 0.980723569387468 | 1.05288600302743 | 0.998735809224318 |
| ebi-fl187-GCST90094775 | prot-a-1017 | FAS-associated factor 2 \|\| id:prot-a-1017 | Green olives liking \|\| id:ebi-fl187-GCST90094775 | Inverse variance weighted | 27 | -0.087820521 | 0.0714225834518848 | 0.218850538064486 | -0.227808784 | 0.0521677427677375 | 0.915925251505229 | 0.796276505797852 | 1.05355245349646 | 0.998735809224318 |
| ebi-fl187-GCST90094775 | prot-a-1019 | Fas apoptotic inhibitory molecule 3 \|\| id:prot-a-1019 | Green olives liking \|\| id:ebi-fl187-GCST90094775 | Inverse variance weighted | 27 | -0.062536796 | 0.0714511668686741 | 0.381444272438909 | -0.202581083 | 0.0775074911607353 | 0.939378496898551 | 0.81662026592162 | 1.08059032730407 | 0.998735809224318 |
| ebi-fl187-GCST90094775 | prot-a-1052 | MIP18 family protein FAM96A \|\| id:prot-a-1052 | Green olives liking \|\| id:ebi-fl187-GCST90094775 | Inverse variance weighted | 27 | -0.010166191 | 0.0714464337374087 | 0.886850087542034 | -0.150201201 | 0.129868819506506 | 0.989885310426134 | 0.860534818760027 | 1.13867900105353 | 0.998735809224318 |
| ebi-fl187-GCST90094775 | prot-a-1066 | F-box/LRR-repeat protein 4 \|\| id:prot-a-1066 | Green olives liking \|\| id:ebi-fl187-GCST90094775 | Inverse variance weighted | 27 | -0.062056886 | 0.0714423403146479 | 0.38505010443404 | -0.202083873 | 0.077970100640174 | 0.939829421780021 | 0.817026398291974 | 1.08109033427794 | 0.998735809224318 |
| ebi-fl187-GCST90094775 | prot-a-1076 | Low affinity immunoglobulin gamma Fc region receptor III-B \|\| id:prot-a-1076 | Green olives liking \|\| id:ebi-fl187-GCST90094775 | Inverse variance weighted | 27 | 0.00186182499640145 | 0.0793794467277265 | 0.981287533634843 | -0.153721891 | 0.157445540582745 | 1.0018635592687 | 0.857510469583339 | 1.1705170105716 | 0.998955228396052 |
| ebi-fl187-GCST90094775 | prot-a-1081 | Fc receptor-like protein 3 \|\| id:prot-a-1081 | Green olives liking \|\| id:ebi-fl187-GCST90094775 | Inverse variance weighted | 27 | 0.0243129668600077 | 0.0815420521843684 | 0.765577412498616 | -0.135509455 | 0.18413538914137 | 1.02461093698411 | 0.873270905745071 | 1.20217857400361 | 0.998735809224318 |
| ebi-fl187-GCST90094775 | prot-a-1105 | Fibroblast growth factor receptor 4 \|\| id:prot-a-1105 | Green olives liking \|\| id:ebi-fl187-GCST90094775 | Inverse variance weighted (multiplicative random effects) | 27 | 0.0697389571016307 | 0.0942535257335418 | 0.459355695065883 | -0.114997953 | 0.254475867539373 | 1.07222824714905 | 0.891367968235597 | 1.28978542527171 | 0.998735809224318 |
| ebi-fl187-GCST90094775 | prot-a-1143 | Follistatin-related protein 1 \|\| id:prot-a-1143 | Green olives liking \|\| id:ebi-fl187-GCST90094775 | Inverse variance weighted (multiplicative random effects) | 27 | -0.053028701 | 0.0944857456085743 | 0.574637395134615 | -0.238220762 | 0.132163360761218 | 0.948352793767554 | 0.788028705077512 | 1.14129474681796 | 0.998735809224318 |
| ebi-fl187-GCST90094775 | prot-a-1151 | Alpha-(1,3)-fucosyltransferase 10 \|\| id:prot-a-1151 | Green olives liking \|\| id:ebi-fl187-GCST90094775 | Inverse variance weighted | 27 | -0.105915658 | 0.0714441892320193 | 0.13820861728622 | -0.245946268 | 0.0341149533511365 | 0.899500510959256 | 0.781964239956445 | 1.0347035425316 | 0.998735809224318 |
| ebi-fl187-GCST90094775 | prot-a-1165 | Growth arrest and DNA damage-inducible proteins-interacting protein 1 \|\| id:prot-a-1165 | Green olives liking \|\| id:ebi-fl187-GCST90094775 | Inverse variance weighted | 27 | 0.122302944403645 | 0.0714342945944342 | 0.0868774969068996 | -0.017708273 | 0.262314161808736 | 1.13009640630995 | 0.982447597045663 | 1.29993486817527 | 0.998735809224318 |
| ebi-fl187-GCST90094775 | prot-a-1194 | Growth/differentiation factor 11/8 \|\| id:prot-a-1194 | Green olives liking \|\| id:ebi-fl187-GCST90094775 | Inverse variance weighted (multiplicative random effects) | 26 | -0.15839774 | 0.0897812921018913 | 0.0776878456972755 | -0.334369072 | 0.0175735930188814 | 0.853510239711582 | 0.715789555573324 | 1.01772891713827 | 0.998735809224318 |
| ebi-fl187-GCST90094775 | prot-a-1216 | D-glucuronyl C5-epimerase \|\| id:prot-a-1216 | Green olives liking \|\| id:ebi-fl187-GCST90094775 | Inverse variance weighted | 27 | 0.0682532425361832 | 0.0742137684534062 | 0.35773772522608 | -0.077205744 | 0.213712228704859 | 1.07063640482925 | 0.925699377260046 | 1.23826626602958 | 0.998735809224318 |
| ebi-fl187-GCST90094775 | prot-a-1246 | Glypican-3 \|\| id:prot-a-1246 | Green olives liking \|\| id:ebi-fl187-GCST90094775 | Inverse variance weighted | 27 | 0.0410819759683072 | 0.0714342945944342 | 0.565222596780617 | -0.098929241 | 0.181093193373398 | 1.04193751588141 | 0.90580679934369 | 1.19852686885077 | 0.998735809224318 |
| ebi-fl187-GCST90094775 | prot-a-1275 | Glutamate receptor 4 \|\| id:prot-a-1275 | Green olives liking \|\| id:ebi-fl187-GCST90094775 | Inverse variance weighted | 26 | 0.0434986149185968 | 0.0729115138245525 | 0.550778082048814 | -0.099407952 | 0.18640518201472 | 1.04445854765091 | 0.90537328367239 | 1.2049103694955 | 0.998735809224318 |
| ebi-fl187-GCST90094775 | prot-a-1289 | Glutathione S-transferase P \|\| id:prot-a-1289 | Green olives liking \|\| id:ebi-fl187-GCST90094775 | Inverse variance weighted | 27 | -0.00736183 | 0.0739075156204886 | 0.920654998098805 | -0.152220561 | 0.137496900137283 | 0.992665201419698 | 0.858798842237453 | 1.14739814918982 | 0.998735809224318 |
| ebi-fl187-GCST90094775 | prot-a-130 | Apolipoprotein D \|\| id:prot-a-130 | Green olives liking \|\| id:ebi-fl187-GCST90094775 | Inverse variance weighted | 27 | 0.0783533880245877 | 0.0714411401806875 | 0.272748749255638 | -0.061671247 | 0.218378022778735 | 1.08150478198569 | 0.940191927160185 | 1.24405726072422 | 0.998735809224318 |
| ebi-fl187-GCST90094775 | prot-a-1313 | Heparin-binding EGF-like growth factor \|\| id:prot-a-1313 | Green olives liking \|\| id:ebi-fl187-GCST90094775 | Inverse variance weighted | 27 | 0.114258036066507 | 0.0714291023121204 | 0.10968721170044 | -0.025743004 | 0.254259076598263 | 1.1210413566685 | 0.974585521554698 | 1.28950584178221 | 0.998735809224318 |
| ebi-fl187-GCST90094775 | prot-a-1347 | HLA class II histocompatibility antigen, DQ alpha 2 chain \|\| id:prot-a-1347 | Green olives liking \|\| id:ebi-fl187-GCST90094775 | Inverse variance weighted | 26 | -0.039547994 | 0.0734109584876373 | 0.590079669867193 | -0.183433473 | 0.104337484364276 | 0.961223819646549 | 0.832407251530297 | 1.10997499091618 | 0.998735809224318 |
| ebi-fl187-GCST90094775 | prot-a-1357 | Non-histone chromosomal protein HMG-14 \|\| id:prot-a-1357 | Green olives liking \|\| id:ebi-fl187-GCST90094775 | Inverse variance weighted | 27 | 0.041031957214707 | 0.0748304787181267 | 0.583463856318442 | -0.105635781 | 0.187699695502235 | 1.04188540076892 | 0.899752295220348 | 1.20647115222925 | 0.998735809224318 |
| ebi-fl187-GCST90094775 | prot-a-137 | Apolipoprotein M \|\| id:prot-a-137 | Green olives liking \|\| id:ebi-fl187-GCST90094775 | Inverse variance weighted | 27 | -0.095395427 | 0.0714345809133229 | 0.181737736117002 | -0.235407206 | 0.0446163514557532 | 0.909013414821287 | 0.790248990189116 | 1.04562662981361 | 0.998735809224318 |
| ebi-fl187-GCST90094775 | prot-a-1381 | 17-beta-hydroxysteroid dehydrogenase 14 \|\| id:prot-a-1381 | Green olives liking \|\| id:ebi-fl187-GCST90094775 | Inverse variance weighted | 26 | -0.116790823 | 0.0729357376200783 | 0.10931405156797 | -0.259744869 | 0.02616322277755 | 0.889771293426172 | 0.771248330299069 | 1.02650848436623 | 0.998735809224318 |
| ebi-fl187-GCST90094775 | prot-a-1392 | Serine protease HTRA2, mitochondrial \|\| id:prot-a-1392 | Green olives liking \|\| id:ebi-fl187-GCST90094775 | Inverse variance weighted (multiplicative random effects) | 27 | -0.07596593 | 0.0891050593979576 | 0.393912564868108 | -0.250611846 | 0.0986799866386344 | 0.926847783928097 | 0.778324422515226 | 1.10371304011807 | 0.998735809224318 |
| ebi-fl187-GCST90094775 | prot-a-1416 | Interferon-induced protein with tetratricopeptide repeats 2 \|\| id:prot-a-1416 | Green olives liking \|\| id:ebi-fl187-GCST90094775 | Inverse variance weighted | 27 | -0.034195587 | 0.0779892346829533 | 0.661048891031143 | -0.187054487 | 0.118663313018666 | 0.9663824743419 | 0.829398543766756 | 1.12599074803513 | 0.998735809224318 |
| ebi-fl187-GCST90094775 | prot-a-1428 | Interferon gamma \|\| id:prot-a-1428 | Green olives liking \|\| id:ebi-fl187-GCST90094775 | Inverse variance weighted | 27 | -0.105178227 | 0.0714379526000722 | 0.140939394148726 | -0.245196614 | 0.0348401600194241 | 0.900164074677014 | 0.782550662564054 | 1.03545418859421 | 0.998735809224318 |
| ebi-fl187-GCST90094775 | prot-a-1483 | Interleukin-17C \|\| id:prot-a-1483 | Green olives liking \|\| id:ebi-fl187-GCST90094775 | Inverse variance weighted | 27 | -0.033313382 | 0.0714553766194466 | 0.641063476374079 | -0.173365921 | 0.106739155742546 | 0.967235397507907 | 0.840829881565543 | 1.1126439898287 | 0.998735809224318 |
| ebi-fl187-GCST90094775 | prot-a-1503 | Interleukin-1 receptor-like 2 \|\| id:prot-a-1503 | Green olives liking \|\| id:ebi-fl187-GCST90094775 | Inverse variance weighted | 27 | 0.00835862389224666 | 0.0714373018070063 | 0.906854955430257 | -0.131658488 | 0.148375735433979 | 1.00839365472409 | 0.876640327462398 | 1.15994864830289 | 0.998735809224318 |
| ebi-fl187-GCST90094775 | prot-a-1522 | Interleukin-31 receptor subunit alpha \|\| id:prot-a-1522 | Green olives liking \|\| id:ebi-fl187-GCST90094775 | Inverse variance weighted | 27 | -0.169895757 | 0.0769656584195015 | 0.0272845277569535 | -0.320748448 | -0.019043067 | 0.843752767212401 | 0.725605755871933 | 0.981137107054916 | 0.998735809224318 |
| ebi-fl187-GCST90094775 | prot-a-1540 | Interleukin-6 receptor subunit alpha \|\| id:prot-a-1540 | Green olives liking \|\| id:ebi-fl187-GCST90094775 | Inverse variance weighted | 27 | -0.027529244 | 0.0714464910621481 | 0.700005719433869 | -0.167564366 | 0.112505878834292 | 0.97284623256515 | 0.845722179673701 | 1.11907883577248 | 0.998735809224318 |
| ebi-fl187-GCST90094775 | prot-a-1541 | Interleukin-6 receptor subunit alpha \|\| id:prot-a-1541 | Green olives liking \|\| id:ebi-fl187-GCST90094775 | Inverse variance weighted | 27 | -0.013188871 | 0.0714373018070063 | 0.853525821311496 | -0.153205982 | 0.126828240622648 | 0.986897721137127 | 0.857952980343041 | 1.13522201600865 | 0.998735809224318 |
| ebi-fl187-GCST90094775 | prot-a-1545 | Interleukin-9 \|\| id:prot-a-1545 | Green olives liking \|\| id:ebi-fl187-GCST90094775 | Inverse variance weighted | 27 | 0.134079961211616 | 0.0838269936915608 | 0.109713032008279 | -0.030220946 | 0.298380868847076 | 1.14348425037527 | 0.970231140763868 | 1.34767497755931 | 0.998735809224318 |
| ebi-fl187-GCST90094775 | prot-a-1548 | Inositol monophosphatase 3 \|\| id:prot-a-1548 | Green olives liking \|\| id:ebi-fl187-GCST90094775 | Inverse variance weighted | 27 | -0.121576037 | 0.0762291095566073 | 0.110739793725715 | -0.270985091 | 0.027833018173201 | 0.88552371861358 | 0.762627866118997 | 1.02822397536791 | 0.998735809224318 |
| ebi-fl187-GCST90094775 | prot-a-1555 | Inhibin beta A chain:Inhibin beta B chain heterodimer \|\| id:prot-a-1555 | Green olives liking \|\| id:ebi-fl187-GCST90094775 | Inverse variance weighted | 27 | 0.00583087644730636 | 0.075312883049267 | 0.938287814321579 | -0.141782374 | 0.15344412722387 | 1.00584790909638 | 0.867810093690974 | 1.16584264643715 | 0.998735809224318 |
| ebi-fl187-GCST90094775 | prot-a-1603 | Junctophilin-3 \|\| id:prot-a-1603 | Green olives liking \|\| id:ebi-fl187-GCST90094775 | Inverse variance weighted | 27 | 0.122388312617689 | 0.0714553766194466 | 0.0867505157585378 | -0.017664226 | 0.262440850791804 | 1.13019288473989 | 0.982490872305254 | 1.30009956603426 | 0.998735809224318 |
| ebi-fl187-GCST90094775 | prot-a-1627 | UPF0258 protein KIAA1024 \|\| id:prot-a-1627 | Green olives liking \|\| id:ebi-fl187-GCST90094775 | Inverse variance weighted | 27 | 0.141633589559344 | 0.0726779986794841 | 0.0513214413376138 | -0.000815288 | 0.284082466971132 | 1.15215440978039 | 0.999185044404395 | 1.32854248711028 | 0.998735809224318 |
| ebi-fl187-GCST90094775 | prot-a-1666 | Kallikrein-7 \|\| id:prot-a-1666 | Green olives liking \|\| id:ebi-fl187-GCST90094775 | Inverse variance weighted | 27 | 0.0230740638908002 | 0.0714494044048378 | 0.746738634108744 | -0.116966769 | 0.163114896524282 | 1.02334232945508 | 0.889614755689069 | 1.17717193488252 | 0.998735809224318 |
| ebi-fl187-GCST90094775 | prot-a-1678 | Importin subunit alpha-3 \|\| id:prot-a-1678 | Green olives liking \|\| id:ebi-fl187-GCST90094775 | Inverse variance weighted | 27 | 0.0877486047115773 | 0.0823958516676331 | 0.286892281869037 | -0.073747265 | 0.249244473980138 | 1.09171363586302 | 0.928906431755293 | 1.28305566845641 | 0.998735809224318 |
| ebi-fl187-GCST90094775 | prot-a-1718 | Protein LDOC1 \|\| id:prot-a-1718 | Green olives liking \|\| id:ebi-fl187-GCST90094775 | Inverse variance weighted | 27 | 0.0591284711790589 | 0.0714329393641894 | 0.407813241230697 | -0.08088009 | 0.19913703233287 | 1.06091152854039 | 0.922304278343539 | 1.22034918173791 | 0.998735809224318 |
| ebi-fl187-GCST90094775 | prot-a-1723 | Leptin \|\| id:prot-a-1723 | Green olives liking \|\| id:ebi-fl187-GCST90094775 | Inverse variance weighted (multiplicative random effects) | 27 | -0.14599159 | 0.0889602117348052 | 0.100779022501286 | -0.320353605 | 0.0283704246737591 | 0.864164970499372 | 0.725892312298519 | 1.0287766981214 | 0.998735809224318 |
| ebi-fl187-GCST90094775 | prot-a-1730 | Galectin-7 \|\| id:prot-a-1730 | Green olives liking \|\| id:ebi-fl187-GCST90094775 | Inverse variance weighted (multiplicative random effects) | 27 | -0.067279858 | 0.0976134575568471 | 0.49066736274644 | -0.258602234 | 0.124042519303528 | 0.934933516557271 | 0.7721300888199 | 1.13206400454889 | 0.998735809224318 |
| ebi-fl187-GCST90094775 | prot-a-1753 | Lipase member N \|\| id:prot-a-1753 | Green olives liking \|\| id:ebi-fl187-GCST90094775 | Inverse variance weighted | 27 | 0.127372401973307 | 0.0714477473419706 | 0.0746294462505635 | -0.012665183 | 0.26740998676357 | 1.13583992806086 | 0.987414683083301 | 1.30657601540695 | 0.998735809224318 |
| ebi-fl187-GCST90094775 | prot-a-1756 | VIP36-like protein \|\| id:prot-a-1756 | Green olives liking \|\| id:ebi-fl187-GCST90094775 | Inverse variance weighted | 27 | -0.109641969 | 0.0717487751686437 | 0.12647849005312 | -0.250269568 | 0.0309856307115772 | 0.896154929452928 | 0.778590871635208 | 1.0314706822803 | 0.998735809224318 |
| ebi-fl187-GCST90094775 | prot-a-1760 | Ig Kappa chain V-I region HK102- like \|\| id:prot-a-1760 | Green olives liking \|\| id:ebi-fl187-GCST90094775 | Inverse variance weighted | 27 | -0.06219842 | 0.0847137561189761 | 0.462815487131701 | -0.228237382 | 0.103840542125467 | 0.939696413853594 | 0.795935296806216 | 1.10942353449151 | 0.998735809224318 |
| ebi-fl187-GCST90094775 | prot-a-179 | Neutral ceramidase \|\| id:prot-a-179 | Green olives liking \|\| id:ebi-fl187-GCST90094775 | Inverse variance weighted | 27 | 0.0258219020369033 | 0.0714464910621481 | 0.717788520075706 | -0.11421322 | 0.165857024518713 | 1.02615817551731 | 0.892067728524985 | 1.18040432078192 | 0.998735809224318 |
| ebi-fl187-GCST90094775 | prot-a-1809 | Lactotransferrin \|\| id:prot-a-1809 | Green olives liking \|\| id:ebi-fl187-GCST90094775 | Inverse variance weighted | 27 | 0.000866343183652089 | 0.076806265959058 | 0.991000380183101 | -0.149673938 | 0.151406624463406 | 1.0008667185673 | 0.860988666265197 | 1.16346965713383 | 0.999107451094609 |
| ebi-fl187-GCST90094775 | prot-a-1818 | Lysophospholipase-like protein 1 \|\| id:prot-a-1818 | Green olives liking \|\| id:ebi-fl187-GCST90094775 | Inverse variance weighted | 27 | -0.056673674 | 0.0767362182049678 | 0.460179185956112 | -0.207076662 | 0.0937293136036411 | 0.944902365196759 | 0.812957324859432 | 1.09826242098109 | 0.998735809224318 |
| ebi-fl187-GCST90094775 | prot-a-1852 | MAP kinase-activated protein kinase 3 \|\| id:prot-a-1852 | Green olives liking \|\| id:ebi-fl187-GCST90094775 | Inverse variance weighted | 27 | 0.185351325745913 | 0.0818338705162588 | 0.0235144725222738 | 0.0249569395340459 | 0.345745711957781 | 1.20364123600886 | 1.02527097092815 | 1.41304325012677 | 0.998735809224318 |
| ebi-fl187-GCST90094775 | prot-a-1856 | Megakaryocyte-associated tyrosine-protein kinase \|\| id:prot-a-1856 | Green olives liking \|\| id:ebi-fl187-GCST90094775 | Inverse variance weighted | 27 | 0.107119539954502 | 0.071466267776965 | 0.133904110360522 | -0.032954345 | 0.247193424797353 | 1.11306730254159 | 0.967582733678375 | 1.28042675511305 | 0.998735809224318 |
| ebi-fl187-GCST90094775 | prot-a-1890 | Beta-1,3-N-acetylglucosaminyltransferase manic fringe \|\| id:prot-a-1890 | Green olives liking \|\| id:ebi-fl187-GCST90094775 | Inverse variance weighted | 27 | 0.0331361577091335 | 0.0714586350107893 | 0.64285478893927 | -0.106922767 | 0.17319508233028 | 1.03369127469589 | 0.898595071582565 | 1.18909805447808 | 0.998735809224318 |
| ebi-fl187-GCST90094775 | prot-a-1919 | Matrilysin \|\| id:prot-a-1919 | Green olives liking \|\| id:ebi-fl187-GCST90094775 | Inverse variance weighted | 27 | 0.0277899822619368 | 0.0779495885108671 | 0.721456988689637 | -0.124991211 | 0.180571175743236 | 1.02817972576432 | 0.882504658690369 | 1.19790137996733 | 0.998735809224318 |
| ebi-fl187-GCST90094775 | prot-a-1923 | Multimerin-2 \|\| id:prot-a-1923 | Green olives liking \|\| id:ebi-fl187-GCST90094775 | Inverse variance weighted | 27 | -0.120544374 | 0.0785800547932317 | 0.125021606379954 | -0.274561282 | 0.0334725331460778 | 0.886437751463433 | 0.759905434568152 | 1.03403904153692 | 0.998735809224318 |
| ebi-fl187-GCST90094775 | prot-a-1948 | Mesothelin \|\| id:prot-a-1948 | Green olives liking \|\| id:ebi-fl187-GCST90094775 | Inverse variance weighted | 27 | 0.102077178778806 | 0.0792827496167497 | 0.197917263750791 | -0.05331701 | 0.257471368027635 | 1.10746894153014 | 0.948079413737772 | 1.29365477056241 | 0.998735809224318 |
| ebi-fl187-GCST90094775 | prot-a-195 | ATPase family AAA domain-containing protein 1 \|\| id:prot-a-195 | Green olives liking \|\| id:ebi-fl187-GCST90094775 | Inverse variance weighted | 27 | 0.0130615031628845 | 0.0714427822952702 | 0.854935590262762 | -0.12696635 | 0.153089356461614 | 1.01314717719953 | 0.880763309664235 | 1.16542911291194 | 0.998735809224318 |
| ebi-fl187-GCST90094775 | prot-a-1966 | Mucin-1 \|\| id:prot-a-1966 | Green olives liking \|\| id:ebi-fl187-GCST90094775 | Inverse variance weighted | 27 | -0.000637696 | 0.0725294030251816 | 0.992984899687792 | -0.142795326 | 0.141519934179675 | 0.99936250753504 | 0.866931489352069 | 1.15202346867473 | 0.999107451094609 |
| ebi-fl187-GCST90094775 | prot-a-1986 | Myocardial zonula adherens protein \|\| id:prot-a-1986 | Green olives liking \|\| id:ebi-fl187-GCST90094775 | Inverse variance weighted | 27 | -0.115953308 | 0.0714392959339513 | 0.104567471492886 | -0.255974328 | 0.0240677122470399 | 0.890516802530088 | 0.774161842963929 | 1.02435967723788 | 0.998735809224318 |
| ebi-fl187-GCST90094775 | prot-a-2071 | Epididymal secretory protein E1 \|\| id:prot-a-2071 | Green olives liking \|\| id:ebi-fl187-GCST90094775 | Inverse variance weighted | 27 | -0.030071301 | 0.0821761983832295 | 0.714412184526873 | -0.19113665 | 0.130994048135046 | 0.970376342573167 | 0.826019705236481 | 1.1399609963979 | 0.998735809224318 |
| ebi-fl187-GCST90094775 | prot-a-2098 | Neuritin \|\| id:prot-a-2098 | Green olives liking \|\| id:ebi-fl187-GCST90094775 | Inverse variance weighted | 27 | -0.004707739 | 0.0735171194169784 | 0.948941598144425 | -0.148801293 | 0.139385815157223 | 0.995303325133717 | 0.861740331760268 | 1.14956753503539 | 0.998735809224318 |
| ebi-fl187-GCST90094775 | prot-a-2115 | Neurotrophin-4 \|\| id:prot-a-2115 | Green olives liking \|\| id:ebi-fl187-GCST90094775 | Inverse variance weighted (multiplicative random effects) | 27 | 0.0681446905042126 | 0.087859040570891 | 0.43797700820142 | -0.104059029 | 0.240348410023159 | 1.07052019137972 | 0.90117210054994 | 1.27169214343445 | 0.998735809224318 |
| ebi-fl187-GCST90094775 | prot-a-2118 | Netrin-1 \|\| id:prot-a-2118 | Green olives liking \|\| id:ebi-fl187-GCST90094775 | Inverse variance weighted | 27 | -0.011470424 | 0.0829467237128956 | 0.890013876614273 | -0.174046003 | 0.151105154001802 | 0.988595110034473 | 0.840258242409088 | 1.16311895826456 | 0.998735809224318 |
| ebi-fl187-GCST90094775 | prot-a-2120 | Netrin-G1 \|\| id:prot-a-2120 | Green olives liking \|\| id:ebi-fl187-GCST90094775 | Inverse variance weighted | 27 | -0.131890202 | 0.0714586350107893 | 0.0649378119025457 | -0.271949126 | 0.00816872276507538 | 0.876437220976697 | 0.761893020285845 | 1.00820217781384 | 0.998735809224318 |
| ebi-fl187-GCST90094775 | prot-a-2145 | Olfactomedin-like protein 3 \|\| id:prot-a-2145 | Green olives liking \|\| id:ebi-fl187-GCST90094775 | Inverse variance weighted | 27 | -0.006659821 | 0.0714533811456273 | 0.9257406063614 | -0.146708448 | 0.133388806030522 | 0.993362306444179 | 0.863545709144589 | 1.14269419836684 | 0.998735809224318 |
| ebi-fl187-GCST90094775 | prot-a-2162 | Prolyl 4-hydroxylase subunit alpha-2 \|\| id:prot-a-2162 | Green olives liking \|\| id:ebi-fl187-GCST90094775 | Inverse variance weighted | 27 | -0.038083103 | 0.0714372651608408 | 0.593965358647989 | -0.178100143 | 0.101933936818344 | 0.962632939981124 | 0.836858614084775 | 1.10731031686893 | 0.998735809224318 |
| ebi-fl187-GCST90094775 | prot-a-2168 | Polyadenylate-binding protein-interacting protein 1 \|\| id:prot-a-2168 | Green olives liking \|\| id:ebi-fl187-GCST90094775 | Inverse variance weighted | 27 | 0.0216626893663107 | 0.0807864041713347 | 0.788585621943179 | -0.136678663 | 0.180004041542127 | 1.02189902891984 | 0.872250467618364 | 1.197222202 | 0.998735809224318 |
| ebi-fl187-GCST90094775 | prot-a-219 | Beta-1,4-galactosyltransferase 5 \|\| id:prot-a-219 | Green olives liking \|\| id:ebi-fl187-GCST90094775 | Inverse variance weighted (multiplicative random effects) | 27 | 0.0737945591505555 | 0.0875119357632829 | 0.39908787765925 | -0.097728835 | 0.24531795324659 | 1.07658560811368 | 0.90689478858952 | 1.27802760163627 | 0.998735809224318 |
| ebi-fl187-GCST90094775 | prot-a-2190 | Pyruvate carboxylase, mitochondrial \|\| id:prot-a-2190 | Green olives liking \|\| id:ebi-fl187-GCST90094775 | Inverse variance weighted | 27 | -0.005468777 | 0.0714593739652894 | 0.938997543761143 | -0.14552915 | 0.134591595737352 | 0.994546149305236 | 0.864564687467381 | 1.14406944608779 | 0.998735809224318 |
| ebi-fl187-GCST90094775 | prot-a-2197 | Protocadherin-9 \|\| id:prot-a-2197 | Green olives liking \|\| id:ebi-fl187-GCST90094775 | Inverse variance weighted (multiplicative random effects) | 27 | -0.141468188 | 0.088357236532854 | 0.109356234558065 | -0.314648372 | 0.0317119956454821 | 0.868082790631048 | 0.730045533911803 | 1.03222017858522 | 0.998735809224318 |
| ebi-fl187-GCST90094775 | prot-a-2198 | Protocadherin alpha-4 \|\| id:prot-a-2198 | Green olives liking \|\| id:ebi-fl187-GCST90094775 | Inverse variance weighted | 27 | 0.0152429272382662 | 0.079084918085192 | 0.847161603703911 | -0.139763512 | 0.170249366685243 | 1.01535969318422 | 0.869563852319723 | 1.18560046371855 | 0.998735809224318 |
| ebi-fl187-GCST90094775 | prot-a-2254 | Placenta growth factor \|\| id:prot-a-2254 | Green olives liking \|\| id:ebi-fl187-GCST90094775 | Inverse variance weighted | 27 | -0.019626979 | 0.076602695730481 | 0.797782613865084 | -0.169768263 | 0.130514304398389 | 0.980564375970441 | 0.843860347718837 | 1.13941423841219 | 0.998735809224318 |
| ebi-fl187-GCST90094775 | prot-a-2261 | Membrane-associated progesterone receptor component 2 \|\| id:prot-a-2261 | Green olives liking \|\| id:ebi-fl187-GCST90094775 | Inverse variance weighted | 27 | -0.124295641 | 0.0758595908104897 | 0.101318254880494 | -0.272980439 | 0.024389157186551 | 0.883118716359548 | 0.761107675669964 | 1.02468900539997 | 0.998735809224318 |
| ebi-fl187-GCST90094775 | prot-a-2268 | Polymeric immunoglobulin receptor \|\| id:prot-a-2268 | Green olives liking \|\| id:ebi-fl187-GCST90094775 | Inverse variance weighted | 27 | 0.0592460535524866 | 0.0788595790509918 | 0.452480889781983 | -0.095318721 | 0.213810828492431 | 1.06103628037008 | 0.909083144048497 | 1.23838836483973 | 0.998735809224318 |
| ebi-fl187-GCST90094775 | prot-a-2283 | Group 10 secretory phospholipase A2 \|\| id:prot-a-2283 | Green olives liking \|\| id:ebi-fl187-GCST90094775 | Inverse variance weighted | 27 | 0.177713877026063 | 0.0743624376037573 | 0.0168559924005725 | 0.031963499322699 | 0.323464254729428 | 1.19448350316389 | 1.03247981840465 | 1.38190675875418 | 0.998735809224318 |
| ebi-fl187-GCST90094775 | prot-a-2298 | Inactive phospholipase D5 \|\| id:prot-a-2298 | Green olives liking \|\| id:ebi-fl187-GCST90094775 | Inverse variance weighted | 27 | 0.169462253624427 | 0.0747905504850088 | 0.0234621372758346 | 0.0228727746738099 | 0.316051732575045 | 1.1846676292799 | 1.02313636240903 | 1.37170121542663 | 0.998735809224318 |
| ebi-fl187-GCST90094775 | prot-a-2304 | Procollagen-lysine,2-oxoglutarate 5-dioxygenase 2 \|\| id:prot-a-2304 | Green olives liking \|\| id:ebi-fl187-GCST90094775 | Inverse variance weighted | 27 | -0.057316476 | 0.0748980821927299 | 0.444116982934462 | -0.204116717 | 0.0894837655633074 | 0.944295175752342 | 0.81536719871986 | 1.09360957903276 | 0.998735809224318 |
| ebi-fl187-GCST90094775 | prot-a-2331 | Periostin \|\| id:prot-a-2331 | Green olives liking \|\| id:ebi-fl187-GCST90094775 | Inverse variance weighted | 27 | -0.139161507 | 0.0822526861035858 | 0.0906692454932231 | -0.300376771 | 0.022053758159296 | 0.870087492233972 | 0.740539154163629 | 1.02229873989178 | 0.998735809224318 |
| ebi-fl187-GCST90094775 | prot-a-2333 | Periostin \|\| id:prot-a-2333 | Green olives liking \|\| id:ebi-fl187-GCST90094775 | Inverse variance weighted | 27 | -0.216196535 | 0.0865768485337033 | 0.0125191264766547 | -0.385887158 | -0.046505912 | 0.805576962201837 | 0.679847236550976 | 0.954558917268881 | 0.998735809224318 |
| ebi-fl187-GCST90094775 | prot-a-2367 | cAMP-dependent protein kinase catalytic subunit alpha \|\| id:prot-a-2367 | Green olives liking \|\| id:ebi-fl187-GCST90094775 | Inverse variance weighted (multiplicative random effects) | 27 | -0.006139501 | 0.0886900149339196 | 0.944811111666783 | -0.17997193 | 0.167692928498077 | 0.993879307451741 | 0.835293657739351 | 1.18257342029166 | 0.998735809224318 |
| ebi-fl187-GCST90094775 | prot-a-2385 | Phosphoribosyl pyrophosphate synthase-associated protein 1 \|\| id:prot-a-2385 | Green olives liking \|\| id:ebi-fl187-GCST90094775 | Inverse variance weighted | 27 | -0.018191135 | 0.0768255182008355 | 0.812823546018776 | -0.16876915 | 0.132386881095319 | 0.98197332536276 | 0.844703880557952 | 1.14154987791351 | 0.998735809224318 |
| ebi-fl187-GCST90094775 | prot-a-2393 | Trypsin-3 \|\| id:prot-a-2393 | Green olives liking \|\| id:ebi-fl187-GCST90094775 | Inverse variance weighted | 26 | 0.00597787757876178 | 0.0728820500779924 | 0.934629807380284 | -0.136870941 | 0.148826695731627 | 1.00599578074547 | 0.87208276937112 | 1.1604718570549 | 0.998735809224318 |
| ebi-fl187-GCST90094775 | prot-a-2405 | Pregnancy-specific beta-1-glycoprotein 6 \|\| id:prot-a-2405 | Green olives liking \|\| id:ebi-fl187-GCST90094775 | Inverse variance weighted | 27 | 0.0751608609371894 | 0.0812726168632166 | 0.355070303590488 | -0.084133468 | 0.234455189989094 | 1.07805755428587 | 0.919308549527653 | 1.26421982146253 | 0.998735809224318 |
| ebi-fl187-GCST90094775 | prot-a-244 | Beclin-1 \|\| id:prot-a-244 | Green olives liking \|\| id:ebi-fl187-GCST90094775 | Inverse variance weighted | 27 | -0.156561798 | 0.0714469118948128 | 0.0284299074791567 | -0.296597746 | -0.016525851 | 0.85507867355176 | 0.743342965102974 | 0.983609951648295 | 0.998735809224318 |
| ebi-fl187-GCST90094775 | prot-a-2454 | tRNA pseudouridine synthase A, mitochondrial \|\| id:prot-a-2454 | Green olives liking \|\| id:ebi-fl187-GCST90094775 | Inverse variance weighted | 27 | 0.0415838176836114 | 0.0804373182931516 | 0.605175909992095 | -0.116073326 | 0.199240961538189 | 1.04246053481701 | 0.890409930552871 | 1.22047601824948 | 0.998735809224318 |
| ebi-fl187-GCST90094775 | prot-a-2466 | Glutaminyl-peptide cyclotransferase-like protein \|\| id:prot-a-2466 | Green olives liking \|\| id:ebi-fl187-GCST90094775 | Inverse variance weighted | 27 | 0.0176216618735334 | 0.0714593739652894 | 0.805220184659673 | -0.122438711 | 0.157682034845501 | 1.01777783937747 | 0.884760129258264 | 1.17079386386488 | 0.998735809224318 |
| ebi-fl187-GCST90094775 | prot-a-2474 | Ras-related protein Rab-27A \|\| id:prot-a-2474 | Green olives liking \|\| id:ebi-fl187-GCST90094775 | Inverse variance weighted | 27 | -0.102997894 | 0.0833402308140125 | 0.216505986191592 | -0.266344746 | 0.060348958744069 | 0.902128873677409 | 0.766174946182004 | 1.06220714835166 | 0.998735809224318 |
| ebi-fl187-GCST90094775 | prot-a-2490 | Rap1 GTPase-GDP dissociation stimulator 1 \|\| id:prot-a-2490 | Green olives liking \|\| id:ebi-fl187-GCST90094775 | Inverse variance weighted (multiplicative random effects) | 27 | 0.072914005554065 | 0.0874757577149398 | 0.404543712451073 | -0.09853848 | 0.244366490675347 | 1.07563803404063 | 0.906160823267088 | 1.27681218451194 | 0.998735809224318 |
| ebi-fl187-GCST90094775 | prot-a-2545 | Receptor-interacting serine/threonine-protein kinase 2 \|\| id:prot-a-2545 | Green olives liking \|\| id:ebi-fl187-GCST90094775 | Inverse variance weighted | 27 | 0.122191434560996 | 0.0714356089424149 | 0.0871713184818251 | -0.017822359 | 0.262205228088129 | 1.1299703964633 | 0.982335519957128 | 1.29979326914613 | 0.998735809224318 |
| ebi-fl187-GCST90094775 | prot-a-2610 | RUN and FYVE domain-containing protein 1 \|\| id:prot-a-2610 | Green olives liking \|\| id:ebi-fl187-GCST90094775 | Inverse variance weighted | 27 | -0.087022582 | 0.0714339100278108 | 0.22313844676905 | -0.227033045 | 0.0529878820168633 | 0.91665639579686 | 0.796894448245175 | 1.05441686763612 | 0.998735809224318 |
| ebi-fl187-GCST90094775 | prot-a-2627 | Serine--tRNA ligase, mitochondrial \|\| id:prot-a-2627 | Green olives liking \|\| id:ebi-fl187-GCST90094775 | Inverse variance weighted | 27 | 0.0487591226519236 | 0.0714464910621481 | 0.494950336139395 | -0.091276 | 0.188794245133734 | 1.04996740691249 | 0.912765752938151 | 1.20779241774777 | 0.998735809224318 |
| ebi-fl187-GCST90094775 | prot-a-264 | BolA-like protein 2 \|\| id:prot-a-264 | Green olives liking \|\| id:ebi-fl187-GCST90094775 | Inverse variance weighted | 27 | 0.0837607790210888 | 0.0714425154715872 | 0.241027659022234 | -0.056266551 | 0.2237881093454 | 1.08736874127837 | 0.94528713478858 | 1.25080595725419 | 0.998735809224318 |
| ebi-fl187-GCST90094775 | prot-a-2646 | Secretoglobin family 1C member 1 \|\| id:prot-a-2646 | Green olives liking \|\| id:ebi-fl187-GCST90094775 | Inverse variance weighted (multiplicative random effects) | 27 | 0.080158834458556 | 0.0984481139464945 | 0.415516538054194 | -0.112799469 | 0.273117137793685 | 1.08345914465533 | 0.893329782580492 | 1.31405416121508 | 0.998735809224318 |
| ebi-fl187-GCST90094775 | prot-a-2672 | Semaphorin-3E \|\| id:prot-a-2672 | Green olives liking \|\| id:ebi-fl187-GCST90094775 | Inverse variance weighted | 27 | 0.0981997507024553 | 0.0823732122412648 | 0.233209451162736 | -0.063251745 | 0.259651246695334 | 1.10318312470538 | 0.938707128842257 | 1.29647785687503 | 0.998735809224318 |
| ebi-fl187-GCST90094775 | prot-a-2701 | Plasma protease C1 inhibitor \|\| id:prot-a-2701 | Green olives liking \|\| id:ebi-fl187-GCST90094775 | Inverse variance weighted | 27 | -0.006985687 | 0.0775725370204884 | 0.928244609936441 | -0.15902786 | 0.145056485243298 | 0.99303865587915 | 0.852972594926508 | 1.15610487128866 | 0.998735809224318 |
| ebi-fl187-GCST90094775 | prot-a-2738 | Signaling threshold-regulating transmembrane adapter 1 \|\| id:prot-a-2738 | Green olives liking \|\| id:ebi-fl187-GCST90094775 | Inverse variance weighted | 27 | -0.10212501 | 0.0714365635370258 | 0.152834673718194 | -0.242140675 | 0.0378906543358772 | 0.902916670822778 | 0.784945747754119 | 1.03861765833155 | 0.998735809224318 |
| ebi-fl187-GCST90094775 | prot-a-274 | Serine/threonine-protein kinase BRSK2 \|\| id:prot-a-274 | Green olives liking \|\| id:ebi-fl187-GCST90094775 | Inverse variance weighted | 27 | -0.067562423 | 0.0714533811456273 | 0.344380454808659 | -0.20761105 | 0.0724862042784544 | 0.934669374146431 | 0.812523006235454 | 1.07517797313191 | 0.998735809224318 |
| ebi-fl187-GCST90094775 | prot-a-2749 | Mitochondrial glutamate carrier 2 \|\| id:prot-a-2749 | Green olives liking \|\| id:ebi-fl187-GCST90094775 | Inverse variance weighted (multiplicative random effects) | 27 | 0.103116745414942 | 0.0931818878290774 | 0.268459163064123 | -0.079519755 | 0.285753245559934 | 1.1086208279199 | 0.923559775114354 | 1.3307640427991 | 0.998735809224318 |
| ebi-fl187-GCST90094775 | prot-a-2760 | Sodium-coupled monocarboxylate transporter 1 \|\| id:prot-a-2760 | Green olives liking \|\| id:ebi-fl187-GCST90094775 | Inverse variance weighted | 27 | -0.110816925 | 0.0790793946004444 | 0.161113157240046 | -0.265812539 | 0.0441786882829864 | 0.895102604719154 | 0.766582818758483 | 1.04516909767507 | 0.998735809224318 |
| ebi-fl187-GCST90094775 | prot-a-2777 | Small integral membrane protein 10 \|\| id:prot-a-2777 | Green olives liking \|\| id:ebi-fl187-GCST90094775 | Inverse variance weighted | 27 | 0.0632232065311409 | 0.0841358758804988 | 0.452386868009206 | -0.10168311 | 0.228129523256919 | 1.06526458671007 | 0.903315757873262 | 1.25624802823133 | 0.998735809224318 |
| ebi-fl187-GCST90094775 | prot-a-2799 | Superoxide dismutase [Mn], mitochondrial \|\| id:prot-a-2799 | Green olives liking \|\| id:ebi-fl187-GCST90094775 | Inverse variance weighted | 27 | 0.0198696347353672 | 0.0740575302297784 | 0.788468412718913 | -0.125283125 | 0.165022393985733 | 1.02006834987792 | 0.882247081443977 | 1.17941953031979 | 0.998735809224318 |
| ebi-fl187-GCST90094775 | prot-a-2805 | Protein SPACA7 \|\| id:prot-a-2805 | Green olives liking \|\| id:ebi-fl187-GCST90094775 | Inverse variance weighted | 27 | 0.0495465915934403 | 0.0719721590150287 | 0.491192549910829 | -0.09151884 | 0.190612023262897 | 1.05079454926675 | 0.912544123589401 | 1.20998991306368 | 0.998735809224318 |
| ebi-fl187-GCST90094775 | prot-a-2811 | SPARC-like protein 1 \|\| id:prot-a-2811 | Green olives liking \|\| id:ebi-fl187-GCST90094775 | Inverse variance weighted | 27 | 0.0877177896627742 | 0.0714441892320193 | 0.21952933942884 | -0.052312821 | 0.227748400557532 | 1.09167999517237 | 0.949031943061659 | 1.25576933481797 | 0.998735809224318 |
| ebi-fl187-GCST90094775 | prot-a-2831 | Sepiapterin reductase \|\| id:prot-a-2831 | Green olives liking \|\| id:ebi-fl187-GCST90094775 | Inverse variance weighted | 27 | 0.0475745606070465 | 0.0714373018070063 | 0.505435101241946 | -0.092442551 | 0.187591672148779 | 1.04872439173355 | 0.911701585863956 | 1.20634083220847 | 0.998735809224318 |
| ebi-fl187-GCST90094775 | prot-a-2834 | SPRY domain-containing SOCS box protein 1 \|\| id:prot-a-2834 | Green olives liking \|\| id:ebi-fl187-GCST90094775 | Inverse variance weighted | 27 | 0.0475198366857582 | 0.0752055857801056 | 0.527474580844308 | -0.099883111 | 0.194922784814765 | 1.04866700299277 | 0.904943189357456 | 1.21521714964965 | 0.998735809224318 |
| ebi-fl187-GCST90094775 | prot-a-2835 | Spectrin alpha chain, non-erythrocytic 1 \|\| id:prot-a-2835 | Green olives liking \|\| id:ebi-fl187-GCST90094775 | Inverse variance weighted | 27 | -0.035929884 | 0.0714102372358564 | 0.614860557702051 | -0.175893949 | 0.10403418110162 | 0.964707932688334 | 0.838706924443837 | 1.1096383829297 | 0.998735809224318 |
| ebi-fl187-GCST90094775 | prot-a-2859 | Sia-alpha-2,3-Gal-beta-1,4-GlcNAc-R:alpha 2,8-sialyltransferase \|\| id:prot-a-2859 | Green olives liking \|\| id:ebi-fl187-GCST90094775 | Inverse variance weighted | 27 | 0.0497665966433164 | 0.0714441892320193 | 0.486065646807846 | -0.090264014 | 0.189797207538074 | 1.05102575480623 | 0.91368992626279 | 1.20900439581764 | 0.998735809224318 |
| ebi-fl187-GCST90094775 | prot-a-2861 | Alpha-2,8-sialyltransferase 8F \|\| id:prot-a-2861 | Green olives liking \|\| id:ebi-fl187-GCST90094775 | Inverse variance weighted | 27 | 0.0364057895833896 | 0.071466267776965 | 0.610463317913938 | -0.103668095 | 0.176479674426241 | 1.0370765959971 | 0.901524468015035 | 1.1930101778968 | 0.998735809224318 |
| ebi-fl187-GCST90094775 | prot-a-289 | Uncharacterized protein C17orf89 \|\| id:prot-a-289 | Green olives liking \|\| id:ebi-fl187-GCST90094775 | Inverse variance weighted | 27 | 0.186264189636933 | 0.0804097477488143 | 0.0205343697801697 | 0.0286610840492565 | 0.343867295224609 | 1.20474049829304 | 1.02907576517522 | 1.41039145740667 | 0.998735809224318 |
| ebi-fl187-GCST90094775 | prot-a-2921 | Tachykinin-3 \|\| id:prot-a-2921 | Green olives liking \|\| id:ebi-fl187-GCST90094775 | Inverse variance weighted | 27 | 0.0487743684387658 | 0.0714188928807451 | 0.49464874085092 | -0.091206662 | 0.188755398485026 | 1.04998341461379 | 0.912829044687163 | 1.20774549997131 | 0.998735809224318 |
| ebi-fl187-GCST90094775 | prot-a-2928 | Tax1-binding protein 1 \|\| id:prot-a-2928 | Green olives liking \|\| id:ebi-fl187-GCST90094775 | Inverse variance weighted | 27 | -0.082964242 | 0.0714356089424148 | 0.245485381906512 | -0.222978035 | 0.0570495515835248 | 0.920384057776752 | 0.800132423633577 | 1.05870826976702 | 0.998735809224318 |
| ebi-fl187-GCST90094775 | prot-a-296 | Uncharacterized protein C1orf185 \|\| id:prot-a-296 | Green olives liking \|\| id:ebi-fl187-GCST90094775 | Inverse variance weighted | 27 | 0.0481342762680054 | 0.0831309170485125 | 0.562577198656673 | -0.114802321 | 0.21107087368309 | 1.04931154350333 | 0.891542365560765 | 1.2349998809499 | 0.998735809224318 |
| ebi-fl187-GCST90094775 | prot-a-2964 | Transforming growth factor beta-2 \|\| id:prot-a-2964 | Green olives liking \|\| id:ebi-fl187-GCST90094775 | Inverse variance weighted | 27 | 0.0875706665619882 | 0.0727219099640338 | 0.228517904746909 | -0.054964277 | 0.230105610091494 | 1.09151939564065 | 0.946518959877097 | 1.25873293781082 | 0.998735809224318 |
| ebi-fl187-GCST90094775 | prot-a-2977 | Thymocyte nuclear protein 1 \|\| id:prot-a-2977 | Green olives liking \|\| id:ebi-fl187-GCST90094775 | Inverse variance weighted (multiplicative random effects) | 27 | 0.129493410793549 | 0.0891730987361814 | 0.146457733764874 | -0.045285863 | 0.304272684316465 | 1.13825161126321 | 0.955724236840816 | 1.35563866709713 | 0.998735809224318 |
| ebi-fl187-GCST90094775 | prot-a-3015 | Transmembrane protein 70, mitochondrial \|\| id:prot-a-3015 | Green olives liking \|\| id:ebi-fl187-GCST90094775 | Inverse variance weighted | 27 | 0.00134797202491482 | 0.078574920307079 | 0.986312766080973 | -0.152658872 | 0.15535481582679 | 1.00134888094756 | 0.85842250401383 | 1.16807233813942 | 0.998955228396052 |
| ebi-fl187-GCST90094775 | prot-a-3030 | Tumor necrosis factor alpha-induced protein 3 \|\| id:prot-a-3030 | Green olives liking \|\| id:ebi-fl187-GCST90094775 | Inverse variance weighted (multiplicative random effects) | 27 | -0.064568489 | 0.0988076488387783 | 0.513449051104041 | -0.258231481 | 0.129094502378892 | 0.937471905226406 | 0.772416411634179 | 1.13779764366925 | 0.998735809224318 |
| ebi-fl187-GCST90094775 | prot-a-3032 | Tumor necrosis factor receptor superfamily member 10A \|\| id:prot-a-3032 | Green olives liking \|\| id:ebi-fl187-GCST90094775 | Inverse variance weighted | 27 | 0.136532819361535 | 0.0714464910621481 | 0.0560071441720187 | -0.003502303 | 0.276567941843346 | 1.14629249774634 | 0.996503822789614 | 1.31859653755384 | 0.998735809224318 |
| ebi-fl187-GCST90094775 | prot-a-3081 | Tumor protein p53-inducible protein 11 \|\| id:prot-a-3081 | Green olives liking \|\| id:ebi-fl187-GCST90094775 | Inverse variance weighted | 27 | 0.0330819357721867 | 0.0714487724483167 | 0.64335278871202 | -0.106957658 | 0.173121529770887 | 1.03363522747228 | 0.898563718966284 | 1.18901059648922 | 0.998735809224318 |
| ebi-fl187-GCST90094775 | prot-a-315 | Complement C4 \|\| id:prot-a-315 | Green olives liking \|\| id:ebi-fl187-GCST90094775 | Inverse variance weighted | 26 | 0.0387832300893129 | 0.0810720521375245 | 0.632379925764918 | -0.120117992 | 0.197684452278861 | 1.03954511712251 | 0.886815793285758 | 1.218577814 | 0.998735809224318 |
| ebi-fl187-GCST90094775 | prot-a-3157 | UDP-glucuronosyltransferase 1-8 \|\| id:prot-a-3157 | Green olives liking \|\| id:ebi-fl187-GCST90094775 | Inverse variance weighted | 27 | 0.0910839028405916 | 0.0765775838809449 | 0.234269334256922 | -0.059008162 | 0.241175967247244 | 1.0953609052945 | 0.942699075224925 | 1.2727449770345 | 0.998735809224318 |
| ebi-fl187-GCST90094775 | prot-a-317 | Glutaredoxin-like protein C5orf63 \|\| id:prot-a-317 | Green olives liking \|\| id:ebi-fl187-GCST90094775 | Inverse variance weighted | 27 | -0.059175106 | 0.0736314088872479 | 0.421589536731495 | -0.203492667 | 0.0851424558907552 | 0.94254171044207 | 0.81587618716385 | 1.08887217190548 | 0.998735809224318 |
| ebi-fl187-GCST90094775 | prot-a-3192 | Guanine nucleotide exchange factor VAV3 \|\| id:prot-a-3192 | Green olives liking \|\| id:ebi-fl187-GCST90094775 | Inverse variance weighted | 27 | 0.0328223016205038 | 0.071466267776965 | 0.646040400604855 | -0.107251583 | 0.172896186463355 | 1.03336689530245 | 0.898299647439443 | 1.1887426910952 | 0.998735809224318 |
| ebi-fl187-GCST90094775 | prot-a-3233 | Serine/threonine-protein kinase WNK3 \|\| id:prot-a-3233 | Green olives liking \|\| id:ebi-fl187-GCST90094775 | Inverse variance weighted | 27 | -0.03003678 | 0.071466267776965 | 0.674271341671199 | -0.170110665 | 0.110037104808384 | 0.970409841184722 | 0.843571457698827 | 1.11631949051122 | 0.998735809224318 |
| ebi-fl187-GCST90094775 | prot-a-3250 | 14-3-3 protein theta \|\| id:prot-a-3250 | Green olives liking \|\| id:ebi-fl187-GCST90094775 | Inverse variance weighted | 27 | 0.0715533254627234 | 0.0805895676439525 | 0.374608907766286 | -0.086402227 | 0.22950887804487 | 1.07417543007593 | 0.917225224153027 | 1.25798203559468 | 0.998735809224318 |
| ebi-fl187-GCST90094775 | prot-a-3278 | E3 ubiquitin-protein ligase ZNRF3 \|\| id:prot-a-3278 | Green olives liking \|\| id:ebi-fl187-GCST90094775 | Inverse variance weighted | 27 | 0.0985399163179863 | 0.0727853195902081 | 0.175786380214444 | -0.04411931 | 0.241199142714794 | 1.10355845350539 | 0.956839790031751 | 1.27277447383621 | 0.998735809224318 |
| ebi-fl187-GCST90094775 | prot-a-355 | Calpain-3 \|\| id:prot-a-355 | Green olives liking \|\| id:ebi-fl187-GCST90094775 | Inverse variance weighted | 27 | -0.021386561 | 0.0714395456633076 | 0.764660906140449 | -0.161408071 | 0.118634948287169 | 0.978840509651516 | 0.85094475461439 | 1.12595881006285 | 0.998735809224318 |
| ebi-fl187-GCST90094775 | prot-a-385 | Coiled-coil domain-containing protein 90B, mitochondrial \|\| id:prot-a-385 | Green olives liking \|\| id:ebi-fl187-GCST90094775 | Inverse variance weighted | 27 | 0.137958105800849 | 0.0776744927893774 | 0.0757155045345308 | -0.0142839 | 0.290200111668029 | 1.14792745776526 | 0.985817630840237 | 1.33669494951949 | 0.998735809224318 |
| ebi-fl187-GCST90094775 | prot-a-390 | C-C motif chemokine 14 \|\| id:prot-a-390 | Green olives liking \|\| id:ebi-fl187-GCST90094775 | Inverse variance weighted | 27 | 0.0208102866494144 | 0.0714305166959465 | 0.770794296592432 | -0.119193526 | 0.16081409937347 | 1.02102833055674 | 0.887636003427401 | 1.17446661443893 | 0.998735809224318 |
| ebi-fl187-GCST90094775 | prot-a-40 | Pituitary adenylate cyclase-activating polypeptide 27 \|\| id:prot-a-40 | Green olives liking \|\| id:ebi-fl187-GCST90094775 | Inverse variance weighted | 27 | 0.0587716196665378 | 0.0870698191014607 | 0.499679473400595 | -0.111885226 | 0.229428465105401 | 1.06053300819858 | 0.894146876628422 | 1.25788088162849 | 0.998735809224318 |
| ebi-fl187-GCST90094775 | prot-a-401 | C-C motif chemokine 24 \|\| id:prot-a-401 | Green olives liking \|\| id:ebi-fl187-GCST90094775 | Inverse variance weighted | 27 | 0.163763617009818 | 0.0714553766194466 | 0.021915421528564 | 0.0237110788357023 | 0.303816155183933 | 1.17793583818474 | 1.02399442148682 | 1.35501991980124 | 0.998735809224318 |
| ebi-fl187-GCST90094775 | prot-a-412 | Cerebral cavernous malformations 2 protein \|\| id:prot-a-412 | Green olives liking \|\| id:ebi-fl187-GCST90094775 | Inverse variance weighted | 27 | -0.126477635 | 0.0715309881758689 | 0.0770355534447092 | -0.266678372 | 0.0137231015196569 | 0.881193856954946 | 0.765919372871233 | 1.01381769548957 | 0.998735809224318 |
| ebi-fl187-GCST90094775 | prot-a-44 | Adhesion G protein-coupled receptor F5 \|\| id:prot-a-44 | Green olives liking \|\| id:ebi-fl187-GCST90094775 | Inverse variance weighted | 27 | -0.009121058 | 0.0714441892320193 | 0.898412590936537 | -0.149151669 | 0.130909553208338 | 0.990920412979018 | 0.861438451842705 | 1.13986467954625 | 0.998735809224318 |
| ebi-fl187-GCST90094775 | prot-a-48 | Adrenomedullin \|\| id:prot-a-48 | Green olives liking \|\| id:ebi-fl187-GCST90094775 | Inverse variance weighted | 27 | -0.04060732 | 0.0768169002559289 | 0.597065736488656 | -0.191168445 | 0.109953804145041 | 0.96020610931951 | 0.825993442084077 | 1.11622650423012 | 0.998735809224318 |
| ebi-fl187-GCST90094775 | prot-a-502 | Carcinoembryonic antigen-related cell adhesion molecule 1 \|\| id:prot-a-502 | Green olives liking \|\| id:ebi-fl187-GCST90094775 | Inverse variance weighted | 27 | -0.008281719 | 0.0724526995567442 | 0.908995873368539 | -0.15028901 | 0.133725571992622 | 0.991752479823496 | 0.860459258923246 | 1.14307908368244 | 0.998735809224318 |
| ebi-fl187-GCST90094775 | prot-a-518 | Complement factor B \|\| id:prot-a-518 | Green olives liking \|\| id:ebi-fl187-GCST90094775 | Inverse variance weighted | 27 | 0.0704744192598719 | 0.0862235225853907 | 0.413730938141983 | -0.098523685 | 0.239472523527238 | 1.07301712050752 | 0.906174229616677 | 1.27057877312323 | 0.998735809224318 |
| ebi-fl187-GCST90094775 | prot-a-525 | Properdin \|\| id:prot-a-525 | Green olives liking \|\| id:ebi-fl187-GCST90094775 | Inverse variance weighted (multiplicative random effects) | 27 | 0.0114131497255831 | 0.0914384908344987 | 0.900667808531083 | -0.167806292 | 0.190632591761201 | 1.01147852820697 | 0.845517602084169 | 1.21001480099511 | 0.998735809224318 |
| ebi-fl187-GCST90094775 | prot-a-533 | Chondroadherin \|\| id:prot-a-533 | Green olives liking \|\| id:ebi-fl187-GCST90094775 | Inverse variance weighted | 27 | -0.031295434 | 0.0737598207830312 | 0.671355519433973 | -0.175864683 | 0.113273814560984 | 0.969189199165519 | 0.838731470361654 | 1.11993854645047 | 0.998735809224318 |
| ebi-fl187-GCST90094775 | prot-a-535 | Coiled-coil-helix-coiled-coil-helix domain-containing protein 2 \|\| id:prot-a-535 | Green olives liking \|\| id:ebi-fl187-GCST90094775 | Inverse variance weighted | 27 | -0.005526802 | 0.0821449563704075 | 0.946357933679051 | -0.166530916 | 0.155477312583899 | 0.994488442769842 | 0.846596642820465 | 1.168215432 | 0.998735809224318 |
| ebi-fl187-GCST90094775 | prot-a-591 | Caseinolytic peptidase B protein homolog \|\| id:prot-a-591 | Green olives liking \|\| id:ebi-fl187-GCST90094775 | Inverse variance weighted (multiplicative random effects) | 27 | -0.024549069 | 0.0987609018734151 | 0.803692854817954 | -0.218120437 | 0.169022298553358 | 0.975749808560676 | 0.804028601210957 | 1.18414654338446 | 0.998735809224318 |
| ebi-fl187-GCST90094775 | prot-a-605 | Ciliary neurotrophic factor receptor subunit alpha \|\| id:prot-a-605 | Green olives liking \|\| id:ebi-fl187-GCST90094775 | Inverse variance weighted | 27 | -0.200921134 | 0.0714382884030206 | 0.00491559955631665 | -0.340940179 | -0.060902089 | 0.817976939622642 | 0.711101445579317 | 0.940915361533733 | 0.998735809224318 |
| ebi-fl187-GCST90094775 | prot-a-606 | Ciliary neurotrophic factor receptor subunit alpha \|\| id:prot-a-606 | Green olives liking \|\| id:ebi-fl187-GCST90094775 | Inverse variance weighted | 27 | -0.196947169 | 0.0714464910621481 | 0.00584113210478346 | -0.336982291 | -0.056912046 | 0.821234018833186 | 0.713921482327664 | 0.94467715341751 | 0.998735809224318 |
| ebi-fl187-GCST90094775 | prot-a-707 | Cancer/testis antigen 55 \|\| id:prot-a-707 | Green olives liking \|\| id:ebi-fl187-GCST90094775 | Inverse variance weighted | 27 | -0.003121273 | 0.0714376359683593 | 0.965149694018776 | -0.14313904 | 0.136896493395176 | 0.996883593005948 | 0.866633564133426 | 1.14670945037556 | 0.998735809224318 |
| ebi-fl187-GCST90094775 | prot-a-719 | Dipeptidyl peptidase 1 \|\| id:prot-a-719 | Green olives liking \|\| id:ebi-fl187-GCST90094775 | Inverse variance weighted | 27 | -0.058096458 | 0.0714451761134246 | 0.416125542223472 | -0.198129003 | 0.0819360876337706 | 0.943558929665933 | 0.820264030012349 | 1.08538643799719 | 0.998735809224318 |
| ebi-fl187-GCST90094775 | prot-a-752 | Cytochrome b5 \|\| id:prot-a-752 | Green olives liking \|\| id:ebi-fl187-GCST90094775 | Inverse variance weighted | 27 | -0.169826108 | 0.079698608432865 | 0.033101162451787 | -0.32603538 | -0.013616835 | 0.843811536057294 | 0.721779650156287 | 0.986475454426009 | 0.998735809224318 |
| ebi-fl187-GCST90094775 | prot-a-755 | Cytoglobin \|\| id:prot-a-755 | Green olives liking \|\| id:ebi-fl187-GCST90094775 | Inverse variance weighted | 27 | 0.0518306327809457 | 0.0834309608209419 | 0.53444186122461 | -0.11169405 | 0.215355315989992 | 1.05319735030056 | 0.894317831806036 | 1.24030251799865 | 0.998735809224318 |
| ebi-fl187-GCST90094775 | prot-a-807 | Desmin \|\| id:prot-a-807 | Green olives liking \|\| id:ebi-fl187-GCST90094775 | Inverse variance weighted | 27 | -0.147948552 | 0.0771446773036262 | 0.0551355703222537 | -0.29915212 | 0.00325501552402061 | 0.862475486447137 | 0.741446612363369 | 1.00326031883962 | 0.998735809224318 |
| ebi-fl187-GCST90094775 | prot-a-82 | Ameloblastin \|\| id:prot-a-82 | Green olives liking \|\| id:ebi-fl187-GCST90094775 | Inverse variance weighted (multiplicative random effects) | 27 | 0.0753852555733044 | 0.0879138064833427 | 0.391173852183798 | -0.096925805 | 0.247696316280656 | 1.07829949176219 | 0.907623344627547 | 1.28107083276019 | 0.998735809224318 |
| ebi-fl187-GCST90094775 | prot-a-84 | Muellerian-inhibiting factor \|\| id:prot-a-84 | Green olives liking \|\| id:ebi-fl187-GCST90094775 | Inverse variance weighted | 27 | -0.009843938 | 0.0714365268919965 | 0.890398656165245 | -0.149859531 | 0.130171654831481 | 0.990204355085416 | 0.860828888062862 | 1.13902388549781 | 0.998735809224318 |
| ebi-fl187-GCST90094775 | prot-a-858 | Inactive dipeptidyl peptidase 10 \|\| id:prot-a-858 | Green olives liking \|\| id:ebi-fl187-GCST90094775 | Inverse variance weighted | 27 | 0.177255007860941 | 0.0714488645866013 | 0.0131063896021961 | 0.0372152332712022 | 0.317294782450679 | 1.19393551725257 | 1.03791639093901 | 1.37340736864895 | 0.998735809224318 |
| ebi-fl187-GCST90094775 | prot-a-863 | Dihydropyrimidinase-related protein 3 \|\| id:prot-a-863 | Green olives liking \|\| id:ebi-fl187-GCST90094775 | Inverse variance weighted | 27 | -0.180011745 | 0.0835887562926543 | 0.031276250002116 | -0.343845707 | -0.016177783 | 0.835260401140965 | 0.709038318955698 | 0.983952374734427 | 0.998735809224318 |
| ebi-fl187-GCST90094775 | prot-a-864 | Dihydropyrimidinase-related protein 5 \|\| id:prot-a-864 | Green olives liking \|\| id:ebi-fl187-GCST90094775 | Inverse variance weighted | 27 | -0.144144042 | 0.0719430589068617 | 0.0451145216505066 | -0.285152437 | -0.003135647 | 0.865763032873817 | 0.751899627946036 | 0.996869264503299 | 0.998735809224318 |
| ebi-fl187-GCST90094775 | prot-a-884 | Endothelin-converting enzyme 1 \|\| id:prot-a-884 | Green olives liking \|\| id:ebi-fl187-GCST90094775 | Inverse variance weighted | 27 | -0.219807793 | 0.072550640632739 | 0.00244784287915355 | -0.362007048 | -0.077608537 | 0.802673062677723 | 0.696277460153781 | 0.925326586625593 | 0.998735809224318 |
| ebi-fl187-GCST90094775 | prot-a-893 | Early endosome antigen 1 \|\| id:prot-a-893 | Green olives liking \|\| id:ebi-fl187-GCST90094775 | Inverse variance weighted | 27 | -0.21244449 | 0.0714372651608408 | 0.00294078351708342 | -0.35246153 | -0.07242745 | 0.808605200906739 | 0.70295561232906 | 0.930133225292975 | 0.998735809224318 |
| ebi-fl187-GCST90094775 | prot-a-897 | Ephrin-A2 \|\| id:prot-a-897 | Green olives liking \|\| id:ebi-fl187-GCST90094775 | Inverse variance weighted | 27 | 0.0198976294998318 | 0.071437550102452 | 0.780604156682074 | -0.120119969 | 0.159915227700638 | 1.02009690685083 | 0.886814040406738 | 1.17341139399346 | 0.998735809224318 |
| ebi-fl187-GCST90094775 | prot-a-90 | Anaphase-promoting complex subunit 10 \|\| id:prot-a-90 | Green olives liking \|\| id:ebi-fl187-GCST90094775 | Inverse variance weighted | 27 | 0.0667052517370876 | 0.0790539184894523 | 0.398784319361112 | -0.088240429 | 0.221650931976414 | 1.06898035163365 | 0.91554072817338 | 1.24813561758052 | 0.998735809224318 |
| ebi-fl187-GCST90094775 | prot-a-914 | Histone-lysine N-methyltransferase EHMT2 \|\| id:prot-a-914 | Green olives liking \|\| id:ebi-fl187-GCST90094775 | Inverse variance weighted | 27 | -0.003703667 | 0.079589031223173 | 0.962883923203583 | -0.159698168 | 0.152290834105154 | 0.996303183023242 | 0.85240103180354 | 1.16449886317245 | 0.998735809224318 |
| ebi-fl187-GCST90094775 | prot-a-932 | RNA polymerase II elongation factor ELL \|\| id:prot-a-932 | Green olives liking \|\| id:ebi-fl187-GCST90094775 | Inverse variance weighted | 27 | -0.147277789 | 0.0760322163810581 | 0.0527399001451742 | -0.296300933 | 0.00174535517110419 | 0.863054197206763 | 0.743563631481961 | 1.00174687918996 | 0.998735809224318 |
| ebi-fl187-GCST90094775 | prot-a-991 | Estrogen receptor \|\| id:prot-a-991 | Green olives liking \|\| id:ebi-fl187-GCST90094775 | Inverse variance weighted (multiplicative random effects) | 27 | 0.152922230034713 | 0.0877679062621798 | 0.0814475808184999 | -0.019102866 | 0.324947326308586 | 1.16523435518353 | 0.98107843720285 | 1.38395774589757 | 0.998735809224318 |
| ebi-fl187-GCST90094775 | prot-a-992 | Persulfide dioxygenase ETHE1, mitochondrial \|\| id:prot-a-992 | Green olives liking \|\| id:ebi-fl187-GCST90094775 | Inverse variance weighted | 27 | -0.04320537 | 0.0714185142605038 | 0.545205340143084 | -0.183185658 | 0.0967749183415722 | 0.957714684378758 | 0.832613560384924 | 1.10161239296976 | 0.998735809224318 |
| ebi-fl187-GCST90094775 | prot-a-993 | Ethanolamine kinase 1 \|\| id:prot-a-993 | Green olives liking \|\| id:ebi-fl187-GCST90094775 | Inverse variance weighted | 27 | 0.117993159470197 | 0.0714441525752534 | 0.0986275498779533 | -0.02203738 | 0.258023698517694 | 1.12523641412779 | 0.978203669528132 | 1.2943694928991 | 0.998735809224318 |
| ebi-fl187-GCST90094775 | prot-b-10 | coagulation factor III, tissue factor \|\| id:prot-b-10 | Green olives liking \|\| id:ebi-fl187-GCST90094775 | Inverse variance weighted | 20 | 0.0235534985721973 | 0.11790466261267 | 0.841662560275628 | -0.20753964 | 0.25464663729303 | 1.02383307288907 | 0.812581030301849 | 1.29000570041868 | 0.998735809224318 |
| ebi-fl187-GCST90094775 | prot-b-28 | prolactin \|\| id:prot-b-28 | Green olives liking \|\| id:ebi-fl187-GCST90094775 | Inverse variance weighted | 20 | 0.0238620512397529 | 0.105612741730585 | 0.82124876139336 | -0.183138923 | 0.230863025031699 | 1.02414902805674 | 0.832652473495133 | 1.25968668208813 | 0.998735809224318 |
| ebi-fl187-GCST90094775 | prot-b-54 | matrix metallopeptidase 7 \|\| id:prot-b-54 | Green olives liking \|\| id:ebi-fl187-GCST90094775 | Inverse variance weighted | 20 | -0.031805132 | 0.10559721985078 | 0.763267409199181 | -0.238775683 | 0.175165418806376 | 0.968695331311853 | 0.787591532722119 | 1.19144328743877 | 0.998735809224318 |
| ebi-fl187-GCST90094775 | prot-c-2421_7_3 | BDNF \|\| id:prot-c-2421_7_3 | Green olives liking \|\| id:ebi-fl187-GCST90094775 | Inverse variance weighted | 3 | -0.035284222 | 0.397493153419171 | 0.929267190465579 | -0.814370802 | 0.743802359044217 | 0.965331009283853 | 0.442917922618518 | 2.103920185 | 0.998735809224318 |
| ebi-fl187-GCST90094775 | prot-c-2443_10_4 | FGF-8B \|\| id:prot-c-2443_10_4 | Green olives liking \|\| id:ebi-fl187-GCST90094775 | Inverse variance weighted | 3 | -0.500616375 | 0.552081700693572 | 0.364523406787113 | -1.582696508 | 0.581463758686858 | 0.606156924768076 | 0.205420432864055 | 1.78865467432565 | 0.998735809224318 |
| ebi-fl187-GCST90094775 | prot-c-2605_49_2 | CD30 \|\| id:prot-c-2605_49_2 | Green olives liking \|\| id:ebi-fl187-GCST90094775 | Inverse variance weighted | 3 | 0.0913352299102223 | 0.396780656687736 | 0.817943563096092 | -0.686354857 | 0.869025317018186 | 1.09563623373831 | 0.503407721753695 | 2.38458550555887 | 0.998735809224318 |
| ebi-fl187-GCST90094775 | prot-c-2624_31_2 | HPV E7 Type18 \|\| id:prot-c-2624_31_2 | Green olives liking \|\| id:ebi-fl187-GCST90094775 | Inverse variance weighted | 3 | 0.140755077915071 | 0.402206013263793 | 0.726370468244739 | -0.647568708 | 0.929078863912106 | 1.15114267319166 | 0.523316566643497 | 2.53217562467417 | 0.998735809224318 |
| ebi-fl187-GCST90094775 | prot-c-2774_10_3 | IL-16 \|\| id:prot-c-2774_10_3 | Green olives liking \|\| id:ebi-fl187-GCST90094775 | Inverse variance weighted | 3 | -0.06444049 | 0.533332823178393 | 0.903828828341788 | -1.109772824 | 0.980891843146439 | 0.93759190843082 | 0.329633837561118 | 2.66683357891604 | 0.998735809224318 |
| ebi-fl187-GCST90094775 | prot-c-2851_63_3 | C5a \|\| id:prot-c-2851_63_3 | Green olives liking \|\| id:ebi-fl187-GCST90094775 | Inverse variance weighted | 3 | -0.349046468 | 0.349237214129622 | 0.31757489933782 | -1.033551407 | 0.33545847205522 | 0.705360353078283 | 0.355741332142751 | 1.39858144876759 | 0.998735809224318 |
| ebi-fl187-GCST90094775 | prot-c-2891_1_3 | CD22 \|\| id:prot-c-2891_1_3 | Green olives liking \|\| id:ebi-fl187-GCST90094775 | Inverse variance weighted (multiplicative random effects) | 3 | -0.069383098 | 0.7516689360879 | 0.926455402195395 | -1.542654213 | 1.40388801678461 | 0.932969193023208 | 0.213812842862379 | 4.07099734271168 | 0.998735809224318 |
| ebi-fl187-GCST90094775 | prot-c-2961_1_2 | Protein C \|\| id:prot-c-2961_1_2 | Green olives liking \|\| id:ebi-fl187-GCST90094775 | Inverse variance weighted | 3 | -0.020250646 | 0.391942056154891 | 0.958793674554697 | -0.788457076 | 0.747955783884989 | 0.979953021042212 | 0.454545583691877 | 2.11267683133123 | 0.998735809224318 |
| ebi-fl187-GCST90094775 | prot-c-2982_82_2 | Galectin-4 \|\| id:prot-c-2982_82_2 | Green olives liking \|\| id:ebi-fl187-GCST90094775 | Inverse variance weighted | 3 | -0.741992896 | 0.370357855965027 | 0.0451291686385283 | -1.467894294 | -0.016091498 | 0.476164023877298 | 0.230410150804095 | 0.984037278061579 | 0.998735809224318 |
| ebi-fl187-GCST90094775 | prot-c-2994_71_2 | IL-1Rrp2 \|\| id:prot-c-2994_71_2 | Green olives liking \|\| id:ebi-fl187-GCST90094775 | Inverse variance weighted | 3 | -0.343105347 | 0.399863814065035 | 0.390861815040046 | -1.126838423 | 0.440627728567943 | 0.709563457241513 | 0.324056167229683 | 1.55368220317089 | 0.998735809224318 |
| ebi-fl187-GCST90094775 | prot-c-3336_50_1 | TFPI \|\| id:prot-c-3336_50_1 | Green olives liking \|\| id:ebi-fl187-GCST90094775 | Inverse variance weighted | 3 | 0.29059953732951 | 0.388841760519264 | 0.454854417257095 | -0.471530313 | 1.05272938794727 | 1.33722896642683 | 0.624046550466714 | 2.86546141039257 | 0.998735809224318 |
| ebi-fl187-GCST90094775 | prot-c-3472_40_2 | Survivin \|\| id:prot-c-3472_40_2 | Green olives liking \|\| id:ebi-fl187-GCST90094775 | Inverse variance weighted | 3 | -0.227032181 | 0.469286477748534 | 0.628540847720951 | -1.146833677 | 0.69276931542112 | 0.796895137022182 | 0.317640932465986 | 1.99924441248643 | 0.998735809224318 |
| ebi-fl187-GCST90094775 | prot-c-3473_78_2 | Thrombopoietin Receptor \|\| id:prot-c-3473_78_2 | Green olives liking \|\| id:ebi-fl187-GCST90094775 | Inverse variance weighted | 3 | 0.0758695035131692 | 0.656593898592466 | 0.908009096783105 | -1.211054538 | 1.3627935447544 | 1.07882178251854 | 0.297882984895171 | 3.90709270905843 | 0.998735809224318 |
| ebi-fl187-GCST90094775 | prot-c-3497_13_2 | IFN-aA \|\| id:prot-c-3497_13_2 | Green olives liking \|\| id:ebi-fl187-GCST90094775 | Inverse variance weighted | 3 | -0.472614401 | 0.411656773350955 | 0.250936048376134 | -1.279461677 | 0.334232874667715 | 0.623370395779037 | 0.278187014657273 | 1.39686840096565 | 0.998735809224318 |
| ebi-fl187-GCST90094775 | prot-c-3665_64_3 | COLEC12 \|\| id:prot-c-3665_64_3 | Green olives liking \|\| id:ebi-fl187-GCST90094775 | Inverse variance weighted | 3 | -0.025996645 | 0.552406352362854 | 0.962464827599863 | -1.108713095 | 1.05671980584657 | 0.974338358718305 | 0.32998334502521 | 2.87691864326472 | 0.998735809224318 |
| ebi-fl187-GCST90094775 | prot-c-3741_4_3 | IL-5 \|\| id:prot-c-3741_4_3 | Green olives liking \|\| id:ebi-fl187-GCST90094775 | Inverse variance weighted | 3 | -0.381979033 | 0.614300985607633 | 0.534066234617672 | -1.586008965 | 0.822050898426089 | 0.68250936298016 | 0.204741112219223 | 2.27516118041215 | 0.998735809224318 |
| ebi-fl187-GCST90094775 | prot-c-3848_14_2 | GAPDH, liver \|\| id:prot-c-3848_14_2 | Green olives liking \|\| id:ebi-fl187-GCST90094775 | Inverse variance weighted | 3 | 0.054431985406351 | 0.517377122783649 | 0.916211110902697 | -0.959627175 | 1.0684911460623 | 1.05594065459675 | 0.383035664533792 | 2.91098393510519 | 0.998735809224318 |
| ebi-fl187-GCST90094775 | prot-c-3859_50_2 | PSA1 \|\| id:prot-c-3859_50_2 | Green olives liking \|\| id:ebi-fl187-GCST90094775 | Inverse variance weighted (multiplicative random effects) | 3 | -0.484421404 | 0.847220006024254 | 0.567472668471448 | -2.144972616 | 1.17612980747764 | 0.616053539575688 | 0.117071242863057 | 3.24180348941607 | 0.998735809224318 |
| ebi-fl187-GCST90094775 | prot-c-3868_8_1 | SGTA \|\| id:prot-c-3868_8_1 | Green olives liking \|\| id:ebi-fl187-GCST90094775 | Inverse variance weighted | 3 | 0.612334730637197 | 0.447682438590453 | 0.171378400677331 | -0.265122849 | 1.48979231027448 | 1.84473333032558 | 0.767111705281089 | 4.43617407554372 | 0.998735809224318 |
| ebi-fl187-GCST90094775 | prot-c-3882_10_1 | EF-1-beta \|\| id:prot-c-3882_10_1 | Green olives liking \|\| id:ebi-fl187-GCST90094775 | Inverse variance weighted | 3 | 0.135270686047329 | 0.399245457965307 | 0.734748466166218 | -0.647250412 | 0.917791783659331 | 1.14484663642392 | 0.523483162996213 | 2.50375544731826 | 0.998735809224318 |
| ebi-fl187-GCST90094775 | prot-c-4134_4_2 | HB-EGF \|\| id:prot-c-4134_4_2 | Green olives liking \|\| id:ebi-fl187-GCST90094775 | Inverse variance weighted | 3 | -0.074023726 | 0.403264570493378 | 0.854357707917738 | -0.864422284 | 0.716374831835415 | 0.928649660130118 | 0.421294870913758 | 2.0469990279951 | 0.998735809224318 |
| ebi-fl187-GCST90094775 | prot-c-4220_39_2 | FER \|\| id:prot-c-4220_39_2 | Green olives liking \|\| id:ebi-fl187-GCST90094775 | Inverse variance weighted | 3 | 0.519199814420922 | 0.419478323730747 | 0.215817156350916 | -0.3029777 | 1.34137732893319 | 1.68068225378498 | 0.738615567246516 | 3.82430720857662 | 0.998735809224318 |
| ebi-fl187-GCST90094775 | prot-c-4228_37_2 | HSP70 protein 8 \|\| id:prot-c-4228_37_2 | Green olives liking \|\| id:ebi-fl187-GCST90094775 | Inverse variance weighted | 3 | 0.228332139081185 | 0.402469042874058 | 0.570491050001498 | -0.560507185 | 1.01717146311434 | 1.25650258974928 | 0.570919428662715 | 2.76536176347112 | 0.998735809224318 |
| ebi-fl187-GCST90094775 | prot-c-4246_40_2 | NCAM-L1 \|\| id:prot-c-4246_40_2 | Green olives liking \|\| id:ebi-fl187-GCST90094775 | Inverse variance weighted | 3 | -0.064691859 | 0.398677735865427 | 0.87109635284347 | -0.846100222 | 0.716716503070936 | 0.937356256563707 | 0.429085009852064 | 2.04769854817826 | 0.998735809224318 |
| ebi-fl187-GCST90094775 | prot-c-4267_81_3 | PESC \|\| id:prot-c-4267_81_3 | Green olives liking \|\| id:ebi-fl187-GCST90094775 | Inverse variance weighted | 3 | -0.02851586 | 0.40305979256705 | 0.943597951872139 | -0.818513053 | 0.761481333788468 | 0.971886880244999 | 0.441087040141691 | 2.14144606853317 | 0.998735809224318 |
| ebi-fl187-GCST90094775 | prot-c-4280_47_2 | PSA2 \|\| id:prot-c-4280_47_2 | Green olives liking \|\| id:ebi-fl187-GCST90094775 | Inverse variance weighted | 3 | 0.185367851965111 | 0.400049870448995 | 0.643105012287839 | -0.598729894 | 0.96946559804514 | 1.20366112781213 | 0.549509127833248 | 2.63653511329045 | 0.998735809224318 |
| ebi-fl187-GCST90094775 | prot-c-4324_33_2 | CYTT \|\| id:prot-c-4324_33_2 | Green olives liking \|\| id:ebi-fl187-GCST90094775 | Inverse variance weighted (multiplicative random effects) | 3 | 0.0735453329125114 | 0.941972922272824 | 0.937767720549213 | -1.772721595 | 1.91981226056725 | 1.07631732816523 | 0.169870041721554 | 6.81967802661544 | 0.998735809224318 |
| ebi-fl187-GCST90094775 | prot-c-4462_8_2 | RASA1 \|\| id:prot-c-4462_8_2 | Green olives liking \|\| id:ebi-fl187-GCST90094775 | Inverse variance weighted | 3 | 0.188247633239403 | 0.401438221100839 | 0.639117520492636 | -0.59857128 | 0.975066546597047 | 1.20713240444834 | 0.549596294584974 | 2.65134364300911 | 0.998735809224318 |
| ebi-fl187-GCST90094775 | prot-c-4499_21_1 | PDGF-AA \|\| id:prot-c-4499_21_1 | Green olives liking \|\| id:ebi-fl187-GCST90094775 | Inverse variance weighted | 3 | -0.039112917 | 0.401195746291183 | 0.922336594205478 | -0.82545658 | 0.747230745349575 | 0.961642116906069 | 0.438034948754534 | 2.11114561437837 | 0.998735809224318 |
| ebi-fl187-GCST90094775 | prot-c-4535_50_2 | BST1 \|\| id:prot-c-4535_50_2 | Green olives liking \|\| id:ebi-fl187-GCST90094775 | Inverse variance weighted | 3 | 0.0778628744836872 | 0.560645900334702 | 0.8895444513702 | -1.02100309 | 1.1767288391397 | 1.08097441933125 | 0.360233412285249 | 3.24374601410724 | 0.998735809224318 |
| ebi-fl187-GCST90094775 | prot-c-4542_24_2 | Clusterin \|\| id:prot-c-4542_24_2 | Green olives liking \|\| id:ebi-fl187-GCST90094775 | Inverse variance weighted | 3 | 0.26944025763028 | 0.399616104130439 | 0.500153818833462 | -0.513807306 | 1.05268782172594 | 1.30923141330254 | 0.598213654861012 | 2.86534230646475 | 0.998735809224318 |
| ebi-fl187-GCST90094775 | prot-c-4696_2_2 | FABP \|\| id:prot-c-4696_2_2 | Green olives liking \|\| id:ebi-fl187-GCST90094775 | Inverse variance weighted (multiplicative random effects) | 3 | -0.373616631 | 0.663366803430086 | 0.573290033138938 | -1.673815565 | 0.926582304079184 | 0.688240711657913 | 0.187530165214669 | 2.52586178144282 | 0.998735809224318 |
| ebi-fl187-GCST90094775 | prot-c-4906_35_1 | Coagulation Factor V \|\| id:prot-c-4906_35_1 | Green olives liking \|\| id:ebi-fl187-GCST90094775 | Inverse variance weighted | 3 | 0.044032840413854 | 0.403040080059557 | 0.913002802864563 | -0.745925717 | 0.833991397330586 | 1.04501667310496 | 0.47429503391325 | 2.30249057861123 | 0.998735809224318 |
| ebi-fl187-GCST90094775 | prot-c-4916_2_1 | IgD \|\| id:prot-c-4916_2_1 | Green olives liking \|\| id:ebi-fl187-GCST90094775 | Inverse variance weighted | 3 | -0.704726781 | 0.567982740788005 | 0.214696688582475 | -1.817972953 | 0.408519390455129 | 0.49424359230877 | 0.162354517776215 | 1.50458842712946 | 0.998735809224318 |
| ebi-fl187-GCST90094775 | prot-c-4922_13_1 | MIP-3b \|\| id:prot-c-4922_13_1 | Green olives liking \|\| id:ebi-fl187-GCST90094775 | Inverse variance weighted | 3 | 0.4408440949627 | 0.40281027332155 | 0.273770298614729 | -0.348664041 | 1.23035223067294 | 1.55401840415778 | 0.705630153431524 | 3.42243481052633 | 0.998735809224318 |
| ebi-fl187-GCST90094775 | prot-c-4973_18_1 | cIAP-2 \|\| id:prot-c-4973_18_1 | Green olives liking \|\| id:ebi-fl187-GCST90094775 | Inverse variance weighted (multiplicative random effects) | 3 | -0.759931961 | 0.773597590338911 | 0.325934855058453 | -2.276183238 | 0.756319316332552 | 0.467698247773924 | 0.102675347180035 | 2.13042036845757 | 0.998735809224318 |
| ebi-fl187-GCST90094775 | prot-c-4997_19_1 | IF4A3 \|\| id:prot-c-4997_19_1 | Green olives liking \|\| id:ebi-fl187-GCST90094775 | Inverse variance weighted | 3 | -0.311837166 | 0.485202780190081 | 0.520422339726331 | -1.262834615 | 0.639160282881441 | 0.732100729200093 | 0.282851114920329 | 1.89488904028635 | 0.998735809224318 |
| ebi-fl187-GCST90094775 | prot-c-5000_52_1 | LG3BP \|\| id:prot-c-5000_52_1 | Green olives liking \|\| id:ebi-fl187-GCST90094775 | Inverse variance weighted | 3 | -0.557304189 | 0.37162560432563 | 0.133707981966769 | -1.285690373 | 0.171081995635253 | 0.572751013084275 | 0.276459657362519 | 1.18658804007307 | 0.998735809224318 |
| ebi-fl187-GCST90094775 | prot-c-5006_71_1 | MK13 \|\| id:prot-c-5006_71_1 | Green olives liking \|\| id:ebi-fl187-GCST90094775 | Inverse variance weighted | 3 | -0.371827173 | 0.401449460388819 | 0.35433599188675 | -1.158668115 | 0.415013769489048 | 0.689473391933246 | 0.313903986523644 | 1.51439159294694 | 0.998735809224318 |
| ebi-fl187-GCST90094775 | prot-c-5109_24_3 | Nr-CAM \|\| id:prot-c-5109_24_3 | Green olives liking \|\| id:ebi-fl187-GCST90094775 | Inverse variance weighted | 3 | -0.116943856 | 0.394213768127875 | 0.766733071934881 | -0.889602841 | 0.655715129562384 | 0.889635139464821 | 0.410818880567238 | 1.92651973608856 | 0.998735809224318 |
| ebi-fl187-GCST90094775 | prot-c-5255_22_3 | PDE4D \|\| id:prot-c-5255_22_3 | Green olives liking \|\| id:ebi-fl187-GCST90094775 | Inverse variance weighted | 3 | 0.245836633022187 | 0.395045482907845 | 0.533744905757573 | -0.528452513 | 1.02012577952156 | 1.27869066064868 | 0.589516533070312 | 2.77354359701248 | 0.998735809224318 |
| ebi-fl187-GCST90094775 | prot-c-5440_26_3 | Troponin I, skeletal, fast twitch \|\| id:prot-c-5440_26_3 | Green olives liking \|\| id:ebi-fl187-GCST90094775 | Inverse variance weighted | 3 | -0.265744326 | 0.507536144100047 | 0.600558973713901 | -1.260515168 | 0.729026516722008 | 0.766635111330586 | 0.283507934613823 | 2.07306153432859 | 0.998735809224318 |
| ebi-fl187-GCST90094775 | ukb-rsfMRI191-edge-pheno716 | Net100_Pair10_33 (OccipitalPrecuneus)&(Temporal\|Frontal\|Supp_Motor_Area) (Default_mode\|Central_executive)&(Default_mode\|Salience) \|\| id:ukb-rsfMRI191-edge-pheno716 | Green olives liking \|\| id:ebi-fl187-GCST90094775 | Inverse variance weighted | 24 | -0.013669159 | 0.0252403759825855 | 0.588122182071611 | -0.063140296 | 0.0358019782626288 | 0.986423840065676 | 0.938811753301465 | 1.03645058642279 | 0.998735809224318 |
| ebi-fl187-GCST90094775 | ukb-rsfMRI191-edge-pheno903 | Net100_Pair27_38 (Precentral\|Frontal\|Supp_Motor_Area)&(Subcortical) (Attention\|Salience\|Motor)&(Subcortical-cerebellum) \|\| id:ukb-rsfMRI191-edge-pheno903 | Green olives liking \|\| id:ebi-fl187-GCST90094775 | Inverse variance weighted (multiplicative random effects) | 24 | 0.0360684396596662 | 0.0298984813336923 | 0.227677323591701 | -0.022532584 | 0.094669463073703 | 1.03672679729215 | 0.977719378906222 | 1.09929543733296 | 0.998735809224318 |
| ebi-fl187-GCST90094775 | ukb-rsfMRI191-node-pheno23 | Net100_Node2 RolandicOper\|SupraMarginal\|Insula Salience\|Motor \|\| id:ukb-rsfMRI191-node-pheno23 | Green olives liking \|\| id:ebi-fl187-GCST90094775 | Inverse variance weighted (multiplicative random effects) | 24 | 0.0384651062203724 | 0.0275654286285515 | 0.162891893551126 | -0.015563134 | 0.0924933463323333 | 1.03921446560477 | 0.984557345852788 | 1.09690584308908 | 0.998735809224318 |
| ebi-fl187-GCST90094775 | ukb-rsfMRI191-node-pheno55 | Net100_Node34 Parietal\|Postcentral\|Precuneus Attention \|\| id:ukb-rsfMRI191-node-pheno55 | Green olives liking \|\| id:ebi-fl187-GCST90094775 | Inverse variance weighted (multiplicative random effects) | 24 | 0.02242026305933 | 0.0310811529359781 | 0.470696706906877 | -0.038498797 | 0.0833393228138471 | 1.02267348605821 | 0.962232862598556 | 1.08691055953135 | 0.998735809224318 |
| ebi-fl187-GCST90094775 | ebi-a-GCST90001414 | IgD- CD24- B cell Absolute Count \|\| id:ebi-a-GCST90001414 | Green olives liking \|\| id:ebi-fl187-GCST90094775 | MR Egger | 25 | -0.202246764 | 0.34252245613304 | 0.560638017259137 | -0.873590778 | 0.46909724998561 | 0.816893323176144 | 0.417449884903298 | 1.59855045020397 | 0.999950574162623 |
| ebi-fl187-GCST90094775 | ebi-a-GCST90001422 | CD20- CD38- B cell %B cell \|\| id:ebi-a-GCST90001422 | Green olives liking \|\| id:ebi-fl187-GCST90094775 | MR Egger | 25 | -0.383654431 | 0.327839280067585 | 0.253883267720075 | -1.02621942 | 0.258910557720659 | 0.681366845615727 | 0.358359208422511 | 1.29551792556968 | 0.999950574162623 |
| ebi-fl187-GCST90094775 | ebi-a-GCST90001444 | CD20- CD38- B cell %lymphocyte \|\| id:ebi-a-GCST90001444 | Green olives liking \|\| id:ebi-fl187-GCST90094775 | MR Egger | 25 | -0.446416584 | 0.328710322516272 | 0.187610885968444 | -1.090688816 | 0.197855648137647 | 0.639917137290576 | 0.33598498210761 | 1.21878644702939 | 0.999950574162623 |
| ebi-fl187-GCST90094775 | ebi-a-GCST90001459 | Myeloid Dendritic Cell %Dendritic Cell \|\| id:ebi-a-GCST90001459 | Green olives liking \|\| id:ebi-fl187-GCST90094775 | MR Egger | 25 | -0.166516091 | 0.35086445936735 | 0.639555763986623 | -0.854210431 | 0.521178249304893 | 0.846609193990638 | 0.425619113929243 | 1.68401066562211 | 0.999950574162623 |
| ebi-fl187-GCST90094775 | ebi-a-GCST90001476 | HLA DR++ monocyte %leukocyte \|\| id:ebi-a-GCST90001476 | Green olives liking \|\| id:ebi-fl187-GCST90094775 | MR Egger | 25 | -0.431289576 | 0.344272838534085 | 0.222882742159366 | -1.106064339 | 0.24348518795895 | 0.649670754755643 | 0.33085854908567 | 1.27568742216626 | 0.999950574162623 |
| ebi-fl187-GCST90094775 | ebi-a-GCST90001478 | CD4 regulatory T cell %CD4+ T cell \|\| id:ebi-a-GCST90001478 | Green olives liking \|\| id:ebi-fl187-GCST90094775 | MR Egger | 25 | -0.316091226 | 0.346785793252525 | 0.371493009354136 | -0.995791381 | 0.363608928393491 | 0.728992943745734 | 0.369430968122394 | 1.43851154312273 | 0.999950574162623 |
| ebi-fl187-GCST90094775 | ebi-a-GCST90001485 | CD39+ resting CD4 regulatory T cell %CD4 regulatory T cell \|\| id:ebi-a-GCST90001485 | Green olives liking \|\| id:ebi-fl187-GCST90094775 | MR Egger | 24 | 0.178059097080762 | 0.382476092858164 | 0.646118330480655 | -0.571594045 | 0.927712239082764 | 1.19489593400979 | 0.564624683858719 | 2.52871745414231 | 0.999950574162623 |
| ebi-fl187-GCST90094775 | ebi-a-GCST90001515 | Immature Myeloid-Derived Suppressor Cells Absolute Count \|\| id:ebi-a-GCST90001515 | Green olives liking \|\| id:ebi-fl187-GCST90094775 | MR Egger | 25 | -0.339524942 | 0.586828734609961 | 0.568498357240185 | -1.489709262 | 0.810659378108694 | 0.712108535472048 | 0.225438189559367 | 2.24939069677281 | 0.999950574162623 |
| ebi-fl187-GCST90094775 | ebi-a-GCST90001517 | CD33+ HLA DR+ Absolute Count \|\| id:ebi-a-GCST90001517 | Green olives liking \|\| id:ebi-fl187-GCST90094775 | MR Egger | 25 | -0.273266294 | 0.448588100380375 | 0.54838257096181 | -1.152498971 | 0.605966382808968 | 0.76089014021751 | 0.31584649122773 | 1.83302275491415 | 0.999950574162623 |
| ebi-fl187-GCST90094775 | ebi-a-GCST90001518 | CD33+ HLA DR+ CD14- Absolute Count \|\| id:ebi-a-GCST90001518 | Green olives liking \|\| id:ebi-fl187-GCST90094775 | MR Egger | 25 | -0.398819722 | 0.453616057178711 | 0.388385809839639 | -1.287907194 | 0.490267749975315 | 0.671111677055684 | 0.275847474631972 | 1.63275333109862 | 0.999950574162623 |
| ebi-fl187-GCST90094775 | ebi-a-GCST90001520 | CD33+ HLA DR+ CD14dim Absolute Count \|\| id:ebi-a-GCST90001520 | Green olives liking \|\| id:ebi-fl187-GCST90094775 | MR Egger | 25 | -0.150920159 | 0.450828181623089 | 0.740838369494051 | -1.034543395 | 0.732703077420045 | 0.859916352877296 | 0.355388616265095 | 2.08069729896528 | 0.999950574162623 |
| ebi-fl187-GCST90094775 | ebi-a-GCST90001531 | CD33dim HLA DR- Absolute Count \|\| id:ebi-a-GCST90001531 | Green olives liking \|\| id:ebi-fl187-GCST90094775 | MR Egger | 25 | -0.296174987 | 0.489701103931864 | 0.551227625486849 | -1.255989151 | 0.663639176868396 | 0.743657286396832 | 0.284794004709769 | 1.94184621328206 | 0.999950574162623 |
| ebi-fl187-GCST90094775 | ebi-a-GCST90001532 | Basophil Absolute Count \|\| id:ebi-a-GCST90001532 | Green olives liking \|\| id:ebi-fl187-GCST90094775 | MR Egger | 25 | -0.312271335 | 0.481570488915071 | 0.523119965526504 | -1.256149494 | 0.631606822887595 | 0.731782942680708 | 0.284748343612747 | 1.88062999209827 | 0.999950574162623 |
| ebi-fl187-GCST90094775 | ebi-a-GCST90001588 | T/B cell \|\| id:ebi-a-GCST90001588 | Green olives liking \|\| id:ebi-fl187-GCST90094775 | MR Egger | 25 | 0.0746026656254516 | 0.360494181540001 | 0.837873580230024 | -0.63196593 | 0.781171261443854 | 1.0774559555337 | 0.53154579123277 | 2.18402883676802 | 0.999950574162623 |
| ebi-fl187-GCST90094775 | ebi-a-GCST90001624 | HLA DR+ CD4+ T cell Absolute Count \|\| id:ebi-a-GCST90001624 | Green olives liking \|\| id:ebi-fl187-GCST90094775 | MR Egger | 25 | -0.157606577 | 0.329886431031661 | 0.637330241075109 | -0.804183982 | 0.488970827713583 | 0.854185772107407 | 0.447452907281252 | 1.63063714951372 | 0.999950574162623 |
| ebi-fl187-GCST90094775 | ebi-a-GCST90001631 | CD8+ Natural Killer T %T cell \|\| id:ebi-a-GCST90001631 | Green olives liking \|\| id:ebi-fl187-GCST90094775 | MR Egger | 25 | 0.225456332705131 | 0.456646328289462 | 0.626184433210275 | -0.669570471 | 1.12048313615248 | 1.25289432241603 | 0.511928418802856 | 3.06633530291826 | 0.999950574162623 |
| ebi-fl187-GCST90094775 | ebi-a-GCST90001632 | CD8+ Natural Killer T %lymphocyte \|\| id:ebi-a-GCST90001632 | Green olives liking \|\| id:ebi-fl187-GCST90094775 | MR Egger | 25 | 0.251442745002713 | 0.455792023854451 | 0.586501031458382 | -0.641909622 | 1.14479511175744 | 1.28587927494182 | 0.52628645577557 | 3.14179757350627 | 0.999950574162623 |
| ebi-fl187-GCST90094775 | ebi-a-GCST90001646 | Natural Killer %CD3- lymphocyte \|\| id:ebi-a-GCST90001646 | Green olives liking \|\| id:ebi-fl187-GCST90094775 | MR Egger | 25 | -0.095141123 | 0.340718035377339 | 0.782558435112411 | -0.762948472 | 0.572666226569136 | 0.909244610295293 | 0.466289556422819 | 1.77298794271383 | 0.999950574162623 |
| ebi-fl187-GCST90094775 | ebi-a-GCST90001684 | CD127- CD8+ T cell Absolute Count \|\| id:ebi-a-GCST90001684 | Green olives liking \|\| id:ebi-fl187-GCST90094775 | MR Egger | 25 | -0.204041169 | 0.3882435195013 | 0.604231735552434 | -0.964998467 | 0.556916129400771 | 0.81542880025331 | 0.380983783770143 | 1.74528196896621 | 0.999950574162623 |
| ebi-fl187-GCST90094775 | ebi-a-GCST90001687 | CD28- CD8+ T cell Absolute Count \|\| id:ebi-a-GCST90001687 | Green olives liking \|\| id:ebi-fl187-GCST90094775 | MR Egger | 25 | -0.190457752 | 0.415681400498787 | 0.651121311596932 | -1.005193297 | 0.624277793059247 | 0.826580678439357 | 0.36597388635026 | 1.866897184 | 0.999950574162623 |
| ebi-fl187-GCST90094775 | ebi-a-GCST90001701 | BAFF-R on CD20- CD38- B cell \|\| id:ebi-a-GCST90001701 | Green olives liking \|\| id:ebi-fl187-GCST90094775 | MR Egger | 25 | 0.129060173432983 | 0.333373981247549 | 0.70221654341713 | -0.52435283 | 0.782473176678179 | 1.13775858494591 | 0.591938325270132 | 2.18687410893252 | 0.999950574162623 |
| ebi-fl187-GCST90094775 | ebi-a-GCST90001732 | CD19 on IgD- CD27- B cell \|\| id:ebi-a-GCST90001732 | Green olives liking \|\| id:ebi-fl187-GCST90094775 | MR Egger | 25 | -0.293045283 | 0.334956744441389 | 0.390686585211827 | -0.949560502 | 0.363469936481433 | 0.745988359622214 | 0.386911032822382 | 1.43831161554732 | 0.999950574162623 |
| ebi-fl187-GCST90094775 | ebi-a-GCST90001798 | CD27 on CD24+ CD27+ B cell \|\| id:ebi-a-GCST90001798 | Green olives liking \|\| id:ebi-fl187-GCST90094775 | MR Egger | 25 | -0.166138406 | 0.354006898417405 | 0.64326772311833 | -0.859991927 | 0.527715114963013 | 0.846929006076003 | 0.423165498589606 | 1.69505487503963 | 0.999950574162623 |
| ebi-fl187-GCST90094775 | ebi-a-GCST90001836 | CD66b on CD66b++ myeloid cell \|\| id:ebi-a-GCST90001836 | Green olives liking \|\| id:ebi-fl187-GCST90094775 | MR Egger | 25 | -0.342731936 | 0.508173110233837 | 0.506755078095061 | -1.338751232 | 0.653287359738754 | 0.709828465289894 | 0.262172857222012 | 1.92184826253441 | 0.999950574162623 |
| ebi-fl187-GCST90094775 | ebi-a-GCST90001943 | CD25 on activated & secreting CD4 regulatory T cell \|\| id:ebi-a-GCST90001943 | Green olives liking \|\| id:ebi-fl187-GCST90094775 | MR Egger | 25 | -0.116029523 | 0.374475400774786 | 0.759468378284876 | -0.850001309 | 0.617942262345706 | 0.890448934031588 | 0.427414372594823 | 1.85510678853478 | 0.999950574162623 |
| ebi-fl187-GCST90094775 | ebi-a-GCST90001960 | CD25 on CD4+ T cell \|\| id:ebi-a-GCST90001960 | Green olives liking \|\| id:ebi-fl187-GCST90094775 | MR Egger | 25 | -0.299959855 | 0.367159184148002 | 0.422324929294225 | -1.019591856 | 0.419672146354863 | 0.740847961740851 | 0.360742145051112 | 1.52146265676224 | 0.999950574162623 |
| ebi-fl187-GCST90094775 | ebi-a-GCST90001988 | HLA DR on CD14+ CD16- monocyte \|\| id:ebi-a-GCST90001988 | Green olives liking \|\| id:ebi-fl187-GCST90094775 | MR Egger | 24 | 0.0754261089991343 | 0.342573716251482 | 0.827765298153557 | -0.596018375 | 0.746870592852039 | 1.07834354489036 | 0.551001154332684 | 2.11038541691441 | 0.999950574162623 |
| ebi-fl187-GCST90094775 | ebi-a-GCST90001991 | HLA DR on CD14+ monocyte \|\| id:ebi-a-GCST90001991 | Green olives liking \|\| id:ebi-fl187-GCST90094775 | MR Egger | 24 | 0.0511815852168161 | 0.343190754300879 | 0.882806150351888 | -0.621472293 | 0.72383546364654 | 1.05251399691421 | 0.537153008401159 | 2.06232804503444 | 0.999950574162623 |
| ebi-fl187-GCST90094775 | ebi-a-GCST90002019 | CD14 on Monocytic Myeloid-Derived Suppressor Cells \|\| id:ebi-a-GCST90002019 | Green olives liking \|\| id:ebi-fl187-GCST90094775 | MR Egger | 25 | -1.010889189 | 0.490250761059226 | 0.050683835650489 | -1.97178068 | -0.049997697 | 0.363895264105951 | 0.139208749090117 | 0.951231615140926 | 0.999950574162623 |
| ebi-fl187-GCST90094775 | ebi-a-GCST90002037 | CD80 on plasmacytoid Dendritic Cell \|\| id:ebi-a-GCST90002037 | Green olives liking \|\| id:ebi-fl187-GCST90094775 | MR Egger | 25 | -0.457308002 | 0.384421342821901 | 0.246339148401527 | -1.210773834 | 0.296157829816512 | 0.632985349209554 | 0.297966613482929 | 1.34468237105664 | 0.999950574162623 |
| ebi-fl187-GCST90094775 | ebi-a-GCST90002042 | CD45 on CD33+ HLA DR+ CD14- \|\| id:ebi-a-GCST90002042 | Green olives liking \|\| id:ebi-fl187-GCST90094775 | MR Egger | 25 | -1.282723019 | 0.593027584918365 | 0.0411780027332371 | -2.445057086 | -0.120388953 | 0.277281229406699 | 0.0867211842248083 | 0.886575533631795 | 0.999950574162623 |
| ebi-fl187-GCST90094775 | ebi-a-GCST90002056 | CD8 on Effector Memory CD8+ T cell \|\| id:ebi-a-GCST90002056 | Green olives liking \|\| id:ebi-fl187-GCST90094775 | MR Egger | 25 | -0.508391882 | 0.389764413823355 | 0.205014393738189 | -1.272330133 | 0.255546369583589 | 0.6014620237263 | 0.280178008658604 | 1.29116688250053 | 0.999950574162623 |
| ebi-fl187-GCST90094775 | ebi-a-GCST90002058 | CD8 on CD8+ T cell \|\| id:ebi-a-GCST90002058 | Green olives liking \|\| id:ebi-fl187-GCST90094775 | MR Egger | 25 | -0.391177568 | 0.362157559666762 | 0.291280631948031 | -1.101006385 | 0.318651249314265 | 0.676260063478843 | 0.332536255883539 | 1.37527161434263 | 0.999950574162623 |
| ebi-fl187-GCST90094775 | ebi-a-GCST90002063 | CD4 on CD4 regulatory T cell \|\| id:ebi-a-GCST90002063 | Green olives liking \|\| id:ebi-fl187-GCST90094775 | MR Egger | 25 | -0.56360439 | 0.434141824248447 | 0.207085099268655 | -1.414522366 | 0.287313585385429 | 0.569153909544268 | 0.243041670776006 | 1.33284210775556 | 0.999950574162623 |
| ebi-fl187-GCST90094775 | ebi-a-GCST90002066 | CD4 on activated CD4 regulatory T cell \|\| id:ebi-a-GCST90002066 | Green olives liking \|\| id:ebi-fl187-GCST90094775 | MR Egger | 25 | -0.644250277 | 0.417278269971618 | 0.136252954641433 | -1.462115686 | 0.173615132462356 | 0.525056041337827 | 0.231745455128059 | 1.18959764019109 | 0.999950574162623 |
| ebi-fl187-GCST90094775 | ebi-a-GCST90002070 | CD4 on activated & secreting CD4 regulatory T cell \|\| id:ebi-a-GCST90002070 | Green olives liking \|\| id:ebi-fl187-GCST90094775 | MR Egger | 25 | -0.605113097 | 0.435380150936078 | 0.177881523682388 | -1.458458193 | 0.24823199911346 | 0.546012670904748 | 0.232594614521405 | 1.28175726425128 | 0.999950574162623 |
| ebi-fl187-GCST90094775 | ebi-a-GCST90002081 | SSC-A on CD4+ T cell \|\| id:ebi-a-GCST90002081 | Green olives liking \|\| id:ebi-fl187-GCST90094775 | MR Egger | 25 | 0.251313198146068 | 0.353587016122553 | 0.484382762373988 | -0.441717353 | 0.944343749746273 | 1.28571270411332 | 0.642931332096603 | 2.57112552304422 | 0.999950574162623 |
| ebi-fl187-GCST90094775 | ebi-a-GCST90002094 | CD11b on Monocytic Myeloid-Derived Suppressor Cells \|\| id:ebi-a-GCST90002094 | Green olives liking \|\| id:ebi-fl187-GCST90094775 | MR Egger | 25 | -0.788461493 | 0.498608000731307 | 0.127459062836685 | -1.765733174 | 0.188810188923164 | 0.454543576301187 | 0.171061322792584 | 1.20781167468927 | 0.999950574162623 |
| ebi-fl187-GCST90094775 | ebi-a-GCST90002104 | HLA DR on myeloid Dendritic Cell \|\| id:ebi-a-GCST90002104 | Green olives liking \|\| id:ebi-fl187-GCST90094775 | MR Egger | 24 | -0.416560931 | 0.381035277837056 | 0.286117518789768 | -1.163390076 | 0.330268213289264 | 0.659310338954352 | 0.312425238311099 | 1.39134125463724 | 0.999950574162623 |
| ebi-fl187-GCST90094775 | ebi-a-GCST90002105 | HLA DR on plasmacytoid Dendritic Cell \|\| id:ebi-a-GCST90002105 | Green olives liking \|\| id:ebi-fl187-GCST90094775 | MR Egger | 24 | -0.129853482 | 0.372566195421929 | 0.730749523597672 | -0.860083225 | 0.600376261238031 | 0.878224097317986 | 0.423126866196787 | 1.82280452206334 | 0.999950574162623 |
| ebi-fl187-GCST90094775 | ebi-a-GCST90002106 | HLA DR on Dendritic Cell \|\| id:ebi-a-GCST90002106 | Green olives liking \|\| id:ebi-fl187-GCST90094775 | MR Egger | 24 | -0.239263458 | 0.380187917440214 | 0.535614556603091 | -0.984431776 | 0.505904860216099 | 0.787207458973685 | 0.373651484311244 | 1.6584855392883 | 0.999950574162623 |
| ebi-fl187-GCST90094775 | ebi-a-GCST90002111 | HLA DR on CD33dim HLA DR+ CD11b- \|\| id:ebi-a-GCST90002111 | Green olives liking \|\| id:ebi-fl187-GCST90094775 | MR Egger | 24 | 0.0733515132697591 | 0.485480088715499 | 0.881281049964586 | -0.878189461 | 1.02489248715214 | 1.07610873694038 | 0.41553457273103 | 2.78679582810308 | 0.999950574162623 |
| ebi-fl187-GCST90094775 | ebi-a-GCST90002112 | HLA DR on CD33- HLA DR+ \|\| id:ebi-a-GCST90002112 | Green olives liking \|\| id:ebi-fl187-GCST90094775 | MR Egger | 24 | 0.465771593913421 | 0.499026888757435 | 0.360765854127045 | -0.512321108 | 1.44386429587799 | 1.59324305137577 | 0.599103380036542 | 4.2370373884426 | 0.999950574162623 |
| ebi-fl187-GCST90094775 | ebi-a-GCST90002115 | HLA DR on HLA DR+ CD8+ T cell \|\| id:ebi-a-GCST90002115 | Green olives liking \|\| id:ebi-fl187-GCST90094775 | MR Egger | 25 | -0.134189031 | 0.366178587464379 | 0.717372999651036 | -0.851899063 | 0.583521000160818 | 0.874424755358048 | 0.42660401442955 | 1.79233815651131 | 0.999950574162623 |
| ebi-fl187-GCST90094775 | ebi-a-GCST90002116 | HLA DR on B cell \|\| id:ebi-a-GCST90002116 | Green olives liking \|\| id:ebi-fl187-GCST90094775 | MR Egger | 25 | -0.268757621 | 0.451182547451367 | 0.557209961170455 | -1.153075414 | 0.615560171525087 | 0.764328490007391 | 0.315664475941256 | 1.85069301477463 | 0.999950574162623 |
| ebi-fl187-GCST90094775 | ebi-cfmet338-GCST90026023 | Sphingomyelin (d18:1/24:1, d18:2/24:0) levels \|\| id:ebi-cfmet338-GCST90026023 | Green olives liking \|\| id:ebi-fl187-GCST90094775 | MR Egger | 23 | -0.304806085 | 0.209446497810325 | 0.160374847065524 | -0.71532122 | 0.105709051051995 | 0.737266327784145 | 0.489034998563216 | 1.11149844015521 | 0.999950574162623 |
| ebi-fl187-GCST90094775 | ebi-cfmet338-GCST90026046 | 1-arachidonoyl-gpc (20:4n6) levels \|\| id:ebi-cfmet338-GCST90026046 | Green olives liking \|\| id:ebi-fl187-GCST90094775 | MR Egger | 23 | -0.060763247 | 0.271555244863409 | 0.825106409908134 | -0.593011527 | 0.471485032950664 | 0.94104600888738 | 0.552660424354639 | 1.6023720024407 | 0.999950574162623 |
| ebi-fl187-GCST90094775 | ebi-cfmet338-GCST90026047 | 1-linoleoyl-gpc (18:2) levels \|\| id:ebi-cfmet338-GCST90026047 | Green olives liking \|\| id:ebi-fl187-GCST90094775 | MR Egger | 23 | -0.295720452 | 0.301681679243056 | 0.338126456801902 | -0.887016543 | 0.295575639268725 | 0.743995381337763 | 0.411882755828087 | 1.34389973753346 | 0.999950574162623 |
| ebi-fl187-GCST90094775 | ebi-cfmet338-GCST90026050 | 1-myristoyl-2-palmitoyl-gpc (14:0/16:0) levels \|\| id:ebi-cfmet338-GCST90026050 | Green olives liking \|\| id:ebi-fl187-GCST90094775 | MR Egger | 23 | -0.120718599 | 0.172743957996548 | 0.492326219269428 | -0.459296757 | 0.217859558691047 | 0.886283325535191 | 0.631727747666811 | 1.24341242888702 | 0.999950574162623 |
| ebi-fl187-GCST90094775 | ebi-cfmet338-GCST90026068 | 2,3-dihydroxyisovalerate levels \|\| id:ebi-cfmet338-GCST90026068 | Green olives liking \|\| id:ebi-fl187-GCST90094775 | MR Egger | 23 | 0.0832441879902755 | 0.544465625056339 | 0.879943354649303 | -0.983908437 | 1.1503968131007 | 1.08680716140549 | 0.373847081893834 | 3.1594463706893 | 0.999950574162623 |
| ebi-fl187-GCST90094775 | ebi-cfmet338-GCST90026082 | 3-hydroxyhexanoate levels \|\| id:ebi-cfmet338-GCST90026082 | Green olives liking \|\| id:ebi-fl187-GCST90094775 | MR Egger | 23 | -0.009141112 | 0.124124771735772 | 0.941990207939691 | -0.252425664 | 0.234143441083477 | 0.990900541426567 | 0.776913963275384 | 1.26382576374345 | 0.999950574162623 |
| ebi-fl187-GCST90094775 | ebi-cfmet338-GCST90026094 | 3-ureidopropionate levels \|\| id:ebi-cfmet338-GCST90026094 | Green olives liking \|\| id:ebi-fl187-GCST90094775 | MR Egger | 23 | 0.0358117441846342 | 0.249983157932347 | 0.887453228165258 | -0.454155245 | 0.525778733732035 | 1.03646070836781 | 0.634984147249326 | 1.69177577840931 | 0.999950574162623 |
| ebi-fl187-GCST90094775 | ebi-cfmet338-GCST90026173 | Glycerol levels \|\| id:ebi-cfmet338-GCST90026173 | Green olives liking \|\| id:ebi-fl187-GCST90094775 | MR Egger | 23 | 0.149351382243211 | 0.174453576856965 | 0.401600544339783 | -0.192577628 | 0.491280392882863 | 1.16108090074991 | 0.82483028546586 | 1.63440756461168 | 0.999950574162623 |
| ebi-fl187-GCST90094775 | ebi-cfmet338-GCST90026179 | Histidine levels \|\| id:ebi-cfmet338-GCST90026179 | Green olives liking \|\| id:ebi-fl187-GCST90094775 | MR Egger | 23 | -0.04195372 | 0.0980432763927352 | 0.673072859152912 | -0.234118542 | 0.150211101302731 | 0.958914157683332 | 0.79126801171911 | 1.16207953334015 | 0.999950574162623 |
| ebi-fl187-GCST90094775 | ebi-cfmet338-GCST90026188 | Isocitrate levels \|\| id:ebi-cfmet338-GCST90026188 | Green olives liking \|\| id:ebi-fl187-GCST90094775 | MR Egger | 23 | -0.119436607 | 0.191855900367646 | 0.540293191620303 | -0.495474172 | 0.256600957768848 | 0.887420262311477 | 0.609281934556554 | 1.29252925008213 | 0.999950574162623 |
| ebi-fl187-GCST90094775 | ebi-cfmet338-GCST90026207 | N-acetyl-isoputreanine levels \|\| id:ebi-cfmet338-GCST90026207 | Green olives liking \|\| id:ebi-fl187-GCST90094775 | MR Egger | 23 | 0.177799328939401 | 0.121508683204608 | 0.158202768787012 | -0.06035769 | 0.415956348020432 | 1.19458557842588 | 0.941427733933397 | 1.51581969889583 | 0.999950574162623 |
| ebi-fl187-GCST90094775 | ebi-cfmet338-GCST90026213 | N-acetylglutamate levels \|\| id:ebi-cfmet338-GCST90026213 | Green olives liking \|\| id:ebi-fl187-GCST90094775 | MR Egger | 23 | 0.325132287701925 | 0.231968350645428 | 0.175635062280667 | -0.12952568 | 0.779790254966965 | 1.38421374832513 | 0.878512028321503 | 2.18101476050719 | 0.999950574162623 |
| ebi-fl187-GCST90094775 | ebi-cfmet338-GCST90026224 | N-acetyltaurine levels \|\| id:ebi-cfmet338-GCST90026224 | Green olives liking \|\| id:ebi-fl187-GCST90094775 | MR Egger | 23 | 0.113273550997195 | 0.166333035748639 | 0.503309435063247 | -0.212739199 | 0.439286301064528 | 1.11993825127527 | 0.808366932575166 | 1.55159944837659 | 0.999950574162623 |
| ebi-fl187-GCST90094775 | ebi-cfmet338-GCST90026250 | Phenyllactate (pla) levels \|\| id:ebi-cfmet338-GCST90026250 | Green olives liking \|\| id:ebi-fl187-GCST90094775 | MR Egger | 23 | 0.110659195140029 | 0.260275533604027 | 0.675043729235211 | -0.399480851 | 0.620799241003922 | 1.11701415812389 | 0.670668132548931 | 1.86041436724771 | 0.999950574162623 |
| ebi-fl187-GCST90094775 | ebi-cfmet338-GCST90026251 | Pipecolate levels \|\| id:ebi-cfmet338-GCST90026251 | Green olives liking \|\| id:ebi-fl187-GCST90094775 | MR Egger | 23 | -0.328042866 | 0.390654759085947 | 0.410520618277417 | -1.093726194 | 0.437640461614043 | 0.720332141145879 | 0.334966017030419 | 1.54904786511727 | 0.999950574162623 |
| ebi-fl187-GCST90094775 | ebi-cfmet338-GCST90026278 | Trigonelline (n'-methylnicotinate) levels \|\| id:ebi-cfmet338-GCST90026278 | Green olives liking \|\| id:ebi-fl187-GCST90094775 | MR Egger | 22 | -0.10281655 | 0.453483953158534 | 0.822938181987093 | -0.991645098 | 0.786011998469452 | 0.902292484107268 | 0.370965913515645 | 2.19462677624727 | 0.999950574162623 |
| ebi-fl187-GCST90094775 | ebi-cfmet338-GCST90026299 | 1-carboxyethylvaline levels \|\| id:ebi-cfmet338-GCST90026299 | Green olives liking \|\| id:ebi-fl187-GCST90094775 | MR Egger | 23 | -0.293614282 | 0.297836226096775 | 0.335442107273337 | -0.877373286 | 0.290144720712631 | 0.74556401312281 | 0.415873860111797 | 1.33662091075972 | 0.999950574162623 |
| ebi-fl187-GCST90094775 | ebi-gm430-GCST90011306 | C_Erysipelotrichia abundance \|\| id:ebi-gm430-GCST90011306 | Green olives liking \|\| id:ebi-fl187-GCST90094775 | MR Egger | 20 | 0.0136289374192735 | 0.290996748532293 | 0.963160172943398 | -0.55672469 | 0.583982564542569 | 1.01372223475288 | 0.573083017992184 | 1.79316562691515 | 0.999950574162623 |
| ebi-fl187-GCST90094775 | ebi-gm430-GCST90011312 | F_Erysipelotrichaceae abundance \|\| id:ebi-gm430-GCST90011312 | Green olives liking \|\| id:ebi-fl187-GCST90094775 | MR Egger | 20 | 0.0136289374192735 | 0.290996748532293 | 0.963160172943398 | -0.55672469 | 0.583982564542569 | 1.01372223475288 | 0.573083017992184 | 1.79316562691515 | 0.999950574162623 |
| ebi-fl187-GCST90094775 | ebi-gm430-GCST90011352 | O_Erysipelotrichales abundance \|\| id:ebi-gm430-GCST90011352 | Green olives liking \|\| id:ebi-fl187-GCST90094775 | MR Egger | 20 | 0.0136289374192735 | 0.290996748532293 | 0.963160172943398 | -0.55672469 | 0.583982564542569 | 1.01372223475288 | 0.573083017992184 | 1.79316562691515 | 0.999950574162623 |
| ebi-fl187-GCST90094775 | ebi-gm430-GCST90011362 | OTU97_11 (Parabacteroides) abundance \|\| id:ebi-gm430-GCST90011362 | Green olives liking \|\| id:ebi-fl187-GCST90094775 | MR Egger | 20 | 0.478322997867883 | 0.306145325731726 | 0.135602515990522 | -0.121721841 | 1.07836783630207 | 1.61336651317721 | 0.885394615117872 | 2.939877272 | 0.999950574162623 |
| ebi-fl187-GCST90094775 | ebi-gm430-GCST90011384 | OTU97_235 (Phascolarctobacterium) abundance \|\| id:ebi-gm430-GCST90011384 | Green olives liking \|\| id:ebi-fl187-GCST90094775 | MR Egger | 20 | -1.483205092 | 1.10914448446179 | 0.197795040647551 | -3.657128281 | 0.690718098030535 | 0.226909256729011 | 0.0258065154772324 | 1.99514773060846 | 0.999950574162623 |
| ebi-fl187-GCST90094775 | ebi-gm430-GCST90011390 | OTU97_3 (Bacteroides) abundance \|\| id:ebi-gm430-GCST90011390 | Green olives liking \|\| id:ebi-fl187-GCST90094775 | MR Egger | 20 | -0.084597501 | 0.245591085929332 | 0.734488515912585 | -0.56595603 | 0.396761027246701 | 0.918882058928784 | 0.567817037291621 | 1.48700053497613 | 0.999950574162623 |
| ebi-fl187-GCST90094775 | ebi-gm430-GCST90011398 | OTU97_4 (Alistipes) abundance \|\| id:ebi-gm430-GCST90011398 | Green olives liking \|\| id:ebi-fl187-GCST90094775 | MR Egger | 20 | 0.0421444122843139 | 0.35001989662268 | 0.905495544253617 | -0.643894585 | 0.728183409664767 | 1.043045096 | 0.525242832571909 | 2.07131445810854 | 0.999950574162623 |
| ebi-fl187-GCST90094775 | ebi-gm430-GCST90011421 | OTU99_108 (Cytophagales) abundance \|\| id:ebi-gm430-GCST90011421 | Green olives liking \|\| id:ebi-fl187-GCST90094775 | MR Egger | 20 | 0.790139164034596 | 1.14049193061569 | 0.497273676555126 | -1.44522502 | 3.02550334804134 | 2.2037030811296 | 0.235693034948217 | 20.6043732724093 | 0.999950574162623 |
| ebi-fl187-GCST90094775 | ebi-gm430-GCST90011428 | OTU99_123 (Phascolarctobacterium) abundance \|\| id:ebi-gm430-GCST90011428 | Green olives liking \|\| id:ebi-fl187-GCST90094775 | MR Egger | 20 | 0.410701121475936 | 0.54638586056004 | 0.461966327150969 | -0.660215165 | 1.48161740817362 | 1.5078746178631 | 0.516740138022941 | 4.40005661626161 | 0.999950574162623 |
| ebi-fl187-GCST90094775 | ebi-gm430-GCST90011450 | OTU99_298 (Phascolarctobacterium) abundance \|\| id:ebi-gm430-GCST90011450 | Green olives liking \|\| id:ebi-fl187-GCST90094775 | MR Egger | 20 | -1.501008573 | 1.11211402860225 | 0.193846745857986 | -3.680752069 | 0.678734922897629 | 0.222905230505163 | 0.0252040125435076 | 1.97138220355944 | 0.999950574162623 |
| ebi-fl187-GCST90094775 | ebi-gm430-GCST90011451 | OTU99_3 (Bacteroides) abundance \|\| id:ebi-gm430-GCST90011451 | Green olives liking \|\| id:ebi-fl187-GCST90094775 | MR Egger | 20 | -0.089570362 | 0.245554978527702 | 0.719535686998228 | -0.570858119 | 0.391717396392271 | 0.914323929637647 | 0.565040358487002 | 1.47951953475773 | 0.999950574162623 |
| ebi-fl187-GCST90094775 | ebi-gm430-GCST90011472 | OTU99_72 (Parabacteroides) abundance \|\| id:ebi-gm430-GCST90011472 | Green olives liking \|\| id:ebi-fl187-GCST90094775 | MR Egger | 20 | -0.40222383 | 0.783441377317857 | 0.613911248077507 | -1.93776893 | 1.13332126945117 | 0.668831024425291 | 0.14402492132332 | 3.10595510223933 | 0.999950574162623 |
| ebi-fl187-GCST90094775 | ebi-gm430-GCST90011507 | TestASV_3 (Bacteroides) abundance \|\| id:ebi-gm430-GCST90011507 | Green olives liking \|\| id:ebi-fl187-GCST90094775 | MR Egger | 20 | 0.279318021246828 | 0.315650480815869 | 0.387880547511903 | -0.339356921 | 0.897992963645931 | 1.32222777353834 | 0.712228194409624 | 2.45467154886412 | 0.999950574162623 |
| ebi-fl187-GCST90094775 | ebi-gm430-GCST90011512 | TestASV_34 (Phascolarctobacterium) abundance \|\| id:ebi-gm430-GCST90011512 | Green olives liking \|\| id:ebi-fl187-GCST90094775 | MR Egger | 20 | -0.742554933 | 0.988546390245143 | 0.46226573033935 | -2.680105858 | 1.19499599140546 | 0.475896477106208 | 0.068555896555677 | 3.30354452790458 | 0.999950574162623 |
| ebi-fl187-GCST90094775 | ebi-gm430-GCST90011519 | TestASV_41 (Barnesiella) abundance \|\| id:ebi-gm430-GCST90011519 | Green olives liking \|\| id:ebi-fl187-GCST90094775 | MR Egger | 20 | -0.855855909 | 1.22351296336303 | 0.493176681368019 | -3.253941317 | 1.54222949909381 | 0.424919343062544 | 0.0386216871396753 | 4.6750015724513 | 0.999950574162623 |
| ebi-fl187-GCST90094775 | ebi-gm430-GCST90011524 | TestASV_46 (Sutterella) abundance \|\| id:ebi-gm430-GCST90011524 | Green olives liking \|\| id:ebi-fl187-GCST90094775 | MR Egger | 20 | -1.51033555 | 1.07521461381937 | 0.177137561909881 | -3.617756193 | 0.597085092908469 | 0.220835864012234 | 0.0268428391139811 | 1.81681522684494 | 0.999950574162623 |
| ebi-fl187-GCST90094775 | ebi-gm430-GCST90011581 | OTU97_19 (Bacteria) prevalence \|\| id:ebi-gm430-GCST90011581 | Green olives liking \|\| id:ebi-fl187-GCST90094775 | MR Egger | 20 | -2.159736537 | 1.24807681062758 | 0.100656935561322 | -4.605967086 | 0.286494011880567 | 0.115355508949123 | 0.00999203417648875 | 1.33175019319112 | 0.999950574162623 |
| ebi-fl187-GCST90094775 | ebi-gm430-GCST90011614 | OTU97_80 (Ruminococcus) prevalence \|\| id:ebi-gm430-GCST90011614 | Green olives liking \|\| id:ebi-fl187-GCST90094775 | MR Egger | 20 | 0.928597381805079 | 0.746841875095735 | 0.229684717455263 | -0.535212693 | 2.39240745699272 | 2.53095672088277 | 0.585544735408864 | 10.9397993622276 | 0.999950574162623 |
| ebi-fl187-GCST90094775 | ebi-gm430-GCST90011623 | OTU99_11 (Parabacteroides) prevalence \|\| id:ebi-gm430-GCST90011623 | Green olives liking \|\| id:ebi-fl187-GCST90094775 | MR Egger | 20 | 0.517001584471602 | 0.563787210771073 | 0.371258372740981 | -0.588021349 | 1.6220245175829 | 1.67699178545452 | 0.555425190999163 | 5.06333074922807 | 0.999950574162623 |
| ebi-fl187-GCST90094775 | ebi-gm430-GCST90011642 | OTU99_19 (Bacteria) prevalence \|\| id:ebi-gm430-GCST90011642 | Green olives liking \|\| id:ebi-fl187-GCST90094775 | MR Egger | 20 | -2.159736537 | 1.24807681062758 | 0.100656935561322 | -4.605967086 | 0.286494011880567 | 0.115355508949123 | 0.00999203417648875 | 1.33175019319112 | 0.999950574162623 |
| ebi-fl187-GCST90094775 | ebi-gm430-GCST90011653 | OTU99_30 (Parasutterella) prevalence \|\| id:ebi-gm430-GCST90011653 | Green olives liking \|\| id:ebi-fl187-GCST90094775 | MR Egger | 20 | 1.07973318084725 | 0.625219538379132 | 0.101294333860528 | -0.145697114 | 2.30516347607035 | 2.94389395897277 | 0.864419483772729 | 10.0258170996467 | 0.999950574162623 |
| ebi-fl187-GCST90094775 | ebi-gm430-GCST90011675 | OTU99_8 (Ruminococcaceae) prevalence \|\| id:ebi-gm430-GCST90011675 | Green olives liking \|\| id:ebi-fl187-GCST90094775 | MR Egger | 20 | -0.488034998 | 0.542403807064301 | 0.380126358784164 | -1.55114646 | 0.575076463926243 | 0.613831389845142 | 0.212004779497269 | 1.77726641848691 | 0.999950574162623 |
| ebi-fl187-GCST90094775 | ebi-gm430-GCST90011679 | OTU99_92 (Ruminococcus) prevalence \|\| id:ebi-gm430-GCST90011679 | Green olives liking \|\| id:ebi-fl187-GCST90094775 | MR Egger | 20 | 1.1577425292317 | 0.718960952183437 | 0.124729175570262 | -0.251420937 | 2.56690599551124 | 3.18274021710752 | 0.777694942037169 | 13.0254611956953 | 0.999950574162623 |
| ebi-fl187-GCST90094775 | ebi-gm430-GCST90011684 | TestASV_11 (Lachnospiraceae) prevalence \|\| id:ebi-gm430-GCST90011684 | Green olives liking \|\| id:ebi-fl187-GCST90094775 | MR Egger | 20 | 0.26474284956507 | 0.570756106212613 | 0.648318373096136 | -0.853939119 | 1.38342481774179 | 1.30309584102792 | 0.425734605510716 | 3.98853827930488 | 0.999950574162623 |
| ebi-fl187-GCST90094775 | ebi-gm430-GCST90011688 | TestASV_15 (Bacteroides) prevalence \|\| id:ebi-gm430-GCST90011688 | Green olives liking \|\| id:ebi-fl187-GCST90094775 | MR Egger | 20 | -0.009958483 | 0.555415598972284 | 0.985892117497101 | -1.098573057 | 1.07865609105991 | 0.990090938574879 | 0.33334641084208 | 2.94072482788028 | 0.999950574162623 |
| ebi-fl187-GCST90094775 | ebi-gm430-GCST90011692 | TestASV_19 (Prevotella) prevalence \|\| id:ebi-gm430-GCST90011692 | Green olives liking \|\| id:ebi-fl187-GCST90094775 | MR Egger | 20 | 0.322611299847732 | 1.31167776755198 | 0.808501202926131 | -2.248277125 | 2.89349972424962 | 1.38072855718607 | 0.105580970815996 | 18.0563915438093 | 0.999950574162623 |
| ebi-fl187-GCST90094775 | ebi-gm430-GCST90011693 | TestASV_2 (Bacteroides) prevalence \|\| id:ebi-gm430-GCST90011693 | Green olives liking \|\| id:ebi-fl187-GCST90094775 | MR Egger | 20 | -0.126152684 | 0.561834115684851 | 0.824868416711325 | -1.22734755 | 0.975042183073784 | 0.881480248870097 | 0.293068898128723 | 2.65127904772347 | 0.999950574162623 |
| ebi-fl187-GCST90094775 | ebi-gm430-GCST90011716 | TestASV_40 (Barnesiella) prevalence \|\| id:ebi-gm430-GCST90011716 | Green olives liking \|\| id:ebi-fl187-GCST90094775 | MR Egger | 20 | -0.118732805 | 1.25951078906406 | 0.925936936030731 | -2.587373952 | 2.34990834132364 | 0.888045050046975 | 0.075217305226517 | 10.4846086753307 | 0.999950574162623 |
| ebi-fl187-GCST90094775 | ebi-gm430-GCST90011718 | TestASV_42 (Alphaproteobacteria) prevalence \|\| id:ebi-gm430-GCST90011718 | Green olives liking \|\| id:ebi-fl187-GCST90094775 | MR Egger | 20 | -0.122578878 | 0.875860591606468 | 0.890251651302147 | -1.839265638 | 1.59410788105325 | 0.884636123424975 | 0.158934098417197 | 4.92393437696495 | 0.999950574162623 |
| ebi-fl187-GCST90094775 | ebi-hbsm44-GCST90104929 | Sulcal depth of the right inferior frontal sulcus \|\| id:ebi-hbsm44-GCST90104929 | Green olives liking \|\| id:ebi-fl187-GCST90094775 | MR Egger | 19 | -0.084451567 | 0.546856535642811 | 0.879088557024055 | -1.156290377 | 0.987387243109082 | 0.919016165237868 | 0.314651256210682 | 2.68421210879578 | 0.999950574162623 |
| ebi-fl187-GCST90094775 | ebi-hbsm44-GCST90104935 | Sulcal opening distance of the left calloso-marginal posterior fissure \|\| id:ebi-hbsm44-GCST90104935 | Green olives liking \|\| id:ebi-fl187-GCST90094775 | MR Egger | 20 | -0.101541314 | 0.101620960393931 | 0.330934074369024 | -0.300718396 | 0.0976357685342906 | 0.903443853838344 | 0.740286210799562 | 1.10256112450982 | 0.999950574162623 |
| ebi-fl187-GCST90094775 | ebi-hbsm44-GCST90104936 | Sulcal opening distance of the left anterior inferior temporal sulcus \|\| id:ebi-hbsm44-GCST90104936 | Green olives liking \|\| id:ebi-fl187-GCST90094775 | MR Egger | 18 | -0.032140725 | 0.0690630814249757 | 0.64792930912381 | -0.167504365 | 0.103222914628858 | 0.968370298614677 | 0.845772925856605 | 1.10873853557009 | 0.999950574162623 |
| ebi-fl187-GCST90094775 | ebi-hbsm44-GCST90104941 | Sulcal opening distance of the right posterior inferior temporal sulcus \|\| id:ebi-hbsm44-GCST90104941 | Green olives liking \|\| id:ebi-fl187-GCST90094775 | MR Egger | 19 | 0.011891818880225 | 0.049168843247583 | 0.811784522214616 | -0.084479114 | 0.108262751645488 | 1.01196280767458 | 0.918990849324916 | 1.11434050172414 | 0.999950574162623 |
| ebi-fl187-GCST90094775 | ebi-IPs91-GCST90274819 | Leukemia inhibitory factor levels \|\| id:ebi-IPs91-GCST90274819 | Green olives liking \|\| id:ebi-fl187-GCST90094775 | MR Egger | 27 | -0.00872624 | 0.172262277011567 | 0.960001645782912 | -0.346360303 | 0.328907822746995 | 0.991311722932922 | 0.707257614055516 | 1.38944977402124 | 0.999950574162623 |
| ebi-fl187-GCST90094775 | ebi-IPs91-GCST90274837 | STAM binding protein levels \|\| id:ebi-IPs91-GCST90274837 | Green olives liking \|\| id:ebi-fl187-GCST90094775 | MR Egger | 27 | 0.0626453207644005 | 0.153893848024449 | 0.687422130011672 | -0.238986621 | 0.364277262892321 | 1.06464916330694 | 0.787425416980628 | 1.43947327135625 | 0.999950574162623 |
| ebi-fl187-GCST90094775 | ebi-IPs91-GCST90274840 | TNF-beta levels \|\| id:ebi-IPs91-GCST90274840 | Green olives liking \|\| id:ebi-fl187-GCST90094775 | MR Egger | 27 | 0.319697714757017 | 0.195001669993058 | 0.113642078267376 | -0.062505558 | 0.701900987943412 | 1.37671154184722 | 0.939407841166822 | 2.01758446799975 | 0.999950574162623 |
| ebi-fl187-GCST90094775 | ebi-IPs91-GCST90274842 | Tumor necrosis factor ligand superfamily member 14 levels \|\| id:ebi-IPs91-GCST90274842 | Green olives liking \|\| id:ebi-fl187-GCST90094775 | MR Egger | 27 | -0.100465774 | 0.18921265882217 | 0.600127185080326 | -0.471322585 | 0.270391037524391 | 0.904416066638013 | 0.624176196016965 | 1.31047679615572 | 0.999950574162623 |
| ebi-fl187-GCST90094775 | ebi-met1400-GCST90199628 | 3-methylhistidine levels \|\| id:ebi-met1400-GCST90199628 | Green olives liking \|\| id:ebi-fl187-GCST90094775 | MR Egger | 24 | 0.0479364490432605 | 0.219199959728945 | 0.828908923947776 | -0.381695472 | 0.477568370111992 | 1.04910398164415 | 0.682702923691151 | 1.61214948128672 | 0.999950574162623 |
| ebi-fl187-GCST90094775 | ebi-met1400-GCST90199651 | Docosahexaenoate DHA; 22:6n3 levels \|\| id:ebi-met1400-GCST90199651 | Green olives liking \|\| id:ebi-fl187-GCST90094775 | MR Egger | 24 | -0.140527651 | 0.210209802445139 | 0.510761244506116 | -0.552538864 | 0.271483561689398 | 0.868899638567132 | 0.575486871224891 | 1.31190930610658 | 0.999950574162623 |
| ebi-fl187-GCST90094775 | ebi-met1400-GCST90199652 | 1-stearoyl-2-oleoyl-GPS (18:0/18:1) levels \|\| id:ebi-met1400-GCST90199652 | Green olives liking \|\| id:ebi-fl187-GCST90094775 | MR Egger | 24 | 0.378160891665381 | 0.246428459312816 | 0.139147248506132 | -0.104838889 | 0.861160671918501 | 1.45959776109971 | 0.900469586827032 | 2.36590514035486 | 0.999950574162623 |
| ebi-fl187-GCST90094775 | ebi-met1400-GCST90199653 | 1-myristoyl-2-palmitoyl-gpc (14:0/16:0) levels \|\| id:ebi-met1400-GCST90199653 | Green olives liking \|\| id:ebi-fl187-GCST90094775 | MR Egger | 23 | -0.061726848 | 0.238024132408157 | 0.797905730847531 | -0.528254148 | 0.404800451315338 | 0.940139652555452 | 0.589633484560238 | 1.49900334606386 | 0.999950574162623 |
| ebi-fl187-GCST90094775 | ebi-met1400-GCST90199667 | Erythritol levels in elite athletes \|\| id:ebi-met1400-GCST90199667 | Green olives liking \|\| id:ebi-fl187-GCST90094775 | MR Egger | 24 | 0.183055530587691 | 0.253790725121301 | 0.478329906937236 | -0.314374291 | 0.68048535182544 | 1.2008810918621 | 0.730245652881592 | 1.97483598991823 | 0.999950574162623 |
| ebi-fl187-GCST90094775 | ebi-met1400-GCST90199674 | 3-hydroxymyristate levels \|\| id:ebi-met1400-GCST90199674 | Green olives liking \|\| id:ebi-fl187-GCST90094775 | MR Egger | 24 | 0.0248477126189711 | 0.214466679843188 | 0.908816018037379 | -0.39550698 | 0.445202405111619 | 1.02515898985866 | 0.673338583590558 | 1.56080607899204 | 0.999950574162623 |
| ebi-fl187-GCST90094775 | ebi-met1400-GCST90199685 | 2-linoleoylglycerol (18:2) levels \|\| id:ebi-met1400-GCST90199685 | Green olives liking \|\| id:ebi-fl187-GCST90094775 | MR Egger | 24 | -0.139295733 | 0.251177840823986 | 0.584783113216304 | -0.631604301 | 0.3530128347296 | 0.869970711114864 | 0.531738048309341 | 1.42334941162119 | 0.999950574162623 |
| ebi-fl187-GCST90094775 | ebi-met1400-GCST90199703 | Gamma-glutamylvaline levels \|\| id:ebi-met1400-GCST90199703 | Green olives liking \|\| id:ebi-fl187-GCST90094775 | MR Egger | 24 | -0.425020648 | 0.234785165038176 | 0.0839380911272328 | -0.885199571 | 0.0351582758662127 | 0.653756286486559 | 0.41263181568271 | 1.03578363537857 | 0.999950574162623 |
| ebi-fl187-GCST90094775 | ebi-met1400-GCST90199779 | Gamma-glutamyl-2-aminobutyrate levels \|\| id:ebi-met1400-GCST90199779 | Green olives liking \|\| id:ebi-fl187-GCST90094775 | MR Egger | 23 | 0.0801997017577666 | 0.229840698298809 | 0.730611660546147 | -0.370288067 | 0.530687470423431 | 1.08350342360915 | 0.690535381591243 | 1.7001006758951 | 0.999950574162623 |
| ebi-fl187-GCST90094775 | ebi-met1400-GCST90199824 | 4-vinylphenol sulfate levels \|\| id:ebi-met1400-GCST90199824 | Green olives liking \|\| id:ebi-fl187-GCST90094775 | MR Egger | 24 | 0.293251690164969 | 0.231939806900368 | 0.219338043588146 | -0.161350331 | 0.747853711689689 | 1.34078020915944 | 0.85099388903265 | 2.11246119677443 | 0.999950574162623 |
| ebi-fl187-GCST90094775 | ebi-met1400-GCST90199830 | 4-ethylphenylsulfate levels \|\| id:ebi-met1400-GCST90199830 | Green olives liking \|\| id:ebi-fl187-GCST90094775 | MR Egger | 24 | 0.0141435389538086 | 0.220496318466653 | 0.949434757409716 | -0.418029245 | 0.446316323148448 | 1.0142440320178 | 0.658342974744443 | 1.56254565772961 | 0.999950574162623 |
| ebi-fl187-GCST90094775 | ebi-met1400-GCST90199833 | Hexanoylglutamine levels \|\| id:ebi-met1400-GCST90199833 | Green olives liking \|\| id:ebi-fl187-GCST90094775 | MR Egger | 24 | 0.189173958061589 | 0.217754384120226 | 0.394363223166808 | -0.237624635 | 0.615972550937233 | 1.20825111922489 | 0.788498610478919 | 1.85145635985521 | 0.999950574162623 |
| ebi-fl187-GCST90094775 | ebi-met1400-GCST90199837 | Carotenoid levels (cryptoxanthin) \|\| id:ebi-met1400-GCST90199837 | Green olives liking \|\| id:ebi-fl187-GCST90094775 | MR Egger | 24 | 0.061033064905571 | 0.218175190047211 | 0.782287693597591 | -0.366590308 | 0.488656437398105 | 1.06293405942046 | 0.693093542050899 | 1.63012457356453 | 0.999950574162623 |
| ebi-fl187-GCST90094775 | ebi-met1400-GCST90199869 | 2-hydroxyhippurate levels \|\| id:ebi-met1400-GCST90199869 | Green olives liking \|\| id:ebi-fl187-GCST90094775 | MR Egger | 24 | -0.092583864 | 0.251336912240852 | 0.716121511760536 | -0.585204212 | 0.400036483535738 | 0.911572759204856 | 0.556992105475739 | 1.4918791256738 | 0.999950574162623 |
| ebi-fl187-GCST90094775 | ebi-met1400-GCST90199902 | Hydantoin-5-propionate levels \|\| id:ebi-met1400-GCST90199902 | Green olives liking \|\| id:ebi-fl187-GCST90094775 | MR Egger | 24 | -0.091195936 | 0.219584950991094 | 0.681937221788654 | -0.52158244 | 0.339190567473353 | 0.912838834957416 | 0.593580498611693 | 1.4038108404089 | 0.999950574162623 |
| ebi-fl187-GCST90094775 | ebi-met1400-GCST90199904 | Pantoate levels \|\| id:ebi-met1400-GCST90199904 | Green olives liking \|\| id:ebi-fl187-GCST90094775 | MR Egger | 24 | -0.337302846 | 0.249557856813185 | 0.190237616878108 | -0.826436246 | 0.151830553023652 | 0.713692667965556 | 0.437606031128051 | 1.16396298971197 | 0.999950574162623 |
| ebi-fl187-GCST90094775 | ebi-met1400-GCST90199936 | 3-methyl catechol sulfate (2) levels \|\| id:ebi-met1400-GCST90199936 | Green olives liking \|\| id:ebi-fl187-GCST90094775 | MR Egger | 24 | 0.435087644225166 | 0.279985578078214 | 0.134461613371847 | -0.113684089 | 0.983859377258466 | 1.54509847197555 | 0.892539874685011 | 2.67475925256975 | 0.999950574162623 |
| ebi-fl187-GCST90094775 | ebi-met1400-GCST90199977 | 4-hydroxychlorothalonil levels \|\| id:ebi-met1400-GCST90199977 | Green olives liking \|\| id:ebi-fl187-GCST90094775 | MR Egger | 24 | 0.306600613178741 | 0.218354948414222 | 0.174237960105049 | -0.121375086 | 0.734576312070616 | 1.35879817360343 | 0.885701683233234 | 2.08459858611538 | 0.999950574162623 |
| ebi-fl187-GCST90094775 | ebi-met1400-GCST90200040 | Sphingomyelin (d18:2/24:1, d18:1/24:2) levels \|\| id:ebi-met1400-GCST90200040 | Green olives liking \|\| id:ebi-fl187-GCST90094775 | MR Egger | 24 | 0.1624444023192 | 0.20040451280695 | 0.426287573213995 | -0.230348443 | 0.555237247420823 | 1.17638291246883 | 0.794256801231653 | 1.74235430480756 | 0.999950574162623 |
| ebi-fl187-GCST90094775 | ebi-met1400-GCST90200077 | 1-stearoyl-2-oleoyl-GPI (18:0/18:1) levels \|\| id:ebi-met1400-GCST90200077 | Green olives liking \|\| id:ebi-fl187-GCST90094775 | MR Egger | 24 | -0.120846344 | 0.217599198799051 | 0.584250687692619 | -0.547340773 | 0.305648085967109 | 0.886170114771645 | 0.578486092462118 | 1.35750449759657 | 0.999950574162623 |
| ebi-fl187-GCST90094775 | ebi-met1400-GCST90200080 | 1-palmitoyl-2-oleoyl-GPI (16:0/18:1) levels \|\| id:ebi-met1400-GCST90200080 | Green olives liking \|\| id:ebi-fl187-GCST90094775 | MR Egger | 24 | 0.178524570147147 | 0.219481586175215 | 0.42470941480337 | -0.251659339 | 0.608708479050569 | 1.19545225535046 | 0.777509560332876 | 1.83805597735757 | 0.999950574162623 |
| ebi-fl187-GCST90094775 | ebi-met1400-GCST90200082 | 1-oleoyl-2-linoleoyl-GPE (18:1/18:2) levels \|\| id:ebi-met1400-GCST90200082 | Green olives liking \|\| id:ebi-fl187-GCST90094775 | MR Egger | 24 | -0.320573675 | 0.2270754137424 | 0.172011697418062 | -0.765641486 | 0.124494136224084 | 0.72573258320064 | 0.465035521659819 | 1.13257537927254 | 0.999950574162623 |
| ebi-fl187-GCST90094775 | ebi-met1400-GCST90200100 | Perfluorooctanesulfonate (PFOS) levels \|\| id:ebi-met1400-GCST90200100 | Green olives liking \|\| id:ebi-fl187-GCST90094775 | MR Egger | 24 | 0.0483598833803895 | 0.202854003673819 | 0.813779355830515 | -0.349233964 | 0.445953730581075 | 1.04954830235688 | 0.705228113103162 | 1.5619791929921 | 0.999950574162623 |
| ebi-fl187-GCST90094775 | ebi-met1400-GCST90200102 | 1-palmitoleoyl-2-linolenoyl-GPC (16:1/18:3) levels \|\| id:ebi-met1400-GCST90200102 | Green olives liking \|\| id:ebi-fl187-GCST90094775 | MR Egger | 24 | -0.352032291 | 0.244755295438349 | 0.164422696202302 | -0.83175267 | 0.127688088148564 | 0.703257412787447 | 0.435285705180397 | 1.13619855362704 | 0.999950574162623 |
| ebi-fl187-GCST90094775 | ebi-met1400-GCST90200114 | Glycosyl-N-tricosanoyl-sphingadienine (d18:2/23:0) levels \|\| id:ebi-met1400-GCST90200114 | Green olives liking \|\| id:ebi-fl187-GCST90094775 | MR Egger | 24 | 0.281119654080222 | 0.229896449249388 | 0.234339780181276 | -0.169477386 | 0.731716694609022 | 1.32461208969458 | 0.844105842495413 | 2.07864594679022 | 0.999950574162623 |
| ebi-fl187-GCST90094775 | ebi-met1400-GCST90200135 | Arachidoylcarnitine (C20) levels \|\| id:ebi-met1400-GCST90200135 | Green olives liking \|\| id:ebi-fl187-GCST90094775 | MR Egger | 24 | 0.447162297299922 | 0.262813086941326 | 0.102951797716062 | -0.067951353 | 0.962275947704922 | 1.56386809025955 | 0.934305923553793 | 2.61764732736519 | 0.999950574162623 |
| ebi-fl187-GCST90094775 | ebi-met1400-GCST90200146 | Docosahexaenoylcarnitine (C22:6) levels \|\| id:ebi-met1400-GCST90200146 | Green olives liking \|\| id:ebi-fl187-GCST90094775 | MR Egger | 24 | -0.228053338 | 0.2464069396433 | 0.364739797044633 | -0.71101094 | 0.254904263774248 | 0.796081797350543 | 0.491147426004412 | 1.29033808286146 | 0.999950574162623 |
| ebi-fl187-GCST90094775 | ebi-met1400-GCST90200162 | 3-hydroxyphenylacetoylglutamine levels \|\| id:ebi-met1400-GCST90200162 | Green olives liking \|\| id:ebi-fl187-GCST90094775 | MR Egger | 24 | 0.392820998966501 | 0.268876704721035 | 0.158159599904722 | -0.134177342 | 0.919819340219729 | 1.48115323763845 | 0.87443497655357 | 2.50883710303241 | 0.999950574162623 |
| ebi-fl187-GCST90094775 | ebi-met1400-GCST90200172 | 3-carboxy-4-methyl-5-pentyl-2-furanpropionate (3-CMPFP) levels \|\| id:ebi-met1400-GCST90200172 | Green olives liking \|\| id:ebi-fl187-GCST90094775 | MR Egger | 24 | 0.0888507066674227 | 0.218890405361164 | 0.688726695013591 | -0.340174488 | 0.517875901175304 | 1.09291747885333 | 0.71164613833074 | 1.67845864854252 | 0.999950574162623 |
| ebi-fl187-GCST90094775 | ebi-met1400-GCST90200222 | Dihydrocaffeate sulfate (2) levels \|\| id:ebi-met1400-GCST90200222 | Green olives liking \|\| id:ebi-fl187-GCST90094775 | MR Egger | 24 | 0.0081929908416857 | 0.326151843613914 | 0.980185574943151 | -0.631064623 | 0.647450604324958 | 1.00822664523842 | 0.532025093435204 | 1.91066357717302 | 0.999950574162623 |
| ebi-fl187-GCST90094775 | ebi-met1400-GCST90200223 | 4-methylhexanoylglutamine levels \|\| id:ebi-met1400-GCST90200223 | Green olives liking \|\| id:ebi-fl187-GCST90094775 | MR Egger | 24 | 0.212003331156326 | 0.261596714433836 | 0.426378735897387 | -0.300726229 | 0.724732891446644 | 1.23615200288721 | 0.740280412216592 | 2.06417966627891 | 0.999950574162623 |
| ebi-fl187-GCST90094775 | ebi-met1400-GCST90200224 | 2-ketocaprylate levels \|\| id:ebi-met1400-GCST90200224 | Green olives liking \|\| id:ebi-fl187-GCST90094775 | MR Egger | 24 | 0.29522410531858 | 0.227157036012249 | 0.207175745159541 | -0.150003685 | 0.740451895902587 | 1.34342739417735 | 0.860704804493554 | 2.09688287320309 | 0.999950574162623 |
| ebi-fl187-GCST90094775 | ebi-met1400-GCST90200243 | Hydroxypalmitoyl sphingomyelin (d18:1/16:0(OH)) levels \|\| id:ebi-met1400-GCST90200243 | Green olives liking \|\| id:ebi-fl187-GCST90094775 | MR Egger | 24 | -0.136675004 | 0.214994699833437 | 0.531521174904416 | -0.558064615 | 0.284714608123633 | 0.872253659415103 | 0.572315643658669 | 1.32938257898957 | 0.999950574162623 |
| ebi-fl187-GCST90094775 | ebi-met1400-GCST90200273 | 2-methoxyhydroquinone sulfate (2) levels \|\| id:ebi-met1400-GCST90200273 | Green olives liking \|\| id:ebi-fl187-GCST90094775 | MR Egger | 24 | 0.486153951434385 | 0.278533455063587 | 0.0948676408932999 | -0.05977162 | 1.03207952335902 | 1.62605030972035 | 0.941979637868504 | 2.80689677722178 | 0.999950574162623 |
| ebi-fl187-GCST90094775 | ebi-met1400-GCST90200276 | 2,4-di-tert-butylphenol levels \|\| id:ebi-met1400-GCST90200276 | Green olives liking \|\| id:ebi-fl187-GCST90094775 | MR Egger | 24 | -0.217198613 | 0.218774979104938 | 0.331604780013237 | -0.645997572 | 0.211600345913322 | 0.804770115517526 | 0.524139414323973 | 1.23565395223221 | 0.999950574162623 |
| ebi-fl187-GCST90094775 | ebi-met1400-GCST90200289 | N-acetylmethionine levels \|\| id:ebi-met1400-GCST90200289 | Green olives liking \|\| id:ebi-fl187-GCST90094775 | MR Egger | 24 | 0.0702268171283206 | 0.218379335471834 | 0.750804972589547 | -0.35779668 | 0.498250314653114 | 1.07275147207021 | 0.699215224717394 | 1.64583904947711 | 0.999950574162623 |
| ebi-fl187-GCST90094775 | ebi-met1400-GCST90200308 | 3-hydroxyisobutyrate levels \|\| id:ebi-met1400-GCST90200308 | Green olives liking \|\| id:ebi-fl187-GCST90094775 | MR Egger | 24 | 0.0211117293972843 | 0.221817569826096 | 0.925036561623988 | -0.413650707 | 0.455874166256433 | 1.02133615853628 | 0.661231874293388 | 1.57755182302481 | 0.999950574162623 |
| ebi-fl187-GCST90094775 | ebi-met1400-GCST90200383 | Cystathionine levels \|\| id:ebi-met1400-GCST90200383 | Green olives liking \|\| id:ebi-fl187-GCST90094775 | MR Egger | 24 | -0.320545617 | 0.240777294285651 | 0.196723776597259 | -0.792469114 | 0.151377879587753 | 0.725752945727451 | 0.452725583038976 | 1.16343621382387 | 0.999950574162623 |
| ebi-fl187-GCST90094775 | ebi-met1400-GCST90200384 | Guanidinoacetate levels \|\| id:ebi-met1400-GCST90200384 | Green olives liking \|\| id:ebi-fl187-GCST90094775 | MR Egger | 24 | 0.315268893288021 | 0.210578283852105 | 0.148562848633241 | -0.097464543 | 0.728002329638146 | 1.37062781403034 | 0.907134505197399 | 2.07093941838845 | 0.999950574162623 |
| ebi-fl187-GCST90094775 | ebi-met1400-GCST90200403 | Citrulline levels \|\| id:ebi-met1400-GCST90200403 | Green olives liking \|\| id:ebi-fl187-GCST90094775 | MR Egger | 24 | 0.297272131096893 | 0.220352251581878 | 0.191034812372987 | -0.134618282 | 0.729162544197373 | 1.34618158747867 | 0.874049488437351 | 2.07334354683567 | 0.999950574162623 |
| ebi-fl187-GCST90094775 | ebi-met1400-GCST90200434 | 12,13-DiHOME levels \|\| id:ebi-met1400-GCST90200434 | Green olives liking \|\| id:ebi-fl187-GCST90094775 | MR Egger | 24 | 0.0337256800685163 | 0.221519963161174 | 0.880379998617875 | -0.400453448 | 0.467904807864417 | 1.03430083847311 | 0.670016159837604 | 1.59664540736669 | 0.999950574162623 |
| ebi-fl187-GCST90094775 | ebi-met1400-GCST90200437 | Fructose levels \|\| id:ebi-met1400-GCST90200437 | Green olives liking \|\| id:ebi-fl187-GCST90094775 | MR Egger | 24 | -0.335286653 | 0.221305974824601 | 0.143999770916506 | -0.769046363 | 0.0984730579600088 | 0.715133062148181 | 0.46345482514575 | 1.10348467386568 | 0.999950574162623 |
| ebi-fl187-GCST90094775 | ebi-met1400-GCST90200448 | N-stearoyl-sphinganine (d18:0/18:0) levels \|\| id:ebi-met1400-GCST90200448 | Green olives liking \|\| id:ebi-fl187-GCST90094775 | MR Egger | 24 | 0.185137060646489 | 0.25265894898977 | 0.471443771003409 | -0.310074479 | 0.680348600666438 | 1.20338336532709 | 0.733392331588753 | 1.97456594727251 | 0.999950574162623 |
| ebi-fl187-GCST90094775 | ebi-met1400-GCST90200468 | X-11847 levels \|\| id:ebi-met1400-GCST90200468 | Green olives liking \|\| id:ebi-fl187-GCST90094775 | MR Egger | 24 | 0.286891615701804 | 0.232831879184002 | 0.230887837670788 | -0.169458867 | 0.743242098902447 | 1.33227980743821 | 0.844121474593828 | 2.10274177204374 | 0.999950574162623 |
| ebi-fl187-GCST90094775 | ebi-met1400-GCST90200476 | X-11843 levels \|\| id:ebi-met1400-GCST90200476 | Green olives liking \|\| id:ebi-fl187-GCST90094775 | MR Egger | 24 | 0.271519557547413 | 0.278086693757149 | 0.339488874682573 | -0.273530362 | 0.816569477311424 | 1.31195653025762 | 0.760689239793772 | 2.26272418123366 | 0.999950574162623 |
| ebi-fl187-GCST90094775 | ebi-met1400-GCST90200511 | X-12906 levels \|\| id:ebi-met1400-GCST90200511 | Green olives liking \|\| id:ebi-fl187-GCST90094775 | MR Egger | 24 | -0.275457662 | 0.236369712307574 | 0.256350372654978 | -0.738742298 | 0.187826974349844 | 0.759224575636021 | 0.47771436020763 | 1.20662472025996 | 0.999950574162623 |
| ebi-fl187-GCST90094775 | ebi-met1400-GCST90200515 | X-12849 levels \|\| id:ebi-met1400-GCST90200515 | Green olives liking \|\| id:ebi-fl187-GCST90094775 | MR Egger | 24 | 0.253812921397627 | 0.298835220834858 | 0.404839740761859 | -0.331904111 | 0.839529954233948 | 1.28893065036651 | 0.717556124980202 | 2.3152784341435 | 0.999950574162623 |
| ebi-fl187-GCST90094775 | ebi-met1400-GCST90200551 | X-18886 levels \|\| id:ebi-met1400-GCST90200551 | Green olives liking \|\| id:ebi-fl187-GCST90094775 | MR Egger | 24 | 0.45468734643784 | 0.263402152022482 | 0.0983285032332717 | -0.061580872 | 0.970955564401905 | 1.57568066384042 | 0.940276901036484 | 2.64046638991535 | 0.999950574162623 |
| ebi-fl187-GCST90094775 | ebi-met1400-GCST90200564 | X-21319 levels \|\| id:ebi-met1400-GCST90200564 | Green olives liking \|\| id:ebi-fl187-GCST90094775 | MR Egger | 24 | 0.0394444633989287 | 0.225763870366611 | 0.862901051677278 | -0.403052723 | 0.481941649317486 | 1.04023272629078 | 0.668276865154611 | 1.61921530022741 | 0.999950574162623 |
| ebi-fl187-GCST90094775 | ebi-met1400-GCST90200579 | X-21442 levels \|\| id:ebi-met1400-GCST90200579 | Green olives liking \|\| id:ebi-fl187-GCST90094775 | MR Egger | 24 | 0.263352269725763 | 0.243842082837193 | 0.291835356633227 | -0.214578213 | 0.741282752086661 | 1.30128504154512 | 0.806881700919829 | 2.09862580526825 | 0.999950574162623 |
| ebi-fl187-GCST90094775 | ebi-met1400-GCST90200581 | X-21441 levels \|\| id:ebi-met1400-GCST90200581 | Green olives liking \|\| id:ebi-fl187-GCST90094775 | MR Egger | 24 | 0.03893675800852 | 0.281362233724134 | 0.891193787209854 | -0.51253322 | 0.590406736107822 | 1.03970472857335 | 0.598976316472985 | 1.80472231186547 | 0.999950574162623 |
| ebi-fl187-GCST90094775 | ebi-met1400-GCST90200648 | X-24585 levels \|\| id:ebi-met1400-GCST90200648 | Green olives liking \|\| id:ebi-fl187-GCST90094775 | MR Egger | 24 | 0.131255138858834 | 0.214899639743856 | 0.547608047439312 | -0.289948155 | 0.552458432756792 | 1.14025866849756 | 0.748302362279597 | 1.73751934595392 | 0.999950574162623 |
| ebi-fl187-GCST90094775 | ebi-met1400-GCST90200720 | Adenosine 5'-diphosphate (ADP) to pantothenate ratio \|\| id:ebi-met1400-GCST90200720 | Green olives liking \|\| id:ebi-fl187-GCST90094775 | MR Egger | 24 | 0.319331830546461 | 0.335824121319978 | 0.35199067828683 | -0.338883447 | 0.977547108333617 | 1.37620791697139 | 0.712565495724153 | 2.65792862845537 | 0.999950574162623 |
| ebi-fl187-GCST90094775 | ebi-met1400-GCST90200724 | Cholate to taurocholate ratio \|\| id:ebi-met1400-GCST90200724 | Green olives liking \|\| id:ebi-fl187-GCST90094775 | MR Egger | 24 | -0.01127839 | 0.237275099131749 | 0.962517324701314 | -0.476337584 | 0.453780804687964 | 0.988784972992745 | 0.621053789089429 | 1.5742528907999 | 0.999950574162623 |
| ebi-fl187-GCST90094775 | ebi-met1400-GCST90200726 | N-acetylputrescine to (N(1) + N(8))-acetylspermidine ratio \|\| id:ebi-met1400-GCST90200726 | Green olives liking \|\| id:ebi-fl187-GCST90094775 | MR Egger | 24 | -0.053736808 | 0.218770580747669 | 0.808244982565046 | -0.482527146 | 0.375053530662014 | 0.94768149624581 | 0.617221610170019 | 1.45506930335655 | 0.999950574162623 |
| ebi-fl187-GCST90094775 | ebi-met1400-GCST90200744 | Arginine to glutamate ratio \|\| id:ebi-met1400-GCST90200744 | Green olives liking \|\| id:ebi-fl187-GCST90094775 | MR Egger | 24 | 0.138621941534218 | 0.266704920736188 | 0.608423070653701 | -0.384119703 | 0.661363586177146 | 1.14868974602014 | 0.681049898509989 | 1.93743239004749 | 0.999950574162623 |
| ebi-fl187-GCST90094775 | ebi-met1400-GCST90200759 | Histidine to trans-urocanate ratio \|\| id:ebi-met1400-GCST90200759 | Green olives liking \|\| id:ebi-fl187-GCST90094775 | MR Egger | 24 | 0.276984798264914 | 0.221611579675695 | 0.224481489069178 | -0.157373898 | 0.711343494429277 | 1.31914631756968 | 0.854384546501657 | 2.03672575104829 | 0.999950574162623 |
| ebi-fl187-GCST90094775 | ebi-met1400-GCST90200795 | Oleoyl-linoleoyl-glycerol (18:1 to 18:2) [2] to linoleoyl-arachidonoyl-glycerol (18:2 to 20:4) [2] ratio \|\| id:ebi-met1400-GCST90200795 | Green olives liking \|\| id:ebi-fl187-GCST90094775 | MR Egger | 24 | 0.243612675021078 | 0.22893115423552 | 0.298810428951175 | -0.205092387 | 0.692317737322698 | 1.27585006617519 | 0.814572056837818 | 1.99834180131142 | 0.999950574162623 |
| ebi-fl187-GCST90094775 | ebi-met1400-GCST90200811 | Choline to taurocholate ratio \|\| id:ebi-met1400-GCST90200811 | Green olives liking \|\| id:ebi-fl187-GCST90094775 | MR Egger | 24 | -0.242129944 | 0.231239783644614 | 0.306424293067742 | -0.69535992 | 0.211100031960484 | 0.784954170852993 | 0.498894853468309 | 1.23503589194401 | 0.999950574162623 |
| ebi-fl187-GCST90094775 | ebi-met1400-GCST90200831 | Cholate to phosphate ratio \|\| id:ebi-met1400-GCST90200831 | Green olives liking \|\| id:ebi-fl187-GCST90094775 | MR Egger | 24 | 0.0397720181114909 | 0.242114114307539 | 0.871018712308679 | -0.434771646 | 0.514315682154267 | 1.04057351523291 | 0.647412489475578 | 1.67249359288892 | 0.999950574162623 |
| ebi-fl187-GCST90094775 | ebi-met1400-GCST90200841 | Adenosine 5'-diphosphate (ADP) to oxalate (ethanedioate) ratio \|\| id:ebi-met1400-GCST90200841 | Green olives liking \|\| id:ebi-fl187-GCST90094775 | MR Egger | 24 | 0.374977041928552 | 0.313456938319816 | 0.244327734622742 | -0.239398557 | 0.989352641035391 | 1.45495801120479 | 0.78710111505029 | 2.68949284137876 | 0.999950574162623 |
| ebi-fl187-GCST90094775 | ebi-met1400-GCST90200844 | Aspartate to phosphate ratio \|\| id:ebi-met1400-GCST90200844 | Green olives liking \|\| id:ebi-fl187-GCST90094775 | MR Egger | 24 | 0.249305886447776 | 0.225692161502798 | 0.281256068891451 | -0.19305075 | 0.69166252299326 | 1.28313446649069 | 0.8244401326601 | 1.99703288798429 | 0.999950574162623 |
| ebi-fl187-GCST90094775 | ebi-met1400-GCST90200870 | Adenosine 5'-monophosphate (AMP) to acetoacetate ratio \|\| id:ebi-met1400-GCST90200870 | Green olives liking \|\| id:ebi-fl187-GCST90094775 | MR Egger | 24 | -0.030572046 | 0.320619612597574 | 0.924897653183589 | -0.658986487 | 0.597842394329036 | 0.969890552463909 | 0.51737543555739 | 1.81819162470538 | 0.999950574162623 |
| ebi-fl187-GCST90094775 | ebi-met1400-GCST90200887 | Cholesterol to cortisol ratio \|\| id:ebi-met1400-GCST90200887 | Green olives liking \|\| id:ebi-fl187-GCST90094775 | MR Egger | 24 | 0.0128510191134765 | 0.27584446180841 | 0.963261985586002 | -0.527804126 | 0.55350616425796 | 1.01293394832045 | 0.589898892134686 | 1.73934075371309 | 0.999950574162623 |
| ebi-fl187-GCST90094775 | ebi-met1400-GCST90200890 | Cortisol to taurocholate ratio \|\| id:ebi-met1400-GCST90200890 | Green olives liking \|\| id:ebi-fl187-GCST90094775 | MR Egger | 24 | 0.0112237784807558 | 0.230365448445453 | 0.961580762475855 | -0.4402925 | 0.462740057433844 | 1.01128700139438 | 0.643848067673849 | 1.58842039067405 | 0.999950574162623 |
| ebi-fl187-GCST90094775 | ebi-met1400-GCST90200948 | Adenosine 5'-diphosphate (ADP) to mannitol to sorbitol ratio \|\| id:ebi-met1400-GCST90200948 | Green olives liking \|\| id:ebi-fl187-GCST90094775 | MR Egger | 24 | -0.148852511 | 0.312993818096684 | 0.639064277084563 | -0.762320394 | 0.464615372686382 | 0.861696196424352 | 0.46658251455898 | 1.59140197449112 | 0.999950574162623 |
| ebi-fl187-GCST90094775 | ebi-met1400-GCST90200972 | Leucine to N-palmitoyl-sphingosine (d18:1 to 16:0) ratio \|\| id:ebi-met1400-GCST90200972 | Green olives liking \|\| id:ebi-fl187-GCST90094775 | MR Egger | 24 | -0.077299257 | 0.236051898121267 | 0.746410241635249 | -0.539960978 | 0.385362463097984 | 0.925612815838001 | 0.58277099308958 | 1.4701470989512 | 0.999950574162623 |
| ebi-fl187-GCST90094775 | ebi-met1400-GCST90200973 | Proline to glutamate ratio \|\| id:ebi-met1400-GCST90200973 | Green olives liking \|\| id:ebi-fl187-GCST90094775 | MR Egger | 24 | 0.206832884496748 | 0.215311978200916 | 0.347183288787942 | -0.215178593 | 0.628844361770544 | 1.22977703979855 | 0.806397410563429 | 1.87544199399028 | 0.999950574162623 |
| ebi-fl187-GCST90094775 | ebi-met1400-GCST90200983 | Cholesterol to linoleoyl-arachidonoyl-glycerol (18:2 to 20:4) [1] ratio \|\| id:ebi-met1400-GCST90200983 | Green olives liking \|\| id:ebi-fl187-GCST90094775 | MR Egger | 24 | 0.0976998981323525 | 0.227603640316607 | 0.671912476675177 | -0.348403237 | 0.543803033152902 | 1.10263183357882 | 0.705814208498899 | 1.72254531827449 | 0.999950574162623 |
| ebi-fl187-GCST90094775 | ebi-met1400-GCST90201001 | Serine to alpha-ketobutyrate ratio \|\| id:ebi-met1400-GCST90201001 | Green olives liking \|\| id:ebi-fl187-GCST90094775 | MR Egger | 24 | 0.107362321690283 | 0.223707171220671 | 0.636018892124385 | -0.331103734 | 0.545828377282798 | 1.11333756775975 | 0.718130670679852 | 1.72603760066637 | 0.999950574162623 |
| ebi-fl187-GCST90094775 | ebi-met1400-GCST90201005 | Fructose to maltose ratio \|\| id:ebi-met1400-GCST90201005 | Green olives liking \|\| id:ebi-fl187-GCST90094775 | MR Egger | 24 | -0.636275119 | 0.2526056877549 | 0.0195447410255229 | -1.131382267 | -0.141167971 | 0.529260188001159 | 0.322587046587114 | 0.868343442697341 | 0.999950574162623 |
| ebi-fl187-GCST90094775 | ebi-met1400-GCST90201020 | Paraxanthine to 5-acetylamino-6-formylamino-3-methyluracil ratio \|\| id:ebi-met1400-GCST90201020 | Green olives liking \|\| id:ebi-fl187-GCST90094775 | MR Egger | 24 | 0.163434597848454 | 0.232564643128959 | 0.48958218425374 | -0.292392103 | 0.619261298381214 | 1.17754833847403 | 0.746475783424128 | 1.85755535575776 | 0.999950574162623 |
| ebi-fl187-GCST90094775 | ebi-pw11-GCST90275197 | Placental weight (fetal genotype, adjusted for sex) \|\| id:ebi-pw11-GCST90275197 | Green olives liking \|\| id:ebi-fl187-GCST90094775 | MR Egger | 23 | 0.0429011876710563 | 0.0779684790451209 | 0.58796202307024 | -0.109917031 | 0.195719406599493 | 1.04383474601222 | 0.895908464611792 | 1.21618560379886 | 0.999950574162623 |
| ebi-fl187-GCST90094775 | prot-a-1017 | FAS-associated factor 2 \|\| id:prot-a-1017 | Green olives liking \|\| id:ebi-fl187-GCST90094775 | MR Egger | 27 | 0.272527516020973 | 0.309190659527074 | 0.386485310533213 | -0.333486177 | 0.878541208694038 | 1.3132795946442 | 0.716421801919333 | 2.40738527092316 | 0.999950574162623 |
| ebi-fl187-GCST90094775 | prot-a-1019 | Fas apoptotic inhibitory molecule 3 \|\| id:prot-a-1019 | Green olives liking \|\| id:ebi-fl187-GCST90094775 | MR Egger | 27 | -0.57126894 | 0.309372381283761 | 0.0766853515801327 | -1.177638807 | 0.035100927208187 | 0.564808275902836 | 0.308005140126888 | 1.03572423628032 | 0.999950574162623 |
| ebi-fl187-GCST90094775 | prot-a-1052 | MIP18 family protein FAM96A \|\| id:prot-a-1052 | Green olives liking \|\| id:ebi-fl187-GCST90094775 | MR Egger | 27 | 0.0218734117395355 | 0.309350407790442 | 0.944193099130619 | -0.584453388 | 0.628200211008802 | 1.02211438859816 | 0.557410466066158 | 1.87423431560649 | 0.999950574162623 |
| ebi-fl187-GCST90094775 | prot-a-1066 | F-box/LRR-repeat protein 4 \|\| id:prot-a-1066 | Green olives liking \|\| id:ebi-fl187-GCST90094775 | MR Egger | 27 | -0.081372284 | 0.309220336506452 | 0.79458931799386 | -0.687444144 | 0.524699575053956 | 0.921850436926625 | 0.50285966489015 | 1.68995106864945 | 0.999950574162623 |
| ebi-fl187-GCST90094775 | prot-a-1076 | Low affinity immunoglobulin gamma Fc region receptor III-B \|\| id:prot-a-1076 | Green olives liking \|\| id:ebi-fl187-GCST90094775 | MR Egger | 27 | -0.702130791 | 0.319283659071197 | 0.0373419723075658 | -1.327926763 | -0.076334819 | 0.495528310792875 | 0.265026154188288 | 0.926505942590066 | 0.999950574162623 |
| ebi-fl187-GCST90094775 | prot-a-1081 | Fc receptor-like protein 3 \|\| id:prot-a-1081 | Green olives liking \|\| id:ebi-fl187-GCST90094775 | MR Egger | 27 | -0.297159045 | 0.353864698994778 | 0.409005880314836 | -0.990733856 | 0.396415764543674 | 0.742925843963131 | 0.371304107448488 | 1.48648721777176 | 0.999950574162623 |
| ebi-fl187-GCST90094775 | prot-a-1105 | Fibroblast growth factor receptor 4 \|\| id:prot-a-1105 | Green olives liking \|\| id:ebi-fl187-GCST90094775 | MR Egger | 27 | -0.099061212 | 0.414700056632673 | 0.813150361757214 | -0.911873323 | 0.713750899404184 | 0.905687267763767 | 0.401770872165212 | 2.04163488151549 | 0.999950574162623 |
| ebi-fl187-GCST90094775 | prot-a-1143 | Follistatin-related protein 1 \|\| id:prot-a-1143 | Green olives liking \|\| id:ebi-fl187-GCST90094775 | MR Egger | 27 | -0.785553054 | 0.38893001647991 | 0.0542466585893403 | -1.547855886 | -0.023250222 | 0.455867512779394 | 0.212703545875018 | 0.977017982247368 | 0.999950574162623 |
| ebi-fl187-GCST90094775 | prot-a-1151 | Alpha-(1,3)-fucosyltransferase 10 \|\| id:prot-a-1151 | Green olives liking \|\| id:ebi-fl187-GCST90094775 | MR Egger | 27 | 0.275367129972038 | 0.309340952591162 | 0.381859387834076 | -0.330941137 | 0.881675397050715 | 1.31701410147224 | 0.718247445919105 | 2.41494230620918 | 0.999950574162623 |
| ebi-fl187-GCST90094775 | prot-a-1165 | Growth arrest and DNA damage-inducible proteins-interacting protein 1 \|\| id:prot-a-1165 | Green olives liking \|\| id:ebi-fl187-GCST90094775 | MR Egger | 27 | 0.16428838144532 | 0.309222534055543 | 0.599903607231363 | -0.441787785 | 0.770364548194184 | 1.17855413923694 | 0.642886050848481 | 2.16055373620152 | 0.999950574162623 |
| ebi-fl187-GCST90094775 | prot-a-1194 | Growth/differentiation factor 11/8 \|\| id:prot-a-1194 | Green olives liking \|\| id:ebi-fl187-GCST90094775 | MR Egger | 26 | -0.602246752 | 0.380116958470282 | 0.126198753341893 | -1.347275991 | 0.14278248614026 | 0.547579976335142 | 0.259947396117071 | 1.15347887673457 | 0.999950574162623 |
| ebi-fl187-GCST90094775 | prot-a-1216 | D-glucuronyl C5-epimerase \|\| id:prot-a-1216 | Green olives liking \|\| id:ebi-fl187-GCST90094775 | MR Egger | 27 | 0.242068519343513 | 0.325742700370505 | 0.464330836950316 | -0.396387173 | 0.880524212069703 | 1.27388147534829 | 0.672746176094785 | 2.41216386045561 | 0.999950574162623 |
| ebi-fl187-GCST90094775 | prot-a-1246 | Glypican-3 \|\| id:prot-a-1246 | Green olives liking \|\| id:ebi-fl187-GCST90094775 | MR Egger | 27 | 0.189718374421139 | 0.309222534055543 | 0.545066072144922 | -0.416357792 | 0.795794541170002 | 1.20890908998941 | 0.659444284164423 | 2.21620116051324 | 0.999950574162623 |
| ebi-fl187-GCST90094775 | prot-a-1275 | Glutamate receptor 4 \|\| id:prot-a-1275 | Green olives liking \|\| id:ebi-fl187-GCST90094775 | MR Egger | 26 | 0.303224034075588 | 0.309593521543178 | 0.337139854898687 | -0.303579268 | 0.910027336300216 | 1.35421782139763 | 0.738171373334171 | 2.48439044649967 | 0.999950574162623 |
| ebi-fl187-GCST90094775 | prot-a-1289 | Glutathione S-transferase P \|\| id:prot-a-1289 | Green olives liking \|\| id:ebi-fl187-GCST90094775 | MR Egger | 27 | 0.0223868099480836 | 0.32634185868803 | 0.945854347525812 | -0.617243233 | 0.662016852976622 | 1.02263927502055 | 0.539429471029822 | 1.93869846380109 | 0.999950574162623 |
| ebi-fl187-GCST90094775 | prot-a-130 | Apolipoprotein D \|\| id:prot-a-130 | Green olives liking \|\| id:ebi-fl187-GCST90094775 | MR Egger | 27 | 0.192498626560512 | 0.309345863194573 | 0.539394791941427 | -0.413819265 | 0.798816518421875 | 1.21227483872017 | 0.661120427870436 | 2.22290859976576 | 0.999950574162623 |
| ebi-fl187-GCST90094775 | prot-a-1313 | Heparin-binding EGF-like growth factor \|\| id:prot-a-1313 | Green olives liking \|\| id:ebi-fl187-GCST90094775 | MR Egger | 27 | 0.213236670726157 | 0.310654879108568 | 0.498772000812031 | -0.395646892 | 0.822120233778951 | 1.23767753862511 | 0.673244381727815 | 2.27531893498434 | 0.999950574162623 |
| ebi-fl187-GCST90094775 | prot-a-1347 | HLA class II histocompatibility antigen, DQ alpha 2 chain \|\| id:prot-a-1347 | Green olives liking \|\| id:ebi-fl187-GCST90094775 | MR Egger | 26 | -0.209725282 | 0.316047684210262 | 0.513278857566502 | -0.829178743 | 0.409728179324129 | 0.810806958863813 | 0.436407542036071 | 1.50640825654578 | 0.999950574162623 |
| ebi-fl187-GCST90094775 | prot-a-1357 | Non-histone chromosomal protein HMG-14 \|\| id:prot-a-1357 | Green olives liking \|\| id:ebi-fl187-GCST90094775 | MR Egger | 27 | -0.006142202 | 0.330240375188259 | 0.98530849859366 | -0.653413338 | 0.641128933051387 | 0.9938766224455 | 0.520266895924699 | 1.89862308822863 | 0.999950574162623 |
| ebi-fl187-GCST90094775 | prot-a-137 | Apolipoprotein M \|\| id:prot-a-137 | Green olives liking \|\| id:ebi-fl187-GCST90094775 | MR Egger | 27 | 0.0296105159384364 | 0.30934244946944 | 0.924505465679234 | -0.576700685 | 0.635921716898538 | 1.03005326648509 | 0.561748698367793 | 1.88876224347196 | 0.999950574162623 |
| ebi-fl187-GCST90094775 | prot-a-1381 | 17-beta-hydroxysteroid dehydrogenase 14 \|\| id:prot-a-1381 | Green olives liking \|\| id:ebi-fl187-GCST90094775 | MR Egger | 26 | 0.100461088154646 | 0.309601631416743 | 0.748382161458763 | -0.506358109 | 0.707280285731462 | 1.10568061679382 | 0.602686505138154 | 2.02846689934318 | 0.999950574162623 |
| ebi-fl187-GCST90094775 | prot-a-1392 | Serine protease HTRA2, mitochondrial \|\| id:prot-a-1392 | Green olives liking \|\| id:ebi-fl187-GCST90094775 | MR Egger | 27 | 0.268546534205173 | 0.387052893013623 | 0.494189652458237 | -0.490077136 | 1.02717020451187 | 1.30806184523262 | 0.612579140395185 | 2.79315059577374 | 0.999950574162623 |
| ebi-fl187-GCST90094775 | prot-a-1416 | Interferon-induced protein with tetratricopeptide repeats 2 \|\| id:prot-a-1416 | Green olives liking \|\| id:ebi-fl187-GCST90094775 | MR Egger | 27 | -0.041261009 | 0.344358046190047 | 0.905583392375556 | -0.71620278 | 0.633680761463121 | 0.959578638531524 | 0.488604075212967 | 1.88453435048545 | 0.999950574162623 |
| ebi-fl187-GCST90094775 | prot-a-1428 | Interferon gamma \|\| id:prot-a-1428 | Green olives liking \|\| id:ebi-fl187-GCST90094775 | MR Egger | 27 | -0.238039864 | 0.309313400188801 | 0.448762782990338 | -0.844294128 | 0.36821440067477 | 0.788171271048137 | 0.429860677734006 | 1.44515184729234 | 0.999950574162623 |
| ebi-fl187-GCST90094775 | prot-a-1483 | Interleukin-17C \|\| id:prot-a-1483 | Green olives liking \|\| id:ebi-fl187-GCST90094775 | MR Egger | 27 | 0.0526796991935311 | 0.309253509638844 | 0.866110132792758 | -0.55345718 | 0.658816578085666 | 1.05409196453623 | 0.574958635117499 | 1.9325040130457 | 0.999950574162623 |
| ebi-fl187-GCST90094775 | prot-a-1503 | Interleukin-1 receptor-like 2 \|\| id:prot-a-1503 | Green olives liking \|\| id:ebi-fl187-GCST90094775 | MR Egger | 27 | 0.0180799477851385 | 0.309340163006164 | 0.953857400762773 | -0.588226772 | 0.62438666727722 | 1.01824437951862 | 0.555311105571095 | 1.8671004523761 | 0.999950574162623 |
| ebi-fl187-GCST90094775 | prot-a-1522 | Interleukin-31 receptor subunit alpha \|\| id:prot-a-1522 | Green olives liking \|\| id:ebi-fl187-GCST90094775 | MR Egger | 27 | -0.124183635 | 0.339766929477999 | 0.717815116849304 | -0.790126817 | 0.541759546642118 | 0.883217636200322 | 0.453787243736388 | 1.71902891423815 | 0.999950574162623 |
| ebi-fl187-GCST90094775 | prot-a-1540 | Interleukin-6 receptor subunit alpha \|\| id:prot-a-1540 | Green olives liking \|\| id:ebi-fl187-GCST90094775 | MR Egger | 27 | 0.0609119949328547 | 0.309345497086839 | 0.845493123373955 | -0.545405179 | 0.667229169223059 | 1.06280537781278 | 0.579606891008684 | 1.94882995463014 | 0.999950574162623 |
| ebi-fl187-GCST90094775 | prot-a-1541 | Interleukin-6 receptor subunit alpha \|\| id:prot-a-1541 | Green olives liking \|\| id:ebi-fl187-GCST90094775 | MR Egger | 27 | 0.465500628511865 | 0.309340163006164 | 0.144901327910713 | -0.140806091 | 1.07180734800395 | 1.59281139611704 | 0.868657735937754 | 2.92065336972041 | 0.999950574162623 |
| ebi-fl187-GCST90094775 | prot-a-1545 | Interleukin-9 \|\| id:prot-a-1545 | Green olives liking \|\| id:ebi-fl187-GCST90094775 | MR Egger | 27 | 0.253256007732604 | 0.369204600462919 | 0.499057033122469 | -0.470385009 | 0.976897024639925 | 1.2882130271197 | 0.624761682991937 | 2.65620131390536 | 0.999950574162623 |
| ebi-fl187-GCST90094775 | prot-a-1548 | Inositol monophosphatase 3 \|\| id:prot-a-1548 | Green olives liking \|\| id:ebi-fl187-GCST90094775 | MR Egger | 27 | 0.00479899225255538 | 0.335639523848272 | 0.988705686563638 | -0.653054474 | 0.662652458995168 | 1.00481052585839 | 0.520453634070739 | 1.93993110390807 | 0.999950574162623 |
| ebi-fl187-GCST90094775 | prot-a-1555 | Inhibin beta A chain:Inhibin beta B chain heterodimer \|\| id:prot-a-1555 | Green olives liking \|\| id:ebi-fl187-GCST90094775 | MR Egger | 27 | -0.13163198 | 0.331343562224769 | 0.694541808885277 | -0.781065362 | 0.517801402114076 | 0.876663565579507 | 0.457917903100757 | 1.67833360960659 | 0.999950574162623 |
| ebi-fl187-GCST90094775 | prot-a-1603 | Junctophilin-3 \|\| id:prot-a-1603 | Green olives liking \|\| id:ebi-fl187-GCST90094775 | MR Egger | 27 | -0.030407197 | 0.309253509638844 | 0.92245874294704 | -0.636544076 | 0.575729681549568 | 0.970050451149852 | 0.529117858927635 | 1.77842773948919 | 0.999950574162623 |
| ebi-fl187-GCST90094775 | prot-a-1627 | UPF0258 protein KIAA1024 \|\| id:prot-a-1627 | Green olives liking \|\| id:ebi-fl187-GCST90094775 | MR Egger | 27 | -0.128867211 | 0.316085593166187 | 0.686966342594426 | -0.748394974 | 0.490660551591353 | 0.87909069135664 | 0.473125322278819 | 1.63339480522345 | 0.999950574162623 |
| ebi-fl187-GCST90094775 | prot-a-1666 | Kallikrein-7 \|\| id:prot-a-1666 | Green olives liking \|\| id:ebi-fl187-GCST90094775 | MR Egger | 27 | 0.0808139840630561 | 0.309345883415948 | 0.796046406323882 | -0.525503947 | 0.687131915558314 | 1.08416920505794 | 0.591257326663912 | 1.98800558096781 | 0.999950574162623 |
| ebi-fl187-GCST90094775 | prot-a-1678 | Importin subunit alpha-3 \|\| id:prot-a-1678 | Green olives liking \|\| id:ebi-fl187-GCST90094775 | MR Egger | 27 | -0.271826584 | 0.356289793326269 | 0.452640594077149 | -0.970154579 | 0.42650141048735 | 0.761986389935421 | 0.379024444221975 | 1.5318886876509 | 0.999950574162623 |
| ebi-fl187-GCST90094775 | prot-a-1718 | Protein LDOC1 \|\| id:prot-a-1718 | Green olives liking \|\| id:ebi-fl187-GCST90094775 | MR Egger | 27 | -0.013707846 | 0.309343788726538 | 0.965007231290912 | -0.620021672 | 0.592605979593659 | 0.986385678386442 | 0.537932779273835 | 1.80869570327968 | 0.999950574162623 |
| ebi-fl187-GCST90094775 | prot-a-1723 | Leptin \|\| id:prot-a-1723 | Green olives liking \|\| id:ebi-fl187-GCST90094775 | MR Egger | 27 | 0.877819949433794 | 0.331822848948775 | 0.0139013126167664 | 0.227447165494195 | 1.52819273337339 | 2.40564954803188 | 1.25539111 | 4.60983808288547 | 0.999950574162623 |
| ebi-fl187-GCST90094775 | prot-a-1730 | Galectin-7 \|\| id:prot-a-1730 | Green olives liking \|\| id:ebi-fl187-GCST90094775 | MR Egger | 27 | -0.796180484 | 0.403985917100768 | 0.0599132632670854 | -1.587992881 | -0.004368086 | 0.451048465052648 | 0.204335325645681 | 0.99564143979257 | 0.999950574162623 |
| ebi-fl187-GCST90094775 | prot-a-1753 | Lipase member N \|\| id:prot-a-1753 | Green olives liking \|\| id:ebi-fl187-GCST90094775 | MR Egger | 27 | -0.20690342 | 0.309247202771538 | 0.509592150870203 | -0.813027938 | 0.399221097175428 | 0.813098175008495 | 0.44351310098104 | 1.49066316359031 | 0.999950574162623 |
| ebi-fl187-GCST90094775 | prot-a-1756 | VIP36-like protein \|\| id:prot-a-1756 | Green olives liking \|\| id:ebi-fl187-GCST90094775 | MR Egger | 27 | 0.0235685543873835 | 0.315701693159166 | 0.941083769594263 | -0.595206764 | 0.642343872979349 | 1.02384848764664 | 0.551448534261855 | 1.90093120305319 | 0.999950574162623 |
| ebi-fl187-GCST90094775 | prot-a-1760 | Ig Kappa chain V-I region HK102- like \|\| id:prot-a-1760 | Green olives liking \|\| id:ebi-fl187-GCST90094775 | MR Egger | 27 | -0.192347366 | 0.373137058574018 | 0.610741354493964 | -0.923696001 | 0.539001268408933 | 0.825020234405471 | 0.397048832849299 | 1.71429388746448 | 0.999950574162623 |
| ebi-fl187-GCST90094775 | prot-a-179 | Neutral ceramidase \|\| id:prot-a-179 | Green olives liking \|\| id:ebi-fl187-GCST90094775 | MR Egger | 27 | 0.306656288932115 | 0.309345497086839 | 0.331034097730584 | -0.299660885 | 0.912973463222319 | 1.35887382782146 | 0.741069485588742 | 2.4917205685121 | 0.999950574162623 |
| ebi-fl187-GCST90094775 | prot-a-1809 | Lactotransferrin \|\| id:prot-a-1809 | Green olives liking \|\| id:ebi-fl187-GCST90094775 | MR Egger | 27 | 0.970362282581149 | 0.309219273388855 | 0.00432276362461119 | 0.364292506738993 | 1.57643205842331 | 2.63890031381607 | 1.43949521463333 | 4.83766447812219 | 0.999950574162623 |
| ebi-fl187-GCST90094775 | prot-a-1818 | Lysophospholipase-like protein 1 \|\| id:prot-a-1818 | Green olives liking \|\| id:ebi-fl187-GCST90094775 | MR Egger | 27 | -0.079912504 | 0.338762926990985 | 0.815435957835575 | -0.743887841 | 0.584062832431734 | 0.923197118473609 | 0.475262573494701 | 1.79330956631172 | 0.999950574162623 |
| ebi-fl187-GCST90094775 | prot-a-1852 | MAP kinase-activated protein kinase 3 \|\| id:prot-a-1852 | Green olives liking \|\| id:ebi-fl187-GCST90094775 | MR Egger | 27 | -0.293291964 | 0.347525051138529 | 0.406703663077235 | -0.974441064 | 0.38785713629754 | 0.745804360931655 | 0.377403238816237 | 1.47381921397741 | 0.999950574162623 |
| ebi-fl187-GCST90094775 | prot-a-1856 | Megakaryocyte-associated tyrosine-protein kinase \|\| id:prot-a-1856 | Green olives liking \|\| id:ebi-fl187-GCST90094775 | MR Egger | 27 | -0.394246773 | 0.309375240787015 | 0.214264437962404 | -1.000622245 | 0.212128699045048 | 0.674187664467378 | 0.367650601292013 | 1.23630698636872 | 0.999950574162623 |
| ebi-fl187-GCST90094775 | prot-a-1890 | Beta-1,3-N-acetylglucosaminyltransferase manic fringe \|\| id:prot-a-1890 | Green olives liking \|\| id:ebi-fl187-GCST90094775 | MR Egger | 27 | -0.292272431 | 0.309368936812711 | 0.353834751956233 | -0.898635548 | 0.314090684687257 | 0.746565120436251 | 0.407124783314767 | 1.36901387951387 | 0.999950574162623 |
| ebi-fl187-GCST90094775 | prot-a-1919 | Matrilysin \|\| id:prot-a-1919 | Green olives liking \|\| id:ebi-fl187-GCST90094775 | MR Egger | 27 | -1.085580423 | 0.309215751395844 | 0.00171850040155305 | -1.691643296 | -0.47951755 | 0.337705716838628 | 0.184216552852633 | 0.619081995724478 | 0.999950574162623 |
| ebi-fl187-GCST90094775 | prot-a-1923 | Multimerin-2 \|\| id:prot-a-1923 | Green olives liking \|\| id:ebi-fl187-GCST90094775 | MR Egger | 27 | -0.12068276 | 0.346937400426738 | 0.730863428972354 | -0.800680065 | 0.55931454480399 | 0.88631508956797 | 0.449023495155309 | 1.74947290391602 | 0.999950574162623 |
| ebi-fl187-GCST90094775 | prot-a-1948 | Mesothelin \|\| id:prot-a-1948 | Green olives liking \|\| id:ebi-fl187-GCST90094775 | MR Egger | 27 | 0.212280775787749 | 0.349308878102865 | 0.548856691988902 | -0.472364625 | 0.896926176869365 | 1.23649501420516 | 0.62352611806928 | 2.45205433396831 | 0.999950574162623 |
| ebi-fl187-GCST90094775 | prot-a-195 | ATPase family AAA domain-containing protein 1 \|\| id:prot-a-195 | Green olives liking \|\| id:ebi-fl187-GCST90094775 | MR Egger | 27 | -0.036970343 | 0.309344525572928 | 0.905825057064152 | -0.643285613 | 0.569344927276496 | 0.963704715663983 | 0.525562788228876 | 1.76710908723726 | 0.999950574162623 |
| ebi-fl187-GCST90094775 | prot-a-1966 | Mucin-1 \|\| id:prot-a-1966 | Green olives liking \|\| id:ebi-fl187-GCST90094775 | MR Egger | 27 | -0.173571716 | 0.318250969904394 | 0.590316944166217 | -0.797343617 | 0.450200185427686 | 0.840656860801534 | 0.450524140840753 | 1.5686261701623 | 0.999950574162623 |
| ebi-fl187-GCST90094775 | prot-a-1986 | Myocardial zonula adherens protein \|\| id:prot-a-1986 | Green olives liking \|\| id:ebi-fl187-GCST90094775 | MR Egger | 27 | -0.503046554 | 0.309247386332018 | 0.116345475884644 | -1.109171431 | 0.103078323449714 | 0.604685643347913 | 0.329832136580055 | 1.10857823334729 | 0.999950574162623 |
| ebi-fl187-GCST90094775 | prot-a-2071 | Epididymal secretory protein E1 \|\| id:prot-a-2071 | Green olives liking \|\| id:ebi-fl187-GCST90094775 | MR Egger | 27 | 0.460179687500666 | 0.348654137891997 | 0.198835969827964 | -0.223182423 | 1.14354179776898 | 1.58435864886422 | 0.799968903441637 | 3.13786238118941 | 0.999950574162623 |
| ebi-fl187-GCST90094775 | prot-a-2098 | Neuritin \|\| id:prot-a-2098 | Green olives liking \|\| id:ebi-fl187-GCST90094775 | MR Egger | 27 | -0.245249614 | 0.320873663153897 | 0.451831904125623 | -0.874161993 | 0.383662766234047 | 0.782509188967289 | 0.417211499244442 | 1.46765041693995 | 0.999950574162623 |
| ebi-fl187-GCST90094775 | prot-a-2115 | Neurotrophin-4 \|\| id:prot-a-2115 | Green olives liking \|\| id:ebi-fl187-GCST90094775 | MR Egger | 27 | -0.387342352 | 0.376541391900307 | 0.313478289458695 | -1.12536348 | 0.350678776266665 | 0.678858646620406 | 0.324534484128754 | 1.42003110494863 | 0.999950574162623 |
| ebi-fl187-GCST90094775 | prot-a-2118 | Netrin-1 \|\| id:prot-a-2118 | Green olives liking \|\| id:ebi-fl187-GCST90094775 | MR Egger | 27 | -0.124598706 | 0.365461605328843 | 0.73600107690786 | -0.840903453 | 0.591706040093721 | 0.882851114053684 | 0.431320669483744 | 1.80706871877655 | 0.999950574162623 |
| ebi-fl187-GCST90094775 | prot-a-2120 | Netrin-G1 \|\| id:prot-a-2120 | Green olives liking \|\| id:ebi-fl187-GCST90094775 | MR Egger | 27 | 0.0793700169337206 | 0.309368936812711 | 0.799622172833545 | -0.526993099 | 0.685733133086633 | 1.08260483008803 | 0.590377510012197 | 1.98522673755936 | 0.999950574162623 |
| ebi-fl187-GCST90094775 | prot-a-2145 | Olfactomedin-like protein 3 \|\| id:prot-a-2145 | Green olives liking \|\| id:ebi-fl187-GCST90094775 | MR Egger | 27 | -0.290092474 | 0.309346287799949 | 0.357339884855943 | -0.896411198 | 0.316226250420047 | 0.748194376101195 | 0.408031379190088 | 1.37194062265654 | 0.999950574162623 |
| ebi-fl187-GCST90094775 | prot-a-2162 | Prolyl 4-hydroxylase subunit alpha-2 \|\| id:prot-a-2162 | Green olives liking \|\| id:ebi-fl187-GCST90094775 | MR Egger | 27 | 0.101846889024836 | 0.30932242731809 | 0.744703300093358 | -0.504425069 | 0.708118846568292 | 1.10721393214421 | 0.603852649543899 | 2.03016860563618 | 0.999950574162623 |
| ebi-fl187-GCST90094775 | prot-a-2168 | Polyadenylate-binding protein-interacting protein 1 \|\| id:prot-a-2168 | Green olives liking \|\| id:ebi-fl187-GCST90094775 | MR Egger | 27 | 0.233655994692463 | 0.354106621241916 | 0.515385904701971 | -0.460392983 | 0.927704972326618 | 1.26320986655667 | 0.631035610542819 | 2.52869907863598 | 0.999950574162623 |
| ebi-fl187-GCST90094775 | prot-a-219 | Beta-1,4-galactosyltransferase 5 \|\| id:prot-a-219 | Green olives liking \|\| id:ebi-fl187-GCST90094775 | MR Egger | 27 | -0.304517484 | 0.378333391863386 | 0.428470873702022 | -1.046050932 | 0.437015963634284 | 0.737479133728472 | 0.351322409172172 | 1.54808078985466 | 0.999950574162623 |
| ebi-fl187-GCST90094775 | prot-a-2190 | Pyruvate carboxylase, mitochondrial \|\| id:prot-a-2190 | Green olives liking \|\| id:ebi-fl187-GCST90094775 | MR Egger | 27 | 0.146302994979957 | 0.309374452156752 | 0.640387727581694 | -0.460070931 | 0.75267692120719 | 1.15754686581072 | 0.631238869358598 | 2.12267465073833 | 0.999950574162623 |
| ebi-fl187-GCST90094775 | prot-a-2197 | Protocadherin-9 \|\| id:prot-a-2197 | Green olives liking \|\| id:ebi-fl187-GCST90094775 | MR Egger | 27 | 0.0867309615286916 | 0.387254082796796 | 0.824605541789484 | -0.672287041 | 0.845748963810412 | 1.09060322602015 | 0.510539616655869 | 2.32972203880367 | 0.999950574162623 |
| ebi-fl187-GCST90094775 | prot-a-2198 | Protocadherin alpha-4 \|\| id:prot-a-2198 | Green olives liking \|\| id:ebi-fl187-GCST90094775 | MR Egger | 27 | -0.439148356 | 0.336427222701443 | 0.203667045239044 | -1.098545713 | 0.220249000056252 | 0.64458514417956 | 0.33335552598364 | 1.24638704239562 | 0.999950574162623 |
| ebi-fl187-GCST90094775 | prot-a-2254 | Placenta growth factor \|\| id:prot-a-2254 | Green olives liking \|\| id:ebi-fl187-GCST90094775 | MR Egger | 27 | 0.0586537286849619 | 0.337881742775603 | 0.863583696273426 | -0.603594487 | 0.720901944525143 | 1.06040798829075 | 0.546842480889176 | 2.05628703132653 | 0.999950574162623 |
| ebi-fl187-GCST90094775 | prot-a-2261 | Membrane-associated progesterone receptor component 2 \|\| id:prot-a-2261 | Green olives liking \|\| id:ebi-fl187-GCST90094775 | MR Egger | 27 | 0.510354439435152 | 0.30922339260127 | 0.111362215044757 | -0.09572341 | 1.11643228893364 | 1.66588154443178 | 0.90871532282623 | 3.0539391714528 | 0.999950574162623 |
| ebi-fl187-GCST90094775 | prot-a-2268 | Polymeric immunoglobulin receptor \|\| id:prot-a-2268 | Green olives liking \|\| id:ebi-fl187-GCST90094775 | MR Egger | 27 | 0.214299396265799 | 0.346797228725244 | 0.542204663866169 | -0.465423172 | 0.894021964567276 | 1.23899354931048 | 0.627869352212076 | 2.44494337846651 | 0.999950574162623 |
| ebi-fl187-GCST90094775 | prot-a-2283 | Group 10 secretory phospholipase A2 \|\| id:prot-a-2283 | Green olives liking \|\| id:ebi-fl187-GCST90094775 | MR Egger | 27 | -0.239994905 | 0.316825889336359 | 0.455832772162343 | -0.860973648 | 0.38098383840199 | 0.78663186918385 | 0.422750272000359 | 1.4637239491005 | 0.999950574162623 |
| ebi-fl187-GCST90094775 | prot-a-2298 | Inactive phospholipase D5 \|\| id:prot-a-2298 | Green olives liking \|\| id:ebi-fl187-GCST90094775 | MR Egger | 27 | 0.0999966287703137 | 0.329951911606162 | 0.764348479984236 | -0.546709118 | 0.746702375518392 | 1.10516719229692 | 0.578851611724767 | 2.1100304433638 | 0.999950574162623 |
| ebi-fl187-GCST90094775 | prot-a-2304 | Procollagen-lysine,2-oxoglutarate 5-dioxygenase 2 \|\| id:prot-a-2304 | Green olives liking \|\| id:ebi-fl187-GCST90094775 | MR Egger | 27 | 0.431600186721176 | 0.31512967869244 | 0.182990904465806 | -0.186053984 | 1.04925435695836 | 1.53971939182363 | 0.830228775103806 | 2.85552112459763 | 0.999950574162623 |
| ebi-fl187-GCST90094775 | prot-a-2331 | Periostin \|\| id:prot-a-2331 | Green olives liking \|\| id:ebi-fl187-GCST90094775 | MR Egger | 27 | -0.970941923 | 0.320385369097292 | 0.00561025607074269 | -1.598897246 | -0.342986599 | 0.378726139246338 | 0.202119282939166 | 0.709647721200392 | 0.999950574162623 |
| ebi-fl187-GCST90094775 | prot-a-2333 | Periostin \|\| id:prot-a-2333 | Green olives liking \|\| id:ebi-fl187-GCST90094775 | MR Egger | 27 | -0.828959267 | 0.360919007639041 | 0.030282567042978 | -1.536360522 | -0.121558012 | 0.436503333297721 | 0.21516275821694 | 0.885539679631324 | 0.999950574162623 |
| ebi-fl187-GCST90094775 | prot-a-2367 | cAMP-dependent protein kinase catalytic subunit alpha \|\| id:prot-a-2367 | Green olives liking \|\| id:ebi-fl187-GCST90094775 | MR Egger | 27 | 0.778681824690137 | 0.356741701711281 | 0.0386563899527934 | 0.0794680893360255 | 1.47789556004425 | 2.17859859703418 | 1.08271100895099 | 4.38371071113231 | 0.999950574162623 |
| ebi-fl187-GCST90094775 | prot-a-2385 | Phosphoribosyl pyrophosphate synthase-associated protein 1 \|\| id:prot-a-2385 | Green olives liking \|\| id:ebi-fl187-GCST90094775 | MR Egger | 27 | -0.064752586 | 0.339095055806157 | 0.850101570881024 | -0.729378895 | 0.599873723854399 | 0.937299336114422 | 0.482208399191554 | 1.82188872477841 | 0.999950574162623 |
| ebi-fl187-GCST90094775 | prot-a-2393 | Trypsin-3 \|\| id:prot-a-2393 | Green olives liking \|\| id:ebi-fl187-GCST90094775 | MR Egger | 26 | 0.342835318085668 | 0.309442170561763 | 0.278879210849927 | -0.263671336 | 0.949341972386723 | 1.40893671642449 | 0.768225986229484 | 2.58400875064391 | 0.999950574162623 |
| ebi-fl187-GCST90094775 | prot-a-2405 | Pregnancy-specific beta-1-glycoprotein 6 \|\| id:prot-a-2405 | Green olives liking \|\| id:ebi-fl187-GCST90094775 | MR Egger | 27 | 0.0757703779437582 | 0.358758805496785 | 0.83444226930583 | -0.627396881 | 0.778937636717456 | 1.07871484899507 | 0.533980006982809 | 2.17915598004761 | 0.999950574162623 |
| ebi-fl187-GCST90094775 | prot-a-244 | Beclin-1 \|\| id:prot-a-244 | Green olives liking \|\| id:ebi-fl187-GCST90094775 | MR Egger | 27 | -0.03454376 | 0.309337001034507 | 0.911976583252177 | -0.640844282 | 0.571756762505421 | 0.966046065047297 | 0.526847428655186 | 1.77137620691352 | 0.999950574162623 |
| ebi-fl187-GCST90094775 | prot-a-2454 | tRNA pseudouridine synthase A, mitochondrial \|\| id:prot-a-2454 | Green olives liking \|\| id:ebi-fl187-GCST90094775 | MR Egger | 27 | 0.648079780986972 | 0.332497841536407 | 0.0625851944800512 | -0.003615988 | 1.29977555039833 | 1.91186610036484 | 0.996390541388809 | 3.6684731878621 | 0.999950574162623 |
| ebi-fl187-GCST90094775 | prot-a-2466 | Glutaminyl-peptide cyclotransferase-like protein \|\| id:prot-a-2466 | Green olives liking \|\| id:ebi-fl187-GCST90094775 | MR Egger | 27 | -0.015048476 | 0.309374452156752 | 0.961591418467363 | -0.621422402 | 0.59132545034589 | 0.985064186591012 | 0.537179808226474 | 1.8063810978076 | 0.999950574162623 |
| ebi-fl187-GCST90094775 | prot-a-2474 | Ras-related protein Rab-27A \|\| id:prot-a-2474 | Green olives liking \|\| id:ebi-fl187-GCST90094775 | MR Egger | 27 | -0.035455716 | 0.36771155595106 | 0.923953733603036 | -0.756170365 | 0.685258934088777 | 0.96516547508142 | 0.469460852419021 | 1.98428556819831 | 0.999950574162623 |
| ebi-fl187-GCST90094775 | prot-a-2490 | Rap1 GTPase-GDP dissociation stimulator 1 \|\| id:prot-a-2490 | Green olives liking \|\| id:ebi-fl187-GCST90094775 | MR Egger | 27 | 0.196514575911673 | 0.385520039202601 | 0.614705038957268 | -0.559104701 | 0.952133852748771 | 1.21715306186442 | 0.571720695792695 | 2.59123307396084 | 0.999950574162623 |
| ebi-fl187-GCST90094775 | prot-a-2545 | Receptor-interacting serine/threonine-protein kinase 2 \|\| id:prot-a-2545 | Green olives liking \|\| id:ebi-fl187-GCST90094775 | MR Egger | 27 | 0.325917773500428 | 0.30922381951972 | 0.301969541227915 | -0.280160913 | 0.93199645975908 | 1.38530145569995 | 0.755662135993465 | 2.53957427765179 | 0.999950574162623 |
| ebi-fl187-GCST90094775 | prot-a-2610 | RUN and FYVE domain-containing protein 1 \|\| id:prot-a-2610 | Green olives liking \|\| id:ebi-fl187-GCST90094775 | MR Egger | 27 | -0.069880461 | 0.30923002839803 | 0.823052669 | -0.675971317 | 0.536210394717159 | 0.932505284045978 | 0.508662108628209 | 1.70951617984436 | 0.999950574162623 |
| ebi-fl187-GCST90094775 | prot-a-2627 | Serine--tRNA ligase, mitochondrial \|\| id:prot-a-2627 | Green olives liking \|\| id:ebi-fl187-GCST90094775 | MR Egger | 27 | 0.0949614110754847 | 0.309345497086839 | 0.761404642355249 | -0.511355763 | 0.701278585365689 | 1.09961642129229 | 0.599682000630216 | 2.01632910893598 | 0.999950574162623 |
| ebi-fl187-GCST90094775 | prot-a-264 | BolA-like protein 2 \|\| id:prot-a-264 | Green olives liking \|\| id:ebi-fl187-GCST90094775 | MR Egger | 27 | 0.558995013664998 | 0.309345098549651 | 0.08280890551584 | -0.047321379 | 1.16531140682231 | 1.7489139821476 | 0.953780822732982 | 3.20692138492251 | 0.999950574162623 |
| ebi-fl187-GCST90094775 | prot-a-2646 | Secretoglobin family 1C member 1 \|\| id:prot-a-2646 | Green olives liking \|\| id:ebi-fl187-GCST90094775 | MR Egger | 27 | -0.396258888 | 0.423414536905839 | 0.358295567641149 | -1.226151381 | 0.433633604048839 | 0.672832484938568 | 0.293419668039057 | 1.54285346927851 | 0.999950574162623 |
| ebi-fl187-GCST90094775 | prot-a-2672 | Semaphorin-3E \|\| id:prot-a-2672 | Green olives liking \|\| id:ebi-fl187-GCST90094775 | MR Egger | 27 | -0.229685105 | 0.357479682403259 | 0.526389608241342 | -0.930345282 | 0.470975073001143 | 0.794783836948798 | 0.394417501586206 | 1.60155506521556 | 0.999950574162623 |
| ebi-fl187-GCST90094775 | prot-a-2701 | Plasma protease C1 inhibitor \|\| id:prot-a-2701 | Green olives liking \|\| id:ebi-fl187-GCST90094775 | MR Egger | 27 | 0.158608476497889 | 0.340825710118477 | 0.645698298889882 | -0.509409915 | 0.826626868330105 | 1.17187903866468 | 0.600850026611949 | 2.28559610624528 | 0.999950574162623 |
| ebi-fl187-GCST90094775 | prot-a-2738 | Signaling threshold-regulating transmembrane adapter 1 \|\| id:prot-a-2738 | Green olives liking \|\| id:ebi-fl187-GCST90094775 | MR Egger | 27 | -0.197816511 | 0.309334646871693 | 0.528320945630644 | -0.804112419 | 0.408479396390111 | 0.820520395400308 | 0.447484929313112 | 1.50452825372537 | 0.999950574162623 |
| ebi-fl187-GCST90094775 | prot-a-274 | Serine/threonine-protein kinase BRSK2 \|\| id:prot-a-274 | Green olives liking \|\| id:ebi-fl187-GCST90094775 | MR Egger | 27 | -0.191060507 | 0.309346287799949 | 0.542407045506782 | -0.797379232 | 0.415258216643898 | 0.826082602492146 | 0.450508095738772 | 1.51476182691264 | 0.999950574162623 |
| ebi-fl187-GCST90094775 | prot-a-2749 | Mitochondrial glutamate carrier 2 \|\| id:prot-a-2749 | Green olives liking \|\| id:ebi-fl187-GCST90094775 | MR Egger | 27 | -0.395946903 | 0.398293282832273 | 0.329695007241363 | -1.176601737 | 0.384707931417498 | 0.673042431567223 | 0.308324728749681 | 1.46918515594539 | 0.999950574162623 |
| ebi-fl187-GCST90094775 | prot-a-2760 | Sodium-coupled monocarboxylate transporter 1 \|\| id:prot-a-2760 | Green olives liking \|\| id:ebi-fl187-GCST90094775 | MR Egger | 27 | -0.270505959 | 0.347718387051227 | 0.44390167254897 | -0.952033998 | 0.411022079423792 | 0.762993353155267 | 0.385955192517174 | 1.50835865988048 | 0.999950574162623 |
| ebi-fl187-GCST90094775 | prot-a-2777 | Small integral membrane protein 10 \|\| id:prot-a-2777 | Green olives liking \|\| id:ebi-fl187-GCST90094775 | MR Egger | 27 | -0.130539245 | 0.369453533217328 | 0.726802704883809 | -0.85466817 | 0.593589680172118 | 0.877622050053593 | 0.425424336203436 | 1.81047579368369 | 0.999950574162623 |
| ebi-fl187-GCST90094775 | prot-a-2799 | Superoxide dismutase [Mn], mitochondrial \|\| id:prot-a-2799 | Green olives liking \|\| id:ebi-fl187-GCST90094775 | MR Egger | 27 | -0.012652001 | 0.326908747643372 | 0.969435435753912 | -0.653393146 | 0.628089144367763 | 0.987427699075619 | 0.520277400891739 | 1.87402616225619 | 0.999950574162623 |
| ebi-fl187-GCST90094775 | prot-a-2805 | Protein SPACA7 \|\| id:prot-a-2805 | Green olives liking \|\| id:ebi-fl187-GCST90094775 | MR Egger | 27 | 0.288339449572401 | 0.313951179833413 | 0.367174831750037 | -0.327004863 | 0.903683762045891 | 1.33421012432042 | 0.721080236476214 | 2.46868041279041 | 0.999950574162623 |
| ebi-fl187-GCST90094775 | prot-a-2811 | SPARC-like protein 1 \|\| id:prot-a-2811 | Green olives liking \|\| id:ebi-fl187-GCST90094775 | MR Egger | 27 | -0.046437362 | 0.309340952591162 | 0.881876827405169 | -0.652745629 | 0.559870905398536 | 0.954624354762926 | 0.520614398778399 | 1.7504465125146 | 0.999950574162623 |
| ebi-fl187-GCST90094775 | prot-a-2831 | Sepiapterin reductase \|\| id:prot-a-2831 | Green olives liking \|\| id:ebi-fl187-GCST90094775 | MR Egger | 27 | -0.258247173 | 0.309340163006164 | 0.411719217005489 | -0.864553893 | 0.348059546237207 | 0.772404290827469 | 0.421239428682173 | 1.41631658355236 | 0.999950574162623 |
| ebi-fl187-GCST90094775 | prot-a-2834 | SPRY domain-containing SOCS box protein 1 \|\| id:prot-a-2834 | Green olives liking \|\| id:ebi-fl187-GCST90094775 | MR Egger | 27 | -0.154884947 | 0.329348206162775 | 0.642233871550615 | -0.800407431 | 0.490637537049056 | 0.856513716261831 | 0.449145930808319 | 1.63335721382219 | 0.999950574162623 |
| ebi-fl187-GCST90094775 | prot-a-2835 | Spectrin alpha chain, non-erythrocytic 1 \|\| id:prot-a-2835 | Green olives liking \|\| id:ebi-fl187-GCST90094775 | MR Egger | 27 | -0.288376576 | 0.309171575731527 | 0.359875888603588 | -0.894352864 | 0.317599712752865 | 0.7494793034118 | 0.408872108858723 | 1.37382622603081 | 0.999950574162623 |
| ebi-fl187-GCST90094775 | prot-a-2859 | Sia-alpha-2,3-Gal-beta-1,4-GlcNAc-R:alpha 2,8-sialyltransferase \|\| id:prot-a-2859 | Green olives liking \|\| id:ebi-fl187-GCST90094775 | MR Egger | 27 | -0.503219804 | 0.309340952591162 | 0.11633094344363 | -1.109528072 | 0.103088462597421 | 0.604580890199241 | 0.329714526026475 | 1.10858947344272 | 0.999950574162623 |
| ebi-fl187-GCST90094775 | prot-a-2861 | Alpha-2,8-sialyltransferase 8F \|\| id:prot-a-2861 | Green olives liking \|\| id:ebi-fl187-GCST90094775 | MR Egger | 27 | -0.219602196 | 0.309375240787016 | 0.484381541544136 | -0.825977668 | 0.386773275690301 | 0.802838106454795 | 0.437806753422924 | 1.47222266476388 | 0.999950574162623 |
| ebi-fl187-GCST90094775 | prot-a-289 | Uncharacterized protein C17orf89 \|\| id:prot-a-289 | Green olives liking \|\| id:ebi-fl187-GCST90094775 | MR Egger | 27 | 0.361317459387582 | 0.353201650912603 | 0.316115880447709 | -0.330957776 | 1.05359269517628 | 1.43521901231389 | 0.718235494887771 | 2.86793625206327 | 0.999950574162623 |
| ebi-fl187-GCST90094775 | prot-a-2921 | Tachykinin-3 \|\| id:prot-a-2921 | Green olives liking \|\| id:ebi-fl187-GCST90094775 | MR Egger | 27 | 0.151522535593769 | 0.30928959090139 | 0.628472776421473 | -0.454685063 | 0.757730133760493 | 1.16360452403307 | 0.634647810826196 | 2.1334281238402 | 0.999950574162623 |
| ebi-fl187-GCST90094775 | prot-a-2928 | Tax1-binding protein 1 \|\| id:prot-a-2928 | Green olives liking \|\| id:ebi-fl187-GCST90094775 | MR Egger | 27 | 0.0891733104483746 | 0.30922381951972 | 0.775434972078487 | -0.516905376 | 0.695251996707026 | 1.0932701150421 | 0.596363215350198 | 2.0042140656551 | 0.999950574162623 |
| ebi-fl187-GCST90094775 | prot-a-296 | Uncharacterized protein C1orf185 \|\| id:prot-a-296 | Green olives liking \|\| id:ebi-fl187-GCST90094775 | MR Egger | 27 | -0.068529056 | 0.366245548460475 | 0.853082811353465 | -0.786370331 | 0.649312218577262 | 0.933766327816493 | 0.455495094698777 | 1.91422380858022 | 0.999950574162623 |
| ebi-fl187-GCST90094775 | prot-a-2964 | Transforming growth factor beta-2 \|\| id:prot-a-2964 | Green olives liking \|\| id:ebi-fl187-GCST90094775 | MR Egger | 27 | -0.632674526 | 0.30920075377366 | 0.0513947072939875 | -1.238708004 | -0.026641049 | 0.531169273459475 | 0.289758342959935 | 0.97371069348805 | 0.999950574162623 |
| ebi-fl187-GCST90094775 | prot-a-2977 | Thymocyte nuclear protein 1 \|\| id:prot-a-2977 | Green olives liking \|\| id:ebi-fl187-GCST90094775 | MR Egger | 27 | 0.381114455065711 | 0.390265895018531 | 0.33814725168362 | -0.383806699 | 1.14603560930203 | 1.46391514832597 | 0.681263103175475 | 3.14569738403442 | 0.999950574162623 |
| ebi-fl187-GCST90094775 | prot-a-3015 | Transmembrane protein 70, mitochondrial \|\| id:prot-a-3015 | Green olives liking \|\| id:ebi-fl187-GCST90094775 | MR Egger | 27 | -0.305844719 | 0.34115746783704 | 0.378542299036197 | -0.974513356 | 0.362823918379318 | 0.73650097549355 | 0.377375956802791 | 1.43738274027462 | 0.999950574162623 |
| ebi-fl187-GCST90094775 | prot-a-3030 | Tumor necrosis factor alpha-induced protein 3 \|\| id:prot-a-3030 | Green olives liking \|\| id:ebi-fl187-GCST90094775 | MR Egger | 27 | -0.129141639 | 0.435970111397425 | 0.769511349162487 | -0.983643057 | 0.725359779462983 | 0.87884947747748 | 0.373946306562531 | 2.06547408145956 | 0.999950574162623 |
| ebi-fl187-GCST90094775 | prot-a-3032 | Tumor necrosis factor receptor superfamily member 10A \|\| id:prot-a-3032 | Green olives liking \|\| id:ebi-fl187-GCST90094775 | MR Egger | 27 | 0.106776203623312 | 0.309345497086839 | 0.732855294180872 | -0.499540971 | 0.713093377913516 | 1.11268521169416 | 0.606809138987059 | 2.04029290394269 | 0.999950574162623 |
| ebi-fl187-GCST90094775 | prot-a-3081 | Tumor protein p53-inducible protein 11 \|\| id:prot-a-3081 | Green olives liking \|\| id:ebi-fl187-GCST90094775 | MR Egger | 27 | 0.154328813342308 | 0.309372684925609 | 0.62225016312529 | -0.452041649 | 0.760699275796501 | 1.16687450761206 | 0.636327666690991 | 2.13977198822018 | 0.999950574162623 |
| ebi-fl187-GCST90094775 | prot-a-315 | Complement C4 \|\| id:prot-a-315 | Green olives liking \|\| id:ebi-fl187-GCST90094775 | MR Egger | 26 | 0.616261795469051 | 0.329607986449636 | 0.073776671401964 | -0.029769858 | 1.26229344891034 | 1.85199196093931 | 0.970668899553401 | 3.53351614022236 | 0.999950574162623 |
| ebi-fl187-GCST90094775 | prot-a-3157 | UDP-glucuronosyltransferase 1-8 \|\| id:prot-a-3157 | Green olives liking \|\| id:ebi-fl187-GCST90094775 | MR Egger | 27 | -0.169660271 | 0.333799958062779 | 0.615720351811165 | -0.823908189 | 0.484587646510574 | 0.843951482445665 | 0.438713723502776 | 1.62350541267654 | 0.999950574162623 |
| ebi-fl187-GCST90094775 | prot-a-317 | Glutaredoxin-like protein C5orf63 \|\| id:prot-a-317 | Green olives liking \|\| id:ebi-fl187-GCST90094775 | MR Egger | 27 | 0.0100201343755273 | 0.324843941489623 | 0.975637299428462 | -0.626673991 | 0.646714259695189 | 1.01007050401827 | 0.534366155283614 | 1.90925718816572 | 0.999950574162623 |
| ebi-fl187-GCST90094775 | prot-a-3192 | Guanine nucleotide exchange factor VAV3 \|\| id:prot-a-3192 | Green olives liking \|\| id:ebi-fl187-GCST90094775 | MR Egger | 27 | -0.055857433 | 0.309375240787016 | 0.858177126152795 | -0.662232905 | 0.550518039096672 | 0.945673948323795 | 0.515698542188706 | 1.73415114330704 | 0.999950574162623 |
| ebi-fl187-GCST90094775 | prot-a-3233 | Serine/threonine-protein kinase WNK3 \|\| id:prot-a-3233 | Green olives liking \|\| id:ebi-fl187-GCST90094775 | MR Egger | 27 | -0.420457028 | 0.309375240787016 | 0.186265352664848 | -1.0268325 | 0.185918443640599 | 0.656746599432526 | 0.358139572857071 | 1.20432403608835 | 0.999950574162623 |
| ebi-fl187-GCST90094775 | prot-a-3250 | 14-3-3 protein theta \|\| id:prot-a-3250 | Green olives liking \|\| id:ebi-fl187-GCST90094775 | MR Egger | 27 | -0.314037111 | 0.346877329631347 | 0.373935364185266 | -0.993916677 | 0.365842454897402 | 0.730491918239293 | 0.37012419138987 | 1.44172808756193 | 0.999950574162623 |
| ebi-fl187-GCST90094775 | prot-a-3278 | E3 ubiquitin-protein ligase ZNRF3 \|\| id:prot-a-3278 | Green olives liking \|\| id:ebi-fl187-GCST90094775 | MR Egger | 27 | 0.40627971022935 | 0.315147778380538 | 0.209139371004122 | -0.211409935 | 1.0239693558552 | 1.50122240099941 | 0.809442179858856 | 2.78422443670454 | 0.999950574162623 |
| ebi-fl187-GCST90094775 | prot-a-355 | Calpain-3 \|\| id:prot-a-355 | Green olives liking \|\| id:ebi-fl187-GCST90094775 | MR Egger | 27 | 0.163221631822513 | 0.309349621210348 | 0.602409195410884 | -0.443103626 | 0.769546889394795 | 1.17729758738572 | 0.642040671694478 | 2.15878786246704 | 0.999950574162623 |
| ebi-fl187-GCST90094775 | prot-a-385 | Coiled-coil domain-containing protein 90B, mitochondrial \|\| id:prot-a-385 | Green olives liking \|\| id:ebi-fl187-GCST90094775 | MR Egger | 27 | 0.63312632931573 | 0.327578272453799 | 0.06467149405649 | -0.008927085 | 1.27517974332518 | 1.88348979365338 | 0.991112643420209 | 3.57934471560605 | 0.999950574162623 |
| ebi-fl187-GCST90094775 | prot-a-390 | C-C motif chemokine 14 \|\| id:prot-a-390 | Green olives liking \|\| id:ebi-fl187-GCST90094775 | MR Egger | 27 | -0.561561938 | 0.309290619668513 | 0.0814386793830931 | -1.167771553 | 0.0446476761994588 | 0.570317566918518 | 0.311059348785564 | 1.04565938431281 | 0.999950574162623 |
| ebi-fl187-GCST90094775 | prot-a-40 | Pituitary adenylate cyclase-activating polypeptide 27 \|\| id:prot-a-40 | Green olives liking \|\| id:ebi-fl187-GCST90094775 | MR Egger | 27 | -0.358643575 | 0.374627029257893 | 0.347565255348693 | -1.092912552 | 0.375625402466605 | 0.698623313879966 | 0.335238670282261 | 1.45590165444124 | 0.999950574162623 |
| ebi-fl187-GCST90094775 | prot-a-401 | C-C motif chemokine 24 \|\| id:prot-a-401 | Green olives liking \|\| id:ebi-fl187-GCST90094775 | MR Egger | 27 | -0.361532227 | 0.309253509638844 | 0.253410675354099 | -0.967669106 | 0.244604651935621 | 0.696608146153318 | 0.379967671132244 | 1.27711630792471 | 0.999950574162623 |
| ebi-fl187-GCST90094775 | prot-a-412 | Cerebral cavernous malformations 2 protein \|\| id:prot-a-412 | Green olives liking \|\| id:ebi-fl187-GCST90094775 | MR Egger | 27 | 0.0443704459911526 | 0.313858729647257 | 0.888710082722686 | -0.570792664 | 0.659533556099776 | 1.04536953612308 | 0.565077344594284 | 1.93389007276303 | 0.999950574162623 |
| ebi-fl187-GCST90094775 | prot-a-44 | Adhesion G protein-coupled receptor F5 \|\| id:prot-a-44 | Green olives liking \|\| id:ebi-fl187-GCST90094775 | MR Egger | 27 | -0.158084814 | 0.309340952591162 | 0.613808597691857 | -0.764393081 | 0.448223453291382 | 0.853777366805637 | 0.465616436761146 | 1.56552847906334 | 0.999950574162623 |
| ebi-fl187-GCST90094775 | prot-a-48 | Adrenomedullin \|\| id:prot-a-48 | Green olives liking \|\| id:ebi-fl187-GCST90094775 | MR Egger | 27 | 0.100043667562757 | 0.338005626816136 | 0.769688328204363 | -0.562447361 | 0.762534696122383 | 1.10521917924979 | 0.569812818320227 | 2.14370297562365 | 0.999950574162623 |
| ebi-fl187-GCST90094775 | prot-a-502 | Carcinoembryonic antigen-related cell adhesion molecule 1 \|\| id:prot-a-502 | Green olives liking \|\| id:ebi-fl187-GCST90094775 | MR Egger | 27 | 0.154517985651022 | 0.318177112158081 | 0.631455011809029 | -0.469109154 | 0.778145125480861 | 1.16709526883694 | 0.625559297018152 | 2.17742965860205 | 0.999950574162623 |
| ebi-fl187-GCST90094775 | prot-a-518 | Complement factor B \|\| id:prot-a-518 | Green olives liking \|\| id:ebi-fl187-GCST90094775 | MR Egger | 27 | 0.559226785008388 | 0.367196368137808 | 0.140318092690191 | -0.160478097 | 1.27893166655849 | 1.74931937726839 | 0.851736479342082 | 3.59279936683049 | 0.999950574162623 |
| ebi-fl187-GCST90094775 | prot-a-525 | Properdin \|\| id:prot-a-525 | Green olives liking \|\| id:ebi-fl187-GCST90094775 | MR Egger | 27 | 0.209307239446661 | 0.401760882929182 | 0.606968721218839 | -0.578144091 | 0.996758569987857 | 1.23282371242459 | 0.560938451783236 | 2.70948497305665 | 0.999950574162623 |
| ebi-fl187-GCST90094775 | prot-a-533 | Chondroadherin \|\| id:prot-a-533 | Green olives liking \|\| id:ebi-fl187-GCST90094775 | MR Egger | 27 | -0.31881962 | 0.320341539756171 | 0.329151212628054 | -0.946689038 | 0.309049797863573 | 0.727006674901241 | 0.388023630454315 | 1.36213019998839 | 0.999950574162623 |
| ebi-fl187-GCST90094775 | prot-a-535 | Coiled-coil-helix-coiled-coil-helix domain-containing protein 2 \|\| id:prot-a-535 | Green olives liking \|\| id:ebi-fl187-GCST90094775 | MR Egger | 27 | -0.420057878 | 0.352483037463056 | 0.244565238368983 | -1.110924632 | 0.270808875130741 | 0.657008792164675 | 0.329254381238496 | 1.31102447705627 | 0.999950574162623 |
| ebi-fl187-GCST90094775 | prot-a-591 | Caseinolytic peptidase B protein homolog \|\| id:prot-a-591 | Green olives liking \|\| id:ebi-fl187-GCST90094775 | MR Egger | 27 | -0.015322307 | 0.436192902372409 | 0.972257157853202 | -0.870260395 | 0.839615781955196 | 0.984794482591883 | 0.418842470417009 | 2.31547715774344 | 0.999950574162623 |
| ebi-fl187-GCST90094775 | prot-a-605 | Ciliary neurotrophic factor receptor subunit alpha \|\| id:prot-a-605 | Green olives liking \|\| id:ebi-fl187-GCST90094775 | MR Egger | 27 | -0.363982779 | 0.309343422650724 | 0.250425416272925 | -0.970295887 | 0.242330329458897 | 0.694903161611326 | 0.378970888827264 | 1.27421503406696 | 0.999950574162623 |
| ebi-fl187-GCST90094775 | prot-a-606 | Ciliary neurotrophic factor receptor subunit alpha \|\| id:prot-a-606 | Green olives liking \|\| id:ebi-fl187-GCST90094775 | MR Egger | 27 | -0.161655671 | 0.309345497086839 | 0.605871800779302 | -0.767972845 | 0.444661503769473 | 0.85073408693847 | 0.463952619641692 | 1.55996206517377 | 0.999950574162623 |
| ebi-fl187-GCST90094775 | prot-a-707 | Cancer/testis antigen 55 \|\| id:prot-a-707 | Green olives liking \|\| id:ebi-fl187-GCST90094775 | MR Egger | 27 | 0.0811142298554131 | 0.314941279651629 | 0.798859692422453 | -0.536170678 | 0.698399137972606 | 1.08449477117244 | 0.584984061007915 | 2.0105315462338 | 0.999950574162623 |
| ebi-fl187-GCST90094775 | prot-a-719 | Dipeptidyl peptidase 1 \|\| id:prot-a-719 | Green olives liking \|\| id:ebi-fl187-GCST90094775 | MR Egger | 27 | -0.07911934 | 0.309344212724201 | 0.800225205758408 | -0.685433997 | 0.527195317143691 | 0.923929656289065 | 0.503871503527583 | 1.69417401816552 | 0.999950574162623 |
| ebi-fl187-GCST90094775 | prot-a-752 | Cytochrome b5 \|\| id:prot-a-752 | Green olives liking \|\| id:ebi-fl187-GCST90094775 | MR Egger | 27 | 0.110342483639048 | 0.347243821415664 | 0.753301029918206 | -0.570255406 | 0.790940373613749 | 1.11666044290905 | 0.56538101836324 | 2.20546941665579 | 0.999950574162623 |
| ebi-fl187-GCST90094775 | prot-a-755 | Cytoglobin \|\| id:prot-a-755 | Green olives liking \|\| id:ebi-fl187-GCST90094775 | MR Egger | 27 | -0.540485789 | 0.347736586612849 | 0.132683101510728 | -1.222049498 | 0.141077921189569 | 0.582465228683261 | 0.294625712814538 | 1.15151437185866 | 0.999950574162623 |
| ebi-fl187-GCST90094775 | prot-a-807 | Desmin \|\| id:prot-a-807 | Green olives liking \|\| id:ebi-fl187-GCST90094775 | MR Egger | 27 | 0.322740890902179 | 0.326757251072159 | 0.332759154150719 | -0.317703321 | 0.963185103003611 | 1.38090749885007 | 0.727818684761633 | 2.62002825745657 | 0.999950574162623 |
| ebi-fl187-GCST90094775 | prot-a-82 | Ameloblastin \|\| id:prot-a-82 | Green olives liking \|\| id:ebi-fl187-GCST90094775 | MR Egger | 27 | -0.386761529 | 0.37628269833892 | 0.313863877210054 | -1.124275618 | 0.350752559418156 | 0.679253057548566 | 0.324887724936983 | 1.42013588318416 | 0.999950574162623 |
| ebi-fl187-GCST90094775 | prot-a-84 | Muellerian-inhibiting factor \|\| id:prot-a-84 | Green olives liking \|\| id:ebi-fl187-GCST90094775 | MR Egger | 27 | 0.0575662873508569 | 0.309316915079328 | 0.853862112083946 | -0.548694866 | 0.66382744090634 | 1.05925548356748 | 0.577703298670809 | 1.94221182750617 | 0.999950574162623 |
| ebi-fl187-GCST90094775 | prot-a-858 | Inactive dipeptidyl peptidase 10 \|\| id:prot-a-858 | Green olives liking \|\| id:ebi-fl187-GCST90094775 | MR Egger | 27 | 0.173984946398994 | 0.309367841860349 | 0.578859723461789 | -0.432376024 | 0.780345916445278 | 1.19003765133354 | 0.648965304498803 | 2.18222700316031 | 0.999950574162623 |
| ebi-fl187-GCST90094775 | prot-a-863 | Dihydropyrimidinase-related protein 3 \|\| id:prot-a-863 | Green olives liking \|\| id:ebi-fl187-GCST90094775 | MR Egger | 27 | 0.326839065400872 | 0.354194204693175 | 0.364948781287637 | -0.367381576 | 1.0210597065995 | 1.38657831079858 | 0.692545336081552 | 2.77613509442599 | 0.999950574162623 |
| ebi-fl187-GCST90094775 | prot-a-864 | Dihydropyrimidinase-related protein 5 \|\| id:prot-a-864 | Green olives liking \|\| id:ebi-fl187-GCST90094775 | MR Egger | 27 | -0.233251944 | 0.317189820620719 | 0.468957307599402 | -0.854943993 | 0.388440104187952 | 0.791954020142471 | 0.425307010735851 | 1.47467865374398 | 0.999950574162623 |
| ebi-fl187-GCST90094775 | prot-a-884 | Endothelin-converting enzyme 1 \|\| id:prot-a-884 | Green olives liking \|\| id:ebi-fl187-GCST90094775 | MR Egger | 27 | -0.306908679 | 0.319786515157306 | 0.346381910829708 | -0.933690249 | 0.3198728905228 | 0.73571778418687 | 0.393100392180531 | 1.37695272947033 | 0.999950574162623 |
| ebi-fl187-GCST90094775 | prot-a-893 | Early endosome antigen 1 \|\| id:prot-a-893 | Green olives liking \|\| id:ebi-fl187-GCST90094775 | MR Egger | 27 | 0.120465821722604 | 0.30932242731809 | 0.700240628692305 | -0.485806136 | 0.72673777926606 | 1.12802218645171 | 0.615201061202367 | 2.06832226628544 | 0.999950574162623 |
| ebi-fl187-GCST90094775 | prot-a-897 | Ephrin-A2 \|\| id:prot-a-897 | Green olives liking \|\| id:ebi-fl187-GCST90094775 | MR Egger | 27 | 0.00231195482951826 | 0.309337905343185 | 0.994096082611466 | -0.60399034 | 0.60861424930216 | 1.00231462945789 | 0.546626054771858 | 1.83788278596529 | 0.999950574162623 |
| ebi-fl187-GCST90094775 | prot-a-90 | Anaphase-promoting complex subunit 10 \|\| id:prot-a-90 | Green olives liking \|\| id:ebi-fl187-GCST90094775 | MR Egger | 27 | 0.152620051762734 | 0.348704859183976 | 0.665378618332992 | -0.530841472 | 0.836081575763328 | 1.16488229987404 | 0.588109883267253 | 2.30730822787957 | 0.999950574162623 |
| ebi-fl187-GCST90094775 | prot-a-914 | Histone-lysine N-methyltransferase EHMT2 \|\| id:prot-a-914 | Green olives liking \|\| id:ebi-fl187-GCST90094775 | MR Egger | 27 | -0.272159895 | 0.347204679801769 | 0.440486127689793 | -0.952681067 | 0.408361277870636 | 0.761732454490945 | 0.385705533606493 | 1.5043505515707 | 0.999950574162623 |
| ebi-fl187-GCST90094775 | prot-a-932 | RNA polymerase II elongation factor ELL \|\| id:prot-a-932 | Green olives liking \|\| id:ebi-fl187-GCST90094775 | MR Egger | 27 | -0.230563206 | 0.335327120436601 | 0.498049228064911 | -0.887804362 | 0.426677950125683 | 0.794086242456517 | 0.411558394708714 | 1.5321591505988 | 0.999950574162623 |
| ebi-fl187-GCST90094775 | prot-a-991 | Estrogen receptor \|\| id:prot-a-991 | Green olives liking \|\| id:ebi-fl187-GCST90094775 | MR Egger | 27 | -0.124299188 | 0.383354351998797 | 0.748452908454704 | -0.875673718 | 0.627075341522498 | 0.883115583419209 | 0.416581266686781 | 1.87212723193392 | 0.999950574162623 |
| ebi-fl187-GCST90094775 | prot-a-992 | Persulfide dioxygenase ETHE1, mitochondrial \|\| id:prot-a-992 | Green olives liking \|\| id:ebi-fl187-GCST90094775 | MR Egger | 27 | 0.0560538712607549 | 0.312636429196791 | 0.859151991225973 | -0.55671353 | 0.668821272486465 | 1.05765465939187 | 0.573089413484814 | 1.95193516441212 | 0.999950574162623 |
| ebi-fl187-GCST90094775 | prot-a-993 | Ethanolamine kinase 1 \|\| id:prot-a-993 | Green olives liking \|\| id:ebi-fl187-GCST90094775 | MR Egger | 27 | 0.463852828356894 | 0.30932321540026 | 0.146248449396765 | -0.142420674 | 1.0701263305414 | 1.59018892250128 | 0.867256347690198 | 2.91574782471247 | 0.999950574162623 |
| ebi-fl187-GCST90094775 | prot-b-10 | coagulation factor III, tissue factor \|\| id:prot-b-10 | Green olives liking \|\| id:ebi-fl187-GCST90094775 | MR Egger | 20 | 0.459326789600421 | 0.500804891102841 | 0.371175934295283 | -0.522250797 | 1.44090437616199 | 1.5830079287944 | 0.593183907744658 | 4.22451464024652 | 0.999950574162623 |
| ebi-fl187-GCST90094775 | prot-b-28 | prolactin \|\| id:prot-b-28 | Green olives liking \|\| id:ebi-fl187-GCST90094775 | MR Egger | 20 | 0.418920832068181 | 0.446162513177034 | 0.360190137581455 | -0.455557694 | 1.29339935789517 | 1.52031998943645 | 0.634094238921619 | 3.64515671079259 | 0.999950574162623 |
| ebi-fl187-GCST90094775 | prot-b-54 | matrix metallopeptidase 7 \|\| id:prot-b-54 | Green olives liking \|\| id:ebi-fl187-GCST90094775 | MR Egger | 20 | 0.820196473349309 | 0.446171205405693 | 0.0825791636273205 | -0.054299089 | 1.69469203594447 | 2.27094597406563 | 0.947148782119321 | 5.44496885229081 | 0.999950574162623 |
| ebi-fl187-GCST90094775 | prot-c-2421_7_3 | BDNF \|\| id:prot-c-2421_7_3 | Green olives liking \|\| id:ebi-fl187-GCST90094775 | MR Egger | 3 | 0.263854362683144 | 2.3614136300226 | 0.929160633474102 | -4.364516352 | 4.89222507752744 | 1.30193857165273 | 0.0127208060780624 | 133.249735429921 | 0.999950574162623 |
| ebi-fl187-GCST90094775 | prot-c-2443_10_4 | FGF-8B \|\| id:prot-c-2443_10_4 | Green olives liking \|\| id:ebi-fl187-GCST90094775 | MR Egger | 3 | -4.393570446 | 2.42725201302721 | 0.321318838700519 | -9.150984391 | 0.363843499902331 | 0.0123565320397447 | 0.000106115292359147 | 1.43884901652516 | 0.999950574162623 |
| ebi-fl187-GCST90094775 | prot-c-2605_49_2 | CD30 \|\| id:prot-c-2605_49_2 | Green olives liking \|\| id:ebi-fl187-GCST90094775 | MR Egger | 3 | 2.18262119293138 | 2.35644560436734 | 0.524367565981502 | -2.436012192 | 6.80125457749135 | 8.86952455067266 | 0.0875091262000177 | 898.974417538741 | 0.999950574162623 |
| ebi-fl187-GCST90094775 | prot-c-2624_31_2 | HPV E7 Type18 \|\| id:prot-c-2624_31_2 | Green olives liking \|\| id:ebi-fl187-GCST90094775 | MR Egger | 3 | -1.871523606 | 2.38951935051143 | 0.577013102860972 | -6.554981533 | 2.81193432146346 | 0.153889016948809 | 0.00142300914612437 | 16.64207823 | 0.999950574162623 |
| ebi-fl187-GCST90094775 | prot-c-2774_10_3 | IL-16 \|\| id:prot-c-2774_10_3 | Green olives liking \|\| id:ebi-fl187-GCST90094775 | MR Egger | 3 | -3.846912664 | 2.31256822578951 | 0.3445793804633 | -8.379546387 | 0.685721058222277 | 0.021345535647693 | 0.000229514032424126 | 1.98520276636052 | 0.999950574162623 |
| ebi-fl187-GCST90094775 | prot-c-2851_63_3 | C5a \|\| id:prot-c-2851_63_3 | Green olives liking \|\| id:ebi-fl187-GCST90094775 | MR Egger | 3 | 1.42366863418044 | 2.07477542273754 | 0.617143105641507 | -2.642891194 | 5.49022846274601 | 4.15232589628094 | 0.0711552482219029 | 242.312559927491 | 0.999950574162623 |
| ebi-fl187-GCST90094775 | prot-c-2891_1_3 | CD22 \|\| id:prot-c-2891_1_3 | Green olives liking \|\| id:ebi-fl187-GCST90094775 | MR Egger | 3 | 2.85065005100057 | 5.58132445113372 | 0.699382441710602 | -8.088745873 | 13.7900459752227 | 17.2990234338594 | 0.00030697450076579 | 974856.905113234 | 0.999950574162623 |
| ebi-fl187-GCST90094775 | prot-c-2961_1_2 | Protein C \|\| id:prot-c-2961_1_2 | Green olives liking \|\| id:ebi-fl187-GCST90094775 | MR Egger | 3 | 1.1835256270526 | 2.32720446760778 | 0.700487587828983 | -3.377795129 | 5.74484638356385 | 3.26586816242012 | 0.0341226077854796 | 312.575607390948 | 0.999950574162623 |
| ebi-fl187-GCST90094775 | prot-c-2982_82_2 | Galectin-4 \|\| id:prot-c-2982_82_2 | Green olives liking \|\| id:ebi-fl187-GCST90094775 | MR Egger | 3 | 0.0396971032034872 | 2.20181805840753 | 0.988523473591967 | -4.275866291 | 4.35526049768224 | 1.04049556368365 | 0.0139000020512262 | 77.8871121065656 | 0.999950574162623 |
| ebi-fl187-GCST90094775 | prot-c-2994_71_2 | IL-1Rrp2 \|\| id:prot-c-2994_71_2 | Green olives liking \|\| id:ebi-fl187-GCST90094775 | MR Egger | 3 | 0.731347431077727 | 2.37540358062268 | 0.809858527748694 | -3.924443587 | 5.38713844909818 | 2.07787852034762 | 0.0197531247108318 | 218.577020523463 | 0.999950574162623 |
| ebi-fl187-GCST90094775 | prot-c-3336_50_1 | TFPI \|\| id:prot-c-3336_50_1 | Green olives liking \|\| id:ebi-fl187-GCST90094775 | MR Egger | 3 | 1.44207935265854 | 2.94379648774738 | 0.710012498796661 | -4.327761763 | 7.21192046864341 | 4.22948126291922 | 0.0131970525851497 | 1355.49295101804 | 0.999950574162623 |
| ebi-fl187-GCST90094775 | prot-c-3472_40_2 | Survivin \|\| id:prot-c-3472_40_2 | Green olives liking \|\| id:ebi-fl187-GCST90094775 | MR Egger | 3 | 2.60830333133467 | 2.69660696372756 | 0.510595965632643 | -2.677046318 | 7.89365298024069 | 13.5759973309959 | 0.0687659673133387 | 2680.21683882366 | 0.999950574162623 |
| ebi-fl187-GCST90094775 | prot-c-3473_78_2 | Thrombopoietin Receptor \|\| id:prot-c-3473_78_2 | Green olives liking \|\| id:ebi-fl187-GCST90094775 | MR Egger | 3 | 2.6054088790887 | 4.88151035490705 | 0.687886871435557 | -6.962351417 | 12.1731691747065 | 13.536759069062 | 0.00094686747496564 | 193526.391959425 | 0.999950574162623 |
| ebi-fl187-GCST90094775 | prot-c-3497_13_2 | IFN-aA \|\| id:prot-c-3497_13_2 | Green olives liking \|\| id:ebi-fl187-GCST90094775 | MR Egger | 3 | 2.81873054816268 | 2.39002186258993 | 0.447720563991596 | -1.865712303 | 7.50317339883894 | 16.7555667805256 | 0.154785916211156 | 1813.7891677022 | 0.999950574162623 |
| ebi-fl187-GCST90094775 | prot-c-3665_64_3 | COLEC12 \|\| id:prot-c-3665_64_3 | Green olives liking \|\| id:ebi-fl187-GCST90094775 | MR Egger | 3 | -3.604207509 | 2.89172747018753 | 0.430452958432359 | -9.27199335 | 2.06357833294135 | 0.0272089991682353 | 9.40209103224557e-05 | 7.87409559424583 | 0.999950574162623 |
| ebi-fl187-GCST90094775 | prot-c-3741_4_3 | IL-5 \|\| id:prot-c-3741_4_3 | Green olives liking \|\| id:ebi-fl187-GCST90094775 | MR Egger | 3 | -5.23574599 | 2.31802665310139 | 0.265338372592891 | -9.77907823 | -0.69241375 | 0.00532285220818291 | 5.66239662716728e-05 | 0.500366849863918 | 0.999950574162623 |
| ebi-fl187-GCST90094775 | prot-c-3848_14_2 | GAPDH, liver \|\| id:prot-c-3848_14_2 | Green olives liking \|\| id:ebi-fl187-GCST90094775 | MR Egger | 3 | 3.02169072411351 | 3.13372317394491 | 0.511585605776799 | -3.120406697 | 9.16378814504554 | 20.5259661093438 | 0.0441392134911351 | 9545.14707894706 | 0.999950574162623 |
| ebi-fl187-GCST90094775 | prot-c-3859_50_2 | PSA1 \|\| id:prot-c-3859_50_2 | Green olives liking \|\| id:ebi-fl187-GCST90094775 | MR Egger | 3 | 0.922656776403314 | 6.97254080948598 | 0.916244494929845 | -12.74352321 | 14.5888367629958 | 2.51596587714524 | 2.92117913397577e-06 | 2166962.03986089 | 0.999950574162623 |
| ebi-fl187-GCST90094775 | prot-c-3868_8_1 | SGTA \|\| id:prot-c-3868_8_1 | Green olives liking \|\| id:ebi-fl187-GCST90094775 | MR Egger | 3 | 3.82670216498422 | 2.29678789324806 | 0.344135504886904 | -0.675002106 | 8.32840643575041 | 45.9108817891251 | 0.509155348436268 | 4139.81523149777 | 0.999950574162623 |
| ebi-fl187-GCST90094775 | prot-c-3882_10_1 | EF-1-beta \|\| id:prot-c-3882_10_1 | Green olives liking \|\| id:ebi-fl187-GCST90094775 | MR Egger | 3 | 2.58736684765159 | 2.37121312326185 | 0.472266118158139 | -2.060210874 | 7.23494456924481 | 13.2947184541893 | 0.127427096007414 | 1387.06401004287 | 0.999950574162623 |
| ebi-fl187-GCST90094775 | prot-c-4134_4_2 | HB-EGF \|\| id:prot-c-4134_4_2 | Green olives liking \|\| id:ebi-fl187-GCST90094775 | MR Egger | 3 | 0.059909964360772 | 2.39537106498978 | 0.984080997390919 | -4.635017323 | 4.75483725174074 | 1.06174094771685 | 0.00970593890072735 | 116.144749270387 | 0.999950574162623 |
| ebi-fl187-GCST90094775 | prot-c-4220_39_2 | FER \|\| id:prot-c-4220_39_2 | Green olives liking \|\| id:ebi-fl187-GCST90094775 | MR Egger | 3 | 2.74030246442931 | 2.70875181383653 | 0.496313939535946 | -2.568851091 | 8.04945601954891 | 15.4916700669317 | 0.0766235283571008 | 3132.0907116701 | 0.999950574162623 |
| ebi-fl187-GCST90094775 | prot-c-4228_37_2 | HSP70 protein 8 \|\| id:prot-c-4228_37_2 | Green olives liking \|\| id:ebi-fl187-GCST90094775 | MR Egger | 3 | 2.59558605997683 | 2.39089092541558 | 0.473881390171268 | -2.090560154 | 7.28173227379136 | 13.4044408661741 | 0.12361787139483 | 1453.50371194203 | 0.999950574162623 |
| ebi-fl187-GCST90094775 | prot-c-4246_40_2 | NCAM-L1 \|\| id:prot-c-4246_40_2 | Green olives liking \|\| id:ebi-fl187-GCST90094775 | MR Egger | 3 | 2.94611127211912 | 2.36803059246284 | 0.431018745133644 | -1.695228689 | 7.58745123334628 | 19.0318001482718 | 0.183557246684429 | 1973.27767454732 | 0.999950574162623 |
| ebi-fl187-GCST90094775 | prot-c-4267_81_3 | PESC \|\| id:prot-c-4267_81_3 | Green olives liking \|\| id:ebi-fl187-GCST90094775 | MR Egger | 3 | -1.118537444 | 2.39494085571329 | 0.721838445695654 | -5.812621521 | 3.57554663343983 | 0.326757346311208 | 0.00298958253730931 | 35.7141380229089 | 0.999950574162623 |
| ebi-fl187-GCST90094775 | prot-c-4280_47_2 | PSA2 \|\| id:prot-c-4280_47_2 | Green olives liking \|\| id:ebi-fl187-GCST90094775 | MR Egger | 3 | -1.296515554 | 2.37672156843121 | 0.682080824892555 | -5.954889828 | 3.36185872017423 | 0.273483071740671 | 0.00259312950893229 | 28.84275169 | 0.999950574162623 |
| ebi-fl187-GCST90094775 | prot-c-4324_33_2 | CYTT \|\| id:prot-c-4324_33_2 | Green olives liking \|\| id:ebi-fl187-GCST90094775 | MR Egger | 3 | -6.578586019 | 4.13224275442478 | 0.357048713599502 | -14.67778182 | 1.5206097797522 | 0.00138981307552738 | 4.22202062004187e-07 | 4.57501409570977 | 0.999950574162623 |
| ebi-fl187-GCST90094775 | prot-c-4462_8_2 | RASA1 \|\| id:prot-c-4462_8_2 | Green olives liking \|\| id:ebi-fl187-GCST90094775 | MR Egger | 3 | -0.073568069 | 2.38499701280537 | 0.980368930783027 | -4.748162214 | 4.60102607626503 | 0.929072902730392 | 0.00866760978735085 | 99.586446525034 | 0.999950574162623 |
| ebi-fl187-GCST90094775 | prot-c-4499_21_1 | PDGF-AA \|\| id:prot-c-4499_21_1 | Green olives liking \|\| id:ebi-fl187-GCST90094775 | MR Egger | 3 | 0.0334864972226116 | 2.38235658429593 | 0.991052236853658 | -4.635932408 | 4.70290540244263 | 1.03405348103805 | 0.00969706120438264 | 110.267077737289 | 0.999950574162623 |
| ebi-fl187-GCST90094775 | prot-c-4535_50_2 | BST1 \|\| id:prot-c-4535_50_2 | Green olives liking \|\| id:ebi-fl187-GCST90094775 | MR Egger | 3 | -4.538278196 | 2.37024716061605 | 0.30641241772627 | -9.183962631 | 0.107406238471799 | 0.0106917998943544 | 0.000102672871259087 | 1.11338646303612 | 0.999950574162623 |
| ebi-fl187-GCST90094775 | prot-c-4542_24_2 | Clusterin \|\| id:prot-c-4542_24_2 | Green olives liking \|\| id:ebi-fl187-GCST90094775 | MR Egger | 3 | 1.38265592907786 | 2.44829490866823 | 0.672719345849815 | -3.416002092 | 6.18131395006759 | 3.98547271612566 | 0.0328434780218034 | 483.627000783454 | 0.999950574162623 |
| ebi-fl187-GCST90094775 | prot-c-4696_2_2 | FABP \|\| id:prot-c-4696_2_2 | Green olives liking \|\| id:ebi-fl187-GCST90094775 | MR Egger | 3 | -3.891641142 | 4.27949630748353 | 0.530195438442148 | -12.2794539 | 4.49617162074964 | 0.0204118198416732 | 4.6462323942267e-06 | 89.6731704093485 | 0.999950574162623 |
| ebi-fl187-GCST90094775 | prot-c-4906_35_1 | Coagulation Factor V \|\| id:prot-c-4906_35_1 | Green olives liking \|\| id:ebi-fl187-GCST90094775 | MR Egger | 3 | 1.82235423432604 | 2.39422406991696 | 0.585817397414718 | -2.870324943 | 6.51503341136329 | 6.18640556784512 | 0.0566805056699538 | 675.216521050779 | 0.999950574162623 |
| ebi-fl187-GCST90094775 | prot-c-4916_2_1 | IgD \|\| id:prot-c-4916_2_1 | Green olives liking \|\| id:ebi-fl187-GCST90094775 | MR Egger | 3 | 3.29414145730173 | 2.5166616380497 | 0.415323798142709 | -1.638515353 | 8.22679826787914 | 26.9542627476532 | 0.194268248014165 | 3739.84059513705 | 0.999950574162623 |
| ebi-fl187-GCST90094775 | prot-c-4922_13_1 | MIP-3b \|\| id:prot-c-4922_13_1 | Green olives liking \|\| id:ebi-fl187-GCST90094775 | MR Egger | 3 | 2.84253969350826 | 2.39246991792371 | 0.445402106088249 | -1.846701346 | 7.53178073263874 | 17.1592895812413 | 0.157756693780329 | 1866.42615205221 | 0.999950574162623 |
| ebi-fl187-GCST90094775 | prot-c-4973_18_1 | cIAP-2 \|\| id:prot-c-4973_18_1 | Green olives liking \|\| id:ebi-fl187-GCST90094775 | MR Egger | 3 | -1.552536315 | 6.45763332024966 | 0.849795349260335 | -14.20949762 | 11.1044249929564 | 0.211710328271086 | 6.74362779432106e-07 | 66464.6158769256 | 0.999950574162623 |
| ebi-fl187-GCST90094775 | prot-c-4997_19_1 | IF4A3 \|\| id:prot-c-4997_19_1 | Green olives liking \|\| id:ebi-fl187-GCST90094775 | MR Egger | 3 | 3.3736370038555 | 2.39059483862757 | 0.392464327290391 | -1.31192888 | 8.05920288756554 | 29.1844783286168 | 0.269300107534215 | 3162.76804755933 | 0.999950574162623 |
| ebi-fl187-GCST90094775 | prot-c-5000_52_1 | LG3BP \|\| id:prot-c-5000_52_1 | Green olives liking \|\| id:ebi-fl187-GCST90094775 | MR Egger | 3 | 0.616562087287325 | 2.2077084193175 | 0.826624181476737 | -3.710546415 | 4.94367058914963 | 1.8525481824829 | 0.0244641520496257 | 140.284231452575 | 0.999950574162623 |
| ebi-fl187-GCST90094775 | prot-c-5006_71_1 | MK13 \|\| id:prot-c-5006_71_1 | Green olives liking \|\| id:ebi-fl187-GCST90094775 | MR Egger | 3 | -1.782839921 | 2.38464818052044 | 0.591300269371915 | -6.456750355 | 2.89107051245932 | 0.168159907594889 | 0.00156988901189581 | 18.012581977482 | 0.999950574162623 |
| ebi-fl187-GCST90094775 | prot-c-5109_24_3 | Nr-CAM \|\| id:prot-c-5109_24_3 | Green olives liking \|\| id:ebi-fl187-GCST90094775 | MR Egger | 3 | 0.354965588730054 | 2.34242137892961 | 0.90425642607217 | -4.236180314 | 4.94611149143209 | 1.42613157843838 | 0.0144627295577137 | 140.627069800554 | 0.999950574162623 |
| ebi-fl187-GCST90094775 | prot-c-5255_22_3 | PDE4D \|\| id:prot-c-5255_22_3 | Green olives liking \|\| id:ebi-fl187-GCST90094775 | MR Egger | 3 | -1.172615356 | 2.34738589268162 | 0.705066604819801 | -5.773491706 | 3.42826099361193 | 0.30955628175348 | 0.00310888323500958 | 30.8229947313363 | 0.999950574162623 |
| ebi-fl187-GCST90094775 | prot-c-5440_26_3 | Troponin I, skeletal, fast twitch \|\| id:prot-c-5440_26_3 | Green olives liking \|\| id:ebi-fl187-GCST90094775 | MR Egger | 3 | -4.230608751 | 2.27720128218748 | 0.314357314424229 | -8.693923264 | 0.232705762205597 | 0.0145435344636848 | 0.000167601190960945 | 1.26201009362562 | 0.999950574162623 |
| ebi-fl187-GCST90094775 | ukb-rsfMRI191-edge-pheno716 | Net100_Pair10_33 (OccipitalPrecuneus)&(Temporal\|Frontal\|Supp_Motor_Area) (Default_mode\|Central_executive)&(Default_mode\|Salience) \|\| id:ukb-rsfMRI191-edge-pheno716 | Green olives liking \|\| id:ebi-fl187-GCST90094775 | MR Egger | 24 | -0.04329208 | 0.116645781343342 | 0.714082311416006 | -0.271917811 | 0.185333651703993 | 0.957631644423859 | 0.761916879579588 | 1.20361996299119 | 0.999950574162623 |
| ebi-fl187-GCST90094775 | ukb-rsfMRI191-edge-pheno903 | Net100_Pair27_38 (Precentral\|Frontal\|Supp_Motor_Area)&(Subcortical) (Attention\|Salience\|Motor)&(Subcortical-cerebellum) \|\| id:ukb-rsfMRI191-edge-pheno903 | Green olives liking \|\| id:ebi-fl187-GCST90094775 | MR Egger | 24 | -0.005391687 | 0.138081456814776 | 0.969204975478403 | -0.276031343 | 0.265247968211514 | 0.994622821911878 | 0.758789148037838 | 1.30375422530241 | 0.999950574162623 |
| ebi-fl187-GCST90094775 | ukb-rsfMRI191-node-pheno23 | Net100_Node2 RolandicOper\|SupraMarginal\|Insula Salience\|Motor \|\| id:ukb-rsfMRI191-node-pheno23 | Green olives liking \|\| id:ebi-fl187-GCST90094775 | MR Egger | 24 | -0.015568457 | 0.127035761584374 | 0.903574686086166 | -0.264558549 | 0.233421635959652 | 0.984552105211598 | 0.767544708230602 | 1.26291385698064 | 0.999950574162623 |
| ebi-fl187-GCST90094775 | ukb-rsfMRI191-node-pheno55 | Net100_Node34 Parietal\|Postcentral\|Precuneus Attention \|\| id:ukb-rsfMRI191-node-pheno55 | Green olives liking \|\| id:ebi-fl187-GCST90094775 | MR Egger | 24 | -0.048487605 | 0.143012431153071 | 0.737790999663057 | -0.32879197 | 0.231816760427405 | 0.952669147916734 | 0.719792739877978 | 1.26088866295908 | 0.999950574162623 |
| ebi-fl187-GCST90094775 | ebi-a-GCST90001414 | IgD- CD24- B cell Absolute Count \|\| id:ebi-a-GCST90001414 | Green olives liking \|\| id:ebi-fl187-GCST90094775 | Weighted median | 25 | -0.057401775 | 0.111815839461867 | 0.6076995449935 | -0.276560821 | 0.161757269912375 | 0.944214630905033 | 0.758387492011541 | 1.17557485929839 | 0.999332001504091 |
| ebi-fl187-GCST90094775 | ebi-a-GCST90001422 | CD20- CD38- B cell %B cell \|\| id:ebi-a-GCST90001422 | Green olives liking \|\| id:ebi-fl187-GCST90094775 | Weighted median | 25 | -0.084719654 | 0.0998221039157291 | 0.396044710041746 | -0.280370977 | 0.110931669874472 | 0.918769821927861 | 0.755503414712202 | 1.11731855772871 | 0.999332001504091 |
| ebi-fl187-GCST90094775 | ebi-a-GCST90001444 | CD20- CD38- B cell %lymphocyte \|\| id:ebi-a-GCST90001444 | Green olives liking \|\| id:ebi-fl187-GCST90094775 | Weighted median | 25 | -0.078327212 | 0.104937528711294 | 0.455415224335199 | -0.284004769 | 0.127350343787099 | 0.92466181614476 | 0.752763054950409 | 1.13581487376855 | 0.999332001504091 |
| ebi-fl187-GCST90094775 | ebi-a-GCST90001459 | Myeloid Dendritic Cell %Dendritic Cell \|\| id:ebi-a-GCST90001459 | Green olives liking \|\| id:ebi-fl187-GCST90094775 | Weighted median | 25 | 0.0680804519039051 | 0.112222499316926 | 0.544079231834767 | -0.151875647 | 0.288036550565081 | 1.07045142486978 | 0.859095105361156 | 1.33380605459745 | 0.999332001504091 |
| ebi-fl187-GCST90094775 | ebi-a-GCST90001476 | HLA DR++ monocyte %leukocyte \|\| id:ebi-a-GCST90001476 | Green olives liking \|\| id:ebi-fl187-GCST90094775 | Weighted median | 25 | -0.009727195 | 0.109342211830916 | 0.9291128839103 | -0.224037931 | 0.204583539824936 | 0.990319960778301 | 0.799284816479884 | 1.22701395609536 | 0.999332001504091 |
| ebi-fl187-GCST90094775 | ebi-a-GCST90001478 | CD4 regulatory T cell %CD4+ T cell \|\| id:ebi-a-GCST90001478 | Green olives liking \|\| id:ebi-fl187-GCST90094775 | Weighted median | 25 | 0.0764248110857492 | 0.117340276736087 | 0.514846859503753 | -0.153562131 | 0.30641175348848 | 1.07942102679088 | 0.857647475776229 | 1.3585415756325 | 0.999332001504091 |
| ebi-fl187-GCST90094775 | ebi-a-GCST90001485 | CD39+ resting CD4 regulatory T cell %CD4 regulatory T cell \|\| id:ebi-a-GCST90001485 | Green olives liking \|\| id:ebi-fl187-GCST90094775 | Weighted median | 24 | -0.021439141 | 0.115951846448478 | 0.853309876214769 | -0.24870476 | 0.205826478193899 | 0.978789043930568 | 0.779810170646429 | 1.2285400070178 | 0.999332001504091 |
| ebi-fl187-GCST90094775 | ebi-a-GCST90001515 | Immature Myeloid-Derived Suppressor Cells Absolute Count \|\| id:ebi-a-GCST90001515 | Green olives liking \|\| id:ebi-fl187-GCST90094775 | Weighted median | 25 | 0.017462724150844 | 0.159083131267669 | 0.912590942073764 | -0.294340213 | 0.329265661435476 | 1.01761608893994 | 0.745022981718711 | 1.3899470618752 | 0.999332001504091 |
| ebi-fl187-GCST90094775 | ebi-a-GCST90001517 | CD33+ HLA DR+ Absolute Count \|\| id:ebi-a-GCST90001517 | Green olives liking \|\| id:ebi-fl187-GCST90094775 | Weighted median | 25 | -0.119390394 | 0.136684658514065 | 0.382405175212196 | -0.387292325 | 0.148511536476459 | 0.887461273381499 | 0.678892608703768 | 1.16010618123488 | 0.999332001504091 |
| ebi-fl187-GCST90094775 | ebi-a-GCST90001518 | CD33+ HLA DR+ CD14- Absolute Count \|\| id:ebi-a-GCST90001518 | Green olives liking \|\| id:ebi-fl187-GCST90094775 | Weighted median | 25 | -0.14957831 | 0.144761401899627 | 0.301475313740065 | -0.433310657 | 0.134154038182286 | 0.861071005304245 | 0.648359043069268 | 1.143568959 | 0.999332001504091 |
| ebi-fl187-GCST90094775 | ebi-a-GCST90001520 | CD33+ HLA DR+ CD14dim Absolute Count \|\| id:ebi-a-GCST90001520 | Green olives liking \|\| id:ebi-fl187-GCST90094775 | Weighted median | 25 | -0.093422363 | 0.138567093895838 | 0.500182204474086 | -0.365013867 | 0.178169140956927 | 0.910808727066591 | 0.694187024440102 | 1.19502743222514 | 0.999332001504091 |
| ebi-fl187-GCST90094775 | ebi-a-GCST90001531 | CD33dim HLA DR- Absolute Count \|\| id:ebi-a-GCST90001531 | Green olives liking \|\| id:ebi-fl187-GCST90094775 | Weighted median | 25 | -0.054717387 | 0.149147126758767 | 0.71371708815129 | -0.347045755 | 0.237610981614181 | 0.946752674920204 | 0.706772988921771 | 1.26821573761044 | 0.999332001504091 |
| ebi-fl187-GCST90094775 | ebi-a-GCST90001532 | Basophil Absolute Count \|\| id:ebi-a-GCST90001532 | Green olives liking \|\| id:ebi-fl187-GCST90094775 | Weighted median | 25 | -0.087129186 | 0.150797501555395 | 0.563406367904531 | -0.382692289 | 0.208433917447241 | 0.91655868180017 | 0.682022733136214 | 1.23174753035026 | 0.999332001504091 |
| ebi-fl187-GCST90094775 | ebi-a-GCST90001588 | T/B cell \|\| id:ebi-a-GCST90001588 | Green olives liking \|\| id:ebi-fl187-GCST90094775 | Weighted median | 25 | -0.010262015 | 0.111319426455459 | 0.926550818781279 | -0.22844809 | 0.207924061349202 | 0.989790460314826 | 0.795767604145243 | 1.23111967643184 | 0.999332001504091 |
| ebi-fl187-GCST90094775 | ebi-a-GCST90001624 | HLA DR+ CD4+ T cell Absolute Count \|\| id:ebi-a-GCST90001624 | Green olives liking \|\| id:ebi-fl187-GCST90094775 | Weighted median | 25 | -0.065891937 | 0.099019158743792 | 0.505765053271632 | -0.259969488 | 0.128185614253265 | 0.936232030975362 | 0.771075112471202 | 1.1367639827138 | 0.999332001504091 |
| ebi-fl187-GCST90094775 | ebi-a-GCST90001631 | CD8+ Natural Killer T %T cell \|\| id:ebi-a-GCST90001631 | Green olives liking \|\| id:ebi-fl187-GCST90094775 | Weighted median | 25 | 0.0665315861609423 | 0.10873864371382 | 0.540637936159118 | -0.146596156 | 0.27965932784003 | 1.06879472266414 | 0.86364268433226 | 1.32267913561721 | 0.999332001504091 |
| ebi-fl187-GCST90094775 | ebi-a-GCST90001632 | CD8+ Natural Killer T %lymphocyte \|\| id:ebi-a-GCST90001632 | Green olives liking \|\| id:ebi-fl187-GCST90094775 | Weighted median | 25 | 0.0703263520100601 | 0.103316268330744 | 0.496067657123433 | -0.132173534 | 0.272826237938318 | 1.07285825357527 | 0.876188933387132 | 1.31367195864367 | 0.999332001504091 |
| ebi-fl187-GCST90094775 | ebi-a-GCST90001646 | Natural Killer %CD3- lymphocyte \|\| id:ebi-a-GCST90001646 | Green olives liking \|\| id:ebi-fl187-GCST90094775 | Weighted median | 25 | -0.100348435 | 0.107604775712912 | 0.351044671805632 | -0.311253795 | 0.110556925300274 | 0.904522195842834 | 0.732527939984839 | 1.11689992710623 | 0.999332001504091 |
| ebi-fl187-GCST90094775 | ebi-a-GCST90001684 | CD127- CD8+ T cell Absolute Count \|\| id:ebi-a-GCST90001684 | Green olives liking \|\| id:ebi-fl187-GCST90094775 | Weighted median | 25 | -0.021147897 | 0.11304469457476 | 0.851601409534294 | -0.242715498 | 0.200419704618361 | 0.979074151977774 | 0.784494672238809 | 1.22191549412995 | 0.999332001504091 |
| ebi-fl187-GCST90094775 | ebi-a-GCST90001687 | CD28- CD8+ T cell Absolute Count \|\| id:ebi-a-GCST90001687 | Green olives liking \|\| id:ebi-fl187-GCST90094775 | Weighted median | 25 | -0.06824558 | 0.114465355973656 | 0.551033834158944 | -0.292597678 | 0.15610651729344 | 0.934031065672661 | 0.746322342109681 | 1.16895070992447 | 0.999332001504091 |
| ebi-fl187-GCST90094775 | ebi-a-GCST90001701 | BAFF-R on CD20- CD38- B cell \|\| id:ebi-a-GCST90001701 | Green olives liking \|\| id:ebi-fl187-GCST90094775 | Weighted median | 25 | -0.011589309 | 0.102578446174025 | 0.910046444763677 | -0.212643063 | 0.189464445973512 | 0.98847758882774 | 0.808444649507761 | 1.20860215255259 | 0.999332001504091 |
| ebi-fl187-GCST90094775 | ebi-a-GCST90001732 | CD19 on IgD- CD27- B cell \|\| id:ebi-a-GCST90001732 | Green olives liking \|\| id:ebi-fl187-GCST90094775 | Weighted median | 25 | -0.157845398 | 0.0995617301591289 | 0.112874953646548 | -0.352986389 | 0.0372955927997793 | 0.853981798790725 | 0.7025867559226 | 1.03799980076223 | 0.999332001504091 |
| ebi-fl187-GCST90094775 | ebi-a-GCST90001798 | CD27 on CD24+ CD27+ B cell \|\| id:ebi-a-GCST90001798 | Green olives liking \|\| id:ebi-fl187-GCST90094775 | Weighted median | 25 | -0.038133121 | 0.110999433572316 | 0.73118970945606 | -0.255692011 | 0.179425768874505 | 0.962584792181674 | 0.774380432933201 | 1.1965300809962 | 0.999332001504091 |
| ebi-fl187-GCST90094775 | ebi-a-GCST90001836 | CD66b on CD66b++ myeloid cell \|\| id:ebi-a-GCST90001836 | Green olives liking \|\| id:ebi-fl187-GCST90094775 | Weighted median | 25 | -0.118231327 | 0.158310564431445 | 0.455165238836469 | -0.428520033 | 0.192057379535289 | 0.888490497220871 | 0.651472539450637 | 1.21174004405078 | 0.999332001504091 |
| ebi-fl187-GCST90094775 | ebi-a-GCST90001943 | CD25 on activated & secreting CD4 regulatory T cell \|\| id:ebi-a-GCST90001943 | Green olives liking \|\| id:ebi-fl187-GCST90094775 | Weighted median | 25 | 0.0464089899560794 | 0.113830822418096 | 0.683492881819603 | -0.176699422 | 0.269517401895547 | 1.04750274145975 | 0.838031640558281 | 1.30933241689386 | 0.999332001504091 |
| ebi-fl187-GCST90094775 | ebi-a-GCST90001960 | CD25 on CD4+ T cell \|\| id:ebi-a-GCST90001960 | Green olives liking \|\| id:ebi-fl187-GCST90094775 | Weighted median | 25 | 0.0780662144869994 | 0.119542765325903 | 0.513730453666134 | -0.156237606 | 0.312370034525769 | 1.08119424702238 | 0.855355928906501 | 1.36666031097572 | 0.999332001504091 |
| ebi-fl187-GCST90094775 | ebi-a-GCST90001988 | HLA DR on CD14+ CD16- monocyte \|\| id:ebi-a-GCST90001988 | Green olives liking \|\| id:ebi-fl187-GCST90094775 | Weighted median | 24 | -0.006240638 | 0.109164306720252 | 0.95441185491825 | -0.22020268 | 0.207721402709146 | 0.993778793877247 | 0.802356160228271 | 1.23087020467213 | 0.999332001504091 |
| ebi-fl187-GCST90094775 | ebi-a-GCST90001991 | HLA DR on CD14+ monocyte \|\| id:ebi-a-GCST90001991 | Green olives liking \|\| id:ebi-fl187-GCST90094775 | Weighted median | 24 | -0.008450944 | 0.104419754573975 | 0.935495692798012 | -0.213113663 | 0.196211774967495 | 0.991584664849669 | 0.808064285015977 | 1.21678456256186 | 0.999332001504091 |
| ebi-fl187-GCST90094775 | ebi-a-GCST90002019 | CD14 on Monocytic Myeloid-Derived Suppressor Cells \|\| id:ebi-a-GCST90002019 | Green olives liking \|\| id:ebi-fl187-GCST90094775 | Weighted median | 25 | -0.022756218 | 0.145365760601708 | 0.875603804566743 | -0.307673109 | 0.262160672889846 | 0.977500751925658 | 0.735155594725703 | 1.29973535788943 | 0.999332001504091 |
| ebi-fl187-GCST90094775 | ebi-a-GCST90002037 | CD80 on plasmacytoid Dendritic Cell \|\| id:ebi-a-GCST90002037 | Green olives liking \|\| id:ebi-fl187-GCST90094775 | Weighted median | 25 | -0.022080739 | 0.113598431503407 | 0.84588195838957 | -0.244733665 | 0.200572186324981 | 0.978161255688769 | 0.782913027486016 | 1.22210182809574 | 0.999332001504091 |
| ebi-fl187-GCST90094775 | ebi-a-GCST90002042 | CD45 on CD33+ HLA DR+ CD14- \|\| id:ebi-a-GCST90002042 | Green olives liking \|\| id:ebi-fl187-GCST90094775 | Weighted median | 25 | 0.00386734461392511 | 0.175527422236589 | 0.982421863864803 | -0.340166403 | 0.34790109219764 | 1.00387483244066 | 0.711651891920989 | 1.41609218024767 | 0.999332001504091 |
| ebi-fl187-GCST90094775 | ebi-a-GCST90002056 | CD8 on Effector Memory CD8+ T cell \|\| id:ebi-a-GCST90002056 | Green olives liking \|\| id:ebi-fl187-GCST90094775 | Weighted median | 25 | 0.0760421493473275 | 0.119575099102492 | 0.524817923897857 | -0.158325045 | 0.310409343588211 | 1.07900805268401 | 0.853572287558502 | 1.36398333770548 | 0.999332001504091 |
| ebi-fl187-GCST90094775 | ebi-a-GCST90002058 | CD8 on CD8+ T cell \|\| id:ebi-a-GCST90002058 | Green olives liking \|\| id:ebi-fl187-GCST90094775 | Weighted median | 25 | -0.054086862 | 0.110355103911016 | 0.624051432032139 | -0.270382866 | 0.162209141564554 | 0.947349814132532 | 0.763087278404832 | 1.17610618828954 | 0.999332001504091 |
| ebi-fl187-GCST90094775 | ebi-a-GCST90002063 | CD4 on CD4 regulatory T cell \|\| id:ebi-a-GCST90002063 | Green olives liking \|\| id:ebi-fl187-GCST90094775 | Weighted median | 25 | -0.03132727 | 0.127829528954468 | 0.806401699445865 | -0.281873147 | 0.219218606290954 | 0.969158344272097 | 0.754369372322975 | 1.2451034343824 | 0.999332001504091 |
| ebi-fl187-GCST90094775 | ebi-a-GCST90002066 | CD4 on activated CD4 regulatory T cell \|\| id:ebi-a-GCST90002066 | Green olives liking \|\| id:ebi-fl187-GCST90094775 | Weighted median | 25 | 0.0482080173462161 | 0.126846469319916 | 0.703908202499636 | -0.200411063 | 0.296827097213251 | 1.04938892372094 | 0.818394272712721 | 1.34558262434804 | 0.999332001504091 |
| ebi-fl187-GCST90094775 | ebi-a-GCST90002070 | CD4 on activated & secreting CD4 regulatory T cell \|\| id:ebi-a-GCST90002070 | Green olives liking \|\| id:ebi-fl187-GCST90094775 | Weighted median | 25 | -0.009861818 | 0.126099128336416 | 0.937663498077332 | -0.25701611 | 0.237292473527535 | 0.990186650256144 | 0.773355755250952 | 1.26781186496417 | 0.999332001504091 |
| ebi-fl187-GCST90094775 | ebi-a-GCST90002081 | SSC-A on CD4+ T cell \|\| id:ebi-a-GCST90002081 | Green olives liking \|\| id:ebi-fl187-GCST90094775 | Weighted median | 25 | 0.107344483004932 | 0.102167881476338 | 0.293411280897704 | -0.092904565 | 0.307593530698554 | 1.11331770745833 | 0.911280464481166 | 1.36014801814714 | 0.999332001504091 |
| ebi-fl187-GCST90094775 | ebi-a-GCST90002094 | CD11b on Monocytic Myeloid-Derived Suppressor Cells \|\| id:ebi-a-GCST90002094 | Green olives liking \|\| id:ebi-fl187-GCST90094775 | Weighted median | 25 | -0.279845984 | 0.14855910577139 | 0.0596008324426166 | -0.571021831 | 0.011329863270259 | 0.755900153177361 | 0.564947862218156 | 1.01139428925375 | 0.999332001504091 |
| ebi-fl187-GCST90094775 | ebi-a-GCST90002104 | HLA DR on myeloid Dendritic Cell \|\| id:ebi-a-GCST90002104 | Green olives liking \|\| id:ebi-fl187-GCST90094775 | Weighted median | 24 | -0.035923806 | 0.119526533654351 | 0.763756986751919 | -0.270195812 | 0.198348200184785 | 0.964713796300252 | 0.763230030303463 | 1.21938691065655 | 0.999332001504091 |
| ebi-fl187-GCST90094775 | ebi-a-GCST90002105 | HLA DR on plasmacytoid Dendritic Cell \|\| id:ebi-a-GCST90002105 | Green olives liking \|\| id:ebi-fl187-GCST90094775 | Weighted median | 24 | 0.101757135179687 | 0.11765603635805 | 0.387110336389004 | -0.128848696 | 0.332362966441465 | 1.10711455989599 | 0.879106967811938 | 1.39425882584506 | 0.999332001504091 |
| ebi-fl187-GCST90094775 | ebi-a-GCST90002106 | HLA DR on Dendritic Cell \|\| id:ebi-a-GCST90002106 | Green olives liking \|\| id:ebi-fl187-GCST90094775 | Weighted median | 24 | 0.146850647183005 | 0.125695977201013 | 0.242685656616314 | -0.099513468 | 0.393214762496989 | 1.15818097252103 | 0.905277757386994 | 1.48173657660777 | 0.999332001504091 |
| ebi-fl187-GCST90094775 | ebi-a-GCST90002111 | HLA DR on CD33dim HLA DR+ CD11b- \|\| id:ebi-a-GCST90002111 | Green olives liking \|\| id:ebi-fl187-GCST90094775 | Weighted median | 24 | 0.086937305995145 | 0.156194495801599 | 0.57780316177371 | -0.219203906 | 0.39307851776628 | 1.0908282891804 | 0.803157932914263 | 1.48153471155874 | 0.999332001504091 |
| ebi-fl187-GCST90094775 | ebi-a-GCST90002112 | HLA DR on CD33- HLA DR+ \|\| id:ebi-a-GCST90002112 | Green olives liking \|\| id:ebi-fl187-GCST90094775 | Weighted median | 24 | -0.037575705 | 0.154024904013809 | 0.807262858018191 | -0.339464517 | 0.264313106814644 | 0.963121501797023 | 0.712151565793146 | 1.302535965 | 0.999332001504091 |
| ebi-fl187-GCST90094775 | ebi-a-GCST90002115 | HLA DR on HLA DR+ CD8+ T cell \|\| id:ebi-a-GCST90002115 | Green olives liking \|\| id:ebi-fl187-GCST90094775 | Weighted median | 25 | -0.024936974 | 0.109603180908918 | 0.82001900849646 | -0.239759208 | 0.189885260959393 | 0.975371384216596 | 0.786817297408789 | 1.20911085747817 | 0.999332001504091 |
| ebi-fl187-GCST90094775 | ebi-a-GCST90002116 | HLA DR on B cell \|\| id:ebi-a-GCST90002116 | Green olives liking \|\| id:ebi-fl187-GCST90094775 | Weighted median | 25 | 0.19708578692105 | 0.122628962807466 | 0.108016729172259 | -0.04326698 | 0.437438554023683 | 1.21784851169885 | 0.957655680846336 | 1.54873513216811 | 0.999332001504091 |
| ebi-fl187-GCST90094775 | ebi-cfmet338-GCST90026023 | Sphingomyelin (d18:1/24:1, d18:2/24:0) levels \|\| id:ebi-cfmet338-GCST90026023 | Green olives liking \|\| id:ebi-fl187-GCST90094775 | Weighted median | 23 | 0.00517631283949265 | 0.0680337146742667 | 0.939351852010256 | -0.128169768 | 0.138522393601055 | 1.00518973309262 | 0.879704020943215 | 1.14857540202153 | 0.999332001504091 |
| ebi-fl187-GCST90094775 | ebi-cfmet338-GCST90026046 | 1-arachidonoyl-gpc (20:4n6) levels \|\| id:ebi-cfmet338-GCST90026046 | Green olives liking \|\| id:ebi-fl187-GCST90094775 | Weighted median | 23 | 0.0331475746922061 | 0.0823977865885907 | 0.687472675127917 | -0.128352087 | 0.194647236405844 | 1.03370307639905 | 0.879543648718316 | 1.21488234462719 | 0.999332001504091 |
| ebi-fl187-GCST90094775 | ebi-cfmet338-GCST90026047 | 1-linoleoyl-gpc (18:2) levels \|\| id:ebi-cfmet338-GCST90026047 | Green olives liking \|\| id:ebi-fl187-GCST90094775 | Weighted median | 23 | -0.081898069 | 0.097901679480773 | 0.402854640488803 | -0.273785361 | 0.109989222665662 | 0.921365869547028 | 0.760495289769482 | 1.11626604002172 | 0.999332001504091 |
| ebi-fl187-GCST90094775 | ebi-cfmet338-GCST90026050 | 1-myristoyl-2-palmitoyl-gpc (14:0/16:0) levels \|\| id:ebi-cfmet338-GCST90026050 | Green olives liking \|\| id:ebi-fl187-GCST90094775 | Weighted median | 23 | -0.013092845 | 0.0526759708822356 | 0.8037054793363 | -0.116337748 | 0.0901520581970105 | 0.986992493712413 | 0.890174518157399 | 1.09434067452423 | 0.999332001504091 |
| ebi-fl187-GCST90094775 | ebi-cfmet338-GCST90026068 | 2,3-dihydroxyisovalerate levels \|\| id:ebi-cfmet338-GCST90026068 | Green olives liking \|\| id:ebi-fl187-GCST90094775 | Weighted median | 23 | 0.134052566407076 | 0.163283963614348 | 0.411658714080354 | -0.185984002 | 0.454089135091198 | 1.14345292527681 | 0.830286877575126 | 1.57473835566645 | 0.999332001504091 |
| ebi-fl187-GCST90094775 | ebi-cfmet338-GCST90026082 | 3-hydroxyhexanoate levels \|\| id:ebi-cfmet338-GCST90026082 | Green olives liking \|\| id:ebi-fl187-GCST90094775 | Weighted median | 23 | -0.037082583 | 0.0369837485365106 | 0.316018963235774 | -0.10957073 | 0.0354055641768385 | 0.963596555412419 | 0.896218772489172 | 1.03603980423641 | 0.999332001504091 |
| ebi-fl187-GCST90094775 | ebi-cfmet338-GCST90026094 | 3-ureidopropionate levels \|\| id:ebi-cfmet338-GCST90026094 | Green olives liking \|\| id:ebi-fl187-GCST90094775 | Weighted median | 23 | -0.034429348 | 0.0740877034833393 | 0.642138650896505 | -0.179641247 | 0.110782550910385 | 0.966156598251368 | 0.835569921076696 | 1.11715195676482 | 0.999332001504091 |
| ebi-fl187-GCST90094775 | ebi-cfmet338-GCST90026173 | Glycerol levels \|\| id:ebi-cfmet338-GCST90026173 | Green olives liking \|\| id:ebi-fl187-GCST90094775 | Weighted median | 23 | 0.0660558019685179 | 0.0488266687023298 | 0.176099346238115 | -0.029644469 | 0.161756072625084 | 1.06828632798282 | 0.970790618662875 | 1.1755734517984 | 0.999332001504091 |
| ebi-fl187-GCST90094775 | ebi-cfmet338-GCST90026179 | Histidine levels \|\| id:ebi-cfmet338-GCST90026179 | Green olives liking \|\| id:ebi-fl187-GCST90094775 | Weighted median | 23 | -0.002075146 | 0.0316495458608163 | 0.947723064247623 | -0.064108256 | 0.0599579640497329 | 0.997927005789086 | 0.937903460615948 | 1.06179191217523 | 0.999332001504091 |
| ebi-fl187-GCST90094775 | ebi-cfmet338-GCST90026188 | Isocitrate levels \|\| id:ebi-cfmet338-GCST90026188 | Green olives liking \|\| id:ebi-fl187-GCST90094775 | Weighted median | 23 | -0.029170597 | 0.0623780853310111 | 0.640040797545239 | -0.151431645 | 0.0930904497592754 | 0.971250757391509 | 0.859476630015026 | 1.09756100490724 | 0.999332001504091 |
| ebi-fl187-GCST90094775 | ebi-cfmet338-GCST90026207 | N-acetyl-isoputreanine levels \|\| id:ebi-cfmet338-GCST90026207 | Green olives liking \|\| id:ebi-fl187-GCST90094775 | Weighted median | 23 | 0.00925388485878877 | 0.0400350366185004 | 0.817202422053515 | -0.069214787 | 0.0877225566310495 | 1.00929683443252 | 0.933126235248561 | 1.09168519918868 | 0.999332001504091 |
| ebi-fl187-GCST90094775 | ebi-cfmet338-GCST90026213 | N-acetylglutamate levels \|\| id:ebi-cfmet338-GCST90026213 | Green olives liking \|\| id:ebi-fl187-GCST90094775 | Weighted median | 23 | 0.105060002837605 | 0.0697441287661456 | 0.131973911252764 | -0.03163849 | 0.241758495219251 | 1.11077725814359 | 0.968856770629883 | 1.27348660257269 | 0.999332001504091 |
| ebi-fl187-GCST90094775 | ebi-cfmet338-GCST90026224 | N-acetyltaurine levels \|\| id:ebi-cfmet338-GCST90026224 | Green olives liking \|\| id:ebi-fl187-GCST90094775 | Weighted median | 23 | 0.0492867958753326 | 0.0519373579570842 | 0.342637816214682 | -0.052510426 | 0.151084017471218 | 1.05052159280024 | 0.948844428617373 | 1.16309437422494 | 0.999332001504091 |
| ebi-fl187-GCST90094775 | ebi-cfmet338-GCST90026250 | Phenyllactate (pla) levels \|\| id:ebi-cfmet338-GCST90026250 | Green olives liking \|\| id:ebi-fl187-GCST90094775 | Weighted median | 23 | 0.0421704683748705 | 0.083927746237174 | 0.615342868169962 | -0.122327914 | 0.206668850999731 | 1.04307227443156 | 0.884858163323047 | 1.22957533171405 | 0.999332001504091 |
| ebi-fl187-GCST90094775 | ebi-cfmet338-GCST90026251 | Pipecolate levels \|\| id:ebi-cfmet338-GCST90026251 | Green olives liking \|\| id:ebi-fl187-GCST90094775 | Weighted median | 23 | 0.0139879465107829 | 0.119254287592742 | 0.906626188445437 | -0.219750457 | 0.247726350192558 | 1.01408623558732 | 0.802719085762807 | 1.28110930890652 | 0.999332001504091 |
| ebi-fl187-GCST90094775 | ebi-cfmet338-GCST90026278 | Trigonelline (n'-methylnicotinate) levels \|\| id:ebi-cfmet338-GCST90026278 | Green olives liking \|\| id:ebi-fl187-GCST90094775 | Weighted median | 22 | -0.066638889 | 0.137957166956696 | 0.629067030905632 | -0.337034936 | 0.203757158233008 | 0.935532971591754 | 0.713883899049794 | 1.22600039320154 | 0.999332001504091 |
| ebi-fl187-GCST90094775 | ebi-cfmet338-GCST90026299 | 1-carboxyethylvaline levels \|\| id:ebi-cfmet338-GCST90026299 | Green olives liking \|\| id:ebi-fl187-GCST90094775 | Weighted median | 23 | -0.02011825 | 0.0883133672819757 | 0.819797631367003 | -0.19321245 | 0.152975949601559 | 0.980082771400742 | 0.824306831430359 | 1.1652969527497 | 0.999332001504091 |
| ebi-fl187-GCST90094775 | ebi-gm430-GCST90011306 | C_Erysipelotrichia abundance \|\| id:ebi-gm430-GCST90011306 | Green olives liking \|\| id:ebi-fl187-GCST90094775 | Weighted median | 20 | 0.117927579670297 | 0.076995953026474 | 0.125619318890387 | -0.032984488 | 0.268839647602186 | 1.12516262376852 | 0.96755356791047 | 1.30844531188044 | 0.999332001504091 |
| ebi-fl187-GCST90094775 | ebi-gm430-GCST90011312 | F_Erysipelotrichaceae abundance \|\| id:ebi-gm430-GCST90011312 | Green olives liking \|\| id:ebi-fl187-GCST90094775 | Weighted median | 20 | 0.117927579670297 | 0.0786563794389002 | 0.133802185226556 | -0.036238924 | 0.272094083370541 | 1.12516262376852 | 0.964409845267659 | 1.3127104997298 | 0.999332001504091 |
| ebi-fl187-GCST90094775 | ebi-gm430-GCST90011352 | O_Erysipelotrichales abundance \|\| id:ebi-gm430-GCST90011352 | Green olives liking \|\| id:ebi-fl187-GCST90094775 | Weighted median | 20 | 0.117927579670297 | 0.0825037011036353 | 0.152900477470186 | -0.043779674 | 0.279634833833422 | 1.12516262376852 | 0.957164822067638 | 1.3226467383025 | 0.999332001504091 |
| ebi-fl187-GCST90094775 | ebi-gm430-GCST90011362 | OTU97_11 (Parabacteroides) abundance \|\| id:ebi-gm430-GCST90011362 | Green olives liking \|\| id:ebi-fl187-GCST90094775 | Weighted median | 20 | 0.0750901509839507 | 0.0917726093402669 | 0.413231784947504 | -0.104784163 | 0.254964465290874 | 1.07798132758164 | 0.900518866611997 | 1.29041576550929 | 0.999332001504091 |
| ebi-fl187-GCST90094775 | ebi-gm430-GCST90011384 | OTU97_235 (Phascolarctobacterium) abundance \|\| id:ebi-gm430-GCST90011384 | Green olives liking \|\| id:ebi-fl187-GCST90094775 | Weighted median | 20 | -0.045558009 | 0.305577351652872 | 0.881483954399085 | -0.644489618 | 0.553373599985123 | 0.955464175199808 | 0.524930388511015 | 1.73911019455315 | 0.999332001504091 |
| ebi-fl187-GCST90094775 | ebi-gm430-GCST90011390 | OTU97_3 (Bacteroides) abundance \|\| id:ebi-gm430-GCST90011390 | Green olives liking \|\| id:ebi-fl187-GCST90094775 | Weighted median | 20 | -0.017505262 | 0.0732587145929581 | 0.811143379061898 | -0.161092343 | 0.126081818650443 | 0.982647065010021 | 0.85121346425258 | 1.13437497751597 | 0.999332001504091 |
| ebi-fl187-GCST90094775 | ebi-gm430-GCST90011398 | OTU97_4 (Alistipes) abundance \|\| id:ebi-gm430-GCST90011398 | Green olives liking \|\| id:ebi-fl187-GCST90094775 | Weighted median | 20 | 0.0358563315419805 | 0.0942283010143617 | 0.703554897364757 | -0.148831138 | 0.220543801530129 | 1.03650692244206 | 0.861714613082612 | 1.24675453329851 | 0.999332001504091 |
| ebi-fl187-GCST90094775 | ebi-gm430-GCST90011421 | OTU99_108 (Cytophagales) abundance \|\| id:ebi-gm430-GCST90011421 | Green olives liking \|\| id:ebi-fl187-GCST90094775 | Weighted median | 20 | 0.140835302550669 | 0.298657253539639 | 0.637239716782255 | -0.444532914 | 0.726203519488363 | 1.15123502689761 | 0.64112366574697 | 2.06721754002293 | 0.999332001504091 |
| ebi-fl187-GCST90094775 | ebi-gm430-GCST90011428 | OTU99_123 (Phascolarctobacterium) abundance \|\| id:ebi-gm430-GCST90011428 | Green olives liking \|\| id:ebi-fl187-GCST90094775 | Weighted median | 20 | 0.151021051750798 | 0.163897666822854 | 0.356823346277919 | -0.170218375 | 0.472260478723591 | 1.16302114145535 | 0.84348060121954 | 1.60361503692728 | 0.999332001504091 |
| ebi-fl187-GCST90094775 | ebi-gm430-GCST90011450 | OTU99_298 (Phascolarctobacterium) abundance \|\| id:ebi-gm430-GCST90011450 | Green olives liking \|\| id:ebi-fl187-GCST90094775 | Weighted median | 20 | -0.037033555 | 0.31454383081799 | 0.906275777496962 | -0.653539463 | 0.579472353658457 | 0.963643799984763 | 0.520201281160292 | 1.78509628268858 | 0.999332001504091 |
| ebi-fl187-GCST90094775 | ebi-gm430-GCST90011451 | OTU99_3 (Bacteroides) abundance \|\| id:ebi-gm430-GCST90011451 | Green olives liking \|\| id:ebi-fl187-GCST90094775 | Weighted median | 20 | -0.018628123 | 0.0728463756293097 | 0.798168662881353 | -0.161407019 | 0.124150773135441 | 0.981544308037587 | 0.85094564928247 | 1.13218656144886 | 0.999332001504091 |
| ebi-fl187-GCST90094775 | ebi-gm430-GCST90011472 | OTU99_72 (Parabacteroides) abundance \|\| id:ebi-gm430-GCST90011472 | Green olives liking \|\| id:ebi-fl187-GCST90094775 | Weighted median | 20 | 0.275959310565272 | 0.236773687820599 | 0.243817382250002 | -0.188117118 | 0.740035738693646 | 1.31779424263358 | 0.828517667580055 | 2.09601042183016 | 0.999332001504091 |
| ebi-fl187-GCST90094775 | ebi-gm430-GCST90011507 | TestASV_3 (Bacteroides) abundance \|\| id:ebi-gm430-GCST90011507 | Green olives liking \|\| id:ebi-fl187-GCST90094775 | Weighted median | 20 | 0.0579753973095969 | 0.104167689139457 | 0.577829216424596 | -0.146193273 | 0.262144068022933 | 1.05968892419105 | 0.863990700623077 | 1.29971377613597 | 0.999332001504091 |
| ebi-fl187-GCST90094775 | ebi-gm430-GCST90011512 | TestASV_34 (Phascolarctobacterium) abundance \|\| id:ebi-gm430-GCST90011512 | Green olives liking \|\| id:ebi-fl187-GCST90094775 | Weighted median | 20 | -0.057667352 | 0.264226640206847 | 0.827234659981359 | -0.575551567 | 0.460216862640254 | 0.943963902764039 | 0.562394584966159 | 1.58441754871295 | 0.999332001504091 |
| ebi-fl187-GCST90094775 | ebi-gm430-GCST90011519 | TestASV_41 (Barnesiella) abundance \|\| id:ebi-gm430-GCST90011519 | Green olives liking \|\| id:ebi-fl187-GCST90094775 | Weighted median | 20 | -0.219621855 | 0.358907920953011 | 0.540592678926054 | -0.92308138 | 0.483837669773611 | 0.802822323581847 | 0.397292942345786 | 1.62228827785315 | 0.999332001504091 |
| ebi-fl187-GCST90094775 | ebi-gm430-GCST90011524 | TestASV_46 (Sutterella) abundance \|\| id:ebi-gm430-GCST90011524 | Green olives liking \|\| id:ebi-fl187-GCST90094775 | Weighted median | 20 | -0.161460924 | 0.340388949792463 | 0.63525573371877 | -0.828623266 | 0.505701417400034 | 0.850899780411187 | 0.436650023725805 | 1.65814816663897 | 0.999332001504091 |
| ebi-fl187-GCST90094775 | ebi-gm430-GCST90011581 | OTU97_19 (Bacteria) prevalence \|\| id:ebi-gm430-GCST90011581 | Green olives liking \|\| id:ebi-fl187-GCST90094775 | Weighted median | 20 | -0.274928881 | 0.374756512589893 | 0.46318035455603 | -1.009451646 | 0.459593883584682 | 0.759626145086257 | 0.364418755359399 | 1.58343079715951 | 0.999332001504091 |
| ebi-fl187-GCST90094775 | ebi-gm430-GCST90011614 | OTU97_80 (Ruminococcus) prevalence \|\| id:ebi-gm430-GCST90011614 | Green olives liking \|\| id:ebi-fl187-GCST90094775 | Weighted median | 20 | -0.045861429 | 0.1988929504234 | 0.817638401901856 | -0.435691612 | 0.343968753932209 | 0.955174312577959 | 0.646817166009806 | 1.41053456116059 | 0.999332001504091 |
| ebi-fl187-GCST90094775 | ebi-gm430-GCST90011623 | OTU99_11 (Parabacteroides) prevalence \|\| id:ebi-gm430-GCST90011623 | Green olives liking \|\| id:ebi-fl187-GCST90094775 | Weighted median | 20 | 0.0183654422022432 | 0.151811000460518 | 0.903710281599537 | -0.279184119 | 0.315915003104859 | 1.01853512410523 | 0.756400622893737 | 1.37151367626755 | 0.999332001504091 |
| ebi-fl187-GCST90094775 | ebi-gm430-GCST90011642 | OTU99_19 (Bacteria) prevalence \|\| id:ebi-gm430-GCST90011642 | Green olives liking \|\| id:ebi-fl187-GCST90094775 | Weighted median | 20 | -0.274928881 | 0.369245954927295 | 0.45653265862661 | -0.998650953 | 0.448793190565988 | 0.759626145086257 | 0.368376062827974 | 1.56642067312629 | 0.999332001504091 |
| ebi-fl187-GCST90094775 | ebi-gm430-GCST90011653 | OTU99_30 (Parasutterella) prevalence \|\| id:ebi-gm430-GCST90011653 | Green olives liking \|\| id:ebi-fl187-GCST90094775 | Weighted median | 20 | 0.150330011271505 | 0.162965526102713 | 0.356286023444276 | -0.16908242 | 0.469742442432823 | 1.16221772439716 | 0.844439301923583 | 1.59958215566943 | 0.999332001504091 |
| ebi-fl187-GCST90094775 | ebi-gm430-GCST90011675 | OTU99_8 (Ruminococcaceae) prevalence \|\| id:ebi-gm430-GCST90011675 | Green olives liking \|\| id:ebi-fl187-GCST90094775 | Weighted median | 20 | 0.0261552844174548 | 0.163355557219905 | 0.872792368982388 | -0.294021608 | 0.346332176568468 | 1.02650033560458 | 0.745260387881416 | 1.41387219303542 | 0.999332001504091 |
| ebi-fl187-GCST90094775 | ebi-gm430-GCST90011679 | OTU99_92 (Ruminococcus) prevalence \|\| id:ebi-gm430-GCST90011679 | Green olives liking \|\| id:ebi-fl187-GCST90094775 | Weighted median | 20 | -0.260921553 | 0.20064756760489 | 0.193464839122589 | -0.654190785 | 0.13234767984285 | 0.77034134847238 | 0.519862572926947 | 1.14150512860566 | 0.999332001504091 |
| ebi-fl187-GCST90094775 | ebi-gm430-GCST90011684 | TestASV_11 (Lachnospiraceae) prevalence \|\| id:ebi-gm430-GCST90011684 | Green olives liking \|\| id:ebi-fl187-GCST90094775 | Weighted median | 20 | -0.176394163 | 0.177620456483224 | 0.320663177862961 | -0.524530258 | 0.171741931583531 | 0.838287496190466 | 0.591833308142775 | 1.18737137062207 | 0.999332001504091 |
| ebi-fl187-GCST90094775 | ebi-gm430-GCST90011688 | TestASV_15 (Bacteroides) prevalence \|\| id:ebi-gm430-GCST90011688 | Green olives liking \|\| id:ebi-fl187-GCST90094775 | Weighted median | 20 | -0.072899744 | 0.167446455630119 | 0.663300034689683 | -0.401094797 | 0.255295309135624 | 0.929694032852115 | 0.669586583273795 | 1.2908427622532 | 0.999332001504091 |
| ebi-fl187-GCST90094775 | ebi-gm430-GCST90011692 | TestASV_19 (Prevotella) prevalence \|\| id:ebi-gm430-GCST90011692 | Green olives liking \|\| id:ebi-fl187-GCST90094775 | Weighted median | 20 | 0.131701833284082 | 0.354498748476249 | 0.710253501031768 | -0.563115714 | 0.82651938029753 | 1.14076812946633 | 0.569432109604119 | 2.28535044521961 | 0.999332001504091 |
| ebi-fl187-GCST90094775 | ebi-gm430-GCST90011693 | TestASV_2 (Bacteroides) prevalence \|\| id:ebi-gm430-GCST90011693 | Green olives liking \|\| id:ebi-fl187-GCST90094775 | Weighted median | 20 | -0.093619882 | 0.165589926018883 | 0.571820870079693 | -0.418176137 | 0.230936372847461 | 0.910628842739172 | 0.658246276592521 | 1.25977908074339 | 0.999332001504091 |
| ebi-fl187-GCST90094775 | ebi-gm430-GCST90011716 | TestASV_40 (Barnesiella) prevalence \|\| id:ebi-gm430-GCST90011716 | Green olives liking \|\| id:ebi-fl187-GCST90094775 | Weighted median | 20 | -0.263957046 | 0.339813845409369 | 0.437294747776117 | -0.929992183 | 0.40207809089893 | 0.768006527821174 | 0.394556794568267 | 1.49492806839468 | 0.999332001504091 |
| ebi-fl187-GCST90094775 | ebi-gm430-GCST90011718 | TestASV_42 (Alphaproteobacteria) prevalence \|\| id:ebi-gm430-GCST90011718 | Green olives liking \|\| id:ebi-fl187-GCST90094775 | Weighted median | 20 | -0.079264893 | 0.271100532585799 | 0.76999463408069 | -0.610621937 | 0.452092150968114 | 0.923795185246006 | 0.543013044254707 | 1.57159676606849 | 0.999332001504091 |
| ebi-fl187-GCST90094775 | ebi-hbsm44-GCST90104929 | Sulcal depth of the right inferior frontal sulcus \|\| id:ebi-hbsm44-GCST90104929 | Green olives liking \|\| id:ebi-fl187-GCST90094775 | Weighted median | 19 | 0.20054842064681 | 0.150135013838867 | 0.181618857726862 | -0.093716206 | 0.494813047770988 | 1.22207278436212 | 0.910541131252445 | 1.64019157292141 | 0.999332001504091 |
| ebi-fl187-GCST90094775 | ebi-hbsm44-GCST90104935 | Sulcal opening distance of the left calloso-marginal posterior fissure \|\| id:ebi-hbsm44-GCST90104935 | Green olives liking \|\| id:ebi-fl187-GCST90094775 | Weighted median | 20 | -0.017173895 | 0.0311700034580043 | 0.581650752242846 | -0.078267102 | 0.0439193113853314 | 0.982972735342205 | 0.924717399530189 | 1.04489804011154 | 0.999332001504091 |
| ebi-fl187-GCST90094775 | ebi-hbsm44-GCST90104936 | Sulcal opening distance of the left anterior inferior temporal sulcus \|\| id:ebi-hbsm44-GCST90104936 | Green olives liking \|\| id:ebi-fl187-GCST90094775 | Weighted median | 18 | -0.014940514 | 0.0185639960071937 | 0.420928561387641 | -0.051325947 | 0.0214449178207281 | 0.985170541366756 | 0.949968980973497 | 1.02167651261862 | 0.999332001504091 |
| ebi-fl187-GCST90094775 | ebi-hbsm44-GCST90104941 | Sulcal opening distance of the right posterior inferior temporal sulcus \|\| id:ebi-hbsm44-GCST90104941 | Green olives liking \|\| id:ebi-fl187-GCST90094775 | Weighted median | 19 | -0.006965351 | 0.0151942005163744 | 0.646649533217384 | -0.036745984 | 0.0228152823894254 | 0.993058851207967 | 0.963920955951125 | 1.02307754165321 | 0.999332001504091 |
| ebi-fl187-GCST90094775 | ebi-IPs91-GCST90274819 | Leukemia inhibitory factor levels \|\| id:ebi-IPs91-GCST90274819 | Green olives liking \|\| id:ebi-fl187-GCST90094775 | Weighted median | 27 | 0.0117912087003281 | 0.0508600212161112 | 0.81666501566479 | -0.087894433 | 0.111476850283906 | 1.01186099903603 | 0.915857556061704 | 1.11792786399331 | 0.999332001504091 |
| ebi-fl187-GCST90094775 | ebi-IPs91-GCST90274837 | STAM binding protein levels \|\| id:ebi-IPs91-GCST90274837 | Green olives liking \|\| id:ebi-fl187-GCST90094775 | Weighted median | 27 | -0.035207152 | 0.0471527819134713 | 0.455268107643304 | -0.127626604 | 0.0572123007213732 | 0.965405410046009 | 0.880181973887258 | 1.05888058764708 | 0.999332001504091 |
| ebi-fl187-GCST90094775 | ebi-IPs91-GCST90274840 | TNF-beta levels \|\| id:ebi-IPs91-GCST90274840 | Green olives liking \|\| id:ebi-fl187-GCST90094775 | Weighted median | 27 | -0.054815482 | 0.0574335818308738 | 0.339873527857306 | -0.167385302 | 0.0577543383339044 | 0.946659807561748 | 0.845873631364045 | 1.05945469633295 | 0.999332001504091 |
| ebi-fl187-GCST90094775 | ebi-IPs91-GCST90274842 | Tumor necrosis factor ligand superfamily member 14 levels \|\| id:ebi-IPs91-GCST90274842 | Green olives liking \|\| id:ebi-fl187-GCST90094775 | Weighted median | 27 | 0.0209919888257768 | 0.0596948982829299 | 0.725097271532642 | -0.096010012 | 0.137993989460319 | 1.02121387048252 | 0.908454920746237 | 1.14796865034232 | 0.999332001504091 |
| ebi-fl187-GCST90094775 | ebi-met1400-GCST90199628 | 3-methylhistidine levels \|\| id:ebi-met1400-GCST90199628 | Green olives liking \|\| id:ebi-fl187-GCST90094775 | Weighted median | 24 | -0.052008583 | 0.0671593541985552 | 0.438690948458053 | -0.183640917 | 0.0796237514501792 | 0.949320718998003 | 0.8322345914657 | 1.08287955915376 | 0.999332001504091 |
| ebi-fl187-GCST90094775 | ebi-met1400-GCST90199651 | Docosahexaenoate DHA; 22:6n3 levels \|\| id:ebi-met1400-GCST90199651 | Green olives liking \|\| id:ebi-fl187-GCST90094775 | Weighted median | 24 | 0.0580181726152522 | 0.0628259900117851 | 0.355760207894237 | -0.065120768 | 0.181157113038351 | 1.05973425367816 | 0.936954302627272 | 1.19860348073514 | 0.999332001504091 |
| ebi-fl187-GCST90094775 | ebi-met1400-GCST90199652 | 1-stearoyl-2-oleoyl-GPS (18:0/18:1) levels \|\| id:ebi-met1400-GCST90199652 | Green olives liking \|\| id:ebi-fl187-GCST90094775 | Weighted median | 24 | 0.0407163689085189 | 0.0701832036964187 | 0.561817471915396 | -0.09684271 | 0.1782754481535 | 1.04155664579839 | 0.907698766539193 | 1.19515447899416 | 0.999332001504091 |
| ebi-fl187-GCST90094775 | ebi-met1400-GCST90199653 | 1-myristoyl-2-palmitoyl-gpc (14:0/16:0) levels \|\| id:ebi-met1400-GCST90199653 | Green olives liking \|\| id:ebi-fl187-GCST90094775 | Weighted median | 23 | 0.0436408889130929 | 0.0695730462321377 | 0.530484109383216 | -0.092722282 | 0.180004059528083 | 1.04460715751198 | 0.911446590546555 | 1.19722222326918 | 0.999332001504091 |
| ebi-fl187-GCST90094775 | ebi-met1400-GCST90199667 | Erythritol levels in elite athletes \|\| id:ebi-met1400-GCST90199667 | Green olives liking \|\| id:ebi-fl187-GCST90094775 | Weighted median | 24 | 0.0349816502565057 | 0.0643724061075759 | 0.586836409648417 | -0.091188266 | 0.161151566227354 | 1.03560070561796 | 0.912845837147191 | 1.17486302487623 | 0.999332001504091 |
| ebi-fl187-GCST90094775 | ebi-met1400-GCST90199674 | 3-hydroxymyristate levels \|\| id:ebi-met1400-GCST90199674 | Green olives liking \|\| id:ebi-fl187-GCST90094775 | Weighted median | 24 | 0.0159823526140919 | 0.0631885058845226 | 0.80032128129508 | -0.107867119 | 0.139831824147756 | 1.01611075354928 | 0.897746882080498 | 1.15008036684654 | 0.999332001504091 |
| ebi-fl187-GCST90094775 | ebi-met1400-GCST90199685 | 2-linoleoylglycerol (18:2) levels \|\| id:ebi-met1400-GCST90199685 | Green olives liking \|\| id:ebi-fl187-GCST90094775 | Weighted median | 24 | 0.000770093117735836 | 0.0741308039983827 | 0.991711483963979 | -0.144526283 | 0.146066468954566 | 1.00077038971557 | 0.865432166193673 | 1.15727310822802 | 0.999332001504091 |
| ebi-fl187-GCST90094775 | ebi-met1400-GCST90199703 | Gamma-glutamylvaline levels \|\| id:ebi-met1400-GCST90199703 | Green olives liking \|\| id:ebi-fl187-GCST90094775 | Weighted median | 24 | -0.022740141 | 0.0689473101611702 | 0.741536638694895 | -0.157876869 | 0.112396586690588 | 0.977516467003322 | 0.853954923698386 | 1.11895653593087 | 0.999332001504091 |
| ebi-fl187-GCST90094775 | ebi-met1400-GCST90199779 | Gamma-glutamyl-2-aminobutyrate levels \|\| id:ebi-met1400-GCST90199779 | Green olives liking \|\| id:ebi-fl187-GCST90094775 | Weighted median | 23 | 0.02765945495312 | 0.0708453610662877 | 0.696225906300407 | -0.111197453 | 0.166516362643044 | 1.0280455289901 | 0.894762058268384 | 1.18118286298581 | 0.999332001504091 |
| ebi-fl187-GCST90094775 | ebi-met1400-GCST90199824 | 4-vinylphenol sulfate levels \|\| id:ebi-met1400-GCST90199824 | Green olives liking \|\| id:ebi-fl187-GCST90094775 | Weighted median | 24 | -0.103017009 | 0.0697360589716064 | 0.139610878887019 | -0.239699685 | 0.0336656663289146 | 0.902111629103889 | 0.786864132814899 | 1.03423876807448 | 0.999332001504091 |
| ebi-fl187-GCST90094775 | ebi-met1400-GCST90199830 | 4-ethylphenylsulfate levels \|\| id:ebi-met1400-GCST90199830 | Green olives liking \|\| id:ebi-fl187-GCST90094775 | Weighted median | 24 | -0.0032789 | 0.0668620081256074 | 0.960887574146206 | -0.134328436 | 0.127770636074417 | 0.996726469869815 | 0.874302865100944 | 1.13629234833223 | 0.999332001504091 |
| ebi-fl187-GCST90094775 | ebi-met1400-GCST90199833 | Hexanoylglutamine levels \|\| id:ebi-met1400-GCST90199833 | Green olives liking \|\| id:ebi-fl187-GCST90094775 | Weighted median | 24 | 0.0893838250554894 | 0.067666311487634 | 0.186517982749963 | -0.043242145 | 0.222009795571252 | 1.09350028859744 | 0.957679464253607 | 1.24858360839408 | 0.999332001504091 |
| ebi-fl187-GCST90094775 | ebi-met1400-GCST90199837 | Carotenoid levels (cryptoxanthin) \|\| id:ebi-met1400-GCST90199837 | Green olives liking \|\| id:ebi-fl187-GCST90094775 | Weighted median | 24 | 0.0792874760643632 | 0.0664108648655483 | 0.232519770829136 | -0.050877819 | 0.209452771200838 | 1.08251547463197 | 0.950394783555399 | 1.23300314047797 | 0.999332001504091 |
| ebi-fl187-GCST90094775 | ebi-met1400-GCST90199869 | 2-hydroxyhippurate levels \|\| id:ebi-met1400-GCST90199869 | Green olives liking \|\| id:ebi-fl187-GCST90094775 | Weighted median | 24 | -0.04583779 | 0.0712167007582983 | 0.519810062073348 | -0.185422524 | 0.0937469431873969 | 0.955196891827176 | 0.830753196700832 | 1.0982817830611 | 0.999332001504091 |
| ebi-fl187-GCST90094775 | ebi-met1400-GCST90199902 | Hydantoin-5-propionate levels \|\| id:ebi-met1400-GCST90199902 | Green olives liking \|\| id:ebi-fl187-GCST90094775 | Weighted median | 24 | 0.0255335657101172 | 0.068694616047805 | 0.710118643064837 | -0.109107882 | 0.160175013163815 | 1.02586233949046 | 0.896633681875372 | 1.17371626881525 | 0.999332001504091 |
| ebi-fl187-GCST90094775 | ebi-met1400-GCST90199904 | Pantoate levels \|\| id:ebi-met1400-GCST90199904 | Green olives liking \|\| id:ebi-fl187-GCST90094775 | Weighted median | 24 | 0.0472374062743838 | 0.0721046850019537 | 0.512388844104062 | -0.094087776 | 0.188562588878213 | 1.04837086936026 | 0.910202864467758 | 1.20751265748419 | 0.999332001504091 |
| ebi-fl187-GCST90094775 | ebi-met1400-GCST90199936 | 3-methyl catechol sulfate (2) levels \|\| id:ebi-met1400-GCST90199936 | Green olives liking \|\| id:ebi-fl187-GCST90094775 | Weighted median | 24 | 0.00669504781937545 | 0.0798287188598189 | 0.933161652845445 | -0.149769241 | 0.163159336784621 | 1.00671750975195 | 0.8609066153294 | 1.1772242498722 | 0.999332001504091 |
| ebi-fl187-GCST90094775 | ebi-met1400-GCST90199977 | 4-hydroxychlorothalonil levels \|\| id:ebi-met1400-GCST90199977 | Green olives liking \|\| id:ebi-fl187-GCST90094775 | Weighted median | 24 | 0.0456423783962246 | 0.0656779611690575 | 0.4870916053906 | -0.083086425 | 0.174371182287577 | 1.04670002147569 | 0.920271608853655 | 1.19049737535849 | 0.999332001504091 |
| ebi-fl187-GCST90094775 | ebi-met1400-GCST90200040 | Sphingomyelin (d18:2/24:1, d18:1/24:2) levels \|\| id:ebi-met1400-GCST90200040 | Green olives liking \|\| id:ebi-fl187-GCST90094775 | Weighted median | 24 | 0.0739120261463772 | 0.0614738432575734 | 0.229234562973985 | -0.046576707 | 0.194400758931221 | 1.07671207881873 | 0.954491341939543 | 1.21458294039463 | 0.999332001504091 |
| ebi-fl187-GCST90094775 | ebi-met1400-GCST90200077 | 1-stearoyl-2-oleoyl-GPI (18:0/18:1) levels \|\| id:ebi-met1400-GCST90200077 | Green olives liking \|\| id:ebi-fl187-GCST90094775 | Weighted median | 24 | -0.012978027 | 0.0661370360943332 | 0.844430669387222 | -0.142606617 | 0.1166505642191 | 0.987105824926369 | 0.867095102050634 | 1.12372669076231 | 0.999332001504091 |
| ebi-fl187-GCST90094775 | ebi-met1400-GCST90200080 | 1-palmitoyl-2-oleoyl-GPI (16:0/18:1) levels \|\| id:ebi-met1400-GCST90200080 | Green olives liking \|\| id:ebi-fl187-GCST90094775 | Weighted median | 24 | -0.00128939 | 0.0660873212984495 | 0.984433939091346 | -0.13082054 | 0.128241759544633 | 0.998711440706057 | 0.877375214067474 | 1.13682780845056 | 0.999332001504091 |
| ebi-fl187-GCST90094775 | ebi-met1400-GCST90200082 | 1-oleoyl-2-linoleoyl-GPE (18:1/18:2) levels \|\| id:ebi-met1400-GCST90200082 | Green olives liking \|\| id:ebi-fl187-GCST90094775 | Weighted median | 24 | -0.007597358 | 0.0663651178535042 | 0.908858746871514 | -0.137672989 | 0.122478273450536 | 0.992431429430707 | 0.871383597586171 | 1.13029456240652 | 0.999332001504091 |
| ebi-fl187-GCST90094775 | ebi-met1400-GCST90200100 | Perfluorooctanesulfonate (PFOS) levels \|\| id:ebi-met1400-GCST90200100 | Green olives liking \|\| id:ebi-fl187-GCST90094775 | Weighted median | 24 | -0.077757385 | 0.0643124043365902 | 0.226640814426427 | -0.203809697 | 0.048294927554227 | 0.925188864063165 | 0.815617570526814 | 1.04948013029392 | 0.999332001504091 |
| ebi-fl187-GCST90094775 | ebi-met1400-GCST90200102 | 1-palmitoleoyl-2-linolenoyl-GPC (16:1/18:3) levels \|\| id:ebi-met1400-GCST90200102 | Green olives liking \|\| id:ebi-fl187-GCST90094775 | Weighted median | 24 | 0.0035383848933373 | 0.070382200735208 | 0.959904154265741 | -0.134410729 | 0.141487498334345 | 1.00354465236723 | 0.874230919256965 | 1.15198610242569 | 0.999332001504091 |
| ebi-fl187-GCST90094775 | ebi-met1400-GCST90200114 | Glycosyl-N-tricosanoyl-sphingadienine (d18:2/23:0) levels \|\| id:ebi-met1400-GCST90200114 | Green olives liking \|\| id:ebi-fl187-GCST90094775 | Weighted median | 24 | 0.0887594332200274 | 0.0697107830478761 | 0.202928433720772 | -0.047873702 | 0.225392567993865 | 1.09281772905964 | 0.953254173996004 | 1.25281443451835 | 0.999332001504091 |
| ebi-fl187-GCST90094775 | ebi-met1400-GCST90200135 | Arachidoylcarnitine (C20) levels \|\| id:ebi-met1400-GCST90200135 | Green olives liking \|\| id:ebi-fl187-GCST90094775 | Weighted median | 24 | 0.100427329702289 | 0.0707782286934472 | 0.155927756188772 | -0.038297999 | 0.239152657941446 | 1.10564329135743 | 0.962426096585105 | 1.27017242369174 | 0.999332001504091 |
| ebi-fl187-GCST90094775 | ebi-met1400-GCST90200146 | Docosahexaenoylcarnitine (C22:6) levels \|\| id:ebi-met1400-GCST90200146 | Green olives liking \|\| id:ebi-fl187-GCST90094775 | Weighted median | 24 | 0.00484374749818406 | 0.0755235864461499 | 0.948862287458278 | -0.143182482 | 0.152869976932638 | 1.00485549740663 | 0.866595916365156 | 1.16517346966457 | 0.999332001504091 |
| ebi-fl187-GCST90094775 | ebi-met1400-GCST90200162 | 3-hydroxyphenylacetoylglutamine levels \|\| id:ebi-met1400-GCST90200162 | Green olives liking \|\| id:ebi-fl187-GCST90094775 | Weighted median | 24 | 0.0621725203594151 | 0.079823631299158 | 0.436054167266309 | -0.094281797 | 0.218626837705765 | 1.06414591572856 | 0.910026283440283 | 1.24436683925305 | 0.999332001504091 |
| ebi-fl187-GCST90094775 | ebi-met1400-GCST90200172 | 3-carboxy-4-methyl-5-pentyl-2-furanpropionate (3-CMPFP) levels \|\| id:ebi-met1400-GCST90200172 | Green olives liking \|\| id:ebi-fl187-GCST90094775 | Weighted median | 24 | 0.0925229944204647 | 0.0658444348160225 | 0.159969062370952 | -0.036532098 | 0.221578086659869 | 1.09693836473229 | 0.964127147021063 | 1.24804470005787 | 0.999332001504091 |
| ebi-fl187-GCST90094775 | ebi-met1400-GCST90200222 | Dihydrocaffeate sulfate (2) levels \|\| id:ebi-met1400-GCST90200222 | Green olives liking \|\| id:ebi-fl187-GCST90094775 | Weighted median | 24 | -0.008738432 | 0.0839037024480507 | 0.917051623761106 | -0.173189689 | 0.155712824847055 | 0.991299637176495 | 0.840978075634346 | 1.16849059343791 | 0.999332001504091 |
| ebi-fl187-GCST90094775 | ebi-met1400-GCST90200223 | 4-methylhexanoylglutamine levels \|\| id:ebi-met1400-GCST90200223 | Green olives liking \|\| id:ebi-fl187-GCST90094775 | Weighted median | 24 | 0.0213850358418504 | 0.0805426602036807 | 0.790615133341191 | -0.136478578 | 0.179248649841065 | 1.02161533443903 | 0.872425009010703 | 1.19631817151193 | 0.999332001504091 |
| ebi-fl187-GCST90094775 | ebi-met1400-GCST90200224 | 2-ketocaprylate levels \|\| id:ebi-met1400-GCST90200224 | Green olives liking \|\| id:ebi-fl187-GCST90094775 | Weighted median | 24 | 0.0226510495059903 | 0.072342119647388 | 0.754197003466865 | -0.119139505 | 0.164441604014871 | 1.02290953247526 | 0.887683955770838 | 1.17873473416576 | 0.999332001504091 |
| ebi-fl187-GCST90094775 | ebi-met1400-GCST90200243 | Hydroxypalmitoyl sphingomyelin (d18:1/16:0(OH)) levels \|\| id:ebi-met1400-GCST90200243 | Green olives liking \|\| id:ebi-fl187-GCST90094775 | Weighted median | 24 | 0.0571790841497167 | 0.0683253624425809 | 0.402668622747176 | -0.076738626 | 0.191096794537175 | 1.05884541584831 | 0.926131888550383 | 1.21057662361442 | 0.999332001504091 |
| ebi-fl187-GCST90094775 | ebi-met1400-GCST90200273 | 2-methoxyhydroquinone sulfate (2) levels \|\| id:ebi-met1400-GCST90200273 | Green olives liking \|\| id:ebi-fl187-GCST90094775 | Weighted median | 24 | 0.0142550206991124 | 0.0817374087345021 | 0.861550954571712 | -0.1459503 | 0.174460341818736 | 1.01435710801549 | 0.864200652526376 | 1.19060352427835 | 0.999332001504091 |
| ebi-fl187-GCST90094775 | ebi-met1400-GCST90200276 | 2,4-di-tert-butylphenol levels \|\| id:ebi-met1400-GCST90200276 | Green olives liking \|\| id:ebi-fl187-GCST90094775 | Weighted median | 24 | -0.016618102 | 0.067750541389483 | 0.80623678366722 | -0.149409164 | 0.116172958689636 | 0.983519216518137 | 0.861216664325069 | 1.12319012082578 | 0.999332001504091 |
| ebi-fl187-GCST90094775 | ebi-met1400-GCST90200289 | N-acetylmethionine levels \|\| id:ebi-met1400-GCST90200289 | Green olives liking \|\| id:ebi-fl187-GCST90094775 | Weighted median | 24 | 0.0316379561856075 | 0.0693744625532235 | 0.64835679608538 | -0.10433599 | 0.167611902789926 | 1.03214375638989 | 0.900922545219939 | 1.18247760532463 | 0.999332001504091 |
| ebi-fl187-GCST90094775 | ebi-met1400-GCST90200308 | 3-hydroxyisobutyrate levels \|\| id:ebi-met1400-GCST90200308 | Green olives liking \|\| id:ebi-fl187-GCST90094775 | Weighted median | 24 | 0.101167795475804 | 0.0684200045558765 | 0.139239526686977 | -0.032935413 | 0.235271004405322 | 1.10646228555356 | 0.967601051581044 | 1.26525161103534 | 0.999332001504091 |
| ebi-fl187-GCST90094775 | ebi-met1400-GCST90200383 | Cystathionine levels \|\| id:ebi-met1400-GCST90200383 | Green olives liking \|\| id:ebi-fl187-GCST90094775 | Weighted median | 24 | 0.0147401951005129 | 0.0678992699550545 | 0.828138865947267 | -0.118342374 | 0.14782276421242 | 1.01484936752438 | 0.88839183826269 | 1.15930740739212 | 0.999332001504091 |
| ebi-fl187-GCST90094775 | ebi-met1400-GCST90200384 | Guanidinoacetate levels \|\| id:ebi-met1400-GCST90200384 | Green olives liking \|\| id:ebi-fl187-GCST90094775 | Weighted median | 24 | 0.0115089160804165 | 0.0647740128972165 | 0.858975875693885 | -0.115448149 | 0.138465981358961 | 1.01157539845699 | 0.890966768380796 | 1.14851061013543 | 0.999332001504091 |
| ebi-fl187-GCST90094775 | ebi-met1400-GCST90200403 | Citrulline levels \|\| id:ebi-met1400-GCST90200403 | Green olives liking \|\| id:ebi-fl187-GCST90094775 | Weighted median | 24 | 0.133011903331367 | 0.0671061604356704 | 0.0474667443673113 | 0.00148382887745324 | 0.264539977785281 | 1.14226359499205 | 1.00148493029623 | 1.30283150646933 | 0.999332001504091 |
| ebi-fl187-GCST90094775 | ebi-met1400-GCST90200434 | 12,13-DiHOME levels \|\| id:ebi-met1400-GCST90200434 | Green olives liking \|\| id:ebi-fl187-GCST90094775 | Weighted median | 24 | 0.00223548662414529 | 0.0638837153050015 | 0.972085275534203 | -0.122976595 | 0.127447568621948 | 1.00223798718735 | 0.884284358663623 | 1.13592530855049 | 0.999332001504091 |
| ebi-fl187-GCST90094775 | ebi-met1400-GCST90200437 | Fructose levels \|\| id:ebi-met1400-GCST90200437 | Green olives liking \|\| id:ebi-fl187-GCST90094775 | Weighted median | 24 | 0.00922178176173236 | 0.0682734715350943 | 0.892555556998247 | -0.124594222 | 0.143037785970517 | 1.00926443339838 | 0.882855072681988 | 1.15377339728993 | 0.999332001504091 |
| ebi-fl187-GCST90094775 | ebi-met1400-GCST90200448 | N-stearoyl-sphinganine (d18:0/18:0) levels \|\| id:ebi-met1400-GCST90200448 | Green olives liking \|\| id:ebi-fl187-GCST90094775 | Weighted median | 24 | 0.0374757661664926 | 0.07442172710189 | 0.614570973069805 | -0.108390819 | 0.183342351286197 | 1.038186838 | 0.897276855095469 | 1.20122557881632 | 0.999332001504091 |
| ebi-fl187-GCST90094775 | ebi-met1400-GCST90200468 | X-11847 levels \|\| id:ebi-met1400-GCST90200468 | Green olives liking \|\| id:ebi-fl187-GCST90094775 | Weighted median | 24 | 0.135255995312516 | 0.0683873992209355 | 0.047952268395725 | 0.00121669283948228 | 0.269295297785549 | 1.14482981790912 | 1.00121743331049 | 1.30904164107516 | 0.999332001504091 |
| ebi-fl187-GCST90094775 | ebi-met1400-GCST90200476 | X-11843 levels \|\| id:ebi-met1400-GCST90200476 | Green olives liking \|\| id:ebi-fl187-GCST90094775 | Weighted median | 24 | 0.132372195231672 | 0.0735577646341035 | 0.0719288866155389 | -0.011801023 | 0.276545413914515 | 1.14153311339076 | 0.988268335522352 | 1.31856683263948 | 0.999332001504091 |
| ebi-fl187-GCST90094775 | ebi-met1400-GCST90200511 | X-12906 levels \|\| id:ebi-met1400-GCST90200511 | Green olives liking \|\| id:ebi-fl187-GCST90094775 | Weighted median | 24 | 0.0155527180777614 | 0.070626326080101 | 0.825706563449483 | -0.122874881 | 0.153980317194759 | 1.01567429104321 | 0.884374307633075 | 1.16646792719146 | 0.999332001504091 |
| ebi-fl187-GCST90094775 | ebi-met1400-GCST90200515 | X-12849 levels \|\| id:ebi-met1400-GCST90200515 | Green olives liking \|\| id:ebi-fl187-GCST90094775 | Weighted median | 24 | 0.0549035687564679 | 0.0759560763534702 | 0.469782407743457 | -0.093970341 | 0.20377747840927 | 1.0564387360624 | 0.910309760811953 | 1.22602530599874 | 0.999332001504091 |
| ebi-fl187-GCST90094775 | ebi-met1400-GCST90200551 | X-18886 levels \|\| id:ebi-met1400-GCST90200551 | Green olives liking \|\| id:ebi-fl187-GCST90094775 | Weighted median | 24 | 0.0612118632428002 | 0.069318950874659 | 0.377211099187176 | -0.07465328 | 0.197077006957132 | 1.06312412725427 | 0.928065208883196 | 1.2178378190798 | 0.999332001504091 |
| ebi-fl187-GCST90094775 | ebi-met1400-GCST90200564 | X-21319 levels \|\| id:ebi-met1400-GCST90200564 | Green olives liking \|\| id:ebi-fl187-GCST90094775 | Weighted median | 24 | 0.000661872335826894 | 0.0663738867035181 | 0.992043723938106 | -0.129430946 | 0.130754690274722 | 1.00066209142165 | 0.878595257187132 | 1.13968817042577 | 0.999332001504091 |
| ebi-fl187-GCST90094775 | ebi-met1400-GCST90200579 | X-21442 levels \|\| id:ebi-met1400-GCST90200579 | Green olives liking \|\| id:ebi-fl187-GCST90094775 | Weighted median | 24 | 0.0687783968464586 | 0.0757566332247916 | 0.36393838435821 | -0.079704604 | 0.21726139796705 | 1.07119880181166 | 0.923389071288739 | 1.24266889080814 | 0.999332001504091 |
| ebi-fl187-GCST90094775 | ebi-met1400-GCST90200581 | X-21441 levels \|\| id:ebi-met1400-GCST90200581 | Green olives liking \|\| id:ebi-fl187-GCST90094775 | Weighted median | 24 | 0.00177306838302172 | 0.0741172634497267 | 0.980914446942425 | -0.143496768 | 0.147042904744486 | 1.0017746411982 | 0.866323600159271 | 1.15840366297684 | 0.999332001504091 |
| ebi-fl187-GCST90094775 | ebi-met1400-GCST90200648 | X-24585 levels \|\| id:ebi-met1400-GCST90200648 | Green olives liking \|\| id:ebi-fl187-GCST90094775 | Weighted median | 24 | 0.0182140164126751 | 0.0645986469081333 | 0.777976770236445 | -0.108399332 | 0.144827364352616 | 1.01838090329665 | 0.897269216992303 | 1.15584001385418 | 0.999332001504091 |
| ebi-fl187-GCST90094775 | ebi-met1400-GCST90200720 | Adenosine 5'-diphosphate (ADP) to pantothenate ratio \|\| id:ebi-met1400-GCST90200720 | Green olives liking \|\| id:ebi-fl187-GCST90094775 | Weighted median | 24 | 0.0768848613353498 | 0.093910239002105 | 0.412954262485097 | -0.107179207 | 0.260948929779476 | 1.0799177289489 | 0.898364665229586 | 1.29816136635349 | 0.999332001504091 |
| ebi-fl187-GCST90094775 | ebi-met1400-GCST90200724 | Cholate to taurocholate ratio \|\| id:ebi-met1400-GCST90200724 | Green olives liking \|\| id:ebi-fl187-GCST90094775 | Weighted median | 24 | 0.125905811027897 | 0.0706563974671876 | 0.0747582085103574 | -0.012580728 | 0.264392350063584 | 1.13417533644279 | 0.98749807852345 | 1.30263918661849 | 0.999332001504091 |
| ebi-fl187-GCST90094775 | ebi-met1400-GCST90200726 | N-acetylputrescine to (N(1) + N(8))-acetylspermidine ratio \|\| id:ebi-met1400-GCST90200726 | Green olives liking \|\| id:ebi-fl187-GCST90094775 | Weighted median | 24 | -0.015733482 | 0.0659171903898275 | 0.811349405216179 | -0.144931175 | 0.113464210862527 | 0.984389642358839 | 0.865081829916086 | 1.12015179890827 | 0.999332001504091 |
| ebi-fl187-GCST90094775 | ebi-met1400-GCST90200744 | Arginine to glutamate ratio \|\| id:ebi-met1400-GCST90200744 | Green olives liking \|\| id:ebi-fl187-GCST90094775 | Weighted median | 24 | 0.0235187222540519 | 0.0721631265660211 | 0.744492054515624 | -0.117921006 | 0.164958450323453 | 1.0237974683635 | 0.888766257207537 | 1.17934411632681 | 0.999332001504091 |
| ebi-fl187-GCST90094775 | ebi-met1400-GCST90200759 | Histidine to trans-urocanate ratio \|\| id:ebi-met1400-GCST90200759 | Green olives liking \|\| id:ebi-fl187-GCST90094775 | Weighted median | 24 | 0.0662398828764306 | 0.0681614581761419 | 0.331145837750615 | -0.067356575 | 0.199836340901669 | 1.06848299720095 | 0.934861793414718 | 1.22120288084239 | 0.999332001504091 |
| ebi-fl187-GCST90094775 | ebi-met1400-GCST90200795 | Oleoyl-linoleoyl-glycerol (18:1 to 18:2) [2] to linoleoyl-arachidonoyl-glycerol (18:2 to 20:4) [2] ratio \|\| id:ebi-met1400-GCST90200795 | Green olives liking \|\| id:ebi-fl187-GCST90094775 | Weighted median | 24 | 0.0405451234952473 | 0.0691806416339164 | 0.557824348702554 | -0.095048934 | 0.176139181097723 | 1.04137829927108 | 0.909328436204295 | 1.19260403503876 | 0.999332001504091 |
| ebi-fl187-GCST90094775 | ebi-met1400-GCST90200811 | Choline to taurocholate ratio \|\| id:ebi-met1400-GCST90200811 | Green olives liking \|\| id:ebi-fl187-GCST90094775 | Weighted median | 24 | 0.0423244917040432 | 0.0693561154619494 | 0.541696955405141 | -0.093613495 | 0.178262478009464 | 1.04323294426897 | 0.910634659443349 | 1.19513897776895 | 0.999332001504091 |
| ebi-fl187-GCST90094775 | ebi-met1400-GCST90200831 | Cholate to phosphate ratio \|\| id:ebi-met1400-GCST90200831 | Green olives liking \|\| id:ebi-fl187-GCST90094775 | Weighted median | 24 | 0.0368027967641541 | 0.0728110117250356 | 0.613238266883901 | -0.105906786 | 0.179512379745224 | 1.03748840459279 | 0.899508490757557 | 1.19663371799634 | 0.999332001504091 |
| ebi-fl187-GCST90094775 | ebi-met1400-GCST90200841 | Adenosine 5'-diphosphate (ADP) to oxalate (ethanedioate) ratio \|\| id:ebi-met1400-GCST90200841 | Green olives liking \|\| id:ebi-fl187-GCST90094775 | Weighted median | 24 | -0.05821322 | 0.0908944878501746 | 0.521881194817791 | -0.236366416 | 0.119939976584533 | 0.943448764219624 | 0.789491338836408 | 1.12742917739845 | 0.999332001504091 |
| ebi-fl187-GCST90094775 | ebi-met1400-GCST90200844 | Aspartate to phosphate ratio \|\| id:ebi-met1400-GCST90200844 | Green olives liking \|\| id:ebi-fl187-GCST90094775 | Weighted median | 24 | -0.124681492 | 0.0641206787673811 | 0.0518373795003692 | -0.250358022 | 0.000995038280923954 | 0.882778029585229 | 0.778522004785434 | 1.00099553349575 | 0.999332001504091 |
| ebi-fl187-GCST90094775 | ebi-met1400-GCST90200870 | Adenosine 5'-monophosphate (AMP) to acetoacetate ratio \|\| id:ebi-met1400-GCST90200870 | Green olives liking \|\| id:ebi-fl187-GCST90094775 | Weighted median | 24 | -0.043918093 | 0.0791277396667431 | 0.578875665712846 | -0.199008463 | 0.111172276817236 | 0.957032341978309 | 0.819542957776734 | 1.11758742467531 | 0.999332001504091 |
| ebi-fl187-GCST90094775 | ebi-met1400-GCST90200887 | Cholesterol to cortisol ratio \|\| id:ebi-met1400-GCST90200887 | Green olives liking \|\| id:ebi-fl187-GCST90094775 | Weighted median | 24 | 0.00739966816039249 | 0.0659144316270578 | 0.910615884565624 | -0.121792618 | 0.136591954149426 | 1.00742711335819 | 0.885331951528528 | 1.14636028551434 | 0.999332001504091 |
| ebi-fl187-GCST90094775 | ebi-met1400-GCST90200890 | Cortisol to taurocholate ratio \|\| id:ebi-met1400-GCST90200890 | Green olives liking \|\| id:ebi-fl187-GCST90094775 | Weighted median | 24 | 0.081051142413822 | 0.0674608285285396 | 0.229574794353434 | -0.051172082 | 0.21327436632976 | 1.08442635533002 | 0.950115159220413 | 1.23772419450635 | 0.999332001504091 |
| ebi-fl187-GCST90094775 | ebi-met1400-GCST90200948 | Adenosine 5'-diphosphate (ADP) to mannitol to sorbitol ratio \|\| id:ebi-met1400-GCST90200948 | Green olives liking \|\| id:ebi-fl187-GCST90094775 | Weighted median | 24 | -0.146165333 | 0.0884444908428838 | 0.0984077687567201 | -0.319516535 | 0.0271858694098623 | 0.86401484151848 | 0.72650018981817 | 1.02755877675826 | 0.999332001504091 |
| ebi-fl187-GCST90094775 | ebi-met1400-GCST90200972 | Leucine to N-palmitoyl-sphingosine (d18:1 to 16:0) ratio \|\| id:ebi-met1400-GCST90200972 | Green olives liking \|\| id:ebi-fl187-GCST90094775 | Weighted median | 24 | -0.059654523 | 0.0665977718776707 | 0.370389756029074 | -0.190186156 | 0.0708771096275714 | 0.94208994754031 | 0.826805204756799 | 1.07344930117798 | 0.999332001504091 |
| ebi-fl187-GCST90094775 | ebi-met1400-GCST90200973 | Proline to glutamate ratio \|\| id:ebi-met1400-GCST90200973 | Green olives liking \|\| id:ebi-fl187-GCST90094775 | Weighted median | 24 | 0.0808624847912135 | 0.0666315621977684 | 0.224909561337342 | -0.049735377 | 0.21146034669884 | 1.08422178932901 | 0.951481174881965 | 1.23548097375823 | 0.999332001504091 |
| ebi-fl187-GCST90094775 | ebi-met1400-GCST90200983 | Cholesterol to linoleoyl-arachidonoyl-glycerol (18:2 to 20:4) [1] ratio \|\| id:ebi-met1400-GCST90200983 | Green olives liking \|\| id:ebi-fl187-GCST90094775 | Weighted median | 24 | 0.000170442576325348 | 0.0673861350598879 | 0.997981879283801 | -0.131906382 | 0.132247267293706 | 1.00017045710249 | 0.876423040087418 | 1.14139051292036 | 0.999381480690152 |
| ebi-fl187-GCST90094775 | ebi-met1400-GCST90201001 | Serine to alpha-ketobutyrate ratio \|\| id:ebi-met1400-GCST90201001 | Green olives liking \|\| id:ebi-fl187-GCST90094775 | Weighted median | 24 | -0.032049021 | 0.0686985966201917 | 0.640845917216076 | -0.166698271 | 0.102600227951796 | 0.968459105671294 | 0.846454972992599 | 1.10804835376145 | 0.999332001504091 |
| ebi-fl187-GCST90094775 | ebi-met1400-GCST90201005 | Fructose to maltose ratio \|\| id:ebi-met1400-GCST90201005 | Green olives liking \|\| id:ebi-fl187-GCST90094775 | Weighted median | 24 | -0.027611692 | 0.078764242652195 | 0.725917522255658 | -0.181989608 | 0.126766223242286 | 0.972766025956219 | 0.833610003283957 | 1.13515161469616 | 0.999332001504091 |
| ebi-fl187-GCST90094775 | ebi-met1400-GCST90201020 | Paraxanthine to 5-acetylamino-6-formylamino-3-methyluracil ratio \|\| id:ebi-met1400-GCST90201020 | Green olives liking \|\| id:ebi-fl187-GCST90094775 | Weighted median | 24 | -0.014427484 | 0.0704186229360425 | 0.837664588738336 | -0.152447985 | 0.123593016977314 | 0.985676093450759 | 0.858603553117134 | 1.13155525349638 | 0.999332001504091 |
| ebi-fl187-GCST90094775 | ebi-pw11-GCST90275197 | Placental weight (fetal genotype, adjusted for sex) \|\| id:ebi-pw11-GCST90275197 | Green olives liking \|\| id:ebi-fl187-GCST90094775 | Weighted median | 23 | 0.0288461569320598 | 0.0252426353557234 | 0.253140176368557 | -0.020629408 | 0.0783217222292778 | 1.02926623681873 | 0.97958192217678 | 1.08147053581886 | 0.999332001504091 |
| ebi-fl187-GCST90094775 | prot-a-1017 | FAS-associated factor 2 \|\| id:prot-a-1017 | Green olives liking \|\| id:ebi-fl187-GCST90094775 | Weighted median | 27 | -0.097343753 | 0.0964849432421089 | 0.313022127687155 | -0.286454242 | 0.0917667354679969 | 0.907244084388582 | 0.750921438377303 | 1.09610910887926 | 0.999332001504091 |
| ebi-fl187-GCST90094775 | prot-a-1019 | Fas apoptotic inhibitory molecule 3 \|\| id:prot-a-1019 | Green olives liking \|\| id:ebi-fl187-GCST90094775 | Weighted median | 27 | -0.049436515 | 0.0998282730970403 | 0.620447964802691 | -0.24509993 | 0.146226900624756 | 0.951765579393772 | 0.782626326551298 | 1.15745878637957 | 0.999332001504091 |
| ebi-fl187-GCST90094775 | prot-a-1052 | MIP18 family protein FAM96A \|\| id:prot-a-1052 | Green olives liking \|\| id:ebi-fl187-GCST90094775 | Weighted median | 27 | 0.0494351639973441 | 0.0964196301030222 | 0.608155242409881 | -0.139547311 | 0.238417638999268 | 1.05067746827928 | 0.869751873396122 | 1.26923916592358 | 0.999332001504091 |
| ebi-fl187-GCST90094775 | prot-a-1066 | F-box/LRR-repeat protein 4 \|\| id:prot-a-1066 | Green olives liking \|\| id:ebi-fl187-GCST90094775 | Weighted median | 27 | -0.034874998 | 0.101850887454384 | 0.732040626504744 | -0.234502737 | 0.164752741862892 | 0.965726126846955 | 0.790964069052748 | 1.17910154 | 0.999332001504091 |
| ebi-fl187-GCST90094775 | prot-a-1076 | Low affinity immunoglobulin gamma Fc region receptor III-B \|\| id:prot-a-1076 | Green olives liking \|\| id:ebi-fl187-GCST90094775 | Weighted median | 27 | -0.073201301 | 0.109459012548269 | 0.503651550899367 | -0.287740965 | 0.141338363941515 | 0.929413719604951 | 0.749955831703672 | 1.15181431448782 | 0.999332001504091 |
| ebi-fl187-GCST90094775 | prot-a-1081 | Fc receptor-like protein 3 \|\| id:prot-a-1081 | Green olives liking \|\| id:ebi-fl187-GCST90094775 | Weighted median | 27 | -0.12879978 | 0.106247477023226 | 0.225412103049947 | -0.337044835 | 0.0794452754227859 | 0.879149971734319 | 0.713876832868456 | 1.08268630835773 | 0.999332001504091 |
| ebi-fl187-GCST90094775 | prot-a-1105 | Fibroblast growth factor receptor 4 \|\| id:prot-a-1105 | Green olives liking \|\| id:ebi-fl187-GCST90094775 | Weighted median | 27 | -0.030253295 | 0.10710642937218 | 0.77759072314271 | -0.240181896 | 0.179675306880094 | 0.97019975597692 | 0.786484789414011 | 1.19682869798284 | 0.999332001504091 |
| ebi-fl187-GCST90094775 | prot-a-1143 | Follistatin-related protein 1 \|\| id:prot-a-1143 | Green olives liking \|\| id:ebi-fl187-GCST90094775 | Weighted median | 27 | -0.103984422 | 0.114105870816557 | 0.362138515514858 | -0.327631929 | 0.119663084305833 | 0.901239336372471 | 0.720628213031723 | 1.12711704418008 | 0.999332001504091 |
| ebi-fl187-GCST90094775 | prot-a-1151 | Alpha-(1,3)-fucosyltransferase 10 \|\| id:prot-a-1151 | Green olives liking \|\| id:ebi-fl187-GCST90094775 | Weighted median | 27 | 0.0211678518956033 | 0.10157444407944 | 0.834918672099467 | -0.177918059 | 0.220253762291307 | 1.02139348008162 | 0.837011006616143 | 1.24639297799782 | 0.999332001504091 |
| ebi-fl187-GCST90094775 | prot-a-1165 | Growth arrest and DNA damage-inducible proteins-interacting protein 1 \|\| id:prot-a-1165 | Green olives liking \|\| id:ebi-fl187-GCST90094775 | Weighted median | 27 | 0.15394190157081 | 0.101645464135067 | 0.129899457452887 | -0.045283208 | 0.353167011275541 | 1.16642311745892 | 0.955726773905389 | 1.42356887563481 | 0.999332001504091 |
| ebi-fl187-GCST90094775 | prot-a-1194 | Growth/differentiation factor 11/8 \|\| id:prot-a-1194 | Green olives liking \|\| id:ebi-fl187-GCST90094775 | Weighted median | 26 | -0.228072276 | 0.106643876386157 | 0.0324652786757386 | -0.437094274 | -0.019050279 | 0.796066720851654 | 0.645910535839937 | 0.981130030993253 | 0.999332001504091 |
| ebi-fl187-GCST90094775 | prot-a-1216 | D-glucuronyl C5-epimerase \|\| id:prot-a-1216 | Green olives liking \|\| id:ebi-fl187-GCST90094775 | Weighted median | 27 | 0.071183198585773 | 0.102485889777358 | 0.48732742333815 | -0.129689145 | 0.272055542549394 | 1.07377792244737 | 0.878368433373792 | 1.31265990776415 | 0.999332001504091 |
| ebi-fl187-GCST90094775 | prot-a-1246 | Glypican-3 \|\| id:prot-a-1246 | Green olives liking \|\| id:ebi-fl187-GCST90094775 | Weighted median | 27 | -0.021138089 | 0.0959442304863134 | 0.825624735925553 | -0.209188781 | 0.166912602731972 | 0.979083754516827 | 0.811242074279917 | 1.18165098772725 | 0.999332001504091 |
| ebi-fl187-GCST90094775 | prot-a-1275 | Glutamate receptor 4 \|\| id:prot-a-1275 | Green olives liking \|\| id:ebi-fl187-GCST90094775 | Weighted median | 26 | 0.152931435118021 | 0.0988484888510707 | 0.121831968092518 | -0.040811603 | 0.34667447326612 | 1.16524508131221 | 0.960009975882403 | 1.41435623965705 | 0.999332001504091 |
| ebi-fl187-GCST90094775 | prot-a-1289 | Glutathione S-transferase P \|\| id:prot-a-1289 | Green olives liking \|\| id:ebi-fl187-GCST90094775 | Weighted median | 27 | 0.0923236430546454 | 0.102307924034874 | 0.366839373943291 | -0.108199888 | 0.292847174162998 | 1.09671971036631 | 0.897448189328496 | 1.340237952 | 0.999332001504091 |
| ebi-fl187-GCST90094775 | prot-a-130 | Apolipoprotein D \|\| id:prot-a-130 | Green olives liking \|\| id:ebi-fl187-GCST90094775 | Weighted median | 27 | 0.0124646956904941 | 0.097689280099923 | 0.898469228999027 | -0.179006293 | 0.203935684686343 | 1.01254270378861 | 0.836100637543859 | 1.22621928624205 | 0.999332001504091 |
| ebi-fl187-GCST90094775 | prot-a-1313 | Heparin-binding EGF-like growth factor \|\| id:prot-a-1313 | Green olives liking \|\| id:ebi-fl187-GCST90094775 | Weighted median | 27 | 0.0657497259032329 | 0.101818493120826 | 0.518438513047993 | -0.133814521 | 0.265313972420052 | 1.06795940114175 | 0.874752298076897 | 1.30384028140818 | 0.999332001504091 |
| ebi-fl187-GCST90094775 | prot-a-1347 | HLA class II histocompatibility antigen, DQ alpha 2 chain \|\| id:prot-a-1347 | Green olives liking \|\| id:ebi-fl187-GCST90094775 | Weighted median | 26 | -0.062906223 | 0.105214141758818 | 0.549914985206981 | -0.269125941 | 0.143313495173734 | 0.939031529426534 | 0.76404702510838 | 1.15409154709039 | 0.999332001504091 |
| ebi-fl187-GCST90094775 | prot-a-1357 | Non-histone chromosomal protein HMG-14 \|\| id:prot-a-1357 | Green olives liking \|\| id:ebi-fl187-GCST90094775 | Weighted median | 27 | 0.0873158610028326 | 0.0988800916929023 | 0.377210371705287 | -0.106489119 | 0.281120840720921 | 1.09124130586163 | 0.898984830217929 | 1.32461366153413 | 0.999332001504091 |
| ebi-fl187-GCST90094775 | prot-a-137 | Apolipoprotein M \|\| id:prot-a-137 | Green olives liking \|\| id:ebi-fl187-GCST90094775 | Weighted median | 27 | -0.109726913 | 0.098294622440724 | 0.264291014627065 | -0.302384373 | 0.0829305465281956 | 0.896078808951892 | 0.739053937583613 | 1.08646634706794 | 0.999332001504091 |
| ebi-fl187-GCST90094775 | prot-a-1381 | 17-beta-hydroxysteroid dehydrogenase 14 \|\| id:prot-a-1381 | Green olives liking \|\| id:ebi-fl187-GCST90094775 | Weighted median | 26 | -0.131412793 | 0.102525867812295 | 0.199929462060874 | -0.332363494 | 0.069537907774353 | 0.876855739641338 | 0.717226567876061 | 1.072012698 | 0.999332001504091 |
| ebi-fl187-GCST90094775 | prot-a-1392 | Serine protease HTRA2, mitochondrial \|\| id:prot-a-1392 | Green olives liking \|\| id:ebi-fl187-GCST90094775 | Weighted median | 27 | -0.166466972 | 0.112329721022058 | 0.138353736745118 | -0.386633225 | 0.0536992810606856 | 0.846650779534923 | 0.679340214010652 | 1.055167246 | 0.999332001504091 |
| ebi-fl187-GCST90094775 | prot-a-1416 | Interferon-induced protein with tetratricopeptide repeats 2 \|\| id:prot-a-1416 | Green olives liking \|\| id:ebi-fl187-GCST90094775 | Weighted median | 27 | 0.00809820619745672 | 0.106209849874637 | 0.93922241281762 | -0.2000731 | 0.216269511951744 | 1.00813108536343 | 0.818670906410177 | 1.24143691600399 | 0.999332001504091 |
| ebi-fl187-GCST90094775 | prot-a-1428 | Interferon gamma \|\| id:prot-a-1428 | Green olives liking \|\| id:ebi-fl187-GCST90094775 | Weighted median | 27 | -0.103492493 | 0.100075214733377 | 0.301067398736827 | -0.299639914 | 0.09265492747939 | 0.901682791290269 | 0.741085026781124 | 1.09708309671346 | 0.999332001504091 |
| ebi-fl187-GCST90094775 | prot-a-1483 | Interleukin-17C \|\| id:prot-a-1483 | Green olives liking \|\| id:ebi-fl187-GCST90094775 | Weighted median | 27 | 0.0759819026530218 | 0.101421398734392 | 0.453754550210958 | -0.122804039 | 0.27476784417243 | 1.07894304797389 | 0.88443696084986 | 1.31622507007462 | 0.999332001504091 |
| ebi-fl187-GCST90094775 | prot-a-1503 | Interleukin-1 receptor-like 2 \|\| id:prot-a-1503 | Green olives liking \|\| id:ebi-fl187-GCST90094775 | Weighted median | 27 | 0.0645136794245242 | 0.102985878027887 | 0.531031435612159 | -0.137338642 | 0.266366000359183 | 1.06664016916854 | 0.871674990810229 | 1.30521267958642 | 0.999332001504091 |
| ebi-fl187-GCST90094775 | prot-a-1522 | Interleukin-31 receptor subunit alpha \|\| id:prot-a-1522 | Green olives liking \|\| id:ebi-fl187-GCST90094775 | Weighted median | 27 | -0.170411204 | 0.107990415671504 | 0.114560656416642 | -0.382072419 | 0.0412500103217329 | 0.843317969233853 | 0.682445629310313 | 1.04211261188887 | 0.999332001504091 |
| ebi-fl187-GCST90094775 | prot-a-1540 | Interleukin-6 receptor subunit alpha \|\| id:prot-a-1540 | Green olives liking \|\| id:ebi-fl187-GCST90094775 | Weighted median | 27 | -0.04045562 | 0.0982120537688986 | 0.680397233179577 | -0.232951246 | 0.152040005273771 | 0.960351783869073 | 0.792192195516958 | 1.16420680991267 | 0.999332001504091 |
| ebi-fl187-GCST90094775 | prot-a-1541 | Interleukin-6 receptor subunit alpha \|\| id:prot-a-1541 | Green olives liking \|\| id:ebi-fl187-GCST90094775 | Weighted median | 27 | -0.040996199 | 0.109711820937556 | 0.70864860965194 | -0.256031368 | 0.17403897037433 | 0.959832778588451 | 0.774117686119373 | 1.19010194363491 | 0.999332001504091 |
| ebi-fl187-GCST90094775 | prot-a-1545 | Interleukin-9 \|\| id:prot-a-1545 | Green olives liking \|\| id:ebi-fl187-GCST90094775 | Weighted median | 27 | 0.118180057873117 | 0.104595853083144 | 0.258529631396881 | -0.086827814 | 0.323187929916079 | 1.12544673867055 | 0.916834948029431 | 1.38152495638016 | 0.999332001504091 |
| ebi-fl187-GCST90094775 | prot-a-1548 | Inositol monophosphatase 3 \|\| id:prot-a-1548 | Green olives liking \|\| id:ebi-fl187-GCST90094775 | Weighted median | 27 | -0.168659591 | 0.105342937027861 | 0.109365252979915 | -0.375131748 | 0.0378125655321839 | 0.844796430717162 | 0.687198736030807 | 1.03853655708771 | 0.999332001504091 |
| ebi-fl187-GCST90094775 | prot-a-1555 | Inhibin beta A chain:Inhibin beta B chain heterodimer \|\| id:prot-a-1555 | Green olives liking \|\| id:ebi-fl187-GCST90094775 | Weighted median | 27 | -0.100265349 | 0.108118328671135 | 0.353736118465441 | -0.312177273 | 0.1116465749474 | 0.904597351959584 | 0.731851778843161 | 1.11811762002652 | 0.999332001504091 |
| ebi-fl187-GCST90094775 | prot-a-1603 | Junctophilin-3 \|\| id:prot-a-1603 | Green olives liking \|\| id:ebi-fl187-GCST90094775 | Weighted median | 27 | 0.0623082454598274 | 0.0940357160547871 | 0.507585543912436 | -0.122001758 | 0.24661824892721 | 1.06429035684175 | 0.885146812406476 | 1.27969049629947 | 0.999332001504091 |
| ebi-fl187-GCST90094775 | prot-a-1627 | UPF0258 protein KIAA1024 \|\| id:prot-a-1627 | Green olives liking \|\| id:ebi-fl187-GCST90094775 | Weighted median | 27 | 0.182897716220121 | 0.103283299578665 | 0.0765880884423792 | -0.019537551 | 0.385332983394305 | 1.20069159052547 | 0.980652070076426 | 1.47010376008917 | 0.999332001504091 |
| ebi-fl187-GCST90094775 | prot-a-1666 | Kallikrein-7 \|\| id:prot-a-1666 | Green olives liking \|\| id:ebi-fl187-GCST90094775 | Weighted median | 27 | 0.0664979223830427 | 0.0938007730846329 | 0.478369635227172 | -0.117351593 | 0.250347437628923 | 1.06875874360158 | 0.889272476336333 | 1.28447161294219 | 0.999332001504091 |
| ebi-fl187-GCST90094775 | prot-a-1678 | Importin subunit alpha-3 \|\| id:prot-a-1678 | Green olives liking \|\| id:ebi-fl187-GCST90094775 | Weighted median | 27 | 0.00911591960938818 | 0.106208205395075 | 0.931601038917032 | -0.199052163 | 0.217284002183735 | 1.00915759614828 | 0.819507144295525 | 1.24269698068249 | 0.999332001504091 |
| ebi-fl187-GCST90094775 | prot-a-1718 | Protein LDOC1 \|\| id:prot-a-1718 | Green olives liking \|\| id:ebi-fl187-GCST90094775 | Weighted median | 27 | 0.120619823278302 | 0.0978834998397488 | 0.21784472142579 | -0.071231836 | 0.31247148296421 | 1.12819591700033 | 0.931245970378928 | 1.36679896356307 | 0.999332001504091 |
| ebi-fl187-GCST90094775 | prot-a-1723 | Leptin \|\| id:prot-a-1723 | Green olives liking \|\| id:ebi-fl187-GCST90094775 | Weighted median | 27 | -0.225356748 | 0.106479750021651 | 0.034308404232885 | -0.434057058 | -0.016656438 | 0.798231400811119 | 0.647875288185479 | 0.983481513896646 | 0.999332001504091 |
| ebi-fl187-GCST90094775 | prot-a-1730 | Galectin-7 \|\| id:prot-a-1730 | Green olives liking \|\| id:ebi-fl187-GCST90094775 | Weighted median | 27 | -0.102678231 | 0.104157389802749 | 0.324231848425113 | -0.306826715 | 0.101470252792962 | 0.902417296482729 | 0.73577808899518 | 1.10679699378291 | 0.999332001504091 |
| ebi-fl187-GCST90094775 | prot-a-1753 | Lipase member N \|\| id:prot-a-1753 | Green olives liking \|\| id:ebi-fl187-GCST90094775 | Weighted median | 27 | 0.0323207666216915 | 0.0976656098982486 | 0.740695146175379 | -0.159103829 | 0.223745362022259 | 1.03284875558131 | 0.852907797996463 | 1.25075248979056 | 0.999332001504091 |
| ebi-fl187-GCST90094775 | prot-a-1756 | VIP36-like protein \|\| id:prot-a-1756 | Green olives liking \|\| id:ebi-fl187-GCST90094775 | Weighted median | 27 | -0.199938058 | 0.102470670708547 | 0.0510364093048926 | -0.400780573 | 0.000904456594397918 | 0.818781468473602 | 0.669797016743517 | 1.00090486573861 | 0.999332001504091 |
| ebi-fl187-GCST90094775 | prot-a-1760 | Ig Kappa chain V-I region HK102- like \|\| id:prot-a-1760 | Green olives liking \|\| id:ebi-fl187-GCST90094775 | Weighted median | 27 | -0.081199614 | 0.105308817559843 | 0.440670259806531 | -0.287604896 | 0.125205668569948 | 0.92200962718536 | 0.750057884373976 | 1.13338153005619 | 0.999332001504091 |
| ebi-fl187-GCST90094775 | prot-a-179 | Neutral ceramidase \|\| id:prot-a-179 | Green olives liking \|\| id:ebi-fl187-GCST90094775 | Weighted median | 27 | 0.0189458074967771 | 0.101354007013251 | 0.851717814998962 | -0.179708046 | 0.21759966124275 | 1.01912641810918 | 0.835514107283854 | 1.24308931116013 | 0.999332001504091 |
| ebi-fl187-GCST90094775 | prot-a-1809 | Lactotransferrin \|\| id:prot-a-1809 | Green olives liking \|\| id:ebi-fl187-GCST90094775 | Weighted median | 27 | -0.011918187 | 0.105460616313962 | 0.910022003411497 | -0.218620995 | 0.19478462075451 | 0.988152553061589 | 0.803626238647505 | 1.21504926191246 | 0.999332001504091 |
| ebi-fl187-GCST90094775 | prot-a-1818 | Lysophospholipase-like protein 1 \|\| id:prot-a-1818 | Green olives liking \|\| id:ebi-fl187-GCST90094775 | Weighted median | 27 | -0.043272775 | 0.0989780544654404 | 0.661969466798411 | -0.237269761 | 0.150724212250253 | 0.957650131898538 | 0.788778477443458 | 1.16267596207457 | 0.999332001504091 |
| ebi-fl187-GCST90094775 | prot-a-1852 | MAP kinase-activated protein kinase 3 \|\| id:prot-a-1852 | Green olives liking \|\| id:ebi-fl187-GCST90094775 | Weighted median | 27 | 0.146107322384953 | 0.105305911792427 | 0.16530274975642 | -0.060292265 | 0.352506909498109 | 1.15732038777014 | 0.941489329247028 | 1.42262948537041 | 0.999332001504091 |
| ebi-fl187-GCST90094775 | prot-a-1856 | Megakaryocyte-associated tyrosine-protein kinase \|\| id:prot-a-1856 | Green olives liking \|\| id:ebi-fl187-GCST90094775 | Weighted median | 27 | 0.132394814922667 | 0.0949737407966724 | 0.163313088903218 | -0.053753717 | 0.318543346884145 | 1.14155893480908 | 0.94766547162226 | 1.37512322719918 | 0.999332001504091 |
| ebi-fl187-GCST90094775 | prot-a-1890 | Beta-1,3-N-acetylglucosaminyltransferase manic fringe \|\| id:prot-a-1890 | Green olives liking \|\| id:ebi-fl187-GCST90094775 | Weighted median | 27 | -0.019736947 | 0.0960229077637921 | 0.837147007904209 | -0.207941846 | 0.16846795228934 | 0.980456551495578 | 0.812254271055822 | 1.18349029808246 | 0.999332001504091 |
| ebi-fl187-GCST90094775 | prot-a-1919 | Matrilysin \|\| id:prot-a-1919 | Green olives liking \|\| id:ebi-fl187-GCST90094775 | Weighted median | 27 | 0.0597630864727921 | 0.106662044690877 | 0.575272635411463 | -0.149294521 | 0.268820694066912 | 1.06158501290084 | 0.861315401960881 | 1.30842051245109 | 0.999332001504091 |
| ebi-fl187-GCST90094775 | prot-a-1923 | Multimerin-2 \|\| id:prot-a-1923 | Green olives liking \|\| id:ebi-fl187-GCST90094775 | Weighted median | 27 | -0.121671876 | 0.103270446314274 | 0.238722145290503 | -0.324081951 | 0.0807381984932297 | 0.885438854330659 | 0.723190973685356 | 1.0840870437903 | 0.999332001504091 |
| ebi-fl187-GCST90094775 | prot-a-1948 | Mesothelin \|\| id:prot-a-1948 | Green olives liking \|\| id:ebi-fl187-GCST90094775 | Weighted median | 27 | 0.15487404379945 | 0.100441897106204 | 0.123091404803381 | -0.041992075 | 0.351740162127609 | 1.16751089660684 | 0.958877380097516 | 1.42153910602947 | 0.999332001504091 |
| ebi-fl187-GCST90094775 | prot-a-195 | ATPase family AAA domain-containing protein 1 \|\| id:prot-a-195 | Green olives liking \|\| id:ebi-fl187-GCST90094775 | Weighted median | 27 | -0.019847163 | 0.0928734945964475 | 0.830780193913126 | -0.201879213 | 0.162184886267711 | 0.980348495241725 | 0.817193628715481 | 1.17607766183078 | 0.999332001504091 |
| ebi-fl187-GCST90094775 | prot-a-1966 | Mucin-1 \|\| id:prot-a-1966 | Green olives liking \|\| id:ebi-fl187-GCST90094775 | Weighted median | 27 | 0.0139774960386781 | 0.103937327083324 | 0.893022992458262 | -0.189739665 | 0.217694657121993 | 1.01407563796278 | 0.8271744483383 | 1.24320740513135 | 0.999332001504091 |
| ebi-fl187-GCST90094775 | prot-a-1986 | Myocardial zonula adherens protein \|\| id:prot-a-1986 | Green olives liking \|\| id:ebi-fl187-GCST90094775 | Weighted median | 27 | -0.076589171 | 0.106218281061988 | 0.470875973982736 | -0.284777002 | 0.131598659648106 | 0.926270313939742 | 0.752181970607164 | 1.14065043834202 | 0.999332001504091 |
| ebi-fl187-GCST90094775 | prot-a-2071 | Epididymal secretory protein E1 \|\| id:prot-a-2071 | Green olives liking \|\| id:ebi-fl187-GCST90094775 | Weighted median | 27 | -0.020867692 | 0.112649660195336 | 0.853037530088539 | -0.241661026 | 0.199925641779008 | 0.979348531443775 | 0.785322336145831 | 1.22131194020053 | 0.999332001504091 |
| ebi-fl187-GCST90094775 | prot-a-2098 | Neuritin \|\| id:prot-a-2098 | Green olives liking \|\| id:ebi-fl187-GCST90094775 | Weighted median | 27 | -0.020296869 | 0.104527671383745 | 0.846037294211742 | -0.225171105 | 0.184577366634584 | 0.9799077256236 | 0.798379600178698 | 1.20271002731269 | 0.999332001504091 |
| ebi-fl187-GCST90094775 | prot-a-2115 | Neurotrophin-4 \|\| id:prot-a-2115 | Green olives liking \|\| id:ebi-fl187-GCST90094775 | Weighted median | 27 | 0.0118741276925667 | 0.113365421200085 | 0.916580486844283 | -0.210322098 | 0.234070353244733 | 1.01194490500901 | 0.810323200562875 | 1.26373339682532 | 0.999332001504091 |
| ebi-fl187-GCST90094775 | prot-a-2118 | Netrin-1 \|\| id:prot-a-2118 | Green olives liking \|\| id:ebi-fl187-GCST90094775 | Weighted median | 27 | 0.0744090239327349 | 0.106783267164671 | 0.485913644512806 | -0.13488618 | 0.283704227575491 | 1.07724733533808 | 0.873815363946197 | 1.32804007502488 | 0.999332001504091 |
| ebi-fl187-GCST90094775 | prot-a-2120 | Netrin-G1 \|\| id:prot-a-2120 | Green olives liking \|\| id:ebi-fl187-GCST90094775 | Weighted median | 27 | -0.089670368 | 0.0989096301036767 | 0.364624257217053 | -0.283533243 | 0.104192506631428 | 0.914232495553883 | 0.753118085536991 | 1.10981408092294 | 0.999332001504091 |
| ebi-fl187-GCST90094775 | prot-a-2145 | Olfactomedin-like protein 3 \|\| id:prot-a-2145 | Green olives liking \|\| id:ebi-fl187-GCST90094775 | Weighted median | 27 | 0.0687537050805346 | 0.0999728721252315 | 0.491626845592873 | -0.127193124 | 0.264700534445988 | 1.07117235234813 | 0.880563597959732 | 1.30304070153885 | 0.999332001504091 |
| ebi-fl187-GCST90094775 | prot-a-2162 | Prolyl 4-hydroxylase subunit alpha-2 \|\| id:prot-a-2162 | Green olives liking \|\| id:ebi-fl187-GCST90094775 | Weighted median | 27 | -0.026983547 | 0.100250396941241 | 0.787805405406495 | -0.223474325 | 0.169507230732292 | 0.973377256103304 | 0.799735424589209 | 1.18472091340192 | 0.999332001504091 |
| ebi-fl187-GCST90094775 | prot-a-2168 | Polyadenylate-binding protein-interacting protein 1 \|\| id:prot-a-2168 | Green olives liking \|\| id:ebi-fl187-GCST90094775 | Weighted median | 27 | -0.015678672 | 0.104891920411481 | 0.881179194804704 | -0.221266836 | 0.189909492055466 | 0.984443598578829 | 0.801502781991283 | 1.20914015591449 | 0.999332001504091 |
| ebi-fl187-GCST90094775 | prot-a-219 | Beta-1,4-galactosyltransferase 5 \|\| id:prot-a-219 | Green olives liking \|\| id:ebi-fl187-GCST90094775 | Weighted median | 27 | 0.0325282760011451 | 0.110202847033261 | 0.767866086148027 | -0.183469304 | 0.248525856186336 | 1.03306310362449 | 0.83237742585002 | 1.28213397303565 | 0.999332001504091 |
| ebi-fl187-GCST90094775 | prot-a-2190 | Pyruvate carboxylase, mitochondrial \|\| id:prot-a-2190 | Green olives liking \|\| id:ebi-fl187-GCST90094775 | Weighted median | 27 | -0.036115908 | 0.0971116285318986 | 0.709966591593078 | -0.2264547 | 0.154222884207228 | 0.964528490710153 | 0.797355461986082 | 1.16675090815123 | 0.999332001504091 |
| ebi-fl187-GCST90094775 | prot-a-2197 | Protocadherin-9 \|\| id:prot-a-2197 | Green olives liking \|\| id:ebi-fl187-GCST90094775 | Weighted median | 27 | -0.054680839 | 0.10844796975146 | 0.614111948622145 | -0.267238859 | 0.157877181980439 | 0.946787277564518 | 0.765490205060807 | 1.17102236322779 | 0.999332001504091 |
| ebi-fl187-GCST90094775 | prot-a-2198 | Protocadherin alpha-4 \|\| id:prot-a-2198 | Green olives liking \|\| id:ebi-fl187-GCST90094775 | Weighted median | 27 | -0.012602783 | 0.106168428082433 | 0.905508631205862 | -0.220692902 | 0.195487336298376 | 0.987476299754782 | 0.801962923860384 | 1.21590339598686 | 0.999332001504091 |
| ebi-fl187-GCST90094775 | prot-a-2254 | Placenta growth factor \|\| id:prot-a-2254 | Green olives liking \|\| id:ebi-fl187-GCST90094775 | Weighted median | 27 | -0.111583422 | 0.102559132568422 | 0.276599043217512 | -0.312599322 | 0.0894324779214696 | 0.894416774332952 | 0.731542967212935 | 1.09355349181466 | 0.999332001504091 |
| ebi-fl187-GCST90094775 | prot-a-2261 | Membrane-associated progesterone receptor component 2 \|\| id:prot-a-2261 | Green olives liking \|\| id:ebi-fl187-GCST90094775 | Weighted median | 27 | -0.004948933 | 0.106608443183107 | 0.96297423187044 | -0.213901482 | 0.204003615619066 | 0.99506329277267 | 0.807427927564551 | 1.22630258729121 | 0.999332001504091 |
| ebi-fl187-GCST90094775 | prot-a-2268 | Polymeric immunoglobulin receptor \|\| id:prot-a-2268 | Green olives liking \|\| id:ebi-fl187-GCST90094775 | Weighted median | 27 | 0.0909503024325746 | 0.102730161024462 | 0.375977577411419 | -0.110400813 | 0.292301418040519 | 1.09521457440578 | 0.895475145120957 | 1.33950670828369 | 0.999332001504091 |
| ebi-fl187-GCST90094775 | prot-a-2283 | Group 10 secretory phospholipase A2 \|\| id:prot-a-2283 | Green olives liking \|\| id:ebi-fl187-GCST90094775 | Weighted median | 27 | 0.216894424836789 | 0.104240507573907 | 0.0374603191062881 | 0.0125830299919315 | 0.421205819681646 | 1.24221294837983 | 1.01266252941174 | 1.52379787372887 | 0.999332001504091 |
| ebi-fl187-GCST90094775 | prot-a-2298 | Inactive phospholipase D5 \|\| id:prot-a-2298 | Green olives liking \|\| id:ebi-fl187-GCST90094775 | Weighted median | 27 | 0.157888676028702 | 0.10841595779336 | 0.14530329135209 | -0.054606601 | 0.370383953303687 | 1.1710358230927 | 0.946857567280947 | 1.44829058387777 | 0.999332001504091 |
| ebi-fl187-GCST90094775 | prot-a-2304 | Procollagen-lysine,2-oxoglutarate 5-dioxygenase 2 \|\| id:prot-a-2304 | Green olives liking \|\| id:ebi-fl187-GCST90094775 | Weighted median | 27 | -0.030712062 | 0.106165682708438 | 0.772363848184681 | -0.2387968 | 0.177372676016735 | 0.969754762037198 | 0.787574901176376 | 1.19407601370884 | 0.999332001504091 |
| ebi-fl187-GCST90094775 | prot-a-2331 | Periostin \|\| id:prot-a-2331 | Green olives liking \|\| id:ebi-fl187-GCST90094775 | Weighted median | 27 | -0.036690334 | 0.106454557231214 | 0.73035206804721 | -0.245341266 | 0.171960598290717 | 0.96397459940617 | 0.782437473324059 | 1.18763103759912 | 0.999332001504091 |
| ebi-fl187-GCST90094775 | prot-a-2333 | Periostin \|\| id:prot-a-2333 | Green olives liking \|\| id:ebi-fl187-GCST90094775 | Weighted median | 27 | -0.155935954 | 0.106121480058276 | 0.14172164132574 | -0.363934055 | 0.0520621465812032 | 0.855613986984732 | 0.694937020681917 | 1.0534412082487 | 0.999332001504091 |
| ebi-fl187-GCST90094775 | prot-a-2367 | cAMP-dependent protein kinase catalytic subunit alpha \|\| id:prot-a-2367 | Green olives liking \|\| id:ebi-fl187-GCST90094775 | Weighted median | 27 | 0.0575795823025707 | 0.110585820799688 | 0.602591239293254 | -0.159168626 | 0.274327791069959 | 1.0592695664116 | 0.852852533335361 | 1.31564598857163 | 0.999332001504091 |
| ebi-fl187-GCST90094775 | prot-a-2385 | Phosphoribosyl pyrophosphate synthase-associated protein 1 \|\| id:prot-a-2385 | Green olives liking \|\| id:ebi-fl187-GCST90094775 | Weighted median | 27 | 0.0141557640798847 | 0.103854192645057 | 0.891580792833258 | -0.189398454 | 0.217709981664196 | 1.01425643135476 | 0.827456737963341 | 1.24322645686168 | 0.999332001504091 |
| ebi-fl187-GCST90094775 | prot-a-2393 | Trypsin-3 \|\| id:prot-a-2393 | Green olives liking \|\| id:ebi-fl187-GCST90094775 | Weighted median | 26 | -0.016526591 | 0.107130091091703 | 0.877399556720944 | -0.22650157 | 0.193448387074006 | 0.983609223431953 | 0.797318090518241 | 1.21342675642987 | 0.999332001504091 |
| ebi-fl187-GCST90094775 | prot-a-2405 | Pregnancy-specific beta-1-glycoprotein 6 \|\| id:prot-a-2405 | Green olives liking \|\| id:ebi-fl187-GCST90094775 | Weighted median | 27 | 0.0628099937602704 | 0.106005725684553 | 0.553505708478749 | -0.144961229 | 0.270581216101994 | 1.06482449671015 | 0.865055831902289 | 1.31072604446889 | 0.999332001504091 |
| ebi-fl187-GCST90094775 | prot-a-244 | Beclin-1 \|\| id:prot-a-244 | Green olives liking \|\| id:ebi-fl187-GCST90094775 | Weighted median | 27 | -0.094404713 | 0.103407149377584 | 0.36127313162154 | -0.297082726 | 0.108273299722203 | 0.909914433459302 | 0.742982545965865 | 1.11435225593523 | 0.999332001504091 |
| ebi-fl187-GCST90094775 | prot-a-2454 | tRNA pseudouridine synthase A, mitochondrial \|\| id:prot-a-2454 | Green olives liking \|\| id:ebi-fl187-GCST90094775 | Weighted median | 27 | 0.14957243524892 | 0.104434682309916 | 0.15208362413164 | -0.055119542 | 0.354264412576355 | 1.16133758954276 | 0.946372009914128 | 1.42513195947899 | 0.999332001504091 |
| ebi-fl187-GCST90094775 | prot-a-2466 | Glutaminyl-peptide cyclotransferase-like protein \|\| id:prot-a-2466 | Green olives liking \|\| id:ebi-fl187-GCST90094775 | Weighted median | 27 | 0.0425969700121931 | 0.0965385133266406 | 0.659036885600377 | -0.146618516 | 0.231812456132409 | 1.04351724134726 | 0.863623372988303 | 1.260883236 | 0.999332001504091 |
| ebi-fl187-GCST90094775 | prot-a-2474 | Ras-related protein Rab-27A \|\| id:prot-a-2474 | Green olives liking \|\| id:ebi-fl187-GCST90094775 | Weighted median | 27 | -0.028552684 | 0.107740924333965 | 0.790999888203678 | -0.239724896 | 0.18261952779008 | 0.971851091887276 | 0.786844295622718 | 1.20035761847267 | 0.999332001504091 |
| ebi-fl187-GCST90094775 | prot-a-2490 | Rap1 GTPase-GDP dissociation stimulator 1 \|\| id:prot-a-2490 | Green olives liking \|\| id:ebi-fl187-GCST90094775 | Weighted median | 27 | 0.158224520345378 | 0.10929201127436 | 0.147694632358284 | -0.055987822 | 0.372436862443122 | 1.17142917486729 | 0.945550650970256 | 1.45126684680738 | 0.999332001504091 |
| ebi-fl187-GCST90094775 | prot-a-2545 | Receptor-interacting serine/threonine-protein kinase 2 \|\| id:prot-a-2545 | Green olives liking \|\| id:ebi-fl187-GCST90094775 | Weighted median | 27 | 0.107163694394218 | 0.106858306669769 | 0.315929439788841 | -0.102278587 | 0.316605975466965 | 1.11311645048974 | 0.902778014704514 | 1.37246168179719 | 0.999332001504091 |
| ebi-fl187-GCST90094775 | prot-a-2610 | RUN and FYVE domain-containing protein 1 \|\| id:prot-a-2610 | Green olives liking \|\| id:ebi-fl187-GCST90094775 | Weighted median | 27 | 0.00936638596280088 | 0.102058728255671 | 0.926877226340746 | -0.190668721 | 0.209401493343916 | 1.00941038782799 | 0.826406313520454 | 1.23293991634036 | 0.999332001504091 |
| ebi-fl187-GCST90094775 | prot-a-2627 | Serine--tRNA ligase, mitochondrial \|\| id:prot-a-2627 | Green olives liking \|\| id:ebi-fl187-GCST90094775 | Weighted median | 27 | -0.000578237 | 0.0983055494375086 | 0.995306837186936 | -0.193257114 | 0.192100639603534 | 0.999421929852983 | 0.824270015372892 | 1.21179246514165 | 0.999364451047625 |
| ebi-fl187-GCST90094775 | prot-a-264 | BolA-like protein 2 \|\| id:prot-a-264 | Green olives liking \|\| id:ebi-fl187-GCST90094775 | Weighted median | 27 | 0.105624536753504 | 0.102084001621042 | 0.300817180474409 | -0.094460106 | 0.305709179930746 | 1.11140450661368 | 0.909864031632151 | 1.35758743546046 | 0.999332001504091 |
| ebi-fl187-GCST90094775 | prot-a-2646 | Secretoglobin family 1C member 1 \|\| id:prot-a-2646 | Green olives liking \|\| id:ebi-fl187-GCST90094775 | Weighted median | 27 | -0.019323804 | 0.110453646885681 | 0.861119359032756 | -0.235812952 | 0.197165343840175 | 0.980861703818204 | 0.789928414683332 | 1.21794540382857 | 0.999332001504091 |
| ebi-fl187-GCST90094775 | prot-a-2672 | Semaphorin-3E \|\| id:prot-a-2672 | Green olives liking \|\| id:ebi-fl187-GCST90094775 | Weighted median | 27 | -0.005209232 | 0.104212780865696 | 0.960133144969416 | -0.209466283 | 0.199047818150371 | 0.994804312175367 | 0.811016984158954 | 1.22024031413964 | 0.999332001504091 |
| ebi-fl187-GCST90094775 | prot-a-2701 | Plasma protease C1 inhibitor \|\| id:prot-a-2701 | Green olives liking \|\| id:ebi-fl187-GCST90094775 | Weighted median | 27 | 0.0169942308378554 | 0.102475650442344 | 0.86828540875531 | -0.183858044 | 0.21784650570485 | 1.01713945426588 | 0.832053910464171 | 1.24339619874768 | 0.999332001504091 |
| ebi-fl187-GCST90094775 | prot-a-2738 | Signaling threshold-regulating transmembrane adapter 1 \|\| id:prot-a-2738 | Green olives liking \|\| id:ebi-fl187-GCST90094775 | Weighted median | 27 | -0.153595346 | 0.101170964097886 | 0.128969994259361 | -0.351890436 | 0.0446997432646314 | 0.85761898944532 | 0.703357180381607 | 1.04571383014553 | 0.999332001504091 |
| ebi-fl187-GCST90094775 | prot-a-274 | Serine/threonine-protein kinase BRSK2 \|\| id:prot-a-274 | Green olives liking \|\| id:ebi-fl187-GCST90094775 | Weighted median | 27 | -0.11052571 | 0.0999562164373464 | 0.268838538506081 | -0.306439894 | 0.0853884744119187 | 0.895363310277206 | 0.736062758621413 | 1.0891400875817 | 0.999332001504091 |
| ebi-fl187-GCST90094775 | prot-a-2749 | Mitochondrial glutamate carrier 2 \|\| id:prot-a-2749 | Green olives liking \|\| id:ebi-fl187-GCST90094775 | Weighted median | 27 | 0.172080579882982 | 0.10628541636349 | 0.105438172433179 | -0.036238836 | 0.380399995955422 | 1.1877735400107 | 0.964409929981896 | 1.46286961435167 | 0.999332001504091 |
| ebi-fl187-GCST90094775 | prot-a-2760 | Sodium-coupled monocarboxylate transporter 1 \|\| id:prot-a-2760 | Green olives liking \|\| id:ebi-fl187-GCST90094775 | Weighted median | 27 | -0.111546894 | 0.102314277614823 | 0.275608401872242 | -0.312082878 | 0.0889890901435352 | 0.89444944612399 | 0.731920865499049 | 1.09306873103832 | 0.999332001504091 |
| ebi-fl187-GCST90094775 | prot-a-2777 | Small integral membrane protein 10 \|\| id:prot-a-2777 | Green olives liking \|\| id:ebi-fl187-GCST90094775 | Weighted median | 27 | 0.145248249858118 | 0.106008375630291 | 0.170637509485009 | -0.062528166 | 0.353024666093489 | 1.15632659255247 | 0.939386603323388 | 1.42336625188564 | 0.999332001504091 |
| ebi-fl187-GCST90094775 | prot-a-2799 | Superoxide dismutase [Mn], mitochondrial \|\| id:prot-a-2799 | Green olives liking \|\| id:ebi-fl187-GCST90094775 | Weighted median | 27 | -0.052139325 | 0.102899157021577 | 0.612363130903758 | -0.253821673 | 0.149543022361835 | 0.949196610431905 | 0.77583014104663 | 1.16130343175371 | 0.999332001504091 |
| ebi-fl187-GCST90094775 | prot-a-2805 | Protein SPACA7 \|\| id:prot-a-2805 | Green olives liking \|\| id:ebi-fl187-GCST90094775 | Weighted median | 27 | -0.015714595 | 0.105211127305529 | 0.881267619177871 | -0.221928404 | 0.190499215017744 | 0.984408235489552 | 0.800972708707912 | 1.20985342392363 | 0.999332001504091 |
| ebi-fl187-GCST90094775 | prot-a-2811 | SPARC-like protein 1 \|\| id:prot-a-2811 | Green olives liking \|\| id:ebi-fl187-GCST90094775 | Weighted median | 27 | 0.142562875463164 | 0.102582692145466 | 0.164609013682185 | -0.058499201 | 0.343624952068278 | 1.15322558827088 | 0.943178993865499 | 1.41004970010216 | 0.999332001504091 |
| ebi-fl187-GCST90094775 | prot-a-2831 | Sepiapterin reductase \|\| id:prot-a-2831 | Green olives liking \|\| id:ebi-fl187-GCST90094775 | Weighted median | 27 | 0.0183102350730293 | 0.0987002836393555 | 0.852826292543492 | -0.175142321 | 0.211762791006166 | 1.01847889525715 | 0.839337557025281 | 1.23585469445755 | 0.999332001504091 |
| ebi-fl187-GCST90094775 | prot-a-2834 | SPRY domain-containing SOCS box protein 1 \|\| id:prot-a-2834 | Green olives liking \|\| id:ebi-fl187-GCST90094775 | Weighted median | 27 | 0.11655051164014 | 0.109020876770634 | 0.285039934911224 | -0.097130407 | 0.330231430110583 | 1.12361426463321 | 0.907437662347777 | 1.3912900776245 | 0.999332001504091 |
| ebi-fl187-GCST90094775 | prot-a-2835 | Spectrin alpha chain, non-erythrocytic 1 \|\| id:prot-a-2835 | Green olives liking \|\| id:ebi-fl187-GCST90094775 | Weighted median | 27 | 0.0384656521576777 | 0.10359013680331 | 0.710395158897661 | -0.164571016 | 0.241502320292165 | 1.03921503295087 | 0.848257514969282 | 1.27316040901822 | 0.999332001504091 |
| ebi-fl187-GCST90094775 | prot-a-2859 | Sia-alpha-2,3-Gal-beta-1,4-GlcNAc-R:alpha 2,8-sialyltransferase \|\| id:prot-a-2859 | Green olives liking \|\| id:ebi-fl187-GCST90094775 | Weighted median | 27 | 0.0924561749668824 | 0.102042816415514 | 0.364907930017005 | -0.107547745 | 0.29246009514129 | 1.09686507035892 | 0.898033644624478 | 1.33971927418885 | 0.999332001504091 |
| ebi-fl187-GCST90094775 | prot-a-2861 | Alpha-2,8-sialyltransferase 8F \|\| id:prot-a-2861 | Green olives liking \|\| id:ebi-fl187-GCST90094775 | Weighted median | 27 | 0.0108679169365458 | 0.101163287677113 | 0.914448287416528 | -0.187412127 | 0.209147960783688 | 1.01092718726622 | 0.829101970731063 | 1.23262736554934 | 0.999332001504091 |
| ebi-fl187-GCST90094775 | prot-a-289 | Uncharacterized protein C17orf89 \|\| id:prot-a-289 | Green olives liking \|\| id:ebi-fl187-GCST90094775 | Weighted median | 27 | 0.17150373904266 | 0.103790117057115 | 0.098451171632884 | -0.03192489 | 0.374932368474604 | 1.18708858129884 | 0.968579328963513 | 1.4548930146569 | 0.999332001504091 |
| ebi-fl187-GCST90094775 | prot-a-2921 | Tachykinin-3 \|\| id:prot-a-2921 | Green olives liking \|\| id:ebi-fl187-GCST90094775 | Weighted median | 27 | 0.0302170004349739 | 0.0951660237715952 | 0.750849986441078 | -0.156308406 | 0.2167424070273 | 1.03067816729899 | 0.855295371332528 | 1.24202412424118 | 0.999332001504091 |
| ebi-fl187-GCST90094775 | prot-a-2928 | Tax1-binding protein 1 \|\| id:prot-a-2928 | Green olives liking \|\| id:ebi-fl187-GCST90094775 | Weighted median | 27 | -0.083337681 | 0.102980025727975 | 0.418365241746243 | -0.285178532 | 0.118503169013095 | 0.920040414210932 | 0.751880007814422 | 1.12581044180436 | 0.999332001504091 |
| ebi-fl187-GCST90094775 | prot-a-296 | Uncharacterized protein C1orf185 \|\| id:prot-a-296 | Green olives liking \|\| id:ebi-fl187-GCST90094775 | Weighted median | 27 | 0.0222421566786558 | 0.107380825464295 | 0.835905669492535 | -0.188224261 | 0.232708574588674 | 1.0224913576046 | 0.828428901913355 | 1.26201364288644 | 0.999332001504091 |
| ebi-fl187-GCST90094775 | prot-a-2964 | Transforming growth factor beta-2 \|\| id:prot-a-2964 | Green olives liking \|\| id:ebi-fl187-GCST90094775 | Weighted median | 27 | -0.04340545 | 0.103898699689433 | 0.676117466178704 | -0.247046902 | 0.160236001265689 | 0.957523083498407 | 0.78110405770709 | 1.17378785372552 | 0.999332001504091 |
| ebi-fl187-GCST90094775 | prot-a-2977 | Thymocyte nuclear protein 1 \|\| id:prot-a-2977 | Green olives liking \|\| id:ebi-fl187-GCST90094775 | Weighted median | 27 | 0.172176144157576 | 0.10144036008025 | 0.0896373102602618 | -0.026646962 | 0.370999249914866 | 1.18788705415129 | 0.973704936085065 | 1.44918198637637 | 0.999332001504091 |
| ebi-fl187-GCST90094775 | prot-a-3015 | Transmembrane protein 70, mitochondrial \|\| id:prot-a-3015 | Green olives liking \|\| id:ebi-fl187-GCST90094775 | Weighted median | 27 | -0.030717061 | 0.102208206119308 | 0.763769717657022 | -0.231045145 | 0.169611023084934 | 0.969749914422621 | 0.793703633553069 | 1.18484388475438 | 0.999332001504091 |
| ebi-fl187-GCST90094775 | prot-a-3030 | Tumor necrosis factor alpha-induced protein 3 \|\| id:prot-a-3030 | Green olives liking \|\| id:ebi-fl187-GCST90094775 | Weighted median | 27 | -0.024857436 | 0.110905143745206 | 0.822654280291296 | -0.242231518 | 0.192516645432684 | 0.975448965722084 | 0.784874443915743 | 1.21229668274233 | 0.999332001504091 |
| ebi-fl187-GCST90094775 | prot-a-3032 | Tumor necrosis factor receptor superfamily member 10A \|\| id:prot-a-3032 | Green olives liking \|\| id:ebi-fl187-GCST90094775 | Weighted median | 27 | 0.131061064876847 | 0.101700049152311 | 0.19750167910675 | -0.068271031 | 0.330393161215376 | 1.14003739542963 | 0.934007293906846 | 1.39151511070277 | 0.999332001504091 |
| ebi-fl187-GCST90094775 | prot-a-3081 | Tumor protein p53-inducible protein 11 \|\| id:prot-a-3081 | Green olives liking \|\| id:ebi-fl187-GCST90094775 | Weighted median | 27 | -0.020109861 | 0.102558662350146 | 0.844546339863821 | -0.221124839 | 0.180905117113724 | 0.980090993524595 | 0.80161660078901 | 1.19830147559638 | 0.999332001504091 |
| ebi-fl187-GCST90094775 | prot-a-315 | Complement C4 \|\| id:prot-a-315 | Green olives liking \|\| id:ebi-fl187-GCST90094775 | Weighted median | 26 | 0.00213353994725658 | 0.110157150234555 | 0.984547420156392 | -0.213774475 | 0.218041554406984 | 1.00213581756312 | 0.807530483193937 | 1.2436387452159 | 0.999332001504091 |
| ebi-fl187-GCST90094775 | prot-a-3157 | UDP-glucuronosyltransferase 1-8 \|\| id:prot-a-3157 | Green olives liking \|\| id:ebi-fl187-GCST90094775 | Weighted median | 27 | 0.159535040800663 | 0.0973590501762001 | 0.101291238607142 | -0.031288698 | 0.350358779146015 | 1.17296536314609 | 0.969195728255642 | 1.41957677178035 | 0.999332001504091 |
| ebi-fl187-GCST90094775 | prot-a-317 | Glutaredoxin-like protein C5orf63 \|\| id:prot-a-317 | Green olives liking \|\| id:ebi-fl187-GCST90094775 | Weighted median | 27 | -0.029883817 | 0.102749921131302 | 0.771173468480126 | -0.231273662 | 0.171506028399532 | 0.970558289354665 | 0.793522279079385 | 1.18709129897135 | 0.999332001504091 |
| ebi-fl187-GCST90094775 | prot-a-3192 | Guanine nucleotide exchange factor VAV3 \|\| id:prot-a-3192 | Green olives liking \|\| id:ebi-fl187-GCST90094775 | Weighted median | 27 | -0.012247844 | 0.0963006471801107 | 0.898795243748021 | -0.200997112 | 0.176501424765143 | 0.987826855849358 | 0.817914793540419 | 1.19303612655467 | 0.999332001504091 |
| ebi-fl187-GCST90094775 | prot-a-3233 | Serine/threonine-protein kinase WNK3 \|\| id:prot-a-3233 | Green olives liking \|\| id:ebi-fl187-GCST90094775 | Weighted median | 27 | -0.064828493 | 0.105678583368075 | 0.539579045344173 | -0.271958517 | 0.142301530236736 | 0.937228190635036 | 0.761885866076243 | 1.15292423764837 | 0.999332001504091 |
| ebi-fl187-GCST90094775 | prot-a-3250 | 14-3-3 protein theta \|\| id:prot-a-3250 | Green olives liking \|\| id:ebi-fl187-GCST90094775 | Weighted median | 27 | 0.0495402210983396 | 0.103403296613802 | 0.63186954199483 | -0.15313024 | 0.252210682461391 | 1.05078785520655 | 0.858017966046977 | 1.28686712906093 | 0.999332001504091 |
| ebi-fl187-GCST90094775 | prot-a-3278 | E3 ubiquitin-protein ligase ZNRF3 \|\| id:prot-a-3278 | Green olives liking \|\| id:ebi-fl187-GCST90094775 | Weighted median | 27 | 0.217616377886236 | 0.105282334189936 | 0.0387361291723631 | 0.0112630028739611 | 0.423969752898511 | 1.24311009161465 | 1.01132666929133 | 1.52801537504894 | 0.999332001504091 |
| ebi-fl187-GCST90094775 | prot-a-355 | Calpain-3 \|\| id:prot-a-355 | Green olives liking \|\| id:ebi-fl187-GCST90094775 | Weighted median | 27 | -0.002907469 | 0.0965297231284689 | 0.975971399897179 | -0.192105727 | 0.186290788077524 | 0.99709675334111 | 0.825219616226536 | 1.20477254293778 | 0.999332001504091 |
| ebi-fl187-GCST90094775 | prot-a-385 | Coiled-coil domain-containing protein 90B, mitochondrial \|\| id:prot-a-385 | Green olives liking \|\| id:ebi-fl187-GCST90094775 | Weighted median | 27 | 0.127010462097083 | 0.100481055086276 | 0.206221423852899 | -0.069932406 | 0.323953330066183 | 1.1354288966867 | 0.932456846383228 | 1.3825827805669 | 0.999332001504091 |
| ebi-fl187-GCST90094775 | prot-a-390 | C-C motif chemokine 14 \|\| id:prot-a-390 | Green olives liking \|\| id:ebi-fl187-GCST90094775 | Weighted median | 27 | -0.042539443 | 0.105336787914941 | 0.68632874506223 | -0.248999547 | 0.163920661687503 | 0.958352664829948 | 0.779580326582013 | 1.17812084126528 | 0.999332001504091 |
| ebi-fl187-GCST90094775 | prot-a-40 | Pituitary adenylate cyclase-activating polypeptide 27 \|\| id:prot-a-40 | Green olives liking \|\| id:ebi-fl187-GCST90094775 | Weighted median | 27 | 0.0241768687418019 | 0.105928689268264 | 0.819461833545907 | -0.183443362 | 0.231797099707599 | 1.02447149885254 | 0.83239901963204 | 1.26086387322406 | 0.999332001504091 |
| ebi-fl187-GCST90094775 | prot-a-401 | C-C motif chemokine 24 \|\| id:prot-a-401 | Green olives liking \|\| id:ebi-fl187-GCST90094775 | Weighted median | 27 | 0.162697185812593 | 0.100129452019118 | 0.104190595640018 | -0.03355654 | 0.358950911770065 | 1.17668032023932 | 0.967000235352321 | 1.43182651401738 | 0.999332001504091 |
| ebi-fl187-GCST90094775 | prot-a-412 | Cerebral cavernous malformations 2 protein \|\| id:prot-a-412 | Green olives liking \|\| id:ebi-fl187-GCST90094775 | Weighted median | 27 | -0.13328967 | 0.101769698626371 | 0.190291039699811 | -0.332758279 | 0.0661789393232281 | 0.875211532875169 | 0.716943473296134 | 1.06841788203476 | 0.999332001504091 |
| ebi-fl187-GCST90094775 | prot-a-44 | Adhesion G protein-coupled receptor F5 \|\| id:prot-a-44 | Green olives liking \|\| id:ebi-fl187-GCST90094775 | Weighted median | 27 | -0.046784639 | 0.0982479975068649 | 0.633939568494015 | -0.239350714 | 0.145781436400558 | 0.954292893207431 | 0.787138773506793 | 1.15694329472432 | 0.999332001504091 |
| ebi-fl187-GCST90094775 | prot-a-48 | Adrenomedullin \|\| id:prot-a-48 | Green olives liking \|\| id:ebi-fl187-GCST90094775 | Weighted median | 27 | -0.067579651 | 0.100715091338044 | 0.50222163593668 | -0.26498123 | 0.129821927654616 | 0.934653271239456 | 0.767220350267073 | 1.13862560753833 | 0.999332001504091 |
| ebi-fl187-GCST90094775 | prot-a-502 | Carcinoembryonic antigen-related cell adhesion molecule 1 \|\| id:prot-a-502 | Green olives liking \|\| id:ebi-fl187-GCST90094775 | Weighted median | 27 | 0.0427769195410203 | 0.0988187722157994 | 0.665100116641914 | -0.150907874 | 0.236461713083987 | 1.04370503867968 | 0.85992691663558 | 1.26675905439413 | 0.999332001504091 |
| ebi-fl187-GCST90094775 | prot-a-518 | Complement factor B \|\| id:prot-a-518 | Green olives liking \|\| id:ebi-fl187-GCST90094775 | Weighted median | 27 | 0.115452703033985 | 0.106312218947133 | 0.277488841663446 | -0.092919246 | 0.323824652170365 | 1.12238142805662 | 0.911267085693876 | 1.38240488416982 | 0.999332001504091 |
| ebi-fl187-GCST90094775 | prot-a-525 | Properdin \|\| id:prot-a-525 | Green olives liking \|\| id:ebi-fl187-GCST90094775 | Weighted median | 27 | 0.0287011242049313 | 0.107839125433819 | 0.790125538581553 | -0.182663562 | 0.240065810055217 | 1.02911697035399 | 0.833048378021328 | 1.27133281405111 | 0.999332001504091 |
| ebi-fl187-GCST90094775 | prot-a-533 | Chondroadherin \|\| id:prot-a-533 | Green olives liking \|\| id:ebi-fl187-GCST90094775 | Weighted median | 27 | -0.117999964 | 0.101332942523798 | 0.244230425878973 | -0.316612532 | 0.080612603115552 | 0.888696084402306 | 0.728613014974136 | 1.08395089601855 | 0.999332001504091 |
| ebi-fl187-GCST90094775 | prot-a-535 | Coiled-coil-helix-coiled-coil-helix domain-containing protein 2 \|\| id:prot-a-535 | Green olives liking \|\| id:ebi-fl187-GCST90094775 | Weighted median | 27 | -0.011925892 | 0.105486409382837 | 0.909986002024146 | -0.218679255 | 0.19482747011381 | 0.988144939320464 | 0.803579421172003 | 1.21510132711032 | 0.999332001504091 |
| ebi-fl187-GCST90094775 | prot-a-591 | Caseinolytic peptidase B protein homolog \|\| id:prot-a-591 | Green olives liking \|\| id:ebi-fl187-GCST90094775 | Weighted median | 27 | 0.0681574958675768 | 0.111669694447899 | 0.541630587804741 | -0.150715105 | 0.287030096985459 | 1.07053389986753 | 0.860092699652182 | 1.33246431603133 | 0.999332001504091 |
| ebi-fl187-GCST90094775 | prot-a-605 | Ciliary neurotrophic factor receptor subunit alpha \|\| id:prot-a-605 | Green olives liking \|\| id:ebi-fl187-GCST90094775 | Weighted median | 27 | -0.211088426 | 0.102387547140702 | 0.0392400100246133 | -0.411768018 | -0.010408833 | 0.809702465162253 | 0.662477941084263 | 0.989645151077473 | 0.999332001504091 |
| ebi-fl187-GCST90094775 | prot-a-606 | Ciliary neurotrophic factor receptor subunit alpha \|\| id:prot-a-606 | Green olives liking \|\| id:ebi-fl187-GCST90094775 | Weighted median | 27 | -0.227730982 | 0.100876787856799 | 0.0239758389827107 | -0.425449486 | -0.030012478 | 0.796338460549783 | 0.653475990788228 | 0.970433424777972 | 0.999332001504091 |
| ebi-fl187-GCST90094775 | prot-a-707 | Cancer/testis antigen 55 \|\| id:prot-a-707 | Green olives liking \|\| id:ebi-fl187-GCST90094775 | Weighted median | 27 | -0.001996382 | 0.104126448742706 | 0.984703363043479 | -0.206084221 | 0.202091457922276 | 0.998005609830897 | 0.813764537212294 | 1.22395994382599 | 0.999332001504091 |
| ebi-fl187-GCST90094775 | prot-a-719 | Dipeptidyl peptidase 1 \|\| id:prot-a-719 | Green olives liking \|\| id:ebi-fl187-GCST90094775 | Weighted median | 27 | -0.081076364 | 0.103913259119815 | 0.435254818864115 | -0.284746352 | 0.122593624206049 | 0.922123272039772 | 0.75220502576758 | 1.13042495025827 | 0.999332001504091 |
| ebi-fl187-GCST90094775 | prot-a-752 | Cytochrome b5 \|\| id:prot-a-752 | Green olives liking \|\| id:ebi-fl187-GCST90094775 | Weighted median | 27 | -0.232732945 | 0.106487024658855 | 0.0288483874471345 | -0.441447513 | -0.024018376 | 0.792365150733165 | 0.643104844488858 | 0.976267769519608 | 0.999332001504091 |
| ebi-fl187-GCST90094775 | prot-a-755 | Cytoglobin \|\| id:prot-a-755 | Green olives liking \|\| id:ebi-fl187-GCST90094775 | Weighted median | 27 | -0.100433851 | 0.104784405383568 | 0.337820319451178 | -0.305811285 | 0.104943583627688 | 0.904444938630913 | 0.736525599419904 | 1.11064794985994 | 0.999332001504091 |
| ebi-fl187-GCST90094775 | prot-a-807 | Desmin \|\| id:prot-a-807 | Green olives liking \|\| id:ebi-fl187-GCST90094775 | Weighted median | 27 | -0.092134758 | 0.099583668933197 | 0.354862156860546 | -0.287318749 | 0.103049232952971 | 0.911982244218406 | 0.750272541897362 | 1.10854598472487 | 0.999332001504091 |
| ebi-fl187-GCST90094775 | prot-a-82 | Ameloblastin \|\| id:prot-a-82 | Green olives liking \|\| id:ebi-fl187-GCST90094775 | Weighted median | 27 | -0.041319403 | 0.107357758626766 | 0.70032943540133 | -0.25174061 | 0.169101803555011 | 0.959522606259907 | 0.777446373527939 | 1.18424069269997 | 0.999332001504091 |
| ebi-fl187-GCST90094775 | prot-a-84 | Muellerian-inhibiting factor \|\| id:prot-a-84 | Green olives liking \|\| id:ebi-fl187-GCST90094775 | Weighted median | 27 | 0.0522586800616617 | 0.0989714365194847 | 0.597486982355132 | -0.141725336 | 0.246242695639852 | 1.05364826506195 | 0.867859593960097 | 1.27920999455944 | 0.999332001504091 |
| ebi-fl187-GCST90094775 | prot-a-858 | Inactive dipeptidyl peptidase 10 \|\| id:prot-a-858 | Green olives liking \|\| id:ebi-fl187-GCST90094775 | Weighted median | 27 | 0.196614693823921 | 0.101999039885525 | 0.0539032580250964 | -0.003303424 | 0.39653281199955 | 1.21727492678819 | 0.996702025951311 | 1.48666121750171 | 0.999332001504091 |
| ebi-fl187-GCST90094775 | prot-a-863 | Dihydropyrimidinase-related protein 3 \|\| id:prot-a-863 | Green olives liking \|\| id:ebi-fl187-GCST90094775 | Weighted median | 27 | -0.097163342 | 0.102969970412663 | 0.345369736217311 | -0.298984484 | 0.104657800508069 | 0.907407776679556 | 0.741570915923761 | 1.11033059077412 | 0.999332001504091 |
| ebi-fl187-GCST90094775 | prot-a-864 | Dihydropyrimidinase-related protein 5 \|\| id:prot-a-864 | Green olives liking \|\| id:ebi-fl187-GCST90094775 | Weighted median | 27 | -0.180560667 | 0.102503296640083 | 0.0781519826116214 | -0.381467128 | 0.0203457948793475 | 0.834802034613667 | 0.682858832659001 | 1.0205541814279 | 0.999332001504091 |
| ebi-fl187-GCST90094775 | prot-a-884 | Endothelin-converting enzyme 1 \|\| id:prot-a-884 | Green olives liking \|\| id:ebi-fl187-GCST90094775 | Weighted median | 27 | -0.121918064 | 0.100964355006601 | 0.227225901193058 | -0.3198082 | 0.0759720716929609 | 0.885220896884449 | 0.726288325865088 | 1.07893244098002 | 0.999332001504091 |
| ebi-fl187-GCST90094775 | prot-a-893 | Early endosome antigen 1 \|\| id:prot-a-893 | Green olives liking \|\| id:ebi-fl187-GCST90094775 | Weighted median | 27 | -0.213897235 | 0.104147182794009 | 0.0399952841229328 | -0.418025714 | -0.009768757 | 0.807431356138327 | 0.658345299744463 | 0.990278802215846 | 0.999332001504091 |
| ebi-fl187-GCST90094775 | prot-a-897 | Ephrin-A2 \|\| id:prot-a-897 | Green olives liking \|\| id:ebi-fl187-GCST90094775 | Weighted median | 27 | 0.0781379779222162 | 0.100663642140912 | 0.437614139991683 | -0.119162761 | 0.275438716518404 | 1.08127184001982 | 0.887663312324777 | 1.31710838533797 | 0.999332001504091 |
| ebi-fl187-GCST90094775 | prot-a-90 | Anaphase-promoting complex subunit 10 \|\| id:prot-a-90 | Green olives liking \|\| id:ebi-fl187-GCST90094775 | Weighted median | 27 | 0.082166378266906 | 0.110173183607398 | 0.455792528691325 | -0.133773062 | 0.298105818137406 | 1.08563642111047 | 0.874788565192958 | 1.34730434957341 | 0.999332001504091 |
| ebi-fl187-GCST90094775 | prot-a-914 | Histone-lysine N-methyltransferase EHMT2 \|\| id:prot-a-914 | Green olives liking \|\| id:ebi-fl187-GCST90094775 | Weighted median | 27 | -0.057803768 | 0.108405917693459 | 0.593884080936104 | -0.270279366 | 0.154671831043323 | 0.943835140266726 | 0.763166261606953 | 1.16727483487878 | 0.999332001504091 |
| ebi-fl187-GCST90094775 | prot-a-932 | RNA polymerase II elongation factor ELL \|\| id:prot-a-932 | Green olives liking \|\| id:ebi-fl187-GCST90094775 | Weighted median | 27 | -0.098032712 | 0.103010766088259 | 0.341262119340751 | -0.299933814 | 0.10386838906725 | 0.906619245517821 | 0.740867254100062 | 1.10945442897425 | 0.999332001504091 |
| ebi-fl187-GCST90094775 | prot-a-991 | Estrogen receptor \|\| id:prot-a-991 | Green olives liking \|\| id:ebi-fl187-GCST90094775 | Weighted median | 27 | 0.116795474933239 | 0.113766787422448 | 0.304598539873815 | -0.106187428 | 0.339778378281237 | 1.12388954259877 | 0.899256086137082 | 1.40463625816409 | 0.999332001504091 |
| ebi-fl187-GCST90094775 | prot-a-992 | Persulfide dioxygenase ETHE1, mitochondrial \|\| id:prot-a-992 | Green olives liking \|\| id:ebi-fl187-GCST90094775 | Weighted median | 27 | 0.00865376666174581 | 0.106744452376527 | 0.935386329056453 | -0.20056536 | 0.217872893319739 | 1.00869131874462 | 0.818268006284082 | 1.24342900944062 | 0.999332001504091 |
| ebi-fl187-GCST90094775 | prot-a-993 | Ethanolamine kinase 1 \|\| id:prot-a-993 | Green olives liking \|\| id:ebi-fl187-GCST90094775 | Weighted median | 27 | 0.175341403504423 | 0.0968128162441673 | 0.070119439651428 | -0.014411716 | 0.365094523342991 | 1.1916529816769 | 0.985691635362225 | 1.44065017678435 | 0.999332001504091 |
| ebi-fl187-GCST90094775 | prot-b-10 | coagulation factor III, tissue factor \|\| id:prot-b-10 | Green olives liking \|\| id:ebi-fl187-GCST90094775 | Weighted median | 20 | 0.149709718195859 | 0.153332484985417 | 0.328879592336498 | -0.150821952 | 0.450241388767276 | 1.16149703233358 | 0.86000080612914 | 1.56869080413063 | 0.999332001504091 |
| ebi-fl187-GCST90094775 | prot-b-28 | prolactin \|\| id:prot-b-28 | Green olives liking \|\| id:ebi-fl187-GCST90094775 | Weighted median | 20 | 0.0272216318547786 | 0.146804051037767 | 0.85289307978065 | -0.260514308 | 0.314957571888801 | 1.02759552542952 | 0.770655129625435 | 1.3702011746759 | 0.999332001504091 |
| ebi-fl187-GCST90094775 | prot-b-54 | matrix metallopeptidase 7 \|\| id:prot-b-54 | Green olives liking \|\| id:ebi-fl187-GCST90094775 | Weighted median | 20 | -0.007408695 | 0.150158480936908 | 0.960649002931271 | -0.301719318 | 0.286901927547646 | 0.992618681642428 | 0.739545613105746 | 1.33229354577304 | 0.999332001504091 |
| ebi-fl187-GCST90094775 | prot-c-2421_7_3 | BDNF \|\| id:prot-c-2421_7_3 | Green olives liking \|\| id:ebi-fl187-GCST90094775 | Weighted median | 3 | -0.038679836 | 0.480419984340787 | 0.935829618538597 | -0.980303005 | 0.902943333325053 | 0.962058676414253 | 0.375197394830069 | 2.46685320745135 | 0.999332001504091 |
| ebi-fl187-GCST90094775 | prot-c-2443_10_4 | FGF-8B \|\| id:prot-c-2443_10_4 | Green olives liking \|\| id:ebi-fl187-GCST90094775 | Weighted median | 3 | -0.289082514 | 0.540331317964504 | 0.592643127660966 | -1.348131897 | 0.7699668695621 | 0.748950404222714 | 0.259725000822493 | 2.15969470096846 | 0.999332001504091 |
| ebi-fl187-GCST90094775 | prot-c-2605_49_2 | CD30 \|\| id:prot-c-2605_49_2 | Green olives liking \|\| id:ebi-fl187-GCST90094775 | Weighted median | 3 | 0.127039611922881 | 0.493399708766424 | 0.796809731152002 | -0.840023817 | 1.09410304110507 | 1.13546199472364 | 0.431700241390042 | 2.98650271148893 | 0.999332001504091 |
| ebi-fl187-GCST90094775 | prot-c-2624_31_2 | HPV E7 Type18 \|\| id:prot-c-2624_31_2 | Green olives liking \|\| id:ebi-fl187-GCST90094775 | Weighted median | 3 | 0.127540860800399 | 0.45463828135049 | 0.779068915128114 | -0.763550171 | 1.01863189224736 | 1.13603128644046 | 0.466009075070192 | 2.7694033288454 | 0.999332001504091 |
| ebi-fl187-GCST90094775 | prot-c-2774_10_3 | IL-16 \|\| id:prot-c-2774_10_3 | Green olives liking \|\| id:ebi-fl187-GCST90094775 | Weighted median | 3 | -0.36986957 | 0.522440199443823 | 0.478966762425067 | -1.39385236 | 0.654113221331209 | 0.690824429282792 | 0.24811762262198 | 1.9234360987772 | 0.999332001504091 |
| ebi-fl187-GCST90094775 | prot-c-2851_63_3 | C5a \|\| id:prot-c-2851_63_3 | Green olives liking \|\| id:ebi-fl187-GCST90094775 | Weighted median | 3 | -0.342691258 | 0.412781471840549 | 0.406425606100556 | -1.151742942 | 0.466360427184671 | 0.709857340774091 | 0.316085370386654 | 1.59418148215612 | 0.999332001504091 |
| ebi-fl187-GCST90094775 | prot-c-2891_1_3 | CD22 \|\| id:prot-c-2891_1_3 | Green olives liking \|\| id:ebi-fl187-GCST90094775 | Weighted median | 3 | 0.290345682382368 | 0.556179345277936 | 0.601645213300807 | -0.799765834 | 1.38045719912712 | 1.33688954732167 | 0.449434193840714 | 3.97671936455147 | 0.999332001504091 |
| ebi-fl187-GCST90094775 | prot-c-2961_1_2 | Protein C \|\| id:prot-c-2961_1_2 | Green olives liking \|\| id:ebi-fl187-GCST90094775 | Weighted median | 3 | -0.067631747 | 0.46108286408185 | 0.883384412670377 | -0.971354161 | 0.836090666442819 | 0.934604581007524 | 0.378570046144344 | 2.3073292029745 | 0.999332001504091 |
| ebi-fl187-GCST90094775 | prot-c-2982_82_2 | Galectin-4 \|\| id:prot-c-2982_82_2 | Green olives liking \|\| id:ebi-fl187-GCST90094775 | Weighted median | 3 | -0.660475969 | 0.454558970893229 | 0.14622266399876 | -1.551411552 | 0.230459613714228 | 0.516605387692835 | 0.211948586085474 | 1.25917861271147 | 0.999332001504091 |
| ebi-fl187-GCST90094775 | prot-c-2994_71_2 | IL-1Rrp2 \|\| id:prot-c-2994_71_2 | Green olives liking \|\| id:ebi-fl187-GCST90094775 | Weighted median | 3 | -0.187666193 | 0.473619224177001 | 0.691929057919787 | -1.115959872 | 0.740627486678047 | 0.828891351033779 | 0.327600673188365 | 2.09725109882041 | 0.999332001504091 |
| ebi-fl187-GCST90094775 | prot-c-3336_50_1 | TFPI \|\| id:prot-c-3336_50_1 | Green olives liking \|\| id:ebi-fl187-GCST90094775 | Weighted median | 3 | 0.385132648909692 | 0.493677300729936 | 0.43531414957312 | -0.582474861 | 1.35274015834037 | 1.46980927710859 | 0.558514409459016 | 3.86800998235125 | 0.999332001504091 |
| ebi-fl187-GCST90094775 | prot-c-3472_40_2 | Survivin \|\| id:prot-c-3472_40_2 | Green olives liking \|\| id:ebi-fl187-GCST90094775 | Weighted median | 3 | 0.182160236618908 | 0.497934628427694 | 0.714490716362695 | -0.793791635 | 1.15811210833719 | 1.19980643140362 | 0.452127239656696 | 3.18391670877992 | 0.999332001504091 |
| ebi-fl187-GCST90094775 | prot-c-3473_78_2 | Thrombopoietin Receptor \|\| id:prot-c-3473_78_2 | Green olives liking \|\| id:ebi-fl187-GCST90094775 | Weighted median | 3 | 0.372463700501389 | 0.527880017209103 | 0.480446869613868 | -0.662181133 | 1.40710853423123 | 1.45130579651424 | 0.515725241397836 | 4.08412919500873 | 0.999332001504091 |
| ebi-fl187-GCST90094775 | prot-c-3497_13_2 | IFN-aA \|\| id:prot-c-3497_13_2 | Green olives liking \|\| id:ebi-fl187-GCST90094775 | Weighted median | 3 | -0.400108177 | 0.472054277434484 | 0.396666709353354 | -1.32533456 | 0.525118207136956 | 0.670247536990895 | 0.265714046817051 | 1.69065868449049 | 0.999332001504091 |
| ebi-fl187-GCST90094775 | prot-c-3665_64_3 | COLEC12 \|\| id:prot-c-3665_64_3 | Green olives liking \|\| id:ebi-fl187-GCST90094775 | Weighted median | 3 | 0.00211058205744297 | 0.529679841235431 | 0.996820727823465 | -1.036061907 | 1.04028307087889 | 1.00211281090353 | 0.354849363842219 | 2.83001799666041 | 0.999364451047625 |
| ebi-fl187-GCST90094775 | prot-c-3741_4_3 | IL-5 \|\| id:prot-c-3741_4_3 | Green olives liking \|\| id:ebi-fl187-GCST90094775 | Weighted median | 3 | 0.153873949332721 | 0.546901960061922 | 0.778437640832131 | -0.918053892 | 1.22580179105409 | 1.16634385909045 | 0.399295357175952 | 3.40689660721137 | 0.999332001504091 |
| ebi-fl187-GCST90094775 | prot-c-3848_14_2 | GAPDH, liver \|\| id:prot-c-3848_14_2 | Green olives liking \|\| id:ebi-fl187-GCST90094775 | Weighted median | 3 | 0.498405109889248 | 0.503874974579102 | 0.322592496436242 | -0.48918984 | 1.48600006006429 | 1.64609383724087 | 0.613122920514502 | 4.41938285185715 | 0.999332001504091 |
| ebi-fl187-GCST90094775 | prot-c-3859_50_2 | PSA1 \|\| id:prot-c-3859_50_2 | Green olives liking \|\| id:ebi-fl187-GCST90094775 | Weighted median | 3 | -0.407804372 | 0.576866098797409 | 0.479609526290786 | -1.538461926 | 0.722853181585025 | 0.665108979997306 | 0.214711089197114 | 2.06030325181268 | 0.999332001504091 |
| ebi-fl187-GCST90094775 | prot-c-3868_8_1 | SGTA \|\| id:prot-c-3868_8_1 | Green olives liking \|\| id:ebi-fl187-GCST90094775 | Weighted median | 3 | 0.836270351714761 | 0.518140244221658 | 0.106530776357134 | -0.179284527 | 1.85182523038921 | 2.30774383330016 | 0.835868038568016 | 6.37143825867385 | 0.999332001504091 |
| ebi-fl187-GCST90094775 | prot-c-3882_10_1 | EF-1-beta \|\| id:prot-c-3882_10_1 | Green olives liking \|\| id:ebi-fl187-GCST90094775 | Weighted median | 3 | 0.275463079049307 | 0.488523085898459 | 0.572843211987418 | -0.682042169 | 1.23296832741029 | 1.31714047382259 | 0.505583450384062 | 3.43139995279457 | 0.999332001504091 |
| ebi-fl187-GCST90094775 | prot-c-4134_4_2 | HB-EGF \|\| id:prot-c-4134_4_2 | Green olives liking \|\| id:ebi-fl187-GCST90094775 | Weighted median | 3 | -0.09131658 | 0.458602073029266 | 0.842169327815481 | -0.990176643 | 0.80754348281107 | 0.912728713202346 | 0.371511060225128 | 2.24239273899191 | 0.999332001504091 |
| ebi-fl187-GCST90094775 | prot-c-4220_39_2 | FER \|\| id:prot-c-4220_39_2 | Green olives liking \|\| id:ebi-fl187-GCST90094775 | Weighted median | 3 | 0.833251309862096 | 0.494225452982392 | 0.0918007893954704 | -0.135430578 | 1.80193319770758 | 2.30078716460312 | 0.873339789833428 | 6.0613539408437 | 0.999332001504091 |
| ebi-fl187-GCST90094775 | prot-c-4228_37_2 | HSP70 protein 8 \|\| id:prot-c-4228_37_2 | Green olives liking \|\| id:ebi-fl187-GCST90094775 | Weighted median | 3 | 0.095147422851886 | 0.519963687106874 | 0.854806969965512 | -0.923981404 | 1.11427624958136 | 1.09982098192093 | 0.39693553021896 | 3.04736185144794 | 0.999332001504091 |
| ebi-fl187-GCST90094775 | prot-c-4246_40_2 | NCAM-L1 \|\| id:prot-c-4246_40_2 | Green olives liking \|\| id:ebi-fl187-GCST90094775 | Weighted median | 3 | -0.127002892 | 0.504717819659003 | 0.801325924323971 | -1.116249818 | 0.8622440348904 | 0.880731125835025 | 0.327505700427475 | 2.36846966328271 | 0.999332001504091 |
| ebi-fl187-GCST90094775 | prot-c-4267_81_3 | PESC \|\| id:prot-c-4267_81_3 | Green olives liking \|\| id:ebi-fl187-GCST90094775 | Weighted median | 3 | -0.248031 | 0.507369707896075 | 0.624943265342986 | -1.242475628 | 0.746413627127013 | 0.780335752222185 | 0.288668696364828 | 2.10942126342161 | 0.999332001504091 |
| ebi-fl187-GCST90094775 | prot-c-4280_47_2 | PSA2 \|\| id:prot-c-4280_47_2 | Green olives liking \|\| id:ebi-fl187-GCST90094775 | Weighted median | 3 | 0.0372755326342959 | 0.485536324126458 | 0.938805027396368 | -0.914375663 | 0.988926727922154 | 1.03797897852331 | 0.400766761654718 | 2.68834759501469 | 0.999332001504091 |
| ebi-fl187-GCST90094775 | prot-c-4324_33_2 | CYTT \|\| id:prot-c-4324_33_2 | Green olives liking \|\| id:ebi-fl187-GCST90094775 | Weighted median | 3 | -0.511275603 | 0.539002479226726 | 0.342844999200532 | -1.567720462 | 0.545169256630524 | 0.599730073402469 | 0.208519970211667 | 1.72490030848473 | 0.999332001504091 |
| ebi-fl187-GCST90094775 | prot-c-4462_8_2 | RASA1 \|\| id:prot-c-4462_8_2 | Green olives liking \|\| id:ebi-fl187-GCST90094775 | Weighted median | 3 | 0.0637967550698539 | 0.471648193394795 | 0.89240359344455 | -0.860633704 | 0.988227214123651 | 1.06587574290424 | 0.422894007769256 | 2.68646771635399 | 0.999332001504091 |
| ebi-fl187-GCST90094775 | prot-c-4499_21_1 | PDGF-AA \|\| id:prot-c-4499_21_1 | Green olives liking \|\| id:ebi-fl187-GCST90094775 | Weighted median | 3 | 0.0188250386422025 | 0.483129271783224 | 0.968918447550625 | -0.928108334 | 0.965758411337322 | 1.01900334681074 | 0.395300780570658 | 2.62677908025504 | 0.999332001504091 |
| ebi-fl187-GCST90094775 | prot-c-4535_50_2 | BST1 \|\| id:prot-c-4535_50_2 | Green olives liking \|\| id:ebi-fl187-GCST90094775 | Weighted median | 3 | 0.143989869847431 | 0.525631406852646 | 0.784133067352969 | -0.886247688 | 1.17422742727862 | 1.15487240943195 | 0.412199556036941 | 3.23564220905574 | 0.999332001504091 |
| ebi-fl187-GCST90094775 | prot-c-4542_24_2 | Clusterin \|\| id:prot-c-4542_24_2 | Green olives liking \|\| id:ebi-fl187-GCST90094775 | Weighted median | 3 | 0.37993180423891 | 0.485900470600564 | 0.43426581386019 | -0.572433118 | 1.33229672661602 | 1.46218487122394 | 0.564151121113553 | 3.78973739060571 | 0.999332001504091 |
| ebi-fl187-GCST90094775 | prot-c-4696_2_2 | FABP \|\| id:prot-c-4696_2_2 | Green olives liking \|\| id:ebi-fl187-GCST90094775 | Weighted median | 3 | -0.875784719 | 0.504991102196964 | 0.0828723651418214 | -1.865567279 | 0.113997841260485 | 0.416535028427205 | 0.154808365381938 | 1.12074970547487 | 0.999332001504091 |
| ebi-fl187-GCST90094775 | prot-c-4906_35_1 | Coagulation Factor V \|\| id:prot-c-4906_35_1 | Green olives liking \|\| id:ebi-fl187-GCST90094775 | Weighted median | 3 | 0.291219222539199 | 0.483844637029807 | 0.547250148681138 | -0.657116266 | 1.23955471111762 | 1.33805788424682 | 0.518343947352473 | 3.45407506104747 | 0.999332001504091 |
| ebi-fl187-GCST90094775 | prot-c-4916_2_1 | IgD \|\| id:prot-c-4916_2_1 | Green olives liking \|\| id:ebi-fl187-GCST90094775 | Weighted median | 3 | -0.781175694 | 0.51322970545279 | 0.127989513127002 | -1.787105917 | 0.224754528706374 | 0.457867382810018 | 0.167444066191773 | 1.25201534464167 | 0.999332001504091 |
| ebi-fl187-GCST90094775 | prot-c-4922_13_1 | MIP-3b \|\| id:prot-c-4922_13_1 | Green olives liking \|\| id:ebi-fl187-GCST90094775 | Weighted median | 3 | 0.590892211549403 | 0.488214116209722 | 0.226158559824602 | -0.366007456 | 1.54779187932046 | 1.80559867293543 | 0.693497630318432 | 4.7010781654859 | 0.999332001504091 |
| ebi-fl187-GCST90094775 | prot-c-4973_18_1 | cIAP-2 \|\| id:prot-c-4973_18_1 | Green olives liking \|\| id:ebi-fl187-GCST90094775 | Weighted median | 3 | -0.528236895 | 0.574338376032238 | 0.357713270404516 | -1.653940112 | 0.597466322118281 | 0.589643657488211 | 0.19129469926672 | 1.81750798191909 | 0.999332001504091 |
| ebi-fl187-GCST90094775 | prot-c-4997_19_1 | IF4A3 \|\| id:prot-c-4997_19_1 | Green olives liking \|\| id:ebi-fl187-GCST90094775 | Weighted median | 3 | -0.548290046 | 0.52373193561454 | 0.295150033026254 | -1.574804639 | 0.478224548120351 | 0.577937212164156 | 0.207047997733053 | 1.61320768546971 | 0.999332001504091 |
| ebi-fl187-GCST90094775 | prot-c-5000_52_1 | LG3BP \|\| id:prot-c-5000_52_1 | Green olives liking \|\| id:ebi-fl187-GCST90094775 | Weighted median | 3 | -0.394106038 | 0.451874201266042 | 0.383121780043926 | -1.279779473 | 0.491567396452969 | 0.674282552856933 | 0.278098622082517 | 1.63487671273811 | 0.999332001504091 |
| ebi-fl187-GCST90094775 | prot-c-5006_71_1 | MK13 \|\| id:prot-c-5006_71_1 | Green olives liking \|\| id:ebi-fl187-GCST90094775 | Weighted median | 3 | -0.561243702 | 0.477763003363054 | 0.240101773859878 | -1.497659189 | 0.375171784605345 | 0.570499091590016 | 0.223653077562692 | 1.45524138121373 | 0.999332001504091 |
| ebi-fl187-GCST90094775 | prot-c-5109_24_3 | Nr-CAM \|\| id:prot-c-5109_24_3 | Green olives liking \|\| id:ebi-fl187-GCST90094775 | Weighted median | 3 | 0.0722526423715771 | 0.462827254939004 | 0.875945158742482 | -0.834888777 | 0.979394062052024 | 1.0749268818381 | 0.433922740804148 | 2.66284223582487 | 0.999332001504091 |
| ebi-fl187-GCST90094775 | prot-c-5255_22_3 | PDE4D \|\| id:prot-c-5255_22_3 | Green olives liking \|\| id:ebi-fl187-GCST90094775 | Weighted median | 3 | 0.0630585052592726 | 0.464004688000018 | 0.89189987859689 | -0.846390683 | 0.972507693739307 | 1.06508915072542 | 0.42896039518976 | 2.64456791749078 | 0.999332001504091 |
| ebi-fl187-GCST90094775 | prot-c-5440_26_3 | Troponin I, skeletal, fast twitch \|\| id:prot-c-5440_26_3 | Green olives liking \|\| id:ebi-fl187-GCST90094775 | Weighted median | 3 | -0.376969616 | 0.501578857354113 | 0.452312095291769 | -1.360064177 | 0.606124944019895 | 0.685936914816254 | 0.256644305812632 | 1.83331342426566 | 0.999332001504091 |
| ebi-fl187-GCST90094775 | ukb-rsfMRI191-edge-pheno716 | Net100_Pair10_33 (OccipitalPrecuneus)&(Temporal\|Frontal\|Supp_Motor_Area) (Default_mode\|Central_executive)&(Default_mode\|Salience) \|\| id:ukb-rsfMRI191-edge-pheno716 | Green olives liking \|\| id:ebi-fl187-GCST90094775 | Weighted median | 24 | -0.028845079 | 0.0361051212687801 | 0.424337189706579 | -0.099611117 | 0.041920958392601 | 0.971566968648704 | 0.905189362370927 | 1.04281204995251 | 0.999332001504091 |
| ebi-fl187-GCST90094775 | ukb-rsfMRI191-edge-pheno903 | Net100_Pair27_38 (Precentral\|Frontal\|Supp_Motor_Area)&(Subcortical) (Attention\|Salience\|Motor)&(Subcortical-cerebellum) \|\| id:ukb-rsfMRI191-edge-pheno903 | Green olives liking \|\| id:ebi-fl187-GCST90094775 | Weighted median | 24 | 0.057506314526503 | 0.0347518598741284 | 0.097971282077894 | -0.010607331 | 0.125619959879795 | 1.05919195892931 | 0.98944872851842 | 1.13385117745353 | 0.999332001504091 |
| ebi-fl187-GCST90094775 | ukb-rsfMRI191-node-pheno23 | Net100_Node2 RolandicOper\|SupraMarginal\|Insula Salience\|Motor \|\| id:ukb-rsfMRI191-node-pheno23 | Green olives liking \|\| id:ebi-fl187-GCST90094775 | Weighted median | 24 | 0.0575707261796942 | 0.0336312363960016 | 0.0869292800726947 | -0.008346497 | 0.123487949515857 | 1.0592601854317 | 0.99168823814438 | 1.13143637010389 | 0.999332001504091 |
| ebi-fl187-GCST90094775 | ukb-rsfMRI191-node-pheno55 | Net100_Node34 Parietal\|Postcentral\|Precuneus Attention \|\| id:ukb-rsfMRI191-node-pheno55 | Green olives liking \|\| id:ebi-fl187-GCST90094775 | Weighted median | 24 | 0.056348676417476 | 0.0356911854822112 | 0.114385585099336 | -0.013606047 | 0.12630339996261 | 1.05796650740443 | 0.986486096753493 | 1.13462636166196 | 0.999332001504091 |
| ebi-fl187-GCST90094775 | ebi-a-GCST90001414 | IgD- CD24- B cell Absolute Count \|\| id:ebi-a-GCST90001414 | Green olives liking \|\| id:ebi-fl187-GCST90094775 | Weighted mode | 25 | -0.127964904 | 0.196411917953603 | 0.520905906812179 | -0.512932263 | 0.257002455045051 | 0.879884258894002 | 0.59873734670441 | 1.29304830124729 | 0.999714303421225 |
| ebi-fl187-GCST90094775 | ebi-a-GCST90001422 | CD20- CD38- B cell %B cell \|\| id:ebi-a-GCST90001422 | Green olives liking \|\| id:ebi-fl187-GCST90094775 | Weighted mode | 25 | -0.132045171 | 0.16324903750942 | 0.426543401374001 | -0.452013285 | 0.187922942406521 | 0.876301410676228 | 0.636345716051288 | 1.2067405232461 | 0.999714303421225 |
| ebi-fl187-GCST90094775 | ebi-a-GCST90001444 | CD20- CD38- B cell %lymphocyte \|\| id:ebi-a-GCST90001444 | Green olives liking \|\| id:ebi-fl187-GCST90094775 | Weighted mode | 25 | -0.161096723 | 0.17077625266389 | 0.354917009750476 | -0.495818179 | 0.173624731758242 | 0.851209735171997 | 0.609072373346183 | 1.18960905954564 | 0.999714303421225 |
| ebi-fl187-GCST90094775 | ebi-a-GCST90001459 | Myeloid Dendritic Cell %Dendritic Cell \|\| id:ebi-a-GCST90001459 | Green olives liking \|\| id:ebi-fl187-GCST90094775 | Weighted mode | 25 | 0.140837862530963 | 0.213109273018112 | 0.51498897712034 | -0.276856313 | 0.558532037646464 | 1.15123797404036 | 0.758163427827777 | 1.74810446432352 | 0.999714303421225 |
| ebi-fl187-GCST90094775 | ebi-a-GCST90001476 | HLA DR++ monocyte %leukocyte \|\| id:ebi-a-GCST90001476 | Green olives liking \|\| id:ebi-fl187-GCST90094775 | Weighted mode | 25 | -0.064645763 | 0.167293564530727 | 0.702589548168786 | -0.39254115 | 0.263249623299332 | 0.93739946597522 | 0.675338555828091 | 1.30115147614096 | 0.999714303421225 |
| ebi-fl187-GCST90094775 | ebi-a-GCST90001478 | CD4 regulatory T cell %CD4+ T cell \|\| id:ebi-a-GCST90001478 | Green olives liking \|\| id:ebi-fl187-GCST90094775 | Weighted mode | 25 | 0.185508346254539 | 0.216471319503878 | 0.399940648790552 | -0.23877544 | 0.60979213248214 | 1.20383024720687 | 0.787591724134937 | 1.84004887263374 | 0.999714303421225 |
| ebi-fl187-GCST90094775 | ebi-a-GCST90001485 | CD39+ resting CD4 regulatory T cell %CD4 regulatory T cell \|\| id:ebi-a-GCST90001485 | Green olives liking \|\| id:ebi-fl187-GCST90094775 | Weighted mode | 24 | -0.047801551 | 0.196235278330887 | 0.809705601543244 | -0.432422696 | 0.336819594805979 | 0.953322954558309 | 0.648935016305083 | 1.40048638592885 | 0.999714303421225 |
| ebi-fl187-GCST90094775 | ebi-a-GCST90001515 | Immature Myeloid-Derived Suppressor Cells Absolute Count \|\| id:ebi-a-GCST90001515 | Green olives liking \|\| id:ebi-fl187-GCST90094775 | Weighted mode | 25 | 0.103170839753783 | 0.334586134607801 | 0.760473811649795 | -0.552617984 | 0.758959663585072 | 1.10868079965266 | 0.575441340400152 | 2.13605285060632 | 0.999714303421225 |
| ebi-fl187-GCST90094775 | ebi-a-GCST90001517 | CD33+ HLA DR+ Absolute Count \|\| id:ebi-a-GCST90001517 | Green olives liking \|\| id:ebi-fl187-GCST90094775 | Weighted mode | 25 | -0.212852368 | 0.24188628655639 | 0.387602911153423 | -0.68694949 | 0.261244753669057 | 0.808275455745716 | 0.503108468176448 | 1.29854545 | 0.999714303421225 |
| ebi-fl187-GCST90094775 | ebi-a-GCST90001518 | CD33+ HLA DR+ CD14- Absolute Count \|\| id:ebi-a-GCST90001518 | Green olives liking \|\| id:ebi-fl187-GCST90094775 | Weighted mode | 25 | -0.108532835 | 0.24329317330832 | 0.659524543333957 | -0.585387454 | 0.36832178487809 | 0.897149436605448 | 0.556890050455557 | 1.44530704210473 | 0.999714303421225 |
| ebi-fl187-GCST90094775 | ebi-a-GCST90001520 | CD33+ HLA DR+ CD14dim Absolute Count \|\| id:ebi-a-GCST90001520 | Green olives liking \|\| id:ebi-fl187-GCST90094775 | Weighted mode | 25 | 0.0390164515274996 | 0.243723873048608 | 0.874154127757941 | -0.43868234 | 0.516715242702772 | 1.03978758960357 | 0.644885601683533 | 1.67651166140342 | 0.999714303421225 |
| ebi-fl187-GCST90094775 | ebi-a-GCST90001531 | CD33dim HLA DR- Absolute Count \|\| id:ebi-a-GCST90001531 | Green olives liking \|\| id:ebi-fl187-GCST90094775 | Weighted mode | 25 | -0.046573772 | 0.262588260379496 | 0.860710688344848 | -0.561246763 | 0.468099217988532 | 0.954494142691548 | 0.570497345458787 | 1.5969558415733 | 0.999714303421225 |
| ebi-fl187-GCST90094775 | ebi-a-GCST90001532 | Basophil Absolute Count \|\| id:ebi-a-GCST90001532 | Green olives liking \|\| id:ebi-fl187-GCST90094775 | Weighted mode | 25 | -0.080847797 | 0.261599816141246 | 0.759949425366952 | -0.593583436 | 0.431887843003535 | 0.922334063111439 | 0.552344443052066 | 1.54016236548878 | 0.999714303421225 |
| ebi-fl187-GCST90094775 | ebi-a-GCST90001588 | T/B cell \|\| id:ebi-a-GCST90001588 | Green olives liking \|\| id:ebi-fl187-GCST90094775 | Weighted mode | 25 | -0.04974252 | 0.194538699578546 | 0.800365307085785 | -0.431038371 | 0.331553331350434 | 0.95147437875489 | 0.649833975518529 | 1.39313044182497 | 0.999714303421225 |
| ebi-fl187-GCST90094775 | ebi-a-GCST90001624 | HLA DR+ CD4+ T cell Absolute Count \|\| id:ebi-a-GCST90001624 | Green olives liking \|\| id:ebi-fl187-GCST90094775 | Weighted mode | 25 | -0.08431664 | 0.178644212550861 | 0.641203604152253 | -0.434459297 | 0.265826016389747 | 0.919140173275864 | 0.647614739782725 | 1.30450807591721 | 0.999714303421225 |
| ebi-fl187-GCST90094775 | ebi-a-GCST90001631 | CD8+ Natural Killer T %T cell \|\| id:ebi-a-GCST90001631 | Green olives liking \|\| id:ebi-fl187-GCST90094775 | Weighted mode | 25 | -0.014434498 | 0.157594197547996 | 0.927781772472977 | -0.323319125 | 0.294450129278806 | 0.985669180004059 | 0.723742852993613 | 1.34238801584193 | 0.999714303421225 |
| ebi-fl187-GCST90094775 | ebi-a-GCST90001632 | CD8+ Natural Killer T %lymphocyte \|\| id:ebi-a-GCST90001632 | Green olives liking \|\| id:ebi-fl187-GCST90094775 | Weighted mode | 25 | -0.026927935 | 0.157386613494895 | 0.86558403518726 | -0.335405697 | 0.281549827780924 | 0.973431389651915 | 0.715047934612612 | 1.32518202555612 | 0.999714303421225 |
| ebi-fl187-GCST90094775 | ebi-a-GCST90001646 | Natural Killer %CD3- lymphocyte \|\| id:ebi-a-GCST90001646 | Green olives liking \|\| id:ebi-fl187-GCST90094775 | Weighted mode | 25 | -0.14694689 | 0.182107704213037 | 0.427635541452614 | -0.50387799 | 0.209984210130185 | 0.863339828067054 | 0.604183094505806 | 1.23365858049457 | 0.999714303421225 |
| ebi-fl187-GCST90094775 | ebi-a-GCST90001684 | CD127- CD8+ T cell Absolute Count \|\| id:ebi-a-GCST90001684 | Green olives liking \|\| id:ebi-fl187-GCST90094775 | Weighted mode | 25 | -0.011528657 | 0.193531160214585 | 0.952991346731933 | -0.390849731 | 0.367792416904207 | 0.988537543206579 | 0.676481802553276 | 1.444542145 | 0.999714303421225 |
| ebi-fl187-GCST90094775 | ebi-a-GCST90001687 | CD28- CD8+ T cell Absolute Count \|\| id:ebi-a-GCST90001687 | Green olives liking \|\| id:ebi-fl187-GCST90094775 | Weighted mode | 25 | -0.080823286 | 0.182335546258132 | 0.661542904949133 | -0.438200956 | 0.27655438488288 | 0.922356670580601 | 0.645196113508908 | 1.31857866151388 | 0.999714303421225 |
| ebi-fl187-GCST90094775 | ebi-a-GCST90001701 | BAFF-R on CD20- CD38- B cell \|\| id:ebi-a-GCST90001701 | Green olives liking \|\| id:ebi-fl187-GCST90094775 | Weighted mode | 25 | 0.0148386652681147 | 0.190527212129896 | 0.938567501134573 | -0.358594671 | 0.38827200104271 | 1.01494930483203 | 0.698657480450105 | 1.47443077645913 | 0.999714303421225 |
| ebi-fl187-GCST90094775 | ebi-a-GCST90001732 | CD19 on IgD- CD27- B cell \|\| id:ebi-a-GCST90001732 | Green olives liking \|\| id:ebi-fl187-GCST90094775 | Weighted mode | 25 | -0.249652849 | 0.183229877945554 | 0.185686622143732 | -0.60878341 | 0.10947771202549 | 0.779071191672091 | 0.544012306836386 | 1.11569520407176 | 0.999714303421225 |
| ebi-fl187-GCST90094775 | ebi-a-GCST90001798 | CD27 on CD24+ CD27+ B cell \|\| id:ebi-a-GCST90001798 | Green olives liking \|\| id:ebi-fl187-GCST90094775 | Weighted mode | 25 | -0.100472357 | 0.170726562211424 | 0.561695738311484 | -0.435096419 | 0.234151705173031 | 0.904410112891801 | 0.647202261671602 | 1.2638362081559 | 0.999714303421225 |
| ebi-fl187-GCST90094775 | ebi-a-GCST90001836 | CD66b on CD66b++ myeloid cell \|\| id:ebi-a-GCST90001836 | Green olives liking \|\| id:ebi-fl187-GCST90094775 | Weighted mode | 25 | -0.29549553 | 0.28857112776255 | 0.316048621126849 | -0.86109494 | 0.270103880776666 | 0.744162741392573 | 0.422698998775733 | 1.31010053792609 | 0.999714303421225 |
| ebi-fl187-GCST90094775 | ebi-a-GCST90001943 | CD25 on activated & secreting CD4 regulatory T cell \|\| id:ebi-a-GCST90001943 | Green olives liking \|\| id:ebi-fl187-GCST90094775 | Weighted mode | 25 | -0.103638037 | 0.191253611818618 | 0.59289159871548 | -0.478495116 | 0.271219042373238 | 0.901551566866869 | 0.619715290082874 | 1.31156232664747 | 0.999714303421225 |
| ebi-fl187-GCST90094775 | ebi-a-GCST90001960 | CD25 on CD4+ T cell \|\| id:ebi-a-GCST90001960 | Green olives liking \|\| id:ebi-fl187-GCST90094775 | Weighted mode | 25 | -0.049422929 | 0.233711509760392 | 0.834306148495373 | -0.507497488 | 0.408651630108169 | 0.951778509810184 | 0.602000208003527 | 1.50478740653722 | 0.999714303421225 |
| ebi-fl187-GCST90094775 | ebi-a-GCST90001988 | HLA DR on CD14+ CD16- monocyte \|\| id:ebi-a-GCST90001988 | Green olives liking \|\| id:ebi-fl187-GCST90094775 | Weighted mode | 24 | 0.0105144601162144 | 0.175950155424459 | 0.952864443685266 | -0.334347845 | 0.355376764748155 | 1.01056993129802 | 0.715804750160826 | 1.42671809011359 | 0.999714303421225 |
| ebi-fl187-GCST90094775 | ebi-a-GCST90001991 | HLA DR on CD14+ monocyte \|\| id:ebi-a-GCST90001991 | Green olives liking \|\| id:ebi-fl187-GCST90094775 | Weighted mode | 24 | 0.00099004857420848 | 0.176298411640793 | 0.99556772524412 | -0.344554838 | 0.346534935390163 | 1.00099053883408 | 0.708535696269651 | 1.41415889716022 | 0.999714303421225 |
| ebi-fl187-GCST90094775 | ebi-a-GCST90002019 | CD14 on Monocytic Myeloid-Derived Suppressor Cells \|\| id:ebi-a-GCST90002019 | Green olives liking \|\| id:ebi-fl187-GCST90094775 | Weighted mode | 25 | -0.049775262 | 0.295598597418236 | 0.867689067599985 | -0.629148513 | 0.529597988597498 | 0.951443225597243 | 0.533045488978806 | 1.69824945572493 | 0.999714303421225 |
| ebi-fl187-GCST90094775 | ebi-a-GCST90002037 | CD80 on plasmacytoid Dendritic Cell \|\| id:ebi-a-GCST90002037 | Green olives liking \|\| id:ebi-fl187-GCST90094775 | Weighted mode | 25 | -0.098949381 | 0.199249846036902 | 0.623982657083156 | -0.489479079 | 0.291580317448354 | 0.905788557169764 | 0.612945607263491 | 1.33854113738185 | 0.999714303421225 |
| ebi-fl187-GCST90094775 | ebi-a-GCST90002042 | CD45 on CD33+ HLA DR+ CD14- \|\| id:ebi-a-GCST90002042 | Green olives liking \|\| id:ebi-fl187-GCST90094775 | Weighted mode | 25 | -0.503016677 | 0.391872189443698 | 0.211529086189108 | -1.271086168 | 0.26505281413019 | 0.604703709557746 | 0.280526756917334 | 1.3034998171695 | 0.999714303421225 |
| ebi-fl187-GCST90094775 | ebi-a-GCST90002056 | CD8 on Effector Memory CD8+ T cell \|\| id:ebi-a-GCST90002056 | Green olives liking \|\| id:ebi-fl187-GCST90094775 | Weighted mode | 25 | -0.287817501 | 0.271749358396205 | 0.300084921277812 | -0.820446243 | 0.244811241655423 | 0.749898435615485 | 0.440235158695424 | 1.27738017428002 | 0.999714303421225 |
| ebi-fl187-GCST90094775 | ebi-a-GCST90002058 | CD8 on CD8+ T cell \|\| id:ebi-a-GCST90002058 | Green olives liking \|\| id:ebi-fl187-GCST90094775 | Weighted mode | 25 | 0.028363485265152 | 0.198388688239999 | 0.887508016015719 | -0.360478344 | 0.41720531421555 | 1.0287695590443 | 0.697342676811979 | 1.51771408922613 | 0.999714303421225 |
| ebi-fl187-GCST90094775 | ebi-a-GCST90002063 | CD4 on CD4 regulatory T cell \|\| id:ebi-a-GCST90002063 | Green olives liking \|\| id:ebi-fl187-GCST90094775 | Weighted mode | 25 | 0.130143938798964 | 0.264572567897402 | 0.627257417096266 | -0.388418294 | 0.648706171877873 | 1.13899231671225 | 0.678128626604138 | 1.91306405102825 | 0.999714303421225 |
| ebi-fl187-GCST90094775 | ebi-a-GCST90002066 | CD4 on activated CD4 regulatory T cell \|\| id:ebi-a-GCST90002066 | Green olives liking \|\| id:ebi-fl187-GCST90094775 | Weighted mode | 25 | 0.0848800854474781 | 0.245948800895461 | 0.733015890673068 | -0.397179564 | 0.566939735202581 | 1.08858652150581 | 0.672213309277351 | 1.76286395768935 | 0.999714303421225 |
| ebi-fl187-GCST90094775 | ebi-a-GCST90002070 | CD4 on activated & secreting CD4 regulatory T cell \|\| id:ebi-a-GCST90002070 | Green olives liking \|\| id:ebi-fl187-GCST90094775 | Weighted mode | 25 | 0.101780510258946 | 0.271463601790878 | 0.711003584097556 | -0.430288149 | 0.633849169769067 | 1.10714043908904 | 0.650321678018077 | 1.88485174844839 | 0.999714303421225 |
| ebi-fl187-GCST90094775 | ebi-a-GCST90002081 | SSC-A on CD4+ T cell \|\| id:ebi-a-GCST90002081 | Green olives liking \|\| id:ebi-fl187-GCST90094775 | Weighted mode | 25 | 0.154271856076957 | 0.173846115934449 | 0.383668042793407 | -0.186466531 | 0.495010243308478 | 1.16680804752381 | 0.829886336824085 | 1.64051504327263 | 0.999714303421225 |
| ebi-fl187-GCST90094775 | ebi-a-GCST90002094 | CD11b on Monocytic Myeloid-Derived Suppressor Cells \|\| id:ebi-a-GCST90002094 | Green olives liking \|\| id:ebi-fl187-GCST90094775 | Weighted mode | 25 | -0.479936182 | 0.272998362695274 | 0.091493117992472 | -1.015012973 | 0.0551406088901043 | 0.618822882589296 | 0.36239772846143 | 1.05668918412352 | 0.999714303421225 |
| ebi-fl187-GCST90094775 | ebi-a-GCST90002104 | HLA DR on myeloid Dendritic Cell \|\| id:ebi-a-GCST90002104 | Green olives liking \|\| id:ebi-fl187-GCST90094775 | Weighted mode | 24 | 0.0178507487145827 | 0.208807060318075 | 0.932612313981333 | -0.39141109 | 0.427112586938009 | 1.01801102559651 | 0.676102160397612 | 1.53282522810841 | 0.999714303421225 |
| ebi-fl187-GCST90094775 | ebi-a-GCST90002105 | HLA DR on plasmacytoid Dendritic Cell \|\| id:ebi-a-GCST90002105 | Green olives liking \|\| id:ebi-fl187-GCST90094775 | Weighted mode | 24 | 0.167797742776973 | 0.215580048136098 | 0.444294153332709 | -0.254739152 | 0.590334637123725 | 1.18269737736807 | 0.775118660078815 | 1.80459219791081 | 0.999714303421225 |
| ebi-fl187-GCST90094775 | ebi-a-GCST90002106 | HLA DR on Dendritic Cell \|\| id:ebi-a-GCST90002106 | Green olives liking \|\| id:ebi-fl187-GCST90094775 | Weighted mode | 24 | 0.204013146636421 | 0.241070523658939 | 0.406115821657221 | -0.26848508 | 0.676511373007942 | 1.22631427525815 | 0.764536829816781 | 1.96700360669651 | 0.999714303421225 |
| ebi-fl187-GCST90094775 | ebi-a-GCST90002111 | HLA DR on CD33dim HLA DR+ CD11b- \|\| id:ebi-a-GCST90002111 | Green olives liking \|\| id:ebi-fl187-GCST90094775 | Weighted mode | 24 | -0.033044553 | 0.268411429741069 | 0.903088236662771 | -0.559130955 | 0.493041849754444 | 0.967495454250772 | 0.571705686088824 | 1.6372890400997 | 0.999714303421225 |
| ebi-fl187-GCST90094775 | ebi-a-GCST90002112 | HLA DR on CD33- HLA DR+ \|\| id:ebi-a-GCST90002112 | Green olives liking \|\| id:ebi-fl187-GCST90094775 | Weighted mode | 24 | -0.059043657 | 0.278517537790617 | 0.833980210555257 | -0.604938031 | 0.486850717399671 | 0.942665614617172 | 0.546108267516616 | 1.62718368103543 | 0.999714303421225 |
| ebi-fl187-GCST90094775 | ebi-a-GCST90002115 | HLA DR on HLA DR+ CD8+ T cell \|\| id:ebi-a-GCST90002115 | Green olives liking \|\| id:ebi-fl187-GCST90094775 | Weighted mode | 25 | -0.101977783 | 0.212333270478796 | 0.63538242612559 | -0.518150993 | 0.314195426937626 | 0.903049614317947 | 0.595620837331044 | 1.36915728061834 | 0.999714303421225 |
| ebi-fl187-GCST90094775 | ebi-a-GCST90002116 | HLA DR on B cell \|\| id:ebi-a-GCST90002116 | Green olives liking \|\| id:ebi-fl187-GCST90094775 | Weighted mode | 25 | 0.225095799525841 | 0.231013760481649 | 0.339585963075821 | -0.227691171 | 0.677882770069872 | 1.25244269386109 | 0.796370164049232 | 1.96970300021064 | 0.999714303421225 |
| ebi-fl187-GCST90094775 | ebi-cfmet338-GCST90026023 | Sphingomyelin (d18:1/24:1, d18:2/24:0) levels \|\| id:ebi-cfmet338-GCST90026023 | Green olives liking \|\| id:ebi-fl187-GCST90094775 | Weighted mode | 23 | 0.115069003048134 | 0.132519515527907 | 0.394594400838653 | -0.144669247 | 0.374807253482833 | 1.12195085292967 | 0.865308448815633 | 1.45471099711614 | 0.999714303421225 |
| ebi-fl187-GCST90094775 | ebi-cfmet338-GCST90026046 | 1-arachidonoyl-gpc (20:4n6) levels \|\| id:ebi-cfmet338-GCST90026046 | Green olives liking \|\| id:ebi-fl187-GCST90094775 | Weighted mode | 23 | 0.0153231444098561 | 0.144004666590652 | 0.916223555320487 | -0.266926002 | 0.297572290927534 | 1.01544114573386 | 0.765729731755105 | 1.34658571776479 | 0.999714303421225 |
| ebi-fl187-GCST90094775 | ebi-cfmet338-GCST90026047 | 1-linoleoyl-gpc (18:2) levels \|\| id:ebi-cfmet338-GCST90026047 | Green olives liking \|\| id:ebi-fl187-GCST90094775 | Weighted mode | 23 | -0.144328438 | 0.161058347668148 | 0.379887100123624 | -0.460002799 | 0.171345923541926 | 0.865603404413464 | 0.631281878346222 | 1.18690125510184 | 0.999714303421225 |
| ebi-fl187-GCST90094775 | ebi-cfmet338-GCST90026050 | 1-myristoyl-2-palmitoyl-gpc (14:0/16:0) levels \|\| id:ebi-cfmet338-GCST90026050 | Green olives liking \|\| id:ebi-fl187-GCST90094775 | Weighted mode | 23 | -0.015488169 | 0.0831185153840875 | 0.853886958772936 | -0.178400459 | 0.147424121095131 | 0.984631155797233 | 0.836607329286888 | 1.15884534957754 | 0.999714303421225 |
| ebi-fl187-GCST90094775 | ebi-cfmet338-GCST90026068 | 2,3-dihydroxyisovalerate levels \|\| id:ebi-cfmet338-GCST90026068 | Green olives liking \|\| id:ebi-fl187-GCST90094775 | Weighted mode | 23 | 0.266334234766193 | 0.263840231059514 | 0.323731863288332 | -0.250792618 | 0.783461087642841 | 1.30517121939019 | 0.778183736039937 | 2.18903561335423 | 0.999714303421225 |
| ebi-fl187-GCST90094775 | ebi-cfmet338-GCST90026082 | 3-hydroxyhexanoate levels \|\| id:ebi-cfmet338-GCST90026082 | Green olives liking \|\| id:ebi-fl187-GCST90094775 | Weighted mode | 23 | -0.020449225 | 0.0564597717428743 | 0.720665201903691 | -0.131110378 | 0.0902119275855413 | 0.979758442416621 | 0.87712095450109 | 1.09440619399254 | 0.999714303421225 |
| ebi-fl187-GCST90094775 | ebi-cfmet338-GCST90026094 | 3-ureidopropionate levels \|\| id:ebi-cfmet338-GCST90026094 | Green olives liking \|\| id:ebi-fl187-GCST90094775 | Weighted mode | 23 | 0.0174464294269169 | 0.129674421008226 | 0.894198666373243 | -0.236715436 | 0.271608294603039 | 1.0175995073018 | 0.789215838680398 | 1.31207295458273 | 0.999714303421225 |
| ebi-fl187-GCST90094775 | ebi-cfmet338-GCST90026173 | Glycerol levels \|\| id:ebi-cfmet338-GCST90026173 | Green olives liking \|\| id:ebi-fl187-GCST90094775 | Weighted mode | 23 | 0.0709816161187228 | 0.0991477714319641 | 0.481572845875177 | -0.123348016 | 0.265311248125372 | 1.07356148945998 | 0.883955978299744 | 1.30383672936788 | 0.999714303421225 |
| ebi-fl187-GCST90094775 | ebi-cfmet338-GCST90026179 | Histidine levels \|\| id:ebi-cfmet338-GCST90026179 | Green olives liking \|\| id:ebi-fl187-GCST90094775 | Weighted mode | 23 | -0.026308961 | 0.0527491700209768 | 0.62290166309244 | -0.129697334 | 0.0770794125849134 | 0.974034104898715 | 0.878361240866224 | 1.08012784873136 | 0.999714303421225 |
| ebi-fl187-GCST90094775 | ebi-cfmet338-GCST90026188 | Isocitrate levels \|\| id:ebi-cfmet338-GCST90026188 | Green olives liking \|\| id:ebi-fl187-GCST90094775 | Weighted mode | 23 | -0.044315686 | 0.0969232177929945 | 0.651992572570451 | -0.234285193 | 0.145653820885396 | 0.956651908195547 | 0.791136157333163 | 1.15679566023018 | 0.999714303421225 |
| ebi-fl187-GCST90094775 | ebi-cfmet338-GCST90026207 | N-acetyl-isoputreanine levels \|\| id:ebi-cfmet338-GCST90026207 | Green olives liking \|\| id:ebi-fl187-GCST90094775 | Weighted mode | 23 | 0.0146231357460893 | 0.0680991201134546 | 0.831953416256852 | -0.11885114 | 0.14809741116846 | 1.01473057686549 | 0.887939969955702 | 1.1596258513705 | 0.999714303421225 |
| ebi-fl187-GCST90094775 | ebi-cfmet338-GCST90026213 | N-acetylglutamate levels \|\| id:ebi-cfmet338-GCST90026213 | Green olives liking \|\| id:ebi-fl187-GCST90094775 | Weighted mode | 23 | 0.176971947696856 | 0.121222776386429 | 0.158453699275028 | -0.060624694 | 0.414568589414256 | 1.19359760949552 | 0.941176402631429 | 1.51371756602714 | 0.999714303421225 |
| ebi-fl187-GCST90094775 | ebi-cfmet338-GCST90026224 | N-acetyltaurine levels \|\| id:ebi-cfmet338-GCST90026224 | Green olives liking \|\| id:ebi-fl187-GCST90094775 | Weighted mode | 23 | 0.0889014432125571 | 0.0905593056566066 | 0.336924476882682 | -0.088594796 | 0.266397682299506 | 1.09297293111704 | 0.915216347889727 | 1.30525403191171 | 0.999714303421225 |
| ebi-fl187-GCST90094775 | ebi-cfmet338-GCST90026250 | Phenyllactate (pla) levels \|\| id:ebi-cfmet338-GCST90026250 | Green olives liking \|\| id:ebi-fl187-GCST90094775 | Weighted mode | 23 | 0.0677480012740995 | 0.150862473319804 | 0.657772792730461 | -0.227942446 | 0.363438448980916 | 1.07009561176783 | 0.796170080945179 | 1.43826632742259 | 0.999714303421225 |
| ebi-fl187-GCST90094775 | ebi-cfmet338-GCST90026251 | Pipecolate levels \|\| id:ebi-cfmet338-GCST90026251 | Green olives liking \|\| id:ebi-fl187-GCST90094775 | Weighted mode | 23 | -0.087484327 | 0.205352024142113 | 0.674231515243482 | -0.489974294 | 0.315005640732572 | 0.916233232041134 | 0.612642142619419 | 1.37026704024512 | 0.999714303421225 |
| ebi-fl187-GCST90094775 | ebi-cfmet338-GCST90026278 | Trigonelline (n'-methylnicotinate) levels \|\| id:ebi-cfmet338-GCST90026278 | Green olives liking \|\| id:ebi-fl187-GCST90094775 | Weighted mode | 22 | -0.162460331 | 0.233416539525832 | 0.494054138435788 | -0.619956748 | 0.295036086763712 | 0.85004981043163 | 0.537967705175157 | 1.34317482864437 | 0.999714303421225 |
| ebi-fl187-GCST90094775 | ebi-cfmet338-GCST90026299 | 1-carboxyethylvaline levels \|\| id:ebi-cfmet338-GCST90026299 | Green olives liking \|\| id:ebi-fl187-GCST90094775 | Weighted mode | 23 | -0.04549664 | 0.155751886645636 | 0.77293966678992 | -0.350770338 | 0.259777058039878 | 0.955522813328107 | 0.704145451013505 | 1.29664097875845 | 0.999714303421225 |
| ebi-fl187-GCST90094775 | ebi-gm430-GCST90011306 | C_Erysipelotrichia abundance \|\| id:ebi-gm430-GCST90011306 | Green olives liking \|\| id:ebi-fl187-GCST90094775 | Weighted mode | 20 | 0.163729625839152 | 0.134349449239736 | 0.237879757707573 | -0.099595295 | 0.427054546349034 | 1.17789579944712 | 0.905203684671192 | 1.53273626461115 | 0.999714303421225 |
| ebi-fl187-GCST90094775 | ebi-gm430-GCST90011312 | F_Erysipelotrichaceae abundance \|\| id:ebi-gm430-GCST90011312 | Green olives liking \|\| id:ebi-fl187-GCST90094775 | Weighted mode | 20 | 0.163729625839152 | 0.136460161543922 | 0.244951656724231 | -0.103732291 | 0.431191542465239 | 1.17789579944712 | 0.901466596033861 | 1.53909032287987 | 0.999714303421225 |
| ebi-fl187-GCST90094775 | ebi-gm430-GCST90011352 | O_Erysipelotrichales abundance \|\| id:ebi-gm430-GCST90011352 | Green olives liking \|\| id:ebi-fl187-GCST90094775 | Weighted mode | 20 | 0.163729625839152 | 0.128021641282879 | 0.216325114121235 | -0.087192791 | 0.414652042753594 | 1.17789579944712 | 0.916500385504807 | 1.51384389608409 | 0.999714303421225 |
| ebi-fl187-GCST90094775 | ebi-gm430-GCST90011362 | OTU97_11 (Parabacteroides) abundance \|\| id:ebi-gm430-GCST90011362 | Green olives liking \|\| id:ebi-fl187-GCST90094775 | Weighted mode | 20 | 0.0961593456789179 | 0.153863959764067 | 0.539428912236349 | -0.205414015 | 0.39773270681649 | 1.10093447916981 | 0.814310109638294 | 1.48844612522775 | 0.999714303421225 |
| ebi-fl187-GCST90094775 | ebi-gm430-GCST90011384 | OTU97_235 (Phascolarctobacterium) abundance \|\| id:ebi-gm430-GCST90011384 | Green olives liking \|\| id:ebi-fl187-GCST90094775 | Weighted mode | 20 | 0.102898129779033 | 0.531976533810259 | 0.848678088123758 | -0.939775876 | 1.14557213604714 | 1.10837849256274 | 0.390715394052061 | 3.14423977523638 | 0.999714303421225 |
| ebi-fl187-GCST90094775 | ebi-gm430-GCST90011390 | OTU97_3 (Bacteroides) abundance \|\| id:ebi-gm430-GCST90011390 | Green olives liking \|\| id:ebi-fl187-GCST90094775 | Weighted mode | 20 | 0.0824432533416372 | 0.117767254641092 | 0.492378080362489 | -0.148380566 | 0.313267072438178 | 1.08593704839188 | 0.862102965638889 | 1.36788680711257 | 0.999714303421225 |
| ebi-fl187-GCST90094775 | ebi-gm430-GCST90011398 | OTU97_4 (Alistipes) abundance \|\| id:ebi-gm430-GCST90011398 | Green olives liking \|\| id:ebi-fl187-GCST90094775 | Weighted mode | 20 | 0.0590814269676207 | 0.144367060747964 | 0.686936775346435 | -0.223878012 | 0.34204086603363 | 1.06086161996809 | 0.799412647092899 | 1.40781782826928 | 0.999714303421225 |
| ebi-fl187-GCST90094775 | ebi-gm430-GCST90011421 | OTU99_108 (Cytophagales) abundance \|\| id:ebi-gm430-GCST90011421 | Green olives liking \|\| id:ebi-fl187-GCST90094775 | Weighted mode | 20 | 0.49183726480436 | 0.501829388433771 | 0.339358167409688 | -0.491748337 | 1.47542286613455 | 1.63531797375982 | 0.611556252838966 | 4.37288452679119 | 0.999714303421225 |
| ebi-fl187-GCST90094775 | ebi-gm430-GCST90011428 | OTU99_123 (Phascolarctobacterium) abundance \|\| id:ebi-gm430-GCST90011428 | Green olives liking \|\| id:ebi-fl187-GCST90094775 | Weighted mode | 20 | 0.268780119262637 | 0.272092269667253 | 0.335651036383793 | -0.264520729 | 0.802080967810453 | 1.30836742462191 | 0.767573737447758 | 2.23017702964111 | 0.999714303421225 |
| ebi-fl187-GCST90094775 | ebi-gm430-GCST90011450 | OTU99_298 (Phascolarctobacterium) abundance \|\| id:ebi-gm430-GCST90011450 | Green olives liking \|\| id:ebi-fl187-GCST90094775 | Weighted mode | 20 | 0.101530632745413 | 0.542092357265158 | 0.853415720556913 | -0.960970387 | 1.16403165298512 | 1.10686382415035 | 0.382521511724513 | 3.20281993995432 | 0.999714303421225 |
| ebi-fl187-GCST90094775 | ebi-gm430-GCST90011451 | OTU99_3 (Bacteroides) abundance \|\| id:ebi-gm430-GCST90011451 | Green olives liking \|\| id:ebi-fl187-GCST90094775 | Weighted mode | 20 | 0.0732982531229299 | 0.110929468500737 | 0.516695755218681 | -0.144123505 | 0.290720011384375 | 1.07605142475729 | 0.865780813076588 | 1.33739007752736 | 0.999714303421225 |
| ebi-fl187-GCST90094775 | ebi-gm430-GCST90011472 | OTU99_72 (Parabacteroides) abundance \|\| id:ebi-gm430-GCST90011472 | Green olives liking \|\| id:ebi-fl187-GCST90094775 | Weighted mode | 20 | 0.595819360470303 | 0.448676184531867 | 0.199928221640059 | -0.283585961 | 1.47522468215276 | 1.81451707960474 | 0.753078383826889 | 4.37201797699475 | 0.999714303421225 |
| ebi-fl187-GCST90094775 | ebi-gm430-GCST90011507 | TestASV_3 (Bacteroides) abundance \|\| id:ebi-gm430-GCST90011507 | Green olives liking \|\| id:ebi-fl187-GCST90094775 | Weighted mode | 20 | 0.0923934184333579 | 0.17312763952918 | 0.599756782501737 | -0.246936755 | 0.431723591910551 | 1.09679623706925 | 0.781190098302582 | 1.53990941291132 | 0.999714303421225 |
| ebi-fl187-GCST90094775 | ebi-gm430-GCST90011512 | TestASV_34 (Phascolarctobacterium) abundance \|\| id:ebi-gm430-GCST90011512 | Green olives liking \|\| id:ebi-fl187-GCST90094775 | Weighted mode | 20 | -0.304855412 | 0.460775306510378 | 0.516162880929196 | -1.207975012 | 0.598264189074422 | 0.737229961523042 | 0.298801737012281 | 1.8189586901395 | 0.999714303421225 |
| ebi-fl187-GCST90094775 | ebi-gm430-GCST90011519 | TestASV_41 (Barnesiella) abundance \|\| id:ebi-gm430-GCST90011519 | Green olives liking \|\| id:ebi-fl187-GCST90094775 | Weighted mode | 20 | 0.983544057773799 | 0.891087196340109 | 0.283481899297497 | -0.762986847 | 2.73007496260041 | 2.67391598181745 | 0.466271662931104 | 15.3340364560719 | 0.999714303421225 |
| ebi-fl187-GCST90094775 | ebi-gm430-GCST90011524 | TestASV_46 (Sutterella) abundance \|\| id:ebi-gm430-GCST90011524 | Green olives liking \|\| id:ebi-fl187-GCST90094775 | Weighted mode | 20 | -0.011858669 | 0.650218897911231 | 0.985639177888798 | -1.286287709 | 1.26257037102507 | 0.988211368011391 | 0.276294567516249 | 3.5344947844822 | 0.999714303421225 |
| ebi-fl187-GCST90094775 | ebi-gm430-GCST90011581 | OTU97_19 (Bacteria) prevalence \|\| id:ebi-gm430-GCST90011581 | Green olives liking \|\| id:ebi-fl187-GCST90094775 | Weighted mode | 20 | -0.416631317 | 0.694046361046527 | 0.55540399696069 | -1.776962185 | 0.943699550254076 | 0.659263934287068 | 0.169151217665691 | 2.56946973867289 | 0.999714303421225 |
| ebi-fl187-GCST90094775 | ebi-gm430-GCST90011614 | OTU97_80 (Ruminococcus) prevalence \|\| id:ebi-gm430-GCST90011614 | Green olives liking \|\| id:ebi-fl187-GCST90094775 | Weighted mode | 20 | -0.192810292 | 0.40418813467888 | 0.638780899023646 | -0.985019036 | 0.59939845242511 | 0.824638400177391 | 0.373432118395902 | 1.82102303885436 | 0.999714303421225 |
| ebi-fl187-GCST90094775 | ebi-gm430-GCST90011623 | OTU99_11 (Parabacteroides) prevalence \|\| id:ebi-gm430-GCST90011623 | Green olives liking \|\| id:ebi-fl187-GCST90094775 | Weighted mode | 20 | -0.03086999 | 0.305333996859382 | 0.920528486261606 | -0.629324624 | 0.56758464409253 | 0.969601623029565 | 0.532951622435904 | 1.76400121100041 | 0.999714303421225 |
| ebi-fl187-GCST90094775 | ebi-gm430-GCST90011642 | OTU99_19 (Bacteria) prevalence \|\| id:ebi-gm430-GCST90011642 | Green olives liking \|\| id:ebi-fl187-GCST90094775 | Weighted mode | 20 | -0.416631317 | 0.672210759768878 | 0.542756500864386 | -1.734164407 | 0.900901771749884 | 0.659263934287068 | 0.176547660739231 | 2.46182211212434 | 0.999714303421225 |
| ebi-fl187-GCST90094775 | ebi-gm430-GCST90011653 | OTU99_30 (Parasutterella) prevalence \|\| id:ebi-gm430-GCST90011653 | Green olives liking \|\| id:ebi-fl187-GCST90094775 | Weighted mode | 20 | 0.340778019198769 | 0.280578105661639 | 0.239414993121887 | -0.209155068 | 0.890711106295581 | 1.40604109208975 | 0.81126942404466 | 2.43686190315007 | 0.999714303421225 |
| ebi-fl187-GCST90094775 | ebi-gm430-GCST90011675 | OTU99_8 (Ruminococcaceae) prevalence \|\| id:ebi-gm430-GCST90011675 | Green olives liking \|\| id:ebi-fl187-GCST90094775 | Weighted mode | 20 | -0.105686348 | 0.287646857808685 | 0.717368706409523 | -0.669474189 | 0.45810149363828 | 0.89970679896171 | 0.511977710550271 | 1.58106946341064 | 0.999714303421225 |
| ebi-fl187-GCST90094775 | ebi-gm430-GCST90011679 | OTU99_92 (Ruminococcus) prevalence \|\| id:ebi-gm430-GCST90011679 | Green olives liking \|\| id:ebi-fl187-GCST90094775 | Weighted mode | 20 | -0.445446552 | 0.462881894143633 | 0.347970860391985 | -1.352695064 | 0.461801960817689 | 0.64053817874337 | 0.25854253212756 | 1.58693099758706 | 0.999714303421225 |
| ebi-fl187-GCST90094775 | ebi-gm430-GCST90011684 | TestASV_11 (Lachnospiraceae) prevalence \|\| id:ebi-gm430-GCST90011684 | Green olives liking \|\| id:ebi-fl187-GCST90094775 | Weighted mode | 20 | -0.210129428 | 0.286636808070835 | 0.472454577614122 | -0.771937571 | 0.351678716272564 | 0.810479340829138 | 0.46211681600519 | 1.42145176102714 | 0.999714303421225 |
| ebi-fl187-GCST90094775 | ebi-gm430-GCST90011688 | TestASV_15 (Bacteroides) prevalence \|\| id:ebi-gm430-GCST90011688 | Green olives liking \|\| id:ebi-fl187-GCST90094775 | Weighted mode | 20 | -0.087246733 | 0.286301682858185 | 0.763879256279251 | -0.648398032 | 0.47390456526447 | 0.916450948917296 | 0.522882747851915 | 1.60625368731667 | 0.999714303421225 |
| ebi-fl187-GCST90094775 | ebi-gm430-GCST90011692 | TestASV_19 (Prevotella) prevalence \|\| id:ebi-gm430-GCST90011692 | Green olives liking \|\| id:ebi-fl187-GCST90094775 | Weighted mode | 20 | 0.57633199212318 | 0.680563722454955 | 0.407622044745494 | -0.757572904 | 1.91023688813489 | 1.77949922797311 | 0.468802876955108 | 6.75468871463461 | 0.999714303421225 |
| ebi-fl187-GCST90094775 | ebi-gm430-GCST90011693 | TestASV_2 (Bacteroides) prevalence \|\| id:ebi-gm430-GCST90011693 | Green olives liking \|\| id:ebi-fl187-GCST90094775 | Weighted mode | 20 | -0.121531001 | 0.242110294853511 | 0.621459130176097 | -0.596067179 | 0.353005176544507 | 0.885563599239953 | 0.550974263692998 | 1.42333851138969 | 0.999714303421225 |
| ebi-fl187-GCST90094775 | ebi-gm430-GCST90011716 | TestASV_40 (Barnesiella) prevalence \|\| id:ebi-gm430-GCST90011716 | Green olives liking \|\| id:ebi-fl187-GCST90094775 | Weighted mode | 20 | -0.249356537 | 0.528694228408139 | 0.642550437667222 | -1.285597225 | 0.786884150834881 | 0.779302073944148 | 0.276485410446215 | 2.19654166009527 | 0.999714303421225 |
| ebi-fl187-GCST90094775 | ebi-gm430-GCST90011718 | TestASV_42 (Alphaproteobacteria) prevalence \|\| id:ebi-gm430-GCST90011718 | Green olives liking \|\| id:ebi-fl187-GCST90094775 | Weighted mode | 20 | -0.034230983 | 0.430885109219118 | 0.93751071357342 | -0.878765797 | 0.810303831312451 | 0.966348269069284 | 0.415295154108087 | 2.24859107527693 | 0.999714303421225 |
| ebi-fl187-GCST90094775 | ebi-hbsm44-GCST90104929 | Sulcal depth of the right inferior frontal sulcus \|\| id:ebi-hbsm44-GCST90104929 | Green olives liking \|\| id:ebi-fl187-GCST90094775 | Weighted mode | 19 | 0.303737777569878 | 0.255445404826445 | 0.249869471466999 | -0.196935216 | 0.80441077102971 | 1.3549137207348 | 0.821243835140314 | 2.23537896064909 | 0.999714303421225 |
| ebi-fl187-GCST90094775 | ebi-hbsm44-GCST90104935 | Sulcal opening distance of the left calloso-marginal posterior fissure \|\| id:ebi-hbsm44-GCST90104935 | Green olives liking \|\| id:ebi-fl187-GCST90094775 | Weighted mode | 20 | -0.045389693 | 0.0594716675073768 | 0.454707765440651 | -0.161954161 | 0.0711747755983592 | 0.955625009157478 | 0.850480188782112 | 1.07376887806753 | 0.999714303421225 |
| ebi-fl187-GCST90094775 | ebi-hbsm44-GCST90104936 | Sulcal opening distance of the left anterior inferior temporal sulcus \|\| id:ebi-hbsm44-GCST90104936 | Green olives liking \|\| id:ebi-fl187-GCST90094775 | Weighted mode | 18 | -0.01297734 | 0.030204438877955 | 0.672846481348399 | -0.07217804 | 0.0462233605421481 | 0.987106502937166 | 0.930365238968513 | 1.04730831218622 | 0.999714303421225 |
| ebi-fl187-GCST90094775 | ebi-hbsm44-GCST90104941 | Sulcal opening distance of the right posterior inferior temporal sulcus \|\| id:ebi-hbsm44-GCST90104941 | Green olives liking \|\| id:ebi-fl187-GCST90094775 | Weighted mode | 19 | 0.00120657433160704 | 0.0267675340538022 | 0.964543016114357 | -0.051257792 | 0.0536709410770594 | 1.00120730253526 | 0.950033727473712 | 1.05513734266626 | 0.999714303421225 |
| ebi-fl187-GCST90094775 | ebi-IPs91-GCST90274819 | Leukemia inhibitory factor levels \|\| id:ebi-IPs91-GCST90274819 | Green olives liking \|\| id:ebi-fl187-GCST90094775 | Weighted mode | 27 | 0.0133473739621521 | 0.0995792819449422 | 0.894404957146204 | -0.181828019 | 0.208522766574239 | 1.01343684779506 | 0.83374471662834 | 1.23185697490497 | 0.999714303421225 |
| ebi-fl187-GCST90094775 | ebi-IPs91-GCST90274837 | STAM binding protein levels \|\| id:ebi-IPs91-GCST90274837 | Green olives liking \|\| id:ebi-fl187-GCST90094775 | Weighted mode | 27 | -0.054621854 | 0.0966393339338255 | 0.576773769818104 | -0.244034949 | 0.13479124011001 | 0.946843124826799 | 0.783460252702068 | 1.14429787591624 | 0.999714303421225 |
| ebi-fl187-GCST90094775 | ebi-IPs91-GCST90274840 | TNF-beta levels \|\| id:ebi-IPs91-GCST90274840 | Green olives liking \|\| id:ebi-fl187-GCST90094775 | Weighted mode | 27 | -0.114310203 | 0.127966974408464 | 0.379901916553967 | -0.365125473 | 0.136505066556699 | 0.891981217459219 | 0.694109553319505 | 1.14626068535581 | 0.999714303421225 |
| ebi-fl187-GCST90094775 | ebi-IPs91-GCST90274842 | Tumor necrosis factor ligand superfamily member 14 levels \|\| id:ebi-IPs91-GCST90274842 | Green olives liking \|\| id:ebi-fl187-GCST90094775 | Weighted mode | 27 | 0.0110639492649421 | 0.11047271355266 | 0.920992738244445 | -0.205462569 | 0.227590467828156 | 1.01112538110215 | 0.814270572715675 | 1.2555710233998 | 0.999714303421225 |
| ebi-fl187-GCST90094775 | ebi-met1400-GCST90199628 | 3-methylhistidine levels \|\| id:ebi-met1400-GCST90199628 | Green olives liking \|\| id:ebi-fl187-GCST90094775 | Weighted mode | 24 | -0.062464434 | 0.126011375614132 | 0.6248061989357 | -0.30944673 | 0.184517862218787 | 0.939446474586801 | 0.733852862560932 | 1.20263846288436 | 0.999714303421225 |
| ebi-fl187-GCST90094775 | ebi-met1400-GCST90199651 | Docosahexaenoate DHA; 22:6n3 levels \|\| id:ebi-met1400-GCST90199651 | Green olives liking \|\| id:ebi-fl187-GCST90094775 | Weighted mode | 24 | 0.0600361162059898 | 0.106272141140161 | 0.577591848021801 | -0.14825728 | 0.268329512840706 | 1.06187489674533 | 0.862209256836145 | 1.30777799866766 | 0.999714303421225 |
| ebi-fl187-GCST90094775 | ebi-met1400-GCST90199652 | 1-stearoyl-2-oleoyl-GPS (18:0/18:1) levels \|\| id:ebi-met1400-GCST90199652 | Green olives liking \|\| id:ebi-fl187-GCST90094775 | Weighted mode | 24 | 0.0853124328285846 | 0.11689721359437 | 0.472876074493626 | -0.143806106 | 0.314430971473551 | 1.08905727079377 | 0.866055654934778 | 1.36947981611886 | 0.999714303421225 |
| ebi-fl187-GCST90094775 | ebi-met1400-GCST90199653 | 1-myristoyl-2-palmitoyl-gpc (14:0/16:0) levels \|\| id:ebi-met1400-GCST90199653 | Green olives liking \|\| id:ebi-fl187-GCST90094775 | Weighted mode | 23 | 0.044834805263598 | 0.127604973862894 | 0.728663784983512 | -0.205270944 | 0.294940554034871 | 1.04585507588403 | 0.814426622909074 | 1.34304651761673 | 0.999714303421225 |
| ebi-fl187-GCST90094775 | ebi-met1400-GCST90199667 | Erythritol levels in elite athletes \|\| id:ebi-met1400-GCST90199667 | Green olives liking \|\| id:ebi-fl187-GCST90094775 | Weighted mode | 24 | 0.0615332361400822 | 0.113553141800461 | 0.593106282893655 | -0.161030922 | 0.284097394068986 | 1.06346584144111 | 0.851265748040483 | 1.328562319 | 0.999714303421225 |
| ebi-fl187-GCST90094775 | ebi-met1400-GCST90199674 | 3-hydroxymyristate levels \|\| id:ebi-met1400-GCST90199674 | Green olives liking \|\| id:ebi-fl187-GCST90094775 | Weighted mode | 24 | -0.013520492 | 0.116796694149412 | 0.90884681178269 | -0.242442012 | 0.215401028998474 | 0.986570499767291 | 0.784709249926651 | 1.24035921725411 | 0.999714303421225 |
| ebi-fl187-GCST90094775 | ebi-met1400-GCST90199685 | 2-linoleoylglycerol (18:2) levels \|\| id:ebi-met1400-GCST90199685 | Green olives liking \|\| id:ebi-fl187-GCST90094775 | Weighted mode | 24 | 0.0626928547105259 | 0.146977562069002 | 0.673675149201098 | -0.225383167 | 0.350768876365771 | 1.0646997714857 | 0.798210312349881 | 1.42015905565602 | 0.999714303421225 |
| ebi-fl187-GCST90094775 | ebi-met1400-GCST90199703 | Gamma-glutamylvaline levels \|\| id:ebi-met1400-GCST90199703 | Green olives liking \|\| id:ebi-fl187-GCST90094775 | Weighted mode | 24 | 0.00460848530078906 | 0.114019108241604 | 0.968108264970816 | -0.218868967 | 0.228085937454332 | 1.00461912070059 | 0.803426986823299 | 1.25619327484602 | 0.999714303421225 |
| ebi-fl187-GCST90094775 | ebi-met1400-GCST90199779 | Gamma-glutamyl-2-aminobutyrate levels \|\| id:ebi-met1400-GCST90199779 | Green olives liking \|\| id:ebi-fl187-GCST90094775 | Weighted mode | 23 | 0.0442679387656001 | 0.13644892100129 | 0.748678092148202 | -0.223171946 | 0.311707823928129 | 1.0452623836843 | 0.799977284256334 | 1.36575559362419 | 0.999714303421225 |
| ebi-fl187-GCST90094775 | ebi-met1400-GCST90199824 | 4-vinylphenol sulfate levels \|\| id:ebi-met1400-GCST90199824 | Green olives liking \|\| id:ebi-fl187-GCST90094775 | Weighted mode | 24 | -0.107886265 | 0.111477063463369 | 0.343217153258777 | -0.326381309 | 0.110608779847296 | 0.897729694323175 | 0.721530009135975 | 1.11695784494766 | 0.999714303421225 |
| ebi-fl187-GCST90094775 | ebi-met1400-GCST90199830 | 4-ethylphenylsulfate levels \|\| id:ebi-met1400-GCST90199830 | Green olives liking \|\| id:ebi-fl187-GCST90094775 | Weighted mode | 24 | 0.0016065529564524 | 0.105947676838508 | 0.988032396445649 | -0.206050894 | 0.209263999559928 | 1.00160784415402 | 0.813791658403563 | 1.23277040641938 | 0.999714303421225 |
| ebi-fl187-GCST90094775 | ebi-met1400-GCST90199833 | Hexanoylglutamine levels \|\| id:ebi-met1400-GCST90199833 | Green olives liking \|\| id:ebi-fl187-GCST90094775 | Weighted mode | 24 | 0.153930511925133 | 0.126066549353818 | 0.234445946155139 | -0.093159925 | 0.401020948658616 | 1.16640983238856 | 0.911047789501933 | 1.49334855182129 | 0.999714303421225 |
| ebi-fl187-GCST90094775 | ebi-met1400-GCST90199837 | Carotenoid levels (cryptoxanthin) \|\| id:ebi-met1400-GCST90199837 | Green olives liking \|\| id:ebi-fl187-GCST90094775 | Weighted mode | 24 | 0.0449004464806385 | 0.13592154743069 | 0.744132612286905 | -0.221505786 | 0.311306679444791 | 1.04592372933728 | 0.801311285359977 | 1.3652078381741 | 0.999714303421225 |
| ebi-fl187-GCST90094775 | ebi-met1400-GCST90199869 | 2-hydroxyhippurate levels \|\| id:ebi-met1400-GCST90199869 | Green olives liking \|\| id:ebi-fl187-GCST90094775 | Weighted mode | 24 | -0.116619457 | 0.131714572509794 | 0.385106847020338 | -0.374780019 | 0.14154110491746 | 0.889923782821955 | 0.687440485783936 | 1.15204785811968 | 0.999714303421225 |
| ebi-fl187-GCST90094775 | ebi-met1400-GCST90199902 | Hydantoin-5-propionate levels \|\| id:ebi-met1400-GCST90199902 | Green olives liking \|\| id:ebi-fl187-GCST90094775 | Weighted mode | 24 | 0.0509768465418172 | 0.111759386366363 | 0.652574072322907 | -0.168071551 | 0.270025243819889 | 1.05229852865116 | 0.845293351159238 | 1.30999751965729 | 0.999714303421225 |
| ebi-fl187-GCST90094775 | ebi-met1400-GCST90199904 | Pantoate levels \|\| id:ebi-met1400-GCST90199904 | Green olives liking \|\| id:ebi-fl187-GCST90094775 | Weighted mode | 24 | 0.0491598499453438 | 0.140500989089874 | 0.729605167007299 | -0.226222089 | 0.324541788561497 | 1.05038824182403 | 0.797540957184414 | 1.3833966125793 | 0.999714303421225 |
| ebi-fl187-GCST90094775 | ebi-met1400-GCST90199936 | 3-methyl catechol sulfate (2) levels \|\| id:ebi-met1400-GCST90199936 | Green olives liking \|\| id:ebi-fl187-GCST90094775 | Weighted mode | 24 | -0.007702843 | 0.160122885736757 | 0.962047197014653 | -0.321543699 | 0.306138012694423 | 0.992326747521519 | 0.72502894600983 | 1.35816973827868 | 0.999714303421225 |
| ebi-fl187-GCST90094775 | ebi-met1400-GCST90199977 | 4-hydroxychlorothalonil levels \|\| id:ebi-met1400-GCST90199977 | Green olives liking \|\| id:ebi-fl187-GCST90094775 | Weighted mode | 24 | 0.0196879333915622 | 0.137496322529189 | 0.88738816426962 | -0.249804859 | 0.289180725548773 | 1.01988301892593 | 0.778952774046898 | 1.33533303551832 | 0.999714303421225 |
| ebi-fl187-GCST90094775 | ebi-met1400-GCST90200040 | Sphingomyelin (d18:2/24:1, d18:1/24:2) levels \|\| id:ebi-met1400-GCST90200040 | Green olives liking \|\| id:ebi-fl187-GCST90094775 | Weighted mode | 24 | -0.057947428 | 0.118642610121803 | 0.629876745893667 | -0.290486944 | 0.17459208744584 | 0.943699557935121 | 0.747899293648338 | 1.19076039141934 | 0.999714303421225 |
| ebi-fl187-GCST90094775 | ebi-met1400-GCST90200077 | 1-stearoyl-2-oleoyl-GPI (18:0/18:1) levels \|\| id:ebi-met1400-GCST90200077 | Green olives liking \|\| id:ebi-fl187-GCST90094775 | Weighted mode | 24 | -0.060840098 | 0.124544312513198 | 0.629819858640129 | -0.304946951 | 0.183266754218959 | 0.940973691033335 | 0.737162479210011 | 1.20113477311784 | 0.999714303421225 |
| ebi-fl187-GCST90094775 | ebi-met1400-GCST90200080 | 1-palmitoyl-2-oleoyl-GPI (16:0/18:1) levels \|\| id:ebi-met1400-GCST90200080 | Green olives liking \|\| id:ebi-fl187-GCST90094775 | Weighted mode | 24 | 0.000824200000937192 | 0.161686921107064 | 0.995976752870035 | -0.316082165 | 0.317730565370783 | 1.00082453974709 | 0.728999549189872 | 1.37400600655117 | 0.999714303421225 |
| ebi-fl187-GCST90094775 | ebi-met1400-GCST90200082 | 1-oleoyl-2-linoleoyl-GPE (18:1/18:2) levels \|\| id:ebi-met1400-GCST90200082 | Green olives liking \|\| id:ebi-fl187-GCST90094775 | Weighted mode | 24 | -0.02877475 | 0.14261237894298 | 0.841871284636948 | -0.308295013 | 0.250745512808371 | 0.971635300748586 | 0.734698540672567 | 1.28498303099467 | 0.999714303421225 |
| ebi-fl187-GCST90094775 | ebi-met1400-GCST90200100 | Perfluorooctanesulfonate (PFOS) levels \|\| id:ebi-met1400-GCST90200100 | Green olives liking \|\| id:ebi-fl187-GCST90094775 | Weighted mode | 24 | -0.099685778 | 0.114461201641264 | 0.392801327651308 | -0.324029734 | 0.124658176903603 | 0.905121782250029 | 0.723228737916657 | 1.13276118294663 | 0.999714303421225 |
| ebi-fl187-GCST90094775 | ebi-met1400-GCST90200102 | 1-palmitoleoyl-2-linolenoyl-GPC (16:1/18:3) levels \|\| id:ebi-met1400-GCST90200102 | Green olives liking \|\| id:ebi-fl187-GCST90094775 | Weighted mode | 24 | -0.01774647 | 0.12143192356057 | 0.885081555261791 | -0.25575304 | 0.220260100620528 | 0.98241007164695 | 0.774333174705693 | 1.24640087807189 | 0.999714303421225 |
| ebi-fl187-GCST90094775 | ebi-met1400-GCST90200114 | Glycosyl-N-tricosanoyl-sphingadienine (d18:2/23:0) levels \|\| id:ebi-met1400-GCST90200114 | Green olives liking \|\| id:ebi-fl187-GCST90094775 | Weighted mode | 24 | -0.032719333 | 0.137871425955264 | 0.814511473307194 | -0.302947328 | 0.237508661839018 | 0.967810153813699 | 0.738638000956383 | 1.26808598069978 | 0.999714303421225 |
| ebi-fl187-GCST90094775 | ebi-met1400-GCST90200135 | Arachidoylcarnitine (C20) levels \|\| id:ebi-met1400-GCST90200135 | Green olives liking \|\| id:ebi-fl187-GCST90094775 | Weighted mode | 24 | 0.165406113710909 | 0.113457201124931 | 0.15839606829773 | -0.05697 | 0.387782227915773 | 1.1798721836983 | 0.944622407147074 | 1.47370881669996 | 0.999714303421225 |
| ebi-fl187-GCST90094775 | ebi-met1400-GCST90200146 | Docosahexaenoylcarnitine (C22:6) levels \|\| id:ebi-met1400-GCST90200146 | Green olives liking \|\| id:ebi-fl187-GCST90094775 | Weighted mode | 24 | 0.0959640022180193 | 0.14843996577704 | 0.524365517248832 | -0.194978331 | 0.386906335141018 | 1.10071943982238 | 0.822852488496163 | 1.47241857093628 | 0.999714303421225 |
| ebi-fl187-GCST90094775 | ebi-met1400-GCST90200162 | 3-hydroxyphenylacetoylglutamine levels \|\| id:ebi-met1400-GCST90200162 | Green olives liking \|\| id:ebi-fl187-GCST90094775 | Weighted mode | 24 | 0.0729112015558893 | 0.148393972761927 | 0.627845289220784 | -0.217940985 | 0.363763388169265 | 1.07563501795777 | 0.80417289848359 | 1.43873375245389 | 0.999714303421225 |
| ebi-fl187-GCST90094775 | ebi-met1400-GCST90200172 | 3-carboxy-4-methyl-5-pentyl-2-furanpropionate (3-CMPFP) levels \|\| id:ebi-met1400-GCST90200172 | Green olives liking \|\| id:ebi-fl187-GCST90094775 | Weighted mode | 24 | 0.0663348682107319 | 0.115543546416366 | 0.57146497179274 | -0.160130483 | 0.292800219186809 | 1.06858449223583 | 0.852032606142034 | 1.34017502242932 | 0.999714303421225 |
| ebi-fl187-GCST90094775 | ebi-met1400-GCST90200222 | Dihydrocaffeate sulfate (2) levels \|\| id:ebi-met1400-GCST90200222 | Green olives liking \|\| id:ebi-fl187-GCST90094775 | Weighted mode | 24 | -0.060360196 | 0.146101545072286 | 0.683331411911049 | -0.346719224 | 0.22599883265091 | 0.941425375142902 | 0.707003810070816 | 1.25357420191863 | 0.999714303421225 |
| ebi-fl187-GCST90094775 | ebi-met1400-GCST90200223 | 4-methylhexanoylglutamine levels \|\| id:ebi-met1400-GCST90200223 | Green olives liking \|\| id:ebi-fl187-GCST90094775 | Weighted mode | 24 | 0.054241032001337 | 0.160672729838174 | 0.738736061891394 | -0.260677518 | 0.369159582484157 | 1.05573903838352 | 0.770529361032451 | 1.44651842426031 | 0.999714303421225 |
| ebi-fl187-GCST90094775 | ebi-met1400-GCST90200224 | 2-ketocaprylate levels \|\| id:ebi-met1400-GCST90200224 | Green olives liking \|\| id:ebi-fl187-GCST90094775 | Weighted mode | 24 | 0.0792557557558526 | 0.113665351975677 | 0.492620718516843 | -0.143528334 | 0.302039845628179 | 1.08248113745175 | 0.866296254100536 | 1.35261512143494 | 0.999714303421225 |
| ebi-fl187-GCST90094775 | ebi-met1400-GCST90200243 | Hydroxypalmitoyl sphingomyelin (d18:1/16:0(OH)) levels \|\| id:ebi-met1400-GCST90200243 | Green olives liking \|\| id:ebi-fl187-GCST90094775 | Weighted mode | 24 | 0.0531286066270572 | 0.140670772121396 | 0.709130881188843 | -0.222586107 | 0.328843319984993 | 1.05456526047981 | 0.80044607998755 | 1.38936015356357 | 0.999714303421225 |
| ebi-fl187-GCST90094775 | ebi-met1400-GCST90200273 | 2-methoxyhydroquinone sulfate (2) levels \|\| id:ebi-met1400-GCST90200273 | Green olives liking \|\| id:ebi-fl187-GCST90094775 | Weighted mode | 24 | 0.0669392809391658 | 0.154560897702567 | 0.668981493232249 | -0.236000079 | 0.369878640436197 | 1.06923055352851 | 0.789780611889322 | 1.44755892888278 | 0.999714303421225 |
| ebi-fl187-GCST90094775 | ebi-met1400-GCST90200276 | 2,4-di-tert-butylphenol levels \|\| id:ebi-met1400-GCST90200276 | Green olives liking \|\| id:ebi-fl187-GCST90094775 | Weighted mode | 24 | 0.00541014853199962 | 0.137745168507088 | 0.969008880342325 | -0.264570382 | 0.275390678805891 | 1.00542480981355 | 0.767535626472145 | 1.31704511598368 | 0.999714303421225 |
| ebi-fl187-GCST90094775 | ebi-met1400-GCST90200289 | N-acetylmethionine levels \|\| id:ebi-met1400-GCST90200289 | Green olives liking \|\| id:ebi-fl187-GCST90094775 | Weighted mode | 24 | 0.00599229159309866 | 0.117483355092885 | 0.959761563339992 | -0.224275084 | 0.236259667575152 | 1.00601028128758 | 0.799095285493928 | 1.26650313727074 | 0.999714303421225 |
| ebi-fl187-GCST90094775 | ebi-met1400-GCST90200308 | 3-hydroxyisobutyrate levels \|\| id:ebi-met1400-GCST90200308 | Green olives liking \|\| id:ebi-fl187-GCST90094775 | Weighted mode | 24 | 0.12194813449699 | 0.105177443435177 | 0.258168028226169 | -0.084199655 | 0.328095923629937 | 1.12969550803506 | 0.919247705706354 | 1.38832213880148 | 0.999714303421225 |
| ebi-fl187-GCST90094775 | ebi-met1400-GCST90200383 | Cystathionine levels \|\| id:ebi-met1400-GCST90200383 | Green olives liking \|\| id:ebi-fl187-GCST90094775 | Weighted mode | 24 | 0.0165380312342445 | 0.126603463821314 | 0.897204958556899 | -0.231604758 | 0.26468082032402 | 1.01667554147649 | 0.793259590976644 | 1.30301501348874 | 0.999714303421225 |
| ebi-fl187-GCST90094775 | ebi-met1400-GCST90200384 | Guanidinoacetate levels \|\| id:ebi-met1400-GCST90200384 | Green olives liking \|\| id:ebi-fl187-GCST90094775 | Weighted mode | 24 | 0.142733704645568 | 0.120419285796201 | 0.247999078708648 | -0.093288096 | 0.378755504806122 | 1.15342260968327 | 0.910931027345877 | 1.46046591519099 | 0.999714303421225 |
| ebi-fl187-GCST90094775 | ebi-met1400-GCST90200403 | Citrulline levels \|\| id:ebi-met1400-GCST90200403 | Green olives liking \|\| id:ebi-fl187-GCST90094775 | Weighted mode | 24 | 0.172777448542439 | 0.120982864491044 | 0.166697865720782 | -0.064348966 | 0.409903862944885 | 1.18860155063908 | 0.937677724916532 | 1.50667293105143 | 0.999714303421225 |
| ebi-fl187-GCST90094775 | ebi-met1400-GCST90200434 | 12,13-DiHOME levels \|\| id:ebi-met1400-GCST90200434 | Green olives liking \|\| id:ebi-fl187-GCST90094775 | Weighted mode | 24 | 0.0254677016093942 | 0.112792737687087 | 0.823357547092503 | -0.195606064 | 0.246541467476086 | 1.02579477421509 | 0.822336118468878 | 1.27959224357822 | 0.999714303421225 |
| ebi-fl187-GCST90094775 | ebi-met1400-GCST90200437 | Fructose levels \|\| id:ebi-met1400-GCST90200437 | Green olives liking \|\| id:ebi-fl187-GCST90094775 | Weighted mode | 24 | 0.135744076628027 | 0.158298365656646 | 0.400004956394674 | -0.17452072 | 0.446008873315053 | 1.14538872433748 | 0.839859452111526 | 1.56206532717003 | 0.999714303421225 |
| ebi-fl187-GCST90094775 | ebi-met1400-GCST90200448 | N-stearoyl-sphinganine (d18:0/18:0) levels \|\| id:ebi-met1400-GCST90200448 | Green olives liking \|\| id:ebi-fl187-GCST90094775 | Weighted mode | 24 | 0.10034428374269 | 0.13148725198035 | 0.453131985176031 | -0.15737073 | 0.358059297624176 | 1.10555147596182 | 0.854387252991692 | 1.43055044620761 | 0.999714303421225 |
| ebi-fl187-GCST90094775 | ebi-met1400-GCST90200468 | X-11847 levels \|\| id:ebi-met1400-GCST90200468 | Green olives liking \|\| id:ebi-fl187-GCST90094775 | Weighted mode | 24 | 0.146993761245711 | 0.127020324517672 | 0.259049521339095 | -0.101966075 | 0.395953597300348 | 1.15834673636664 | 0.903060187638625 | 1.4858003708034 | 0.999714303421225 |
| ebi-fl187-GCST90094775 | ebi-met1400-GCST90200476 | X-11843 levels \|\| id:ebi-met1400-GCST90200476 | Green olives liking \|\| id:ebi-fl187-GCST90094775 | Weighted mode | 24 | 0.172949373088886 | 0.142634097090845 | 0.237614465557132 | -0.106613457 | 0.452512203386941 | 1.18880591798896 | 0.898873058746981 | 1.57225705776035 | 0.999714303421225 |
| ebi-fl187-GCST90094775 | ebi-met1400-GCST90200511 | X-12906 levels \|\| id:ebi-met1400-GCST90200511 | Green olives liking \|\| id:ebi-fl187-GCST90094775 | Weighted mode | 24 | -0.145924255 | 0.156234807450703 | 0.360003611701838 | -0.452144478 | 0.160295967668424 | 0.864223161345117 | 0.636262237482371 | 1.17385824367117 | 0.999714303421225 |
| ebi-fl187-GCST90094775 | ebi-met1400-GCST90200515 | X-12849 levels \|\| id:ebi-met1400-GCST90200515 | Green olives liking \|\| id:ebi-fl187-GCST90094775 | Weighted mode | 24 | 0.0673693745337933 | 0.133252044094081 | 0.617963623780877 | -0.193804632 | 0.328543380958192 | 1.06969052164835 | 0.82381883647612 | 1.38894349272078 | 0.999714303421225 |
| ebi-fl187-GCST90094775 | ebi-met1400-GCST90200551 | X-18886 levels \|\| id:ebi-met1400-GCST90200551 | Green olives liking \|\| id:ebi-fl187-GCST90094775 | Weighted mode | 24 | 0.104182884682804 | 0.115388944922686 | 0.375946470179519 | -0.121979447 | 0.330345216731268 | 1.10980340240025 | 0.88516656082033 | 1.39144839682795 | 0.999714303421225 |
| ebi-fl187-GCST90094775 | ebi-met1400-GCST90200564 | X-21319 levels \|\| id:ebi-met1400-GCST90200564 | Green olives liking \|\| id:ebi-fl187-GCST90094775 | Weighted mode | 24 | -0.000847319 | 0.110507411712742 | 0.993948371992323 | -0.217441846 | 0.215747207618522 | 0.999153039535212 | 0.80457439254091 | 1.24078867742698 | 0.999714303421225 |
| ebi-fl187-GCST90094775 | ebi-met1400-GCST90200579 | X-21442 levels \|\| id:ebi-met1400-GCST90200579 | Green olives liking \|\| id:ebi-fl187-GCST90094775 | Weighted mode | 24 | 0.111497351620136 | 0.128115372549322 | 0.393135425594021 | -0.139608779 | 0.362603481816807 | 1.11795078324327 | 0.869698413503302 | 1.43706592348462 | 0.999714303421225 |
| ebi-fl187-GCST90094775 | ebi-met1400-GCST90200581 | X-21441 levels \|\| id:ebi-met1400-GCST90200581 | Green olives liking \|\| id:ebi-fl187-GCST90094775 | Weighted mode | 24 | -0.039258136 | 0.124045275720916 | 0.754490956940388 | -0.282386877 | 0.203870604249152 | 0.961502478547759 | 0.753981930152124 | 1.22613948595155 | 0.999714303421225 |
| ebi-fl187-GCST90094775 | ebi-met1400-GCST90200648 | X-24585 levels \|\| id:ebi-met1400-GCST90200648 | Green olives liking \|\| id:ebi-fl187-GCST90094775 | Weighted mode | 24 | 0.0455179022492345 | 0.115589791556227 | 0.697364407168338 | -0.181038089 | 0.27207389369944 | 1.04656974039858 | 0.834403576325001 | 1.31268399680411 | 0.999714303421225 |
| ebi-fl187-GCST90094775 | ebi-met1400-GCST90200720 | Adenosine 5'-diphosphate (ADP) to pantothenate ratio \|\| id:ebi-met1400-GCST90200720 | Green olives liking \|\| id:ebi-fl187-GCST90094775 | Weighted mode | 24 | 0.104037151582518 | 0.181733165677001 | 0.572556311384135 | -0.252159853 | 0.460234156309441 | 1.10964167909421 | 0.777120502983543 | 1.58444494934282 | 0.999714303421225 |
| ebi-fl187-GCST90094775 | ebi-met1400-GCST90200724 | Cholate to taurocholate ratio \|\| id:ebi-met1400-GCST90200724 | Green olives liking \|\| id:ebi-fl187-GCST90094775 | Weighted mode | 24 | 0.176460240468555 | 0.130177315863085 | 0.188408986362385 | -0.078687299 | 0.431607779560201 | 1.19298699321277 | 0.924328918183795 | 1.5397310827095 | 0.999714303421225 |
| ebi-fl187-GCST90094775 | ebi-met1400-GCST90200726 | N-acetylputrescine to (N(1) + N(8))-acetylspermidine ratio \|\| id:ebi-met1400-GCST90200726 | Green olives liking \|\| id:ebi-fl187-GCST90094775 | Weighted mode | 24 | 0.0108874110763041 | 0.117470061732126 | 0.926958440604116 | -0.21935391 | 0.24112873207127 | 1.01094689461419 | 0.80303746493268 | 1.27268486012137 | 0.999714303421225 |
| ebi-fl187-GCST90094775 | ebi-met1400-GCST90200744 | Arginine to glutamate ratio \|\| id:ebi-met1400-GCST90200744 | Green olives liking \|\| id:ebi-fl187-GCST90094775 | Weighted mode | 24 | -0.017750726 | 0.143005358305856 | 0.902293707363923 | -0.298041228 | 0.262539776272073 | 0.982405890077271 | 0.742270736566049 | 1.30022818536998 | 0.999714303421225 |
| ebi-fl187-GCST90094775 | ebi-met1400-GCST90200759 | Histidine to trans-urocanate ratio \|\| id:ebi-met1400-GCST90200759 | Green olives liking \|\| id:ebi-fl187-GCST90094775 | Weighted mode | 24 | 0.0874093318038829 | 0.109845189833971 | 0.434313647921934 | -0.12788724 | 0.302705903878466 | 1.09134330982775 | 0.879952596767271 | 1.35351634199541 | 0.999714303421225 |
| ebi-fl187-GCST90094775 | ebi-met1400-GCST90200795 | Oleoyl-linoleoyl-glycerol (18:1 to 18:2) [2] to linoleoyl-arachidonoyl-glycerol (18:2 to 20:4) [2] ratio \|\| id:ebi-met1400-GCST90200795 | Green olives liking \|\| id:ebi-fl187-GCST90094775 | Weighted mode | 24 | 0.0341252893346476 | 0.118475670012894 | 0.775898609103066 | -0.198087024 | 0.266337602559921 | 1.03471423726562 | 0.820298464467764 | 1.30517561494503 | 0.999714303421225 |
| ebi-fl187-GCST90094775 | ebi-met1400-GCST90200811 | Choline to taurocholate ratio \|\| id:ebi-met1400-GCST90200811 | Green olives liking \|\| id:ebi-fl187-GCST90094775 | Weighted mode | 24 | 0.00246005323970544 | 0.124582067470928 | 0.984415944873998 | -0.241720799 | 0.246640905482724 | 1.00246308165352 | 0.785275396620724 | 1.27971949000669 | 0.999714303421225 |
| ebi-fl187-GCST90094775 | ebi-met1400-GCST90200831 | Cholate to phosphate ratio \|\| id:ebi-met1400-GCST90200831 | Green olives liking \|\| id:ebi-fl187-GCST90094775 | Weighted mode | 24 | 0.0562417085306444 | 0.108963549777628 | 0.610675488030227 | -0.157326849 | 0.269810266094794 | 1.05785334501527 | 0.854424745271295 | 1.30971592963951 | 0.999714303421225 |
| ebi-fl187-GCST90094775 | ebi-met1400-GCST90200841 | Adenosine 5'-diphosphate (ADP) to oxalate (ethanedioate) ratio \|\| id:ebi-met1400-GCST90200841 | Green olives liking \|\| id:ebi-fl187-GCST90094775 | Weighted mode | 24 | -0.113297373 | 0.173315487260849 | 0.519783994371547 | -0.452995728 | 0.226400981865699 | 0.892885100563989 | 0.635720849294953 | 1.25407842717971 | 0.999714303421225 |
| ebi-fl187-GCST90094775 | ebi-met1400-GCST90200844 | Aspartate to phosphate ratio \|\| id:ebi-met1400-GCST90200844 | Green olives liking \|\| id:ebi-fl187-GCST90094775 | Weighted mode | 24 | -0.12321113 | 0.107180142498865 | 0.262134704679601 | -0.33328421 | 0.0868619489524811 | 0.884076987375505 | 0.716566510100513 | 1.09074609068363 | 0.999714303421225 |
| ebi-fl187-GCST90094775 | ebi-met1400-GCST90200870 | Adenosine 5'-monophosphate (AMP) to acetoacetate ratio \|\| id:ebi-met1400-GCST90200870 | Green olives liking \|\| id:ebi-fl187-GCST90094775 | Weighted mode | 24 | -0.0537513 | 0.13386866551004 | 0.691741796364727 | -0.316133884 | 0.208631284504961 | 0.947667762269025 | 0.72896184709147 | 1.23199066072835 | 0.999714303421225 |
| ebi-fl187-GCST90094775 | ebi-met1400-GCST90200887 | Cholesterol to cortisol ratio \|\| id:ebi-met1400-GCST90200887 | Green olives liking \|\| id:ebi-fl187-GCST90094775 | Weighted mode | 24 | 0.0151526744305713 | 0.142537153872355 | 0.916260687809823 | -0.264220147 | 0.294525496020387 | 1.01526805825631 | 0.767804491072041 | 1.3424891910652 | 0.999714303421225 |
| ebi-fl187-GCST90094775 | ebi-met1400-GCST90200890 | Cortisol to taurocholate ratio \|\| id:ebi-met1400-GCST90200890 | Green olives liking \|\| id:ebi-fl187-GCST90094775 | Weighted mode | 24 | 0.144779271175236 | 0.122017289122904 | 0.247518532593694 | -0.094374616 | 0.383933157856127 | 1.15578442717205 | 0.9099418200686 | 1.46804731097282 | 0.999714303421225 |
| ebi-fl187-GCST90094775 | ebi-met1400-GCST90200948 | Adenosine 5'-diphosphate (ADP) to mannitol to sorbitol ratio \|\| id:ebi-met1400-GCST90200948 | Green olives liking \|\| id:ebi-fl187-GCST90094775 | Weighted mode | 24 | -0.195823372 | 0.136677866390372 | 0.165386111976963 | -0.46371199 | 0.0720652459789655 | 0.822157437758102 | 0.628944670568189 | 1.07472546329126 | 0.999714303421225 |
| ebi-fl187-GCST90094775 | ebi-met1400-GCST90200972 | Leucine to N-palmitoyl-sphingosine (d18:1 to 16:0) ratio \|\| id:ebi-met1400-GCST90200972 | Green olives liking \|\| id:ebi-fl187-GCST90094775 | Weighted mode | 24 | -0.133727523 | 0.116272959894141 | 0.261914094011215 | -0.361622525 | 0.094167478143217 | 0.87482840253163 | 0.696545246890029 | 1.0987437460712 | 0.999714303421225 |
| ebi-fl187-GCST90094775 | ebi-met1400-GCST90200973 | Proline to glutamate ratio \|\| id:ebi-met1400-GCST90200973 | Green olives liking \|\| id:ebi-fl187-GCST90094775 | Weighted mode | 24 | 0.0959637251270337 | 0.10303318351184 | 0.361327815119714 | -0.105981315 | 0.29790876481024 | 1.10071913482298 | 0.899441454381679 | 1.34703888492482 | 0.999714303421225 |
| ebi-fl187-GCST90094775 | ebi-met1400-GCST90200983 | Cholesterol to linoleoyl-arachidonoyl-glycerol (18:2 to 20:4) [1] ratio \|\| id:ebi-met1400-GCST90200983 | Green olives liking \|\| id:ebi-fl187-GCST90094775 | Weighted mode | 24 | -0.014455407 | 0.120403128367606 | 0.905479476003411 | -0.250445539 | 0.221534724511162 | 0.985648570691052 | 0.778453874477199 | 1.24799058333131 | 0.999714303421225 |
| ebi-fl187-GCST90094775 | ebi-met1400-GCST90201001 | Serine to alpha-ketobutyrate ratio \|\| id:ebi-met1400-GCST90201001 | Green olives liking \|\| id:ebi-fl187-GCST90094775 | Weighted mode | 24 | -0.005743836 | 0.128810305389177 | 0.964817863524701 | -0.258212034 | 0.246724362666499 | 0.994272628391309 | 0.772431432660982 | 1.27982629624816 | 0.999714303421225 |
| ebi-fl187-GCST90094775 | ebi-met1400-GCST90201005 | Fructose to maltose ratio \|\| id:ebi-met1400-GCST90201005 | Green olives liking \|\| id:ebi-fl187-GCST90094775 | Weighted mode | 24 | 0.0357311986102286 | 0.157533949681295 | 0.822570760938417 | -0.273035343 | 0.344497739985567 | 1.03637722940668 | 0.761065888980653 | 1.41128091165832 | 0.999714303421225 |
| ebi-fl187-GCST90094775 | ebi-met1400-GCST90201020 | Paraxanthine to 5-acetylamino-6-formylamino-3-methyluracil ratio \|\| id:ebi-met1400-GCST90201020 | Green olives liking \|\| id:ebi-fl187-GCST90094775 | Weighted mode | 24 | -0.036934813 | 0.114364155895197 | 0.749644867281778 | -0.261088558 | 0.187218932774612 | 0.963738956764877 | 0.7702127078401 | 1.2058912652725 | 0.999714303421225 |
| ebi-fl187-GCST90094775 | ebi-pw11-GCST90275197 | Placental weight (fetal genotype, adjusted for sex) \|\| id:ebi-pw11-GCST90275197 | Green olives liking \|\| id:ebi-fl187-GCST90094775 | Weighted mode | 23 | 0.0507356446908387 | 0.0420211288258804 | 0.24010533466637 | -0.031625768 | 0.133097057189564 | 1.0520447429063 | 0.96886909624849 | 1.14236086728574 | 0.999714303421225 |
| ebi-fl187-GCST90094775 | prot-a-1017 | FAS-associated factor 2 \|\| id:prot-a-1017 | Green olives liking \|\| id:ebi-fl187-GCST90094775 | Weighted mode | 27 | -0.114693907 | 0.195157829768325 | 0.561804934587524 | -0.497203253 | 0.267815439585129 | 0.891639026818819 | 0.608229346742477 | 1.30710587774881 | 0.999714303421225 |
| ebi-fl187-GCST90094775 | prot-a-1019 | Fas apoptotic inhibitory molecule 3 \|\| id:prot-a-1019 | Green olives liking \|\| id:ebi-fl187-GCST90094775 | Weighted mode | 27 | 0.019521560844707 | 0.194996304037156 | 0.921023007057103 | -0.362671195 | 0.401714316757532 | 1.01971335250488 | 0.695815183353959 | 1.49438435112129 | 0.999714303421225 |
| ebi-fl187-GCST90094775 | prot-a-1052 | MIP18 family protein FAM96A \|\| id:prot-a-1052 | Green olives liking \|\| id:ebi-fl187-GCST90094775 | Weighted mode | 27 | 0.165078288937786 | 0.188898101368631 | 0.390172691620876 | -0.20516199 | 0.535318567620303 | 1.17948545576032 | 0.814515362588455 | 1.70799226662721 | 0.999714303421225 |
| ebi-fl187-GCST90094775 | prot-a-1066 | F-box/LRR-repeat protein 4 \|\| id:prot-a-1066 | Green olives liking \|\| id:ebi-fl187-GCST90094775 | Weighted mode | 27 | 0.0640505652139127 | 0.236935304064812 | 0.789041198428946 | -0.400342631 | 0.528443761180945 | 1.06614630731464 | 0.670090413115332 | 1.69629042044659 | 0.999714303421225 |
| ebi-fl187-GCST90094775 | prot-a-1076 | Low affinity immunoglobulin gamma Fc region receptor III-B \|\| id:prot-a-1076 | Green olives liking \|\| id:ebi-fl187-GCST90094775 | Weighted mode | 27 | -0.123318571 | 0.221321815721303 | 0.58216227021313 | -0.557109329 | 0.310472188265856 | 0.883982007067307 | 0.572862629924063 | 1.3640690594922 | 0.999714303421225 |
| ebi-fl187-GCST90094775 | prot-a-1081 | Fc receptor-like protein 3 \|\| id:prot-a-1081 | Green olives liking \|\| id:ebi-fl187-GCST90094775 | Weighted mode | 27 | -0.236780249 | 0.180013182921089 | 0.199874909988373 | -0.589606088 | 0.116045589486784 | 0.789164688663508 | 0.554545684157943 | 1.12304707010576 | 0.999714303421225 |
| ebi-fl187-GCST90094775 | prot-a-1105 | Fibroblast growth factor receptor 4 \|\| id:prot-a-1105 | Green olives liking \|\| id:ebi-fl187-GCST90094775 | Weighted mode | 27 | -0.082528207 | 0.218351703232738 | 0.708528522051929 | -0.510497545 | 0.345441131274254 | 0.92078546483788 | 0.600196879314903 | 1.41261292998472 | 0.999714303421225 |
| ebi-fl187-GCST90094775 | prot-a-1143 | Follistatin-related protein 1 \|\| id:prot-a-1143 | Green olives liking \|\| id:ebi-fl187-GCST90094775 | Weighted mode | 27 | -0.072614701 | 0.289951066068091 | 0.804217235844098 | -0.640918791 | 0.495689388051991 | 0.929959072896412 | 0.526808175039646 | 1.64162956886018 | 0.999714303421225 |
| ebi-fl187-GCST90094775 | prot-a-1151 | Alpha-(1,3)-fucosyltransferase 10 \|\| id:prot-a-1151 | Green olives liking \|\| id:ebi-fl187-GCST90094775 | Weighted mode | 27 | 0.0654702513527972 | 0.155050364696531 | 0.676312831278827 | -0.238428463 | 0.369368966157998 | 1.06766097537137 | 0.78786504738671 | 1.44682133331326 | 0.999714303421225 |
| ebi-fl187-GCST90094775 | prot-a-1165 | Growth arrest and DNA damage-inducible proteins-interacting protein 1 \|\| id:prot-a-1165 | Green olives liking \|\| id:ebi-fl187-GCST90094775 | Weighted mode | 27 | 0.200635663343611 | 0.193438174841647 | 0.309189500202466 | -0.178503159 | 0.579774486033239 | 1.22217940593843 | 0.83652141401262 | 1.78563569955123 | 0.999714303421225 |
| ebi-fl187-GCST90094775 | prot-a-1194 | Growth/differentiation factor 11/8 \|\| id:prot-a-1194 | Green olives liking \|\| id:ebi-fl187-GCST90094775 | Weighted mode | 26 | -0.275340799 | 0.179168114686789 | 0.136909925453752 | -0.626510304 | 0.0758287059609042 | 0.759313306042518 | 0.534453631413497 | 1.07877777012829 | 0.999714303421225 |
| ebi-fl187-GCST90094775 | prot-a-1216 | D-glucuronyl C5-epimerase \|\| id:prot-a-1216 | Green olives liking \|\| id:ebi-fl187-GCST90094775 | Weighted mode | 27 | 0.0759605351290094 | 0.176334101770044 | 0.670181368682977 | -0.269654304 | 0.421575374598296 | 1.07891999387871 | 0.763643436934043 | 1.52436110479108 | 0.999714303421225 |
| ebi-fl187-GCST90094775 | prot-a-1246 | Glypican-3 \|\| id:prot-a-1246 | Green olives liking \|\| id:ebi-fl187-GCST90094775 | Weighted mode | 27 | -0.011818984 | 0.180645862528243 | 0.948335092115064 | -0.365884875 | 0.342246906594781 | 0.988250585879015 | 0.693582645651425 | 1.40810792572951 | 0.999714303421225 |
| ebi-fl187-GCST90094775 | prot-a-1275 | Glutamate receptor 4 \|\| id:prot-a-1275 | Green olives liking \|\| id:ebi-fl187-GCST90094775 | Weighted mode | 26 | 0.260322707527553 | 0.179724761824346 | 0.159917748329907 | -0.091937826 | 0.61258324070327 | 1.29734868330596 | 0.912161860854545 | 1.84519182209494 | 0.999714303421225 |
| ebi-fl187-GCST90094775 | prot-a-1289 | Glutathione S-transferase P \|\| id:prot-a-1289 | Green olives liking \|\| id:ebi-fl187-GCST90094775 | Weighted mode | 27 | 0.182216807573022 | 0.167220547328422 | 0.285851253676754 | -0.145535465 | 0.509969080336729 | 1.19987430751809 | 0.864559227772362 | 1.66523970549891 | 0.999714303421225 |
| ebi-fl187-GCST90094775 | prot-a-130 | Apolipoprotein D \|\| id:prot-a-130 | Green olives liking \|\| id:ebi-fl187-GCST90094775 | Weighted mode | 27 | 0.00997368351142125 | 0.165808624333001 | 0.952494733652205 | -0.31501122 | 0.334958587204103 | 1.01002358646024 | 0.729780685951543 | 1.39788249380135 | 0.999714303421225 |
| ebi-fl187-GCST90094775 | prot-a-1313 | Heparin-binding EGF-like growth factor \|\| id:prot-a-1313 | Green olives liking \|\| id:ebi-fl187-GCST90094775 | Weighted mode | 27 | 0.022079044468442 | 0.164947831605537 | 0.894548192235309 | -0.301218705 | 0.345376794415294 | 1.02232459038059 | 0.7399159313818 | 1.41252204982938 | 0.999714303421225 |
| ebi-fl187-GCST90094775 | prot-a-1347 | HLA class II histocompatibility antigen, DQ alpha 2 chain \|\| id:prot-a-1347 | Green olives liking \|\| id:ebi-fl187-GCST90094775 | Weighted mode | 26 | -0.116006377 | 0.163616962515283 | 0.484877058916276 | -0.436695623 | 0.20468286967088 | 0.89046954488057 | 0.646168079931789 | 1.22713584125591 | 0.999714303421225 |
| ebi-fl187-GCST90094775 | prot-a-1357 | Non-histone chromosomal protein HMG-14 \|\| id:prot-a-1357 | Green olives liking \|\| id:ebi-fl187-GCST90094775 | Weighted mode | 27 | 0.16465729129112 | 0.20985348615398 | 0.439761643362516 | -0.246655542 | 0.57597012415292 | 1.17898899966993 | 0.781409810374869 | 1.77885540069667 | 0.999714303421225 |
| ebi-fl187-GCST90094775 | prot-a-137 | Apolipoprotein M \|\| id:prot-a-137 | Green olives liking \|\| id:ebi-fl187-GCST90094775 | Weighted mode | 27 | -0.126267901 | 0.179982206669862 | 0.489187670708098 | -0.479033027 | 0.226497223591502 | 0.881378692494368 | 0.619382028300773 | 1.25419912765995 | 0.999714303421225 |
| ebi-fl187-GCST90094775 | prot-a-1381 | 17-beta-hydroxysteroid dehydrogenase 14 \|\| id:prot-a-1381 | Green olives liking \|\| id:ebi-fl187-GCST90094775 | Weighted mode | 26 | -0.137704864 | 0.16901820121985 | 0.422918342448774 | -0.468980539 | 0.193570809965619 | 0.87135582190108 | 0.625639758728177 | 1.21357531673556 | 0.999714303421225 |
| ebi-fl187-GCST90094775 | prot-a-1392 | Serine protease HTRA2, mitochondrial \|\| id:prot-a-1392 | Green olives liking \|\| id:ebi-fl187-GCST90094775 | Weighted mode | 27 | -0.298001112 | 0.270401894703287 | 0.28052992469337 | -0.827988826 | 0.231986601504789 | 0.742300514224779 | 0.436927139888079 | 1.26110283183488 | 0.999714303421225 |
| ebi-fl187-GCST90094775 | prot-a-1416 | Interferon-induced protein with tetratricopeptide repeats 2 \|\| id:prot-a-1416 | Green olives liking \|\| id:ebi-fl187-GCST90094775 | Weighted mode | 27 | 0.101855205596035 | 0.192141812389309 | 0.600537980840946 | -0.274742747 | 0.478453157879081 | 1.10722314 | 0.759767550806241 | 1.61357652264775 | 0.999714303421225 |
| ebi-fl187-GCST90094775 | prot-a-1428 | Interferon gamma \|\| id:prot-a-1428 | Green olives liking \|\| id:ebi-fl187-GCST90094775 | Weighted mode | 27 | -0.106829713 | 0.169523965406043 | 0.534080113445633 | -0.439096685 | 0.225437259086961 | 0.898678693162043 | 0.644618451485328 | 1.25287042541602 | 0.999714303421225 |
| ebi-fl187-GCST90094775 | prot-a-1483 | Interleukin-17C \|\| id:prot-a-1483 | Green olives liking \|\| id:ebi-fl187-GCST90094775 | Weighted mode | 27 | 0.182398277384777 | 0.177404087449644 | 0.313349169107344 | -0.165313734 | 0.530110288786078 | 1.20009206824071 | 0.847627732714862 | 1.69911969213346 | 0.999714303421225 |
| ebi-fl187-GCST90094775 | prot-a-1503 | Interleukin-1 receptor-like 2 \|\| id:prot-a-1503 | Green olives liking \|\| id:ebi-fl187-GCST90094775 | Weighted mode | 27 | 0.135732064400583 | 0.177618497607261 | 0.45164139394312 | -0.212400191 | 0.483864319710815 | 1.14537496575024 | 0.808641022018633 | 1.62233151230998 | 0.999714303421225 |
| ebi-fl187-GCST90094775 | prot-a-1522 | Interleukin-31 receptor subunit alpha \|\| id:prot-a-1522 | Green olives liking \|\| id:ebi-fl187-GCST90094775 | Weighted mode | 27 | -0.105661687 | 0.175264408215878 | 0.55181791216679 | -0.449179927 | 0.237856552713709 | 0.899728986254461 | 0.638151267406025 | 1.26852721298668 | 0.999714303421225 |
| ebi-fl187-GCST90094775 | prot-a-1540 | Interleukin-6 receptor subunit alpha \|\| id:prot-a-1540 | Green olives liking \|\| id:ebi-fl187-GCST90094775 | Weighted mode | 27 | -0.040742869 | 0.177323039995676 | 0.820071373532593 | -0.388296028 | 0.306810289170505 | 0.960075963292535 | 0.678211544200306 | 1.35908311082929 | 0.999714303421225 |
| ebi-fl187-GCST90094775 | prot-a-1541 | Interleukin-6 receptor subunit alpha \|\| id:prot-a-1541 | Green olives liking \|\| id:ebi-fl187-GCST90094775 | Weighted mode | 27 | -0.183978164 | 0.202543198307126 | 0.3720415056672 | -0.580962832 | 0.21300650494309 | 0.831953970392466 | 0.559359537708268 | 1.23739270038652 | 0.999714303421225 |
| ebi-fl187-GCST90094775 | prot-a-1545 | Interleukin-9 \|\| id:prot-a-1545 | Green olives liking \|\| id:ebi-fl187-GCST90094775 | Weighted mode | 27 | 0.116557377897386 | 0.177847114829691 | 0.517980729385236 | -0.232022967 | 0.465137722963579 | 1.12362197968428 | 0.792927911788348 | 1.5922334608983 | 0.999714303421225 |
| ebi-fl187-GCST90094775 | prot-a-1548 | Inositol monophosphatase 3 \|\| id:prot-a-1548 | Green olives liking \|\| id:ebi-fl187-GCST90094775 | Weighted mode | 27 | -0.253099084 | 0.195026921680029 | 0.205764235641747 | -0.63535185 | 0.129153682973854 | 0.776390950464992 | 0.529749063308464 | 1.13786498120327 | 0.999714303421225 |
| ebi-fl187-GCST90094775 | prot-a-1555 | Inhibin beta A chain:Inhibin beta B chain heterodimer \|\| id:prot-a-1555 | Green olives liking \|\| id:ebi-fl187-GCST90094775 | Weighted mode | 27 | -0.204231467 | 0.197167117302807 | 0.309820340222295 | -0.590679017 | 0.182216082569259 | 0.815273640121286 | 0.553951014703113 | 1.19987343760502 | 0.999714303421225 |
| ebi-fl187-GCST90094775 | prot-a-1603 | Junctophilin-3 \|\| id:prot-a-1603 | Green olives liking \|\| id:ebi-fl187-GCST90094775 | Weighted mode | 27 | -0.011114685 | 0.216386693946932 | 0.959427336071508 | -0.435232605 | 0.413003234909407 | 0.988946854677354 | 0.647114127354324 | 1.51134991500614 | 0.999714303421225 |
| ebi-fl187-GCST90094775 | prot-a-1627 | UPF0258 protein KIAA1024 \|\| id:prot-a-1627 | Green olives liking \|\| id:ebi-fl187-GCST90094775 | Weighted mode | 27 | 0.302314149188012 | 0.210932297900647 | 0.16370892400007 | -0.111113155 | 0.71574145307328 | 1.35298619946952 | 0.894837488135009 | 2.04570291279391 | 0.999714303421225 |
| ebi-fl187-GCST90094775 | prot-a-1666 | Kallikrein-7 \|\| id:prot-a-1666 | Green olives liking \|\| id:ebi-fl187-GCST90094775 | Weighted mode | 27 | 0.0765523196865225 | 0.153345400808144 | 0.621825971918022 | -0.224004666 | 0.377109305270485 | 1.07955867103088 | 0.799311404855627 | 1.45806367470568 | 0.999714303421225 |
| ebi-fl187-GCST90094775 | prot-a-1678 | Importin subunit alpha-3 \|\| id:prot-a-1678 | Green olives liking \|\| id:ebi-fl187-GCST90094775 | Weighted mode | 27 | -0.03289101 | 0.200604990029421 | 0.871031286092843 | -0.42607679 | 0.360294770809187 | 0.967644017703606 | 0.653066191173012 | 1.4337519805087 | 0.999714303421225 |
| ebi-fl187-GCST90094775 | prot-a-1718 | Protein LDOC1 \|\| id:prot-a-1718 | Green olives liking \|\| id:ebi-fl187-GCST90094775 | Weighted mode | 27 | 0.137257650403591 | 0.19387718799109 | 0.485267723980781 | -0.242741638 | 0.517256938866128 | 1.14712366732435 | 0.784474165859834 | 1.67742006735602 | 0.999714303421225 |
| ebi-fl187-GCST90094775 | prot-a-1723 | Leptin \|\| id:prot-a-1723 | Green olives liking \|\| id:ebi-fl187-GCST90094775 | Weighted mode | 27 | -0.245269699 | 0.183615627442282 | 0.19319774695613 | -0.605156329 | 0.114616930529251 | 0.782493471872468 | 0.545989066018716 | 1.12144376441055 | 0.999714303421225 |
| ebi-fl187-GCST90094775 | prot-a-1730 | Galectin-7 \|\| id:prot-a-1730 | Green olives liking \|\| id:ebi-fl187-GCST90094775 | Weighted mode | 27 | -0.270162544 | 0.177399330231366 | 0.139853509039408 | -0.617865231 | 0.0775401430552292 | 0.763255421512919 | 0.539094051103562 | 1.08062561120147 | 0.999714303421225 |
| ebi-fl187-GCST90094775 | prot-a-1753 | Lipase member N \|\| id:prot-a-1753 | Green olives liking \|\| id:ebi-fl187-GCST90094775 | Weighted mode | 27 | -0.009924576 | 0.159062190587114 | 0.950726020304427 | -0.321686469 | 0.301837317750552 | 0.990124510281823 | 0.724925441047065 | 1.35234120690375 | 0.999714303421225 |
| ebi-fl187-GCST90094775 | prot-a-1756 | VIP36-like protein \|\| id:prot-a-1756 | Green olives liking \|\| id:ebi-fl187-GCST90094775 | Weighted mode | 27 | -0.272853948 | 0.21487418915146 | 0.215392629576502 | -0.694007359 | 0.148299462870786 | 0.761203954972598 | 0.499570095902045 | 1.15986017942023 | 0.999714303421225 |
| ebi-fl187-GCST90094775 | prot-a-1760 | Ig Kappa chain V-I region HK102- like \|\| id:prot-a-1760 | Green olives liking \|\| id:ebi-fl187-GCST90094775 | Weighted mode | 27 | -0.057165533 | 0.17606516802827 | 0.748018284675148 | -0.402253262 | 0.287922196823915 | 0.94443772127858 | 0.668811339844074 | 1.33365353760573 | 0.999714303421225 |
| ebi-fl187-GCST90094775 | prot-a-179 | Neutral ceramidase \|\| id:prot-a-179 | Green olives liking \|\| id:ebi-fl187-GCST90094775 | Weighted mode | 27 | 0.0613085674810356 | 0.221219629567992 | 0.783864992397285 | -0.372281906 | 0.494899041434301 | 1.06322694083432 | 0.689159936490947 | 1.64033262506801 | 0.999714303421225 |
| ebi-fl187-GCST90094775 | prot-a-1809 | Lactotransferrin \|\| id:prot-a-1809 | Green olives liking \|\| id:ebi-fl187-GCST90094775 | Weighted mode | 27 | -0.051610587 | 0.237213004140684 | 0.829462104123438 | -0.516548075 | 0.41332690146819 | 0.949698620168272 | 0.596576334623029 | 1.51183916760533 | 0.999714303421225 |
| ebi-fl187-GCST90094775 | prot-a-1818 | Lysophospholipase-like protein 1 \|\| id:prot-a-1818 | Green olives liking \|\| id:ebi-fl187-GCST90094775 | Weighted mode | 27 | -0.047523732 | 0.181962751923583 | 0.796016911433186 | -0.404170726 | 0.309123261925463 | 0.953587842465451 | 0.667530147045173 | 1.36223027128148 | 0.999714303421225 |
| ebi-fl187-GCST90094775 | prot-a-1852 | MAP kinase-activated protein kinase 3 \|\| id:prot-a-1852 | Green olives liking \|\| id:ebi-fl187-GCST90094775 | Weighted mode | 27 | -0.135204367 | 0.179376098962163 | 0.457772745605571 | -0.486781521 | 0.216372787333447 | 0.873537371553939 | 0.614601296000716 | 1.24156513249604 | 0.999714303421225 |
| ebi-fl187-GCST90094775 | prot-a-1856 | Megakaryocyte-associated tyrosine-protein kinase \|\| id:prot-a-1856 | Green olives liking \|\| id:ebi-fl187-GCST90094775 | Weighted mode | 27 | -0.073153544 | 0.165149336990075 | 0.661462729313848 | -0.396846244 | 0.250539156827565 | 0.929458106657359 | 0.672437408854116 | 1.28471789441819 | 0.999714303421225 |
| ebi-fl187-GCST90094775 | prot-a-1890 | Beta-1,3-N-acetylglucosaminyltransferase manic fringe \|\| id:prot-a-1890 | Green olives liking \|\| id:ebi-fl187-GCST90094775 | Weighted mode | 27 | -0.059946355 | 0.161131413250579 | 0.712882684622001 | -0.375763925 | 0.255871214823395 | 0.941815055758631 | 0.686764441740596 | 1.29158638004831 | 0.999714303421225 |
| ebi-fl187-GCST90094775 | prot-a-1919 | Matrilysin \|\| id:prot-a-1919 | Green olives liking \|\| id:ebi-fl187-GCST90094775 | Weighted mode | 27 | 0.114049241064151 | 0.241464916487106 | 0.640637301151891 | -0.359221995 | 0.587320477378879 | 1.12080731327019 | 0.69821933276978 | 1.7991610580255 | 0.999714303421225 |
| ebi-fl187-GCST90094775 | prot-a-1923 | Multimerin-2 \|\| id:prot-a-1923 | Green olives liking \|\| id:ebi-fl187-GCST90094775 | Weighted mode | 27 | -0.187543318 | 0.17897432468087 | 0.304338564870097 | -0.538332994 | 0.163246358280794 | 0.828993206997177 | 0.583720507086175 | 1.1773266981453 | 0.999714303421225 |
| ebi-fl187-GCST90094775 | prot-a-1948 | Mesothelin \|\| id:prot-a-1948 | Green olives liking \|\| id:ebi-fl187-GCST90094775 | Weighted mode | 27 | 0.159623218152055 | 0.173980481884772 | 0.367325183615722 | -0.181378526 | 0.500624962646208 | 1.17306879668527 | 0.834119562704067 | 1.64975198195279 | 0.999714303421225 |
| ebi-fl187-GCST90094775 | prot-a-195 | ATPase family AAA domain-containing protein 1 \|\| id:prot-a-195 | Green olives liking \|\| id:ebi-fl187-GCST90094775 | Weighted mode | 27 | -0.045169003 | 0.186345701817016 | 0.810377086657437 | -0.410406578 | 0.32006857292273 | 0.955835929387934 | 0.663380479257403 | 1.3772222012496 | 0.999714303421225 |
| ebi-fl187-GCST90094775 | prot-a-1966 | Mucin-1 \|\| id:prot-a-1966 | Green olives liking \|\| id:ebi-fl187-GCST90094775 | Weighted mode | 27 | 0.0930966754706559 | 0.21684849776652 | 0.671229516953769 | -0.33192638 | 0.518119731093035 | 1.09756783802655 | 0.717540146106689 | 1.67886795687549 | 0.999714303421225 |
| ebi-fl187-GCST90094775 | prot-a-1986 | Myocardial zonula adherens protein \|\| id:prot-a-1986 | Green olives liking \|\| id:ebi-fl187-GCST90094775 | Weighted mode | 27 | 0.0581034999575139 | 0.185058072983082 | 0.756045855317088 | -0.304610323 | 0.420817323004355 | 1.05982468184348 | 0.737410670323606 | 1.52320599829632 | 0.999714303421225 |
| ebi-fl187-GCST90094775 | prot-a-2071 | Epididymal secretory protein E1 \|\| id:prot-a-2071 | Green olives liking \|\| id:ebi-fl187-GCST90094775 | Weighted mode | 27 | -0.361018024 | 0.251898563477717 | 0.16372031810082 | -0.854739209 | 0.132703159927878 | 0.696966435890185 | 0.425394115614895 | 1.14191098307815 | 0.999714303421225 |
| ebi-fl187-GCST90094775 | prot-a-2098 | Neuritin \|\| id:prot-a-2098 | Green olives liking \|\| id:ebi-fl187-GCST90094775 | Weighted mode | 27 | -0.064134792 | 0.191560273407779 | 0.740460407452019 | -0.439592928 | 0.311323343787721 | 0.937878572395861 | 0.644298643664085 | 1.36523058865525 | 0.999714303421225 |
| ebi-fl187-GCST90094775 | prot-a-2115 | Neurotrophin-4 \|\| id:prot-a-2115 | Green olives liking \|\| id:ebi-fl187-GCST90094775 | Weighted mode | 27 | 0.0120257368311958 | 0.169779858399875 | 0.944074001532892 | -0.320742786 | 0.344794259294951 | 1.01209833673493 | 0.725609864271551 | 1.41169944574824 | 0.999714303421225 |
| ebi-fl187-GCST90094775 | prot-a-2118 | Netrin-1 \|\| id:prot-a-2118 | Green olives liking \|\| id:ebi-fl187-GCST90094775 | Weighted mode | 27 | 0.109150110525241 | 0.202185769799204 | 0.593894165236508 | -0.287133998 | 0.50543421933168 | 1.11532976051229 | 0.750411168293005 | 1.65770517183811 | 0.999714303421225 |
| ebi-fl187-GCST90094775 | prot-a-2120 | Netrin-G1 \|\| id:prot-a-2120 | Green olives liking \|\| id:ebi-fl187-GCST90094775 | Weighted mode | 27 | 0.00613741325010175 | 0.176504113645309 | 0.972527078753336 | -0.339810649 | 0.352085475994908 | 1.00615628576051 | 0.711905109593454 | 1.42203006795879 | 0.999714303421225 |
| ebi-fl187-GCST90094775 | prot-a-2145 | Olfactomedin-like protein 3 \|\| id:prot-a-2145 | Green olives liking \|\| id:ebi-fl187-GCST90094775 | Weighted mode | 27 | 0.11374707952887 | 0.180159891162836 | 0.533312304741465 | -0.239366307 | 0.466860466208028 | 1.12046869957225 | 0.787126499492612 | 1.59497883444453 | 0.999714303421225 |
| ebi-fl187-GCST90094775 | prot-a-2162 | Prolyl 4-hydroxylase subunit alpha-2 \|\| id:prot-a-2162 | Green olives liking \|\| id:ebi-fl187-GCST90094775 | Weighted mode | 27 | -0.005596526 | 0.213080080248922 | 0.979246611141686 | -0.423233484 | 0.41204043104303 | 0.994419105134079 | 0.654925700892595 | 1.50989548174387 | 0.999714303421225 |
| ebi-fl187-GCST90094775 | prot-a-2168 | Polyadenylate-binding protein-interacting protein 1 \|\| id:prot-a-2168 | Green olives liking \|\| id:ebi-fl187-GCST90094775 | Weighted mode | 27 | -0.061560123 | 0.175447940481957 | 0.72851001690257 | -0.405438086 | 0.282317840630554 | 0.940296410867667 | 0.666684681609696 | 1.32620017330507 | 0.999714303421225 |
| ebi-fl187-GCST90094775 | prot-a-219 | Beta-1,4-galactosyltransferase 5 \|\| id:prot-a-219 | Green olives liking \|\| id:ebi-fl187-GCST90094775 | Weighted mode | 27 | 0.0883471633286259 | 0.23030753966985 | 0.704390075915622 | -0.363055614 | 0.539749941081533 | 1.09236728607158 | 0.69554774993575 | 1.71557781301084 | 0.999714303421225 |
| ebi-fl187-GCST90094775 | prot-a-2190 | Pyruvate carboxylase, mitochondrial \|\| id:prot-a-2190 | Green olives liking \|\| id:ebi-fl187-GCST90094775 | Weighted mode | 27 | -0.127450079 | 0.186724947257391 | 0.500924609536489 | -0.493430976 | 0.238530817660797 | 0.88033736209071 | 0.610528089655471 | 1.26938282484295 | 0.999714303421225 |
| ebi-fl187-GCST90094775 | prot-a-2197 | Protocadherin-9 \|\| id:prot-a-2197 | Green olives liking \|\| id:ebi-fl187-GCST90094775 | Weighted mode | 27 | 0.0295703635073452 | 0.220812819292222 | 0.894500229008832 | -0.403222762 | 0.4623634893201 | 1.03001190817261 | 0.668163241160191 | 1.58782235481139 | 0.999714303421225 |
| ebi-fl187-GCST90094775 | prot-a-2198 | Protocadherin alpha-4 \|\| id:prot-a-2198 | Green olives liking \|\| id:ebi-fl187-GCST90094775 | Weighted mode | 27 | -0.045920048 | 0.184457557382248 | 0.805358292618382 | -0.40745686 | 0.315616764956402 | 0.955118323223554 | 0.665340153565111 | 1.37110469955741 | 0.999714303421225 |
| ebi-fl187-GCST90094775 | prot-a-2254 | Placenta growth factor \|\| id:prot-a-2254 | Green olives liking \|\| id:ebi-fl187-GCST90094775 | Weighted mode | 27 | -0.261876033 | 0.209521616725053 | 0.222478729911521 | -0.672538401 | 0.148786336194674 | 0.769606423912035 | 0.510411303231244 | 1.16042502189324 | 0.999714303421225 |
| ebi-fl187-GCST90094775 | prot-a-2261 | Membrane-associated progesterone receptor component 2 \|\| id:prot-a-2261 | Green olives liking \|\| id:ebi-fl187-GCST90094775 | Weighted mode | 27 | 0.0651056183952523 | 0.234371909165533 | 0.783372397511982 | -0.394263324 | 0.524474560359697 | 1.06727174196025 | 0.674176506301024 | 1.68957084760569 | 0.999714303421225 |
| ebi-fl187-GCST90094775 | prot-a-2268 | Polymeric immunoglobulin receptor \|\| id:prot-a-2268 | Green olives liking \|\| id:ebi-fl187-GCST90094775 | Weighted mode | 27 | 0.202603980880588 | 0.217567955488889 | 0.360305771550962 | -0.223829212 | 0.629037173638811 | 1.22458741217949 | 0.799451659558451 | 1.87580363632832 | 0.999714303421225 |
| ebi-fl187-GCST90094775 | prot-a-2283 | Group 10 secretory phospholipase A2 \|\| id:prot-a-2283 | Green olives liking \|\| id:ebi-fl187-GCST90094775 | Weighted mode | 27 | 0.206000771284182 | 0.206510727657247 | 0.327700894662555 | -0.198760255 | 0.610761797492385 | 1.22875415171359 | 0.819746399939175 | 1.84183396897553 | 0.999714303421225 |
| ebi-fl187-GCST90094775 | prot-a-2298 | Inactive phospholipase D5 \|\| id:prot-a-2298 | Green olives liking \|\| id:ebi-fl187-GCST90094775 | Weighted mode | 27 | 0.136527461914117 | 0.209116199196219 | 0.519566380916525 | -0.273340289 | 0.546395212338707 | 1.14628635656101 | 0.760833840558756 | 1.7270162566283 | 0.999714303421225 |
| ebi-fl187-GCST90094775 | prot-a-2304 | Procollagen-lysine,2-oxoglutarate 5-dioxygenase 2 \|\| id:prot-a-2304 | Green olives liking \|\| id:ebi-fl187-GCST90094775 | Weighted mode | 27 | -0.107983517 | 0.211865070784061 | 0.614574502879322 | -0.523239056 | 0.307272021722248 | 0.897642392135018 | 0.592597978106412 | 1.35971078864057 | 0.999714303421225 |
| ebi-fl187-GCST90094775 | prot-a-2331 | Periostin \|\| id:prot-a-2331 | Green olives liking \|\| id:ebi-fl187-GCST90094775 | Weighted mode | 27 | 0.139857000732049 | 0.231750756608335 | 0.551418287438807 | -0.314374482 | 0.594088483684386 | 1.15010932230634 | 0.730245512988278 | 1.81137909063357 | 0.999714303421225 |
| ebi-fl187-GCST90094775 | prot-a-2333 | Periostin \|\| id:prot-a-2333 | Green olives liking \|\| id:ebi-fl187-GCST90094775 | Weighted mode | 27 | -0.059758996 | 0.21465922195662 | 0.782915031059118 | -0.480491071 | 0.360973078678033 | 0.941991529620081 | 0.61847959958257 | 1.4347248356694 | 0.999714303421225 |
| ebi-fl187-GCST90094775 | prot-a-2367 | cAMP-dependent protein kinase catalytic subunit alpha \|\| id:prot-a-2367 | Green olives liking \|\| id:ebi-fl187-GCST90094775 | Weighted mode | 27 | 0.145096062092552 | 0.197969061741772 | 0.470162259227613 | -0.242923299 | 0.533115423106425 | 1.15615062718231 | 0.784331670549656 | 1.70423345495829 | 0.999714303421225 |
| ebi-fl187-GCST90094775 | prot-a-2385 | Phosphoribosyl pyrophosphate synthase-associated protein 1 \|\| id:prot-a-2385 | Green olives liking \|\| id:ebi-fl187-GCST90094775 | Weighted mode | 27 | 0.0106761938913191 | 0.178741233792156 | 0.952827511084733 | -0.339656624 | 0.361009012123945 | 1.01073338780588 | 0.712014769332125 | 1.43477639120296 | 0.999714303421225 |
| ebi-fl187-GCST90094775 | prot-a-2393 | Trypsin-3 \|\| id:prot-a-2393 | Green olives liking \|\| id:ebi-fl187-GCST90094775 | Weighted mode | 26 | -0.051598283 | 0.231590514727859 | 0.825501764809222 | -0.505515692 | 0.402319125381358 | 0.949710304536415 | 0.603194432451416 | 1.49528844103729 | 0.999714303421225 |
| ebi-fl187-GCST90094775 | prot-a-2405 | Pregnancy-specific beta-1-glycoprotein 6 \|\| id:prot-a-2405 | Green olives liking \|\| id:ebi-fl187-GCST90094775 | Weighted mode | 27 | -0.021070345 | 0.226172032528687 | 0.926490066264176 | -0.464367529 | 0.422226838262908 | 0.979150083351101 | 0.628532507930566 | 1.52535449420602 | 0.999714303421225 |
| ebi-fl187-GCST90094775 | prot-a-244 | Beclin-1 \|\| id:prot-a-244 | Green olives liking \|\| id:ebi-fl187-GCST90094775 | Weighted mode | 27 | -0.058752873 | 0.160669422859024 | 0.717565582501575 | -0.373664942 | 0.25615919566903 | 0.942939766114616 | 0.68820746266132 | 1.29195838574893 | 0.999714303421225 |
| ebi-fl187-GCST90094775 | prot-a-2454 | tRNA pseudouridine synthase A, mitochondrial \|\| id:prot-a-2454 | Green olives liking \|\| id:ebi-fl187-GCST90094775 | Weighted mode | 27 | 0.226257543208838 | 0.191635864765281 | 0.248424976782425 | -0.149348752 | 0.601863838148789 | 1.25389855675501 | 0.861268693567939 | 1.82551810181207 | 0.999714303421225 |
| ebi-fl187-GCST90094775 | prot-a-2466 | Glutaminyl-peptide cyclotransferase-like protein \|\| id:prot-a-2466 | Green olives liking \|\| id:ebi-fl187-GCST90094775 | Weighted mode | 27 | 0.0426207720479006 | 0.169456013207612 | 0.803393127037529 | -0.289513014 | 0.374754557934819 | 1.0435420794775 | 0.748628050322566 | 1.45463434234265 | 0.999714303421225 |
| ebi-fl187-GCST90094775 | prot-a-2474 | Ras-related protein Rab-27A \|\| id:prot-a-2474 | Green olives liking \|\| id:ebi-fl187-GCST90094775 | Weighted mode | 27 | 0.0325899405097005 | 0.186228298332177 | 0.862435368421907 | -0.332417524 | 0.397597405240767 | 1.03312680891724 | 0.717187817048444 | 1.48824474974514 | 0.999714303421225 |
| ebi-fl187-GCST90094775 | prot-a-2490 | Rap1 GTPase-GDP dissociation stimulator 1 \|\| id:prot-a-2490 | Green olives liking \|\| id:ebi-fl187-GCST90094775 | Weighted mode | 27 | 0.17677199164153 | 0.163409644549066 | 0.289285513256861 | -0.143510912 | 0.4970548949577 | 1.19335896628575 | 0.866311347228113 | 1.64387275656867 | 0.999714303421225 |
| ebi-fl187-GCST90094775 | prot-a-2545 | Receptor-interacting serine/threonine-protein kinase 2 \|\| id:prot-a-2545 | Green olives liking \|\| id:ebi-fl187-GCST90094775 | Weighted mode | 27 | 0.0230084365717271 | 0.190641810171607 | 0.904864948659243 | -0.350649511 | 0.396666384508077 | 1.02327517244519 | 0.704230535405375 | 1.48685980783267 | 0.999714303421225 |
| ebi-fl187-GCST90094775 | prot-a-2610 | RUN and FYVE domain-containing protein 1 \|\| id:prot-a-2610 | Green olives liking \|\| id:ebi-fl187-GCST90094775 | Weighted mode | 27 | 0.131843935084559 | 0.181798994361022 | 0.474795329463224 | -0.224482094 | 0.488169964032163 | 1.14093024618972 | 0.798929882319733 | 1.62933175423471 | 0.999714303421225 |
| ebi-fl187-GCST90094775 | prot-a-2627 | Serine--tRNA ligase, mitochondrial \|\| id:prot-a-2627 | Green olives liking \|\| id:ebi-fl187-GCST90094775 | Weighted mode | 27 | -0.046155911 | 0.175214767326493 | 0.794299698263374 | -0.389576855 | 0.297265032753995 | 0.954893072053796 | 0.677343428239621 | 1.34617203185408 | 0.999714303421225 |
| ebi-fl187-GCST90094775 | prot-a-264 | BolA-like protein 2 \|\| id:prot-a-264 | Green olives liking \|\| id:ebi-fl187-GCST90094775 | Weighted mode | 27 | 0.167123552722672 | 0.188349009599239 | 0.383047883872518 | -0.202040506 | 0.536287611537181 | 1.18190028328564 | 0.817061831290359 | 1.70964818834411 | 0.999714303421225 |
| ebi-fl187-GCST90094775 | prot-a-2646 | Secretoglobin family 1C member 1 \|\| id:prot-a-2646 | Green olives liking \|\| id:ebi-fl187-GCST90094775 | Weighted mode | 27 | 0.00654388755721036 | 0.241440755108568 | 0.978584155021888 | -0.466679992 | 0.479767767570005 | 1.00656534557013 | 0.627080728871952 | 1.61569914088303 | 0.999714303421225 |
| ebi-fl187-GCST90094775 | prot-a-2672 | Semaphorin-3E \|\| id:prot-a-2672 | Green olives liking \|\| id:ebi-fl187-GCST90094775 | Weighted mode | 27 | -0.021919004 | 0.174149483205803 | 0.900808474275089 | -0.363251991 | 0.319413983487061 | 0.97831947220111 | 0.695411174283709 | 1.37632098114286 | 0.999714303421225 |
| ebi-fl187-GCST90094775 | prot-a-2701 | Plasma protease C1 inhibitor \|\| id:prot-a-2701 | Green olives liking \|\| id:ebi-fl187-GCST90094775 | Weighted mode | 27 | 0.0867898865235444 | 0.174121072733593 | 0.622360589440264 | -0.254487416 | 0.428067189081386 | 1.09066749170304 | 0.775313809551771 | 1.53428916498406 | 0.999714303421225 |
| ebi-fl187-GCST90094775 | prot-a-2738 | Signaling threshold-regulating transmembrane adapter 1 \|\| id:prot-a-2738 | Green olives liking \|\| id:ebi-fl187-GCST90094775 | Weighted mode | 27 | -0.203484597 | 0.171088287151352 | 0.245048934660395 | -0.53881764 | 0.131848445464543 | 0.815882770980936 | 0.583437677993586 | 1.14093539223027 | 0.999714303421225 |
| ebi-fl187-GCST90094775 | prot-a-274 | Serine/threonine-protein kinase BRSK2 \|\| id:prot-a-274 | Green olives liking \|\| id:ebi-fl187-GCST90094775 | Weighted mode | 27 | -0.163903452 | 0.18772768273111 | 0.390605725748437 | -0.53184971 | 0.204042806648416 | 0.848823970601385 | 0.587517227695029 | 1.22635064829367 | 0.999714303421225 |
| ebi-fl187-GCST90094775 | prot-a-2749 | Mitochondrial glutamate carrier 2 \|\| id:prot-a-2749 | Green olives liking \|\| id:ebi-fl187-GCST90094775 | Weighted mode | 27 | 0.313297971023793 | 0.189637377263376 | 0.110543942983935 | -0.058391288 | 0.68498723046001 | 1.36792907353318 | 0.94328078037709 | 1.98374650384511 | 0.999714303421225 |
| ebi-fl187-GCST90094775 | prot-a-2760 | Sodium-coupled monocarboxylate transporter 1 \|\| id:prot-a-2760 | Green olives liking \|\| id:ebi-fl187-GCST90094775 | Weighted mode | 27 | -0.114859657 | 0.176207307372996 | 0.520223031045028 | -0.460225979 | 0.230506665425156 | 0.891491249661116 | 0.631141004476462 | 1.25923786061341 | 0.999714303421225 |
| ebi-fl187-GCST90094775 | prot-a-2777 | Small integral membrane protein 10 \|\| id:prot-a-2777 | Green olives liking \|\| id:ebi-fl187-GCST90094775 | Weighted mode | 27 | 0.220705744279123 | 0.205637295182614 | 0.293007729591462 | -0.182343354 | 0.623754842837045 | 1.24695645250418 | 0.833315168960599 | 1.86592114527479 | 0.999714303421225 |
| ebi-fl187-GCST90094775 | prot-a-2799 | Superoxide dismutase [Mn], mitochondrial \|\| id:prot-a-2799 | Green olives liking \|\| id:ebi-fl187-GCST90094775 | Weighted mode | 27 | -0.077668755 | 0.192159511553011 | 0.689379379969041 | -0.454301398 | 0.298963887684619 | 0.925270867173339 | 0.634891349675027 | 1.34846092654738 | 0.999714303421225 |
| ebi-fl187-GCST90094775 | prot-a-2805 | Protein SPACA7 \|\| id:prot-a-2805 | Green olives liking \|\| id:ebi-fl187-GCST90094775 | Weighted mode | 27 | -0.184226495 | 0.236655330173181 | 0.443325765197839 | -0.648070942 | 0.279617952309294 | 0.831747396005601 | 0.523053805319124 | 1.32262441019814 | 0.999714303421225 |
| ebi-fl187-GCST90094775 | prot-a-2811 | SPARC-like protein 1 \|\| id:prot-a-2811 | Green olives liking \|\| id:ebi-fl187-GCST90094775 | Weighted mode | 27 | 0.294371448917918 | 0.18419974328661 | 0.122102128153542 | -0.066660048 | 0.655402945759674 | 1.34228240042338 | 0.935513176932257 | 1.92591840169966 | 0.999714303421225 |
| ebi-fl187-GCST90094775 | prot-a-2831 | Sepiapterin reductase \|\| id:prot-a-2831 | Green olives liking \|\| id:ebi-fl187-GCST90094775 | Weighted mode | 27 | -0.033847033 | 0.173021311266144 | 0.846425697816022 | -0.372968803 | 0.305274737041716 | 0.966719369451193 | 0.688686717384629 | 1.35699776935028 | 0.999714303421225 |
| ebi-fl187-GCST90094775 | prot-a-2834 | SPRY domain-containing SOCS box protein 1 \|\| id:prot-a-2834 | Green olives liking \|\| id:ebi-fl187-GCST90094775 | Weighted mode | 27 | 0.184625840549908 | 0.204344321532754 | 0.374554033680311 | -0.21588903 | 0.585140710754106 | 1.20276832878975 | 0.805824719559744 | 1.79524357794591 | 0.999714303421225 |
| ebi-fl187-GCST90094775 | prot-a-2835 | Spectrin alpha chain, non-erythrocytic 1 \|\| id:prot-a-2835 | Green olives liking \|\| id:ebi-fl187-GCST90094775 | Weighted mode | 27 | 0.130894910093305 | 0.17536323355633 | 0.462109173420889 | -0.212817028 | 0.474606847863712 | 1.13984798849884 | 0.808304020951082 | 1.60738212752683 | 0.999714303421225 |
| ebi-fl187-GCST90094775 | prot-a-2859 | Sia-alpha-2,3-Gal-beta-1,4-GlcNAc-R:alpha 2,8-sialyltransferase \|\| id:prot-a-2859 | Green olives liking \|\| id:ebi-fl187-GCST90094775 | Weighted mode | 27 | 0.111482653475164 | 0.221431812531739 | 0.618878058214653 | -0.322523699 | 0.545489006037373 | 1.11793435156134 | 0.724318765910027 | 1.72545193252127 | 0.999714303421225 |
| ebi-fl187-GCST90094775 | prot-a-2861 | Alpha-2,8-sialyltransferase 8F \|\| id:prot-a-2861 | Green olives liking \|\| id:ebi-fl187-GCST90094775 | Weighted mode | 27 | -0.07087756 | 0.179933120511947 | 0.696859170239299 | -0.423546476 | 0.28179135595227 | 0.931575947070019 | 0.65472074586031 | 1.32550213300338 | 0.999714303421225 |
| ebi-fl187-GCST90094775 | prot-a-289 | Uncharacterized protein C17orf89 \|\| id:prot-a-289 | Green olives liking \|\| id:ebi-fl187-GCST90094775 | Weighted mode | 27 | 0.164488577409076 | 0.176805514817694 | 0.360755253751473 | -0.182050232 | 0.511027386451756 | 1.17879010463755 | 0.833559468310292 | 1.66700297173537 | 0.999714303421225 |
| ebi-fl187-GCST90094775 | prot-a-2921 | Tachykinin-3 \|\| id:prot-a-2921 | Green olives liking \|\| id:ebi-fl187-GCST90094775 | Weighted mode | 27 | -0.039558573 | 0.186921547394215 | 0.834044357615811 | -0.405924806 | 0.326807659574925 | 0.961213650869099 | 0.666360271695166 | 1.38653476484529 | 0.999714303421225 |
| ebi-fl187-GCST90094775 | prot-a-2928 | Tax1-binding protein 1 \|\| id:prot-a-2928 | Green olives liking \|\| id:ebi-fl187-GCST90094775 | Weighted mode | 27 | -0.330314274 | 0.234760493167558 | 0.171261678825755 | -0.79044484 | 0.129816292906814 | 0.718697830108649 | 0.453642951720329 | 1.13861919168827 | 0.999714303421225 |
| ebi-fl187-GCST90094775 | prot-a-296 | Uncharacterized protein C1orf185 \|\| id:prot-a-296 | Green olives liking \|\| id:ebi-fl187-GCST90094775 | Weighted mode | 27 | -0.012716113 | 0.178968178388399 | 0.943899797548404 | -0.363493743 | 0.338061516495529 | 0.987364395009457 | 0.695243077486576 | 1.40222676082843 | 0.999714303421225 |
| ebi-fl187-GCST90094775 | prot-a-2964 | Transforming growth factor beta-2 \|\| id:prot-a-2964 | Green olives liking \|\| id:ebi-fl187-GCST90094775 | Weighted mode | 27 | -0.079878735 | 0.162114659036118 | 0.626339040187774 | -0.397623466 | 0.237865996962547 | 0.923228295110387 | 0.671914978562876 | 1.26853919332991 | 0.999714303421225 |
| ebi-fl187-GCST90094775 | prot-a-2977 | Thymocyte nuclear protein 1 \|\| id:prot-a-2977 | Green olives liking \|\| id:ebi-fl187-GCST90094775 | Weighted mode | 27 | 0.176985592639271 | 0.172981821051281 | 0.315664981481291 | -0.162058777 | 0.516029961899782 | 1.19361389617728 | 0.850391219948644 | 1.6753631737091 | 0.999714303421225 |
| ebi-fl187-GCST90094775 | prot-a-3015 | Transmembrane protein 70, mitochondrial \|\| id:prot-a-3015 | Green olives liking \|\| id:ebi-fl187-GCST90094775 | Weighted mode | 27 | -0.079444324 | 0.178191285651939 | 0.659405387680945 | -0.428699244 | 0.26981059568961 | 0.923629442356115 | 0.651355798846728 | 1.30971636131516 | 0.999714303421225 |
| ebi-fl187-GCST90094775 | prot-a-3030 | Tumor necrosis factor alpha-induced protein 3 \|\| id:prot-a-3030 | Green olives liking \|\| id:ebi-fl187-GCST90094775 | Weighted mode | 27 | -0.155943256 | 0.223008675685958 | 0.490592378177943 | -0.59304026 | 0.281153748526181 | 0.85560773975461 | 0.552644544853259 | 1.32465725237978 | 0.999714303421225 |
| ebi-fl187-GCST90094775 | prot-a-3032 | Tumor necrosis factor receptor superfamily member 10A \|\| id:prot-a-3032 | Green olives liking \|\| id:ebi-fl187-GCST90094775 | Weighted mode | 27 | 0.335442451484882 | 0.208038207642731 | 0.118947203817592 | -0.072312435 | 0.743197338464636 | 1.39855904287459 | 0.930240210342874 | 2.10264765450781 | 0.999714303421225 |
| ebi-fl187-GCST90094775 | prot-a-3081 | Tumor protein p53-inducible protein 11 \|\| id:prot-a-3081 | Green olives liking \|\| id:ebi-fl187-GCST90094775 | Weighted mode | 27 | -0.123263972 | 0.228998191858968 | 0.594965894167389 | -0.572100428 | 0.325572484170112 | 0.884030272630721 | 0.56433883989917 | 1.38482320845962 | 0.999714303421225 |
| ebi-fl187-GCST90094775 | prot-a-315 | Complement C4 \|\| id:prot-a-315 | Green olives liking \|\| id:ebi-fl187-GCST90094775 | Weighted mode | 26 | -0.105788565 | 0.26569980133882 | 0.693898933115474 | -0.626560175 | 0.414983045791166 | 0.899614838182395 | 0.534426977889092 | 1.51434506595191 | 0.999714303421225 |
| ebi-fl187-GCST90094775 | prot-a-3157 | UDP-glucuronosyltransferase 1-8 \|\| id:prot-a-3157 | Green olives liking \|\| id:ebi-fl187-GCST90094775 | Weighted mode | 27 | 0.196812710092626 | 0.166686642864737 | 0.24839730946501 | -0.12989311 | 0.52351853010751 | 1.2175159908937 | 0.878189295625951 | 1.68795633864484 | 0.999714303421225 |
| ebi-fl187-GCST90094775 | prot-a-317 | Glutaredoxin-like protein C5orf63 \|\| id:prot-a-317 | Green olives liking \|\| id:ebi-fl187-GCST90094775 | Weighted mode | 27 | 0.0427110308587027 | 0.190424189334503 | 0.824281705301325 | -0.33052038 | 0.415942441954329 | 1.04363627259543 | 0.718549717052999 | 1.51579861995346 | 0.999714303421225 |
| ebi-fl187-GCST90094775 | prot-a-3192 | Guanine nucleotide exchange factor VAV3 \|\| id:prot-a-3192 | Green olives liking \|\| id:ebi-fl187-GCST90094775 | Weighted mode | 27 | -0.040591792 | 0.181151605873793 | 0.824449384487179 | -0.395648939 | 0.314465355708076 | 0.960221020045801 | 0.673243003604335 | 1.3695269054436 | 0.999714303421225 |
| ebi-fl187-GCST90094775 | prot-a-3233 | Serine/threonine-protein kinase WNK3 \|\| id:prot-a-3233 | Green olives liking \|\| id:ebi-fl187-GCST90094775 | Weighted mode | 27 | -0.107156866 | 0.186237039542259 | 0.56998167164456 | -0.472181463 | 0.257867731800298 | 0.898384736183735 | 0.62364033487508 | 1.29416763008054 | 0.999714303421225 |
| ebi-fl187-GCST90094775 | prot-a-3250 | 14-3-3 protein theta \|\| id:prot-a-3250 | Green olives liking \|\| id:ebi-fl187-GCST90094775 | Weighted mode | 27 | 0.0328572505251814 | 0.166176767264255 | 0.844798212479441 | -0.292849213 | 0.358563714363121 | 1.03340301097467 | 0.746134639385865 | 1.43127222182113 | 0.999714303421225 |
| ebi-fl187-GCST90094775 | prot-a-3278 | E3 ubiquitin-protein ligase ZNRF3 \|\| id:prot-a-3278 | Green olives liking \|\| id:ebi-fl187-GCST90094775 | Weighted mode | 27 | 0.303627602553782 | 0.200514178401391 | 0.142026756194732 | -0.089380187 | 0.696635392220507 | 1.35476445131684 | 0.914497827185582 | 2.0069886051018 | 0.999714303421225 |
| ebi-fl187-GCST90094775 | prot-a-355 | Calpain-3 \|\| id:prot-a-355 | Green olives liking \|\| id:ebi-fl187-GCST90094775 | Weighted mode | 27 | -0.002820373 | 0.167835031614704 | 0.986720920710376 | -0.331777035 | 0.326136288475836 | 0.997183600027847 | 0.717647314925403 | 1.38560419788913 | 0.999714303421225 |
| ebi-fl187-GCST90094775 | prot-a-385 | Coiled-coil domain-containing protein 90B, mitochondrial \|\| id:prot-a-385 | Green olives liking \|\| id:ebi-fl187-GCST90094775 | Weighted mode | 27 | 0.258873674508343 | 0.183184165262833 | 0.169461231737757 | -0.100167289 | 0.617914638423496 | 1.29547014358816 | 0.904686060981606 | 1.85505554391695 | 0.999714303421225 |
| ebi-fl187-GCST90094775 | prot-a-390 | C-C motif chemokine 14 \|\| id:prot-a-390 | Green olives liking \|\| id:ebi-fl187-GCST90094775 | Weighted mode | 27 | -0.045483058 | 0.209042312762023 | 0.829456249634817 | -0.455205991 | 0.36423987473033 | 0.955535790851553 | 0.634317290648214 | 1.43941945310249 | 0.999714303421225 |
| ebi-fl187-GCST90094775 | prot-a-40 | Pituitary adenylate cyclase-activating polypeptide 27 \|\| id:prot-a-40 | Green olives liking \|\| id:ebi-fl187-GCST90094775 | Weighted mode | 27 | -0.007688086 | 0.183612478034395 | 0.966921220458669 | -0.367568543 | 0.352192370607256 | 0.992341391404773 | 0.692415864722323 | 1.42218208343633 | 0.999714303421225 |
| ebi-fl187-GCST90094775 | prot-a-401 | C-C motif chemokine 24 \|\| id:prot-a-401 | Green olives liking \|\| id:ebi-fl187-GCST90094775 | Weighted mode | 27 | 0.258820906175949 | 0.189633520081748 | 0.183996703257001 | -0.112860793 | 0.630502605536174 | 1.2954017855926 | 0.89327500142975 | 1.87855451392978 | 0.999714303421225 |
| ebi-fl187-GCST90094775 | prot-a-412 | Cerebral cavernous malformations 2 protein \|\| id:prot-a-412 | Green olives liking \|\| id:ebi-fl187-GCST90094775 | Weighted mode | 27 | 0.133022433560288 | 0.205949789548492 | 0.524006094599089 | -0.270639154 | 0.536684021075332 | 1.14227562335252 | 0.7628917332079 | 1.71032604353811 | 0.999714303421225 |
| ebi-fl187-GCST90094775 | prot-a-44 | Adhesion G protein-coupled receptor F5 \|\| id:prot-a-44 | Green olives liking \|\| id:ebi-fl187-GCST90094775 | Weighted mode | 27 | -0.147487393 | 0.188877186968108 | 0.441934650320581 | -0.517686679 | 0.222711893665764 | 0.862873316676419 | 0.5958974566923 | 1.24946054437789 | 0.999714303421225 |
| ebi-fl187-GCST90094775 | prot-a-48 | Adrenomedullin \|\| id:prot-a-48 | Green olives liking \|\| id:ebi-fl187-GCST90094775 | Weighted mode | 27 | -0.041899756 | 0.190042672231509 | 0.827222949025117 | -0.414383394 | 0.33058388137877 | 0.958965906145716 | 0.660747576194377 | 1.39178052600127 | 0.999714303421225 |
| ebi-fl187-GCST90094775 | prot-a-502 | Carcinoembryonic antigen-related cell adhesion molecule 1 \|\| id:prot-a-502 | Green olives liking \|\| id:ebi-fl187-GCST90094775 | Weighted mode | 27 | 0.0801682413403595 | 0.169129414379347 | 0.639452599700378 | -0.251325411 | 0.411661893523879 | 1.08346933667538 | 0.77776923583182 | 1.50932403781733 | 0.999714303421225 |
| ebi-fl187-GCST90094775 | prot-a-518 | Complement factor B \|\| id:prot-a-518 | Green olives liking \|\| id:ebi-fl187-GCST90094775 | Weighted mode | 27 | 0.184909823073775 | 0.214356226542675 | 0.39622898611978 | -0.235228381 | 0.605048027097418 | 1.20310994247925 | 0.790390318922832 | 1.83134016072602 | 0.999714303421225 |
| ebi-fl187-GCST90094775 | prot-a-525 | Properdin \|\| id:prot-a-525 | Green olives liking \|\| id:ebi-fl187-GCST90094775 | Weighted mode | 27 | 0.092971086561374 | 0.22552057147002 | 0.683534819243528 | -0.349049234 | 0.534991406642613 | 1.09743000433428 | 0.705358402138164 | 1.70743356960429 | 0.999714303421225 |
| ebi-fl187-GCST90094775 | prot-a-533 | Chondroadherin \|\| id:prot-a-533 | Green olives liking \|\| id:ebi-fl187-GCST90094775 | Weighted mode | 27 | -0.123961551 | 0.179536646717116 | 0.496027623888717 | -0.475853378 | 0.22793027692797 | 0.883413806927391 | 0.621354579693834 | 1.25599775035776 | 0.999714303421225 |
| ebi-fl187-GCST90094775 | prot-a-535 | Coiled-coil-helix-coiled-coil-helix domain-containing protein 2 \|\| id:prot-a-535 | Green olives liking \|\| id:ebi-fl187-GCST90094775 | Weighted mode | 27 | 0.037435049642967 | 0.194049419327712 | 0.848524557362448 | -0.342901812 | 0.417771911525283 | 1.03814456703177 | 0.709707892767939 | 1.51857426560983 | 0.999714303421225 |
| ebi-fl187-GCST90094775 | prot-a-591 | Caseinolytic peptidase B protein homolog \|\| id:prot-a-591 | Green olives liking \|\| id:ebi-fl187-GCST90094775 | Weighted mode | 27 | 0.120786522948933 | 0.194255970660419 | 0.539494144307519 | -0.25995518 | 0.501528225443354 | 1.12838400256456 | 0.771086145460578 | 1.65124281474815 | 0.999714303421225 |
| ebi-fl187-GCST90094775 | prot-a-605 | Ciliary neurotrophic factor receptor subunit alpha \|\| id:prot-a-605 | Green olives liking \|\| id:ebi-fl187-GCST90094775 | Weighted mode | 27 | -0.42453524 | 0.181632890581367 | 0.0273945283679858 | -0.780535706 | -0.068534775 | 0.654073701710528 | 0.458160506372292 | 0.933760988385326 | 0.999714303421225 |
| ebi-fl187-GCST90094775 | prot-a-606 | Ciliary neurotrophic factor receptor subunit alpha \|\| id:prot-a-606 | Green olives liking \|\| id:ebi-fl187-GCST90094775 | Weighted mode | 27 | -0.288012468 | 0.178138686392723 | 0.117994373822451 | -0.637164294 | 0.0611393571238625 | 0.74975224411543 | 0.528789792624023 | 1.06304704704428 | 0.999714303421225 |
| ebi-fl187-GCST90094775 | prot-a-707 | Cancer/testis antigen 55 \|\| id:prot-a-707 | Green olives liking \|\| id:ebi-fl187-GCST90094775 | Weighted mode | 27 | 0.174011433802477 | 0.242240056594781 | 0.478951912359404 | -0.300779077 | 0.648801944728248 | 1.19006917275843 | 0.740241290919036 | 1.91324727939938 | 0.999714303421225 |
| ebi-fl187-GCST90094775 | prot-a-719 | Dipeptidyl peptidase 1 \|\| id:prot-a-719 | Green olives liking \|\| id:ebi-fl187-GCST90094775 | Weighted mode | 27 | -0.114953728 | 0.163628473558112 | 0.488592053023998 | -0.435665536 | 0.205758080170311 | 0.891407390152109 | 0.646834032343077 | 1.22845598018309 | 0.999714303421225 |
| ebi-fl187-GCST90094775 | prot-a-752 | Cytochrome b5 \|\| id:prot-a-752 | Green olives liking \|\| id:ebi-fl187-GCST90094775 | Weighted mode | 27 | -0.254477204 | 0.224376818715567 | 0.26708026781621 | -0.694255768 | 0.185301361023364 | 0.775321727387682 | 0.499446013237357 | 1.20358109791086 | 0.999714303421225 |
| ebi-fl187-GCST90094775 | prot-a-755 | Cytoglobin \|\| id:prot-a-755 | Green olives liking \|\| id:ebi-fl187-GCST90094775 | Weighted mode | 27 | -0.158077409 | 0.171179514756107 | 0.364258915833555 | -0.493589258 | 0.1774344397571 | 0.853783688728079 | 0.610431461391418 | 1.19414976658733 | 0.999714303421225 |
| ebi-fl187-GCST90094775 | prot-a-807 | Desmin \|\| id:prot-a-807 | Green olives liking \|\| id:ebi-fl187-GCST90094775 | Weighted mode | 27 | 0.0682260029194377 | 0.215865751067116 | 0.754482166223142 | -0.354870869 | 0.491322875010985 | 1.07060724150111 | 0.701263992161994 | 1.6344769991981 | 0.999714303421225 |
| ebi-fl187-GCST90094775 | prot-a-82 | Ameloblastin \|\| id:prot-a-82 | Green olives liking \|\| id:ebi-fl187-GCST90094775 | Weighted mode | 27 | -0.076395788 | 0.251627360177883 | 0.763843764355662 | -0.569585413 | 0.416793838447959 | 0.926449456871647 | 0.565759946549019 | 1.5170897151225 | 0.999714303421225 |
| ebi-fl187-GCST90094775 | prot-a-84 | Muellerian-inhibiting factor \|\| id:prot-a-84 | Green olives liking \|\| id:ebi-fl187-GCST90094775 | Weighted mode | 27 | 0.0633998057112353 | 0.152230506494333 | 0.680482500416023 | -0.234971987 | 0.361771598440128 | 1.06545272817499 | 0.790592996186181 | 1.43587094934016 | 0.999714303421225 |
| ebi-fl187-GCST90094775 | prot-a-858 | Inactive dipeptidyl peptidase 10 \|\| id:prot-a-858 | Green olives liking \|\| id:ebi-fl187-GCST90094775 | Weighted mode | 27 | 0.232791165477051 | 0.187759604777517 | 0.226109985638701 | -0.13521766 | 0.600799990840985 | 1.26211787801873 | 0.873525759476534 | 1.82357706196219 | 0.999714303421225 |
| ebi-fl187-GCST90094775 | prot-a-863 | Dihydropyrimidinase-related protein 3 \|\| id:prot-a-863 | Green olives liking \|\| id:ebi-fl187-GCST90094775 | Weighted mode | 27 | -0.095542237 | 0.175077405937592 | 0.589914271520307 | -0.438693952 | 0.247609479137028 | 0.908879972933405 | 0.644878112999053 | 1.28095959305823 | 0.999714303421225 |
| ebi-fl187-GCST90094775 | prot-a-864 | Dihydropyrimidinase-related protein 5 \|\| id:prot-a-864 | Green olives liking \|\| id:ebi-fl187-GCST90094775 | Weighted mode | 27 | -0.215680017 | 0.190104917143348 | 0.266923641963807 | -0.588285655 | 0.156925620600572 | 0.805993164725929 | 0.555278408208568 | 1.16990859356612 | 0.999714303421225 |
| ebi-fl187-GCST90094775 | prot-a-884 | Endothelin-converting enzyme 1 \|\| id:prot-a-884 | Green olives liking \|\| id:ebi-fl187-GCST90094775 | Weighted mode | 27 | -0.033291291 | 0.177554072239378 | 0.852724631820752 | -0.381297273 | 0.314714690558572 | 0.967256765328919 | 0.68297482972244 | 1.36986841880364 | 0.999714303421225 |
| ebi-fl187-GCST90094775 | prot-a-893 | Early endosome antigen 1 \|\| id:prot-a-893 | Green olives liking \|\| id:ebi-fl187-GCST90094775 | Weighted mode | 27 | -0.383595615 | 0.182366132321024 | 0.0452547757381487 | -0.741033235 | -0.026157996 | 0.681406922028809 | 0.476621199496925 | 0.974181160802042 | 0.999714303421225 |
| ebi-fl187-GCST90094775 | prot-a-897 | Ephrin-A2 \|\| id:prot-a-897 | Green olives liking \|\| id:ebi-fl187-GCST90094775 | Weighted mode | 27 | 0.146706769513327 | 0.195206218315836 | 0.459072565705885 | -0.235897418 | 0.529310957412365 | 1.15801434812869 | 0.789861695064837 | 1.69776207512106 | 0.999714303421225 |
| ebi-fl187-GCST90094775 | prot-a-90 | Anaphase-promoting complex subunit 10 \|\| id:prot-a-90 | Green olives liking \|\| id:ebi-fl187-GCST90094775 | Weighted mode | 27 | 0.0777470217519714 | 0.205286011086664 | 0.707966883479117 | -0.32461356 | 0.480107603481833 | 1.08084919274591 | 0.722806621085172 | 1.61624830678167 | 0.999714303421225 |
| ebi-fl187-GCST90094775 | prot-a-914 | Histone-lysine N-methyltransferase EHMT2 \|\| id:prot-a-914 | Green olives liking \|\| id:ebi-fl187-GCST90094775 | Weighted mode | 27 | -0.254865627 | 0.219671536119242 | 0.256505526882861 | -0.685421838 | 0.175690583713879 | 0.775020632750086 | 0.503877630068718 | 1.19206915597032 | 0.999714303421225 |
| ebi-fl187-GCST90094775 | prot-a-932 | RNA polymerase II elongation factor ELL \|\| id:prot-a-932 | Green olives liking \|\| id:ebi-fl187-GCST90094775 | Weighted mode | 27 | -0.031963828 | 0.180461388546325 | 0.860784535230286 | -0.38566815 | 0.321740493416643 | 0.968541615402963 | 0.67999614517731 | 1.37952673323284 | 0.999714303421225 |
| ebi-fl187-GCST90094775 | prot-a-991 | Estrogen receptor \|\| id:prot-a-991 | Green olives liking \|\| id:ebi-fl187-GCST90094775 | Weighted mode | 27 | 0.0156206286085472 | 0.209761176564499 | 0.941207500645659 | -0.395511277 | 0.426752534674966 | 1.01574326836554 | 0.673335689867521 | 1.53227343026017 | 0.999714303421225 |
| ebi-fl187-GCST90094775 | prot-a-992 | Persulfide dioxygenase ETHE1, mitochondrial \|\| id:prot-a-992 | Green olives liking \|\| id:ebi-fl187-GCST90094775 | Weighted mode | 27 | 0.0736351330248672 | 0.207261361037331 | 0.72524835393298 | -0.332597135 | 0.479867400658035 | 1.0764139859221 | 0.717059014234627 | 1.61586012599735 | 0.999714303421225 |
| ebi-fl187-GCST90094775 | prot-a-993 | Ethanolamine kinase 1 \|\| id:prot-a-993 | Green olives liking \|\| id:ebi-fl187-GCST90094775 | Weighted mode | 27 | 0.277828374006964 | 0.190824848415088 | 0.157380945517473 | -0.096188329 | 0.651845076900536 | 1.32025958690087 | 0.908292942161621 | 1.91907841170201 | 0.999714303421225 |
| ebi-fl187-GCST90094775 | prot-b-10 | coagulation factor III, tissue factor \|\| id:prot-b-10 | Green olives liking \|\| id:ebi-fl187-GCST90094775 | Weighted mode | 20 | 0.258215337654086 | 0.195939056620549 | 0.203228316074891 | -0.125825213 | 0.642255888630363 | 1.29461756852063 | 0.881768954781213 | 1.90076395821639 | 0.999714303421225 |
| ebi-fl187-GCST90094775 | prot-b-28 | prolactin \|\| id:prot-b-28 | Green olives liking \|\| id:ebi-fl187-GCST90094775 | Weighted mode | 20 | -0.016556555 | 0.216310775329294 | 0.939789257834671 | -0.440525675 | 0.40741256420145 | 0.983579751028149 | 0.64369795614901 | 1.50292403042623 | 0.999714303421225 |
| ebi-fl187-GCST90094775 | prot-b-54 | matrix metallopeptidase 7 \|\| id:prot-b-54 | Green olives liking \|\| id:ebi-fl187-GCST90094775 | Weighted mode | 20 | 0.217147572404084 | 0.237878237872228 | 0.372758852729125 | -0.249093774 | 0.683388918633651 | 1.24252745137193 | 0.779506872615849 | 1.9805783908382 | 0.999714303421225 |
| ebi-fl187-GCST90094775 | prot-c-2421_7_3 | BDNF \|\| id:prot-c-2421_7_3 | Green olives liking \|\| id:ebi-fl187-GCST90094775 | Weighted mode | 3 | -0.049592295 | 0.535423845245981 | 0.934646018664614 | -1.099023032 | 0.999838441367911 | 0.951617324263244 | 0.333196447005089 | 2.71784270203849 | 0.999714303421225 |
| ebi-fl187-GCST90094775 | prot-c-2443_10_4 | FGF-8B \|\| id:prot-c-2443_10_4 | Green olives liking \|\| id:ebi-fl187-GCST90094775 | Weighted mode | 3 | 0.0149258886726842 | 0.76218096071691 | 0.986153963554368 | -1.478948794 | 1.50880057167783 | 1.0150378360268 | 0.22787710822969 | 4.52130456003277 | 0.999714303421225 |
| ebi-fl187-GCST90094775 | prot-c-2605_49_2 | CD30 \|\| id:prot-c-2605_49_2 | Green olives liking \|\| id:ebi-fl187-GCST90094775 | Weighted mode | 3 | 0.30103300807334 | 0.639992019457125 | 0.684397055371169 | -0.95335135 | 1.5554173662093 | 1.35125394309116 | 0.385447088327187 | 4.73706320274368 | 0.999714303421225 |
| ebi-fl187-GCST90094775 | prot-c-2624_31_2 | HPV E7 Type18 \|\| id:prot-c-2624_31_2 | Green olives liking \|\| id:ebi-fl187-GCST90094775 | Weighted mode | 3 | 0.0362676952463312 | 0.563698440158489 | 0.954552588510521 | -1.068581247 | 1.14111663795697 | 1.03693339148019 | 0.343495506999723 | 3.13026178350413 | 0.999714303421225 |
| ebi-fl187-GCST90094775 | prot-c-2774_10_3 | IL-16 \|\| id:prot-c-2774_10_3 | Green olives liking \|\| id:ebi-fl187-GCST90094775 | Weighted mode | 3 | -0.533058063 | 0.57930389613215 | 0.454623728158152 | -1.668493699 | 0.602377573706113 | 0.58680772820713 | 0.188530836025268 | 1.82645617631199 | 0.999714303421225 |
| ebi-fl187-GCST90094775 | prot-c-2851_63_3 | C5a \|\| id:prot-c-2851_63_3 | Green olives liking \|\| id:ebi-fl187-GCST90094775 | Weighted mode | 3 | -0.27512913 | 0.497223914791183 | 0.635633471286648 | -1.249688003 | 0.69942974344856 | 0.759474046356907 | 0.286594199577114 | 2.0126046791625 | 0.999714303421225 |
| ebi-fl187-GCST90094775 | prot-c-2891_1_3 | CD22 \|\| id:prot-c-2891_1_3 | Green olives liking \|\| id:ebi-fl187-GCST90094775 | Weighted mode | 3 | 0.510545917783698 | 0.585378836688704 | 0.475082997391255 | -0.636796602 | 1.65788843769356 | 1.66620055521963 | 0.528984259837946 | 5.24821719849415 | 0.999714303421225 |
| ebi-fl187-GCST90094775 | prot-c-2961_1_2 | Protein C \|\| id:prot-c-2961_1_2 | Green olives liking \|\| id:ebi-fl187-GCST90094775 | Weighted mode | 3 | -0.178224269 | 0.509176603322118 | 0.759744797914792 | -1.176210411 | 0.81976187389333 | 0.836754744614116 | 0.308445407891384 | 2.26995923661405 | 0.999714303421225 |
| ebi-fl187-GCST90094775 | prot-c-2982_82_2 | Galectin-4 \|\| id:prot-c-2982_82_2 | Green olives liking \|\| id:ebi-fl187-GCST90094775 | Weighted mode | 3 | -0.592084051 | 0.527435526486033 | 0.378281076648285 | -1.625857683 | 0.441689580540691 | 0.553173241157001 | 0.19674285975728 | 1.55533285990481 | 0.999714303421225 |
| ebi-fl187-GCST90094775 | prot-c-2994_71_2 | IL-1Rrp2 \|\| id:prot-c-2994_71_2 | Green olives liking \|\| id:ebi-fl187-GCST90094775 | Weighted mode | 3 | -0.164850409 | 0.531440181353539 | 0.785751852411388 | -1.206473165 | 0.876772346235694 | 0.848020550658107 | 0.299250828885905 | 2.40313070147807 | 0.999714303421225 |
| ebi-fl187-GCST90094775 | prot-c-3336_50_1 | TFPI \|\| id:prot-c-3336_50_1 | Green olives liking \|\| id:ebi-fl187-GCST90094775 | Weighted mode | 3 | 0.4900888495274 | 0.565337757895235 | 0.477385527142944 | -0.617973156 | 1.59815085500206 | 1.63246125692323 | 0.539035872789541 | 4.94388201208984 | 0.999714303421225 |
| ebi-fl187-GCST90094775 | prot-c-3472_40_2 | Survivin \|\| id:prot-c-3472_40_2 | Green olives liking \|\| id:ebi-fl187-GCST90094775 | Weighted mode | 3 | 0.196429080716278 | 0.565772350953475 | 0.761580970005512 | -0.912484727 | 1.30534288858509 | 1.21704900557379 | 0.401525302702039 | 3.68895377701095 | 0.999714303421225 |
| ebi-fl187-GCST90094775 | prot-c-3473_78_2 | Thrombopoietin Receptor \|\| id:prot-c-3473_78_2 | Green olives liking \|\| id:ebi-fl187-GCST90094775 | Weighted mode | 3 | 0.578710291890701 | 0.648806326096834 | 0.466531264677929 | -0.692950107 | 1.8503706910405 | 1.78373644726488 | 0.500098546360535 | 6.3621774877293 | 0.999714303421225 |
| ebi-fl187-GCST90094775 | prot-c-3497_13_2 | IFN-aA \|\| id:prot-c-3497_13_2 | Green olives liking \|\| id:ebi-fl187-GCST90094775 | Weighted mode | 3 | -0.229335592 | 0.651584482938146 | 0.758489658220906 | -1.506441179 | 1.04776999442521 | 0.795061672286428 | 0.221697557692549 | 2.85128564030246 | 0.999714303421225 |
| ebi-fl187-GCST90094775 | prot-c-3665_64_3 | COLEC12 \|\| id:prot-c-3665_64_3 | Green olives liking \|\| id:ebi-fl187-GCST90094775 | Weighted mode | 3 | 0.0589989150934449 | 0.759814917413922 | 0.945176398056136 | -1.430238323 | 1.54823615322473 | 1.06077408989877 | 0.239251896210001 | 4.70316719585326 | 0.999714303421225 |
| ebi-fl187-GCST90094775 | prot-c-3741_4_3 | IL-5 \|\| id:prot-c-3741_4_3 | Green olives liking \|\| id:ebi-fl187-GCST90094775 | Weighted mode | 3 | 0.325114334042191 | 0.742623272019405 | 0.704279921470369 | -1.130427279 | 1.78065594720022 | 1.38418889684558 | 0.32289526054154 | 5.93374736729559 | 0.999714303421225 |
| ebi-fl187-GCST90094775 | prot-c-3848_14_2 | GAPDH, liver \|\| id:prot-c-3848_14_2 | Green olives liking \|\| id:ebi-fl187-GCST90094775 | Weighted mode | 3 | 0.519665088048633 | 0.622865097001091 | 0.491883653497522 | -0.701150502 | 1.74048067817077 | 1.68146441285911 | 0.496014309897538 | 5.70008266958201 | 0.999714303421225 |
| ebi-fl187-GCST90094775 | prot-c-3859_50_2 | PSA1 \|\| id:prot-c-3859_50_2 | Green olives liking \|\| id:ebi-fl187-GCST90094775 | Weighted mode | 3 | -0.415265297 | 0.815113258576822 | 0.661080131442924 | -2.012887283 | 1.18235669014192 | 0.660165117864421 | 0.133602369336899 | 3.26205279897368 | 0.999714303421225 |
| ebi-fl187-GCST90094775 | prot-c-3868_8_1 | SGTA \|\| id:prot-c-3868_8_1 | Green olives liking \|\| id:ebi-fl187-GCST90094775 | Weighted mode | 3 | 0.993292619403836 | 0.636778952729083 | 0.259150378957926 | -0.254794128 | 2.24137936675284 | 2.700110288 | 0.775076048035729 | 9.4062970765525 | 0.999714303421225 |
| ebi-fl187-GCST90094775 | prot-c-3882_10_1 | EF-1-beta \|\| id:prot-c-3882_10_1 | Green olives liking \|\| id:ebi-fl187-GCST90094775 | Weighted mode | 3 | 0.397499920304204 | 0.61603113384499 | 0.584899185811429 | -0.809921102 | 1.60492094264038 | 1.48809967537152 | 0.444893166005058 | 4.97746608185852 | 0.999714303421225 |
| ebi-fl187-GCST90094775 | prot-c-4134_4_2 | HB-EGF \|\| id:prot-c-4134_4_2 | Green olives liking \|\| id:ebi-fl187-GCST90094775 | Weighted mode | 3 | -0.12210938 | 0.583743491567608 | 0.853677108136608 | -1.266246623 | 1.02202786392398 | 0.88505155666841 | 0.281887669355072 | 2.77882413144682 | 0.999714303421225 |
| ebi-fl187-GCST90094775 | prot-c-4220_39_2 | FER \|\| id:prot-c-4220_39_2 | Green olives liking \|\| id:ebi-fl187-GCST90094775 | Weighted mode | 3 | 0.893258089587717 | 0.611863507606403 | 0.281744465869629 | -0.305994385 | 2.09251056449627 | 2.44307646052999 | 0.736390754042318 | 8.1052383659515 | 0.999714303421225 |
| ebi-fl187-GCST90094775 | prot-c-4228_37_2 | HSP70 protein 8 \|\| id:prot-c-4228_37_2 | Green olives liking \|\| id:ebi-fl187-GCST90094775 | Weighted mode | 3 | -0.127027849 | 0.627972328728653 | 0.858405744771941 | -1.357853613 | 1.10379791549275 | 0.880709145549208 | 0.257212261915222 | 3.01559728637538 | 0.999714303421225 |
| ebi-fl187-GCST90094775 | prot-c-4246_40_2 | NCAM-L1 \|\| id:prot-c-4246_40_2 | Green olives liking \|\| id:ebi-fl187-GCST90094775 | Weighted mode | 3 | -0.310557861 | 0.617259844581267 | 0.664817702145434 | -1.520387156 | 0.899271434513565 | 0.733037908976666 | 0.218627227668568 | 2.45781178184942 | 0.999714303421225 |
| ebi-fl187-GCST90094775 | prot-c-4267_81_3 | PESC \|\| id:prot-c-4267_81_3 | Green olives liking \|\| id:ebi-fl187-GCST90094775 | Weighted mode | 3 | -0.313503323 | 0.584081098161533 | 0.645160607005481 | -1.458302276 | 0.831295629249742 | 0.73088195017151 | 0.232630882806173 | 2.29629195678028 | 0.999714303421225 |
| ebi-fl187-GCST90094775 | prot-c-4280_47_2 | PSA2 \|\| id:prot-c-4280_47_2 | Green olives liking \|\| id:ebi-fl187-GCST90094775 | Weighted mode | 3 | -0.012277876 | 0.573150929405013 | 0.984854297365243 | -1.135653698 | 1.11109794565348 | 0.98779718960954 | 0.321212077120713 | 3.03769178465173 | 0.999714303421225 |
| ebi-fl187-GCST90094775 | prot-c-4324_33_2 | CYTT \|\| id:prot-c-4324_33_2 | Green olives liking \|\| id:ebi-fl187-GCST90094775 | Weighted mode | 3 | -0.754699866 | 0.5513629121751 | 0.304527171799048 | -1.835371174 | 0.325971442012022 | 0.470151702137845 | 0.159554268425413 | 1.38537580476227 | 0.999714303421225 |
| ebi-fl187-GCST90094775 | prot-c-4462_8_2 | RASA1 \|\| id:prot-c-4462_8_2 | Green olives liking \|\| id:ebi-fl187-GCST90094775 | Weighted mode | 3 | -0.003161582 | 0.535459954228607 | 0.995824978338333 | -1.052663093 | 1.04633992783493 | 0.99684341008584 | 0.349007072221093 | 2.84721102614811 | 0.999714303421225 |
| ebi-fl187-GCST90094775 | prot-c-4499_21_1 | PDGF-AA \|\| id:prot-c-4499_21_1 | Green olives liking \|\| id:ebi-fl187-GCST90094775 | Weighted mode | 3 | 0.0754088362811749 | 0.524702058067791 | 0.898897145340295 | -0.953007198 | 1.10382487009405 | 1.0783249191273 | 0.385579763747155 | 3.01567857169341 | 0.999714303421225 |
| ebi-fl187-GCST90094775 | prot-c-4535_50_2 | BST1 \|\| id:prot-c-4535_50_2 | Green olives liking \|\| id:ebi-fl187-GCST90094775 | Weighted mode | 3 | 0.377277798696976 | 0.817237425642579 | 0.689679522840163 | -1.224507556 | 1.97906315295643 | 1.45830936954867 | 0.293902395293228 | 7.23596082022967 | 0.999714303421225 |
| ebi-fl187-GCST90094775 | prot-c-4542_24_2 | Clusterin \|\| id:prot-c-4542_24_2 | Green olives liking \|\| id:ebi-fl187-GCST90094775 | Weighted mode | 3 | 0.478965521903251 | 0.585162271383296 | 0.499072724391547 | -0.66795253 | 1.62588357381451 | 1.61440347303992 | 0.512757359051903 | 5.08290817821212 | 0.999714303421225 |
| ebi-fl187-GCST90094775 | prot-c-4696_2_2 | FABP \|\| id:prot-c-4696_2_2 | Green olives liking \|\| id:ebi-fl187-GCST90094775 | Weighted mode | 3 | -0.962782783 | 0.54727509955869 | 0.220609316997289 | -2.035441978 | 0.10987641260762 | 0.381828859505925 | 0.130622737411801 | 1.11614012108755 | 0.999714303421225 |
| ebi-fl187-GCST90094775 | prot-c-4906_35_1 | Coagulation Factor V \|\| id:prot-c-4906_35_1 | Green olives liking \|\| id:ebi-fl187-GCST90094775 | Weighted mode | 3 | 0.354878741844936 | 0.577200384233327 | 0.601299977525968 | -0.776434011 | 1.48619149494226 | 1.42600772873108 | 0.460043600059578 | 4.42022895685851 | 0.999714303421225 |
| ebi-fl187-GCST90094775 | prot-c-4916_2_1 | IgD \|\| id:prot-c-4916_2_1 | Green olives liking \|\| id:ebi-fl187-GCST90094775 | Weighted mode | 3 | -1.170981678 | 0.91319231408614 | 0.328289713423814 | -2.960838613 | 0.618875257950438 | 0.310062410473333 | 0.0517754793576009 | 1.85683840268338 | 0.999714303421225 |
| ebi-fl187-GCST90094775 | prot-c-4922_13_1 | MIP-3b \|\| id:prot-c-4922_13_1 | Green olives liking \|\| id:ebi-fl187-GCST90094775 | Weighted mode | 3 | 0.7176813093536 | 0.613389718931847 | 0.36254825836072 | -0.48456254 | 1.91992515846002 | 2.04967513396041 | 0.6159665987342 | 6.82044799735726 | 0.999714303421225 |
| ebi-fl187-GCST90094775 | prot-c-4973_18_1 | cIAP-2 \|\| id:prot-c-4973_18_1 | Green olives liking \|\| id:ebi-fl187-GCST90094775 | Weighted mode | 3 | -0.361442709 | 0.74126860665568 | 0.674044963881447 | -1.814329178 | 1.09144376025742 | 0.696670508030151 | 0.162947180315715 | 2.97857131260945 | 0.999714303421225 |
| ebi-fl187-GCST90094775 | prot-c-4997_19_1 | IF4A3 \|\| id:prot-c-4997_19_1 | Green olives liking \|\| id:ebi-fl187-GCST90094775 | Weighted mode | 3 | -0.860021986 | 0.794594968672747 | 0.392237192152334 | -2.417428124 | 0.697384152753239 | 0.423152778843924 | 0.0891506071339879 | 2.00849192170078 | 0.999714303421225 |
| ebi-fl187-GCST90094775 | prot-c-5000_52_1 | LG3BP \|\| id:prot-c-5000_52_1 | Green olives liking \|\| id:ebi-fl187-GCST90094775 | Weighted mode | 3 | -0.348107034 | 0.50793465908528 | 0.563902231973336 | -1.343658966 | 0.647444897388922 | 0.706023303376477 | 0.260889334786208 | 1.91065267316932 | 0.999714303421225 |
| ebi-fl187-GCST90094775 | prot-c-5006_71_1 | MK13 \|\| id:prot-c-5006_71_1 | Green olives liking \|\| id:ebi-fl187-GCST90094775 | Weighted mode | 3 | -0.625706761 | 0.576015540271056 | 0.390849361412467 | -1.75469722 | 0.503283697950381 | 0.534883260279758 | 0.17295960308823 | 1.65414407190539 | 0.999714303421225 |
| ebi-fl187-GCST90094775 | prot-c-5109_24_3 | Nr-CAM \|\| id:prot-c-5109_24_3 | Green olives liking \|\| id:ebi-fl187-GCST90094775 | Weighted mode | 3 | 0.13920451706573 | 0.556380532300869 | 0.825789602957344 | -0.951301326 | 1.22971036037543 | 1.14935913952668 | 0.386238074532352 | 3.42023875614274 | 0.999714303421225 |
| ebi-fl187-GCST90094775 | prot-c-5255_22_3 | PDE4D \|\| id:prot-c-5255_22_3 | Green olives liking \|\| id:ebi-fl187-GCST90094775 | Weighted mode | 3 | -0.033055798 | 0.538708094632205 | 0.956651833980277 | -1.088923663 | 1.02281206759196 | 0.967484574487802 | 0.336578570659158 | 2.78100415020101 | 0.999714303421225 |
| ebi-fl187-GCST90094775 | prot-c-5440_26_3 | Troponin I, skeletal, fast twitch \|\| id:prot-c-5440_26_3 | Green olives liking \|\| id:ebi-fl187-GCST90094775 | Weighted mode | 3 | -0.574059258 | 0.662186550131918 | 0.477377181859865 | -1.871944897 | 0.723826379874102 | 0.563234477767847 | 0.153824198529624 | 2.06230931140088 | 0.999714303421225 |
| ebi-fl187-GCST90094775 | ukb-rsfMRI191-edge-pheno716 | Net100_Pair10_33 (OccipitalPrecuneus)&(Temporal\|Frontal\|Supp_Motor_Area) (Default_mode\|Central_executive)&(Default_mode\|Salience) \|\| id:ukb-rsfMRI191-edge-pheno716 | Green olives liking \|\| id:ebi-fl187-GCST90094775 | Weighted mode | 24 | -0.026319006 | 0.058284626970672 | 0.655815270151257 | -0.140556875 | 0.0879188630687127 | 0.974024320641243 | 0.868874246603303 | 1.09189952505727 | 0.999714303421225 |
| ebi-fl187-GCST90094775 | ukb-rsfMRI191-edge-pheno903 | Net100_Pair27_38 (Precentral\|Frontal\|Supp_Motor_Area)&(Subcortical) (Attention\|Salience\|Motor)&(Subcortical-cerebellum) \|\| id:ukb-rsfMRI191-edge-pheno903 | Green olives liking \|\| id:ebi-fl187-GCST90094775 | Weighted mode | 24 | 0.0695318856007906 | 0.0663694593648556 | 0.305685622791338 | -0.060552255 | 0.199616025955907 | 1.07200624222291 | 0.941244583228864 | 1.22093386123154 | 0.999714303421225 |
| ebi-fl187-GCST90094775 | ukb-rsfMRI191-node-pheno23 | Net100_Node2 RolandicOper\|SupraMarginal\|Insula Salience\|Motor \|\| id:ukb-rsfMRI191-node-pheno23 | Green olives liking \|\| id:ebi-fl187-GCST90094775 | Weighted mode | 24 | 0.0728250232266991 | 0.0626070883313561 | 0.2566719158164 | -0.04988487 | 0.195534916356157 | 1.0755423255232 | 0.951338945941341 | 1.21596125011704 | 0.999714303421225 |
| ebi-fl187-GCST90094775 | ukb-rsfMRI191-node-pheno55 | Net100_Node34 Parietal\|Postcentral\|Precuneus Attention \|\| id:ukb-rsfMRI191-node-pheno55 | Green olives liking \|\| id:ebi-fl187-GCST90094775 | Weighted mode | 24 | 0.0576149050797516 | 0.0574061704207807 | 0.325996845753289 | -0.054901189 | 0.170130999104482 | 1.0593069834153 | 0.94657867577023 | 1.18546013536524 | 0.999714303421225 |
| ebi-fl187-GCST90094780 | ebi-a-GCST90001414 | IgD- CD24- B cell Absolute Count \|\| id:ebi-a-GCST90001414 | F-healthy breakfast food liking (derived food-liking factor) \|\| id:ebi-fl187-GCST90094780 | Inverse variance weighted | 8 | 0.00138995060024897 | 0.137341201493934 | 0.991925211218947 | -0.267798804 | 0.270578705528359 | 1.0013909170293 | 0.765061692720664 | 1.31072275379877 | 0.999107451094609 |
| ebi-fl187-GCST90094780 | ebi-a-GCST90001422 | CD20- CD38- B cell %B cell \|\| id:ebi-a-GCST90001422 | F-healthy breakfast food liking (derived food-liking factor) \|\| id:ebi-fl187-GCST90094780 | Inverse variance weighted | 8 | 0.0993938657514702 | 0.136090869386253 | 0.465176724949587 | -0.167344238 | 0.366131969748525 | 1.1045012391099 | 0.845908367199128 | 1.44214554968238 | 0.998735809224318 |
| ebi-fl187-GCST90094780 | ebi-a-GCST90001444 | CD20- CD38- B cell %lymphocyte \|\| id:ebi-a-GCST90001444 | F-healthy breakfast food liking (derived food-liking factor) \|\| id:ebi-fl187-GCST90094780 | Inverse variance weighted (multiplicative random effects) | 8 | 0.0878063462328664 | 0.150571702576229 | 0.559790280050371 | -0.207314191 | 0.382926883282275 | 1.09177667488913 | 0.812764246804645 | 1.46657079530525 | 0.998735809224318 |
| ebi-fl187-GCST90094780 | ebi-a-GCST90001459 | Myeloid Dendritic Cell %Dendritic Cell \|\| id:ebi-a-GCST90001459 | F-healthy breakfast food liking (derived food-liking factor) \|\| id:ebi-fl187-GCST90094780 | Inverse variance weighted | 8 | 0.00867319402852987 | 0.123297354640425 | 0.943920088776149 | -0.232989621 | 0.250336009123762 | 1.0087109151512 | 0.792161795276082 | 1.28445693343561 | 0.998735809224318 |
| ebi-fl187-GCST90094780 | ebi-a-GCST90001476 | HLA DR++ monocyte %leukocyte \|\| id:ebi-a-GCST90001476 | F-healthy breakfast food liking (derived food-liking factor) \|\| id:ebi-fl187-GCST90094780 | Inverse variance weighted | 8 | 0.111510367592752 | 0.138327795716692 | 0.420167138217371 | -0.159612112 | 0.38263284719747 | 1.11796533455475 | 0.852474389419858 | 1.46613963396212 | 0.998735809224318 |
| ebi-fl187-GCST90094780 | ebi-a-GCST90001478 | CD4 regulatory T cell %CD4+ T cell \|\| id:ebi-a-GCST90001478 | F-healthy breakfast food liking (derived food-liking factor) \|\| id:ebi-fl187-GCST90094780 | Inverse variance weighted | 8 | -0.145858706 | 0.111471328206265 | 0.19070841745222 | -0.364342509 | 0.0726250971906247 | 0.864279812028639 | 0.694653228747187 | 1.07532731810294 | 0.998735809224318 |
| ebi-fl187-GCST90094780 | ebi-a-GCST90001485 | CD39+ resting CD4 regulatory T cell %CD4 regulatory T cell \|\| id:ebi-a-GCST90001485 | F-healthy breakfast food liking (derived food-liking factor) \|\| id:ebi-fl187-GCST90094780 | Inverse variance weighted | 8 | 0.135857499925418 | 0.0998356745789871 | 0.17357337487427 | -0.059820422 | 0.331535422100233 | 1.14551864547129 | 0.941933668726788 | 1.39310549212674 | 0.998735809224318 |
| ebi-fl187-GCST90094780 | ebi-a-GCST90001515 | Immature Myeloid-Derived Suppressor Cells Absolute Count \|\| id:ebi-a-GCST90001515 | F-healthy breakfast food liking (derived food-liking factor) \|\| id:ebi-fl187-GCST90094780 | Inverse variance weighted | 8 | -0.087754626 | 0.136509245111073 | 0.520323062807355 | -0.355312746 | 0.179803494460082 | 0.915985608242013 | 0.700954188043152 | 1.19698212639089 | 0.998735809224318 |
| ebi-fl187-GCST90094780 | ebi-a-GCST90001517 | CD33+ HLA DR+ Absolute Count \|\| id:ebi-a-GCST90001517 | F-healthy breakfast food liking (derived food-liking factor) \|\| id:ebi-fl187-GCST90094780 | Inverse variance weighted | 8 | -0.015463252 | 0.130008989401743 | 0.905322997912467 | -0.270280871 | 0.23935436752998 | 0.984655690512119 | 0.763165113340369 | 1.27042865549002 | 0.998735809224318 |
| ebi-fl187-GCST90094780 | ebi-a-GCST90001518 | CD33+ HLA DR+ CD14- Absolute Count \|\| id:ebi-a-GCST90001518 | F-healthy breakfast food liking (derived food-liking factor) \|\| id:ebi-fl187-GCST90094780 | Inverse variance weighted | 8 | -0.003257154 | 0.148921810949358 | 0.982550400196896 | -0.295143904 | 0.288629595087017 | 0.996748144399048 | 0.744424454224219 | 1.33459729556883 | 0.998955228396052 |
| ebi-fl187-GCST90094780 | ebi-a-GCST90001520 | CD33+ HLA DR+ CD14dim Absolute Count \|\| id:ebi-a-GCST90001520 | F-healthy breakfast food liking (derived food-liking factor) \|\| id:ebi-fl187-GCST90094780 | Inverse variance weighted | 8 | -0.074014991 | 0.130424415591888 | 0.570379028673237 | -0.329646846 | 0.181616863211094 | 0.928657771904166 | 0.719177669126002 | 1.19915466558642 | 0.998735809224318 |
| ebi-fl187-GCST90094780 | ebi-a-GCST90001531 | CD33dim HLA DR- Absolute Count \|\| id:ebi-a-GCST90001531 | F-healthy breakfast food liking (derived food-liking factor) \|\| id:ebi-fl187-GCST90094780 | Inverse variance weighted (multiplicative random effects) | 8 | -0.104255918 | 0.202576408580973 | 0.606797725407905 | -0.501305679 | 0.29279384303445 | 0.90099468734984 | 0.605739242388972 | 1.34016647729643 | 0.998735809224318 |
| ebi-fl187-GCST90094780 | ebi-a-GCST90001532 | Basophil Absolute Count \|\| id:ebi-a-GCST90001532 | F-healthy breakfast food liking (derived food-liking factor) \|\| id:ebi-fl187-GCST90094780 | Inverse variance weighted (multiplicative random effects) | 8 | -0.105905564 | 0.209627549250407 | 0.613412586346529 | -0.516775561 | 0.30496443218531 | 0.899509589841952 | 0.596440637226783 | 1.35657675167762 | 0.998735809224318 |
| ebi-fl187-GCST90094780 | ebi-a-GCST90001588 | T/B cell \|\| id:ebi-a-GCST90001588 | F-healthy breakfast food liking (derived food-liking factor) \|\| id:ebi-fl187-GCST90094780 | Inverse variance weighted | 8 | 0.16134985609766 | 0.0993699855798126 | 0.104433811926709 | -0.033415316 | 0.356115027834092 | 1.17509601141173 | 0.967136809126518 | 1.42777177231304 | 0.998735809224318 |
| ebi-fl187-GCST90094780 | ebi-a-GCST90001624 | HLA DR+ CD4+ T cell Absolute Count \|\| id:ebi-a-GCST90001624 | F-healthy breakfast food liking (derived food-liking factor) \|\| id:ebi-fl187-GCST90094780 | Inverse variance weighted | 8 | -0.081995268 | 0.109658549308354 | 0.454621485935591 | -0.296926025 | 0.132935488553665 | 0.921276318082005 | 0.743098981272746 | 1.14217631250824 | 0.998735809224318 |
| ebi-fl187-GCST90094780 | ebi-a-GCST90001631 | CD8+ Natural Killer T %T cell \|\| id:ebi-a-GCST90001631 | F-healthy breakfast food liking (derived food-liking factor) \|\| id:ebi-fl187-GCST90094780 | Inverse variance weighted | 8 | 0.109903475759694 | 0.135208163318487 | 0.416306099235734 | -0.155104524 | 0.374911475863929 | 1.11617032776612 | 0.856325665926682 | 1.4548626184611 | 0.998735809224318 |
| ebi-fl187-GCST90094780 | ebi-a-GCST90001632 | CD8+ Natural Killer T %lymphocyte \|\| id:ebi-a-GCST90001632 | F-healthy breakfast food liking (derived food-liking factor) \|\| id:ebi-fl187-GCST90094780 | Inverse variance weighted | 8 | 0.127895694040126 | 0.130471191144308 | 0.326957778150869 | -0.127827841 | 0.383619228682971 | 1.13643445962765 | 0.880004867211767 | 1.46758652042591 | 0.998735809224318 |
| ebi-fl187-GCST90094780 | ebi-a-GCST90001646 | Natural Killer %CD3- lymphocyte \|\| id:ebi-a-GCST90001646 | F-healthy breakfast food liking (derived food-liking factor) \|\| id:ebi-fl187-GCST90094780 | Inverse variance weighted | 8 | 0.00584815371308432 | 0.0958613625727401 | 0.951354136319121 | -0.182040117 | 0.193736424355655 | 1.00586528754816 | 0.833567899560319 | 1.21377631891537 | 0.998735809224318 |
| ebi-fl187-GCST90094780 | ebi-a-GCST90001684 | CD127- CD8+ T cell Absolute Count \|\| id:ebi-a-GCST90001684 | F-healthy breakfast food liking (derived food-liking factor) \|\| id:ebi-fl187-GCST90094780 | Inverse variance weighted | 8 | 0.0609349663931087 | 0.126190599647488 | 0.629180664030115 | -0.186398609 | 0.308268541702185 | 1.06282979228469 | 0.829942706476216 | 1.36106644296451 | 0.998735809224318 |
| ebi-fl187-GCST90094780 | ebi-a-GCST90001687 | CD28- CD8+ T cell Absolute Count \|\| id:ebi-a-GCST90001687 | F-healthy breakfast food liking (derived food-liking factor) \|\| id:ebi-fl187-GCST90094780 | Inverse variance weighted | 8 | 0.114968090705886 | 0.103778356976002 | 0.267938098677883 | -0.088437489 | 0.318373670378849 | 1.1218376399536 | 0.915360329067257 | 1.3748899208895 | 0.998735809224318 |
| ebi-fl187-GCST90094780 | ebi-a-GCST90001701 | BAFF-R on CD20- CD38- B cell \|\| id:ebi-a-GCST90001701 | F-healthy breakfast food liking (derived food-liking factor) \|\| id:ebi-fl187-GCST90094780 | Inverse variance weighted | 8 | -0.190476663 | 0.0967321525806583 | 0.0489399698197511 | -0.380071682 | -0.000881644 | 0.8265650473714 | 0.683812390639841 | 0.999118744977416 | 0.998735809224318 |
| ebi-fl187-GCST90094780 | ebi-a-GCST90001732 | CD19 on IgD- CD27- B cell \|\| id:ebi-a-GCST90001732 | F-healthy breakfast food liking (derived food-liking factor) \|\| id:ebi-fl187-GCST90094780 | Inverse variance weighted | 8 | 0.0689963925100979 | 0.105358509762441 | 0.512549836025924 | -0.137506287 | 0.275499071644482 | 1.07143234396001 | 0.871528871005369 | 1.31718788197962 | 0.998735809224318 |
| ebi-fl187-GCST90094780 | ebi-a-GCST90001798 | CD27 on CD24+ CD27+ B cell \|\| id:ebi-a-GCST90001798 | F-healthy breakfast food liking (derived food-liking factor) \|\| id:ebi-fl187-GCST90094780 | Inverse variance weighted | 8 | 0.0144607116994267 | 0.112451407656614 | 0.897677961510231 | -0.205944047 | 0.23486547070639 | 1.0145657736033 | 0.813878613708714 | 1.26473861289548 | 0.998735809224318 |
| ebi-fl187-GCST90094780 | ebi-a-GCST90001836 | CD66b on CD66b++ myeloid cell \|\| id:ebi-a-GCST90001836 | F-healthy breakfast food liking (derived food-liking factor) \|\| id:ebi-fl187-GCST90094780 | Inverse variance weighted | 8 | -0.003021096 | 0.146985903729348 | 0.983601720110513 | -0.291113467 | 0.285071275506935 | 0.996983463115205 | 0.74743086438472 | 1.32985681096194 | 0.998955228396052 |
| ebi-fl187-GCST90094780 | ebi-a-GCST90001943 | CD25 on activated & secreting CD4 regulatory T cell \|\| id:ebi-a-GCST90001943 | F-healthy breakfast food liking (derived food-liking factor) \|\| id:ebi-fl187-GCST90094780 | Inverse variance weighted | 8 | -0.112737818 | 0.0997470937650976 | 0.258376532056973 | -0.308242122 | 0.0827664854494206 | 0.89338485854742 | 0.734737400301551 | 1.08628811484787 | 0.998735809224318 |
| ebi-fl187-GCST90094780 | ebi-a-GCST90001960 | CD25 on CD4+ T cell \|\| id:ebi-a-GCST90001960 | F-healthy breakfast food liking (derived food-liking factor) \|\| id:ebi-fl187-GCST90094780 | Inverse variance weighted | 8 | -0.056033713 | 0.105935554882563 | 0.596846020314699 | -0.263667401 | 0.151599974464668 | 0.94550725936741 | 0.768229009619843 | 1.16369463574261 | 0.998735809224318 |
| ebi-fl187-GCST90094780 | ebi-a-GCST90001988 | HLA DR on CD14+ CD16- monocyte \|\| id:ebi-a-GCST90001988 | F-healthy breakfast food liking (derived food-liking factor) \|\| id:ebi-fl187-GCST90094780 | Inverse variance weighted | 8 | -0.179805924 | 0.0992579036781012 | 0.0700631287666681 | -0.374351415 | 0.0147395674546536 | 0.835432333249434 | 0.687735188922629 | 1.01484873055858 | 0.998735809224318 |
| ebi-fl187-GCST90094780 | ebi-a-GCST90001991 | HLA DR on CD14+ monocyte \|\| id:ebi-a-GCST90001991 | F-healthy breakfast food liking (derived food-liking factor) \|\| id:ebi-fl187-GCST90094780 | Inverse variance weighted | 8 | -0.188200343 | 0.0994441487331881 | 0.0584213266578289 | -0.383110874 | 0.00671018871861215 | 0.82844871687145 | 0.68173730793814 | 1.00673275247572 | 0.998735809224318 |
| ebi-fl187-GCST90094780 | ebi-a-GCST90002019 | CD14 on Monocytic Myeloid-Derived Suppressor Cells \|\| id:ebi-a-GCST90002019 | F-healthy breakfast food liking (derived food-liking factor) \|\| id:ebi-fl187-GCST90094780 | Inverse variance weighted | 8 | -0.107500636 | 0.141976008458492 | 0.448945227597131 | -0.385773613 | 0.17077234021181 | 0.898075950944871 | 0.679924434348187 | 1.18622066353407 | 0.998735809224318 |
| ebi-fl187-GCST90094780 | ebi-a-GCST90002037 | CD80 on plasmacytoid Dendritic Cell \|\| id:ebi-a-GCST90002037 | F-healthy breakfast food liking (derived food-liking factor) \|\| id:ebi-fl187-GCST90094780 | Inverse variance weighted | 8 | -0.128789535 | 0.11043162589671 | 0.243518320031993 | -0.345235521 | 0.0876564521055066 | 0.879158978575817 | 0.708053572052913 | 1.09161303624172 | 0.998735809224318 |
| ebi-fl187-GCST90094780 | ebi-a-GCST90002042 | CD45 on CD33+ HLA DR+ CD14- \|\| id:ebi-a-GCST90002042 | F-healthy breakfast food liking (derived food-liking factor) \|\| id:ebi-fl187-GCST90094780 | Inverse variance weighted | 8 | -0.101757303 | 0.146690370515288 | 0.487878094553352 | -0.389270429 | 0.185755823577083 | 0.903248741160778 | 0.67755101589929 | 1.20412820476059 | 0.998735809224318 |
| ebi-fl187-GCST90094780 | ebi-a-GCST90002056 | CD8 on Effector Memory CD8+ T cell \|\| id:ebi-a-GCST90002056 | F-healthy breakfast food liking (derived food-liking factor) \|\| id:ebi-fl187-GCST90094780 | Inverse variance weighted | 8 | -0.01812669 | 0.108268977664544 | 0.867037428551077 | -0.230333886 | 0.19408050660772 | 0.98203661063709 | 0.794268363268481 | 1.21419402966399 | 0.998735809224318 |
| ebi-fl187-GCST90094780 | ebi-a-GCST90002058 | CD8 on CD8+ T cell \|\| id:ebi-a-GCST90002058 | F-healthy breakfast food liking (derived food-liking factor) \|\| id:ebi-fl187-GCST90094780 | Inverse variance weighted | 8 | -0.106569474 | 0.104409131041301 | 0.307400797408324 | -0.311211371 | 0.0980724227070499 | 0.898912594817834 | 0.732559017789933 | 1.1030426675518 | 0.998735809224318 |
| ebi-fl187-GCST90094780 | ebi-a-GCST90002063 | CD4 on CD4 regulatory T cell \|\| id:ebi-a-GCST90002063 | F-healthy breakfast food liking (derived food-liking factor) \|\| id:ebi-fl187-GCST90094780 | Inverse variance weighted | 8 | 0.00423708943620368 | 0.107704818802881 | 0.968619450142458 | -0.206864355 | 0.21533853428985 | 1.00424607859112 | 0.813129939178501 | 1.24028170378836 | 0.998735809224318 |
| ebi-fl187-GCST90094780 | ebi-a-GCST90002066 | CD4 on activated CD4 regulatory T cell \|\| id:ebi-a-GCST90002066 | F-healthy breakfast food liking (derived food-liking factor) \|\| id:ebi-fl187-GCST90094780 | Inverse variance weighted | 8 | -0.02413034 | 0.126139720608666 | 0.848291729744086 | -0.271364193 | 0.223103512007823 | 0.976158468594982 | 0.762338807553784 | 1.24994995186897 | 0.998735809224318 |
| ebi-fl187-GCST90094780 | ebi-a-GCST90002070 | CD4 on activated & secreting CD4 regulatory T cell \|\| id:ebi-a-GCST90002070 | F-healthy breakfast food liking (derived food-liking factor) \|\| id:ebi-fl187-GCST90094780 | Inverse variance weighted | 8 | 0.0165337577330185 | 0.11093349942108 | 0.881520441260439 | -0.200895901 | 0.233963416598335 | 1.0166711967216 | 0.817997579743702 | 1.26359826463934 | 0.998735809224318 |
| ebi-fl187-GCST90094780 | ebi-a-GCST90002081 | SSC-A on CD4+ T cell \|\| id:ebi-a-GCST90002081 | F-healthy breakfast food liking (derived food-liking factor) \|\| id:ebi-fl187-GCST90094780 | Inverse variance weighted | 8 | 0.0540079925030461 | 0.102130538570105 | 0.596934934079941 | -0.146167863 | 0.254183848100451 | 1.05549303815269 | 0.864012655173016 | 1.28940883784359 | 0.998735809224318 |
| ebi-fl187-GCST90094780 | ebi-a-GCST90002094 | CD11b on Monocytic Myeloid-Derived Suppressor Cells \|\| id:ebi-a-GCST90002094 | F-healthy breakfast food liking (derived food-liking factor) \|\| id:ebi-fl187-GCST90094780 | Inverse variance weighted | 8 | -0.068467768 | 0.143765711440327 | 0.633899515177482 | -0.350248562 | 0.213313026868711 | 0.933823559035571 | 0.704512952820792 | 1.23777204651577 | 0.998735809224318 |
| ebi-fl187-GCST90094780 | ebi-a-GCST90002104 | HLA DR on myeloid Dendritic Cell \|\| id:ebi-a-GCST90002104 | F-healthy breakfast food liking (derived food-liking factor) \|\| id:ebi-fl187-GCST90094780 | Inverse variance weighted | 8 | -0.111817137 | 0.1095530598075 | 0.307412476276327 | -0.326541134 | 0.102906860255317 | 0.894207760093412 | 0.721414699628896 | 1.10838816927712 | 0.998735809224318 |
| ebi-fl187-GCST90094780 | ebi-a-GCST90002105 | HLA DR on plasmacytoid Dendritic Cell \|\| id:ebi-a-GCST90002105 | F-healthy breakfast food liking (derived food-liking factor) \|\| id:ebi-fl187-GCST90094780 | Inverse variance weighted | 8 | 0.0263692805761039 | 0.130948867420991 | 0.840408631518281 | -0.2302905 | 0.283029060721247 | 1.02672002623889 | 0.794302824356319 | 1.32714372901072 | 0.998735809224318 |
| ebi-fl187-GCST90094780 | ebi-a-GCST90002106 | HLA DR on Dendritic Cell \|\| id:ebi-a-GCST90002106 | F-healthy breakfast food liking (derived food-liking factor) \|\| id:ebi-fl187-GCST90094780 | Inverse variance weighted | 8 | -0.042846801 | 0.109310070450736 | 0.695076780229074 | -0.257094539 | 0.171400937184407 | 0.95805815247244 | 0.773295103777365 | 1.18696655265926 | 0.998735809224318 |
| ebi-fl187-GCST90094780 | ebi-a-GCST90002111 | HLA DR on CD33dim HLA DR+ CD11b- \|\| id:ebi-a-GCST90002111 | F-healthy breakfast food liking (derived food-liking factor) \|\| id:ebi-fl187-GCST90094780 | Inverse variance weighted | 8 | -0.259938775 | 0.145619173693399 | 0.0742517942246927 | -0.545352355 | 0.0254748059232555 | 0.771098795255442 | 0.579637509205063 | 1.02580206180901 | 0.998735809224318 |
| ebi-fl187-GCST90094780 | ebi-a-GCST90002112 | HLA DR on CD33- HLA DR+ \|\| id:ebi-a-GCST90002112 | F-healthy breakfast food liking (derived food-liking factor) \|\| id:ebi-fl187-GCST90094780 | Inverse variance weighted | 8 | 0.170214060397311 | 0.200762795974466 | 0.396528937898944 | -0.22328102 | 0.563709140507264 | 1.18555860530614 | 0.799890032839933 | 1.75717804811892 | 0.998735809224318 |
| ebi-fl187-GCST90094780 | ebi-a-GCST90002115 | HLA DR on HLA DR+ CD8+ T cell \|\| id:ebi-a-GCST90002115 | F-healthy breakfast food liking (derived food-liking factor) \|\| id:ebi-fl187-GCST90094780 | Inverse variance weighted | 8 | -0.215514679 | 0.100728157999177 | 0.0323897468425109 | -0.412941869 | -0.018087489 | 0.806126437031004 | 0.661700747240343 | 0.982075107502132 | 0.998735809224318 |
| ebi-fl187-GCST90094780 | ebi-a-GCST90002116 | HLA DR on B cell \|\| id:ebi-a-GCST90002116 | F-healthy breakfast food liking (derived food-liking factor) \|\| id:ebi-fl187-GCST90094780 | Inverse variance weighted | 8 | -0.004338856 | 0.10606025744136 | 0.967368166640524 | -0.21221696 | 0.203539248814531 | 0.995670543465275 | 0.808789203336383 | 1.22573326527471 | 0.998735809224318 |
| ebi-fl187-GCST90094780 | ebi-cfmet338-GCST90026023 | Sphingomyelin (d18:1/24:1, d18:2/24:0) levels \|\| id:ebi-cfmet338-GCST90026023 | F-healthy breakfast food liking (derived food-liking factor) \|\| id:ebi-fl187-GCST90094780 | Inverse variance weighted | 6 | 0.0948754061647307 | 0.081984467162099 | 0.247175807197196 | -0.065814149 | 0.255564961802445 | 1.09952185294685 | 0.936304860874282 | 1.29119088838095 | 0.998735809224318 |
| ebi-fl187-GCST90094780 | ebi-cfmet338-GCST90026046 | 1-arachidonoyl-gpc (20:4n6) levels \|\| id:ebi-cfmet338-GCST90026046 | F-healthy breakfast food liking (derived food-liking factor) \|\| id:ebi-fl187-GCST90094780 | Inverse variance weighted | 6 | 0.00237102834262655 | 0.111644228813341 | 0.983056314430865 | -0.21645166 | 0.221193716816776 | 1.00237384145321 | 0.805371465532433 | 1.24756508149369 | 0.998955228396052 |
| ebi-fl187-GCST90094780 | ebi-cfmet338-GCST90026047 | 1-linoleoyl-gpc (18:2) levels \|\| id:ebi-cfmet338-GCST90026047 | F-healthy breakfast food liking (derived food-liking factor) \|\| id:ebi-fl187-GCST90094780 | Inverse variance weighted | 6 | -0.060551734 | 0.113586625771584 | 0.593972256617677 | -0.283181521 | 0.162078052269494 | 0.941245073157635 | 0.753383020792236 | 1.1759520234633 | 0.998735809224318 |
| ebi-fl187-GCST90094780 | ebi-cfmet338-GCST90026050 | 1-myristoyl-2-palmitoyl-gpc (14:0/16:0) levels \|\| id:ebi-cfmet338-GCST90026050 | F-healthy breakfast food liking (derived food-liking factor) \|\| id:ebi-fl187-GCST90094780 | Inverse variance weighted | 6 | 0.0183491413529227 | 0.05189292009719 | 0.723641493693173 | -0.083360982 | 0.120059264743415 | 1.01851852125296 | 0.92001897694508 | 1.12756367437108 | 0.998735809224318 |
| ebi-fl187-GCST90094780 | ebi-cfmet338-GCST90026068 | 2,3-dihydroxyisovalerate levels \|\| id:ebi-cfmet338-GCST90026068 | F-healthy breakfast food liking (derived food-liking factor) \|\| id:ebi-fl187-GCST90094780 | Inverse variance weighted | 6 | -0.329774373 | 0.165938883313171 | 0.0468864717350682 | -0.655014585 | -0.004534162 | 0.719085960144724 | 0.519434486807142 | 0.995476101819256 | 0.998735809224318 |
| ebi-fl187-GCST90094780 | ebi-cfmet338-GCST90026082 | 3-hydroxyhexanoate levels \|\| id:ebi-cfmet338-GCST90026082 | F-healthy breakfast food liking (derived food-liking factor) \|\| id:ebi-fl187-GCST90094780 | Inverse variance weighted | 6 | 0.00780750958904533 | 0.0359204119662005 | 0.827931080531494 | -0.062596498 | 0.0782115170427982 | 1.00783806766776 | 0.939322415832526 | 1.08135135872387 | 0.998735809224318 |
| ebi-fl187-GCST90094780 | ebi-cfmet338-GCST90026094 | 3-ureidopropionate levels \|\| id:ebi-cfmet338-GCST90026094 | F-healthy breakfast food liking (derived food-liking factor) \|\| id:ebi-fl187-GCST90094780 | Inverse variance weighted | 6 | 0.00216508514505504 | 0.110253580423583 | 0.984332690304349 | -0.213931932 | 0.218262102775278 | 1.00216743063432 | 0.807403341091194 | 1.2439130579604 | 0.998955228396052 |
| ebi-fl187-GCST90094780 | ebi-cfmet338-GCST90026173 | Glycerol levels \|\| id:ebi-cfmet338-GCST90026173 | F-healthy breakfast food liking (derived food-liking factor) \|\| id:ebi-fl187-GCST90094780 | Inverse variance weighted | 6 | -0.037851847 | 0.045841697448633 | 0.408969872580337 | -0.127701574 | 0.0519978803390242 | 0.962855580594582 | 0.880115989751668 | 1.05337350971628 | 0.998735809224318 |
| ebi-fl187-GCST90094780 | ebi-cfmet338-GCST90026179 | Histidine levels \|\| id:ebi-cfmet338-GCST90026179 | F-healthy breakfast food liking (derived food-liking factor) \|\| id:ebi-fl187-GCST90094780 | Inverse variance weighted | 6 | 0.0412967343316461 | 0.0317300158654251 | 0.193086085044547 | -0.020894097 | 0.103487565427879 | 1.04216130470643 | 0.979322672517392 | 1.1090320029409 | 0.998735809224318 |
| ebi-fl187-GCST90094780 | ebi-cfmet338-GCST90026188 | Isocitrate levels \|\| id:ebi-cfmet338-GCST90026188 | F-healthy breakfast food liking (derived food-liking factor) \|\| id:ebi-fl187-GCST90094780 | Inverse variance weighted | 6 | -0.055889624 | 0.0620258889382942 | 0.367551480138425 | -0.177460366 | 0.0656811186357167 | 0.945643506777405 | 0.837394187957289 | 1.06788613387878 | 0.998735809224318 |
| ebi-fl187-GCST90094780 | ebi-cfmet338-GCST90026207 | N-acetyl-isoputreanine levels \|\| id:ebi-cfmet338-GCST90026207 | F-healthy breakfast food liking (derived food-liking factor) \|\| id:ebi-fl187-GCST90094780 | Inverse variance weighted | 6 | -0.001662555 | 0.0394791698562726 | 0.966409245353271 | -0.079041728 | 0.0757166177632512 | 0.998338826124188 | 0.924001366843826 | 1.07865685864887 | 0.998735809224318 |
| ebi-fl187-GCST90094780 | ebi-cfmet338-GCST90026213 | N-acetylglutamate levels \|\| id:ebi-cfmet338-GCST90026213 | F-healthy breakfast food liking (derived food-liking factor) \|\| id:ebi-fl187-GCST90094780 | Inverse variance weighted | 6 | -0.006515263 | 0.0675441067668806 | 0.923155724422745 | -0.138901713 | 0.125871185829111 | 0.993505914875782 | 0.870313565027621 | 1.13413606607618 | 0.998735809224318 |
| ebi-fl187-GCST90094780 | ebi-cfmet338-GCST90026224 | N-acetyltaurine levels \|\| id:ebi-cfmet338-GCST90026224 | F-healthy breakfast food liking (derived food-liking factor) \|\| id:ebi-fl187-GCST90094780 | Inverse variance weighted | 6 | 0.001162578907262 | 0.0540885728087195 | 0.982851602594539 | -0.104851024 | 0.107176181612352 | 1.00116325496408 | 0.900458659505719 | 1.11313035030445 | 0.998955228396052 |
| ebi-fl187-GCST90094780 | ebi-cfmet338-GCST90026250 | Phenyllactate (pla) levels \|\| id:ebi-cfmet338-GCST90026250 | F-healthy breakfast food liking (derived food-liking factor) \|\| id:ebi-fl187-GCST90094780 | Inverse variance weighted | 6 | 0.0840952596433277 | 0.0856318867676383 | 0.32607253977768 | -0.083743238 | 0.251933757707899 | 1.08773250588427 | 0.919667361026038 | 1.28651081303708 | 0.998735809224318 |
| ebi-fl187-GCST90094780 | ebi-cfmet338-GCST90026251 | Pipecolate levels \|\| id:ebi-cfmet338-GCST90026251 | F-healthy breakfast food liking (derived food-liking factor) \|\| id:ebi-fl187-GCST90094780 | Inverse variance weighted | 6 | -0.011372626 | 0.128249971757034 | 0.929339830260036 | -0.262742571 | 0.239997318513838 | 0.988691797728501 | 0.768939819417358 | 1.27124574148897 | 0.998735809224318 |
| ebi-fl187-GCST90094780 | ebi-cfmet338-GCST90026278 | Trigonelline (n'-methylnicotinate) levels \|\| id:ebi-cfmet338-GCST90026278 | F-healthy breakfast food liking (derived food-liking factor) \|\| id:ebi-fl187-GCST90094780 | Inverse variance weighted | 6 | 0.167693735743523 | 0.143764226337123 | 0.243432682464474 | -0.114084148 | 0.449471619364283 | 1.18257437491905 | 0.892182877428364 | 1.56748373858752 | 0.998735809224318 |
| ebi-fl187-GCST90094780 | ebi-cfmet338-GCST90026299 | 1-carboxyethylvaline levels \|\| id:ebi-cfmet338-GCST90026299 | F-healthy breakfast food liking (derived food-liking factor) \|\| id:ebi-fl187-GCST90094780 | Inverse variance weighted | 6 | -0.150221933 | 0.087751744648285 | 0.086915683653681 | -0.322215353 | 0.0217714863391562 | 0.86051697796936 | 0.724542141434264 | 1.02201021448896 | 0.998735809224318 |
| ebi-fl187-GCST90094780 | ebi-gm430-GCST90011306 | C_Erysipelotrichia abundance \|\| id:ebi-gm430-GCST90011306 | F-healthy breakfast food liking (derived food-liking factor) \|\| id:ebi-fl187-GCST90094780 | Inverse variance weighted | 6 | -0.06879105 | 0.0690238218432499 | 0.31894527851134 | -0.20407774 | 0.0664956412897579 | 0.933521719509272 | 0.815398979332823 | 1.06875630566597 | 0.998735809224318 |
| ebi-fl187-GCST90094780 | ebi-gm430-GCST90011312 | F_Erysipelotrichaceae abundance \|\| id:ebi-gm430-GCST90011312 | F-healthy breakfast food liking (derived food-liking factor) \|\| id:ebi-fl187-GCST90094780 | Inverse variance weighted | 6 | -0.06879105 | 0.0690238218432499 | 0.31894527851134 | -0.20407774 | 0.0664956412897579 | 0.933521719509272 | 0.815398979332823 | 1.06875630566597 | 0.998735809224318 |
| ebi-fl187-GCST90094780 | ebi-gm430-GCST90011352 | O_Erysipelotrichales abundance \|\| id:ebi-gm430-GCST90011352 | F-healthy breakfast food liking (derived food-liking factor) \|\| id:ebi-fl187-GCST90094780 | Inverse variance weighted | 6 | -0.06879105 | 0.0690238218432499 | 0.31894527851134 | -0.20407774 | 0.0664956412897579 | 0.933521719509272 | 0.815398979332823 | 1.06875630566597 | 0.998735809224318 |
| ebi-fl187-GCST90094780 | ebi-gm430-GCST90011362 | OTU97_11 (Parabacteroides) abundance \|\| id:ebi-gm430-GCST90011362 | F-healthy breakfast food liking (derived food-liking factor) \|\| id:ebi-fl187-GCST90094780 | Inverse variance weighted | 6 | -0.053033581 | 0.0894446695935372 | 0.553234339254946 | -0.228345133 | 0.12227797169203 | 0.948348165741615 | 0.795849538400479 | 1.13006818509068 | 0.998735809224318 |
| ebi-fl187-GCST90094780 | ebi-gm430-GCST90011384 | OTU97_235 (Phascolarctobacterium) abundance \|\| id:ebi-gm430-GCST90011384 | F-healthy breakfast food liking (derived food-liking factor) \|\| id:ebi-fl187-GCST90094780 | Inverse variance weighted | 6 | 0.107286325328295 | 0.277299246334141 | 0.698832251760232 | -0.436220197 | 0.650792848143211 | 1.11325296136986 | 0.646475358012489 | 1.91706016422489 | 0.998735809224318 |
| ebi-fl187-GCST90094780 | ebi-gm430-GCST90011390 | OTU97_3 (Bacteroides) abundance \|\| id:ebi-gm430-GCST90011390 | F-healthy breakfast food liking (derived food-liking factor) \|\| id:ebi-fl187-GCST90094780 | Inverse variance weighted | 6 | -0.062467996 | 0.0899810180321008 | 0.487534677065824 | -0.238830792 | 0.11389479896648 | 0.939443127916601 | 0.787548130764136 | 1.1206342268039 | 0.998735809224318 |
| ebi-fl187-GCST90094780 | ebi-gm430-GCST90011398 | OTU97_4 (Alistipes) abundance \|\| id:ebi-gm430-GCST90011398 | F-healthy breakfast food liking (derived food-liking factor) \|\| id:ebi-fl187-GCST90094780 | Inverse variance weighted | 6 | 0.0210039265174699 | 0.0836068820406433 | 0.801642121273363 | -0.142865562 | 0.184873415317131 | 1.02122606149163 | 0.866870601167404 | 1.20306614074262 | 0.998735809224318 |
| ebi-fl187-GCST90094780 | ebi-gm430-GCST90011421 | OTU99_108 (Cytophagales) abundance \|\| id:ebi-gm430-GCST90011421 | F-healthy breakfast food liking (derived food-liking factor) \|\| id:ebi-fl187-GCST90094780 | Inverse variance weighted | 6 | 0.057878106777059 | 0.325288467932596 | 0.85877908568084 | -0.57968729 | 0.695443503924948 | 1.05958583150634 | 0.560073479554315 | 2.00459792386957 | 0.998735809224318 |
| ebi-fl187-GCST90094780 | ebi-gm430-GCST90011428 | OTU99_123 (Phascolarctobacterium) abundance \|\| id:ebi-gm430-GCST90011428 | F-healthy breakfast food liking (derived food-liking factor) \|\| id:ebi-fl187-GCST90094780 | Inverse variance weighted | 6 | -0.114166722 | 0.157859639359888 | 0.469546780597764 | -0.423571615 | 0.195238170900711 | 0.892109209033228 | 0.654704287084594 | 1.21560047267425 | 0.998735809224318 |
| ebi-fl187-GCST90094780 | ebi-gm430-GCST90011450 | OTU99_298 (Phascolarctobacterium) abundance \|\| id:ebi-gm430-GCST90011450 | F-healthy breakfast food liking (derived food-liking factor) \|\| id:ebi-fl187-GCST90094780 | Inverse variance weighted | 6 | 0.110683811914145 | 0.277282209577877 | 0.689765097312882 | -0.432789319 | 0.654156942686784 | 1.11704165574755 | 0.648697145664416 | 1.92352019584921 | 0.998735809224318 |
| ebi-fl187-GCST90094780 | ebi-gm430-GCST90011451 | OTU99_3 (Bacteroides) abundance \|\| id:ebi-gm430-GCST90011451 | F-healthy breakfast food liking (derived food-liking factor) \|\| id:ebi-fl187-GCST90094780 | Inverse variance weighted | 6 | -0.06391003 | 0.0898464593051176 | 0.476882881992227 | -0.24000909 | 0.112189030322401 | 0.938089395716186 | 0.786620710530923 | 1.11872431347657 | 0.998735809224318 |
| ebi-fl187-GCST90094780 | ebi-gm430-GCST90011472 | OTU99_72 (Parabacteroides) abundance \|\| id:ebi-gm430-GCST90011472 | F-healthy breakfast food liking (derived food-liking factor) \|\| id:ebi-fl187-GCST90094780 | Inverse variance weighted | 6 | 0.0231099642430588 | 0.210439809042165 | 0.912554147746619 | -0.389352061 | 0.435571989965703 | 1.02337906846465 | 0.677495707880837 | 1.54584701510136 | 0.998735809224318 |
| ebi-fl187-GCST90094780 | ebi-gm430-GCST90011507 | TestASV_3 (Bacteroides) abundance \|\| id:ebi-gm430-GCST90011507 | F-healthy breakfast food liking (derived food-liking factor) \|\| id:ebi-fl187-GCST90094780 | Inverse variance weighted | 6 | -0.129625286 | 0.0916320571452002 | 0.157177554151361 | -0.309224118 | 0.0499735455860195 | 0.878424526858825 | 0.734016245026997 | 1.05124328598309 | 0.998735809224318 |
| ebi-fl187-GCST90094780 | ebi-gm430-GCST90011512 | TestASV_34 (Phascolarctobacterium) abundance \|\| id:ebi-gm430-GCST90011512 | F-healthy breakfast food liking (derived food-liking factor) \|\| id:ebi-fl187-GCST90094780 | Inverse variance weighted (multiplicative random effects) | 6 | 0.0750324089376355 | 0.422037148368144 | 0.858890833259337 | -0.752160402 | 0.902225219739197 | 1.07791908453093 | 0.471347152713367 | 2.46508236255875 | 0.998735809224318 |
| ebi-fl187-GCST90094780 | ebi-gm430-GCST90011519 | TestASV_41 (Barnesiella) abundance \|\| id:ebi-gm430-GCST90011519 | F-healthy breakfast food liking (derived food-liking factor) \|\| id:ebi-fl187-GCST90094780 | Inverse variance weighted | 6 | -0.4531734 | 0.411117355345199 | 0.270332602698651 | -1.258963416 | 0.352616616952579 | 0.63560790996128 | 0.283948210171707 | 1.42278556699141 | 0.998735809224318 |
| ebi-fl187-GCST90094780 | ebi-gm430-GCST90011524 | TestASV_46 (Sutterella) abundance \|\| id:ebi-gm430-GCST90011524 | F-healthy breakfast food liking (derived food-liking factor) \|\| id:ebi-fl187-GCST90094780 | Inverse variance weighted | 6 | 0.738381802236156 | 0.29515616465588 | 0.0123610880041012 | 0.159875719510631 | 1.31688788496168 | 2.09254661902269 | 1.17336503554892 | 3.73178952851175 | 0.998735809224318 |
| ebi-fl187-GCST90094780 | ebi-gm430-GCST90011581 | OTU97_19 (Bacteria) prevalence \|\| id:ebi-gm430-GCST90011581 | F-healthy breakfast food liking (derived food-liking factor) \|\| id:ebi-fl187-GCST90094780 | Inverse variance weighted | 6 | 0.196080675019085 | 0.338164614846826 | 0.562023906973277 | -0.46672197 | 0.858883320118864 | 1.21662505262459 | 0.627054406064689 | 2.36052327255488 | 0.998735809224318 |
| ebi-fl187-GCST90094780 | ebi-gm430-GCST90011614 | OTU97_80 (Ruminococcus) prevalence \|\| id:ebi-gm430-GCST90011614 | F-healthy breakfast food liking (derived food-liking factor) \|\| id:ebi-fl187-GCST90094780 | Inverse variance weighted | 6 | 0.0392189290423119 | 0.157514239913932 | 0.80337117621451 | -0.269508981 | 0.347946839273619 | 1.03999814452625 | 0.763754420068769 | 1.41615696380605 | 0.998735809224318 |
| ebi-fl187-GCST90094780 | ebi-gm430-GCST90011623 | OTU99_11 (Parabacteroides) prevalence \|\| id:ebi-gm430-GCST90011623 | F-healthy breakfast food liking (derived food-liking factor) \|\| id:ebi-fl187-GCST90094780 | Inverse variance weighted | 6 | -0.012061543 | 0.160723645764847 | 0.940178726403101 | -0.327079888 | 0.302956803021213 | 0.988010906153899 | 0.721026139117676 | 1.35385598069106 | 0.998735809224318 |
| ebi-fl187-GCST90094780 | ebi-gm430-GCST90011642 | OTU99_19 (Bacteria) prevalence \|\| id:ebi-gm430-GCST90011642 | F-healthy breakfast food liking (derived food-liking factor) \|\| id:ebi-fl187-GCST90094780 | Inverse variance weighted | 6 | 0.196080675019085 | 0.338164614846826 | 0.562023906973277 | -0.46672197 | 0.858883320118864 | 1.21662505262459 | 0.627054406064689 | 2.36052327255488 | 0.998735809224318 |
| ebi-fl187-GCST90094780 | ebi-gm430-GCST90011653 | OTU99_30 (Parasutterella) prevalence \|\| id:ebi-gm430-GCST90011653 | F-healthy breakfast food liking (derived food-liking factor) \|\| id:ebi-fl187-GCST90094780 | Inverse variance weighted | 6 | 0.0828696007611966 | 0.151943531166463 | 0.585480255783562 | -0.21493972 | 0.380678921847465 | 1.08640013356083 | 0.806590059698527 | 1.46327770347446 | 0.998735809224318 |
| ebi-fl187-GCST90094780 | ebi-gm430-GCST90011675 | OTU99_8 (Ruminococcaceae) prevalence \|\| id:ebi-gm430-GCST90011675 | F-healthy breakfast food liking (derived food-liking factor) \|\| id:ebi-fl187-GCST90094780 | Inverse variance weighted | 6 | 0.0430590082165029 | 0.158389265693494 | 0.785733055024416 | -0.267383953 | 0.353501968975752 | 1.04399949758144 | 0.765379145773042 | 1.42404579086024 | 0.998735809224318 |
| ebi-fl187-GCST90094780 | ebi-gm430-GCST90011679 | OTU99_92 (Ruminococcus) prevalence \|\| id:ebi-gm430-GCST90011679 | F-healthy breakfast food liking (derived food-liking factor) \|\| id:ebi-fl187-GCST90094780 | Inverse variance weighted | 6 | 0.0894375526945144 | 0.164634451685836 | 0.586958158249197 | -0.233245973 | 0.412121077998752 | 1.09355904136453 | 0.791958749404213 | 1.51001725512819 | 0.998735809224318 |
| ebi-fl187-GCST90094780 | ebi-gm430-GCST90011684 | TestASV_11 (Lachnospiraceae) prevalence \|\| id:ebi-gm430-GCST90011684 | F-healthy breakfast food liking (derived food-liking factor) \|\| id:ebi-fl187-GCST90094780 | Inverse variance weighted | 6 | -0.085008998 | 0.167088231812474 | 0.610915469763597 | -0.412501933 | 0.242483936077779 | 0.91850401941282 | 0.661991917306066 | 1.27441077696339 | 0.998735809224318 |
| ebi-fl187-GCST90094780 | ebi-gm430-GCST90011688 | TestASV_15 (Bacteroides) prevalence \|\| id:ebi-gm430-GCST90011688 | F-healthy breakfast food liking (derived food-liking factor) \|\| id:ebi-fl187-GCST90094780 | Inverse variance weighted | 6 | -0.202569625 | 0.15721298515731 | 0.197570433015057 | -0.510707076 | 0.105567825453718 | 0.816629622409968 | 0.600071132658046 | 1.11134147920672 | 0.998735809224318 |
| ebi-fl187-GCST90094780 | ebi-gm430-GCST90011692 | TestASV_19 (Prevotella) prevalence \|\| id:ebi-gm430-GCST90011692 | F-healthy breakfast food liking (derived food-liking factor) \|\| id:ebi-fl187-GCST90094780 | Inverse variance weighted | 6 | 0.305901429575252 | 0.30743112471279 | 0.319724461880902 | -0.296663575 | 0.908466434012319 | 1.35784845625208 | 0.743294033100748 | 2.48051558069788 | 0.998735809224318 |
| ebi-fl187-GCST90094780 | ebi-gm430-GCST90011693 | TestASV_2 (Bacteroides) prevalence \|\| id:ebi-gm430-GCST90011693 | F-healthy breakfast food liking (derived food-liking factor) \|\| id:ebi-fl187-GCST90094780 | Inverse variance weighted | 6 | 0.0175693138013268 | 0.172465439000859 | 0.9188586681225 | -0.320462947 | 0.355601574243011 | 1.01772456206414 | 0.725812946618555 | 1.42703886594211 | 0.998735809224318 |
| ebi-fl187-GCST90094780 | ebi-gm430-GCST90011716 | TestASV_40 (Barnesiella) prevalence \|\| id:ebi-gm430-GCST90011716 | F-healthy breakfast food liking (derived food-liking factor) \|\| id:ebi-fl187-GCST90094780 | Inverse variance weighted (multiplicative random effects) | 6 | -0.042365838 | 0.477349535332329 | 0.929278818417783 | -0.977970928 | 0.893239250906518 | 0.958519053397673 | 0.376073405370933 | 2.44303043662492 | 0.998735809224318 |
| ebi-fl187-GCST90094780 | ebi-gm430-GCST90011718 | TestASV_42 (Alphaproteobacteria) prevalence \|\| id:ebi-gm430-GCST90011718 | F-healthy breakfast food liking (derived food-liking factor) \|\| id:ebi-fl187-GCST90094780 | Inverse variance weighted | 6 | -0.091040601 | 0.246172052468603 | 0.711512819961873 | -0.573537824 | 0.391456621541769 | 0.912980641948829 | 0.5635282440982 | 1.47913376357414 | 0.998735809224318 |
| ebi-fl187-GCST90094780 | ebi-hbsm44-GCST90104929 | Sulcal depth of the right inferior frontal sulcus \|\| id:ebi-hbsm44-GCST90104929 | F-healthy breakfast food liking (derived food-liking factor) \|\| id:ebi-fl187-GCST90094780 | Inverse variance weighted | 7 | 0.107928166301228 | 0.166046206196294 | 0.515699487374791 | -0.217522398 | 0.433378730445964 | 1.11396772209053 | 0.804509585438235 | 1.54246028676414 | 0.998735809224318 |
| ebi-fl187-GCST90094780 | ebi-hbsm44-GCST90104935 | Sulcal opening distance of the left calloso-marginal posterior fissure \|\| id:ebi-hbsm44-GCST90104935 | F-healthy breakfast food liking (derived food-liking factor) \|\| id:ebi-fl187-GCST90094780 | Inverse variance weighted | 6 | 0.0388506827316731 | 0.0366341464818129 | 0.288915075149253 | -0.032952244 | 0.110653609836026 | 1.03961523955245 | 0.967584766103203 | 1.11700791927766 | 0.998735809224318 |
| ebi-fl187-GCST90094780 | ebi-hbsm44-GCST90104936 | Sulcal opening distance of the left anterior inferior temporal sulcus \|\| id:ebi-hbsm44-GCST90104936 | F-healthy breakfast food liking (derived food-liking factor) \|\| id:ebi-fl187-GCST90094780 | Inverse variance weighted | 7 | 0.0133319489333057 | 0.0158139048436605 | 0.399199191480517 | -0.017663305 | 0.0443272024268803 | 1.01342121562301 | 0.982491777175989 | 1.04532433159576 | 0.998735809224318 |
| ebi-fl187-GCST90094780 | ebi-hbsm44-GCST90104941 | Sulcal opening distance of the right posterior inferior temporal sulcus \|\| id:ebi-hbsm44-GCST90104941 | F-healthy breakfast food liking (derived food-liking factor) \|\| id:ebi-fl187-GCST90094780 | Inverse variance weighted | 6 | -0.020378728 | 0.0140010423435448 | 0.145526715769797 | -0.047820771 | 0.00706331458796541 | 0.979827514514881 | 0.953304631222607 | 1.00708831863025 | 0.998735809224318 |
| ebi-fl187-GCST90094780 | ebi-IPs91-GCST90274819 | Leukemia inhibitory factor levels \|\| id:ebi-IPs91-GCST90274819 | F-healthy breakfast food liking (derived food-liking factor) \|\| id:ebi-fl187-GCST90094780 | Inverse variance weighted | 9 | 0.0619951584539034 | 0.0578878060648006 | 0.284190261121541 | -0.051464941 | 0.175455258340913 | 1.06395719351777 | 0.949836949300708 | 1.19178866485623 | 0.998735809224318 |
| ebi-fl187-GCST90094780 | ebi-IPs91-GCST90274837 | STAM binding protein levels \|\| id:ebi-IPs91-GCST90274837 | F-healthy breakfast food liking (derived food-liking factor) \|\| id:ebi-fl187-GCST90094780 | Inverse variance weighted | 9 | 0.0203930458351446 | 0.0533815328733319 | 0.702443297217576 | -0.084234759 | 0.125020850266875 | 1.0206024047277 | 0.919215437037467 | 1.13317207976079 | 0.998735809224318 |
| ebi-fl187-GCST90094780 | ebi-IPs91-GCST90274840 | TNF-beta levels \|\| id:ebi-IPs91-GCST90274840 | F-healthy breakfast food liking (derived food-liking factor) \|\| id:ebi-fl187-GCST90094780 | Inverse variance weighted | 8 | -0.050619907 | 0.051777044397376 | 0.328246697563295 | -0.152102914 | 0.0508630998819229 | 0.950639933325376 | 0.858899883236132 | 1.05217884001554 | 0.998735809224318 |
| ebi-fl187-GCST90094780 | ebi-IPs91-GCST90274842 | Tumor necrosis factor ligand superfamily member 14 levels \|\| id:ebi-IPs91-GCST90274842 | F-healthy breakfast food liking (derived food-liking factor) \|\| id:ebi-fl187-GCST90094780 | Inverse variance weighted | 9 | 0.0311456590474035 | 0.0603971446500686 | 0.606077237381243 | -0.087232744 | 0.149524062561538 | 1.03163576002525 | 0.916463768937632 | 1.16128141388129 | 0.998735809224318 |
| ebi-fl187-GCST90094780 | ebi-met1400-GCST90199628 | 3-methylhistidine levels \|\| id:ebi-met1400-GCST90199628 | F-healthy breakfast food liking (derived food-liking factor) \|\| id:ebi-fl187-GCST90094780 | Inverse variance weighted | 8 | -0.063447559 | 0.0662288527934224 | 0.338060340887284 | -0.19325611 | 0.0663609929612112 | 0.938523335568626 | 0.824270843107314 | 1.06861240910372 | 0.998735809224318 |
| ebi-fl187-GCST90094780 | ebi-met1400-GCST90199651 | Docosahexaenoate DHA; 22:6n3 levels \|\| id:ebi-met1400-GCST90199651 | F-healthy breakfast food liking (derived food-liking factor) \|\| id:ebi-fl187-GCST90094780 | Inverse variance weighted | 8 | 0.0850149502297737 | 0.0572535761737992 | 0.137574411546064 | -0.027202059 | 0.19723195953042 | 1.08873334339037 | 0.973164584925045 | 1.21802654080479 | 0.998735809224318 |
| ebi-fl187-GCST90094780 | ebi-met1400-GCST90199652 | 1-stearoyl-2-oleoyl-GPS (18:0/18:1) levels \|\| id:ebi-met1400-GCST90199652 | F-healthy breakfast food liking (derived food-liking factor) \|\| id:ebi-fl187-GCST90094780 | Inverse variance weighted | 8 | 0.0145974294303622 | 0.0643733030199379 | 0.820608686861074 | -0.111574244 | 0.140769103349441 | 1.01470449221618 | 0.894424982812519 | 1.15115881858089 | 0.998735809224318 |
| ebi-fl187-GCST90094780 | ebi-met1400-GCST90199653 | 1-myristoyl-2-palmitoyl-gpc (14:0/16:0) levels \|\| id:ebi-met1400-GCST90199653 | F-healthy breakfast food liking (derived food-liking factor) \|\| id:ebi-fl187-GCST90094780 | Inverse variance weighted | 8 | 0.128491693378632 | 0.0768219162219701 | 0.0944077175357716 | -0.022079262 | 0.279062649173694 | 1.13711197569335 | 0.978162700439163 | 1.32189015660146 | 0.998735809224318 |
| ebi-fl187-GCST90094780 | ebi-met1400-GCST90199667 | Erythritol levels in elite athletes \|\| id:ebi-met1400-GCST90199667 | F-healthy breakfast food liking (derived food-liking factor) \|\| id:ebi-fl187-GCST90094780 | Inverse variance weighted | 8 | 0.0163690803259313 | 0.056209847822896 | 0.770887850450623 | -0.093802221 | 0.126540382058807 | 1.01650378772968 | 0.910462814489419 | 1.13489527965866 | 0.998735809224318 |
| ebi-fl187-GCST90094780 | ebi-met1400-GCST90199674 | 3-hydroxymyristate levels \|\| id:ebi-met1400-GCST90199674 | F-healthy breakfast food liking (derived food-liking factor) \|\| id:ebi-fl187-GCST90094780 | Inverse variance weighted | 8 | 0.00838086862798172 | 0.0583596131892747 | 0.885810546771275 | -0.106003973 | 0.12276571047896 | 1.00841608642395 | 0.899421074468328 | 1.13061949761375 | 0.998735809224318 |
| ebi-fl187-GCST90094780 | ebi-met1400-GCST90199685 | 2-linoleoylglycerol (18:2) levels \|\| id:ebi-met1400-GCST90199685 | F-healthy breakfast food liking (derived food-liking factor) \|\| id:ebi-fl187-GCST90094780 | Inverse variance weighted | 7 | 0.00778636769174617 | 0.0727215336819401 | 0.914732649616159 | -0.134747838 | 0.150320573708349 | 1.00781676028408 | 0.873936257136096 | 1.16220675594574 | 0.998735809224318 |
| ebi-fl187-GCST90094780 | ebi-met1400-GCST90199703 | Gamma-glutamylvaline levels \|\| id:ebi-met1400-GCST90199703 | F-healthy breakfast food liking (derived food-liking factor) \|\| id:ebi-fl187-GCST90094780 | Inverse variance weighted | 8 | 0.0457947021072585 | 0.0584251185906851 | 0.433146483454757 | -0.06871853 | 0.160307934545001 | 1.04685947085095 | 0.933589420205348 | 1.17387229117195 | 0.998735809224318 |
| ebi-fl187-GCST90094780 | ebi-met1400-GCST90199779 | Gamma-glutamyl-2-aminobutyrate levels \|\| id:ebi-met1400-GCST90199779 | F-healthy breakfast food liking (derived food-liking factor) \|\| id:ebi-fl187-GCST90094780 | Inverse variance weighted | 8 | -0.016573355 | 0.0658968593308742 | 0.801424100514065 | -0.145731199 | 0.112584489082132 | 0.983563227260813 | 0.864390020433848 | 1.11916681029491 | 0.998735809224318 |
| ebi-fl187-GCST90094780 | ebi-met1400-GCST90199824 | 4-vinylphenol sulfate levels \|\| id:ebi-met1400-GCST90199824 | F-healthy breakfast food liking (derived food-liking factor) \|\| id:ebi-fl187-GCST90094780 | Inverse variance weighted | 8 | -0.021272567 | 0.0687587131394488 | 0.757032521398334 | -0.156039645 | 0.113494510615144 | 0.978952098029816 | 0.855525272492088 | 1.12018573974487 | 0.998735809224318 |
| ebi-fl187-GCST90094780 | ebi-met1400-GCST90199830 | 4-ethylphenylsulfate levels \|\| id:ebi-met1400-GCST90199830 | F-healthy breakfast food liking (derived food-liking factor) \|\| id:ebi-fl187-GCST90094780 | Inverse variance weighted | 8 | 0.0565352862926798 | 0.0599800905320364 | 0.345902150271535 | -0.061025691 | 0.174096263735471 | 1.0581639528244 | 0.940799069255414 | 1.19017013052867 | 0.998735809224318 |
| ebi-fl187-GCST90094780 | ebi-met1400-GCST90199833 | Hexanoylglutamine levels \|\| id:ebi-met1400-GCST90199833 | F-healthy breakfast food liking (derived food-liking factor) \|\| id:ebi-fl187-GCST90094780 | Inverse variance weighted | 8 | -0.009145077 | 0.0591026710916028 | 0.877032573881235 | -0.124986312 | 0.106696158769435 | 0.990896612462708 | 0.882508982364608 | 1.11259615053347 | 0.998735809224318 |
| ebi-fl187-GCST90094780 | ebi-met1400-GCST90199837 | Carotenoid levels (cryptoxanthin) \|\| id:ebi-met1400-GCST90199837 | F-healthy breakfast food liking (derived food-liking factor) \|\| id:ebi-fl187-GCST90094780 | Inverse variance weighted | 8 | -0.055799119 | 0.073444302270576 | 0.447405576537026 | -0.199749952 | 0.0881517131375518 | 0.945729095520782 | 0.818935500856624 | 1.09215380354007 | 0.998735809224318 |
| ebi-fl187-GCST90094780 | ebi-met1400-GCST90199869 | 2-hydroxyhippurate levels \|\| id:ebi-met1400-GCST90199869 | F-healthy breakfast food liking (derived food-liking factor) \|\| id:ebi-fl187-GCST90094780 | Inverse variance weighted | 8 | 0.0694522101717426 | 0.0811450753102459 | 0.392052139113066 | -0.089592137 | 0.228496557779824 | 1.07192083306817 | 0.91430401961488 | 1.25670919925469 | 0.998735809224318 |
| ebi-fl187-GCST90094780 | ebi-met1400-GCST90199902 | Hydantoin-5-propionate levels \|\| id:ebi-met1400-GCST90199902 | F-healthy breakfast food liking (derived food-liking factor) \|\| id:ebi-fl187-GCST90094780 | Inverse variance weighted | 8 | -0.023080701 | 0.0597895524625691 | 0.699472717680963 | -0.140268224 | 0.0941068219703559 | 0.977183621036215 | 0.869125084200869 | 1.09867710250181 | 0.998735809224318 |
| ebi-fl187-GCST90094780 | ebi-met1400-GCST90199904 | Pantoate levels \|\| id:ebi-met1400-GCST90199904 | F-healthy breakfast food liking (derived food-liking factor) \|\| id:ebi-fl187-GCST90094780 | Inverse variance weighted | 8 | 0.0507047042349772 | 0.0607269699724895 | 0.403739179056557 | -0.068320157 | 0.169729565381057 | 1.05201219266593 | 0.933961411505777 | 1.18498434719423 | 0.998735809224318 |
| ebi-fl187-GCST90094780 | ebi-met1400-GCST90199936 | 3-methyl catechol sulfate (2) levels \|\| id:ebi-met1400-GCST90199936 | F-healthy breakfast food liking (derived food-liking factor) \|\| id:ebi-fl187-GCST90094780 | Inverse variance weighted (multiplicative random effects) | 8 | 0.0329883988985106 | 0.130931016001236 | 0.801077766721378 | -0.223636392 | 0.289613190260933 | 1.03353854898616 | 0.799605824221239 | 1.33591064482398 | 0.998735809224318 |
| ebi-fl187-GCST90094780 | ebi-met1400-GCST90199977 | 4-hydroxychlorothalonil levels \|\| id:ebi-met1400-GCST90199977 | F-healthy breakfast food liking (derived food-liking factor) \|\| id:ebi-fl187-GCST90094780 | Inverse variance weighted | 8 | -0.062374073 | 0.0744855426370677 | 0.40236867773213 | -0.208365736 | 0.0836175910534815 | 0.939531368186484 | 0.811910037606033 | 1.08721305430478 | 0.998735809224318 |
| ebi-fl187-GCST90094780 | ebi-met1400-GCST90200040 | Sphingomyelin (d18:2/24:1, d18:1/24:2) levels \|\| id:ebi-met1400-GCST90200040 | F-healthy breakfast food liking (derived food-liking factor) \|\| id:ebi-fl187-GCST90094780 | Inverse variance weighted | 8 | 0.0233855808616356 | 0.0545769260576663 | 0.668295561981397 | -0.083585194 | 0.130356355934662 | 1.02366116761685 | 0.919812720613757 | 1.13923428389582 | 0.998735809224318 |
| ebi-fl187-GCST90094780 | ebi-met1400-GCST90200077 | 1-stearoyl-2-oleoyl-GPI (18:0/18:1) levels \|\| id:ebi-met1400-GCST90200077 | F-healthy breakfast food liking (derived food-liking factor) \|\| id:ebi-fl187-GCST90094780 | Inverse variance weighted | 7 | -0.019933984 | 0.062831203255383 | 0.751044442733576 | -0.143083142 | 0.10321517487102 | 0.980263384719116 | 0.866682008319633 | 1.10872995423552 | 0.998735809224318 |
| ebi-fl187-GCST90094780 | ebi-met1400-GCST90200080 | 1-palmitoyl-2-oleoyl-GPI (16:0/18:1) levels \|\| id:ebi-met1400-GCST90200080 | F-healthy breakfast food liking (derived food-liking factor) \|\| id:ebi-fl187-GCST90094780 | Inverse variance weighted | 8 | 0.0501116124834386 | 0.0740550585486209 | 0.498608704755462 | -0.095036302 | 0.195259527238736 | 1.05138843790213 | 0.909339922763947 | 1.21562643372606 | 0.998735809224318 |
| ebi-fl187-GCST90094780 | ebi-met1400-GCST90200082 | 1-oleoyl-2-linoleoyl-GPE (18:1/18:2) levels \|\| id:ebi-met1400-GCST90200082 | F-healthy breakfast food liking (derived food-liking factor) \|\| id:ebi-fl187-GCST90094780 | Inverse variance weighted | 8 | -0.023042408 | 0.0706993343629794 | 0.74448401884323 | -0.161613103 | 0.115528287511336 | 0.97722104106088 | 0.850770301187166 | 1.12246626587641 | 0.998735809224318 |
| ebi-fl187-GCST90094780 | ebi-met1400-GCST90200100 | Perfluorooctanesulfonate (PFOS) levels \|\| id:ebi-met1400-GCST90200100 | F-healthy breakfast food liking (derived food-liking factor) \|\| id:ebi-fl187-GCST90094780 | Inverse variance weighted | 8 | 0.0282306112024702 | 0.0718211735827699 | 0.694269194927029 | -0.112538889 | 0.169000111424699 | 1.02863287133475 | 0.893562596659337 | 1.18412027086421 | 0.998735809224318 |
| ebi-fl187-GCST90094780 | ebi-met1400-GCST90200102 | 1-palmitoleoyl-2-linolenoyl-GPC (16:1/18:3) levels \|\| id:ebi-met1400-GCST90200102 | F-healthy breakfast food liking (derived food-liking factor) \|\| id:ebi-fl187-GCST90094780 | Inverse variance weighted | 8 | 0.0143442496586563 | 0.0668675813583518 | 0.830143716595243 | -0.11671621 | 0.145404709121026 | 1.01444762208302 | 0.889837684545588 | 1.1565075247127 | 0.998735809224318 |
| ebi-fl187-GCST90094780 | ebi-met1400-GCST90200114 | Glycosyl-N-tricosanoyl-sphingadienine (d18:2/23:0) levels \|\| id:ebi-met1400-GCST90200114 | F-healthy breakfast food liking (derived food-liking factor) \|\| id:ebi-fl187-GCST90094780 | Inverse variance weighted | 8 | 0.0151176092290775 | 0.0625218347681212 | 0.808937251074264 | -0.107425187 | 0.137660405374595 | 1.01523245830144 | 0.898143712838049 | 1.14758577013458 | 0.998735809224318 |
| ebi-fl187-GCST90094780 | ebi-met1400-GCST90200135 | Arachidoylcarnitine (C20) levels \|\| id:ebi-met1400-GCST90200135 | F-healthy breakfast food liking (derived food-liking factor) \|\| id:ebi-fl187-GCST90094780 | Inverse variance weighted | 8 | 0.00852508639645657 | 0.0661062574238335 | 0.897389019282237 | -0.121043178 | 0.13809335094717 | 1.00856152842904 | 0.885995703107819 | 1.14808271988124 | 0.998735809224318 |
| ebi-fl187-GCST90094780 | ebi-met1400-GCST90200146 | Docosahexaenoylcarnitine (C22:6) levels \|\| id:ebi-met1400-GCST90200146 | F-healthy breakfast food liking (derived food-liking factor) \|\| id:ebi-fl187-GCST90094780 | Inverse variance weighted | 8 | 0.115249510038644 | 0.067057794462198 | 0.0856764054664611 | -0.016183767 | 0.246682787184552 | 1.12215339118087 | 0.983946486440643 | 1.27977308795917 | 0.998735809224318 |
| ebi-fl187-GCST90094780 | ebi-met1400-GCST90200162 | 3-hydroxyphenylacetoylglutamine levels \|\| id:ebi-met1400-GCST90200162 | F-healthy breakfast food liking (derived food-liking factor) \|\| id:ebi-fl187-GCST90094780 | Inverse variance weighted | 7 | -0.030738723 | 0.086832250538384 | 0.723337968493953 | -0.200929934 | 0.139452488377875 | 0.96972890815204 | 0.817969741632735 | 1.14964418296047 | 0.998735809224318 |
| ebi-fl187-GCST90094780 | ebi-met1400-GCST90200172 | 3-carboxy-4-methyl-5-pentyl-2-furanpropionate (3-CMPFP) levels \|\| id:ebi-met1400-GCST90200172 | F-healthy breakfast food liking (derived food-liking factor) \|\| id:ebi-fl187-GCST90094780 | Inverse variance weighted | 8 | -0.003205418 | 0.0594678660650494 | 0.957013500651625 | -0.119762435 | 0.113351599786014 | 0.996799714165114 | 0.88713116283266 | 1.12002566451053 | 0.998735809224318 |
| ebi-fl187-GCST90094780 | ebi-met1400-GCST90200222 | Dihydrocaffeate sulfate (2) levels \|\| id:ebi-met1400-GCST90200222 | F-healthy breakfast food liking (derived food-liking factor) \|\| id:ebi-fl187-GCST90094780 | Inverse variance weighted | 8 | 0.0592923115822368 | 0.0678897669194008 | 0.382466045561631 | -0.073771632 | 0.192356254744262 | 1.06108536295313 | 0.928883797346854 | 1.21210225723525 | 0.998735809224318 |
| ebi-fl187-GCST90094780 | ebi-met1400-GCST90200223 | 4-methylhexanoylglutamine levels \|\| id:ebi-met1400-GCST90200223 | F-healthy breakfast food liking (derived food-liking factor) \|\| id:ebi-fl187-GCST90094780 | Inverse variance weighted | 8 | 0.0327380207122379 | 0.0719021690297384 | 0.648883790674278 | -0.108190231 | 0.173666272010525 | 1.03327980587199 | 0.89745685644721 | 1.18965847723249 | 0.998735809224318 |
| ebi-fl187-GCST90094780 | ebi-met1400-GCST90200224 | 2-ketocaprylate levels \|\| id:ebi-met1400-GCST90200224 | F-healthy breakfast food liking (derived food-liking factor) \|\| id:ebi-fl187-GCST90094780 | Inverse variance weighted | 8 | 0.0111590819517574 | 0.0606269793618251 | 0.853965348835361 | -0.107669798 | 0.129987961500935 | 1.01122157675196 | 0.897924044160581 | 1.13881467362272 | 0.998735809224318 |
| ebi-fl187-GCST90094780 | ebi-met1400-GCST90200243 | Hydroxypalmitoyl sphingomyelin (d18:1/16:0(OH)) levels \|\| id:ebi-met1400-GCST90200243 | F-healthy breakfast food liking (derived food-liking factor) \|\| id:ebi-fl187-GCST90094780 | Inverse variance weighted | 8 | -0.016855268 | 0.0584864476817118 | 0.773200534455485 | -0.131488706 | 0.0977781693689052 | 0.983285987198508 | 0.876789177939186 | 1.10271814131356 | 0.998735809224318 |
| ebi-fl187-GCST90094780 | ebi-met1400-GCST90200273 | 2-methoxyhydroquinone sulfate (2) levels \|\| id:ebi-met1400-GCST90200273 | F-healthy breakfast food liking (derived food-liking factor) \|\| id:ebi-fl187-GCST90094780 | Inverse variance weighted | 8 | 0.132603049404702 | 0.0743429915434169 | 0.0744779911165338 | -0.013109214 | 0.278315312829799 | 1.14179667149421 | 0.986976337480042 | 1.32090262909836 | 0.998735809224318 |
| ebi-fl187-GCST90094780 | ebi-met1400-GCST90200276 | 2,4-di-tert-butylphenol levels \|\| id:ebi-met1400-GCST90200276 | F-healthy breakfast food liking (derived food-liking factor) \|\| id:ebi-fl187-GCST90094780 | Inverse variance weighted | 8 | 0.0339081342682868 | 0.0595743393571032 | 0.569238413675179 | -0.082857571 | 0.150673839408209 | 1.03448956822166 | 0.920482241367404 | 1.16261739625706 | 0.998735809224318 |
| ebi-fl187-GCST90094780 | ebi-met1400-GCST90200289 | N-acetylmethionine levels \|\| id:ebi-met1400-GCST90200289 | F-healthy breakfast food liking (derived food-liking factor) \|\| id:ebi-fl187-GCST90094780 | Inverse variance weighted | 8 | -0.08705824 | 0.0809595681842102 | 0.282227080302967 | -0.245738993 | 0.071622514013165 | 0.916623710254797 | 0.782126338525702 | 1.07424975328799 | 0.998735809224318 |
| ebi-fl187-GCST90094780 | ebi-met1400-GCST90200308 | 3-hydroxyisobutyrate levels \|\| id:ebi-met1400-GCST90200308 | F-healthy breakfast food liking (derived food-liking factor) \|\| id:ebi-fl187-GCST90094780 | Inverse variance weighted | 8 | 0.00352395530951693 | 0.0604746977108124 | 0.953532316450638 | -0.115006452 | 0.122054362822709 | 1.00353017174002 | 0.891360392649476 | 1.12981552007168 | 0.998735809224318 |
| ebi-fl187-GCST90094780 | ebi-met1400-GCST90200383 | Cystathionine levels \|\| id:ebi-met1400-GCST90200383 | F-healthy breakfast food liking (derived food-liking factor) \|\| id:ebi-fl187-GCST90094780 | Inverse variance weighted | 8 | 0.0115999901516174 | 0.0757373971525318 | 0.87827157600376 | -0.136845308 | 0.16004528857058 | 1.01166753094223 | 0.872105123150671 | 1.1735640188252 | 0.998735809224318 |
| ebi-fl187-GCST90094780 | ebi-met1400-GCST90200384 | Guanidinoacetate levels \|\| id:ebi-met1400-GCST90200384 | F-healthy breakfast food liking (derived food-liking factor) \|\| id:ebi-fl187-GCST90094780 | Inverse variance weighted | 8 | 0.0164269937704115 | 0.0572356563283206 | 0.774107498795547 | -0.095754893 | 0.12860888017392 | 1.01656265867005 | 0.908686714583019 | 1.1372452380098 | 0.998735809224318 |
| ebi-fl187-GCST90094780 | ebi-met1400-GCST90200403 | Citrulline levels \|\| id:ebi-met1400-GCST90200403 | F-healthy breakfast food liking (derived food-liking factor) \|\| id:ebi-fl187-GCST90094780 | Inverse variance weighted | 8 | -0.034376262 | 0.059930529584725 | 0.566237265142432 | -0.1518401 | 0.083087575836329 | 0.966207888777035 | 0.859125643832447 | 1.08663696752258 | 0.998735809224318 |
| ebi-fl187-GCST90094780 | ebi-met1400-GCST90200434 | 12,13-DiHOME levels \|\| id:ebi-met1400-GCST90200434 | F-healthy breakfast food liking (derived food-liking factor) \|\| id:ebi-fl187-GCST90094780 | Inverse variance weighted | 8 | 0.00168589095623457 | 0.0604820421556539 | 0.977762453993257 | -0.116858912 | 0.120230693581316 | 1.00168731286934 | 0.889710712108154 | 1.12775698787072 | 0.998955228396052 |
| ebi-fl187-GCST90094780 | ebi-met1400-GCST90200437 | Fructose levels \|\| id:ebi-met1400-GCST90200437 | F-healthy breakfast food liking (derived food-liking factor) \|\| id:ebi-fl187-GCST90094780 | Inverse variance weighted | 8 | 0.0120202771331666 | 0.0738010226221705 | 0.870617542423116 | -0.132629727 | 0.156670281472621 | 1.01209281099872 | 0.875789313035614 | 1.1696099082607 | 0.998735809224318 |
| ebi-fl187-GCST90094780 | ebi-met1400-GCST90200448 | N-stearoyl-sphinganine (d18:0/18:0) levels \|\| id:ebi-met1400-GCST90200448 | F-healthy breakfast food liking (derived food-liking factor) \|\| id:ebi-fl187-GCST90094780 | Inverse variance weighted | 8 | 0.117282477528745 | 0.0667934533214555 | 0.0791058612419495 | -0.013632691 | 0.248197646038798 | 1.12443701302202 | 0.986459813312451 | 1.28171323270459 | 0.998735809224318 |
| ebi-fl187-GCST90094780 | ebi-met1400-GCST90200468 | X-11847 levels \|\| id:ebi-met1400-GCST90200468 | F-healthy breakfast food liking (derived food-liking factor) \|\| id:ebi-fl187-GCST90094780 | Inverse variance weighted (multiplicative random effects) | 8 | 0.0903577510432875 | 0.101144773897784 | 0.371669413617325 | -0.107886006 | 0.288601507882945 | 1.09456579572449 | 0.897729926605859 | 1.33455981098866 | 0.998735809224318 |
| ebi-fl187-GCST90094780 | ebi-met1400-GCST90200476 | X-11843 levels \|\| id:ebi-met1400-GCST90200476 | F-healthy breakfast food liking (derived food-liking factor) \|\| id:ebi-fl187-GCST90094780 | Inverse variance weighted (multiplicative random effects) | 8 | -0.073493264 | 0.0988252719224184 | 0.457076914882952 | -0.267190797 | 0.120204269036619 | 0.929142404537489 | 0.765526997353177 | 1.12772718779952 | 0.998735809224318 |
| ebi-fl187-GCST90094780 | ebi-met1400-GCST90200511 | X-12906 levels \|\| id:ebi-met1400-GCST90200511 | F-healthy breakfast food liking (derived food-liking factor) \|\| id:ebi-fl187-GCST90094780 | Inverse variance weighted | 8 | -0.093596658 | 0.0638149953194283 | 0.142461076866077 | -0.218674049 | 0.03148073301556 | 0.910649991737731 | 0.803583604641719 | 1.03198149223275 | 0.998735809224318 |
| ebi-fl187-GCST90094780 | ebi-met1400-GCST90200515 | X-12849 levels \|\| id:ebi-met1400-GCST90200515 | F-healthy breakfast food liking (derived food-liking factor) \|\| id:ebi-fl187-GCST90094780 | Inverse variance weighted | 8 | 0.0368754072570939 | 0.0629851651199349 | 0.558237254477692 | -0.086575516 | 0.160326330892166 | 1.0375637398723 | 0.917066292644965 | 1.17389388633278 | 0.998735809224318 |
| ebi-fl187-GCST90094780 | ebi-met1400-GCST90200551 | X-18886 levels \|\| id:ebi-met1400-GCST90200551 | F-healthy breakfast food liking (derived food-liking factor) \|\| id:ebi-fl187-GCST90094780 | Inverse variance weighted | 8 | -0.011245005 | 0.0641510166775832 | 0.860852091834588 | -0.136980998 | 0.114490987335748 | 0.988817983396351 | 0.871986795432562 | 1.12130253509515 | 0.998735809224318 |
| ebi-fl187-GCST90094780 | ebi-met1400-GCST90200564 | X-21319 levels \|\| id:ebi-met1400-GCST90200564 | F-healthy breakfast food liking (derived food-liking factor) \|\| id:ebi-fl187-GCST90094780 | Inverse variance weighted | 8 | -0.135437576 | 0.0729342549462103 | 0.0633137588816246 | -0.278388716 | 0.00751356356141042 | 0.873333678092165 | 0.757002505459881 | 1.00754186120776 | 0.998735809224318 |
| ebi-fl187-GCST90094780 | ebi-met1400-GCST90200579 | X-21442 levels \|\| id:ebi-met1400-GCST90200579 | F-healthy breakfast food liking (derived food-liking factor) \|\| id:ebi-fl187-GCST90094780 | Inverse variance weighted | 8 | -0.002169067 | 0.0684066038154454 | 0.974704562740111 | -0.13624601 | 0.131907876666722 | 0.997833283913931 | 0.872627930630246 | 1.14100320140721 | 0.998955228396052 |
| ebi-fl187-GCST90094780 | ebi-met1400-GCST90200581 | X-21441 levels \|\| id:ebi-met1400-GCST90200581 | F-healthy breakfast food liking (derived food-liking factor) \|\| id:ebi-fl187-GCST90094780 | Inverse variance weighted | 8 | 0.0515869575941251 | 0.0600032276239663 | 0.389934376966103 | -0.066019369 | 0.169193283737099 | 1.05294074350509 | 0.936112732970834 | 1.18434903220956 | 0.998735809224318 |
| ebi-fl187-GCST90094780 | ebi-met1400-GCST90200648 | X-24585 levels \|\| id:ebi-met1400-GCST90200648 | F-healthy breakfast food liking (derived food-liking factor) \|\| id:ebi-fl187-GCST90094780 | Inverse variance weighted | 8 | 0.0108982254777694 | 0.0585161138265261 | 0.852254085548245 | -0.103793358 | 0.125589808577761 | 1.01095782745888 | 0.901411548002547 | 1.13381699087961 | 0.998735809224318 |
| ebi-fl187-GCST90094780 | ebi-met1400-GCST90200720 | Adenosine 5'-diphosphate (ADP) to pantothenate ratio \|\| id:ebi-met1400-GCST90200720 | F-healthy breakfast food liking (derived food-liking factor) \|\| id:ebi-fl187-GCST90094780 | Inverse variance weighted | 7 | -0.078195982 | 0.0845504022113118 | 0.355046752953748 | -0.24391477 | 0.0875228063351527 | 0.924783167928497 | 0.783554413498627 | 1.09146715652488 | 0.998735809224318 |
| ebi-fl187-GCST90094780 | ebi-met1400-GCST90200724 | Cholate to taurocholate ratio \|\| id:ebi-met1400-GCST90200724 | F-healthy breakfast food liking (derived food-liking factor) \|\| id:ebi-fl187-GCST90094780 | Inverse variance weighted | 8 | 0.0714456490158073 | 0.0815784065004693 | 0.381143564950188 | -0.088448028 | 0.231339325756727 | 1.07405977290914 | 0.915350682357066 | 1.26028681467857 | 0.998735809224318 |
| ebi-fl187-GCST90094780 | ebi-met1400-GCST90200726 | N-acetylputrescine to (N(1) + N(8))-acetylspermidine ratio \|\| id:ebi-met1400-GCST90200726 | F-healthy breakfast food liking (derived food-liking factor) \|\| id:ebi-fl187-GCST90094780 | Inverse variance weighted | 8 | 0.00194970618377811 | 0.0596090807445694 | 0.973907278730631 | -0.114884092 | 0.118783504443134 | 1.00195160809674 | 0.891469466294298 | 1.12612609060041 | 0.998955228396052 |
| ebi-fl187-GCST90094780 | ebi-met1400-GCST90200744 | Arginine to glutamate ratio \|\| id:ebi-met1400-GCST90200744 | F-healthy breakfast food liking (derived food-liking factor) \|\| id:ebi-fl187-GCST90094780 | Inverse variance weighted (multiplicative random effects) | 8 | 0.0221687103145016 | 0.0941619373934878 | 0.813873640550681 | -0.162388687 | 0.206726107605738 | 1.02241626208978 | 0.850110713352521 | 1.22964573503988 | 0.998735809224318 |
| ebi-fl187-GCST90094780 | ebi-met1400-GCST90200759 | Histidine to trans-urocanate ratio \|\| id:ebi-met1400-GCST90200759 | F-healthy breakfast food liking (derived food-liking factor) \|\| id:ebi-fl187-GCST90094780 | Inverse variance weighted | 8 | -0.020636122 | 0.0603654057770698 | 0.732461182858132 | -0.138952318 | 0.0976800731003855 | 0.97957534542541 | 0.870269524055723 | 1.10260997408416 | 0.998735809224318 |
| ebi-fl187-GCST90094780 | ebi-met1400-GCST90200795 | Oleoyl-linoleoyl-glycerol (18:1 to 18:2) [2] to linoleoyl-arachidonoyl-glycerol (18:2 to 20:4) [2] ratio \|\| id:ebi-met1400-GCST90200795 | F-healthy breakfast food liking (derived food-liking factor) \|\| id:ebi-fl187-GCST90094780 | Inverse variance weighted | 8 | 0.0481275069088336 | 0.0682176737454793 | 0.480499696601681 | -0.085579134 | 0.181834147449973 | 1.04930444036065 | 0.917980497048708 | 1.19941525130479 | 0.998735809224318 |
| ebi-fl187-GCST90094780 | ebi-met1400-GCST90200811 | Choline to taurocholate ratio \|\| id:ebi-met1400-GCST90200811 | F-healthy breakfast food liking (derived food-liking factor) \|\| id:ebi-fl187-GCST90094780 | Inverse variance weighted | 8 | 0.00952881188662317 | 0.0626663327283086 | 0.879142515810979 | -0.1132972 | 0.132354824034108 | 1.00957435555869 | 0.892885254948036 | 1.14151328376575 | 0.998735809224318 |
| ebi-fl187-GCST90094780 | ebi-met1400-GCST90200831 | Cholate to phosphate ratio \|\| id:ebi-met1400-GCST90200831 | F-healthy breakfast food liking (derived food-liking factor) \|\| id:ebi-fl187-GCST90094780 | Inverse variance weighted | 8 | 0.104058841072829 | 0.0624991808660483 | 0.0959200822766156 | -0.018439553 | 0.226557235570283 | 1.10966574691767 | 0.981729414979391 | 1.2542743968899 | 0.998735809224318 |
| ebi-fl187-GCST90094780 | ebi-met1400-GCST90200841 | Adenosine 5'-diphosphate (ADP) to oxalate (ethanedioate) ratio \|\| id:ebi-met1400-GCST90200841 | F-healthy breakfast food liking (derived food-liking factor) \|\| id:ebi-fl187-GCST90094780 | Inverse variance weighted (multiplicative random effects) | 7 | -0.154937311 | 0.129726286013207 | 0.232344940785119 | -0.409200832 | 0.0993262095784267 | 0.85646886697114 | 0.664180830432564 | 1.10442651531074 | 0.998735809224318 |
| ebi-fl187-GCST90094780 | ebi-met1400-GCST90200844 | Aspartate to phosphate ratio \|\| id:ebi-met1400-GCST90200844 | F-healthy breakfast food liking (derived food-liking factor) \|\| id:ebi-fl187-GCST90094780 | Inverse variance weighted | 8 | 0.0328746198904537 | 0.0722780655645073 | 0.64922769010574 | -0.108790389 | 0.174539628396888 | 1.03342096068493 | 0.896918402103308 | 1.19069792690011 | 0.998735809224318 |
| ebi-fl187-GCST90094780 | ebi-met1400-GCST90200870 | Adenosine 5'-monophosphate (AMP) to acetoacetate ratio \|\| id:ebi-met1400-GCST90200870 | F-healthy breakfast food liking (derived food-liking factor) \|\| id:ebi-fl187-GCST90094780 | Inverse variance weighted | 8 | 0.0265661866028193 | 0.0659257168142689 | 0.68696917277938 | -0.102648218 | 0.155780591558786 | 1.0269222135051 | 0.902444381019718 | 1.16856978088623 | 0.998735809224318 |
| ebi-fl187-GCST90094780 | ebi-met1400-GCST90200887 | Cholesterol to cortisol ratio \|\| id:ebi-met1400-GCST90200887 | F-healthy breakfast food liking (derived food-liking factor) \|\| id:ebi-fl187-GCST90094780 | Inverse variance weighted (multiplicative random effects) | 8 | -0.039850328 | 0.0851822953209556 | 0.63991040838676 | -0.206807627 | 0.127106971007179 | 0.960933253362607 | 0.813176068345348 | 1.13553848097985 | 0.998735809224318 |
| ebi-fl187-GCST90094780 | ebi-met1400-GCST90200890 | Cortisol to taurocholate ratio \|\| id:ebi-met1400-GCST90200890 | F-healthy breakfast food liking (derived food-liking factor) \|\| id:ebi-fl187-GCST90094780 | Inverse variance weighted | 8 | 0.0503707058334414 | 0.0624602224188167 | 0.419986569408983 | -0.07205133 | 0.172792741774322 | 1.05166088094722 | 0.930483132786199 | 1.18861972833721 | 0.998735809224318 |
| ebi-fl187-GCST90094780 | ebi-met1400-GCST90200948 | Adenosine 5'-diphosphate (ADP) to mannitol to sorbitol ratio \|\| id:ebi-met1400-GCST90200948 | F-healthy breakfast food liking (derived food-liking factor) \|\| id:ebi-fl187-GCST90094780 | Inverse variance weighted | 8 | -0.070977092 | 0.0805557225164457 | 0.378267428709747 | -0.228866308 | 0.0869121244311774 | 0.931483230579485 | 0.795434869807992 | 1.09080082076391 | 0.998735809224318 |
| ebi-fl187-GCST90094780 | ebi-met1400-GCST90200972 | Leucine to N-palmitoyl-sphingosine (d18:1 to 16:0) ratio \|\| id:ebi-met1400-GCST90200972 | F-healthy breakfast food liking (derived food-liking factor) \|\| id:ebi-fl187-GCST90094780 | Inverse variance weighted | 8 | -0.026622032 | 0.0576947026675679 | 0.644490564853017 | -0.139703649 | 0.0864595853809816 | 0.973729210610475 | 0.869615908694227 | 1.0903073024731 | 0.998735809224318 |
| ebi-fl187-GCST90094780 | ebi-met1400-GCST90200973 | Proline to glutamate ratio \|\| id:ebi-met1400-GCST90200973 | F-healthy breakfast food liking (derived food-liking factor) \|\| id:ebi-fl187-GCST90094780 | Inverse variance weighted | 8 | 0.000553392965707147 | 0.0604692943725837 | 0.992698152927703 | -0.117966424 | 0.119073209935971 | 1.00055354611584 | 0.88872589197017 | 1.12645238277652 | 0.999107451094609 |
| ebi-fl187-GCST90094780 | ebi-met1400-GCST90200983 | Cholesterol to linoleoyl-arachidonoyl-glycerol (18:2 to 20:4) [1] ratio \|\| id:ebi-met1400-GCST90200983 | F-healthy breakfast food liking (derived food-liking factor) \|\| id:ebi-fl187-GCST90094780 | Inverse variance weighted (multiplicative random effects) | 8 | -0.005770506 | 0.09675103610264 | 0.952440104684977 | -0.195402536 | 0.183861525044253 | 0.994246111672249 | 0.822503503746026 | 1.20184938553237 | 0.998735809224318 |
| ebi-fl187-GCST90094780 | ebi-met1400-GCST90201001 | Serine to alpha-ketobutyrate ratio \|\| id:ebi-met1400-GCST90201001 | F-healthy breakfast food liking (derived food-liking factor) \|\| id:ebi-fl187-GCST90094780 | Inverse variance weighted | 8 | -0.045321785 | 0.073726429035431 | 0.538733602576097 | -0.189825586 | 0.0991820155090637 | 0.955689905290119 | 0.827103379516372 | 1.10426727503821 | 0.998735809224318 |
| ebi-fl187-GCST90094780 | ebi-met1400-GCST90201005 | Fructose to maltose ratio \|\| id:ebi-met1400-GCST90201005 | F-healthy breakfast food liking (derived food-liking factor) \|\| id:ebi-fl187-GCST90094780 | Inverse variance weighted | 8 | 0.00587070169975945 | 0.0684472836506381 | 0.931649508511443 | -0.128285974 | 0.14002737765501 | 1.00588796804096 | 0.879601799703938 | 1.15030529108755 | 0.998735809224318 |
| ebi-fl187-GCST90094780 | ebi-met1400-GCST90201020 | Paraxanthine to 5-acetylamino-6-formylamino-3-methyluracil ratio \|\| id:ebi-met1400-GCST90201020 | F-healthy breakfast food liking (derived food-liking factor) \|\| id:ebi-fl187-GCST90094780 | Inverse variance weighted | 8 | 0.00793154495682452 | 0.0749791982780866 | 0.915754308994949 | -0.139027684 | 0.154890773581874 | 1.00796308298615 | 0.870203937687694 | 1.1675304289735 | 0.998735809224318 |
| ebi-fl187-GCST90094780 | ebi-pw11-GCST90275197 | Placental weight (fetal genotype, adjusted for sex) \|\| id:ebi-pw11-GCST90275197 | F-healthy breakfast food liking (derived food-liking factor) \|\| id:ebi-fl187-GCST90094780 | Inverse variance weighted (multiplicative random effects) | 7 | -0.056961346 | 0.0371920009368373 | 0.125633934925121 | -0.129857668 | 0.015934975443956 | 0.944630582040837 | 0.878220420693635 | 1.01606261423759 | 0.998735809224318 |
| ebi-fl187-GCST90094780 | prot-a-1017 | FAS-associated factor 2 \|\| id:prot-a-1017 | F-healthy breakfast food liking (derived food-liking factor) \|\| id:ebi-fl187-GCST90094780 | Inverse variance weighted | 8 | 0.0183534463120091 | 0.12049242969905 | 0.878934293230685 | -0.217811716 | 0.254518608522148 | 1.01852290594296 | 0.804276859957516 | 1.28984055314646 | 0.998735809224318 |
| ebi-fl187-GCST90094780 | prot-a-1019 | Fas apoptotic inhibitory molecule 3 \|\| id:prot-a-1019 | F-healthy breakfast food liking (derived food-liking factor) \|\| id:ebi-fl187-GCST90094780 | Inverse variance weighted | 8 | -0.016780352 | 0.12206264904199 | 0.890656718291919 | -0.256023144 | 0.222462440415199 | 0.983359654184717 | 0.774124052389917 | 1.24914890125545 | 0.998735809224318 |
| ebi-fl187-GCST90094780 | prot-a-1052 | MIP18 family protein FAM96A \|\| id:prot-a-1052 | F-healthy breakfast food liking (derived food-liking factor) \|\| id:ebi-fl187-GCST90094780 | Inverse variance weighted | 8 | -0.019634117 | 0.0954722468858269 | 0.837062274240012 | -0.206759721 | 0.167491486567537 | 0.98055737663343 | 0.813215024824472 | 1.18233522441099 | 0.998735809224318 |
| ebi-fl187-GCST90094780 | prot-a-1066 | F-box/LRR-repeat protein 4 \|\| id:prot-a-1066 | F-healthy breakfast food liking (derived food-liking factor) \|\| id:ebi-fl187-GCST90094780 | Inverse variance weighted | 8 | 0.192397604895035 | 0.0954722468858269 | 0.0438815770412821 | 0.00527200099881428 | 0.379523208791256 | 1.2121523788826 | 1.00528592244996 | 1.46158755118136 | 0.998735809224318 |
| ebi-fl187-GCST90094780 | prot-a-1076 | Low affinity immunoglobulin gamma Fc region receptor III-B \|\| id:prot-a-1076 | F-healthy breakfast food liking (derived food-liking factor) \|\| id:ebi-fl187-GCST90094780 | Inverse variance weighted | 8 | 0.0187215348750366 | 0.0956176791076867 | 0.844770039313377 | -0.168689116 | 0.206132185926102 | 1.01889788158371 | 0.844771488357891 | 1.22891563861108 | 0.998735809224318 |
| ebi-fl187-GCST90094780 | prot-a-1081 | Fc receptor-like protein 3 \|\| id:prot-a-1081 | F-healthy breakfast food liking (derived food-liking factor) \|\| id:ebi-fl187-GCST90094780 | Inverse variance weighted | 8 | 0.0890350715321646 | 0.0955234255800558 | 0.351297363184874 | -0.098190843 | 0.276260985669074 | 1.09311899301199 | 0.906475893024974 | 1.31819184831937 | 0.998735809224318 |
| ebi-fl187-GCST90094780 | prot-a-1105 | Fibroblast growth factor receptor 4 \|\| id:prot-a-1105 | F-healthy breakfast food liking (derived food-liking factor) \|\| id:ebi-fl187-GCST90094780 | Inverse variance weighted (multiplicative random effects) | 8 | -0.056647758 | 0.160456623387775 | 0.72405774553848 | -0.37114274 | 0.257847224064788 | 0.944926853889941 | 0.689945451981386 | 1.29414108990522 | 0.998735809224318 |
| ebi-fl187-GCST90094780 | prot-a-1143 | Follistatin-related protein 1 \|\| id:prot-a-1143 | F-healthy breakfast food liking (derived food-liking factor) \|\| id:ebi-fl187-GCST90094780 | Inverse variance weighted | 8 | -0.01094561 | 0.126203420832177 | 0.930886212637024 | -0.258304314 | 0.236413095308745 | 0.98911407569939 | 0.77236015605876 | 1.26669746888423 | 0.998735809224318 |
| ebi-fl187-GCST90094780 | prot-a-1151 | Alpha-(1,3)-fucosyltransferase 10 \|\| id:prot-a-1151 | F-healthy breakfast food liking (derived food-liking factor) \|\| id:ebi-fl187-GCST90094780 | Inverse variance weighted | 8 | -0.022123608 | 0.0955234255800558 | 0.816845598852183 | -0.209349522 | 0.165102306484683 | 0.978119324545328 | 0.811111684885424 | 1.17951378444776 | 0.998735809224318 |
| ebi-fl187-GCST90094780 | prot-a-1165 | Growth arrest and DNA damage-inducible proteins-interacting protein 1 \|\| id:prot-a-1165 | F-healthy breakfast food liking (derived food-liking factor) \|\| id:ebi-fl187-GCST90094780 | Inverse variance weighted | 8 | 0.193348956519215 | 0.115558405028812 | 0.094293664106876 | -0.033145517 | 0.419843430375687 | 1.21330611073232 | 0.967397776197608 | 1.5217232813234 | 0.998735809224318 |
| ebi-fl187-GCST90094780 | prot-a-1194 | Growth/differentiation factor 11/8 \|\| id:prot-a-1194 | F-healthy breakfast food liking (derived food-liking factor) \|\| id:ebi-fl187-GCST90094780 | Inverse variance weighted | 8 | 0.0602167158678566 | 0.0956176791076867 | 0.528848018358318 | -0.127193935 | 0.247627366918923 | 1.06206668831085 | 0.880562883912506 | 1.28098250678898 | 0.998735809224318 |
| ebi-fl187-GCST90094780 | prot-a-1216 | D-glucuronyl C5-epimerase \|\| id:prot-a-1216 | F-healthy breakfast food liking (derived food-liking factor) \|\| id:ebi-fl187-GCST90094780 | Inverse variance weighted | 8 | 0.0690875419267677 | 0.104330153883711 | 0.507842353874924 | -0.13539956 | 0.273574643538841 | 1.07153000884415 | 0.873366879767499 | 1.31465548608758 | 0.998735809224318 |
| ebi-fl187-GCST90094780 | prot-a-1246 | Glypican-3 \|\| id:prot-a-1246 | F-healthy breakfast food liking (derived food-liking factor) \|\| id:ebi-fl187-GCST90094780 | Inverse variance weighted | 8 | -0.029889181 | 0.0955234255800558 | 0.754357425932683 | -0.217115095 | 0.157336733390606 | 0.970553083557486 | 0.804837331315372 | 1.17038965683096 | 0.998735809224318 |
| ebi-fl187-GCST90094780 | prot-a-1275 | Glutamate receptor 4 \|\| id:prot-a-1275 | F-healthy breakfast food liking (derived food-liking factor) \|\| id:ebi-fl187-GCST90094780 | Inverse variance weighted | 7 | 0.0355744438560098 | 0.10457723967684 | 0.733725598213637 | -0.169396946 | 0.240545833622617 | 1.0362147850811 | 0.844173745554539 | 1.27194323025922 | 0.998735809224318 |
| ebi-fl187-GCST90094780 | prot-a-1289 | Glutathione S-transferase P \|\| id:prot-a-1289 | F-healthy breakfast food liking (derived food-liking factor) \|\| id:ebi-fl187-GCST90094780 | Inverse variance weighted (multiplicative random effects) | 8 | 0.058803177347995 | 0.169726644683242 | 0.728998439102081 | -0.273861046 | 0.391467400927148 | 1.06056647668952 | 0.760437733608954 | 1.47914970781294 | 0.998735809224318 |
| ebi-fl187-GCST90094780 | prot-a-130 | Apolipoprotein D \|\| id:prot-a-130 | F-healthy breakfast food liking (derived food-liking factor) \|\| id:ebi-fl187-GCST90094780 | Inverse variance weighted | 8 | 0.182198610281844 | 0.11264409799258 | 0.105776557851633 | -0.038583822 | 0.4029810423473 | 1.1998524732546 | 0.962151052142347 | 1.49627852546608 | 0.998735809224318 |
| ebi-fl187-GCST90094780 | prot-a-1313 | Heparin-binding EGF-like growth factor \|\| id:prot-a-1313 | F-healthy breakfast food liking (derived food-liking factor) \|\| id:ebi-fl187-GCST90094780 | Inverse variance weighted | 8 | 0.107576249527751 | 0.11680551027864 | 0.357057692380048 | -0.121362551 | 0.336515049673884 | 1.11357576713582 | 0.885712785657339 | 1.40005993955683 | 0.998735809224318 |
| ebi-fl187-GCST90094780 | prot-a-1347 | HLA class II histocompatibility antigen, DQ alpha 2 chain \|\| id:prot-a-1347 | F-healthy breakfast food liking (derived food-liking factor) \|\| id:ebi-fl187-GCST90094780 | Inverse variance weighted | 8 | -0.042916934 | 0.0954928938475475 | 0.653124793881226 | -0.230083006 | 0.144249137566325 | 0.957990962880304 | 0.794467653933115 | 1.15517186938563 | 0.998735809224318 |
| ebi-fl187-GCST90094780 | prot-a-1357 | Non-histone chromosomal protein HMG-14 \|\| id:prot-a-1357 | F-healthy breakfast food liking (derived food-liking factor) \|\| id:ebi-fl187-GCST90094780 | Inverse variance weighted | 8 | -0.18330388 | 0.0955188045749545 | 0.0549799698752423 | -0.370520737 | 0.00391297714264696 | 0.832515132742512 | 0.690374733494322 | 1.003920643 | 0.998735809224318 |
| ebi-fl187-GCST90094780 | prot-a-137 | Apolipoprotein M \|\| id:prot-a-137 | F-healthy breakfast food liking (derived food-liking factor) \|\| id:ebi-fl187-GCST90094780 | Inverse variance weighted (multiplicative random effects) | 8 | 0.0508731384930239 | 0.163446049441409 | 0.755607990852526 | -0.269481118 | 0.371227395398186 | 1.05218940248274 | 0.763775700684216 | 1.44951264841916 | 0.998735809224318 |
| ebi-fl187-GCST90094780 | prot-a-1381 | 17-beta-hydroxysteroid dehydrogenase 14 \|\| id:prot-a-1381 | F-healthy breakfast food liking (derived food-liking factor) \|\| id:ebi-fl187-GCST90094780 | Inverse variance weighted (multiplicative random effects) | 8 | 0.237519801794836 | 0.136811590514137 | 0.0825446044135093 | -0.030630916 | 0.505670519202544 | 1.26810010720026 | 0.969833457414449 | 1.65809693364096 | 0.998735809224318 |
| ebi-fl187-GCST90094780 | prot-a-1392 | Serine protease HTRA2, mitochondrial \|\| id:prot-a-1392 | F-healthy breakfast food liking (derived food-liking factor) \|\| id:ebi-fl187-GCST90094780 | Inverse variance weighted | 8 | -0.000607982 | 0.10607757181442 | 0.995426958376111 | -0.208520023 | 0.207304058309923 | 0.999392202337537 | 0.811784780008688 | 1.23035661506537 | 0.999107451094609 |
| ebi-fl187-GCST90094780 | prot-a-1416 | Interferon-induced protein with tetratricopeptide repeats 2 \|\| id:prot-a-1416 | F-healthy breakfast food liking (derived food-liking factor) \|\| id:ebi-fl187-GCST90094780 | Inverse variance weighted | 8 | -0.141889393 | 0.0955188045749545 | 0.137421930899526 | -0.32910625 | 0.0453274643175345 | 0.867717227081868 | 0.719566559014557 | 1.0463704527984 | 0.998735809224318 |
| ebi-fl187-GCST90094780 | prot-a-1428 | Interferon gamma \|\| id:prot-a-1428 | F-healthy breakfast food liking (derived food-liking factor) \|\| id:ebi-fl187-GCST90094780 | Inverse variance weighted | 8 | 0.0214095454766016 | 0.0956006640496768 | 0.822798026265759 | -0.165967756 | 0.208786847013968 | 1.02164037416459 | 0.847073546737034 | 1.23218232719428 | 0.998735809224318 |
| ebi-fl187-GCST90094780 | prot-a-1483 | Interleukin-17C \|\| id:prot-a-1483 | F-healthy breakfast food liking (derived food-liking factor) \|\| id:ebi-fl187-GCST90094780 | Inverse variance weighted | 8 | -0.082550474 | 0.0955234255800558 | 0.387483034949601 | -0.269776388 | 0.104675439810823 | 0.920764961692962 | 0.763550213885618 | 1.11035017640431 | 0.998735809224318 |
| ebi-fl187-GCST90094780 | prot-a-1503 | Interleukin-1 receptor-like 2 \|\| id:prot-a-1503 | F-healthy breakfast food liking (derived food-liking factor) \|\| id:ebi-fl187-GCST90094780 | Inverse variance weighted | 8 | -0.03597111 | 0.0955663488504015 | 0.706620039016949 | -0.223281154 | 0.151338933425562 | 0.96466816203389 | 0.799889925370418 | 1.16339090333073 | 0.998735809224318 |
| ebi-fl187-GCST90094780 | prot-a-1522 | Interleukin-31 receptor subunit alpha \|\| id:prot-a-1522 | F-healthy breakfast food liking (derived food-liking factor) \|\| id:ebi-fl187-GCST90094780 | Inverse variance weighted (multiplicative random effects) | 8 | -0.086033602 | 0.155673357509569 | 0.580499924341056 | -0.391153383 | 0.219086178504356 | 0.917563398540088 | 0.676276418822991 | 1.24493855900778 | 0.998735809224318 |
| ebi-fl187-GCST90094780 | prot-a-1540 | Interleukin-6 receptor subunit alpha \|\| id:prot-a-1540 | F-healthy breakfast food liking (derived food-liking factor) \|\| id:ebi-fl187-GCST90094780 | Inverse variance weighted | 8 | -0.013159874 | 0.095549361175301 | 0.890454964346722 | -0.200436622 | 0.174116874091433 | 0.986926338730786 | 0.818373355481292 | 1.1901946606115 | 0.998735809224318 |
| ebi-fl187-GCST90094780 | prot-a-1541 | Interleukin-6 receptor subunit alpha \|\| id:prot-a-1541 | F-healthy breakfast food liking (derived food-liking factor) \|\| id:ebi-fl187-GCST90094780 | Inverse variance weighted | 8 | -0.106156898 | 0.0954722468858269 | 0.266175264705364 | -0.293282502 | 0.080968706178861 | 0.899283541471645 | 0.745811418004859 | 1.08433696298883 | 0.998735809224318 |
| ebi-fl187-GCST90094780 | prot-a-1545 | Interleukin-9 \|\| id:prot-a-1545 | F-healthy breakfast food liking (derived food-liking factor) \|\| id:ebi-fl187-GCST90094780 | Inverse variance weighted | 8 | 0.122814947109186 | 0.0956006640496768 | 0.198908935 | -0.064562354 | 0.310192248646552 | 1.13067516687835 | 0.937477656556314 | 1.36368725596244 | 0.998735809224318 |
| ebi-fl187-GCST90094780 | prot-a-1548 | Inositol monophosphatase 3 \|\| id:prot-a-1548 | F-healthy breakfast food liking (derived food-liking factor) \|\| id:ebi-fl187-GCST90094780 | Inverse variance weighted | 8 | -0.020415186 | 0.0954928938475475 | 0.830712778095217 | -0.207581258 | 0.166750885501927 | 0.979791792581336 | 0.81254721281987 | 1.18145990985334 | 0.998735809224318 |
| ebi-fl187-GCST90094780 | prot-a-1555 | Inhibin beta A chain:Inhibin beta B chain heterodimer \|\| id:prot-a-1555 | F-healthy breakfast food liking (derived food-liking factor) \|\| id:ebi-fl187-GCST90094780 | Inverse variance weighted | 8 | -0.093088891 | 0.102587412297262 | 0.364189780968628 | -0.294160219 | 0.107982437295646 | 0.911112507170402 | 0.745157093621612 | 1.11402817986709 | 0.998735809224318 |
| ebi-fl187-GCST90094780 | prot-a-1603 | Junctophilin-3 \|\| id:prot-a-1603 | F-healthy breakfast food liking (derived food-liking factor) \|\| id:ebi-fl187-GCST90094780 | Inverse variance weighted | 8 | -0.04510393 | 0.0955700582188264 | 0.636965155617013 | -0.232421245 | 0.142213383650909 | 0.955898129739918 | 0.792612169403476 | 1.15282261579198 | 0.998735809224318 |
| ebi-fl187-GCST90094780 | prot-a-1627 | UPF0258 protein KIAA1024 \|\| id:prot-a-1627 | F-healthy breakfast food liking (derived food-liking factor) \|\| id:ebi-fl187-GCST90094780 | Inverse variance weighted | 8 | -0.117058178 | 0.121810840810563 | 0.336560586768784 | -0.355807426 | 0.1216910695436 | 0.889533439985561 | 0.700607525734953 | 1.12940514023524 | 0.998735809224318 |
| ebi-fl187-GCST90094780 | prot-a-1666 | Kallikrein-7 \|\| id:prot-a-1666 | F-healthy breakfast food liking (derived food-liking factor) \|\| id:ebi-fl187-GCST90094780 | Inverse variance weighted | 8 | 0.0575057711257441 | 0.11977442586633 | 0.631144004250901 | -0.177252104 | 0.292263645823752 | 1.05919138336375 | 0.837568603867363 | 1.33945611310149 | 0.998735809224318 |
| ebi-fl187-GCST90094780 | prot-a-1678 | Importin subunit alpha-3 \|\| id:prot-a-1678 | F-healthy breakfast food liking (derived food-liking factor) \|\| id:ebi-fl187-GCST90094780 | Inverse variance weighted | 8 | 0.0850033808820852 | 0.0955197216386407 | 0.373517444274998 | -0.102215274 | 0.272222035293821 | 1.08872074752865 | 0.902835174232819 | 1.31287847430906 | 0.998735809224318 |
| ebi-fl187-GCST90094780 | prot-a-1718 | Protein LDOC1 \|\| id:prot-a-1718 | F-healthy breakfast food liking (derived food-liking factor) \|\| id:ebi-fl187-GCST90094780 | Inverse variance weighted | 8 | 0.0205232192594388 | 0.0955709767595579 | 0.829967524109251 | -0.166795895 | 0.207842333708172 | 1.02073526868506 | 0.846372342375697 | 1.23101906403633 | 0.998735809224318 |
| ebi-fl187-GCST90094780 | prot-a-1723 | Leptin \|\| id:prot-a-1723 | F-healthy breakfast food liking (derived food-liking factor) \|\| id:ebi-fl187-GCST90094780 | Inverse variance weighted (multiplicative random effects) | 8 | -0.070662258 | 0.149528616017322 | 0.63652228299523 | -0.363738345 | 0.222413829829346 | 0.931776539467405 | 0.695073040316638 | 1.24908818087139 | 0.998735809224318 |
| ebi-fl187-GCST90094780 | prot-a-1730 | Galectin-7 \|\| id:prot-a-1730 | F-healthy breakfast food liking (derived food-liking factor) \|\| id:ebi-fl187-GCST90094780 | Inverse variance weighted | 8 | -0.000202211 | 0.0955188045749545 | 0.998310901138323 | -0.187419068 | 0.187014646230531 | 0.999797809706833 | 0.829096216126132 | 1.20564494307439 | 0.999882738470636 |
| ebi-fl187-GCST90094780 | prot-a-1753 | Lipase member N \|\| id:prot-a-1753 | F-healthy breakfast food liking (derived food-liking factor) \|\| id:ebi-fl187-GCST90094780 | Inverse variance weighted | 8 | 0.0415353635465033 | 0.0955709767595579 | 0.663851146283831 | -0.145783751 | 0.228854477995237 | 1.04241002451505 | 0.864344596715338 | 1.25715908138815 | 0.998735809224318 |
| ebi-fl187-GCST90094780 | prot-a-1756 | VIP36-like protein \|\| id:prot-a-1756 | F-healthy breakfast food liking (derived food-liking factor) \|\| id:ebi-fl187-GCST90094780 | Inverse variance weighted | 8 | 0.0788795434174051 | 0.0956006640496768 | 0.409318171731657 | -0.108497758 | 0.266256844954772 | 1.08207397128697 | 0.897180906186681 | 1.305070216 | 0.998735809224318 |
| ebi-fl187-GCST90094780 | prot-a-1760 | Ig Kappa chain V-I region HK102- like \|\| id:prot-a-1760 | F-healthy breakfast food liking (derived food-liking factor) \|\| id:ebi-fl187-GCST90094780 | Inverse variance weighted | 8 | -0.27366324 | 0.0954417641854396 | 0.00413948306248402 | -0.460729098 | -0.086597382 | 0.760588168021654 | 0.630823545913255 | 0.917046240715443 | 0.998735809224318 |
| ebi-fl187-GCST90094780 | prot-a-179 | Neutral ceramidase \|\| id:prot-a-179 | F-healthy breakfast food liking (derived food-liking factor) \|\| id:ebi-fl187-GCST90094780 | Inverse variance weighted | 8 | 0.0485575696074143 | 0.0954891934564833 | 0.611093085834112 | -0.13860125 | 0.235716388782122 | 1.0497558041103 | 0.870575101454308 | 1.26581525984648 | 0.998735809224318 |
| ebi-fl187-GCST90094780 | prot-a-1809 | Lactotransferrin \|\| id:prot-a-1809 | F-healthy breakfast food liking (derived food-liking factor) \|\| id:ebi-fl187-GCST90094780 | Inverse variance weighted | 8 | -0.105884813 | 0.0955234255800558 | 0.267659062111541 | -0.293110727 | 0.0813411010338649 | 0.899528255977203 | 0.745939540297779 | 1.08474083969108 | 0.998735809224318 |
| ebi-fl187-GCST90094780 | prot-a-1818 | Lysophospholipase-like protein 1 \|\| id:prot-a-1818 | F-healthy breakfast food liking (derived food-liking factor) \|\| id:ebi-fl187-GCST90094780 | Inverse variance weighted | 8 | -0.004575079 | 0.0955197216386407 | 0.961798575576456 | -0.191793733 | 0.182643575701472 | 0.995435371020152 | 0.825477119520809 | 1.20038648491341 | 0.998735809224318 |
| ebi-fl187-GCST90094780 | prot-a-1852 | MAP kinase-activated protein kinase 3 \|\| id:prot-a-1852 | F-healthy breakfast food liking (derived food-liking factor) \|\| id:ebi-fl187-GCST90094780 | Inverse variance weighted | 8 | -0.022153691 | 0.0954417641854396 | 0.816446854246951 | -0.209219549 | 0.164912166546694 | 0.97808989963298 | 0.811217114135872 | 1.17928953309018 | 0.998735809224318 |
| ebi-fl187-GCST90094780 | prot-a-1856 | Megakaryocyte-associated tyrosine-protein kinase \|\| id:prot-a-1856 | F-healthy breakfast food liking (derived food-liking factor) \|\| id:ebi-fl187-GCST90094780 | Inverse variance weighted | 8 | 0.0237081961011599 | 0.0954722468858269 | 0.803882549107432 | -0.163417408 | 0.210833799997381 | 1.02399146958699 | 0.84923663643146 | 1.23470712967945 | 0.998735809224318 |
| ebi-fl187-GCST90094780 | prot-a-1890 | Beta-1,3-N-acetylglucosaminyltransferase manic fringe \|\| id:prot-a-1890 | F-healthy breakfast food liking (derived food-liking factor) \|\| id:ebi-fl187-GCST90094780 | Inverse variance weighted | 8 | -0.121855367 | 0.128991608614513 | 0.344823956013411 | -0.37467892 | 0.130968185753828 | 0.885276399309501 | 0.687509989053182 | 1.13993151467327 | 0.998735809224318 |
| ebi-fl187-GCST90094780 | prot-a-1919 | Matrilysin \|\| id:prot-a-1919 | F-healthy breakfast food liking (derived food-liking factor) \|\| id:ebi-fl187-GCST90094780 | Inverse variance weighted | 8 | -0.130901389 | 0.0954417641854396 | 0.170209322538109 | -0.317967246 | 0.0561644692433785 | 0.877304282364165 | 0.727626620442102 | 1.05777164033232 | 0.998735809224318 |
| ebi-fl187-GCST90094780 | prot-a-1923 | Multimerin-2 \|\| id:prot-a-1923 | F-healthy breakfast food liking (derived food-liking factor) \|\| id:ebi-fl187-GCST90094780 | Inverse variance weighted | 8 | 0.0463702271906307 | 0.0955234255800558 | 0.627369197750516 | -0.140855687 | 0.23359614132754 | 1.04746213814363 | 0.868614655086489 | 1.26313426145819 | 0.998735809224318 |
| ebi-fl187-GCST90094780 | prot-a-1948 | Mesothelin \|\| id:prot-a-1948 | F-healthy breakfast food liking (derived food-liking factor) \|\| id:ebi-fl187-GCST90094780 | Inverse variance weighted | 8 | 0.0999027419168223 | 0.0954722468858269 | 0.295373575696382 | -0.087222862 | 0.287028345813043 | 1.10506343649738 | 0.916472825923972 | 1.33246198265862 | 0.998735809224318 |
| ebi-fl187-GCST90094780 | prot-a-195 | ATPase family AAA domain-containing protein 1 \|\| id:prot-a-195 | F-healthy breakfast food liking (derived food-liking factor) \|\| id:ebi-fl187-GCST90094780 | Inverse variance weighted (multiplicative random effects) | 8 | 0.00976421582526866 | 0.141953373198771 | 0.945161021343812 | -0.268464396 | 0.287992827294861 | 1.00981204131333 | 0.764552643729518 | 1.33374773750982 | 0.998735809224318 |
| ebi-fl187-GCST90094780 | prot-a-1966 | Mucin-1 \|\| id:prot-a-1966 | F-healthy breakfast food liking (derived food-liking factor) \|\| id:ebi-fl187-GCST90094780 | Inverse variance weighted | 8 | 0.0914283767462451 | 0.0955234255800558 | 0.338501429598157 | -0.095797537 | 0.278654290883155 | 1.09573829354012 | 0.908647964684617 | 1.32135046199871 | 0.998735809224318 |
| ebi-fl187-GCST90094780 | prot-a-1986 | Myocardial zonula adherens protein \|\| id:prot-a-1986 | F-healthy breakfast food liking (derived food-liking factor) \|\| id:ebi-fl187-GCST90094780 | Inverse variance weighted | 8 | -0.108491573 | 0.106024242360068 | 0.306179551591391 | -0.316299088 | 0.0993159422074581 | 0.897186455538416 | 0.728841429953743 | 1.10441517581221 | 0.998735809224318 |
| ebi-fl187-GCST90094780 | prot-a-2071 | Epididymal secretory protein E1 \|\| id:prot-a-2071 | F-healthy breakfast food liking (derived food-liking factor) \|\| id:ebi-fl187-GCST90094780 | Inverse variance weighted | 8 | -0.181509043 | 0.115621672281241 | 0.11644928643295 | -0.40812752 | 0.0451094348549083 | 0.834010703463776 | 0.664894085798098 | 1.04614233807963 | 0.998735809224318 |
| ebi-fl187-GCST90094780 | prot-a-2098 | Neuritin \|\| id:prot-a-2098 | F-healthy breakfast food liking (derived food-liking factor) \|\| id:ebi-fl187-GCST90094780 | Inverse variance weighted | 8 | -0.097183077 | 0.130922750987128 | 0.457909670088199 | -0.353791669 | 0.159425515329947 | 0.907389869069351 | 0.702021205224811 | 1.17283690059765 | 0.998735809224318 |
| ebi-fl187-GCST90094780 | prot-a-2115 | Neurotrophin-4 \|\| id:prot-a-2115 | F-healthy breakfast food liking (derived food-liking factor) \|\| id:ebi-fl187-GCST90094780 | Inverse variance weighted | 8 | 0.113942499341752 | 0.09791047561014 | 0.244528721345804 | -0.077962033 | 0.305847031537626 | 1.12068768275199 | 0.924999545469532 | 1.35777459396967 | 0.998735809224318 |
| ebi-fl187-GCST90094780 | prot-a-2118 | Netrin-1 \|\| id:prot-a-2118 | F-healthy breakfast food liking (derived food-liking factor) \|\| id:ebi-fl187-GCST90094780 | Inverse variance weighted | 8 | 0.186887845869802 | 0.125626682178456 | 0.13684466480985 | -0.059340451 | 0.433116142939576 | 1.20549207655266 | 0.942385878133285 | 1.54205530913709 | 0.998735809224318 |
| ebi-fl187-GCST90094780 | prot-a-2120 | Netrin-G1 \|\| id:prot-a-2120 | F-healthy breakfast food liking (derived food-liking factor) \|\| id:ebi-fl187-GCST90094780 | Inverse variance weighted | 8 | -0.027833246 | 0.0955403994232226 | 0.770803255991912 | -0.215092429 | 0.159425936847324 | 0.972550529949684 | 0.80646689589097 | 1.17283739496889 | 0.998735809224318 |
| ebi-fl187-GCST90094780 | prot-a-2145 | Olfactomedin-like protein 3 \|\| id:prot-a-2145 | F-healthy breakfast food liking (derived food-liking factor) \|\| id:ebi-fl187-GCST90094780 | Inverse variance weighted (multiplicative random effects) | 8 | 0.0351947818647042 | 0.154870817650799 | 0.820227399404888 | -0.268352021 | 0.338741584460269 | 1.0358214483846 | 0.764638565094307 | 1.40318069466093 | 0.998735809224318 |
| ebi-fl187-GCST90094780 | prot-a-2162 | Prolyl 4-hydroxylase subunit alpha-2 \|\| id:prot-a-2162 | F-healthy breakfast food liking (derived food-liking factor) \|\| id:ebi-fl187-GCST90094780 | Inverse variance weighted | 8 | 0.0613814354016152 | 0.095549361175301 | 0.520609927756323 | -0.125895313 | 0.248658183305205 | 1.06330441879339 | 0.881707145667133 | 1.28230364535618 | 0.998735809224318 |
| ebi-fl187-GCST90094780 | prot-a-2168 | Polyadenylate-binding protein-interacting protein 1 \|\| id:prot-a-2168 | F-healthy breakfast food liking (derived food-liking factor) \|\| id:ebi-fl187-GCST90094780 | Inverse variance weighted | 8 | 0.022666251972671 | 0.0955197216386407 | 0.812428755922087 | -0.164552402 | 0.209884906384407 | 1.02292508334155 | 0.8482733041895 | 1.23353607965901 | 0.998735809224318 |
| ebi-fl187-GCST90094780 | prot-a-219 | Beta-1,4-galactosyltransferase 5 \|\| id:prot-a-219 | F-healthy breakfast food liking (derived food-liking factor) \|\| id:ebi-fl187-GCST90094780 | Inverse variance weighted | 8 | 0.0492622067483103 | 0.104639422210472 | 0.637797441738304 | -0.155831061 | 0.254355474280835 | 1.05049576170893 | 0.855703740079411 | 1.28963015314859 | 0.998735809224318 |
| ebi-fl187-GCST90094780 | prot-a-2190 | Pyruvate carboxylase, mitochondrial \|\| id:prot-a-2190 | F-healthy breakfast food liking (derived food-liking factor) \|\| id:ebi-fl187-GCST90094780 | Inverse variance weighted | 8 | -0.194195717 | 0.0955403994232226 | 0.0420926323298143 | -0.3814549 | -0.006936535 | 0.82349671580615 | 0.682867182445475 | 0.993087467631619 | 0.998735809224318 |
| ebi-fl187-GCST90094780 | prot-a-2197 | Protocadherin-9 \|\| id:prot-a-2197 | F-healthy breakfast food liking (derived food-liking factor) \|\| id:ebi-fl187-GCST90094780 | Inverse variance weighted | 8 | 0.112323760797798 | 0.120112965758392 | 0.349710483426841 | -0.123097652 | 0.347745173684246 | 1.11887504988933 | 0.884177316583256 | 1.41587140247221 | 0.998735809224318 |
| ebi-fl187-GCST90094780 | prot-a-2198 | Protocadherin alpha-4 \|\| id:prot-a-2198 | F-healthy breakfast food liking (derived food-liking factor) \|\| id:ebi-fl187-GCST90094780 | Inverse variance weighted | 8 | 0.112351224139896 | 0.0955197216386407 | 0.239511085061968 | -0.07486743 | 0.299569878551632 | 1.11890577835954 | 0.92786648518312 | 1.34927832919765 | 0.998735809224318 |
| ebi-fl187-GCST90094780 | prot-a-2254 | Placenta growth factor \|\| id:prot-a-2254 | F-healthy breakfast food liking (derived food-liking factor) \|\| id:ebi-fl187-GCST90094780 | Inverse variance weighted | 8 | 0.129039699779776 | 0.0954417641854396 | 0.176367155143271 | -0.058026158 | 0.316105557583237 | 1.13773529110967 | 0.943625263741962 | 1.37177504924282 | 0.998735809224318 |
| ebi-fl187-GCST90094780 | prot-a-2261 | Membrane-associated progesterone receptor component 2 \|\| id:prot-a-2261 | F-healthy breakfast food liking (derived food-liking factor) \|\| id:ebi-fl187-GCST90094780 | Inverse variance weighted | 8 | 0.00159107343124871 | 0.0954722468858269 | 0.986703631614579 | -0.18553453 | 0.188716677327469 | 1.00159233986015 | 0.830660152004414 | 1.20769873557291 | 0.998955228396052 |
| ebi-fl187-GCST90094780 | prot-a-2268 | Polymeric immunoglobulin receptor \|\| id:prot-a-2268 | F-healthy breakfast food liking (derived food-liking factor) \|\| id:ebi-fl187-GCST90094780 | Inverse variance weighted | 8 | -0.062331867 | 0.0955234255800558 | 0.514059552433876 | -0.249557782 | 0.124894046749625 | 0.939571022064825 | 0.779145259327921 | 1.13302839866526 | 0.998735809224318 |
| ebi-fl187-GCST90094780 | prot-a-2283 | Group 10 secretory phospholipase A2 \|\| id:prot-a-2283 | F-healthy breakfast food liking (derived food-liking factor) \|\| id:ebi-fl187-GCST90094780 | Inverse variance weighted | 8 | 0.174995730378046 | 0.0954722468858269 | 0.0668102011107515 | -0.012129874 | 0.362121334274267 | 1.19124113045222 | 0.987943396844979 | 1.43637321268897 | 0.998735809224318 |
| ebi-fl187-GCST90094780 | prot-a-2298 | Inactive phospholipase D5 \|\| id:prot-a-2298 | F-healthy breakfast food liking (derived food-liking factor) \|\| id:ebi-fl187-GCST90094780 | Inverse variance weighted | 8 | 0.207109499115075 | 0.0955234255800558 | 0.0301469699215941 | 0.0198835849781659 | 0.394335413251985 | 1.23011726115797 | 1.02008258017833 | 1.48339801659416 | 0.998735809224318 |
| ebi-fl187-GCST90094780 | prot-a-2304 | Procollagen-lysine,2-oxoglutarate 5-dioxygenase 2 \|\| id:prot-a-2304 | F-healthy breakfast food liking (derived food-liking factor) \|\| id:ebi-fl187-GCST90094780 | Inverse variance weighted | 8 | 0.0770247540965641 | 0.0967972299579504 | 0.426187218274593 | -0.112697817 | 0.266747324814147 | 1.08006881218936 | 0.893420596183471 | 1.30571048400655 | 0.998735809224318 |
| ebi-fl187-GCST90094780 | prot-a-2331 | Periostin \|\| id:prot-a-2331 | F-healthy breakfast food liking (derived food-liking factor) \|\| id:ebi-fl187-GCST90094780 | Inverse variance weighted (multiplicative random effects) | 8 | -0.110945954 | 0.197646801981916 | 0.574569577709913 | -0.498333686 | 0.276441777881016 | 0.894987118092556 | 0.607542172825455 | 1.31843018868378 | 0.998735809224318 |
| ebi-fl187-GCST90094780 | prot-a-2333 | Periostin \|\| id:prot-a-2333 | F-healthy breakfast food liking (derived food-liking factor) \|\| id:ebi-fl187-GCST90094780 | Inverse variance weighted (multiplicative random effects) | 8 | -0.085409764 | 0.17699252070602 | 0.629407170511493 | -0.432315105 | 0.261495576607496 | 0.918135988257085 | 0.649004840076994 | 1.2988711961421 | 0.998735809224318 |
| ebi-fl187-GCST90094780 | prot-a-2367 | cAMP-dependent protein kinase catalytic subunit alpha \|\| id:prot-a-2367 | F-healthy breakfast food liking (derived food-liking factor) \|\| id:ebi-fl187-GCST90094780 | Inverse variance weighted | 8 | 0.0679037803976106 | 0.132791693721832 | 0.609102056928388 | -0.192367939 | 0.328175500092401 | 1.07026232330906 | 0.825003261520425 | 1.38843262096188 | 0.998735809224318 |
| ebi-fl187-GCST90094780 | prot-a-2385 | Phosphoribosyl pyrophosphate synthase-associated protein 1 \|\| id:prot-a-2385 | F-healthy breakfast food liking (derived food-liking factor) \|\| id:ebi-fl187-GCST90094780 | Inverse variance weighted | 8 | 0.086860613691567 | 0.126125360875787 | 0.491021669678226 | -0.160345094 | 0.33406632100811 | 1.09074463425398 | 0.851849770311975 | 1.39663576679506 | 0.998735809224318 |
| ebi-fl187-GCST90094780 | prot-a-2393 | Trypsin-3 \|\| id:prot-a-2393 | F-healthy breakfast food liking (derived food-liking factor) \|\| id:ebi-fl187-GCST90094780 | Inverse variance weighted | 8 | -0.077355937 | 0.126380708289596 | 0.540480466192527 | -0.325062126 | 0.170350250861199 | 0.925560353436093 | 0.722482467566434 | 1.18572007807784 | 0.998735809224318 |
| ebi-fl187-GCST90094780 | prot-a-2405 | Pregnancy-specific beta-1-glycoprotein 6 \|\| id:prot-a-2405 | F-healthy breakfast food liking (derived food-liking factor) \|\| id:ebi-fl187-GCST90094780 | Inverse variance weighted | 8 | -0.146750163 | 0.104851344717252 | 0.161632502624399 | -0.352258799 | 0.0587584726389796 | 0.863509687132867 | 0.703098137578015 | 1.06051906543355 | 0.998735809224318 |
| ebi-fl187-GCST90094780 | prot-a-244 | Beclin-1 \|\| id:prot-a-244 | F-healthy breakfast food liking (derived food-liking factor) \|\| id:ebi-fl187-GCST90094780 | Inverse variance weighted | 8 | 0.0171388074021764 | 0.124208509828018 | 0.890252933043173 | -0.226309872 | 0.260587486665091 | 1.01728651942444 | 0.797470949567884 | 1.29769223965268 | 0.998735809224318 |
| ebi-fl187-GCST90094780 | prot-a-2454 | tRNA pseudouridine synthase A, mitochondrial \|\| id:prot-a-2454 | F-healthy breakfast food liking (derived food-liking factor) \|\| id:ebi-fl187-GCST90094780 | Inverse variance weighted | 8 | 0.0857165905500152 | 0.0955188045749545 | 0.369516595887533 | -0.101500266 | 0.272933447516926 | 1.08949751065602 | 0.903480938639602 | 1.31381280441066 | 0.998735809224318 |
| ebi-fl187-GCST90094780 | prot-a-2466 | Glutaminyl-peptide cyclotransferase-like protein \|\| id:prot-a-2466 | F-healthy breakfast food liking (derived food-liking factor) \|\| id:ebi-fl187-GCST90094780 | Inverse variance weighted | 8 | 0.0906723616590449 | 0.125272990746199 | 0.469189651986624 | -0.1548627 | 0.336207423521595 | 1.09491021191912 | 0.856532771185808 | 1.39962931074439 | 0.998735809224318 |
| ebi-fl187-GCST90094780 | prot-a-2474 | Ras-related protein Rab-27A \|\| id:prot-a-2474 | F-healthy breakfast food liking (derived food-liking factor) \|\| id:ebi-fl187-GCST90094780 | Inverse variance weighted | 8 | -0.138198081 | 0.113658301020416 | 0.224019822994366 | -0.360968351 | 0.0845721885943485 | 0.870926160380655 | 0.697001057221506 | 1.08825140073544 | 0.998735809224318 |
| ebi-fl187-GCST90094780 | prot-a-2490 | Rap1 GTPase-GDP dissociation stimulator 1 \|\| id:prot-a-2490 | F-healthy breakfast food liking (derived food-liking factor) \|\| id:ebi-fl187-GCST90094780 | Inverse variance weighted | 8 | 0.092769085465592 | 0.0954417641854396 | 0.331052160899588 | -0.094296772 | 0.279834943269054 | 1.09720834465937 | 0.91001265557937 | 1.3229114388785 | 0.998735809224318 |
| ebi-fl187-GCST90094780 | prot-a-2545 | Receptor-interacting serine/threonine-protein kinase 2 \|\| id:prot-a-2545 | F-healthy breakfast food liking (derived food-liking factor) \|\| id:ebi-fl187-GCST90094780 | Inverse variance weighted | 8 | -0.046525816 | 0.122363651508223 | 0.703777818521901 | -0.286358573 | 0.193306941198698 | 0.954539918080918 | 0.750993281962903 | 1.21325513435809 | 0.998735809224318 |
| ebi-fl187-GCST90094780 | prot-a-2610 | RUN and FYVE domain-containing protein 1 \|\| id:prot-a-2610 | F-healthy breakfast food liking (derived food-liking factor) \|\| id:ebi-fl187-GCST90094780 | Inverse variance weighted | 8 | -0.15312072 | 0.103030486958209 | 0.137234414966716 | -0.355060474 | 0.048819034753526 | 0.858026134914677 | 0.701131041641879 | 1.0500303145509 | 0.998735809224318 |
| ebi-fl187-GCST90094780 | prot-a-2627 | Serine--tRNA ligase, mitochondrial \|\| id:prot-a-2627 | F-healthy breakfast food liking (derived food-liking factor) \|\| id:ebi-fl187-GCST90094780 | Inverse variance weighted | 8 | -0.086469787 | 0.0955234255800558 | 0.365348452024682 | -0.273695701 | 0.100756127347588 | 0.917163258812864 | 0.760563478813299 | 1.1060068840391 | 0.998735809224318 |
| ebi-fl187-GCST90094780 | prot-a-264 | BolA-like protein 2 \|\| id:prot-a-264 | F-healthy breakfast food liking (derived food-liking factor) \|\| id:ebi-fl187-GCST90094780 | Inverse variance weighted | 8 | -0.044554663 | 0.0983313854177045 | 0.650471522732103 | -0.237284178 | 0.148174852833951 | 0.956423318094017 | 0.788767105903963 | 1.15971565820513 | 0.998735809224318 |
| ebi-fl187-GCST90094780 | prot-a-2646 | Secretoglobin family 1C member 1 \|\| id:prot-a-2646 | F-healthy breakfast food liking (derived food-liking factor) \|\| id:ebi-fl187-GCST90094780 | Inverse variance weighted | 8 | 0.0407321697121775 | 0.095549361175301 | 0.669893245535557 | -0.146544578 | 0.228008917615767 | 1.04157310336047 | 0.86368722986193 | 1.25609652676859 | 0.998735809224318 |
| ebi-fl187-GCST90094780 | prot-a-2672 | Semaphorin-3E \|\| id:prot-a-2672 | F-healthy breakfast food liking (derived food-liking factor) \|\| id:ebi-fl187-GCST90094780 | Inverse variance weighted | 8 | -0.00558705 | 0.130696293750867 | 0.965902144818795 | -0.261751785 | 0.250577686117178 | 0.9944285289011 | 0.769702051296062 | 1.28476739463961 | 0.998735809224318 |
| ebi-fl187-GCST90094780 | prot-a-2701 | Plasma protease C1 inhibitor \|\| id:prot-a-2701 | F-healthy breakfast food liking (derived food-liking factor) \|\| id:ebi-fl187-GCST90094780 | Inverse variance weighted | 8 | 0.0448395833044299 | 0.11449422683386 | 0.695330267672174 | -0.179569101 | 0.269248267898796 | 1.04586007303422 | 0.835630205822918 | 1.30898007844268 | 0.998735809224318 |
| ebi-fl187-GCST90094780 | prot-a-2738 | Signaling threshold-regulating transmembrane adapter 1 \|\| id:prot-a-2738 | F-healthy breakfast food liking (derived food-liking factor) \|\| id:ebi-fl187-GCST90094780 | Inverse variance weighted | 8 | -0.039724658 | 0.113579712332238 | 0.726525312417146 | -0.262340894 | 0.182891578393714 | 0.961054021475551 | 0.769248746763018 | 1.20068422091153 | 0.998735809224318 |
| ebi-fl187-GCST90094780 | prot-a-274 | Serine/threonine-protein kinase BRSK2 \|\| id:prot-a-274 | F-healthy breakfast food liking (derived food-liking factor) \|\| id:ebi-fl187-GCST90094780 | Inverse variance weighted | 8 | 0.119030386473701 | 0.095549361175301 | 0.212857075860583 | -0.068246361 | 0.306307134377291 | 1.12640414521827 | 0.934030336180707 | 1.35839945365482 | 0.998735809224318 |
| ebi-fl187-GCST90094780 | prot-a-2749 | Mitochondrial glutamate carrier 2 \|\| id:prot-a-2749 | F-healthy breakfast food liking (derived food-liking factor) \|\| id:ebi-fl187-GCST90094780 | Inverse variance weighted | 8 | 0.0388567090970025 | 0.0979127957433272 | 0.691477955790973 | -0.153052371 | 0.230765788753924 | 1.03962150467257 | 0.858084782254145 | 1.25956420079891 | 0.998735809224318 |
| ebi-fl187-GCST90094780 | prot-a-2760 | Sodium-coupled monocarboxylate transporter 1 \|\| id:prot-a-2760 | F-healthy breakfast food liking (derived food-liking factor) \|\| id:ebi-fl187-GCST90094780 | Inverse variance weighted | 8 | -0.170569058 | 0.0955188045749545 | 0.0741459317493569 | -0.357785915 | 0.0166477993123549 | 0.843184859249273 | 0.699222752351705 | 1.01678714612194 | 0.998735809224318 |
| ebi-fl187-GCST90094780 | prot-a-2777 | Small integral membrane protein 10 \|\| id:prot-a-2777 | F-healthy breakfast food liking (derived food-liking factor) \|\| id:ebi-fl187-GCST90094780 | Inverse variance weighted | 8 | 0.0978721509018617 | 0.0955188045749545 | 0.305534254614815 | -0.089344706 | 0.285089007868773 | 1.10282178132495 | 0.914530275102427 | 1.32988039267318 | 0.998735809224318 |
| ebi-fl187-GCST90094780 | prot-a-2799 | Superoxide dismutase [Mn], mitochondrial \|\| id:prot-a-2799 | F-healthy breakfast food liking (derived food-liking factor) \|\| id:ebi-fl187-GCST90094780 | Inverse variance weighted | 8 | 0.0633117706535692 | 0.122439258888276 | 0.605095304994327 | -0.176669177 | 0.303292718074591 | 1.06535893511123 | 0.838056987389519 | 1.35431083768746 | 0.998735809224318 |
| ebi-fl187-GCST90094780 | prot-a-2805 | Protein SPACA7 \|\| id:prot-a-2805 | F-healthy breakfast food liking (derived food-liking factor) \|\| id:ebi-fl187-GCST90094780 | Inverse variance weighted (multiplicative random effects) | 8 | -0.155804588 | 0.135575007493636 | 0.250468081017933 | -0.421531602 | 0.109922427081596 | 0.855726393576934 | 0.656041255657676 | 1.11619148086974 | 0.998735809224318 |
| ebi-fl187-GCST90094780 | prot-a-2811 | SPARC-like protein 1 \|\| id:prot-a-2811 | F-healthy breakfast food liking (derived food-liking factor) \|\| id:ebi-fl187-GCST90094780 | Inverse variance weighted | 8 | 0.0313095418207059 | 0.0955234255800558 | 0.743086928527113 | -0.155916372 | 0.218535455957615 | 1.03180484120901 | 0.855630741796258 | 1.2442531320314 | 0.998735809224318 |
| ebi-fl187-GCST90094780 | prot-a-2831 | Sepiapterin reductase \|\| id:prot-a-2831 | F-healthy breakfast food liking (derived food-liking factor) \|\| id:ebi-fl187-GCST90094780 | Inverse variance weighted | 8 | -0.091126113 | 0.0956006640496768 | 0.340491075659748 | -0.278503415 | 0.0962511881413956 | 0.912902574395443 | 0.756915682928893 | 1.10103559634676 | 0.998735809224318 |
| ebi-fl187-GCST90094780 | prot-a-2834 | SPRY domain-containing SOCS box protein 1 \|\| id:prot-a-2834 | F-healthy breakfast food liking (derived food-liking factor) \|\| id:ebi-fl187-GCST90094780 | Inverse variance weighted | 8 | -0.058834718 | 0.112057415565291 | 0.599554808944056 | -0.278467253 | 0.160797816249824 | 0.942862594251131 | 0.756943055135309 | 1.17444749060953 | 0.998735809224318 |
| ebi-fl187-GCST90094780 | prot-a-2835 | Spectrin alpha chain, non-erythrocytic 1 \|\| id:prot-a-2835 | F-healthy breakfast food liking (derived food-liking factor) \|\| id:ebi-fl187-GCST90094780 | Inverse variance weighted | 8 | -0.042555016 | 0.0959273216352896 | 0.657319317906031 | -0.230572567 | 0.145462533913029 | 0.958337739689844 | 0.794078809075999 | 1.15657440145332 | 0.998735809224318 |
| ebi-fl187-GCST90094780 | prot-a-2859 | Sia-alpha-2,3-Gal-beta-1,4-GlcNAc-R:alpha 2,8-sialyltransferase \|\| id:prot-a-2859 | F-healthy breakfast food liking (derived food-liking factor) \|\| id:ebi-fl187-GCST90094780 | Inverse variance weighted | 8 | -0.077883015 | 0.0955197216386407 | 0.414865946542264 | -0.265101669 | 0.109335639590237 | 0.925072640001647 | 0.767127952700217 | 1.11553670579651 | 0.998735809224318 |
| ebi-fl187-GCST90094780 | prot-a-2861 | Alpha-2,8-sialyltransferase 8F \|\| id:prot-a-2861 | F-healthy breakfast food liking (derived food-liking factor) \|\| id:ebi-fl187-GCST90094780 | Inverse variance weighted | 8 | -0.026838402 | 0.0997317324392714 | 0.787848153045985 | -0.222312598 | 0.168635793133967 | 0.973518547028897 | 0.800665038898849 | 1.18368895276441 | 0.998735809224318 |
| ebi-fl187-GCST90094780 | prot-a-289 | Uncharacterized protein C17orf89 \|\| id:prot-a-289 | F-healthy breakfast food liking (derived food-liking factor) \|\| id:ebi-fl187-GCST90094780 | Inverse variance weighted | 8 | 0.00660759736074417 | 0.0954928938475475 | 0.944834681638512 | -0.180558475 | 0.193773669301937 | 1.00662947569338 | 0.834803864463971 | 1.21382152679105 | 0.998735809224318 |
| ebi-fl187-GCST90094780 | prot-a-2921 | Tachykinin-3 \|\| id:prot-a-2921 | F-healthy breakfast food liking (derived food-liking factor) \|\| id:ebi-fl187-GCST90094780 | Inverse variance weighted | 8 | 0.0536999602271005 | 0.0956176791076867 | 0.574380942719676 | -0.133710691 | 0.241110611278166 | 1.0551679622994 | 0.874843128139329 | 1.27266179827128 | 0.998735809224318 |
| ebi-fl187-GCST90094780 | prot-a-2928 | Tax1-binding protein 1 \|\| id:prot-a-2928 | F-healthy breakfast food liking (derived food-liking factor) \|\| id:ebi-fl187-GCST90094780 | Inverse variance weighted | 8 | -0.077068101 | 0.116218797395847 | 0.507247687312607 | -0.304856943 | 0.150720742345569 | 0.925826802144899 | 0.737228832264368 | 1.16267192770681 | 0.998735809224318 |
| ebi-fl187-GCST90094780 | prot-a-296 | Uncharacterized protein C1orf185 \|\| id:prot-a-296 | F-healthy breakfast food liking (derived food-liking factor) \|\| id:ebi-fl187-GCST90094780 | Inverse variance weighted | 8 | -0.136076247 | 0.0955234255800558 | 0.154292315136904 | -0.323302161 | 0.0511496671489651 | 0.872776083404159 | 0.72375513066028 | 1.05248040323717 | 0.998735809224318 |
| ebi-fl187-GCST90094780 | prot-a-2964 | Transforming growth factor beta-2 \|\| id:prot-a-2964 | F-healthy breakfast food liking (derived food-liking factor) \|\| id:ebi-fl187-GCST90094780 | Inverse variance weighted | 8 | 0.165862008053921 | 0.0956483307270675 | 0.0829036100859992 | -0.02160872 | 0.353332736278973 | 1.18041020338307 | 0.978623075616984 | 1.42380481614169 | 0.998735809224318 |
| ebi-fl187-GCST90094780 | prot-a-2977 | Thymocyte nuclear protein 1 \|\| id:prot-a-2977 | F-healthy breakfast food liking (derived food-liking factor) \|\| id:ebi-fl187-GCST90094780 | Inverse variance weighted | 8 | -0.040039553 | 0.0955663488504015 | 0.675237144146368 | -0.227349596 | 0.147270491143943 | 0.960751438180744 | 0.796642230386908 | 1.15866732989798 | 0.998735809224318 |
| ebi-fl187-GCST90094780 | prot-a-3015 | Transmembrane protein 70, mitochondrial \|\| id:prot-a-3015 | F-healthy breakfast food liking (derived food-liking factor) \|\| id:ebi-fl187-GCST90094780 | Inverse variance weighted | 8 | -0.182778896 | 0.0954417641854396 | 0.0554819248206786 | -0.369844754 | 0.00428696173144846 | 0.832952304404673 | 0.690841572789512 | 1.00429616389697 | 0.998735809224318 |
| ebi-fl187-GCST90094780 | prot-a-3030 | Tumor necrosis factor alpha-induced protein 3 \|\| id:prot-a-3030 | F-healthy breakfast food liking (derived food-liking factor) \|\| id:ebi-fl187-GCST90094780 | Inverse variance weighted | 8 | -0.13800929 | 0.119309765777949 | 0.247382331350943 | -0.371856431 | 0.0958378511904296 | 0.871090599507946 | 0.689453219763325 | 1.10058059169206 | 0.998735809224318 |
| ebi-fl187-GCST90094780 | prot-a-3032 | Tumor necrosis factor receptor superfamily member 10A \|\| id:prot-a-3032 | F-healthy breakfast food liking (derived food-liking factor) \|\| id:ebi-fl187-GCST90094780 | Inverse variance weighted | 8 | 0.0802256201554526 | 0.111674722677146 | 0.472518969816008 | -0.138656836 | 0.299108076602659 | 1.08353150664571 | 0.870526710380984 | 1.34865537368762 | 0.998735809224318 |
| ebi-fl187-GCST90094780 | prot-a-3081 | Tumor protein p53-inducible protein 11 \|\| id:prot-a-3081 | F-healthy breakfast food liking (derived food-liking factor) \|\| id:ebi-fl187-GCST90094780 | Inverse variance weighted | 8 | -0.054216805 | 0.0956006640496768 | 0.570634386023411 | -0.241594107 | 0.133160496445763 | 0.947226720662312 | 0.785374891327713 | 1.14243334010826 | 0.998735809224318 |
| ebi-fl187-GCST90094780 | prot-a-315 | Complement C4 \|\| id:prot-a-315 | F-healthy breakfast food liking (derived food-liking factor) \|\| id:ebi-fl187-GCST90094780 | Inverse variance weighted | 7 | -0.067510724 | 0.103368423404564 | 0.513686780262545 | -0.270112834 | 0.135091385660484 | 0.934717696451109 | 0.763293363969104 | 1.14464138338065 | 0.998735809224318 |
| ebi-fl187-GCST90094780 | prot-a-3157 | UDP-glucuronosyltransferase 1-8 \|\| id:prot-a-3157 | F-healthy breakfast food liking (derived food-liking factor) \|\| id:ebi-fl187-GCST90094780 | Inverse variance weighted | 8 | 0.12117488704363 | 0.111129222060448 | 0.27553863285315 | -0.096638388 | 0.338988162282109 | 1.12882231150241 | 0.907884248443497 | 1.4035267305609 | 0.998735809224318 |
| ebi-fl187-GCST90094780 | prot-a-317 | Glutaredoxin-like protein C5orf63 \|\| id:prot-a-317 | F-healthy breakfast food liking (derived food-liking factor) \|\| id:ebi-fl187-GCST90094780 | Inverse variance weighted | 8 | -0.002472599 | 0.1255067617096 | 0.984281952707491 | -0.248465852 | 0.243520653624163 | 0.997530455029144 | 0.779996495484276 | 1.27573266607159 | 0.998955228396052 |
| ebi-fl187-GCST90094780 | prot-a-3192 | Guanine nucleotide exchange factor VAV3 \|\| id:prot-a-3192 | F-healthy breakfast food liking (derived food-liking factor) \|\| id:ebi-fl187-GCST90094780 | Inverse variance weighted | 8 | 0.225834918277237 | 0.108724094987404 | 0.0377888504700863 | 0.0127356921019244 | 0.438934144452549 | 1.25336873992802 | 1.01281713641121 | 1.55105313857065 | 0.998735809224318 |
| ebi-fl187-GCST90094780 | prot-a-3233 | Serine/threonine-protein kinase WNK3 \|\| id:prot-a-3233 | F-healthy breakfast food liking (derived food-liking factor) \|\| id:ebi-fl187-GCST90094780 | Inverse variance weighted | 8 | -0.004431536 | 0.127606919541626 | 0.972296614350611 | -0.254541098 | 0.245678026116442 | 0.995578268582593 | 0.775272189922198 | 1.27848786756221 | 0.998955228396052 |
| ebi-fl187-GCST90094780 | prot-a-3250 | 14-3-3 protein theta \|\| id:prot-a-3250 | F-healthy breakfast food liking (derived food-liking factor) \|\| id:ebi-fl187-GCST90094780 | Inverse variance weighted | 8 | 0.112940746935681 | 0.116215657063908 | 0.331139902670901 | -0.114841941 | 0.340723434780942 | 1.11956559329108 | 0.891507043563712 | 1.40596434624988 | 0.998735809224318 |
| ebi-fl187-GCST90094780 | prot-a-3278 | E3 ubiquitin-protein ligase ZNRF3 \|\| id:prot-a-3278 | F-healthy breakfast food liking (derived food-liking factor) \|\| id:ebi-fl187-GCST90094780 | Inverse variance weighted | 8 | -0.069461646 | 0.107793475725479 | 0.519318912426886 | -0.280736858 | 0.141813566865528 | 0.932895913402074 | 0.755227041305588 | 1.15236179008869 | 0.998735809224318 |
| ebi-fl187-GCST90094780 | prot-a-355 | Calpain-3 \|\| id:prot-a-355 | F-healthy breakfast food liking (derived food-liking factor) \|\| id:ebi-fl187-GCST90094780 | Inverse variance weighted | 8 | -0.032364165 | 0.0955709767595579 | 0.734880872789219 | -0.219683279 | 0.154954949653102 | 0.968153950289606 | 0.802773012577978 | 1.1676053588938 | 0.998735809224318 |
| ebi-fl187-GCST90094780 | prot-a-385 | Coiled-coil domain-containing protein 90B, mitochondrial \|\| id:prot-a-385 | F-healthy breakfast food liking (derived food-liking factor) \|\| id:ebi-fl187-GCST90094780 | Inverse variance weighted | 8 | -0.026334297 | 0.0955197216386407 | 0.782782436005286 | -0.213552952 | 0.160884357155685 | 0.974009426498994 | 0.807709389458925 | 1.17454913275729 | 0.998735809224318 |
| ebi-fl187-GCST90094780 | prot-a-390 | C-C motif chemokine 14 \|\| id:prot-a-390 | F-healthy breakfast food liking (derived food-liking factor) \|\| id:ebi-fl187-GCST90094780 | Inverse variance weighted | 8 | -0.01335635 | 0.132529364640857 | 0.919724863173047 | -0.273113904 | 0.246401205174987 | 0.986732450726481 | 0.761006100887849 | 1.27941277761211 | 0.998735809224318 |
| ebi-fl187-GCST90094780 | prot-a-40 | Pituitary adenylate cyclase-activating polypeptide 27 \|\| id:prot-a-40 | F-healthy breakfast food liking (derived food-liking factor) \|\| id:ebi-fl187-GCST90094780 | Inverse variance weighted | 8 | 0.203544669758826 | 0.111124126285173 | 0.0669979964802954 | -0.014258618 | 0.421347957277765 | 1.22573990992447 | 0.985842554898522 | 1.52401447808906 | 0.998735809224318 |
| ebi-fl187-GCST90094780 | prot-a-401 | C-C motif chemokine 24 \|\| id:prot-a-401 | F-healthy breakfast food liking (derived food-liking factor) \|\| id:ebi-fl187-GCST90094780 | Inverse variance weighted | 8 | 0.362185495111328 | 0.122405463612935 | 0.00308739525289293 | 0.122270786429976 | 0.602100203792681 | 1.43646537455319 | 1.1300600652838 | 1.82594964257234 | 0.998735809224318 |
| ebi-fl187-GCST90094780 | prot-a-412 | Cerebral cavernous malformations 2 protein \|\| id:prot-a-412 | F-healthy breakfast food liking (derived food-liking factor) \|\| id:ebi-fl187-GCST90094780 | Inverse variance weighted | 8 | 0.13990314039729 | 0.0954722468858269 | 0.142817215950376 | -0.047222463 | 0.327028744293511 | 1.1501623891897 | 0.953875171576764 | 1.38684134038192 | 0.998735809224318 |
| ebi-fl187-GCST90094780 | prot-a-44 | Adhesion G protein-coupled receptor F5 \|\| id:prot-a-44 | F-healthy breakfast food liking (derived food-liking factor) \|\| id:ebi-fl187-GCST90094780 | Inverse variance weighted | 8 | -0.069053885 | 0.0962560227771212 | 0.473128512723742 | -0.257715689 | 0.119607920079346 | 0.933276389532107 | 0.772814920498471 | 1.12705486935511 | 0.998735809224318 |
| ebi-fl187-GCST90094780 | prot-a-48 | Adrenomedullin \|\| id:prot-a-48 | F-healthy breakfast food liking (derived food-liking factor) \|\| id:ebi-fl187-GCST90094780 | Inverse variance weighted | 8 | -0.024436044 | 0.113923349889713 | 0.830160690989234 | -0.247725809 | 0.198853722108428 | 0.975860099347884 | 0.780573939929451 | 1.22000349330818 | 0.998735809224318 |
| ebi-fl187-GCST90094780 | prot-a-502 | Carcinoembryonic antigen-related cell adhesion molecule 1 \|\| id:prot-a-502 | F-healthy breakfast food liking (derived food-liking factor) \|\| id:ebi-fl187-GCST90094780 | Inverse variance weighted | 8 | -0.170934938 | 0.126171014158174 | 0.175485206504899 | -0.418230126 | 0.0763602496522488 | 0.842876410830321 | 0.658210739690262 | 1.0793513400716 | 0.998735809224318 |
| ebi-fl187-GCST90094780 | prot-a-518 | Complement factor B \|\| id:prot-a-518 | F-healthy breakfast food liking (derived food-liking factor) \|\| id:ebi-fl187-GCST90094780 | Inverse variance weighted | 8 | 0.0282881331451412 | 0.09565739933378 | 0.767441311219242 | -0.15920037 | 0.21577663583935 | 1.0286920419976 | 0.852825461595215 | 1.24082519216746 | 0.998735809224318 |
| ebi-fl187-GCST90094780 | prot-a-525 | Properdin \|\| id:prot-a-525 | F-healthy breakfast food liking (derived food-liking factor) \|\| id:ebi-fl187-GCST90094780 | Inverse variance weighted | 8 | -0.043816149 | 0.134495221303899 | 0.744589148188023 | -0.307426783 | 0.219794484879171 | 0.957129910707398 | 0.735336704994909 | 1.24582066929067 | 0.998735809224318 |
| ebi-fl187-GCST90094780 | prot-a-533 | Chondroadherin \|\| id:prot-a-533 | F-healthy breakfast food liking (derived food-liking factor) \|\| id:ebi-fl187-GCST90094780 | Inverse variance weighted (multiplicative random effects) | 8 | -0.050833309 | 0.1366074507087 | 0.709809256593058 | -0.318583913 | 0.216917293972114 | 0.950437086240926 | 0.727178055844228 | 1.24224135704069 | 0.998735809224318 |
| ebi-fl187-GCST90094780 | prot-a-535 | Coiled-coil-helix-coiled-coil-helix domain-containing protein 2 \|\| id:prot-a-535 | F-healthy breakfast food liking (derived food-liking factor) \|\| id:ebi-fl187-GCST90094780 | Inverse variance weighted (multiplicative random effects) | 8 | -0.090249607 | 0.146828142278959 | 0.538778286560403 | -0.378032766 | 0.197533551844734 | 0.91370309 | 0.685208048847323 | 1.21839394364828 | 0.998735809224318 |
| ebi-fl187-GCST90094780 | prot-a-591 | Caseinolytic peptidase B protein homolog \|\| id:prot-a-591 | F-healthy breakfast food liking (derived food-liking factor) \|\| id:ebi-fl187-GCST90094780 | Inverse variance weighted | 8 | -0.233301224 | 0.0954722468858269 | 0.0145393266196519 | -0.420426828 | -0.04617562 | 0.79191499380668 | 0.656766433758748 | 0.954874252368079 | 0.998735809224318 |
| ebi-fl187-GCST90094780 | prot-a-605 | Ciliary neurotrophic factor receptor subunit alpha \|\| id:prot-a-605 | F-healthy breakfast food liking (derived food-liking factor) \|\| id:ebi-fl187-GCST90094780 | Inverse variance weighted (multiplicative random effects) | 8 | 0.0384375634791131 | 0.150628349485918 | 0.798582943065743 | -0.256794002 | 0.333669128471512 | 1.0391858431838 | 0.77352754285737 | 1.39608114364552 | 0.998735809224318 |
| ebi-fl187-GCST90094780 | prot-a-606 | Ciliary neurotrophic factor receptor subunit alpha \|\| id:prot-a-606 | F-healthy breakfast food liking (derived food-liking factor) \|\| id:ebi-fl187-GCST90094780 | Inverse variance weighted | 8 | -0.033138803 | 0.122476650896189 | 0.786719915363875 | -0.273193039 | 0.206915432711516 | 0.967404271610798 | 0.760945881369107 | 1.22987855988784 | 0.998735809224318 |
| ebi-fl187-GCST90094780 | prot-a-707 | Cancer/testis antigen 55 \|\| id:prot-a-707 | F-healthy breakfast food liking (derived food-liking factor) \|\| id:ebi-fl187-GCST90094780 | Inverse variance weighted | 8 | 0.0168009074942512 | 0.0955188045749545 | 0.860379498446808 | -0.170415949 | 0.204017764461162 | 1.01694283647169 | 0.843313967633598 | 1.22631993817563 | 0.998735809224318 |
| ebi-fl187-GCST90094780 | prot-a-719 | Dipeptidyl peptidase 1 \|\| id:prot-a-719 | F-healthy breakfast food liking (derived food-liking factor) \|\| id:ebi-fl187-GCST90094780 | Inverse variance weighted | 8 | 0.0695933860825301 | 0.0955709767595579 | 0.466500126662178 | -0.117725728 | 0.256912500531264 | 1.07207217315061 | 0.888939830162022 | 1.29293199094744 | 0.998735809224318 |
| ebi-fl187-GCST90094780 | prot-a-752 | Cytochrome b5 \|\| id:prot-a-752 | F-healthy breakfast food liking (derived food-liking factor) \|\| id:ebi-fl187-GCST90094780 | Inverse variance weighted | 8 | -0.123061311 | 0.116625641484134 | 0.291341965692138 | -0.351647568 | 0.105524946739184 | 0.884209449513821 | 0.703528024163433 | 1.11129382733432 | 0.998735809224318 |
| ebi-fl187-GCST90094780 | prot-a-755 | Cytoglobin \|\| id:prot-a-755 | F-healthy breakfast food liking (derived food-liking factor) \|\| id:ebi-fl187-GCST90094780 | Inverse variance weighted | 8 | -0.09707442 | 0.0955403994232226 | 0.309602601437054 | -0.284333603 | 0.0901847629719637 | 0.907488468421379 | 0.752515561553911 | 1.09437646527497 | 0.998735809224318 |
| ebi-fl187-GCST90094780 | prot-a-807 | Desmin \|\| id:prot-a-807 | F-healthy breakfast food liking (derived food-liking factor) \|\| id:ebi-fl187-GCST90094780 | Inverse variance weighted | 8 | 0.0302390836843288 | 0.0955234255800558 | 0.751575997013617 | -0.15698683 | 0.217464997821238 | 1.03070092827329 | 0.854715314957436 | 1.24292192377098 | 0.998735809224318 |
| ebi-fl187-GCST90094780 | prot-a-82 | Ameloblastin \|\| id:prot-a-82 | F-healthy breakfast food liking (derived food-liking factor) \|\| id:ebi-fl187-GCST90094780 | Inverse variance weighted | 8 | 0.0266589746522021 | 0.095549361175301 | 0.780239179390742 | -0.160617773 | 0.213935722555792 | 1.027017504 | 0.851617519901265 | 1.23854304185355 | 0.998735809224318 |
| ebi-fl187-GCST90094780 | prot-a-84 | Muellerian-inhibiting factor \|\| id:prot-a-84 | F-healthy breakfast food liking (derived food-liking factor) \|\| id:ebi-fl187-GCST90094780 | Inverse variance weighted | 8 | -0.060363946 | 0.0955403994232226 | 0.527507166051469 | -0.247623129 | 0.126895236991824 | 0.941421844628393 | 0.780654093932467 | 1.13529807430972 | 0.998735809224318 |
| ebi-fl187-GCST90094780 | prot-a-858 | Inactive dipeptidyl peptidase 10 \|\| id:prot-a-858 | F-healthy breakfast food liking (derived food-liking factor) \|\| id:ebi-fl187-GCST90094780 | Inverse variance weighted | 8 | -0.053430189 | 0.118472223720089 | 0.651994317825231 | -0.285635747 | 0.178775369486411 | 0.947972117570645 | 0.751536315080424 | 1.19575211158652 | 0.998735809224318 |
| ebi-fl187-GCST90094780 | prot-a-863 | Dihydropyrimidinase-related protein 3 \|\| id:prot-a-863 | F-healthy breakfast food liking (derived food-liking factor) \|\| id:ebi-fl187-GCST90094780 | Inverse variance weighted | 8 | 0.0174209783537486 | 0.104124417092934 | 0.867126724592741 | -0.186662879 | 0.221504835855899 | 1.01757360863186 | 0.829723406302799 | 1.24795328312842 | 0.998735809224318 |
| ebi-fl187-GCST90094780 | prot-a-864 | Dihydropyrimidinase-related protein 5 \|\| id:prot-a-864 | F-healthy breakfast food liking (derived food-liking factor) \|\| id:ebi-fl187-GCST90094780 | Inverse variance weighted | 8 | -0.056167773 | 0.0954928938475475 | 0.556406116530792 | -0.243333845 | 0.13099829847121 | 0.945380512815307 | 0.784009732025316 | 1.13996584162564 | 0.998735809224318 |
| ebi-fl187-GCST90094780 | prot-a-884 | Endothelin-converting enzyme 1 \|\| id:prot-a-884 | F-healthy breakfast food liking (derived food-liking factor) \|\| id:ebi-fl187-GCST90094780 | Inverse variance weighted | 8 | 0.076923700298641 | 0.0955709767595579 | 0.420885714974338 | -0.110395414 | 0.264242814747374 | 1.07995967264845 | 0.895479979826953 | 1.30244441061912 | 0.998735809224318 |
| ebi-fl187-GCST90094780 | prot-a-893 | Early endosome antigen 1 \|\| id:prot-a-893 | F-healthy breakfast food liking (derived food-liking factor) \|\| id:ebi-fl187-GCST90094780 | Inverse variance weighted | 8 | 0.111137741779211 | 0.0954891934564833 | 0.244473582524322 | -0.076021077 | 0.298296560953918 | 1.11754882941755 | 0.926796671893515 | 1.34756136271067 | 0.998735809224318 |
| ebi-fl187-GCST90094780 | prot-a-897 | Ephrin-A2 \|\| id:prot-a-897 | F-healthy breakfast food liking (derived food-liking factor) \|\| id:ebi-fl187-GCST90094780 | Inverse variance weighted | 8 | 0.00490691563410983 | 0.095549361175301 | 0.959042824099658 | -0.182369832 | 0.1921836635377 | 1.00491897426012 | 0.833293104741431 | 1.21189307709604 | 0.998735809224318 |
| ebi-fl187-GCST90094780 | prot-a-90 | Anaphase-promoting complex subunit 10 \|\| id:prot-a-90 | F-healthy breakfast food liking (derived food-liking factor) \|\| id:ebi-fl187-GCST90094780 | Inverse variance weighted (multiplicative random effects) | 8 | 0.0555956237818558 | 0.173470565060339 | 0.748596724577746 | -0.284406684 | 0.395597931300121 | 1.05717010284254 | 0.752460568996708 | 1.48527201609285 | 0.998735809224318 |
| ebi-fl187-GCST90094780 | prot-a-914 | Histone-lysine N-methyltransferase EHMT2 \|\| id:prot-a-914 | F-healthy breakfast food liking (derived food-liking factor) \|\| id:ebi-fl187-GCST90094780 | Inverse variance weighted (multiplicative random effects) | 8 | 0.0740390426128209 | 0.161689088582654 | 0.647017137464894 | -0.242871571 | 0.390949656234823 | 1.07684884766812 | 0.784372243438764 | 1.47838408411847 | 0.998735809224318 |
| ebi-fl187-GCST90094780 | prot-a-932 | RNA polymerase II elongation factor ELL \|\| id:prot-a-932 | F-healthy breakfast food liking (derived food-liking factor) \|\| id:ebi-fl187-GCST90094780 | Inverse variance weighted | 8 | 0.0291508276894451 | 0.0955197216386407 | 0.760228114871996 | -0.158067827 | 0.216369482101181 | 1.02957987191795 | 0.85379187010045 | 1.24156102884168 | 0.998735809224318 |
| ebi-fl187-GCST90094780 | prot-a-991 | Estrogen receptor \|\| id:prot-a-991 | F-healthy breakfast food liking (derived food-liking factor) \|\| id:ebi-fl187-GCST90094780 | Inverse variance weighted | 8 | -0.221078953 | 0.0954417641854396 | 0.0205377816100813 | -0.408144811 | -0.034013095 | 0.801653384747522 | 0.664882589582168 | 0.966558847150769 | 0.998735809224318 |
| ebi-fl187-GCST90094780 | prot-a-992 | Persulfide dioxygenase ETHE1, mitochondrial \|\| id:prot-a-992 | F-healthy breakfast food liking (derived food-liking factor) \|\| id:ebi-fl187-GCST90094780 | Inverse variance weighted | 8 | -0.188972076 | 0.102864533636814 | 0.0661956493628912 | -0.390586562 | 0.0126424102235876 | 0.827809622372443 | 0.676659855361891 | 1.01272266333269 | 0.998735809224318 |
| ebi-fl187-GCST90094780 | prot-a-993 | Ethanolamine kinase 1 \|\| id:prot-a-993 | F-healthy breakfast food liking (derived food-liking factor) \|\| id:ebi-fl187-GCST90094780 | Inverse variance weighted | 8 | 0.10800218858199 | 0.109215693764545 | 0.322717496070423 | -0.106060571 | 0.322064948360498 | 1.11405018357397 | 0.899370170498715 | 1.37997440012156 | 0.998735809224318 |
| ebi-fl187-GCST90094780 | prot-b-10 | coagulation factor III, tissue factor \|\| id:prot-b-10 | F-healthy breakfast food liking (derived food-liking factor) \|\| id:ebi-fl187-GCST90094780 | Inverse variance weighted | 8 | -0.040578672 | 0.102376933911341 | 0.691835502621687 | -0.241237463 | 0.160080118258329 | 0.960233617840925 | 0.785655040488772 | 1.17360489440537 | 0.998735809224318 |
| ebi-fl187-GCST90094780 | prot-b-28 | prolactin \|\| id:prot-b-28 | F-healthy breakfast food liking (derived food-liking factor) \|\| id:ebi-fl187-GCST90094780 | Inverse variance weighted (multiplicative random effects) | 8 | 0.0464219105962779 | 0.163168624268665 | 0.776025181924537 | -0.273388593 | 0.366232414162862 | 1.04751627595322 | 0.760797089778685 | 1.44229041242271 | 0.998735809224318 |
| ebi-fl187-GCST90094780 | prot-b-54 | matrix metallopeptidase 7 \|\| id:prot-b-54 | F-healthy breakfast food liking (derived food-liking factor) \|\| id:ebi-fl187-GCST90094780 | Inverse variance weighted | 8 | -0.043610814 | 0.102376933911341 | 0.670120368411839 | -0.244269605 | 0.157047975981691 | 0.957326462574352 | 0.783276430589857 | 1.17005174693557 | 0.998735809224318 |
| ebi-fl187-GCST90094780 | ukb-rsfMRI191-edge-pheno716 | Net100_Pair10_33 (OccipitalPrecuneus)&(Temporal\|Frontal\|Supp_Motor_Area) (Default_mode\|Central_executive)&(Default_mode\|Salience) \|\| id:ukb-rsfMRI191-edge-pheno716 | F-healthy breakfast food liking (derived food-liking factor) \|\| id:ebi-fl187-GCST90094780 | Inverse variance weighted | 8 | 0.053243169200466 | 0.0297805962218596 | 0.0738005895393502 | -0.005126799 | 0.111613137795311 | 1.05468608111051 | 0.994886320211532 | 1.11808023398265 | 0.998735809224318 |
| ebi-fl187-GCST90094780 | ukb-rsfMRI191-edge-pheno903 | Net100_Pair27_38 (Precentral\|Frontal\|Supp_Motor_Area)&(Subcortical) (Attention\|Salience\|Motor)&(Subcortical-cerebellum) \|\| id:ukb-rsfMRI191-edge-pheno903 | F-healthy breakfast food liking (derived food-liking factor) \|\| id:ebi-fl187-GCST90094780 | Inverse variance weighted | 8 | -0.029281663 | 0.0295696115502342 | 0.322046064645304 | -0.087238102 | 0.0286747753337104 | 0.971142890624601 | 0.9164588590177 | 1.0290898546407 | 0.998735809224318 |
| ebi-fl187-GCST90094780 | ukb-rsfMRI191-node-pheno23 | Net100_Node2 RolandicOper\|SupraMarginal\|Insula Salience\|Motor \|\| id:ukb-rsfMRI191-node-pheno23 | F-healthy breakfast food liking (derived food-liking factor) \|\| id:ebi-fl187-GCST90094780 | Inverse variance weighted (multiplicative random effects) | 8 | -7.59E-05 | 0.0433488330116491 | 0.998603273154704 | -0.085039596 | 0.0848878289062569 | 0.999924119082527 | 0.918475915250382 | 1.08859495096331 | 0.999882738470636 |
| ebi-fl187-GCST90094780 | ukb-rsfMRI191-node-pheno55 | Net100_Node34 Parietal\|Postcentral\|Precuneus Attention \|\| id:ukb-rsfMRI191-node-pheno55 | F-healthy breakfast food liking (derived food-liking factor) \|\| id:ebi-fl187-GCST90094780 | Inverse variance weighted | 8 | 0.00128739877991308 | 0.0283681377545459 | 0.963802941851293 | -0.054314151 | 0.056888948778823 | 1.00128822783346 | 0.947134516297234 | 1.05853825190258 | 0.998735809224318 |
| ebi-fl187-GCST90094780 | ebi-a-GCST90001414 | IgD- CD24- B cell Absolute Count \|\| id:ebi-a-GCST90001414 | F-healthy breakfast food liking (derived food-liking factor) \|\| id:ebi-fl187-GCST90094780 | MR Egger | 8 | -0.868533886 | 1.01401804497409 | 0.424587997490699 | -2.856009254 | 1.11894148197078 | 0.419566230390711 | 0.0574977619624984 | 3.06161171628013 | 0.999950574162623 |
| ebi-fl187-GCST90094780 | ebi-a-GCST90001422 | CD20- CD38- B cell %B cell \|\| id:ebi-a-GCST90001422 | F-healthy breakfast food liking (derived food-liking factor) \|\| id:ebi-fl187-GCST90094780 | MR Egger | 8 | -0.872019689 | 0.986798059010373 | 0.410872514054972 | -2.806143884 | 1.06210450682076 | 0.418106251385972 | 0.0604375979855078 | 2.89245177298323 | 0.999950574162623 |
| ebi-fl187-GCST90094780 | ebi-a-GCST90001444 | CD20- CD38- B cell %lymphocyte \|\| id:ebi-a-GCST90001444 | F-healthy breakfast food liking (derived food-liking factor) \|\| id:ebi-fl187-GCST90094780 | MR Egger | 8 | -1.036310029 | 1.08333315311845 | 0.375711129546789 | -3.159643009 | 1.08702295089685 | 0.354761328670418 | 0.0424408893780914 | 2.96543267976304 | 0.999950574162623 |
| ebi-fl187-GCST90094780 | ebi-a-GCST90001459 | Myeloid Dendritic Cell %Dendritic Cell \|\| id:ebi-a-GCST90001459 | F-healthy breakfast food liking (derived food-liking factor) \|\| id:ebi-fl187-GCST90094780 | MR Egger | 8 | 0.892068335216241 | 0.892447830110232 | 0.356107497344062 | -0.857129412 | 2.64126608223229 | 2.44017152805195 | 0.424378551553797 | 14.0309567119123 | 0.999950574162623 |
| ebi-fl187-GCST90094780 | ebi-a-GCST90001476 | HLA DR++ monocyte %leukocyte \|\| id:ebi-a-GCST90001476 | F-healthy breakfast food liking (derived food-liking factor) \|\| id:ebi-fl187-GCST90094780 | MR Egger | 8 | 0.0912278607613033 | 1.08108462401494 | 0.935495106816131 | -2.027698002 | 2.21015372383058 | 1.09551860252347 | 0.131638203468394 | 9.1171178035951 | 0.999950574162623 |
| ebi-fl187-GCST90094780 | ebi-a-GCST90001478 | CD4 regulatory T cell %CD4+ T cell \|\| id:ebi-a-GCST90001478 | F-healthy breakfast food liking (derived food-liking factor) \|\| id:ebi-fl187-GCST90094780 | MR Egger | 8 | -0.033650817 | 0.869316263164486 | 0.9703778246038 | -1.737510693 | 1.67020905833081 | 0.966909073452528 | 0.1759578690014 | 5.31327846620709 | 0.999950574162623 |
| ebi-fl187-GCST90094780 | ebi-a-GCST90001485 | CD39+ resting CD4 regulatory T cell %CD4 regulatory T cell \|\| id:ebi-a-GCST90001485 | F-healthy breakfast food liking (derived food-liking factor) \|\| id:ebi-fl187-GCST90094780 | MR Egger | 8 | 0.221650677799124 | 0.72222456303216 | 0.769291735904524 | -1.193909466 | 1.63721082134216 | 1.24813530033283 | 0.303034243276685 | 5.14081085718932 | 0.999950574162623 |
| ebi-fl187-GCST90094780 | ebi-a-GCST90001515 | Immature Myeloid-Derived Suppressor Cells Absolute Count \|\| id:ebi-a-GCST90001515 | F-healthy breakfast food liking (derived food-liking factor) \|\| id:ebi-fl187-GCST90094780 | MR Egger | 8 | -0.172690938 | 1.06815447656746 | 0.876870751812089 | -2.266273712 | 1.92089183653033 | 0.841397619080174 | 0.103697869208036 | 6.82704436263312 | 0.999950574162623 |
| ebi-fl187-GCST90094780 | ebi-a-GCST90001517 | CD33+ HLA DR+ Absolute Count \|\| id:ebi-a-GCST90001517 | F-healthy breakfast food liking (derived food-liking factor) \|\| id:ebi-fl187-GCST90094780 | MR Egger | 8 | -1.08354586 | 0.94243630369927 | 0.294011475081472 | -2.930721015 | 0.763629295162639 | 0.338393499791699 | 0.0533585519379042 | 2.14605075554777 | 0.999950574162623 |
| ebi-fl187-GCST90094780 | ebi-a-GCST90001518 | CD33+ HLA DR+ CD14- Absolute Count \|\| id:ebi-a-GCST90001518 | F-healthy breakfast food liking (derived food-liking factor) \|\| id:ebi-fl187-GCST90094780 | MR Egger | 8 | -1.085438291 | 1.07752132145845 | 0.352650694318795 | -3.197380081 | 1.02650349879672 | 0.337753718945192 | 0.0408691376555946 | 2.79128900694315 | 0.999950574162623 |
| ebi-fl187-GCST90094780 | ebi-a-GCST90001520 | CD33+ HLA DR+ CD14dim Absolute Count \|\| id:ebi-a-GCST90001520 | F-healthy breakfast food liking (derived food-liking factor) \|\| id:ebi-fl187-GCST90094780 | MR Egger | 8 | -0.606305997 | 0.945408397429239 | 0.545020303562036 | -2.459306456 | 1.2466944614989 | 0.545361720321852 | 0.085494224380726 | 3.47882454220452 | 0.999950574162623 |
| ebi-fl187-GCST90094780 | ebi-a-GCST90001531 | CD33dim HLA DR- Absolute Count \|\| id:ebi-a-GCST90001531 | F-healthy breakfast food liking (derived food-liking factor) \|\| id:ebi-fl187-GCST90094780 | MR Egger | 8 | -0.568049527 | 1.57458335207527 | 0.730638762999565 | -3.654232897 | 2.51813384304166 | 0.566629557210522 | 0.0258813435240584 | 12.4054245795292 | 0.999950574162623 |
| ebi-fl187-GCST90094780 | ebi-a-GCST90001532 | Basophil Absolute Count \|\| id:ebi-a-GCST90001532 | F-healthy breakfast food liking (derived food-liking factor) \|\| id:ebi-fl187-GCST90094780 | MR Egger | 8 | -0.589176843 | 1.62956969018654 | 0.730076591777526 | -3.783133436 | 2.60477974977272 | 0.554783770977315 | 0.0227512899121241 | 13.5282453754762 | 0.999950574162623 |
| ebi-fl187-GCST90094780 | ebi-a-GCST90001588 | T/B cell \|\| id:ebi-a-GCST90001588 | F-healthy breakfast food liking (derived food-liking factor) \|\| id:ebi-fl187-GCST90094780 | MR Egger | 8 | -0.29780244 | 0.719023829757008 | 0.693143999934057 | -1.707089146 | 1.11148426638819 | 0.742448003335256 | 0.181393033474361 | 3.03886553468119 | 0.999950574162623 |
| ebi-fl187-GCST90094780 | ebi-a-GCST90001624 | HLA DR+ CD4+ T cell Absolute Count \|\| id:ebi-a-GCST90001624 | F-healthy breakfast food liking (derived food-liking factor) \|\| id:ebi-fl187-GCST90094780 | MR Egger | 8 | -0.322931375 | 0.851409443355657 | 0.717532948570439 | -1.991693884 | 1.34583113404799 | 0.724023538829783 | 0.136464075259807 | 3.84137791416228 | 0.999950574162623 |
| ebi-fl187-GCST90094780 | ebi-a-GCST90001631 | CD8+ Natural Killer T %T cell \|\| id:ebi-a-GCST90001631 | F-healthy breakfast food liking (derived food-liking factor) \|\| id:ebi-fl187-GCST90094780 | MR Egger | 8 | -1.584290029 | 0.792011677030617 | 0.0923831990608516 | -3.136632916 | -0.031947142 | 0.205093351818301 | 0.0434287803586897 | 0.96855777695468 | 0.999950574162623 |
| ebi-fl187-GCST90094780 | ebi-a-GCST90001632 | CD8+ Natural Killer T %lymphocyte \|\| id:ebi-a-GCST90001632 | F-healthy breakfast food liking (derived food-liking factor) \|\| id:ebi-fl187-GCST90094780 | MR Egger | 8 | -1.574610255 | 0.738831299666242 | 0.0770796161205497 | -3.022719602 | -0.126500907 | 0.20708824864125 | 0.0486686787813864 | 0.881173350070514 | 0.999950574162623 |
| ebi-fl187-GCST90094780 | ebi-a-GCST90001646 | Natural Killer %CD3- lymphocyte \|\| id:ebi-a-GCST90001646 | F-healthy breakfast food liking (derived food-liking factor) \|\| id:ebi-fl187-GCST90094780 | MR Egger | 8 | 0.239220267936433 | 0.693394469201714 | 0.741867634298254 | -1.119832892 | 1.59827342757179 | 1.27025830294605 | 0.326334323241159 | 4.94448803355256 | 0.999950574162623 |
| ebi-fl187-GCST90094780 | ebi-a-GCST90001684 | CD127- CD8+ T cell Absolute Count \|\| id:ebi-a-GCST90001684 | F-healthy breakfast food liking (derived food-liking factor) \|\| id:ebi-fl187-GCST90094780 | MR Egger | 8 | -1.074517092 | 0.867166014520898 | 0.261574132723818 | -2.77416248 | 0.625128296780452 | 0.341462610628253 | 0.0624017174878695 | 1.86848566275003 | 0.999950574162623 |
| ebi-fl187-GCST90094780 | ebi-a-GCST90001687 | CD28- CD8+ T cell Absolute Count \|\| id:ebi-a-GCST90001687 | F-healthy breakfast food liking (derived food-liking factor) \|\| id:ebi-fl187-GCST90094780 | MR Egger | 8 | -0.850052888 | 0.729470064201175 | 0.288123856183681 | -2.279814214 | 0.579708437549258 | 0.427392327303739 | 0.102303211442309 | 1.78551776491508 | 0.999950574162623 |
| ebi-fl187-GCST90094780 | ebi-a-GCST90001701 | BAFF-R on CD20- CD38- B cell \|\| id:ebi-a-GCST90001701 | F-healthy breakfast food liking (derived food-liking factor) \|\| id:ebi-fl187-GCST90094780 | MR Egger | 8 | -0.65035364 | 0.700355347931082 | 0.388924287348709 | -2.023050122 | 0.722342842029829 | 0.521861193176834 | 0.132251466182374 | 2.0592520658213 | 0.999950574162623 |
| ebi-fl187-GCST90094780 | ebi-a-GCST90001732 | CD19 on IgD- CD27- B cell \|\| id:ebi-a-GCST90001732 | F-healthy breakfast food liking (derived food-liking factor) \|\| id:ebi-fl187-GCST90094780 | MR Egger | 8 | 0.577061543280198 | 0.797983922032438 | 0.496798578705586 | -0.986986944 | 2.14111003046378 | 1.78079793737366 | 0.372697960812392 | 8.50887750188316 | 0.999950574162623 |
| ebi-fl187-GCST90094780 | ebi-a-GCST90001798 | CD27 on CD24+ CD27+ B cell \|\| id:ebi-a-GCST90001798 | F-healthy breakfast food liking (derived food-liking factor) \|\| id:ebi-fl187-GCST90094780 | MR Egger | 8 | -0.091690862 | 0.880777372077531 | 0.920480863703849 | -1.818014511 | 1.6346327871231 | 0.912387159358741 | 0.162347770789478 | 5.12757473979844 | 0.999950574162623 |
| ebi-fl187-GCST90094780 | ebi-a-GCST90001836 | CD66b on CD66b++ myeloid cell \|\| id:ebi-a-GCST90001836 | F-healthy breakfast food liking (derived food-liking factor) \|\| id:ebi-fl187-GCST90094780 | MR Egger | 8 | -0.309992154 | 1.06401913689497 | 0.780600319749083 | -2.395469662 | 1.77548535429879 | 0.733452710860417 | 0.0911298686038117 | 5.90314555820608 | 0.999950574162623 |
| ebi-fl187-GCST90094780 | ebi-a-GCST90001943 | CD25 on activated & secreting CD4 regulatory T cell \|\| id:ebi-a-GCST90001943 | F-healthy breakfast food liking (derived food-liking factor) \|\| id:ebi-fl187-GCST90094780 | MR Egger | 8 | 0.385283594354132 | 0.751907005896332 | 0.626675025329614 | -1.088454137 | 1.85902132591094 | 1.47003115486847 | 0.336736640210182 | 6.41745310202977 | 0.999950574162623 |
| ebi-fl187-GCST90094780 | ebi-a-GCST90001960 | CD25 on CD4+ T cell \|\| id:ebi-a-GCST90001960 | F-healthy breakfast food liking (derived food-liking factor) \|\| id:ebi-fl187-GCST90094780 | MR Egger | 8 | -0.296060814 | 0.804042991936015 | 0.725354467794541 | -1.871985078 | 1.27986345036979 | 0.743742196837196 | 0.153818017785783 | 3.59614863927433 | 0.999950574162623 |
| ebi-fl187-GCST90094780 | ebi-a-GCST90001988 | HLA DR on CD14+ CD16- monocyte \|\| id:ebi-a-GCST90001988 | F-healthy breakfast food liking (derived food-liking factor) \|\| id:ebi-fl187-GCST90094780 | MR Egger | 8 | -0.300237064 | 0.720875000192677 | 0.691540184741816 | -1.713152064 | 1.11267793643989 | 0.740642620212254 | 0.180296589559936 | 3.04249510328379 | 0.999950574162623 |
| ebi-fl187-GCST90094780 | ebi-a-GCST90001991 | HLA DR on CD14+ monocyte \|\| id:ebi-a-GCST90001991 | F-healthy breakfast food liking (derived food-liking factor) \|\| id:ebi-fl187-GCST90094780 | MR Egger | 8 | -0.357353144 | 0.722277100650117 | 0.638362724219689 | -1.773016261 | 1.05830997372109 | 0.699525421217681 | 0.169819994155399 | 2.88149706613458 | 0.999950574162623 |
| ebi-fl187-GCST90094780 | ebi-a-GCST90002019 | CD14 on Monocytic Myeloid-Derived Suppressor Cells \|\| id:ebi-a-GCST90002019 | F-healthy breakfast food liking (derived food-liking factor) \|\| id:ebi-fl187-GCST90094780 | MR Egger | 8 | -1.10016869 | 1.02967975415721 | 0.326404502531369 | -3.118341008 | 0.918003628004087 | 0.332814936362903 | 0.044230485595419 | 2.50428590993619 | 0.999950574162623 |
| ebi-fl187-GCST90094780 | ebi-a-GCST90002037 | CD80 on plasmacytoid Dendritic Cell \|\| id:ebi-a-GCST90002037 | F-healthy breakfast food liking (derived food-liking factor) \|\| id:ebi-fl187-GCST90094780 | MR Egger | 8 | 0.401707377786368 | 0.799647377948694 | 0.633317936198232 | -1.165601483 | 1.96901623856581 | 1.49437398166734 | 0.311735102268939 | 7.16362572206492 | 0.999950574162623 |
| ebi-fl187-GCST90094780 | ebi-a-GCST90002042 | CD45 on CD33+ HLA DR+ CD14- \|\| id:ebi-a-GCST90002042 | F-healthy breakfast food liking (derived food-liking factor) \|\| id:ebi-fl187-GCST90094780 | MR Egger | 8 | -2.118035863 | 1.04639214278725 | 0.0893873327047642 | -4.168964463 | -0.067107263 | 0.120267618790546 | 0.0154682698053219 | 0.935094895007037 | 0.999950574162623 |
| ebi-fl187-GCST90094780 | ebi-a-GCST90002056 | CD8 on Effector Memory CD8+ T cell \|\| id:ebi-a-GCST90002056 | F-healthy breakfast food liking (derived food-liking factor) \|\| id:ebi-fl187-GCST90094780 | MR Egger | 8 | 0.458607966025916 | 0.783344925792476 | 0.579583559380314 | -1.076748089 | 1.99396402057917 | 1.58187043425448 | 0.340701657777964 | 7.34459024088234 | 0.999950574162623 |
| ebi-fl187-GCST90094780 | ebi-a-GCST90002058 | CD8 on CD8+ T cell \|\| id:ebi-a-GCST90002058 | F-healthy breakfast food liking (derived food-liking factor) \|\| id:ebi-fl187-GCST90094780 | MR Egger | 8 | 0.835955246780623 | 0.756186685027043 | 0.311294155881949 | -0.646170656 | 2.31808114943363 | 2.30701676638876 | 0.524048702188413 | 10.1561674290442 | 0.999950574162623 |
| ebi-fl187-GCST90094780 | ebi-a-GCST90002063 | CD4 on CD4 regulatory T cell \|\| id:ebi-a-GCST90002063 | F-healthy breakfast food liking (derived food-liking factor) \|\| id:ebi-fl187-GCST90094780 | MR Egger | 8 | -0.362580509 | 0.796409104356005 | 0.664925658300339 | -1.923542354 | 1.19838133539007 | 0.695878286855556 | 0.146088547164415 | 3.31474711410491 | 0.999950574162623 |
| ebi-fl187-GCST90094780 | ebi-a-GCST90002066 | CD4 on activated CD4 regulatory T cell \|\| id:ebi-a-GCST90002066 | F-healthy breakfast food liking (derived food-liking factor) \|\| id:ebi-fl187-GCST90094780 | MR Egger | 8 | -0.61859124 | 0.955555622662512 | 0.541358279011754 | -2.49148026 | 1.25429778039825 | 0.538702806243954 | 0.082787329021822 | 3.50537596615318 | 0.999950574162623 |
| ebi-fl187-GCST90094780 | ebi-a-GCST90002070 | CD4 on activated & secreting CD4 regulatory T cell \|\| id:ebi-a-GCST90002070 | F-healthy breakfast food liking (derived food-liking factor) \|\| id:ebi-fl187-GCST90094780 | MR Egger | 8 | -0.471299972 | 0.843865667718811 | 0.596712485167915 | -2.125276681 | 1.18267673656108 | 0.624190310604665 | 0.119399928016983 | 3.26309697437449 | 0.999950574162623 |
| ebi-fl187-GCST90094780 | ebi-a-GCST90002081 | SSC-A on CD4+ T cell \|\| id:ebi-a-GCST90002081 | F-healthy breakfast food liking (derived food-liking factor) \|\| id:ebi-fl187-GCST90094780 | MR Egger | 8 | -0.678567351 | 0.740258526685356 | 0.394670186874765 | -2.129474063 | 0.772339361201514 | 0.507343316801241 | 0.11889981117272 | 2.16482464155452 | 0.999950574162623 |
| ebi-fl187-GCST90094780 | ebi-a-GCST90002094 | CD11b on Monocytic Myeloid-Derived Suppressor Cells \|\| id:ebi-a-GCST90002094 | F-healthy breakfast food liking (derived food-liking factor) \|\| id:ebi-fl187-GCST90094780 | MR Egger | 8 | -0.82322748 | 1.04215049729111 | 0.459638760431165 | -2.865842455 | 1.2193874945779 | 0.43901246154056 | 0.0569351456351526 | 3.38511369801268 | 0.999950574162623 |
| ebi-fl187-GCST90094780 | ebi-a-GCST90002104 | HLA DR on myeloid Dendritic Cell \|\| id:ebi-a-GCST90002104 | F-healthy breakfast food liking (derived food-liking factor) \|\| id:ebi-fl187-GCST90094780 | MR Egger | 8 | -0.542523401 | 0.793680047287452 | 0.519766862333594 | -2.098136294 | 1.01308949154576 | 0.581279598551664 | 0.122684863857272 | 2.75409664296868 | 0.999950574162623 |
| ebi-fl187-GCST90094780 | ebi-a-GCST90002105 | HLA DR on plasmacytoid Dendritic Cell \|\| id:ebi-a-GCST90002105 | F-healthy breakfast food liking (derived food-liking factor) \|\| id:ebi-fl187-GCST90094780 | MR Egger | 8 | -1.493495299 | 0.812018283984808 | 0.115499978648822 | -3.085051136 | 0.0980605372341858 | 0.224586285736694 | 0.0457276955096215 | 1.10302955744601 | 0.999950574162623 |
| ebi-fl187-GCST90094780 | ebi-a-GCST90002106 | HLA DR on Dendritic Cell \|\| id:ebi-a-GCST90002106 | F-healthy breakfast food liking (derived food-liking factor) \|\| id:ebi-fl187-GCST90094780 | MR Egger | 8 | -1.057621696 | 0.791994273066451 | 0.230175229701881 | -2.609930471 | 0.494687079678585 | 0.34728076834366 | 0.0735396567430783 | 1.6399849741305 | 0.999950574162623 |
| ebi-fl187-GCST90094780 | ebi-a-GCST90002111 | HLA DR on CD33dim HLA DR+ CD11b- \|\| id:ebi-a-GCST90002111 | F-healthy breakfast food liking (derived food-liking factor) \|\| id:ebi-fl187-GCST90094780 | MR Egger | 8 | -2.186123429 | 1.01742015653868 | 0.0752419144948362 | -4.180266936 | -0.191979923 | 0.112351443822446 | 0.015294424387211 | 0.825323438752179 | 0.999950574162623 |
| ebi-fl187-GCST90094780 | ebi-a-GCST90002112 | HLA DR on CD33- HLA DR+ \|\| id:ebi-a-GCST90002112 | F-healthy breakfast food liking (derived food-liking factor) \|\| id:ebi-fl187-GCST90094780 | MR Egger | 8 | -0.69610804 | 1.53003688290979 | 0.665134715642185 | -3.69498033 | 2.30276425059485 | 0.498521759836341 | 0.0248479424064842 | 10.0017917365048 | 0.999950574162623 |
| ebi-fl187-GCST90094780 | ebi-a-GCST90002115 | HLA DR on HLA DR+ CD8+ T cell \|\| id:ebi-a-GCST90002115 | F-healthy breakfast food liking (derived food-liking factor) \|\| id:ebi-fl187-GCST90094780 | MR Egger | 8 | 0.332505244692463 | 0.730151718765802 | 0.664842180146573 | -1.098592124 | 1.76360261347344 | 1.39445721266497 | 0.333340054927636 | 5.83341512431052 | 0.999950574162623 |
| ebi-fl187-GCST90094780 | ebi-a-GCST90002116 | HLA DR on B cell \|\| id:ebi-a-GCST90002116 | F-healthy breakfast food liking (derived food-liking factor) \|\| id:ebi-fl187-GCST90094780 | MR Egger | 8 | -0.065945194 | 0.769172623104038 | 0.934466277118624 | -1.573523536 | 1.44163314683392 | 0.936182170864445 | 0.207313417679304 | 4.22759446472605 | 0.999950574162623 |
| ebi-fl187-GCST90094780 | ebi-cfmet338-GCST90026023 | Sphingomyelin (d18:1/24:1, d18:2/24:0) levels \|\| id:ebi-cfmet338-GCST90026023 | F-healthy breakfast food liking (derived food-liking factor) \|\| id:ebi-fl187-GCST90094780 | MR Egger | 6 | -0.86106162 | 0.480571511922066 | 0.147650300709361 | -1.802981783 | 0.0808585432653666 | 0.422713083320156 | 0.164806736838295 | 1.08421751584922 | 0.999950574162623 |
| ebi-fl187-GCST90094780 | ebi-cfmet338-GCST90026046 | 1-arachidonoyl-gpc (20:4n6) levels \|\| id:ebi-cfmet338-GCST90026046 | F-healthy breakfast food liking (derived food-liking factor) \|\| id:ebi-fl187-GCST90094780 | MR Egger | 6 | -1.54904621 | 0.563172235791867 | 0.0513442031667293 | -2.652863792 | -0.445228627 | 0.212450510488991 | 0.0704491721418205 | 0.640677782787448 | 0.999950574162623 |
| ebi-fl187-GCST90094780 | ebi-cfmet338-GCST90026047 | 1-linoleoyl-gpc (18:2) levels \|\| id:ebi-cfmet338-GCST90026047 | F-healthy breakfast food liking (derived food-liking factor) \|\| id:ebi-fl187-GCST90094780 | MR Egger | 6 | -1.182125285 | 0.697453169354742 | 0.165337003853763 | -2.549133497 | 0.184882927223723 | 0.306626377268126 | 0.0781493533479829 | 1.2030775842498 | 0.999950574162623 |
| ebi-fl187-GCST90094780 | ebi-cfmet338-GCST90026050 | 1-myristoyl-2-palmitoyl-gpc (14:0/16:0) levels \|\| id:ebi-cfmet338-GCST90026050 | F-healthy breakfast food liking (derived food-liking factor) \|\| id:ebi-fl187-GCST90094780 | MR Egger | 6 | -0.68655329 | 0.359966821684312 | 0.129151900551863 | -1.39208826 | 0.0189816806595043 | 0.503307839138768 | 0.248555713260434 | 1.01916297805278 | 0.999950574162623 |
| ebi-fl187-GCST90094780 | ebi-cfmet338-GCST90026068 | 2,3-dihydroxyisovalerate levels \|\| id:ebi-cfmet338-GCST90026068 | F-healthy breakfast food liking (derived food-liking factor) \|\| id:ebi-fl187-GCST90094780 | MR Egger | 6 | -0.032762969 | 1.22300047365824 | 0.979911247909156 | -2.429843897 | 2.36431795978504 | 0.967767923804965 | 0.0880505764726927 | 10.636781630115 | 0.999950574162623 |
| ebi-fl187-GCST90094780 | ebi-cfmet338-GCST90026082 | 3-hydroxyhexanoate levels \|\| id:ebi-cfmet338-GCST90026082 | F-healthy breakfast food liking (derived food-liking factor) \|\| id:ebi-fl187-GCST90094780 | MR Egger | 6 | -0.475214523 | 0.248770450801369 | 0.128708372618182 | -0.962804606 | 0.0123755609317432 | 0.62175166235013 | 0.381820526685163 | 1.01245245506119 | 0.999950574162623 |
| ebi-fl187-GCST90094780 | ebi-cfmet338-GCST90026094 | 3-ureidopropionate levels \|\| id:ebi-cfmet338-GCST90026094 | F-healthy breakfast food liking (derived food-liking factor) \|\| id:ebi-fl187-GCST90094780 | MR Egger | 6 | -1.201964579 | 0.620784085276441 | 0.124921135781186 | -2.418701386 | 0.014772228 | 0.300603072897535 | 0.0890371673001623 | 1.01488187658544 | 0.999950574162623 |
| ebi-fl187-GCST90094780 | ebi-cfmet338-GCST90026173 | Glycerol levels \|\| id:ebi-cfmet338-GCST90026173 | F-healthy breakfast food liking (derived food-liking factor) \|\| id:ebi-fl187-GCST90094780 | MR Egger | 6 | -0.552866403 | 0.32360197293746 | 0.162730828533646 | -1.18712627 | 0.0813934640281945 | 0.575298407677465 | 0.305096771257546 | 1.08479764145666 | 0.999950574162623 |
| ebi-fl187-GCST90094780 | ebi-cfmet338-GCST90026179 | Histidine levels \|\| id:ebi-cfmet338-GCST90026179 | F-healthy breakfast food liking (derived food-liking factor) \|\| id:ebi-fl187-GCST90094780 | MR Egger | 6 | 0.0984624733182201 | 0.222948660132769 | 0.681576199646788 | -0.338516901 | 0.535441847178448 | 1.1034729939375 | 0.712826732128955 | 1.70820284013859 | 0.999950574162623 |
| ebi-fl187-GCST90094780 | ebi-cfmet338-GCST90026188 | Isocitrate levels \|\| id:ebi-cfmet338-GCST90026188 | F-healthy breakfast food liking (derived food-liking factor) \|\| id:ebi-fl187-GCST90094780 | MR Egger | 6 | -0.571849416 | 0.437904470119525 | 0.261631329406679 | -1.430142177 | 0.286443345508392 | 0.564480513495208 | 0.239274900351559 | 1.33168271994952 | 0.999950574162623 |
| ebi-fl187-GCST90094780 | ebi-cfmet338-GCST90026207 | N-acetyl-isoputreanine levels \|\| id:ebi-cfmet338-GCST90026207 | F-healthy breakfast food liking (derived food-liking factor) \|\| id:ebi-fl187-GCST90094780 | MR Egger | 6 | -0.134102716 | 0.279138960821056 | 0.656024703071463 | -0.681215079 | 0.413009647301273 | 0.874500234904273 | 0.506001786475321 | 1.51135960640512 | 0.999950574162623 |
| ebi-fl187-GCST90094780 | ebi-cfmet338-GCST90026213 | N-acetylglutamate levels \|\| id:ebi-cfmet338-GCST90026213 | F-healthy breakfast food liking (derived food-liking factor) \|\| id:ebi-fl187-GCST90094780 | MR Egger | 6 | -0.002371491 | 0.476347399591002 | 0.996266151157992 | -0.936012394 | 0.931269412037826 | 0.997631318603075 | 0.392188614936929 | 2.53772855700507 | 0.999950574162623 |
| ebi-fl187-GCST90094780 | ebi-cfmet338-GCST90026224 | N-acetyltaurine levels \|\| id:ebi-cfmet338-GCST90026224 | F-healthy breakfast food liking (derived food-liking factor) \|\| id:ebi-fl187-GCST90094780 | MR Egger | 6 | 0.259755918737646 | 0.383697178393124 | 0.535548030470381 | -0.492290551 | 1.01180238838817 | 1.29661356896263 | 0.611224748121624 | 2.75055411677058 | 0.999950574162623 |
| ebi-fl187-GCST90094780 | ebi-cfmet338-GCST90026250 | Phenyllactate (pla) levels \|\| id:ebi-cfmet338-GCST90026250 | F-healthy breakfast food liking (derived food-liking factor) \|\| id:ebi-fl187-GCST90094780 | MR Egger | 6 | 0.247765675808718 | 0.599339450572395 | 0.700516910687476 | -0.926939647 | 1.42247099893061 | 1.28115969031009 | 0.395763033412891 | 4.14735590113351 | 0.999950574162623 |
| ebi-fl187-GCST90094780 | ebi-cfmet338-GCST90026251 | Pipecolate levels \|\| id:ebi-cfmet338-GCST90026251 | F-healthy breakfast food liking (derived food-liking factor) \|\| id:ebi-fl187-GCST90094780 | MR Egger | 6 | 0.720898364125445 | 0.904094431561666 | 0.469889385116912 | -1.051126722 | 2.49292344998631 | 2.05627966901025 | 0.349543688683101 | 12.0965882494256 | 0.999950574162623 |
| ebi-fl187-GCST90094780 | ebi-cfmet338-GCST90026278 | Trigonelline (n'-methylnicotinate) levels \|\| id:ebi-cfmet338-GCST90026278 | F-healthy breakfast food liking (derived food-liking factor) \|\| id:ebi-fl187-GCST90094780 | MR Egger | 6 | -0.626573556 | 1.00931228494353 | 0.56835955772555 | -2.604825634 | 1.35167852249789 | 0.534419827018359 | 0.0739160244799637 | 3.86390574330403 | 0.999950574162623 |
| ebi-fl187-GCST90094780 | ebi-cfmet338-GCST90026299 | 1-carboxyethylvaline levels \|\| id:ebi-cfmet338-GCST90026299 | F-healthy breakfast food liking (derived food-liking factor) \|\| id:ebi-fl187-GCST90094780 | MR Egger | 6 | 0.120382531870965 | 0.67493168440513 | 0.867107357642447 | -1.20248357 | 1.44324863330502 | 1.1279282375637 | 0.300447103267085 | 4.23442960594166 | 0.999950574162623 |
| ebi-fl187-GCST90094780 | ebi-gm430-GCST90011306 | C_Erysipelotrichia abundance \|\| id:ebi-gm430-GCST90011306 | F-healthy breakfast food liking (derived food-liking factor) \|\| id:ebi-fl187-GCST90094780 | MR Egger | 6 | -0.241783337 | 0.470707401120063 | 0.634561636181131 | -1.164369843 | 0.680803169206279 | 0.78522628861475 | 0.31211928416896 | 1.9754637268677 | 0.999950574162623 |
| ebi-fl187-GCST90094780 | ebi-gm430-GCST90011312 | F_Erysipelotrichaceae abundance \|\| id:ebi-gm430-GCST90011312 | F-healthy breakfast food liking (derived food-liking factor) \|\| id:ebi-fl187-GCST90094780 | MR Egger | 6 | -0.241783337 | 0.470707401120063 | 0.634561636181131 | -1.164369843 | 0.680803169206279 | 0.78522628861475 | 0.31211928416896 | 1.9754637268677 | 0.999950574162623 |
| ebi-fl187-GCST90094780 | ebi-gm430-GCST90011352 | O_Erysipelotrichales abundance \|\| id:ebi-gm430-GCST90011352 | F-healthy breakfast food liking (derived food-liking factor) \|\| id:ebi-fl187-GCST90094780 | MR Egger | 6 | -0.241783337 | 0.470707401120063 | 0.634561636181131 | -1.164369843 | 0.680803169206279 | 0.78522628861475 | 0.31211928416896 | 1.9754637268677 | 0.999950574162623 |
| ebi-fl187-GCST90094780 | ebi-gm430-GCST90011362 | OTU97_11 (Parabacteroides) abundance \|\| id:ebi-gm430-GCST90011362 | F-healthy breakfast food liking (derived food-liking factor) \|\| id:ebi-fl187-GCST90094780 | MR Egger | 6 | -0.114886284 | 0.609943898391364 | 0.859767878178317 | -1.310376325 | 1.08060375676676 | 0.891467512191147 | 0.269718535475182 | 2.94645795807903 | 0.999950574162623 |
| ebi-fl187-GCST90094780 | ebi-gm430-GCST90011384 | OTU97_235 (Phascolarctobacterium) abundance \|\| id:ebi-gm430-GCST90011384 | F-healthy breakfast food liking (derived food-liking factor) \|\| id:ebi-fl187-GCST90094780 | MR Egger | 6 | 0.261332699668591 | 1.86190723652539 | 0.895161673653725 | -3.388005484 | 3.91067088325835 | 1.29865965720551 | 0.0337759764903925 | 49.9324395767761 | 0.999950574162623 |
| ebi-fl187-GCST90094780 | ebi-gm430-GCST90011390 | OTU97_3 (Bacteroides) abundance \|\| id:ebi-gm430-GCST90011390 | F-healthy breakfast food liking (derived food-liking factor) \|\| id:ebi-fl187-GCST90094780 | MR Egger | 6 | -0.327401396 | 0.673874786469428 | 0.65248836144051 | -1.648195977 | 0.993393185564628 | 0.720794361039717 | 0.192396683698083 | 2.70038184089464 | 0.999950574162623 |
| ebi-fl187-GCST90094780 | ebi-gm430-GCST90011398 | OTU97_4 (Alistipes) abundance \|\| id:ebi-gm430-GCST90011398 | F-healthy breakfast food liking (derived food-liking factor) \|\| id:ebi-fl187-GCST90094780 | MR Egger | 6 | -0.575543013 | 0.569952790165277 | 0.369710675260895 | -1.692650481 | 0.541564456221537 | 0.562399395973319 | 0.184031106016021 | 1.71869358087551 | 0.999950574162623 |
| ebi-fl187-GCST90094780 | ebi-gm430-GCST90011421 | OTU99_108 (Cytophagales) abundance \|\| id:ebi-gm430-GCST90011421 | F-healthy breakfast food liking (derived food-liking factor) \|\| id:ebi-fl187-GCST90094780 | MR Egger | 6 | 3.47412980674281 | 1.75900602162297 | 0.119476194301878 | 0.0264780043617887 | 6.92178160912383 | 32.269735408994 | 1.02683166119556 | 1014.12516064613 | 0.999950574162623 |
| ebi-fl187-GCST90094780 | ebi-gm430-GCST90011428 | OTU99_123 (Phascolarctobacterium) abundance \|\| id:ebi-gm430-GCST90011428 | F-healthy breakfast food liking (derived food-liking factor) \|\| id:ebi-fl187-GCST90094780 | MR Egger | 6 | -0.202682816 | 1.08086518183119 | 0.860381570663687 | -2.321178573 | 1.9158129400514 | 0.816537192613021 | 0.09815783126245 | 6.79245841462892 | 0.999950574162623 |
| ebi-fl187-GCST90094780 | ebi-gm430-GCST90011450 | OTU99_298 (Phascolarctobacterium) abundance \|\| id:ebi-gm430-GCST90011450 | F-healthy breakfast food liking (derived food-liking factor) \|\| id:ebi-fl187-GCST90094780 | MR Egger | 6 | 0.281821589638263 | 1.86181346825225 | 0.887011628553293 | -3.367332808 | 3.93097598741267 | 1.32554220842446 | 0.0344814835356558 | 50.9566864922704 | 0.999950574162623 |
| ebi-fl187-GCST90094780 | ebi-gm430-GCST90011451 | OTU99_3 (Bacteroides) abundance \|\| id:ebi-gm430-GCST90011451 | F-healthy breakfast food liking (derived food-liking factor) \|\| id:ebi-fl187-GCST90094780 | MR Egger | 6 | -0.317755477 | 0.67392299504722 | 0.661850338517126 | -1.638644547 | 1.00313359352785 | 0.727780725956475 | 0.194243151385764 | 2.72681317871447 | 0.999950574162623 |
| ebi-fl187-GCST90094780 | ebi-gm430-GCST90011472 | OTU99_72 (Parabacteroides) abundance \|\| id:ebi-gm430-GCST90011472 | F-healthy breakfast food liking (derived food-liking factor) \|\| id:ebi-fl187-GCST90094780 | MR Egger | 6 | 0.576089440076522 | 1.44562059003203 | 0.710613020976505 | -2.257326916 | 3.4095057965393 | 1.77906765913441 | 0.104629795469782 | 30.2502907662864 | 0.999950574162623 |
| ebi-fl187-GCST90094780 | ebi-gm430-GCST90011507 | TestASV_3 (Bacteroides) abundance \|\| id:ebi-gm430-GCST90011507 | F-healthy breakfast food liking (derived food-liking factor) \|\| id:ebi-fl187-GCST90094780 | MR Egger | 6 | -0.391071329 | 0.624212189213284 | 0.564962948028088 | -1.61452722 | 0.83238456144622 | 0.676331911961226 | 0.198984724149911 | 2.29879382495971 | 0.999950574162623 |
| ebi-fl187-GCST90094780 | ebi-gm430-GCST90011512 | TestASV_34 (Phascolarctobacterium) abundance \|\| id:ebi-gm430-GCST90011512 | F-healthy breakfast food liking (derived food-liking factor) \|\| id:ebi-fl187-GCST90094780 | MR Egger | 6 | -5.044390433 | 1.92435794742458 | 0.0587169745934031 | -8.816132009 | -1.272648856 | 0.00644538806547527 | 0.000148320956171207 | 0.280088723717623 | 0.999950574162623 |
| ebi-fl187-GCST90094780 | ebi-gm430-GCST90011519 | TestASV_41 (Barnesiella) abundance \|\| id:ebi-gm430-GCST90011519 | F-healthy breakfast food liking (derived food-liking factor) \|\| id:ebi-fl187-GCST90094780 | MR Egger | 6 | 4.62642714680876 | 2.02183994970791 | 0.0840211137500266 | 0.663620845381245 | 8.58923344823626 | 102.148449941195 | 1.94181061667943 | 5373.49303570701 | 0.999950574162623 |
| ebi-fl187-GCST90094780 | ebi-gm430-GCST90011524 | TestASV_46 (Sutterella) abundance \|\| id:ebi-gm430-GCST90011524 | F-healthy breakfast food liking (derived food-liking factor) \|\| id:ebi-fl187-GCST90094780 | MR Egger | 6 | 2.13937354604698 | 1.97823270304355 | 0.340337060777607 | -1.737962552 | 6.01670964401234 | 8.49411479001588 | 0.175878378877744 | 410.226581154238 | 0.999950574162623 |
| ebi-fl187-GCST90094780 | ebi-gm430-GCST90011581 | OTU97_19 (Bacteria) prevalence \|\| id:ebi-gm430-GCST90011581 | F-healthy breakfast food liking (derived food-liking factor) \|\| id:ebi-fl187-GCST90094780 | MR Egger | 6 | 1.0400683250325 | 2.30689985577143 | 0.675456317057372 | -3.481455392 | 5.5615920423445 | 2.82941032729999 | 0.0307626067763755 | 260.236814728581 | 0.999950574162623 |
| ebi-fl187-GCST90094780 | ebi-gm430-GCST90011614 | OTU97_80 (Ruminococcus) prevalence \|\| id:ebi-gm430-GCST90011614 | F-healthy breakfast food liking (derived food-liking factor) \|\| id:ebi-fl187-GCST90094780 | MR Egger | 6 | 0.33381434978617 | 1.07545352225193 | 0.771761896263567 | -1.774074554 | 2.44170325339995 | 1.39628389910642 | 0.16964036990859 | 11.4925988899598 | 0.999950574162623 |
| ebi-fl187-GCST90094780 | ebi-gm430-GCST90011623 | OTU99_11 (Parabacteroides) prevalence \|\| id:ebi-gm430-GCST90011623 | F-healthy breakfast food liking (derived food-liking factor) \|\| id:ebi-fl187-GCST90094780 | MR Egger | 6 | -0.553451256 | 1.19510248491502 | 0.667367537687405 | -2.895852127 | 1.78894961416606 | 0.57496204085552 | 0.0552519233900395 | 5.98316453331541 | 0.999950574162623 |
| ebi-fl187-GCST90094780 | ebi-gm430-GCST90011642 | OTU99_19 (Bacteria) prevalence \|\| id:ebi-gm430-GCST90011642 | F-healthy breakfast food liking (derived food-liking factor) \|\| id:ebi-fl187-GCST90094780 | MR Egger | 6 | 1.0400683250325 | 2.30689985577143 | 0.675456317057372 | -3.481455392 | 5.5615920423445 | 2.82941032729999 | 0.0307626067763755 | 260.236814728581 | 0.999950574162623 |
| ebi-fl187-GCST90094780 | ebi-gm430-GCST90011653 | OTU99_30 (Parasutterella) prevalence \|\| id:ebi-gm430-GCST90011653 | F-healthy breakfast food liking (derived food-liking factor) \|\| id:ebi-fl187-GCST90094780 | MR Egger | 6 | 1.60735432913341 | 1.0205495705306 | 0.190379020343021 | -0.392922829 | 3.60763148737339 | 4.98959292926098 | 0.675080842167745 | 36.8786018572054 | 0.999950574162623 |
| ebi-fl187-GCST90094780 | ebi-gm430-GCST90011675 | OTU99_8 (Ruminococcaceae) prevalence \|\| id:ebi-gm430-GCST90011675 | F-healthy breakfast food liking (derived food-liking factor) \|\| id:ebi-fl187-GCST90094780 | MR Egger | 6 | -0.520784778 | 1.08070363734512 | 0.655061298490215 | -2.638963907 | 1.59739435112735 | 0.594054164310301 | 0.0714352447652683 | 4.94014336052209 | 0.999950574162623 |
| ebi-fl187-GCST90094780 | ebi-gm430-GCST90011679 | OTU99_92 (Ruminococcus) prevalence \|\| id:ebi-gm430-GCST90011679 | F-healthy breakfast food liking (derived food-liking factor) \|\| id:ebi-fl187-GCST90094780 | MR Egger | 6 | 0.32149050174256 | 1.12375742899682 | 0.789017627680939 | -1.881074059 | 2.52405506257633 | 1.37918190613911 | 0.152426302949261 | 12.4790977240633 | 0.999950574162623 |
| ebi-fl187-GCST90094780 | ebi-gm430-GCST90011684 | TestASV_11 (Lachnospiraceae) prevalence \|\| id:ebi-gm430-GCST90011684 | F-healthy breakfast food liking (derived food-liking factor) \|\| id:ebi-fl187-GCST90094780 | MR Egger | 6 | -1.05864854 | 1.1415611243882 | 0.406225708808233 | -3.296108344 | 1.17881126351394 | 0.346924347933613 | 0.0370269836579028 | 3.25050790799359 | 0.999950574162623 |
| ebi-fl187-GCST90094780 | ebi-gm430-GCST90011688 | TestASV_15 (Bacteroides) prevalence \|\| id:ebi-gm430-GCST90011688 | F-healthy breakfast food liking (derived food-liking factor) \|\| id:ebi-fl187-GCST90094780 | MR Egger | 6 | 0.276809394272044 | 1.07336579675053 | 0.809217073658907 | -1.826987567 | 2.38060635590308 | 1.31891495433001 | 0.160897531430769 | 10.8114564672722 | 0.999950574162623 |
| ebi-fl187-GCST90094780 | ebi-gm430-GCST90011692 | TestASV_19 (Prevotella) prevalence \|\| id:ebi-gm430-GCST90011692 | F-healthy breakfast food liking (derived food-liking factor) \|\| id:ebi-fl187-GCST90094780 | MR Egger | 6 | -1.218169889 | 2.12004219492063 | 0.596334171548662 | -5.373452591 | 2.93711281349474 | 0.295770965743092 | 0.00463809020791183 | 18.8613115000165 | 0.999950574162623 |
| ebi-fl187-GCST90094780 | ebi-gm430-GCST90011693 | TestASV_2 (Bacteroides) prevalence \|\| id:ebi-gm430-GCST90011693 | F-healthy breakfast food liking (derived food-liking factor) \|\| id:ebi-fl187-GCST90094780 | MR Egger | 6 | 1.25660024814118 | 1.15843492096149 | 0.339042693921209 | -1.013932197 | 3.52713269322571 | 3.51345628000339 | 0.362789610934599 | 34.0262638714883 | 0.999950574162623 |
| ebi-fl187-GCST90094780 | ebi-gm430-GCST90011716 | TestASV_40 (Barnesiella) prevalence \|\| id:ebi-gm430-GCST90011716 | F-healthy breakfast food liking (derived food-liking factor) \|\| id:ebi-fl187-GCST90094780 | MR Egger | 6 | 1.355508204 | 3.55319381257398 | 0.722238468648488 | -5.608751668 | 8.319768077 | 3.87873164395815 | 0.00366564244029138 | 4104.20803744471 | 0.999950574162623 |
| ebi-fl187-GCST90094780 | ebi-gm430-GCST90011718 | TestASV_42 (Alphaproteobacteria) prevalence \|\| id:ebi-gm430-GCST90011718 | F-healthy breakfast food liking (derived food-liking factor) \|\| id:ebi-fl187-GCST90094780 | MR Egger | 6 | 1.72129622828398 | 1.68035968040436 | 0.363568822251903 | -1.572208745 | 5.01480120187653 | 5.59177198163597 | 0.207586170643114 | 150.626189585459 | 0.999950574162623 |
| ebi-fl187-GCST90094780 | ebi-hbsm44-GCST90104929 | Sulcal depth of the right inferior frontal sulcus \|\| id:ebi-hbsm44-GCST90104929 | F-healthy breakfast food liking (derived food-liking factor) \|\| id:ebi-fl187-GCST90094780 | MR Egger | 7 | -0.736097322 | 1.22113206908562 | 0.572936870215515 | -3.129516177 | 1.6573215335455 | 0.478979575736018 | 0.0437389540344151 | 5.24524280557191 | 0.999950574162623 |
| ebi-fl187-GCST90094780 | ebi-hbsm44-GCST90104935 | Sulcal opening distance of the left calloso-marginal posterior fissure \|\| id:ebi-hbsm44-GCST90104935 | F-healthy breakfast food liking (derived food-liking factor) \|\| id:ebi-fl187-GCST90094780 | MR Egger | 6 | -0.609204891 | 0.275383352463388 | 0.0914013039065691 | -1.148956262 | -0.06945352 | 0.543783064140056 | 0.316967427851161 | 0.932903493744477 | 0.999950574162623 |
| ebi-fl187-GCST90094780 | ebi-hbsm44-GCST90104936 | Sulcal opening distance of the left anterior inferior temporal sulcus \|\| id:ebi-hbsm44-GCST90104936 | F-healthy breakfast food liking (derived food-liking factor) \|\| id:ebi-fl187-GCST90094780 | MR Egger | 7 | 0.0791285525198097 | 0.111204336316365 | 0.508548318790862 | -0.138831947 | 0.297089051699885 | 1.08234345110537 | 0.87037428547391 | 1.3459351519247 | 0.999950574162623 |
| ebi-fl187-GCST90094780 | ebi-hbsm44-GCST90104941 | Sulcal opening distance of the right posterior inferior temporal sulcus \|\| id:ebi-hbsm44-GCST90104941 | F-healthy breakfast food liking (derived food-liking factor) \|\| id:ebi-fl187-GCST90094780 | MR Egger | 6 | 0.00505646950681384 | 0.09606047220107 | 0.960543972997564 | -0.183222056 | 0.193334995020911 | 1.00506927502322 | 0.832583255095344 | 1.21328917127936 | 0.999950574162623 |
| ebi-fl187-GCST90094780 | ebi-IPs91-GCST90274819 | Leukemia inhibitory factor levels \|\| id:ebi-IPs91-GCST90274819 | F-healthy breakfast food liking (derived food-liking factor) \|\| id:ebi-fl187-GCST90094780 | MR Egger | 9 | 0.643075165625105 | 0.418816254766225 | 0.168547803211667 | -0.177804694 | 1.46395502496691 | 1.90232184848448 | 0.837105899566272 | 4.32302342761702 | 0.999950574162623 |
| ebi-fl187-GCST90094780 | ebi-IPs91-GCST90274837 | STAM binding protein levels \|\| id:ebi-IPs91-GCST90274837 | F-healthy breakfast food liking (derived food-liking factor) \|\| id:ebi-fl187-GCST90094780 | MR Egger | 9 | 0.0424615842529285 | 0.429984186557747 | 0.924103975837927 | -0.800307421 | 0.885230589906112 | 1.04337597353628 | 0.449190852008294 | 2.42354317165095 | 0.999950574162623 |
| ebi-fl187-GCST90094780 | ebi-IPs91-GCST90274840 | TNF-beta levels \|\| id:ebi-IPs91-GCST90274840 | F-healthy breakfast food liking (derived food-liking factor) \|\| id:ebi-fl187-GCST90094780 | MR Egger | 8 | -0.089997664 | 0.404884557622664 | 0.8314691200768 | -0.883571397 | 0.703576068948031 | 0.913933320223925 | 0.41330419936906 | 2.02096691756492 | 0.999950574162623 |
| ebi-fl187-GCST90094780 | ebi-IPs91-GCST90274842 | Tumor necrosis factor ligand superfamily member 14 levels \|\| id:ebi-IPs91-GCST90274842 | F-healthy breakfast food liking (derived food-liking factor) \|\| id:ebi-fl187-GCST90094780 | MR Egger | 9 | -0.061293759 | 0.491638923406958 | 0.904287716456354 | -1.024906049 | 0.902318530909451 | 0.940546905101728 | 0.358830176331556 | 2.46531239301081 | 0.999950574162623 |
| ebi-fl187-GCST90094780 | ebi-met1400-GCST90199628 | 3-methylhistidine levels \|\| id:ebi-met1400-GCST90199628 | F-healthy breakfast food liking (derived food-liking factor) \|\| id:ebi-fl187-GCST90094780 | MR Egger | 8 | -0.227498298 | 0.517980389573824 | 0.675890451388003 | -1.242739861 | 0.787743265677383 | 0.796523777269567 | 0.288592430464809 | 2.1984295524797 | 0.999950574162623 |
| ebi-fl187-GCST90094780 | ebi-met1400-GCST90199651 | Docosahexaenoate DHA; 22:6n3 levels \|\| id:ebi-met1400-GCST90199651 | F-healthy breakfast food liking (derived food-liking factor) \|\| id:ebi-fl187-GCST90094780 | MR Egger | 8 | -0.345472332 | 0.417944777831019 | 0.440091126965855 | -1.164644097 | 0.473699432217611 | 0.707885917086028 | 0.31203369603877 | 1.60592422539669 | 0.999950574162623 |
| ebi-fl187-GCST90094780 | ebi-met1400-GCST90199652 | 1-stearoyl-2-oleoyl-GPS (18:0/18:1) levels \|\| id:ebi-met1400-GCST90199652 | F-healthy breakfast food liking (derived food-liking factor) \|\| id:ebi-fl187-GCST90094780 | MR Egger | 8 | -0.716367057 | 0.442491130148419 | 0.156587234118644 | -1.583649673 | 0.150915557659445 | 0.488523814988628 | 0.20522472668675 | 1.16289845606822 | 0.999950574162623 |
| ebi-fl187-GCST90094780 | ebi-met1400-GCST90199653 | 1-myristoyl-2-palmitoyl-gpc (14:0/16:0) levels \|\| id:ebi-met1400-GCST90199653 | F-healthy breakfast food liking (derived food-liking factor) \|\| id:ebi-fl187-GCST90094780 | MR Egger | 8 | -0.320083781 | 0.576883514336106 | 0.599055743990113 | -1.450775469 | 0.810607907531729 | 0.726088202444026 | 0.234388456696839 | 2.24927492231535 | 0.999950574162623 |
| ebi-fl187-GCST90094780 | ebi-met1400-GCST90199667 | Erythritol levels in elite athletes \|\| id:ebi-met1400-GCST90199667 | F-healthy breakfast food liking (derived food-liking factor) \|\| id:ebi-fl187-GCST90094780 | MR Egger | 8 | 0.313953727764767 | 0.410495733045588 | 0.473379093078304 | -0.490617909 | 1.11852536453412 | 1.36882639642493 | 0.61224796374883 | 3.06033799128867 | 0.999950574162623 |
| ebi-fl187-GCST90094780 | ebi-met1400-GCST90199674 | 3-hydroxymyristate levels \|\| id:ebi-met1400-GCST90199674 | F-healthy breakfast food liking (derived food-liking factor) \|\| id:ebi-fl187-GCST90094780 | MR Egger | 8 | -0.039760962 | 0.426038423652207 | 0.92868201689863 | -0.874796272 | 0.795274348670874 | 0.961019132090185 | 0.416946954776504 | 2.21504860909328 | 0.999950574162623 |
| ebi-fl187-GCST90094780 | ebi-met1400-GCST90199685 | 2-linoleoylglycerol (18:2) levels \|\| id:ebi-met1400-GCST90199685 | F-healthy breakfast food liking (derived food-liking factor) \|\| id:ebi-fl187-GCST90094780 | MR Egger | 7 | 0.571959805983168 | 0.510988296642721 | 0.313872794742886 | -0.429577255 | 1.5734968674029 | 1.7717359098154 | 0.650784152041972 | 4.82348582748978 | 0.999950574162623 |
| ebi-fl187-GCST90094780 | ebi-met1400-GCST90199703 | Gamma-glutamylvaline levels \|\| id:ebi-met1400-GCST90199703 | F-healthy breakfast food liking (derived food-liking factor) \|\| id:ebi-fl187-GCST90094780 | MR Egger | 8 | -0.048061206 | 0.458527116862251 | 0.919937560614982 | -0.946774355 | 0.850651943203781 | 0.953075451502753 | 0.387990526887143 | 2.3411726661084 | 0.999950574162623 |
| ebi-fl187-GCST90094780 | ebi-met1400-GCST90199779 | Gamma-glutamyl-2-aminobutyrate levels \|\| id:ebi-met1400-GCST90199779 | F-healthy breakfast food liking (derived food-liking factor) \|\| id:ebi-fl187-GCST90094780 | MR Egger | 8 | 0.102323919059945 | 0.517329353524885 | 0.849738257502256 | -0.911641614 | 1.11628945196872 | 1.10774223244267 | 0.401863976776768 | 3.05350298720286 | 0.999950574162623 |
| ebi-fl187-GCST90094780 | ebi-met1400-GCST90199824 | 4-vinylphenol sulfate levels \|\| id:ebi-met1400-GCST90199824 | F-healthy breakfast food liking (derived food-liking factor) \|\| id:ebi-fl187-GCST90094780 | MR Egger | 8 | 1.18577544621958 | 0.437424696576249 | 0.035068280322751 | 0.328423040930134 | 2.04312785150903 | 3.27322404681086 | 1.38877635727864 | 7.71470194208616 | 0.999950574162623 |
| ebi-fl187-GCST90094780 | ebi-met1400-GCST90199830 | 4-ethylphenylsulfate levels \|\| id:ebi-met1400-GCST90199830 | F-healthy breakfast food liking (derived food-liking factor) \|\| id:ebi-fl187-GCST90094780 | MR Egger | 8 | -0.043853164 | 0.437968723083322 | 0.923504139530694 | -0.902271861 | 0.814565533055191 | 0.957094482901153 | 0.405647038242374 | 2.2581943483894 | 0.999950574162623 |
| ebi-fl187-GCST90094780 | ebi-met1400-GCST90199833 | Hexanoylglutamine levels \|\| id:ebi-met1400-GCST90199833 | F-healthy breakfast food liking (derived food-liking factor) \|\| id:ebi-fl187-GCST90094780 | MR Egger | 8 | 0.545001236034311 | 0.431595688570643 | 0.253531313345423 | -0.300926314 | 1.39092878563277 | 1.72461051405289 | 0.74013230844928 | 4.01858072026805 | 0.999950574162623 |
| ebi-fl187-GCST90094780 | ebi-met1400-GCST90199837 | Carotenoid levels (cryptoxanthin) \|\| id:ebi-met1400-GCST90199837 | F-healthy breakfast food liking (derived food-liking factor) \|\| id:ebi-fl187-GCST90094780 | MR Egger | 8 | -0.778838608 | 0.496603271874812 | 0.167851562451789 | -1.752181021 | 0.194503805216174 | 0.458938709812176 | 0.17339535191969 | 1.21470810510318 | 0.999950574162623 |
| ebi-fl187-GCST90094780 | ebi-met1400-GCST90199869 | 2-hydroxyhippurate levels \|\| id:ebi-met1400-GCST90199869 | F-healthy breakfast food liking (derived food-liking factor) \|\| id:ebi-fl187-GCST90094780 | MR Egger | 8 | 1.19060874844768 | 0.442635211391366 | 0.0360613405222688 | 0.323043734120598 | 2.05817376277475 | 3.28908282214362 | 1.38132576065204 | 7.83165428393497 | 0.999950574162623 |
| ebi-fl187-GCST90094780 | ebi-met1400-GCST90199902 | Hydantoin-5-propionate levels \|\| id:ebi-met1400-GCST90199902 | F-healthy breakfast food liking (derived food-liking factor) \|\| id:ebi-fl187-GCST90094780 | MR Egger | 8 | 0.508134753466809 | 0.436838812428081 | 0.288909296889135 | -0.348069319 | 1.36433882582585 | 1.66218791141192 | 0.706049931918883 | 3.9131349327307 | 0.999950574162623 |
| ebi-fl187-GCST90094780 | ebi-met1400-GCST90199904 | Pantoate levels \|\| id:ebi-met1400-GCST90199904 | F-healthy breakfast food liking (derived food-liking factor) \|\| id:ebi-fl187-GCST90094780 | MR Egger | 8 | 0.558643109313281 | 0.44361384781791 | 0.254693928808186 | -0.310840032 | 1.42812625103639 | 1.74829863998365 | 0.732831095717783 | 4.17087668963459 | 0.999950574162623 |
| ebi-fl187-GCST90094780 | ebi-met1400-GCST90199936 | 3-methyl catechol sulfate (2) levels \|\| id:ebi-met1400-GCST90199936 | F-healthy breakfast food liking (derived food-liking factor) \|\| id:ebi-fl187-GCST90094780 | MR Egger | 8 | 1.00486824526544 | 0.95217763013898 | 0.331901040827695 | -0.86139991 | 2.87113640033784 | 2.73154735481932 | 0.422570108020766 | 17.6570723058664 | 0.999950574162623 |
| ebi-fl187-GCST90094780 | ebi-met1400-GCST90199977 | 4-hydroxychlorothalonil levels \|\| id:ebi-met1400-GCST90199977 | F-healthy breakfast food liking (derived food-liking factor) \|\| id:ebi-fl187-GCST90094780 | MR Egger | 8 | -0.07242714 | 0.587543345022333 | 0.905918044656147 | -1.224012097 | 1.07915781594632 | 0.930133513442632 | 0.29404804796583 | 2.94220063290361 | 0.999950574162623 |
| ebi-fl187-GCST90094780 | ebi-met1400-GCST90200040 | Sphingomyelin (d18:2/24:1, d18:1/24:2) levels \|\| id:ebi-met1400-GCST90200040 | F-healthy breakfast food liking (derived food-liking factor) \|\| id:ebi-fl187-GCST90094780 | MR Egger | 8 | -0.044355556 | 0.398549506546001 | 0.915014056465337 | -0.825512589 | 0.736801476798901 | 0.956613767203762 | 0.438010415651829 | 2.08924232598886 | 0.999950574162623 |
| ebi-fl187-GCST90094780 | ebi-met1400-GCST90200077 | 1-stearoyl-2-oleoyl-GPI (18:0/18:1) levels \|\| id:ebi-met1400-GCST90200077 | F-healthy breakfast food liking (derived food-liking factor) \|\| id:ebi-fl187-GCST90094780 | MR Egger | 7 | -0.144853157 | 0.441164105611229 | 0.755956975497388 | -1.009534804 | 0.719828489826113 | 0.86514932475725 | 0.364388452137971 | 2.0540808846613 | 0.999950574162623 |
| ebi-fl187-GCST90094780 | ebi-met1400-GCST90200080 | 1-palmitoyl-2-oleoyl-GPI (16:0/18:1) levels \|\| id:ebi-met1400-GCST90200080 | F-healthy breakfast food liking (derived food-liking factor) \|\| id:ebi-fl187-GCST90094780 | MR Egger | 8 | -0.090716114 | 0.580882119769699 | 0.881021382061901 | -1.229245068 | 1.04781284099919 | 0.913276940868095 | 0.292513321868429 | 2.85140781074084 | 0.999950574162623 |
| ebi-fl187-GCST90094780 | ebi-met1400-GCST90200082 | 1-oleoyl-2-linoleoyl-GPE (18:1/18:2) levels \|\| id:ebi-met1400-GCST90200082 | F-healthy breakfast food liking (derived food-liking factor) \|\| id:ebi-fl187-GCST90094780 | MR Egger | 8 | 0.478017295594714 | 0.517995109204334 | 0.391698669638922 | -0.537253118 | 1.49328770963521 | 1.61287337874667 | 0.584351193335772 | 4.4517074073552 | 0.999950574162623 |
| ebi-fl187-GCST90094780 | ebi-met1400-GCST90200100 | Perfluorooctanesulfonate (PFOS) levels \|\| id:ebi-met1400-GCST90200100 | F-healthy breakfast food liking (derived food-liking factor) \|\| id:ebi-fl187-GCST90094780 | MR Egger | 8 | -0.431457147 | 0.533709385513534 | 0.449710190636981 | -1.477527543 | 0.614613248576067 | 0.649561897597989 | 0.228201209216103 | 1.84894138054956 | 0.999950574162623 |
| ebi-fl187-GCST90094780 | ebi-met1400-GCST90200102 | 1-palmitoleoyl-2-linolenoyl-GPC (16:1/18:3) levels \|\| id:ebi-met1400-GCST90200102 | F-healthy breakfast food liking (derived food-liking factor) \|\| id:ebi-fl187-GCST90094780 | MR Egger | 8 | -0.534249632 | 0.476586457419834 | 0.305142867217001 | -1.468359089 | 0.399859824056535 | 0.586108922276074 | 0.230303082154608 | 1.49161559436276 | 0.999950574162623 |
| ebi-fl187-GCST90094780 | ebi-met1400-GCST90200114 | Glycosyl-N-tricosanoyl-sphingadienine (d18:2/23:0) levels \|\| id:ebi-met1400-GCST90200114 | F-healthy breakfast food liking (derived food-liking factor) \|\| id:ebi-fl187-GCST90094780 | MR Egger | 8 | -0.615926337 | 0.456135978061029 | 0.225621856354097 | -1.509952854 | 0.278100180427285 | 0.540140311765712 | 0.220920393321282 | 1.32061849070706 | 0.999950574162623 |
| ebi-fl187-GCST90094780 | ebi-met1400-GCST90200135 | Arachidoylcarnitine (C20) levels \|\| id:ebi-met1400-GCST90200135 | F-healthy breakfast food liking (derived food-liking factor) \|\| id:ebi-fl187-GCST90094780 | MR Egger | 8 | 0.885343449725565 | 0.447824268428524 | 0.0954213884009631 | 0.00760788360565801 | 1.76307901584547 | 2.42381670773107 | 1.00763689708252 | 5.83036156147739 | 0.999950574162623 |
| ebi-fl187-GCST90094780 | ebi-met1400-GCST90200146 | Docosahexaenoylcarnitine (C22:6) levels \|\| id:ebi-met1400-GCST90200146 | F-healthy breakfast food liking (derived food-liking factor) \|\| id:ebi-fl187-GCST90094780 | MR Egger | 8 | -0.521539706 | 0.490371618387696 | 0.328447247920164 | -1.482668078 | 0.439588666058958 | 0.593605865478455 | 0.227031142786032 | 1.55206866866948 | 0.999950574162623 |
| ebi-fl187-GCST90094780 | ebi-met1400-GCST90200162 | 3-hydroxyphenylacetoylglutamine levels \|\| id:ebi-met1400-GCST90200162 | F-healthy breakfast food liking (derived food-liking factor) \|\| id:ebi-fl187-GCST90094780 | MR Egger | 7 | 0.330112779960913 | 0.647443487334302 | 0.631843313169461 | -0.938876455 | 1.59910201513614 | 1.39112501064136 | 0.391066969873532 | 4.94858667265546 | 0.999950574162623 |
| ebi-fl187-GCST90094780 | ebi-met1400-GCST90200172 | 3-carboxy-4-methyl-5-pentyl-2-furanpropionate (3-CMPFP) levels \|\| id:ebi-met1400-GCST90200172 | F-healthy breakfast food liking (derived food-liking factor) \|\| id:ebi-fl187-GCST90094780 | MR Egger | 8 | 0.533747210959072 | 0.434139969442345 | 0.264929745802905 | -0.317167129 | 1.38466155106607 | 1.70531050915226 | 0.728209039998546 | 3.99347408902112 | 0.999950574162623 |
| ebi-fl187-GCST90094780 | ebi-met1400-GCST90200222 | Dihydrocaffeate sulfate (2) levels \|\| id:ebi-met1400-GCST90200222 | F-healthy breakfast food liking (derived food-liking factor) \|\| id:ebi-fl187-GCST90094780 | MR Egger | 8 | 0.47822196206692 | 0.496062952776507 | 0.372257950311924 | -0.494061425 | 1.45050534950887 | 1.61320351363384 | 0.610143303658346 | 4.26526942244014 | 0.999950574162623 |
| ebi-fl187-GCST90094780 | ebi-met1400-GCST90200223 | 4-methylhexanoylglutamine levels \|\| id:ebi-met1400-GCST90200223 | F-healthy breakfast food liking (derived food-liking factor) \|\| id:ebi-fl187-GCST90094780 | MR Egger | 8 | 0.0163995765880448 | 0.525770411793554 | 0.976128484479183 | -1.014110431 | 1.04690958370341 | 1.01653478776832 | 0.362724955404076 | 2.84883341867679 | 0.999950574162623 |
| ebi-fl187-GCST90094780 | ebi-met1400-GCST90200224 | 2-ketocaprylate levels \|\| id:ebi-met1400-GCST90200224 | F-healthy breakfast food liking (derived food-liking factor) \|\| id:ebi-fl187-GCST90094780 | MR Egger | 8 | 0.0760816339438611 | 0.442692113139265 | 0.869196657843708 | -0.791594908 | 0.94375817569682 | 1.07905065772275 | 0.453121531597007 | 2.56962037938959 | 0.999950574162623 |
| ebi-fl187-GCST90094780 | ebi-met1400-GCST90200243 | Hydroxypalmitoyl sphingomyelin (d18:1/16:0(OH)) levels \|\| id:ebi-met1400-GCST90200243 | F-healthy breakfast food liking (derived food-liking factor) \|\| id:ebi-fl187-GCST90094780 | MR Egger | 8 | -0.194760243 | 0.435457739400025 | 0.670384505480615 | -1.048257412 | 0.658736926285901 | 0.823031962127601 | 0.350548077998148 | 1.93235009175314 | 0.999950574162623 |
| ebi-fl187-GCST90094780 | ebi-met1400-GCST90200273 | 2-methoxyhydroquinone sulfate (2) levels \|\| id:ebi-met1400-GCST90200273 | F-healthy breakfast food liking (derived food-liking factor) \|\| id:ebi-fl187-GCST90094780 | MR Egger | 8 | 0.992139443151838 | 0.520295724252669 | 0.105162442132896 | -0.027640176 | 2.01191906268707 | 2.69699837908797 | 0.972738318056723 | 7.47765367291614 | 0.999950574162623 |
| ebi-fl187-GCST90094780 | ebi-met1400-GCST90200276 | 2,4-di-tert-butylphenol levels \|\| id:ebi-met1400-GCST90200276 | F-healthy breakfast food liking (derived food-liking factor) \|\| id:ebi-fl187-GCST90094780 | MR Egger | 8 | 0.210836188238382 | 0.434844964087082 | 0.644972704761538 | -0.641459941 | 1.06313231784906 | 1.23471007846116 | 0.526523169687652 | 2.89542619512443 | 0.999950574162623 |
| ebi-fl187-GCST90094780 | ebi-met1400-GCST90200289 | N-acetylmethionine levels \|\| id:ebi-met1400-GCST90200289 | F-healthy breakfast food liking (derived food-liking factor) \|\| id:ebi-fl187-GCST90094780 | MR Egger | 8 | 0.961573848138148 | 0.469856281715037 | 0.0866576257762245 | 0.0406555359766755 | 1.88249216029962 | 2.61581012333623 | 1.04149328678112 | 6.56985761520928 | 0.999950574162623 |
| ebi-fl187-GCST90094780 | ebi-met1400-GCST90200308 | 3-hydroxyisobutyrate levels \|\| id:ebi-met1400-GCST90200308 | F-healthy breakfast food liking (derived food-liking factor) \|\| id:ebi-fl187-GCST90094780 | MR Egger | 8 | -0.605373622 | 0.441633341191037 | 0.219514435875819 | -1.47097497 | 0.260227727203802 | 0.54587043958594 | 0.229701423919772 | 1.29722546655968 | 0.999950574162623 |
| ebi-fl187-GCST90094780 | ebi-met1400-GCST90200383 | Cystathionine levels \|\| id:ebi-met1400-GCST90200383 | F-healthy breakfast food liking (derived food-liking factor) \|\| id:ebi-fl187-GCST90094780 | MR Egger | 8 | -0.19502572 | 0.591122485523655 | 0.752671589767919 | -1.353625791 | 0.963574352080002 | 0.82281349539403 | 0.25830201157271 | 2.62104829954824 | 0.999950574162623 |
| ebi-fl187-GCST90094780 | ebi-met1400-GCST90200384 | Guanidinoacetate levels \|\| id:ebi-met1400-GCST90200384 | F-healthy breakfast food liking (derived food-liking factor) \|\| id:ebi-fl187-GCST90094780 | MR Egger | 8 | -0.068455682 | 0.417813917178606 | 0.875234907754648 | -0.88737096 | 0.750459595236967 | 0.933834844474681 | 0.411736803550362 | 2.11797320335583 | 0.999950574162623 |
| ebi-fl187-GCST90094780 | ebi-met1400-GCST90200403 | Citrulline levels \|\| id:ebi-met1400-GCST90200403 | F-healthy breakfast food liking (derived food-liking factor) \|\| id:ebi-fl187-GCST90094780 | MR Egger | 8 | -0.054667911 | 0.437776883001298 | 0.904700404346863 | -0.912710601 | 0.803374779991467 | 0.946799517748709 | 0.401434618729016 | 2.23306432725552 | 0.999950574162623 |
| ebi-fl187-GCST90094780 | ebi-met1400-GCST90200434 | 12,13-DiHOME levels \|\| id:ebi-met1400-GCST90200434 | F-healthy breakfast food liking (derived food-liking factor) \|\| id:ebi-fl187-GCST90094780 | MR Egger | 8 | -0.459014571 | 0.441664227571327 | 0.338728826702924 | -1.324676457 | 0.406647314909819 | 0.631906037247408 | 0.265888971644213 | 1.50177435882536 | 0.999950574162623 |
| ebi-fl187-GCST90094780 | ebi-met1400-GCST90200437 | Fructose levels \|\| id:ebi-met1400-GCST90200437 | F-healthy breakfast food liking (derived food-liking factor) \|\| id:ebi-fl187-GCST90094780 | MR Egger | 8 | -0.160495282 | 0.577258087196488 | 0.790321495031075 | -1.291921132 | 0.970930569381128 | 0.851721842391551 | 0.274742459114227 | 2.64040039222788 | 0.999950574162623 |
| ebi-fl187-GCST90094780 | ebi-met1400-GCST90200448 | N-stearoyl-sphinganine (d18:0/18:0) levels \|\| id:ebi-met1400-GCST90200448 | F-healthy breakfast food liking (derived food-liking factor) \|\| id:ebi-fl187-GCST90094780 | MR Egger | 8 | -0.013064996 | 0.487738876572765 | 0.97949843729221 | -0.969033194 | 0.942903202107787 | 0.987019980609603 | 0.379449715061526 | 2.56742436073411 | 0.999950574162623 |
| ebi-fl187-GCST90094780 | ebi-met1400-GCST90200468 | X-11847 levels \|\| id:ebi-met1400-GCST90200468 | F-healthy breakfast food liking (derived food-liking factor) \|\| id:ebi-fl187-GCST90094780 | MR Egger | 8 | 1.32925613656042 | 0.612893832645992 | 0.0731810045675002 | 0.127984224574277 | 2.53052804854657 | 3.77823185345767 | 1.13653507323101 | 12.5601367478262 | 0.999950574162623 |
| ebi-fl187-GCST90094780 | ebi-met1400-GCST90200476 | X-11843 levels \|\| id:ebi-met1400-GCST90200476 | F-healthy breakfast food liking (derived food-liking factor) \|\| id:ebi-fl187-GCST90094780 | MR Egger | 8 | -0.807178932 | 0.718545913017946 | 0.304216120146195 | -2.215528921 | 0.601171057550548 | 0.446114812944367 | 0.109095795875772 | 1.82425385626235 | 0.999950574162623 |
| ebi-fl187-GCST90094780 | ebi-met1400-GCST90200511 | X-12906 levels \|\| id:ebi-met1400-GCST90200511 | F-healthy breakfast food liking (derived food-liking factor) \|\| id:ebi-fl187-GCST90094780 | MR Egger | 8 | -0.885866886 | 0.438801784129919 | 0.0900460469208612 | -1.745918383 | -0.025815389 | 0.412356552163626 | 0.174484671620812 | 0.974514979068176 | 0.999950574162623 |
| ebi-fl187-GCST90094780 | ebi-met1400-GCST90200515 | X-12849 levels \|\| id:ebi-met1400-GCST90200515 | F-healthy breakfast food liking (derived food-liking factor) \|\| id:ebi-fl187-GCST90094780 | MR Egger | 8 | -0.641306643 | 0.459975385537303 | 0.212692499660391 | -1.542858399 | 0.260245112460853 | 0.526603890918364 | 0.213769189694716 | 1.29724801935391 | 0.999950574162623 |
| ebi-fl187-GCST90094780 | ebi-met1400-GCST90200551 | X-18886 levels \|\| id:ebi-met1400-GCST90200551 | F-healthy breakfast food liking (derived food-liking factor) \|\| id:ebi-fl187-GCST90094780 | MR Egger | 8 | 1.14072643440585 | 0.431137307606165 | 0.0382399263439205 | 0.295697311497767 | 1.98575555731393 | 3.12904058251425 | 1.34406326275817 | 7.28454920115076 | 0.999950574162623 |
| ebi-fl187-GCST90094780 | ebi-met1400-GCST90200564 | X-21319 levels \|\| id:ebi-met1400-GCST90200564 | F-healthy breakfast food liking (derived food-liking factor) \|\| id:ebi-fl187-GCST90094780 | MR Egger | 8 | 0.978665264059047 | 0.441574117876271 | 0.0685451771089828 | 0.113179993021557 | 1.84415053509654 | 2.6609022687557 | 1.11983347702095 | 6.3227265742269 | 0.999950574162623 |
| ebi-fl187-GCST90094780 | ebi-met1400-GCST90200579 | X-21442 levels \|\| id:ebi-met1400-GCST90200579 | F-healthy breakfast food liking (derived food-liking factor) \|\| id:ebi-fl187-GCST90094780 | MR Egger | 8 | 0.421820683404897 | 0.51030341443431 | 0.440086104756738 | -0.578374009 | 1.42201537569615 | 1.52473508986356 | 0.560809496878139 | 4.14546669983804 | 0.999950574162623 |
| ebi-fl187-GCST90094780 | ebi-met1400-GCST90200581 | X-21441 levels \|\| id:ebi-met1400-GCST90200581 | F-healthy breakfast food liking (derived food-liking factor) \|\| id:ebi-fl187-GCST90094780 | MR Egger | 8 | 0.0368019795632489 | 0.437831873548099 | 0.935747007805368 | -0.821348493 | 0.894952451717523 | 1.03748755675667 | 0.439838135950672 | 2.44721942561535 | 0.999950574162623 |
| ebi-fl187-GCST90094780 | ebi-met1400-GCST90200648 | X-24585 levels \|\| id:ebi-met1400-GCST90200648 | F-healthy breakfast food liking (derived food-liking factor) \|\| id:ebi-fl187-GCST90094780 | MR Egger | 8 | 0.181465380509367 | 0.427182491800548 | 0.68579707853968 | -0.655812303 | 1.01874306443844 | 1.1989730281557 | 0.519020289350383 | 2.76971122659597 | 0.999950574162623 |
| ebi-fl187-GCST90094780 | ebi-met1400-GCST90200720 | Adenosine 5'-diphosphate (ADP) to pantothenate ratio \|\| id:ebi-met1400-GCST90200720 | F-healthy breakfast food liking (derived food-liking factor) \|\| id:ebi-fl187-GCST90094780 | MR Egger | 7 | 0.618852031360616 | 0.594085727374741 | 0.345281318349596 | -0.545555994 | 1.78326005701511 | 1.85679527516029 | 0.579519484223496 | 5.94921963405105 | 0.999950574162623 |
| ebi-fl187-GCST90094780 | ebi-met1400-GCST90200724 | Cholate to taurocholate ratio \|\| id:ebi-met1400-GCST90200724 | F-healthy breakfast food liking (derived food-liking factor) \|\| id:ebi-fl187-GCST90094780 | MR Egger | 8 | -0.914844665 | 0.49977510293734 | 0.116905054199972 | -1.894403867 | 0.0647145367941452 | 0.400578845188013 | 0.15040797152347 | 1.06685443322479 | 0.999950574162623 |
| ebi-fl187-GCST90094780 | ebi-met1400-GCST90200726 | N-acetylputrescine to (N(1) + N(8))-acetylspermidine ratio \|\| id:ebi-met1400-GCST90200726 | F-healthy breakfast food liking (derived food-liking factor) \|\| id:ebi-fl187-GCST90094780 | MR Egger | 8 | -0.147832338 | 0.435313178491772 | 0.745729785892401 | -1.001046168 | 0.705381492035275 | 0.862575724155121 | 0.367494778845805 | 2.02461891360344 | 0.999950574162623 |
| ebi-fl187-GCST90094780 | ebi-met1400-GCST90200744 | Arginine to glutamate ratio \|\| id:ebi-met1400-GCST90200744 | F-healthy breakfast food liking (derived food-liking factor) \|\| id:ebi-fl187-GCST90094780 | MR Egger | 8 | -1.00871505 | 0.608734198914539 | 0.148581704932116 | -2.20183408 | 0.184403979698649 | 0.364687283495093 | 0.110600122749208 | 1.20250151118375 | 0.999950574162623 |
| ebi-fl187-GCST90094780 | ebi-met1400-GCST90200759 | Histidine to trans-urocanate ratio \|\| id:ebi-met1400-GCST90200759 | F-healthy breakfast food liking (derived food-liking factor) \|\| id:ebi-fl187-GCST90094780 | MR Egger | 8 | 0.71555283978951 | 0.440473777827491 | 0.155393308501376 | -0.147775765 | 1.57888144433139 | 2.04531710243553 | 0.862624524080429 | 4.84952830894156 | 0.999950574162623 |
| ebi-fl187-GCST90094780 | ebi-met1400-GCST90200795 | Oleoyl-linoleoyl-glycerol (18:1 to 18:2) [2] to linoleoyl-arachidonoyl-glycerol (18:2 to 20:4) [2] ratio \|\| id:ebi-met1400-GCST90200795 | F-healthy breakfast food liking (derived food-liking factor) \|\| id:ebi-fl187-GCST90094780 | MR Egger | 8 | 0.0866425754151122 | 0.537562501482129 | 0.877244332610902 | -0.966979927 | 1.14026507832009 | 1.09050683609937 | 0.380229626884977 | 3.12759731355496 | 0.999950574162623 |
| ebi-fl187-GCST90094780 | ebi-met1400-GCST90200811 | Choline to taurocholate ratio \|\| id:ebi-met1400-GCST90200811 | F-healthy breakfast food liking (derived food-liking factor) \|\| id:ebi-fl187-GCST90094780 | MR Egger | 8 | -0.87705604 | 0.457972724688473 | 0.103972476836742 | -1.77468258 | 0.020570500380299 | 0.416005815184739 | 0.169537255407542 | 1.02078353133361 | 0.999950574162623 |
| ebi-fl187-GCST90094780 | ebi-met1400-GCST90200831 | Cholate to phosphate ratio \|\| id:ebi-met1400-GCST90200831 | F-healthy breakfast food liking (derived food-liking factor) \|\| id:ebi-fl187-GCST90094780 | MR Egger | 8 | -0.46348484 | 0.456516601701428 | 0.349157538478273 | -1.358257379 | 0.4312877 | 0.629087551878849 | 0.257108429311748 | 1.53923832442331 | 0.999950574162623 |
| ebi-fl187-GCST90094780 | ebi-met1400-GCST90200841 | Adenosine 5'-diphosphate (ADP) to oxalate (ethanedioate) ratio \|\| id:ebi-met1400-GCST90200841 | F-healthy breakfast food liking (derived food-liking factor) \|\| id:ebi-fl187-GCST90094780 | MR Egger | 7 | 1.1491035996244 | 0.806903375001359 | 0.213720718734006 | -0.432427015 | 2.73063421462707 | 3.15536317285737 | 0.648932213478277 | 15.3426144454421 | 0.999950574162623 |
| ebi-fl187-GCST90094780 | ebi-met1400-GCST90200844 | Aspartate to phosphate ratio \|\| id:ebi-met1400-GCST90200844 | F-healthy breakfast food liking (derived food-liking factor) \|\| id:ebi-fl187-GCST90094780 | MR Egger | 8 | 0.661842596230231 | 0.507623260711449 | 0.240085397696033 | -0.333098995 | 1.65678418722467 | 1.93836066194754 | 0.716699241171381 | 5.24242505077138 | 0.999950574162623 |
| ebi-fl187-GCST90094780 | ebi-met1400-GCST90200870 | Adenosine 5'-monophosphate (AMP) to acetoacetate ratio \|\| id:ebi-met1400-GCST90200870 | F-healthy breakfast food liking (derived food-liking factor) \|\| id:ebi-fl187-GCST90094780 | MR Egger | 8 | -0.120041259 | 0.480490868026086 | 0.811052134478686 | -1.06180336 | 0.821720842791006 | 0.886883844429612 | 0.345831588841708 | 2.2744103745545 | 0.999950574162623 |
| ebi-fl187-GCST90094780 | ebi-met1400-GCST90200887 | Cholesterol to cortisol ratio \|\| id:ebi-met1400-GCST90200887 | F-healthy breakfast food liking (derived food-liking factor) \|\| id:ebi-fl187-GCST90094780 | MR Egger | 8 | -0.797504124 | 0.594741724322356 | 0.228476883267351 | -1.963197903 | 0.368189656168473 | 0.450451834407912 | 0.140408688812671 | 1.44511608816578 | 0.999950574162623 |
| ebi-fl187-GCST90094780 | ebi-met1400-GCST90200890 | Cortisol to taurocholate ratio \|\| id:ebi-met1400-GCST90200890 | F-healthy breakfast food liking (derived food-liking factor) \|\| id:ebi-fl187-GCST90094780 | MR Egger | 8 | -0.556512164 | 0.456247511179901 | 0.268322328358208 | -1.450757286 | 0.337732958040178 | 0.573204825880358 | 0.234392718592949 | 1.4017661230472 | 0.999950574162623 |
| ebi-fl187-GCST90094780 | ebi-met1400-GCST90200948 | Adenosine 5'-diphosphate (ADP) to mannitol to sorbitol ratio \|\| id:ebi-met1400-GCST90200948 | F-healthy breakfast food liking (derived food-liking factor) \|\| id:ebi-fl187-GCST90094780 | MR Egger | 8 | -0.216894552 | 0.616993484362399 | 0.737203173655676 | -1.426201782 | 0.992412676929905 | 0.805014851697257 | 0.240219598132717 | 2.69773539082819 | 0.999950574162623 |
| ebi-fl187-GCST90094780 | ebi-met1400-GCST90200972 | Leucine to N-palmitoyl-sphingosine (d18:1 to 16:0) ratio \|\| id:ebi-met1400-GCST90200972 | F-healthy breakfast food liking (derived food-liking factor) \|\| id:ebi-fl187-GCST90094780 | MR Egger | 8 | -0.269400971 | 0.421423624105981 | 0.546266913481945 | -1.095391274 | 0.556589332442809 | 0.763836917932257 | 0.334408735885725 | 1.7447117093126 | 0.999950574162623 |
| ebi-fl187-GCST90094780 | ebi-met1400-GCST90200973 | Proline to glutamate ratio \|\| id:ebi-met1400-GCST90200973 | F-healthy breakfast food liking (derived food-liking factor) \|\| id:ebi-fl187-GCST90094780 | MR Egger | 8 | -0.743501704 | 0.428304936368624 | 0.133260546457139 | -1.582979379 | 0.0959759715095982 | 0.475446125680131 | 0.205362333593658 | 1.10073261473314 | 0.999950574162623 |
| ebi-fl187-GCST90094780 | ebi-met1400-GCST90200983 | Cholesterol to linoleoyl-arachidonoyl-glycerol (18:2 to 20:4) [1] ratio \|\| id:ebi-met1400-GCST90200983 | F-healthy breakfast food liking (derived food-liking factor) \|\| id:ebi-fl187-GCST90094780 | MR Egger | 8 | -1.474483339 | 0.465665574565077 | 0.019407031711105 | -2.387187866 | -0.561778813 | 0.228896958527032 | 0.0918877215115159 | 0.570193892732006 | 0.999950574162623 |
| ebi-fl187-GCST90094780 | ebi-met1400-GCST90201001 | Serine to alpha-ketobutyrate ratio \|\| id:ebi-met1400-GCST90201001 | F-healthy breakfast food liking (derived food-liking factor) \|\| id:ebi-fl187-GCST90094780 | MR Egger | 8 | 0.343100383945239 | 0.55885488358205 | 0.561797799875382 | -0.752255188 | 1.43845595576606 | 1.40931022694657 | 0.471302477713973 | 4.21418390459123 | 0.999950574162623 |
| ebi-fl187-GCST90094780 | ebi-met1400-GCST90201005 | Fructose to maltose ratio \|\| id:ebi-met1400-GCST90201005 | F-healthy breakfast food liking (derived food-liking factor) \|\| id:ebi-fl187-GCST90094780 | MR Egger | 8 | 0.161461106437553 | 0.499329803459968 | 0.757398910756485 | -0.817225308 | 1.14014752121909 | 1.17522674851454 | 0.441655413534651 | 3.12722966389204 | 0.999950574162623 |
| ebi-fl187-GCST90094780 | ebi-met1400-GCST90201020 | Paraxanthine to 5-acetylamino-6-formylamino-3-methyluracil ratio \|\| id:ebi-met1400-GCST90201020 | F-healthy breakfast food liking (derived food-liking factor) \|\| id:ebi-fl187-GCST90094780 | MR Egger | 8 | 0.377652363135502 | 0.570508228259062 | 0.532583182659514 | -0.740543764 | 1.49584849052326 | 1.45885570269097 | 0.476854548553152 | 4.46312186332589 | 0.999950574162623 |
| ebi-fl187-GCST90094780 | ebi-pw11-GCST90275197 | Placental weight (fetal genotype, adjusted for sex) \|\| id:ebi-pw11-GCST90275197 | F-healthy breakfast food liking (derived food-liking factor) \|\| id:ebi-fl187-GCST90094780 | MR Egger | 7 | -0.042576778 | 0.305514939205256 | 0.894604553220594 | -0.641386059 | 0.556232502454091 | 0.958316884670474 | 0.526562071784196 | 1.74408925491486 | 0.999950574162623 |
| ebi-fl187-GCST90094780 | prot-a-1017 | FAS-associated factor 2 \|\| id:prot-a-1017 | F-healthy breakfast food liking (derived food-liking factor) \|\| id:ebi-fl187-GCST90094780 | MR Egger | 8 | 0.386525726905676 | 0.954892984417746 | 0.699671888603849 | -1.485064523 | 2.25811597636446 | 1.471858263 | 0.226487726638636 | 9.56505139740009 | 0.999950574162623 |
| ebi-fl187-GCST90094780 | prot-a-1019 | Fas apoptotic inhibitory molecule 3 \|\| id:prot-a-1019 | F-healthy breakfast food liking (derived food-liking factor) \|\| id:ebi-fl187-GCST90094780 | MR Egger | 8 | -1.508999166 | 0.764959823043907 | 0.0959967444110215 | -3.008320419 | -0.009677913 | 0.221131182843092 | 0.0493745376819354 | 0.990368767411783 | 0.999950574162623 |
| ebi-fl187-GCST90094780 | prot-a-1052 | MIP18 family protein FAM96A \|\| id:prot-a-1052 | F-healthy breakfast food liking (derived food-liking factor) \|\| id:ebi-fl187-GCST90094780 | MR Egger | 8 | -0.024186515 | 0.711020740696452 | 0.973967314854317 | -1.417787166 | 1.3694141371526 | 0.976103635187425 | 0.242249482029783 | 3.93304579495021 | 0.999950574162623 |
| ebi-fl187-GCST90094780 | prot-a-1066 | F-box/LRR-repeat protein 4 \|\| id:prot-a-1066 | F-healthy breakfast food liking (derived food-liking factor) \|\| id:ebi-fl187-GCST90094780 | MR Egger | 8 | 0.0248323471560388 | 0.711020740696452 | 0.973272510886438 | -1.368768305 | 1.41843299892108 | 1.02514323793722 | 0.254420134752333 | 4.13064264473971 | 0.999950574162623 |
| ebi-fl187-GCST90094780 | prot-a-1076 | Low affinity immunoglobulin gamma Fc region receptor III-B \|\| id:prot-a-1076 | F-healthy breakfast food liking (derived food-liking factor) \|\| id:ebi-fl187-GCST90094780 | MR Egger | 8 | -0.548953014 | 0.710879005802792 | 0.46930046830638 | -1.942275866 | 0.844369837072116 | 0.577554184911327 | 0.143377270770841 | 2.32651127138366 | 0.999950574162623 |
| ebi-fl187-GCST90094780 | prot-a-1081 | Fc receptor-like protein 3 \|\| id:prot-a-1081 | F-healthy breakfast food liking (derived food-liking factor) \|\| id:ebi-fl187-GCST90094780 | MR Egger | 8 | -1.010041933 | 0.711033977786455 | 0.205272880990678 | -2.403668529 | 0.38358466373844 | 0.364203707198145 | 0.0903857615303528 | 1.46753579425592 | 0.999950574162623 |
| ebi-fl187-GCST90094780 | prot-a-1105 | Fibroblast growth factor receptor 4 \|\| id:prot-a-1105 | F-healthy breakfast food liking (derived food-liking factor) \|\| id:ebi-fl187-GCST90094780 | MR Egger | 8 | -1.350752726 | 1.17474205451222 | 0.293973069309859 | -3.653247153 | 0.951741701048596 | 0.259045197238351 | 0.0259068684933841 | 2.59021711672303 | 0.999950574162623 |
| ebi-fl187-GCST90094780 | prot-a-1143 | Follistatin-related protein 1 \|\| id:prot-a-1143 | F-healthy breakfast food liking (derived food-liking factor) \|\| id:ebi-fl187-GCST90094780 | MR Egger | 8 | -1.151167753 | 0.899382505165771 | 0.247819936383722 | -2.913957463 | 0.611621957328969 | 0.316267231712628 | 0.054260570212139 | 1.84341892214011 | 0.999950574162623 |
| ebi-fl187-GCST90094780 | prot-a-1151 | Alpha-(1,3)-fucosyltransferase 10 \|\| id:prot-a-1151 | F-healthy breakfast food liking (derived food-liking factor) \|\| id:ebi-fl187-GCST90094780 | MR Egger | 8 | 0.610921897960958 | 0.711033977786455 | 0.423222165222325 | -0.782704699 | 2.00454849442241 | 1.84212887106335 | 0.457167836453473 | 7.4227417307615 | 0.999950574162623 |
| ebi-fl187-GCST90094780 | prot-a-1165 | Growth arrest and DNA damage-inducible proteins-interacting protein 1 \|\| id:prot-a-1165 | F-healthy breakfast food liking (derived food-liking factor) \|\| id:ebi-fl187-GCST90094780 | MR Egger | 8 | -0.083608576 | 0.920709722406704 | 0.930600216645711 | -1.888199632 | 1.72098247972273 | 0.919791213819782 | 0.151344038653229 | 5.59001784641499 | 0.999950574162623 |
| ebi-fl187-GCST90094780 | prot-a-1194 | Growth/differentiation factor 11/8 \|\| id:prot-a-1194 | F-healthy breakfast food liking (derived food-liking factor) \|\| id:ebi-fl187-GCST90094780 | MR Egger | 8 | -1.412204157 | 0.710879005802792 | 0.0941638250821677 | -2.805527008 | -0.018881306 | 0.243605745648506 | 0.0604748919974336 | 0.981295829606159 | 0.999950574162623 |
| ebi-fl187-GCST90094780 | prot-a-1216 | D-glucuronyl C5-epimerase \|\| id:prot-a-1216 | F-healthy breakfast food liking (derived food-liking factor) \|\| id:ebi-fl187-GCST90094780 | MR Egger | 8 | -0.627468026 | 0.788657850195783 | 0.456568870947912 | -2.173237413 | 0.918301359915792 | 0.533942017985924 | 0.113808574520088 | 2.50503162677395 | 0.999950574162623 |
| ebi-fl187-GCST90094780 | prot-a-1246 | Glypican-3 \|\| id:prot-a-1246 | F-healthy breakfast food liking (derived food-liking factor) \|\| id:ebi-fl187-GCST90094780 | MR Egger | 8 | -0.132804949 | 0.758941942292247 | 0.866845561843137 | -1.620331156 | 1.35472125822064 | 0.875635869392087 | 0.197833174678098 | 3.87568049197829 | 0.999950574162623 |
| ebi-fl187-GCST90094780 | prot-a-1275 | Glutamate receptor 4 \|\| id:prot-a-1275 | F-healthy breakfast food liking (derived food-liking factor) \|\| id:ebi-fl187-GCST90094780 | MR Egger | 7 | 0.182852209746984 | 0.800168239812714 | 0.82829621845044 | -1.38547754 | 1.7511819597799 | 1.20063695252906 | 0.250204288630735 | 5.76140840617544 | 0.999950574162623 |
| ebi-fl187-GCST90094780 | prot-a-1289 | Glutathione S-transferase P \|\| id:prot-a-1289 | F-healthy breakfast food liking (derived food-liking factor) \|\| id:ebi-fl187-GCST90094780 | MR Egger | 8 | 1.19314704539347 | 1.28022073177098 | 0.387309729950965 | -1.316085589 | 3.7023796796646 | 3.2974420956966 | 0.268183028641686 | 40.543670602659 | 0.999950574162623 |
| ebi-fl187-GCST90094780 | prot-a-130 | Apolipoprotein D \|\| id:prot-a-130 | F-healthy breakfast food liking (derived food-liking factor) \|\| id:ebi-fl187-GCST90094780 | MR Egger | 8 | 1.3399592389308 | 0.770425027583405 | 0.132648452215152 | -0.170073815 | 2.84999229299427 | 3.81888784024228 | 0.843602543664381 | 17.2876486040474 | 0.999950574162623 |
| ebi-fl187-GCST90094780 | prot-a-1313 | Heparin-binding EGF-like growth factor \|\| id:prot-a-1313 | F-healthy breakfast food liking (derived food-liking factor) \|\| id:ebi-fl187-GCST90094780 | MR Egger | 8 | 0.670214320054751 | 0.911285205649046 | 0.489798222132277 | -1.115904683 | 2.45633332312688 | 1.95465619777078 | 0.327618753666525 | 11.6619723649052 | 0.999950574162623 |
| ebi-fl187-GCST90094780 | prot-a-1347 | HLA class II histocompatibility antigen, DQ alpha 2 chain \|\| id:prot-a-1347 | F-healthy breakfast food liking (derived food-liking factor) \|\| id:ebi-fl187-GCST90094780 | MR Egger | 8 | -0.678656329 | 0.709773950587505 | 0.375914072082069 | -2.069813272 | 0.712500614594834 | 0.507298176692411 | 0.126209346360533 | 2.03908385152623 | 0.999950574162623 |
| ebi-fl187-GCST90094780 | prot-a-1357 | Non-histone chromosomal protein HMG-14 \|\| id:prot-a-1357 | F-healthy breakfast food liking (derived food-liking factor) \|\| id:ebi-fl187-GCST90094780 | MR Egger | 8 | 0.95436972065281 | 0.710865234564549 | 0.227981444761135 | -0.438926139 | 2.34766558039933 | 2.59703321057615 | 0.644728398095005 | 10.461120553653 | 0.999950574162623 |
| ebi-fl187-GCST90094780 | prot-a-137 | Apolipoprotein M \|\| id:prot-a-137 | F-healthy breakfast food liking (derived food-liking factor) \|\| id:ebi-fl187-GCST90094780 | MR Egger | 8 | -1.313403999 | 1.18574219686736 | 0.31042457661004 | -3.637458704 | 1.01065070734094 | 0.268903150770372 | 0.0263191437755272 | 2.74738817915003 | 0.999950574162623 |
| ebi-fl187-GCST90094780 | prot-a-1381 | 17-beta-hydroxysteroid dehydrogenase 14 \|\| id:prot-a-1381 | F-healthy breakfast food liking (derived food-liking factor) \|\| id:ebi-fl187-GCST90094780 | MR Egger | 8 | 0.0292368184269308 | 1.09660750980166 | 0.979594627770632 | -2.120113901 | 2.17858753763818 | 1.02966841005711 | 0.120017957593405 | 8.83382000434735 | 0.999950574162623 |
| ebi-fl187-GCST90094780 | prot-a-1392 | Serine protease HTRA2, mitochondrial \|\| id:prot-a-1392 | F-healthy breakfast food liking (derived food-liking factor) \|\| id:ebi-fl187-GCST90094780 | MR Egger | 8 | 0.448582729315427 | 0.833212427813699 | 0.609692607574683 | -1.184513629 | 2.08167908783028 | 1.56609103696156 | 0.305894921681803 | 8.01792041059969 | 0.999950574162623 |
| ebi-fl187-GCST90094780 | prot-a-1416 | Interferon-induced protein with tetratricopeptide repeats 2 \|\| id:prot-a-1416 | F-healthy breakfast food liking (derived food-liking factor) \|\| id:ebi-fl187-GCST90094780 | MR Egger | 8 | -0.819983683 | 0.710865234564549 | 0.292576001788979 | -2.213279542 | 0.57331217723545 | 0.440438841303283 | 0.109341469895677 | 1.77413357542807 | 0.999950574162623 |
| ebi-fl187-GCST90094780 | prot-a-1428 | Interferon gamma \|\| id:prot-a-1428 | F-healthy breakfast food liking (derived food-liking factor) \|\| id:ebi-fl187-GCST90094780 | MR Egger | 8 | -0.468238865 | 0.712142303138471 | 0.535249242568366 | -1.864037779 | 0.927560048944575 | 0.626103951344623 | 0.155045325945161 | 2.52833263756691 | 0.999950574162623 |
| ebi-fl187-GCST90094780 | prot-a-1483 | Interleukin-17C \|\| id:prot-a-1483 | F-healthy breakfast food liking (derived food-liking factor) \|\| id:ebi-fl187-GCST90094780 | MR Egger | 8 | -0.532479119 | 0.711033977786455 | 0.482244613207047 | -1.926105716 | 0.861147477198591 | 0.587147555058582 | 0.145714548879597 | 2.36587392310515 | 0.999950574162623 |
| ebi-fl187-GCST90094780 | prot-a-1503 | Interleukin-1 receptor-like 2 \|\| id:prot-a-1503 | F-healthy breakfast food liking (derived food-liking factor) \|\| id:ebi-fl187-GCST90094780 | MR Egger | 8 | 0.581334761094965 | 0.710865235065668 | 0.444733980810806 | -0.8119611 | 1.97463062182367 | 1.78842395706124 | 0.443986510115712 | 7.20395817737118 | 0.999950574162623 |
| ebi-fl187-GCST90094780 | prot-a-1522 | Interleukin-31 receptor subunit alpha \|\| id:prot-a-1522 | F-healthy breakfast food liking (derived food-liking factor) \|\| id:ebi-fl187-GCST90094780 | MR Egger | 8 | -0.51706287 | 1.24054985812212 | 0.691324333020091 | -2.948540592 | 1.91441485225692 | 0.596269299205932 | 0.0524161467214871 | 6.78296859676996 | 0.999950574162623 |
| ebi-fl187-GCST90094780 | prot-a-1540 | Interleukin-6 receptor subunit alpha \|\| id:prot-a-1540 | F-healthy breakfast food liking (derived food-liking factor) \|\| id:ebi-fl187-GCST90094780 | MR Egger | 8 | -0.651724038 | 0.712128744340258 | 0.395389375601272 | -2.047496377 | 0.74404830108055 | 0.521146525489132 | 0.129057611088107 | 2.1044376905751 | 0.999950574162623 |
| ebi-fl187-GCST90094780 | prot-a-1541 | Interleukin-6 receptor subunit alpha \|\| id:prot-a-1541 | F-healthy breakfast food liking (derived food-liking factor) \|\| id:ebi-fl187-GCST90094780 | MR Egger | 8 | -0.746807276 | 0.711020740696452 | 0.334017987671487 | -2.140407928 | 0.646793375807912 | 0.47387709888635 | 0.117606858137509 | 1.90940824714816 | 0.999950574162623 |
| ebi-fl187-GCST90094780 | prot-a-1545 | Interleukin-9 \|\| id:prot-a-1545 | F-healthy breakfast food liking (derived food-liking factor) \|\| id:ebi-fl187-GCST90094780 | MR Egger | 8 | 0.418800533846032 | 0.712142303138471 | 0.577923027421191 | -0.97699838 | 1.81459944799743 | 1.52013710864496 | 0.376439332454337 | 6.13861684966137 | 0.999950574162623 |
| ebi-fl187-GCST90094780 | prot-a-1548 | Inositol monophosphatase 3 \|\| id:prot-a-1548 | F-healthy breakfast food liking (derived food-liking factor) \|\| id:ebi-fl187-GCST90094780 | MR Egger | 8 | -1.051675642 | 0.709773950587505 | 0.188928607657294 | -2.442832585 | 0.33948130140843 | 0.349351869810256 | 0.0869143102899746 | 1.4042190351938 | 0.999950574162623 |
| ebi-fl187-GCST90094780 | prot-a-1555 | Inhibin beta A chain:Inhibin beta B chain heterodimer \|\| id:prot-a-1555 | F-healthy breakfast food liking (derived food-liking factor) \|\| id:ebi-fl187-GCST90094780 | MR Egger | 8 | -1.000182101 | 0.735298169975526 | 0.22263783765579 | -2.441366514 | 0.441002311872824 | 0.367812455953843 | 0.0870418262439804 | 1.55426429559954 | 0.999950574162623 |
| ebi-fl187-GCST90094780 | prot-a-1603 | Junctophilin-3 \|\| id:prot-a-1603 | F-healthy breakfast food liking (derived food-liking factor) \|\| id:ebi-fl187-GCST90094780 | MR Egger | 8 | 0.412012429450554 | 0.71087900511671 | 0.583287767191952 | -0.981310421 | 1.8053352794793 | 1.50985320285784 | 0.374819605566029 | 6.08201027995186 | 0.999950574162623 |
| ebi-fl187-GCST90094780 | prot-a-1627 | UPF0258 protein KIAA1024 \|\| id:prot-a-1627 | F-healthy breakfast food liking (derived food-liking factor) \|\| id:ebi-fl187-GCST90094780 | MR Egger | 8 | 0.65858226240294 | 0.924505450842242 | 0.502987861924935 | -1.153448421 | 2.47061294605374 | 1.93205125009531 | 0.315546752913815 | 11.829695595107 | 0.999950574162623 |
| ebi-fl187-GCST90094780 | prot-a-1666 | Kallikrein-7 \|\| id:prot-a-1666 | F-healthy breakfast food liking (derived food-liking factor) \|\| id:ebi-fl187-GCST90094780 | MR Egger | 8 | -1.066813816 | 0.844262184837842 | 0.253247577519318 | -2.721567698 | 0.587940066232265 | 0.344103148573724 | 0.0657715635909472 | 1.80027614357412 | 0.999950574162623 |
| ebi-fl187-GCST90094780 | prot-a-1678 | Importin subunit alpha-3 \|\| id:prot-a-1678 | F-healthy breakfast food liking (derived food-liking factor) \|\| id:ebi-fl187-GCST90094780 | MR Egger | 8 | -0.106954435 | 0.737054678100897 | 0.889375500751164 | -1.551581604 | 1.3376727342591 | 0.898566615408299 | 0.211912546930496 | 3.81016591052132 | 0.999950574162623 |
| ebi-fl187-GCST90094780 | prot-a-1718 | Protein LDOC1 \|\| id:prot-a-1718 | F-healthy breakfast food liking (derived food-liking factor) \|\| id:ebi-fl187-GCST90094780 | MR Egger | 8 | 0.091157580514908 | 0.711033979977604 | 0.902176543503985 | -1.30246902 | 1.48478418127101 | 1.09544161191164 | 0.271859736522615 | 4.41401268336679 | 0.999950574162623 |
| ebi-fl187-GCST90094780 | prot-a-1723 | Leptin \|\| id:prot-a-1723 | F-healthy breakfast food liking (derived food-liking factor) \|\| id:ebi-fl187-GCST90094780 | MR Egger | 8 | 0.970327320725078 | 1.12219462368873 | 0.420440829142475 | -1.229174142 | 3.16982878315498 | 2.6388080545759 | 0.292534069636097 | 23.8034084630101 | 0.999950574162623 |
| ebi-fl187-GCST90094780 | prot-a-1730 | Galectin-7 \|\| id:prot-a-1730 | F-healthy breakfast food liking (derived food-liking factor) \|\| id:ebi-fl187-GCST90094780 | MR Egger | 8 | 0.0834797235726956 | 0.710865234564549 | 0.910348647137566 | -1.309816136 | 1.47677558331921 | 1.087063173 | 0.269869671093669 | 4.37880380548951 | 0.999950574162623 |
| ebi-fl187-GCST90094780 | prot-a-1753 | Lipase member N \|\| id:prot-a-1753 | F-healthy breakfast food liking (derived food-liking factor) \|\| id:ebi-fl187-GCST90094780 | MR Egger | 8 | 0.95381425662376 | 0.711033979977604 | 0.228317384367017 | -0.439812344 | 2.34744085737986 | 2.59559105261594 | 0.644157289637035 | 10.458769963181 | 0.999950574162623 |
| ebi-fl187-GCST90094780 | prot-a-1756 | VIP36-like protein \|\| id:prot-a-1756 | F-healthy breakfast food liking (derived food-liking factor) \|\| id:ebi-fl187-GCST90094780 | MR Egger | 8 | 0.332051257092297 | 0.715741012704762 | 0.659054567223452 | -1.070801128 | 1.73490364199363 | 1.39382429006184 | 0.3427338337997 | 5.66838158354033 | 0.999950574162623 |
| ebi-fl187-GCST90094780 | prot-a-1760 | Ig Kappa chain V-I region HK102- like \|\| id:prot-a-1760 | F-healthy breakfast food liking (derived food-liking factor) \|\| id:ebi-fl187-GCST90094780 | MR Egger | 8 | 0.830934393786096 | 0.709760504001355 | 0.286097335099143 | -0.560196194 | 2.22206498162875 | 2.29546260449535 | 0.571097007018236 | 9.22636347920541 | 0.999950574162623 |
| ebi-fl187-GCST90094780 | prot-a-179 | Neutral ceramidase \|\| id:prot-a-179 | F-healthy breakfast food liking (derived food-liking factor) \|\| id:ebi-fl187-GCST90094780 | MR Egger | 8 | 0.904576395535567 | 0.709760504157883 | 0.249625170234841 | -0.486554193 | 2.29570698368502 | 2.47088502341043 | 0.614741027956404 | 9.93145490745888 | 0.999950574162623 |
| ebi-fl187-GCST90094780 | prot-a-1809 | Lactotransferrin \|\| id:prot-a-1809 | F-healthy breakfast food liking (derived food-liking factor) \|\| id:ebi-fl187-GCST90094780 | MR Egger | 8 | -0.366839745 | 0.711033977786455 | 0.624360795242366 | -1.760466341 | 1.02678685190776 | 0.692920680460039 | 0.171964650951334 | 2.79208003943254 | 0.999950574162623 |
| ebi-fl187-GCST90094780 | prot-a-1818 | Lysophospholipase-like protein 1 \|\| id:prot-a-1818 | F-healthy breakfast food liking (derived food-liking factor) \|\| id:ebi-fl187-GCST90094780 | MR Egger | 8 | -0.477360867 | 0.711020743250819 | 0.526970331989504 | -1.870961524 | 0.916239789790117 | 0.620418600304028 | 0.153975539476368 | 2.49987264803372 | 0.999950574162623 |
| ebi-fl187-GCST90094780 | prot-a-1852 | MAP kinase-activated protein kinase 3 \|\| id:prot-a-1852 | F-healthy breakfast food liking (derived food-liking factor) \|\| id:ebi-fl187-GCST90094780 | MR Egger | 8 | 0.138676447112393 | 0.714760964616296 | 0.852563866338821 | -1.262255044 | 1.53960793776033 | 1.14875235772522 | 0.283015095000912 | 4.66276181973612 | 0.999950574162623 |
| ebi-fl187-GCST90094780 | prot-a-1856 | Megakaryocyte-associated tyrosine-protein kinase \|\| id:prot-a-1856 | F-healthy breakfast food liking (derived food-liking factor) \|\| id:ebi-fl187-GCST90094780 | MR Egger | 8 | -0.389002415 | 0.711020740696452 | 0.604044175894322 | -1.782603067 | 1.00459823685999 | 0.677732633352033 | 0.168199741775049 | 2.73080991363578 | 0.999950574162623 |
| ebi-fl187-GCST90094780 | prot-a-1890 | Beta-1,3-N-acetylglucosaminyltransferase manic fringe \|\| id:prot-a-1890 | F-healthy breakfast food liking (derived food-liking factor) \|\| id:ebi-fl187-GCST90094780 | MR Egger | 8 | 1.53287855634634 | 0.780239868486654 | 0.0970708232359032 | 0.00360841411250279 | 3.06214869858019 | 4.63148965617877 | 1.00361493227643 | 21.3734329227604 | 0.999950574162623 |
| ebi-fl187-GCST90094780 | prot-a-1919 | Matrilysin \|\| id:prot-a-1919 | F-healthy breakfast food liking (derived food-liking factor) \|\| id:ebi-fl187-GCST90094780 | MR Egger | 8 | -0.476161254 | 0.709760504001355 | 0.527266271935007 | -1.867291841 | 0.914969334186901 | 0.621163309316605 | 0.154541618811623 | 2.49669868743562 | 0.999950574162623 |
| ebi-fl187-GCST90094780 | prot-a-1923 | Multimerin-2 \|\| id:prot-a-1923 | F-healthy breakfast food liking (derived food-liking factor) \|\| id:ebi-fl187-GCST90094780 | MR Egger | 8 | -0.036853095 | 0.711033977786455 | 0.960346412908043 | -1.430479691 | 1.35677350166793 | 0.963817714789819 | 0.23919415537505 | 3.88364249906571 | 0.999950574162623 |
| ebi-fl187-GCST90094780 | prot-a-1948 | Mesothelin \|\| id:prot-a-1948 | F-healthy breakfast food liking (derived food-liking factor) \|\| id:ebi-fl187-GCST90094780 | MR Egger | 8 | 0.250021475565299 | 0.727765271811328 | 0.742905504915753 | -1.176398457 | 1.6764414083155 | 1.28405299215552 | 0.30838741140226 | 5.34649608155654 | 0.999950574162623 |
| ebi-fl187-GCST90094780 | prot-a-195 | ATPase family AAA domain-containing protein 1 \|\| id:prot-a-195 | F-healthy breakfast food liking (derived food-liking factor) \|\| id:ebi-fl187-GCST90094780 | MR Egger | 8 | 1.54710937201958 | 0.948457040998242 | 0.153972342491498 | -0.311866428 | 3.40608517237613 | 4.69787073998612 | 0.732079306748416 | 30.1469926634632 | 0.999950574162623 |
| ebi-fl187-GCST90094780 | prot-a-1966 | Mucin-1 \|\| id:prot-a-1966 | F-healthy breakfast food liking (derived food-liking factor) \|\| id:ebi-fl187-GCST90094780 | MR Egger | 8 | 0.134469137680697 | 0.711033977786455 | 0.856235590310208 | -1.259157459 | 1.52809573414215 | 1.14392935414462 | 0.283893117416934 | 4.60939095382121 | 0.999950574162623 |
| ebi-fl187-GCST90094780 | prot-a-1986 | Myocardial zonula adherens protein \|\| id:prot-a-1986 | F-healthy breakfast food liking (derived food-liking factor) \|\| id:ebi-fl187-GCST90094780 | MR Egger | 8 | -0.974846248 | 0.773225863487755 | 0.254205288229602 | -2.490368941 | 0.540676444000189 | 0.377250351936073 | 0.0828793833418719 | 1.7171680374218 | 0.999950574162623 |
| ebi-fl187-GCST90094780 | prot-a-2071 | Epididymal secretory protein E1 \|\| id:prot-a-2071 | F-healthy breakfast food liking (derived food-liking factor) \|\| id:ebi-fl187-GCST90094780 | MR Egger | 8 | -0.75806451 | 0.899232314438062 | 0.43153778929022 | -2.520559846 | 1.0044308264958 | 0.468572467328179 | 0.0804145743591537 | 2.73035278601862 | 0.999950574162623 |
| ebi-fl187-GCST90094780 | prot-a-2098 | Neuritin \|\| id:prot-a-2098 | F-healthy breakfast food liking (derived food-liking factor) \|\| id:ebi-fl187-GCST90094780 | MR Egger | 8 | 0.308586416185273 | 1.03905278457823 | 0.776488723365434 | -1.727957042 | 2.34512987395861 | 1.36149916002773 | 0.177646964862716 | 10.4346278259734 | 0.999950574162623 |
| ebi-fl187-GCST90094780 | prot-a-2115 | Neurotrophin-4 \|\| id:prot-a-2115 | F-healthy breakfast food liking (derived food-liking factor) \|\| id:ebi-fl187-GCST90094780 | MR Egger | 8 | -0.180298627 | 0.778817090275565 | 0.824616969198722 | -1.706780124 | 1.34618287016587 | 0.835020814602555 | 0.181449096673149 | 3.84272930316933 | 0.999950574162623 |
| ebi-fl187-GCST90094780 | prot-a-2118 | Netrin-1 \|\| id:prot-a-2118 | F-healthy breakfast food liking (derived food-liking factor) \|\| id:ebi-fl187-GCST90094780 | MR Egger | 8 | -0.062914394 | 1.00530214448564 | 0.952131647339445 | -2.033306597 | 1.90747780907405 | 0.939023856214084 | 0.130901964660984 | 6.73607768090269 | 0.999950574162623 |
| ebi-fl187-GCST90094780 | prot-a-2120 | Netrin-G1 \|\| id:prot-a-2120 | F-healthy breakfast food liking (derived food-liking factor) \|\| id:ebi-fl187-GCST90094780 | MR Egger | 8 | -0.946047753 | 0.709773950664136 | 0.230947517629208 | -2.337204696 | 0.445109190668387 | 0.388272544126747 | 0.0965972799589425 | 1.56066059610305 | 0.999950574162623 |
| ebi-fl187-GCST90094780 | prot-a-2145 | Olfactomedin-like protein 3 \|\| id:prot-a-2145 | F-healthy breakfast food liking (derived food-liking factor) \|\| id:ebi-fl187-GCST90094780 | MR Egger | 8 | -1.940449845 | 0.939887380429967 | 0.0845218440568449 | -3.782629111 | -0.098270579 | 0.143639319824769 | 0.0227627668548401 | 0.906403616541686 | 0.999950574162623 |
| ebi-fl187-GCST90094780 | prot-a-2162 | Prolyl 4-hydroxylase subunit alpha-2 \|\| id:prot-a-2162 | F-healthy breakfast food liking (derived food-liking factor) \|\| id:ebi-fl187-GCST90094780 | MR Egger | 8 | -0.31914446 | 0.712128744340258 | 0.669769408331617 | -1.714916799 | 1.07662787855734 | 0.726770552194539 | 0.179978694451389 | 2.93476645748099 | 0.999950574162623 |
| ebi-fl187-GCST90094780 | prot-a-2168 | Polyadenylate-binding protein-interacting protein 1 \|\| id:prot-a-2168 | F-healthy breakfast food liking (derived food-liking factor) \|\| id:ebi-fl187-GCST90094780 | MR Egger | 8 | -0.365507216 | 0.729791420041531 | 0.634324128210436 | -1.795898399 | 1.06488396776258 | 0.693844632845588 | 0.165978270665316 | 2.90050241274885 | 0.999950574162623 |
| ebi-fl187-GCST90094780 | prot-a-219 | Beta-1,4-galactosyltransferase 5 \|\| id:prot-a-219 | F-healthy breakfast food liking (derived food-liking factor) \|\| id:ebi-fl187-GCST90094780 | MR Egger | 8 | 0.767409245732809 | 0.786247098926919 | 0.366738680520655 | -0.773635068 | 2.30845355962957 | 2.15417807207525 | 0.461333039606497 | 10.0588571981925 | 0.999950574162623 |
| ebi-fl187-GCST90094780 | prot-a-2190 | Pyruvate carboxylase, mitochondrial \|\| id:prot-a-2190 | F-healthy breakfast food liking (derived food-liking factor) \|\| id:ebi-fl187-GCST90094780 | MR Egger | 8 | -0.433622666 | 0.709773950664136 | 0.56365756143359 | -1.82477961 | 0.957534277081412 | 0.64815678079656 | 0.161253178879045 | 2.60526468633328 | 0.999950574162623 |
| ebi-fl187-GCST90094780 | prot-a-2197 | Protocadherin-9 \|\| id:prot-a-2197 | F-healthy breakfast food liking (derived food-liking factor) \|\| id:ebi-fl187-GCST90094780 | MR Egger | 8 | -1.899624282 | 0.709773950587505 | 0.0367129196950087 | -3.290781225 | -0.508467339 | 0.149624825370661 | 0.0372247571092493 | 0.601416640584017 | 0.999950574162623 |
| ebi-fl187-GCST90094780 | prot-a-2198 | Protocadherin alpha-4 \|\| id:prot-a-2198 | F-healthy breakfast food liking (derived food-liking factor) \|\| id:ebi-fl187-GCST90094780 | MR Egger | 8 | 0.765445744096323 | 0.711020743250819 | 0.323053639792889 | -0.628154913 | 2.15904640086793 | 2.14995248973196 | 0.533575386509718 | 8.66287281042053 | 0.999950574162623 |
| ebi-fl187-GCST90094780 | prot-a-2254 | Placenta growth factor \|\| id:prot-a-2254 | F-healthy breakfast food liking (derived food-liking factor) \|\| id:ebi-fl187-GCST90094780 | MR Egger | 8 | 0.417374853341712 | 0.709760504001355 | 0.577945169138937 | -0.973755735 | 1.80850544118437 | 1.51797142296003 | 0.377661973100308 | 6.10132183022644 | 0.999950574162623 |
| ebi-fl187-GCST90094780 | prot-a-2261 | Membrane-associated progesterone receptor component 2 \|\| id:prot-a-2261 | F-healthy breakfast food liking (derived food-liking factor) \|\| id:ebi-fl187-GCST90094780 | MR Egger | 8 | -0.229804088 | 0.711020740696452 | 0.757508843672705 | -1.62340474 | 1.1637965633324 | 0.794689276075341 | 0.197226051173255 | 3.20206707862531 | 0.999950574162623 |
| ebi-fl187-GCST90094780 | prot-a-2268 | Polymeric immunoglobulin receptor \|\| id:prot-a-2268 | F-healthy breakfast food liking (derived food-liking factor) \|\| id:ebi-fl187-GCST90094780 | MR Egger | 8 | 0.492068893832585 | 0.711033977786455 | 0.514782470677388 | -0.901557703 | 1.88569549029404 | 1.63569680474535 | 0.405936838114717 | 6.59093678090398 | 0.999950574162623 |
| ebi-fl187-GCST90094780 | prot-a-2283 | Group 10 secretory phospholipase A2 \|\| id:prot-a-2283 | F-healthy breakfast food liking (derived food-liking factor) \|\| id:ebi-fl187-GCST90094780 | MR Egger | 8 | 0.861674895229617 | 0.711020740696452 | 0.271111097477593 | -0.531925757 | 2.25527554699466 | 2.36712205678651 | 0.587472550542955 | 9.53792109358389 | 0.999950574162623 |
| ebi-fl187-GCST90094780 | prot-a-2298 | Inactive phospholipase D5 \|\| id:prot-a-2298 | F-healthy breakfast food liking (derived food-liking factor) \|\| id:ebi-fl187-GCST90094780 | MR Egger | 8 | 0.732780753867034 | 0.711033977786455 | 0.342476289649396 | -0.660845843 | 2.12640735032849 | 2.08085892641596 | 0.51641434445645 | 8.38468938387545 | 0.999950574162623 |
| ebi-fl187-GCST90094780 | prot-a-2304 | Procollagen-lysine,2-oxoglutarate 5-dioxygenase 2 \|\| id:prot-a-2304 | F-healthy breakfast food liking (derived food-liking factor) \|\| id:ebi-fl187-GCST90094780 | MR Egger | 8 | 0.00599962241774679 | 0.778098505481474 | 0.994097853782626 | -1.519073448 | 1.53107269316144 | 1.00601765619958 | 0.218914628728068 | 4.62313336694586 | 0.999950574162623 |
| ebi-fl187-GCST90094780 | prot-a-2331 | Periostin \|\| id:prot-a-2331 | F-healthy breakfast food liking (derived food-liking factor) \|\| id:ebi-fl187-GCST90094780 | MR Egger | 8 | -1.692330365 | 1.44683746197485 | 0.286488570609242 | -4.52813179 | 1.14347106089673 | 0.184090026867882 | 0.0108008354642319 | 3.1376404264693 | 0.999950574162623 |
| ebi-fl187-GCST90094780 | prot-a-2333 | Periostin \|\| id:prot-a-2333 | F-healthy breakfast food liking (derived food-liking factor) \|\| id:ebi-fl187-GCST90094780 | MR Egger | 8 | -1.621509697 | 1.27222960849017 | 0.249605135474372 | -4.11507973 | 0.872060335471614 | 0.197600157388313 | 0.0163246387972056 | 2.39183376030163 | 0.999950574162623 |
| ebi-fl187-GCST90094780 | prot-a-2367 | cAMP-dependent protein kinase catalytic subunit alpha \|\| id:prot-a-2367 | F-healthy breakfast food liking (derived food-liking factor) \|\| id:ebi-fl187-GCST90094780 | MR Egger | 8 | -0.032837376 | 1.06818746759563 | 0.976472985827858 | -2.126484812 | 2.06081006097286 | 0.967695917844214 | 0.119255764351806 | 7.85232809920917 | 0.999950574162623 |
| ebi-fl187-GCST90094780 | prot-a-2385 | Phosphoribosyl pyrophosphate synthase-associated protein 1 \|\| id:prot-a-2385 | F-healthy breakfast food liking (derived food-liking factor) \|\| id:ebi-fl187-GCST90094780 | MR Egger | 8 | 1.82028243454173 | 0.717108335558249 | 0.0441782509180923 | 0.414750096847563 | 3.2258147722359 | 6.17360184208198 | 1.51399234195348 | 25.1740769410901 | 0.999950574162623 |
| ebi-fl187-GCST90094780 | prot-a-2393 | Trypsin-3 \|\| id:prot-a-2393 | F-healthy breakfast food liking (derived food-liking factor) \|\| id:ebi-fl187-GCST90094780 | MR Egger | 8 | 0.26038439092683 | 1.00601306730204 | 0.804418517728503 | -1.711401221 | 2.23217000283882 | 1.29742871065095 | 0.180612537147885 | 9.32006872725052 | 0.999950574162623 |
| ebi-fl187-GCST90094780 | prot-a-2405 | Pregnancy-specific beta-1-glycoprotein 6 \|\| id:prot-a-2405 | F-healthy breakfast food liking (derived food-liking factor) \|\| id:ebi-fl187-GCST90094780 | MR Egger | 8 | 1.07755514357906 | 0.709773950587505 | 0.179771312694409 | -0.3136018 | 2.46871208673057 | 2.93748902584004 | 0.730809979073313 | 11.807230366329 | 0.999950574162623 |
| ebi-fl187-GCST90094780 | prot-a-244 | Beclin-1 \|\| id:prot-a-244 | F-healthy breakfast food liking (derived food-liking factor) \|\| id:ebi-fl187-GCST90094780 | MR Egger | 8 | 1.57025192683679 | 0.764807728788126 | 0.085868799984015 | 0.0712287784120675 | 3.06927507526152 | 4.80785926999512 | 1.07382686617397 | 21.5262920757778 | 0.999950574162623 |
| ebi-fl187-GCST90094780 | prot-a-2454 | tRNA pseudouridine synthase A, mitochondrial \|\| id:prot-a-2454 | F-healthy breakfast food liking (derived food-liking factor) \|\| id:ebi-fl187-GCST90094780 | MR Egger | 8 | 0.974165966588615 | 0.710865234564549 | 0.219622415517099 | -0.419129893 | 2.36746182633513 | 2.64895697078469 | 0.657618769541158 | 10.6702748736396 | 0.999950574162623 |
| ebi-fl187-GCST90094780 | prot-a-2466 | Glutaminyl-peptide cyclotransferase-like protein \|\| id:prot-a-2466 | F-healthy breakfast food liking (derived food-liking factor) \|\| id:ebi-fl187-GCST90094780 | MR Egger | 8 | -1.575266234 | 0.73511895400108 | 0.0758489344491914 | -3.016099383 | -0.134433084 | 0.206952447658814 | 0.0489919449380671 | 0.874211375892844 | 0.999950574162623 |
| ebi-fl187-GCST90094780 | prot-a-2474 | Ras-related protein Rab-27A \|\| id:prot-a-2474 | F-healthy breakfast food liking (derived food-liking factor) \|\| id:ebi-fl187-GCST90094780 | MR Egger | 8 | -0.542196125 | 0.899003799657939 | 0.568518289030389 | -2.304243572 | 1.21985132217963 | 0.581469868540118 | 0.0998342895031682 | 3.38668417136711 | 0.999950574162623 |
| ebi-fl187-GCST90094780 | prot-a-2490 | Rap1 GTPase-GDP dissociation stimulator 1 \|\| id:prot-a-2490 | F-healthy breakfast food liking (derived food-liking factor) \|\| id:ebi-fl187-GCST90094780 | MR Egger | 8 | 0.712039998575133 | 0.718797506183628 | 0.360133001357251 | -0.696803114 | 2.12088311069504 | 2.03814483311877 | 0.498175370900748 | 8.33849805392396 | 0.999950574162623 |
| ebi-fl187-GCST90094780 | prot-a-2545 | Receptor-interacting serine/threonine-protein kinase 2 \|\| id:prot-a-2545 | F-healthy breakfast food liking (derived food-liking factor) \|\| id:ebi-fl187-GCST90094780 | MR Egger | 8 | 1.35384668704688 | 0.795731052960708 | 0.139771702072946 | -0.205786177 | 2.91347955084987 | 3.87229241531436 | 0.814007111316985 | 18.4207832354698 | 0.999950574162623 |
| ebi-fl187-GCST90094780 | prot-a-2610 | RUN and FYVE domain-containing protein 1 \|\| id:prot-a-2610 | F-healthy breakfast food liking (derived food-liking factor) \|\| id:ebi-fl187-GCST90094780 | MR Egger | 8 | -0.771414784 | 0.788223430036632 | 0.365536250207767 | -2.316332707 | 0.773503138882011 | 0.462358468003325 | 0.0986346453017523 | 2.16734548271938 | 0.999950574162623 |
| ebi-fl187-GCST90094780 | prot-a-2627 | Serine--tRNA ligase, mitochondrial \|\| id:prot-a-2627 | F-healthy breakfast food liking (derived food-liking factor) \|\| id:ebi-fl187-GCST90094780 | MR Egger | 8 | -0.795015001 | 0.711033977786455 | 0.306278121066673 | -2.188641598 | 0.598611595272787 | 0.4515744607181 | 0.112068879895992 | 1.81959071744177 | 0.999950574162623 |
| ebi-fl187-GCST90094780 | prot-a-264 | BolA-like protein 2 \|\| id:prot-a-264 | F-healthy breakfast food liking (derived food-liking factor) \|\| id:ebi-fl187-GCST90094780 | MR Egger | 8 | 1.15581167695621 | 0.710865235065668 | 0.155091162525175 | -0.237484184 | 2.54910753768492 | 3.17660074503126 | 0.788609363707548 | 12.7956790240133 | 0.999950574162623 |
| ebi-fl187-GCST90094780 | prot-a-2646 | Secretoglobin family 1C member 1 \|\| id:prot-a-2646 | F-healthy breakfast food liking (derived food-liking factor) \|\| id:ebi-fl187-GCST90094780 | MR Egger | 8 | -0.327409574 | 0.712128744340258 | 0.661875665360657 | -1.723181913 | 1.06836276506773 | 0.720788466462515 | 0.178497280576136 | 2.91061024408034 | 0.999950574162623 |
| ebi-fl187-GCST90094780 | prot-a-2672 | Semaphorin-3E \|\| id:prot-a-2672 | F-healthy breakfast food liking (derived food-liking factor) \|\| id:ebi-fl187-GCST90094780 | MR Egger | 8 | -0.706304506 | 1.01015327559718 | 0.51060570001576 | -2.686204926 | 1.27359591408086 | 0.493464426850547 | 0.0681390419916701 | 3.57368013182086 | 0.999950574162623 |
| ebi-fl187-GCST90094780 | prot-a-2701 | Plasma protease C1 inhibitor \|\| id:prot-a-2701 | F-healthy breakfast food liking (derived food-liking factor) \|\| id:ebi-fl187-GCST90094780 | MR Egger | 8 | -0.740221834 | 0.861858189809347 | 0.423392926284026 | -2.429463886 | 0.949020217823428 | 0.477008087074515 | 0.0880840429946019 | 2.5831774677785 | 0.999950574162623 |
| ebi-fl187-GCST90094780 | prot-a-2738 | Signaling threshold-regulating transmembrane adapter 1 \|\| id:prot-a-2738 | F-healthy breakfast food liking (derived food-liking factor) \|\| id:ebi-fl187-GCST90094780 | MR Egger | 8 | -0.98820275 | 0.82587291704896 | 0.276611462343744 | -2.606913668 | 0.630508167327752 | 0.372245107673171 | 0.0737618464003671 | 1.87856496208751 | 0.999950574162623 |
| ebi-fl187-GCST90094780 | prot-a-274 | Serine/threonine-protein kinase BRSK2 \|\| id:prot-a-274 | F-healthy breakfast food liking (derived food-liking factor) \|\| id:ebi-fl187-GCST90094780 | MR Egger | 8 | -0.57681594 | 0.712128744340259 | 0.448870581490291 | -1.972588279 | 0.818956398774252 | 0.561683957690133 | 0.139096369678307 | 2.26813157709359 | 0.999950574162623 |
| ebi-fl187-GCST90094780 | prot-a-2749 | Mitochondrial glutamate carrier 2 \|\| id:prot-a-2749 | F-healthy breakfast food liking (derived food-liking factor) \|\| id:ebi-fl187-GCST90094780 | MR Egger | 8 | -0.290798264 | 0.774265045239739 | 0.720149331078625 | -1.808357752 | 1.22676122498787 | 0.747666494290655 | 0.163923118230574 | 3.41016686797393 | 0.999950574162623 |
| ebi-fl187-GCST90094780 | prot-a-2760 | Sodium-coupled monocarboxylate transporter 1 \|\| id:prot-a-2760 | F-healthy breakfast food liking (derived food-liking factor) \|\| id:ebi-fl187-GCST90094780 | MR Egger | 8 | 0.587414717209266 | 0.710865234564549 | 0.440227785773383 | -0.805881143 | 1.98071057695578 | 1.79933061864801 | 0.446694151880661 | 7.24789134035758 | 0.999950574162623 |
| ebi-fl187-GCST90094780 | prot-a-2777 | Small integral membrane protein 10 \|\| id:prot-a-2777 | F-healthy breakfast food liking (derived food-liking factor) \|\| id:ebi-fl187-GCST90094780 | MR Egger | 8 | -0.062883401 | 0.738980553622611 | 0.934954427300335 | -1.511285286 | 1.385518484 | 0.939052959570864 | 0.220626227737181 | 3.99689769400063 | 0.999950574162623 |
| ebi-fl187-GCST90094780 | prot-a-2799 | Superoxide dismutase [Mn], mitochondrial \|\| id:prot-a-2799 | F-healthy breakfast food liking (derived food-liking factor) \|\| id:ebi-fl187-GCST90094780 | MR Egger | 8 | -0.846119947 | 0.90931771143354 | 0.38801870192882 | -2.628382661 | 0.936142767594541 | 0.429076546107715 | 0.072195131852305 | 2.5501259945943 | 0.999950574162623 |
| ebi-fl187-GCST90094780 | prot-a-2805 | Protein SPACA7 \|\| id:prot-a-2805 | F-healthy breakfast food liking (derived food-liking factor) \|\| id:ebi-fl187-GCST90094780 | MR Egger | 8 | 0.841131478737521 | 1.00857105665702 | 0.436227693864004 | -1.135667792 | 2.81793074978528 | 2.31898938008063 | 0.32120754976602 | 16.7421710630527 | 0.999950574162623 |
| ebi-fl187-GCST90094780 | prot-a-2811 | SPARC-like protein 1 \|\| id:prot-a-2811 | F-healthy breakfast food liking (derived food-liking factor) \|\| id:ebi-fl187-GCST90094780 | MR Egger | 8 | -0.268947102 | 0.711033977786455 | 0.718267567248077 | -1.662573698 | 1.12467949461023 | 0.764183678480959 | 0.189650248922341 | 3.07922978100503 | 0.999950574162623 |
| ebi-fl187-GCST90094780 | prot-a-2831 | Sepiapterin reductase \|\| id:prot-a-2831 | F-healthy breakfast food liking (derived food-liking factor) \|\| id:ebi-fl187-GCST90094780 | MR Egger | 8 | 0.125582346067551 | 0.712142303138471 | 0.865824942322517 | -1.270216568 | 1.52138126021895 | 1.13380852979031 | 0.280770809197436 | 4.57854499155321 | 0.999950574162623 |
| ebi-fl187-GCST90094780 | prot-a-2834 | SPRY domain-containing SOCS box protein 1 \|\| id:prot-a-2834 | F-healthy breakfast food liking (derived food-liking factor) \|\| id:ebi-fl187-GCST90094780 | MR Egger | 8 | -0.707532263 | 0.860874758567771 | 0.442574345992258 | -2.39484679 | 0.979782263715725 | 0.492858944221226 | 0.0911866485707123 | 2.66387615628282 | 0.999950574162623 |
| ebi-fl187-GCST90094780 | prot-a-2835 | Spectrin alpha chain, non-erythrocytic 1 \|\| id:prot-a-2835 | F-healthy breakfast food liking (derived food-liking factor) \|\| id:ebi-fl187-GCST90094780 | MR Egger | 8 | 0.780328202020471 | 0.711033977786455 | 0.314521473994005 | -0.613298394 | 2.17395479848192 | 2.18218834660654 | 0.54156163600879 | 8.79298987121775 | 0.999950574162623 |
| ebi-fl187-GCST90094780 | prot-a-2859 | Sia-alpha-2,3-Gal-beta-1,4-GlcNAc-R:alpha 2,8-sialyltransferase \|\| id:prot-a-2859 | F-healthy breakfast food liking (derived food-liking factor) \|\| id:ebi-fl187-GCST90094780 | MR Egger | 8 | -1.023171176 | 0.711020743250819 | 0.200200147673529 | -2.416771833 | 0.370429480860474 | 0.359453241405718 | 0.0892091351465809 | 1.44835652251057 | 0.999950574162623 |
| ebi-fl187-GCST90094780 | prot-a-2861 | Alpha-2,8-sialyltransferase 8F \|\| id:prot-a-2861 | F-healthy breakfast food liking (derived food-liking factor) \|\| id:ebi-fl187-GCST90094780 | MR Egger | 8 | 0.665693462010833 | 0.748326887184269 | 0.407942468546769 | -0.801027237 | 2.132414161 | 1.94583941929449 | 0.44886763382668 | 8.43520619520166 | 0.999950574162623 |
| ebi-fl187-GCST90094780 | prot-a-289 | Uncharacterized protein C17orf89 \|\| id:prot-a-289 | F-healthy breakfast food liking (derived food-liking factor) \|\| id:ebi-fl187-GCST90094780 | MR Egger | 8 | 1.27182528063131 | 0.709773950587505 | 0.123332497244342 | -0.119331663 | 2.66298222378282 | 3.56735805293535 | 0.887513397013341 | 14.3389874684353 | 0.999950574162623 |
| ebi-fl187-GCST90094780 | prot-a-2921 | Tachykinin-3 \|\| id:prot-a-2921 | F-healthy breakfast food liking (derived food-liking factor) \|\| id:ebi-fl187-GCST90094780 | MR Egger | 8 | 0.908700343859224 | 0.710879005802792 | 0.248371720597581 | -0.484622508 | 2.3020231952327 | 2.48109586558213 | 0.615929661703686 | 9.99438260073633 | 0.999950574162623 |
| ebi-fl187-GCST90094780 | prot-a-2928 | Tax1-binding protein 1 \|\| id:prot-a-2928 | F-healthy breakfast food liking (derived food-liking factor) \|\| id:ebi-fl187-GCST90094780 | MR Egger | 8 | 0.310042855321176 | 0.919304865810792 | 0.747407722854489 | -1.491794682 | 2.11188039231033 | 1.36348354540538 | 0.224968546098447 | 8.26376580563257 | 0.999950574162623 |
| ebi-fl187-GCST90094780 | prot-a-296 | Uncharacterized protein C1orf185 \|\| id:prot-a-296 | F-healthy breakfast food liking (derived food-liking factor) \|\| id:ebi-fl187-GCST90094780 | MR Egger | 8 | 0.00985147992756941 | 0.711033977786455 | 0.989394758594508 | -1.383775117 | 1.40347807638902 | 1.00990016549959 | 0.250630605137275 | 4.06932881847167 | 0.999950574162623 |
| ebi-fl187-GCST90094780 | prot-a-2964 | Transforming growth factor beta-2 \|\| id:prot-a-2964 | F-healthy breakfast food liking (derived food-liking factor) \|\| id:ebi-fl187-GCST90094780 | MR Egger | 8 | 0.296593099327148 | 0.712142303279138 | 0.691546883346048 | -1.099205815 | 1.69239201375426 | 1.34526779769419 | 0.333135549890039 | 5.4324596942906 | 0.999950574162623 |
| ebi-fl187-GCST90094780 | prot-a-2977 | Thymocyte nuclear protein 1 \|\| id:prot-a-2977 | F-healthy breakfast food liking (derived food-liking factor) \|\| id:ebi-fl187-GCST90094780 | MR Egger | 8 | 0.813273488240358 | 0.710865235065668 | 0.296179113596588 | -0.580022372 | 2.20656934896907 | 2.25527854417117 | 0.55988584038584 | 9.08449713301136 | 0.999950574162623 |
| ebi-fl187-GCST90094780 | prot-a-3015 | Transmembrane protein 70, mitochondrial \|\| id:prot-a-3015 | F-healthy breakfast food liking (derived food-liking factor) \|\| id:ebi-fl187-GCST90094780 | MR Egger | 8 | -1.421072976 | 0.709760504001355 | 0.0921466960117788 | -2.812203564 | -0.029942388 | 0.241454802702011 | 0.0600724729225606 | 0.970501444530545 | 0.999950574162623 |
| ebi-fl187-GCST90094780 | prot-a-3030 | Tumor necrosis factor alpha-induced protein 3 \|\| id:prot-a-3030 | F-healthy breakfast food liking (derived food-liking factor) \|\| id:ebi-fl187-GCST90094780 | MR Egger | 8 | -1.21781942 | 0.84773254810333 | 0.200867301787743 | -2.879375215 | 0.443736373977748 | 0.295874642240951 | 0.0561698459736732 | 1.55851956514607 | 0.999950574162623 |
| ebi-fl187-GCST90094780 | prot-a-3032 | Tumor necrosis factor receptor superfamily member 10A \|\| id:prot-a-3032 | F-healthy breakfast food liking (derived food-liking factor) \|\| id:ebi-fl187-GCST90094780 | MR Egger | 8 | 0.75968569958404 | 0.853598503938823 | 0.40774188540357 | -0.913367368 | 2.43273876730413 | 2.13760426499476 | 0.401171056373116 | 11.3900340543859 | 0.999950574162623 |
| ebi-fl187-GCST90094780 | prot-a-3081 | Tumor protein p53-inducible protein 11 \|\| id:prot-a-3081 | F-healthy breakfast food liking (derived food-liking factor) \|\| id:ebi-fl187-GCST90094780 | MR Egger | 8 | 0.0686707829895409 | 0.712142303138471 | 0.926320516111846 | -1.327128131 | 1.46446969714094 | 1.07108353217949 | 0.26523789700515 | 4.32524894013848 | 0.999950574162623 |
| ebi-fl187-GCST90094780 | prot-a-315 | Complement C4 \|\| id:prot-a-315 | F-healthy breakfast food liking (derived food-liking factor) \|\| id:ebi-fl187-GCST90094780 | MR Egger | 7 | -0.039691761 | 0.793454281386678 | 0.962040102557662 | -1.594862152 | 1.51547863076783 | 0.961085637816085 | 0.202936500910935 | 4.5515991409635 | 0.999950574162623 |
| ebi-fl187-GCST90094780 | prot-a-3157 | UDP-glucuronosyltransferase 1-8 \|\| id:prot-a-3157 | F-healthy breakfast food liking (derived food-liking factor) \|\| id:ebi-fl187-GCST90094780 | MR Egger | 8 | -0.469568568 | 0.859688645713677 | 0.604623039933648 | -2.154558314 | 1.21542117743122 | 0.625271972331842 | 0.115954394763856 | 3.371713855 | 0.999950574162623 |
| ebi-fl187-GCST90094780 | prot-a-317 | Glutaredoxin-like protein C5orf63 \|\| id:prot-a-317 | F-healthy breakfast food liking (derived food-liking factor) \|\| id:ebi-fl187-GCST90094780 | MR Egger | 8 | -0.114183461 | 1.0065514886653 | 0.913382044871319 | -2.087024379 | 1.8586574566682 | 0.892094276257129 | 0.124055729994954 | 6.4151184130157 | 0.999950574162623 |
| ebi-fl187-GCST90094780 | prot-a-3192 | Guanine nucleotide exchange factor VAV3 \|\| id:prot-a-3192 | F-healthy breakfast food liking (derived food-liking factor) \|\| id:ebi-fl187-GCST90094780 | MR Egger | 8 | 0.252782030563644 | 0.873247742266265 | 0.781961541753422 | -1.458783544 | 1.96434760540552 | 1.28760258823457 | 0.23251895177215 | 7.13025932979856 | 0.999950574162623 |
| ebi-fl187-GCST90094780 | prot-a-3233 | Serine/threonine-protein kinase WNK3 \|\| id:prot-a-3233 | F-healthy breakfast food liking (derived food-liking factor) \|\| id:ebi-fl187-GCST90094780 | MR Egger | 8 | 2.04865229179548 | 0.709760504001355 | 0.0278240932714268 | 0.657521703952819 | 3.43978287963813 | 7.75743929357279 | 1.93000328300232 | 31.1801875797093 | 0.999950574162623 |
| ebi-fl187-GCST90094780 | prot-a-3250 | 14-3-3 protein theta \|\| id:prot-a-3250 | F-healthy breakfast food liking (derived food-liking factor) \|\| id:ebi-fl187-GCST90094780 | MR Egger | 8 | 0.82510410050646 | 0.885676529316069 | 0.387489335837467 | -0.910821897 | 2.56103009796595 | 2.28211832263736 | 0.402193526518636 | 12.9491493401146 | 0.999950574162623 |
| ebi-fl187-GCST90094780 | prot-a-3278 | E3 ubiquitin-protein ligase ZNRF3 \|\| id:prot-a-3278 | F-healthy breakfast food liking (derived food-liking factor) \|\| id:ebi-fl187-GCST90094780 | MR Egger | 8 | 0.965676510884473 | 0.753971527380369 | 0.247546162230516 | -0.512107683 | 2.443460705 | 2.6265639546684 | 0.599231257482898 | 11.5128143297167 | 0.999950574162623 |
| ebi-fl187-GCST90094780 | prot-a-355 | Calpain-3 \|\| id:prot-a-355 | F-healthy breakfast food liking (derived food-liking factor) \|\| id:ebi-fl187-GCST90094780 | MR Egger | 8 | -0.663015618 | 0.711033979977604 | 0.38708018850855 | -2.056642218 | 0.730610983175812 | 0.515295056270384 | 0.127882651805245 | 2.07634883440701 | 0.999950574162623 |
| ebi-fl187-GCST90094780 | prot-a-385 | Coiled-coil domain-containing protein 90B, mitochondrial \|\| id:prot-a-385 | F-healthy breakfast food liking (derived food-liking factor) \|\| id:ebi-fl187-GCST90094780 | MR Egger | 8 | -0.07175802 | 0.711020743250818 | 0.922899920933225 | -1.465358676 | 1.32184263722546 | 0.930756093345301 | 0.230995124136399 | 3.75032550378798 | 0.999950574162623 |
| ebi-fl187-GCST90094780 | prot-a-390 | C-C motif chemokine 14 \|\| id:prot-a-390 | F-healthy breakfast food liking (derived food-liking factor) \|\| id:ebi-fl187-GCST90094780 | MR Egger | 8 | 0.151348105293047 | 1.06261430677648 | 0.891402880662446 | -1.931375936 | 2.23407214657494 | 1.16340157384687 | 0.144948621187893 | 9.33781370900286 | 0.999950574162623 |
| ebi-fl187-GCST90094780 | prot-a-40 | Pituitary adenylate cyclase-activating polypeptide 27 \|\| id:prot-a-40 | F-healthy breakfast food liking (derived food-liking factor) \|\| id:ebi-fl187-GCST90094780 | MR Egger | 8 | 0.888582896680163 | 0.846802870608846 | 0.334439093156239 | -0.77115073 | 2.5483165230735 | 2.43168126491377 | 0.462480571854441 | 12.7855614570415 | 0.999950574162623 |
| ebi-fl187-GCST90094780 | prot-a-401 | C-C motif chemokine 24 \|\| id:prot-a-401 | F-healthy breakfast food liking (derived food-liking factor) \|\| id:ebi-fl187-GCST90094780 | MR Egger | 8 | 1.58146552043627 | 0.846507738381401 | 0.110951747650279 | -0.077689647 | 3.24062068766381 | 4.86207606435983 | 0.925251536771757 | 25.5495751329428 | 0.999950574162623 |
| ebi-fl187-GCST90094780 | prot-a-412 | Cerebral cavernous malformations 2 protein \|\| id:prot-a-412 | F-healthy breakfast food liking (derived food-liking factor) \|\| id:ebi-fl187-GCST90094780 | MR Egger | 8 | 1.12593046212921 | 0.711020740696452 | 0.164387824434275 | -0.26767019 | 2.51953111389426 | 3.08308420782328 | 0.765160097222653 | 12.4227704333141 | 0.999950574162623 |
| ebi-fl187-GCST90094780 | prot-a-44 | Adhesion G protein-coupled receptor F5 \|\| id:prot-a-44 | F-healthy breakfast food liking (derived food-liking factor) \|\| id:ebi-fl187-GCST90094780 | MR Egger | 8 | -0.723655841 | 0.724609220055887 | 0.356505253579303 | -2.143889912 | 0.696578230765301 | 0.484976016130737 | 0.117198065042267 | 2.00687388599134 | 0.999950574162623 |
| ebi-fl187-GCST90094780 | prot-a-48 | Adrenomedullin \|\| id:prot-a-48 | F-healthy breakfast food liking (derived food-liking factor) \|\| id:ebi-fl187-GCST90094780 | MR Egger | 8 | 0.264710334283179 | 0.906817080324262 | 0.780184444761344 | -1.512651143 | 2.04207181171873 | 1.30305347118815 | 0.220325089637931 | 7.70655921014623 | 0.999950574162623 |
| ebi-fl187-GCST90094780 | prot-a-502 | Carcinoembryonic antigen-related cell adhesion molecule 1 \|\| id:prot-a-502 | F-healthy breakfast food liking (derived food-liking factor) \|\| id:ebi-fl187-GCST90094780 | MR Egger | 8 | 0.0652380616565809 | 1.00877772487034 | 0.950537253836824 | -1.911966279 | 2.04244240240244 | 1.06741310427151 | 0.147789505298392 | 7.70941571845793 | 0.999950574162623 |
| ebi-fl187-GCST90094780 | prot-a-518 | Complement factor B \|\| id:prot-a-518 | F-healthy breakfast food liking (derived food-liking factor) \|\| id:ebi-fl187-GCST90094780 | MR Egger | 8 | 0.688832935810915 | 0.719943823410351 | 0.375621231385141 | -0.722256958 | 2.0999228296952 | 1.99139009647195 | 0.485654915317869 | 8.16553975106167 | 0.999950574162623 |
| ebi-fl187-GCST90094780 | prot-a-525 | Properdin \|\| id:prot-a-525 | F-healthy breakfast food liking (derived food-liking factor) \|\| id:ebi-fl187-GCST90094780 | MR Egger | 8 | -0.287064228 | 1.07668238975895 | 0.79868868869234 | -2.397361712 | 1.82323325557714 | 0.750463526254771 | 0.0909576093535605 | 6.19184594055845 | 0.999950574162623 |
| ebi-fl187-GCST90094780 | prot-a-533 | Chondroadherin \|\| id:prot-a-533 | F-healthy breakfast food liking (derived food-liking factor) \|\| id:ebi-fl187-GCST90094780 | MR Egger | 8 | -1.381199354 | 0.950596003711847 | 0.196445984810133 | -3.244367522 | 0.481968812963145 | 0.251277002106684 | 0.0389932189289356 | 1.61925928461546 | 0.999950574162623 |
| ebi-fl187-GCST90094780 | prot-a-535 | Coiled-coil-helix-coiled-coil-helix domain-containing protein 2 \|\| id:prot-a-535 | F-healthy breakfast food liking (derived food-liking factor) \|\| id:ebi-fl187-GCST90094780 | MR Egger | 8 | -1.026625198 | 1.11632804381031 | 0.393229372984143 | -3.214628164 | 1.16137776752139 | 0.358213823566047 | 0.0401702677900162 | 3.19433129160515 | 0.999950574162623 |
| ebi-fl187-GCST90094780 | prot-a-591 | Caseinolytic peptidase B protein homolog \|\| id:prot-a-591 | F-healthy breakfast food liking (derived food-liking factor) \|\| id:ebi-fl187-GCST90094780 | MR Egger | 8 | -0.358492116 | 0.740848423800846 | 0.645615021043832 | -1.810555027 | 1.09357079444657 | 0.69872913445547 | 0.163563329564774 | 2.98491357834303 | 0.999950574162623 |
| ebi-fl187-GCST90094780 | prot-a-605 | Ciliary neurotrophic factor receptor subunit alpha \|\| id:prot-a-605 | F-healthy breakfast food liking (derived food-liking factor) \|\| id:ebi-fl187-GCST90094780 | MR Egger | 8 | -1.474370523 | 1.0370291536034 | 0.204940229069496 | -3.506947664 | 0.558206618458455 | 0.228922783417893 | 0.029988309288064 | 1.74753569013822 | 0.999950574162623 |
| ebi-fl187-GCST90094780 | prot-a-606 | Ciliary neurotrophic factor receptor subunit alpha \|\| id:prot-a-606 | F-healthy breakfast food liking (derived food-liking factor) \|\| id:ebi-fl187-GCST90094780 | MR Egger | 8 | -1.066945514 | 0.886240995646055 | 0.273964170126954 | -2.803977866 | 0.670086836971948 | 0.344057833708353 | 0.0605686488212491 | 1.95440702805566 | 0.999950574162623 |
| ebi-fl187-GCST90094780 | prot-a-707 | Cancer/testis antigen 55 \|\| id:prot-a-707 | F-healthy breakfast food liking (derived food-liking factor) \|\| id:ebi-fl187-GCST90094780 | MR Egger | 8 | -0.046100893 | 0.710865234564549 | 0.950398736305822 | -1.439396753 | 1.34719496666948 | 0.954945609929172 | 0.237070727802093 | 3.84662048485494 | 0.999950574162623 |
| ebi-fl187-GCST90094780 | prot-a-719 | Dipeptidyl peptidase 1 \|\| id:prot-a-719 | F-healthy breakfast food liking (derived food-liking factor) \|\| id:ebi-fl187-GCST90094780 | MR Egger | 8 | -0.003574906 | 0.711033979977604 | 0.996151444507926 | -1.397201507 | 1.39005169500336 | 0.996431476615107 | 0.247288030461703 | 4.01505760604586 | 0.999950574162623 |
| ebi-fl187-GCST90094780 | prot-a-752 | Cytochrome b5 \|\| id:prot-a-752 | F-healthy breakfast food liking (derived food-liking factor) \|\| id:ebi-fl187-GCST90094780 | MR Egger | 8 | 0.987094253219871 | 0.818351878388298 | 0.273140427298592 | -0.616875428 | 2.59106393486094 | 2.68342577698667 | 0.539627912194165 | 13.3439611589357 | 0.999950574162623 |
| ebi-fl187-GCST90094780 | prot-a-755 | Cytoglobin \|\| id:prot-a-755 | F-healthy breakfast food liking (derived food-liking factor) \|\| id:ebi-fl187-GCST90094780 | MR Egger | 8 | -0.45208756 | 0.709773950664136 | 0.547677093038161 | -1.843244504 | 0.939069382828715 | 0.636298452692337 | 0.158302977385131 | 2.55760016385321 | 0.999950574162623 |
| ebi-fl187-GCST90094780 | prot-a-807 | Desmin \|\| id:prot-a-807 | F-healthy breakfast food liking (derived food-liking factor) \|\| id:ebi-fl187-GCST90094780 | MR Egger | 8 | 0.0933537467647051 | 0.76245865109872 | 0.906550369130045 | -1.401065209 | 1.5877727029182 | 1.09785002748083 | 0.246334426393924 | 4.89283897701025 | 0.999950574162623 |
| ebi-fl187-GCST90094780 | prot-a-82 | Ameloblastin \|\| id:prot-a-82 | F-healthy breakfast food liking (derived food-liking factor) \|\| id:ebi-fl187-GCST90094780 | MR Egger | 8 | -0.078273924 | 0.712128744340258 | 0.916060653679328 | -1.474046263 | 1.31749841474856 | 0.924711091140428 | 0.22899702585036 | 3.73406859282482 | 0.999950574162623 |
| ebi-fl187-GCST90094780 | prot-a-84 | Muellerian-inhibiting factor \|\| id:prot-a-84 | F-healthy breakfast food liking (derived food-liking factor) \|\| id:ebi-fl187-GCST90094780 | MR Egger | 8 | -0.430633237 | 0.709773950664136 | 0.566271381765219 | -1.821790181 | 0.960523706048916 | 0.650097298527365 | 0.161735955056097 | 2.61306459287913 | 0.999950574162623 |
| ebi-fl187-GCST90094780 | prot-a-858 | Inactive dipeptidyl peptidase 10 \|\| id:prot-a-858 | F-healthy breakfast food liking (derived food-liking factor) \|\| id:ebi-fl187-GCST90094780 | MR Egger | 8 | 0.994186798527054 | 0.847558519266867 | 0.285250560825872 | -0.667027899 | 2.65540149629011 | 2.7025257495314 | 0.513231689541143 | 14.2306984851424 | 0.999950574162623 |
| ebi-fl187-GCST90094780 | prot-a-863 | Dihydropyrimidinase-related protein 3 \|\| id:prot-a-863 | F-healthy breakfast food liking (derived food-liking factor) \|\| id:ebi-fl187-GCST90094780 | MR Egger | 8 | -1.476001713 | 0.709773950587505 | 0.082787717247547 | -2.867158656 | -0.08484477 | 0.228549671228818 | 0.0568602568311486 | 0.918654876532073 | 0.999950574162623 |
| ebi-fl187-GCST90094780 | prot-a-864 | Dihydropyrimidinase-related protein 5 \|\| id:prot-a-864 | F-healthy breakfast food liking (derived food-liking factor) \|\| id:ebi-fl187-GCST90094780 | MR Egger | 8 | 0.0639698721003533 | 0.710082315261045 | 0.931149390213403 | -1.327791466 | 1.45573121 | 1.06606028012054 | 0.265062013858866 | 4.28761746847592 | 0.999950574162623 |
| ebi-fl187-GCST90094780 | prot-a-884 | Endothelin-converting enzyme 1 \|\| id:prot-a-884 | F-healthy breakfast food liking (derived food-liking factor) \|\| id:ebi-fl187-GCST90094780 | MR Egger | 8 | -0.01317879 | 0.711033979977604 | 0.985813286290728 | -1.40680539 | 1.38044781121262 | 0.986907670473881 | 0.244924472787709 | 3.9766820316253 | 0.999950574162623 |
| ebi-fl187-GCST90094780 | prot-a-893 | Early endosome antigen 1 \|\| id:prot-a-893 | F-healthy breakfast food liking (derived food-liking factor) \|\| id:ebi-fl187-GCST90094780 | MR Egger | 8 | 0.219014486269514 | 0.709760504157883 | 0.768077784635373 | -1.172116102 | 1.61014507441897 | 1.24484930977952 | 0.309710867601778 | 5.00353706041426 | 0.999950574162623 |
| ebi-fl187-GCST90094780 | prot-a-897 | Ephrin-A2 \|\| id:prot-a-897 | F-healthy breakfast food liking (derived food-liking factor) \|\| id:ebi-fl187-GCST90094780 | MR Egger | 8 | -0.694437119 | 0.712128744340258 | 0.367142897528814 | -2.090209458 | 0.701335219881116 | 0.499355446573582 | 0.123661231278736 | 2.01644330599173 | 0.999950574162623 |
| ebi-fl187-GCST90094780 | prot-a-90 | Anaphase-promoting complex subunit 10 \|\| id:prot-a-90 | F-healthy breakfast food liking (derived food-liking factor) \|\| id:ebi-fl187-GCST90094780 | MR Egger | 8 | 1.13206104761592 | 1.32129640764766 | 0.424458432575434 | -1.457679911 | 3.72180200660534 | 3.10204337513987 | 0.232775708995102 | 41.3388198570651 | 0.999950574162623 |
| ebi-fl187-GCST90094780 | prot-a-914 | Histone-lysine N-methyltransferase EHMT2 \|\| id:prot-a-914 | F-healthy breakfast food liking (derived food-liking factor) \|\| id:ebi-fl187-GCST90094780 | MR Egger | 8 | -0.461033157 | 1.27922937988131 | 0.73089630689688 | -2.968322741 | 2.04625642766613 | 0.63063176725617 | 0.0513894314503624 | 7.73887576975394 | 0.999950574162623 |
| ebi-fl187-GCST90094780 | prot-a-932 | RNA polymerase II elongation factor ELL \|\| id:prot-a-932 | F-healthy breakfast food liking (derived food-liking factor) \|\| id:ebi-fl187-GCST90094780 | MR Egger | 8 | -0.267616217 | 0.711020743250819 | 0.719582493420395 | -1.661216874 | 1.12598443969235 | 0.765201395983783 | 0.189907745668708 | 3.08325062968725 | 0.999950574162623 |
| ebi-fl187-GCST90094780 | prot-a-991 | Estrogen receptor \|\| id:prot-a-991 | F-healthy breakfast food liking (derived food-liking factor) \|\| id:ebi-fl187-GCST90094780 | MR Egger | 8 | 0.5279024451869 | 0.709760504001355 | 0.485108405370707 | -0.863228143 | 1.91903303302956 | 1.69537243979268 | 0.421798257244824 | 6.81436601560034 | 0.999950574162623 |
| ebi-fl187-GCST90094780 | prot-a-992 | Persulfide dioxygenase ETHE1, mitochondrial \|\| id:prot-a-992 | F-healthy breakfast food liking (derived food-liking factor) \|\| id:ebi-fl187-GCST90094780 | MR Egger | 8 | -0.080847232 | 0.826876253327974 | 0.925295979309894 | -1.701524688 | 1.53983022475001 | 0.922334584101654 | 0.182405200853783 | 4.66379840623019 | 0.999950574162623 |
| ebi-fl187-GCST90094780 | prot-a-993 | Ethanolamine kinase 1 \|\| id:prot-a-993 | F-healthy breakfast food liking (derived food-liking factor) \|\| id:ebi-fl187-GCST90094780 | MR Egger | 8 | 0.689278049542458 | 0.843475522613391 | 0.445049650794796 | -0.963933975 | 2.34249007386471 | 1.99227668885133 | 0.381389553992195 | 10.4071188195722 | 0.999950574162623 |
| ebi-fl187-GCST90094780 | prot-b-10 | coagulation factor III, tissue factor \|\| id:prot-b-10 | F-healthy breakfast food liking (derived food-liking factor) \|\| id:ebi-fl187-GCST90094780 | MR Egger | 8 | -0.409880714 | 0.729010379876636 | 0.594316827816259 | -1.838741058 | 1.01897963091217 | 0.663729419276774 | 0.159017493912979 | 2.77036652492186 | 0.999950574162623 |
| ebi-fl187-GCST90094780 | prot-b-28 | prolactin \|\| id:prot-b-28 | F-healthy breakfast food liking (derived food-liking factor) \|\| id:ebi-fl187-GCST90094780 | MR Egger | 8 | -0.301968172 | 1.24674521088899 | 0.816687515448086 | -2.745588785 | 2.14165244121156 | 0.739361596819194 | 0.0642104836124639 | 8.51349406041408 | 0.999950574162623 |
| ebi-fl187-GCST90094780 | prot-b-54 | matrix metallopeptidase 7 \|\| id:prot-b-54 | F-healthy breakfast food liking (derived food-liking factor) \|\| id:ebi-fl187-GCST90094780 | MR Egger | 8 | 0.919490182441063 | 0.729010379876635 | 0.254026037494488 | -0.509370162 | 2.34835052699927 | 2.50801143567941 | 0.600873912808325 | 10.4682883170952 | 0.999950574162623 |
| ebi-fl187-GCST90094780 | ukb-rsfMRI191-edge-pheno716 | Net100_Pair10_33 (OccipitalPrecuneus)&(Temporal\|Frontal\|Supp_Motor_Area) (Default_mode\|Central_executive)&(Default_mode\|Salience) \|\| id:ukb-rsfMRI191-edge-pheno716 | F-healthy breakfast food liking (derived food-liking factor) \|\| id:ebi-fl187-GCST90094780 | MR Egger | 8 | -0.210271709 | 0.217575419039447 | 0.371151057280497 | -0.63671953 | 0.21617611251939 | 0.810364033017351 | 0.529025031289746 | 1.24132097191537 | 0.999950574162623 |
| ebi-fl187-GCST90094780 | ukb-rsfMRI191-edge-pheno903 | Net100_Pair27_38 (Precentral\|Frontal\|Supp_Motor_Area)&(Subcortical) (Attention\|Salience\|Motor)&(Subcortical-cerebellum) \|\| id:ukb-rsfMRI191-edge-pheno903 | F-healthy breakfast food liking (derived food-liking factor) \|\| id:ebi-fl187-GCST90094780 | MR Egger | 8 | -0.160368165 | 0.216033568654419 | 0.485921555065843 | -0.583793959 | 0.263057629779661 | 0.85183011737802 | 0.557778159457945 | 1.3009016874691 | 0.999950574162623 |
| ebi-fl187-GCST90094780 | ukb-rsfMRI191-node-pheno23 | Net100_Node2 RolandicOper\|SupraMarginal\|Insula Salience\|Motor \|\| id:ukb-rsfMRI191-node-pheno23 | F-healthy breakfast food liking (derived food-liking factor) \|\| id:ebi-fl187-GCST90094780 | MR Egger | 8 | -0.641487603 | 0.21709844608673 | 0.0254531964887565 | -1.067000557 | -0.215974648 | 0.526508605616477 | 0.344038896451413 | 0.805755728923389 | 0.999950574162623 |
| ebi-fl187-GCST90094780 | ukb-rsfMRI191-node-pheno55 | Net100_Node34 Parietal\|Postcentral\|Precuneus Attention \|\| id:ukb-rsfMRI191-node-pheno55 | F-healthy breakfast food liking (derived food-liking factor) \|\| id:ebi-fl187-GCST90094780 | MR Egger | 8 | -0.350737179 | 0.207246360064177 | 0.141522639017209 | -0.756940045 | 0.0554656862699436 | 0.704168799564728 | 0.46909965683757 | 1.05703274571383 | 0.999950574162623 |
| ebi-fl187-GCST90094780 | ebi-a-GCST90001414 | IgD- CD24- B cell Absolute Count \|\| id:ebi-a-GCST90001414 | F-healthy breakfast food liking (derived food-liking factor) \|\| id:ebi-fl187-GCST90094780 | Weighted median | 8 | -0.064542491 | 0.140218962062818 | 0.645302422972674 | -0.339371657 | 0.210286674728511 | 0.937496278341412 | 0.712217699515723 | 1.23403177497781 | 0.999332001504091 |
| ebi-fl187-GCST90094780 | ebi-a-GCST90001422 | CD20- CD38- B cell %B cell \|\| id:ebi-a-GCST90001422 | F-healthy breakfast food liking (derived food-liking factor) \|\| id:ebi-fl187-GCST90094780 | Weighted median | 8 | -0.045876188 | 0.138938561403087 | 0.741256049679726 | -0.318195769 | 0.226443391905531 | 0.955160214741967 | 0.727460360435713 | 1.25413161382358 | 0.999332001504091 |
| ebi-fl187-GCST90094780 | ebi-a-GCST90001444 | CD20- CD38- B cell %lymphocyte \|\| id:ebi-a-GCST90001444 | F-healthy breakfast food liking (derived food-liking factor) \|\| id:ebi-fl187-GCST90094780 | Weighted median | 8 | -0.079469492 | 0.144410422511295 | 0.582111552526114 | -0.36251392 | 0.203574936351898 | 0.923606197128856 | 0.695924626415316 | 1.22577700945697 | 0.999332001504091 |
| ebi-fl187-GCST90094780 | ebi-a-GCST90001459 | Myeloid Dendritic Cell %Dendritic Cell \|\| id:ebi-a-GCST90001459 | F-healthy breakfast food liking (derived food-liking factor) \|\| id:ebi-fl187-GCST90094780 | Weighted median | 8 | 0.0339017097798679 | 0.139209281675716 | 0.807594439388456 | -0.238948482 | 0.306751901864271 | 1.03448292217676 | 0.787455449217749 | 1.35900375994407 | 0.999332001504091 |
| ebi-fl187-GCST90094780 | ebi-a-GCST90001476 | HLA DR++ monocyte %leukocyte \|\| id:ebi-a-GCST90001476 | F-healthy breakfast food liking (derived food-liking factor) \|\| id:ebi-fl187-GCST90094780 | Weighted median | 8 | -0.019650629 | 0.147169371605441 | 0.893779063315474 | -0.308102597 | 0.268801339755391 | 0.980541186526759 | 0.734839921815572 | 1.30839518911795 | 0.999332001504091 |
| ebi-fl187-GCST90094780 | ebi-a-GCST90001478 | CD4 regulatory T cell %CD4+ T cell \|\| id:ebi-a-GCST90001478 | F-healthy breakfast food liking (derived food-liking factor) \|\| id:ebi-fl187-GCST90094780 | Weighted median | 8 | -0.19671537 | 0.141277047547841 | 0.16379871534571 | -0.473618383 | 0.0801876435710847 | 0.821424402379777 | 0.622744857366303 | 1.08349035860137 | 0.999332001504091 |
| ebi-fl187-GCST90094780 | ebi-a-GCST90001485 | CD39+ resting CD4 regulatory T cell %CD4 regulatory T cell \|\| id:ebi-a-GCST90001485 | F-healthy breakfast food liking (derived food-liking factor) \|\| id:ebi-fl187-GCST90094780 | Weighted median | 8 | 0.147945070931282 | 0.123003853029079 | 0.229066197347798 | -0.093142481 | 0.389032622868277 | 1.15944920714862 | 0.911063681778375 | 1.47555268730884 | 0.999332001504091 |
| ebi-fl187-GCST90094780 | ebi-a-GCST90001515 | Immature Myeloid-Derived Suppressor Cells Absolute Count \|\| id:ebi-a-GCST90001515 | F-healthy breakfast food liking (derived food-liking factor) \|\| id:ebi-fl187-GCST90094780 | Weighted median | 8 | -0.03106041 | 0.170550355340848 | 0.85548956854206 | -0.365339107 | 0.303218286356394 | 0.969417008717355 | 0.693961284135501 | 1.35421003775624 | 0.999332001504091 |
| ebi-fl187-GCST90094780 | ebi-a-GCST90001517 | CD33+ HLA DR+ Absolute Count \|\| id:ebi-a-GCST90001517 | F-healthy breakfast food liking (derived food-liking factor) \|\| id:ebi-fl187-GCST90094780 | Weighted median | 8 | -0.12998065 | 0.173830797815603 | 0.454615578391715 | -0.470689014 | 0.210727713629959 | 0.878112422153719 | 0.624571781413048 | 1.23457615103288 | 0.999332001504091 |
| ebi-fl187-GCST90094780 | ebi-a-GCST90001518 | CD33+ HLA DR+ CD14- Absolute Count \|\| id:ebi-a-GCST90001518 | F-healthy breakfast food liking (derived food-liking factor) \|\| id:ebi-fl187-GCST90094780 | Weighted median | 8 | -0.09138582 | 0.178885960179394 | 0.609448551871023 | -0.442002302 | 0.259230662371512 | 0.912665518735108 | 0.642748156148564 | 1.29593269326391 | 0.999332001504091 |
| ebi-fl187-GCST90094780 | ebi-a-GCST90001520 | CD33+ HLA DR+ CD14dim Absolute Count \|\| id:ebi-a-GCST90001520 | F-healthy breakfast food liking (derived food-liking factor) \|\| id:ebi-fl187-GCST90094780 | Weighted median | 8 | -0.076474187 | 0.164429932028608 | 0.641869317370413 | -0.398756853 | 0.245808480219688 | 0.926376826956177 | 0.671153870342628 | 1.27865466242978 | 0.999332001504091 |
| ebi-fl187-GCST90094780 | ebi-a-GCST90001531 | CD33dim HLA DR- Absolute Count \|\| id:ebi-a-GCST90001531 | F-healthy breakfast food liking (derived food-liking factor) \|\| id:ebi-fl187-GCST90094780 | Weighted median | 8 | -0.241195227 | 0.188103609397862 | 0.199756128200484 | -0.609878302 | 0.12748784705801 | 0.785688223575535 | 0.543416997931029 | 1.13597106276685 | 0.999332001504091 |
| ebi-fl187-GCST90094780 | ebi-a-GCST90001532 | Basophil Absolute Count \|\| id:ebi-a-GCST90001532 | F-healthy breakfast food liking (derived food-liking factor) \|\| id:ebi-fl187-GCST90094780 | Weighted median | 8 | -0.23930621 | 0.178247046490363 | 0.179416570817988 | -0.588670421 | 0.110058001228811 | 0.787173805058373 | 0.555064796825625 | 1.11634281783635 | 0.999332001504091 |
| ebi-fl187-GCST90094780 | ebi-a-GCST90001588 | T/B cell \|\| id:ebi-a-GCST90001588 | F-healthy breakfast food liking (derived food-liking factor) \|\| id:ebi-fl187-GCST90094780 | Weighted median | 8 | 0.134857266378275 | 0.131428718827973 | 0.304850697332311 | -0.122743023 | 0.392457555281102 | 1.14437343212909 | 0.884490927604204 | 1.48061501965901 | 0.999332001504091 |
| ebi-fl187-GCST90094780 | ebi-a-GCST90001624 | HLA DR+ CD4+ T cell Absolute Count \|\| id:ebi-a-GCST90001624 | F-healthy breakfast food liking (derived food-liking factor) \|\| id:ebi-fl187-GCST90094780 | Weighted median | 8 | -0.115277373 | 0.13684599974812 | 0.399571033312544 | -0.383495532 | 0.152940786636573 | 0.891118937407278 | 0.681475122616531 | 1.16525597817415 | 0.999332001504091 |
| ebi-fl187-GCST90094780 | ebi-a-GCST90001631 | CD8+ Natural Killer T %T cell \|\| id:ebi-a-GCST90001631 | F-healthy breakfast food liking (derived food-liking factor) \|\| id:ebi-fl187-GCST90094780 | Weighted median | 8 | 0.0194358774826944 | 0.132307904074626 | 0.883211868423077 | -0.239887615 | 0.278759369468961 | 1.01962598377963 | 0.786716271597161 | 1.32148931493172 | 0.999332001504091 |
| ebi-fl187-GCST90094780 | ebi-a-GCST90001632 | CD8+ Natural Killer T %lymphocyte \|\| id:ebi-a-GCST90001632 | F-healthy breakfast food liking (derived food-liking factor) \|\| id:ebi-fl187-GCST90094780 | Weighted median | 8 | 0.0384437238679268 | 0.140276985180882 | 0.784041726767559 | -0.236499167 | 0.313386614822456 | 1.03919224499236 | 0.78938653979232 | 1.36805033733712 | 0.999332001504091 |
| ebi-fl187-GCST90094780 | ebi-a-GCST90001646 | Natural Killer %CD3- lymphocyte \|\| id:ebi-a-GCST90001646 | F-healthy breakfast food liking (derived food-liking factor) \|\| id:ebi-fl187-GCST90094780 | Weighted median | 8 | 0.0404205978845979 | 0.121558503714309 | 0.739496862396751 | -0.197834069 | 0.278675265164643 | 1.04124862907624 | 0.820505988897751 | 1.3213781766659 | 0.999332001504091 |
| ebi-fl187-GCST90094780 | ebi-a-GCST90001684 | CD127- CD8+ T cell Absolute Count \|\| id:ebi-a-GCST90001684 | F-healthy breakfast food liking (derived food-liking factor) \|\| id:ebi-fl187-GCST90094780 | Weighted median | 8 | 0.0716192336167975 | 0.137491473578115 | 0.602436815285216 | -0.197864055 | 0.341102521829904 | 1.07424622932878 | 0.820481386229726 | 1.40649743016106 | 0.999332001504091 |
| ebi-fl187-GCST90094780 | ebi-a-GCST90001687 | CD28- CD8+ T cell Absolute Count \|\| id:ebi-a-GCST90001687 | F-healthy breakfast food liking (derived food-liking factor) \|\| id:ebi-fl187-GCST90094780 | Weighted median | 8 | 0.108566190449284 | 0.147174159625331 | 0.460714064523086 | -0.179895162 | 0.397027543314933 | 1.11467868717971 | 0.835357783712273 | 1.48739689732829 | 0.999332001504091 |
| ebi-fl187-GCST90094780 | ebi-a-GCST90001701 | BAFF-R on CD20- CD38- B cell \|\| id:ebi-a-GCST90001701 | F-healthy breakfast food liking (derived food-liking factor) \|\| id:ebi-fl187-GCST90094780 | Weighted median | 8 | -0.268943927 | 0.126721487148685 | 0.0338106229404685 | -0.517318042 | -0.020569813 | 0.764186104373287 | 0.596117167079007 | 0.979640302893378 | 0.999332001504091 |
| ebi-fl187-GCST90094780 | ebi-a-GCST90001732 | CD19 on IgD- CD27- B cell \|\| id:ebi-a-GCST90001732 | F-healthy breakfast food liking (derived food-liking factor) \|\| id:ebi-fl187-GCST90094780 | Weighted median | 8 | -0.076909067 | 0.140509149199187 | 0.584131531715196 | -0.352307 | 0.198488865278118 | 0.925974051235658 | 0.703064248410619 | 1.21955844789451 | 0.999332001504091 |
| ebi-fl187-GCST90094780 | ebi-a-GCST90001798 | CD27 on CD24+ CD27+ B cell \|\| id:ebi-a-GCST90001798 | F-healthy breakfast food liking (derived food-liking factor) \|\| id:ebi-fl187-GCST90094780 | Weighted median | 8 | -0.027054138 | 0.135836216920535 | 0.842131848674887 | -0.293293123 | 0.239184847543519 | 0.973308547488988 | 0.745803496656026 | 1.27021331069469 | 0.999332001504091 |
| ebi-fl187-GCST90094780 | ebi-a-GCST90001836 | CD66b on CD66b++ myeloid cell \|\| id:ebi-a-GCST90001836 | F-healthy breakfast food liking (derived food-liking factor) \|\| id:ebi-fl187-GCST90094780 | Weighted median | 8 | 0.0403960002546072 | 0.201386884125777 | 0.84101993453388 | -0.354322293 | 0.43511429314113 | 1.04122301714274 | 0.701648794673742 | 1.54513964772353 | 0.999332001504091 |
| ebi-fl187-GCST90094780 | ebi-a-GCST90001943 | CD25 on activated & secreting CD4 regulatory T cell \|\| id:ebi-a-GCST90001943 | F-healthy breakfast food liking (derived food-liking factor) \|\| id:ebi-fl187-GCST90094780 | Weighted median | 8 | -0.022711 | 0.13389443984083 | 0.865310017488396 | -0.285144103 | 0.239722101657591 | 0.977544953025218 | 0.751905894979029 | 1.27089592137288 | 0.999332001504091 |
| ebi-fl187-GCST90094780 | ebi-a-GCST90001960 | CD25 on CD4+ T cell \|\| id:ebi-a-GCST90001960 | F-healthy breakfast food liking (derived food-liking factor) \|\| id:ebi-fl187-GCST90094780 | Weighted median | 8 | -0.119905162 | 0.140839273619684 | 0.394568465727083 | -0.395950138 | 0.156139814229672 | 0.887004554408677 | 0.673040253991944 | 1.16898963304973 | 0.999332001504091 |
| ebi-fl187-GCST90094780 | ebi-a-GCST90001988 | HLA DR on CD14+ CD16- monocyte \|\| id:ebi-a-GCST90001988 | F-healthy breakfast food liking (derived food-liking factor) \|\| id:ebi-fl187-GCST90094780 | Weighted median | 8 | -0.150085145 | 0.121053594614312 | 0.215040521371629 | -0.38735019 | 0.0871799008177052 | 0.860634694885821 | 0.678853325602956 | 1.09109295057711 | 0.999332001504091 |
| ebi-fl187-GCST90094780 | ebi-a-GCST90001991 | HLA DR on CD14+ monocyte \|\| id:ebi-a-GCST90001991 | F-healthy breakfast food liking (derived food-liking factor) \|\| id:ebi-fl187-GCST90094780 | Weighted median | 8 | -0.146823118 | 0.129165342151476 | 0.255660890393267 | -0.399987189 | 0.106340952406077 | 0.863446691905444 | 0.670328633676247 | 1.11220101948164 | 0.999332001504091 |
| ebi-fl187-GCST90094780 | ebi-a-GCST90002019 | CD14 on Monocytic Myeloid-Derived Suppressor Cells \|\| id:ebi-a-GCST90002019 | F-healthy breakfast food liking (derived food-liking factor) \|\| id:ebi-fl187-GCST90094780 | Weighted median | 8 | 0.0804527776743475 | 0.197988437313503 | 0.684484798467583 | -0.307604559 | 0.468510114808814 | 1.08377766693193 | 0.735205990787302 | 1.59761216048118 | 0.999332001504091 |
| ebi-fl187-GCST90094780 | ebi-a-GCST90002037 | CD80 on plasmacytoid Dendritic Cell \|\| id:ebi-a-GCST90002037 | F-healthy breakfast food liking (derived food-liking factor) \|\| id:ebi-fl187-GCST90094780 | Weighted median | 8 | -0.054977371 | 0.141193673122937 | 0.696998319147489 | -0.33171697 | 0.221762228150714 | 0.946506566047098 | 0.717690421679127 | 1.24827453803028 | 0.999332001504091 |
| ebi-fl187-GCST90094780 | ebi-a-GCST90002042 | CD45 on CD33+ HLA DR+ CD14- \|\| id:ebi-a-GCST90002042 | F-healthy breakfast food liking (derived food-liking factor) \|\| id:ebi-fl187-GCST90094780 | Weighted median | 8 | -0.130036847 | 0.196154454673764 | 0.507374816371814 | -0.514499579 | 0.254425883765695 | 0.878063075987575 | 0.597799671554925 | 1.28972095854008 | 0.999332001504091 |
| ebi-fl187-GCST90094780 | ebi-a-GCST90002056 | CD8 on Effector Memory CD8+ T cell \|\| id:ebi-a-GCST90002056 | F-healthy breakfast food liking (derived food-liking factor) \|\| id:ebi-fl187-GCST90094780 | Weighted median | 8 | 0.0371313325553836 | 0.135898626447229 | 0.784677829565388 | -0.229229975 | 0.303492640391952 | 1.03782931266387 | 0.795145648632273 | 1.35458162171554 | 0.999332001504091 |
| ebi-fl187-GCST90094780 | ebi-a-GCST90002058 | CD8 on CD8+ T cell \|\| id:ebi-a-GCST90002058 | F-healthy breakfast food liking (derived food-liking factor) \|\| id:ebi-fl187-GCST90094780 | Weighted median | 8 | -0.055249455 | 0.134702484664497 | 0.681689215454052 | -0.319266325 | 0.208767415295069 | 0.946249072280994 | 0.726681990249774 | 1.2321583840063 | 0.999332001504091 |
| ebi-fl187-GCST90094780 | ebi-a-GCST90002063 | CD4 on CD4 regulatory T cell \|\| id:ebi-a-GCST90002063 | F-healthy breakfast food liking (derived food-liking factor) \|\| id:ebi-fl187-GCST90094780 | Weighted median | 8 | -0.081114965 | 0.154309998589264 | 0.599122788794915 | -0.383562562 | 0.221332632160684 | 0.922087677472445 | 0.681429444915505 | 1.24773839946402 | 0.999332001504091 |
| ebi-fl187-GCST90094780 | ebi-a-GCST90002066 | CD4 on activated CD4 regulatory T cell \|\| id:ebi-a-GCST90002066 | F-healthy breakfast food liking (derived food-liking factor) \|\| id:ebi-fl187-GCST90094780 | Weighted median | 8 | -0.076442777 | 0.151243214489432 | 0.613257229860717 | -0.372879477 | 0.219993923805355 | 0.926405924874507 | 0.68874823785039 | 1.24606915920563 | 0.999332001504091 |
| ebi-fl187-GCST90094780 | ebi-a-GCST90002070 | CD4 on activated & secreting CD4 regulatory T cell \|\| id:ebi-a-GCST90002070 | F-healthy breakfast food liking (derived food-liking factor) \|\| id:ebi-fl187-GCST90094780 | Weighted median | 8 | -0.006636326 | 0.148464086592771 | 0.964346533585975 | -0.297625936 | 0.284353283655768 | 0.99338564571493 | 0.742579060052948 | 1.3289023273052 | 0.999332001504091 |
| ebi-fl187-GCST90094780 | ebi-a-GCST90002081 | SSC-A on CD4+ T cell \|\| id:ebi-a-GCST90002081 | F-healthy breakfast food liking (derived food-liking factor) \|\| id:ebi-fl187-GCST90094780 | Weighted median | 8 | 0.0823257954147076 | 0.13247655907884 | 0.534312332640213 | -0.17732826 | 0.341979851209234 | 1.0858095039681 | 0.837504819745212 | 1.40773193313217 | 0.999332001504091 |
| ebi-fl187-GCST90094780 | ebi-a-GCST90002094 | CD11b on Monocytic Myeloid-Derived Suppressor Cells \|\| id:ebi-a-GCST90002094 | F-healthy breakfast food liking (derived food-liking factor) \|\| id:ebi-fl187-GCST90094780 | Weighted median | 8 | 0.0953847550633735 | 0.197417521229865 | 0.628980316446518 | -0.291553587 | 0.48232309667391 | 1.10008203584384 | 0.747101977914918 | 1.61983306343775 | 0.999332001504091 |
| ebi-fl187-GCST90094780 | ebi-a-GCST90002104 | HLA DR on myeloid Dendritic Cell \|\| id:ebi-a-GCST90002104 | F-healthy breakfast food liking (derived food-liking factor) \|\| id:ebi-fl187-GCST90094780 | Weighted median | 8 | -0.097036707 | 0.140475124885067 | 0.489706937279014 | -0.372367952 | 0.178294537848427 | 0.907522693153252 | 0.68910064011765 | 1.19517729434632 | 0.999332001504091 |
| ebi-fl187-GCST90094780 | ebi-a-GCST90002105 | HLA DR on plasmacytoid Dendritic Cell \|\| id:ebi-a-GCST90002105 | F-healthy breakfast food liking (derived food-liking factor) \|\| id:ebi-fl187-GCST90094780 | Weighted median | 8 | 0.187327205479235 | 0.153088916191089 | 0.22108440448333 | -0.11272707 | 0.48738148121377 | 1.20602183744984 | 0.893394460766371 | 1.62804756048991 | 0.999332001504091 |
| ebi-fl187-GCST90094780 | ebi-a-GCST90002106 | HLA DR on Dendritic Cell \|\| id:ebi-a-GCST90002106 | F-healthy breakfast food liking (derived food-liking factor) \|\| id:ebi-fl187-GCST90094780 | Weighted median | 8 | 0.0565218240026538 | 0.148330861545268 | 0.703164400728785 | -0.234206665 | 0.347250312631379 | 1.05814970761026 | 0.791198286300288 | 1.41517091619524 | 0.999332001504091 |
| ebi-fl187-GCST90094780 | ebi-a-GCST90002111 | HLA DR on CD33dim HLA DR+ CD11b- \|\| id:ebi-a-GCST90002111 | F-healthy breakfast food liking (derived food-liking factor) \|\| id:ebi-fl187-GCST90094780 | Weighted median | 8 | -0.109234678 | 0.195234971724935 | 0.575818045601587 | -0.491895223 | 0.273425866639991 | 0.896519999341546 | 0.611466430386696 | 1.31445991027025 | 0.999332001504091 |
| ebi-fl187-GCST90094780 | ebi-a-GCST90002112 | HLA DR on CD33- HLA DR+ \|\| id:ebi-a-GCST90002112 | F-healthy breakfast food liking (derived food-liking factor) \|\| id:ebi-fl187-GCST90094780 | Weighted median | 8 | 0.0264958331048364 | 0.202265510322488 | 0.895779017990911 | -0.369944567 | 0.422936233336913 | 1.0268499684766 | 0.69077262108688 | 1.52643695707184 | 0.999332001504091 |
| ebi-fl187-GCST90094780 | ebi-a-GCST90002115 | HLA DR on HLA DR+ CD8+ T cell \|\| id:ebi-a-GCST90002115 | F-healthy breakfast food liking (derived food-liking factor) \|\| id:ebi-fl187-GCST90094780 | Weighted median | 8 | -0.215895074 | 0.126741001674554 | 0.0884866688090051 | -0.464307437 | 0.0325172890688808 | 0.80581984871944 | 0.628570278686366 | 1.0330517534925 | 0.999332001504091 |
| ebi-fl187-GCST90094780 | ebi-a-GCST90002116 | HLA DR on B cell \|\| id:ebi-a-GCST90002116 | F-healthy breakfast food liking (derived food-liking factor) \|\| id:ebi-fl187-GCST90094780 | Weighted median | 8 | -0.005623948 | 0.133340941893513 | 0.96635743276928 | -0.266972195 | 0.255724297637272 | 0.994391836319387 | 0.76569436161864 | 1.29139663775026 | 0.999332001504091 |
| ebi-fl187-GCST90094780 | ebi-cfmet338-GCST90026023 | Sphingomyelin (d18:1/24:1, d18:2/24:0) levels \|\| id:ebi-cfmet338-GCST90026023 | F-healthy breakfast food liking (derived food-liking factor) \|\| id:ebi-fl187-GCST90094780 | Weighted median | 6 | 0.100450715696021 | 0.09884651302974 | 0.309520240772236 | -0.09328845 | 0.294189881234312 | 1.10566914822685 | 0.910930704578217 | 1.34203870744124 | 0.999332001504091 |
| ebi-fl187-GCST90094780 | ebi-cfmet338-GCST90026046 | 1-arachidonoyl-gpc (20:4n6) levels \|\| id:ebi-cfmet338-GCST90026046 | F-healthy breakfast food liking (derived food-liking factor) \|\| id:ebi-fl187-GCST90094780 | Weighted median | 6 | -0.020265469 | 0.118587556744113 | 0.864309982179912 | -0.252697081 | 0.212166141771046 | 0.979938495042813 | 0.776703124585458 | 1.2363532779391 | 0.999332001504091 |
| ebi-fl187-GCST90094780 | ebi-cfmet338-GCST90026047 | 1-linoleoyl-gpc (18:2) levels \|\| id:ebi-cfmet338-GCST90026047 | F-healthy breakfast food liking (derived food-liking factor) \|\| id:ebi-fl187-GCST90094780 | Weighted median | 6 | -0.178204008 | 0.129916059406734 | 0.170161283997342 | -0.432839485 | 0.0764314680583341 | 0.836771697637094 | 0.648664603967181 | 1.07942821249098 | 0.999332001504091 |
| ebi-fl187-GCST90094780 | ebi-cfmet338-GCST90026050 | 1-myristoyl-2-palmitoyl-gpc (14:0/16:0) levels \|\| id:ebi-cfmet338-GCST90026050 | F-healthy breakfast food liking (derived food-liking factor) \|\| id:ebi-fl187-GCST90094780 | Weighted median | 6 | 0.0352910406415756 | 0.0674958759390294 | 0.601070167003008 | -0.097000876 | 0.167582957482073 | 1.03592116008926 | 0.907555210934027 | 1.18244337864167 | 0.999332001504091 |
| ebi-fl187-GCST90094780 | ebi-cfmet338-GCST90026068 | 2,3-dihydroxyisovalerate levels \|\| id:ebi-cfmet338-GCST90026068 | F-healthy breakfast food liking (derived food-liking factor) \|\| id:ebi-fl187-GCST90094780 | Weighted median | 6 | -0.397151533 | 0.212220228154563 | 0.0612879635116939 | -0.81310318 | 0.0188001144228308 | 0.672232152720772 | 0.443479731311039 | 1.01897794926429 | 0.999332001504091 |
| ebi-fl187-GCST90094780 | ebi-cfmet338-GCST90026082 | 3-hydroxyhexanoate levels \|\| id:ebi-cfmet338-GCST90026082 | F-healthy breakfast food liking (derived food-liking factor) \|\| id:ebi-fl187-GCST90094780 | Weighted median | 6 | 0.00141323638676105 | 0.0468315307059199 | 0.975925869640217 | -0.090376564 | 0.093203036570364 | 1.0014142354759 | 0.913587096663773 | 1.09768458275725 | 0.999332001504091 |
| ebi-fl187-GCST90094780 | ebi-cfmet338-GCST90026094 | 3-ureidopropionate levels \|\| id:ebi-cfmet338-GCST90026094 | F-healthy breakfast food liking (derived food-liking factor) \|\| id:ebi-fl187-GCST90094780 | Weighted median | 6 | -0.025389724 | 0.122559006345723 | 0.835882356954102 | -0.265605376 | 0.214825928796275 | 0.974929884755595 | 0.766741642400634 | 1.23964609149651 | 0.999332001504091 |
| ebi-fl187-GCST90094780 | ebi-cfmet338-GCST90026173 | Glycerol levels \|\| id:ebi-cfmet338-GCST90026173 | F-healthy breakfast food liking (derived food-liking factor) \|\| id:ebi-fl187-GCST90094780 | Weighted median | 6 | 0.000195639407779065 | 0.0596083421352991 | 0.997381282936055 | -0.116636711 | 0.117027989992965 | 1.00019565854642 | 0.88990842823112 | 1.12415089422581 | 0.999364451047625 |
| ebi-fl187-GCST90094780 | ebi-cfmet338-GCST90026179 | Histidine levels \|\| id:ebi-cfmet338-GCST90026179 | F-healthy breakfast food liking (derived food-liking factor) \|\| id:ebi-fl187-GCST90094780 | Weighted median | 6 | 0.0530712212438252 | 0.0381697482518139 | 0.16440742044313 | -0.021741485 | 0.12788392781738 | 1.05450474558455 | 0.978493157193653 | 1.13642108816533 | 0.999332001504091 |
| ebi-fl187-GCST90094780 | ebi-cfmet338-GCST90026188 | Isocitrate levels \|\| id:ebi-cfmet338-GCST90026188 | F-healthy breakfast food liking (derived food-liking factor) \|\| id:ebi-fl187-GCST90094780 | Weighted median | 6 | -0.052168398 | 0.0868941664756192 | 0.548261614151633 | -0.222480964 | 0.118144168237481 | 0.949169015168145 | 0.800530245221198 | 1.12540634752198 | 0.999332001504091 |
| ebi-fl187-GCST90094780 | ebi-cfmet338-GCST90026207 | N-acetyl-isoputreanine levels \|\| id:ebi-cfmet338-GCST90026207 | F-healthy breakfast food liking (derived food-liking factor) \|\| id:ebi-fl187-GCST90094780 | Weighted median | 6 | 0.00759782518497473 | 0.0502529044128091 | 0.879824448233446 | -0.090897867 | 0.106093517834081 | 1.00762676189768 | 0.913110964475404 | 1.11192585654222 | 0.999332001504091 |
| ebi-fl187-GCST90094780 | ebi-cfmet338-GCST90026213 | N-acetylglutamate levels \|\| id:ebi-cfmet338-GCST90026213 | F-healthy breakfast food liking (derived food-liking factor) \|\| id:ebi-fl187-GCST90094780 | Weighted median | 6 | 0.0105011668467691 | 0.0809260565047163 | 0.896754338143929 | -0.148113904 | 0.169116237596013 | 1.01055649760892 | 0.862332886266859 | 1.18425778620205 | 0.999332001504091 |
| ebi-fl187-GCST90094780 | ebi-cfmet338-GCST90026224 | N-acetyltaurine levels \|\| id:ebi-cfmet338-GCST90026224 | F-healthy breakfast food liking (derived food-liking factor) \|\| id:ebi-fl187-GCST90094780 | Weighted median | 6 | -0.00929598 | 0.0683644381161092 | 0.891839589219553 | -0.143290279 | 0.124698318385856 | 0.990747093728181 | 0.866502504879381 | 1.13280665457219 | 0.999332001504091 |
| ebi-fl187-GCST90094780 | ebi-cfmet338-GCST90026250 | Phenyllactate (pla) levels \|\| id:ebi-cfmet338-GCST90026250 | F-healthy breakfast food liking (derived food-liking factor) \|\| id:ebi-fl187-GCST90094780 | Weighted median | 6 | 0.0821633532994339 | 0.108014033795283 | 0.446852437754442 | -0.129544153 | 0.293870859538188 | 1.08563313710058 | 0.878495799388204 | 1.34161063626216 | 0.999332001504091 |
| ebi-fl187-GCST90094780 | ebi-cfmet338-GCST90026251 | Pipecolate levels \|\| id:ebi-cfmet338-GCST90026251 | F-healthy breakfast food liking (derived food-liking factor) \|\| id:ebi-fl187-GCST90094780 | Weighted median | 6 | 0.0442555491138874 | 0.151299286660704 | 0.769901794286044 | -0.252291053 | 0.340802150968868 | 1.04524943332764 | 0.777018551775098 | 1.40607502275953 | 0.999332001504091 |
| ebi-fl187-GCST90094780 | ebi-cfmet338-GCST90026278 | Trigonelline (n'-methylnicotinate) levels \|\| id:ebi-cfmet338-GCST90026278 | F-healthy breakfast food liking (derived food-liking factor) \|\| id:ebi-fl187-GCST90094780 | Weighted median | 6 | 0.172371816262688 | 0.17615964709687 | 0.327828209394899 | -0.172901092 | 0.517644724572554 | 1.18811951325395 | 0.841220814158618 | 1.67807067302145 | 0.999332001504091 |
| ebi-fl187-GCST90094780 | ebi-cfmet338-GCST90026299 | 1-carboxyethylvaline levels \|\| id:ebi-cfmet338-GCST90026299 | F-healthy breakfast food liking (derived food-liking factor) \|\| id:ebi-fl187-GCST90094780 | Weighted median | 6 | -0.211732501 | 0.11228426363777 | 0.0593377169811562 | -0.431809657 | 0.00834465617776201 | 0.809181124122036 | 0.649332960723553 | 1.0083795698678 | 0.999332001504091 |
| ebi-fl187-GCST90094780 | ebi-gm430-GCST90011306 | C_Erysipelotrichia abundance \|\| id:ebi-gm430-GCST90011306 | F-healthy breakfast food liking (derived food-liking factor) \|\| id:ebi-fl187-GCST90094780 | Weighted median | 6 | -0.085740073 | 0.0837459212042758 | 0.305924114919657 | -0.249882079 | 0.0784019324364356 | 0.917832769622086 | 0.778892625699388 | 1.08155728427358 | 0.999332001504091 |
| ebi-fl187-GCST90094780 | ebi-gm430-GCST90011312 | F_Erysipelotrichaceae abundance \|\| id:ebi-gm430-GCST90011312 | F-healthy breakfast food liking (derived food-liking factor) \|\| id:ebi-fl187-GCST90094780 | Weighted median | 6 | -0.085740073 | 0.0802320397234105 | 0.285226825852133 | -0.242994871 | 0.0715147247339396 | 0.917832769622086 | 0.784275536324717 | 1.07413396692175 | 0.999332001504091 |
| ebi-fl187-GCST90094780 | ebi-gm430-GCST90011352 | O_Erysipelotrichales abundance \|\| id:ebi-gm430-GCST90011352 | F-healthy breakfast food liking (derived food-liking factor) \|\| id:ebi-fl187-GCST90094780 | Weighted median | 6 | -0.085740073 | 0.0808955584629457 | 0.289196447275597 | -0.244295368 | 0.0728152214634287 | 0.917832769622086 | 0.783256251486667 | 1.07553178336361 | 0.999332001504091 |
| ebi-fl187-GCST90094780 | ebi-gm430-GCST90011362 | OTU97_11 (Parabacteroides) abundance \|\| id:ebi-gm430-GCST90011362 | F-healthy breakfast food liking (derived food-liking factor) \|\| id:ebi-fl187-GCST90094780 | Weighted median | 6 | -0.000251571 | 0.11011266673056 | 0.99817710254905 | -0.216072397 | 0.21556925622144 | 0.999748461070765 | 0.805676970874399 | 1.24056789699302 | 0.999381480690152 |
| ebi-fl187-GCST90094780 | ebi-gm430-GCST90011384 | OTU97_235 (Phascolarctobacterium) abundance \|\| id:ebi-gm430-GCST90011384 | F-healthy breakfast food liking (derived food-liking factor) \|\| id:ebi-fl187-GCST90094780 | Weighted median | 6 | 0.191539827608353 | 0.351740610750767 | 0.586064071995501 | -0.497871769 | 0.880951424679855 | 1.21111306791639 | 0.607822871358571 | 2.41319458742865 | 0.999332001504091 |
| ebi-fl187-GCST90094780 | ebi-gm430-GCST90011390 | OTU97_3 (Bacteroides) abundance \|\| id:ebi-gm430-GCST90011390 | F-healthy breakfast food liking (derived food-liking factor) \|\| id:ebi-fl187-GCST90094780 | Weighted median | 6 | -0.114805368 | 0.0904255443521312 | 0.204222827966355 | -0.292039435 | 0.0624286993428695 | 0.891539649534374 | 0.746739088097103 | 1.06441856246919 | 0.999332001504091 |
| ebi-fl187-GCST90094780 | ebi-gm430-GCST90011398 | OTU97_4 (Alistipes) abundance \|\| id:ebi-gm430-GCST90011398 | F-healthy breakfast food liking (derived food-liking factor) \|\| id:ebi-fl187-GCST90094780 | Weighted median | 6 | 0.0252697329544586 | 0.10456421771168 | 0.809038111824423 | -0.179676134 | 0.230215599669352 | 1.02559171910334 | 0.835540771043864 | 1.25887139292946 | 0.999332001504091 |
| ebi-fl187-GCST90094780 | ebi-gm430-GCST90011421 | OTU99_108 (Cytophagales) abundance \|\| id:ebi-gm430-GCST90011421 | F-healthy breakfast food liking (derived food-liking factor) \|\| id:ebi-fl187-GCST90094780 | Weighted median | 6 | 0.0427990141176748 | 0.378923382583853 | 0.910070994794743 | -0.699890816 | 0.785488843982026 | 1.04372809915542 | 0.49663952604707 | 2.19347894767307 | 0.999332001504091 |
| ebi-fl187-GCST90094780 | ebi-gm430-GCST90011428 | OTU99_123 (Phascolarctobacterium) abundance \|\| id:ebi-gm430-GCST90011428 | F-healthy breakfast food liking (derived food-liking factor) \|\| id:ebi-fl187-GCST90094780 | Weighted median | 6 | -0.13375312 | 0.184568426848402 | 0.468648127561576 | -0.495507236 | 0.228000996874236 | 0.874806010273591 | 0.609261789165727 | 1.25608657759207 | 0.999332001504091 |
| ebi-fl187-GCST90094780 | ebi-gm430-GCST90011450 | OTU99_298 (Phascolarctobacterium) abundance \|\| id:ebi-gm430-GCST90011450 | F-healthy breakfast food liking (derived food-liking factor) \|\| id:ebi-fl187-GCST90094780 | Weighted median | 6 | 0.197845447364798 | 0.362423283469384 | 0.585137377615627 | -0.512504188 | 0.908195082964791 | 1.2187740145291 | 0.598993706119331 | 2.47984258151029 | 0.999332001504091 |
| ebi-fl187-GCST90094780 | ebi-gm430-GCST90011451 | OTU99_3 (Bacteroides) abundance \|\| id:ebi-gm430-GCST90011451 | F-healthy breakfast food liking (derived food-liking factor) \|\| id:ebi-fl187-GCST90094780 | Weighted median | 6 | -0.113408726 | 0.0920280373969594 | 0.21782659134833 | -0.293783679 | 0.066966227724571 | 0.892785681193456 | 0.745437727933756 | 1.06925936624299 | 0.999332001504091 |
| ebi-fl187-GCST90094780 | ebi-gm430-GCST90011472 | OTU99_72 (Parabacteroides) abundance \|\| id:ebi-gm430-GCST90011472 | F-healthy breakfast food liking (derived food-liking factor) \|\| id:ebi-fl187-GCST90094780 | Weighted median | 6 | 0.0446554961784698 | 0.254967233175148 | 0.860967979926183 | -0.455080281 | 0.544391273201759 | 1.04566756137923 | 0.634397035973826 | 1.72355888649815 | 0.999332001504091 |
| ebi-fl187-GCST90094780 | ebi-gm430-GCST90011507 | TestASV_3 (Bacteroides) abundance \|\| id:ebi-gm430-GCST90011507 | F-healthy breakfast food liking (derived food-liking factor) \|\| id:ebi-fl187-GCST90094780 | Weighted median | 6 | -0.057223681 | 0.11446375987964 | 0.61712551266322 | -0.281572651 | 0.167125288045526 | 0.944382804948409 | 0.754596091762608 | 1.181902334 | 0.999332001504091 |
| ebi-fl187-GCST90094780 | ebi-gm430-GCST90011512 | TestASV_34 (Phascolarctobacterium) abundance \|\| id:ebi-gm430-GCST90011512 | F-healthy breakfast food liking (derived food-liking factor) \|\| id:ebi-fl187-GCST90094780 | Weighted median | 6 | -0.382037808 | 0.307299827509167 | 0.213790950839476 | -0.98434547 | 0.220269853901831 | 0.682469249909195 | 0.373683734148408 | 1.24641303462955 | 0.999332001504091 |
| ebi-fl187-GCST90094780 | ebi-gm430-GCST90011519 | TestASV_41 (Barnesiella) abundance \|\| id:ebi-gm430-GCST90011519 | F-healthy breakfast food liking (derived food-liking factor) \|\| id:ebi-fl187-GCST90094780 | Weighted median | 6 | 0.0423724210094715 | 0.383822082090858 | 0.912095328512119 | -0.70991886 | 0.794663701907552 | 1.04328294689767 | 0.491684091144379 | 2.21369640972963 | 0.999332001504091 |
| ebi-fl187-GCST90094780 | ebi-gm430-GCST90011524 | TestASV_46 (Sutterella) abundance \|\| id:ebi-gm430-GCST90011524 | F-healthy breakfast food liking (derived food-liking factor) \|\| id:ebi-fl187-GCST90094780 | Weighted median | 6 | 0.885850070944912 | 0.377338492476517 | 0.0188934258765269 | 0.146266625690938 | 1.62543351619889 | 2.42504497581408 | 1.15750476741975 | 5.08062109137603 | 0.999332001504091 |
| ebi-fl187-GCST90094780 | ebi-gm430-GCST90011581 | OTU97_19 (Bacteria) prevalence \|\| id:ebi-gm430-GCST90011581 | F-healthy breakfast food liking (derived food-liking factor) \|\| id:ebi-fl187-GCST90094780 | Weighted median | 6 | 0.0798007369648905 | 0.428942274721517 | 0.852412815352801 | -0.760926121 | 0.920527595419064 | 1.0830712301109 | 0.467233511579123 | 2.51061462935174 | 0.999332001504091 |
| ebi-fl187-GCST90094780 | ebi-gm430-GCST90011614 | OTU97_80 (Ruminococcus) prevalence \|\| id:ebi-gm430-GCST90011614 | F-healthy breakfast food liking (derived food-liking factor) \|\| id:ebi-fl187-GCST90094780 | Weighted median | 6 | 0.00237235225429438 | 0.192068574824538 | 0.990145107758744 | -0.374082054 | 0.378826758910388 | 1.00237516850851 | 0.687920462611012 | 1.46056998308918 | 0.999332001504091 |
| ebi-fl187-GCST90094780 | ebi-gm430-GCST90011623 | OTU99_11 (Parabacteroides) prevalence \|\| id:ebi-gm430-GCST90011623 | F-healthy breakfast food liking (derived food-liking factor) \|\| id:ebi-fl187-GCST90094780 | Weighted median | 6 | -0.124540543 | 0.183882335957837 | 0.498225851534382 | -0.484949922 | 0.235868835037806 | 0.882902464738022 | 0.6157280304717 | 1.26600824335267 | 0.999332001504091 |
| ebi-fl187-GCST90094780 | ebi-gm430-GCST90011642 | OTU99_19 (Bacteria) prevalence \|\| id:ebi-gm430-GCST90011642 | F-healthy breakfast food liking (derived food-liking factor) \|\| id:ebi-fl187-GCST90094780 | Weighted median | 6 | 0.0798007369648905 | 0.414101323502836 | 0.847187470339942 | -0.731837857 | 0.891439331030448 | 1.0830712301109 | 0.481024123609032 | 2.43863713256793 | 0.999332001504091 |
| ebi-fl187-GCST90094780 | ebi-gm430-GCST90011653 | OTU99_30 (Parasutterella) prevalence \|\| id:ebi-gm430-GCST90011653 | F-healthy breakfast food liking (derived food-liking factor) \|\| id:ebi-fl187-GCST90094780 | Weighted median | 6 | 0.0300682159766909 | 0.200892562859402 | 0.881022519099891 | -0.363681207 | 0.423817639181118 | 1.03052482981361 | 0.695112756347547 | 1.52778296062717 | 0.999332001504091 |
| ebi-fl187-GCST90094780 | ebi-gm430-GCST90011675 | OTU99_8 (Ruminococcaceae) prevalence \|\| id:ebi-gm430-GCST90011675 | F-healthy breakfast food liking (derived food-liking factor) \|\| id:ebi-fl187-GCST90094780 | Weighted median | 6 | 0.0903031633755282 | 0.208384040394462 | 0.66476072993343 | -0.318129556 | 0.498735882548674 | 1.09450604756126 | 0.727508529361029 | 1.64663841013702 | 0.999332001504091 |
| ebi-fl187-GCST90094780 | ebi-gm430-GCST90011679 | OTU99_92 (Ruminococcus) prevalence \|\| id:ebi-gm430-GCST90011679 | F-healthy breakfast food liking (derived food-liking factor) \|\| id:ebi-fl187-GCST90094780 | Weighted median | 6 | 0.0417514229577089 | 0.188709254277965 | 0.824899840497232 | -0.328118715 | 0.411621561342521 | 1.04263527134365 | 0.720277506577712 | 1.50926316471409 | 0.999332001504091 |
| ebi-fl187-GCST90094780 | ebi-gm430-GCST90011684 | TestASV_11 (Lachnospiraceae) prevalence \|\| id:ebi-gm430-GCST90011684 | F-healthy breakfast food liking (derived food-liking factor) \|\| id:ebi-fl187-GCST90094780 | Weighted median | 6 | -0.078602298 | 0.213695598045185 | 0.713004723067398 | -0.49744567 | 0.340241074386984 | 0.924407490258974 | 0.608081919574293 | 1.40528632827159 | 0.999332001504091 |
| ebi-fl187-GCST90094780 | ebi-gm430-GCST90011688 | TestASV_15 (Bacteroides) prevalence \|\| id:ebi-gm430-GCST90011688 | F-healthy breakfast food liking (derived food-liking factor) \|\| id:ebi-fl187-GCST90094780 | Weighted median | 6 | -0.206533242 | 0.196607255167082 | 0.293494554704444 | -0.591883462 | 0.178816978505971 | 0.813399222298466 | 0.553284213098403 | 1.19580186669465 | 0.999332001504091 |
| ebi-fl187-GCST90094780 | ebi-gm430-GCST90011692 | TestASV_19 (Prevotella) prevalence \|\| id:ebi-gm430-GCST90011692 | F-healthy breakfast food liking (derived food-liking factor) \|\| id:ebi-fl187-GCST90094780 | Weighted median | 6 | 0.244085323673761 | 0.397113741679698 | 0.538786947266108 | -0.53425761 | 1.02242825736597 | 1.27645323752282 | 0.586104246591979 | 2.77993697717869 | 0.999332001504091 |
| ebi-fl187-GCST90094780 | ebi-gm430-GCST90011693 | TestASV_2 (Bacteroides) prevalence \|\| id:ebi-gm430-GCST90011693 | F-healthy breakfast food liking (derived food-liking factor) \|\| id:ebi-fl187-GCST90094780 | Weighted median | 6 | -0.187717244 | 0.218805005533058 | 0.390936505199734 | -0.616575054 | 0.24114056720945 | 0.828849036442475 | 0.539790026703122 | 1.27269992261168 | 0.999332001504091 |
| ebi-fl187-GCST90094780 | ebi-gm430-GCST90011716 | TestASV_40 (Barnesiella) prevalence \|\| id:ebi-gm430-GCST90011716 | F-healthy breakfast food liking (derived food-liking factor) \|\| id:ebi-fl187-GCST90094780 | Weighted median | 6 | 0.0634935340371719 | 0.451558946411178 | 0.88817834428234 | -0.821562001 | 0.94854906900308 | 1.06555259595572 | 0.439744236865878 | 2.58196069342521 | 0.999332001504091 |
| ebi-fl187-GCST90094780 | ebi-gm430-GCST90011718 | TestASV_42 (Alphaproteobacteria) prevalence \|\| id:ebi-gm430-GCST90011718 | F-healthy breakfast food liking (derived food-liking factor) \|\| id:ebi-fl187-GCST90094780 | Weighted median | 6 | 0.168344788154493 | 0.330311736914586 | 0.610293693835944 | -0.479066216 | 0.815755792507082 | 1.18334454349984 | 0.619361471573053 | 2.26088378580341 | 0.999332001504091 |
| ebi-fl187-GCST90094780 | ebi-hbsm44-GCST90104929 | Sulcal depth of the right inferior frontal sulcus \|\| id:ebi-hbsm44-GCST90104929 | F-healthy breakfast food liking (derived food-liking factor) \|\| id:ebi-fl187-GCST90094780 | Weighted median | 7 | 0.0963758075194495 | 0.169469042522648 | 0.569564702064772 | -0.235783516 | 0.428535130863839 | 1.10117281526788 | 0.789951667458506 | 1.5350072909982 | 0.999332001504091 |
| ebi-fl187-GCST90094780 | ebi-hbsm44-GCST90104935 | Sulcal opening distance of the left calloso-marginal posterior fissure \|\| id:ebi-hbsm44-GCST90104935 | F-healthy breakfast food liking (derived food-liking factor) \|\| id:ebi-fl187-GCST90094780 | Weighted median | 6 | 0.0382823394558932 | 0.0433977299943925 | 0.377707757890054 | -0.046777211 | 0.123341890244902 | 1.03902454909503 | 0.954299981129493 | 1.1312711254006 | 0.999332001504091 |
| ebi-fl187-GCST90094780 | ebi-hbsm44-GCST90104936 | Sulcal opening distance of the left anterior inferior temporal sulcus \|\| id:ebi-hbsm44-GCST90104936 | F-healthy breakfast food liking (derived food-liking factor) \|\| id:ebi-fl187-GCST90094780 | Weighted median | 7 | 0.0222749399390342 | 0.0186581034856199 | 0.232537422932569 | -0.014294943 | 0.0588448227708492 | 1.02252487875447 | 0.985806744687335 | 1.06061064534861 | 0.999332001504091 |
| ebi-fl187-GCST90094780 | ebi-hbsm44-GCST90104941 | Sulcal opening distance of the right posterior inferior temporal sulcus \|\| id:ebi-hbsm44-GCST90104941 | F-healthy breakfast food liking (derived food-liking factor) \|\| id:ebi-fl187-GCST90094780 | Weighted median | 6 | -0.017902565 | 0.0176585529252399 | 0.31066944875045 | -0.052513328 | 0.0167081991556513 | 0.982256734295442 | 0.948841674514321 | 1.01684856176096 | 0.999332001504091 |
| ebi-fl187-GCST90094780 | ebi-IPs91-GCST90274819 | Leukemia inhibitory factor levels \|\| id:ebi-IPs91-GCST90274819 | F-healthy breakfast food liking (derived food-liking factor) \|\| id:ebi-fl187-GCST90094780 | Weighted median | 9 | 0.149719402459073 | 0.0681313507397457 | 0.0279839615202512 | 0.0161819550091718 | 0.283256849908975 | 1.16150828063103 | 1.01631359193219 | 1.32744607243673 | 0.999332001504091 |
| ebi-fl187-GCST90094780 | ebi-IPs91-GCST90274837 | STAM binding protein levels \|\| id:ebi-IPs91-GCST90274837 | F-healthy breakfast food liking (derived food-liking factor) \|\| id:ebi-fl187-GCST90094780 | Weighted median | 9 | 0.037086713557086 | 0.0604465704396203 | 0.53951579161296 | -0.081388565 | 0.155561991618742 | 1.0377830067926 | 0.921835429318255 | 1.16831435952075 | 0.999332001504091 |
| ebi-fl187-GCST90094780 | ebi-IPs91-GCST90274840 | TNF-beta levels \|\| id:ebi-IPs91-GCST90274840 | F-healthy breakfast food liking (derived food-liking factor) \|\| id:ebi-fl187-GCST90094780 | Weighted median | 8 | -0.078179138 | 0.0676061377507486 | 0.247521206264072 | -0.210687168 | 0.0543288916656485 | 0.924798744805144 | 0.810027429493097 | 1.05583179933595 | 0.999332001504091 |
| ebi-fl187-GCST90094780 | ebi-IPs91-GCST90274842 | Tumor necrosis factor ligand superfamily member 14 levels \|\| id:ebi-IPs91-GCST90274842 | F-healthy breakfast food liking (derived food-liking factor) \|\| id:ebi-fl187-GCST90094780 | Weighted median | 9 | 0.0619957580604505 | 0.0672684757646582 | 0.356728486957437 | -0.069850454 | 0.193841970559181 | 1.06395783147366 | 0.93253326568998 | 1.21390443515874 | 0.999332001504091 |
| ebi-fl187-GCST90094780 | ebi-met1400-GCST90199628 | 3-methylhistidine levels \|\| id:ebi-met1400-GCST90199628 | F-healthy breakfast food liking (derived food-liking factor) \|\| id:ebi-fl187-GCST90094780 | Weighted median | 8 | -0.025500095 | 0.0849224878579901 | 0.763967444963395 | -0.191948171 | 0.140947981057473 | 0.974822286217063 | 0.825349644144432 | 1.15136475364999 | 0.999332001504091 |
| ebi-fl187-GCST90094780 | ebi-met1400-GCST90199651 | Docosahexaenoate DHA; 22:6n3 levels \|\| id:ebi-met1400-GCST90199651 | F-healthy breakfast food liking (derived food-liking factor) \|\| id:ebi-fl187-GCST90094780 | Weighted median | 8 | 0.0314990605204899 | 0.0706519223804605 | 0.655716839373851 | -0.106978707 | 0.169976828386192 | 1.03200040605195 | 0.898544805190965 | 1.18527738621231 | 0.999332001504091 |
| ebi-fl187-GCST90094780 | ebi-met1400-GCST90199652 | 1-stearoyl-2-oleoyl-GPS (18:0/18:1) levels \|\| id:ebi-met1400-GCST90199652 | F-healthy breakfast food liking (derived food-liking factor) \|\| id:ebi-fl187-GCST90094780 | Weighted median | 8 | 0.0361781915006797 | 0.0831395622063115 | 0.66345345652701 | -0.12677535 | 0.19913173342505 | 1.03684058621093 | 0.880931551269395 | 1.22034271523722 | 0.999332001504091 |
| ebi-fl187-GCST90094780 | ebi-met1400-GCST90199653 | 1-myristoyl-2-palmitoyl-gpc (14:0/16:0) levels \|\| id:ebi-met1400-GCST90199653 | F-healthy breakfast food liking (derived food-liking factor) \|\| id:ebi-fl187-GCST90094780 | Weighted median | 8 | 0.163087020324971 | 0.083677491363427 | 0.0512960103518257 | -0.000920863 | 0.327094903397287 | 1.17713912026041 | 0.999079561116637 | 1.38693309559727 | 0.999332001504091 |
| ebi-fl187-GCST90094780 | ebi-met1400-GCST90199667 | Erythritol levels in elite athletes \|\| id:ebi-met1400-GCST90199667 | F-healthy breakfast food liking (derived food-liking factor) \|\| id:ebi-fl187-GCST90094780 | Weighted median | 8 | 0.0752682213088981 | 0.0722898672167906 | 0.297782661508432 | -0.066419918 | 0.216956361053808 | 1.07817330115882 | 0.935737848206309 | 1.24228988873326 | 0.999332001504091 |
| ebi-fl187-GCST90094780 | ebi-met1400-GCST90199674 | 3-hydroxymyristate levels \|\| id:ebi-met1400-GCST90199674 | F-healthy breakfast food liking (derived food-liking factor) \|\| id:ebi-fl187-GCST90094780 | Weighted median | 8 | 0.0104245149822694 | 0.077744232726137 | 0.893333534704778 | -0.141954181 | 0.162803211125498 | 1.01047903953789 | 0.867661010795355 | 1.17680508475244 | 0.999332001504091 |
| ebi-fl187-GCST90094780 | ebi-met1400-GCST90199685 | 2-linoleoylglycerol (18:2) levels \|\| id:ebi-met1400-GCST90199685 | F-healthy breakfast food liking (derived food-liking factor) \|\| id:ebi-fl187-GCST90094780 | Weighted median | 7 | 0.0520983904368498 | 0.0939678177080472 | 0.579285959250638 | -0.132078532 | 0.236275313144622 | 1.0534793897117 | 0.876272176733193 | 1.26652295258857 | 0.999332001504091 |
| ebi-fl187-GCST90094780 | ebi-met1400-GCST90199703 | Gamma-glutamylvaline levels \|\| id:ebi-met1400-GCST90199703 | F-healthy breakfast food liking (derived food-liking factor) \|\| id:ebi-fl187-GCST90094780 | Weighted median | 8 | 0.0816581000666177 | 0.075755653447615 | 0.281072015470008 | -0.066822981 | 0.230139180823943 | 1.08508475599573 | 0.935360763598797 | 1.2587751951068 | 0.999332001504091 |
| ebi-fl187-GCST90094780 | ebi-met1400-GCST90199779 | Gamma-glutamyl-2-aminobutyrate levels \|\| id:ebi-met1400-GCST90199779 | F-healthy breakfast food liking (derived food-liking factor) \|\| id:ebi-fl187-GCST90094780 | Weighted median | 8 | -0.048724881 | 0.0834454838686126 | 0.559279023425093 | -0.212278029 | 0.114828267513824 | 0.952443128982644 | 0.808739812981134 | 1.12168079199955 | 0.999332001504091 |
| ebi-fl187-GCST90094780 | ebi-met1400-GCST90199824 | 4-vinylphenol sulfate levels \|\| id:ebi-met1400-GCST90199824 | F-healthy breakfast food liking (derived food-liking factor) \|\| id:ebi-fl187-GCST90094780 | Weighted median | 8 | 0.00203229807991722 | 0.0800064955235555 | 0.97973458422806 | -0.154780433 | 0.158845029306086 | 1.00203436459735 | 0.856603238514865 | 1.17215628273228 | 0.999332001504091 |
| ebi-fl187-GCST90094780 | ebi-met1400-GCST90199830 | 4-ethylphenylsulfate levels \|\| id:ebi-met1400-GCST90199830 | F-healthy breakfast food liking (derived food-liking factor) \|\| id:ebi-fl187-GCST90094780 | Weighted median | 8 | 0.0362262323862471 | 0.077585534842899 | 0.640557166695119 | -0.115841416 | 0.188293880678329 | 1.03689039814738 | 0.890616449701971 | 1.20718823252144 | 0.999332001504091 |
| ebi-fl187-GCST90094780 | ebi-met1400-GCST90199833 | Hexanoylglutamine levels \|\| id:ebi-met1400-GCST90199833 | F-healthy breakfast food liking (derived food-liking factor) \|\| id:ebi-fl187-GCST90094780 | Weighted median | 8 | 0.0126336342253786 | 0.0775038183806661 | 0.870513235040104 | -0.13927385 | 0.164541118251484 | 1.01271377571941 | 0.869989749313909 | 1.17885204088975 | 0.999332001504091 |
| ebi-fl187-GCST90094780 | ebi-met1400-GCST90199837 | Carotenoid levels (cryptoxanthin) \|\| id:ebi-met1400-GCST90199837 | F-healthy breakfast food liking (derived food-liking factor) \|\| id:ebi-fl187-GCST90094780 | Weighted median | 8 | -0.050379888 | 0.0925631884837352 | 0.586251816009682 | -0.231803737 | 0.131043961924005 | 0.950868132958215 | 0.793101764618235 | 1.14001789759056 | 0.999332001504091 |
| ebi-fl187-GCST90094780 | ebi-met1400-GCST90199869 | 2-hydroxyhippurate levels \|\| id:ebi-met1400-GCST90199869 | F-healthy breakfast food liking (derived food-liking factor) \|\| id:ebi-fl187-GCST90094780 | Weighted median | 8 | 0.081897479371091 | 0.0846089064395247 | 0.333067639095123 | -0.083935977 | 0.24773093599256 | 1.08534453392141 | 0.919490122496603 | 1.28111518383106 | 0.999332001504091 |
| ebi-fl187-GCST90094780 | ebi-met1400-GCST90199902 | Hydantoin-5-propionate levels \|\| id:ebi-met1400-GCST90199902 | F-healthy breakfast food liking (derived food-liking factor) \|\| id:ebi-fl187-GCST90094780 | Weighted median | 8 | 0.0155003643881621 | 0.0794575026278335 | 0.845332341414523 | -0.140236341 | 0.171237069538716 | 1.01562111813856 | 0.8691527948886 | 1.18677206318046 | 0.999332001504091 |
| ebi-fl187-GCST90094780 | ebi-met1400-GCST90199904 | Pantoate levels \|\| id:ebi-met1400-GCST90199904 | F-healthy breakfast food liking (derived food-liking factor) \|\| id:ebi-fl187-GCST90094780 | Weighted median | 8 | 0.0416473056259334 | 0.076748678874554 | 0.587374080888931 | -0.108780105 | 0.192074716220059 | 1.04252672059229 | 0.896927625743676 | 1.21176105178805 | 0.999332001504091 |
| ebi-fl187-GCST90094780 | ebi-met1400-GCST90199936 | 3-methyl catechol sulfate (2) levels \|\| id:ebi-met1400-GCST90199936 | F-healthy breakfast food liking (derived food-liking factor) \|\| id:ebi-fl187-GCST90094780 | Weighted median | 8 | -0.037808611 | 0.0979271450682414 | 0.699430620177495 | -0.229745816 | 0.154128592836376 | 0.962897210712413 | 0.794735586036146 | 1.16664089879521 | 0.999332001504091 |
| ebi-fl187-GCST90094780 | ebi-met1400-GCST90199977 | 4-hydroxychlorothalonil levels \|\| id:ebi-met1400-GCST90199977 | F-healthy breakfast food liking (derived food-liking factor) \|\| id:ebi-fl187-GCST90094780 | Weighted median | 8 | -0.04169631 | 0.0814085016058371 | 0.608520711352428 | -0.201256973 | 0.117864353344058 | 0.959161024146348 | 0.817702277185958 | 1.1250914861183 | 0.999332001504091 |
| ebi-fl187-GCST90094780 | ebi-met1400-GCST90200040 | Sphingomyelin (d18:2/24:1, d18:1/24:2) levels \|\| id:ebi-met1400-GCST90200040 | F-healthy breakfast food liking (derived food-liking factor) \|\| id:ebi-fl187-GCST90094780 | Weighted median | 8 | -0.03245465 | 0.0706040228272609 | 0.645752097180573 | -0.170838535 | 0.105929234344926 | 0.968066350261006 | 0.842957670527875 | 1.11174320048693 | 0.999332001504091 |
| ebi-fl187-GCST90094780 | ebi-met1400-GCST90200077 | 1-stearoyl-2-oleoyl-GPI (18:0/18:1) levels \|\| id:ebi-met1400-GCST90200077 | F-healthy breakfast food liking (derived food-liking factor) \|\| id:ebi-fl187-GCST90094780 | Weighted median | 7 | -0.038732407 | 0.0790706981969894 | 0.624243399307986 | -0.193710975 | 0.116246161578452 | 0.962008101448603 | 0.82389599610854 | 1.12327234459679 | 0.999332001504091 |
| ebi-fl187-GCST90094780 | ebi-met1400-GCST90200080 | 1-palmitoyl-2-oleoyl-GPI (16:0/18:1) levels \|\| id:ebi-met1400-GCST90200080 | F-healthy breakfast food liking (derived food-liking factor) \|\| id:ebi-fl187-GCST90094780 | Weighted median | 8 | 0.0595433879830408 | 0.0811395012836917 | 0.463046436220422 | -0.099490035 | 0.218576810499077 | 1.06135180989487 | 0.905298971550586 | 1.24430458861311 | 0.999332001504091 |
| ebi-fl187-GCST90094780 | ebi-met1400-GCST90200082 | 1-oleoyl-2-linoleoyl-GPE (18:1/18:2) levels \|\| id:ebi-met1400-GCST90200082 | F-healthy breakfast food liking (derived food-liking factor) \|\| id:ebi-fl187-GCST90094780 | Weighted median | 8 | 0.0770699957101301 | 0.0894925679992179 | 0.389134077022593 | -0.098335438 | 0.252475428988597 | 1.08011767735055 | 0.906344830651932 | 1.28720786776703 | 0.999332001504091 |
| ebi-fl187-GCST90094780 | ebi-met1400-GCST90200100 | Perfluorooctanesulfonate (PFOS) levels \|\| id:ebi-met1400-GCST90200100 | F-healthy breakfast food liking (derived food-liking factor) \|\| id:ebi-fl187-GCST90094780 | Weighted median | 8 | -0.043204791 | 0.0760645412171711 | 0.57003379059774 | -0.192291292 | 0.105881709487543 | 0.957715238235762 | 0.825066498144901 | 1.11169036630536 | 0.999332001504091 |
| ebi-fl187-GCST90094780 | ebi-met1400-GCST90200102 | 1-palmitoleoyl-2-linolenoyl-GPC (16:1/18:3) levels \|\| id:ebi-met1400-GCST90200102 | F-healthy breakfast food liking (derived food-liking factor) \|\| id:ebi-fl187-GCST90094780 | Weighted median | 8 | 0.0137238236434145 | 0.0793609216224936 | 0.862707049858008 | -0.141823583 | 0.169271230023502 | 1.01381842759168 | 0.867774333355892 | 1.18444135141635 | 0.999332001504091 |
| ebi-fl187-GCST90094780 | ebi-met1400-GCST90200114 | Glycosyl-N-tricosanoyl-sphingadienine (d18:2/23:0) levels \|\| id:ebi-met1400-GCST90200114 | F-healthy breakfast food liking (derived food-liking factor) \|\| id:ebi-fl187-GCST90094780 | Weighted median | 8 | -0.02385722 | 0.0749541250039267 | 0.75026424399794 | -0.170767305 | 0.123052864909082 | 0.976425113687076 | 0.843017716567922 | 1.13094420662985 | 0.999332001504091 |
| ebi-fl187-GCST90094780 | ebi-met1400-GCST90200135 | Arachidoylcarnitine (C20) levels \|\| id:ebi-met1400-GCST90200135 | F-healthy breakfast food liking (derived food-liking factor) \|\| id:ebi-fl187-GCST90094780 | Weighted median | 8 | -0.005447031 | 0.0869453453540195 | 0.950046078633495 | -0.175859908 | 0.164965845483029 | 0.994567776765678 | 0.838735474981655 | 1.17935283779699 | 0.999332001504091 |
| ebi-fl187-GCST90094780 | ebi-met1400-GCST90200146 | Docosahexaenoylcarnitine (C22:6) levels \|\| id:ebi-met1400-GCST90200146 | F-healthy breakfast food liking (derived food-liking factor) \|\| id:ebi-fl187-GCST90094780 | Weighted median | 8 | 0.095543591201416 | 0.0876309610798359 | 0.27558325473833 | -0.076213093 | 0.267300274917894 | 1.10025678250362 | 0.926618730004061 | 1.30643267640392 | 0.999332001504091 |
| ebi-fl187-GCST90094780 | ebi-met1400-GCST90200162 | 3-hydroxyphenylacetoylglutamine levels \|\| id:ebi-met1400-GCST90200162 | F-healthy breakfast food liking (derived food-liking factor) \|\| id:ebi-fl187-GCST90094780 | Weighted median | 7 | 0.00155703717014116 | 0.0986422273036551 | 0.987406160980015 | -0.191781728 | 0.194895802685305 | 1.0015582499819 | 0.82548702924901 | 1.21518436094555 | 0.999332001504091 |
| ebi-fl187-GCST90094780 | ebi-met1400-GCST90200172 | 3-carboxy-4-methyl-5-pentyl-2-furanpropionate (3-CMPFP) levels \|\| id:ebi-met1400-GCST90200172 | F-healthy breakfast food liking (derived food-liking factor) \|\| id:ebi-fl187-GCST90094780 | Weighted median | 8 | 0.0272626141465203 | 0.0747087745713092 | 0.715172242282734 | -0.119166584 | 0.173691812306286 | 1.0276376395121 | 0.887659918493261 | 1.18968886184987 | 0.999332001504091 |
| ebi-fl187-GCST90094780 | ebi-met1400-GCST90200222 | Dihydrocaffeate sulfate (2) levels \|\| id:ebi-met1400-GCST90200222 | F-healthy breakfast food liking (derived food-liking factor) \|\| id:ebi-fl187-GCST90094780 | Weighted median | 8 | 0.0618877857376682 | 0.0898981870136149 | 0.491187801725066 | -0.114312661 | 0.238088232284353 | 1.06384295967685 | 0.891979025395659 | 1.26882113887373 | 0.999332001504091 |
| ebi-fl187-GCST90094780 | ebi-met1400-GCST90200223 | 4-methylhexanoylglutamine levels \|\| id:ebi-met1400-GCST90200223 | F-healthy breakfast food liking (derived food-liking factor) \|\| id:ebi-fl187-GCST90094780 | Weighted median | 8 | 0.054736864016949 | 0.0899671237854937 | 0.542915829605833 | -0.121598699 | 0.231072426636517 | 1.05626263739673 | 0.885503651062723 | 1.25995049012088 | 0.999332001504091 |
| ebi-fl187-GCST90094780 | ebi-met1400-GCST90200224 | 2-ketocaprylate levels \|\| id:ebi-met1400-GCST90200224 | F-healthy breakfast food liking (derived food-liking factor) \|\| id:ebi-fl187-GCST90094780 | Weighted median | 8 | 0.0366028301386542 | 0.0764258646886256 | 0.63198665214649 | -0.113191865 | 0.18639752492836 | 1.03728096227899 | 0.892979312514575 | 1.20490114342807 | 0.999332001504091 |
| ebi-fl187-GCST90094780 | ebi-met1400-GCST90200243 | Hydroxypalmitoyl sphingomyelin (d18:1/16:0(OH)) levels \|\| id:ebi-met1400-GCST90200243 | F-healthy breakfast food liking (derived food-liking factor) \|\| id:ebi-fl187-GCST90094780 | Weighted median | 8 | -0.077546241 | 0.078762306389027 | 0.324840091341079 | -0.231920362 | 0.0768278793863795 | 0.925384232589017 | 0.793009274735363 | 1.07985619488515 | 0.999332001504091 |
| ebi-fl187-GCST90094780 | ebi-met1400-GCST90200273 | 2-methoxyhydroquinone sulfate (2) levels \|\| id:ebi-met1400-GCST90200273 | F-healthy breakfast food liking (derived food-liking factor) \|\| id:ebi-fl187-GCST90094780 | Weighted median | 8 | 0.210842561596019 | 0.0924741108893235 | 0.0226066695250342 | 0.0295933042529453 | 0.392091818939094 | 1.23471794773515 | 1.0300355376848 | 1.48007360395153 | 0.999332001504091 |
| ebi-fl187-GCST90094780 | ebi-met1400-GCST90200276 | 2,4-di-tert-butylphenol levels \|\| id:ebi-met1400-GCST90200276 | F-healthy breakfast food liking (derived food-liking factor) \|\| id:ebi-fl187-GCST90094780 | Weighted median | 8 | 0.021817715684607 | 0.0764312080755455 | 0.775295122691999 | -0.127987452 | 0.171622883512676 | 1.02205746244433 | 0.879864419487886 | 1.18723002476465 | 0.999332001504091 |
| ebi-fl187-GCST90094780 | ebi-met1400-GCST90200289 | N-acetylmethionine levels \|\| id:ebi-met1400-GCST90200289 | F-healthy breakfast food liking (derived food-liking factor) \|\| id:ebi-fl187-GCST90094780 | Weighted median | 8 | 0.0129338359960991 | 0.0905092635444473 | 0.886368615508203 | -0.164464321 | 0.190331992543216 | 1.01301783982612 | 0.84834802499444 | 1.20965112615511 | 0.999332001504091 |
| ebi-fl187-GCST90094780 | ebi-met1400-GCST90200308 | 3-hydroxyisobutyrate levels \|\| id:ebi-met1400-GCST90200308 | F-healthy breakfast food liking (derived food-liking factor) \|\| id:ebi-fl187-GCST90094780 | Weighted median | 8 | -0.008526016 | 0.080488662855645 | 0.915639348820981 | -0.166283795 | 0.149231762987408 | 0.991510227189469 | 0.846805880466112 | 1.16094202142312 | 0.999332001504091 |
| ebi-fl187-GCST90094780 | ebi-met1400-GCST90200383 | Cystathionine levels \|\| id:ebi-met1400-GCST90200383 | F-healthy breakfast food liking (derived food-liking factor) \|\| id:ebi-fl187-GCST90094780 | Weighted median | 8 | 0.0592073155624569 | 0.0823551804148561 | 0.472186252853186 | -0.102208838 | 0.220623469175575 | 1.06099517875333 | 0.90284098442831 | 1.24685386325326 | 0.999332001504091 |
| ebi-fl187-GCST90094780 | ebi-met1400-GCST90200384 | Guanidinoacetate levels \|\| id:ebi-met1400-GCST90200384 | F-healthy breakfast food liking (derived food-liking factor) \|\| id:ebi-fl187-GCST90094780 | Weighted median | 8 | 0.0296726055632216 | 0.0760174806142066 | 0.696285711139073 | -0.119321656 | 0.178666867567067 | 1.03011722409145 | 0.887522277587445 | 1.1956223772257 | 0.999332001504091 |
| ebi-fl187-GCST90094780 | ebi-met1400-GCST90200403 | Citrulline levels \|\| id:ebi-met1400-GCST90200403 | F-healthy breakfast food liking (derived food-liking factor) \|\| id:ebi-fl187-GCST90094780 | Weighted median | 8 | -0.02280095 | 0.0821085696294462 | 0.781248271391803 | -0.183733746 | 0.138131846780257 | 0.977457027531599 | 0.832157339414148 | 1.14812691713269 | 0.999332001504091 |
| ebi-fl187-GCST90094780 | ebi-met1400-GCST90200434 | 12,13-DiHOME levels \|\| id:ebi-met1400-GCST90200434 | F-healthy breakfast food liking (derived food-liking factor) \|\| id:ebi-fl187-GCST90094780 | Weighted median | 8 | -0.005310039 | 0.0761435433643933 | 0.944402814616218 | -0.154551384 | 0.143931305768581 | 0.99470403411165 | 0.856799465038887 | 1.15480477737353 | 0.999332001504091 |
| ebi-fl187-GCST90094780 | ebi-met1400-GCST90200437 | Fructose levels \|\| id:ebi-met1400-GCST90200437 | F-healthy breakfast food liking (derived food-liking factor) \|\| id:ebi-fl187-GCST90094780 | Weighted median | 8 | -0.001314184 | 0.0817237577372532 | 0.987169922158786 | -0.161492749 | 0.158864380864499 | 0.998686678861512 | 0.850872700724852 | 1.17217896600253 | 0.999332001504091 |
| ebi-fl187-GCST90094780 | ebi-met1400-GCST90200448 | N-stearoyl-sphinganine (d18:0/18:0) levels \|\| id:ebi-met1400-GCST90200448 | F-healthy breakfast food liking (derived food-liking factor) \|\| id:ebi-fl187-GCST90094780 | Weighted median | 8 | 0.127237246076856 | 0.0853446087618879 | 0.135997077667536 | -0.040038187 | 0.294512679250156 | 1.13568642297095 | 0.960752750093877 | 1.34247198479996 | 0.999332001504091 |
| ebi-fl187-GCST90094780 | ebi-met1400-GCST90200468 | X-11847 levels \|\| id:ebi-met1400-GCST90200468 | F-healthy breakfast food liking (derived food-liking factor) \|\| id:ebi-fl187-GCST90094780 | Weighted median | 8 | 0.162271562623876 | 0.0974718301765232 | 0.0959517226238395 | -0.028773225 | 0.353316349769862 | 1.17617960437502 | 0.971636782880028 | 1.42378148514226 | 0.999332001504091 |
| ebi-fl187-GCST90094780 | ebi-met1400-GCST90200476 | X-11843 levels \|\| id:ebi-met1400-GCST90200476 | F-healthy breakfast food liking (derived food-liking factor) \|\| id:ebi-fl187-GCST90094780 | Weighted median | 8 | -0.22403603 | 0.0950599531119149 | 0.018433833538037 | -0.410353538 | -0.037718522 | 0.799286335623263 | 0.663415666007198 | 0.962983961712973 | 0.999332001504091 |
| ebi-fl187-GCST90094780 | ebi-met1400-GCST90200511 | X-12906 levels \|\| id:ebi-met1400-GCST90200511 | F-healthy breakfast food liking (derived food-liking factor) \|\| id:ebi-fl187-GCST90094780 | Weighted median | 8 | -0.11904333 | 0.0789045554002539 | 0.131375376556103 | -0.273696259 | 0.0356095983872991 | 0.887769332708289 | 0.760563054528924 | 1.03625121336649 | 0.999332001504091 |
| ebi-fl187-GCST90094780 | ebi-met1400-GCST90200515 | X-12849 levels \|\| id:ebi-met1400-GCST90200515 | F-healthy breakfast food liking (derived food-liking factor) \|\| id:ebi-fl187-GCST90094780 | Weighted median | 8 | 0.0568113108030278 | 0.0800446749794151 | 0.477862258305206 | -0.100076252 | 0.213698873762681 | 1.05845607232557 | 0.904768424861911 | 1.23824972916562 | 0.999332001504091 |
| ebi-fl187-GCST90094780 | ebi-met1400-GCST90200551 | X-18886 levels \|\| id:ebi-met1400-GCST90200551 | F-healthy breakfast food liking (derived food-liking factor) \|\| id:ebi-fl187-GCST90094780 | Weighted median | 8 | 0.0476696924470314 | 0.0779098861060104 | 0.540632515552874 | -0.105033684 | 0.200373069214812 | 1.04882416356024 | 0.900294196277032 | 1.2218585109365 | 0.999332001504091 |
| ebi-fl187-GCST90094780 | ebi-met1400-GCST90200564 | X-21319 levels \|\| id:ebi-met1400-GCST90200564 | F-healthy breakfast food liking (derived food-liking factor) \|\| id:ebi-fl187-GCST90094780 | Weighted median | 8 | -0.089374612 | 0.0819713855692272 | 0.275574510840876 | -0.250038528 | 0.0712893032486013 | 0.914502925201318 | 0.778770777870521 | 1.0738918613364 | 0.999332001504091 |
| ebi-fl187-GCST90094780 | ebi-met1400-GCST90200579 | X-21442 levels \|\| id:ebi-met1400-GCST90200579 | F-healthy breakfast food liking (derived food-liking factor) \|\| id:ebi-fl187-GCST90094780 | Weighted median | 8 | 0.00997831400345177 | 0.0880662770605487 | 0.909789073087235 | -0.162631589 | 0.182588217042127 | 1.01002826337723 | 0.849904244787139 | 1.20032003496621 | 0.999332001504091 |
| ebi-fl187-GCST90094780 | ebi-met1400-GCST90200581 | X-21441 levels \|\| id:ebi-met1400-GCST90200581 | F-healthy breakfast food liking (derived food-liking factor) \|\| id:ebi-fl187-GCST90094780 | Weighted median | 8 | 0.0771937660740328 | 0.0749439142190609 | 0.303000394451584 | -0.069696306 | 0.224083837943392 | 1.08025137218209 | 0.9326770255073 | 1.25117591104656 | 0.999332001504091 |
| ebi-fl187-GCST90094780 | ebi-met1400-GCST90200648 | X-24585 levels \|\| id:ebi-met1400-GCST90200648 | F-healthy breakfast food liking (derived food-liking factor) \|\| id:ebi-fl187-GCST90094780 | Weighted median | 8 | -0.008789652 | 0.076068275601074 | 0.908009574888288 | -0.157883473 | 0.140304167721023 | 0.991248863607859 | 0.853949284630799 | 1.15062372823309 | 0.999332001504091 |
| ebi-fl187-GCST90094780 | ebi-met1400-GCST90200720 | Adenosine 5'-diphosphate (ADP) to pantothenate ratio \|\| id:ebi-met1400-GCST90200720 | F-healthy breakfast food liking (derived food-liking factor) \|\| id:ebi-fl187-GCST90094780 | Weighted median | 7 | -0.133363581 | 0.1057034514991 | 0.207065146290885 | -0.340542346 | 0.0738151836451269 | 0.875146847235957 | 0.711384401471414 | 1.07660781237668 | 0.999332001504091 |
| ebi-fl187-GCST90094780 | ebi-met1400-GCST90200724 | Cholate to taurocholate ratio \|\| id:ebi-met1400-GCST90200724 | F-healthy breakfast food liking (derived food-liking factor) \|\| id:ebi-fl187-GCST90094780 | Weighted median | 8 | 0.0315992490767598 | 0.0877544075643591 | 0.718781837920948 | -0.14039939 | 0.203597887902904 | 1.03210380586236 | 0.869011091958425 | 1.22580514326338 | 0.999332001504091 |
| ebi-fl187-GCST90094780 | ebi-met1400-GCST90200726 | N-acetylputrescine to (N(1) + N(8))-acetylspermidine ratio \|\| id:ebi-met1400-GCST90200726 | F-healthy breakfast food liking (derived food-liking factor) \|\| id:ebi-fl187-GCST90094780 | Weighted median | 8 | -0.054084864 | 0.0803935113129941 | 0.501105328743589 | -0.211656146 | 0.103486418135261 | 0.947351706998874 | 0.809242910972004 | 1.1090307305574 | 0.999332001504091 |
| ebi-fl187-GCST90094780 | ebi-met1400-GCST90200744 | Arginine to glutamate ratio \|\| id:ebi-met1400-GCST90200744 | F-healthy breakfast food liking (derived food-liking factor) \|\| id:ebi-fl187-GCST90094780 | Weighted median | 8 | 0.0231019262352034 | 0.0932319588623772 | 0.804297065588661 | -0.159632713 | 0.205836565605463 | 1.02337084256872 | 0.852456827670926 | 1.22855239986898 | 0.999332001504091 |
| ebi-fl187-GCST90094780 | ebi-met1400-GCST90200759 | Histidine to trans-urocanate ratio \|\| id:ebi-met1400-GCST90200759 | F-healthy breakfast food liking (derived food-liking factor) \|\| id:ebi-fl187-GCST90094780 | Weighted median | 8 | 0.0141275003240056 | 0.0762892820699807 | 0.853085274256362 | -0.135399493 | 0.163654493181168 | 1.01422776506369 | 0.873366938415965 | 1.17780730432936 | 0.999332001504091 |
| ebi-fl187-GCST90094780 | ebi-met1400-GCST90200795 | Oleoyl-linoleoyl-glycerol (18:1 to 18:2) [2] to linoleoyl-arachidonoyl-glycerol (18:2 to 20:4) [2] ratio \|\| id:ebi-met1400-GCST90200795 | F-healthy breakfast food liking (derived food-liking factor) \|\| id:ebi-fl187-GCST90094780 | Weighted median | 8 | 0.000331815678984312 | 0.0811027688391966 | 0.99673562478215 | -0.158629611 | 0.15929324260381 | 1.0003318707359 | 0.853312357745416 | 1.17268177652306 | 0.999364451047625 |
| ebi-fl187-GCST90094780 | ebi-met1400-GCST90200811 | Choline to taurocholate ratio \|\| id:ebi-met1400-GCST90200811 | F-healthy breakfast food liking (derived food-liking factor) \|\| id:ebi-fl187-GCST90094780 | Weighted median | 8 | -0.0296399 | 0.0830767246789134 | 0.721258441081871 | -0.19247028 | 0.133190480829902 | 0.970795054358323 | 0.824918834500105 | 1.14246759578195 | 0.999332001504091 |
| ebi-fl187-GCST90094780 | ebi-met1400-GCST90200831 | Cholate to phosphate ratio \|\| id:ebi-met1400-GCST90200831 | F-healthy breakfast food liking (derived food-liking factor) \|\| id:ebi-fl187-GCST90094780 | Weighted median | 8 | 0.109287854652454 | 0.080357291170041 | 0.173822289509661 | -0.048212436 | 0.266788145345734 | 1.11548340121801 | 0.952931328614899 | 1.30576378489048 | 0.999332001504091 |
| ebi-fl187-GCST90094780 | ebi-met1400-GCST90200841 | Adenosine 5'-diphosphate (ADP) to oxalate (ethanedioate) ratio \|\| id:ebi-met1400-GCST90200841 | F-healthy breakfast food liking (derived food-liking factor) \|\| id:ebi-fl187-GCST90094780 | Weighted median | 7 | -0.132634395 | 0.118099206797468 | 0.261405180485547 | -0.364108841 | 0.0988400500894561 | 0.875785224836739 | 0.694815566514269 | 1.10388971837548 | 0.999332001504091 |
| ebi-fl187-GCST90094780 | ebi-met1400-GCST90200844 | Aspartate to phosphate ratio \|\| id:ebi-met1400-GCST90200844 | F-healthy breakfast food liking (derived food-liking factor) \|\| id:ebi-fl187-GCST90094780 | Weighted median | 8 | -0.007832993 | 0.082547977625223 | 0.924402052908571 | -0.169627029 | 0.153961043311724 | 0.992197605111371 | 0.843979537811583 | 1.16644544504172 | 0.999332001504091 |
| ebi-fl187-GCST90094780 | ebi-met1400-GCST90200870 | Adenosine 5'-monophosphate (AMP) to acetoacetate ratio \|\| id:ebi-met1400-GCST90200870 | F-healthy breakfast food liking (derived food-liking factor) \|\| id:ebi-fl187-GCST90094780 | Weighted median | 8 | 0.0675836305690401 | 0.0861456040479369 | 0.432730412620726 | -0.101261753 | 0.236429014502996 | 1.06991973385263 | 0.903696456336552 | 1.2667176338478 | 0.999332001504091 |
| ebi-fl187-GCST90094780 | ebi-met1400-GCST90200887 | Cholesterol to cortisol ratio \|\| id:ebi-met1400-GCST90200887 | F-healthy breakfast food liking (derived food-liking factor) \|\| id:ebi-fl187-GCST90094780 | Weighted median | 8 | 0.0122091168216925 | 0.0783510802271449 | 0.876170363109772 | -0.141359 | 0.165777234066897 | 1.01228395233687 | 0.868177579626274 | 1.18031013954527 | 0.999332001504091 |
| ebi-fl187-GCST90094780 | ebi-met1400-GCST90200890 | Cortisol to taurocholate ratio \|\| id:ebi-met1400-GCST90200890 | F-healthy breakfast food liking (derived food-liking factor) \|\| id:ebi-fl187-GCST90094780 | Weighted median | 8 | 0.0481462105498226 | 0.0801202573052845 | 0.547890398945594 | -0.108889494 | 0.20518191486818 | 1.04932406635773 | 0.896829517272784 | 1.22774839033605 | 0.999332001504091 |
| ebi-fl187-GCST90094780 | ebi-met1400-GCST90200948 | Adenosine 5'-diphosphate (ADP) to mannitol to sorbitol ratio \|\| id:ebi-met1400-GCST90200948 | F-healthy breakfast food liking (derived food-liking factor) \|\| id:ebi-fl187-GCST90094780 | Weighted median | 8 | -0.122965536 | 0.103141741196485 | 0.233183015005816 | -0.325123349 | 0.079192276662202 | 0.884294138275528 | 0.722438236236166 | 1.08241242471118 | 0.999332001504091 |
| ebi-fl187-GCST90094780 | ebi-met1400-GCST90200972 | Leucine to N-palmitoyl-sphingosine (d18:1 to 16:0) ratio \|\| id:ebi-met1400-GCST90200972 | F-healthy breakfast food liking (derived food-liking factor) \|\| id:ebi-fl187-GCST90094780 | Weighted median | 8 | -0.091902465 | 0.0751880634237529 | 0.221593721690784 | -0.239271069 | 0.0554661393988755 | 0.91219411614002 | 0.787201467359374 | 1.05703322468605 | 0.999332001504091 |
| ebi-fl187-GCST90094780 | ebi-met1400-GCST90200973 | Proline to glutamate ratio \|\| id:ebi-met1400-GCST90200973 | F-healthy breakfast food liking (derived food-liking factor) \|\| id:ebi-fl187-GCST90094780 | Weighted median | 8 | 0.0290728745409991 | 0.0835664047557595 | 0.727914185895862 | -0.134717279 | 0.192863027862288 | 1.02949961605349 | 0.873962964638178 | 1.21271667374725 | 0.999332001504091 |
| ebi-fl187-GCST90094780 | ebi-met1400-GCST90200983 | Cholesterol to linoleoyl-arachidonoyl-glycerol (18:2 to 20:4) [1] ratio \|\| id:ebi-met1400-GCST90200983 | F-healthy breakfast food liking (derived food-liking factor) \|\| id:ebi-fl187-GCST90094780 | Weighted median | 8 | -0.011196749 | 0.0913644543042028 | 0.902463141725562 | -0.19027108 | 0.167877580983963 | 0.988865700849431 | 0.826734992335043 | 1.18279180557553 | 0.999332001504091 |
| ebi-fl187-GCST90094780 | ebi-met1400-GCST90201001 | Serine to alpha-ketobutyrate ratio \|\| id:ebi-met1400-GCST90201001 | F-healthy breakfast food liking (derived food-liking factor) \|\| id:ebi-fl187-GCST90094780 | Weighted median | 8 | -0.088504241 | 0.0813578349466793 | 0.276666108260062 | -0.247965597 | 0.0709571157820729 | 0.915299229206044 | 0.780386790299942 | 1.07353518716428 | 0.999332001504091 |
| ebi-fl187-GCST90094780 | ebi-met1400-GCST90201005 | Fructose to maltose ratio \|\| id:ebi-met1400-GCST90201005 | F-healthy breakfast food liking (derived food-liking factor) \|\| id:ebi-fl187-GCST90094780 | Weighted median | 8 | -0.012992605 | 0.0883571237500366 | 0.883095322087382 | -0.186172568 | 0.16018735726378 | 0.98709143425186 | 0.830130328825978 | 1.17373075737563 | 0.999332001504091 |
| ebi-fl187-GCST90094780 | ebi-met1400-GCST90201020 | Paraxanthine to 5-acetylamino-6-formylamino-3-methyluracil ratio \|\| id:ebi-met1400-GCST90201020 | F-healthy breakfast food liking (derived food-liking factor) \|\| id:ebi-fl187-GCST90094780 | Weighted median | 8 | 0.00994656395387811 | 0.0903411354158514 | 0.912330024752175 | -0.167122061 | 0.187015189368947 | 1.00999619543888 | 0.846096329279677 | 1.20564559790665 | 0.999332001504091 |
| ebi-fl187-GCST90094780 | ebi-pw11-GCST90275197 | Placental weight (fetal genotype, adjusted for sex) \|\| id:ebi-pw11-GCST90275197 | F-healthy breakfast food liking (derived food-liking factor) \|\| id:ebi-fl187-GCST90094780 | Weighted median | 7 | -0.027262932 | 0.0339773566584621 | 0.422330111712197 | -0.093858551 | 0.0393326874174926 | 0.973105347706277 | 0.910411530222002 | 1.04011645975491 | 0.999332001504091 |
| ebi-fl187-GCST90094780 | prot-a-1017 | FAS-associated factor 2 \|\| id:prot-a-1017 | F-healthy breakfast food liking (derived food-liking factor) \|\| id:ebi-fl187-GCST90094780 | Weighted median | 8 | -0.03593976 | 0.129377648695845 | 0.781173908953386 | -0.289519951 | 0.217640431868755 | 0.964698405574608 | 0.748622856973087 | 1.2431399937227 | 0.999332001504091 |
| ebi-fl187-GCST90094780 | prot-a-1019 | Fas apoptotic inhibitory molecule 3 \|\| id:prot-a-1019 | F-healthy breakfast food liking (derived food-liking factor) \|\| id:ebi-fl187-GCST90094780 | Weighted median | 8 | -0.081722925 | 0.142987511090862 | 0.567634398015356 | -0.361978447 | 0.198532596656609 | 0.921527255415764 | 0.696297375103002 | 1.21961178203277 | 0.999332001504091 |
| ebi-fl187-GCST90094780 | prot-a-1052 | MIP18 family protein FAM96A \|\| id:prot-a-1052 | F-healthy breakfast food liking (derived food-liking factor) \|\| id:ebi-fl187-GCST90094780 | Weighted median | 8 | -0.005752676 | 0.123504737997576 | 0.962849100046496 | -0.247821962 | 0.236316610515362 | 0.994263838996888 | 0.780498889030467 | 1.26657525773652 | 0.999332001504091 |
| ebi-fl187-GCST90094780 | prot-a-1066 | F-box/LRR-repeat protein 4 \|\| id:prot-a-1066 | F-healthy breakfast food liking (derived food-liking factor) \|\| id:ebi-fl187-GCST90094780 | Weighted median | 8 | 0.137829797700937 | 0.124087056813253 | 0.266675629052944 | -0.105380834 | 0.381040429054913 | 1.14778017882306 | 0.899981713990077 | 1.46380678453832 | 0.999332001504091 |
| ebi-fl187-GCST90094780 | prot-a-1076 | Low affinity immunoglobulin gamma Fc region receptor III-B \|\| id:prot-a-1076 | F-healthy breakfast food liking (derived food-liking factor) \|\| id:ebi-fl187-GCST90094780 | Weighted median | 8 | 0.0275610450191311 | 0.124419376636658 | 0.824689663717232 | -0.216300933 | 0.271423023226981 | 1.02794436407535 | 0.805492865860174 | 1.31182988753832 | 0.999332001504091 |
| ebi-fl187-GCST90094780 | prot-a-1081 | Fc receptor-like protein 3 \|\| id:prot-a-1081 | F-healthy breakfast food liking (derived food-liking factor) \|\| id:ebi-fl187-GCST90094780 | Weighted median | 8 | 0.0829469168647444 | 0.125114203637951 | 0.507350007097721 | -0.162276922 | 0.328170755995128 | 1.08648413303327 | 0.850205731040511 | 1.38842603411809 | 0.999332001504091 |
| ebi-fl187-GCST90094780 | prot-a-1105 | Fibroblast growth factor receptor 4 \|\| id:prot-a-1105 | F-healthy breakfast food liking (derived food-liking factor) \|\| id:ebi-fl187-GCST90094780 | Weighted median | 8 | -0.108566454 | 0.136961874130498 | 0.427966346010309 | -0.377011727 | 0.159878819137445 | 0.897119275529641 | 0.685908029893911 | 1.17336867254829 | 0.999332001504091 |
| ebi-fl187-GCST90094780 | prot-a-1143 | Follistatin-related protein 1 \|\| id:prot-a-1143 | F-healthy breakfast food liking (derived food-liking factor) \|\| id:ebi-fl187-GCST90094780 | Weighted median | 8 | 0.0340948959742058 | 0.135651206500822 | 0.801549418394646 | -0.231781469 | 0.299971260715818 | 1.03468278930076 | 0.793119425735697 | 1.34982001415758 | 0.999332001504091 |
| ebi-fl187-GCST90094780 | prot-a-1151 | Alpha-(1,3)-fucosyltransferase 10 \|\| id:prot-a-1151 | F-healthy breakfast food liking (derived food-liking factor) \|\| id:ebi-fl187-GCST90094780 | Weighted median | 8 | -0.083804502 | 0.122845881412307 | 0.495117425547645 | -0.32458243 | 0.156973425348632 | 0.919611020436216 | 0.722829122543057 | 1.1699645220885 | 0.999332001504091 |
| ebi-fl187-GCST90094780 | prot-a-1165 | Growth arrest and DNA damage-inducible proteins-interacting protein 1 \|\| id:prot-a-1165 | F-healthy breakfast food liking (derived food-liking factor) \|\| id:ebi-fl187-GCST90094780 | Weighted median | 8 | 0.119514294294492 | 0.138869180467125 | 0.38944458906239 | -0.152669299 | 0.391697888010057 | 1.126949353 | 0.858413552736131 | 1.47949067200669 | 0.999332001504091 |
| ebi-fl187-GCST90094780 | prot-a-1194 | Growth/differentiation factor 11/8 \|\| id:prot-a-1194 | F-healthy breakfast food liking (derived food-liking factor) \|\| id:ebi-fl187-GCST90094780 | Weighted median | 8 | 0.0477911445364218 | 0.124761746069727 | 0.701675811408633 | -0.196741878 | 0.292324166833086 | 1.04895155318202 | 0.821402628237322 | 1.33953718079054 | 0.999332001504091 |
| ebi-fl187-GCST90094780 | prot-a-1216 | D-glucuronyl C5-epimerase \|\| id:prot-a-1216 | F-healthy breakfast food liking (derived food-liking factor) \|\| id:ebi-fl187-GCST90094780 | Weighted median | 8 | 0.16523046744928 | 0.134224836342352 | 0.218323717182053 | -0.097850212 | 0.428311146680291 | 1.17966496175944 | 0.906784719249337 | 1.53466351214533 | 0.999332001504091 |
| ebi-fl187-GCST90094780 | prot-a-1246 | Glypican-3 \|\| id:prot-a-1246 | F-healthy breakfast food liking (derived food-liking factor) \|\| id:ebi-fl187-GCST90094780 | Weighted median | 8 | 0.00707656791352623 | 0.129645781776351 | 0.95646999306958 | -0.247029164 | 0.261182300195174 | 1.00710166598806 | 0.781117912388836 | 1.29846435416402 | 0.999332001504091 |
| ebi-fl187-GCST90094780 | prot-a-1275 | Glutamate receptor 4 \|\| id:prot-a-1275 | F-healthy breakfast food liking (derived food-liking factor) \|\| id:ebi-fl187-GCST90094780 | Weighted median | 7 | 0.0131716468912279 | 0.127632248672943 | 0.917804247199525 | -0.236987561 | 0.263330854290196 | 1.01325877515278 | 0.789001102729675 | 1.30125717425756 | 0.999332001504091 |
| ebi-fl187-GCST90094780 | prot-a-1289 | Glutathione S-transferase P \|\| id:prot-a-1289 | F-healthy breakfast food liking (derived food-liking factor) \|\| id:ebi-fl187-GCST90094780 | Weighted median | 8 | -0.025062385 | 0.14055481178385 | 0.858479212829079 | -0.300549816 | 0.250425046591034 | 0.975249069699774 | 0.740411019219112 | 1.28457130331931 | 0.999332001504091 |
| ebi-fl187-GCST90094780 | prot-a-130 | Apolipoprotein D \|\| id:prot-a-130 | F-healthy breakfast food liking (derived food-liking factor) \|\| id:ebi-fl187-GCST90094780 | Weighted median | 8 | 0.29979576226566 | 0.130197337221158 | 0.0212998999854128 | 0.0446089813121904 | 0.554982543219131 | 1.34958314362291 | 1.04561892342364 | 1.74191057635743 | 0.999332001504091 |
| ebi-fl187-GCST90094780 | prot-a-1313 | Heparin-binding EGF-like growth factor \|\| id:prot-a-1313 | F-healthy breakfast food liking (derived food-liking factor) \|\| id:ebi-fl187-GCST90094780 | Weighted median | 8 | 0.157226175854182 | 0.128090561553924 | 0.219649350699631 | -0.093831325 | 0.408283676499874 | 1.1702602685864 | 0.910436317325577 | 1.50423381643525 | 0.999332001504091 |
| ebi-fl187-GCST90094780 | prot-a-1347 | HLA class II histocompatibility antigen, DQ alpha 2 chain \|\| id:prot-a-1347 | F-healthy breakfast food liking (derived food-liking factor) \|\| id:ebi-fl187-GCST90094780 | Weighted median | 8 | -0.133798169 | 0.126387424334775 | 0.289765976313506 | -0.381517521 | 0.113921182872692 | 0.874766601959829 | 0.682824422514596 | 1.12066379390229 | 0.999332001504091 |
| ebi-fl187-GCST90094780 | prot-a-1357 | Non-histone chromosomal protein HMG-14 \|\| id:prot-a-1357 | F-healthy breakfast food liking (derived food-liking factor) \|\| id:ebi-fl187-GCST90094780 | Weighted median | 8 | -0.215243494 | 0.127371070309903 | 0.0910484067483281 | -0.464890791 | 0.0344038041754397 | 0.806345076380361 | 0.628203706666219 | 1.03500246067207 | 0.999332001504091 |
| ebi-fl187-GCST90094780 | prot-a-137 | Apolipoprotein M \|\| id:prot-a-137 | F-healthy breakfast food liking (derived food-liking factor) \|\| id:ebi-fl187-GCST90094780 | Weighted median | 8 | -0.052751739 | 0.140589341153696 | 0.707497878960009 | -0.328306848 | 0.222803369247604 | 0.948615487088612 | 0.720142011609175 | 1.24957484473594 | 0.999332001504091 |
| ebi-fl187-GCST90094780 | prot-a-1381 | 17-beta-hydroxysteroid dehydrogenase 14 \|\| id:prot-a-1381 | F-healthy breakfast food liking (derived food-liking factor) \|\| id:ebi-fl187-GCST90094780 | Weighted median | 8 | 0.0615251674928657 | 0.128848129857984 | 0.633005095993707 | -0.191017167 | 0.314067502014514 | 1.06345726074503 | 0.826118406031008 | 1.368982142 | 0.999332001504091 |
| ebi-fl187-GCST90094780 | prot-a-1392 | Serine protease HTRA2, mitochondrial \|\| id:prot-a-1392 | F-healthy breakfast food liking (derived food-liking factor) \|\| id:ebi-fl187-GCST90094780 | Weighted median | 8 | -0.05994956 | 0.128602156956101 | 0.641098870356064 | -0.312009788 | 0.19211066735463 | 0.941812037122284 | 0.73197436369165 | 1.21180461675579 | 0.999332001504091 |
| ebi-fl187-GCST90094780 | prot-a-1416 | Interferon-induced protein with tetratricopeptide repeats 2 \|\| id:prot-a-1416 | F-healthy breakfast food liking (derived food-liking factor) \|\| id:ebi-fl187-GCST90094780 | Weighted median | 8 | -0.196920265 | 0.126767654063918 | 0.120328749878078 | -0.445384867 | 0.0515443371236966 | 0.821256113688406 | 0.640577691493589 | 1.05289586763159 | 0.999332001504091 |
| ebi-fl187-GCST90094780 | prot-a-1428 | Interferon gamma \|\| id:prot-a-1428 | F-healthy breakfast food liking (derived food-liking factor) \|\| id:ebi-fl187-GCST90094780 | Weighted median | 8 | 0.031523894498974 | 0.133008826296766 | 0.812652108622499 | -0.229173405 | 0.292221194040634 | 1.03202603504607 | 0.79519063148359 | 1.33939925200802 | 0.999332001504091 |
| ebi-fl187-GCST90094780 | prot-a-1483 | Interleukin-17C \|\| id:prot-a-1483 | F-healthy breakfast food liking (derived food-liking factor) \|\| id:ebi-fl187-GCST90094780 | Weighted median | 8 | -0.086931192 | 0.121008758879472 | 0.472518329798755 | -0.324108359 | 0.150245975423872 | 0.916740172539254 | 0.723171875675308 | 1.16212006054925 | 0.999332001504091 |
| ebi-fl187-GCST90094780 | prot-a-1503 | Interleukin-1 receptor-like 2 \|\| id:prot-a-1503 | F-healthy breakfast food liking (derived food-liking factor) \|\| id:ebi-fl187-GCST90094780 | Weighted median | 8 | -0.048835077 | 0.123731378305901 | 0.693074434547761 | -0.291348578 | 0.193678424913864 | 0.952338179630739 | 0.747255155873027 | 1.21370592260791 | 0.999332001504091 |
| ebi-fl187-GCST90094780 | prot-a-1522 | Interleukin-31 receptor subunit alpha \|\| id:prot-a-1522 | F-healthy breakfast food liking (derived food-liking factor) \|\| id:ebi-fl187-GCST90094780 | Weighted median | 8 | -0.062826833 | 0.130992223998943 | 0.631495761791645 | -0.319571592 | 0.193917925866546 | 0.939106081631468 | 0.726460191624133 | 1.21399664114494 | 0.999332001504091 |
| ebi-fl187-GCST90094780 | prot-a-1540 | Interleukin-6 receptor subunit alpha \|\| id:prot-a-1540 | F-healthy breakfast food liking (derived food-liking factor) \|\| id:ebi-fl187-GCST90094780 | Weighted median | 8 | -0.069438173 | 0.122008965696161 | 0.569272313534611 | -0.308575746 | 0.169699399952042 | 0.932917811286009 | 0.734492315547905 | 1.18494860217215 | 0.999332001504091 |
| ebi-fl187-GCST90094780 | prot-a-1541 | Interleukin-6 receptor subunit alpha \|\| id:prot-a-1541 | F-healthy breakfast food liking (derived food-liking factor) \|\| id:ebi-fl187-GCST90094780 | Weighted median | 8 | -0.135814217 | 0.121678687763588 | 0.26434896555641 | -0.374304445 | 0.102676011164925 | 0.873004807005127 | 0.687767492668549 | 1.10813232840789 | 0.999332001504091 |
| ebi-fl187-GCST90094780 | prot-a-1545 | Interleukin-9 \|\| id:prot-a-1545 | F-healthy breakfast food liking (derived food-liking factor) \|\| id:ebi-fl187-GCST90094780 | Weighted median | 8 | 0.0796778100632811 | 0.119624905232708 | 0.505370369093821 | -0.154787004 | 0.314142624319389 | 1.08293809970318 | 0.856597609753581 | 1.3690849874378 | 0.999332001504091 |
| ebi-fl187-GCST90094780 | prot-a-1548 | Inositol monophosphatase 3 \|\| id:prot-a-1548 | F-healthy breakfast food liking (derived food-liking factor) \|\| id:ebi-fl187-GCST90094780 | Weighted median | 8 | -0.052286961 | 0.122361406369789 | 0.669149330903335 | -0.292115318 | 0.187541395023268 | 0.949056485127195 | 0.746682425124581 | 1.2062801823837 | 0.999332001504091 |
| ebi-fl187-GCST90094780 | prot-a-1555 | Inhibin beta A chain:Inhibin beta B chain heterodimer \|\| id:prot-a-1555 | F-healthy breakfast food liking (derived food-liking factor) \|\| id:ebi-fl187-GCST90094780 | Weighted median | 8 | -0.128418033 | 0.130215475579599 | 0.324036744795297 | -0.383640366 | 0.126804298699907 | 0.879485647880627 | 0.681376429543674 | 1.13519483693621 | 0.999332001504091 |
| ebi-fl187-GCST90094780 | prot-a-1603 | Junctophilin-3 \|\| id:prot-a-1603 | F-healthy breakfast food liking (derived food-liking factor) \|\| id:ebi-fl187-GCST90094780 | Weighted median | 8 | -0.049215369 | 0.117478530826384 | 0.675266741368714 | -0.27947329 | 0.181042551098541 | 0.951976081176343 | 0.756181925360592 | 1.19846617426053 | 0.999332001504091 |
| ebi-fl187-GCST90094780 | prot-a-1627 | UPF0258 protein KIAA1024 \|\| id:prot-a-1627 | F-healthy breakfast food liking (derived food-liking factor) \|\| id:ebi-fl187-GCST90094780 | Weighted median | 8 | 0.0049234775801304 | 0.138037550541705 | 0.971547351015206 | -0.265630121 | 0.275477076641872 | 1.00493561781175 | 0.766722669304713 | 1.31715891074733 | 0.999332001504091 |
| ebi-fl187-GCST90094780 | prot-a-1666 | Kallikrein-7 \|\| id:prot-a-1666 | F-healthy breakfast food liking (derived food-liking factor) \|\| id:ebi-fl187-GCST90094780 | Weighted median | 8 | 0.00357011251387472 | 0.128390284812254 | 0.977816307410807 | -0.248074846 | 0.255215070745893 | 1.00357649295626 | 0.780301538863357 | 1.2907391912638 | 0.999332001504091 |
| ebi-fl187-GCST90094780 | prot-a-1678 | Importin subunit alpha-3 \|\| id:prot-a-1678 | F-healthy breakfast food liking (derived food-liking factor) \|\| id:ebi-fl187-GCST90094780 | Weighted median | 8 | 0.118769206949216 | 0.129278144107897 | 0.358246941263955 | -0.134615956 | 0.372154369400695 | 1.12610998993458 | 0.87405152191701 | 1.45085693192223 | 0.999332001504091 |
| ebi-fl187-GCST90094780 | prot-a-1718 | Protein LDOC1 \|\| id:prot-a-1718 | F-healthy breakfast food liking (derived food-liking factor) \|\| id:ebi-fl187-GCST90094780 | Weighted median | 8 | 0.0157495529352458 | 0.114649537592702 | 0.890737336531706 | -0.208963541 | 0.240462646616942 | 1.01587423082445 | 0.811424819047328 | 1.27183742551136 | 0.999332001504091 |
| ebi-fl187-GCST90094780 | prot-a-1723 | Leptin \|\| id:prot-a-1723 | F-healthy breakfast food liking (derived food-liking factor) \|\| id:ebi-fl187-GCST90094780 | Weighted median | 8 | -0.087783577 | 0.139579048389198 | 0.529403676750285 | -0.361358511 | 0.185791358341686 | 0.915959090344652 | 0.69672916837555 | 1.20417099393315 | 0.999332001504091 |
| ebi-fl187-GCST90094780 | prot-a-1730 | Galectin-7 \|\| id:prot-a-1730 | F-healthy breakfast food liking (derived food-liking factor) \|\| id:ebi-fl187-GCST90094780 | Weighted median | 8 | 0.0456423580041537 | 0.120895030858058 | 0.705774507169317 | -0.191311902 | 0.282596618485948 | 1.04670000013131 | 0.825874955530305 | 1.32656994008421 | 0.999332001504091 |
| ebi-fl187-GCST90094780 | prot-a-1753 | Lipase member N \|\| id:prot-a-1753 | F-healthy breakfast food liking (derived food-liking factor) \|\| id:ebi-fl187-GCST90094780 | Weighted median | 8 | 0.00153757991743367 | 0.128938215945431 | 0.990485503522113 | -0.251181323 | 0.254256483170478 | 1.00153876259951 | 0.777881310736562 | 1.28950249754627 | 0.999332001504091 |
| ebi-fl187-GCST90094780 | prot-a-1756 | VIP36-like protein \|\| id:prot-a-1756 | F-healthy breakfast food liking (derived food-liking factor) \|\| id:ebi-fl187-GCST90094780 | Weighted median | 8 | 0.101760176693075 | 0.126711424234788 | 0.421925002315498 | -0.146594215 | 0.35011456819326 | 1.10711792720487 | 0.863644360414776 | 1.41923013791202 | 0.999332001504091 |
| ebi-fl187-GCST90094780 | prot-a-1760 | Ig Kappa chain V-I region HK102- like \|\| id:prot-a-1760 | F-healthy breakfast food liking (derived food-liking factor) \|\| id:ebi-fl187-GCST90094780 | Weighted median | 8 | -0.241357937 | 0.127619700084459 | 0.0585939290951481 | -0.491492549 | 0.00877667533787596 | 0.785560395064077 | 0.611712701311874 | 1.00881530327847 | 0.999332001504091 |
| ebi-fl187-GCST90094780 | prot-a-179 | Neutral ceramidase \|\| id:prot-a-179 | F-healthy breakfast food liking (derived food-liking factor) \|\| id:ebi-fl187-GCST90094780 | Weighted median | 8 | 0.0616441563259048 | 0.129202994675401 | 0.633283157444938 | -0.191593713 | 0.314882025889691 | 1.06358380781216 | 0.825642247872524 | 1.37009766536908 | 0.999332001504091 |
| ebi-fl187-GCST90094780 | prot-a-1809 | Lactotransferrin \|\| id:prot-a-1809 | F-healthy breakfast food liking (derived food-liking factor) \|\| id:ebi-fl187-GCST90094780 | Weighted median | 8 | -0.064901109 | 0.124972953247637 | 0.603536746029511 | -0.309848097 | 0.180045879661981 | 0.93716013577604 | 0.73355837742924 | 1.1972722923098 | 0.999332001504091 |
| ebi-fl187-GCST90094780 | prot-a-1818 | Lysophospholipase-like protein 1 \|\| id:prot-a-1818 | F-healthy breakfast food liking (derived food-liking factor) \|\| id:ebi-fl187-GCST90094780 | Weighted median | 8 | 0.0885216212728267 | 0.124267474273333 | 0.476249633682311 | -0.155042628 | 0.332085870848559 | 1.092557875 | 0.856378670736131 | 1.39387253639108 | 0.999332001504091 |
| ebi-fl187-GCST90094780 | prot-a-1852 | MAP kinase-activated protein kinase 3 \|\| id:prot-a-1852 | F-healthy breakfast food liking (derived food-liking factor) \|\| id:ebi-fl187-GCST90094780 | Weighted median | 8 | -0.084690522 | 0.129921031895225 | 0.514490509573277 | -0.339335744 | 0.169954701003538 | 0.918796588185951 | 0.712243277515923 | 1.1852511594162 | 0.999332001504091 |
| ebi-fl187-GCST90094780 | prot-a-1856 | Megakaryocyte-associated tyrosine-protein kinase \|\| id:prot-a-1856 | F-healthy breakfast food liking (derived food-liking factor) \|\| id:ebi-fl187-GCST90094780 | Weighted median | 8 | -0.099286983 | 0.123024327579571 | 0.419636745148117 | -0.340414665 | 0.141840699480126 | 0.905482812942621 | 0.711475237968643 | 1.15239305710119 | 0.999332001504091 |
| ebi-fl187-GCST90094780 | prot-a-1890 | Beta-1,3-N-acetylglucosaminyltransferase manic fringe \|\| id:prot-a-1890 | F-healthy breakfast food liking (derived food-liking factor) \|\| id:ebi-fl187-GCST90094780 | Weighted median | 8 | -0.157289913 | 0.151689946935027 | 0.299774452714757 | -0.454602209 | 0.140022382732497 | 0.854456304692863 | 0.634700395682831 | 1.1502995454161 | 0.999332001504091 |
| ebi-fl187-GCST90094780 | prot-a-1919 | Matrilysin \|\| id:prot-a-1919 | F-healthy breakfast food liking (derived food-liking factor) \|\| id:ebi-fl187-GCST90094780 | Weighted median | 8 | -0.203554171 | 0.124699270004869 | 0.102603551688905 | -0.44796474 | 0.040856398527971 | 0.81582600927468 | 0.638927212277815 | 1.04170250479119 | 0.999332001504091 |
| ebi-fl187-GCST90094780 | prot-a-1923 | Multimerin-2 \|\| id:prot-a-1923 | F-healthy breakfast food liking (derived food-liking factor) \|\| id:ebi-fl187-GCST90094780 | Weighted median | 8 | 0.107783389556017 | 0.116604353864007 | 0.35530342600783 | -0.120761144 | 0.33632792312947 | 1.11380645714344 | 0.886245619381975 | 1.39979797568933 | 0.999332001504091 |
| ebi-fl187-GCST90094780 | prot-a-1948 | Mesothelin \|\| id:prot-a-1948 | F-healthy breakfast food liking (derived food-liking factor) \|\| id:ebi-fl187-GCST90094780 | Weighted median | 8 | 0.213605372188781 | 0.124612802987984 | 0.086500685972259 | -0.030635722 | 0.457846466045231 | 1.23813395627962 | 0.96982879635291 | 1.58066629848222 | 0.999332001504091 |
| ebi-fl187-GCST90094780 | prot-a-195 | ATPase family AAA domain-containing protein 1 \|\| id:prot-a-195 | F-healthy breakfast food liking (derived food-liking factor) \|\| id:ebi-fl187-GCST90094780 | Weighted median | 8 | 0.0744737182323661 | 0.126094879013876 | 0.554777395543775 | -0.172672245 | 0.321619681099563 | 1.07731702935435 | 0.841413347394678 | 1.37936007947884 | 0.999332001504091 |
| ebi-fl187-GCST90094780 | prot-a-1966 | Mucin-1 \|\| id:prot-a-1966 | F-healthy breakfast food liking (derived food-liking factor) \|\| id:ebi-fl187-GCST90094780 | Weighted median | 8 | 0.0380417607113446 | 0.11951705524339 | 0.75026067144349 | -0.196211668 | 0.272295188988388 | 1.03877461193944 | 0.82183825976257 | 1.31297451973297 | 0.999332001504091 |
| ebi-fl187-GCST90094780 | prot-a-1986 | Myocardial zonula adherens protein \|\| id:prot-a-1986 | F-healthy breakfast food liking (derived food-liking factor) \|\| id:ebi-fl187-GCST90094780 | Weighted median | 8 | -0.232135764 | 0.129466675047317 | 0.0729704342706549 | -0.485890447 | 0.0216189187615798 | 0.792838476814049 | 0.615149194801201 | 1.02185430076027 | 0.999332001504091 |
| ebi-fl187-GCST90094780 | prot-a-2071 | Epididymal secretory protein E1 \|\| id:prot-a-2071 | F-healthy breakfast food liking (derived food-liking factor) \|\| id:ebi-fl187-GCST90094780 | Weighted median | 8 | -0.290237295 | 0.131449646513068 | 0.0272461422861551 | -0.547878602 | -0.032595988 | 0.748086029419736 | 0.578175049593089 | 0.967929535885105 | 0.999332001504091 |
| ebi-fl187-GCST90094780 | prot-a-2098 | Neuritin \|\| id:prot-a-2098 | F-healthy breakfast food liking (derived food-liking factor) \|\| id:ebi-fl187-GCST90094780 | Weighted median | 8 | 0.0187767750449557 | 0.142334559306417 | 0.895047569273588 | -0.260198961 | 0.297752511285534 | 1.01895416723042 | 0.77089819171841 | 1.34682842179437 | 0.999332001504091 |
| ebi-fl187-GCST90094780 | prot-a-2115 | Neurotrophin-4 \|\| id:prot-a-2115 | F-healthy breakfast food liking (derived food-liking factor) \|\| id:ebi-fl187-GCST90094780 | Weighted median | 8 | 0.118598628707238 | 0.129786710982178 | 0.36082384069132 | -0.135783325 | 0.372980582232306 | 1.12591791645447 | 0.873031776315765 | 1.45205614387015 | 0.999332001504091 |
| ebi-fl187-GCST90094780 | prot-a-2118 | Netrin-1 \|\| id:prot-a-2118 | F-healthy breakfast food liking (derived food-liking factor) \|\| id:ebi-fl187-GCST90094780 | Weighted median | 8 | 0.111570641505317 | 0.144297792149745 | 0.439405713138896 | -0.171253031 | 0.394394314118818 | 1.11803272073037 | 0.842608340373324 | 1.48348539259643 | 0.999332001504091 |
| ebi-fl187-GCST90094780 | prot-a-2120 | Netrin-G1 \|\| id:prot-a-2120 | F-healthy breakfast food liking (derived food-liking factor) \|\| id:ebi-fl187-GCST90094780 | Weighted median | 8 | -0.039260669 | 0.129942570310001 | 0.762546389214444 | -0.293948107 | 0.215426768317057 | 0.961500042750943 | 0.745315166857535 | 1.24039114366604 | 0.999332001504091 |
| ebi-fl187-GCST90094780 | prot-a-2145 | Olfactomedin-like protein 3 \|\| id:prot-a-2145 | F-healthy breakfast food liking (derived food-liking factor) \|\| id:ebi-fl187-GCST90094780 | Weighted median | 8 | 0.0600550778941369 | 0.161322402174792 | 0.709693737390563 | -0.25613683 | 0.376246986156729 | 1.06189503187687 | 0.774036049908047 | 1.4568069 | 0.999332001504091 |
| ebi-fl187-GCST90094780 | prot-a-2162 | Prolyl 4-hydroxylase subunit alpha-2 \|\| id:prot-a-2162 | F-healthy breakfast food liking (derived food-liking factor) \|\| id:ebi-fl187-GCST90094780 | Weighted median | 8 | 0.0258467772634816 | 0.122664726572382 | 0.833112981012592 | -0.214576087 | 0.26626964134535 | 1.02618370175191 | 0.806883416204287 | 1.30508691663907 | 0.999332001504091 |
| ebi-fl187-GCST90094780 | prot-a-2168 | Polyadenylate-binding protein-interacting protein 1 \|\| id:prot-a-2168 | F-healthy breakfast food liking (derived food-liking factor) \|\| id:ebi-fl187-GCST90094780 | Weighted median | 8 | 0.153715986064289 | 0.129933185630262 | 0.236793813120946 | -0.100953058 | 0.408385029899603 | 1.16615963415312 | 0.903975466513229 | 1.50438628337293 | 0.999332001504091 |
| ebi-fl187-GCST90094780 | prot-a-219 | Beta-1,4-galactosyltransferase 5 \|\| id:prot-a-219 | F-healthy breakfast food liking (derived food-liking factor) \|\| id:ebi-fl187-GCST90094780 | Weighted median | 8 | 0.122184748584083 | 0.12887653300671 | 0.343090762578192 | -0.130413256 | 0.374782753277234 | 1.12996284153258 | 0.877732627589898 | 1.45467535683423 | 0.999332001504091 |
| ebi-fl187-GCST90094780 | prot-a-2190 | Pyruvate carboxylase, mitochondrial \|\| id:prot-a-2190 | F-healthy breakfast food liking (derived food-liking factor) \|\| id:ebi-fl187-GCST90094780 | Weighted median | 8 | -0.153120004 | 0.12323666724536 | 0.21405679230347 | -0.394663872 | 0.0884238634682336 | 0.858026748705515 | 0.673906519944216 | 1.09245107400816 | 0.999332001504091 |
| ebi-fl187-GCST90094780 | prot-a-2197 | Protocadherin-9 \|\| id:prot-a-2197 | F-healthy breakfast food liking (derived food-liking factor) \|\| id:ebi-fl187-GCST90094780 | Weighted median | 8 | 0.0943405149547817 | 0.137853809651205 | 0.493752988809649 | -0.175852952 | 0.364533981871144 | 1.09893388563585 | 0.838741309533722 | 1.43984285890255 | 0.999332001504091 |
| ebi-fl187-GCST90094780 | prot-a-2198 | Protocadherin alpha-4 \|\| id:prot-a-2198 | F-healthy breakfast food liking (derived food-liking factor) \|\| id:ebi-fl187-GCST90094780 | Weighted median | 8 | 0.185438968682612 | 0.125208730432053 | 0.138596275356446 | -0.059970143 | 0.430848080329436 | 1.20374673128441 | 0.9417926523014 | 1.53856179440033 | 0.999332001504091 |
| ebi-fl187-GCST90094780 | prot-a-2254 | Placenta growth factor \|\| id:prot-a-2254 | F-healthy breakfast food liking (derived food-liking factor) \|\| id:ebi-fl187-GCST90094780 | Weighted median | 8 | 0.201766833076152 | 0.125428964610708 | 0.107700693259729 | -0.044073938 | 0.44760760371314 | 1.22356268050182 | 0.956883205247254 | 1.56456464582839 | 0.999332001504091 |
| ebi-fl187-GCST90094780 | prot-a-2261 | Membrane-associated progesterone receptor component 2 \|\| id:prot-a-2261 | F-healthy breakfast food liking (derived food-liking factor) \|\| id:ebi-fl187-GCST90094780 | Weighted median | 8 | 0.0584277264873307 | 0.12333739314282 | 0.63569764289378 | -0.183313564 | 0.300169017047258 | 1.0601683608342 | 0.832507070498264 | 1.35008697600751 | 0.999332001504091 |
| ebi-fl187-GCST90094780 | prot-a-2268 | Polymeric immunoglobulin receptor \|\| id:prot-a-2268 | F-healthy breakfast food liking (derived food-liking factor) \|\| id:ebi-fl187-GCST90094780 | Weighted median | 8 | -0.057344101 | 0.120761528913502 | 0.634891005337587 | -0.294036698 | 0.179348495655174 | 0.944269089504373 | 0.745249142022638 | 1.19643762483706 | 0.999332001504091 |
| ebi-fl187-GCST90094780 | prot-a-2283 | Group 10 secretory phospholipase A2 \|\| id:prot-a-2283 | F-healthy breakfast food liking (derived food-liking factor) \|\| id:ebi-fl187-GCST90094780 | Weighted median | 8 | 0.241102900456901 | 0.122546379149317 | 0.0491322695963491 | 0.000911997324240632 | 0.481293803589562 | 1.27265198504146 | 1.00091241332025 | 1.61816663823485 | 0.999332001504091 |
| ebi-fl187-GCST90094780 | prot-a-2298 | Inactive phospholipase D5 \|\| id:prot-a-2298 | F-healthy breakfast food liking (derived food-liking factor) \|\| id:ebi-fl187-GCST90094780 | Weighted median | 8 | 0.181215524088314 | 0.12527608656123 | 0.148028812814775 | -0.064325606 | 0.426756653748325 | 1.19867349446771 | 0.937699629594376 | 1.53227974181984 | 0.999332001504091 |
| ebi-fl187-GCST90094780 | prot-a-2304 | Procollagen-lysine,2-oxoglutarate 5-dioxygenase 2 \|\| id:prot-a-2304 | F-healthy breakfast food liking (derived food-liking factor) \|\| id:ebi-fl187-GCST90094780 | Weighted median | 8 | -0.042180945 | 0.130301622955445 | 0.746151750974398 | -0.297572126 | 0.21321023616981 | 0.958696293746136 | 0.742619019286737 | 1.23764482160091 | 0.999332001504091 |
| ebi-fl187-GCST90094780 | prot-a-2331 | Periostin \|\| id:prot-a-2331 | F-healthy breakfast food liking (derived food-liking factor) \|\| id:ebi-fl187-GCST90094780 | Weighted median | 8 | -0.152576321 | 0.156810342245216 | 0.330553752380608 | -0.459924592 | 0.154771949864748 | 0.858493370438544 | 0.631331251305323 | 1.16739170690997 | 0.999332001504091 |
| ebi-fl187-GCST90094780 | prot-a-2333 | Periostin \|\| id:prot-a-2333 | F-healthy breakfast food liking (derived food-liking factor) \|\| id:ebi-fl187-GCST90094780 | Weighted median | 8 | -0.1088481 | 0.155047457095204 | 0.482659916545328 | -0.412741116 | 0.195044915814002 | 0.896866641111534 | 0.661833598781331 | 1.21536557439788 | 0.999332001504091 |
| ebi-fl187-GCST90094780 | prot-a-2367 | cAMP-dependent protein kinase catalytic subunit alpha \|\| id:prot-a-2367 | F-healthy breakfast food liking (derived food-liking factor) \|\| id:ebi-fl187-GCST90094780 | Weighted median | 8 | -0.072277436 | 0.137742843028067 | 0.599772745347438 | -0.342253409 | 0.197698536062955 | 0.930272768597021 | 0.710168219165996 | 1.21859497600369 | 0.999332001504091 |
| ebi-fl187-GCST90094780 | prot-a-2385 | Phosphoribosyl pyrophosphate synthase-associated protein 1 \|\| id:prot-a-2385 | F-healthy breakfast food liking (derived food-liking factor) \|\| id:ebi-fl187-GCST90094780 | Weighted median | 8 | -0.032378534 | 0.143918816795612 | 0.821996571174716 | -0.314459415 | 0.249702347052802 | 0.968140038916736 | 0.730183493997016 | 1.28364327961312 | 0.999332001504091 |
| ebi-fl187-GCST90094780 | prot-a-2393 | Trypsin-3 \|\| id:prot-a-2393 | F-healthy breakfast food liking (derived food-liking factor) \|\| id:ebi-fl187-GCST90094780 | Weighted median | 8 | 0.0205980399551728 | 0.130305508050091 | 0.874397719552338 | -0.234800756 | 0.27599683573335 | 1.0208116436652 | 0.790728381960205 | 1.31784369401186 | 0.999332001504091 |
| ebi-fl187-GCST90094780 | prot-a-2405 | Pregnancy-specific beta-1-glycoprotein 6 \|\| id:prot-a-2405 | F-healthy breakfast food liking (derived food-liking factor) \|\| id:ebi-fl187-GCST90094780 | Weighted median | 8 | -0.060772147 | 0.127204862424308 | 0.632828018202878 | -0.310093677 | 0.188549383362513 | 0.941037633608101 | 0.733378252081893 | 1.20749671176212 | 0.999332001504091 |
| ebi-fl187-GCST90094780 | prot-a-244 | Beclin-1 \|\| id:prot-a-244 | F-healthy breakfast food liking (derived food-liking factor) \|\| id:ebi-fl187-GCST90094780 | Weighted median | 8 | 0.0680791432781965 | 0.132503298817227 | 0.607397377548247 | -0.191627322 | 0.327785608959962 | 1.07045002405044 | 0.825614499191724 | 1.38789138891262 | 0.999332001504091 |
| ebi-fl187-GCST90094780 | prot-a-2454 | tRNA pseudouridine synthase A, mitochondrial \|\| id:prot-a-2454 | F-healthy breakfast food liking (derived food-liking factor) \|\| id:ebi-fl187-GCST90094780 | Weighted median | 8 | 0.0736440206441459 | 0.122320278444989 | 0.547134885917568 | -0.166103725 | 0.313391766396324 | 1.07642355272231 | 0.846958378783734 | 1.36805738496764 | 0.999332001504091 |
| ebi-fl187-GCST90094780 | prot-a-2466 | Glutaminyl-peptide cyclotransferase-like protein \|\| id:prot-a-2466 | F-healthy breakfast food liking (derived food-liking factor) \|\| id:ebi-fl187-GCST90094780 | Weighted median | 8 | -0.037754911 | 0.135672467551425 | 0.78079756043175 | -0.303672947 | 0.228163125592312 | 0.962948920344373 | 0.738102225372535 | 1.25629024180815 | 0.999332001504091 |
| ebi-fl187-GCST90094780 | prot-a-2474 | Ras-related protein Rab-27A \|\| id:prot-a-2474 | F-healthy breakfast food liking (derived food-liking factor) \|\| id:ebi-fl187-GCST90094780 | Weighted median | 8 | -0.06895409 | 0.135165475357843 | 0.609949370210628 | -0.333878422 | 0.195970241737715 | 0.933369530123621 | 0.716140844146116 | 1.21649070414624 | 0.999332001504091 |
| ebi-fl187-GCST90094780 | prot-a-2490 | Rap1 GTPase-GDP dissociation stimulator 1 \|\| id:prot-a-2490 | F-healthy breakfast food liking (derived food-liking factor) \|\| id:ebi-fl187-GCST90094780 | Weighted median | 8 | 0.0735400560864723 | 0.120476895100236 | 0.541591594749144 | -0.162594658 | 0.309674770482935 | 1.07631164864091 | 0.849935632946868 | 1.36298176014056 | 0.999332001504091 |
| ebi-fl187-GCST90094780 | prot-a-2545 | Receptor-interacting serine/threonine-protein kinase 2 \|\| id:prot-a-2545 | F-healthy breakfast food liking (derived food-liking factor) \|\| id:ebi-fl187-GCST90094780 | Weighted median | 8 | 0.057849719917338 | 0.133191006551294 | 0.664044078817024 | -0.203204653 | 0.318904092757874 | 1.05955575361889 | 0.81611120479021 | 1.37561938671763 | 0.999332001504091 |
| ebi-fl187-GCST90094780 | prot-a-2610 | RUN and FYVE domain-containing protein 1 \|\| id:prot-a-2610 | F-healthy breakfast food liking (derived food-liking factor) \|\| id:ebi-fl187-GCST90094780 | Weighted median | 8 | -0.118077604 | 0.124053932191258 | 0.341185862057327 | -0.361223311 | 0.125068102699226 | 0.888627088570537 | 0.696823372425062 | 1.13322562616292 | 0.999332001504091 |
| ebi-fl187-GCST90094780 | prot-a-2627 | Serine--tRNA ligase, mitochondrial \|\| id:prot-a-2627 | F-healthy breakfast food liking (derived food-liking factor) \|\| id:ebi-fl187-GCST90094780 | Weighted median | 8 | -0.090005361 | 0.125521453338009 | 0.473342247636691 | -0.33602741 | 0.156016687393624 | 0.913926285563217 | 0.714603518485722 | 1.16884570791554 | 0.999332001504091 |
| ebi-fl187-GCST90094780 | prot-a-264 | BolA-like protein 2 \|\| id:prot-a-264 | F-healthy breakfast food liking (derived food-liking factor) \|\| id:ebi-fl187-GCST90094780 | Weighted median | 8 | 0.0987834532515699 | 0.128496362949633 | 0.44203307586022 | -0.153069418 | 0.35063632463285 | 1.103827243 | 0.85807015411862 | 1.41997082358769 | 0.999332001504091 |
| ebi-fl187-GCST90094780 | prot-a-2646 | Secretoglobin family 1C member 1 \|\| id:prot-a-2646 | F-healthy breakfast food liking (derived food-liking factor) \|\| id:ebi-fl187-GCST90094780 | Weighted median | 8 | -0.045356585 | 0.125572711403086 | 0.717951598417477 | -0.291479099 | 0.200765929231518 | 0.95565664812945 | 0.74715762943129 | 1.22233862459408 | 0.999332001504091 |
| ebi-fl187-GCST90094780 | prot-a-2672 | Semaphorin-3E \|\| id:prot-a-2672 | F-healthy breakfast food liking (derived food-liking factor) \|\| id:ebi-fl187-GCST90094780 | Weighted median | 8 | -0.073479307 | 0.128101441751286 | 0.566236820053864 | -0.324558133 | 0.177599518669381 | 0.929155372453131 | 0.722846685185219 | 1.19434691180371 | 0.999332001504091 |
| ebi-fl187-GCST90094780 | prot-a-2701 | Plasma protease C1 inhibitor \|\| id:prot-a-2701 | F-healthy breakfast food liking (derived food-liking factor) \|\| id:ebi-fl187-GCST90094780 | Weighted median | 8 | -0.140867137 | 0.127030832955664 | 0.267464337311899 | -0.389847569 | 0.108113295967793 | 0.868604709784423 | 0.677160086672881 | 1.11417396965416 | 0.999332001504091 |
| ebi-fl187-GCST90094780 | prot-a-2738 | Signaling threshold-regulating transmembrane adapter 1 \|\| id:prot-a-2738 | F-healthy breakfast food liking (derived food-liking factor) \|\| id:ebi-fl187-GCST90094780 | Weighted median | 8 | -0.121518745 | 0.127204233964303 | 0.339423858300798 | -0.370839044 | 0.127801553098779 | 0.885574452682828 | 0.690155017181858 | 1.13632747965352 | 0.999332001504091 |
| ebi-fl187-GCST90094780 | prot-a-274 | Serine/threonine-protein kinase BRSK2 \|\| id:prot-a-274 | F-healthy breakfast food liking (derived food-liking factor) \|\| id:ebi-fl187-GCST90094780 | Weighted median | 8 | 0.120681603806218 | 0.1208256686994 | 0.317887873071419 | -0.116136707 | 0.357499914457043 | 1.12826561969278 | 0.890353497560056 | 1.42975044414298 | 0.999332001504091 |
| ebi-fl187-GCST90094780 | prot-a-2749 | Mitochondrial glutamate carrier 2 \|\| id:prot-a-2749 | F-healthy breakfast food liking (derived food-liking factor) \|\| id:ebi-fl187-GCST90094780 | Weighted median | 8 | -0.017782426 | 0.12766551916181 | 0.889221635921262 | -0.268006844 | 0.232441991477447 | 0.982374748233133 | 0.764902546369699 | 1.26167725620261 | 0.999332001504091 |
| ebi-fl187-GCST90094780 | prot-a-2760 | Sodium-coupled monocarboxylate transporter 1 \|\| id:prot-a-2760 | F-healthy breakfast food liking (derived food-liking factor) \|\| id:ebi-fl187-GCST90094780 | Weighted median | 8 | -0.14052459 | 0.117584852288303 | 0.232051506052576 | -0.370990901 | 0.089941720239667 | 0.868902298149324 | 0.690050220483159 | 1.09411051734836 | 0.999332001504091 |
| ebi-fl187-GCST90094780 | prot-a-2777 | Small integral membrane protein 10 \|\| id:prot-a-2777 | F-healthy breakfast food liking (derived food-liking factor) \|\| id:ebi-fl187-GCST90094780 | Weighted median | 8 | 0.0503013704378089 | 0.124646481423704 | 0.686541990798703 | -0.194005733 | 0.294608474028268 | 1.05158796615179 | 0.823653182125616 | 1.34260059276576 | 0.999332001504091 |
| ebi-fl187-GCST90094780 | prot-a-2799 | Superoxide dismutase [Mn], mitochondrial \|\| id:prot-a-2799 | F-healthy breakfast food liking (derived food-liking factor) \|\| id:ebi-fl187-GCST90094780 | Weighted median | 8 | 0.0327468074817906 | 0.138283567749403 | 0.812804890107837 | -0.238288985 | 0.303782600270621 | 1.03328888510341 | 0.787974945006297 | 1.35497445298812 | 0.999332001504091 |
| ebi-fl187-GCST90094780 | prot-a-2805 | Protein SPACA7 \|\| id:prot-a-2805 | F-healthy breakfast food liking (derived food-liking factor) \|\| id:ebi-fl187-GCST90094780 | Weighted median | 8 | -0.12172445 | 0.124556653274959 | 0.328439594906335 | -0.365855491 | 0.122406590126802 | 0.8853923044837 | 0.693603026047987 | 1.13021354203942 | 0.999332001504091 |
| ebi-fl187-GCST90094780 | prot-a-2811 | SPARC-like protein 1 \|\| id:prot-a-2811 | F-healthy breakfast food liking (derived food-liking factor) \|\| id:ebi-fl187-GCST90094780 | Weighted median | 8 | 0.0331759045590609 | 0.125993488467541 | 0.792308232625026 | -0.213771333 | 0.280123141955441 | 1.03373236148439 | 0.807533020196376 | 1.32329275516217 | 0.999332001504091 |
| ebi-fl187-GCST90094780 | prot-a-2831 | Sepiapterin reductase \|\| id:prot-a-2831 | F-healthy breakfast food liking (derived food-liking factor) \|\| id:ebi-fl187-GCST90094780 | Weighted median | 8 | -0.115369733 | 0.12374079994519 | 0.351155684442923 | -0.357901701 | 0.127162235166856 | 0.891036637591222 | 0.699141796835172 | 1.13560123729385 | 0.999332001504091 |
| ebi-fl187-GCST90094780 | prot-a-2834 | SPRY domain-containing SOCS box protein 1 \|\| id:prot-a-2834 | F-healthy breakfast food liking (derived food-liking factor) \|\| id:ebi-fl187-GCST90094780 | Weighted median | 8 | -0.181575202 | 0.132533339650625 | 0.170676241016724 | -0.441340547 | 0.0781901441113959 | 0.833955528152055 | 0.643173638215322 | 1.08132824732244 | 0.999332001504091 |
| ebi-fl187-GCST90094780 | prot-a-2835 | Spectrin alpha chain, non-erythrocytic 1 \|\| id:prot-a-2835 | F-healthy breakfast food liking (derived food-liking factor) \|\| id:ebi-fl187-GCST90094780 | Weighted median | 8 | -0.050954076 | 0.135869548576615 | 0.707644070884953 | -0.317258391 | 0.215350239113396 | 0.950322312040221 | 0.7281425851018 | 1.24029622115181 | 0.999332001504091 |
| ebi-fl187-GCST90094780 | prot-a-2859 | Sia-alpha-2,3-Gal-beta-1,4-GlcNAc-R:alpha 2,8-sialyltransferase \|\| id:prot-a-2859 | F-healthy breakfast food liking (derived food-liking factor) \|\| id:ebi-fl187-GCST90094780 | Weighted median | 8 | -0.173226788 | 0.126807275063072 | 0.17191903783363 | -0.421769047 | 0.0753154709293528 | 0.840946876393254 | 0.655885500418083 | 1.07822424564163 | 0.999332001504091 |
| ebi-fl187-GCST90094780 | prot-a-2861 | Alpha-2,8-sialyltransferase 8F \|\| id:prot-a-2861 | F-healthy breakfast food liking (derived food-liking factor) \|\| id:ebi-fl187-GCST90094780 | Weighted median | 8 | -0.096219575 | 0.121337160773415 | 0.427781634420603 | -0.33404041 | 0.14160126043388 | 0.90826456226956 | 0.716024847223211 | 1.15211716223802 | 0.999332001504091 |
| ebi-fl187-GCST90094780 | prot-a-289 | Uncharacterized protein C17orf89 \|\| id:prot-a-289 | F-healthy breakfast food liking (derived food-liking factor) \|\| id:ebi-fl187-GCST90094780 | Weighted median | 8 | 0.118498813697709 | 0.124237834455967 | 0.340181763021273 | -0.125007342 | 0.362004969231403 | 1.1258055385555 | 0.882490423460863 | 1.43620607878297 | 0.999332001504091 |
| ebi-fl187-GCST90094780 | prot-a-2921 | Tachykinin-3 \|\| id:prot-a-2921 | F-healthy breakfast food liking (derived food-liking factor) \|\| id:ebi-fl187-GCST90094780 | Weighted median | 8 | 0.0783088784553156 | 0.125468909705036 | 0.532542381692477 | -0.167610185 | 0.324227941477185 | 1.08145664574495 | 0.845683430892812 | 1.38296250571114 | 0.999332001504091 |
| ebi-fl187-GCST90094780 | prot-a-2928 | Tax1-binding protein 1 \|\| id:prot-a-2928 | F-healthy breakfast food liking (derived food-liking factor) \|\| id:ebi-fl187-GCST90094780 | Weighted median | 8 | 0.105641971039441 | 0.130310279247604 | 0.417540496281948 | -0.149766176 | 0.361050118364746 | 1.11142388332655 | 0.860909253891698 | 1.434835371 | 0.999332001504091 |
| ebi-fl187-GCST90094780 | prot-a-296 | Uncharacterized protein C1orf185 \|\| id:prot-a-296 | F-healthy breakfast food liking (derived food-liking factor) \|\| id:ebi-fl187-GCST90094780 | Weighted median | 8 | -0.077336806 | 0.129280314402568 | 0.549699669347178 | -0.330726222 | 0.176052610199773 | 0.925578060831161 | 0.718401824548554 | 1.19250079470542 | 0.999332001504091 |
| ebi-fl187-GCST90094780 | prot-a-2964 | Transforming growth factor beta-2 \|\| id:prot-a-2964 | F-healthy breakfast food liking (derived food-liking factor) \|\| id:ebi-fl187-GCST90094780 | Weighted median | 8 | 0.165349062682731 | 0.11601146457499 | 0.154075675185111 | -0.062033408 | 0.39273153324971 | 1.1798048726972 | 0.939851487816889 | 1.48102073112985 | 0.999332001504091 |
| ebi-fl187-GCST90094780 | prot-a-2977 | Thymocyte nuclear protein 1 \|\| id:prot-a-2977 | F-healthy breakfast food liking (derived food-liking factor) \|\| id:ebi-fl187-GCST90094780 | Weighted median | 8 | -0.019360934 | 0.123112993234568 | 0.875038760167253 | -0.260662401 | 0.221940532591549 | 0.980825285008585 | 0.770541009670179 | 1.24849713076784 | 0.999332001504091 |
| ebi-fl187-GCST90094780 | prot-a-3015 | Transmembrane protein 70, mitochondrial \|\| id:prot-a-3015 | F-healthy breakfast food liking (derived food-liking factor) \|\| id:ebi-fl187-GCST90094780 | Weighted median | 8 | -0.268297319 | 0.121330232089454 | 0.0270151964229822 | -0.506104574 | -0.030490065 | 0.764680392954011 | 0.602839326684891 | 0.969970069112541 | 0.999332001504091 |
| ebi-fl187-GCST90094780 | prot-a-3030 | Tumor necrosis factor alpha-induced protein 3 \|\| id:prot-a-3030 | F-healthy breakfast food liking (derived food-liking factor) \|\| id:ebi-fl187-GCST90094780 | Weighted median | 8 | -0.204209765 | 0.133131677303374 | 0.125055947069726 | -0.465147852 | 0.0567283226829902 | 0.815291333799748 | 0.628042240805864 | 1.05836823669069 | 0.999332001504091 |
| ebi-fl187-GCST90094780 | prot-a-3032 | Tumor necrosis factor receptor superfamily member 10A \|\| id:prot-a-3032 | F-healthy breakfast food liking (derived food-liking factor) \|\| id:ebi-fl187-GCST90094780 | Weighted median | 8 | 0.102334195599154 | 0.129815505055643 | 0.430518396308016 | -0.152104194 | 0.356772585508213 | 1.10775361625765 | 0.858898783712616 | 1.42871092333796 | 0.999332001504091 |
| ebi-fl187-GCST90094780 | prot-a-3081 | Tumor protein p53-inducible protein 11 \|\| id:prot-a-3081 | F-healthy breakfast food liking (derived food-liking factor) \|\| id:ebi-fl187-GCST90094780 | Weighted median | 8 | -0.007687293 | 0.126252384081117 | 0.951448169241634 | -0.255141966 | 0.239767380052505 | 0.992342178920936 | 0.7748064943263 | 1.27095346680308 | 0.999332001504091 |
| ebi-fl187-GCST90094780 | prot-a-315 | Complement C4 \|\| id:prot-a-315 | F-healthy breakfast food liking (derived food-liking factor) \|\| id:ebi-fl187-GCST90094780 | Weighted median | 7 | -6.51E-05 | 0.134360978644009 | 0.999613648904691 | -0.263412578 | 0.26328245796207 | 0.99993494193618 | 0.768424796487736 | 1.30119419971226 | 0.99994023009904 |
| ebi-fl187-GCST90094780 | prot-a-3157 | UDP-glucuronosyltransferase 1-8 \|\| id:prot-a-3157 | F-healthy breakfast food liking (derived food-liking factor) \|\| id:ebi-fl187-GCST90094780 | Weighted median | 8 | 0.0444815292303382 | 0.138710579358286 | 0.748453885147527 | -0.227391206 | 0.316354264772578 | 1.0454856656072 | 0.796609082823397 | 1.372116262 | 0.999332001504091 |
| ebi-fl187-GCST90094780 | prot-a-317 | Glutaredoxin-like protein C5orf63 \|\| id:prot-a-317 | F-healthy breakfast food liking (derived food-liking factor) \|\| id:ebi-fl187-GCST90094780 | Weighted median | 8 | 0.148602493417574 | 0.127531075825256 | 0.243927625252166 | -0.101358415 | 0.398563402035076 | 1.16021170574351 | 0.903609107600501 | 1.48968308400384 | 0.999332001504091 |
| ebi-fl187-GCST90094780 | prot-a-3192 | Guanine nucleotide exchange factor VAV3 \|\| id:prot-a-3192 | F-healthy breakfast food liking (derived food-liking factor) \|\| id:ebi-fl187-GCST90094780 | Weighted median | 8 | 0.115734451304219 | 0.130190276431994 | 0.374022500319525 | -0.139438491 | 0.370907393110928 | 1.12269770163519 | 0.869846525381623 | 1.44904887526445 | 0.999332001504091 |
| ebi-fl187-GCST90094780 | prot-a-3233 | Serine/threonine-protein kinase WNK3 \|\| id:prot-a-3233 | F-healthy breakfast food liking (derived food-liking factor) \|\| id:ebi-fl187-GCST90094780 | Weighted median | 8 | 0.0713765578008228 | 0.138381641998144 | 0.605997601306279 | -0.199851461 | 0.342604576117185 | 1.07398556737797 | 0.818852375954632 | 1.4086116530974 | 0.999332001504091 |
| ebi-fl187-GCST90094780 | prot-a-3250 | 14-3-3 protein theta \|\| id:prot-a-3250 | F-healthy breakfast food liking (derived food-liking factor) \|\| id:ebi-fl187-GCST90094780 | Weighted median | 8 | 0.0590413543218841 | 0.128321361664945 | 0.645440552127512 | -0.192468515 | 0.310551223185176 | 1.06081910928798 | 0.824920290788379 | 1.36417687284072 | 0.999332001504091 |
| ebi-fl187-GCST90094780 | prot-a-3278 | E3 ubiquitin-protein ligase ZNRF3 \|\| id:prot-a-3278 | F-healthy breakfast food liking (derived food-liking factor) \|\| id:ebi-fl187-GCST90094780 | Weighted median | 8 | -0.186532088 | 0.135418649698075 | 0.168373918010361 | -0.451952642 | 0.0788884651480584 | 0.829831933661137 | 0.636384307110497 | 1.08208362530256 | 0.999332001504091 |
| ebi-fl187-GCST90094780 | prot-a-355 | Calpain-3 \|\| id:prot-a-355 | F-healthy breakfast food liking (derived food-liking factor) \|\| id:ebi-fl187-GCST90094780 | Weighted median | 8 | -0.03859541 | 0.120188786413036 | 0.748117016026743 | -0.274165431 | 0.196974611526582 | 0.962139902743445 | 0.760206303007369 | 1.21771312443616 | 0.999332001504091 |
| ebi-fl187-GCST90094780 | prot-a-385 | Coiled-coil domain-containing protein 90B, mitochondrial \|\| id:prot-a-385 | F-healthy breakfast food liking (derived food-liking factor) \|\| id:ebi-fl187-GCST90094780 | Weighted median | 8 | 0.0414235450132228 | 0.122892263513518 | 0.736062599991918 | -0.199445291 | 0.282292381499718 | 1.04229347027162 | 0.819185035993463 | 1.32616640983114 | 0.999332001504091 |
| ebi-fl187-GCST90094780 | prot-a-390 | C-C motif chemokine 14 \|\| id:prot-a-390 | F-healthy breakfast food liking (derived food-liking factor) \|\| id:ebi-fl187-GCST90094780 | Weighted median | 8 | -0.009518587 | 0.132453807316784 | 0.942710578247025 | -0.26912805 | 0.250090874907856 | 0.990526570925477 | 0.76404541354152 | 1.28414210768123 | 0.999332001504091 |
| ebi-fl187-GCST90094780 | prot-a-40 | Pituitary adenylate cyclase-activating polypeptide 27 \|\| id:prot-a-40 | F-healthy breakfast food liking (derived food-liking factor) \|\| id:ebi-fl187-GCST90094780 | Weighted median | 8 | 0.170196995236258 | 0.131139713815827 | 0.194345914370411 | -0.086836844 | 0.427230834315279 | 1.18553837373024 | 0.916826669347107 | 1.53300649138819 | 0.999332001504091 |
| ebi-fl187-GCST90094780 | prot-a-401 | C-C motif chemokine 24 \|\| id:prot-a-401 | F-healthy breakfast food liking (derived food-liking factor) \|\| id:ebi-fl187-GCST90094780 | Weighted median | 8 | 0.326785370412671 | 0.126367342926769 | 0.00970982199352879 | 0.0791053782762035 | 0.574465362549138 | 1.38650386049136 | 1.0823183689052 | 1.77618066031903 | 0.999332001504091 |
| ebi-fl187-GCST90094780 | prot-a-412 | Cerebral cavernous malformations 2 protein \|\| id:prot-a-412 | F-healthy breakfast food liking (derived food-liking factor) \|\| id:ebi-fl187-GCST90094780 | Weighted median | 8 | 0.159459874011558 | 0.125855306878293 | 0.205151880049102 | -0.087216527 | 0.406136275493013 | 1.17287719841959 | 0.916478631348182 | 1.50100708900211 | 0.999332001504091 |
| ebi-fl187-GCST90094780 | prot-a-44 | Adhesion G protein-coupled receptor F5 \|\| id:prot-a-44 | F-healthy breakfast food liking (derived food-liking factor) \|\| id:ebi-fl187-GCST90094780 | Weighted median | 8 | -0.116733367 | 0.12946852597037 | 0.367250742019801 | -0.370491678 | 0.137024944268726 | 0.889822417883128 | 0.690394795561965 | 1.14685675566671 | 0.999332001504091 |
| ebi-fl187-GCST90094780 | prot-a-48 | Adrenomedullin \|\| id:prot-a-48 | F-healthy breakfast food liking (derived food-liking factor) \|\| id:ebi-fl187-GCST90094780 | Weighted median | 8 | -0.037670244 | 0.130167195208357 | 0.7722761305182 | -0.292797947 | 0.21745745845165 | 0.963030453456786 | 0.746172892113444 | 1.24291255295855 | 0.999332001504091 |
| ebi-fl187-GCST90094780 | prot-a-502 | Carcinoembryonic antigen-related cell adhesion molecule 1 \|\| id:prot-a-502 | F-healthy breakfast food liking (derived food-liking factor) \|\| id:ebi-fl187-GCST90094780 | Weighted median | 8 | -0.208224215 | 0.13414265 | 0.120600122384532 | -0.471143809 | 0.0546953788675454 | 0.812024948006764 | 0.624287793652492 | 1.05621881909232 | 0.999332001504091 |
| ebi-fl187-GCST90094780 | prot-a-518 | Complement factor B \|\| id:prot-a-518 | F-healthy breakfast food liking (derived food-liking factor) \|\| id:ebi-fl187-GCST90094780 | Weighted median | 8 | 0.0997515713244195 | 0.127438616179407 | 0.43377856851183 | -0.150028116 | 0.349531259036056 | 1.10489639602917 | 0.860683776766517 | 1.41840252937568 | 0.999332001504091 |
| ebi-fl187-GCST90094780 | prot-a-525 | Properdin \|\| id:prot-a-525 | F-healthy breakfast food liking (derived food-liking factor) \|\| id:ebi-fl187-GCST90094780 | Weighted median | 8 | -0.161699514 | 0.13867710657949 | 0.243608593044405 | -0.433506643 | 0.110107615248648 | 0.850696788913981 | 0.648231986692508 | 1.11639820546505 | 0.999332001504091 |
| ebi-fl187-GCST90094780 | prot-a-533 | Chondroadherin \|\| id:prot-a-533 | F-healthy breakfast food liking (derived food-liking factor) \|\| id:ebi-fl187-GCST90094780 | Weighted median | 8 | -0.172513401 | 0.119290698713065 | 0.148132413317829 | -0.406323171 | 0.0612963680496138 | 0.841547010804534 | 0.666094870155449 | 1.06321397014928 | 0.999332001504091 |
| ebi-fl187-GCST90094780 | prot-a-535 | Coiled-coil-helix-coiled-coil-helix domain-containing protein 2 \|\| id:prot-a-535 | F-healthy breakfast food liking (derived food-liking factor) \|\| id:ebi-fl187-GCST90094780 | Weighted median | 8 | -0.160694416 | 0.137849157663026 | 0.24372504167938 | -0.430878765 | 0.109489932704498 | 0.851552251826691 | 0.649937700978335 | 1.11570883870804 | 0.999332001504091 |
| ebi-fl187-GCST90094780 | prot-a-591 | Caseinolytic peptidase B protein homolog \|\| id:prot-a-591 | F-healthy breakfast food liking (derived food-liking factor) \|\| id:ebi-fl187-GCST90094780 | Weighted median | 8 | -0.22468724 | 0.125834681795975 | 0.0741681261822781 | -0.471323216 | 0.0219487364534439 | 0.798766001857486 | 0.624175802081851 | 1.02219138197178 | 0.999332001504091 |
| ebi-fl187-GCST90094780 | prot-a-605 | Ciliary neurotrophic factor receptor subunit alpha \|\| id:prot-a-605 | F-healthy breakfast food liking (derived food-liking factor) \|\| id:ebi-fl187-GCST90094780 | Weighted median | 8 | -0.203084698 | 0.151627567213968 | 0.180451788986484 | -0.50027473 | 0.0941053338417621 | 0.816209107302492 | 0.606364050651938 | 1.09867546753021 | 0.999332001504091 |
| ebi-fl187-GCST90094780 | prot-a-606 | Ciliary neurotrophic factor receptor subunit alpha \|\| id:prot-a-606 | F-healthy breakfast food liking (derived food-liking factor) \|\| id:ebi-fl187-GCST90094780 | Weighted median | 8 | -0.111793357 | 0.133463050596351 | 0.40223558467895 | -0.373380936 | 0.149794222373077 | 0.894229024760241 | 0.688402945449705 | 1.16159518783185 | 0.999332001504091 |
| ebi-fl187-GCST90094780 | prot-a-707 | Cancer/testis antigen 55 \|\| id:prot-a-707 | F-healthy breakfast food liking (derived food-liking factor) \|\| id:ebi-fl187-GCST90094780 | Weighted median | 8 | 0.0422780355289873 | 0.117915894414482 | 0.719936252096558 | -0.188837118 | 0.273393188581372 | 1.04318448078241 | 0.827921349592517 | 1.31441695697407 | 0.999332001504091 |
| ebi-fl187-GCST90094780 | prot-a-719 | Dipeptidyl peptidase 1 \|\| id:prot-a-719 | F-healthy breakfast food liking (derived food-liking factor) \|\| id:ebi-fl187-GCST90094780 | Weighted median | 8 | 0.151753941556823 | 0.116393395951099 | 0.192301103096535 | -0.076377115 | 0.379884997620977 | 1.16387382021574 | 0.926466756617721 | 1.46211643289702 | 0.999332001504091 |
| ebi-fl187-GCST90094780 | prot-a-752 | Cytochrome b5 \|\| id:prot-a-752 | F-healthy breakfast food liking (derived food-liking factor) \|\| id:ebi-fl187-GCST90094780 | Weighted median | 8 | -0.073041749 | 0.134979977993944 | 0.588417812992958 | -0.337602505 | 0.191519008350053 | 0.929562021378843 | 0.713478835534399 | 1.21108785370307 | 0.999332001504091 |
| ebi-fl187-GCST90094780 | prot-a-755 | Cytoglobin \|\| id:prot-a-755 | F-healthy breakfast food liking (derived food-liking factor) \|\| id:ebi-fl187-GCST90094780 | Weighted median | 8 | 0.0376481136061187 | 0.12814936107925 | 0.768923667396986 | -0.213524634 | 0.288820861321449 | 1.03836578179315 | 0.807732262140719 | 1.33485258338123 | 0.999332001504091 |
| ebi-fl187-GCST90094780 | prot-a-807 | Desmin \|\| id:prot-a-807 | F-healthy breakfast food liking (derived food-liking factor) \|\| id:ebi-fl187-GCST90094780 | Weighted median | 8 | 0.0424343326171317 | 0.126809049926928 | 0.737902901307854 | -0.206111405 | 0.290980070473911 | 1.04334754022168 | 0.81374241606413 | 1.33773792320153 | 0.999332001504091 |
| ebi-fl187-GCST90094780 | prot-a-82 | Ameloblastin \|\| id:prot-a-82 | F-healthy breakfast food liking (derived food-liking factor) \|\| id:ebi-fl187-GCST90094780 | Weighted median | 8 | -0.050124139 | 0.12643762522212 | 0.691785090425436 | -0.297941884 | 0.197693606624783 | 0.951111347340506 | 0.742344480403446 | 1.2185889690299 | 0.999332001504091 |
| ebi-fl187-GCST90094780 | prot-a-84 | Muellerian-inhibiting factor \|\| id:prot-a-84 | F-healthy breakfast food liking (derived food-liking factor) \|\| id:ebi-fl187-GCST90094780 | Weighted median | 8 | -0.1085886 | 0.116749734334519 | 0.352320766392593 | -0.337418079 | 0.120240879644722 | 0.897099408601342 | 0.713610432030343 | 1.12776847533341 | 0.999332001504091 |
| ebi-fl187-GCST90094780 | prot-a-858 | Inactive dipeptidyl peptidase 10 \|\| id:prot-a-858 | F-healthy breakfast food liking (derived food-liking factor) \|\| id:ebi-fl187-GCST90094780 | Weighted median | 8 | -0.042712377 | 0.129082517933085 | 0.74072603998653 | -0.295714112 | 0.210289358352195 | 0.958186947235984 | 0.744000098359447 | 1.23403508665915 | 0.999332001504091 |
| ebi-fl187-GCST90094780 | prot-a-863 | Dihydropyrimidinase-related protein 3 \|\| id:prot-a-863 | F-healthy breakfast food liking (derived food-liking factor) \|\| id:ebi-fl187-GCST90094780 | Weighted median | 8 | 0.0481558257241786 | 0.133543383287053 | 0.718398031851212 | -0.213589206 | 0.309900856966803 | 1.04933415584009 | 0.80768010741414 | 1.36328994673139 | 0.999332001504091 |
| ebi-fl187-GCST90094780 | prot-a-864 | Dihydropyrimidinase-related protein 5 \|\| id:prot-a-864 | F-healthy breakfast food liking (derived food-liking factor) \|\| id:ebi-fl187-GCST90094780 | Weighted median | 8 | -0.021740008 | 0.128168825331836 | 0.865308917357365 | -0.272950906 | 0.2294708892119 | 0.978494602322684 | 0.761130153567803 | 1.25793424723297 | 0.999332001504091 |
| ebi-fl187-GCST90094780 | prot-a-884 | Endothelin-converting enzyme 1 \|\| id:prot-a-884 | F-healthy breakfast food liking (derived food-liking factor) \|\| id:ebi-fl187-GCST90094780 | Weighted median | 8 | 0.0709668854362524 | 0.122751524842258 | 0.563173349276915 | -0.169626103 | 0.311559874127079 | 1.07354567528304 | 0.843980319104545 | 1.3655535453027 | 0.999332001504091 |
| ebi-fl187-GCST90094780 | prot-a-893 | Early endosome antigen 1 \|\| id:prot-a-893 | F-healthy breakfast food liking (derived food-liking factor) \|\| id:ebi-fl187-GCST90094780 | Weighted median | 8 | 0.150887379069359 | 0.120069698446757 | 0.208874992663907 | -0.08444923 | 0.386223988025003 | 1.162865688 | 0.919018312856669 | 1.47141421307015 | 0.999332001504091 |
| ebi-fl187-GCST90094780 | prot-a-897 | Ephrin-A2 \|\| id:prot-a-897 | F-healthy breakfast food liking (derived food-liking factor) \|\| id:ebi-fl187-GCST90094780 | Weighted median | 8 | -0.036441482 | 0.130160874640988 | 0.779498504806507 | -0.291556796 | 0.218673832108537 | 0.964214515969427 | 0.747099579768506 | 1.24442531890358 | 0.999332001504091 |
| ebi-fl187-GCST90094780 | prot-a-90 | Anaphase-promoting complex subunit 10 \|\| id:prot-a-90 | F-healthy breakfast food liking (derived food-liking factor) \|\| id:ebi-fl187-GCST90094780 | Weighted median | 8 | 0.10776286680516 | 0.134209347464061 | 0.422005911920931 | -0.155287454 | 0.370813187834719 | 1.11378359900558 | 0.85616903270238 | 1.44891237364461 | 0.999332001504091 |
| ebi-fl187-GCST90094780 | prot-a-914 | Histone-lysine N-methyltransferase EHMT2 \|\| id:prot-a-914 | F-healthy breakfast food liking (derived food-liking factor) \|\| id:ebi-fl187-GCST90094780 | Weighted median | 8 | 0.188810327637108 | 0.138898877988998 | 0.174039953342528 | -0.083431473 | 0.461052128495544 | 1.2078118422296 | 0.919954126004052 | 1.58574151144537 | 0.999332001504091 |
| ebi-fl187-GCST90094780 | prot-a-932 | RNA polymerase II elongation factor ELL \|\| id:prot-a-932 | F-healthy breakfast food liking (derived food-liking factor) \|\| id:ebi-fl187-GCST90094780 | Weighted median | 8 | 0.0189784490122269 | 0.122731701226898 | 0.877110152528344 | -0.221575685 | 0.259532583416947 | 1.01915968448284 | 0.801255276532879 | 1.29632402168965 | 0.999332001504091 |
| ebi-fl187-GCST90094780 | prot-a-991 | Estrogen receptor \|\| id:prot-a-991 | F-healthy breakfast food liking (derived food-liking factor) \|\| id:ebi-fl187-GCST90094780 | Weighted median | 8 | -0.260188893 | 0.129376704727606 | 0.0443153828746046 | -0.513767235 | -0.006610552 | 0.770905952989339 | 0.59823762682827 | 0.993411249481637 | 0.999332001504091 |
| ebi-fl187-GCST90094780 | prot-a-992 | Persulfide dioxygenase ETHE1, mitochondrial \|\| id:prot-a-992 | F-healthy breakfast food liking (derived food-liking factor) \|\| id:ebi-fl187-GCST90094780 | Weighted median | 8 | -0.170392954 | 0.13550258385075 | 0.208576767042742 | -0.435978018 | 0.0951921108417594 | 0.843333360676706 | 0.646631940137457 | 1.09987013180803 | 0.999332001504091 |
| ebi-fl187-GCST90094780 | prot-a-993 | Ethanolamine kinase 1 \|\| id:prot-a-993 | F-healthy breakfast food liking (derived food-liking factor) \|\| id:ebi-fl187-GCST90094780 | Weighted median | 8 | 0.17677746921086 | 0.127140428344156 | 0.164404062500232 | -0.07241777 | 0.425972708765406 | 1.19336550301013 | 0.930142228791483 | 1.53107898952717 | 0.999332001504091 |
| ebi-fl187-GCST90094780 | prot-b-10 | coagulation factor III, tissue factor \|\| id:prot-b-10 | F-healthy breakfast food liking (derived food-liking factor) \|\| id:ebi-fl187-GCST90094780 | Weighted median | 8 | 0.00193504898628521 | 0.126183454386044 | 0.987764757228743 | -0.245384522 | 0.249254619582931 | 1.00193692240176 | 0.782403629289092 | 1.28306868589562 | 0.999332001504091 |
| ebi-fl187-GCST90094780 | prot-b-28 | prolactin \|\| id:prot-b-28 | F-healthy breakfast food liking (derived food-liking factor) \|\| id:ebi-fl187-GCST90094780 | Weighted median | 8 | 0.10275317419752 | 0.165798142276338 | 0.535423283192447 | -0.222211185 | 0.427717533059142 | 1.10821783855794 | 0.800746241151208 | 1.53375278531731 | 0.999332001504091 |
| ebi-fl187-GCST90094780 | prot-b-54 | matrix metallopeptidase 7 \|\| id:prot-b-54 | F-healthy breakfast food liking (derived food-liking factor) \|\| id:ebi-fl187-GCST90094780 | Weighted median | 8 | -0.011033063 | 0.141992951655748 | 0.938065516008917 | -0.289339248 | 0.267273122611229 | 0.989027578377914 | 0.748758147697119 | 1.30639720422483 | 0.999332001504091 |
| ebi-fl187-GCST90094780 | ukb-rsfMRI191-edge-pheno716 | Net100_Pair10_33 (OccipitalPrecuneus)&(Temporal\|Frontal\|Supp_Motor_Area) (Default_mode\|Central_executive)&(Default_mode\|Salience) \|\| id:ukb-rsfMRI191-edge-pheno716 | F-healthy breakfast food liking (derived food-liking factor) \|\| id:ebi-fl187-GCST90094780 | Weighted median | 8 | 0.045393300916947 | 0.0394470859210832 | 0.24983961937674 | -0.031922987 | 0.12270958932227 | 1.04643934453851 | 0.968581172075752 | 1.13055604772022 | 0.999332001504091 |
| ebi-fl187-GCST90094780 | ukb-rsfMRI191-edge-pheno903 | Net100_Pair27_38 (Precentral\|Frontal\|Supp_Motor_Area)&(Subcortical) (Attention\|Salience\|Motor)&(Subcortical-cerebellum) \|\| id:ukb-rsfMRI191-edge-pheno903 | F-healthy breakfast food liking (derived food-liking factor) \|\| id:ebi-fl187-GCST90094780 | Weighted median | 8 | -0.039983453 | 0.0379589273639635 | 0.292187651349643 | -0.11438295 | 0.0344160448084275 | 0.960805337634905 | 0.891916330706207 | 1.03501512983487 | 0.999332001504091 |
| ebi-fl187-GCST90094780 | ukb-rsfMRI191-node-pheno23 | Net100_Node2 RolandicOper\|SupraMarginal\|Insula Salience\|Motor \|\| id:ukb-rsfMRI191-node-pheno23 | F-healthy breakfast food liking (derived food-liking factor) \|\| id:ebi-fl187-GCST90094780 | Weighted median | 8 | -0.048891389 | 0.0404259831096833 | 0.226507259739417 | -0.128126316 | 0.030343537746964 | 0.952284552518564 | 0.879742246566437 | 1.03080859479561 | 0.999332001504091 |
| ebi-fl187-GCST90094780 | ukb-rsfMRI191-node-pheno55 | Net100_Node34 Parietal\|Postcentral\|Precuneus Attention \|\| id:ukb-rsfMRI191-node-pheno55 | F-healthy breakfast food liking (derived food-liking factor) \|\| id:ebi-fl187-GCST90094780 | Weighted median | 8 | -0.016073251 | 0.0385340001569753 | 0.676591598547597 | -0.091599892 | 0.0594533888348414 | 0.984055233919879 | 0.912470163330108 | 1.06125629343431 | 0.999332001504091 |
| ebi-fl187-GCST90094780 | ebi-a-GCST90001414 | IgD- CD24- B cell Absolute Count \|\| id:ebi-a-GCST90001414 | F-healthy breakfast food liking (derived food-liking factor) \|\| id:ebi-fl187-GCST90094780 | Weighted mode | 8 | -0.094357387 | 0.197534255659892 | 0.647436198720223 | -0.481524528 | 0.292809754422041 | 0.9099574974405 | 0.617840758063204 | 1.34018780137434 | 0.999714303421225 |
| ebi-fl187-GCST90094780 | ebi-a-GCST90001422 | CD20- CD38- B cell %B cell \|\| id:ebi-a-GCST90001422 | F-healthy breakfast food liking (derived food-liking factor) \|\| id:ebi-fl187-GCST90094780 | Weighted mode | 8 | -0.150969072 | 0.194025751102551 | 0.462001755105859 | -0.531259544 | 0.229321400591147 | 0.859874292809947 | 0.587864062679183 | 1.25774621443208 | 0.999714303421225 |
| ebi-fl187-GCST90094780 | ebi-a-GCST90001444 | CD20- CD38- B cell %lymphocyte \|\| id:ebi-a-GCST90001444 | F-healthy breakfast food liking (derived food-liking factor) \|\| id:ebi-fl187-GCST90094780 | Weighted mode | 8 | -0.191399479 | 0.191235561514419 | 0.350229954355889 | -0.56622118 | 0.183422221601963 | 0.82580263146862 | 0.567666500597511 | 1.20132152491418 | 0.999714303421225 |
| ebi-fl187-GCST90094780 | ebi-a-GCST90001459 | Myeloid Dendritic Cell %Dendritic Cell \|\| id:ebi-a-GCST90001459 | F-healthy breakfast food liking (derived food-liking factor) \|\| id:ebi-fl187-GCST90094780 | Weighted mode | 8 | 0.0386535593960899 | 0.199935596805304 | 0.852190531630156 | -0.35322021 | 0.430527329134487 | 1.03941032732586 | 0.702422495646636 | 1.53806837800246 | 0.999714303421225 |
| ebi-fl187-GCST90094780 | ebi-a-GCST90001476 | HLA DR++ monocyte %leukocyte \|\| id:ebi-a-GCST90001476 | F-healthy breakfast food liking (derived food-liking factor) \|\| id:ebi-fl187-GCST90094780 | Weighted mode | 8 | -0.03526915 | 0.170487429990203 | 0.841998418900787 | -0.369424513 | 0.298886212549703 | 0.965345558308616 | 0.691131953659339 | 1.34835618873079 | 0.999714303421225 |
| ebi-fl187-GCST90094780 | ebi-a-GCST90001478 | CD4 regulatory T cell %CD4+ T cell \|\| id:ebi-a-GCST90001478 | F-healthy breakfast food liking (derived food-liking factor) \|\| id:ebi-fl187-GCST90094780 | Weighted mode | 8 | -0.302275793 | 0.210733564894914 | 0.19458434374263 | -0.71531358 | 0.110761994114141 | 0.739134188682651 | 0.489038734849163 | 1.11712899193571 | 0.999714303421225 |
| ebi-fl187-GCST90094780 | ebi-a-GCST90001485 | CD39+ resting CD4 regulatory T cell %CD4 regulatory T cell \|\| id:ebi-a-GCST90001485 | F-healthy breakfast food liking (derived food-liking factor) \|\| id:ebi-fl187-GCST90094780 | Weighted mode | 8 | 0.20885513009002 | 0.188580091790623 | 0.304670934821272 | -0.16076185 | 0.578472109999642 | 1.23226646726648 | 0.851494830610105 | 1.78331164413694 | 0.999714303421225 |
| ebi-fl187-GCST90094780 | ebi-a-GCST90001515 | Immature Myeloid-Derived Suppressor Cells Absolute Count \|\| id:ebi-a-GCST90001515 | F-healthy breakfast food liking (derived food-liking factor) \|\| id:ebi-fl187-GCST90094780 | Weighted mode | 8 | 0.0715507466061707 | 0.269798677774587 | 0.79849505016531 | -0.457254662 | 0.600356155044362 | 1.07417265993516 | 0.633019113727328 | 1.82276787277104 | 0.999714303421225 |
| ebi-fl187-GCST90094780 | ebi-a-GCST90001517 | CD33+ HLA DR+ Absolute Count \|\| id:ebi-a-GCST90001517 | F-healthy breakfast food liking (derived food-liking factor) \|\| id:ebi-fl187-GCST90094780 | Weighted mode | 8 | -0.175326934 | 0.23872569093925 | 0.486555422325575 | -0.643229288 | 0.2925754201004 | 0.839182618467794 | 0.525592391170028 | 1.33987378616873 | 0.999714303421225 |
| ebi-fl187-GCST90094780 | ebi-a-GCST90001518 | CD33+ HLA DR+ CD14- Absolute Count \|\| id:ebi-a-GCST90001518 | F-healthy breakfast food liking (derived food-liking factor) \|\| id:ebi-fl187-GCST90094780 | Weighted mode | 8 | -0.131276252 | 0.242495131570531 | 0.605055010591248 | -0.60656671 | 0.344014205825283 | 0.876975474649419 | 0.545219556254144 | 1.41059867408365 | 0.999714303421225 |
| ebi-fl187-GCST90094780 | ebi-a-GCST90001520 | CD33+ HLA DR+ CD14dim Absolute Count \|\| id:ebi-a-GCST90001520 | F-healthy breakfast food liking (derived food-liking factor) \|\| id:ebi-fl187-GCST90094780 | Weighted mode | 8 | -0.081604199 | 0.226101646163731 | 0.728806899438776 | -0.524763425 | 0.361555027472913 | 0.921636671223532 | 0.591695327843165 | 1.43556001505075 | 0.999714303421225 |
| ebi-fl187-GCST90094780 | ebi-a-GCST90001531 | CD33dim HLA DR- Absolute Count \|\| id:ebi-a-GCST90001531 | F-healthy breakfast food liking (derived food-liking factor) \|\| id:ebi-fl187-GCST90094780 | Weighted mode | 8 | -0.259590278 | 0.222451806124056 | 0.281440810953778 | -0.695595818 | 0.176415261511558 | 0.771367566950079 | 0.498777178766818 | 1.19293333510885 | 0.999714303421225 |
| ebi-fl187-GCST90094780 | ebi-a-GCST90001532 | Basophil Absolute Count \|\| id:ebi-a-GCST90001532 | F-healthy breakfast food liking (derived food-liking factor) \|\| id:ebi-fl187-GCST90094780 | Weighted mode | 8 | -0.247521786 | 0.244317859387319 | 0.344736533418658 | -0.726384791 | 0.231341218174585 | 0.780733211396576 | 0.483654345000472 | 1.2602891996701 | 0.999714303421225 |
| ebi-fl187-GCST90094780 | ebi-a-GCST90001588 | T/B cell \|\| id:ebi-a-GCST90001588 | F-healthy breakfast food liking (derived food-liking factor) \|\| id:ebi-fl187-GCST90094780 | Weighted mode | 8 | 0.114651986317112 | 0.191445277143818 | 0.568121896365209 | -0.260580757 | 0.489884729518995 | 1.12148307819434 | 0.770603922291013 | 1.63212807292366 | 0.999714303421225 |
| ebi-fl187-GCST90094780 | ebi-a-GCST90001624 | HLA DR+ CD4+ T cell Absolute Count \|\| id:ebi-a-GCST90001624 | F-healthy breakfast food liking (derived food-liking factor) \|\| id:ebi-fl187-GCST90094780 | Weighted mode | 8 | -0.273526609 | 0.197501144104872 | 0.208602618144582 | -0.660628851 | 0.113575633540967 | 0.760692094903194 | 0.516526414006238 | 1.12027661617557 | 0.999714303421225 |
| ebi-fl187-GCST90094780 | ebi-a-GCST90001631 | CD8+ Natural Killer T %T cell \|\| id:ebi-a-GCST90001631 | F-healthy breakfast food liking (derived food-liking factor) \|\| id:ebi-fl187-GCST90094780 | Weighted mode | 8 | 0.00898660192946188 | 0.153566843538903 | 0.954970620734847 | -0.292004411 | 0.309977615265713 | 1.00902710266701 | 0.746765241680834 | 1.36339459456486 | 0.999714303421225 |
| ebi-fl187-GCST90094780 | ebi-a-GCST90001632 | CD8+ Natural Killer T %lymphocyte \|\| id:ebi-a-GCST90001632 | F-healthy breakfast food liking (derived food-liking factor) \|\| id:ebi-fl187-GCST90094780 | Weighted mode | 8 | -0.008650948 | 0.163444105240644 | 0.959267240286706 | -0.329001394 | 0.311699498589722 | 0.991386364094363 | 0.7196420135988 | 1.36574422329402 | 0.999714303421225 |
| ebi-fl187-GCST90094780 | ebi-a-GCST90001646 | Natural Killer %CD3- lymphocyte \|\| id:ebi-a-GCST90001646 | F-healthy breakfast food liking (derived food-liking factor) \|\| id:ebi-fl187-GCST90094780 | Weighted mode | 8 | 0.0413446744203221 | 0.170746816823847 | 0.815612508746687 | -0.293319087 | 0.376008435395062 | 1.04221126720938 | 0.745784133037368 | 1.45645941952986 | 0.999714303421225 |
| ebi-fl187-GCST90094780 | ebi-a-GCST90001684 | CD127- CD8+ T cell Absolute Count \|\| id:ebi-a-GCST90001684 | F-healthy breakfast food liking (derived food-liking factor) \|\| id:ebi-fl187-GCST90094780 | Weighted mode | 8 | 0.0264871534612074 | 0.204681833962227 | 0.900675920900894 | -0.374689241 | 0.427663548027173 | 1.02684105582349 | 0.687502893237537 | 1.5336699878591 | 0.999714303421225 |
| ebi-fl187-GCST90094780 | ebi-a-GCST90001687 | CD28- CD8+ T cell Absolute Count \|\| id:ebi-a-GCST90001687 | F-healthy breakfast food liking (derived food-liking factor) \|\| id:ebi-fl187-GCST90094780 | Weighted mode | 8 | 0.152669945346934 | 0.210852546504162 | 0.492511783966048 | -0.260601046 | 0.565940936495092 | 1.16494042147709 | 0.77058828773102 | 1.76110409046979 | 0.999714303421225 |
| ebi-fl187-GCST90094780 | ebi-a-GCST90001701 | BAFF-R on CD20- CD38- B cell \|\| id:ebi-a-GCST90001701 | F-healthy breakfast food liking (derived food-liking factor) \|\| id:ebi-fl187-GCST90094780 | Weighted mode | 8 | -0.299557016 | 0.178576782237871 | 0.137350718358776 | -0.64956751 | 0.0504534767573739 | 0.741146463680446 | 0.522271605370784 | 1.05174793149258 | 0.999714303421225 |
| ebi-fl187-GCST90094780 | ebi-a-GCST90001732 | CD19 on IgD- CD27- B cell \|\| id:ebi-a-GCST90001732 | F-healthy breakfast food liking (derived food-liking factor) \|\| id:ebi-fl187-GCST90094780 | Weighted mode | 8 | -0.178617142 | 0.275231782017479 | 0.537060128856703 | -0.718071435 | 0.360837150403679 | 0.836426070222359 | 0.487691895059709 | 1.43452982925206 | 0.999714303421225 |
| ebi-fl187-GCST90094780 | ebi-a-GCST90001798 | CD27 on CD24+ CD27+ B cell \|\| id:ebi-a-GCST90001798 | F-healthy breakfast food liking (derived food-liking factor) \|\| id:ebi-fl187-GCST90094780 | Weighted mode | 8 | -0.145597699 | 0.215156888106059 | 0.520320224658067 | -0.5673052 | 0.276109801515959 | 0.864505424483822 | 0.567051471984894 | 1.3179925736651 | 0.999714303421225 |
| ebi-fl187-GCST90094780 | ebi-a-GCST90001836 | CD66b on CD66b++ myeloid cell \|\| id:ebi-a-GCST90001836 | F-healthy breakfast food liking (derived food-liking factor) \|\| id:ebi-fl187-GCST90094780 | Weighted mode | 8 | 0.0993303103041077 | 0.27801103992225 | 0.731404485713999 | -0.445571328 | 0.644231948551718 | 1.1044310442702 | 0.640458259784158 | 1.90452369520354 | 0.999714303421225 |
| ebi-fl187-GCST90094780 | ebi-a-GCST90001943 | CD25 on activated & secreting CD4 regulatory T cell \|\| id:ebi-a-GCST90001943 | F-healthy breakfast food liking (derived food-liking factor) \|\| id:ebi-fl187-GCST90094780 | Weighted mode | 8 | 0.00746761828902165 | 0.17403638153339 | 0.966972866875888 | -0.33364369 | 0.348578926094466 | 1.00749557048594 | 0.716308965156098 | 1.41705238091999 | 0.999714303421225 |
| ebi-fl187-GCST90094780 | ebi-a-GCST90001960 | CD25 on CD4+ T cell \|\| id:ebi-a-GCST90001960 | F-healthy breakfast food liking (derived food-liking factor) \|\| id:ebi-fl187-GCST90094780 | Weighted mode | 8 | -0.162513878 | 0.202777896427646 | 0.449222964825889 | -0.559958555 | 0.234930798952143 | 0.850004293744817 | 0.571232738073788 | 1.26482123874927 | 0.999714303421225 |
| ebi-fl187-GCST90094780 | ebi-a-GCST90001988 | HLA DR on CD14+ CD16- monocyte \|\| id:ebi-a-GCST90001988 | F-healthy breakfast food liking (derived food-liking factor) \|\| id:ebi-fl187-GCST90094780 | Weighted mode | 8 | -0.064964069 | 0.202707891907593 | 0.757958468771073 | -0.462271537 | 0.332343399593182 | 0.937101134179057 | 0.629851288988868 | 1.39423154486105 | 0.999714303421225 |
| ebi-fl187-GCST90094780 | ebi-a-GCST90001991 | HLA DR on CD14+ monocyte \|\| id:ebi-a-GCST90001991 | F-healthy breakfast food liking (derived food-liking factor) \|\| id:ebi-fl187-GCST90094780 | Weighted mode | 8 | -0.058690933 | 0.206883186380848 | 0.784854403919541 | -0.464181978 | 0.346800112218017 | 0.942998173656169 | 0.628649143495158 | 1.41453394905599 | 0.999714303421225 |
| ebi-fl187-GCST90094780 | ebi-a-GCST90002019 | CD14 on Monocytic Myeloid-Derived Suppressor Cells \|\| id:ebi-a-GCST90002019 | F-healthy breakfast food liking (derived food-liking factor) \|\| id:ebi-fl187-GCST90094780 | Weighted mode | 8 | 0.146735473701455 | 0.288671966721374 | 0.626857876363118 | -0.419061581 | 0.712532528475347 | 1.15804758846746 | 0.657663694385182 | 2.03914892764303 | 0.999714303421225 |
| ebi-fl187-GCST90094780 | ebi-a-GCST90002037 | CD80 on plasmacytoid Dendritic Cell \|\| id:ebi-a-GCST90002037 | F-healthy breakfast food liking (derived food-liking factor) \|\| id:ebi-fl187-GCST90094780 | Weighted mode | 8 | -0.014585528 | 0.191718836112165 | 0.941485970797153 | -0.390354447 | 0.361183390584453 | 0.985520325352755 | 0.67681693626214 | 1.43502660711673 | 0.999714303421225 |
| ebi-fl187-GCST90094780 | ebi-a-GCST90002042 | CD45 on CD33+ HLA DR+ CD14- \|\| id:ebi-a-GCST90002042 | F-healthy breakfast food liking (derived food-liking factor) \|\| id:ebi-fl187-GCST90094780 | Weighted mode | 8 | -0.145321849 | 0.323146959238532 | 0.66651597088211 | -0.778689889 | 0.488046191024655 | 0.864743931276696 | 0.459006967549503 | 1.62913009942322 | 0.999714303421225 |
| ebi-fl187-GCST90094780 | ebi-a-GCST90002056 | CD8 on Effector Memory CD8+ T cell \|\| id:ebi-a-GCST90002056 | F-healthy breakfast food liking (derived food-liking factor) \|\| id:ebi-fl187-GCST90094780 | Weighted mode | 8 | 0.044187439350768 | 0.189207460419808 | 0.822025003739375 | -0.326659183 | 0.415034061773592 | 1.0451782440607 | 0.72132954245662 | 1.51442232372385 | 0.999714303421225 |
| ebi-fl187-GCST90094780 | ebi-a-GCST90002058 | CD8 on CD8+ T cell \|\| id:ebi-a-GCST90002058 | F-healthy breakfast food liking (derived food-liking factor) \|\| id:ebi-fl187-GCST90094780 | Weighted mode | 8 | -0.059163206 | 0.189645791734122 | 0.764152725317139 | -0.430868958 | 0.312542546003284 | 0.942552926503173 | 0.649944075429637 | 1.36689609590245 | 0.999714303421225 |
| ebi-fl187-GCST90094780 | ebi-a-GCST90002063 | CD4 on CD4 regulatory T cell \|\| id:ebi-a-GCST90002063 | F-healthy breakfast food liking (derived food-liking factor) \|\| id:ebi-fl187-GCST90094780 | Weighted mode | 8 | -0.188313054 | 0.233321102102792 | 0.446162467039635 | -0.645622414 | 0.268996306392383 | 0.82835534690758 | 0.524336086479306 | 1.3086503073968 | 0.999714303421225 |
| ebi-fl187-GCST90094780 | ebi-a-GCST90002066 | CD4 on activated CD4 regulatory T cell \|\| id:ebi-a-GCST90002066 | F-healthy breakfast food liking (derived food-liking factor) \|\| id:ebi-fl187-GCST90094780 | Weighted mode | 8 | -0.149582401 | 0.238992882199166 | 0.551242899520484 | -0.61800845 | 0.318843648085749 | 0.861067482253526 | 0.539016848291988 | 1.37553624036773 | 0.999714303421225 |
| ebi-fl187-GCST90094780 | ebi-a-GCST90002070 | CD4 on activated & secreting CD4 regulatory T cell \|\| id:ebi-a-GCST90002070 | F-healthy breakfast food liking (derived food-liking factor) \|\| id:ebi-fl187-GCST90094780 | Weighted mode | 8 | -0.09199585 | 0.233196424747868 | 0.704955696129139 | -0.549060842 | 0.365069143005145 | 0.912108935244752 | 0.577491911926409 | 1.44061361306015 | 0.999714303421225 |
| ebi-fl187-GCST90094780 | ebi-a-GCST90002081 | SSC-A on CD4+ T cell \|\| id:ebi-a-GCST90002081 | F-healthy breakfast food liking (derived food-liking factor) \|\| id:ebi-fl187-GCST90094780 | Weighted mode | 8 | 0.249505983094968 | 0.211629343945605 | 0.276923605872591 | -0.165287531 | 0.664299497228353 | 1.28339124308454 | 0.847649943376777 | 1.94312887731058 | 0.999714303421225 |
| ebi-fl187-GCST90094780 | ebi-a-GCST90002094 | CD11b on Monocytic Myeloid-Derived Suppressor Cells \|\| id:ebi-a-GCST90002094 | F-healthy breakfast food liking (derived food-liking factor) \|\| id:ebi-fl187-GCST90094780 | Weighted mode | 8 | 0.126798744757345 | 0.276640457820547 | 0.660589852935902 | -0.415416553 | 0.669014042085617 | 1.1351885321468 | 0.660065271545231 | 1.95231147444074 | 0.999714303421225 |
| ebi-fl187-GCST90094780 | ebi-a-GCST90002104 | HLA DR on myeloid Dendritic Cell \|\| id:ebi-a-GCST90002104 | F-healthy breakfast food liking (derived food-liking factor) \|\| id:ebi-fl187-GCST90094780 | Weighted mode | 8 | -0.041323632 | 0.201636418562381 | 0.843449869498398 | -0.436531012 | 0.353883748869419 | 0.959518549253959 | 0.646274455379817 | 1.42458956670559 | 0.999714303421225 |
| ebi-fl187-GCST90094780 | ebi-a-GCST90002105 | HLA DR on plasmacytoid Dendritic Cell \|\| id:ebi-a-GCST90002105 | F-healthy breakfast food liking (derived food-liking factor) \|\| id:ebi-fl187-GCST90094780 | Weighted mode | 8 | 0.26512212247227 | 0.307767917251399 | 0.417527094465696 | -0.338102995 | 0.868347240285011 | 1.30359016371158 | 0.713121835889455 | 2.38296912168737 | 0.999714303421225 |
| ebi-fl187-GCST90094780 | ebi-a-GCST90002106 | HLA DR on Dendritic Cell \|\| id:ebi-a-GCST90002106 | F-healthy breakfast food liking (derived food-liking factor) \|\| id:ebi-fl187-GCST90094780 | Weighted mode | 8 | 0.150835709240528 | 0.24995119001166 | 0.565235638423917 | -0.339068623 | 0.640740041663383 | 1.16280560417223 | 0.712433557953735 | 1.89788487361251 | 0.999714303421225 |
| ebi-fl187-GCST90094780 | ebi-a-GCST90002111 | HLA DR on CD33dim HLA DR+ CD11b- \|\| id:ebi-a-GCST90002111 | F-healthy breakfast food liking (derived food-liking factor) \|\| id:ebi-fl187-GCST90094780 | Weighted mode | 8 | -0.0543822 | 0.28906354554275 | 0.856110555254058 | -0.620946749 | 0.512182349669399 | 0.947070067524962 | 0.537435380325335 | 1.6689294111206 | 0.999714303421225 |
| ebi-fl187-GCST90094780 | ebi-a-GCST90002112 | HLA DR on CD33- HLA DR+ \|\| id:ebi-a-GCST90002112 | F-healthy breakfast food liking (derived food-liking factor) \|\| id:ebi-fl187-GCST90094780 | Weighted mode | 8 | -0.157646461 | 0.305220624833701 | 0.621416813569887 | -0.755878886 | 0.440585963624595 | 0.854151704491859 | 0.469597710585709 | 1.55361731507673 | 0.999714303421225 |
| ebi-fl187-GCST90094780 | ebi-a-GCST90002115 | HLA DR on HLA DR+ CD8+ T cell \|\| id:ebi-a-GCST90002115 | F-healthy breakfast food liking (derived food-liking factor) \|\| id:ebi-fl187-GCST90094780 | Weighted mode | 8 | -0.255576546 | 0.176161124697853 | 0.19012244174104 | -0.60085235 | 0.0896992585142726 | 0.774469851804738 | 0.548344055630122 | 1.09384526958206 | 0.999714303421225 |
| ebi-fl187-GCST90094780 | ebi-a-GCST90002116 | HLA DR on B cell \|\| id:ebi-a-GCST90002116 | F-healthy breakfast food liking (derived food-liking factor) \|\| id:ebi-fl187-GCST90094780 | Weighted mode | 8 | -0.010190369 | 0.171107660229036 | 0.954174389872066 | -0.345561383 | 0.325180645118611 | 0.989861376960102 | 0.707822882193076 | 1.38428068694462 | 0.999714303421225 |
| ebi-fl187-GCST90094780 | ebi-cfmet338-GCST90026023 | Sphingomyelin (d18:1/24:1, d18:2/24:0) levels \|\| id:ebi-cfmet338-GCST90026023 | F-healthy breakfast food liking (derived food-liking factor) \|\| id:ebi-fl187-GCST90094780 | Weighted mode | 6 | -0.07109194 | 0.146404475141388 | 0.647790912216474 | -0.358044711 | 0.215860831770896 | 0.931376257917807 | 0.69904181960046 | 1.2409296689986 | 0.999714303421225 |
| ebi-fl187-GCST90094780 | ebi-cfmet338-GCST90026046 | 1-arachidonoyl-gpc (20:4n6) levels \|\| id:ebi-cfmet338-GCST90026046 | F-healthy breakfast food liking (derived food-liking factor) \|\| id:ebi-fl187-GCST90094780 | Weighted mode | 6 | -0.105640333 | 0.169062574084466 | 0.55946859864199 | -0.437002979 | 0.225722311807499 | 0.899748199264594 | 0.645969507320653 | 1.25322761044513 | 0.999714303421225 |
| ebi-fl187-GCST90094780 | ebi-cfmet338-GCST90026047 | 1-linoleoyl-gpc (18:2) levels \|\| id:ebi-cfmet338-GCST90026047 | F-healthy breakfast food liking (derived food-liking factor) \|\| id:ebi-fl187-GCST90094780 | Weighted mode | 6 | -0.195411844 | 0.152293504336161 | 0.255701559962433 | -0.493907113 | 0.103083424419676 | 0.822495848247134 | 0.610237463842954 | 1.10858388818598 | 0.999714303421225 |
| ebi-fl187-GCST90094780 | ebi-cfmet338-GCST90026050 | 1-myristoyl-2-palmitoyl-gpc (14:0/16:0) levels \|\| id:ebi-cfmet338-GCST90026050 | F-healthy breakfast food liking (derived food-liking factor) \|\| id:ebi-fl187-GCST90094780 | Weighted mode | 6 | 0.115071531585097 | 0.106884748231725 | 0.330841698919249 | -0.094422575 | 0.324565638119279 | 1.12195368982746 | 0.909898180811822 | 1.38342960637019 | 0.999714303421225 |
| ebi-fl187-GCST90094780 | ebi-cfmet338-GCST90026068 | 2,3-dihydroxyisovalerate levels \|\| id:ebi-cfmet338-GCST90026068 | F-healthy breakfast food liking (derived food-liking factor) \|\| id:ebi-fl187-GCST90094780 | Weighted mode | 6 | -0.459519919 | 0.309295088552151 | 0.197496465103131 | -1.065738293 | 0.146698454446036 | 0.631586785477433 | 0.344473438644344 | 1.1580047192015 | 0.999714303421225 |
| ebi-fl187-GCST90094780 | ebi-cfmet338-GCST90026082 | 3-hydroxyhexanoate levels \|\| id:ebi-cfmet338-GCST90026082 | F-healthy breakfast food liking (derived food-liking factor) \|\| id:ebi-fl187-GCST90094780 | Weighted mode | 6 | -0.003857057 | 0.0680015906753885 | 0.956965027188667 | -0.137140175 | 0.129426060647043 | 0.996150371813641 | 0.871848006445898 | 1.13817495243196 | 0.999714303421225 |
| ebi-fl187-GCST90094780 | ebi-cfmet338-GCST90026094 | 3-ureidopropionate levels \|\| id:ebi-cfmet338-GCST90026094 | F-healthy breakfast food liking (derived food-liking factor) \|\| id:ebi-fl187-GCST90094780 | Weighted mode | 6 | -0.135883679 | 0.206178317242478 | 0.538995381397983 | -0.539993181 | 0.268225822746833 | 0.872944168279562 | 0.582752226238967 | 1.30764240207435 | 0.999714303421225 |
| ebi-fl187-GCST90094780 | ebi-cfmet338-GCST90026173 | Glycerol levels \|\| id:ebi-cfmet338-GCST90026173 | F-healthy breakfast food liking (derived food-liking factor) \|\| id:ebi-fl187-GCST90094780 | Weighted mode | 6 | 0.0209029643259476 | 0.0870017252048633 | 0.819669108105478 | -0.149620417 | 0.19142634572748 | 1.02112296147511 | 0.861034748490441 | 1.21097563632564 | 0.999714303421225 |
| ebi-fl187-GCST90094780 | ebi-cfmet338-GCST90026179 | Histidine levels \|\| id:ebi-cfmet338-GCST90026179 | F-healthy breakfast food liking (derived food-liking factor) \|\| id:ebi-fl187-GCST90094780 | Weighted mode | 6 | 0.0552513692661434 | 0.0523074654716255 | 0.339181149854318 | -0.047271263 | 0.157774001590529 | 1.05680622989681 | 0.953828624024511 | 1.17090154291701 | 0.999714303421225 |
| ebi-fl187-GCST90094780 | ebi-cfmet338-GCST90026188 | Isocitrate levels \|\| id:ebi-cfmet338-GCST90026188 | F-healthy breakfast food liking (derived food-liking factor) \|\| id:ebi-fl187-GCST90094780 | Weighted mode | 6 | -0.162010514 | 0.121336679603343 | 0.239366229222462 | -0.399830406 | 0.0758093783092697 | 0.850432263292248 | 0.670433738111092 | 1.07875692008885 | 0.999714303421225 |
| ebi-fl187-GCST90094780 | ebi-cfmet338-GCST90026207 | N-acetyl-isoputreanine levels \|\| id:ebi-cfmet338-GCST90026207 | F-healthy breakfast food liking (derived food-liking factor) \|\| id:ebi-fl187-GCST90094780 | Weighted mode | 6 | 0.0118048585472417 | 0.0694303720981756 | 0.871656369612528 | -0.124278671 | 0.147888387859666 | 1.01187481087803 | 0.883133703043848 | 1.15938348786878 | 0.999714303421225 |
| ebi-fl187-GCST90094780 | ebi-cfmet338-GCST90026213 | N-acetylglutamate levels \|\| id:ebi-cfmet338-GCST90026213 | F-healthy breakfast food liking (derived food-liking factor) \|\| id:ebi-fl187-GCST90094780 | Weighted mode | 6 | 0.0257746936168847 | 0.110534811823511 | 0.824865748849957 | -0.190873538 | 0.242422924790965 | 1.0261097333546 | 0.826237069502716 | 1.27433302589383 | 0.999714303421225 |
| ebi-fl187-GCST90094780 | ebi-cfmet338-GCST90026224 | N-acetyltaurine levels \|\| id:ebi-cfmet338-GCST90026224 | F-healthy breakfast food liking (derived food-liking factor) \|\| id:ebi-fl187-GCST90094780 | Weighted mode | 6 | -0.01867154 | 0.090611265579495 | 0.844870136699797 | -0.196269621 | 0.158926540489168 | 0.981501693303889 | 0.821790633136201 | 1.17225183047169 | 0.999714303421225 |
| ebi-fl187-GCST90094780 | ebi-cfmet338-GCST90026250 | Phenyllactate (pla) levels \|\| id:ebi-cfmet338-GCST90026250 | F-healthy breakfast food liking (derived food-liking factor) \|\| id:ebi-fl187-GCST90094780 | Weighted mode | 6 | 0.0804074845779944 | 0.132058429117728 | 0.569205227453175 | -0.178427036 | 0.339242005648741 | 1.08372858039728 | 0.836585094833224 | 1.40388305173431 | 0.999714303421225 |
| ebi-fl187-GCST90094780 | ebi-cfmet338-GCST90026251 | Pipecolate levels \|\| id:ebi-cfmet338-GCST90026251 | F-healthy breakfast food liking (derived food-liking factor) \|\| id:ebi-fl187-GCST90094780 | Weighted mode | 6 | 0.0643067633952292 | 0.205053006436821 | 0.766478184063915 | -0.337597129 | 0.466210656011397 | 1.06641948705217 | 0.713482671324846 | 1.5939427376041 | 0.999714303421225 |
| ebi-fl187-GCST90094780 | ebi-cfmet338-GCST90026278 | Trigonelline (n'-methylnicotinate) levels \|\| id:ebi-cfmet338-GCST90026278 | F-healthy breakfast food liking (derived food-liking factor) \|\| id:ebi-fl187-GCST90094780 | Weighted mode | 6 | -0.010423957 | 0.238634898231873 | 0.966848985161939 | -0.478148358 | 0.457300443318822 | 0.989630183941365 | 0.619930218706839 | 1.57980345434807 | 0.999714303421225 |
| ebi-fl187-GCST90094780 | ebi-cfmet338-GCST90026299 | 1-carboxyethylvaline levels \|\| id:ebi-cfmet338-GCST90026299 | F-healthy breakfast food liking (derived food-liking factor) \|\| id:ebi-fl187-GCST90094780 | Weighted mode | 6 | -0.220049096 | 0.148713001027579 | 0.199025838239844 | -0.511526578 | 0.0714283855883809 | 0.802479398125166 | 0.599579574562558 | 1.07404123111625 | 0.999714303421225 |
| ebi-fl187-GCST90094780 | ebi-gm430-GCST90011306 | C_Erysipelotrichia abundance \|\| id:ebi-gm430-GCST90011306 | F-healthy breakfast food liking (derived food-liking factor) \|\| id:ebi-fl187-GCST90094780 | Weighted mode | 6 | -0.090593861 | 0.106961608731603 | 0.435662505073857 | -0.300238614 | 0.119050892280795 | 0.913388598462577 | 0.740641472210168 | 1.1264272432812 | 0.999714303421225 |
| ebi-fl187-GCST90094780 | ebi-gm430-GCST90011312 | F_Erysipelotrichaceae abundance \|\| id:ebi-gm430-GCST90011312 | F-healthy breakfast food liking (derived food-liking factor) \|\| id:ebi-fl187-GCST90094780 | Weighted mode | 6 | -0.090593861 | 0.106026360907829 | 0.431881346637261 | -0.298405528 | 0.117217806546197 | 0.913388598462577 | 0.742000376640975 | 1.12436429692691 | 0.999714303421225 |
| ebi-fl187-GCST90094780 | ebi-gm430-GCST90011352 | O_Erysipelotrichales abundance \|\| id:ebi-gm430-GCST90011352 | F-healthy breakfast food liking (derived food-liking factor) \|\| id:ebi-fl187-GCST90094780 | Weighted mode | 6 | -0.090593861 | 0.108088901819078 | 0.440165627097328 | -0.302448108 | 0.121260386732246 | 0.913388598462577 | 0.739006835512169 | 1.12891882958462 | 0.999714303421225 |
| ebi-fl187-GCST90094780 | ebi-gm430-GCST90011362 | OTU97_11 (Parabacteroides) abundance \|\| id:ebi-gm430-GCST90011362 | F-healthy breakfast food liking (derived food-liking factor) \|\| id:ebi-fl187-GCST90094780 | Weighted mode | 6 | 0.0123170875381186 | 0.152839115474607 | 0.938895418921746 | -0.287247579 | 0.311881753868348 | 1.01239325526109 | 0.750325941049284 | 1.3659931600723 | 0.999714303421225 |
| ebi-fl187-GCST90094780 | ebi-gm430-GCST90011384 | OTU97_235 (Phascolarctobacterium) abundance \|\| id:ebi-gm430-GCST90011384 | F-healthy breakfast food liking (derived food-liking factor) \|\| id:ebi-fl187-GCST90094780 | Weighted mode | 6 | 0.304859578650033 | 0.482004414234153 | 0.554861752705216 | -0.639869073 | 1.24958823054897 | 1.35643451726635 | 0.527361465246576 | 3.48890603671868 | 0.999714303421225 |
| ebi-fl187-GCST90094780 | ebi-gm430-GCST90011390 | OTU97_3 (Bacteroides) abundance \|\| id:ebi-gm430-GCST90011390 | F-healthy breakfast food liking (derived food-liking factor) \|\| id:ebi-fl187-GCST90094780 | Weighted mode | 6 | -0.196585302 | 0.119389416565438 | 0.160560179870576 | -0.430588559 | 0.037417954146363 | 0.821531249783123 | 0.650126344524089 | 1.03812681958655 | 0.999714303421225 |
| ebi-fl187-GCST90094780 | ebi-gm430-GCST90011398 | OTU97_4 (Alistipes) abundance \|\| id:ebi-gm430-GCST90011398 | F-healthy breakfast food liking (derived food-liking factor) \|\| id:ebi-fl187-GCST90094780 | Weighted mode | 6 | -0.084846423 | 0.15295113634923 | 0.602978992320636 | -0.38463065 | 0.214937804085993 | 0.918653357449477 | 0.680702006791811 | 1.23978478502019 | 0.999714303421225 |
| ebi-fl187-GCST90094780 | ebi-gm430-GCST90011421 | OTU99_108 (Cytophagales) abundance \|\| id:ebi-gm430-GCST90011421 | F-healthy breakfast food liking (derived food-liking factor) \|\| id:ebi-fl187-GCST90094780 | Weighted mode | 6 | -0.017922235 | 0.707257202215334 | 0.980763640857323 | -1.404146352 | 1.36830188112168 | 0.982237412864357 | 0.245576603084151 | 3.92867367295517 | 0.999714303421225 |
| ebi-fl187-GCST90094780 | ebi-gm430-GCST90011428 | OTU99_123 (Phascolarctobacterium) abundance \|\| id:ebi-gm430-GCST90011428 | F-healthy breakfast food liking (derived food-liking factor) \|\| id:ebi-fl187-GCST90094780 | Weighted mode | 6 | -0.011294742 | 0.250636219169059 | 0.965800481647036 | -0.502541731 | 0.479952247719625 | 0.988768804274307 | 0.60499097923182 | 1.61599723279746 | 0.999714303421225 |
| ebi-fl187-GCST90094780 | ebi-gm430-GCST90011450 | OTU99_298 (Phascolarctobacterium) abundance \|\| id:ebi-gm430-GCST90011450 | F-healthy breakfast food liking (derived food-liking factor) \|\| id:ebi-fl187-GCST90094780 | Weighted mode | 6 | 0.31205342461203 | 0.514209651599878 | 0.570442163804222 | -0.695797493 | 1.31990434174779 | 1.36622768126359 | 0.498676598506161 | 3.74306330524118 | 0.999714303421225 |
| ebi-fl187-GCST90094780 | ebi-gm430-GCST90011451 | OTU99_3 (Bacteroides) abundance \|\| id:ebi-gm430-GCST90011451 | F-healthy breakfast food liking (derived food-liking factor) \|\| id:ebi-fl187-GCST90094780 | Weighted mode | 6 | -0.192474953 | 0.120572662893103 | 0.171301000549544 | -0.428797372 | 0.0438474662279456 | 0.824914979555255 | 0.651291885579898 | 1.04482297194407 | 0.999714303421225 |
| ebi-fl187-GCST90094780 | ebi-gm430-GCST90011472 | OTU99_72 (Parabacteroides) abundance \|\| id:ebi-gm430-GCST90011472 | F-healthy breakfast food liking (derived food-liking factor) \|\| id:ebi-fl187-GCST90094780 | Weighted mode | 6 | 0.153020360097322 | 0.35067831334325 | 0.680777488955594 | -0.534309134 | 0.840349854250091 | 1.16534870531426 | 0.58607404891305 | 2.31717750938856 | 0.999714303421225 |
| ebi-fl187-GCST90094780 | ebi-gm430-GCST90011507 | TestASV_3 (Bacteroides) abundance \|\| id:ebi-gm430-GCST90011507 | F-healthy breakfast food liking (derived food-liking factor) \|\| id:ebi-fl187-GCST90094780 | Weighted mode | 6 | 0.00229653475785296 | 0.181059488457856 | 0.990370547100117 | -0.352580063 | 0.357173132135251 | 1.00229917381364 | 0.7028722937609 | 1.4292833033041 | 0.999714303421225 |
| ebi-fl187-GCST90094780 | ebi-gm430-GCST90011512 | TestASV_34 (Phascolarctobacterium) abundance \|\| id:ebi-gm430-GCST90011512 | F-healthy breakfast food liking (derived food-liking factor) \|\| id:ebi-fl187-GCST90094780 | Weighted mode | 6 | -0.464440753 | 0.301928052284871 | 0.184601464703623 | -1.056219736 | 0.127338229172146 | 0.628486485915574 | 0.347767983450006 | 1.13580111389204 | 0.999714303421225 |
| ebi-fl187-GCST90094780 | ebi-gm430-GCST90011519 | TestASV_41 (Barnesiella) abundance \|\| id:ebi-gm430-GCST90011519 | F-healthy breakfast food liking (derived food-liking factor) \|\| id:ebi-fl187-GCST90094780 | Weighted mode | 6 | 0.0549200626011519 | 0.466015282115236 | 0.910774148586639 | -0.85846989 | 0.968310015547014 | 1.05645616094253 | 0.423810062319461 | 2.63349013915609 | 0.999714303421225 |
| ebi-fl187-GCST90094780 | ebi-gm430-GCST90011524 | TestASV_46 (Sutterella) abundance \|\| id:ebi-gm430-GCST90011524 | F-healthy breakfast food liking (derived food-liking factor) \|\| id:ebi-fl187-GCST90094780 | Weighted mode | 6 | 0.855363097156582 | 0.48400185127846 | 0.137429662310885 | -0.093280531 | 1.80400672566236 | 2.35222831294738 | 0.910937917805248 | 6.07393536714559 | 0.999714303421225 |
| ebi-fl187-GCST90094780 | ebi-gm430-GCST90011581 | OTU97_19 (Bacteria) prevalence \|\| id:ebi-gm430-GCST90011581 | F-healthy breakfast food liking (derived food-liking factor) \|\| id:ebi-fl187-GCST90094780 | Weighted mode | 6 | 0.0270753058231055 | 0.542983399676511 | 0.962161424400369 | -1.037172158 | 1.09132276918907 | 1.02744517245467 | 0.354455610682461 | 2.97821095388471 | 0.999714303421225 |
| ebi-fl187-GCST90094780 | ebi-gm430-GCST90011614 | OTU97_80 (Ruminococcus) prevalence \|\| id:ebi-gm430-GCST90011614 | F-healthy breakfast food liking (derived food-liking factor) \|\| id:ebi-fl187-GCST90094780 | Weighted mode | 6 | -0.001802147 | 0.251281887197186 | 0.994555118700772 | -0.494314646 | 0.490710351895197 | 0.998199475880594 | 0.609988822399584 | 1.63347615080655 | 0.999714303421225 |
| ebi-fl187-GCST90094780 | ebi-gm430-GCST90011623 | OTU99_11 (Parabacteroides) prevalence \|\| id:ebi-gm430-GCST90011623 | F-healthy breakfast food liking (derived food-liking factor) \|\| id:ebi-fl187-GCST90094780 | Weighted mode | 6 | -0.182814399 | 0.235577369596987 | 0.472824454022551 | -0.644546044 | 0.278917245015041 | 0.83292273235489 | 0.52490076998621 | 1.32169796224868 | 0.999714303421225 |
| ebi-fl187-GCST90094780 | ebi-gm430-GCST90011642 | OTU99_19 (Bacteria) prevalence \|\| id:ebi-gm430-GCST90011642 | F-healthy breakfast food liking (derived food-liking factor) \|\| id:ebi-fl187-GCST90094780 | Weighted mode | 6 | 0.0270753058231055 | 0.561968894892607 | 0.96343855177632 | -1.074383728 | 1.12853433981261 | 1.02744517245467 | 0.341508152318674 | 3.09112264299722 | 0.999714303421225 |
| ebi-fl187-GCST90094780 | ebi-gm430-GCST90011653 | OTU99_30 (Parasutterella) prevalence \|\| id:ebi-gm430-GCST90011653 | F-healthy breakfast food liking (derived food-liking factor) \|\| id:ebi-fl187-GCST90094780 | Weighted mode | 6 | -0.162493378 | 0.351956619291959 | 0.663697329897019 | -0.852328351 | 0.527341596134778 | 0.85002171932475 | 0.426420917411882 | 1.69442185835809 | 0.999714303421225 |
| ebi-fl187-GCST90094780 | ebi-gm430-GCST90011675 | OTU99_8 (Ruminococcaceae) prevalence \|\| id:ebi-gm430-GCST90011675 | F-healthy breakfast food liking (derived food-liking factor) \|\| id:ebi-fl187-GCST90094780 | Weighted mode | 6 | 0.210204622097654 | 0.26582785657814 | 0.46492385815895 | -0.310817977 | 0.731227220990809 | 1.23393052357814 | 0.732847258936038 | 2.07762875340264 | 0.999714303421225 |
| ebi-fl187-GCST90094780 | ebi-gm430-GCST90011679 | OTU99_92 (Ruminococcus) prevalence \|\| id:ebi-gm430-GCST90011679 | F-healthy breakfast food liking (derived food-liking factor) \|\| id:ebi-fl187-GCST90094780 | Weighted mode | 6 | 0.0117087547200527 | 0.241721562946678 | 0.963241698615254 | -0.462065509 | 0.485483018095542 | 1.01177757050873 | 0.629981069377332 | 1.62495970425961 | 0.999714303421225 |
| ebi-fl187-GCST90094780 | ebi-gm430-GCST90011684 | TestASV_11 (Lachnospiraceae) prevalence \|\| id:ebi-gm430-GCST90011684 | F-healthy breakfast food liking (derived food-liking factor) \|\| id:ebi-fl187-GCST90094780 | Weighted mode | 6 | -0.058661079 | 0.266798787720789 | 0.834667115808499 | -0.581586703 | 0.464264544709305 | 0.943026326216573 | 0.559010678494998 | 1.59084376407934 | 0.999714303421225 |
| ebi-fl187-GCST90094780 | ebi-gm430-GCST90011688 | TestASV_15 (Bacteroides) prevalence \|\| id:ebi-gm430-GCST90011688 | F-healthy breakfast food liking (derived food-liking factor) \|\| id:ebi-fl187-GCST90094780 | Weighted mode | 6 | -0.037664531 | 0.300101599235914 | 0.905013268581356 | -0.625863666 | 0.550534603038115 | 0.963035954969303 | 0.534799341013258 | 1.73417986792292 | 0.999714303421225 |
| ebi-fl187-GCST90094780 | ebi-gm430-GCST90011692 | TestASV_19 (Prevotella) prevalence \|\| id:ebi-gm430-GCST90011692 | F-healthy breakfast food liking (derived food-liking factor) \|\| id:ebi-fl187-GCST90094780 | Weighted mode | 6 | 0.25244683324863 | 0.496454282253377 | 0.632738064667795 | -0.72060356 | 1.22549722646525 | 1.28717105963184 | 0.486458560424296 | 3.4058591451421 | 0.999714303421225 |
| ebi-fl187-GCST90094780 | ebi-gm430-GCST90011693 | TestASV_2 (Bacteroides) prevalence \|\| id:ebi-gm430-GCST90011693 | F-healthy breakfast food liking (derived food-liking factor) \|\| id:ebi-fl187-GCST90094780 | Weighted mode | 6 | -0.296906111 | 0.4048160394152 | 0.496219432222696 | -1.090345548 | 0.496533326347344 | 0.743113779365859 | 0.336100334786297 | 1.64301558769505 | 0.999714303421225 |
| ebi-fl187-GCST90094780 | ebi-gm430-GCST90011716 | TestASV_40 (Barnesiella) prevalence \|\| id:ebi-gm430-GCST90011716 | F-healthy breakfast food liking (derived food-liking factor) \|\| id:ebi-fl187-GCST90094780 | Weighted mode | 6 | 0.128811122082016 | 0.617644149424241 | 0.843027389070099 | -1.081771411 | 1.33939365495353 | 1.13747525991596 | 0.338994494960031 | 3.81672854915664 | 0.999714303421225 |
| ebi-fl187-GCST90094780 | ebi-gm430-GCST90011718 | TestASV_42 (Alphaproteobacteria) prevalence \|\| id:ebi-gm430-GCST90011718 | F-healthy breakfast food liking (derived food-liking factor) \|\| id:ebi-fl187-GCST90094780 | Weighted mode | 6 | 0.285478839130385 | 0.430073565522005 | 0.536202214093615 | -0.557465349 | 1.12842302755352 | 1.33039892268749 | 0.572658715710947 | 3.09077858230212 | 0.999714303421225 |
| ebi-fl187-GCST90094780 | ebi-hbsm44-GCST90104929 | Sulcal depth of the right inferior frontal sulcus \|\| id:ebi-hbsm44-GCST90104929 | F-healthy breakfast food liking (derived food-liking factor) \|\| id:ebi-fl187-GCST90094780 | Weighted mode | 7 | -0.120232974 | 0.251314112869361 | 0.649287099796917 | -0.612808636 | 0.372342686754214 | 0.886713830966585 | 0.541826935518303 | 1.45113017918777 | 0.999714303421225 |
| ebi-fl187-GCST90094780 | ebi-hbsm44-GCST90104935 | Sulcal opening distance of the left calloso-marginal posterior fissure \|\| id:ebi-hbsm44-GCST90104935 | F-healthy breakfast food liking (derived food-liking factor) \|\| id:ebi-fl187-GCST90094780 | Weighted mode | 6 | 0.114598022592582 | 0.0711207612120267 | 0.168027581398032 | -0.024798669 | 0.253994714568154 | 1.12142256042334 | 0.975506291542638 | 1.2891649904561 | 0.999714303421225 |
| ebi-fl187-GCST90094780 | ebi-hbsm44-GCST90104936 | Sulcal opening distance of the left anterior inferior temporal sulcus \|\| id:ebi-hbsm44-GCST90104936 | F-healthy breakfast food liking (derived food-liking factor) \|\| id:ebi-fl187-GCST90094780 | Weighted mode | 7 | 0.0272408872265339 | 0.027255820664287 | 0.356162269629204 | -0.026180521 | 0.0806622957285365 | 1.02761531235388 | 0.974159217270466 | 1.08400476170927 | 0.999714303421225 |
| ebi-fl187-GCST90094780 | ebi-hbsm44-GCST90104941 | Sulcal opening distance of the right posterior inferior temporal sulcus \|\| id:ebi-hbsm44-GCST90104941 | F-healthy breakfast food liking (derived food-liking factor) \|\| id:ebi-fl187-GCST90094780 | Weighted mode | 6 | -0.016692951 | 0.0237372196649858 | 0.513293285434552 | -0.063217902 | 0.029831999524582 | 0.983445604251343 | 0.938738898728845 | 1.03028143164296 | 0.999714303421225 |
| ebi-fl187-GCST90094780 | ebi-IPs91-GCST90274819 | Leukemia inhibitory factor levels \|\| id:ebi-IPs91-GCST90274819 | F-healthy breakfast food liking (derived food-liking factor) \|\| id:ebi-fl187-GCST90094780 | Weighted mode | 9 | 0.173461264280844 | 0.103642842418213 | 0.1327361038525 | -0.029678707 | 0.376601235420542 | 1.18941461304685 | 0.970757381136857 | 1.45732306467001 | 0.999714303421225 |
| ebi-fl187-GCST90094780 | ebi-IPs91-GCST90274837 | STAM binding protein levels \|\| id:ebi-IPs91-GCST90274837 | F-healthy breakfast food liking (derived food-liking factor) \|\| id:ebi-fl187-GCST90094780 | Weighted mode | 9 | 0.0700558114261035 | 0.11159706803224 | 0.547659619977281 | -0.148674442 | 0.288786064769295 | 1.07256804113574 | 0.861849651351279 | 1.3348061359218 | 0.999714303421225 |
| ebi-fl187-GCST90094780 | ebi-IPs91-GCST90274840 | TNF-beta levels \|\| id:ebi-IPs91-GCST90274840 | F-healthy breakfast food liking (derived food-liking factor) \|\| id:ebi-fl187-GCST90094780 | Weighted mode | 8 | -0.072589073 | 0.0941054786714211 | 0.465730269621337 | -0.257035811 | 0.111857664918568 | 0.929982906345494 | 0.773340518806133 | 1.11835366835554 | 0.999714303421225 |
| ebi-fl187-GCST90094780 | ebi-IPs91-GCST90274842 | Tumor necrosis factor ligand superfamily member 14 levels \|\| id:ebi-IPs91-GCST90274842 | F-healthy breakfast food liking (derived food-liking factor) \|\| id:ebi-fl187-GCST90094780 | Weighted mode | 9 | 0.0644618289779799 | 0.125967443351395 | 0.622659932095739 | -0.18243436 | 0.311358017946713 | 1.06658486483326 | 0.833239335971035 | 1.36527792769846 | 0.999714303421225 |
| ebi-fl187-GCST90094780 | ebi-met1400-GCST90199628 | 3-methylhistidine levels \|\| id:ebi-met1400-GCST90199628 | F-healthy breakfast food liking (derived food-liking factor) \|\| id:ebi-fl187-GCST90094780 | Weighted mode | 8 | 0.0755757412095306 | 0.140712967009023 | 0.607845443662064 | -0.200221674 | 0.351373156547215 | 1.07850491189109 | 0.818549281766591 | 1.42101748896885 | 0.999714303421225 |
| ebi-fl187-GCST90094780 | ebi-met1400-GCST90199651 | Docosahexaenoate DHA; 22:6n3 levels \|\| id:ebi-met1400-GCST90199651 | F-healthy breakfast food liking (derived food-liking factor) \|\| id:ebi-fl187-GCST90094780 | Weighted mode | 8 | 0.0258986319274148 | 0.0983680860827985 | 0.799914126821789 | -0.166902817 | 0.2187000806497 | 1.02623691554258 | 0.846281851723725 | 1.24445798368149 | 0.999714303421225 |
| ebi-fl187-GCST90094780 | ebi-met1400-GCST90199652 | 1-stearoyl-2-oleoyl-GPS (18:0/18:1) levels \|\| id:ebi-met1400-GCST90199652 | F-healthy breakfast food liking (derived food-liking factor) \|\| id:ebi-fl187-GCST90094780 | Weighted mode | 8 | 0.057475348281292 | 0.152566528270762 | 0.717539158298192 | -0.241555047 | 0.356505743691985 | 1.05915916023922 | 0.785405568277052 | 1.42832973438115 | 0.999714303421225 |
| ebi-fl187-GCST90094780 | ebi-met1400-GCST90199653 | 1-myristoyl-2-palmitoyl-gpc (14:0/16:0) levels \|\| id:ebi-met1400-GCST90199653 | F-healthy breakfast food liking (derived food-liking factor) \|\| id:ebi-fl187-GCST90094780 | Weighted mode | 8 | 0.184837926342976 | 0.134086337701208 | 0.210491685760968 | -0.077971296 | 0.447647148237344 | 1.20302344591704 | 0.924990977518448 | 1.56462651701622 | 0.999714303421225 |
| ebi-fl187-GCST90094780 | ebi-met1400-GCST90199667 | Erythritol levels in elite athletes \|\| id:ebi-met1400-GCST90199667 | F-healthy breakfast food liking (derived food-liking factor) \|\| id:ebi-fl187-GCST90094780 | Weighted mode | 8 | 0.0866836918123248 | 0.111515284318699 | 0.462423021175103 | -0.131886265 | 0.305253649076974 | 1.0905516747334 | 0.876440670994248 | 1.3569691533309 | 0.999714303421225 |
| ebi-fl187-GCST90094780 | ebi-met1400-GCST90199674 | 3-hydroxymyristate levels \|\| id:ebi-met1400-GCST90199674 | F-healthy breakfast food liking (derived food-liking factor) \|\| id:ebi-fl187-GCST90094780 | Weighted mode | 8 | 0.013365978758816 | 0.11126060942877 | 0.907753769687086 | -0.204704816 | 0.231436773239205 | 1.01345570275694 | 0.814887822986815 | 1.2604096324399 | 0.999714303421225 |
| ebi-fl187-GCST90094780 | ebi-met1400-GCST90199685 | 2-linoleoylglycerol (18:2) levels \|\| id:ebi-met1400-GCST90199685 | F-healthy breakfast food liking (derived food-liking factor) \|\| id:ebi-fl187-GCST90094780 | Weighted mode | 7 | 0.0841304624689799 | 0.127392309149709 | 0.533512700840127 | -0.165558463 | 0.333819388402409 | 1.08777079781602 | 0.847420318628991 | 1.39629093446287 | 0.999714303421225 |
| ebi-fl187-GCST90094780 | ebi-met1400-GCST90199703 | Gamma-glutamylvaline levels \|\| id:ebi-met1400-GCST90199703 | F-healthy breakfast food liking (derived food-liking factor) \|\| id:ebi-fl187-GCST90094780 | Weighted mode | 8 | 0.101762450690443 | 0.105773687478656 | 0.368058626491309 | -0.105553977 | 0.309078878148608 | 1.10712044479098 | 0.899825901842247 | 1.36216981169882 | 0.999714303421225 |
| ebi-fl187-GCST90094780 | ebi-met1400-GCST90199779 | Gamma-glutamyl-2-aminobutyrate levels \|\| id:ebi-met1400-GCST90199779 | F-healthy breakfast food liking (derived food-liking factor) \|\| id:ebi-fl187-GCST90094780 | Weighted mode | 8 | -0.051002179 | 0.124631611142813 | 0.694607058627762 | -0.295280137 | 0.193275779030711 | 0.95027660005877 | 0.744323046093239 | 1.21321732728686 | 0.999714303421225 |
| ebi-fl187-GCST90094780 | ebi-met1400-GCST90199824 | 4-vinylphenol sulfate levels \|\| id:ebi-met1400-GCST90199824 | F-healthy breakfast food liking (derived food-liking factor) \|\| id:ebi-fl187-GCST90094780 | Weighted mode | 8 | 0.0495729017754766 | 0.11238890808442 | 0.672454454240508 | -0.170709358 | 0.269855161620939 | 1.05082219622632 | 0.843066568361561 | 1.30977473134523 | 0.999714303421225 |
| ebi-fl187-GCST90094780 | ebi-met1400-GCST90199830 | 4-ethylphenylsulfate levels \|\| id:ebi-met1400-GCST90199830 | F-healthy breakfast food liking (derived food-liking factor) \|\| id:ebi-fl187-GCST90094780 | Weighted mode | 8 | -0.001293306 | 0.109260362403996 | 0.990886014896637 | -0.215443617 | 0.212857003812328 | 0.998707529460924 | 0.806183724185705 | 1.23720772260615 | 0.999714303421225 |
| ebi-fl187-GCST90094780 | ebi-met1400-GCST90199833 | Hexanoylglutamine levels \|\| id:ebi-met1400-GCST90199833 | F-healthy breakfast food liking (derived food-liking factor) \|\| id:ebi-fl187-GCST90094780 | Weighted mode | 8 | 0.0543028446693205 | 0.116481255452422 | 0.655236180166843 | -0.174000416 | 0.282606105356068 | 1.05580429844711 | 0.840296548080351 | 1.32658252514063 | 0.999714303421225 |
| ebi-fl187-GCST90094780 | ebi-met1400-GCST90199837 | Carotenoid levels (cryptoxanthin) \|\| id:ebi-met1400-GCST90199837 | F-healthy breakfast food liking (derived food-liking factor) \|\| id:ebi-fl187-GCST90094780 | Weighted mode | 8 | 0.108162336367291 | 0.171960246134862 | 0.549319122977218 | -0.228879746 | 0.445204418791621 | 1.11422861053054 | 0.795424180648056 | 1.56080922195919 | 0.999714303421225 |
| ebi-fl187-GCST90094780 | ebi-met1400-GCST90199869 | 2-hydroxyhippurate levels \|\| id:ebi-met1400-GCST90199869 | F-healthy breakfast food liking (derived food-liking factor) \|\| id:ebi-fl187-GCST90094780 | Weighted mode | 8 | 0.11177886583054 | 0.145877021257332 | 0.468572233142867 | -0.174140096 | 0.397697827494912 | 1.11826554657838 | 0.840179183809691 | 1.48839421014208 | 0.999714303421225 |
| ebi-fl187-GCST90094780 | ebi-met1400-GCST90199902 | Hydantoin-5-propionate levels \|\| id:ebi-met1400-GCST90199902 | F-healthy breakfast food liking (derived food-liking factor) \|\| id:ebi-fl187-GCST90094780 | Weighted mode | 8 | 0.0291776076644368 | 0.108221205082592 | 0.795234473956193 | -0.182935954 | 0.241291169626317 | 1.02960744441036 | 0.832821492666674 | 1.2728916087298 | 0.999714303421225 |
| ebi-fl187-GCST90094780 | ebi-met1400-GCST90199904 | Pantoate levels \|\| id:ebi-met1400-GCST90199904 | F-healthy breakfast food liking (derived food-liking factor) \|\| id:ebi-fl187-GCST90094780 | Weighted mode | 8 | 0.070701395422382 | 0.0985898381981076 | 0.496521697616976 | -0.122534687 | 0.263937478290673 | 1.07326069745788 | 0.884675217287502 | 1.30204678756523 | 0.999714303421225 |
| ebi-fl187-GCST90094780 | ebi-met1400-GCST90199936 | 3-methyl catechol sulfate (2) levels \|\| id:ebi-met1400-GCST90199936 | F-healthy breakfast food liking (derived food-liking factor) \|\| id:ebi-fl187-GCST90094780 | Weighted mode | 8 | -0.067708963 | 0.137263438128054 | 0.63691380560641 | -0.336745301 | 0.201327376204278 | 0.934532417955923 | 0.714090694744481 | 1.22302509560505 | 0.999714303421225 |
| ebi-fl187-GCST90094780 | ebi-met1400-GCST90199977 | 4-hydroxychlorothalonil levels \|\| id:ebi-met1400-GCST90199977 | F-healthy breakfast food liking (derived food-liking factor) \|\| id:ebi-fl187-GCST90094780 | Weighted mode | 8 | -0.033058987 | 0.126555882196489 | 0.801441233503932 | -0.281108516 | 0.21499054230304 | 0.96748148926673 | 0.75494640734076 | 1.23985017078342 | 0.999714303421225 |
| ebi-fl187-GCST90094780 | ebi-met1400-GCST90200040 | Sphingomyelin (d18:2/24:1, d18:1/24:2) levels \|\| id:ebi-met1400-GCST90200040 | F-healthy breakfast food liking (derived food-liking factor) \|\| id:ebi-fl187-GCST90094780 | Weighted mode | 8 | -0.039245311 | 0.0966743965707498 | 0.696899377737941 | -0.228727128 | 0.15023650635235 | 0.961514810124505 | 0.795545585842039 | 1.16210905640338 | 0.999714303421225 |
| ebi-fl187-GCST90094780 | ebi-met1400-GCST90200077 | 1-stearoyl-2-oleoyl-GPI (18:0/18:1) levels \|\| id:ebi-met1400-GCST90200077 | F-healthy breakfast food liking (derived food-liking factor) \|\| id:ebi-fl187-GCST90094780 | Weighted mode | 7 | -0.034367244 | 0.115579130295838 | 0.776226909556009 | -0.260902339 | 0.192167851577288 | 0.966216602414513 | 0.770356149552923 | 1.21187391484218 | 0.999714303421225 |
| ebi-fl187-GCST90094780 | ebi-met1400-GCST90200080 | 1-palmitoyl-2-oleoyl-GPI (16:0/18:1) levels \|\| id:ebi-met1400-GCST90200080 | F-healthy breakfast food liking (derived food-liking factor) \|\| id:ebi-fl187-GCST90094780 | Weighted mode | 8 | 0.0402824667963077 | 0.127177259545776 | 0.760675961373407 | -0.208984962 | 0.289549895506029 | 1.04110481020309 | 0.811407437566967 | 1.33582609136307 | 0.999714303421225 |
| ebi-fl187-GCST90094780 | ebi-met1400-GCST90200082 | 1-oleoyl-2-linoleoyl-GPE (18:1/18:2) levels \|\| id:ebi-met1400-GCST90200082 | F-healthy breakfast food liking (derived food-liking factor) \|\| id:ebi-fl187-GCST90094780 | Weighted mode | 8 | 0.11663985305663 | 0.12249792830433 | 0.372719009262657 | -0.123456086 | 0.356735792533116 | 1.12371465440762 | 0.883860453868749 | 1.42865835777957 | 0.999714303421225 |
| ebi-fl187-GCST90094780 | ebi-met1400-GCST90200100 | Perfluorooctanesulfonate (PFOS) levels \|\| id:ebi-met1400-GCST90200100 | F-healthy breakfast food liking (derived food-liking factor) \|\| id:ebi-fl187-GCST90094780 | Weighted mode | 8 | -0.050537756 | 0.113249194553887 | 0.668892240342232 | -0.272506178 | 0.171430664986164 | 0.95071803236192 | 0.761468725061929 | 1.18700183909012 | 0.999714303421225 |
| ebi-fl187-GCST90094780 | ebi-met1400-GCST90200102 | 1-palmitoleoyl-2-linolenoyl-GPC (16:1/18:3) levels \|\| id:ebi-met1400-GCST90200102 | F-healthy breakfast food liking (derived food-liking factor) \|\| id:ebi-fl187-GCST90094780 | Weighted mode | 8 | 0.00432190092373475 | 0.11488418952246 | 0.971041332744425 | -0.220851111 | 0.229494912387756 | 1.00433125380676 | 0.801836056340264 | 1.25796446717159 | 0.999714303421225 |
| ebi-fl187-GCST90094780 | ebi-met1400-GCST90200114 | Glycosyl-N-tricosanoyl-sphingadienine (d18:2/23:0) levels \|\| id:ebi-met1400-GCST90200114 | F-healthy breakfast food liking (derived food-liking factor) \|\| id:ebi-fl187-GCST90094780 | Weighted mode | 8 | -0.046615207 | 0.112571640412392 | 0.691199235822778 | -0.267255622 | 0.174025208126941 | 0.954454594307556 | 0.765477373375195 | 1.19008556527025 | 0.999714303421225 |
| ebi-fl187-GCST90094780 | ebi-met1400-GCST90200135 | Arachidoylcarnitine (C20) levels \|\| id:ebi-met1400-GCST90200135 | F-healthy breakfast food liking (derived food-liking factor) \|\| id:ebi-fl187-GCST90094780 | Weighted mode | 8 | -0.03795114 | 0.150087178634982 | 0.807640748681524 | -0.33212201 | 0.256219730469325 | 0.962759980526573 | 0.717399787724348 | 1.29203659655902 | 0.999714303421225 |
| ebi-fl187-GCST90094780 | ebi-met1400-GCST90200146 | Docosahexaenoylcarnitine (C22:6) levels \|\| id:ebi-met1400-GCST90200146 | F-healthy breakfast food liking (derived food-liking factor) \|\| id:ebi-fl187-GCST90094780 | Weighted mode | 8 | 0.11442903102893 | 0.151146692447571 | 0.47371357006382 | -0.181818486 | 0.410676548226169 | 1.12123306548331 | 0.833752664322412 | 1.50783756493875 | 0.999714303421225 |
| ebi-fl187-GCST90094780 | ebi-met1400-GCST90200162 | 3-hydroxyphenylacetoylglutamine levels \|\| id:ebi-met1400-GCST90200162 | F-healthy breakfast food liking (derived food-liking factor) \|\| id:ebi-fl187-GCST90094780 | Weighted mode | 7 | 0.00738473624465552 | 0.132978985682802 | 0.95751679273402 | -0.253254076 | 0.268023548182947 | 1.00741207065373 | 0.776270625268187 | 1.30737792602704 | 0.999714303421225 |
| ebi-fl187-GCST90094780 | ebi-met1400-GCST90200172 | 3-carboxy-4-methyl-5-pentyl-2-furanpropionate (3-CMPFP) levels \|\| id:ebi-met1400-GCST90200172 | F-healthy breakfast food liking (derived food-liking factor) \|\| id:ebi-fl187-GCST90094780 | Weighted mode | 8 | 0.0405640576569116 | 0.103792711842197 | 0.707553755264774 | -0.162869658 | 0.243997772867617 | 1.04139801708282 | 0.849701933425595 | 1.27634148790483 | 0.999714303421225 |
| ebi-fl187-GCST90094780 | ebi-met1400-GCST90200222 | Dihydrocaffeate sulfate (2) levels \|\| id:ebi-met1400-GCST90200222 | F-healthy breakfast food liking (derived food-liking factor) \|\| id:ebi-fl187-GCST90094780 | Weighted mode | 8 | 0.0185436270483395 | 0.133852497068315 | 0.893716650565625 | -0.243807267 | 0.280894521302236 | 1.01871662779968 | 0.783638652576649 | 1.32431390966138 | 0.999714303421225 |
| ebi-fl187-GCST90094780 | ebi-met1400-GCST90200223 | 4-methylhexanoylglutamine levels \|\| id:ebi-met1400-GCST90200223 | F-healthy breakfast food liking (derived food-liking factor) \|\| id:ebi-fl187-GCST90094780 | Weighted mode | 8 | 0.0286342063553954 | 0.128010662456361 | 0.829389594761343 | -0.222266692 | 0.279535104769863 | 1.02904810636355 | 0.800701795046885 | 1.32251483855909 | 0.999714303421225 |
| ebi-fl187-GCST90094780 | ebi-met1400-GCST90200224 | 2-ketocaprylate levels \|\| id:ebi-met1400-GCST90200224 | F-healthy breakfast food liking (derived food-liking factor) \|\| id:ebi-fl187-GCST90094780 | Weighted mode | 8 | 0.0684336216012256 | 0.104636528723304 | 0.533991869498421 | -0.136653975 | 0.273521217898902 | 1.07082954264144 | 0.872272002102342 | 1.31458525165312 | 0.999714303421225 |
| ebi-fl187-GCST90094780 | ebi-met1400-GCST90200243 | Hydroxypalmitoyl sphingomyelin (d18:1/16:0(OH)) levels \|\| id:ebi-met1400-GCST90200243 | F-healthy breakfast food liking (derived food-liking factor) \|\| id:ebi-fl187-GCST90094780 | Weighted mode | 8 | -0.087687869 | 0.101439661830276 | 0.415986190975177 | -0.286509606 | 0.111133868628999 | 0.916046759100109 | 0.750879865735419 | 1.11754450099145 | 0.999714303421225 |
| ebi-fl187-GCST90094780 | ebi-met1400-GCST90200273 | 2-methoxyhydroquinone sulfate (2) levels \|\| id:ebi-met1400-GCST90200273 | F-healthy breakfast food liking (derived food-liking factor) \|\| id:ebi-fl187-GCST90094780 | Weighted mode | 8 | 0.250823673222845 | 0.115436303876345 | 0.0663390861008206 | 0.0245685176252076 | 0.477078828820482 | 1.28508346972608 | 1.02487281055266 | 1.61136046069238 | 0.999714303421225 |
| ebi-fl187-GCST90094780 | ebi-met1400-GCST90200276 | 2,4-di-tert-butylphenol levels \|\| id:ebi-met1400-GCST90200276 | F-healthy breakfast food liking (derived food-liking factor) \|\| id:ebi-fl187-GCST90094780 | Weighted mode | 8 | 0.0198002198856895 | 0.10555479696039 | 0.856525578923168 | -0.187087182 | 0.226687621928055 | 1.01999754444426 | 0.829371426843722 | 1.25443794782232 | 0.999714303421225 |
| ebi-fl187-GCST90094780 | ebi-met1400-GCST90200289 | N-acetylmethionine levels \|\| id:ebi-met1400-GCST90200289 | F-healthy breakfast food liking (derived food-liking factor) \|\| id:ebi-fl187-GCST90094780 | Weighted mode | 8 | 0.0898242792282432 | 0.132667733461999 | 0.520106803160464 | -0.170204478 | 0.349853036813761 | 1.09398203144749 | 0.843492323036797 | 1.41885901322871 | 0.999714303421225 |
| ebi-fl187-GCST90094780 | ebi-met1400-GCST90200308 | 3-hydroxyisobutyrate levels \|\| id:ebi-met1400-GCST90200308 | F-healthy breakfast food liking (derived food-liking factor) \|\| id:ebi-fl187-GCST90094780 | Weighted mode | 8 | -0.034213735 | 0.130273810991598 | 0.800397877221072 | -0.289550404 | 0.221122934936401 | 0.96636493693282 | 0.748600059409757 | 1.24747677961646 | 0.999714303421225 |
| ebi-fl187-GCST90094780 | ebi-met1400-GCST90200383 | Cystathionine levels \|\| id:ebi-met1400-GCST90200383 | F-healthy breakfast food liking (derived food-liking factor) \|\| id:ebi-fl187-GCST90094780 | Weighted mode | 8 | 0.118053668674791 | 0.120810341587699 | 0.361033363540153 | -0.118734601 | 0.35484193818668 | 1.12530450334818 | 0.888043455478989 | 1.42595524739572 | 0.999714303421225 |
| ebi-fl187-GCST90094780 | ebi-met1400-GCST90200384 | Guanidinoacetate levels \|\| id:ebi-met1400-GCST90200384 | F-healthy breakfast food liking (derived food-liking factor) \|\| id:ebi-fl187-GCST90094780 | Weighted mode | 8 | 0.113001661218853 | 0.118084309931474 | 0.370463381959026 | -0.118443586 | 0.344446908684541 | 1.11963379290381 | 0.888301926688944 | 1.41120917623668 | 0.999714303421225 |
| ebi-fl187-GCST90094780 | ebi-met1400-GCST90200403 | Citrulline levels \|\| id:ebi-met1400-GCST90200403 | F-healthy breakfast food liking (derived food-liking factor) \|\| id:ebi-fl187-GCST90094780 | Weighted mode | 8 | -0.152277469 | 0.122827824528603 | 0.255009592438234 | -0.393020005 | 0.0884650674972229 | 0.858749971546813 | 0.675015244004057 | 1.09249608832126 | 0.999714303421225 |
| ebi-fl187-GCST90094780 | ebi-met1400-GCST90200434 | 12,13-DiHOME levels \|\| id:ebi-met1400-GCST90200434 | F-healthy breakfast food liking (derived food-liking factor) \|\| id:ebi-fl187-GCST90094780 | Weighted mode | 8 | -0.053726049 | 0.1130681905918 | 0.649137970645863 | -0.275339703 | 0.167887604286095 | 0.947691691770531 | 0.759314138243858 | 1.18280366111457 | 0.999714303421225 |
| ebi-fl187-GCST90094780 | ebi-met1400-GCST90200437 | Fructose levels \|\| id:ebi-met1400-GCST90200437 | F-healthy breakfast food liking (derived food-liking factor) \|\| id:ebi-fl187-GCST90094780 | Weighted mode | 8 | 0.023627903470006 | 0.126290373691008 | 0.856895774201397 | -0.223901229 | 0.271157035904382 | 1.02390925391832 | 0.799394087452056 | 1.31148100382021 | 0.999714303421225 |
| ebi-fl187-GCST90094780 | ebi-met1400-GCST90200448 | N-stearoyl-sphinganine (d18:0/18:0) levels \|\| id:ebi-met1400-GCST90200448 | F-healthy breakfast food liking (derived food-liking factor) \|\| id:ebi-fl187-GCST90094780 | Weighted mode | 8 | 0.138121319290943 | 0.125627697940404 | 0.307940295176397 | -0.108108969 | 0.384351607254136 | 1.14811483030246 | 0.897529788472176 | 1.46866174303174 | 0.999714303421225 |
| ebi-fl187-GCST90094780 | ebi-met1400-GCST90200468 | X-11847 levels \|\| id:ebi-met1400-GCST90200468 | F-healthy breakfast food liking (derived food-liking factor) \|\| id:ebi-fl187-GCST90094780 | Weighted mode | 8 | 0.238774244852749 | 0.132352113130605 | 0.114199841454272 | -0.020635897 | 0.498184386588736 | 1.26969186475234 | 0.979575566162389 | 1.64573054607105 | 0.999714303421225 |
| ebi-fl187-GCST90094780 | ebi-met1400-GCST90200476 | X-11843 levels \|\| id:ebi-met1400-GCST90200476 | F-healthy breakfast food liking (derived food-liking factor) \|\| id:ebi-fl187-GCST90094780 | Weighted mode | 8 | -0.220124907 | 0.137265801923959 | 0.152828062655776 | -0.489165879 | 0.0489160647457925 | 0.802418563986878 | 0.613137612029038 | 1.05013220392728 | 0.999714303421225 |
| ebi-fl187-GCST90094780 | ebi-met1400-GCST90200511 | X-12906 levels \|\| id:ebi-met1400-GCST90200511 | F-healthy breakfast food liking (derived food-liking factor) \|\| id:ebi-fl187-GCST90094780 | Weighted mode | 8 | -0.116561502 | 0.109998158215096 | 0.324484657303449 | -0.332157892 | 0.0990348884818573 | 0.889975360367317 | 0.717374046488949 | 1.10410481942789 | 0.999714303421225 |
| ebi-fl187-GCST90094780 | ebi-met1400-GCST90200515 | X-12849 levels \|\| id:ebi-met1400-GCST90200515 | F-healthy breakfast food liking (derived food-liking factor) \|\| id:ebi-fl187-GCST90094780 | Weighted mode | 8 | 0.0676204657717376 | 0.135938766482139 | 0.634125865690126 | -0.198819517 | 0.33406044807673 | 1.06995914528875 | 0.819697821887772 | 1.39662756447312 | 0.999714303421225 |
| ebi-fl187-GCST90094780 | ebi-met1400-GCST90200551 | X-18886 levels \|\| id:ebi-met1400-GCST90200551 | F-healthy breakfast food liking (derived food-liking factor) \|\| id:ebi-fl187-GCST90094780 | Weighted mode | 8 | 0.0934348006023423 | 0.11849181608072 | 0.456254409833038 | -0.138809159 | 0.325678760120553 | 1.09793901604509 | 0.870394119566644 | 1.38497038968306 | 0.999714303421225 |
| ebi-fl187-GCST90094780 | ebi-met1400-GCST90200564 | X-21319 levels \|\| id:ebi-met1400-GCST90200564 | F-healthy breakfast food liking (derived food-liking factor) \|\| id:ebi-fl187-GCST90094780 | Weighted mode | 8 | -0.000972299 | 0.134111173862443 | 0.994417722354061 | -0.263830199 | 0.261885602162781 | 0.999028173921525 | 0.768103953113252 | 1.29937788790656 | 0.999714303421225 |
| ebi-fl187-GCST90094780 | ebi-met1400-GCST90200579 | X-21442 levels \|\| id:ebi-met1400-GCST90200579 | F-healthy breakfast food liking (derived food-liking factor) \|\| id:ebi-fl187-GCST90094780 | Weighted mode | 8 | 0.013639584791462 | 0.128683052247468 | 0.918560938972227 | -0.238579198 | 0.2658583671965 | 1.01373302828827 | 0.787746298159683 | 1.30455027848852 | 0.999714303421225 |
| ebi-fl187-GCST90094780 | ebi-met1400-GCST90200581 | X-21441 levels \|\| id:ebi-met1400-GCST90200581 | F-healthy breakfast food liking (derived food-liking factor) \|\| id:ebi-fl187-GCST90094780 | Weighted mode | 8 | 0.087081021791401 | 0.105513662712105 | 0.43641699381918 | -0.119725757 | 0.293887800707128 | 1.09098506970221 | 0.887163701683534 | 1.34163336490712 | 0.999714303421225 |
| ebi-fl187-GCST90094780 | ebi-met1400-GCST90200648 | X-24585 levels \|\| id:ebi-met1400-GCST90200648 | F-healthy breakfast food liking (derived food-liking factor) \|\| id:ebi-fl187-GCST90094780 | Weighted mode | 8 | -0.047866311 | 0.109167246088951 | 0.674260605044515 | -0.261834113 | 0.166101491520899 | 0.953261219276141 | 0.7696386860575 | 1.18069292596337 | 0.999714303421225 |
| ebi-fl187-GCST90094780 | ebi-met1400-GCST90200720 | Adenosine 5'-diphosphate (ADP) to pantothenate ratio \|\| id:ebi-met1400-GCST90200720 | F-healthy breakfast food liking (derived food-liking factor) \|\| id:ebi-fl187-GCST90094780 | Weighted mode | 7 | -0.133081507 | 0.152159085759447 | 0.415413183795763 | -0.431313315 | 0.16515030145549 | 0.875393738804704 | 0.649655332304809 | 1.17957039653595 | 0.999714303421225 |
| ebi-fl187-GCST90094780 | ebi-met1400-GCST90200724 | Cholate to taurocholate ratio \|\| id:ebi-met1400-GCST90200724 | F-healthy breakfast food liking (derived food-liking factor) \|\| id:ebi-fl187-GCST90094780 | Weighted mode | 8 | 0.00974221698220401 | 0.128585113500359 | 0.94172615720006 | -0.242284605 | 0.261769039442908 | 1.00978982686106 | 0.784832778054636 | 1.2992264377127 | 0.999714303421225 |
| ebi-fl187-GCST90094780 | ebi-met1400-GCST90200726 | N-acetylputrescine to (N(1) + N(8))-acetylspermidine ratio \|\| id:ebi-met1400-GCST90200726 | F-healthy breakfast food liking (derived food-liking factor) \|\| id:ebi-fl187-GCST90094780 | Weighted mode | 8 | -0.072260206 | 0.131174788868582 | 0.598855822629369 | -0.329362792 | 0.184842379874086 | 0.930288797301162 | 0.719381983017977 | 1.20302880363132 | 0.999714303421225 |
| ebi-fl187-GCST90094780 | ebi-met1400-GCST90200744 | Arginine to glutamate ratio \|\| id:ebi-met1400-GCST90200744 | F-healthy breakfast food liking (derived food-liking factor) \|\| id:ebi-fl187-GCST90094780 | Weighted mode | 8 | 0.227024280471701 | 0.211809927726478 | 0.31935261620708 | -0.188123178 | 0.642171738815598 | 1.25486033617127 | 0.828512646522113 | 1.90060401601104 | 0.999714303421225 |
| ebi-fl187-GCST90094780 | ebi-met1400-GCST90200759 | Histidine to trans-urocanate ratio \|\| id:ebi-met1400-GCST90200759 | F-healthy breakfast food liking (derived food-liking factor) \|\| id:ebi-fl187-GCST90094780 | Weighted mode | 8 | 0.0157596723669352 | 0.100488620518234 | 0.879806027323723 | -0.181198024 | 0.212717368582674 | 1.01588451094635 | 0.834270136953945 | 1.23703497688265 | 0.999714303421225 |
| ebi-fl187-GCST90094780 | ebi-met1400-GCST90200795 | Oleoyl-linoleoyl-glycerol (18:1 to 18:2) [2] to linoleoyl-arachidonoyl-glycerol (18:2 to 20:4) [2] ratio \|\| id:ebi-met1400-GCST90200795 | F-healthy breakfast food liking (derived food-liking factor) \|\| id:ebi-fl187-GCST90094780 | Weighted mode | 8 | -0.004481257 | 0.106004493242855 | 0.967460648247027 | -0.212250064 | 0.203287549328442 | 0.995528768424779 | 0.808762429760902 | 1.22542478766522 | 0.999714303421225 |
| ebi-fl187-GCST90094780 | ebi-met1400-GCST90200811 | Choline to taurocholate ratio \|\| id:ebi-met1400-GCST90200811 | F-healthy breakfast food liking (derived food-liking factor) \|\| id:ebi-fl187-GCST90094780 | Weighted mode | 8 | -0.070105307 | 0.111914747234699 | 0.550914347268436 | -0.289458212 | 0.149247597649476 | 0.932295637544456 | 0.748669078007079 | 1.16096040469325 | 0.999714303421225 |
| ebi-fl187-GCST90094780 | ebi-met1400-GCST90200831 | Cholate to phosphate ratio \|\| id:ebi-met1400-GCST90200831 | F-healthy breakfast food liking (derived food-liking factor) \|\| id:ebi-fl187-GCST90094780 | Weighted mode | 8 | 0.126812364343344 | 0.114220928757882 | 0.303573293405391 | -0.097060656 | 0.350685384708793 | 1.13520399304992 | 0.907500959065589 | 1.42004048917302 | 0.999714303421225 |
| ebi-fl187-GCST90094780 | ebi-met1400-GCST90200841 | Adenosine 5'-diphosphate (ADP) to oxalate (ethanedioate) ratio \|\| id:ebi-met1400-GCST90200841 | F-healthy breakfast food liking (derived food-liking factor) \|\| id:ebi-fl187-GCST90094780 | Weighted mode | 7 | -0.153715021 | 0.152245464717369 | 0.351630672052329 | -0.452116132 | 0.144686089650247 | 0.85751636018097 | 0.636280273 | 1.15567673443413 | 0.999714303421225 |
| ebi-fl187-GCST90094780 | ebi-met1400-GCST90200844 | Aspartate to phosphate ratio \|\| id:ebi-met1400-GCST90200844 | F-healthy breakfast food liking (derived food-liking factor) \|\| id:ebi-fl187-GCST90094780 | Weighted mode | 8 | 0.196997428911357 | 0.130856621759599 | 0.175928364758145 | -0.05948155 | 0.45347640756017 | 1.21774090978206 | 0.942252918244577 | 1.57377376566737 | 0.999714303421225 |
| ebi-fl187-GCST90094780 | ebi-met1400-GCST90200870 | Adenosine 5'-monophosphate (AMP) to acetoacetate ratio \|\| id:ebi-met1400-GCST90200870 | F-healthy breakfast food liking (derived food-liking factor) \|\| id:ebi-fl187-GCST90094780 | Weighted mode | 8 | 0.116994947105882 | 0.130782385500553 | 0.400724818653212 | -0.139338528 | 0.373328422686966 | 1.12411374964843 | 0.869933481349804 | 1.45256131559391 | 0.999714303421225 |
| ebi-fl187-GCST90094780 | ebi-met1400-GCST90200887 | Cholesterol to cortisol ratio \|\| id:ebi-met1400-GCST90200887 | F-healthy breakfast food liking (derived food-liking factor) \|\| id:ebi-fl187-GCST90094780 | Weighted mode | 8 | 0.0350660687494934 | 0.103921829221832 | 0.745686947182512 | -0.168620717 | 0.238752854024284 | 1.03568813315907 | 0.844829272408826 | 1.26966470528194 | 0.999714303421225 |
| ebi-fl187-GCST90094780 | ebi-met1400-GCST90200890 | Cortisol to taurocholate ratio \|\| id:ebi-met1400-GCST90200890 | F-healthy breakfast food liking (derived food-liking factor) \|\| id:ebi-fl187-GCST90094780 | Weighted mode | 8 | -0.000355276 | 0.113758742388842 | 0.99759529850077 | -0.223322411 | 0.222611859236398 | 0.999644787257257 | 0.799856925104618 | 1.24933556155672 | 0.999714303421225 |
| ebi-fl187-GCST90094780 | ebi-met1400-GCST90200948 | Adenosine 5'-diphosphate (ADP) to mannitol to sorbitol ratio \|\| id:ebi-met1400-GCST90200948 | F-healthy breakfast food liking (derived food-liking factor) \|\| id:ebi-fl187-GCST90094780 | Weighted mode | 8 | -0.147028565 | 0.157620642326973 | 0.381971359365355 | -0.455965024 | 0.161907894067258 | 0.863269317867902 | 0.633836005851237 | 1.17575194260425 | 0.999714303421225 |
| ebi-fl187-GCST90094780 | ebi-met1400-GCST90200972 | Leucine to N-palmitoyl-sphingosine (d18:1 to 16:0) ratio \|\| id:ebi-met1400-GCST90200972 | F-healthy breakfast food liking (derived food-liking factor) \|\| id:ebi-fl187-GCST90094780 | Weighted mode | 8 | -0.12075607 | 0.113653300992274 | 0.323285819391171 | -0.34351654 | 0.102004399518408 | 0.88625011584117 | 0.709271749429307 | 1.10738834369262 | 0.999714303421225 |
| ebi-fl187-GCST90094780 | ebi-met1400-GCST90200973 | Proline to glutamate ratio \|\| id:ebi-met1400-GCST90200973 | F-healthy breakfast food liking (derived food-liking factor) \|\| id:ebi-fl187-GCST90094780 | Weighted mode | 8 | 0.109060950195108 | 0.143997988795855 | 0.473541826333762 | -0.173175108 | 0.391297008234983 | 1.11523032177569 | 0.840990337944781 | 1.47889769298346 | 0.999714303421225 |
| ebi-fl187-GCST90094780 | ebi-met1400-GCST90200983 | Cholesterol to linoleoyl-arachidonoyl-glycerol (18:2 to 20:4) [1] ratio \|\| id:ebi-met1400-GCST90200983 | F-healthy breakfast food liking (derived food-liking factor) \|\| id:ebi-fl187-GCST90094780 | Weighted mode | 8 | 0.0859130395862696 | 0.150324389222884 | 0.585523553628559 | -0.208722763 | 0.380548842463122 | 1.08971156241643 | 0.811620215373457 | 1.46308737359094 | 0.999714303421225 |
| ebi-fl187-GCST90094780 | ebi-met1400-GCST90201001 | Serine to alpha-ketobutyrate ratio \|\| id:ebi-met1400-GCST90201001 | F-healthy breakfast food liking (derived food-liking factor) \|\| id:ebi-fl187-GCST90094780 | Weighted mode | 8 | -0.101230391 | 0.113463107078763 | 0.401919422175194 | -0.323618081 | 0.121157299053637 | 0.903724799001034 | 0.723526518363879 | 1.12880245796148 | 0.999714303421225 |
| ebi-fl187-GCST90094780 | ebi-met1400-GCST90201005 | Fructose to maltose ratio \|\| id:ebi-met1400-GCST90201005 | F-healthy breakfast food liking (derived food-liking factor) \|\| id:ebi-fl187-GCST90094780 | Weighted mode | 8 | -0.082578933 | 0.132343947097747 | 0.552429164391563 | -0.341973069 | 0.176815203568037 | 0.92073875855215 | 0.710367335315116 | 1.19341053473988 | 0.999714303421225 |
| ebi-fl187-GCST90094780 | ebi-met1400-GCST90201020 | Paraxanthine to 5-acetylamino-6-formylamino-3-methyluracil ratio \|\| id:ebi-met1400-GCST90201020 | F-healthy breakfast food liking (derived food-liking factor) \|\| id:ebi-fl187-GCST90094780 | Weighted mode | 8 | -0.015379713 | 0.136551910550731 | 0.913486523501495 | -0.283021458 | 0.252262031184629 | 0.984737950314586 | 0.753503618874298 | 1.28693320974155 | 0.999714303421225 |
| ebi-fl187-GCST90094780 | ebi-pw11-GCST90275197 | Placental weight (fetal genotype, adjusted for sex) \|\| id:ebi-pw11-GCST90275197 | F-healthy breakfast food liking (derived food-liking factor) \|\| id:ebi-fl187-GCST90094780 | Weighted mode | 7 | -0.035260086 | 0.0407727671884694 | 0.420377072161055 | -0.115174709 | 0.044654537946282 | 0.965354308711482 | 0.891210427436628 | 1.04566655938739 | 0.999714303421225 |
| ebi-fl187-GCST90094780 | prot-a-1017 | FAS-associated factor 2 \|\| id:prot-a-1017 | F-healthy breakfast food liking (derived food-liking factor) \|\| id:ebi-fl187-GCST90094780 | Weighted mode | 8 | -0.057297853 | 0.198227979807524 | 0.780915824436521 | -0.445824693 | 0.331228987656815 | 0.94431276130655 | 0.640296010475177 | 1.3926786620217 | 0.999714303421225 |
| ebi-fl187-GCST90094780 | prot-a-1019 | Fas apoptotic inhibitory molecule 3 \|\| id:prot-a-1019 | F-healthy breakfast food liking (derived food-liking factor) \|\| id:ebi-fl187-GCST90094780 | Weighted mode | 8 | -0.190129441 | 0.200584982064275 | 0.374758948872793 | -0.583276006 | 0.203017124079357 | 0.826852098646614 | 0.558067137570041 | 1.22509344666528 | 0.999714303421225 |
| ebi-fl187-GCST90094780 | prot-a-1052 | MIP18 family protein FAM96A \|\| id:prot-a-1052 | F-healthy breakfast food liking (derived food-liking factor) \|\| id:ebi-fl187-GCST90094780 | Weighted mode | 8 | -0.015962989 | 0.189381413413766 | 0.93518572085225 | -0.387150559 | 0.355224581747528 | 0.984163744714889 | 0.67898885945933 | 1.42650098439391 | 0.999714303421225 |
| ebi-fl187-GCST90094780 | prot-a-1066 | F-box/LRR-repeat protein 4 \|\| id:prot-a-1066 | F-healthy breakfast food liking (derived food-liking factor) \|\| id:ebi-fl187-GCST90094780 | Weighted mode | 8 | 0.0488692622847189 | 0.176226892611474 | 0.789553859164024 | -0.296535447 | 0.394273971803208 | 1.05008305630581 | 0.743389275703624 | 1.4833068772708 | 0.999714303421225 |
| ebi-fl187-GCST90094780 | prot-a-1076 | Low affinity immunoglobulin gamma Fc region receptor III-B \|\| id:prot-a-1076 | F-healthy breakfast food liking (derived food-liking factor) \|\| id:ebi-fl187-GCST90094780 | Weighted mode | 8 | 0.108367436719075 | 0.177651947197851 | 0.561134672712063 | -0.23983038 | 0.456565253226864 | 1.11445716264779 | 0.786761300367233 | 1.578642425 | 0.999714303421225 |
| ebi-fl187-GCST90094780 | prot-a-1081 | Fc receptor-like protein 3 \|\| id:prot-a-1081 | F-healthy breakfast food liking (derived food-liking factor) \|\| id:ebi-fl187-GCST90094780 | Weighted mode | 8 | 0.142220080812071 | 0.191467356594727 | 0.481786609701163 | -0.233055938 | 0.517496099737736 | 1.15283033645669 | 0.792109263187065 | 1.67782128857768 | 0.999714303421225 |
| ebi-fl187-GCST90094780 | prot-a-1105 | Fibroblast growth factor receptor 4 \|\| id:prot-a-1105 | F-healthy breakfast food liking (derived food-liking factor) \|\| id:ebi-fl187-GCST90094780 | Weighted mode | 8 | -0.148145538 | 0.175967091192721 | 0.427670141685996 | -0.493041037 | 0.196749960868468 | 0.86230560768844 | 0.610766204778898 | 1.21743959510679 | 0.999714303421225 |
| ebi-fl187-GCST90094780 | prot-a-1143 | Follistatin-related protein 1 \|\| id:prot-a-1143 | F-healthy breakfast food liking (derived food-liking factor) \|\| id:ebi-fl187-GCST90094780 | Weighted mode | 8 | 0.183435373829104 | 0.256882575562568 | 0.498288860877742 | -0.320054474 | 0.686925221931737 | 1.20133732507165 | 0.726109481709807 | 1.98759471534774 | 0.999714303421225 |
| ebi-fl187-GCST90094780 | prot-a-1151 | Alpha-(1,3)-fucosyltransferase 10 \|\| id:prot-a-1151 | F-healthy breakfast food liking (derived food-liking factor) \|\| id:ebi-fl187-GCST90094780 | Weighted mode | 8 | -0.094788521 | 0.181798292524728 | 0.618176746850352 | -0.451113174 | 0.261536132323635 | 0.909565268060997 | 0.636918755217897 | 1.29892387386181 | 0.999714303421225 |
| ebi-fl187-GCST90094780 | prot-a-1165 | Growth arrest and DNA damage-inducible proteins-interacting protein 1 \|\| id:prot-a-1165 | F-healthy breakfast food liking (derived food-liking factor) \|\| id:ebi-fl187-GCST90094780 | Weighted mode | 8 | 0.0781211035148886 | 0.180215518405595 | 0.677705846247279 | -0.275101313 | 0.431343519589854 | 1.0812535943523 | 0.759495172926683 | 1.53932424717678 | 0.999714303421225 |
| ebi-fl187-GCST90094780 | prot-a-1194 | Growth/differentiation factor 11/8 \|\| id:prot-a-1194 | F-healthy breakfast food liking (derived food-liking factor) \|\| id:ebi-fl187-GCST90094780 | Weighted mode | 8 | 0.0171904575863203 | 0.18081002923682 | 0.926919912533376 | -0.3371972 | 0.371578114890487 | 1.01733906381745 | 0.713768071161158 | 1.45002110991777 | 0.999714303421225 |
| ebi-fl187-GCST90094780 | prot-a-1216 | D-glucuronyl C5-epimerase \|\| id:prot-a-1216 | F-healthy breakfast food liking (derived food-liking factor) \|\| id:ebi-fl187-GCST90094780 | Weighted mode | 8 | 0.231908564601297 | 0.258361135800538 | 0.399210441990105 | -0.274479262 | 0.738296390770351 | 1.2610044231147 | 0.759967764625493 | 2.09236789918116 | 0.999714303421225 |
| ebi-fl187-GCST90094780 | prot-a-1246 | Glypican-3 \|\| id:prot-a-1246 | F-healthy breakfast food liking (derived food-liking factor) \|\| id:ebi-fl187-GCST90094780 | Weighted mode | 8 | 0.00315709368220418 | 0.200562233728726 | 0.987880103931805 | -0.389944884 | 0.396259071790508 | 1.00316208255119 | 0.677094191904742 | 1.48625431424469 | 0.999714303421225 |
| ebi-fl187-GCST90094780 | prot-a-1275 | Glutamate receptor 4 \|\| id:prot-a-1275 | F-healthy breakfast food liking (derived food-liking factor) \|\| id:ebi-fl187-GCST90094780 | Weighted mode | 7 | 0.0156981737616341 | 0.17596463046231 | 0.931816704228544 | -0.329192502 | 0.360588849467762 | 1.01582203738682 | 0.71950449740006 | 1.43417367837098 | 0.999714303421225 |
| ebi-fl187-GCST90094780 | prot-a-1289 | Glutathione S-transferase P \|\| id:prot-a-1289 | F-healthy breakfast food liking (derived food-liking factor) \|\| id:ebi-fl187-GCST90094780 | Weighted mode | 8 | -0.015083491 | 0.228228874938854 | 0.949154652419607 | -0.462412085 | 0.432245104273734 | 0.985029695443188 | 0.629762770365505 | 1.54071270415328 | 0.999714303421225 |
| ebi-fl187-GCST90094780 | prot-a-130 | Apolipoprotein D \|\| id:prot-a-130 | F-healthy breakfast food liking (derived food-liking factor) \|\| id:ebi-fl187-GCST90094780 | Weighted mode | 8 | 0.300838157023632 | 0.158403958815954 | 0.0993252302723497 | -0.009633602 | 0.611309916302903 | 1.35099067549174 | 0.990412652238938 | 1.84284378954532 | 0.999714303421225 |
| ebi-fl187-GCST90094780 | prot-a-1313 | Heparin-binding EGF-like growth factor \|\| id:prot-a-1313 | F-healthy breakfast food liking (derived food-liking factor) \|\| id:ebi-fl187-GCST90094780 | Weighted mode | 8 | 0.288276003880879 | 0.222950858048798 | 0.237045860049273 | -0.148707678 | 0.725259685656523 | 1.33412547712172 | 0.861821007411512 | 2.06526735064302 | 0.999714303421225 |
| ebi-fl187-GCST90094780 | prot-a-1347 | HLA class II histocompatibility antigen, DQ alpha 2 chain \|\| id:prot-a-1347 | F-healthy breakfast food liking (derived food-liking factor) \|\| id:ebi-fl187-GCST90094780 | Weighted mode | 8 | -0.131174526 | 0.189931838523801 | 0.512034972909447 | -0.50344093 | 0.241091877227168 | 0.877064690195581 | 0.6044472168454 | 1.27263795638358 | 0.999714303421225 |
| ebi-fl187-GCST90094780 | prot-a-1357 | Non-histone chromosomal protein HMG-14 \|\| id:prot-a-1357 | F-healthy breakfast food liking (derived food-liking factor) \|\| id:ebi-fl187-GCST90094780 | Weighted mode | 8 | -0.277492891 | 0.193839158092666 | 0.195364579018314 | -0.65741764 | 0.102431859277845 | 0.75768095125307 | 0.518187755290468 | 1.10786180883406 | 0.999714303421225 |
| ebi-fl187-GCST90094780 | prot-a-137 | Apolipoprotein M \|\| id:prot-a-137 | F-healthy breakfast food liking (derived food-liking factor) \|\| id:ebi-fl187-GCST90094780 | Weighted mode | 8 | -0.083750232 | 0.195694077613296 | 0.681535300661922 | -0.467310624 | 0.299810159767607 | 0.919660928956432 | 0.626685396352391 | 1.34960257438873 | 0.999714303421225 |
| ebi-fl187-GCST90094780 | prot-a-1381 | 17-beta-hydroxysteroid dehydrogenase 14 \|\| id:prot-a-1381 | F-healthy breakfast food liking (derived food-liking factor) \|\| id:ebi-fl187-GCST90094780 | Weighted mode | 8 | 0.0490227945437432 | 0.146010228565656 | 0.746898843150936 | -0.237157253 | 0.335202842532429 | 1.0502442903066 | 0.788867226174371 | 1.39822397575153 | 0.999714303421225 |
| ebi-fl187-GCST90094780 | prot-a-1392 | Serine protease HTRA2, mitochondrial \|\| id:prot-a-1392 | F-healthy breakfast food liking (derived food-liking factor) \|\| id:ebi-fl187-GCST90094780 | Weighted mode | 8 | -0.095139265 | 0.178109791302724 | 0.60976711808802 | -0.444234456 | 0.253955925482422 | 0.90924629903645 | 0.64131504276771 | 1.28911498589459 | 0.999714303421225 |
| ebi-fl187-GCST90094780 | prot-a-1416 | Interferon-induced protein with tetratricopeptide repeats 2 \|\| id:prot-a-1416 | F-healthy breakfast food liking (derived food-liking factor) \|\| id:ebi-fl187-GCST90094780 | Weighted mode | 8 | -0.206840861 | 0.18271052421486 | 0.2948903321315 | -0.564953489 | 0.151271766217304 | 0.813149043218884 | 0.568386582538798 | 1.16331276423587 | 0.999714303421225 |
| ebi-fl187-GCST90094780 | prot-a-1428 | Interferon gamma \|\| id:prot-a-1428 | F-healthy breakfast food liking (derived food-liking factor) \|\| id:ebi-fl187-GCST90094780 | Weighted mode | 8 | 0.158418507919874 | 0.196493971975792 | 0.446633242755932 | -0.226709677 | 0.543546692992427 | 1.17165643961418 | 0.797152180189167 | 1.72210381732081 | 0.999714303421225 |
| ebi-fl187-GCST90094780 | prot-a-1483 | Interleukin-17C \|\| id:prot-a-1483 | F-healthy breakfast food liking (derived food-liking factor) \|\| id:ebi-fl187-GCST90094780 | Weighted mode | 8 | -0.025529515 | 0.178640473192905 | 0.890387920805702 | -0.375664843 | 0.32460581226495 | 0.974793607319548 | 0.686832491427381 | 1.38348518558913 | 0.999714303421225 |
| ebi-fl187-GCST90094780 | prot-a-1503 | Interleukin-1 receptor-like 2 \|\| id:prot-a-1503 | F-healthy breakfast food liking (derived food-liking factor) \|\| id:ebi-fl187-GCST90094780 | Weighted mode | 8 | -0.057828249 | 0.157375037250534 | 0.724137721333371 | -0.366283322 | 0.250626824174964 | 0.943812034332509 | 0.693306344546674 | 1.28483052716518 | 0.999714303421225 |
| ebi-fl187-GCST90094780 | prot-a-1522 | Interleukin-31 receptor subunit alpha \|\| id:prot-a-1522 | F-healthy breakfast food liking (derived food-liking factor) \|\| id:ebi-fl187-GCST90094780 | Weighted mode | 8 | -0.095128568 | 0.183692453889527 | 0.620509764475669 | -0.455165778 | 0.264908641229664 | 0.909256025366242 | 0.634342799139612 | 1.30331190136653 | 0.999714303421225 |
| ebi-fl187-GCST90094780 | prot-a-1540 | Interleukin-6 receptor subunit alpha \|\| id:prot-a-1540 | F-healthy breakfast food liking (derived food-liking factor) \|\| id:ebi-fl187-GCST90094780 | Weighted mode | 8 | -0.09682339 | 0.16491109939465 | 0.575559227132403 | -0.420049145 | 0.226402364580317 | 0.907716303542504 | 0.657014530011855 | 1.25408016121348 | 0.999714303421225 |
| ebi-fl187-GCST90094780 | prot-a-1541 | Interleukin-6 receptor subunit alpha \|\| id:prot-a-1541 | F-healthy breakfast food liking (derived food-liking factor) \|\| id:ebi-fl187-GCST90094780 | Weighted mode | 8 | -0.143797294 | 0.157893933698315 | 0.392722659498976 | -0.453269404 | 0.165674816079814 | 0.866063286518758 | 0.635546891674838 | 1.18018926074683 | 0.999714303421225 |
| ebi-fl187-GCST90094780 | prot-a-1545 | Interleukin-9 \|\| id:prot-a-1545 | F-healthy breakfast food liking (derived food-liking factor) \|\| id:ebi-fl187-GCST90094780 | Weighted mode | 8 | 0.0319775927143157 | 0.184936303523529 | 0.867614575722629 | -0.330497562 | 0.394452747620433 | 1.03249436964961 | 0.718566113139928 | 1.48357208037522 | 0.999714303421225 |
| ebi-fl187-GCST90094780 | prot-a-1548 | Inositol monophosphatase 3 \|\| id:prot-a-1548 | F-healthy breakfast food liking (derived food-liking factor) \|\| id:ebi-fl187-GCST90094780 | Weighted mode | 8 | -0.064051489 | 0.169901823646235 | 0.717348734708055 | -0.397059064 | 0.268956084993874 | 0.937956703503808 | 0.672294316270597 | 1.3085976727098 | 0.999714303421225 |
| ebi-fl187-GCST90094780 | prot-a-1555 | Inhibin beta A chain:Inhibin beta B chain heterodimer \|\| id:prot-a-1555 | F-healthy breakfast food liking (derived food-liking factor) \|\| id:ebi-fl187-GCST90094780 | Weighted mode | 8 | -0.190865519 | 0.164970621396424 | 0.285237444878658 | -0.514207937 | 0.13247689853739 | 0.826243694428309 | 0.597974040005053 | 1.14165264193872 | 0.999714303421225 |
| ebi-fl187-GCST90094780 | prot-a-1603 | Junctophilin-3 \|\| id:prot-a-1603 | F-healthy breakfast food liking (derived food-liking factor) \|\| id:ebi-fl187-GCST90094780 | Weighted mode | 8 | -0.027906644 | 0.167332367177607 | 0.872263349114147 | -0.355878084 | 0.300064795493139 | 0.972479149156944 | 0.700558024371106 | 1.34994627517683 | 0.999714303421225 |
| ebi-fl187-GCST90094780 | prot-a-1627 | UPF0258 protein KIAA1024 \|\| id:prot-a-1627 | F-healthy breakfast food liking (derived food-liking factor) \|\| id:ebi-fl187-GCST90094780 | Weighted mode | 8 | 0.0510969830864754 | 0.176818194538695 | 0.780967175745324 | -0.295466678 | 0.397660644382318 | 1.05242495575445 | 0.744184211860471 | 1.48833886804148 | 0.999714303421225 |
| ebi-fl187-GCST90094780 | prot-a-1666 | Kallikrein-7 \|\| id:prot-a-1666 | F-healthy breakfast food liking (derived food-liking factor) \|\| id:ebi-fl187-GCST90094780 | Weighted mode | 8 | 0.0175612050093417 | 0.179094578033724 | 0.924636944822955 | -0.333464168 | 0.368586577955442 | 1.01771630958083 | 0.716437569616343 | 1.44568980007772 | 0.999714303421225 |
| ebi-fl187-GCST90094780 | prot-a-1678 | Importin subunit alpha-3 \|\| id:prot-a-1678 | F-healthy breakfast food liking (derived food-liking factor) \|\| id:ebi-fl187-GCST90094780 | Weighted mode | 8 | 0.229652130488518 | 0.210412530929571 | 0.311216440817768 | -0.18275643 | 0.642060691110478 | 1.25816225750365 | 0.832971017670182 | 1.90039297001503 | 0.999714303421225 |
| ebi-fl187-GCST90094780 | prot-a-1718 | Protein LDOC1 \|\| id:prot-a-1718 | F-healthy breakfast food liking (derived food-liking factor) \|\| id:ebi-fl187-GCST90094780 | Weighted mode | 8 | 0.00894301754761401 | 0.169602488742362 | 0.959420843153243 | -0.32347786 | 0.341363895482644 | 1.00898312580283 | 0.723627978588137 | 1.40686509957941 | 0.999714303421225 |
| ebi-fl187-GCST90094780 | prot-a-1723 | Leptin \|\| id:prot-a-1723 | F-healthy breakfast food liking (derived food-liking factor) \|\| id:ebi-fl187-GCST90094780 | Weighted mode | 8 | -0.053187445 | 0.210782693461091 | 0.808032484736944 | -0.466321524 | 0.359946634324842 | 0.948202260184576 | 0.627305557800248 | 1.43325292600936 | 0.999714303421225 |
| ebi-fl187-GCST90094780 | prot-a-1730 | Galectin-7 \|\| id:prot-a-1730 | F-healthy breakfast food liking (derived food-liking factor) \|\| id:ebi-fl187-GCST90094780 | Weighted mode | 8 | 0.0655214272415341 | 0.174483678819002 | 0.718397120327371 | -0.276466583 | 0.407509437726779 | 1.06771561526877 | 0.758458963946551 | 1.50306963102766 | 0.999714303421225 |
| ebi-fl187-GCST90094780 | prot-a-1753 | Lipase member N \|\| id:prot-a-1753 | F-healthy breakfast food liking (derived food-liking factor) \|\| id:ebi-fl187-GCST90094780 | Weighted mode | 8 | 0.0186879542401878 | 0.213967904760504 | 0.932847258090477 | -0.400689139 | 0.438065047570776 | 1.01886366692048 | 0.669858261424026 | 1.5497057087328 | 0.999714303421225 |
| ebi-fl187-GCST90094780 | prot-a-1756 | VIP36-like protein \|\| id:prot-a-1756 | F-healthy breakfast food liking (derived food-liking factor) \|\| id:ebi-fl187-GCST90094780 | Weighted mode | 8 | 0.12011593094479 | 0.196038969828148 | 0.559436259929146 | -0.26412045 | 0.50435231180796 | 1.12762757093169 | 0.767881042877264 | 1.65591265798255 | 0.999714303421225 |
| ebi-fl187-GCST90094780 | prot-a-1760 | Ig Kappa chain V-I region HK102- like \|\| id:prot-a-1760 | F-healthy breakfast food liking (derived food-liking factor) \|\| id:ebi-fl187-GCST90094780 | Weighted mode | 8 | -0.231644422 | 0.214008037763135 | 0.314941516783498 | -0.651100176 | 0.187811331978766 | 0.793228127608281 | 0.521471750321969 | 1.20660584593595 | 0.999714303421225 |
| ebi-fl187-GCST90094780 | prot-a-179 | Neutral ceramidase \|\| id:prot-a-179 | F-healthy breakfast food liking (derived food-liking factor) \|\| id:ebi-fl187-GCST90094780 | Weighted mode | 8 | 0.0190266573305172 | 0.216264376054337 | 0.932357619064107 | -0.40485152 | 0.442904834397018 | 1.0192088176416 | 0.667075851104023 | 1.55722413311646 | 0.999714303421225 |
| ebi-fl187-GCST90094780 | prot-a-1809 | Lactotransferrin \|\| id:prot-a-1809 | F-healthy breakfast food liking (derived food-liking factor) \|\| id:ebi-fl187-GCST90094780 | Weighted mode | 8 | 0.0350848711548006 | 0.199589717904706 | 0.8654399911257 | -0.356110976 | 0.426280718248024 | 1.03570760677019 | 0.700394888942114 | 1.53155064900865 | 0.999714303421225 |
| ebi-fl187-GCST90094780 | prot-a-1818 | Lysophospholipase-like protein 1 \|\| id:prot-a-1818 | F-healthy breakfast food liking (derived food-liking factor) \|\| id:ebi-fl187-GCST90094780 | Weighted mode | 8 | 0.0881580976230545 | 0.20628980986531 | 0.681961984049582 | -0.31616993 | 0.492486124959062 | 1.09216077640246 | 0.728935571830134 | 1.63637941075811 | 0.999714303421225 |
| ebi-fl187-GCST90094780 | prot-a-1852 | MAP kinase-activated protein kinase 3 \|\| id:prot-a-1852 | F-healthy breakfast food liking (derived food-liking factor) \|\| id:ebi-fl187-GCST90094780 | Weighted mode | 8 | -0.154709663 | 0.181899824628665 | 0.423170382253857 | -0.511233319 | 0.201813993757534 | 0.85666386301228 | 0.599755432845289 | 1.22362038591225 | 0.999714303421225 |
| ebi-fl187-GCST90094780 | prot-a-1856 | Megakaryocyte-associated tyrosine-protein kinase \|\| id:prot-a-1856 | F-healthy breakfast food liking (derived food-liking factor) \|\| id:ebi-fl187-GCST90094780 | Weighted mode | 8 | -0.124048309 | 0.171924890890096 | 0.493974830964207 | -0.461021095 | 0.212924477246405 | 0.883337166806737 | 0.630639373893169 | 1.23729120407622 | 0.999714303421225 |
| ebi-fl187-GCST90094780 | prot-a-1890 | Beta-1,3-N-acetylglucosaminyltransferase manic fringe \|\| id:prot-a-1890 | F-healthy breakfast food liking (derived food-liking factor) \|\| id:ebi-fl187-GCST90094780 | Weighted mode | 8 | -0.210738011 | 0.288118612558284 | 0.4882742203306 | -0.775450492 | 0.35397446945694 | 0.809986246445036 | 0.460496284477759 | 1.42471881217058 | 0.999714303421225 |
| ebi-fl187-GCST90094780 | prot-a-1919 | Matrilysin \|\| id:prot-a-1919 | F-healthy breakfast food liking (derived food-liking factor) \|\| id:ebi-fl187-GCST90094780 | Weighted mode | 8 | -0.238057537 | 0.150616417341957 | 0.157991880674214 | -0.533265715 | 0.0571506407003332 | 0.788157341351694 | 0.586685888726471 | 1.05881529906063 | 0.999714303421225 |
| ebi-fl187-GCST90094780 | prot-a-1923 | Multimerin-2 \|\| id:prot-a-1923 | F-healthy breakfast food liking (derived food-liking factor) \|\| id:ebi-fl187-GCST90094780 | Weighted mode | 8 | 0.118426475391135 | 0.181396345678983 | 0.534692086277462 | -0.237110362 | 0.473963312921942 | 1.12572410263482 | 0.788904218055584 | 1.60634805372999 | 0.999714303421225 |
| ebi-fl187-GCST90094780 | prot-a-1948 | Mesothelin \|\| id:prot-a-1948 | F-healthy breakfast food liking (derived food-liking factor) \|\| id:ebi-fl187-GCST90094780 | Weighted mode | 8 | 0.256628759323842 | 0.184474950547159 | 0.206799262842332 | -0.104942144 | 0.618199662396273 | 1.29256518490467 | 0.900376613495074 | 1.85558435457608 | 0.999714303421225 |
| ebi-fl187-GCST90094780 | prot-a-195 | ATPase family AAA domain-containing protein 1 \|\| id:prot-a-195 | F-healthy breakfast food liking (derived food-liking factor) \|\| id:ebi-fl187-GCST90094780 | Weighted mode | 8 | 0.25289378781828 | 0.155272229088664 | 0.147397724156707 | -0.051439781 | 0.557227356832061 | 1.28774649520606 | 0.949860847724682 | 1.74582523312526 | 0.999714303421225 |
| ebi-fl187-GCST90094780 | prot-a-1966 | Mucin-1 \|\| id:prot-a-1966 | F-healthy breakfast food liking (derived food-liking factor) \|\| id:ebi-fl187-GCST90094780 | Weighted mode | 8 | 0.0220742388371212 | 0.171340522648969 | 0.901113633306636 | -0.313753186 | 0.357901663229101 | 1.02231967747732 | 0.730699353060505 | 1.43032496002606 | 0.999714303421225 |
| ebi-fl187-GCST90094780 | prot-a-1986 | Myocardial zonula adherens protein \|\| id:prot-a-1986 | F-healthy breakfast food liking (derived food-liking factor) \|\| id:ebi-fl187-GCST90094780 | Weighted mode | 8 | -0.307918889 | 0.190106284501375 | 0.149324360280718 | -0.680527207 | 0.0646894283233568 | 0.734974929922989 | 0.506349970774395 | 1.0668276464777 | 0.999714303421225 |
| ebi-fl187-GCST90094780 | prot-a-2071 | Epididymal secretory protein E1 \|\| id:prot-a-2071 | F-healthy breakfast food liking (derived food-liking factor) \|\| id:ebi-fl187-GCST90094780 | Weighted mode | 8 | -0.386929038 | 0.195312215901175 | 0.0880415750196313 | -0.769740981 | -0.004117095 | 0.679139286286237 | 0.463133012940909 | 0.9958913687637 | 0.999714303421225 |
| ebi-fl187-GCST90094780 | prot-a-2098 | Neuritin \|\| id:prot-a-2098 | F-healthy breakfast food liking (derived food-liking factor) \|\| id:ebi-fl187-GCST90094780 | Weighted mode | 8 | 0.0932725966414005 | 0.226957297498699 | 0.693384220856403 | -0.351563706 | 0.538108899738851 | 1.09776094043049 | 0.703587025498292 | 1.71276478766984 | 0.999714303421225 |
| ebi-fl187-GCST90094780 | prot-a-2115 | Neurotrophin-4 \|\| id:prot-a-2115 | F-healthy breakfast food liking (derived food-liking factor) \|\| id:ebi-fl187-GCST90094780 | Weighted mode | 8 | 0.113949080333665 | 0.184528340654493 | 0.556441626220918 | -0.247726467 | 0.475624628016472 | 1.12069505801284 | 0.780573426397913 | 1.60901892196128 | 0.999714303421225 |
| ebi-fl187-GCST90094780 | prot-a-2118 | Netrin-1 \|\| id:prot-a-2118 | F-healthy breakfast food liking (derived food-liking factor) \|\| id:ebi-fl187-GCST90094780 | Weighted mode | 8 | 0.0156324933859995 | 0.191046532051194 | 0.937075937566935 | -0.358818709 | 0.39008369620634 | 1.01575532000486 | 0.698500971509945 | 1.47710441674524 | 0.999714303421225 |
| ebi-fl187-GCST90094780 | prot-a-2120 | Netrin-G1 \|\| id:prot-a-2120 | F-healthy breakfast food liking (derived food-liking factor) \|\| id:ebi-fl187-GCST90094780 | Weighted mode | 8 | -0.161223647 | 0.171588934638165 | 0.378708825248529 | -0.497537959 | 0.175090664849339 | 0.851101703442401 | 0.608025802902399 | 1.19135422566735 | 0.999714303421225 |
| ebi-fl187-GCST90094780 | prot-a-2145 | Olfactomedin-like protein 3 \|\| id:prot-a-2145 | F-healthy breakfast food liking (derived food-liking factor) \|\| id:ebi-fl187-GCST90094780 | Weighted mode | 8 | -0.219588087 | 0.230162569536648 | 0.371831291397206 | -0.670706723 | 0.231530549513584 | 0.8028494341581 | 0.511347069287709 | 1.26052783450161 | 0.999714303421225 |
| ebi-fl187-GCST90094780 | prot-a-2162 | Prolyl 4-hydroxylase subunit alpha-2 \|\| id:prot-a-2162 | F-healthy breakfast food liking (derived food-liking factor) \|\| id:ebi-fl187-GCST90094780 | Weighted mode | 8 | -0.008429218 | 0.169588777355073 | 0.961746871489902 | -0.340823222 | 0.323964785502832 | 0.991606208137478 | 0.711184619081893 | 1.38259861874707 | 0.999714303421225 |
| ebi-fl187-GCST90094780 | prot-a-2168 | Polyadenylate-binding protein-interacting protein 1 \|\| id:prot-a-2168 | F-healthy breakfast food liking (derived food-liking factor) \|\| id:ebi-fl187-GCST90094780 | Weighted mode | 8 | 0.18744067213632 | 0.206772164426794 | 0.394799271401066 | -0.21783277 | 0.592714114412835 | 1.20615868847998 | 0.804259926696077 | 1.80889129683754 | 0.999714303421225 |
| ebi-fl187-GCST90094780 | prot-a-219 | Beta-1,4-galactosyltransferase 5 \|\| id:prot-a-219 | F-healthy breakfast food liking (derived food-liking factor) \|\| id:ebi-fl187-GCST90094780 | Weighted mode | 8 | 0.195787374644021 | 0.179674675838257 | 0.311940052621718 | -0.15637499 | 0.547949739287005 | 1.21626826836537 | 0.855238424376887 | 1.72970303773513 | 0.999714303421225 |
| ebi-fl187-GCST90094780 | prot-a-2190 | Pyruvate carboxylase, mitochondrial \|\| id:prot-a-2190 | F-healthy breakfast food liking (derived food-liking factor) \|\| id:ebi-fl187-GCST90094780 | Weighted mode | 8 | -0.159446717 | 0.182794476888699 | 0.411977112652931 | -0.517723892 | 0.198830457742236 | 0.852615396126709 | 0.595875282323071 | 1.21997511103031 | 0.999714303421225 |
| ebi-fl187-GCST90094780 | prot-a-2197 | Protocadherin-9 \|\| id:prot-a-2197 | F-healthy breakfast food liking (derived food-liking factor) \|\| id:ebi-fl187-GCST90094780 | Weighted mode | 8 | 0.0846999050997216 | 0.20934679519644 | 0.697855286591235 | -0.325619813 | 0.495019623684745 | 1.0883903972772 | 0.722079660202334 | 1.64053043199318 | 0.999714303421225 |
| ebi-fl187-GCST90094780 | prot-a-2198 | Protocadherin alpha-4 \|\| id:prot-a-2198 | F-healthy breakfast food liking (derived food-liking factor) \|\| id:ebi-fl187-GCST90094780 | Weighted mode | 8 | 0.30551981434164 | 0.212300145776287 | 0.193298291066104 | -0.110588471 | 0.721628100063164 | 1.35733037945553 | 0.8953071176294 | 2.05778075781533 | 0.999714303421225 |
| ebi-fl187-GCST90094780 | prot-a-2254 | Placenta growth factor \|\| id:prot-a-2254 | F-healthy breakfast food liking (derived food-liking factor) \|\| id:ebi-fl187-GCST90094780 | Weighted mode | 8 | 0.24102161953571 | 0.1873189974886 | 0.239116982842817 | -0.126123616 | 0.608166854613366 | 1.27254854691959 | 0.881505872221957 | 1.83706071087795 | 0.999714303421225 |
| ebi-fl187-GCST90094780 | prot-a-2261 | Membrane-associated progesterone receptor component 2 \|\| id:prot-a-2261 | F-healthy breakfast food liking (derived food-liking factor) \|\| id:ebi-fl187-GCST90094780 | Weighted mode | 8 | 0.112086675683798 | 0.1968196474248 | 0.586825755691244 | -0.273679833 | 0.497853184636406 | 1.11860981271371 | 0.760575547269776 | 1.6451855671552 | 0.999714303421225 |
| ebi-fl187-GCST90094780 | prot-a-2268 | Polymeric immunoglobulin receptor \|\| id:prot-a-2268 | F-healthy breakfast food liking (derived food-liking factor) \|\| id:ebi-fl187-GCST90094780 | Weighted mode | 8 | -0.053678694 | 0.179723940990784 | 0.773861110196372 | -0.405937619 | 0.298580229864209 | 0.947736570579971 | 0.666351733936071 | 1.34794367819692 | 0.999714303421225 |
| ebi-fl187-GCST90094780 | prot-a-2283 | Group 10 secretory phospholipase A2 \|\| id:prot-a-2283 | F-healthy breakfast food liking (derived food-liking factor) \|\| id:ebi-fl187-GCST90094780 | Weighted mode | 8 | 0.282678845029256 | 0.183573945534021 | 0.167488867390832 | -0.077126088 | 0.642483778275936 | 1.32667902382957 | 0.925773117165022 | 1.90119717200174 | 0.999714303421225 |
| ebi-fl187-GCST90094780 | prot-a-2298 | Inactive phospholipase D5 \|\| id:prot-a-2298 | F-healthy breakfast food liking (derived food-liking factor) \|\| id:ebi-fl187-GCST90094780 | Weighted mode | 8 | 0.148299036815046 | 0.181472702526411 | 0.440738203869002 | -0.20738746 | 0.50398553376681 | 1.15985968525525 | 0.812704698302383 | 1.65530541694969 | 0.999714303421225 |
| ebi-fl187-GCST90094780 | prot-a-2304 | Procollagen-lysine,2-oxoglutarate 5-dioxygenase 2 \|\| id:prot-a-2304 | F-healthy breakfast food liking (derived food-liking factor) \|\| id:ebi-fl187-GCST90094780 | Weighted mode | 8 | -0.113193914 | 0.213425756938514 | 0.612259796489043 | -0.531508397 | 0.30512056983539 | 0.892977482700891 | 0.587717788772782 | 1.35678858092061 | 0.999714303421225 |
| ebi-fl187-GCST90094780 | prot-a-2331 | Periostin \|\| id:prot-a-2331 | F-healthy breakfast food liking (derived food-liking factor) \|\| id:ebi-fl187-GCST90094780 | Weighted mode | 8 | 0.0641511604821883 | 0.212385942170673 | 0.771390787353092 | -0.352125286 | 0.480427607136707 | 1.06625356198301 | 0.703192016221114 | 1.61676559490966 | 0.999714303421225 |
| ebi-fl187-GCST90094780 | prot-a-2333 | Periostin \|\| id:prot-a-2333 | F-healthy breakfast food liking (derived food-liking factor) \|\| id:ebi-fl187-GCST90094780 | Weighted mode | 8 | -0.200366931 | 0.286376514130028 | 0.506715782169889 | -0.761664899 | 0.360931036468534 | 0.818430390308338 | 0.466888457480129 | 1.43466451793524 | 0.999714303421225 |
| ebi-fl187-GCST90094780 | prot-a-2367 | cAMP-dependent protein kinase catalytic subunit alpha \|\| id:prot-a-2367 | F-healthy breakfast food liking (derived food-liking factor) \|\| id:ebi-fl187-GCST90094780 | Weighted mode | 8 | -0.121557145 | 0.211311147966647 | 0.583130475648943 | -0.535726995 | 0.292612704919153 | 0.885540447609521 | 0.585243666166743 | 1.33992374405133 | 0.999714303421225 |
| ebi-fl187-GCST90094780 | prot-a-2385 | Phosphoribosyl pyrophosphate synthase-associated protein 1 \|\| id:prot-a-2385 | F-healthy breakfast food liking (derived food-liking factor) \|\| id:ebi-fl187-GCST90094780 | Weighted mode | 8 | -0.152120819 | 0.292764324116259 | 0.619362300368679 | -0.725938894 | 0.421697256620041 | 0.858884505207681 | 0.483870052968885 | 1.52454690832722 | 0.999714303421225 |
| ebi-fl187-GCST90094780 | prot-a-2393 | Trypsin-3 \|\| id:prot-a-2393 | F-healthy breakfast food liking (derived food-liking factor) \|\| id:ebi-fl187-GCST90094780 | Weighted mode | 8 | 0.0920972589789222 | 0.167180858569461 | 0.598846484356507 | -0.235577224 | 0.419771741775065 | 1.09647145858953 | 0.79011464498389 | 1.52161419502099 | 0.999714303421225 |
| ebi-fl187-GCST90094780 | prot-a-2405 | Pregnancy-specific beta-1-glycoprotein 6 \|\| id:prot-a-2405 | F-healthy breakfast food liking (derived food-liking factor) \|\| id:ebi-fl187-GCST90094780 | Weighted mode | 8 | -0.044995205 | 0.179786937002039 | 0.80956525751949 | -0.397377601 | 0.307387191752611 | 0.956002066070571 | 0.672080199359229 | 1.35986739559143 | 0.999714303421225 |
| ebi-fl187-GCST90094780 | prot-a-244 | Beclin-1 \|\| id:prot-a-244 | F-healthy breakfast food liking (derived food-liking factor) \|\| id:ebi-fl187-GCST90094780 | Weighted mode | 8 | 0.170232319194296 | 0.202074443722455 | 0.427392552819966 | -0.225833591 | 0.566298228890308 | 1.18558025237766 | 0.79785086058061 | 1.76173343199127 | 0.999714303421225 |
| ebi-fl187-GCST90094780 | prot-a-2454 | tRNA pseudouridine synthase A, mitochondrial \|\| id:prot-a-2454 | F-healthy breakfast food liking (derived food-liking factor) \|\| id:ebi-fl187-GCST90094780 | Weighted mode | 8 | 0.103492024566524 | 0.169815131720856 | 0.561484762891442 | -0.229345634 | 0.436329682739403 | 1.10903694827939 | 0.795053688736353 | 1.54701873606015 | 0.999714303421225 |
| ebi-fl187-GCST90094780 | prot-a-2466 | Glutaminyl-peptide cyclotransferase-like protein \|\| id:prot-a-2466 | F-healthy breakfast food liking (derived food-liking factor) \|\| id:ebi-fl187-GCST90094780 | Weighted mode | 8 | -0.109741365 | 0.163680534344518 | 0.524060058994219 | -0.430555212 | 0.211072482451181 | 0.896065859444589 | 0.650148024395758 | 1.23500186777989 | 0.999714303421225 |
| ebi-fl187-GCST90094780 | prot-a-2474 | Ras-related protein Rab-27A \|\| id:prot-a-2474 | F-healthy breakfast food liking (derived food-liking factor) \|\| id:ebi-fl187-GCST90094780 | Weighted mode | 8 | -0.025410163 | 0.199538367360619 | 0.902248550286102 | -0.416505363 | 0.365685037406414 | 0.974909958387738 | 0.659346976940731 | 1.44150115220596 | 0.999714303421225 |
| ebi-fl187-GCST90094780 | prot-a-2490 | Rap1 GTPase-GDP dissociation stimulator 1 \|\| id:prot-a-2490 | F-healthy breakfast food liking (derived food-liking factor) \|\| id:ebi-fl187-GCST90094780 | Weighted mode | 8 | 0.0584187225811763 | 0.183151677202353 | 0.759061142180287 | -0.300558565 | 0.417396009897788 | 1.06015881522074 | 0.740404541292392 | 1.51800353834729 | 0.999714303421225 |
| ebi-fl187-GCST90094780 | prot-a-2545 | Receptor-interacting serine/threonine-protein kinase 2 \|\| id:prot-a-2545 | F-healthy breakfast food liking (derived food-liking factor) \|\| id:ebi-fl187-GCST90094780 | Weighted mode | 8 | 0.0873837957854633 | 0.164892969718783 | 0.612538820593223 | -0.235806425 | 0.410574016434277 | 1.09131544162072 | 0.789933570632705 | 1.50768297157684 | 0.999714303421225 |
| ebi-fl187-GCST90094780 | prot-a-2610 | RUN and FYVE domain-containing protein 1 \|\| id:prot-a-2610 | F-healthy breakfast food liking (derived food-liking factor) \|\| id:ebi-fl187-GCST90094780 | Weighted mode | 8 | -0.078446634 | 0.180350374345129 | 0.676680621554251 | -0.431933368 | 0.275040099529263 | 0.924551398051899 | 0.649252636308114 | 1.31658346818643 | 0.999714303421225 |
| ebi-fl187-GCST90094780 | prot-a-2627 | Serine--tRNA ligase, mitochondrial \|\| id:prot-a-2627 | F-healthy breakfast food liking (derived food-liking factor) \|\| id:ebi-fl187-GCST90094780 | Weighted mode | 8 | -0.067362842 | 0.199743077135696 | 0.745816826549433 | -0.458859274 | 0.324133588687821 | 0.934855934327639 | 0.632004178261353 | 1.38283202549683 | 0.999714303421225 |
| ebi-fl187-GCST90094780 | prot-a-264 | BolA-like protein 2 \|\| id:prot-a-264 | F-healthy breakfast food liking (derived food-liking factor) \|\| id:ebi-fl187-GCST90094780 | Weighted mode | 8 | 0.119310866249049 | 0.168954018429526 | 0.502899461122777 | -0.21183901 | 0.450460742370919 | 1.1267201231105 | 0.809094943379908 | 1.56903493985385 | 0.999714303421225 |
| ebi-fl187-GCST90094780 | prot-a-2646 | Secretoglobin family 1C member 1 \|\| id:prot-a-2646 | F-healthy breakfast food liking (derived food-liking factor) \|\| id:ebi-fl187-GCST90094780 | Weighted mode | 8 | -0.038511936 | 0.185465829435586 | 0.841414357399991 | -0.402024962 | 0.325001089267737 | 0.962220219200816 | 0.668964046721277 | 1.3840321535615 | 0.999714303421225 |
| ebi-fl187-GCST90094780 | prot-a-2672 | Semaphorin-3E \|\| id:prot-a-2672 | F-healthy breakfast food liking (derived food-liking factor) \|\| id:ebi-fl187-GCST90094780 | Weighted mode | 8 | -0.12096479 | 0.189105394996705 | 0.542747811145227 | -0.491611364 | 0.24968178389055 | 0.886065157129357 | 0.611640024677016 | 1.28361688411948 | 0.999714303421225 |
| ebi-fl187-GCST90094780 | prot-a-2701 | Plasma protease C1 inhibitor \|\| id:prot-a-2701 | F-healthy breakfast food liking (derived food-liking factor) \|\| id:ebi-fl187-GCST90094780 | Weighted mode | 8 | -0.155393474 | 0.174352467663348 | 0.402383654324568 | -0.497124311 | 0.186337362385513 | 0.856078266464362 | 0.608277363632035 | 1.20482865569195 | 0.999714303421225 |
| ebi-fl187-GCST90094780 | prot-a-2738 | Signaling threshold-regulating transmembrane adapter 1 \|\| id:prot-a-2738 | F-healthy breakfast food liking (derived food-liking factor) \|\| id:ebi-fl187-GCST90094780 | Weighted mode | 8 | -0.203828209 | 0.194361274000971 | 0.329165384287933 | -0.584776306 | 0.177119887689533 | 0.81560247202982 | 0.557230496770677 | 1.19377420337946 | 0.999714303421225 |
| ebi-fl187-GCST90094780 | prot-a-274 | Serine/threonine-protein kinase BRSK2 \|\| id:prot-a-274 | F-healthy breakfast food liking (derived food-liking factor) \|\| id:ebi-fl187-GCST90094780 | Weighted mode | 8 | 0.10269493413061 | 0.175622209453718 | 0.577069593723789 | -0.241524596 | 0.446914464659897 | 1.10815329775632 | 0.785429484814657 | 1.56348056072531 | 0.999714303421225 |
| ebi-fl187-GCST90094780 | prot-a-2749 | Mitochondrial glutamate carrier 2 \|\| id:prot-a-2749 | F-healthy breakfast food liking (derived food-liking factor) \|\| id:ebi-fl187-GCST90094780 | Weighted mode | 8 | -0.100695988 | 0.191847598381066 | 0.615877623733452 | -0.476717281 | 0.275325304458145 | 0.904207880818104 | 0.620818021413549 | 1.3169590178326 | 0.999714303421225 |
| ebi-fl187-GCST90094780 | prot-a-2760 | Sodium-coupled monocarboxylate transporter 1 \|\| id:prot-a-2760 | F-healthy breakfast food liking (derived food-liking factor) \|\| id:ebi-fl187-GCST90094780 | Weighted mode | 8 | -0.130576406 | 0.171120027327494 | 0.470351441643221 | -0.46597166 | 0.204818847496017 | 0.877589437230691 | 0.627525068089357 | 1.22730271586411 | 0.999714303421225 |
| ebi-fl187-GCST90094780 | prot-a-2777 | Small integral membrane protein 10 \|\| id:prot-a-2777 | F-healthy breakfast food liking (derived food-liking factor) \|\| id:ebi-fl187-GCST90094780 | Weighted mode | 8 | 0.00443097770223078 | 0.177380017766916 | 0.980768005062497 | -0.343233857 | 0.352095812525387 | 1.00444080899932 | 0.709472277014399 | 1.4220447668919 | 0.999714303421225 |
| ebi-fl187-GCST90094780 | prot-a-2799 | Superoxide dismutase [Mn], mitochondrial \|\| id:prot-a-2799 | F-healthy breakfast food liking (derived food-liking factor) \|\| id:ebi-fl187-GCST90094780 | Weighted mode | 8 | -0.100089596 | 0.225609096616297 | 0.670690446266998 | -0.542283426 | 0.342104233186049 | 0.904756351689741 | 0.581419108203792 | 1.40790704050272 | 0.999714303421225 |
| ebi-fl187-GCST90094780 | prot-a-2805 | Protein SPACA7 \|\| id:prot-a-2805 | F-healthy breakfast food liking (derived food-liking factor) \|\| id:ebi-fl187-GCST90094780 | Weighted mode | 8 | -0.110529245 | 0.189340400388906 | 0.577699492274499 | -0.48163643 | 0.260577939861434 | 0.895360145087954 | 0.61777162437767 | 1.2976798508988 | 0.999714303421225 |
| ebi-fl187-GCST90094780 | prot-a-2811 | SPARC-like protein 1 \|\| id:prot-a-2811 | F-healthy breakfast food liking (derived food-liking factor) \|\| id:ebi-fl187-GCST90094780 | Weighted mode | 8 | 0.200134865120467 | 0.199386012853032 | 0.348924669341864 | -0.19066172 | 0.590931450312411 | 1.22156749389859 | 0.826412099497939 | 1.80566952378389 | 0.999714303421225 |
| ebi-fl187-GCST90094780 | prot-a-2831 | Sepiapterin reductase \|\| id:prot-a-2831 | F-healthy breakfast food liking (derived food-liking factor) \|\| id:ebi-fl187-GCST90094780 | Weighted mode | 8 | -0.168666914 | 0.172314556735415 | 0.360272366092042 | -0.506403445 | 0.169069617695111 | 0.844790244748463 | 0.602659182792688 | 1.18420257750833 | 0.999714303421225 |
| ebi-fl187-GCST90094780 | prot-a-2834 | SPRY domain-containing SOCS box protein 1 \|\| id:prot-a-2834 | F-healthy breakfast food liking (derived food-liking factor) \|\| id:ebi-fl187-GCST90094780 | Weighted mode | 8 | -0.273541452 | 0.204609878432595 | 0.223069395070213 | -0.674576813 | 0.127493910055139 | 0.760680804210577 | 0.509371934379785 | 1.13597795017702 | 0.999714303421225 |
| ebi-fl187-GCST90094780 | prot-a-2835 | Spectrin alpha chain, non-erythrocytic 1 \|\| id:prot-a-2835 | F-healthy breakfast food liking (derived food-liking factor) \|\| id:ebi-fl187-GCST90094780 | Weighted mode | 8 | -0.201017327 | 0.236460535036137 | 0.423384387390534 | -0.664479975 | 0.262445321884379 | 0.817898259882599 | 0.514541032111111 | 1.30010537891277 | 0.999714303421225 |
| ebi-fl187-GCST90094780 | prot-a-2859 | Sia-alpha-2,3-Gal-beta-1,4-GlcNAc-R:alpha 2,8-sialyltransferase \|\| id:prot-a-2859 | F-healthy breakfast food liking (derived food-liking factor) \|\| id:ebi-fl187-GCST90094780 | Weighted mode | 8 | -0.22110498 | 0.187078999438299 | 0.275841895509464 | -0.587779819 | 0.145569859241782 | 0.801632520765456 | 0.555559359094004 | 1.15669853784255 | 0.999714303421225 |
| ebi-fl187-GCST90094780 | prot-a-2861 | Alpha-2,8-sialyltransferase 8F \|\| id:prot-a-2861 | F-healthy breakfast food liking (derived food-liking factor) \|\| id:ebi-fl187-GCST90094780 | Weighted mode | 8 | -0.091711872 | 0.165475483287563 | 0.596673021234714 | -0.416043819 | 0.232620075455063 | 0.912367990634621 | 0.659651364566704 | 1.2619019607144 | 0.999714303421225 |
| ebi-fl187-GCST90094780 | prot-a-289 | Uncharacterized protein C17orf89 \|\| id:prot-a-289 | F-healthy breakfast food liking (derived food-liking factor) \|\| id:ebi-fl187-GCST90094780 | Weighted mode | 8 | 0.127018499838861 | 0.157664485120081 | 0.446957459781834 | -0.182003891 | 0.43604089067422 | 1.13543802300765 | 0.83359809688215 | 1.54657203382962 | 0.999714303421225 |
| ebi-fl187-GCST90094780 | prot-a-2921 | Tachykinin-3 \|\| id:prot-a-2921 | F-healthy breakfast food liking (derived food-liking factor) \|\| id:ebi-fl187-GCST90094780 | Weighted mode | 8 | 0.073279346832797 | 0.16690638504422 | 0.673862080566195 | -0.253857168 | 0.400415861519468 | 1.07603108080917 | 0.775802603684125 | 1.49244521914338 | 0.999714303421225 |
| ebi-fl187-GCST90094780 | prot-a-2928 | Tax1-binding protein 1 \|\| id:prot-a-2928 | F-healthy breakfast food liking (derived food-liking factor) \|\| id:ebi-fl187-GCST90094780 | Weighted mode | 8 | 0.142374128036774 | 0.223106373305207 | 0.543683252840628 | -0.294914364 | 0.579662619714979 | 1.15300794044992 | 0.744595349170021 | 1.78543595823214 | 0.999714303421225 |
| ebi-fl187-GCST90094780 | prot-a-296 | Uncharacterized protein C1orf185 \|\| id:prot-a-296 | F-healthy breakfast food liking (derived food-liking factor) \|\| id:ebi-fl187-GCST90094780 | Weighted mode | 8 | -0.11342786 | 0.194576013309329 | 0.578215700102311 | -0.494796846 | 0.26794112624144 | 0.892768598553385 | 0.60969475668622 | 1.30727017384073 | 0.999714303421225 |
| ebi-fl187-GCST90094780 | prot-a-2964 | Transforming growth factor beta-2 \|\| id:prot-a-2964 | F-healthy breakfast food liking (derived food-liking factor) \|\| id:ebi-fl187-GCST90094780 | Weighted mode | 8 | 0.183392124960373 | 0.162607226590493 | 0.296562976420036 | -0.135318039 | 0.502102289077739 | 1.20128536971489 | 0.873438079999049 | 1.65219100533446 | 0.999714303421225 |
| ebi-fl187-GCST90094780 | prot-a-2977 | Thymocyte nuclear protein 1 \|\| id:prot-a-2977 | F-healthy breakfast food liking (derived food-liking factor) \|\| id:ebi-fl187-GCST90094780 | Weighted mode | 8 | 0.0237732746191657 | 0.165091383635735 | 0.889557927245822 | -0.299805837 | 0.347352386545206 | 1.02405811160274 | 0.740962073907575 | 1.41531537560204 | 0.999714303421225 |
| ebi-fl187-GCST90094780 | prot-a-3015 | Transmembrane protein 70, mitochondrial \|\| id:prot-a-3015 | F-healthy breakfast food liking (derived food-liking factor) \|\| id:ebi-fl187-GCST90094780 | Weighted mode | 8 | -0.312115961 | 0.176241804155939 | 0.11986934845835 | -0.657549898 | 0.0333179747740849 | 0.731896651567649 | 0.51811922582717 | 1.03387923449616 | 0.999714303421225 |
| ebi-fl187-GCST90094780 | prot-a-3030 | Tumor necrosis factor alpha-induced protein 3 \|\| id:prot-a-3030 | F-healthy breakfast food liking (derived food-liking factor) \|\| id:ebi-fl187-GCST90094780 | Weighted mode | 8 | -0.200907936 | 0.183275105782573 | 0.309261736094973 | -0.560127144 | 0.158311270879972 | 0.817987734939027 | 0.571136442781618 | 1.17153080138247 | 0.999714303421225 |
| ebi-fl187-GCST90094780 | prot-a-3032 | Tumor necrosis factor receptor superfamily member 10A \|\| id:prot-a-3032 | F-healthy breakfast food liking (derived food-liking factor) \|\| id:ebi-fl187-GCST90094780 | Weighted mode | 8 | 0.288085227290166 | 0.233417250171959 | 0.25694982392467 | -0.169412583 | 0.745583037627204 | 1.33387098148829 | 0.844160545197847 | 2.10766992769076 | 0.999714303421225 |
| ebi-fl187-GCST90094780 | prot-a-3081 | Tumor protein p53-inducible protein 11 \|\| id:prot-a-3081 | F-healthy breakfast food liking (derived food-liking factor) \|\| id:ebi-fl187-GCST90094780 | Weighted mode | 8 | -0.036319618 | 0.158229245049895 | 0.825013606367189 | -0.346448938 | 0.273809702665512 | 0.964332026702889 | 0.707194929202264 | 1.31496454418006 | 0.999714303421225 |
| ebi-fl187-GCST90094780 | prot-a-315 | Complement C4 \|\| id:prot-a-315 | F-healthy breakfast food liking (derived food-liking factor) \|\| id:ebi-fl187-GCST90094780 | Weighted mode | 7 | 0.0108113773557431 | 0.202054845358643 | 0.959064912661461 | -0.38521612 | 0.406838874258684 | 1.01087003148263 | 0.680303593411184 | 1.50206206529923 | 0.999714303421225 |
| ebi-fl187-GCST90094780 | prot-a-3157 | UDP-glucuronosyltransferase 1-8 \|\| id:prot-a-3157 | F-healthy breakfast food liking (derived food-liking factor) \|\| id:ebi-fl187-GCST90094780 | Weighted mode | 8 | -0.052669376 | 0.194315743763044 | 0.794171064655996 | -0.433528234 | 0.328189481664434 | 0.948693621410594 | 0.648217990643954 | 1.38845203356829 | 0.999714303421225 |
| ebi-fl187-GCST90094780 | prot-a-317 | Glutaredoxin-like protein C5orf63 \|\| id:prot-a-317 | F-healthy breakfast food liking (derived food-liking factor) \|\| id:ebi-fl187-GCST90094780 | Weighted mode | 8 | 0.168391464889883 | 0.160926974538567 | 0.330164606005451 | -0.147025405 | 0.483808334985475 | 1.18339977944908 | 0.863272045533829 | 1.62224068906823 | 0.999714303421225 |
| ebi-fl187-GCST90094780 | prot-a-3192 | Guanine nucleotide exchange factor VAV3 \|\| id:prot-a-3192 | F-healthy breakfast food liking (derived food-liking factor) \|\| id:ebi-fl187-GCST90094780 | Weighted mode | 8 | 0.0505353685421699 | 0.16642221464714 | 0.770215730381056 | -0.275652172 | 0.376722909250565 | 1.05183406453459 | 0.7590769129267 | 1.45750039353679 | 0.999714303421225 |
| ebi-fl187-GCST90094780 | prot-a-3233 | Serine/threonine-protein kinase WNK3 \|\| id:prot-a-3233 | F-healthy breakfast food liking (derived food-liking factor) \|\| id:ebi-fl187-GCST90094780 | Weighted mode | 8 | 0.082137030981905 | 0.24823096750147 | 0.750412157590191 | -0.404395665 | 0.568669727284787 | 1.08560456109652 | 0.66738000989669 | 1.76591633791369 | 0.999714303421225 |
| ebi-fl187-GCST90094780 | prot-a-3250 | 14-3-3 protein theta \|\| id:prot-a-3250 | F-healthy breakfast food liking (derived food-liking factor) \|\| id:ebi-fl187-GCST90094780 | Weighted mode | 8 | 0.0772327663803866 | 0.172902949628378 | 0.668596266529524 | -0.261657015 | 0.416122547652008 | 1.0802935031381 | 0.769774999797084 | 1.51607164850771 | 0.999714303421225 |
| ebi-fl187-GCST90094780 | prot-a-3278 | E3 ubiquitin-protein ligase ZNRF3 \|\| id:prot-a-3278 | F-healthy breakfast food liking (derived food-liking factor) \|\| id:ebi-fl187-GCST90094780 | Weighted mode | 8 | -0.203037309 | 0.244798160366517 | 0.434242621177476 | -0.682841703 | 0.276767085650431 | 0.81624778773985 | 0.505179380945226 | 1.31885915403669 | 0.999714303421225 |
| ebi-fl187-GCST90094780 | prot-a-355 | Calpain-3 \|\| id:prot-a-355 | F-healthy breakfast food liking (derived food-liking factor) \|\| id:ebi-fl187-GCST90094780 | Weighted mode | 8 | -0.034740237 | 0.182701162441158 | 0.854589950434966 | -0.392834515 | 0.323354041303953 | 0.96585627732016 | 0.675140463647663 | 1.38175446246931 | 0.999714303421225 |
| ebi-fl187-GCST90094780 | prot-a-385 | Coiled-coil domain-containing protein 90B, mitochondrial \|\| id:prot-a-385 | F-healthy breakfast food liking (derived food-liking factor) \|\| id:ebi-fl187-GCST90094780 | Weighted mode | 8 | 0.0878613077789355 | 0.214168334676036 | 0.693892220721668 | -0.331908628 | 0.507631243743967 | 1.09183668227218 | 0.717552883967759 | 1.66135119430261 | 0.999714303421225 |
| ebi-fl187-GCST90094780 | prot-a-390 | C-C motif chemokine 14 \|\| id:prot-a-390 | F-healthy breakfast food liking (derived food-liking factor) \|\| id:ebi-fl187-GCST90094780 | Weighted mode | 8 | -0.028956081 | 0.229190397342034 | 0.903014560664046 | -0.47816926 | 0.420257097406026 | 0.971459128669803 | 0.619917260797594 | 1.52235289829109 | 0.999714303421225 |
| ebi-fl187-GCST90094780 | prot-a-40 | Pituitary adenylate cyclase-activating polypeptide 27 \|\| id:prot-a-40 | F-healthy breakfast food liking (derived food-liking factor) \|\| id:ebi-fl187-GCST90094780 | Weighted mode | 8 | 0.136244644734826 | 0.19306068719822 | 0.503171216459332 | -0.242154302 | 0.514643591643337 | 1.14596221292584 | 0.784935051022452 | 1.6730421093354 | 0.999714303421225 |
| ebi-fl187-GCST90094780 | prot-a-401 | C-C motif chemokine 24 \|\| id:prot-a-401 | F-healthy breakfast food liking (derived food-liking factor) \|\| id:ebi-fl187-GCST90094780 | Weighted mode | 8 | 0.327772524081621 | 0.208658905449721 | 0.160209934543008 | -0.081198931 | 0.736743978763073 | 1.38787322864146 | 0.92201025714636 | 2.08912220211222 | 0.999714303421225 |
| ebi-fl187-GCST90094780 | prot-a-412 | Cerebral cavernous malformations 2 protein \|\| id:prot-a-412 | F-healthy breakfast food liking (derived food-liking factor) \|\| id:ebi-fl187-GCST90094780 | Weighted mode | 8 | 0.216724728368226 | 0.173545470019813 | 0.25187863 | -0.123424393 | 0.556873849607059 | 1.24200216711421 | 0.883888466987487 | 1.74520818036449 | 0.999714303421225 |
| ebi-fl187-GCST90094780 | prot-a-44 | Adhesion G protein-coupled receptor F5 \|\| id:prot-a-44 | F-healthy breakfast food liking (derived food-liking factor) \|\| id:ebi-fl187-GCST90094780 | Weighted mode | 8 | -0.178563816 | 0.189011761106828 | 0.376258180357048 | -0.549026868 | 0.191899235504797 | 0.836470674740189 | 0.577511531954045 | 1.21154842974804 | 0.999714303421225 |
| ebi-fl187-GCST90094780 | prot-a-48 | Adrenomedullin \|\| id:prot-a-48 | F-healthy breakfast food liking (derived food-liking factor) \|\| id:ebi-fl187-GCST90094780 | Weighted mode | 8 | -0.004996059 | 0.181657261696428 | 0.978826466471413 | -0.361044292 | 0.351052173578002 | 0.995016400199322 | 0.696948128367155 | 1.42056143975187 | 0.999714303421225 |
| ebi-fl187-GCST90094780 | prot-a-502 | Carcinoembryonic antigen-related cell adhesion molecule 1 \|\| id:prot-a-502 | F-healthy breakfast food liking (derived food-liking factor) \|\| id:ebi-fl187-GCST90094780 | Weighted mode | 8 | -0.276412364 | 0.180801919755973 | 0.17015713715637 | -0.630784127 | 0.0779593984550469 | 0.758500087930551 | 0.532174345092558 | 1.08107876431095 | 0.999714303421225 |
| ebi-fl187-GCST90094780 | prot-a-518 | Complement factor B \|\| id:prot-a-518 | F-healthy breakfast food liking (derived food-liking factor) \|\| id:ebi-fl187-GCST90094780 | Weighted mode | 8 | 0.159365472770921 | 0.192594519527568 | 0.435270550953141 | -0.218119786 | 0.536850731044954 | 1.17276648258288 | 0.804029124864757 | 1.71061119570907 | 0.999714303421225 |
| ebi-fl187-GCST90094780 | prot-a-525 | Properdin \|\| id:prot-a-525 | F-healthy breakfast food liking (derived food-liking factor) \|\| id:ebi-fl187-GCST90094780 | Weighted mode | 8 | -0.220673216 | 0.179233460759257 | 0.258003146818733 | -0.571970799 | 0.130624366693824 | 0.801978710969048 | 0.564411999001207 | 1.13953965186024 | 0.999714303421225 |
| ebi-fl187-GCST90094780 | prot-a-533 | Chondroadherin \|\| id:prot-a-533 | F-healthy breakfast food liking (derived food-liking factor) \|\| id:ebi-fl187-GCST90094780 | Weighted mode | 8 | -0.170334197 | 0.142996797379936 | 0.272403411775834 | -0.45060792 | 0.109939526139199 | 0.843382913685416 | 0.637240642776214 | 1.11621056685535 | 0.999714303421225 |
| ebi-fl187-GCST90094780 | prot-a-535 | Coiled-coil-helix-coiled-coil-helix domain-containing protein 2 \|\| id:prot-a-535 | F-healthy breakfast food liking (derived food-liking factor) \|\| id:ebi-fl187-GCST90094780 | Weighted mode | 8 | -0.17188639 | 0.178528598657344 | 0.367722551088757 | -0.521802444 | 0.178029662961996 | 0.842074835514162 | 0.593449923269786 | 1.19486076381856 | 0.999714303421225 |
| ebi-fl187-GCST90094780 | prot-a-591 | Caseinolytic peptidase B protein homolog \|\| id:prot-a-591 | F-healthy breakfast food liking (derived food-liking factor) \|\| id:ebi-fl187-GCST90094780 | Weighted mode | 8 | -0.186744241 | 0.199895047837623 | 0.381291745123561 | -0.578538534 | 0.205050053218209 | 0.829655901595065 | 0.560717237050481 | 1.22758650808081 | 0.999714303421225 |
| ebi-fl187-GCST90094780 | prot-a-605 | Ciliary neurotrophic factor receptor subunit alpha \|\| id:prot-a-605 | F-healthy breakfast food liking (derived food-liking factor) \|\| id:ebi-fl187-GCST90094780 | Weighted mode | 8 | -0.291230884 | 0.236198964611806 | 0.257376597805257 | -0.754180855 | 0.171719086537625 | 0.747343108454955 | 0.470395779428818 | 1.18734424537844 | 0.999714303421225 |
| ebi-fl187-GCST90094780 | prot-a-606 | Ciliary neurotrophic factor receptor subunit alpha \|\| id:prot-a-606 | F-healthy breakfast food liking (derived food-liking factor) \|\| id:ebi-fl187-GCST90094780 | Weighted mode | 8 | -0.129125114 | 0.163445643403532 | 0.455441433433185 | -0.449478575 | 0.191228347512937 | 0.878864000864556 | 0.637960713812997 | 1.21073588904734 | 0.999714303421225 |
| ebi-fl187-GCST90094780 | prot-a-707 | Cancer/testis antigen 55 \|\| id:prot-a-707 | F-healthy breakfast food liking (derived food-liking factor) \|\| id:ebi-fl187-GCST90094780 | Weighted mode | 8 | 0.113955820375442 | 0.162292053173828 | 0.505246750253835 | -0.204136604 | 0.432048244596144 | 1.1207026115698 | 0.81535098349989 | 1.54040942979931 | 0.999714303421225 |
| ebi-fl187-GCST90094780 | prot-a-719 | Dipeptidyl peptidase 1 \|\| id:prot-a-719 | F-healthy breakfast food liking (derived food-liking factor) \|\| id:ebi-fl187-GCST90094780 | Weighted mode | 8 | 0.164810274976663 | 0.172730039789792 | 0.371787378357172 | -0.173740603 | 0.503361152964656 | 1.17916937954955 | 0.840514896416161 | 1.65427219862005 | 0.999714303421225 |
| ebi-fl187-GCST90094780 | prot-a-752 | Cytochrome b5 \|\| id:prot-a-752 | F-healthy breakfast food liking (derived food-liking factor) \|\| id:ebi-fl187-GCST90094780 | Weighted mode | 8 | -0.066330038 | 0.186593145187954 | 0.732701135046163 | -0.432052603 | 0.299392526289196 | 0.935821956250612 | 0.649175227321442 | 1.34903905285203 | 0.999714303421225 |
| ebi-fl187-GCST90094780 | prot-a-755 | Cytoglobin \|\| id:prot-a-755 | F-healthy breakfast food liking (derived food-liking factor) \|\| id:ebi-fl187-GCST90094780 | Weighted mode | 8 | 0.0554008293221656 | 0.178077026520345 | 0.764780073552017 | -0.293630143 | 0.404431801302041 | 1.05696419201935 | 0.745552188406815 | 1.49845084030728 | 0.999714303421225 |
| ebi-fl187-GCST90094780 | prot-a-807 | Desmin \|\| id:prot-a-807 | F-healthy breakfast food liking (derived food-liking factor) \|\| id:ebi-fl187-GCST90094780 | Weighted mode | 8 | 0.0684936827529818 | 0.186024079276026 | 0.723607860237825 | -0.296113513 | 0.433100878133992 | 1.07089385982857 | 0.743703003546243 | 1.54203177014226 | 0.999714303421225 |
| ebi-fl187-GCST90094780 | prot-a-82 | Ameloblastin \|\| id:prot-a-82 | F-healthy breakfast food liking (derived food-liking factor) \|\| id:ebi-fl187-GCST90094780 | Weighted mode | 8 | -0.072402193 | 0.171782322861185 | 0.686048022861308 | -0.409095545 | 0.264291160223894 | 0.930156718436428 | 0.664250763190652 | 1.30250737943691 | 0.999714303421225 |
| ebi-fl187-GCST90094780 | prot-a-84 | Muellerian-inhibiting factor \|\| id:prot-a-84 | F-healthy breakfast food liking (derived food-liking factor) \|\| id:ebi-fl187-GCST90094780 | Weighted mode | 8 | -0.127878564 | 0.168604561575874 | 0.472938444688701 | -0.458343504 | 0.202586376972753 | 0.879960231757268 | 0.632330230677656 | 1.22456585484529 | 0.999714303421225 |
| ebi-fl187-GCST90094780 | prot-a-858 | Inactive dipeptidyl peptidase 10 \|\| id:prot-a-858 | F-healthy breakfast food liking (derived food-liking factor) \|\| id:ebi-fl187-GCST90094780 | Weighted mode | 8 | -0.065264483 | 0.186067451939946 | 0.73608934229642 | -0.429956688 | 0.299427723284046 | 0.936819658186437 | 0.650537269974867 | 1.34908653580825 | 0.999714303421225 |
| ebi-fl187-GCST90094780 | prot-a-863 | Dihydropyrimidinase-related protein 3 \|\| id:prot-a-863 | F-healthy breakfast food liking (derived food-liking factor) \|\| id:ebi-fl187-GCST90094780 | Weighted mode | 8 | 0.0891004755594249 | 0.191444957079885 | 0.655770078927596 | -0.28613164 | 0.464332591435999 | 1.09319048973447 | 0.751163726006955 | 1.59095201947334 | 0.999714303421225 |
| ebi-fl187-GCST90094780 | prot-a-864 | Dihydropyrimidinase-related protein 5 \|\| id:prot-a-864 | F-healthy breakfast food liking (derived food-liking factor) \|\| id:ebi-fl187-GCST90094780 | Weighted mode | 8 | -0.018150359 | 0.169963484137109 | 0.91795188965751 | -0.351278788 | 0.314978069763401 | 0.982013366566625 | 0.703787518950033 | 1.37022926117561 | 0.999714303421225 |
| ebi-fl187-GCST90094780 | prot-a-884 | Endothelin-converting enzyme 1 \|\| id:prot-a-884 | F-healthy breakfast food liking (derived food-liking factor) \|\| id:ebi-fl187-GCST90094780 | Weighted mode | 8 | 0.0436731099388431 | 0.161895009142549 | 0.795122834059128 | -0.273641108 | 0.36098732785824 | 1.04464081636836 | 0.76060500134739 | 1.43474527946779 | 0.999714303421225 |
| ebi-fl187-GCST90094780 | prot-a-893 | Early endosome antigen 1 \|\| id:prot-a-893 | F-healthy breakfast food liking (derived food-liking factor) \|\| id:ebi-fl187-GCST90094780 | Weighted mode | 8 | 0.163723704557894 | 0.183645615660157 | 0.402253799108351 | -0.196221702 | 0.523669111251801 | 1.17788882481545 | 0.821830013010142 | 1.68821053217972 | 0.999714303421225 |
| ebi-fl187-GCST90094780 | prot-a-897 | Ephrin-A2 \|\| id:prot-a-897 | F-healthy breakfast food liking (derived food-liking factor) \|\| id:ebi-fl187-GCST90094780 | Weighted mode | 8 | -0.145569583 | 0.217781831387168 | 0.525286377787255 | -0.572421972 | 0.281282806855127 | 0.86452973169939 | 0.564157409151958 | 1.32482822146345 | 0.999714303421225 |
| ebi-fl187-GCST90094780 | prot-a-90 | Anaphase-promoting complex subunit 10 \|\| id:prot-a-90 | F-healthy breakfast food liking (derived food-liking factor) \|\| id:ebi-fl187-GCST90094780 | Weighted mode | 8 | 0.127079185412994 | 0.19209992801018 | 0.529440404171816 | -0.249436673 | 0.503595044312946 | 1.13550692980677 | 0.779239625795152 | 1.65465916382717 | 0.999714303421225 |
| ebi-fl187-GCST90094780 | prot-a-914 | Histone-lysine N-methyltransferase EHMT2 \|\| id:prot-a-914 | F-healthy breakfast food liking (derived food-liking factor) \|\| id:ebi-fl187-GCST90094780 | Weighted mode | 8 | 0.239375047980924 | 0.176504116261696 | 0.217152548920409 | -0.10657302 | 0.585323115853849 | 1.27045492879918 | 0.898909407496869 | 1.79557106939696 | 0.999714303421225 |
| ebi-fl187-GCST90094780 | prot-a-932 | RNA polymerase II elongation factor ELL \|\| id:prot-a-932 | F-healthy breakfast food liking (derived food-liking factor) \|\| id:ebi-fl187-GCST90094780 | Weighted mode | 8 | 0.0340440868013521 | 0.152373413806712 | 0.829584720796044 | -0.264607804 | 0.332695977862508 | 1.0346302192596 | 0.767506903893854 | 1.39472320727583 | 0.999714303421225 |
| ebi-fl187-GCST90094780 | prot-a-991 | Estrogen receptor \|\| id:prot-a-991 | F-healthy breakfast food liking (derived food-liking factor) \|\| id:ebi-fl187-GCST90094780 | Weighted mode | 8 | -0.441559719 | 0.220397353636046 | 0.0851821406902417 | -0.873538532 | -0.009580906 | 0.643032688288984 | 0.41747169557851 | 0.990464844892452 | 0.999714303421225 |
| ebi-fl187-GCST90094780 | prot-a-992 | Persulfide dioxygenase ETHE1, mitochondrial \|\| id:prot-a-992 | F-healthy breakfast food liking (derived food-liking factor) \|\| id:ebi-fl187-GCST90094780 | Weighted mode | 8 | -0.166460329 | 0.204951589711158 | 0.443420072491081 | -0.568165445 | 0.235244786838018 | 0.846656403978933 | 0.56656387856339 | 1.26521843965091 | 0.999714303421225 |
| ebi-fl187-GCST90094780 | prot-a-993 | Ethanolamine kinase 1 \|\| id:prot-a-993 | F-healthy breakfast food liking (derived food-liking factor) \|\| id:ebi-fl187-GCST90094780 | Weighted mode | 8 | 0.292178305321748 | 0.216570084611504 | 0.219307263750693 | -0.132299061 | 0.716655671160296 | 1.33934180812188 | 0.876078955273325 | 2.04757398655186 | 0.999714303421225 |
| ebi-fl187-GCST90094780 | prot-b-10 | coagulation factor III, tissue factor \|\| id:prot-b-10 | F-healthy breakfast food liking (derived food-liking factor) \|\| id:ebi-fl187-GCST90094780 | Weighted mode | 8 | 0.00206016388856767 | 0.175000378605939 | 0.990935739063802 | -0.340940578 | 0.345060905956208 | 1.00206228748426 | 0.711101161876904 | 1.41207592088284 | 0.999714303421225 |
| ebi-fl187-GCST90094780 | prot-b-28 | prolactin \|\| id:prot-b-28 | F-healthy breakfast food liking (derived food-liking factor) \|\| id:ebi-fl187-GCST90094780 | Weighted mode | 8 | 0.351093507510319 | 0.376811550770396 | 0.382478526005427 | -0.387457132 | 1.08964414702029 | 1.4206201583558 | 0.678780731600373 | 2.97321585656771 | 0.999714303421225 |
| ebi-fl187-GCST90094780 | prot-b-54 | matrix metallopeptidase 7 \|\| id:prot-b-54 | F-healthy breakfast food liking (derived food-liking factor) \|\| id:ebi-fl187-GCST90094780 | Weighted mode | 8 | 0.0647500296879278 | 0.179175048370038 | 0.728477627374643 | -0.286433065 | 0.415933124493203 | 1.06689229964786 | 0.750937340751706 | 1.51578449662454 | 0.999714303421225 |
| ebi-fl187-GCST90094780 | ukb-rsfMRI191-edge-pheno716 | Net100_Pair10_33 (OccipitalPrecuneus)&(Temporal\|Frontal\|Supp_Motor_Area) (Default_mode\|Central_executive)&(Default_mode\|Salience) \|\| id:ukb-rsfMRI191-edge-pheno716 | F-healthy breakfast food liking (derived food-liking factor) \|\| id:ebi-fl187-GCST90094780 | Weighted mode | 8 | 0.0374250525588372 | 0.0567877777843273 | 0.530947788447188 | -0.073878992 | 0.148729097016119 | 1.03813418866507 | 0.928784077439453 | 1.16035860201914 | 0.999714303421225 |
| ebi-fl187-GCST90094780 | ukb-rsfMRI191-edge-pheno903 | Net100_Pair27_38 (Precentral\|Frontal\|Supp_Motor_Area)&(Subcortical) (Attention\|Salience\|Motor)&(Subcortical-cerebellum) \|\| id:ukb-rsfMRI191-edge-pheno903 | F-healthy breakfast food liking (derived food-liking factor) \|\| id:ebi-fl187-GCST90094780 | Weighted mode | 8 | -0.036592346 | 0.0532124235261844 | 0.513796403983371 | -0.140888696 | 0.0677040045958015 | 0.964069062330996 | 0.868585983736012 | 1.07004853215113 | 0.999714303421225 |
| ebi-fl187-GCST90094780 | ukb-rsfMRI191-node-pheno23 | Net100_Node2 RolandicOper\|SupraMarginal\|Insula Salience\|Motor \|\| id:ukb-rsfMRI191-node-pheno23 | F-healthy breakfast food liking (derived food-liking factor) \|\| id:ebi-fl187-GCST90094780 | Weighted mode | 8 | -0.063147191 | 0.0470360466686154 | 0.221328183415048 | -0.155337842 | 0.0290434608665884 | 0.938805280202856 | 0.856125893272459 | 1.02946933513232 | 0.999714303421225 |
| ebi-fl187-GCST90094780 | ukb-rsfMRI191-node-pheno55 | Net100_Node34 Parietal\|Postcentral\|Precuneus Attention \|\| id:ukb-rsfMRI191-node-pheno55 | F-healthy breakfast food liking (derived food-liking factor) \|\| id:ebi-fl187-GCST90094780 | Weighted mode | 8 | -0.018933044 | 0.0566929188850692 | 0.748193110890881 | -0.130051165 | 0.0921850772261001 | 0.981245060494999 | 0.878050504489833 | 1.09656775301925 | 0.999714303421225 |
| ebi-fl187-GCST90094784 | ebi-a-GCST90001414 | IgD- CD24- B cell Absolute Count \|\| id:ebi-a-GCST90001414 | Ice cream liking \|\| id:ebi-fl187-GCST90094784 | Inverse variance weighted | 8 | 0.430413020038393 | 0.21566128145016 | 0.0459575981210155 | 0.0077169083960793 | 0.853109131680706 | 1.53789257284469 | 1.00774676047285 | 2.34693244213568 | 0.998735809224318 |
| ebi-fl187-GCST90094784 | ebi-a-GCST90001422 | CD20- CD38- B cell %B cell \|\| id:ebi-a-GCST90001422 | Ice cream liking \|\| id:ebi-fl187-GCST90094784 | Inverse variance weighted | 8 | 0.25011115791304 | 0.197959041145008 | 0.206427854306699 | -0.137888563 | 0.638110878557256 | 1.28416815420641 | 0.871195770013851 | 1.89290157854149 | 0.998735809224318 |
| ebi-fl187-GCST90094784 | ebi-a-GCST90001444 | CD20- CD38- B cell %lymphocyte \|\| id:ebi-a-GCST90001444 | Ice cream liking \|\| id:ebi-fl187-GCST90094784 | Inverse variance weighted | 8 | 0.318470723230621 | 0.22238875898145 | 0.152130926666053 | -0.117411244 | 0.754352690834263 | 1.37502336435262 | 0.889219431472274 | 2.1262347465633 | 0.998735809224318 |
| ebi-fl187-GCST90094784 | ebi-a-GCST90001459 | Myeloid Dendritic Cell %Dendritic Cell \|\| id:ebi-a-GCST90001459 | Ice cream liking \|\| id:ebi-fl187-GCST90094784 | Inverse variance weighted | 8 | -0.237828012 | 0.193065262124872 | 0.218004021876983 | -0.616235925 | 0.140579902236278 | 0.788338264528067 | 0.539973116299743 | 1.15094103865374 | 0.998735809224318 |
| ebi-fl187-GCST90094784 | ebi-a-GCST90001476 | HLA DR++ monocyte %leukocyte \|\| id:ebi-a-GCST90001476 | Ice cream liking \|\| id:ebi-fl187-GCST90094784 | Inverse variance weighted | 8 | -0.120289163 | 0.187921333534155 | 0.522105106604931 | -0.488614977 | 0.248036650533927 | 0.886664009048234 | 0.613475483748392 | 1.28150689924542 | 0.998735809224318 |
| ebi-fl187-GCST90094784 | ebi-a-GCST90001478 | CD4 regulatory T cell %CD4+ T cell \|\| id:ebi-a-GCST90001478 | Ice cream liking \|\| id:ebi-fl187-GCST90094784 | Inverse variance weighted | 8 | 0.0642725147947716 | 0.189409613695719 | 0.734360546335429 | -0.306970328 | 0.435515357638381 | 1.06638296430267 | 0.735672429419802 | 1.5457594726661 | 0.998735809224318 |
| ebi-fl187-GCST90094784 | ebi-a-GCST90001485 | CD39+ resting CD4 regulatory T cell %CD4 regulatory T cell \|\| id:ebi-a-GCST90001485 | Ice cream liking \|\| id:ebi-fl187-GCST90094784 | Inverse variance weighted | 8 | -0.007187422 | 0.199164168113397 | 0.971212249253055 | -0.397549191 | 0.383174347626336 | 0.992838345869179 | 0.67196488695569 | 1.46693376419421 | 0.998955228396052 |
| ebi-fl187-GCST90094784 | ebi-a-GCST90001515 | Immature Myeloid-Derived Suppressor Cells Absolute Count \|\| id:ebi-a-GCST90001515 | Ice cream liking \|\| id:ebi-fl187-GCST90094784 | Inverse variance weighted | 8 | 0.00823998475668735 | 0.2460501427284 | 0.973284600283475 | -0.474018295 | 0.490498264504351 | 1.008274027 | 0.622495863907296 | 1.63312974784686 | 0.998955228396052 |
| ebi-fl187-GCST90094784 | ebi-a-GCST90001517 | CD33+ HLA DR+ Absolute Count \|\| id:ebi-a-GCST90001517 | Ice cream liking \|\| id:ebi-fl187-GCST90094784 | Inverse variance weighted | 8 | -0.188018432 | 0.24021598087536 | 0.433799918386195 | -0.658841755 | 0.282804890389375 | 0.828599434242475 | 0.517450321970902 | 1.32684625610409 | 0.998735809224318 |
| ebi-fl187-GCST90094784 | ebi-a-GCST90001518 | CD33+ HLA DR+ CD14- Absolute Count \|\| id:ebi-a-GCST90001518 | Ice cream liking \|\| id:ebi-fl187-GCST90094784 | Inverse variance weighted | 8 | -0.15041995 | 0.254650191836083 | 0.554726495117072 | -0.649534326 | 0.348694425545947 | 0.860346597606561 | 0.522288936282445 | 1.41721605914489 | 0.998735809224318 |
| ebi-fl187-GCST90094784 | ebi-a-GCST90001520 | CD33+ HLA DR+ CD14dim Absolute Count \|\| id:ebi-a-GCST90001520 | Ice cream liking \|\| id:ebi-fl187-GCST90094784 | Inverse variance weighted | 7 | -0.227357628 | 0.24537934064219 | 0.354157261881354 | -0.708301136 | 0.253585879586248 | 0.796635832003026 | 0.492480144099908 | 1.28863804243529 | 0.998735809224318 |
| ebi-fl187-GCST90094784 | ebi-a-GCST90001531 | CD33dim HLA DR- Absolute Count \|\| id:ebi-a-GCST90001531 | Ice cream liking \|\| id:ebi-fl187-GCST90094784 | Inverse variance weighted | 8 | 0.0987674430503935 | 0.246054218668502 | 0.688121820952453 | -0.383498826 | 0.581033711640657 | 1.10380957112124 | 0.681472878411013 | 1.78788563403988 | 0.998735809224318 |
| ebi-fl187-GCST90094784 | ebi-a-GCST90001532 | Basophil Absolute Count \|\| id:ebi-a-GCST90001532 | Ice cream liking \|\| id:ebi-fl187-GCST90094784 | Inverse variance weighted | 8 | 0.0704651997785489 | 0.245201354115892 | 0.773823763070225 | -0.410129454 | 0.551059853845698 | 1.07300722789182 | 0.663564343325931 | 1.735090987 | 0.998735809224318 |
| ebi-fl187-GCST90094784 | ebi-a-GCST90001588 | T/B cell \|\| id:ebi-a-GCST90001588 | Ice cream liking \|\| id:ebi-fl187-GCST90094784 | Inverse variance weighted | 8 | -0.001516881 | 0.185120936001604 | 0.993462207684208 | -0.364353915 | 0.361320153756394 | 0.998484269075456 | 0.69464530558323 | 1.43522287932844 | 0.999107451094609 |
| ebi-fl187-GCST90094784 | ebi-a-GCST90001624 | HLA DR+ CD4+ T cell Absolute Count \|\| id:ebi-a-GCST90001624 | Ice cream liking \|\| id:ebi-fl187-GCST90094784 | Inverse variance weighted | 8 | 0.170982532081961 | 0.179378807902116 | 0.340492552129255 | -0.180599931 | 0.522564995570108 | 1.18647002367957 | 0.834769256863003 | 1.68634758110315 | 0.998735809224318 |
| ebi-fl187-GCST90094784 | ebi-a-GCST90001631 | CD8+ Natural Killer T %T cell \|\| id:ebi-a-GCST90001631 | Ice cream liking \|\| id:ebi-fl187-GCST90094784 | Inverse variance weighted | 8 | -0.153818833 | 0.181331531484335 | 0.396285364375175 | -0.509228635 | 0.201590968569407 | 0.857427344361061 | 0.600958958869616 | 1.22334751817481 | 0.998735809224318 |
| ebi-fl187-GCST90094784 | ebi-a-GCST90001632 | CD8+ Natural Killer T %lymphocyte \|\| id:ebi-a-GCST90001632 | Ice cream liking \|\| id:ebi-fl187-GCST90094784 | Inverse variance weighted | 8 | -0.097375436 | 0.182429036595277 | 0.59349957415405 | -0.454936348 | 0.260185475418623 | 0.907215340610022 | 0.634488353093448 | 1.29717065762583 | 0.998735809224318 |
| ebi-fl187-GCST90094784 | ebi-a-GCST90001646 | Natural Killer %CD3- lymphocyte \|\| id:ebi-a-GCST90001646 | Ice cream liking \|\| id:ebi-fl187-GCST90094784 | Inverse variance weighted | 8 | -0.397705234 | 0.185105393471091 | 0.0316713572316462 | -0.760511805 | -0.034898663 | 0.671860039846901 | 0.467427134051973 | 0.965703272786233 | 0.998735809224318 |
| ebi-fl187-GCST90094784 | ebi-a-GCST90001684 | CD127- CD8+ T cell Absolute Count \|\| id:ebi-a-GCST90001684 | Ice cream liking \|\| id:ebi-fl187-GCST90094784 | Inverse variance weighted | 8 | 0.3380915739806 | 0.197557820498128 | 0.0870155895188388 | -0.049121754 | 0.725304902156931 | 1.40226890887179 | 0.952065204708189 | 2.0653607369163 | 0.998735809224318 |
| ebi-fl187-GCST90094784 | ebi-a-GCST90001687 | CD28- CD8+ T cell Absolute Count \|\| id:ebi-a-GCST90001687 | Ice cream liking \|\| id:ebi-fl187-GCST90094784 | Inverse variance weighted | 8 | 0.182859722733136 | 0.186957889736475 | 0.328034891138646 | -0.183577741 | 0.549297186616628 | 1.20064597293174 | 0.832287170260746 | 1.73203529241666 | 0.998735809224318 |
| ebi-fl187-GCST90094784 | ebi-a-GCST90001701 | BAFF-R on CD20- CD38- B cell \|\| id:ebi-a-GCST90001701 | Ice cream liking \|\| id:ebi-fl187-GCST90094784 | Inverse variance weighted | 8 | -0.070681514 | 0.176653545525185 | 0.689072732874214 | -0.416922463 | 0.275559435634068 | 0.931758597322514 | 0.659072020545818 | 1.31726739509505 | 0.998735809224318 |
| ebi-fl187-GCST90094784 | ebi-a-GCST90001732 | CD19 on IgD- CD27- B cell \|\| id:ebi-a-GCST90001732 | Ice cream liking \|\| id:ebi-fl187-GCST90094784 | Inverse variance weighted (multiplicative random effects) | 8 | -0.461794265 | 0.273375086846181 | 0.091174754677058 | -0.997609435 | 0.0740209055872975 | 0.630151971154767 | 0.36875993296018 | 1.0768293170102 | 0.998735809224318 |
| ebi-fl187-GCST90094784 | ebi-a-GCST90001798 | CD27 on CD24+ CD27+ B cell \|\| id:ebi-a-GCST90001798 | Ice cream liking \|\| id:ebi-fl187-GCST90094784 | Inverse variance weighted | 8 | -0.092750944 | 0.227470466006321 | 0.683457673848644 | -0.538593057 | 0.353091169861819 | 0.911420467213135 | 0.583568723059085 | 1.42346091425278 | 0.998735809224318 |
| ebi-fl187-GCST90094784 | ebi-a-GCST90001836 | CD66b on CD66b++ myeloid cell \|\| id:ebi-a-GCST90001836 | Ice cream liking \|\| id:ebi-fl187-GCST90094784 | Inverse variance weighted | 8 | 0.177550094898872 | 0.269325313386038 | 0.509741485101929 | -0.350327519 | 0.705427709135506 | 1.19428788413473 | 0.704457328533678 | 2.02471248778105 | 0.998735809224318 |
| ebi-fl187-GCST90094784 | ebi-a-GCST90001943 | CD25 on activated & secreting CD4 regulatory T cell \|\| id:ebi-a-GCST90001943 | Ice cream liking \|\| id:ebi-fl187-GCST90094784 | Inverse variance weighted | 8 | 0.0239483508752396 | 0.207392674748985 | 0.908069851220906 | -0.382541292 | 0.430437993383251 | 1.02423741555839 | 0.68212572431002 | 1.53793097964584 | 0.998735809224318 |
| ebi-fl187-GCST90094784 | ebi-a-GCST90001960 | CD25 on CD4+ T cell \|\| id:ebi-a-GCST90001960 | Ice cream liking \|\| id:ebi-fl187-GCST90094784 | Inverse variance weighted | 8 | -0.139865047 | 0.237199482934342 | 0.555424270785176 | -0.604776034 | 0.325045939248292 | 0.869475565554148 | 0.54619674252109 | 1.3840942287687 | 0.998735809224318 |
| ebi-fl187-GCST90094784 | ebi-a-GCST90001988 | HLA DR on CD14+ CD16- monocyte \|\| id:ebi-a-GCST90001988 | Ice cream liking \|\| id:ebi-fl187-GCST90094784 | Inverse variance weighted | 8 | -0.192042906 | 0.18220163526231 | 0.291876929636073 | -0.549158112 | 0.165072298718465 | 0.825271458308307 | 0.577435742306821 | 1.17947839040492 | 0.998735809224318 |
| ebi-fl187-GCST90094784 | ebi-a-GCST90001991 | HLA DR on CD14+ monocyte \|\| id:ebi-a-GCST90001991 | Ice cream liking \|\| id:ebi-fl187-GCST90094784 | Inverse variance weighted | 8 | -0.18660744 | 0.182541881403678 | 0.306652218952588 | -0.544389527 | 0.171174647748218 | 0.829769406900427 | 0.580195868956264 | 1.18669798505544 | 0.998735809224318 |
| ebi-fl187-GCST90094784 | ebi-a-GCST90002019 | CD14 on Monocytic Myeloid-Derived Suppressor Cells \|\| id:ebi-a-GCST90002019 | Ice cream liking \|\| id:ebi-fl187-GCST90094784 | Inverse variance weighted | 8 | -0.27181342 | 0.344385411523104 | 0.429953628749497 | -0.946808827 | 0.403181986284963 | 0.761996420890725 | 0.387977152312353 | 1.49657922377556 | 0.998735809224318 |
| ebi-fl187-GCST90094784 | ebi-a-GCST90002037 | CD80 on plasmacytoid Dendritic Cell \|\| id:ebi-a-GCST90002037 | Ice cream liking \|\| id:ebi-fl187-GCST90094784 | Inverse variance weighted | 8 | -0.256212655 | 0.202330500356417 | 0.205402968343112 | -0.652780436 | 0.14035512545381 | 0.773977360945371 | 0.520596277972323 | 1.1506823629035 | 0.998735809224318 |
| ebi-fl187-GCST90094784 | ebi-a-GCST90002042 | CD45 on CD33+ HLA DR+ CD14- \|\| id:ebi-a-GCST90002042 | Ice cream liking \|\| id:ebi-fl187-GCST90094784 | Inverse variance weighted (multiplicative random effects) | 8 | -0.162689711 | 0.479871266744215 | 0.734588770931957 | -1.103237393 | 0.777857972298156 | 0.84985484852559 | 0.331795191551624 | 2.17680449250898 | 0.998735809224318 |
| ebi-fl187-GCST90094784 | ebi-a-GCST90002056 | CD8 on Effector Memory CD8+ T cell \|\| id:ebi-a-GCST90002056 | Ice cream liking \|\| id:ebi-fl187-GCST90094784 | Inverse variance weighted | 8 | -0.254212846 | 0.198117059636144 | 0.199441401726722 | -0.642522283 | 0.134096590838503 | 0.775526716681188 | 0.52596411924451 | 1.14350326624981 | 0.998735809224318 |
| ebi-fl187-GCST90094784 | ebi-a-GCST90002058 | CD8 on CD8+ T cell \|\| id:ebi-a-GCST90002058 | Ice cream liking \|\| id:ebi-fl187-GCST90094784 | Inverse variance weighted | 8 | -0.276516753 | 0.268104140032521 | 0.30236352163834 | -0.802000867 | 0.248967361626986 | 0.75842091332349 | 0.448430815323673 | 1.28270016714008 | 0.998735809224318 |
| ebi-fl187-GCST90094784 | ebi-a-GCST90002063 | CD4 on CD4 regulatory T cell \|\| id:ebi-a-GCST90002063 | Ice cream liking \|\| id:ebi-fl187-GCST90094784 | Inverse variance weighted | 8 | -0.262758507 | 0.197248021236615 | 0.182819971667156 | -0.649364629 | 0.12384761474521 | 0.768927565609433 | 0.522377575164516 | 1.131843382 | 0.998735809224318 |
| ebi-fl187-GCST90094784 | ebi-a-GCST90002066 | CD4 on activated CD4 regulatory T cell \|\| id:ebi-a-GCST90002066 | Ice cream liking \|\| id:ebi-fl187-GCST90094784 | Inverse variance weighted | 8 | -0.28239907 | 0.197269861142477 | 0.152276422408974 | -0.669047997 | 0.10424985825597 | 0.753972736901637 | 0.512195957628127 | 1.10987773238866 | 0.998735809224318 |
| ebi-fl187-GCST90094784 | ebi-a-GCST90002070 | CD4 on activated & secreting CD4 regulatory T cell \|\| id:ebi-a-GCST90002070 | Ice cream liking \|\| id:ebi-fl187-GCST90094784 | Inverse variance weighted | 8 | -0.263557883 | 0.198183045724618 | 0.183560781732142 | -0.651996652 | 0.124880886788213 | 0.76831314901116 | 0.521004472687745 | 1.13301348815336 | 0.998735809224318 |
| ebi-fl187-GCST90094784 | ebi-a-GCST90002081 | SSC-A on CD4+ T cell \|\| id:ebi-a-GCST90002081 | Ice cream liking \|\| id:ebi-fl187-GCST90094784 | Inverse variance weighted | 8 | -0.123167749 | 0.187346387265769 | 0.510902360914813 | -0.490366668 | 0.244031169851746 | 0.884115340489223 | 0.612401804726193 | 1.27638411457304 | 0.998735809224318 |
| ebi-fl187-GCST90094784 | ebi-a-GCST90002094 | CD11b on Monocytic Myeloid-Derived Suppressor Cells \|\| id:ebi-a-GCST90002094 | Ice cream liking \|\| id:ebi-fl187-GCST90094784 | Inverse variance weighted | 8 | 0.197345885145456 | 0.331828788141537 | 0.552029304284865 | -0.45303854 | 0.847730309902869 | 1.21816531313229 | 0.635693633768341 | 2.33434260041602 | 0.998735809224318 |
| ebi-fl187-GCST90094784 | ebi-a-GCST90002104 | HLA DR on myeloid Dendritic Cell \|\| id:ebi-a-GCST90002104 | Ice cream liking \|\| id:ebi-fl187-GCST90094784 | Inverse variance weighted | 8 | 0.0963271709489807 | 0.200949931367057 | 0.631683210526395 | -0.297534695 | 0.490189036428412 | 1.10111925930105 | 0.742646816991205 | 1.6326248163507 | 0.998735809224318 |
| ebi-fl187-GCST90094784 | ebi-a-GCST90002105 | HLA DR on plasmacytoid Dendritic Cell \|\| id:ebi-a-GCST90002105 | Ice cream liking \|\| id:ebi-fl187-GCST90094784 | Inverse variance weighted | 8 | -0.134400462 | 0.196870309512164 | 0.494805735204057 | -0.520266268 | 0.251465344912082 | 0.874239894870951 | 0.594362267023165 | 1.28590833602528 | 0.998735809224318 |
| ebi-fl187-GCST90094784 | ebi-a-GCST90002106 | HLA DR on Dendritic Cell \|\| id:ebi-a-GCST90002106 | Ice cream liking \|\| id:ebi-fl187-GCST90094784 | Inverse variance weighted | 8 | -0.027595747 | 0.200604035426416 | 0.890585586498932 | -0.420779656 | 0.365588162531851 | 0.972781537273949 | 0.656534748742176 | 1.4413615137265 | 0.998735809224318 |
| ebi-fl187-GCST90094784 | ebi-a-GCST90002111 | HLA DR on CD33dim HLA DR+ CD11b- \|\| id:ebi-a-GCST90002111 | Ice cream liking \|\| id:ebi-fl187-GCST90094784 | Inverse variance weighted | 8 | 0.520062917380398 | 0.307489194573977 | 0.0907755204650324 | -0.082615904 | 1.12274173874539 | 1.68213348180167 | 0.920704718326823 | 3.07326876280196 | 0.998735809224318 |
| ebi-fl187-GCST90094784 | ebi-a-GCST90002112 | HLA DR on CD33- HLA DR+ \|\| id:ebi-a-GCST90002112 | Ice cream liking \|\| id:ebi-fl187-GCST90094784 | Inverse variance weighted | 8 | 0.0450346145144285 | 0.26122067925053 | 0.863122562353093 | -0.466957917 | 0.557027145845467 | 1.04606406828183 | 0.626906472077294 | 1.74547573472079 | 0.998735809224318 |
| ebi-fl187-GCST90094784 | ebi-a-GCST90002115 | HLA DR on HLA DR+ CD8+ T cell \|\| id:ebi-a-GCST90002115 | Ice cream liking \|\| id:ebi-fl187-GCST90094784 | Inverse variance weighted | 8 | 0.145446980056705 | 0.184542794138751 | 0.430610312637866 | -0.216256896 | 0.507150856568656 | 1.15655641240115 | 0.805528337915854 | 1.66055329416102 | 0.998735809224318 |
| ebi-fl187-GCST90094784 | ebi-a-GCST90002116 | HLA DR on B cell \|\| id:ebi-a-GCST90002116 | Ice cream liking \|\| id:ebi-fl187-GCST90094784 | Inverse variance weighted | 8 | -0.166329917 | 0.19490063406415 | 0.393432640991605 | -0.54833516 | 0.215675325776105 | 0.846766825339137 | 0.577911139651731 | 1.2406994904563 | 0.998735809224318 |
| ebi-fl187-GCST90094784 | ebi-cfmet338-GCST90026023 | Sphingomyelin (d18:1/24:1, d18:2/24:0) levels \|\| id:ebi-cfmet338-GCST90026023 | Ice cream liking \|\| id:ebi-fl187-GCST90094784 | Inverse variance weighted | 8 | -0.086939557 | 0.112806922158177 | 0.440888649477628 | -0.308041125 | 0.134162010309021 | 0.916732503910422 | 0.734885095568016 | 1.14357807607501 | 0.998735809224318 |
| ebi-fl187-GCST90094784 | ebi-cfmet338-GCST90026046 | 1-arachidonoyl-gpc (20:4n6) levels \|\| id:ebi-cfmet338-GCST90026046 | Ice cream liking \|\| id:ebi-fl187-GCST90094784 | Inverse variance weighted | 8 | -0.044593206 | 0.126202105225713 | 0.723827893364484 | -0.291949332 | 0.202762920126148 | 0.956386454872154 | 0.746806373932284 | 1.22478206264729 | 0.998735809224318 |
| ebi-fl187-GCST90094784 | ebi-cfmet338-GCST90026047 | 1-linoleoyl-gpc (18:2) levels \|\| id:ebi-cfmet338-GCST90026047 | Ice cream liking \|\| id:ebi-fl187-GCST90094784 | Inverse variance weighted | 8 | -0.066969166 | 0.153685817779656 | 0.663015427064027 | -0.368193369 | 0.234255036454524 | 0.935224037222074 | 0.691983360452992 | 1.26396680871833 | 0.998735809224318 |
| ebi-fl187-GCST90094784 | ebi-cfmet338-GCST90026050 | 1-myristoyl-2-palmitoyl-gpc (14:0/16:0) levels \|\| id:ebi-cfmet338-GCST90026050 | Ice cream liking \|\| id:ebi-fl187-GCST90094784 | Inverse variance weighted | 8 | 0.0380994549993823 | 0.0808563652062036 | 0.637498486426502 | -0.120379021 | 0.196577930803541 | 1.03883454502999 | 0.886584339117475 | 1.21723017690782 | 0.998735809224318 |
| ebi-fl187-GCST90094784 | ebi-cfmet338-GCST90026068 | 2,3-dihydroxyisovalerate levels \|\| id:ebi-cfmet338-GCST90026068 | Ice cream liking \|\| id:ebi-fl187-GCST90094784 | Inverse variance weighted | 8 | -0.080165506 | 0.261293117183543 | 0.758993342832421 | -0.592300016 | 0.431969003689727 | 0.922963577744238 | 0.553053788385628 | 1.54028737119592 | 0.998735809224318 |
| ebi-fl187-GCST90094784 | ebi-cfmet338-GCST90026082 | 3-hydroxyhexanoate levels \|\| id:ebi-cfmet338-GCST90026082 | Ice cream liking \|\| id:ebi-fl187-GCST90094784 | Inverse variance weighted | 8 | 0.00578954976431041 | 0.0594045094093471 | 0.922361281779865 | -0.110643289 | 0.122222388206631 | 1.00580634159762 | 0.895258040657363 | 1.13000537370787 | 0.998735809224318 |
| ebi-fl187-GCST90094784 | ebi-cfmet338-GCST90026094 | 3-ureidopropionate levels \|\| id:ebi-cfmet338-GCST90026094 | Ice cream liking \|\| id:ebi-fl187-GCST90094784 | Inverse variance weighted | 8 | -0.120835584 | 0.162405295215928 | 0.456853960281989 | -0.439149962 | 0.197478794856859 | 0.88617964993599 | 0.644584109008232 | 1.21832722989242 | 0.998735809224318 |
| ebi-fl187-GCST90094784 | ebi-cfmet338-GCST90026173 | Glycerol levels \|\| id:ebi-cfmet338-GCST90026173 | Ice cream liking \|\| id:ebi-fl187-GCST90094784 | Inverse variance weighted | 7 | -0.054549112 | 0.0811923159478559 | 0.501678763427194 | -0.213686052 | 0.104587826875259 | 0.946912002611315 | 0.807601890515571 | 1.11025289962726 | 0.998735809224318 |
| ebi-fl187-GCST90094784 | ebi-cfmet338-GCST90026179 | Histidine levels \|\| id:ebi-cfmet338-GCST90026179 | Ice cream liking \|\| id:ebi-fl187-GCST90094784 | Inverse variance weighted | 8 | -0.054431142 | 0.065156565652623 | 0.403498170717263 | -0.182138011 | 0.0732757267276048 | 0.947023716817742 | 0.833486302507421 | 1.07602718547052 | 0.998735809224318 |
| ebi-fl187-GCST90094784 | ebi-cfmet338-GCST90026188 | Isocitrate levels \|\| id:ebi-cfmet338-GCST90026188 | Ice cream liking \|\| id:ebi-fl187-GCST90094784 | Inverse variance weighted | 8 | 0.195971813684483 | 0.108006471065061 | 0.0696092255466167 | -0.01572087 | 0.407664496972003 | 1.21649261640637 | 0.9844020582469 | 1.50330271394053 | 0.998735809224318 |
| ebi-fl187-GCST90094784 | ebi-cfmet338-GCST90026207 | N-acetyl-isoputreanine levels \|\| id:ebi-cfmet338-GCST90026207 | Ice cream liking \|\| id:ebi-fl187-GCST90094784 | Inverse variance weighted | 7 | -0.009055839 | 0.0755787119404685 | 0.90462576000392 | -0.157190115 | 0.139078436202215 | 0.990985041414883 | 0.854541582538692 | 1.14921423646882 | 0.998735809224318 |
| ebi-fl187-GCST90094784 | ebi-cfmet338-GCST90026213 | N-acetylglutamate levels \|\| id:ebi-cfmet338-GCST90026213 | Ice cream liking \|\| id:ebi-fl187-GCST90094784 | Inverse variance weighted | 8 | 0.0443724405890059 | 0.120848628160265 | 0.713489439351846 | -0.192490871 | 0.281235751783125 | 1.04537162121699 | 0.82490184902395 | 1.32476588304278 | 0.998735809224318 |
| ebi-fl187-GCST90094784 | ebi-cfmet338-GCST90026224 | N-acetyltaurine levels \|\| id:ebi-cfmet338-GCST90026224 | Ice cream liking \|\| id:ebi-fl187-GCST90094784 | Inverse variance weighted | 8 | 0.00523657081431475 | 0.115349883523114 | 0.963790650008272 | -0.220849201 | 0.231322342519618 | 1.00525030561521 | 0.801837587567389 | 1.26026541111052 | 0.998735809224318 |
| ebi-fl187-GCST90094784 | ebi-cfmet338-GCST90026250 | Phenyllactate (pla) levels \|\| id:ebi-cfmet338-GCST90026250 | Ice cream liking \|\| id:ebi-fl187-GCST90094784 | Inverse variance weighted | 8 | 0.0152746988099875 | 0.146816847897153 | 0.917138286690154 | -0.272486323 | 0.303035720688406 | 1.01539195327001 | 0.761483843866408 | 1.35396282806279 | 0.998735809224318 |
| ebi-fl187-GCST90094784 | ebi-cfmet338-GCST90026251 | Pipecolate levels \|\| id:ebi-cfmet338-GCST90026251 | Ice cream liking \|\| id:ebi-fl187-GCST90094784 | Inverse variance weighted | 8 | 0.0201641341209929 | 0.212843674630447 | 0.924523866770499 | -0.397009468 | 0.437337736396668 | 1.02036880361981 | 0.67232765990024 | 1.54857900023777 | 0.998735809224318 |
| ebi-fl187-GCST90094784 | ebi-cfmet338-GCST90026278 | Trigonelline (n'-methylnicotinate) levels \|\| id:ebi-cfmet338-GCST90026278 | Ice cream liking \|\| id:ebi-fl187-GCST90094784 | Inverse variance weighted | 8 | -0.03300279 | 0.293991576766839 | 0.910619156778701 | -0.60922628 | 0.543220700611462 | 0.967535860303855 | 0.543771433016331 | 1.72154251609206 | 0.998735809224318 |
| ebi-fl187-GCST90094784 | ebi-cfmet338-GCST90026299 | 1-carboxyethylvaline levels \|\| id:ebi-cfmet338-GCST90026299 | Ice cream liking \|\| id:ebi-fl187-GCST90094784 | Inverse variance weighted | 8 | -0.196082765 | 0.136524065038153 | 0.150931056096975 | -0.463669933 | 0.0715044022983715 | 0.821944203505918 | 0.628971123040471 | 1.07412287930031 | 0.998735809224318 |
| ebi-fl187-GCST90094784 | ebi-gm430-GCST90011306 | C_Erysipelotrichia abundance \|\| id:ebi-gm430-GCST90011306 | Ice cream liking \|\| id:ebi-fl187-GCST90094784 | Inverse variance weighted | 8 | -0.19545733 | 0.137446660089071 | 0.155008975769452 | -0.464852784 | 0.0739381236634389 | 0.82245843702556 | 0.628227583606061 | 1.07674017869724 | 0.998735809224318 |
| ebi-fl187-GCST90094784 | ebi-gm430-GCST90011312 | F_Erysipelotrichaceae abundance \|\| id:ebi-gm430-GCST90011312 | Ice cream liking \|\| id:ebi-fl187-GCST90094784 | Inverse variance weighted | 8 | -0.19545733 | 0.137446660089071 | 0.155008975769452 | -0.464852784 | 0.0739381236634389 | 0.82245843702556 | 0.628227583606061 | 1.07674017869724 | 0.998735809224318 |
| ebi-fl187-GCST90094784 | ebi-gm430-GCST90011352 | O_Erysipelotrichales abundance \|\| id:ebi-gm430-GCST90011352 | Ice cream liking \|\| id:ebi-fl187-GCST90094784 | Inverse variance weighted | 8 | -0.19545733 | 0.137446660089071 | 0.155008975769452 | -0.464852784 | 0.0739381236634389 | 0.82245843702556 | 0.628227583606061 | 1.07674017869724 | 0.998735809224318 |
| ebi-fl187-GCST90094784 | ebi-gm430-GCST90011362 | OTU97_11 (Parabacteroides) abundance \|\| id:ebi-gm430-GCST90011362 | Ice cream liking \|\| id:ebi-fl187-GCST90094784 | Inverse variance weighted (multiplicative random effects) | 8 | 0.103130624419879 | 0.218375866371181 | 0.6367397870106 | -0.324886074 | 0.531147322507394 | 1.10863621458062 | 0.722609673222601 | 1.7008826505164 | 0.998735809224318 |
| ebi-fl187-GCST90094784 | ebi-gm430-GCST90011384 | OTU97_235 (Phascolarctobacterium) abundance \|\| id:ebi-gm430-GCST90011384 | Ice cream liking \|\| id:ebi-fl187-GCST90094784 | Inverse variance weighted | 8 | 0.548542411102464 | 0.453046507620371 | 0.225977396316362 | -0.339428744 | 1.43651356603839 | 1.73072848782238 | 0.712177042108052 | 4.20600626172032 | 0.998735809224318 |
| ebi-fl187-GCST90094784 | ebi-gm430-GCST90011390 | OTU97_3 (Bacteroides) abundance \|\| id:ebi-gm430-GCST90011390 | Ice cream liking \|\| id:ebi-fl187-GCST90094784 | Inverse variance weighted | 8 | 0.117289764459118 | 0.114340418993942 | 0.304988494059728 | -0.106817457 | 0.341396985687243 | 1.12444520674609 | 0.898689707741044 | 1.40691165380364 | 0.998735809224318 |
| ebi-fl187-GCST90094784 | ebi-gm430-GCST90011398 | OTU97_4 (Alistipes) abundance \|\| id:ebi-gm430-GCST90011398 | Ice cream liking \|\| id:ebi-fl187-GCST90094784 | Inverse variance weighted | 8 | 0.185899940971937 | 0.133919247158856 | 0.165091514798427 | -0.076581783 | 0.448381665403295 | 1.2043017530859 | 0.926277157040733 | 1.56577618422473 | 0.998735809224318 |
| ebi-fl187-GCST90094784 | ebi-gm430-GCST90011421 | OTU99_108 (Cytophagales) abundance \|\| id:ebi-gm430-GCST90011421 | Ice cream liking \|\| id:ebi-fl187-GCST90094784 | Inverse variance weighted | 8 | 0.721378256695252 | 0.424156879256873 | 0.0889928453584527 | -0.109969227 | 1.55272574003872 | 2.05726669916013 | 0.895861703539692 | 4.72432994372962 | 0.998735809224318 |
| ebi-fl187-GCST90094784 | ebi-gm430-GCST90011428 | OTU99_123 (Phascolarctobacterium) abundance \|\| id:ebi-gm430-GCST90011428 | Ice cream liking \|\| id:ebi-fl187-GCST90094784 | Inverse variance weighted | 8 | 0.0629918131788979 | 0.256085485956037 | 0.805698000747458 | -0.438935739 | 0.56491936565273 | 1.06501812008279 | 0.644722208602356 | 1.75930591651802 | 0.998735809224318 |
| ebi-fl187-GCST90094784 | ebi-gm430-GCST90011450 | OTU99_298 (Phascolarctobacterium) abundance \|\| id:ebi-gm430-GCST90011450 | Ice cream liking \|\| id:ebi-fl187-GCST90094784 | Inverse variance weighted | 8 | 0.565607953171215 | 0.453034525935125 | 0.21185257477271 | -0.322339718 | 1.45355562400406 | 1.76051776979897 | 0.724452039368578 | 4.27829952757031 | 0.998735809224318 |
| ebi-fl187-GCST90094784 | ebi-gm430-GCST90011451 | OTU99_3 (Bacteroides) abundance \|\| id:ebi-gm430-GCST90011451 | Ice cream liking \|\| id:ebi-fl187-GCST90094784 | Inverse variance weighted | 8 | 0.121693348710943 | 0.114341373399198 | 0.287193511104732 | -0.102415743 | 0.345802440573371 | 1.12940771434148 | 0.902654201347251 | 1.41312341238784 | 0.998735809224318 |
| ebi-fl187-GCST90094784 | ebi-gm430-GCST90011472 | OTU99_72 (Parabacteroides) abundance \|\| id:ebi-gm430-GCST90011472 | Ice cream liking \|\| id:ebi-fl187-GCST90094784 | Inverse variance weighted (multiplicative random effects) | 8 | 0.451042032609823 | 0.49612532427599 | 0.363281916515039 | -0.521363603 | 1.42344766819076 | 1.56994726968741 | 0.593710410463079 | 4.151408475 | 0.998735809224318 |
| ebi-fl187-GCST90094784 | ebi-gm430-GCST90011507 | TestASV_3 (Bacteroides) abundance \|\| id:ebi-gm430-GCST90011507 | Ice cream liking \|\| id:ebi-fl187-GCST90094784 | Inverse variance weighted | 8 | 0.0825360398489036 | 0.180652877876583 | 0.647759874457593 | -0.271543601 | 0.436615680487006 | 1.08603781337245 | 0.76220205013263 | 1.54746124320917 | 0.998735809224318 |
| ebi-fl187-GCST90094784 | ebi-gm430-GCST90011512 | TestASV_34 (Phascolarctobacterium) abundance \|\| id:ebi-gm430-GCST90011512 | Ice cream liking \|\| id:ebi-fl187-GCST90094784 | Inverse variance weighted | 8 | 0.537607274371934 | 0.465175632430058 | 0.247800549201978 | -0.374136965 | 1.44935151393485 | 1.71190583685855 | 0.687882689392606 | 4.26035084101052 | 0.998735809224318 |
| ebi-fl187-GCST90094784 | ebi-gm430-GCST90011519 | TestASV_41 (Barnesiella) abundance \|\| id:ebi-gm430-GCST90011519 | Ice cream liking \|\| id:ebi-fl187-GCST90094784 | Inverse variance weighted (multiplicative random effects) | 8 | 0.315802421747112 | 0.682981401537309 | 0.643803084862016 | -1.022841125 | 1.65444596876024 | 1.37135927808704 | 0.359571898760324 | 5.23018143542125 | 0.998735809224318 |
| ebi-fl187-GCST90094784 | ebi-gm430-GCST90011524 | TestASV_46 (Sutterella) abundance \|\| id:ebi-gm430-GCST90011524 | Ice cream liking \|\| id:ebi-fl187-GCST90094784 | Inverse variance weighted | 8 | 0.127112949572324 | 0.479900874560263 | 0.791107033309701 | -0.813492765 | 1.06771866371044 | 1.13554526989093 | 0.443306992077686 | 2.9087361196994 | 0.998735809224318 |
| ebi-fl187-GCST90094784 | ebi-gm430-GCST90011581 | OTU97_19 (Bacteria) prevalence \|\| id:ebi-gm430-GCST90011581 | Ice cream liking \|\| id:ebi-fl187-GCST90094784 | Inverse variance weighted | 8 | -0.64960644 | 0.542432386455069 | 0.231080377348016 | -1.712773918 | 0.413561037316218 | 0.522251273461042 | 0.180364781017678 | 1.51219318479336 | 0.998735809224318 |
| ebi-fl187-GCST90094784 | ebi-gm430-GCST90011614 | OTU97_80 (Ruminococcus) prevalence \|\| id:ebi-gm430-GCST90011614 | Ice cream liking \|\| id:ebi-fl187-GCST90094784 | Inverse variance weighted | 8 | 0.0487367108995114 | 0.258220442767866 | 0.850296039387374 | -0.457375357 | 0.554848778724528 | 1.04994387556661 | 0.632942716036725 | 1.7416775861528 | 0.998735809224318 |
| ebi-fl187-GCST90094784 | ebi-gm430-GCST90011623 | OTU99_11 (Parabacteroides) prevalence \|\| id:ebi-gm430-GCST90011623 | Ice cream liking \|\| id:ebi-fl187-GCST90094784 | Inverse variance weighted | 8 | -0.165674901 | 0.287440199136944 | 0.564358079112773 | -0.729057692 | 0.397707888940064 | 0.84732165252772 | 0.482363310964164 | 1.48840918561413 | 0.998735809224318 |
| ebi-fl187-GCST90094784 | ebi-gm430-GCST90011642 | OTU99_19 (Bacteria) prevalence \|\| id:ebi-gm430-GCST90011642 | Ice cream liking \|\| id:ebi-fl187-GCST90094784 | Inverse variance weighted | 8 | -0.64960644 | 0.542432386455069 | 0.231080377348016 | -1.712773918 | 0.413561037316218 | 0.522251273461042 | 0.180364781017678 | 1.51219318479336 | 0.998735809224318 |
| ebi-fl187-GCST90094784 | ebi-gm430-GCST90011653 | OTU99_30 (Parasutterella) prevalence \|\| id:ebi-gm430-GCST90011653 | Ice cream liking \|\| id:ebi-fl187-GCST90094784 | Inverse variance weighted | 8 | 0.27374108111911 | 0.321214872993293 | 0.394100045108492 | -0.35584007 | 0.903322232185965 | 1.31487431237553 | 0.700584655816699 | 2.46778807242014 | 0.998735809224318 |
| ebi-fl187-GCST90094784 | ebi-gm430-GCST90011675 | OTU99_8 (Ruminococcaceae) prevalence \|\| id:ebi-gm430-GCST90011675 | Ice cream liking \|\| id:ebi-fl187-GCST90094784 | Inverse variance weighted | 8 | -0.054849557 | 0.347900516661243 | 0.874725715420232 | -0.736734569 | 0.627035455963011 | 0.94662755102067 | 0.478674444437579 | 1.87205256258097 | 0.998735809224318 |
| ebi-fl187-GCST90094784 | ebi-gm430-GCST90011679 | OTU99_92 (Ruminococcus) prevalence \|\| id:ebi-gm430-GCST90011679 | Ice cream liking \|\| id:ebi-fl187-GCST90094784 | Inverse variance weighted | 8 | -0.194298413 | 0.350201663378377 | 0.579018711498112 | -0.880693673 | 0.492096847253043 | 0.823412150738117 | 0.414495287666064 | 1.63574252870494 | 0.998735809224318 |
| ebi-fl187-GCST90094784 | ebi-gm430-GCST90011684 | TestASV_11 (Lachnospiraceae) prevalence \|\| id:ebi-gm430-GCST90011684 | Ice cream liking \|\| id:ebi-fl187-GCST90094784 | Inverse variance weighted | 8 | -0.605869633 | 0.268781312158185 | 0.0241876133035505 | -1.132681005 | -0.079058261 | 0.545599748841856 | 0.322168362680495 | 0.923986090563199 | 0.998735809224318 |
| ebi-fl187-GCST90094784 | ebi-gm430-GCST90011688 | TestASV_15 (Bacteroides) prevalence \|\| id:ebi-gm430-GCST90011688 | Ice cream liking \|\| id:ebi-fl187-GCST90094784 | Inverse variance weighted | 8 | -0.174808787 | 0.252107813253053 | 0.488065458017099 | -0.668940101 | 0.319322527233346 | 0.839617551427871 | 0.512251224865239 | 1.37619511373778 | 0.998735809224318 |
| ebi-fl187-GCST90094784 | ebi-gm430-GCST90011692 | TestASV_19 (Prevotella) prevalence \|\| id:ebi-gm430-GCST90011692 | Ice cream liking \|\| id:ebi-fl187-GCST90094784 | Inverse variance weighted | 8 | 0.0131015705227033 | 0.49570681785902 | 0.978914302685849 | -0.958483792 | 0.984686933526383 | 1.01318777214529 | 0.383473871383729 | 2.67697368251069 | 0.998955228396052 |
| ebi-fl187-GCST90094784 | ebi-gm430-GCST90011693 | TestASV_2 (Bacteroides) prevalence \|\| id:ebi-gm430-GCST90011693 | Ice cream liking \|\| id:ebi-fl187-GCST90094784 | Inverse variance weighted | 8 | 0.586412968715915 | 0.247491203041858 | 0.0178155490457641 | 0.101330210753873 | 1.07149572667796 | 1.79752904442452 | 1.10664200652766 | 2.91974337363904 | 0.998735809224318 |
| ebi-fl187-GCST90094784 | ebi-gm430-GCST90011716 | TestASV_40 (Barnesiella) prevalence \|\| id:ebi-gm430-GCST90011716 | Ice cream liking \|\| id:ebi-fl187-GCST90094784 | Inverse variance weighted | 8 | -0.731836534 | 0.495613261480678 | 0.139775319755654 | -1.703238526 | 0.239565458672437 | 0.481024760134714 | 0.182092855613594 | 1.27069686003313 | 0.998735809224318 |
| ebi-fl187-GCST90094784 | ebi-gm430-GCST90011718 | TestASV_42 (Alphaproteobacteria) prevalence \|\| id:ebi-gm430-GCST90011718 | Ice cream liking \|\| id:ebi-fl187-GCST90094784 | Inverse variance weighted | 8 | 0.392432814074226 | 0.478187839058653 | 0.411836169548969 | -0.54481535 | 1.32968097862919 | 1.48057838790966 | 0.579948860731981 | 3.77983734631086 | 0.998735809224318 |
| ebi-fl187-GCST90094784 | ebi-hbsm44-GCST90104929 | Sulcal depth of the right inferior frontal sulcus \|\| id:ebi-hbsm44-GCST90104929 | Ice cream liking \|\| id:ebi-fl187-GCST90094784 | Inverse variance weighted (multiplicative random effects) | 7 | 0.113343332064867 | 0.41768200439839 | 0.786112058407321 | -0.705313397 | 0.932000060685711 | 1.12001640448894 | 0.493953746593187 | 2.5395834224889 | 0.998735809224318 |
| ebi-fl187-GCST90094784 | ebi-hbsm44-GCST90104935 | Sulcal opening distance of the left calloso-marginal posterior fissure \|\| id:ebi-hbsm44-GCST90104935 | Ice cream liking \|\| id:ebi-fl187-GCST90094784 | Inverse variance weighted | 8 | 0.0429362823755003 | 0.0670326814124889 | 0.521829653264291 | -0.088447773 | 0.174320337943979 | 1.04387137972694 | 0.915350915343259 | 1.19043684683967 | 0.998735809224318 |
| ebi-fl187-GCST90094784 | ebi-hbsm44-GCST90104936 | Sulcal opening distance of the left anterior inferior temporal sulcus \|\| id:ebi-hbsm44-GCST90104936 | Ice cream liking \|\| id:ebi-fl187-GCST90094784 | Inverse variance weighted | 7 | 0.0371272775019027 | 0.0366169959943654 | 0.310613460702315 | -0.034642035 | 0.108896589650859 | 1.03782510421903 | 0.965951131414537 | 1.11504703697585 | 0.998735809224318 |
| ebi-fl187-GCST90094784 | ebi-hbsm44-GCST90104941 | Sulcal opening distance of the right posterior inferior temporal sulcus \|\| id:ebi-hbsm44-GCST90104941 | Ice cream liking \|\| id:ebi-fl187-GCST90094784 | Inverse variance weighted | 8 | -0.008102502 | 0.0224015898890666 | 0.717581335878536 | -0.052009618 | 0.0358046142523596 | 0.991930234862245 | 0.949319736134692 | 1.0364533184995 | 0.998735809224318 |
| ebi-fl187-GCST90094784 | ebi-IPs91-GCST90274819 | Leukemia inhibitory factor levels \|\| id:ebi-IPs91-GCST90274819 | Ice cream liking \|\| id:ebi-fl187-GCST90094784 | Inverse variance weighted | 9 | 0.297212998908157 | 0.0923213545894972 | 0.00128490073911366 | 0.116263143912742 | 0.478162853903571 | 1.34610198716846 | 1.12329142054522 | 1.61310816295507 | 0.998735809224318 |
| ebi-fl187-GCST90094784 | ebi-IPs91-GCST90274837 | STAM binding protein levels \|\| id:ebi-IPs91-GCST90274837 | Ice cream liking \|\| id:ebi-fl187-GCST90094784 | Inverse variance weighted | 9 | 0.085642650268442 | 0.0966389189469236 | 0.375502848939232 | -0.103769631 | 0.275054931404412 | 1.08941695588147 | 0.901432935826955 | 1.31660299573287 | 0.998735809224318 |
| ebi-fl187-GCST90094784 | ebi-IPs91-GCST90274840 | TNF-beta levels \|\| id:ebi-IPs91-GCST90274840 | Ice cream liking \|\| id:ebi-fl187-GCST90094784 | Inverse variance weighted | 9 | 0.163706890127989 | 0.0987744019039694 | 0.097442320702577 | -0.029890938 | 0.357304717859769 | 1.17786901945288 | 0.970551378435531 | 1.42947138895753 | 0.998735809224318 |
| ebi-fl187-GCST90094784 | ebi-IPs91-GCST90274842 | Tumor necrosis factor ligand superfamily member 14 levels \|\| id:ebi-IPs91-GCST90274842 | Ice cream liking \|\| id:ebi-fl187-GCST90094784 | Inverse variance weighted | 9 | 0.0242350702240499 | 0.0929340256490803 | 0.794264367574506 | -0.15791562 | 0.206385760496247 | 1.02453112634748 | 0.853921832811754 | 1.2292272998789 | 0.998735809224318 |
| ebi-fl187-GCST90094784 | ebi-met1400-GCST90199628 | 3-methylhistidine levels \|\| id:ebi-met1400-GCST90199628 | Ice cream liking \|\| id:ebi-fl187-GCST90094784 | Inverse variance weighted | 8 | -0.185897529 | 0.111382760463395 | 0.0951179100741068 | -0.40420774 | 0.0324126813142334 | 0.830358678145589 | 0.667505439483306 | 1.03294369392017 | 0.998735809224318 |
| ebi-fl187-GCST90094784 | ebi-met1400-GCST90199651 | Docosahexaenoate DHA; 22:6n3 levels \|\| id:ebi-met1400-GCST90199651 | Ice cream liking \|\| id:ebi-fl187-GCST90094784 | Inverse variance weighted | 8 | 0.0181439041319255 | 0.113859258181423 | 0.873390145132476 | -0.205020242 | 0.241308050167514 | 1.01830950479184 | 0.81463082656581 | 1.2729130960104 | 0.998735809224318 |
| ebi-fl187-GCST90094784 | ebi-met1400-GCST90199652 | 1-stearoyl-2-oleoyl-GPS (18:0/18:1) levels \|\| id:ebi-met1400-GCST90199652 | Ice cream liking \|\| id:ebi-fl187-GCST90094784 | Inverse variance weighted | 8 | 0.0758883480124213 | 0.110941106626801 | 0.493948806731713 | -0.141556221 | 0.293332917000951 | 1.07884211256637 | 0.868006374047453 | 1.3408891209168 | 0.998735809224318 |
| ebi-fl187-GCST90094784 | ebi-met1400-GCST90199653 | 1-myristoyl-2-palmitoyl-gpc (14:0/16:0) levels \|\| id:ebi-met1400-GCST90199653 | Ice cream liking \|\| id:ebi-fl187-GCST90094784 | Inverse variance weighted | 8 | -0.133679095 | 0.108810801652663 | 0.219242005442141 | -0.346948266 | 0.0795900766832469 | 0.874870770353951 | 0.706841895215235 | 1.08284309405097 | 0.998735809224318 |
| ebi-fl187-GCST90094784 | ebi-met1400-GCST90199667 | Erythritol levels in elite athletes \|\| id:ebi-met1400-GCST90199667 | Ice cream liking \|\| id:ebi-fl187-GCST90094784 | Inverse variance weighted | 8 | -0.12172282 | 0.102840354928107 | 0.236567683103682 | -0.323289915 | 0.0798442758902671 | 0.885393748137656 | 0.723763993600536 | 1.08311838689493 | 0.998735809224318 |
| ebi-fl187-GCST90094784 | ebi-met1400-GCST90199674 | 3-hydroxymyristate levels \|\| id:ebi-met1400-GCST90199674 | Ice cream liking \|\| id:ebi-fl187-GCST90094784 | Inverse variance weighted | 8 | 0.0866355478192696 | 0.114015685073042 | 0.447340426677972 | -0.136835195 | 0.310106290562432 | 1.09049917248499 | 0.872113943093907 | 1.36357004105644 | 0.998735809224318 |
| ebi-fl187-GCST90094784 | ebi-met1400-GCST90199685 | 2-linoleoylglycerol (18:2) levels \|\| id:ebi-met1400-GCST90199685 | Ice cream liking \|\| id:ebi-fl187-GCST90094784 | Inverse variance weighted | 8 | 0.168675051615337 | 0.125340104910698 | 0.178386744840462 | -0.076991554 | 0.414341657240305 | 1.18373542350729 | 0.925897673696306 | 1.51337409378305 | 0.998735809224318 |
| ebi-fl187-GCST90094784 | ebi-met1400-GCST90199703 | Gamma-glutamylvaline levels \|\| id:ebi-met1400-GCST90199703 | Ice cream liking \|\| id:ebi-fl187-GCST90094784 | Inverse variance weighted | 8 | -0.030595812 | 0.111868018806375 | 0.784469517477385 | -0.249857129 | 0.188665504705089 | 0.969867502519516 | 0.778912059054564 | 1.2076369360428 | 0.998735809224318 |
| ebi-fl187-GCST90094784 | ebi-met1400-GCST90199779 | Gamma-glutamyl-2-aminobutyrate levels \|\| id:ebi-met1400-GCST90199779 | Ice cream liking \|\| id:ebi-fl187-GCST90094784 | Inverse variance weighted | 8 | 0.0543199344127283 | 0.113433561815905 | 0.632031085060746 | -0.168009847 | 0.276649715571902 | 1.05582234202584 | 0.845345510740774 | 1.31870436851798 | 0.998735809224318 |
| ebi-fl187-GCST90094784 | ebi-met1400-GCST90199824 | 4-vinylphenol sulfate levels \|\| id:ebi-met1400-GCST90199824 | Ice cream liking \|\| id:ebi-fl187-GCST90094784 | Inverse variance weighted | 8 | 0.200646384258032 | 0.10961080163136 | 0.0671700114904926 | -0.014190787 | 0.415483555455499 | 1.22219250888948 | 0.985909427676073 | 1.51510320000343 | 0.998735809224318 |
| ebi-fl187-GCST90094784 | ebi-met1400-GCST90199830 | 4-ethylphenylsulfate levels \|\| id:ebi-met1400-GCST90199830 | Ice cream liking \|\| id:ebi-fl187-GCST90094784 | Inverse variance weighted | 8 | -0.146413385 | 0.125986688040392 | 0.245181130184311 | -0.393347294 | 0.100520523258704 | 0.863800546919524 | 0.674794354950938 | 1.1057463349893 | 0.998735809224318 |
| ebi-fl187-GCST90094784 | ebi-met1400-GCST90199833 | Hexanoylglutamine levels \|\| id:ebi-met1400-GCST90199833 | Ice cream liking \|\| id:ebi-fl187-GCST90094784 | Inverse variance weighted | 8 | 0.138989278209582 | 0.108081724329322 | 0.198455394479109 | -0.072850901 | 0.350829457895053 | 1.14911177940189 | 0.929739442470761 | 1.42024509366957 | 0.998735809224318 |
| ebi-fl187-GCST90094784 | ebi-met1400-GCST90199837 | Carotenoid levels (cryptoxanthin) \|\| id:ebi-met1400-GCST90199837 | Ice cream liking \|\| id:ebi-fl187-GCST90094784 | Inverse variance weighted | 8 | -0.199399989 | 0.14757291699469 | 0.176632538291359 | -0.488642907 | 0.089842928105715 | 0.819222147775265 | 0.613458349866774 | 1.09400243317459 | 0.998735809224318 |
| ebi-fl187-GCST90094784 | ebi-met1400-GCST90199869 | 2-hydroxyhippurate levels \|\| id:ebi-met1400-GCST90199869 | Ice cream liking \|\| id:ebi-fl187-GCST90094784 | Inverse variance weighted (multiplicative random effects) | 8 | -0.07468669 | 0.160280733908043 | 0.641233952266297 | -0.388836929 | 0.239463548171667 | 0.92803420291276 | 0.677844798001722 | 1.27056736927814 | 0.998735809224318 |
| ebi-fl187-GCST90094784 | ebi-met1400-GCST90199902 | Hydantoin-5-propionate levels \|\| id:ebi-met1400-GCST90199902 | Ice cream liking \|\| id:ebi-fl187-GCST90094784 | Inverse variance weighted (multiplicative random effects) | 8 | 0.185989465270497 | 0.161486441720301 | 0.249430294945316 | -0.130523961 | 0.502502891042288 | 1.20440957218174 | 0.877635464111136 | 1.65285300888812 | 0.998735809224318 |
| ebi-fl187-GCST90094784 | ebi-met1400-GCST90199904 | Pantoate levels \|\| id:ebi-met1400-GCST90199904 | Ice cream liking \|\| id:ebi-fl187-GCST90094784 | Inverse variance weighted | 8 | -0.023647315 | 0.110998887589717 | 0.831294976383158 | -0.241205135 | 0.193910504682556 | 0.976630091815631 | 0.785680439559392 | 1.21398763188594 | 0.998735809224318 |
| ebi-fl187-GCST90094784 | ebi-met1400-GCST90199936 | 3-methyl catechol sulfate (2) levels \|\| id:ebi-met1400-GCST90199936 | Ice cream liking \|\| id:ebi-fl187-GCST90094784 | Inverse variance weighted | 7 | 0.128225418953107 | 0.129839477269872 | 0.323363864444462 | -0.126259956 | 0.382710794402056 | 1.13680923216348 | 0.881385695063193 | 1.46625391990219 | 0.998735809224318 |
| ebi-fl187-GCST90094784 | ebi-met1400-GCST90199977 | 4-hydroxychlorothalonil levels \|\| id:ebi-met1400-GCST90199977 | Ice cream liking \|\| id:ebi-fl187-GCST90094784 | Inverse variance weighted | 8 | -0.039062859 | 0.13354521741028 | 0.769899306337997 | -0.300811485 | 0.222685767230466 | 0.961690256460806 | 0.740217301646072 | 1.24942790085425 | 0.998735809224318 |
| ebi-fl187-GCST90094784 | ebi-met1400-GCST90200040 | Sphingomyelin (d18:2/24:1, d18:1/24:2) levels \|\| id:ebi-met1400-GCST90200040 | Ice cream liking \|\| id:ebi-fl187-GCST90094784 | Inverse variance weighted | 8 | 0.0968889430462754 | 0.120239625079624 | 0.420358882350698 | -0.138780722 | 0.332558608202338 | 1.10173801115921 | 0.870418871147362 | 1.39453162778172 | 0.998735809224318 |
| ebi-fl187-GCST90094784 | ebi-met1400-GCST90200077 | 1-stearoyl-2-oleoyl-GPI (18:0/18:1) levels \|\| id:ebi-met1400-GCST90200077 | Ice cream liking \|\| id:ebi-fl187-GCST90094784 | Inverse variance weighted | 8 | 0.146634348102072 | 0.112832140228163 | 0.193745093930681 | -0.074516647 | 0.367785342949272 | 1.15793048613209 | 0.928192022554347 | 1.44453192672811 | 0.998735809224318 |
| ebi-fl187-GCST90094784 | ebi-met1400-GCST90200080 | 1-palmitoyl-2-oleoyl-GPI (16:0/18:1) levels \|\| id:ebi-met1400-GCST90200080 | Ice cream liking \|\| id:ebi-fl187-GCST90094784 | Inverse variance weighted | 8 | 0.0832900118486026 | 0.142473995974088 | 0.558818058709021 | -0.19595902 | 0.362539043957814 | 1.08685696424395 | 0.822045921215568 | 1.43697332501674 | 0.998735809224318 |
| ebi-fl187-GCST90094784 | ebi-met1400-GCST90200082 | 1-oleoyl-2-linoleoyl-GPE (18:1/18:2) levels \|\| id:ebi-met1400-GCST90200082 | Ice cream liking \|\| id:ebi-fl187-GCST90094784 | Inverse variance weighted | 8 | -0.047240102 | 0.123830197611607 | 0.702838971891265 | -0.289947289 | 0.195467085360398 | 0.953858346836639 | 0.748303010131645 | 1.21587877305192 | 0.998735809224318 |
| ebi-fl187-GCST90094784 | ebi-met1400-GCST90200100 | Perfluorooctanesulfonate (PFOS) levels \|\| id:ebi-met1400-GCST90200100 | Ice cream liking \|\| id:ebi-fl187-GCST90094784 | Inverse variance weighted | 8 | 0.139733036303849 | 0.116154943134676 | 0.228980852433381 | -0.087930652 | 0.367396724847813 | 1.14996675849844 | 0.915824384890765 | 1.44397066453863 | 0.998735809224318 |
| ebi-fl187-GCST90094784 | ebi-met1400-GCST90200102 | 1-palmitoleoyl-2-linolenoyl-GPC (16:1/18:3) levels \|\| id:ebi-met1400-GCST90200102 | Ice cream liking \|\| id:ebi-fl187-GCST90094784 | Inverse variance weighted | 8 | -0.084071854 | 0.107187807705427 | 0.432839711598247 | -0.294159957 | 0.126016249372272 | 0.919365193902996 | 0.745157288909891 | 1.13430059980599 | 0.998735809224318 |
| ebi-fl187-GCST90094784 | ebi-met1400-GCST90200114 | Glycosyl-N-tricosanoyl-sphingadienine (d18:2/23:0) levels \|\| id:ebi-met1400-GCST90200114 | Ice cream liking \|\| id:ebi-fl187-GCST90094784 | Inverse variance weighted | 8 | -0.112547278 | 0.114716069807832 | 0.326546258695494 | -0.337390775 | 0.112296218721665 | 0.89355510052095 | 0.713629916730965 | 1.11884423417189 | 0.998735809224318 |
| ebi-fl187-GCST90094784 | ebi-met1400-GCST90200135 | Arachidoylcarnitine (C20) levels \|\| id:ebi-met1400-GCST90200135 | Ice cream liking \|\| id:ebi-fl187-GCST90094784 | Inverse variance weighted (multiplicative random effects) | 8 | 0.286184156065224 | 0.18432500196849 | 0.120517223848963 | -0.075092848 | 0.647461159923464 | 1.33133760657366 | 0.927657351392144 | 1.91068374547707 | 0.998735809224318 |
| ebi-fl187-GCST90094784 | ebi-met1400-GCST90200146 | Docosahexaenoylcarnitine (C22:6) levels \|\| id:ebi-met1400-GCST90200146 | Ice cream liking \|\| id:ebi-fl187-GCST90094784 | Inverse variance weighted (multiplicative random effects) | 8 | 0.184525949089502 | 0.192573459044362 | 0.337956522516817 | -0.192918031 | 0.561969928816452 | 1.20264818850545 | 0.824549559170877 | 1.75412459958118 | 0.998735809224318 |
| ebi-fl187-GCST90094784 | ebi-met1400-GCST90200162 | 3-hydroxyphenylacetoylglutamine levels \|\| id:ebi-met1400-GCST90200162 | Ice cream liking \|\| id:ebi-fl187-GCST90094784 | Inverse variance weighted | 8 | -0.057883673 | 0.133959195356814 | 0.66566925312394 | -0.320443696 | 0.204676350143488 | 0.943759726019606 | 0.725826919367296 | 1.22712784093626 | 0.998735809224318 |
| ebi-fl187-GCST90094784 | ebi-met1400-GCST90200172 | 3-carboxy-4-methyl-5-pentyl-2-furanpropionate (3-CMPFP) levels \|\| id:ebi-met1400-GCST90200172 | Ice cream liking \|\| id:ebi-fl187-GCST90094784 | Inverse variance weighted | 8 | -0.186071818 | 0.108812718890917 | 0.0872623568236105 | -0.399344747 | 0.0272011106131156 | 0.830213968191094 | 0.670759418896682 | 1.02757443810978 | 0.998735809224318 |
| ebi-fl187-GCST90094784 | ebi-met1400-GCST90200222 | Dihydrocaffeate sulfate (2) levels \|\| id:ebi-met1400-GCST90200222 | Ice cream liking \|\| id:ebi-fl187-GCST90094784 | Inverse variance weighted | 8 | 0.0548254487354778 | 0.16171673820317 | 0.734593544924086 | -0.262139358 | 0.371790255613691 | 1.05635621026966 | 0.769403793552369 | 1.45032875107514 | 0.998735809224318 |
| ebi-fl187-GCST90094784 | ebi-met1400-GCST90200223 | 4-methylhexanoylglutamine levels \|\| id:ebi-met1400-GCST90200223 | Ice cream liking \|\| id:ebi-fl187-GCST90094784 | Inverse variance weighted | 8 | -0.018930307 | 0.158733648773468 | 0.905070709916732 | -0.330048259 | 0.292187644582597 | 0.981247745945855 | 0.718889040009422 | 1.3393543166428 | 0.998735809224318 |
| ebi-fl187-GCST90094784 | ebi-met1400-GCST90200224 | 2-ketocaprylate levels \|\| id:ebi-met1400-GCST90200224 | Ice cream liking \|\| id:ebi-fl187-GCST90094784 | Inverse variance weighted | 8 | 0.157940641493248 | 0.110915730085086 | 0.154455236629619 | -0.059454189 | 0.375335472460016 | 1.17109667809442 | 0.942278698885798 | 1.45547960605018 | 0.998735809224318 |
| ebi-fl187-GCST90094784 | ebi-met1400-GCST90200243 | Hydroxypalmitoyl sphingomyelin (d18:1/16:0(OH)) levels \|\| id:ebi-met1400-GCST90200243 | Ice cream liking \|\| id:ebi-fl187-GCST90094784 | Inverse variance weighted | 8 | 0.143113586743345 | 0.138251082798055 | 0.300588805810641 | -0.127858536 | 0.414085709027532 | 1.15386085751984 | 0.879977855931376 | 1.51298679795445 | 0.998735809224318 |
| ebi-fl187-GCST90094784 | ebi-met1400-GCST90200273 | 2-methoxyhydroquinone sulfate (2) levels \|\| id:ebi-met1400-GCST90200273 | Ice cream liking \|\| id:ebi-fl187-GCST90094784 | Inverse variance weighted | 8 | 0.129712655794568 | 0.130195212368071 | 0.319107516364895 | -0.12546996 | 0.384895272035988 | 1.13850119459784 | 0.882082261386025 | 1.46946041978442 | 0.998735809224318 |
| ebi-fl187-GCST90094784 | ebi-met1400-GCST90200276 | 2,4-di-tert-butylphenol levels \|\| id:ebi-met1400-GCST90200276 | Ice cream liking \|\| id:ebi-fl187-GCST90094784 | Inverse variance weighted | 8 | 0.0534470408581547 | 0.108990334525785 | 0.623862685611419 | -0.160174015 | 0.267068096528693 | 1.054901124 | 0.851995516225852 | 1.30612938617959 | 0.998735809224318 |
| ebi-fl187-GCST90094784 | ebi-met1400-GCST90200289 | N-acetylmethionine levels \|\| id:ebi-met1400-GCST90200289 | Ice cream liking \|\| id:ebi-fl187-GCST90094784 | Inverse variance weighted | 8 | 0.163016102415531 | 0.14835307898925 | 0.271838434567128 | -0.127755932 | 0.453788137234461 | 1.17705564297494 | 0.880068149052374 | 1.57426443412474 | 0.998735809224318 |
| ebi-fl187-GCST90094784 | ebi-met1400-GCST90200308 | 3-hydroxyisobutyrate levels \|\| id:ebi-met1400-GCST90200308 | Ice cream liking \|\| id:ebi-fl187-GCST90094784 | Inverse variance weighted | 8 | 0.0598383780853155 | 0.125619274282062 | 0.633827109193451 | -0.1863754 | 0.306052155678157 | 1.06166494435734 | 0.829961969179011 | 1.35805313488305 | 0.998735809224318 |
| ebi-fl187-GCST90094784 | ebi-met1400-GCST90200383 | Cystathionine levels \|\| id:ebi-met1400-GCST90200383 | Ice cream liking \|\| id:ebi-fl187-GCST90094784 | Inverse variance weighted (multiplicative random effects) | 8 | 0.117645909422292 | 0.16819114856746 | 0.484253724073599 | -0.212008742 | 0.447300560614512 | 1.12484574356319 | 0.808957625814143 | 1.56408433079401 | 0.998735809224318 |
| ebi-fl187-GCST90094784 | ebi-met1400-GCST90200384 | Guanidinoacetate levels \|\| id:ebi-met1400-GCST90200384 | Ice cream liking \|\| id:ebi-fl187-GCST90094784 | Inverse variance weighted | 8 | 0.0982216600717421 | 0.1281078524715 | 0.443253545878247 | -0.152869731 | 0.349313050915882 | 1.10320729501662 | 0.858241516989014 | 1.4180930561921 | 0.998735809224318 |
| ebi-fl187-GCST90094784 | ebi-met1400-GCST90200403 | Citrulline levels \|\| id:ebi-met1400-GCST90200403 | Ice cream liking \|\| id:ebi-fl187-GCST90094784 | Inverse variance weighted | 8 | 0.0225945438770153 | 0.125961105987504 | 0.857641574077684 | -0.224289224 | 0.269478311612524 | 1.02285173396173 | 0.799083986790359 | 1.30928123571946 | 0.998735809224318 |
| ebi-fl187-GCST90094784 | ebi-met1400-GCST90200434 | 12,13-DiHOME levels \|\| id:ebi-met1400-GCST90200434 | Ice cream liking \|\| id:ebi-fl187-GCST90094784 | Inverse variance weighted | 8 | -0.044970634 | 0.112706151956296 | 0.689887454814812 | -0.265874692 | 0.175933423767048 | 0.95602555600303 | 0.766535174548212 | 1.19235867325932 | 0.998735809224318 |
| ebi-fl187-GCST90094784 | ebi-met1400-GCST90200437 | Fructose levels \|\| id:ebi-met1400-GCST90200437 | Ice cream liking \|\| id:ebi-fl187-GCST90094784 | Inverse variance weighted | 7 | 0.0614034562536531 | 0.127688566179365 | 0.630598567571868 | -0.188866133 | 0.311673045965209 | 1.06332783392048 | 0.827897327029381 | 1.36570809625273 | 0.998735809224318 |
| ebi-fl187-GCST90094784 | ebi-met1400-GCST90200448 | N-stearoyl-sphinganine (d18:0/18:0) levels \|\| id:ebi-met1400-GCST90200448 | Ice cream liking \|\| id:ebi-fl187-GCST90094784 | Inverse variance weighted | 8 | 0.131443814109995 | 0.150348067020992 | 0.381974819570581 | -0.163238397 | 0.42612602547114 | 1.14047382738516 | 0.849388672351267 | 1.53131374750977 | 0.998735809224318 |
| ebi-fl187-GCST90094784 | ebi-met1400-GCST90200468 | X-11847 levels \|\| id:ebi-met1400-GCST90200468 | Ice cream liking \|\| id:ebi-fl187-GCST90094784 | Inverse variance weighted | 8 | -0.312205055 | 0.129664268433186 | 0.0160491638838174 | -0.566347021 | -0.058063088 | 0.731831447463704 | 0.567595069282516 | 0.943590415917162 | 0.998735809224318 |
| ebi-fl187-GCST90094784 | ebi-met1400-GCST90200476 | X-11843 levels \|\| id:ebi-met1400-GCST90200476 | Ice cream liking \|\| id:ebi-fl187-GCST90094784 | Inverse variance weighted | 8 | -0.006143316 | 0.131652887785721 | 0.962781835531589 | -0.264182976 | 0.251896343934814 | 0.993875515458783 | 0.767833031643278 | 1.28646268071386 | 0.998735809224318 |
| ebi-fl187-GCST90094784 | ebi-met1400-GCST90200511 | X-12906 levels \|\| id:ebi-met1400-GCST90200511 | Ice cream liking \|\| id:ebi-fl187-GCST90094784 | Inverse variance weighted | 8 | -0.017644386 | 0.137936420204106 | 0.898214809961661 | -0.28799977 | 0.252710997108029 | 0.982510364198299 | 0.749761764615199 | 1.28751112862165 | 0.998735809224318 |
| ebi-fl187-GCST90094784 | ebi-met1400-GCST90200515 | X-12849 levels \|\| id:ebi-met1400-GCST90200515 | Ice cream liking \|\| id:ebi-fl187-GCST90094784 | Inverse variance weighted | 8 | 0.136134526717347 | 0.115355413530147 | 0.237948023058987 | -0.089962084 | 0.362231137236436 | 1.14583602878655 | 0.913965838724203 | 1.43653093938177 | 0.998735809224318 |
| ebi-fl187-GCST90094784 | ebi-met1400-GCST90200551 | X-18886 levels \|\| id:ebi-met1400-GCST90200551 | Ice cream liking \|\| id:ebi-fl187-GCST90094784 | Inverse variance weighted | 8 | 0.15988039411414 | 0.126597543736115 | 0.206624108861538 | -0.088250792 | 0.408011579836925 | 1.17337052057805 | 0.915531240376555 | 1.50382457511263 | 0.998735809224318 |
| ebi-fl187-GCST90094784 | ebi-met1400-GCST90200564 | X-21319 levels \|\| id:ebi-met1400-GCST90200564 | Ice cream liking \|\| id:ebi-fl187-GCST90094784 | Inverse variance weighted | 8 | 0.173049795604332 | 0.1107139691495 | 0.118044390659758 | -0.043949584 | 0.390049175137352 | 1.18892530686419 | 0.957002204548212 | 1.47705342640189 | 0.998735809224318 |
| ebi-fl187-GCST90094784 | ebi-met1400-GCST90200579 | X-21442 levels \|\| id:ebi-met1400-GCST90200579 | Ice cream liking \|\| id:ebi-fl187-GCST90094784 | Inverse variance weighted | 7 | -0.061764949 | 0.124433163996956 | 0.619633050444949 | -0.30565395 | 0.182124052439979 | 0.940103833174917 | 0.736641489826872 | 1.19976301817847 | 0.998735809224318 |
| ebi-fl187-GCST90094784 | ebi-met1400-GCST90200581 | X-21441 levels \|\| id:ebi-met1400-GCST90200581 | Ice cream liking \|\| id:ebi-fl187-GCST90094784 | Inverse variance weighted | 8 | 0.312596115442919 | 0.109778468219287 | 0.00440616639482415 | 0.097430317733116 | 0.527761913152723 | 1.36696932172166 | 1.10233462571142 | 1.69513420239541 | 0.998735809224318 |
| ebi-fl187-GCST90094784 | ebi-met1400-GCST90200648 | X-24585 levels \|\| id:ebi-met1400-GCST90200648 | Ice cream liking \|\| id:ebi-fl187-GCST90094784 | Inverse variance weighted (multiplicative random effects) | 8 | 0.0336187118134251 | 0.186942671645242 | 0.857282624338449 | -0.332788925 | 0.400026348238099 | 1.03419020703431 | 0.716921502671266 | 1.49186400511144 | 0.998735809224318 |
| ebi-fl187-GCST90094784 | ebi-met1400-GCST90200720 | Adenosine 5'-diphosphate (ADP) to pantothenate ratio \|\| id:ebi-met1400-GCST90200720 | Ice cream liking \|\| id:ebi-fl187-GCST90094784 | Inverse variance weighted | 8 | -0.005650207 | 0.14569093709407 | 0.969064083256043 | -0.291204443 | 0.279904030187794 | 0.994365725879025 | 0.747362869126062 | 1.32300283791078 | 0.998735809224318 |
| ebi-fl187-GCST90094784 | ebi-met1400-GCST90200724 | Cholate to taurocholate ratio \|\| id:ebi-met1400-GCST90200724 | Ice cream liking \|\| id:ebi-fl187-GCST90094784 | Inverse variance weighted | 8 | 0.00604195997920764 | 0.117285730934826 | 0.958915247621175 | -0.223838073 | 0.235921992611466 | 1.00606024943557 | 0.799444575828223 | 1.26607554306785 | 0.998735809224318 |
| ebi-fl187-GCST90094784 | ebi-met1400-GCST90200726 | N-acetylputrescine to (N(1) + N(8))-acetylspermidine ratio \|\| id:ebi-met1400-GCST90200726 | Ice cream liking \|\| id:ebi-fl187-GCST90094784 | Inverse variance weighted | 8 | -0.011122706 | 0.109095486235572 | 0.918793298698958 | -0.224949859 | 0.202704447111431 | 0.988938922679236 | 0.798556258219193 | 1.2247104480415 | 0.998735809224318 |
| ebi-fl187-GCST90094784 | ebi-met1400-GCST90200744 | Arginine to glutamate ratio \|\| id:ebi-met1400-GCST90200744 | Ice cream liking \|\| id:ebi-fl187-GCST90094784 | Inverse variance weighted | 8 | 0.015717470933813 | 0.145863359707222 | 0.914190232239193 | -0.270174714 | 0.301609655959968 | 1.01584164006872 | 0.763246132831794 | 1.35203336552629 | 0.998735809224318 |
| ebi-fl187-GCST90094784 | ebi-met1400-GCST90200759 | Histidine to trans-urocanate ratio \|\| id:ebi-met1400-GCST90200759 | Ice cream liking \|\| id:ebi-fl187-GCST90094784 | Inverse variance weighted | 8 | 0.102766652583993 | 0.121165491475544 | 0.396353790906546 | -0.134717711 | 0.340251015876059 | 1.10823277564693 | 0.873962587149373 | 1.40530029897971 | 0.998735809224318 |
| ebi-fl187-GCST90094784 | ebi-met1400-GCST90200795 | Oleoyl-linoleoyl-glycerol (18:1 to 18:2) [2] to linoleoyl-arachidonoyl-glycerol (18:2 to 20:4) [2] ratio \|\| id:ebi-met1400-GCST90200795 | Ice cream liking \|\| id:ebi-fl187-GCST90094784 | Inverse variance weighted | 8 | 0.0303956201964958 | 0.128278090188111 | 0.812694813479739 | -0.221029437 | 0.281820676965193 | 1.03086228323033 | 0.801693080846597 | 1.32554099863833 | 0.998735809224318 |
| ebi-fl187-GCST90094784 | ebi-met1400-GCST90200811 | Choline to taurocholate ratio \|\| id:ebi-met1400-GCST90200811 | Ice cream liking \|\| id:ebi-fl187-GCST90094784 | Inverse variance weighted | 8 | 0.00680075445197628 | 0.114615329245299 | 0.952684909744266 | -0.217845291 | 0.231446799772762 | 1.00682393209456 | 0.804249856838842 | 1.26042227004273 | 0.998735809224318 |
| ebi-fl187-GCST90094784 | ebi-met1400-GCST90200831 | Cholate to phosphate ratio \|\| id:ebi-met1400-GCST90200831 | Ice cream liking \|\| id:ebi-fl187-GCST90094784 | Inverse variance weighted | 8 | 0.0671856342218451 | 0.131723845115191 | 0.610017033927267 | -0.190993102 | 0.325364370647619 | 1.06949399443375 | 0.826138286664968 | 1.3845350380108 | 0.998735809224318 |
| ebi-fl187-GCST90094784 | ebi-met1400-GCST90200841 | Adenosine 5'-diphosphate (ADP) to oxalate (ethanedioate) ratio \|\| id:ebi-met1400-GCST90200841 | Ice cream liking \|\| id:ebi-fl187-GCST90094784 | Inverse variance weighted | 8 | 0.0966849962459674 | 0.147647660943149 | 0.512573382128539 | -0.192704419 | 0.38607441169454 | 1.10151333812851 | 0.824725711198685 | 1.47119414079078 | 0.998735809224318 |
| ebi-fl187-GCST90094784 | ebi-met1400-GCST90200844 | Aspartate to phosphate ratio \|\| id:ebi-met1400-GCST90200844 | Ice cream liking \|\| id:ebi-fl187-GCST90094784 | Inverse variance weighted (multiplicative random effects) | 8 | 0.0315749680767839 | 0.165208539214088 | 0.84843009886586 | -0.292233769 | 0.355383704936397 | 1.03207874565412 | 0.746593985204703 | 1.42672799184007 | 0.998735809224318 |
| ebi-fl187-GCST90094784 | ebi-met1400-GCST90200870 | Adenosine 5'-monophosphate (AMP) to acetoacetate ratio \|\| id:ebi-met1400-GCST90200870 | Ice cream liking \|\| id:ebi-fl187-GCST90094784 | Inverse variance weighted | 8 | -0.067721924 | 0.15797615558873 | 0.668152865550244 | -0.377355189 | 0.24191134077339 | 0.934520304948747 | 0.685672487221671 | 1.27368126421436 | 0.998735809224318 |
| ebi-fl187-GCST90094784 | ebi-met1400-GCST90200887 | Cholesterol to cortisol ratio \|\| id:ebi-met1400-GCST90200887 | Ice cream liking \|\| id:ebi-fl187-GCST90094784 | Inverse variance weighted | 8 | 0.0601165441738767 | 0.106528595191367 | 0.572534281801159 | -0.148679502 | 0.268912590748956 | 1.06196030461997 | 0.861845289985844 | 1.30854075747988 | 0.998735809224318 |
| ebi-fl187-GCST90094784 | ebi-met1400-GCST90200890 | Cortisol to taurocholate ratio \|\| id:ebi-met1400-GCST90200890 | Ice cream liking \|\| id:ebi-fl187-GCST90094784 | Inverse variance weighted | 8 | -0.077164134 | 0.114178646728589 | 0.499155375745867 | -0.300954281 | 0.146626013813136 | 0.925737896280689 | 0.740111608867266 | 1.15792083564506 | 0.998735809224318 |
| ebi-fl187-GCST90094784 | ebi-met1400-GCST90200948 | Adenosine 5'-diphosphate (ADP) to mannitol to sorbitol ratio \|\| id:ebi-met1400-GCST90200948 | Ice cream liking \|\| id:ebi-fl187-GCST90094784 | Inverse variance weighted | 8 | 0.151387565008156 | 0.147167805376492 | 0.303633306857972 | -0.137061334 | 0.43983646354608 | 1.16344748224729 | 0.871916746760345 | 1.55245331504065 | 0.998735809224318 |
| ebi-fl187-GCST90094784 | ebi-met1400-GCST90200972 | Leucine to N-palmitoyl-sphingosine (d18:1 to 16:0) ratio \|\| id:ebi-met1400-GCST90200972 | Ice cream liking \|\| id:ebi-fl187-GCST90094784 | Inverse variance weighted (multiplicative random effects) | 8 | -0.083218854 | 0.167483051739434 | 0.619274202041417 | -0.411485635 | 0.245047927592904 | 0.920149746898559 | 0.662665039959865 | 1.27768254798648 | 0.998735809224318 |
| ebi-fl187-GCST90094784 | ebi-met1400-GCST90200973 | Proline to glutamate ratio \|\| id:ebi-met1400-GCST90200973 | Ice cream liking \|\| id:ebi-fl187-GCST90094784 | Inverse variance weighted | 8 | 0.0920969345932424 | 0.120162229238982 | 0.443415551612052 | -0.143421035 | 0.327614903901647 | 1.09647110290995 | 0.866389212157042 | 1.38765448905272 | 0.998735809224318 |
| ebi-fl187-GCST90094784 | ebi-met1400-GCST90200983 | Cholesterol to linoleoyl-arachidonoyl-glycerol (18:2 to 20:4) [1] ratio \|\| id:ebi-met1400-GCST90200983 | Ice cream liking \|\| id:ebi-fl187-GCST90094784 | Inverse variance weighted | 8 | -0.028719792 | 0.119306552299218 | 0.80977006682518 | -0.262560634 | 0.205121050594501 | 0.971688701356505 | 0.769079730252595 | 1.22767366659603 | 0.998735809224318 |
| ebi-fl187-GCST90094784 | ebi-met1400-GCST90201001 | Serine to alpha-ketobutyrate ratio \|\| id:ebi-met1400-GCST90201001 | Ice cream liking \|\| id:ebi-fl187-GCST90094784 | Inverse variance weighted | 8 | 0.0836373862028208 | 0.111594204147102 | 0.45356923331294 | -0.135087254 | 0.30236202633114 | 1.08723457606257 | 0.87363967987078 | 1.35305097813414 | 0.998735809224318 |
| ebi-fl187-GCST90094784 | ebi-met1400-GCST90201005 | Fructose to maltose ratio \|\| id:ebi-met1400-GCST90201005 | Ice cream liking \|\| id:ebi-fl187-GCST90094784 | Inverse variance weighted | 8 | -0.126908572 | 0.150745628739792 | 0.399859427008311 | -0.422370004 | 0.168552860518004 | 0.880814200162151 | 0.655491459963292 | 1.18359079041356 | 0.998735809224318 |
| ebi-fl187-GCST90094784 | ebi-met1400-GCST90201020 | Paraxanthine to 5-acetylamino-6-formylamino-3-methyluracil ratio \|\| id:ebi-met1400-GCST90201020 | Ice cream liking \|\| id:ebi-fl187-GCST90094784 | Inverse variance weighted | 8 | 0.13175489399795 | 0.151199961255555 | 0.383538501347197 | -0.16459703 | 0.428106818058838 | 1.14082866104355 | 0.848235448612247 | 1.53434996849956 | 0.998735809224318 |
| ebi-fl187-GCST90094784 | ebi-pw11-GCST90275197 | Placental weight (fetal genotype, adjusted for sex) \|\| id:ebi-pw11-GCST90275197 | Ice cream liking \|\| id:ebi-fl187-GCST90094784 | Inverse variance weighted | 8 | -0.045401825 | 0.0404109542292866 | 0.261223963334041 | -0.124607295 | 0.0338036454216121 | 0.955613415440236 | 0.882843531448966 | 1.03438148124682 | 0.998735809224318 |
| ebi-fl187-GCST90094784 | prot-a-1017 | FAS-associated factor 2 \|\| id:prot-a-1017 | Ice cream liking \|\| id:ebi-fl187-GCST90094784 | Inverse variance weighted | 9 | 0.137933888285553 | 0.169774396827928 | 0.416531169803691 | -0.194823929 | 0.470691706068291 | 1.14789965815111 | 0.822979547723019 | 1.60110130176275 | 0.998735809224318 |
| ebi-fl187-GCST90094784 | prot-a-1019 | Fas apoptotic inhibitory molecule 3 \|\| id:prot-a-1019 | Ice cream liking \|\| id:ebi-fl187-GCST90094784 | Inverse variance weighted | 9 | 0.074533909264199 | 0.169774396827928 | 0.6606489499577 | -0.258223909 | 0.407291727046937 | 1.07738187612954 | 0.772422260818671 | 1.50274243233506 | 0.998735809224318 |
| ebi-fl187-GCST90094784 | prot-a-1052 | MIP18 family protein FAM96A \|\| id:prot-a-1052 | Ice cream liking \|\| id:ebi-fl187-GCST90094784 | Inverse variance weighted | 9 | 0.0580953011956713 | 0.16993569644673 | 0.732451452546939 | -0.274978664 | 0.391169266231263 | 1.05981599262894 | 0.759588329750274 | 1.47870878769469 | 0.998735809224318 |
| ebi-fl187-GCST90094784 | prot-a-1066 | F-box/LRR-repeat protein 4 \|\| id:prot-a-1066 | Ice cream liking \|\| id:ebi-fl187-GCST90094784 | Inverse variance weighted | 9 | -0.054132633 | 0.16993569644673 | 0.750069484906009 | -0.387206598 | 0.278941332348347 | 0.947306454368506 | 0.678950810744522 | 1.32172979881145 | 0.998735809224318 |
| ebi-fl187-GCST90094784 | prot-a-1076 | Low affinity immunoglobulin gamma Fc region receptor III-B \|\| id:prot-a-1076 | Ice cream liking \|\| id:ebi-fl187-GCST90094784 | Inverse variance weighted | 9 | -0.051361585 | 0.17069199682926 | 0.763489129778952 | -0.385917898 | 0.283194729201604 | 0.949935126528751 | 0.67982633824024 | 1.32736361310895 | 0.998735809224318 |
| ebi-fl187-GCST90094784 | prot-a-1081 | Fc receptor-like protein 3 \|\| id:prot-a-1081 | Ice cream liking \|\| id:ebi-fl187-GCST90094784 | Inverse variance weighted | 9 | 0.0228019090381233 | 0.19529312567205 | 0.90705233736606 | -0.359972617 | 0.405576435355341 | 1.02306385976932 | 0.697695430608716 | 1.50016700016643 | 0.998735809224318 |
| ebi-fl187-GCST90094784 | prot-a-1105 | Fibroblast growth factor receptor 4 \|\| id:prot-a-1105 | Ice cream liking \|\| id:ebi-fl187-GCST90094784 | Inverse variance weighted | 9 | 0.163789961926299 | 0.195163013770542 | 0.401330751997713 | -0.218729545 | 0.546309468916561 | 1.1779668712148 | 0.803539009860009 | 1.72686818269263 | 0.998735809224318 |
| ebi-fl187-GCST90094784 | prot-a-1143 | Follistatin-related protein 1 \|\| id:prot-a-1143 | Ice cream liking \|\| id:ebi-fl187-GCST90094784 | Inverse variance weighted | 9 | -0.114754412 | 0.205445791989433 | 0.576460016966969 | -0.517428164 | 0.287919340264402 | 0.891585079587168 | 0.59605152498631 | 1.33364972795047 | 0.998735809224318 |
| ebi-fl187-GCST90094784 | prot-a-1151 | Alpha-(1,3)-fucosyltransferase 10 \|\| id:prot-a-1151 | Ice cream liking \|\| id:ebi-fl187-GCST90094784 | Inverse variance weighted | 9 | 0.00508383817966558 | 0.219042484080135 | 0.981483264002221 | -0.424239431 | 0.43440710697673 | 1.00509678281183 | 0.654267211551361 | 1.54404733262318 | 0.998955228396052 |
| ebi-fl187-GCST90094784 | prot-a-1165 | Growth arrest and DNA damage-inducible proteins-interacting protein 1 \|\| id:prot-a-1165 | Ice cream liking \|\| id:ebi-fl187-GCST90094784 | Inverse variance weighted | 9 | -0.409271835 | 0.193804962276771 | 0.0347060358043865 | -0.789129561 | -0.029414108 | 0.664133673312301 | 0.454240011654653 | 0.971014275956445 | 0.998735809224318 |
| ebi-fl187-GCST90094784 | prot-a-1194 | Growth/differentiation factor 11/8 \|\| id:prot-a-1194 | Ice cream liking \|\| id:ebi-fl187-GCST90094784 | Inverse variance weighted | 9 | -0.209250557 | 0.169774396827928 | 0.217754421452246 | -0.542008375 | 0.1235072604224 | 0.811191960062137 | 0.581579049760988 | 1.13145821937686 | 0.998735809224318 |
| ebi-fl187-GCST90094784 | prot-a-1216 | D-glucuronyl C5-epimerase \|\| id:prot-a-1216 | Ice cream liking \|\| id:ebi-fl187-GCST90094784 | Inverse variance weighted (multiplicative random effects) | 9 | -0.274610452 | 0.301250179480166 | 0.36199542475819 | -0.865060804 | 0.315839899516352 | 0.759868070464358 | 0.421025951767715 | 1.37141067453672 | 0.998735809224318 |
| ebi-fl187-GCST90094784 | prot-a-1246 | Glypican-3 \|\| id:prot-a-1246 | Ice cream liking \|\| id:ebi-fl187-GCST90094784 | Inverse variance weighted | 9 | 0.234343876401014 | 0.16993569644673 | 0.167890125556198 | -0.098730089 | 0.567417841436606 | 1.26407910445008 | 0.905987211270211 | 1.76370699545201 | 0.998735809224318 |
| ebi-fl187-GCST90094784 | prot-a-1275 | Glutamate receptor 4 \|\| id:prot-a-1275 | Ice cream liking \|\| id:ebi-fl187-GCST90094784 | Inverse variance weighted | 9 | 0.283307435696226 | 0.176733202435748 | 0.108929517 | -0.063089641 | 0.629704512470292 | 1.32751322403978 | 0.93885930955646 | 1.87705585071424 | 0.998735809224318 |
| ebi-fl187-GCST90094784 | prot-a-1289 | Glutathione S-transferase P \|\| id:prot-a-1289 | Ice cream liking \|\| id:ebi-fl187-GCST90094784 | Inverse variance weighted | 9 | -0.138584026 | 0.16993569644673 | 0.414781231199293 | -0.471657991 | 0.194489939367302 | 0.870590096281065 | 0.623966878902152 | 1.21469126226094 | 0.998735809224318 |
| ebi-fl187-GCST90094784 | prot-a-130 | Apolipoprotein D \|\| id:prot-a-130 | Ice cream liking \|\| id:ebi-fl187-GCST90094784 | Inverse variance weighted | 9 | 0.0683585641311101 | 0.169774396827928 | 0.687210474461862 | -0.264399254 | 0.401116381913849 | 1.07074917190129 | 0.767666984617326 | 1.49349107373535 | 0.998735809224318 |
| ebi-fl187-GCST90094784 | prot-a-1313 | Heparin-binding EGF-like growth factor \|\| id:prot-a-1313 | Ice cream liking \|\| id:ebi-fl187-GCST90094784 | Inverse variance weighted | 9 | -0.065064313 | 0.169774396827928 | 0.701541941493968 | -0.39782213 | 0.267693505265682 | 0.937007200148043 | 0.671781506611037 | 1.30694650639978 | 0.998735809224318 |
| ebi-fl187-GCST90094784 | prot-a-1347 | HLA class II histocompatibility antigen, DQ alpha 2 chain \|\| id:prot-a-1347 | Ice cream liking \|\| id:ebi-fl187-GCST90094784 | Inverse variance weighted | 9 | -0.031073988 | 0.169774396827928 | 0.854773645932434 | -0.363831806 | 0.301683829543774 | 0.969403845939171 | 0.695008081086952 | 1.35213365440579 | 0.998735809224318 |
| ebi-fl187-GCST90094784 | prot-a-1357 | Non-histone chromosomal protein HMG-14 \|\| id:prot-a-1357 | Ice cream liking \|\| id:ebi-fl187-GCST90094784 | Inverse variance weighted | 9 | -0.033124167 | 0.169774396827928 | 0.845309210835887 | -0.365881985 | 0.29963365064678 | 0.967418430555352 | 0.693584649828816 | 1.34936437824737 | 0.998735809224318 |
| ebi-fl187-GCST90094784 | prot-a-137 | Apolipoprotein M \|\| id:prot-a-137 | Ice cream liking \|\| id:ebi-fl187-GCST90094784 | Inverse variance weighted | 9 | -0.06380838 | 0.16993569644673 | 0.7072994719594 | -0.396882345 | 0.269265585079168 | 0.938184757311662 | 0.672413133751454 | 1.30900274648307 | 0.998735809224318 |
| ebi-fl187-GCST90094784 | prot-a-1381 | 17-beta-hydroxysteroid dehydrogenase 14 \|\| id:prot-a-1381 | Ice cream liking \|\| id:ebi-fl187-GCST90094784 | Inverse variance weighted | 9 | -0.04086662 | 0.16993569644673 | 0.809955958703084 | -0.373940585 | 0.292207344823472 | 0.959957160291693 | 0.688017789021118 | 1.33938070250536 | 0.998735809224318 |
| ebi-fl187-GCST90094784 | prot-a-1392 | Serine protease HTRA2, mitochondrial \|\| id:prot-a-1392 | Ice cream liking \|\| id:ebi-fl187-GCST90094784 | Inverse variance weighted | 9 | 0.0332970798491528 | 0.169774396827928 | 0.844511977442692 | -0.299460738 | 0.366054897631891 | 1.03385763189286 | 0.741217823581899 | 1.44203440475552 | 0.998735809224318 |
| ebi-fl187-GCST90094784 | prot-a-1416 | Interferon-induced protein with tetratricopeptide repeats 2 \|\| id:prot-a-1416 | Ice cream liking \|\| id:ebi-fl187-GCST90094784 | Inverse variance weighted | 9 | -0.177345324 | 0.16993569644673 | 0.296669317873071 | -0.510419289 | 0.155728640808974 | 0.837490528813205 | 0.600243850241298 | 1.16850907438679 | 0.998735809224318 |
| ebi-fl187-GCST90094784 | prot-a-1428 | Interferon gamma \|\| id:prot-a-1428 | Ice cream liking \|\| id:ebi-fl187-GCST90094784 | Inverse variance weighted | 9 | -0.298071428 | 0.16993569644673 | 0.0794262558032778 | -0.631145393 | 0.0350025371139137 | 0.742248320599364 | 0.531982123335834 | 1.03562233628813 | 0.998735809224318 |
| ebi-fl187-GCST90094784 | prot-a-1483 | Interleukin-17C \|\| id:prot-a-1483 | Ice cream liking \|\| id:ebi-fl187-GCST90094784 | Inverse variance weighted | 9 | -0.232886651 | 0.16993569644673 | 0.170549638008624 | -0.565960616 | 0.100187313765638 | 0.792243368214836 | 0.56781443288593 | 1.10537795119146 | 0.998735809224318 |
| ebi-fl187-GCST90094784 | prot-a-1503 | Interleukin-1 receptor-like 2 \|\| id:prot-a-1503 | Ice cream liking \|\| id:ebi-fl187-GCST90094784 | Inverse variance weighted | 9 | 0.0632560831721188 | 0.169774396827928 | 0.709454315268641 | -0.269501735 | 0.396013900954857 | 1.06529960960715 | 0.763759954695087 | 1.48588997269726 | 0.998735809224318 |
| ebi-fl187-GCST90094784 | prot-a-1522 | Interleukin-31 receptor subunit alpha \|\| id:prot-a-1522 | Ice cream liking \|\| id:ebi-fl187-GCST90094784 | Inverse variance weighted | 9 | 0.048866958390459 | 0.18113711318491 | 0.787330410679535 | -0.306161783 | 0.403895700232882 | 1.05008063702827 | 0.736267493923354 | 1.49764773450189 | 0.998735809224318 |
| ebi-fl187-GCST90094784 | prot-a-1540 | Interleukin-6 receptor subunit alpha \|\| id:prot-a-1540 | Ice cream liking \|\| id:ebi-fl187-GCST90094784 | Inverse variance weighted | 9 | -0.161224122 | 0.169774396827928 | 0.34229651362391 | -0.493981939 | 0.171533696198264 | 0.851101299558133 | 0.610191803441254 | 1.18712414362885 | 0.998735809224318 |
| ebi-fl187-GCST90094784 | prot-a-1541 | Interleukin-6 receptor subunit alpha \|\| id:prot-a-1541 | Ice cream liking \|\| id:ebi-fl187-GCST90094784 | Inverse variance weighted | 9 | -0.087072786 | 0.16993569644673 | 0.608380417827477 | -0.420146751 | 0.246001179291983 | 0.916610377037208 | 0.656950404756944 | 1.27890108173864 | 0.998735809224318 |
| ebi-fl187-GCST90094784 | prot-a-1545 | Interleukin-9 \|\| id:prot-a-1545 | Ice cream liking \|\| id:ebi-fl187-GCST90094784 | Inverse variance weighted | 9 | -0.036476583 | 0.231937088455023 | 0.875032615640469 | -0.491073276 | 0.418120110251609 | 0.964180671734832 | 0.61196922939995 | 1.51910312330339 | 0.998735809224318 |
| ebi-fl187-GCST90094784 | prot-a-1548 | Inositol monophosphatase 3 \|\| id:prot-a-1548 | Ice cream liking \|\| id:ebi-fl187-GCST90094784 | Inverse variance weighted | 9 | -0.031567227 | 0.16993569644673 | 0.852633104676951 | -0.364641192 | 0.301506738407417 | 0.968925816648595 | 0.694445779115271 | 1.35189422472149 | 0.998735809224318 |
| ebi-fl187-GCST90094784 | prot-a-1555 | Inhibin beta A chain:Inhibin beta B chain heterodimer \|\| id:prot-a-1555 | Ice cream liking \|\| id:ebi-fl187-GCST90094784 | Inverse variance weighted | 9 | -0.160545613 | 0.169774396827928 | 0.344331776926848 | -0.493303431 | 0.172212204753383 | 0.851678975027868 | 0.610605964290103 | 1.18792989083857 | 0.998735809224318 |
| ebi-fl187-GCST90094784 | prot-a-1603 | Junctophilin-3 \|\| id:prot-a-1603 | Ice cream liking \|\| id:ebi-fl187-GCST90094784 | Inverse variance weighted (multiplicative random effects) | 9 | -0.163970276 | 0.270004967377128 | 0.543661030225268 | -0.693180012 | 0.365239459794226 | 0.848767250038132 | 0.499983584387394 | 1.44085899464074 | 0.998735809224318 |
| ebi-fl187-GCST90094784 | prot-a-1627 | UPF0258 protein KIAA1024 \|\| id:prot-a-1627 | Ice cream liking \|\| id:ebi-fl187-GCST90094784 | Inverse variance weighted | 9 | 0.21112299461802 | 0.16993569644673 | 0.214100115049095 | -0.12195097 | 0.544196959653612 | 1.23506425197585 | 0.885191768021444 | 1.72322400819223 | 0.998735809224318 |
| ebi-fl187-GCST90094784 | prot-a-1666 | Kallikrein-7 \|\| id:prot-a-1666 | Ice cream liking \|\| id:ebi-fl187-GCST90094784 | Inverse variance weighted | 9 | 0.0544357271466034 | 0.169774396827928 | 0.748486504393151 | -0.278322091 | 0.387193544929342 | 1.05594460565979 | 0.75705294257702 | 1.47284152469769 | 0.998735809224318 |
| ebi-fl187-GCST90094784 | prot-a-1678 | Importin subunit alpha-3 \|\| id:prot-a-1678 | Ice cream liking \|\| id:ebi-fl187-GCST90094784 | Inverse variance weighted | 9 | -0.017582618 | 0.171343014534767 | 0.918267356293089 | -0.353414927 | 0.318249690071918 | 0.982571053847265 | 0.702285735668317 | 1.37471947218147 | 0.998735809224318 |
| ebi-fl187-GCST90094784 | prot-a-1718 | Protein LDOC1 \|\| id:prot-a-1718 | Ice cream liking \|\| id:ebi-fl187-GCST90094784 | Inverse variance weighted | 9 | 0.0878324560663952 | 0.224229630860165 | 0.695274255746327 | -0.35165762 | 0.527322532552318 | 1.09180518136851 | 0.703520951954739 | 1.69438955691517 | 0.998735809224318 |
| ebi-fl187-GCST90094784 | prot-a-1723 | Leptin \|\| id:prot-a-1723 | Ice cream liking \|\| id:ebi-fl187-GCST90094784 | Inverse variance weighted | 9 | -0.323170768 | 0.16993569644673 | 0.0572072651273902 | -0.656244733 | 0.00990319677950896 | 0.723850233170944 | 0.51879589799337 | 1.00995239570751 | 0.998735809224318 |
| ebi-fl187-GCST90094784 | prot-a-1730 | Galectin-7 \|\| id:prot-a-1730 | Ice cream liking \|\| id:ebi-fl187-GCST90094784 | Inverse variance weighted | 9 | -0.079891535 | 0.209452214276226 | 0.702883576535711 | -0.490417875 | 0.330634804809339 | 0.923216477472563 | 0.612370446316779 | 1.39185140204486 | 0.998735809224318 |
| ebi-fl187-GCST90094784 | prot-a-1753 | Lipase member N \|\| id:prot-a-1753 | Ice cream liking \|\| id:ebi-fl187-GCST90094784 | Inverse variance weighted | 9 | 0.129837484019673 | 0.16993569644673 | 0.44484405640044 | -0.203236481 | 0.462911449055264 | 1.13864332055172 | 0.816085229940481 | 1.58869265595163 | 0.998735809224318 |
| ebi-fl187-GCST90094784 | prot-a-1756 | VIP36-like protein \|\| id:prot-a-1756 | Ice cream liking \|\| id:ebi-fl187-GCST90094784 | Inverse variance weighted | 9 | -0.27568538 | 0.169774396827928 | 0.104411314475341 | -0.608443198 | 0.0570724378898108 | 0.759051706126561 | 0.544197417753903 | 1.05873249996602 | 0.998735809224318 |
| ebi-fl187-GCST90094784 | prot-a-1760 | Ig Kappa chain V-I region HK102- like \|\| id:prot-a-1760 | Ice cream liking \|\| id:ebi-fl187-GCST90094784 | Inverse variance weighted | 9 | -0.287149154 | 0.169774396827928 | 0.090768594861843 | -0.619906972 | 0.0456086634598526 | 0.750399795116242 | 0.537994483760804 | 1.04666473264595 | 0.998735809224318 |
| ebi-fl187-GCST90094784 | prot-a-179 | Neutral ceramidase \|\| id:prot-a-179 | Ice cream liking \|\| id:ebi-fl187-GCST90094784 | Inverse variance weighted | 9 | -0.486928274 | 0.169774396827928 | 0.00412957858747339 | -0.819686092 | -0.154170456 | 0.614511107848261 | 0.440569931366908 | 0.85712590620355 | 0.998735809224318 |
| ebi-fl187-GCST90094784 | prot-a-1809 | Lactotransferrin \|\| id:prot-a-1809 | Ice cream liking \|\| id:ebi-fl187-GCST90094784 | Inverse variance weighted | 9 | -0.264435939 | 0.169774396827928 | 0.119334611101943 | -0.597193757 | 0.0683218783876554 | 0.767638822699843 | 0.550353897776823 | 1.07070989139239 | 0.998735809224318 |
| ebi-fl187-GCST90094784 | prot-a-1818 | Lysophospholipase-like protein 1 \|\| id:prot-a-1818 | Ice cream liking \|\| id:ebi-fl187-GCST90094784 | Inverse variance weighted | 9 | -0.018937738 | 0.169774396827928 | 0.911183002689684 | -0.351695556 | 0.313820079892408 | 0.981240454441709 | 0.703494264215261 | 1.3686434679137 | 0.998735809224318 |
| ebi-fl187-GCST90094784 | prot-a-1852 | MAP kinase-activated protein kinase 3 \|\| id:prot-a-1852 | Ice cream liking \|\| id:ebi-fl187-GCST90094784 | Inverse variance weighted | 9 | 0.152114239913504 | 0.16993569644673 | 0.370718542445526 | -0.180959725 | 0.485188204949096 | 1.1642932375937 | 0.834468966154751 | 1.62448071538593 | 0.998735809224318 |
| ebi-fl187-GCST90094784 | prot-a-1856 | Megakaryocyte-associated tyrosine-protein kinase \|\| id:prot-a-1856 | Ice cream liking \|\| id:ebi-fl187-GCST90094784 | Inverse variance weighted | 9 | 0.0129648723394422 | 0.188433757008421 | 0.945146157391352 | -0.356365291 | 0.382295036075948 | 1.01304928068351 | 0.700216790342277 | 1.4656444393339 | 0.998735809224318 |
| ebi-fl187-GCST90094784 | prot-a-1890 | Beta-1,3-N-acetylglucosaminyltransferase manic fringe \|\| id:prot-a-1890 | Ice cream liking \|\| id:ebi-fl187-GCST90094784 | Inverse variance weighted | 9 | -0.290637525 | 0.169774396827928 | 0.0869143005367504 | -0.623395343 | 0.0421202926721421 | 0.747786682794054 | 0.536121029071805 | 1.04301993886019 | 0.998735809224318 |
| ebi-fl187-GCST90094784 | prot-a-1919 | Matrilysin \|\| id:prot-a-1919 | Ice cream liking \|\| id:ebi-fl187-GCST90094784 | Inverse variance weighted (multiplicative random effects) | 9 | -0.166124101 | 0.239256436041386 | 0.487472190622113 | -0.635066715 | 0.302818513974701 | 0.846941121709652 | 0.529900134687821 | 1.35366877018325 | 0.998735809224318 |
| ebi-fl187-GCST90094784 | prot-a-1923 | Multimerin-2 \|\| id:prot-a-1923 | Ice cream liking \|\| id:ebi-fl187-GCST90094784 | Inverse variance weighted | 9 | 0.117983429549051 | 0.169774396827928 | 0.487091389421116 | -0.214774388 | 0.45074124733179 | 1.12522546571947 | 0.806723425944543 | 1.56947512367224 | 0.998735809224318 |
| ebi-fl187-GCST90094784 | prot-a-1948 | Mesothelin \|\| id:prot-a-1948 | Ice cream liking \|\| id:ebi-fl187-GCST90094784 | Inverse variance weighted | 9 | 0.174817774341895 | 0.169774396827928 | 0.303147874202095 | -0.157940043 | 0.507575592124633 | 1.19102916076374 | 0.85390097739818 | 1.66125874 | 0.998735809224318 |
| ebi-fl187-GCST90094784 | prot-a-195 | ATPase family AAA domain-containing protein 1 \|\| id:prot-a-195 | Ice cream liking \|\| id:ebi-fl187-GCST90094784 | Inverse variance weighted | 9 | 0.234021890439893 | 0.169774396827928 | 0.168070986826148 | -0.098735927 | 0.566779708222632 | 1.26367215424435 | 0.905981921490633 | 1.76258187446521 | 0.998735809224318 |
| ebi-fl187-GCST90094784 | prot-a-1966 | Mucin-1 \|\| id:prot-a-1966 | Ice cream liking \|\| id:ebi-fl187-GCST90094784 | Inverse variance weighted | 9 | -0.221089734 | 0.16993569644673 | 0.193251619820925 | -0.554163699 | 0.111984230626225 | 0.801644741945131 | 0.574552558955757 | 1.11849522253675 | 0.998735809224318 |
| ebi-fl187-GCST90094784 | prot-a-1986 | Myocardial zonula adherens protein \|\| id:prot-a-1986 | Ice cream liking \|\| id:ebi-fl187-GCST90094784 | Inverse variance weighted | 9 | -0.121938323 | 0.169774396827928 | 0.472610916733708 | -0.45469614 | 0.210819495195948 | 0.885202963847935 | 0.634640780365793 | 1.23468946756546 | 0.998735809224318 |
| ebi-fl187-GCST90094784 | prot-a-2071 | Epididymal secretory protein E1 \|\| id:prot-a-2071 | Ice cream liking \|\| id:ebi-fl187-GCST90094784 | Inverse variance weighted | 9 | -0.274426904 | 0.169774396827928 | 0.10600331638487 | -0.607184722 | 0.0583309138300856 | 0.76000755576688 | 0.54488270823125 | 1.06006572808621 | 0.998735809224318 |
| ebi-fl187-GCST90094784 | prot-a-2098 | Neuritin \|\| id:prot-a-2098 | Ice cream liking \|\| id:ebi-fl187-GCST90094784 | Inverse variance weighted | 9 | -0.152168625 | 0.169774396827928 | 0.370093082957434 | -0.484926443 | 0.180589192468172 | 0.858843445783838 | 0.615742487208769 | 1.19792296242141 | 0.998735809224318 |
| ebi-fl187-GCST90094784 | prot-a-2115 | Neurotrophin-4 \|\| id:prot-a-2115 | Ice cream liking \|\| id:ebi-fl187-GCST90094784 | Inverse variance weighted | 9 | -0.131923399 | 0.224717441965037 | 0.557161087433781 | -0.572369585 | 0.308522787370773 | 0.876408126351617 | 0.564186964468735 | 1.36141253220628 | 0.998735809224318 |
| ebi-fl187-GCST90094784 | prot-a-2118 | Netrin-1 \|\| id:prot-a-2118 | Ice cream liking \|\| id:ebi-fl187-GCST90094784 | Inverse variance weighted | 9 | -0.285624742 | 0.210707021283392 | 0.17524112148284 | -0.698610504 | 0.12736101967113 | 0.751544586122793 | 0.497275786804665 | 1.13582699966115 | 0.998735809224318 |
| ebi-fl187-GCST90094784 | prot-a-2120 | Netrin-G1 \|\| id:prot-a-2120 | Ice cream liking \|\| id:ebi-fl187-GCST90094784 | Inverse variance weighted | 9 | -0.019852149 | 0.16993569644673 | 0.907001476773816 | -0.352926114 | 0.313221815833577 | 0.980343607176818 | 0.702629105746584 | 1.36782490260101 | 0.998735809224318 |
| ebi-fl187-GCST90094784 | prot-a-2145 | Olfactomedin-like protein 3 \|\| id:prot-a-2145 | Ice cream liking \|\| id:ebi-fl187-GCST90094784 | Inverse variance weighted | 9 | -0.122434617 | 0.16993569644673 | 0.471231876508851 | -0.455508582 | 0.210639348007337 | 0.88476375153574 | 0.634125381129174 | 1.23446706176256 | 0.998735809224318 |
| ebi-fl187-GCST90094784 | prot-a-2162 | Prolyl 4-hydroxylase subunit alpha-2 \|\| id:prot-a-2162 | Ice cream liking \|\| id:ebi-fl187-GCST90094784 | Inverse variance weighted | 9 | -0.216871993 | 0.218615963376894 | 0.32118646367317 | -0.645359281 | 0.211615295113705 | 0.805033012486037 | 0.524474074512429 | 1.23567242440882 | 0.998735809224318 |
| ebi-fl187-GCST90094784 | prot-a-2168 | Polyadenylate-binding protein-interacting protein 1 \|\| id:prot-a-2168 | Ice cream liking \|\| id:ebi-fl187-GCST90094784 | Inverse variance weighted | 9 | 0.361461977754658 | 0.169774396827928 | 0.0332482731640835 | 0.0287041599719199 | 0.694219795537397 | 1.43542644281034 | 1.02912009451805 | 2.00214638086925 | 0.998735809224318 |
| ebi-fl187-GCST90094784 | prot-a-219 | Beta-1,4-galactosyltransferase 5 \|\| id:prot-a-219 | Ice cream liking \|\| id:ebi-fl187-GCST90094784 | Inverse variance weighted | 9 | -0.194021753 | 0.169721270927029 | 0.252964376883756 | -0.526675444 | 0.138631938091098 | 0.823639987494108 | 0.590565076332421 | 1.14870122901992 | 0.998735809224318 |
| ebi-fl187-GCST90094784 | prot-a-2190 | Pyruvate carboxylase, mitochondrial \|\| id:prot-a-2190 | Ice cream liking \|\| id:ebi-fl187-GCST90094784 | Inverse variance weighted | 9 | -0.133076654 | 0.16993569644673 | 0.433568355336769 | -0.466150619 | 0.199997310775258 | 0.875397986551684 | 0.627412776459542 | 1.22139947353807 | 0.998735809224318 |
| ebi-fl187-GCST90094784 | prot-a-2197 | Protocadherin-9 \|\| id:prot-a-2197 | Ice cream liking \|\| id:ebi-fl187-GCST90094784 | Inverse variance weighted | 9 | 0.0148800841028215 | 0.169774396827928 | 0.930157865406325 | -0.317877734 | 0.34763790188556 | 1.01499134372012 | 0.727691755168725 | 1.41571952754626 | 0.998735809224318 |
| ebi-fl187-GCST90094784 | prot-a-2198 | Protocadherin alpha-4 \|\| id:prot-a-2198 | Ice cream liking \|\| id:ebi-fl187-GCST90094784 | Inverse variance weighted | 9 | 0.0497654122711888 | 0.213952630666426 | 0.816071917046888 | -0.369581744 | 0.469112568377384 | 1.05102451000136 | 0.691023294955858 | 1.59857493761359 | 0.998735809224318 |
| ebi-fl187-GCST90094784 | prot-a-2254 | Placenta growth factor \|\| id:prot-a-2254 | Ice cream liking \|\| id:ebi-fl187-GCST90094784 | Inverse variance weighted | 9 | 0.207466420913356 | 0.16993569644673 | 0.222141869471008 | -0.125607544 | 0.540540385948947 | 1.23055639518651 | 0.881960909614715 | 1.71693441877818 | 0.998735809224318 |
| ebi-fl187-GCST90094784 | prot-a-2261 | Membrane-associated progesterone receptor component 2 \|\| id:prot-a-2261 | Ice cream liking \|\| id:ebi-fl187-GCST90094784 | Inverse variance weighted | 9 | -0.343240925 | 0.198452363987102 | 0.0837038237130056 | -0.732207558 | 0.0457257085997067 | 0.7094672626991 | 0.480846321316304 | 1.04678724683567 | 0.998735809224318 |
| ebi-fl187-GCST90094784 | prot-a-2268 | Polymeric immunoglobulin receptor \|\| id:prot-a-2268 | Ice cream liking \|\| id:ebi-fl187-GCST90094784 | Inverse variance weighted | 9 | -0.369308805 | 0.216072077610657 | 0.0874152716476159 | -0.792810077 | 0.0541924672371354 | 0.691211927873504 | 0.452571246685947 | 1.05568776791104 | 0.998735809224318 |
| ebi-fl187-GCST90094784 | prot-a-2283 | Group 10 secretory phospholipase A2 \|\| id:prot-a-2283 | Ice cream liking \|\| id:ebi-fl187-GCST90094784 | Inverse variance weighted (multiplicative random effects) | 9 | 0.0825228602958196 | 0.262149649155543 | 0.752918868093656 | -0.431290452 | 0.596336172640684 | 1.08602349997376 | 0.649670185331698 | 1.81545508648065 | 0.998735809224318 |
| ebi-fl187-GCST90094784 | prot-a-2298 | Inactive phospholipase D5 \|\| id:prot-a-2298 | Ice cream liking \|\| id:ebi-fl187-GCST90094784 | Inverse variance weighted | 9 | -0.080831207 | 0.225958216915697 | 0.720548114368356 | -0.523709313 | 0.362046897729274 | 0.922349364029802 | 0.592319370374095 | 1.43626629800893 | 0.998735809224318 |
| ebi-fl187-GCST90094784 | prot-a-2304 | Procollagen-lysine,2-oxoglutarate 5-dioxygenase 2 \|\| id:prot-a-2304 | Ice cream liking \|\| id:ebi-fl187-GCST90094784 | Inverse variance weighted | 9 | -0.075719675 | 0.217966857652068 | 0.728297766188551 | -0.502934716 | 0.351495366372637 | 0.927076053078741 | 0.604753274045856 | 1.42119116188015 | 0.998735809224318 |
| ebi-fl187-GCST90094784 | prot-a-2331 | Periostin \|\| id:prot-a-2331 | Ice cream liking \|\| id:ebi-fl187-GCST90094784 | Inverse variance weighted | 9 | 0.0425257872455588 | 0.169774396827928 | 0.802212998078735 | -0.290232031 | 0.375283605028297 | 1.04344296354667 | 0.748089967722041 | 1.45540411601885 | 0.998735809224318 |
| ebi-fl187-GCST90094784 | prot-a-2333 | Periostin \|\| id:prot-a-2333 | Ice cream liking \|\| id:ebi-fl187-GCST90094784 | Inverse variance weighted | 9 | 0.149234788840598 | 0.169774396827928 | 0.379391394586972 | -0.183523029 | 0.481992606623337 | 1.16094553426855 | 0.832332707775418 | 1.61929781317901 | 0.998735809224318 |
| ebi-fl187-GCST90094784 | prot-a-2367 | cAMP-dependent protein kinase catalytic subunit alpha \|\| id:prot-a-2367 | Ice cream liking \|\| id:ebi-fl187-GCST90094784 | Inverse variance weighted | 9 | 0.0386841794551676 | 0.16993569644673 | 0.819926040595943 | -0.294389786 | 0.371758144490759 | 1.03944215461877 | 0.744986050022111 | 1.45028218013805 | 0.998735809224318 |
| ebi-fl187-GCST90094784 | prot-a-2385 | Phosphoribosyl pyrophosphate synthase-associated protein 1 \|\| id:prot-a-2385 | Ice cream liking \|\| id:ebi-fl187-GCST90094784 | Inverse variance weighted | 9 | -0.227184753 | 0.169774396827928 | 0.180845349281139 | -0.55994257 | 0.105573065190561 | 0.79677356270987 | 0.571241869113185 | 1.11134730235887 | 0.998735809224318 |
| ebi-fl187-GCST90094784 | prot-a-2393 | Trypsin-3 \|\| id:prot-a-2393 | Ice cream liking \|\| id:ebi-fl187-GCST90094784 | Inverse variance weighted | 9 | 0.0134260899793389 | 0.198670460345891 | 0.946120216022271 | -0.375968012 | 0.402820192257286 | 1.01351662464721 | 0.686624296223979 | 1.49603786828595 | 0.998735809224318 |
| ebi-fl187-GCST90094784 | prot-a-2405 | Pregnancy-specific beta-1-glycoprotein 6 \|\| id:prot-a-2405 | Ice cream liking \|\| id:ebi-fl187-GCST90094784 | Inverse variance weighted | 9 | -0.065589691 | 0.181281944833142 | 0.717493729210262 | -0.420902303 | 0.289722921165532 | 0.936515046295636 | 0.656454232160994 | 1.33605724355056 | 0.998735809224318 |
| ebi-fl187-GCST90094784 | prot-a-244 | Beclin-1 \|\| id:prot-a-244 | Ice cream liking \|\| id:ebi-fl187-GCST90094784 | Inverse variance weighted | 9 | -0.080530966 | 0.16993569644673 | 0.635577766731432 | -0.413604931 | 0.25254299871409 | 0.922626332797746 | 0.661262143605744 | 1.28729484698785 | 0.998735809224318 |
| ebi-fl187-GCST90094784 | prot-a-2454 | tRNA pseudouridine synthase A, mitochondrial \|\| id:prot-a-2454 | Ice cream liking \|\| id:ebi-fl187-GCST90094784 | Inverse variance weighted | 9 | 0.0652528919241143 | 0.169774396827928 | 0.700718606470228 | -0.267504926 | 0.398010709706853 | 1.0674289344108 | 0.765286560920135 | 1.48885997507577 | 0.998735809224318 |
| ebi-fl187-GCST90094784 | prot-a-2466 | Glutaminyl-peptide cyclotransferase-like protein \|\| id:prot-a-2466 | Ice cream liking \|\| id:ebi-fl187-GCST90094784 | Inverse variance weighted | 9 | 0.130959818611462 | 0.186038465648344 | 0.481470470868646 | -0.233675574 | 0.495595211282216 | 1.1399219767439 | 0.791618595847934 | 1.64147497277001 | 0.998735809224318 |
| ebi-fl187-GCST90094784 | prot-a-2474 | Ras-related protein Rab-27A \|\| id:prot-a-2474 | Ice cream liking \|\| id:ebi-fl187-GCST90094784 | Inverse variance weighted | 9 | -0.239056635 | 0.16993569644673 | 0.159502242741046 | -0.5721306 | 0.0940173302862436 | 0.787370288590983 | 0.564321812999113 | 1.098578784 | 0.998735809224318 |
| ebi-fl187-GCST90094784 | prot-a-2490 | Rap1 GTPase-GDP dissociation stimulator 1 \|\| id:prot-a-2490 | Ice cream liking \|\| id:ebi-fl187-GCST90094784 | Inverse variance weighted | 9 | 0.176533045168481 | 0.169774396827928 | 0.298428374927763 | -0.156224773 | 0.50929086295122 | 1.19307385143463 | 0.85536690570612 | 1.66411069388053 | 0.998735809224318 |
| ebi-fl187-GCST90094784 | prot-a-2545 | Receptor-interacting serine/threonine-protein kinase 2 \|\| id:prot-a-2545 | Ice cream liking \|\| id:ebi-fl187-GCST90094784 | Inverse variance weighted | 9 | -0.209892467 | 0.16993569644673 | 0.216782584452222 | -0.542966432 | 0.123181497699636 | 0.810671414940261 | 0.581022130571264 | 1.13108969249604 | 0.998735809224318 |
| ebi-fl187-GCST90094784 | prot-a-2610 | RUN and FYVE domain-containing protein 1 \|\| id:prot-a-2610 | Ice cream liking \|\| id:ebi-fl187-GCST90094784 | Inverse variance weighted | 9 | -0.341341202 | 0.169774396827928 | 0.0443722077034321 | -0.67409902 | -0.008583384 | 0.710816335060863 | 0.509615367326395 | 0.99145334812039 | 0.998735809224318 |
| ebi-fl187-GCST90094784 | prot-a-2627 | Serine--tRNA ligase, mitochondrial \|\| id:prot-a-2627 | Ice cream liking \|\| id:ebi-fl187-GCST90094784 | Inverse variance weighted | 9 | 0.159806465132616 | 0.169774396827928 | 0.346557704938545 | -0.172951353 | 0.492564282915354 | 1.17328377769696 | 0.841178534955787 | 1.63650731182675 | 0.998735809224318 |
| ebi-fl187-GCST90094784 | prot-a-264 | BolA-like protein 2 \|\| id:prot-a-264 | Ice cream liking \|\| id:ebi-fl187-GCST90094784 | Inverse variance weighted | 9 | -0.162008109 | 0.169774396827928 | 0.339954456134894 | -0.494765927 | 0.170749709040161 | 0.850434308559387 | 0.609713608377264 | 1.18619381827439 | 0.998735809224318 |
| ebi-fl187-GCST90094784 | prot-a-2646 | Secretoglobin family 1C member 1 \|\| id:prot-a-2646 | Ice cream liking \|\| id:ebi-fl187-GCST90094784 | Inverse variance weighted (multiplicative random effects) | 9 | 0.0418511326501544 | 0.254293255874289 | 0.869275971509308 | -0.456563649 | 0.540265914163761 | 1.04273923736902 | 0.633456689312056 | 1.71646323338972 | 0.998735809224318 |
| ebi-fl187-GCST90094784 | prot-a-2672 | Semaphorin-3E \|\| id:prot-a-2672 | Ice cream liking \|\| id:ebi-fl187-GCST90094784 | Inverse variance weighted | 9 | -0.262273392 | 0.174087165958596 | 0.131922546254351 | -0.603484238 | 0.078937452924387 | 0.769300674032327 | 0.546902773334799 | 1.08213663547157 | 0.998735809224318 |
| ebi-fl187-GCST90094784 | prot-a-2701 | Plasma protease C1 inhibitor \|\| id:prot-a-2701 | Ice cream liking \|\| id:ebi-fl187-GCST90094784 | Inverse variance weighted | 9 | 0.159466346540037 | 0.169774396827928 | 0.347585035844222 | -0.173291471 | 0.492224164322775 | 1.17288478992522 | 0.840892483144898 | 1.63595079990849 | 0.998735809224318 |
| ebi-fl187-GCST90094784 | prot-a-2738 | Signaling threshold-regulating transmembrane adapter 1 \|\| id:prot-a-2738 | Ice cream liking \|\| id:ebi-fl187-GCST90094784 | Inverse variance weighted | 9 | -0.010726593 | 0.16993569644673 | 0.949669768238061 | -0.343800558 | 0.322347372029702 | 0.989330731743365 | 0.709070332323876 | 1.38036419259577 | 0.998735809224318 |
| ebi-fl187-GCST90094784 | prot-a-274 | Serine/threonine-protein kinase BRSK2 \|\| id:prot-a-274 | Ice cream liking \|\| id:ebi-fl187-GCST90094784 | Inverse variance weighted | 9 | 0.352837738993912 | 0.170046565822593 | 0.0379913776422655 | 0.0195464699816288 | 0.686129008006195 | 1.42310021102644 | 1.01973875300075 | 1.98601279461428 | 0.998735809224318 |
| ebi-fl187-GCST90094784 | prot-a-2749 | Mitochondrial glutamate carrier 2 \|\| id:prot-a-2749 | Ice cream liking \|\| id:ebi-fl187-GCST90094784 | Inverse variance weighted | 9 | 0.188216247425675 | 0.211853101591394 | 0.374310518451858 | -0.227015832 | 0.603448326544807 | 1.2070945182101 | 0.796908165784475 | 1.82841290684796 | 0.998735809224318 |
| ebi-fl187-GCST90094784 | prot-a-2760 | Sodium-coupled monocarboxylate transporter 1 \|\| id:prot-a-2760 | Ice cream liking \|\| id:ebi-fl187-GCST90094784 | Inverse variance weighted | 9 | -0.019668432 | 0.16993569644673 | 0.907858255786634 | -0.352742397 | 0.31340553289952 | 0.980523729573218 | 0.702758202562619 | 1.36807621846364 | 0.998735809224318 |
| ebi-fl187-GCST90094784 | prot-a-2777 | Small integral membrane protein 10 \|\| id:prot-a-2777 | Ice cream liking \|\| id:ebi-fl187-GCST90094784 | Inverse variance weighted | 9 | -0.297182611 | 0.175074614485168 | 0.0896092354640374 | -0.640328855 | 0.0459636334700485 | 0.742908336798884 | 0.527119049637635 | 1.04703633318639 | 0.998735809224318 |
| ebi-fl187-GCST90094784 | prot-a-2799 | Superoxide dismutase [Mn], mitochondrial \|\| id:prot-a-2799 | Ice cream liking \|\| id:ebi-fl187-GCST90094784 | Inverse variance weighted | 9 | -0.018743601 | 0.228256385050657 | 0.934554123197472 | -0.466126116 | 0.428638913856384 | 0.981430968058626 | 0.627428150616067 | 1.53516660691543 | 0.998735809224318 |
| ebi-fl187-GCST90094784 | prot-a-2805 | Protein SPACA7 \|\| id:prot-a-2805 | Ice cream liking \|\| id:ebi-fl187-GCST90094784 | Inverse variance weighted | 9 | 0.0846275383033937 | 0.169774396827928 | 0.618152415978988 | -0.248130279 | 0.417385356086132 | 1.08831163680085 | 0.780258285013059 | 1.51798736590965 | 0.998735809224318 |
| ebi-fl187-GCST90094784 | prot-a-2811 | SPARC-like protein 1 \|\| id:prot-a-2811 | Ice cream liking \|\| id:ebi-fl187-GCST90094784 | Inverse variance weighted | 9 | -0.197923865 | 0.169774396827928 | 0.243693237202519 | -0.530681683 | 0.134833952737826 | 0.820432314337465 | 0.588203864507042 | 1.14434675292935 | 0.998735809224318 |
| ebi-fl187-GCST90094784 | prot-a-2831 | Sepiapterin reductase \|\| id:prot-a-2831 | Ice cream liking \|\| id:ebi-fl187-GCST90094784 | Inverse variance weighted | 9 | -0.393014312 | 0.227960865000181 | 0.0847000978907521 | -0.839817607 | 0.0537889833756836 | 0.675019086627177 | 0.431789271404395 | 1.05526190085498 | 0.998735809224318 |
| ebi-fl187-GCST90094784 | prot-a-2834 | SPRY domain-containing SOCS box protein 1 \|\| id:prot-a-2834 | Ice cream liking \|\| id:ebi-fl187-GCST90094784 | Inverse variance weighted | 9 | -0.049453782 | 0.171124497157098 | 0.772586732169874 | -0.384857797 | 0.285950232141088 | 0.951749144788963 | 0.680547405400802 | 1.3310262112792 | 0.998735809224318 |
| ebi-fl187-GCST90094784 | prot-a-2835 | Spectrin alpha chain, non-erythrocytic 1 \|\| id:prot-a-2835 | Ice cream liking \|\| id:ebi-fl187-GCST90094784 | Inverse variance weighted | 9 | -0.079041933 | 0.169700177696978 | 0.641377268742119 | -0.411654281 | 0.253570415651505 | 0.924001177828984 | 0.662553293776688 | 1.28861811517477 | 0.998735809224318 |
| ebi-fl187-GCST90094784 | prot-a-2859 | Sia-alpha-2,3-Gal-beta-1,4-GlcNAc-R:alpha 2,8-sialyltransferase \|\| id:prot-a-2859 | Ice cream liking \|\| id:ebi-fl187-GCST90094784 | Inverse variance weighted | 9 | -0.10151642 | 0.16993569644673 | 0.550252785609457 | -0.434590385 | 0.231557544991032 | 0.903466344262794 | 0.647529850650627 | 1.26056186351165 | 0.998735809224318 |
| ebi-fl187-GCST90094784 | prot-a-2861 | Alpha-2,8-sialyltransferase 8F \|\| id:prot-a-2861 | Ice cream liking \|\| id:ebi-fl187-GCST90094784 | Inverse variance weighted (multiplicative random effects) | 9 | 0.0362694363957726 | 0.241968334441234 | 0.880848783961682 | -0.437988499 | 0.51052737190059 | 1.03693519693776 | 0.645333204721433 | 1.66616965434544 | 0.998735809224318 |
| ebi-fl187-GCST90094784 | prot-a-289 | Uncharacterized protein C17orf89 \|\| id:prot-a-289 | Ice cream liking \|\| id:ebi-fl187-GCST90094784 | Inverse variance weighted (multiplicative random effects) | 9 | 0.143705487901739 | 0.257554096724186 | 0.576870096987187 | -0.361100542 | 0.648511517481143 | 1.15454403126376 | 0.696908926551703 | 1.91269170094046 | 0.998735809224318 |
| ebi-fl187-GCST90094784 | prot-a-2921 | Tachykinin-3 \|\| id:prot-a-2921 | Ice cream liking \|\| id:ebi-fl187-GCST90094784 | Inverse variance weighted | 9 | 0.0714228456849826 | 0.217544532562965 | 0.742674503054233 | -0.354964438 | 0.497810129508395 | 1.07403528104807 | 0.70119837868487 | 1.64511473500486 | 0.998735809224318 |
| ebi-fl187-GCST90094784 | prot-a-2928 | Tax1-binding protein 1 \|\| id:prot-a-2928 | Ice cream liking \|\| id:ebi-fl187-GCST90094784 | Inverse variance weighted (multiplicative random effects) | 9 | -0.148278038 | 0.246354407924592 | 0.547247946097077 | -0.631132678 | 0.33457660141261 | 0.862191359548685 | 0.531988887694048 | 1.39734862452227 | 0.998735809224318 |
| ebi-fl187-GCST90094784 | prot-a-296 | Uncharacterized protein C1orf185 \|\| id:prot-a-296 | Ice cream liking \|\| id:ebi-fl187-GCST90094784 | Inverse variance weighted | 9 | -0.11899208 | 0.194088396916308 | 0.539822199041049 | -0.499405338 | 0.261421178179074 | 0.887814832425657 | 0.606891447872753 | 1.298774566 | 0.998735809224318 |
| ebi-fl187-GCST90094784 | prot-a-2964 | Transforming growth factor beta-2 \|\| id:prot-a-2964 | Ice cream liking \|\| id:ebi-fl187-GCST90094784 | Inverse variance weighted | 9 | 0.134345158578755 | 0.202010655834855 | 0.506024934457441 | -0.261595727 | 0.530286044015071 | 1.1437875396018 | 0.769822179238945 | 1.69941834754839 | 0.998735809224318 |
| ebi-fl187-GCST90094784 | prot-a-2977 | Thymocyte nuclear protein 1 \|\| id:prot-a-2977 | Ice cream liking \|\| id:ebi-fl187-GCST90094784 | Inverse variance weighted | 9 | 0.050023695379093 | 0.16993569644673 | 0.768476427456362 | -0.28305027 | 0.383097660414684 | 1.05129600693831 | 0.753481909630888 | 1.46682127344751 | 0.998735809224318 |
| ebi-fl187-GCST90094784 | prot-a-3015 | Transmembrane protein 70, mitochondrial \|\| id:prot-a-3015 | Ice cream liking \|\| id:ebi-fl187-GCST90094784 | Inverse variance weighted | 9 | 0.0827756709428625 | 0.169774396827928 | 0.625859589339046 | -0.249982147 | 0.415533488725601 | 1.08629809298605 | 0.778814687250606 | 1.51517885594961 | 0.998735809224318 |
| ebi-fl187-GCST90094784 | prot-a-3030 | Tumor necrosis factor alpha-induced protein 3 \|\| id:prot-a-3030 | Ice cream liking \|\| id:ebi-fl187-GCST90094784 | Inverse variance weighted | 9 | -0.09769397 | 0.169774396827928 | 0.564997778025233 | -0.430451787 | 0.235063848203995 | 0.906926408360129 | 0.650215269314093 | 1.26498953346431 | 0.998735809224318 |
| ebi-fl187-GCST90094784 | prot-a-3032 | Tumor necrosis factor receptor superfamily member 10A \|\| id:prot-a-3032 | Ice cream liking \|\| id:ebi-fl187-GCST90094784 | Inverse variance weighted | 9 | 0.0153272712796789 | 0.169774396827928 | 0.928064531633273 | -0.317430547 | 0.348085089062417 | 1.01544533633592 | 0.728017242361767 | 1.41635276074133 | 0.998735809224318 |
| ebi-fl187-GCST90094784 | prot-a-3081 | Tumor protein p53-inducible protein 11 \|\| id:prot-a-3081 | Ice cream liking \|\| id:ebi-fl187-GCST90094784 | Inverse variance weighted | 9 | -0.01569558 | 0.169861265664699 | 0.926378371667636 | -0.348623661 | 0.317232500427334 | 0.984426953427793 | 0.705658647189578 | 1.37332183272286 | 0.998735809224318 |
| ebi-fl187-GCST90094784 | prot-a-315 | Complement C4 \|\| id:prot-a-315 | Ice cream liking \|\| id:ebi-fl187-GCST90094784 | Inverse variance weighted | 9 | 0.431834162150282 | 0.169774396827928 | 0.010972403451954 | 0.0990763443675438 | 0.764591979933021 | 1.54007969047791 | 1.10415059201983 | 2.1481177206849 | 0.998735809224318 |
| ebi-fl187-GCST90094784 | prot-a-3157 | UDP-glucuronosyltransferase 1-8 \|\| id:prot-a-3157 | Ice cream liking \|\| id:ebi-fl187-GCST90094784 | Inverse variance weighted | 9 | -0.139496526 | 0.18100970737824 | 0.440909845389885 | -0.494275553 | 0.215282500060544 | 0.86979604452199 | 0.610012669192662 | 1.24021220750607 | 0.998735809224318 |
| ebi-fl187-GCST90094784 | prot-a-317 | Glutaredoxin-like protein C5orf63 \|\| id:prot-a-317 | Ice cream liking \|\| id:ebi-fl187-GCST90094784 | Inverse variance weighted | 9 | -0.062616066 | 0.169774396827928 | 0.712262455280896 | -0.395373883 | 0.270141752081352 | 0.939304035504736 | 0.673428208489222 | 1.31015015408224 | 0.998735809224318 |
| ebi-fl187-GCST90094784 | prot-a-3192 | Guanine nucleotide exchange factor VAV3 \|\| id:prot-a-3192 | Ice cream liking \|\| id:ebi-fl187-GCST90094784 | Inverse variance weighted | 9 | 0.122413601917173 | 0.169774396827928 | 0.470886822386472 | -0.210344216 | 0.455171419699911 | 1.13022146688764 | 0.810305278027697 | 1.57644359336134 | 0.998735809224318 |
| ebi-fl187-GCST90094784 | prot-a-3233 | Serine/threonine-protein kinase WNK3 \|\| id:prot-a-3233 | Ice cream liking \|\| id:ebi-fl187-GCST90094784 | Inverse variance weighted | 9 | 0.0411558330453217 | 0.16993569644673 | 0.808637016191297 | -0.291918132 | 0.374229798080913 | 1.04201447318265 | 0.746829674929755 | 1.45387120888609 | 0.998735809224318 |
| ebi-fl187-GCST90094784 | prot-a-3250 | 14-3-3 protein theta \|\| id:prot-a-3250 | Ice cream liking \|\| id:ebi-fl187-GCST90094784 | Inverse variance weighted | 9 | -0.157748894 | 0.16993569644673 | 0.353259337145381 | -0.490822859 | 0.175325070842903 | 0.854064215528935 | 0.612122496249439 | 1.19163351897104 | 0.998735809224318 |
| ebi-fl187-GCST90094784 | prot-a-3278 | E3 ubiquitin-protein ligase ZNRF3 \|\| id:prot-a-3278 | Ice cream liking \|\| id:ebi-fl187-GCST90094784 | Inverse variance weighted | 9 | 0.0479728675027915 | 0.16993569644673 | 0.777713393552994 | -0.285101098 | 0.381046832538383 | 1.04914218909078 | 0.751938231376571 | 1.46381615803088 | 0.998735809224318 |
| ebi-fl187-GCST90094784 | prot-a-355 | Calpain-3 \|\| id:prot-a-355 | Ice cream liking \|\| id:ebi-fl187-GCST90094784 | Inverse variance weighted | 9 | -0.11525332 | 0.17394542036403 | 0.507597507457075 | -0.456186344 | 0.225679703795544 | 0.891140371527667 | 0.633695740676577 | 1.25317421404569 | 0.998735809224318 |
| ebi-fl187-GCST90094784 | prot-a-385 | Coiled-coil domain-containing protein 90B, mitochondrial \|\| id:prot-a-385 | Ice cream liking \|\| id:ebi-fl187-GCST90094784 | Inverse variance weighted | 9 | 0.159296706238015 | 0.16993569644673 | 0.348555914024843 | -0.173777259 | 0.492370671273606 | 1.17268583827081 | 0.840484087246452 | 1.63619049563001 | 0.998735809224318 |
| ebi-fl187-GCST90094784 | prot-a-390 | C-C motif chemokine 14 \|\| id:prot-a-390 | Ice cream liking \|\| id:ebi-fl187-GCST90094784 | Inverse variance weighted | 9 | 0.16968978405053 | 0.169774396827928 | 0.317551756453277 | -0.163068034 | 0.502447601833269 | 1.18493720787788 | 0.849533389521351 | 1.65276162647888 | 0.998735809224318 |
| ebi-fl187-GCST90094784 | prot-a-40 | Pituitary adenylate cyclase-activating polypeptide 27 \|\| id:prot-a-40 | Ice cream liking \|\| id:ebi-fl187-GCST90094784 | Inverse variance weighted | 9 | 0.0322526972215288 | 0.206394493777877 | 0.875822380332953 | -0.372280511 | 0.436785905026168 | 1.03277845257883 | 0.689160898482475 | 1.54772468150736 | 0.998735809224318 |
| ebi-fl187-GCST90094784 | prot-a-401 | C-C motif chemokine 24 \|\| id:prot-a-401 | Ice cream liking \|\| id:ebi-fl187-GCST90094784 | Inverse variance weighted | 9 | -0.031262908 | 0.208964700462473 | 0.881073452168902 | -0.440833721 | 0.378307904415803 | 0.969220723218955 | 0.643499697908265 | 1.45981235635481 | 0.998735809224318 |
| ebi-fl187-GCST90094784 | prot-a-412 | Cerebral cavernous malformations 2 protein \|\| id:prot-a-412 | Ice cream liking \|\| id:ebi-fl187-GCST90094784 | Inverse variance weighted | 9 | 0.127773540562807 | 0.179018992659487 | 0.475386154323256 | -0.223103685 | 0.478650766175402 | 1.13629564868495 | 0.800031893647273 | 1.61389541026175 | 0.998735809224318 |
| ebi-fl187-GCST90094784 | prot-a-44 | Adhesion G protein-coupled receptor F5 \|\| id:prot-a-44 | Ice cream liking \|\| id:ebi-fl187-GCST90094784 | Inverse variance weighted | 9 | -0.212565017 | 0.169774396827928 | 0.210553827154563 | -0.545322835 | 0.120192800879299 | 0.808507747942377 | 0.579654620512641 | 1.12771425492087 | 0.998735809224318 |
| ebi-fl187-GCST90094784 | prot-a-48 | Adrenomedullin \|\| id:prot-a-48 | Ice cream liking \|\| id:ebi-fl187-GCST90094784 | Inverse variance weighted | 9 | -0.40313598 | 0.169774396827928 | 0.0175708648640754 | -0.735893798 | -0.070378162 | 0.668221228387105 | 0.479077069508583 | 0.932041290402799 | 0.998735809224318 |
| ebi-fl187-GCST90094784 | prot-a-502 | Carcinoembryonic antigen-related cell adhesion molecule 1 \|\| id:prot-a-502 | Ice cream liking \|\| id:ebi-fl187-GCST90094784 | Inverse variance weighted | 9 | -0.033371281 | 0.16993569644673 | 0.844315932104967 | -0.366445246 | 0.299702684077799 | 0.967179397625052 | 0.693194090597298 | 1.34945753271545 | 0.998735809224318 |
| ebi-fl187-GCST90094784 | prot-a-518 | Complement factor B \|\| id:prot-a-518 | Ice cream liking \|\| id:ebi-fl187-GCST90094784 | Inverse variance weighted | 9 | 0.036087696371123 | 0.169774396827928 | 0.831668103496135 | -0.296670121 | 0.368845514153861 | 1.03674676143316 | 0.743289167105273 | 1.4460641899681 | 0.998735809224318 |
| ebi-fl187-GCST90094784 | prot-a-525 | Properdin \|\| id:prot-a-525 | Ice cream liking \|\| id:ebi-fl187-GCST90094784 | Inverse variance weighted | 9 | -0.245852188 | 0.182600775433281 | 0.178176190928254 | -0.603749708 | 0.112045331366767 | 0.782037810578373 | 0.546757605943309 | 1.11856356551102 | 0.998735809224318 |
| ebi-fl187-GCST90094784 | prot-a-533 | Chondroadherin \|\| id:prot-a-533 | Ice cream liking \|\| id:ebi-fl187-GCST90094784 | Inverse variance weighted | 9 | 0.251553374304957 | 0.175487161054961 | 0.151727157133007 | -0.092401461 | 0.595508209972681 | 1.2860215387379 | 0.911739048061484 | 1.81395257953927 | 0.998735809224318 |
| ebi-fl187-GCST90094784 | prot-a-535 | Coiled-coil-helix-coiled-coil-helix domain-containing protein 2 \|\| id:prot-a-535 | Ice cream liking \|\| id:ebi-fl187-GCST90094784 | Inverse variance weighted | 9 | -0.083458086 | 0.214713635770259 | 0.697501888824165 | -0.504296812 | 0.337380640596685 | 0.919929644242264 | 0.603930102777155 | 1.40127234337906 | 0.998735809224318 |
| ebi-fl187-GCST90094784 | prot-a-591 | Caseinolytic peptidase B protein homolog \|\| id:prot-a-591 | Ice cream liking \|\| id:ebi-fl187-GCST90094784 | Inverse variance weighted | 9 | -0.38706183 | 0.197738898236589 | 0.05029587750323 | -0.774630071 | 0.00050641015329056 | 0.67904910775716 | 0.460874240245281 | 1.00050653840056 | 0.998735809224318 |
| ebi-fl187-GCST90094784 | prot-a-605 | Ciliary neurotrophic factor receptor subunit alpha \|\| id:prot-a-605 | Ice cream liking \|\| id:ebi-fl187-GCST90094784 | Inverse variance weighted | 9 | -0.141212863 | 0.169774396827928 | 0.40554006633534 | -0.473970681 | 0.191544954427105 | 0.868304461823028 | 0.622525504039242 | 1.21111927708949 | 0.998735809224318 |
| ebi-fl187-GCST90094784 | prot-a-606 | Ciliary neurotrophic factor receptor subunit alpha \|\| id:prot-a-606 | Ice cream liking \|\| id:ebi-fl187-GCST90094784 | Inverse variance weighted | 9 | -0.189263823 | 0.169774396827928 | 0.264937840348083 | -0.52202164 | 0.14349399508785 | 0.827568146633182 | 0.593319855259069 | 1.15429987931693 | 0.998735809224318 |
| ebi-fl187-GCST90094784 | prot-a-707 | Cancer/testis antigen 55 \|\| id:prot-a-707 | Ice cream liking \|\| id:ebi-fl187-GCST90094784 | Inverse variance weighted | 9 | -0.148427266 | 0.217685210783118 | 0.495337855994239 | -0.575090279 | 0.278235746856824 | 0.862062705919438 | 0.562654070434766 | 1.3207975343765 | 0.998735809224318 |
| ebi-fl187-GCST90094784 | prot-a-719 | Dipeptidyl peptidase 1 \|\| id:prot-a-719 | Ice cream liking \|\| id:ebi-fl187-GCST90094784 | Inverse variance weighted (multiplicative random effects) | 9 | 0.10904098950942 | 0.246427235906043 | 0.658136420982893 | -0.373956393 | 0.592038371885264 | 1.11520806123594 | 0.688006913184202 | 1.80766936496268 | 0.998735809224318 |
| ebi-fl187-GCST90094784 | prot-a-752 | Cytochrome b5 \|\| id:prot-a-752 | Ice cream liking \|\| id:ebi-fl187-GCST90094784 | Inverse variance weighted | 9 | -0.062280957 | 0.16993569644673 | 0.713994102697454 | -0.395354922 | 0.270793008364266 | 0.93961885751591 | 0.673440978005943 | 1.31100367550208 | 0.998735809224318 |
| ebi-fl187-GCST90094784 | prot-a-755 | Cytoglobin \|\| id:prot-a-755 | Ice cream liking \|\| id:ebi-fl187-GCST90094784 | Inverse variance weighted | 9 | 0.02630605653367 | 0.185133922814606 | 0.88700730653256 | -0.336556432 | 0.389168545250298 | 1.02665511490039 | 0.71422557713043 | 1.47575326157585 | 0.998735809224318 |
| ebi-fl187-GCST90094784 | prot-a-807 | Desmin \|\| id:prot-a-807 | Ice cream liking \|\| id:ebi-fl187-GCST90094784 | Inverse variance weighted | 9 | -0.289169707 | 0.169774396827928 | 0.088519678905849 | -0.621927524 | 0.0435881112142418 | 0.748885103896814 | 0.5369085352758 | 1.04455202700485 | 0.998735809224318 |
| ebi-fl187-GCST90094784 | prot-a-82 | Ameloblastin \|\| id:prot-a-82 | Ice cream liking \|\| id:ebi-fl187-GCST90094784 | Inverse variance weighted (multiplicative random effects) | 9 | 0.0910473128432292 | 0.257130067348383 | 0.723271065080504 | -0.412927619 | 0.59502224484606 | 1.09532082677511 | 0.661710176233201 | 1.81307127600301 | 0.998735809224318 |
| ebi-fl187-GCST90094784 | prot-a-84 | Muellerian-inhibiting factor \|\| id:prot-a-84 | Ice cream liking \|\| id:ebi-fl187-GCST90094784 | Inverse variance weighted | 9 | -0.286998079 | 0.169774396827928 | 0.0909385748756346 | -0.619755897 | 0.04575973893774 | 0.750513170687815 | 0.538075767674387 | 1.04682286996566 | 0.998735809224318 |
| ebi-fl187-GCST90094784 | prot-a-858 | Inactive dipeptidyl peptidase 10 \|\| id:prot-a-858 | Ice cream liking \|\| id:ebi-fl187-GCST90094784 | Inverse variance weighted | 9 | 0.0729340526315455 | 0.16993569644673 | 0.667787738548213 | -0.260139912 | 0.406008017667137 | 1.07565959765578 | 0.770943713669054 | 1.50081458544156 | 0.998735809224318 |
| ebi-fl187-GCST90094784 | prot-a-863 | Dihydropyrimidinase-related protein 3 \|\| id:prot-a-863 | Ice cream liking \|\| id:ebi-fl187-GCST90094784 | Inverse variance weighted | 9 | -0.040005331 | 0.169774396827928 | 0.8137134392988 | -0.372763149 | 0.292752486831047 | 0.96078431724389 | 0.688828363393985 | 1.34011105424505 | 0.998735809224318 |
| ebi-fl187-GCST90094784 | prot-a-864 | Dihydropyrimidinase-related protein 5 \|\| id:prot-a-864 | Ice cream liking \|\| id:ebi-fl187-GCST90094784 | Inverse variance weighted | 9 | -0.433485858 | 0.16993569644673 | 0.0107450792456171 | -0.766559823 | -0.100411893 | 0.64824546027208 | 0.46460865835289 | 0.904464798940927 | 0.998735809224318 |
| ebi-fl187-GCST90094784 | prot-a-884 | Endothelin-converting enzyme 1 \|\| id:prot-a-884 | Ice cream liking \|\| id:ebi-fl187-GCST90094784 | Inverse variance weighted | 9 | -0.110891731 | 0.169774396827928 | 0.513645974044914 | -0.443649549 | 0.221866086860061 | 0.895035648367149 | 0.641690262610225 | 1.24840418894528 | 0.998735809224318 |
| ebi-fl187-GCST90094784 | prot-a-893 | Early endosome antigen 1 \|\| id:prot-a-893 | Ice cream liking \|\| id:ebi-fl187-GCST90094784 | Inverse variance weighted (multiplicative random effects) | 9 | -0.109836584 | 0.23905375223836 | 0.645901025265248 | -0.578381939 | 0.358708770144617 | 0.895980540672428 | 0.560805049820364 | 1.43147985119036 | 0.998735809224318 |
| ebi-fl187-GCST90094784 | prot-a-897 | Ephrin-A2 \|\| id:prot-a-897 | Ice cream liking \|\| id:ebi-fl187-GCST90094784 | Inverse variance weighted | 9 | -0.268745035 | 0.189884356142254 | 0.156977922744363 | -0.640918373 | 0.103428303320004 | 0.764338110487796 | 0.52680839533891 | 1.10896628131413 | 0.998735809224318 |
| ebi-fl187-GCST90094784 | prot-a-90 | Anaphase-promoting complex subunit 10 \|\| id:prot-a-90 | Ice cream liking \|\| id:ebi-fl187-GCST90094784 | Inverse variance weighted | 9 | -0.171191407 | 0.169774396827928 | 0.31328818137382 | -0.503949224 | 0.161566411102605 | 0.842660267230336 | 0.60414005761302 | 1.17535051188997 | 0.998735809224318 |
| ebi-fl187-GCST90094784 | prot-a-914 | Histone-lysine N-methyltransferase EHMT2 \|\| id:prot-a-914 | Ice cream liking \|\| id:ebi-fl187-GCST90094784 | Inverse variance weighted (multiplicative random effects) | 9 | -0.014520768 | 0.253145961212636 | 0.954257433907424 | -0.510686852 | 0.481645316395902 | 0.985584150321304 | 0.600083269102602 | 1.61873554451404 | 0.998735809224318 |
| ebi-fl187-GCST90094784 | prot-a-932 | RNA polymerase II elongation factor ELL \|\| id:prot-a-932 | Ice cream liking \|\| id:ebi-fl187-GCST90094784 | Inverse variance weighted | 9 | -0.098018461 | 0.195843860947551 | 0.616728058465577 | -0.481872428 | 0.285835506722553 | 0.906632166503583 | 0.617625848385339 | 1.33087351749913 | 0.998735809224318 |
| ebi-fl187-GCST90094784 | prot-a-991 | Estrogen receptor \|\| id:prot-a-991 | Ice cream liking \|\| id:ebi-fl187-GCST90094784 | Inverse variance weighted (multiplicative random effects) | 9 | -0.118369937 | 0.258412544287524 | 0.646905201534127 | -0.624858524 | 0.388118649806043 | 0.888367351868251 | 0.535337160628359 | 1.47420468801209 | 0.998735809224318 |
| ebi-fl187-GCST90094784 | prot-a-992 | Persulfide dioxygenase ETHE1, mitochondrial \|\| id:prot-a-992 | Ice cream liking \|\| id:ebi-fl187-GCST90094784 | Inverse variance weighted | 9 | 0.0333628196811321 | 0.169774396827928 | 0.844208918332958 | -0.299394998 | 0.366120637463871 | 1.03392559975394 | 0.741266552718787 | 1.4421292069711 | 0.998735809224318 |
| ebi-fl187-GCST90094784 | prot-a-993 | Ethanolamine kinase 1 \|\| id:prot-a-993 | Ice cream liking \|\| id:ebi-fl187-GCST90094784 | Inverse variance weighted | 9 | 0.00788124312758298 | 0.16993569644673 | 0.9630091333882 | -0.325192722 | 0.340955208163174 | 1.00791238187446 | 0.722388120208987 | 1.40629024912805 | 0.998735809224318 |
| ebi-fl187-GCST90094784 | prot-b-10 | coagulation factor III, tissue factor \|\| id:prot-b-10 | Ice cream liking \|\| id:ebi-fl187-GCST90094784 | Inverse variance weighted | 7 | -0.142340355 | 0.224805492469804 | 0.526621876478787 | -0.58295912 | 0.298278410677392 | 0.8673260078794 | 0.558244009148616 | 1.34753690432126 | 0.998735809224318 |
| ebi-fl187-GCST90094784 | prot-b-28 | prolactin \|\| id:prot-b-28 | Ice cream liking \|\| id:ebi-fl187-GCST90094784 | Inverse variance weighted | 8 | -0.18204194 | 0.187946722932576 | 0.332753449234363 | -0.550417517 | 0.186333636661561 | 0.833566379670006 | 0.576708974171554 | 1.20482416684133 | 0.998735809224318 |
| ebi-fl187-GCST90094784 | prot-b-54 | matrix metallopeptidase 7 \|\| id:prot-b-54 | Ice cream liking \|\| id:ebi-fl187-GCST90094784 | Inverse variance weighted | 8 | 0.00337764860926255 | 0.187865609424863 | 0.985655551848337 | -0.364838946 | 0.371594243081995 | 1.00338335929208 | 0.694308463123839 | 1.45004449632451 | 0.998955228396052 |
| ebi-fl187-GCST90094784 | ukb-rsfMRI191-edge-pheno716 | Net100_Pair10_33 (OccipitalPrecuneus)&(Temporal\|Frontal\|Supp_Motor_Area) (Default_mode\|Central_executive)&(Default_mode\|Salience) \|\| id:ukb-rsfMRI191-edge-pheno716 | Ice cream liking \|\| id:ebi-fl187-GCST90094784 | Inverse variance weighted | 8 | 0.0145212346442313 | 0.0628641609318879 | 0.817319578800574 | -0.108692521 | 0.137734990070732 | 1.01462717996984 | 0.897006185859871 | 1.14767136566255 | 0.998735809224318 |
| ebi-fl187-GCST90094784 | ukb-rsfMRI191-edge-pheno903 | Net100_Pair27_38 (Precentral\|Frontal\|Supp_Motor_Area)&(Subcortical) (Attention\|Salience\|Motor)&(Subcortical-cerebellum) \|\| id:ukb-rsfMRI191-edge-pheno903 | Ice cream liking \|\| id:ebi-fl187-GCST90094784 | Inverse variance weighted | 8 | -0.052608294 | 0.0578985736116502 | 0.363546256507898 | -0.166089498 | 0.0608729100876426 | 0.94875157120825 | 0.846970428239707 | 1.06276383904087 | 0.998735809224318 |
| ebi-fl187-GCST90094784 | ukb-rsfMRI191-node-pheno23 | Net100_Node2 RolandicOper\|SupraMarginal\|Insula Salience\|Motor \|\| id:ukb-rsfMRI191-node-pheno23 | Ice cream liking \|\| id:ebi-fl187-GCST90094784 | Inverse variance weighted | 8 | -0.091448421 | 0.0553770828403427 | 0.0986616912913598 | -0.199987503 | 0.0170906612561389 | 0.912608386264806 | 0.818740984428774 | 1.01723754217816 | 0.998735809224318 |
| ebi-fl187-GCST90094784 | ukb-rsfMRI191-node-pheno55 | Net100_Node34 Parietal\|Postcentral\|Precuneus Attention \|\| id:ukb-rsfMRI191-node-pheno55 | Ice cream liking \|\| id:ebi-fl187-GCST90094784 | Inverse variance weighted | 8 | -0.03590244 | 0.0521188466430905 | 0.490913166664133 | -0.13805538 | 0.0662504992265849 | 0.96473440819397 | 0.871050451971969 | 1.0684943406508 | 0.998735809224318 |
| ebi-fl187-GCST90094784 | ebi-a-GCST90001414 | IgD- CD24- B cell Absolute Count \|\| id:ebi-a-GCST90001414 | Ice cream liking \|\| id:ebi-fl187-GCST90094784 | MR Egger | 8 | -1.874581881 | 0.882298687192298 | 0.0777813726080264 | -3.603887308 | -0.145276454 | 0.153419100911129 | 0.0272177129106289 | 0.864783187392189 | 0.999950574162623 |
| ebi-fl187-GCST90094784 | ebi-a-GCST90001422 | CD20- CD38- B cell %B cell \|\| id:ebi-a-GCST90001422 | Ice cream liking \|\| id:ebi-fl187-GCST90094784 | MR Egger | 8 | -0.35605653 | 0.97565121536802 | 0.727672749242342 | -2.268332912 | 1.55621985193264 | 0.700433023505083 | 0.103484554188197 | 4.7408661540374 | 0.999950574162623 |
| ebi-fl187-GCST90094784 | ebi-a-GCST90001444 | CD20- CD38- B cell %lymphocyte \|\| id:ebi-a-GCST90001444 | Ice cream liking \|\| id:ebi-fl187-GCST90094784 | MR Egger | 8 | -0.58981276 | 1.06860639528371 | 0.600923327621658 | -2.684281295 | 1.50465577475052 | 0.554431086689859 | 0.0682702425494266 | 4.50260345370165 | 0.999950574162623 |
| ebi-fl187-GCST90094784 | ebi-a-GCST90001459 | Myeloid Dendritic Cell %Dendritic Cell \|\| id:ebi-a-GCST90001459 | Ice cream liking \|\| id:ebi-fl187-GCST90094784 | MR Egger | 8 | 0.175058825225298 | 0.902639742400843 | 0.852621507702921 | -1.59411507 | 1.94423272033095 | 1.19131629400057 | 0.203088167843358 | 6.98826784161017 | 0.999950574162623 |
| ebi-fl187-GCST90094784 | ebi-a-GCST90001476 | HLA DR++ monocyte %leukocyte \|\| id:ebi-a-GCST90001476 | Ice cream liking \|\| id:ebi-fl187-GCST90094784 | MR Egger | 8 | 0.609960461210542 | 0.880879364724824 | 0.51454965778199 | -1.116563094 | 2.3364840160712 | 1.84035863179062 | 0.327403116992157 | 10.344800393844 | 0.999950574162623 |
| ebi-fl187-GCST90094784 | ebi-a-GCST90001478 | CD4 regulatory T cell %CD4+ T cell \|\| id:ebi-a-GCST90001478 | Ice cream liking \|\| id:ebi-fl187-GCST90094784 | MR Egger | 8 | -0.469131908 | 0.888368207327716 | 0.616393893758518 | -2.210333594 | 1.27206977869728 | 0.625545063525237 | 0.109664059137707 | 3.5682303717157 | 0.999950574162623 |
| ebi-fl187-GCST90094784 | ebi-a-GCST90001485 | CD39+ resting CD4 regulatory T cell %CD4 regulatory T cell \|\| id:ebi-a-GCST90001485 | Ice cream liking \|\| id:ebi-fl187-GCST90094784 | MR Egger | 8 | 0.477149056998566 | 0.985096943588703 | 0.64529799706284 | -1.453640952 | 2.40793906643242 | 1.61147362757547 | 0.233717781741429 | 11.1110384200217 | 0.999950574162623 |
| ebi-fl187-GCST90094784 | ebi-a-GCST90001515 | Immature Myeloid-Derived Suppressor Cells Absolute Count \|\| id:ebi-a-GCST90001515 | Ice cream liking \|\| id:ebi-fl187-GCST90094784 | MR Egger | 8 | 0.192947974706401 | 1.16055938751419 | 0.87341781690806 | -2.081748425 | 2.46764437423422 | 1.21281969457707 | 0.124711971958552 | 11.7946303667051 | 0.999950574162623 |
| ebi-fl187-GCST90094784 | ebi-a-GCST90001517 | CD33+ HLA DR+ Absolute Count \|\| id:ebi-a-GCST90001517 | Ice cream liking \|\| id:ebi-fl187-GCST90094784 | MR Egger | 8 | -0.313706295 | 1.13382860375336 | 0.791310988836596 | -2.536010358 | 1.90859776879076 | 0.730733617079189 | 0.0791816770012058 | 6.7436260426955 | 0.999950574162623 |
| ebi-fl187-GCST90094784 | ebi-a-GCST90001518 | CD33+ HLA DR+ CD14- Absolute Count \|\| id:ebi-a-GCST90001518 | Ice cream liking \|\| id:ebi-fl187-GCST90094784 | MR Egger | 8 | 0.0373376809043874 | 1.29467738965767 | 0.977928024978654 | -2.500230003 | 2.57490536463342 | 1.0380434891258 | 0.0820661210133891 | 13.1300745302763 | 0.999950574162623 |
| ebi-fl187-GCST90094784 | ebi-a-GCST90001520 | CD33+ HLA DR+ CD14dim Absolute Count \|\| id:ebi-a-GCST90001520 | Ice cream liking \|\| id:ebi-fl187-GCST90094784 | MR Egger | 7 | -0.745700714 | 1.15542515330363 | 0.547117906507663 | -3.010334015 | 1.5189325863558 | 0.474401763433146 | 0.0492752173696986 | 4.56734734339042 | 0.999950574162623 |
| ebi-fl187-GCST90094784 | ebi-a-GCST90001531 | CD33dim HLA DR- Absolute Count \|\| id:ebi-a-GCST90001531 | Ice cream liking \|\| id:ebi-fl187-GCST90094784 | MR Egger | 8 | 1.63393486034581 | 1.15939599913232 | 0.208410057489917 | -0.638481298 | 3.90635101864516 | 5.12399731661994 | 0.528093832522844 | 49.7172034282234 | 0.999950574162623 |
| ebi-fl187-GCST90094784 | ebi-a-GCST90001532 | Basophil Absolute Count \|\| id:ebi-a-GCST90001532 | Ice cream liking \|\| id:ebi-fl187-GCST90094784 | MR Egger | 8 | 1.76886015863764 | 1.15535193126947 | 0.176644898356948 | -0.495629627 | 4.03334994392581 | 5.86416533225917 | 0.609187226008345 | 56.449698181293 | 0.999950574162623 |
| ebi-fl187-GCST90094784 | ebi-a-GCST90001588 | T/B cell \|\| id:ebi-a-GCST90001588 | Ice cream liking \|\| id:ebi-fl187-GCST90094784 | MR Egger | 8 | -0.337312433 | 0.86721606096178 | 0.710734483314734 | -2.037055912 | 1.36243104654355 | 0.71368582610412 | 0.130412090865515 | 3.9056766516164 | 0.999950574162623 |
| ebi-fl187-GCST90094784 | ebi-a-GCST90001624 | HLA DR+ CD4+ T cell Absolute Count \|\| id:ebi-a-GCST90001624 | Ice cream liking \|\| id:ebi-fl187-GCST90094784 | MR Egger | 8 | -0.0189317 | 0.842194005862632 | 0.982794771386631 | -1.669631952 | 1.6317685514259 | 0.981246379018199 | 0.18831636242992 | 5.11290917003902 | 0.999950574162623 |
| ebi-fl187-GCST90094784 | ebi-a-GCST90001631 | CD8+ Natural Killer T %T cell \|\| id:ebi-a-GCST90001631 | Ice cream liking \|\| id:ebi-fl187-GCST90094784 | MR Egger | 8 | -0.044171692 | 0.85251878006436 | 0.960359502358514 | -1.715108501 | 1.62676511720192 | 0.956789670501842 | 0.179944195591634 | 5.08739095789751 | 0.999950574162623 |
| ebi-fl187-GCST90094784 | ebi-a-GCST90001632 | CD8+ Natural Killer T %lymphocyte \|\| id:ebi-a-GCST90001632 | Ice cream liking \|\| id:ebi-fl187-GCST90094784 | MR Egger | 8 | 0.00196312896075945 | 0.857654820839566 | 0.998247889463558 | -1.67904032 | 1.68296657780631 | 1.00196505715998 | 0.186552921289573 | 5.38149694376141 | 0.999974376584515 |
| ebi-fl187-GCST90094784 | ebi-a-GCST90001646 | Natural Killer %CD3- lymphocyte \|\| id:ebi-a-GCST90001646 | Ice cream liking \|\| id:ebi-fl187-GCST90094784 | MR Egger | 8 | -1.423049284 | 0.836659146106128 | 0.139871361214913 | -3.06290121 | 0.216802642399461 | 0.240978084823508 | 0.0467518612906351 | 1.2420989402798 | 0.999950574162623 |
| ebi-fl187-GCST90094784 | ebi-a-GCST90001684 | CD127- CD8+ T cell Absolute Count \|\| id:ebi-a-GCST90001684 | Ice cream liking \|\| id:ebi-fl187-GCST90094784 | MR Egger | 8 | 0.152749575610061 | 0.999375182002486 | 0.883531047348358 | -1.806025781 | 2.11152493233493 | 1.1650331896829 | 0.164305828288372 | 8.2608288896516 | 0.999950574162623 |
| ebi-fl187-GCST90094784 | ebi-a-GCST90001687 | CD28- CD8+ T cell Absolute Count \|\| id:ebi-a-GCST90001687 | Ice cream liking \|\| id:ebi-fl187-GCST90094784 | MR Egger | 8 | 0.3824162629595 | 0.910382710417525 | 0.689068157740913 | -1.401933849 | 2.16676637537785 | 1.46582212561163 | 0.246120543347664 | 8.73000877824934 | 0.999950574162623 |
| ebi-fl187-GCST90094784 | ebi-a-GCST90001701 | BAFF-R on CD20- CD38- B cell \|\| id:ebi-a-GCST90001701 | Ice cream liking \|\| id:ebi-fl187-GCST90094784 | MR Egger | 8 | -1.012032678 | 0.8408724973215 | 0.274089570323471 | -2.660142773 | 0.636077416423759 | 0.363479391472692 | 0.0699382357347233 | 1.88905634575172 | 0.999950574162623 |
| ebi-fl187-GCST90094784 | ebi-a-GCST90001732 | CD19 on IgD- CD27- B cell \|\| id:ebi-a-GCST90001732 | Ice cream liking \|\| id:ebi-fl187-GCST90094784 | MR Egger | 8 | -1.675309596 | 1.31095417858425 | 0.248485616957135 | -4.244779786 | 0.894160594012021 | 0.187250198592158 | 0.0143388909553454 | 2.4452823431042 | 0.999950574162623 |
| ebi-fl187-GCST90094784 | ebi-a-GCST90001798 | CD27 on CD24+ CD27+ B cell \|\| id:ebi-a-GCST90001798 | Ice cream liking \|\| id:ebi-fl187-GCST90094784 | MR Egger | 8 | -0.882844057 | 1.12103040265249 | 0.460940766550491 | -3.080063646 | 1.31437553221042 | 0.413604921372731 | 0.0459563316106695 | 3.72242572433754 | 0.999950574162623 |
| ebi-fl187-GCST90094784 | ebi-a-GCST90001836 | CD66b on CD66b++ myeloid cell \|\| id:ebi-a-GCST90001836 | Ice cream liking \|\| id:ebi-fl187-GCST90094784 | MR Egger | 8 | -0.976641427 | 1.28011023542205 | 0.474419401077759 | -3.485657488 | 1.53237463473523 | 0.376573727819308 | 0.0306336105714442 | 4.62915633640388 | 0.999950574162623 |
| ebi-fl187-GCST90094784 | ebi-a-GCST90001943 | CD25 on activated & secreting CD4 regulatory T cell \|\| id:ebi-a-GCST90001943 | Ice cream liking \|\| id:ebi-fl187-GCST90094784 | MR Egger | 8 | 0.198747903849488 | 1.06464358921469 | 0.858063499028226 | -1.887953531 | 2.28544933871028 | 1.21987440149288 | 0.151381289171166 | 9.83010227727037 | 0.999950574162623 |
| ebi-fl187-GCST90094784 | ebi-a-GCST90001960 | CD25 on CD4+ T cell \|\| id:ebi-a-GCST90001960 | Ice cream liking \|\| id:ebi-fl187-GCST90094784 | MR Egger | 8 | 0.123306317955073 | 1.21656079231752 | 0.922569730477957 | -2.261152835 | 2.50776547089741 | 1.13123088421199 | 0.104230255180747 | 12.2774650333141 | 0.999950574162623 |
| ebi-fl187-GCST90094784 | ebi-a-GCST90001988 | HLA DR on CD14+ CD16- monocyte \|\| id:ebi-a-GCST90001988 | Ice cream liking \|\| id:ebi-fl187-GCST90094784 | MR Egger | 8 | 0.290472237593986 | 0.866806312547953 | 0.748950601403002 | -1.408468135 | 1.98941261018797 | 1.33705874836766 | 0.244517564304792 | 7.31123795408856 | 0.999950574162623 |
| ebi-fl187-GCST90094784 | ebi-a-GCST90001991 | HLA DR on CD14+ monocyte \|\| id:ebi-a-GCST90001991 | Ice cream liking \|\| id:ebi-fl187-GCST90094784 | MR Egger | 8 | 0.144352790463907 | 0.868413572788774 | 0.87343923257774 | -1.557737812 | 1.8464433931299 | 1.15529161250285 | 0.210611976547617 | 6.33724032126768 | 0.999950574162623 |
| ebi-fl187-GCST90094784 | ebi-a-GCST90002019 | CD14 on Monocytic Myeloid-Derived Suppressor Cells \|\| id:ebi-a-GCST90002019 | Ice cream liking \|\| id:ebi-fl187-GCST90094784 | MR Egger | 8 | 0.309059135030961 | 1.75310810889543 | 0.865864288905902 | -3.127032758 | 3.745151028 | 1.36214291848545 | 0.043847711166907 | 42.3153975658462 | 0.999950574162623 |
| ebi-fl187-GCST90094784 | ebi-a-GCST90002037 | CD80 on plasmacytoid Dendritic Cell \|\| id:ebi-a-GCST90002037 | Ice cream liking \|\| id:ebi-fl187-GCST90094784 | MR Egger | 8 | -0.217612904 | 0.964703487481092 | 0.829019248352979 | -2.10843174 | 1.67320593118566 | 0.804436775439655 | 0.121428248284849 | 5.32922556999853 | 0.999950574162623 |
| ebi-fl187-GCST90094784 | ebi-a-GCST90002042 | CD45 on CD33+ HLA DR+ CD14- \|\| id:ebi-a-GCST90002042 | Ice cream liking \|\| id:ebi-fl187-GCST90094784 | MR Egger | 8 | -0.495403794 | 2.45990328574607 | 0.847046444323138 | -5.316814234 | 4.32600664595888 | 0.609324815846811 | 0.00490836574781301 | 75.6416188773581 | 0.999950574162623 |
| ebi-fl187-GCST90094784 | ebi-a-GCST90002056 | CD8 on Effector Memory CD8+ T cell \|\| id:ebi-a-GCST90002056 | Ice cream liking \|\| id:ebi-fl187-GCST90094784 | MR Egger | 8 | 1.72278509821764 | 0.944569624108378 | 0.117983780840928 | -0.128571365 | 3.57414156147006 | 5.60010360362696 | 0.879350805278306 | 35.6639923260548 | 0.999950574162623 |
| ebi-fl187-GCST90094784 | ebi-a-GCST90002058 | CD8 on CD8+ T cell \|\| id:ebi-a-GCST90002058 | Ice cream liking \|\| id:ebi-fl187-GCST90094784 | MR Egger | 8 | 1.68699298279992 | 1.11052414907779 | 0.179543385274282 | -0.489634349 | 3.86362031499239 | 5.40320871077995 | 0.612850442356761 | 47.6375023243487 | 0.999950574162623 |
| ebi-fl187-GCST90094784 | ebi-a-GCST90002063 | CD4 on CD4 regulatory T cell \|\| id:ebi-a-GCST90002063 | Ice cream liking \|\| id:ebi-fl187-GCST90094784 | MR Egger | 8 | 1.06265690112652 | 0.940352512239063 | 0.301591708176093 | -0.780434023 | 2.90574782511508 | 2.89404998825417 | 0.458207095786281 | 18.2789079687681 | 0.999950574162623 |
| ebi-fl187-GCST90094784 | ebi-a-GCST90002066 | CD4 on activated CD4 regulatory T cell \|\| id:ebi-a-GCST90002066 | Ice cream liking \|\| id:ebi-fl187-GCST90094784 | MR Egger | 8 | 1.16090716482974 | 0.940450018774277 | 0.263198035325355 | -0.682374872 | 3.00418920162733 | 3.19282838436515 | 0.505415269405886 | 20.1698557781821 | 0.999950574162623 |
| ebi-fl187-GCST90094784 | ebi-a-GCST90002070 | CD4 on activated & secreting CD4 regulatory T cell \|\| id:ebi-a-GCST90002070 | Ice cream liking \|\| id:ebi-fl187-GCST90094784 | MR Egger | 8 | 0.988051797438847 | 0.944807118803999 | 0.335956127122578 | -0.863770155 | 2.83987375029469 | 2.68599650642361 | 0.421569699153027 | 17.1136048131888 | 0.999950574162623 |
| ebi-fl187-GCST90094784 | ebi-a-GCST90002081 | SSC-A on CD4+ T cell \|\| id:ebi-a-GCST90002081 | Ice cream liking \|\| id:ebi-fl187-GCST90094784 | MR Egger | 8 | -0.170524207 | 0.892986560576602 | 0.85485513788888 | -1.920777866 | 1.57972945138776 | 0.843222677201496 | 0.146492965891165 | 4.85364248734719 | 0.999950574162623 |
| ebi-fl187-GCST90094784 | ebi-a-GCST90002094 | CD11b on Monocytic Myeloid-Derived Suppressor Cells \|\| id:ebi-a-GCST90002094 | Ice cream liking \|\| id:ebi-fl187-GCST90094784 | MR Egger | 8 | -0.613011504 | 1.67008284524775 | 0.726176237540906 | -3.886373881 | 2.66035087256052 | 0.541717027086663 | 0.0205196178774281 | 14.3013061543617 | 0.999950574162623 |
| ebi-fl187-GCST90094784 | ebi-a-GCST90002104 | HLA DR on myeloid Dendritic Cell \|\| id:ebi-a-GCST90002104 | Ice cream liking \|\| id:ebi-fl187-GCST90094784 | MR Egger | 8 | 0.514797019275286 | 0.958225020568631 | 0.610430828806425 | -1.363324021 | 2.3929180595898 | 1.67329881991713 | 0.255809047498569 | 10.9453866785213 | 0.999950574162623 |
| ebi-fl187-GCST90094784 | ebi-a-GCST90002105 | HLA DR on plasmacytoid Dendritic Cell \|\| id:ebi-a-GCST90002105 | Ice cream liking \|\| id:ebi-fl187-GCST90094784 | MR Egger | 8 | 0.716311012796619 | 0.938801782753096 | 0.474380548411228 | -1.123740481 | 2.55636250699269 | 2.04686839465321 | 0.325061630800094 | 12.8888488460416 | 0.999950574162623 |
| ebi-fl187-GCST90094784 | ebi-a-GCST90002106 | HLA DR on Dendritic Cell \|\| id:ebi-a-GCST90002106 | Ice cream liking \|\| id:ebi-fl187-GCST90094784 | MR Egger | 8 | 0.75507245123413 | 0.956580445254895 | 0.459956108558746 | -1.119825221 | 2.62997012393373 | 2.12776567702016 | 0.326336826311204 | 13.8733554146525 | 0.999950574162623 |
| ebi-fl187-GCST90094784 | ebi-a-GCST90002111 | HLA DR on CD33dim HLA DR+ CD11b- \|\| id:ebi-a-GCST90002111 | Ice cream liking \|\| id:ebi-fl187-GCST90094784 | MR Egger | 8 | 1.29811855548482 | 1.54594795951076 | 0.433258894253482 | -1.731939445 | 4.3281765561259 | 3.66239957980793 | 0.176940909787234 | 75.8059326037503 | 0.999950574162623 |
| ebi-fl187-GCST90094784 | ebi-a-GCST90002112 | HLA DR on CD33- HLA DR+ \|\| id:ebi-a-GCST90002112 | Ice cream liking \|\| id:ebi-fl187-GCST90094784 | MR Egger | 8 | 0.866108628555887 | 1.24241250505868 | 0.511821036988959 | -1.569019881 | 3.3012371384709 | 2.37764054559319 | 0.208249191278534 | 27.1462017659773 | 0.999950574162623 |
| ebi-fl187-GCST90094784 | ebi-a-GCST90002115 | HLA DR on HLA DR+ CD8+ T cell \|\| id:ebi-a-GCST90002115 | Ice cream liking \|\| id:ebi-fl187-GCST90094784 | MR Egger | 8 | 0.184076383883161 | 0.879298958189223 | 0.841106219551562 | -1.539349574 | 1.90750234193404 | 1.20210764123916 | 0.214520585790499 | 6.73624293818042 | 0.999950574162623 |
| ebi-fl187-GCST90094784 | ebi-a-GCST90002116 | HLA DR on B cell \|\| id:ebi-a-GCST90002116 | Ice cream liking \|\| id:ebi-fl187-GCST90094784 | MR Egger | 8 | 1.25162708346942 | 0.929309673365493 | 0.226675716721533 | -0.569819876 | 3.07307404326579 | 3.49602665946541 | 0.565627312393419 | 21.608225302939 | 0.999950574162623 |
| ebi-fl187-GCST90094784 | ebi-cfmet338-GCST90026023 | Sphingomyelin (d18:1/24:1, d18:2/24:0) levels \|\| id:ebi-cfmet338-GCST90026023 | Ice cream liking \|\| id:ebi-fl187-GCST90094784 | MR Egger | 8 | 0.62434517575755 | 0.50804766550663 | 0.26511290249882 | -0.371428249 | 1.62011860015055 | 1.86702298514809 | 0.689748494449346 | 5.05368964937591 | 0.999950574162623 |
| ebi-fl187-GCST90094784 | ebi-cfmet338-GCST90026046 | 1-arachidonoyl-gpc (20:4n6) levels \|\| id:ebi-cfmet338-GCST90026046 | Ice cream liking \|\| id:ebi-fl187-GCST90094784 | MR Egger | 8 | 0.0151918702425919 | 0.603655607989137 | 0.980738319715712 | -1.167973121 | 1.1983568619013 | 1.01530785329216 | 0.310996655333276 | 3.31466599167133 | 0.999950574162623 |
| ebi-fl187-GCST90094784 | ebi-cfmet338-GCST90026047 | 1-linoleoyl-gpc (18:2) levels \|\| id:ebi-cfmet338-GCST90026047 | Ice cream liking \|\| id:ebi-fl187-GCST90094784 | MR Egger | 8 | -0.227906787 | 0.734016135259052 | 0.766689038426218 | -1.666578412 | 1.21076483787316 | 0.796198472238049 | 0.188892272661856 | 3.35605050572412 | 0.999950574162623 |
| ebi-fl187-GCST90094784 | ebi-cfmet338-GCST90026050 | 1-myristoyl-2-palmitoyl-gpc (14:0/16:0) levels \|\| id:ebi-cfmet338-GCST90026050 | Ice cream liking \|\| id:ebi-fl187-GCST90094784 | MR Egger | 8 | 0.27736464990011 | 0.389547251699383 | 0.503185901367858 | -0.486147963 | 1.0408772632309 | 1.31964749263553 | 0.614990804431884 | 2.83170007139857 | 0.999950574162623 |
| ebi-fl187-GCST90094784 | ebi-cfmet338-GCST90026068 | 2,3-dihydroxyisovalerate levels \|\| id:ebi-cfmet338-GCST90026068 | Ice cream liking \|\| id:ebi-fl187-GCST90094784 | MR Egger | 8 | 0.0967475745372307 | 1.25106903402104 | 0.94087381789037 | -2.355347732 | 2.54884288121847 | 1.10158227110785 | 0.0948605147486284 | 12.7922930129015 | 0.999950574162623 |
| ebi-fl187-GCST90094784 | ebi-cfmet338-GCST90026082 | 3-hydroxyhexanoate levels \|\| id:ebi-cfmet338-GCST90026082 | Ice cream liking \|\| id:ebi-fl187-GCST90094784 | MR Egger | 8 | 0.389078148710336 | 0.267938958502934 | 0.196677313022404 | -0.13608221 | 0.914238507376086 | 1.47561986461657 | 0.872770879084282 | 2.49487469968708 | 0.999950574162623 |
| ebi-fl187-GCST90094784 | ebi-cfmet338-GCST90026094 | 3-ureidopropionate levels \|\| id:ebi-cfmet338-GCST90026094 | Ice cream liking \|\| id:ebi-fl187-GCST90094784 | MR Egger | 8 | 0.830937018044367 | 0.739446752741871 | 0.304068016473275 | -0.618378617 | 2.28025265341843 | 2.29546862838998 | 0.538817358861849 | 9.77915083332266 | 0.999950574162623 |
| ebi-fl187-GCST90094784 | ebi-cfmet338-GCST90026173 | Glycerol levels \|\| id:ebi-cfmet338-GCST90026173 | Ice cream liking \|\| id:ebi-fl187-GCST90094784 | MR Egger | 7 | 0.315823365361897 | 0.433967774270892 | 0.499398718659941 | -0.534753472 | 1.16640020293284 | 1.37138799960826 | 0.585813691699887 | 3.21041497000899 | 0.999950574162623 |
| ebi-fl187-GCST90094784 | ebi-cfmet338-GCST90026179 | Histidine levels \|\| id:ebi-cfmet338-GCST90026179 | Ice cream liking \|\| id:ebi-fl187-GCST90094784 | MR Egger | 8 | 0.0994374460496876 | 0.330904648140254 | 0.773934637324377 | -0.549135664 | 0.748010556404585 | 1.10454937465216 | 0.57744870427054 | 2.11279255113348 | 0.999950574162623 |
| ebi-fl187-GCST90094784 | ebi-cfmet338-GCST90026188 | Isocitrate levels \|\| id:ebi-cfmet338-GCST90026188 | Ice cream liking \|\| id:ebi-fl187-GCST90094784 | MR Egger | 8 | 0.1776222046392 | 0.55912034102106 | 0.761491677177246 | -0.918253664 | 1.27349807304048 | 1.19437400702905 | 0.399215597361172 | 3.57333049634339 | 0.999950574162623 |
| ebi-fl187-GCST90094784 | ebi-cfmet338-GCST90026207 | N-acetyl-isoputreanine levels \|\| id:ebi-cfmet338-GCST90026207 | Ice cream liking \|\| id:ebi-fl187-GCST90094784 | MR Egger | 7 | 0.395673789006016 | 0.418046389625989 | 0.387363332991296 | -0.423697135 | 1.21504471267295 | 1.48538468969415 | 0.654622114237089 | 3.3704447625469 | 0.999950574162623 |
| ebi-fl187-GCST90094784 | ebi-cfmet338-GCST90026213 | N-acetylglutamate levels \|\| id:ebi-cfmet338-GCST90026213 | Ice cream liking \|\| id:ebi-fl187-GCST90094784 | MR Egger | 8 | 0.391437625915925 | 0.608362818266965 | 0.5437397353395 | -0.800953498 | 1.58382874971918 | 1.47910566676945 | 0.448900734089878 | 4.87357985258154 | 0.999950574162623 |
| ebi-fl187-GCST90094784 | ebi-cfmet338-GCST90026224 | N-acetyltaurine levels \|\| id:ebi-cfmet338-GCST90026224 | Ice cream liking \|\| id:ebi-fl187-GCST90094784 | MR Egger | 8 | 0.497218354264713 | 0.560091992574278 | 0.408852011468909 | -0.600561951 | 1.5949986597103 | 1.64414148483275 | 0.548503317385193 | 4.92832246673484 | 0.999950574162623 |
| ebi-fl187-GCST90094784 | ebi-cfmet338-GCST90026250 | Phenyllactate (pla) levels \|\| id:ebi-cfmet338-GCST90026250 | Ice cream liking \|\| id:ebi-fl187-GCST90094784 | MR Egger | 8 | 0.23827387890159 | 0.759447992656097 | 0.764335277129456 | -1.250244187 | 1.72679194450754 | 1.26905671309214 | 0.286434844739098 | 5.62258737239589 | 0.999950574162623 |
| ebi-fl187-GCST90094784 | ebi-cfmet338-GCST90026251 | Pipecolate levels \|\| id:ebi-cfmet338-GCST90026251 | Ice cream liking \|\| id:ebi-fl187-GCST90094784 | MR Egger | 8 | 0.707719191237855 | 1.05737832133312 | 0.528194402970222 | -1.364742319 | 2.78018070105077 | 2.02935740004337 | 0.255446491323899 | 16.1219339352322 | 0.999950574162623 |
| ebi-fl187-GCST90094784 | ebi-cfmet338-GCST90026278 | Trigonelline (n'-methylnicotinate) levels \|\| id:ebi-cfmet338-GCST90026278 | Ice cream liking \|\| id:ebi-fl187-GCST90094784 | MR Egger | 8 | -0.426391094 | 1.51449786224403 | 0.787754461466626 | -3.394806904 | 2.54202471577192 | 0.652860962032209 | 0.0335470313367974 | 12.7053697081714 | 0.999950574162623 |
| ebi-fl187-GCST90094784 | ebi-cfmet338-GCST90026299 | 1-carboxyethylvaline levels \|\| id:ebi-cfmet338-GCST90026299 | Ice cream liking \|\| id:ebi-fl187-GCST90094784 | MR Egger | 8 | 0.32192481441683 | 0.666832498076949 | 0.646369667241343 | -0.985066882 | 1.62891651064765 | 1.37978103241572 | 0.37341425147896 | 5.09834771938652 | 0.999950574162623 |
| ebi-fl187-GCST90094784 | ebi-gm430-GCST90011306 | C_Erysipelotrichia abundance \|\| id:ebi-gm430-GCST90011306 | Ice cream liking \|\| id:ebi-fl187-GCST90094784 | MR Egger | 8 | -0.119400721 | 0.705948316959377 | 0.871248364137081 | -1.503059422 | 1.26425798042964 | 0.887452108971559 | 0.222448554008286 | 3.54046466711908 | 0.999950574162623 |
| ebi-fl187-GCST90094784 | ebi-gm430-GCST90011312 | F_Erysipelotrichaceae abundance \|\| id:ebi-gm430-GCST90011312 | Ice cream liking \|\| id:ebi-fl187-GCST90094784 | MR Egger | 8 | -0.119400721 | 0.705948316959377 | 0.871248364137081 | -1.503059422 | 1.26425798042964 | 0.887452108971559 | 0.222448554008286 | 3.54046466711908 | 0.999950574162623 |
| ebi-fl187-GCST90094784 | ebi-gm430-GCST90011352 | O_Erysipelotrichales abundance \|\| id:ebi-gm430-GCST90011352 | Ice cream liking \|\| id:ebi-fl187-GCST90094784 | MR Egger | 8 | -0.119400721 | 0.705948316959377 | 0.871248364137081 | -1.503059422 | 1.26425798042964 | 0.887452108971559 | 0.222448554008286 | 3.54046466711908 | 0.999950574162623 |
| ebi-fl187-GCST90094784 | ebi-gm430-GCST90011362 | OTU97_11 (Parabacteroides) abundance \|\| id:ebi-gm430-GCST90011362 | Ice cream liking \|\| id:ebi-fl187-GCST90094784 | MR Egger | 8 | -0.499231504 | 1.09691437434919 | 0.665024671252199 | -2.649183678 | 1.6507206693407 | 0.606996955015767 | 0.0707089107390738 | 5.21073369038352 | 0.999950574162623 |
| ebi-fl187-GCST90094784 | ebi-gm430-GCST90011384 | OTU97_235 (Phascolarctobacterium) abundance \|\| id:ebi-gm430-GCST90011384 | Ice cream liking \|\| id:ebi-fl187-GCST90094784 | MR Egger | 8 | 1.83961158 | 2.17178432420123 | 0.429452595540365 | -2.417085695 | 6.09630885578941 | 6.2940930367879 | 0.0891811401471371 | 444.215078326891 | 0.999950574162623 |
| ebi-fl187-GCST90094784 | ebi-gm430-GCST90011390 | OTU97_3 (Bacteroides) abundance \|\| id:ebi-gm430-GCST90011390 | Ice cream liking \|\| id:ebi-fl187-GCST90094784 | MR Egger | 8 | -0.708994755 | 0.544184965699057 | 0.240389445799318 | -1.775597287 | 0.357607778179676 | 0.492138669024577 | 0.169382249403026 | 1.42990467066589 | 0.999950574162623 |
| ebi-fl187-GCST90094784 | ebi-gm430-GCST90011398 | OTU97_4 (Alistipes) abundance \|\| id:ebi-gm430-GCST90011398 | Ice cream liking \|\| id:ebi-fl187-GCST90094784 | MR Egger | 8 | 0.160781628768344 | 0.637348835439165 | 0.809254554091685 | -1.088422089 | 1.40998534622911 | 1.1744284794164 | 0.336747432290785 | 4.09589538331887 | 0.999950574162623 |
| ebi-fl187-GCST90094784 | ebi-gm430-GCST90011421 | OTU99_108 (Cytophagales) abundance \|\| id:ebi-gm430-GCST90011421 | Ice cream liking \|\| id:ebi-fl187-GCST90094784 | MR Egger | 8 | -0.217681607 | 2.01856654069859 | 0.917638601730881 | -4.174072027 | 3.73870881240021 | 0.804381510044536 | 0.0153894660377166 | 42.0436688391776 | 0.999950574162623 |
| ebi-fl187-GCST90094784 | ebi-gm430-GCST90011428 | OTU99_123 (Phascolarctobacterium) abundance \|\| id:ebi-gm430-GCST90011428 | Ice cream liking \|\| id:ebi-fl187-GCST90094784 | MR Egger | 8 | 1.42018249658886 | 1.19856915720825 | 0.28085846226731 | -0.929013052 | 3.76937804471702 | 4.13787551951703 | 0.394943306772872 | 43.3530927639319 | 0.999950574162623 |
| ebi-fl187-GCST90094784 | ebi-gm430-GCST90011450 | OTU99_298 (Phascolarctobacterium) abundance \|\| id:ebi-gm430-GCST90011450 | Ice cream liking \|\| id:ebi-fl187-GCST90094784 | MR Egger | 8 | 1.8640408870631 | 2.14372543149781 | 0.417977803200528 | -2.337660959 | 6.0657427327988 | 6.44974688281412 | 0.0965532162726195 | 430.84255976429 | 0.999950574162623 |
| ebi-fl187-GCST90094784 | ebi-gm430-GCST90011451 | OTU99_3 (Bacteroides) abundance \|\| id:ebi-gm430-GCST90011451 | Ice cream liking \|\| id:ebi-fl187-GCST90094784 | MR Egger | 8 | -0.684782391 | 0.544191247468169 | 0.255014674930257 | -1.751397236 | 0.38183245437135 | 0.504199936249993 | 0.173531309839899 | 1.46496661581728 | 0.999950574162623 |
| ebi-fl187-GCST90094784 | ebi-gm430-GCST90011472 | OTU99_72 (Parabacteroides) abundance \|\| id:ebi-gm430-GCST90011472 | Ice cream liking \|\| id:ebi-fl187-GCST90094784 | MR Egger | 8 | -1.369230087 | 2.42734652141382 | 0.593139118123101 | -6.126829269 | 3.38836909457064 | 0.254302675034833 | 0.00218349326536953 | 29.6176093398333 | 0.999950574162623 |
| ebi-fl187-GCST90094784 | ebi-gm430-GCST90011507 | TestASV_3 (Bacteroides) abundance \|\| id:ebi-gm430-GCST90011507 | Ice cream liking \|\| id:ebi-fl187-GCST90094784 | MR Egger | 8 | -0.089109329 | 0.925969920789607 | 0.926468941339303 | -1.904010374 | 1.72579171579031 | 0.914745559929081 | 0.148969994322111 | 5.61696631068321 | 0.999950574162623 |
| ebi-fl187-GCST90094784 | ebi-gm430-GCST90011512 | TestASV_34 (Phascolarctobacterium) abundance \|\| id:ebi-gm430-GCST90011512 | Ice cream liking \|\| id:ebi-fl187-GCST90094784 | MR Egger | 8 | 2.82139735311294 | 2.19586966500586 | 0.24620933682986 | -1.48250719 | 7.12530189652442 | 16.8003102434872 | 0.227067672247971 | 1243.02337485182 | 0.999950574162623 |
| ebi-fl187-GCST90094784 | ebi-gm430-GCST90011519 | TestASV_41 (Barnesiella) abundance \|\| id:ebi-gm430-GCST90011519 | Ice cream liking \|\| id:ebi-fl187-GCST90094784 | MR Egger | 8 | 2.48593593453181 | 3.37268127531266 | 0.488882391848296 | -4.124519365 | 9.09639123414462 | 12.0123577756696 | 0.0161712651953409 | 8923.03339210987 | 0.999950574162623 |
| ebi-fl187-GCST90094784 | ebi-gm430-GCST90011524 | TestASV_46 (Sutterella) abundance \|\| id:ebi-gm430-GCST90011524 | Ice cream liking \|\| id:ebi-fl187-GCST90094784 | MR Egger | 8 | 0.474162716693308 | 2.35253268868109 | 0.846924903303948 | -4.136801353 | 5.08512678650825 | 1.60666839752785 | 0.0159738646267591 | 161.600426692637 | 0.999950574162623 |
| ebi-fl187-GCST90094784 | ebi-gm430-GCST90011581 | OTU97_19 (Bacteria) prevalence \|\| id:ebi-gm430-GCST90011581 | Ice cream liking \|\| id:ebi-fl187-GCST90094784 | MR Egger | 8 | 2.53375419984739 | 2.58500201692712 | 0.364853164380535 | -2.532849753 | 7.60035815302455 | 12.6007230829606 | 0.0794323348797488 | 1998.91168318077 | 0.999950574162623 |
| ebi-fl187-GCST90094784 | ebi-gm430-GCST90011614 | OTU97_80 (Ruminococcus) prevalence \|\| id:ebi-gm430-GCST90011614 | Ice cream liking \|\| id:ebi-fl187-GCST90094784 | MR Egger | 8 | 0.813608402871454 | 1.28761731952774 | 0.550771912025679 | -1.710121543 | 3.33733834914583 | 2.25603399645176 | 0.180843810909108 | 28.1441171116669 | 0.999950574162623 |
| ebi-fl187-GCST90094784 | ebi-gm430-GCST90011623 | OTU99_11 (Parabacteroides) prevalence \|\| id:ebi-gm430-GCST90011623 | Ice cream liking \|\| id:ebi-fl187-GCST90094784 | MR Egger | 8 | 0.341091405185432 | 1.46275548627249 | 0.823369946031772 | -2.525909348 | 3.20809215827952 | 1.40648179471609 | 0.0799855450180413 | 24.7318567176306 | 0.999950574162623 |
| ebi-fl187-GCST90094784 | ebi-gm430-GCST90011642 | OTU99_19 (Bacteria) prevalence \|\| id:ebi-gm430-GCST90011642 | Ice cream liking \|\| id:ebi-fl187-GCST90094784 | MR Egger | 8 | 2.53375419984739 | 2.58500201692712 | 0.364853164380535 | -2.532849753 | 7.60035815302455 | 12.6007230829606 | 0.0794323348797488 | 1998.91168318077 | 0.999950574162623 |
| ebi-fl187-GCST90094784 | ebi-gm430-GCST90011653 | OTU99_30 (Parasutterella) prevalence \|\| id:ebi-gm430-GCST90011653 | Ice cream liking \|\| id:ebi-fl187-GCST90094784 | MR Egger | 8 | -0.49411408 | 1.62002370750774 | 0.770666308717657 | -3.669360547 | 2.68113238666661 | 0.610111177607696 | 0.025492766167618 | 14.6016186158205 | 0.999950574162623 |
| ebi-fl187-GCST90094784 | ebi-gm430-GCST90011675 | OTU99_8 (Ruminococcaceae) prevalence \|\| id:ebi-gm430-GCST90011675 | Ice cream liking \|\| id:ebi-fl187-GCST90094784 | MR Egger | 8 | 0.146980249559341 | 1.78413476074225 | 0.937022654213979 | -3.349923881 | 3.64388438061416 | 1.15833108525457 | 0.0350870247710538 | 38.2400876626603 | 0.999950574162623 |
| ebi-fl187-GCST90094784 | ebi-gm430-GCST90011679 | OTU99_92 (Ruminococcus) prevalence \|\| id:ebi-gm430-GCST90011679 | Ice cream liking \|\| id:ebi-fl187-GCST90094784 | MR Egger | 8 | 0.352058064350918 | 1.78431088738797 | 0.850101195078552 | -3.145191275 | 3.84930740363133 | 1.42199108831108 | 0.0430586872117454 | 46.9605272750757 | 0.999950574162623 |
| ebi-fl187-GCST90094784 | ebi-gm430-GCST90011684 | TestASV_11 (Lachnospiraceae) prevalence \|\| id:ebi-gm430-GCST90011684 | Ice cream liking \|\| id:ebi-fl187-GCST90094784 | MR Egger | 8 | -0.761656695 | 1.27854598274905 | 0.573131183415539 | -3.267606821 | 1.74429343135959 | 0.466892287891988 | 0.0380974921817671 | 5.72185716196147 | 0.999950574162623 |
| ebi-fl187-GCST90094784 | ebi-gm430-GCST90011688 | TestASV_15 (Bacteroides) prevalence \|\| id:ebi-gm430-GCST90011688 | Ice cream liking \|\| id:ebi-fl187-GCST90094784 | MR Egger | 8 | 0.92224045846717 | 1.20075511237998 | 0.471593678882734 | -1.431239562 | 3.27572047873193 | 2.51491865342799 | 0.239012467820667 | 26.4622841269753 | 0.999950574162623 |
| ebi-fl187-GCST90094784 | ebi-gm430-GCST90011692 | TestASV_19 (Prevotella) prevalence \|\| id:ebi-gm430-GCST90011692 | Ice cream liking \|\| id:ebi-fl187-GCST90094784 | MR Egger | 8 | -0.885256733 | 2.35122821575301 | 0.719494262980511 | -5.493664036 | 3.72315056986078 | 0.412608229525177 | 0.00411274727972007 | 41.3946054773123 | 0.999950574162623 |
| ebi-fl187-GCST90094784 | ebi-gm430-GCST90011693 | TestASV_2 (Bacteroides) prevalence \|\| id:ebi-gm430-GCST90011693 | Ice cream liking \|\| id:ebi-fl187-GCST90094784 | MR Egger | 8 | -1.090822556 | 1.17827880981211 | 0.3902795350821 | -3.400249023 | 1.2186039112003 | 0.335940050512561 | 0.0333649602744331 | 3.38246221815109 | 0.999950574162623 |
| ebi-fl187-GCST90094784 | ebi-gm430-GCST90011716 | TestASV_40 (Barnesiella) prevalence \|\| id:ebi-gm430-GCST90011716 | Ice cream liking \|\| id:ebi-fl187-GCST90094784 | MR Egger | 8 | 1.88576216151369 | 2.36411451940492 | 0.455467287130446 | -2.747902297 | 6.51942661954733 | 6.59137622134669 | 0.0640621036548324 | 678.189413282828 | 0.999950574162623 |
| ebi-fl187-GCST90094784 | ebi-gm430-GCST90011718 | TestASV_42 (Alphaproteobacteria) prevalence \|\| id:ebi-gm430-GCST90011718 | Ice cream liking \|\| id:ebi-fl187-GCST90094784 | MR Egger | 8 | 4.17849891020564 | 1.88294785358385 | 0.0682800014254191 | 0.487921117181286 | 7.86907670322999 | 65.2678068054928 | 1.62892635060243 | 2615.14991369848 | 0.999950574162623 |
| ebi-fl187-GCST90094784 | ebi-hbsm44-GCST90104929 | Sulcal depth of the right inferior frontal sulcus \|\| id:ebi-hbsm44-GCST90104929 | Ice cream liking \|\| id:ebi-fl187-GCST90094784 | MR Egger | 7 | -0.113444381 | 2.53946013549028 | 0.966097466768592 | -5.090786246 | 4.86389748502787 | 0.89275384952353 | 0.0061531800709782 | 129.528053241643 | 0.999950574162623 |
| ebi-fl187-GCST90094784 | ebi-hbsm44-GCST90104935 | Sulcal opening distance of the left calloso-marginal posterior fissure \|\| id:ebi-hbsm44-GCST90104935 | Ice cream liking \|\| id:ebi-fl187-GCST90094784 | MR Egger | 8 | -0.31273678 | 0.311921062262012 | 0.354752112467644 | -0.924102062 | 0.298628502314885 | 0.731442417711116 | 0.396887639710765 | 1.34800874831218 | 0.999950574162623 |
| ebi-fl187-GCST90094784 | ebi-hbsm44-GCST90104936 | Sulcal opening distance of the left anterior inferior temporal sulcus \|\| id:ebi-hbsm44-GCST90104936 | Ice cream liking \|\| id:ebi-fl187-GCST90094784 | MR Egger | 7 | 0.0681109838125407 | 0.189333698096471 | 0.733736326944355 | -0.302983064 | 0.439205032081624 | 1.07048410829383 | 0.738611605053497 | 1.55147335659129 | 0.999950574162623 |
| ebi-fl187-GCST90094784 | ebi-hbsm44-GCST90104941 | Sulcal opening distance of the right posterior inferior temporal sulcus \|\| id:ebi-hbsm44-GCST90104941 | Ice cream liking \|\| id:ebi-fl187-GCST90094784 | MR Egger | 8 | 0.00424172610066312 | 0.106905224879872 | 0.96963757743445 | -0.205292515 | 0.213775966865212 | 1.00425073495401 | 0.81440905497465 | 1.23834519335872 | 0.999950574162623 |
| ebi-fl187-GCST90094784 | ebi-IPs91-GCST90274819 | Leukemia inhibitory factor levels \|\| id:ebi-IPs91-GCST90274819 | Ice cream liking \|\| id:ebi-fl187-GCST90094784 | MR Egger | 9 | 0.0271129478193095 | 0.433218113148252 | 0.951846556551267 | -0.821994554 | 0.876220449589883 | 1.02748384826986 | 0.439554065299691 | 2.40180478762184 | 0.999950574162623 |
| ebi-fl187-GCST90094784 | ebi-IPs91-GCST90274837 | STAM binding protein levels \|\| id:ebi-IPs91-GCST90274837 | Ice cream liking \|\| id:ebi-fl187-GCST90094784 | MR Egger | 9 | 0.581370769218313 | 0.444574611973012 | 0.232282687254102 | -0.28999547 | 1.45273700868542 | 1.78848835601112 | 0.748266957034042 | 4.27479867915887 | 0.999950574162623 |
| ebi-fl187-GCST90094784 | ebi-IPs91-GCST90274840 | TNF-beta levels \|\| id:ebi-IPs91-GCST90274840 | Ice cream liking \|\| id:ebi-fl187-GCST90094784 | MR Egger | 9 | -0.270356808 | 0.466354933012602 | 0.580272438085886 | -1.184412476 | 0.643698860961963 | 0.763107163210444 | 0.305925865359942 | 1.90350868782519 | 0.999950574162623 |
| ebi-fl187-GCST90094784 | ebi-IPs91-GCST90274842 | Tumor necrosis factor ligand superfamily member 14 levels \|\| id:ebi-IPs91-GCST90274842 | Ice cream liking \|\| id:ebi-fl187-GCST90094784 | MR Egger | 9 | -0.231666523 | 0.436008903922533 | 0.611623509185711 | -1.086243975 | 0.622910928663952 | 0.793210596677286 | 0.337481705956376 | 1.86434713223379 | 0.999950574162623 |
| ebi-fl187-GCST90094784 | ebi-met1400-GCST90199628 | 3-methylhistidine levels \|\| id:ebi-met1400-GCST90199628 | Ice cream liking \|\| id:ebi-fl187-GCST90094784 | MR Egger | 8 | -0.328431212 | 0.570184394300473 | 0.58555051963338 | -1.445992625 | 0.789130200359899 | 0.720052457153012 | 0.235512185138854 | 2.20148074608712 | 0.999950574162623 |
| ebi-fl187-GCST90094784 | ebi-met1400-GCST90199651 | Docosahexaenoate DHA; 22:6n3 levels \|\| id:ebi-met1400-GCST90199651 | Ice cream liking \|\| id:ebi-fl187-GCST90094784 | MR Egger | 8 | 0.493319015270941 | 0.551312309871483 | 0.405352426882679 | -0.587253112 | 1.57389114261905 | 1.63774290305683 | 0.555852052883101 | 4.82538798336881 | 0.999950574162623 |
| ebi-fl187-GCST90094784 | ebi-met1400-GCST90199652 | 1-stearoyl-2-oleoyl-GPS (18:0/18:1) levels \|\| id:ebi-met1400-GCST90199652 | Ice cream liking \|\| id:ebi-fl187-GCST90094784 | MR Egger | 8 | -0.102656221 | 0.528380782771315 | 0.852364209685376 | -1.138282555 | 0.932970113279241 | 0.902437159147762 | 0.320368765279182 | 2.54204814723742 | 0.999950574162623 |
| ebi-fl187-GCST90094784 | ebi-met1400-GCST90199653 | 1-myristoyl-2-palmitoyl-gpc (14:0/16:0) levels \|\| id:ebi-met1400-GCST90199653 | Ice cream liking \|\| id:ebi-fl187-GCST90094784 | MR Egger | 8 | -0.685883935 | 0.518440407311801 | 0.234026248296391 | -1.702027133 | 0.330259263554612 | 0.503644843565493 | 0.182313575327245 | 1.39132880255792 | 0.999950574162623 |
| ebi-fl187-GCST90094784 | ebi-met1400-GCST90199667 | Erythritol levels in elite athletes \|\| id:ebi-met1400-GCST90199667 | Ice cream liking \|\| id:ebi-fl187-GCST90094784 | MR Egger | 8 | -0.321114 | 0.489733849276808 | 0.536339956677956 | -1.280992345 | 0.638764344525467 | 0.72534055741175 | 0.277761528481525 | 1.8941389295436 | 0.999950574162623 |
| ebi-fl187-GCST90094784 | ebi-met1400-GCST90199674 | 3-hydroxymyristate levels \|\| id:ebi-met1400-GCST90199674 | Ice cream liking \|\| id:ebi-fl187-GCST90094784 | MR Egger | 8 | 0.28911296043883 | 0.580605920468407 | 0.636240784437381 | -0.848874644 | 1.42710056455691 | 1.33524254959429 | 0.427896196790404 | 4.166600871 | 0.999950574162623 |
| ebi-fl187-GCST90094784 | ebi-met1400-GCST90199685 | 2-linoleoylglycerol (18:2) levels \|\| id:ebi-met1400-GCST90199685 | Ice cream liking \|\| id:ebi-fl187-GCST90094784 | MR Egger | 8 | -0.175754879 | 0.597038637263192 | 0.778388291473583 | -1.345950609 | 0.994440849560183 | 0.838823571012708 | 0.260292154278005 | 2.70321241621062 | 0.999950574162623 |
| ebi-fl187-GCST90094784 | ebi-met1400-GCST90199703 | Gamma-glutamylvaline levels \|\| id:ebi-met1400-GCST90199703 | Ice cream liking \|\| id:ebi-fl187-GCST90094784 | MR Egger | 8 | -0.587564895 | 0.526647116661545 | 0.307242453288889 | -1.619793244 | 0.444663453502329 | 0.555678774633638 | 0.19793962009465 | 1.55996510668602 | 0.999950574162623 |
| ebi-fl187-GCST90094784 | ebi-met1400-GCST90199779 | Gamma-glutamyl-2-aminobutyrate levels \|\| id:ebi-met1400-GCST90199779 | Ice cream liking \|\| id:ebi-fl187-GCST90094784 | MR Egger | 8 | 0.0374715679685814 | 0.540372261068469 | 0.946969142663456 | -1.021658064 | 1.09660119966278 | 1.03818247902534 | 0.359997546178654 | 2.99397279563764 | 0.999950574162623 |
| ebi-fl187-GCST90094784 | ebi-met1400-GCST90199824 | 4-vinylphenol sulfate levels \|\| id:ebi-met1400-GCST90199824 | Ice cream liking \|\| id:ebi-fl187-GCST90094784 | MR Egger | 8 | 0.676321713254868 | 0.522207467207966 | 0.242876701048275 | -0.347204922 | 1.69984834898248 | 1.9666305806533 | 0.706660502801635 | 5.47311732497722 | 0.999950574162623 |
| ebi-fl187-GCST90094784 | ebi-met1400-GCST90199830 | 4-ethylphenylsulfate levels \|\| id:ebi-met1400-GCST90199830 | Ice cream liking \|\| id:ebi-fl187-GCST90094784 | MR Egger | 8 | -0.667041818 | 0.611018487317787 | 0.316855767523739 | -1.864638053 | 0.530554417015156 | 0.513224545974555 | 0.154952284208823 | 1.69987448675371 | 0.999950574162623 |
| ebi-fl187-GCST90094784 | ebi-met1400-GCST90199833 | Hexanoylglutamine levels \|\| id:ebi-met1400-GCST90199833 | Ice cream liking \|\| id:ebi-fl187-GCST90094784 | MR Egger | 8 | 0.21285023045278 | 0.543803394131345 | 0.709016435327953 | -0.853004422 | 1.27870488295022 | 1.23719934258179 | 0.426132724216046 | 3.59198467120957 | 0.999950574162623 |
| ebi-fl187-GCST90094784 | ebi-met1400-GCST90199837 | Carotenoid levels (cryptoxanthin) \|\| id:ebi-met1400-GCST90199837 | Ice cream liking \|\| id:ebi-fl187-GCST90094784 | MR Egger | 8 | 0.202611211227079 | 0.740700661038548 | 0.793610516961403 | -1.249162084 | 1.65438450686263 | 1.22459626640279 | 0.286744964302497 | 5.22985998842389 | 0.999950574162623 |
| ebi-fl187-GCST90094784 | ebi-met1400-GCST90199869 | 2-hydroxyhippurate levels \|\| id:ebi-met1400-GCST90199869 | Ice cream liking \|\| id:ebi-fl187-GCST90094784 | MR Egger | 8 | 0.639314604102419 | 0.769290414070008 | 0.437762084568784 | -0.868494607 | 2.14712381567963 | 1.89518148444126 | 0.419582710731994 | 8.56020223689138 | 0.999950574162623 |
| ebi-fl187-GCST90094784 | ebi-met1400-GCST90199902 | Hydantoin-5-propionate levels \|\| id:ebi-met1400-GCST90199902 | Ice cream liking \|\| id:ebi-fl187-GCST90094784 | MR Egger | 8 | -0.116281311 | 0.821749716429924 | 0.892102990215776 | -1.726910756 | 1.49434813277425 | 0.890224757671525 | 0.177832931657616 | 4.4564306047495 | 0.999950574162623 |
| ebi-fl187-GCST90094784 | ebi-met1400-GCST90199904 | Pantoate levels \|\| id:ebi-met1400-GCST90199904 | Ice cream liking \|\| id:ebi-fl187-GCST90094784 | MR Egger | 8 | -0.158717952 | 0.528955373260704 | 0.77425617171831 | -1.195470483 | 0.878034579969225 | 0.853236979140498 | 0.302561570550453 | 2.40616592929605 | 0.999950574162623 |
| ebi-fl187-GCST90094784 | ebi-met1400-GCST90199936 | 3-methyl catechol sulfate (2) levels \|\| id:ebi-met1400-GCST90199936 | Ice cream liking \|\| id:ebi-fl187-GCST90094784 | MR Egger | 7 | -0.35613859 | 0.615559649915473 | 0.587967566195823 | -1.562635504 | 0.850358324019541 | 0.700375548591277 | 0.209582985967519 | 2.34048535380899 | 0.999950574162623 |
| ebi-fl187-GCST90094784 | ebi-met1400-GCST90199977 | 4-hydroxychlorothalonil levels \|\| id:ebi-met1400-GCST90199977 | Ice cream liking \|\| id:ebi-fl187-GCST90094784 | MR Egger | 8 | 0.57529076846271 | 0.637581112505775 | 0.40166518005672 | -0.674368212 | 1.82494974897403 | 1.77764733555757 | 0.509478201137289 | 6.20248333012268 | 0.999950574162623 |
| ebi-fl187-GCST90094784 | ebi-met1400-GCST90200040 | Sphingomyelin (d18:2/24:1, d18:1/24:2) levels \|\| id:ebi-met1400-GCST90200040 | Ice cream liking \|\| id:ebi-fl187-GCST90094784 | MR Egger | 8 | 1.02976210698493 | 0.480803486802597 | 0.0759663163645361 | 0.0873872728518442 | 1.97213694111802 | 2.80039955995646 | 1.09131923620355 | 7.18601618595648 | 0.999950574162623 |
| ebi-fl187-GCST90094784 | ebi-met1400-GCST90200077 | 1-stearoyl-2-oleoyl-GPI (18:0/18:1) levels \|\| id:ebi-met1400-GCST90200077 | Ice cream liking \|\| id:ebi-fl187-GCST90094784 | MR Egger | 8 | 0.561602013630137 | 0.554070855524126 | 0.349892772658509 | -0.524376863 | 1.64758089045742 | 1.75347934920829 | 0.591924099159454 | 5.19439879617344 | 0.999950574162623 |
| ebi-fl187-GCST90094784 | ebi-met1400-GCST90200080 | 1-palmitoyl-2-oleoyl-GPI (16:0/18:1) levels \|\| id:ebi-met1400-GCST90200080 | Ice cream liking \|\| id:ebi-fl187-GCST90094784 | MR Egger | 8 | 0.00717894883345877 | 0.732296395669309 | 0.992496023027165 | -1.428121987 | 1.4424798843453 | 1.00720477926141 | 0.239758769865407 | 4.23117564348748 | 0.999950574162623 |
| ebi-fl187-GCST90094784 | ebi-met1400-GCST90200082 | 1-oleoyl-2-linoleoyl-GPE (18:1/18:2) levels \|\| id:ebi-met1400-GCST90200082 | Ice cream liking \|\| id:ebi-fl187-GCST90094784 | MR Egger | 8 | -0.741764503 | 0.567136365251944 | 0.238776053320051 | -1.853351779 | 0.369822773020673 | 0.476272788949645 | 0.156711024366481 | 1.4474780597656 | 0.999950574162623 |
| ebi-fl187-GCST90094784 | ebi-met1400-GCST90200100 | Perfluorooctanesulfonate (PFOS) levels \|\| id:ebi-met1400-GCST90200100 | Ice cream liking \|\| id:ebi-fl187-GCST90094784 | MR Egger | 8 | 0.588480488405175 | 0.567780034242157 | 0.339942299183258 | -0.524368379 | 1.7013293555198 | 1.80124931565767 | 0.591929121353497 | 5.48122905279333 | 0.999950574162623 |
| ebi-fl187-GCST90094784 | ebi-met1400-GCST90200102 | 1-palmitoleoyl-2-linolenoyl-GPC (16:1/18:3) levels \|\| id:ebi-met1400-GCST90200102 | Ice cream liking \|\| id:ebi-fl187-GCST90094784 | MR Egger | 8 | 0.0820051637144932 | 0.510793375421553 | 0.87772091855957 | -0.919149852 | 1.08316017954074 | 1.08546141482792 | 0.398857985261644 | 2.95399998650479 | 0.999950574162623 |
| ebi-fl187-GCST90094784 | ebi-met1400-GCST90200114 | Glycosyl-N-tricosanoyl-sphingadienine (d18:2/23:0) levels \|\| id:ebi-met1400-GCST90200114 | Ice cream liking \|\| id:ebi-fl187-GCST90094784 | MR Egger | 8 | 0.771404615406109 | 0.546526273323235 | 0.207800258208425 | -0.29978688 | 1.84259611111965 | 2.16280202628587 | 0.740976120458336 | 6.31290601107743 | 0.999950574162623 |
| ebi-fl187-GCST90094784 | ebi-met1400-GCST90200135 | Arachidoylcarnitine (C20) levels \|\| id:ebi-met1400-GCST90200135 | Ice cream liking \|\| id:ebi-fl187-GCST90094784 | MR Egger | 8 | 0.651663355157961 | 0.936217980202498 | 0.512438486332809 | -1.183323886 | 2.48665059635486 | 1.91872970511364 | 0.306259074654001 | 12.0209456175126 | 0.999950574162623 |
| ebi-fl187-GCST90094784 | ebi-met1400-GCST90200146 | Docosahexaenoylcarnitine (C22:6) levels \|\| id:ebi-met1400-GCST90200146 | Ice cream liking \|\| id:ebi-fl187-GCST90094784 | MR Egger | 8 | 0.8155405488001 | 0.955083692288571 | 0.425935391077739 | -1.056423488 | 2.6875045856857 | 2.26039719716575 | 0.347697132141484 | 14.6949601151029 | 0.999950574162623 |
| ebi-fl187-GCST90094784 | ebi-met1400-GCST90200162 | 3-hydroxyphenylacetoylglutamine levels \|\| id:ebi-met1400-GCST90200162 | Ice cream liking \|\| id:ebi-fl187-GCST90094784 | MR Egger | 8 | -0.123925724 | 0.643443751423772 | 0.853627712111963 | -1.385075476 | 1.1372240291151 | 0.883445457527325 | 0.250304906948902 | 3.11810058356145 | 0.999950574162623 |
| ebi-fl187-GCST90094784 | ebi-met1400-GCST90200172 | 3-carboxy-4-methyl-5-pentyl-2-furanpropionate (3-CMPFP) levels \|\| id:ebi-met1400-GCST90200172 | Ice cream liking \|\| id:ebi-fl187-GCST90094784 | MR Egger | 8 | -0.499031559 | 0.518506727534437 | 0.372996038346288 | -1.515304745 | 0.517241627165584 | 0.60711833350923 | 0.219741209658844 | 1.6773943833989 | 0.999950574162623 |
| ebi-fl187-GCST90094784 | ebi-met1400-GCST90200222 | Dihydrocaffeate sulfate (2) levels \|\| id:ebi-met1400-GCST90200222 | Ice cream liking \|\| id:ebi-fl187-GCST90094784 | MR Egger | 8 | 0.730226819507683 | 0.784865258233583 | 0.388073554671844 | -0.808109087 | 2.26856272564551 | 2.07555132981842 | 0.445700050096455 | 9.66549885237559 | 0.999950574162623 |
| ebi-fl187-GCST90094784 | ebi-met1400-GCST90200223 | 4-methylhexanoylglutamine levels \|\| id:ebi-met1400-GCST90200223 | Ice cream liking \|\| id:ebi-fl187-GCST90094784 | MR Egger | 8 | -0.938565324 | 0.720850262459583 | 0.240655501297572 | -2.351431838 | 0.474301190382486 | 0.391188661929887 | 0.095232706695723 | 1.60689089423275 | 0.999950574162623 |
| ebi-fl187-GCST90094784 | ebi-met1400-GCST90200224 | 2-ketocaprylate levels \|\| id:ebi-met1400-GCST90200224 | Ice cream liking \|\| id:ebi-fl187-GCST90094784 | MR Egger | 8 | -0.238865245 | 0.528492216604116 | 0.667166550964027 | -1.274709989 | 0.796979499922844 | 0.787520997913077 | 0.279512017981832 | 2.21882882400541 | 0.999950574162623 |
| ebi-fl187-GCST90094784 | ebi-met1400-GCST90200243 | Hydroxypalmitoyl sphingomyelin (d18:1/16:0(OH)) levels \|\| id:ebi-met1400-GCST90200243 | Ice cream liking \|\| id:ebi-fl187-GCST90094784 | MR Egger | 8 | 1.49479456204088 | 0.509866230534241 | 0.0262277154408954 | 0.495456750193763 | 2.49413237388799 | 4.45842052994352 | 1.64124770809263 | 12.111220947216 | 0.999950574162623 |
| ebi-fl187-GCST90094784 | ebi-met1400-GCST90200273 | 2-methoxyhydroquinone sulfate (2) levels \|\| id:ebi-met1400-GCST90200273 | Ice cream liking \|\| id:ebi-fl187-GCST90094784 | MR Egger | 8 | 0.00886497657500885 | 0.620132763238353 | 0.989057884220126 | -1.206595239 | 1.22432519252218 | 1.00890438685082 | 0.299214300159812 | 3.40186970095737 | 0.999950574162623 |
| ebi-fl187-GCST90094784 | ebi-met1400-GCST90200276 | 2,4-di-tert-butylphenol levels \|\| id:ebi-met1400-GCST90200276 | Ice cream liking \|\| id:ebi-fl187-GCST90094784 | MR Egger | 8 | 0.0513265530692914 | 0.519191297535723 | 0.92447059281785 | -0.96628839 | 1.06894149623931 | 1.05266658866823 | 0.380492660826845 | 2.91229519247595 | 0.999950574162623 |
| ebi-fl187-GCST90094784 | ebi-met1400-GCST90200289 | N-acetylmethionine levels \|\| id:ebi-met1400-GCST90200289 | Ice cream liking \|\| id:ebi-fl187-GCST90094784 | MR Egger | 8 | -0.944270431 | 0.60821196145877 | 0.171522122384845 | -2.136365875 | 0.247825013582625 | 0.388963242982206 | 0.118083193256319 | 1.28123571372964 | 0.999950574162623 |
| ebi-fl187-GCST90094784 | ebi-met1400-GCST90200308 | 3-hydroxyisobutyrate levels \|\| id:ebi-met1400-GCST90200308 | Ice cream liking \|\| id:ebi-fl187-GCST90094784 | MR Egger | 8 | -0.341861695 | 0.624135234483559 | 0.603636011928385 | -1.565166754 | 0.881443364784419 | 0.710446456351515 | 0.20905314973585 | 2.41438202667687 | 0.999950574162623 |
| ebi-fl187-GCST90094784 | ebi-met1400-GCST90200383 | Cystathionine levels \|\| id:ebi-met1400-GCST90200383 | Ice cream liking \|\| id:ebi-fl187-GCST90094784 | MR Egger | 8 | 0.482564096260252 | 0.852099918096998 | 0.591710284139013 | -1.187551743 | 2.15267993573037 | 1.62022348958036 | 0.30496698833192 | 8.60789612195935 | 0.999950574162623 |
| ebi-fl187-GCST90094784 | ebi-met1400-GCST90200384 | Guanidinoacetate levels \|\| id:ebi-met1400-GCST90200384 | Ice cream liking \|\| id:ebi-fl187-GCST90094784 | MR Egger | 8 | 0.286848263951783 | 0.654555980718735 | 0.67655470297909 | -0.996081458 | 1.5697779861605 | 1.33222205202895 | 0.369323820199645 | 4.80558117 | 0.999950574162623 |
| ebi-fl187-GCST90094784 | ebi-met1400-GCST90200403 | Citrulline levels \|\| id:ebi-met1400-GCST90200403 | Ice cream liking \|\| id:ebi-fl187-GCST90094784 | MR Egger | 8 | -0.661614068 | 0.581907825559827 | 0.298909040627433 | -1.802153406 | 0.478925270452524 | 0.516017774367737 | 0.164943315629978 | 1.61433849226587 | 0.999950574162623 |
| ebi-fl187-GCST90094784 | ebi-met1400-GCST90200434 | 12,13-DiHOME levels \|\| id:ebi-met1400-GCST90200434 | Ice cream liking \|\| id:ebi-fl187-GCST90094784 | MR Egger | 8 | -0.934464268 | 0.526287196631762 | 0.126146298928766 | -1.965987173 | 0.0970586377551205 | 0.392796242830464 | 0.14001759677219 | 1.10192498613412 | 0.999950574162623 |
| ebi-fl187-GCST90094784 | ebi-met1400-GCST90200437 | Fructose levels \|\| id:ebi-met1400-GCST90200437 | Ice cream liking \|\| id:ebi-fl187-GCST90094784 | MR Egger | 7 | 0.382762230370575 | 0.709042125382335 | 0.612478866726667 | -1.006960335 | 1.77248479611995 | 1.4663293400323 | 0.365327767435992 | 5.88545937400246 | 0.999950574162623 |
| ebi-fl187-GCST90094784 | ebi-met1400-GCST90200448 | N-stearoyl-sphinganine (d18:0/18:0) levels \|\| id:ebi-met1400-GCST90200448 | Ice cream liking \|\| id:ebi-fl187-GCST90094784 | MR Egger | 8 | 0.485569738791021 | 0.759412950762633 | 0.546183403678103 | -1.002879645 | 1.97401912228578 | 1.62510062800571 | 0.366821602917219 | 7.19955430689433 | 0.999950574162623 |
| ebi-fl187-GCST90094784 | ebi-met1400-GCST90200468 | X-11847 levels \|\| id:ebi-met1400-GCST90200468 | Ice cream liking \|\| id:ebi-fl187-GCST90094784 | MR Egger | 8 | -0.375635713 | 0.666436475788342 | 0.593418285961465 | -1.681851206 | 0.930579779441694 | 0.686852498838659 | 0.186029278633544 | 2.53597905999644 | 0.999950574162623 |
| ebi-fl187-GCST90094784 | ebi-met1400-GCST90200476 | X-11843 levels \|\| id:ebi-met1400-GCST90200476 | Ice cream liking \|\| id:ebi-fl187-GCST90094784 | MR Egger | 8 | -0.040853482 | 0.677994849477313 | 0.953908391341875 | -1.369723387 | 1.28801642271973 | 0.95996977224978 | 0.254177258504765 | 3.62558778489626 | 0.999950574162623 |
| ebi-fl187-GCST90094784 | ebi-met1400-GCST90200511 | X-12906 levels \|\| id:ebi-met1400-GCST90200511 | Ice cream liking \|\| id:ebi-fl187-GCST90094784 | MR Egger | 8 | 0.188246517770009 | 0.70441774381635 | 0.798234911930365 | -1.19241226 | 1.56890529565006 | 1.20713105792984 | 0.303488287667069 | 4.80138921412793 | 0.999950574162623 |
| ebi-fl187-GCST90094784 | ebi-met1400-GCST90200515 | X-12849 levels \|\| id:ebi-met1400-GCST90200515 | Ice cream liking \|\| id:ebi-fl187-GCST90094784 | MR Egger | 8 | 0.548897572112646 | 0.549426553032691 | 0.356347567750099 | -0.527978472 | 1.62577361605672 | 1.73134328426986 | 0.589796054705066 | 5.08234930375265 | 0.999950574162623 |
| ebi-fl187-GCST90094784 | ebi-met1400-GCST90200551 | X-18886 levels \|\| id:ebi-met1400-GCST90200551 | Ice cream liking \|\| id:ebi-fl187-GCST90094784 | MR Egger | 8 | 0.0841140886179702 | 0.650720580000294 | 0.901373902314661 | -1.191298248 | 1.35952642541855 | 1.08775298696486 | 0.303826565627223 | 3.89434860051935 | 0.999950574162623 |
| ebi-fl187-GCST90094784 | ebi-met1400-GCST90200564 | X-21319 levels \|\| id:ebi-met1400-GCST90200564 | Ice cream liking \|\| id:ebi-fl187-GCST90094784 | MR Egger | 8 | -0.484536892 | 0.527407353717249 | 0.393678288353141 | -1.518255305 | 0.549181521330533 | 0.615982397123415 | 0.219093805503862 | 1.73183496764458 | 0.999950574162623 |
| ebi-fl187-GCST90094784 | ebi-met1400-GCST90200579 | X-21442 levels \|\| id:ebi-met1400-GCST90200579 | Ice cream liking \|\| id:ebi-fl187-GCST90094784 | MR Egger | 7 | 0.0348322851977363 | 0.589399177242112 | 0.955163357587518 | -1.120390102 | 1.19005467259228 | 1.03544603460921 | 0.326152536981741 | 3.28726092554653 | 0.999950574162623 |
| ebi-fl187-GCST90094784 | ebi-met1400-GCST90200581 | X-21441 levels \|\| id:ebi-met1400-GCST90200581 | Ice cream liking \|\| id:ebi-fl187-GCST90094784 | MR Egger | 8 | 0.793090035307926 | 0.524029097916886 | 0.180932921032319 | -0.234006997 | 1.82018706722502 | 2.21021552921898 | 0.791356279065616 | 6.1730131103132 | 0.999950574162623 |
| ebi-fl187-GCST90094784 | ebi-met1400-GCST90200648 | X-24585 levels \|\| id:ebi-met1400-GCST90200648 | Ice cream liking \|\| id:ebi-fl187-GCST90094784 | MR Egger | 8 | 0.281115635619997 | 0.956357396610864 | 0.778703979992988 | -1.593344862 | 2.15557613297729 | 1.32460676680428 | 0.203244648257498 | 8.63286242322471 | 0.999950574162623 |
| ebi-fl187-GCST90094784 | ebi-met1400-GCST90200720 | Adenosine 5'-diphosphate (ADP) to pantothenate ratio \|\| id:ebi-met1400-GCST90200720 | Ice cream liking \|\| id:ebi-fl187-GCST90094784 | MR Egger | 8 | -0.65639923 | 0.692808530425364 | 0.379990463482688 | -2.01430395 | 0.701505489310659 | 0.518715751758574 | 0.133413233341882 | 2.01678667387484 | 0.999950574162623 |
| ebi-fl187-GCST90094784 | ebi-met1400-GCST90200724 | Cholate to taurocholate ratio \|\| id:ebi-met1400-GCST90200724 | Ice cream liking \|\| id:ebi-fl187-GCST90094784 | MR Egger | 8 | -0.486500241 | 0.558800437373995 | 0.417431398388574 | -1.581749099 | 0.608748615934794 | 0.614774194926043 | 0.205615142345806 | 1.83812975267808 | 0.999950574162623 |
| ebi-fl187-GCST90094784 | ebi-met1400-GCST90200726 | N-acetylputrescine to (N(1) + N(8))-acetylspermidine ratio \|\| id:ebi-met1400-GCST90200726 | Ice cream liking \|\| id:ebi-fl187-GCST90094784 | MR Egger | 8 | 0.325700915375433 | 0.519916175160534 | 0.55409087656147 | -0.693334788 | 1.34473661869008 | 1.38500107439496 | 0.499906205108947 | 3.83717576711643 | 0.999950574162623 |
| ebi-fl187-GCST90094784 | ebi-met1400-GCST90200744 | Arginine to glutamate ratio \|\| id:ebi-met1400-GCST90200744 | Ice cream liking \|\| id:ebi-fl187-GCST90094784 | MR Egger | 8 | 0.741338695747952 | 0.686895527281579 | 0.321935205990907 | -0.604976538 | 2.08765392921985 | 2.09874321336356 | 0.546087238938957 | 8.06596961356933 | 0.999950574162623 |
| ebi-fl187-GCST90094784 | ebi-met1400-GCST90200759 | Histidine to trans-urocanate ratio \|\| id:ebi-met1400-GCST90200759 | Ice cream liking \|\| id:ebi-fl187-GCST90094784 | MR Egger | 8 | 0.292719842176602 | 0.618554631521619 | 0.652773540360828 | -0.919647236 | 1.50508691995897 | 1.34006730749682 | 0.398659649211944 | 4.50454514815235 | 0.999950574162623 |
| ebi-fl187-GCST90094784 | ebi-met1400-GCST90200795 | Oleoyl-linoleoyl-glycerol (18:1 to 18:2) [2] to linoleoyl-arachidonoyl-glycerol (18:2 to 20:4) [2] ratio \|\| id:ebi-met1400-GCST90200795 | Ice cream liking \|\| id:ebi-fl187-GCST90094784 | MR Egger | 8 | 0.158375314940134 | 0.657887825352597 | 0.817776921349981 | -1.131084823 | 1.44783545263122 | 1.17160583337424 | 0.322683012606949 | 4.25389678157168 | 0.999950574162623 |
| ebi-fl187-GCST90094784 | ebi-met1400-GCST90200811 | Choline to taurocholate ratio \|\| id:ebi-met1400-GCST90200811 | Ice cream liking \|\| id:ebi-fl187-GCST90094784 | MR Egger | 8 | 0.189570858110516 | 0.546025822720093 | 0.740307504217812 | -0.880639754 | 1.2597814706419 | 1.2087307693335 | 0.414517637344396 | 3.52465116344297 | 0.999950574162623 |
| ebi-fl187-GCST90094784 | ebi-met1400-GCST90200831 | Cholate to phosphate ratio \|\| id:ebi-met1400-GCST90200831 | Ice cream liking \|\| id:ebi-fl187-GCST90094784 | MR Egger | 8 | -0.574788998 | 0.622630801852591 | 0.391535229395374 | -1.79514537 | 0.64556737345873 | 0.562823613089893 | 0.166103304186991 | 1.9070687425637 | 0.999950574162623 |
| ebi-fl187-GCST90094784 | ebi-met1400-GCST90200841 | Adenosine 5'-diphosphate (ADP) to oxalate (ethanedioate) ratio \|\| id:ebi-met1400-GCST90200841 | Ice cream liking \|\| id:ebi-fl187-GCST90094784 | MR Egger | 8 | -0.521062935 | 0.701858012896853 | 0.485879226498917 | -1.89670464 | 0.854578770450317 | 0.593888947108827 | 0.150062314657218 | 2.35038412078142 | 0.999950574162623 |
| ebi-fl187-GCST90094784 | ebi-met1400-GCST90200844 | Aspartate to phosphate ratio \|\| id:ebi-met1400-GCST90200844 | Ice cream liking \|\| id:ebi-fl187-GCST90094784 | MR Egger | 8 | -0.960989284 | 0.742407077834918 | 0.24310195457539 | -2.416107157 | 0.494128588211851 | 0.38251428334107 | 0.0892684500086124 | 1.63906931223535 | 0.999950574162623 |
| ebi-fl187-GCST90094784 | ebi-met1400-GCST90200870 | Adenosine 5'-monophosphate (AMP) to acetoacetate ratio \|\| id:ebi-met1400-GCST90200870 | Ice cream liking \|\| id:ebi-fl187-GCST90094784 | MR Egger | 8 | -0.380449755 | 0.802367197785129 | 0.652149412811723 | -1.953089463 | 1.19218995216979 | 0.683553907945181 | 0.141835199374285 | 3.29428764600335 | 0.999950574162623 |
| ebi-fl187-GCST90094784 | ebi-met1400-GCST90200887 | Cholesterol to cortisol ratio \|\| id:ebi-met1400-GCST90200887 | Ice cream liking \|\| id:ebi-fl187-GCST90094784 | MR Egger | 8 | 0.736789230032774 | 0.507527202859601 | 0.196781714315232 | -0.257964088 | 1.73154254763759 | 2.08921673968338 | 0.772622978375755 | 5.6493616000772 | 0.999950574162623 |
| ebi-fl187-GCST90094784 | ebi-met1400-GCST90200890 | Cortisol to taurocholate ratio \|\| id:ebi-met1400-GCST90200890 | Ice cream liking \|\| id:ebi-fl187-GCST90094784 | MR Egger | 8 | 0.17104756322578 | 0.569491816127961 | 0.774044038572741 | -0.945156396 | 1.28725152283658 | 1.18654718369119 | 0.388618787575202 | 3.62281563356752 | 0.999950574162623 |
| ebi-fl187-GCST90094784 | ebi-met1400-GCST90200948 | Adenosine 5'-diphosphate (ADP) to mannitol to sorbitol ratio \|\| id:ebi-met1400-GCST90200948 | Ice cream liking \|\| id:ebi-fl187-GCST90094784 | MR Egger | 8 | 0.514904836646502 | 0.699751626605837 | 0.48958385611829 | -0.856608352 | 1.88641802479394 | 1.6734792403232 | 0.424599735988915 | 6.59570068094869 | 0.999950574162623 |
| ebi-fl187-GCST90094784 | ebi-met1400-GCST90200972 | Leucine to N-palmitoyl-sphingosine (d18:1 to 16:0) ratio \|\| id:ebi-met1400-GCST90200972 | Ice cream liking \|\| id:ebi-fl187-GCST90094784 | MR Egger | 8 | -1.043928158 | 0.762483430834925 | 0.220000131395944 | -2.538395682 | 0.450539366889604 | 0.352068979692612 | 0.0789930281256708 | 1.56915830932066 | 0.999950574162623 |
| ebi-fl187-GCST90094784 | ebi-met1400-GCST90200973 | Proline to glutamate ratio \|\| id:ebi-met1400-GCST90200973 | Ice cream liking \|\| id:ebi-fl187-GCST90094784 | MR Egger | 8 | 0.696335489299465 | 0.564679478966468 | 0.263636868647939 | -0.410436289 | 1.80310726807374 | 2.00638679360366 | 0.663360769670461 | 6.06847457612993 | 0.999950574162623 |
| ebi-fl187-GCST90094784 | ebi-met1400-GCST90200983 | Cholesterol to linoleoyl-arachidonoyl-glycerol (18:2 to 20:4) [1] ratio \|\| id:ebi-met1400-GCST90200983 | Ice cream liking \|\| id:ebi-fl187-GCST90094784 | MR Egger | 8 | 1.1683537004955 | 0.53765436852683 | 0.0727545285109882 | 0.11455113818291 | 2.22215626280809 | 3.21669263753573 | 1.12136998442111 | 9.22720571098412 | 0.999950574162623 |
| ebi-fl187-GCST90094784 | ebi-met1400-GCST90201001 | Serine to alpha-ketobutyrate ratio \|\| id:ebi-met1400-GCST90201001 | Ice cream liking \|\| id:ebi-fl187-GCST90094784 | MR Egger | 8 | 0.637277625049445 | 0.53168236511956 | 0.275870179386916 | -0.404819811 | 1.67937506068378 | 1.89132496861033 | 0.66709700384834 | 5.36220387178069 | 0.999950574162623 |
| ebi-fl187-GCST90094784 | ebi-met1400-GCST90201005 | Fructose to maltose ratio \|\| id:ebi-met1400-GCST90201005 | Ice cream liking \|\| id:ebi-fl187-GCST90094784 | MR Egger | 8 | -0.383728592 | 0.767536931590329 | 0.634914472638651 | -1.888100977 | 1.12064379437202 | 0.681316317097042 | 0.151358970182833 | 3.06682797446336 | 0.999950574162623 |
| ebi-fl187-GCST90094784 | ebi-met1400-GCST90201020 | Paraxanthine to 5-acetylamino-6-formylamino-3-methyluracil ratio \|\| id:ebi-met1400-GCST90201020 | Ice cream liking \|\| id:ebi-fl187-GCST90094784 | MR Egger | 8 | -0.445344989 | 0.740467712607061 | 0.569559395565945 | -1.896661706 | 1.00597172721231 | 0.640603236517674 | 0.150068757555634 | 2.7345632316892 | 0.999950574162623 |
| ebi-fl187-GCST90094784 | ebi-pw11-GCST90275197 | Placental weight (fetal genotype, adjusted for sex) \|\| id:ebi-pw11-GCST90275197 | Ice cream liking \|\| id:ebi-fl187-GCST90094784 | MR Egger | 8 | -0.408428586 | 0.187884368604418 | 0.0726770791123216 | -0.776681949 | -0.040175224 | 0.664693939111056 | 0.459929552174946 | 0.960621100778744 | 0.999950574162623 |
| ebi-fl187-GCST90094784 | prot-a-1017 | FAS-associated factor 2 \|\| id:prot-a-1017 | Ice cream liking \|\| id:ebi-fl187-GCST90094784 | MR Egger | 9 | -0.299106158 | 0.794806502192534 | 0.717821586138237 | -1.856926902 | 1.25871458616322 | 0.741480691050118 | 0.15615176338404 | 3.52089277306462 | 0.999950574162623 |
| ebi-fl187-GCST90094784 | prot-a-1019 | Fas apoptotic inhibitory molecule 3 \|\| id:prot-a-1019 | Ice cream liking \|\| id:ebi-fl187-GCST90094784 | MR Egger | 9 | 0.722316642581721 | 0.794806502192534 | 0.393670707209884 | -0.835504102 | 2.28013738687909 | 2.0591981152604 | 0.433655819681038 | 9.77802368941061 | 0.999950574162623 |
| ebi-fl187-GCST90094784 | prot-a-1052 | MIP18 family protein FAM96A \|\| id:prot-a-1052 | Ice cream liking \|\| id:ebi-fl187-GCST90094784 | MR Egger | 9 | 0.24088589760747 | 0.796041975110031 | 0.770985363607294 | -1.319356374 | 1.80112816882313 | 1.27237584589694 | 0.26730729263931 | 6.05647633941087 | 0.999950574162623 |
| ebi-fl187-GCST90094784 | prot-a-1066 | F-box/LRR-repeat protein 4 \|\| id:prot-a-1066 | Ice cream liking \|\| id:ebi-fl187-GCST90094784 | MR Egger | 9 | 0.831329644955621 | 0.796041975110031 | 0.331050509497936 | -0.728912626 | 2.39157191617128 | 2.29637006810059 | 0.482433290274543 | 10.9306625309115 | 0.999950574162623 |
| ebi-fl187-GCST90094784 | prot-a-1076 | Low affinity immunoglobulin gamma Fc region receptor III-B \|\| id:prot-a-1076 | Ice cream liking \|\| id:ebi-fl187-GCST90094784 | MR Egger | 9 | -0.008942569 | 0.854118621394117 | 0.991938494891042 | -1.683015067 | 1.66512992871034 | 0.991097296627141 | 0.185812892263464 | 5.28636005508628 | 0.999950574162623 |
| ebi-fl187-GCST90094784 | prot-a-1081 | Fc receptor-like protein 3 \|\| id:prot-a-1081 | Ice cream liking \|\| id:ebi-fl187-GCST90094784 | MR Egger | 9 | 0.581649109846638 | 0.953184756636949 | 0.560998260671838 | -1.286593013 | 2.44989123285506 | 1.78898623427054 | 0.276210226451549 | 11.5870863563804 | 0.999950574162623 |
| ebi-fl187-GCST90094784 | prot-a-1105 | Fibroblast growth factor receptor 4 \|\| id:prot-a-1105 | Ice cream liking \|\| id:ebi-fl187-GCST90094784 | MR Egger | 9 | -1.180292052 | 0.827518927920483 | 0.196819589002432 | -2.802229151 | 0.441645046905841 | 0.307189010390077 | 0.0606746588149386 | 1.55526359682144 | 0.999950574162623 |
| ebi-fl187-GCST90094784 | prot-a-1143 | Follistatin-related protein 1 \|\| id:prot-a-1143 | Ice cream liking \|\| id:ebi-fl187-GCST90094784 | MR Egger | 9 | 1.92982913952068 | 0.794806502192534 | 0.0455507853204907 | 0.372008395223311 | 3.48764988381804 | 6.88833319711718 | 1.4506451597321 | 32.7089873882523 | 0.999950574162623 |
| ebi-fl187-GCST90094784 | prot-a-1151 | Alpha-(1,3)-fucosyltransferase 10 \|\| id:prot-a-1151 | Ice cream liking \|\| id:ebi-fl187-GCST90094784 | MR Egger | 9 | 0.873724643855564 | 1.04347571854462 | 0.430068402641649 | -1.171487764 | 2.91893705220302 | 2.39581782371736 | 0.309905531670328 | 18.5215895098892 | 0.999950574162623 |
| ebi-fl187-GCST90094784 | prot-a-1165 | Growth arrest and DNA damage-inducible proteins-interacting protein 1 \|\| id:prot-a-1165 | Ice cream liking \|\| id:ebi-fl187-GCST90094784 | MR Egger | 9 | -1.213187311 | 0.918953053445318 | 0.228303042247296 | -3.014335295 | 0.587960674178317 | 0.297248345165327 | 0.0490784473204584 | 1.80031324395004 | 0.999950574162623 |
| ebi-fl187-GCST90094784 | prot-a-1194 | Growth/differentiation factor 11/8 \|\| id:prot-a-1194 | Ice cream liking \|\| id:ebi-fl187-GCST90094784 | MR Egger | 9 | 0.604491229186546 | 0.794806502192534 | 0.471761097473256 | -0.953329515 | 2.16231197348391 | 1.83032075817738 | 0.385455504637668 | 8.69120829125042 | 0.999950574162623 |
| ebi-fl187-GCST90094784 | prot-a-1216 | D-glucuronyl C5-epimerase \|\| id:prot-a-1216 | Ice cream liking \|\| id:ebi-fl187-GCST90094784 | MR Egger | 9 | -0.523875751 | 1.50460403702341 | 0.737939731087267 | -3.472899664 | 2.42514816111373 | 0.592220793610025 | 0.0310269324173524 | 11.3039040942358 | 0.999950574162623 |
| ebi-fl187-GCST90094784 | prot-a-1246 | Glypican-3 \|\| id:prot-a-1246 | Ice cream liking \|\| id:ebi-fl187-GCST90094784 | MR Egger | 9 | -0.129921518 | 0.796041975110031 | 0.874965560661174 | -1.690163789 | 1.43032075349902 | 0.878164348559359 | 0.184489304212066 | 4.18003973929154 | 0.999950574162623 |
| ebi-fl187-GCST90094784 | prot-a-1275 | Glutamate receptor 4 \|\| id:prot-a-1275 | Ice cream liking \|\| id:ebi-fl187-GCST90094784 | MR Egger | 9 | -0.646920547 | 0.807980430835091 | 0.449642695912891 | -2.230562191 | 0.936721097599847 | 0.523655870110678 | 0.107467995580179 | 2.55160123552121 | 0.999950574162623 |
| ebi-fl187-GCST90094784 | prot-a-1289 | Glutathione S-transferase P \|\| id:prot-a-1289 | Ice cream liking \|\| id:ebi-fl187-GCST90094784 | MR Egger | 9 | -0.780460969 | 0.796041975110031 | 0.359537570083959 | -2.34070324 | 0.77978130257209 | 0.45819474920433 | 0.0962599206093996 | 2.18099523528922 | 0.999950574162623 |
| ebi-fl187-GCST90094784 | prot-a-130 | Apolipoprotein D \|\| id:prot-a-130 | Ice cream liking \|\| id:ebi-fl187-GCST90094784 | MR Egger | 9 | -0.584797585 | 0.794806502192534 | 0.485786192053161 | -2.142618329 | 0.973023159403207 | 0.557218639867763 | 0.117347186860091 | 2.64593145284574 | 0.999950574162623 |
| ebi-fl187-GCST90094784 | prot-a-1313 | Heparin-binding EGF-like growth factor \|\| id:prot-a-1313 | Ice cream liking \|\| id:ebi-fl187-GCST90094784 | MR Egger | 9 | 0.445297493361457 | 0.794806502192534 | 0.592772233840553 | -1.112523251 | 2.00311823765882 | 1.56095450036688 | 0.328728449353599 | 7.41213289268641 | 0.999950574162623 |
| ebi-fl187-GCST90094784 | prot-a-1347 | HLA class II histocompatibility antigen, DQ alpha 2 chain \|\| id:prot-a-1347 | Ice cream liking \|\| id:ebi-fl187-GCST90094784 | MR Egger | 9 | -0.360650719 | 0.794806502192534 | 0.663735518849464 | -1.918471463 | 1.19717002522095 | 0.697222482454857 | 0.14683122759695 | 3.31073435805431 | 0.999950574162623 |
| ebi-fl187-GCST90094784 | prot-a-1357 | Non-histone chromosomal protein HMG-14 \|\| id:prot-a-1357 | Ice cream liking \|\| id:ebi-fl187-GCST90094784 | MR Egger | 9 | -0.828999328 | 0.794806502192534 | 0.331615501967914 | -2.386820073 | 0.728821415928969 | 0.436485846862784 | 0.0919215233821815 | 2.07263639136395 | 0.999950574162623 |
| ebi-fl187-GCST90094784 | prot-a-137 | Apolipoprotein M \|\| id:prot-a-137 | Ice cream liking \|\| id:ebi-fl187-GCST90094784 | MR Egger | 9 | 0.168585425418412 | 0.796041975110031 | 0.838313160020153 | -1.391656846 | 1.72882769663407 | 1.18362933455736 | 0.2486629669443 | 5.63404522531303 | 0.999950574162623 |
| ebi-fl187-GCST90094784 | prot-a-1381 | 17-beta-hydroxysteroid dehydrogenase 14 \|\| id:prot-a-1381 | Ice cream liking \|\| id:ebi-fl187-GCST90094784 | MR Egger | 9 | 0.0984157505760169 | 0.796041975110031 | 0.905082208573365 | -1.461826521 | 1.65865802179168 | 1.10342143785771 | 0.231812477535674 | 5.25225769754608 | 0.999950574162623 |
| ebi-fl187-GCST90094784 | prot-a-1392 | Serine protease HTRA2, mitochondrial \|\| id:prot-a-1392 | Ice cream liking \|\| id:ebi-fl187-GCST90094784 | MR Egger | 9 | -0.255861311 | 0.794806502192534 | 0.756916149016012 | -1.813682055 | 1.3019594333768 | 0.774249341274776 | 0.163052661247001 | 3.67649345849269 | 0.999950574162623 |
| ebi-fl187-GCST90094784 | prot-a-1416 | Interferon-induced protein with tetratricopeptide repeats 2 \|\| id:prot-a-1416 | Ice cream liking \|\| id:ebi-fl187-GCST90094784 | MR Egger | 9 | 0.176887115887308 | 0.796041975110031 | 0.830495741756652 | -1.383355155 | 1.73712938710297 | 1.19349635874514 | 0.250735882372981 | 5.68101200696514 | 0.999950574162623 |
| ebi-fl187-GCST90094784 | prot-a-1428 | Interferon gamma \|\| id:prot-a-1428 | Ice cream liking \|\| id:ebi-fl187-GCST90094784 | MR Egger | 9 | -0.785291486 | 0.796041975110031 | 0.356753314330224 | -2.345533757 | 0.774950785633864 | 0.455986768843954 | 0.0957960566856728 | 2.17048530549637 | 0.999950574162623 |
| ebi-fl187-GCST90094784 | prot-a-1483 | Interleukin-17C \|\| id:prot-a-1483 | Ice cream liking \|\| id:ebi-fl187-GCST90094784 | MR Egger | 9 | -0.729056927 | 0.796041975110031 | 0.390204019154604 | -2.289299198 | 0.831185344321195 | 0.482363679867222 | 0.1013374544547 | 2.29603872435001 | 0.999950574162623 |
| ebi-fl187-GCST90094784 | prot-a-1503 | Interleukin-1 receptor-like 2 \|\| id:prot-a-1503 | Ice cream liking \|\| id:ebi-fl187-GCST90094784 | MR Egger | 9 | 0.473825893173824 | 0.794806502192534 | 0.569840571545591 | -1.083994851 | 2.03164663747119 | 1.60612732495165 | 0.33824159824755 | 7.62663432682918 | 0.999950574162623 |
| ebi-fl187-GCST90094784 | prot-a-1522 | Interleukin-31 receptor subunit alpha \|\| id:prot-a-1522 | Ice cream liking \|\| id:ebi-fl187-GCST90094784 | MR Egger | 9 | -1.034183469 | 0.804522563992714 | 0.239521877358009 | -2.611047695 | 0.542680756080754 | 0.355516552605368 | 0.0734575423501241 | 1.72061322953029 | 0.999950574162623 |
| ebi-fl187-GCST90094784 | prot-a-1540 | Interleukin-6 receptor subunit alpha \|\| id:prot-a-1540 | Ice cream liking \|\| id:ebi-fl187-GCST90094784 | MR Egger | 9 | -0.17027828 | 0.794806502192534 | 0.836468008941468 | -1.728099024 | 1.38754246465358 | 0.843430074515229 | 0.177621743919132 | 4.00499553095617 | 0.999950574162623 |
| ebi-fl187-GCST90094784 | prot-a-1541 | Interleukin-6 receptor subunit alpha \|\| id:prot-a-1541 | Ice cream liking \|\| id:ebi-fl187-GCST90094784 | MR Egger | 9 | -0.246850034 | 0.796041975110031 | 0.765515762194902 | -1.807092305 | 1.31339223704739 | 0.781257846729448 | 0.164130685548538 | 3.71876728008811 | 0.999950574162623 |
| ebi-fl187-GCST90094784 | prot-a-1545 | Interleukin-9 \|\| id:prot-a-1545 | Ice cream liking \|\| id:ebi-fl187-GCST90094784 | MR Egger | 9 | 1.79017039599347 | 0.920865347123463 | 0.0929844408191932 | -0.014725684 | 3.59506647635546 | 5.99047313204268 | 0.985382208274623 | 36.41812085 | 0.999950574162623 |
| ebi-fl187-GCST90094784 | prot-a-1548 | Inositol monophosphatase 3 \|\| id:prot-a-1548 | Ice cream liking \|\| id:ebi-fl187-GCST90094784 | MR Egger | 9 | 0.0276091946640118 | 0.796041975110031 | 0.973300731846885 | -1.532633077 | 1.58785146587967 | 1.02799386042304 | 0.215966262300277 | 4.89322436667512 | 0.999950574162623 |
| ebi-fl187-GCST90094784 | prot-a-1555 | Inhibin beta A chain:Inhibin beta B chain heterodimer \|\| id:prot-a-1555 | Ice cream liking \|\| id:ebi-fl187-GCST90094784 | MR Egger | 9 | -0.626490516 | 0.794806502192534 | 0.456420726502894 | -2.18431126 | 0.931330228349869 | 0.534464207106911 | 0.112555228224757 | 2.53788289699002 | 0.999950574162623 |
| ebi-fl187-GCST90094784 | prot-a-1603 | Junctophilin-3 \|\| id:prot-a-1603 | Ice cream liking \|\| id:ebi-fl187-GCST90094784 | MR Egger | 9 | 1.13290347632458 | 1.25559537310099 | 0.396889848912942 | -1.328063455 | 3.59387040760252 | 3.10465772658232 | 0.264989929672623 | 36.3745883141124 | 0.999950574162623 |
| ebi-fl187-GCST90094784 | prot-a-1627 | UPF0258 protein KIAA1024 \|\| id:prot-a-1627 | Ice cream liking \|\| id:ebi-fl187-GCST90094784 | MR Egger | 9 | 0.669532396848177 | 0.796041975110031 | 0.428097838904969 | -0.890709874 | 2.22977466806384 | 1.95332372672174 | 0.410364342204283 | 9.29777075873405 | 0.999950574162623 |
| ebi-fl187-GCST90094784 | prot-a-1666 | Kallikrein-7 \|\| id:prot-a-1666 | Ice cream liking \|\| id:ebi-fl187-GCST90094784 | MR Egger | 9 | -0.141405546 | 0.794806502192534 | 0.863831220307583 | -1.69922629 | 1.41641519843846 | 0.868137170863261 | 0.182824922787324 | 4.12231623535914 | 0.999950574162623 |
| ebi-fl187-GCST90094784 | prot-a-1678 | Importin subunit alpha-3 \|\| id:prot-a-1678 | Ice cream liking \|\| id:ebi-fl187-GCST90094784 | MR Egger | 9 | 1.00110722038837 | 0.794806502192534 | 0.248197918009655 | -0.556713524 | 2.55892796468574 | 2.7212932323571 | 0.57308941695542 | 12.9219570932133 | 0.999950574162623 |
| ebi-fl187-GCST90094784 | prot-a-1718 | Protein LDOC1 \|\| id:prot-a-1718 | Ice cream liking \|\| id:ebi-fl187-GCST90094784 | MR Egger | 9 | 1.34701596625422 | 1.01096180574489 | 0.224463018004571 | -0.634469173 | 3.32850110551421 | 3.8459319998122 | 0.530216867055076 | 27.896496445563 | 0.999950574162623 |
| ebi-fl187-GCST90094784 | prot-a-1723 | Leptin \|\| id:prot-a-1723 | Ice cream liking \|\| id:ebi-fl187-GCST90094784 | MR Egger | 9 | -1.505167693 | 0.796041975110031 | 0.100554903441373 | -3.065409965 | 0.0550745778338768 | 0.22198006612264 | 0.0466347193610149 | 1.05661941212417 | 0.999950574162623 |
| ebi-fl187-GCST90094784 | prot-a-1730 | Galectin-7 \|\| id:prot-a-1730 | Ice cream liking \|\| id:ebi-fl187-GCST90094784 | MR Egger | 9 | 0.688181351933086 | 1.00549755027745 | 0.515723418693267 | -1.282593847 | 2.6589565504769 | 1.99009296143265 | 0.277317048858661 | 14.2813794227353 | 0.999950574162623 |
| ebi-fl187-GCST90094784 | prot-a-1753 | Lipase member N \|\| id:prot-a-1753 | Ice cream liking \|\| id:ebi-fl187-GCST90094784 | MR Egger | 9 | 0.0650939757106048 | 0.796041975110031 | 0.937117006083891 | -1.495148296 | 1.62533624692627 | 1.06725931612426 | 0.224215352136105 | 5.0801269270919 | 0.999950574162623 |
| ebi-fl187-GCST90094784 | prot-a-1756 | VIP36-like protein \|\| id:prot-a-1756 | Ice cream liking \|\| id:ebi-fl187-GCST90094784 | MR Egger | 9 | -0.528155941 | 0.794806502192534 | 0.527640545250551 | -2.085976685 | 1.02966480329906 | 0.589691393377961 | 0.124185770499229 | 2.80012708401402 | 0.999950574162623 |
| ebi-fl187-GCST90094784 | prot-a-1760 | Ig Kappa chain V-I region HK102- like \|\| id:prot-a-1760 | Ice cream liking \|\| id:ebi-fl187-GCST90094784 | MR Egger | 9 | -0.959910914 | 0.794806502192534 | 0.266368310729762 | -2.517731658 | 0.597909830719918 | 0.382926998051983 | 0.0806423238189653 | 1.81831424112084 | 0.999950574162623 |
| ebi-fl187-GCST90094784 | prot-a-179 | Neutral ceramidase \|\| id:prot-a-179 | Ice cream liking \|\| id:ebi-fl187-GCST90094784 | MR Egger | 9 | -1.054995401 | 0.794806502192534 | 0.226041942933867 | -2.612816145 | 0.502825343590791 | 0.348194028749022 | 0.0733277511406937 | 1.6533860614906 | 0.999950574162623 |
| ebi-fl187-GCST90094784 | prot-a-1809 | Lactotransferrin \|\| id:prot-a-1809 | Ice cream liking \|\| id:ebi-fl187-GCST90094784 | MR Egger | 9 | 0.460173745339016 | 0.794806502192534 | 0.58074964191985 | -1.097646999 | 2.01799448963638 | 1.58434923437699 | 0.333655251917271 | 7.52322189459596 | 0.999950574162623 |
| ebi-fl187-GCST90094784 | prot-a-1818 | Lysophospholipase-like protein 1 \|\| id:prot-a-1818 | Ice cream liking \|\| id:ebi-fl187-GCST90094784 | MR Egger | 9 | 0.843470259759947 | 0.794806502192534 | 0.323822991055949 | -0.714350485 | 2.40129100405731 | 2.32441933569743 | 0.48950995284728 | 11.0374165361449 | 0.999950574162623 |
| ebi-fl187-GCST90094784 | prot-a-1852 | MAP kinase-activated protein kinase 3 \|\| id:prot-a-1852 | Ice cream liking \|\| id:ebi-fl187-GCST90094784 | MR Egger | 9 | 0.408317159361092 | 0.816787748635664 | 0.632469985401934 | -1.192586828 | 2.00922114668699 | 1.50428418333058 | 0.303435312991714 | 7.45750678096037 | 0.999950574162623 |
| ebi-fl187-GCST90094784 | prot-a-1856 | Megakaryocyte-associated tyrosine-protein kinase \|\| id:prot-a-1856 | Ice cream liking \|\| id:ebi-fl187-GCST90094784 | MR Egger | 9 | 0.346763819131618 | 0.934185480303707 | 0.721472825912915 | -1.484239722 | 2.17776736052688 | 1.41448261218475 | 0.226674610841605 | 8.82657767777572 | 0.999950574162623 |
| ebi-fl187-GCST90094784 | prot-a-1890 | Beta-1,3-N-acetylglucosaminyltransferase manic fringe \|\| id:prot-a-1890 | Ice cream liking \|\| id:ebi-fl187-GCST90094784 | MR Egger | 9 | -0.131900655 | 0.794806502192534 | 0.872885280888199 | -1.689721399 | 1.42592008970822 | 0.876428059860249 | 0.184570938499571 | 4.16168520545388 | 0.999950574162623 |
| ebi-fl187-GCST90094784 | prot-a-1919 | Matrilysin \|\| id:prot-a-1919 | Ice cream liking \|\| id:ebi-fl187-GCST90094784 | MR Egger | 9 | 0.971908685203707 | 1.11488388656791 | 0.412239824122676 | -1.213263732 | 3.15708110287681 | 2.64298427309084 | 0.297225629751526 | 23.5018960970665 | 0.999950574162623 |
| ebi-fl187-GCST90094784 | prot-a-1923 | Multimerin-2 \|\| id:prot-a-1923 | Ice cream liking \|\| id:ebi-fl187-GCST90094784 | MR Egger | 9 | -0.375392391 | 0.794806502192534 | 0.651077872296029 | -1.933213135 | 1.18242835376425 | 0.68701964588862 | 0.144682566221721 | 3.26228657787011 | 0.999950574162623 |
| ebi-fl187-GCST90094784 | prot-a-1948 | Mesothelin \|\| id:prot-a-1948 | Ice cream liking \|\| id:ebi-fl187-GCST90094784 | MR Egger | 9 | 0.503219023220769 | 0.794806502192534 | 0.546765647658195 | -1.054601721 | 2.06103976751814 | 1.65403709404422 | 0.348331132631152 | 7.85413203754951 | 0.999950574162623 |
| ebi-fl187-GCST90094784 | prot-a-195 | ATPase family AAA domain-containing protein 1 \|\| id:prot-a-195 | Ice cream liking \|\| id:ebi-fl187-GCST90094784 | MR Egger | 9 | 1.69038384597975 | 0.794806502192534 | 0.0710063288078536 | 0.132563101682382 | 3.24820459027712 | 5.42156135031029 | 1.14175106022887 | 25.7440772328132 | 0.999950574162623 |
| ebi-fl187-GCST90094784 | prot-a-1966 | Mucin-1 \|\| id:prot-a-1966 | Ice cream liking \|\| id:ebi-fl187-GCST90094784 | MR Egger | 9 | 0.619239007071427 | 0.796041975110031 | 0.462106959019489 | -0.941003264 | 2.17948127828709 | 1.85751394887739 | 0.390236128982903 | 8.84171867752727 | 0.999950574162623 |
| ebi-fl187-GCST90094784 | prot-a-1986 | Myocardial zonula adherens protein \|\| id:prot-a-1986 | Ice cream liking \|\| id:ebi-fl187-GCST90094784 | MR Egger | 9 | -0.58855774 | 0.794806502192534 | 0.483087115157744 | -2.146378485 | 0.969263004014178 | 0.555127345456199 | 0.116906771736592 | 2.63600102111784 | 0.999950574162623 |
| ebi-fl187-GCST90094784 | prot-a-2071 | Epididymal secretory protein E1 \|\| id:prot-a-2071 | Ice cream liking \|\| id:ebi-fl187-GCST90094784 | MR Egger | 9 | -1.201366851 | 0.794806502192534 | 0.174410754355416 | -2.759187595 | 0.356453893485555 | 0.300782805588722 | 0.0633432077937996 | 1.42825567710953 | 0.999950574162623 |
| ebi-fl187-GCST90094784 | prot-a-2098 | Neuritin \|\| id:prot-a-2098 | Ice cream liking \|\| id:ebi-fl187-GCST90094784 | MR Egger | 9 | -0.436642608 | 0.794806502192534 | 0.599830868164672 | -1.994463352 | 1.12117813654988 | 0.646202337855185 | 0.13608666520509 | 3.0684671479031 | 0.999950574162623 |
| ebi-fl187-GCST90094784 | prot-a-2115 | Neurotrophin-4 \|\| id:prot-a-2115 | Ice cream liking \|\| id:ebi-fl187-GCST90094784 | MR Egger | 9 | 1.29245295229157 | 0.98124089011236 | 0.229261139160275 | -0.630779192 | 3.2156850969118 | 3.64170854599413 | 0.53217697119834 | 24.9203589251591 | 0.999950574162623 |
| ebi-fl187-GCST90094784 | prot-a-2118 | Netrin-1 \|\| id:prot-a-2118 | Ice cream liking \|\| id:ebi-fl187-GCST90094784 | MR Egger | 9 | 1.27660144372205 | 0.864142335718936 | 0.183114649347515 | -0.417117534 | 2.97032042173116 | 3.58443709046671 | 0.658943466941915 | 19.4981662313772 | 0.999950574162623 |
| ebi-fl187-GCST90094784 | prot-a-2120 | Netrin-G1 \|\| id:prot-a-2120 | Ice cream liking \|\| id:ebi-fl187-GCST90094784 | MR Egger | 9 | -0.052770465 | 0.796041975110031 | 0.948999704238585 | -1.613012736 | 1.5074718066609 | 0.948597724296052 | 0.199286311747485 | 4.51530079838011 | 0.999950574162623 |
| ebi-fl187-GCST90094784 | prot-a-2145 | Olfactomedin-like protein 3 \|\| id:prot-a-2145 | Ice cream liking \|\| id:ebi-fl187-GCST90094784 | MR Egger | 9 | -0.210747406 | 0.796041975110031 | 0.798832733491439 | -1.770989678 | 1.34949486478173 | 0.809978636435931 | 0.170164497463201 | 3.85547750126013 | 0.999950574162623 |
| ebi-fl187-GCST90094784 | prot-a-2162 | Prolyl 4-hydroxylase subunit alpha-2 \|\| id:prot-a-2162 | Ice cream liking \|\| id:ebi-fl187-GCST90094784 | MR Egger | 9 | -0.227378408 | 1.09459189985541 | 0.841354880680119 | -2.372778532 | 1.91802171554379 | 0.796619278002479 | 0.0932213476118695 | 6.80747801166082 | 0.999950574162623 |
| ebi-fl187-GCST90094784 | prot-a-2168 | Polyadenylate-binding protein-interacting protein 1 \|\| id:prot-a-2168 | Ice cream liking \|\| id:ebi-fl187-GCST90094784 | MR Egger | 9 | 0.913257872266982 | 0.794806502192534 | 0.288285096781713 | -0.644562872 | 2.47107861656435 | 2.49242933716389 | 0.524891936912147 | 11.8352056183232 | 0.999950574162623 |
| ebi-fl187-GCST90094784 | prot-a-219 | Beta-1,4-galactosyltransferase 5 \|\| id:prot-a-219 | Ice cream liking \|\| id:ebi-fl187-GCST90094784 | MR Egger | 9 | 0.3512200253718 | 0.79472930066694 | 0.671865928851933 | -1.206449404 | 1.908889455 | 1.42079990355046 | 0.299257939389957 | 6.74559335015171 | 0.999950574162623 |
| ebi-fl187-GCST90094784 | prot-a-2190 | Pyruvate carboxylase, mitochondrial \|\| id:prot-a-2190 | Ice cream liking \|\| id:ebi-fl187-GCST90094784 | MR Egger | 9 | 0.294495070200847 | 0.796041975110031 | 0.722359484460153 | -1.265747201 | 1.85473734141651 | 1.34244834535272 | 0.282028485420827 | 6.39001963667311 | 0.999950574162623 |
| ebi-fl187-GCST90094784 | prot-a-2197 | Protocadherin-9 \|\| id:prot-a-2197 | Ice cream liking \|\| id:ebi-fl187-GCST90094784 | MR Egger | 9 | 0.931744709461683 | 0.794806502192534 | 0.279427203543401 | -0.626076035 | 2.48956545375905 | 2.53893501954222 | 0.534685778341071 | 12.0560360768486 | 0.999950574162623 |
| ebi-fl187-GCST90094784 | prot-a-2198 | Protocadherin alpha-4 \|\| id:prot-a-2198 | Ice cream liking \|\| id:ebi-fl187-GCST90094784 | MR Egger | 9 | -1.062477523 | 0.980514175219527 | 0.314451629268772 | -2.984285306 | 0.859330260614268 | 0.345598520571208 | 0.0505756367271778 | 2.36157852179497 | 0.999950574162623 |
| ebi-fl187-GCST90094784 | prot-a-2254 | Placenta growth factor \|\| id:prot-a-2254 | Ice cream liking \|\| id:ebi-fl187-GCST90094784 | MR Egger | 9 | 0.755240139791108 | 0.796041975110031 | 0.374346166837723 | -0.805002131 | 2.31548241100677 | 2.1281225088936 | 0.447086973626188 | 10.1298084713294 | 0.999950574162623 |
| ebi-fl187-GCST90094784 | prot-a-2261 | Membrane-associated progesterone receptor component 2 \|\| id:prot-a-2261 | Ice cream liking \|\| id:ebi-fl187-GCST90094784 | MR Egger | 9 | -0.732082583 | 0.981751242860141 | 0.4801398503086 | -2.656315019 | 1.19214985299989 | 0.48090641894818 | 0.0702064551122494 | 3.29415555045181 | 0.999950574162623 |
| ebi-fl187-GCST90094784 | prot-a-2268 | Polymeric immunoglobulin receptor \|\| id:prot-a-2268 | Ice cream liking \|\| id:ebi-fl187-GCST90094784 | MR Egger | 9 | -2.326687105 | 0.794806502192534 | 0.0221070828493146 | -3.884507849 | -0.768866361 | 0.0976186121868621 | 0.0205579438764521 | 0.463538255700968 | 0.999950574162623 |
| ebi-fl187-GCST90094784 | prot-a-2283 | Group 10 secretory phospholipase A2 \|\| id:prot-a-2283 | Ice cream liking \|\| id:ebi-fl187-GCST90094784 | MR Egger | 9 | 0.870122996506998 | 1.27612544534777 | 0.517252739045231 | -1.631082876 | 3.37132886938862 | 2.38720445327728 | 0.195717521454133 | 29.1171943084432 | 0.999950574162623 |
| ebi-fl187-GCST90094784 | prot-a-2298 | Inactive phospholipase D5 \|\| id:prot-a-2298 | Ice cream liking \|\| id:ebi-fl187-GCST90094784 | MR Egger | 9 | -1.294897081 | 1.0287073703013 | 0.248469985224268 | -3.311163527 | 0.721369364692143 | 0.273926055049446 | 0.036473710911816 | 2.05724840601957 | 0.999950574162623 |
| ebi-fl187-GCST90094784 | prot-a-2304 | Procollagen-lysine,2-oxoglutarate 5-dioxygenase 2 \|\| id:prot-a-2304 | Ice cream liking \|\| id:ebi-fl187-GCST90094784 | MR Egger | 9 | 0.332540846945707 | 1.07938064942955 | 0.766983118105712 | -1.783045226 | 2.44812691982762 | 1.39450685936756 | 0.168125387140173 | 11.5666611325144 | 0.999950574162623 |
| ebi-fl187-GCST90094784 | prot-a-2331 | Periostin \|\| id:prot-a-2331 | Ice cream liking \|\| id:ebi-fl187-GCST90094784 | MR Egger | 9 | 1.06034295119083 | 0.794806502192534 | 0.223940078461377 | -0.497477793 | 2.6181636954882 | 2.88736104339633 | 0.608062386377426 | 13.7105237582449 | 0.999950574162623 |
| ebi-fl187-GCST90094784 | prot-a-2333 | Periostin \|\| id:prot-a-2333 | Ice cream liking \|\| id:ebi-fl187-GCST90094784 | MR Egger | 9 | 1.39550173084735 | 0.794806502192534 | 0.12255576332675 | -0.162319013 | 2.95332247514471 | 4.03699955179833 | 0.850169945627455 | 19.1695383552895 | 0.999950574162623 |
| ebi-fl187-GCST90094784 | prot-a-2367 | cAMP-dependent protein kinase catalytic subunit alpha \|\| id:prot-a-2367 | Ice cream liking \|\| id:ebi-fl187-GCST90094784 | MR Egger | 9 | -0.663306269 | 0.796041975110031 | 0.432209347849104 | -2.22354854 | 0.896936002536367 | 0.515145306959423 | 0.108224388071513 | 2.45207842715603 | 0.999950574162623 |
| ebi-fl187-GCST90094784 | prot-a-2385 | Phosphoribosyl pyrophosphate synthase-associated protein 1 \|\| id:prot-a-2385 | Ice cream liking \|\| id:ebi-fl187-GCST90094784 | MR Egger | 9 | -1.449529227 | 0.794806502192534 | 0.110956982132565 | -3.007349972 | 0.108291516929958 | 0.23468074336347 | 0.049422476337966 | 1.1143725565067 | 0.999950574162623 |
| ebi-fl187-GCST90094784 | prot-a-2393 | Trypsin-3 \|\| id:prot-a-2393 | Ice cream liking \|\| id:ebi-fl187-GCST90094784 | MR Egger | 9 | 0.868031927204192 | 0.93771704660752 | 0.385410988260424 | -0.969893484 | 2.70595733855493 | 2.38221785879985 | 0.379123418607276 | 14.9686398894379 | 0.999950574162623 |
| ebi-fl187-GCST90094784 | prot-a-2405 | Pregnancy-specific beta-1-glycoprotein 6 \|\| id:prot-a-2405 | Ice cream liking \|\| id:ebi-fl187-GCST90094784 | MR Egger | 9 | -0.626299452 | 0.881525426987053 | 0.500390040211918 | -2.354089289 | 1.10149038447133 | 0.534566333477886 | 0.0949799664286836 | 3.00864672449169 | 0.999950574162623 |
| ebi-fl187-GCST90094784 | prot-a-244 | Beclin-1 \|\| id:prot-a-244 | Ice cream liking \|\| id:ebi-fl187-GCST90094784 | MR Egger | 9 | 0.0964222739355923 | 0.797080131414432 | 0.907114474774133 | -1.465854784 | 1.65869933150788 | 1.10122398401096 | 0.23088055418652 | 5.25247467130251 | 0.999950574162623 |
| ebi-fl187-GCST90094784 | prot-a-2454 | tRNA pseudouridine synthase A, mitochondrial \|\| id:prot-a-2454 | Ice cream liking \|\| id:ebi-fl187-GCST90094784 | MR Egger | 9 | -0.948828516 | 0.794806502192534 | 0.27144410423471 | -2.506649261 | 0.608992228066218 | 0.387194349766591 | 0.0815410046656266 | 1.83857759793302 | 0.999950574162623 |
| ebi-fl187-GCST90094784 | prot-a-2466 | Glutaminyl-peptide cyclotransferase-like protein \|\| id:prot-a-2466 | Ice cream liking \|\| id:ebi-fl187-GCST90094784 | MR Egger | 9 | 0.870385773486376 | 0.886636438517894 | 0.358965125609091 | -0.867421646 | 2.60819319298145 | 2.3878318380802 | 0.420033148420379 | 13.5745021753451 | 0.999950574162623 |
| ebi-fl187-GCST90094784 | prot-a-2474 | Ras-related protein Rab-27A \|\| id:prot-a-2474 | Ice cream liking \|\| id:ebi-fl187-GCST90094784 | MR Egger | 9 | 0.678225986445672 | 0.796041975110031 | 0.422403719230513 | -0.882016285 | 2.23846825766133 | 1.97037915056207 | 0.413947433777639 | 9.3789541380639 | 0.999950574162623 |
| ebi-fl187-GCST90094784 | prot-a-2490 | Rap1 GTPase-GDP dissociation stimulator 1 \|\| id:prot-a-2490 | Ice cream liking \|\| id:ebi-fl187-GCST90094784 | MR Egger | 9 | 1.12670598374081 | 0.794806502192534 | 0.199252023301011 | -0.431114761 | 2.68452672803818 | 3.08547613363201 | 0.649784336883617 | 14.6512657058982 | 0.999950574162623 |
| ebi-fl187-GCST90094784 | prot-a-2545 | Receptor-interacting serine/threonine-protein kinase 2 \|\| id:prot-a-2545 | Ice cream liking \|\| id:ebi-fl187-GCST90094784 | MR Egger | 9 | 1.43991010447665 | 0.796041975110031 | 0.11340780703855 | -0.120332167 | 3.00015237569231 | 4.22031641239083 | 0.886625880171584 | 20.0885977039702 | 0.999950574162623 |
| ebi-fl187-GCST90094784 | prot-a-2610 | RUN and FYVE domain-containing protein 1 \|\| id:prot-a-2610 | Ice cream liking \|\| id:ebi-fl187-GCST90094784 | MR Egger | 9 | 0.252899288443327 | 0.794806502192534 | 0.759623665510718 | -1.304921456 | 1.81072003274069 | 1.28775357863617 | 0.271193834897251 | 6.11484873879437 | 0.999950574162623 |
| ebi-fl187-GCST90094784 | prot-a-2627 | Serine--tRNA ligase, mitochondrial \|\| id:prot-a-2627 | Ice cream liking \|\| id:ebi-fl187-GCST90094784 | MR Egger | 9 | 0.562804139203105 | 0.794806502192534 | 0.501772691284134 | -0.995016605 | 2.12062488350047 | 1.75558851906516 | 0.369717305302383 | 8.33634510495151 | 0.999950574162623 |
| ebi-fl187-GCST90094784 | prot-a-264 | BolA-like protein 2 \|\| id:prot-a-264 | Ice cream liking \|\| id:ebi-fl187-GCST90094784 | MR Egger | 9 | -1.3071344 | 0.794806502192534 | 0.144051658731122 | -2.864955144 | 0.250686344742562 | 0.270594361753429 | 0.0569856872331159 | 1.28490700328336 | 0.999950574162623 |
| ebi-fl187-GCST90094784 | prot-a-2646 | Secretoglobin family 1C member 1 \|\| id:prot-a-2646 | Ice cream liking \|\| id:ebi-fl187-GCST90094784 | MR Egger | 9 | 0.605301100132958 | 1.25387338219734 | 0.644007802241965 | -1.852290729 | 3.06289292923975 | 1.83180368218952 | 0.156877390812397 | 21.3893456074611 | 0.999950574162623 |
| ebi-fl187-GCST90094784 | prot-a-2672 | Semaphorin-3E \|\| id:prot-a-2672 | Ice cream liking \|\| id:ebi-fl187-GCST90094784 | MR Egger | 9 | 0.180996276831386 | 0.854222967721872 | 0.838234692530161 | -1.49328074 | 1.85527329356626 | 1.19841071739972 | 0.224634478021575 | 6.39344529934795 | 0.999950574162623 |
| ebi-fl187-GCST90094784 | prot-a-2701 | Plasma protease C1 inhibitor \|\| id:prot-a-2701 | Ice cream liking \|\| id:ebi-fl187-GCST90094784 | MR Egger | 9 | 0.621080898108435 | 0.794806502192534 | 0.460160808152972 | -0.936739846 | 2.1789016424058 | 1.86093843997252 | 0.391903420356514 | 8.83659518515299 | 0.999950574162623 |
| ebi-fl187-GCST90094784 | prot-a-2738 | Signaling threshold-regulating transmembrane adapter 1 \|\| id:prot-a-2738 | Ice cream liking \|\| id:ebi-fl187-GCST90094784 | MR Egger | 9 | -0.138624427 | 0.796041975110031 | 0.866683073409715 | -1.698866699 | 1.42161784381779 | 0.87055492364594 | 0.182890676904964 | 4.14381907218814 | 0.999950574162623 |
| ebi-fl187-GCST90094784 | prot-a-274 | Serine/threonine-protein kinase BRSK2 \|\| id:prot-a-274 | Ice cream liking \|\| id:ebi-fl187-GCST90094784 | MR Egger | 9 | -0.189601915 | 0.825295570372026 | 0.824863986247839 | -1.807181233 | 1.42797740325266 | 0.827288399771313 | 0.164116090511627 | 4.17025591008514 | 0.999950574162623 |
| ebi-fl187-GCST90094784 | prot-a-2749 | Mitochondrial glutamate carrier 2 \|\| id:prot-a-2749 | Ice cream liking \|\| id:ebi-fl187-GCST90094784 | MR Egger | 9 | 1.38200465009798 | 0.954393735746781 | 0.190868053576217 | -0.488607072 | 3.25261637216167 | 3.98287790658496 | 0.613480333263192 | 25.8579053942664 | 0.999950574162623 |
| ebi-fl187-GCST90094784 | prot-a-2760 | Sodium-coupled monocarboxylate transporter 1 \|\| id:prot-a-2760 | Ice cream liking \|\| id:ebi-fl187-GCST90094784 | MR Egger | 9 | -0.631506704 | 0.796041975110031 | 0.453643957386674 | -2.191748975 | 0.928735567570432 | 0.5317899472286 | 0.111721180109539 | 2.53130648724013 | 0.999950574162623 |
| ebi-fl187-GCST90094784 | prot-a-2777 | Small integral membrane protein 10 \|\| id:prot-a-2777 | Ice cream liking \|\| id:ebi-fl187-GCST90094784 | MR Egger | 9 | -1.652710484 | 0.794806502192534 | 0.0761540038494027 | -3.210531229 | -0.09488974 | 0.19153006519927 | 0.0403351803801787 | 0.909473207494655 | 0.999950574162623 |
| ebi-fl187-GCST90094784 | prot-a-2799 | Superoxide dismutase [Mn], mitochondrial \|\| id:prot-a-2799 | Ice cream liking \|\| id:ebi-fl187-GCST90094784 | MR Egger | 9 | 0.76508134547227 | 1.10164318791704 | 0.509760251356269 | -1.394139303 | 2.92430199378967 | 2.14916919272777 | 0.248046437380161 | 18.621223782751 | 0.999950574162623 |
| ebi-fl187-GCST90094784 | prot-a-2805 | Protein SPACA7 \|\| id:prot-a-2805 | Ice cream liking \|\| id:ebi-fl187-GCST90094784 | MR Egger | 9 | -0.443001177 | 0.794806502192534 | 0.594640478309643 | -2.000821921 | 1.11481956714291 | 0.642106451228882 | 0.135224093964816 | 3.04901798652114 | 0.999950574162623 |
| ebi-fl187-GCST90094784 | prot-a-2811 | SPARC-like protein 1 \|\| id:prot-a-2811 | Ice cream liking \|\| id:ebi-fl187-GCST90094784 | MR Egger | 9 | 0.446269961222468 | 0.794806502192534 | 0.591982045820149 | -1.111550783 | 2.00409070551983 | 1.56247321678275 | 0.329048282694213 | 7.41934445964631 | 0.999950574162623 |
| ebi-fl187-GCST90094784 | prot-a-2831 | Sepiapterin reductase \|\| id:prot-a-2831 | Ice cream liking \|\| id:ebi-fl187-GCST90094784 | MR Egger | 9 | -0.429616934 | 1.14174310408083 | 0.717852960870335 | -2.667433418 | 1.80819954996209 | 0.65075833035035 | 0.069430195111223 | 6.0994557748538 | 0.999950574162623 |
| ebi-fl187-GCST90094784 | prot-a-2834 | SPRY domain-containing SOCS box protein 1 \|\| id:prot-a-2834 | Ice cream liking \|\| id:ebi-fl187-GCST90094784 | MR Egger | 9 | -0.379246379 | 0.846882818900251 | 0.667817744724772 | -2.039136704 | 1.28064394628349 | 0.684376975941486 | 0.13014101263757 | 3.59895651421727 | 0.999950574162623 |
| ebi-fl187-GCST90094784 | prot-a-2835 | Spectrin alpha chain, non-erythrocytic 1 \|\| id:prot-a-2835 | Ice cream liking \|\| id:ebi-fl187-GCST90094784 | MR Egger | 9 | 0.21141591593469 | 0.794803328171235 | 0.797905304265978 | -1.346398607 | 1.76923043915031 | 1.23542608161393 | 0.260175569829999 | 5.8663371204654 | 0.999950574162623 |
| ebi-fl187-GCST90094784 | prot-a-2859 | Sia-alpha-2,3-Gal-beta-1,4-GlcNAc-R:alpha 2,8-sialyltransferase \|\| id:prot-a-2859 | Ice cream liking \|\| id:ebi-fl187-GCST90094784 | MR Egger | 9 | 0.707590523548547 | 0.796041975110031 | 0.40357438993862 | -0.852651748 | 2.26783279476421 | 2.02909630411359 | 0.426283036813452 | 9.65844628053821 | 0.999950574162623 |
| ebi-fl187-GCST90094784 | prot-a-2861 | Alpha-2,8-sialyltransferase 8F \|\| id:prot-a-2861 | Ice cream liking \|\| id:ebi-fl187-GCST90094784 | MR Egger | 9 | 0.34736350652421 | 1.20600208815955 | 0.781665908905116 | -2.016400586 | 2.71112759931692 | 1.4153311139668 | 0.133133807342102 | 15.0462320739853 | 0.999950574162623 |
| ebi-fl187-GCST90094784 | prot-a-289 | Uncharacterized protein C17orf89 \|\| id:prot-a-289 | Ice cream liking \|\| id:ebi-fl187-GCST90094784 | MR Egger | 9 | 0.584430713126977 | 1.27846012237269 | 0.661421749446066 | -1.921351127 | 3.09021255297744 | 1.7939694116465 | 0.146409011304406 | 21.9817497656064 | 0.999950574162623 |
| ebi-fl187-GCST90094784 | prot-a-2921 | Tachykinin-3 \|\| id:prot-a-2921 | Ice cream liking \|\| id:ebi-fl187-GCST90094784 | MR Egger | 9 | -0.16534547 | 1.08556249736518 | 0.88323713949983 | -2.293047965 | 1.96235702455708 | 0.847600832605778 | 0.100958275120685 | 7.11608008927651 | 0.999950574162623 |
| ebi-fl187-GCST90094784 | prot-a-2928 | Tax1-binding protein 1 \|\| id:prot-a-2928 | Ice cream liking \|\| id:ebi-fl187-GCST90094784 | MR Egger | 9 | 0.822404316735131 | 1.17571260231697 | 0.506814638277415 | -1.481992384 | 3.12680101727639 | 2.27596540613501 | 0.227184598254412 | 22.8009229926866 | 0.999950574162623 |
| ebi-fl187-GCST90094784 | prot-a-296 | Uncharacterized protein C1orf185 \|\| id:prot-a-296 | Ice cream liking \|\| id:ebi-fl187-GCST90094784 | MR Egger | 9 | -0.0614725 | 0.971115216592126 | 0.951296532165984 | -1.964858325 | 1.84191332445827 | 0.940378805742428 | 0.140175744666181 | 6.30859711425456 | 0.999950574162623 |
| ebi-fl187-GCST90094784 | prot-a-2964 | Transforming growth factor beta-2 \|\| id:prot-a-2964 | Ice cream liking \|\| id:ebi-fl187-GCST90094784 | MR Egger | 9 | 1.29191546447206 | 0.907100086937811 | 0.197396537844491 | -0.486000706 | 3.06983163487017 | 3.63975169794655 | 0.615081373111413 | 21.5382760750668 | 0.999950574162623 |
| ebi-fl187-GCST90094784 | prot-a-2977 | Thymocyte nuclear protein 1 \|\| id:prot-a-2977 | Ice cream liking \|\| id:ebi-fl187-GCST90094784 | MR Egger | 9 | -0.138722469 | 0.796041975110031 | 0.866589868225459 | -1.69896474 | 1.42151980234577 | 0.870469577343559 | 0.182872746912737 | 4.14341282598133 | 0.999950574162623 |
| ebi-fl187-GCST90094784 | prot-a-3015 | Transmembrane protein 70, mitochondrial \|\| id:prot-a-3015 | Ice cream liking \|\| id:ebi-fl187-GCST90094784 | MR Egger | 9 | 0.824073823625832 | 0.794806502192534 | 0.334301516639035 | -0.733746921 | 2.3818945679232 | 2.27976831967542 | 0.480106693972323 | 10.8253928900548 | 0.999950574162623 |
| ebi-fl187-GCST90094784 | prot-a-3030 | Tumor necrosis factor alpha-induced protein 3 \|\| id:prot-a-3030 | Ice cream liking \|\| id:ebi-fl187-GCST90094784 | MR Egger | 9 | 0.850927295326077 | 0.794806502192534 | 0.319864806004681 | -0.706893449 | 2.40874803962344 | 2.34181740173126 | 0.493173890052182 | 11.1200305889493 | 0.999950574162623 |
| ebi-fl187-GCST90094784 | prot-a-3032 | Tumor necrosis factor receptor superfamily member 10A \|\| id:prot-a-3032 | Ice cream liking \|\| id:ebi-fl187-GCST90094784 | MR Egger | 9 | 1.10860366723719 | 0.794806502192534 | 0.205734716420134 | -0.449217077 | 2.66642441153455 | 3.03012437713188 | 0.638127560802694 | 14.3884300018939 | 0.999950574162623 |
| ebi-fl187-GCST90094784 | prot-a-3081 | Tumor protein p53-inducible protein 11 \|\| id:prot-a-3081 | Ice cream liking \|\| id:ebi-fl187-GCST90094784 | MR Egger | 9 | -1.239820178 | 0.79603891090998 | 0.163312948501814 | -2.800056443 | 0.320416087286822 | 0.289436260238927 | 0.0608066303905108 | 1.37770088891768 | 0.999950574162623 |
| ebi-fl187-GCST90094784 | prot-a-315 | Complement C4 \|\| id:prot-a-315 | Ice cream liking \|\| id:ebi-fl187-GCST90094784 | MR Egger | 9 | 0.0407821195418545 | 0.81766811154658 | 0.96161442141092 | -1.561847379 | 1.64341161817315 | 1.04162513105895 | 0.209748228573373 | 5.17278701724071 | 0.999950574162623 |
| ebi-fl187-GCST90094784 | prot-a-3157 | UDP-glucuronosyltransferase 1-8 \|\| id:prot-a-3157 | Ice cream liking \|\| id:ebi-fl187-GCST90094784 | MR Egger | 9 | -1.032522994 | 0.837439995140038 | 0.257389948997624 | -2.673905385 | 0.608859396385243 | 0.35610736942658 | 0.0689822961692495 | 1.83833339279953 | 0.999950574162623 |
| ebi-fl187-GCST90094784 | prot-a-317 | Glutaredoxin-like protein C5orf63 \|\| id:prot-a-317 | Ice cream liking \|\| id:ebi-fl187-GCST90094784 | MR Egger | 9 | -0.347758339 | 0.799599063804392 | 0.676716656661035 | -1.914972504 | 1.2194558257723 | 0.706269533193873 | 0.147345883902656 | 3.38534501477784 | 0.999950574162623 |
| ebi-fl187-GCST90094784 | prot-a-3192 | Guanine nucleotide exchange factor VAV3 \|\| id:prot-a-3192 | Ice cream liking \|\| id:ebi-fl187-GCST90094784 | MR Egger | 9 | 0.341005729197047 | 0.794806502192534 | 0.680787364879374 | -1.216815015 | 1.89882647349441 | 1.40636129816009 | 0.296171969565045 | 6.67805296992545 | 0.999950574162623 |
| ebi-fl187-GCST90094784 | prot-a-3233 | Serine/threonine-protein kinase WNK3 \|\| id:prot-a-3233 | Ice cream liking \|\| id:ebi-fl187-GCST90094784 | MR Egger | 9 | -0.767346983 | 0.796041975110031 | 0.367180568934727 | -2.327589255 | 0.792895287847722 | 0.464243080582373 | 0.0975305853197236 | 2.20978513726839 | 0.999950574162623 |
| ebi-fl187-GCST90094784 | prot-a-3250 | 14-3-3 protein theta \|\| id:prot-a-3250 | Ice cream liking \|\| id:ebi-fl187-GCST90094784 | MR Egger | 9 | -0.405788988 | 0.796041975110031 | 0.625895068834638 | -1.966031259 | 1.1544532832831 | 0.666450781746005 | 0.140011424077525 | 3.17228860013544 | 0.999950574162623 |
| ebi-fl187-GCST90094784 | prot-a-3278 | E3 ubiquitin-protein ligase ZNRF3 \|\| id:prot-a-3278 | Ice cream liking \|\| id:ebi-fl187-GCST90094784 | MR Egger | 9 | -0.109396455 | 0.796041975110031 | 0.89456383890319 | -1.669638727 | 1.45084581576612 | 0.896374974300802 | 0.188315086570251 | 4.2667218499938 | 0.999950574162623 |
| ebi-fl187-GCST90094784 | prot-a-355 | Calpain-3 \|\| id:prot-a-355 | Ice cream liking \|\| id:ebi-fl187-GCST90094784 | MR Egger | 9 | -0.321143038 | 0.867434952073155 | 0.722165461633596 | -2.021315544 | 1.37902946829817 | 0.725319495490137 | 0.132481065741844 | 3.97104573089121 | 0.999950574162623 |
| ebi-fl187-GCST90094784 | prot-a-385 | Coiled-coil domain-containing protein 90B, mitochondrial \|\| id:prot-a-385 | Ice cream liking \|\| id:ebi-fl187-GCST90094784 | MR Egger | 9 | 0.414165242063398 | 0.796229753895099 | 0.618994168303417 | -1.146445076 | 1.97477555969779 | 1.51310713517304 | 0.317764392285221 | 7.20500237942503 | 0.999950574162623 |
| ebi-fl187-GCST90094784 | prot-a-390 | C-C motif chemokine 14 \|\| id:prot-a-390 | Ice cream liking \|\| id:ebi-fl187-GCST90094784 | MR Egger | 9 | 0.936352799429766 | 0.794806502192534 | 0.277254828006688 | -0.621467945 | 2.49417354372713 | 2.55066165848826 | 0.537155344133001 | 12.1117195744984 | 0.999950574162623 |
| ebi-fl187-GCST90094784 | prot-a-40 | Pituitary adenylate cyclase-activating polypeptide 27 \|\| id:prot-a-40 | Ice cream liking \|\| id:ebi-fl187-GCST90094784 | MR Egger | 9 | 1.48560368643063 | 0.86650580285946 | 0.130159240411113 | -0.212747687 | 3.18395506003517 | 4.41763147214092 | 0.808360071101873 | 24.1420482298791 | 0.999950574162623 |
| ebi-fl187-GCST90094784 | prot-a-401 | C-C motif chemokine 24 \|\| id:prot-a-401 | Ice cream liking \|\| id:ebi-fl187-GCST90094784 | MR Egger | 9 | -0.14262855 | 1.04493489048449 | 0.895272550337449 | -2.190700936 | 1.90544383493822 | 0.867076084140331 | 0.111838329652511 | 6.72239059742835 | 0.999950574162623 |
| ebi-fl187-GCST90094784 | prot-a-412 | Cerebral cavernous malformations 2 protein \|\| id:prot-a-412 | Ice cream liking \|\| id:ebi-fl187-GCST90094784 | MR Egger | 9 | -1.001804797 | 0.794806502192534 | 0.247899574313195 | -2.559625542 | 0.556015946935121 | 0.367216092111768 | 0.0773336932685854 | 1.74371160365384 | 0.999950574162623 |
| ebi-fl187-GCST90094784 | prot-a-44 | Adhesion G protein-coupled receptor F5 \|\| id:prot-a-44 | Ice cream liking \|\| id:ebi-fl187-GCST90094784 | MR Egger | 9 | 0.317193785221973 | 0.794806502192534 | 0.701726825580351 | -1.240626959 | 1.87501452951934 | 1.37326866531528 | 0.289202842740693 | 6.52091386538593 | 0.999950574162623 |
| ebi-fl187-GCST90094784 | prot-a-48 | Adrenomedullin \|\| id:prot-a-48 | Ice cream liking \|\| id:ebi-fl187-GCST90094784 | MR Egger | 9 | -0.965709722 | 0.794806502192534 | 0.263744430842504 | -2.523530467 | 0.592111021993747 | 0.380712903394196 | 0.0801760476376896 | 1.80780069710874 | 0.999950574162623 |
| ebi-fl187-GCST90094784 | prot-a-502 | Carcinoembryonic antigen-related cell adhesion molecule 1 \|\| id:prot-a-502 | Ice cream liking \|\| id:ebi-fl187-GCST90094784 | MR Egger | 9 | -0.516743213 | 0.796041975110031 | 0.536956054917931 | -2.076985484 | 1.04349905843957 | 0.596459931260336 | 0.125307384533566 | 2.83913394987337 | 0.999950574162623 |
| ebi-fl187-GCST90094784 | prot-a-518 | Complement factor B \|\| id:prot-a-518 | Ice cream liking \|\| id:ebi-fl187-GCST90094784 | MR Egger | 9 | -1.072806584 | 0.794806502192534 | 0.219107523974051 | -2.630627328 | 0.485014160349221 | 0.342047184880904 | 0.0720332595634505 | 1.62419800789239 | 0.999950574162623 |
| ebi-fl187-GCST90094784 | prot-a-525 | Properdin \|\| id:prot-a-525 | Ice cream liking \|\| id:ebi-fl187-GCST90094784 | MR Egger | 9 | -1.030441884 | 0.862578181235092 | 0.271142860823837 | -2.721095119 | 0.660211351109359 | 0.356849239715341 | 0.065802653196258 | 1.93520129812423 | 0.999950574162623 |
| ebi-fl187-GCST90094784 | prot-a-533 | Chondroadherin \|\| id:prot-a-533 | Ice cream liking \|\| id:ebi-fl187-GCST90094784 | MR Egger | 9 | 0.276682270636453 | 0.878221220020982 | 0.761908652984503 | -1.444631321 | 1.99799586187758 | 1.31874729972263 | 0.235833007300622 | 7.37426223933478 | 0.999950574162623 |
| ebi-fl187-GCST90094784 | prot-a-535 | Coiled-coil-helix-coiled-coil-helix domain-containing protein 2 \|\| id:prot-a-535 | Ice cream liking \|\| id:ebi-fl187-GCST90094784 | MR Egger | 9 | 0.938295425624289 | 0.999241833629866 | 0.378988985780509 | -1.020218568 | 2.89680941953883 | 2.5556214565965 | 0.360516134166093 | 18.116251702642 | 0.999950574162623 |
| ebi-fl187-GCST90094784 | prot-a-591 | Caseinolytic peptidase B protein homolog \|\| id:prot-a-591 | Ice cream liking \|\| id:ebi-fl187-GCST90094784 | MR Egger | 9 | -1.318884239 | 0.922283046653927 | 0.195789944294033 | -3.12655901 | 0.488790532398132 | 0.267433527449106 | 0.0438684888501209 | 1.63034317977594 | 0.999950574162623 |
| ebi-fl187-GCST90094784 | prot-a-605 | Ciliary neurotrophic factor receptor subunit alpha \|\| id:prot-a-605 | Ice cream liking \|\| id:ebi-fl187-GCST90094784 | MR Egger | 9 | 0.30768720580812 | 0.794806502192534 | 0.710166185137325 | -1.250133538 | 1.86550795010549 | 1.36027543612962 | 0.286466539996894 | 6.45921601230526 | 0.999950574162623 |
| ebi-fl187-GCST90094784 | prot-a-606 | Ciliary neurotrophic factor receptor subunit alpha \|\| id:prot-a-606 | Ice cream liking \|\| id:ebi-fl187-GCST90094784 | MR Egger | 9 | 0.229336419202331 | 0.794806502192534 | 0.781287822699962 | -1.328484325 | 1.7871571634997 | 1.25776510417529 | 0.264878426789143 | 5.97244961191352 | 0.999950574162623 |
| ebi-fl187-GCST90094784 | prot-a-707 | Cancer/testis antigen 55 \|\| id:prot-a-707 | Ice cream liking \|\| id:ebi-fl187-GCST90094784 | MR Egger | 9 | -1.457210943 | 0.965394293757789 | 0.174927596489867 | -3.349383759 | 0.434961872742628 | 0.232884899036803 | 0.0351059811888087 | 1.54490415487009 | 0.999950574162623 |
| ebi-fl187-GCST90094784 | prot-a-719 | Dipeptidyl peptidase 1 \|\| id:prot-a-719 | Ice cream liking \|\| id:ebi-fl187-GCST90094784 | MR Egger | 9 | 1.48396120094868 | 1.11299535893527 | 0.22418455134421 | -0.697509703 | 3.66543210446181 | 4.41038153217378 | 0.497823489986031 | 39.0730161404906 | 0.999950574162623 |
| ebi-fl187-GCST90094784 | prot-a-752 | Cytochrome b5 \|\| id:prot-a-752 | Ice cream liking \|\| id:ebi-fl187-GCST90094784 | MR Egger | 9 | -0.97168766 | 0.796041975110031 | 0.261736807184182 | -2.531929931 | 0.588554610974053 | 0.37844381427976 | 0.0795054320918083 | 1.80138283383243 | 0.999950574162623 |
| ebi-fl187-GCST90094784 | prot-a-755 | Cytoglobin \|\| id:prot-a-755 | Ice cream liking \|\| id:ebi-fl187-GCST90094784 | MR Egger | 9 | 0.826349372278511 | 0.873323611182984 | 0.375549353659878 | -0.885364906 | 2.53806365019716 | 2.28496195034221 | 0.412563599023824 | 12.6551424480136 | 0.999950574162623 |
| ebi-fl187-GCST90094784 | prot-a-807 | Desmin \|\| id:prot-a-807 | Ice cream liking \|\| id:ebi-fl187-GCST90094784 | MR Egger | 9 | -1.184395483 | 0.794806502192534 | 0.179797121541186 | -2.742216228 | 0.373425260799238 | 0.305931063986834 | 0.0644274027525157 | 1.45270198570067 | 0.999950574162623 |
| ebi-fl187-GCST90094784 | prot-a-82 | Ameloblastin \|\| id:prot-a-82 | Ice cream liking \|\| id:ebi-fl187-GCST90094784 | MR Egger | 9 | 0.768079844914823 | 1.25993956181391 | 0.561373903309924 | -1.701401696 | 3.23756138607009 | 2.15562314660704 | 0.182427636623998 | 25.4715307185905 | 0.999950574162623 |
| ebi-fl187-GCST90094784 | prot-a-84 | Muellerian-inhibiting factor \|\| id:prot-a-84 | Ice cream liking \|\| id:ebi-fl187-GCST90094784 | MR Egger | 9 | -0.909817661 | 0.794806502192534 | 0.289958822712534 | -2.467638406 | 0.648003083063476 | 0.402597626495829 | 0.0847848501928197 | 1.91171946982814 | 0.999950574162623 |
| ebi-fl187-GCST90094784 | prot-a-858 | Inactive dipeptidyl peptidase 10 \|\| id:prot-a-858 | Ice cream liking \|\| id:ebi-fl187-GCST90094784 | MR Egger | 9 | 0.26759346316205 | 0.796041975110031 | 0.74660566014093 | -1.292648808 | 1.82783573437771 | 1.30681576326196 | 0.274542608445762 | 6.22040946131438 | 0.999950574162623 |
| ebi-fl187-GCST90094784 | prot-a-863 | Dihydropyrimidinase-related protein 3 \|\| id:prot-a-863 | Ice cream liking \|\| id:ebi-fl187-GCST90094784 | MR Egger | 9 | 0.695967021896114 | 0.794806502192534 | 0.410262255758858 | -0.861853722 | 2.25378776619348 | 2.00564764165676 | 0.422378383890479 | 9.52374130851922 | 0.999950574162623 |
| ebi-fl187-GCST90094784 | prot-a-864 | Dihydropyrimidinase-related protein 5 \|\| id:prot-a-864 | Ice cream liking \|\| id:ebi-fl187-GCST90094784 | MR Egger | 9 | -0.515922077 | 0.796041975110031 | 0.537584937149599 | -2.076164348 | 1.04432019406874 | 0.596949906901963 | 0.125410321148282 | 2.84146622134015 | 0.999950574162623 |
| ebi-fl187-GCST90094784 | prot-a-884 | Endothelin-converting enzyme 1 \|\| id:prot-a-884 | Ice cream liking \|\| id:ebi-fl187-GCST90094784 | MR Egger | 9 | -0.982908723 | 0.794806502192534 | 0.256090319402785 | -2.540729467 | 0.574912021422614 | 0.374221009026201 | 0.0788088903192303 | 1.77697418437595 | 0.999950574162623 |
| ebi-fl187-GCST90094784 | prot-a-893 | Early endosome antigen 1 \|\| id:prot-a-893 | Ice cream liking \|\| id:ebi-fl187-GCST90094784 | MR Egger | 9 | -1.854712984 | 0.987757985606342 | 0.102511747878765 | -3.790718636 | 0.0812926675788628 | 0.156497853584724 | 0.0225793696714481 | 1.0846883032167 | 0.999950574162623 |
| ebi-fl187-GCST90094784 | prot-a-897 | Ephrin-A2 \|\| id:prot-a-897 | Ice cream liking \|\| id:ebi-fl187-GCST90094784 | MR Egger | 9 | -1.206272698 | 0.878383927392328 | 0.212032487466763 | -2.927905195 | 0.515359799940548 | 0.299310824791804 | 0.0535090117447909 | 1.67424078517524 | 0.999950574162623 |
| ebi-fl187-GCST90094784 | prot-a-90 | Anaphase-promoting complex subunit 10 \|\| id:prot-a-90 | Ice cream liking \|\| id:ebi-fl187-GCST90094784 | MR Egger | 9 | 0.246654584575655 | 0.794806502192534 | 0.765343620062547 | -1.31116616 | 1.80447532887302 | 1.27973699552825 | 0.269505586499516 | 6.07678229974875 | 0.999950574162623 |
| ebi-fl187-GCST90094784 | prot-a-914 | Histone-lysine N-methyltransferase EHMT2 \|\| id:prot-a-914 | Ice cream liking \|\| id:ebi-fl187-GCST90094784 | MR Egger | 9 | 1.96563635878189 | 1.0100421175938 | 0.0926994039994229 | -0.014046192 | 3.94531890926573 | 7.13945439940004 | 0.98605199579099 | 51.6928208032519 | 0.999950574162623 |
| ebi-fl187-GCST90094784 | prot-a-932 | RNA polymerase II elongation factor ELL \|\| id:prot-a-932 | Ice cream liking \|\| id:ebi-fl187-GCST90094784 | MR Egger | 9 | -0.489221483 | 0.968399453407248 | 0.628942168048194 | -2.387284412 | 1.40884144584402 | 0.61310352004976 | 0.0918788505561263 | 4.09121276574724 | 0.999950574162623 |
| ebi-fl187-GCST90094784 | prot-a-991 | Estrogen receptor \|\| id:prot-a-991 | Ice cream liking \|\| id:ebi-fl187-GCST90094784 | MR Egger | 9 | 0.445394294898412 | 1.2747729439466 | 0.737070621362237 | -2.053160675 | 2.94394926503375 | 1.56110561047536 | 0.128328656711323 | 18.9906977093964 | 0.999950574162623 |
| ebi-fl187-GCST90094784 | prot-a-992 | Persulfide dioxygenase ETHE1, mitochondrial \|\| id:prot-a-992 | Ice cream liking \|\| id:ebi-fl187-GCST90094784 | MR Egger | 9 | -0.701269656 | 0.794806502192534 | 0.406882874012922 | -2.2590904 | 0.856551088172239 | 0.495955211303839 | 0.104445445093005 | 2.35502439958398 | 0.999950574162623 |
| ebi-fl187-GCST90094784 | prot-a-993 | Ethanolamine kinase 1 \|\| id:prot-a-993 | Ice cream liking \|\| id:ebi-fl187-GCST90094784 | MR Egger | 9 | 0.351469412776176 | 0.796041975110031 | 0.672152517964662 | -1.208772858 | 1.91171168399184 | 1.42115427733689 | 0.298563434320702 | 6.76465785097948 | 0.999950574162623 |
| ebi-fl187-GCST90094784 | prot-b-10 | coagulation factor III, tissue factor \|\| id:prot-b-10 | Ice cream liking \|\| id:ebi-fl187-GCST90094784 | MR Egger | 7 | -1.678255765 | 1.40108258334129 | 0.284663789856246 | -4.424377628 | 1.06786609825239 | 0.186699339706357 | 0.0119816660288699 | 2.90916499949193 | 0.999950574162623 |
| ebi-fl187-GCST90094784 | prot-b-28 | prolactin \|\| id:prot-b-28 | Ice cream liking \|\| id:ebi-fl187-GCST90094784 | MR Egger | 8 | -0.18270728 | 0.917640813996252 | 0.84875607341667 | -1.981283275 | 1.61586871571917 | 0.833011959551663 | 0.137892170127463 | 5.03225762648215 | 0.999950574162623 |
| ebi-fl187-GCST90094784 | prot-b-54 | matrix metallopeptidase 7 \|\| id:prot-b-54 | Ice cream liking \|\| id:ebi-fl187-GCST90094784 | MR Egger | 8 | 0.227955778378157 | 0.955688148724339 | 0.819411455538245 | -1.645192993 | 2.10110454987786 | 1.25602978053023 | 0.192975316291045 | 8.17519483786969 | 0.999950574162623 |
| ebi-fl187-GCST90094784 | ukb-rsfMRI191-edge-pheno716 | Net100_Pair10_33 (OccipitalPrecuneus)&(Temporal\|Frontal\|Supp_Motor_Area) (Default_mode\|Central_executive)&(Default_mode\|Salience) \|\| id:ukb-rsfMRI191-edge-pheno716 | Ice cream liking \|\| id:ebi-fl187-GCST90094784 | MR Egger | 8 | -0.281011705 | 0.299685276099555 | 0.384596932375835 | -0.868394846 | 0.306371436380823 | 0.755019498095668 | 0.419624571039615 | 1.35848680426966 | 0.999950574162623 |
| ebi-fl187-GCST90094784 | ukb-rsfMRI191-edge-pheno903 | Net100_Pair27_38 (Precentral\|Frontal\|Supp_Motor_Area)&(Subcortical) (Attention\|Salience\|Motor)&(Subcortical-cerebellum) \|\| id:ukb-rsfMRI191-edge-pheno903 | Ice cream liking \|\| id:ebi-fl187-GCST90094784 | MR Egger | 8 | 0.189219222568552 | 0.280893037731875 | 0.525628655699354 | -0.361331131 | 0.739769576523026 | 1.20830581135388 | 0.69674824505211 | 2.09545261738315 | 0.999950574162623 |
| ebi-fl187-GCST90094784 | ukb-rsfMRI191-node-pheno23 | Net100_Node2 RolandicOper\|SupraMarginal\|Insula Salience\|Motor \|\| id:ukb-rsfMRI191-node-pheno23 | Ice cream liking \|\| id:ebi-fl187-GCST90094784 | MR Egger | 8 | 0.054104353805321 | 0.278951286688966 | 0.852610040518809 | -0.492640168 | 0.600848875715695 | 1.05559475173694 | 0.611011090792712 | 1.82366620947735 | 0.999950574162623 |
| ebi-fl187-GCST90094784 | ukb-rsfMRI191-node-pheno55 | Net100_Node34 Parietal\|Postcentral\|Precuneus Attention \|\| id:ukb-rsfMRI191-node-pheno55 | Ice cream liking \|\| id:ebi-fl187-GCST90094784 | MR Egger | 8 | -0.029452953 | 0.248762634024927 | 0.909616805195896 | -0.517027716 | 0.458121809862374 | 0.970976558269232 | 0.596290260913025 | 1.58110158509847 | 0.999950574162623 |
| ebi-fl187-GCST90094784 | ebi-a-GCST90001414 | IgD- CD24- B cell Absolute Count \|\| id:ebi-a-GCST90001414 | Ice cream liking \|\| id:ebi-fl187-GCST90094784 | Weighted median | 8 | 0.252614752030126 | 0.264400667134023 | 0.339363304623659 | -0.265610556 | 0.77084005961281 | 1.28738721797576 | 0.766737671092825 | 2.16158134847496 | 0.999332001504091 |
| ebi-fl187-GCST90094784 | ebi-a-GCST90001422 | CD20- CD38- B cell %B cell \|\| id:ebi-a-GCST90001422 | Ice cream liking \|\| id:ebi-fl187-GCST90094784 | Weighted median | 8 | 0.164971924948713 | 0.235771064391057 | 0.48410696375067 | -0.297139361 | 0.627083211155184 | 1.1793600076539 | 0.742940468029009 | 1.87214196494555 | 0.999332001504091 |
| ebi-fl187-GCST90094784 | ebi-a-GCST90001444 | CD20- CD38- B cell %lymphocyte \|\| id:ebi-a-GCST90001444 | Ice cream liking \|\| id:ebi-fl187-GCST90094784 | Weighted median | 8 | 0.276603651192924 | 0.25537485764429 | 0.278751625555652 | -0.22393107 | 0.777138372175733 | 1.31864362461927 | 0.799370233228492 | 2.17523862719568 | 0.999332001504091 |
| ebi-fl187-GCST90094784 | ebi-a-GCST90001459 | Myeloid Dendritic Cell %Dendritic Cell \|\| id:ebi-a-GCST90001459 | Ice cream liking \|\| id:ebi-fl187-GCST90094784 | Weighted median | 8 | -0.348417294 | 0.248708504038252 | 0.161242113884181 | -0.835885962 | 0.139051373777466 | 0.70580428676234 | 0.433490255336904 | 1.14918313636585 | 0.999332001504091 |
| ebi-fl187-GCST90094784 | ebi-a-GCST90001476 | HLA DR++ monocyte %leukocyte \|\| id:ebi-a-GCST90001476 | Ice cream liking \|\| id:ebi-fl187-GCST90094784 | Weighted median | 8 | -0.22337638 | 0.25756550275284 | 0.385799308353664 | -0.728204765 | 0.281452005415313 | 0.799813758749157 | 0.482774906824205 | 1.32505239945578 | 0.999332001504091 |
| ebi-fl187-GCST90094784 | ebi-a-GCST90001478 | CD4 regulatory T cell %CD4+ T cell \|\| id:ebi-a-GCST90001478 | Ice cream liking \|\| id:ebi-fl187-GCST90094784 | Weighted median | 8 | 0.0503251215900454 | 0.242003833252999 | 0.835266638536024 | -0.424002392 | 0.524652634765923 | 1.05161294287427 | 0.654422316799846 | 1.6898717437213 | 0.999332001504091 |
| ebi-fl187-GCST90094784 | ebi-a-GCST90001485 | CD39+ resting CD4 regulatory T cell %CD4 regulatory T cell \|\| id:ebi-a-GCST90001485 | Ice cream liking \|\| id:ebi-fl187-GCST90094784 | Weighted median | 8 | 0.00665993483165541 | 0.231844824940154 | 0.977083256178497 | -0.447755922 | 0.461075791714358 | 1.00668216151299 | 0.639060645609388 | 1.5857790356377 | 0.999332001504091 |
| ebi-fl187-GCST90094784 | ebi-a-GCST90001515 | Immature Myeloid-Derived Suppressor Cells Absolute Count \|\| id:ebi-a-GCST90001515 | Ice cream liking \|\| id:ebi-fl187-GCST90094784 | Weighted median | 8 | -0.049447575 | 0.301905900676588 | 0.869900616980222 | -0.64118314 | 0.54228799041103 | 0.951755052668044 | 0.526668932069239 | 1.71993756442057 | 0.999332001504091 |
| ebi-fl187-GCST90094784 | ebi-a-GCST90001517 | CD33+ HLA DR+ Absolute Count \|\| id:ebi-a-GCST90001517 | Ice cream liking \|\| id:ebi-fl187-GCST90094784 | Weighted median | 8 | -0.28904291 | 0.315029417458575 | 0.358875008142152 | -0.906500568 | 0.328414748515149 | 0.748980066200447 | 0.403935297753521 | 1.38876484101651 | 0.999332001504091 |
| ebi-fl187-GCST90094784 | ebi-a-GCST90001518 | CD33+ HLA DR+ CD14- Absolute Count \|\| id:ebi-a-GCST90001518 | Ice cream liking \|\| id:ebi-fl187-GCST90094784 | Weighted median | 8 | -0.349163189 | 0.330649156190785 | 0.290971420317389 | -0.997235535 | 0.29890915706895 | 0.705278027216612 | 0.368897837950052 | 1.34838712647024 | 0.999332001504091 |
| ebi-fl187-GCST90094784 | ebi-a-GCST90001520 | CD33+ HLA DR+ CD14dim Absolute Count \|\| id:ebi-a-GCST90001520 | Ice cream liking \|\| id:ebi-fl187-GCST90094784 | Weighted median | 7 | -0.247618756 | 0.295370994880563 | 0.401844649519738 | -0.826545906 | 0.331308393511629 | 0.780657507188307 | 0.437558045559649 | 1.39278925325208 | 0.999332001504091 |
| ebi-fl187-GCST90094784 | ebi-a-GCST90001531 | CD33dim HLA DR- Absolute Count \|\| id:ebi-a-GCST90001531 | Ice cream liking \|\| id:ebi-fl187-GCST90094784 | Weighted median | 8 | -0.072331742 | 0.320973242311843 | 0.821705983607507 | -0.701439297 | 0.556775813143177 | 0.930222251026025 | 0.495871084303017 | 1.7450370947122 | 0.999332001504091 |
| ebi-fl187-GCST90094784 | ebi-a-GCST90001532 | Basophil Absolute Count \|\| id:ebi-a-GCST90001532 | Ice cream liking \|\| id:ebi-fl187-GCST90094784 | Weighted median | 8 | 0.064832113846004 | 0.320739484005189 | 0.839812555709715 | -0.563817275 | 0.693481502496175 | 1.06697987819839 | 0.569032758302322 | 2.00066875565607 | 0.999332001504091 |
| ebi-fl187-GCST90094784 | ebi-a-GCST90001588 | T/B cell \|\| id:ebi-a-GCST90001588 | Ice cream liking \|\| id:ebi-fl187-GCST90094784 | Weighted median | 8 | -0.013034558 | 0.235266471409403 | 0.955817102925788 | -0.474156842 | 0.448087726186853 | 0.987050024177676 | 0.622409625104504 | 1.56531600883513 | 0.999332001504091 |
| ebi-fl187-GCST90094784 | ebi-a-GCST90001624 | HLA DR+ CD4+ T cell Absolute Count \|\| id:ebi-a-GCST90001624 | Ice cream liking \|\| id:ebi-fl187-GCST90094784 | Weighted median | 8 | 0.105835207350773 | 0.217525883 | 0.626583817201215 | -0.320515524 | 0.532185938228732 | 1.11163867152986 | 0.725774786636577 | 1.70265013168536 | 0.999332001504091 |
| ebi-fl187-GCST90094784 | ebi-a-GCST90001631 | CD8+ Natural Killer T %T cell \|\| id:ebi-a-GCST90001631 | Ice cream liking \|\| id:ebi-fl187-GCST90094784 | Weighted median | 8 | -0.164213285 | 0.217471841045906 | 0.450188162735311 | -0.590458093 | 0.262031523887868 | 0.848561017613122 | 0.554073409432653 | 1.2995675092041 | 0.999332001504091 |
| ebi-fl187-GCST90094784 | ebi-a-GCST90001632 | CD8+ Natural Killer T %lymphocyte \|\| id:ebi-a-GCST90001632 | Ice cream liking \|\| id:ebi-fl187-GCST90094784 | Weighted median | 8 | -0.10440631 | 0.224332458908228 | 0.641638703196252 | -0.544097929 | 0.335285309465918 | 0.90085919495641 | 0.580365077522392 | 1.39833928774979 | 0.999332001504091 |
| ebi-fl187-GCST90094784 | ebi-a-GCST90001646 | Natural Killer %CD3- lymphocyte \|\| id:ebi-a-GCST90001646 | Ice cream liking \|\| id:ebi-fl187-GCST90094784 | Weighted median | 8 | -0.367184076 | 0.249879385661285 | 0.14171206161955 | -0.856947672 | 0.122579519587458 | 0.692682126939219 | 0.424455684948782 | 1.13040900615794 | 0.999332001504091 |
| ebi-fl187-GCST90094784 | ebi-a-GCST90001684 | CD127- CD8+ T cell Absolute Count \|\| id:ebi-a-GCST90001684 | Ice cream liking \|\| id:ebi-fl187-GCST90094784 | Weighted median | 8 | 0.417607056455394 | 0.247230557107682 | 0.0911925781880151 | -0.066964835 | 0.902178948386451 | 1.5183239415773 | 0.935228087609406 | 2.46496830250208 | 0.999332001504091 |
| ebi-fl187-GCST90094784 | ebi-a-GCST90001687 | CD28- CD8+ T cell Absolute Count \|\| id:ebi-a-GCST90001687 | Ice cream liking \|\| id:ebi-fl187-GCST90094784 | Weighted median | 8 | 0.238609179100999 | 0.241935537115307 | 0.324009930585535 | -0.235584474 | 0.712802831847001 | 1.2694822994067 | 0.79010891680955 | 2.03970019097429 | 0.999332001504091 |
| ebi-fl187-GCST90094784 | ebi-a-GCST90001701 | BAFF-R on CD20- CD38- B cell \|\| id:ebi-a-GCST90001701 | Ice cream liking \|\| id:ebi-fl187-GCST90094784 | Weighted median | 8 | -0.161557141 | 0.217365349669754 | 0.457329375627519 | -0.587593227 | 0.264478944018184 | 0.850817913205324 | 0.555663031625136 | 1.30275199218115 | 0.999332001504091 |
| ebi-fl187-GCST90094784 | ebi-a-GCST90001732 | CD19 on IgD- CD27- B cell \|\| id:ebi-a-GCST90001732 | Ice cream liking \|\| id:ebi-fl187-GCST90094784 | Weighted median | 8 | -0.445038338 | 0.248546031841492 | 0.0733627964817265 | -0.932188561 | 0.0421118841551588 | 0.640799708419292 | 0.393691149868732 | 1.04301116864619 | 0.999332001504091 |
| ebi-fl187-GCST90094784 | ebi-a-GCST90001798 | CD27 on CD24+ CD27+ B cell \|\| id:ebi-a-GCST90001798 | Ice cream liking \|\| id:ebi-fl187-GCST90094784 | Weighted median | 8 | -0.163389993 | 0.263529167722899 | 0.53525301760025 | -0.679907162 | 0.353127175690876 | 0.849259918359868 | 0.506664027967237 | 1.42351216806584 | 0.999332001504091 |
| ebi-fl187-GCST90094784 | ebi-a-GCST90001836 | CD66b on CD66b++ myeloid cell \|\| id:ebi-a-GCST90001836 | Ice cream liking \|\| id:ebi-fl187-GCST90094784 | Weighted median | 8 | 0.09652734322506 | 0.350606819353654 | 0.783074182654232 | -0.590662023 | 0.783716709158221 | 1.10133969491123 | 0.553960428931128 | 2.1895952494794 | 0.999332001504091 |
| ebi-fl187-GCST90094784 | ebi-a-GCST90001943 | CD25 on activated & secreting CD4 regulatory T cell \|\| id:ebi-a-GCST90001943 | Ice cream liking \|\| id:ebi-fl187-GCST90094784 | Weighted median | 8 | -0.086112485 | 0.251850169040004 | 0.732411472019042 | -0.579738816 | 0.407513846700363 | 0.917491021788378 | 0.560044622194944 | 1.50307625803657 | 0.999332001504091 |
| ebi-fl187-GCST90094784 | ebi-a-GCST90001960 | CD25 on CD4+ T cell \|\| id:ebi-a-GCST90001960 | Ice cream liking \|\| id:ebi-fl187-GCST90094784 | Weighted median | 8 | 0.185931259546526 | 0.257661016232284 | 0.470533111067372 | -0.319084332 | 0.690946851361803 | 1.20433947069081 | 0.726814252826838 | 1.99560417950332 | 0.999332001504091 |
| ebi-fl187-GCST90094784 | ebi-a-GCST90001988 | HLA DR on CD14+ CD16- monocyte \|\| id:ebi-a-GCST90001988 | Ice cream liking \|\| id:ebi-fl187-GCST90094784 | Weighted median | 8 | -0.182176334 | 0.22712458437474 | 0.422495866350852 | -0.627340519 | 0.262987851281857 | 0.833454361038874 | 0.534010103671857 | 1.30081091567056 | 0.999332001504091 |
| ebi-fl187-GCST90094784 | ebi-a-GCST90001991 | HLA DR on CD14+ monocyte \|\| id:ebi-a-GCST90001991 | Ice cream liking \|\| id:ebi-fl187-GCST90094784 | Weighted median | 8 | -0.177474951 | 0.21010137842057 | 0.39827220054232 | -0.589273653 | 0.234323750303432 | 0.837381974318436 | 0.554730064897827 | 1.26405366372669 | 0.999332001504091 |
| ebi-fl187-GCST90094784 | ebi-a-GCST90002019 | CD14 on Monocytic Myeloid-Derived Suppressor Cells \|\| id:ebi-a-GCST90002019 | Ice cream liking \|\| id:ebi-fl187-GCST90094784 | Weighted median | 8 | 0.0721937124262764 | 0.349047653397143 | 0.836142209299948 | -0.611939688 | 0.756327113084677 | 1.07486353832218 | 0.542297959276335 | 2.13043697888186 | 0.999332001504091 |
| ebi-fl187-GCST90094784 | ebi-a-GCST90002037 | CD80 on plasmacytoid Dendritic Cell \|\| id:ebi-a-GCST90002037 | Ice cream liking \|\| id:ebi-fl187-GCST90094784 | Weighted median | 8 | -0.057191784 | 0.25824634867509 | 0.824732627767268 | -0.563354627 | 0.448971059405033 | 0.944412928709773 | 0.56929608073811 | 1.56669931533302 | 0.999332001504091 |
| ebi-fl187-GCST90094784 | ebi-a-GCST90002042 | CD45 on CD33+ HLA DR+ CD14- \|\| id:ebi-a-GCST90002042 | Ice cream liking \|\| id:ebi-fl187-GCST90094784 | Weighted median | 8 | -0.142057543 | 0.423066959338034 | 0.737036560079341 | -0.971268783 | 0.687153697194231 | 0.867571332298385 | 0.378602368830396 | 1.98804888345317 | 0.999332001504091 |
| ebi-fl187-GCST90094784 | ebi-a-GCST90002056 | CD8 on Effector Memory CD8+ T cell \|\| id:ebi-a-GCST90002056 | Ice cream liking \|\| id:ebi-fl187-GCST90094784 | Weighted median | 8 | -0.022685912 | 0.267860483414292 | 0.93250544931291 | -0.547692459 | 0.502320635577318 | 0.977569478484814 | 0.57828268272532 | 1.65255179484424 | 0.999332001504091 |
| ebi-fl187-GCST90094784 | ebi-a-GCST90002058 | CD8 on CD8+ T cell \|\| id:ebi-a-GCST90002058 | Ice cream liking \|\| id:ebi-fl187-GCST90094784 | Weighted median | 8 | -0.032789743 | 0.278166639512136 | 0.906164280117666 | -0.577996356 | 0.512416870500719 | 0.967742012787027 | 0.561021327983134 | 1.66932085573268 | 0.999332001504091 |
| ebi-fl187-GCST90094784 | ebi-a-GCST90002063 | CD4 on CD4 regulatory T cell \|\| id:ebi-a-GCST90002063 | Ice cream liking \|\| id:ebi-fl187-GCST90094784 | Weighted median | 8 | -0.105544426 | 0.249824385960901 | 0.672678728541785 | -0.595200223 | 0.384111370420438 | 0.899834495854842 | 0.551452141661528 | 1.46830895876241 | 0.999332001504091 |
| ebi-fl187-GCST90094784 | ebi-a-GCST90002066 | CD4 on activated CD4 regulatory T cell \|\| id:ebi-a-GCST90002066 | Ice cream liking \|\| id:ebi-fl187-GCST90094784 | Weighted median | 8 | -0.232437332 | 0.244418198451377 | 0.341613461855627 | -0.711497001 | 0.246622336955686 | 0.792599418403641 | 0.490908756234107 | 1.27969572772135 | 0.999332001504091 |
| ebi-fl187-GCST90094784 | ebi-a-GCST90002070 | CD4 on activated & secreting CD4 regulatory T cell \|\| id:ebi-a-GCST90002070 | Ice cream liking \|\| id:ebi-fl187-GCST90094784 | Weighted median | 8 | -0.146391427 | 0.247105067824086 | 0.553565469499781 | -0.630717359 | 0.337934506402498 | 0.863819515123378 | 0.532209878240244 | 1.40204867518666 | 0.999332001504091 |
| ebi-fl187-GCST90094784 | ebi-a-GCST90002081 | SSC-A on CD4+ T cell \|\| id:ebi-a-GCST90002081 | Ice cream liking \|\| id:ebi-fl187-GCST90094784 | Weighted median | 8 | -0.123669016 | 0.230490518942623 | 0.591580504777717 | -0.575430433 | 0.328092401466619 | 0.88367227416829 | 0.562462714300552 | 1.38831724891278 | 0.999332001504091 |
| ebi-fl187-GCST90094784 | ebi-a-GCST90002094 | CD11b on Monocytic Myeloid-Derived Suppressor Cells \|\| id:ebi-a-GCST90002094 | Ice cream liking \|\| id:ebi-fl187-GCST90094784 | Weighted median | 8 | -0.080604579 | 0.39073738822435 | 0.836565661772618 | -0.84644986 | 0.685240701609121 | 0.9225584180153 | 0.428935011347427 | 1.98424939008187 | 0.999332001504091 |
| ebi-fl187-GCST90094784 | ebi-a-GCST90002104 | HLA DR on myeloid Dendritic Cell \|\| id:ebi-a-GCST90002104 | Ice cream liking \|\| id:ebi-fl187-GCST90094784 | Weighted median | 8 | 0.131933001260204 | 0.25238378400775 | 0.601149953271673 | -0.362739215 | 0.626605217915393 | 1.14103186900894 | 0.695767855387398 | 1.87124730757949 | 0.999332001504091 |
| ebi-fl187-GCST90094784 | ebi-a-GCST90002105 | HLA DR on plasmacytoid Dendritic Cell \|\| id:ebi-a-GCST90002105 | Ice cream liking \|\| id:ebi-fl187-GCST90094784 | Weighted median | 8 | -0.038037795 | 0.252246263956322 | 0.880136430157078 | -0.532440473 | 0.456364882085156 | 0.962676555584003 | 0.587170246776796 | 1.57832614264491 | 0.999332001504091 |
| ebi-fl187-GCST90094784 | ebi-a-GCST90002106 | HLA DR on Dendritic Cell \|\| id:ebi-a-GCST90002106 | Ice cream liking \|\| id:ebi-fl187-GCST90094784 | Weighted median | 8 | 0.079550484318453 | 0.247110868054052 | 0.747511603799698 | -0.404786817 | 0.563887785704394 | 1.08280022258087 | 0.667119014088043 | 1.75749198757877 | 0.999332001504091 |
| ebi-fl187-GCST90094784 | ebi-a-GCST90002111 | HLA DR on CD33dim HLA DR+ CD11b- \|\| id:ebi-a-GCST90002111 | Ice cream liking \|\| id:ebi-fl187-GCST90094784 | Weighted median | 8 | 0.424234564291244 | 0.334140490143502 | 0.2042166992204 | -0.230680796 | 1.07914992497251 | 1.52842006450935 | 0.79399287097992 | 2.94217741616707 | 0.999332001504091 |
| ebi-fl187-GCST90094784 | ebi-a-GCST90002112 | HLA DR on CD33- HLA DR+ \|\| id:ebi-a-GCST90002112 | Ice cream liking \|\| id:ebi-fl187-GCST90094784 | Weighted median | 8 | 0.267348502955688 | 0.33781630368911 | 0.428709435346443 | -0.394771452 | 0.929468458186343 | 1.30649568460778 | 0.673834024885101 | 2.53316233799535 | 0.999332001504091 |
| ebi-fl187-GCST90094784 | ebi-a-GCST90002115 | HLA DR on HLA DR+ CD8+ T cell \|\| id:ebi-a-GCST90002115 | Ice cream liking \|\| id:ebi-fl187-GCST90094784 | Weighted median | 8 | 0.192704049557143 | 0.230345509726397 | 0.40282477829915 | -0.25877315 | 0.644181248620882 | 1.21252389343018 | 0.771998131338253 | 1.90442713843165 | 0.999332001504091 |
| ebi-fl187-GCST90094784 | ebi-a-GCST90002116 | HLA DR on B cell \|\| id:ebi-a-GCST90002116 | Ice cream liking \|\| id:ebi-fl187-GCST90094784 | Weighted median | 8 | -0.172908359 | 0.256876635115474 | 0.500872044722106 | -0.676386564 | 0.330569845831883 | 0.841214701073533 | 0.508450931951172 | 1.39176099173753 | 0.999332001504091 |
| ebi-fl187-GCST90094784 | ebi-cfmet338-GCST90026023 | Sphingomyelin (d18:1/24:1, d18:2/24:0) levels \|\| id:ebi-cfmet338-GCST90026023 | Ice cream liking \|\| id:ebi-fl187-GCST90094784 | Weighted median | 8 | -0.012955007 | 0.148754920954564 | 0.93060029074238 | -0.304514652 | 0.278604638276502 | 0.987128548098813 | 0.737481222679843 | 1.32128485513276 | 0.999332001504091 |
| ebi-fl187-GCST90094784 | ebi-cfmet338-GCST90026046 | 1-arachidonoyl-gpc (20:4n6) levels \|\| id:ebi-cfmet338-GCST90026046 | Ice cream liking \|\| id:ebi-fl187-GCST90094784 | Weighted median | 8 | -0.010819197 | 0.15869857475233 | 0.945646718186588 | -0.321868404 | 0.300230009015309 | 0.989239119514096 | 0.724793563978197 | 1.35016932298044 | 0.999332001504091 |
| ebi-fl187-GCST90094784 | ebi-cfmet338-GCST90026047 | 1-linoleoyl-gpc (18:2) levels \|\| id:ebi-cfmet338-GCST90026047 | Ice cream liking \|\| id:ebi-fl187-GCST90094784 | Weighted median | 8 | -0.117425398 | 0.199352770426021 | 0.555839008941083 | -0.508156828 | 0.273306031642882 | 0.88920684553253 | 0.601603415846001 | 1.31430240140843 | 0.999332001504091 |
| ebi-fl187-GCST90094784 | ebi-cfmet338-GCST90026050 | 1-myristoyl-2-palmitoyl-gpc (14:0/16:0) levels \|\| id:ebi-cfmet338-GCST90026050 | Ice cream liking \|\| id:ebi-fl187-GCST90094784 | Weighted median | 8 | 0.0586269949536175 | 0.0996926261104863 | 0.556480243602597 | -0.136770552 | 0.254024542130171 | 1.06037964000741 | 0.872170320716913 | 1.28920344367829 | 0.999332001504091 |
| ebi-fl187-GCST90094784 | ebi-cfmet338-GCST90026068 | 2,3-dihydroxyisovalerate levels \|\| id:ebi-cfmet338-GCST90026068 | Ice cream liking \|\| id:ebi-fl187-GCST90094784 | Weighted median | 8 | -0.127846234 | 0.340453046181496 | 0.707275289790481 | -0.795134205 | 0.539441736148484 | 0.879988680758319 | 0.451520634582325 | 1.71504914493908 | 0.999332001504091 |
| ebi-fl187-GCST90094784 | ebi-cfmet338-GCST90026082 | 3-hydroxyhexanoate levels \|\| id:ebi-cfmet338-GCST90026082 | Ice cream liking \|\| id:ebi-fl187-GCST90094784 | Weighted median | 8 | 0.0428124519737272 | 0.0739731095296497 | 0.56275317632147 | -0.102174843 | 0.187799746651841 | 1.04374212471761 | 0.902871677341912 | 1.20659186709373 | 0.999332001504091 |
| ebi-fl187-GCST90094784 | ebi-cfmet338-GCST90026094 | 3-ureidopropionate levels \|\| id:ebi-cfmet338-GCST90026094 | Ice cream liking \|\| id:ebi-fl187-GCST90094784 | Weighted median | 8 | -0.10917687 | 0.190385698558039 | 0.566339366212844 | -0.482332839 | 0.263979099641171 | 0.896571827233744 | 0.61734155240217 | 1.30210098163875 | 0.999332001504091 |
| ebi-fl187-GCST90094784 | ebi-cfmet338-GCST90026173 | Glycerol levels \|\| id:ebi-cfmet338-GCST90026173 | Ice cream liking \|\| id:ebi-fl187-GCST90094784 | Weighted median | 7 | -0.006339283 | 0.108730278602089 | 0.953507412730806 | -0.219450629 | 0.206772063028051 | 0.993680767830899 | 0.802959799568727 | 1.2297022452274 | 0.999332001504091 |
| ebi-fl187-GCST90094784 | ebi-cfmet338-GCST90026179 | Histidine levels \|\| id:ebi-cfmet338-GCST90026179 | Ice cream liking \|\| id:ebi-fl187-GCST90094784 | Weighted median | 8 | -0.035681844 | 0.071268720804844 | 0.616606025405439 | -0.175368536 | 0.104004849269523 | 0.964947248882305 | 0.839147707397067 | 1.1096058356803 | 0.999332001504091 |
| ebi-fl187-GCST90094784 | ebi-cfmet338-GCST90026188 | Isocitrate levels \|\| id:ebi-cfmet338-GCST90026188 | Ice cream liking \|\| id:ebi-fl187-GCST90094784 | Weighted median | 8 | 0.0478279090029399 | 0.129940309792947 | 0.71281656849627 | -0.206855098 | 0.302510916197115 | 1.04899011803518 | 0.813137466541125 | 1.35325244871102 | 0.999332001504091 |
| ebi-fl187-GCST90094784 | ebi-cfmet338-GCST90026207 | N-acetyl-isoputreanine levels \|\| id:ebi-cfmet338-GCST90026207 | Ice cream liking \|\| id:ebi-fl187-GCST90094784 | Weighted median | 7 | -0.017107502 | 0.0931177762485265 | 0.854233926815811 | -0.199618343 | 0.165403339566395 | 0.983038000520519 | 0.819043286769096 | 1.17986891056689 | 0.999332001504091 |
| ebi-fl187-GCST90094784 | ebi-cfmet338-GCST90026213 | N-acetylglutamate levels \|\| id:ebi-cfmet338-GCST90026213 | Ice cream liking \|\| id:ebi-fl187-GCST90094784 | Weighted median | 8 | 0.0251689928643463 | 0.14295631285701 | 0.860246231514549 | -0.25502538 | 0.305363366064086 | 1.0254884061051 | 0.774896830570044 | 1.35711804406578 | 0.999332001504091 |
| ebi-fl187-GCST90094784 | ebi-cfmet338-GCST90026224 | N-acetyltaurine levels \|\| id:ebi-cfmet338-GCST90026224 | Ice cream liking \|\| id:ebi-fl187-GCST90094784 | Weighted median | 8 | 0.0411224908488239 | 0.121068787049327 | 0.734110929245107 | -0.196172332 | 0.278417313465504 | 1.04197973071053 | 0.821870588062041 | 1.32103736887788 | 0.999332001504091 |
| ebi-fl187-GCST90094784 | ebi-cfmet338-GCST90026250 | Phenyllactate (pla) levels \|\| id:ebi-cfmet338-GCST90026250 | Ice cream liking \|\| id:ebi-fl187-GCST90094784 | Weighted median | 8 | 0.047510789792589 | 0.183472285010559 | 0.795671120942512 | -0.312094889 | 0.407116468413285 | 1.04865751585734 | 0.731912074654139 | 1.5024790868271 | 0.999332001504091 |
| ebi-fl187-GCST90094784 | ebi-cfmet338-GCST90026251 | Pipecolate levels \|\| id:ebi-cfmet338-GCST90026251 | Ice cream liking \|\| id:ebi-fl187-GCST90094784 | Weighted median | 8 | -0.153157091 | 0.289647933208231 | 0.596965122220688 | -0.72086704 | 0.414552857795229 | 0.857994927691684 | 0.486330405005828 | 1.51369375298638 | 0.999332001504091 |
| ebi-fl187-GCST90094784 | ebi-cfmet338-GCST90026278 | Trigonelline (n'-methylnicotinate) levels \|\| id:ebi-cfmet338-GCST90026278 | Ice cream liking \|\| id:ebi-fl187-GCST90094784 | Weighted median | 8 | 0.225936802428938 | 0.327796688908601 | 0.490660324057936 | -0.416544708 | 0.868418312689796 | 1.2534964448443 | 0.659321035322479 | 2.38313849105205 | 0.999332001504091 |
| ebi-fl187-GCST90094784 | ebi-cfmet338-GCST90026299 | 1-carboxyethylvaline levels \|\| id:ebi-cfmet338-GCST90026299 | Ice cream liking \|\| id:ebi-fl187-GCST90094784 | Weighted median | 8 | -0.12482437 | 0.180562283546367 | 0.489371244876102 | -0.478726445 | 0.229077706062198 | 0.882651909401884 | 0.619571948244942 | 1.25743974590469 | 0.999332001504091 |
| ebi-fl187-GCST90094784 | ebi-gm430-GCST90011306 | C_Erysipelotrichia abundance \|\| id:ebi-gm430-GCST90011306 | Ice cream liking \|\| id:ebi-fl187-GCST90094784 | Weighted median | 8 | -0.057901622 | 0.154276748816002 | 0.707429855138044 | -0.36028405 | 0.244480805570601 | 0.943742786295265 | 0.697478179401522 | 1.27695815150602 | 0.999332001504091 |
| ebi-fl187-GCST90094784 | ebi-gm430-GCST90011312 | F_Erysipelotrichaceae abundance \|\| id:ebi-gm430-GCST90011312 | Ice cream liking \|\| id:ebi-fl187-GCST90094784 | Weighted median | 8 | -0.057901622 | 0.151406332225434 | 0.702145877851709 | -0.354658033 | 0.238854789053088 | 0.943742786295265 | 0.701413262200401 | 1.26979413518686 | 0.999332001504091 |
| ebi-fl187-GCST90094784 | ebi-gm430-GCST90011352 | O_Erysipelotrichales abundance \|\| id:ebi-gm430-GCST90011352 | Ice cream liking \|\| id:ebi-fl187-GCST90094784 | Weighted median | 8 | -0.057901622 | 0.146849919784697 | 0.693366104697186 | -0.345727465 | 0.229924220669244 | 0.943742786295265 | 0.707705335380057 | 1.2585046376767 | 0.999332001504091 |
| ebi-fl187-GCST90094784 | ebi-gm430-GCST90011362 | OTU97_11 (Parabacteroides) abundance \|\| id:ebi-gm430-GCST90011362 | Ice cream liking \|\| id:ebi-fl187-GCST90094784 | Weighted median | 8 | 0.307011806804456 | 0.226059154385636 | 0.174430735283342 | -0.136064136 | 0.750087749400304 | 1.35935701763973 | 0.872786653830862 | 2.11718579024522 | 0.999332001504091 |
| ebi-fl187-GCST90094784 | ebi-gm430-GCST90011384 | OTU97_235 (Phascolarctobacterium) abundance \|\| id:ebi-gm430-GCST90011384 | Ice cream liking \|\| id:ebi-fl187-GCST90094784 | Weighted median | 8 | 0.568177389550123 | 0.59330079799744 | 0.338236828503472 | -0.594692175 | 1.7310469536251 | 1.76504712465508 | 0.551732377011496 | 5.64656250395876 | 0.999332001504091 |
| ebi-fl187-GCST90094784 | ebi-gm430-GCST90011390 | OTU97_3 (Bacteroides) abundance \|\| id:ebi-gm430-GCST90011390 | Ice cream liking \|\| id:ebi-fl187-GCST90094784 | Weighted median | 8 | 0.0443157099538925 | 0.155342366187179 | 0.775431410857913 | -0.260155328 | 0.348786747680764 | 1.04531231830315 | 0.770931829378882 | 1.41734690559689 | 0.999332001504091 |
| ebi-fl187-GCST90094784 | ebi-gm430-GCST90011398 | OTU97_4 (Alistipes) abundance \|\| id:ebi-gm430-GCST90011398 | Ice cream liking \|\| id:ebi-fl187-GCST90094784 | Weighted median | 8 | 0.176832266622838 | 0.171120458575091 | 0.301426629473415 | -0.158563832 | 0.512228365430016 | 1.19343089814296 | 0.853368489677608 | 1.66900620994383 | 0.999332001504091 |
| ebi-fl187-GCST90094784 | ebi-gm430-GCST90011421 | OTU99_108 (Cytophagales) abundance \|\| id:ebi-gm430-GCST90011421 | Ice cream liking \|\| id:ebi-fl187-GCST90094784 | Weighted median | 8 | 0.437471059428915 | 0.552386597258751 | 0.428380864977273 | -0.645206671 | 1.52014879005607 | 1.54878547524939 | 0.524554120674526 | 4.57290554739886 | 0.999332001504091 |
| ebi-fl187-GCST90094784 | ebi-gm430-GCST90011428 | OTU99_123 (Phascolarctobacterium) abundance \|\| id:ebi-gm430-GCST90011428 | Ice cream liking \|\| id:ebi-fl187-GCST90094784 | Weighted median | 8 | 0.197824807843184 | 0.33883814001572 | 0.559332832524641 | -0.466297947 | 0.861947562273996 | 1.21874885987607 | 0.627320348243173 | 2.367767581 | 0.999332001504091 |
| ebi-fl187-GCST90094784 | ebi-gm430-GCST90011450 | OTU99_298 (Phascolarctobacterium) abundance \|\| id:ebi-gm430-GCST90011450 | Ice cream liking \|\| id:ebi-fl187-GCST90094784 | Weighted median | 8 | 0.573918397631887 | 0.604777316482548 | 0.342633458676379 | -0.611445143 | 1.75928193793768 | 1.77520941745324 | 0.542566216650605 | 5.80826520174597 | 0.999332001504091 |
| ebi-fl187-GCST90094784 | ebi-gm430-GCST90011451 | OTU99_3 (Bacteroides) abundance \|\| id:ebi-gm430-GCST90011451 | Ice cream liking \|\| id:ebi-fl187-GCST90094784 | Weighted median | 8 | 0.0412902859936896 | 0.162877591752079 | 0.799877987945947 | -0.277949794 | 0.360530365827765 | 1.0421545845198 | 0.757334843433891 | 1.43408980512652 | 0.999332001504091 |
| ebi-fl187-GCST90094784 | ebi-gm430-GCST90011472 | OTU99_72 (Parabacteroides) abundance \|\| id:ebi-gm430-GCST90011472 | Ice cream liking \|\| id:ebi-fl187-GCST90094784 | Weighted median | 8 | 0.618425707378855 | 0.54460133459263 | 0.256142129275555 | -0.448992908 | 1.68584432318041 | 1.85600384751954 | 0.638270625023195 | 5.3970058263016 | 0.999332001504091 |
| ebi-fl187-GCST90094784 | ebi-gm430-GCST90011507 | TestASV_3 (Bacteroides) abundance \|\| id:ebi-gm430-GCST90011507 | Ice cream liking \|\| id:ebi-fl187-GCST90094784 | Weighted median | 8 | -0.044840486 | 0.196488732515294 | 0.819483808112491 | -0.429958402 | 0.34027742945655 | 0.956149988717169 | 0.650536155161251 | 1.4053374184825 | 0.999332001504091 |
| ebi-fl187-GCST90094784 | ebi-gm430-GCST90011512 | TestASV_34 (Phascolarctobacterium) abundance \|\| id:ebi-gm430-GCST90011512 | Ice cream liking \|\| id:ebi-fl187-GCST90094784 | Weighted median | 8 | 1.04146095978149 | 0.479737653988746 | 0.0299389708399552 | 0.101175157963544 | 1.98174676159943 | 2.83335340743887 | 1.10647043189856 | 7.25540538636051 | 0.999332001504091 |
| ebi-fl187-GCST90094784 | ebi-gm430-GCST90011519 | TestASV_41 (Barnesiella) abundance \|\| id:ebi-gm430-GCST90011519 | Ice cream liking \|\| id:ebi-fl187-GCST90094784 | Weighted median | 8 | -0.267434969 | 0.663444401040546 | 0.686874072170914 | -1.567785995 | 1.03291605727991 | 0.76534010002059 | 0.208506305749229 | 2.80924582398004 | 0.999332001504091 |
| ebi-fl187-GCST90094784 | ebi-gm430-GCST90011524 | TestASV_46 (Sutterella) abundance \|\| id:ebi-gm430-GCST90011524 | Ice cream liking \|\| id:ebi-fl187-GCST90094784 | Weighted median | 8 | 0.354766680184692 | 0.588711236247526 | 0.546764321006459 | -0.799107343 | 1.50864070322984 | 1.42584793689092 | 0.449730239899244 | 4.52058180386438 | 0.999332001504091 |
| ebi-fl187-GCST90094784 | ebi-gm430-GCST90011581 | OTU97_19 (Bacteria) prevalence \|\| id:ebi-gm430-GCST90011581 | Ice cream liking \|\| id:ebi-fl187-GCST90094784 | Weighted median | 8 | -0.530016716 | 0.680577665749929 | 0.436112401270581 | -1.863948941 | 0.80391550843783 | 0.588595130385626 | 0.155059100482405 | 2.23427213517845 | 0.999332001504091 |
| ebi-fl187-GCST90094784 | ebi-gm430-GCST90011614 | OTU97_80 (Ruminococcus) prevalence \|\| id:ebi-gm430-GCST90011614 | Ice cream liking \|\| id:ebi-fl187-GCST90094784 | Weighted median | 8 | 0.0901587717951467 | 0.348680896906117 | 0.795966253117104 | -0.593255786 | 0.773573329731136 | 1.0943480215124 | 0.552525448431852 | 2.16749761587828 | 0.999332001504091 |
| ebi-fl187-GCST90094784 | ebi-gm430-GCST90011623 | OTU99_11 (Parabacteroides) prevalence \|\| id:ebi-gm430-GCST90011623 | Ice cream liking \|\| id:ebi-fl187-GCST90094784 | Weighted median | 8 | -0.071952971 | 0.293794516305423 | 0.806526761657553 | -0.647790223 | 0.503884280974379 | 0.930574658792418 | 0.523200657085341 | 1.65513782113881 | 0.999332001504091 |
| ebi-fl187-GCST90094784 | ebi-gm430-GCST90011642 | OTU99_19 (Bacteria) prevalence \|\| id:ebi-gm430-GCST90011642 | Ice cream liking \|\| id:ebi-fl187-GCST90094784 | Weighted median | 8 | -0.530016716 | 0.70099469817788 | 0.449593838756195 | -1.903966325 | 0.843932891996614 | 0.588595130385626 | 0.148976556422718 | 2.32549493579811 | 0.999332001504091 |
| ebi-fl187-GCST90094784 | ebi-gm430-GCST90011653 | OTU99_30 (Parasutterella) prevalence \|\| id:ebi-gm430-GCST90011653 | Ice cream liking \|\| id:ebi-fl187-GCST90094784 | Weighted median | 8 | 0.179686849457378 | 0.345598121428135 | 0.603112328261248 | -0.497685469 | 0.857059167456522 | 1.19684251255031 | 0.607936119868366 | 2.35622124271525 | 0.999332001504091 |
| ebi-fl187-GCST90094784 | ebi-gm430-GCST90011675 | OTU99_8 (Ruminococcaceae) prevalence \|\| id:ebi-gm430-GCST90011675 | Ice cream liking \|\| id:ebi-fl187-GCST90094784 | Weighted median | 8 | -0.12543707 | 0.343855786518539 | 0.715264007258385 | -0.799394411 | 0.548520271664064 | 0.882111274020262 | 0.449601154985239 | 1.73069017088979 | 0.999332001504091 |
| ebi-fl187-GCST90094784 | ebi-gm430-GCST90011679 | OTU99_92 (Ruminococcus) prevalence \|\| id:ebi-gm430-GCST90011679 | Ice cream liking \|\| id:ebi-fl187-GCST90094784 | Weighted median | 8 | -0.239756266 | 0.410684472579024 | 0.559357055427031 | -1.044697833 | 0.565185299991333 | 0.786819612181483 | 0.351798105266674 | 1.75977383858885 | 0.999332001504091 |
| ebi-fl187-GCST90094784 | ebi-gm430-GCST90011684 | TestASV_11 (Lachnospiraceae) prevalence \|\| id:ebi-gm430-GCST90011684 | Ice cream liking \|\| id:ebi-fl187-GCST90094784 | Weighted median | 8 | -0.554722821 | 0.350175531811389 | 0.113164622386291 | -1.241066863 | 0.131621221598269 | 0.574231404168608 | 0.289075649224078 | 1.14067617393069 | 0.999332001504091 |
| ebi-fl187-GCST90094784 | ebi-gm430-GCST90011688 | TestASV_15 (Bacteroides) prevalence \|\| id:ebi-gm430-GCST90011688 | Ice cream liking \|\| id:ebi-fl187-GCST90094784 | Weighted median | 8 | 0.0501804826934666 | 0.324999894571339 | 0.877293143183531 | -0.586819311 | 0.687180276053292 | 1.05146084973815 | 0.556093234596543 | 1.98810172422647 | 0.999332001504091 |
| ebi-fl187-GCST90094784 | ebi-gm430-GCST90011692 | TestASV_19 (Prevotella) prevalence \|\| id:ebi-gm430-GCST90011692 | Ice cream liking \|\| id:ebi-fl187-GCST90094784 | Weighted median | 8 | -0.432916528 | 0.634944187300964 | 0.495353889551591 | -1.677407135 | 0.811574079183021 | 0.648614630753292 | 0.186857845625105 | 2.25144915815463 | 0.999332001504091 |
| ebi-fl187-GCST90094784 | ebi-gm430-GCST90011693 | TestASV_2 (Bacteroides) prevalence \|\| id:ebi-gm430-GCST90011693 | Ice cream liking \|\| id:ebi-fl187-GCST90094784 | Weighted median | 8 | 0.547567515550944 | 0.333030681322753 | 0.100135846051019 | -0.10517262 | 1.20030765094354 | 1.72904203051214 | 0.90016912212273 | 3.32113851697965 | 0.999332001504091 |
| ebi-fl187-GCST90094784 | ebi-gm430-GCST90011716 | TestASV_40 (Barnesiella) prevalence \|\| id:ebi-gm430-GCST90011716 | Ice cream liking \|\| id:ebi-fl187-GCST90094784 | Weighted median | 8 | -0.526458661 | 0.623929774936138 | 0.39879307438468 | -1.74936102 | 0.69644369808064 | 0.590693114766946 | 0.173885017065495 | 2.00660391402012 | 0.999332001504091 |
| ebi-fl187-GCST90094784 | ebi-gm430-GCST90011718 | TestASV_42 (Alphaproteobacteria) prevalence \|\| id:ebi-gm430-GCST90011718 | Ice cream liking \|\| id:ebi-fl187-GCST90094784 | Weighted median | 8 | 0.158674550284006 | 0.58120706576596 | 0.784846574418377 | -0.980491299 | 1.29784039918529 | 1.17195647170775 | 0.375126754315301 | 3.66138100196191 | 0.999332001504091 |
| ebi-fl187-GCST90094784 | ebi-hbsm44-GCST90104929 | Sulcal depth of the right inferior frontal sulcus \|\| id:ebi-hbsm44-GCST90104929 | Ice cream liking \|\| id:ebi-fl187-GCST90094784 | Weighted median | 7 | -0.172279265 | 0.357441937847427 | 0.629821346534374 | -0.872865463 | 0.52830693303518 | 0.841744070561475 | 0.417752777286386 | 1.69605833605188 | 0.999332001504091 |
| ebi-fl187-GCST90094784 | ebi-hbsm44-GCST90104935 | Sulcal opening distance of the left calloso-marginal posterior fissure \|\| id:ebi-hbsm44-GCST90104935 | Ice cream liking \|\| id:ebi-fl187-GCST90094784 | Weighted median | 8 | 0.0153117725391192 | 0.0705926641186065 | 0.828283808072592 | -0.123049849 | 0.153673394211588 | 1.01542959833406 | 0.884219583882241 | 1.16610996631149 | 0.999332001504091 |
| ebi-fl187-GCST90094784 | ebi-hbsm44-GCST90104936 | Sulcal opening distance of the left anterior inferior temporal sulcus \|\| id:ebi-hbsm44-GCST90104936 | Ice cream liking \|\| id:ebi-fl187-GCST90094784 | Weighted median | 7 | 0.0414801748990298 | 0.0369197399588169 | 0.261215462065939 | -0.030882515 | 0.113842865218311 | 1.04235249690313 | 0.969589478197222 | 1.12057602957939 | 0.999332001504091 |
| ebi-fl187-GCST90094784 | ebi-hbsm44-GCST90104941 | Sulcal opening distance of the right posterior inferior temporal sulcus \|\| id:ebi-hbsm44-GCST90104941 | Ice cream liking \|\| id:ebi-fl187-GCST90094784 | Weighted median | 8 | -0.005842248 | 0.0271413844535936 | 0.829570540590597 | -0.059039362 | 0.0473548654415903 | 0.994174784657921 | 0.942669663425059 | 1.04849401736179 | 0.999332001504091 |
| ebi-fl187-GCST90094784 | ebi-IPs91-GCST90274819 | Leukemia inhibitory factor levels \|\| id:ebi-IPs91-GCST90274819 | Ice cream liking \|\| id:ebi-fl187-GCST90094784 | Weighted median | 9 | 0.270889725591015 | 0.117712584268066 | 0.0213757568608076 | 0.0401730604256057 | 0.501606390756424 | 1.31113047827374 | 1.04099091293494 | 1.65137188970424 | 0.999332001504091 |
| ebi-fl187-GCST90094784 | ebi-IPs91-GCST90274837 | STAM binding protein levels \|\| id:ebi-IPs91-GCST90274837 | Ice cream liking \|\| id:ebi-fl187-GCST90094784 | Weighted median | 9 | 0.198451078499012 | 0.105790565469838 | 0.0606708863768685 | -0.00889843 | 0.405800586819895 | 1.21951236557949 | 0.991141044032898 | 1.50050330248651 | 0.999332001504091 |
| ebi-fl187-GCST90094784 | ebi-IPs91-GCST90274840 | TNF-beta levels \|\| id:ebi-IPs91-GCST90274840 | Ice cream liking \|\| id:ebi-fl187-GCST90094784 | Weighted median | 9 | 0.140803626850825 | 0.124664867899164 | 0.25870503442774 | -0.103539514 | 0.385146767933186 | 1.15119856129999 | 0.90164039441064 | 1.46983002952681 | 0.999332001504091 |
| ebi-fl187-GCST90094784 | ebi-IPs91-GCST90274842 | Tumor necrosis factor ligand superfamily member 14 levels \|\| id:ebi-IPs91-GCST90274842 | Ice cream liking \|\| id:ebi-fl187-GCST90094784 | Weighted median | 9 | 0.0170130634230553 | 0.125322001619316 | 0.892015123544494 | -0.22861806 | 0.262644186596915 | 1.01715860981168 | 0.795632359501369 | 1.30036394970466 | 0.999332001504091 |
| ebi-fl187-GCST90094784 | ebi-met1400-GCST90199628 | 3-methylhistidine levels \|\| id:ebi-met1400-GCST90199628 | Ice cream liking \|\| id:ebi-fl187-GCST90094784 | Weighted median | 8 | -0.14782426 | 0.14140534762972 | 0.295841046467985 | -0.424978741 | 0.129330221561781 | 0.862582692083874 | 0.65378368367352 | 1.13806587601262 | 0.999332001504091 |
| ebi-fl187-GCST90094784 | ebi-met1400-GCST90199651 | Docosahexaenoate DHA; 22:6n3 levels \|\| id:ebi-met1400-GCST90199651 | Ice cream liking \|\| id:ebi-fl187-GCST90094784 | Weighted median | 8 | -0.06606255 | 0.146792709806284 | 0.652681801681873 | -0.353776261 | 0.221651161427434 | 0.936072311331204 | 0.702032021718389 | 1.24813590396655 | 0.999332001504091 |
| ebi-fl187-GCST90094784 | ebi-met1400-GCST90199652 | 1-stearoyl-2-oleoyl-GPS (18:0/18:1) levels \|\| id:ebi-met1400-GCST90199652 | Ice cream liking \|\| id:ebi-fl187-GCST90094784 | Weighted median | 8 | 0.0515922754756639 | 0.140700597078818 | 0.713856769438248 | -0.224180895 | 0.327365445750147 | 1.05294634293412 | 0.799170555496118 | 1.3873083705018 | 0.999332001504091 |
| ebi-fl187-GCST90094784 | ebi-met1400-GCST90199653 | 1-myristoyl-2-palmitoyl-gpc (14:0/16:0) levels \|\| id:ebi-met1400-GCST90199653 | Ice cream liking \|\| id:ebi-fl187-GCST90094784 | Weighted median | 8 | -0.168524519 | 0.135903012156023 | 0.214962374890588 | -0.434894423 | 0.0978453849816881 | 0.844910546934973 | 0.647333007160958 | 1.10279226368022 | 0.999332001504091 |
| ebi-fl187-GCST90094784 | ebi-met1400-GCST90199667 | Erythritol levels in elite athletes \|\| id:ebi-met1400-GCST90199667 | Ice cream liking \|\| id:ebi-fl187-GCST90094784 | Weighted median | 8 | -0.090470015 | 0.138055523844825 | 0.512264222196119 | -0.361058842 | 0.180118811787866 | 0.913501724887022 | 0.696937988255563 | 1.19735961510763 | 0.999332001504091 |
| ebi-fl187-GCST90094784 | ebi-met1400-GCST90199674 | 3-hydroxymyristate levels \|\| id:ebi-met1400-GCST90199674 | Ice cream liking \|\| id:ebi-fl187-GCST90094784 | Weighted median | 8 | 0.203958662955433 | 0.144535259416998 | 0.158205016266508 | -0.079330446 | 0.487247771412749 | 1.22624746296249 | 0.923734630052851 | 1.62782988912727 | 0.999332001504091 |
| ebi-fl187-GCST90094784 | ebi-met1400-GCST90199685 | 2-linoleoylglycerol (18:2) levels \|\| id:ebi-met1400-GCST90199685 | Ice cream liking \|\| id:ebi-fl187-GCST90094784 | Weighted median | 8 | 0.0862096326584247 | 0.167116390957172 | 0.605948133734737 | -0.241338494 | 0.413757758934482 | 1.09003481125081 | 0.785575669028317 | 1.5124906951462 | 0.999332001504091 |
| ebi-fl187-GCST90094784 | ebi-met1400-GCST90199703 | Gamma-glutamylvaline levels \|\| id:ebi-met1400-GCST90199703 | Ice cream liking \|\| id:ebi-fl187-GCST90094784 | Weighted median | 8 | -0.136402229 | 0.143641474480799 | 0.34231453041278 | -0.417939519 | 0.14513506095015 | 0.872491620439727 | 0.658402048025316 | 1.15619571661519 | 0.999332001504091 |
| ebi-fl187-GCST90094784 | ebi-met1400-GCST90199779 | Gamma-glutamyl-2-aminobutyrate levels \|\| id:ebi-met1400-GCST90199779 | Ice cream liking \|\| id:ebi-fl187-GCST90094784 | Weighted median | 8 | 0.0697217121618024 | 0.141385143985139 | 0.621918455323676 | -0.20739317 | 0.346836594372674 | 1.07220975679688 | 0.812700057843038 | 1.41458555524363 | 0.999332001504091 |
| ebi-fl187-GCST90094784 | ebi-met1400-GCST90199824 | 4-vinylphenol sulfate levels \|\| id:ebi-met1400-GCST90199824 | Ice cream liking \|\| id:ebi-fl187-GCST90094784 | Weighted median | 8 | 0.256664730665572 | 0.141367242597558 | 0.0694335290697881 | -0.020415065 | 0.533744526156787 | 1.29261168104491 | 0.979791911737374 | 1.70530593073685 | 0.999332001504091 |
| ebi-fl187-GCST90094784 | ebi-met1400-GCST90199830 | 4-ethylphenylsulfate levels \|\| id:ebi-met1400-GCST90199830 | Ice cream liking \|\| id:ebi-fl187-GCST90094784 | Weighted median | 8 | -0.107700987 | 0.139721164947969 | 0.440808883486653 | -0.381554471 | 0.16615249601345 | 0.897896038627304 | 0.682799192575318 | 1.18075314814271 | 0.999332001504091 |
| ebi-fl187-GCST90094784 | ebi-met1400-GCST90199833 | Hexanoylglutamine levels \|\| id:ebi-met1400-GCST90199833 | Ice cream liking \|\| id:ebi-fl187-GCST90094784 | Weighted median | 8 | 0.216118165488991 | 0.148520351695223 | 0.145630021128647 | -0.074981724 | 0.507218054811628 | 1.24124904313532 | 0.927760442077785 | 1.66066488417403 | 0.999332001504091 |
| ebi-fl187-GCST90094784 | ebi-met1400-GCST90199837 | Carotenoid levels (cryptoxanthin) \|\| id:ebi-met1400-GCST90199837 | Ice cream liking \|\| id:ebi-fl187-GCST90094784 | Weighted median | 8 | -0.044610153 | 0.148958362116404 | 0.764573305589014 | -0.336568542 | 0.247348237093048 | 0.956370247569266 | 0.714216927753723 | 1.28062499626316 | 0.999332001504091 |
| ebi-fl187-GCST90094784 | ebi-met1400-GCST90199869 | 2-hydroxyhippurate levels \|\| id:ebi-met1400-GCST90199869 | Ice cream liking \|\| id:ebi-fl187-GCST90094784 | Weighted median | 8 | 0.0433318622591739 | 0.154620953933944 | 0.779288983480057 | -0.259725207 | 0.346388931969704 | 1.04428439593097 | 0.771263494148049 | 1.41395244019624 | 0.999332001504091 |
| ebi-fl187-GCST90094784 | ebi-met1400-GCST90199902 | Hydantoin-5-propionate levels \|\| id:ebi-met1400-GCST90199902 | Ice cream liking \|\| id:ebi-fl187-GCST90094784 | Weighted median | 8 | 0.0882236221175527 | 0.160985258955748 | 0.583676120640604 | -0.227307485 | 0.403754729670818 | 1.09223234202987 | 0.796675778425659 | 1.49743662513943 | 0.999332001504091 |
| ebi-fl187-GCST90094784 | ebi-met1400-GCST90199904 | Pantoate levels \|\| id:ebi-met1400-GCST90199904 | Ice cream liking \|\| id:ebi-fl187-GCST90094784 | Weighted median | 8 | -0.039580468 | 0.137182044570703 | 0.77294479548313 | -0.308457275 | 0.229296339833411 | 0.961192606088423 | 0.734579336516268 | 1.25771469475386 | 0.999332001504091 |
| ebi-fl187-GCST90094784 | ebi-met1400-GCST90199936 | 3-methyl catechol sulfate (2) levels \|\| id:ebi-met1400-GCST90199936 | Ice cream liking \|\| id:ebi-fl187-GCST90094784 | Weighted median | 7 | 0.216623411445221 | 0.162468355325443 | 0.182424584266652 | -0.101814565 | 0.535061387883089 | 1.24187633765071 | 0.903197020487242 | 1.70755306210459 | 0.999332001504091 |
| ebi-fl187-GCST90094784 | ebi-met1400-GCST90199977 | 4-hydroxychlorothalonil levels \|\| id:ebi-met1400-GCST90199977 | Ice cream liking \|\| id:ebi-fl187-GCST90094784 | Weighted median | 8 | 0.0109910938236463 | 0.152440271641442 | 0.942521541909191 | -0.287791839 | 0.309774026240872 | 1.01105171779972 | 0.749917679911713 | 1.36311705064229 | 0.999332001504091 |
| ebi-fl187-GCST90094784 | ebi-met1400-GCST90200040 | Sphingomyelin (d18:2/24:1, d18:1/24:2) levels \|\| id:ebi-met1400-GCST90200040 | Ice cream liking \|\| id:ebi-fl187-GCST90094784 | Weighted median | 8 | 0.183972397061354 | 0.146856098689665 | 0.210300621379379 | -0.103865556 | 0.471810350493099 | 1.2019826443852 | 0.901346469566515 | 1.60289336696251 | 0.999332001504091 |
| ebi-fl187-GCST90094784 | ebi-met1400-GCST90200077 | 1-stearoyl-2-oleoyl-GPI (18:0/18:1) levels \|\| id:ebi-met1400-GCST90200077 | Ice cream liking \|\| id:ebi-fl187-GCST90094784 | Weighted median | 8 | 0.0784002294124419 | 0.149488046890198 | 0.599959897722459 | -0.214596342 | 0.371396801317231 | 1.08155544235714 | 0.806867072402413 | 1.44975822524203 | 0.999332001504091 |
| ebi-fl187-GCST90094784 | ebi-met1400-GCST90200080 | 1-palmitoyl-2-oleoyl-GPI (16:0/18:1) levels \|\| id:ebi-met1400-GCST90200080 | Ice cream liking \|\| id:ebi-fl187-GCST90094784 | Weighted median | 8 | 0.0377434014140501 | 0.163032322295603 | 0.816919625378527 | -0.28179995 | 0.357286753113432 | 1.03846473010654 | 0.754424591862442 | 1.42944570909729 | 0.999332001504091 |
| ebi-fl187-GCST90094784 | ebi-met1400-GCST90200082 | 1-oleoyl-2-linoleoyl-GPE (18:1/18:2) levels \|\| id:ebi-met1400-GCST90200082 | Ice cream liking \|\| id:ebi-fl187-GCST90094784 | Weighted median | 8 | -0.180518992 | 0.142695798259492 | 0.205849203434603 | -0.460202757 | 0.0991647724515516 | 0.834836825210979 | 0.631155661477253 | 1.10424823425824 | 0.999332001504091 |
| ebi-fl187-GCST90094784 | ebi-met1400-GCST90200100 | Perfluorooctanesulfonate (PFOS) levels \|\| id:ebi-met1400-GCST90200100 | Ice cream liking \|\| id:ebi-fl187-GCST90094784 | Weighted median | 8 | 0.191788883643111 | 0.137664130349169 | 0.163569823044663 | -0.078032812 | 0.461610579127483 | 1.21141474049986 | 0.924934077255515 | 1.58662731711087 | 0.999332001504091 |
| ebi-fl187-GCST90094784 | ebi-met1400-GCST90200102 | 1-palmitoleoyl-2-linolenoyl-GPC (16:1/18:3) levels \|\| id:ebi-met1400-GCST90200102 | Ice cream liking \|\| id:ebi-fl187-GCST90094784 | Weighted median | 8 | -0.021865822 | 0.13710468338372 | 0.873288606199793 | -0.290591001 | 0.246859357637825 | 0.978371502377136 | 0.747821473544414 | 1.2799990780244 | 0.999332001504091 |
| ebi-fl187-GCST90094784 | ebi-met1400-GCST90200114 | Glycosyl-N-tricosanoyl-sphingadienine (d18:2/23:0) levels \|\| id:ebi-met1400-GCST90200114 | Ice cream liking \|\| id:ebi-fl187-GCST90094784 | Weighted median | 8 | -0.054666741 | 0.152993674895576 | 0.720856951028446 | -0.354534344 | 0.245200861855398 | 0.946800625269177 | 0.70150002504661 | 1.2778779643672 | 0.999332001504091 |
| ebi-fl187-GCST90094784 | ebi-met1400-GCST90200135 | Arachidoylcarnitine (C20) levels \|\| id:ebi-met1400-GCST90200135 | Ice cream liking \|\| id:ebi-fl187-GCST90094784 | Weighted median | 8 | 0.277898722633341 | 0.163550552350332 | 0.0892888495109213 | -0.04266036 | 0.598457805239991 | 1.32035246861629 | 0.958236790373477 | 1.81931090404247 | 0.999332001504091 |
| ebi-fl187-GCST90094784 | ebi-met1400-GCST90200146 | Docosahexaenoylcarnitine (C22:6) levels \|\| id:ebi-met1400-GCST90200146 | Ice cream liking \|\| id:ebi-fl187-GCST90094784 | Weighted median | 8 | 0.344457599165153 | 0.176188146416779 | 0.0505765235770504 | -0.000871168 | 0.68978636614204 | 1.41122426282166 | 0.999129211544775 | 1.99328965359482 | 0.999332001504091 |
| ebi-fl187-GCST90094784 | ebi-met1400-GCST90200162 | 3-hydroxyphenylacetoylglutamine levels \|\| id:ebi-met1400-GCST90200162 | Ice cream liking \|\| id:ebi-fl187-GCST90094784 | Weighted median | 8 | -0.05769484 | 0.176599322471941 | 0.743895479228877 | -0.403829512 | 0.288439831912709 | 0.94393795547193 | 0.66775795616496 | 1.33434406217755 | 0.999332001504091 |
| ebi-fl187-GCST90094784 | ebi-met1400-GCST90200172 | 3-carboxy-4-methyl-5-pentyl-2-furanpropionate (3-CMPFP) levels \|\| id:ebi-met1400-GCST90200172 | Ice cream liking \|\| id:ebi-fl187-GCST90094784 | Weighted median | 8 | -0.140215276 | 0.14572646367229 | 0.335958548480028 | -0.425839145 | 0.145408592752093 | 0.869171103538909 | 0.653221407702185 | 1.15651201616998 | 0.999332001504091 |
| ebi-fl187-GCST90094784 | ebi-met1400-GCST90200222 | Dihydrocaffeate sulfate (2) levels \|\| id:ebi-met1400-GCST90200222 | Ice cream liking \|\| id:ebi-fl187-GCST90094784 | Weighted median | 8 | 0.165317012383783 | 0.16270489016329 | 0.309603503015563 | -0.153584572 | 0.484218597103832 | 1.17976706020428 | 0.85762822950864 | 1.62290636951221 | 0.999332001504091 |
| ebi-fl187-GCST90094784 | ebi-met1400-GCST90200223 | 4-methylhexanoylglutamine levels \|\| id:ebi-met1400-GCST90200223 | Ice cream liking \|\| id:ebi-fl187-GCST90094784 | Weighted median | 8 | -0.179299863 | 0.184529136512719 | 0.331218961746355 | -0.54097697 | 0.182377244689759 | 0.835855219864717 | 0.582179202575078 | 1.20006682733569 | 0.999332001504091 |
| ebi-fl187-GCST90094784 | ebi-met1400-GCST90200224 | 2-ketocaprylate levels \|\| id:ebi-met1400-GCST90200224 | Ice cream liking \|\| id:ebi-fl187-GCST90094784 | Weighted median | 8 | 0.10155602699614 | 0.139540202082341 | 0.466741869089759 | -0.171942769 | 0.375054823077529 | 1.10689193248472 | 0.842027361785708 | 1.45507118391191 | 0.999332001504091 |
| ebi-fl187-GCST90094784 | ebi-met1400-GCST90200243 | Hydroxypalmitoyl sphingomyelin (d18:1/16:0(OH)) levels \|\| id:ebi-met1400-GCST90200243 | Ice cream liking \|\| id:ebi-fl187-GCST90094784 | Weighted median | 8 | 0.0320012583172806 | 0.152168463562942 | 0.833432353469036 | -0.26624893 | 0.330251446900647 | 1.03251880454056 | 0.766248361349791 | 1.39131792706463 | 0.999332001504091 |
| ebi-fl187-GCST90094784 | ebi-met1400-GCST90200273 | 2-methoxyhydroquinone sulfate (2) levels \|\| id:ebi-met1400-GCST90200273 | Ice cream liking \|\| id:ebi-fl187-GCST90094784 | Weighted median | 8 | 0.101081893928992 | 0.172391484742711 | 0.557639764857479 | -0.236805416 | 0.438969204024706 | 1.10636724281396 | 0.78914482790456 | 1.55110751878335 | 0.999332001504091 |
| ebi-fl187-GCST90094784 | ebi-met1400-GCST90200276 | 2,4-di-tert-butylphenol levels \|\| id:ebi-met1400-GCST90200276 | Ice cream liking \|\| id:ebi-fl187-GCST90094784 | Weighted median | 8 | 0.088528325997203 | 0.142683133452525 | 0.534958906384372 | -0.191130616 | 0.368187267564153 | 1.09256520017097 | 0.826024689419287 | 1.44511263635937 | 0.999332001504091 |
| ebi-fl187-GCST90094784 | ebi-met1400-GCST90200289 | N-acetylmethionine levels \|\| id:ebi-met1400-GCST90200289 | Ice cream liking \|\| id:ebi-fl187-GCST90094784 | Weighted median | 8 | 0.0980369256127426 | 0.152265362820459 | 0.519668994317684 | -0.200403186 | 0.396477036740843 | 1.10300351343713 | 0.818400719234266 | 1.48657830090006 | 0.999332001504091 |
| ebi-fl187-GCST90094784 | ebi-met1400-GCST90200308 | 3-hydroxyisobutyrate levels \|\| id:ebi-met1400-GCST90200308 | Ice cream liking \|\| id:ebi-fl187-GCST90094784 | Weighted median | 8 | -0.04999593 | 0.145732980745648 | 0.731549330102534 | -0.335632572 | 0.235640712493889 | 0.951233296233434 | 0.714885726577972 | 1.26571947127054 | 0.999332001504091 |
| ebi-fl187-GCST90094784 | ebi-met1400-GCST90200383 | Cystathionine levels \|\| id:ebi-met1400-GCST90200383 | Ice cream liking \|\| id:ebi-fl187-GCST90094784 | Weighted median | 8 | 0.167545965314869 | 0.147094820836814 | 0.254689077854819 | -0.120759884 | 0.455851814155025 | 1.18239963830752 | 0.886246736488326 | 1.57751656182057 | 0.999332001504091 |
| ebi-fl187-GCST90094784 | ebi-met1400-GCST90200384 | Guanidinoacetate levels \|\| id:ebi-met1400-GCST90200384 | Ice cream liking \|\| id:ebi-fl187-GCST90094784 | Weighted median | 8 | -0.100279803 | 0.153869988883595 | 0.514583269976119 | -0.401864981 | 0.201305375300397 | 0.904584277308412 | 0.669071076817232 | 1.22299818824347 | 0.999332001504091 |
| ebi-fl187-GCST90094784 | ebi-met1400-GCST90200403 | Citrulline levels \|\| id:ebi-met1400-GCST90200403 | Ice cream liking \|\| id:ebi-fl187-GCST90094784 | Weighted median | 8 | 0.0526313881089771 | 0.150877077654141 | 0.727212276465014 | -0.243087684 | 0.348350460311093 | 1.05404104144029 | 0.784202748649944 | 1.41672866991755 | 0.999332001504091 |
| ebi-fl187-GCST90094784 | ebi-met1400-GCST90200434 | 12,13-DiHOME levels \|\| id:ebi-met1400-GCST90200434 | Ice cream liking \|\| id:ebi-fl187-GCST90094784 | Weighted median | 8 | -0.096362776 | 0.146696984863352 | 0.511256060863703 | -0.383888866 | 0.191163314499803 | 0.90813450705167 | 0.681207128133896 | 1.21065715380459 | 0.999332001504091 |
| ebi-fl187-GCST90094784 | ebi-met1400-GCST90200437 | Fructose levels \|\| id:ebi-met1400-GCST90200437 | Ice cream liking \|\| id:ebi-fl187-GCST90094784 | Weighted median | 7 | 0.221435793701753 | 0.160247149876181 | 0.167021079200952 | -0.09264862 | 0.535520207459068 | 1.24786712471979 | 0.911513731675795 | 1.70833670063711 | 0.999332001504091 |
| ebi-fl187-GCST90094784 | ebi-met1400-GCST90200448 | N-stearoyl-sphinganine (d18:0/18:0) levels \|\| id:ebi-met1400-GCST90200448 | Ice cream liking \|\| id:ebi-fl187-GCST90094784 | Weighted median | 8 | 0.186099147163447 | 0.175648775675025 | 0.289374022705661 | -0.158172453 | 0.530370747486495 | 1.20454168134837 | 0.853702545571812 | 1.69956230027839 | 0.999332001504091 |
| ebi-fl187-GCST90094784 | ebi-met1400-GCST90200468 | X-11847 levels \|\| id:ebi-met1400-GCST90200468 | Ice cream liking \|\| id:ebi-fl187-GCST90094784 | Weighted median | 8 | -0.194626694 | 0.152714906890455 | 0.202505881244107 | -0.493947912 | 0.104694523321286 | 0.823141884360588 | 0.610212567204678 | 1.1103713659857 | 0.999332001504091 |
| ebi-fl187-GCST90094784 | ebi-met1400-GCST90200476 | X-11843 levels \|\| id:ebi-met1400-GCST90200476 | Ice cream liking \|\| id:ebi-fl187-GCST90094784 | Weighted median | 8 | -0.014631306 | 0.151568247576316 | 0.923097430096369 | -0.311705072 | 0.282442458906835 | 0.985475211090712 | 0.7321974422129 | 1.32636545238286 | 0.999332001504091 |
| ebi-fl187-GCST90094784 | ebi-met1400-GCST90200511 | X-12906 levels \|\| id:ebi-met1400-GCST90200511 | Ice cream liking \|\| id:ebi-fl187-GCST90094784 | Weighted median | 8 | 0.02213655139522 | 0.163924417137376 | 0.892579293039313 | -0.299155306 | 0.343428408984477 | 1.02238338281642 | 0.741444249608228 | 1.40977259181854 | 0.999332001504091 |
| ebi-fl187-GCST90094784 | ebi-met1400-GCST90200515 | X-12849 levels \|\| id:ebi-met1400-GCST90200515 | Ice cream liking \|\| id:ebi-fl187-GCST90094784 | Weighted median | 8 | 0.184270676495226 | 0.139658113586626 | 0.187021637661664 | -0.089459226 | 0.458000579125012 | 1.20234122456378 | 0.914425549028485 | 1.58090991860563 | 0.999332001504091 |
| ebi-fl187-GCST90094784 | ebi-met1400-GCST90200551 | X-18886 levels \|\| id:ebi-met1400-GCST90200551 | Ice cream liking \|\| id:ebi-fl187-GCST90094784 | Weighted median | 8 | 0.218098656323906 | 0.158765255633327 | 0.169529311328872 | -0.093081245 | 0.529278557365227 | 1.24370976139977 | 0.911119473644877 | 1.69770706844091 | 0.999332001504091 |
| ebi-fl187-GCST90094784 | ebi-met1400-GCST90200564 | X-21319 levels \|\| id:ebi-met1400-GCST90200564 | Ice cream liking \|\| id:ebi-fl187-GCST90094784 | Weighted median | 8 | 0.107783366379811 | 0.139519528478305 | 0.439799121549084 | -0.165674909 | 0.381241642197289 | 1.11380643132963 | 0.847321645690409 | 1.46410135133563 | 0.999332001504091 |
| ebi-fl187-GCST90094784 | ebi-met1400-GCST90200579 | X-21442 levels \|\| id:ebi-met1400-GCST90200579 | Ice cream liking \|\| id:ebi-fl187-GCST90094784 | Weighted median | 7 | -0.076865339 | 0.159493588637081 | 0.629853027009402 | -0.389472773 | 0.23574209432289 | 0.926014542879533 | 0.677413931188562 | 1.26584779873046 | 0.999332001504091 |
| ebi-fl187-GCST90094784 | ebi-met1400-GCST90200581 | X-21441 levels \|\| id:ebi-met1400-GCST90200581 | Ice cream liking \|\| id:ebi-fl187-GCST90094784 | Weighted median | 8 | 0.201722067082212 | 0.159752894792026 | 0.206692317782205 | -0.111393607 | 0.514837740874583 | 1.22350790772827 | 0.894586564347925 | 1.67336696070857 | 0.999332001504091 |
| ebi-fl187-GCST90094784 | ebi-met1400-GCST90200648 | X-24585 levels \|\| id:ebi-met1400-GCST90200648 | Ice cream liking \|\| id:ebi-fl187-GCST90094784 | Weighted median | 8 | 0.140202465643957 | 0.182058089728319 | 0.441242104851434 | -0.21663139 | 0.497036321511462 | 1.15050671336044 | 0.805226729051959 | 1.64384222446995 | 0.999332001504091 |
| ebi-fl187-GCST90094784 | ebi-met1400-GCST90200720 | Adenosine 5'-diphosphate (ADP) to pantothenate ratio \|\| id:ebi-met1400-GCST90200720 | Ice cream liking \|\| id:ebi-fl187-GCST90094784 | Weighted median | 8 | -0.094864917 | 0.196481925644035 | 0.62922530513171 | -0.479969492 | 0.290239656910137 | 0.909495783269268 | 0.618802270176385 | 1.33674781049009 | 0.999332001504091 |
| ebi-fl187-GCST90094784 | ebi-met1400-GCST90200724 | Cholate to taurocholate ratio \|\| id:ebi-met1400-GCST90200724 | Ice cream liking \|\| id:ebi-fl187-GCST90094784 | Weighted median | 8 | -0.062603573 | 0.146686745894461 | 0.669536578987699 | -0.350109595 | 0.224902449210446 | 0.939315770264549 | 0.704610863873706 | 1.25220055708058 | 0.999332001504091 |
| ebi-fl187-GCST90094784 | ebi-met1400-GCST90200726 | N-acetylputrescine to (N(1) + N(8))-acetylspermidine ratio \|\| id:ebi-met1400-GCST90200726 | Ice cream liking \|\| id:ebi-fl187-GCST90094784 | Weighted median | 8 | 0.0557299499823407 | 0.143009531479983 | 0.696762866753064 | -0.224568732 | 0.336028631683108 | 1.05731211802369 | 0.798860667746473 | 1.39937909081603 | 0.999332001504091 |
| ebi-fl187-GCST90094784 | ebi-met1400-GCST90200744 | Arginine to glutamate ratio \|\| id:ebi-met1400-GCST90200744 | Ice cream liking \|\| id:ebi-fl187-GCST90094784 | Weighted median | 8 | 0.163164175604088 | 0.141289282381223 | 0.248162734790314 | -0.113762818 | 0.440091169071286 | 1.1772299462616 | 0.892469608630175 | 1.55284878383952 | 0.999332001504091 |
| ebi-fl187-GCST90094784 | ebi-met1400-GCST90200759 | Histidine to trans-urocanate ratio \|\| id:ebi-met1400-GCST90200759 | Ice cream liking \|\| id:ebi-fl187-GCST90094784 | Weighted median | 8 | -0.076447208 | 0.155548992679236 | 0.623096136281518 | -0.381323234 | 0.228428817769603 | 0.926401819712359 | 0.682957099302308 | 1.25662407264396 | 0.999332001504091 |
| ebi-fl187-GCST90094784 | ebi-met1400-GCST90200795 | Oleoyl-linoleoyl-glycerol (18:1 to 18:2) [2] to linoleoyl-arachidonoyl-glycerol (18:2 to 20:4) [2] ratio \|\| id:ebi-met1400-GCST90200795 | Ice cream liking \|\| id:ebi-fl187-GCST90094784 | Weighted median | 8 | -0.05745918 | 0.156495436766739 | 0.713498169025278 | -0.364190236 | 0.249271875696661 | 0.944160429882879 | 0.694759013696734 | 1.28309082686582 | 0.999332001504091 |
| ebi-fl187-GCST90094784 | ebi-met1400-GCST90200811 | Choline to taurocholate ratio \|\| id:ebi-met1400-GCST90200811 | Ice cream liking \|\| id:ebi-fl187-GCST90094784 | Weighted median | 8 | 0.0887362894143474 | 0.149006890100363 | 0.551497251781726 | -0.203317215 | 0.380789794011059 | 1.09279243739115 | 0.816019346639246 | 1.46343994923349 | 0.999332001504091 |
| ebi-fl187-GCST90094784 | ebi-met1400-GCST90200831 | Cholate to phosphate ratio \|\| id:ebi-met1400-GCST90200831 | Ice cream liking \|\| id:ebi-fl187-GCST90094784 | Weighted median | 8 | 0.0383454451166783 | 0.154577127949712 | 0.804082764517192 | -0.264625726 | 0.341316615898113 | 1.03909011949468 | 0.767493149215077 | 1.40679858515441 | 0.999332001504091 |
| ebi-fl187-GCST90094784 | ebi-met1400-GCST90200841 | Adenosine 5'-diphosphate (ADP) to oxalate (ethanedioate) ratio \|\| id:ebi-met1400-GCST90200841 | Ice cream liking \|\| id:ebi-fl187-GCST90094784 | Weighted median | 8 | 0.0406979694764352 | 0.187163877068239 | 0.827861064540866 | -0.32614323 | 0.407539168530184 | 1.04153748192392 | 0.721701810983277 | 1.50311431915967 | 0.999332001504091 |
| ebi-fl187-GCST90094784 | ebi-met1400-GCST90200844 | Aspartate to phosphate ratio \|\| id:ebi-met1400-GCST90200844 | Ice cream liking \|\| id:ebi-fl187-GCST90094784 | Weighted median | 8 | -0.147774765 | 0.137958762956401 | 0.284101219424027 | -0.418173941 | 0.122624410194614 | 0.862625386319308 | 0.658247722466338 | 1.13045975204356 | 0.999332001504091 |
| ebi-fl187-GCST90094784 | ebi-met1400-GCST90200870 | Adenosine 5'-monophosphate (AMP) to acetoacetate ratio \|\| id:ebi-met1400-GCST90200870 | Ice cream liking \|\| id:ebi-fl187-GCST90094784 | Weighted median | 8 | -0.083259313 | 0.162827881994598 | 0.609117714390225 | -0.402401962 | 0.235883335857024 | 0.920112519279922 | 0.668711895182399 | 1.26602660164244 | 0.999332001504091 |
| ebi-fl187-GCST90094784 | ebi-met1400-GCST90200887 | Cholesterol to cortisol ratio \|\| id:ebi-met1400-GCST90200887 | Ice cream liking \|\| id:ebi-fl187-GCST90094784 | Weighted median | 8 | 0.0608417097989573 | 0.138456010226691 | 0.66035007014632 | -0.21053207 | 0.332215489843271 | 1.06273068101956 | 0.810153072928897 | 1.39405322045779 | 0.999332001504091 |
| ebi-fl187-GCST90094784 | ebi-met1400-GCST90200890 | Cortisol to taurocholate ratio \|\| id:ebi-met1400-GCST90200890 | Ice cream liking \|\| id:ebi-fl187-GCST90094784 | Weighted median | 8 | -0.046501477 | 0.157083195257113 | 0.767206498866616 | -0.354384539 | 0.26138158617215 | 0.954563151126091 | 0.701605120778492 | 1.29872314568732 | 0.999332001504091 |
| ebi-fl187-GCST90094784 | ebi-met1400-GCST90200948 | Adenosine 5'-diphosphate (ADP) to mannitol to sorbitol ratio \|\| id:ebi-met1400-GCST90200948 | Ice cream liking \|\| id:ebi-fl187-GCST90094784 | Weighted median | 8 | 0.0870831733632016 | 0.185386956553504 | 0.638542696309055 | -0.276275261 | 0.45044160820807 | 1.09098741703745 | 0.758604087534179 | 1.56900491797101 | 0.999332001504091 |
| ebi-fl187-GCST90094784 | ebi-met1400-GCST90200972 | Leucine to N-palmitoyl-sphingosine (d18:1 to 16:0) ratio \|\| id:ebi-met1400-GCST90200972 | Ice cream liking \|\| id:ebi-fl187-GCST90094784 | Weighted median | 8 | -0.238304419 | 0.145458633105633 | 0.101359785910808 | -0.52340334 | 0.0467945014642341 | 0.787962783403627 | 0.592500631405667 | 1.04790664367088 | 0.999332001504091 |
| ebi-fl187-GCST90094784 | ebi-met1400-GCST90200973 | Proline to glutamate ratio \|\| id:ebi-met1400-GCST90200973 | Ice cream liking \|\| id:ebi-fl187-GCST90094784 | Weighted median | 8 | 0.231783147640963 | 0.14436666981514 | 0.108380067379783 | -0.051175525 | 0.514741820478639 | 1.26084628169001 | 0.950111887319607 | 1.67320645838498 | 0.999332001504091 |
| ebi-fl187-GCST90094784 | ebi-met1400-GCST90200983 | Cholesterol to linoleoyl-arachidonoyl-glycerol (18:2 to 20:4) [1] ratio \|\| id:ebi-met1400-GCST90200983 | Ice cream liking \|\| id:ebi-fl187-GCST90094784 | Weighted median | 8 | 0.00967727104526148 | 0.146316186817462 | 0.947266808896785 | -0.277102455 | 0.296456997207486 | 1.00972424724422 | 0.757976834526875 | 1.34508471635457 | 0.999332001504091 |
| ebi-fl187-GCST90094784 | ebi-met1400-GCST90201001 | Serine to alpha-ketobutyrate ratio \|\| id:ebi-met1400-GCST90201001 | Ice cream liking \|\| id:ebi-fl187-GCST90094784 | Weighted median | 8 | 0.18441595795254 | 0.146745164869583 | 0.208859056211909 | -0.103204565 | 0.472036481096924 | 1.20251591513842 | 0.901942448578449 | 1.60325587119256 | 0.999332001504091 |
| ebi-fl187-GCST90094784 | ebi-met1400-GCST90201005 | Fructose to maltose ratio \|\| id:ebi-met1400-GCST90201005 | Ice cream liking \|\| id:ebi-fl187-GCST90094784 | Weighted median | 8 | -0.126596382 | 0.169155304416692 | 0.454216953944951 | -0.458140779 | 0.204948014592008 | 0.881089224252318 | 0.632458433250246 | 1.22746125323051 | 0.999332001504091 |
| ebi-fl187-GCST90094784 | ebi-met1400-GCST90201020 | Paraxanthine to 5-acetylamino-6-formylamino-3-methyluracil ratio \|\| id:ebi-met1400-GCST90201020 | Ice cream liking \|\| id:ebi-fl187-GCST90094784 | Weighted median | 8 | 0.0932542557780407 | 0.157717427207671 | 0.55433667020619 | -0.215871902 | 0.402380413105075 | 1.09774080673172 | 0.805838521928594 | 1.49538008667049 | 0.999332001504091 |
| ebi-fl187-GCST90094784 | ebi-pw11-GCST90275197 | Placental weight (fetal genotype, adjusted for sex) \|\| id:ebi-pw11-GCST90275197 | Ice cream liking \|\| id:ebi-fl187-GCST90094784 | Weighted median | 8 | -0.108343195 | 0.0517670609241613 | 0.0363582326936427 | -0.209806634 | -0.006879755 | 0.897319588354355 | 0.810741000559644 | 0.993143856161983 | 0.999332001504091 |
| ebi-fl187-GCST90094784 | prot-a-1017 | FAS-associated factor 2 \|\| id:prot-a-1017 | Ice cream liking \|\| id:ebi-fl187-GCST90094784 | Weighted median | 9 | 0.0321117819740776 | 0.218650952852299 | 0.883239986932071 | -0.396444086 | 0.460667649564583 | 1.03263292860113 | 0.672707889696649 | 1.58513194443461 | 0.999332001504091 |
| ebi-fl187-GCST90094784 | prot-a-1019 | Fas apoptotic inhibitory molecule 3 \|\| id:prot-a-1019 | Ice cream liking \|\| id:ebi-fl187-GCST90094784 | Weighted median | 9 | -0.033963807 | 0.224485832564333 | 0.879742230899174 | -0.473956039 | 0.406028425123687 | 0.966606488680711 | 0.622534619504072 | 1.50084521356252 | 0.999332001504091 |
| ebi-fl187-GCST90094784 | prot-a-1052 | MIP18 family protein FAM96A \|\| id:prot-a-1052 | Ice cream liking \|\| id:ebi-fl187-GCST90094784 | Weighted median | 9 | 0.0500400736860205 | 0.214552363910637 | 0.815582717929587 | -0.37048256 | 0.470562706950868 | 1.05131322552799 | 0.690401090580238 | 1.60089477442912 | 0.999332001504091 |
| ebi-fl187-GCST90094784 | prot-a-1066 | F-box/LRR-repeat protein 4 \|\| id:prot-a-1066 | Ice cream liking \|\| id:ebi-fl187-GCST90094784 | Weighted median | 9 | 0.0643208970282503 | 0.233954899020033 | 0.783371080164067 | -0.394230705 | 0.522872499107514 | 1.06643455954036 | 0.674198497298305 | 1.68686621868699 | 0.999332001504091 |
| ebi-fl187-GCST90094784 | prot-a-1076 | Low affinity immunoglobulin gamma Fc region receptor III-B \|\| id:prot-a-1076 | Ice cream liking \|\| id:ebi-fl187-GCST90094784 | Weighted median | 9 | -0.01595741 | 0.220910475157485 | 0.942415101432365 | -0.448941941 | 0.41702712128857 | 0.984169234910635 | 0.638303156651106 | 1.51744366740459 | 0.999332001504091 |
| ebi-fl187-GCST90094784 | prot-a-1081 | Fc receptor-like protein 3 \|\| id:prot-a-1081 | Ice cream liking \|\| id:ebi-fl187-GCST90094784 | Weighted median | 9 | 0.155590537855391 | 0.232110617124924 | 0.502647919552893 | -0.299346272 | 0.610527347420241 | 1.16834771097497 | 0.741302672843514 | 1.84140220148457 | 0.999332001504091 |
| ebi-fl187-GCST90094784 | prot-a-1105 | Fibroblast growth factor receptor 4 \|\| id:prot-a-1105 | Ice cream liking \|\| id:ebi-fl187-GCST90094784 | Weighted median | 9 | -0.173944623 | 0.235963711509429 | 0.461020898476293 | -0.636433497 | 0.288544251899793 | 0.840343432355008 | 0.529176371495705 | 1.33448340164207 | 0.999332001504091 |
| ebi-fl187-GCST90094784 | prot-a-1143 | Follistatin-related protein 1 \|\| id:prot-a-1143 | Ice cream liking \|\| id:ebi-fl187-GCST90094784 | Weighted median | 9 | 0.221870545980803 | 0.229206713347803 | 0.33304775256589 | -0.227374612 | 0.671115704142498 | 1.24840975574271 | 0.796622301968561 | 1.95641888807561 | 0.999332001504091 |
| ebi-fl187-GCST90094784 | prot-a-1151 | Alpha-(1,3)-fucosyltransferase 10 \|\| id:prot-a-1151 | Ice cream liking \|\| id:ebi-fl187-GCST90094784 | Weighted median | 9 | 0.0720110287411823 | 0.248769603517112 | 0.772222682709125 | -0.415577394 | 0.559599451634721 | 1.07466719622482 | 0.659959114140601 | 1.74997141170729 | 0.999332001504091 |
| ebi-fl187-GCST90094784 | prot-a-1165 | Growth arrest and DNA damage-inducible proteins-interacting protein 1 \|\| id:prot-a-1165 | Ice cream liking \|\| id:ebi-fl187-GCST90094784 | Weighted median | 9 | -0.528499755 | 0.223827717068882 | 0.0182163975235347 | -0.967202081 | -0.08979743 | 0.589488683899892 | 0.380145167018465 | 0.914116339217193 | 0.999332001504091 |
| ebi-fl187-GCST90094784 | prot-a-1194 | Growth/differentiation factor 11/8 \|\| id:prot-a-1194 | Ice cream liking \|\| id:ebi-fl187-GCST90094784 | Weighted median | 9 | -0.021242111 | 0.216891935580565 | 0.921980979639045 | -0.446350305 | 0.40386608228672 | 0.978981913142507 | 0.6399595516394 | 1.49760337790879 | 0.999332001504091 |
| ebi-fl187-GCST90094784 | prot-a-1216 | D-glucuronyl C5-epimerase \|\| id:prot-a-1216 | Ice cream liking \|\| id:ebi-fl187-GCST90094784 | Weighted median | 9 | -0.677235254 | 0.292169509544797 | 0.020451863875438 | -1.249887493 | -0.104583015 | 0.508019597775099 | 0.286537032595311 | 0.900700022562449 | 0.999332001504091 |
| ebi-fl187-GCST90094784 | prot-a-1246 | Glypican-3 \|\| id:prot-a-1246 | Ice cream liking \|\| id:ebi-fl187-GCST90094784 | Weighted median | 9 | 0.19790634194224 | 0.212618581526713 | 0.351954678468513 | -0.218826078 | 0.614638761734597 | 1.21884823351745 | 0.803461445744404 | 1.84898855348588 | 0.999332001504091 |
| ebi-fl187-GCST90094784 | prot-a-1275 | Glutamate receptor 4 \|\| id:prot-a-1275 | Ice cream liking \|\| id:ebi-fl187-GCST90094784 | Weighted median | 9 | 0.33264128110442 | 0.240002009013936 | 0.165748816245457 | -0.137762657 | 0.803045218771735 | 1.39464692252424 | 0.871305465840621 | 2.232328517 | 0.999332001504091 |
| ebi-fl187-GCST90094784 | prot-a-1289 | Glutathione S-transferase P \|\| id:prot-a-1289 | Ice cream liking \|\| id:ebi-fl187-GCST90094784 | Weighted median | 9 | -0.228519454 | 0.219482497443272 | 0.297794833414312 | -0.658705149 | 0.201666240877319 | 0.795710817206956 | 0.517521013380762 | 1.22343960583166 | 0.999332001504091 |
| ebi-fl187-GCST90094784 | prot-a-130 | Apolipoprotein D \|\| id:prot-a-130 | Ice cream liking \|\| id:ebi-fl187-GCST90094784 | Weighted median | 9 | 0.173986103782984 | 0.228364318066288 | 0.446131896588608 | -0.27360796 | 0.621580167192909 | 1.19003902866486 | 0.76063021456882 | 1.86186778098002 | 0.999332001504091 |
| ebi-fl187-GCST90094784 | prot-a-1313 | Heparin-binding EGF-like growth factor \|\| id:prot-a-1313 | Ice cream liking \|\| id:ebi-fl187-GCST90094784 | Weighted median | 9 | -0.250871073 | 0.233630552639699 | 0.282915044120518 | -0.708786956 | 0.207044810256823 | 0.778122686180338 | 0.492240945327697 | 1.23003768885058 | 0.999332001504091 |
| ebi-fl187-GCST90094784 | prot-a-1347 | HLA class II histocompatibility antigen, DQ alpha 2 chain \|\| id:prot-a-1347 | Ice cream liking \|\| id:ebi-fl187-GCST90094784 | Weighted median | 9 | 0.0155293037773436 | 0.239586542570561 | 0.948319629941503 | -0.45406032 | 0.485118927215643 | 1.01565051001864 | 0.635044426426119 | 1.62436817894211 | 0.999332001504091 |
| ebi-fl187-GCST90094784 | prot-a-1357 | Non-histone chromosomal protein HMG-14 \|\| id:prot-a-1357 | Ice cream liking \|\| id:ebi-fl187-GCST90094784 | Weighted median | 9 | -0.166370669 | 0.217232447973484 | 0.443756797159165 | -0.592146267 | 0.259404929074691 | 0.846732318631313 | 0.553138826217263 | 1.29615855086107 | 0.999332001504091 |
| ebi-fl187-GCST90094784 | prot-a-137 | Apolipoprotein M \|\| id:prot-a-137 | Ice cream liking \|\| id:ebi-fl187-GCST90094784 | Weighted median | 9 | -0.134377538 | 0.224528407912736 | 0.549514422767593 | -0.574453217 | 0.305698141652296 | 0.874259936066814 | 0.563012630189118 | 1.35757245011503 | 0.999332001504091 |
| ebi-fl187-GCST90094784 | prot-a-1381 | 17-beta-hydroxysteroid dehydrogenase 14 \|\| id:prot-a-1381 | Ice cream liking \|\| id:ebi-fl187-GCST90094784 | Weighted median | 9 | 0.0277698881421526 | 0.215674165203872 | 0.897548721773148 | -0.394951476 | 0.450491251941742 | 1.02815906560532 | 0.67371272992301 | 1.56908281116673 | 0.999332001504091 |
| ebi-fl187-GCST90094784 | prot-a-1392 | Serine protease HTRA2, mitochondrial \|\| id:prot-a-1392 | Ice cream liking \|\| id:ebi-fl187-GCST90094784 | Weighted median | 9 | -0.018590702 | 0.202508127526881 | 0.926855157062246 | -0.415506632 | 0.378325228378392 | 0.981581039608455 | 0.660005816232519 | 1.45983764630852 | 0.999332001504091 |
| ebi-fl187-GCST90094784 | prot-a-1416 | Interferon-induced protein with tetratricopeptide repeats 2 \|\| id:prot-a-1416 | Ice cream liking \|\| id:ebi-fl187-GCST90094784 | Weighted median | 9 | -0.181018221 | 0.213054574534047 | 0.395529096626933 | -0.598605187 | 0.236568744876224 | 0.834420154411984 | 0.549577659640968 | 1.26689464514219 | 0.999332001504091 |
| ebi-fl187-GCST90094784 | prot-a-1428 | Interferon gamma \|\| id:prot-a-1428 | Ice cream liking \|\| id:ebi-fl187-GCST90094784 | Weighted median | 9 | -0.294002512 | 0.219384928619416 | 0.180205769301719 | -0.723996972 | 0.135991947856204 | 0.745274619133829 | 0.484810603610717 | 1.14567266843665 | 0.999332001504091 |
| ebi-fl187-GCST90094784 | prot-a-1483 | Interleukin-17C \|\| id:prot-a-1483 | Ice cream liking \|\| id:ebi-fl187-GCST90094784 | Weighted median | 9 | -0.194521428 | 0.233516215516532 | 0.40483888337909 | -0.65221321 | 0.263170354380106 | 0.823228537899827 | 0.520891657221089 | 1.30104833935752 | 0.999332001504091 |
| ebi-fl187-GCST90094784 | prot-a-1503 | Interleukin-1 receptor-like 2 \|\| id:prot-a-1503 | Ice cream liking \|\| id:ebi-fl187-GCST90094784 | Weighted median | 9 | 0.00228721504429286 | 0.227412903720369 | 0.991975377056083 | -0.443442076 | 0.448016506336215 | 1.00228983271597 | 0.641823409477823 | 1.56520453123253 | 0.999332001504091 |
| ebi-fl187-GCST90094784 | prot-a-1522 | Interleukin-31 receptor subunit alpha \|\| id:prot-a-1522 | Ice cream liking \|\| id:ebi-fl187-GCST90094784 | Weighted median | 9 | -0.081506654 | 0.221815968913243 | 0.713282118051078 | -0.516265953 | 0.353252645554213 | 0.921726577111194 | 0.596744665781514 | 1.42369078714844 | 0.999332001504091 |
| ebi-fl187-GCST90094784 | prot-a-1540 | Interleukin-6 receptor subunit alpha \|\| id:prot-a-1540 | Ice cream liking \|\| id:ebi-fl187-GCST90094784 | Weighted median | 9 | -0.184590182 | 0.210830508189957 | 0.381281087404947 | -0.597817978 | 0.228637614087332 | 0.831444955178486 | 0.55001046260559 | 1.25688647851683 | 0.999332001504091 |
| ebi-fl187-GCST90094784 | prot-a-1541 | Interleukin-6 receptor subunit alpha \|\| id:prot-a-1541 | Ice cream liking \|\| id:ebi-fl187-GCST90094784 | Weighted median | 9 | -0.065978123 | 0.221028958390744 | 0.76531819619712 | -0.499194881 | 0.367238635408771 | 0.936151344215836 | 0.607019185412083 | 1.44374240606938 | 0.999332001504091 |
| ebi-fl187-GCST90094784 | prot-a-1545 | Interleukin-9 \|\| id:prot-a-1545 | Ice cream liking \|\| id:ebi-fl187-GCST90094784 | Weighted median | 9 | 0.0517251610365008 | 0.238751275909957 | 0.828482105409094 | -0.41622734 | 0.519677661820016 | 1.05308627359661 | 0.659530315984542 | 1.68148555534115 | 0.999332001504091 |
| ebi-fl187-GCST90094784 | prot-a-1548 | Inositol monophosphatase 3 \|\| id:prot-a-1548 | Ice cream liking \|\| id:ebi-fl187-GCST90094784 | Weighted median | 9 | -0.057788689 | 0.218019330899444 | 0.790961526122296 | -0.485106578 | 0.369529199409717 | 0.943849371975823 | 0.615631580659913 | 1.44705318077449 | 0.999332001504091 |
| ebi-fl187-GCST90094784 | prot-a-1555 | Inhibin beta A chain:Inhibin beta B chain heterodimer \|\| id:prot-a-1555 | Ice cream liking \|\| id:ebi-fl187-GCST90094784 | Weighted median | 9 | -0.248509669 | 0.213734824532252 | 0.244950884141233 | -0.667429925 | 0.170410587345095 | 0.779962319547266 | 0.513025398740617 | 1.18579162241659 | 0.999332001504091 |
| ebi-fl187-GCST90094784 | prot-a-1603 | Junctophilin-3 \|\| id:prot-a-1603 | Ice cream liking \|\| id:ebi-fl187-GCST90094784 | Weighted median | 9 | -0.153720389 | 0.261182778418468 | 0.556159933848813 | -0.665638635 | 0.358197856595824 | 0.8575117571239 | 0.513945199584811 | 1.43074867553924 | 0.999332001504091 |
| ebi-fl187-GCST90094784 | prot-a-1627 | UPF0258 protein KIAA1024 \|\| id:prot-a-1627 | Ice cream liking \|\| id:ebi-fl187-GCST90094784 | Weighted median | 9 | 0.225939507000386 | 0.221777061020781 | 0.30831283387838 | -0.208743533 | 0.660622546601116 | 1.25349983501957 | 0.811603358756846 | 1.93599720779969 | 0.999332001504091 |
| ebi-fl187-GCST90094784 | prot-a-1666 | Kallikrein-7 \|\| id:prot-a-1666 | Ice cream liking \|\| id:ebi-fl187-GCST90094784 | Weighted median | 9 | 0.0699828909772335 | 0.228437260063893 | 0.759334386383435 | -0.377754139 | 0.517719920702464 | 1.0724898318443 | 0.685398993006695 | 1.67819686218619 | 0.999332001504091 |
| ebi-fl187-GCST90094784 | prot-a-1678 | Importin subunit alpha-3 \|\| id:prot-a-1678 | Ice cream liking \|\| id:ebi-fl187-GCST90094784 | Weighted median | 9 | 0.0883973654918557 | 0.230709807887104 | 0.701605647850783 | -0.363793858 | 0.54058858895058 | 1.09242212664893 | 0.695034455792155 | 1.71701718216548 | 0.999332001504091 |
| ebi-fl187-GCST90094784 | prot-a-1718 | Protein LDOC1 \|\| id:prot-a-1718 | Ice cream liking \|\| id:ebi-fl187-GCST90094784 | Weighted median | 9 | 0.154870269910544 | 0.232105343537907 | 0.504618017918174 | -0.300056203 | 0.609796743244842 | 1.16750649055873 | 0.740776585331371 | 1.84005735668201 | 0.999332001504091 |
| ebi-fl187-GCST90094784 | prot-a-1723 | Leptin \|\| id:prot-a-1723 | Ice cream liking \|\| id:ebi-fl187-GCST90094784 | Weighted median | 9 | -0.293340374 | 0.230030721618501 | 0.202230465720288 | -0.744200588 | 0.157519840435544 | 0.745768257414391 | 0.475113959821467 | 1.17060398304417 | 0.999332001504091 |
| ebi-fl187-GCST90094784 | prot-a-1730 | Galectin-7 \|\| id:prot-a-1730 | Ice cream liking \|\| id:ebi-fl187-GCST90094784 | Weighted median | 9 | -0.040495652 | 0.224442456929508 | 0.856816755066207 | -0.480402867 | 0.399411563674525 | 0.960313340033749 | 0.618534154302973 | 1.49094711202489 | 0.999332001504091 |
| ebi-fl187-GCST90094784 | prot-a-1753 | Lipase member N \|\| id:prot-a-1753 | Ice cream liking \|\| id:ebi-fl187-GCST90094784 | Weighted median | 9 | 0.0378711736309089 | 0.215658589873565 | 0.860602645638774 | -0.384819663 | 0.460562009783097 | 1.03859742552445 | 0.680573358022054 | 1.58496450028691 | 0.999332001504091 |
| ebi-fl187-GCST90094784 | prot-a-1756 | VIP36-like protein \|\| id:prot-a-1756 | Ice cream liking \|\| id:ebi-fl187-GCST90094784 | Weighted median | 9 | -0.38826614 | 0.228685376019045 | 0.0895421239214206 | -0.836489477 | 0.0599571969958661 | 0.678231814626087 | 0.433228716417991 | 1.06179109772395 | 0.999332001504091 |
| ebi-fl187-GCST90094784 | prot-a-1760 | Ig Kappa chain V-I region HK102- like \|\| id:prot-a-1760 | Ice cream liking \|\| id:ebi-fl187-GCST90094784 | Weighted median | 9 | -0.064837332 | 0.229966834172545 | 0.777987907202711 | -0.515572327 | 0.385897662787763 | 0.937219906487552 | 0.597158726634175 | 1.47093413181357 | 0.999332001504091 |
| ebi-fl187-GCST90094784 | prot-a-179 | Neutral ceramidase \|\| id:prot-a-179 | Ice cream liking \|\| id:ebi-fl187-GCST90094784 | Weighted median | 9 | -0.676705662 | 0.226792518856763 | 0.00284683999700934 | -1.121218998 | -0.232192325 | 0.508288712312473 | 0.325882302370365 | 0.792793634956736 | 0.999332001504091 |
| ebi-fl187-GCST90094784 | prot-a-1809 | Lactotransferrin \|\| id:prot-a-1809 | Ice cream liking \|\| id:ebi-fl187-GCST90094784 | Weighted median | 9 | -0.076308818 | 0.235739186926343 | 0.74616503226407 | -0.538357624 | 0.385739988417505 | 0.926530033260914 | 0.583706130305621 | 1.47070222148428 | 0.999332001504091 |
| ebi-fl187-GCST90094784 | prot-a-1818 | Lysophospholipase-like protein 1 \|\| id:prot-a-1818 | Ice cream liking \|\| id:ebi-fl187-GCST90094784 | Weighted median | 9 | 0.18222791609856 | 0.216510430942777 | 0.399978948916698 | -0.242132529 | 0.606588360746403 | 1.19988763642651 | 0.784952142089499 | 1.83416320925848 | 0.999332001504091 |
| ebi-fl187-GCST90094784 | prot-a-1852 | MAP kinase-activated protein kinase 3 \|\| id:prot-a-1852 | Ice cream liking \|\| id:ebi-fl187-GCST90094784 | Weighted median | 9 | 0.232107389860631 | 0.221512617030482 | 0.29471730332688 | -0.20205734 | 0.666272119240375 | 1.26125516757249 | 0.817048077455203 | 1.94696571918135 | 0.999332001504091 |
| ebi-fl187-GCST90094784 | prot-a-1856 | Megakaryocyte-associated tyrosine-protein kinase \|\| id:prot-a-1856 | Ice cream liking \|\| id:ebi-fl187-GCST90094784 | Weighted median | 9 | -0.051754585 | 0.222799746801843 | 0.816311274602339 | -0.488442088 | 0.384932919005867 | 0.949561875237843 | 0.613581555750669 | 1.46951574155792 | 0.999332001504091 |
| ebi-fl187-GCST90094784 | prot-a-1890 | Beta-1,3-N-acetylglucosaminyltransferase manic fringe \|\| id:prot-a-1890 | Ice cream liking \|\| id:ebi-fl187-GCST90094784 | Weighted median | 9 | -0.255396044 | 0.214344370840119 | 0.233448705182025 | -0.675511011 | 0.164718922432571 | 0.774609657375959 | 0.508896302410487 | 1.17906166434692 | 0.999332001504091 |
| ebi-fl187-GCST90094784 | prot-a-1919 | Matrilysin \|\| id:prot-a-1919 | Ice cream liking \|\| id:ebi-fl187-GCST90094784 | Weighted median | 9 | -0.236157467 | 0.245946573314991 | 0.336955451360037 | -0.718212751 | 0.245897816319288 | 0.789656319031985 | 0.48762298127561 | 1.27876889755264 | 0.999332001504091 |
| ebi-fl187-GCST90094784 | prot-a-1923 | Multimerin-2 \|\| id:prot-a-1923 | Ice cream liking \|\| id:ebi-fl187-GCST90094784 | Weighted median | 9 | 0.11190275620083 | 0.219220970549557 | 0.609731724569954 | -0.317770346 | 0.541575858477962 | 1.11840409749341 | 0.72776990443854 | 1.71871317797216 | 0.999332001504091 |
| ebi-fl187-GCST90094784 | prot-a-1948 | Mesothelin \|\| id:prot-a-1948 | Ice cream liking \|\| id:ebi-fl187-GCST90094784 | Weighted median | 9 | 0.193591341396964 | 0.222380994941731 | 0.38400588521813 | -0.242275409 | 0.629458091482756 | 1.21360023342965 | 0.784839996029815 | 1.87659336174369 | 0.999332001504091 |
| ebi-fl187-GCST90094784 | prot-a-195 | ATPase family AAA domain-containing protein 1 \|\| id:prot-a-195 | Ice cream liking \|\| id:ebi-fl187-GCST90094784 | Weighted median | 9 | 0.417207482707284 | 0.228540666847067 | 0.0679213263205514 | -0.030732224 | 0.865147189727535 | 1.51771738038015 | 0.96973520982432 | 2.3753556882041 | 0.999332001504091 |
| ebi-fl187-GCST90094784 | prot-a-1966 | Mucin-1 \|\| id:prot-a-1966 | Ice cream liking \|\| id:ebi-fl187-GCST90094784 | Weighted median | 9 | -0.097998834 | 0.216929677292245 | 0.651446261610982 | -0.523181002 | 0.32718333310499 | 0.90664996055555 | 0.592632381711409 | 1.38705574710845 | 0.999332001504091 |
| ebi-fl187-GCST90094784 | prot-a-1986 | Myocardial zonula adherens protein \|\| id:prot-a-1986 | Ice cream liking \|\| id:ebi-fl187-GCST90094784 | Weighted median | 9 | -0.100264143 | 0.216016517032016 | 0.642540027689369 | -0.523656516 | 0.323128230601109 | 0.904598443326536 | 0.592350643539444 | 1.38144248274849 | 0.999332001504091 |
| ebi-fl187-GCST90094784 | prot-a-2071 | Epididymal secretory protein E1 \|\| id:prot-a-2071 | Ice cream liking \|\| id:ebi-fl187-GCST90094784 | Weighted median | 9 | -0.355460769 | 0.213389937903106 | 0.0957571681431001 | -0.773705047 | 0.062783509440519 | 0.700850438748708 | 0.461300757122488 | 1.06479629593114 | 0.999332001504091 |
| ebi-fl187-GCST90094784 | prot-a-2098 | Neuritin \|\| id:prot-a-2098 | Ice cream liking \|\| id:ebi-fl187-GCST90094784 | Weighted median | 9 | -0.197177677 | 0.22324493998974 | 0.377109478965946 | -0.634737759 | 0.240382405639339 | 0.821044739798886 | 0.530074477289633 | 1.27173537612731 | 0.999332001504091 |
| ebi-fl187-GCST90094784 | prot-a-2115 | Neurotrophin-4 \|\| id:prot-a-2115 | Ice cream liking \|\| id:ebi-fl187-GCST90094784 | Weighted median | 9 | 0.176060045892345 | 0.230018288160016 | 0.44402291645681 | -0.274775799 | 0.626895890685977 | 1.19250966180768 | 0.759742439221351 | 1.87179130727796 | 0.999332001504091 |
| ebi-fl187-GCST90094784 | prot-a-2118 | Netrin-1 \|\| id:prot-a-2118 | Ice cream liking \|\| id:ebi-fl187-GCST90094784 | Weighted median | 9 | -0.33982419 | 0.243000835512252 | 0.161978383272046 | -0.816105827 | 0.136457447825849 | 0.711895470261733 | 0.442150115214673 | 1.14620610317633 | 0.999332001504091 |
| ebi-fl187-GCST90094784 | prot-a-2120 | Netrin-G1 \|\| id:prot-a-2120 | Ice cream liking \|\| id:ebi-fl187-GCST90094784 | Weighted median | 9 | -0.074917734 | 0.22131958306094 | 0.734982836031529 | -0.508704116 | 0.358868649100146 | 0.927819811492626 | 0.601274255553629 | 1.43170873299003 | 0.999332001504091 |
| ebi-fl187-GCST90094784 | prot-a-2145 | Olfactomedin-like protein 3 \|\| id:prot-a-2145 | Ice cream liking \|\| id:ebi-fl187-GCST90094784 | Weighted median | 9 | -0.018204603 | 0.23137443990913 | 0.937286938831253 | -0.471698506 | 0.435289298746309 | 0.981960099353991 | 0.62394159940015 | 1.54541007948551 | 0.999332001504091 |
| ebi-fl187-GCST90094784 | prot-a-2162 | Prolyl 4-hydroxylase subunit alpha-2 \|\| id:prot-a-2162 | Ice cream liking \|\| id:ebi-fl187-GCST90094784 | Weighted median | 9 | -0.045733411 | 0.229205057285206 | 0.841847747413307 | -0.494975324 | 0.403508500867453 | 0.955296599419507 | 0.609585949442239 | 1.49706795850114 | 0.999332001504091 |
| ebi-fl187-GCST90094784 | prot-a-2168 | Polyadenylate-binding protein-interacting protein 1 \|\| id:prot-a-2168 | Ice cream liking \|\| id:ebi-fl187-GCST90094784 | Weighted median | 9 | 0.378697455277313 | 0.225752950867112 | 0.0934474495934497 | -0.063778328 | 0.821173238976852 | 1.46038113829343 | 0.938212951626618 | 2.27316523970985 | 0.999332001504091 |
| ebi-fl187-GCST90094784 | prot-a-219 | Beta-1,4-galactosyltransferase 5 \|\| id:prot-a-219 | Ice cream liking \|\| id:ebi-fl187-GCST90094784 | Weighted median | 9 | -0.041840765 | 0.211749589179145 | 0.843361558754971 | -0.45686996 | 0.373188429623493 | 0.959022478198324 | 0.633262684214198 | 1.45235798131848 | 0.999332001504091 |
| ebi-fl187-GCST90094784 | prot-a-2190 | Pyruvate carboxylase, mitochondrial \|\| id:prot-a-2190 | Ice cream liking \|\| id:ebi-fl187-GCST90094784 | Weighted median | 9 | -0.205359379 | 0.223579082363501 | 0.358352496563187 | -0.643574381 | 0.23285562225215 | 0.814354601727531 | 0.525411044611441 | 1.26219923268884 | 0.999332001504091 |
| ebi-fl187-GCST90094784 | prot-a-2197 | Protocadherin-9 \|\| id:prot-a-2197 | Ice cream liking \|\| id:ebi-fl187-GCST90094784 | Weighted median | 9 | 0.0377693210494122 | 0.219669762111154 | 0.863487196920877 | -0.392783413 | 0.468322054787273 | 1.03849164708252 | 0.675174966081756 | 1.59731174175313 | 0.999332001504091 |
| ebi-fl187-GCST90094784 | prot-a-2198 | Protocadherin alpha-4 \|\| id:prot-a-2198 | Ice cream liking \|\| id:ebi-fl187-GCST90094784 | Weighted median | 9 | 0.293154223214265 | 0.254988764670423 | 0.250277730316713 | -0.206623756 | 0.792932201968294 | 1.34064953376926 | 0.813325601679648 | 2.20986671104898 | 0.999332001504091 |
| ebi-fl187-GCST90094784 | prot-a-2254 | Placenta growth factor \|\| id:prot-a-2254 | Ice cream liking \|\| id:ebi-fl187-GCST90094784 | Weighted median | 9 | 0.195735997314722 | 0.217381984 | 0.36789455114335 | -0.230332691 | 0.621804685835161 | 1.21620578135526 | 0.794269312127114 | 1.86228585193686 | 0.999332001504091 |
| ebi-fl187-GCST90094784 | prot-a-2261 | Membrane-associated progesterone receptor component 2 \|\| id:prot-a-2261 | Ice cream liking \|\| id:ebi-fl187-GCST90094784 | Weighted median | 9 | -0.458137352 | 0.226211653664326 | 0.0428407015496375 | -0.901512193 | -0.014762511 | 0.632460600528214 | 0.405955312505906 | 0.985345920838738 | 0.999332001504091 |
| ebi-fl187-GCST90094784 | prot-a-2268 | Polymeric immunoglobulin receptor \|\| id:prot-a-2268 | Ice cream liking \|\| id:ebi-fl187-GCST90094784 | Weighted median | 9 | -0.48616643 | 0.242375525867001 | 0.0448733630945607 | -0.961222461 | -0.0111104 | 0.614979447620872 | 0.38242510036241 | 0.988951093005315 | 0.999332001504091 |
| ebi-fl187-GCST90094784 | prot-a-2283 | Group 10 secretory phospholipase A2 \|\| id:prot-a-2283 | Ice cream liking \|\| id:ebi-fl187-GCST90094784 | Weighted median | 9 | 0.142291866362681 | 0.22391704371216 | 0.525123957415388 | -0.296585539 | 0.581169272038515 | 1.15291309598758 | 0.743352038721659 | 1.78812801695615 | 0.999332001504091 |
| ebi-fl187-GCST90094784 | prot-a-2298 | Inactive phospholipase D5 \|\| id:prot-a-2298 | Ice cream liking \|\| id:ebi-fl187-GCST90094784 | Weighted median | 9 | -0.051394132 | 0.254689867060935 | 0.840080091329637 | -0.550586271 | 0.447798007858974 | 0.94990420949644 | 0.576611660560134 | 1.56486257378584 | 0.999332001504091 |
| ebi-fl187-GCST90094784 | prot-a-2304 | Procollagen-lysine,2-oxoglutarate 5-dioxygenase 2 \|\| id:prot-a-2304 | Ice cream liking \|\| id:ebi-fl187-GCST90094784 | Weighted median | 9 | -0.026152893 | 0.223538980305619 | 0.906864192397063 | -0.464289295 | 0.411983508244218 | 0.974186131830675 | 0.628581682903654 | 1.50980953671327 | 0.999332001504091 |
| ebi-fl187-GCST90094784 | prot-a-2331 | Periostin \|\| id:prot-a-2331 | Ice cream liking \|\| id:ebi-fl187-GCST90094784 | Weighted median | 9 | -0.017646113 | 0.220258160172279 | 0.936145320936241 | -0.449352107 | 0.414059880751782 | 0.982508667705145 | 0.638041400214987 | 1.51294772061887 | 0.999332001504091 |
| ebi-fl187-GCST90094784 | prot-a-2333 | Periostin \|\| id:prot-a-2333 | Ice cream liking \|\| id:ebi-fl187-GCST90094784 | Weighted median | 9 | 0.0795247759108045 | 0.226845838970892 | 0.72591285124374 | -0.365093068 | 0.524142620293752 | 1.08277238586917 | 0.694132046062697 | 1.68901010441883 | 0.999332001504091 |
| ebi-fl187-GCST90094784 | prot-a-2367 | cAMP-dependent protein kinase catalytic subunit alpha \|\| id:prot-a-2367 | Ice cream liking \|\| id:ebi-fl187-GCST90094784 | Weighted median | 9 | 0.0300359440201474 | 0.221686901659504 | 0.892225947649836 | -0.404470383 | 0.464542271272776 | 1.03049157329771 | 0.667330146519038 | 1.59128564500915 | 0.999332001504091 |
| ebi-fl187-GCST90094784 | prot-a-2385 | Phosphoribosyl pyrophosphate synthase-associated protein 1 \|\| id:prot-a-2385 | Ice cream liking \|\| id:ebi-fl187-GCST90094784 | Weighted median | 9 | -0.419942802 | 0.229055109812455 | 0.0667475427704781 | -0.868890818 | 0.0290052129563253 | 0.657084402472466 | 0.419416500781234 | 1.02942996083457 | 0.999332001504091 |
| ebi-fl187-GCST90094784 | prot-a-2393 | Trypsin-3 \|\| id:prot-a-2393 | Ice cream liking \|\| id:ebi-fl187-GCST90094784 | Weighted median | 9 | 0.0938434268288589 | 0.23215203575534 | 0.686041664770775 | -0.361174563 | 0.548861416909326 | 1.09838775439905 | 0.696857342165267 | 1.73128068833299 | 0.999332001504091 |
| ebi-fl187-GCST90094784 | prot-a-2405 | Pregnancy-specific beta-1-glycoprotein 6 \|\| id:prot-a-2405 | Ice cream liking \|\| id:ebi-fl187-GCST90094784 | Weighted median | 9 | -0.139802023 | 0.221954360636184 | 0.528780733503909 | -0.57483257 | 0.295228523868474 | 0.869530365391243 | 0.562799090468963 | 1.34343333019144 | 0.999332001504091 |
| ebi-fl187-GCST90094784 | prot-a-244 | Beclin-1 \|\| id:prot-a-244 | Ice cream liking \|\| id:ebi-fl187-GCST90094784 | Weighted median | 9 | -0.092115572 | 0.226057304071197 | 0.683649824057707 | -0.535187888 | 0.3509567444095 | 0.911999742212073 | 0.58555926051392 | 1.42042588322299 | 0.999332001504091 |
| ebi-fl187-GCST90094784 | prot-a-2454 | tRNA pseudouridine synthase A, mitochondrial \|\| id:prot-a-2454 | Ice cream liking \|\| id:ebi-fl187-GCST90094784 | Weighted median | 9 | 0.151256837593095 | 0.223554266983604 | 0.498659720772173 | -0.286909526 | 0.589423200880958 | 1.1632953977064 | 0.750579633936089 | 1.80294817650237 | 0.999332001504091 |
| ebi-fl187-GCST90094784 | prot-a-2466 | Glutaminyl-peptide cyclotransferase-like protein \|\| id:prot-a-2466 | Ice cream liking \|\| id:ebi-fl187-GCST90094784 | Weighted median | 9 | 0.175908595931574 | 0.228986769668487 | 0.442365884563557 | -0.272905473 | 0.624722664481808 | 1.19232906994184 | 0.761164735137557 | 1.86772789831292 | 0.999332001504091 |
| ebi-fl187-GCST90094784 | prot-a-2474 | Ras-related protein Rab-27A \|\| id:prot-a-2474 | Ice cream liking \|\| id:ebi-fl187-GCST90094784 | Weighted median | 9 | -0.30878062 | 0.218162871327664 | 0.15696079710947 | -0.736379848 | 0.118818607647396 | 0.734341852158362 | 0.478844270621541 | 1.12616562192843 | 0.999332001504091 |
| ebi-fl187-GCST90094784 | prot-a-2490 | Rap1 GTPase-GDP dissociation stimulator 1 \|\| id:prot-a-2490 | Ice cream liking \|\| id:ebi-fl187-GCST90094784 | Weighted median | 9 | 0.185311062320021 | 0.219889191610178 | 0.399369686146216 | -0.245671753 | 0.61629387787597 | 1.20359277426478 | 0.782178930494647 | 1.85205137825213 | 0.999332001504091 |
| ebi-fl187-GCST90094784 | prot-a-2545 | Receptor-interacting serine/threonine-protein kinase 2 \|\| id:prot-a-2545 | Ice cream liking \|\| id:ebi-fl187-GCST90094784 | Weighted median | 9 | -0.096336345 | 0.227146029884713 | 0.671480961197467 | -0.541542564 | 0.348869873177592 | 0.908158509759765 | 0.581850018885436 | 1.41746472815956 | 0.999332001504091 |
| ebi-fl187-GCST90094784 | prot-a-2610 | RUN and FYVE domain-containing protein 1 \|\| id:prot-a-2610 | Ice cream liking \|\| id:ebi-fl187-GCST90094784 | Weighted median | 9 | -0.240567832 | 0.22266133406174 | 0.279955047236629 | -0.676984047 | 0.19584838296861 | 0.786181315551621 | 0.508147232035701 | 1.21634247311821 | 0.999332001504091 |
| ebi-fl187-GCST90094784 | prot-a-2627 | Serine--tRNA ligase, mitochondrial \|\| id:prot-a-2627 | Ice cream liking \|\| id:ebi-fl187-GCST90094784 | Weighted median | 9 | 0.23600292606996 | 0.207070066887605 | 0.254400900270746 | -0.169854405 | 0.641860257169666 | 1.26617801508691 | 0.843787658892654 | 1.90001210493338 | 0.999332001504091 |
| ebi-fl187-GCST90094784 | prot-a-264 | BolA-like protein 2 \|\| id:prot-a-264 | Ice cream liking \|\| id:ebi-fl187-GCST90094784 | Weighted median | 9 | -0.350539326 | 0.22764085561413 | 0.123589900472927 | -0.796715403 | 0.0956367505287563 | 0.704308135244318 | 0.450807254935096 | 1.1003592864599 | 0.999332001504091 |
| ebi-fl187-GCST90094784 | prot-a-2646 | Secretoglobin family 1C member 1 \|\| id:prot-a-2646 | Ice cream liking \|\| id:ebi-fl187-GCST90094784 | Weighted median | 9 | 0.206615236253363 | 0.228657124908279 | 0.366206014413421 | -0.241552729 | 0.654783201073589 | 1.2295094101117 | 0.785407389291089 | 1.9247251937847 | 0.999332001504091 |
| ebi-fl187-GCST90094784 | prot-a-2672 | Semaphorin-3E \|\| id:prot-a-2672 | Ice cream liking \|\| id:ebi-fl187-GCST90094784 | Weighted median | 9 | -0.179422682 | 0.215112992525142 | 0.404232663421909 | -0.601044147 | 0.242198783212965 | 0.835752567048191 | 0.548238894869326 | 1.27404742688701 | 0.999332001504091 |
| ebi-fl187-GCST90094784 | prot-a-2701 | Plasma protease C1 inhibitor \|\| id:prot-a-2701 | Ice cream liking \|\| id:ebi-fl187-GCST90094784 | Weighted median | 9 | 0.157577695579155 | 0.222095674263255 | 0.478011975968922 | -0.277729826 | 0.592885217135135 | 1.17067171046489 | 0.757501451084893 | 1.80920082954297 | 0.999332001504091 |
| ebi-fl187-GCST90094784 | prot-a-2738 | Signaling threshold-regulating transmembrane adapter 1 \|\| id:prot-a-2738 | Ice cream liking \|\| id:ebi-fl187-GCST90094784 | Weighted median | 9 | -0.080010875 | 0.223314320238341 | 0.720127682538536 | -0.517706943 | 0.357685192200798 | 0.923106307120463 | 0.595885381617539 | 1.43001536962104 | 0.999332001504091 |
| ebi-fl187-GCST90094784 | prot-a-274 | Serine/threonine-protein kinase BRSK2 \|\| id:prot-a-274 | Ice cream liking \|\| id:ebi-fl187-GCST90094784 | Weighted median | 9 | 0.447078134619982 | 0.233221764093124 | 0.0552422826071571 | -0.010036523 | 0.904192792242505 | 1.56373647646856 | 0.990013674816894 | 2.46993736555263 | 0.999332001504091 |
| ebi-fl187-GCST90094784 | prot-a-2749 | Mitochondrial glutamate carrier 2 \|\| id:prot-a-2749 | Ice cream liking \|\| id:ebi-fl187-GCST90094784 | Weighted median | 9 | 0.315488367900181 | 0.238168962796401 | 0.185290257637864 | -0.151322799 | 0.782299534981126 | 1.37092866505162 | 0.859570185319446 | 2.18649440936779 | 0.999332001504091 |
| ebi-fl187-GCST90094784 | prot-a-2760 | Sodium-coupled monocarboxylate transporter 1 \|\| id:prot-a-2760 | Ice cream liking \|\| id:ebi-fl187-GCST90094784 | Weighted median | 9 | -0.041327645 | 0.222774018986244 | 0.852826090874483 | -0.477964722 | 0.395309431948119 | 0.959514697992124 | 0.62004407021453 | 1.4848435778837 | 0.999332001504091 |
| ebi-fl187-GCST90094784 | prot-a-2777 | Small integral membrane protein 10 \|\| id:prot-a-2777 | Ice cream liking \|\| id:ebi-fl187-GCST90094784 | Weighted median | 9 | -0.326809584 | 0.239044509485717 | 0.17157844701182 | -0.795336823 | 0.141717654324837 | 0.72122106178938 | 0.451429157652885 | 1.15225126944185 | 0.999332001504091 |
| ebi-fl187-GCST90094784 | prot-a-2799 | Superoxide dismutase [Mn], mitochondrial \|\| id:prot-a-2799 | Ice cream liking \|\| id:ebi-fl187-GCST90094784 | Weighted median | 9 | -0.205470379 | 0.258519334061161 | 0.426731623400602 | -0.712168274 | 0.301227515614695 | 0.814264213411992 | 0.49057933305309 | 1.35151679773615 | 0.999332001504091 |
| ebi-fl187-GCST90094784 | prot-a-2805 | Protein SPACA7 \|\| id:prot-a-2805 | Ice cream liking \|\| id:ebi-fl187-GCST90094784 | Weighted median | 9 | 0.00470175681788934 | 0.20893781534189 | 0.982046607628947 | -0.404816361 | 0.414219874887994 | 1.0047128274201 | 0.667099304891803 | 1.51318980274791 | 0.999332001504091 |
| ebi-fl187-GCST90094784 | prot-a-2811 | SPARC-like protein 1 \|\| id:prot-a-2811 | Ice cream liking \|\| id:ebi-fl187-GCST90094784 | Weighted median | 9 | -0.087457796 | 0.217481527892635 | 0.687581761070719 | -0.51372159 | 0.338805999083284 | 0.916257540947197 | 0.598264933665681 | 1.40327108292753 | 0.999332001504091 |
| ebi-fl187-GCST90094784 | prot-a-2831 | Sepiapterin reductase \|\| id:prot-a-2831 | Ice cream liking \|\| id:ebi-fl187-GCST90094784 | Weighted median | 9 | -0.14924392 | 0.250808439858083 | 0.551809094389378 | -0.640828463 | 0.342340621670611 | 0.86135898620007 | 0.526855762908341 | 1.40823989285411 | 0.999332001504091 |
| ebi-fl187-GCST90094784 | prot-a-2834 | SPRY domain-containing SOCS box protein 1 \|\| id:prot-a-2834 | Ice cream liking \|\| id:ebi-fl187-GCST90094784 | Weighted median | 9 | -0.048899927 | 0.239965999238119 | 0.838526543864062 | -0.519233286 | 0.421433431484893 | 0.952276422067936 | 0.594976550267348 | 1.52414474758548 | 0.999332001504091 |
| ebi-fl187-GCST90094784 | prot-a-2835 | Spectrin alpha chain, non-erythrocytic 1 \|\| id:prot-a-2835 | Ice cream liking \|\| id:ebi-fl187-GCST90094784 | Weighted median | 9 | -0.162893927 | 0.227527719237793 | 0.47403455556584 | -0.608848256 | 0.283060403199484 | 0.849681312299709 | 0.543977030581895 | 1.32718532563603 | 0.999332001504091 |
| ebi-fl187-GCST90094784 | prot-a-2859 | Sia-alpha-2,3-Gal-beta-1,4-GlcNAc-R:alpha 2,8-sialyltransferase \|\| id:prot-a-2859 | Ice cream liking \|\| id:ebi-fl187-GCST90094784 | Weighted median | 9 | 0.110151580977679 | 0.224107985667131 | 0.623064945140507 | -0.329100071 | 0.549403232885256 | 1.11644728980505 | 0.719571005004396 | 1.73221897803597 | 0.999332001504091 |
| ebi-fl187-GCST90094784 | prot-a-2861 | Alpha-2,8-sialyltransferase 8F \|\| id:prot-a-2861 | Ice cream liking \|\| id:ebi-fl187-GCST90094784 | Weighted median | 9 | -0.065797166 | 0.251848533212189 | 0.793894403851058 | -0.559420291 | 0.427825958840047 | 0.936320762478074 | 0.57154029468251 | 1.53391909267661 | 0.999332001504091 |
| ebi-fl187-GCST90094784 | prot-a-289 | Uncharacterized protein C17orf89 \|\| id:prot-a-289 | Ice cream liking \|\| id:ebi-fl187-GCST90094784 | Weighted median | 9 | 0.0797810102077599 | 0.243084930834968 | 0.74275878105034 | -0.396665454 | 0.556227474644297 | 1.08304986483852 | 0.672558989766083 | 1.74408048598786 | 0.999332001504091 |
| ebi-fl187-GCST90094784 | prot-a-2921 | Tachykinin-3 \|\| id:prot-a-2921 | Ice cream liking \|\| id:ebi-fl187-GCST90094784 | Weighted median | 9 | 0.0330352605952005 | 0.234007710615359 | 0.887734321604938 | -0.425619852 | 0.491690373401303 | 1.03358698349101 | 0.653364670051823 | 1.63507777725004 | 0.999332001504091 |
| ebi-fl187-GCST90094784 | prot-a-2928 | Tax1-binding protein 1 \|\| id:prot-a-2928 | Ice cream liking \|\| id:ebi-fl187-GCST90094784 | Weighted median | 9 | -0.148959394 | 0.250838086511648 | 0.552614585473536 | -0.640602044 | 0.342683255110076 | 0.861604100094651 | 0.526975066335931 | 1.40872248560367 | 0.999332001504091 |
| ebi-fl187-GCST90094784 | prot-a-296 | Uncharacterized protein C1orf185 \|\| id:prot-a-296 | Ice cream liking \|\| id:ebi-fl187-GCST90094784 | Weighted median | 9 | -0.147760832 | 0.247026011752686 | 0.549733846842876 | -0.631931815 | 0.336410151302211 | 0.862637405765293 | 0.531563925452476 | 1.39991308325155 | 0.999332001504091 |
| ebi-fl187-GCST90094784 | prot-a-2964 | Transforming growth factor beta-2 \|\| id:prot-a-2964 | Ice cream liking \|\| id:ebi-fl187-GCST90094784 | Weighted median | 9 | 0.203622779621304 | 0.228706632561216 | 0.373292464276155 | -0.24464222 | 0.651887779441287 | 1.22583565603958 | 0.782984624217618 | 1.91916036297584 | 0.999332001504091 |
| ebi-fl187-GCST90094784 | prot-a-2977 | Thymocyte nuclear protein 1 \|\| id:prot-a-2977 | Ice cream liking \|\| id:ebi-fl187-GCST90094784 | Weighted median | 9 | 0.0455915399207731 | 0.221672742563999 | 0.837048317156123 | -0.388887036 | 0.480070115346211 | 1.04664681019495 | 0.677810834248194 | 1.61618771778164 | 0.999332001504091 |
| ebi-fl187-GCST90094784 | prot-a-3015 | Transmembrane protein 70, mitochondrial \|\| id:prot-a-3015 | Ice cream liking \|\| id:ebi-fl187-GCST90094784 | Weighted median | 9 | 0.0916670756615082 | 0.23241295558443 | 0.693274925890194 | -0.363862317 | 0.547196468606991 | 1.09599987630072 | 0.69498687583661 | 1.72840059375969 | 0.999332001504091 |
| ebi-fl187-GCST90094784 | prot-a-3030 | Tumor necrosis factor alpha-induced protein 3 \|\| id:prot-a-3030 | Ice cream liking \|\| id:ebi-fl187-GCST90094784 | Weighted median | 9 | 0.0778106097150469 | 0.218005208468416 | 0.721151041756008 | -0.349479599 | 0.505100818313142 | 1.08091792392969 | 0.705054905625131 | 1.65715258336728 | 0.999332001504091 |
| ebi-fl187-GCST90094784 | prot-a-3032 | Tumor necrosis factor receptor superfamily member 10A \|\| id:prot-a-3032 | Ice cream liking \|\| id:ebi-fl187-GCST90094784 | Weighted median | 9 | 0.237159218763685 | 0.215869955219543 | 0.271933509574022 | -0.185945893 | 0.660264330993989 | 1.26764293424916 | 0.830318519423256 | 1.93530382758172 | 0.999332001504091 |
| ebi-fl187-GCST90094784 | prot-a-3081 | Tumor protein p53-inducible protein 11 \|\| id:prot-a-3081 | Ice cream liking \|\| id:ebi-fl187-GCST90094784 | Weighted median | 9 | -0.173517579 | 0.220411236773158 | 0.431138632815185 | -0.605523603 | 0.258488445336058 | 0.840702372544125 | 0.545788575375668 | 1.29497118680955 | 0.999332001504091 |
| ebi-fl187-GCST90094784 | prot-a-315 | Complement C4 \|\| id:prot-a-315 | Ice cream liking \|\| id:ebi-fl187-GCST90094784 | Weighted median | 9 | 0.310106150992879 | 0.230566908643191 | 0.178634116208101 | -0.14180499 | 0.762017291933534 | 1.36356985074359 | 0.867790467850875 | 2.14259410161718 | 0.999332001504091 |
| ebi-fl187-GCST90094784 | prot-a-3157 | UDP-glucuronosyltransferase 1-8 \|\| id:prot-a-3157 | Ice cream liking \|\| id:ebi-fl187-GCST90094784 | Weighted median | 9 | -0.305116974 | 0.221700022943207 | 0.16874101180591 | -0.739649019 | 0.129415070904108 | 0.737037155117238 | 0.477281402815055 | 1.13816244425055 | 0.999332001504091 |
| ebi-fl187-GCST90094784 | prot-a-317 | Glutaredoxin-like protein C5orf63 \|\| id:prot-a-317 | Ice cream liking \|\| id:ebi-fl187-GCST90094784 | Weighted median | 9 | 0.231574065122015 | 0.232317170289253 | 0.318860950911331 | -0.223767589 | 0.68691571888895 | 1.26058268833076 | 0.799500925872071 | 1.98757582723986 | 0.999332001504091 |
| ebi-fl187-GCST90094784 | prot-a-3192 | Guanine nucleotide exchange factor VAV3 \|\| id:prot-a-3192 | Ice cream liking \|\| id:ebi-fl187-GCST90094784 | Weighted median | 9 | 0.0901794696365737 | 0.215004077477733 | 0.674900851391243 | -0.331228522 | 0.51158746149293 | 1.09437067238863 | 0.718041061952854 | 1.66793687999835 | 0.999332001504091 |
| ebi-fl187-GCST90094784 | prot-a-3233 | Serine/threonine-protein kinase WNK3 \|\| id:prot-a-3233 | Ice cream liking \|\| id:ebi-fl187-GCST90094784 | Weighted median | 9 | -0.148063417 | 0.221124358683681 | 0.503117004651584 | -0.58146716 | 0.285340325665186 | 0.862376423576224 | 0.559077508180732 | 1.33021465728453 | 0.999332001504091 |
| ebi-fl187-GCST90094784 | prot-a-3250 | 14-3-3 protein theta \|\| id:prot-a-3250 | Ice cream liking \|\| id:ebi-fl187-GCST90094784 | Weighted median | 9 | -0.276996968 | 0.221928451179131 | 0.211981127259731 | -0.711976732 | 0.157982796567695 | 0.758056795729744 | 0.490673308526219 | 1.17114604680263 | 0.999332001504091 |
| ebi-fl187-GCST90094784 | prot-a-3278 | E3 ubiquitin-protein ligase ZNRF3 \|\| id:prot-a-3278 | Ice cream liking \|\| id:ebi-fl187-GCST90094784 | Weighted median | 9 | 0.0618335281248318 | 0.218075213651205 | 0.776761229379632 | -0.365593891 | 0.489260946881193 | 1.06378523966331 | 0.693784496390042 | 1.6311102972375 | 0.999332001504091 |
| ebi-fl187-GCST90094784 | prot-a-355 | Calpain-3 \|\| id:prot-a-355 | Ice cream liking \|\| id:ebi-fl187-GCST90094784 | Weighted median | 9 | -0.081466257 | 0.215296462342852 | 0.705140108420482 | -0.503447323 | 0.340514809580235 | 0.921763812763331 | 0.604443352616048 | 1.40567105725076 | 0.999332001504091 |
| ebi-fl187-GCST90094784 | prot-a-385 | Coiled-coil domain-containing protein 90B, mitochondrial \|\| id:prot-a-385 | Ice cream liking \|\| id:ebi-fl187-GCST90094784 | Weighted median | 9 | -0.068749252 | 0.236068189585907 | 0.77087829280071 | -0.531442904 | 0.39394439958524 | 0.933560739217353 | 0.587756281888198 | 1.48281810108128 | 0.999332001504091 |
| ebi-fl187-GCST90094784 | prot-a-390 | C-C motif chemokine 14 \|\| id:prot-a-390 | Ice cream liking \|\| id:ebi-fl187-GCST90094784 | Weighted median | 9 | 0.266517338278091 | 0.234869664499196 | 0.25648188975291 | -0.193827204 | 0.726861880696515 | 1.30541022270459 | 0.823800241241482 | 2.06857896396527 | 0.999332001504091 |
| ebi-fl187-GCST90094784 | prot-a-40 | Pituitary adenylate cyclase-activating polypeptide 27 \|\| id:prot-a-40 | Ice cream liking \|\| id:ebi-fl187-GCST90094784 | Weighted median | 9 | 0.343184885916934 | 0.231954737113639 | 0.138997690524619 | -0.111446399 | 0.797816170659667 | 1.40942932147127 | 0.894539338477158 | 2.22068602997908 | 0.999332001504091 |
| ebi-fl187-GCST90094784 | prot-a-401 | C-C motif chemokine 24 \|\| id:prot-a-401 | Ice cream liking \|\| id:ebi-fl187-GCST90094784 | Weighted median | 9 | 0.0273342284024313 | 0.244887087896354 | 0.91112493472256 | -0.452644464 | 0.507312920679285 | 1.02771123565223 | 0.635944194572638 | 1.660822432 | 0.999332001504091 |
| ebi-fl187-GCST90094784 | prot-a-412 | Cerebral cavernous malformations 2 protein \|\| id:prot-a-412 | Ice cream liking \|\| id:ebi-fl187-GCST90094784 | Weighted median | 9 | 0.0936913245170836 | 0.237391048730262 | 0.693085825979189 | -0.371595131 | 0.558977780028397 | 1.0982206997874 | 0.689633397197741 | 1.7488838422593 | 0.999332001504091 |
| ebi-fl187-GCST90094784 | prot-a-44 | Adhesion G protein-coupled receptor F5 \|\| id:prot-a-44 | Ice cream liking \|\| id:ebi-fl187-GCST90094784 | Weighted median | 9 | -0.22997372 | 0.218385157260181 | 0.292311299074764 | -0.658008628 | 0.198061188599919 | 0.794554483414735 | 0.517881603323591 | 1.21903698270585 | 0.999332001504091 |
| ebi-fl187-GCST90094784 | prot-a-48 | Adrenomedullin \|\| id:prot-a-48 | Ice cream liking \|\| id:ebi-fl187-GCST90094784 | Weighted median | 9 | -0.451978264 | 0.232860496918063 | 0.0522603402980894 | -0.908384838 | 0.00442830976325564 | 0.636368001544823 | 0.403174891126641 | 1.00443812921611 | 0.999332001504091 |
| ebi-fl187-GCST90094784 | prot-a-502 | Carcinoembryonic antigen-related cell adhesion molecule 1 \|\| id:prot-a-502 | Ice cream liking \|\| id:ebi-fl187-GCST90094784 | Weighted median | 9 | -0.082854786 | 0.213479542780253 | 0.697930332831161 | -0.501274689 | 0.335565118265351 | 0.92048480517883 | 0.605758014036019 | 1.39873061013226 | 0.999332001504091 |
| ebi-fl187-GCST90094784 | prot-a-518 | Complement factor B \|\| id:prot-a-518 | Ice cream liking \|\| id:ebi-fl187-GCST90094784 | Weighted median | 9 | -0.13073821 | 0.218503367316454 | 0.549616433741655 | -0.55900481 | 0.297528389746042 | 0.877447451124041 | 0.571777808277669 | 1.34652660235842 | 0.999332001504091 |
| ebi-fl187-GCST90094784 | prot-a-525 | Properdin \|\| id:prot-a-525 | Ice cream liking \|\| id:ebi-fl187-GCST90094784 | Weighted median | 9 | -0.192825276 | 0.240182522884277 | 0.422074103691198 | -0.663583021 | 0.277932468639904 | 0.824626043337505 | 0.51500275899214 | 1.32039702599117 | 0.999332001504091 |
| ebi-fl187-GCST90094784 | prot-a-533 | Chondroadherin \|\| id:prot-a-533 | Ice cream liking \|\| id:ebi-fl187-GCST90094784 | Weighted median | 9 | 0.0306428422164422 | 0.23832834462445 | 0.897694695237892 | -0.436480713 | 0.497766397680365 | 1.03111716659136 | 0.646306962928458 | 1.64504279270327 | 0.999332001504091 |
| ebi-fl187-GCST90094784 | prot-a-535 | Coiled-coil-helix-coiled-coil-helix domain-containing protein 2 \|\| id:prot-a-535 | Ice cream liking \|\| id:ebi-fl187-GCST90094784 | Weighted median | 9 | -0.066163517 | 0.247570409376378 | 0.789275670045388 | -0.551401519 | 0.419074485320381 | 0.935977803441794 | 0.576141770382354 | 1.520553609 | 0.999332001504091 |
| ebi-fl187-GCST90094784 | prot-a-591 | Caseinolytic peptidase B protein homolog \|\| id:prot-a-591 | Ice cream liking \|\| id:ebi-fl187-GCST90094784 | Weighted median | 9 | -0.172591921 | 0.245036210633238 | 0.481213142681527 | -0.652862894 | 0.307679051597341 | 0.841480935282402 | 0.520553352340522 | 1.36026434420222 | 0.999332001504091 |
| ebi-fl187-GCST90094784 | prot-a-605 | Ciliary neurotrophic factor receptor subunit alpha \|\| id:prot-a-605 | Ice cream liking \|\| id:ebi-fl187-GCST90094784 | Weighted median | 9 | -0.229776228 | 0.222996425256344 | 0.302820775669279 | -0.666849222 | 0.207296765216385 | 0.794711416543492 | 0.513323400662529 | 1.23034764199221 | 0.999332001504091 |
| ebi-fl187-GCST90094784 | prot-a-606 | Ciliary neurotrophic factor receptor subunit alpha \|\| id:prot-a-606 | Ice cream liking \|\| id:ebi-fl187-GCST90094784 | Weighted median | 9 | -0.191225553 | 0.234336362728008 | 0.414482853068143 | -0.650524824 | 0.268073717807911 | 0.825946272365564 | 0.521771866447092 | 1.30744351833263 | 0.999332001504091 |
| ebi-fl187-GCST90094784 | prot-a-707 | Cancer/testis antigen 55 \|\| id:prot-a-707 | Ice cream liking \|\| id:ebi-fl187-GCST90094784 | Weighted median | 9 | -0.002719927 | 0.250159351322759 | 0.991324948793599 | -0.493032256 | 0.487592401365907 | 0.997283768423966 | 0.610771567810817 | 1.62839098474533 | 0.999332001504091 |
| ebi-fl187-GCST90094784 | prot-a-719 | Dipeptidyl peptidase 1 \|\| id:prot-a-719 | Ice cream liking \|\| id:ebi-fl187-GCST90094784 | Weighted median | 9 | 0.334633264943714 | 0.236439150769313 | 0.156979396225949 | -0.128787471 | 0.798054000451566 | 1.39742780547283 | 0.879160793239106 | 2.22121423808479 | 0.999332001504091 |
| ebi-fl187-GCST90094784 | prot-a-752 | Cytochrome b5 \|\| id:prot-a-752 | Ice cream liking \|\| id:ebi-fl187-GCST90094784 | Weighted median | 9 | 0.0876020542624195 | 0.221968980316419 | 0.693094569952006 | -0.347457147 | 0.522661255682601 | 1.09155365646214 | 0.706482288054927 | 1.68650991692411 | 0.999332001504091 |
| ebi-fl187-GCST90094784 | prot-a-755 | Cytoglobin \|\| id:prot-a-755 | Ice cream liking \|\| id:ebi-fl187-GCST90094784 | Weighted median | 9 | -0.125516337 | 0.224460983783549 | 0.576032059116331 | -0.565459865 | 0.314427191343961 | 0.882041354512801 | 0.568098837856917 | 1.36947463931747 | 0.999332001504091 |
| ebi-fl187-GCST90094784 | prot-a-807 | Desmin \|\| id:prot-a-807 | Ice cream liking \|\| id:ebi-fl187-GCST90094784 | Weighted median | 9 | -0.266545827 | 0.2255656522503 | 0.23733374060127 | -0.708654505 | 0.175562851496476 | 0.766020898547894 | 0.492306147335944 | 1.19191690005793 | 0.999332001504091 |
| ebi-fl187-GCST90094784 | prot-a-82 | Ameloblastin \|\| id:prot-a-82 | Ice cream liking \|\| id:ebi-fl187-GCST90094784 | Weighted median | 9 | 0.187988244410704 | 0.239570711219222 | 0.432636563879364 | -0.28157035 | 0.65754683840038 | 1.20681932839384 | 0.754597828168458 | 1.93005179317826 | 0.999332001504091 |
| ebi-fl187-GCST90094784 | prot-a-84 | Muellerian-inhibiting factor \|\| id:prot-a-84 | Ice cream liking \|\| id:ebi-fl187-GCST90094784 | Weighted median | 9 | -0.420292132 | 0.217662332310582 | 0.0534909112133574 | -0.846910304 | 0.00632603893006301 | 0.656854903185438 | 0.428737556472672 | 1.00634609057453 | 0.999332001504091 |
| ebi-fl187-GCST90094784 | prot-a-858 | Inactive dipeptidyl peptidase 10 \|\| id:prot-a-858 | Ice cream liking \|\| id:ebi-fl187-GCST90094784 | Weighted median | 9 | -0.01838263 | 0.212931117586555 | 0.931203021776841 | -0.43572762 | 0.398962360695569 | 0.981785300192121 | 0.646793875502731 | 1.49027752454231 | 0.999332001504091 |
| ebi-fl187-GCST90094784 | prot-a-863 | Dihydropyrimidinase-related protein 3 \|\| id:prot-a-863 | Ice cream liking \|\| id:ebi-fl187-GCST90094784 | Weighted median | 9 | 0.0527384848307263 | 0.226474628177359 | 0.815864623904927 | -0.391151786 | 0.49662875605835 | 1.0541539318254 | 0.676277498523676 | 1.64317238767933 | 0.999332001504091 |
| ebi-fl187-GCST90094784 | prot-a-864 | Dihydropyrimidinase-related protein 5 \|\| id:prot-a-864 | Ice cream liking \|\| id:ebi-fl187-GCST90094784 | Weighted median | 9 | -0.426416305 | 0.210935440559551 | 0.0432229771548309 | -0.839849768 | -0.012982841 | 0.652844503211594 | 0.431775384889334 | 0.987101072199453 | 0.999332001504091 |
| ebi-fl187-GCST90094784 | prot-a-884 | Endothelin-converting enzyme 1 \|\| id:prot-a-884 | Ice cream liking \|\| id:ebi-fl187-GCST90094784 | Weighted median | 9 | -0.19509571 | 0.228208301927365 | 0.392605461511932 | -0.642383982 | 0.252192562032838 | 0.822755908529494 | 0.526036865855621 | 1.28684381068833 | 0.999332001504091 |
| ebi-fl187-GCST90094784 | prot-a-893 | Early endosome antigen 1 \|\| id:prot-a-893 | Ice cream liking \|\| id:ebi-fl187-GCST90094784 | Weighted median | 9 | -0.222213255 | 0.249841879952182 | 0.373779786001308 | -0.71190334 | 0.267476829345887 | 0.800744583050655 | 0.490709321337216 | 1.30666335324073 | 0.999332001504091 |
| ebi-fl187-GCST90094784 | prot-a-897 | Ephrin-A2 \|\| id:prot-a-897 | Ice cream liking \|\| id:ebi-fl187-GCST90094784 | Weighted median | 9 | -0.225561521 | 0.231395329840628 | 0.329665151698327 | -0.679096367 | 0.227973325547219 | 0.79806796104611 | 0.50707499488323 | 1.25605182049051 | 0.999332001504091 |
| ebi-fl187-GCST90094784 | prot-a-90 | Anaphase-promoting complex subunit 10 \|\| id:prot-a-90 | Ice cream liking \|\| id:ebi-fl187-GCST90094784 | Weighted median | 9 | -0.195847798 | 0.213626704228256 | 0.359260122948753 | -0.614556138 | 0.222860542192443 | 0.822137356027902 | 0.540880918316253 | 1.24964628865192 | 0.999332001504091 |
| ebi-fl187-GCST90094784 | prot-a-914 | Histone-lysine N-methyltransferase EHMT2 \|\| id:prot-a-914 | Ice cream liking \|\| id:ebi-fl187-GCST90094784 | Weighted median | 9 | 0.0945237431154736 | 0.253735653763423 | 0.709499453400714 | -0.402798138 | 0.591845624491783 | 1.09913525971857 | 0.668447019583395 | 1.80732097498102 | 0.999332001504091 |
| ebi-fl187-GCST90094784 | prot-a-932 | RNA polymerase II elongation factor ELL \|\| id:prot-a-932 | Ice cream liking \|\| id:ebi-fl187-GCST90094784 | Weighted median | 9 | -0.096130854 | 0.225106077473979 | 0.66934516347055 | -0.537338766 | 0.345077057949787 | 0.908345147787268 | 0.584301147375573 | 1.41209872890824 | 0.999332001504091 |
| ebi-fl187-GCST90094784 | prot-a-991 | Estrogen receptor \|\| id:prot-a-991 | Ice cream liking \|\| id:ebi-fl187-GCST90094784 | Weighted median | 9 | -0.109457334 | 0.240171836193114 | 0.648572697927708 | -0.580194133 | 0.361279464836673 | 0.896320405861464 | 0.559789682343164 | 1.43516448284797 | 0.999332001504091 |
| ebi-fl187-GCST90094784 | prot-a-992 | Persulfide dioxygenase ETHE1, mitochondrial \|\| id:prot-a-992 | Ice cream liking \|\| id:ebi-fl187-GCST90094784 | Weighted median | 9 | -0.024766463 | 0.22436095649254 | 0.912102710179667 | -0.464513938 | 0.414981011859505 | 0.975537709708647 | 0.628440492264466 | 1.51434198588066 | 0.999332001504091 |
| ebi-fl187-GCST90094784 | prot-a-993 | Ethanolamine kinase 1 \|\| id:prot-a-993 | Ice cream liking \|\| id:ebi-fl187-GCST90094784 | Weighted median | 9 | 0.0610357045145262 | 0.211620913090538 | 0.773025257356293 | -0.353741285 | 0.47581269417198 | 1.06293686515442 | 0.702056576328713 | 1.60932155242045 | 0.999332001504091 |
| ebi-fl187-GCST90094784 | prot-b-10 | coagulation factor III, tissue factor \|\| id:prot-b-10 | Ice cream liking \|\| id:ebi-fl187-GCST90094784 | Weighted median | 7 | -0.14491564 | 0.2841539088968 | 0.610058497012135 | -0.701857302 | 0.412026021348357 | 0.865095269392173 | 0.495663851120954 | 1.50987372476774 | 0.999332001504091 |
| ebi-fl187-GCST90094784 | prot-b-28 | prolactin \|\| id:prot-b-28 | Ice cream liking \|\| id:ebi-fl187-GCST90094784 | Weighted median | 8 | -0.136625029 | 0.236205772565504 | 0.562983778262496 | -0.599588343 | 0.326338285464022 | 0.872297251193902 | 0.549037604757283 | 1.38588411403407 | 0.999332001504091 |
| ebi-fl187-GCST90094784 | prot-b-54 | matrix metallopeptidase 7 \|\| id:prot-b-54 | Ice cream liking \|\| id:ebi-fl187-GCST90094784 | Weighted median | 8 | 0.031582420116893 | 0.235868486837141 | 0.893482891641541 | -0.430719814 | 0.493884654317689 | 1.03208643677498 | 0.650041017599692 | 1.63866953643663 | 0.999332001504091 |
| ebi-fl187-GCST90094784 | ukb-rsfMRI191-edge-pheno716 | Net100_Pair10_33 (OccipitalPrecuneus)&(Temporal\|Frontal\|Supp_Motor_Area) (Default_mode\|Central_executive)&(Default_mode\|Salience) \|\| id:ukb-rsfMRI191-edge-pheno716 | Ice cream liking \|\| id:ebi-fl187-GCST90094784 | Weighted median | 8 | -0.020249968 | 0.0728498593275195 | 0.781036522671216 | -0.163035692 | 0.122535756558205 | 0.979953685896331 | 0.849560865342224 | 1.130359537 | 0.999332001504091 |
| ebi-fl187-GCST90094784 | ukb-rsfMRI191-edge-pheno903 | Net100_Pair27_38 (Precentral\|Frontal\|Supp_Motor_Area)&(Subcortical) (Attention\|Salience\|Motor)&(Subcortical-cerebellum) \|\| id:ukb-rsfMRI191-edge-pheno903 | Ice cream liking \|\| id:ebi-fl187-GCST90094784 | Weighted median | 8 | -0.074118344 | 0.0740049132310618 | 0.316569318374591 | -0.219167974 | 0.0709312861765756 | 0.928561797847544 | 0.803186792573315 | 1.07350745853202 | 0.999332001504091 |
| ebi-fl187-GCST90094784 | ukb-rsfMRI191-node-pheno23 | Net100_Node2 RolandicOper\|SupraMarginal\|Insula Salience\|Motor \|\| id:ukb-rsfMRI191-node-pheno23 | Ice cream liking \|\| id:ebi-fl187-GCST90094784 | Weighted median | 8 | -0.046237074 | 0.0672358034091804 | 0.491650979425822 | -0.178019248 | 0.0855451010888456 | 0.954815573797546 | 0.836926313945764 | 1.08931068933437 | 0.999332001504091 |
| ebi-fl187-GCST90094784 | ukb-rsfMRI191-node-pheno55 | Net100_Node34 Parietal\|Postcentral\|Precuneus Attention \|\| id:ukb-rsfMRI191-node-pheno55 | Ice cream liking \|\| id:ebi-fl187-GCST90094784 | Weighted median | 8 | -0.079485095 | 0.0703184761970746 | 0.258325149955405 | -0.217309308 | 0.0583391187940979 | 0.923591786415199 | 0.804681036608218 | 1.06007442592305 | 0.999332001504091 |
| ebi-fl187-GCST90094784 | ebi-a-GCST90001414 | IgD- CD24- B cell Absolute Count \|\| id:ebi-a-GCST90001414 | Ice cream liking \|\| id:ebi-fl187-GCST90094784 | Weighted mode | 8 | -0.05731871 | 0.350928893560853 | 0.874870859794979 | -0.745139341 | 0.630501921728944 | 0.944293066089846 | 0.47466815458226 | 1.87855322936106 | 0.999714303421225 |
| ebi-fl187-GCST90094784 | ebi-a-GCST90001422 | CD20- CD38- B cell %B cell \|\| id:ebi-a-GCST90001422 | Ice cream liking \|\| id:ebi-fl187-GCST90094784 | Weighted mode | 8 | 0.120665456769085 | 0.265973075005265 | 0.663792902277071 | -0.40064177 | 0.641972683779403 | 1.12824740169301 | 0.669889992590503 | 1.90022572886107 | 0.999714303421225 |
| ebi-fl187-GCST90094784 | ebi-a-GCST90001444 | CD20- CD38- B cell %lymphocyte \|\| id:ebi-a-GCST90001444 | Ice cream liking \|\| id:ebi-fl187-GCST90094784 | Weighted mode | 8 | 0.118670661828463 | 0.34050696974643 | 0.737702596511101 | -0.548722999 | 0.786064322531465 | 1.12599902275737 | 0.577687046563209 | 2.1947416110391 | 0.999714303421225 |
| ebi-fl187-GCST90094784 | ebi-a-GCST90001459 | Myeloid Dendritic Cell %Dendritic Cell \|\| id:ebi-a-GCST90001459 | Ice cream liking \|\| id:ebi-fl187-GCST90094784 | Weighted mode | 8 | -0.401111872 | 0.364295559054496 | 0.307284500069059 | -1.115131167 | 0.312907424127394 | 0.669575150391428 | 0.327872269936856 | 1.36739493738841 | 0.999714303421225 |
| ebi-fl187-GCST90094784 | ebi-a-GCST90001476 | HLA DR++ monocyte %leukocyte \|\| id:ebi-a-GCST90001476 | Ice cream liking \|\| id:ebi-fl187-GCST90094784 | Weighted mode | 8 | -0.347385602 | 0.380315323713006 | 0.391398968727181 | -1.092803636 | 0.398032432789008 | 0.706532835469959 | 0.335275185145329 | 1.48889231805462 | 0.999714303421225 |
| ebi-fl187-GCST90094784 | ebi-a-GCST90001478 | CD4 regulatory T cell %CD4+ T cell \|\| id:ebi-a-GCST90001478 | Ice cream liking \|\| id:ebi-fl187-GCST90094784 | Weighted mode | 8 | 0.0445082081097215 | 0.288773288547064 | 0.881857927817411 | -0.521487437 | 0.610503853661967 | 1.04551355836524 | 0.59363689320031 | 1.84135894053455 | 0.999714303421225 |
| ebi-fl187-GCST90094784 | ebi-a-GCST90001485 | CD39+ resting CD4 regulatory T cell %CD4 regulatory T cell \|\| id:ebi-a-GCST90001485 | Ice cream liking \|\| id:ebi-fl187-GCST90094784 | Weighted mode | 8 | 0.0385176632228661 | 0.269224654036106 | 0.890267045227621 | -0.489162659 | 0.566197985133635 | 1.03926908503733 | 0.613139586401685 | 1.76155683806516 | 0.999714303421225 |
| ebi-fl187-GCST90094784 | ebi-a-GCST90001515 | Immature Myeloid-Derived Suppressor Cells Absolute Count \|\| id:ebi-a-GCST90001515 | Ice cream liking \|\| id:ebi-fl187-GCST90094784 | Weighted mode | 8 | -0.245466802 | 0.407175798328866 | 0.56561823087418 | -1.043531367 | 0.55259776227075 | 0.78233925510696 | 0.352208704991776 | 1.73776145054565 | 0.999714303421225 |
| ebi-fl187-GCST90094784 | ebi-a-GCST90001517 | CD33+ HLA DR+ Absolute Count \|\| id:ebi-a-GCST90001517 | Ice cream liking \|\| id:ebi-fl187-GCST90094784 | Weighted mode | 8 | -0.541844863 | 0.495743922376457 | 0.310578019298441 | -1.513502951 | 0.429813225021555 | 0.58167415286804 | 0.220137494973552 | 1.53697042911932 | 0.999714303421225 |
| ebi-fl187-GCST90094784 | ebi-a-GCST90001518 | CD33+ HLA DR+ CD14- Absolute Count \|\| id:ebi-a-GCST90001518 | Ice cream liking \|\| id:ebi-fl187-GCST90094784 | Weighted mode | 8 | -0.67282799 | 0.641740863073509 | 0.329279677247922 | -1.930640082 | 0.584984101598798 | 0.510263515306928 | 0.14505532151293 | 1.7949624483799 | 0.999714303421225 |
| ebi-fl187-GCST90094784 | ebi-a-GCST90001520 | CD33+ HLA DR+ CD14dim Absolute Count \|\| id:ebi-a-GCST90001520 | Ice cream liking \|\| id:ebi-fl187-GCST90094784 | Weighted mode | 7 | 0.0513173628984236 | 0.41778900817766 | 0.906252143767483 | -0.767549093 | 0.870183818926637 | 1.05265691452687 | 0.46414926200506 | 2.38734965324396 | 0.999714303421225 |
| ebi-fl187-GCST90094784 | ebi-a-GCST90001531 | CD33dim HLA DR- Absolute Count \|\| id:ebi-a-GCST90001531 | Ice cream liking \|\| id:ebi-fl187-GCST90094784 | Weighted mode | 8 | 0.0688937605648325 | 0.508263654919461 | 0.895994725792415 | -0.927303003 | 1.06509052420698 | 1.07132238641701 | 0.395619256756089 | 2.90110159209424 | 0.999714303421225 |
| ebi-fl187-GCST90094784 | ebi-a-GCST90001532 | Basophil Absolute Count \|\| id:ebi-a-GCST90001532 | Ice cream liking \|\| id:ebi-fl187-GCST90094784 | Weighted mode | 8 | 0.10259159033964 | 0.471062593819669 | 0.833807053146494 | -0.820691094 | 1.02587427422619 | 1.10803878291085 | 0.440127380184835 | 2.78953321176921 | 0.999714303421225 |
| ebi-fl187-GCST90094784 | ebi-a-GCST90001588 | T/B cell \|\| id:ebi-a-GCST90001588 | Ice cream liking \|\| id:ebi-fl187-GCST90094784 | Weighted mode | 8 | -0.0673932 | 0.28113083730183 | 0.817414127908822 | -0.618409641 | 0.483623240959799 | 0.934827554725747 | 0.538800642887143 | 1.62194044979558 | 0.999714303421225 |
| ebi-fl187-GCST90094784 | ebi-a-GCST90001624 | HLA DR+ CD4+ T cell Absolute Count \|\| id:ebi-a-GCST90001624 | Ice cream liking \|\| id:ebi-fl187-GCST90094784 | Weighted mode | 8 | 0.0139676228448827 | 0.290258303504169 | 0.96296371452473 | -0.554938652 | 0.582873897713053 | 1.01406562584691 | 0.574107480448451 | 1.79117870528533 | 0.999714303421225 |
| ebi-fl187-GCST90094784 | ebi-a-GCST90001631 | CD8+ Natural Killer T %T cell \|\| id:ebi-a-GCST90001631 | Ice cream liking \|\| id:ebi-fl187-GCST90094784 | Weighted mode | 8 | -0.154445186 | 0.26147308820142 | 0.573307396060516 | -0.666932439 | 0.358042066501866 | 0.856890460128974 | 0.513280684970739 | 1.43052579643028 | 0.999714303421225 |
| ebi-fl187-GCST90094784 | ebi-a-GCST90001632 | CD8+ Natural Killer T %lymphocyte \|\| id:ebi-a-GCST90001632 | Ice cream liking \|\| id:ebi-fl187-GCST90094784 | Weighted mode | 8 | -0.100758232 | 0.252291968631887 | 0.701523806466967 | -0.595250491 | 0.393734026242876 | 0.904151601138525 | 0.551424421825327 | 1.48250618849144 | 0.999714303421225 |
| ebi-fl187-GCST90094784 | ebi-a-GCST90001646 | Natural Killer %CD3- lymphocyte \|\| id:ebi-a-GCST90001646 | Ice cream liking \|\| id:ebi-fl187-GCST90094784 | Weighted mode | 8 | -0.415941894 | 0.293721715130045 | 0.199666689 | -0.991636456 | 0.159752667164692 | 0.659718602656457 | 0.370969119330455 | 1.17322065911177 | 0.999714303421225 |
| ebi-fl187-GCST90094784 | ebi-a-GCST90001684 | CD127- CD8+ T cell Absolute Count \|\| id:ebi-a-GCST90001684 | Ice cream liking \|\| id:ebi-fl187-GCST90094784 | Weighted mode | 8 | 0.582729639985502 | 0.34610847482105 | 0.136121697275531 | -0.095642971 | 1.26110225063476 | 1.79092033255225 | 0.908788422281186 | 3.52930952784152 | 0.999714303421225 |
| ebi-fl187-GCST90094784 | ebi-a-GCST90001687 | CD28- CD8+ T cell Absolute Count \|\| id:ebi-a-GCST90001687 | Ice cream liking \|\| id:ebi-fl187-GCST90094784 | Weighted mode | 8 | 0.285851008387485 | 0.355110251057395 | 0.447314649924932 | -0.410165084 | 0.981867100459978 | 1.33089414841437 | 0.663540701350077 | 2.66943569652874 | 0.999714303421225 |
| ebi-fl187-GCST90094784 | ebi-a-GCST90001701 | BAFF-R on CD20- CD38- B cell \|\| id:ebi-a-GCST90001701 | Ice cream liking \|\| id:ebi-fl187-GCST90094784 | Weighted mode | 8 | -0.137557 | 0.255969614903016 | 0.60764610772326 | -0.639257445 | 0.364143445379777 | 0.871484674103025 | 0.527684113055181 | 1.43928065751154 | 0.999714303421225 |
| ebi-fl187-GCST90094784 | ebi-a-GCST90001732 | CD19 on IgD- CD27- B cell \|\| id:ebi-a-GCST90001732 | Ice cream liking \|\| id:ebi-fl187-GCST90094784 | Weighted mode | 8 | -0.466236238 | 0.314398257981879 | 0.181652473666775 | -1.082456824 | 0.149984347446289 | 0.627359060365941 | 0.33876222331833 | 1.16181605719772 | 0.999714303421225 |
| ebi-fl187-GCST90094784 | ebi-a-GCST90001798 | CD27 on CD24+ CD27+ B cell \|\| id:ebi-a-GCST90001798 | Ice cream liking \|\| id:ebi-fl187-GCST90094784 | Weighted mode | 8 | 0.277219220905646 | 0.465898896822258 | 0.570556059091571 | -0.635942617 | 1.19038105867727 | 1.31945559158195 | 0.529436197545321 | 3.28833401688189 | 0.999714303421225 |
| ebi-fl187-GCST90094784 | ebi-a-GCST90001836 | CD66b on CD66b++ myeloid cell \|\| id:ebi-a-GCST90001836 | Ice cream liking \|\| id:ebi-fl187-GCST90094784 | Weighted mode | 8 | -0.103863288 | 0.444260804461124 | 0.821838867703368 | -0.974614464 | 0.766887889158799 | 0.901348514530533 | 0.377337802706472 | 2.15305526989137 | 0.999714303421225 |
| ebi-fl187-GCST90094784 | ebi-a-GCST90001943 | CD25 on activated & secreting CD4 regulatory T cell \|\| id:ebi-a-GCST90001943 | Ice cream liking \|\| id:ebi-fl187-GCST90094784 | Weighted mode | 8 | -0.085301757 | 0.422900543717052 | 0.845882690161094 | -0.914186823 | 0.743583308207267 | 0.918235158265407 | 0.400842449391785 | 2.1034593695205 | 0.999714303421225 |
| ebi-fl187-GCST90094784 | ebi-a-GCST90001960 | CD25 on CD4+ T cell \|\| id:ebi-a-GCST90001960 | Ice cream liking \|\| id:ebi-fl187-GCST90094784 | Weighted mode | 8 | 0.208367047446175 | 0.336855311326649 | 0.555787882518733 | -0.451869363 | 0.868603457646408 | 1.23166516614547 | 0.636437306711539 | 2.38357975797249 | 0.999714303421225 |
| ebi-fl187-GCST90094784 | ebi-a-GCST90001988 | HLA DR on CD14+ CD16- monocyte \|\| id:ebi-a-GCST90001988 | Ice cream liking \|\| id:ebi-fl187-GCST90094784 | Weighted mode | 8 | -0.317653044 | 0.273176965673005 | 0.283010083563672 | -0.853079897 | 0.217773808739747 | 0.727855278381608 | 0.42610056320991 | 1.24330581090311 | 0.999714303421225 |
| ebi-fl187-GCST90094784 | ebi-a-GCST90001991 | HLA DR on CD14+ monocyte \|\| id:ebi-a-GCST90001991 | Ice cream liking \|\| id:ebi-fl187-GCST90094784 | Weighted mode | 8 | -0.104393881 | 0.281049365425794 | 0.721295161406087 | -0.655250637 | 0.446462875053892 | 0.900870391636956 | 0.519311887285207 | 1.56277466855367 | 0.999714303421225 |
| ebi-fl187-GCST90094784 | ebi-a-GCST90002019 | CD14 on Monocytic Myeloid-Derived Suppressor Cells \|\| id:ebi-a-GCST90002019 | Ice cream liking \|\| id:ebi-fl187-GCST90094784 | Weighted mode | 8 | 0.0745450927302649 | 0.442955162161752 | 0.871114102762709 | -0.793647025 | 0.942737210567299 | 1.07739392506056 | 0.45219262650116 | 2.5669982253778 | 0.999714303421225 |
| ebi-fl187-GCST90094784 | ebi-a-GCST90002037 | CD80 on plasmacytoid Dendritic Cell \|\| id:ebi-a-GCST90002037 | Ice cream liking \|\| id:ebi-fl187-GCST90094784 | Weighted mode | 8 | -0.019792888 | 0.34024488999282 | 0.955237039260761 | -0.686672872 | 0.64708709678287 | 0.980401705627994 | 0.503247656105123 | 1.90996916277241 | 0.999714303421225 |
| ebi-fl187-GCST90094784 | ebi-a-GCST90002042 | CD45 on CD33+ HLA DR+ CD14- \|\| id:ebi-a-GCST90002042 | Ice cream liking \|\| id:ebi-fl187-GCST90094784 | Weighted mode | 8 | -0.173549792 | 0.519991195780149 | 0.748339536673694 | -1.192732536 | 0.845632951503504 | 0.840675291025998 | 0.303391103263136 | 2.32945177805258 | 0.999714303421225 |
| ebi-fl187-GCST90094784 | ebi-a-GCST90002056 | CD8 on Effector Memory CD8+ T cell \|\| id:ebi-a-GCST90002056 | Ice cream liking \|\| id:ebi-fl187-GCST90094784 | Weighted mode | 8 | 0.152908437966933 | 0.394381245928436 | 0.709745206112204 | -0.620078804 | 0.925895679986668 | 1.16521828430315 | 0.537902047063104 | 2.52412805916517 | 0.999714303421225 |
| ebi-fl187-GCST90094784 | ebi-a-GCST90002058 | CD8 on CD8+ T cell \|\| id:ebi-a-GCST90002058 | Ice cream liking \|\| id:ebi-fl187-GCST90094784 | Weighted mode | 8 | 0.127734755770906 | 0.349069507695415 | 0.725226423974581 | -0.556441479 | 0.811910990853918 | 1.13625157854931 | 0.573245344043436 | 2.2522078254472 | 0.999714303421225 |
| ebi-fl187-GCST90094784 | ebi-a-GCST90002063 | CD4 on CD4 regulatory T cell \|\| id:ebi-a-GCST90002063 | Ice cream liking \|\| id:ebi-fl187-GCST90094784 | Weighted mode | 8 | -0.059252645 | 0.293072311723749 | 0.845528258155765 | -0.633674376 | 0.515169086397825 | 0.942468629484276 | 0.530638449581266 | 1.67392151522925 | 0.999714303421225 |
| ebi-fl187-GCST90094784 | ebi-a-GCST90002066 | CD4 on activated CD4 regulatory T cell \|\| id:ebi-a-GCST90002066 | Ice cream liking \|\| id:ebi-fl187-GCST90094784 | Weighted mode | 8 | -0.079619622 | 0.308882518826156 | 0.804000674990822 | -0.685029359 | 0.525790114687948 | 0.923467546131227 | 0.504075430151282 | 1.69179503254442 | 0.999714303421225 |
| ebi-fl187-GCST90094784 | ebi-a-GCST90002070 | CD4 on activated & secreting CD4 regulatory T cell \|\| id:ebi-a-GCST90002070 | Ice cream liking \|\| id:ebi-fl187-GCST90094784 | Weighted mode | 8 | -0.072463649 | 0.328107936553657 | 0.831510462014117 | -0.715555205 | 0.570627906623227 | 0.930099556074327 | 0.488920585436075 | 1.76937770668437 | 0.999714303421225 |
| ebi-fl187-GCST90094784 | ebi-a-GCST90002081 | SSC-A on CD4+ T cell \|\| id:ebi-a-GCST90002081 | Ice cream liking \|\| id:ebi-fl187-GCST90094784 | Weighted mode | 8 | -0.074242569 | 0.31201462240075 | 0.818737963666253 | -0.685791229 | 0.537306091157478 | 0.928446454430342 | 0.503691536690673 | 1.71139031719259 | 0.999714303421225 |
| ebi-fl187-GCST90094784 | ebi-a-GCST90002094 | CD11b on Monocytic Myeloid-Derived Suppressor Cells \|\| id:ebi-a-GCST90002094 | Ice cream liking \|\| id:ebi-fl187-GCST90094784 | Weighted mode | 8 | -0.439733592 | 0.547391823174075 | 0.448200929065837 | -1.512621566 | 0.633154380927263 | 0.644208020076519 | 0.220331606341755 | 1.88354262931846 | 0.999714303421225 |
| ebi-fl187-GCST90094784 | ebi-a-GCST90002104 | HLA DR on myeloid Dendritic Cell \|\| id:ebi-a-GCST90002104 | Ice cream liking \|\| id:ebi-fl187-GCST90094784 | Weighted mode | 8 | 0.154236260060437 | 0.325775201550262 | 0.650305744909974 | -0.484283135 | 0.792755655098951 | 1.16676651454448 | 0.616138726788548 | 2.2094766004369 | 0.999714303421225 |
| ebi-fl187-GCST90094784 | ebi-a-GCST90002105 | HLA DR on plasmacytoid Dendritic Cell \|\| id:ebi-a-GCST90002105 | Ice cream liking \|\| id:ebi-fl187-GCST90094784 | Weighted mode | 8 | 0.00639323901840982 | 0.331414667532305 | 0.985147501579753 | -0.643179509 | 0.655965987381728 | 1.00641371939303 | 0.525618555304087 | 1.92700307925114 | 0.999714303421225 |
| ebi-fl187-GCST90094784 | ebi-a-GCST90002106 | HLA DR on Dendritic Cell \|\| id:ebi-a-GCST90002106 | Ice cream liking \|\| id:ebi-fl187-GCST90094784 | Weighted mode | 8 | 0.16690165289468 | 0.323832354231281 | 0.622149508663858 | -0.467809761 | 0.80161306718799 | 1.18163804891204 | 0.626372672585003 | 2.2291337725101 | 0.999714303421225 |
| ebi-fl187-GCST90094784 | ebi-a-GCST90002111 | HLA DR on CD33dim HLA DR+ CD11b- \|\| id:ebi-a-GCST90002111 | Ice cream liking \|\| id:ebi-fl187-GCST90094784 | Weighted mode | 8 | 0.636406425681314 | 0.416942527690688 | 0.17075357232574 | -0.180800929 | 1.45361377995506 | 1.88967796503116 | 0.834601487452361 | 4.27854834338301 | 0.999714303421225 |
| ebi-fl187-GCST90094784 | ebi-a-GCST90002112 | HLA DR on CD33- HLA DR+ \|\| id:ebi-a-GCST90002112 | Ice cream liking \|\| id:ebi-fl187-GCST90094784 | Weighted mode | 8 | 0.300463695079839 | 0.397125134775758 | 0.473980955419484 | -0.477901569 | 1.07882895924032 | 1.35048487560467 | 0.620083229340549 | 2.94123322957235 | 0.999714303421225 |
| ebi-fl187-GCST90094784 | ebi-a-GCST90002115 | HLA DR on HLA DR+ CD8+ T cell \|\| id:ebi-a-GCST90002115 | Ice cream liking \|\| id:ebi-fl187-GCST90094784 | Weighted mode | 8 | 0.266980978112967 | 0.305375324644806 | 0.410959607829187 | -0.331554658 | 0.865516614416787 | 1.30601560321311 | 0.717806921116796 | 2.3762333653489 | 0.999714303421225 |
| ebi-fl187-GCST90094784 | ebi-a-GCST90002116 | HLA DR on B cell \|\| id:ebi-a-GCST90002116 | Ice cream liking \|\| id:ebi-fl187-GCST90094784 | Weighted mode | 8 | -0.092395573 | 0.325574183982832 | 0.784779730099303 | -0.730520974 | 0.545729827192935 | 0.911744416350625 | 0.481657993413524 | 1.72586750788719 | 0.999714303421225 |
| ebi-fl187-GCST90094784 | ebi-cfmet338-GCST90026023 | Sphingomyelin (d18:1/24:1, d18:2/24:0) levels \|\| id:ebi-cfmet338-GCST90026023 | Ice cream liking \|\| id:ebi-fl187-GCST90094784 | Weighted mode | 8 | 0.0612211299242973 | 0.166143033376765 | 0.723403533842354 | -0.264419215 | 0.386861475342756 | 1.06313397893259 | 0.767651660722808 | 1.47235252001776 | 0.999714303421225 |
| ebi-fl187-GCST90094784 | ebi-cfmet338-GCST90026046 | 1-arachidonoyl-gpc (20:4n6) levels \|\| id:ebi-cfmet338-GCST90026046 | Ice cream liking \|\| id:ebi-fl187-GCST90094784 | Weighted mode | 8 | 0.0099589225979197 | 0.210501788547276 | 0.963587328658291 | -0.402624583 | 0.422542428150581 | 1.01000867769948 | 0.668563042178364 | 1.52583595662785 | 0.999714303421225 |
| ebi-fl187-GCST90094784 | ebi-cfmet338-GCST90026047 | 1-linoleoyl-gpc (18:2) levels \|\| id:ebi-cfmet338-GCST90026047 | Ice cream liking \|\| id:ebi-fl187-GCST90094784 | Weighted mode | 8 | -0.125976814 | 0.233822014014059 | 0.606744885065841 | -0.584267962 | 0.332314333129243 | 0.881635287843962 | 0.557513833888548 | 1.39419102006909 | 0.999714303421225 |
| ebi-fl187-GCST90094784 | ebi-cfmet338-GCST90026050 | 1-myristoyl-2-palmitoyl-gpc (14:0/16:0) levels \|\| id:ebi-cfmet338-GCST90026050 | Ice cream liking \|\| id:ebi-fl187-GCST90094784 | Weighted mode | 8 | 0.0923328459273828 | 0.129401970661743 | 0.498607141301966 | -0.161295017 | 0.3459607084244 | 1.09672980338467 | 0.851040962882941 | 1.41334708209296 | 0.999714303421225 |
| ebi-fl187-GCST90094784 | ebi-cfmet338-GCST90026068 | 2,3-dihydroxyisovalerate levels \|\| id:ebi-cfmet338-GCST90026068 | Ice cream liking \|\| id:ebi-fl187-GCST90094784 | Weighted mode | 8 | -0.586894533 | 0.556786502558214 | 0.326866702793984 | -1.678196078 | 0.504407011903695 | 0.556051405477583 | 0.186710483557194 | 1.65600323904085 | 0.999714303421225 |
| ebi-fl187-GCST90094784 | ebi-cfmet338-GCST90026082 | 3-hydroxyhexanoate levels \|\| id:ebi-cfmet338-GCST90026082 | Ice cream liking \|\| id:ebi-fl187-GCST90094784 | Weighted mode | 8 | 0.0662288155519917 | 0.0973027165567167 | 0.517967714017669 | -0.124484509 | 0.256942140003156 | 1.0684711720184 | 0.882951939157961 | 1.29297031333677 | 0.999714303421225 |
| ebi-fl187-GCST90094784 | ebi-cfmet338-GCST90026094 | 3-ureidopropionate levels \|\| id:ebi-cfmet338-GCST90026094 | Ice cream liking \|\| id:ebi-fl187-GCST90094784 | Weighted mode | 8 | -0.082300047 | 0.25260245538838 | 0.754092385287362 | -0.57740086 | 0.412800765276905 | 0.920995575013182 | 0.561355513736902 | 1.51104394352028 | 0.999714303421225 |
| ebi-fl187-GCST90094784 | ebi-cfmet338-GCST90026173 | Glycerol levels \|\| id:ebi-cfmet338-GCST90026173 | Ice cream liking \|\| id:ebi-fl187-GCST90094784 | Weighted mode | 7 | 0.0344050758400017 | 0.12834742243737 | 0.797629081163248 | -0.217155872 | 0.285966023817247 | 1.03500377684885 | 0.804804512928186 | 1.33104723058006 | 0.999714303421225 |
| ebi-fl187-GCST90094784 | ebi-cfmet338-GCST90026179 | Histidine levels \|\| id:ebi-cfmet338-GCST90026179 | Ice cream liking \|\| id:ebi-fl187-GCST90094784 | Weighted mode | 8 | -0.014619523 | 0.111091270241688 | 0.899003883119549 | -0.232358413 | 0.203119366697742 | 0.985486823374991 | 0.792661972310277 | 1.22521870983054 | 0.999714303421225 |
| ebi-fl187-GCST90094784 | ebi-cfmet338-GCST90026188 | Isocitrate levels \|\| id:ebi-cfmet338-GCST90026188 | Ice cream liking \|\| id:ebi-fl187-GCST90094784 | Weighted mode | 8 | 0.0592895059031298 | 0.192263852722244 | 0.766770917755822 | -0.317547645 | 0.436126657238729 | 1.06108238589227 | 0.727931997313244 | 1.5467046836881 | 0.999714303421225 |
| ebi-fl187-GCST90094784 | ebi-cfmet338-GCST90026207 | N-acetyl-isoputreanine levels \|\| id:ebi-cfmet338-GCST90026207 | Ice cream liking \|\| id:ebi-fl187-GCST90094784 | Weighted mode | 7 | 0.100118761411236 | 0.144879927932694 | 0.515368025422045 | -0.183845897 | 0.384083420159316 | 1.10530217752766 | 0.832064017228376 | 1.46826791971713 | 0.999714303421225 |
| ebi-fl187-GCST90094784 | ebi-cfmet338-GCST90026213 | N-acetylglutamate levels \|\| id:ebi-cfmet338-GCST90026213 | Ice cream liking \|\| id:ebi-fl187-GCST90094784 | Weighted mode | 8 | 0.0346217868381864 | 0.190514470256559 | 0.860946678289792 | -0.338786575 | 0.408030148541041 | 1.035228098 | 0.712634526980355 | 1.50385249944547 | 0.999714303421225 |
| ebi-fl187-GCST90094784 | ebi-cfmet338-GCST90026224 | N-acetyltaurine levels \|\| id:ebi-cfmet338-GCST90026224 | Ice cream liking \|\| id:ebi-fl187-GCST90094784 | Weighted mode | 8 | 0.0359655569905717 | 0.170732213807672 | 0.839157495816803 | -0.298669582 | 0.370600696053609 | 1.03662014155873 | 0.741804474442926 | 1.44860452438254 | 0.999714303421225 |
| ebi-fl187-GCST90094784 | ebi-cfmet338-GCST90026250 | Phenyllactate (pla) levels \|\| id:ebi-cfmet338-GCST90026250 | Ice cream liking \|\| id:ebi-fl187-GCST90094784 | Weighted mode | 8 | 0.0684642714767816 | 0.245732388499821 | 0.788592661553117 | -0.41317121 | 0.550099752936431 | 1.07086236393665 | 0.661549009336794 | 1.73342592356929 | 0.999714303421225 |
| ebi-fl187-GCST90094784 | ebi-cfmet338-GCST90026251 | Pipecolate levels \|\| id:ebi-cfmet338-GCST90026251 | Ice cream liking \|\| id:ebi-fl187-GCST90094784 | Weighted mode | 8 | -0.354868848 | 0.550793452625669 | 0.53991934287574 | -1.434424015 | 0.724686319400249 | 0.701265409716726 | 0.23825255442428 | 2.06408353544625 | 0.999714303421225 |
| ebi-fl187-GCST90094784 | ebi-cfmet338-GCST90026278 | Trigonelline (n'-methylnicotinate) levels \|\| id:ebi-cfmet338-GCST90026278 | Ice cream liking \|\| id:ebi-fl187-GCST90094784 | Weighted mode | 8 | 0.0929010935228418 | 0.507529266683539 | 0.859950959841843 | -0.901856269 | 1.08765845622258 | 1.09735319456181 | 0.405815657045451 | 2.96731782697122 | 0.999714303421225 |
| ebi-fl187-GCST90094784 | ebi-cfmet338-GCST90026299 | 1-carboxyethylvaline levels \|\| id:ebi-cfmet338-GCST90026299 | Ice cream liking \|\| id:ebi-fl187-GCST90094784 | Weighted mode | 8 | -0.07886116 | 0.235947815980273 | 0.747995406616391 | -0.541318879 | 0.383596559715136 | 0.924168227418696 | 0.581980184589418 | 1.46755325213137 | 0.999714303421225 |
| ebi-fl187-GCST90094784 | ebi-gm430-GCST90011306 | C_Erysipelotrichia abundance \|\| id:ebi-gm430-GCST90011306 | Ice cream liking \|\| id:ebi-fl187-GCST90094784 | Weighted mode | 8 | -0.086580868 | 0.202751817456537 | 0.682185595678338 | -0.48397443 | 0.310812694068438 | 0.917061384731696 | 0.616328961019735 | 1.36453361200901 | 0.999714303421225 |
| ebi-fl187-GCST90094784 | ebi-gm430-GCST90011312 | F_Erysipelotrichaceae abundance \|\| id:ebi-gm430-GCST90011312 | Ice cream liking \|\| id:ebi-fl187-GCST90094784 | Weighted mode | 8 | -0.086580868 | 0.196530255548264 | 0.672824560983767 | -0.471780169 | 0.298618432728222 | 0.917061384731696 | 0.623890648335924 | 1.3479951744896 | 0.999714303421225 |
| ebi-fl187-GCST90094784 | ebi-gm430-GCST90011352 | O_Erysipelotrichales abundance \|\| id:ebi-gm430-GCST90011352 | Ice cream liking \|\| id:ebi-fl187-GCST90094784 | Weighted mode | 8 | -0.086580868 | 0.194817577284594 | 0.670154022655521 | -0.46842332 | 0.29526158333143 | 0.917061384731696 | 0.625988474355524 | 1.34347774410999 | 0.999714303421225 |
| ebi-fl187-GCST90094784 | ebi-gm430-GCST90011362 | OTU97_11 (Parabacteroides) abundance \|\| id:ebi-gm430-GCST90011362 | Ice cream liking \|\| id:ebi-fl187-GCST90094784 | Weighted mode | 8 | 0.51431201104564 | 0.44103772565126 | 0.281747267492622 | -0.350121931 | 1.37874595332211 | 1.67248745299453 | 0.704602171470749 | 3.96992003953861 | 0.999714303421225 |
| ebi-fl187-GCST90094784 | ebi-gm430-GCST90011384 | OTU97_235 (Phascolarctobacterium) abundance \|\| id:ebi-gm430-GCST90011384 | Ice cream liking \|\| id:ebi-fl187-GCST90094784 | Weighted mode | 8 | 0.564649064776067 | 0.859991840148059 | 0.532437100742847 | -1.120934942 | 2.25023307146626 | 1.75883043885038 | 0.325974884526139 | 9.4899474145793 | 0.999714303421225 |
| ebi-fl187-GCST90094784 | ebi-gm430-GCST90011390 | OTU97_3 (Bacteroides) abundance \|\| id:ebi-gm430-GCST90011390 | Ice cream liking \|\| id:ebi-fl187-GCST90094784 | Weighted mode | 8 | 0.0559153063383433 | 0.224754777842861 | 0.81066961990072 | -0.384604058 | 0.496434670910352 | 1.05750811570919 | 0.680720108375513 | 1.64285350326965 | 0.999714303421225 |
| ebi-fl187-GCST90094784 | ebi-gm430-GCST90011398 | OTU97_4 (Alistipes) abundance \|\| id:ebi-gm430-GCST90011398 | Ice cream liking \|\| id:ebi-fl187-GCST90094784 | Weighted mode | 8 | 0.180399941355656 | 0.228208525745565 | 0.455175494751764 | -0.266888769 | 0.627688651816964 | 1.19769627561911 | 0.76575824270264 | 1.87327577900968 | 0.999714303421225 |
| ebi-fl187-GCST90094784 | ebi-gm430-GCST90011421 | OTU99_108 (Cytophagales) abundance \|\| id:ebi-gm430-GCST90011421 | Ice cream liking \|\| id:ebi-fl187-GCST90094784 | Weighted mode | 8 | 0.309794838729367 | 0.660955886655223 | 0.653524782889023 | -0.985678699 | 1.60526837657361 | 1.36314542079545 | 0.373185860053547 | 4.97919572292741 | 0.999714303421225 |
| ebi-fl187-GCST90094784 | ebi-gm430-GCST90011428 | OTU99_123 (Phascolarctobacterium) abundance \|\| id:ebi-gm430-GCST90011428 | Ice cream liking \|\| id:ebi-fl187-GCST90094784 | Weighted mode | 8 | 0.38875904880743 | 0.496095205114611 | 0.458941910747781 | -0.583587553 | 1.36110565083207 | 1.47514906958036 | 0.557893300170841 | 3.90050351351674 | 0.999714303421225 |
| ebi-fl187-GCST90094784 | ebi-gm430-GCST90011450 | OTU99_298 (Phascolarctobacterium) abundance \|\| id:ebi-gm430-GCST90011450 | Ice cream liking \|\| id:ebi-fl187-GCST90094784 | Weighted mode | 8 | 0.560159253275106 | 0.809627851179234 | 0.511306258186783 | -1.026711335 | 2.14702984158641 | 1.75095132282652 | 0.35818296954205 | 8.55939783744527 | 0.999714303421225 |
| ebi-fl187-GCST90094784 | ebi-gm430-GCST90011451 | OTU99_3 (Bacteroides) abundance \|\| id:ebi-gm430-GCST90011451 | Ice cream liking \|\| id:ebi-fl187-GCST90094784 | Weighted mode | 8 | 0.0466950748808468 | 0.250701203922821 | 0.857525309943009 | -0.444679285 | 0.538069434569576 | 1.04780245907304 | 0.64102983107367 | 1.71269719445136 | 0.999714303421225 |
| ebi-fl187-GCST90094784 | ebi-gm430-GCST90011472 | OTU99_72 (Parabacteroides) abundance \|\| id:ebi-gm430-GCST90011472 | Ice cream liking \|\| id:ebi-fl187-GCST90094784 | Weighted mode | 8 | 0.553902437416856 | 1.01730388740382 | 0.603015065007179 | -1.440013182 | 2.54781805672834 | 1.74003014435244 | 0.236924635545958 | 12.7791898730931 | 0.999714303421225 |
| ebi-fl187-GCST90094784 | ebi-gm430-GCST90011507 | TestASV_3 (Bacteroides) abundance \|\| id:ebi-gm430-GCST90011507 | Ice cream liking \|\| id:ebi-fl187-GCST90094784 | Weighted mode | 8 | -0.058144289 | 0.272939381389026 | 0.837374740137578 | -0.593105477 | 0.476816898235805 | 0.943513798681642 | 0.552608504404622 | 1.61093845137577 | 0.999714303421225 |
| ebi-fl187-GCST90094784 | ebi-gm430-GCST90011512 | TestASV_34 (Phascolarctobacterium) abundance \|\| id:ebi-gm430-GCST90011512 | Ice cream liking \|\| id:ebi-fl187-GCST90094784 | Weighted mode | 8 | 1.15292220883251 | 0.560308304456879 | 0.0786366151615253 | 0.0547179320970295 | 2.251126486 | 3.16743530643497 | 1.05624264050636 | 9.498429656 | 0.999714303421225 |
| ebi-fl187-GCST90094784 | ebi-gm430-GCST90011519 | TestASV_41 (Barnesiella) abundance \|\| id:ebi-gm430-GCST90011519 | Ice cream liking \|\| id:ebi-fl187-GCST90094784 | Weighted mode | 8 | -1.036134348 | 0.999298524820695 | 0.334284786323984 | -2.994759457 | 0.922490760271012 | 0.354823658912801 | 0.0500486644933679 | 2.51554822089111 | 0.999714303421225 |
| ebi-fl187-GCST90094784 | ebi-gm430-GCST90011524 | TestASV_46 (Sutterella) abundance \|\| id:ebi-gm430-GCST90011524 | Ice cream liking \|\| id:ebi-fl187-GCST90094784 | Weighted mode | 8 | 0.405378322811687 | 0.725790086728609 | 0.593887395592471 | -1.017170247 | 1.82792689279976 | 1.49986982770389 | 0.36161677981816 | 6.22097652987152 | 0.999714303421225 |
| ebi-fl187-GCST90094784 | ebi-gm430-GCST90011581 | OTU97_19 (Bacteria) prevalence \|\| id:ebi-gm430-GCST90011581 | Ice cream liking \|\| id:ebi-fl187-GCST90094784 | Weighted mode | 8 | -0.309265863 | 0.869062811660632 | 0.73242744688558 | -2.012628974 | 1.39409724739908 | 0.733985604134481 | 0.133636884511449 | 4.03133363252346 | 0.999714303421225 |
| ebi-fl187-GCST90094784 | ebi-gm430-GCST90011614 | OTU97_80 (Ruminococcus) prevalence \|\| id:ebi-gm430-GCST90011614 | Ice cream liking \|\| id:ebi-fl187-GCST90094784 | Weighted mode | 8 | 0.14615231595998 | 0.639461577 | 0.825747990889976 | -1.107192375 | 1.39949700695446 | 1.15737246092331 | 0.330485539175145 | 4.05316074236394 | 0.999714303421225 |
| ebi-fl187-GCST90094784 | ebi-gm430-GCST90011623 | OTU99_11 (Parabacteroides) prevalence \|\| id:ebi-gm430-GCST90011623 | Ice cream liking \|\| id:ebi-fl187-GCST90094784 | Weighted mode | 8 | 0.034347193362807 | 0.377514887294172 | 0.930055403977078 | -0.705581986 | 0.774276372459385 | 1.03494387000014 | 0.493821093777874 | 2.16902199510469 | 0.999714303421225 |
| ebi-fl187-GCST90094784 | ebi-gm430-GCST90011642 | OTU99_19 (Bacteria) prevalence \|\| id:ebi-gm430-GCST90011642 | Ice cream liking \|\| id:ebi-fl187-GCST90094784 | Weighted mode | 8 | -0.309265863 | 0.944932728882447 | 0.753019550946133 | -2.161334012 | 1.54280228515384 | 0.733985604134481 | 0.115171378505156 | 4.67768011522533 | 0.999714303421225 |
| ebi-fl187-GCST90094784 | ebi-gm430-GCST90011653 | OTU99_30 (Parasutterella) prevalence \|\| id:ebi-gm430-GCST90011653 | Ice cream liking \|\| id:ebi-fl187-GCST90094784 | Weighted mode | 8 | 0.215484108125688 | 0.534256227252311 | 0.698737646116924 | -0.831658097 | 1.26262631354022 | 1.240462269 | 0.435326873263161 | 3.53469251854105 | 0.999714303421225 |
| ebi-fl187-GCST90094784 | ebi-gm430-GCST90011675 | OTU99_8 (Ruminococcaceae) prevalence \|\| id:ebi-gm430-GCST90011675 | Ice cream liking \|\| id:ebi-fl187-GCST90094784 | Weighted mode | 8 | -0.150844812 | 0.382070245673019 | 0.704737322241631 | -0.899702493 | 0.59801286991481 | 0.85998114739868 | 0.406690635004773 | 1.81850160840923 | 0.999714303421225 |
| ebi-fl187-GCST90094784 | ebi-gm430-GCST90011679 | OTU99_92 (Ruminococcus) prevalence \|\| id:ebi-gm430-GCST90011679 | Ice cream liking \|\| id:ebi-fl187-GCST90094784 | Weighted mode | 8 | -0.791552166 | 0.907328779022301 | 0.411913301090436 | -2.569916573 | 0.986812240817535 | 0.453140899214924 | 0.0765419308250491 | 2.68266912433454 | 0.999714303421225 |
| ebi-fl187-GCST90094784 | ebi-gm430-GCST90011684 | TestASV_11 (Lachnospiraceae) prevalence \|\| id:ebi-gm430-GCST90011684 | Ice cream liking \|\| id:ebi-fl187-GCST90094784 | Weighted mode | 8 | -0.666277269 | 0.493849741689893 | 0.219297142198208 | -1.634222762 | 0.301668225079442 | 0.513617081579308 | 0.195103954523564 | 1.35211255524903 | 0.999714303421225 |
| ebi-fl187-GCST90094784 | ebi-gm430-GCST90011688 | TestASV_15 (Bacteroides) prevalence \|\| id:ebi-gm430-GCST90011688 | Ice cream liking \|\| id:ebi-fl187-GCST90094784 | Weighted mode | 8 | 0.140598252447335 | 0.431837067089718 | 0.754256869025734 | -0.705802399 | 0.986998903943183 | 1.1509621588585 | 0.493712261028239 | 2.68316992647756 | 0.999714303421225 |
| ebi-fl187-GCST90094784 | ebi-gm430-GCST90011692 | TestASV_19 (Prevotella) prevalence \|\| id:ebi-gm430-GCST90011692 | Ice cream liking \|\| id:ebi-fl187-GCST90094784 | Weighted mode | 8 | -0.44550563 | 0.781606826434696 | 0.586506141445049 | -1.977455009 | 1.08644375016662 | 0.64050033818407 | 0.138421069729263 | 2.96371559630549 | 0.999714303421225 |
| ebi-fl187-GCST90094784 | ebi-gm430-GCST90011693 | TestASV_2 (Bacteroides) prevalence \|\| id:ebi-gm430-GCST90011693 | Ice cream liking \|\| id:ebi-fl187-GCST90094784 | Weighted mode | 8 | 0.388096810339221 | 0.465265865691241 | 0.431742994183134 | -0.523824286 | 1.30001790709405 | 1.47417249251929 | 0.592251273059108 | 3.66936237464809 | 0.999714303421225 |
| ebi-fl187-GCST90094784 | ebi-gm430-GCST90011716 | TestASV_40 (Barnesiella) prevalence \|\| id:ebi-gm430-GCST90011716 | Ice cream liking \|\| id:ebi-fl187-GCST90094784 | Weighted mode | 8 | -0.512491595 | 0.782226240045696 | 0.533288938893228 | -2.045655026 | 1.02067183529212 | 0.599001249317182 | 0.129295470379332 | 2.77505852007712 | 0.999714303421225 |
| ebi-fl187-GCST90094784 | ebi-gm430-GCST90011718 | TestASV_42 (Alphaproteobacteria) prevalence \|\| id:ebi-gm430-GCST90011718 | Ice cream liking \|\| id:ebi-fl187-GCST90094784 | Weighted mode | 8 | -0.005332242 | 0.969345550062017 | 0.995764449859448 | -1.90524952 | 1.8945850358911 | 0.994681948938363 | 0.148785512976461 | 6.64978840850152 | 0.999714303421225 |
| ebi-fl187-GCST90094784 | ebi-hbsm44-GCST90104929 | Sulcal depth of the right inferior frontal sulcus \|\| id:ebi-hbsm44-GCST90104929 | Ice cream liking \|\| id:ebi-fl187-GCST90094784 | Weighted mode | 7 | -0.169275495 | 0.477620144269731 | 0.735151724608132 | -1.105410978 | 0.766859987732407 | 0.84427627742019 | 0.33107478988801 | 2.1529951974163 | 0.999714303421225 |
| ebi-fl187-GCST90094784 | ebi-hbsm44-GCST90104935 | Sulcal opening distance of the left calloso-marginal posterior fissure \|\| id:ebi-hbsm44-GCST90104935 | Ice cream liking \|\| id:ebi-fl187-GCST90094784 | Weighted mode | 8 | -0.014090225 | 0.0930939153507501 | 0.883965312491796 | -0.196554299 | 0.168373848757586 | 0.98600857730023 | 0.821556720032861 | 1.18337893270563 | 0.999714303421225 |
| ebi-fl187-GCST90094784 | ebi-hbsm44-GCST90104936 | Sulcal opening distance of the left anterior inferior temporal sulcus \|\| id:ebi-hbsm44-GCST90104936 | Ice cream liking \|\| id:ebi-fl187-GCST90094784 | Weighted mode | 7 | 0.0457935686699968 | 0.0454032182218753 | 0.352097268632628 | -0.043196739 | 0.134783876384872 | 1.04685828430209 | 0.957722950032434 | 1.14428944965223 | 0.999714303421225 |
| ebi-fl187-GCST90094784 | ebi-hbsm44-GCST90104941 | Sulcal opening distance of the right posterior inferior temporal sulcus \|\| id:ebi-hbsm44-GCST90104941 | Ice cream liking \|\| id:ebi-fl187-GCST90094784 | Weighted mode | 8 | -0.001429847 | 0.0349661186798094 | 0.968523602339913 | -0.06996344 | 0.0671037452794008 | 0.998571174411635 | 0.932427908898008 | 1.0694064182874 | 0.999714303421225 |
| ebi-fl187-GCST90094784 | ebi-IPs91-GCST90274819 | Leukemia inhibitory factor levels \|\| id:ebi-IPs91-GCST90274819 | Ice cream liking \|\| id:ebi-fl187-GCST90094784 | Weighted mode | 9 | 0.23512563027128 | 0.159404726864559 | 0.178441013507466 | -0.077307634 | 0.547558894925816 | 1.26506768954708 | 0.925605061860531 | 1.72902712515321 | 0.999714303421225 |
| ebi-fl187-GCST90094784 | ebi-IPs91-GCST90274837 | STAM binding protein levels \|\| id:ebi-IPs91-GCST90274837 | Ice cream liking \|\| id:ebi-fl187-GCST90094784 | Weighted mode | 9 | 0.194735246862052 | 0.131351913083842 | 0.176479778012373 | -0.062714503 | 0.452184996506383 | 1.21498927168186 | 0.939211577708123 | 1.5717426885902 | 0.999714303421225 |
| ebi-fl187-GCST90094784 | ebi-IPs91-GCST90274840 | TNF-beta levels \|\| id:ebi-IPs91-GCST90274840 | Ice cream liking \|\| id:ebi-fl187-GCST90094784 | Weighted mode | 9 | 0.13149632031558 | 0.159246519473298 | 0.432884220735085 | -0.180626858 | 0.443619498483243 | 1.14053371091052 | 0.834746779796386 | 1.55833742304538 | 0.999714303421225 |
| ebi-fl187-GCST90094784 | ebi-IPs91-GCST90274842 | Tumor necrosis factor ligand superfamily member 14 levels \|\| id:ebi-IPs91-GCST90274842 | Ice cream liking \|\| id:ebi-fl187-GCST90094784 | Weighted mode | 9 | -0.059027235 | 0.147131662667333 | 0.698785332278646 | -0.347405294 | 0.229350824067234 | 0.94268109511341 | 0.706518922632842 | 1.25778322224219 | 0.999714303421225 |
| ebi-fl187-GCST90094784 | ebi-met1400-GCST90199628 | 3-methylhistidine levels \|\| id:ebi-met1400-GCST90199628 | Ice cream liking \|\| id:ebi-fl187-GCST90094784 | Weighted mode | 8 | -0.172898352 | 0.179668396511955 | 0.367945232226631 | -0.525048409 | 0.179251705572963 | 0.841223119491001 | 0.591526728601879 | 1.19632182714511 | 0.999714303421225 |
| ebi-fl187-GCST90094784 | ebi-met1400-GCST90199651 | Docosahexaenoate DHA; 22:6n3 levels \|\| id:ebi-met1400-GCST90199651 | Ice cream liking \|\| id:ebi-fl187-GCST90094784 | Weighted mode | 8 | -0.127873842 | 0.232755073531339 | 0.599816178328848 | -0.584073786 | 0.328326102410293 | 0.879964386943542 | 0.557622100190895 | 1.38864173787918 | 0.999714303421225 |
| ebi-fl187-GCST90094784 | ebi-met1400-GCST90199652 | 1-stearoyl-2-oleoyl-GPS (18:0/18:1) levels \|\| id:ebi-met1400-GCST90199652 | Ice cream liking \|\| id:ebi-fl187-GCST90094784 | Weighted mode | 8 | 0.0429803216075839 | 0.172876661271645 | 0.810792423174716 | -0.295857934 | 0.381818577700009 | 1.04391735203319 | 0.743893102070452 | 1.46494628709807 | 0.999714303421225 |
| ebi-fl187-GCST90094784 | ebi-met1400-GCST90199653 | 1-myristoyl-2-palmitoyl-gpc (14:0/16:0) levels \|\| id:ebi-met1400-GCST90199653 | Ice cream liking \|\| id:ebi-fl187-GCST90094784 | Weighted mode | 8 | -0.256114314 | 0.190488777483939 | 0.220718051362898 | -0.629472317 | 0.117243690344104 | 0.774053478953286 | 0.532872914599806 | 1.12439340012179 | 0.999714303421225 |
| ebi-fl187-GCST90094784 | ebi-met1400-GCST90199667 | Erythritol levels in elite athletes \|\| id:ebi-met1400-GCST90199667 | Ice cream liking \|\| id:ebi-fl187-GCST90094784 | Weighted mode | 8 | -0.03804539 | 0.222928608437287 | 0.869317841412874 | -0.474985462 | 0.398894682878994 | 0.962669244671661 | 0.621894097374314 | 1.49017666922623 | 0.999714303421225 |
| ebi-fl187-GCST90094784 | ebi-met1400-GCST90199674 | 3-hydroxymyristate levels \|\| id:ebi-met1400-GCST90199674 | Ice cream liking \|\| id:ebi-fl187-GCST90094784 | Weighted mode | 8 | 0.288977896439139 | 0.216039398136881 | 0.2228450188723 | -0.134459324 | 0.712415116787426 | 1.33506221857337 | 0.874188436721665 | 2.03890952178032 | 0.999714303421225 |
| ebi-fl187-GCST90094784 | ebi-met1400-GCST90199685 | 2-linoleoylglycerol (18:2) levels \|\| id:ebi-met1400-GCST90199685 | Ice cream liking \|\| id:ebi-fl187-GCST90094784 | Weighted mode | 8 | 0.0683771124821347 | 0.203098422996249 | 0.746233854466886 | -0.329695797 | 0.466450021554782 | 1.07076903271699 | 0.719142465750669 | 1.59432431824042 | 0.999714303421225 |
| ebi-fl187-GCST90094784 | ebi-met1400-GCST90199703 | Gamma-glutamylvaline levels \|\| id:ebi-met1400-GCST90199703 | Ice cream liking \|\| id:ebi-fl187-GCST90094784 | Weighted mode | 8 | -0.160327542 | 0.172007489782023 | 0.382310676524855 | -0.497462222 | 0.176807137488686 | 0.851864721378522 | 0.608071854377545 | 1.19340090863463 | 0.999714303421225 |
| ebi-fl187-GCST90094784 | ebi-met1400-GCST90199779 | Gamma-glutamyl-2-aminobutyrate levels \|\| id:ebi-met1400-GCST90199779 | Ice cream liking \|\| id:ebi-fl187-GCST90094784 | Weighted mode | 8 | 0.0616336205478689 | 0.179377479900079 | 0.741238348617676 | -0.28994624 | 0.413213481152024 | 1.06357260218827 | 0.748303795267151 | 1.51166770405286 | 0.999714303421225 |
| ebi-fl187-GCST90094784 | ebi-met1400-GCST90199824 | 4-vinylphenol sulfate levels \|\| id:ebi-met1400-GCST90199824 | Ice cream liking \|\| id:ebi-fl187-GCST90094784 | Weighted mode | 8 | 0.263753504472889 | 0.163617440210817 | 0.15099407032221 | -0.056936678 | 0.584443687286091 | 1.30180726708021 | 0.944653884524503 | 1.79399268704208 | 0.999714303421225 |
| ebi-fl187-GCST90094784 | ebi-met1400-GCST90199830 | 4-ethylphenylsulfate levels \|\| id:ebi-met1400-GCST90199830 | Ice cream liking \|\| id:ebi-fl187-GCST90094784 | Weighted mode | 8 | -0.122892801 | 0.178242486505043 | 0.512727505663827 | -0.472248074 | 0.22646247279345 | 0.884358460037578 | 0.623598794889299 | 1.25415554399664 | 0.999714303421225 |
| ebi-fl187-GCST90094784 | ebi-met1400-GCST90199833 | Hexanoylglutamine levels \|\| id:ebi-met1400-GCST90199833 | Ice cream liking \|\| id:ebi-fl187-GCST90094784 | Weighted mode | 8 | 0.26739107986605 | 0.211064245523664 | 0.245722539650257 | -0.146294841 | 0.681077001092431 | 1.30655131234166 | 0.863902951309436 | 1.97600474589681 | 0.999714303421225 |
| ebi-fl187-GCST90094784 | ebi-met1400-GCST90199837 | Carotenoid levels (cryptoxanthin) \|\| id:ebi-met1400-GCST90199837 | Ice cream liking \|\| id:ebi-fl187-GCST90094784 | Weighted mode | 8 | 0.0246217670279933 | 0.166420643434999 | 0.886554546135685 | -0.301562694 | 0.350806228160591 | 1.02492738587079 | 0.739661452488497 | 1.42021210213637 | 0.999714303421225 |
| ebi-fl187-GCST90094784 | ebi-met1400-GCST90199869 | 2-hydroxyhippurate levels \|\| id:ebi-met1400-GCST90199869 | Ice cream liking \|\| id:ebi-fl187-GCST90094784 | Weighted mode | 8 | 0.134382630285968 | 0.174688584850507 | 0.46689188402642 | -0.208006996 | 0.476772256592961 | 1.14383040007663 | 0.81220135451431 | 1.61086653804196 | 0.999714303421225 |
| ebi-fl187-GCST90094784 | ebi-met1400-GCST90199902 | Hydantoin-5-propionate levels \|\| id:ebi-met1400-GCST90199902 | Ice cream liking \|\| id:ebi-fl187-GCST90094784 | Weighted mode | 8 | -0.012274796 | 0.23961247862787 | 0.960575252624869 | -0.481915254 | 0.457365661939793 | 0.98780023184141 | 0.617599398451788 | 1.57990649031067 | 0.999714303421225 |
| ebi-fl187-GCST90094784 | ebi-met1400-GCST90199904 | Pantoate levels \|\| id:ebi-met1400-GCST90199904 | Ice cream liking \|\| id:ebi-fl187-GCST90094784 | Weighted mode | 8 | -0.047199721 | 0.171367783149661 | 0.790939484693015 | -0.383080576 | 0.288681133491585 | 0.953896864868976 | 0.681757963432831 | 1.3346660803567 | 0.999714303421225 |
| ebi-fl187-GCST90094784 | ebi-met1400-GCST90199936 | 3-methyl catechol sulfate (2) levels \|\| id:ebi-met1400-GCST90199936 | Ice cream liking \|\| id:ebi-fl187-GCST90094784 | Weighted mode | 7 | 0.274566170226799 | 0.247687476802292 | 0.310083658842936 | -0.210901284 | 0.760033624759291 | 1.3159596485366 | 0.809854008236189 | 2.13834812072884 | 0.999714303421225 |
| ebi-fl187-GCST90094784 | ebi-met1400-GCST90199977 | 4-hydroxychlorothalonil levels \|\| id:ebi-met1400-GCST90199977 | Ice cream liking \|\| id:ebi-fl187-GCST90094784 | Weighted mode | 8 | 0.0751636377387099 | 0.207215699081164 | 0.727510514023703 | -0.330979132 | 0.481306407937792 | 1.07806054784188 | 0.71822015637177 | 1.61818703429919 | 0.999714303421225 |
| ebi-fl187-GCST90094784 | ebi-met1400-GCST90200040 | Sphingomyelin (d18:2/24:1, d18:1/24:2) levels \|\| id:ebi-met1400-GCST90200040 | Ice cream liking \|\| id:ebi-fl187-GCST90094784 | Weighted mode | 8 | 0.216134578269996 | 0.243727829962019 | 0.404629245367755 | -0.261571968 | 0.693841124995553 | 1.24126941565123 | 0.769840469201018 | 2.00138837054151 | 0.999714303421225 |
| ebi-fl187-GCST90094784 | ebi-met1400-GCST90200077 | 1-stearoyl-2-oleoyl-GPI (18:0/18:1) levels \|\| id:ebi-met1400-GCST90200077 | Ice cream liking \|\| id:ebi-fl187-GCST90094784 | Weighted mode | 8 | 0.0599478610094886 | 0.222874238587528 | 0.795703399610729 | -0.376885647 | 0.496781368641043 | 1.061781185 | 0.685994515200978 | 1.64342317559743 | 0.999714303421225 |
| ebi-fl187-GCST90094784 | ebi-met1400-GCST90200080 | 1-palmitoyl-2-oleoyl-GPI (16:0/18:1) levels \|\| id:ebi-met1400-GCST90200080 | Ice cream liking \|\| id:ebi-fl187-GCST90094784 | Weighted mode | 8 | 0.133110951922117 | 0.286690812586846 | 0.656526199886181 | -0.428803041 | 0.695024944592336 | 1.14237674019474 | 0.651288193784585 | 2.00375905627058 | 0.999714303421225 |
| ebi-fl187-GCST90094784 | ebi-met1400-GCST90200082 | 1-oleoyl-2-linoleoyl-GPE (18:1/18:2) levels \|\| id:ebi-met1400-GCST90200082 | Ice cream liking \|\| id:ebi-fl187-GCST90094784 | Weighted mode | 8 | -0.217880041 | 0.163151997658196 | 0.223519583796317 | -0.537657956 | 0.101897874646203 | 0.804221909726304 | 0.58411467380547 | 1.10727038557367 | 0.999714303421225 |
| ebi-fl187-GCST90094784 | ebi-met1400-GCST90200100 | Perfluorooctanesulfonate (PFOS) levels \|\| id:ebi-met1400-GCST90200100 | Ice cream liking \|\| id:ebi-fl187-GCST90094784 | Weighted mode | 8 | 0.285501950727114 | 0.18768689012304 | 0.172027670427672 | -0.082364354 | 0.653368255368273 | 1.3304296706862 | 0.920936350796037 | 1.92200373794801 | 0.999714303421225 |
| ebi-fl187-GCST90094784 | ebi-met1400-GCST90200102 | 1-palmitoleoyl-2-linolenoyl-GPC (16:1/18:3) levels \|\| id:ebi-met1400-GCST90200102 | Ice cream liking \|\| id:ebi-fl187-GCST90094784 | Weighted mode | 8 | -0.019633072 | 0.174600547953108 | 0.913626728226125 | -0.361850146 | 0.322584001570919 | 0.980558401229654 | 0.696386716075826 | 1.38069086619016 | 0.999714303421225 |
| ebi-fl187-GCST90094784 | ebi-met1400-GCST90200114 | Glycosyl-N-tricosanoyl-sphingadienine (d18:2/23:0) levels \|\| id:ebi-met1400-GCST90200114 | Ice cream liking \|\| id:ebi-fl187-GCST90094784 | Weighted mode | 8 | 0.0571748155647449 | 0.192117678138644 | 0.774644395034398 | -0.319375834 | 0.433725464716487 | 1.05884089608632 | 0.726602416390818 | 1.54299520333808 | 0.999714303421225 |
| ebi-fl187-GCST90094784 | ebi-met1400-GCST90200135 | Arachidoylcarnitine (C20) levels \|\| id:ebi-met1400-GCST90200135 | Ice cream liking \|\| id:ebi-fl187-GCST90094784 | Weighted mode | 8 | 0.244510974601358 | 0.222816337250112 | 0.308789754901512 | -0.192209046 | 0.681230995611578 | 1.27699667667689 | 0.825134359086479 | 1.97630906322848 | 0.999714303421225 |
| ebi-fl187-GCST90094784 | ebi-met1400-GCST90200146 | Docosahexaenoylcarnitine (C22:6) levels \|\| id:ebi-met1400-GCST90200146 | Ice cream liking \|\| id:ebi-fl187-GCST90094784 | Weighted mode | 8 | 0.394398771482609 | 0.196547998703215 | 0.0847863947483459 | 0.00916469402430814 | 0.77963284894091 | 1.48349200504524 | 1.00920681841998 | 2.18067148265868 | 0.999714303421225 |
| ebi-fl187-GCST90094784 | ebi-met1400-GCST90200162 | 3-hydroxyphenylacetoylglutamine levels \|\| id:ebi-met1400-GCST90200162 | Ice cream liking \|\| id:ebi-fl187-GCST90094784 | Weighted mode | 8 | -0.066594145 | 0.245849367433431 | 0.794301568412109 | -0.548458905 | 0.415270615192144 | 0.935574832038671 | 0.57783963023605 | 1.51478060787666 | 0.999714303421225 |
| ebi-fl187-GCST90094784 | ebi-met1400-GCST90200172 | 3-carboxy-4-methyl-5-pentyl-2-furanpropionate (3-CMPFP) levels \|\| id:ebi-met1400-GCST90200172 | Ice cream liking \|\| id:ebi-fl187-GCST90094784 | Weighted mode | 8 | -0.153468206 | 0.205553009053874 | 0.479617800862447 | -0.556352104 | 0.249415691876199 | 0.857728034482461 | 0.573296580535296 | 1.28327536935632 | 0.999714303421225 |
| ebi-fl187-GCST90094784 | ebi-met1400-GCST90200222 | Dihydrocaffeate sulfate (2) levels \|\| id:ebi-met1400-GCST90200222 | Ice cream liking \|\| id:ebi-fl187-GCST90094784 | Weighted mode | 8 | 0.169419175655711 | 0.168861406107851 | 0.349128261541956 | -0.16154918 | 0.500387531627098 | 1.18461659730401 | 0.850824686609738 | 1.6493603261559 | 0.999714303421225 |
| ebi-fl187-GCST90094784 | ebi-met1400-GCST90200223 | 4-methylhexanoylglutamine levels \|\| id:ebi-met1400-GCST90200223 | Ice cream liking \|\| id:ebi-fl187-GCST90094784 | Weighted mode | 8 | -0.264158163 | 0.224354536575435 | 0.2775070355852 | -0.703893055 | 0.175576728574592 | 0.767852084175914 | 0.494655828204328 | 1.19193344049663 | 0.999714303421225 |
| ebi-fl187-GCST90094784 | ebi-met1400-GCST90200224 | 2-ketocaprylate levels \|\| id:ebi-met1400-GCST90200224 | Ice cream liking \|\| id:ebi-fl187-GCST90094784 | Weighted mode | 8 | 0.0583540457677349 | 0.174201788990309 | 0.74745470601053 | -0.283081461 | 0.39978955218874 | 1.06009024974416 | 0.753458408145596 | 1.49151077943173 | 0.999714303421225 |
| ebi-fl187-GCST90094784 | ebi-met1400-GCST90200243 | Hydroxypalmitoyl sphingomyelin (d18:1/16:0(OH)) levels \|\| id:ebi-met1400-GCST90200243 | Ice cream liking \|\| id:ebi-fl187-GCST90094784 | Weighted mode | 8 | -0.028151468 | 0.21277675854174 | 0.898465666134011 | -0.445193915 | 0.388890978257498 | 0.972241091763209 | 0.640700022495642 | 1.47534369802421 | 0.999714303421225 |
| ebi-fl187-GCST90094784 | ebi-met1400-GCST90200273 | 2-methoxyhydroquinone sulfate (2) levels \|\| id:ebi-met1400-GCST90200273 | Ice cream liking \|\| id:ebi-fl187-GCST90094784 | Weighted mode | 8 | 0.0316851233138546 | 0.243614870510207 | 0.900175729656648 | -0.445800023 | 0.50917026951386 | 1.03219244079496 | 0.640311806966316 | 1.66391002515172 | 0.999714303421225 |
| ebi-fl187-GCST90094784 | ebi-met1400-GCST90200276 | 2,4-di-tert-butylphenol levels \|\| id:ebi-met1400-GCST90200276 | Ice cream liking \|\| id:ebi-fl187-GCST90094784 | Weighted mode | 8 | 0.086152974965822 | 0.165638442826139 | 0.619014855117691 | -0.238498373 | 0.410804322905055 | 1.08997305414307 | 0.787809970043866 | 1.5080302407087 | 0.999714303421225 |
| ebi-fl187-GCST90094784 | ebi-met1400-GCST90200289 | N-acetylmethionine levels \|\| id:ebi-met1400-GCST90200289 | Ice cream liking \|\| id:ebi-fl187-GCST90094784 | Weighted mode | 8 | 0.086558920677744 | 0.208632660699714 | 0.690645062939038 | -0.322361094 | 0.495478935649183 | 1.09041561385204 | 0.724436553189406 | 1.64128412032441 | 0.999714303421225 |
| ebi-fl187-GCST90094784 | ebi-met1400-GCST90200308 | 3-hydroxyisobutyrate levels \|\| id:ebi-met1400-GCST90200308 | Ice cream liking \|\| id:ebi-fl187-GCST90094784 | Weighted mode | 8 | -0.107020834 | 0.172452611434645 | 0.554534273720735 | -0.445027953 | 0.230986284024096 | 0.898506952952989 | 0.640806363450034 | 1.25984195936877 | 0.999714303421225 |
| ebi-fl187-GCST90094784 | ebi-met1400-GCST90200383 | Cystathionine levels \|\| id:ebi-met1400-GCST90200383 | Ice cream liking \|\| id:ebi-fl187-GCST90094784 | Weighted mode | 8 | 0.151817155374976 | 0.165442622059425 | 0.389328305738394 | -0.172450384 | 0.476084694611449 | 1.16394739544922 | 0.841600044720265 | 1.60975934812757 | 0.999714303421225 |
| ebi-fl187-GCST90094784 | ebi-met1400-GCST90200384 | Guanidinoacetate levels \|\| id:ebi-met1400-GCST90200384 | Ice cream liking \|\| id:ebi-fl187-GCST90094784 | Weighted mode | 8 | -0.17077092 | 0.271964627078955 | 0.549986935051158 | -0.703821589 | 0.362279749384333 | 0.843014669414988 | 0.494691180558758 | 1.43660077393365 | 0.999714303421225 |
| ebi-fl187-GCST90094784 | ebi-met1400-GCST90200403 | Citrulline levels \|\| id:ebi-met1400-GCST90200403 | Ice cream liking \|\| id:ebi-fl187-GCST90094784 | Weighted mode | 8 | 0.103037303090496 | 0.237895265894431 | 0.677959807325198 | -0.363237418 | 0.56931202424358 | 1.10853276000261 | 0.695421308318317 | 1.76705094494533 | 0.999714303421225 |
| ebi-fl187-GCST90094784 | ebi-met1400-GCST90200434 | 12,13-DiHOME levels \|\| id:ebi-met1400-GCST90200434 | Ice cream liking \|\| id:ebi-fl187-GCST90094784 | Weighted mode | 8 | -0.167438375 | 0.195408243261755 | 0.419883863539959 | -0.550438532 | 0.21556178142078 | 0.845828739564028 | 0.576696854799505 | 1.24055862402995 | 0.999714303421225 |
| ebi-fl187-GCST90094784 | ebi-met1400-GCST90200437 | Fructose levels \|\| id:ebi-met1400-GCST90200437 | Ice cream liking \|\| id:ebi-fl187-GCST90094784 | Weighted mode | 7 | 0.231675715400583 | 0.227816839549394 | 0.34842220806165 | -0.21484529 | 0.678196720917397 | 1.26071083342507 | 0.806666229762635 | 1.97032148721911 | 0.999714303421225 |
| ebi-fl187-GCST90094784 | ebi-met1400-GCST90200448 | N-stearoyl-sphinganine (d18:0/18:0) levels \|\| id:ebi-met1400-GCST90200448 | Ice cream liking \|\| id:ebi-fl187-GCST90094784 | Weighted mode | 8 | 0.42991755150211 | 0.324713518880978 | 0.227101510658903 | -0.206520946 | 1.06635604850883 | 1.5371307841994 | 0.813409224011884 | 2.90477533077366 | 0.999714303421225 |
| ebi-fl187-GCST90094784 | ebi-met1400-GCST90200468 | X-11847 levels \|\| id:ebi-met1400-GCST90200468 | Ice cream liking \|\| id:ebi-fl187-GCST90094784 | Weighted mode | 8 | -0.207787609 | 0.194820806366677 | 0.321569410220237 | -0.58963639 | 0.174061171176084 | 0.812379560251446 | 0.554528880448656 | 1.19012836514552 | 0.999714303421225 |
| ebi-fl187-GCST90094784 | ebi-met1400-GCST90200476 | X-11843 levels \|\| id:ebi-met1400-GCST90200476 | Ice cream liking \|\| id:ebi-fl187-GCST90094784 | Weighted mode | 8 | -0.053862187 | 0.187893066407179 | 0.782668699016161 | -0.422132597 | 0.314408223536901 | 0.947562684319086 | 0.655647096936052 | 1.36944866363309 | 0.999714303421225 |
| ebi-fl187-GCST90094784 | ebi-met1400-GCST90200511 | X-12906 levels \|\| id:ebi-met1400-GCST90200511 | Ice cream liking \|\| id:ebi-fl187-GCST90094784 | Weighted mode | 8 | -0.253640442 | 0.242580442520707 | 0.330505400984272 | -0.729098109 | 0.221817225261189 | 0.775970758325313 | 0.482343815321706 | 1.2483431914108 | 0.999714303421225 |
| ebi-fl187-GCST90094784 | ebi-met1400-GCST90200515 | X-12849 levels \|\| id:ebi-met1400-GCST90200515 | Ice cream liking \|\| id:ebi-fl187-GCST90094784 | Weighted mode | 8 | 0.188674369882997 | 0.164377364594181 | 0.288755797279952 | -0.133505265 | 0.510854004487591 | 1.20764764200654 | 0.875022862213729 | 1.66671396854056 | 0.999714303421225 |
| ebi-fl187-GCST90094784 | ebi-met1400-GCST90200551 | X-18886 levels \|\| id:ebi-met1400-GCST90200551 | Ice cream liking \|\| id:ebi-fl187-GCST90094784 | Weighted mode | 8 | 0.424724977981464 | 0.251368050712342 | 0.134942436030189 | -0.067956401 | 0.917406357377654 | 1.52916980646023 | 0.93430120690009 | 2.50279062011279 | 0.999714303421225 |
| ebi-fl187-GCST90094784 | ebi-met1400-GCST90200564 | X-21319 levels \|\| id:ebi-met1400-GCST90200564 | Ice cream liking \|\| id:ebi-fl187-GCST90094784 | Weighted mode | 8 | 0.0981105051933483 | 0.1741657496931 | 0.590798486004944 | -0.243254364 | 0.439475374591824 | 1.10308467495894 | 0.784072048540835 | 1.5518928424929 | 0.999714303421225 |
| ebi-fl187-GCST90094784 | ebi-met1400-GCST90200579 | X-21442 levels \|\| id:ebi-met1400-GCST90200579 | Ice cream liking \|\| id:ebi-fl187-GCST90094784 | Weighted mode | 7 | -0.106688891 | 0.211115097118277 | 0.631329446827639 | -0.520474481 | 0.307096699611846 | 0.898805256135739 | 0.594238526123804 | 1.35947242217164 | 0.999714303421225 |
| ebi-fl187-GCST90094784 | ebi-met1400-GCST90200581 | X-21441 levels \|\| id:ebi-met1400-GCST90200581 | Ice cream liking \|\| id:ebi-fl187-GCST90094784 | Weighted mode | 8 | 0.143292303315464 | 0.266826831802939 | 0.60788994657337 | -0.379688287 | 0.666272893649224 | 1.15406709000504 | 0.684074610918437 | 1.94696722692942 | 0.999714303421225 |
| ebi-fl187-GCST90094784 | ebi-met1400-GCST90200648 | X-24585 levels \|\| id:ebi-met1400-GCST90200648 | Ice cream liking \|\| id:ebi-fl187-GCST90094784 | Weighted mode | 8 | 0.38558128866492 | 0.305668700906245 | 0.247561143183387 | -0.213529365 | 0.984691942441159 | 1.47046883992489 | 0.807728440766724 | 2.6769870912773 | 0.999714303421225 |
| ebi-fl187-GCST90094784 | ebi-met1400-GCST90200720 | Adenosine 5'-diphosphate (ADP) to pantothenate ratio \|\| id:ebi-met1400-GCST90200720 | Ice cream liking \|\| id:ebi-fl187-GCST90094784 | Weighted mode | 8 | -0.146860758 | 0.221989438765834 | 0.529416263949376 | -0.581960058 | 0.288238541944155 | 0.863414192533785 | 0.558802008096903 | 1.33407549913366 | 0.999714303421225 |
| ebi-fl187-GCST90094784 | ebi-met1400-GCST90200724 | Cholate to taurocholate ratio \|\| id:ebi-met1400-GCST90200724 | Ice cream liking \|\| id:ebi-fl187-GCST90094784 | Weighted mode | 8 | -0.098704185 | 0.176769558406986 | 0.593988363279759 | -0.445172519 | 0.247764149606483 | 0.906010680052509 | 0.640713730981311 | 1.2811577350028 | 0.999714303421225 |
| ebi-fl187-GCST90094784 | ebi-met1400-GCST90200726 | N-acetylputrescine to (N(1) + N(8))-acetylspermidine ratio \|\| id:ebi-met1400-GCST90200726 | Ice cream liking \|\| id:ebi-fl187-GCST90094784 | Weighted mode | 8 | 0.0771400976995575 | 0.184469925048727 | 0.688351668365359 | -0.284420955 | 0.438701150795062 | 1.08019339840261 | 0.752449830212282 | 1.55069179512394 | 0.999714303421225 |
| ebi-fl187-GCST90094784 | ebi-met1400-GCST90200744 | Arginine to glutamate ratio \|\| id:ebi-met1400-GCST90200744 | Ice cream liking \|\| id:ebi-fl187-GCST90094784 | Weighted mode | 8 | 0.168852100610877 | 0.157910310874287 | 0.320418586918377 | -0.140652109 | 0.47835630992448 | 1.18394502122899 | 0.868791504132995 | 1.61342025862899 | 0.999714303421225 |
| ebi-fl187-GCST90094784 | ebi-met1400-GCST90200759 | Histidine to trans-urocanate ratio \|\| id:ebi-met1400-GCST90200759 | Ice cream liking \|\| id:ebi-fl187-GCST90094784 | Weighted mode | 8 | -0.149309614 | 0.232281851803719 | 0.540831949943025 | -0.604582043 | 0.305962815693179 | 0.861302402466112 | 0.546302709765656 | 1.35793181185596 | 0.999714303421225 |
| ebi-fl187-GCST90094784 | ebi-met1400-GCST90200795 | Oleoyl-linoleoyl-glycerol (18:1 to 18:2) [2] to linoleoyl-arachidonoyl-glycerol (18:2 to 20:4) [2] ratio \|\| id:ebi-met1400-GCST90200795 | Ice cream liking \|\| id:ebi-fl187-GCST90094784 | Weighted mode | 8 | -0.080158696 | 0.221781674971871 | 0.728440407935093 | -0.514850779 | 0.354533386520705 | 0.922969862746901 | 0.597589760686515 | 1.42551533439997 | 0.999714303421225 |
| ebi-fl187-GCST90094784 | ebi-met1400-GCST90200811 | Choline to taurocholate ratio \|\| id:ebi-met1400-GCST90200811 | Ice cream liking \|\| id:ebi-fl187-GCST90094784 | Weighted mode | 8 | 0.137556414877683 | 0.198684937134918 | 0.511033343118252 | -0.251866062 | 0.526978891662123 | 1.14746643832489 | 0.777348847719116 | 1.69380739541247 | 0.999714303421225 |
| ebi-fl187-GCST90094784 | ebi-met1400-GCST90200831 | Cholate to phosphate ratio \|\| id:ebi-met1400-GCST90200831 | Ice cream liking \|\| id:ebi-fl187-GCST90094784 | Weighted mode | 8 | -0.023540932 | 0.209174709314766 | 0.913553105619043 | -0.433523362 | 0.386441498279913 | 0.976733994197195 | 0.648221148544589 | 1.47173429556007 | 0.999714303421225 |
| ebi-fl187-GCST90094784 | ebi-met1400-GCST90200841 | Adenosine 5'-diphosphate (ADP) to oxalate (ethanedioate) ratio \|\| id:ebi-met1400-GCST90200841 | Ice cream liking \|\| id:ebi-fl187-GCST90094784 | Weighted mode | 8 | -0.072644663 | 0.243777551811149 | 0.774356940700283 | -0.550448664 | 0.405159338896717 | 0.929931210613183 | 0.57669101171484 | 1.49954141629679 | 0.999714303421225 |
| ebi-fl187-GCST90094784 | ebi-met1400-GCST90200844 | Aspartate to phosphate ratio \|\| id:ebi-met1400-GCST90200844 | Ice cream liking \|\| id:ebi-fl187-GCST90094784 | Weighted mode | 8 | -0.194329383 | 0.158816834302595 | 0.26068597822459 | -0.505610379 | 0.116951611878288 | 0.823386649740678 | 0.603137320944889 | 1.12406503697875 | 0.999714303421225 |
| ebi-fl187-GCST90094784 | ebi-met1400-GCST90200870 | Adenosine 5'-monophosphate (AMP) to acetoacetate ratio \|\| id:ebi-met1400-GCST90200870 | Ice cream liking \|\| id:ebi-fl187-GCST90094784 | Weighted mode | 8 | -0.035806013 | 0.192039394849815 | 0.857379229819821 | -0.412203226 | 0.340591201386825 | 0.96482743977534 | 0.662189687934068 | 1.40577844310394 | 0.999714303421225 |
| ebi-fl187-GCST90094784 | ebi-met1400-GCST90200887 | Cholesterol to cortisol ratio \|\| id:ebi-met1400-GCST90200887 | Ice cream liking \|\| id:ebi-fl187-GCST90094784 | Weighted mode | 8 | 0.0432560360070612 | 0.172402777708681 | 0.809096121605554 | -0.294653408 | 0.381165480316075 | 1.04420521476113 | 0.744789680656954 | 1.46398984686867 | 0.999714303421225 |
| ebi-fl187-GCST90094784 | ebi-met1400-GCST90200890 | Cortisol to taurocholate ratio \|\| id:ebi-met1400-GCST90200890 | Ice cream liking \|\| id:ebi-fl187-GCST90094784 | Weighted mode | 8 | -0.155026125 | 0.240897424940839 | 0.54037857265669 | -0.627185078 | 0.317132828031942 | 0.856392804056016 | 0.534093117578476 | 1.37318495726761 | 0.999714303421225 |
| ebi-fl187-GCST90094784 | ebi-met1400-GCST90200948 | Adenosine 5'-diphosphate (ADP) to mannitol to sorbitol ratio \|\| id:ebi-met1400-GCST90200948 | Ice cream liking \|\| id:ebi-fl187-GCST90094784 | Weighted mode | 8 | 0.116129861790337 | 0.259548920752254 | 0.668082473618841 | -0.392586023 | 0.624845746464754 | 1.12314171585731 | 0.675308251890423 | 1.8679577961141 | 0.999714303421225 |
| ebi-fl187-GCST90094784 | ebi-met1400-GCST90200972 | Leucine to N-palmitoyl-sphingosine (d18:1 to 16:0) ratio \|\| id:ebi-met1400-GCST90200972 | Ice cream liking \|\| id:ebi-fl187-GCST90094784 | Weighted mode | 8 | -0.274375267 | 0.170965293477374 | 0.152559655521407 | -0.609467242 | 0.0607167084510047 | 0.760046801433182 | 0.543640420731145 | 1.06259784655434 | 0.999714303421225 |
| ebi-fl187-GCST90094784 | ebi-met1400-GCST90200973 | Proline to glutamate ratio \|\| id:ebi-met1400-GCST90200973 | Ice cream liking \|\| id:ebi-fl187-GCST90094784 | Weighted mode | 8 | 0.232594404937133 | 0.178094062636523 | 0.232823261158353 | -0.116469958 | 0.581658767704718 | 1.26186956745327 | 0.890056835813398 | 1.78900351212913 | 0.999714303421225 |
| ebi-fl187-GCST90094784 | ebi-met1400-GCST90200983 | Cholesterol to linoleoyl-arachidonoyl-glycerol (18:2 to 20:4) [1] ratio \|\| id:ebi-met1400-GCST90200983 | Ice cream liking \|\| id:ebi-fl187-GCST90094784 | Weighted mode | 8 | 0.131150566235939 | 0.176498046278538 | 0.481627066469339 | -0.214785604 | 0.477086736941874 | 1.14013943489221 | 0.806714377594706 | 1.61137320357689 | 0.999714303421225 |
| ebi-fl187-GCST90094784 | ebi-met1400-GCST90201001 | Serine to alpha-ketobutyrate ratio \|\| id:ebi-met1400-GCST90201001 | Ice cream liking \|\| id:ebi-fl187-GCST90094784 | Weighted mode | 8 | 0.222319575447376 | 0.185483503349549 | 0.269684287372211 | -0.141228091 | 0.585867242012492 | 1.24897045438508 | 0.86829123958992 | 1.79654835244404 | 0.999714303421225 |
| ebi-fl187-GCST90094784 | ebi-met1400-GCST90201005 | Fructose to maltose ratio \|\| id:ebi-met1400-GCST90201005 | Ice cream liking \|\| id:ebi-fl187-GCST90094784 | Weighted mode | 8 | -0.077498347 | 0.2229667754267 | 0.738373839542411 | -0.514513227 | 0.359516532807784 | 0.925428554102353 | 0.597791512655714 | 1.43263661429936 | 0.999714303421225 |
| ebi-fl187-GCST90094784 | ebi-met1400-GCST90201020 | Paraxanthine to 5-acetylamino-6-formylamino-3-methyluracil ratio \|\| id:ebi-met1400-GCST90201020 | Ice cream liking \|\| id:ebi-fl187-GCST90094784 | Weighted mode | 8 | 0.0768897226017653 | 0.196205730850507 | 0.706801598225671 | -0.30767351 | 0.461452955068759 | 1.07992297872944 | 0.7351553 | 1.58637724618258 | 0.999714303421225 |
| ebi-fl187-GCST90094784 | ebi-pw11-GCST90275197 | Placental weight (fetal genotype, adjusted for sex) \|\| id:ebi-pw11-GCST90275197 | Ice cream liking \|\| id:ebi-fl187-GCST90094784 | Weighted mode | 8 | -0.122990663 | 0.0656003181107596 | 0.102942052877237 | -0.251567287 | 0.00558596027898461 | 0.88427191877632 | 0.77758113496911 | 1.00560159084546 | 0.999714303421225 |
| ebi-fl187-GCST90094784 | prot-a-1017 | FAS-associated factor 2 \|\| id:prot-a-1017 | Ice cream liking \|\| id:ebi-fl187-GCST90094784 | Weighted mode | 9 | 0.0462369954166111 | 0.280621236588574 | 0.873214904908629 | -0.503780628 | 0.596254619130216 | 1.04732259220093 | 0.60424192189711 | 1.81530703578236 | 0.999714303421225 |
| ebi-fl187-GCST90094784 | prot-a-1019 | Fas apoptotic inhibitory molecule 3 \|\| id:prot-a-1019 | Ice cream liking \|\| id:ebi-fl187-GCST90094784 | Weighted mode | 9 | -0.271428088 | 0.38763246101098 | 0.50364072044892 | -1.031187712 | 0.488331535479923 | 0.762290099225231 | 0.356583190939011 | 1.62959502899339 | 0.999714303421225 |
| ebi-fl187-GCST90094784 | prot-a-1052 | MIP18 family protein FAM96A \|\| id:prot-a-1052 | Ice cream liking \|\| id:ebi-fl187-GCST90094784 | Weighted mode | 9 | 0.0456237516260414 | 0.263184725623857 | 0.866680229528932 | -0.470218311 | 0.5614658138488 | 1.04668052501652 | 0.624865838557132 | 1.75324054196746 | 0.999714303421225 |
| ebi-fl187-GCST90094784 | prot-a-1066 | F-box/LRR-repeat protein 4 \|\| id:prot-a-1066 | Ice cream liking \|\| id:ebi-fl187-GCST90094784 | Weighted mode | 9 | 0.166594584650372 | 0.310220157355794 | 0.60586265949265 | -0.441436924 | 0.774626093067728 | 1.18127526109411 | 0.643111654411606 | 2.16978067945237 | 0.999714303421225 |
| ebi-fl187-GCST90094784 | prot-a-1076 | Low affinity immunoglobulin gamma Fc region receptor III-B \|\| id:prot-a-1076 | Ice cream liking \|\| id:ebi-fl187-GCST90094784 | Weighted mode | 9 | -0.067838898 | 0.291466995226942 | 0.821799913333418 | -0.639114209 | 0.503436412309315 | 0.934410996619022 | 0.527759701861718 | 1.6543967027466 | 0.999714303421225 |
| ebi-fl187-GCST90094784 | prot-a-1081 | Fc receptor-like protein 3 \|\| id:prot-a-1081 | Ice cream liking \|\| id:ebi-fl187-GCST90094784 | Weighted mode | 9 | 0.153198868504302 | 0.293142834351794 | 0.615406744412739 | -0.421361087 | 0.727758823833819 | 1.16555674842339 | 0.656153130377485 | 2.07043519401293 | 0.999714303421225 |
| ebi-fl187-GCST90094784 | prot-a-1105 | Fibroblast growth factor receptor 4 \|\| id:prot-a-1105 | Ice cream liking \|\| id:ebi-fl187-GCST90094784 | Weighted mode | 9 | -0.188659551 | 0.290557464625931 | 0.534336555241553 | -0.758152182 | 0.38083307945629 | 0.828068373586773 | 0.468531388408651 | 1.46350329625426 | 0.999714303421225 |
| ebi-fl187-GCST90094784 | prot-a-1143 | Follistatin-related protein 1 \|\| id:prot-a-1143 | Ice cream liking \|\| id:ebi-fl187-GCST90094784 | Weighted mode | 9 | 0.201753000800808 | 0.253877015676686 | 0.449725522795778 | -0.29584595 | 0.699351951527112 | 1.22354575596298 | 0.743902017354902 | 2.01244812086696 | 0.999714303421225 |
| ebi-fl187-GCST90094784 | prot-a-1151 | Alpha-(1,3)-fucosyltransferase 10 \|\| id:prot-a-1151 | Ice cream liking \|\| id:ebi-fl187-GCST90094784 | Weighted mode | 9 | 0.253463316212498 | 0.315238212970762 | 0.444608542431068 | -0.364403581 | 0.871330213635192 | 1.28848011228769 | 0.694610806297142 | 2.39008806760587 | 0.999714303421225 |
| ebi-fl187-GCST90094784 | prot-a-1165 | Growth arrest and DNA damage-inducible proteins-interacting protein 1 \|\| id:prot-a-1165 | Ice cream liking \|\| id:ebi-fl187-GCST90094784 | Weighted mode | 9 | -0.503629842 | 0.259870637428717 | 0.0886192562762471 | -1.012976291 | 0.00571660750491321 | 0.604333040255925 | 0.36313656940501 | 1.00573297848622 | 0.999714303421225 |
| ebi-fl187-GCST90094784 | prot-a-1194 | Growth/differentiation factor 11/8 \|\| id:prot-a-1194 | Ice cream liking \|\| id:ebi-fl187-GCST90094784 | Weighted mode | 9 | -0.016599827 | 0.260035837134899 | 0.950666521379665 | -0.526270068 | 0.493070413765217 | 0.983537190903797 | 0.590804525853335 | 1.63733580830942 | 0.999714303421225 |
| ebi-fl187-GCST90094784 | prot-a-1216 | D-glucuronyl C5-epimerase \|\| id:prot-a-1216 | Ice cream liking \|\| id:ebi-fl187-GCST90094784 | Weighted mode | 9 | -1.043006154 | 0.731568869836801 | 0.191781461013641 | -2.476881139 | 0.390868830947186 | 0.352393738255511 | 0.0840048168094629 | 1.47826459812844 | 0.999714303421225 |
| ebi-fl187-GCST90094784 | prot-a-1246 | Glypican-3 \|\| id:prot-a-1246 | Ice cream liking \|\| id:ebi-fl187-GCST90094784 | Weighted mode | 9 | 0.159532848658504 | 0.282095079290488 | 0.587220462409877 | -0.393373507 | 0.71243920406786 | 1.17296279184209 | 0.674776666871746 | 2.03895863415724 | 0.999714303421225 |
| ebi-fl187-GCST90094784 | prot-a-1275 | Glutamate receptor 4 \|\| id:prot-a-1275 | Ice cream liking \|\| id:ebi-fl187-GCST90094784 | Weighted mode | 9 | 0.287652183624749 | 0.3447592261233 | 0.428289495377625 | -0.3880759 | 0.963380266826416 | 1.33329348215951 | 0.678360854008305 | 2.62053964208742 | 0.999714303421225 |
| ebi-fl187-GCST90094784 | prot-a-1289 | Glutathione S-transferase P \|\| id:prot-a-1289 | Ice cream liking \|\| id:ebi-fl187-GCST90094784 | Weighted mode | 9 | -0.234828096 | 0.273651267628942 | 0.415789150310339 | -0.771184581 | 0.301528388510225 | 0.790706763568207 | 0.462464916744123 | 1.35192349368727 | 0.999714303421225 |
| ebi-fl187-GCST90094784 | prot-a-130 | Apolipoprotein D \|\| id:prot-a-130 | Ice cream liking \|\| id:ebi-fl187-GCST90094784 | Weighted mode | 9 | 0.297588666201426 | 0.363472095721942 | 0.43664320553533 | -0.414816641 | 1.00999397381643 | 1.34660776865528 | 0.66046137086653 | 2.74558446957105 | 0.999714303421225 |
| ebi-fl187-GCST90094784 | prot-a-1313 | Heparin-binding EGF-like growth factor \|\| id:prot-a-1313 | Ice cream liking \|\| id:ebi-fl187-GCST90094784 | Weighted mode | 9 | -0.312100085 | 0.36563878108925 | 0.418163767598753 | -1.028752096 | 0.40455192600014 | 0.731908271570798 | 0.357452749122618 | 1.4986308520738 | 0.999714303421225 |
| ebi-fl187-GCST90094784 | prot-a-1347 | HLA class II histocompatibility antigen, DQ alpha 2 chain \|\| id:prot-a-1347 | Ice cream liking \|\| id:ebi-fl187-GCST90094784 | Weighted mode | 9 | -0.347257128 | 0.403286385664763 | 0.41426161095359 | -1.137698444 | 0.443184187476444 | 0.706623611879084 | 0.320555950815779 | 1.5576592092405 | 0.999714303421225 |
| ebi-fl187-GCST90094784 | prot-a-1357 | Non-histone chromosomal protein HMG-14 \|\| id:prot-a-1357 | Ice cream liking \|\| id:ebi-fl187-GCST90094784 | Weighted mode | 9 | -0.163899609 | 0.257769336791875 | 0.542639226840685 | -0.669127509 | 0.341328291597358 | 0.84882723262955 | 0.512155233929739 | 1.40681501060748 | 0.999714303421225 |
| ebi-fl187-GCST90094784 | prot-a-137 | Apolipoprotein M \|\| id:prot-a-137 | Ice cream liking \|\| id:ebi-fl187-GCST90094784 | Weighted mode | 9 | -0.182284648 | 0.276922981798806 | 0.528861077104234 | -0.725053693 | 0.360484395835892 | 0.833364090821088 | 0.484298564903796 | 1.43402388154509 | 0.999714303421225 |
| ebi-fl187-GCST90094784 | prot-a-1381 | 17-beta-hydroxysteroid dehydrogenase 14 \|\| id:prot-a-1381 | Ice cream liking \|\| id:ebi-fl187-GCST90094784 | Weighted mode | 9 | 0.0127220491258957 | 0.258917546528762 | 0.962015876453179 | -0.494756342 | 0.520200440322269 | 1.01280331866555 | 0.609719452177813 | 1.68236482965416 | 0.999714303421225 |
| ebi-fl187-GCST90094784 | prot-a-1392 | Serine protease HTRA2, mitochondrial \|\| id:prot-a-1392 | Ice cream liking \|\| id:ebi-fl187-GCST90094784 | Weighted mode | 9 | -0.035810077 | 0.281632351752986 | 0.901958117728963 | -0.587809486 | 0.516189332448912 | 0.964823518272931 | 0.55554287707763 | 1.67563019853547 | 0.999714303421225 |
| ebi-fl187-GCST90094784 | prot-a-1416 | Interferon-induced protein with tetratricopeptide repeats 2 \|\| id:prot-a-1416 | Ice cream liking \|\| id:ebi-fl187-GCST90094784 | Weighted mode | 9 | -0.184106612 | 0.272876826205564 | 0.518892657417586 | -0.718945191 | 0.350731967334446 | 0.831847114190853 | 0.487265957310505 | 1.42010663992826 | 0.999714303421225 |
| ebi-fl187-GCST90094784 | prot-a-1428 | Interferon gamma \|\| id:prot-a-1428 | Ice cream liking \|\| id:ebi-fl187-GCST90094784 | Weighted mode | 9 | -0.275187356 | 0.287067245812302 | 0.36582286027569 | -0.837839158 | 0.287464445664827 | 0.759429826064106 | 0.432644390300791 | 1.33304319585605 | 0.999714303421225 |
| ebi-fl187-GCST90094784 | prot-a-1483 | Interleukin-17C \|\| id:prot-a-1483 | Ice cream liking \|\| id:ebi-fl187-GCST90094784 | Weighted mode | 9 | -0.189569624 | 0.305447589116119 | 0.552110247815131 | -0.788246899 | 0.409107650666306 | 0.82731511390365 | 0.454641129020003 | 1.50547377701786 | 0.999714303421225 |
| ebi-fl187-GCST90094784 | prot-a-1503 | Interleukin-1 receptor-like 2 \|\| id:prot-a-1503 | Ice cream liking \|\| id:ebi-fl187-GCST90094784 | Weighted mode | 9 | 0.00447432185101959 | 0.413509194826787 | 0.99163173192213 | -0.8060037 | 0.814952343711523 | 1.00448434657474 | 0.446639409529156 | 2.25906801098753 | 0.999714303421225 |
| ebi-fl187-GCST90094784 | prot-a-1522 | Interleukin-31 receptor subunit alpha \|\| id:prot-a-1522 | Ice cream liking \|\| id:ebi-fl187-GCST90094784 | Weighted mode | 9 | -0.105089701 | 0.247004651163082 | 0.681717920278393 | -0.589218817 | 0.379039415492416 | 0.900243766389833 | 0.554760484930983 | 1.46088061593749 | 0.999714303421225 |
| ebi-fl187-GCST90094784 | prot-a-1540 | Interleukin-6 receptor subunit alpha \|\| id:prot-a-1540 | Ice cream liking \|\| id:ebi-fl187-GCST90094784 | Weighted mode | 9 | -0.204744214 | 0.272794544640043 | 0.474421560418743 | -0.739421522 | 0.329933093018449 | 0.814855718054015 | 0.477389995284063 | 1.39087506609817 | 0.999714303421225 |
| ebi-fl187-GCST90094784 | prot-a-1541 | Interleukin-6 receptor subunit alpha \|\| id:prot-a-1541 | Ice cream liking \|\| id:ebi-fl187-GCST90094784 | Weighted mode | 9 | -0.047343366 | 0.293724979875161 | 0.875945665697919 | -0.623044327 | 0.528357594063666 | 0.95375985218521 | 0.536309249080752 | 1.6961442622881 | 0.999714303421225 |
| ebi-fl187-GCST90094784 | prot-a-1545 | Interleukin-9 \|\| id:prot-a-1545 | Ice cream liking \|\| id:ebi-fl187-GCST90094784 | Weighted mode | 9 | 0.416886399362351 | 0.290249684235362 | 0.188844226235008 | -0.152002982 | 0.985775780463661 | 1.51723014483279 | 0.85898571946612 | 2.67989008457566 | 0.999714303421225 |
| ebi-fl187-GCST90094784 | prot-a-1548 | Inositol monophosphatase 3 \|\| id:prot-a-1548 | Ice cream liking \|\| id:ebi-fl187-GCST90094784 | Weighted mode | 9 | -0.087281887 | 0.286952790778003 | 0.768761282703876 | -0.649709357 | 0.475145582509056 | 0.916418732311909 | 0.522197527585405 | 1.60824831326842 | 0.999714303421225 |
| ebi-fl187-GCST90094784 | prot-a-1555 | Inhibin beta A chain:Inhibin beta B chain heterodimer \|\| id:prot-a-1555 | Ice cream liking \|\| id:ebi-fl187-GCST90094784 | Weighted mode | 9 | -0.28778708 | 0.271248151346248 | 0.319679362044936 | -0.819433457 | 0.243859296379753 | 0.749921248279512 | 0.44068124871823 | 1.2761647568551 | 0.999714303421225 |
| ebi-fl187-GCST90094784 | prot-a-1603 | Junctophilin-3 \|\| id:prot-a-1603 | Ice cream liking \|\| id:ebi-fl187-GCST90094784 | Weighted mode | 9 | 0.0447876403440226 | 0.387116793585592 | 0.910745340265309 | -0.713961275 | 0.803536555771783 | 1.04580574937674 | 0.489700511829873 | 2.23342561220235 | 0.999714303421225 |
| ebi-fl187-GCST90094784 | prot-a-1627 | UPF0258 protein KIAA1024 \|\| id:prot-a-1627 | Ice cream liking \|\| id:ebi-fl187-GCST90094784 | Weighted mode | 9 | 0.276153091994789 | 0.277284956657573 | 0.34845546841583 | -0.267325423 | 0.819631607043633 | 1.31804963142973 | 0.765423944334105 | 2.2696635554345 | 0.999714303421225 |
| ebi-fl187-GCST90094784 | prot-a-1666 | Kallikrein-7 \|\| id:prot-a-1666 | Ice cream liking \|\| id:ebi-fl187-GCST90094784 | Weighted mode | 9 | 0.186805759337829 | 0.389605366448733 | 0.644434491020863 | -0.576820759 | 0.950432277577346 | 1.20539312595009 | 0.561681251071393 | 2.58682764524581 | 0.999714303421225 |
| ebi-fl187-GCST90094784 | prot-a-1678 | Importin subunit alpha-3 \|\| id:prot-a-1678 | Ice cream liking \|\| id:ebi-fl187-GCST90094784 | Weighted mode | 9 | 0.0340180032568775 | 0.299844733279487 | 0.912467595494089 | -0.553677674 | 0.621713680484672 | 1.03460323278822 | 0.574831874007202 | 1.86211638167167 | 0.999714303421225 |
| ebi-fl187-GCST90094784 | prot-a-1718 | Protein LDOC1 \|\| id:prot-a-1718 | Ice cream liking \|\| id:ebi-fl187-GCST90094784 | Weighted mode | 9 | 0.279623777098313 | 0.308640371349127 | 0.391413054361136 | -0.325311351 | 0.884558904942602 | 1.32263211422871 | 0.722302429228536 | 2.42191586072546 | 0.999714303421225 |
| ebi-fl187-GCST90094784 | prot-a-1723 | Leptin \|\| id:prot-a-1723 | Ice cream liking \|\| id:ebi-fl187-GCST90094784 | Weighted mode | 9 | -0.2314733 | 0.328921404152377 | 0.50156386382703 | -0.876159253 | 0.413212651735686 | 0.793363877716176 | 0.416379051317924 | 1.51166645025149 | 0.999714303421225 |
| ebi-fl187-GCST90094784 | prot-a-1730 | Galectin-7 \|\| id:prot-a-1730 | Ice cream liking \|\| id:ebi-fl187-GCST90094784 | Weighted mode | 9 | -0.005641842 | 0.263148418228357 | 0.983419950218127 | -0.521412742 | 0.510129057274581 | 0.994374042851963 | 0.593681236719652 | 1.66550612675785 | 0.999714303421225 |
| ebi-fl187-GCST90094784 | prot-a-1753 | Lipase member N \|\| id:prot-a-1753 | Ice cream liking \|\| id:ebi-fl187-GCST90094784 | Weighted mode | 9 | 0.0561329477485789 | 0.280796311166628 | 0.846541861627094 | -0.494227822 | 0.606493717635171 | 1.05773829831455 | 0.610041786233983 | 1.83398962656018 | 0.999714303421225 |
| ebi-fl187-GCST90094784 | prot-a-1756 | VIP36-like protein \|\| id:prot-a-1756 | Ice cream liking \|\| id:ebi-fl187-GCST90094784 | Weighted mode | 9 | -0.458055927 | 0.283570379942718 | 0.144905275364013 | -1.013853872 | 0.097742017579833 | 0.632512100645649 | 0.362818027597322 | 1.1026782768005 | 0.999714303421225 |
| ebi-fl187-GCST90094784 | prot-a-1760 | Ig Kappa chain V-I region HK102- like \|\| id:prot-a-1760 | Ice cream liking \|\| id:ebi-fl187-GCST90094784 | Weighted mode | 9 | -0.026401581 | 0.345232697261925 | 0.940919364451636 | -0.703057668 | 0.650254505665992 | 0.973943893734578 | 0.495069230003038 | 1.91602840705137 | 0.999714303421225 |
| ebi-fl187-GCST90094784 | prot-a-179 | Neutral ceramidase \|\| id:prot-a-179 | Ice cream liking \|\| id:ebi-fl187-GCST90094784 | Weighted mode | 9 | -0.687150922 | 0.271058669035188 | 0.0349785756156322 | -1.218425913 | -0.155875931 | 0.503007135987649 | 0.295695250707555 | 0.855665345483456 | 0.999714303421225 |
| ebi-fl187-GCST90094784 | prot-a-1809 | Lactotransferrin \|\| id:prot-a-1809 | Ice cream liking \|\| id:ebi-fl187-GCST90094784 | Weighted mode | 9 | 0.0636729148198008 | 0.435380202450736 | 0.887344690293205 | -0.789672282 | 0.917018111623244 | 1.06574375275873 | 0.453993552772323 | 2.50181911088473 | 0.999714303421225 |
| ebi-fl187-GCST90094784 | prot-a-1818 | Lysophospholipase-like protein 1 \|\| id:prot-a-1818 | Ice cream liking \|\| id:ebi-fl187-GCST90094784 | Weighted mode | 9 | 0.250557430211459 | 0.292825636627555 | 0.417078491888041 | -0.323380818 | 0.824495678001467 | 1.28474137077597 | 0.723698204887178 | 2.28073025280009 | 0.999714303421225 |
| ebi-fl187-GCST90094784 | prot-a-1852 | MAP kinase-activated protein kinase 3 \|\| id:prot-a-1852 | Ice cream liking \|\| id:ebi-fl187-GCST90094784 | Weighted mode | 9 | 0.189536522244998 | 0.303804267774386 | 0.550079467105799 | -0.405919843 | 0.784992887082795 | 1.20868926722887 | 0.666363579260947 | 2.19239134637963 | 0.999714303421225 |
| ebi-fl187-GCST90094784 | prot-a-1856 | Megakaryocyte-associated tyrosine-protein kinase \|\| id:prot-a-1856 | Ice cream liking \|\| id:ebi-fl187-GCST90094784 | Weighted mode | 9 | -0.058396283 | 0.26853983569842 | 0.833294765255468 | -0.584734361 | 0.467941795105001 | 0.943276069218765 | 0.557253870606941 | 1.59670446396666 | 0.999714303421225 |
| ebi-fl187-GCST90094784 | prot-a-1890 | Beta-1,3-N-acetylglucosaminyltransferase manic fringe \|\| id:prot-a-1890 | Ice cream liking \|\| id:ebi-fl187-GCST90094784 | Weighted mode | 9 | -0.138464084 | 0.319098562383153 | 0.675809086072533 | -0.763897266 | 0.486969098454136 | 0.870694522731488 | 0.465847353512679 | 1.62737632015759 | 0.999714303421225 |
| ebi-fl187-GCST90094784 | prot-a-1919 | Matrilysin \|\| id:prot-a-1919 | Ice cream liking \|\| id:ebi-fl187-GCST90094784 | Weighted mode | 9 | 0.117252772599673 | 0.334947905334837 | 0.735329339405107 | -0.539245122 | 0.773750667055954 | 1.12440361219638 | 0.583188322371521 | 2.16788202819134 | 0.999714303421225 |
| ebi-fl187-GCST90094784 | prot-a-1923 | Multimerin-2 \|\| id:prot-a-1923 | Ice cream liking \|\| id:ebi-fl187-GCST90094784 | Weighted mode | 9 | -0.185542585 | 0.339525851684453 | 0.599644617634371 | -0.851013254 | 0.47992808423894 | 0.830653461398079 | 0.426982071239677 | 1.6159581851513 | 0.999714303421225 |
| ebi-fl187-GCST90094784 | prot-a-1948 | Mesothelin \|\| id:prot-a-1948 | Ice cream liking \|\| id:ebi-fl187-GCST90094784 | Weighted mode | 9 | 0.262598369151776 | 0.289112837760127 | 0.390265870955219 | -0.304062793 | 0.829259531161625 | 1.3003043717156 | 0.737814535512568 | 2.29162123775197 | 0.999714303421225 |
| ebi-fl187-GCST90094784 | prot-a-195 | ATPase family AAA domain-containing protein 1 \|\| id:prot-a-195 | Ice cream liking \|\| id:ebi-fl187-GCST90094784 | Weighted mode | 9 | 0.459762754744144 | 0.316243195 | 0.184071345405036 | -0.160073908 | 1.0795994176811 | 1.58369821553314 | 0.852080810886076 | 2.94350020073177 | 0.999714303421225 |
| ebi-fl187-GCST90094784 | prot-a-1966 | Mucin-1 \|\| id:prot-a-1966 | Ice cream liking \|\| id:ebi-fl187-GCST90094784 | Weighted mode | 9 | -0.031981713 | 0.272759451738452 | 0.909550285139303 | -0.566590239 | 0.502626811964683 | 0.968524292892258 | 0.567457036642064 | 1.65305784464902 | 0.999714303421225 |
| ebi-fl187-GCST90094784 | prot-a-1986 | Myocardial zonula adherens protein \|\| id:prot-a-1986 | Ice cream liking \|\| id:ebi-fl187-GCST90094784 | Weighted mode | 9 | -0.169422752 | 0.303530828433066 | 0.591999953433587 | -0.764343176 | 0.425497671901422 | 0.84415196115819 | 0.465639674073488 | 1.53035184328979 | 0.999714303421225 |
| ebi-fl187-GCST90094784 | prot-a-2071 | Epididymal secretory protein E1 \|\| id:prot-a-2071 | Ice cream liking \|\| id:ebi-fl187-GCST90094784 | Weighted mode | 9 | -0.382507786 | 0.271338556648047 | 0.196296165602004 | -0.914331357 | 0.149315785511589 | 0.682148580075331 | 0.400784518462227 | 1.1610395708003 | 0.999714303421225 |
| ebi-fl187-GCST90094784 | prot-a-2098 | Neuritin \|\| id:prot-a-2098 | Ice cream liking \|\| id:ebi-fl187-GCST90094784 | Weighted mode | 9 | -0.20069784 | 0.275938984649905 | 0.487761937722895 | -0.74153825 | 0.340142570049492 | 0.818159609426921 | 0.476380559333442 | 1.40514790829043 | 0.999714303421225 |
| ebi-fl187-GCST90094784 | prot-a-2115 | Neurotrophin-4 \|\| id:prot-a-2115 | Ice cream liking \|\| id:ebi-fl187-GCST90094784 | Weighted mode | 9 | 0.194973848794332 | 0.241206706477356 | 0.442274286629387 | -0.277791296 | 0.667738993489949 | 1.21527920505774 | 0.757454888959018 | 1.9498237687468 | 0.999714303421225 |
| ebi-fl187-GCST90094784 | prot-a-2118 | Netrin-1 \|\| id:prot-a-2118 | Ice cream liking \|\| id:ebi-fl187-GCST90094784 | Weighted mode | 9 | -0.435531165 | 0.435079191443229 | 0.346120861503526 | -1.28828638 | 0.417224050443542 | 0.646920954172363 | 0.275742897001627 | 1.51774252572973 | 0.999714303421225 |
| ebi-fl187-GCST90094784 | prot-a-2120 | Netrin-G1 \|\| id:prot-a-2120 | Ice cream liking \|\| id:ebi-fl187-GCST90094784 | Weighted mode | 9 | -0.012316438 | 0.270535055105511 | 0.964803789894578 | -0.542565146 | 0.517932270235831 | 0.987759099115561 | 0.581255333750475 | 1.67855326434635 | 0.999714303421225 |
| ebi-fl187-GCST90094784 | prot-a-2145 | Olfactomedin-like protein 3 \|\| id:prot-a-2145 | Ice cream liking \|\| id:ebi-fl187-GCST90094784 | Weighted mode | 9 | 0.134898606559851 | 0.389705659306702 | 0.738153775973706 | -0.628924486 | 0.898721698800987 | 1.14442074171245 | 0.533164919258107 | 2.45646100625696 | 0.999714303421225 |
| ebi-fl187-GCST90094784 | prot-a-2162 | Prolyl 4-hydroxylase subunit alpha-2 \|\| id:prot-a-2162 | Ice cream liking \|\| id:ebi-fl187-GCST90094784 | Weighted mode | 9 | -0.000635541 | 0.257486598273281 | 0.998091062873574 | -0.505309274 | 0.504038191455935 | 0.999364660753811 | 0.603318955839058 | 1.65539258380272 | 0.999714303421225 |
| ebi-fl187-GCST90094784 | prot-a-2168 | Polyadenylate-binding protein-interacting protein 1 \|\| id:prot-a-2168 | Ice cream liking \|\| id:ebi-fl187-GCST90094784 | Weighted mode | 9 | 0.536607486880331 | 0.31707107674856 | 0.129030166012836 | -0.084851824 | 1.15806679730751 | 1.71019515012015 | 0.918648396377983 | 3.18377244550382 | 0.999714303421225 |
| ebi-fl187-GCST90094784 | prot-a-219 | Beta-1,4-galactosyltransferase 5 \|\| id:prot-a-219 | Ice cream liking \|\| id:ebi-fl187-GCST90094784 | Weighted mode | 9 | -0.036237307 | 0.24215224096884 | 0.884747192622147 | -0.510855699 | 0.438381085203791 | 0.964411404656771 | 0.599981954894517 | 1.55019555145715 | 0.999714303421225 |
| ebi-fl187-GCST90094784 | prot-a-2190 | Pyruvate carboxylase, mitochondrial \|\| id:prot-a-2190 | Ice cream liking \|\| id:ebi-fl187-GCST90094784 | Weighted mode | 9 | -0.301373028 | 0.289280051781781 | 0.327960244855351 | -0.86836193 | 0.265615873314796 | 0.739801754368453 | 0.419638383738279 | 1.30423397138043 | 0.999714303421225 |
| ebi-fl187-GCST90094784 | prot-a-2197 | Protocadherin-9 \|\| id:prot-a-2197 | Ice cream liking \|\| id:ebi-fl187-GCST90094784 | Weighted mode | 9 | -0.006422612 | 0.28488307152625 | 0.98256561119004 | -0.564793432 | 0.551948208171037 | 0.993597968867579 | 0.568477563782792 | 1.73663304699038 | 0.999714303421225 |
| ebi-fl187-GCST90094784 | prot-a-2198 | Protocadherin alpha-4 \|\| id:prot-a-2198 | Ice cream liking \|\| id:ebi-fl187-GCST90094784 | Weighted mode | 9 | 0.394358163205141 | 0.447146168297084 | 0.403526677295277 | -0.482048327 | 1.27076465306743 | 1.48343176421342 | 0.617517218500675 | 3.56357642046049 | 0.999714303421225 |
| ebi-fl187-GCST90094784 | prot-a-2254 | Placenta growth factor \|\| id:prot-a-2254 | Ice cream liking \|\| id:ebi-fl187-GCST90094784 | Weighted mode | 9 | 0.257095616407005 | 0.292399493357568 | 0.40489514852784 | -0.316007391 | 0.83019862338784 | 1.29316876899949 | 0.729054062019842 | 2.29377429224191 | 0.999714303421225 |
| ebi-fl187-GCST90094784 | prot-a-2261 | Membrane-associated progesterone receptor component 2 \|\| id:prot-a-2261 | Ice cream liking \|\| id:ebi-fl187-GCST90094784 | Weighted mode | 9 | -0.694597299 | 0.295634928162474 | 0.0467169579207062 | -1.274041759 | -0.11515284 | 0.499275466047311 | 0.279698858881205 | 0.891229917754627 | 0.999714303421225 |
| ebi-fl187-GCST90094784 | prot-a-2268 | Polymeric immunoglobulin receptor \|\| id:prot-a-2268 | Ice cream liking \|\| id:ebi-fl187-GCST90094784 | Weighted mode | 9 | -0.843609629 | 0.382570695821671 | 0.0585258598461635 | -1.593448193 | -0.093771065 | 0.430155017806041 | 0.2032236479086 | 0.910491181749349 | 0.999714303421225 |
| ebi-fl187-GCST90094784 | prot-a-2283 | Group 10 secretory phospholipase A2 \|\| id:prot-a-2283 | Ice cream liking \|\| id:ebi-fl187-GCST90094784 | Weighted mode | 9 | 0.226890009302561 | 0.301886162695217 | 0.473834706111912 | -0.36480687 | 0.818586888185187 | 1.2546918559181 | 0.694330734316059 | 2.26729363328255 | 0.999714303421225 |
| ebi-fl187-GCST90094784 | prot-a-2298 | Inactive phospholipase D5 \|\| id:prot-a-2298 | Ice cream liking \|\| id:ebi-fl187-GCST90094784 | Weighted mode | 9 | -0.475099762 | 0.452014632527683 | 0.323936107035052 | -1.361048442 | 0.410848917623344 | 0.62182301898115 | 0.256391824060092 | 1.50809749239198 | 0.999714303421225 |
| ebi-fl187-GCST90094784 | prot-a-2304 | Procollagen-lysine,2-oxoglutarate 5-dioxygenase 2 \|\| id:prot-a-2304 | Ice cream liking \|\| id:ebi-fl187-GCST90094784 | Weighted mode | 9 | 0.0116876803177477 | 0.269274404938517 | 0.966443014467503 | -0.516090153 | 0.539465513997242 | 1.01175624812584 | 0.596849582252502 | 1.7150899256031 | 0.999714303421225 |
| ebi-fl187-GCST90094784 | prot-a-2331 | Periostin \|\| id:prot-a-2331 | Ice cream liking \|\| id:ebi-fl187-GCST90094784 | Weighted mode | 9 | -0.041608022 | 0.334963209260286 | 0.904208107306358 | -0.698135912 | 0.614919868317036 | 0.959245710264647 | 0.497511845815127 | 1.84950838940034 | 0.999714303421225 |
| ebi-fl187-GCST90094784 | prot-a-2333 | Periostin \|\| id:prot-a-2333 | Ice cream liking \|\| id:ebi-fl187-GCST90094784 | Weighted mode | 9 | 0.0621321850991111 | 0.357096717997834 | 0.866193555093251 | -0.637777382 | 0.762041752374865 | 1.06410299399169 | 0.528465696968505 | 2.14264651105548 | 0.999714303421225 |
| ebi-fl187-GCST90094784 | prot-a-2367 | cAMP-dependent protein kinase catalytic subunit alpha \|\| id:prot-a-2367 | Ice cream liking \|\| id:ebi-fl187-GCST90094784 | Weighted mode | 9 | 0.0220967356655915 | 0.319131443293101 | 0.94649782849771 | -0.603400893 | 0.64759436452007 | 1.02234267668645 | 0.546948356542123 | 1.91093827428645 | 0.999714303421225 |
| ebi-fl187-GCST90094784 | prot-a-2385 | Phosphoribosyl pyrophosphate synthase-associated protein 1 \|\| id:prot-a-2385 | Ice cream liking \|\| id:ebi-fl187-GCST90094784 | Weighted mode | 9 | -0.529236021 | 0.27249366188303 | 0.0880472490797758 | -1.063323598 | 0.00485155612695498 | 0.589054823235499 | 0.345306241744678 | 1.00486334398082 | 0.999714303421225 |
| ebi-fl187-GCST90094784 | prot-a-2393 | Trypsin-3 \|\| id:prot-a-2393 | Ice cream liking \|\| id:ebi-fl187-GCST90094784 | Weighted mode | 9 | 0.121358268399074 | 0.264464000982042 | 0.658527809326566 | -0.396991174 | 0.639707710323877 | 1.12902933544953 | 0.672339959997825 | 1.89592663852634 | 0.999714303421225 |
| ebi-fl187-GCST90094784 | prot-a-2405 | Pregnancy-specific beta-1-glycoprotein 6 \|\| id:prot-a-2405 | Ice cream liking \|\| id:ebi-fl187-GCST90094784 | Weighted mode | 9 | -0.129453826 | 0.282284674561322 | 0.658727849745516 | -0.682731788 | 0.423824136585601 | 0.878575155200207 | 0.5052349109359 | 1.52779288728328 | 0.999714303421225 |
| ebi-fl187-GCST90094784 | prot-a-244 | Beclin-1 \|\| id:prot-a-244 | Ice cream liking \|\| id:ebi-fl187-GCST90094784 | Weighted mode | 9 | -0.046949178 | 0.29707781904791 | 0.8783442153246 | -0.629221703 | 0.535323347474389 | 0.954135887586298 | 0.533006476854343 | 1.70800043060054 | 0.999714303421225 |
| ebi-fl187-GCST90094784 | prot-a-2454 | tRNA pseudouridine synthase A, mitochondrial \|\| id:prot-a-2454 | Ice cream liking \|\| id:ebi-fl187-GCST90094784 | Weighted mode | 9 | 0.213276328887696 | 0.346667610260383 | 0.55550273275635 | -0.466192187 | 0.892744844998047 | 1.23772662361418 | 0.627386696753264 | 2.44182288647704 | 0.999714303421225 |
| ebi-fl187-GCST90094784 | prot-a-2466 | Glutaminyl-peptide cyclotransferase-like protein \|\| id:prot-a-2466 | Ice cream liking \|\| id:ebi-fl187-GCST90094784 | Weighted mode | 9 | 0.27194217347592 | 0.299161961470956 | 0.389906481793915 | -0.314415271 | 0.858299617958994 | 1.31251110116181 | 0.730215727767144 | 2.35914583206886 | 0.999714303421225 |
| ebi-fl187-GCST90094784 | prot-a-2474 | Ras-related protein Rab-27A \|\| id:prot-a-2474 | Ice cream liking \|\| id:ebi-fl187-GCST90094784 | Weighted mode | 9 | -0.352366324 | 0.308351849642962 | 0.286190014320585 | -0.95673595 | 0.252003301007732 | 0.703022540565292 | 0.384144709551754 | 1.28660028435532 | 0.999714303421225 |
| ebi-fl187-GCST90094784 | prot-a-2490 | Rap1 GTPase-GDP dissociation stimulator 1 \|\| id:prot-a-2490 | Ice cream liking \|\| id:ebi-fl187-GCST90094784 | Weighted mode | 9 | 0.159267556716849 | 0.347906384766564 | 0.659281680172676 | -0.522628957 | 0.841164070859315 | 1.17265165553835 | 0.592959631451475 | 2.31906496209662 | 0.999714303421225 |
| ebi-fl187-GCST90094784 | prot-a-2545 | Receptor-interacting serine/threonine-protein kinase 2 \|\| id:prot-a-2545 | Ice cream liking \|\| id:ebi-fl187-GCST90094784 | Weighted mode | 9 | 0.013129131361129 | 0.311686133536636 | 0.967433047246203 | -0.59777569 | 0.624033953092935 | 1.01321569683458 | 0.550033721745455 | 1.86644201568987 | 0.999714303421225 |
| ebi-fl187-GCST90094784 | prot-a-2610 | RUN and FYVE domain-containing protein 1 \|\| id:prot-a-2610 | Ice cream liking \|\| id:ebi-fl187-GCST90094784 | Weighted mode | 9 | -0.179505819 | 0.299680800625566 | 0.565751000762819 | -0.766880188 | 0.407868550155414 | 0.835683088030041 | 0.464459837550804 | 1.50360949894411 | 0.999714303421225 |
| ebi-fl187-GCST90094784 | prot-a-2627 | Serine--tRNA ligase, mitochondrial \|\| id:prot-a-2627 | Ice cream liking \|\| id:ebi-fl187-GCST90094784 | Weighted mode | 9 | 0.246516846528322 | 0.265727372120295 | 0.380697817822959 | -0.274308803 | 0.767342495884101 | 1.27956073919226 | 0.760097318814828 | 2.15403428581376 | 0.999714303421225 |
| ebi-fl187-GCST90094784 | prot-a-264 | BolA-like protein 2 \|\| id:prot-a-264 | Ice cream liking \|\| id:ebi-fl187-GCST90094784 | Weighted mode | 9 | -0.461380248 | 0.315194656536528 | 0.181404238562239 | -1.079161775 | 0.156401278833164 | 0.630412918579174 | 0.33988030251263 | 1.16929532242235 | 0.999714303421225 |
| ebi-fl187-GCST90094784 | prot-a-2646 | Secretoglobin family 1C member 1 \|\| id:prot-a-2646 | Ice cream liking \|\| id:ebi-fl187-GCST90094784 | Weighted mode | 9 | 0.153362346957132 | 0.254886349420564 | 0.564039014971108 | -0.346214898 | 0.652939591821438 | 1.16574730741303 | 0.70736046048941 | 1.92118002156987 | 0.999714303421225 |
| ebi-fl187-GCST90094784 | prot-a-2672 | Semaphorin-3E \|\| id:prot-a-2672 | Ice cream liking \|\| id:ebi-fl187-GCST90094784 | Weighted mode | 9 | -0.180295287 | 0.271984652737813 | 0.526036951071199 | -0.713385206 | 0.352794632820214 | 0.835023603767404 | 0.489982694468673 | 1.4230388679437 | 0.999714303421225 |
| ebi-fl187-GCST90094784 | prot-a-2701 | Plasma protease C1 inhibitor \|\| id:prot-a-2701 | Ice cream liking \|\| id:ebi-fl187-GCST90094784 | Weighted mode | 9 | 0.189793841434772 | 0.282012168107484 | 0.519911484866007 | -0.362950008 | 0.742537690925441 | 1.2090003261908 | 0.695621208086414 | 2.10126110552381 | 0.999714303421225 |
| ebi-fl187-GCST90094784 | prot-a-2738 | Signaling threshold-regulating transmembrane adapter 1 \|\| id:prot-a-2738 | Ice cream liking \|\| id:ebi-fl187-GCST90094784 | Weighted mode | 9 | -0.03329723 | 0.276116485394428 | 0.906988511212216 | -0.574485542 | 0.507891081158454 | 0.967251020630057 | 0.562994431537958 | 1.66178293158981 | 0.999714303421225 |
| ebi-fl187-GCST90094784 | prot-a-274 | Serine/threonine-protein kinase BRSK2 \|\| id:prot-a-274 | Ice cream liking \|\| id:ebi-fl187-GCST90094784 | Weighted mode | 9 | 0.54182210288145 | 0.343299141211318 | 0.153153936070762 | -0.131044214 | 1.21468841965563 | 1.71913645358592 | 0.877178990035873 | 3.3692441105174 | 0.999714303421225 |
| ebi-fl187-GCST90094784 | prot-a-2749 | Mitochondrial glutamate carrier 2 \|\| id:prot-a-2749 | Ice cream liking \|\| id:ebi-fl187-GCST90094784 | Weighted mode | 9 | 0.345257605731237 | 0.301737660481568 | 0.285606930560966 | -0.246148209 | 0.936663420275111 | 1.41235370321777 | 0.781806345748317 | 2.55145407023224 | 0.999714303421225 |
| ebi-fl187-GCST90094784 | prot-a-2760 | Sodium-coupled monocarboxylate transporter 1 \|\| id:prot-a-2760 | Ice cream liking \|\| id:ebi-fl187-GCST90094784 | Weighted mode | 9 | -0.089892617 | 0.31524505673314 | 0.782771097237196 | -0.707772928 | 0.527987693965954 | 0.914029331002064 | 0.4927403444225 | 1.69551697438382 | 0.999714303421225 |
| ebi-fl187-GCST90094784 | prot-a-2777 | Small integral membrane protein 10 \|\| id:prot-a-2777 | Ice cream liking \|\| id:ebi-fl187-GCST90094784 | Weighted mode | 9 | -0.603146645 | 0.310192550767592 | 0.0877444049906858 | -1.211124045 | 0.0048307543727707 | 0.547087434778587 | 0.297862280689459 | 1.00484244127795 | 0.999714303421225 |
| ebi-fl187-GCST90094784 | prot-a-2799 | Superoxide dismutase [Mn], mitochondrial \|\| id:prot-a-2799 | Ice cream liking \|\| id:ebi-fl187-GCST90094784 | Weighted mode | 9 | -0.270617236 | 0.465042238834151 | 0.576647263925323 | -1.182100025 | 0.640865551691594 | 0.762908454094669 | 0.306634122801379 | 1.89812309214564 | 0.999714303421225 |
| ebi-fl187-GCST90094784 | prot-a-2805 | Protein SPACA7 \|\| id:prot-a-2805 | Ice cream liking \|\| id:ebi-fl187-GCST90094784 | Weighted mode | 9 | -0.023978727 | 0.260979117994377 | 0.929052570501004 | -0.535497799 | 0.487540343876519 | 0.976306478121557 | 0.585377817309526 | 1.62830621700534 | 0.999714303421225 |
| ebi-fl187-GCST90094784 | prot-a-2811 | SPARC-like protein 1 \|\| id:prot-a-2811 | Ice cream liking \|\| id:ebi-fl187-GCST90094784 | Weighted mode | 9 | -0.017024771 | 0.282519267948803 | 0.953426304919571 | -0.570762536 | 0.536712994166265 | 0.983119331471162 | 0.565094369458279 | 1.71037559768796 | 0.999714303421225 |
| ebi-fl187-GCST90094784 | prot-a-2831 | Sepiapterin reductase \|\| id:prot-a-2831 | Ice cream liking \|\| id:ebi-fl187-GCST90094784 | Weighted mode | 9 | 0.0396408844689806 | 0.400846730345658 | 0.923656412785146 | -0.746018707 | 0.825300475946469 | 1.04043706998403 | 0.474250931028835 | 2.28256651863302 | 0.999714303421225 |
| ebi-fl187-GCST90094784 | prot-a-2834 | SPRY domain-containing SOCS box protein 1 \|\| id:prot-a-2834 | Ice cream liking \|\| id:ebi-fl187-GCST90094784 | Weighted mode | 9 | 0.415148462181236 | 0.499632628547161 | 0.430124827713939 | -0.56413149 | 1.39442841413367 | 1.51459558416537 | 0.568853987780589 | 4.03266889720401 | 0.999714303421225 |
| ebi-fl187-GCST90094784 | prot-a-2835 | Spectrin alpha chain, non-erythrocytic 1 \|\| id:prot-a-2835 | Ice cream liking \|\| id:ebi-fl187-GCST90094784 | Weighted mode | 9 | -0.322539672 | 0.379386518374656 | 0.419950029688753 | -1.066137248 | 0.421057904472568 | 0.724307196853735 | 0.344336036696174 | 1.52357249751648 | 0.999714303421225 |
| ebi-fl187-GCST90094784 | prot-a-2859 | Sia-alpha-2,3-Gal-beta-1,4-GlcNAc-R:alpha 2,8-sialyltransferase \|\| id:prot-a-2859 | Ice cream liking \|\| id:ebi-fl187-GCST90094784 | Weighted mode | 9 | 0.172165239920698 | 0.30344315506692 | 0.586025847479763 | -0.422583344 | 0.766913823851862 | 1.18787410122009 | 0.655351632417357 | 2.15311110944303 | 0.999714303421225 |
| ebi-fl187-GCST90094784 | prot-a-2861 | Alpha-2,8-sialyltransferase 8F \|\| id:prot-a-2861 | Ice cream liking \|\| id:ebi-fl187-GCST90094784 | Weighted mode | 9 | 0.14540314708292 | 0.372912928556034 | 0.706777294304266 | -0.585506193 | 0.876312487052746 | 1.15650571820529 | 0.556823930149592 | 2.40202585381382 | 0.999714303421225 |
| ebi-fl187-GCST90094784 | prot-a-289 | Uncharacterized protein C17orf89 \|\| id:prot-a-289 | Ice cream liking \|\| id:ebi-fl187-GCST90094784 | Weighted mode | 9 | 0.199345442330153 | 0.275583891647403 | 0.490067309195802 | -0.340798985 | 0.739489869959062 | 1.22060354121217 | 0.711201855867218 | 2.09486658749362 | 0.999714303421225 |
| ebi-fl187-GCST90094784 | prot-a-2921 | Tachykinin-3 \|\| id:prot-a-2921 | Ice cream liking \|\| id:ebi-fl187-GCST90094784 | Weighted mode | 9 | 0.0398486972195529 | 0.26920740621761 | 0.885988044642452 | -0.487797819 | 0.567495213406068 | 1.04065330854113 | 0.613976994997952 | 1.76384346221508 | 0.999714303421225 |
| ebi-fl187-GCST90094784 | prot-a-2928 | Tax1-binding protein 1 \|\| id:prot-a-2928 | Ice cream liking \|\| id:ebi-fl187-GCST90094784 | Weighted mode | 9 | -0.197366403 | 0.365002049991703 | 0.603421107579678 | -0.912770421 | 0.518037615359071 | 0.820889802025312 | 0.401410605935528 | 1.67873010106113 | 0.999714303421225 |
| ebi-fl187-GCST90094784 | prot-a-296 | Uncharacterized protein C1orf185 \|\| id:prot-a-296 | Ice cream liking \|\| id:ebi-fl187-GCST90094784 | Weighted mode | 9 | 0.43129526525666 | 0.573948189741133 | 0.473903788073383 | -0.693643187 | 1.55623371714928 | 1.53924996990378 | 0.499752058457331 | 4.74093188762939 | 0.999714303421225 |
| ebi-fl187-GCST90094784 | prot-a-2964 | Transforming growth factor beta-2 \|\| id:prot-a-2964 | Ice cream liking \|\| id:ebi-fl187-GCST90094784 | Weighted mode | 9 | 0.294405053139592 | 0.295796588583058 | 0.348740044789782 | -0.28535626 | 0.874166366762385 | 1.3423275075366 | 0.751746389075498 | 2.39687634510003 | 0.999714303421225 |
| ebi-fl187-GCST90094784 | prot-a-2977 | Thymocyte nuclear protein 1 \|\| id:prot-a-2977 | Ice cream liking \|\| id:ebi-fl187-GCST90094784 | Weighted mode | 9 | -0.003295236 | 0.28356363987886 | 0.991012724697881 | -0.55907997 | 0.552489498220026 | 0.996710187388731 | 0.571734835089333 | 1.7375733236357 | 0.999714303421225 |
| ebi-fl187-GCST90094784 | prot-a-3015 | Transmembrane protein 70, mitochondrial \|\| id:prot-a-3015 | Ice cream liking \|\| id:ebi-fl187-GCST90094784 | Weighted mode | 9 | 0.19622084076027 | 0.311045629941823 | 0.54573937189361 | -0.413428594 | 0.805870275446243 | 1.21679559372856 | 0.661378759155164 | 2.23864388812325 | 0.999714303421225 |
| ebi-fl187-GCST90094784 | prot-a-3030 | Tumor necrosis factor alpha-induced protein 3 \|\| id:prot-a-3030 | Ice cream liking \|\| id:ebi-fl187-GCST90094784 | Weighted mode | 9 | 0.120333314927672 | 0.27256424569427 | 0.670550477510213 | -0.413892607 | 0.654559236488441 | 1.12787272574967 | 0.661071942195505 | 1.92429417177378 | 0.999714303421225 |
| ebi-fl187-GCST90094784 | prot-a-3032 | Tumor necrosis factor receptor superfamily member 10A \|\| id:prot-a-3032 | Ice cream liking \|\| id:ebi-fl187-GCST90094784 | Weighted mode | 9 | 0.257786608703803 | 0.268193792190378 | 0.364601636228351 | -0.267873224 | 0.783446441396944 | 1.29406264745332 | 0.765004759206996 | 2.18900355243514 | 0.999714303421225 |
| ebi-fl187-GCST90094784 | prot-a-3081 | Tumor protein p53-inducible protein 11 \|\| id:prot-a-3081 | Ice cream liking \|\| id:ebi-fl187-GCST90094784 | Weighted mode | 9 | -0.254235719 | 0.280641628005273 | 0.391451403310582 | -0.80429331 | 0.295821872016213 | 0.775508978396571 | 0.447403990898122 | 1.34423069040222 | 0.999714303421225 |
| ebi-fl187-GCST90094784 | prot-a-315 | Complement C4 \|\| id:prot-a-315 | Ice cream liking \|\| id:ebi-fl187-GCST90094784 | Weighted mode | 9 | 0.256801413546171 | 0.286340632581531 | 0.395990511144758 | -0.304426226 | 0.818029053405971 | 1.29278837100794 | 0.737546437747167 | 2.26602921074143 | 0.999714303421225 |
| ebi-fl187-GCST90094784 | prot-a-3157 | UDP-glucuronosyltransferase 1-8 \|\| id:prot-a-3157 | Ice cream liking \|\| id:ebi-fl187-GCST90094784 | Weighted mode | 9 | -0.335333795 | 0.283604591830936 | 0.270995938243007 | -0.891198795 | 0.220531205090697 | 0.71509934999574 | 0.410163755696503 | 1.24673882872945 | 0.999714303421225 |
| ebi-fl187-GCST90094784 | prot-a-317 | Glutaredoxin-like protein C5orf63 \|\| id:prot-a-317 | Ice cream liking \|\| id:ebi-fl187-GCST90094784 | Weighted mode | 9 | 0.301659454680483 | 0.413145655519095 | 0.48612529638439 | -0.50810603 | 1.11142493949791 | 1.35210069673448 | 0.601633977047112 | 3.03868525358685 | 0.999714303421225 |
| ebi-fl187-GCST90094784 | prot-a-3192 | Guanine nucleotide exchange factor VAV3 \|\| id:prot-a-3192 | Ice cream liking \|\| id:ebi-fl187-GCST90094784 | Weighted mode | 9 | 0.00260865579647351 | 0.332890062632872 | 0.993939422405172 | -0.649855867 | 0.655073178556903 | 1.00261206129962 | 0.522121026226611 | 1.92528340168241 | 0.999714303421225 |
| ebi-fl187-GCST90094784 | prot-a-3233 | Serine/threonine-protein kinase WNK3 \|\| id:prot-a-3233 | Ice cream liking \|\| id:ebi-fl187-GCST90094784 | Weighted mode | 9 | -0.211989475 | 0.287323681067693 | 0.481710108820116 | -0.77514389 | 0.351164939412764 | 0.808973211576512 | 0.460637494929342 | 1.42072163958082 | 0.999714303421225 |
| ebi-fl187-GCST90094784 | prot-a-3250 | 14-3-3 protein theta \|\| id:prot-a-3250 | Ice cream liking \|\| id:ebi-fl187-GCST90094784 | Weighted mode | 9 | -0.302594513 | 0.25980551238879 | 0.277695092449843 | -0.811813317 | 0.206624291207974 | 0.738898649375885 | 0.444052128301911 | 1.22952054331401 | 0.999714303421225 |
| ebi-fl187-GCST90094784 | prot-a-3278 | E3 ubiquitin-protein ligase ZNRF3 \|\| id:prot-a-3278 | Ice cream liking \|\| id:ebi-fl187-GCST90094784 | Weighted mode | 9 | 0.0553092509535442 | 0.261578438261731 | 0.837827771629735 | -0.457384488 | 0.568002989946537 | 1.05686740139499 | 0.632936936591052 | 1.76473932797681 | 0.999714303421225 |
| ebi-fl187-GCST90094784 | prot-a-355 | Calpain-3 \|\| id:prot-a-355 | Ice cream liking \|\| id:ebi-fl187-GCST90094784 | Weighted mode | 9 | -0.105054039 | 0.259136145109416 | 0.695808113624053 | -0.612960884 | 0.402852805100262 | 0.900275870981076 | 0.541744449711388 | 1.49608665912964 | 0.999714303421225 |
| ebi-fl187-GCST90094784 | prot-a-385 | Coiled-coil domain-containing protein 90B, mitochondrial \|\| id:prot-a-385 | Ice cream liking \|\| id:ebi-fl187-GCST90094784 | Weighted mode | 9 | -0.1425862 | 0.359098783994793 | 0.701700978472208 | -0.846419817 | 0.561247416661411 | 0.867112805974191 | 0.42894789830671 | 1.75285768097368 | 0.999714303421225 |
| ebi-fl187-GCST90094784 | prot-a-390 | C-C motif chemokine 14 \|\| id:prot-a-390 | Ice cream liking \|\| id:ebi-fl187-GCST90094784 | Weighted mode | 9 | 0.327839035353652 | 0.276399397892673 | 0.269609938601536 | -0.213903785 | 0.869581855223291 | 1.38796554092518 | 0.807426068175416 | 2.3859129878591 | 0.999714303421225 |
| ebi-fl187-GCST90094784 | prot-a-40 | Pituitary adenylate cyclase-activating polypeptide 27 \|\| id:prot-a-40 | Ice cream liking \|\| id:ebi-fl187-GCST90094784 | Weighted mode | 9 | 0.353446863750082 | 0.259222378253957 | 0.209858386494378 | -0.154628998 | 0.861522725127838 | 1.42396732065765 | 0.85673296849312 | 2.36676187898704 | 0.999714303421225 |
| ebi-fl187-GCST90094784 | prot-a-401 | C-C motif chemokine 24 \|\| id:prot-a-401 | Ice cream liking \|\| id:ebi-fl187-GCST90094784 | Weighted mode | 9 | 0.299770073900325 | 0.466375102135601 | 0.538357498000854 | -0.614325126 | 1.2138652740861 | 1.34954847548336 | 0.541005882784944 | 3.36647187328909 | 0.999714303421225 |
| ebi-fl187-GCST90094784 | prot-a-412 | Cerebral cavernous malformations 2 protein \|\| id:prot-a-412 | Ice cream liking \|\| id:ebi-fl187-GCST90094784 | Weighted mode | 9 | -0.088167806 | 0.327167743248765 | 0.794374598217515 | -0.729416582 | 0.553080971212891 | 0.915607219854044 | 0.482190226345203 | 1.73860135532627 | 0.999714303421225 |
| ebi-fl187-GCST90094784 | prot-a-44 | Adhesion G protein-coupled receptor F5 \|\| id:prot-a-44 | Ice cream liking \|\| id:ebi-fl187-GCST90094784 | Weighted mode | 9 | -0.106902369 | 0.288656045908229 | 0.720738679756294 | -0.672668219 | 0.458863480769942 | 0.898613401043838 | 0.510345047047845 | 1.58227467711638 | 0.999714303421225 |
| ebi-fl187-GCST90094784 | prot-a-48 | Adrenomedullin \|\| id:prot-a-48 | Ice cream liking \|\| id:ebi-fl187-GCST90094784 | Weighted mode | 9 | -0.583838102 | 0.337589881535836 | 0.121987388256916 | -1.24551427 | 0.0778380659838265 | 0.557753538289703 | 0.287792866934378 | 1.08094760231016 | 0.999714303421225 |
| ebi-fl187-GCST90094784 | prot-a-502 | Carcinoembryonic antigen-related cell adhesion molecule 1 \|\| id:prot-a-502 | Ice cream liking \|\| id:ebi-fl187-GCST90094784 | Weighted mode | 9 | -0.083847692 | 0.263592491639357 | 0.7585571541663 | -0.600488976 | 0.432793591648356 | 0.919571303528162 | 0.548543346206044 | 1.54155799741458 | 0.999714303421225 |
| ebi-fl187-GCST90094784 | prot-a-518 | Complement factor B \|\| id:prot-a-518 | Ice cream liking \|\| id:ebi-fl187-GCST90094784 | Weighted mode | 9 | -0.230006433 | 0.289476561234296 | 0.449795937427563 | -0.797380493 | 0.337367627052957 | 0.794528491311915 | 0.45050752744408 | 1.4012541079788 | 0.999714303421225 |
| ebi-fl187-GCST90094784 | prot-a-525 | Properdin \|\| id:prot-a-525 | Ice cream liking \|\| id:ebi-fl187-GCST90094784 | Weighted mode | 9 | -0.215601804 | 0.345084886481145 | 0.549516332979269 | -0.891968181 | 0.46076457353062 | 0.806056206357168 | 0.409848302571882 | 1.58528558915517 | 0.999714303421225 |
| ebi-fl187-GCST90094784 | prot-a-533 | Chondroadherin \|\| id:prot-a-533 | Ice cream liking \|\| id:ebi-fl187-GCST90094784 | Weighted mode | 9 | -0.015493598 | 0.379660719984339 | 0.968448203814732 | -0.759628609 | 0.728641413526704 | 0.984625810657898 | 0.467840146456808 | 2.07226334541007 | 0.999714303421225 |
| ebi-fl187-GCST90094784 | prot-a-535 | Coiled-coil-helix-coiled-coil-helix domain-containing protein 2 \|\| id:prot-a-535 | Ice cream liking \|\| id:ebi-fl187-GCST90094784 | Weighted mode | 9 | 0.393062217721623 | 0.361415451935073 | 0.308465105777007 | -0.315312068 | 1.10143650351437 | 1.4815105626736 | 0.729561165994827 | 3.00848462009421 | 0.999714303421225 |
| ebi-fl187-GCST90094784 | prot-a-591 | Caseinolytic peptidase B protein homolog \|\| id:prot-a-591 | Ice cream liking \|\| id:ebi-fl187-GCST90094784 | Weighted mode | 9 | -0.107034761 | 0.360134586600717 | 0.773878992703748 | -0.81289855 | 0.598829029070146 | 0.898494440181202 | 0.443570489649346 | 1.81998640097703 | 0.999714303421225 |
| ebi-fl187-GCST90094784 | prot-a-605 | Ciliary neurotrophic factor receptor subunit alpha \|\| id:prot-a-605 | Ice cream liking \|\| id:ebi-fl187-GCST90094784 | Weighted mode | 9 | -0.350081902 | 0.364712374458558 | 0.365221772190016 | -1.064918156 | 0.364754352119882 | 0.704630376845841 | 0.344756069911913 | 1.44016019239566 | 0.999714303421225 |
| ebi-fl187-GCST90094784 | prot-a-606 | Ciliary neurotrophic factor receptor subunit alpha \|\| id:prot-a-606 | Ice cream liking \|\| id:ebi-fl187-GCST90094784 | Weighted mode | 9 | -0.493969476 | 0.47317986682326 | 0.327030999174721 | -1.421402015 | 0.433463063118325 | 0.610199408621481 | 0.241375367712067 | 1.54259037204727 | 0.999714303421225 |
| ebi-fl187-GCST90094784 | prot-a-707 | Cancer/testis antigen 55 \|\| id:prot-a-707 | Ice cream liking \|\| id:ebi-fl187-GCST90094784 | Weighted mode | 9 | 0.0494154135497316 | 0.360172666554778 | 0.894263439777862 | -0.656523013 | 0.755353839997096 | 1.05065671713391 | 0.518651547761097 | 2.12836449061765 | 0.999714303421225 |
| ebi-fl187-GCST90094784 | prot-a-719 | Dipeptidyl peptidase 1 \|\| id:prot-a-719 | Ice cream liking \|\| id:ebi-fl187-GCST90094784 | Weighted mode | 9 | 0.413174660106771 | 0.291167522946942 | 0.193655779620966 | -0.157513685 | 0.983863005082777 | 1.51160902067159 | 0.854265123021987 | 2.67476895614399 | 0.999714303421225 |
| ebi-fl187-GCST90094784 | prot-a-752 | Cytochrome b5 \|\| id:prot-a-752 | Ice cream liking \|\| id:ebi-fl187-GCST90094784 | Weighted mode | 9 | 0.104887867529357 | 0.350239317072368 | 0.772210723852352 | -0.581581194 | 0.791356928991199 | 1.1105860706134 | 0.559013758218362 | 2.20638830817241 | 0.999714303421225 |
| ebi-fl187-GCST90094784 | prot-a-755 | Cytoglobin \|\| id:prot-a-755 | Ice cream liking \|\| id:ebi-fl187-GCST90094784 | Weighted mode | 9 | -0.198042559 | 0.332600433927266 | 0.568009284105339 | -0.849939409 | 0.453854291928381 | 0.82033494011371 | 0.427440830203036 | 1.57436858255144 | 0.999714303421225 |
| ebi-fl187-GCST90094784 | prot-a-807 | Desmin \|\| id:prot-a-807 | Ice cream liking \|\| id:ebi-fl187-GCST90094784 | Weighted mode | 9 | -0.242570931 | 0.32198978693688 | 0.472825522599501 | -0.873670913 | 0.388529051424371 | 0.784608092590335 | 0.417416433766732 | 1.47480982816855 | 0.999714303421225 |
| ebi-fl187-GCST90094784 | prot-a-82 | Ameloblastin \|\| id:prot-a-82 | Ice cream liking \|\| id:ebi-fl187-GCST90094784 | Weighted mode | 9 | 0.261512772379653 | 0.272067428356204 | 0.364596694389244 | -0.271739387 | 0.794764931957814 | 1.29889353142728 | 0.762052835937599 | 2.21392051367129 | 0.999714303421225 |
| ebi-fl187-GCST90094784 | prot-a-84 | Muellerian-inhibiting factor \|\| id:prot-a-84 | Ice cream liking \|\| id:ebi-fl187-GCST90094784 | Weighted mode | 9 | -0.471284274 | 0.281488961464659 | 0.132613868304417 | -1.023002638 | 0.0804340906003708 | 0.624200109406725 | 0.359513827886972 | 1.08375741448772 | 0.999714303421225 |
| ebi-fl187-GCST90094784 | prot-a-858 | Inactive dipeptidyl peptidase 10 \|\| id:prot-a-858 | Ice cream liking \|\| id:ebi-fl187-GCST90094784 | Weighted mode | 9 | -0.072460296 | 0.324813946029136 | 0.829061996469743 | -0.709095631 | 0.564175037762342 | 0.930102674300798 | 0.492089026508057 | 1.7579969032846 | 0.999714303421225 |
| ebi-fl187-GCST90094784 | prot-a-863 | Dihydropyrimidinase-related protein 3 \|\| id:prot-a-863 | Ice cream liking \|\| id:ebi-fl187-GCST90094784 | Weighted mode | 9 | 0.0893853054606579 | 0.256196524594953 | 0.736173997761302 | -0.412759883 | 0.591530493666766 | 1.09350190742211 | 0.661821178434665 | 1.80675152216188 | 0.999714303421225 |
| ebi-fl187-GCST90094784 | prot-a-864 | Dihydropyrimidinase-related protein 5 \|\| id:prot-a-864 | Ice cream liking \|\| id:ebi-fl187-GCST90094784 | Weighted mode | 9 | -0.369147325 | 0.282735962635114 | 0.227973215402517 | -0.923309812 | 0.185015161478907 | 0.691323553507259 | 0.397202198412906 | 1.20323668283699 | 0.999714303421225 |
| ebi-fl187-GCST90094784 | prot-a-884 | Endothelin-converting enzyme 1 \|\| id:prot-a-884 | Ice cream liking \|\| id:ebi-fl187-GCST90094784 | Weighted mode | 9 | -0.206863825 | 0.294676949610113 | 0.502586983090391 | -0.784430646 | 0.370702996493682 | 0.813130370686593 | 0.45637946931521 | 1.44875272484323 | 0.999714303421225 |
| ebi-fl187-GCST90094784 | prot-a-893 | Early endosome antigen 1 \|\| id:prot-a-893 | Ice cream liking \|\| id:ebi-fl187-GCST90094784 | Weighted mode | 9 | -0.412441541 | 0.314159377396332 | 0.225643515008574 | -1.028193921 | 0.203310838696116 | 0.662031897281848 | 0.357652326066684 | 1.22545332736599 | 0.999714303421225 |
| ebi-fl187-GCST90094784 | prot-a-897 | Ephrin-A2 \|\| id:prot-a-897 | Ice cream liking \|\| id:ebi-fl187-GCST90094784 | Weighted mode | 9 | -0.220230102 | 0.297204116870968 | 0.479872596412004 | -0.802750171 | 0.362289967432809 | 0.802334158319277 | 0.448094930444244 | 1.43661545326499 | 0.999714303421225 |
| ebi-fl187-GCST90094784 | prot-a-90 | Anaphase-promoting complex subunit 10 \|\| id:prot-a-90 | Ice cream liking \|\| id:ebi-fl187-GCST90094784 | Weighted mode | 9 | -0.115146757 | 0.289385820762372 | 0.701110519461825 | -0.682342966 | 0.452049451683674 | 0.891235339274707 | 0.505431395575665 | 1.57152966144382 | 0.999714303421225 |
| ebi-fl187-GCST90094784 | prot-a-914 | Histone-lysine N-methyltransferase EHMT2 \|\| id:prot-a-914 | Ice cream liking \|\| id:ebi-fl187-GCST90094784 | Weighted mode | 9 | 0.265859592391635 | 0.358533374731667 | 0.479578036677737 | -0.436865822 | 0.968585006865702 | 1.30455187681815 | 0.646058112327302 | 2.6342144256639 | 0.999714303421225 |
| ebi-fl187-GCST90094784 | prot-a-932 | RNA polymerase II elongation factor ELL \|\| id:prot-a-932 | Ice cream liking \|\| id:ebi-fl187-GCST90094784 | Weighted mode | 9 | -0.153916563 | 0.265923876989554 | 0.578651786691776 | -0.675127362 | 0.367294235816218 | 0.857343552129783 | 0.509091577565605 | 1.44382268096705 | 0.999714303421225 |
| ebi-fl187-GCST90094784 | prot-a-991 | Estrogen receptor \|\| id:prot-a-991 | Ice cream liking \|\| id:ebi-fl187-GCST90094784 | Weighted mode | 9 | -0.064720297 | 0.31937841466591 | 0.844471685480382 | -0.690701989 | 0.561261396071684 | 0.937329600922727 | 0.501224091529613 | 1.75288218506163 | 0.999714303421225 |
| ebi-fl187-GCST90094784 | prot-a-992 | Persulfide dioxygenase ETHE1, mitochondrial \|\| id:prot-a-992 | Ice cream liking \|\| id:ebi-fl187-GCST90094784 | Weighted mode | 9 | -0.108912185 | 0.282018809921973 | 0.709425692897714 | -0.661669052 | 0.443844682658389 | 0.896809167527021 | 0.515989402121485 | 1.55868837548557 | 0.999714303421225 |
| ebi-fl187-GCST90094784 | prot-a-993 | Ethanolamine kinase 1 \|\| id:prot-a-993 | Ice cream liking \|\| id:ebi-fl187-GCST90094784 | Weighted mode | 9 | 0.100142587525239 | 0.277042968412836 | 0.72710905014758 | -0.442861631 | 0.643146805614397 | 1.10532851289708 | 0.642196061247151 | 1.90245813568309 | 0.999714303421225 |
| ebi-fl187-GCST90094784 | prot-b-10 | coagulation factor III, tissue factor \|\| id:prot-b-10 | Ice cream liking \|\| id:ebi-fl187-GCST90094784 | Weighted mode | 7 | -0.185523366 | 0.45179519640558 | 0.695601235196212 | -1.071041951 | 0.699995218723409 | 0.830669425740031 | 0.342651305418066 | 2.01374307918481 | 0.999714303421225 |
| ebi-fl187-GCST90094784 | prot-b-28 | prolactin \|\| id:prot-b-28 | Ice cream liking \|\| id:ebi-fl187-GCST90094784 | Weighted mode | 8 | -0.188890732 | 0.256822980259154 | 0.485948273248675 | -0.692263773 | 0.314482309795277 | 0.827876962616058 | 0.500441899029442 | 1.36955012471901 | 0.999714303421225 |
| ebi-fl187-GCST90094784 | prot-b-54 | matrix metallopeptidase 7 \|\| id:prot-b-54 | Ice cream liking \|\| id:ebi-fl187-GCST90094784 | Weighted mode | 8 | 0.0241235294602253 | 0.283621445948564 | 0.934598918924453 | -0.531774505 | 0.580021563519411 | 1.02441685573612 | 0.587561413624034 | 1.78607694443969 | 0.999714303421225 |
| ebi-fl187-GCST90094784 | ukb-rsfMRI191-edge-pheno716 | Net100_Pair10_33 (OccipitalPrecuneus)&(Temporal\|Frontal\|Supp_Motor_Area) (Default_mode\|Central_executive)&(Default_mode\|Salience) \|\| id:ukb-rsfMRI191-edge-pheno716 | Ice cream liking \|\| id:ebi-fl187-GCST90094784 | Weighted mode | 8 | -0.038648515 | 0.091966374662255 | 0.686905174113573 | -0.21890261 | 0.141605578866348 | 0.962088809055703 | 0.803399957618453 | 1.15212213758892 | 0.999714303421225 |
| ebi-fl187-GCST90094784 | ukb-rsfMRI191-edge-pheno903 | Net100_Pair27_38 (Precentral\|Frontal\|Supp_Motor_Area)&(Subcortical) (Attention\|Salience\|Motor)&(Subcortical-cerebellum) \|\| id:ukb-rsfMRI191-edge-pheno903 | Ice cream liking \|\| id:ebi-fl187-GCST90094784 | Weighted mode | 8 | 0.0188688197946909 | 0.100014297308179 | 0.855711474783944 | -0.177159203 | 0.214896842518721 | 1.01904796092828 | 0.837646418143597 | 1.23973400253238 | 0.999714303421225 |
| ebi-fl187-GCST90094784 | ukb-rsfMRI191-node-pheno23 | Net100_Node2 RolandicOper\|SupraMarginal\|Insula Salience\|Motor \|\| id:ukb-rsfMRI191-node-pheno23 | Ice cream liking \|\| id:ebi-fl187-GCST90094784 | Weighted mode | 8 | -0.029209924 | 0.0817235933178886 | 0.731308282752235 | -0.189388167 | 0.130968318524827 | 0.971212561872107 | 0.827465249411753 | 1.13993166602313 | 0.999714303421225 |
| ebi-fl187-GCST90094784 | ukb-rsfMRI191-node-pheno55 | Net100_Node34 Parietal\|Postcentral\|Precuneus Attention \|\| id:ukb-rsfMRI191-node-pheno55 | Ice cream liking \|\| id:ebi-fl187-GCST90094784 | Weighted mode | 8 | -0.128909501 | 0.10954057279277 | 0.277729229291772 | -0.343609024 | 0.0857900216881997 | 0.879053515422661 | 0.70920615667889 | 1.08957751663571 | 0.999714303421225 |
| ebi-fl187-GCST90094798 | ebi-a-GCST90001414 | IgD- CD24- B cell Absolute Count \|\| id:ebi-a-GCST90001414 | Mayonnaise liking \|\| id:ebi-fl187-GCST90094798 | Inverse variance weighted | 9 | 0.0206527916399372 | 0.19376489847531 | 0.91511674530744 | -0.359126409 | 0.400431992651545 | 1.02086753635262 | 0.69828607586817 | 1.4924692941685 | 0.998735809224318 |
| ebi-fl187-GCST90094798 | ebi-a-GCST90001422 | CD20- CD38- B cell %B cell \|\| id:ebi-a-GCST90001422 | Mayonnaise liking \|\| id:ebi-fl187-GCST90094798 | Inverse variance weighted | 9 | -0.004531385 | 0.17923325599608 | 0.979829989062832 | -0.355828566 | 0.346765797091796 | 0.995478866573011 | 0.700592715063098 | 1.4144854099778 | 0.998955228396052 |
| ebi-fl187-GCST90094798 | ebi-a-GCST90001444 | CD20- CD38- B cell %lymphocyte \|\| id:ebi-a-GCST90001444 | Mayonnaise liking \|\| id:ebi-fl187-GCST90094798 | Inverse variance weighted | 9 | -0.057145176 | 0.160725836182159 | 0.722181765299233 | -0.372167815 | 0.257877462964431 | 0.944456946976359 | 0.689238568338077 | 1.29418022389944 | 0.998735809224318 |
| ebi-fl187-GCST90094798 | ebi-a-GCST90001459 | Myeloid Dendritic Cell %Dendritic Cell \|\| id:ebi-a-GCST90001459 | Mayonnaise liking \|\| id:ebi-fl187-GCST90094798 | Inverse variance weighted | 9 | -0.057429685 | 0.172175581417472 | 0.738716874677753 | -0.394893825 | 0.280034454510286 | 0.944188278586996 | 0.673751571262781 | 1.32317540091253 | 0.998735809224318 |
| ebi-fl187-GCST90094798 | ebi-a-GCST90001476 | HLA DR++ monocyte %leukocyte \|\| id:ebi-a-GCST90001476 | Mayonnaise liking \|\| id:ebi-fl187-GCST90094798 | Inverse variance weighted | 9 | -0.045723245 | 0.168116100778548 | 0.785641843223233 | -0.375230803 | 0.283784312442131 | 0.955306311327181 | 0.687130668936244 | 1.32814643519604 | 0.998735809224318 |
| ebi-fl187-GCST90094798 | ebi-a-GCST90001478 | CD4 regulatory T cell %CD4+ T cell \|\| id:ebi-a-GCST90001478 | Mayonnaise liking \|\| id:ebi-fl187-GCST90094798 | Inverse variance weighted | 9 | -0.15517842 | 0.184537167783501 | 0.400400572509935 | -0.516871268 | 0.206514429225203 | 0.856262389834691 | 0.596383556050582 | 1.22938547316894 | 0.998735809224318 |
| ebi-fl187-GCST90094798 | ebi-a-GCST90001485 | CD39+ resting CD4 regulatory T cell %CD4 regulatory T cell \|\| id:ebi-a-GCST90001485 | Mayonnaise liking \|\| id:ebi-fl187-GCST90094798 | Inverse variance weighted (multiplicative random effects) | 9 | -0.113361293 | 0.25101534285129 | 0.651549234297532 | -0.605351365 | 0.378628778552031 | 0.892828028930487 | 0.545882588671286 | 1.46028084754305 | 0.998735809224318 |
| ebi-fl187-GCST90094798 | ebi-a-GCST90001515 | Immature Myeloid-Derived Suppressor Cells Absolute Count \|\| id:ebi-a-GCST90001515 | Mayonnaise liking \|\| id:ebi-fl187-GCST90094798 | Inverse variance weighted (multiplicative random effects) | 9 | 0.202207858976948 | 0.330553047007055 | 0.540719125319183 | -0.445676113 | 0.850091831110775 | 1.22410242234649 | 0.640391152744722 | 2.33986171416059 | 0.998735809224318 |
| ebi-fl187-GCST90094798 | ebi-a-GCST90001517 | CD33+ HLA DR+ Absolute Count \|\| id:ebi-a-GCST90001517 | Mayonnaise liking \|\| id:ebi-fl187-GCST90094798 | Inverse variance weighted | 9 | -0.081576799 | 0.218845014881548 | 0.709326629998085 | -0.510513028 | 0.347359430301294 | 0.921661924544668 | 0.600187586756851 | 1.41532534477344 | 0.998735809224318 |
| ebi-fl187-GCST90094798 | ebi-a-GCST90001518 | CD33+ HLA DR+ CD14- Absolute Count \|\| id:ebi-a-GCST90001518 | Mayonnaise liking \|\| id:ebi-fl187-GCST90094798 | Inverse variance weighted | 9 | -0.069972942 | 0.221700893765101 | 0.75229196387414 | -0.504506693 | 0.364560810246278 | 0.93241904939439 | 0.603803362207155 | 1.43988148806508 | 0.998735809224318 |
| ebi-fl187-GCST90094798 | ebi-a-GCST90001520 | CD33+ HLA DR+ CD14dim Absolute Count \|\| id:ebi-a-GCST90001520 | Mayonnaise liking \|\| id:ebi-fl187-GCST90094798 | Inverse variance weighted | 9 | -0.063751569 | 0.219590208740626 | 0.771570953952226 | -0.494148378 | 0.366645240307657 | 0.938238058164201 | 0.610090252430083 | 1.44288595053174 | 0.998735809224318 |
| ebi-fl187-GCST90094798 | ebi-a-GCST90001531 | CD33dim HLA DR- Absolute Count \|\| id:ebi-a-GCST90001531 | Mayonnaise liking \|\| id:ebi-fl187-GCST90094798 | Inverse variance weighted | 9 | -0.229370625 | 0.223411771832065 | 0.304574907065823 | -0.667257698 | 0.208516447442163 | 0.795033819207713 | 0.513113763011844 | 1.23184919066264 | 0.998735809224318 |
| ebi-fl187-GCST90094798 | ebi-a-GCST90001532 | Basophil Absolute Count \|\| id:ebi-a-GCST90001532 | Mayonnaise liking \|\| id:ebi-fl187-GCST90094798 | Inverse variance weighted | 9 | -0.274955032 | 0.236498282353628 | 0.244988176848692 | -0.738491665 | 0.188581601440131 | 0.759606280452715 | 0.477834105962137 | 1.2075356156116 | 0.998735809224318 |
| ebi-fl187-GCST90094798 | ebi-a-GCST90001588 | T/B cell \|\| id:ebi-a-GCST90001588 | Mayonnaise liking \|\| id:ebi-fl187-GCST90094798 | Inverse variance weighted | 9 | 0.0330932480521375 | 0.185618971872271 | 0.858498440609059 | -0.330719937 | 0.396906432921789 | 1.03364692030948 | 0.718406340034869 | 1.48721676901322 | 0.998735809224318 |
| ebi-fl187-GCST90094798 | ebi-a-GCST90001624 | HLA DR+ CD4+ T cell Absolute Count \|\| id:ebi-a-GCST90001624 | Mayonnaise liking \|\| id:ebi-fl187-GCST90094798 | Inverse variance weighted | 9 | 0.0655859933309128 | 0.160854394820844 | 0.683468117091243 | -0.249688621 | 0.380860607179768 | 1.0677845557162 | 0.779043323414947 | 1.46354358372281 | 0.998735809224318 |
| ebi-fl187-GCST90094798 | ebi-a-GCST90001631 | CD8+ Natural Killer T %T cell \|\| id:ebi-a-GCST90001631 | Mayonnaise liking \|\| id:ebi-fl187-GCST90094798 | Inverse variance weighted | 9 | -0.064348848 | 0.163073122017362 | 0.693137233269746 | -0.383972168 | 0.255274470730071 | 0.937677835033706 | 0.681150384980694 | 1.29081586342848 | 0.998735809224318 |
| ebi-fl187-GCST90094798 | ebi-a-GCST90001632 | CD8+ Natural Killer T %lymphocyte \|\| id:ebi-a-GCST90001632 | Mayonnaise liking \|\| id:ebi-fl187-GCST90094798 | Inverse variance weighted | 9 | -0.10143911 | 0.164057625412594 | 0.536368439772664 | -0.422992056 | 0.220113835311533 | 0.903536193536941 | 0.655083836876791 | 1.24621858619414 | 0.998735809224318 |
| ebi-fl187-GCST90094798 | ebi-a-GCST90001646 | Natural Killer %CD3- lymphocyte \|\| id:ebi-a-GCST90001646 | Mayonnaise liking \|\| id:ebi-fl187-GCST90094798 | Inverse variance weighted | 9 | 0.435260524351919 | 0.159792683060836 | 0.00645145280081032 | 0.122066865552681 | 0.748454183151157 | 1.54536561188616 | 1.12982964593835 | 2.11373005035361 | 0.998735809224318 |
| ebi-fl187-GCST90094798 | ebi-a-GCST90001684 | CD127- CD8+ T cell Absolute Count \|\| id:ebi-a-GCST90001684 | Mayonnaise liking \|\| id:ebi-fl187-GCST90094798 | Inverse variance weighted | 9 | -0.0552516 | 0.172826790512018 | 0.749200692476668 | -0.39399211 | 0.283488908949739 | 0.946247041815791 | 0.674359377011122 | 1.32775415404414 | 0.998735809224318 |
| ebi-fl187-GCST90094798 | ebi-a-GCST90001687 | CD28- CD8+ T cell Absolute Count \|\| id:ebi-a-GCST90001687 | Mayonnaise liking \|\| id:ebi-fl187-GCST90094798 | Inverse variance weighted | 9 | -0.09748072 | 0.168276239121768 | 0.56239360145488 | -0.427302149 | 0.232340708462001 | 0.90711983 | 0.65226644086634 | 1.26154947619666 | 0.998735809224318 |
| ebi-fl187-GCST90094798 | ebi-a-GCST90001701 | BAFF-R on CD20- CD38- B cell \|\| id:ebi-a-GCST90001701 | Mayonnaise liking \|\| id:ebi-fl187-GCST90094798 | Inverse variance weighted | 9 | -0.222909929 | 0.193411596102036 | 0.249109161911965 | -0.601996658 | 0.156176798994394 | 0.800186919392277 | 0.547716940330984 | 1.16903286865579 | 0.998735809224318 |
| ebi-fl187-GCST90094798 | ebi-a-GCST90001732 | CD19 on IgD- CD27- B cell \|\| id:ebi-a-GCST90001732 | Mayonnaise liking \|\| id:ebi-fl187-GCST90094798 | Inverse variance weighted | 9 | 0.152493579090358 | 0.166137529685393 | 0.358684015017845 | -0.173135979 | 0.478123137273729 | 1.16473498341252 | 0.841023245490754 | 1.61304409700751 | 0.998735809224318 |
| ebi-fl187-GCST90094798 | ebi-a-GCST90001798 | CD27 on CD24+ CD27+ B cell \|\| id:ebi-a-GCST90001798 | Mayonnaise liking \|\| id:ebi-fl187-GCST90094798 | Inverse variance weighted | 9 | 0.441138741058341 | 0.162780274552334 | 0.00672779779940909 | 0.122089402935766 | 0.760188079180916 | 1.55447635707682 | 1.12985510962884 | 2.13867842355877 | 0.998735809224318 |
| ebi-fl187-GCST90094798 | ebi-a-GCST90001836 | CD66b on CD66b++ myeloid cell \|\| id:ebi-a-GCST90001836 | Mayonnaise liking \|\| id:ebi-fl187-GCST90094798 | Inverse variance weighted | 9 | 0.126380571181412 | 0.341570983766851 | 0.711384001687485 | -0.543098557 | 0.795859699364441 | 1.13471392553984 | 0.580945368308404 | 2.21634556888402 | 0.998735809224318 |
| ebi-fl187-GCST90094798 | ebi-a-GCST90001943 | CD25 on activated & secreting CD4 regulatory T cell \|\| id:ebi-a-GCST90001943 | Mayonnaise liking \|\| id:ebi-fl187-GCST90094798 | Inverse variance weighted | 9 | -0.147029188 | 0.165934481363576 | 0.375581036561819 | -0.472260771 | 0.178202395972534 | 0.863268780391011 | 0.623590877313719 | 1.19506717354184 | 0.998735809224318 |
| ebi-fl187-GCST90094798 | ebi-a-GCST90001960 | CD25 on CD4+ T cell \|\| id:ebi-a-GCST90001960 | Mayonnaise liking \|\| id:ebi-fl187-GCST90094798 | Inverse variance weighted | 9 | -0.182147529 | 0.177490890684822 | 0.304780388953374 | -0.530029675 | 0.165734616403971 | 0.833478368832775 | 0.588587503037642 | 1.18025983855746 | 0.998735809224318 |
| ebi-fl187-GCST90094798 | ebi-a-GCST90001988 | HLA DR on CD14+ CD16- monocyte \|\| id:ebi-a-GCST90001988 | Mayonnaise liking \|\| id:ebi-fl187-GCST90094798 | Inverse variance weighted | 9 | -0.209836594 | 0.165995284656726 | 0.206189910198789 | -0.535187352 | 0.115514163699477 | 0.810716710937384 | 0.585559574019329 | 1.12245041248601 | 0.998735809224318 |
| ebi-fl187-GCST90094798 | ebi-a-GCST90001991 | HLA DR on CD14+ monocyte \|\| id:ebi-a-GCST90001991 | Mayonnaise liking \|\| id:ebi-fl187-GCST90094798 | Inverse variance weighted | 9 | -0.211348152 | 0.166300074107335 | 0.20376981614393 | -0.537296297 | 0.114599993656572 | 0.809492191819743 | 0.584325962531857 | 1.12142477082114 | 0.998735809224318 |
| ebi-fl187-GCST90094798 | ebi-a-GCST90002019 | CD14 on Monocytic Myeloid-Derived Suppressor Cells \|\| id:ebi-a-GCST90002019 | Mayonnaise liking \|\| id:ebi-fl187-GCST90094798 | Inverse variance weighted | 9 | -0.220337535 | 0.285678968038755 | 0.440542933948225 | -0.780268312 | 0.339593242725111 | 0.80224796578645 | 0.458283031976717 | 1.40437623411987 | 0.998735809224318 |
| ebi-fl187-GCST90094798 | ebi-a-GCST90002037 | CD80 on plasmacytoid Dendritic Cell \|\| id:ebi-a-GCST90002037 | Mayonnaise liking \|\| id:ebi-fl187-GCST90094798 | Inverse variance weighted | 9 | -0.014442295 | 0.185043279891756 | 0.937789713564186 | -0.377127123 | 0.348242534070865 | 0.985661495164002 | 0.685828883657126 | 1.41657577596957 | 0.998735809224318 |
| ebi-fl187-GCST90094798 | ebi-a-GCST90002042 | CD45 on CD33+ HLA DR+ CD14- \|\| id:ebi-a-GCST90002042 | Mayonnaise liking \|\| id:ebi-fl187-GCST90094798 | Inverse variance weighted | 9 | -0.651620536 | 0.244485771293816 | 0.00769265850336652 | -1.130812647 | -0.172428424 | 0.521200468063199 | 0.322770850916314 | 0.841618526388336 | 0.998735809224318 |
| ebi-fl187-GCST90094798 | ebi-a-GCST90002056 | CD8 on Effector Memory CD8+ T cell \|\| id:ebi-a-GCST90002056 | Mayonnaise liking \|\| id:ebi-fl187-GCST90094798 | Inverse variance weighted | 9 | -0.268268225 | 0.181483940561158 | 0.139356223917858 | -0.623976748 | 0.0874402986806874 | 0.7647026413646 | 0.535809415992206 | 1.09137710584486 | 0.998735809224318 |
| ebi-fl187-GCST90094798 | ebi-a-GCST90002058 | CD8 on CD8+ T cell \|\| id:ebi-a-GCST90002058 | Mayonnaise liking \|\| id:ebi-fl187-GCST90094798 | Inverse variance weighted | 9 | -0.037308218 | 0.202215961130207 | 0.85362365722451 | -0.433651502 | 0.35903506543512 | 0.963379158420914 | 0.648138090833307 | 1.43194701253638 | 0.998735809224318 |
| ebi-fl187-GCST90094798 | ebi-a-GCST90002063 | CD4 on CD4 regulatory T cell \|\| id:ebi-a-GCST90002063 | Mayonnaise liking \|\| id:ebi-fl187-GCST90094798 | Inverse variance weighted | 9 | -0.267170641 | 0.180475937183733 | 0.138775302771964 | -0.620903478 | 0.0865621955717554 | 0.765542427157657 | 0.537458636017305 | 1.09041918485345 | 0.998735809224318 |
| ebi-fl187-GCST90094798 | ebi-a-GCST90002066 | CD4 on activated CD4 regulatory T cell \|\| id:ebi-a-GCST90002066 | Mayonnaise liking \|\| id:ebi-fl187-GCST90094798 | Inverse variance weighted | 9 | -0.261984856 | 0.180472903420142 | 0.146596843544857 | -0.615711747 | 0.0917420348740846 | 0.769522677402045 | 0.540256232933847 | 1.09608203466765 | 0.998735809224318 |
| ebi-fl187-GCST90094798 | ebi-a-GCST90002070 | CD4 on activated & secreting CD4 regulatory T cell \|\| id:ebi-a-GCST90002070 | Mayonnaise liking \|\| id:ebi-fl187-GCST90094798 | Inverse variance weighted | 9 | -0.213463872 | 0.181323748590898 | 0.239094661408941 | -0.568858419 | 0.141930675336763 | 0.807781343227413 | 0.566171400355029 | 1.15249674931852 | 0.998735809224318 |
| ebi-fl187-GCST90094798 | ebi-a-GCST90002081 | SSC-A on CD4+ T cell \|\| id:ebi-a-GCST90002081 | Mayonnaise liking \|\| id:ebi-fl187-GCST90094798 | Inverse variance weighted | 9 | 0.0028651346466452 | 0.17104261553187 | 0.986635261701735 | -0.332378392 | 0.338108661089111 | 1.00286924306771 | 0.717215882896435 | 1.40229286979751 | 0.998955228396052 |
| ebi-fl187-GCST90094798 | ebi-a-GCST90002094 | CD11b on Monocytic Myeloid-Derived Suppressor Cells \|\| id:ebi-a-GCST90002094 | Mayonnaise liking \|\| id:ebi-fl187-GCST90094798 | Inverse variance weighted | 9 | -0.402387326 | 0.299626457660749 | 0.179283868651427 | -0.989655183 | 0.184880530747479 | 0.668721682049345 | 0.371704838969392 | 1.2030747011064 | 0.998735809224318 |
| ebi-fl187-GCST90094798 | ebi-a-GCST90002104 | HLA DR on myeloid Dendritic Cell \|\| id:ebi-a-GCST90002104 | Mayonnaise liking \|\| id:ebi-fl187-GCST90094798 | Inverse variance weighted | 9 | -0.364889829 | 0.183416534061254 | 0.0466564827897769 | -0.724386236 | -0.005393422 | 0.694273135535063 | 0.484621921372213 | 0.994621096298855 | 0.998735809224318 |
| ebi-fl187-GCST90094798 | ebi-a-GCST90002105 | HLA DR on plasmacytoid Dendritic Cell \|\| id:ebi-a-GCST90002105 | Mayonnaise liking \|\| id:ebi-fl187-GCST90094798 | Inverse variance weighted | 9 | -0.290383026 | 0.179279585078767 | 0.105292082517641 | -0.641771013 | 0.0610049607025009 | 0.747977018019984 | 0.526359408842221 | 1.06290418692558 | 0.998735809224318 |
| ebi-fl187-GCST90094798 | ebi-a-GCST90002106 | HLA DR on Dendritic Cell \|\| id:ebi-a-GCST90002106 | Mayonnaise liking \|\| id:ebi-fl187-GCST90094798 | Inverse variance weighted | 9 | -0.46324833 | 0.182977288163635 | 0.0113502695146509 | -0.821883815 | -0.104612846 | 0.629236354571333 | 0.439602743650535 | 0.900673154644764 | 0.998735809224318 |
| ebi-fl187-GCST90094798 | ebi-a-GCST90002111 | HLA DR on CD33dim HLA DR+ CD11b- \|\| id:ebi-a-GCST90002111 | Mayonnaise liking \|\| id:ebi-fl187-GCST90094798 | Inverse variance weighted (multiplicative random effects) | 9 | -0.220632452 | 0.357445876462088 | 0.53707171339406 | -0.92122637 | 0.479961465585691 | 0.802011403587241 | 0.398030608790645 | 1.61601212891217 | 0.998735809224318 |
| ebi-fl187-GCST90094798 | ebi-a-GCST90002112 | HLA DR on CD33- HLA DR+ \|\| id:ebi-a-GCST90002112 | Mayonnaise liking \|\| id:ebi-fl187-GCST90094798 | Inverse variance weighted | 9 | -0.266874839 | 0.239486735494429 | 0.265124059501644 | -0.73626884 | 0.202519162737314 | 0.765768909998989 | 0.478897428904254 | 1.2244835493537 | 0.998735809224318 |
| ebi-fl187-GCST90094798 | ebi-a-GCST90002115 | HLA DR on HLA DR+ CD8+ T cell \|\| id:ebi-a-GCST90002115 | Mayonnaise liking \|\| id:ebi-fl187-GCST90094798 | Inverse variance weighted | 9 | 0.0852538527288296 | 0.169224912786091 | 0.614408814754366 | -0.246426976 | 0.416934681789567 | 1.08899347557879 | 0.781588433907545 | 1.51730340215534 | 0.998735809224318 |
| ebi-fl187-GCST90094798 | ebi-a-GCST90002116 | HLA DR on B cell \|\| id:ebi-a-GCST90002116 | Mayonnaise liking \|\| id:ebi-fl187-GCST90094798 | Inverse variance weighted | 9 | 0.0231990948514882 | 0.177372953830476 | 0.895939265327713 | -0.324451895 | 0.370850084359221 | 1.0234702869288 | 0.722923483296086 | 1.44896583446175 | 0.998735809224318 |
| ebi-fl187-GCST90094798 | ebi-cfmet338-GCST90026023 | Sphingomyelin (d18:1/24:1, d18:2/24:0) levels \|\| id:ebi-cfmet338-GCST90026023 | Mayonnaise liking \|\| id:ebi-fl187-GCST90094798 | Inverse variance weighted | 9 | -0.075015628 | 0.0982724211124191 | 0.445258480764817 | -0.267629573 | 0.11759831746336 | 0.92772898774366 | 0.765191175855321 | 1.12479221122462 | 0.998735809224318 |
| ebi-fl187-GCST90094798 | ebi-cfmet338-GCST90026046 | 1-arachidonoyl-gpc (20:4n6) levels \|\| id:ebi-cfmet338-GCST90026046 | Mayonnaise liking \|\| id:ebi-fl187-GCST90094798 | Inverse variance weighted | 9 | 0.0012740308240838 | 0.159756475513943 | 0.993637073261068 | -0.311848661 | 0.314396722831413 | 1.00127484274612 | 0.732092313829551 | 1.36943291409789 | 0.999107451094609 |
| ebi-fl187-GCST90094798 | ebi-cfmet338-GCST90026047 | 1-linoleoyl-gpc (18:2) levels \|\| id:ebi-cfmet338-GCST90026047 | Mayonnaise liking \|\| id:ebi-fl187-GCST90094798 | Inverse variance weighted | 9 | 0.0405756719167395 | 0.191308127947219 | 0.832032208717981 | -0.334388259 | 0.415539602693288 | 1.04141011222021 | 0.715775821965918 | 1.5151881197325 | 0.998735809224318 |
| ebi-fl187-GCST90094798 | ebi-cfmet338-GCST90026050 | 1-myristoyl-2-palmitoyl-gpc (14:0/16:0) levels \|\| id:ebi-cfmet338-GCST90026050 | Mayonnaise liking \|\| id:ebi-fl187-GCST90094798 | Inverse variance weighted | 9 | -0.043994894 | 0.0742709388443155 | 0.553611702618985 | -0.189565934 | 0.101576146082175 | 0.956958843642012 | 0.82731816654796 | 1.10691420236276 | 0.998735809224318 |
| ebi-fl187-GCST90094798 | ebi-cfmet338-GCST90026068 | 2,3-dihydroxyisovalerate levels \|\| id:ebi-cfmet338-GCST90026068 | Mayonnaise liking \|\| id:ebi-fl187-GCST90094798 | Inverse variance weighted | 9 | -0.243061577 | 0.283489144858854 | 0.391227788156759 | -0.798700301 | 0.312577147135957 | 0.784223222338123 | 0.449913336324334 | 1.36694339287387 | 0.998735809224318 |
| ebi-fl187-GCST90094798 | ebi-cfmet338-GCST90026082 | 3-hydroxyhexanoate levels \|\| id:ebi-cfmet338-GCST90026082 | Mayonnaise liking \|\| id:ebi-fl187-GCST90094798 | Inverse variance weighted | 9 | -0.003722216 | 0.0544790962569674 | 0.945527915868459 | -0.110501245 | 0.103056812328245 | 0.996284702524652 | 0.895385215435034 | 1.10855438684274 | 0.998735809224318 |
| ebi-fl187-GCST90094798 | ebi-cfmet338-GCST90026094 | 3-ureidopropionate levels \|\| id:ebi-cfmet338-GCST90026094 | Mayonnaise liking \|\| id:ebi-fl187-GCST90094798 | Inverse variance weighted | 9 | -0.225582355 | 0.151144136542441 | 0.135568582582075 | -0.521824863 | 0.070660152320617 | 0.798051333982392 | 0.593436618775508 | 1.0732164337705 | 0.998735809224318 |
| ebi-fl187-GCST90094798 | ebi-cfmet338-GCST90026173 | Glycerol levels \|\| id:ebi-cfmet338-GCST90026173 | Mayonnaise liking \|\| id:ebi-fl187-GCST90094798 | Inverse variance weighted | 8 | -0.070965684 | 0.0746287790981957 | 0.341647131667582 | -0.217238091 | 0.0753067234981114 | 0.931493857156076 | 0.804738345885132 | 1.07821481399043 | 0.998735809224318 |
| ebi-fl187-GCST90094798 | ebi-cfmet338-GCST90026179 | Histidine levels \|\| id:ebi-cfmet338-GCST90026179 | Mayonnaise liking \|\| id:ebi-fl187-GCST90094798 | Inverse variance weighted | 9 | -0.015344524 | 0.0492508999935653 | 0.7553762698287 | -0.111876288 | 0.0811872399214518 | 0.984772603290348 | 0.894154868297664 | 1.08457395309781 | 0.998735809224318 |
| ebi-fl187-GCST90094798 | ebi-cfmet338-GCST90026188 | Isocitrate levels \|\| id:ebi-cfmet338-GCST90026188 | Mayonnaise liking \|\| id:ebi-fl187-GCST90094798 | Inverse variance weighted | 9 | 0.0943020884712289 | 0.0902173354603038 | 0.295895053108991 | -0.082523889 | 0.271128065973424 | 1.0988916582823 | 0.920789440826596 | 1.3114430108564 | 0.998735809224318 |
| ebi-fl187-GCST90094798 | ebi-cfmet338-GCST90026207 | N-acetyl-isoputreanine levels \|\| id:ebi-cfmet338-GCST90026207 | Mayonnaise liking \|\| id:ebi-fl187-GCST90094798 | Inverse variance weighted | 9 | 0.000724997531109312 | 0.0579513713680223 | 0.990018368262448 | -0.11285969 | 0.114309685412433 | 1.00072526040534 | 0.893275986564392 | 1.12109925921663 | 0.999107451094609 |
| ebi-fl187-GCST90094798 | ebi-cfmet338-GCST90026213 | N-acetylglutamate levels \|\| id:ebi-cfmet338-GCST90026213 | Mayonnaise liking \|\| id:ebi-fl187-GCST90094798 | Inverse variance weighted | 9 | 0.148468990036313 | 0.0995364256513597 | 0.135802832465178 | -0.046622404 | 0.343560384312979 | 1.16005682389667 | 0.9544477249708 | 1.40995865929735 | 0.998735809224318 |
| ebi-fl187-GCST90094798 | ebi-cfmet338-GCST90026224 | N-acetyltaurine levels \|\| id:ebi-cfmet338-GCST90026224 | Mayonnaise liking \|\| id:ebi-fl187-GCST90094798 | Inverse variance weighted | 9 | -0.020381368 | 0.0786203507512772 | 0.795451755721023 | -0.174477255 | 0.133714519505386 | 0.97982492820308 | 0.839895957076275 | 1.14306644988527 | 0.998735809224318 |
| ebi-fl187-GCST90094798 | ebi-cfmet338-GCST90026250 | Phenyllactate (pla) levels \|\| id:ebi-cfmet338-GCST90026250 | Mayonnaise liking \|\| id:ebi-fl187-GCST90094798 | Inverse variance weighted | 9 | -0.002545642 | 0.124118099080967 | 0.983636663459734 | -0.245817116 | 0.240725831988005 | 0.997457595188772 | 0.782065238746593 | 1.27217219856788 | 0.998955228396052 |
| ebi-fl187-GCST90094798 | ebi-cfmet338-GCST90026251 | Pipecolate levels \|\| id:ebi-cfmet338-GCST90026251 | Mayonnaise liking \|\| id:ebi-fl187-GCST90094798 | Inverse variance weighted | 9 | 0.0690524793797294 | 0.23121055005191 | 0.765202330006891 | -0.384120199 | 0.522225157481474 | 1.07149243893146 | 0.681049560972681 | 1.68577459333146 | 0.998735809224318 |
| ebi-fl187-GCST90094798 | ebi-cfmet338-GCST90026278 | Trigonelline (n'-methylnicotinate) levels \|\| id:ebi-cfmet338-GCST90026278 | Mayonnaise liking \|\| id:ebi-fl187-GCST90094798 | Inverse variance weighted | 9 | 0.0155679539963213 | 0.234663231996087 | 0.947105797226736 | -0.444371981 | 0.475507888708652 | 1.01568976589188 | 0.641226852434957 | 1.60883109716954 | 0.998735809224318 |
| ebi-fl187-GCST90094798 | ebi-cfmet338-GCST90026299 | 1-carboxyethylvaline levels \|\| id:ebi-cfmet338-GCST90026299 | Mayonnaise liking \|\| id:ebi-fl187-GCST90094798 | Inverse variance weighted | 9 | 0.0669846100153676 | 0.125680046624023 | 0.594049304496286 | -0.179348281 | 0.313317501398453 | 1.06927902186026 | 0.835814749994736 | 1.36795578996138 | 0.998735809224318 |
| ebi-fl187-GCST90094798 | ebi-gm430-GCST90011306 | C_Erysipelotrichia abundance \|\| id:ebi-gm430-GCST90011306 | Mayonnaise liking \|\| id:ebi-fl187-GCST90094798 | Inverse variance weighted | 8 | -0.061806293 | 0.105984171160085 | 0.55978201505465 | -0.269535269 | 0.145922682435849 | 0.940064966284266 | 0.763734343273756 | 1.15710671991906 | 0.998735809224318 |
| ebi-fl187-GCST90094798 | ebi-gm430-GCST90011312 | F_Erysipelotrichaceae abundance \|\| id:ebi-gm430-GCST90011312 | Mayonnaise liking \|\| id:ebi-fl187-GCST90094798 | Inverse variance weighted | 8 | -0.061806293 | 0.105984171160085 | 0.55978201505465 | -0.269535269 | 0.145922682435849 | 0.940064966284266 | 0.763734343273756 | 1.15710671991906 | 0.998735809224318 |
| ebi-fl187-GCST90094798 | ebi-gm430-GCST90011352 | O_Erysipelotrichales abundance \|\| id:ebi-gm430-GCST90011352 | Mayonnaise liking \|\| id:ebi-fl187-GCST90094798 | Inverse variance weighted | 8 | -0.061806293 | 0.105984171160085 | 0.55978201505465 | -0.269535269 | 0.145922682435849 | 0.940064966284266 | 0.763734343273756 | 1.15710671991906 | 0.998735809224318 |
| ebi-fl187-GCST90094798 | ebi-gm430-GCST90011362 | OTU97_11 (Parabacteroides) abundance \|\| id:ebi-gm430-GCST90011362 | Mayonnaise liking \|\| id:ebi-fl187-GCST90094798 | Inverse variance weighted | 8 | -0.050290107 | 0.141642097608562 | 0.722551709423716 | -0.327908618 | 0.227328404355664 | 0.950953506251689 | 0.720428850732174 | 1.25524202720827 | 0.998735809224318 |
| ebi-fl187-GCST90094798 | ebi-gm430-GCST90011384 | OTU97_235 (Phascolarctobacterium) abundance \|\| id:ebi-gm430-GCST90011384 | Mayonnaise liking \|\| id:ebi-fl187-GCST90094798 | Inverse variance weighted | 8 | -0.328063185 | 0.43084712019473 | 0.446395753342548 | -1.172523541 | 0.516397170571182 | 0.720317504998285 | 0.309584705108209 | 1.6759784945629 | 0.998735809224318 |
| ebi-fl187-GCST90094798 | ebi-gm430-GCST90011390 | OTU97_3 (Bacteroides) abundance \|\| id:ebi-gm430-GCST90011390 | Mayonnaise liking \|\| id:ebi-fl187-GCST90094798 | Inverse variance weighted (multiplicative random effects) | 8 | 0.0190822910331733 | 0.17484560967149 | 0.913093076596786 | -0.323615104 | 0.361779685989294 | 1.01926552157921 | 0.723528672140686 | 1.43588256206402 | 0.998735809224318 |
| ebi-fl187-GCST90094798 | ebi-gm430-GCST90011398 | OTU97_4 (Alistipes) abundance \|\| id:ebi-gm430-GCST90011398 | Mayonnaise liking \|\| id:ebi-fl187-GCST90094798 | Inverse variance weighted | 8 | -0.129250005 | 0.1461521583463 | 0.376506035781347 | -0.415708235 | 0.157208225378509 | 0.878754245143438 | 0.659872769955789 | 1.17023926204646 | 0.998735809224318 |
| ebi-fl187-GCST90094798 | ebi-gm430-GCST90011421 | OTU99_108 (Cytophagales) abundance \|\| id:ebi-gm430-GCST90011421 | Mayonnaise liking \|\| id:ebi-fl187-GCST90094798 | Inverse variance weighted | 8 | 0.201407055258189 | 0.408863064770862 | 0.622293330185825 | -0.599964552 | 1.00277866220908 | 1.22312254897002 | 0.54883109088237 | 2.72584551905344 | 0.998735809224318 |
| ebi-fl187-GCST90094798 | ebi-gm430-GCST90011428 | OTU99_123 (Phascolarctobacterium) abundance \|\| id:ebi-gm430-GCST90011428 | Mayonnaise liking \|\| id:ebi-fl187-GCST90094798 | Inverse variance weighted | 8 | -0.302270404 | 0.324841076863723 | 0.35210305333658 | -0.938958914 | 0.334418107079651 | 0.739138172262008 | 0.391034724207131 | 1.3971271702342 | 0.998735809224318 |
| ebi-fl187-GCST90094798 | ebi-gm430-GCST90011450 | OTU99_298 (Phascolarctobacterium) abundance \|\| id:ebi-gm430-GCST90011450 | Mayonnaise liking \|\| id:ebi-fl187-GCST90094798 | Inverse variance weighted | 8 | -0.317111534 | 0.430833227862257 | 0.461704879496879 | -1.16154466 | 0.527321592796595 | 0.728249526149117 | 0.313002324978435 | 1.69438796460363 | 0.998735809224318 |
| ebi-fl187-GCST90094798 | ebi-gm430-GCST90011451 | OTU99_3 (Bacteroides) abundance \|\| id:ebi-gm430-GCST90011451 | Mayonnaise liking \|\| id:ebi-fl187-GCST90094798 | Inverse variance weighted (multiplicative random effects) | 8 | 0.0193023784115526 | 0.173895046678979 | 0.911616217999437 | -0.321531913 | 0.360136669902351 | 1.01948987374336 | 0.725037491479311 | 1.43352532093841 | 0.998735809224318 |
| ebi-fl187-GCST90094798 | ebi-gm430-GCST90011472 | OTU99_72 (Parabacteroides) abundance \|\| id:ebi-gm430-GCST90011472 | Mayonnaise liking \|\| id:ebi-fl187-GCST90094798 | Inverse variance weighted | 8 | -0.101312816 | 0.353554198535341 | 0.774452873441373 | -0.794279045 | 0.59165341283745 | 0.903650312128599 | 0.451906921870302 | 1.80697362021039 | 0.998735809224318 |
| ebi-fl187-GCST90094798 | ebi-gm430-GCST90011507 | TestASV_3 (Bacteroides) abundance \|\| id:ebi-gm430-GCST90011507 | Mayonnaise liking \|\| id:ebi-fl187-GCST90094798 | Inverse variance weighted | 8 | -0.165460549 | 0.179061944523596 | 0.355465054781642 | -0.51642196 | 0.185500862284411 | 0.847503297413392 | 0.596651576302689 | 1.20382123781097 | 0.998735809224318 |
| ebi-fl187-GCST90094798 | ebi-gm430-GCST90011512 | TestASV_34 (Phascolarctobacterium) abundance \|\| id:ebi-gm430-GCST90011512 | Mayonnaise liking \|\| id:ebi-fl187-GCST90094798 | Inverse variance weighted | 8 | -0.332204401 | 0.437976836301413 | 0.448153119560522 | -1.190639 | 0.526230198234575 | 0.717340682775487 | 0.304026928755344 | 1.6925397275545 | 0.998735809224318 |
| ebi-fl187-GCST90094798 | ebi-gm430-GCST90011519 | TestASV_41 (Barnesiella) abundance \|\| id:ebi-gm430-GCST90011519 | Mayonnaise liking \|\| id:ebi-fl187-GCST90094798 | Inverse variance weighted | 8 | 0.450048710720643 | 0.467972326149412 | 0.336200618000807 | -0.467177049 | 1.36727446997349 | 1.56838858096754 | 0.626769112037071 | 3.92463938261828 | 0.998735809224318 |
| ebi-fl187-GCST90094798 | ebi-gm430-GCST90011524 | TestASV_46 (Sutterella) abundance \|\| id:ebi-gm430-GCST90011524 | Mayonnaise liking \|\| id:ebi-fl187-GCST90094798 | Inverse variance weighted | 8 | -0.308867459 | 0.461454092613196 | 0.503281689425053 | -1.21331748 | 0.595582562812951 | 0.734278085742447 | 0.297209654968609 | 1.81408745707988 | 0.998735809224318 |
| ebi-fl187-GCST90094798 | ebi-gm430-GCST90011581 | OTU97_19 (Bacteria) prevalence \|\| id:ebi-gm430-GCST90011581 | Mayonnaise liking \|\| id:ebi-fl187-GCST90094798 | Inverse variance weighted | 8 | -0.720740985 | 0.51122636806028 | 0.158590628457608 | -1.722744667 | 0.281262696030494 | 0.486391713255563 | 0.178575344859962 | 1.32480157834333 | 0.998735809224318 |
| ebi-fl187-GCST90094798 | ebi-gm430-GCST90011614 | OTU97_80 (Ruminococcus) prevalence \|\| id:ebi-gm430-GCST90011614 | Mayonnaise liking \|\| id:ebi-fl187-GCST90094798 | Inverse variance weighted | 8 | -0.241741177 | 0.297655586660526 | 0.416705194578523 | -0.825146127 | 0.341663772564882 | 0.785259394216812 | 0.438170959115552 | 1.40728704944396 | 0.998735809224318 |
| ebi-fl187-GCST90094798 | ebi-gm430-GCST90011623 | OTU99_11 (Parabacteroides) prevalence \|\| id:ebi-gm430-GCST90011623 | Mayonnaise liking \|\| id:ebi-fl187-GCST90094798 | Inverse variance weighted | 8 | -0.095248257 | 0.270911464250312 | 0.725149931658336 | -0.626234727 | 0.4357382128558 | 0.909147204224332 | 0.534600934626972 | 1.54610399161696 | 0.998735809224318 |
| ebi-fl187-GCST90094798 | ebi-gm430-GCST90011642 | OTU99_19 (Bacteria) prevalence \|\| id:ebi-gm430-GCST90011642 | Mayonnaise liking \|\| id:ebi-fl187-GCST90094798 | Inverse variance weighted | 8 | -0.720740985 | 0.51122636806028 | 0.158590628457608 | -1.722744667 | 0.281262696030494 | 0.486391713255563 | 0.178575344859962 | 1.32480157834333 | 0.998735809224318 |
| ebi-fl187-GCST90094798 | ebi-gm430-GCST90011653 | OTU99_30 (Parasutterella) prevalence \|\| id:ebi-gm430-GCST90011653 | Mayonnaise liking \|\| id:ebi-fl187-GCST90094798 | Inverse variance weighted | 8 | 0.509543779806691 | 0.229290985797741 | 0.0262658593828251 | 0.0601334476431179 | 0.958954111970263 | 1.6645316287529 | 1.06197825558503 | 2.60896635928997 | 0.998735809224318 |
| ebi-fl187-GCST90094798 | ebi-gm430-GCST90011675 | OTU99_8 (Ruminococcaceae) prevalence \|\| id:ebi-gm430-GCST90011675 | Mayonnaise liking \|\| id:ebi-fl187-GCST90094798 | Inverse variance weighted | 8 | 0.0367080682960213 | 0.24203390027568 | 0.879451174112109 | -0.437678376 | 0.511094512836354 | 1.03739012956031 | 0.645533368339642 | 1.66711487537378 | 0.998735809224318 |
| ebi-fl187-GCST90094798 | ebi-gm430-GCST90011679 | OTU99_92 (Ruminococcus) prevalence \|\| id:ebi-gm430-GCST90011679 | Mayonnaise liking \|\| id:ebi-fl187-GCST90094798 | Inverse variance weighted | 8 | -0.245217616 | 0.261792357835011 | 0.348919354070347 | -0.758330637 | 0.267895405463203 | 0.782534227826294 | 0.468447783925175 | 1.30721040579734 | 0.998735809224318 |
| ebi-fl187-GCST90094798 | ebi-gm430-GCST90011684 | TestASV_11 (Lachnospiraceae) prevalence \|\| id:ebi-gm430-GCST90011684 | Mayonnaise liking \|\| id:ebi-fl187-GCST90094798 | Inverse variance weighted | 8 | -0.059174371 | 0.255387792679596 | 0.816767931087582 | -0.559734445 | 0.441385702743794 | 0.94254240285235 | 0.571360771665498 | 1.55486030058568 | 0.998735809224318 |
| ebi-fl187-GCST90094798 | ebi-gm430-GCST90011688 | TestASV_15 (Bacteroides) prevalence \|\| id:ebi-gm430-GCST90011688 | Mayonnaise liking \|\| id:ebi-fl187-GCST90094798 | Inverse variance weighted | 8 | 0.0182059488083805 | 0.240705023482037 | 0.939708748661423 | -0.453575897 | 0.489987794833173 | 1.01837268743565 | 0.635352130722978 | 1.63229629738518 | 0.998735809224318 |
| ebi-fl187-GCST90094798 | ebi-gm430-GCST90011692 | TestASV_19 (Prevotella) prevalence \|\| id:ebi-gm430-GCST90011692 | Mayonnaise liking \|\| id:ebi-fl187-GCST90094798 | Inverse variance weighted | 8 | -0.046827632 | 0.470451623464825 | 0.920711423900472 | -0.968912814 | 0.875257549521184 | 0.954251865452672 | 0.379495395814586 | 2.39949320271808 | 0.998735809224318 |
| ebi-fl187-GCST90094798 | ebi-gm430-GCST90011693 | TestASV_2 (Bacteroides) prevalence \|\| id:ebi-gm430-GCST90011693 | Mayonnaise liking \|\| id:ebi-fl187-GCST90094798 | Inverse variance weighted | 8 | -0.126315098 | 0.31859681408659 | 0.691755975806563 | -0.750764854 | 0.498134657663865 | 0.881337095517888 | 0.472005399635844 | 1.64564870769525 | 0.998735809224318 |
| ebi-fl187-GCST90094798 | ebi-gm430-GCST90011716 | TestASV_40 (Barnesiella) prevalence \|\| id:ebi-gm430-GCST90011716 | Mayonnaise liking \|\| id:ebi-fl187-GCST90094798 | Inverse variance weighted | 8 | 0.166137695909757 | 0.469636852305943 | 0.723520407694517 | -0.754350535 | 1.08662592642941 | 1.180735673 | 0.470315969505734 | 2.96425556411994 | 0.998735809224318 |
| ebi-fl187-GCST90094798 | ebi-gm430-GCST90011718 | TestASV_42 (Alphaproteobacteria) prevalence \|\| id:ebi-gm430-GCST90011718 | Mayonnaise liking \|\| id:ebi-fl187-GCST90094798 | Inverse variance weighted | 8 | 0.597776593875454 | 0.376014520766202 | 0.111886664932758 | -0.139211867 | 1.33476505457721 | 1.8180719908078 | 0.870043675537527 | 3.79910325963548 | 0.998735809224318 |
| ebi-fl187-GCST90094798 | ebi-hbsm44-GCST90104929 | Sulcal depth of the right inferior frontal sulcus \|\| id:ebi-hbsm44-GCST90104929 | Mayonnaise liking \|\| id:ebi-fl187-GCST90094798 | Inverse variance weighted | 8 | -0.087951716 | 0.261874762325275 | 0.736980882220028 | -0.60122625 | 0.425322818369326 | 0.915805094582946 | 0.548139068307421 | 1.53008427925758 | 0.998735809224318 |
| ebi-fl187-GCST90094798 | ebi-hbsm44-GCST90104935 | Sulcal opening distance of the left calloso-marginal posterior fissure \|\| id:ebi-hbsm44-GCST90104935 | Mayonnaise liking \|\| id:ebi-fl187-GCST90094798 | Inverse variance weighted | 8 | -0.040831636 | 0.0677325603609319 | 0.546617694937334 | -0.173587454 | 0.0919241823397791 | 0.959990744255123 | 0.840643630067798 | 1.09628170141631 | 0.998735809224318 |
| ebi-fl187-GCST90094798 | ebi-hbsm44-GCST90104936 | Sulcal opening distance of the left anterior inferior temporal sulcus \|\| id:ebi-hbsm44-GCST90104936 | Mayonnaise liking \|\| id:ebi-fl187-GCST90094798 | Inverse variance weighted | 8 | 0.00985220459468254 | 0.0281917988510623 | 0.726736045972549 | -0.045403721 | 0.0651081303427646 | 1.0099008973413 | 0.955611603325987 | 1.06727442289421 | 0.998735809224318 |
| ebi-fl187-GCST90094798 | ebi-hbsm44-GCST90104941 | Sulcal opening distance of the right posterior inferior temporal sulcus \|\| id:ebi-hbsm44-GCST90104941 | Mayonnaise liking \|\| id:ebi-fl187-GCST90094798 | Inverse variance weighted | 8 | -0.007115818 | 0.0230039506282221 | 0.757070300759522 | -0.052203562 | 0.0379719247781196 | 0.992909439038184 | 0.949135639527106 | 1.03870207067803 | 0.998735809224318 |
| ebi-fl187-GCST90094798 | ebi-IPs91-GCST90274819 | Leukemia inhibitory factor levels \|\| id:ebi-IPs91-GCST90274819 | Mayonnaise liking \|\| id:ebi-fl187-GCST90094798 | Inverse variance weighted | 12 | -0.033656149 | 0.0981817360883356 | 0.731753142455354 | -0.226092352 | 0.158780053851797 | 0.9669039184778 | 0.797644434512779 | 1.17208012381956 | 0.998735809224318 |
| ebi-fl187-GCST90094798 | ebi-IPs91-GCST90274837 | STAM binding protein levels \|\| id:ebi-IPs91-GCST90274837 | Mayonnaise liking \|\| id:ebi-fl187-GCST90094798 | Inverse variance weighted | 12 | -0.226117852 | 0.0761357409712389 | 0.0029786722059805 | -0.375343904 | -0.0768918 | 0.797624094437669 | 0.68705295760756 | 0.925990040480849 | 0.998735809224318 |
| ebi-fl187-GCST90094798 | ebi-IPs91-GCST90274840 | TNF-beta levels \|\| id:ebi-IPs91-GCST90274840 | Mayonnaise liking \|\| id:ebi-fl187-GCST90094798 | Inverse variance weighted | 12 | -0.121542147 | 0.0920264297118162 | 0.18659111616567 | -0.301913949 | 0.0588296553934656 | 0.885553729269488 | 0.739401688349922 | 1.06059455878869 | 0.998735809224318 |
| ebi-fl187-GCST90094798 | ebi-IPs91-GCST90274842 | Tumor necrosis factor ligand superfamily member 14 levels \|\| id:ebi-IPs91-GCST90274842 | Mayonnaise liking \|\| id:ebi-fl187-GCST90094798 | Inverse variance weighted | 12 | 0.0769037442613511 | 0.0837808917413453 | 0.358663324122055 | -0.087306804 | 0.241114292074388 | 1.07993812114799 | 0.916395898982734 | 1.27266648268864 | 0.998735809224318 |
| ebi-fl187-GCST90094798 | ebi-met1400-GCST90199628 | 3-methylhistidine levels \|\| id:ebi-met1400-GCST90199628 | Mayonnaise liking \|\| id:ebi-fl187-GCST90094798 | Inverse variance weighted | 9 | 0.0723944007671047 | 0.132508478291725 | 0.584833704136401 | -0.187322217 | 0.332111018218886 | 1.07507927254925 | 0.82917651882783 | 1.39390758906068 | 0.998735809224318 |
| ebi-fl187-GCST90094798 | ebi-met1400-GCST90199651 | Docosahexaenoate DHA; 22:6n3 levels \|\| id:ebi-met1400-GCST90199651 | Mayonnaise liking \|\| id:ebi-fl187-GCST90094798 | Inverse variance weighted (multiplicative random effects) | 9 | -0.138374166 | 0.1576517881459 | 0.38009555607303 | -0.447371671 | 0.17062333892525 | 0.870772817340793 | 0.639306252769933 | 1.18604392829627 | 0.998735809224318 |
| ebi-fl187-GCST90094798 | ebi-met1400-GCST90199652 | 1-stearoyl-2-oleoyl-GPS (18:0/18:1) levels \|\| id:ebi-met1400-GCST90199652 | Mayonnaise liking \|\| id:ebi-fl187-GCST90094798 | Inverse variance weighted | 9 | -0.109757058 | 0.108868644546031 | 0.313377458831006 | -0.323139601 | 0.103625485626056 | 0.896051797754601 | 0.723872793952167 | 1.10918497140301 | 0.998735809224318 |
| ebi-fl187-GCST90094798 | ebi-met1400-GCST90199653 | 1-myristoyl-2-palmitoyl-gpc (14:0/16:0) levels \|\| id:ebi-met1400-GCST90199653 | Mayonnaise liking \|\| id:ebi-fl187-GCST90094798 | Inverse variance weighted | 9 | 0.144773636216211 | 0.106753037931568 | 0.175049945601629 | -0.064462318 | 0.354009590562084 | 1.15577791439251 | 0.937571443041967 | 1.42476885074848 | 0.998735809224318 |
| ebi-fl187-GCST90094798 | ebi-met1400-GCST90199667 | Erythritol levels in elite athletes \|\| id:ebi-met1400-GCST90199667 | Mayonnaise liking \|\| id:ebi-fl187-GCST90094798 | Inverse variance weighted | 9 | 0.0385485935641533 | 0.135614741803907 | 0.776218217362451 | -0.2272563 | 0.304353487499812 | 1.03930123048195 | 0.796716557370158 | 1.35574821144261 | 0.998735809224318 |
| ebi-fl187-GCST90094798 | ebi-met1400-GCST90199674 | 3-hydroxymyristate levels \|\| id:ebi-met1400-GCST90199674 | Mayonnaise liking \|\| id:ebi-fl187-GCST90094798 | Inverse variance weighted | 9 | -0.220601776 | 0.138549544852062 | 0.111334600851158 | -0.492158884 | 0.0509553321259869 | 0.802036006864181 | 0.611305231682667 | 1.05227588930658 | 0.998735809224318 |
| ebi-fl187-GCST90094798 | ebi-met1400-GCST90199685 | 2-linoleoylglycerol (18:2) levels \|\| id:ebi-met1400-GCST90199685 | Mayonnaise liking \|\| id:ebi-fl187-GCST90094798 | Inverse variance weighted | 9 | 0.0469562185764943 | 0.123156514123745 | 0.703000897204064 | -0.194430549 | 0.288342986259035 | 1.0480761218106 | 0.823303355425027 | 1.33421484301188 | 0.998735809224318 |
| ebi-fl187-GCST90094798 | ebi-met1400-GCST90199703 | Gamma-glutamylvaline levels \|\| id:ebi-met1400-GCST90199703 | Mayonnaise liking \|\| id:ebi-fl187-GCST90094798 | Inverse variance weighted | 9 | 0.00574702262859093 | 0.113338591309548 | 0.959559261029218 | -0.216396616 | 0.227890661595304 | 1.00576356844434 | 0.805415797453079 | 1.25594799457461 | 0.998735809224318 |
| ebi-fl187-GCST90094798 | ebi-met1400-GCST90199779 | Gamma-glutamyl-2-aminobutyrate levels \|\| id:ebi-met1400-GCST90199779 | Mayonnaise liking \|\| id:ebi-fl187-GCST90094798 | Inverse variance weighted | 9 | 0.0672495631371162 | 0.150861810831155 | 0.655763903226787 | -0.228439586 | 0.36293871236618 | 1.06956236821021 | 0.795774371592005 | 1.43754775264105 | 0.998735809224318 |
| ebi-fl187-GCST90094798 | ebi-met1400-GCST90199824 | 4-vinylphenol sulfate levels \|\| id:ebi-met1400-GCST90199824 | Mayonnaise liking \|\| id:ebi-fl187-GCST90094798 | Inverse variance weighted | 9 | 0.293837574629774 | 0.144307116837903 | 0.0417306773871273 | 0.0109956256274839 | 0.576679523632064 | 1.34156598161817 | 1.01105629969816 | 1.78011776749963 | 0.998735809224318 |
| ebi-fl187-GCST90094798 | ebi-met1400-GCST90199830 | 4-ethylphenylsulfate levels \|\| id:ebi-met1400-GCST90199830 | Mayonnaise liking \|\| id:ebi-fl187-GCST90094798 | Inverse variance weighted | 9 | 0.00512873241572536 | 0.118930137513781 | 0.965602760477829 | -0.227974337 | 0.238231801942736 | 1.00514190687695 | 0.796144690945897 | 1.26900331616844 | 0.998735809224318 |
| ebi-fl187-GCST90094798 | ebi-met1400-GCST90199833 | Hexanoylglutamine levels \|\| id:ebi-met1400-GCST90199833 | Mayonnaise liking \|\| id:ebi-fl187-GCST90094798 | Inverse variance weighted (multiplicative random effects) | 9 | -0.144685337 | 0.149645375775846 | 0.333616668331602 | -0.437990273 | 0.1486195996045 | 0.865294526521739 | 0.645332059689821 | 1.16023155271158 | 0.998735809224318 |
| ebi-fl187-GCST90094798 | ebi-met1400-GCST90199837 | Carotenoid levels (cryptoxanthin) \|\| id:ebi-met1400-GCST90199837 | Mayonnaise liking \|\| id:ebi-fl187-GCST90094798 | Inverse variance weighted | 9 | -0.353129874 | 0.129333847471887 | 0.00632623638766892 | -0.606624215 | -0.099635532 | 0.702485953138257 | 0.545188204511871 | 0.905167262007078 | 0.998735809224318 |
| ebi-fl187-GCST90094798 | ebi-met1400-GCST90199869 | 2-hydroxyhippurate levels \|\| id:ebi-met1400-GCST90199869 | Mayonnaise liking \|\| id:ebi-fl187-GCST90094798 | Inverse variance weighted | 9 | 0.00956184636684988 | 0.124879435955209 | 0.938966721411701 | -0.235201848 | 0.25432554083906 | 1.00960770687365 | 0.79041129050448 | 1.28959155065725 | 0.998735809224318 |
| ebi-fl187-GCST90094798 | ebi-met1400-GCST90199902 | Hydantoin-5-propionate levels \|\| id:ebi-met1400-GCST90199902 | Mayonnaise liking \|\| id:ebi-fl187-GCST90094798 | Inverse variance weighted | 9 | 0.100447358432034 | 0.135318154736506 | 0.457902493363025 | -0.164776225 | 0.365670941715586 | 1.10566543620987 | 0.848083462858303 | 1.4414808333946 | 0.998735809224318 |
| ebi-fl187-GCST90094798 | ebi-met1400-GCST90199904 | Pantoate levels \|\| id:ebi-met1400-GCST90199904 | Mayonnaise liking \|\| id:ebi-fl187-GCST90094798 | Inverse variance weighted | 9 | 0.0126716911838816 | 0.111753577026524 | 0.909721700237368 | -0.20636532 | 0.231708702155869 | 1.01275231725893 | 0.813535821255817 | 1.26075242087074 | 0.998735809224318 |
| ebi-fl187-GCST90094798 | ebi-met1400-GCST90199936 | 3-methyl catechol sulfate (2) levels \|\| id:ebi-met1400-GCST90199936 | Mayonnaise liking \|\| id:ebi-fl187-GCST90094798 | Inverse variance weighted | 9 | 0.013892222382362 | 0.163702511417606 | 0.932370617415695 | -0.3069647 | 0.33474914476087 | 1.01398916771222 | 0.73567656983465 | 1.39758974853421 | 0.998735809224318 |
| ebi-fl187-GCST90094798 | ebi-met1400-GCST90199977 | 4-hydroxychlorothalonil levels \|\| id:ebi-met1400-GCST90199977 | Mayonnaise liking \|\| id:ebi-fl187-GCST90094798 | Inverse variance weighted | 9 | 0.0635395660933689 | 0.141983744436508 | 0.654504730777024 | -0.214748573 | 0.341827705188924 | 1.06560164666164 | 0.806744251965354 | 1.40751776861344 | 0.998735809224318 |
| ebi-fl187-GCST90094798 | ebi-met1400-GCST90200040 | Sphingomyelin (d18:2/24:1, d18:1/24:2) levels \|\| id:ebi-met1400-GCST90200040 | Mayonnaise liking \|\| id:ebi-fl187-GCST90094798 | Inverse variance weighted | 9 | -0.145131938 | 0.0994768135725049 | 0.144578202439306 | -0.340106493 | 0.0498426162141659 | 0.864908170992563 | 0.711694528248572 | 1.05110565637007 | 0.998735809224318 |
| ebi-fl187-GCST90094798 | ebi-met1400-GCST90200077 | 1-stearoyl-2-oleoyl-GPI (18:0/18:1) levels \|\| id:ebi-met1400-GCST90200077 | Mayonnaise liking \|\| id:ebi-fl187-GCST90094798 | Inverse variance weighted | 9 | 0.0112124988191392 | 0.106373411264223 | 0.916052886741411 | -0.197279387 | 0.219704384897016 | 1.01127559448354 | 0.820961235159669 | 1.24570842592723 | 0.998735809224318 |
| ebi-fl187-GCST90094798 | ebi-met1400-GCST90200080 | 1-palmitoyl-2-oleoyl-GPI (16:0/18:1) levels \|\| id:ebi-met1400-GCST90200080 | Mayonnaise liking \|\| id:ebi-fl187-GCST90094798 | Inverse variance weighted | 9 | 0.0240510035667956 | 0.107241988929025 | 0.822548359387028 | -0.186143295 | 0.234245301867684 | 1.02434256168257 | 0.83015462967167 | 1.26395450458356 | 0.998735809224318 |
| ebi-fl187-GCST90094798 | ebi-met1400-GCST90200082 | 1-oleoyl-2-linoleoyl-GPE (18:1/18:2) levels \|\| id:ebi-met1400-GCST90200082 | Mayonnaise liking \|\| id:ebi-fl187-GCST90094798 | Inverse variance weighted | 9 | 0.188143466200336 | 0.136119030753027 | 0.166910882182617 | -0.078649834 | 0.454936766476269 | 1.20700666758894 | 0.924363548397148 | 1.57607371918805 | 0.998735809224318 |
| ebi-fl187-GCST90094798 | ebi-met1400-GCST90200100 | Perfluorooctanesulfonate (PFOS) levels \|\| id:ebi-met1400-GCST90200100 | Mayonnaise liking \|\| id:ebi-fl187-GCST90094798 | Inverse variance weighted | 9 | -0.019532318 | 0.0990583773942934 | 0.843686778952512 | -0.213686738 | 0.174622101231026 | 0.980657201344231 | 0.807601336085541 | 1.19079613118228 | 0.998735809224318 |
| ebi-fl187-GCST90094798 | ebi-met1400-GCST90200102 | 1-palmitoleoyl-2-linolenoyl-GPC (16:1/18:3) levels \|\| id:ebi-met1400-GCST90200102 | Mayonnaise liking \|\| id:ebi-fl187-GCST90094798 | Inverse variance weighted | 9 | 0.0441540737022761 | 0.105340230297446 | 0.675101542654557 | -0.162312778 | 0.250620925085271 | 1.04514337159258 | 0.850175247107632 | 1.28482294785702 | 0.998735809224318 |
| ebi-fl187-GCST90094798 | ebi-met1400-GCST90200114 | Glycosyl-N-tricosanoyl-sphingadienine (d18:2/23:0) levels \|\| id:ebi-met1400-GCST90200114 | Mayonnaise liking \|\| id:ebi-fl187-GCST90094798 | Inverse variance weighted | 9 | -0.086333707 | 0.112371732417553 | 0.442316874377108 | -0.306582303 | 0.133914888052267 | 0.917288074242235 | 0.735957944121943 | 1.14329550739602 | 0.998735809224318 |
| ebi-fl187-GCST90094798 | ebi-met1400-GCST90200135 | Arachidoylcarnitine (C20) levels \|\| id:ebi-met1400-GCST90200135 | Mayonnaise liking \|\| id:ebi-fl187-GCST90094798 | Inverse variance weighted | 9 | 0.275009974708814 | 0.153002827602499 | 0.0722693492135011 | -0.024875567 | 0.574895516809712 | 1.31654380694324 | 0.975431279935119 | 1.77694485634692 | 0.998735809224318 |
| ebi-fl187-GCST90094798 | ebi-met1400-GCST90200146 | Docosahexaenoylcarnitine (C22:6) levels \|\| id:ebi-met1400-GCST90200146 | Mayonnaise liking \|\| id:ebi-fl187-GCST90094798 | Inverse variance weighted | 9 | -0.052144476 | 0.137519390797887 | 0.704554884813794 | -0.321682482 | 0.217393529765653 | 0.949191721324731 | 0.724928331467477 | 1.24283309773199 | 0.998735809224318 |
| ebi-fl187-GCST90094798 | ebi-met1400-GCST90200162 | 3-hydroxyphenylacetoylglutamine levels \|\| id:ebi-met1400-GCST90200162 | Mayonnaise liking \|\| id:ebi-fl187-GCST90094798 | Inverse variance weighted | 9 | 0.0453271711169981 | 0.13127355944101 | 0.729877945492488 | -0.211969005 | 0.302623347621377 | 1.04637014600206 | 0.8089897715025 | 1.35340460536465 | 0.998735809224318 |
| ebi-fl187-GCST90094798 | ebi-met1400-GCST90200172 | 3-carboxy-4-methyl-5-pentyl-2-furanpropionate (3-CMPFP) levels \|\| id:ebi-met1400-GCST90200172 | Mayonnaise liking \|\| id:ebi-fl187-GCST90094798 | Inverse variance weighted | 9 | 0.210423358784482 | 0.1138834546062 | 0.0646446104781493 | -0.012788212 | 0.433634929812633 | 1.23420045897395 | 0.987293209493479 | 1.54285551473914 | 0.998735809224318 |
| ebi-fl187-GCST90094798 | ebi-met1400-GCST90200222 | Dihydrocaffeate sulfate (2) levels \|\| id:ebi-met1400-GCST90200222 | Mayonnaise liking \|\| id:ebi-fl187-GCST90094798 | Inverse variance weighted | 9 | 0.0112723302250984 | 0.122121949663602 | 0.926456602889244 | -0.228086691 | 0.250631351565759 | 1.01133610233428 | 0.796055245926732 | 1.28483634410825 | 0.998735809224318 |
| ebi-fl187-GCST90094798 | ebi-met1400-GCST90200223 | 4-methylhexanoylglutamine levels \|\| id:ebi-met1400-GCST90200223 | Mayonnaise liking \|\| id:ebi-fl187-GCST90094798 | Inverse variance weighted | 9 | -0.036157338 | 0.129413265307021 | 0.779942191373579 | -0.289807338 | 0.217492662298199 | 0.964488531133866 | 0.748407743642718 | 1.24295630903152 | 0.998735809224318 |
| ebi-fl187-GCST90094798 | ebi-met1400-GCST90200224 | 2-ketocaprylate levels \|\| id:ebi-met1400-GCST90200224 | Mayonnaise liking \|\| id:ebi-fl187-GCST90094798 | Inverse variance weighted | 9 | 0.152677209276246 | 0.121417868314224 | 0.20858980729428 | -0.085301813 | 0.390656231172125 | 1.16494888355269 | 0.918235107632565 | 1.47795035281282 | 0.998735809224318 |
| ebi-fl187-GCST90094798 | ebi-met1400-GCST90200243 | Hydroxypalmitoyl sphingomyelin (d18:1/16:0(OH)) levels \|\| id:ebi-met1400-GCST90200243 | Mayonnaise liking \|\| id:ebi-fl187-GCST90094798 | Inverse variance weighted | 9 | 0.0501772096429549 | 0.105030409238199 | 0.632835393092319 | -0.155682392 | 0.256036811749824 | 1.05145740825931 | 0.855830965574089 | 1.2918002804932 | 0.998735809224318 |
| ebi-fl187-GCST90094798 | ebi-met1400-GCST90200273 | 2-methoxyhydroquinone sulfate (2) levels \|\| id:ebi-met1400-GCST90200273 | Mayonnaise liking \|\| id:ebi-fl187-GCST90094798 | Inverse variance weighted | 9 | 0.216249969108824 | 0.156539456537984 | 0.167143513406307 | -0.090567366 | 0.523067303923272 | 1.24141265503441 | 0.913412799130571 | 1.68719486035938 | 0.998735809224318 |
| ebi-fl187-GCST90094798 | ebi-met1400-GCST90200276 | 2,4-di-tert-butylphenol levels \|\| id:ebi-met1400-GCST90200276 | Mayonnaise liking \|\| id:ebi-fl187-GCST90094798 | Inverse variance weighted | 9 | 0.0119411744636452 | 0.106979299942013 | 0.911123638644959 | -0.197738253 | 0.22162060234999 | 1.01201275492193 | 0.820584610243753 | 1.24809776266758 | 0.998735809224318 |
| ebi-fl187-GCST90094798 | ebi-met1400-GCST90200289 | N-acetylmethionine levels \|\| id:ebi-met1400-GCST90200289 | Mayonnaise liking \|\| id:ebi-fl187-GCST90094798 | Inverse variance weighted | 9 | 0.083203325326647 | 0.107092023806674 | 0.437198311495332 | -0.126697041 | 0.293103691987727 | 1.08676275247736 | 0.881000538918018 | 1.34058179081554 | 0.998735809224318 |
| ebi-fl187-GCST90094798 | ebi-met1400-GCST90200308 | 3-hydroxyisobutyrate levels \|\| id:ebi-met1400-GCST90200308 | Mayonnaise liking \|\| id:ebi-fl187-GCST90094798 | Inverse variance weighted | 9 | -0.142093157 | 0.127156950208752 | 0.263796511977233 | -0.391320779 | 0.10713446534752 | 0.867540435203644 | 0.676163221966643 | 1.11308391563251 | 0.998735809224318 |
| ebi-fl187-GCST90094798 | ebi-met1400-GCST90200383 | Cystathionine levels \|\| id:ebi-met1400-GCST90200383 | Mayonnaise liking \|\| id:ebi-fl187-GCST90094798 | Inverse variance weighted | 9 | 0.160745243469564 | 0.113719918050175 | 0.15750323406142 | -0.062145796 | 0.383636282847907 | 1.17438574826267 | 0.939745865700263 | 1.46761154910191 | 0.998735809224318 |
| ebi-fl187-GCST90094798 | ebi-met1400-GCST90200384 | Guanidinoacetate levels \|\| id:ebi-met1400-GCST90200384 | Mayonnaise liking \|\| id:ebi-fl187-GCST90094798 | Inverse variance weighted | 9 | -0.073694509 | 0.102785454109591 | 0.473390077308195 | -0.275153999 | 0.127764981500689 | 0.928955438438364 | 0.759455159181009 | 1.13628592310153 | 0.998735809224318 |
| ebi-fl187-GCST90094798 | ebi-met1400-GCST90200403 | Citrulline levels \|\| id:ebi-met1400-GCST90200403 | Mayonnaise liking \|\| id:ebi-fl187-GCST90094798 | Inverse variance weighted | 9 | 0.00994776235202859 | 0.131464010560462 | 0.939682366225556 | -0.247721698 | 0.267617223050533 | 1.00999740581718 | 0.780577148963541 | 1.30684681342764 | 0.998735809224318 |
| ebi-fl187-GCST90094798 | ebi-met1400-GCST90200434 | 12,13-DiHOME levels \|\| id:ebi-met1400-GCST90200434 | Mayonnaise liking \|\| id:ebi-fl187-GCST90094798 | Inverse variance weighted | 9 | -0.024390158 | 0.118225398492711 | 0.83655487248116 | -0.256111939 | 0.207331623107764 | 0.975904878435553 | 0.774055316976999 | 1.23039053006416 | 0.998735809224318 |
| ebi-fl187-GCST90094798 | ebi-met1400-GCST90200437 | Fructose levels \|\| id:ebi-met1400-GCST90200437 | Mayonnaise liking \|\| id:ebi-fl187-GCST90094798 | Inverse variance weighted | 9 | 0.0132551758108465 | 0.138243471384806 | 0.923613628284881 | -0.257702028 | 0.284212379725065 | 1.01334341509846 | 0.772825478075275 | 1.3287150929351 | 0.998735809224318 |
| ebi-fl187-GCST90094798 | ebi-met1400-GCST90200448 | N-stearoyl-sphinganine (d18:0/18:0) levels \|\| id:ebi-met1400-GCST90200448 | Mayonnaise liking \|\| id:ebi-fl187-GCST90094798 | Inverse variance weighted (multiplicative random effects) | 9 | 0.0364359345853624 | 0.187618303564669 | 0.846017363033064 | -0.33129594 | 0.404167809572114 | 1.03710785914434 | 0.717992654561926 | 1.49805531388789 | 0.998735809224318 |
| ebi-fl187-GCST90094798 | ebi-met1400-GCST90200468 | X-11847 levels \|\| id:ebi-met1400-GCST90200468 | Mayonnaise liking \|\| id:ebi-fl187-GCST90094798 | Inverse variance weighted | 9 | -0.050655046 | 0.113995331099967 | 0.656781817485348 | -0.274085895 | 0.172775803093505 | 0.950606529636599 | 0.760266769480136 | 1.18859959485754 | 0.998735809224318 |
| ebi-fl187-GCST90094798 | ebi-met1400-GCST90200476 | X-11843 levels \|\| id:ebi-met1400-GCST90200476 | Mayonnaise liking \|\| id:ebi-fl187-GCST90094798 | Inverse variance weighted | 9 | -0.087993438 | 0.137196243833598 | 0.521282873783571 | -0.356898076 | 0.180911200097483 | 0.915766886134078 | 0.69984382517039 | 1.19830876486697 | 0.998735809224318 |
| ebi-fl187-GCST90094798 | ebi-met1400-GCST90200511 | X-12906 levels \|\| id:ebi-met1400-GCST90200511 | Mayonnaise liking \|\| id:ebi-fl187-GCST90094798 | Inverse variance weighted | 9 | 0.0184118192156813 | 0.107875448940624 | 0.864478031487604 | -0.193024061 | 0.229847699139304 | 1.01858236181773 | 0.824462136757778 | 1.25840833866091 | 0.998735809224318 |
| ebi-fl187-GCST90094798 | ebi-met1400-GCST90200515 | X-12849 levels \|\| id:ebi-met1400-GCST90200515 | Mayonnaise liking \|\| id:ebi-fl187-GCST90094798 | Inverse variance weighted | 9 | -0.03640631 | 0.152735646068111 | 0.811600660679056 | -0.335768177 | 0.262955555833786 | 0.964248429656598 | 0.714788791268807 | 1.30076890607754 | 0.998735809224318 |
| ebi-fl187-GCST90094798 | ebi-met1400-GCST90200551 | X-18886 levels \|\| id:ebi-met1400-GCST90200551 | Mayonnaise liking \|\| id:ebi-fl187-GCST90094798 | Inverse variance weighted | 9 | -0.00433581 | 0.130783979122916 | 0.973553010919975 | -0.260672409 | 0.252000788810675 | 0.995673575784824 | 0.770533297777433 | 1.28659705216593 | 0.998955228396052 |
| ebi-fl187-GCST90094798 | ebi-met1400-GCST90200564 | X-21319 levels \|\| id:ebi-met1400-GCST90200564 | Mayonnaise liking \|\| id:ebi-fl187-GCST90094798 | Inverse variance weighted | 9 | 0.0420772537108554 | 0.108634705019925 | 0.698513462632393 | -0.170846768 | 0.255001275549909 | 1.04297504933143 | 0.842950730494151 | 1.29046326692214 | 0.998735809224318 |
| ebi-fl187-GCST90094798 | ebi-met1400-GCST90200579 | X-21442 levels \|\| id:ebi-met1400-GCST90200579 | Mayonnaise liking \|\| id:ebi-fl187-GCST90094798 | Inverse variance weighted | 9 | -0.155146337 | 0.159356770084845 | 0.330265828772697 | -0.467485606 | 0.157192932298261 | 0.85628986136694 | 0.626575747308384 | 1.17022136562036 | 0.998735809224318 |
| ebi-fl187-GCST90094798 | ebi-met1400-GCST90200581 | X-21441 levels \|\| id:ebi-met1400-GCST90200581 | Mayonnaise liking \|\| id:ebi-fl187-GCST90094798 | Inverse variance weighted | 9 | 0.141281733217867 | 0.113961384309949 | 0.21507402297276 | -0.08208258 | 0.364646046465368 | 1.15174908825661 | 0.921195883171902 | 1.44000422334973 | 0.998735809224318 |
| ebi-fl187-GCST90094798 | ebi-met1400-GCST90200648 | X-24585 levels \|\| id:ebi-met1400-GCST90200648 | Mayonnaise liking \|\| id:ebi-fl187-GCST90094798 | Inverse variance weighted | 9 | 0.0755932260885472 | 0.125002205216623 | 0.545354995492455 | -0.169411096 | 0.320597548313128 | 1.07852376958386 | 0.844161800390245 | 1.37795091061884 | 0.998735809224318 |
| ebi-fl187-GCST90094798 | ebi-met1400-GCST90200720 | Adenosine 5'-diphosphate (ADP) to pantothenate ratio \|\| id:ebi-met1400-GCST90200720 | Mayonnaise liking \|\| id:ebi-fl187-GCST90094798 | Inverse variance weighted | 9 | -0.039012381 | 0.174617019306006 | 0.82321110550838 | -0.381261739 | 0.303236976610678 | 0.961738801564221 | 0.68299909867451 | 1.35423534852273 | 0.998735809224318 |
| ebi-fl187-GCST90094798 | ebi-met1400-GCST90200724 | Cholate to taurocholate ratio \|\| id:ebi-met1400-GCST90200724 | Mayonnaise liking \|\| id:ebi-fl187-GCST90094798 | Inverse variance weighted | 9 | -0.046776031 | 0.115261260024303 | 0.684870360396817 | -0.2726881 | 0.179136038944672 | 0.954301107805493 | 0.761330209226492 | 1.19618346063536 | 0.998735809224318 |
| ebi-fl187-GCST90094798 | ebi-met1400-GCST90200726 | N-acetylputrescine to (N(1) + N(8))-acetylspermidine ratio \|\| id:ebi-met1400-GCST90200726 | Mayonnaise liking \|\| id:ebi-fl187-GCST90094798 | Inverse variance weighted | 9 | 0.215839533610654 | 0.106995009513853 | 0.0436656768904719 | 0.0061293149635018 | 0.425549752257806 | 1.24090323976111 | 1.00614813765154 | 1.53043154663465 | 0.998735809224318 |
[truncated: 163,661 more chars]
